# The evolutionary dynamics of microRNAs in domestic mammals

**DOI:** 10.1038/s41598-018-34243-8

**Published:** 2018-11-19

**Authors:** Luca Penso-Dolfin, Simon Moxon, Wilfried Haerty, Federica Di Palma

**Affiliations:** 1 Earlham Institute, Norwich Research Park, Colney Lane, Norwich, NR47UZ United Kingdom; 2 University of East Anglia, Norwich Research Park, Norwich, NR47TJ United Kingdom

## Abstract

MiRNAs are crucial regulators of gene expression found across both the plant and animal kingdoms. While the number of annotated miRNAs deposited in miRBase has greatly increased in recent years, few studies provided comparative analyses across sets of related species, or investigated the role of miRNAs in the evolution of gene regulation. We generated small RNA libraries across 5 mammalian species (cow, dog, horse, pig and rabbit) from 4 different tissues (brain, heart, kidney and testis). We identified 1676 miRBase and 413 novel miRNAs by manually curating the set of computational predictions obtained from *miRCat* and *miRDeep2*. Our dataset spanning five species has enabled us to investigate the molecular mechanisms and selective pressures driving the evolution of miRNAs in mammals. We highlight the important contributions of intronic sequences (366 orthogroups), duplication events (135 orthogroups) and repetitive elements (37 orthogroups) in the emergence of new miRNA loci. We use this framework to estimate the patterns of gains and losses across the phylogeny, and observe high levels of miRNA turnover. Additionally, the identification of lineage-specific losses enables the characterisation of the selective constraints acting on the associated target sites. Compared to the miRBase subset, novel miRNAs tend to be more tissue specific. 20 percent of novel orthogroups are restricted to the brain, and their target repertoires appear to be enriched for neuron activity and differentiation processes. These findings may reflect an important role for young miRNAs in the evolution of brain expression plasticity. Many seed sequences appear to be specific to either the cow or the dog. Analyses on the associated targets highlight the presence of several genes under artificial positive selection, suggesting an involvement of these miRNAs in the domestication process. Altogether, we provide an overview on the evolutionary mechanisms responsible for miRNA turnover in 5 domestic species, and their possible contribution to the evolution of gene regulation.

## Introduction

MiRNAs are short, ~22 nt non-coding RNA molecules found across the plant and animal kingdoms. They represent important regulators of gene expression which have been shown to be implicated in fundamental processes such as embryonic development or tissue differentiation^1–4^. miRNAs typically act by binding to complementary RNA molecules, resulting in translational repression or mRNA degradation^5–7^. Their biogenesis starts with the transcription of a long RNA molecule, the *pri-miRNA*, located inside the nucleus of a cell. This precursor, characterized by one or more stem-loop structures, is processed by the enzyme *Drosha* which cleaves the double-stranded stem region. The resulting *pre-miRNA* is then exported out of the nucleus; following the excision of the loop region operated by *Dicer*, a ~22 bp, double stranded RNA molecule will be generated^8,9^. One of these two strands (referred as 5p- and 3p-miRNA) will be typically degraded^9^, while the other will be loaded into the miRNA-induced silencing complex and guide the targeting of mRNA molecules, by partial base-pairing^8^.

The recent advent of *RNA-Seq* technology^10^ and the increasing number of assembled genomes provide us with greater power to study miRNA function and evolution. Computational tools based on this technology have been recently developed, allowing for an *in silico* identification of putative miRNA loci from a genome assembly and small RNA reads data for the same species^11–15^. Studies based on homology analyses and computational miRNA prediction have allowed for the recent identification of thousands of miRNAs, available online from databases such as *miRBase*^16^.

While many studies have been focusing on the functional role of miRNAs, especially in disease, few have tried to clarify their evolutionary history. As miRNAs represent a relatively easy path to phenotypic diversification, through both temporal and tissue specific variations in gene expression, there is a great interest in elucidating their evolution including gains and losses, and how in turn this relates to gene regulation and target sites evolution.

Meunier *et al*.^17^ highlighted the high rates of miRNA family gains in placentals and marsupials, and the key role of introns and duplication events in the emergence of novel miRNA loci. Their analyses also suggested a gradual increase in expression levels for selectively retained miRNA families, along with changes in target repertoires, while many novel miRNAs with neutral or deleterious regulatory effects seem to be rapidly lost. Mohammed *et al*.^18^ provided an overview of the miRNA diversity and evolution in the *Drosophila* genus. The authors generated a new miRNA annotation across 11 species, supported by deep sequencing from multiple tissues. They inferred gain and loss patterns across the *Drosophila* phylogeny, described cases of clade specific, 5′ end shift in miRNA processing, and compared different subpopulations of their large set of novel miRNA loci.

Other studies focused on the evolution of 3′UTR target sites, looking at their conservation across different evolutionary timescales. Xu *et al*.^19^ used high confidence CLIP data to define the evolvability of miRNA targets in vertebrates. They found that the conservation levels progressively decrease as larger taxonomic groups are considered, with 94% of target sites being conserved among Human and Chimpanzee, 80% among Human and 10 other Mammalian species, and only 6% between Human and Zebrafish. Chen and Rajewsky^20^ observed small numbers of conserved target sites across vertebrates, flies and nematodes. However, they were able to identify a small subset of deeply conserved target sites, and pointed out the enrichment for developmental processes in the corresponding genes. Comparative analyses performed by Friedman *et al*.^21^, on the contrary, suggest that a high number of predicted 3′ UTR target sites are conserved above background levels in mammals. However, results might have been influenced by the use of sequence conservation (P_ct_ score) as one of the criteria for *in silico* target identification.

In this study, we focus on the evolution of miRNAs in five domestic species of great economic and biomedical interest: cow, dog, horse, pig and rabbit, none of which have been previously included in a comparative study across domestic mammals.

The cow (*Bos taurus*) and the pig (*Sus scrofa*) represent invaluable resources for food production^22–26^. There is a great economical interest in gaining more understanding about the genetic basis of agro-economically relevant traits (for example, milk productivity, resistance to pathogens, stress, meat quality)^25^. Moreover, the pig’s high resemblance to humans in anatomy, physiology and genetics has also encouraged recent biomedical research^24,26–28^.

The dog (*Canis familiaris*) is a model system for several human diseases, and a unique example of great phenotypic diversification following a domestication event. Abundant polymorphism data have been generated^29^, while GWAS studies on this organism have successfully identified the genetic base of heritable diseases^30–36^. Genetic and genomic studies on the horse (*Equus caballus*) have mainly aimed at understanding the biology of infectious, respiratory and allergic diseases these animals are subject to, and the development of adequate therapies (https://www.uky.edu/Ag/Horsemap/welcome.html). However, the similarity with the corresponding human diseases means these studies have an even broader range of potential applications.

The rabbit (*Oryctolagus cuniculus*) represent yet another important model system for biomedical studies, which has been used in research fields such as embryology, toxicology, pulmonary and cardiovascular research, as well as neurology^37^.

To the best of our knowledge, this is the first comprehensive, comparative analysis of miRNA and target evolution across five domestic mammals, enabling us to investigate their potential role in the process or aftermath of domestication. We generate an improved miRNA annotation in these species, supported by deep sequencing from four different tissues (brain, heart, kidney and testis) and use this data to elucidate: 1) the relative contributions of different evolutionary mechanisms by which miRNAs newly arise; 2) the patterns of expression and gain/loss evolution of miRNA orthogroups, and their variation across different miRNA subpopulations; 3) the association of miRNA evolution with the regulation of specific biological processes, and their potential involvement in domestication; 4) the effect of branch specific miRNA loss on the conservation of the associated target sites; 5) the levels of target site conservation compared to the surrounding 3′ UTR regions.

## Results

### Improved genomic annotations of conserved and novel miRNA loci across five mammals

Adapter-trimmed reads were mapped against the corresponding genome using *patman*^38^. Despite some technical variability, we observed generally high proportions of reads perfectly matching to the genome (Figs S1–S3), providing us with a robust dataset across several tissues and organisms.

For the identification of miRNA loci, we ran *miRCat*^11^ and *miRDeep2*^12^ using the combined set of small RNA libraries of each species. By running both tools, we were able to generate two independent sets of putative miRNA loci per species. Predictions were then filtered based on the following two main criteria: evidence of both miRNA-3p and miRNA-5p expression in small RNA reads alignments against the predicted loci (Supplementary Files 1–5), and miRNA-like hairpin secondary structure (as predicted using the *Vienna-RNA* package)^39^. This lead to the identification of a final set (union of miRCat and miRDeep2 high confidence predictions) of 2088 loci: 1676 miRBase annotated and 412 representing novel miRNA loci (Table 1, Supplementary Tables S1–S5). Tissue specific expression plots (Supplementary Figs S4–S8) for all novel and conserved miRNAs were also generated. The number of novel miRNA loci is particularly high in the dog. The computational predictions are highly dependent on sequencing depth, and in our study more brain samples were available for the dog compared to all the other species.

**Table 1:** Counts of annotated miRNA loci, either belonging to a miRBase family or representing a novel gene.

	Novel	miRBase	Total
Cow	47	386	433
Dog	166	381	547
Horse	26	323	349
Pig	96	324	420
Rabbit	77	262	339

Therefore, we sampled 40,680,089 reads (the total number of reads available for the horse) from the combined set of genome matching dog reads (n = 99,597,072), and recalled dog miRNA loci. Based on our filtering criteria (coverage of at least one read at both the 3′ and 5′ ends, and a total coverage of at least 10 reads), we could still confidently annotate 111 novel loci. This corresponds to 66% of the original novel annotation, and still represents the highest count across all our species.

**Figure 1:**
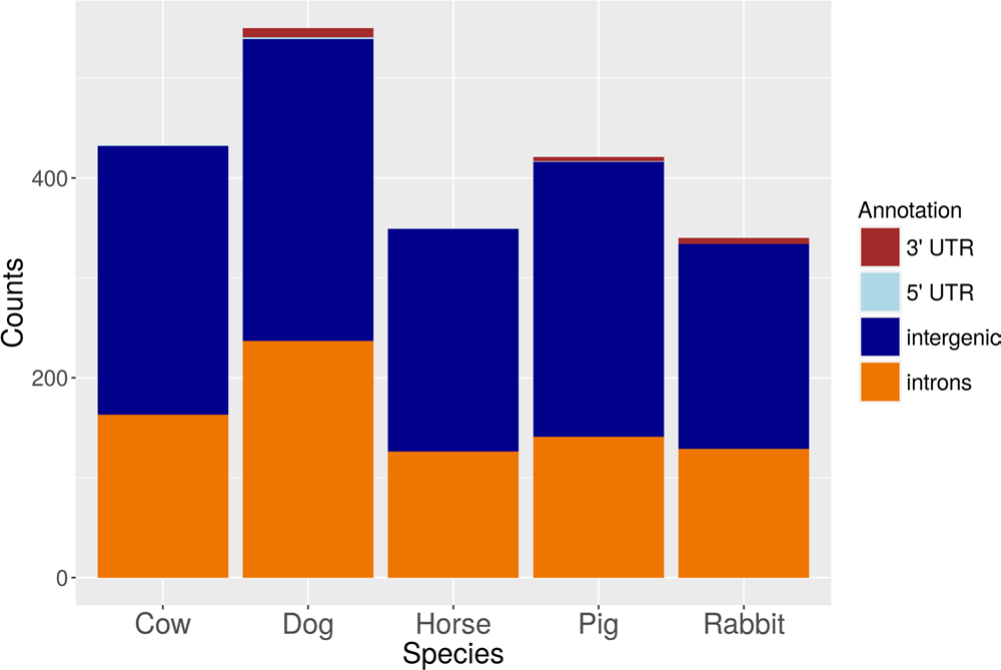
Genomic annotation for all predicted miRNA loci. Colour labelling indicate different genomic features (3′UTR, 5′UTR, intergenic regions, introns).

We compared each of the five miRNA annotations with the corresponding latest *Ensembl* gene annotation (*B. taurus* UMD3.1.8, *C. familiaris* 3.1.86, *E. caballus* 2.86, *S. scrofa* 10.2.84, *O. cuniculus* 2.0.84). Unsurprisingly, we observed very high proportions of intronic and intergenic miRNAs, and very low numbers of miRNA overlapping UTR sequences. These patterns appear to be consistent across all 5 species (see Fig. 1). Analyses performed with *GAT*^40^ indicate a significantly higher than expected overlap with introns in all species (q-value < 0.001), while intergenic regions, despite containing a high number of miRNAs, are significantly underrepresented in all five genomes (q-value < 0.001). We additionally looked at the representation of different non-coding RNA classes in our dataset, in a species specific manner. When considering the set of genome matching reads, we observed the expected enrichment for miRNA sequences (Supplementary Table S6), while other non-coding RNA classes represent, altogether, 5–10% of the reads.

### The fate of mammalian miRNA families

In order to generate clusters of homologous miRNA loci, we used *CD-hit*^41^ with 80% minimum identity on our set of annotated miRNAs. We thus obtained a total of 732 clusters (Supplementary Table S7), of which 432 grouped together only miRBase loci, 291 consisted of only novel (: absent in miRBase) sequences, and 9 represented mixed orthogroups. In order to limit potential biases in our annotations resulting from different genome assembly quality and sequencing depth across our species, we decided to look for evidence of sequence homology between genome assemblies. We thus aligned all annotated miRNA loci against the genome assemblies of human, mouse and all species considered in this study. This allowed for the identification of loci missing in the annotation, but showing high sequence homology to a miRNA annotated in another species, as well as evidence of synteny conservation in the surrounding region (see Materials and Methods). As an additional strategy to overcome differences in annotation and assembly quality, we looked for annotated miRNA sequences in the set of unaligned reads of horse and pig (for which the initial estimates of miRNA gain rates were surprisingly low). This analysis allowed us to further improve the presence-absence information used for the gain/loss inference.

**Figure 2:**
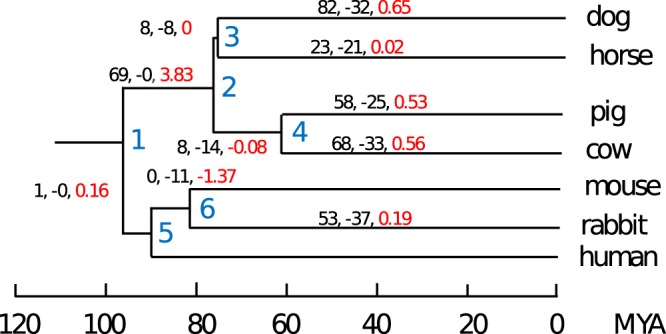
Gain and loss of miRNA clusters across the phylogenetic tree, as inferred by Dollo parsimony and synteny analyses. For each branch of the tree, the (black labelled) number of gained (+) and lost (−) orthogroups is provided. Red labelled numbers represent the branch specific net gain rate of orthogroups per million year.

We used this curated set to characterise the most parsimonious patterns of gain and loss of miRNA orthogroups across the phylogeny. Despite the high proportion of broadly conserved miRNA families, we observed high levels of miRNA turnover across the phylogeny, with many families being gained or lost in internal and terminal branches (Fig. 2). We observed a positive net gain rate in all terminal branches, with the only exception of the horse lineage. In this case, the virtually equal rate of gain and loss is likely due to a lack of sequencing depth, resulting in a limited number of predicted novel, horse specific miRNAs.

### Genomic sources of miRNAs

Various mechanisms can contribute to the appearance of new miRNA genes. While introns represent a crucial source of newly processed miRNA hairpins (sometimes not requiring Drosha processing, see the case of miRtrons), other miRNAs could arise by gene duplication, transcription of the opposite strand of an existing miRNA locus, or evolution from repetitive elements^8^. Our dataset allows us to determine the extent of the contribution of different evolutionary processes in mammals.

**Figure 3:**
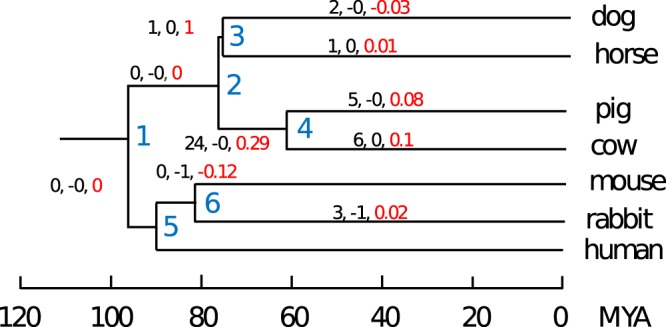
Gain and loss of repeat-derived miRNA clusters across the phylogenetic tree, as inferred by Dollo parsimony. The tree labelling is equivalent to Fig. 2.

For the identification of miRNAs derived from repetitive elements, we used BLASTN^42^ to align all hairpin sequences against the *Repbase* (www.girinst.org/repbase) database. A bit score threshold, determined through the alignment of miRNAs against shuffled Repbase sequences (see Materials and Methods) was used in combination with other parameters to select high confidence BLAST hits. Following these conservative approach, we identified 72 novel and 45 miRBase miRNA loci showing a significant similarity with one or more Repbase sequences (Supplementary Table S8, Fig. S9). Interestingly, 49 out of the 72 novel, putatively repeat-derived miRNA loci are part of a single, large orthogroup specific to the dog: cluster 508. We performed GO:term enrichment analyses on repeat-derived miRNAs, and found significant enrichment for immunological processes (including “positive regulation of memory T cell differentiation”, GO:0043382; “positive regulation of activated T cell proliferation”, GO:0042104), as well as cognitive and behavioural (including “cognition”, GO:0050890; “behaviour”, GO:0007610, “exploration behaviour”, GO:0035640). These results might reflect an important role of these novel miRNAs in the evolution of immune response and neural expression plasticity. While we find 16 broadly conserved orthogroups, there are also 18 orthogroups appearing in terminal branches (Fig. 3), suggesting an important role for repetitive elements in the emergence of novel miRNA loci.

Next, we asked the question whether we could find any case of reverse complement miRNA sequences, lying on the opposite strand of exactly the same genomic interval. Our analyses lead to the identification of at least one of such cases in every species (Table 2, Supplementary Table S9), with numbers ranging from 1 (in rabbit) to 22 coupled loci (in dog). It must be noted here that single sequencing errors in reads representing very abundant miRNAs might map to the reverse complementary strand, creating an artificial reverse complementary miRNA couple. Therefore, we looked for evidence of 1 mismatch differences in reads mapping to reverse complementary miRNA couples. We observed a single mismatch difference between the most abundant read of “X/81951230-81951286(−)_mir_cow_317” at the 5′ end, TTACAATACAACCTGATAAGT, and read TTATAATACAACCTGATAAGT (mapping to “X/81951229-81951289(+)_mir_cow_318” on the opposite strand). Given that *mir_cow_317* has low expression levels (24 reads), while its reverse complementary partner *mir_cow_318* is much more abundant (13,607 reads), we cannot completely rule out the possibility of 1-error reads of *mir_cow_318* mapping to the opposite strand.

**Table 2:** The number of reverse complement miRNA gene couples and number of miRNA orthogroups containing paralogous duplicated genes.

	Reverse complement miRNA couples		Duplicated miRNAs		
	Novel	Conserved	Novel	Conserved	Total
Cow	0	12	11	89	100
Dog	4	19	14	87	101
Horse	0	7	6	79	85
Pig	1	8	21	81	102
Rabbit	0	5	10	72	82

**Figure 4:**
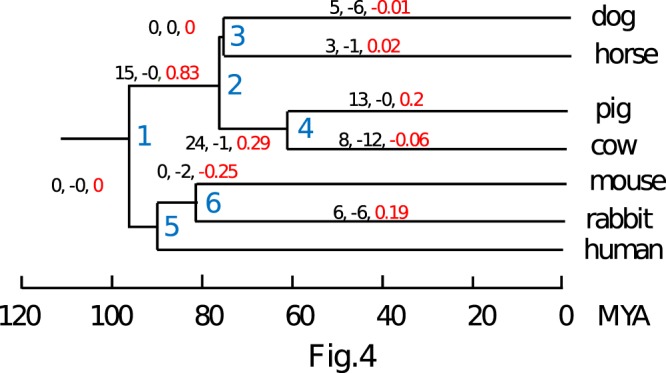
Gain and loss of clusters of duplicated miRNAs across the phylogenetic tree. The tree labelling is equivalent to Fig. 2.

We also investigated miRNA clusters containing multiple paralogous gene copies, and found a total of 135 orthogroups associated with duplication events (58 in tandem) in at least one species (Table 2 and Supplementary Table S10, Fig. S10). We identified both old duplication events (many duplicated loci have orthologous counterparts in other species) as well as more recent duplications, with genes arising in terminal branches (Fig. 4). Thus, duplication seems to be an important mechanism for the evolution of miRNAs in our set of species, as previously suggested by other studies in mammals^17^. When we compared the set of clusters with duplicated loci and with high similarity to repetitive elements, we identified 10 common clusters, 5 of which represents species specific groups.

Additionally, we looked for miRtrons, representing intronic miRNAs which do not require *Drosha* processing, as the pre-miRNA is generated by intron splicing^43^. We were able to identify only a few putative miRtrons, with numbers ranging from 0 to 8 loci per species (Table 3, Supplementary File 6).

**Table 3:** The number of miRtrons and putatively repeat-derived miRNA genes.

	Novel Repeat-Derived	Conserved Repeat-Derived	Total RD	Species specific repeat	novel miRtrons	miRBase miRtrons	Total miRtrons
Cow	4	13	17	8	0	1	1
Dog	58	7	65	49	6	2	8
Horse	2	6	8	0	0	0	0
Pig	3	14	17	4	4	2	6
Rabbit	5	5	10	5	1	1	2

### Expression patterns across the phylogeny

**Figure 5:**
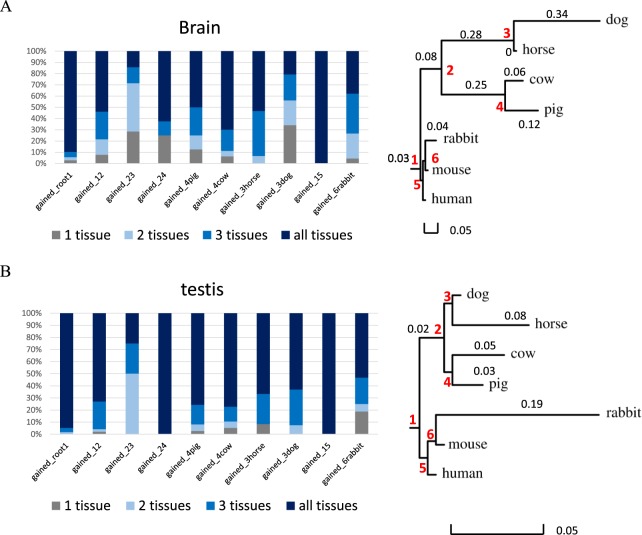
Expression patterns across the phylogeny for all miRNA orthogroups expressed in brain (**A**) and testis (**B**). Colour labelling indicate the number of miRNA families expressed in a specific tissue, divided in the following categories: tissue specific; expressed in the tissue + *n* additional ones (n = 3 indicated as “all tissues”). Branch lengths in the phylogenetic trees are proportional to the fraction of tissue specific orthogroups.

Our dataset provides us with a great opportunity to investigate how the gain and loss of miRNAs relates to the observed patterns of miRNA expression across 4 different tissues. Our data suggests that young orthogroups tend to be more tissue restricted than the older, conserved ones, particularly when we consider brain and testis. Fig. 5 shows the number of gains across the phylogeny when considering only the orthogroups with evidence of expression in brain (Fig. 5A) and testis (Fig. 5B). By constructing trees where branch lengths are proportional to the fraction of tissue specific orthogroups, we can clearly visualise the higher tissue restriction of young miRNAs. This pattern is particularly evident in the case of brain tissues, and is in line with previous studies on the evolution of novel mammalian miRNAs^17^.

**Figure 6:**
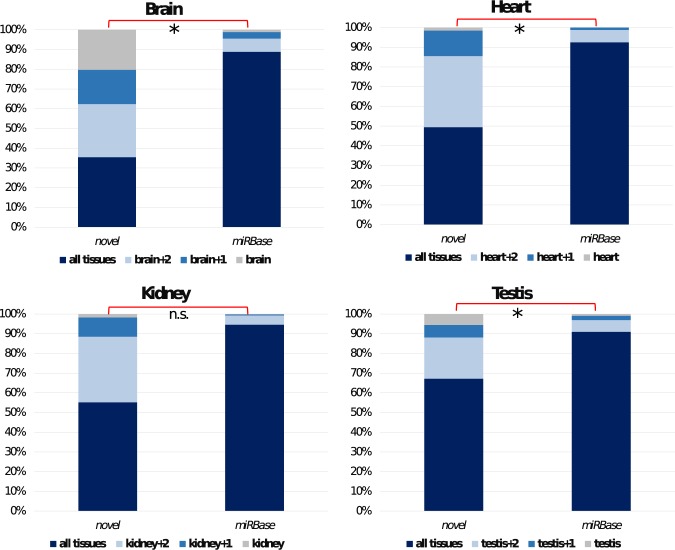
Expression patterns of novel and miRBase loci across 4 tissues. Colour labelling indicate the number of miRNA families expressed in a specific tissue, divided in the following categories: not expressed in the tissue considered; tissue specific; expressed in the tissue + *n* additional ones (n = 3 indicated as “all tissues”).

Next, we asked the question whether we can see differences in tissue specificity between novel and miRBase miRNAs. Fig. 6 shows the difference in the proportions of orthogroups expressed in a particular tissue, divided into sub-sets depending on the total number of tissues showing evidence of expression. We observed a significantly higher proportion of tissue specific families in the novel set compared to the miRBase set (z test, α = 0.05) for all four tissues except the kidney, with a particularly striking difference in the cases of brain and testis (brain: p < 10e^−4^, heart: p = 0.012, kidney: p = 0.051, testis: p = 10e^−3^). This result suggests that young miRNAs are expressed in a single or a few tissues when they first appear, and become more broadly expressed over time. Interestingly, we find that as much as 20% of novel orthogroups are restricted to the brain tissue. When we performed GO term enrichment analyses of the mRNA targets of novel, brain specific miRNA orthogroups (see Materials and Methods), results highlighted several neuronal (for example: “regulation of neuron projection development”,GO:0010975; “forebrain generation of neurons”, GO:0021872), behavioural (including “locomotory behaviour”, GO:0007626; “aggressive behaviour”, GO:0002118) and immune related processes (for instance, “negative regulation of innate immune response”, GO:0045824). Moreover, we find that the vast majority of these brain restricted orthogroups (32 out of the 55) have a species specific miRNA seed sequence (nt 2–8), potentially leading to novel regulatory interactions restricted to a particular lineage. Thus, our results suggest that the emergence of novel, tissue restricted miRNAs might play an important role in the lineage specific evolution of neuronal regulation, especially through the acquisition of novel seeds and associated targets.

### The co-evolution of miRNAs and their UTR target sites

**Figure 7:**
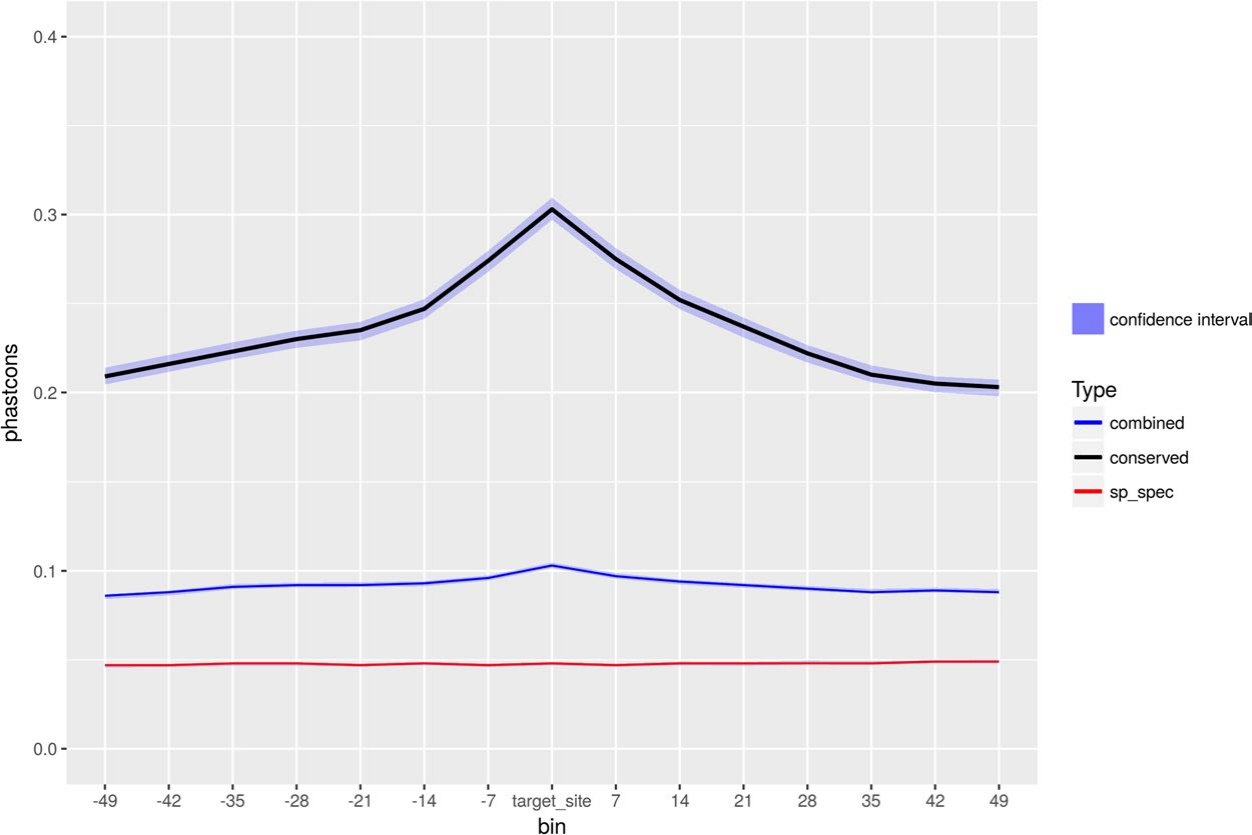
Median and confidence interval of 20-way Phastcons scores, calculated across 7nt bins, centred around the predicted targets of conserved (black line), species specific (red) and combined (blue) seed families.

Homology analyses and Dollo parsimony provided an overview of the evolutionary patterns of miRNA families in our species. Next, we asked the question whether we can detect signatures of selection acting on the predicted target repertoires. As a result of the selective constraints acting on miRNA target sites, we would expect these loci to show increased conservation compared to the surrounding 3′UTR regions. We compared 20-way *phastcons* scores of the targets associated with species specific and conserved seed families (defined as groups of miRNA loci sharing exactly the same miRNA seed sequence), and observed an evident increase in scores corresponding to the targets of conserved families (Fig. 7). Additionally, the 3′UTR of genes targeted by conserved families show substantially higher conservation across all bins, as compared to those targeted by species specific seeds. These results suggest increased levels of purifying selection at the binding sites of conserved seed families.

We next asked the question whether 3′UTR target site conservation reflects the loss of a miRNA orthogroup (or seed family) during evolution. Based on homology analyses and Dollo parsimony inference, we first identified all miRNA orthogroups which appear to be lost in a terminal branch of our phylogenetic tree (see Table 4) to test the hypothesis that the loss of a miRNA might be lead to relaxed selective constraints on the associated target sites.

**Table 4:** Summary of the number of lost orthogroups (also absent in the miRBase annotation) and seed families.

	Total orthogroup losses	Total mature seed losses
Cow	9	13
Dog	2	1
Horse	4	7
Pig	3	4
Rabbit	18	15

We tested our hypothesis of relaxation of selection by calculating pairwise target sequence similarity between the rabbit (chosen as an outgroup) and all other species.

However, we could not find any example where the species missing the miRNA family has a significantly lower conservation level in all comparisons (Supplementary Figs S11–S16). The observed lack of evidence for differential target site conservation between the species retaining and losing the miRNA orthogroup could be explained by: 1) a very recent miRNA loss; 2) a shared seed sequence between the lost and a more conserved orthogroup, leading to continued purifying selection; 3) the seed/orthogroup loss having a very weak or null effect on target site conservation; 4) the presence of false positive target predictions in our data.

**Figure 8:**
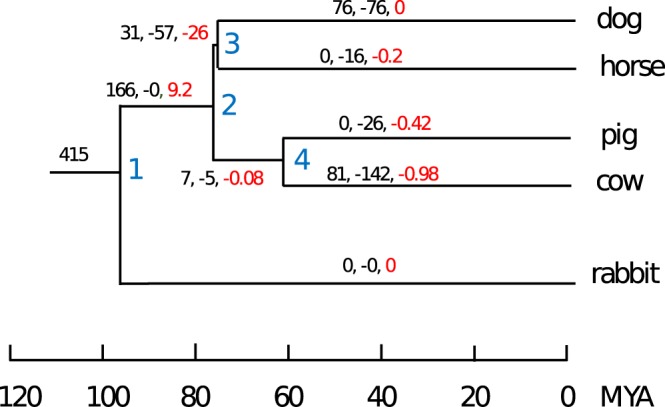
Gain and loss of miRNA seed sequences across the phylogeny. The tree labelling is equivalent to Fig. 2.

**Figure 9:**
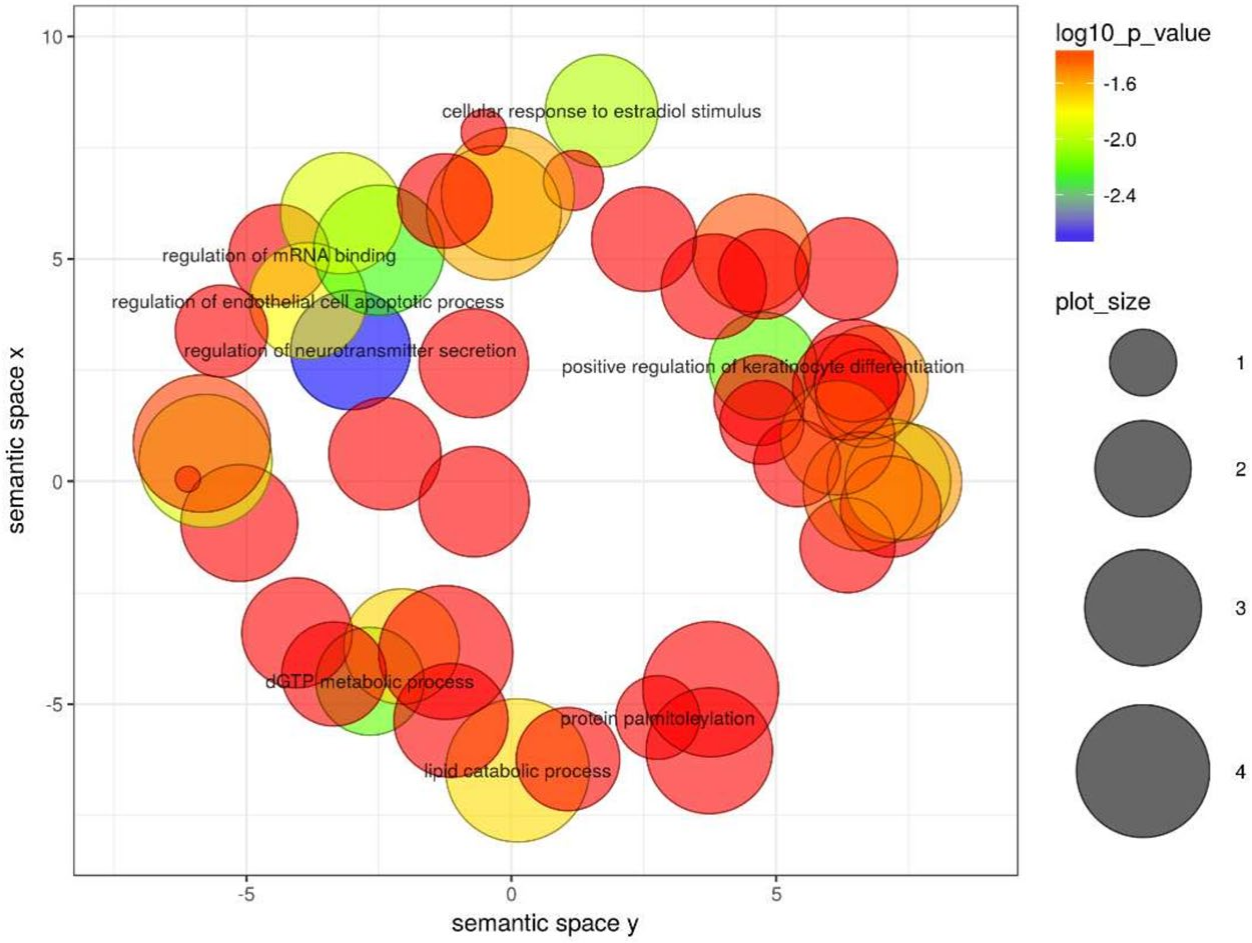
GO term results for dog specific seed family GGACCGA (orthogroup 490) as summarised by *Revigo*^62^.

Seed sharing appears to be widespread in our set of species, as clearly shown in Fig. 8. However, we also observe a considerable number of species specific seeds in the cow and the dog. Among the significantly enriched GO:term accessions (adjusted p-value < 0.05) for dog specific seed families (Fig. 9) we find: “extracellular structure organisation” (GO:0043062), “positive regulation of axon extension” (GO:0045773), “positive regulation of neuroblast proliferation” (GO:0002052), and “behaviour” (GO:0007610). The significantly enriched GO:term accessions for cow specific seed families include “lactation” (GO:0007595) and “mRNA 3′ end processing” (GO:0031124), while terms found significant only for the dog include “forebrain development” (GO:0030900) and “DNA repair” (GO:0006281). Among the accessions enriched in both the cow and dog specific seed targets we find “behaviour” (GO:0007610), “behavioural fear response” (GO:0001662) and several immune-related processes: “T-cell activation” (GO:0042110), “T cell receptor V(D)J recombination” (GO:0033153), “negative regulation of innate immune response” (GO:0045824) and additional related accessions.

Next, we searched for domestication genes described in the literature^44–46^ in the target genes corresponding to significantly enriched GO accessions. 12 genes located in the candidate domestication regions identified by^44^ are found among the targets of dog specific seed families. This set includes genes associated with behavioural (POLR1E), immunological (TLX3) and body weight (TNKS2) phenotypes in mouse (http://www.informatics.jax.org/). When we considered the set of genes lying in the top 100 genomic regions under selection in the dog identified by^45^, we found 25 genes belonging to one or more significant GO term accessions for dog specific seed families. Once again, we observed genes associated with behaviour and body weight phenotypes (for instance GLRA1 and HTR2B). This overlap is significantly higher than expected (hypergeometric test, 1.24 fold enrichment, p = 0.01).

We also looked for positively selected genes^47–49^ in the set of targets of cow specific seed families belonging to a significant GO accession. We found 24 positively selected targets among the 35 common between our set of 1:1 orthologs and the gene list provided by^47^ (1.33 fold under-enrichment, p = 10e^−4^). We observed genes associated with immune system, cardiovascular and muscular phenotypes, including TNFRSF8, CREBBP and MTMR14. Only 3 targets were found in the gene set from Xu *et al*.^48^: WIF1 (Increased osteosarcoma incidence), KIT (coat/hair pigmentation) and LRIG3 (Abnormal craniofacial morphology) (2.43 fold under-enrichment, p = 2 × 10e^−4^). Additionally, we considered the dataset from Braud *et al*.^49^, representing genes with high miRNA binding sites divergence between *B. taurus* and *B. primigenius*. Among the top 200 scoring genes, we identified 43 targets of cow specific seed families (1.31 fold under-enrichment, p < 10e^−4^). Even in this case, we observed many genes involved in immunity (for example CIITA, RHOH), brain morphology/behaviour/body size (ASPA), and neurological (FOXI1) phenotypes. Interestingly, FOXI1 (nervous system phenotype) appears as a positively selected^45,49^ target of both dog and cow specific seed families. Altogether, we clearly highlight a trend among positively selected miRNA targets towards a few biological processes and phenotypic traits. Thus, our results suggest that species specific seed families might have played a role in domestication, by modulating the expression of genes under artificial positive selection.

## Discussion

With this study, we provide the first comprehensive analysis of miRNA and target evolution in domestic species, providing new insights into the possible role of miRNAs in domestication. We based our analyses on our improved annotation of high confidence, manually curated miRNAs. This includes not only 1676 loci corresponding to a known miRBase family, but also 413 novel ones, representing previously undescribed pre-miRNA sequences.

While a vast proportion of miRNAs are conserved, our data suggests the emergence of species specific seed sequences in the cow and the dog lineages. We compared the target sites of conserved and species specific miRNA seeds, and showed that the former have higher conservation levels. Moreover, the target repertoires of species specific seeds are enriched for neurological, behavioural and immune related processes, and include several genes previously described as positively selected during domestication^45–49^. Altogether, these results suggest that lineage specific miRNAs might have played an important regulatory role in the domestication process.

Our data highlights the importance of intronic sequences (366 orthogroups), duplication events (135 orthogroups) and repetitive elements (37 orthogroups) for the emergence of new miRNA genes.

It also provides evidence for high levels of miRNA turnover across the phylogeny, and a general increase in net gain rates along terminal branches. With the only exception of the horse, these rates are comparable to previous studies on mammalian miRNA evolution^17^.

Novel miRNAs tend to be more tissue specific compared to the miRBase set, with the most striking difference being represented by the brain tissues. Indeed, we observe that 20% of novel orthogroups are restricted to the brain tissues, and the associated target repertoires are enriched for behaviour, neuron activity and differentiation processes. Similar results are observed for the targets of repeat-derived miRNAs.

Branch specific losses of a seed or an orthogroup appear to be rare events, and do not result in a detectable inter species difference in sequence conservation. Our dataset highlighted examples of miRNA loss compensation, through the retention of loci with exactly the same seed sequence. The lack of evidence for differential target site conservation between the species retaining and losing a miRNA family can be explained in many ways: a very recent miRNA loss; the lack of orthogroup seed specificity (when considering orthogroups rather than seed families); the presence of false positives in our target predictions; the absence of any effect on target site conservation.

An additional factor to consider is represented by the demographic history of our five species. Population bottlenecks associated with the domestication process can have a severe impact on genetic diversity, affect the strength of natural selection, as well as determine an increased accumulation of mildly deleterious mutations^50–54^.

Compared to protein-coding genes, miRNAs represent a relatively simple source of innovation, as they can rapidly evolve from already transcribed genomic regions. Short sequence similarity is sufficient for mRNA targeting and down-regulation, while the turnover of miRNA seed sequences and target sites provides the organism with a wide space of possible regulatory changes.

While a significant fraction of miRNAs appear to be conserved over long evolutionary times, our data confirms previous observations of high evolutionary turnover in animals, with many orthogroups appearing to be lineage specific^8,17,18^. The gain of novel miRNAs might result in the acquisition of novel regulatory pathways, and spatio-temporal changes in protein coding gene expression. It represents an additional layer of regulatory complexity which we are still trying to fully uncover. Further research is needed to better clarify the extent of the contribution of miRNAs to lineage specific adaptations and phenotypic diversity.

## Materials and Methods

### Small RNA library sequencing

Tissue samples were obtained commercially from *Zyagen*. Heart, kidney and testis were obtained for all five species. As for the brain tissues, we obtained four different brain regions (cortex, cerebellum, hypothalamus) for the cow, the dog and the pig, and whole brain for the horse and the rabbit.

Small RNA libraries were prepared using the TruSeq Small RNA Library Prep Kits. six-base indexes distinguish samples and allow multiplexed sequencing and analysis using unique indexes ((Set A: indexes 1–12 (RS-200-0012), Set B: indexes 13–24(RS-200-0024), Set C: indexes 25–36 (RS-200-0036), (TruSeq Small RNA Library Prep Kit Reference Guide, Part 15004197 Rev.G).

The TruSeq Small RNA Library Prep Kit protocol was followed using an input of 1 µg of total RNA. Quantification of total RNA was done using the Qubit RNA HS Assay kit (ThermoFisher Q32852). RNA quality was established using the Bioanalyzer RNA Nano kit (Agilent Technologies 5067-1511). An RNA Integrity Number (RIN) value ≥8 was required for the RNA to pass the QC step.

This protocol generates small RNA libraries directly from RNA by ligating adapters to each end of the RNA molecule. Reverse transcription is used to create cDNA, and PCR amplification of the cDNA (14 cycles of PCR in the standard protocol) is used to generate libraries. Library purification combines the use of *BluePippin* cassettes (*Sage Science Pippin Prep 3%* Cassettes Dye-Free (CDF3010), set to collection mode range 125–160 bp) to extract the library molecules with a concentration step (*Qiagen MinElute PCR Purification* (cat. no. 28004)) to produce libraries ready for sequencing. Library concentration and size are established using *HS DNA Qubit* and *HS DNA Bioanalyser*.

All libraries were pooled together and sequenced on 2 lanes of an Illumina *HiSeq* 2500 machine. With the only exception of dog cortex, all libraries were later sequenced on a second run of the Illumina *HiSeq*, using the same pooling strategy. This run, however, was loaded with 10% *phix* to increase sequence diversity, which lead to an improved read quality.

In the case of dog cortex, two additional libraries were also constructed (as part of the development work establishing the protocol) with a different mix of conditions: 11 PCR cycles + PAGE, and 14 PCR cycles + PAGE. All 3 available dog cortex libraries (11 PCR cycles + PAGE, 14 PCR cycles + PAGE and 14 PCR cycles + Pippin) were sequenced on an Illumina *MiSeq* machine, and resulting sequencing data was included in the study.

### Initial data quality control

Raw sequencing data quality was initially assessed using *FASTQC* (www.bioinformatics.babraham.ac.uk/projects/fastqc/). Small RNA reads were then adapter trimmed and filtered for a minimum length of 16nt after adapter removal, using in house *Perl* scripts. Filtered reads were then mapped to the corresponding genome assembly, using *patman*^38^ with parameters *-e 0*–*g 0*. For all downstream analyses, we used the resulting set of trimmed genome matching reads across all samples.

### Annotation of conserved and novel miRNAs

For each organism, we ran *miRCat*^11^ and *miRDeep2*^12^ on the corresponding combined set of small RNA libraries, thus generating two independent sets of putative miRNA loci. Genomic coordinates of miRCat and MiRDeep2 predictions were then merged using *Bedtools merge*^55^ in order to generate a non-overlapping set of loci.

We then aligned small RNA reads of each library to our predicted hairpins. We looked for evidence of 3p-miRNA and 5p-miRNA expression in the alignments, as well as miRNA-like hairpin secondary structures, generated using the *Vienna-RNA* package^39^. Based on the consistency of both the alignments and predicted secondary structure, a set of high confidence miRNA loci was derived.

Initially, all loci covered by less than 10 reads were discarded. However, some of these genes were rescued at a later stage, when we generated BLAST alignments of our final set of miRNAs against the 5 genomes (see *Homology and synteny analyses*). Specifically, when the low-coverage prediction showed both evidence of Dicer and Drosha processing and sequence homology (as identified by the BLAST analysis) with a miRNA gene present in our annotation, the gene was rescued and added to the final dataset.

The identification of novel miRNA loci was performed as follows. First, we aligned the complete set of miRBase hairpin sequences to the organism’s genome sequence, using the command-line version of *BLASTN* (e-value ≤ 10^−6^)^42^. We then mapped all mature miRBase sequences to these putative pre-miRNA hairpins, and selected those for which at least one alignment without gaps and no more than one mismatch was observed. The subset of novel miRNA genes was then identified by removing all predicted miRNA loci overlapping at least one miRBase genomic hit.

Tissue specific expression plots were generated using *Rstudio* (https://www.rstudio.com/).

In order to measure representation of different non-coding RNA (ncRNA) classes in our data, we mapped all read dataset to the corresponding genome assembly, using *bowtie*^56^ with parameters *–best –strata –M 1*. We then used *samtools view*^57^ to identify reads mapping to each different ncRNA class, using the latest *Ensembl* gene annotations (*B. taurus* UMD3.1.8, *C. familiaris* 3.1.86, *E. caballus* 2.86, *S. scrofa* 10.2.84, *O. cuniculus* 2.0.84) converted to BED format.

### Homology and synteny analyses

The combined set of annotated miRNA loci was aligned, using BLASTN^42^, against the latest genome assemblies for our five species, as well as human and mouse. We selected BLAST hits with an e-value ≤ 10^−6^ and an alignment length of at least 40 nucleotides. We then identified the closest protein coding gene upstream and downstream of the selected hits, as well as the gene containing the hit for all intragenic hits. Genes surrounding or containing the query (miRNA) and the subject (BLAST hit) sequences were compared, looking for the presence of at least one homologous pair of genes with conserved synteny structure (i.e. same upstream or downstream gene, both on the same or on the opposite strand with respect to the miRNA/BLAST hit). Any BLAST hit supported by synteny conservation of at least one protein coding gene was then flagged as an orthologous locus. Predicted miRNA loci across all five species were grouped into orthogroups, using *CD-hit*^41^ with 80% minimum identity.

To characterise the most likely patterns of gain and loss of miRNA clusters across the phylogeny, we ran *dollop* from the package *phylip-3.696 (*http://evolution.genetics.washington.edu/phylip) on the 732 miRNA orthogroups.

### Identifying the genomic sources of miRNAs

For the identification of putative miRtrons, we used *Bedtools intersect*^55^ to identify miRNA genes with a minimum, reciprocal overlap of 90% with an intronic sequence on the same strand. Small RNA reads were then aligned to the identified introns. When alignments provided evidence of Dicer-Drosha processing, and we observed a hairpin-like secondary structure of the intron sequence (similar to the criteria used for genome-wide miRNA discovery) we considered the locus a putative miRtron.

In order to find putative repeat-derived miRNAs, we aligned the hairpin sequences of our annotated miRNAs against all sequences in the *Repbase* database (http://www.girinst.org/repbase), using BLASTN^42^. We then selected all repetitive elements which had been returned by our BLASTN search, and generated 1000 shuffled sequences for each of these elements. All miRNAs were subsequently aligned (using BLASTN) to all of these shuffled sequences. Finally, we selected all original BLASTN results having a minimum alignment length of 30 nucleotides, as well as a bit score higher than the maximum value (42.8) observed in the alignments against the shuffled repetitive sequences.

Cases of reverse strand transcription were identified as follows: for each species, we used *Bedtools intersect*^55^ to identify pairs of miRNA loci lying on opposite strands, with a minimum overlap of 95% of at least one locus.

Duplicated orthogroups were defined as *CD-hit* sequence clusters containing two or more paralogous copies in at least one species.

### Generation of 3′ UTR multiple alignments

The latest genome annotation for our 5 species was downloaded from the *Ensembl* website (www.ensembl.org). Genes representing one to one orthologs across all 5 species plus human and mouse were selected. The corresponding UTRs were defined as the region starting from the end position of the last annotated exon, plus one, and ending 5 kb downstream. Any overlap with downstream coding sequences, as determined using *bedtools subtract*^55^, was then removed by trimming the 5 kb window up to the starting position of the first overlapping coding sequence. Resulting UTR sequences were then filtered for a minimum length of 500 bp. We used *mafft*^58^ to generate gene specific multiple alignments across our 5 species, as well as human and mouse. The final alignments were obtained by extracting the alignment region corresponding to the human or the mouse homologous sequence, depending on which one was the longest sequence. The final dataset used for our analyses includes 3355 one to one orthologs across 7 species.

### Target prediction and Gene Ontology analyses

Target sites were identified *in silico* using *TargetScan7*^59^, limiting the set of targeted genes to the one to one orthologs across all 7 species. The sequences of all predicted 7mer and 8mer miRNA-target interactions were then extracted. For the analyses of branch specific miRNA and seed losses scenarios, we predicted target sites across all our five species. We then selected all 7mer and 8mer sites which were independently predicted in the outgroup (rabbit) and at least one additional species, with an associated *context*++ score (calculated without *Pct* contribution)^59^ smaller or equal to −0.1. Target sites sequences plus 2 nucleotides upstream and downstream were aligned using *mafft*^58^. Multiple alignments were then used to calculate the pairwise sequence similarity across target sites between pairs of species. In order to consider all target sites at once, sequence similarity was calculated on the 6mer sequence complementary to positions 2–7 of the miRNA.

We adapted the above described pipeline for the calculation of *Phastcons* scores across 7nt bins in the 3′UTR regions. We downloaded human-centred, 20-way *Phastcons* scores from the UCSC database (ftp://hgdownload.soe.ucsc.edu/goldenPath/hg38/phastCons20way/). For conserved seed families, we selected targets sites predicted in at least 2 species, and with an associated *context*++ score smaller or equal to −0.1. For the species specific seeds, we only used the *context*++ score as a filtering criterion. Sequences corresponding to the target site ±49 nt were identified, and the genomic coordinates of the homologous human counterparts inferred from multiple alignments. We then used a combination of *UCSCtools*^60^ and in-house developed *perl* scripts to calculate single nucleotide *Phastcons* scores across bins, for each target site of every seed family.

Gene Ontology analyses were performed in *Rstudio* using the package *topGO* (https://bioconductor.org/packages/release/bioc/vignettes/topGO/). Significantly enriched GO terms were independently identified for the targets of each seed family, using the *elim* algorithm coupled with Fisher exact test. The gene background was defined as the complete set of 1:1 orthologs used for all target sites analyses. For the GO analysis of brain specific novel miRNAs, this background was restricted to the target genes with evidence of expression of the human homologue in brain tissues (https://www.proteinatlas.org/about/download). The raw p-values obtained were not corrected, as the *elim* method already includes an adjustment equivalent to a Bonferroni correction^61^. Results were filtered for p ≤ 0.05. For the visualisation of significant GO terms, we used the tool REVIGO^62^ (http://revigo.irb.hr/).

### Data access

Raw sequencing data used in this study has been deposited in the Short Read Archive database (https://www.ncbi.nlm.nih.gov/sra), under Bioproject PRJNA432546.

## Electronic supplementary material

Supplementary Information

Supplementary Dataset 1 Genomic coordinates of all miRNA loci annotated in the cow genome (B. taurusUMD3.1).

Supplementary Dataset 2 Genomic coordinates of all miRNA loci annotated in the dog genome (C. familiaris3.1).

Supplementary Dataset 3 Genomic coordinates of all miRNA loci annotated in the horse genome (E.caballus 2.86).

Supplementary Dataset 4 Genomic coordinates of all miRNA loci annotated in the pig genome (S. scrofa10.2).

Supplementary Dataset 5 Genomic coordinates of all miRNA loci annotated in the rabbit genome (O.cuniculus 2.0).

Supplementary Dataset 6 Representation of different non coding RNA classes in our sequencing data (columnsB-N) and an overview on the number and proportion of adapter-trimmed, genome matching reads (columns O-R). Each row provides the calculations for a single species.

Supplementary Dataset 7 Presence-absence of CD-hit miRNA orthogroups across seven mammalianspecies (cow, dog, horse, pig, rabbit, human and mouse)

Supplementary Dataset 8 List of miRNA loci showing a significant similarity with one or more Repbasesequences. Columns 3-5 show a summary of BLASTN results.

Supplementary Dataset 9 List of reverse complement miRNA gene couples, lying on the opposite strand ofexactly the same genomic interval.

Supplementary Dataset 10 Presence-absence of CD-hit miRNA orthogroups across seven mammalianspecies (cow, dog, horse, pig, rabbit, human and mouse), limited to orthogroups containing multiple paralogousgene copies in at least one domestic species.
