# Supplementary material for: The evolutionary dynamics of microRNAs in domestic mammals
Source: Sci Rep. 2018 Nov 19;8:17050. doi: 10.1038/s41598-018-34243-8 (PMC6242877; doi:10.1038/s41598-018-34243-8)
Supplement: Supplementary file 1 — Supplementary Information [file 41598_2018_34243_MOESM1_ESM.pdf]

The evolutionary dynamics of miRNAs in domestic mammals

Luca Penso-Dolfin[1], Simon Moxon[2], Wilfried Haerty[1] and Federica Di Palma[1*]*

1. Earlham Institute, Norwich Research Park, Colney Lane, Norwich NR47UZ, United Kingdom

2. University of East Anglia, Norwich Research Park, Norwich NR47TJ, United Kingdom

[*] **Correspondence**: Luca Penso-Dolfin. Earlham Institute, Norwich Research Park, Colney Lane, Norwich NR47UZ, United Kingdom. Tel: +44 (0)1603 450 981. Fax: +44 (0)1603 450 981. E-mail: luca.penso-dolfin@earlham.ac.uk

Supplementary Information

Target predictions, combined with the inference of gain and loss patterns across the phylogeny, enable us to investigate the co-evolution patterns of miRNAs and associated targets. One particularly interesting question is whether the loss of a miRNA orthogroup (or seed family) in a single species results in a detectable relaxation of selection on its target sites. For this analysis, we identified all orthogroups and seed families lost in a single species (see Table 4). We then calculated, for each of them, the pairwise sequence similarity across target sites between the rabbit (chosen as an outgroup) and all 4 other species (see Material and Methods).

Interestingly, we identified 24 cases of lost orthogroups sharing the seed sequence with at least one broadly conserved miRNA. For example, orthogroup 306 (miR-384), appears to be missing in the cow, but the corresponding seed sequence is shared with conserved orthogroups 16, 179, 185, 207, 236 and 380). While we observe significantly lower values compared to the horse and the pig, the difference with the dog is not significant (MW test, α=0.05, Supplementary Fig. S11).

In the case of orthogroup 566 (miR-350, lost in the pig), we observe a significant shift down in the distribution of the pig. However, the comparison against the cow does not reach significance after Bonferroni correction (MW test, Supplementary Fig. S12, raw p-values shown). The closer similarity between the pig and the cow might reflect the more recent divergence time compared to the other species.

Given the observation of seed redundancy across miRNA families, we asked the question whether the loss of a *seed sequence*, rather than a *miRNA orthogroup*, has a greater effect on the observed patterns of target site conservation (see Table 4). For this purpose, we grouped all miRNA loci in seed specific orthogroups. In the case of TTATTGA, appearing to be lost in the cow, we find a significant decrease in target conservation levels compared to the dog and the horse, but not pig (MW test, Supplementary Fig. S13). An interesting example is represented by seed CAGCAAT, missing in the dog but showing extended similarity with conserved seed ACAGCAA. Our data does not support the hypothesis of relaxation of selection in the dog (Supplementary Fig. S14). We then decided to investigate the possibility of a family loss compensation through the retention of similar seed ACAGCAA. We compared the targets of the two families, but found a limited overlap in target repertoires (Supplementary Fig. S15). Thus, the retention of family ACAGCAA is unlikely to have a strong compensation effect upon the seed loss.

We also looked at target site conservation levels of broadly conserved seed GAGGTAG (the miRNA seed found in all let-7 families). *Let-7* is a broadly conserved miRNA implicated in developmental timing [1], and this is reflected in the consistently high levels of target site conservation across species (Supplementary Fig. S16).

Supplementary figures

Supplementary Fig. S1. Read counts across tissues and organisms for the first Illumina *HiSeq* run (id: 1099). Colour labelling correspond to the number of reads in the raw data (red), after adapter trimming (green) and the number of genome matching reads (blue).

Supplementary Fig. S2. Read counts across tissues and organisms for the second Illumina *HiSeq* run (id: 1610). Colour labelling is identical to figure S1.

Supplementary Fig. S3. Read counts for all dog cortex samples. Colour labelling is identical to figure S1.

Supplementary Fig. S4. Abundance plots for each miRNA locus annotated in the cow genome (*B. taurus* UMD3.1)

Supplementary Fig. S5. Abundance plots for each miRNA locus annotated in the dog genome (*C. familiaris* 3.1)

Supplementary Fig. S6. Abundance plots for each miRNA locus annotated in the horse genome (*E. caballus* 2.86)

Supplementary Fig. S7. Abundance plots for each miRNA locus annotated in the pig genome (*S. scrofa* 10.2)

Supplementary Fig. S8. Abundance plots for each miRNA locus annotated in the rabbit genome (*O. cuniculus* 2.0)

Supplementary Fig. S9. Venn diagram of cross species relationships for the orthogroups of repeat-derived miRNAs

Supplementary Fig. S10. Venn diagram of cross species relationships for the orthogroups of duplicated miRNAs

Supplementary Fig. S11. Pairwise target site similarity against the rabbit for orthogroup 306 (absent in the cow). Raw p-values of pairwise MW tests are shown. Only target predicted in rabbit and at least one other species were considered. The associated seed is not group specific and is conserved in all five species.

Supplementary Fig. S12. Pairwise target site similarity against the rabbit for orthogroup 566 (absent in the pig). Raw p-values of pairwise MW tests are shown. Only target predicted in rabbit and at least one other species (n=932) were considered

Supplementary Fig. S13. Pairwise target site similarity against the rabbit for seed family TTATTGA (absent in the cow). Raw p-values of pairwise MW tests are shown. Only target predicted in rabbit and at least one other species (n=267) were considered.

Supplementary Fig S14. Pairwise target site similarity against the rabbit for seed family CAGCAAT (absent in the dog). Raw p-values of pairwise MW tests are shown. Only target predicted in rabbit and 1 additional species (n=111) were considered.

Supplementary Fig. S15. Venn diagram of the counts of predicted targeted sites in dog for seed families ACAGCAA and CAGCAAT

Supplementary Fig. S16. Pairwise target site similarity against the rabbit for the broadly conserved let-7-5p seed family. Raw p-values of pairwise MW tests are shown. Only targets predicted in rabbit and at least one other species (n=161) were considered.

Supplementary data

Supplementary file 1. Small RNA reads alignments showing the miRNA-5p and miRNA-3p expression of each miRNA locus in the cow genome (*B. taurus* UMD3.1).
Supplementary file 2. Small RNA reads alignments showing the miRNA-5p and miRNA-3p expression of each miRNA locus in the dog genome (*C. familiaris* 3.1).
Supplementary file 3. Small RNA reads alignments showing the miRNA-5p and miRNA-3p expression of each miRNA locus in the horse genome (*E. caballus* 2.86).
Supplementary file 4. Small RNA reads alignments showing the miRNA-5p and miRNA-3p expression of each miRNA locus in the pig genome (*S. scrofa* 10.2).
Supplementary file 5. Small RNA reads alignments showing the miRNA-5p and miRNA-3p expression of each miRNA locus in the rabbit genome (*O. cuniculus* 2.0).
Supplementary file 6. Small RNA reads alignments against 17 intronic sequences, which were identified as putative miRtrons.

Supplementary Table S1. Genomic coordinates of all miRNA loci annotated in the cow genome (*B. taurus* UMD3.1).
Supplementary Table S2. Genomic coordinates of all miRNA loci annotated in the dog genome (*C. familiaris* 3.1).
Supplementary Table S3. Genomic coordinates of all miRNA loci annotated in the horse genome (*E. caballus* 2.86).
Supplementary Table S4. Genomic coordinates of all miRNA loci annotated in the pig genome (*S. scrofa* 10.2).
Supplementary Table S5. Genomic coordinates of all miRNA loci annotated in the rabbit genome (*O. cuniculus* 2.0).
Supplementary Table S6. Representation of different non coding RNA classes in our sequencing data (columns B-N) and an overview on the number and proportion of adapter-trimmed, genome matching reads (columns O-R). Each row provides the calculations for a single species.
Supplementary Table S7 Presence-absence of *CD-hit* miRNA orthogroups across seven mammalian species (cow, dog, horse, pig, rabbit, human and mouse)
Supplementary Table S8. List of miRNA loci showing a significant similarity with one or more *Repbase* sequences. Columns 3-5 show a summary of BLASTN results.
Supplementary Table S9. List of reverse complement miRNA gene couples, lying on the opposite strand of exactly the same genomic interval.
Supplementary Table S10. Presence-absence of *CD-hit* miRNA orthogroups across seven mammalian species (cow, dog, horse, pig, rabbit, human and mouse), limited to orthogroups containing multiple paralogous gene copies in at least one domestic species.

References

1 Reinhart, B. J. *et al.* The 21-nucleotide let-7 RNA regulates developmental timing in Caenorhabditis elegans. *Nature* **403**, 901-906, doi:10.1038/35002607 (2000).

Fig. S1

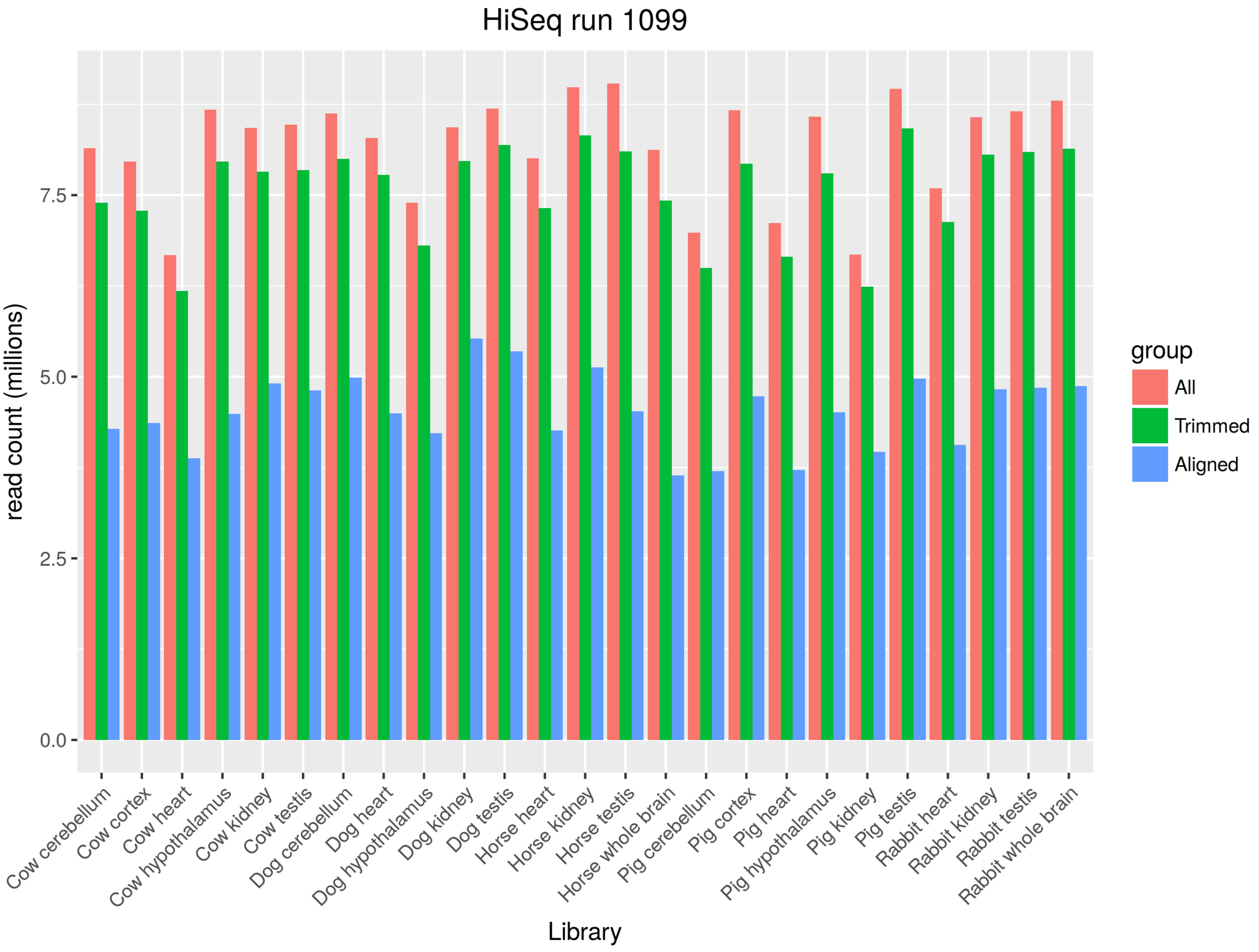
HiSeq run 1099
read count (millions)
0.0
2.5
5.0
7.5
Library
group
All
Trimmed
Aligned
Cow cerebellum
Cow cortex
Cow heart
Cow hypothalamus
Cow kidney
Cow testis
Dog cerebellum
Dog heart
Dog hypothalamus
Dog kidney
Dog testis
Horse heart
Horse kidney
Horse testis
Horse whole brain
Pig cerebellum
Pig cortex
Pig heart
Pig hypothalamus
Pig kidney
Pig testis
Rabbit heart
Rabbit kidney
Rabbit testis
Rabbit whole brain

Fig. S2

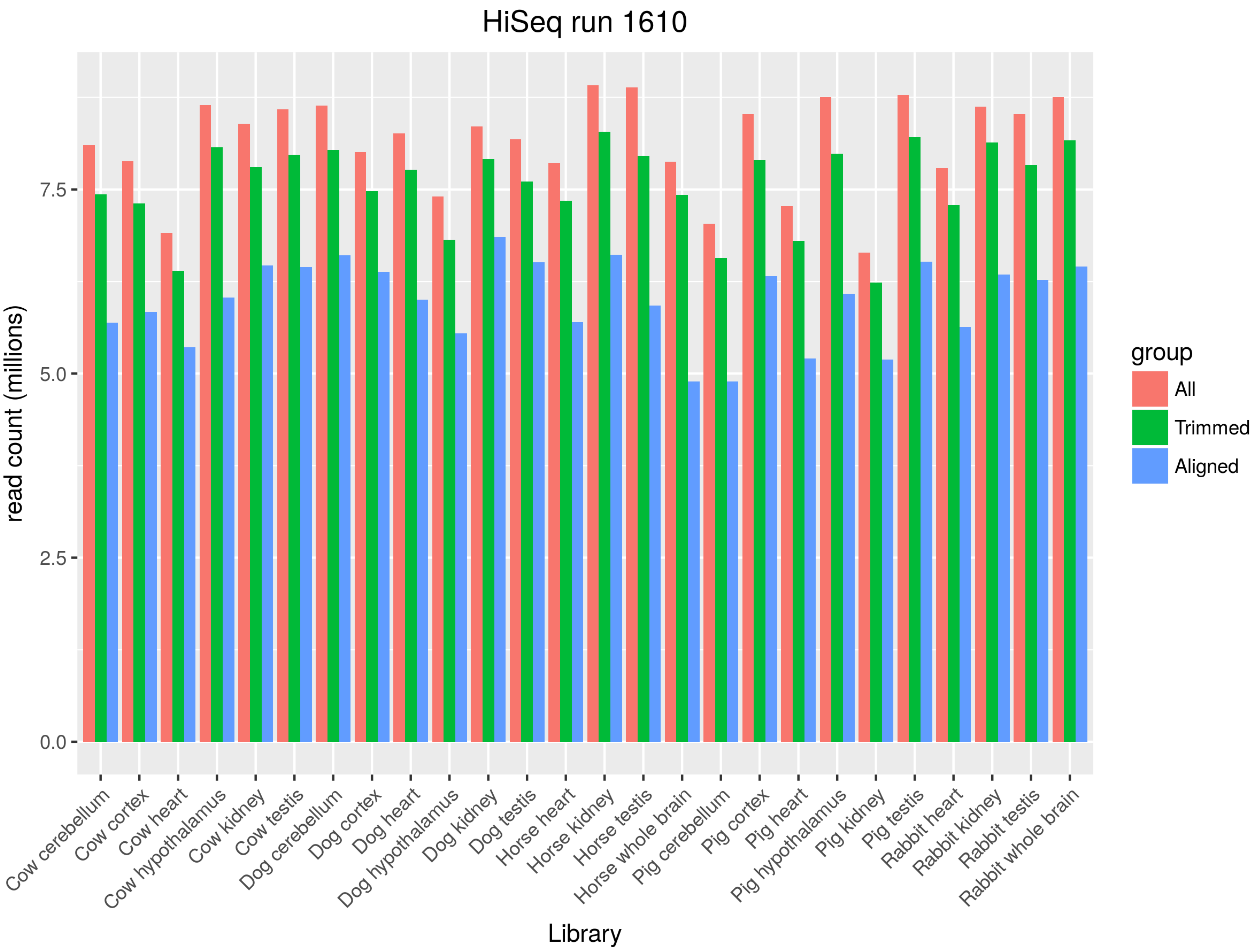
HiSeq run 1610
read count (millions)
0.0
2.5
5.0
7.5
Library
Cow cerebellum
Cow cortex
Cow heart
Cow hypothalamus
Cow kidney
Cow testis
Dog cerebellum
Dog cortex
Dog heart
Dog hypothalamus
Dog kidney
Dog testis
Horse heart
Horse kidney
Horse testis
Horse whole brain
Pig cerebellum
Pig cortex
Pig heart
Pig hypothalamus
Pig kidney
Pig testis
Rabbit heart
Rabbit kidney
Rabbit testis
Rabbit whole brain
group
All
Trimmed
Aligned

Fig. S3

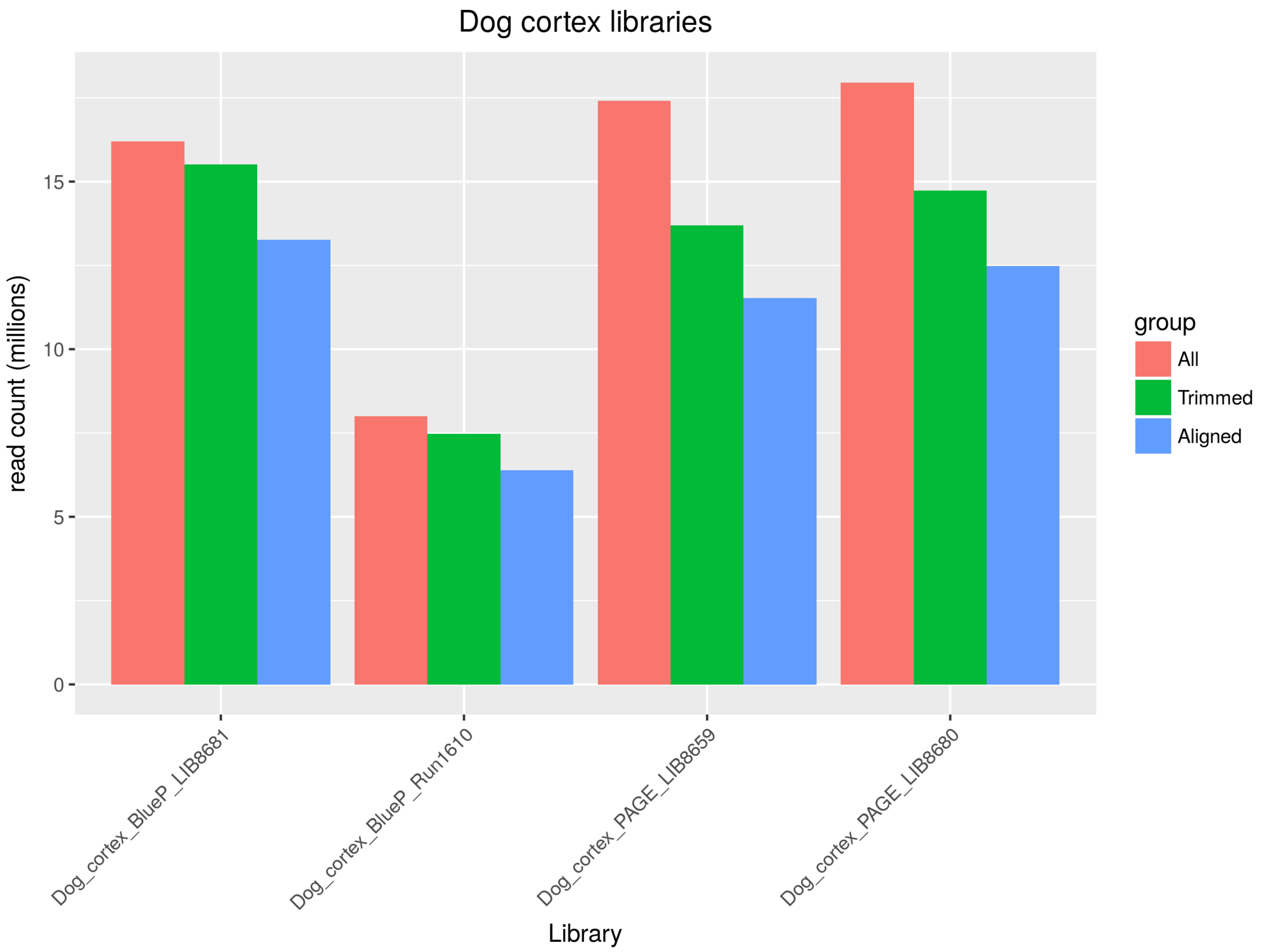
Dog cortex libraries
read count (millions)
0
5
10
15
Dog_cortex_BlueP_LIB8681
Dog_cortex_BlueP_Run1610
Dog_cortex_PAGE_LIB8659
Dog_cortex_PAGE_LIB8680
Library
group
All
Trimmed
Aligned

Fig. S4

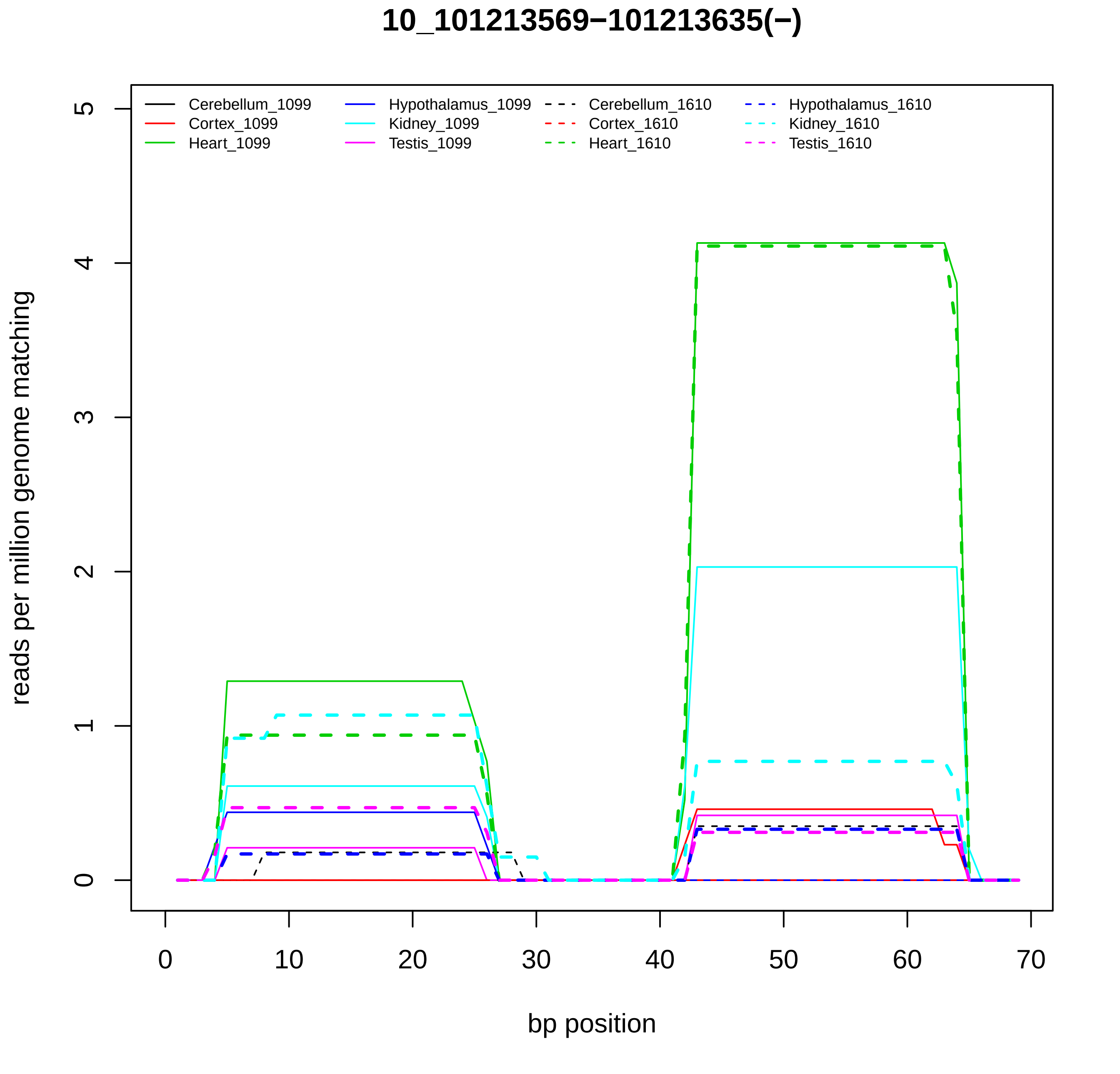

10_101213569−101213635(−)
Cerebellum_1099
Cortex_1099
Heart_1099
Hypothalamus_1099
Kidney_1099
Testis_1099
Cerebellum_1610
Cortex_1610
Heart_1610
Hypothalamus_1610
Kidney_1610
Testis_1610
reads per million genome matching
bp position
0
1
2
3
4
5
0
10
20
30
40
50
60
70

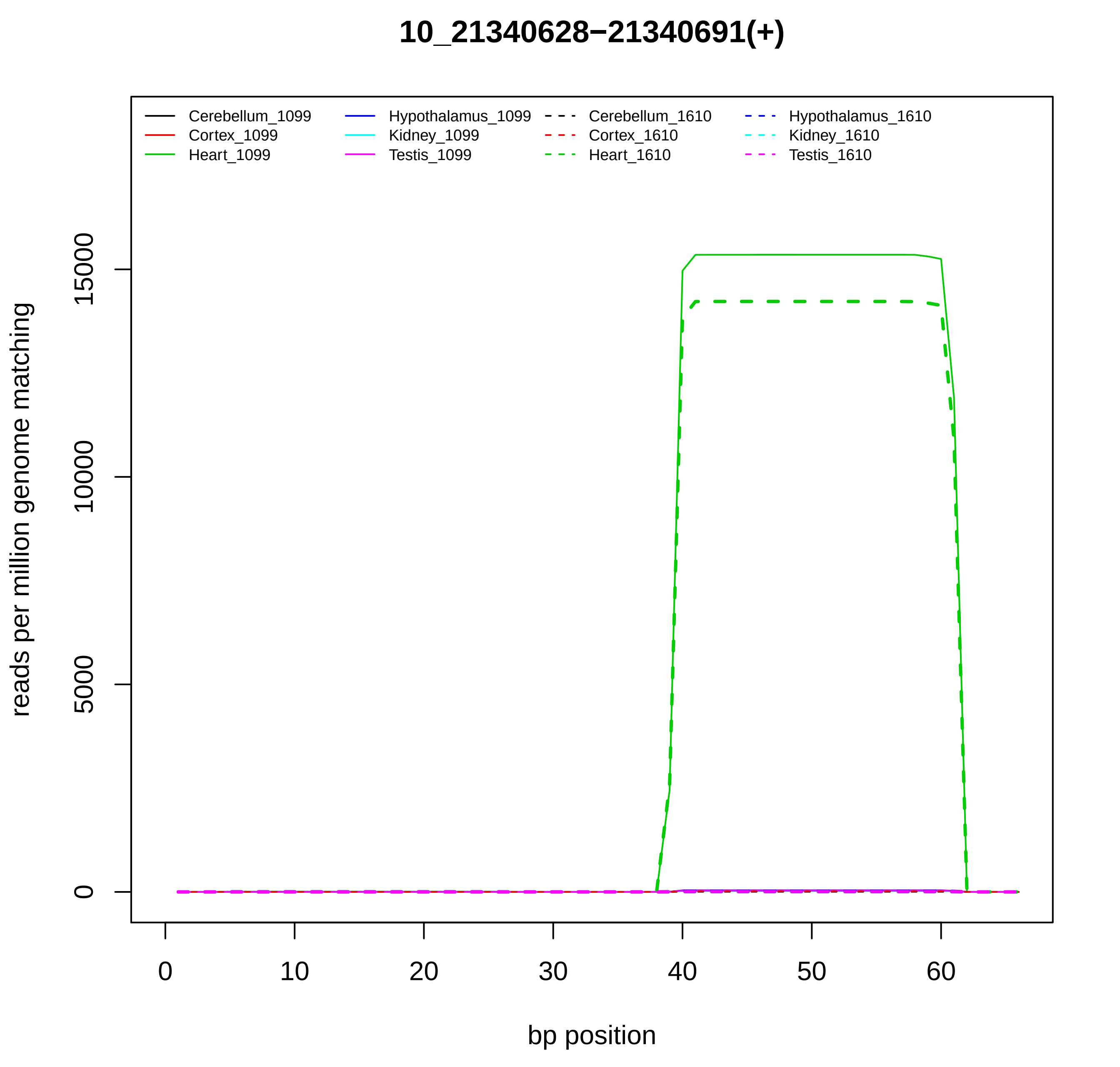

10_21340628−21340691(+)
Cerebellum_1099
Cortex_1099
Heart_1099
Hypothalamus_1099
Kidney_1099
Testis_1099
Cerebellum_1610
Cortex_1610
Heart_1610
Hypothalamus_1610
Kidney_1610
Testis_1610
reads per million genome matching
0
5000
10000
15000
0
10
20
30
40
50
60
bp position

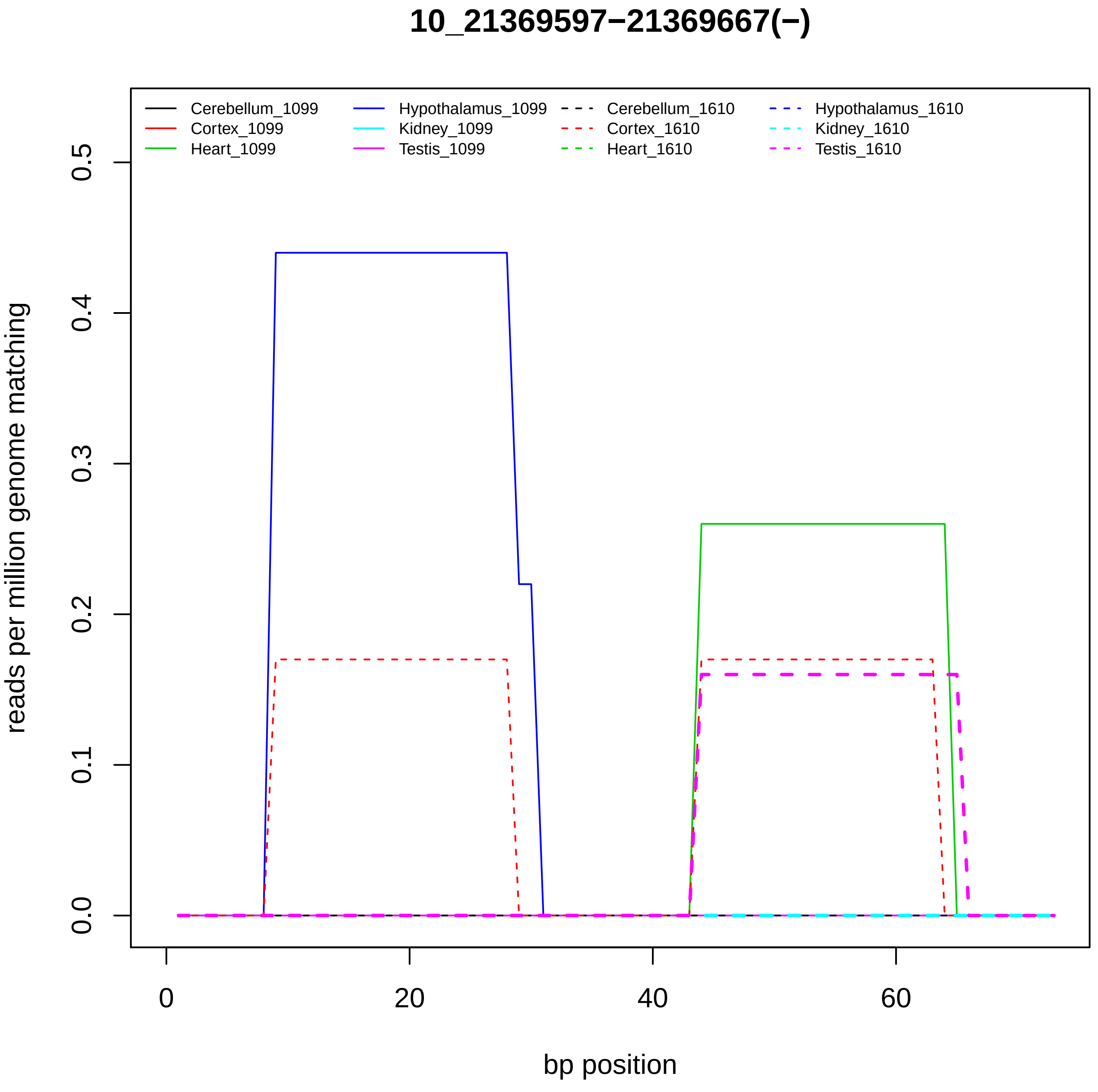
10_21369597−21369667(−)
Cerebellum_1099
Cortex_1099
Heart_1099
Hypothalamus_1099
Kidney_1099
Testis_1099
Cerebellum_1610
Cortex_1610
Heart_1610
Hypothalamus_1610
Kidney_1610
Testis_1610
reads per million genome matching
0.0
0.1
0.2
0.3
0.4
0.5
0
20
40
60
bp position

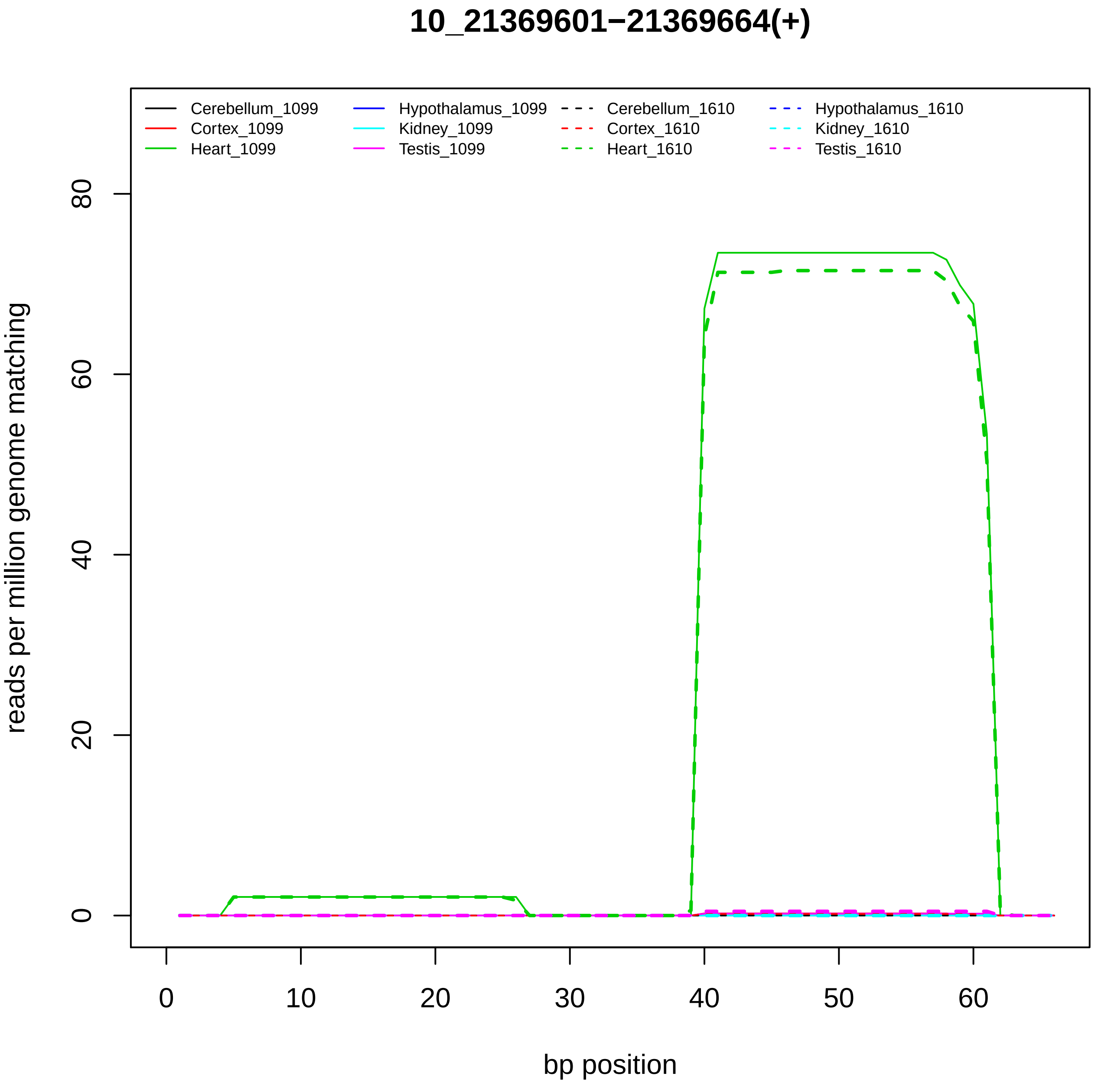

10_21369601−21369664(+)
Cerebellum_1099
Cortex_1099
Heart_1099
Hypothalamus_1099
Kidney_1099
Testis_1099
Cerebellum_1610
Cortex_1610
Heart_1610
Hypothalamus_1610
Kidney_1610
Testis_1610
reads per million genome matching
bp position
0
20
40
60
80
0
10
20
30
40
50
60

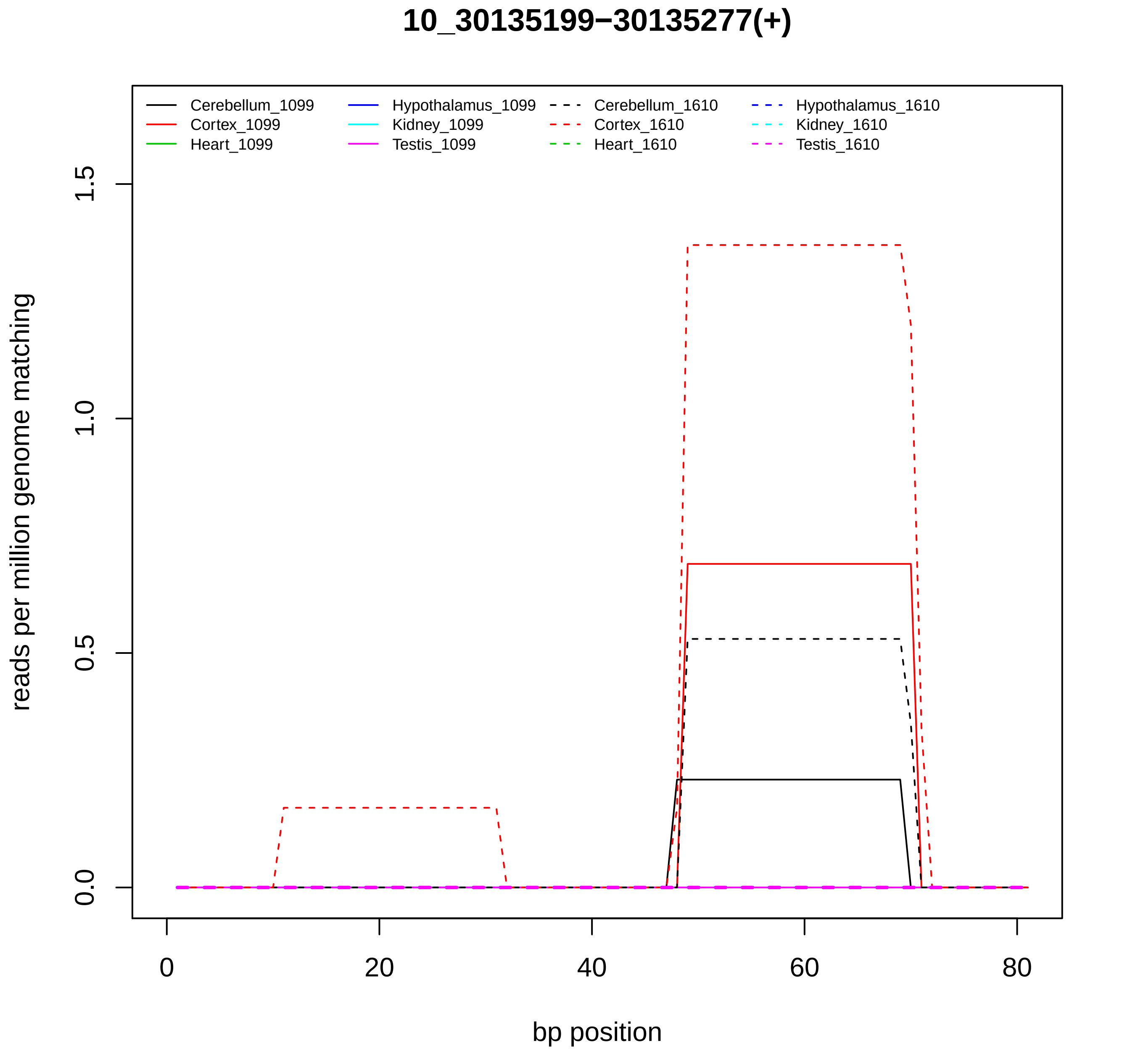

10_30135199−30135277(+)
Cerebellum_1099
Cortex_1099
Heart_1099
Hypothalamus_1099
Kidney_1099
Testis_1099
Cerebellum_1610
Cortex_1610
Heart_1610
Hypothalamus_1610
Kidney_1610
Testis_1610
reads per million genome matching
0.0
0.5
1.0
1.5
0
20
40
60
80
bp position

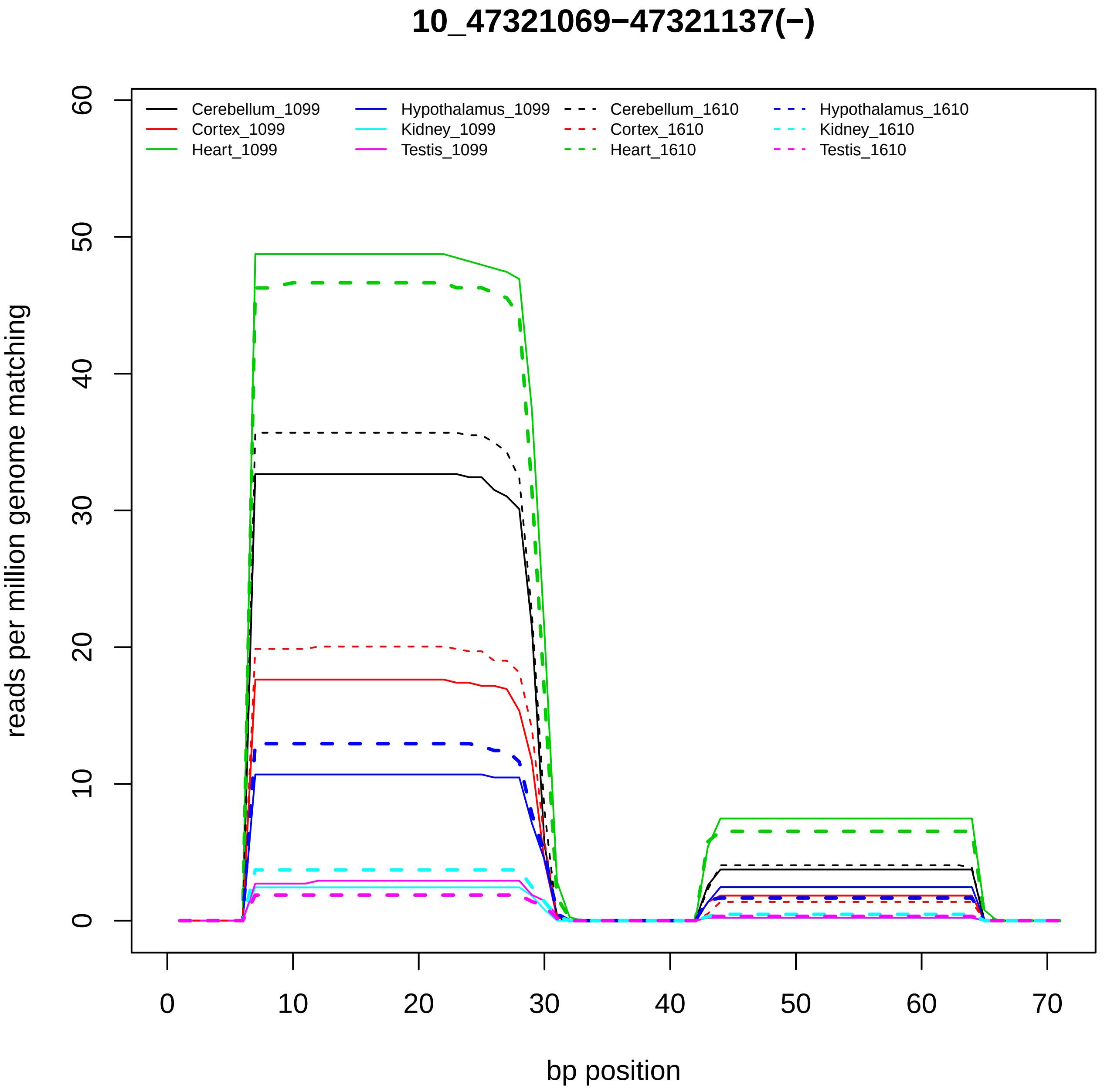
10_47321069−47321137(−)
Cerebellum_1099
Cortex_1099
Heart_1099
Hypothalamus_1099
Kidney_1099
Testis_1099
Cerebellum_1610
Cortex_1610
Heart_1610
Hypothalamus_1610
Kidney_1610
Testis_1610
reads per million genome matching
bp position
0
10
20
30
40
50
60
70

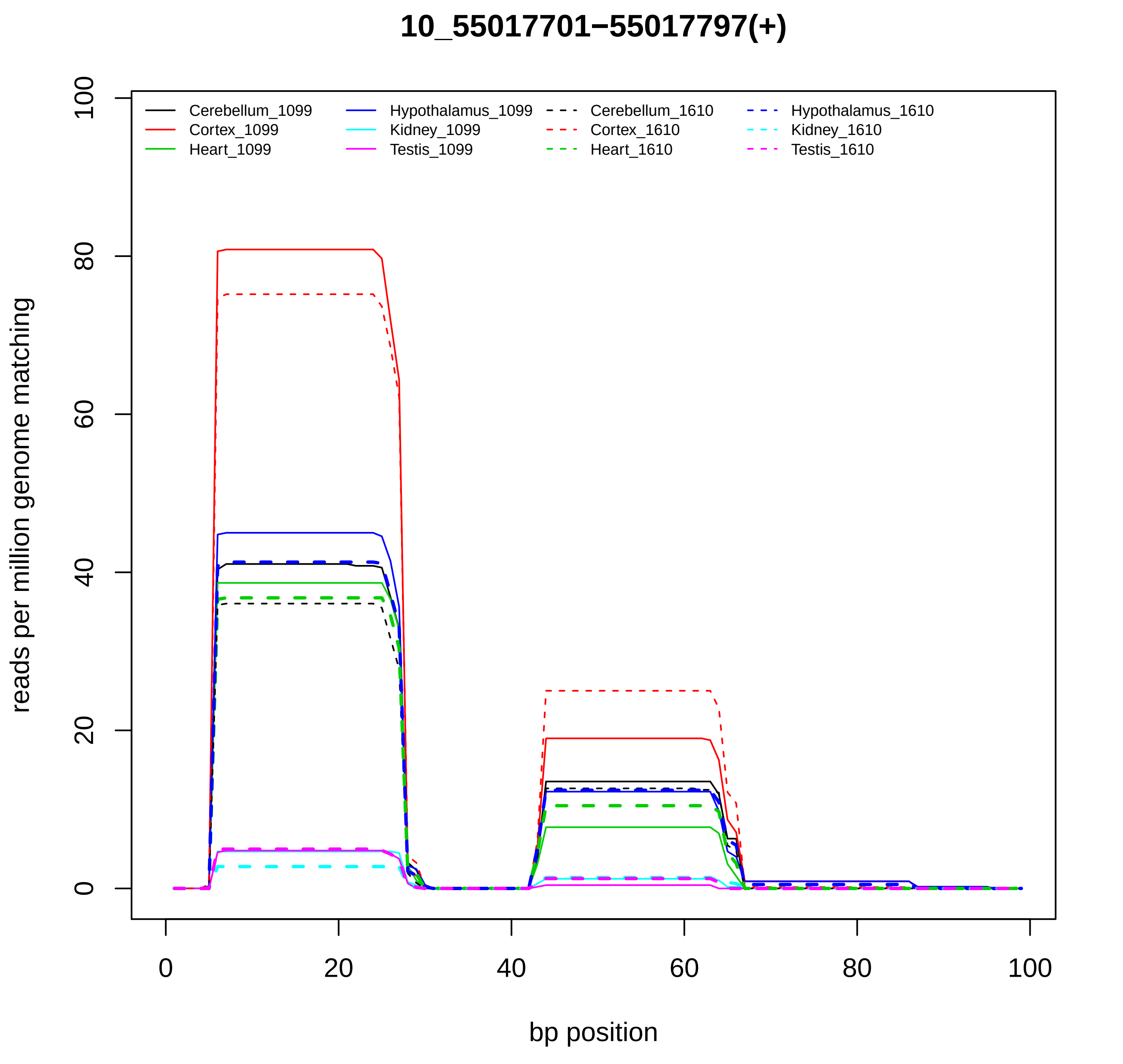
10_55017701−55017797(+)
Cerebellum_1099
Cortex_1099
Heart_1099
Hypothalamus_1099
Kidney_1099
Testis_1099
Cerebellum_1610
Cortex_1610
Heart_1610
Hypothalamus_1610
Kidney_1610
Testis_1610
reads per million genome matching
bp position
0
20
40
60
80
100
0
20
40
60
80
100

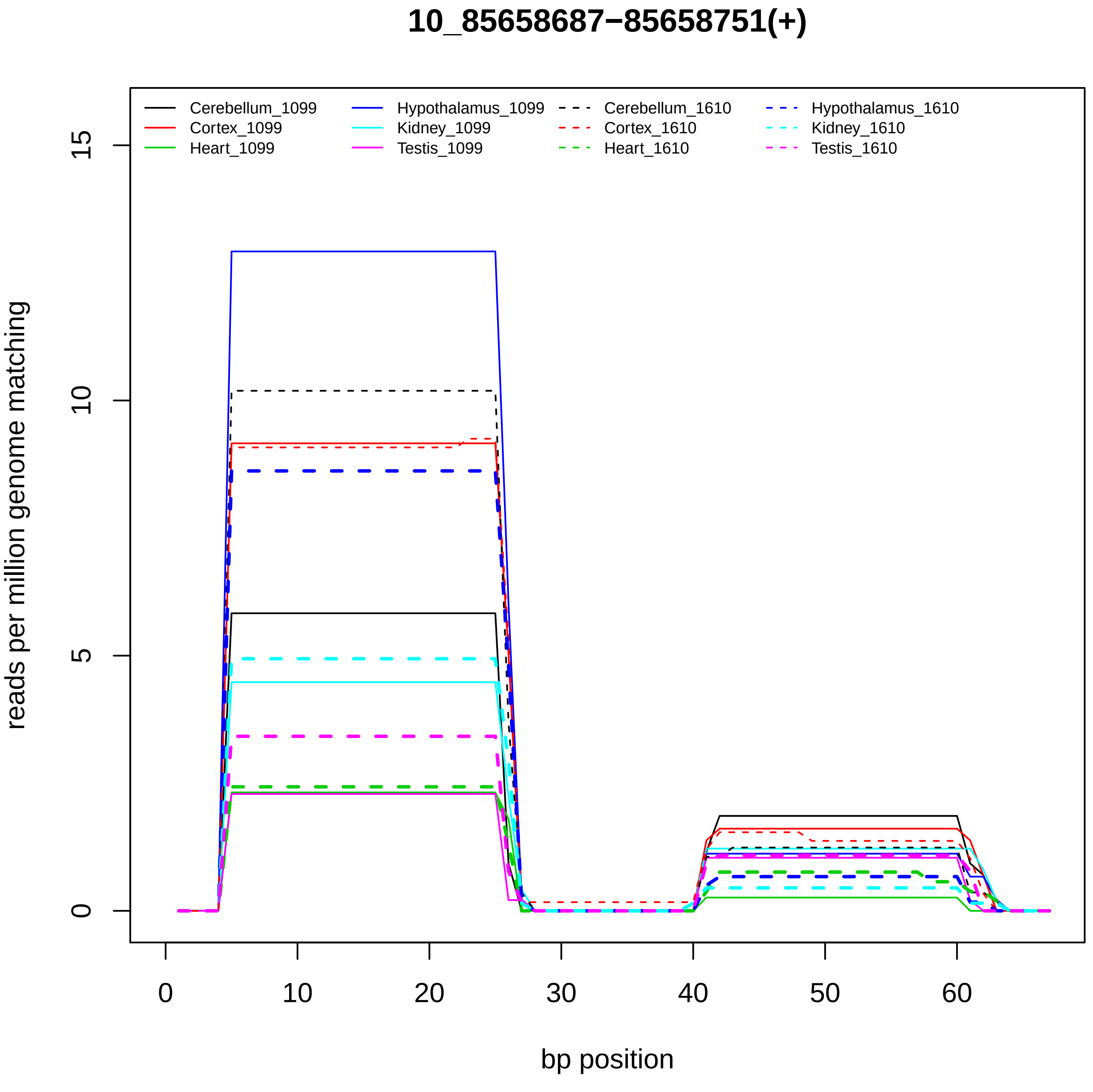
10_85658687−85658751(+)
Cerebellum_1099
Cortex_1099
Heart_1099
Hypothalamus_1099
Kidney_1099
Testis_1099
Cerebellum_1610
Cortex_1610
Heart_1610
Hypothalamus_1610
Kidney_1610
Testis_1610
reads per million genome matching
0
5
10
15
0
10
20
30
40
50
60
bp position

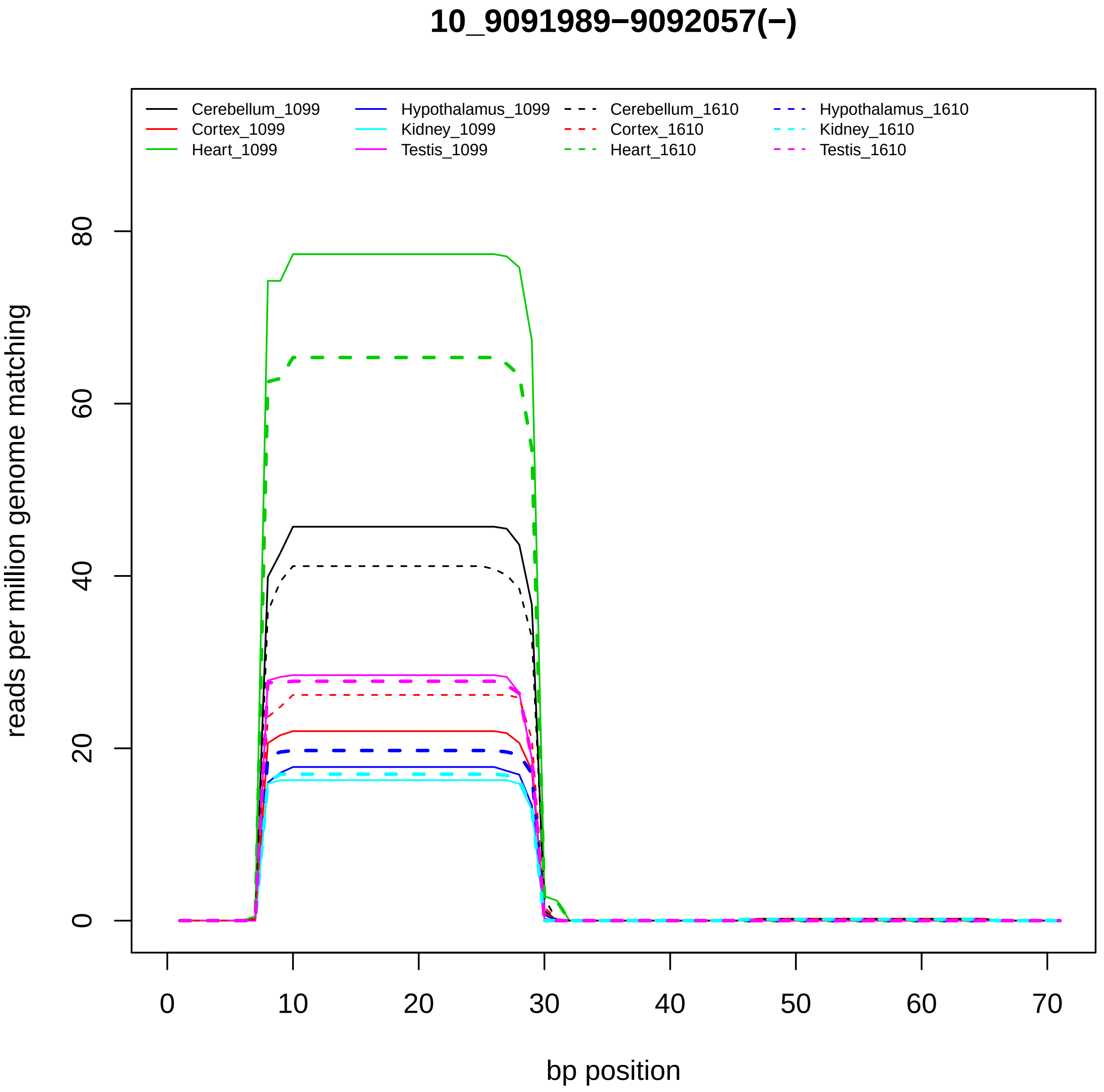

10_9091989−9092057(−)
Cerebellum_1099
Cortex_1099
Heart_1099
Hypothalamus_1099
Kidney_1099
Testis_1099
Cerebellum_1610
Cortex_1610
Heart_1610
Hypothalamus_1610
Kidney_1610
Testis_1610
reads per million genome matching
bp position
0
20
40
60
80
0
10
20
30
40
50
60
70

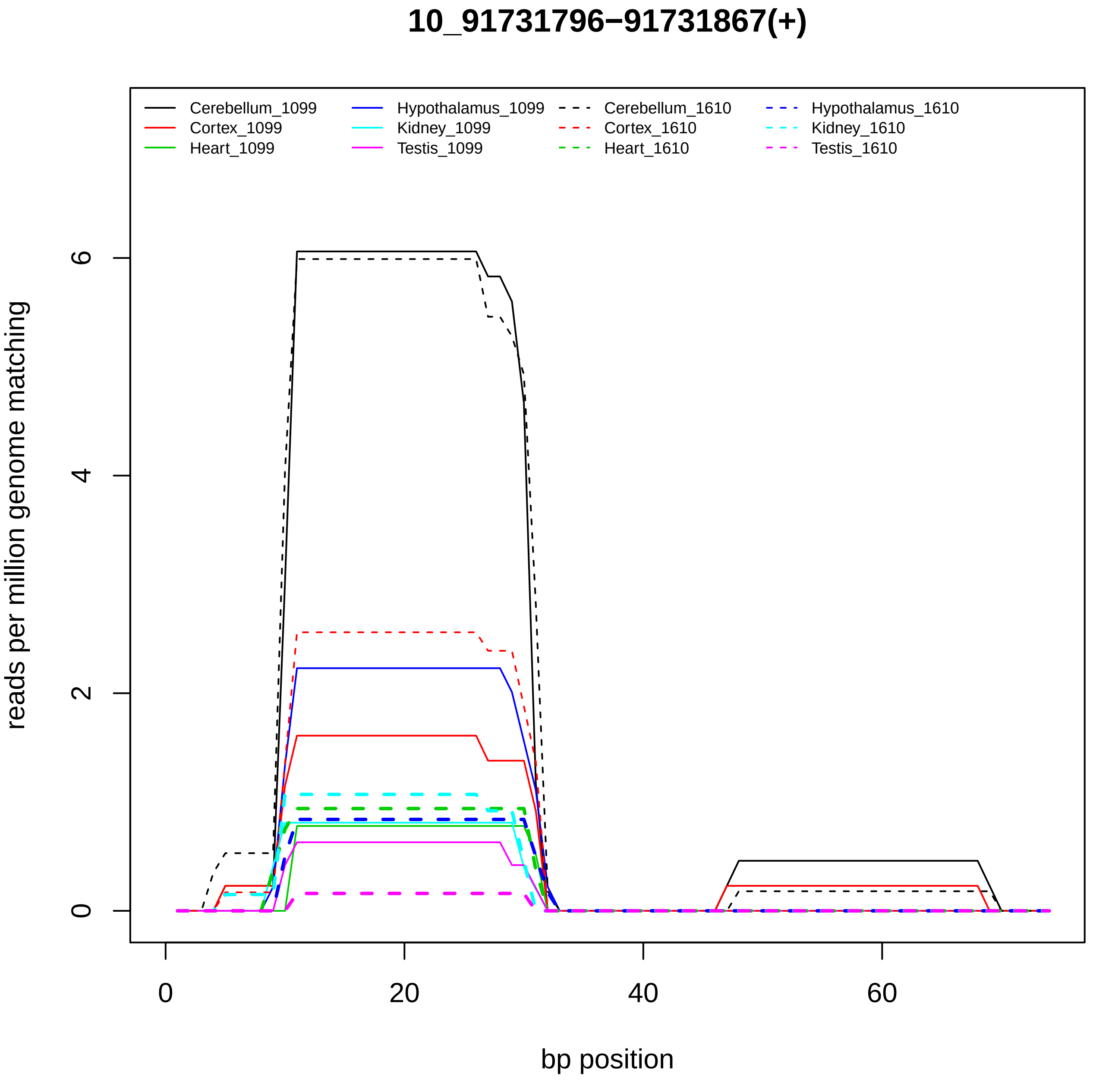

10_91731796−91731867(+)
Cerebellum_1099
Cortex_1099
Heart_1099
Hypothalamus_1099
Kidney_1099
Testis_1099
Cerebellum_1610
Cortex_1610
Heart_1610
Hypothalamus_1610
Kidney_1610
Testis_1610
reads per million genome matching
bp position
0
2
4
6
0
20
40
60

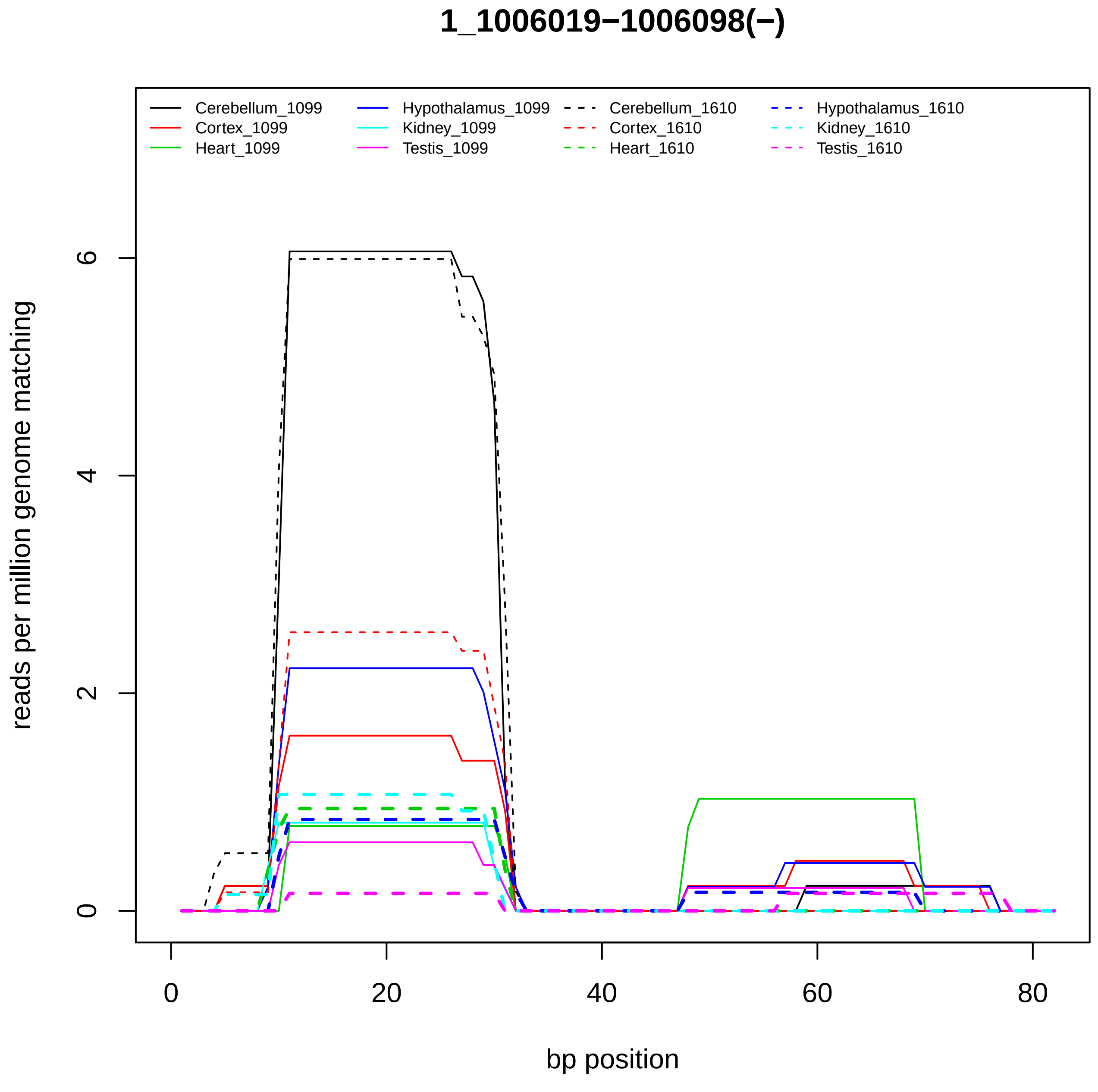
1_1006019−1006098(−)
Cerebellum_1099
Cortex_1099
Heart_1099
Hypothalamus_1099
Kidney_1099
Testis_1099
Cerebellum_1610
Cortex_1610
Heart_1610
Hypothalamus_1610
Kidney_1610
Testis_1610
reads per million genome matching
bp position
0
2
4
6
0
20
40
60
80

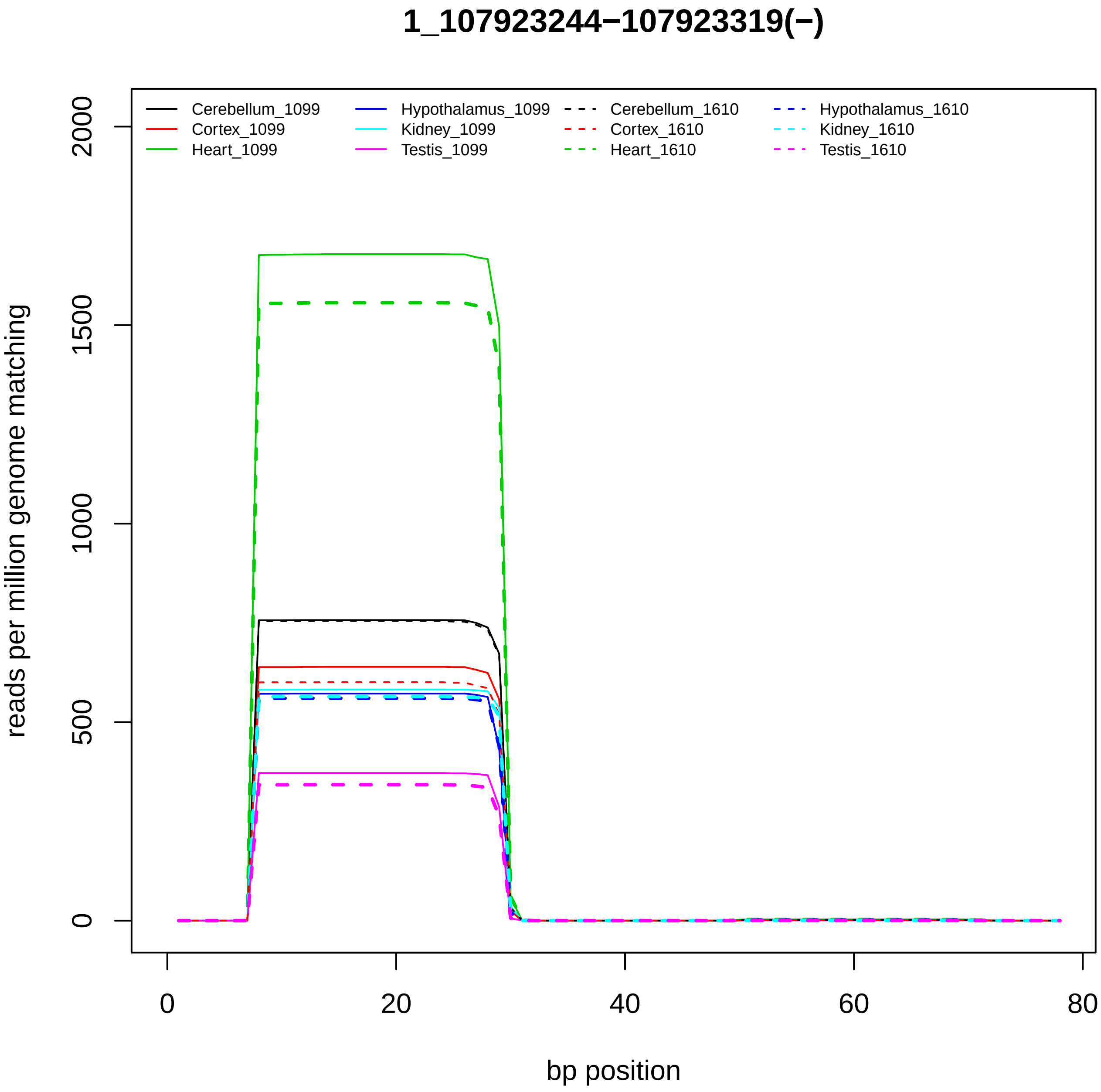

1_107923244−107923319(−)
Cerebellum_1099
Cortex_1099
Heart_1099
Hypothalamus_1099
Kidney_1099
Testis_1099
Cerebellum_1610
Cortex_1610
Heart_1610
Hypothalamus_1610
Kidney_1610
Testis_1610
reads per million genome matching
0
500
1000
1500
2000
bp position
0
20
40
60
80

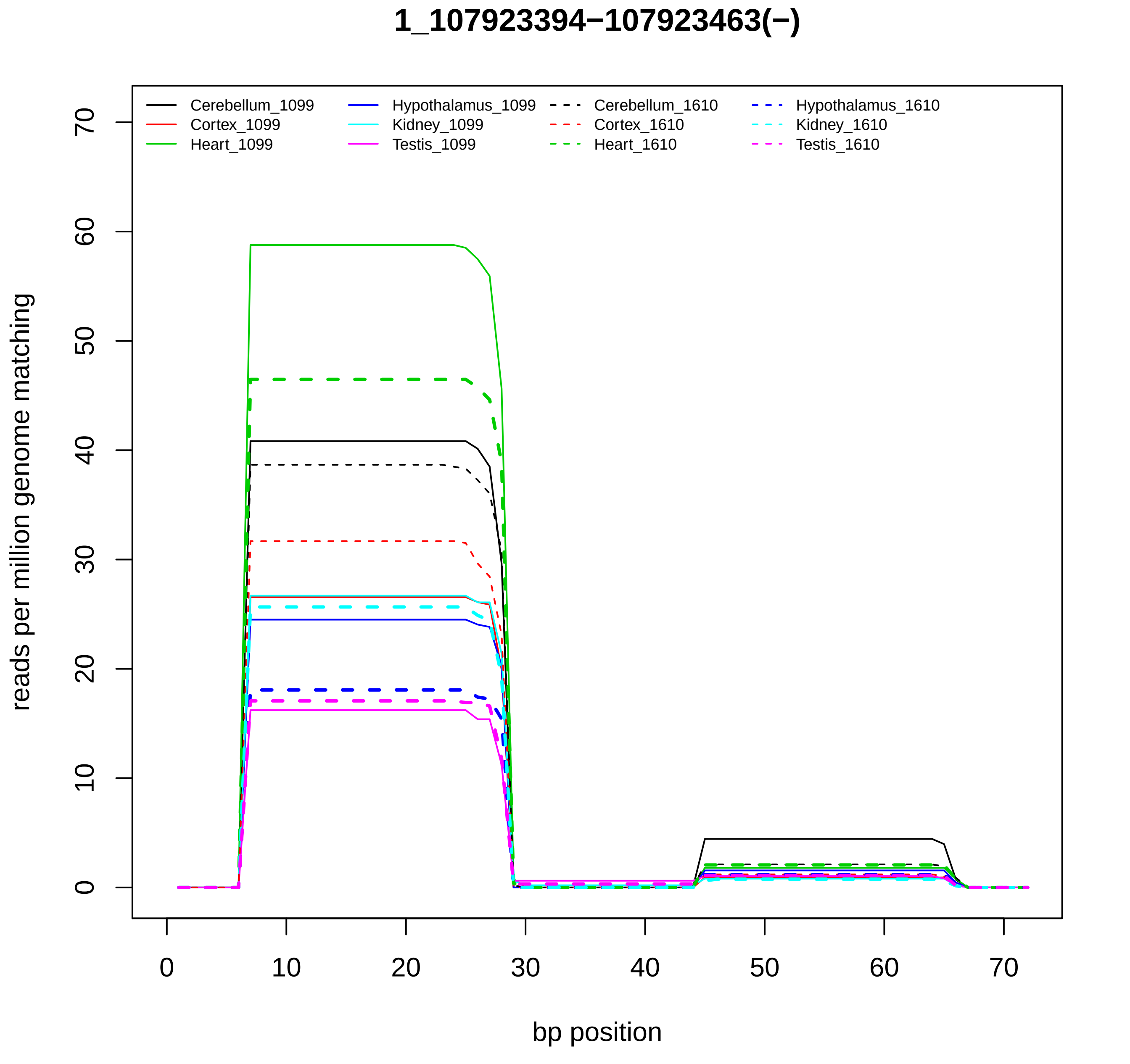

1_107923394−107923463(−)
Cerebellum_1099
Cortex_1099
Heart_1099
Hypothalamus_1099
Kidney_1099
Testis_1099
Cerebellum_1610
Cortex_1610
Heart_1610
Hypothalamus_1610
Kidney_1610
Testis_1610
reads per million genome matching
bp position
0
10
20
30
40
50
60
70

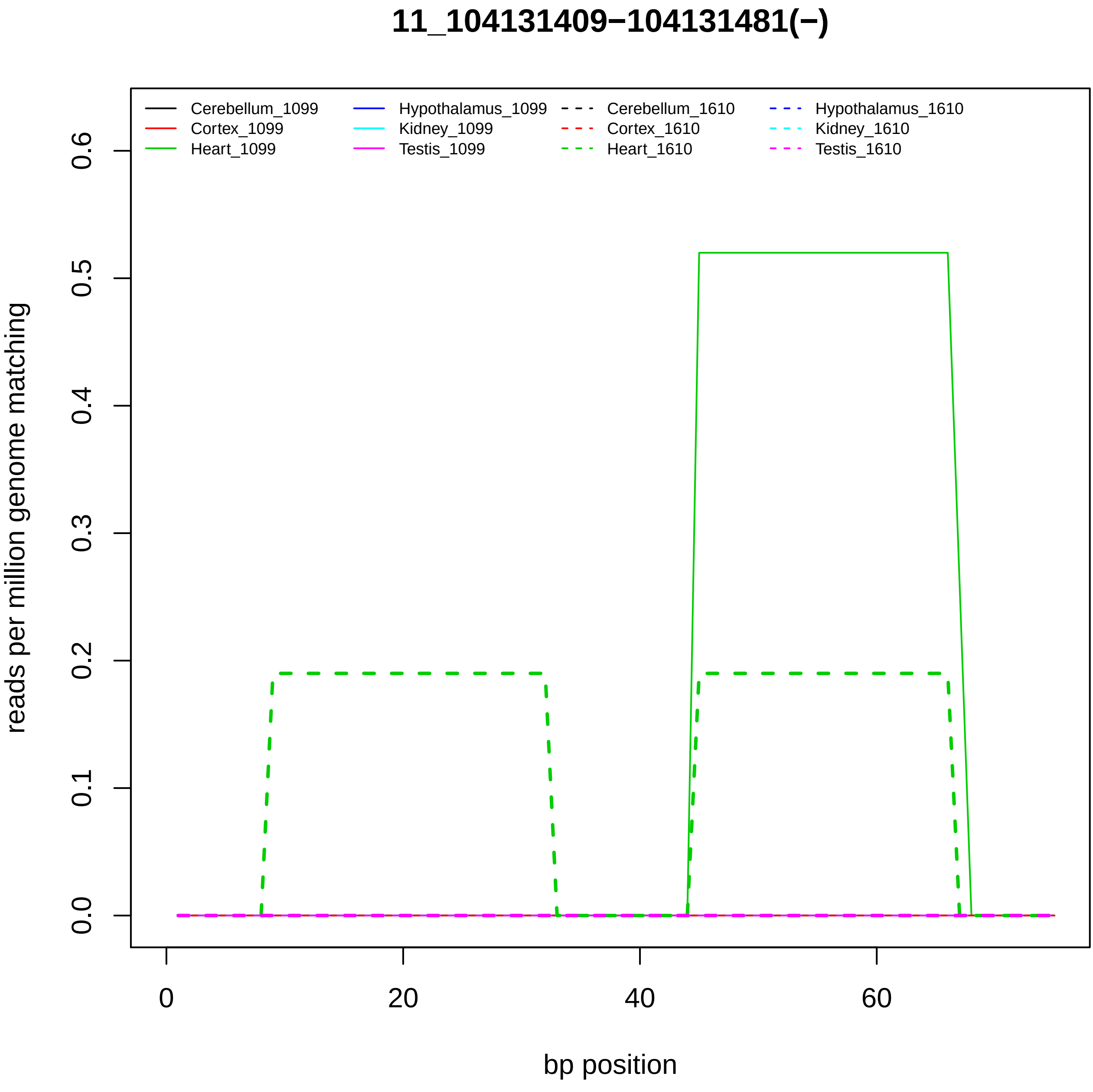

11_104131409−104131481(−)
Cerebellum_1099
Cortex_1099
Heart_1099
Hypothalamus_1099
Kidney_1099
Testis_1099
Cerebellum_1610
Cortex_1610
Heart_1610
Hypothalamus_1610
Kidney_1610
Testis_1610
reads per million genome matching
0.0
0.1
0.2
0.3
0.4
0.5
0.6
0
20
40
60
bp position

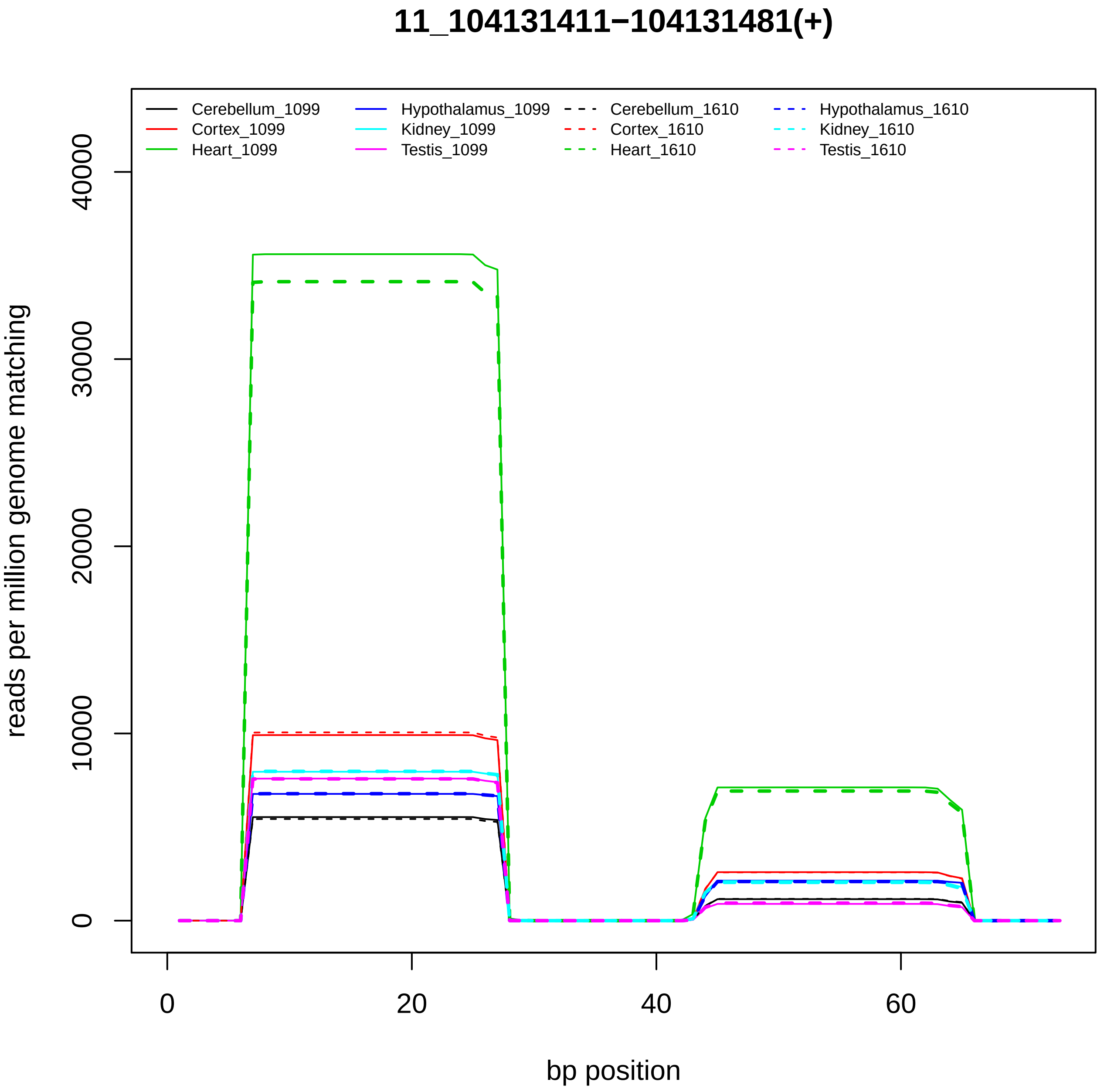

11_104131411−104131481(+)
Cerebellum_1099
Cortex_1099
Heart_1099
Hypothalamus_1099
Kidney_1099
Testis_1099
Cerebellum_1610
Cortex_1610
Heart_1610
Hypothalamus_1610
Kidney_1610
Testis_1610
reads per million genome matching
0
10000
20000
30000
40000
0
20
40
60
bp position

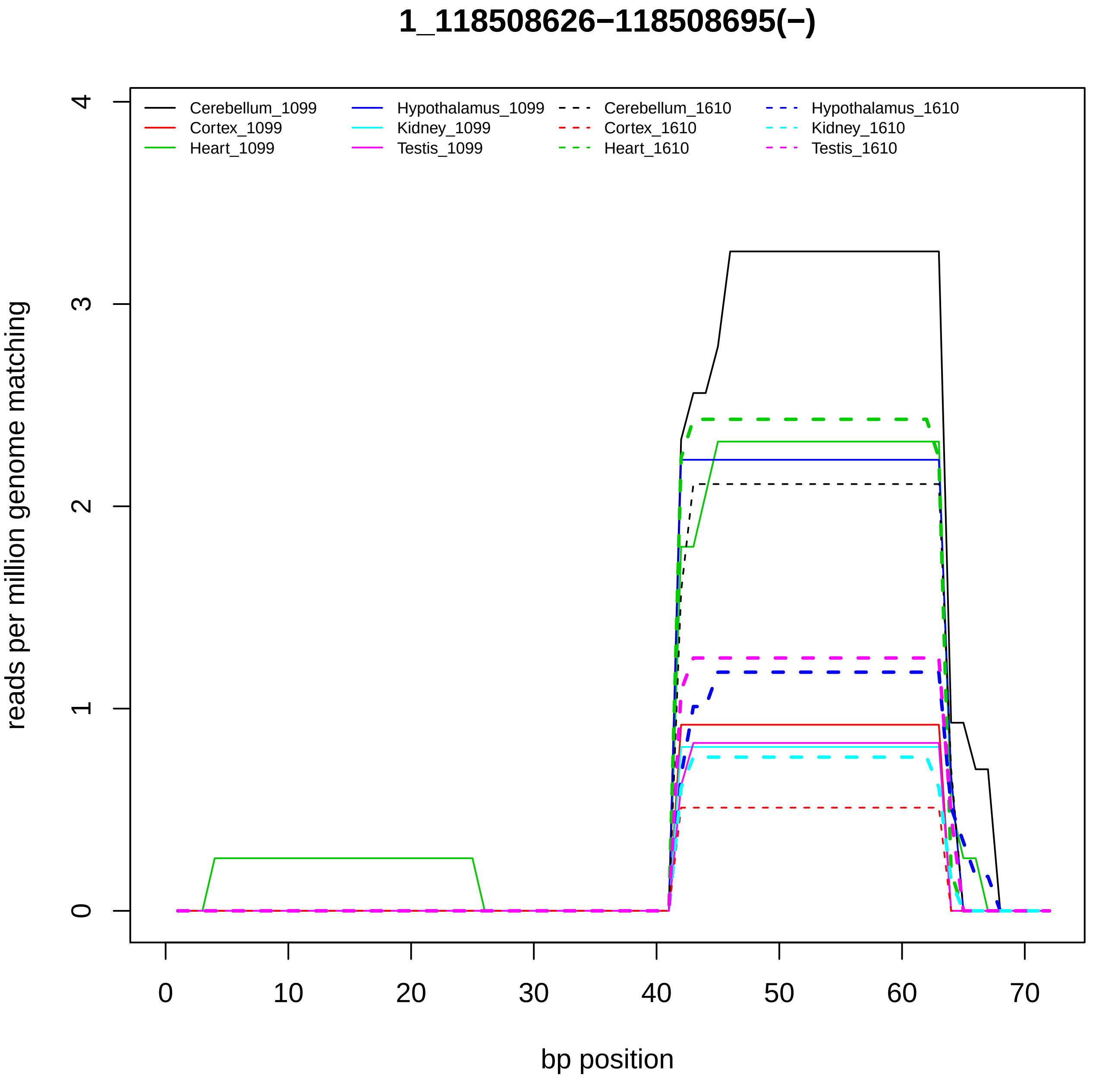
1_118508626−118508695(−)
Cerebellum_1099
Cortex_1099
Heart_1099
Hypothalamus_1099
Kidney_1099
Testis_1099
Cerebellum_1610
Cortex_1610
Heart_1610
Hypothalamus_1610
Kidney_1610
Testis_1610
reads per million genome matching
bp position
0
1
2
3
4
0
10
20
30
40
50
60
70

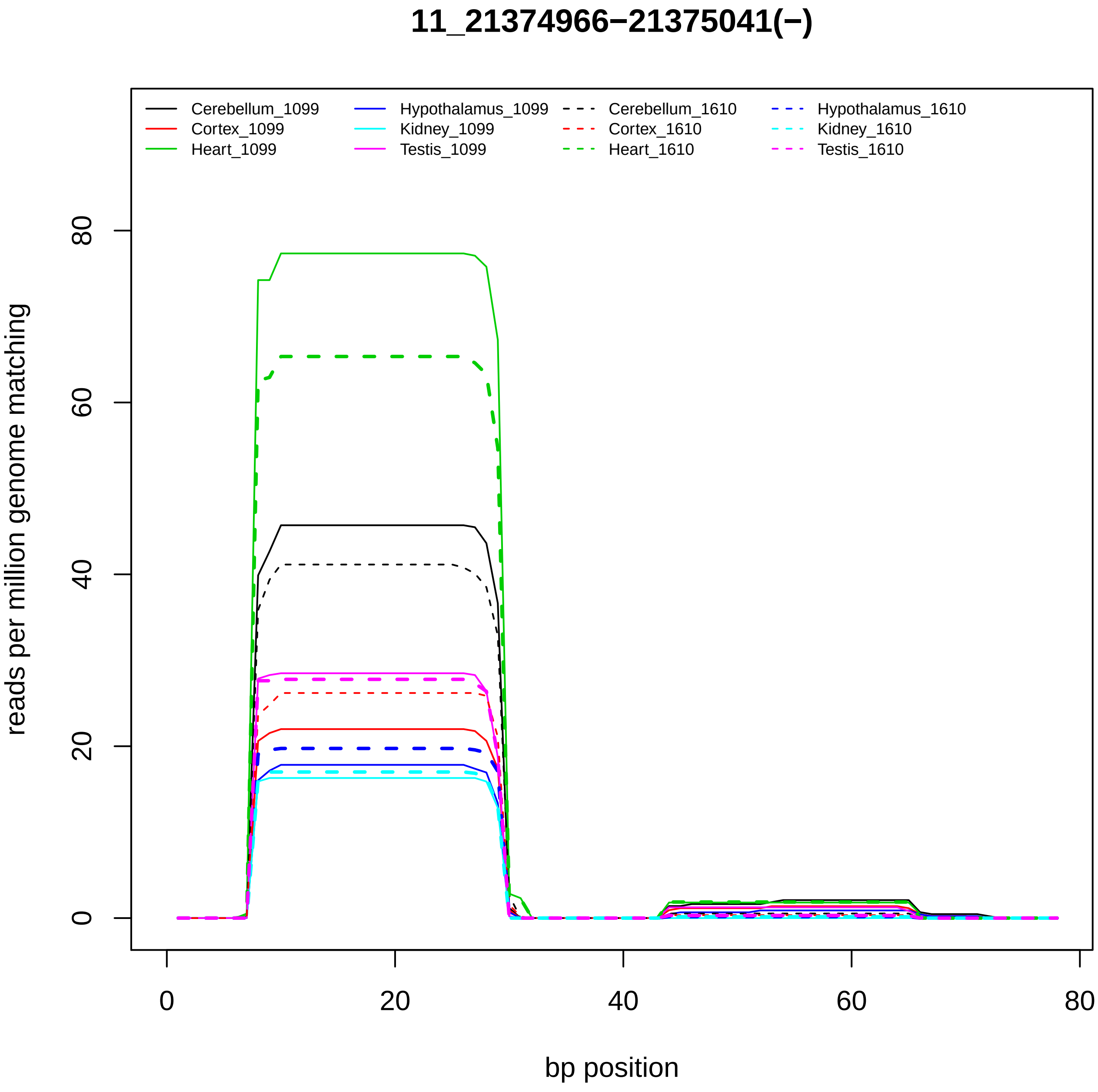
11_21374966−21375041(−)
Cerebellum_1099
Cortex_1099
Heart_1099
Hypothalamus_1099
Kidney_1099
Testis_1099
Cerebellum_1610
Cortex_1610
Heart_1610
Hypothalamus_1610
Kidney_1610
Testis_1610
reads per million genome matching
0
20
40
60
80
bp position
0
20
40
60
80

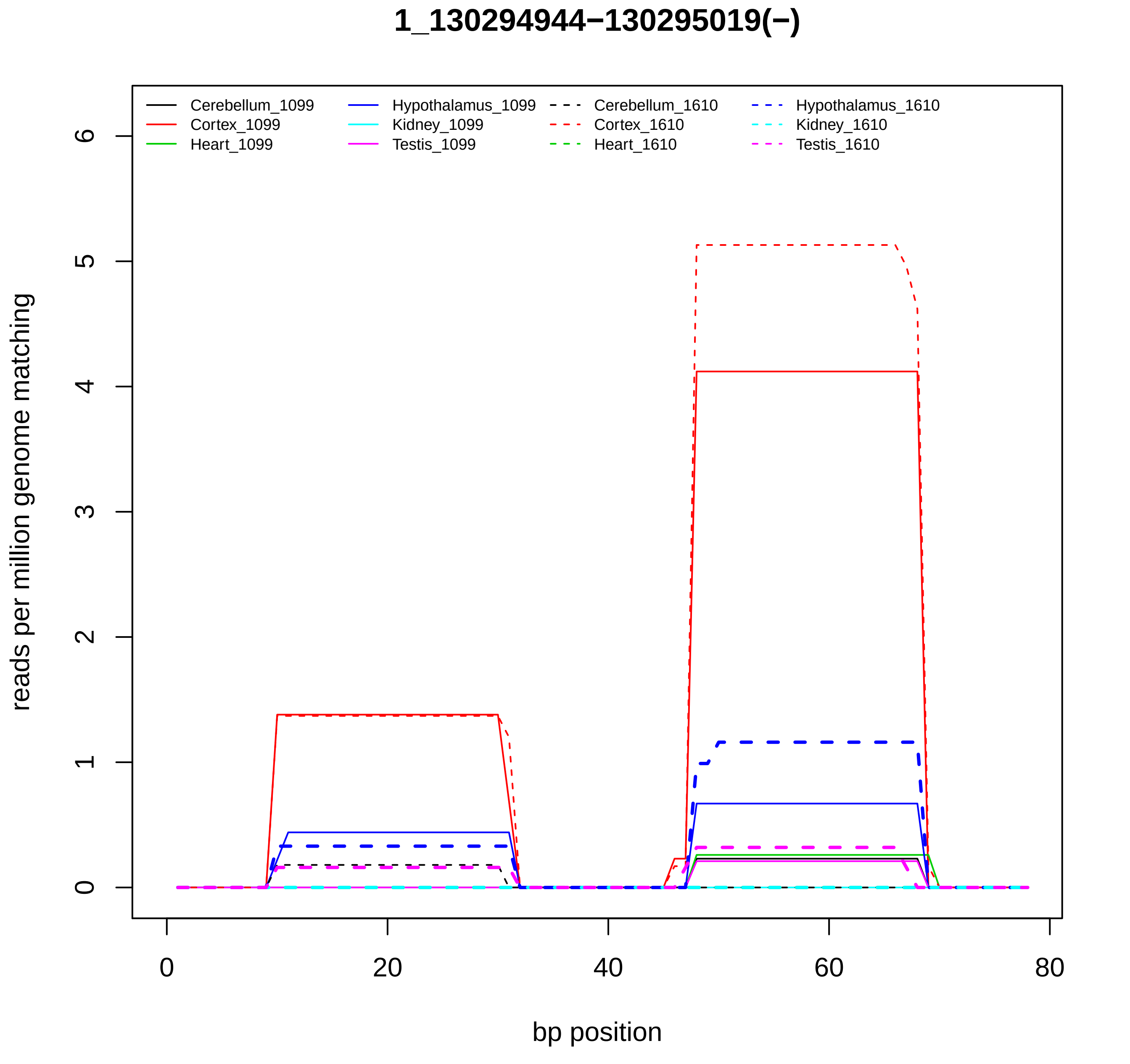

1_130294944−130295019(−)
Cerebellum_1099
Cortex_1099
Heart_1099
Hypothalamus_1099
Kidney_1099
Testis_1099
Cerebellum_1610
Cortex_1610
Heart_1610
Hypothalamus_1610
Kidney_1610
Testis_1610
reads per million genome matching
bp position
0
1
2
3
4
5
6
0
20
40
60
80

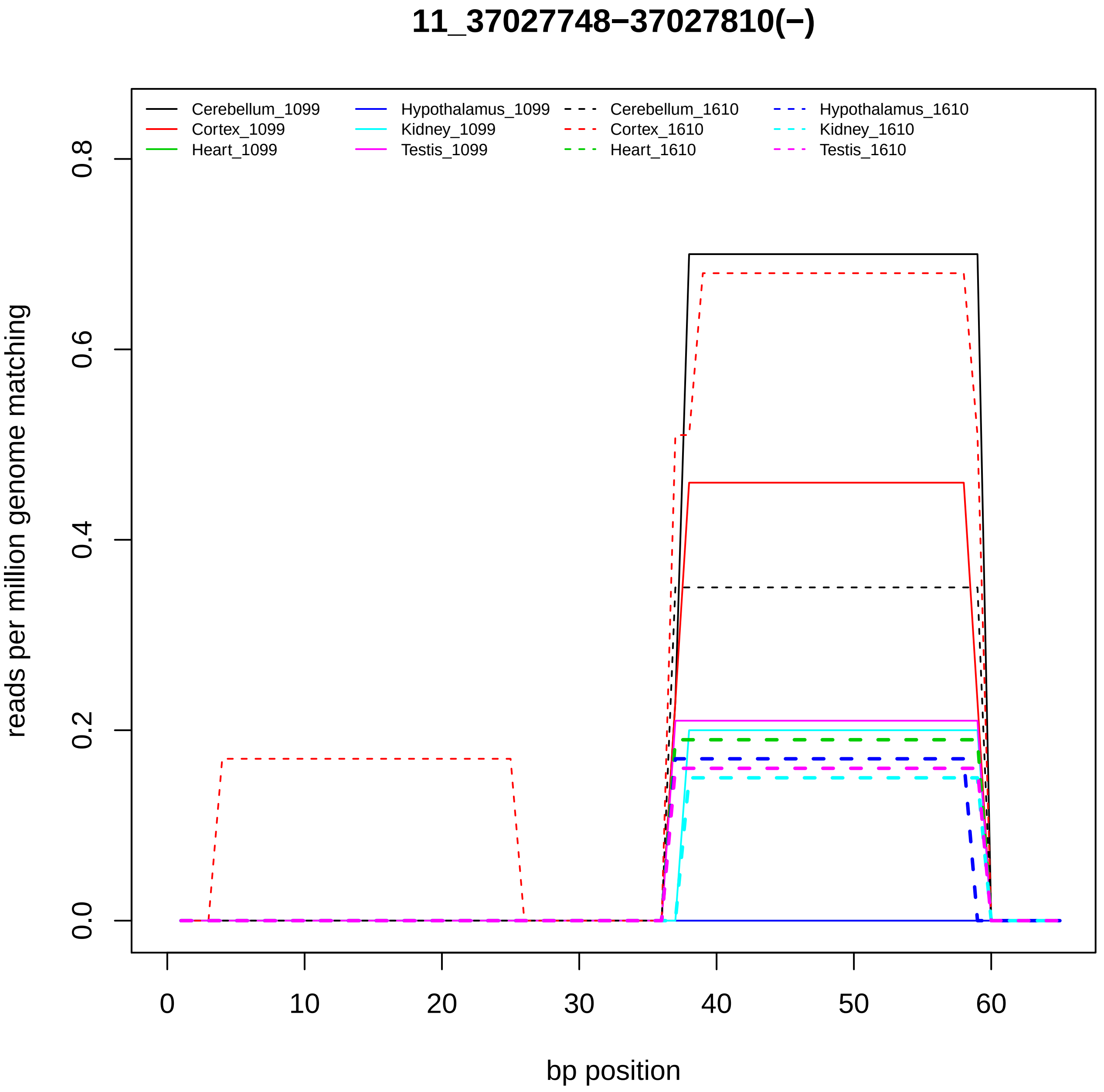

11_37027748−37027810(−)
Cerebellum_1099
Cortex_1099
Heart_1099
Hypothalamus_1099
Kidney_1099
Testis_1099
Cerebellum_1610
Cortex_1610
Heart_1610
Hypothalamus_1610
Kidney_1610
Testis_1610
reads per million genome matching
0.0
0.2
0.4
0.6
0.8
0
10
20
30
40
50
60
bp position

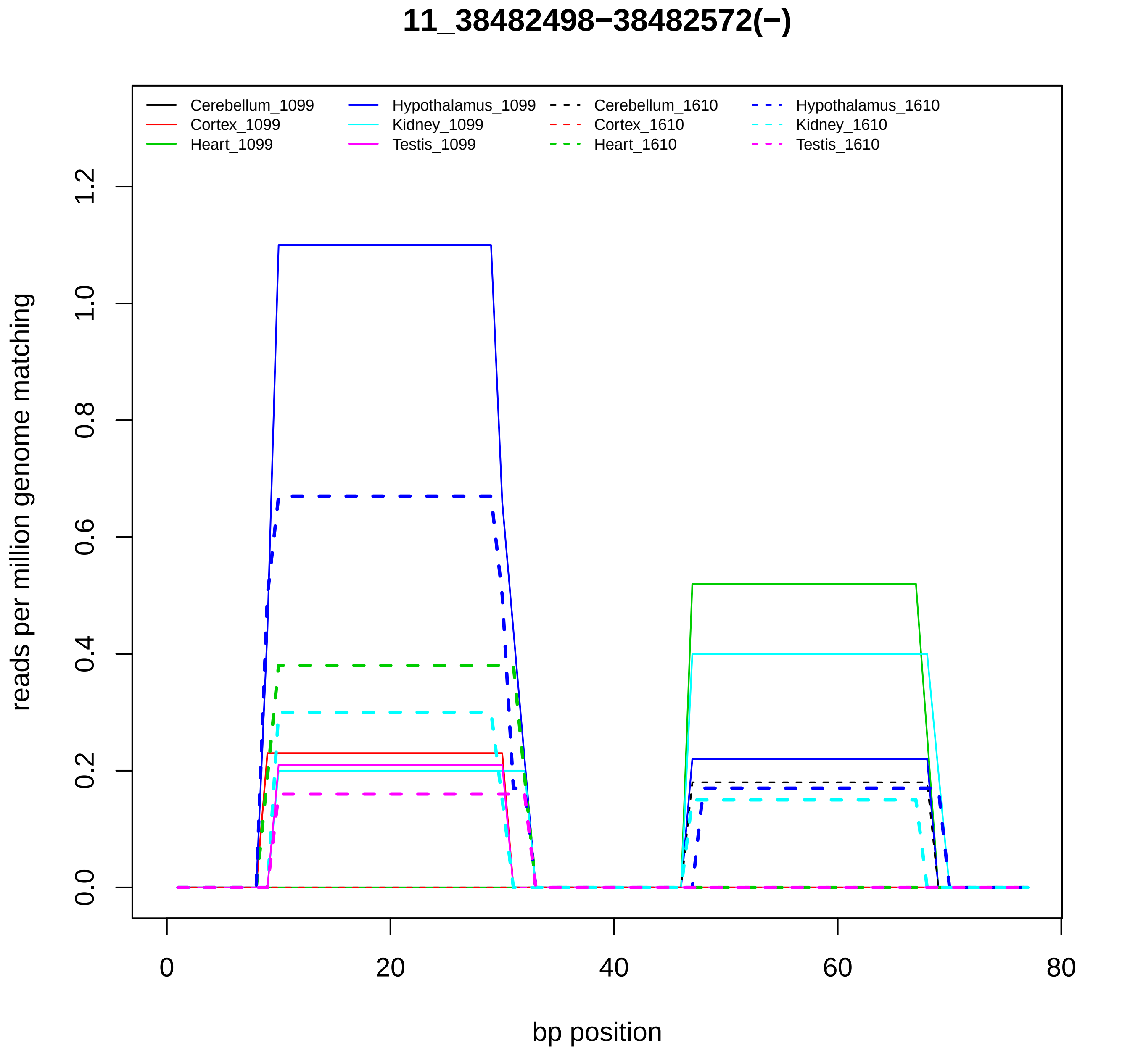

11_38482498−38482572(−)
Cerebellum_1099
Cortex_1099
Heart_1099
Hypothalamus_1099
Kidney_1099
Testis_1099
Cerebellum_1610
Cortex_1610
Heart_1610
Hypothalamus_1610
Kidney_1610
Testis_1610
reads per million genome matching
0.0
0.2
0.4
0.6
0.8
1.0
1.2
0
20
40
60
80
bp position

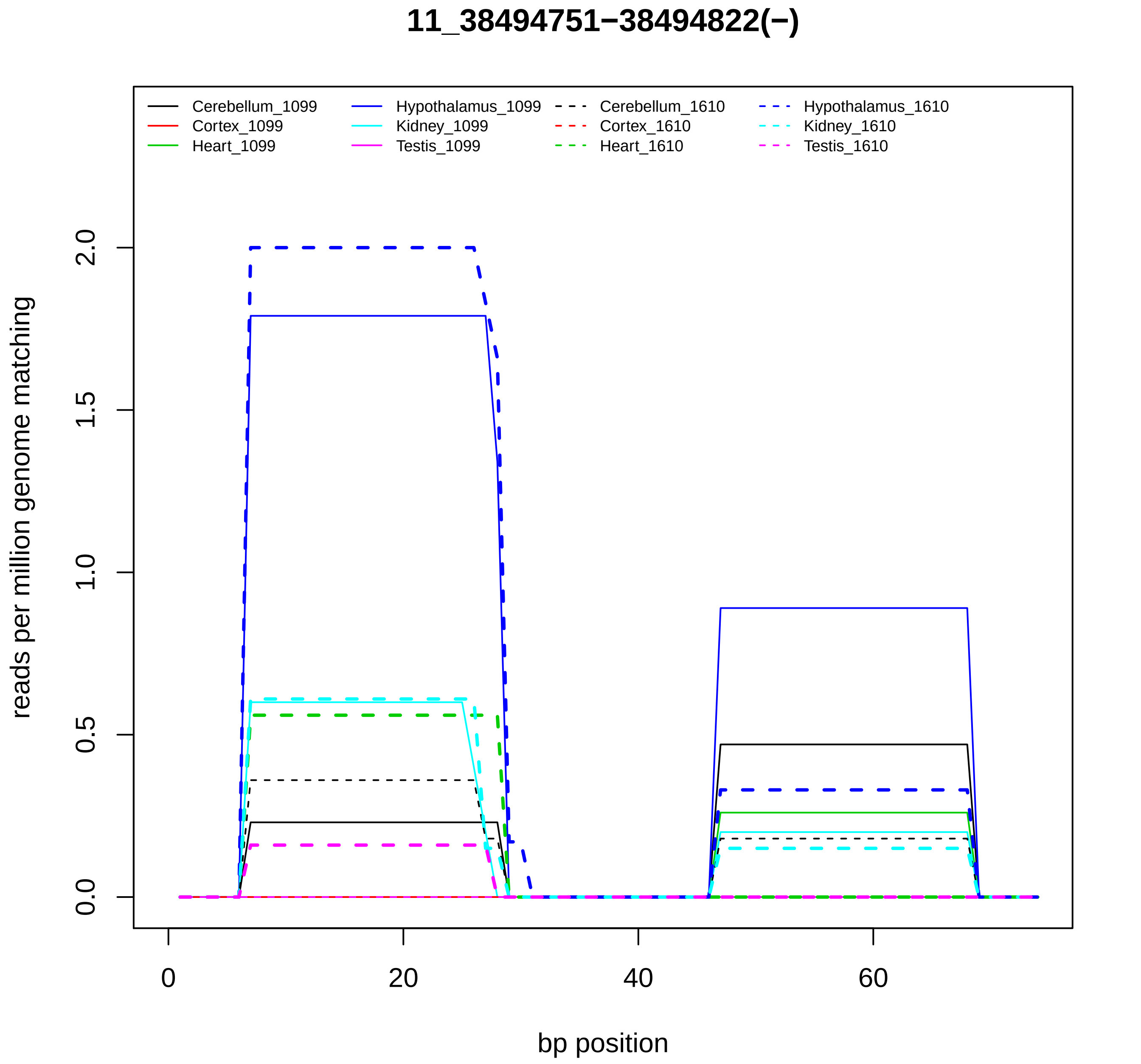

11_38494751−38494822(−)
Cerebellum_1099
Cortex_1099
Heart_1099
Hypothalamus_1099
Kidney_1099
Testis_1099
Cerebellum_1610
Cortex_1610
Heart_1610
Hypothalamus_1610
Kidney_1610
Testis_1610
reads per million genome matching
0.0
0.5
1.0
1.5
2.0
0
20
40
60
bp position

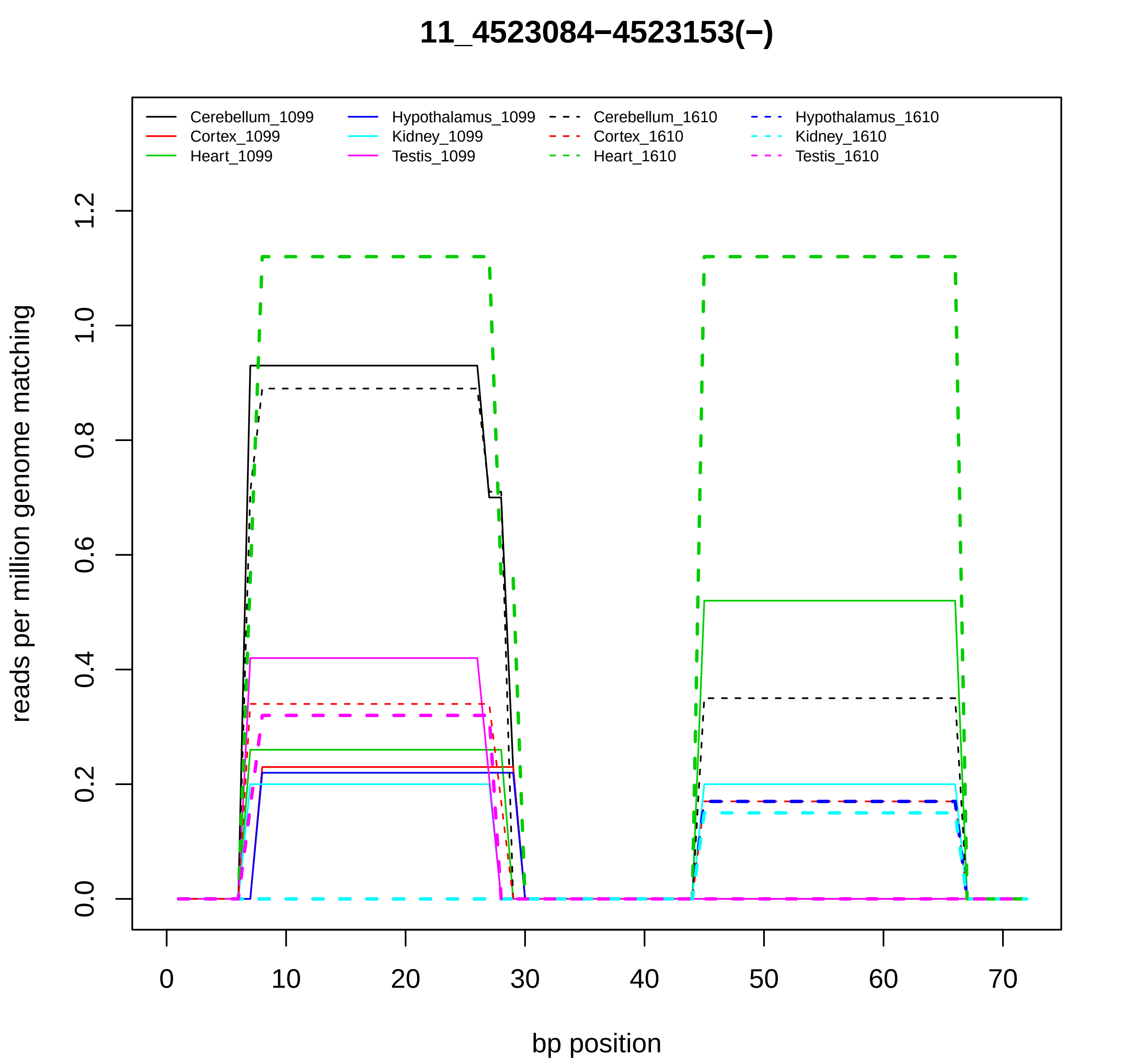

11_4523084−4523153(−)
Cerebellum_1099
Cortex_1099
Heart_1099
Hypothalamus_1099
Kidney_1099
Testis_1099
Cerebellum_1610
Cortex_1610
Heart_1610
Hypothalamus_1610
Kidney_1610
Testis_1610
reads per million genome matching
0.0
0.2
0.4
0.6
0.8
1.0
1.2
0
10
20
30
40
50
60
70
bp position

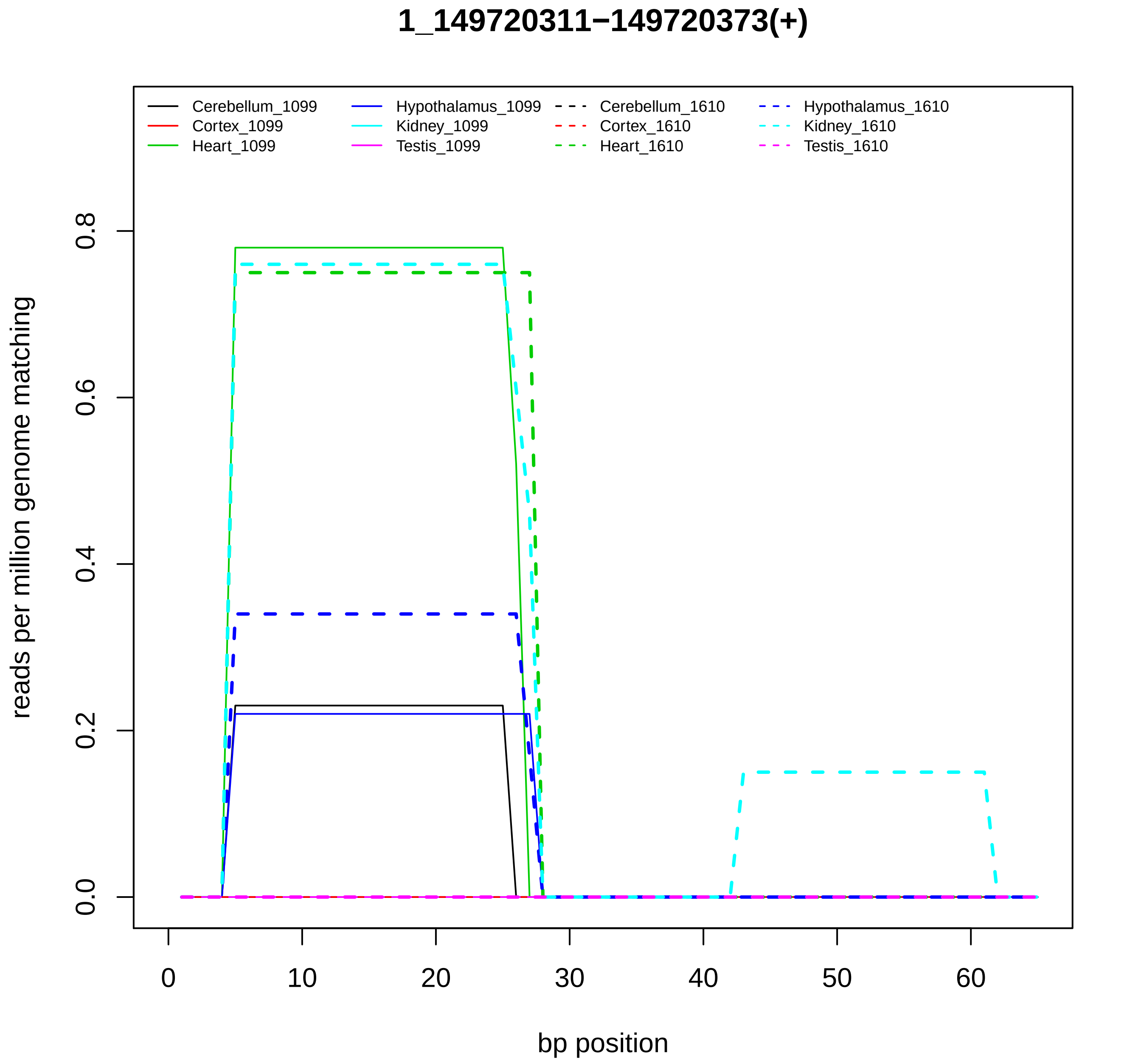

1_149720311−149720373(+)
Cerebellum_1099
Cortex_1099
Heart_1099
Hypothalamus_1099
Kidney_1099
Testis_1099
Cerebellum_1610
Cortex_1610
Heart_1610
Hypothalamus_1610
Kidney_1610
Testis_1610
reads per million genome matching
0.0
0.2
0.4
0.6
0.8
0
10
20
30
40
50
60
bp position

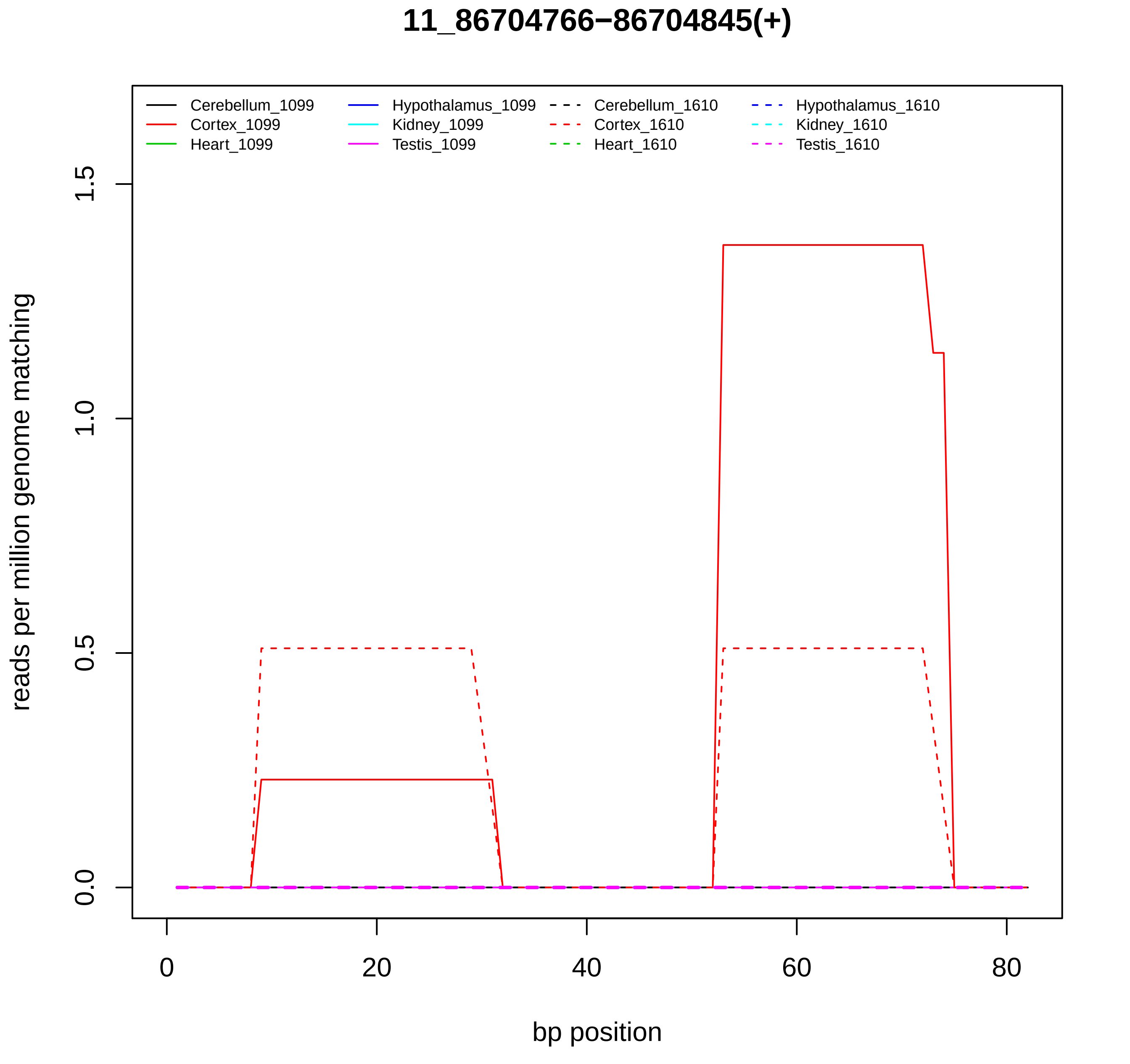

11_86704766−86704845(+)
Cerebellum_1099
Cortex_1099
Heart_1099
Hypothalamus_1099
Kidney_1099
Testis_1099
Cerebellum_1610
Cortex_1610
Heart_1610
Hypothalamus_1610
Kidney_1610
Testis_1610
reads per million genome matching
0.0
0.5
1.0
1.5
0
20
40
60
80
bp position

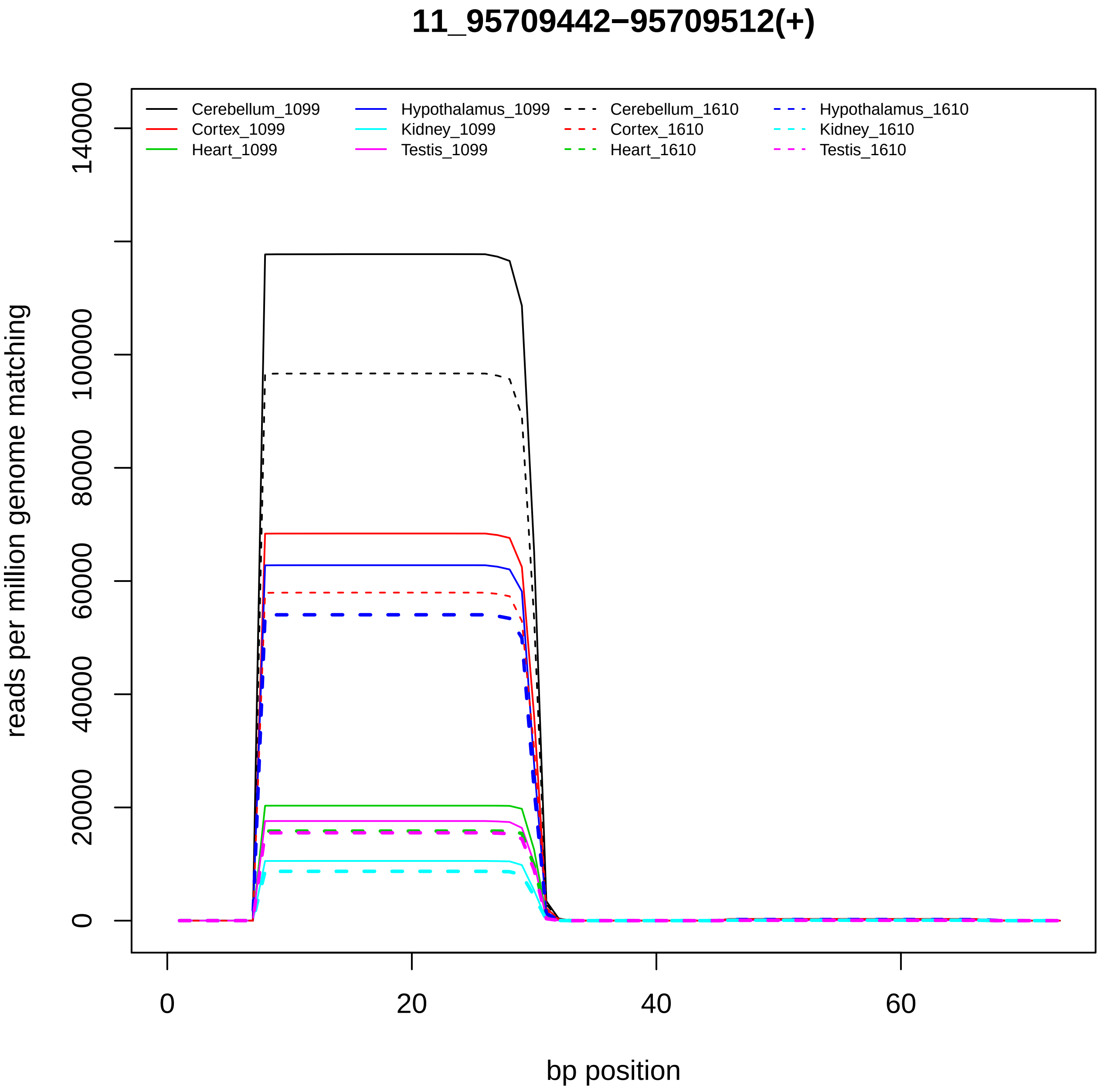
11_95709442−95709512(+)
Cerebellum_1099
Cortex_1099
Heart_1099
Hypothalamus_1099
Kidney_1099
Testis_1099
Cerebellum_1610
Cortex_1610
Heart_1610
Hypothalamus_1610
Kidney_1610
Testis_1610
reads per million genome matching
0
20000
40000
60000
80000
100000
140000
bp position
0
20
40
60

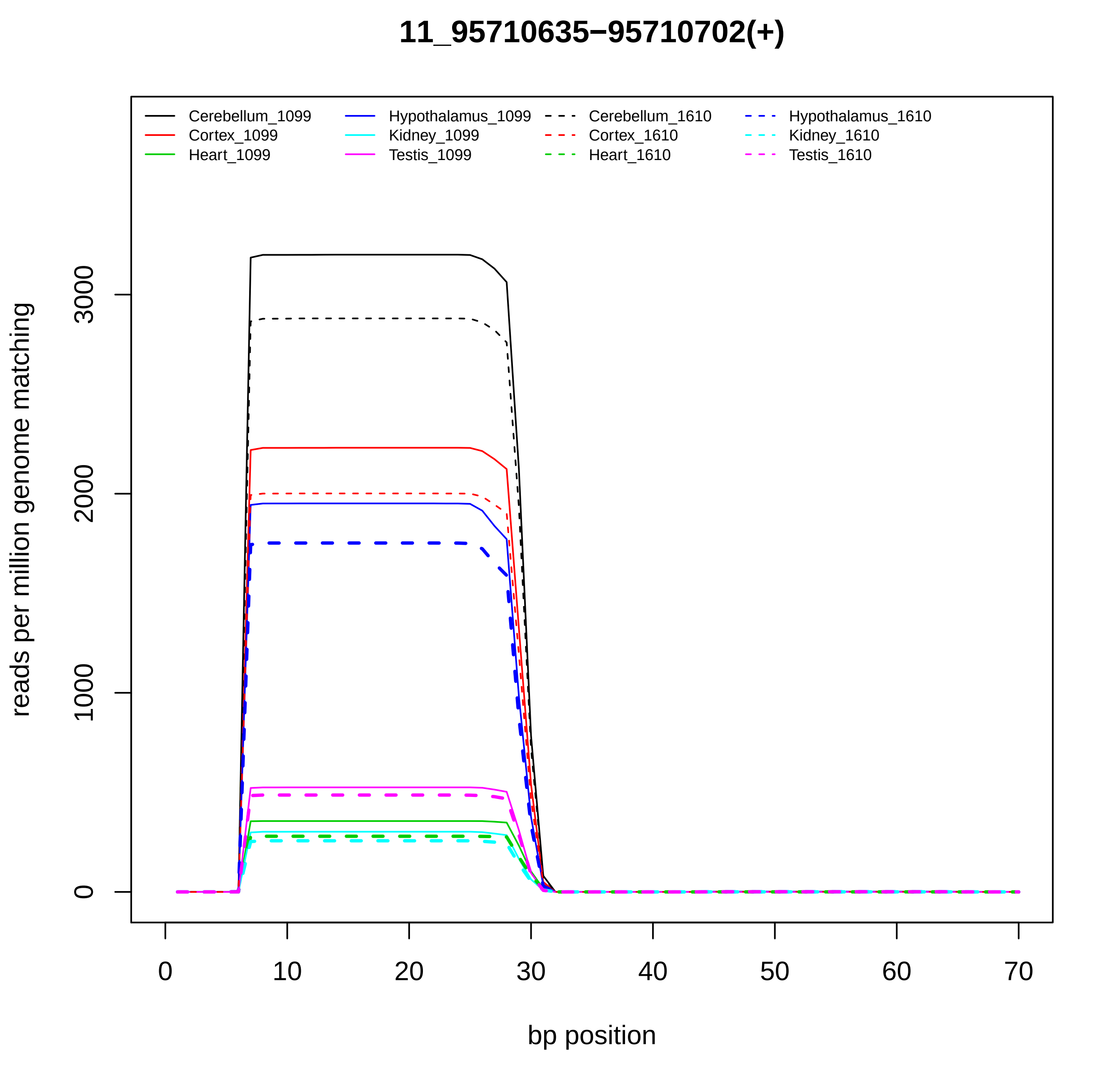

11_95710635−95710702(+)
Cerebellum_1099
Cortex_1099
Heart_1099
Hypothalamus_1099
Kidney_1099
Testis_1099
Cerebellum_1610
Cortex_1610
Heart_1610
Hypothalamus_1610
Kidney_1610
Testis_1610
reads per million genome matching
0
1000
2000
3000
0
10
20
30
40
50
60
70
bp position

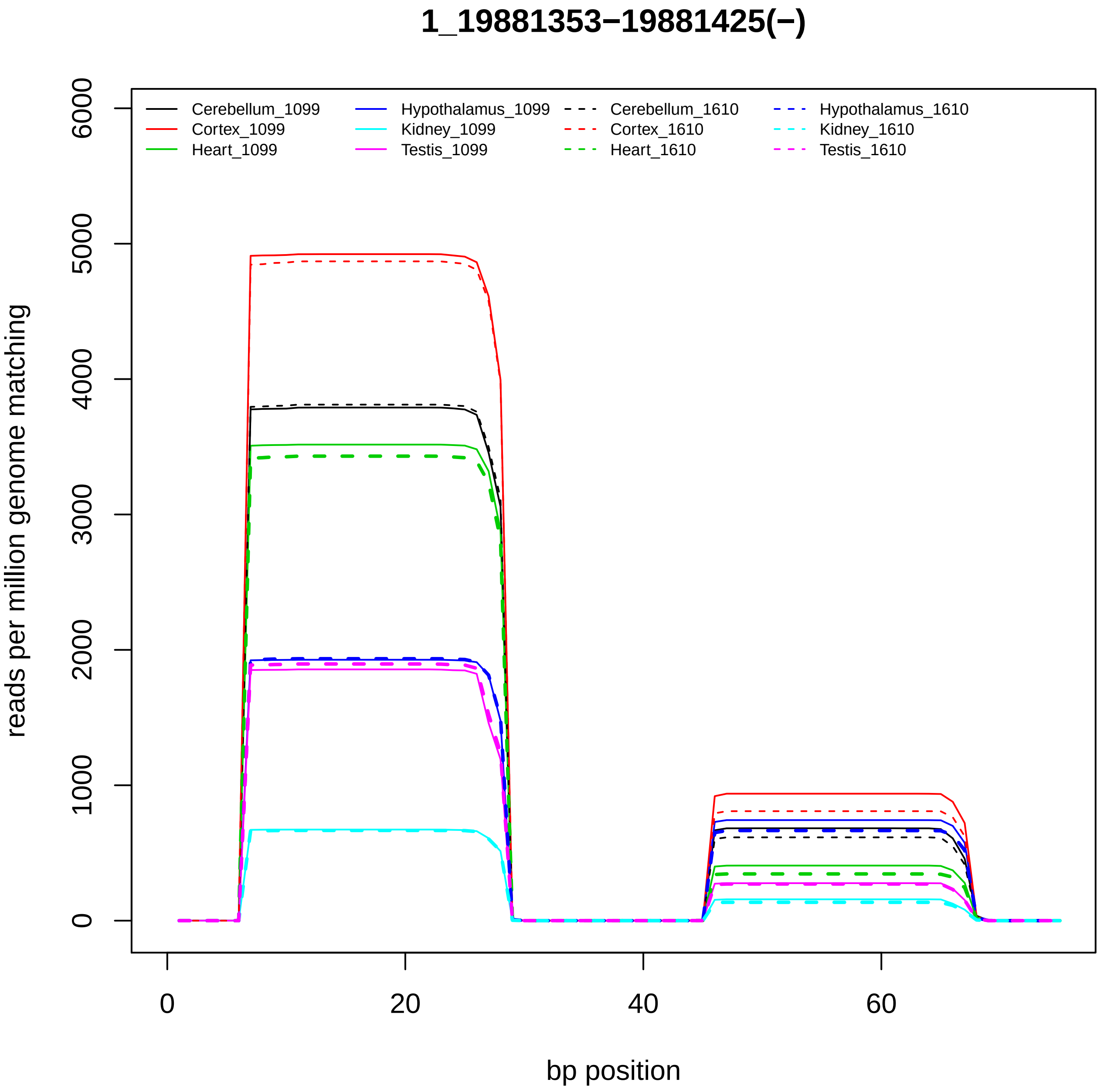
1_19881353−19881425(−)
Cerebellum_1099
Cortex_1099
Heart_1099
Hypothalamus_1099
Kidney_1099
Testis_1099
Cerebellum_1610
Cortex_1610
Heart_1610
Hypothalamus_1610
Kidney_1610
Testis_1610
reads per million genome matching
0
1000
2000
3000
4000
5000
6000
0
20
40
60
bp position

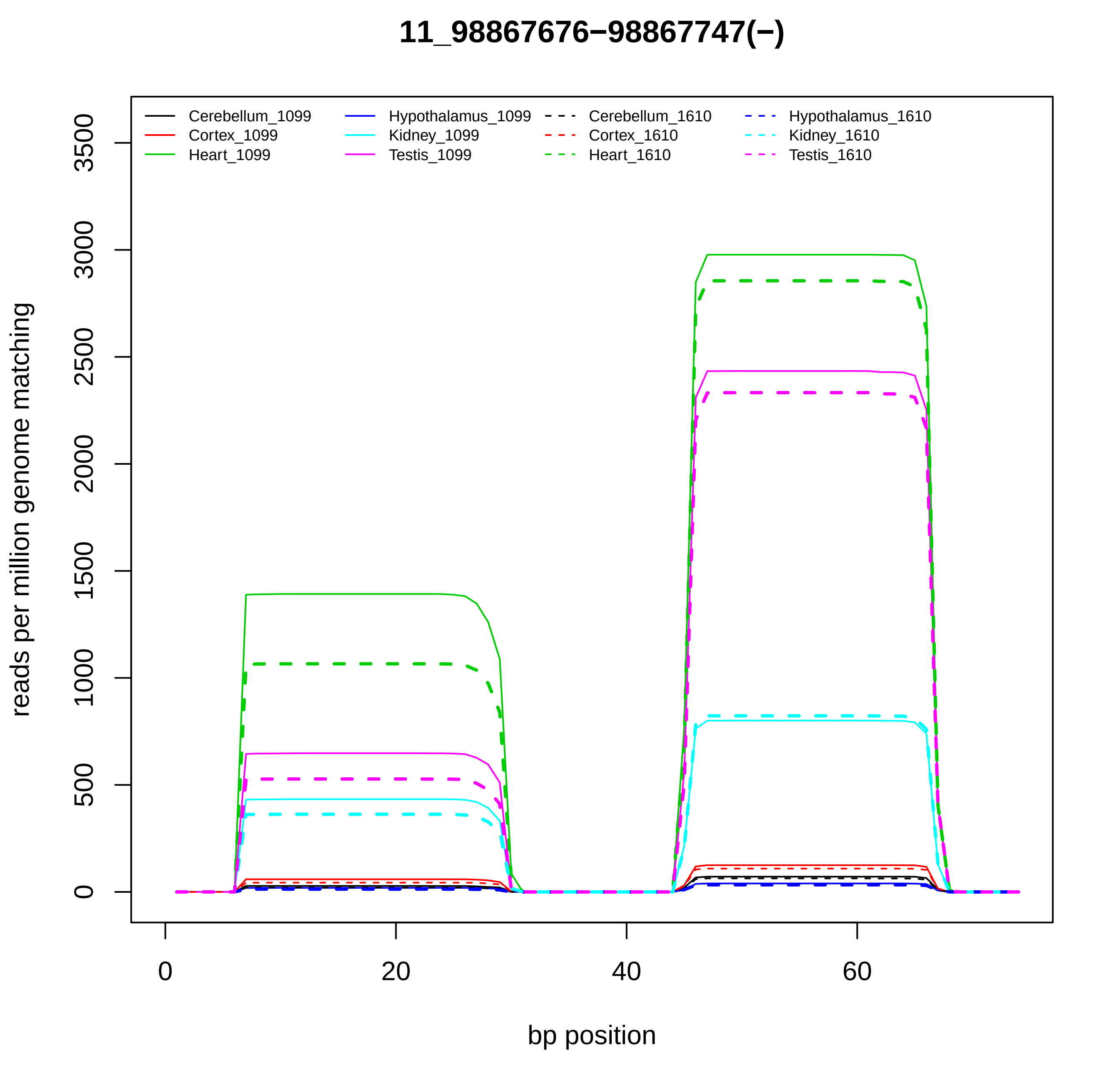

11_98867676−98867747(−)
Cerebellum_1099
Cortex_1099
Heart_1099
Hypothalamus_1099
Kidney_1099
Testis_1099
Cerebellum_1610
Cortex_1610
Heart_1610
Hypothalamus_1610
Kidney_1610
Testis_1610
reads per million genome matching
0
500
1000
1500
2000
2500
3000
3500
bp position
0
20
40
60

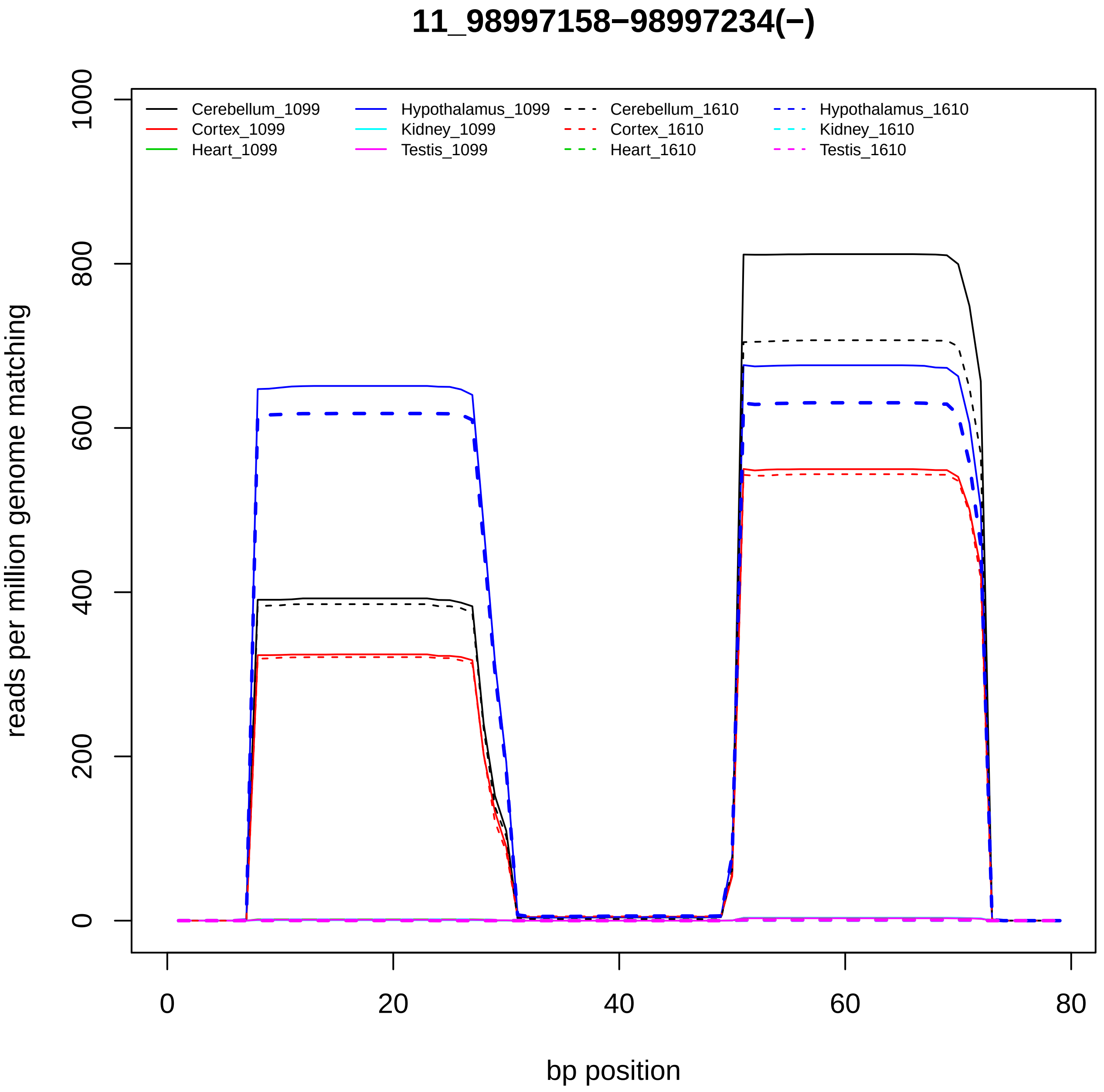

11_98997158−98997234(−)
Cerebellum_1099
Cortex_1099
Heart_1099
Hypothalamus_1099
Kidney_1099
Testis_1099
Cerebellum_1610
Cortex_1610
Heart_1610
Hypothalamus_1610
Kidney_1610
Testis_1610
reads per million genome matching
0
200
400
600
800
1000
bp position
0
20
40
60
80

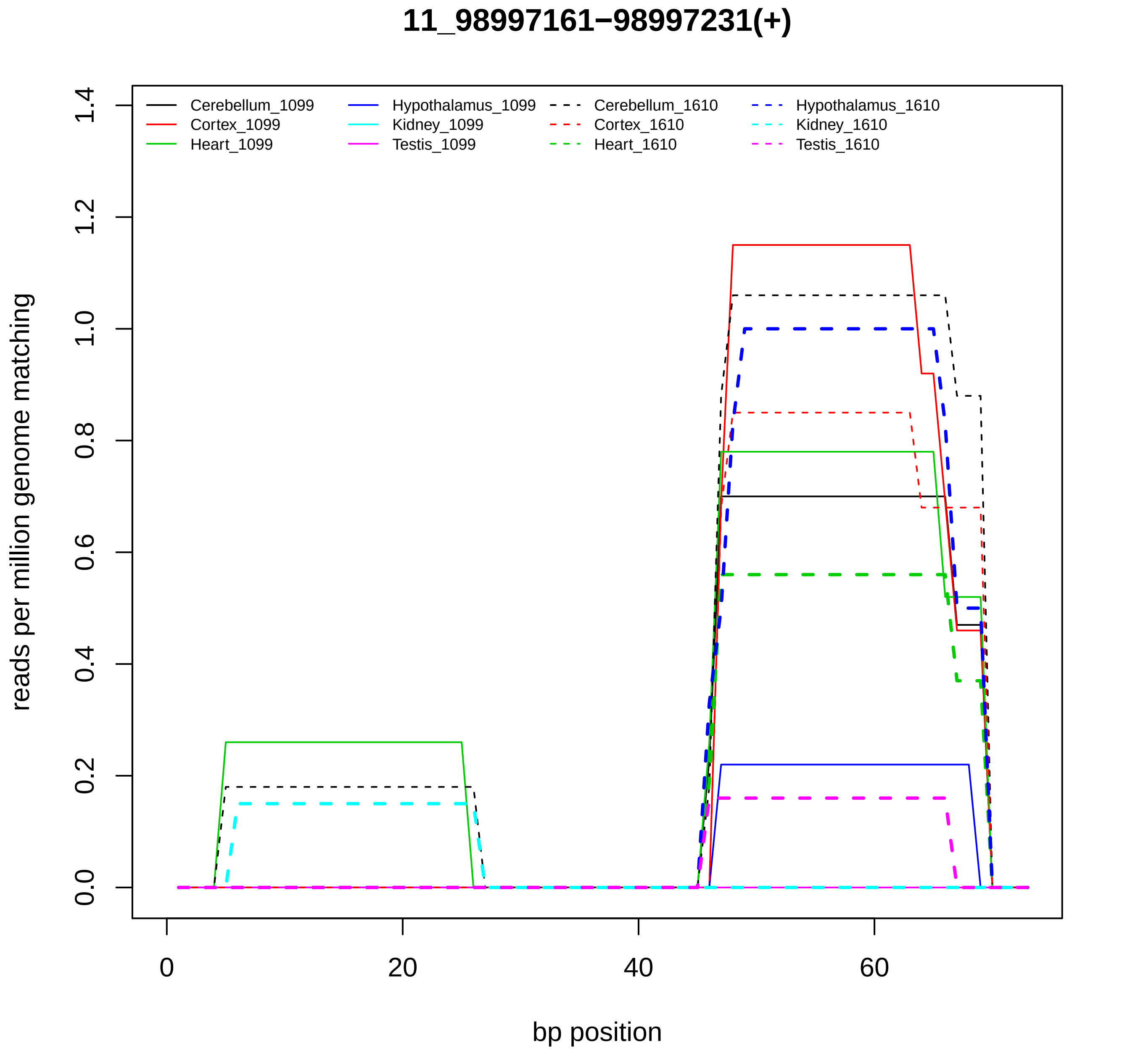

11_98997161−98997231(+)
Cerebellum_1099
Cortex_1099
Heart_1099
Hypothalamus_1099
Kidney_1099
Testis_1099
Cerebellum_1610
Cortex_1610
Heart_1610
Hypothalamus_1610
Kidney_1610
Testis_1610
reads per million genome matching
bp position
0.0
0.2
0.4
0.6
0.8
1.0
1.2
1.4
0
20
40
60

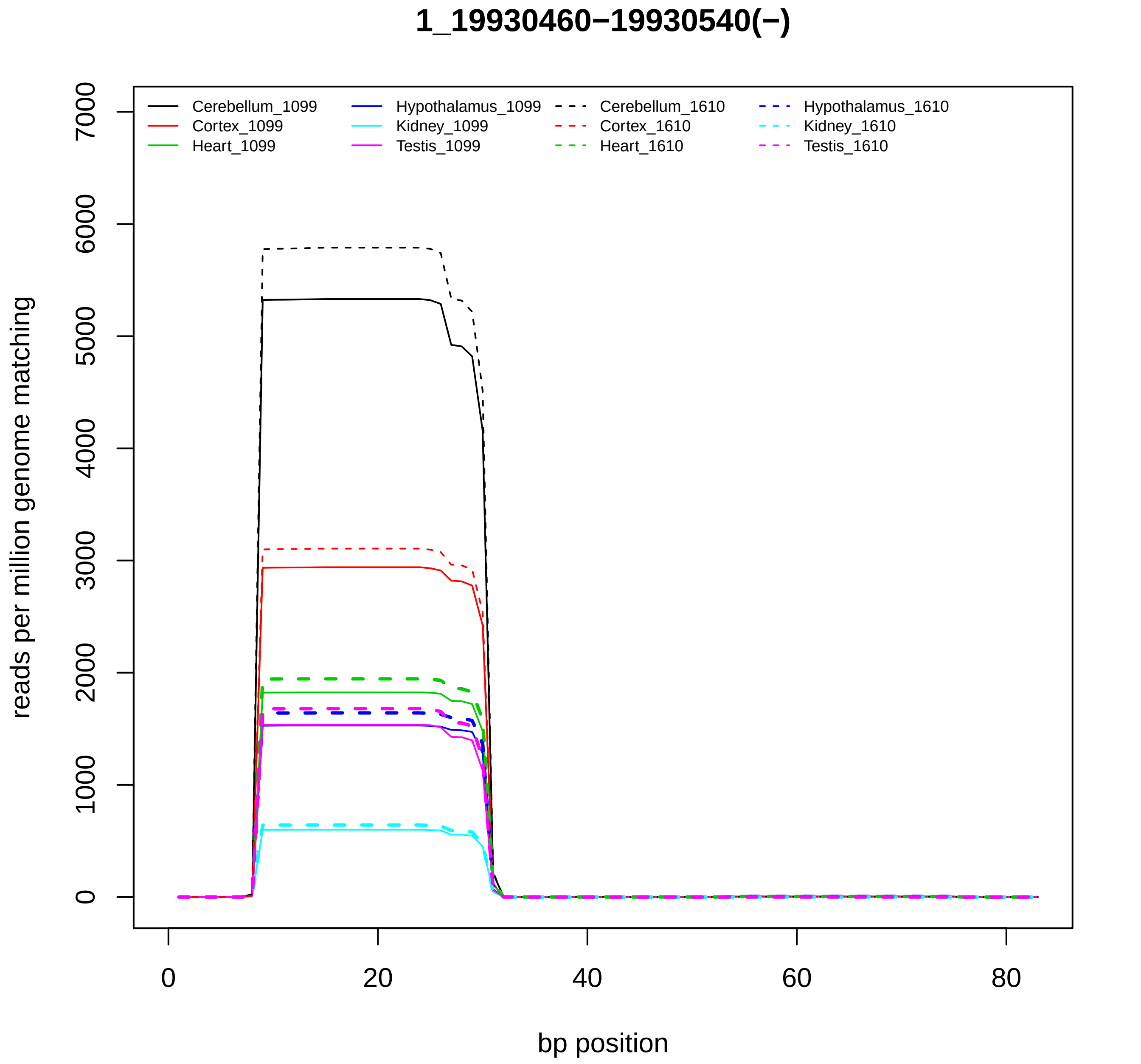
1_19930460−19930540(−)
reads per million genome matching
bp position
0
1000
2000
3000
4000
5000
6000
7000
0
20
40
60
80
Cerebellum_1099
Cortex_1099
Heart_1099
Hypothalamus_1099
Kidney_1099
Testis_1099
Cerebellum_1610
Cortex_1610
Heart_1610
Hypothalamus_1610
Kidney_1610
Testis_1610

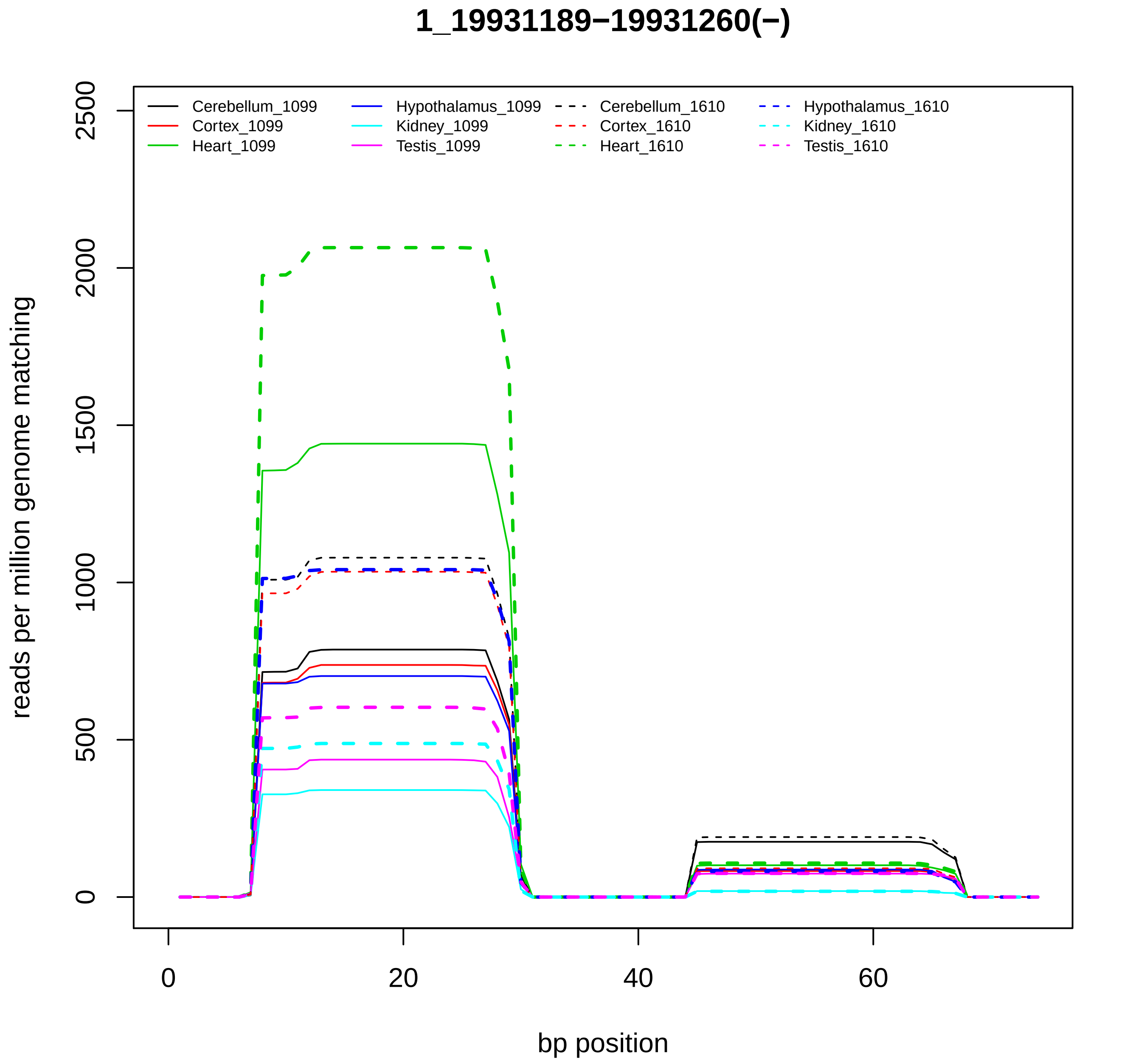
1_19931189−19931260(−)
Cerebellum_1099
Cortex_1099
Heart_1099
Hypothalamus_1099
Kidney_1099
Testis_1099
Cerebellum_1610
Cortex_1610
Heart_1610
Hypothalamus_1610
Kidney_1610
Testis_1610
reads per million genome matching
0
500
1000
1500
2000
2500
0
20
40
60
bp position

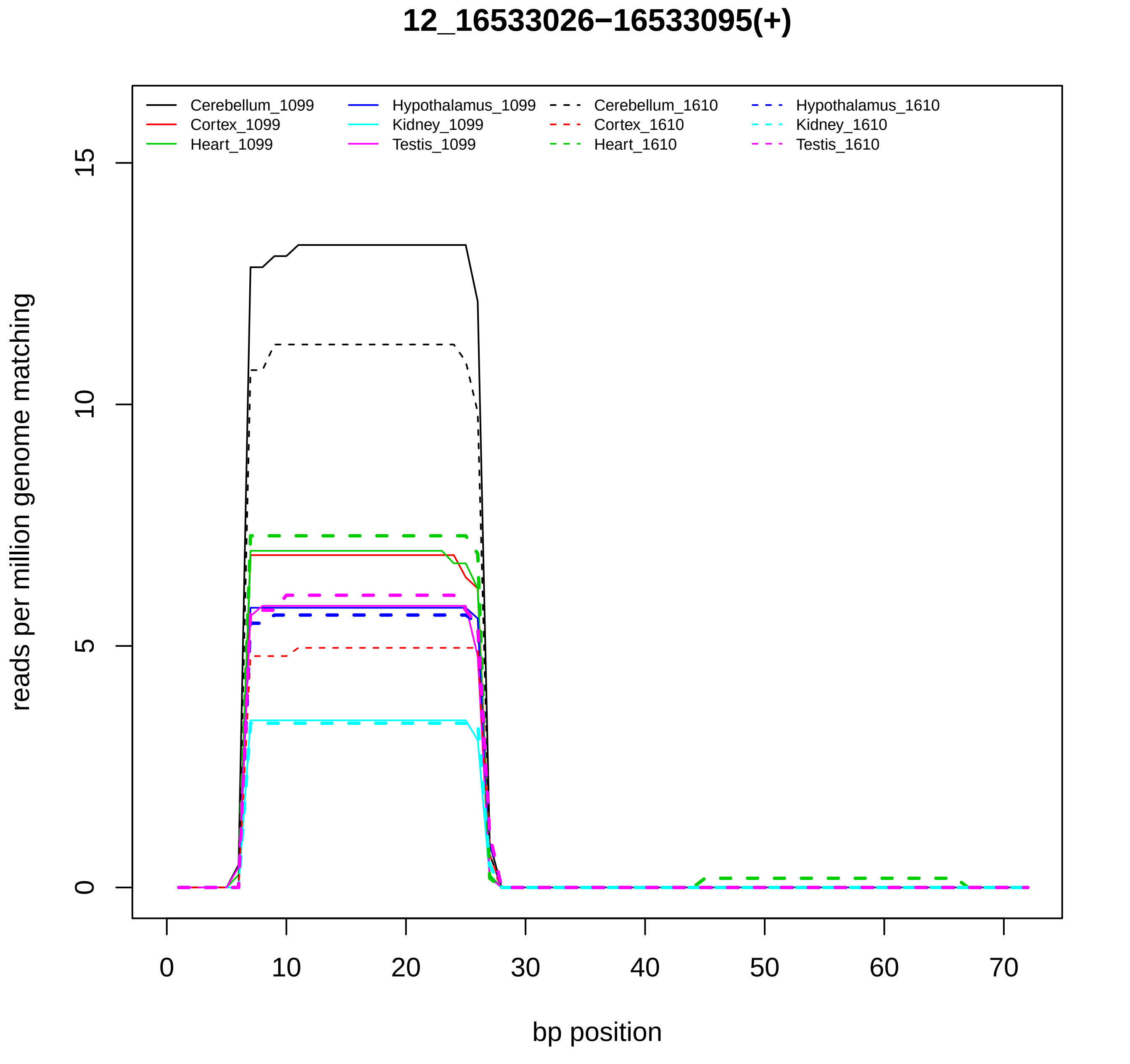

12_16533026−16533095(+)
Cerebellum_1099
Cortex_1099
Heart_1099
Hypothalamus_1099
Kidney_1099
Testis_1099
Cerebellum_1610
Cortex_1610
Heart_1610
Hypothalamus_1610
Kidney_1610
Testis_1610
reads per million genome matching
bp position
0
5
10
15
0
10
20
30
40
50
60
70

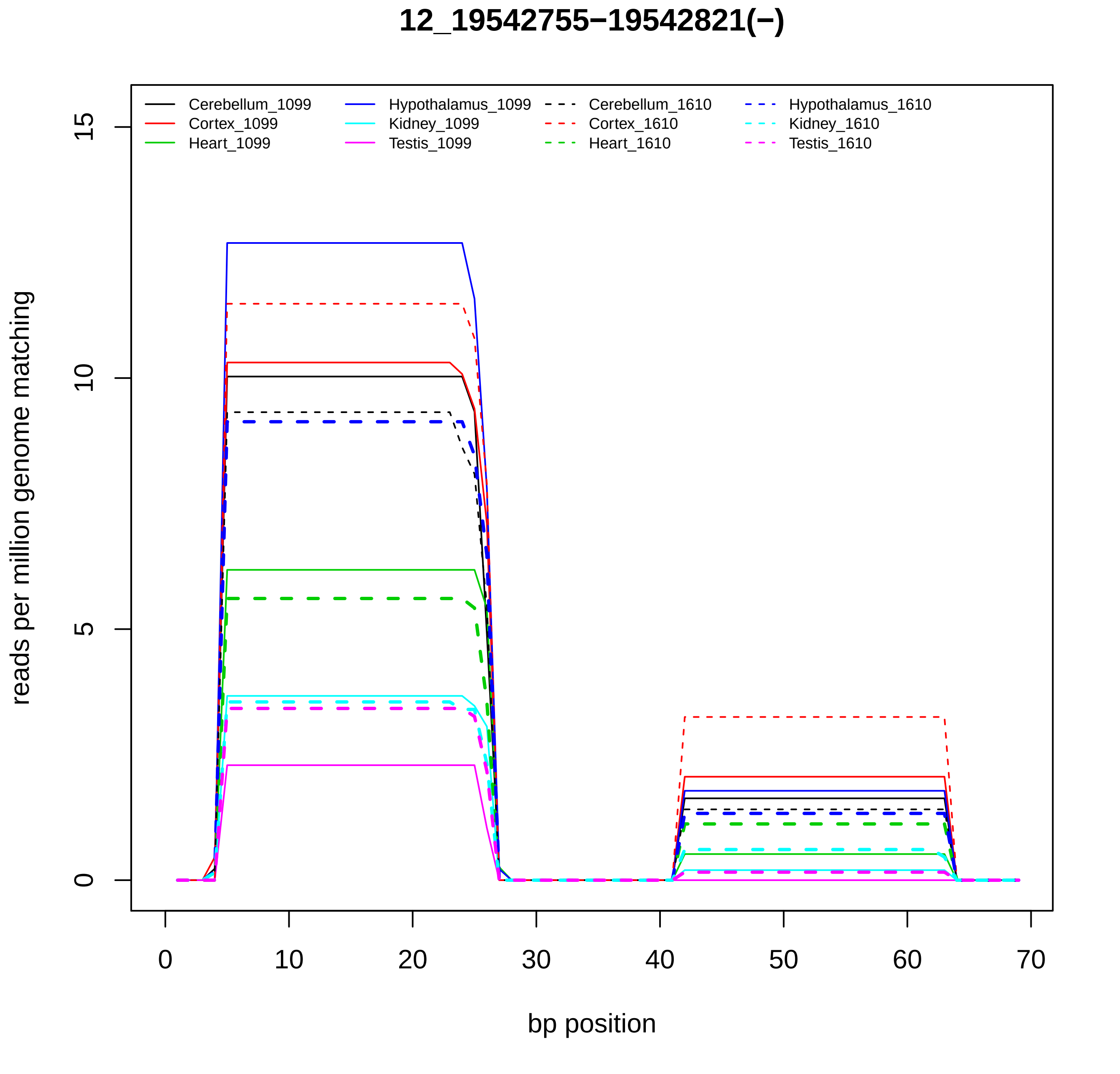

12_19542755−19542821(−)
Cerebellum_1099
Cortex_1099
Heart_1099
Hypothalamus_1099
Kidney_1099
Testis_1099
Cerebellum_1610
Cortex_1610
Heart_1610
Hypothalamus_1610
Kidney_1610
Testis_1610
reads per million genome matching
0
5
10
15
0
10
20
30
40
50
60
70
bp position

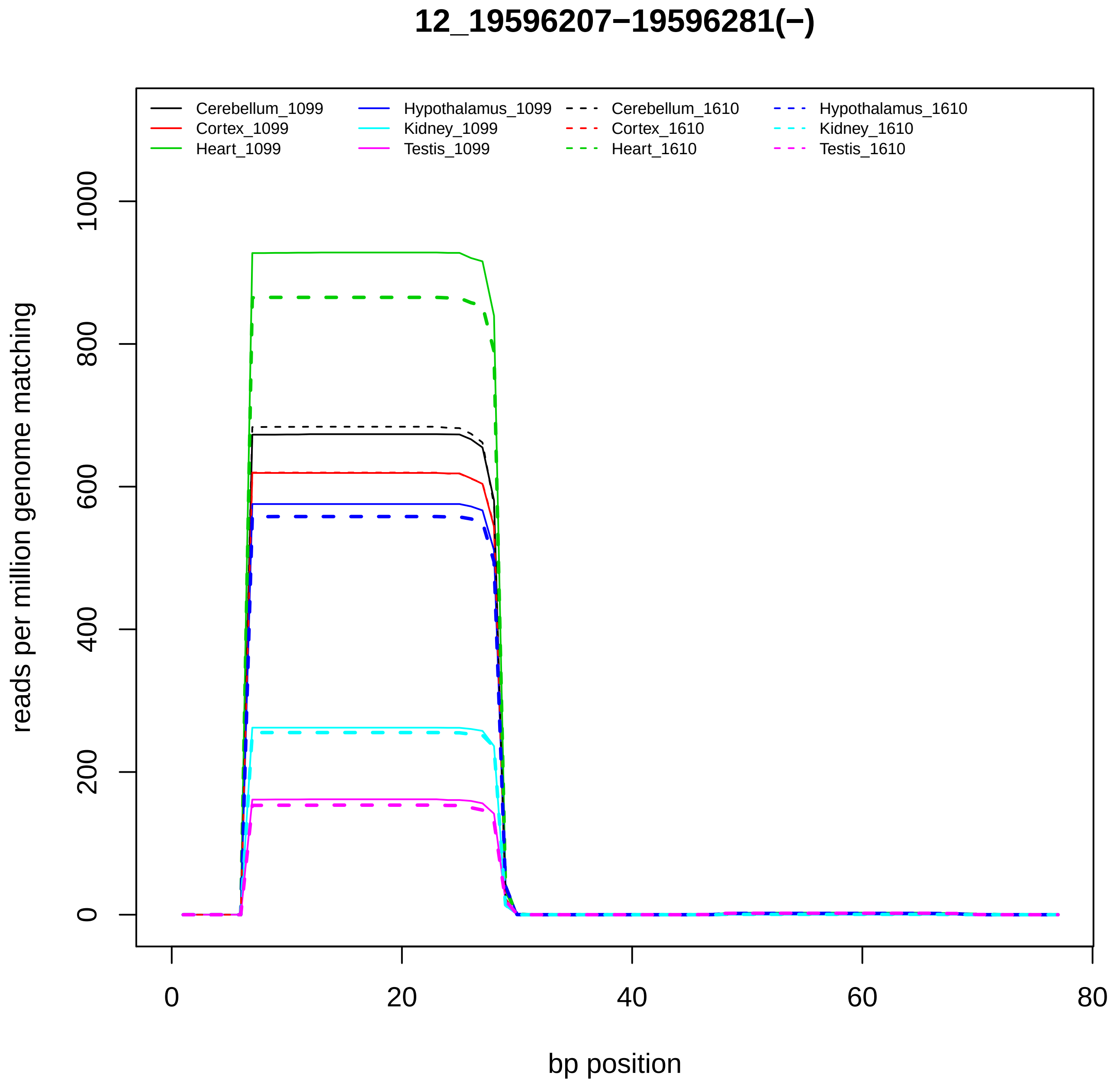

12_19596207−19596281(−)
Cerebellum_1099
Cortex_1099
Heart_1099
Hypothalamus_1099
Kidney_1099
Testis_1099
Cerebellum_1610
Cortex_1610
Heart_1610
Hypothalamus_1610
Kidney_1610
Testis_1610
reads per million genome matching
0
200
400
600
800
1000
0
20
40
60
80
bp position

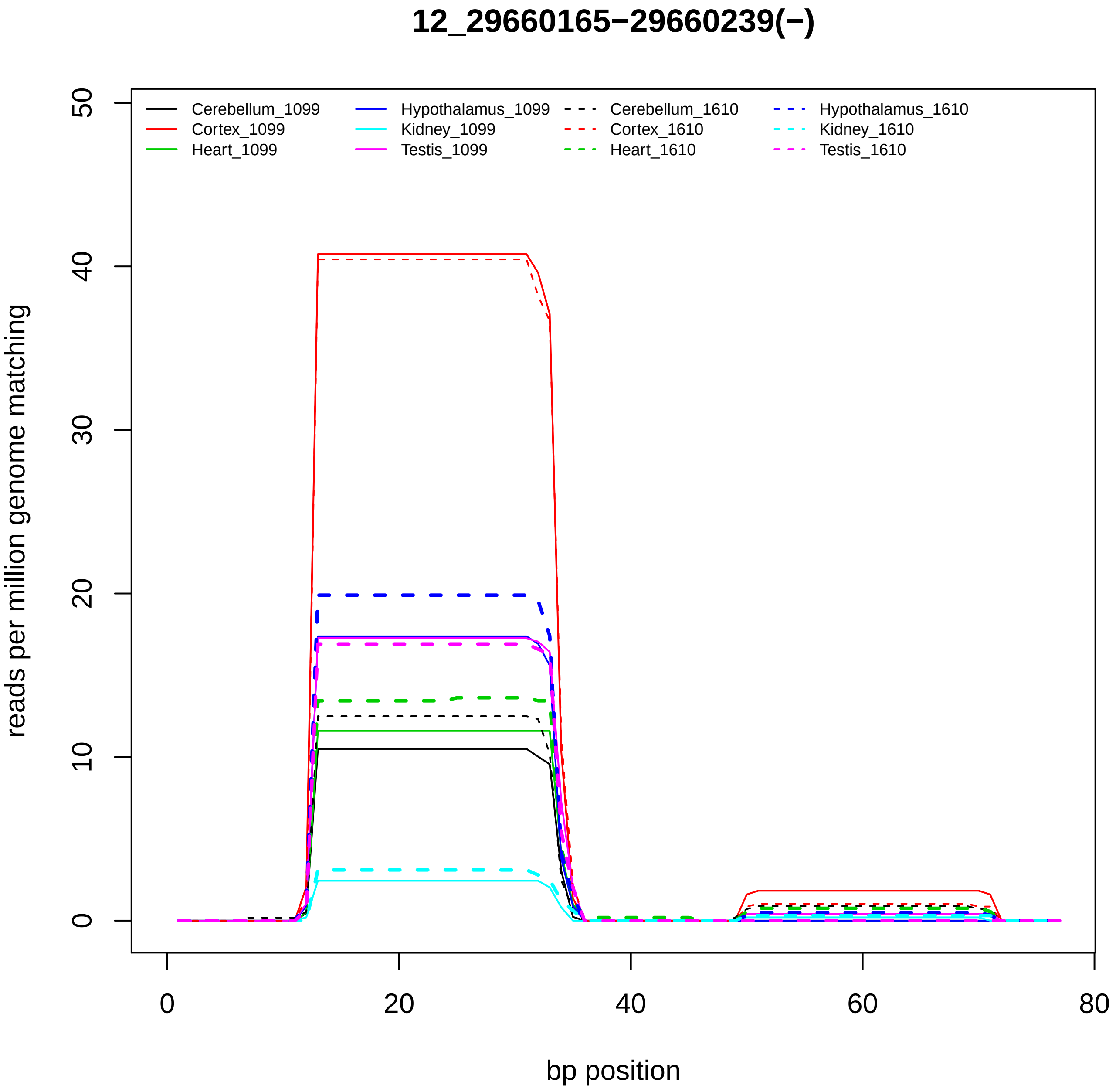

12_29660165−29660239(−)
Cerebellum_1099
Cortex_1099
Heart_1099
Hypothalamus_1099
Kidney_1099
Testis_1099
Cerebellum_1610
Cortex_1610
Heart_1610
Hypothalamus_1610
Kidney_1610
Testis_1610
reads per million genome matching
bp position
0
10
20
30
40
50
0
20
40
60
80

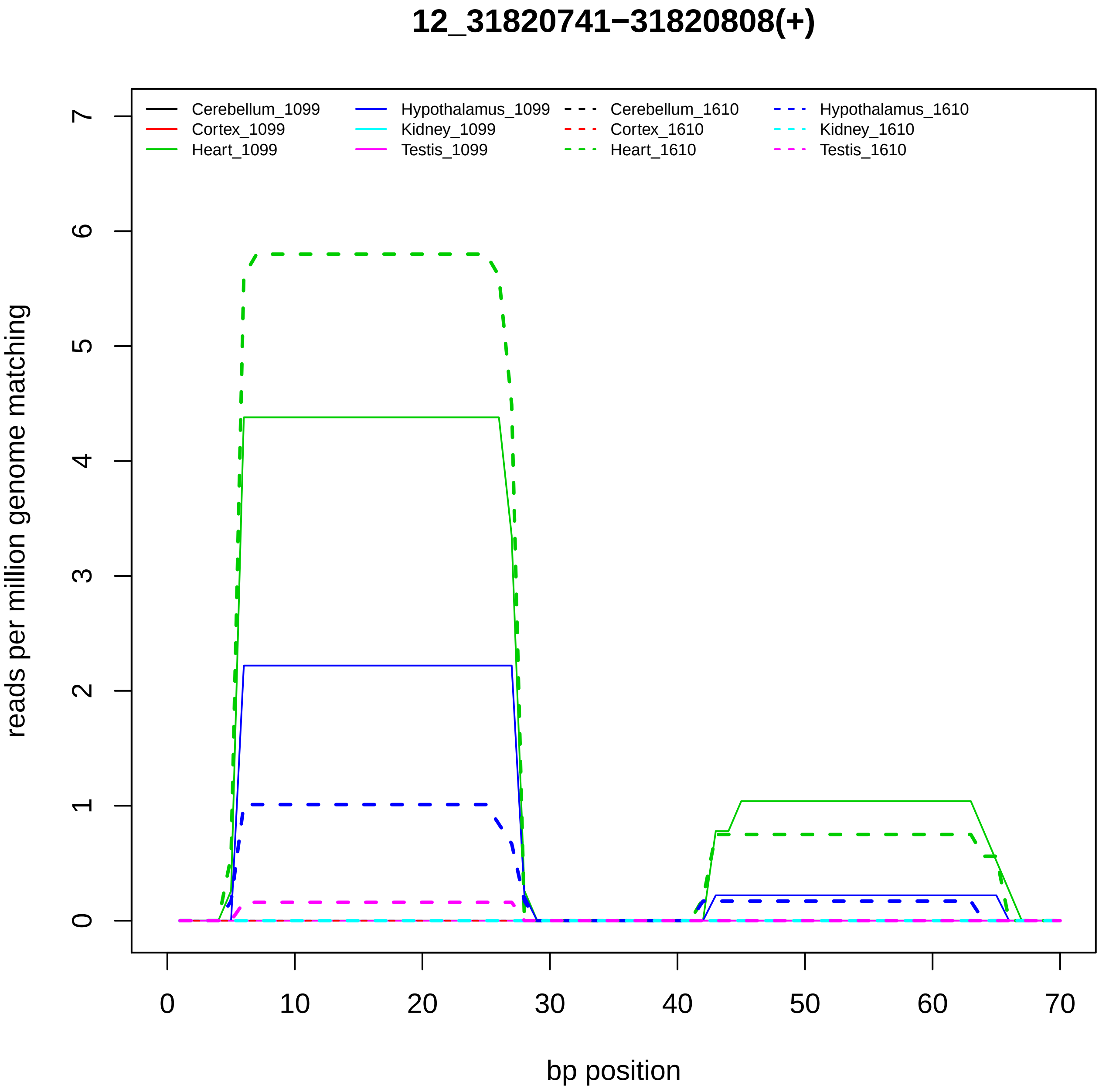

12_31820741−31820808(+)
Cerebellum_1099
Cortex_1099
Heart_1099
Hypothalamus_1099
Kidney_1099
Testis_1099
Cerebellum_1610
Cortex_1610
Heart_1610
Hypothalamus_1610
Kidney_1610
Testis_1610
reads per million genome matching
0
1
2
3
4
5
6
7
0
10
20
30
40
50
60
70
bp position

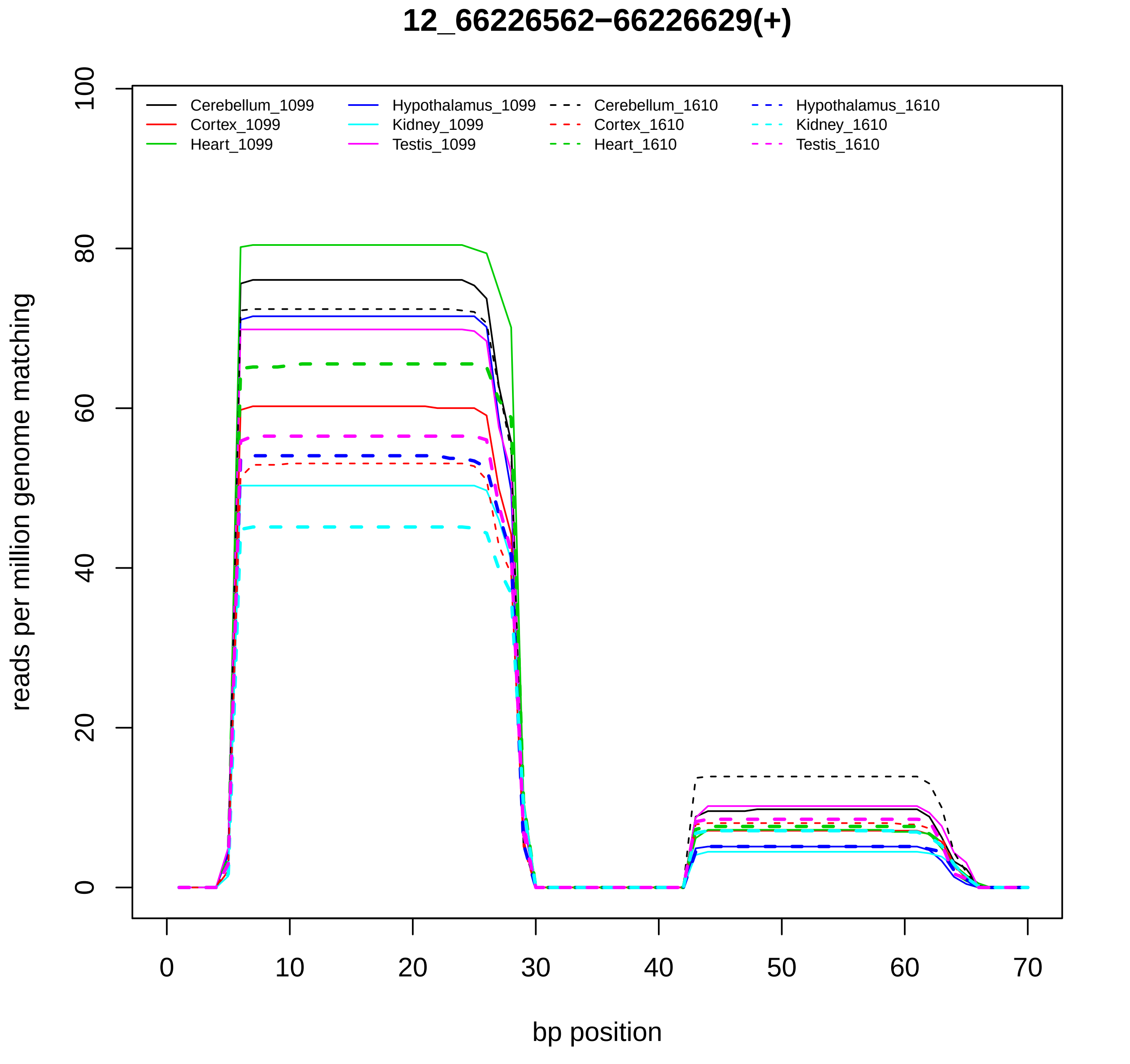
12_66226562−66226629(+)
Cerebellum_1099
Cortex_1099
Heart_1099
Hypothalamus_1099
Kidney_1099
Testis_1099
Cerebellum_1610
Cortex_1610
Heart_1610
Hypothalamus_1610
Kidney_1610
Testis_1610
reads per million genome matching
bp position
0
20
40
60
80
100
0
10
20
30
40
50
60
70

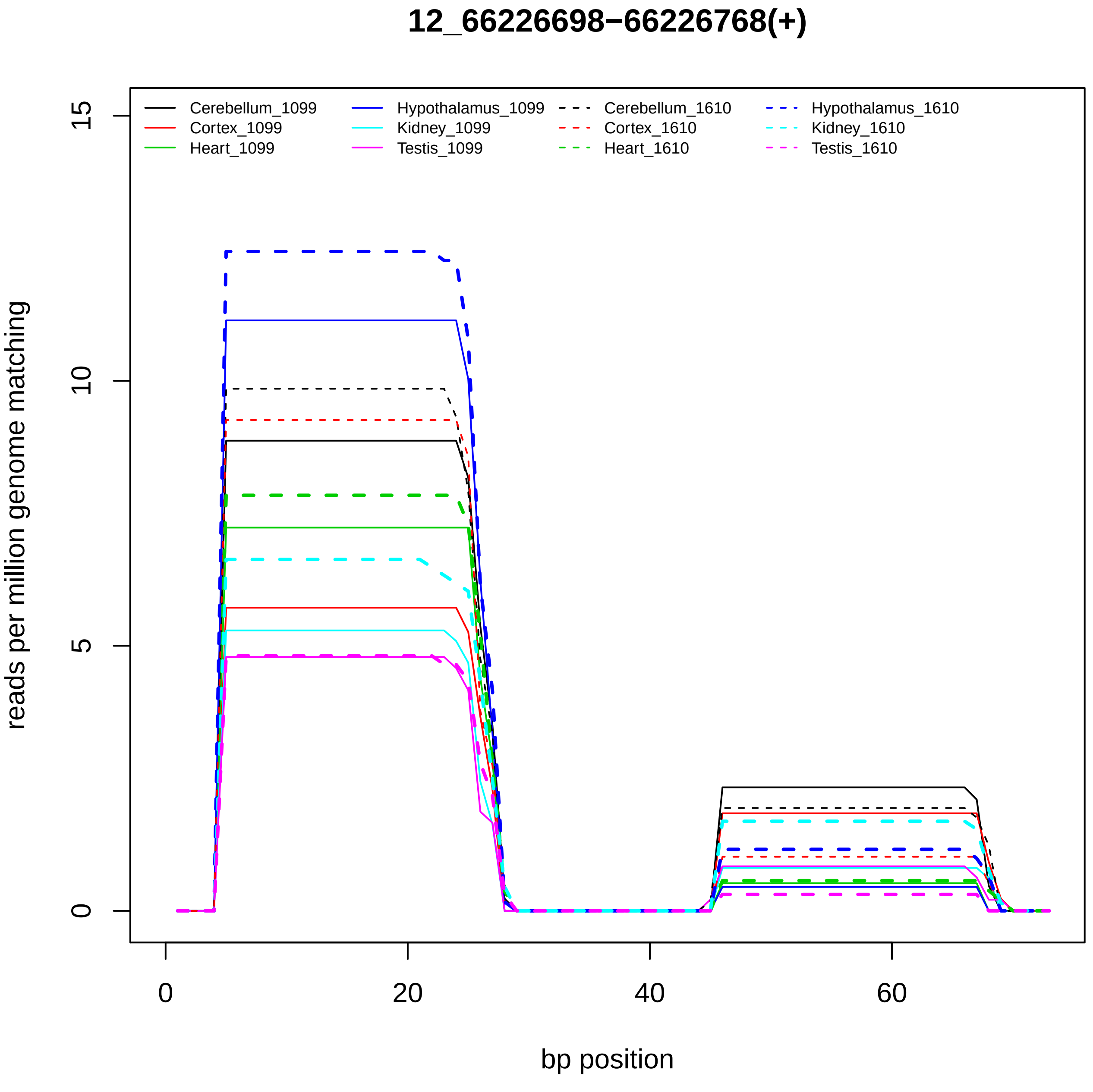
12_66226698−66226768(+)
Cerebellum_1099
Cortex_1099
Heart_1099
Hypothalamus_1099
Kidney_1099
Testis_1099
Cerebellum_1610
Cortex_1610
Heart_1610
Hypothalamus_1610
Kidney_1610
Testis_1610
0
5
10
15
0
20
40
60
reads per million genome matching
bp position

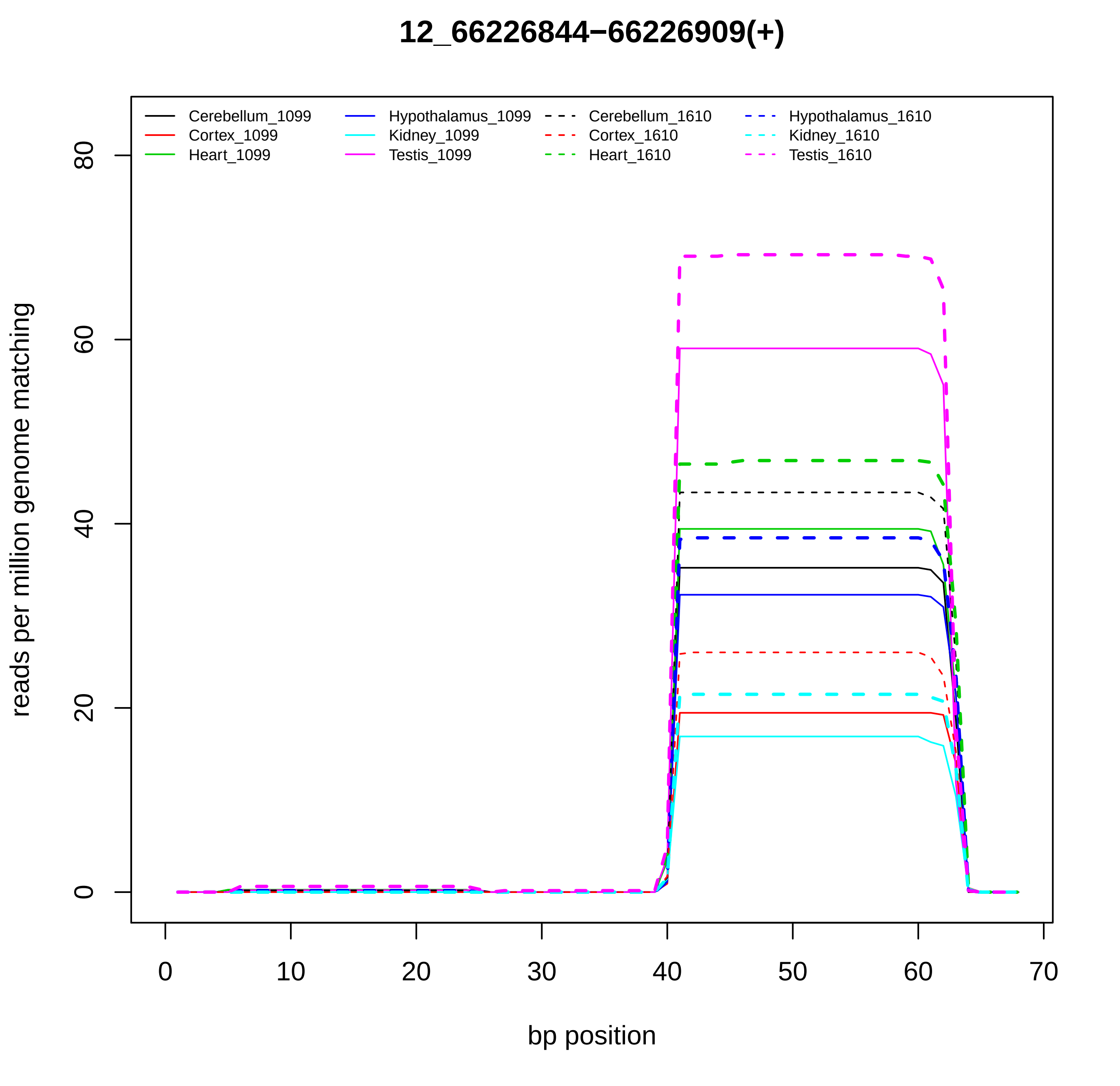
12_66226844−66226909(+)
Cerebellum_1099
Cortex_1099
Heart_1099
Hypothalamus_1099
Kidney_1099
Testis_1099
Cerebellum_1610
Cortex_1610
Heart_1610
Hypothalamus_1610
Kidney_1610
Testis_1610
reads per million genome matching
bp position
0
20
40
60
80
0
10
20
30
40
50
60
70

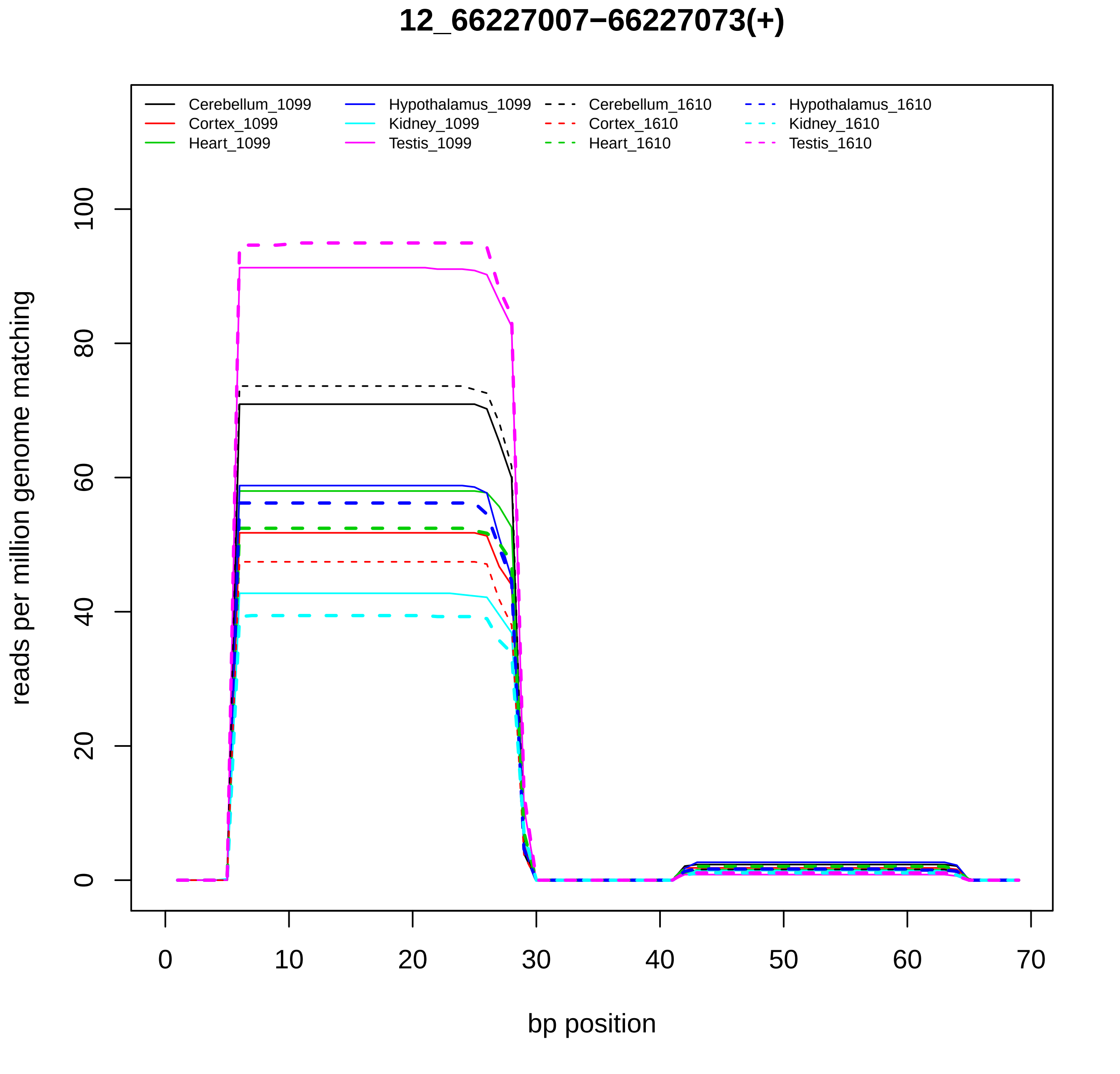
12_66227007−66227073(+)
Cerebellum_1099
Cortex_1099
Heart_1099
Hypothalamus_1099
Kidney_1099
Testis_1099
Cerebellum_1610
Cortex_1610
Heart_1610
Hypothalamus_1610
Kidney_1610
Testis_1610
reads per million genome matching
0
20
40
60
80
100
0
10
20
30
40
50
60
70
bp position

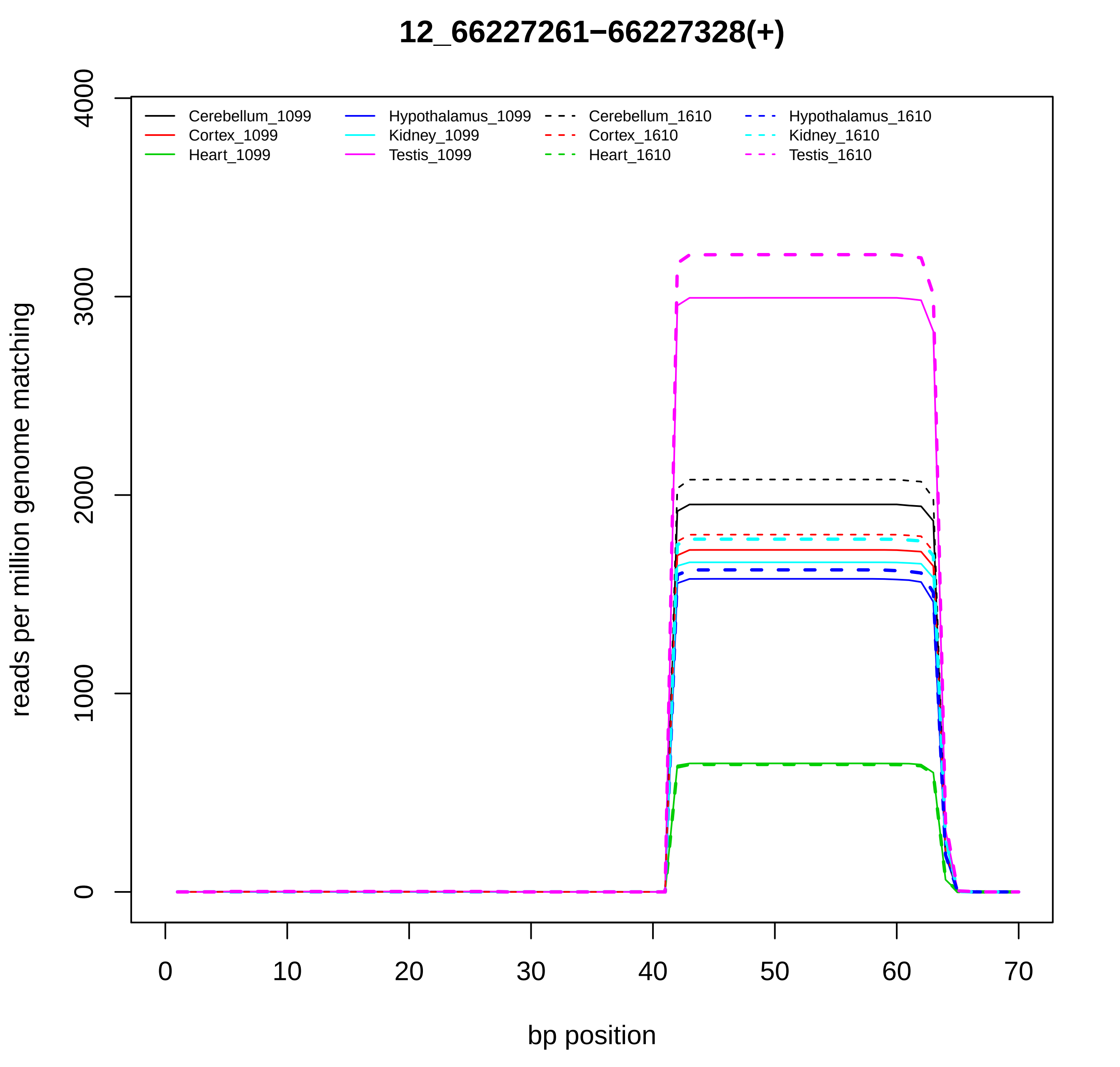

12_66227261−66227328(+)
Cerebellum_1099
Cortex_1099
Heart_1099
Hypothalamus_1099
Kidney_1099
Testis_1099
Cerebellum_1610
Cortex_1610
Heart_1610
Hypothalamus_1610
Kidney_1610
Testis_1610
reads per million genome matching
0
1000
2000
3000
4000
0
10
20
30
40
50
60
70
bp position

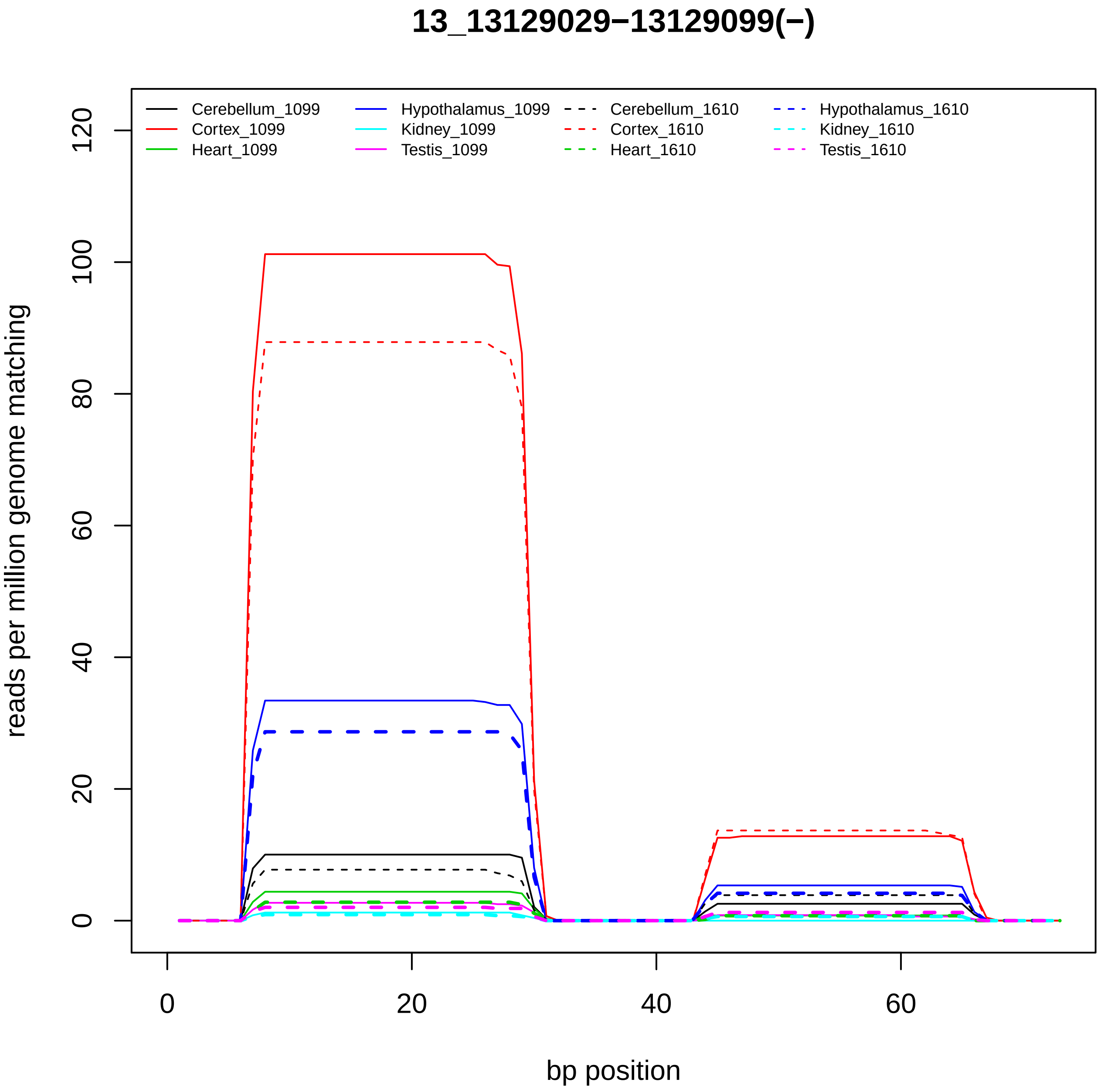
13_13129029−13129099(−)
Cerebellum_1099
Cortex_1099
Heart_1099
Hypothalamus_1099
Kidney_1099
Testis_1099
Cerebellum_1610
Cortex_1610
Heart_1610
Hypothalamus_1610
Kidney_1610
Testis_1610
reads per million genome matching
bp position
0
20
40
60
80
100
120
0
20
40
60

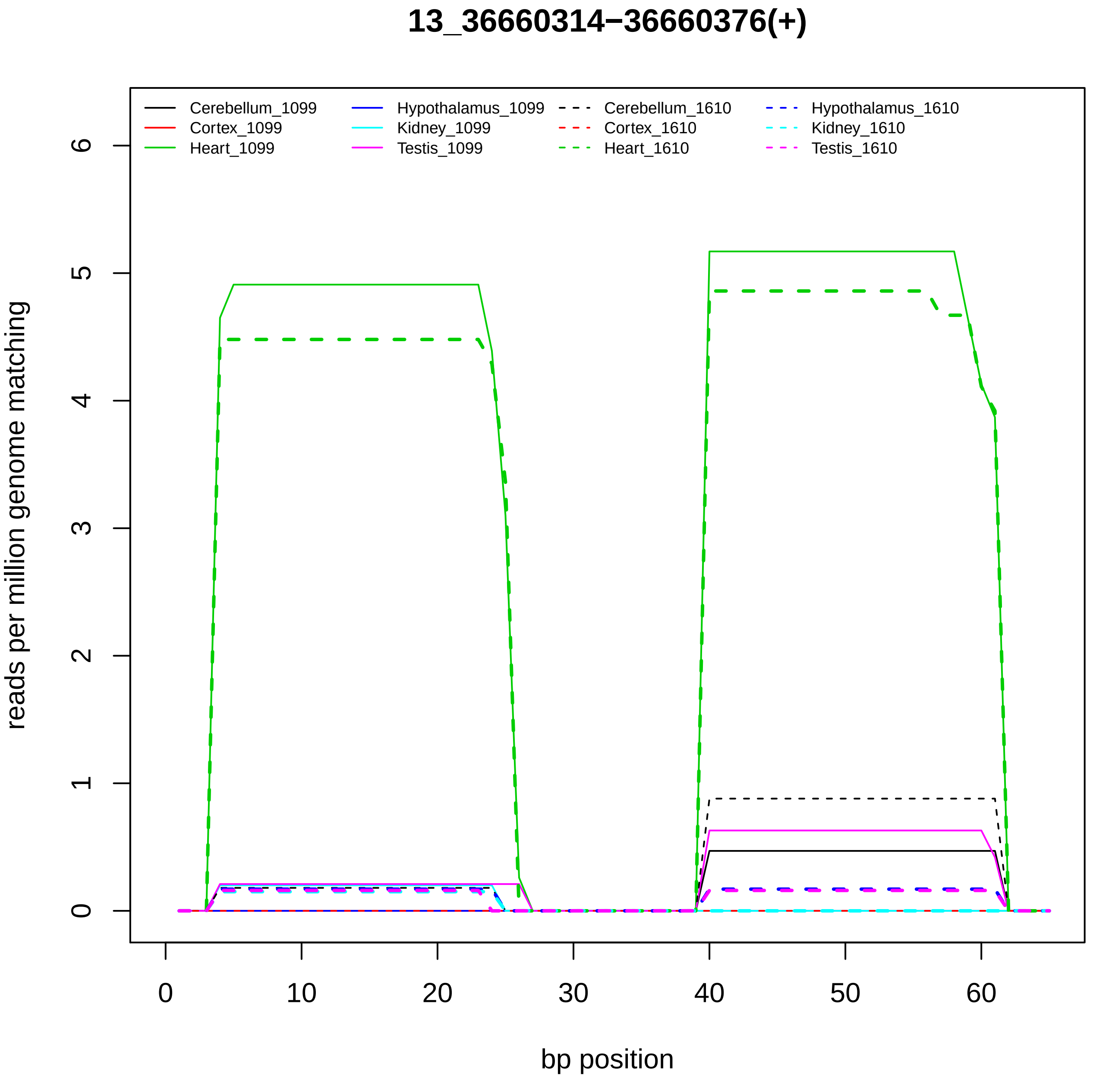

13_36660314−36660376(+)
Cerebellum_1099
Cortex_1099
Heart_1099
Hypothalamus_1099
Kidney_1099
Testis_1099
Cerebellum_1610
Cortex_1610
Heart_1610
Hypothalamus_1610
Kidney_1610
Testis_1610
reads per million genome matching
bp position
0
1
2
3
4
5
6
0
10
20
30
40
50
60

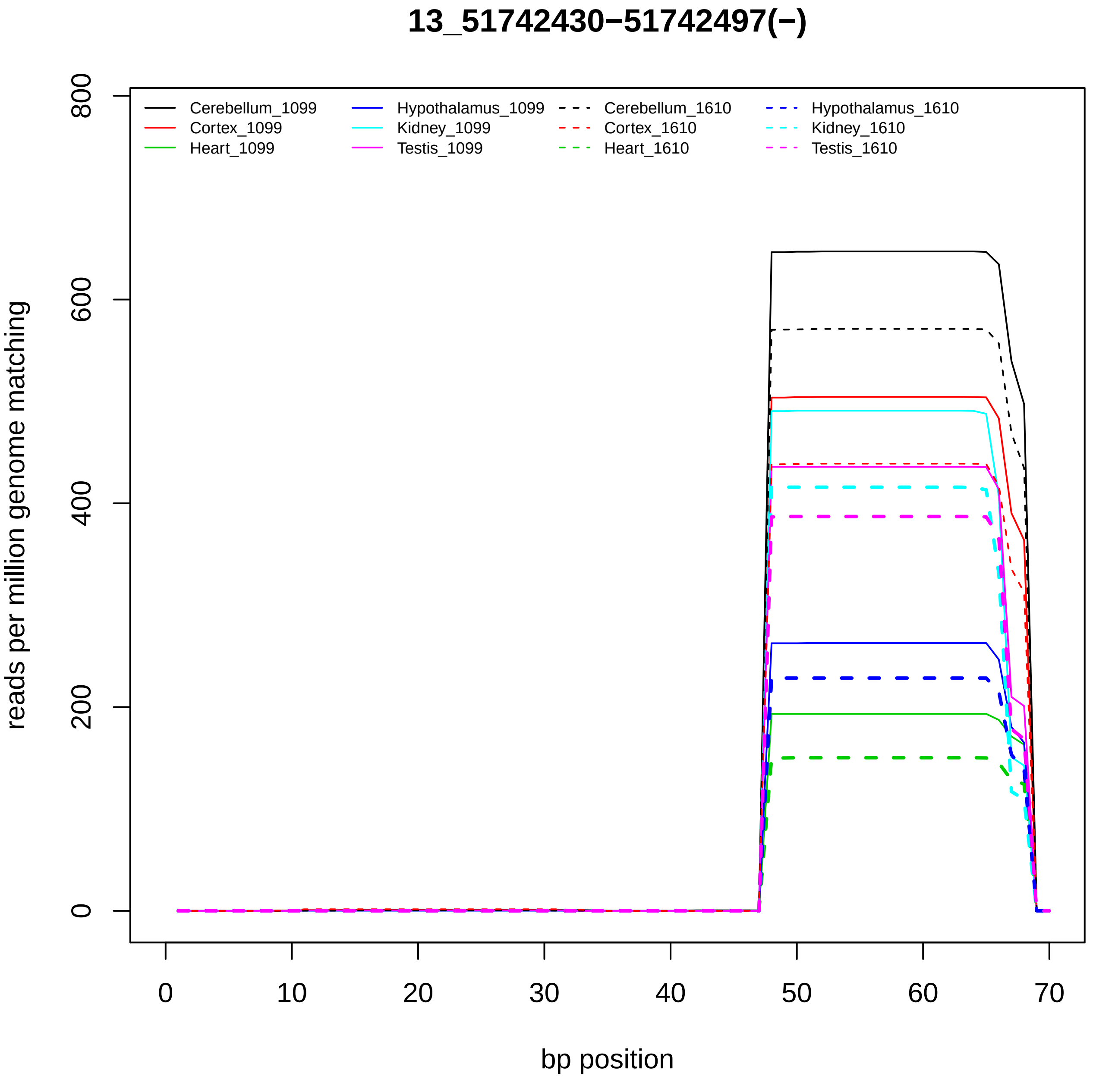

13_51742430−51742497(−)
Cerebellum_1099
Cortex_1099
Heart_1099
Hypothalamus_1099
Kidney_1099
Testis_1099
Cerebellum_1610
Cortex_1610
Heart_1610
Hypothalamus_1610
Kidney_1610
Testis_1610
reads per million genome matching
bp position
0
200
400
600
800
0
10
20
30
40
50
60
70

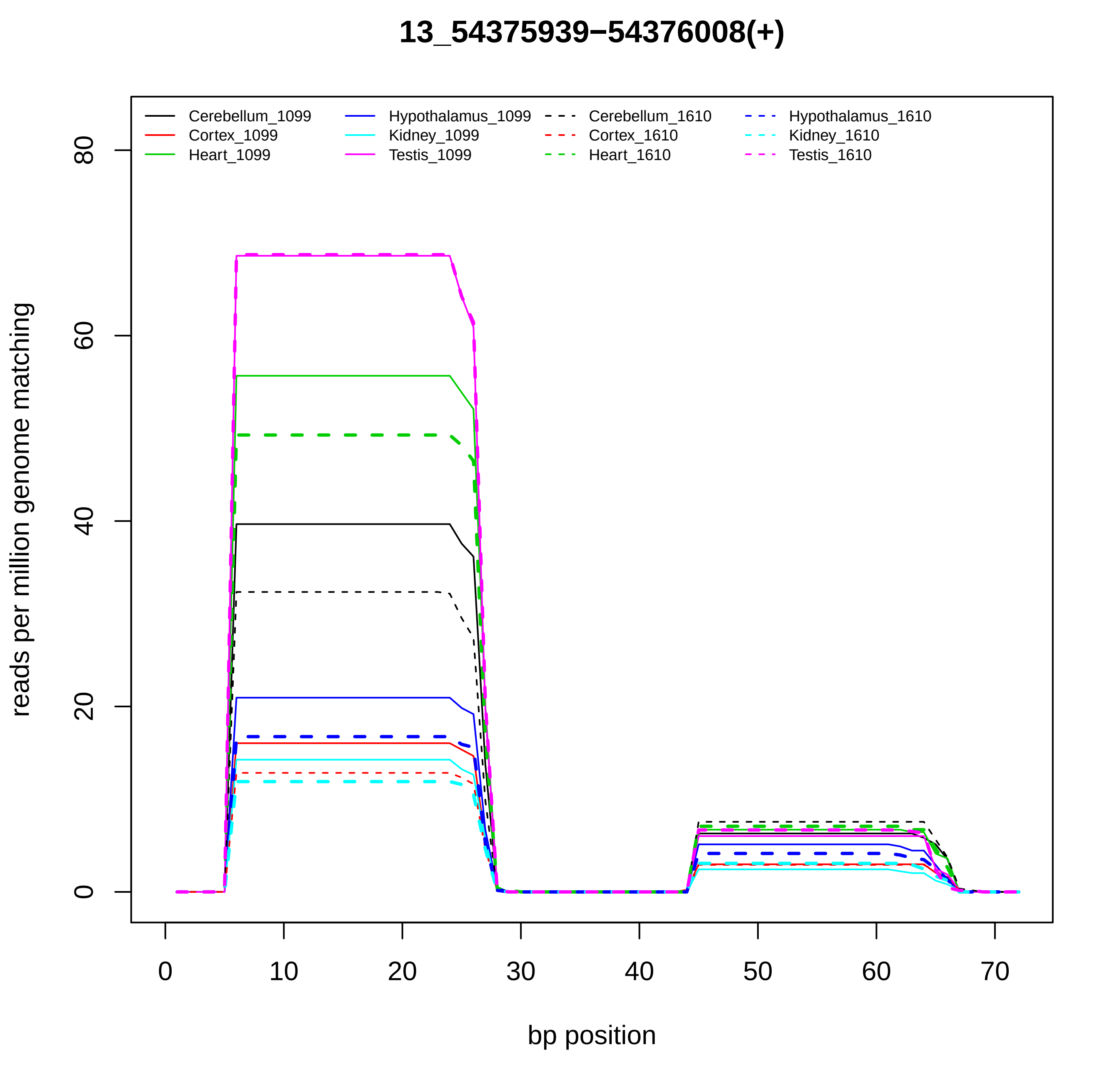

13_54375939−54376008(+)
Cerebellum_1099
Cortex_1099
Heart_1099
Hypothalamus_1099
Kidney_1099
Testis_1099
Cerebellum_1610
Cortex_1610
Heart_1610
Hypothalamus_1610
Kidney_1610
Testis_1610
reads per million genome matching
bp position
0
20
40
60
80
0
10
20
30
40
50
60
70

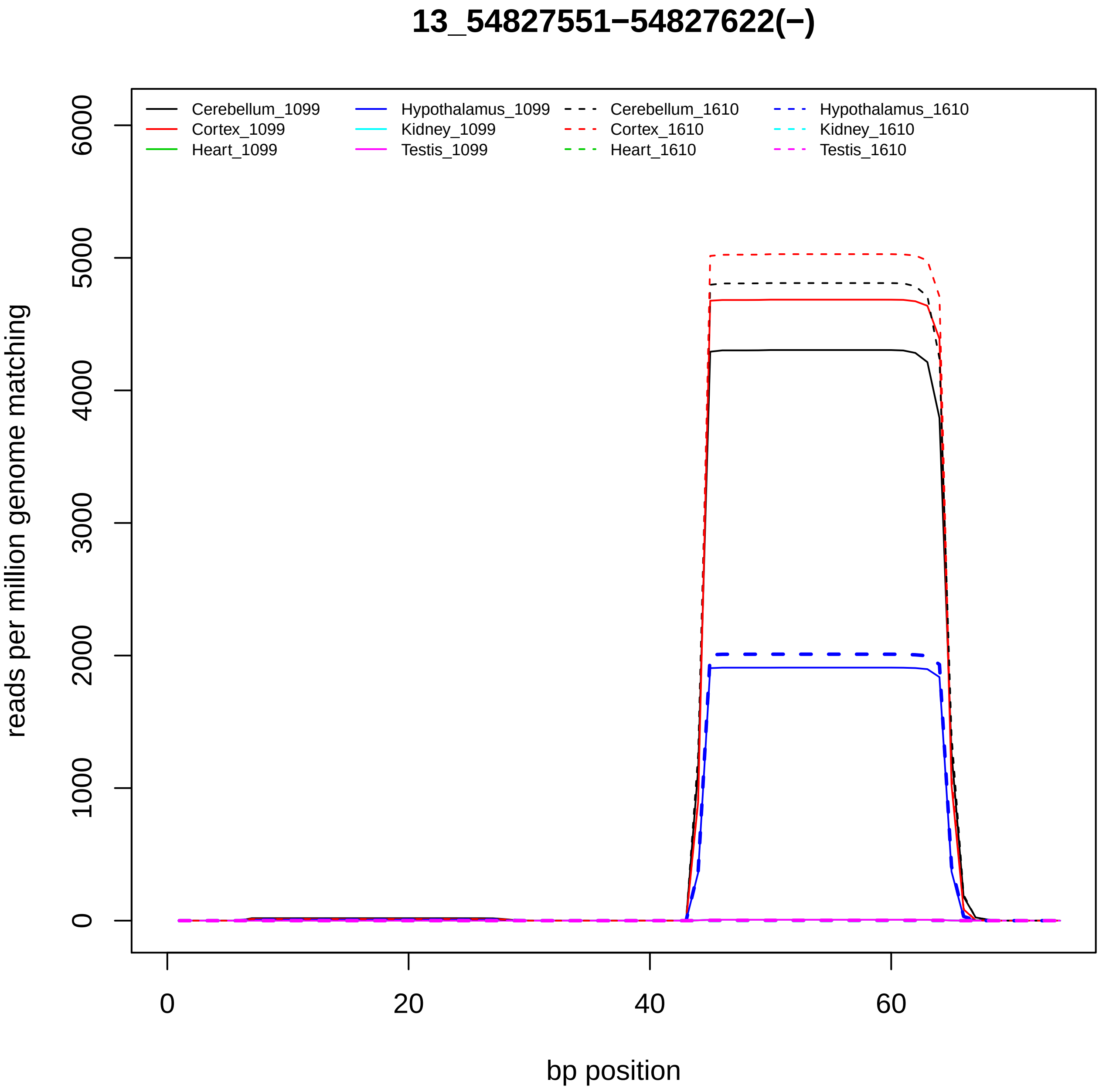
13_54827551−54827622(−)
Cerebellum_1099
Cortex_1099
Heart_1099
Hypothalamus_1099
Kidney_1099
Testis_1099
Cerebellum_1610
Cortex_1610
Heart_1610
Hypothalamus_1610
Kidney_1610
Testis_1610
reads per million genome matching
0
1000
2000
3000
4000
5000
6000
0
20
40
60
bp position

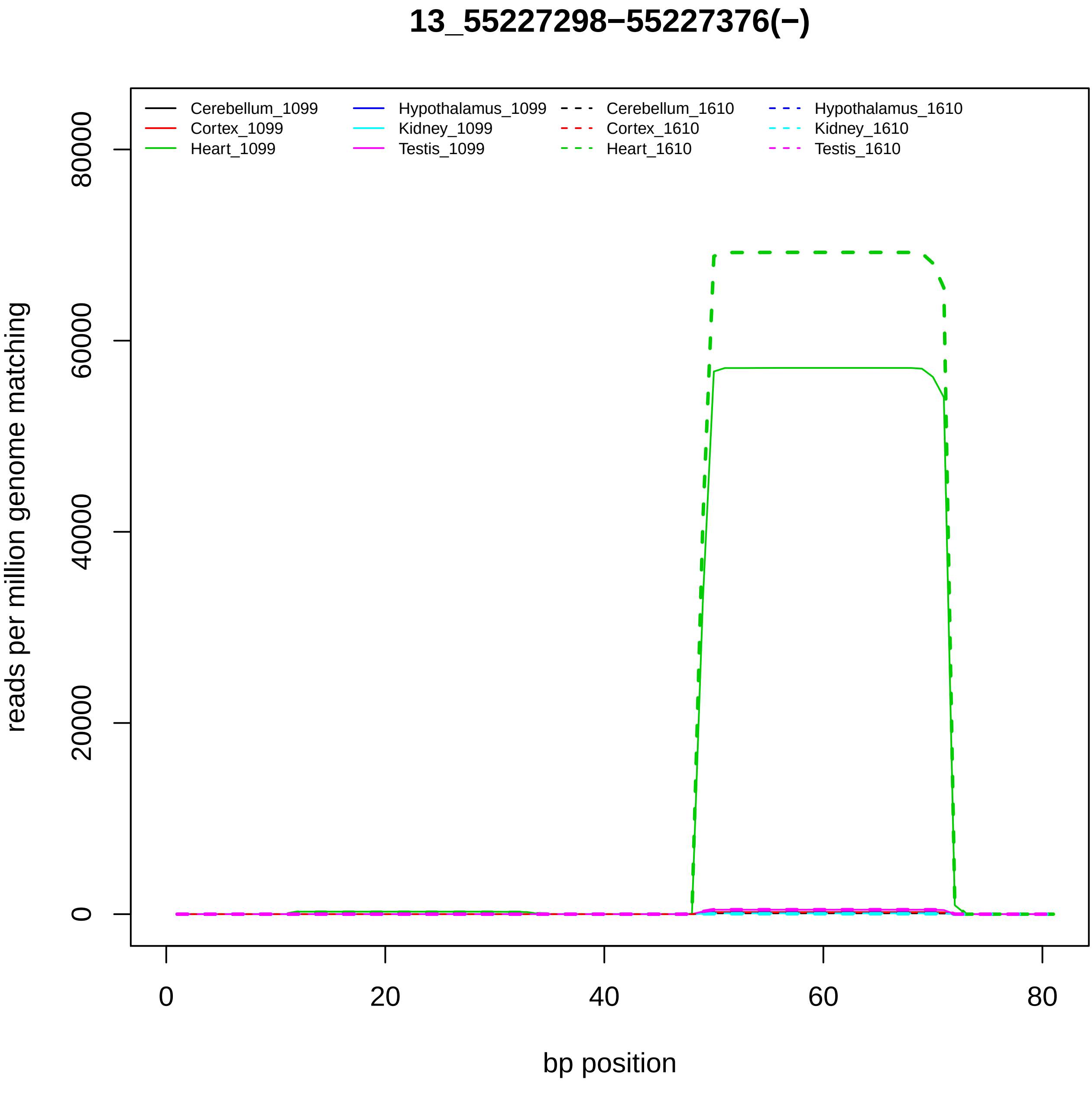

13_55227298−55227376(−)
Cerebellum_1099
Cortex_1099
Heart_1099
Hypothalamus_1099
Kidney_1099
Testis_1099
Cerebellum_1610
Cortex_1610
Heart_1610
Hypothalamus_1610
Kidney_1610
Testis_1610
reads per million genome matching
0
20000
40000
60000
80000
bp position
0
20
40
60
80

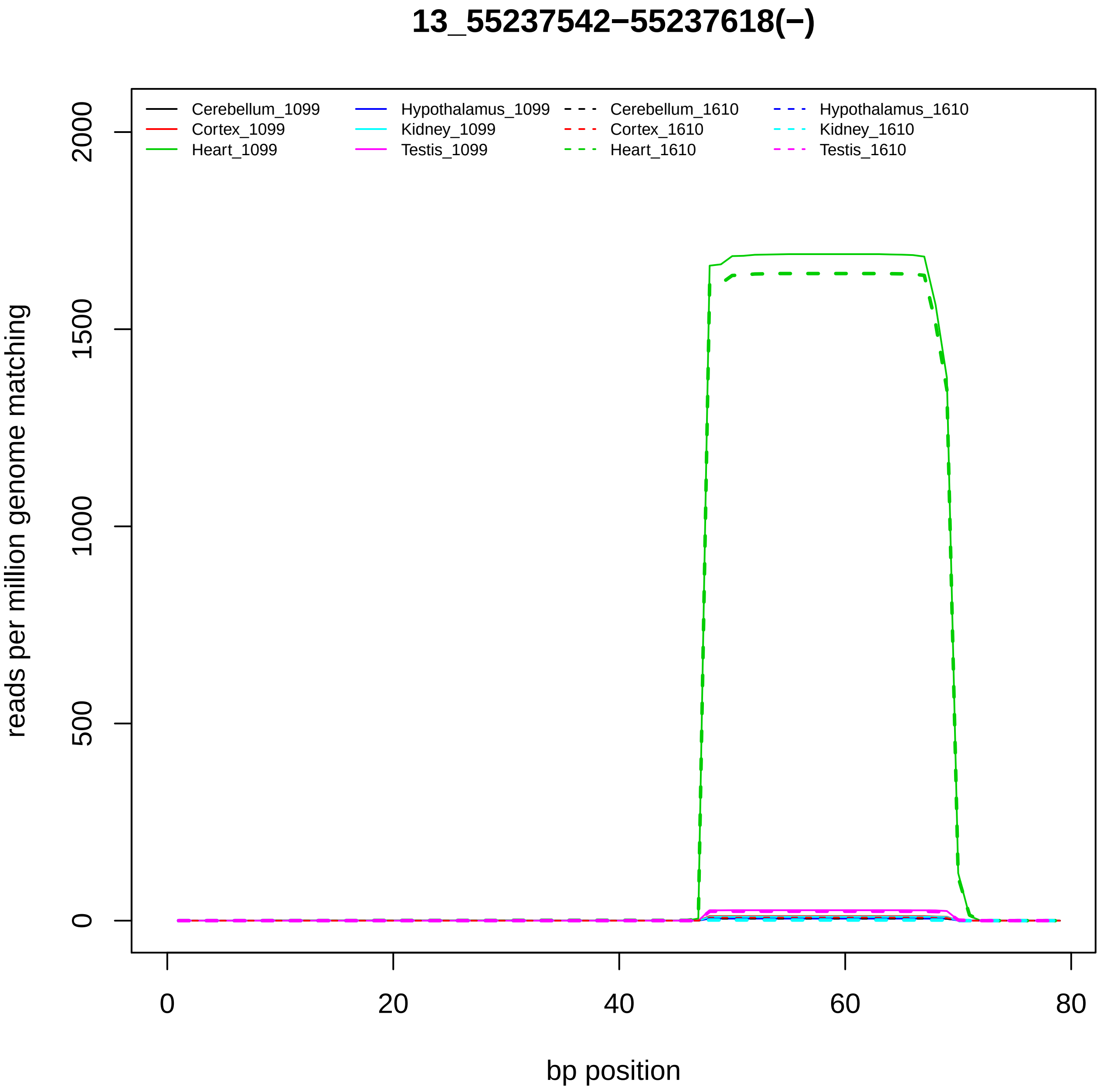
13_55237542−55237618(−)
Cerebellum_1099
Cortex_1099
Heart_1099
Hypothalamus_1099
Kidney_1099
Testis_1099
Cerebellum_1610
Cortex_1610
Heart_1610
Hypothalamus_1610
Kidney_1610
Testis_1610
reads per million genome matching
0
500
1000
1500
2000
bp position
0
20
40
60
80

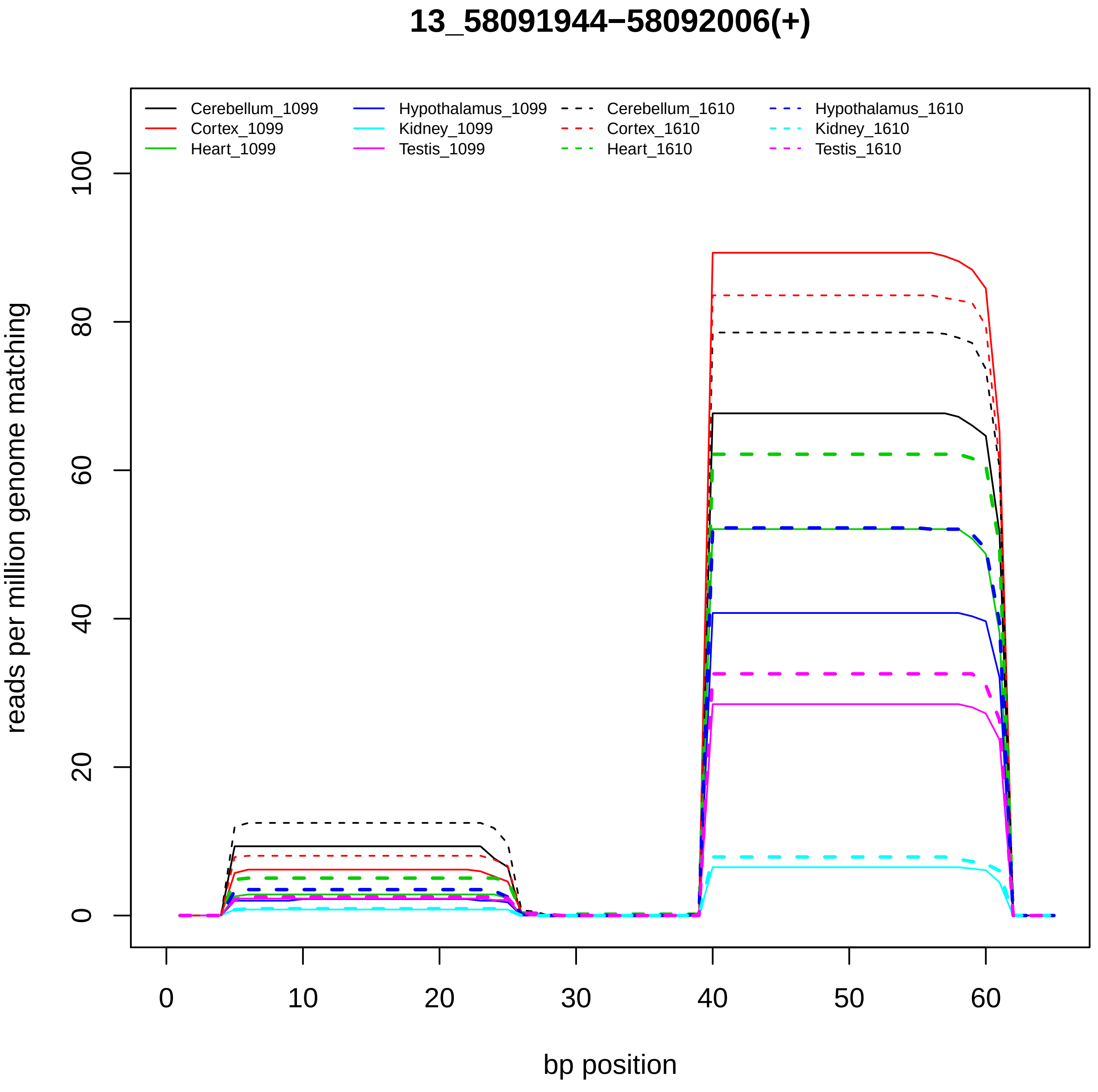

13_58091944−58092006(+)
Cerebellum_1099
Cortex_1099
Heart_1099
Hypothalamus_1099
Kidney_1099
Testis_1099
Cerebellum_1610
Cortex_1610
Heart_1610
Hypothalamus_1610
Kidney_1610
Testis_1610
reads per million genome matching
0
20
40
60
80
100
bp position
0
10
20
30
40
50
60

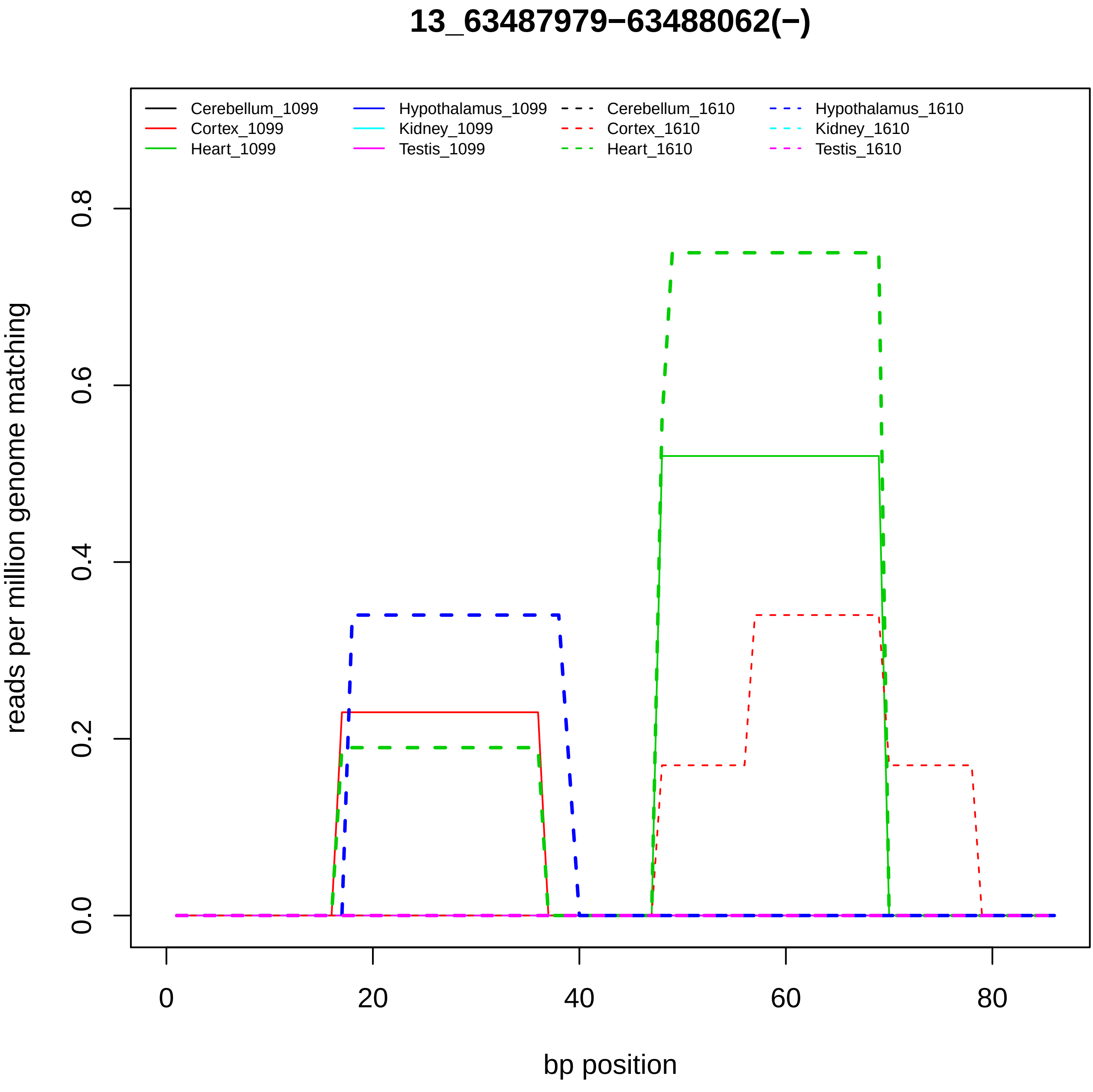

13_63487979−63488062(−)
Cerebellum_1099
Cortex_1099
Heart_1099
Hypothalamus_1099
Kidney_1099
Testis_1099
Cerebellum_1610
Cortex_1610
Heart_1610
Hypothalamus_1610
Kidney_1610
Testis_1610
reads per million genome matching
0.0
0.2
0.4
0.6
0.8
0
20
40
60
80
bp position

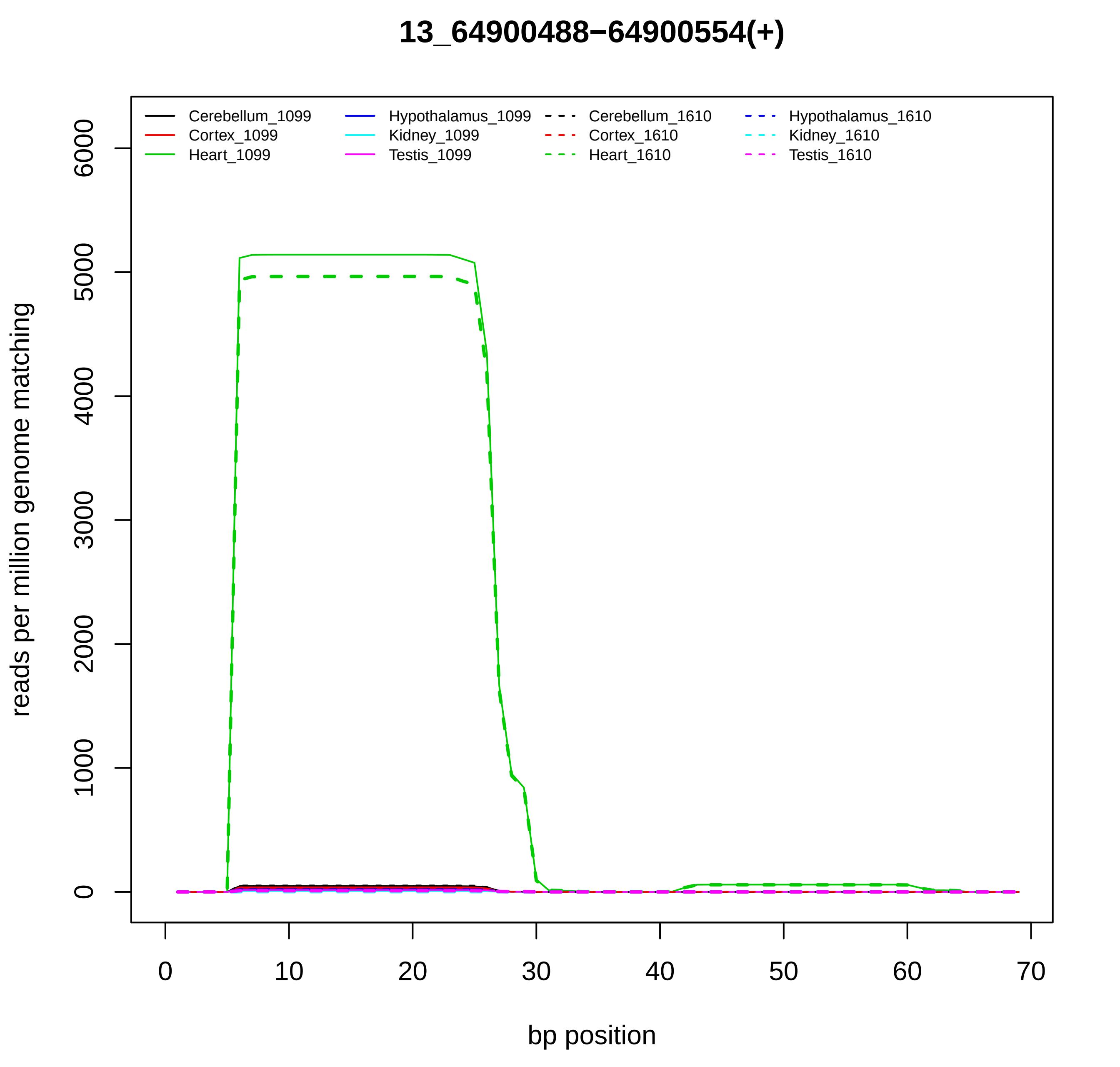
13_64900488−64900554(+)
Cerebellum_1099
Cortex_1099
Heart_1099
Hypothalamus_1099
Kidney_1099
Testis_1099
Cerebellum_1610
Cortex_1610
Heart_1610
Hypothalamus_1610
Kidney_1610
Testis_1610
reads per million genome matching
0
1000
2000
3000
4000
5000
6000
bp position
0
10
20
30
40
50
60
70

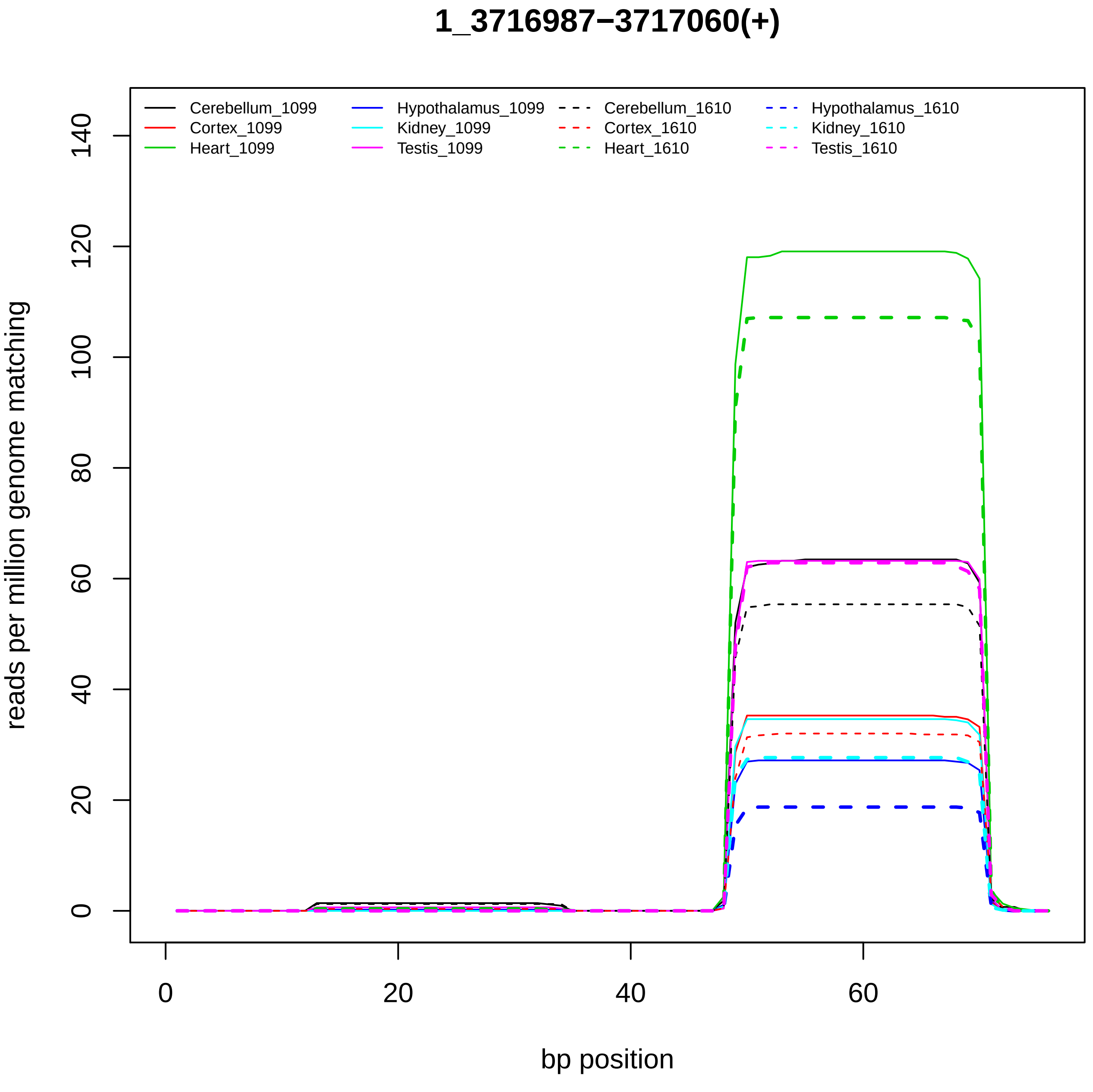

1_3716987−3717060(+)
Cerebellum_1099
Cortex_1099
Heart_1099
Hypothalamus_1099
Kidney_1099
Testis_1099
Cerebellum_1610
Cortex_1610
Heart_1610
Hypothalamus_1610
Kidney_1610
Testis_1610
reads per million genome matching
bp position
0
20
40
60
80
100
120
140

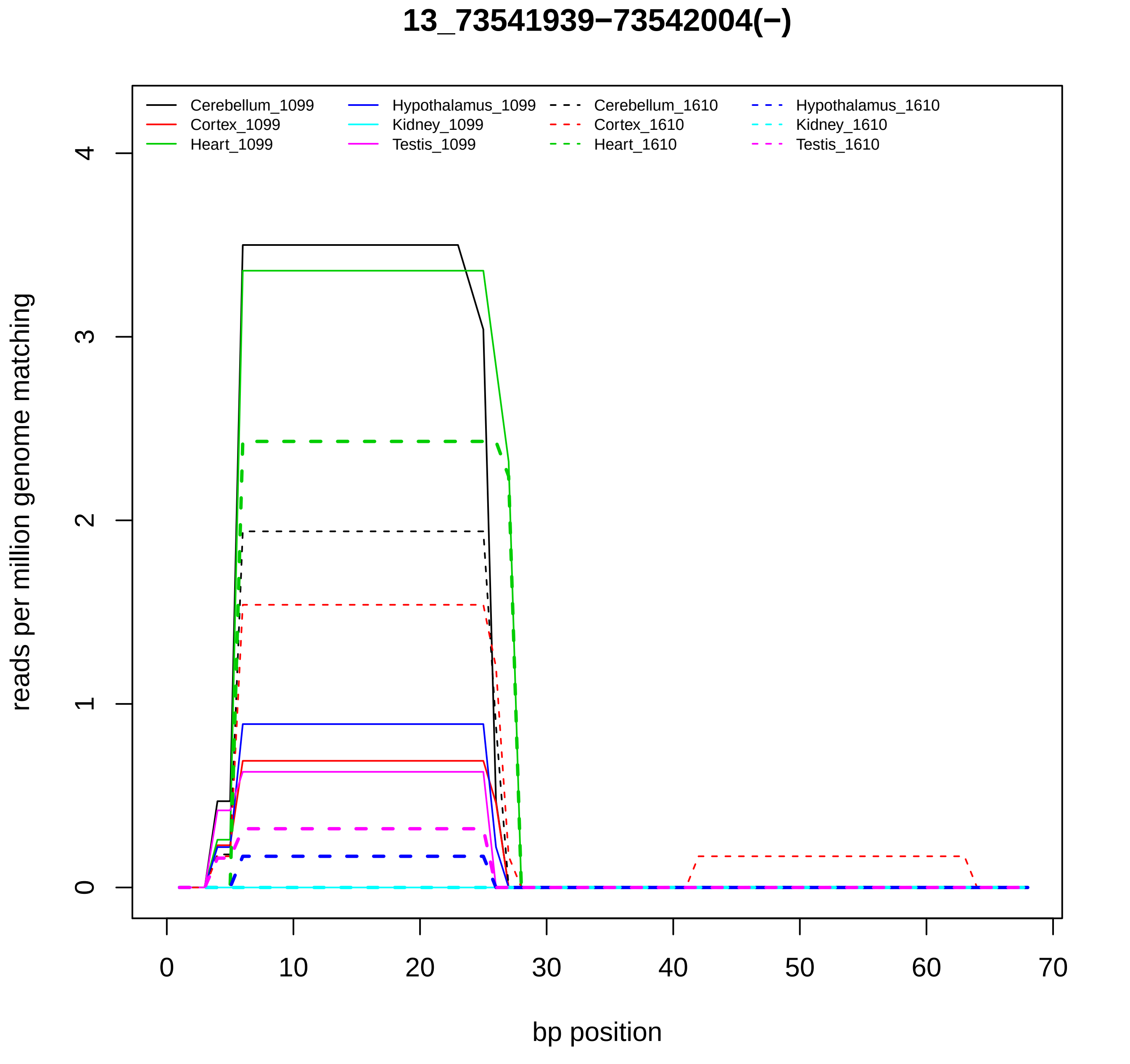

13_73541939−73542004(−)
Cerebellum_1099
Cortex_1099
Heart_1099
Hypothalamus_1099
Kidney_1099
Testis_1099
Cerebellum_1610
Cortex_1610
Heart_1610
Hypothalamus_1610
Kidney_1610
Testis_1610
reads per million genome matching
bp position
0
1
2
3
4
0
10
20
30
40
50
60
70

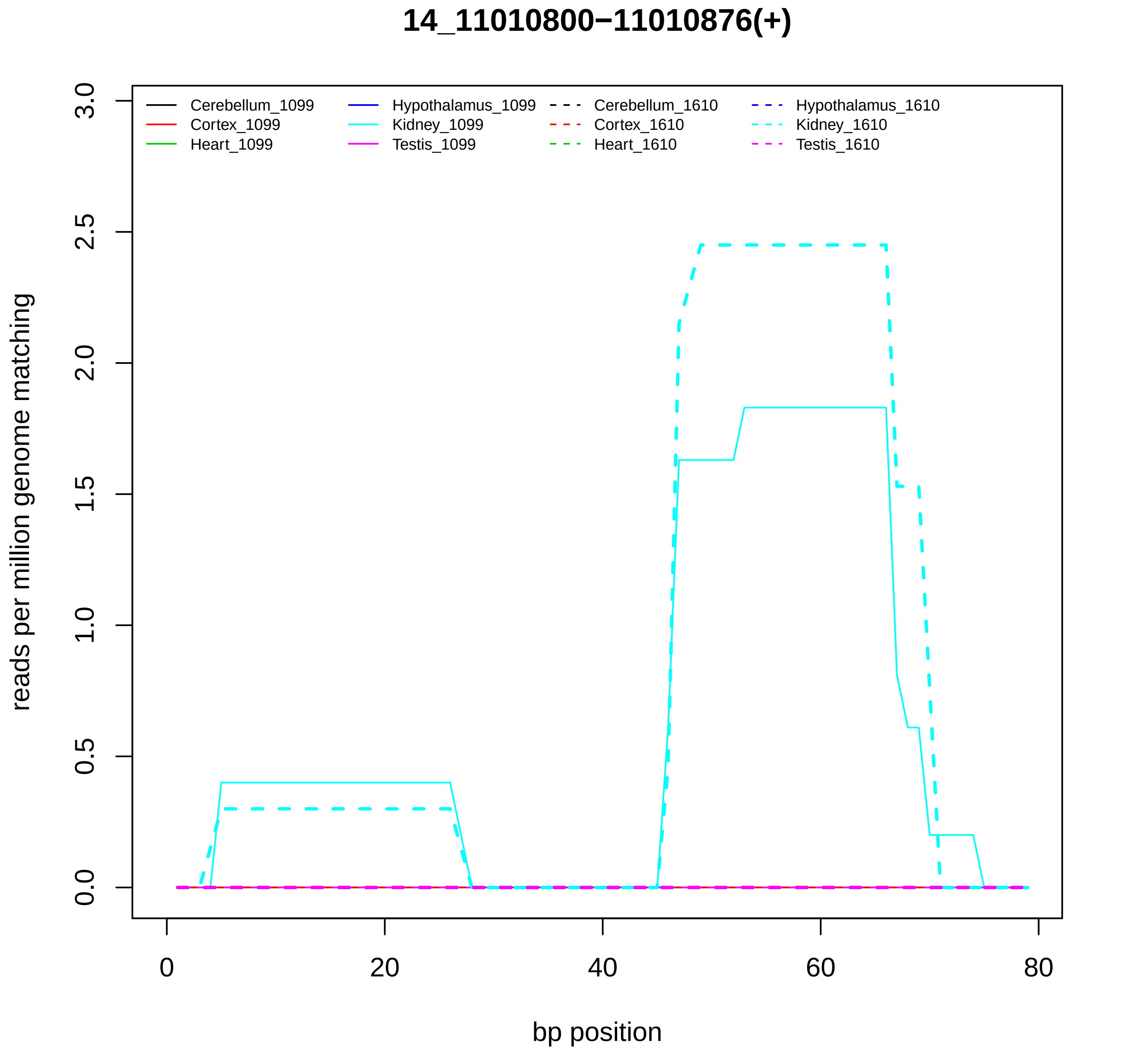
14_11010800−11010876(+)
Cerebellum_1099
Cortex_1099
Heart_1099
Hypothalamus_1099
Kidney_1099
Testis_1099
Cerebellum_1610
Cortex_1610
Heart_1610
Hypothalamus_1610
Kidney_1610
Testis_1610
reads per million genome matching
0.0
0.5
1.0
1.5
2.0
2.5
3.0
0
20
40
60
80
bp position

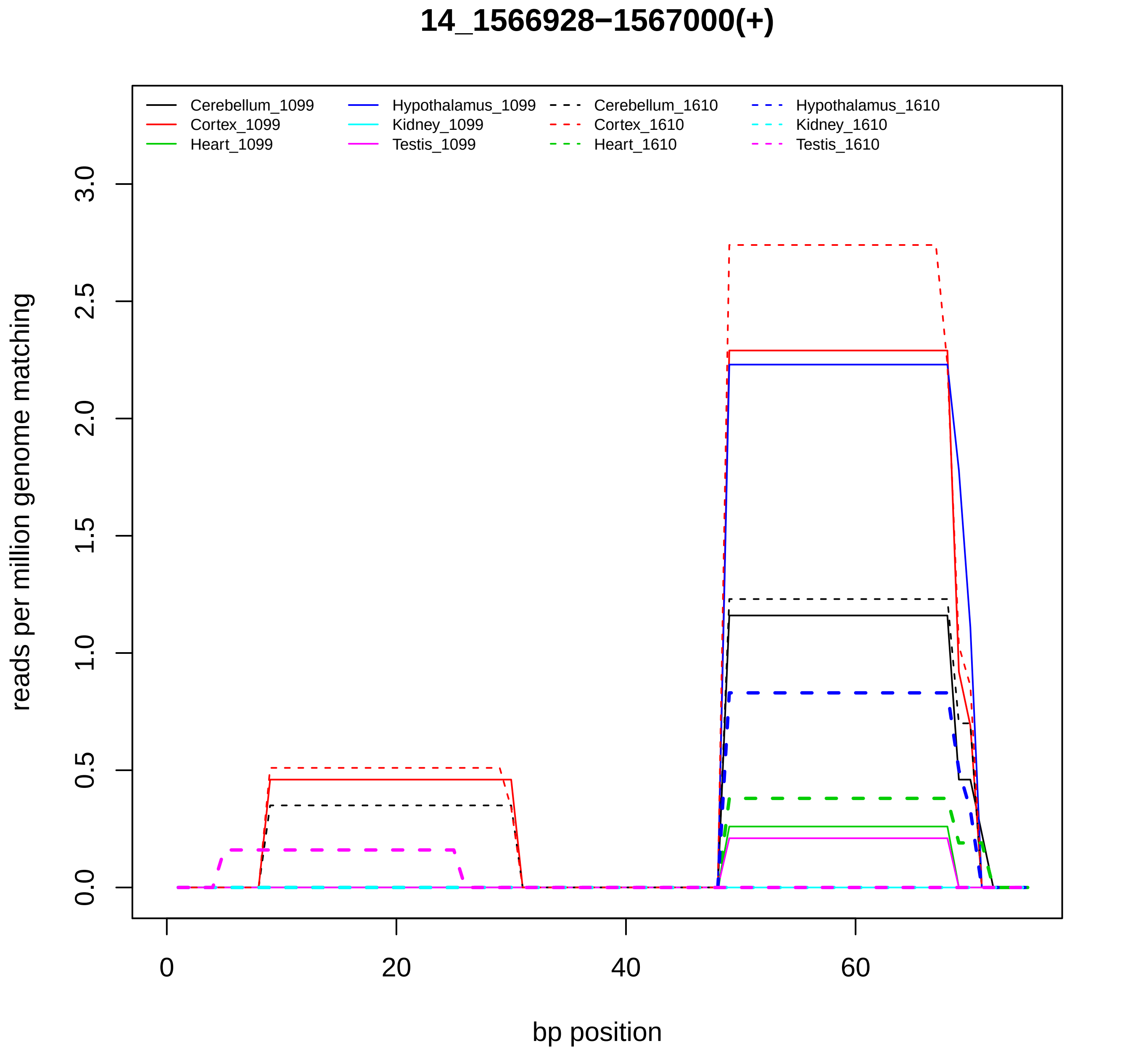

14_1566928−1567000(+)
Cerebellum_1099
Cortex_1099
Heart_1099
Hypothalamus_1099
Kidney_1099
Testis_1099
Cerebellum_1610
Cortex_1610
Heart_1610
Hypothalamus_1610
Kidney_1610
Testis_1610
reads per million genome matching
0.0
0.5
1.0
1.5
2.0
2.5
3.0
0
20
40
60
bp position

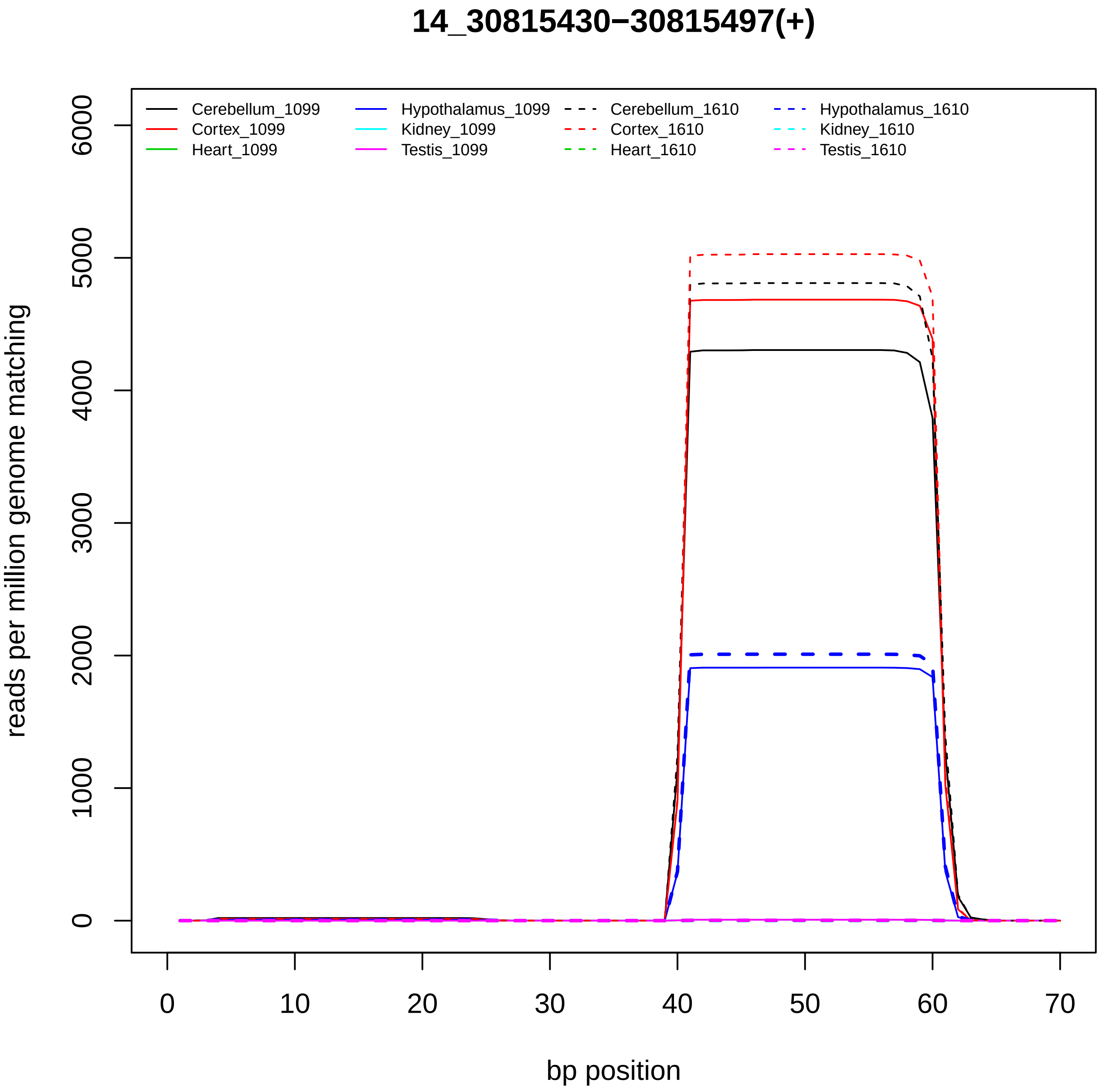

14_30815430−30815497(+)
Cerebellum_1099
Cortex_1099
Heart_1099
Hypothalamus_1099
Kidney_1099
Testis_1099
Cerebellum_1610
Cortex_1610
Heart_1610
Hypothalamus_1610
Kidney_1610
Testis_1610
reads per million genome matching
0
1000
2000
3000
4000
5000
6000
0
10
20
30
40
50
60
70
bp position

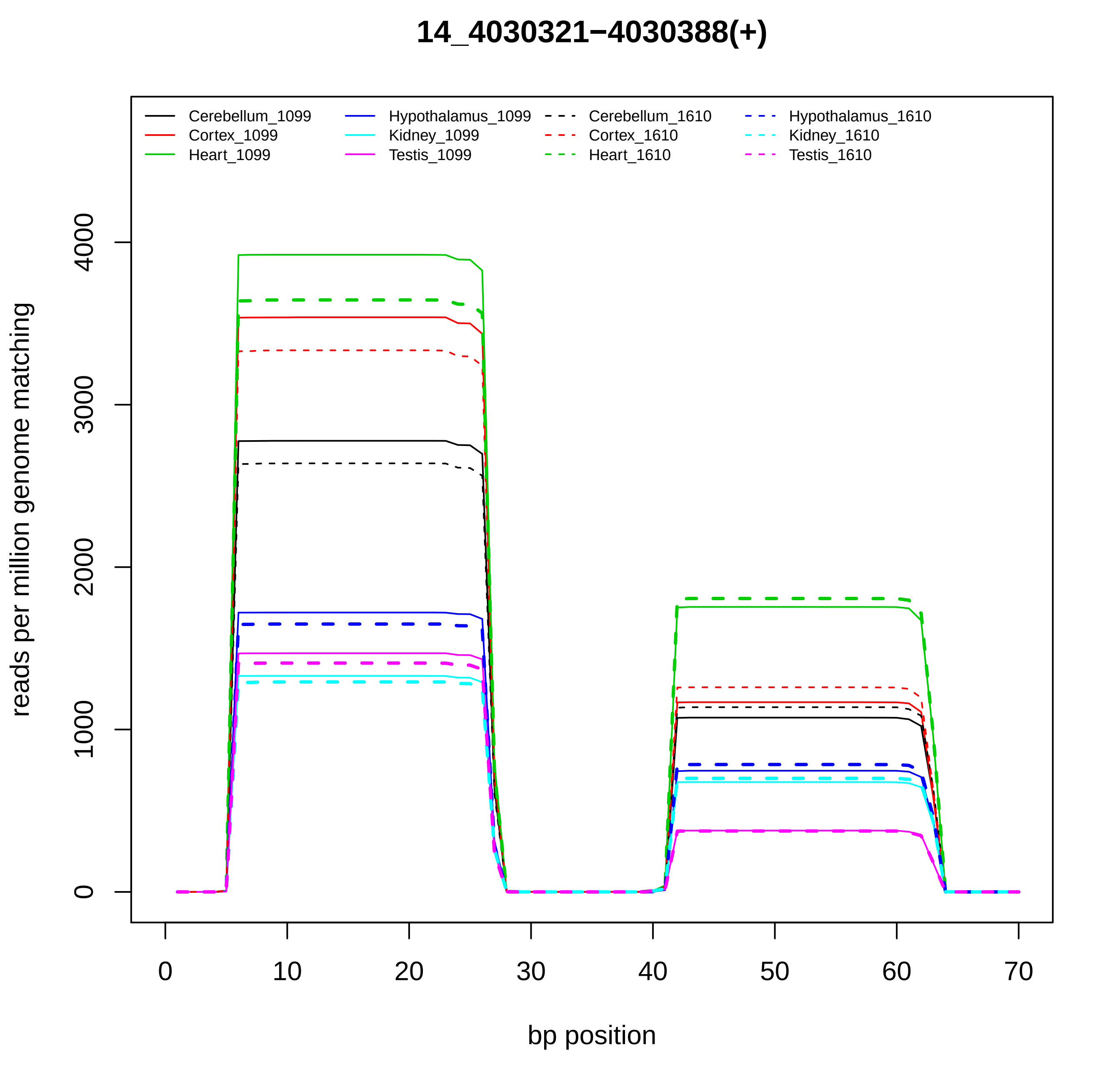
14_4030321−4030388(+)
Cerebellum_1099
Cortex_1099
Heart_1099
Hypothalamus_1099
Kidney_1099
Testis_1099
Cerebellum_1610
Cortex_1610
Heart_1610
Hypothalamus_1610
Kidney_1610
Testis_1610
reads per million genome matching
0
1000
2000
3000
4000
bp position
0
10
20
30
40
50
60
70

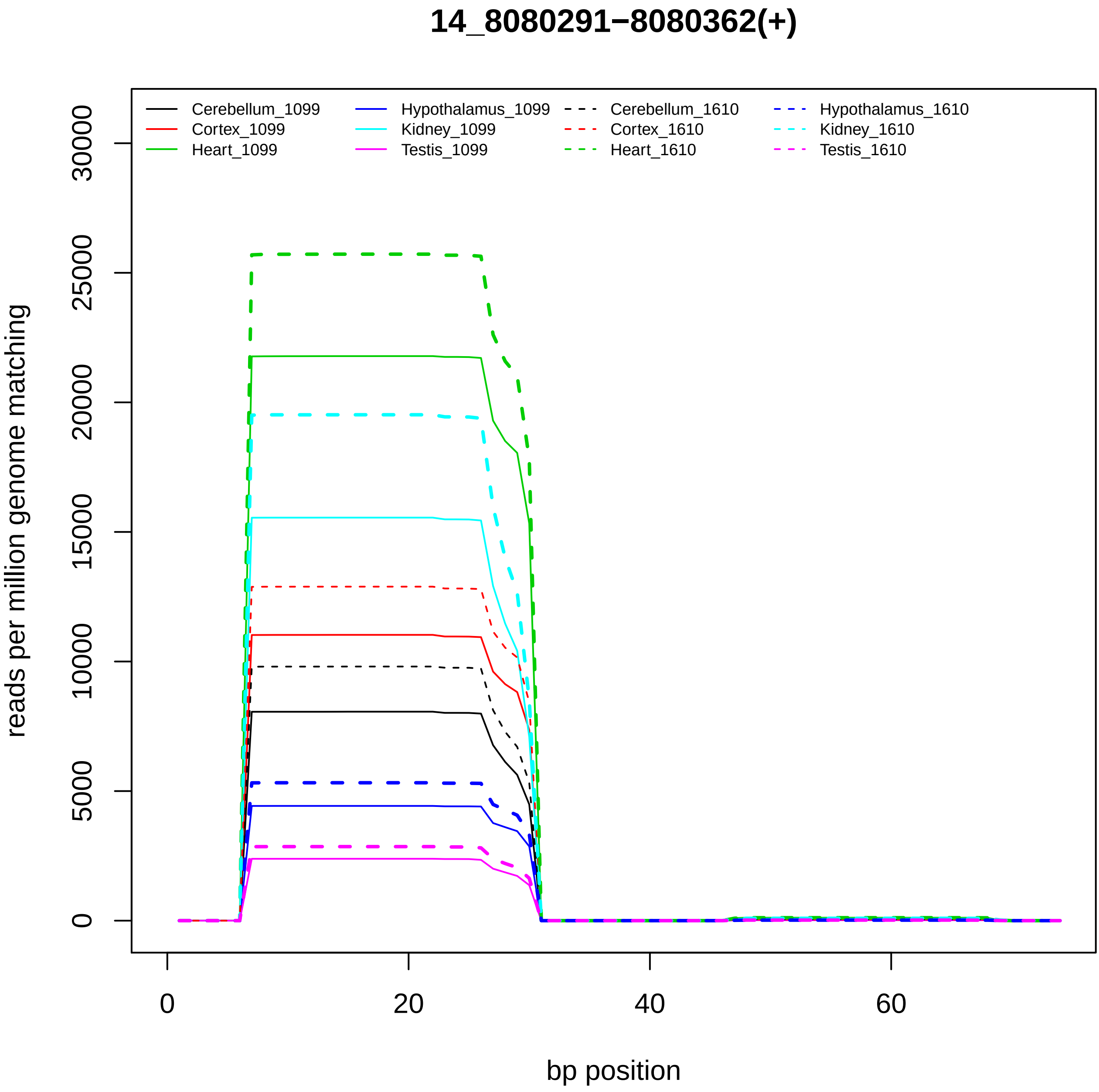
14_8080291−8080362(+)
Cerebellum_1099
Cortex_1099
Heart_1099
Hypothalamus_1099
Kidney_1099
Testis_1099
Cerebellum_1610
Cortex_1610
Heart_1610
Hypothalamus_1610
Kidney_1610
Testis_1610
reads per million genome matching
0
5000
10000
15000
20000
25000
30000
0
20
40
60
bp position

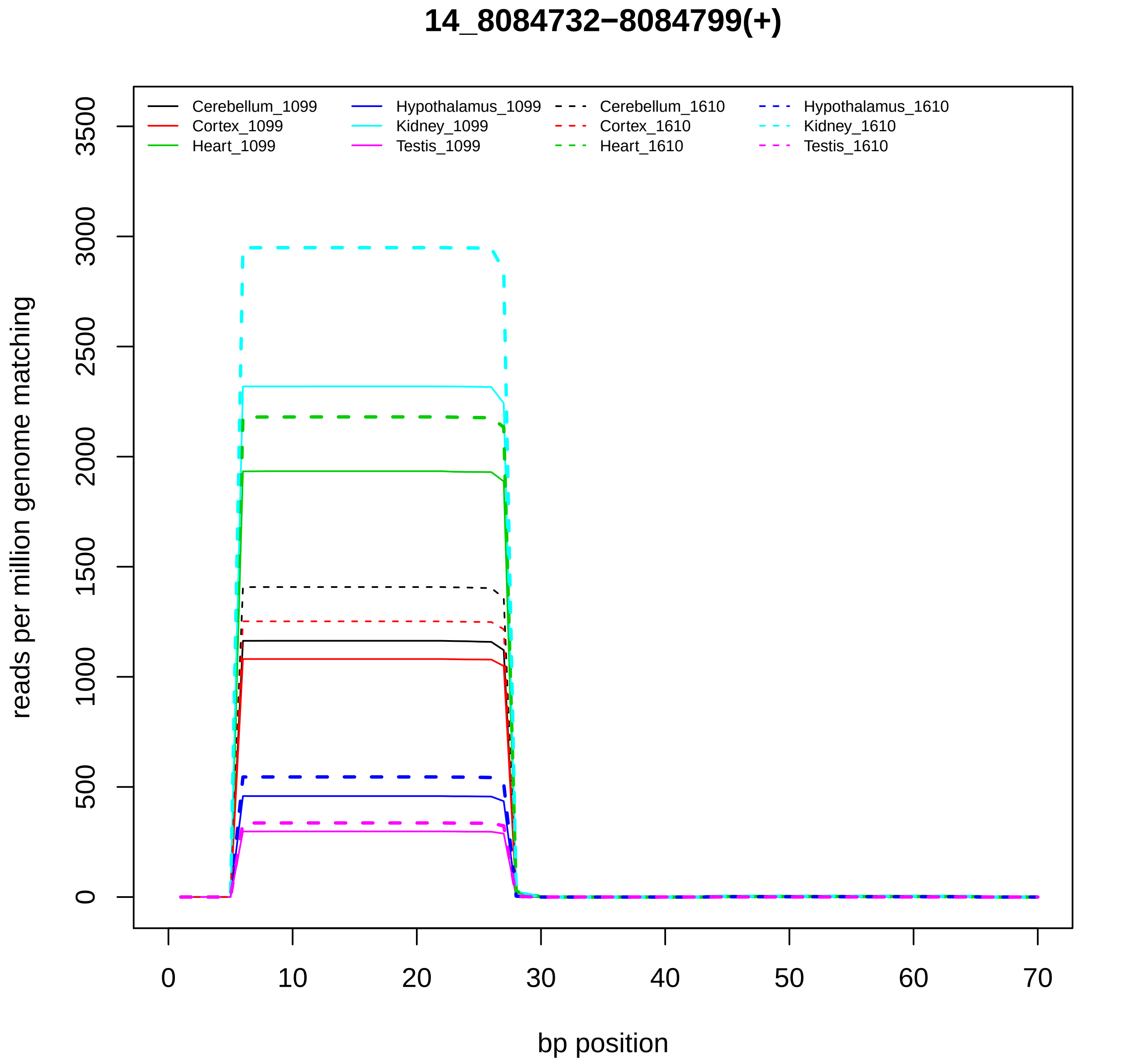
14_8084732−8084799(+)
Cerebellum_1099
Cortex_1099
Heart_1099
Hypothalamus_1099
Kidney_1099
Testis_1099
Cerebellum_1610
Cortex_1610
Heart_1610
Hypothalamus_1610
Kidney_1610
Testis_1610
reads per million genome matching
0
500
1000
1500
2000
2500
3000
3500
bp position
0
10
20
30
40
50
60
70

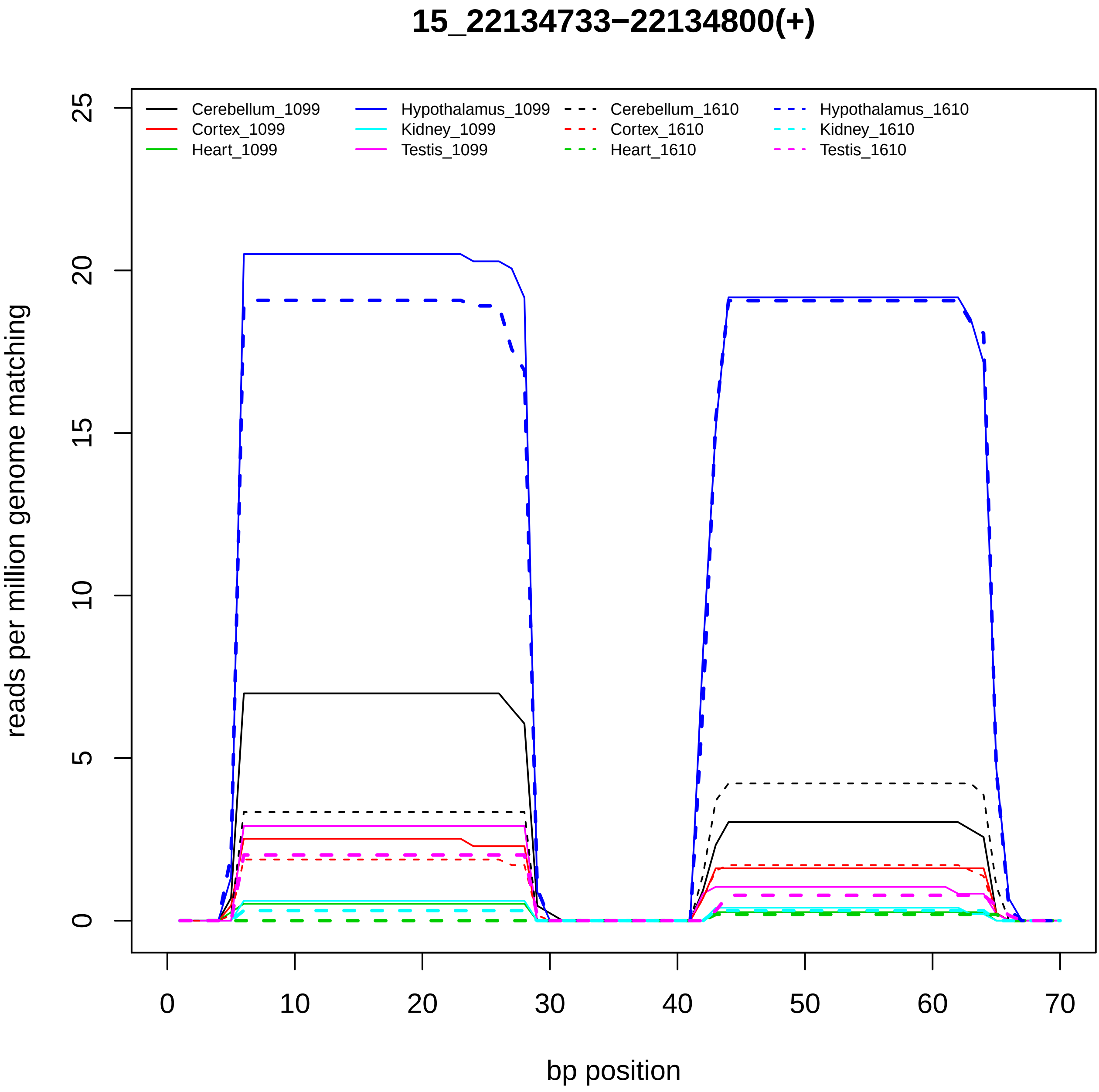
15_22134733−22134800(+)
Cerebellum_1099
Cortex_1099
Heart_1099
Hypothalamus_1099
Kidney_1099
Testis_1099
Cerebellum_1610
Cortex_1610
Heart_1610
Hypothalamus_1610
Kidney_1610
Testis_1610
reads per million genome matching
bp position
0
5
10
15
20
25
0
10
20
30
40
50
60
70

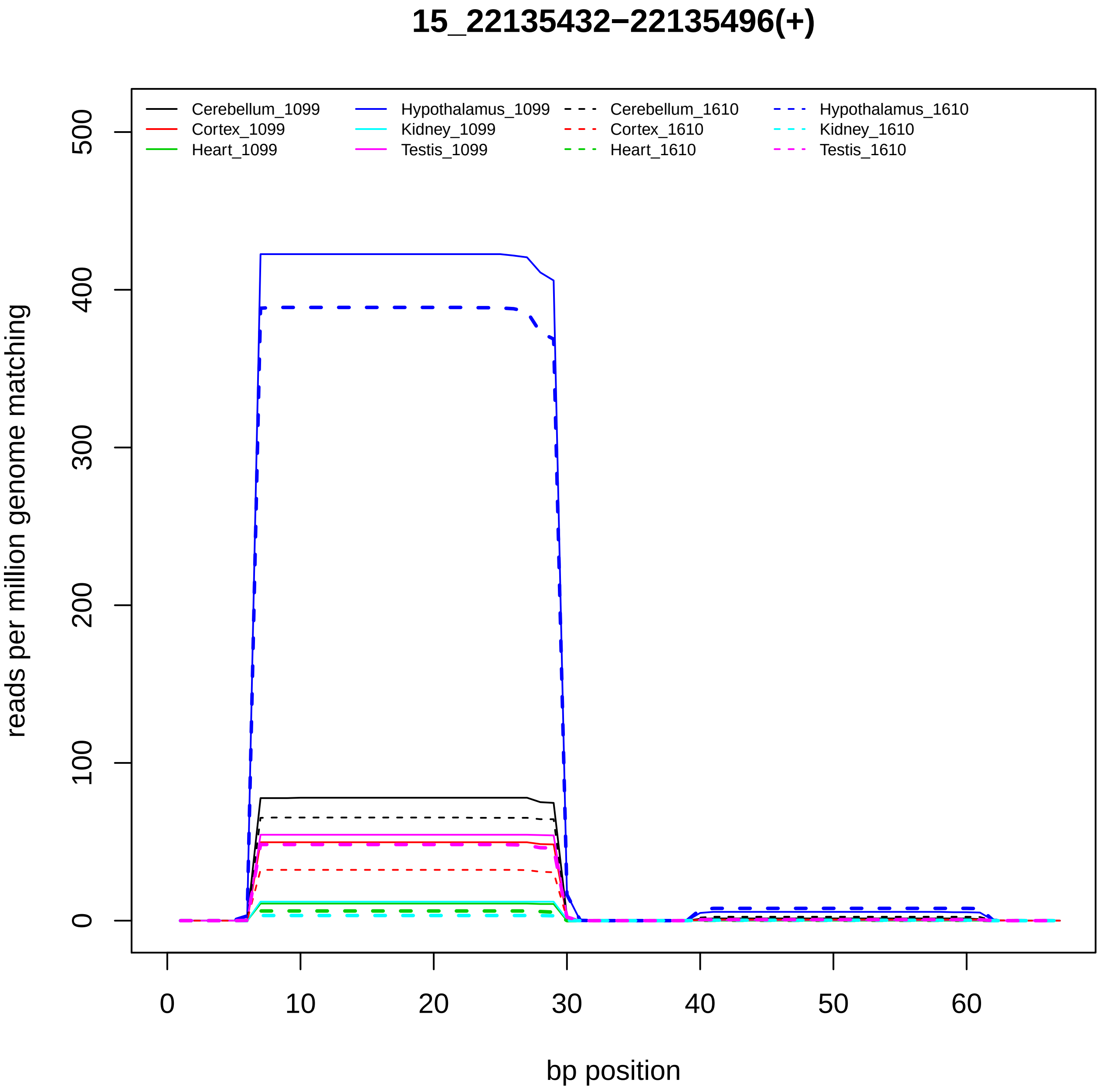
15_22135432−22135496(+)
Cerebellum_1099
Cortex_1099
Heart_1099
Hypothalamus_1099
Kidney_1099
Testis_1099
Cerebellum_1610
Cortex_1610
Heart_1610
Hypothalamus_1610
Kidney_1610
Testis_1610
reads per million genome matching
0
100
200
300
400
500
0
10
20
30
40
50
60
bp position

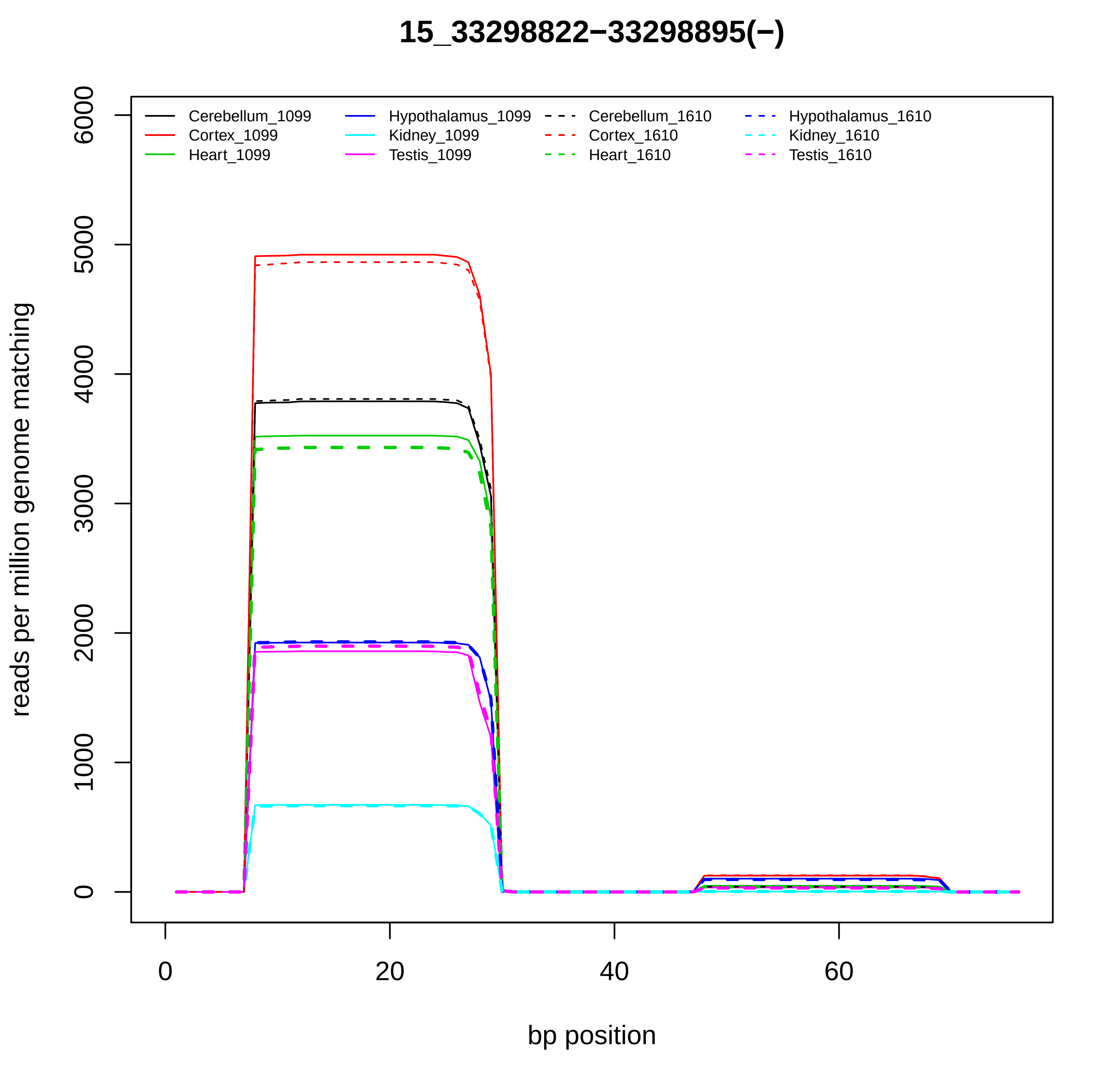

15_33298822−33298895(−)
Cerebellum_1099
Cortex_1099
Heart_1099
Hypothalamus_1099
Kidney_1099
Testis_1099
Cerebellum_1610
Cortex_1610
Heart_1610
Hypothalamus_1610
Kidney_1610
Testis_1610
reads per million genome matching
0
1000
2000
3000
4000
5000
6000
0
20
40
60
bp position

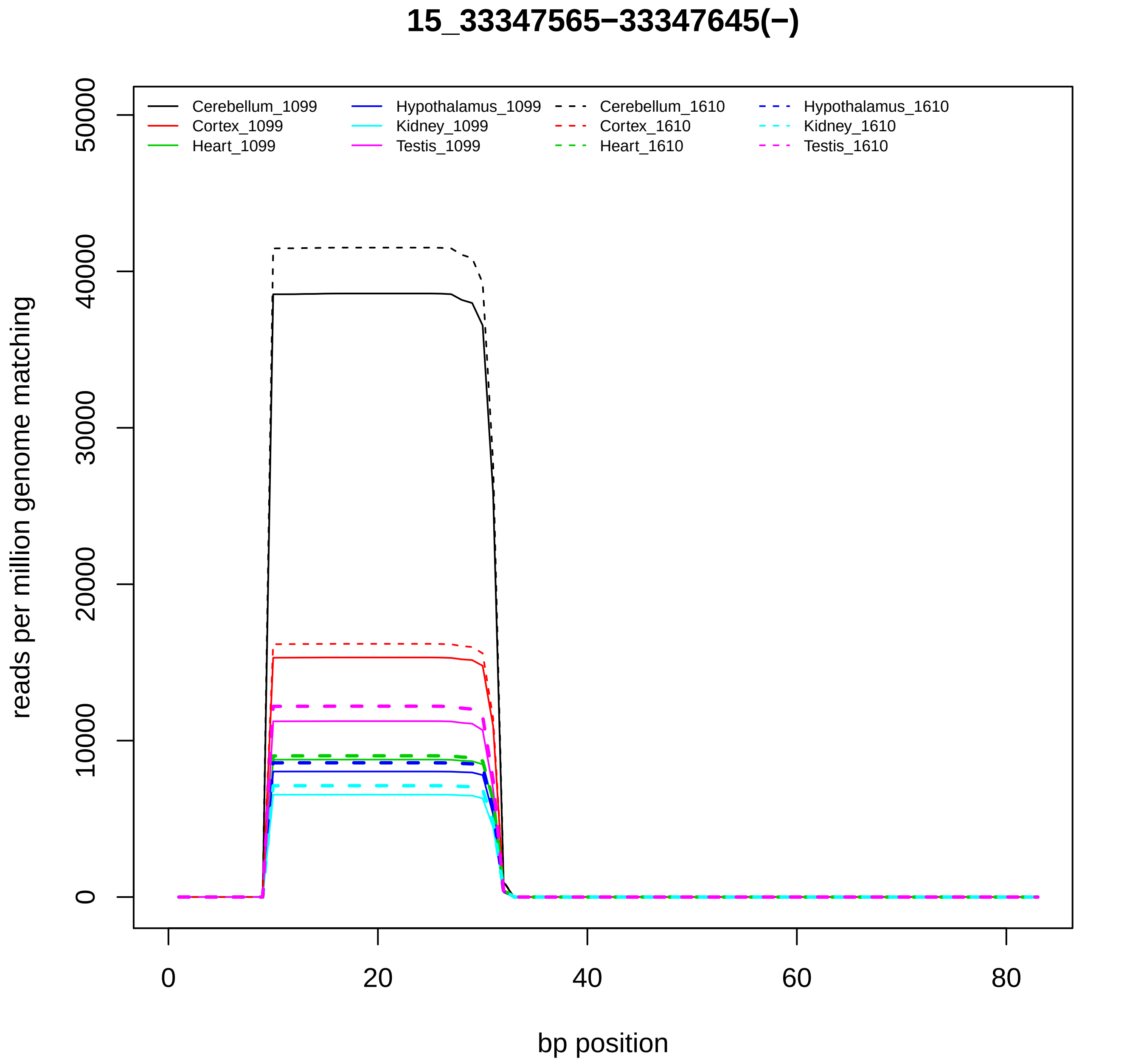
15_33347565−33347645(−)
Cerebellum_1099
Cortex_1099
Heart_1099
Hypothalamus_1099
Kidney_1099
Testis_1099
Cerebellum_1610
Cortex_1610
Heart_1610
Hypothalamus_1610
Kidney_1610
Testis_1610
reads per million genome matching
0
10000
20000
30000
40000
50000
bp position
0
20
40
60
80

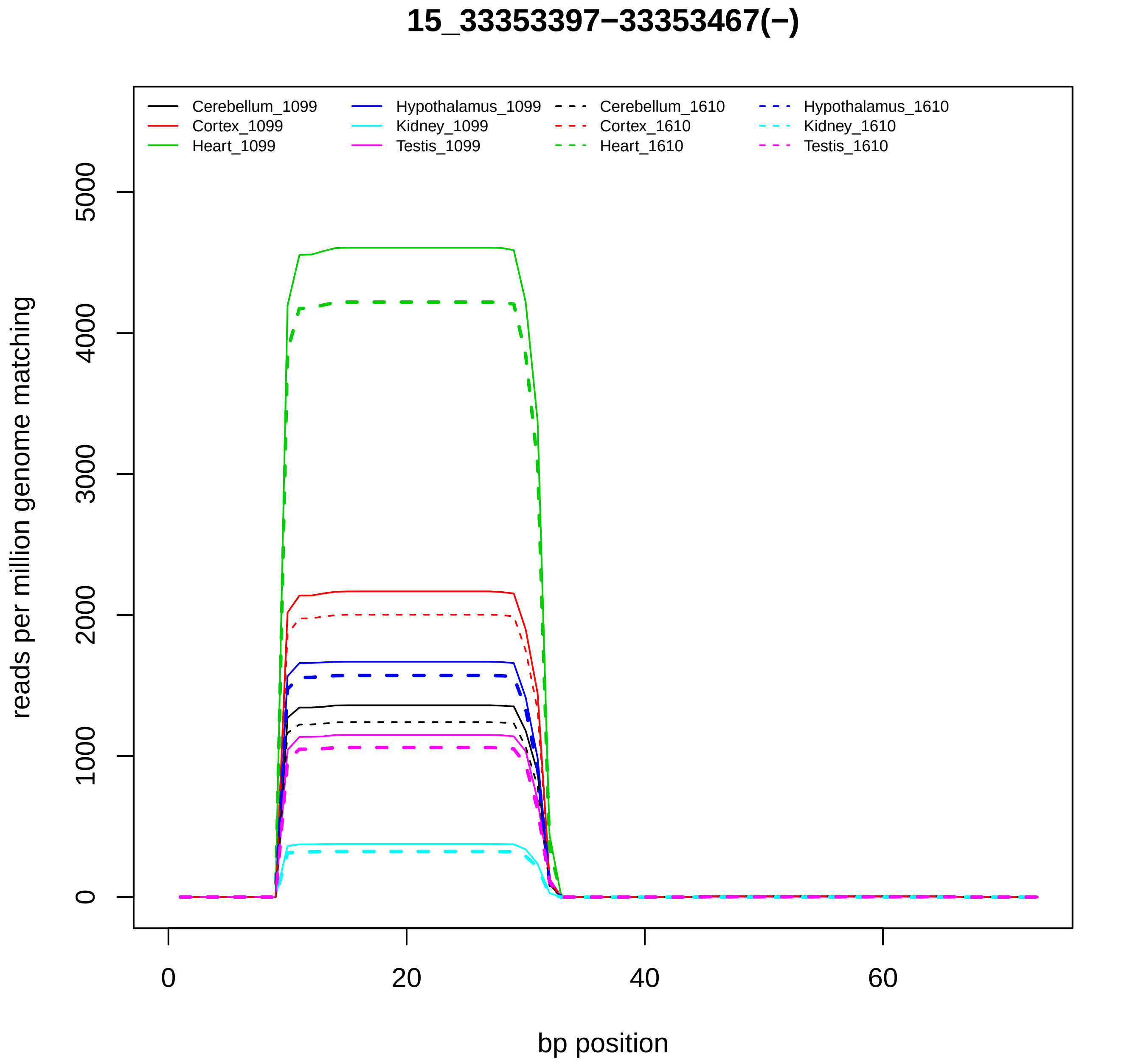
15_33353397−33353467(−)
Cerebellum_1099
Cortex_1099
Heart_1099
Hypothalamus_1099
Kidney_1099
Testis_1099
Cerebellum_1610
Cortex_1610
Heart_1610
Hypothalamus_1610
Kidney_1610
Testis_1610
reads per million genome matching
0
1000
2000
3000
4000
5000
0
20
40
60
bp position

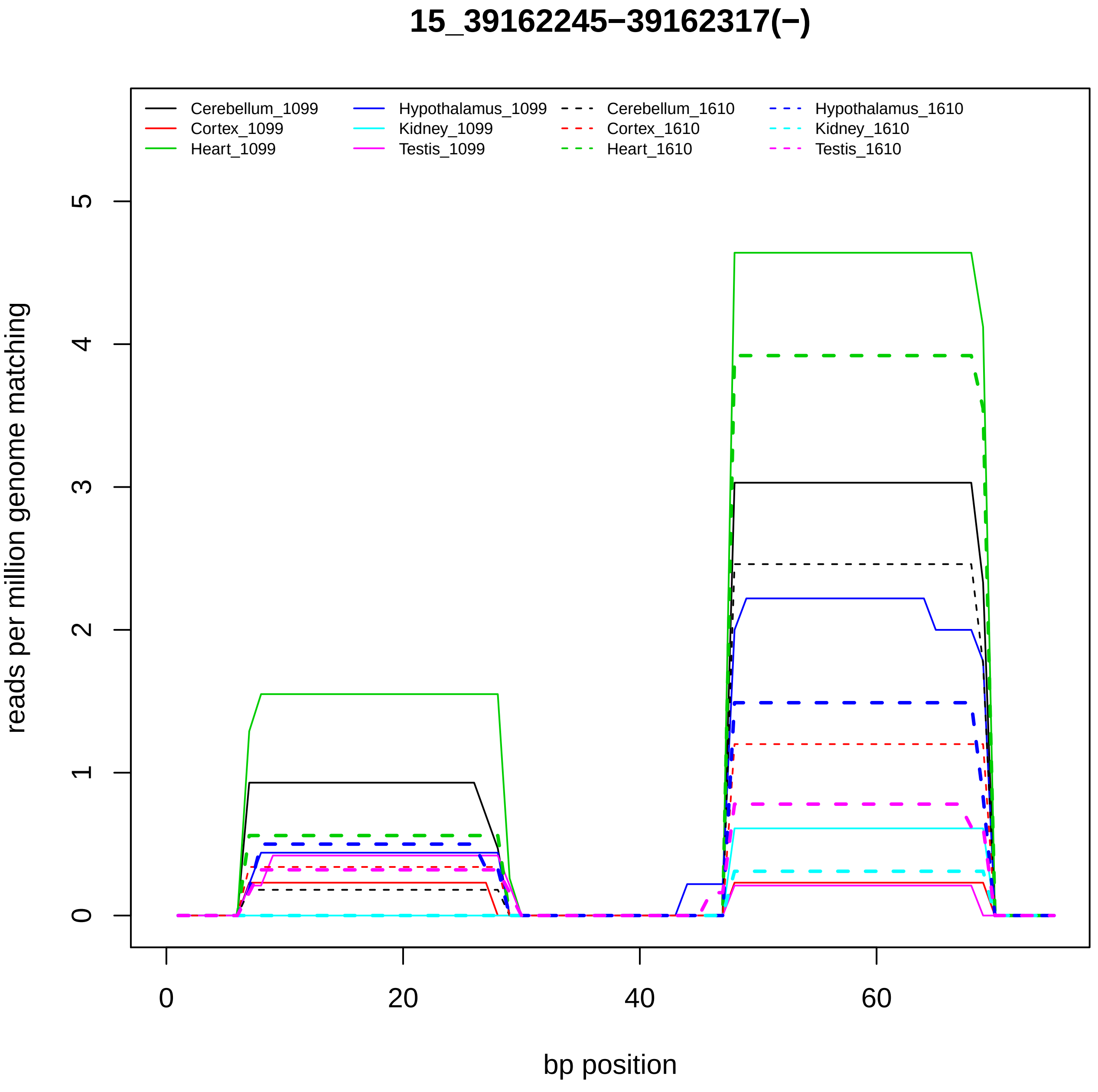

15_39162245−39162317(−)
Cerebellum_1099
Cortex_1099
Heart_1099
Hypothalamus_1099
Kidney_1099
Testis_1099
Cerebellum_1610
Cortex_1610
Heart_1610
Hypothalamus_1610
Kidney_1610
Testis_1610
reads per million genome matching
0
1
2
3
4
5
0
20
40
60
bp position

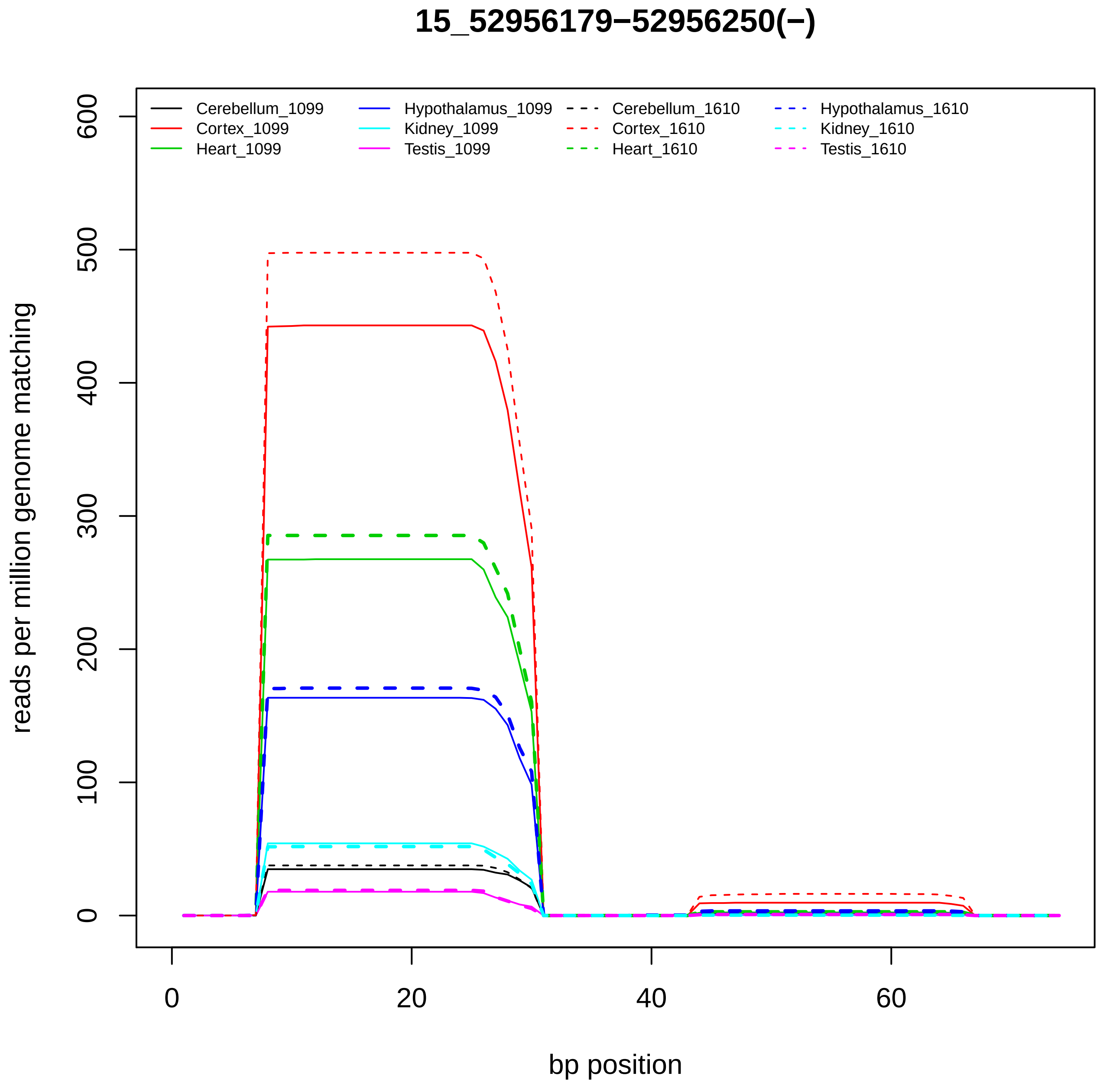

15_52956179−52956250(−)
Cerebellum_1099
Cortex_1099
Heart_1099
Hypothalamus_1099
Kidney_1099
Testis_1099
Cerebellum_1610
Cortex_1610
Heart_1610
Hypothalamus_1610
Kidney_1610
Testis_1610
reads per million genome matching
0
100
200
300
400
500
600
0
20
40
60
bp position

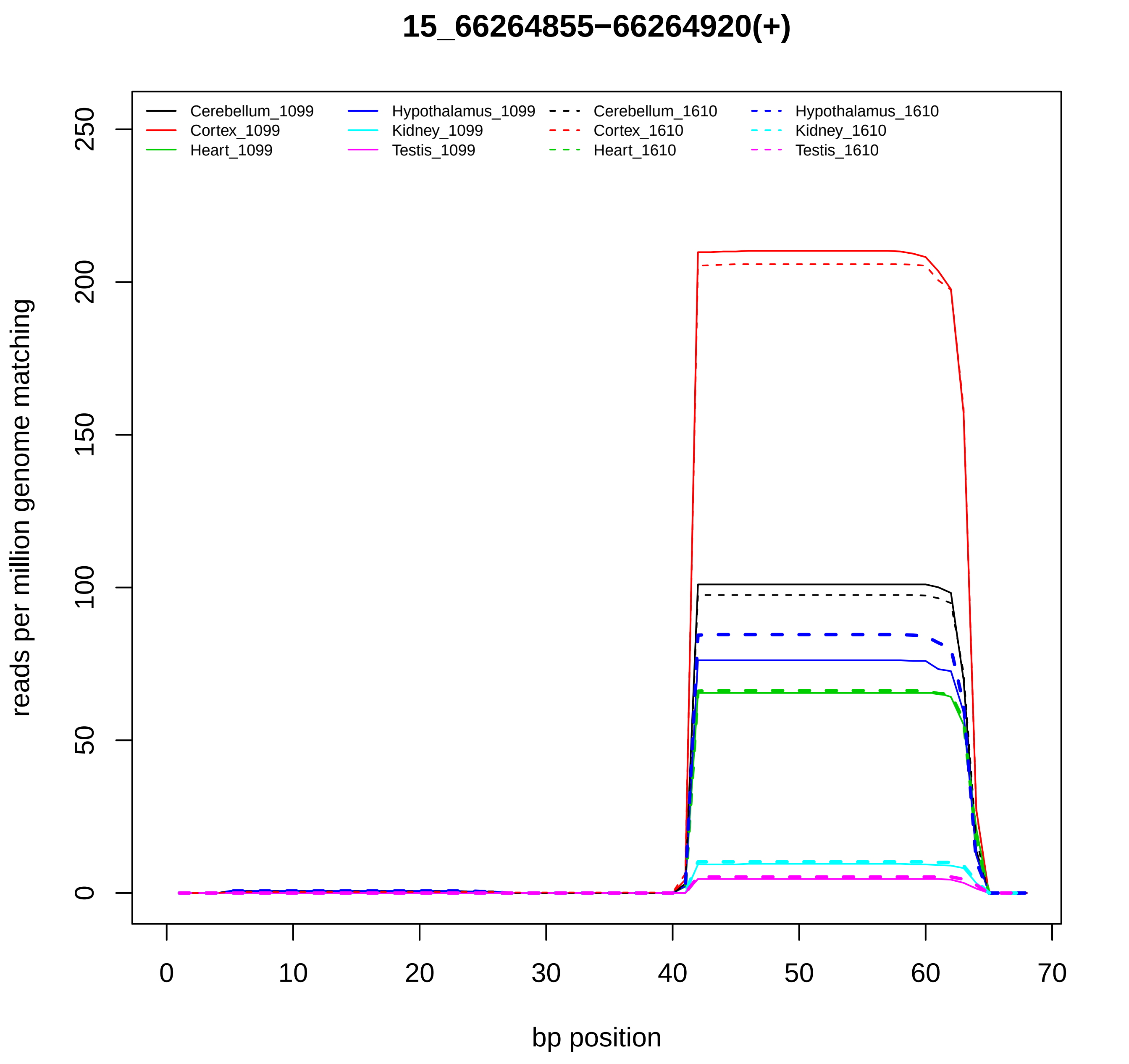

15_66264855−66264920(+)
Cerebellum_1099
Cortex_1099
Heart_1099
Hypothalamus_1099
Kidney_1099
Testis_1099
Cerebellum_1610
Cortex_1610
Heart_1610
Hypothalamus_1610
Kidney_1610
Testis_1610
reads per million genome matching
0
50
100
150
200
250
bp position
0
10
20
30
40
50
60
70

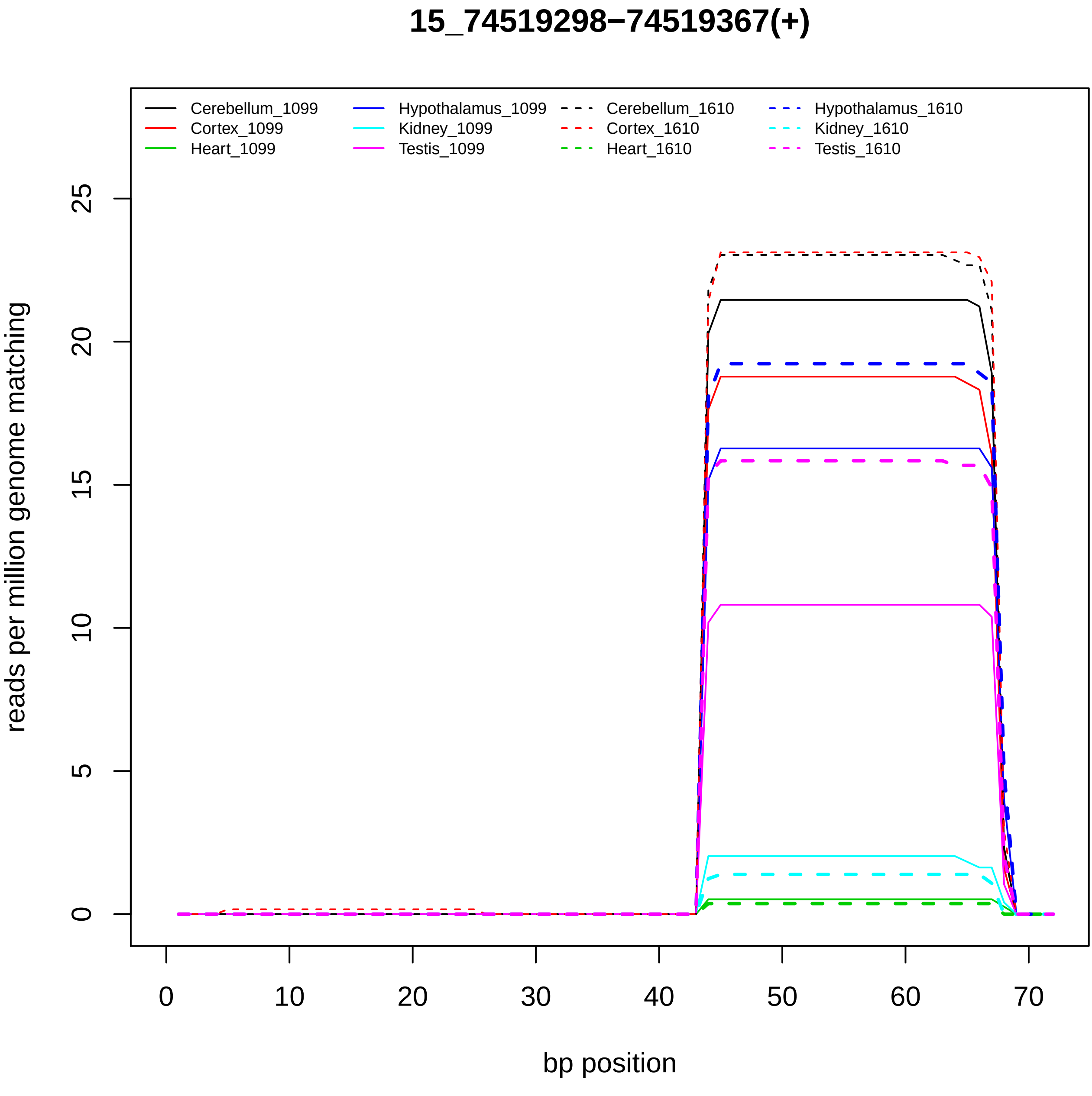
15_74519298−74519367(+)
Cerebellum_1099
Cortex_1099
Heart_1099
Hypothalamus_1099
Kidney_1099
Testis_1099
Cerebellum_1610
Cortex_1610
Heart_1610
Hypothalamus_1610
Kidney_1610
Testis_1610
reads per million genome matching
0
5
10
15
20
25
0
10
20
30
40
50
60
70
bp position

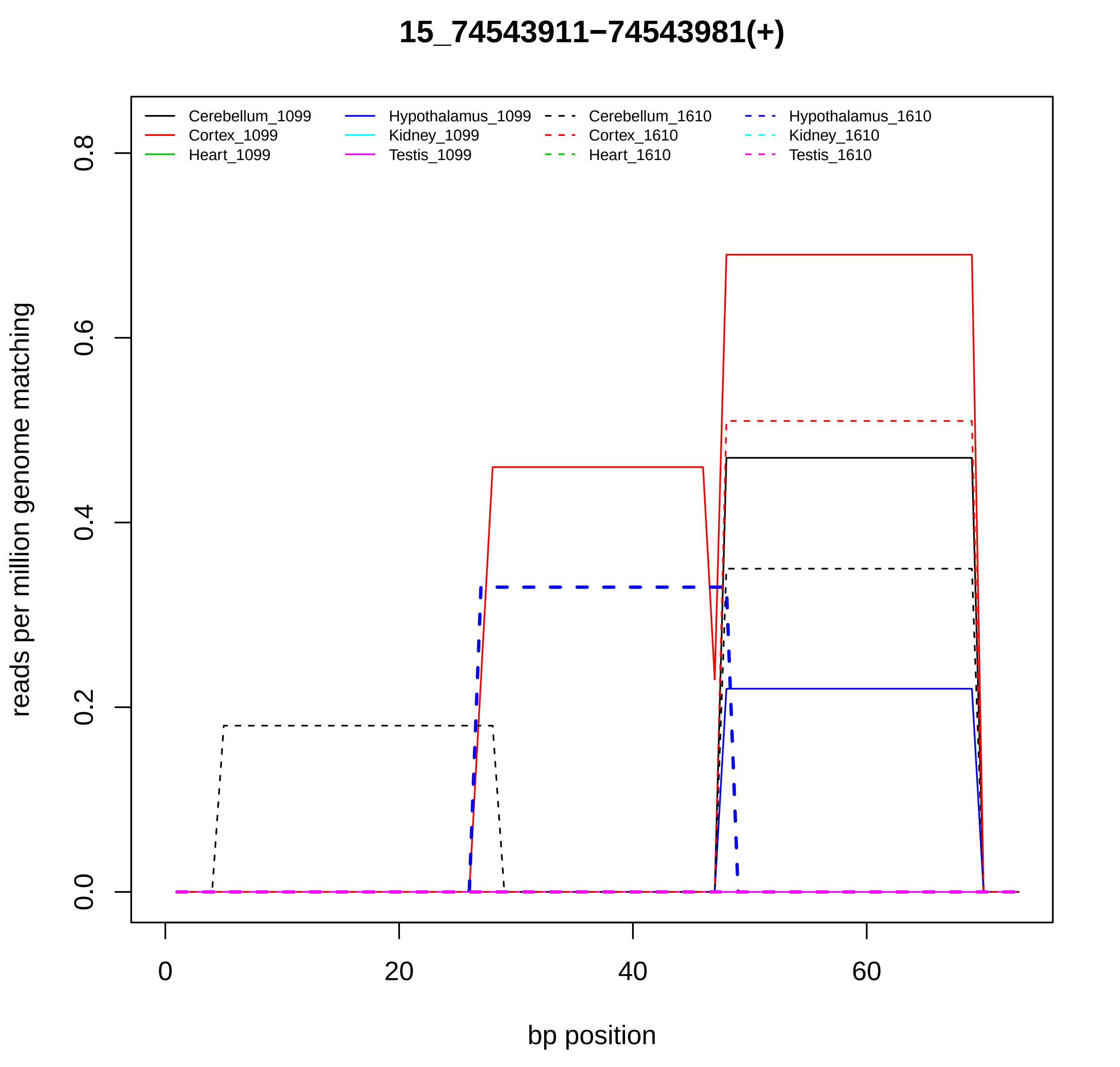
15_74543911−74543981(+)
Cerebellum_1099
Cortex_1099
Heart_1099
Hypothalamus_1099
Kidney_1099
Testis_1099
Cerebellum_1610
Cortex_1610
Heart_1610
Hypothalamus_1610
Kidney_1610
Testis_1610
reads per million genome matching
0.0
0.2
0.4
0.6
0.8
0
20
40
60
bp position

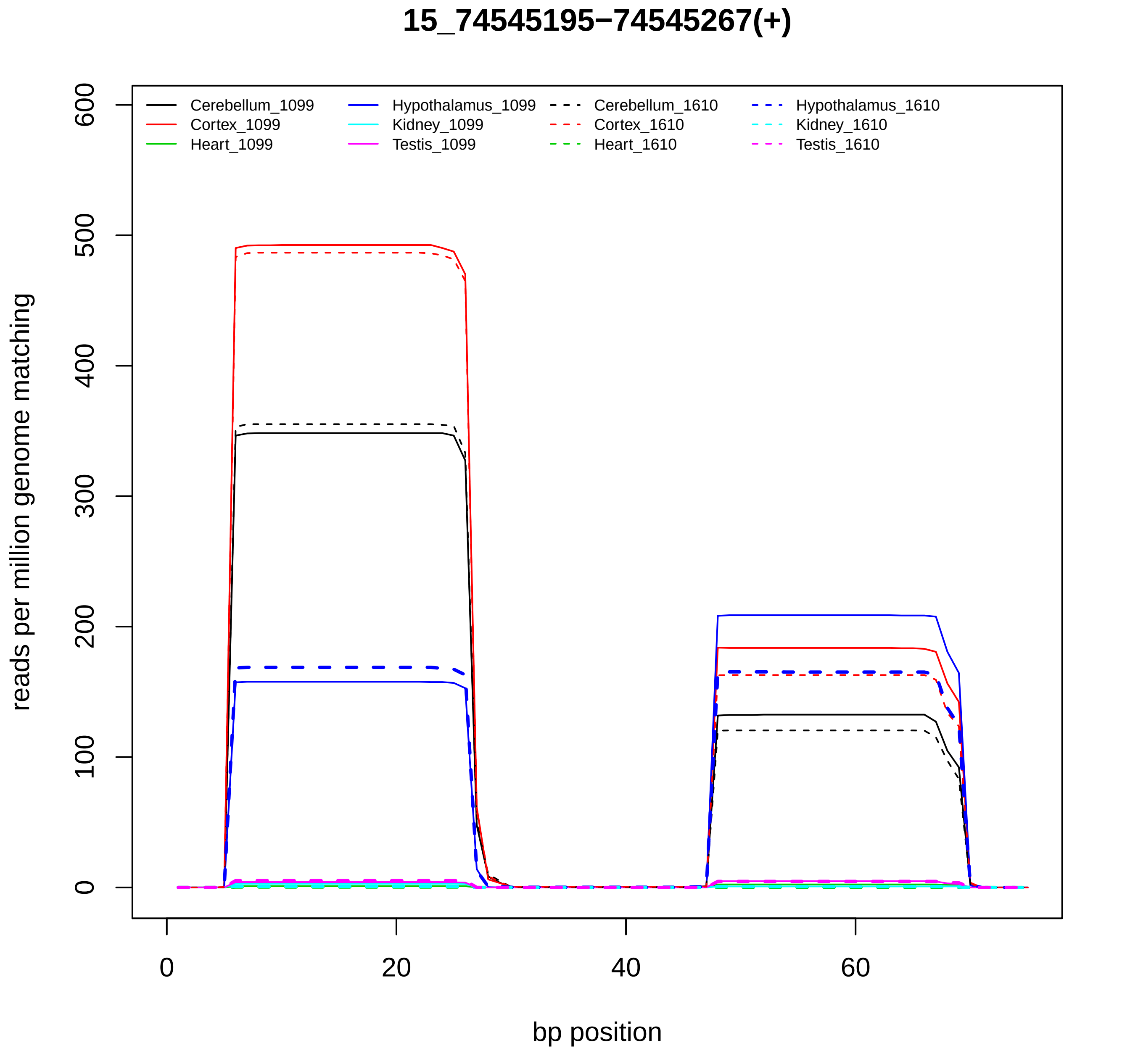
15_74545195−74545267(+)
Cerebellum_1099
Cortex_1099
Heart_1099
Hypothalamus_1099
Kidney_1099
Testis_1099
Cerebellum_1610
Cortex_1610
Heart_1610
Hypothalamus_1610
Kidney_1610
Testis_1610
reads per million genome matching
0
100
200
300
400
500
600
bp position
0
20
40
60

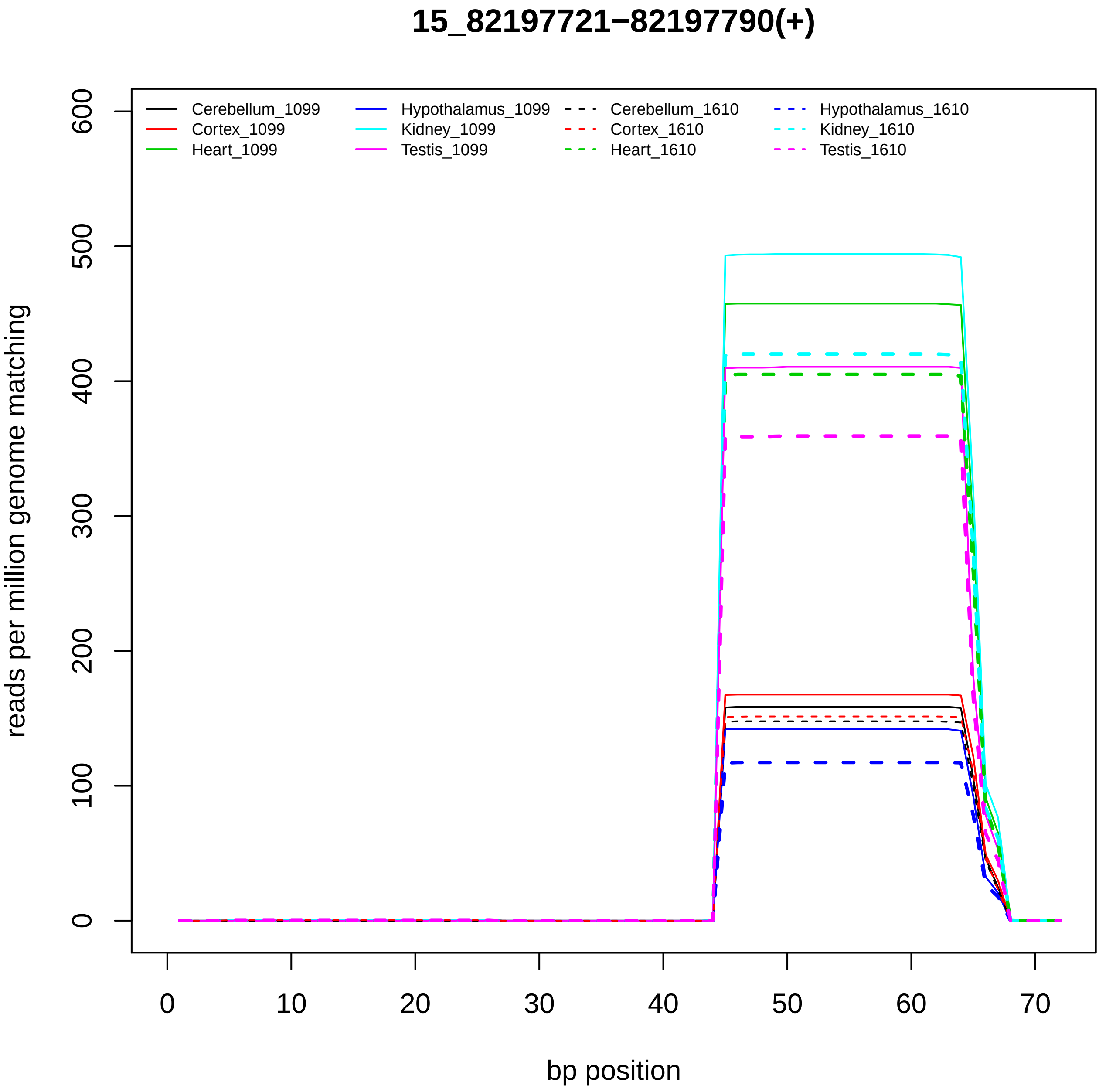

15_82197721−82197790(+)
Cerebellum_1099
Cortex_1099
Heart_1099
Hypothalamus_1099
Kidney_1099
Testis_1099
Cerebellum_1610
Cortex_1610
Heart_1610
Hypothalamus_1610
Kidney_1610
Testis_1610
reads per million genome matching
0
100
200
300
400
500
600
0
10
20
30
40
50
60
70
bp position

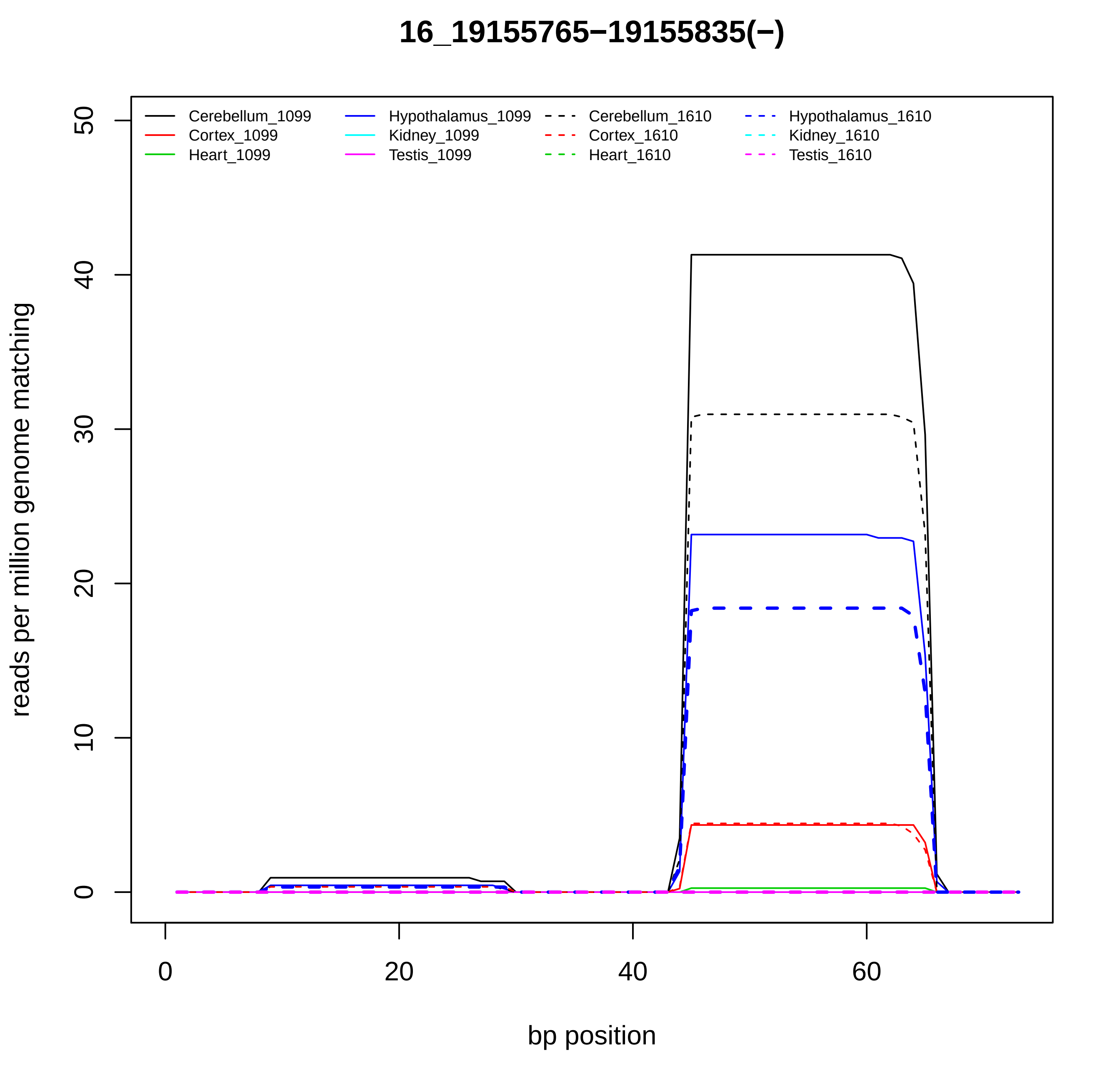
16_19155765−19155835(−)
Cerebellum_1099
Cortex_1099
Heart_1099
Hypothalamus_1099
Kidney_1099
Testis_1099
Cerebellum_1610
Cortex_1610
Heart_1610
Hypothalamus_1610
Kidney_1610
Testis_1610
reads per million genome matching
bp position
0
10
20
30
40
50
0
20
40
60

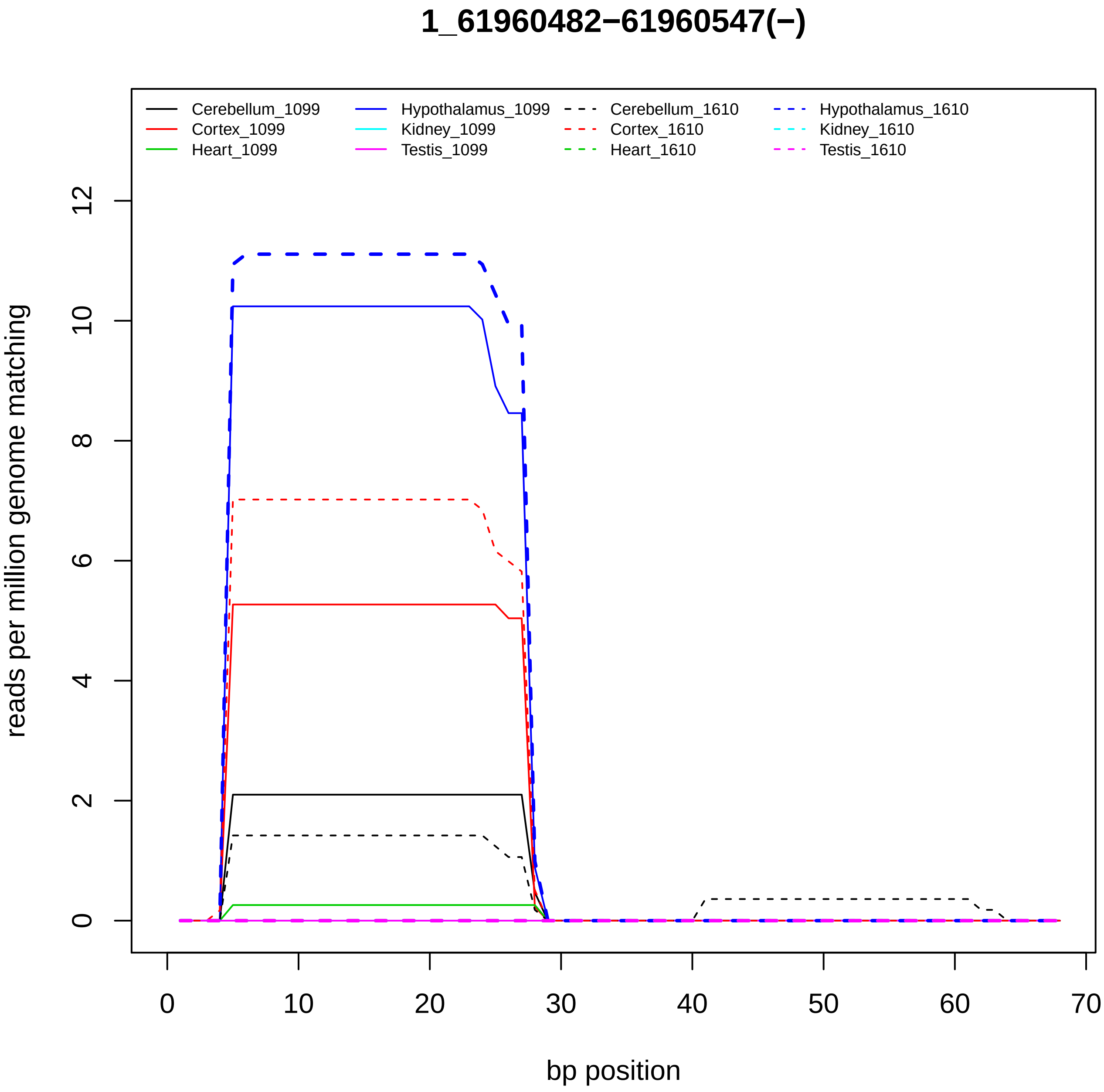

1_61960482−61960547(−)
Cerebellum_1099
Cortex_1099
Heart_1099
Hypothalamus_1099
Kidney_1099
Testis_1099
Cerebellum_1610
Cortex_1610
Heart_1610
Hypothalamus_1610
Kidney_1610
Testis_1610
reads per million genome matching
0
2
4
6
8
10
12
bp position
0
10
20
30
40
50
60
70

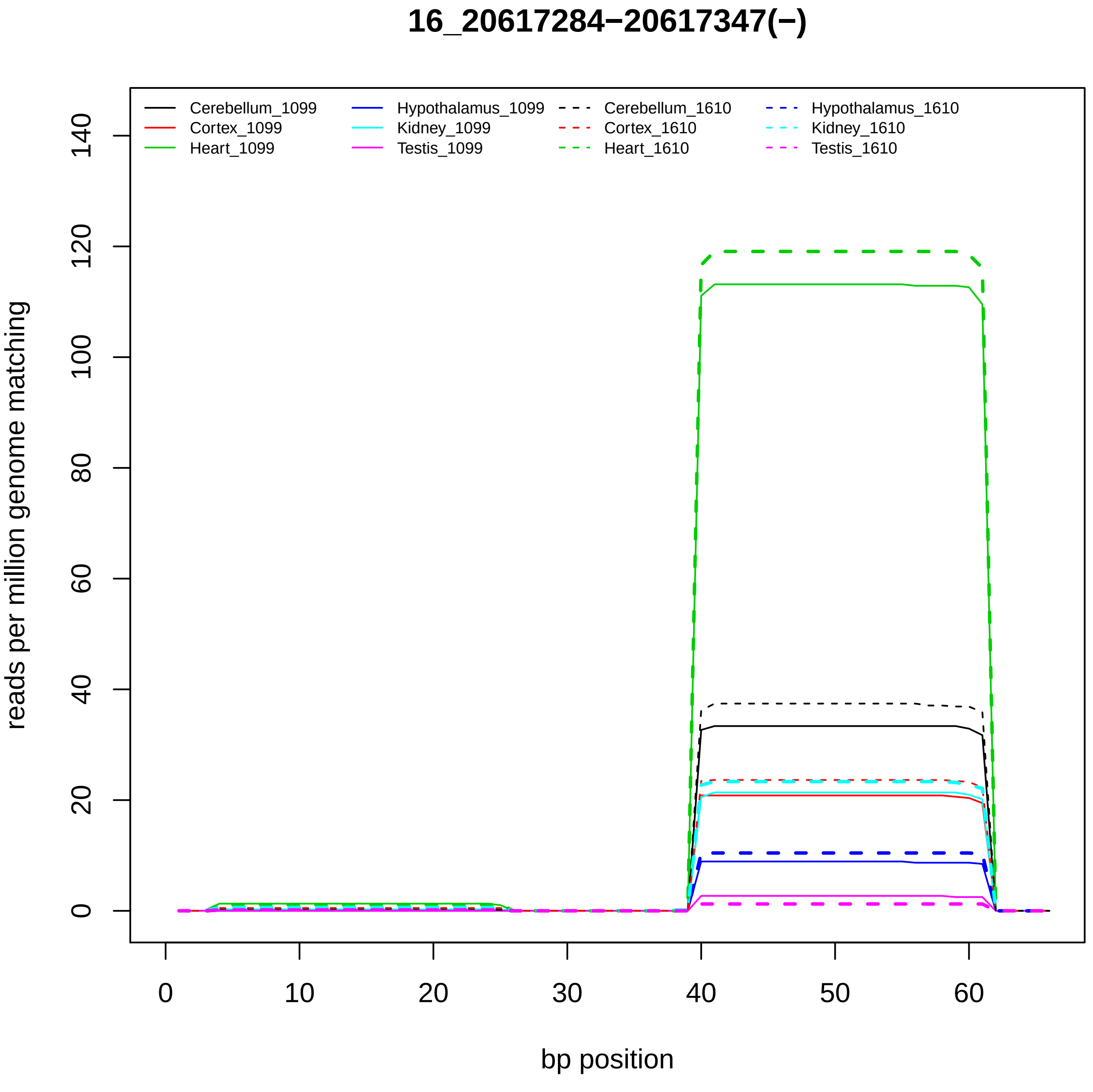
16_20617284−20617347(−)
Cerebellum_1099
Cortex_1099
Heart_1099
Hypothalamus_1099
Kidney_1099
Testis_1099
Cerebellum_1610
Cortex_1610
Heart_1610
Hypothalamus_1610
Kidney_1610
Testis_1610
reads per million genome matching
0
20
40
60
80
100
120
140
bp position
0
10
20
30
40
50
60

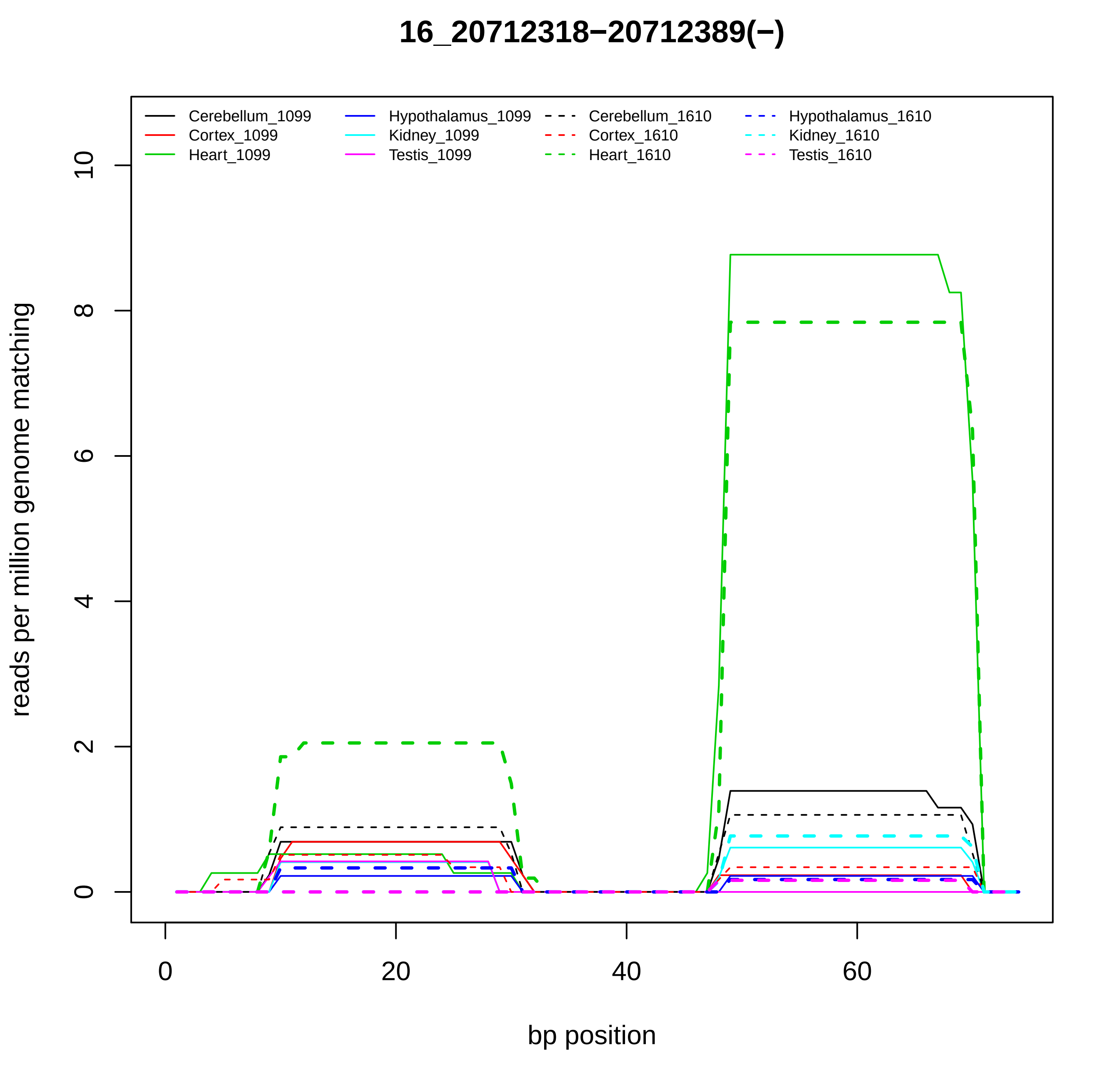

16_20712318−20712389(−)
Cerebellum_1099
Cortex_1099
Heart_1099
Hypothalamus_1099
Kidney_1099
Testis_1099
Cerebellum_1610
Cortex_1610
Heart_1610
Hypothalamus_1610
Kidney_1610
Testis_1610
reads per million genome matching
bp position
0
2
4
6
8
10
0
20
40
60

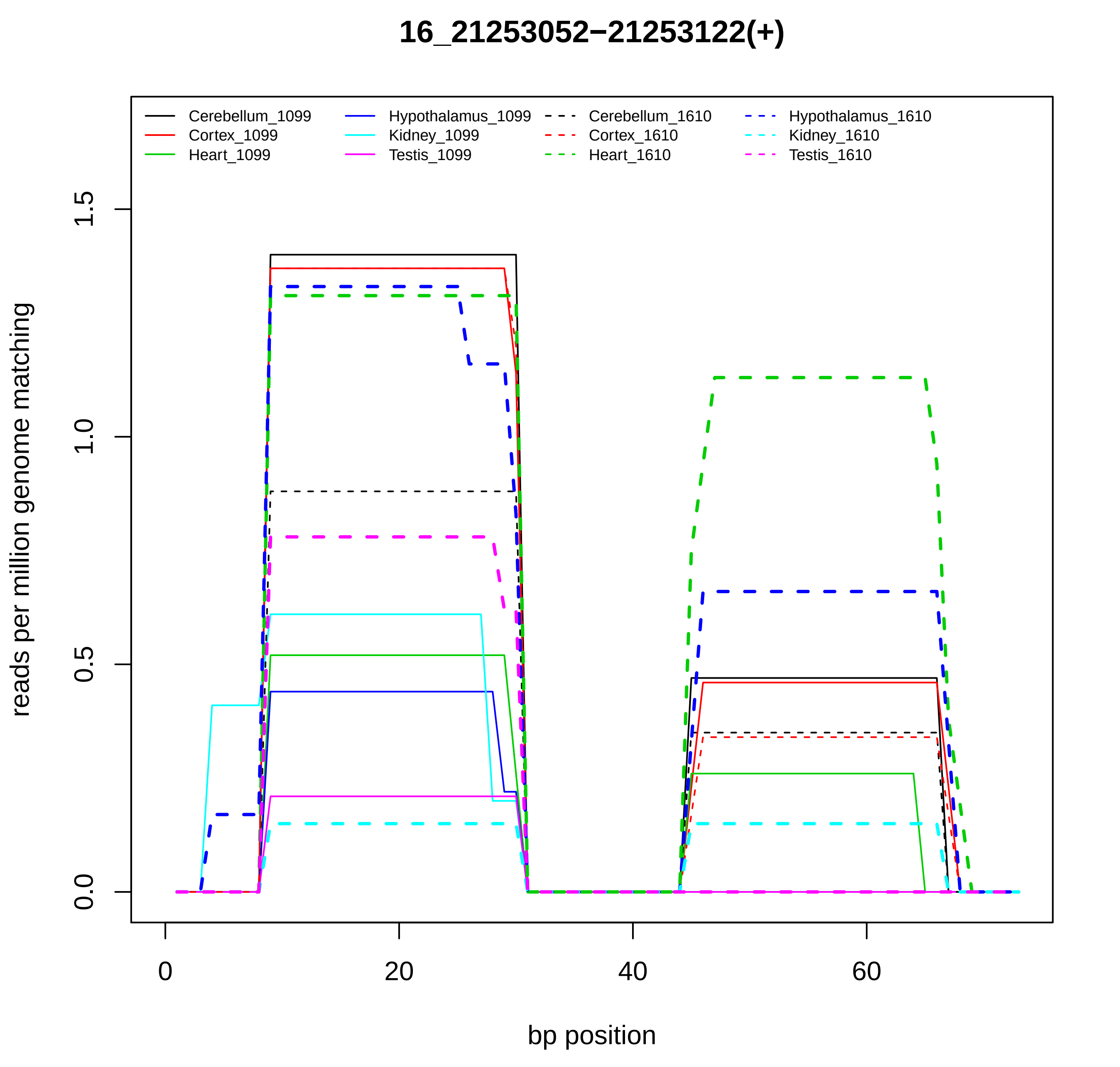
16_21253052−21253122(+)
Cerebellum_1099
Cortex_1099
Heart_1099
Hypothalamus_1099
Kidney_1099
Testis_1099
Cerebellum_1610
Cortex_1610
Heart_1610
Hypothalamus_1610
Kidney_1610
Testis_1610
0.0
0.5
1.0
1.5
reads per million genome matching
0
20
40
60
bp position

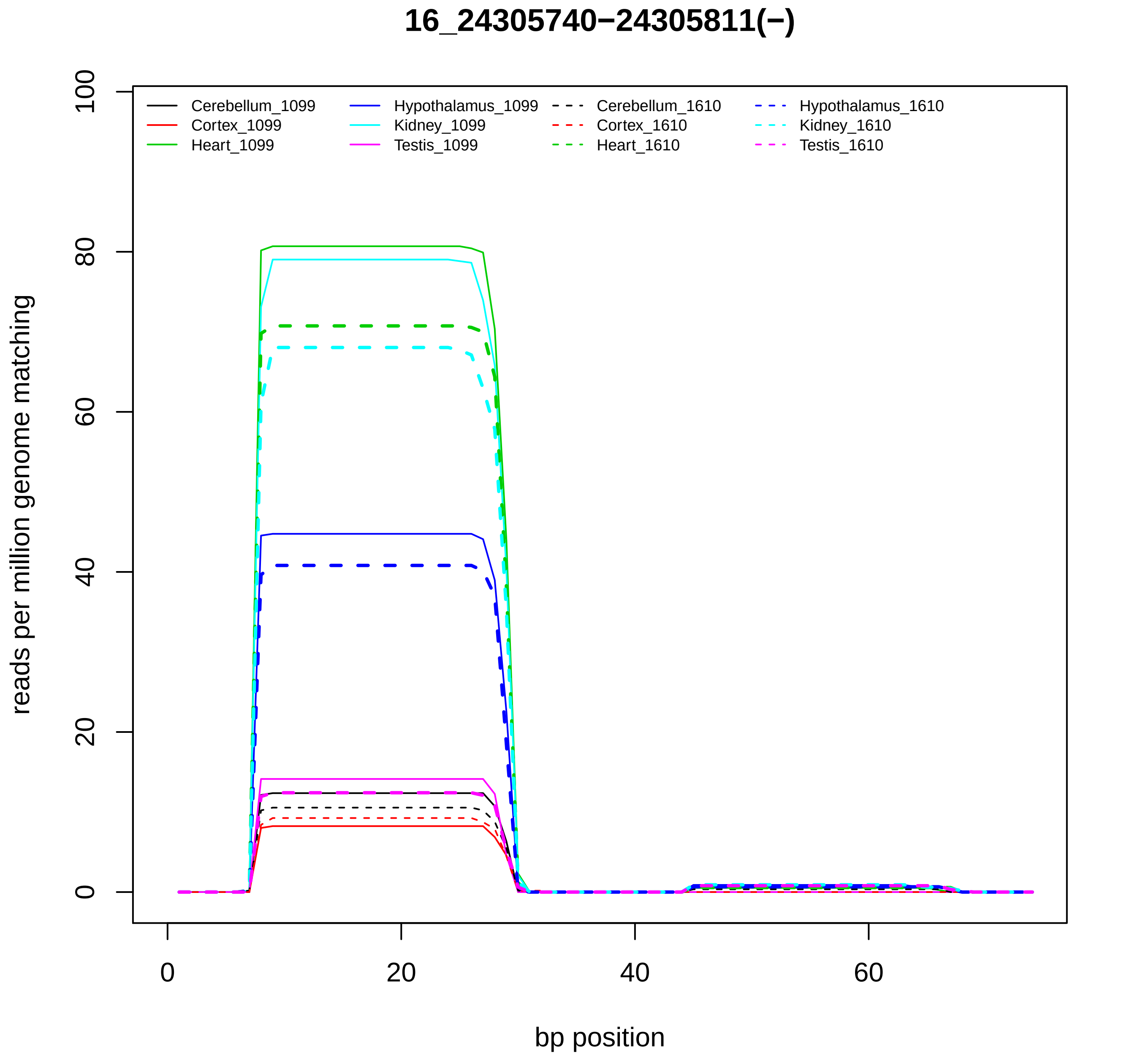
16_24305740−24305811(−)
Cerebellum_1099
Cortex_1099
Heart_1099
Hypothalamus_1099
Kidney_1099
Testis_1099
Cerebellum_1610
Cortex_1610
Heart_1610
Hypothalamus_1610
Kidney_1610
Testis_1610
reads per million genome matching
0
20
40
60
80
100
0
20
40
60
bp position

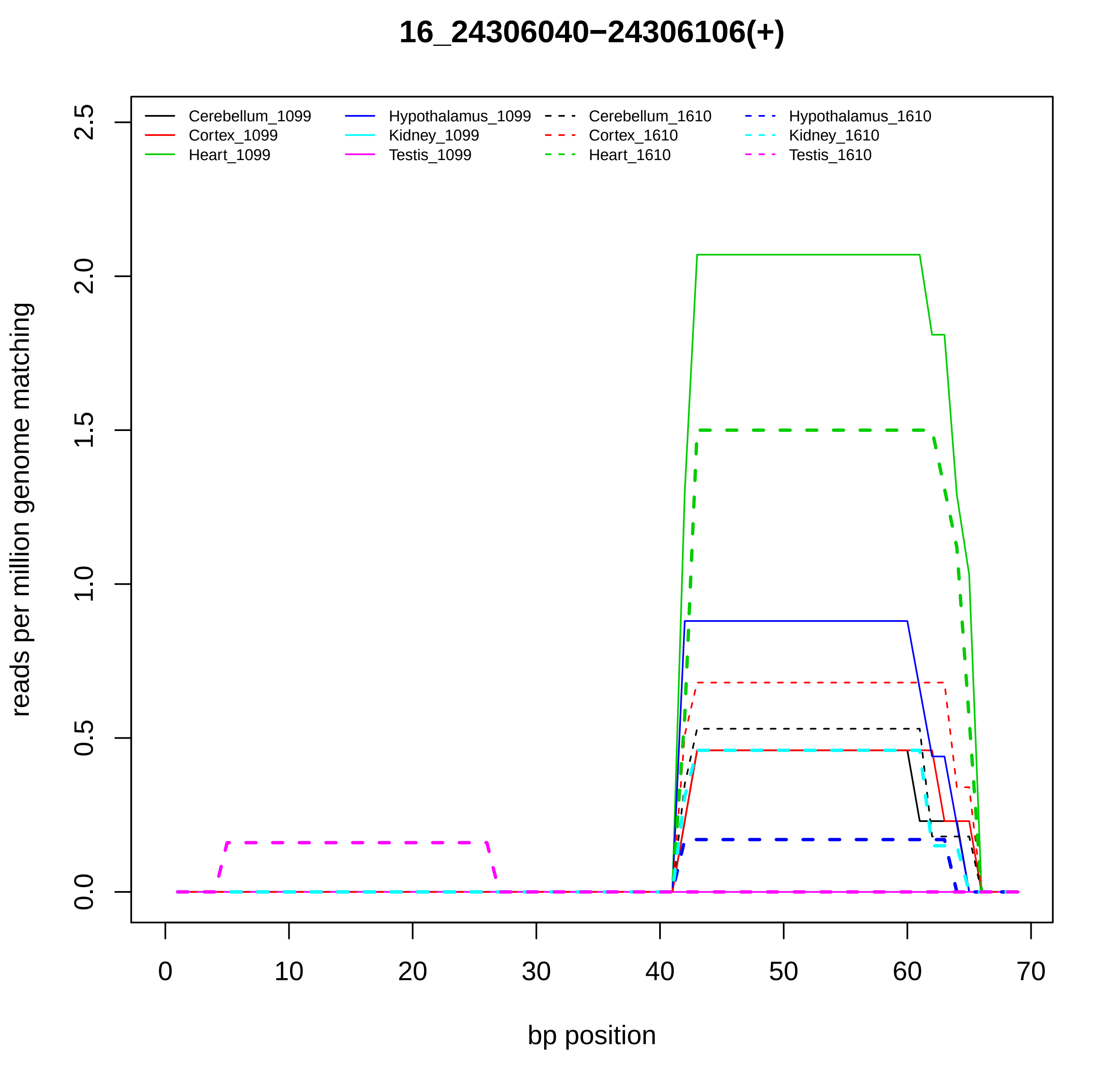

16_24306040−24306106(+)
Cerebellum_1099
Cortex_1099
Heart_1099
Hypothalamus_1099
Kidney_1099
Testis_1099
Cerebellum_1610
Cortex_1610
Heart_1610
Hypothalamus_1610
Kidney_1610
Testis_1610
reads per million genome matching
0.0
0.5
1.0
1.5
2.0
2.5
0
10
20
30
40
50
60
70
bp position

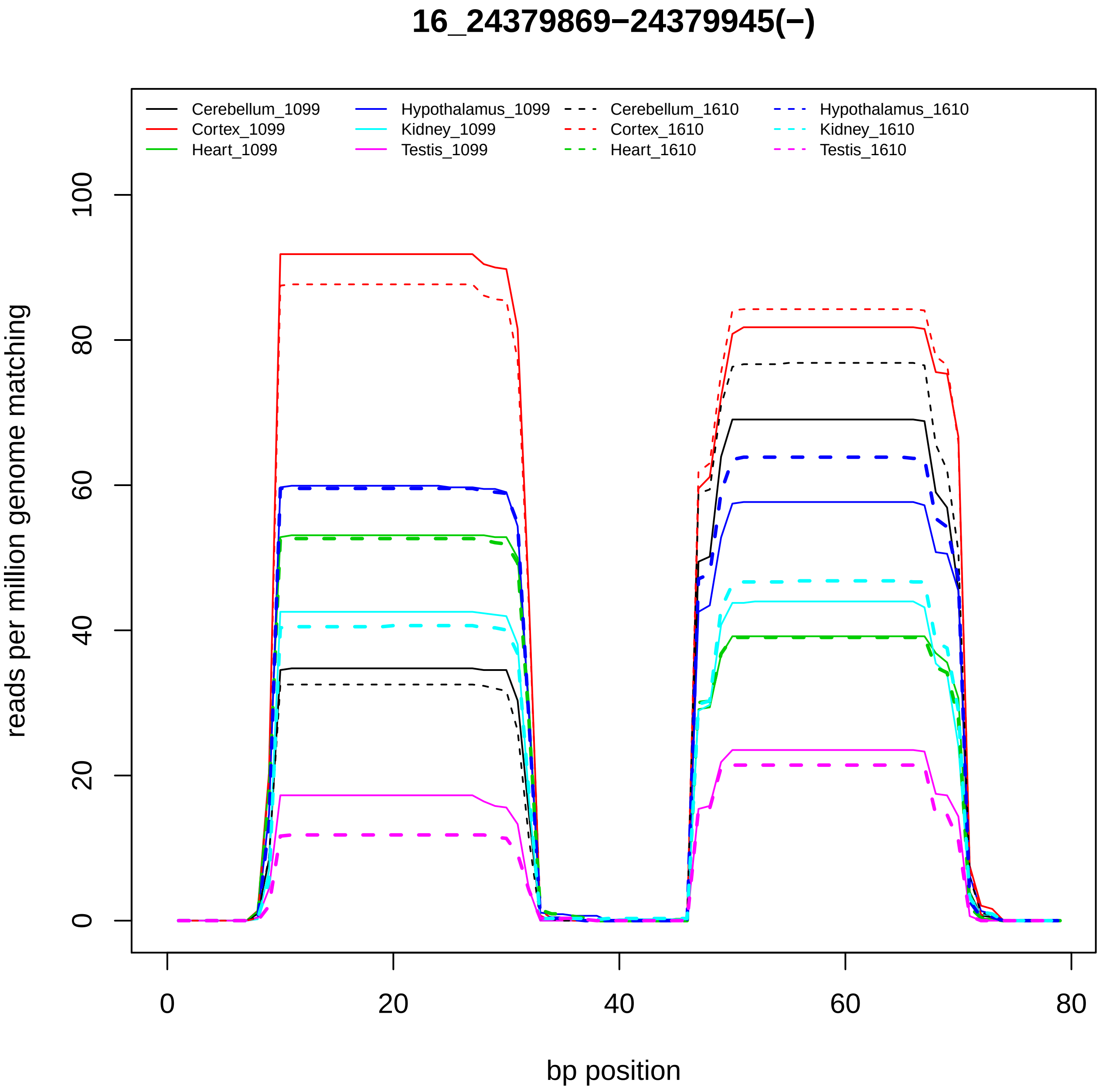

16_24379869−24379945(−)
Cerebellum_1099
Cortex_1099
Heart_1099
Hypothalamus_1099
Kidney_1099
Testis_1099
Cerebellum_1610
Cortex_1610
Heart_1610
Hypothalamus_1610
Kidney_1610
Testis_1610
reads per million genome matching
0
20
40
60
80
100
bp position
0
20
40
60
80

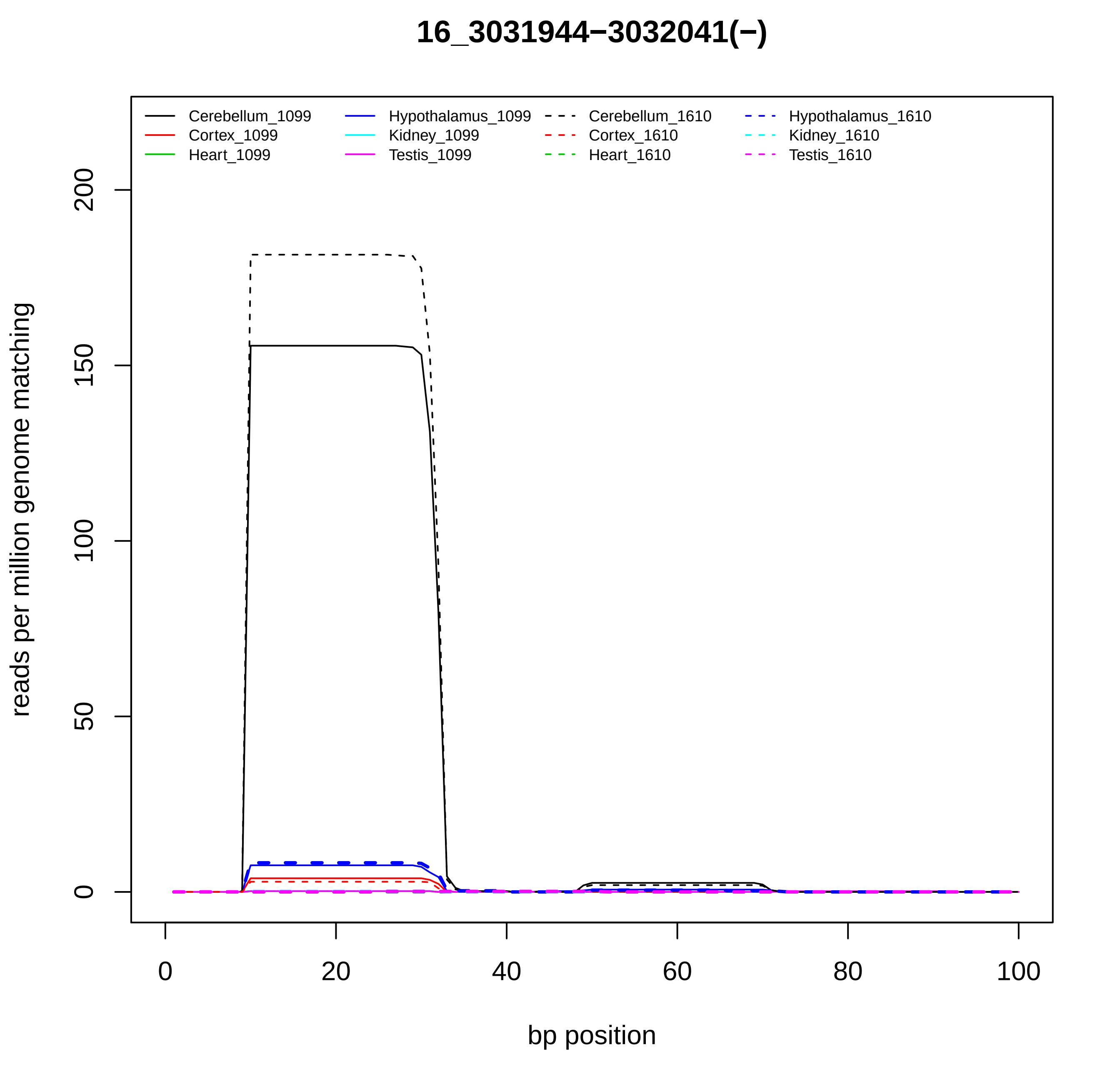

16_3031944−3032041(−)
Cerebellum_1099
Cortex_1099
Heart_1099
Hypothalamus_1099
Kidney_1099
Testis_1099
Cerebellum_1610
Cortex_1610
Heart_1610
Hypothalamus_1610
Kidney_1610
Testis_1610
reads per million genome matching
0
50
100
150
200
0
20
40
60
80
100
bp position

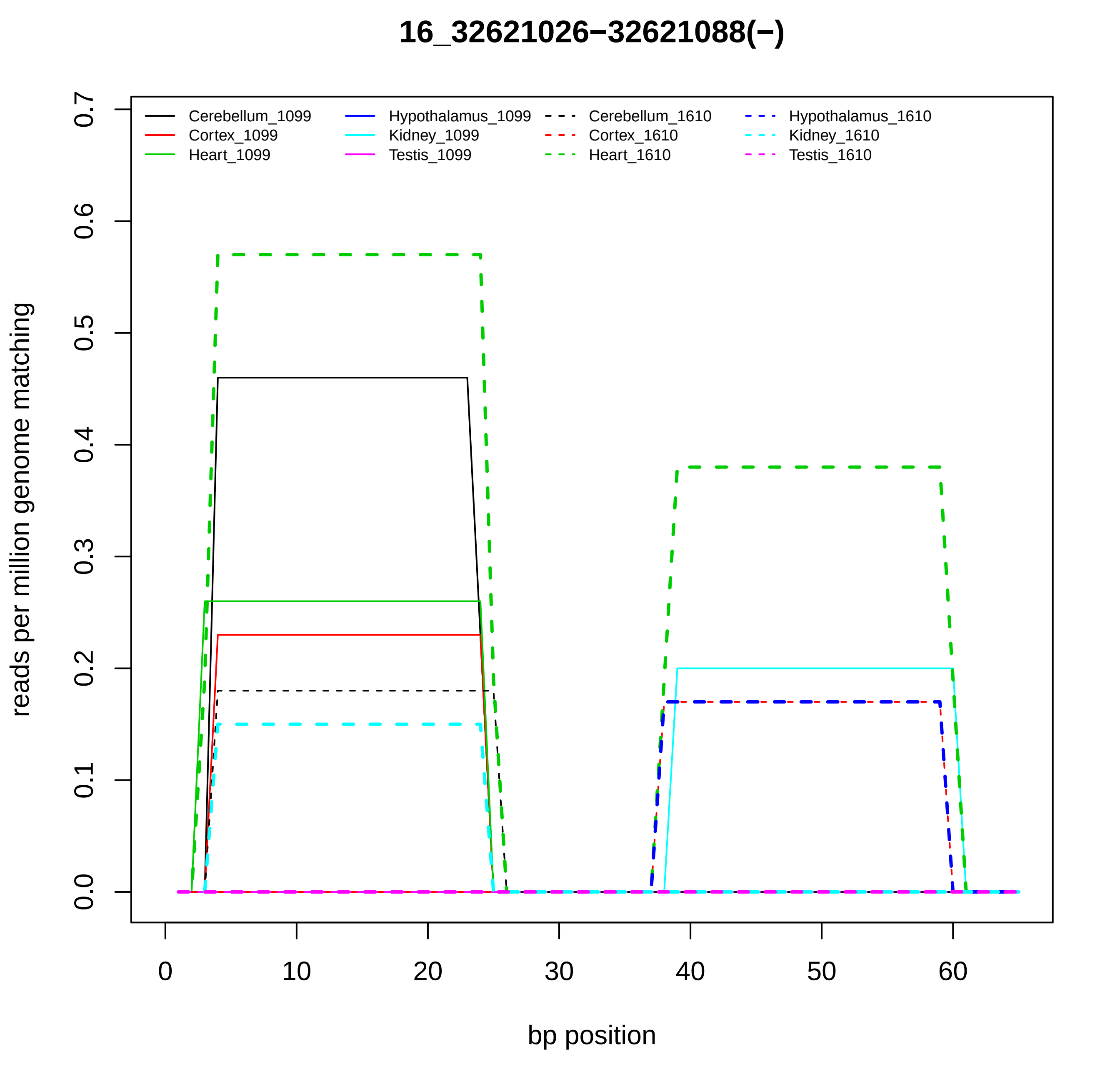

16_32621026−32621088(−)
Cerebellum_1099
Cortex_1099
Heart_1099
Hypothalamus_1099
Kidney_1099
Testis_1099
Cerebellum_1610
Cortex_1610
Heart_1610
Hypothalamus_1610
Kidney_1610
Testis_1610
reads per million genome matching
bp position

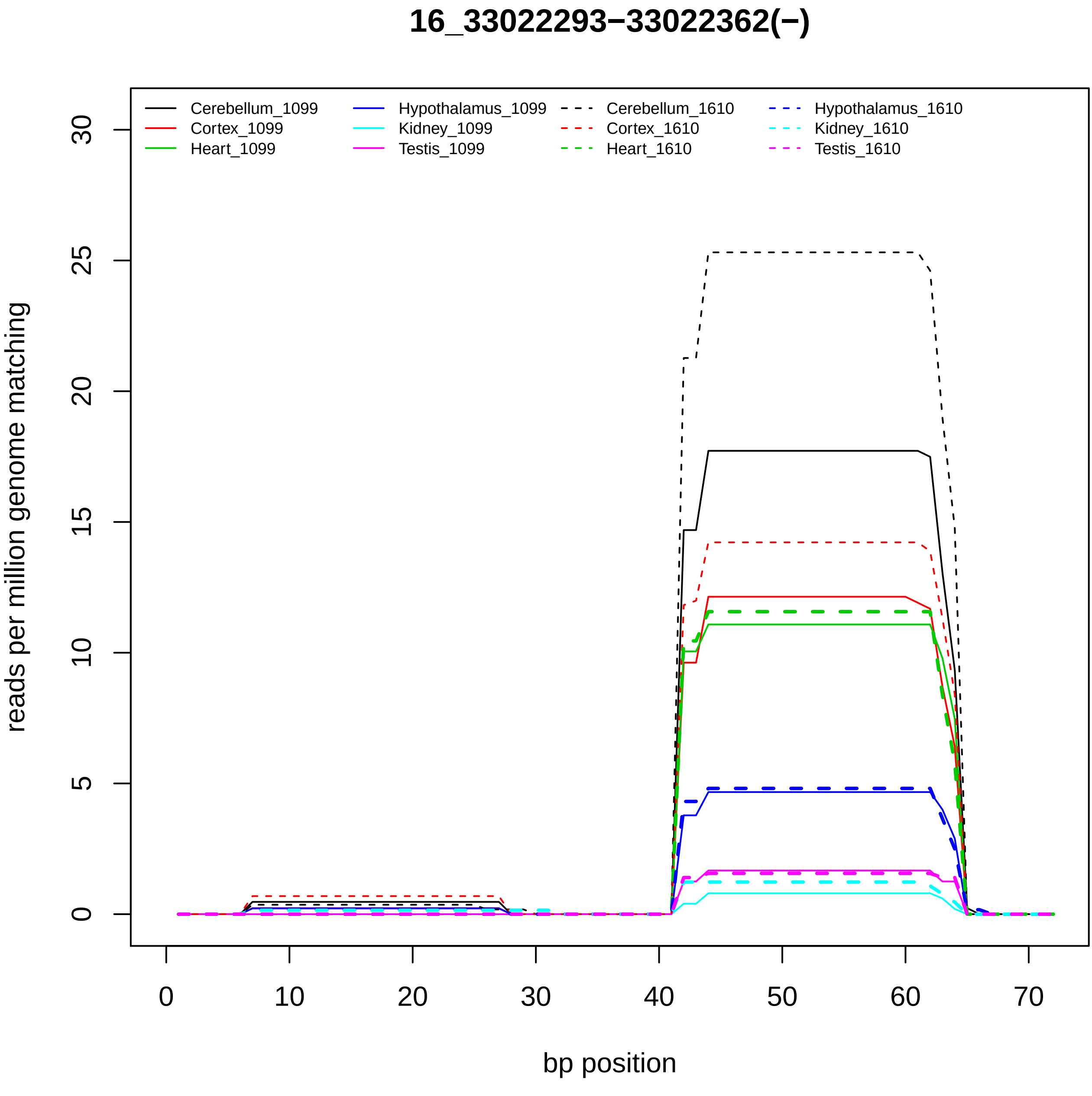
16_33022293−33022362(−)
Cerebellum_1099
Cortex_1099
Heart_1099
Hypothalamus_1099
Kidney_1099
Testis_1099
Cerebellum_1610
Cortex_1610
Heart_1610
Hypothalamus_1610
Kidney_1610
Testis_1610
reads per million genome matching
0
5
10
15
20
25
30
bp position
0
10
20
30
40
50
60
70

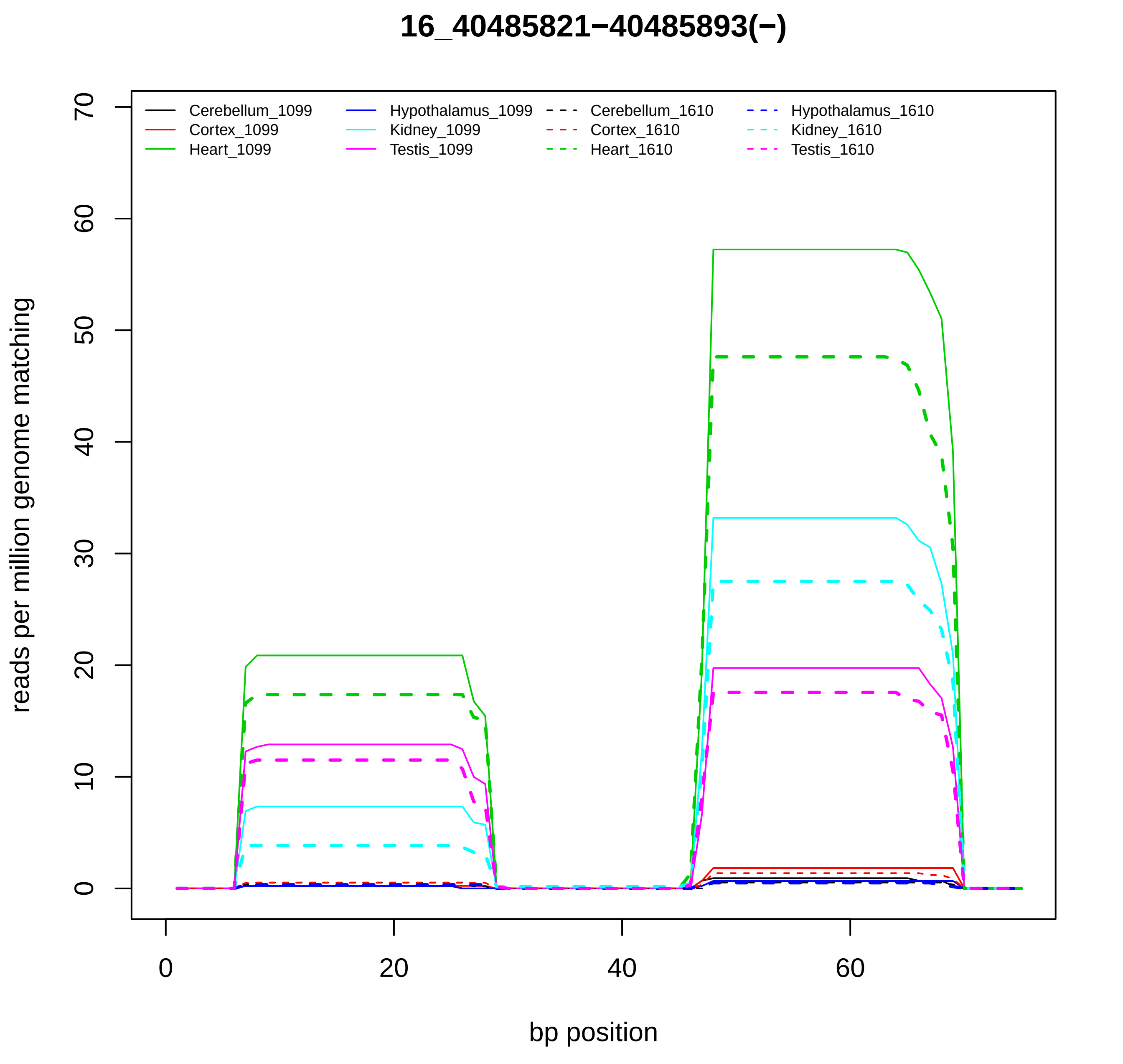

16_40485821−40485893(−)
Cerebellum_1099
Cortex_1099
Heart_1099
Hypothalamus_1099
Kidney_1099
Testis_1099
Cerebellum_1610
Cortex_1610
Heart_1610
Hypothalamus_1610
Kidney_1610
Testis_1610
reads per million genome matching
bp position
0
10
20
30
40
50
60
70

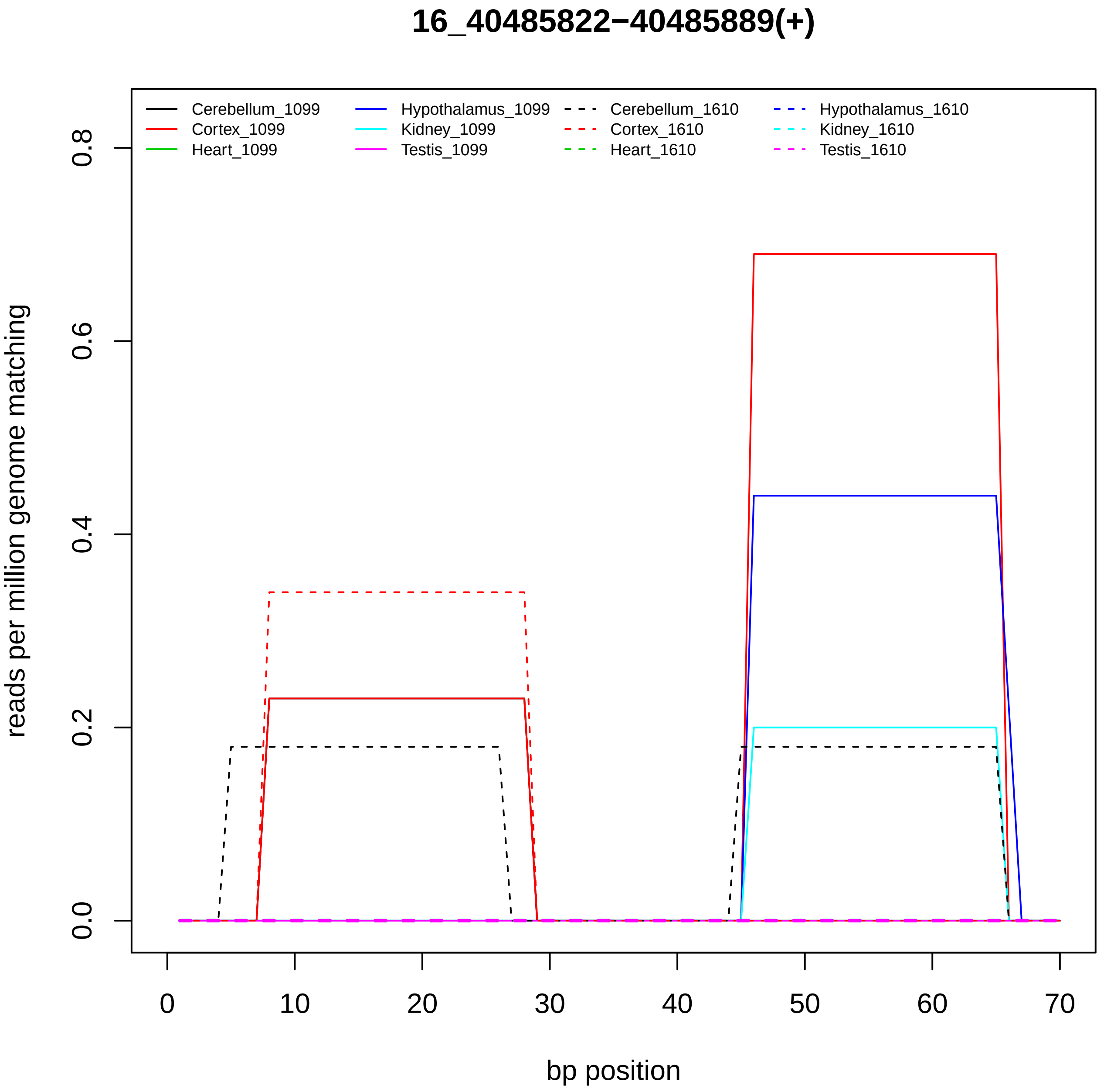
16_40485822−40485889(+)
Cerebellum_1099
Cortex_1099
Heart_1099
Hypothalamus_1099
Kidney_1099
Testis_1099
Cerebellum_1610
Cortex_1610
Heart_1610
Hypothalamus_1610
Kidney_1610
Testis_1610
reads per million genome matching
0.0
0.2
0.4
0.6
0.8
0
10
20
30
40
50
60
70
bp position

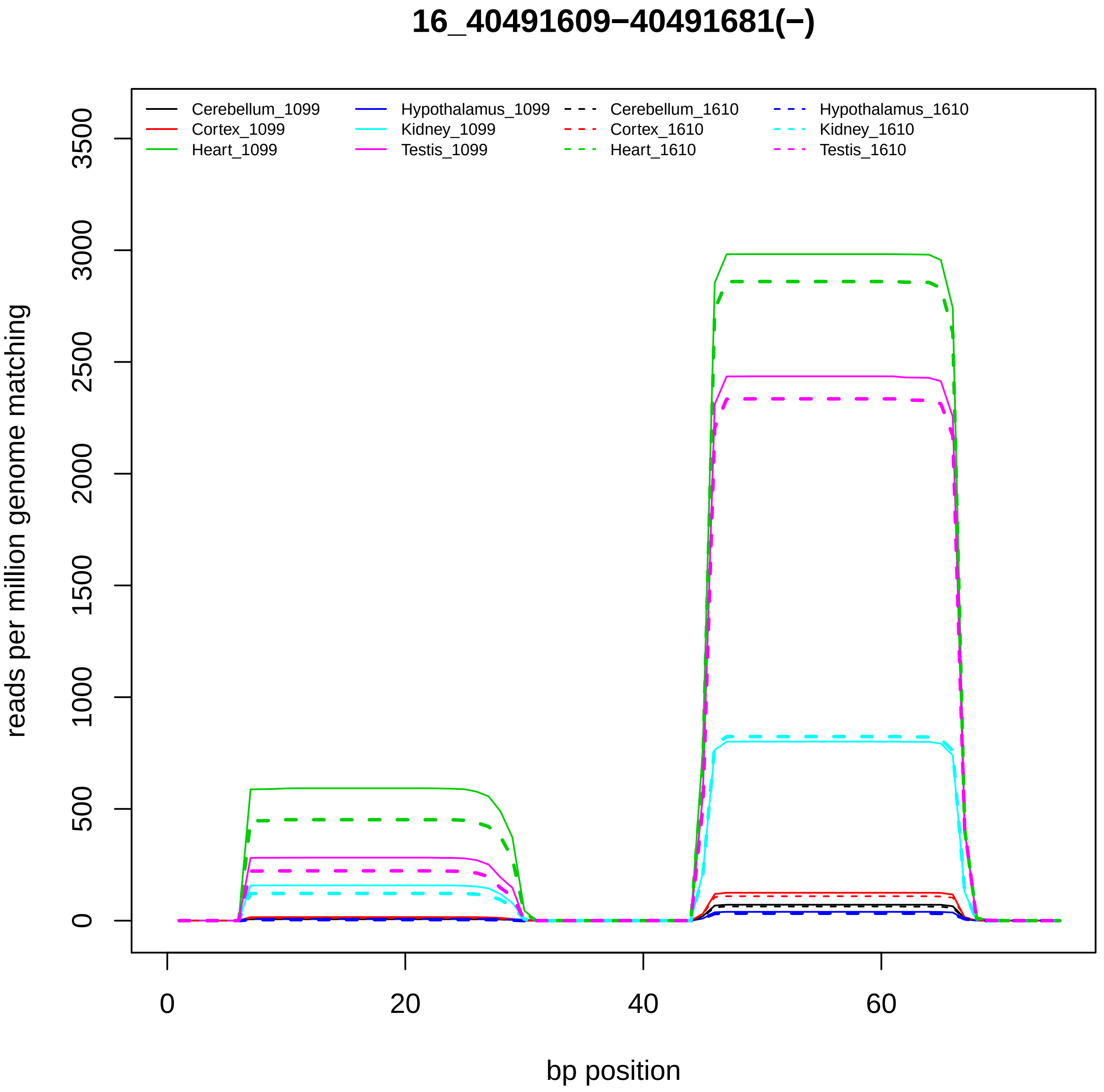

16_40491609−40491681(−)
Cerebellum_1099
Cortex_1099
Heart_1099
Hypothalamus_1099
Kidney_1099
Testis_1099
Cerebellum_1610
Cortex_1610
Heart_1610
Hypothalamus_1610
Kidney_1610
Testis_1610
reads per million genome matching
0
500
1000
1500
2000
2500
3000
3500
0
20
40
60
bp position

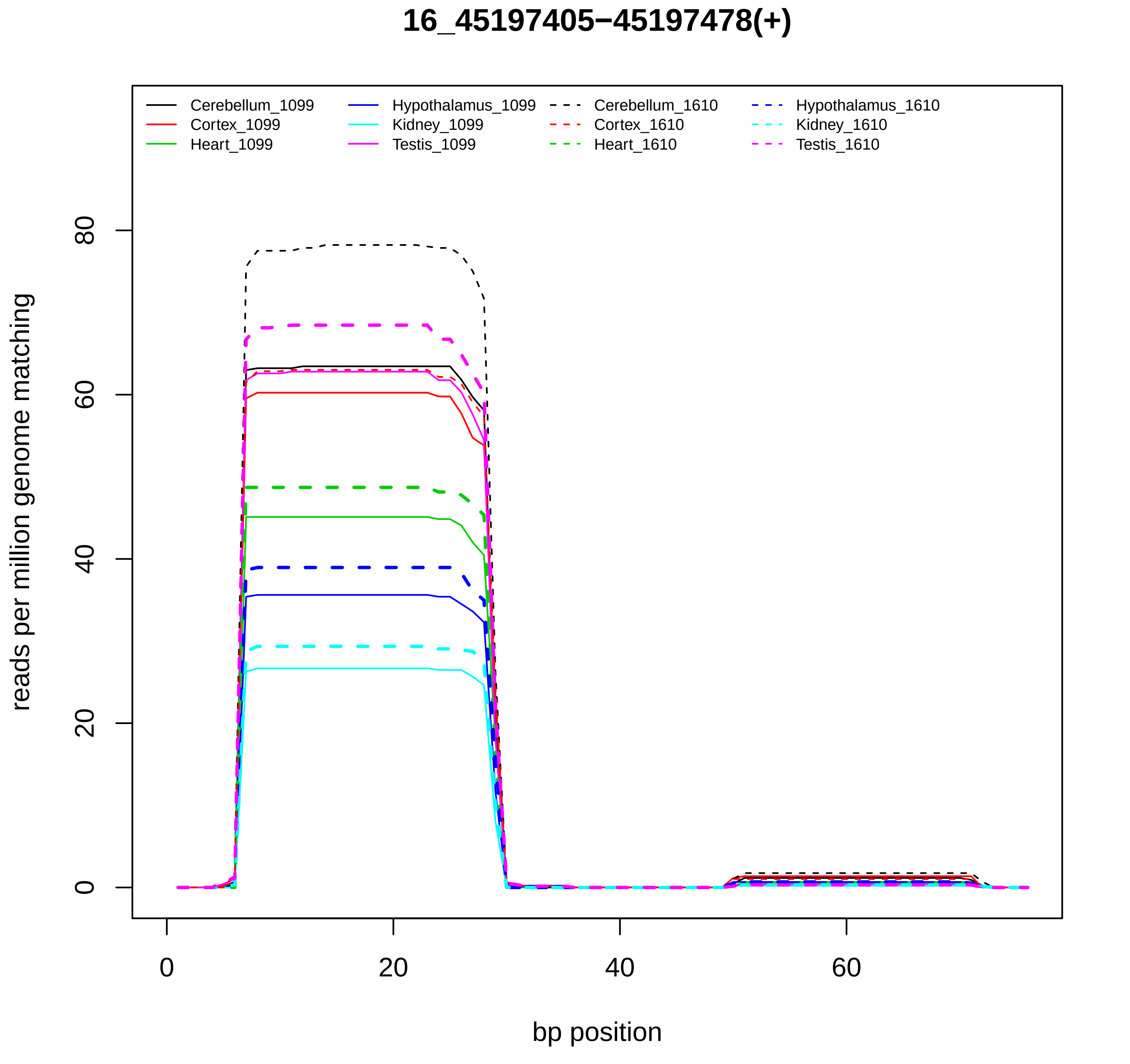
16_45197405−45197478(+)
Cerebellum_1099
Cortex_1099
Heart_1099
Hypothalamus_1099
Kidney_1099
Testis_1099
Cerebellum_1610
Cortex_1610
Heart_1610
Hypothalamus_1610
Kidney_1610
Testis_1610
reads per million genome matching
bp position
0
20
40
60
80
0
20
40
60

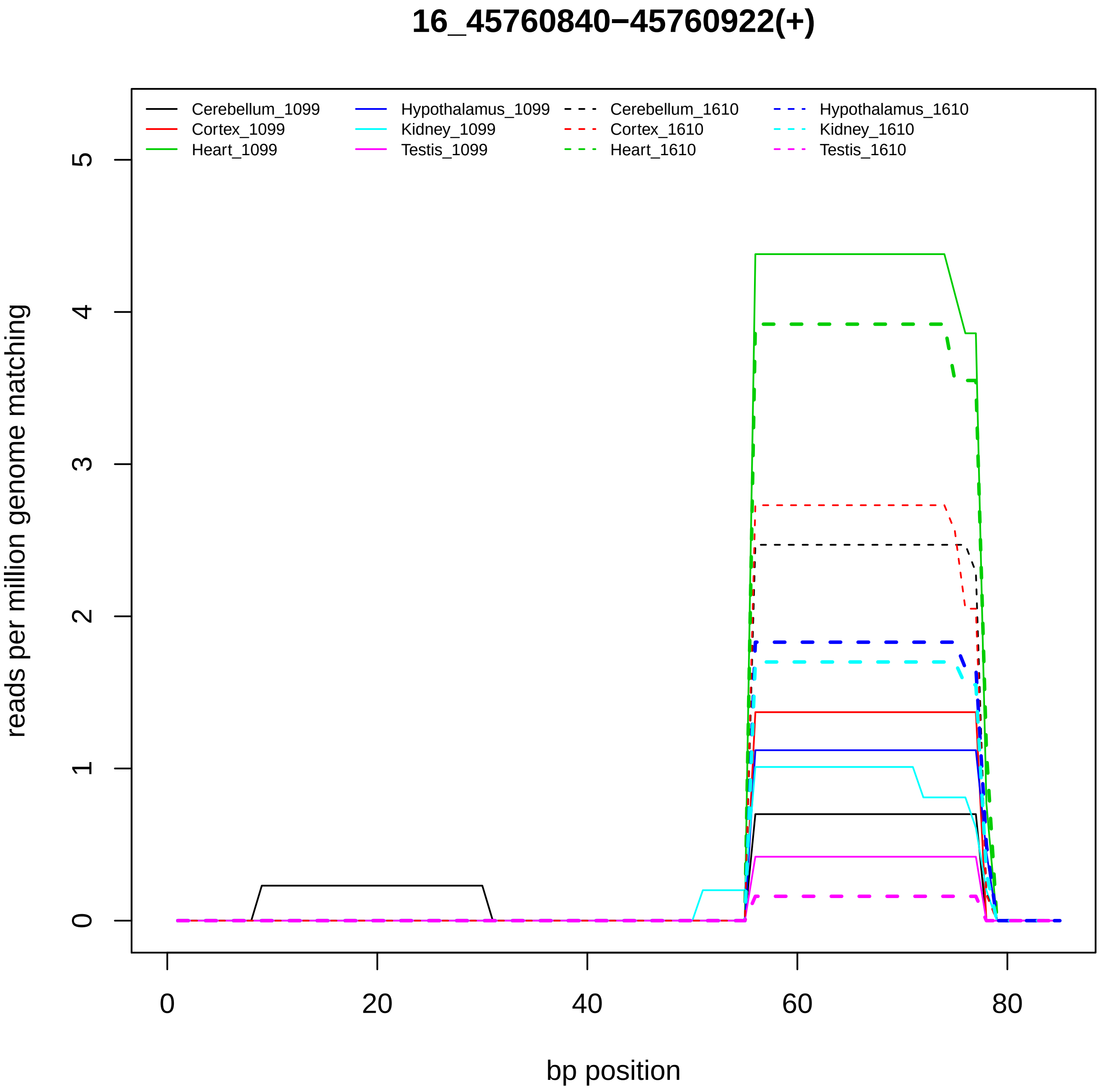

16_45760840−45760922(+)
Cerebellum_1099
Cortex_1099
Heart_1099
Hypothalamus_1099
Kidney_1099
Testis_1099
Cerebellum_1610
Cortex_1610
Heart_1610
Hypothalamus_1610
Kidney_1610
Testis_1610
reads per million genome matching
0
1
2
3
4
5
0
20
40
60
80
bp position

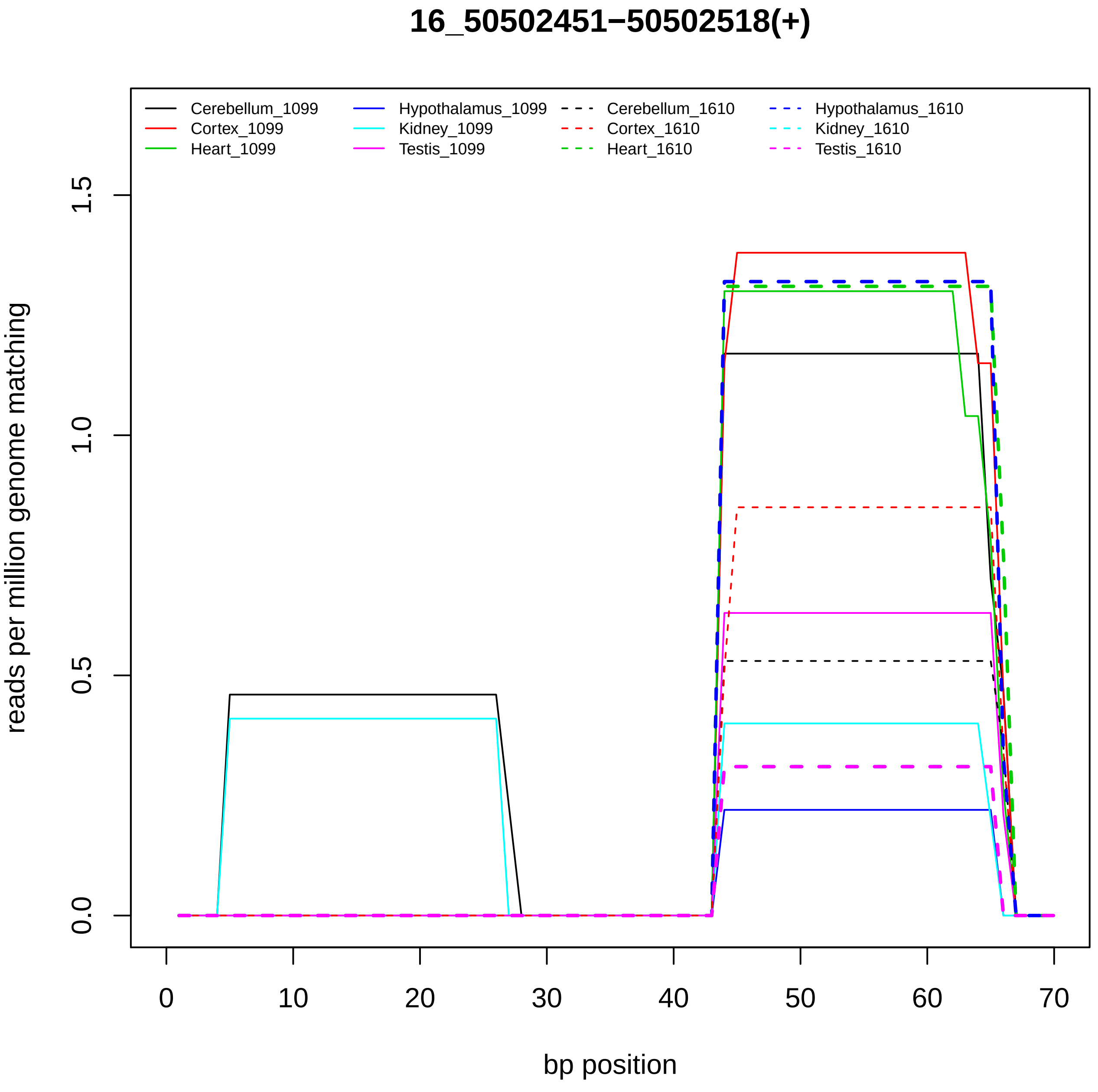

16_50502451−50502518(+)
Cerebellum_1099
Cortex_1099
Heart_1099
Hypothalamus_1099
Kidney_1099
Testis_1099
Cerebellum_1610
Cortex_1610
Heart_1610
Hypothalamus_1610
Kidney_1610
Testis_1610
reads per million genome matching
0.0
0.5
1.0
1.5
0
10
20
30
40
50
60
70
bp position

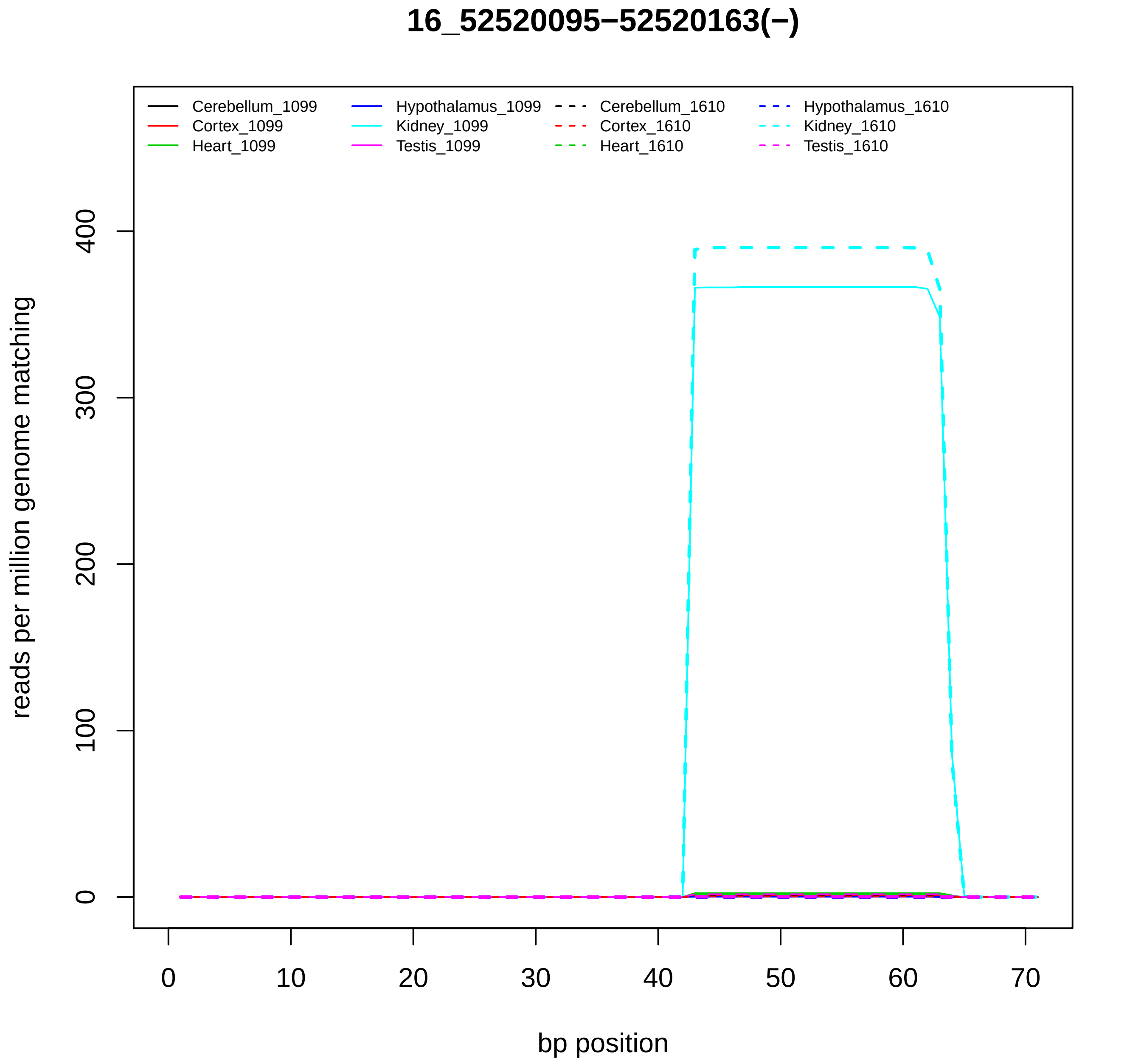

16_52520095−52520163(−)
Cerebellum_1099
Cortex_1099
Heart_1099
Hypothalamus_1099
Kidney_1099
Testis_1099
Cerebellum_1610
Cortex_1610
Heart_1610
Hypothalamus_1610
Kidney_1610
Testis_1610
reads per million genome matching
0
100
200
300
400
0
10
20
30
40
50
60
70
bp position

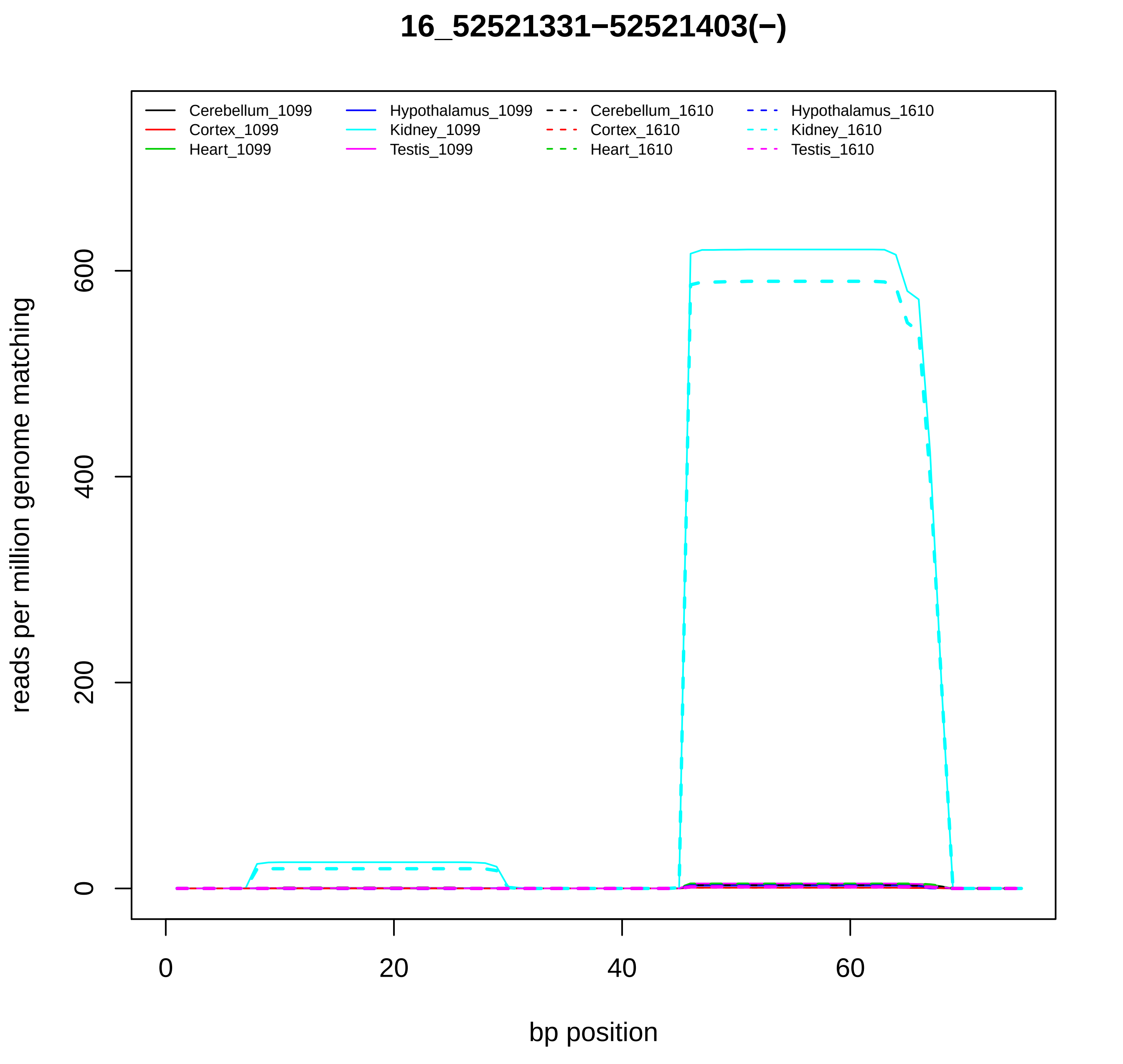

16_52521331−52521403(−)
Cerebellum_1099
Cortex_1099
Heart_1099
Hypothalamus_1099
Kidney_1099
Testis_1099
Cerebellum_1610
Cortex_1610
Heart_1610
Hypothalamus_1610
Kidney_1610
Testis_1610
reads per million genome matching
0
200
400
600
0
20
40
60
bp position

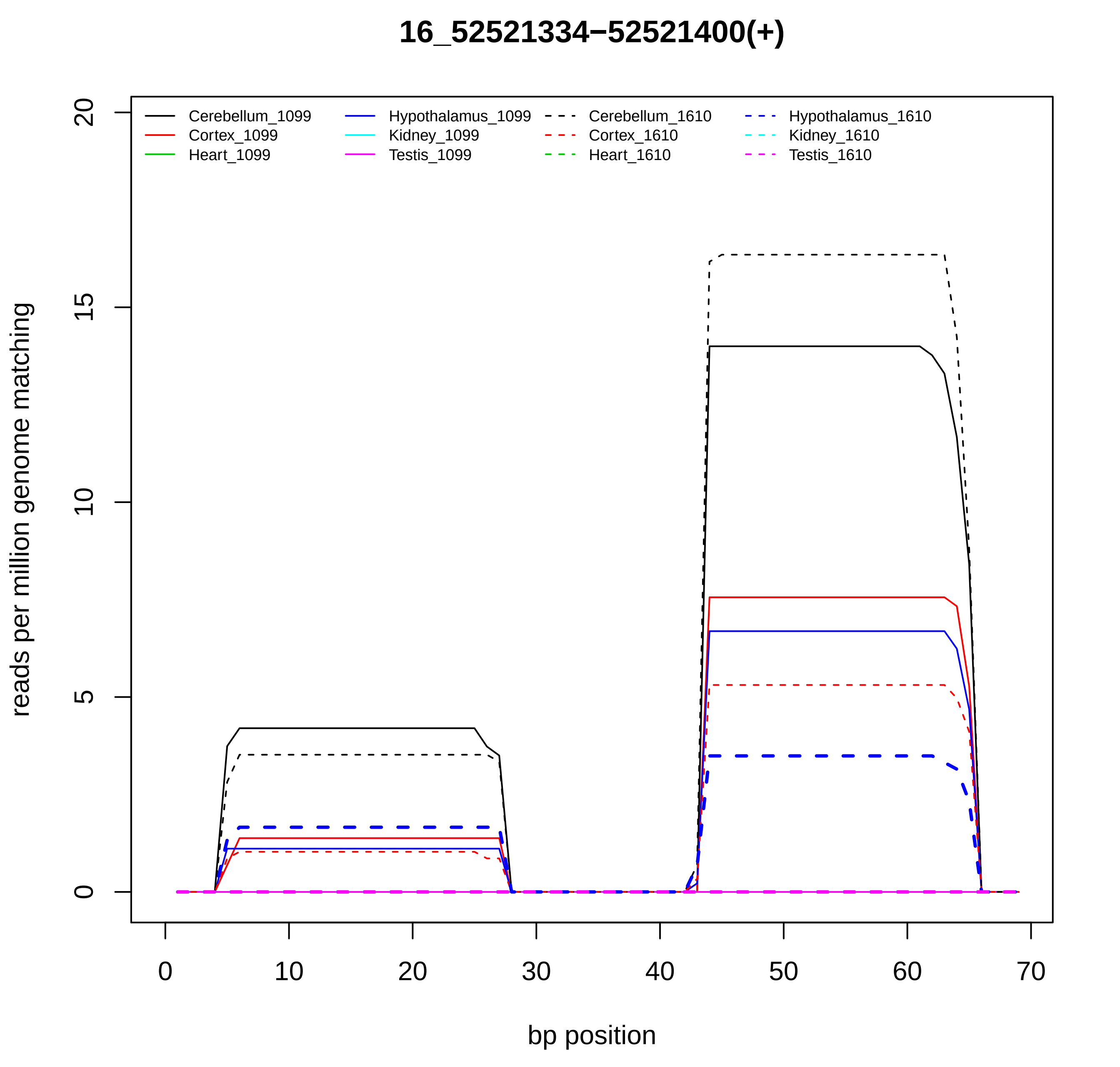

16_52521334−52521400(+)
Cerebellum_1099
Cortex_1099
Heart_1099
Hypothalamus_1099
Kidney_1099
Testis_1099
Cerebellum_1610
Cortex_1610
Heart_1610
Hypothalamus_1610
Kidney_1610
Testis_1610
reads per million genome matching
0
5
10
15
20
0
10
20
30
40
50
60
70
bp position

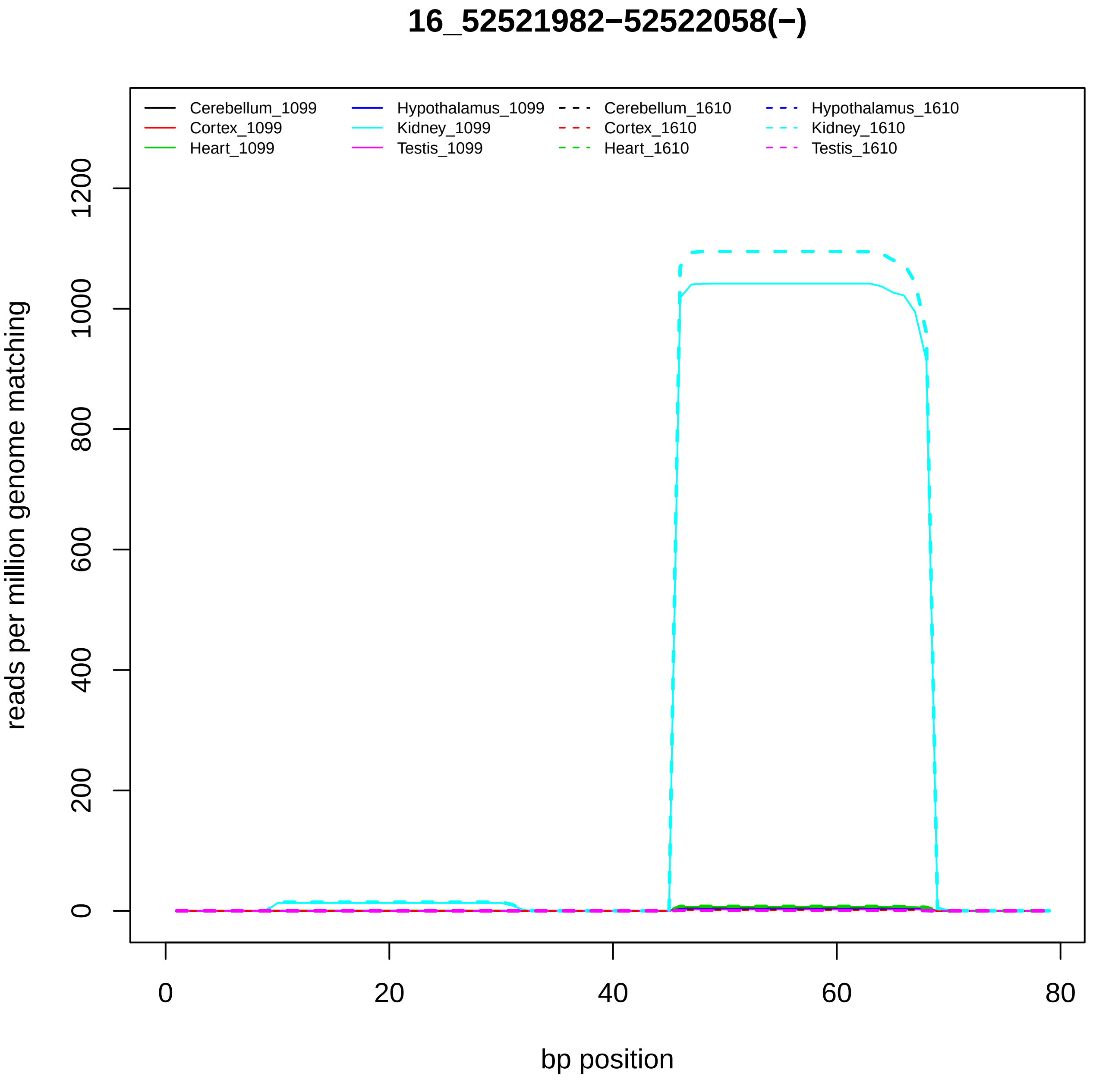
16_52521982−52522058(−)
Cerebellum_1099
Cortex_1099
Heart_1099
Hypothalamus_1099
Kidney_1099
Testis_1099
Cerebellum_1610
Cortex_1610
Heart_1610
Hypothalamus_1610
Kidney_1610
Testis_1610
reads per million genome matching
0
200
400
600
800
1000
1200
0
20
40
60
80
bp position

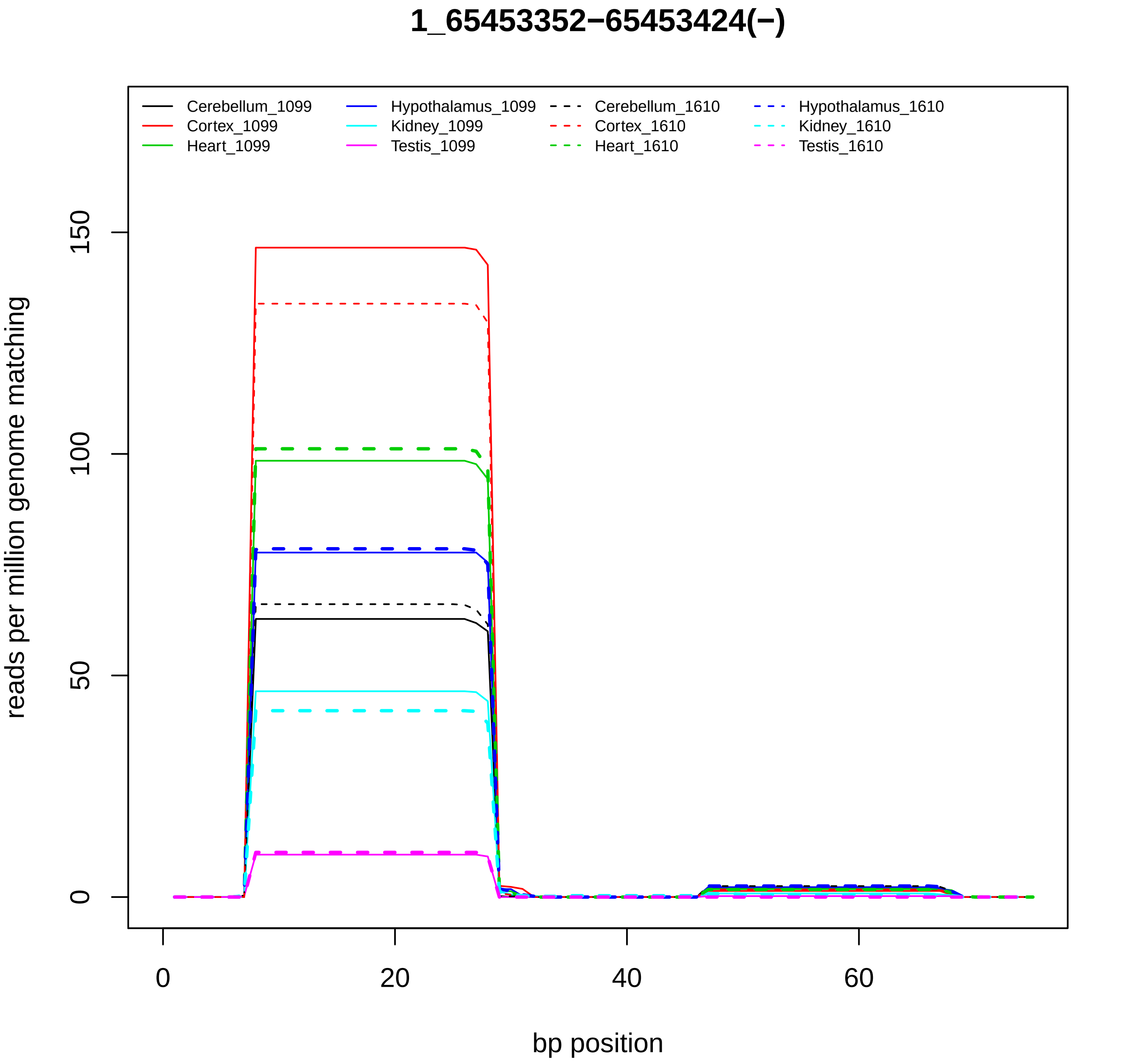

1_65453352−65453424(−)
Cerebellum_1099
Cortex_1099
Heart_1099
Hypothalamus_1099
Kidney_1099
Testis_1099
Cerebellum_1610
Cortex_1610
Heart_1610
Hypothalamus_1610
Kidney_1610
Testis_1610
reads per million genome matching
bp position
0
50
100
150
0
20
40
60

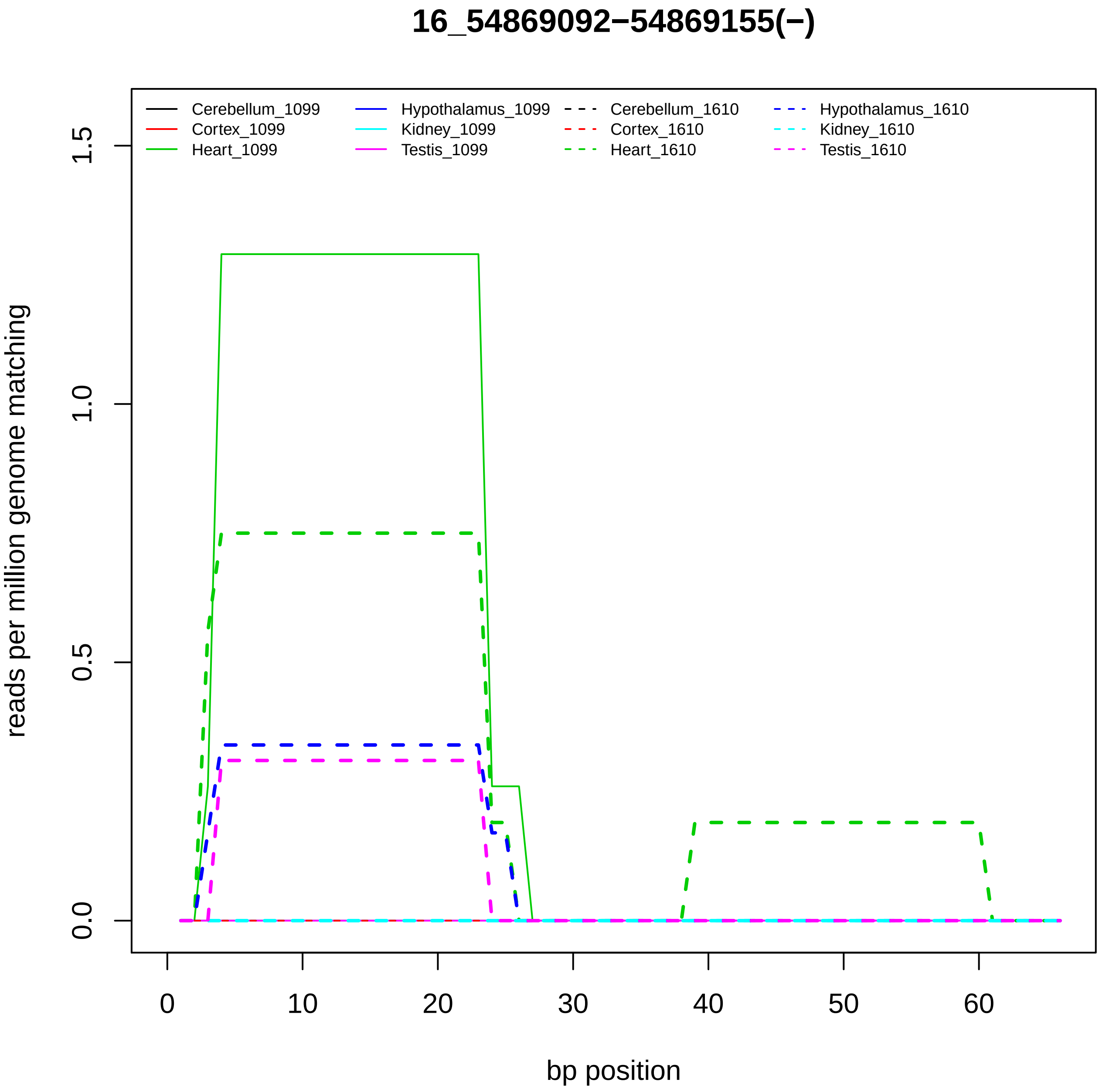

16_54869092−54869155(−)
Cerebellum_1099
Cortex_1099
Heart_1099
Hypothalamus_1099
Kidney_1099
Testis_1099
Cerebellum_1610
Cortex_1610
Heart_1610
Hypothalamus_1610
Kidney_1610
Testis_1610
reads per million genome matching
0.0
0.5
1.0
1.5
0
10
20
30
40
50
60
bp position

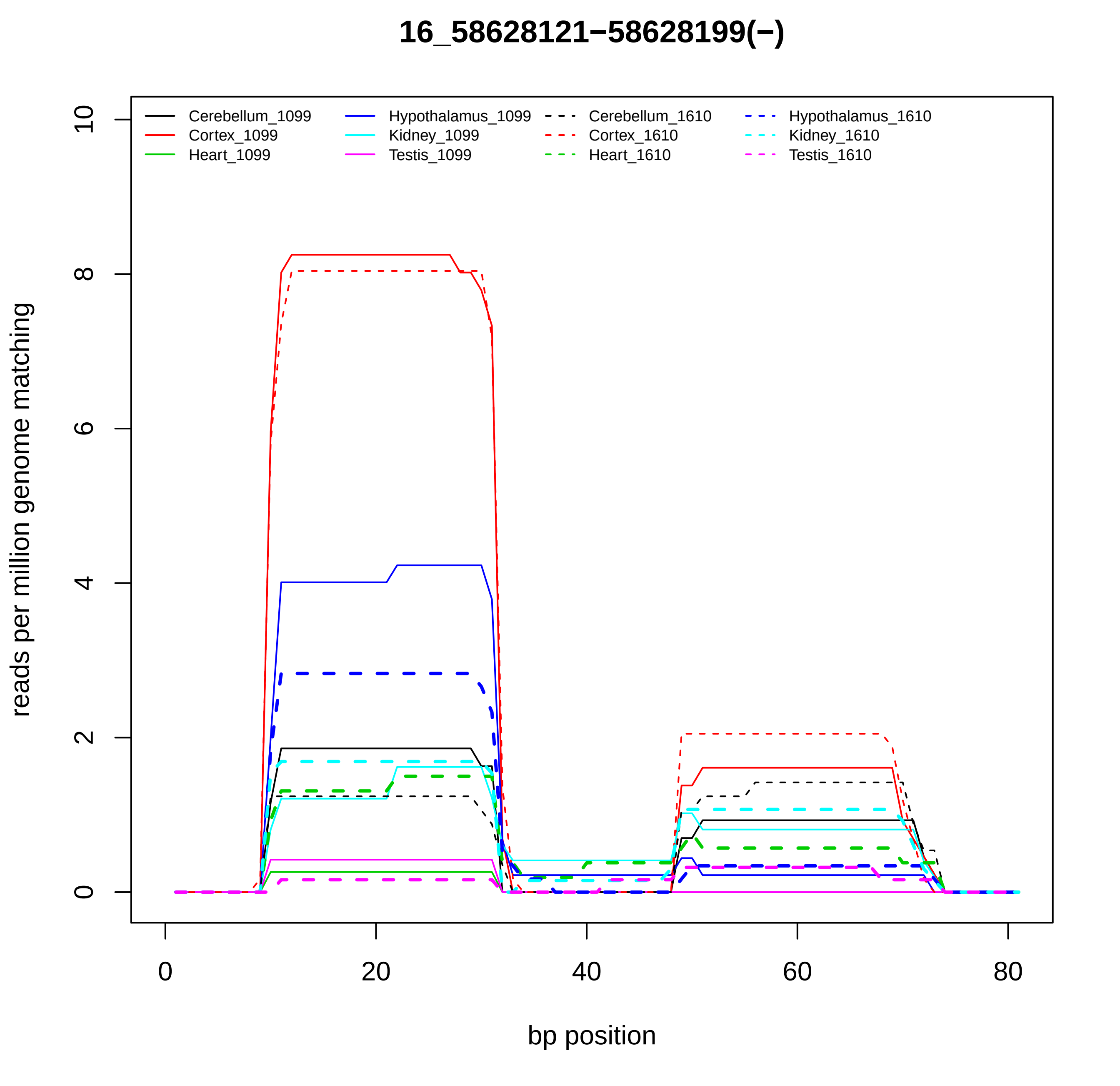

16_58628121−58628199(−)
Cerebellum_1099
Cortex_1099
Heart_1099
Hypothalamus_1099
Kidney_1099
Testis_1099
Cerebellum_1610
Cortex_1610
Heart_1610
Hypothalamus_1610
Kidney_1610
Testis_1610
reads per million genome matching
bp position
0
2
4
6
8
10
20
40
60
80

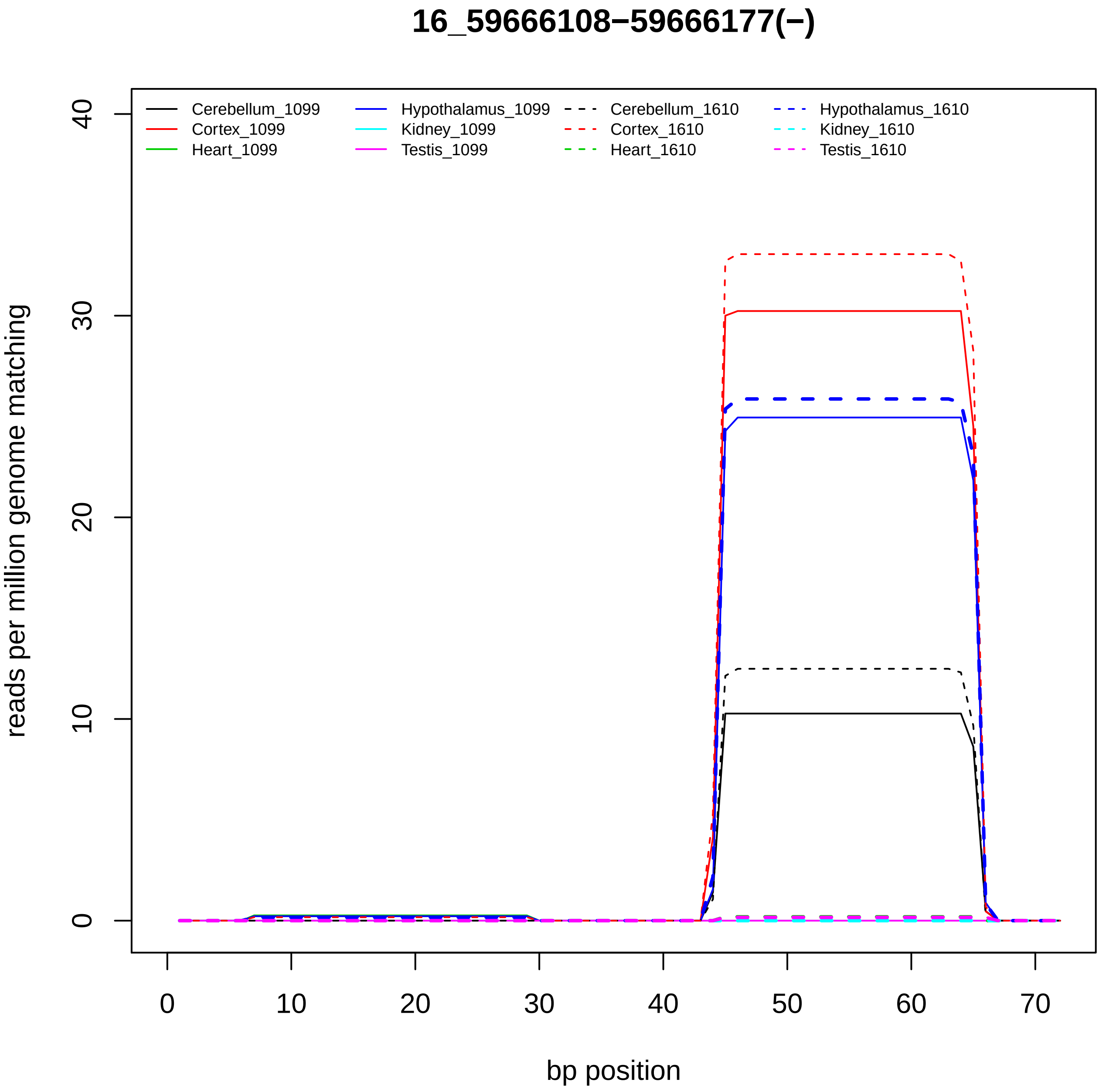

16_59666108−59666177(−)
Cerebellum_1099
Cortex_1099
Heart_1099
Hypothalamus_1099
Kidney_1099
Testis_1099
Cerebellum_1610
Cortex_1610
Heart_1610
Hypothalamus_1610
Kidney_1610
Testis_1610
reads per million genome matching
bp position
0
10
20
30
40
50
60
70

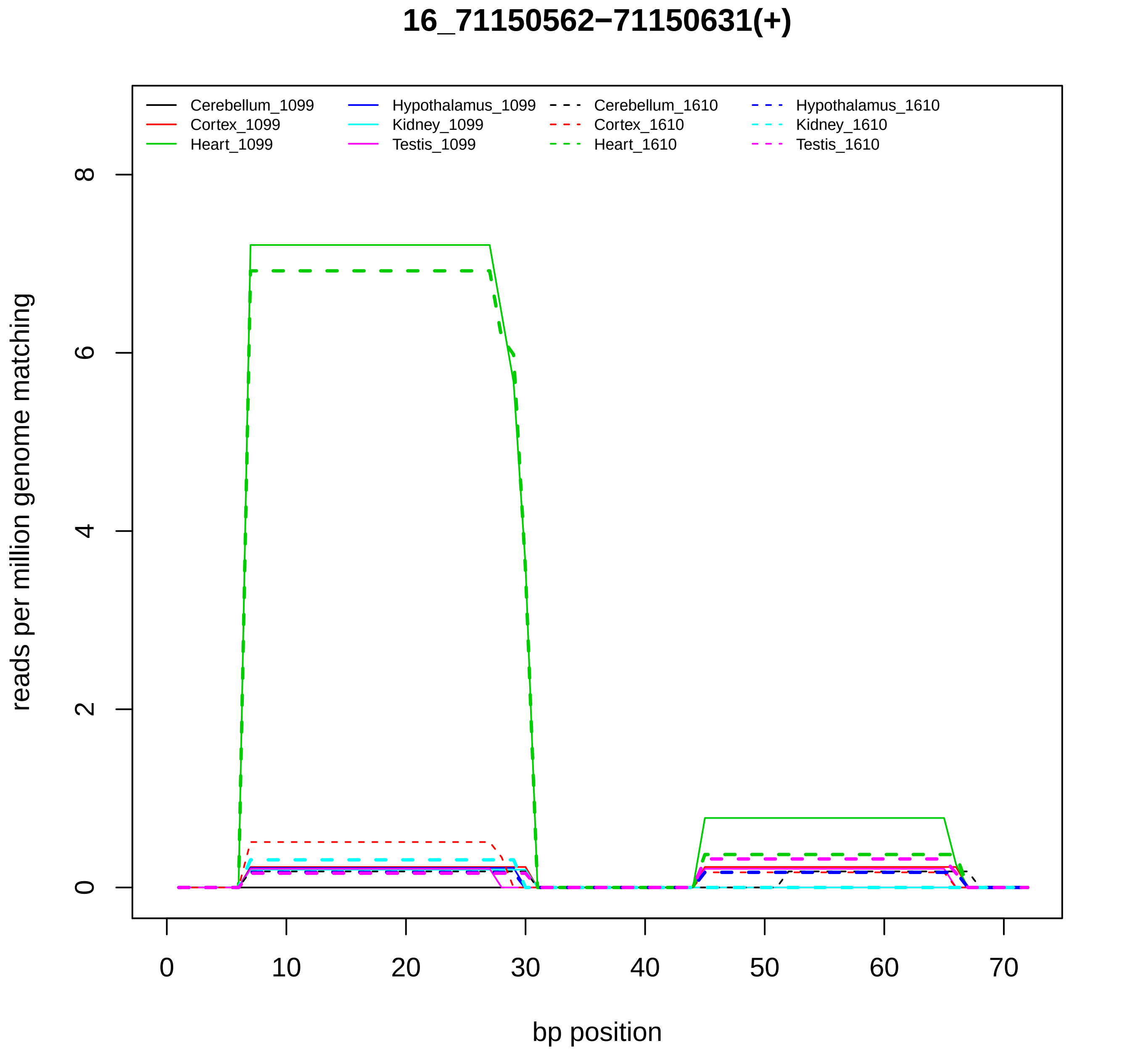

16_71150562−71150631(+)
Cerebellum_1099
Cortex_1099
Heart_1099
Hypothalamus_1099
Kidney_1099
Testis_1099
Cerebellum_1610
Cortex_1610
Heart_1610
Hypothalamus_1610
Kidney_1610
Testis_1610
reads per million genome matching
bp position
0
2
4
6
8
0
10
20
30
40
50
60
70

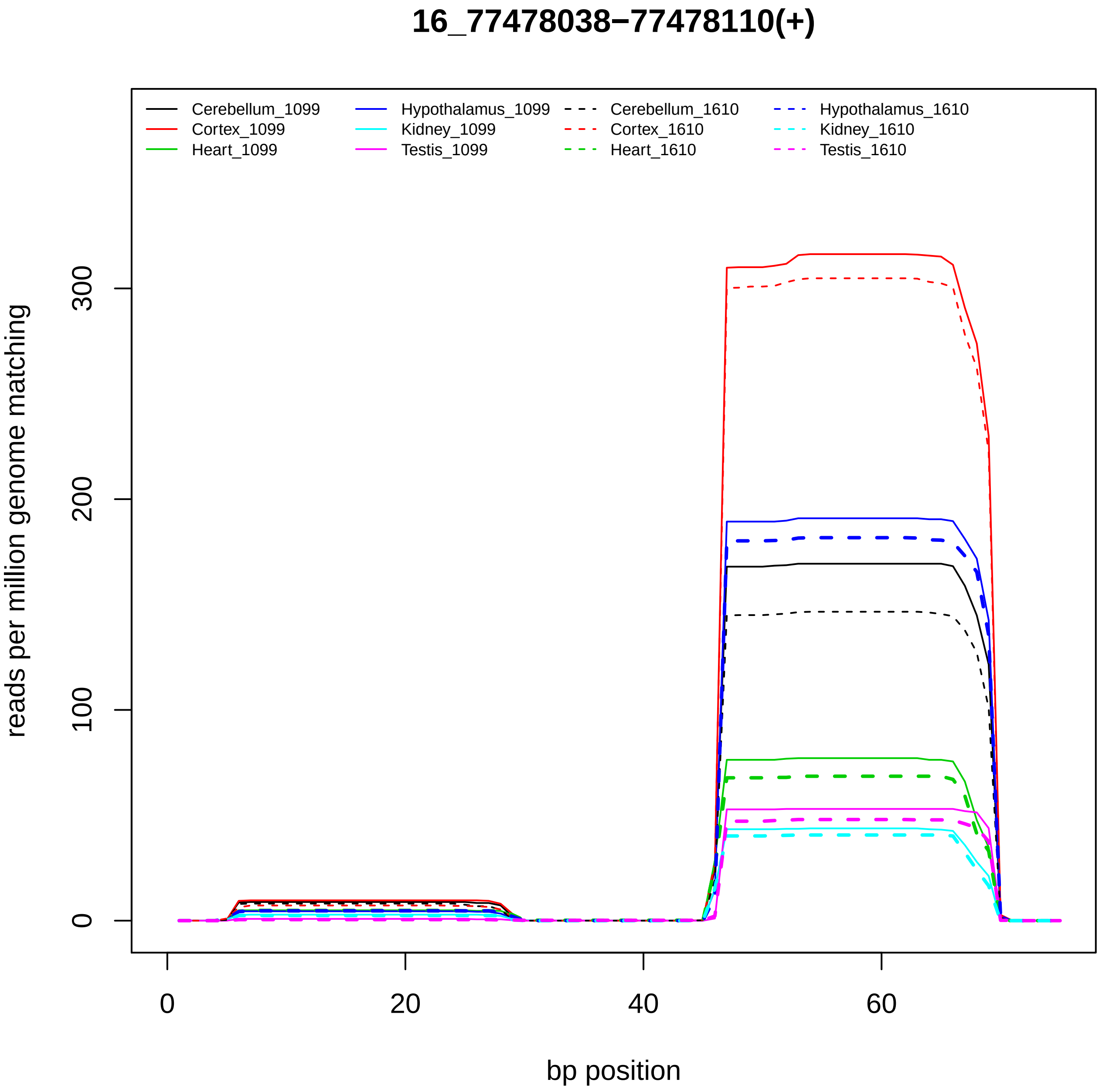

16_77478038−77478110(+)
Cerebellum_1099
Cortex_1099
Heart_1099
Hypothalamus_1099
Kidney_1099
Testis_1099
Cerebellum_1610
Cortex_1610
Heart_1610
Hypothalamus_1610
Kidney_1610
Testis_1610
reads per million genome matching
0
100
200
300
0
20
40
60
bp position

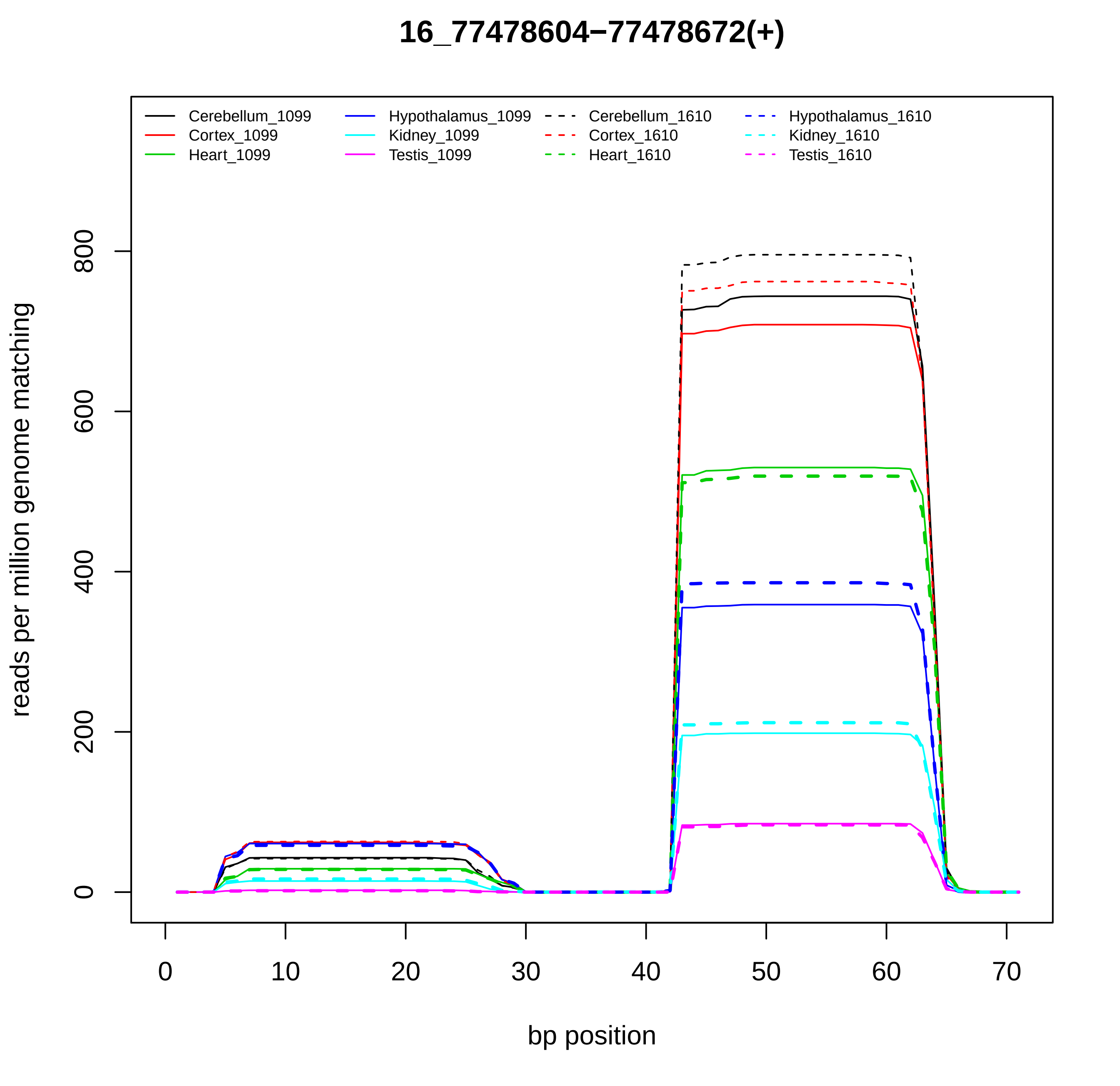
16_77478604−77478672(+)
Cerebellum_1099
Cortex_1099
Heart_1099
Hypothalamus_1099
Kidney_1099
Testis_1099
Cerebellum_1610
Cortex_1610
Heart_1610
Hypothalamus_1610
Kidney_1610
Testis_1610
reads per million genome matching
0
200
400
600
800
0
10
20
30
40
50
60
70
bp position

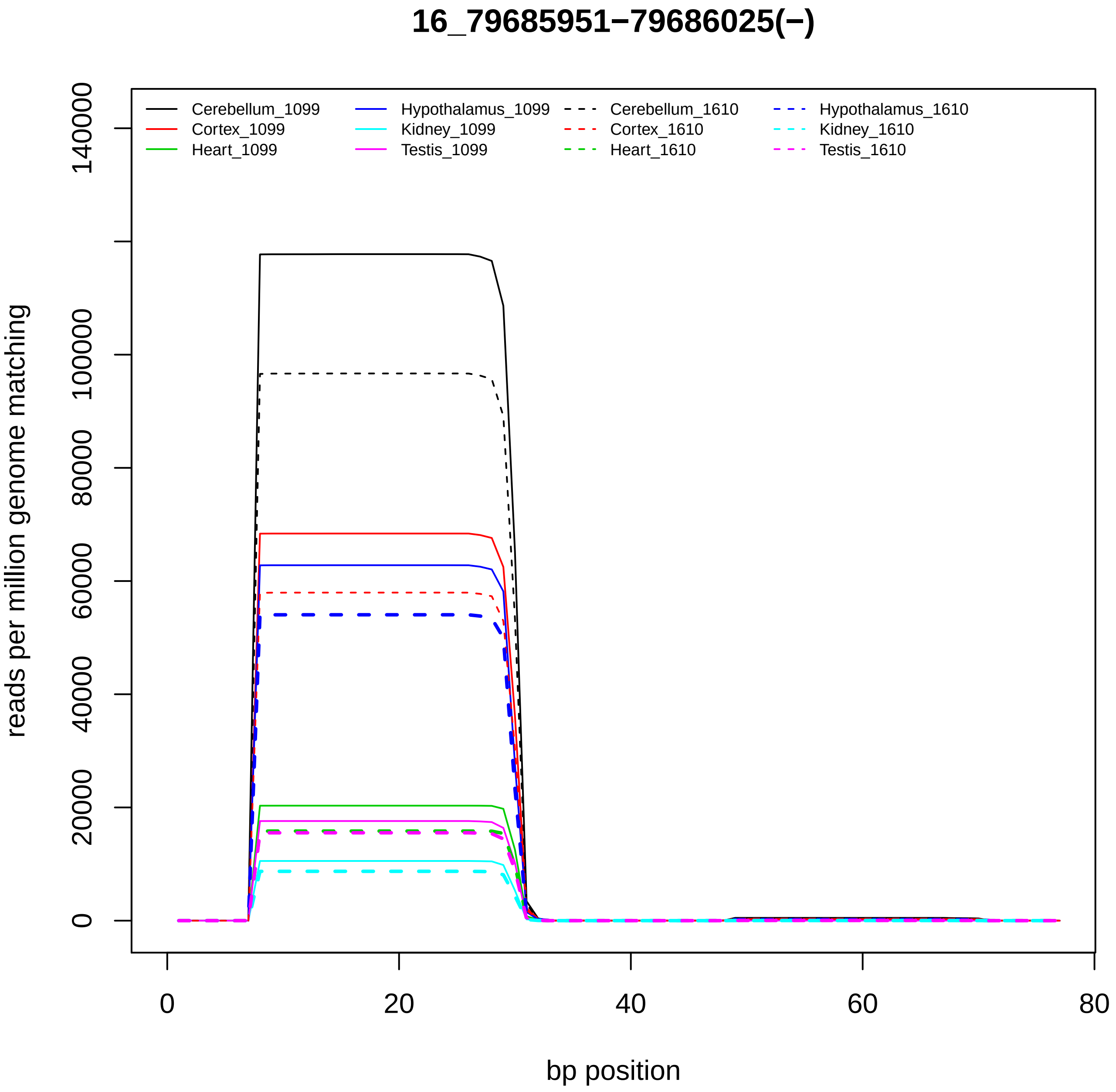

16_79685951−79686025(−)
Cerebellum_1099
Cortex_1099
Heart_1099
Hypothalamus_1099
Kidney_1099
Testis_1099
Cerebellum_1610
Cortex_1610
Heart_1610
Hypothalamus_1610
Kidney_1610
Testis_1610
reads per million genome matching
bp position
0
20000
40000
60000
80000
100000
140000
0
20
40
60
80

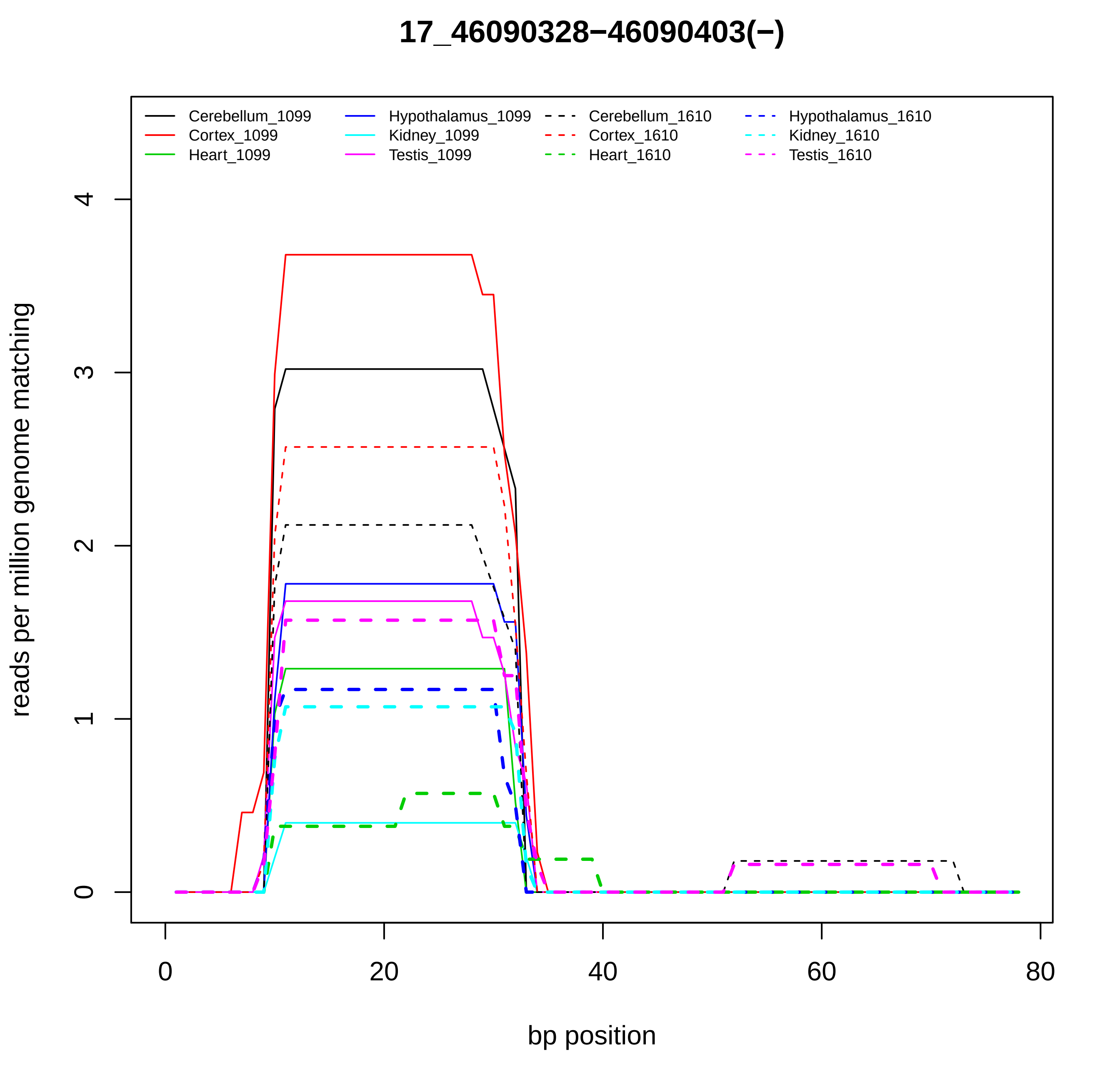

17_46090328−46090403(−)
Cerebellum_1099
Cortex_1099
Heart_1099
Hypothalamus_1099
Kidney_1099
Testis_1099
Cerebellum_1610
Cortex_1610
Heart_1610
Hypothalamus_1610
Kidney_1610
Testis_1610
reads per million genome matching
0
1
2
3
4
0
20
40
60
80
bp position

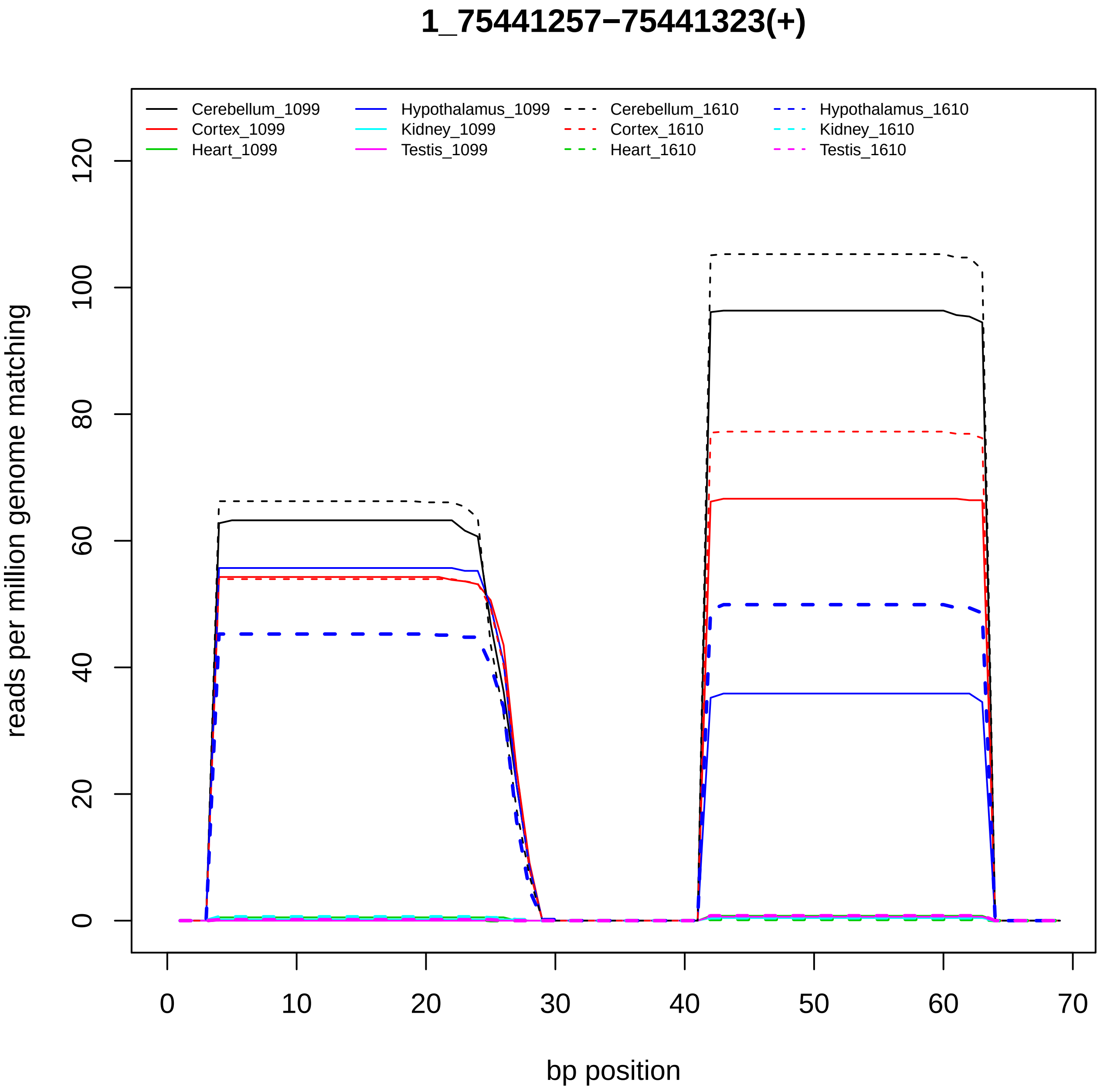
1_75441257−75441323(+)
Cerebellum_1099
Cortex_1099
Heart_1099
Hypothalamus_1099
Kidney_1099
Testis_1099
Cerebellum_1610
Cortex_1610
Heart_1610
Hypothalamus_1610
Kidney_1610
Testis_1610
reads per million genome matching
bp position
0
20
40
60
80
100
120
0
10
20
30
40
50
60
70

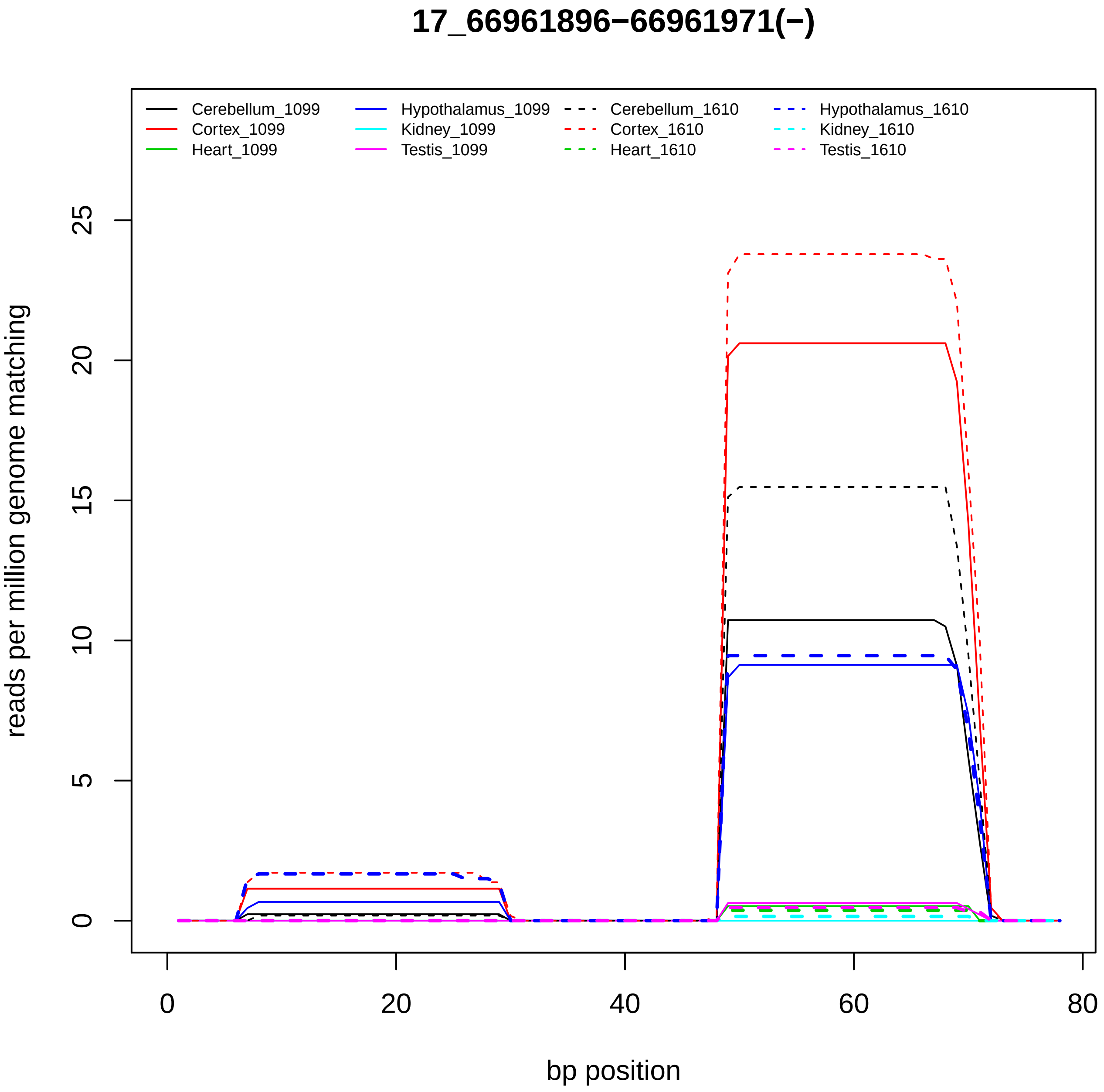
17_66961896−66961971(−)
Cerebellum_1099
Cortex_1099
Heart_1099
Hypothalamus_1099
Kidney_1099
Testis_1099
Cerebellum_1610
Cortex_1610
Heart_1610
Hypothalamus_1610
Kidney_1610
Testis_1610
reads per million genome matching
0
5
10
15
20
25
0
20
40
60
80
bp position

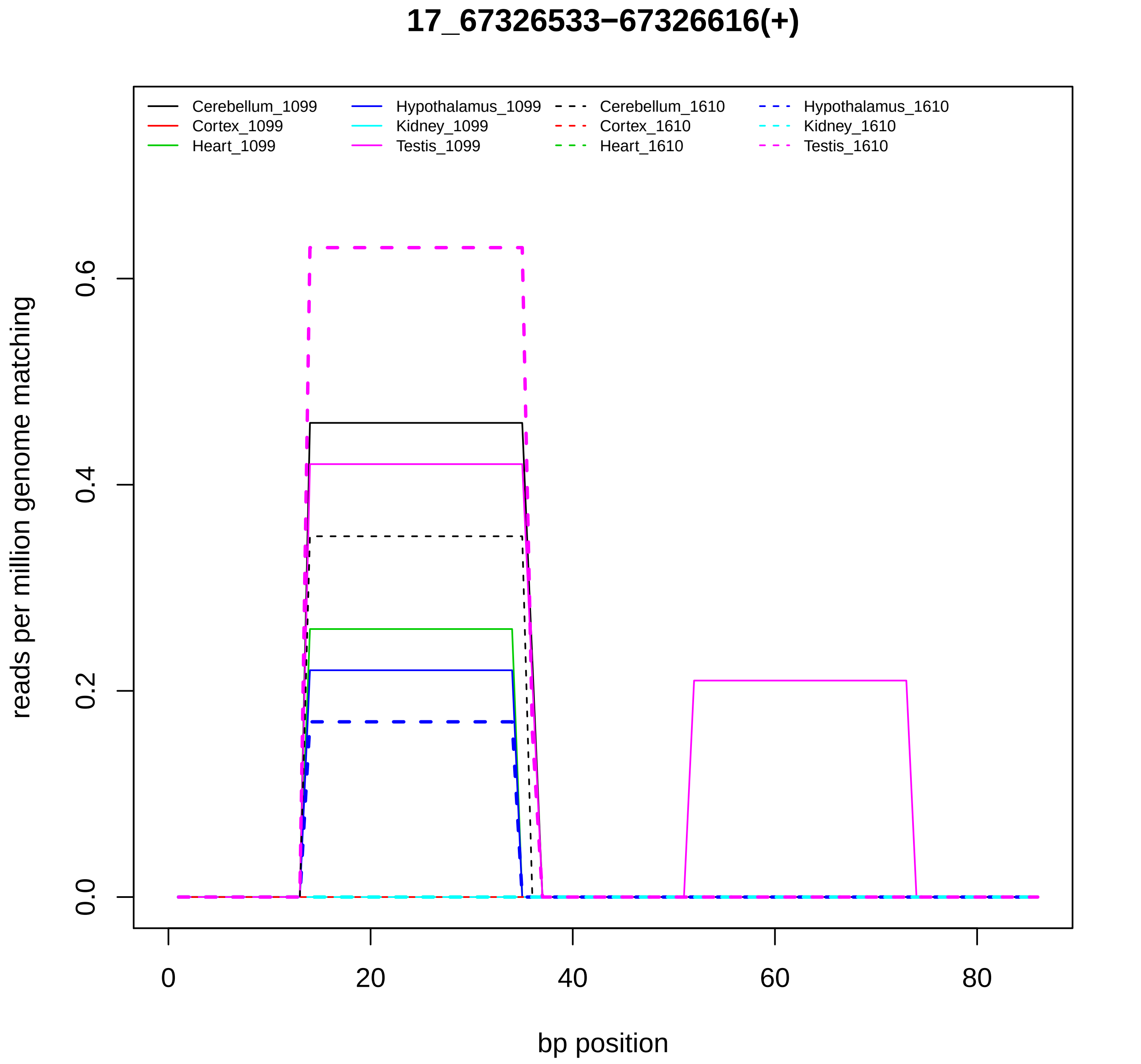

17_67326533−67326616(+)
Cerebellum_1099
Cortex_1099
Heart_1099
Hypothalamus_1099
Kidney_1099
Testis_1099
Cerebellum_1610
Cortex_1610
Heart_1610
Hypothalamus_1610
Kidney_1610
Testis_1610
reads per million genome matching
0.0
0.2
0.4
0.6
0
20
40
60
80
bp position

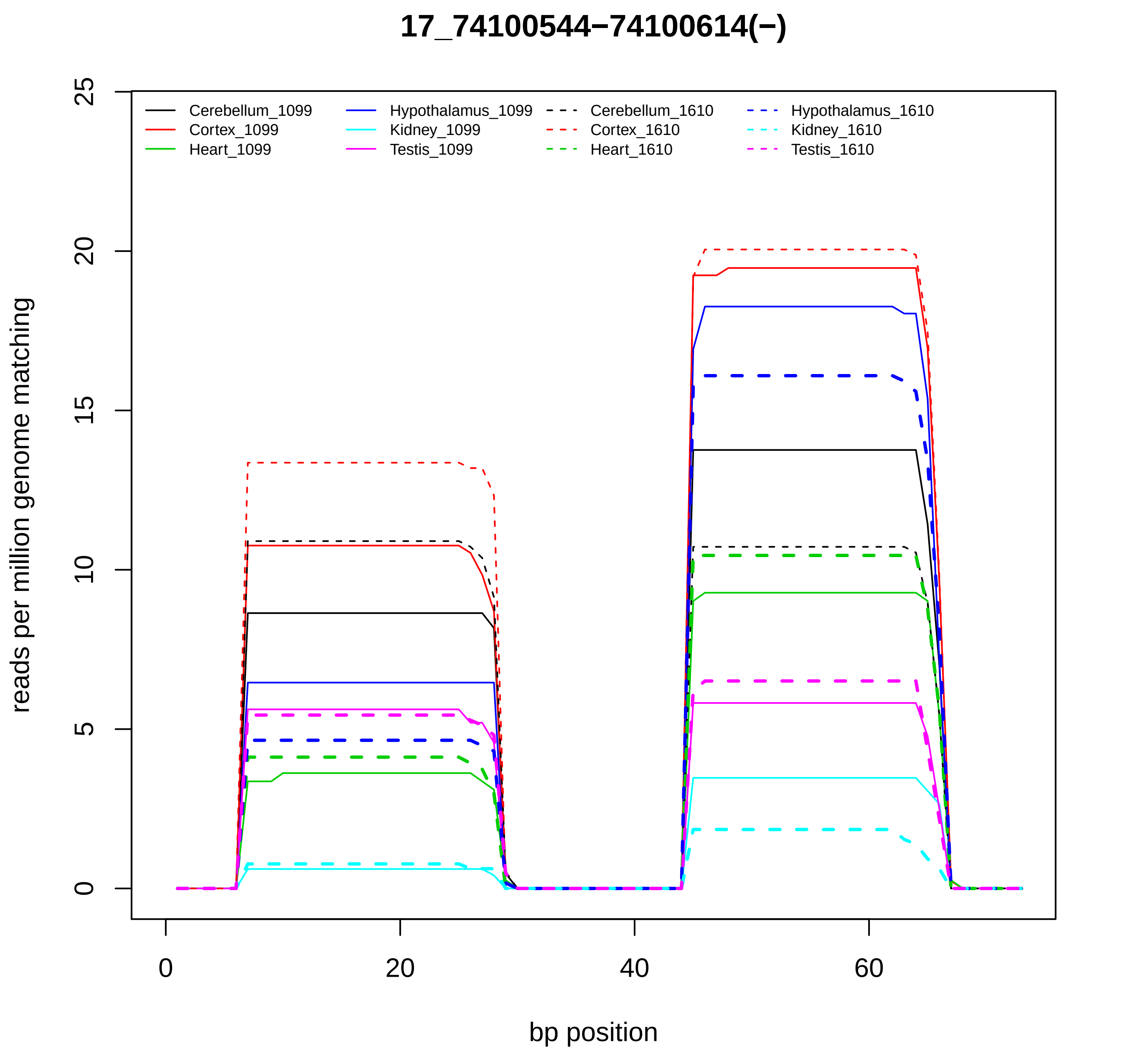

17_74100544−74100614(−)
Cerebellum_1099
Cortex_1099
Heart_1099
Hypothalamus_1099
Kidney_1099
Testis_1099
Cerebellum_1610
Cortex_1610
Heart_1610
Hypothalamus_1610
Kidney_1610
Testis_1610
reads per million genome matching
bp position
0
5
10
15
20
25
0
20
40
60

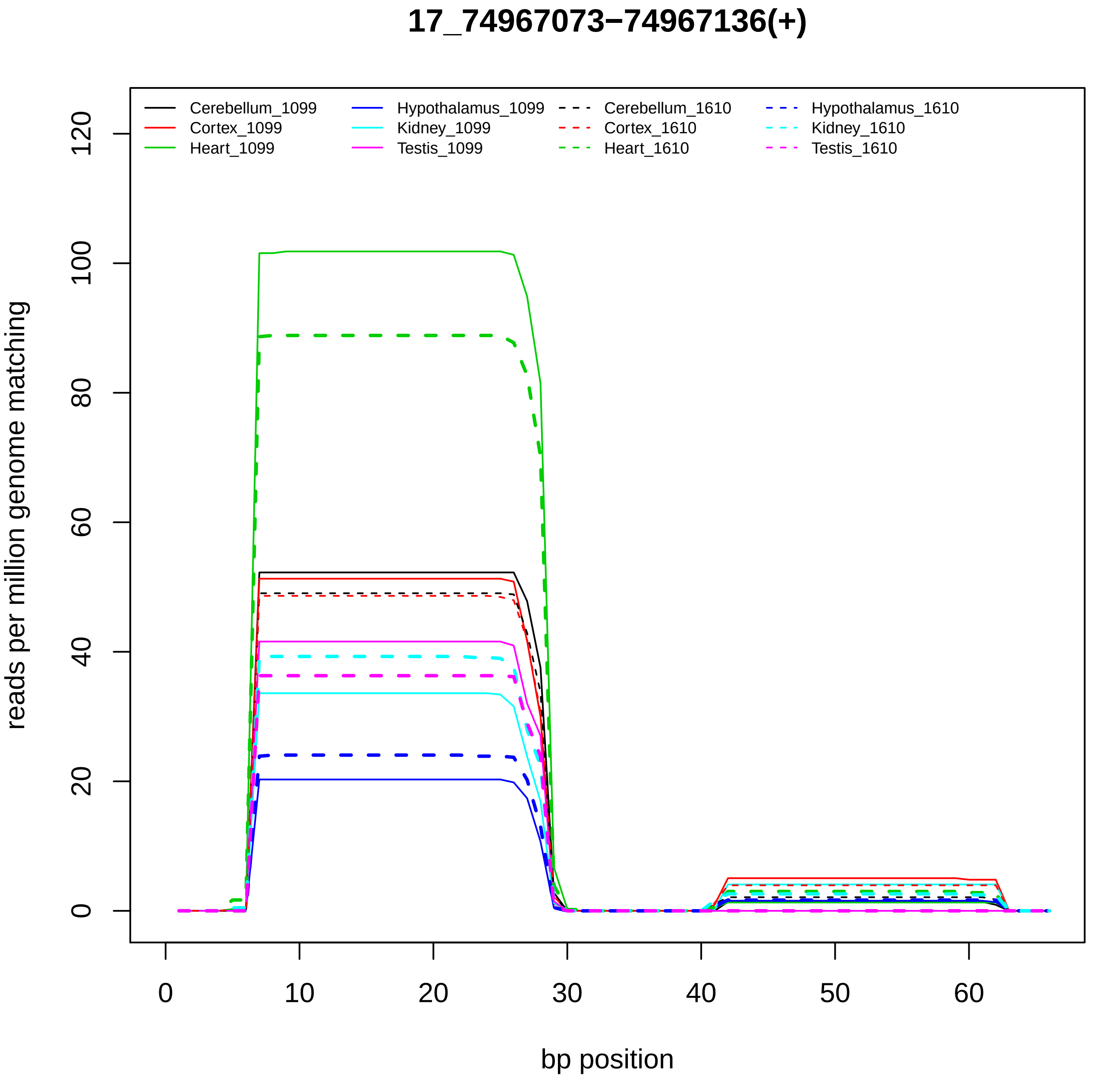

17_74967073−74967136(+)
Cerebellum_1099
Cortex_1099
Heart_1099
Hypothalamus_1099
Kidney_1099
Testis_1099
Cerebellum_1610
Cortex_1610
Heart_1610
Hypothalamus_1610
Kidney_1610
Testis_1610
reads per million genome matching
bp position
0
20
40
60
80
100
120
0
10
20
30
40
50
60

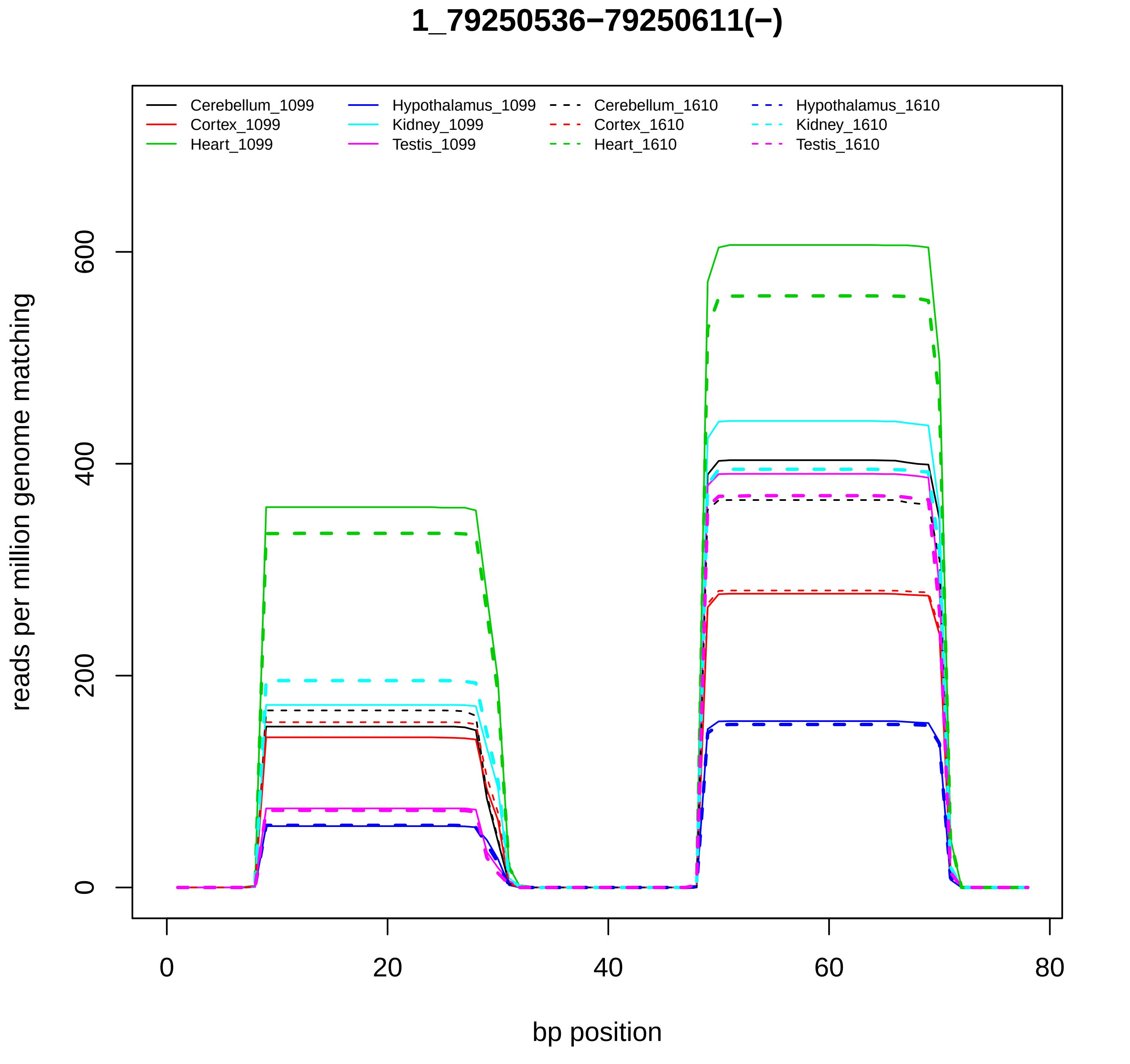
1_79250536−79250611(−)
Cerebellum_1099
Cortex_1099
Heart_1099
Hypothalamus_1099
Kidney_1099
Testis_1099
Cerebellum_1610
Cortex_1610
Heart_1610
Hypothalamus_1610
Kidney_1610
Testis_1610
reads per million genome matching
0
200
400
600
0
20
40
60
80
bp position

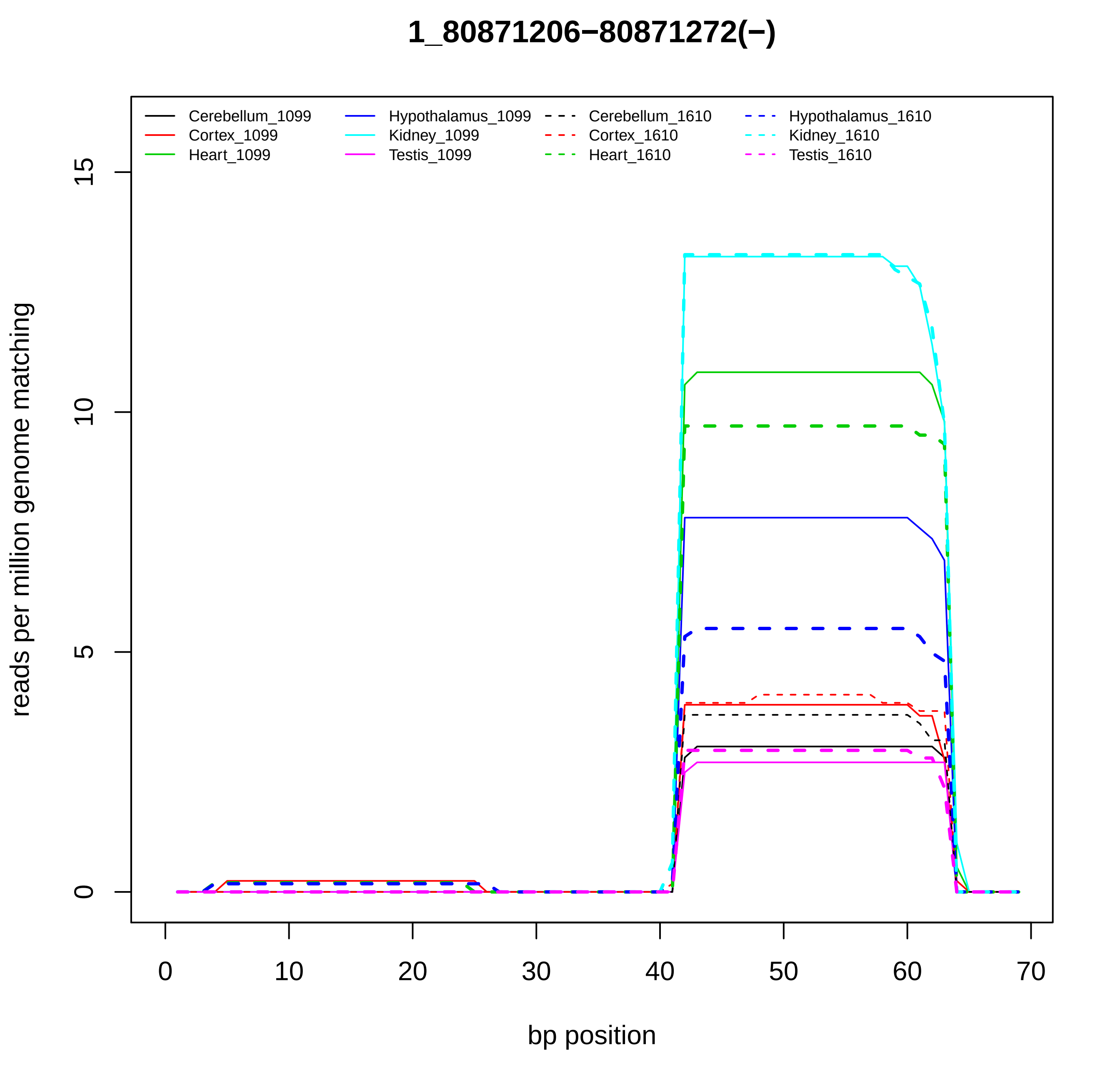

1_80871206−80871272(−)
Cerebellum_1099
Cortex_1099
Heart_1099
Hypothalamus_1099
Kidney_1099
Testis_1099
Cerebellum_1610
Cortex_1610
Heart_1610
Hypothalamus_1610
Kidney_1610
Testis_1610
reads per million genome matching
bp position
0
5
10
15
0
10
20
30
40
50
60
70

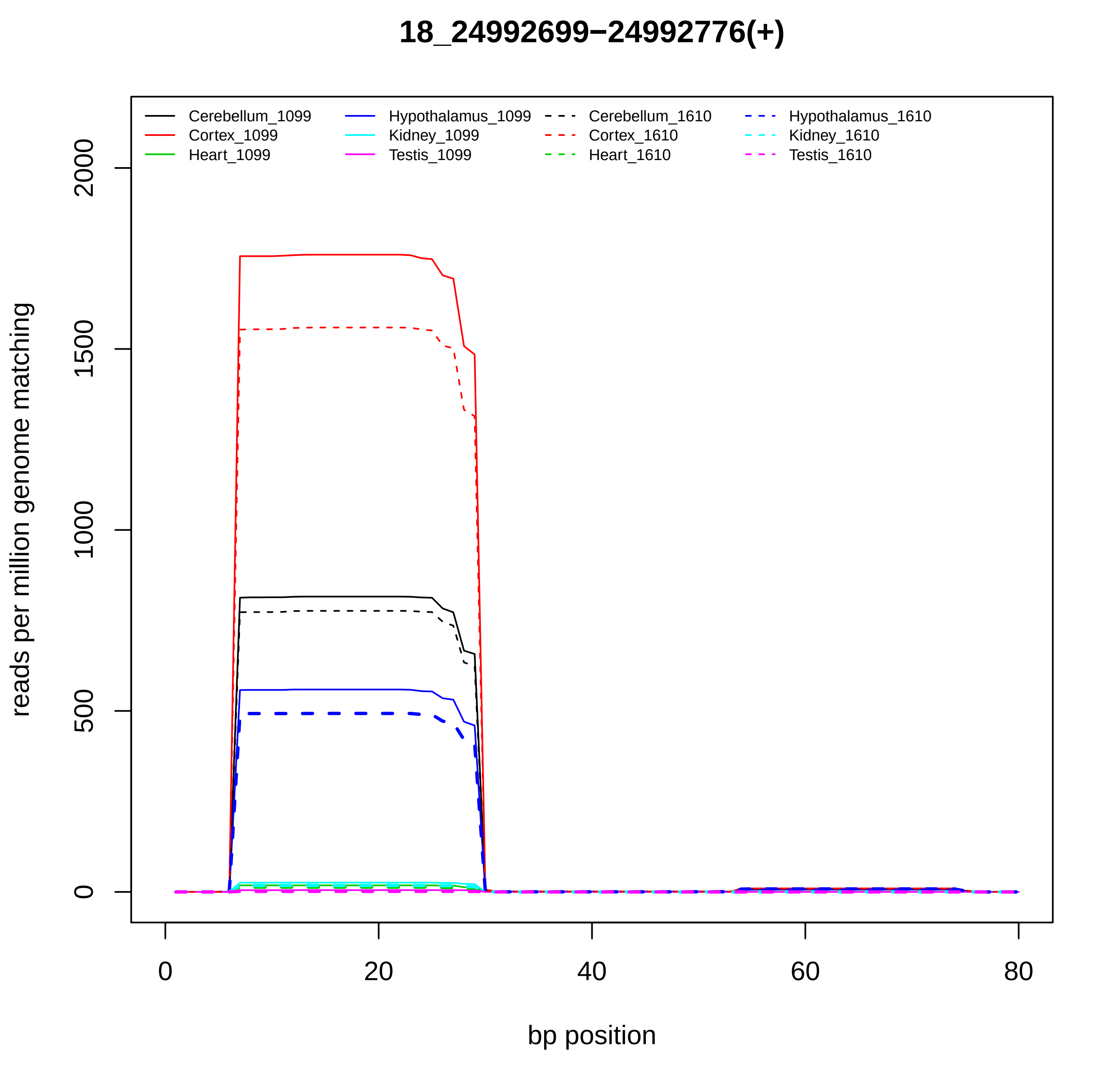

18_24992699−24992776(+)
Cerebellum_1099
Cortex_1099
Heart_1099
Hypothalamus_1099
Kidney_1099
Testis_1099
Cerebellum_1610
Cortex_1610
Heart_1610
Hypothalamus_1610
Kidney_1610
Testis_1610
reads per million genome matching
0
500
1000
1500
2000
bp position
0
20
40
60
80

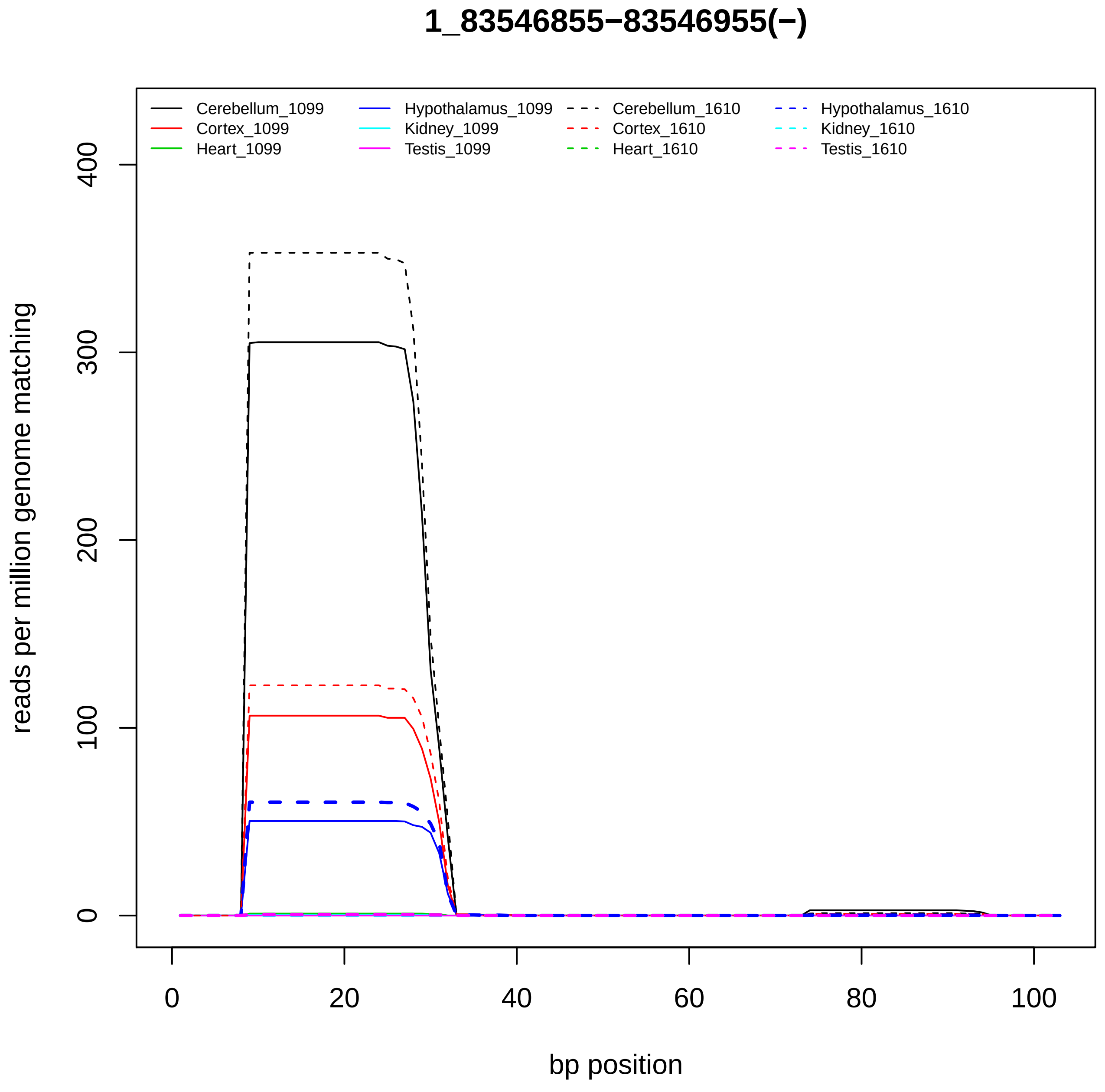
1_83546855−83546955(−)
Cerebellum_1099
Cortex_1099
Heart_1099
Hypothalamus_1099
Kidney_1099
Testis_1099
Cerebellum_1610
Cortex_1610
Heart_1610
Hypothalamus_1610
Kidney_1610
Testis_1610
reads per million genome matching
0
100
200
300
400
0
20
40
60
80
100
bp position

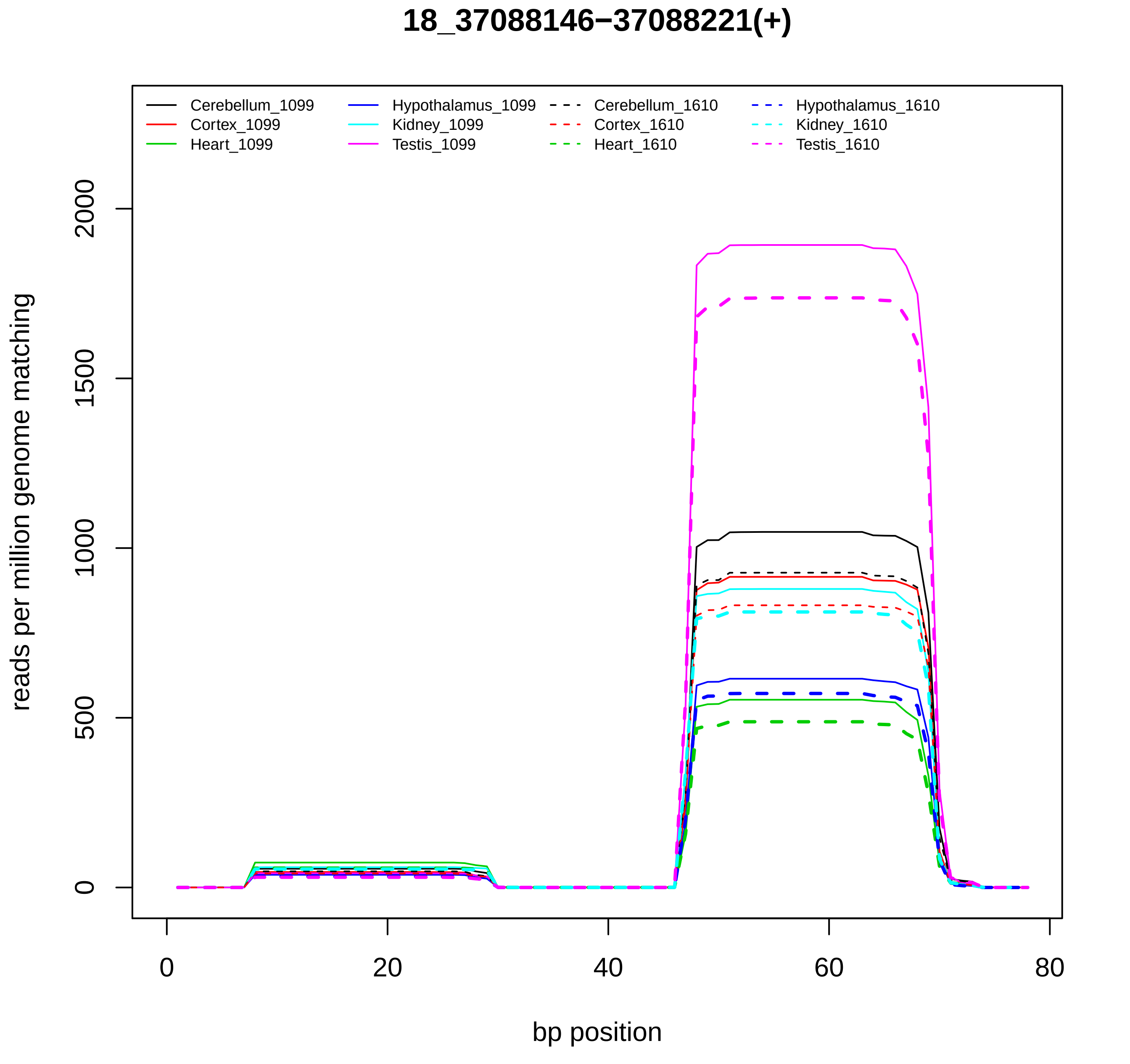
18_37088146−37088221(+)
Cerebellum_1099
Cortex_1099
Heart_1099
Hypothalamus_1099
Kidney_1099
Testis_1099
Cerebellum_1610
Cortex_1610
Heart_1610
Hypothalamus_1610
Kidney_1610
Testis_1610
reads per million genome matching
0
500
1000
1500
2000
bp position
0
20
40
60
80

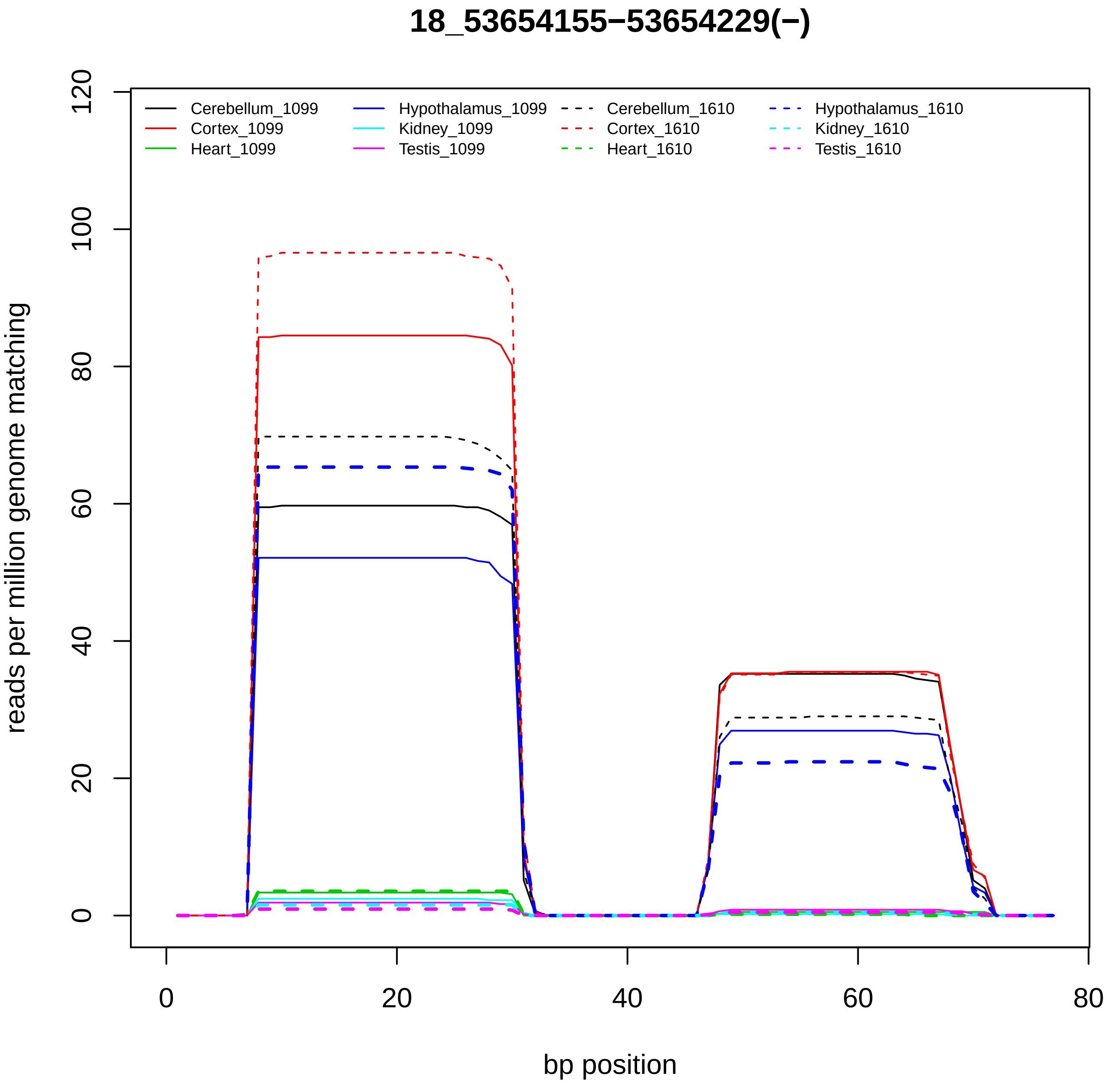

18_53654155−53654229(−)
Cerebellum_1099
Cortex_1099
Heart_1099
Hypothalamus_1099
Kidney_1099
Testis_1099
Cerebellum_1610
Cortex_1610
Heart_1610
Hypothalamus_1610
Kidney_1610
Testis_1610
reads per million genome matching
bp position
0
20
40
60
80
100
120

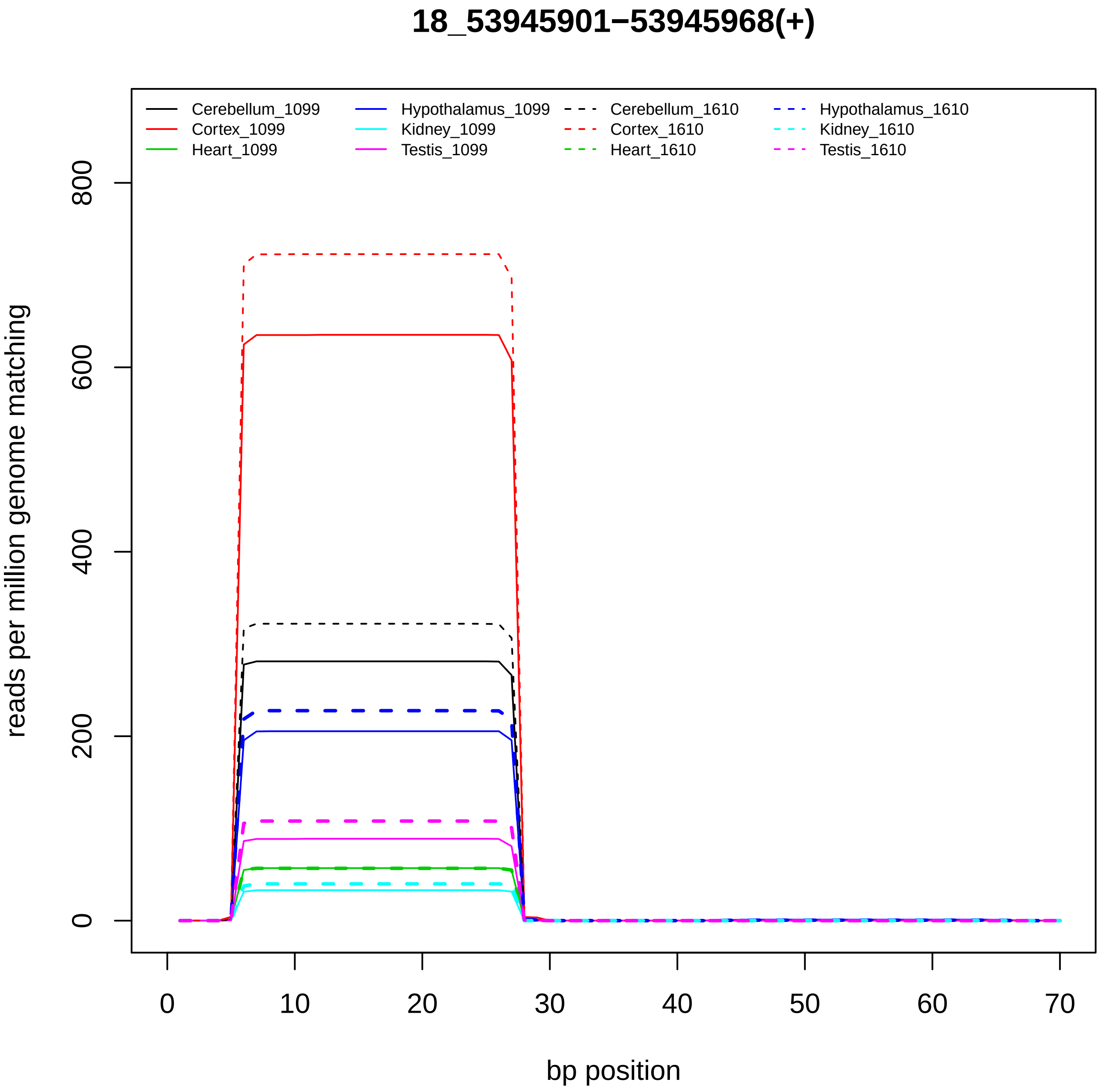

18_53945901−53945968(+)
Cerebellum_1099
Cortex_1099
Heart_1099
Hypothalamus_1099
Kidney_1099
Testis_1099
Cerebellum_1610
Cortex_1610
Heart_1610
Hypothalamus_1610
Kidney_1610
Testis_1610
reads per million genome matching
0
200
400
600
800
bp position
0
10
20
30
40
50
60
70

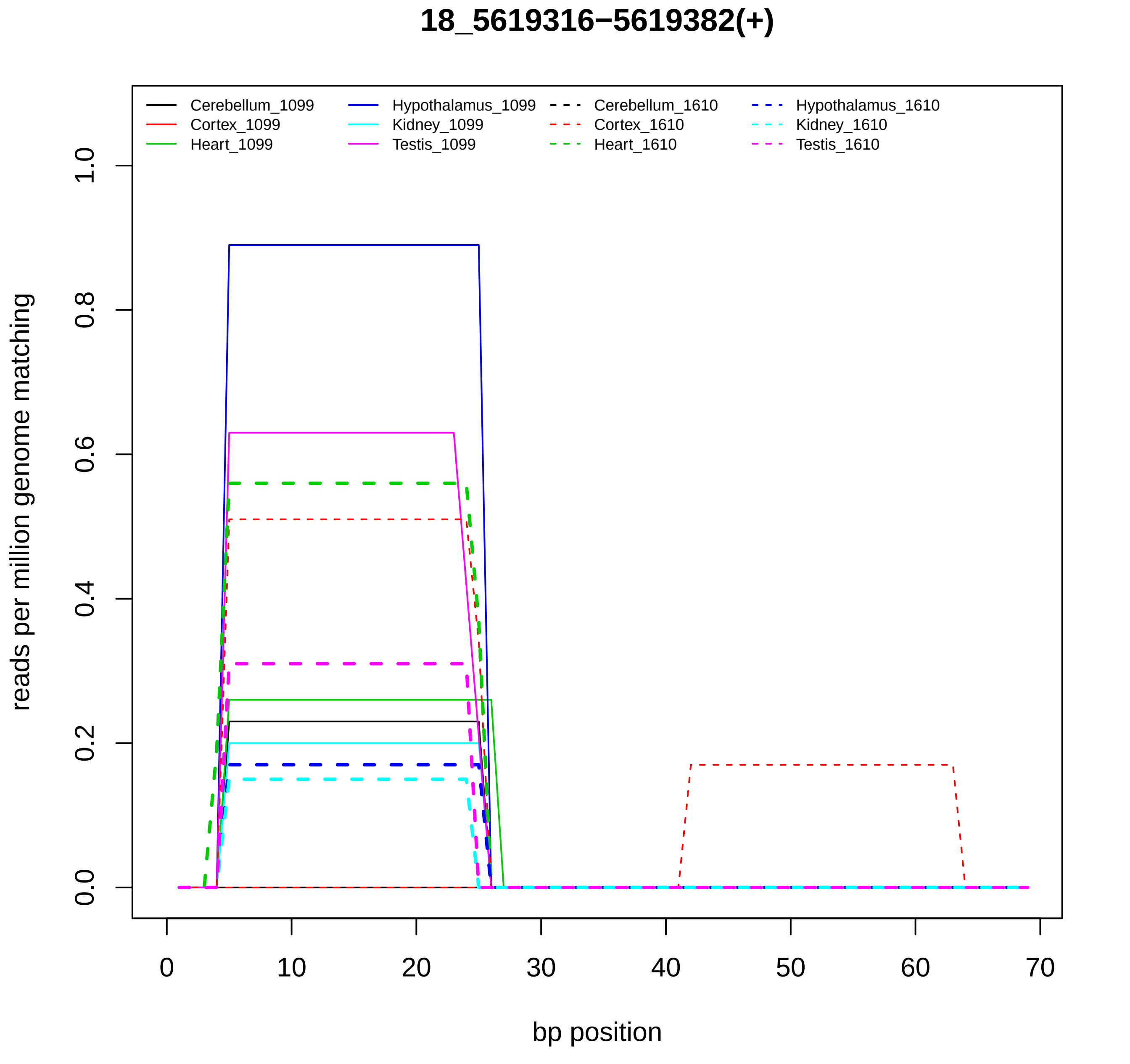

18_5619316−5619382(+)
Cerebellum_1099
Cortex_1099
Heart_1099
Hypothalamus_1099
Kidney_1099
Testis_1099
Cerebellum_1610
Cortex_1610
Heart_1610
Hypothalamus_1610
Kidney_1610
Testis_1610
reads per million genome matching
0.0
0.2
0.4
0.6
0.8
1.0
0
10
20
30
40
50
60
70
bp position

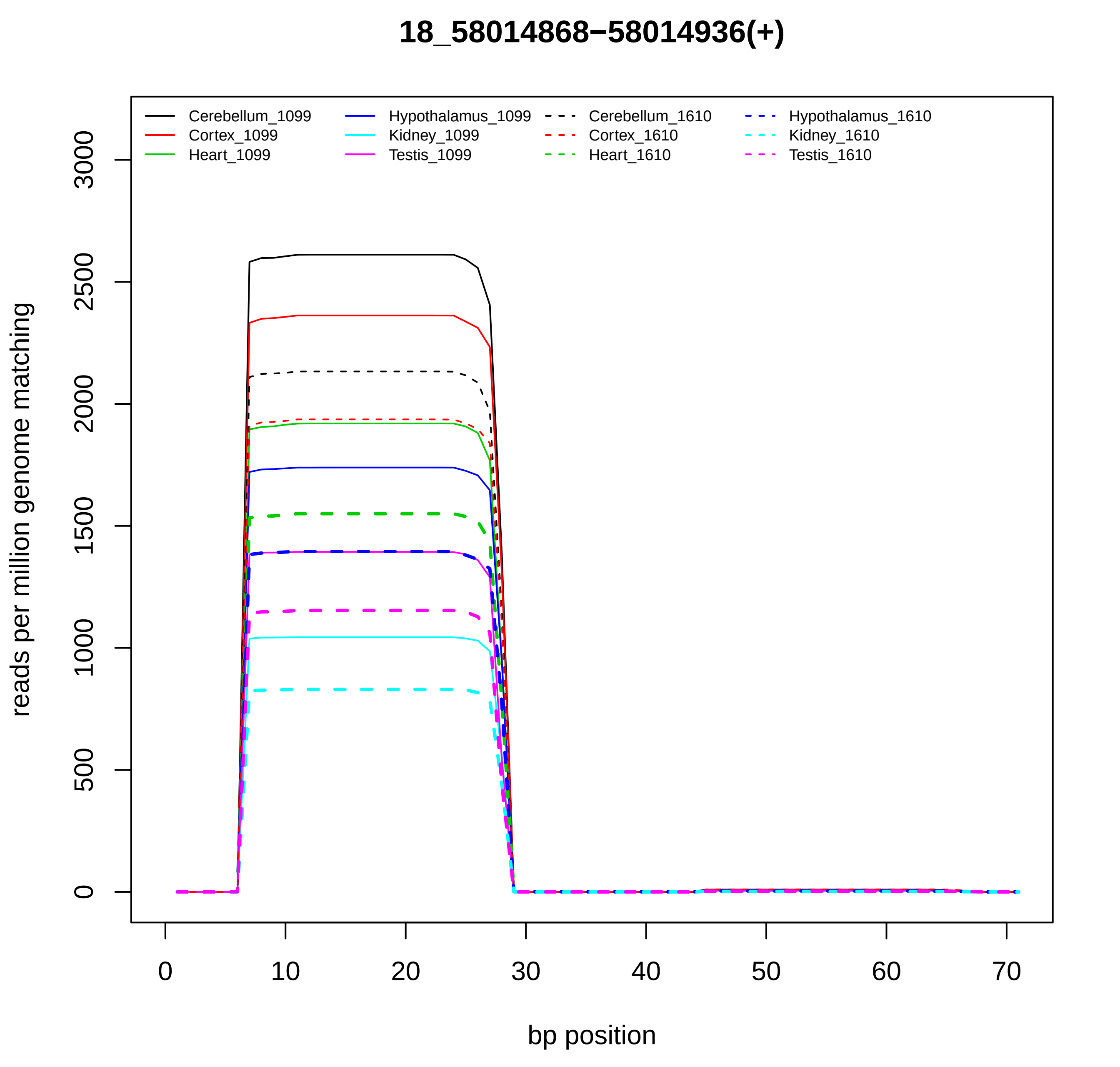
18_58014868−58014936(+)
Cerebellum_1099
Cortex_1099
Heart_1099
Hypothalamus_1099
Kidney_1099
Testis_1099
Cerebellum_1610
Cortex_1610
Heart_1610
Hypothalamus_1610
Kidney_1610
Testis_1610
reads per million genome matching
0
500
1000
1500
2000
2500
3000
bp position
0
10
20
30
40
50
60
70

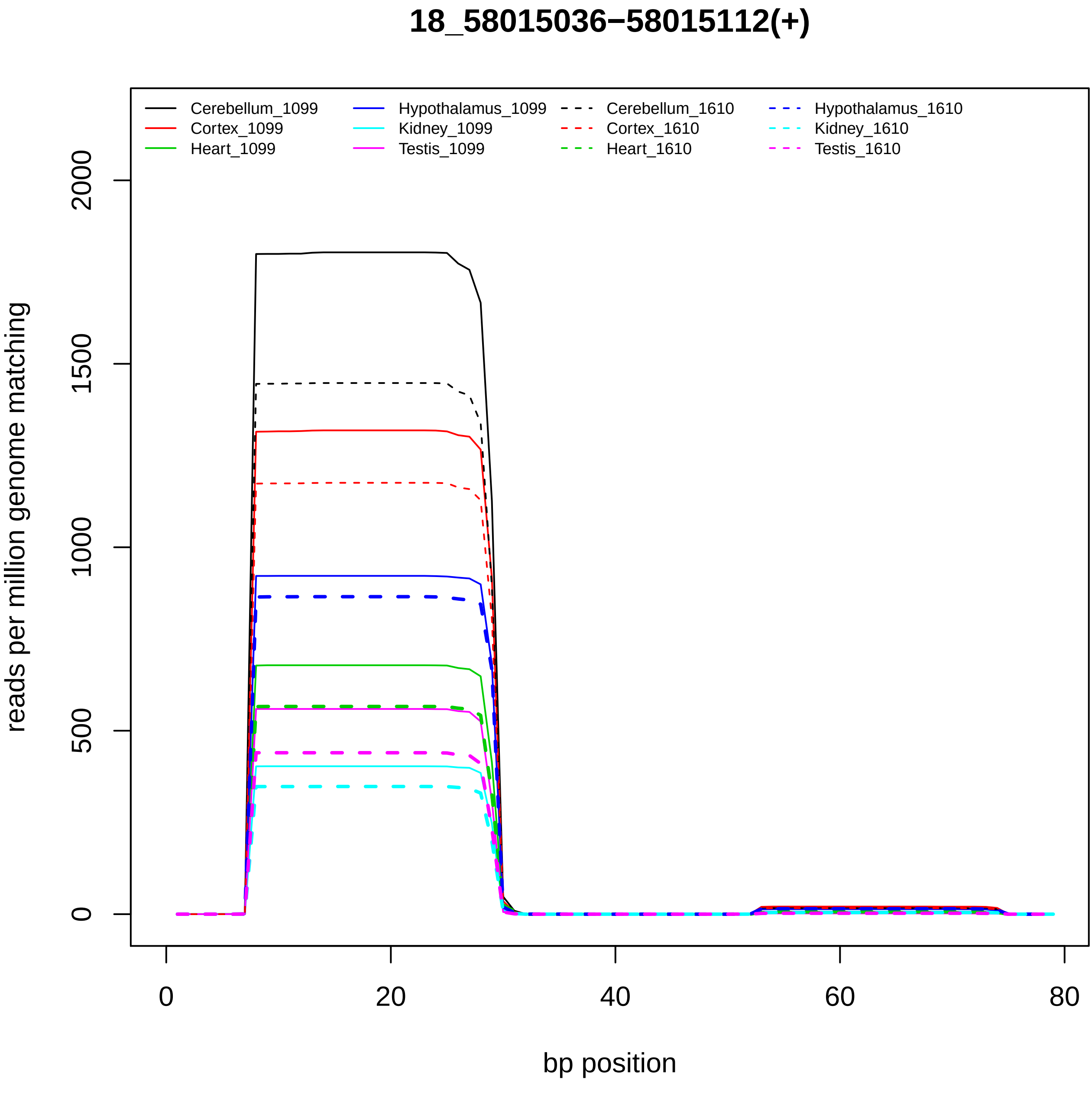

18_58015036−58015112(+)
Cerebellum_1099
Cortex_1099
Heart_1099
Hypothalamus_1099
Kidney_1099
Testis_1099
Cerebellum_1610
Cortex_1610
Heart_1610
Hypothalamus_1610
Kidney_1610
Testis_1610
reads per million genome matching
0
500
1000
1500
2000
0
20
40
60
80
bp position

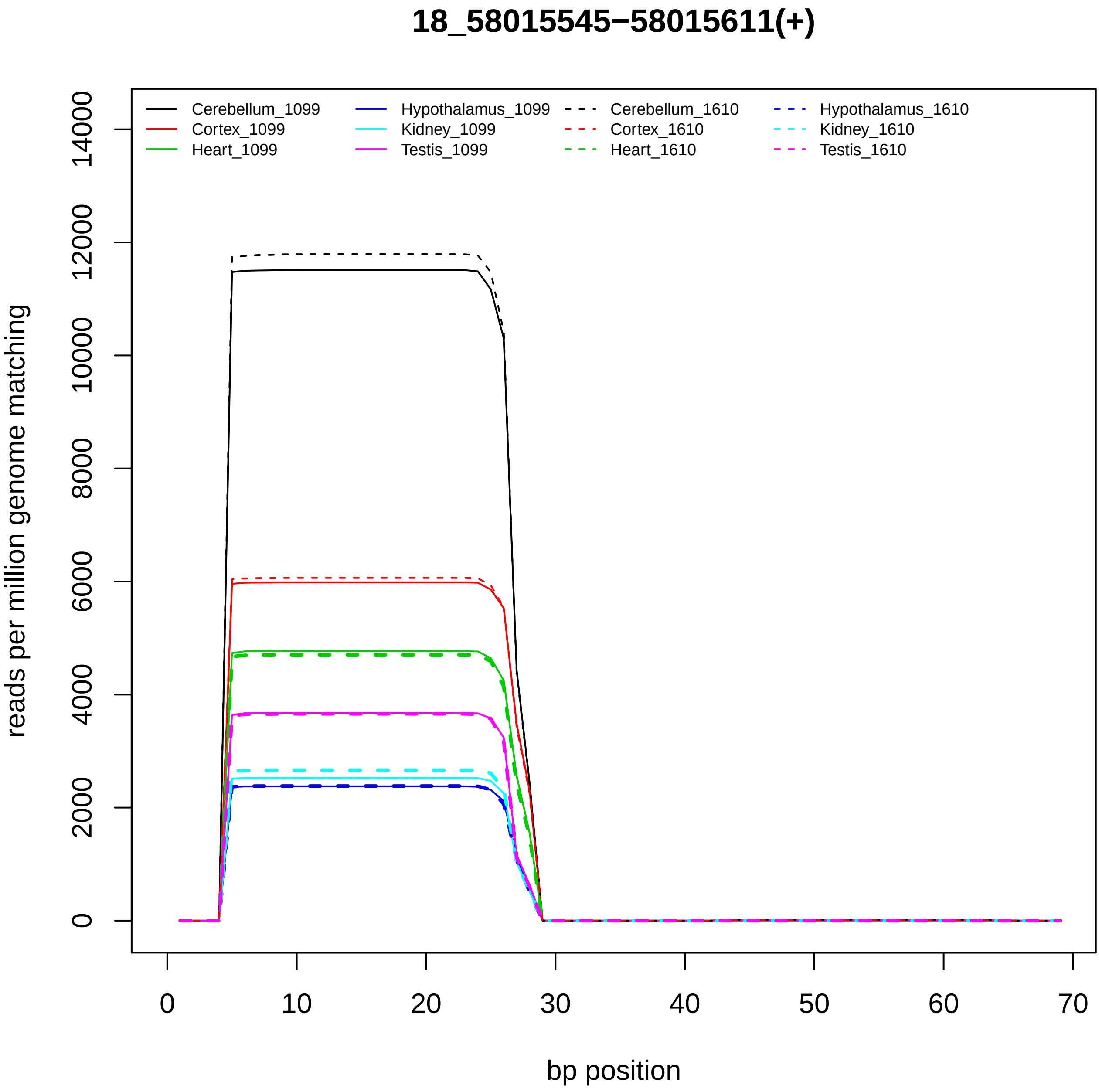

18_58015545−58015611(+)
Cerebellum_1099
Cortex_1099
Heart_1099
Hypothalamus_1099
Kidney_1099
Testis_1099
Cerebellum_1610
Cortex_1610
Heart_1610
Hypothalamus_1610
Kidney_1610
Testis_1610
reads per million genome matching
0
2000
4000
6000
8000
10000
12000
14000
bp position
0
10
20
30
40
50
60
70

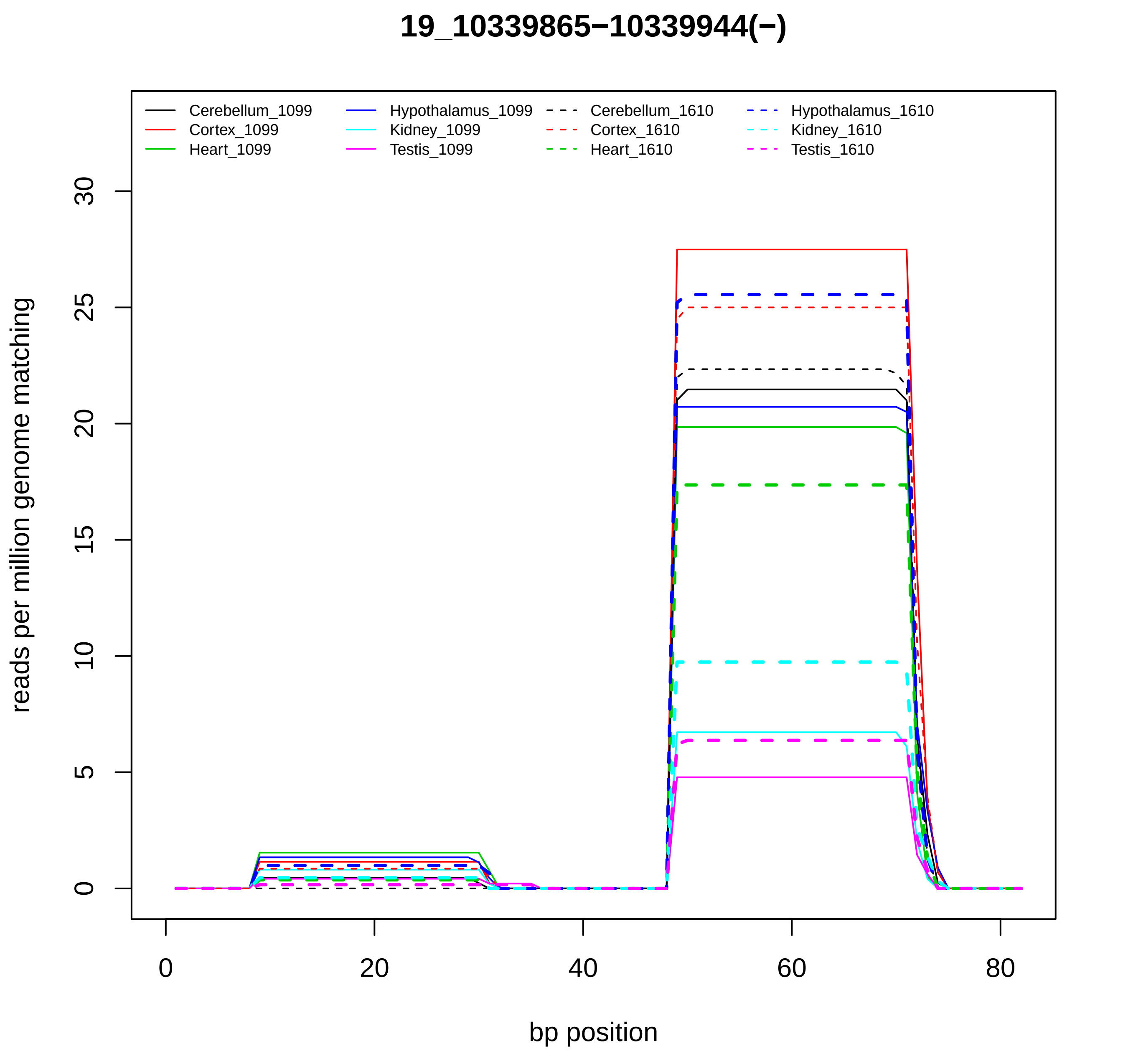

19_10339865−10339944(−)
Cerebellum_1099
Cortex_1099
Heart_1099
Hypothalamus_1099
Kidney_1099
Testis_1099
Cerebellum_1610
Cortex_1610
Heart_1610
Hypothalamus_1610
Kidney_1610
Testis_1610
reads per million genome matching
0
5
10
15
20
25
30
0
20
40
60
80
bp position

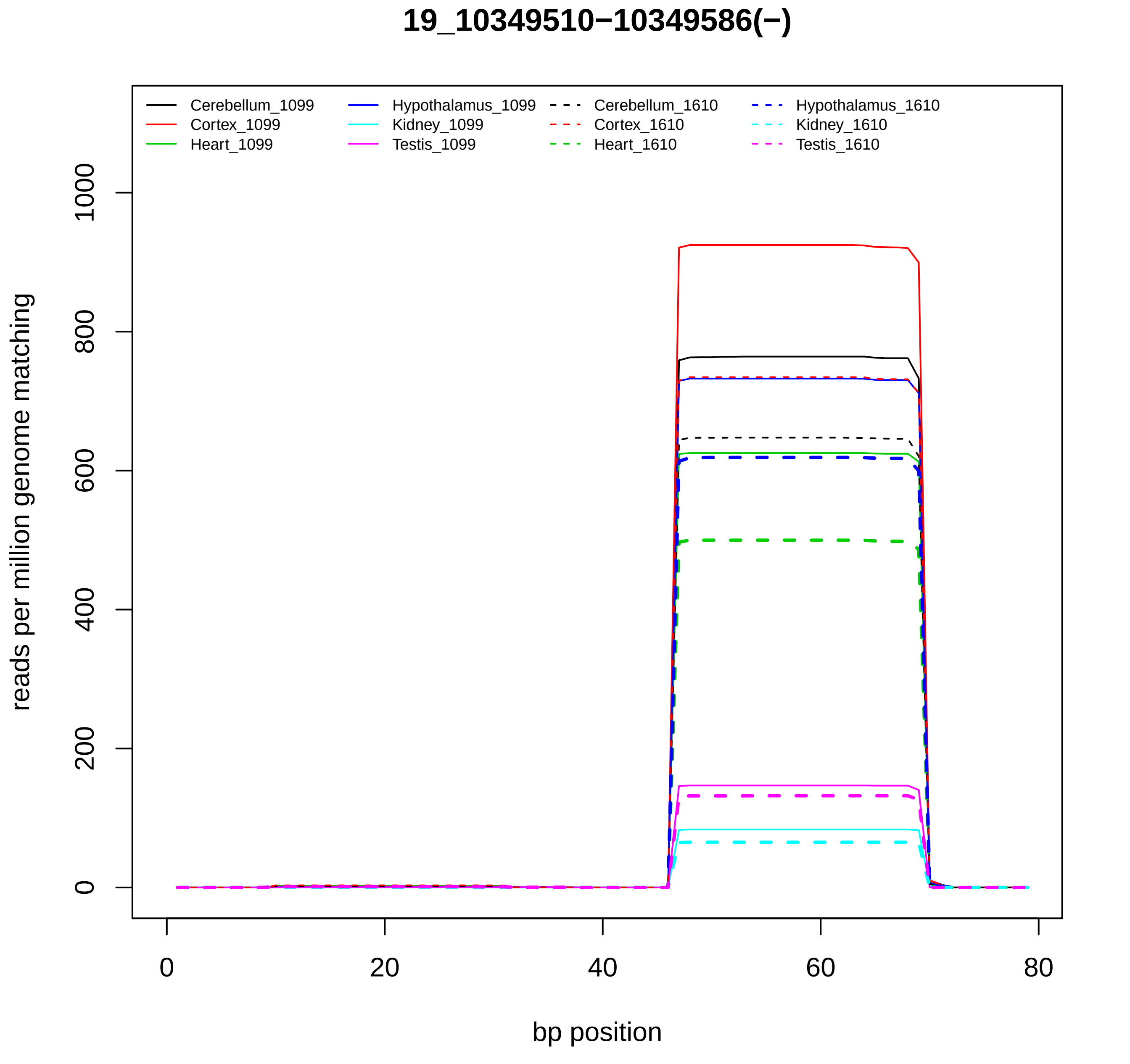

19_10349510−10349586(−)
Cerebellum_1099
Cortex_1099
Heart_1099
Hypothalamus_1099
Kidney_1099
Testis_1099
Cerebellum_1610
Cortex_1610
Heart_1610
Hypothalamus_1610
Kidney_1610
Testis_1610
reads per million genome matching
0
200
400
600
800
1000
0
20
40
60
80
bp position

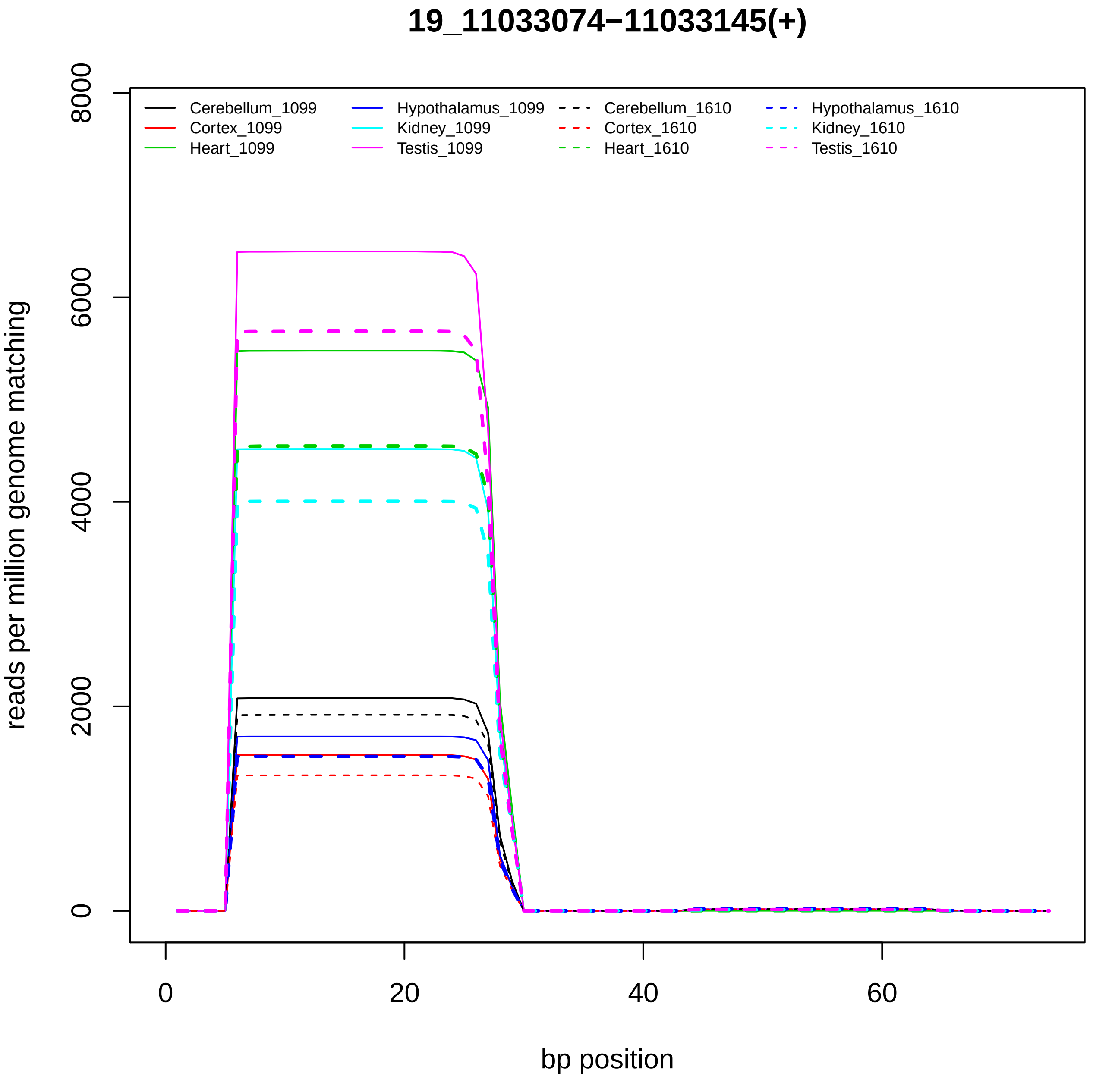

19_11033074−11033145(+)
Cerebellum_1099
Cortex_1099
Heart_1099
Hypothalamus_1099
Kidney_1099
Testis_1099
Cerebellum_1610
Cortex_1610
Heart_1610
Hypothalamus_1610
Kidney_1610
Testis_1610
reads per million genome matching
0
2000
4000
6000
8000
0
20
40
60
bp position

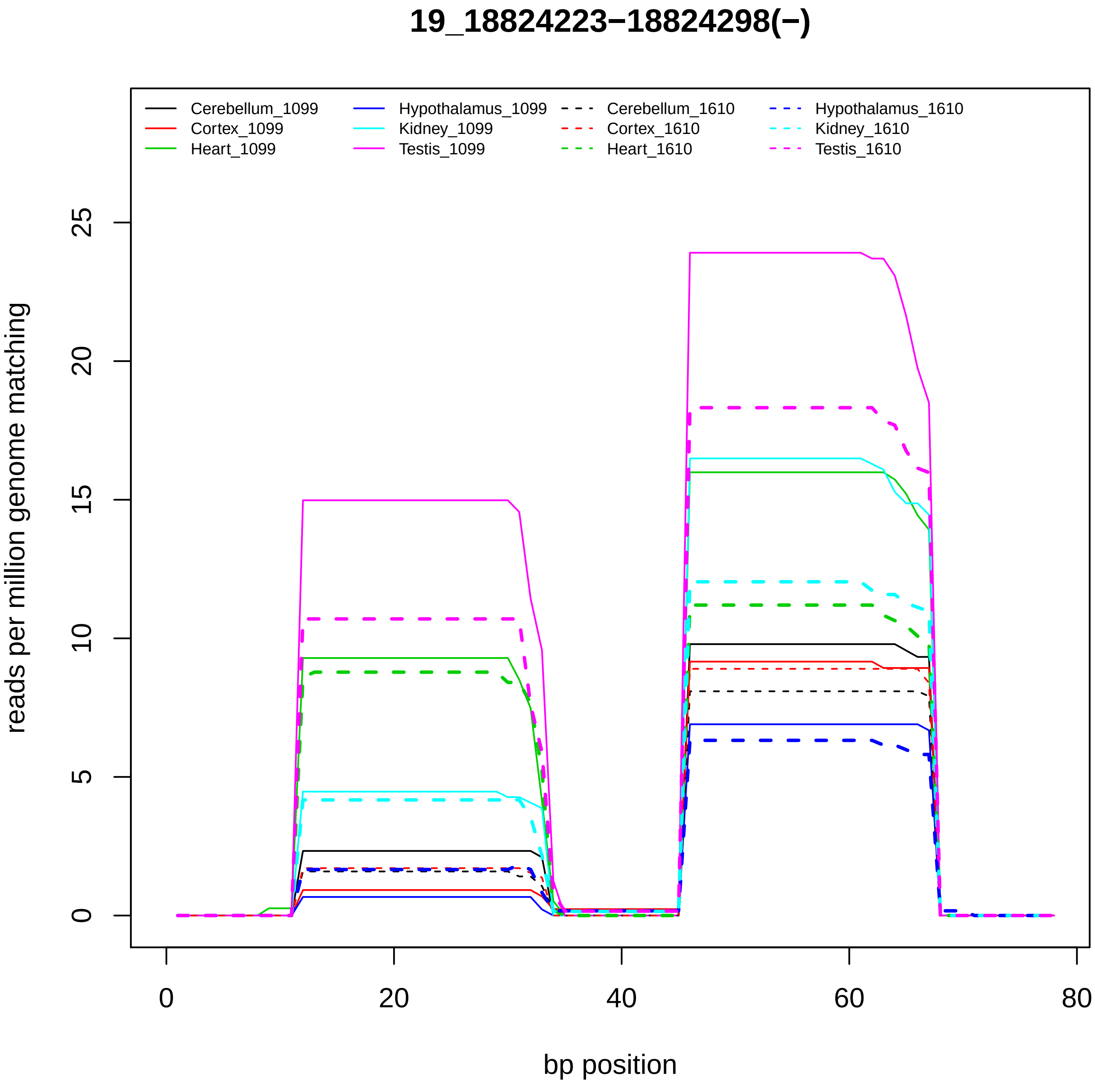
19_18824223−18824298(−)
Cerebellum_1099
Cortex_1099
Heart_1099
Hypothalamus_1099
Kidney_1099
Testis_1099
Cerebellum_1610
Cortex_1610
Heart_1610
Hypothalamus_1610
Kidney_1610
Testis_1610
reads per million genome matching
bp position
0
5
10
15
20
25
0
20
40
60
80

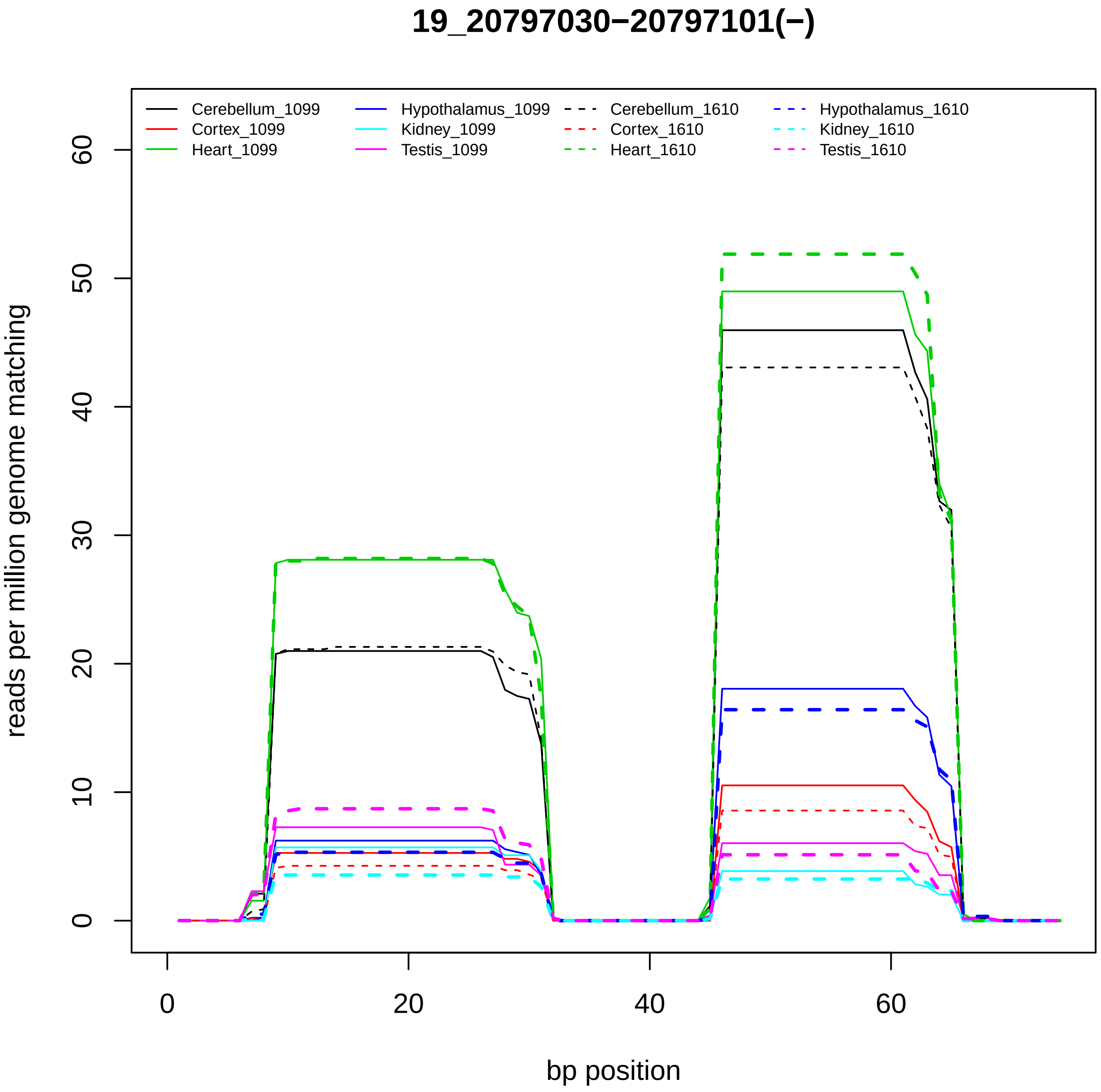

19_20797030−20797101(−)
Cerebellum_1099
Cortex_1099
Heart_1099
Hypothalamus_1099
Kidney_1099
Testis_1099
Cerebellum_1610
Cortex_1610
Heart_1610
Hypothalamus_1610
Kidney_1610
Testis_1610
reads per million genome matching
0
10
20
30
40
50
60
0
20
40
60
bp position

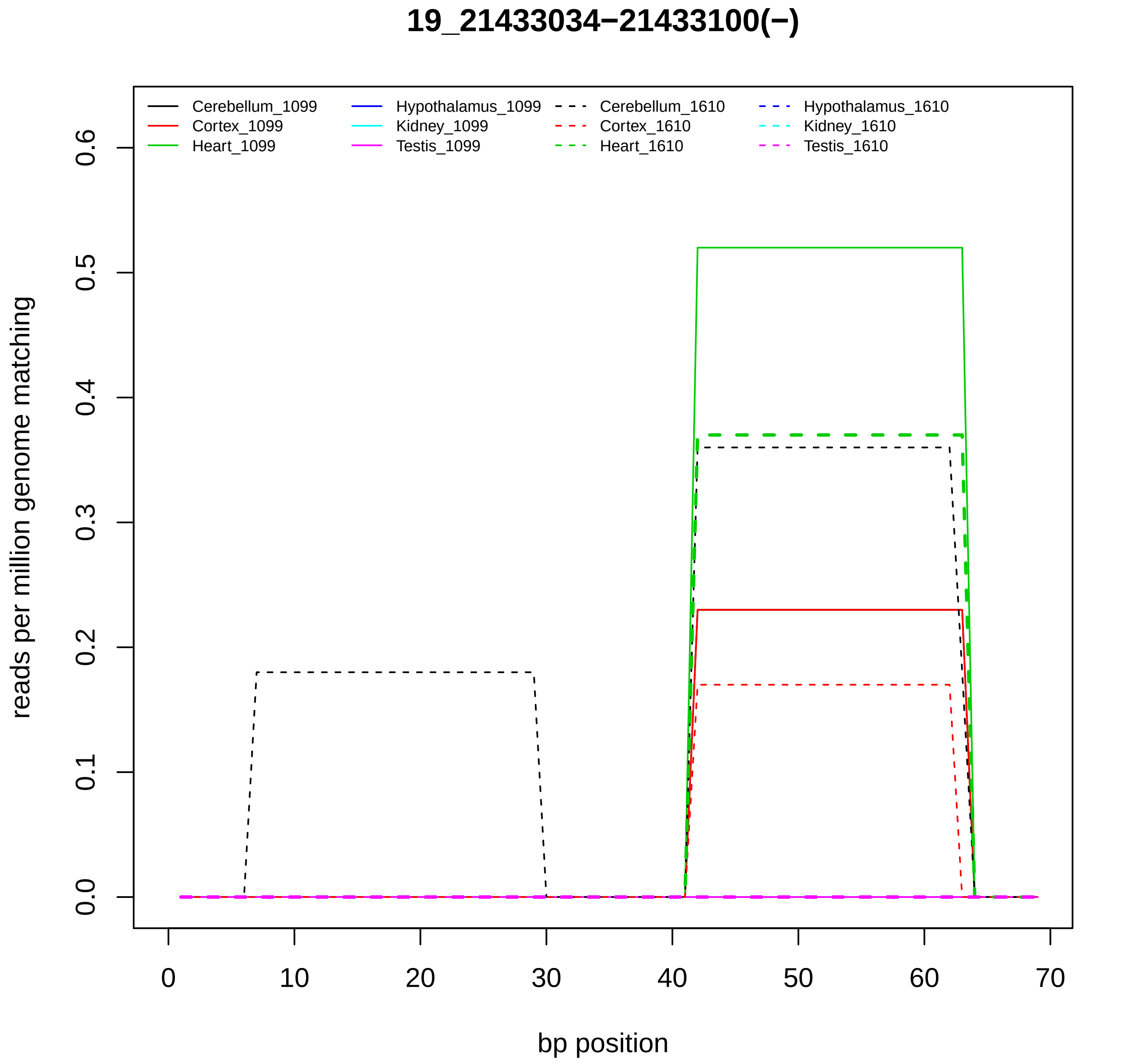
19_21433034−21433100(−)
Cerebellum_1099
Cortex_1099
Heart_1099
Hypothalamus_1099
Kidney_1099
Testis_1099
Cerebellum_1610
Cortex_1610
Heart_1610
Hypothalamus_1610
Kidney_1610
Testis_1610
reads per million genome matching
0.0
0.1
0.2
0.3
0.4
0.5
0.6
0
10
20
30
40
50
60
70
bp position

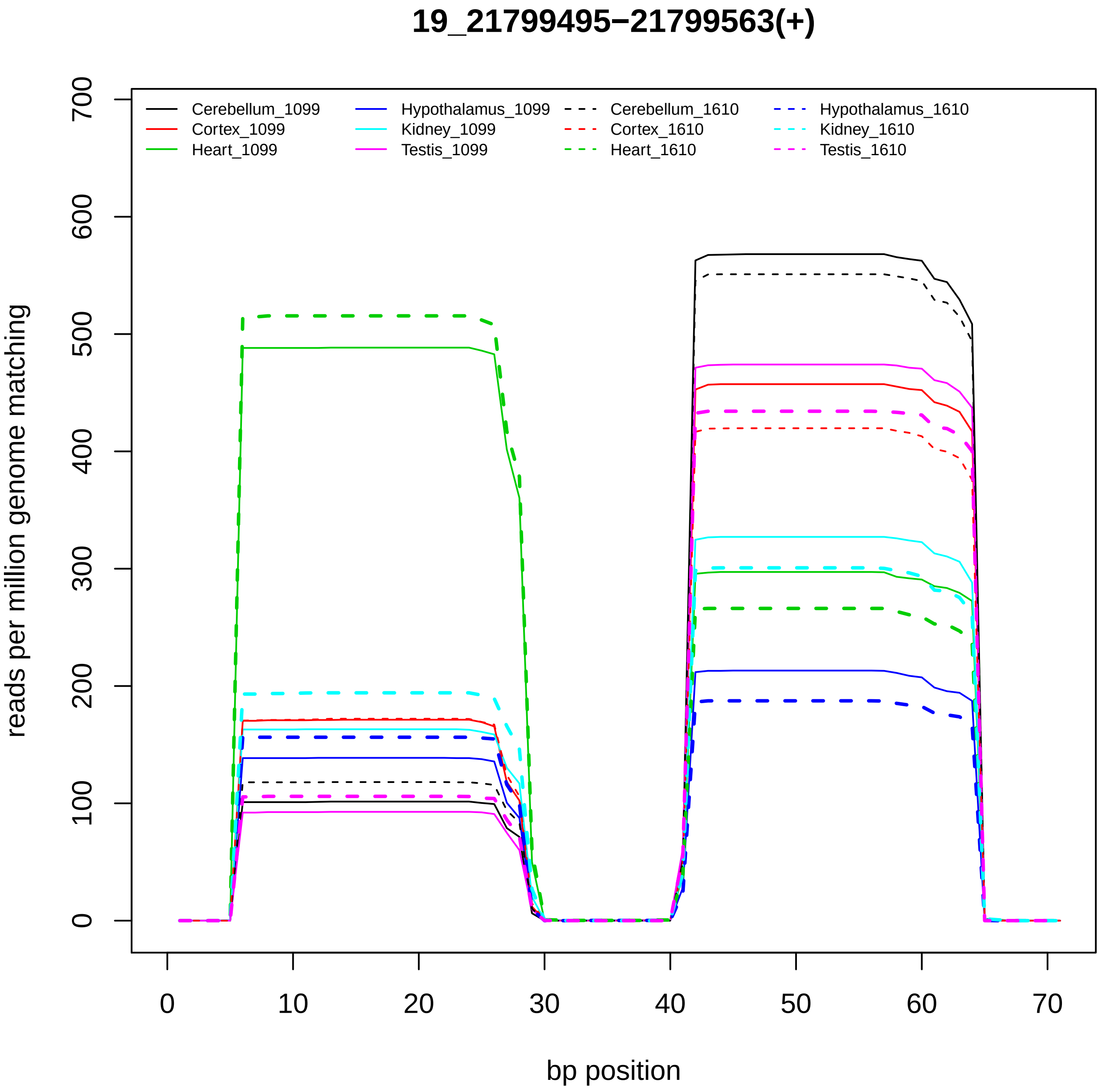
19_21799495−21799563(+)
Cerebellum_1099
Cortex_1099
Heart_1099
Hypothalamus_1099
Kidney_1099
Testis_1099
Cerebellum_1610
Cortex_1610
Heart_1610
Hypothalamus_1610
Kidney_1610
Testis_1610
reads per million genome matching
bp position
0
100
200
300
400
500
600
700
0
10
20
30
40
50
60
70

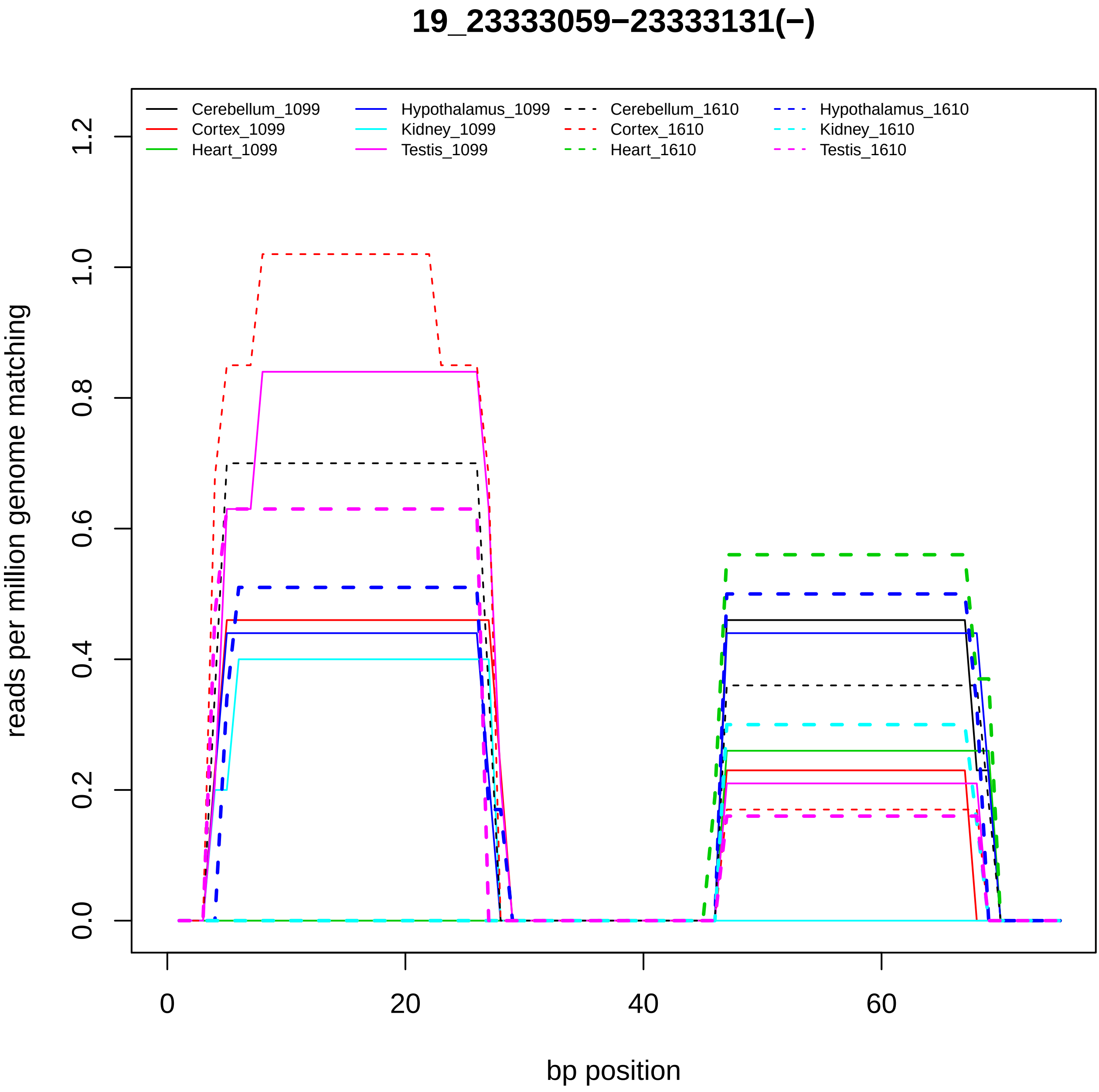

19_23333059−23333131(−)
Cerebellum_1099
Cortex_1099
Heart_1099
Hypothalamus_1099
Kidney_1099
Testis_1099
Cerebellum_1610
Cortex_1610
Heart_1610
Hypothalamus_1610
Kidney_1610
Testis_1610
reads per million genome matching
0.0
0.2
0.4
0.6
0.8
1.0
1.2
0
20
40
60
bp position

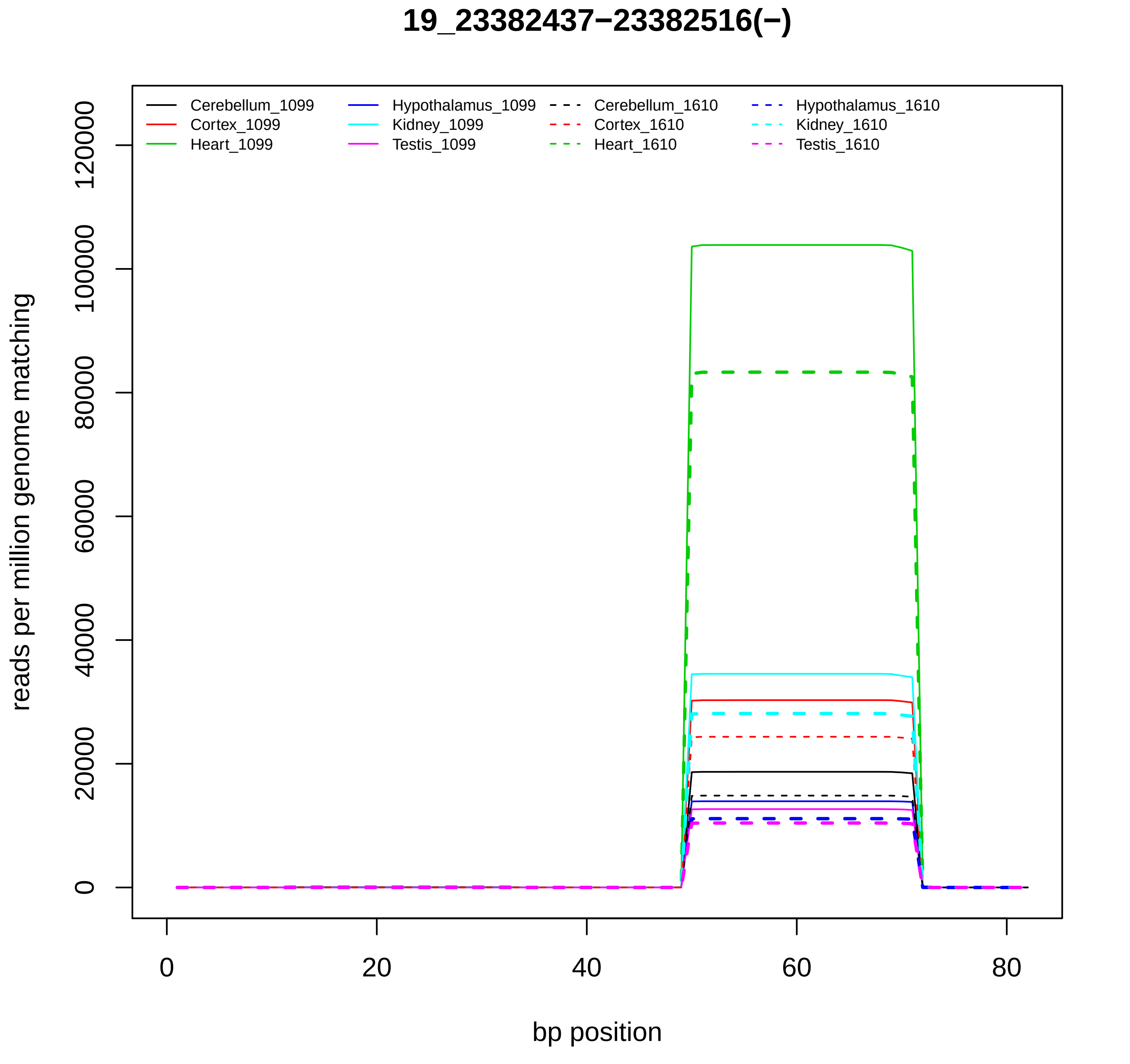

19_23382437−23382516(−)
Cerebellum_1099
Cortex_1099
Heart_1099
Hypothalamus_1099
Kidney_1099
Testis_1099
Cerebellum_1610
Cortex_1610
Heart_1610
Hypothalamus_1610
Kidney_1610
Testis_1610
reads per million genome matching
0
20000
40000
60000
80000
100000
120000
0
20
40
60
80
bp position

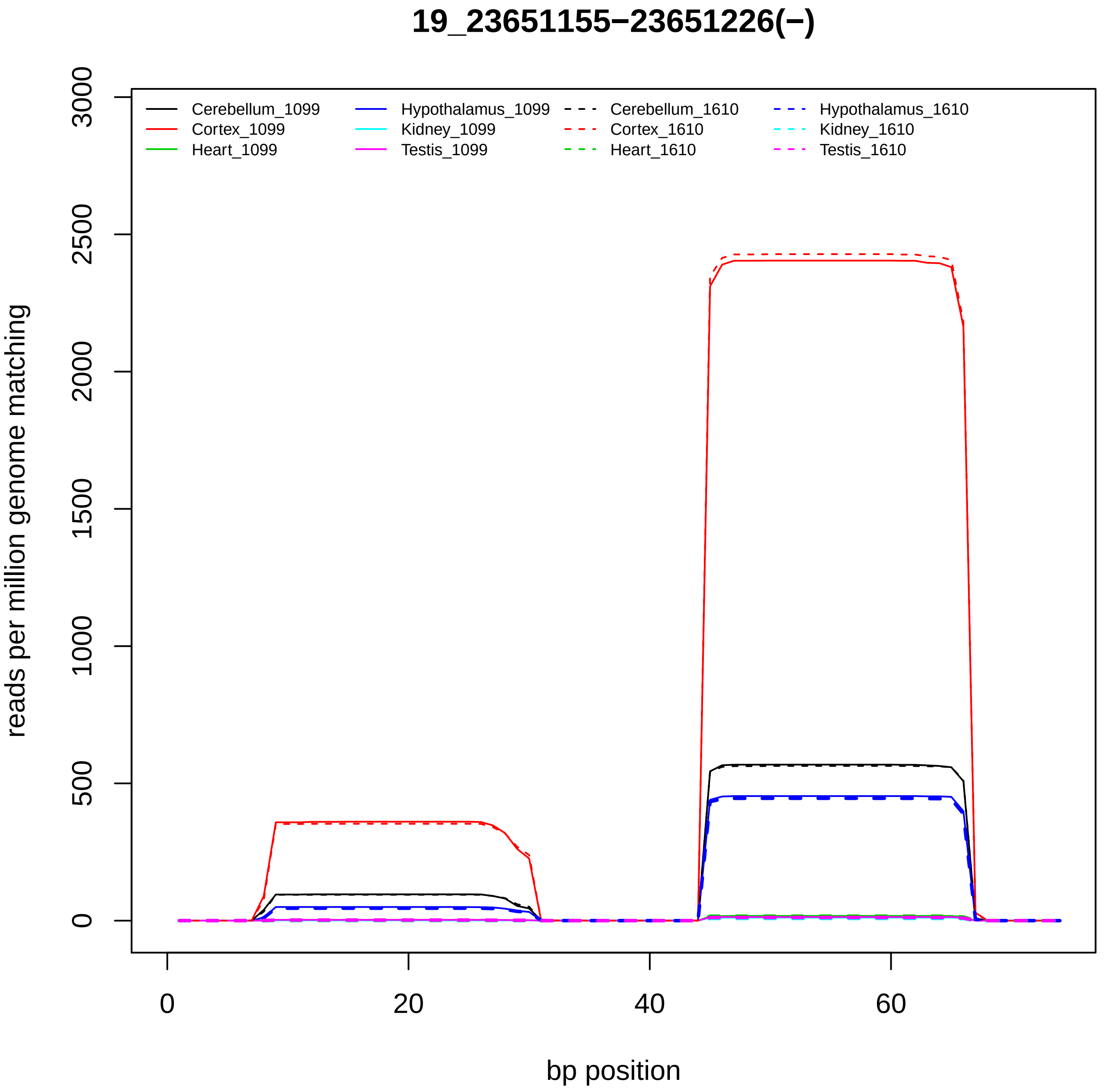

19_23651155−23651226(−)
Cerebellum_1099
Cortex_1099
Heart_1099
Hypothalamus_1099
Kidney_1099
Testis_1099
Cerebellum_1610
Cortex_1610
Heart_1610
Hypothalamus_1610
Kidney_1610
Testis_1610
reads per million genome matching
0
500
1000
1500
2000
2500
3000
bp position
0
20
40
60

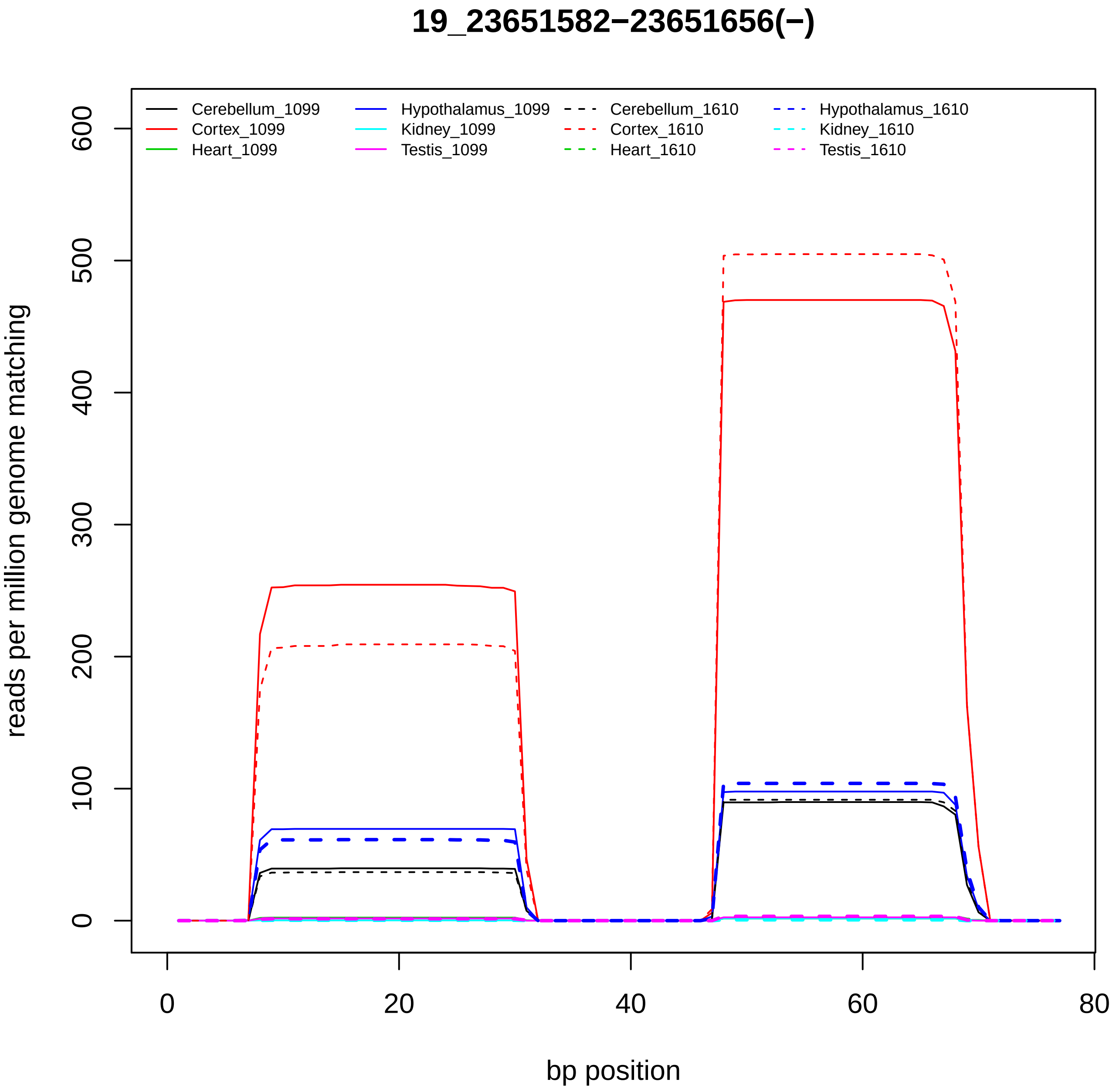

19_23651582−23651656(−)
Cerebellum_1099
Cortex_1099
Heart_1099
Hypothalamus_1099
Kidney_1099
Testis_1099
Cerebellum_1610
Cortex_1610
Heart_1610
Hypothalamus_1610
Kidney_1610
Testis_1610
0
100
200
300
400
500
600
reads per million genome matching
0
20
40
60
80
bp position

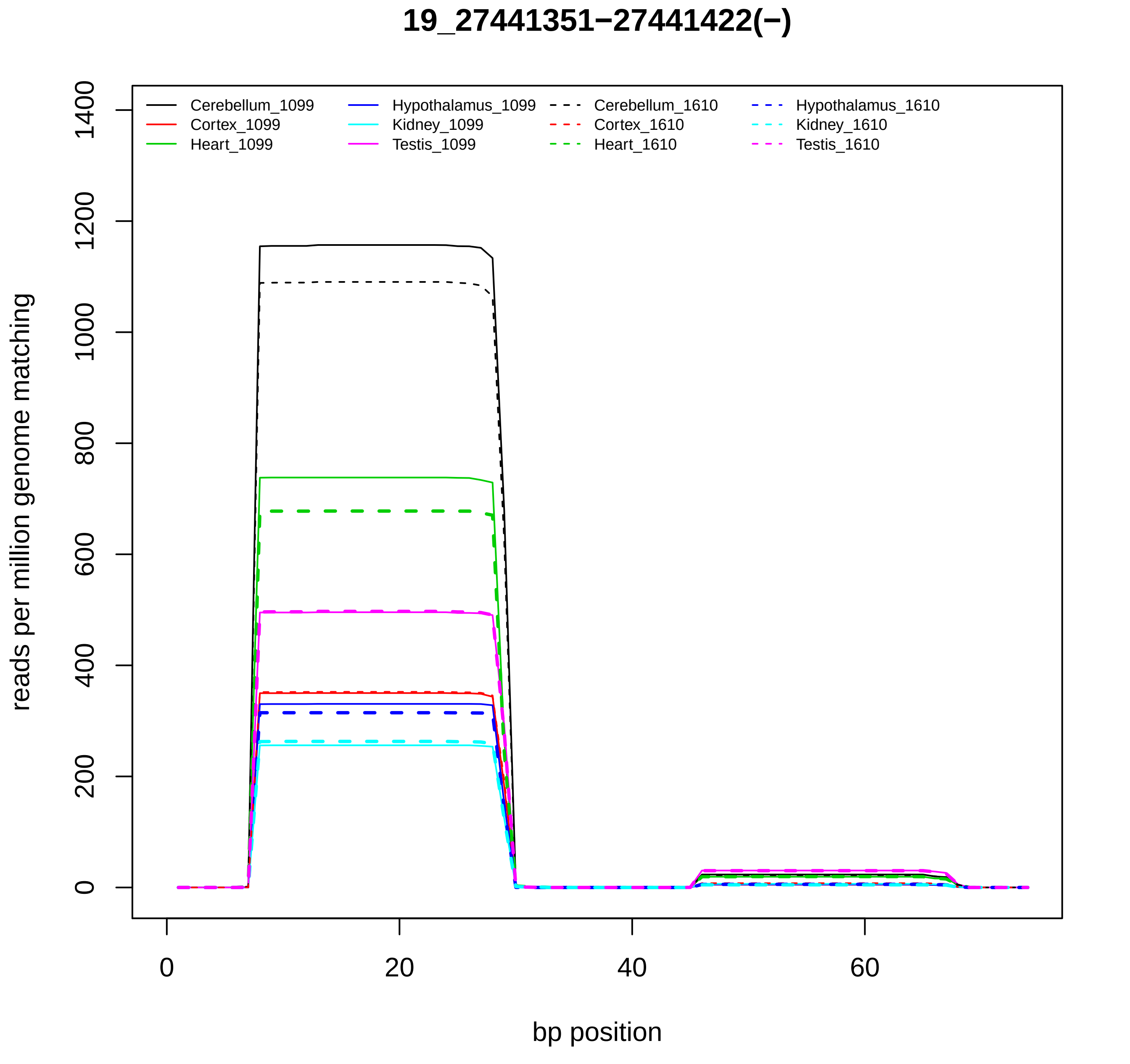

19_27441351−27441422(−)
Cerebellum_1099
Cortex_1099
Heart_1099
Hypothalamus_1099
Kidney_1099
Testis_1099
Cerebellum_1610
Cortex_1610
Heart_1610
Hypothalamus_1610
Kidney_1610
Testis_1610
reads per million genome matching
bp position
0
200
400
600
800
1000
1200
1400
0
20
40
60

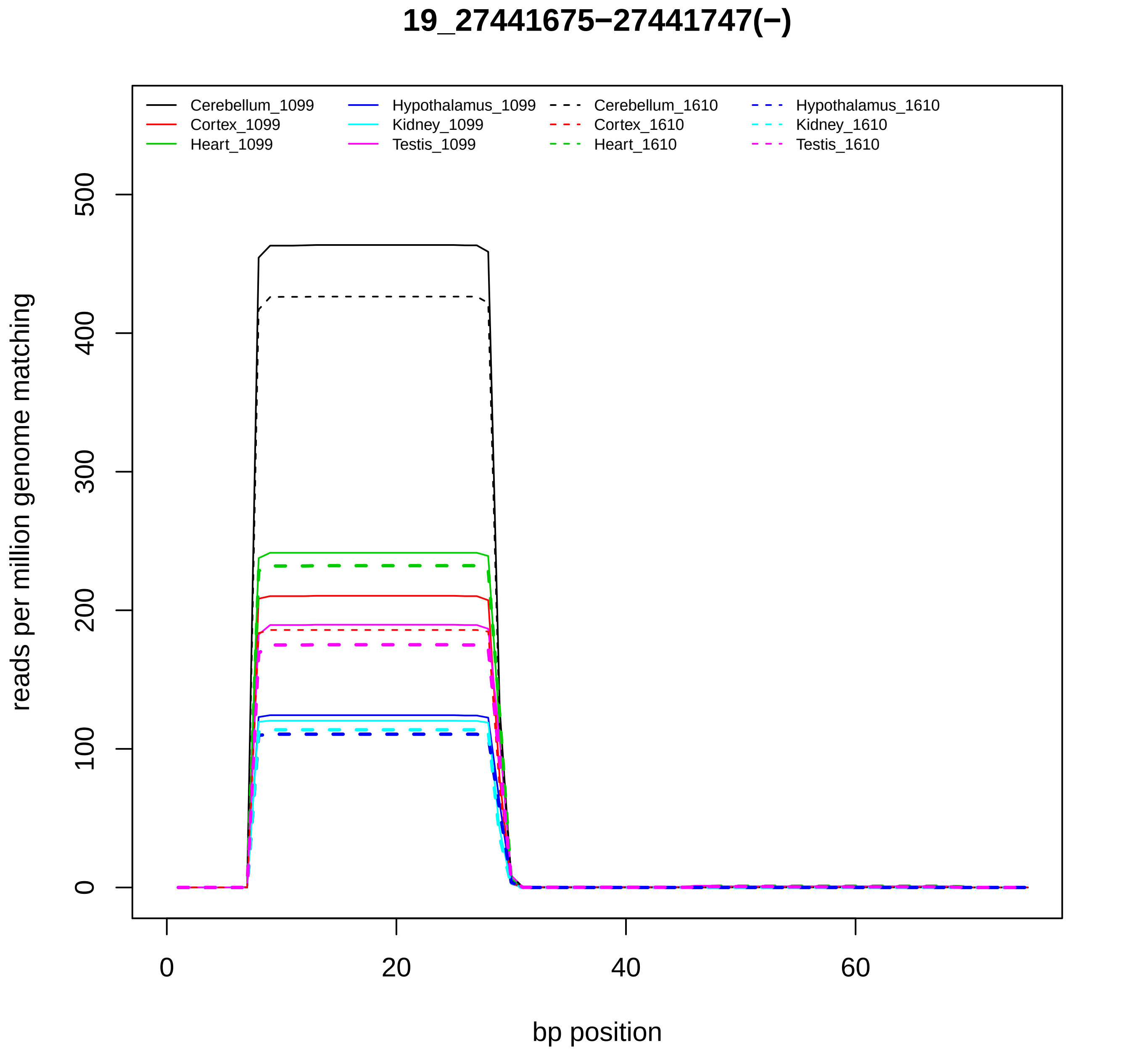

19_27441675−27441747(−)
Cerebellum_1099
Cortex_1099
Heart_1099
Hypothalamus_1099
Kidney_1099
Testis_1099
Cerebellum_1610
Cortex_1610
Heart_1610
Hypothalamus_1610
Kidney_1610
Testis_1610
reads per million genome matching
0
100
200
300
400
500
0
20
40
60
bp position

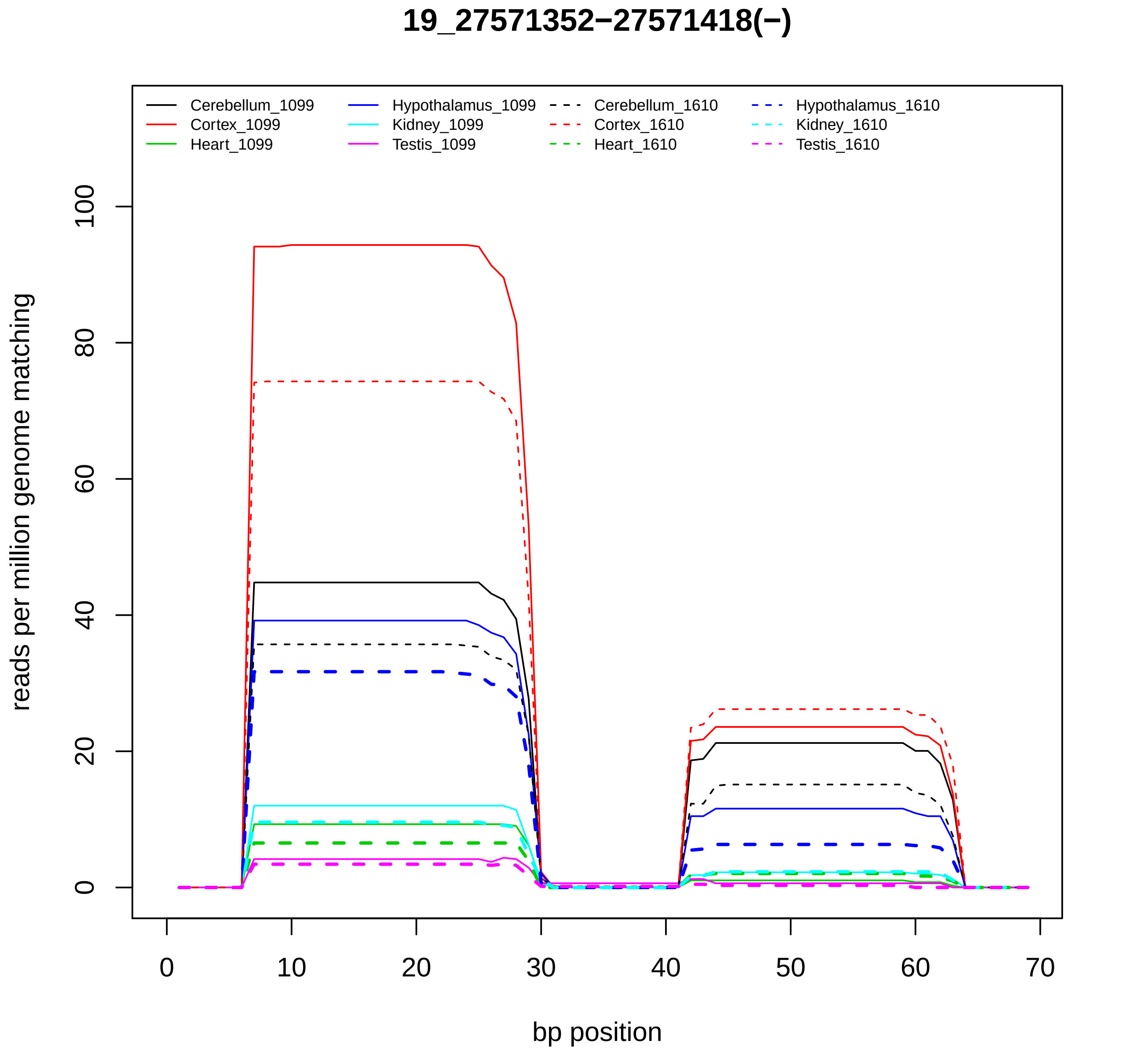
19_27571352−27571418(−)
Cerebellum_1099
Cortex_1099
Heart_1099
Hypothalamus_1099
Kidney_1099
Testis_1099
Cerebellum_1610
Cortex_1610
Heart_1610
Hypothalamus_1610
Kidney_1610
Testis_1610
reads per million genome matching
0
20
40
60
80
100
0
10
20
30
40
50
60
70
bp position

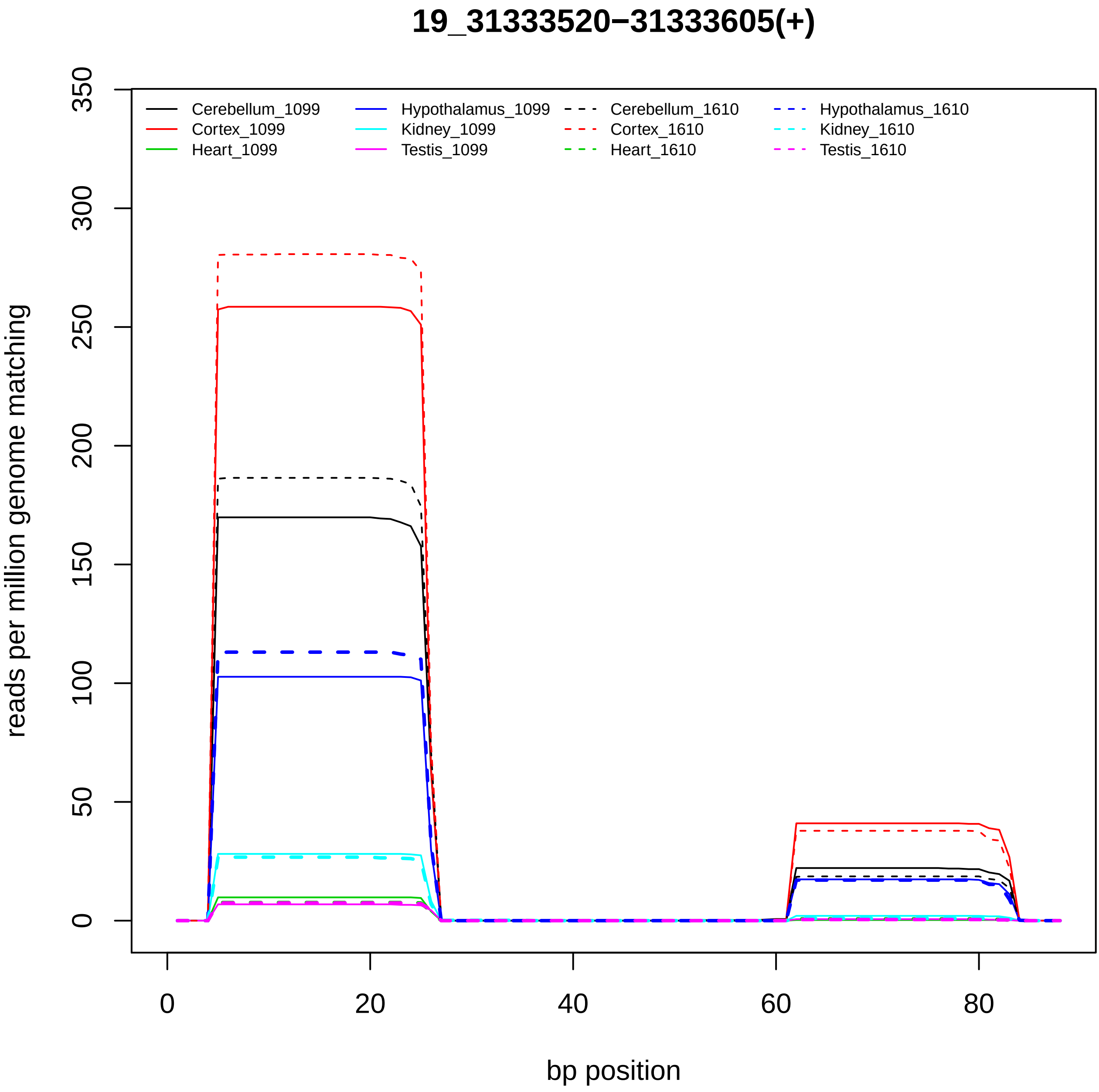

19_31333520−31333605(+)
Cerebellum_1099
Cortex_1099
Heart_1099
Hypothalamus_1099
Kidney_1099
Testis_1099
Cerebellum_1610
Cortex_1610
Heart_1610
Hypothalamus_1610
Kidney_1610
Testis_1610
reads per million genome matching
0
50
100
150
200
250
300
350
0
20
40
60
80
bp position

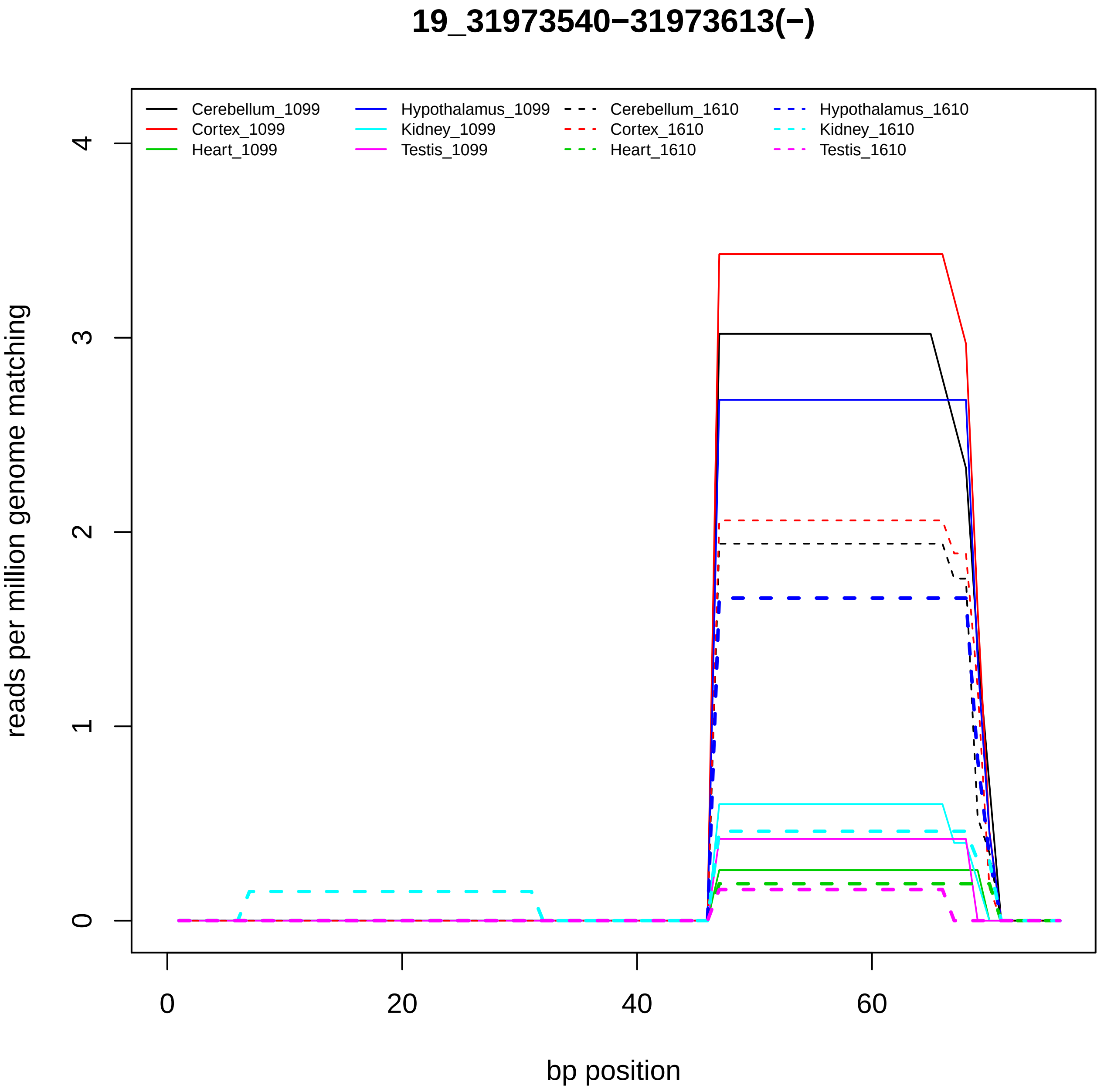

19_31973540−31973613(−)
Cerebellum_1099
Cortex_1099
Heart_1099
Hypothalamus_1099
Kidney_1099
Testis_1099
Cerebellum_1610
Cortex_1610
Heart_1610
Hypothalamus_1610
Kidney_1610
Testis_1610
reads per million genome matching
0
1
2
3
4
0
20
40
60
bp position

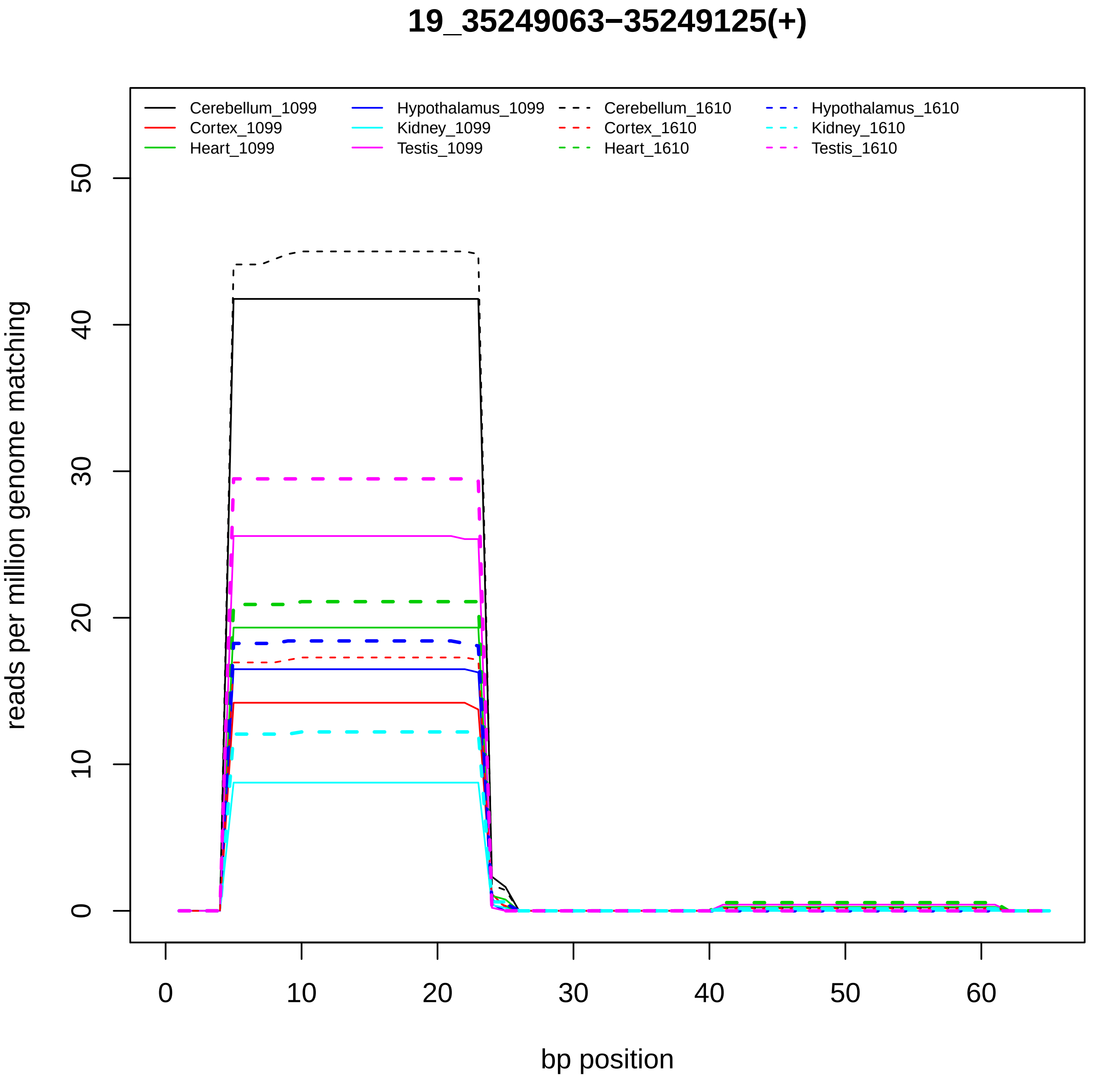

19_35249063−35249125(+)
Cerebellum_1099
Cortex_1099
Heart_1099
Hypothalamus_1099
Kidney_1099
Testis_1099
Cerebellum_1610
Cortex_1610
Heart_1610
Hypothalamus_1610
Kidney_1610
Testis_1610
reads per million genome matching
bp position
0
10
20
30
40
50
60

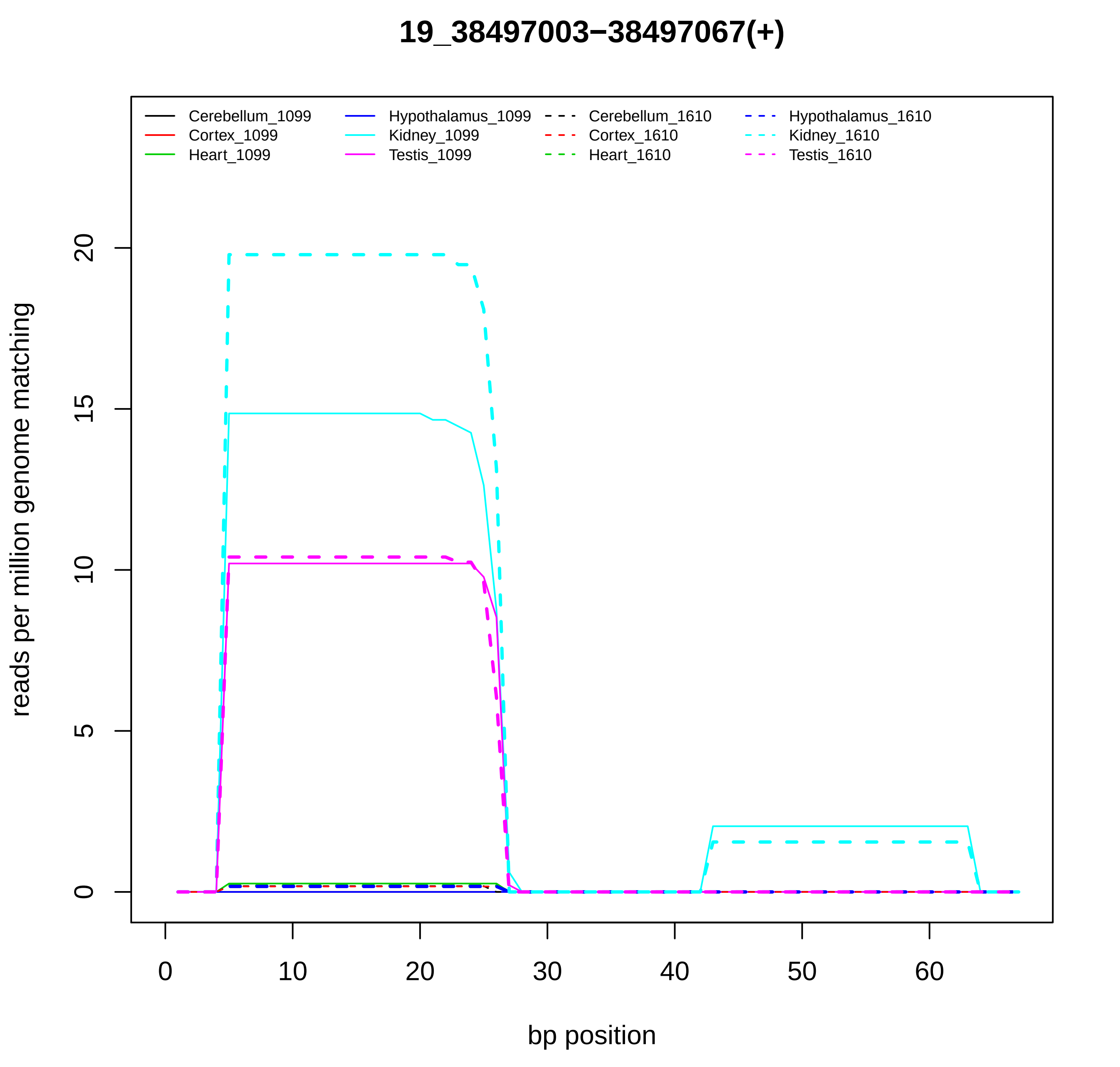

19_38497003−38497067(+)
Cerebellum_1099
Cortex_1099
Heart_1099
Hypothalamus_1099
Kidney_1099
Testis_1099
Cerebellum_1610
Cortex_1610
Heart_1610
Hypothalamus_1610
Kidney_1610
Testis_1610
reads per million genome matching
0
5
10
15
20
0
10
20
30
40
50
60
bp position

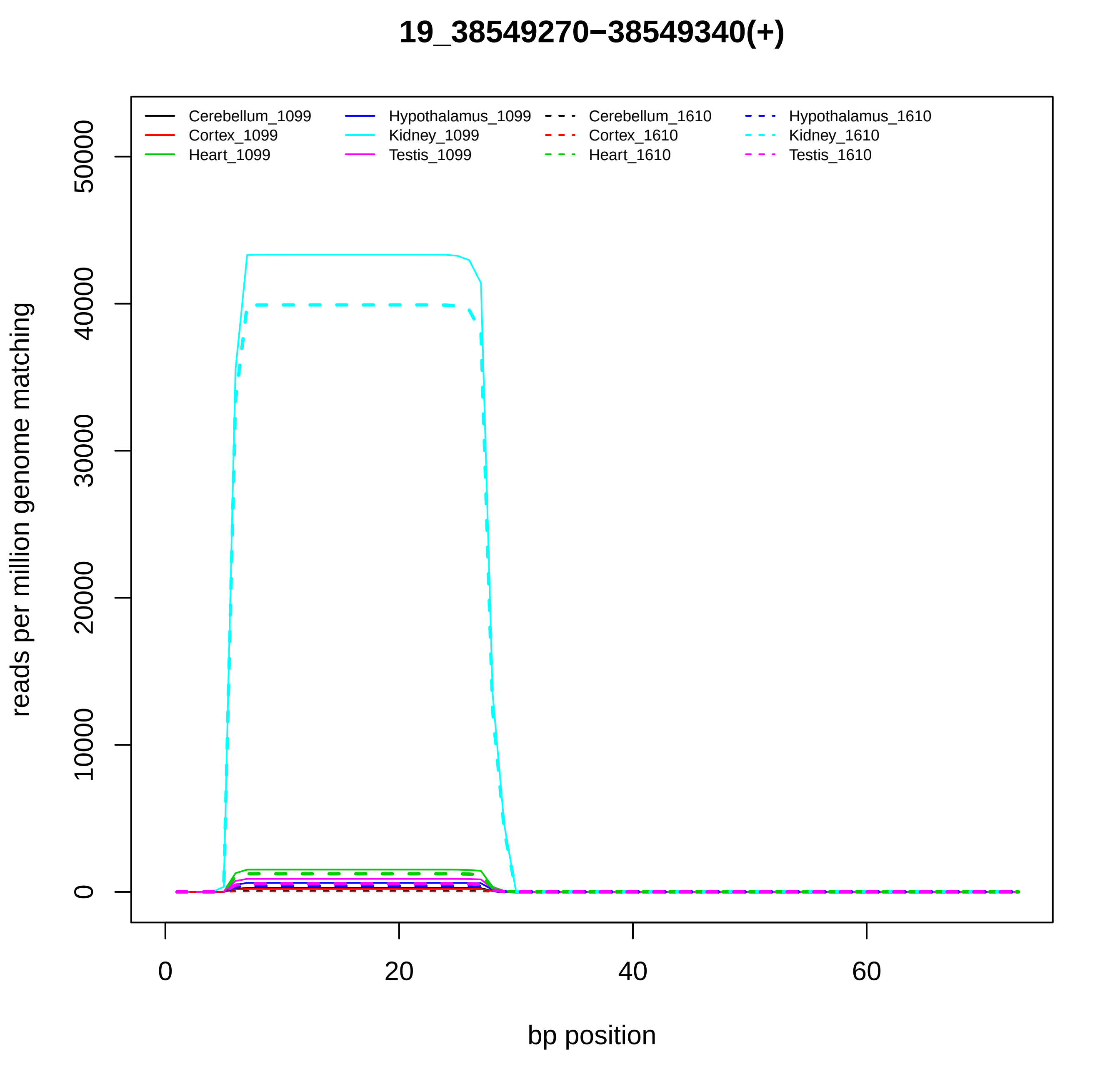

19_38549270−38549340(+)
Cerebellum_1099
Cortex_1099
Heart_1099
Hypothalamus_1099
Kidney_1099
Testis_1099
Cerebellum_1610
Cortex_1610
Heart_1610
Hypothalamus_1610
Kidney_1610
Testis_1610
reads per million genome matching
0
10000
20000
30000
40000
50000
0
20
40
60
bp position

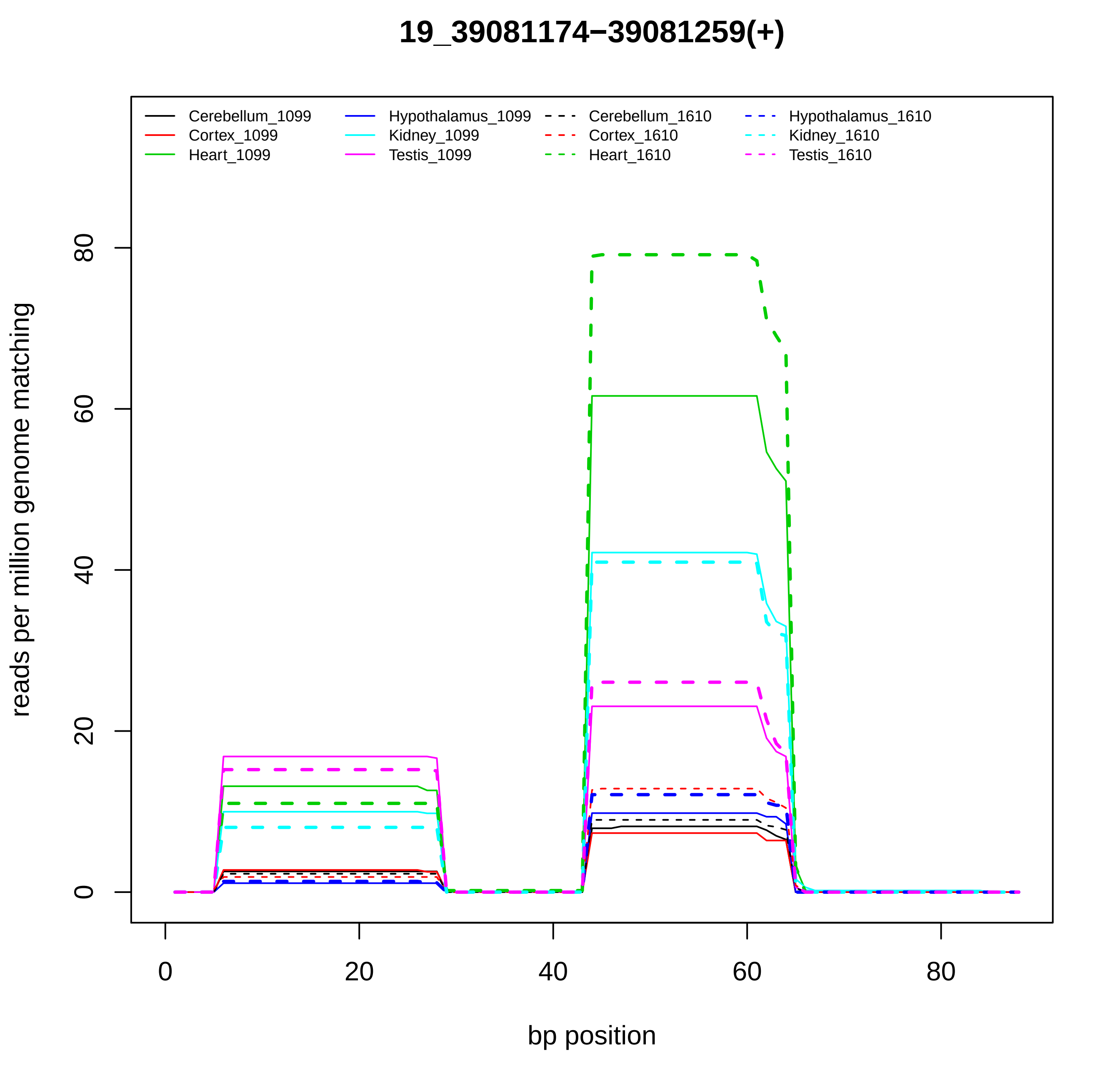

19_39081174−39081259(+)
Cerebellum_1099
Cortex_1099
Heart_1099
Hypothalamus_1099
Kidney_1099
Testis_1099
Cerebellum_1610
Cortex_1610
Heart_1610
Hypothalamus_1610
Kidney_1610
Testis_1610
reads per million genome matching
0
20
40
60
80
bp position

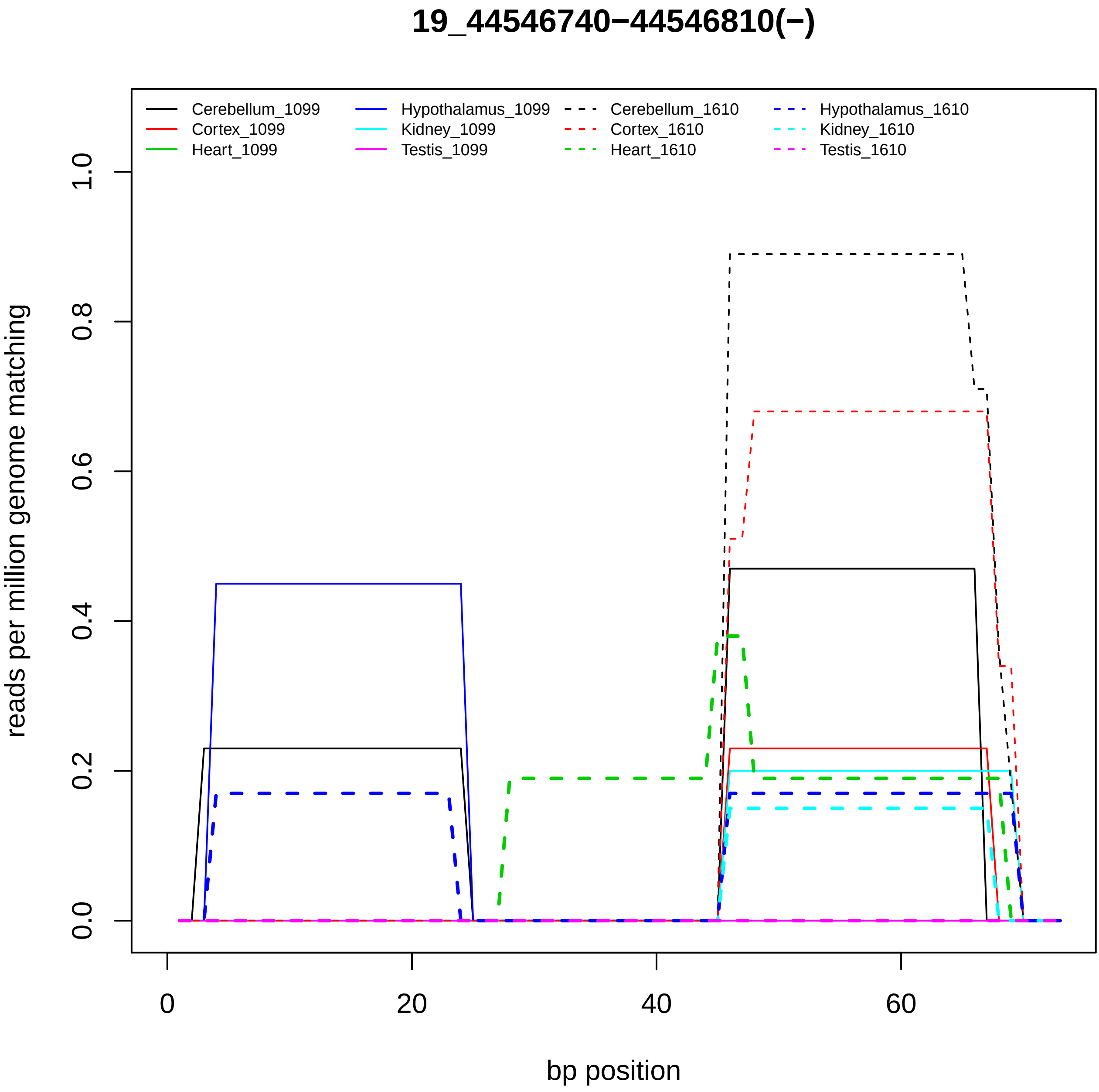

19_44546740−44546810(−)
Cerebellum_1099
Cortex_1099
Heart_1099
Hypothalamus_1099
Kidney_1099
Testis_1099
Cerebellum_1610
Cortex_1610
Heart_1610
Hypothalamus_1610
Kidney_1610
Testis_1610
reads per million genome matching
0.0
0.2
0.4
0.6
0.8
1.0
0
20
40
60
bp position

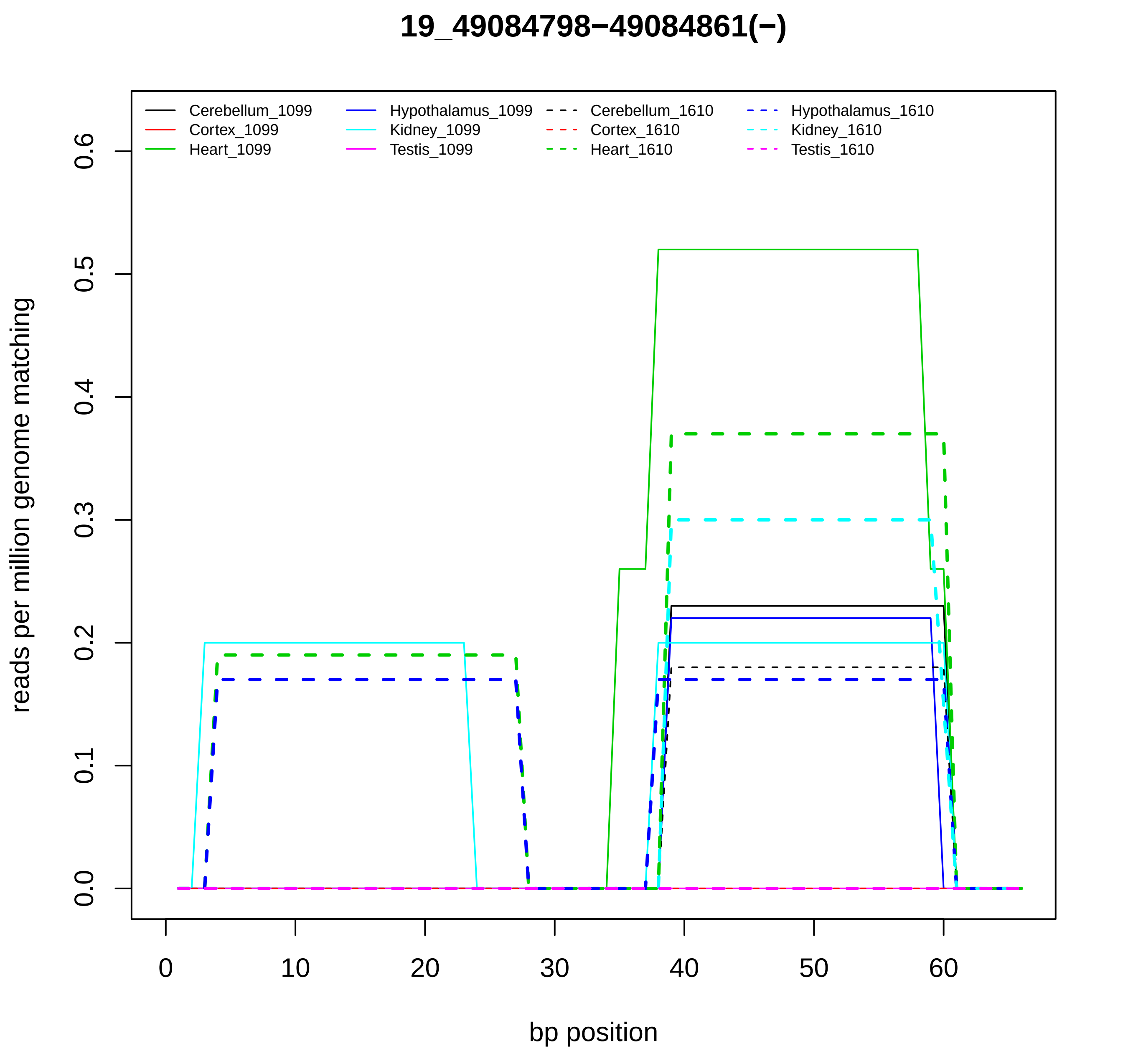
19_49084798−49084861(−)
Cerebellum_1099
Cortex_1099
Heart_1099
Hypothalamus_1099
Kidney_1099
Testis_1099
Cerebellum_1610
Cortex_1610
Heart_1610
Hypothalamus_1610
Kidney_1610
Testis_1610
reads per million genome matching
0.0
0.1
0.2
0.3
0.4
0.5
0.6
0
10
20
30
40
50
60
bp position

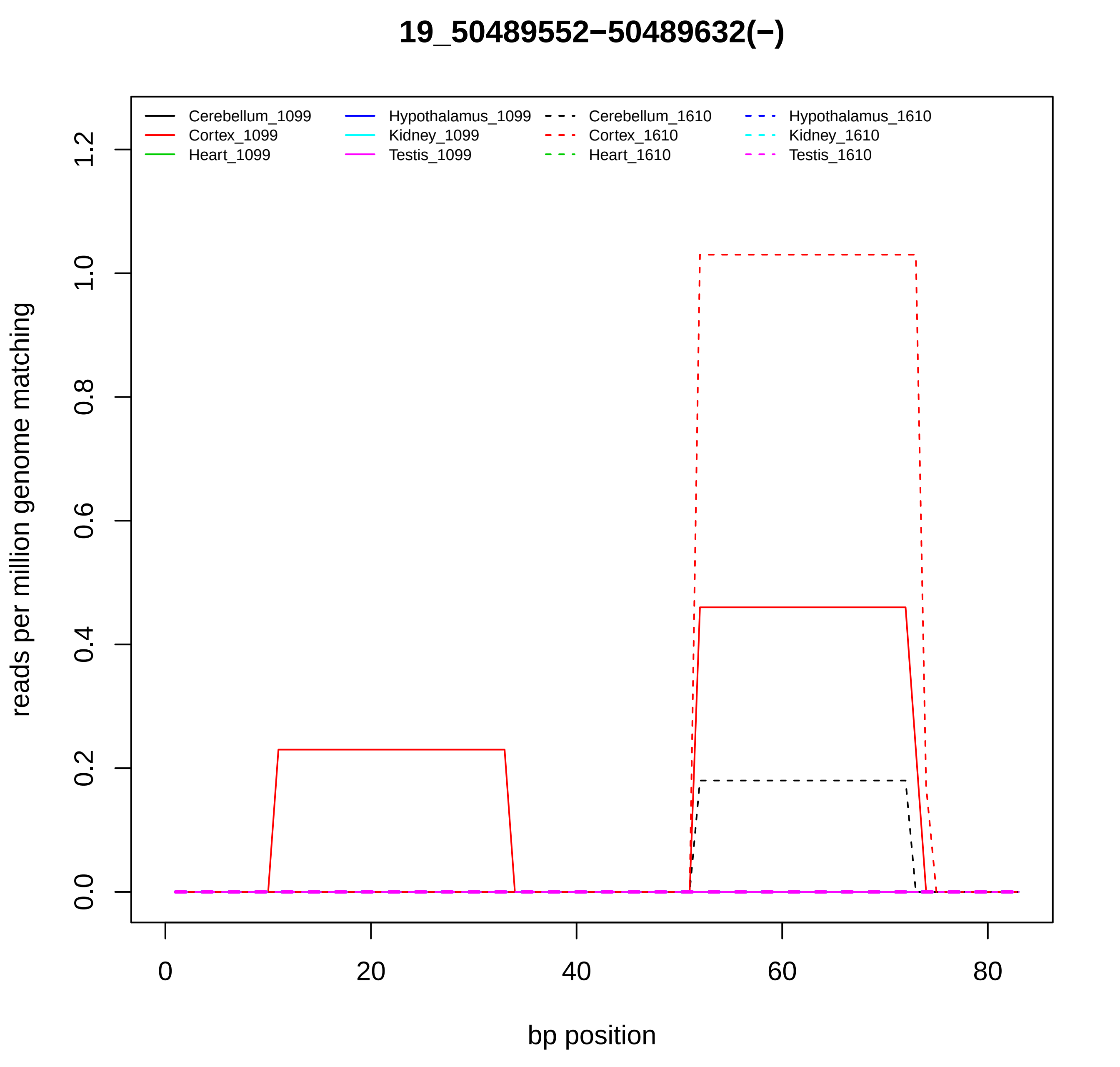

19_50489552−50489632(−)
Cerebellum_1099
Cortex_1099
Heart_1099
Hypothalamus_1099
Kidney_1099
Testis_1099
Cerebellum_1610
Cortex_1610
Heart_1610
Hypothalamus_1610
Kidney_1610
Testis_1610
reads per million genome matching
0.0
0.2
0.4
0.6
0.8
1.0
1.2
0
20
40
60
80
bp position

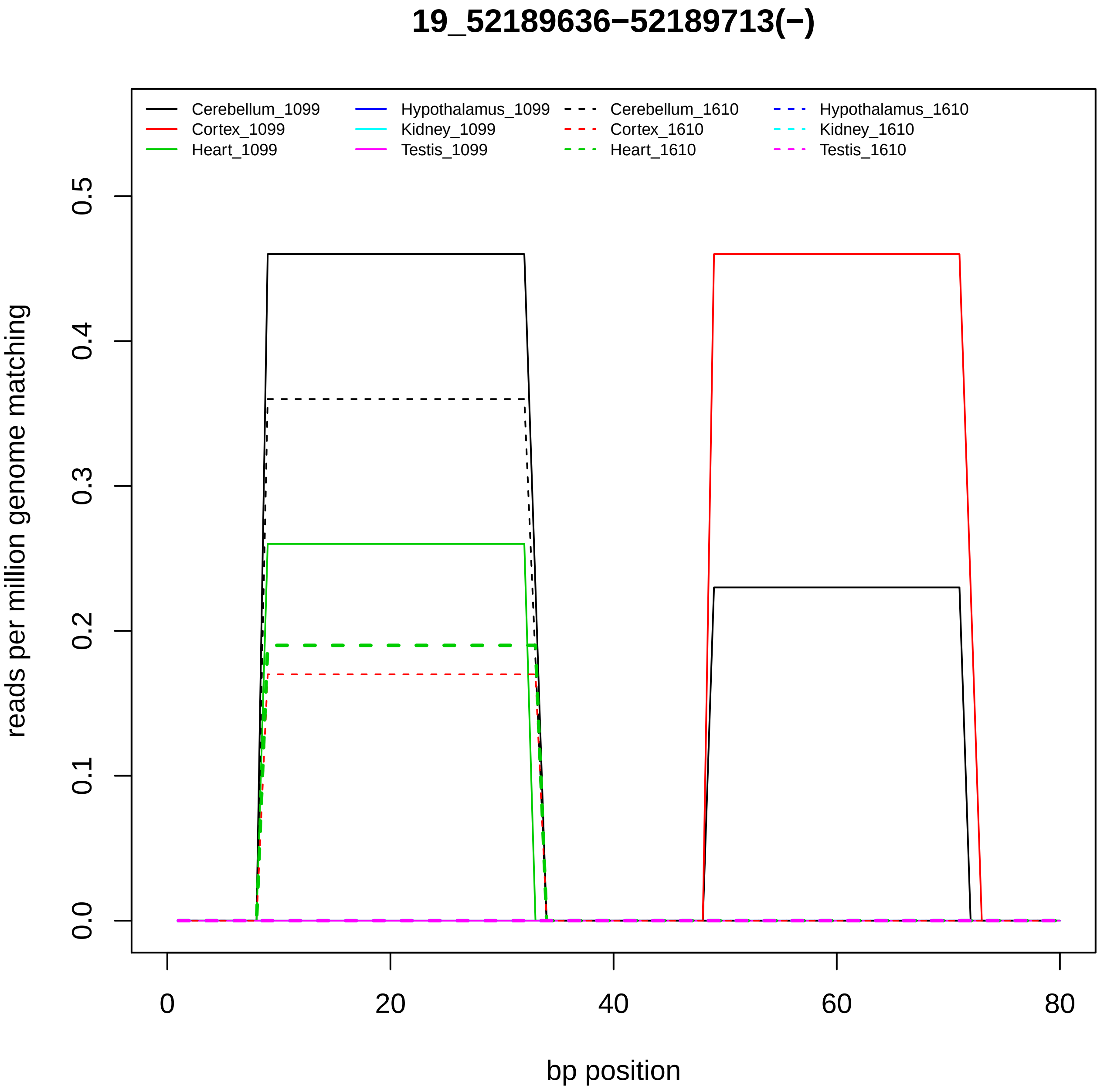

19_52189636−52189713(−)
Cerebellum_1099
Cortex_1099
Heart_1099
Hypothalamus_1099
Kidney_1099
Testis_1099
Cerebellum_1610
Cortex_1610
Heart_1610
Hypothalamus_1610
Kidney_1610
Testis_1610
reads per million genome matching
0.0
0.1
0.2
0.3
0.4
0.5
0
20
40
60
80
bp position

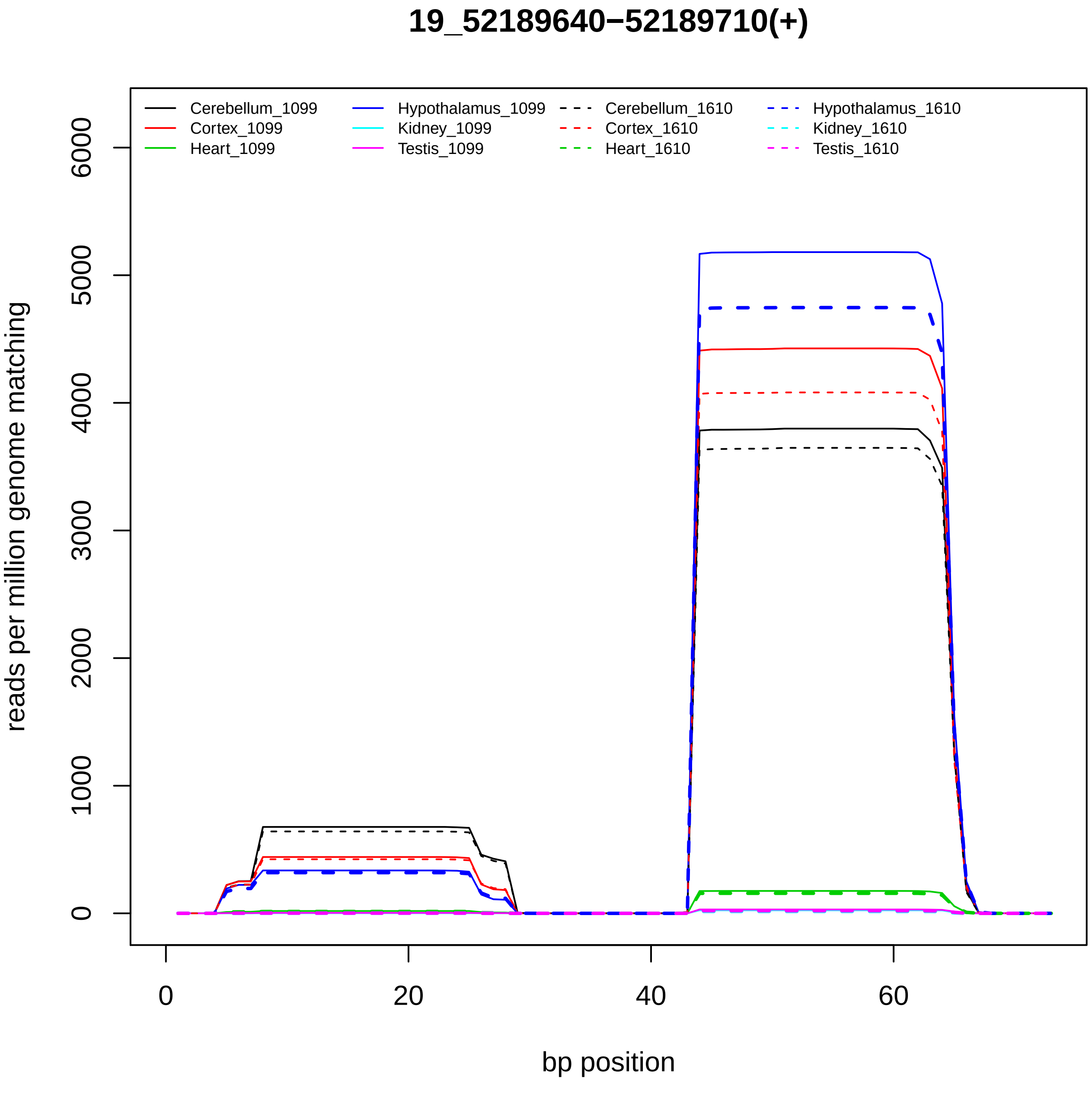

19_52189640−52189710(+)
Cerebellum_1099
Cortex_1099
Heart_1099
Hypothalamus_1099
Kidney_1099
Testis_1099
Cerebellum_1610
Cortex_1610
Heart_1610
Hypothalamus_1610
Kidney_1610
Testis_1610
reads per million genome matching
0
1000
2000
3000
4000
5000
6000
bp position
0
20
40
60

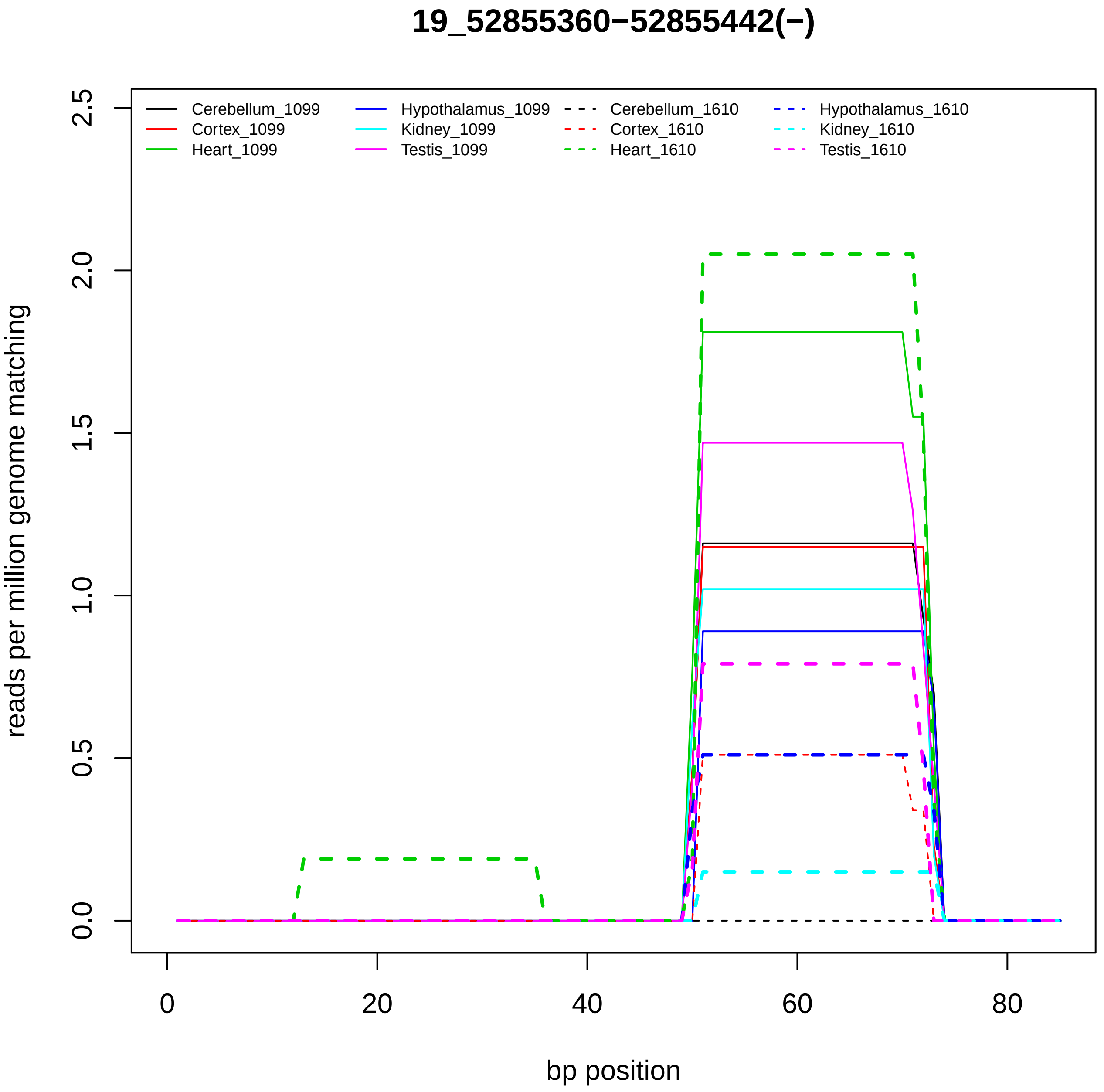
19_52855360−52855442(−)
Cerebellum_1099
Cortex_1099
Heart_1099
Hypothalamus_1099
Kidney_1099
Testis_1099
Cerebellum_1610
Cortex_1610
Heart_1610
Hypothalamus_1610
Kidney_1610
Testis_1610
reads per million genome matching
0.0
0.5
1.0
1.5
2.0
2.5
0
20
40
60
80
bp position

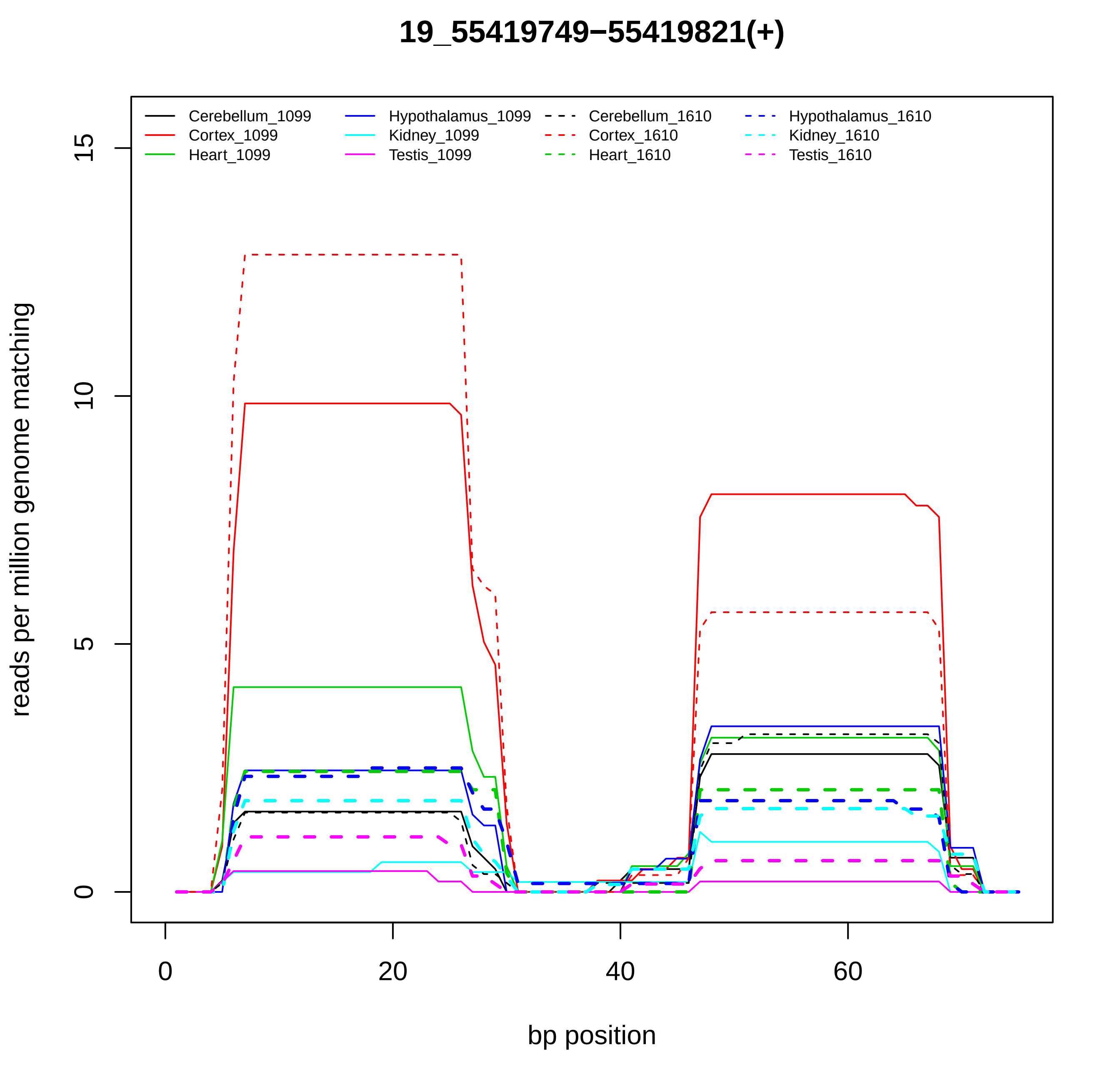

19_55419749−55419821(+)
Cerebellum_1099
Cortex_1099
Heart_1099
Hypothalamus_1099
Kidney_1099
Testis_1099
Cerebellum_1610
Cortex_1610
Heart_1610
Hypothalamus_1610
Kidney_1610
Testis_1610
reads per million genome matching
0
5
10
15
0
20
40
60
bp position

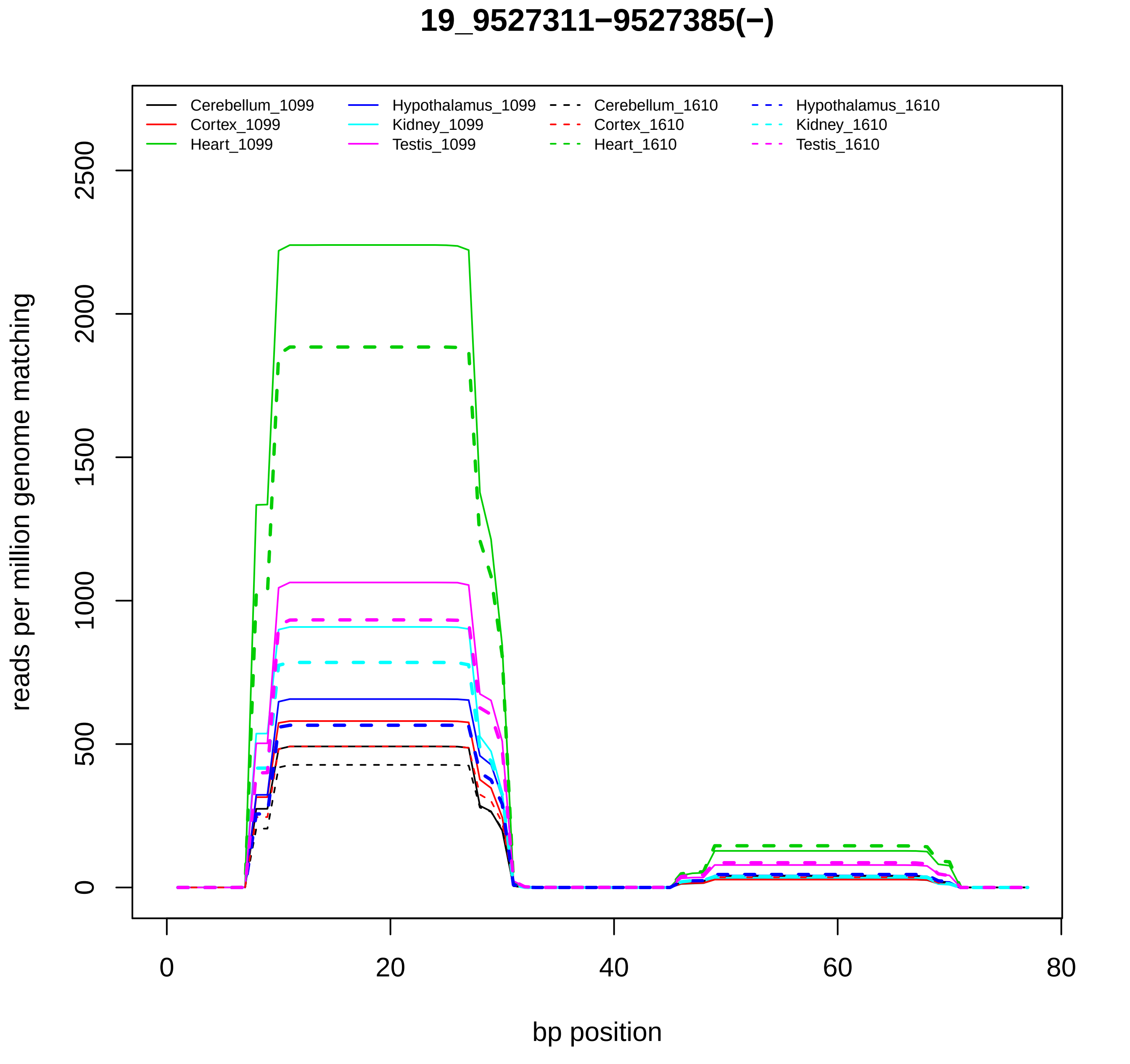
19_9527311−9527385(−)
Cerebellum_1099
Cortex_1099
Heart_1099
Hypothalamus_1099
Kidney_1099
Testis_1099
Cerebellum_1610
Cortex_1610
Heart_1610
Hypothalamus_1610
Kidney_1610
Testis_1610
reads per million genome matching
0
500
1000
1500
2000
2500
0
20
40
60
80
bp position

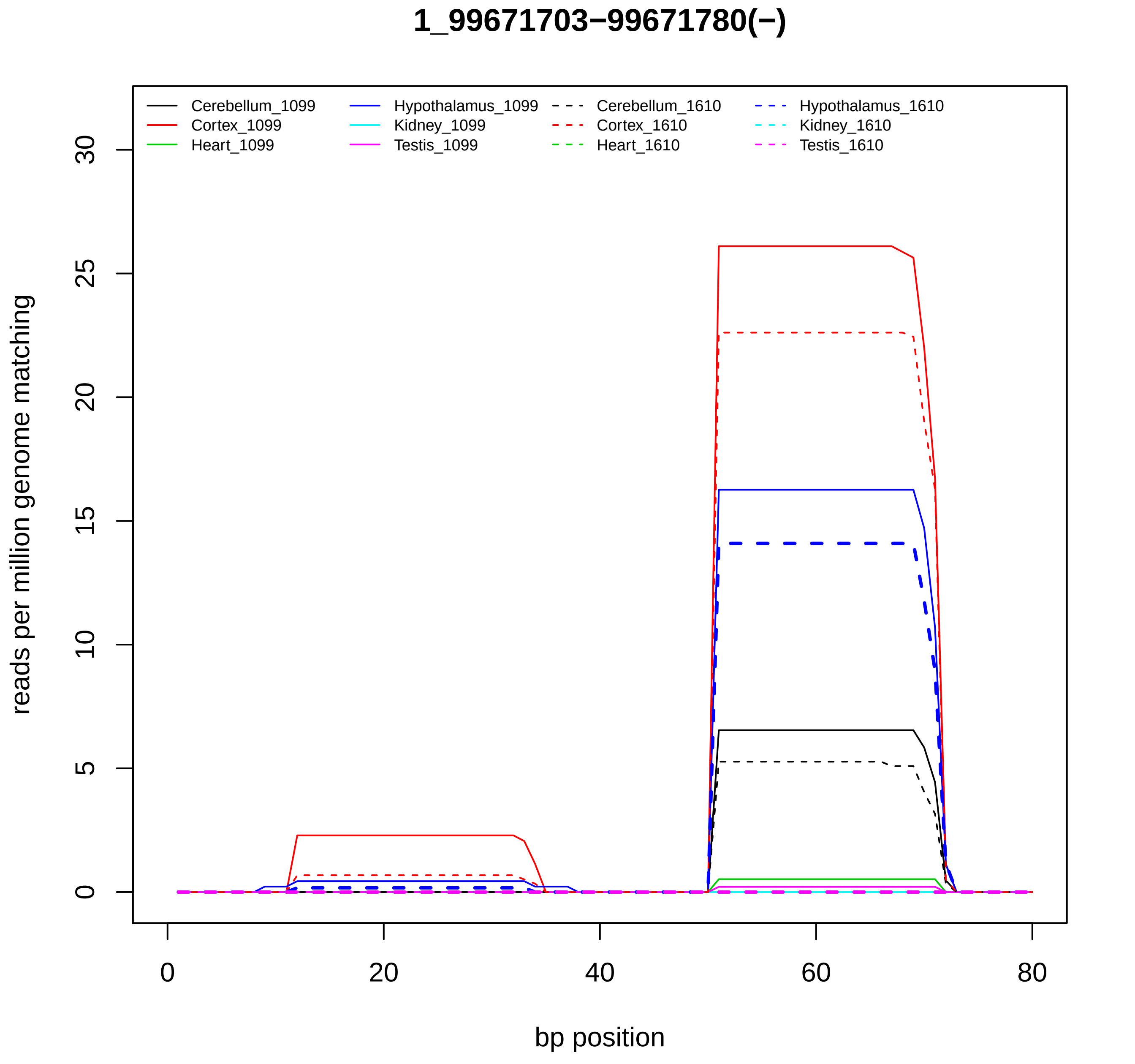
1_99671703−99671780(−)
Cerebellum_1099
Cortex_1099
Heart_1099
Hypothalamus_1099
Kidney_1099
Testis_1099
Cerebellum_1610
Cortex_1610
Heart_1610
Hypothalamus_1610
Kidney_1610
Testis_1610
reads per million genome matching
bp position
0
5
10
15
20
25
30
0
20
40
60
80

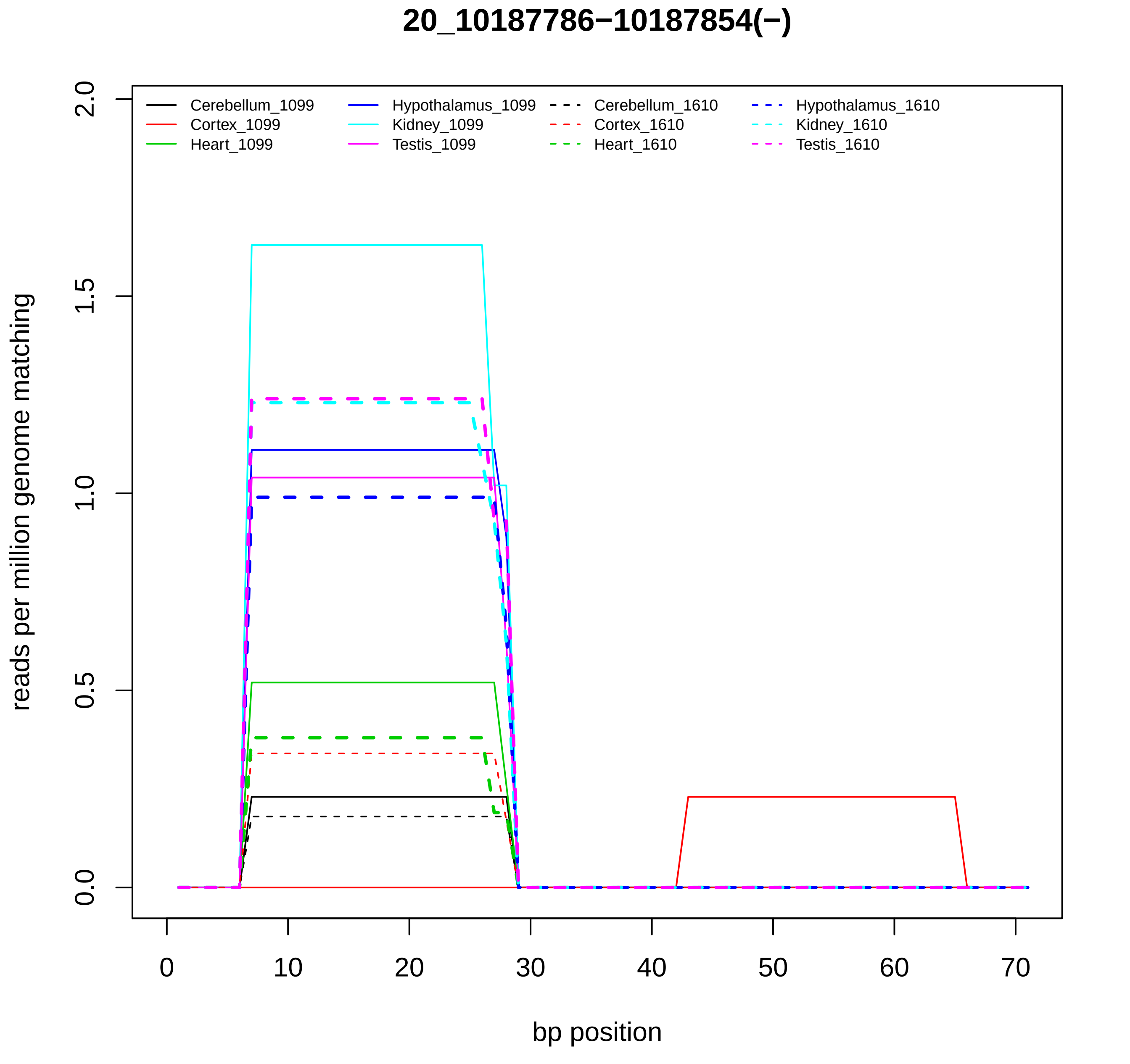

20_10187786−10187854(−)
Cerebellum_1099
Cortex_1099
Heart_1099
Hypothalamus_1099
Kidney_1099
Testis_1099
Cerebellum_1610
Cortex_1610
Heart_1610
Hypothalamus_1610
Kidney_1610
Testis_1610
reads per million genome matching
bp position
0.0
0.5
1.0
1.5
2.0
0
10
20
30
40
50
60
70

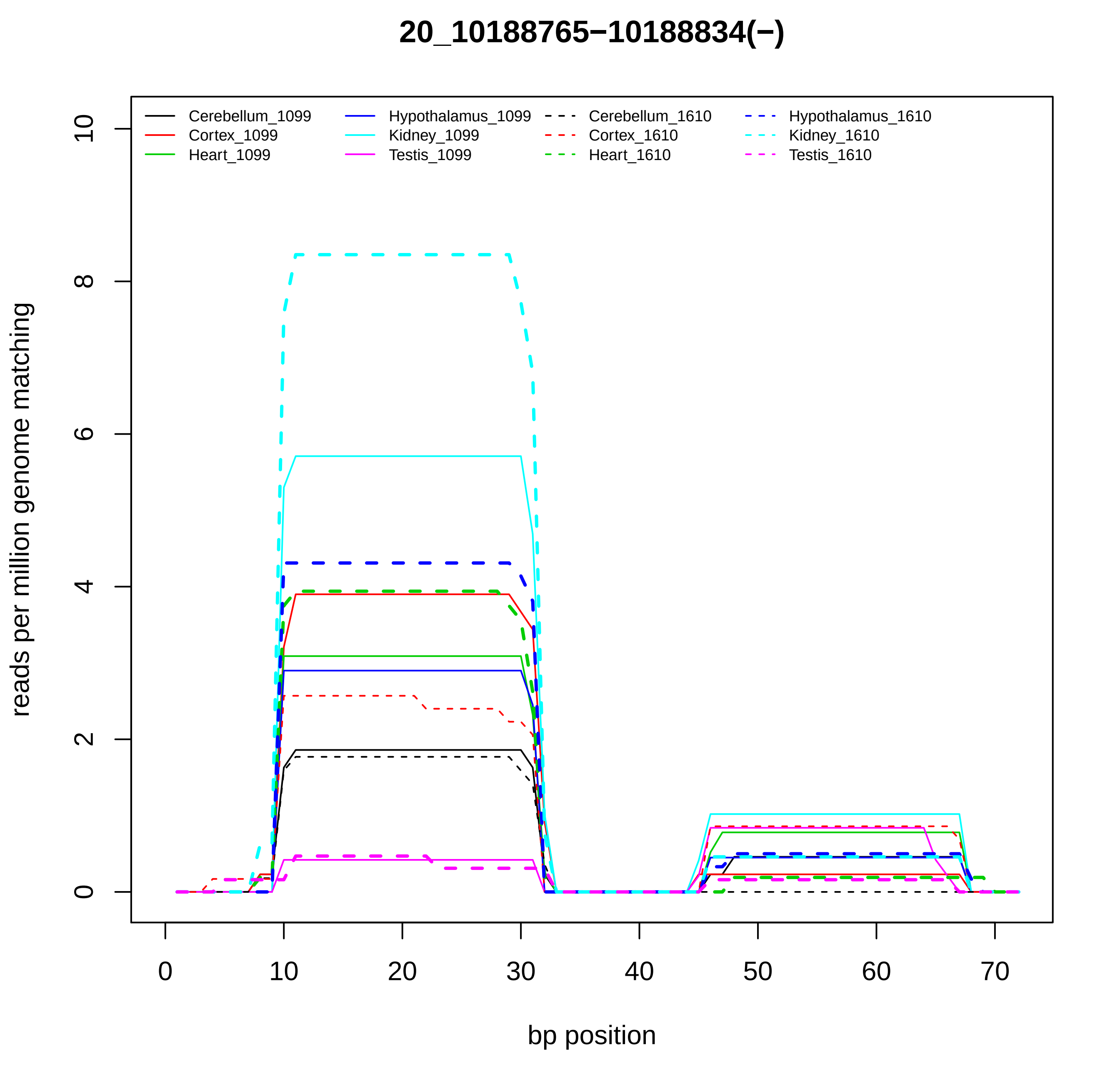
20_10188765−10188834(−)
Cerebellum_1099
Cortex_1099
Heart_1099
Hypothalamus_1099
Kidney_1099
Testis_1099
Cerebellum_1610
Cortex_1610
Heart_1610
Hypothalamus_1610
Kidney_1610
Testis_1610
reads per million genome matching
bp position
0
2
4
6
8
10
0
10
20
30
40
50
60
70

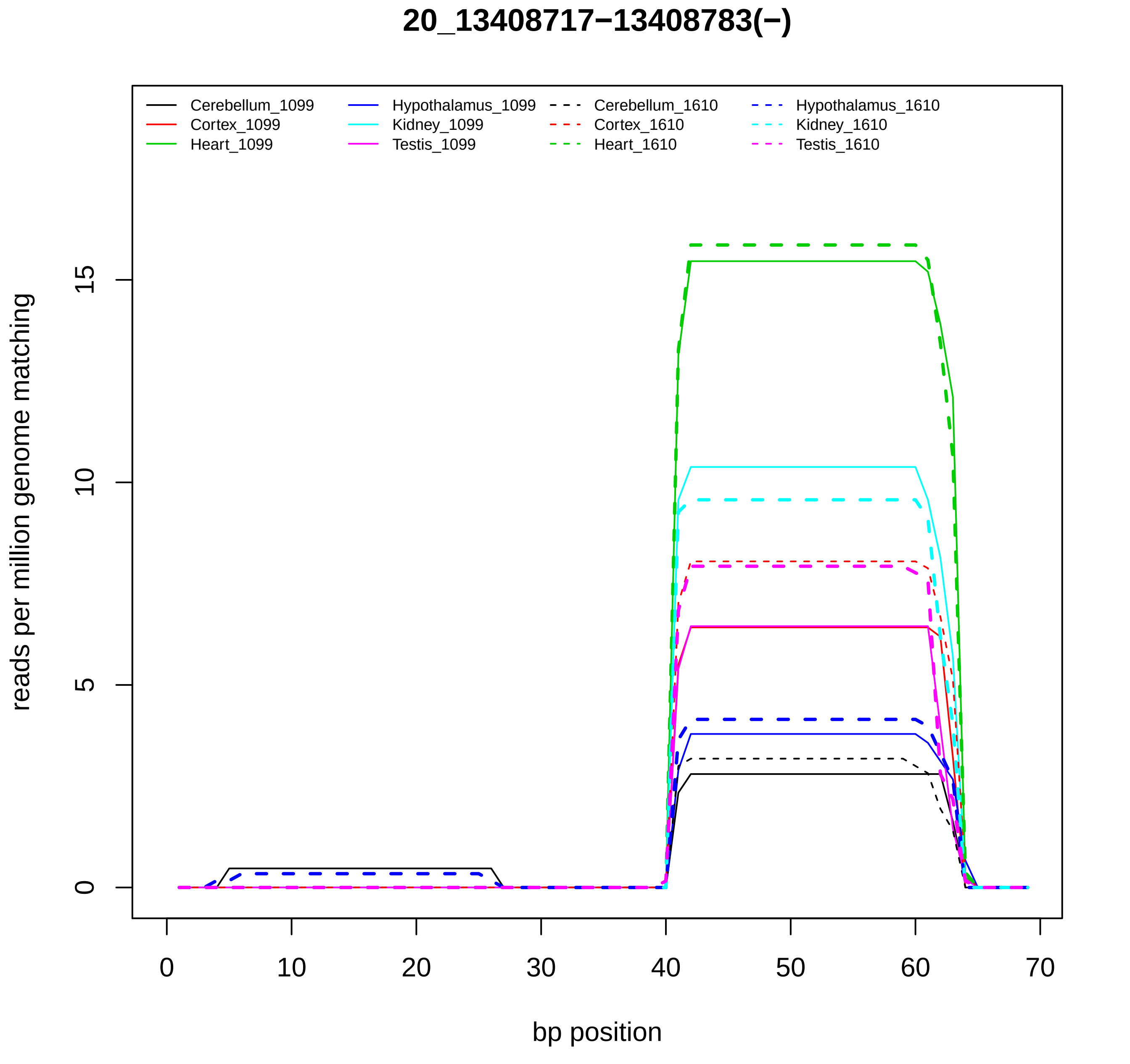

20_13408717−13408783(−)
Cerebellum_1099
Cortex_1099
Heart_1099
Hypothalamus_1099
Kidney_1099
Testis_1099
Cerebellum_1610
Cortex_1610
Heart_1610
Hypothalamus_1610
Kidney_1610
Testis_1610
reads per million genome matching
bp position
0
5
10
15
0
10
20
30
40
50
60
70

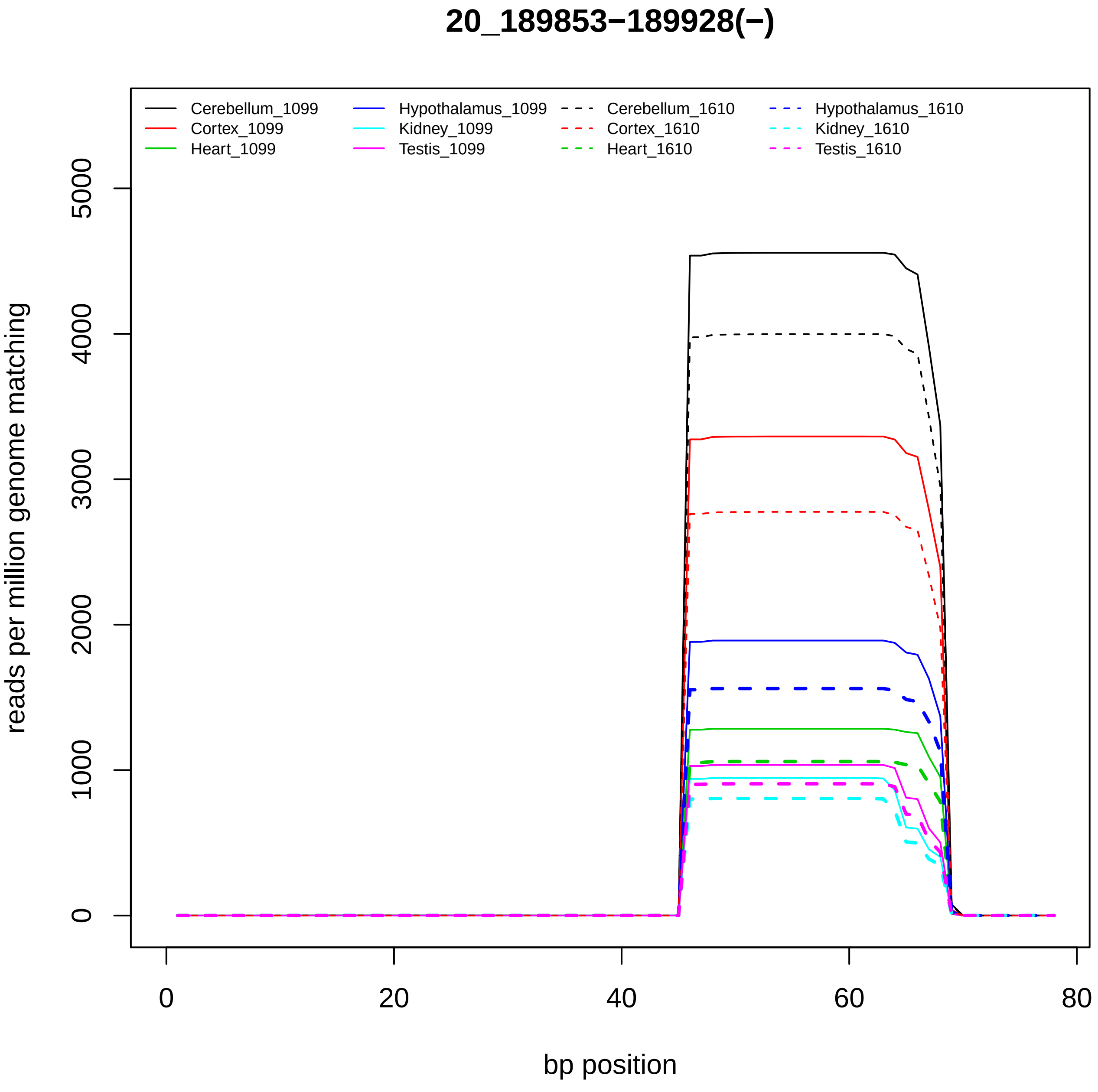

20_189853−189928(−)
Cerebellum_1099
Cortex_1099
Heart_1099
Hypothalamus_1099
Kidney_1099
Testis_1099
Cerebellum_1610
Cortex_1610
Heart_1610
Hypothalamus_1610
Kidney_1610
Testis_1610
reads per million genome matching
0
1000
2000
3000
4000
5000
bp position
0
20
40
60
80

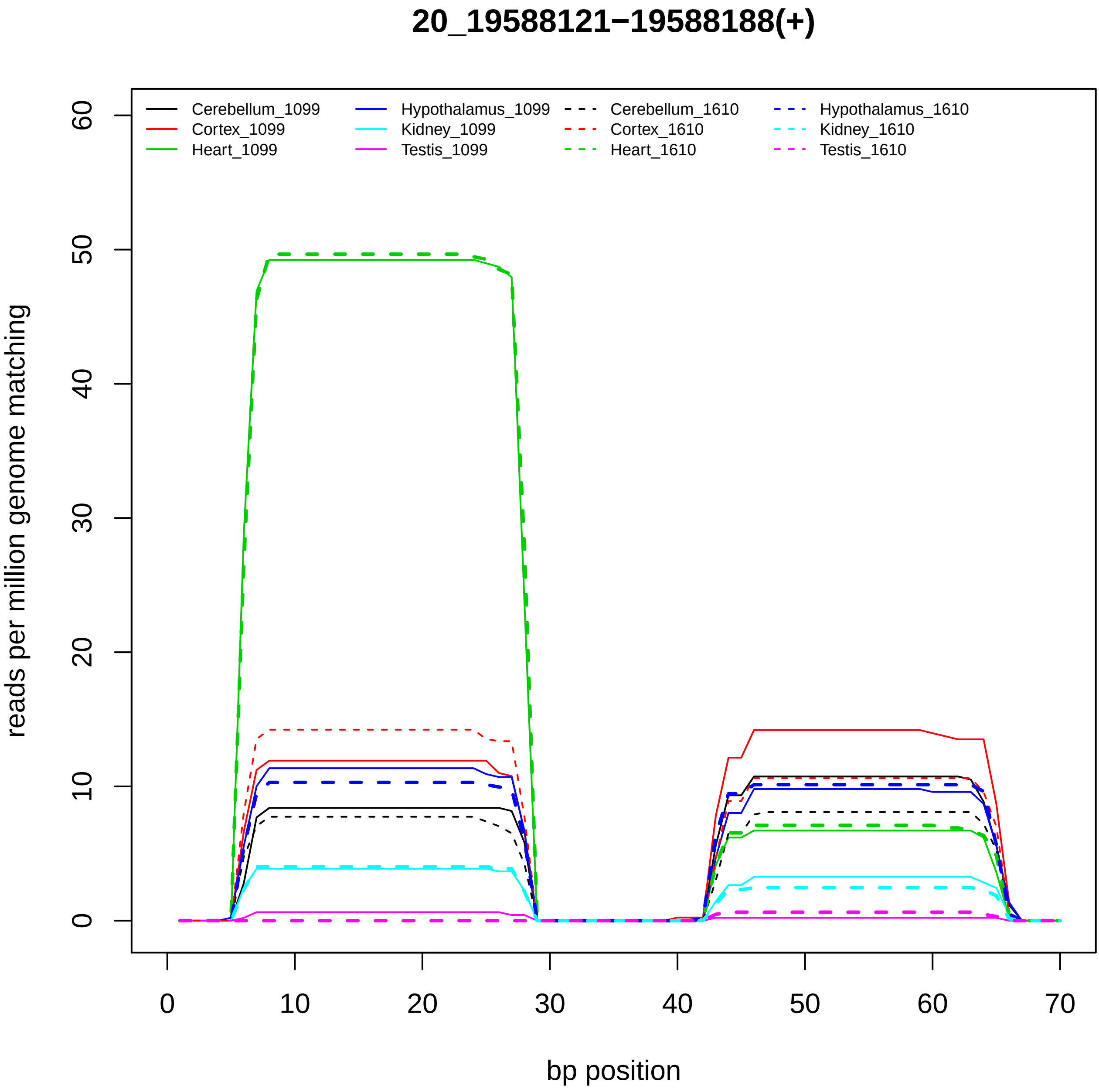

20_19588121−19588188(+)
Cerebellum_1099
Cortex_1099
Heart_1099
Hypothalamus_1099
Kidney_1099
Testis_1099
Cerebellum_1610
Cortex_1610
Heart_1610
Hypothalamus_1610
Kidney_1610
Testis_1610
reads per million genome matching
bp position
0
10
20
30
40
50
60
70

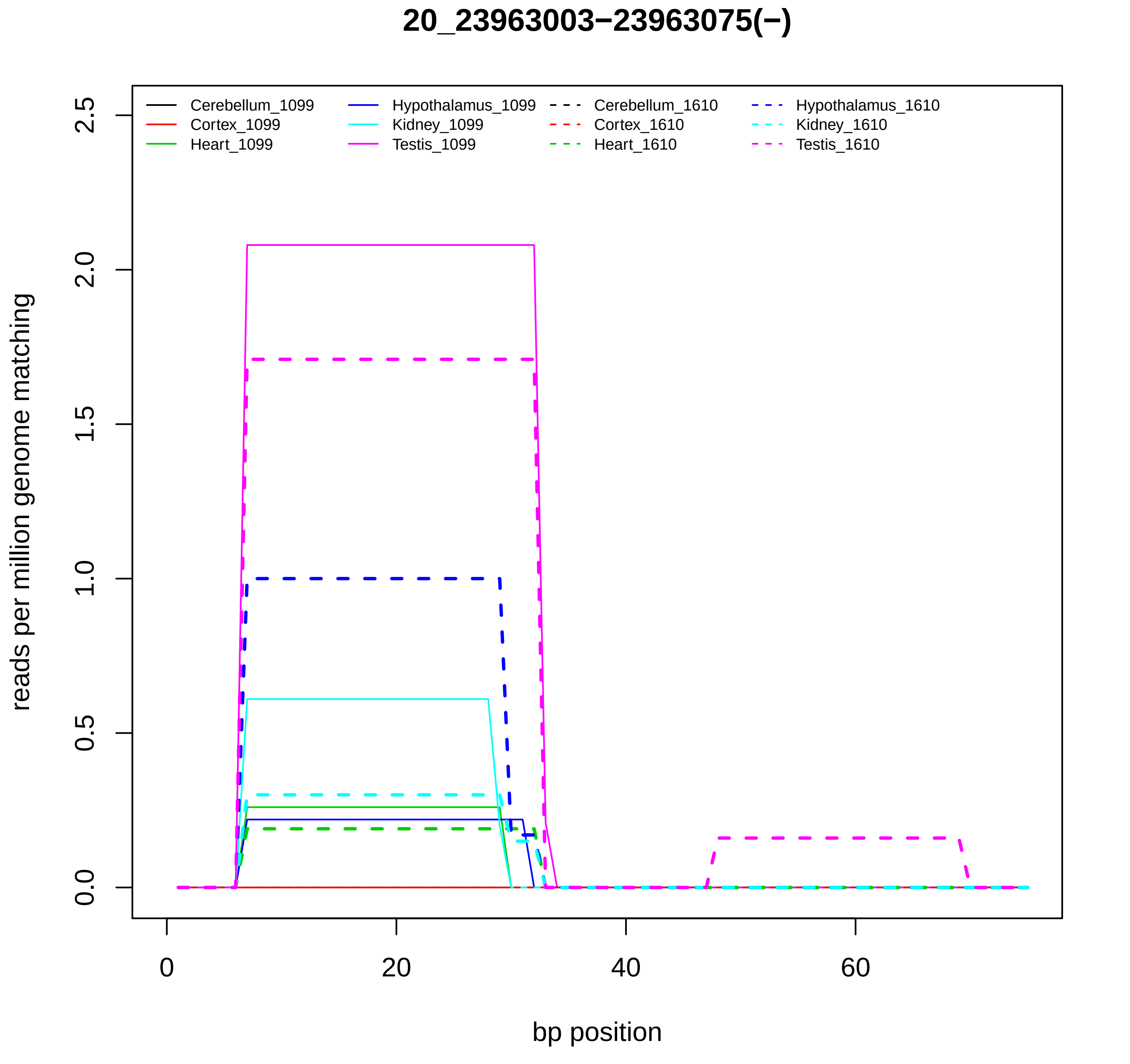

20_23963003−23963075(−)
Cerebellum_1099
Cortex_1099
Heart_1099
Hypothalamus_1099
Kidney_1099
Testis_1099
Cerebellum_1610
Cortex_1610
Heart_1610
Hypothalamus_1610
Kidney_1610
Testis_1610
reads per million genome matching
bp position
0.0
0.5
1.0
1.5
2.0
2.5
0
20
40
60

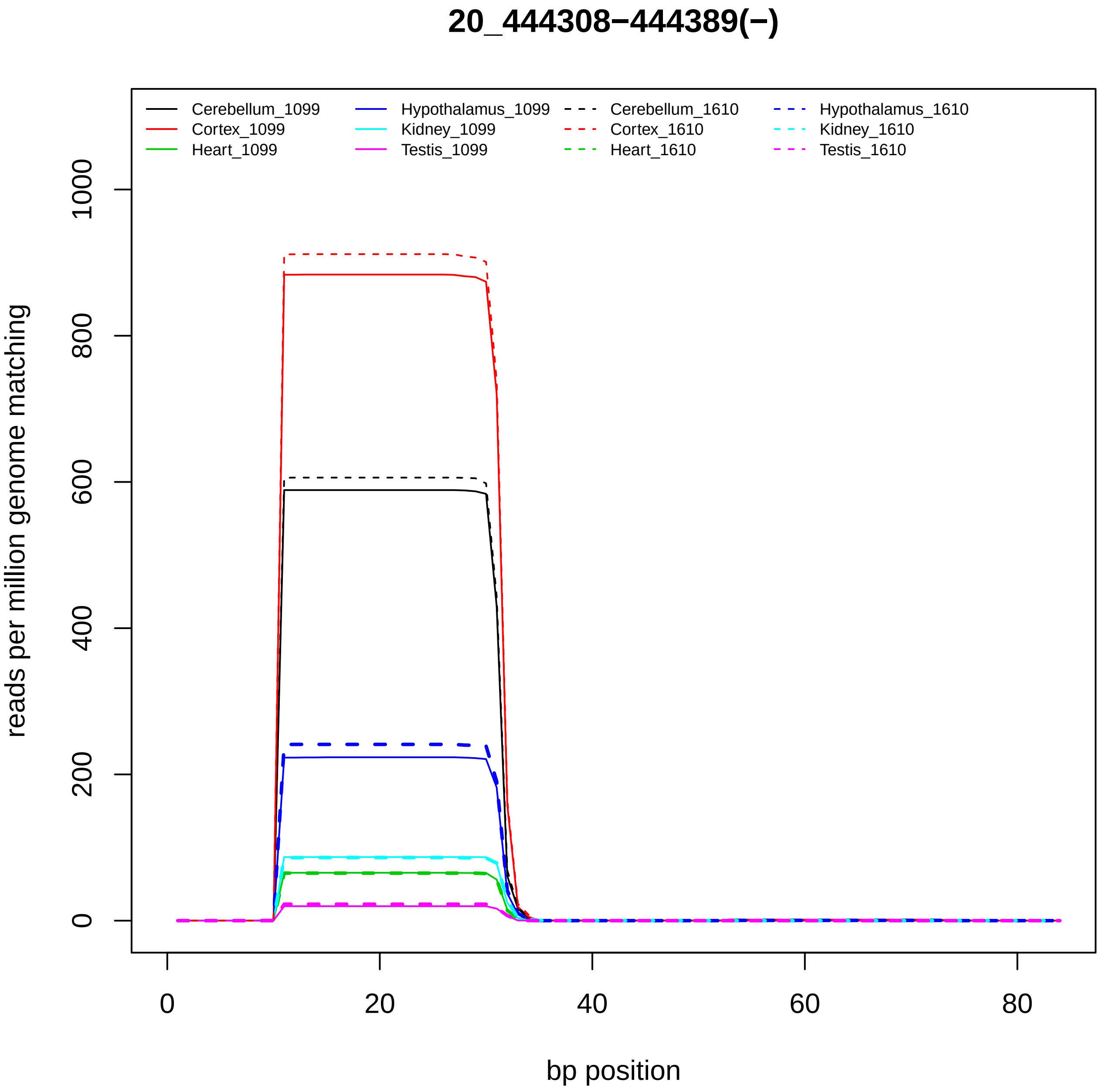
20_444308−444389(−)
Cerebellum_1099
Cortex_1099
Heart_1099
Hypothalamus_1099
Kidney_1099
Testis_1099
Cerebellum_1610
Cortex_1610
Heart_1610
Hypothalamus_1610
Kidney_1610
Testis_1610
reads per million genome matching
0
200
400
600
800
1000
0
20
40
60
80
bp position

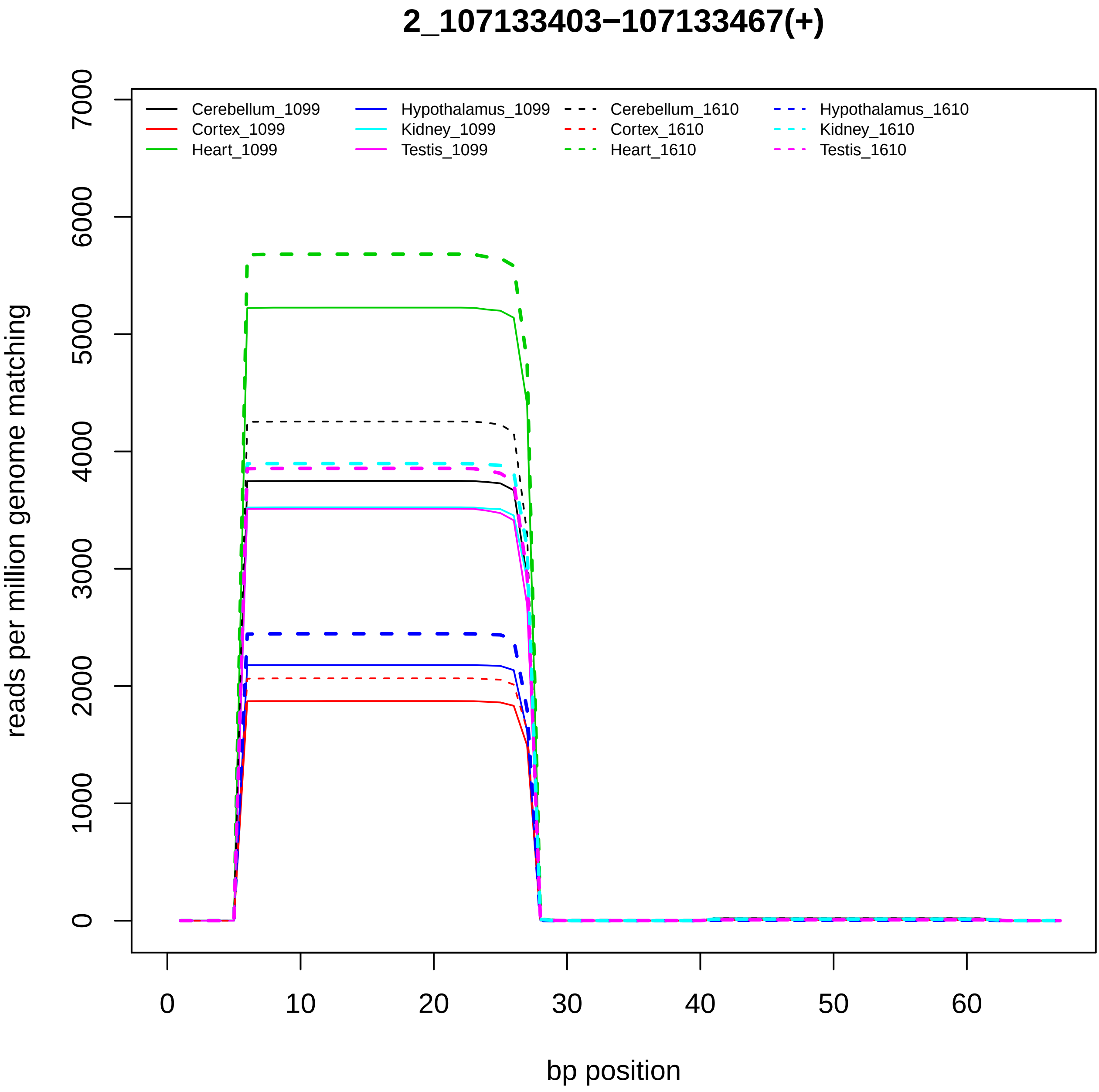

2_107133403−107133467(+)
Cerebellum_1099
Cortex_1099
Heart_1099
Hypothalamus_1099
Kidney_1099
Testis_1099
Cerebellum_1610
Cortex_1610
Heart_1610
Hypothalamus_1610
Kidney_1610
Testis_1610
reads per million genome matching
0
1000
2000
3000
4000
5000
6000
7000
bp position
0
10
20
30
40
50
60

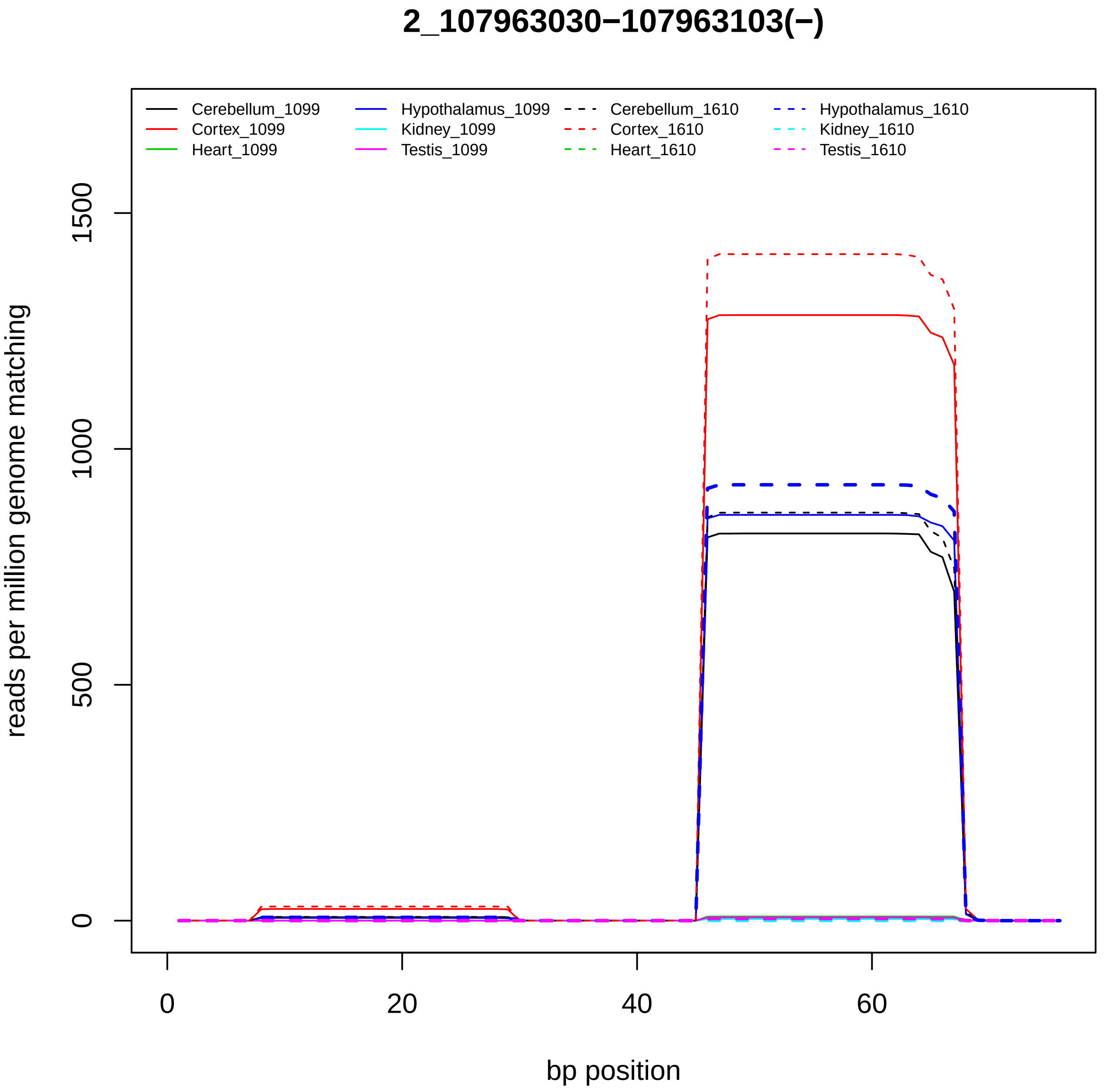

2_107963030−107963103(−)
Cerebellum_1099
Cortex_1099
Heart_1099
Hypothalamus_1099
Kidney_1099
Testis_1099
Cerebellum_1610
Cortex_1610
Heart_1610
Hypothalamus_1610
Kidney_1610
Testis_1610
reads per million genome matching
0
500
1000
1500
0
20
40
60
bp position

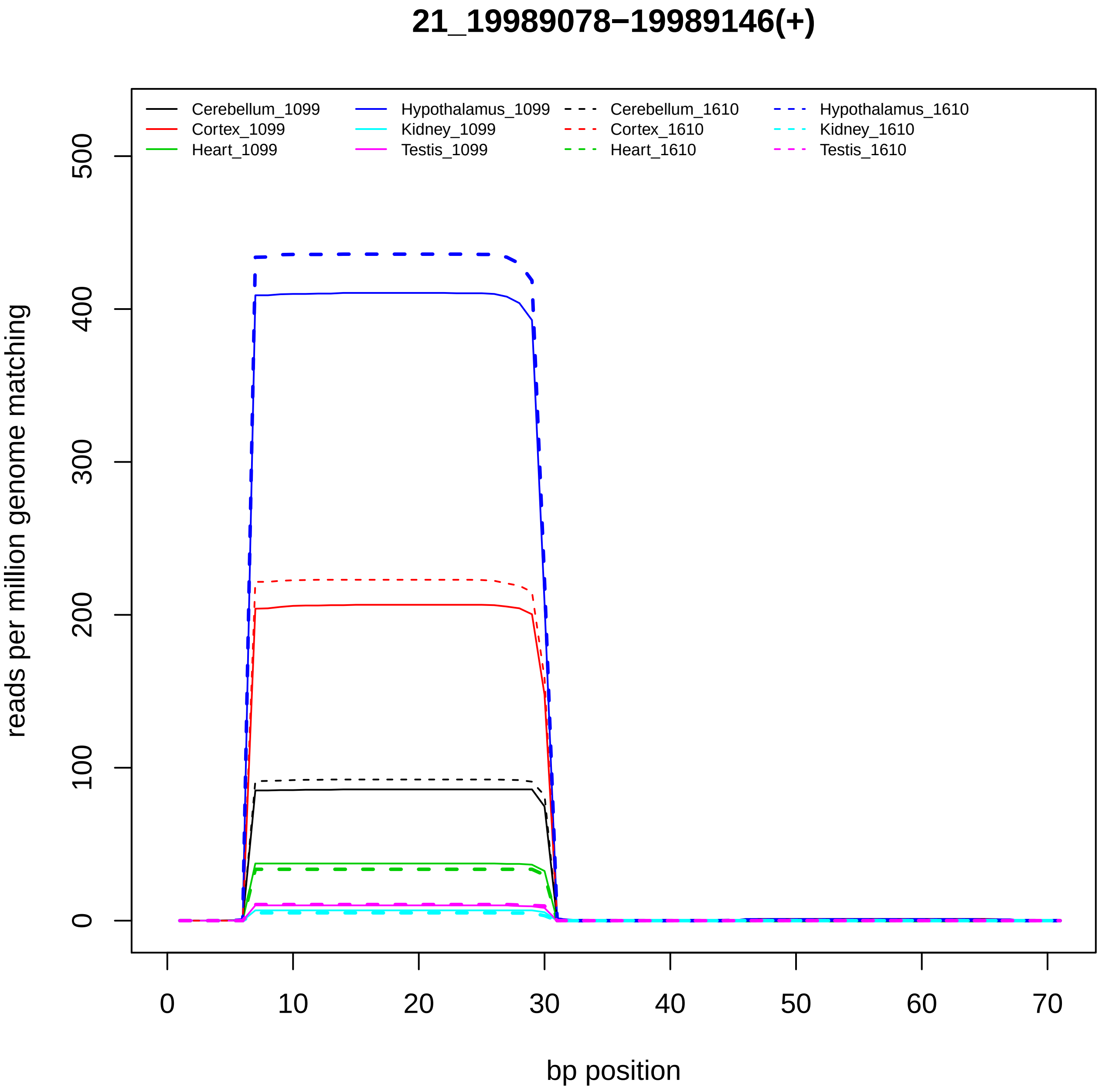

21_19989078−19989146(+)
Cerebellum_1099
Cortex_1099
Heart_1099
Hypothalamus_1099
Kidney_1099
Testis_1099
Cerebellum_1610
Cortex_1610
Heart_1610
Hypothalamus_1610
Kidney_1610
Testis_1610
reads per million genome matching
0
100
200
300
400
500
0
10
20
30
40
50
60
70
bp position

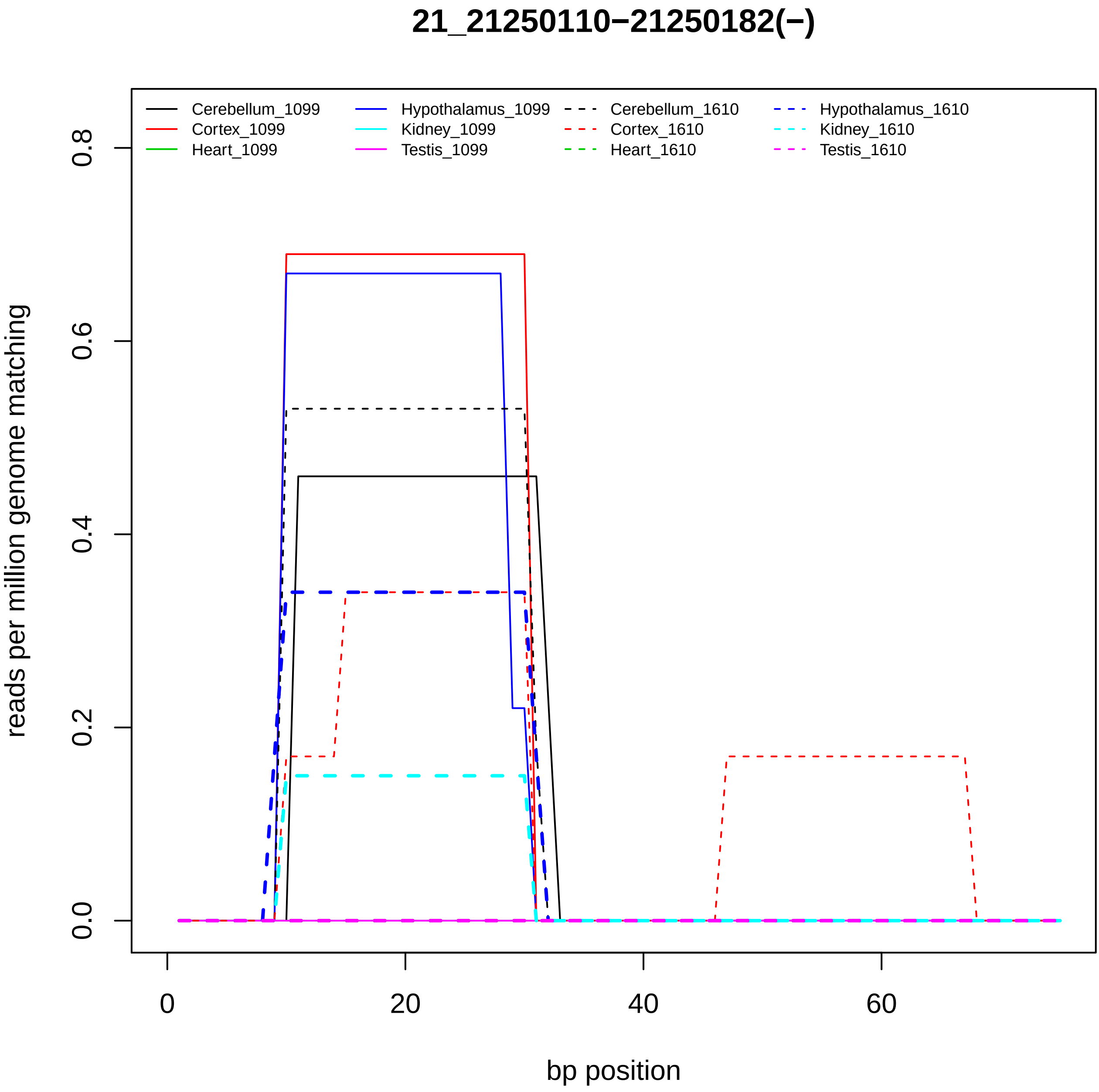

21_21250110−21250182(−)
Cerebellum_1099
Cortex_1099
Heart_1099
Hypothalamus_1099
Kidney_1099
Testis_1099
Cerebellum_1610
Cortex_1610
Heart_1610
Hypothalamus_1610
Kidney_1610
Testis_1610
reads per million genome matching
0.0
0.2
0.4
0.6
0.8
0
20
40
60
bp position

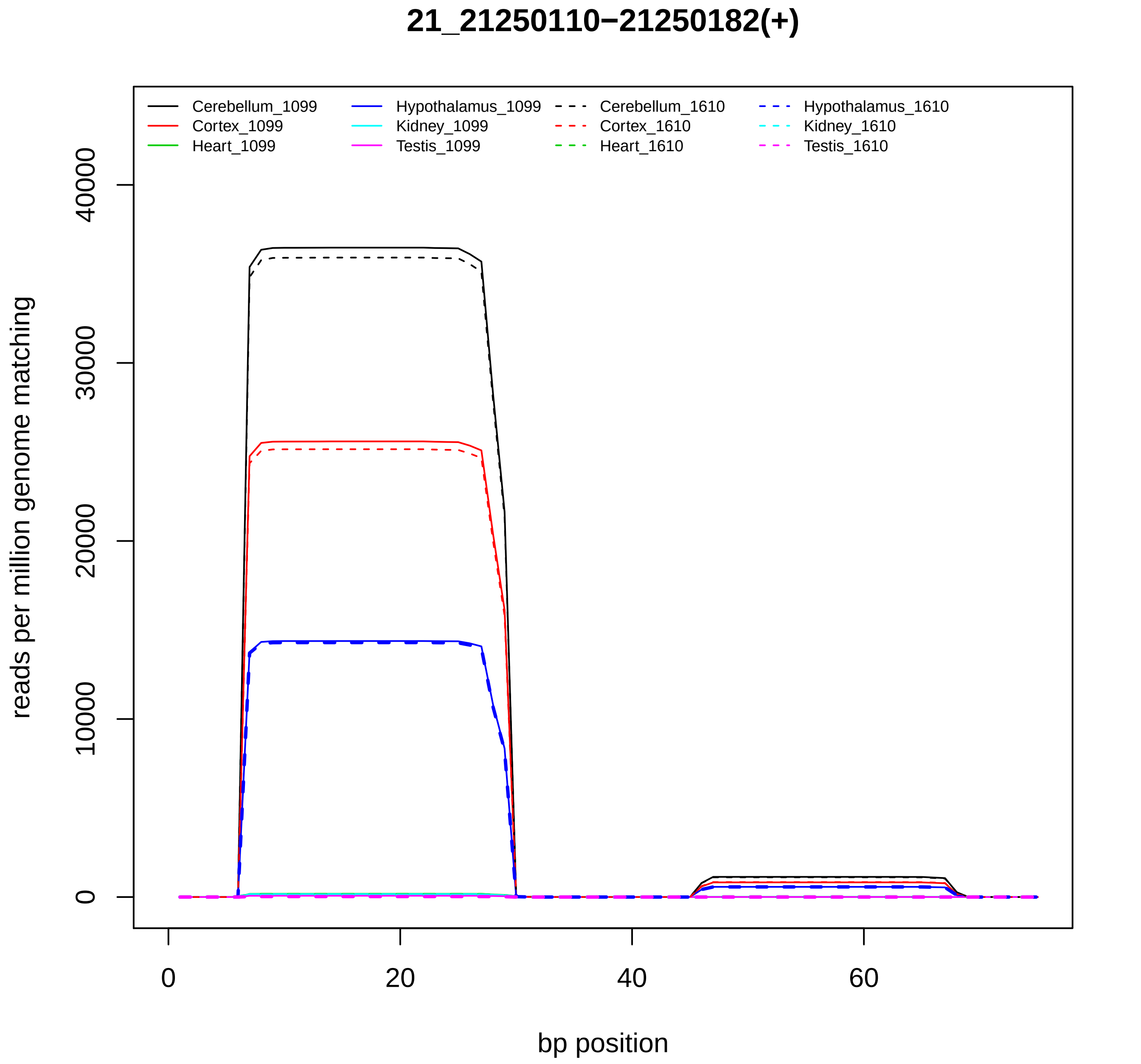

21_21250110−21250182(+)
Cerebellum_1099
Cortex_1099
Heart_1099
Hypothalamus_1099
Kidney_1099
Testis_1099
Cerebellum_1610
Cortex_1610
Heart_1610
Hypothalamus_1610
Kidney_1610
Testis_1610
reads per million genome matching
0
10000
20000
30000
40000
bp position
0
20
40
60

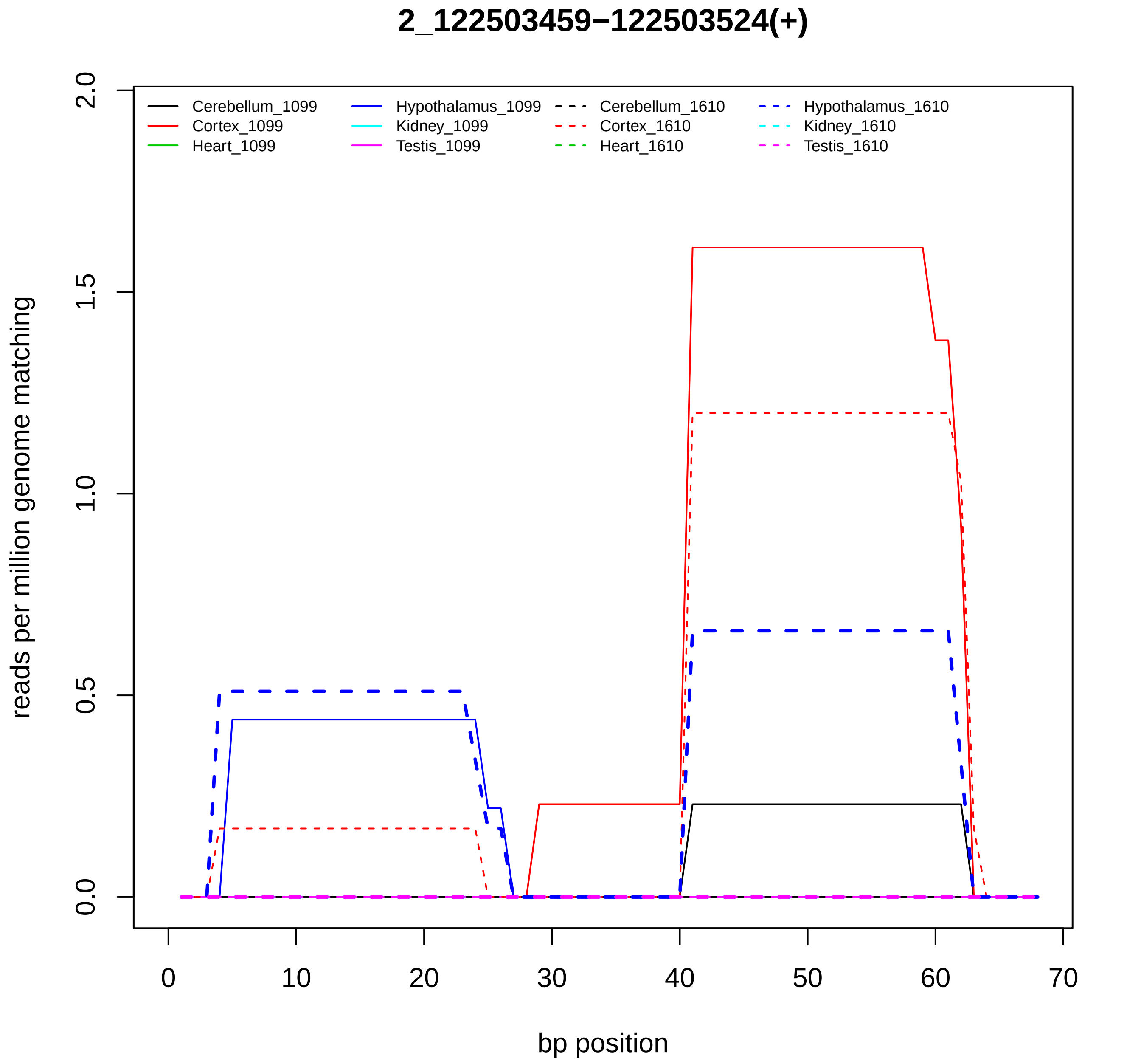

2_122503459−122503524(+)
Cerebellum_1099
Cortex_1099
Heart_1099
Hypothalamus_1099
Kidney_1099
Testis_1099
Cerebellum_1610
Cortex_1610
Heart_1610
Hypothalamus_1610
Kidney_1610
Testis_1610
reads per million genome matching
bp position
0.0
0.5
1.0
1.5
2.0
0
10
20
30
40
50
60
70

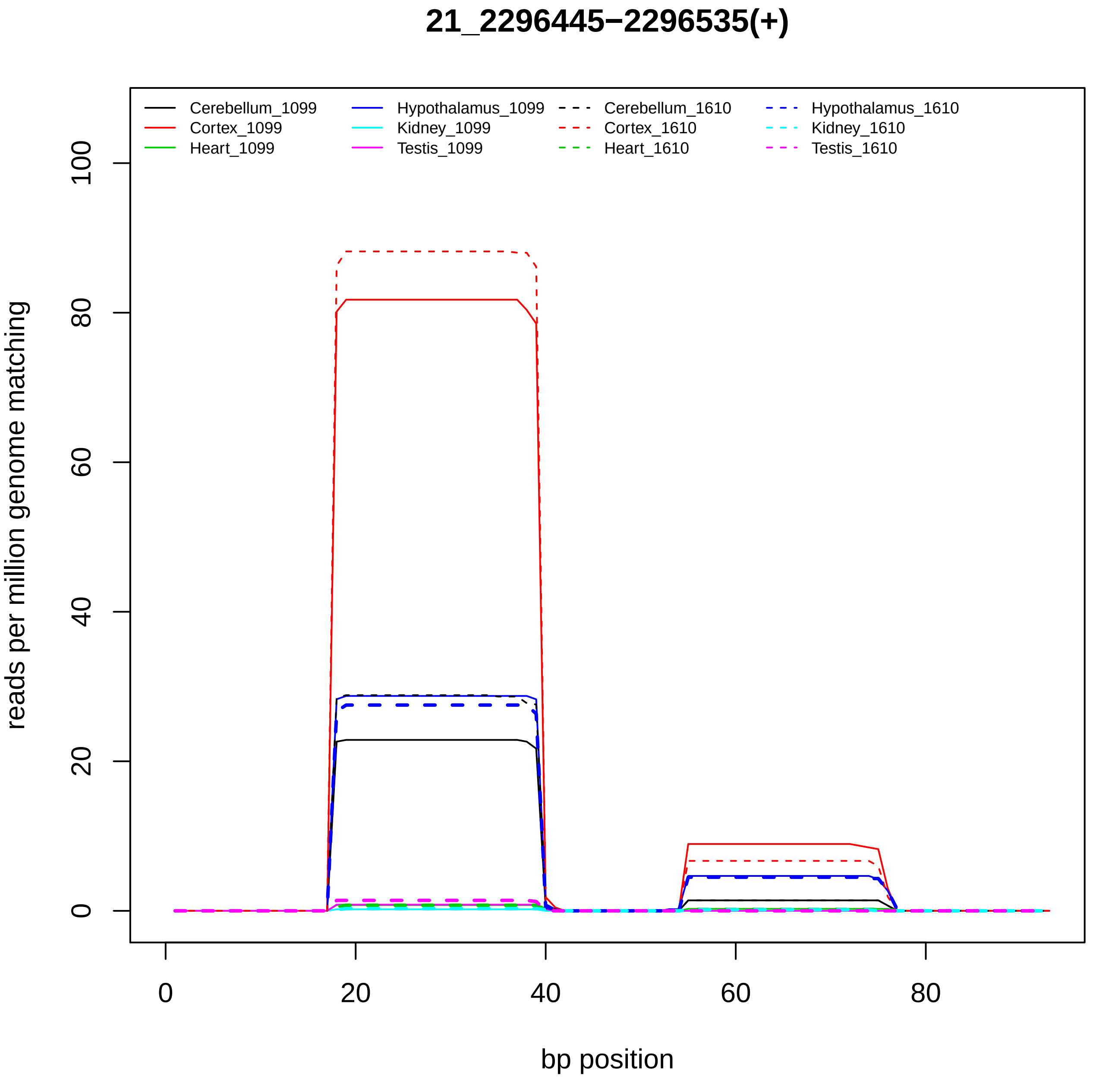

21_2296445−2296535(+)
Cerebellum_1099
Cortex_1099
Heart_1099
Hypothalamus_1099
Kidney_1099
Testis_1099
Cerebellum_1610
Cortex_1610
Heart_1610
Hypothalamus_1610
Kidney_1610
Testis_1610
reads per million genome matching
0
20
40
60
80
100
bp position

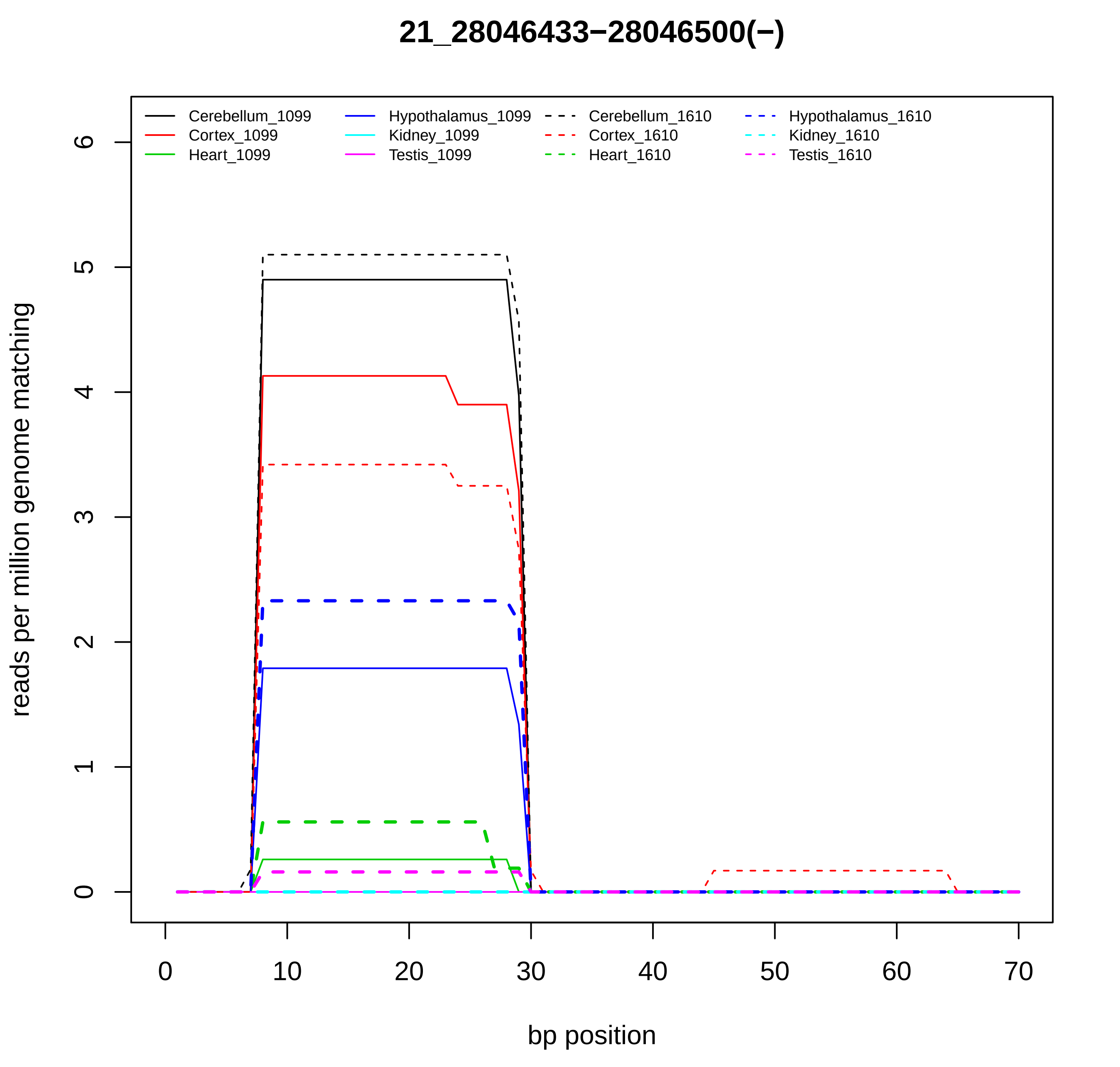

21_28046433−28046500(−)
Cerebellum_1099
Cortex_1099
Heart_1099
Hypothalamus_1099
Kidney_1099
Testis_1099
Cerebellum_1610
Cortex_1610
Heart_1610
Hypothalamus_1610
Kidney_1610
Testis_1610
reads per million genome matching
0
1
2
3
4
5
6
0
10
20
30
40
50
60
70
bp position

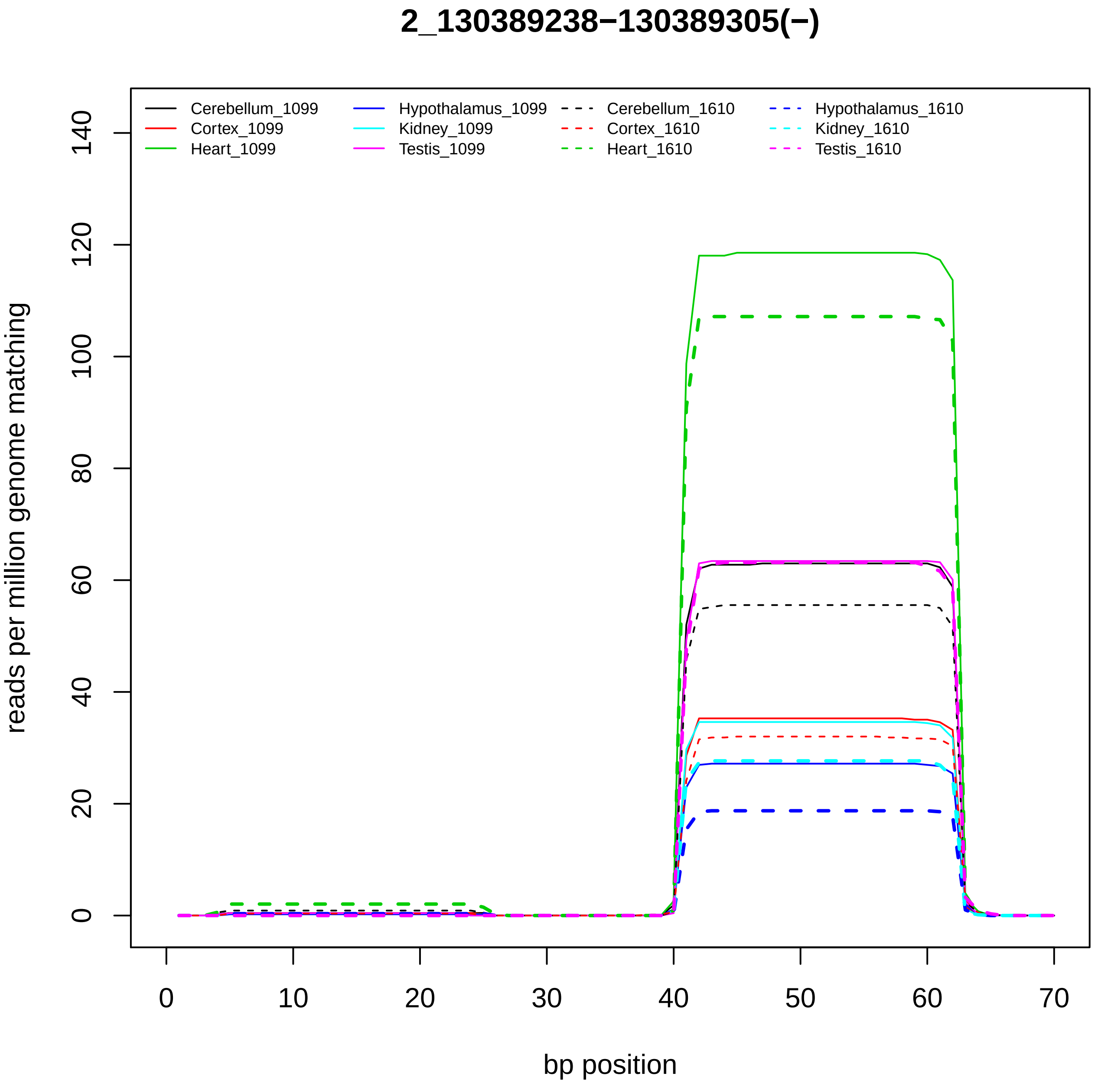
2_130389238−130389305(−)
Cerebellum_1099
Cortex_1099
Heart_1099
Hypothalamus_1099
Kidney_1099
Testis_1099
Cerebellum_1610
Cortex_1610
Heart_1610
Hypothalamus_1610
Kidney_1610
Testis_1610
reads per million genome matching
bp position
0
20
40
60
80
100
120
140
0
10
20
30
40
50
60
70

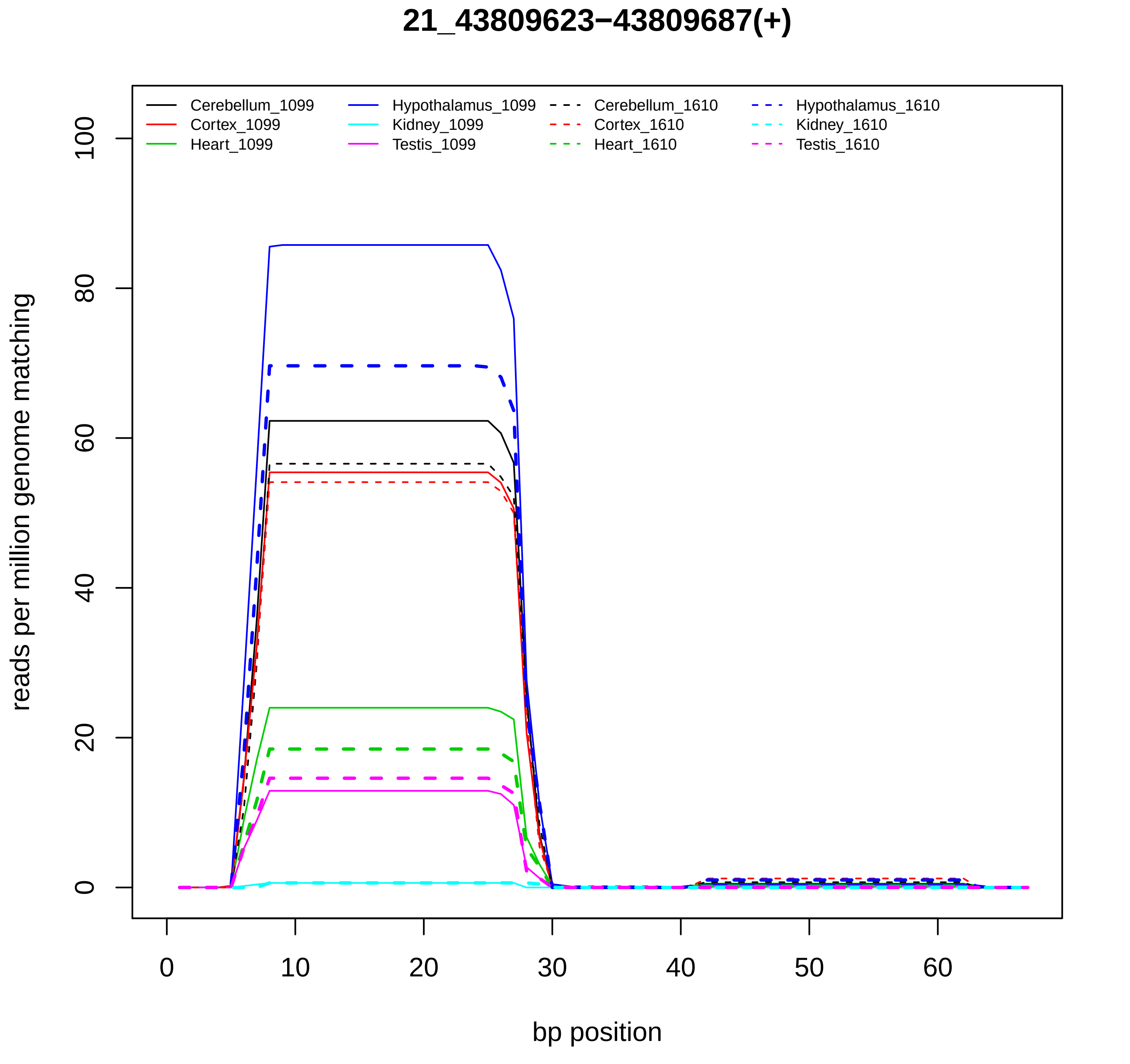

21_43809623−43809687(+)
Cerebellum_1099
Cortex_1099
Heart_1099
Hypothalamus_1099
Kidney_1099
Testis_1099
Cerebellum_1610
Cortex_1610
Heart_1610
Hypothalamus_1610
Kidney_1610
Testis_1610
reads per million genome matching
0
20
40
60
80
100
0
10
20
30
40
50
60
bp position

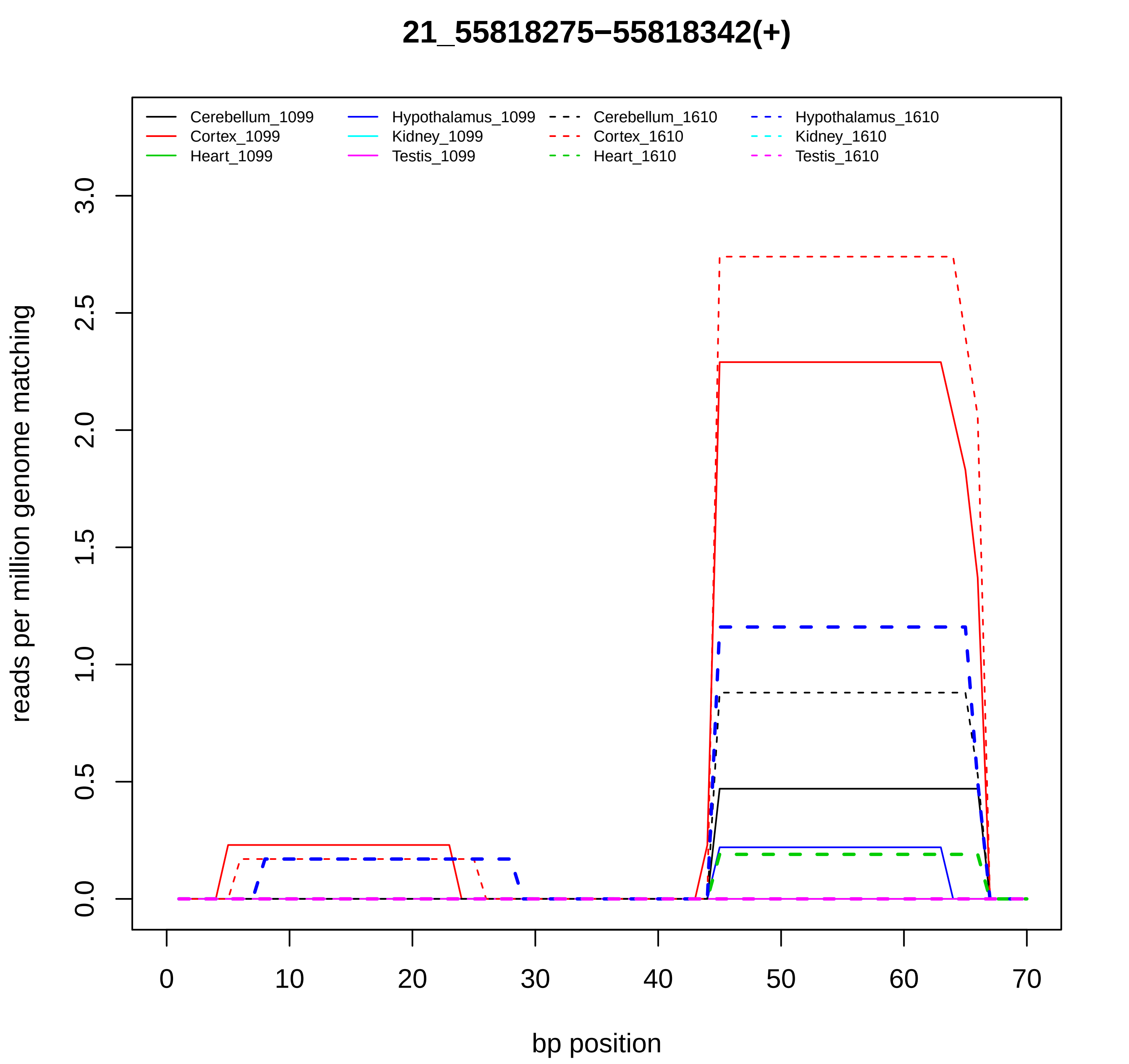
21_55818275−55818342(+)
Cerebellum_1099
Cortex_1099
Heart_1099
Hypothalamus_1099
Kidney_1099
Testis_1099
Cerebellum_1610
Cortex_1610
Heart_1610
Hypothalamus_1610
Kidney_1610
Testis_1610
reads per million genome matching
bp position
0.0
0.5
1.0
1.5
2.0
2.5
3.0
0
10
20
30
40
50
60
70

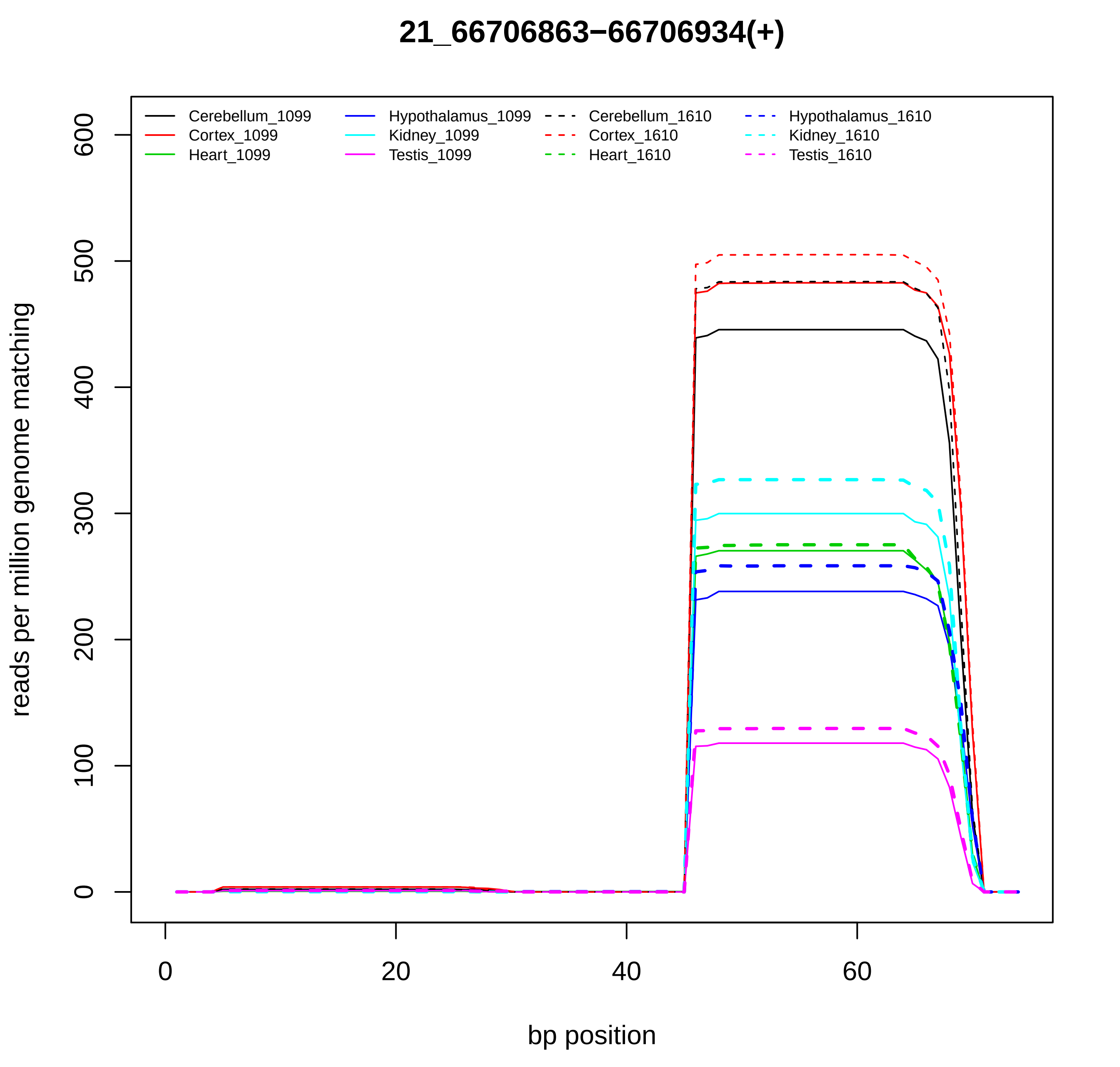
21_66706863−66706934(+)
Cerebellum_1099
Cortex_1099
Heart_1099
Hypothalamus_1099
Kidney_1099
Testis_1099
Cerebellum_1610
Cortex_1610
Heart_1610
Hypothalamus_1610
Kidney_1610
Testis_1610
reads per million genome matching
0
100
200
300
400
500
600
0
20
40
60
bp position

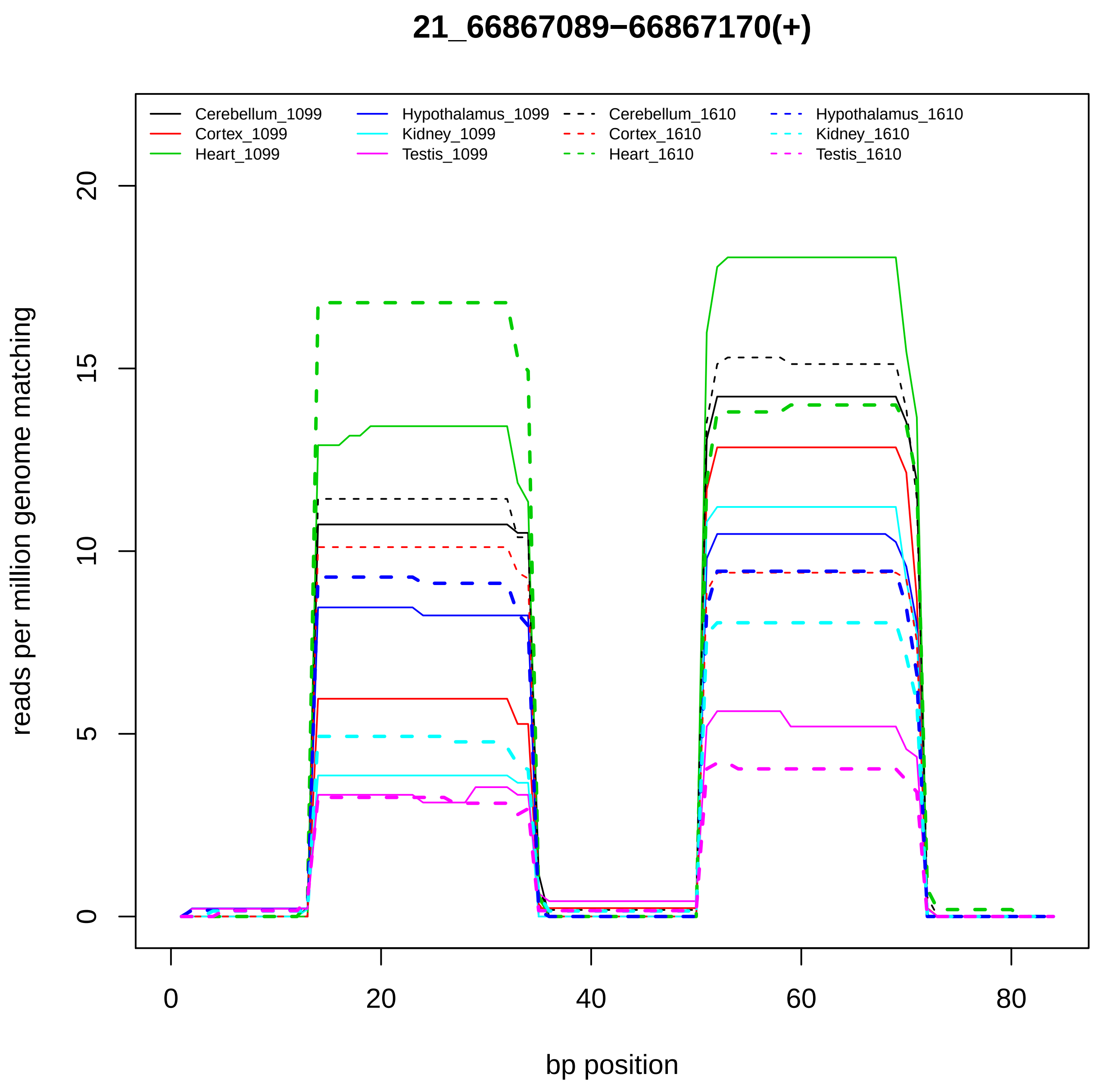

21_66867089−66867170(+)
Cerebellum_1099
Cortex_1099
Heart_1099
Hypothalamus_1099
Kidney_1099
Testis_1099
Cerebellum_1610
Cortex_1610
Heart_1610
Hypothalamus_1610
Kidney_1610
Testis_1610
reads per million genome matching
bp position
0
5
10
15
20
0
20
40
60
80

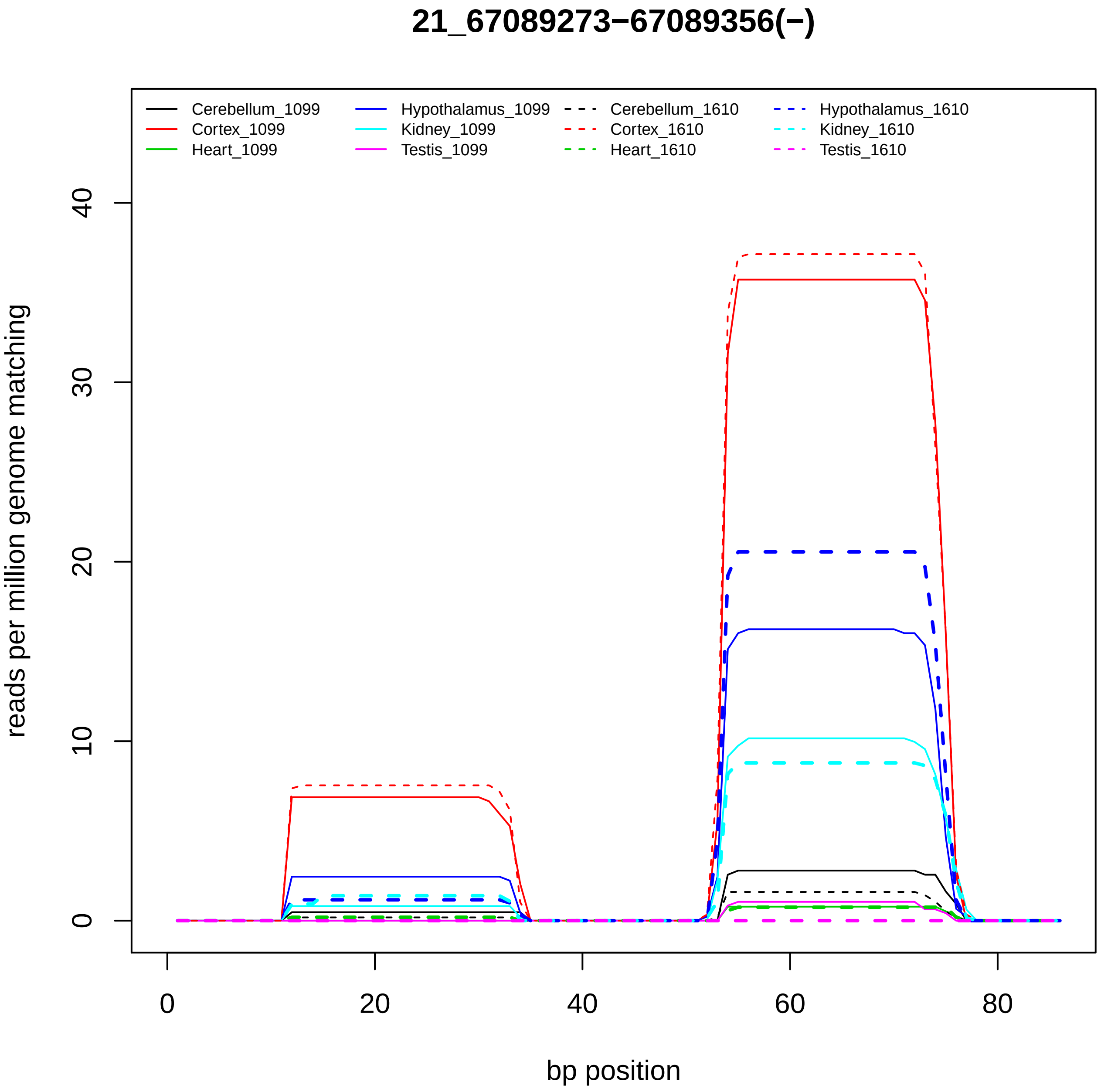

21_67089273−67089356(−)
Cerebellum_1099
Cortex_1099
Heart_1099
Hypothalamus_1099
Kidney_1099
Testis_1099
Cerebellum_1610
Cortex_1610
Heart_1610
Hypothalamus_1610
Kidney_1610
Testis_1610
reads per million genome matching
0
10
20
30
40
bp position
0
20
40
60
80

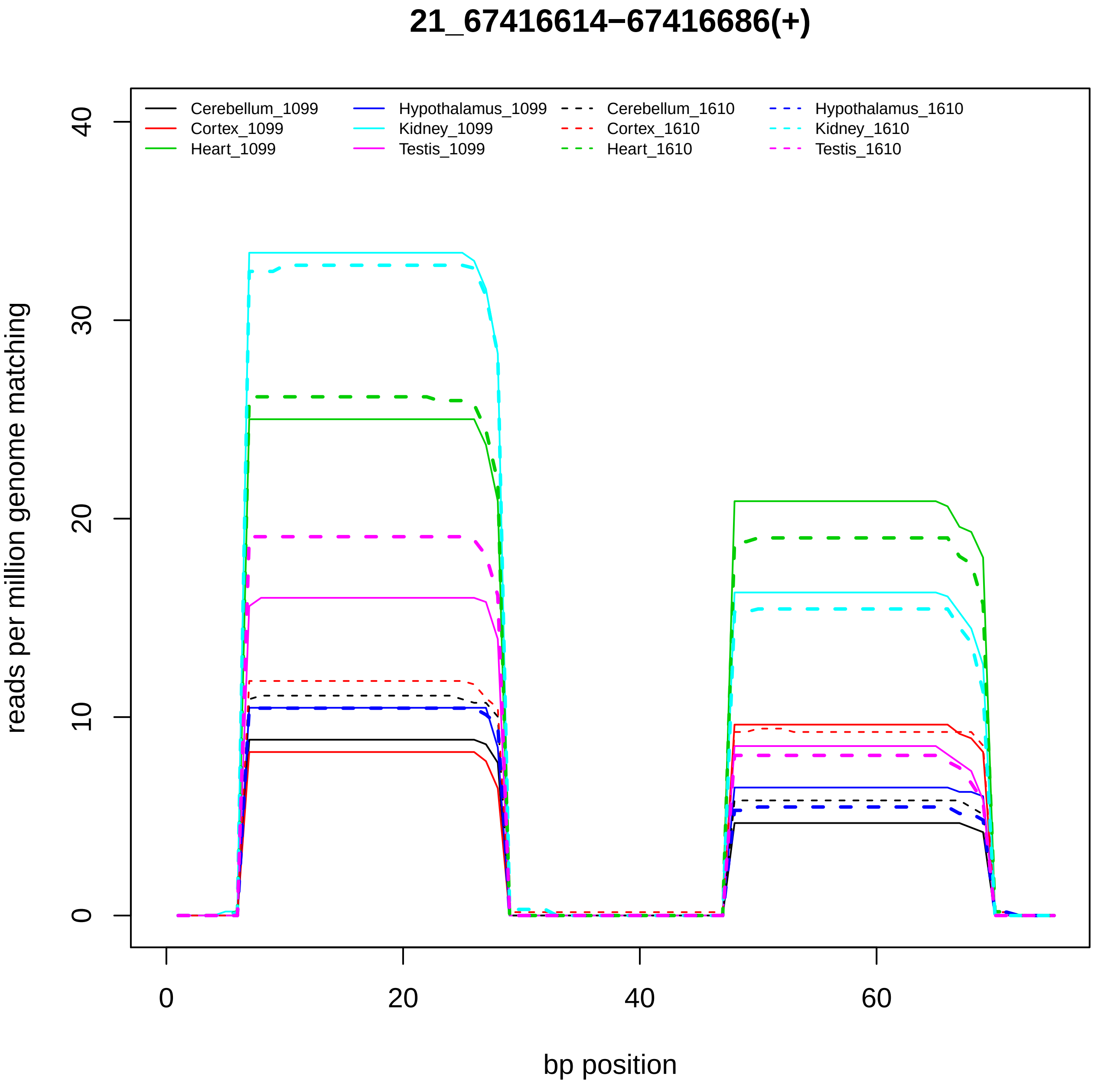

21_67416614−67416686(+)
Cerebellum_1099
Cortex_1099
Heart_1099
Hypothalamus_1099
Kidney_1099
Testis_1099
Cerebellum_1610
Cortex_1610
Heart_1610
Hypothalamus_1610
Kidney_1610
Testis_1610
reads per million genome matching
bp position
0
10
20
30
40
0
20
40
60

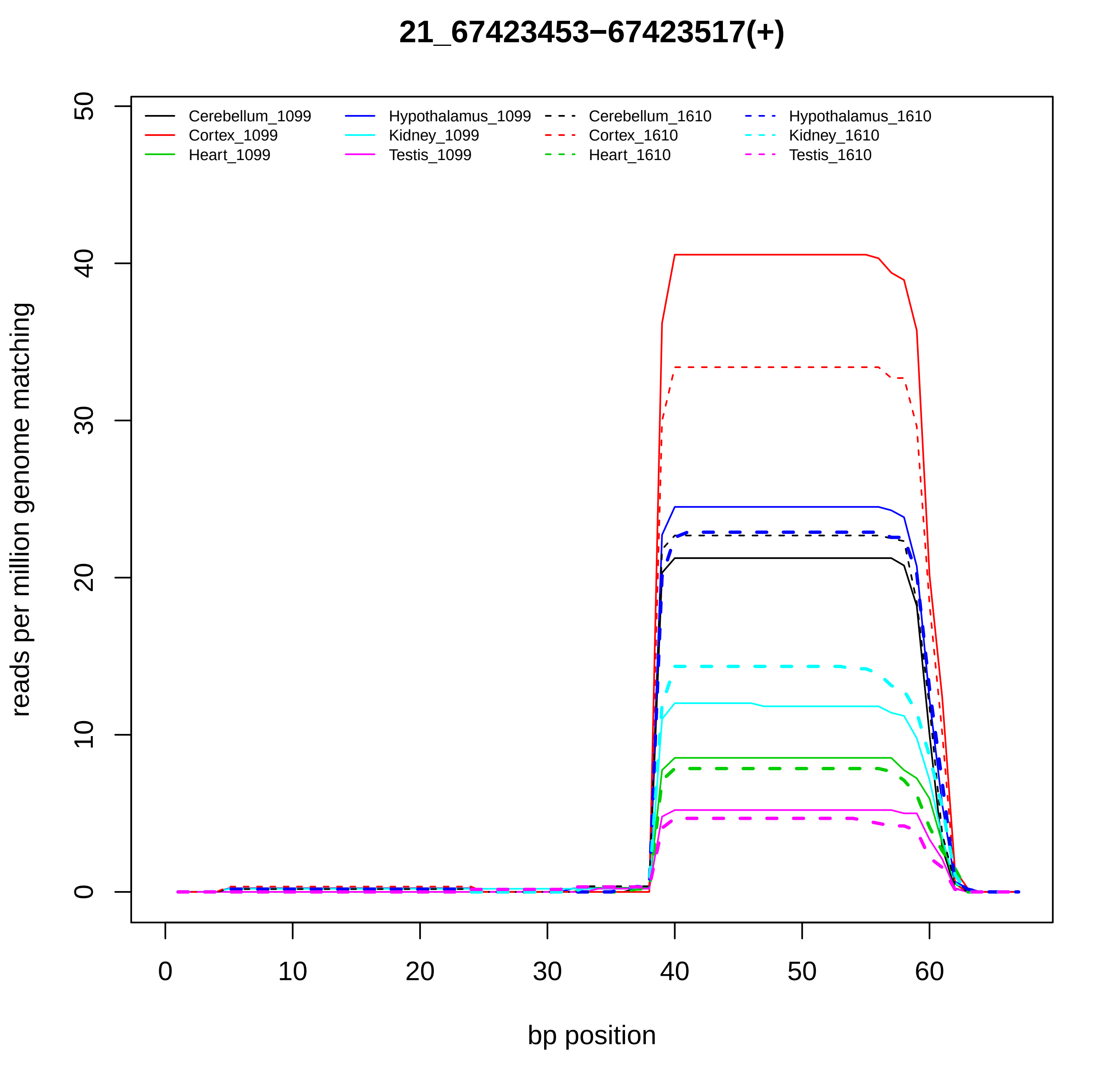

21_67423453−67423517(+)
Cerebellum_1099
Cortex_1099
Heart_1099
Hypothalamus_1099
Kidney_1099
Testis_1099
Cerebellum_1610
Cortex_1610
Heart_1610
Hypothalamus_1610
Kidney_1610
Testis_1610
reads per million genome matching
bp position
0
10
20
30
40
50
60

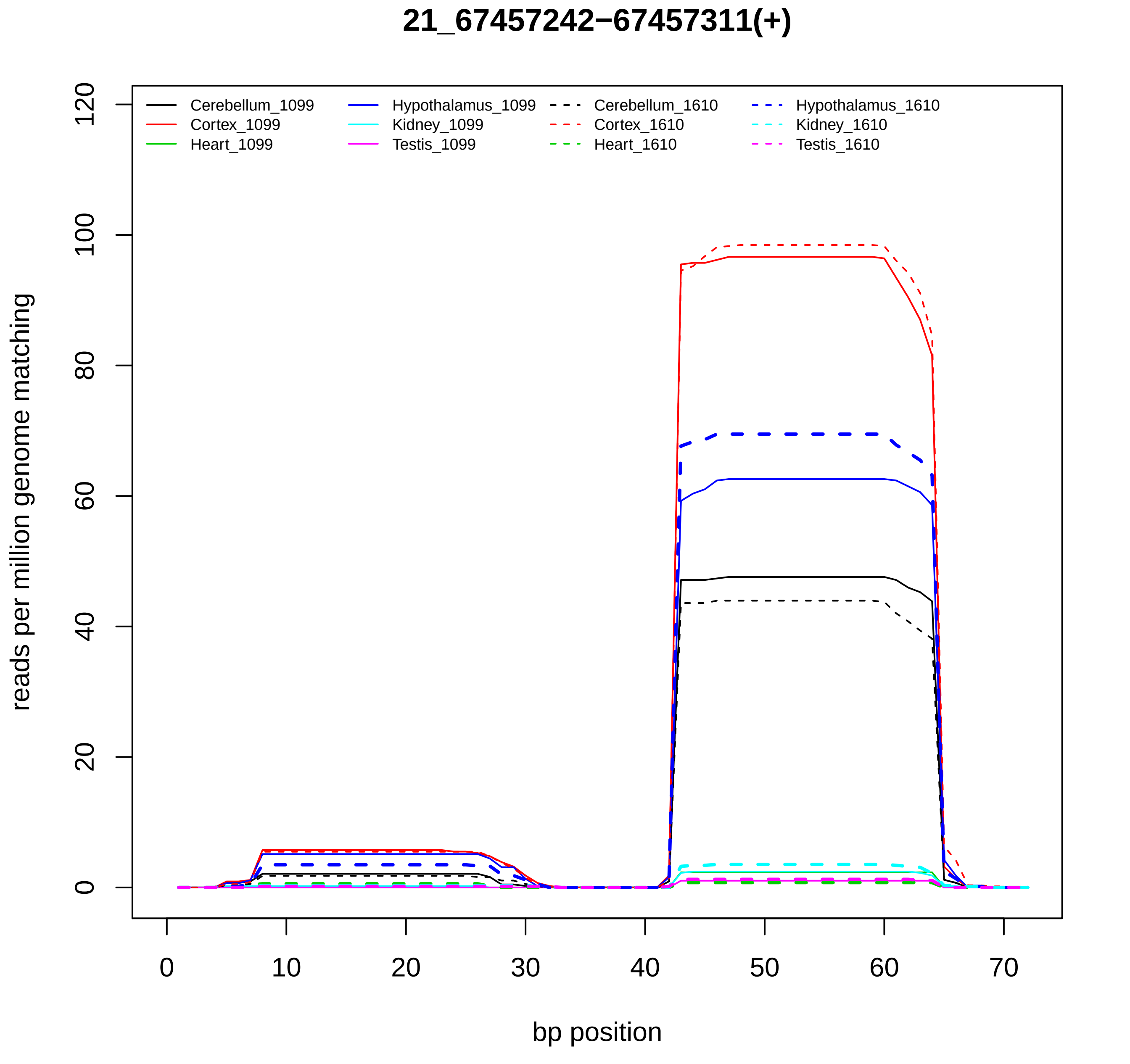
21_67457242−67457311(+)
Cerebellum_1099
Cortex_1099
Heart_1099
Hypothalamus_1099
Kidney_1099
Testis_1099
Cerebellum_1610
Cortex_1610
Heart_1610
Hypothalamus_1610
Kidney_1610
Testis_1610
reads per million genome matching
0
20
40
60
80
100
120
bp position
0
10
20
30
40
50
60
70

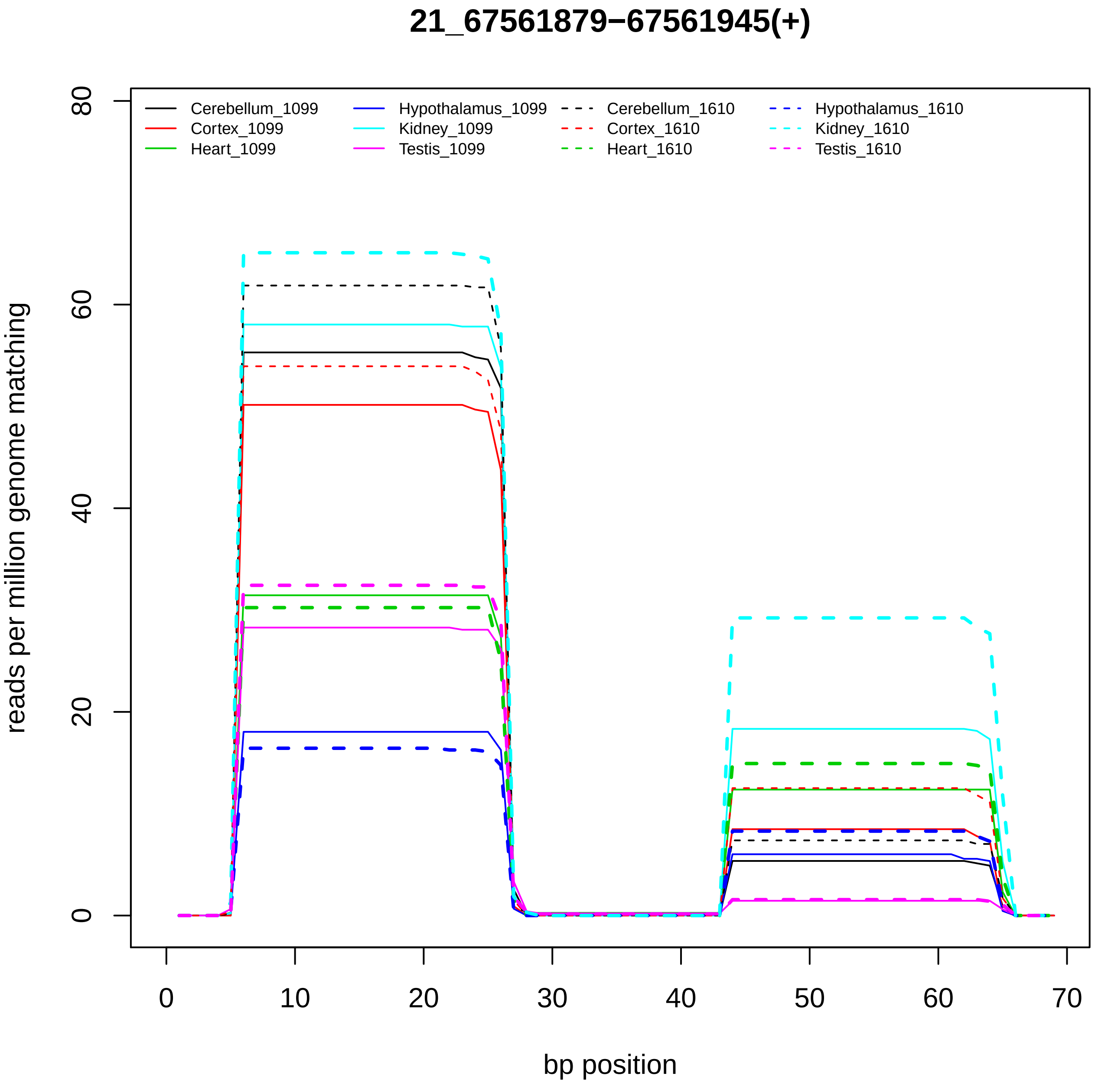

21_67561879−67561945(+)
Cerebellum_1099
Cortex_1099
Heart_1099
Hypothalamus_1099
Kidney_1099
Testis_1099
Cerebellum_1610
Cortex_1610
Heart_1610
Hypothalamus_1610
Kidney_1610
Testis_1610
reads per million genome matching
bp position
0
20
40
60
80
0
10
20
30
40
50
60
70

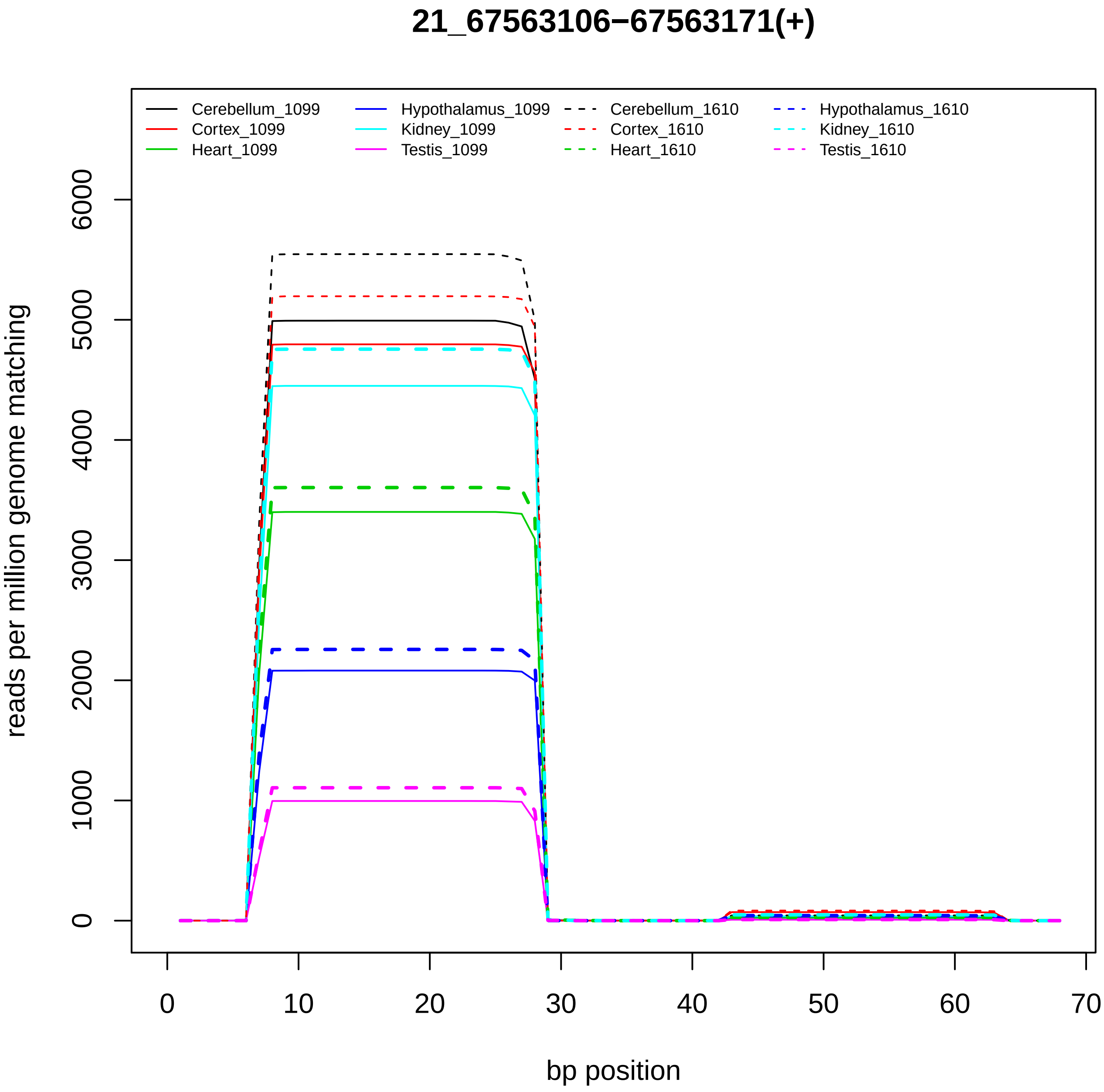

21_67563106−67563171(+)
Cerebellum_1099
Cortex_1099
Heart_1099
Hypothalamus_1099
Kidney_1099
Testis_1099
Cerebellum_1610
Cortex_1610
Heart_1610
Hypothalamus_1610
Kidney_1610
Testis_1610
reads per million genome matching
0
1000
2000
3000
4000
5000
6000
bp position
0
10
20
30
40
50
60
70

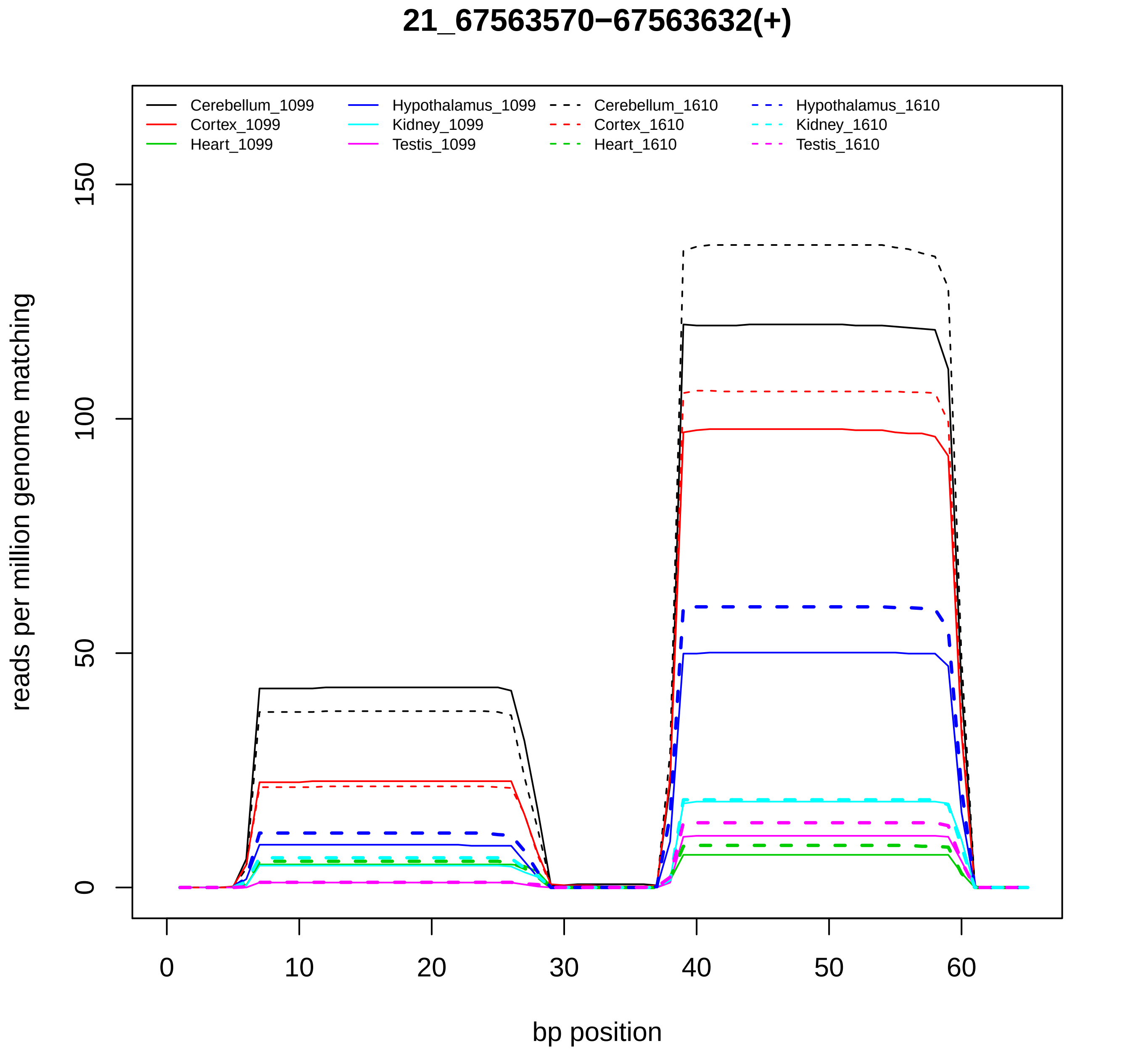

21_67563570−67563632(+)
Cerebellum_1099
Cortex_1099
Heart_1099
Hypothalamus_1099
Kidney_1099
Testis_1099
Cerebellum_1610
Cortex_1610
Heart_1610
Hypothalamus_1610
Kidney_1610
Testis_1610
reads per million genome matching
0
50
100
150
0
10
20
30
40
50
60
bp position

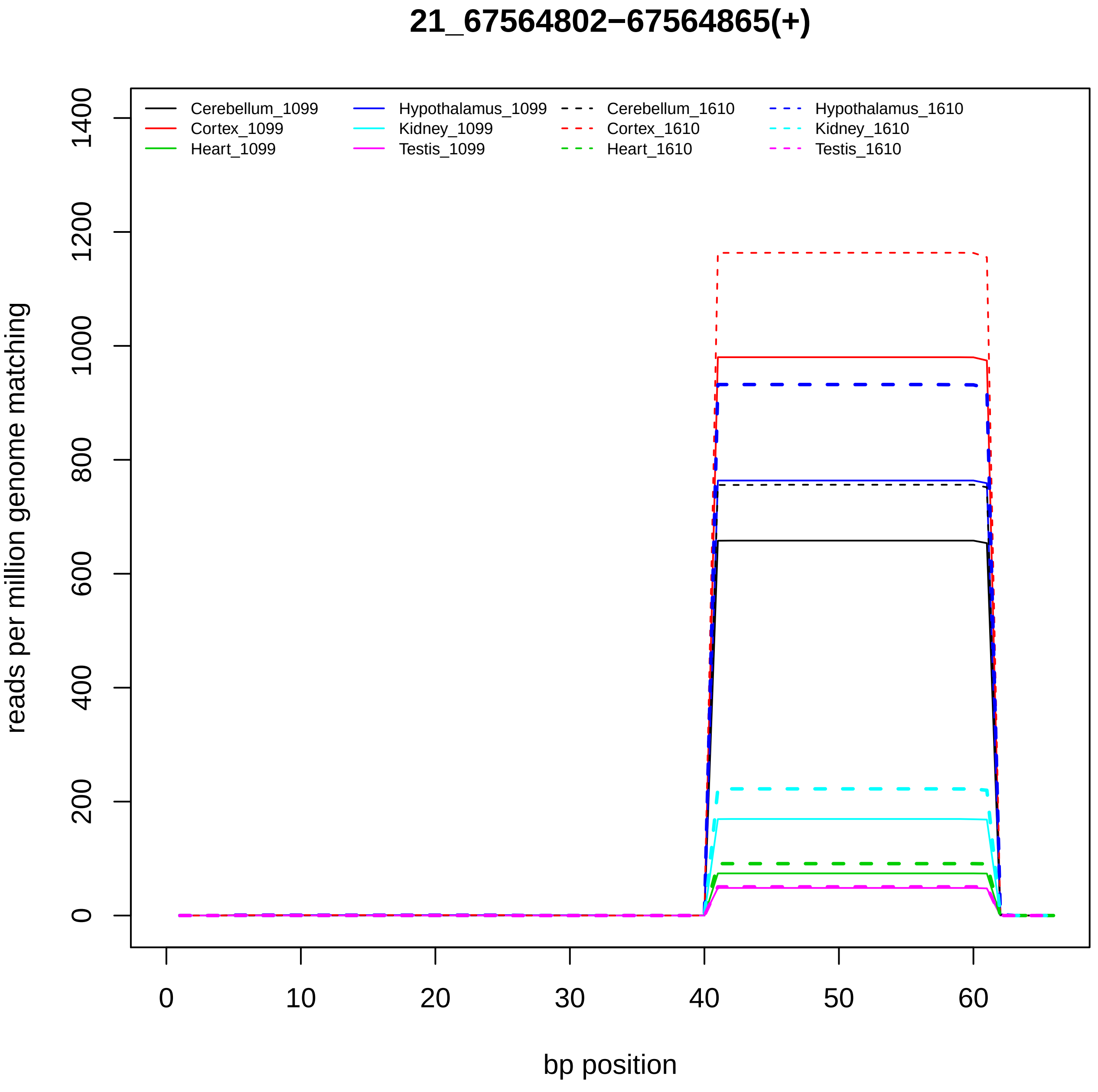

21_67564802−67564865(+)
Cerebellum_1099
Cortex_1099
Heart_1099
Hypothalamus_1099
Kidney_1099
Testis_1099
Cerebellum_1610
Cortex_1610
Heart_1610
Hypothalamus_1610
Kidney_1610
Testis_1610
reads per million genome matching
0
200
400
600
800
1000
1200
1400
0
10
20
30
40
50
60
bp position

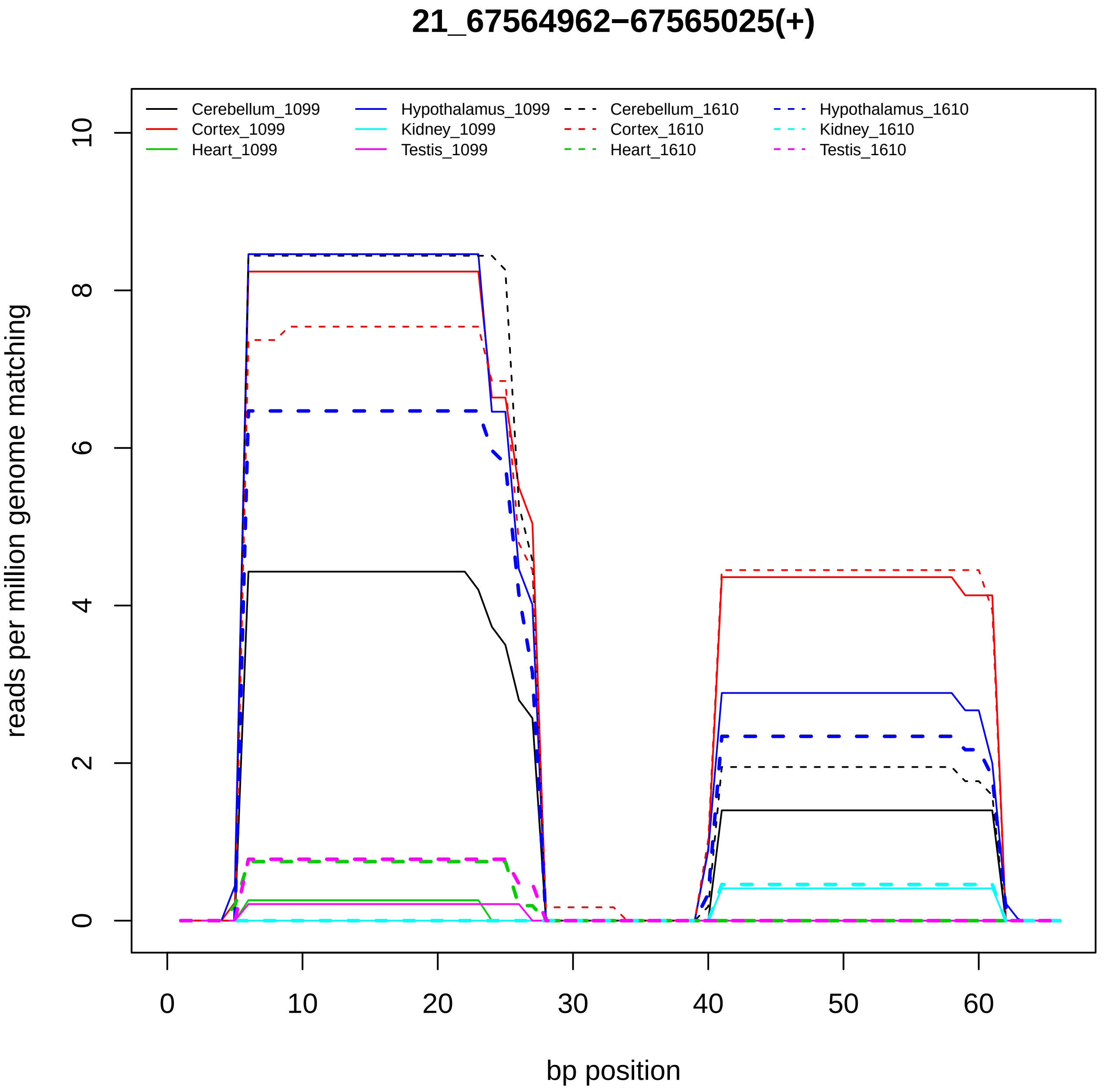

21_67564962−67565025(+)
Cerebellum_1099
Cortex_1099
Heart_1099
Hypothalamus_1099
Kidney_1099
Testis_1099
Cerebellum_1610
Cortex_1610
Heart_1610
Hypothalamus_1610
Kidney_1610
Testis_1610
reads per million genome matching
0
2
4
6
8
10
0
10
20
30
40
50
60
bp position

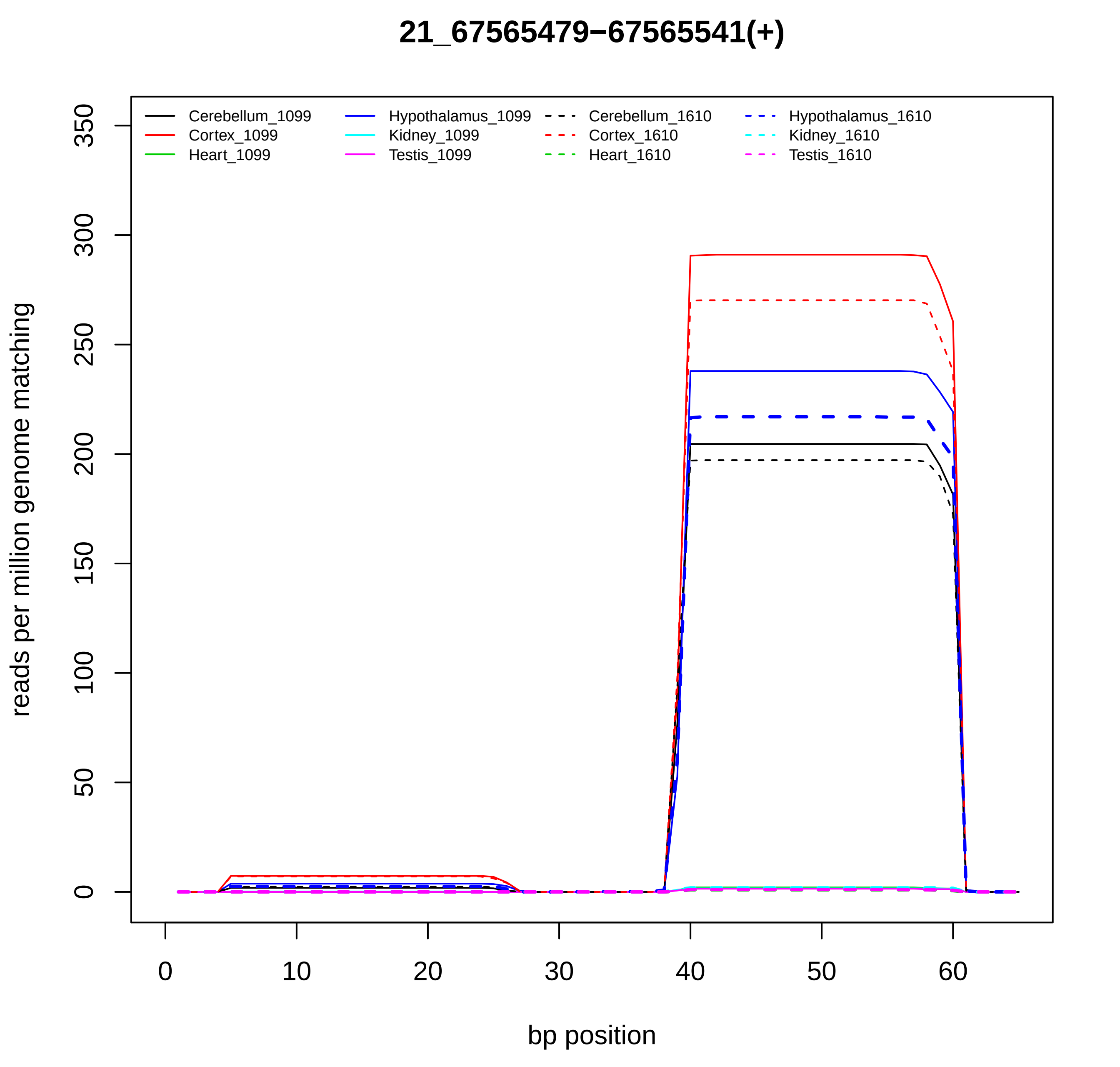

21_67565479−67565541(+)
Cerebellum_1099
Cortex_1099
Heart_1099
Hypothalamus_1099
Kidney_1099
Testis_1099
Cerebellum_1610
Cortex_1610
Heart_1610
Hypothalamus_1610
Kidney_1610
Testis_1610
reads per million genome matching
0
50
100
150
200
250
300
350
bp position
0
10
20
30
40
50
60

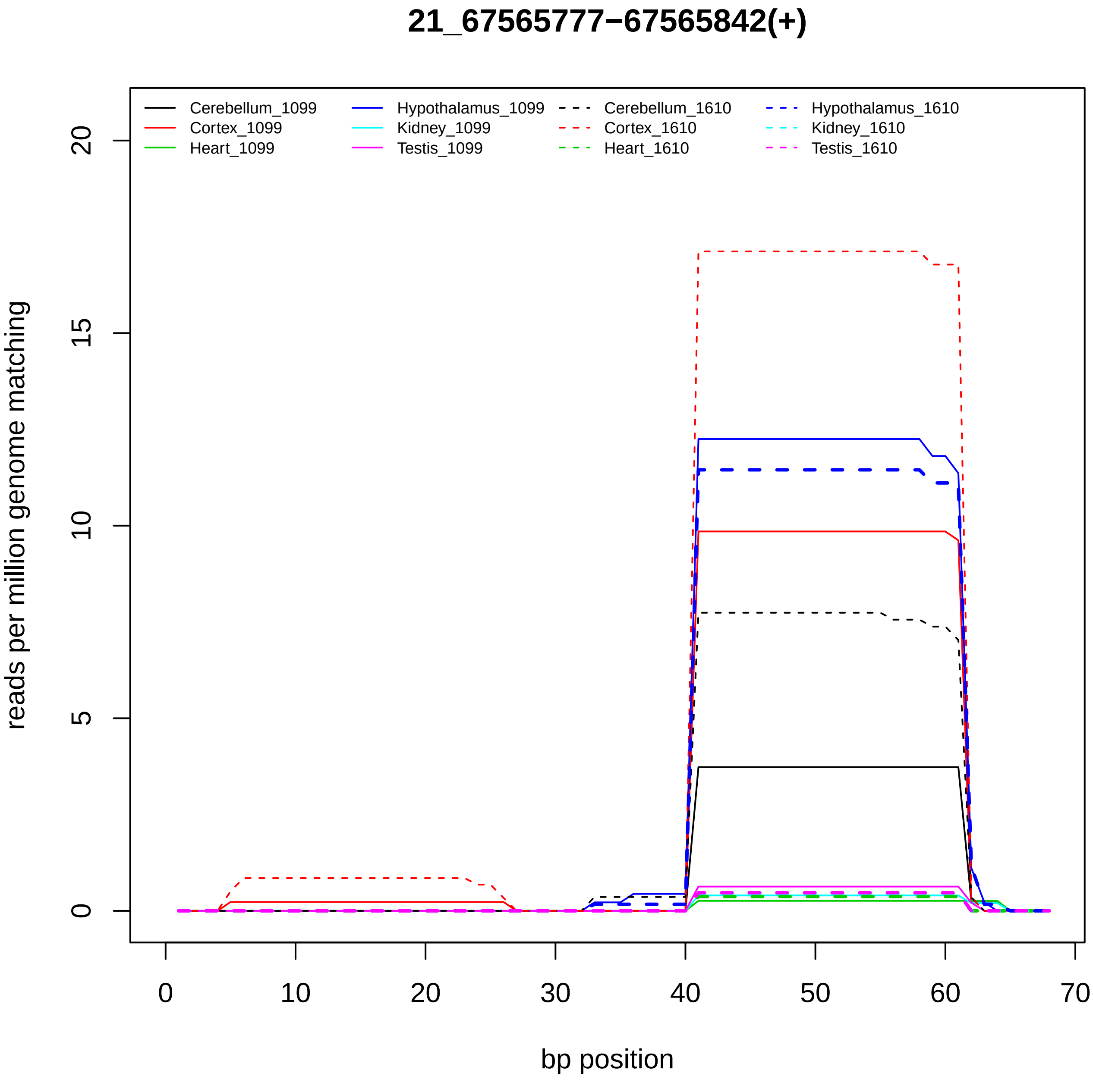

21_67565777−67565842(+)
Cerebellum_1099
Cortex_1099
Heart_1099
Hypothalamus_1099
Kidney_1099
Testis_1099
Cerebellum_1610
Cortex_1610
Heart_1610
Hypothalamus_1610
Kidney_1610
Testis_1610
reads per million genome matching
0
5
10
15
20
0
10
20
30
40
50
60
70
bp position

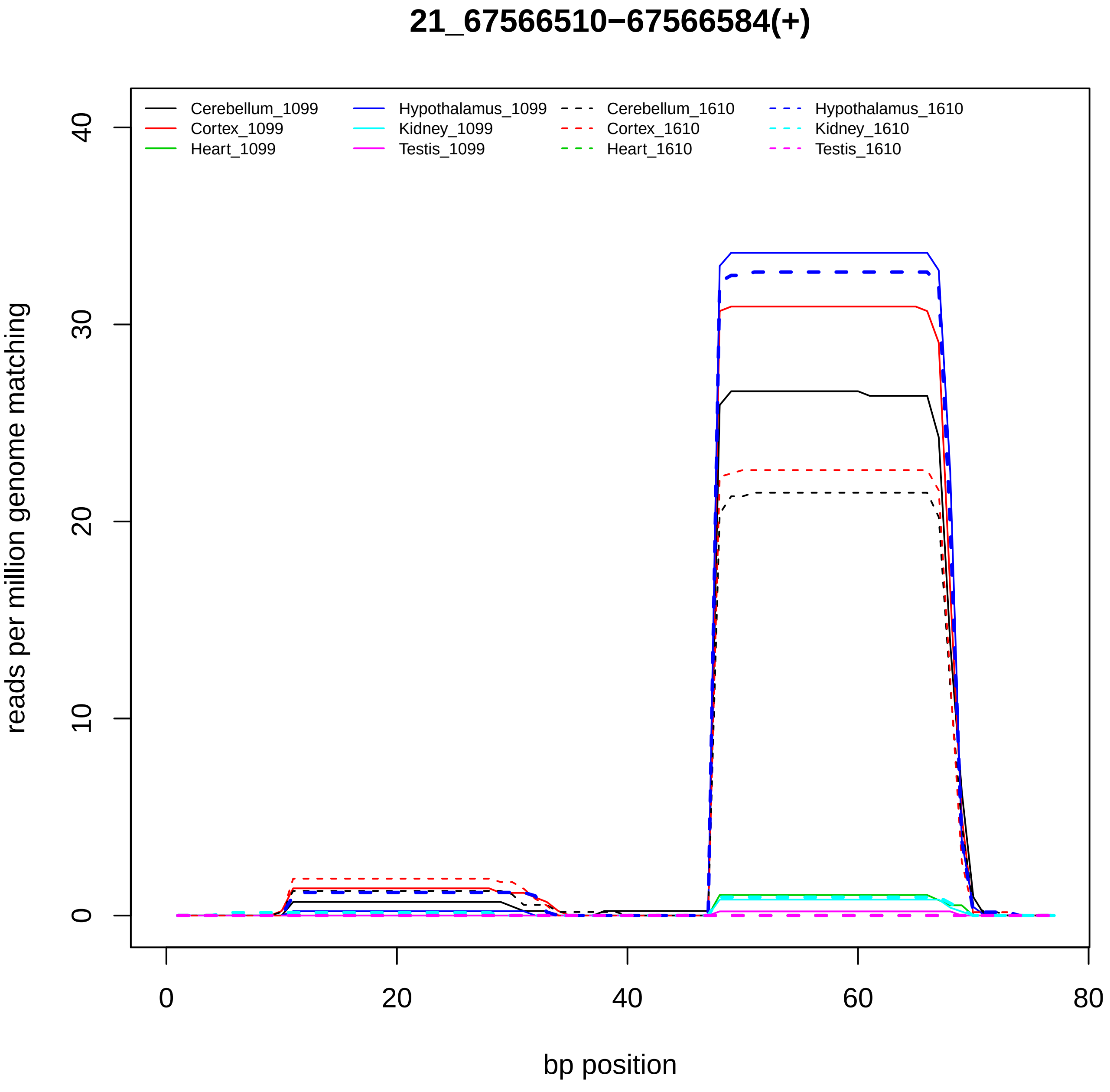

21_67566510−67566584(+)
Cerebellum_1099
Cortex_1099
Heart_1099
Hypothalamus_1099
Kidney_1099
Testis_1099
Cerebellum_1610
Cortex_1610
Heart_1610
Hypothalamus_1610
Kidney_1610
Testis_1610
reads per million genome matching
0
10
20
30
40
0
20
40
60
80
bp position

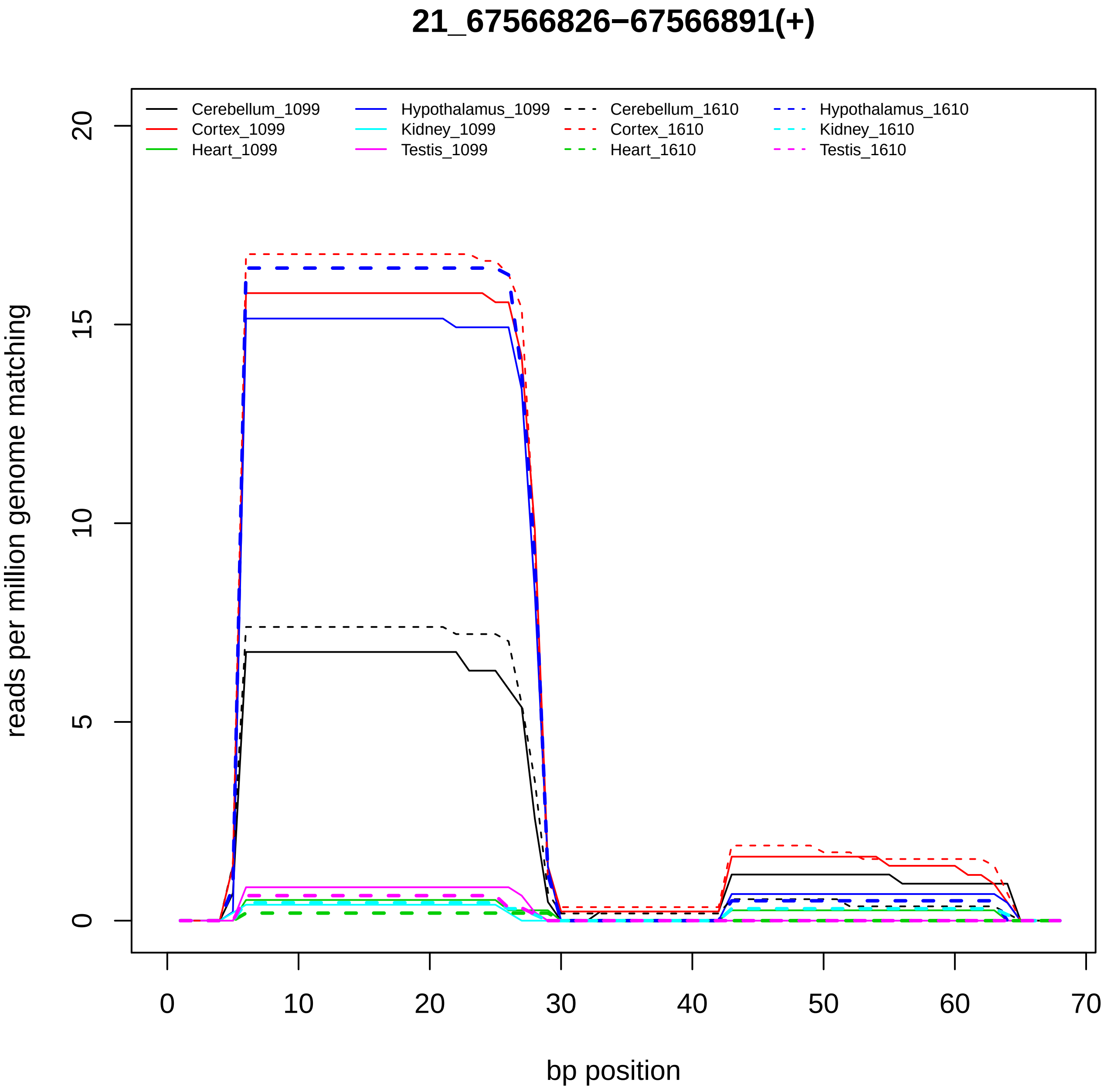

21_67566826−67566891(+)
Cerebellum_1099
Cortex_1099
Heart_1099
Hypothalamus_1099
Kidney_1099
Testis_1099
Cerebellum_1610
Cortex_1610
Heart_1610
Hypothalamus_1610
Kidney_1610
Testis_1610
reads per million genome matching
bp position
0
5
10
15
20
0
10
20
30
40
50
60
70

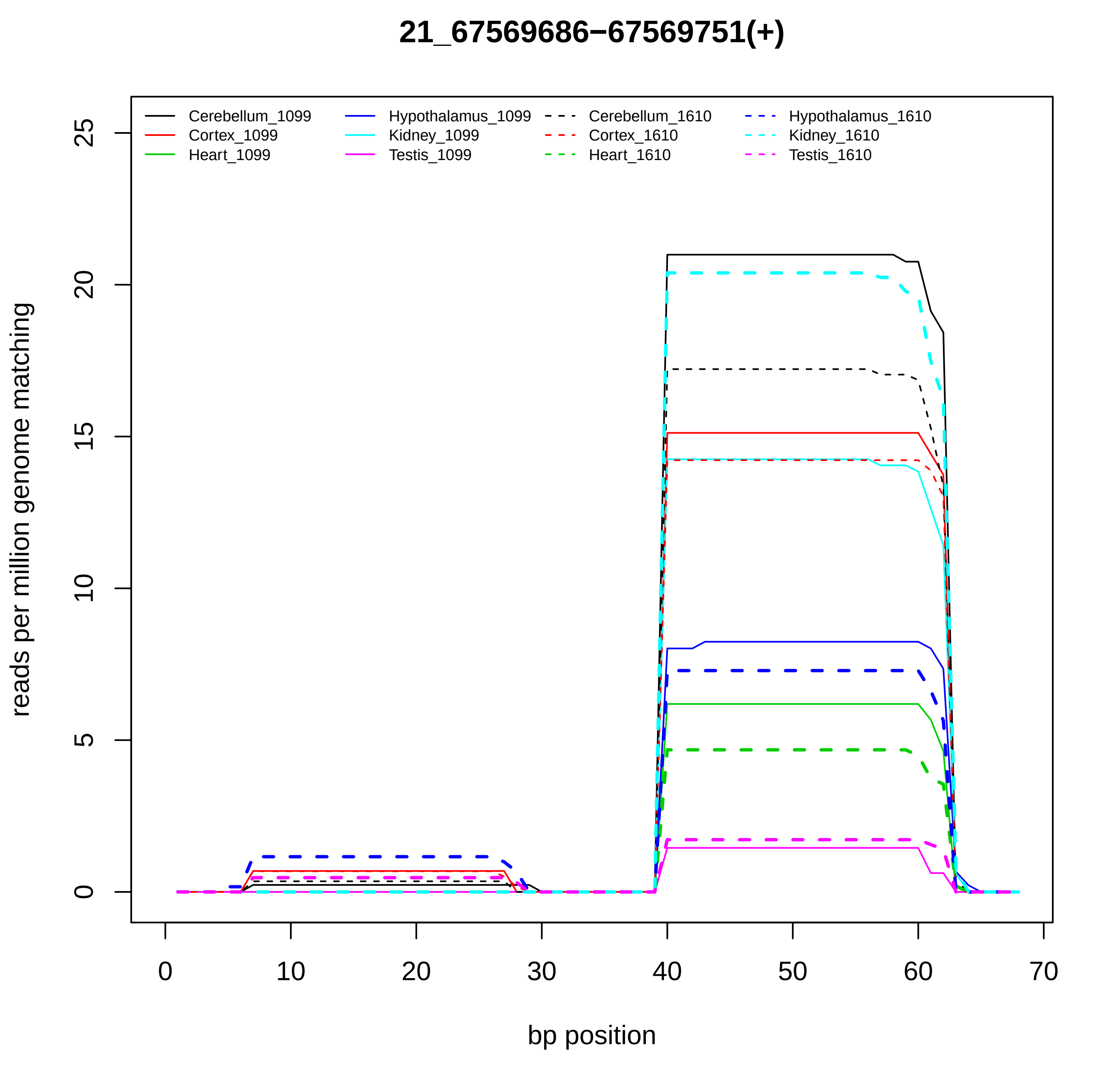

21_67569686−67569751(+)
Cerebellum_1099
Cortex_1099
Heart_1099
Hypothalamus_1099
Kidney_1099
Testis_1099
Cerebellum_1610
Cortex_1610
Heart_1610
Hypothalamus_1610
Kidney_1610
Testis_1610
reads per million genome matching
bp position
0
5
10
15
20
25
0
10
20
30
40
50
60
70

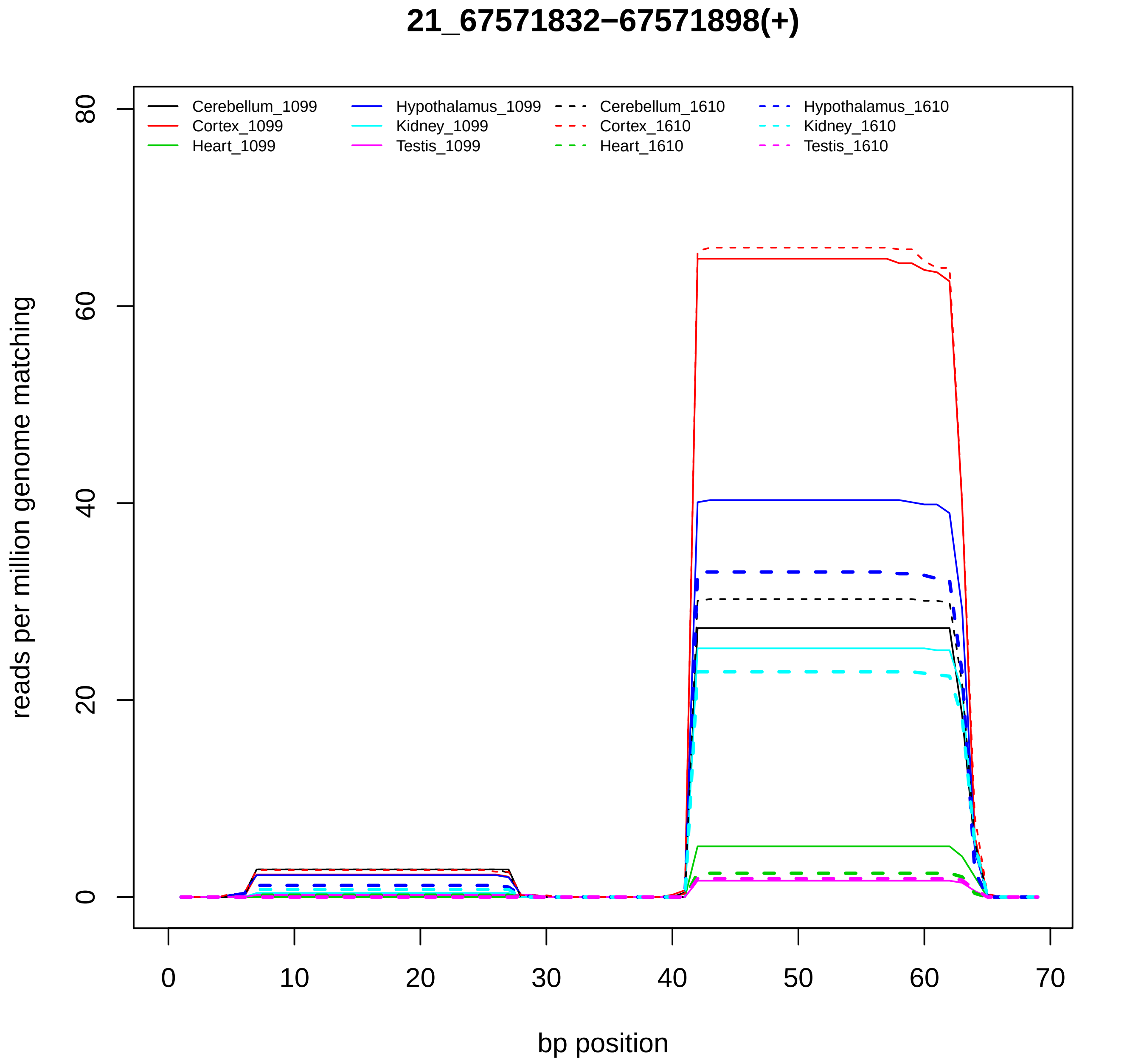

21_67571832−67571898(+)
Cerebellum_1099
Cortex_1099
Heart_1099
Hypothalamus_1099
Kidney_1099
Testis_1099
Cerebellum_1610
Cortex_1610
Heart_1610
Hypothalamus_1610
Kidney_1610
Testis_1610
reads per million genome matching
0
20
40
60
80
bp position
0
10
20
30
40
50
60
70

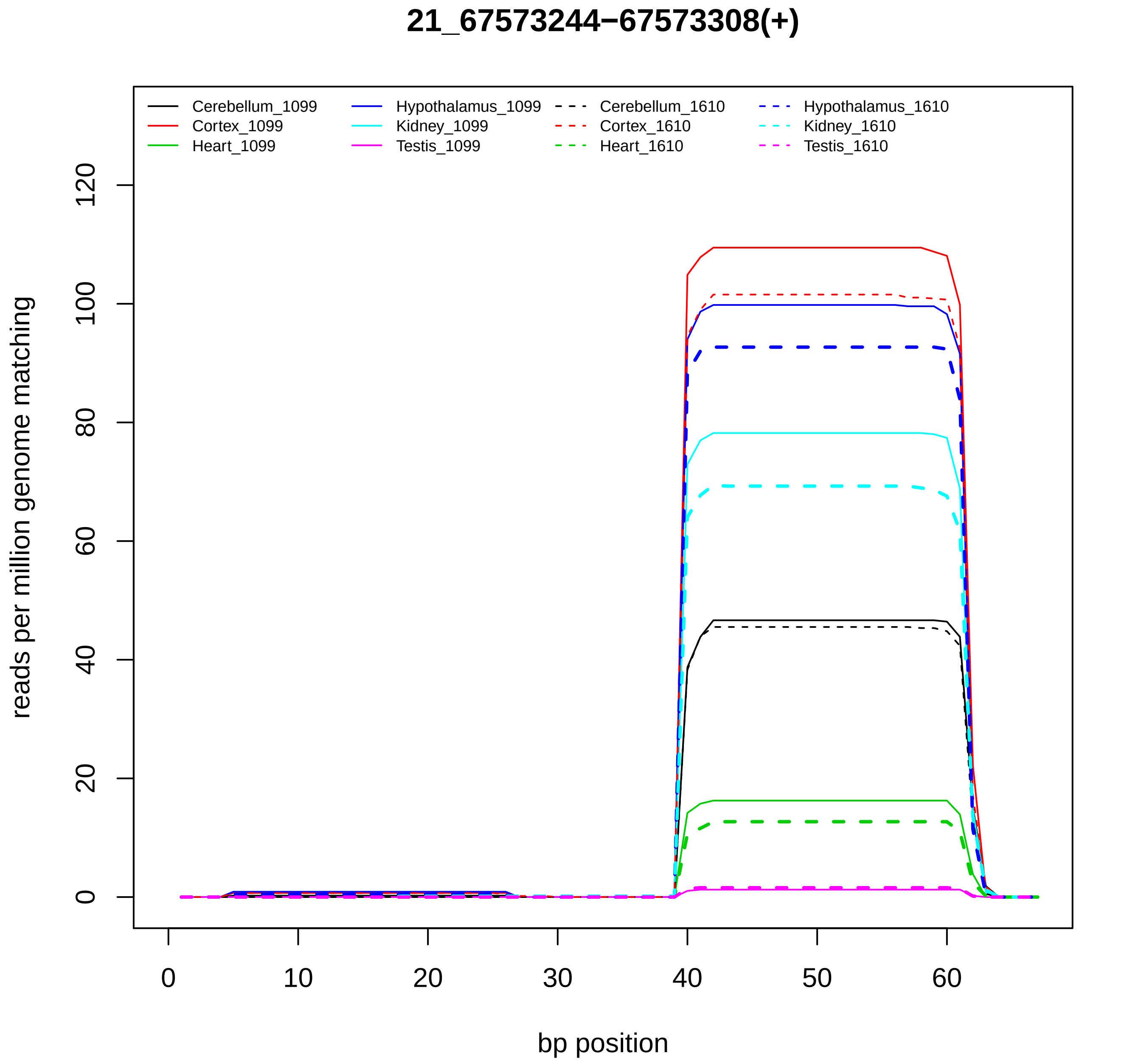
21_67573244−67573308(+)
Cerebellum_1099
Cortex_1099
Heart_1099
Hypothalamus_1099
Kidney_1099
Testis_1099
Cerebellum_1610
Cortex_1610
Heart_1610
Hypothalamus_1610
Kidney_1610
Testis_1610
reads per million genome matching
0
20
40
60
80
100
120
0
10
20
30
40
50
60
bp position

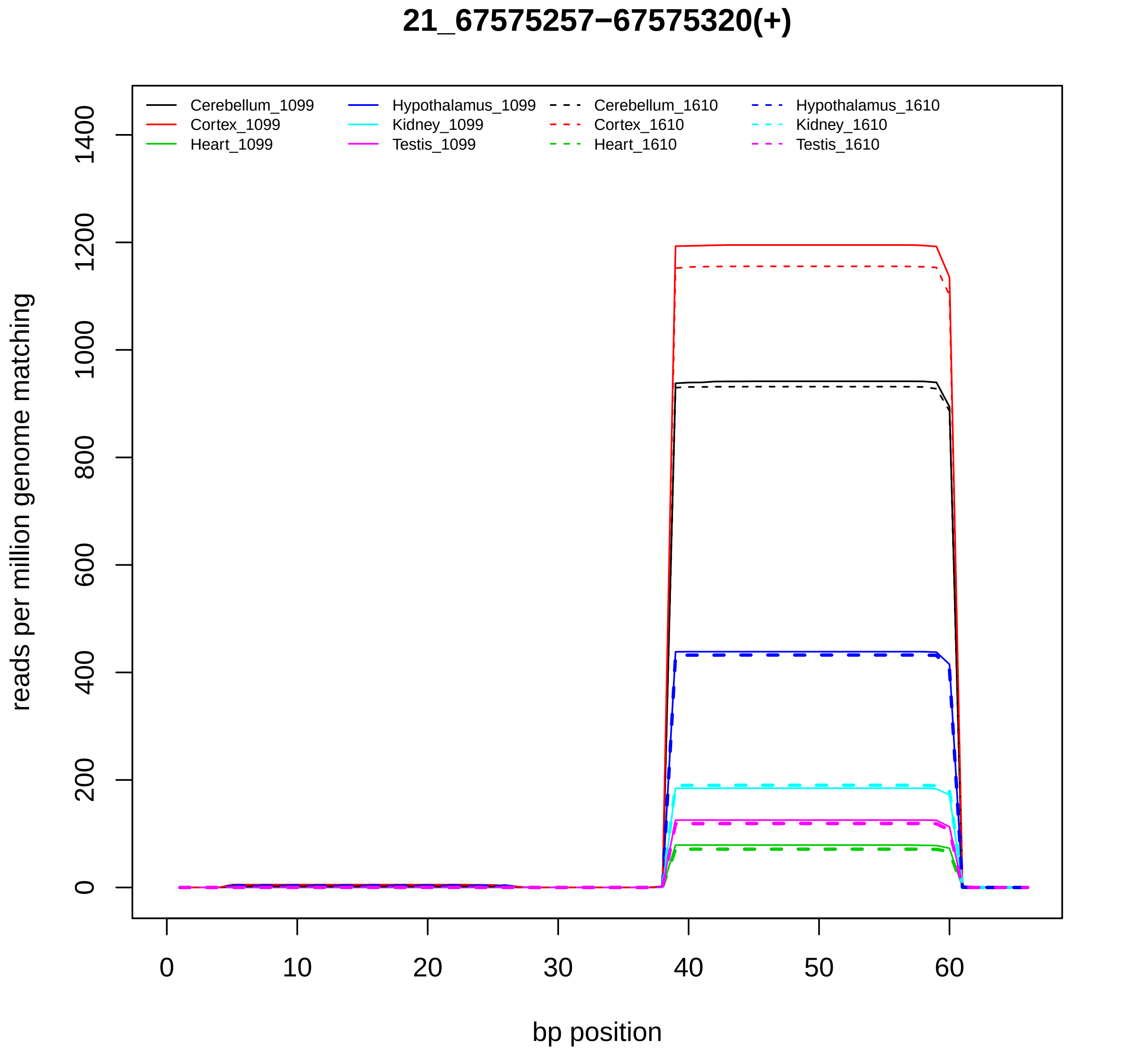

21_67575257−67575320(+)
Cerebellum_1099
Cortex_1099
Heart_1099
Hypothalamus_1099
Kidney_1099
Testis_1099
Cerebellum_1610
Cortex_1610
Heart_1610
Hypothalamus_1610
Kidney_1610
Testis_1610
reads per million genome matching
0
200
400
600
800
1000
1200
1400
0
10
20
30
40
50
60
bp position

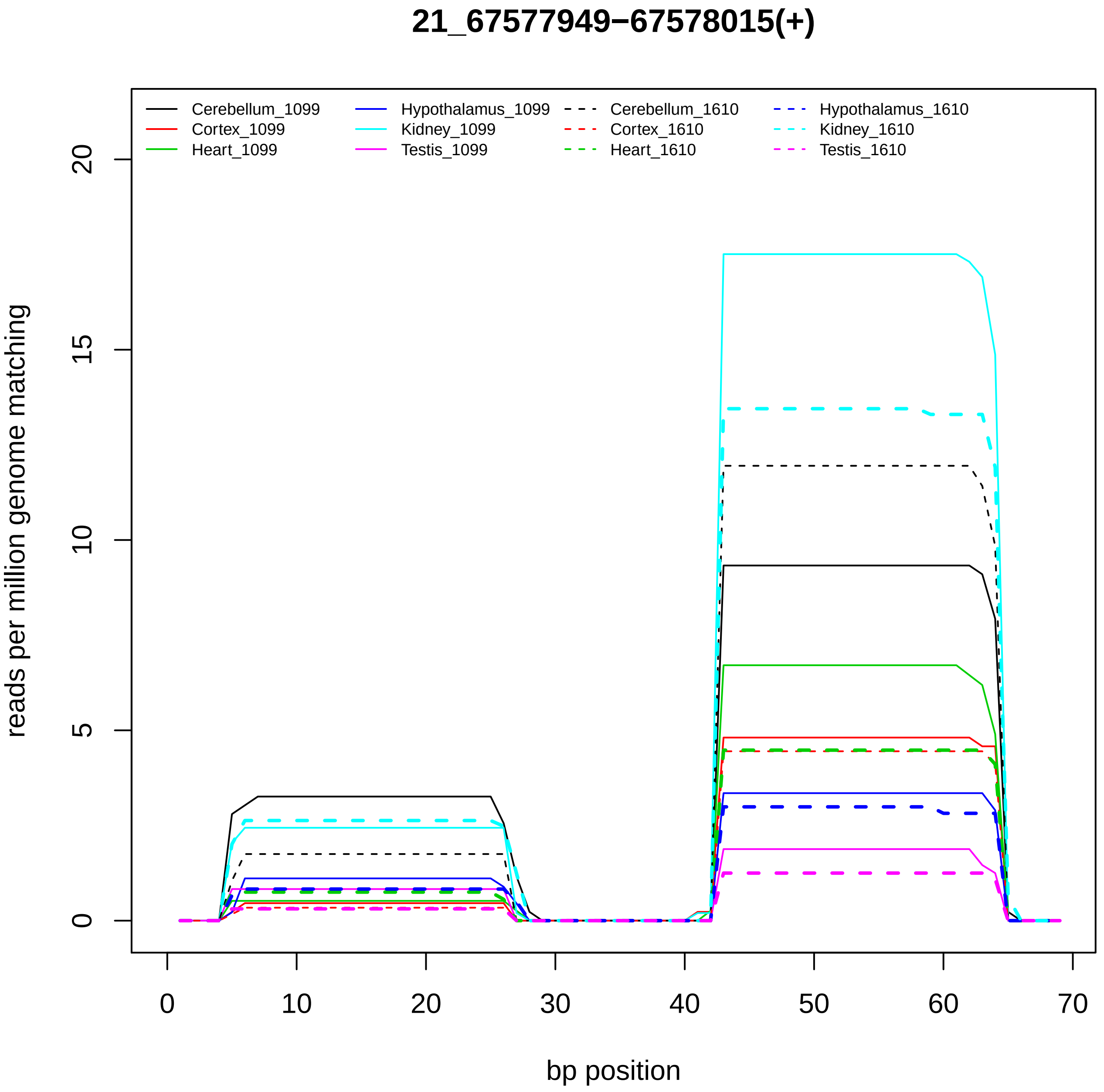
21_67577949−67578015(+)
Cerebellum_1099
Cortex_1099
Heart_1099
Hypothalamus_1099
Kidney_1099
Testis_1099
Cerebellum_1610
Cortex_1610
Heart_1610
Hypothalamus_1610
Kidney_1610
Testis_1610
reads per million genome matching
bp position
0
5
10
15
20
0
10
20
30
40
50
60
70

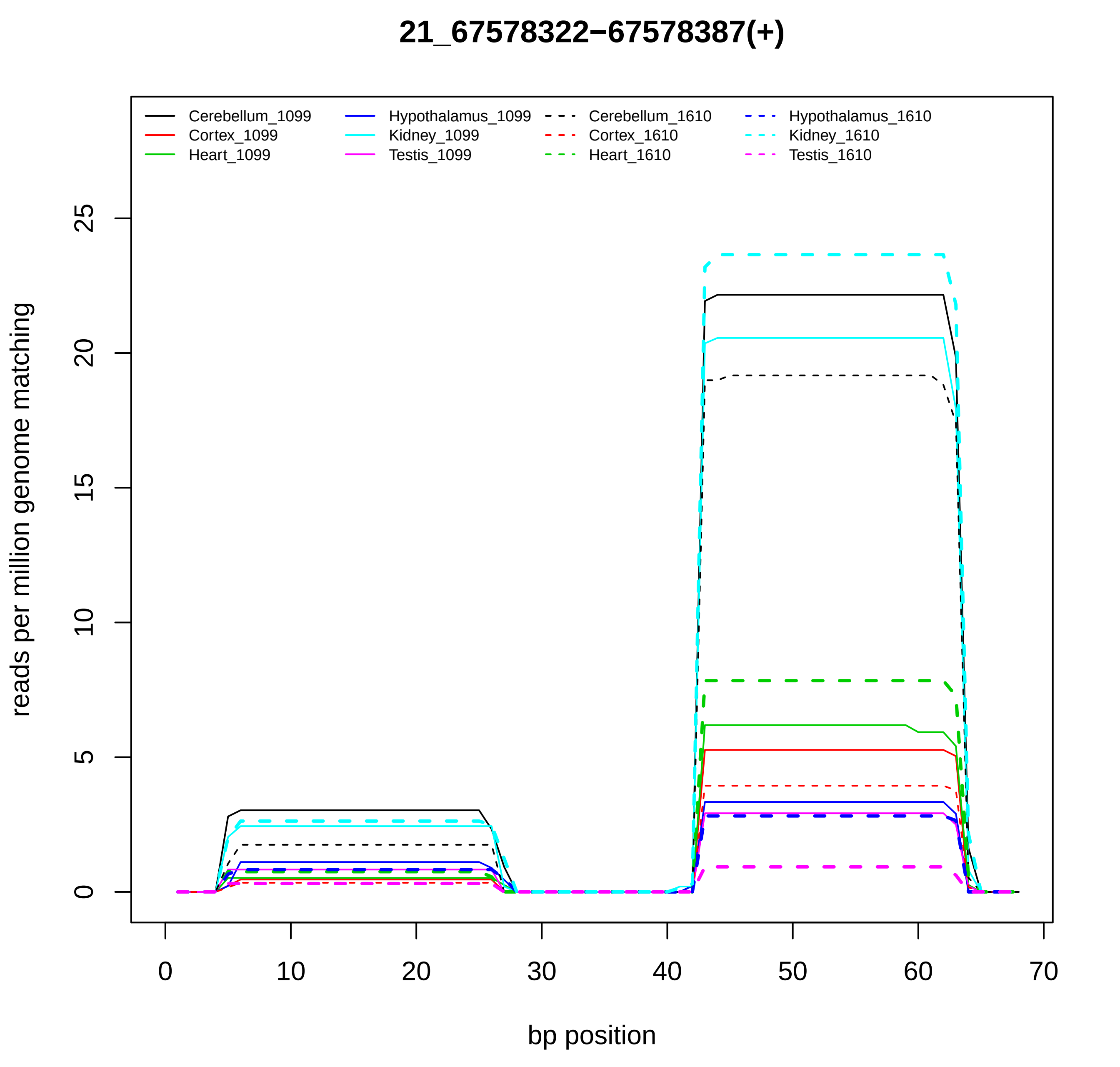

21_67578322−67578387(+)
Cerebellum_1099
Cortex_1099
Heart_1099
Hypothalamus_1099
Kidney_1099
Testis_1099
Cerebellum_1610
Cortex_1610
Heart_1610
Hypothalamus_1610
Kidney_1610
Testis_1610
reads per million genome matching
0
5
10
15
20
25
0
10
20
30
40
50
60
70
bp position

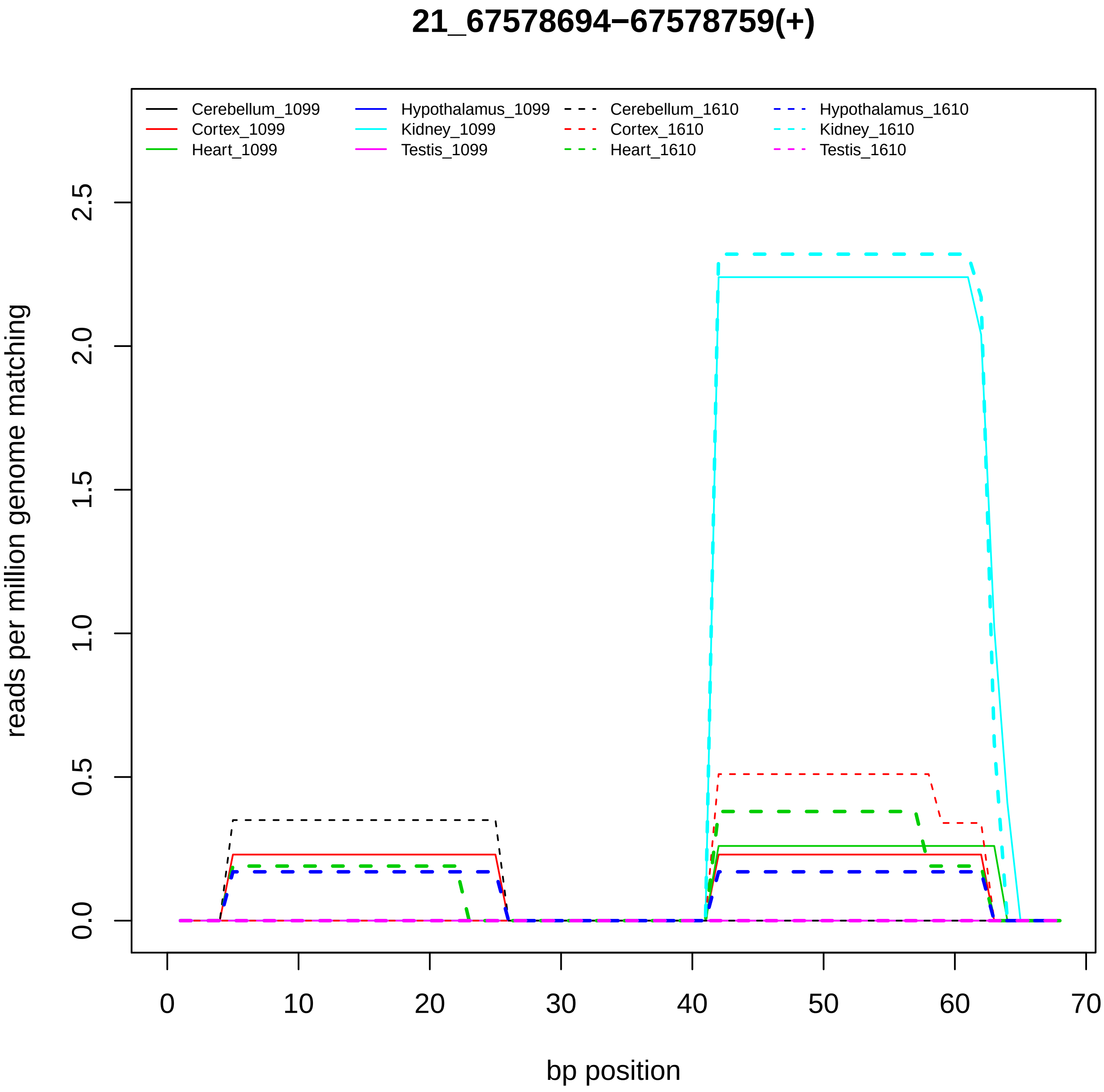
21_67578694−67578759(+)
Cerebellum_1099
Cortex_1099
Heart_1099
Hypothalamus_1099
Kidney_1099
Testis_1099
Cerebellum_1610
Cortex_1610
Heart_1610
Hypothalamus_1610
Kidney_1610
Testis_1610
reads per million genome matching
0.0
0.5
1.0
1.5
2.0
2.5
bp position
0
10
20
30
40
50
60
70

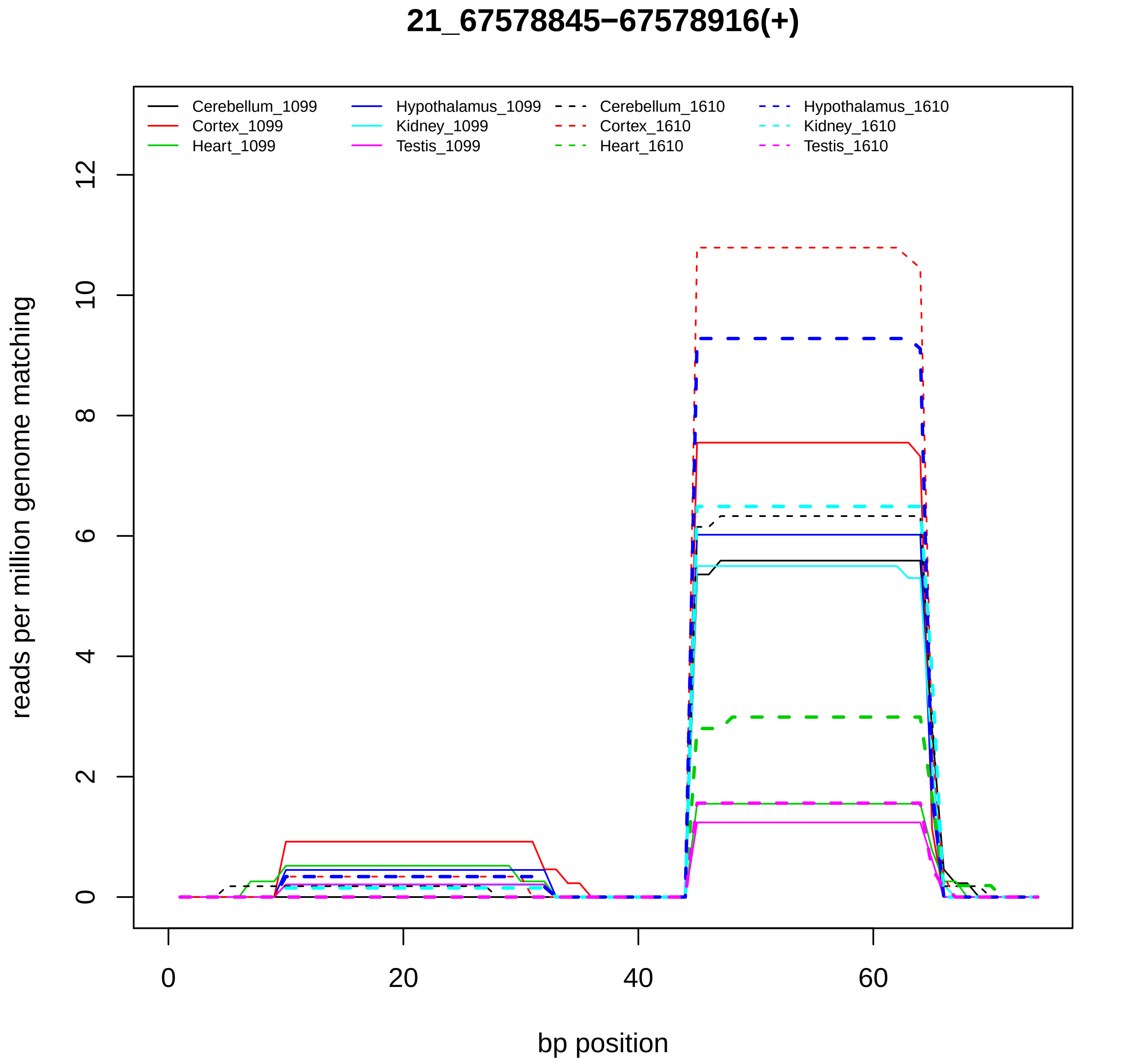
21_67578845−67578916(+)
Cerebellum_1099
Cortex_1099
Heart_1099
Hypothalamus_1099
Kidney_1099
Testis_1099
Cerebellum_1610
Cortex_1610
Heart_1610
Hypothalamus_1610
Kidney_1610
Testis_1610
reads per million genome matching
0
2
4
6
8
10
12
0
20
40
60
bp position

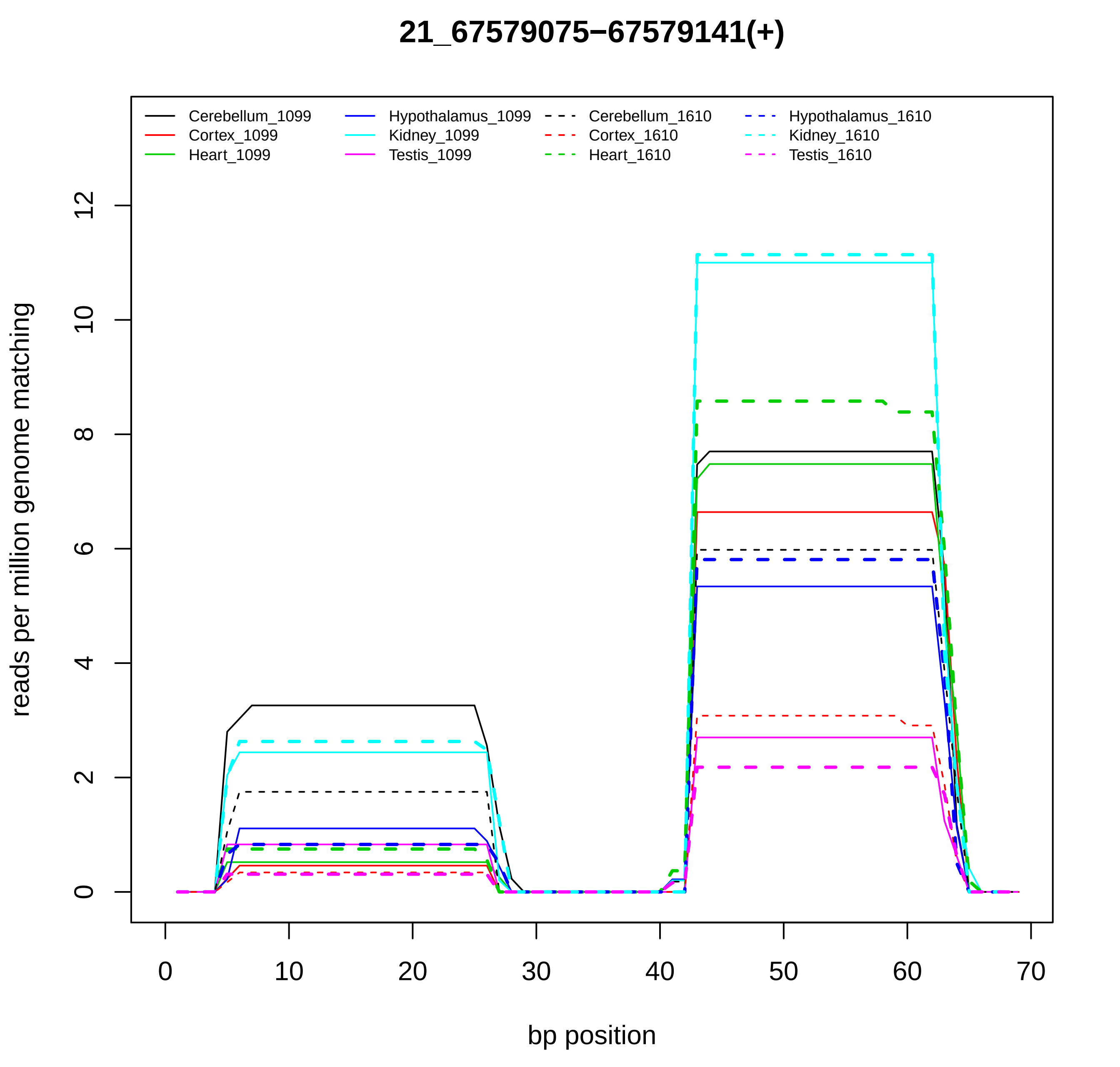
21_67579075−67579141(+)
Cerebellum_1099
Cortex_1099
Heart_1099
Hypothalamus_1099
Kidney_1099
Testis_1099
Cerebellum_1610
Cortex_1610
Heart_1610
Hypothalamus_1610
Kidney_1610
Testis_1610
reads per million genome matching
bp position
0
2
4
6
8
10
12
0
10
20
30
40
50
60
70

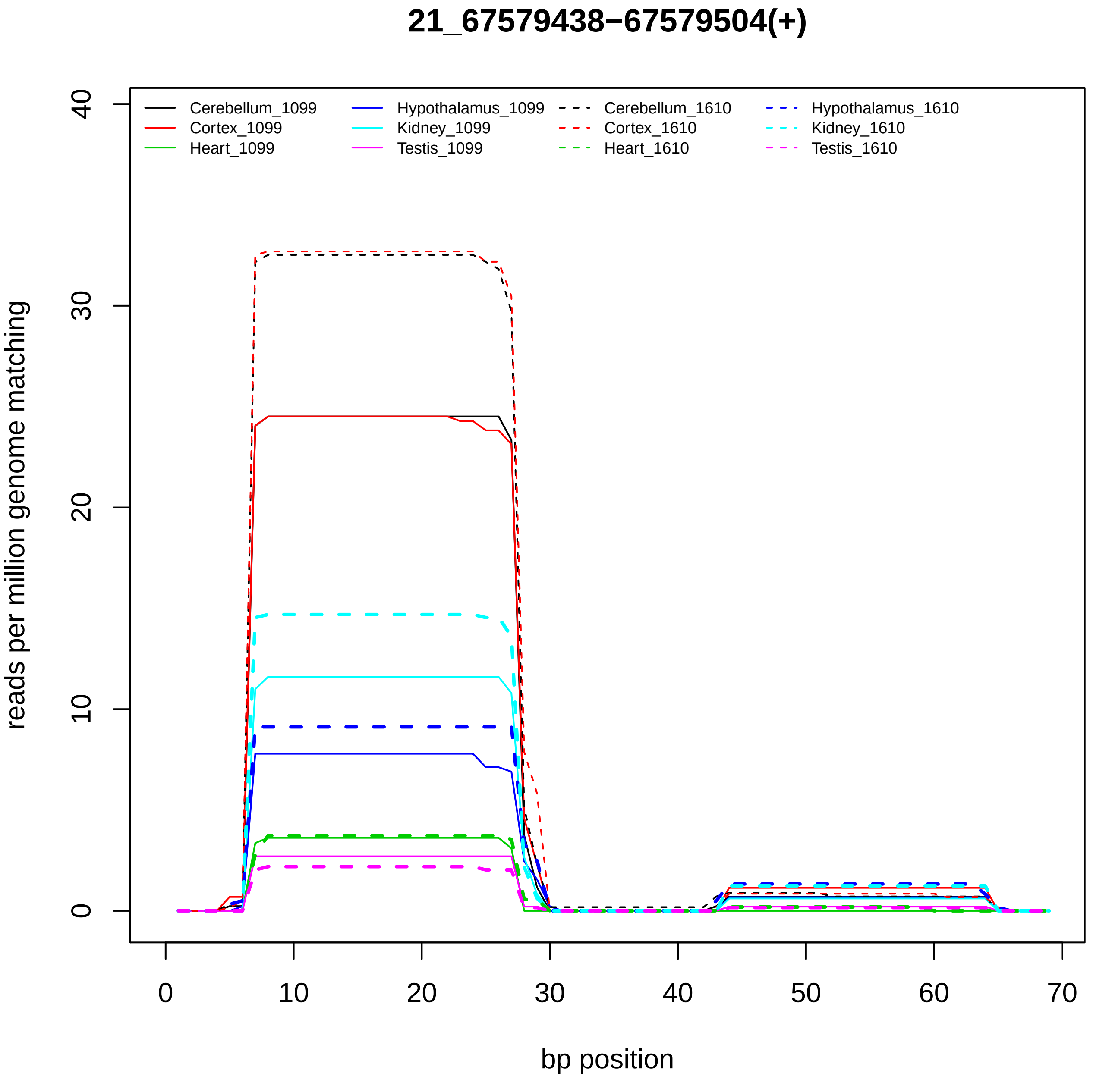
21_67579438−67579504(+)
Cerebellum_1099
Cortex_1099
Heart_1099
Hypothalamus_1099
Kidney_1099
Testis_1099
Cerebellum_1610
Cortex_1610
Heart_1610
Hypothalamus_1610
Kidney_1610
Testis_1610
reads per million genome matching
0
10
20
30
40
0
10
20
30
40
50
60
70
bp position

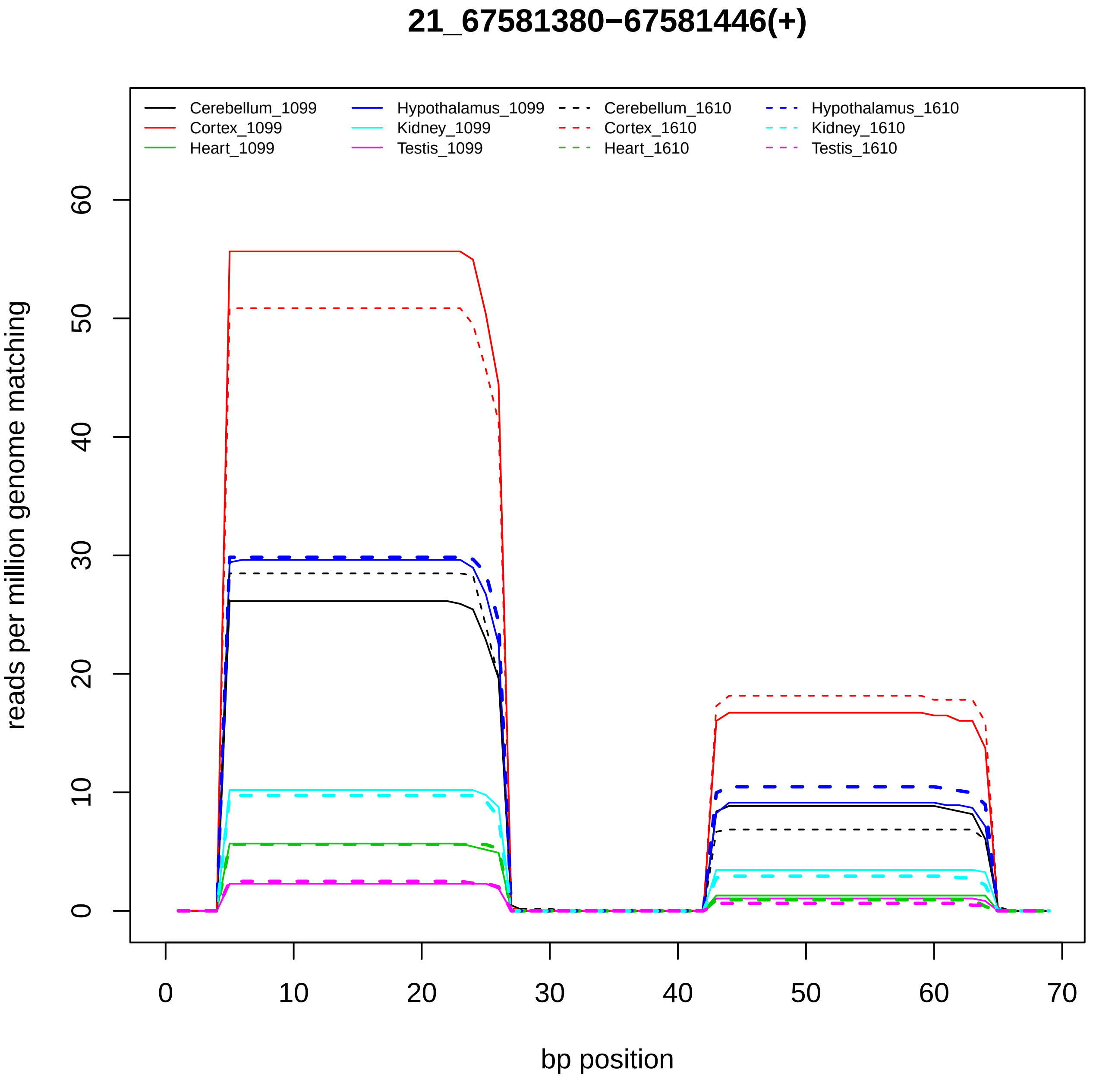

21_67581380−67581446(+)
Cerebellum_1099
Cortex_1099
Heart_1099
Hypothalamus_1099
Kidney_1099
Testis_1099
Cerebellum_1610
Cortex_1610
Heart_1610
Hypothalamus_1610
Kidney_1610
Testis_1610
reads per million genome matching
0
10
20
30
40
50
60
0
10
20
30
40
50
60
70
bp position

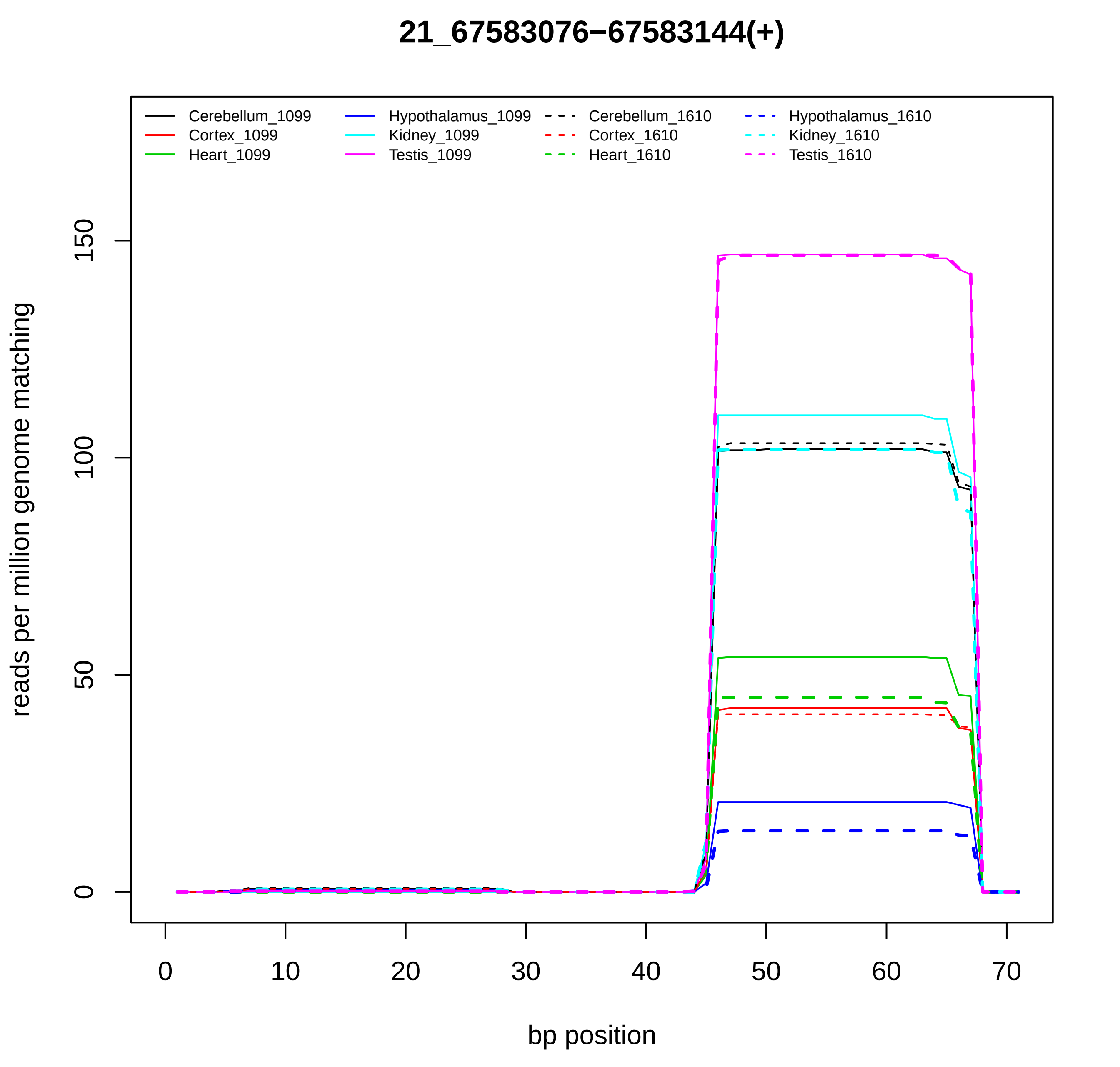

21_67583076−67583144(+)
Cerebellum_1099
Cortex_1099
Heart_1099
Hypothalamus_1099
Kidney_1099
Testis_1099
Cerebellum_1610
Cortex_1610
Heart_1610
Hypothalamus_1610
Kidney_1610
Testis_1610
reads per million genome matching
0
50
100
150
0
10
20
30
40
50
60
70
bp position

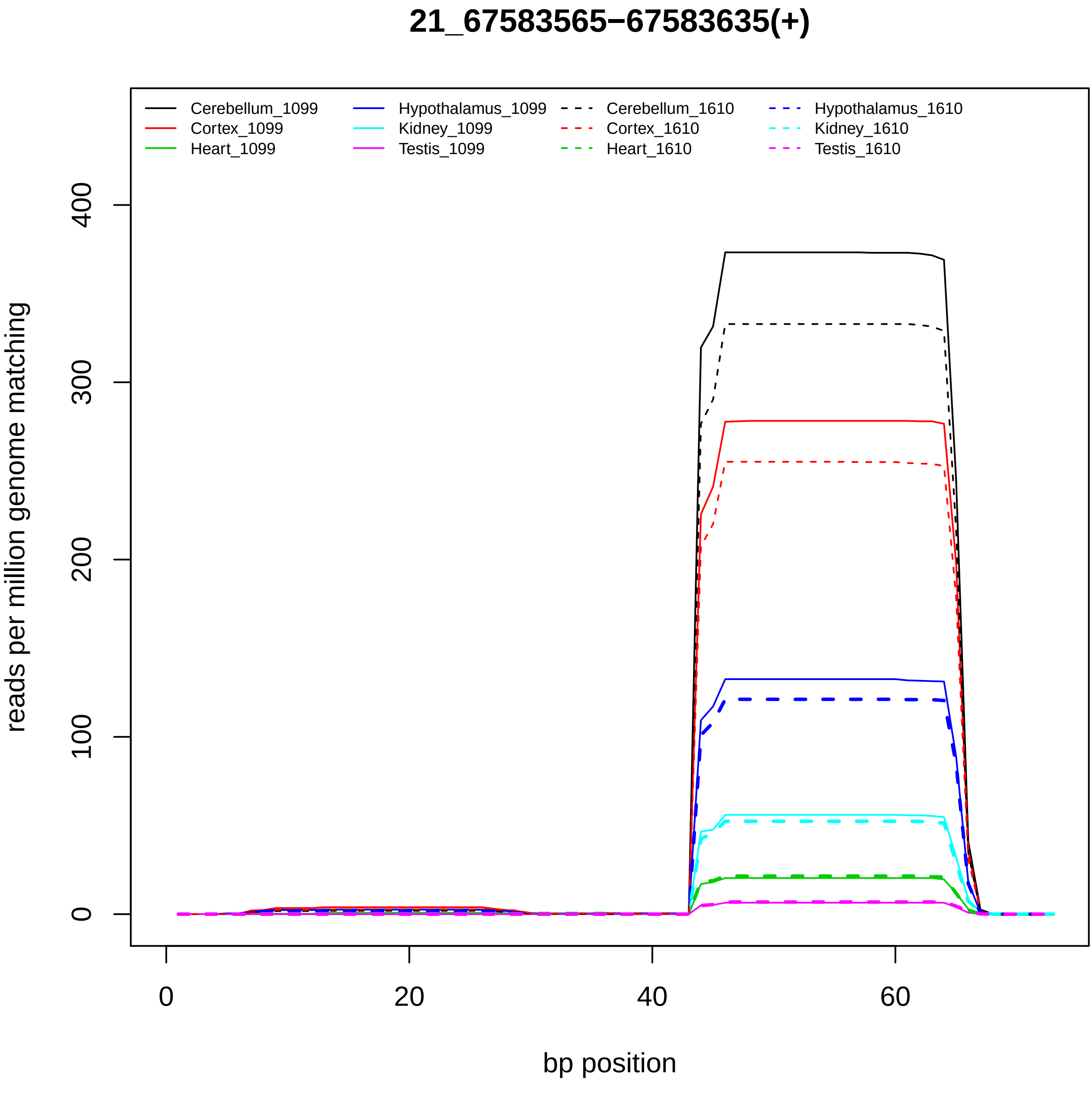

21_67583565−67583635(+)
Cerebellum_1099
Cortex_1099
Heart_1099
Hypothalamus_1099
Kidney_1099
Testis_1099
Cerebellum_1610
Cortex_1610
Heart_1610
Hypothalamus_1610
Kidney_1610
Testis_1610
reads per million genome matching
0
100
200
300
400
0
20
40
60
bp position

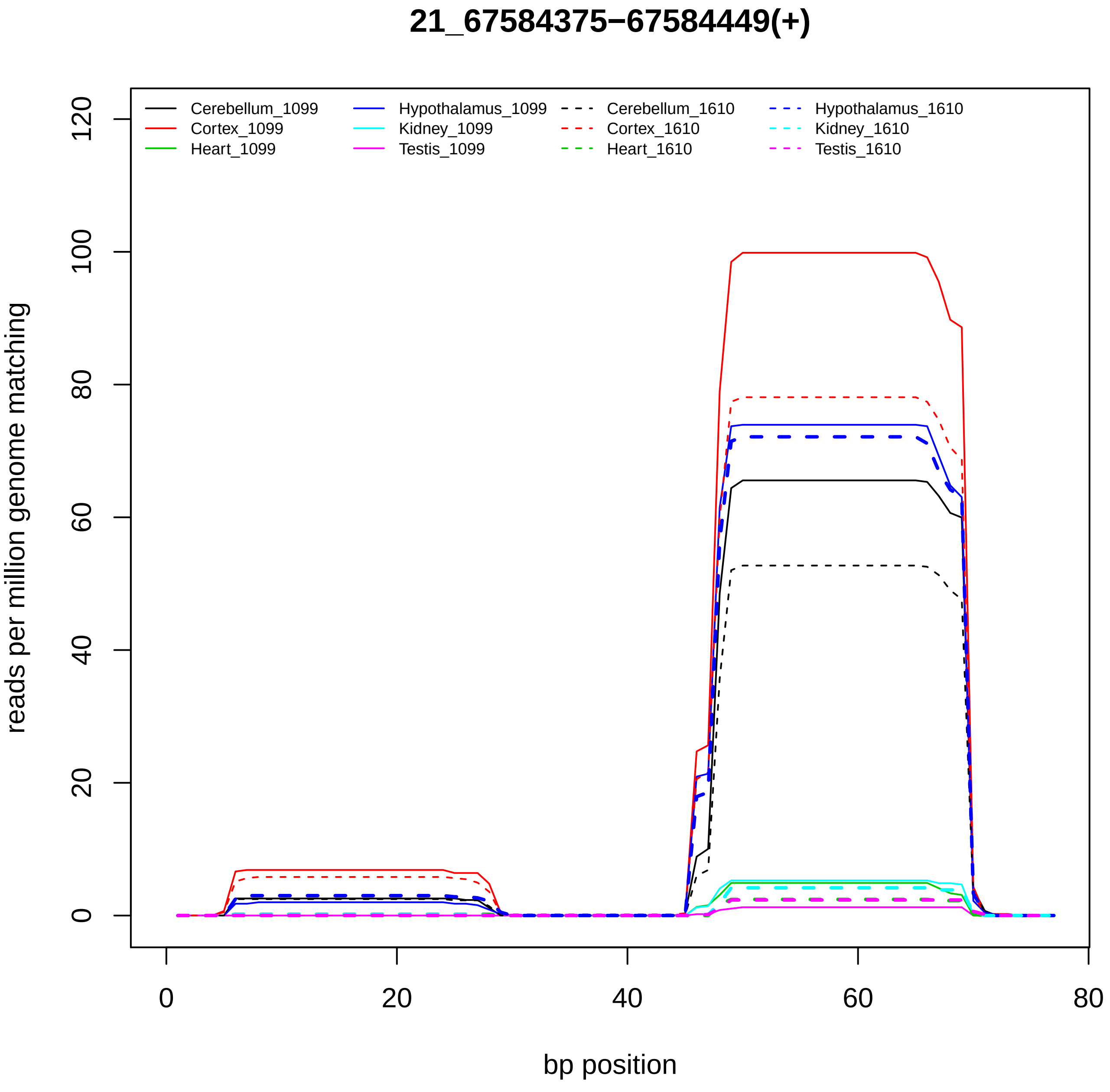

21_67584375−67584449(+)
Cerebellum_1099
Cortex_1099
Heart_1099
Hypothalamus_1099
Kidney_1099
Testis_1099
Cerebellum_1610
Cortex_1610
Heart_1610
Hypothalamus_1610
Kidney_1610
Testis_1610
reads per million genome matching
bp position
0
20
40
60
80
100
120

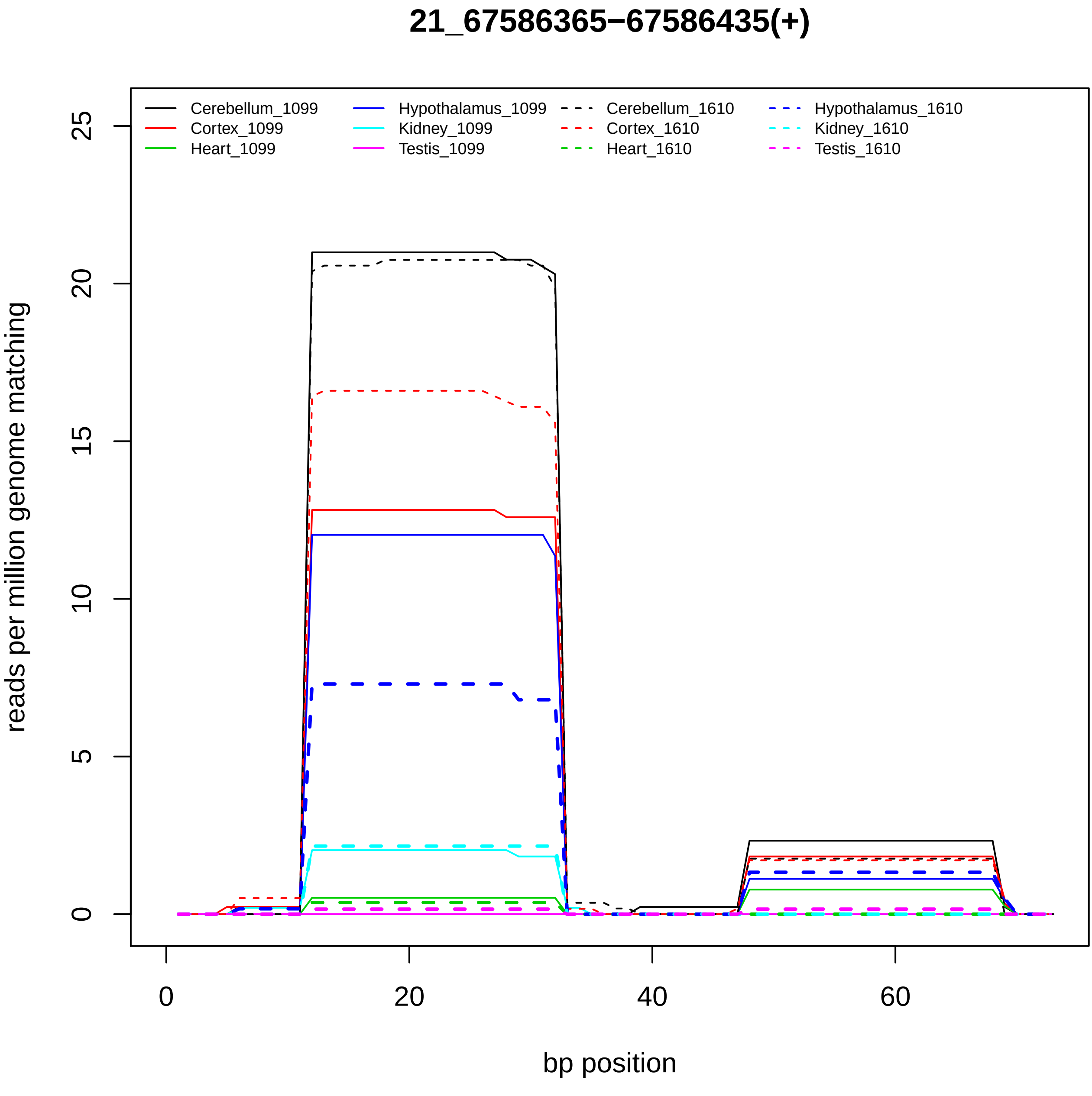

21_67586365−67586435(+)
Cerebellum_1099
Cortex_1099
Heart_1099
Hypothalamus_1099
Kidney_1099
Testis_1099
Cerebellum_1610
Cortex_1610
Heart_1610
Hypothalamus_1610
Kidney_1610
Testis_1610
reads per million genome matching
0
5
10
15
20
25
0
20
40
60
bp position

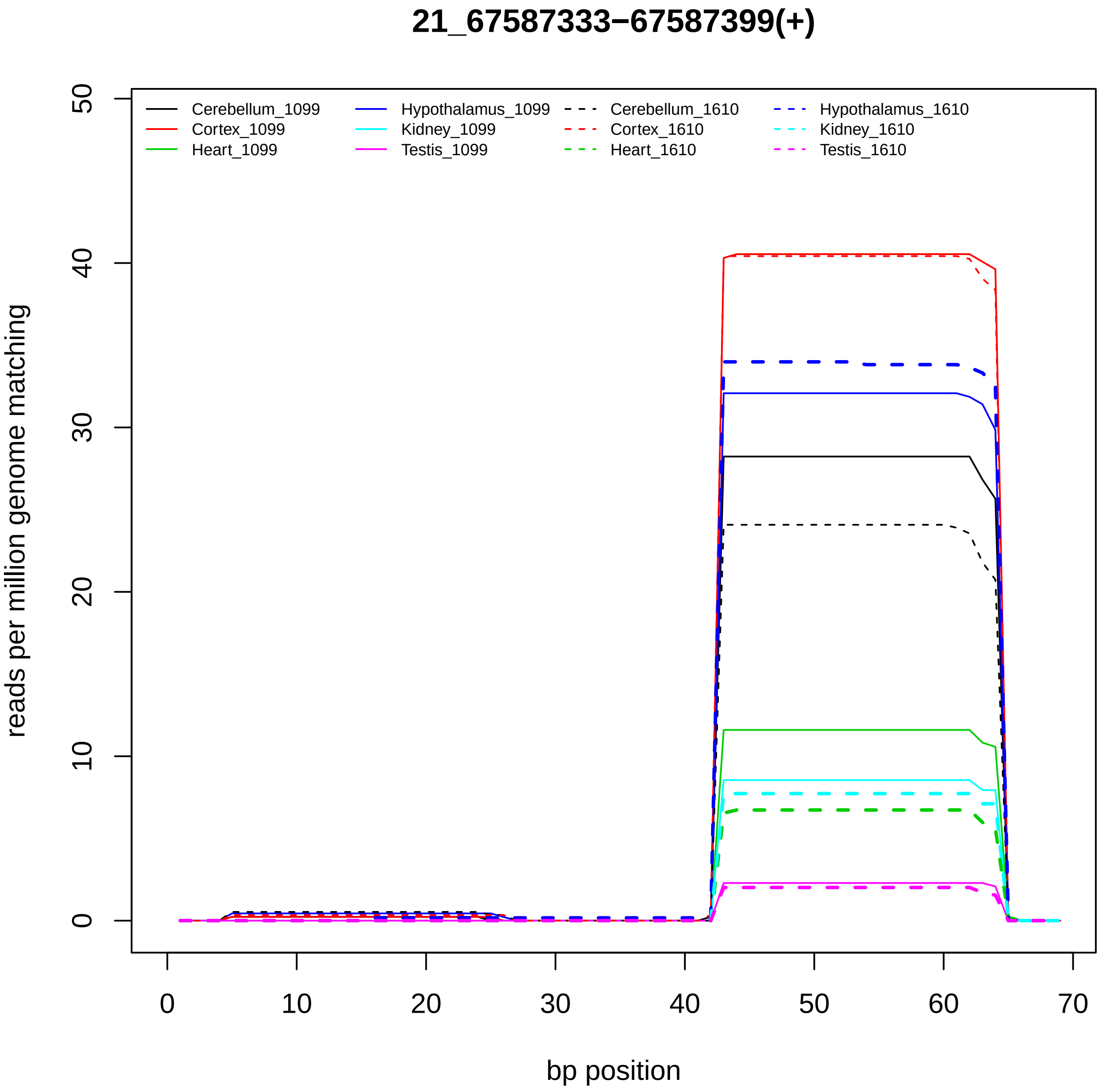

21_67587333−67587399(+)
Cerebellum_1099
Cortex_1099
Heart_1099
Hypothalamus_1099
Kidney_1099
Testis_1099
Cerebellum_1610
Cortex_1610
Heart_1610
Hypothalamus_1610
Kidney_1610
Testis_1610
reads per million genome matching
bp position
0
10
20
30
40
50
60
70

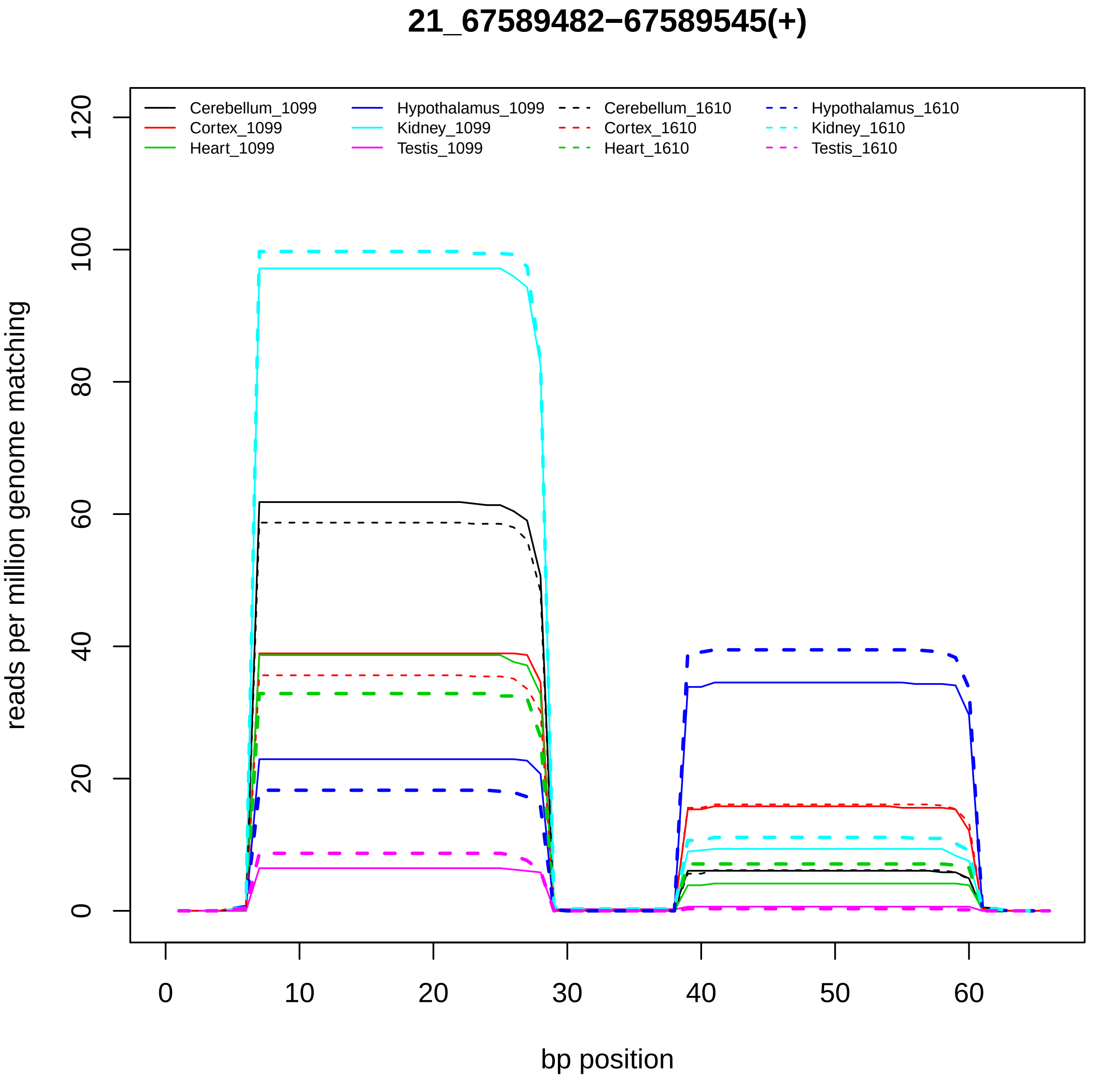
21_67589482−67589545(+)
Cerebellum_1099
Cortex_1099
Heart_1099
Hypothalamus_1099
Kidney_1099
Testis_1099
Cerebellum_1610
Cortex_1610
Heart_1610
Hypothalamus_1610
Kidney_1610
Testis_1610
reads per million genome matching
bp position
0
20
40
60
80
100
120
0
10
20
30
40
50
60

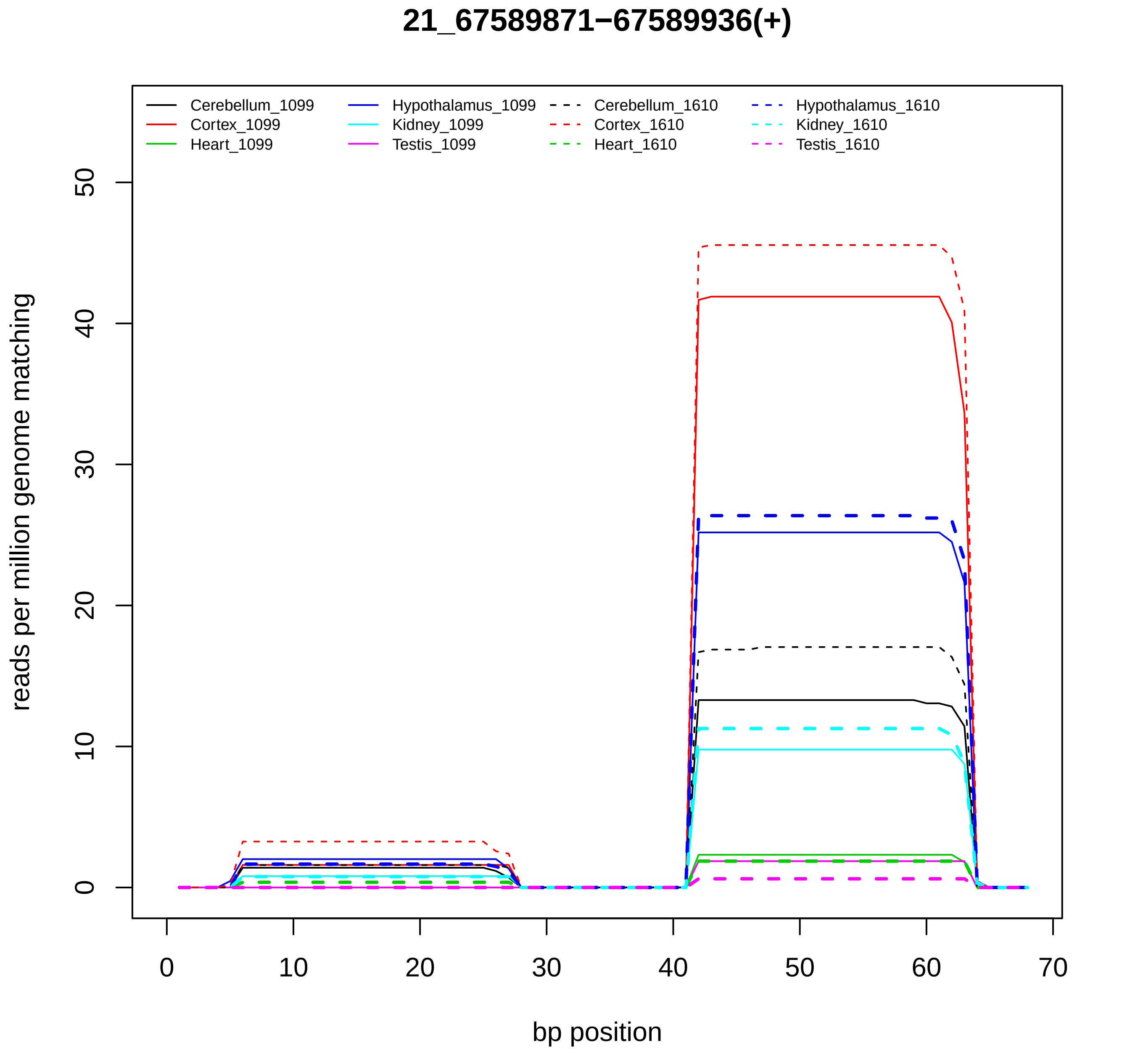
21_67589871−67589936(+)
Cerebellum_1099
Cortex_1099
Heart_1099
Hypothalamus_1099
Kidney_1099
Testis_1099
Cerebellum_1610
Cortex_1610
Heart_1610
Hypothalamus_1610
Kidney_1610
Testis_1610
reads per million genome matching
bp position
0
10
20
30
40
50
60
70

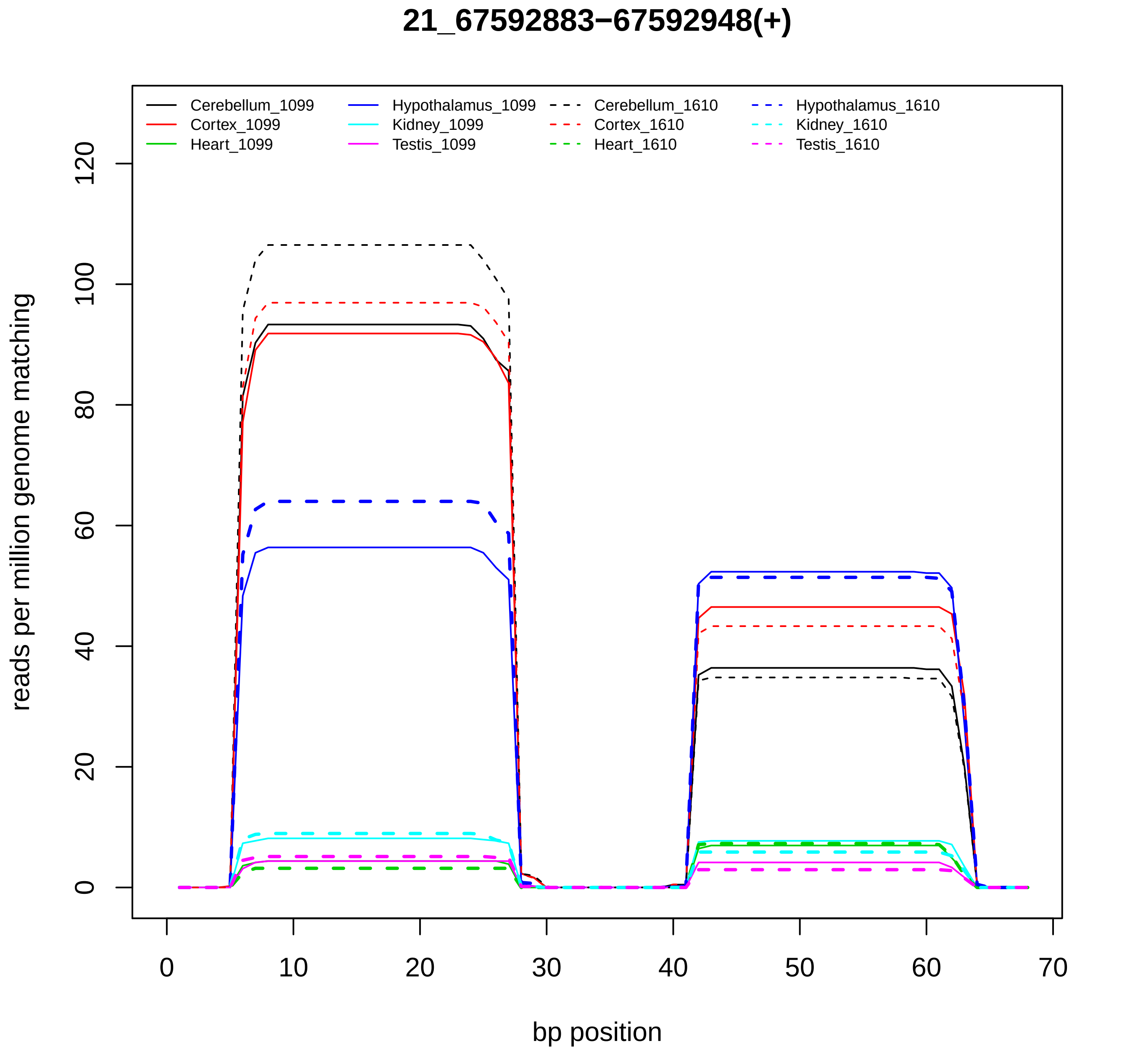

21_67592883−67592948(+)
Cerebellum_1099
Cortex_1099
Heart_1099
Hypothalamus_1099
Kidney_1099
Testis_1099
Cerebellum_1610
Cortex_1610
Heart_1610
Hypothalamus_1610
Kidney_1610
Testis_1610
reads per million genome matching
bp position
0
20
40
60
80
100
120
0
10
20
30
40
50
60
70

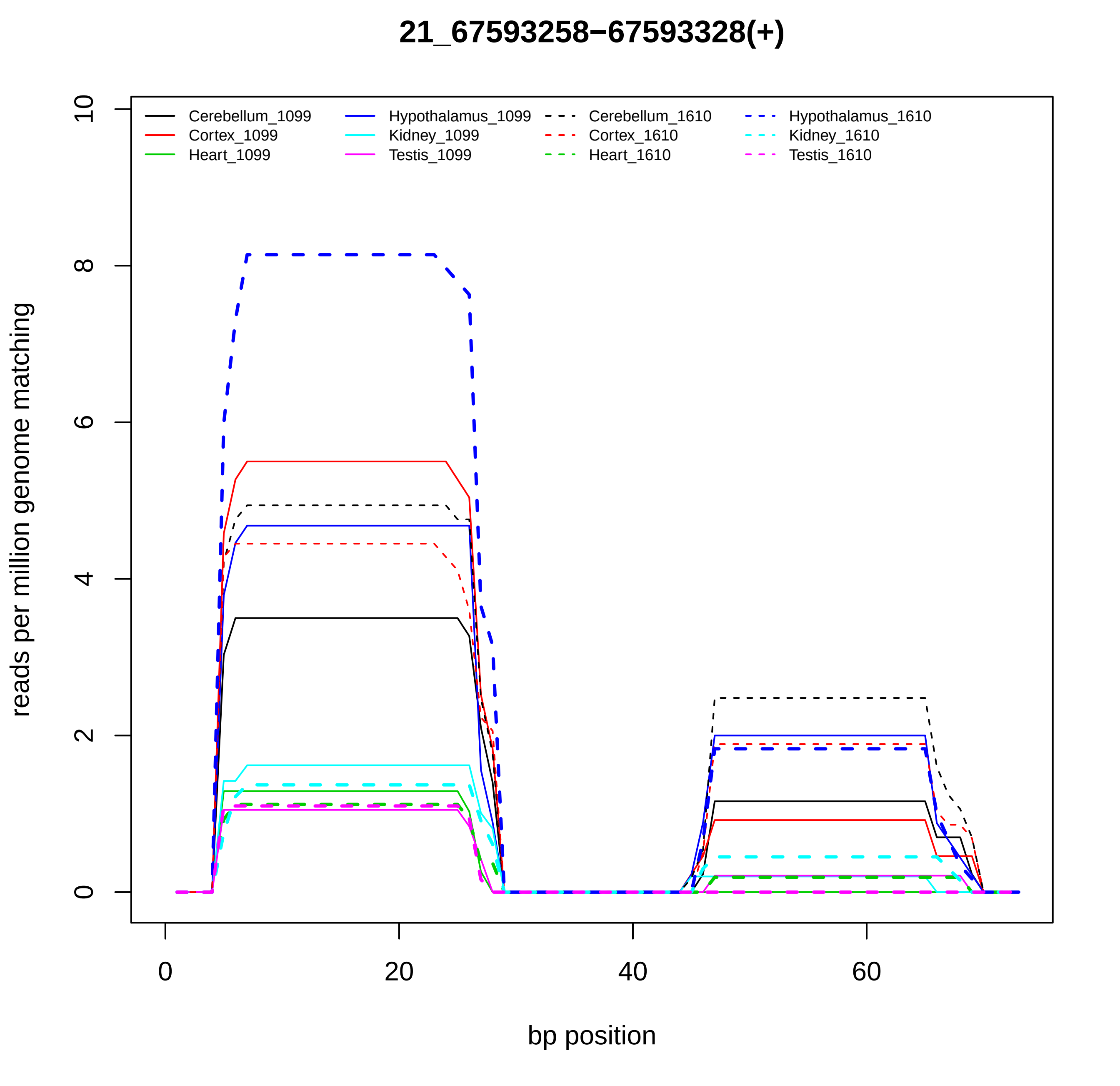

21_67593258−67593328(+)
Cerebellum_1099
Cortex_1099
Heart_1099
Hypothalamus_1099
Kidney_1099
Testis_1099
Cerebellum_1610
Cortex_1610
Heart_1610
Hypothalamus_1610
Kidney_1610
Testis_1610
reads per million genome matching
bp position
0
2
4
6
8
10
0
20
40
60

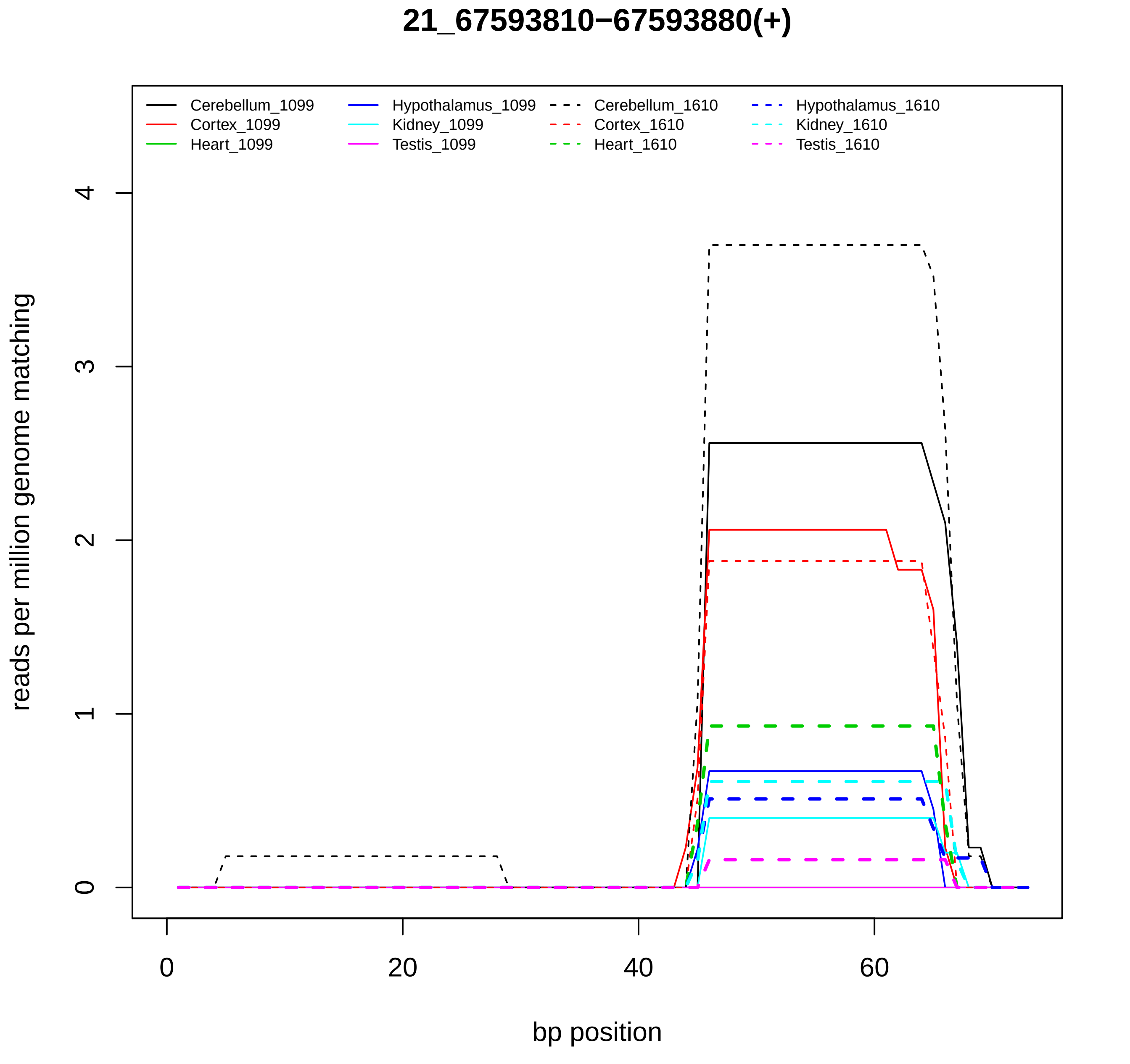

21_67593810−67593880(+)
Cerebellum_1099
Cortex_1099
Heart_1099
Hypothalamus_1099
Kidney_1099
Testis_1099
Cerebellum_1610
Cortex_1610
Heart_1610
Hypothalamus_1610
Kidney_1610
Testis_1610
reads per million genome matching
0
1
2
3
4
0
20
40
60
bp position

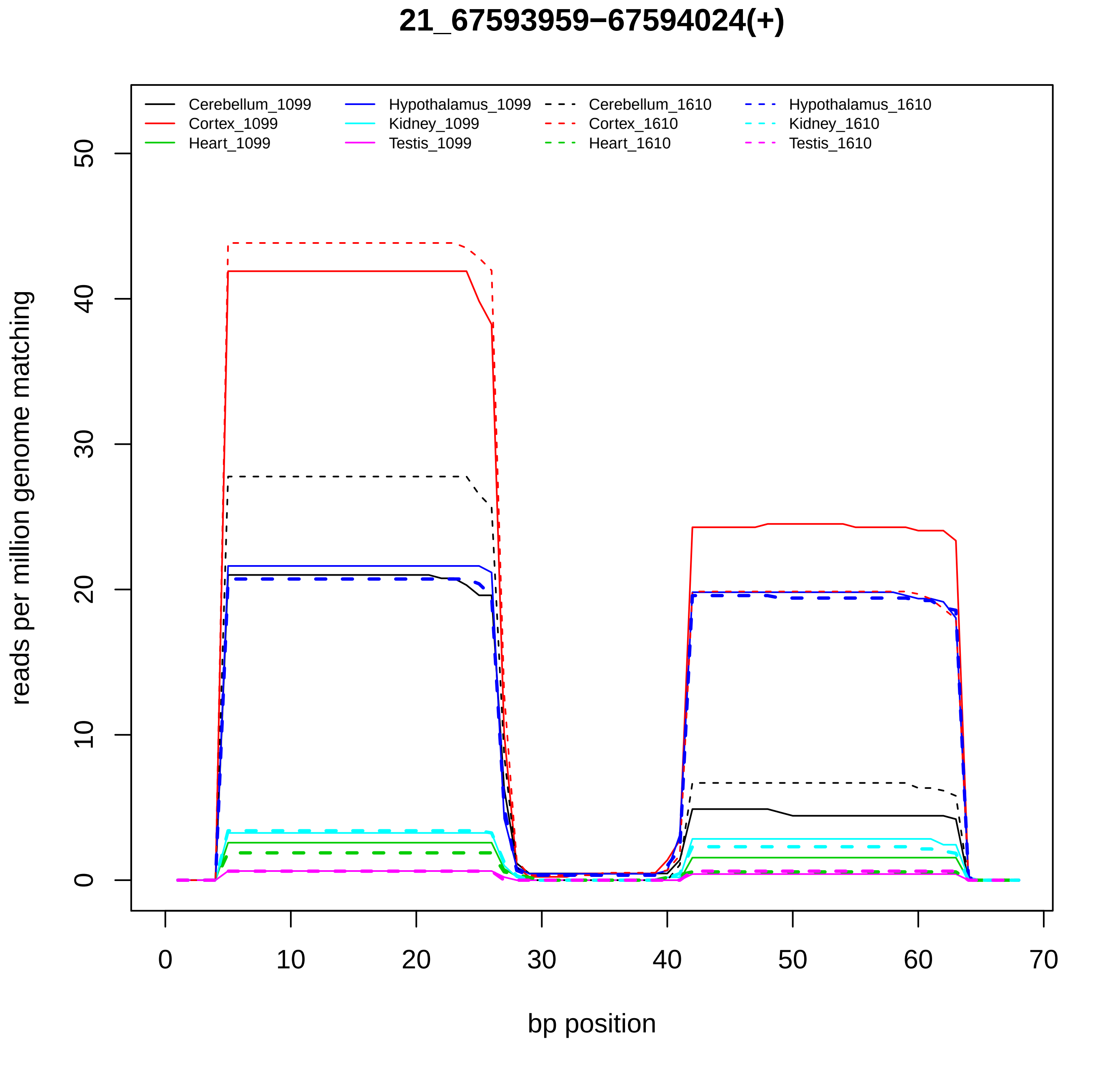
21_67593959−67594024(+)
Cerebellum_1099
Cortex_1099
Heart_1099
Hypothalamus_1099
Kidney_1099
Testis_1099
Cerebellum_1610
Cortex_1610
Heart_1610
Hypothalamus_1610
Kidney_1610
Testis_1610
reads per million genome matching
bp position
0
10
20
30
40
50
0
10
20
30
40
50
60
70

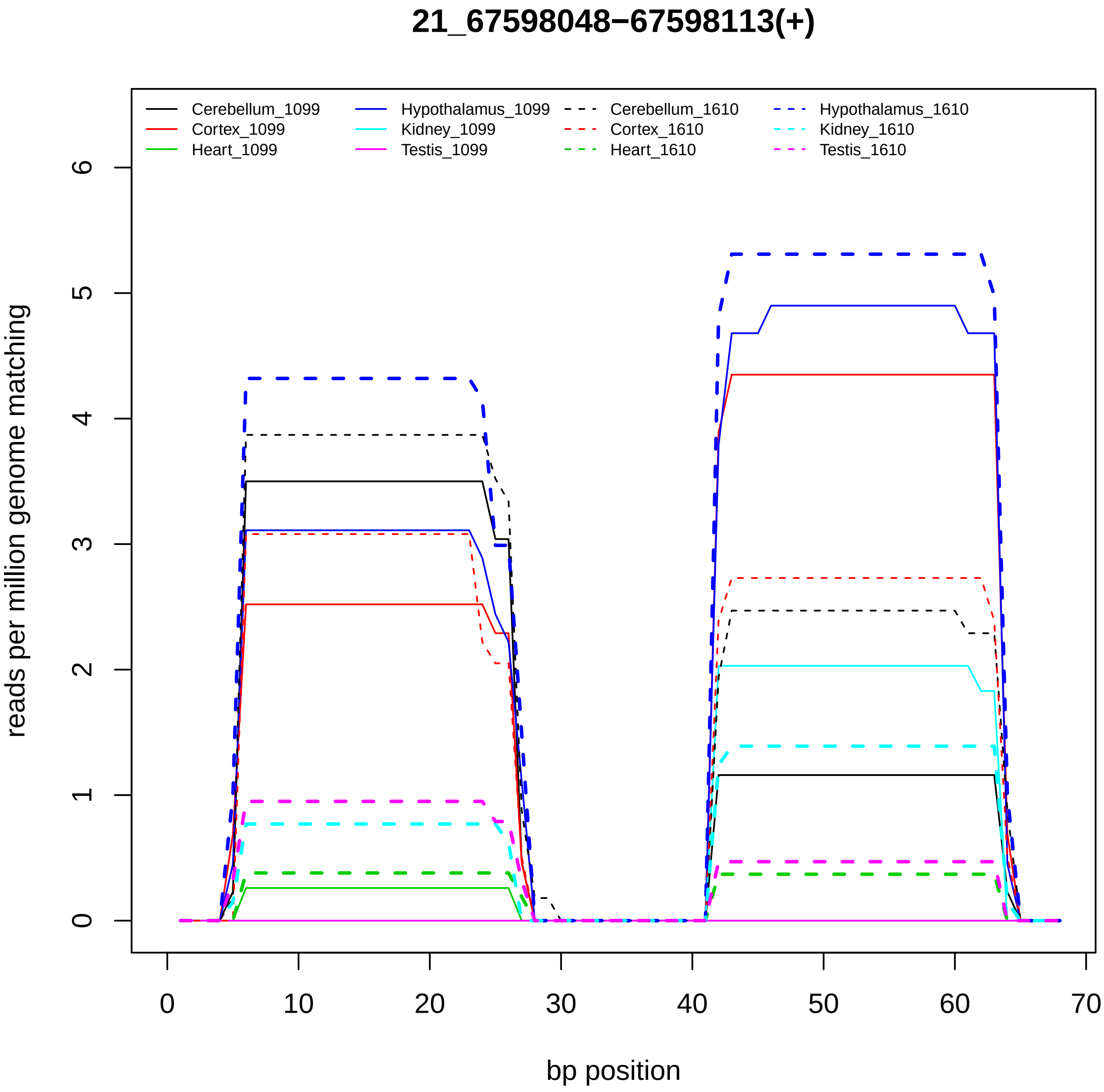

21_67598048−67598113(+)
Cerebellum_1099
Cortex_1099
Heart_1099
Hypothalamus_1099
Kidney_1099
Testis_1099
Cerebellum_1610
Cortex_1610
Heart_1610
Hypothalamus_1610
Kidney_1610
Testis_1610
reads per million genome matching
bp position
0
1
2
3
4
5
6
0
10
20
30
40
50
60
70

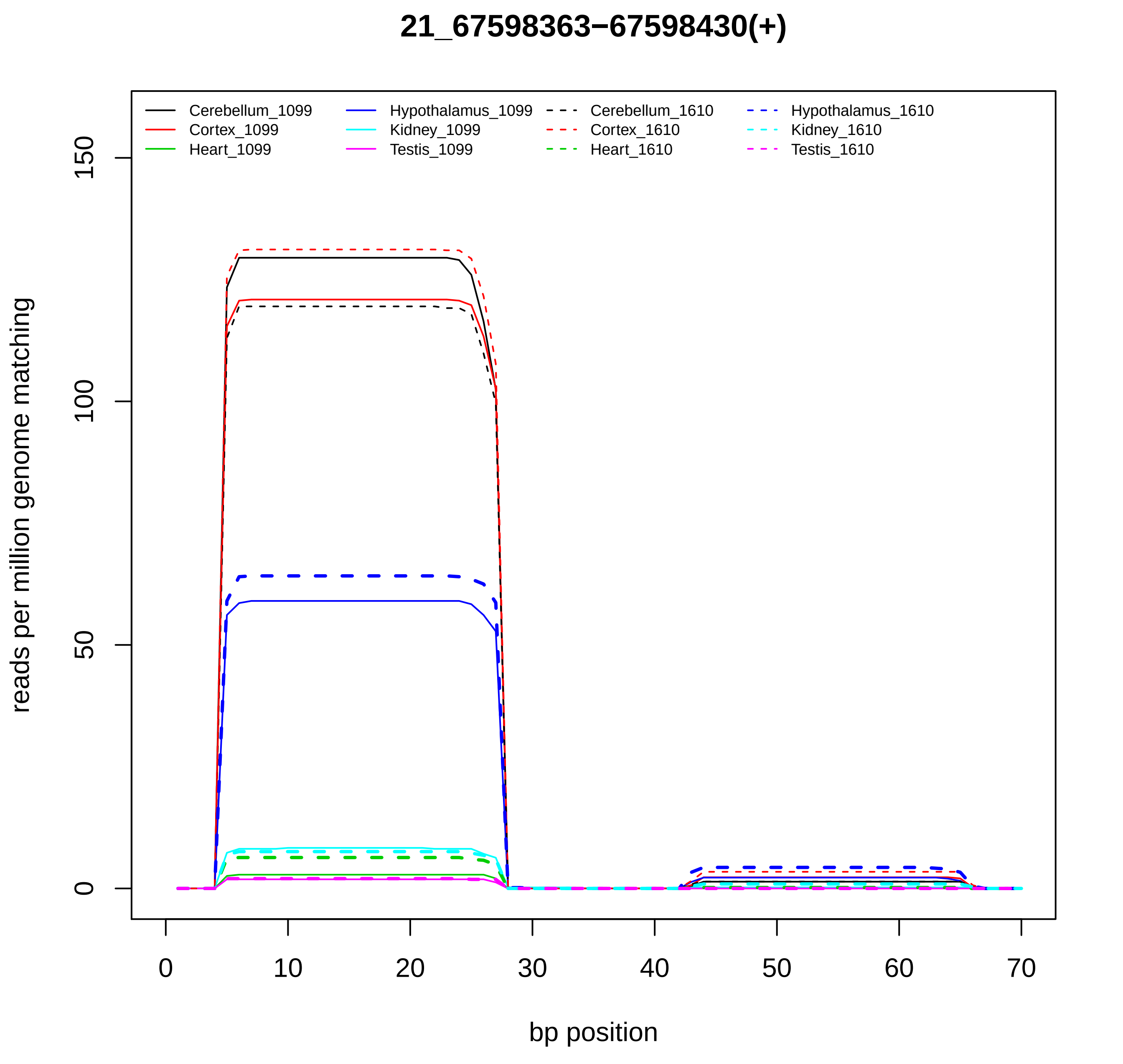

21_67598363−67598430(+)
Cerebellum_1099
Cortex_1099
Heart_1099
Hypothalamus_1099
Kidney_1099
Testis_1099
Cerebellum_1610
Cortex_1610
Heart_1610
Hypothalamus_1610
Kidney_1610
Testis_1610
reads per million genome matching
0
50
100
150
0
10
20
30
40
50
60
70
bp position

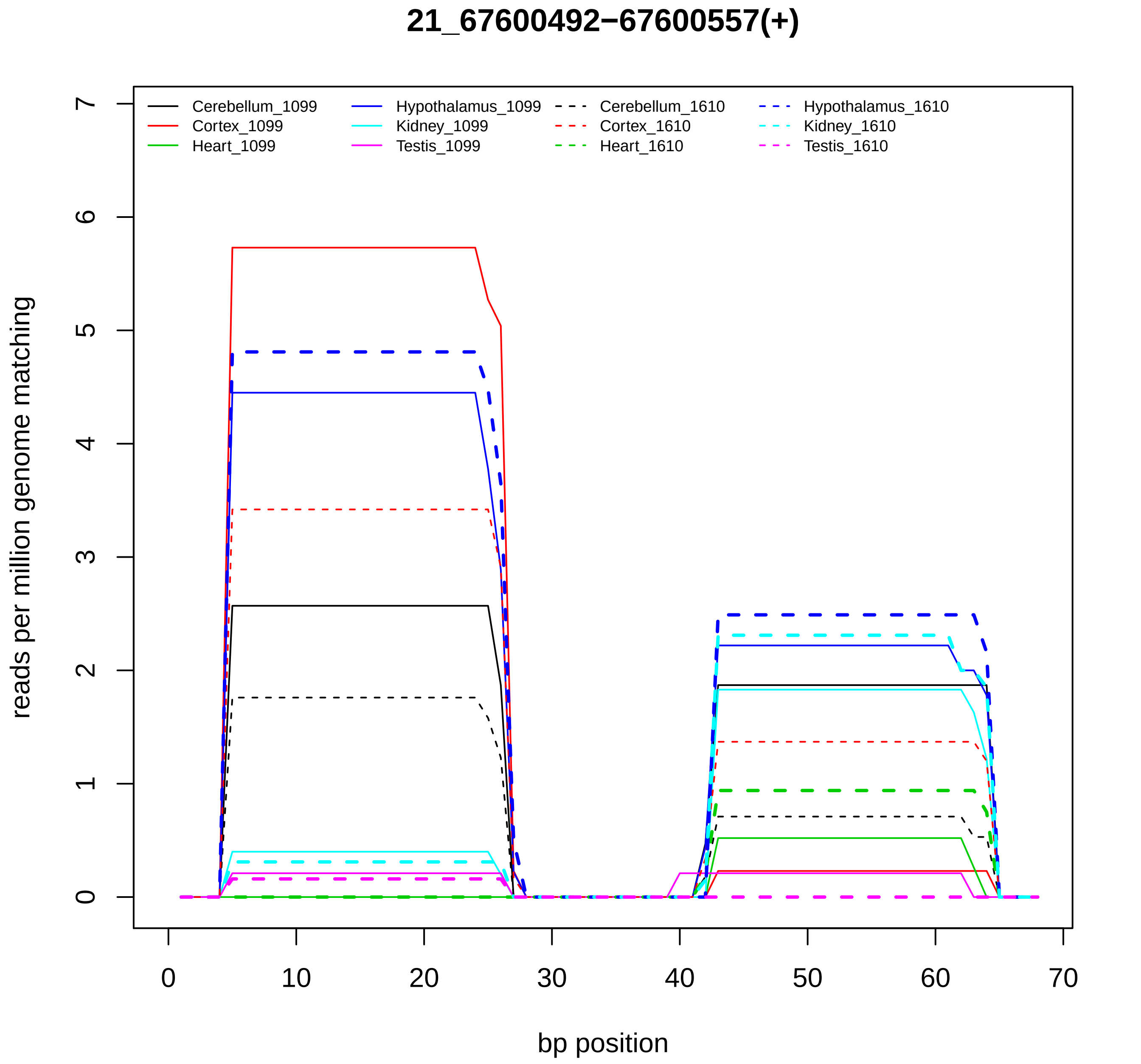

21_67600492−67600557(+)
Cerebellum_1099
Cortex_1099
Heart_1099
Hypothalamus_1099
Kidney_1099
Testis_1099
Cerebellum_1610
Cortex_1610
Heart_1610
Hypothalamus_1610
Kidney_1610
Testis_1610
reads per million genome matching
bp position
0
1
2
3
4
5
6
7
0
10
20
30
40
50
60
70

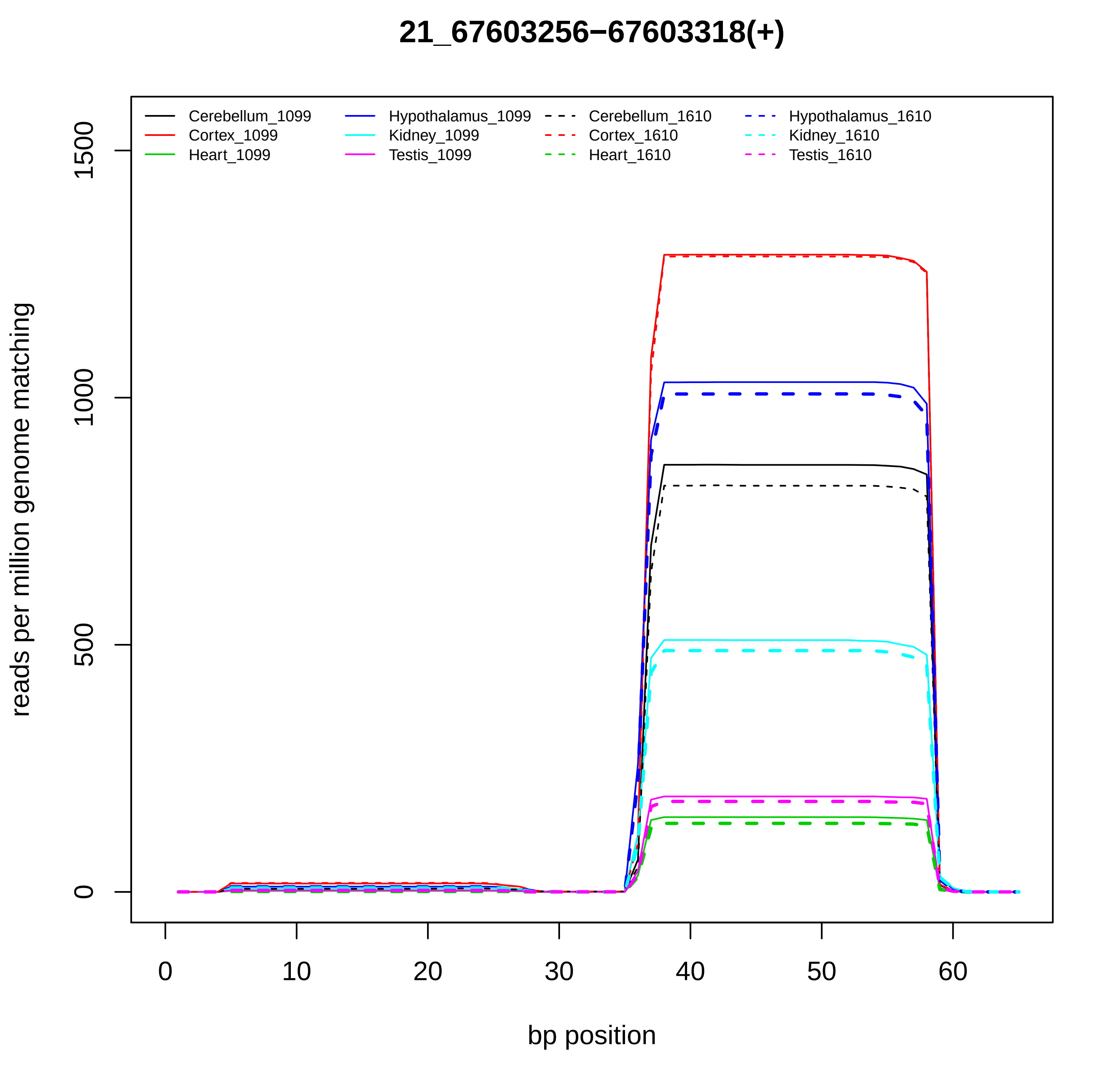

21_67603256−67603318(+)
Cerebellum_1099
Cortex_1099
Heart_1099
Hypothalamus_1099
Kidney_1099
Testis_1099
Cerebellum_1610
Cortex_1610
Heart_1610
Hypothalamus_1610
Kidney_1610
Testis_1610
reads per million genome matching
0
500
1000
1500
0
10
20
30
40
50
60
bp position

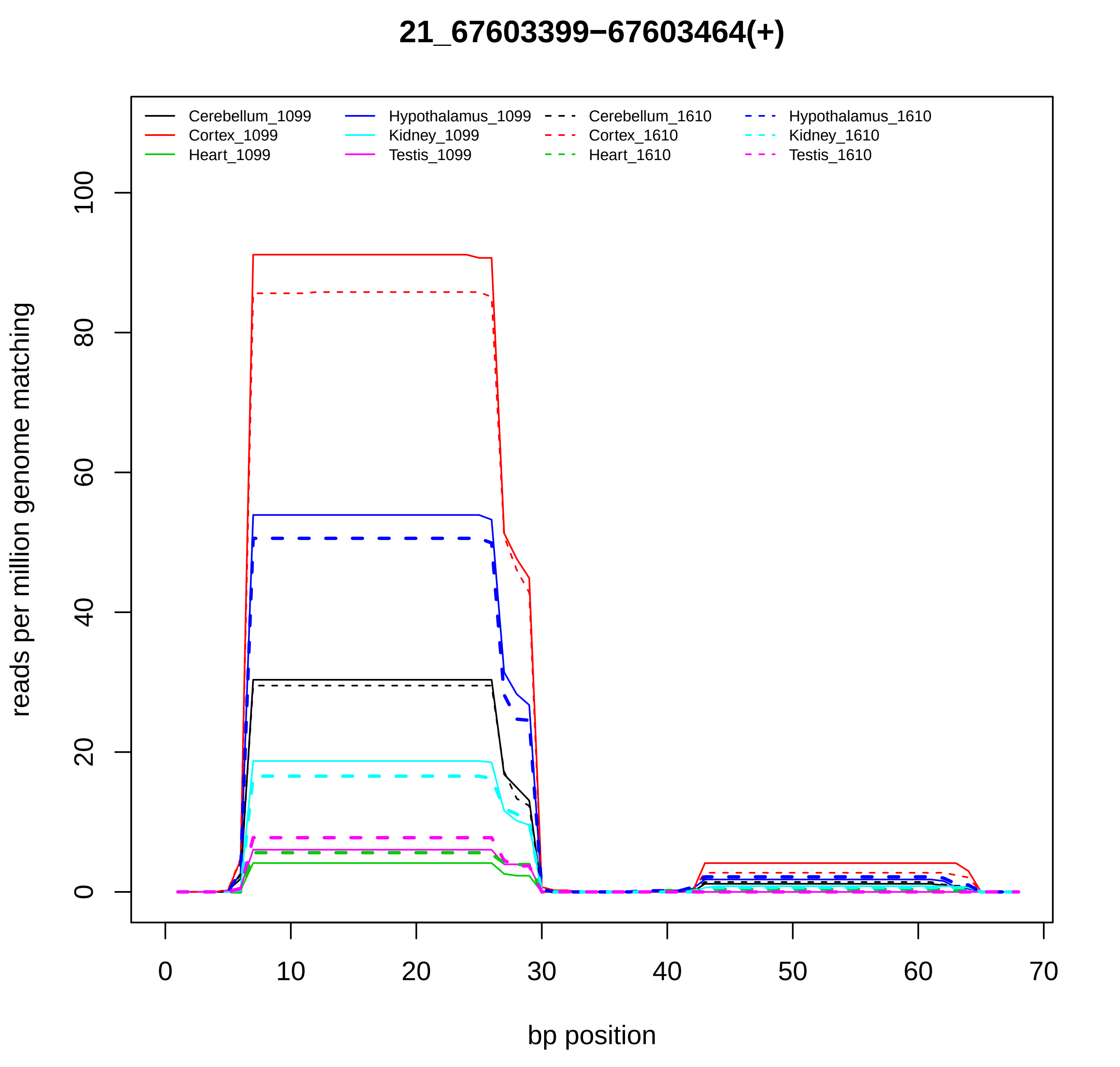

21_67603399−67603464(+)
Cerebellum_1099
Cortex_1099
Heart_1099
Hypothalamus_1099
Kidney_1099
Testis_1099
Cerebellum_1610
Cortex_1610
Heart_1610
Hypothalamus_1610
Kidney_1610
Testis_1610
reads per million genome matching
0
20
40
60
80
100
0
10
20
30
40
50
60
70
bp position

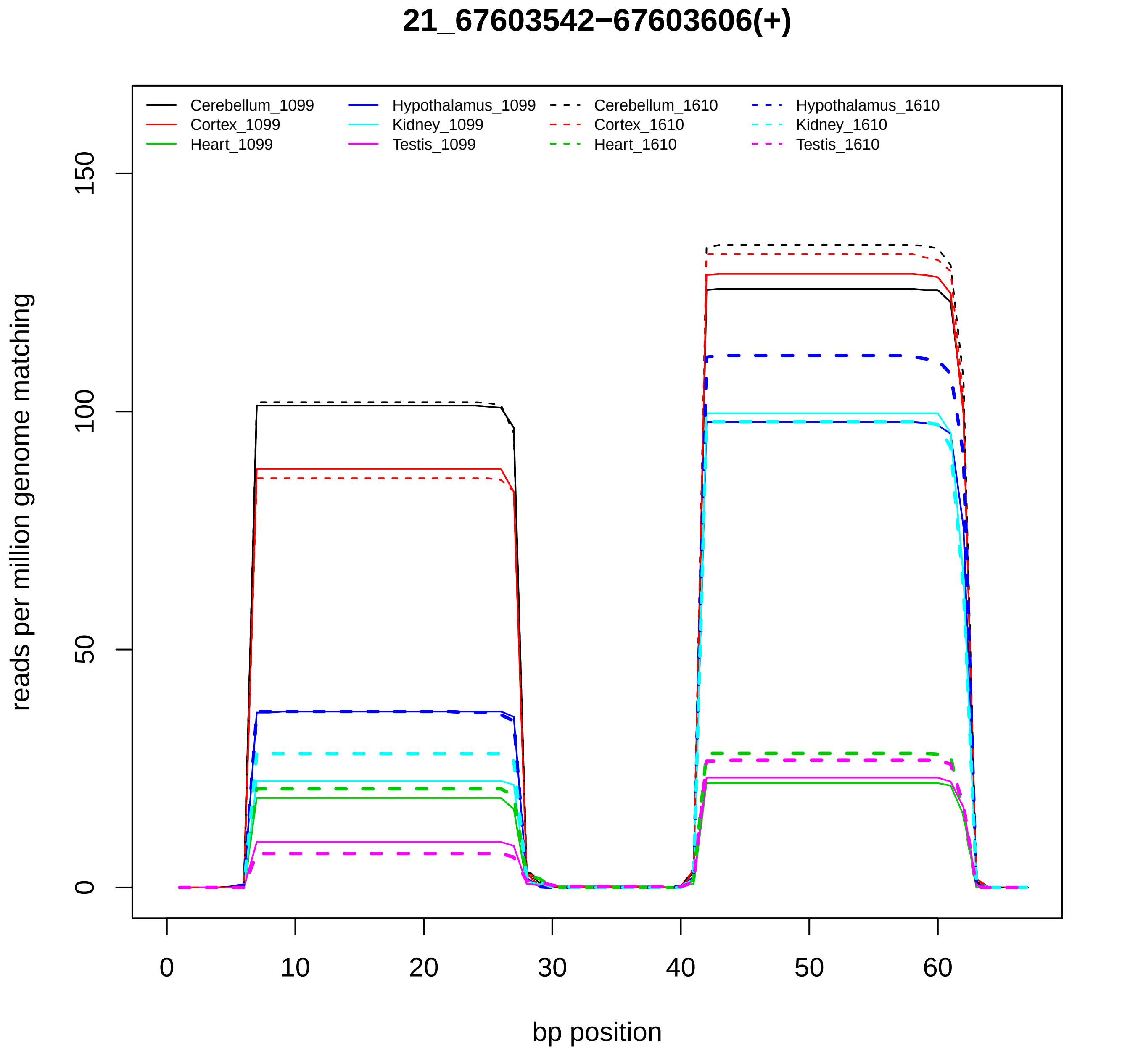

21_67603542−67603606(+)
Cerebellum_1099
Cortex_1099
Heart_1099
Hypothalamus_1099
Kidney_1099
Testis_1099
Cerebellum_1610
Cortex_1610
Heart_1610
Hypothalamus_1610
Kidney_1610
Testis_1610
reads per million genome matching
0
50
100
150
bp position
0
10
20
30
40
50
60

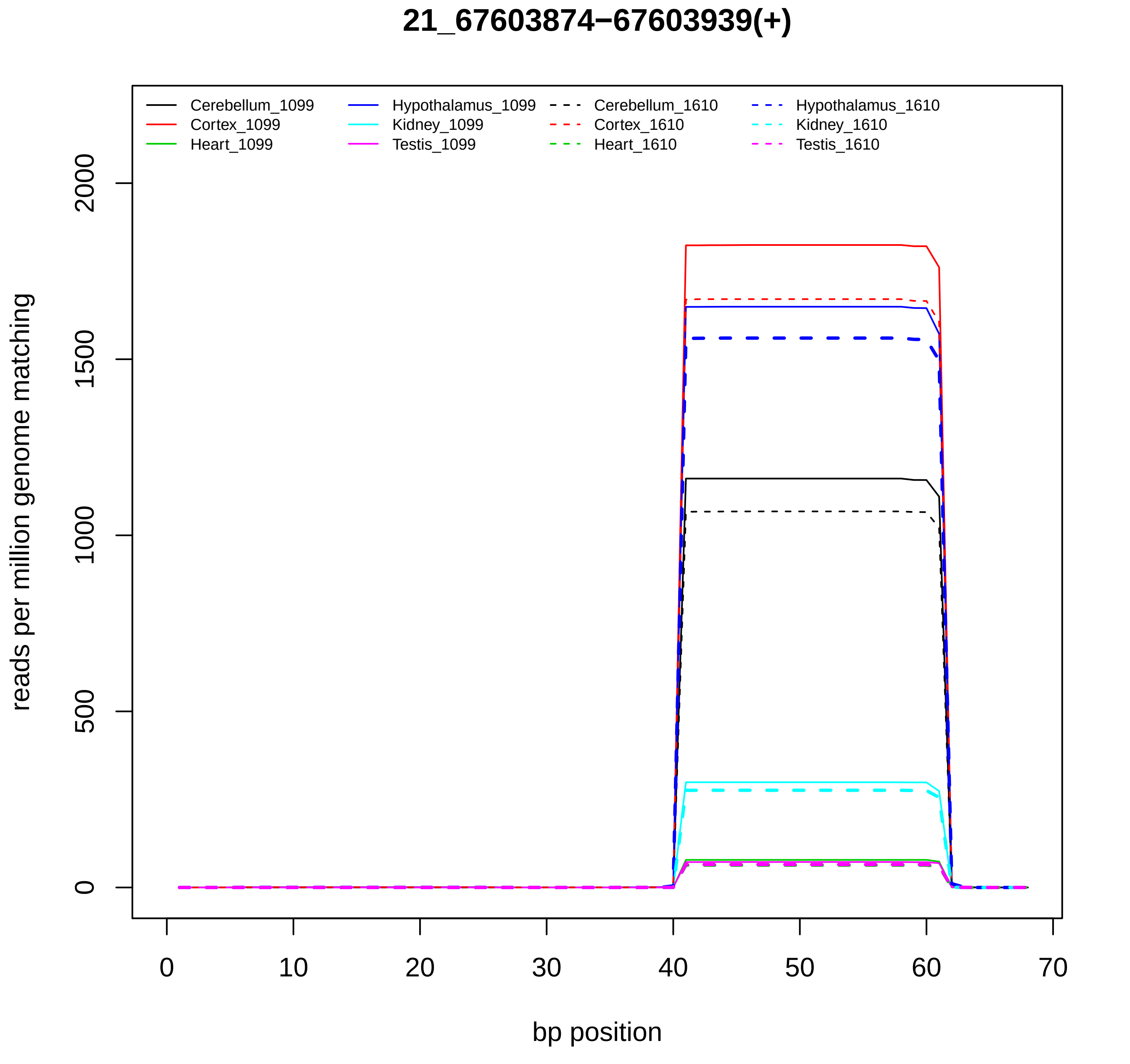

21_67603874−67603939(+)
Cerebellum_1099
Cortex_1099
Heart_1099
Hypothalamus_1099
Kidney_1099
Testis_1099
Cerebellum_1610
Cortex_1610
Heart_1610
Hypothalamus_1610
Kidney_1610
Testis_1610
reads per million genome matching
0
500
1000
1500
2000
0
10
20
30
40
50
60
70
bp position

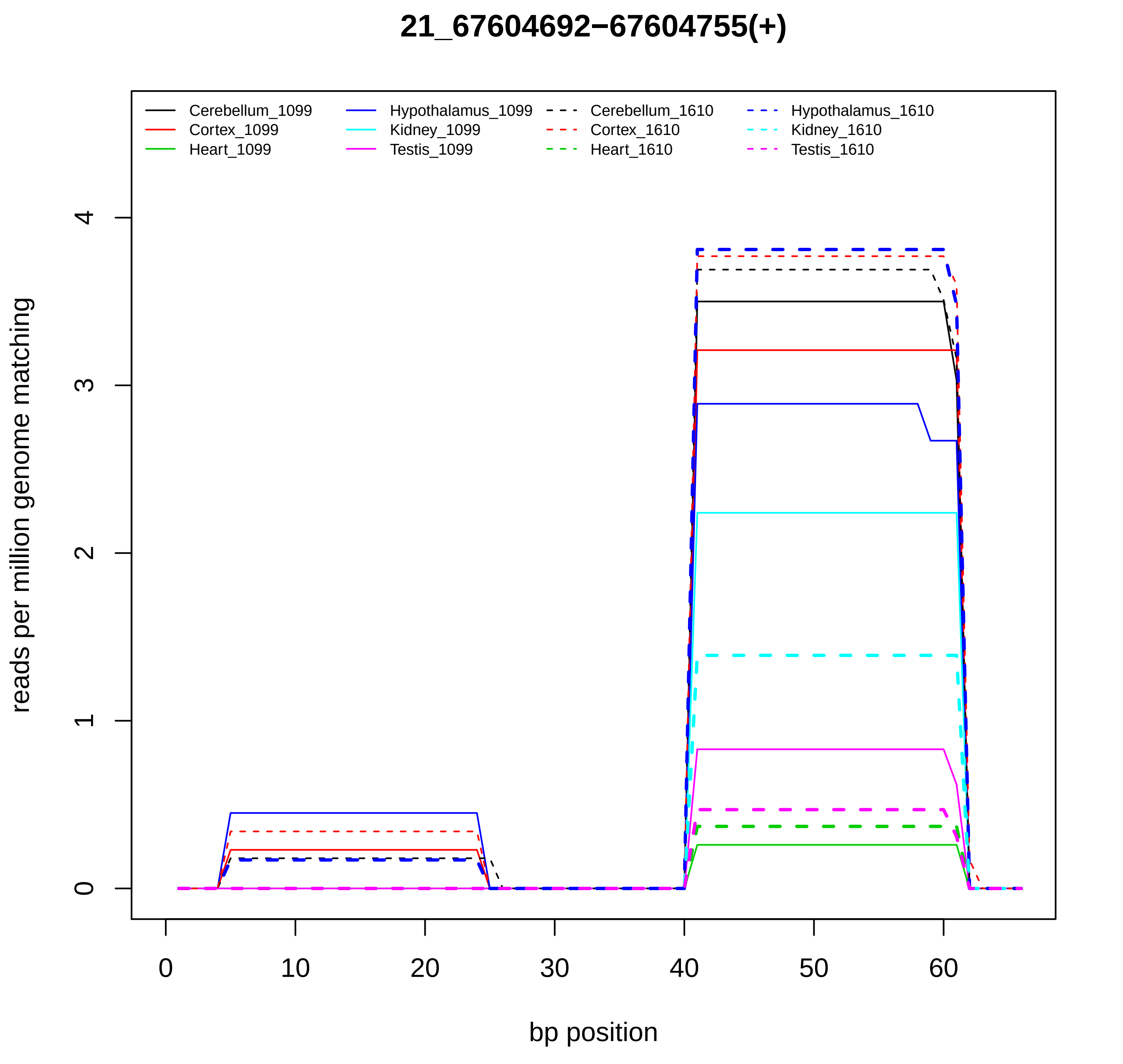

21_67604692−67604755(+)
Cerebellum_1099
Cortex_1099
Heart_1099
Hypothalamus_1099
Kidney_1099
Testis_1099
Cerebellum_1610
Cortex_1610
Heart_1610
Hypothalamus_1610
Kidney_1610
Testis_1610
reads per million genome matching
bp position
0
1
2
3
4
0
10
20
30
40
50
60

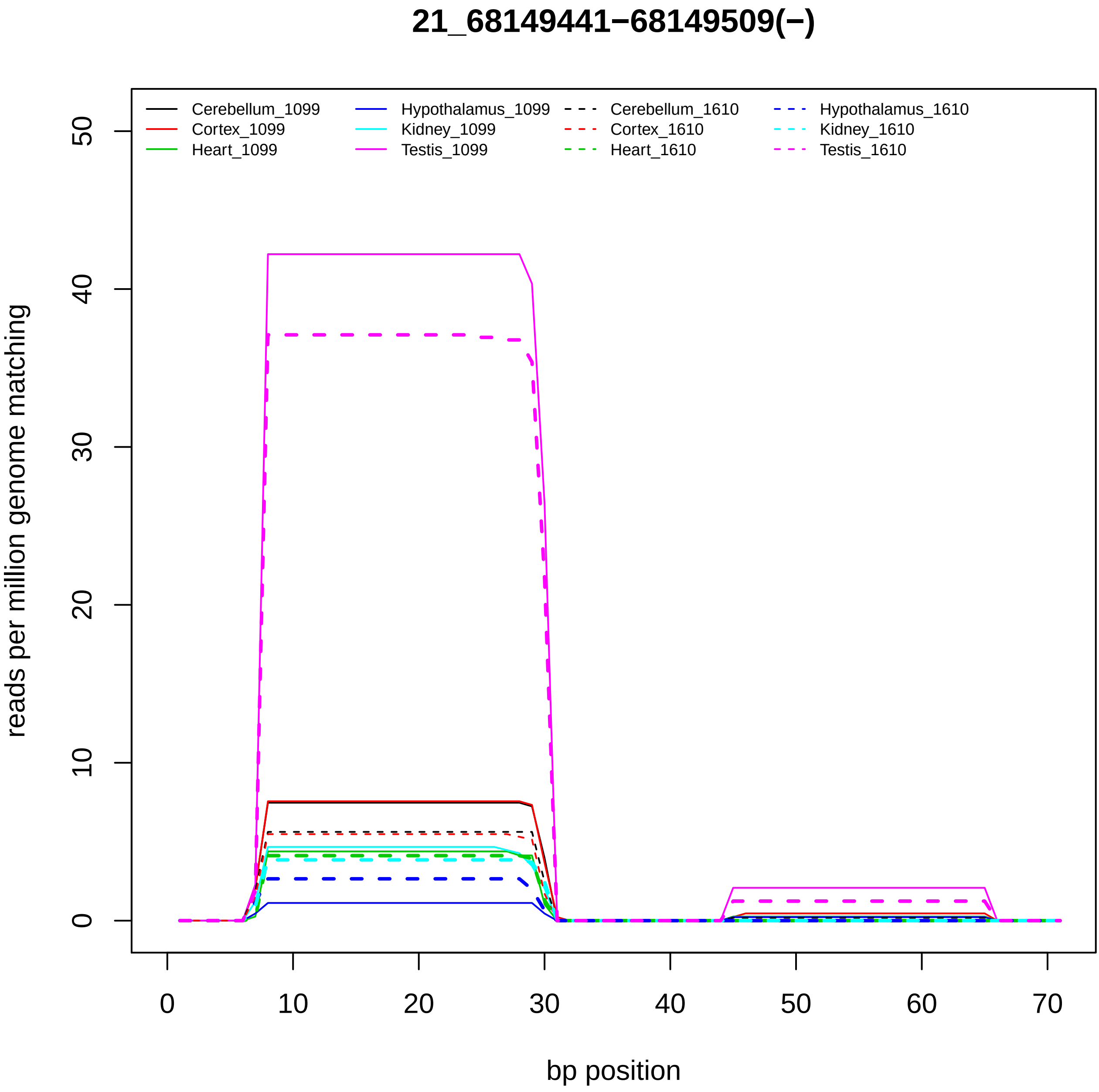

21_68149441−68149509(−)
Cerebellum_1099
Cortex_1099
Heart_1099
Hypothalamus_1099
Kidney_1099
Testis_1099
Cerebellum_1610
Cortex_1610
Heart_1610
Hypothalamus_1610
Kidney_1610
Testis_1610
reads per million genome matching
bp position
0
10
20
30
40
50
60
70

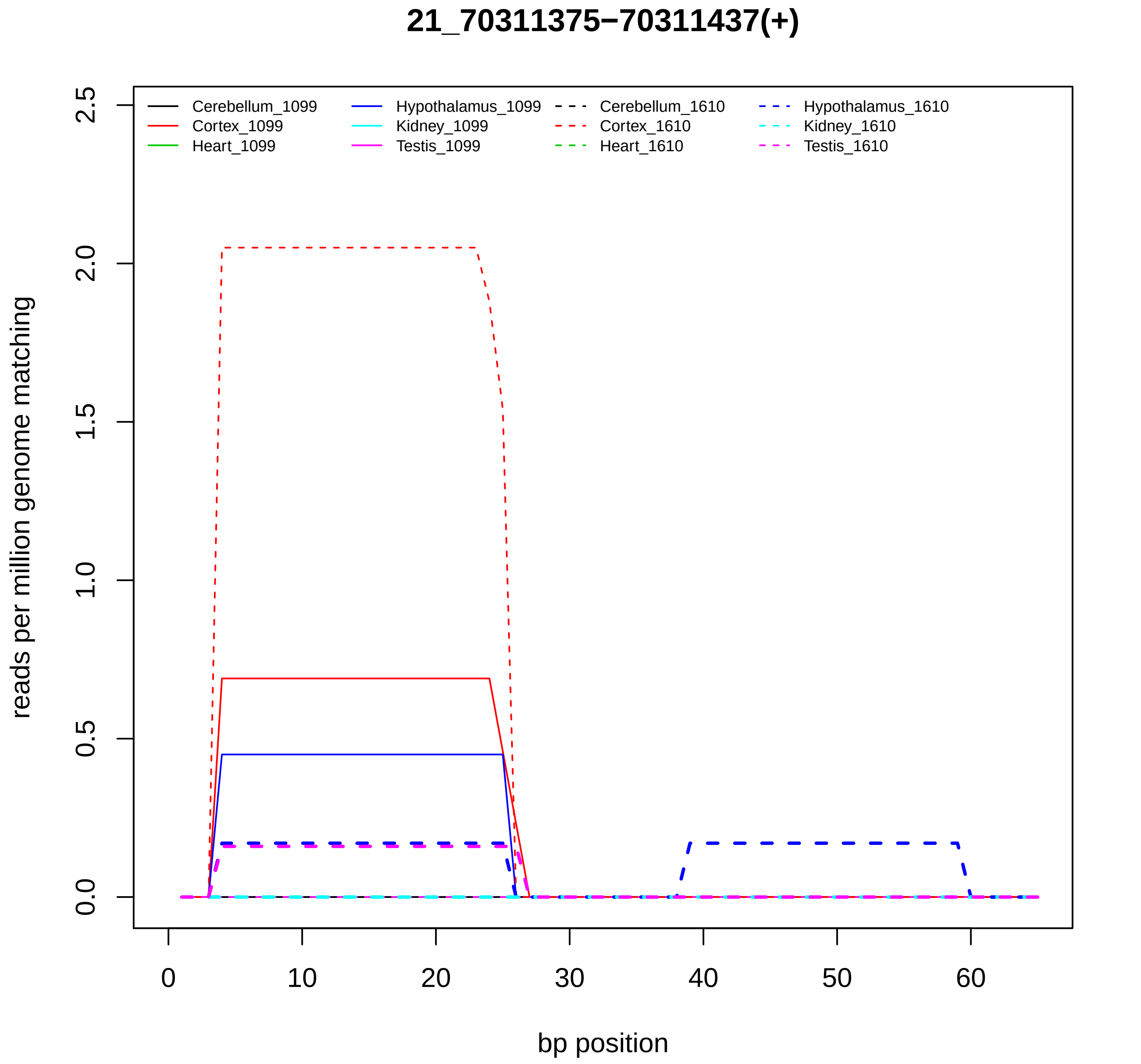

21_70311375−70311437(+)
Cerebellum_1099
Cortex_1099
Heart_1099
Hypothalamus_1099
Kidney_1099
Testis_1099
Cerebellum_1610
Cortex_1610
Heart_1610
Hypothalamus_1610
Kidney_1610
Testis_1610
reads per million genome matching
bp position
0.0
0.5
1.0
1.5
2.0
2.5
0
10
20
30
40
50
60

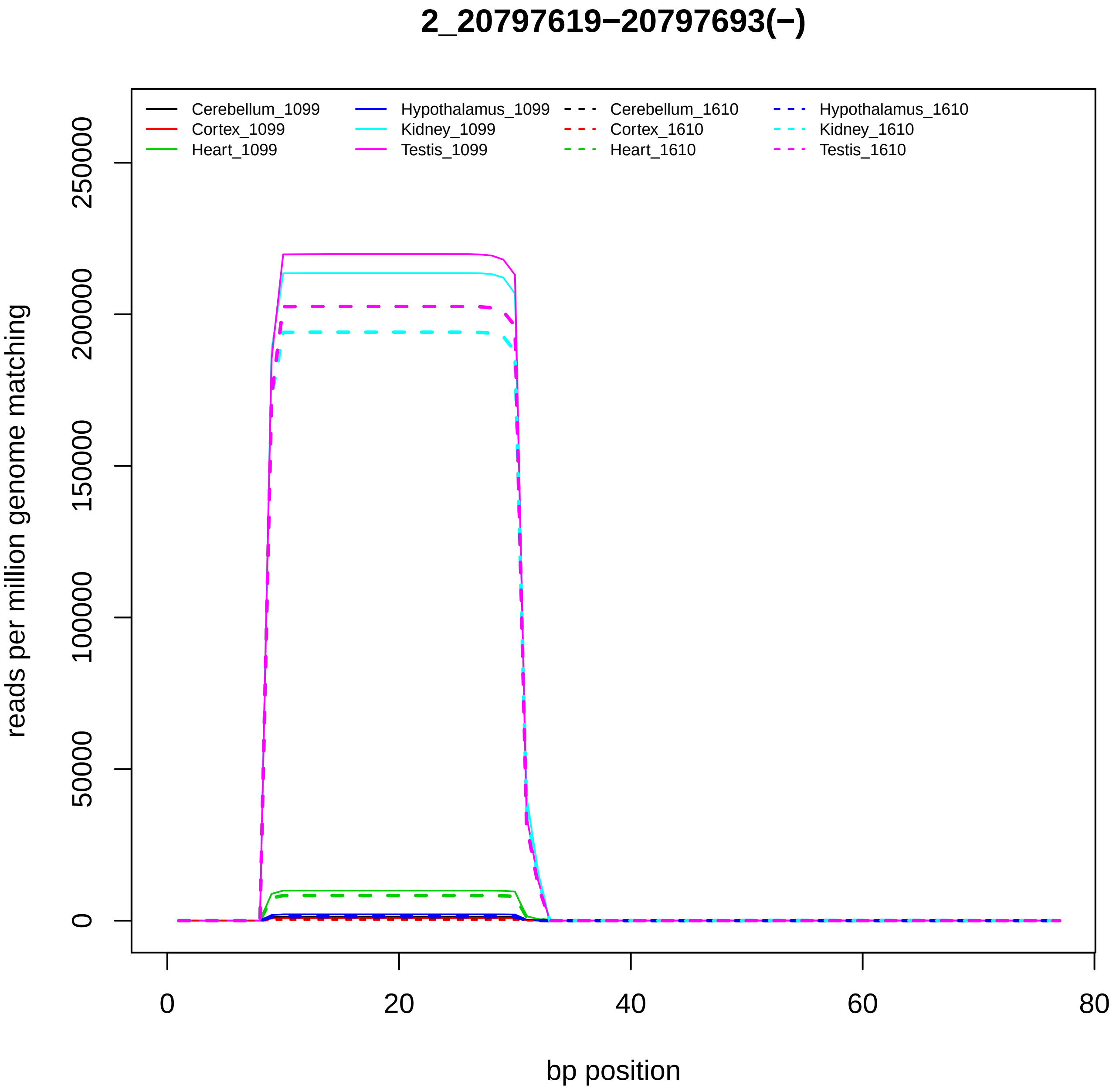
2_20797619−20797693(−)
Cerebellum_1099
Cortex_1099
Heart_1099
Hypothalamus_1099
Kidney_1099
Testis_1099
Cerebellum_1610
Cortex_1610
Heart_1610
Hypothalamus_1610
Kidney_1610
Testis_1610
reads per million genome matching
0
50000
100000
150000
200000
250000
0
20
40
60
80
bp position

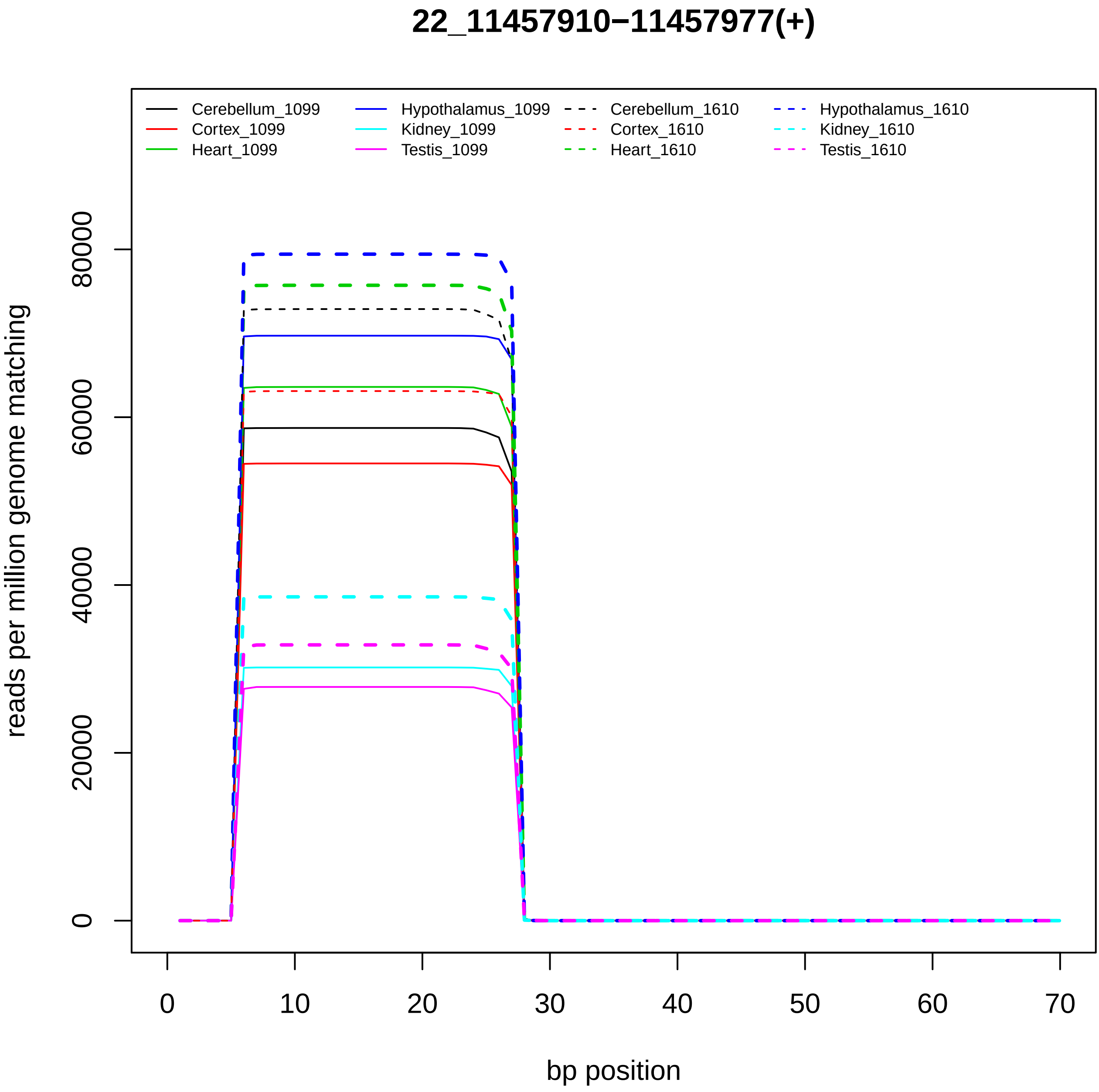
22_11457910−11457977(+)
Cerebellum_1099
Cortex_1099
Heart_1099
Hypothalamus_1099
Kidney_1099
Testis_1099
Cerebellum_1610
Cortex_1610
Heart_1610
Hypothalamus_1610
Kidney_1610
Testis_1610
reads per million genome matching
0
20000
40000
60000
80000
0
10
20
30
40
50
60
70
bp position

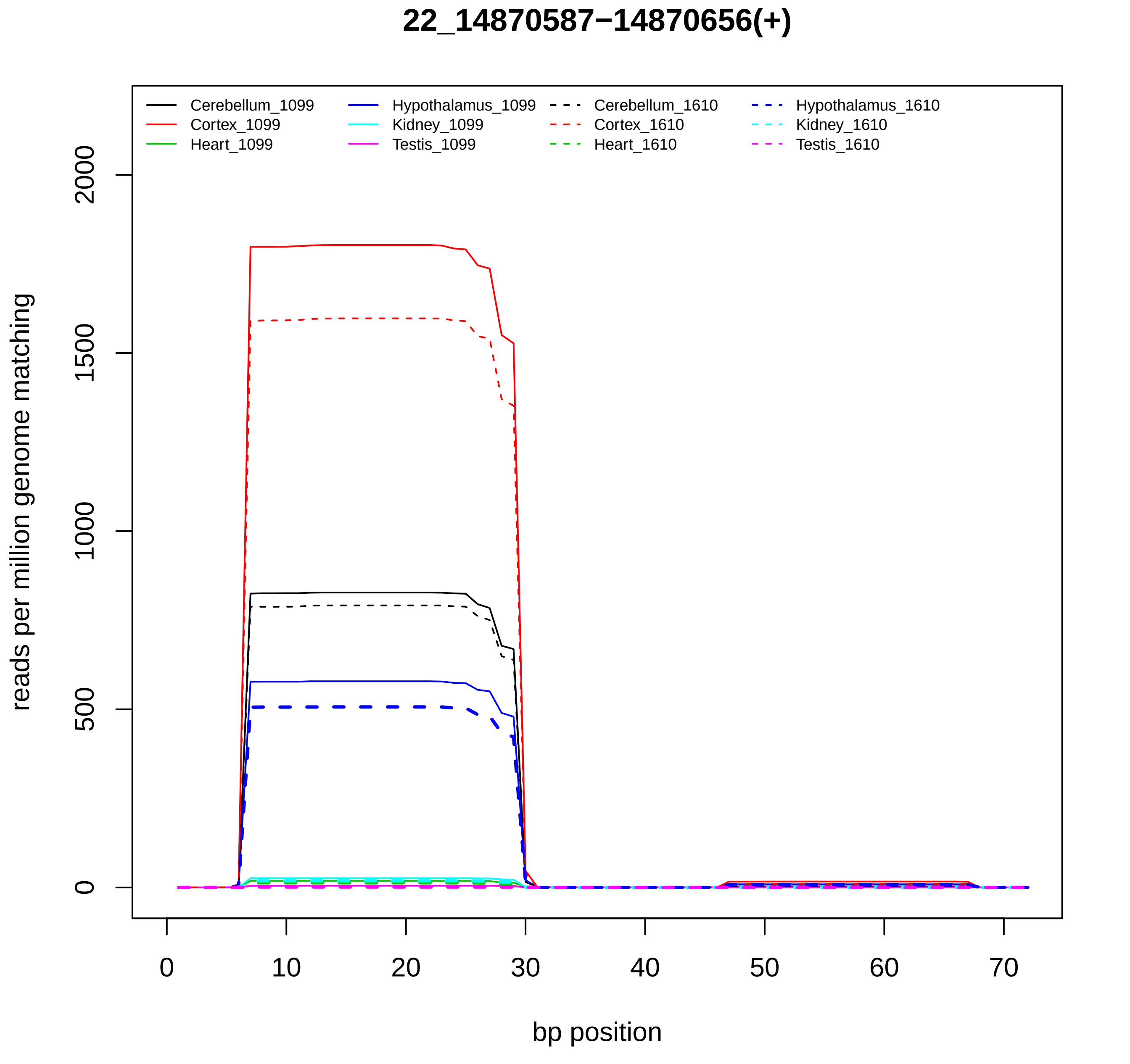
22_14870587−14870656(+)
Cerebellum_1099
Cortex_1099
Heart_1099
Hypothalamus_1099
Kidney_1099
Testis_1099
Cerebellum_1610
Cortex_1610
Heart_1610
Hypothalamus_1610
Kidney_1610
Testis_1610
reads per million genome matching
bp position
0
500
1000
1500
2000
0
10
20
30
40
50
60
70

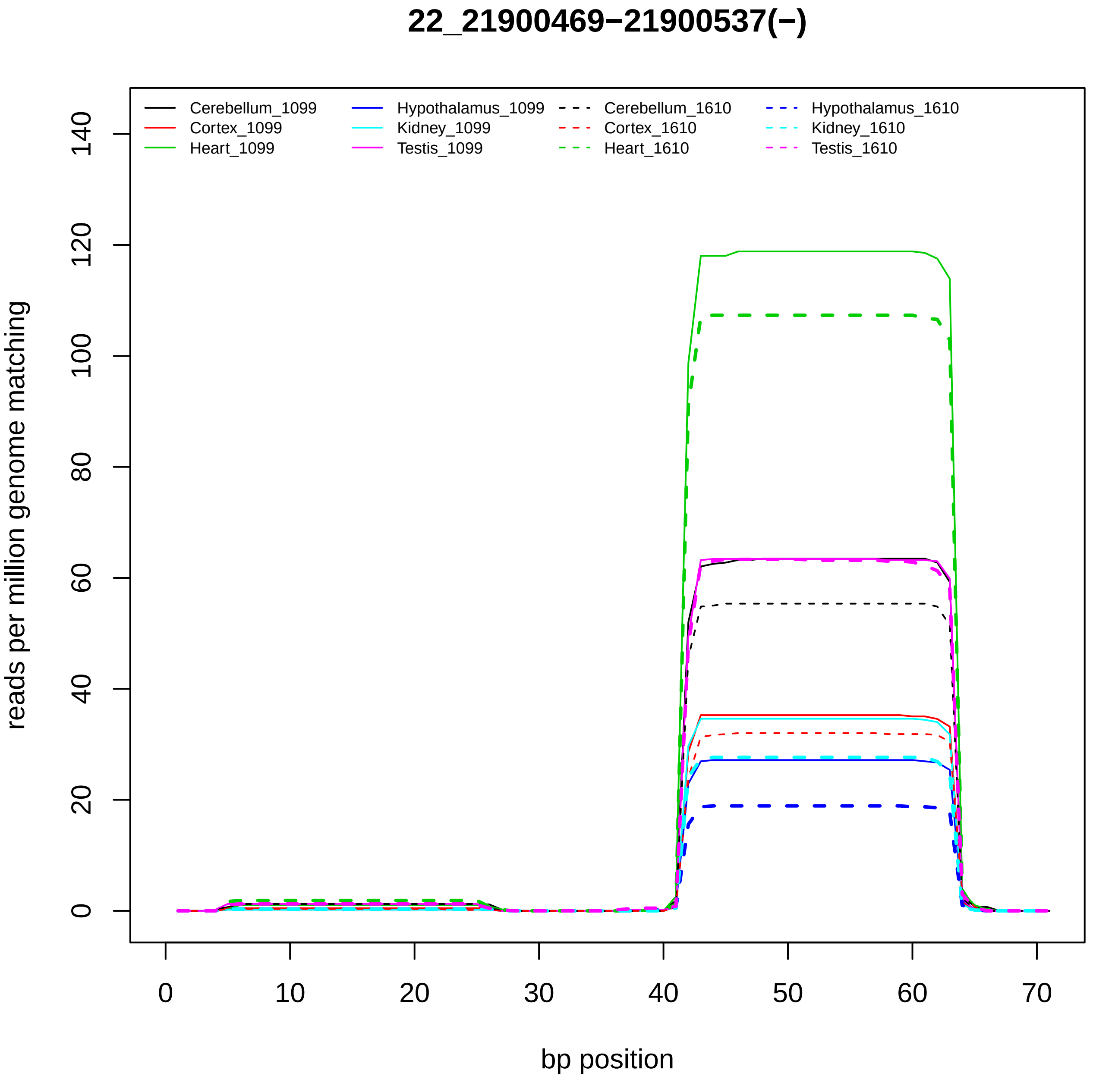

22_21900469−21900537(−)
Cerebellum_1099
Cortex_1099
Heart_1099
Hypothalamus_1099
Kidney_1099
Testis_1099
Cerebellum_1610
Cortex_1610
Heart_1610
Hypothalamus_1610
Kidney_1610
Testis_1610
reads per million genome matching
bp position
0
20
40
60
80
100
120
140
0
10
20
30
40
50
60
70

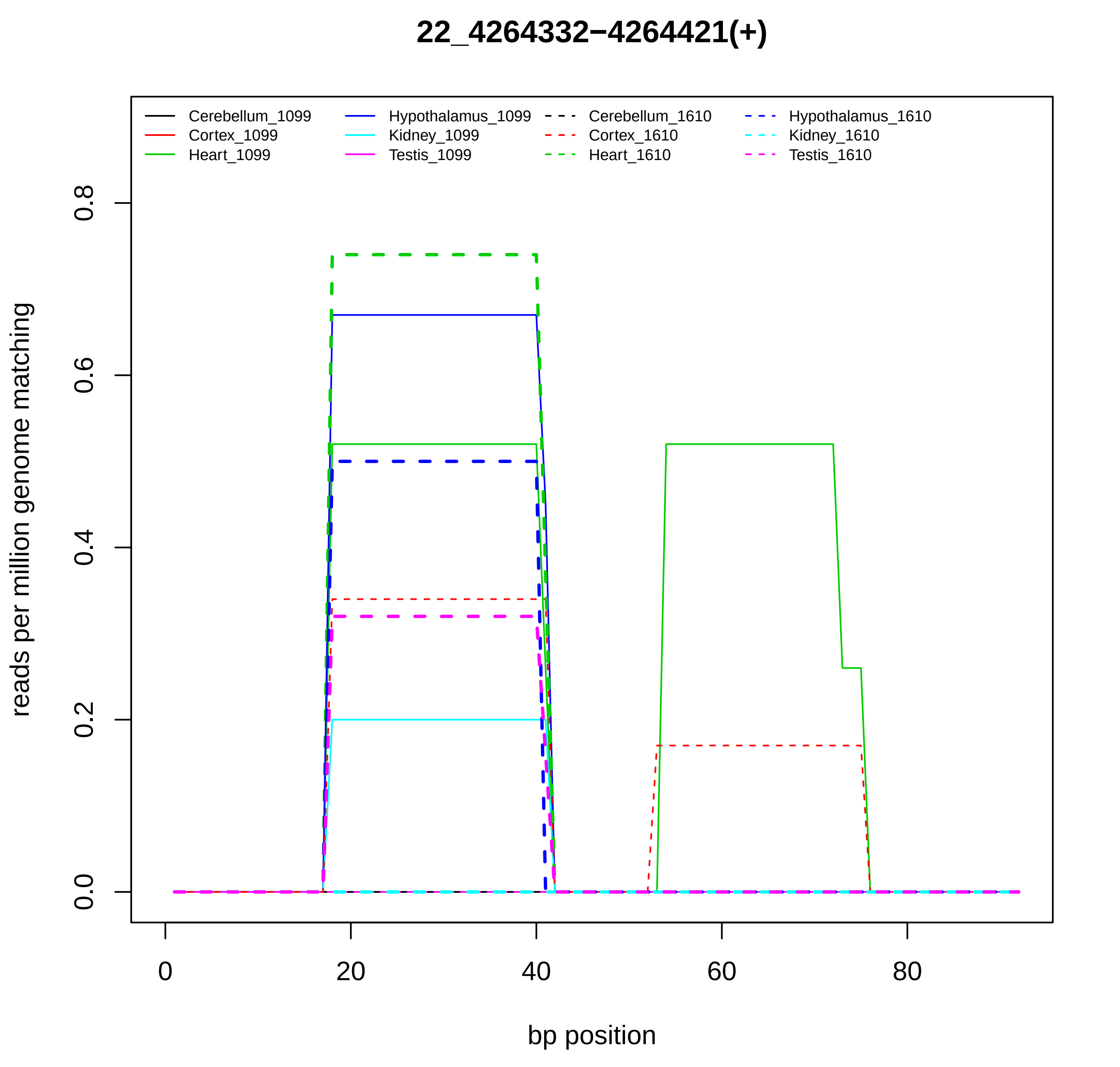
22_4264332−4264421(+)
Cerebellum_1099
Cortex_1099
Heart_1099
Hypothalamus_1099
Kidney_1099
Testis_1099
Cerebellum_1610
Cortex_1610
Heart_1610
Hypothalamus_1610
Kidney_1610
Testis_1610
reads per million genome matching
0.0
0.2
0.4
0.6
0.8
0
20
40
60
80
bp position

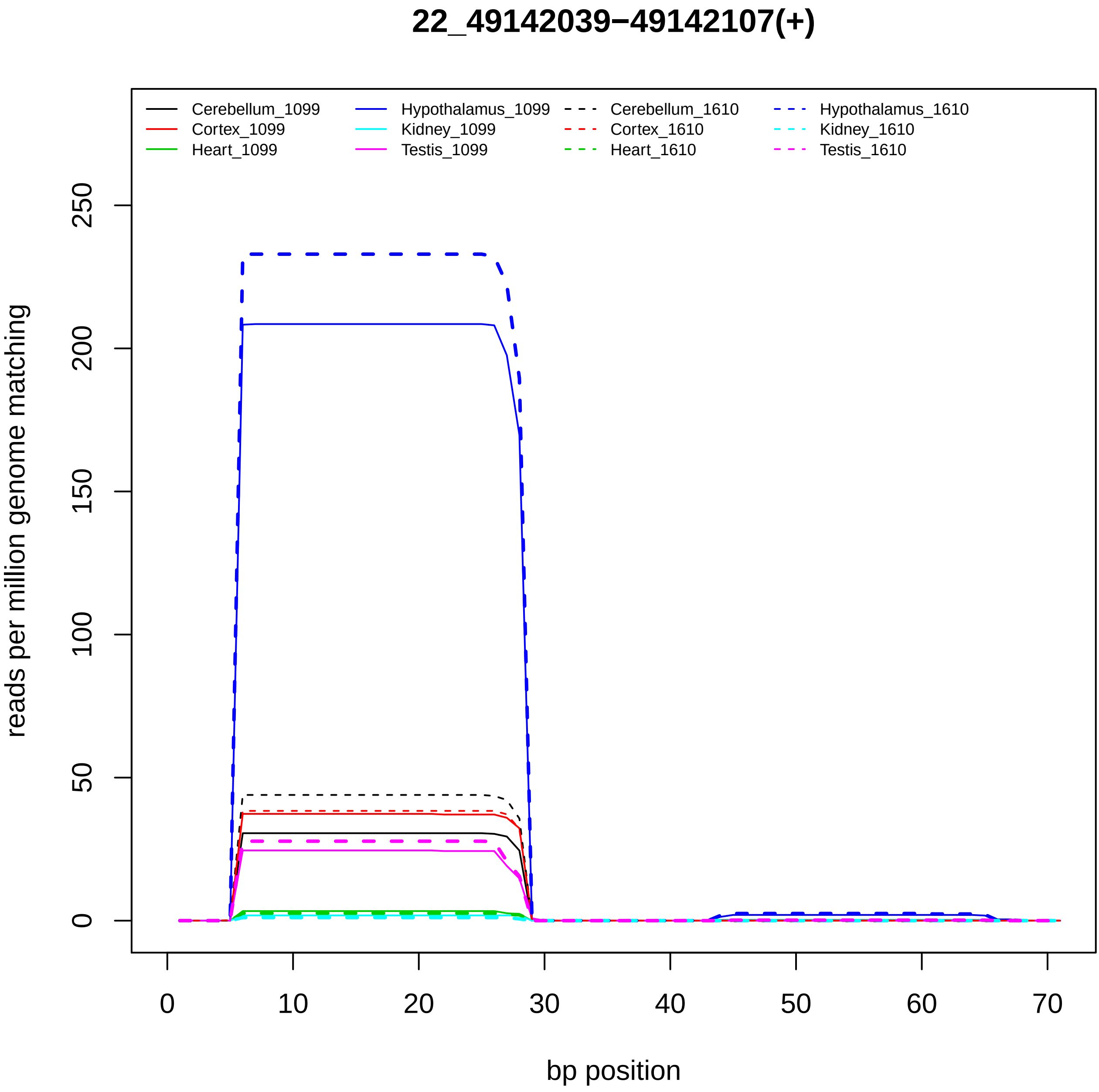

22_49142039−49142107(+)
Cerebellum_1099
Cortex_1099
Heart_1099
Hypothalamus_1099
Kidney_1099
Testis_1099
Cerebellum_1610
Cortex_1610
Heart_1610
Hypothalamus_1610
Kidney_1610
Testis_1610
reads per million genome matching
bp position
0
50
100
150
200
250
0
10
20
30
40
50
60
70

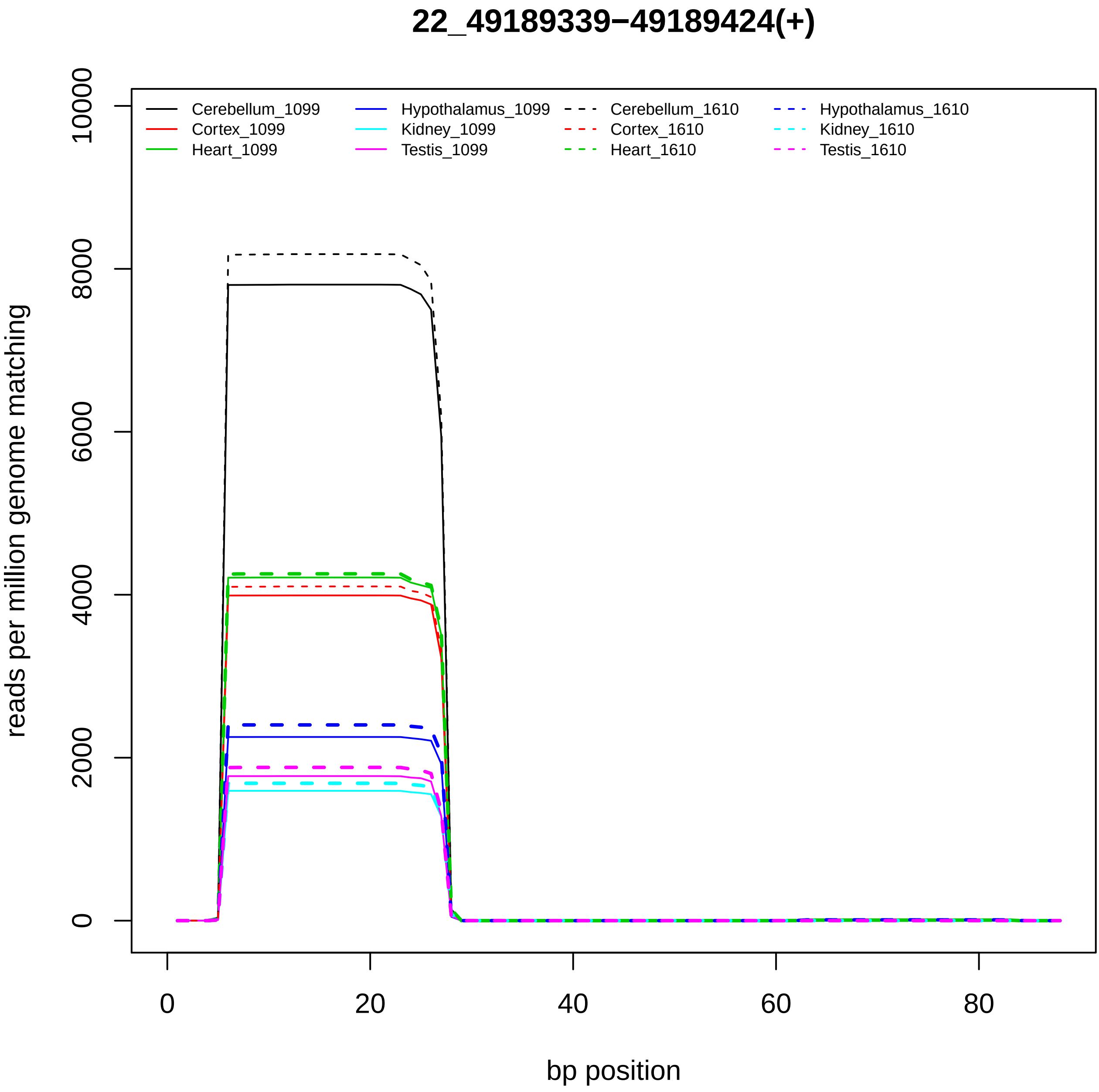

22_49189339−49189424(+)
Cerebellum_1099
Cortex_1099
Heart_1099
Hypothalamus_1099
Kidney_1099
Testis_1099
Cerebellum_1610
Cortex_1610
Heart_1610
Hypothalamus_1610
Kidney_1610
Testis_1610
reads per million genome matching
0
2000
4000
6000
8000
10000
bp position
0
20
40
60
80

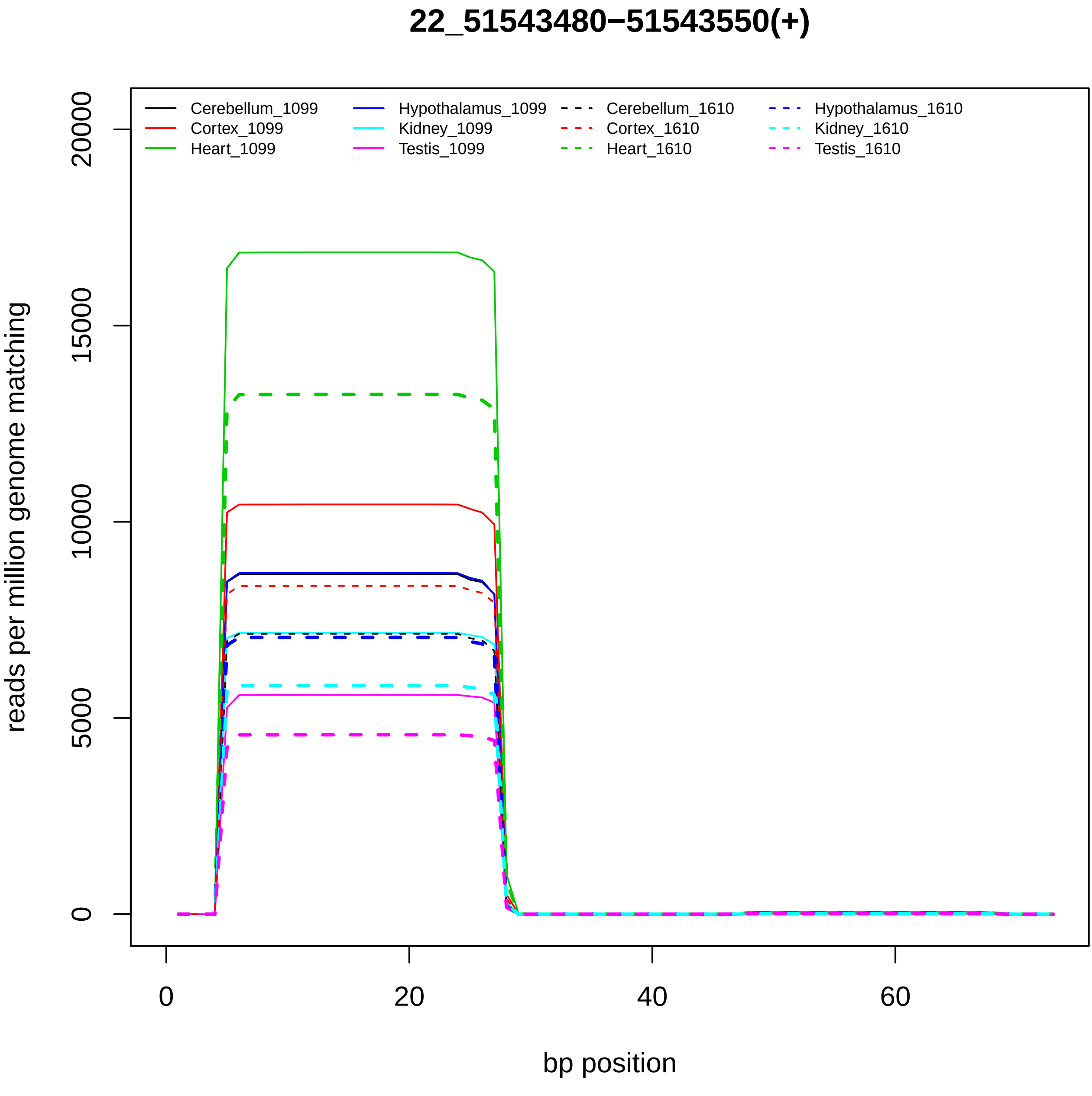

22_51543480−51543550(+)
Cerebellum_1099
Cortex_1099
Heart_1099
Hypothalamus_1099
Kidney_1099
Testis_1099
Cerebellum_1610
Cortex_1610
Heart_1610
Hypothalamus_1610
Kidney_1610
Testis_1610
reads per million genome matching
0
5000
10000
15000
20000
0
20
40
60
bp position

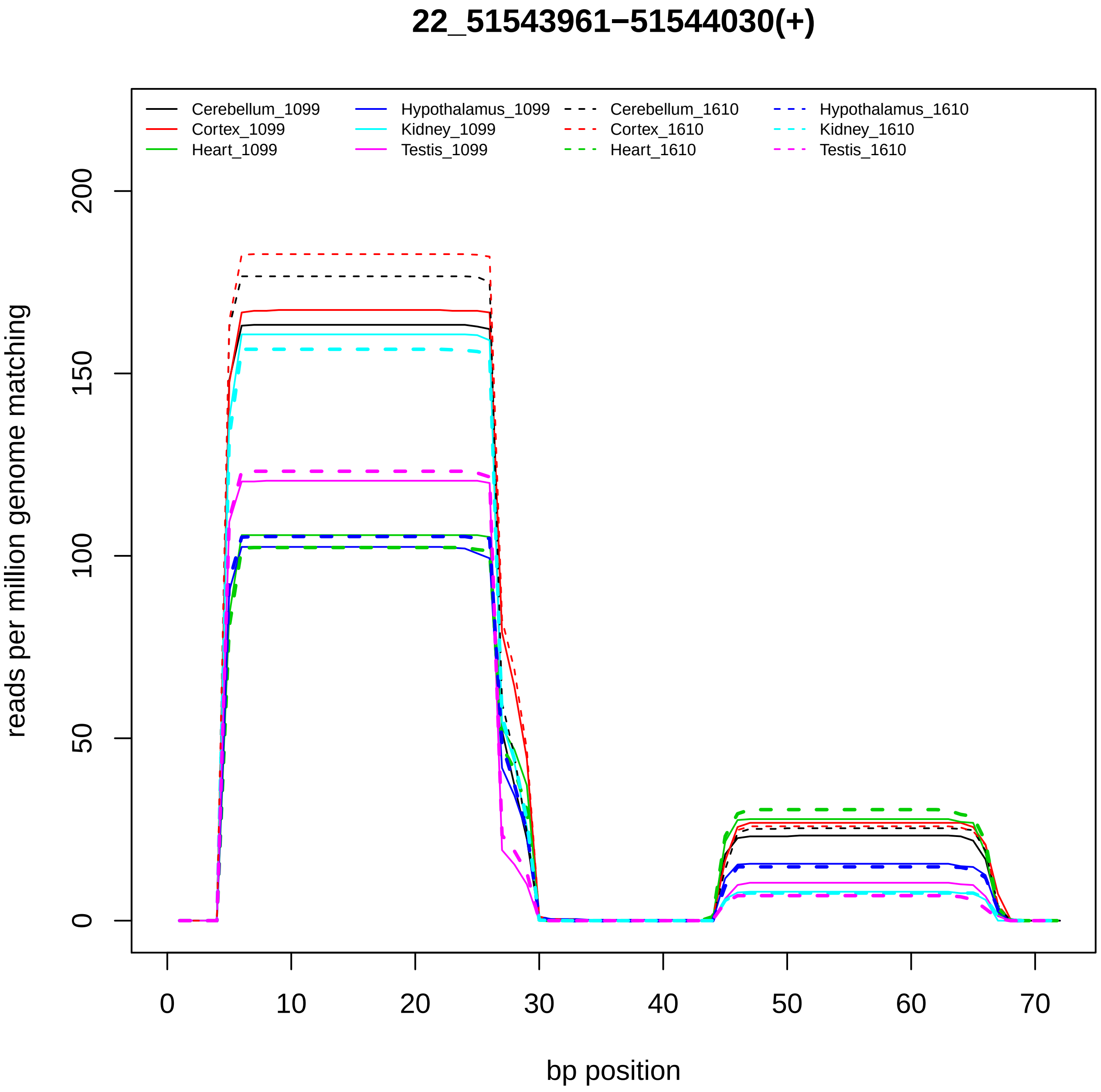
22_51543961−51544030(+)
Cerebellum_1099
Cortex_1099
Heart_1099
Hypothalamus_1099
Kidney_1099
Testis_1099
Cerebellum_1610
Cortex_1610
Heart_1610
Hypothalamus_1610
Kidney_1610
Testis_1610
reads per million genome matching
bp position
0
50
100
150
200
0
10
20
30
40
50
60
70

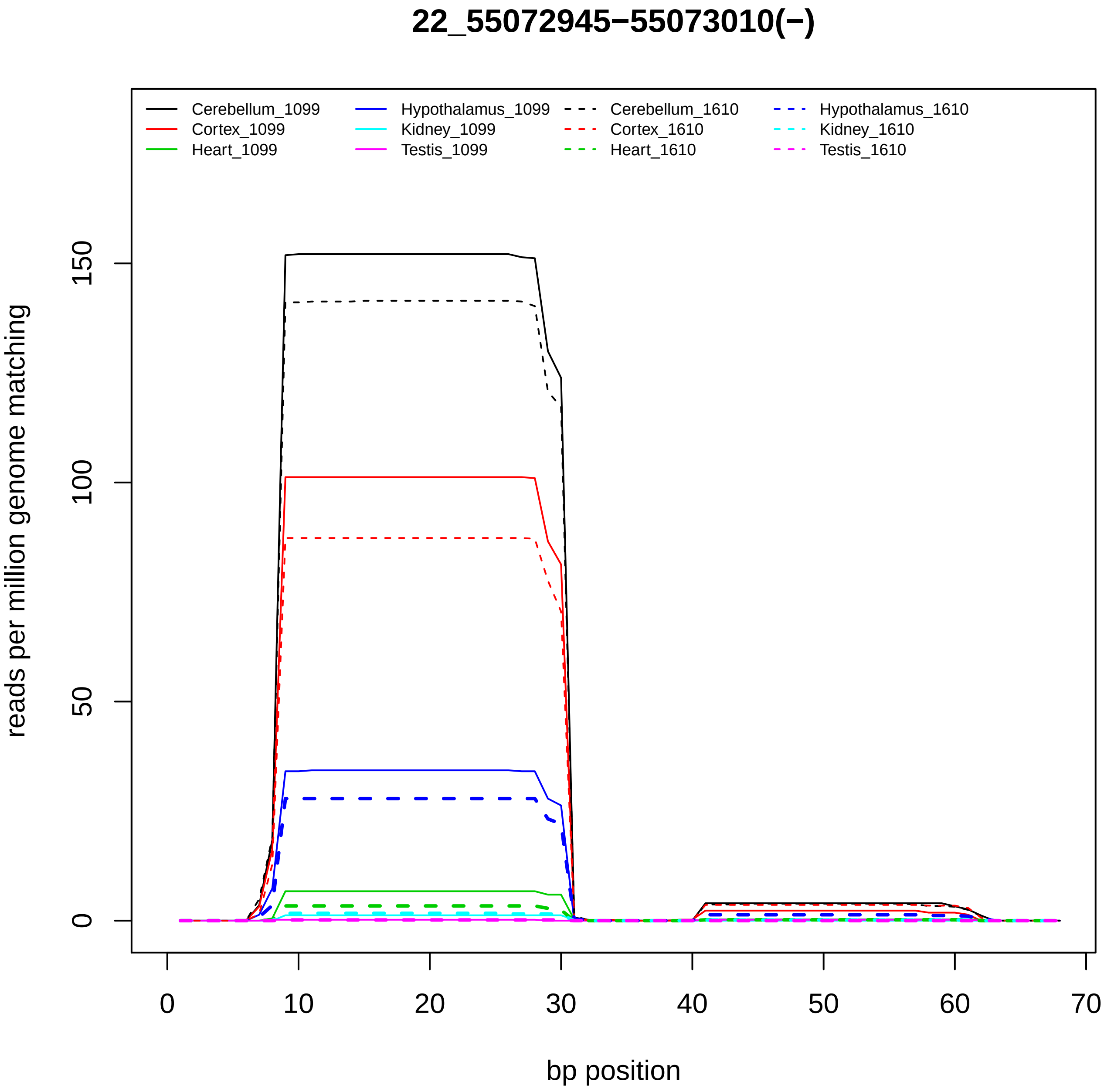

22_55072945−55073010(−)
Cerebellum_1099
Cortex_1099
Heart_1099
Hypothalamus_1099
Kidney_1099
Testis_1099
Cerebellum_1610
Cortex_1610
Heart_1610
Hypothalamus_1610
Kidney_1610
Testis_1610
reads per million genome matching
0
50
100
150
0
10
20
30
40
50
60
70
bp position

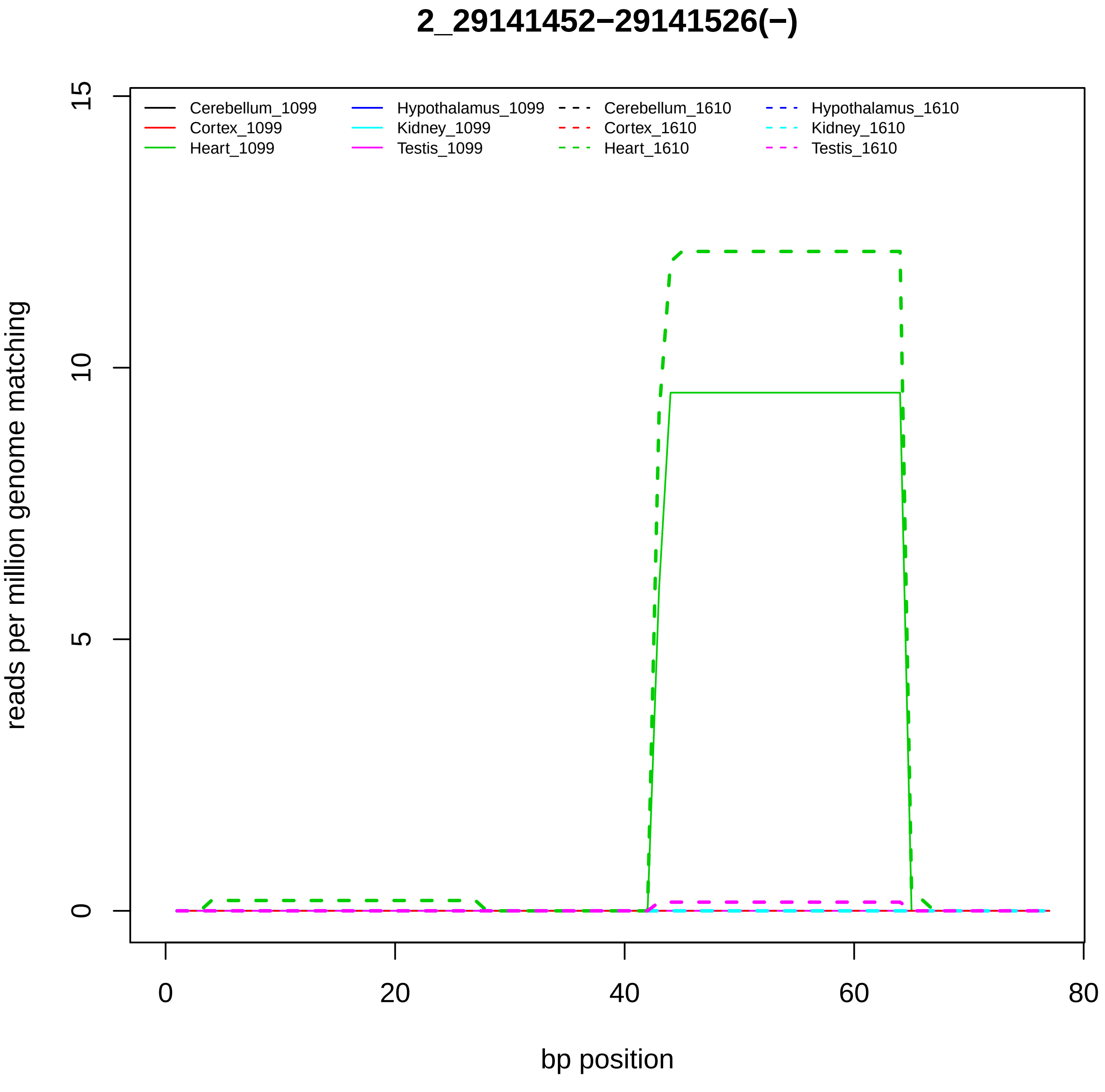

2_29141452−29141526(−)
Cerebellum_1099
Cortex_1099
Heart_1099
Hypothalamus_1099
Kidney_1099
Testis_1099
Cerebellum_1610
Cortex_1610
Heart_1610
Hypothalamus_1610
Kidney_1610
Testis_1610
reads per million genome matching
bp position
0
5
10
15
0
20
40
60
80

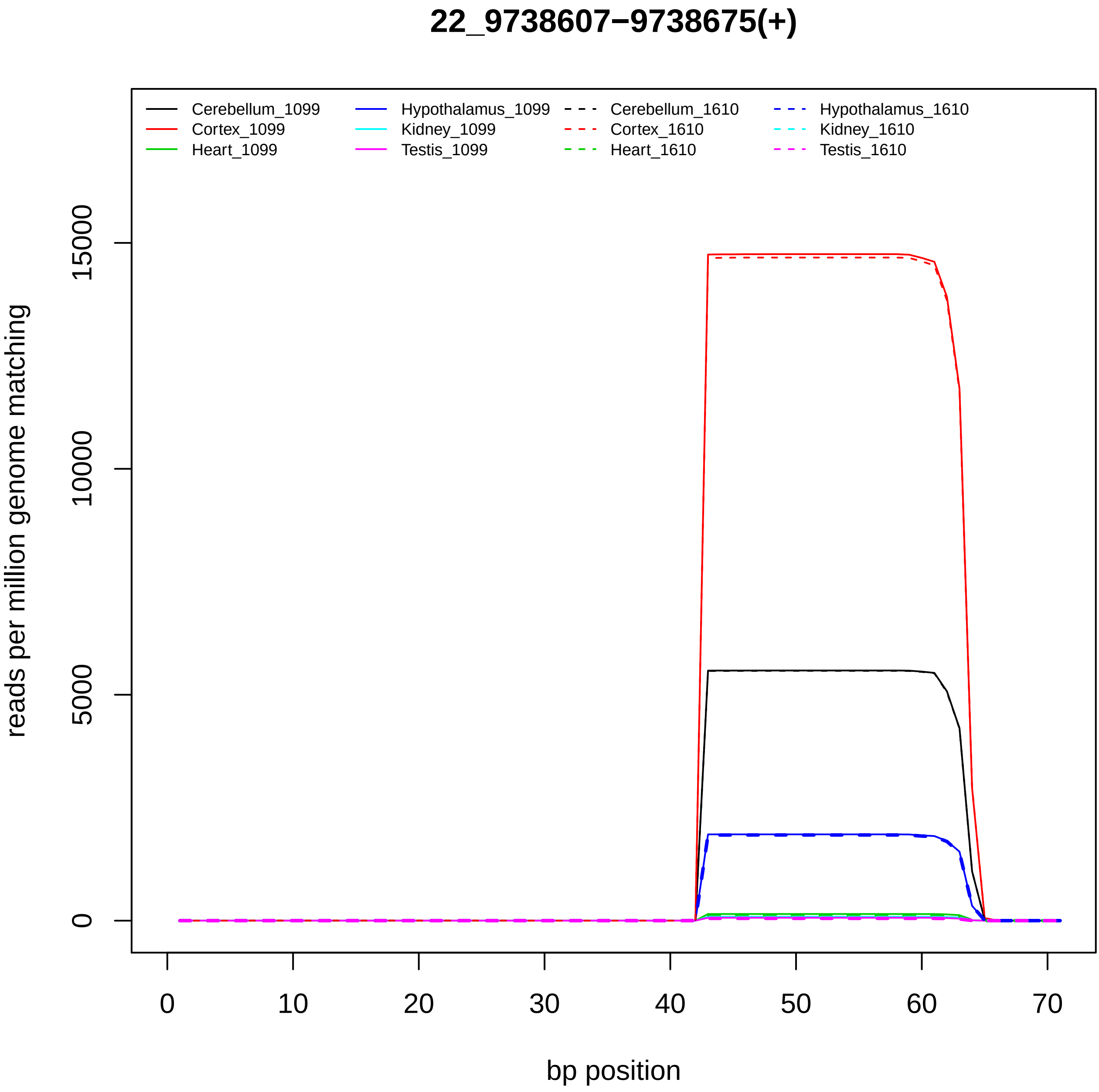

22_9738607−9738675(+)
Cerebellum_1099
Cortex_1099
Heart_1099
Hypothalamus_1099
Kidney_1099
Testis_1099
Cerebellum_1610
Cortex_1610
Heart_1610
Hypothalamus_1610
Kidney_1610
Testis_1610
reads per million genome matching
0
5000
10000
15000
0
10
20
30
40
50
60
70
bp position

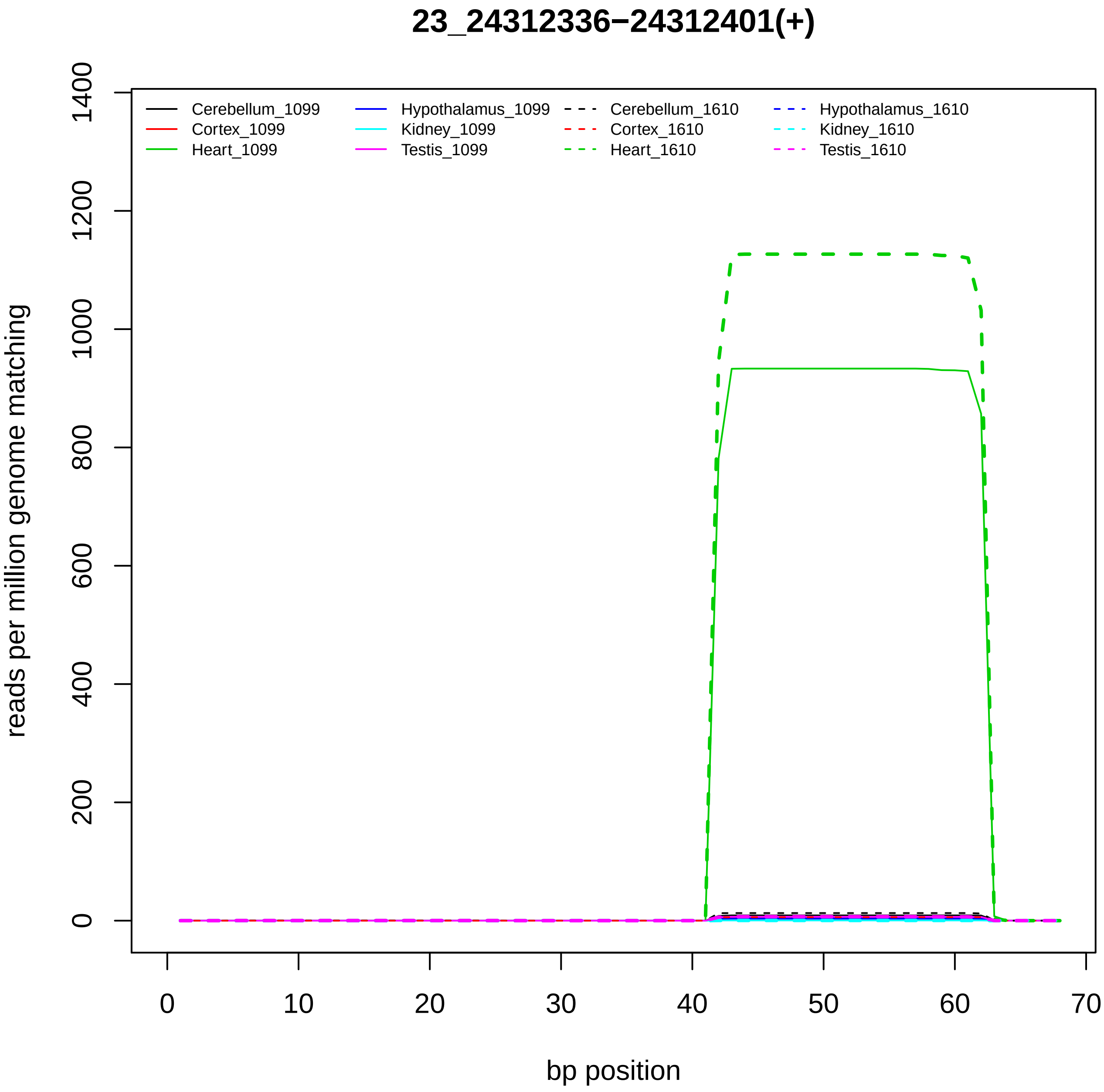
23_24312336−24312401(+)
Cerebellum_1099
Cortex_1099
Heart_1099
Hypothalamus_1099
Kidney_1099
Testis_1099
Cerebellum_1610
Cortex_1610
Heart_1610
Hypothalamus_1610
Kidney_1610
Testis_1610
reads per million genome matching
0
200
400
600
800
1000
1200
1400
0
10
20
30
40
50
60
70
bp position

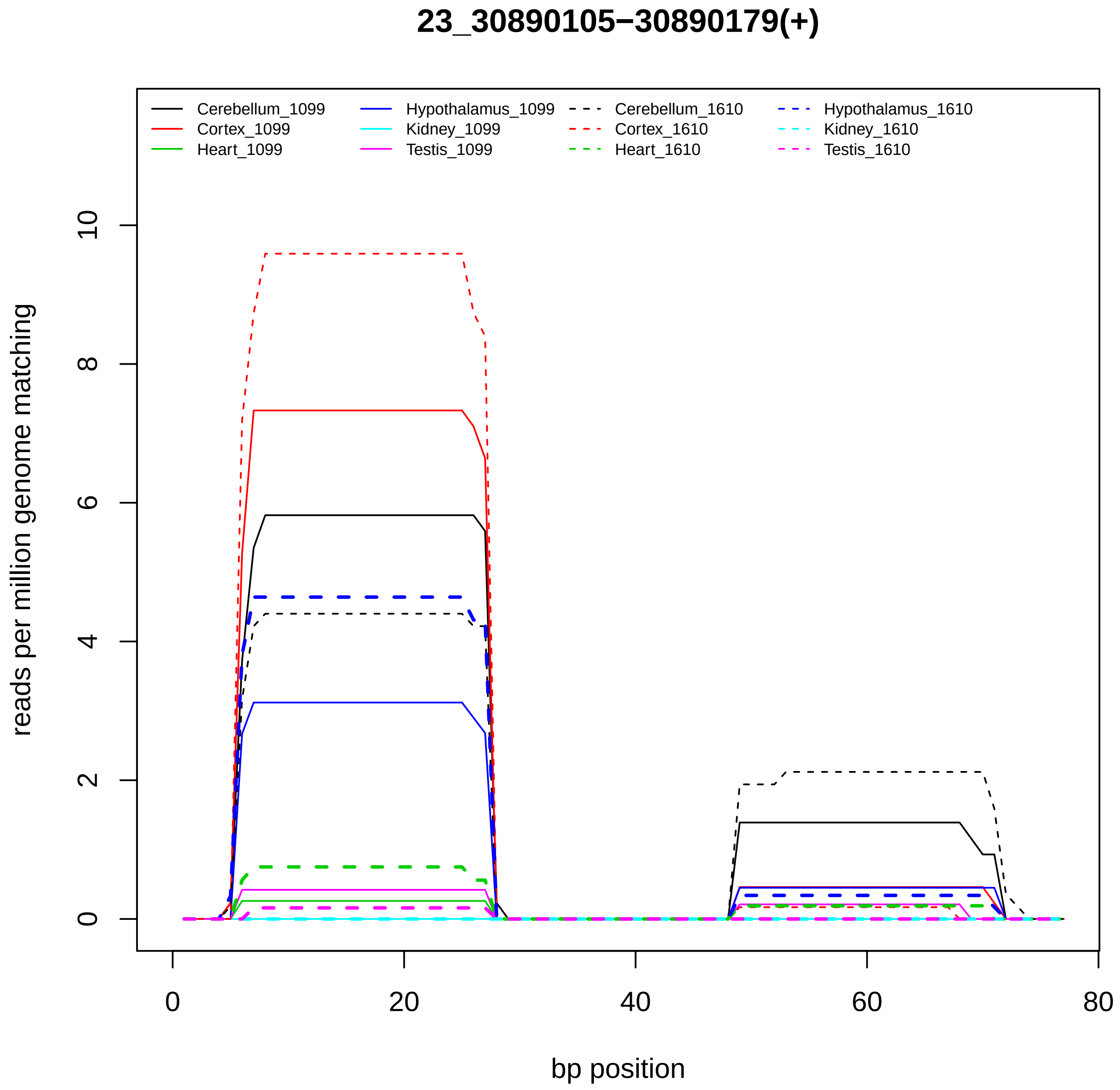

23_30890105−30890179(+)
Cerebellum_1099
Cortex_1099
Heart_1099
Hypothalamus_1099
Kidney_1099
Testis_1099
Cerebellum_1610
Cortex_1610
Heart_1610
Hypothalamus_1610
Kidney_1610
Testis_1610
reads per million genome matching
0
2
4
6
8
10
0
20
40
60
80
bp position

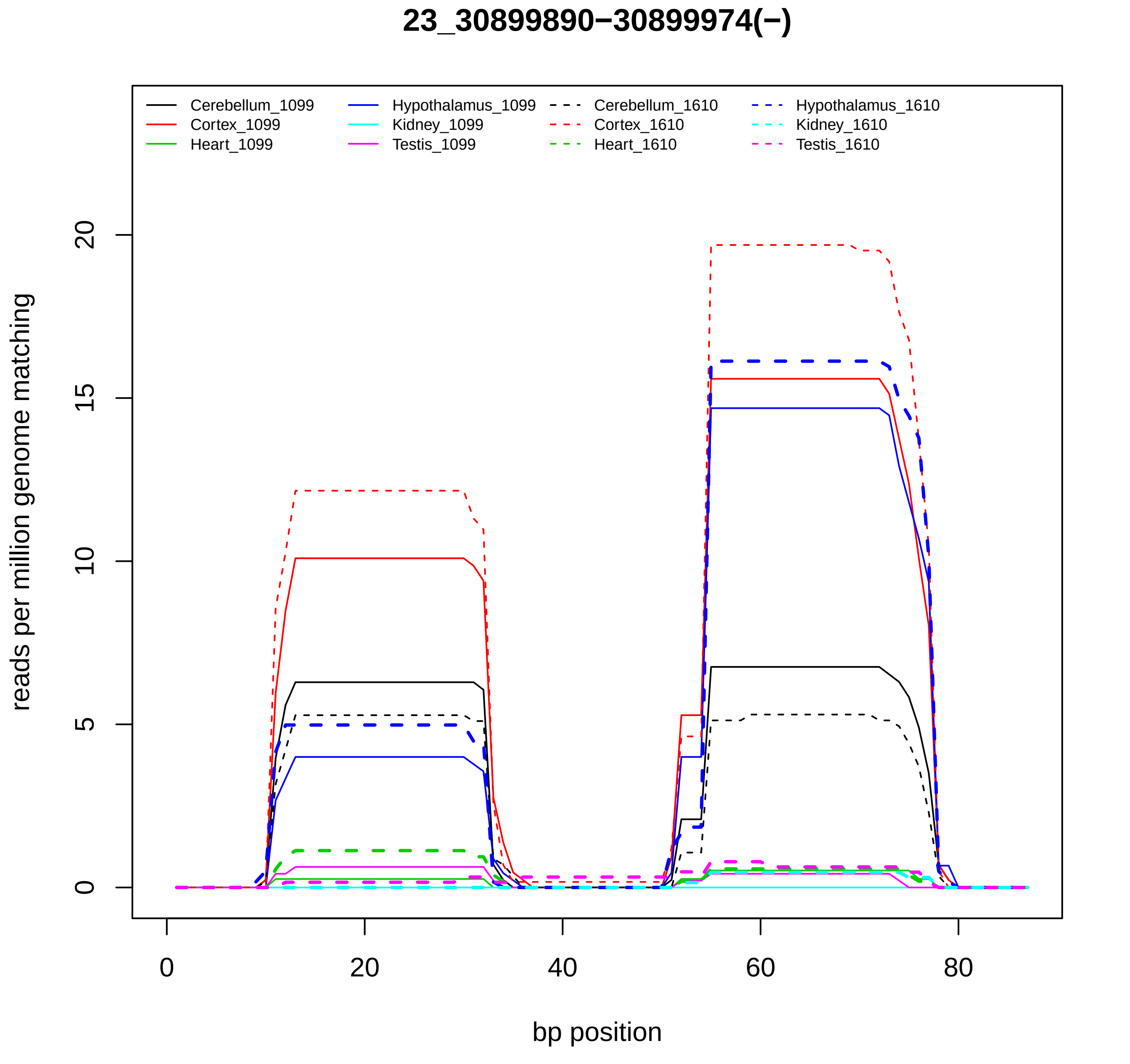

23_30899890−30899974(−)
Cerebellum_1099
Cortex_1099
Heart_1099
Hypothalamus_1099
Kidney_1099
Testis_1099
Cerebellum_1610
Cortex_1610
Heart_1610
Hypothalamus_1610
Kidney_1610
Testis_1610
reads per million genome matching
bp position
0
5
10
15
20
0
20
40
60
80

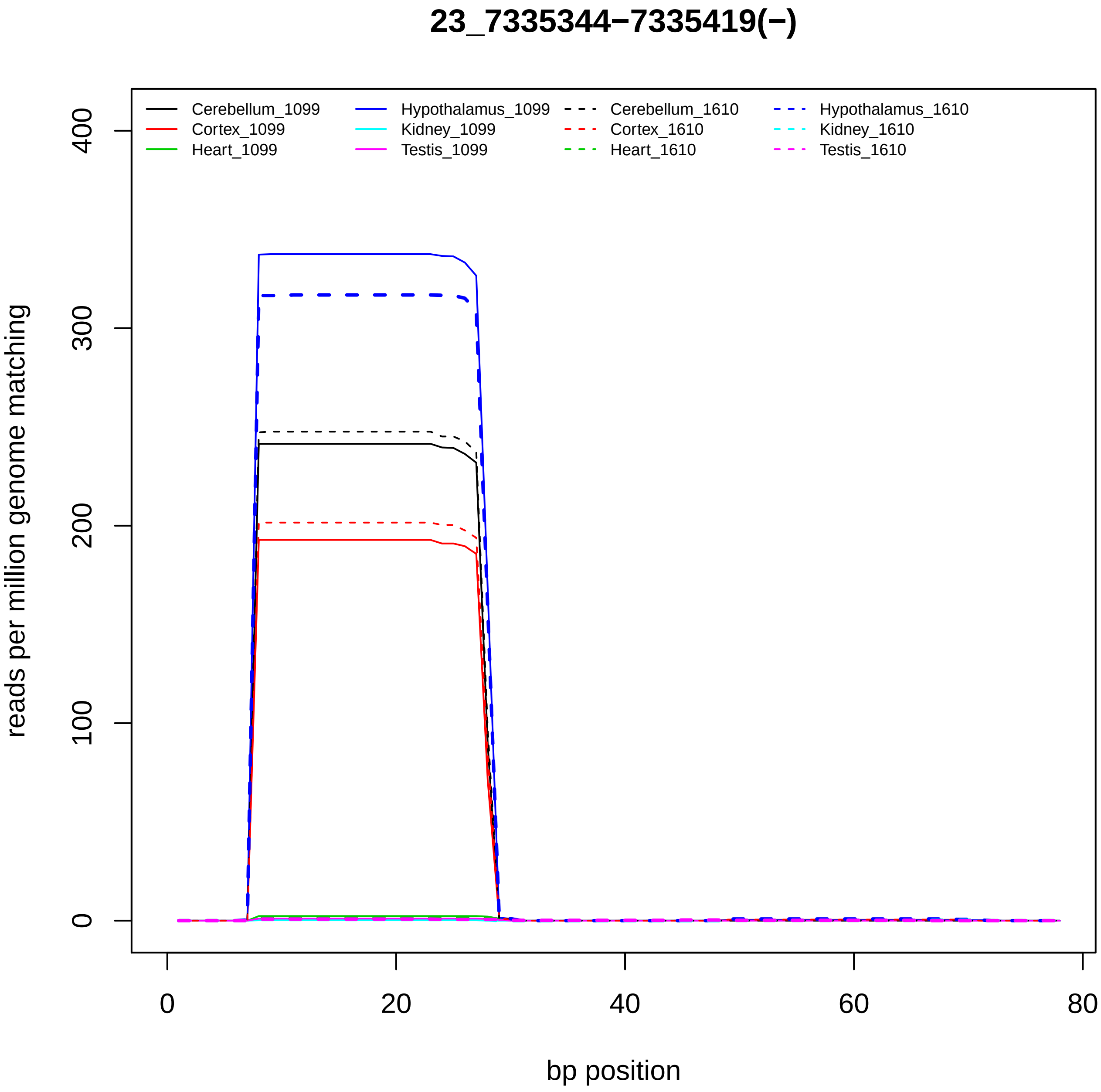
23_7335344−7335419(−)
Cerebellum_1099
Cortex_1099
Heart_1099
Hypothalamus_1099
Kidney_1099
Testis_1099
Cerebellum_1610
Cortex_1610
Heart_1610
Hypothalamus_1610
Kidney_1610
Testis_1610
reads per million genome matching
0
100
200
300
400
0
20
40
60
80
bp position

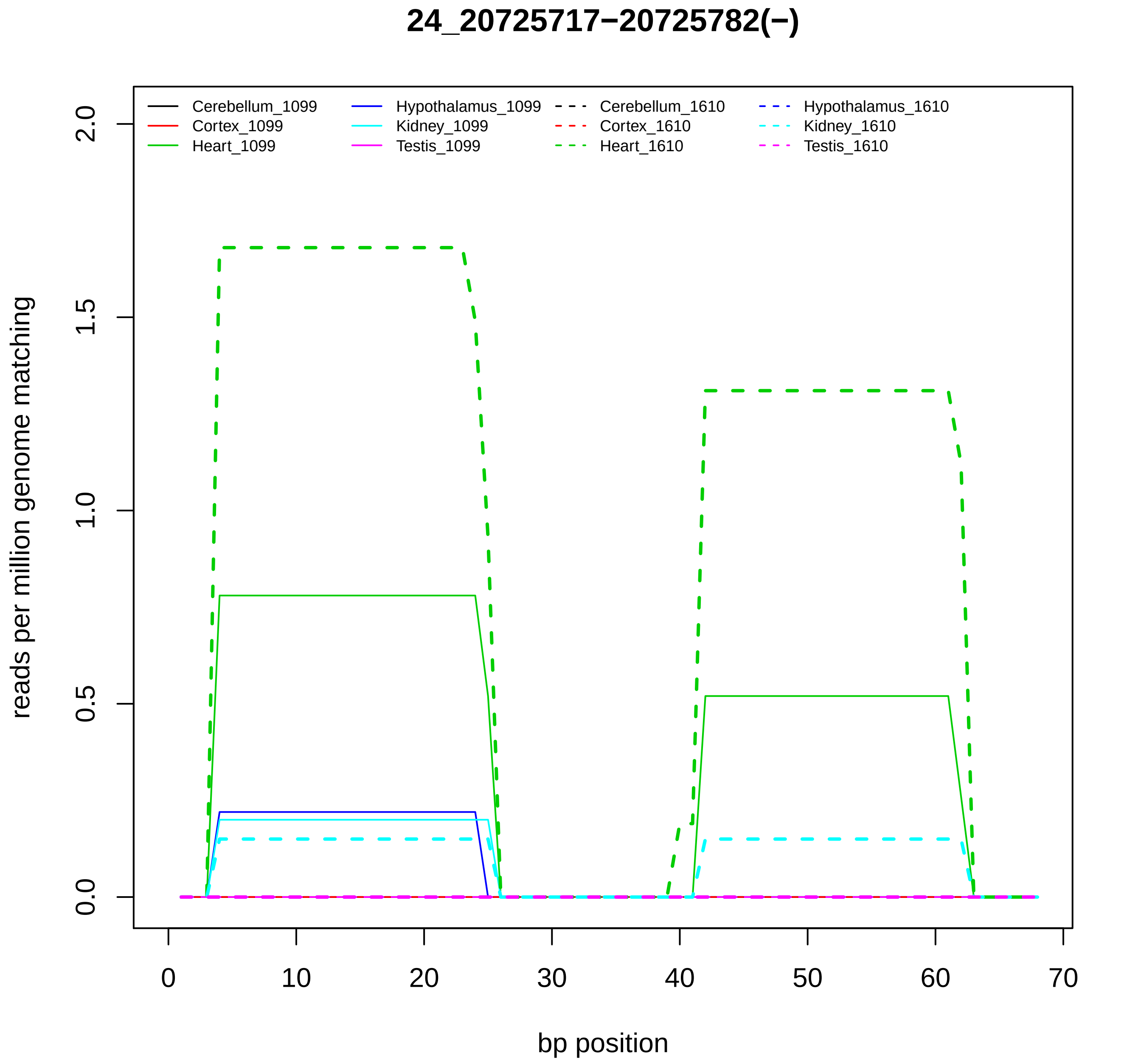
24_20725717−20725782(−)
Cerebellum_1099
Cortex_1099
Heart_1099
Hypothalamus_1099
Kidney_1099
Testis_1099
Cerebellum_1610
Cortex_1610
Heart_1610
Hypothalamus_1610
Kidney_1610
Testis_1610
reads per million genome matching
0.0
0.5
1.0
1.5
2.0
0
10
20
30
40
50
60
70
bp position

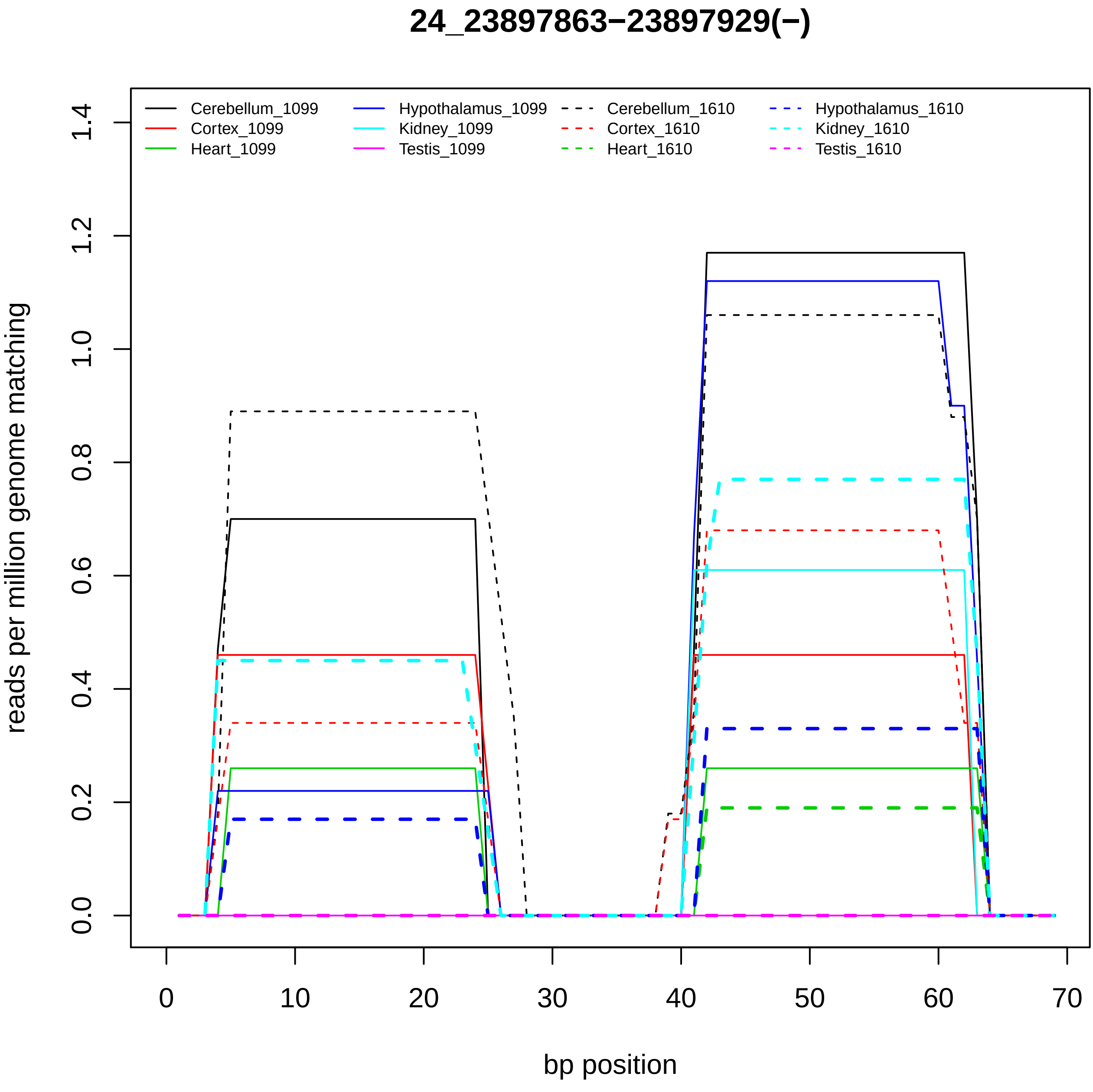

24_23897863−23897929(−)
Cerebellum_1099
Cortex_1099
Heart_1099
Hypothalamus_1099
Kidney_1099
Testis_1099
Cerebellum_1610
Cortex_1610
Heart_1610
Hypothalamus_1610
Kidney_1610
Testis_1610
reads per million genome matching
0.0
0.2
0.4
0.6
0.8
1.0
1.2
1.4
0
10
20
30
40
50
60
70
bp position

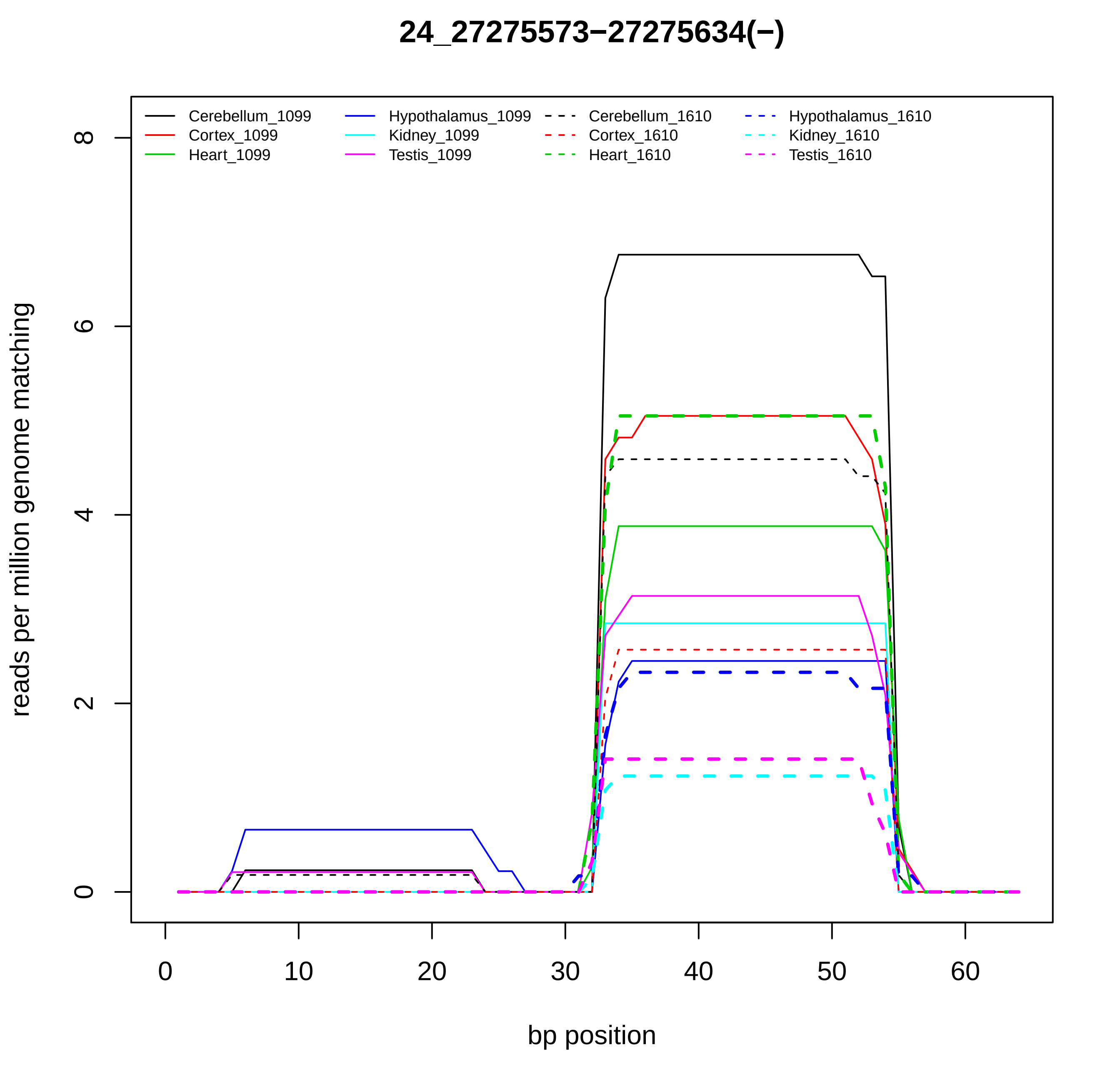

24_27275573−27275634(−)
Cerebellum_1099
Cortex_1099
Heart_1099
Hypothalamus_1099
Kidney_1099
Testis_1099
Cerebellum_1610
Cortex_1610
Heart_1610
Hypothalamus_1610
Kidney_1610
Testis_1610
reads per million genome matching
0
2
4
6
8
0
10
20
30
40
50
60
bp position

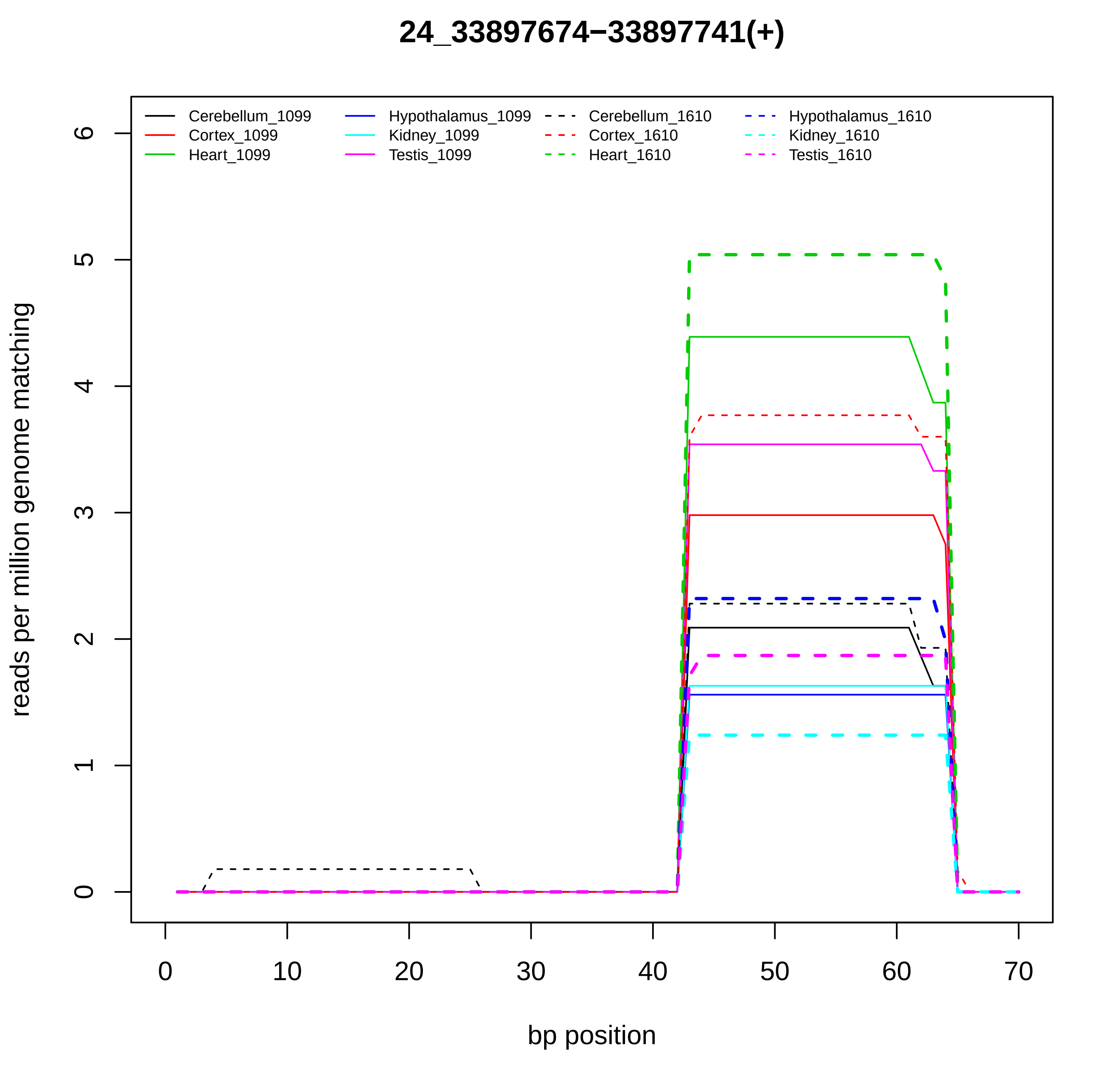

24_33897674−33897741(+)
Cerebellum_1099
Cortex_1099
Heart_1099
Hypothalamus_1099
Kidney_1099
Testis_1099
Cerebellum_1610
Cortex_1610
Heart_1610
Hypothalamus_1610
Kidney_1610
Testis_1610
reads per million genome matching
bp position
0
1
2
3
4
5
6
10
20
30
40
50
60
70

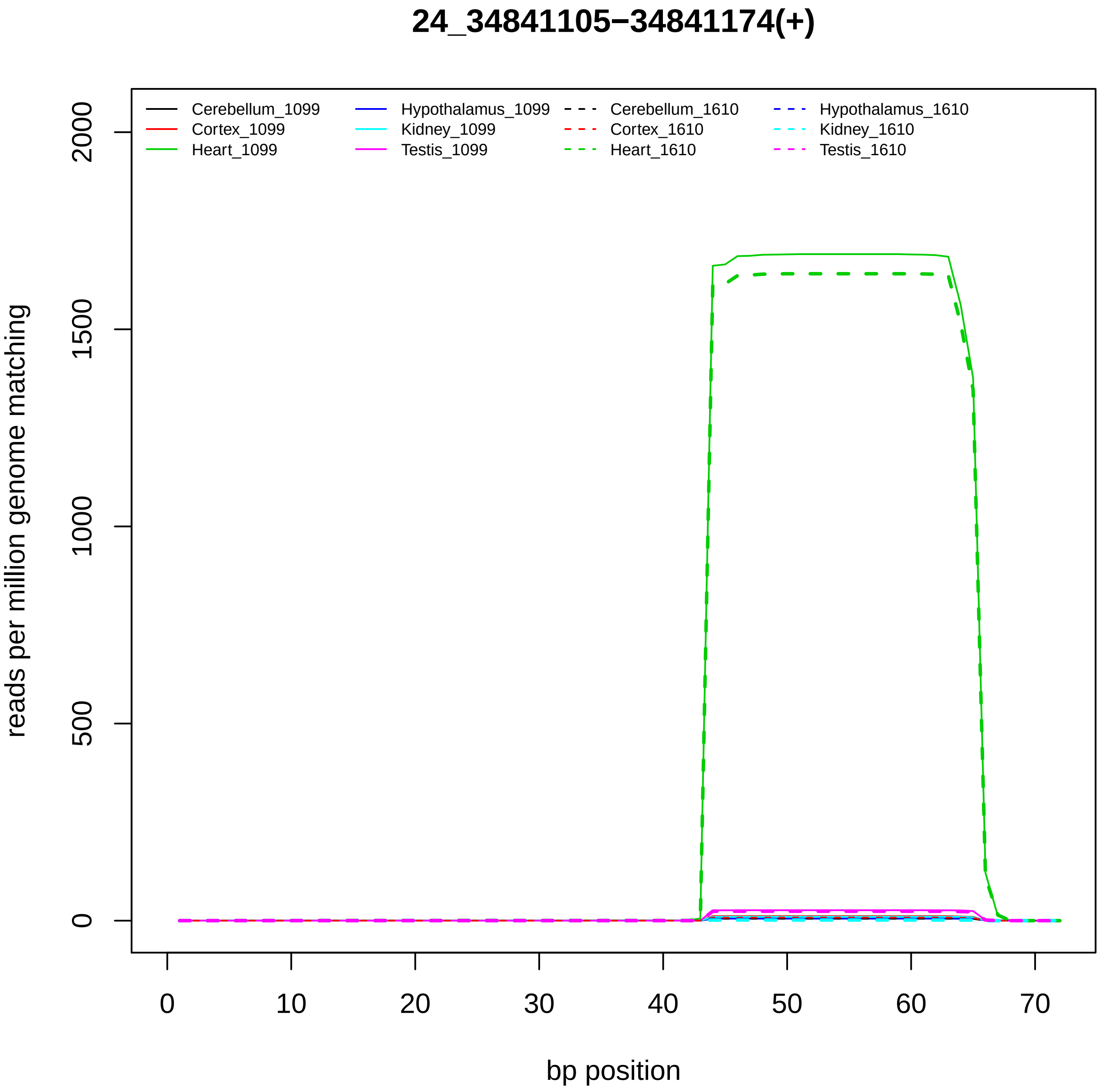

24_34841105−34841174(+)
Cerebellum_1099
Cortex_1099
Heart_1099
Hypothalamus_1099
Kidney_1099
Testis_1099
Cerebellum_1610
Cortex_1610
Heart_1610
Hypothalamus_1610
Kidney_1610
Testis_1610
reads per million genome matching
0
500
1000
1500
2000
0
10
20
30
40
50
60
70
bp position

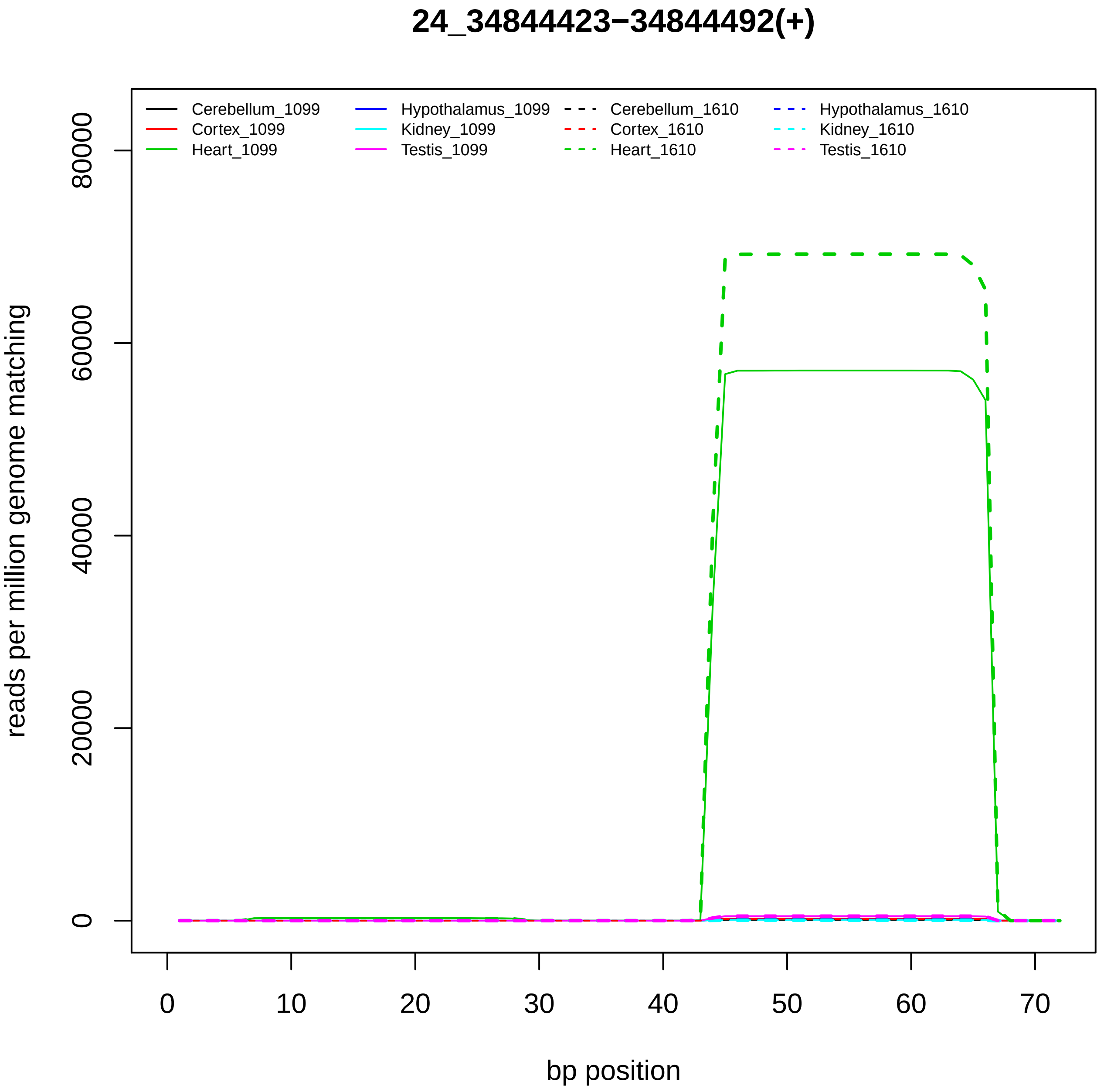
24_34844423−34844492(+)
Cerebellum_1099
Cortex_1099
Heart_1099
Hypothalamus_1099
Kidney_1099
Testis_1099
Cerebellum_1610
Cortex_1610
Heart_1610
Hypothalamus_1610
Kidney_1610
Testis_1610
reads per million genome matching
0
20000
40000
60000
80000
0
10
20
30
40
50
60
70
bp position

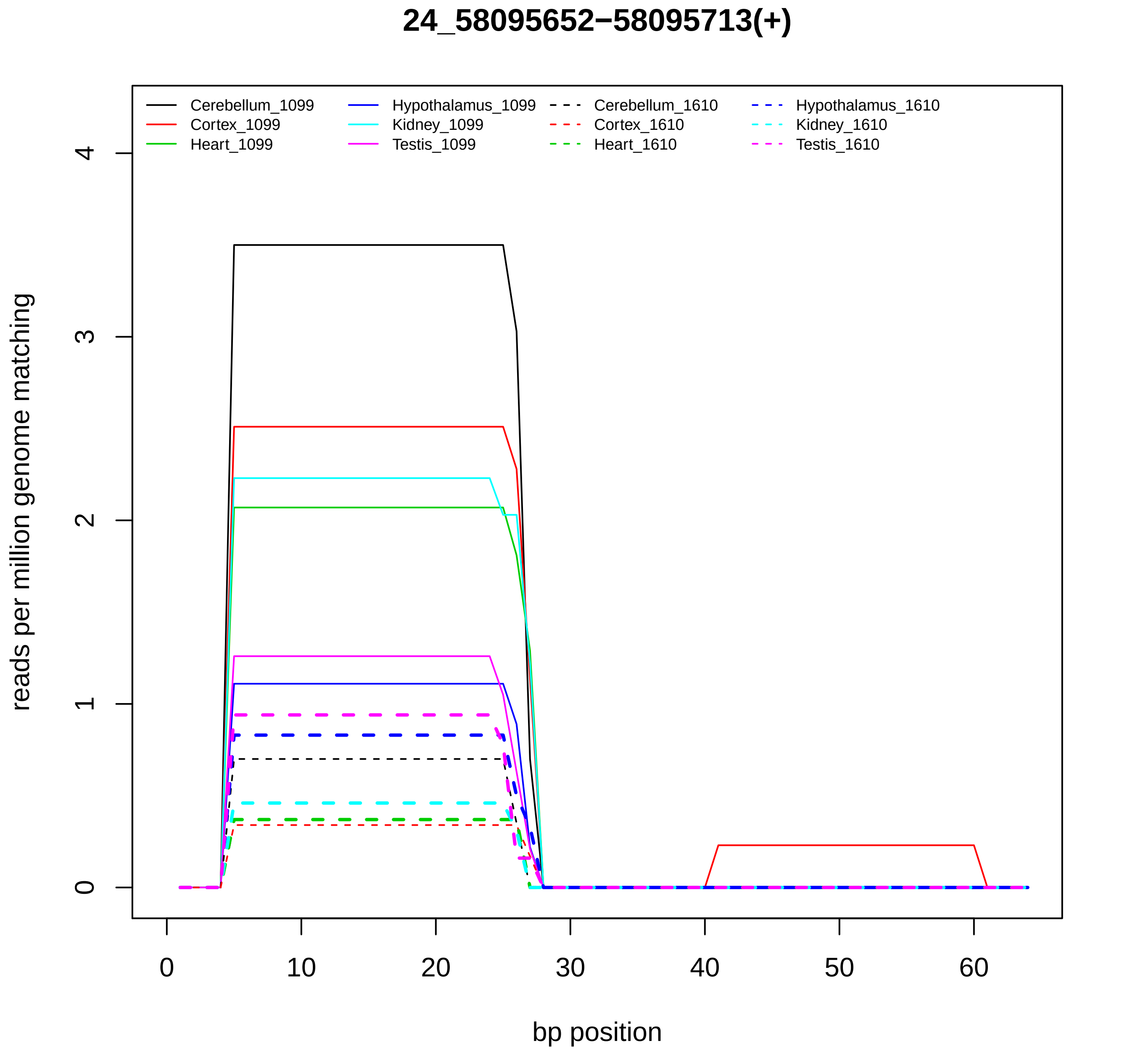

24_58095652−58095713(+)
Cerebellum_1099
Cortex_1099
Heart_1099
Hypothalamus_1099
Kidney_1099
Testis_1099
Cerebellum_1610
Cortex_1610
Heart_1610
Hypothalamus_1610
Kidney_1610
Testis_1610
reads per million genome matching
0
1
2
3
4
0
10
20
30
40
50
60
bp position

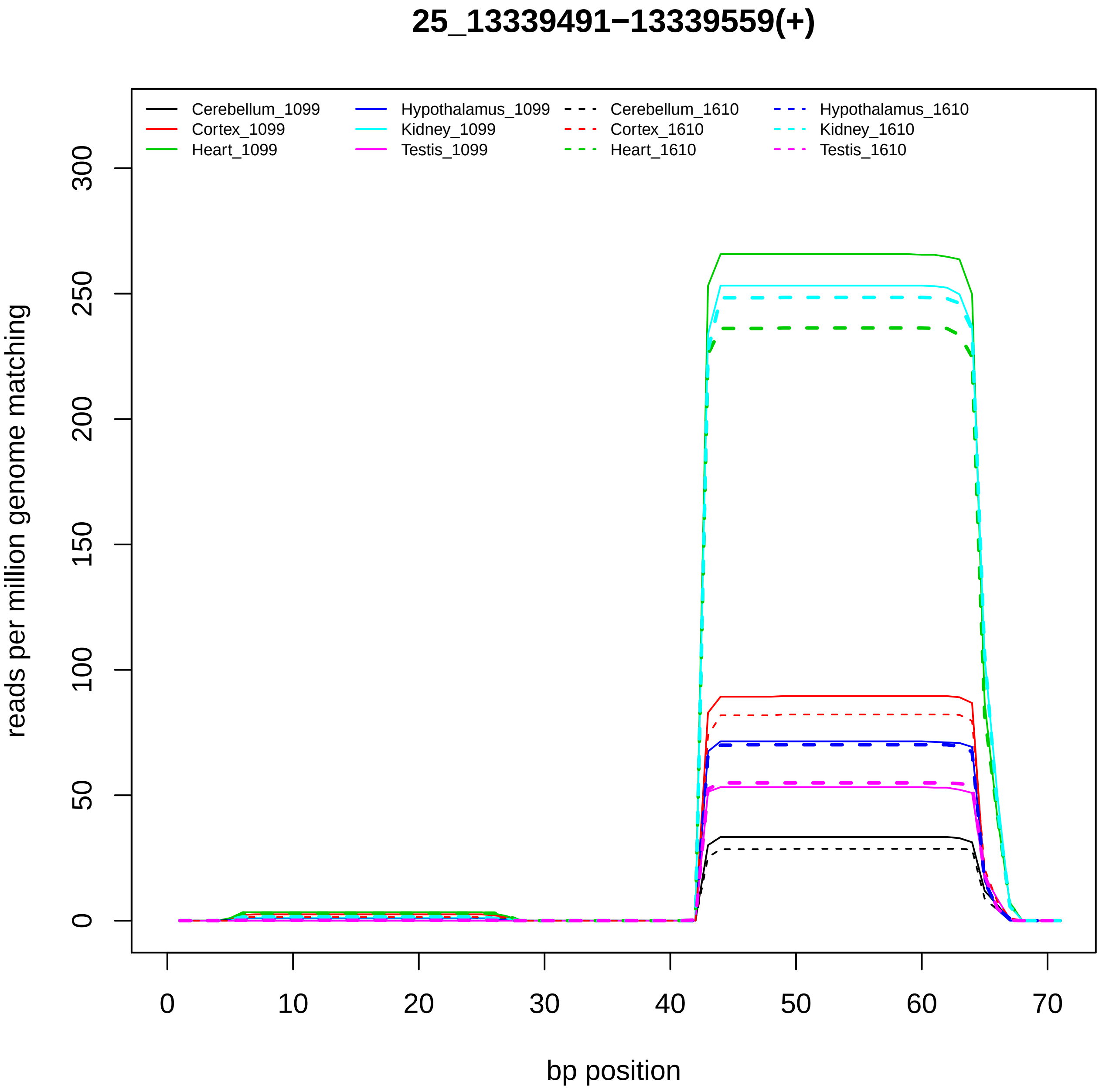

25_13339491−13339559(+)
Cerebellum_1099
Cortex_1099
Heart_1099
Hypothalamus_1099
Kidney_1099
Testis_1099
Cerebellum_1610
Cortex_1610
Heart_1610
Hypothalamus_1610
Kidney_1610
Testis_1610
reads per million genome matching
bp position
0
50
100
150
200
250
300
0
10
20
30
40
50
60
70

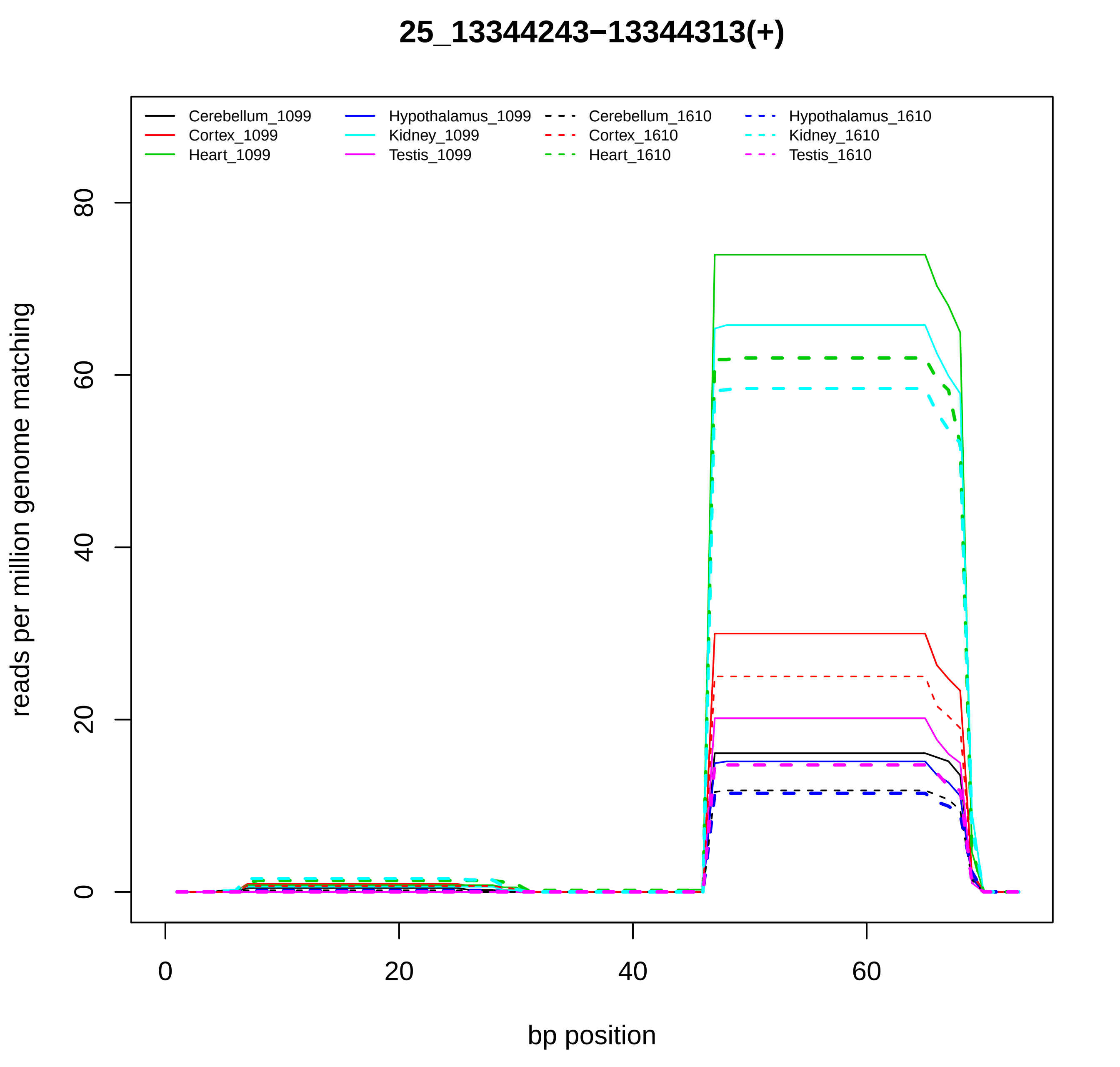

25_13344243−13344313(+)
Cerebellum_1099
Cortex_1099
Heart_1099
Hypothalamus_1099
Kidney_1099
Testis_1099
Cerebellum_1610
Cortex_1610
Heart_1610
Hypothalamus_1610
Kidney_1610
Testis_1610
reads per million genome matching
bp position
0
20
40
60
80

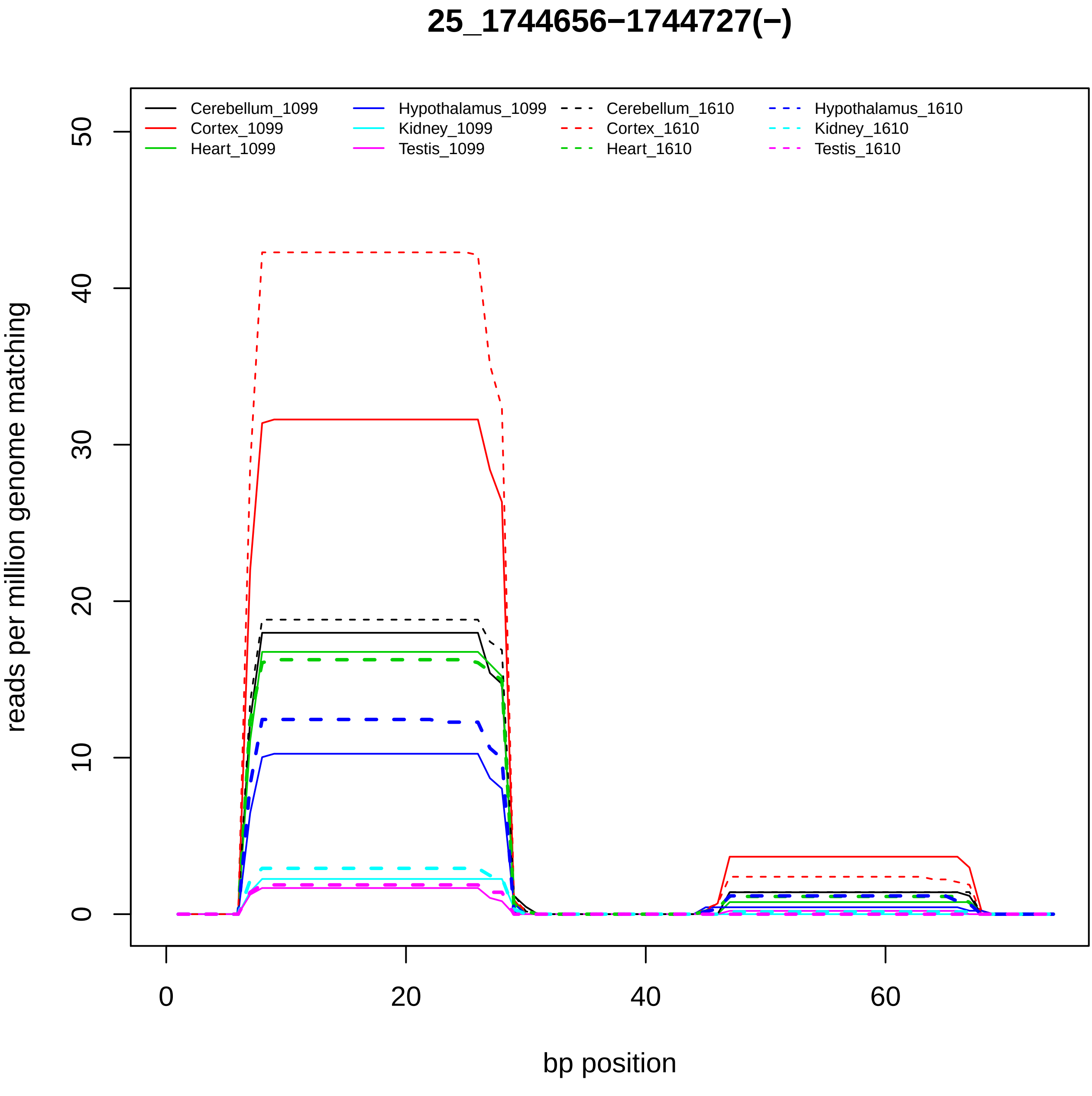

25_1744656−1744727(−)
Cerebellum_1099
Cortex_1099
Heart_1099
Hypothalamus_1099
Kidney_1099
Testis_1099
Cerebellum_1610
Cortex_1610
Heart_1610
Hypothalamus_1610
Kidney_1610
Testis_1610
reads per million genome matching
bp position
0
10
20
30
40
50
0
20
40
60

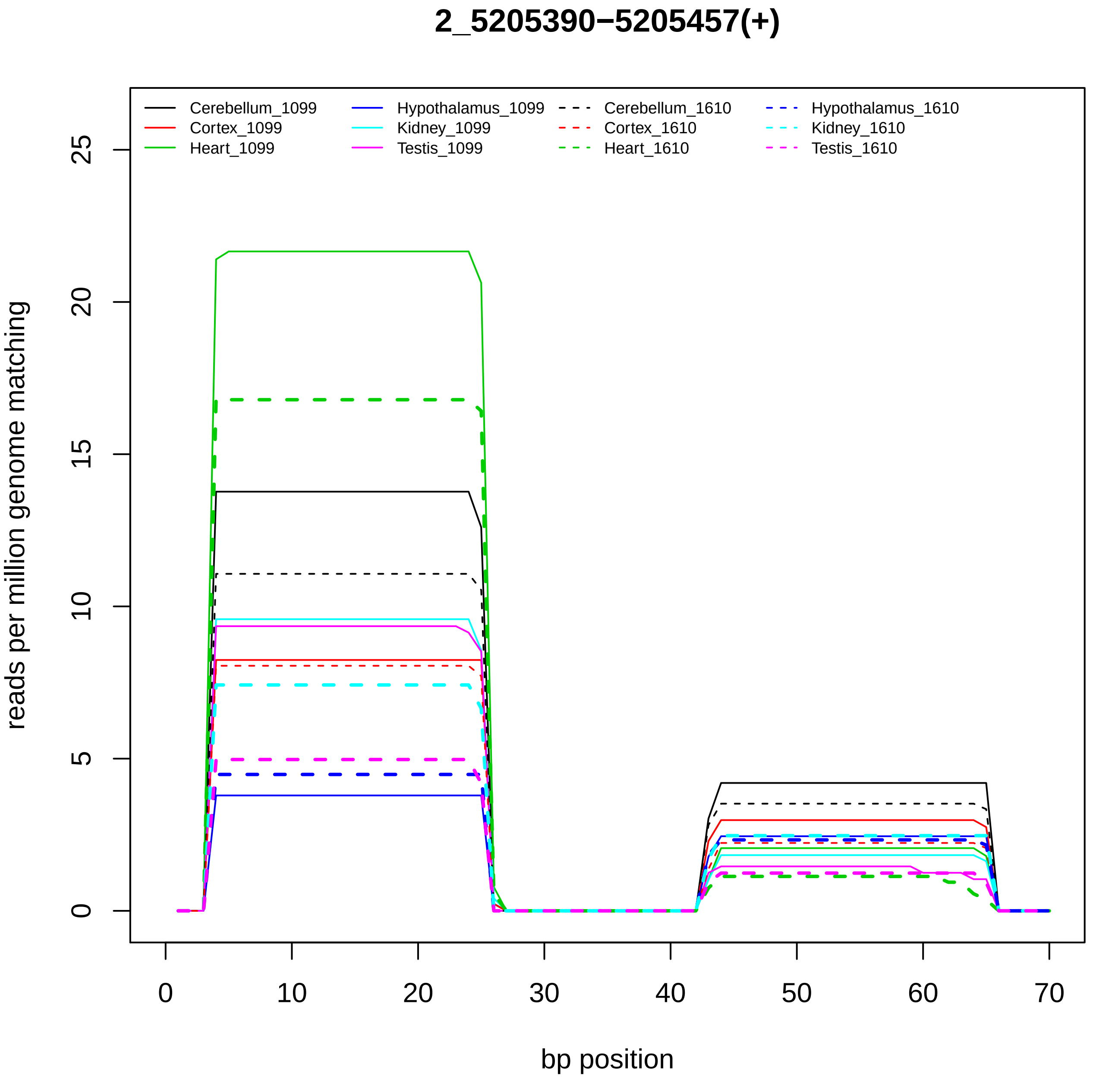

2_5205390−5205457(+)
Cerebellum_1099
Cortex_1099
Heart_1099
Hypothalamus_1099
Kidney_1099
Testis_1099
Cerebellum_1610
Cortex_1610
Heart_1610
Hypothalamus_1610
Kidney_1610
Testis_1610
reads per million genome matching
bp position
0
5
10
15
20
25
0
10
20
30
40
50
60
70

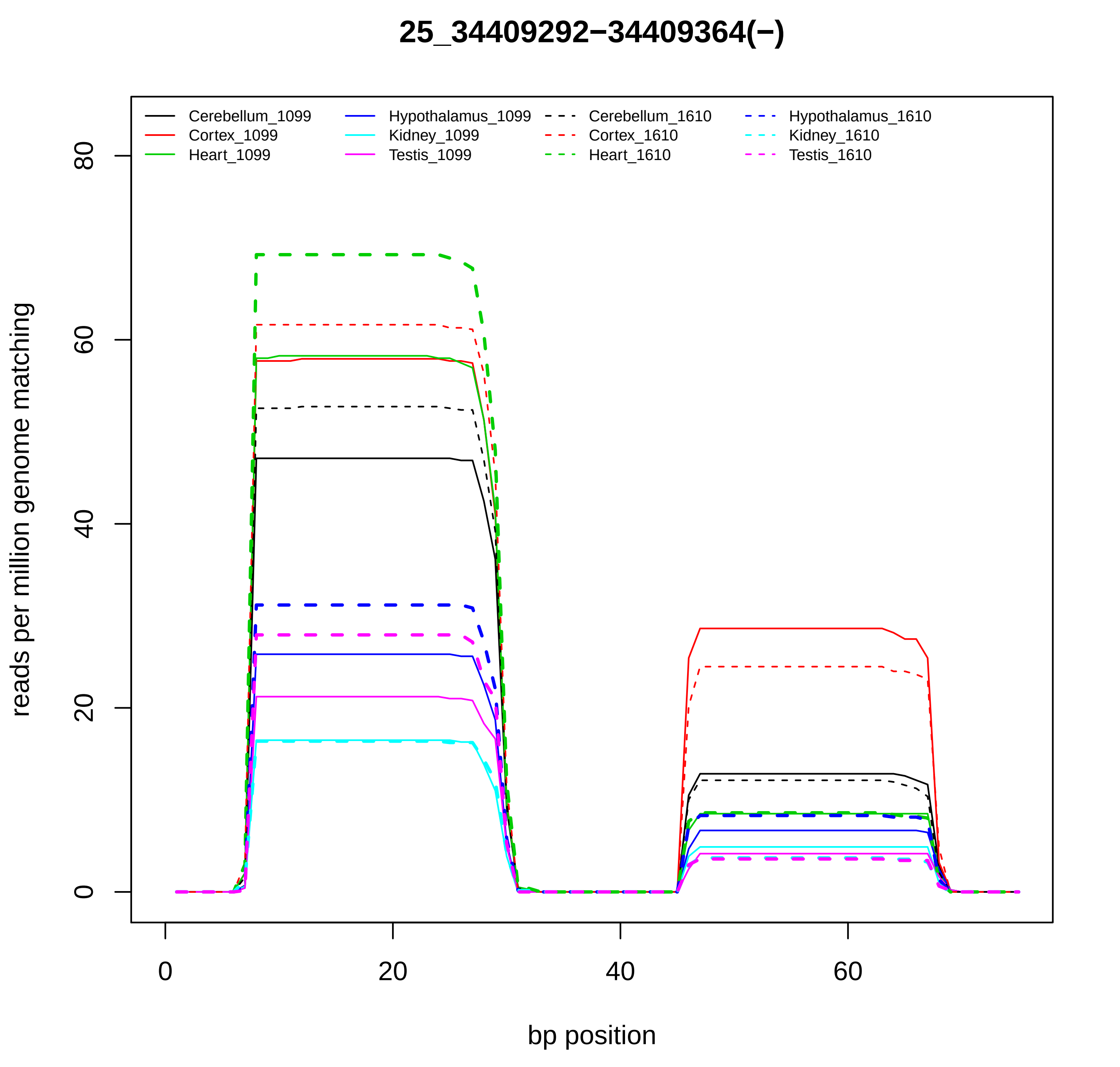

25_34409292−34409364(−)
Cerebellum_1099
Cortex_1099
Heart_1099
Hypothalamus_1099
Kidney_1099
Testis_1099
Cerebellum_1610
Cortex_1610
Heart_1610
Hypothalamus_1610
Kidney_1610
Testis_1610
reads per million genome matching
bp position
0
20
40
60
80

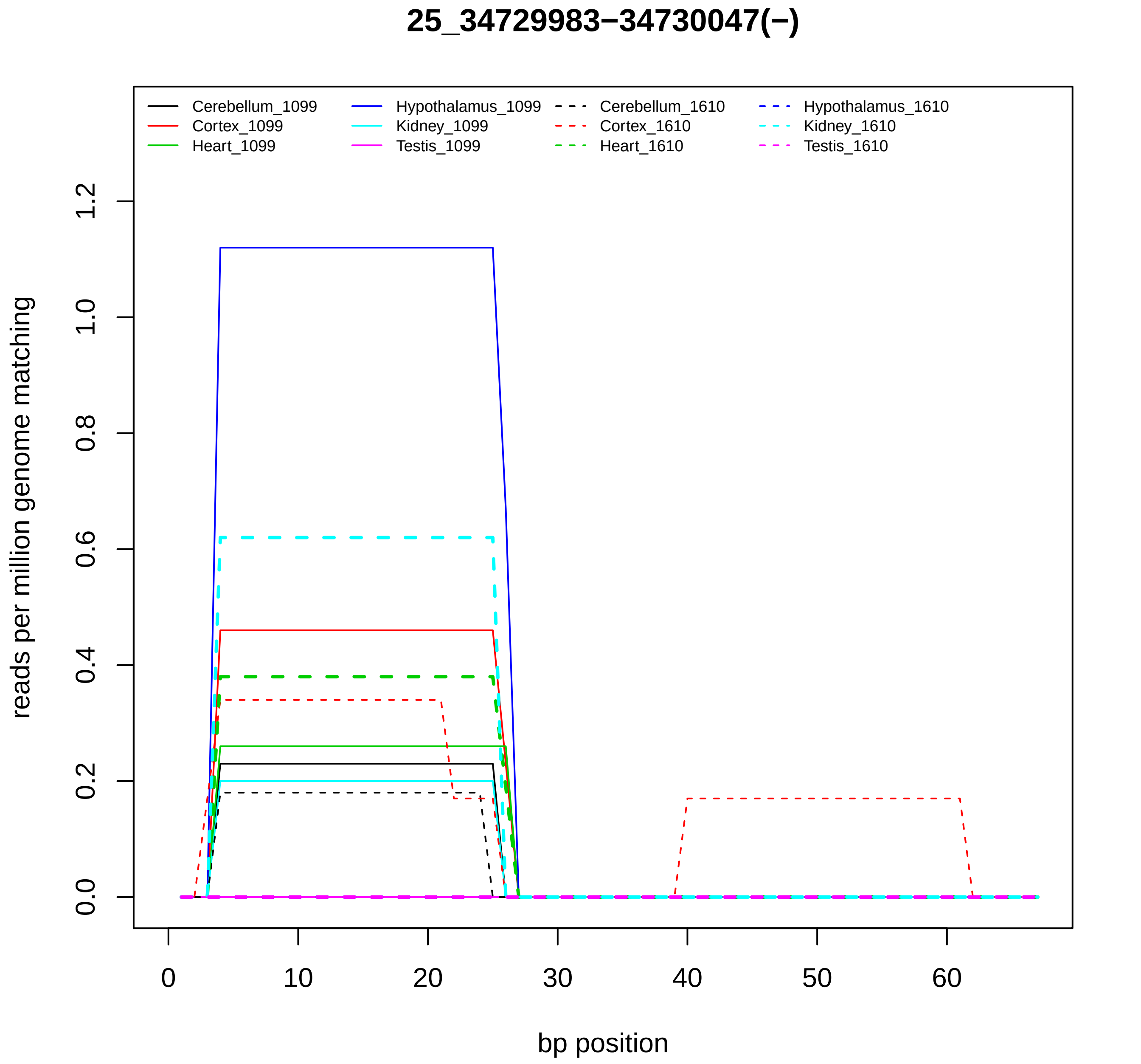

25_34729983−34730047(−)
Cerebellum_1099
Cortex_1099
Heart_1099
Hypothalamus_1099
Kidney_1099
Testis_1099
Cerebellum_1610
Cortex_1610
Heart_1610
Hypothalamus_1610
Kidney_1610
Testis_1610
reads per million genome matching
0.0
0.2
0.4
0.6
0.8
1.0
1.2
0
10
20
30
40
50
60
bp position

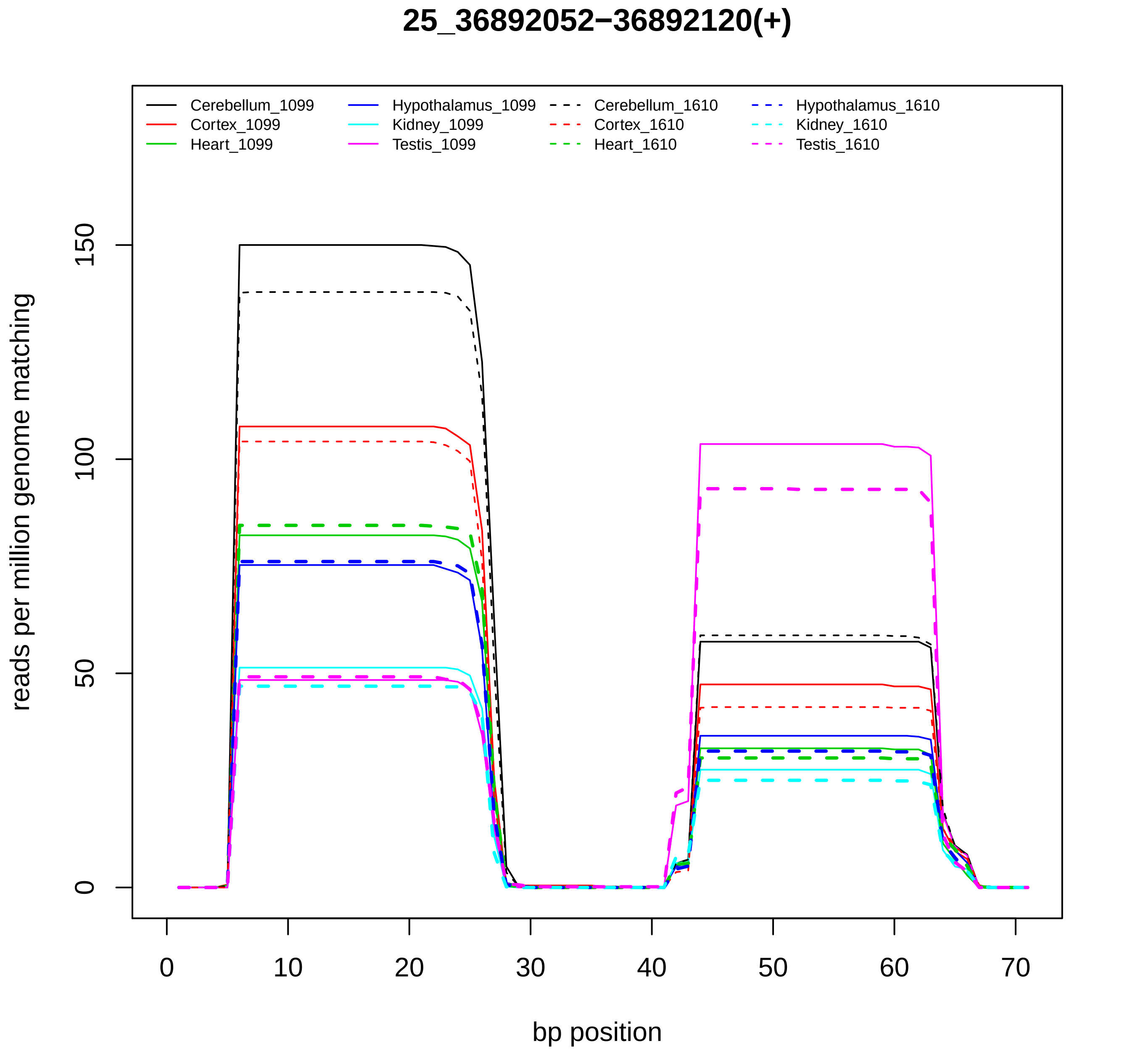
25_36892052−36892120(+)
Cerebellum_1099
Cortex_1099
Heart_1099
Hypothalamus_1099
Kidney_1099
Testis_1099
Cerebellum_1610
Cortex_1610
Heart_1610
Hypothalamus_1610
Kidney_1610
Testis_1610
reads per million genome matching
0
50
100
150
bp position
0
10
20
30
40
50
60
70

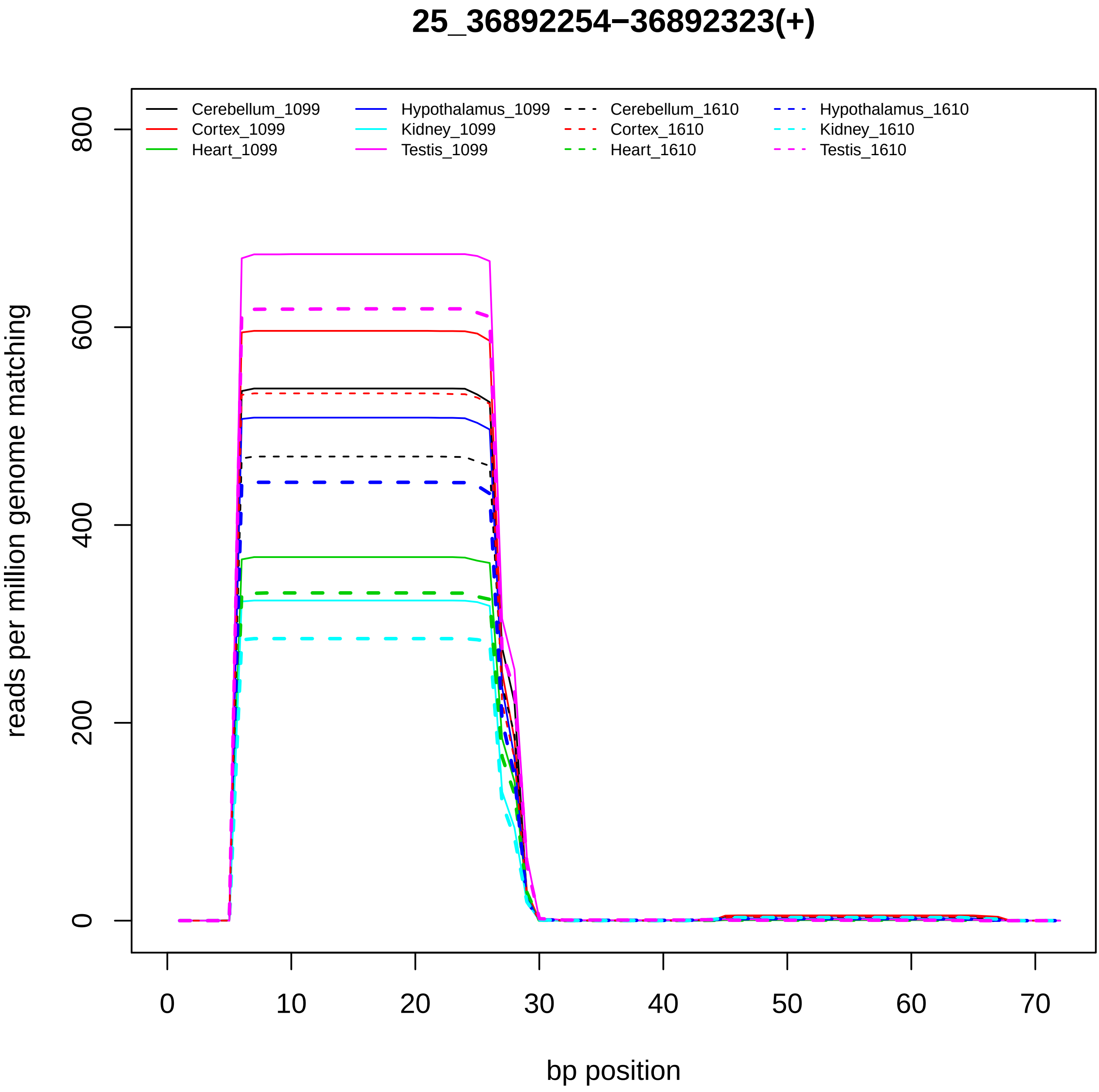
25_36892254−36892323(+)
Cerebellum_1099
Cortex_1099
Heart_1099
Hypothalamus_1099
Kidney_1099
Testis_1099
Cerebellum_1610
Cortex_1610
Heart_1610
Hypothalamus_1610
Kidney_1610
Testis_1610
reads per million genome matching
0
200
400
600
800
bp position
0
10
20
30
40
50
60
70

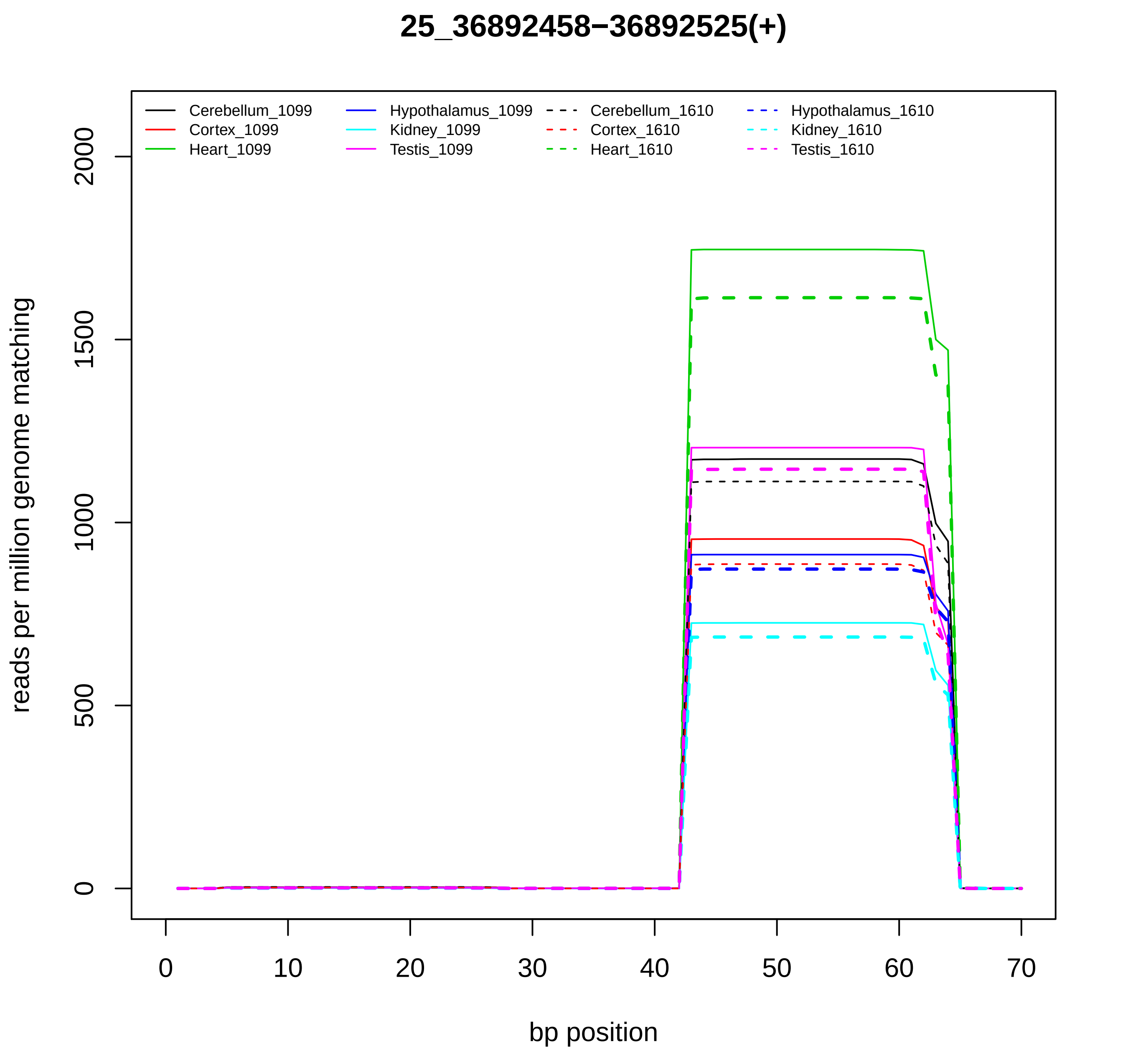
25_36892458−36892525(+)
Cerebellum_1099
Cortex_1099
Heart_1099
Hypothalamus_1099
Kidney_1099
Testis_1099
Cerebellum_1610
Cortex_1610
Heart_1610
Hypothalamus_1610
Kidney_1610
Testis_1610
reads per million genome matching
0
500
1000
1500
2000
bp position
0
10
20
30
40
50
60
70

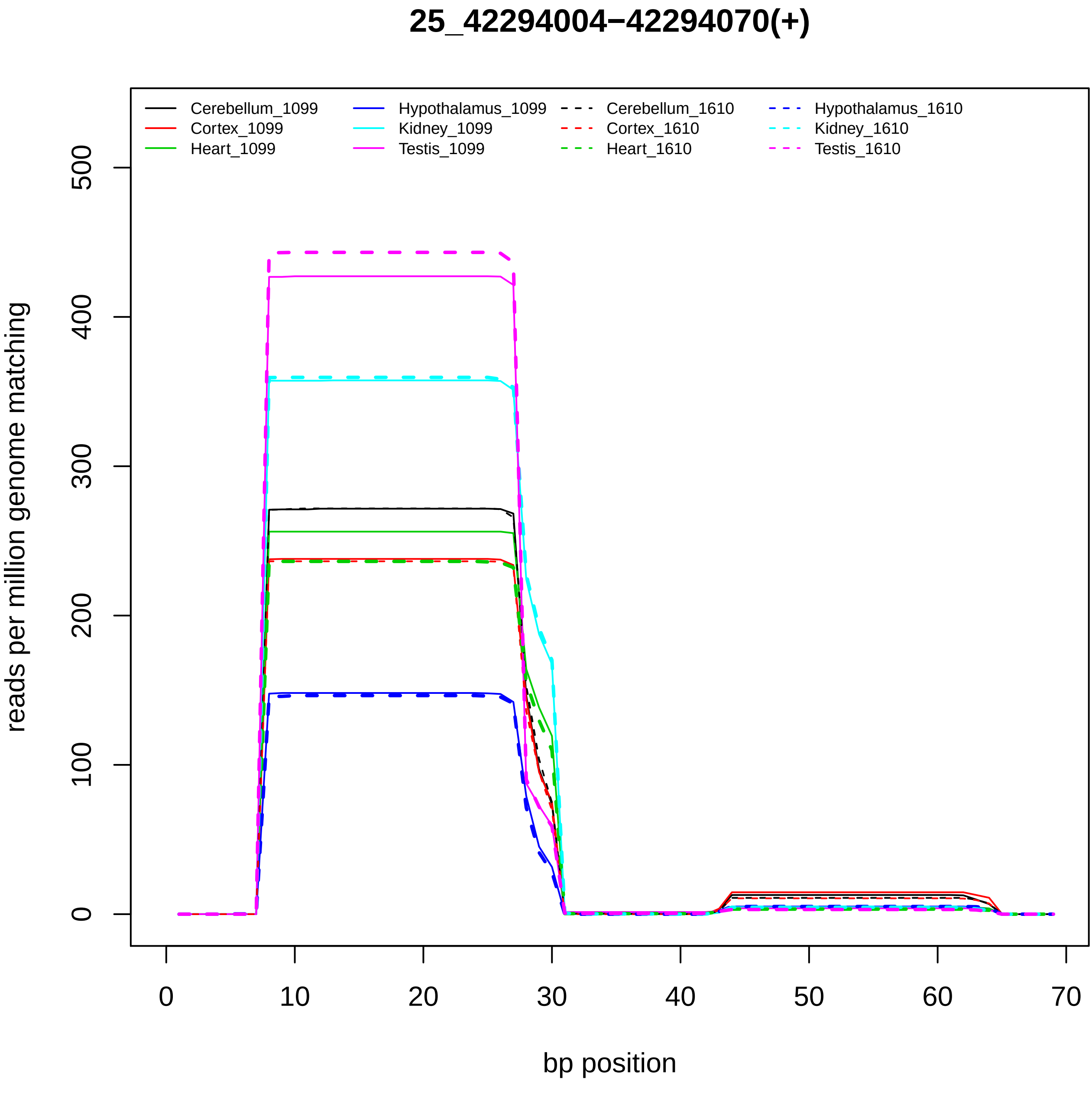

25_42294004−42294070(+)
Cerebellum_1099
Cortex_1099
Heart_1099
Hypothalamus_1099
Kidney_1099
Testis_1099
Cerebellum_1610
Cortex_1610
Heart_1610
Hypothalamus_1610
Kidney_1610
Testis_1610
reads per million genome matching
0
100
200
300
400
500
0
10
20
30
40
50
60
70
bp position

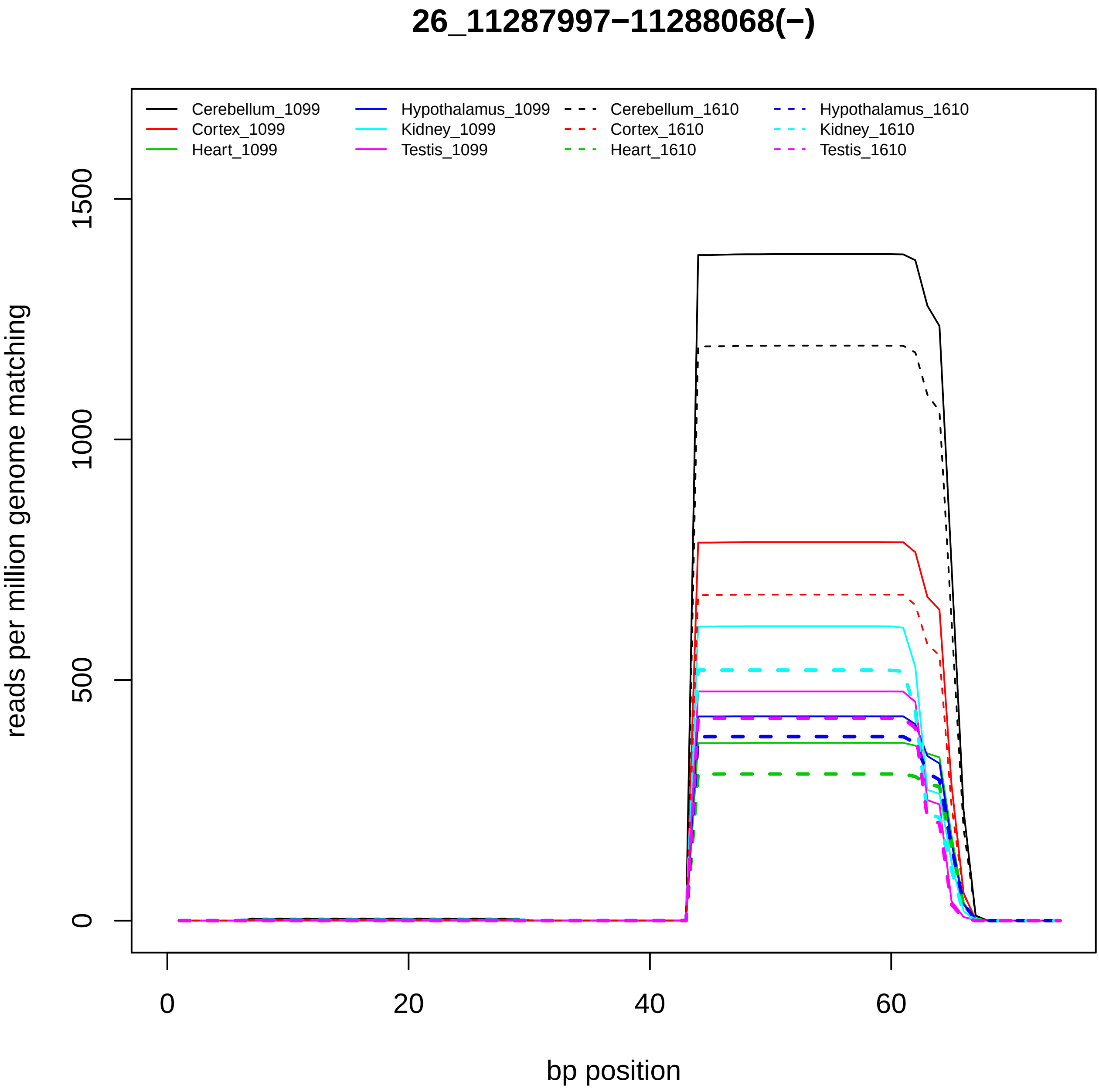

26_11287997−11288068(−)
Cerebellum_1099
Cortex_1099
Heart_1099
Hypothalamus_1099
Kidney_1099
Testis_1099
Cerebellum_1610
Cortex_1610
Heart_1610
Hypothalamus_1610
Kidney_1610
Testis_1610
reads per million genome matching
0
500
1000
1500
0
20
40
60
bp position

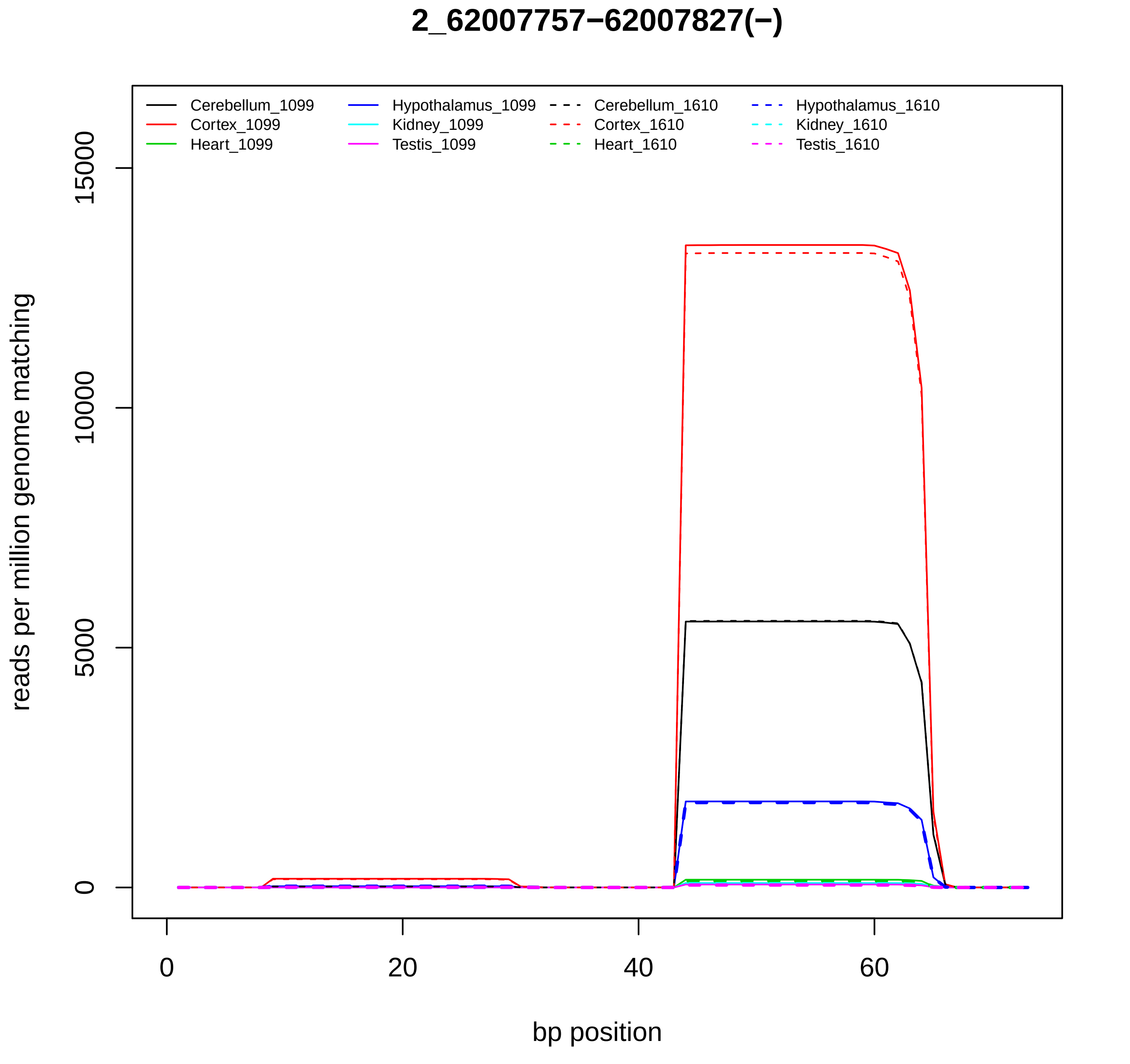
2_62007757−62007827(−)
Cerebellum_1099
Cortex_1099
Heart_1099
Hypothalamus_1099
Kidney_1099
Testis_1099
Cerebellum_1610
Cortex_1610
Heart_1610
Hypothalamus_1610
Kidney_1610
Testis_1610
reads per million genome matching
0
5000
10000
15000
0
20
40
60
bp position

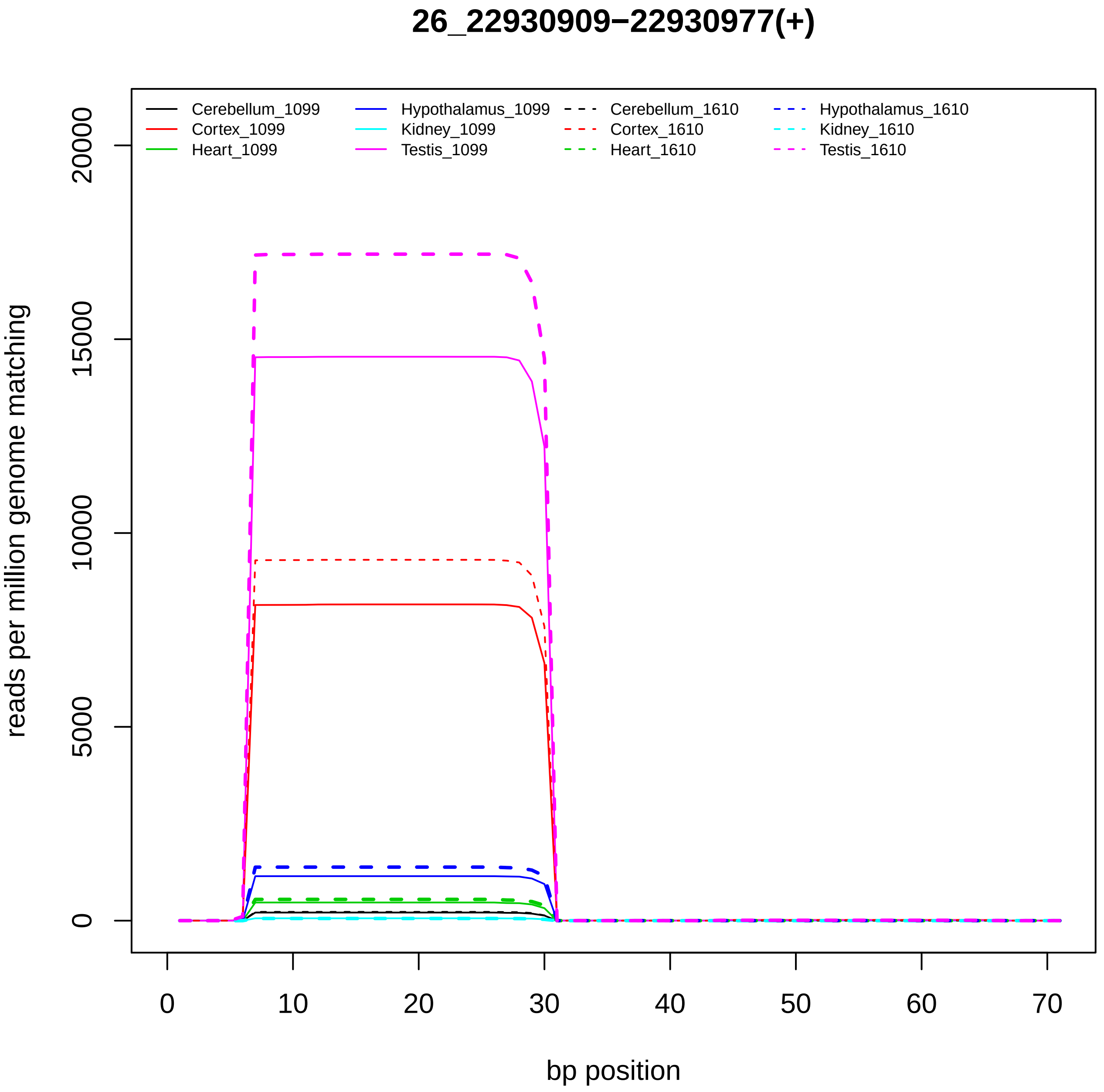

26_22930909−22930977(+)
Cerebellum_1099
Cortex_1099
Heart_1099
Hypothalamus_1099
Kidney_1099
Testis_1099
Cerebellum_1610
Cortex_1610
Heart_1610
Hypothalamus_1610
Kidney_1610
Testis_1610
reads per million genome matching
0
5000
10000
15000
20000
0
10
20
30
40
50
60
70
bp position

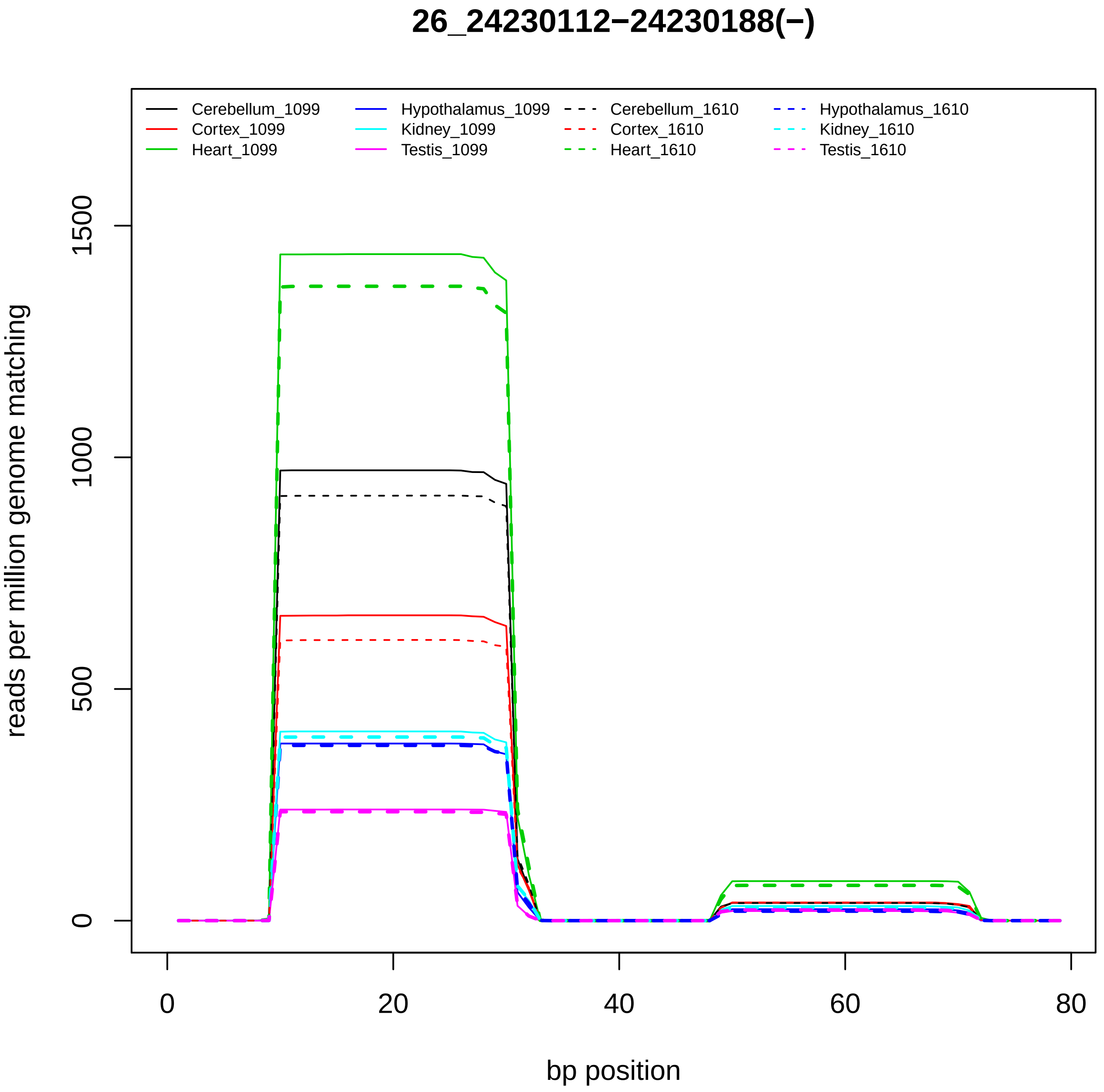
26_24230112−24230188(−)
Cerebellum_1099
Cortex_1099
Heart_1099
Hypothalamus_1099
Kidney_1099
Testis_1099
Cerebellum_1610
Cortex_1610
Heart_1610
Hypothalamus_1610
Kidney_1610
Testis_1610
reads per million genome matching
0
500
1000
1500
0
20
40
60
80
bp position

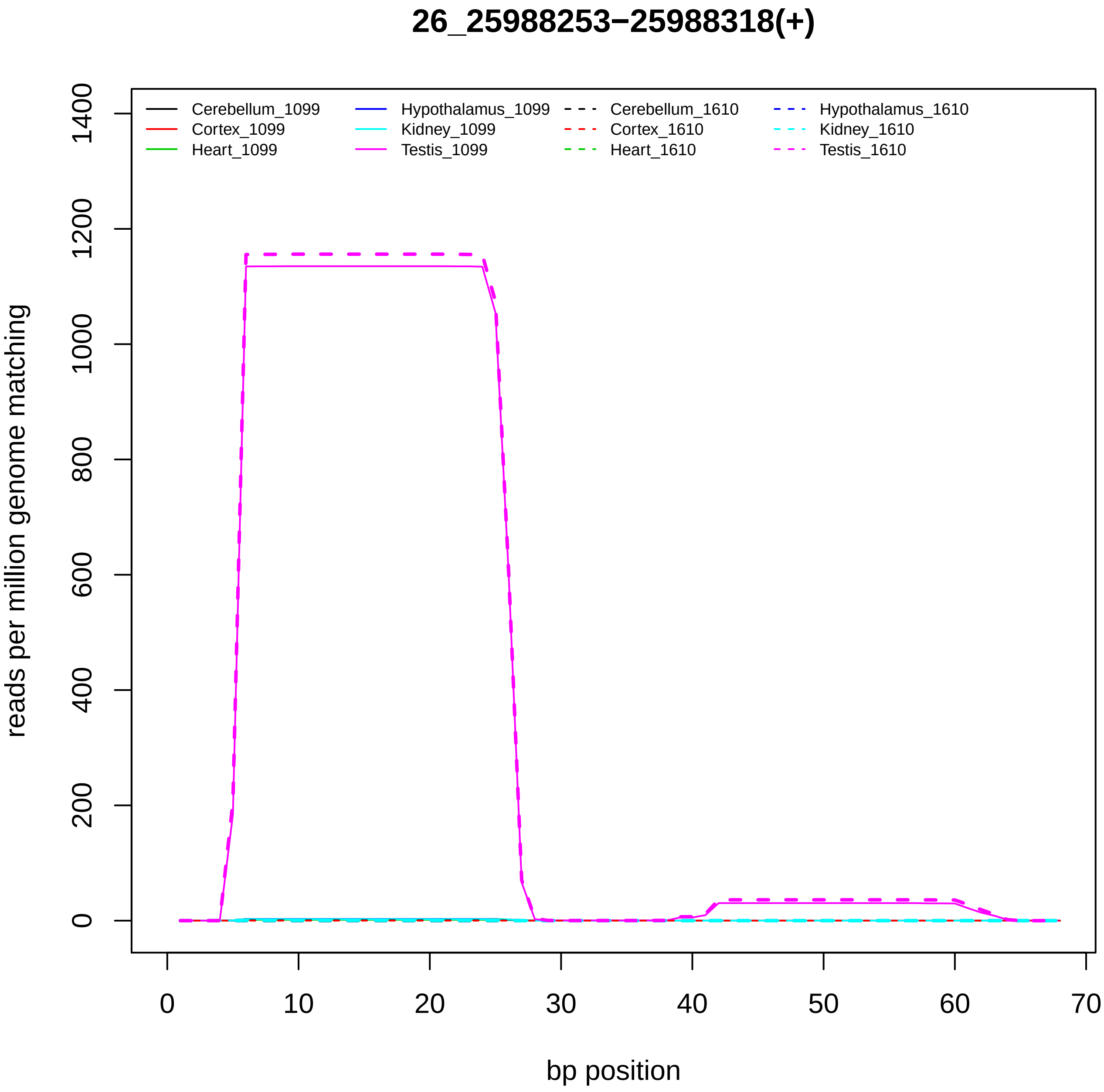

26_25988253−25988318(+)
Cerebellum_1099
Cortex_1099
Heart_1099
Hypothalamus_1099
Kidney_1099
Testis_1099
Cerebellum_1610
Cortex_1610
Heart_1610
Hypothalamus_1610
Kidney_1610
Testis_1610
reads per million genome matching
bp position
0
200
400
600
800
1000
1200
1400
0
10
20
30
40
50
60
70

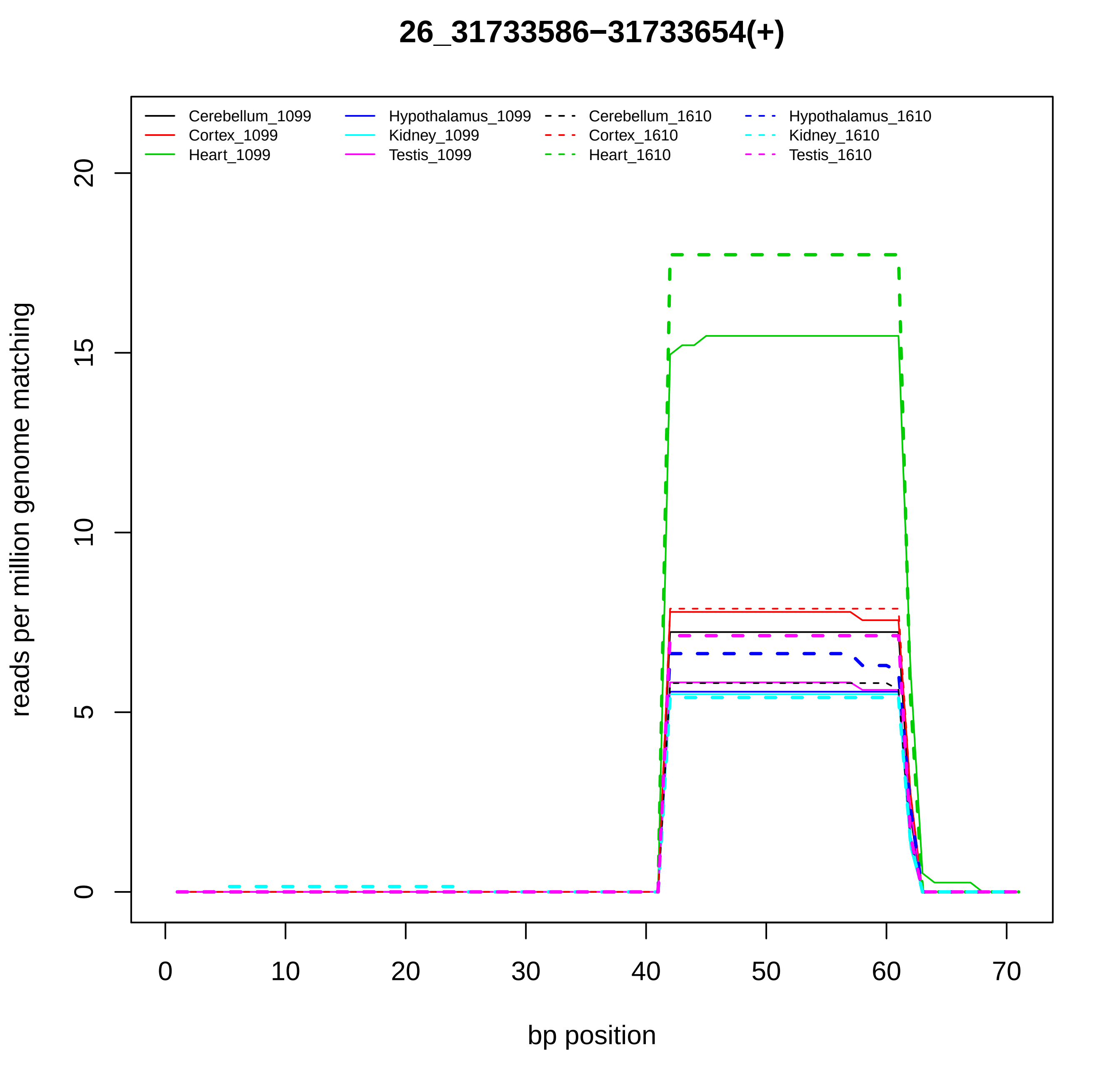
26_31733586−31733654(+)
Cerebellum_1099
Cortex_1099
Heart_1099
Hypothalamus_1099
Kidney_1099
Testis_1099
Cerebellum_1610
Cortex_1610
Heart_1610
Hypothalamus_1610
Kidney_1610
Testis_1610
reads per million genome matching
0
5
10
15
20
0
10
20
30
40
50
60
70
bp position

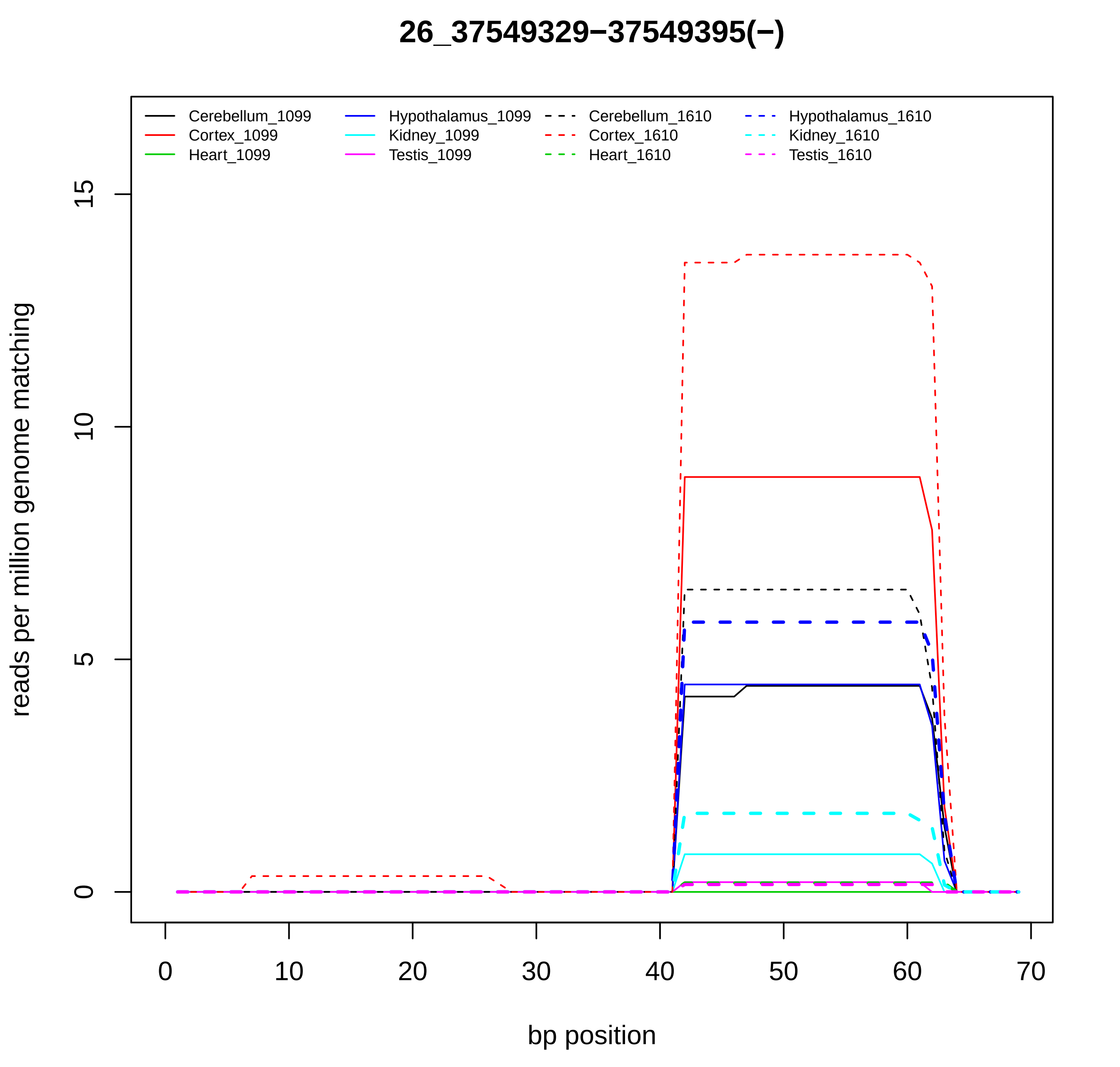

26_37549329−37549395(−)
Cerebellum_1099
Cortex_1099
Heart_1099
Hypothalamus_1099
Kidney_1099
Testis_1099
Cerebellum_1610
Cortex_1610
Heart_1610
Hypothalamus_1610
Kidney_1610
Testis_1610
reads per million genome matching
0
5
10
15
0
10
20
30
40
50
60
70
bp position

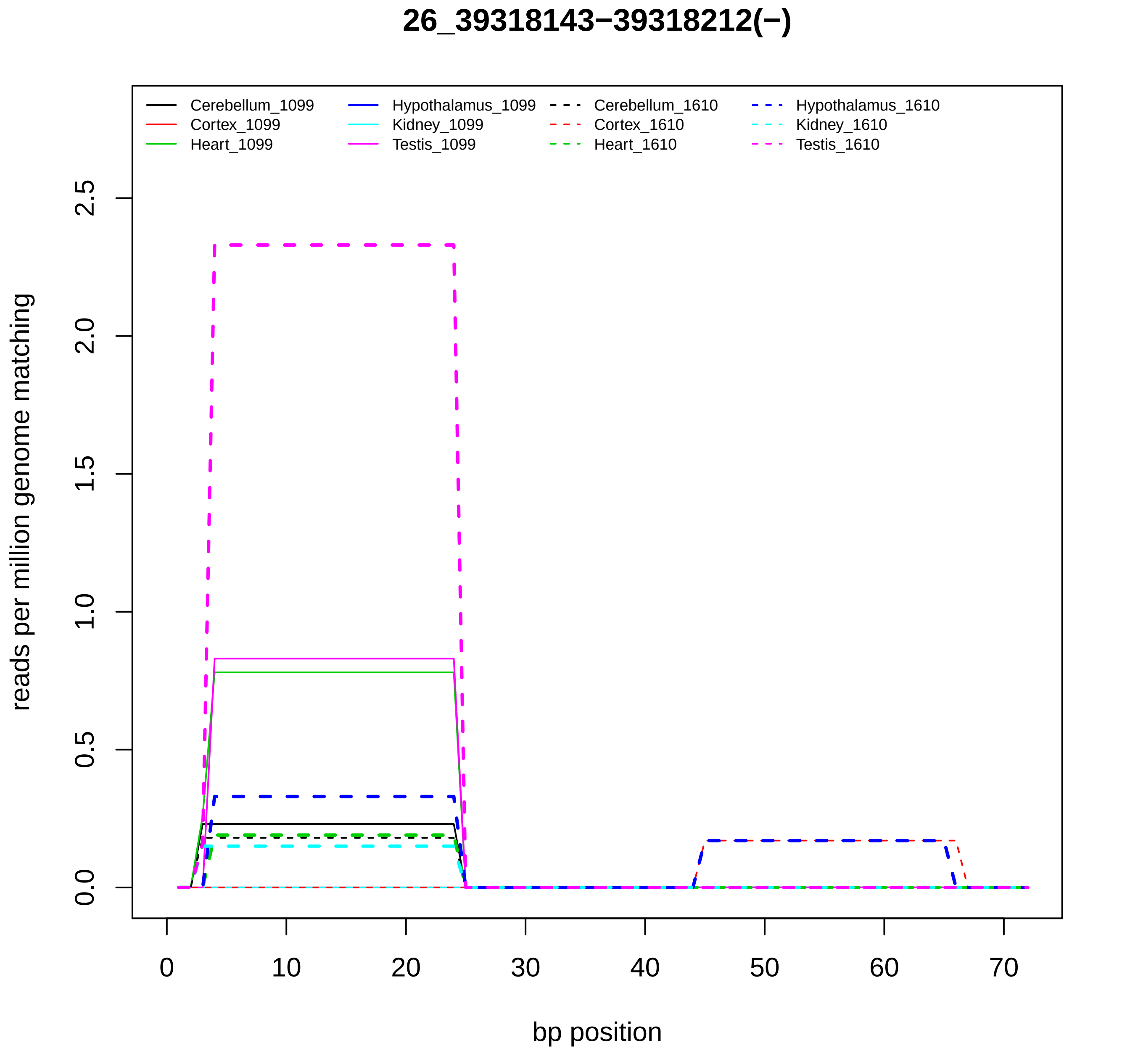

26_39318143−39318212(−)
Cerebellum_1099
Cortex_1099
Heart_1099
Hypothalamus_1099
Kidney_1099
Testis_1099
Cerebellum_1610
Cortex_1610
Heart_1610
Hypothalamus_1610
Kidney_1610
Testis_1610
reads per million genome matching
bp position
0.0
0.5
1.0
1.5
2.0
2.5
0
10
20
30
40
50
60
70

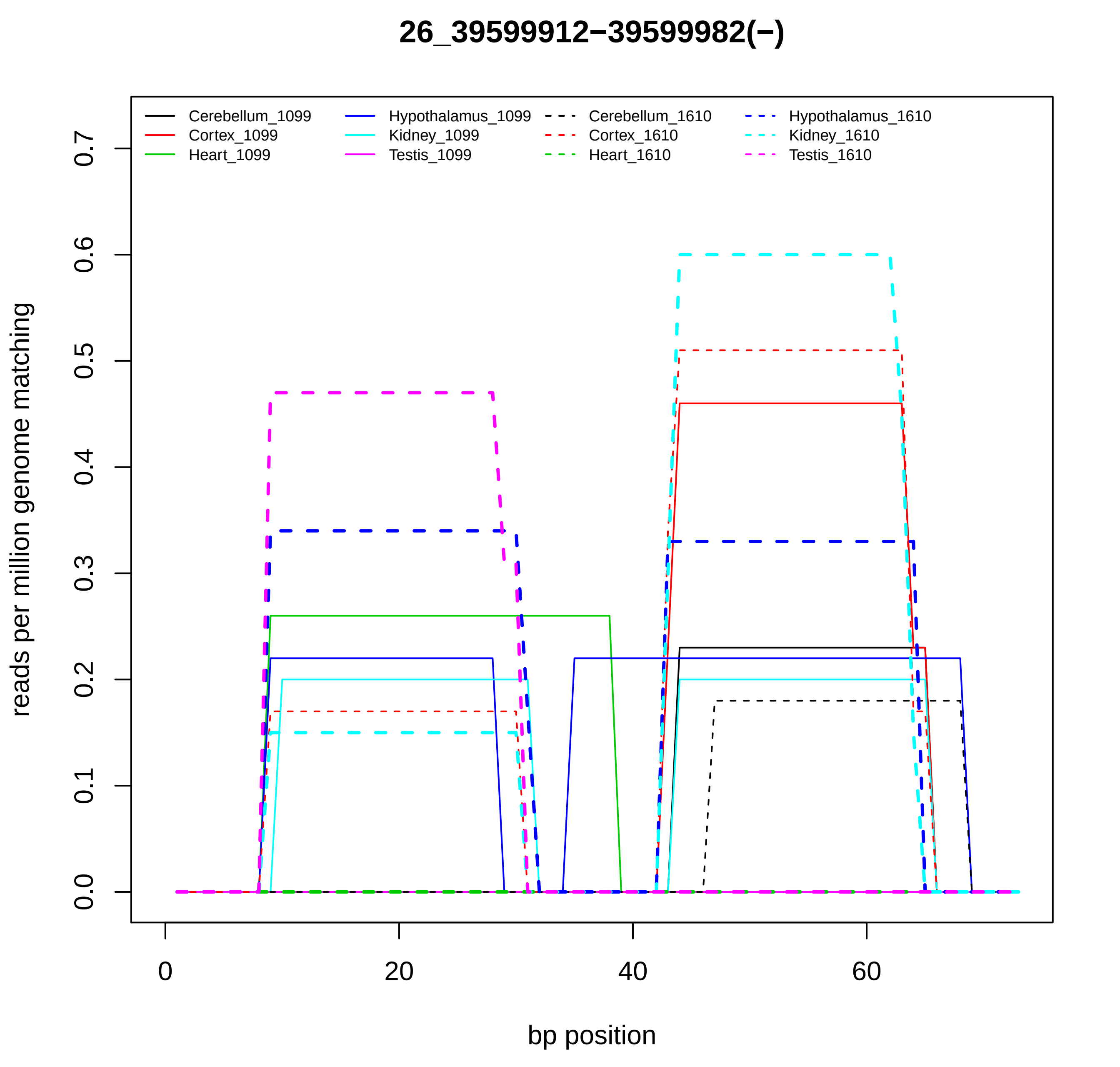
26_39599912−39599982(−)
Cerebellum_1099
Cortex_1099
Heart_1099
Hypothalamus_1099
Kidney_1099
Testis_1099
Cerebellum_1610
Cortex_1610
Heart_1610
Hypothalamus_1610
Kidney_1610
Testis_1610
reads per million genome matching
0.0
0.1
0.2
0.3
0.4
0.5
0.6
0.7
0
20
40
60
bp position

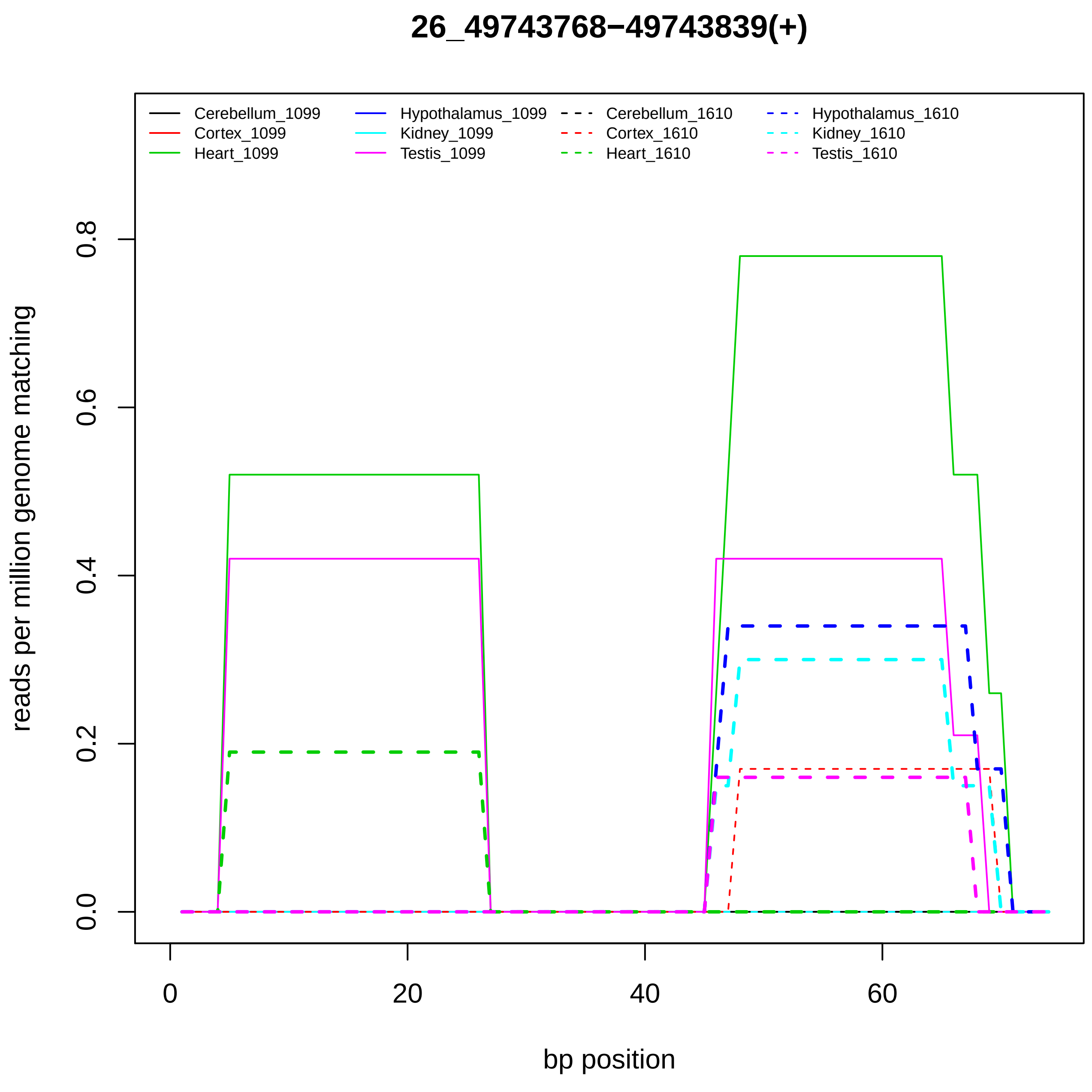
26_49743768−49743839(+)
Cerebellum_1099
Cortex_1099
Heart_1099
Hypothalamus_1099
Kidney_1099
Testis_1099
Cerebellum_1610
Cortex_1610
Heart_1610
Hypothalamus_1610
Kidney_1610
Testis_1610
reads per million genome matching
0.0
0.2
0.4
0.6
0.8
0
20
40
60
bp position

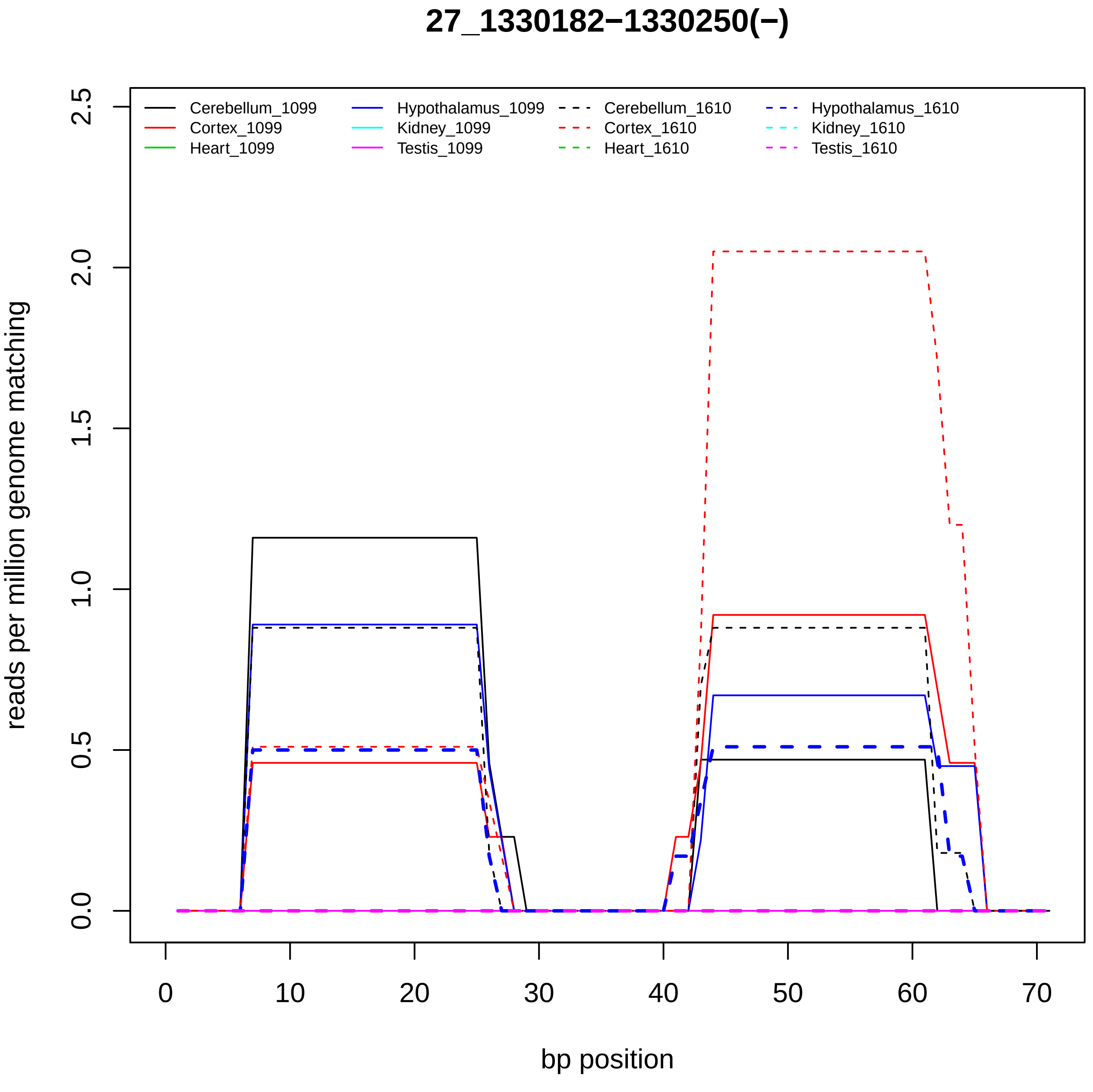

27_1330182−1330250(−)
Cerebellum_1099
Cortex_1099
Heart_1099
Hypothalamus_1099
Kidney_1099
Testis_1099
Cerebellum_1610
Cortex_1610
Heart_1610
Hypothalamus_1610
Kidney_1610
Testis_1610
reads per million genome matching
0.0
0.5
1.0
1.5
2.0
2.5
bp position
0
10
20
30
40
50
60
70

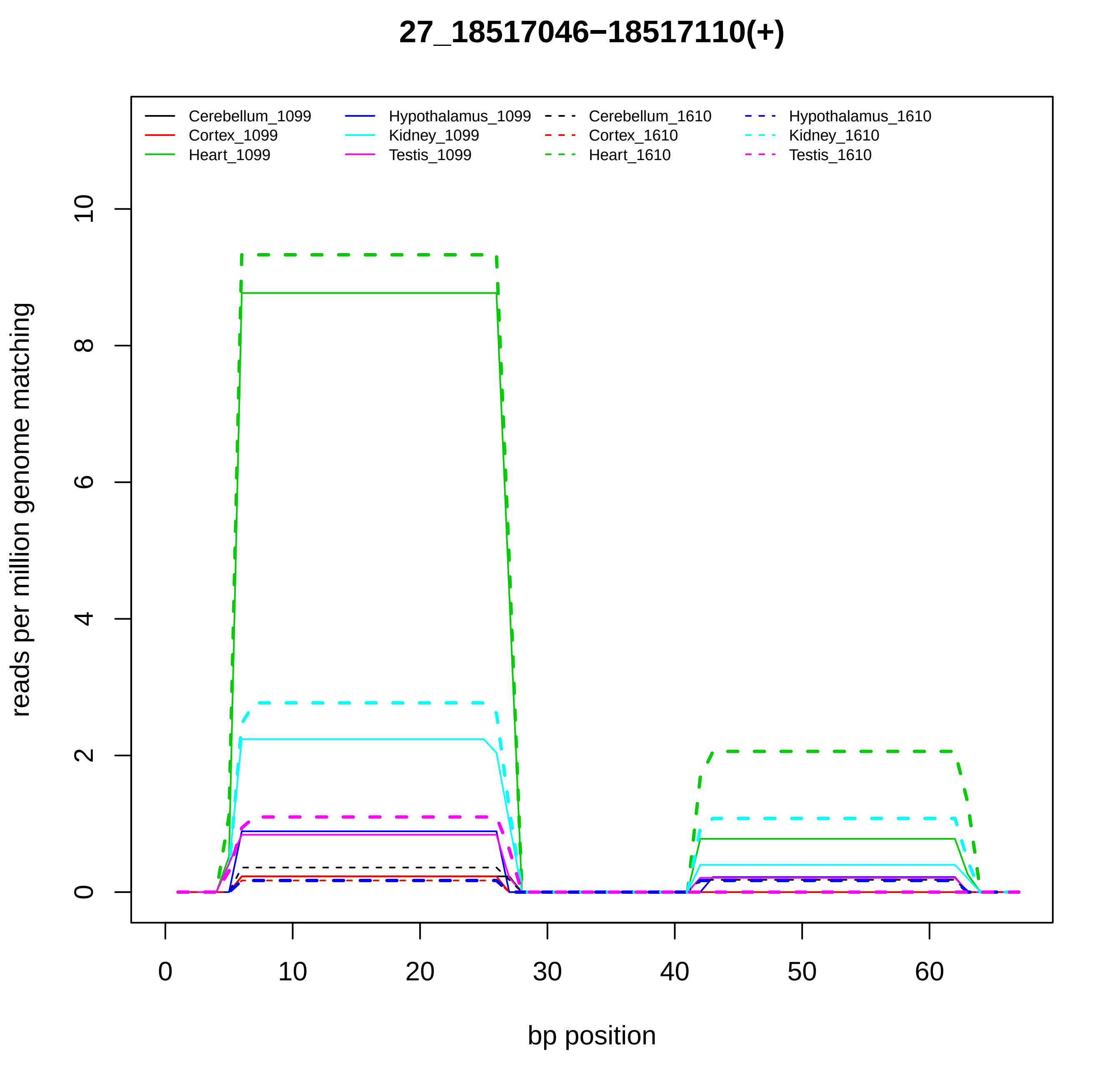

27_18517046−18517110(+)
Cerebellum_1099
Cortex_1099
Heart_1099
Hypothalamus_1099
Kidney_1099
Testis_1099
Cerebellum_1610
Cortex_1610
Heart_1610
Hypothalamus_1610
Kidney_1610
Testis_1610
reads per million genome matching
bp position
0
2
4
6
8
10
0
10
20
30
40
50
60

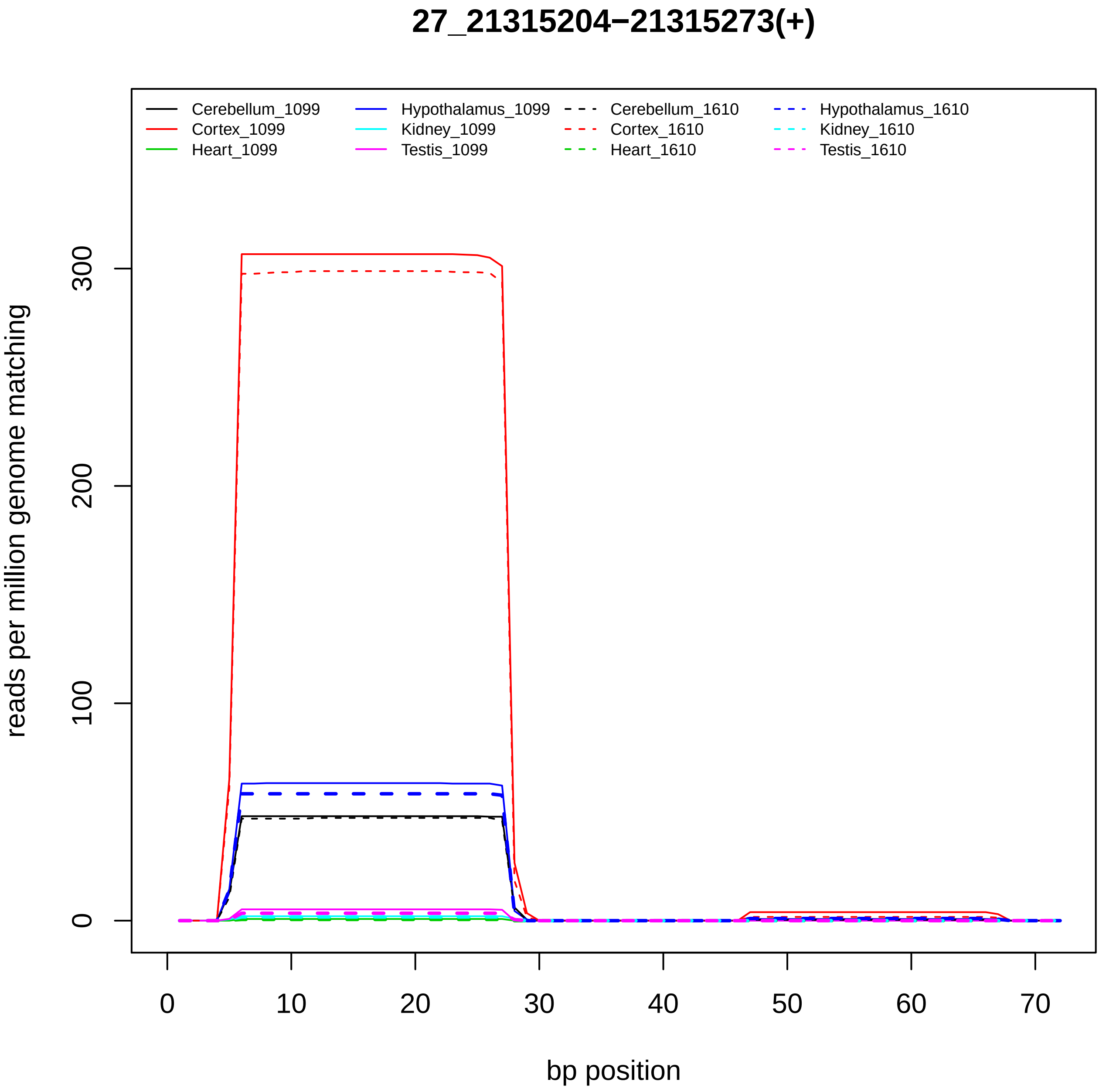
27_21315204−21315273(+)
Cerebellum_1099
Cortex_1099
Heart_1099
Hypothalamus_1099
Kidney_1099
Testis_1099
Cerebellum_1610
Cortex_1610
Heart_1610
Hypothalamus_1610
Kidney_1610
Testis_1610
reads per million genome matching
0
100
200
300
0
10
20
30
40
50
60
70
bp position

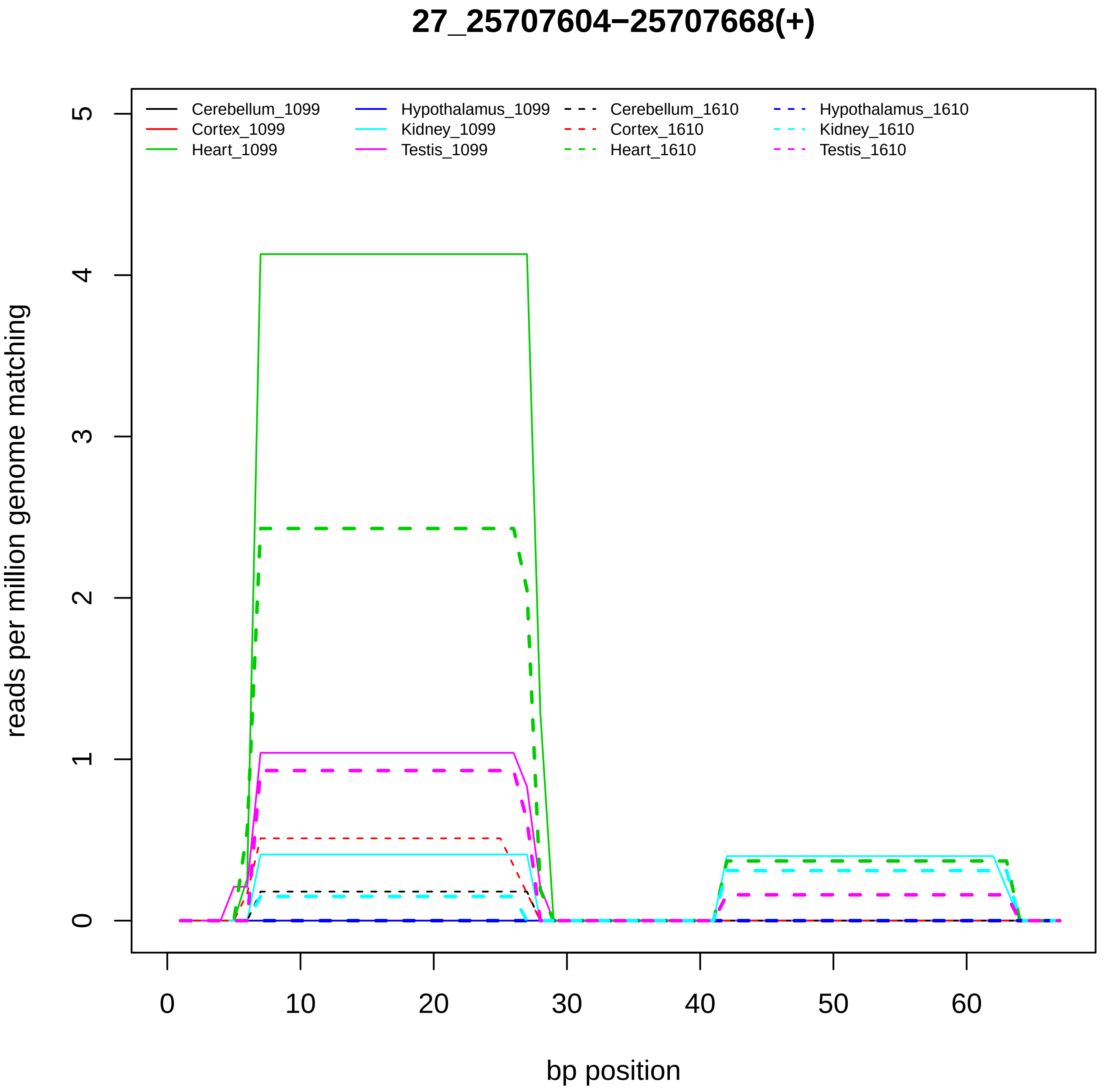
27_25707604−25707668(+)
Cerebellum_1099
Cortex_1099
Heart_1099
Hypothalamus_1099
Kidney_1099
Testis_1099
Cerebellum_1610
Cortex_1610
Heart_1610
Hypothalamus_1610
Kidney_1610
Testis_1610
reads per million genome matching
bp position
0
1
2
3
4
5
0
10
20
30
40
50
60

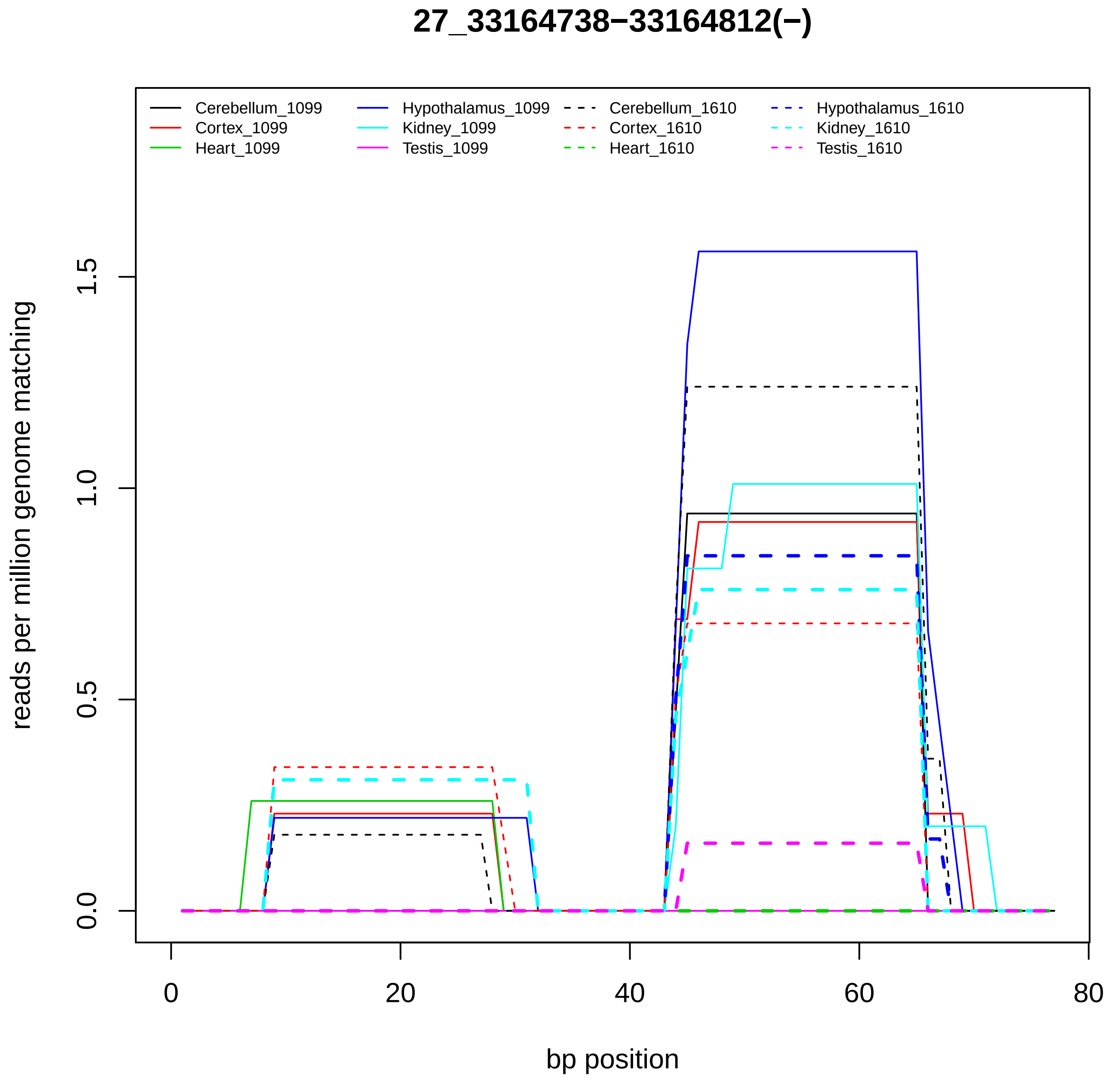

27_33164738−33164812(−)
Cerebellum_1099
Cortex_1099
Heart_1099
Hypothalamus_1099
Kidney_1099
Testis_1099
Cerebellum_1610
Cortex_1610
Heart_1610
Hypothalamus_1610
Kidney_1610
Testis_1610
reads per million genome matching
0.0
0.5
1.0
1.5
0
20
40
60
80
bp position

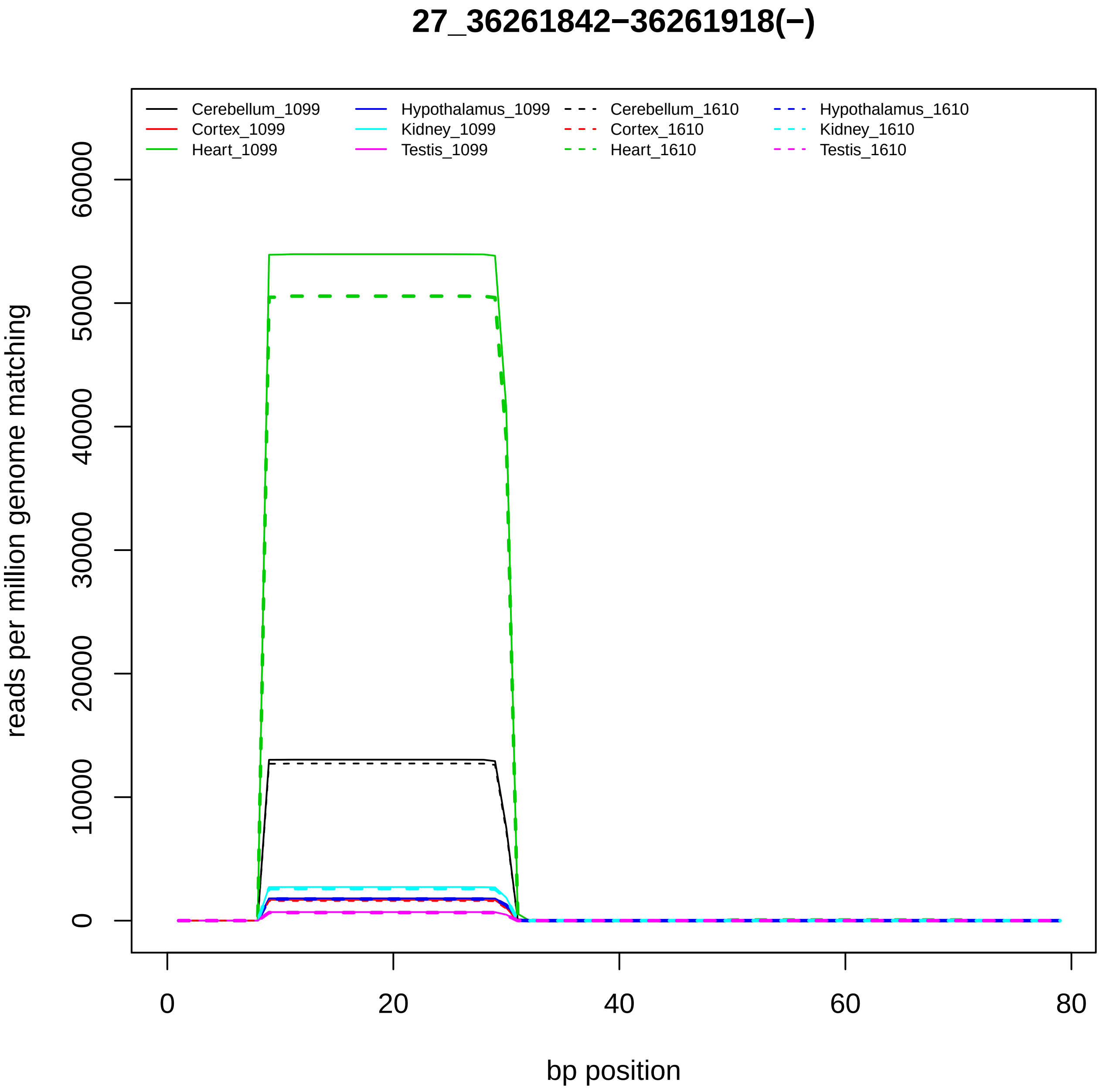

27_36261842−36261918(−)
Cerebellum_1099
Cortex_1099
Heart_1099
Hypothalamus_1099
Kidney_1099
Testis_1099
Cerebellum_1610
Cortex_1610
Heart_1610
Hypothalamus_1610
Kidney_1610
Testis_1610
reads per million genome matching
0
10000
20000
30000
40000
50000
60000
bp position
0
20
40
60
80

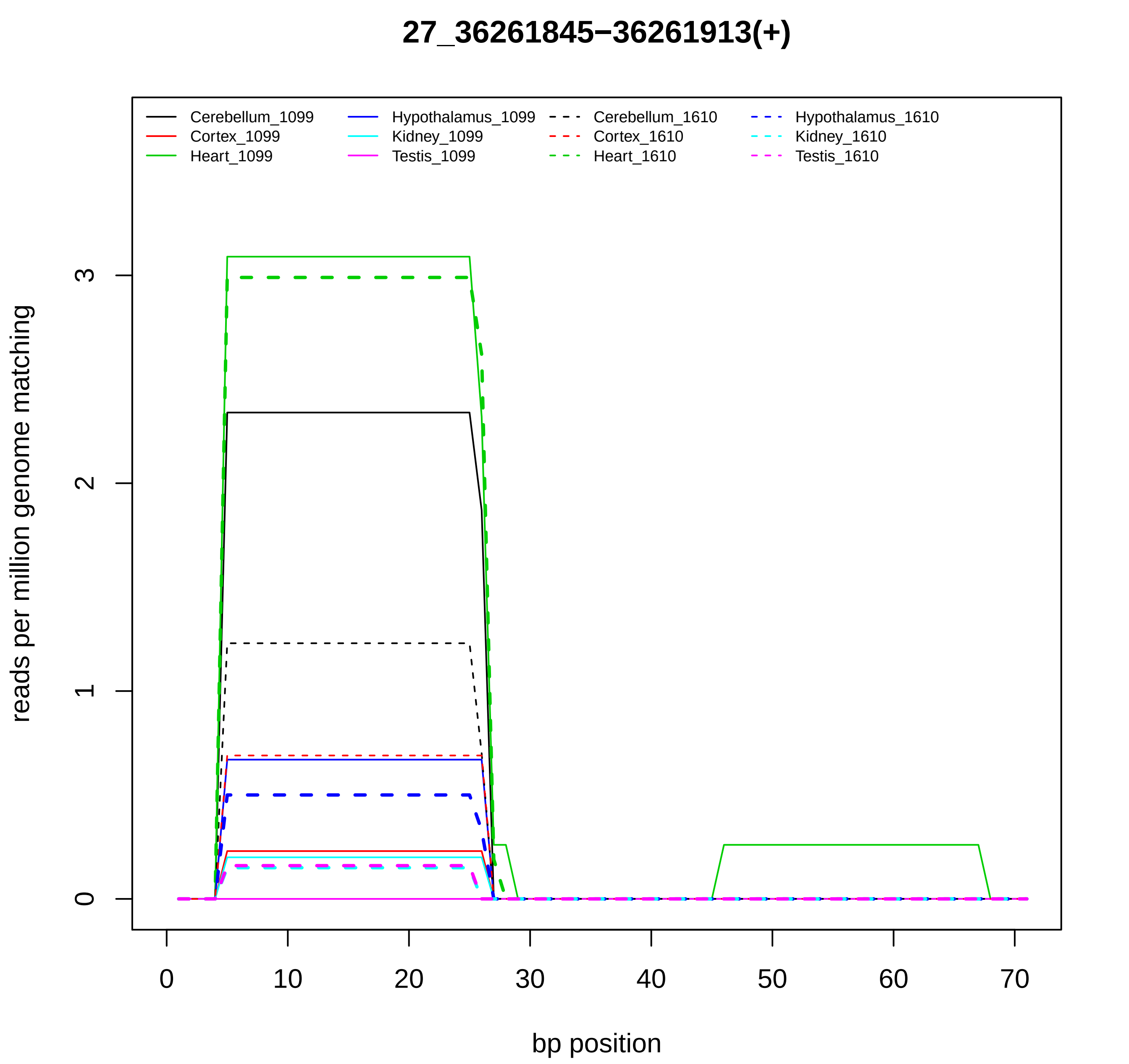
27_36261845−36261913(+)
Cerebellum_1099
Cortex_1099
Heart_1099
Hypothalamus_1099
Kidney_1099
Testis_1099
Cerebellum_1610
Cortex_1610
Heart_1610
Hypothalamus_1610
Kidney_1610
Testis_1610
reads per million genome matching
bp position
0
1
2
3
0
10
20
30
40
50
60
70

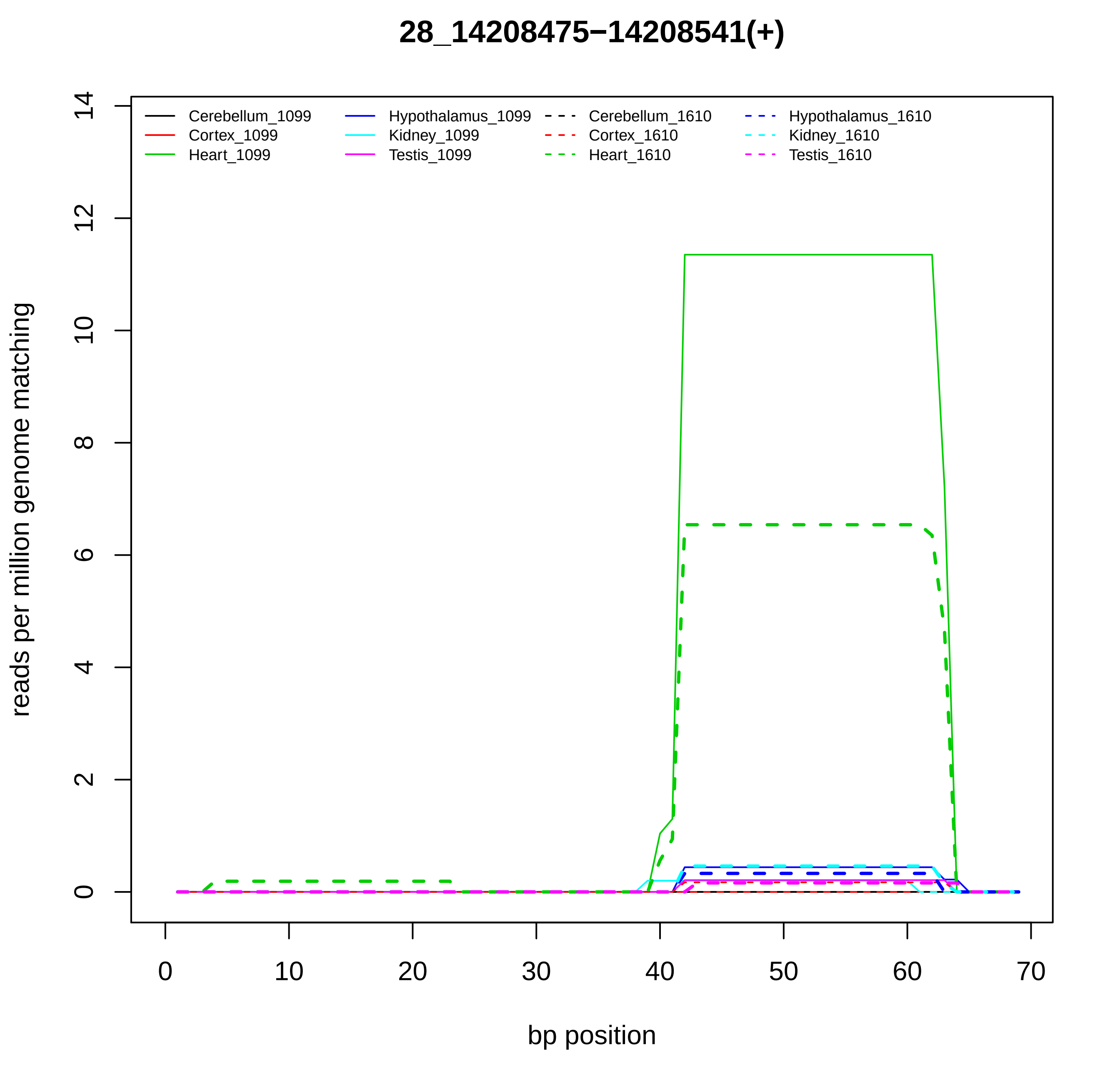

28_14208475−14208541(+)
Cerebellum_1099
Cortex_1099
Heart_1099
Hypothalamus_1099
Kidney_1099
Testis_1099
Cerebellum_1610
Cortex_1610
Heart_1610
Hypothalamus_1610
Kidney_1610
Testis_1610
reads per million genome matching
bp position
0
2
4
6
8
10
12
14
0
10
20
30
40
50
60
70

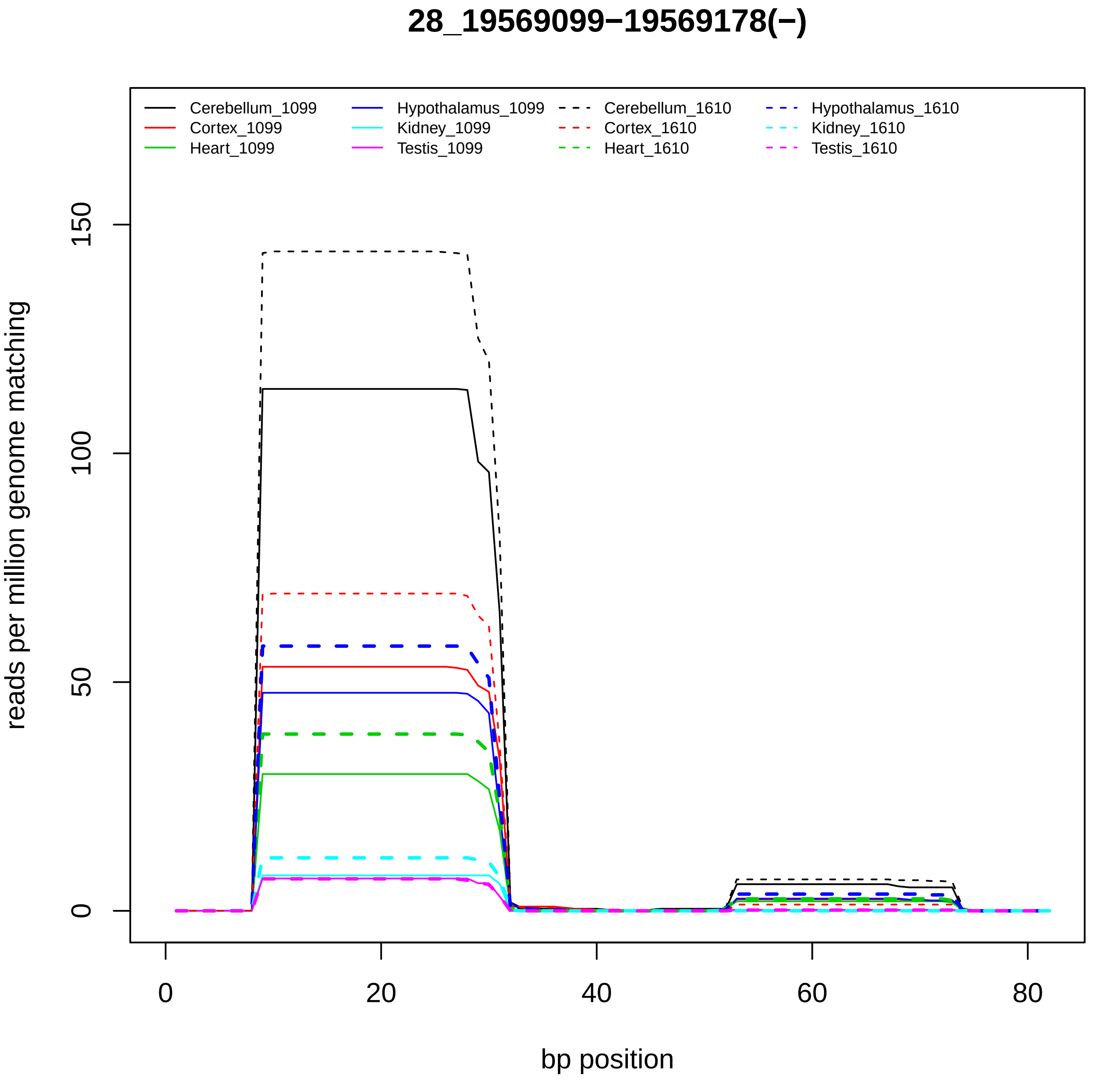
28_19569099−19569178(−)
Cerebellum_1099
Cortex_1099
Heart_1099
Hypothalamus_1099
Kidney_1099
Testis_1099
Cerebellum_1610
Cortex_1610
Heart_1610
Hypothalamus_1610
Kidney_1610
Testis_1610
reads per million genome matching
0
50
100
150
0
20
40
60
80
bp position

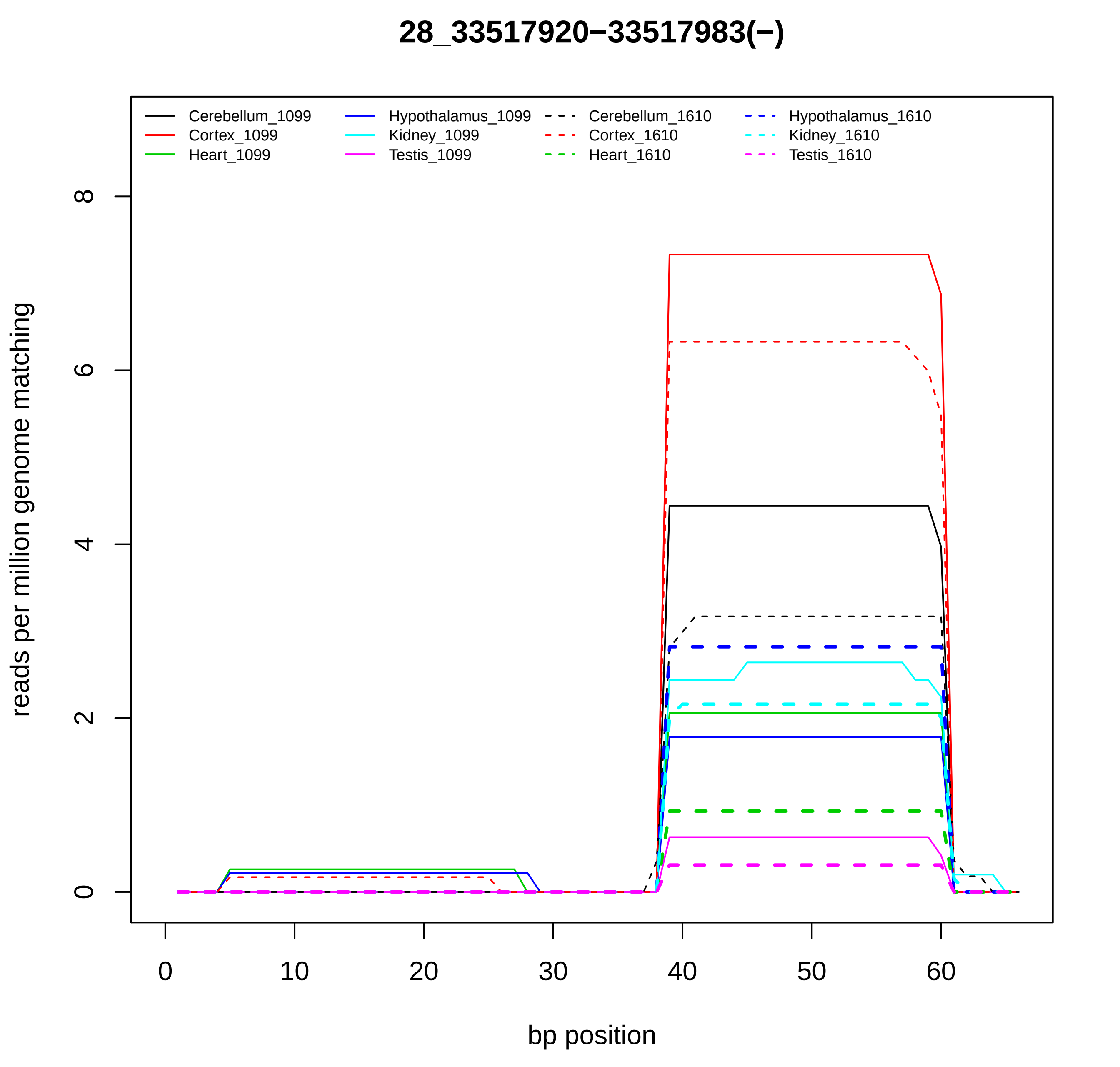

28_33517920−33517983(−)
Cerebellum_1099
Cortex_1099
Heart_1099
Hypothalamus_1099
Kidney_1099
Testis_1099
Cerebellum_1610
Cortex_1610
Heart_1610
Hypothalamus_1610
Kidney_1610
Testis_1610
reads per million genome matching
0
2
4
6
8
0
10
20
30
40
50
60
bp position

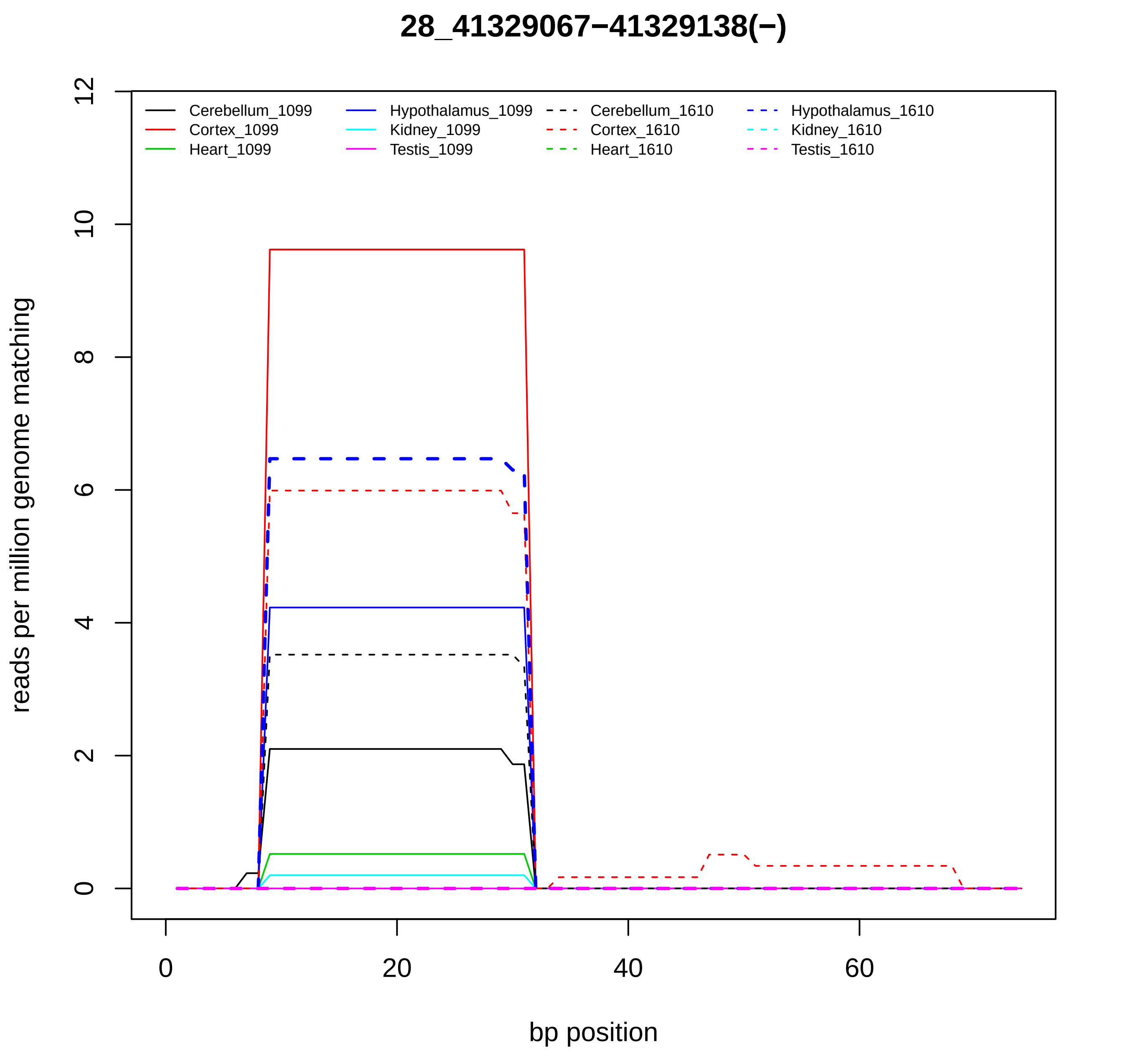

28_41329067−41329138(−)
reads per million genome matching
bp position
0
2
4
6
8
10
12
0
20
40
60
Cerebellum_1099
Cortex_1099
Heart_1099
Hypothalamus_1099
Kidney_1099
Testis_1099
Cerebellum_1610
Cortex_1610
Heart_1610
Hypothalamus_1610
Kidney_1610
Testis_1610

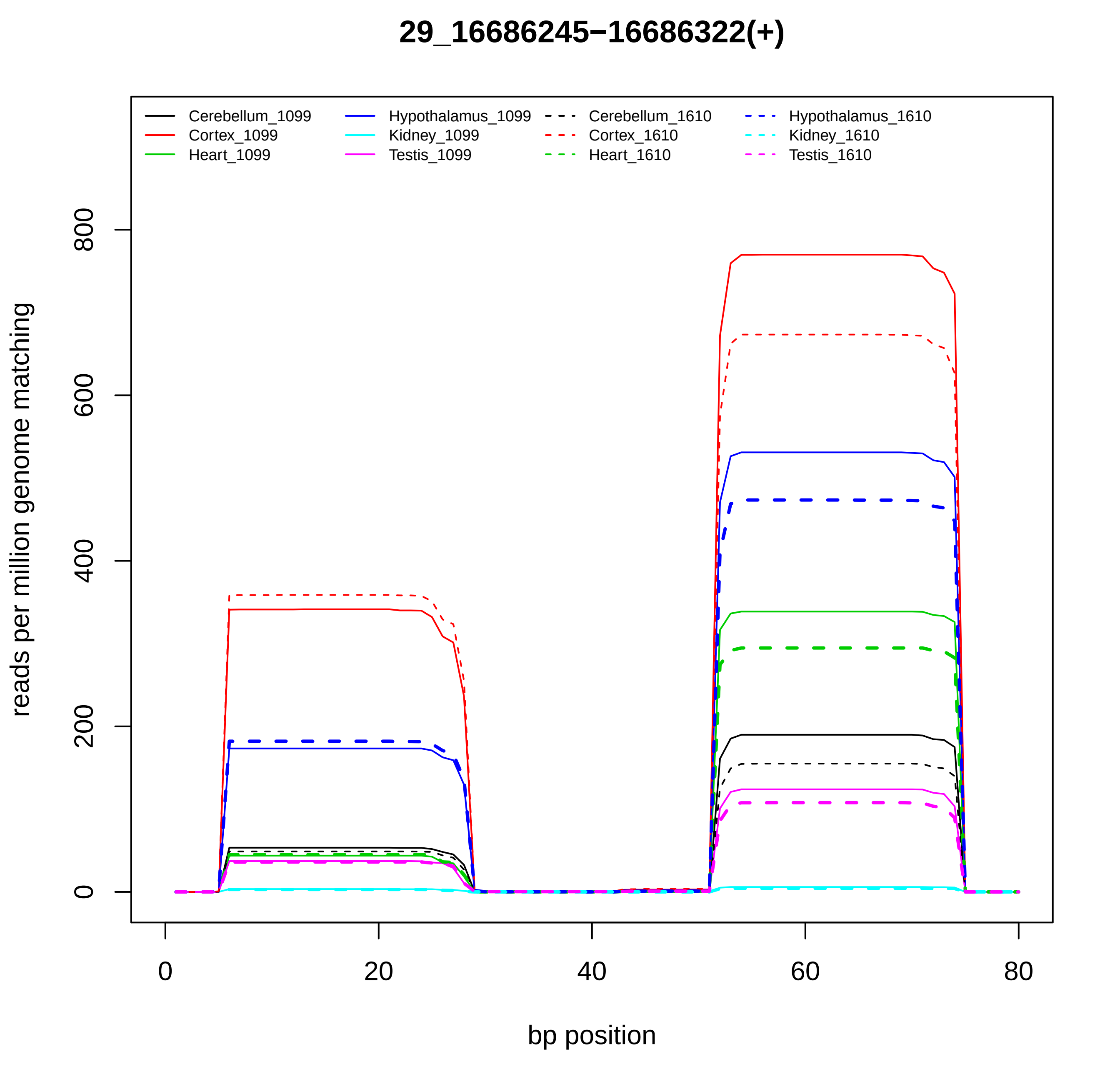
29_16686245−16686322(+)
Cerebellum_1099
Cortex_1099
Heart_1099
Hypothalamus_1099
Kidney_1099
Testis_1099
Cerebellum_1610
Cortex_1610
Heart_1610
Hypothalamus_1610
Kidney_1610
Testis_1610
reads per million genome matching
0
200
400
600
800
bp position
0
20
40
60
80

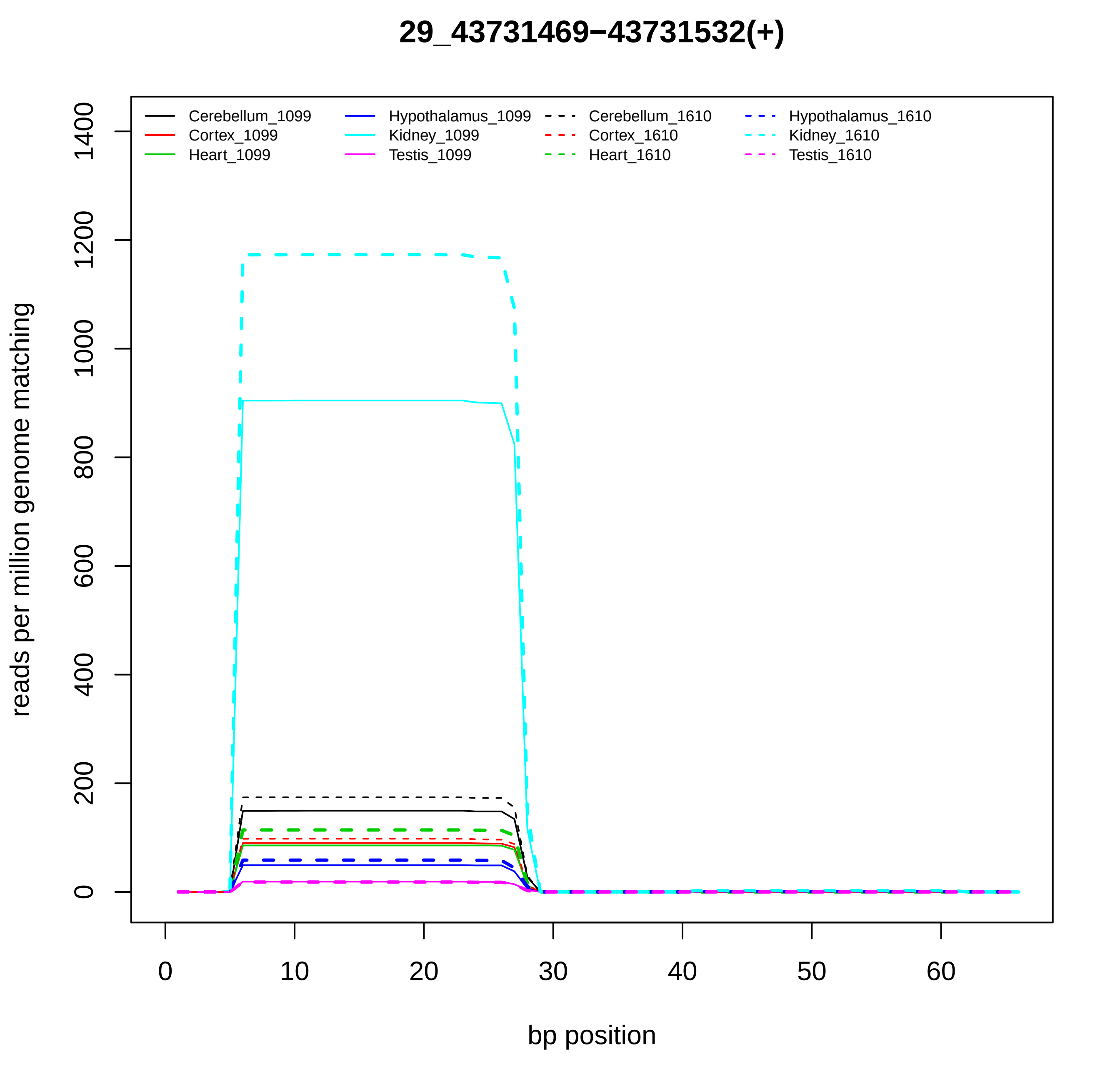

29_43731469−43731532(+)
Cerebellum_1099
Cortex_1099
Heart_1099
Hypothalamus_1099
Kidney_1099
Testis_1099
Cerebellum_1610
Cortex_1610
Heart_1610
Hypothalamus_1610
Kidney_1610
Testis_1610
reads per million genome matching
0
200
400
600
800
1000
1200
1400
bp position
0
10
20
30
40
50
60

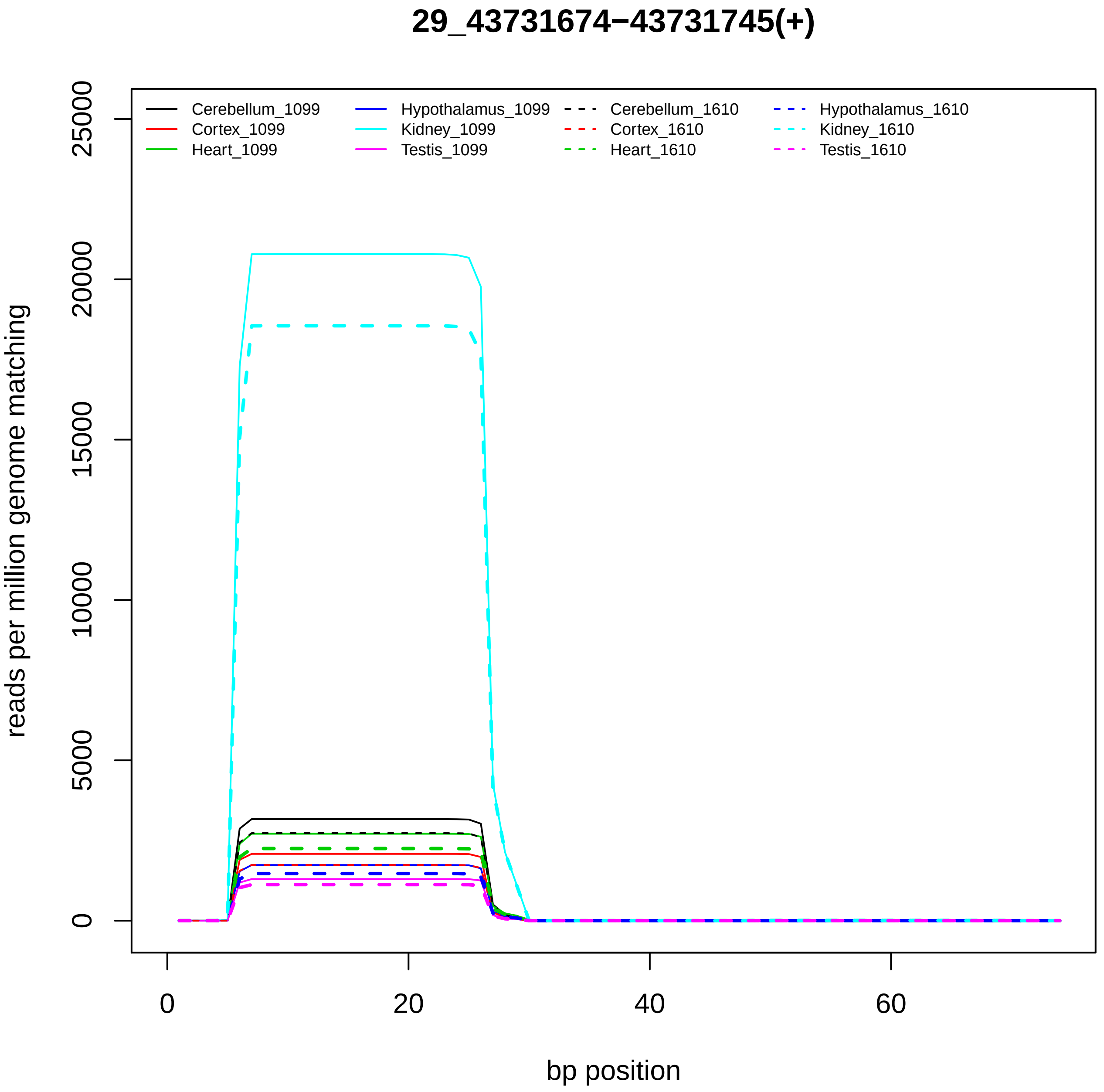

29_43731674−43731745(+)
Cerebellum_1099
Cortex_1099
Heart_1099
Hypothalamus_1099
Kidney_1099
Testis_1099
Cerebellum_1610
Cortex_1610
Heart_1610
Hypothalamus_1610
Kidney_1610
Testis_1610
reads per million genome matching
0
5000
10000
15000
20000
25000
bp position
0
20
40
60

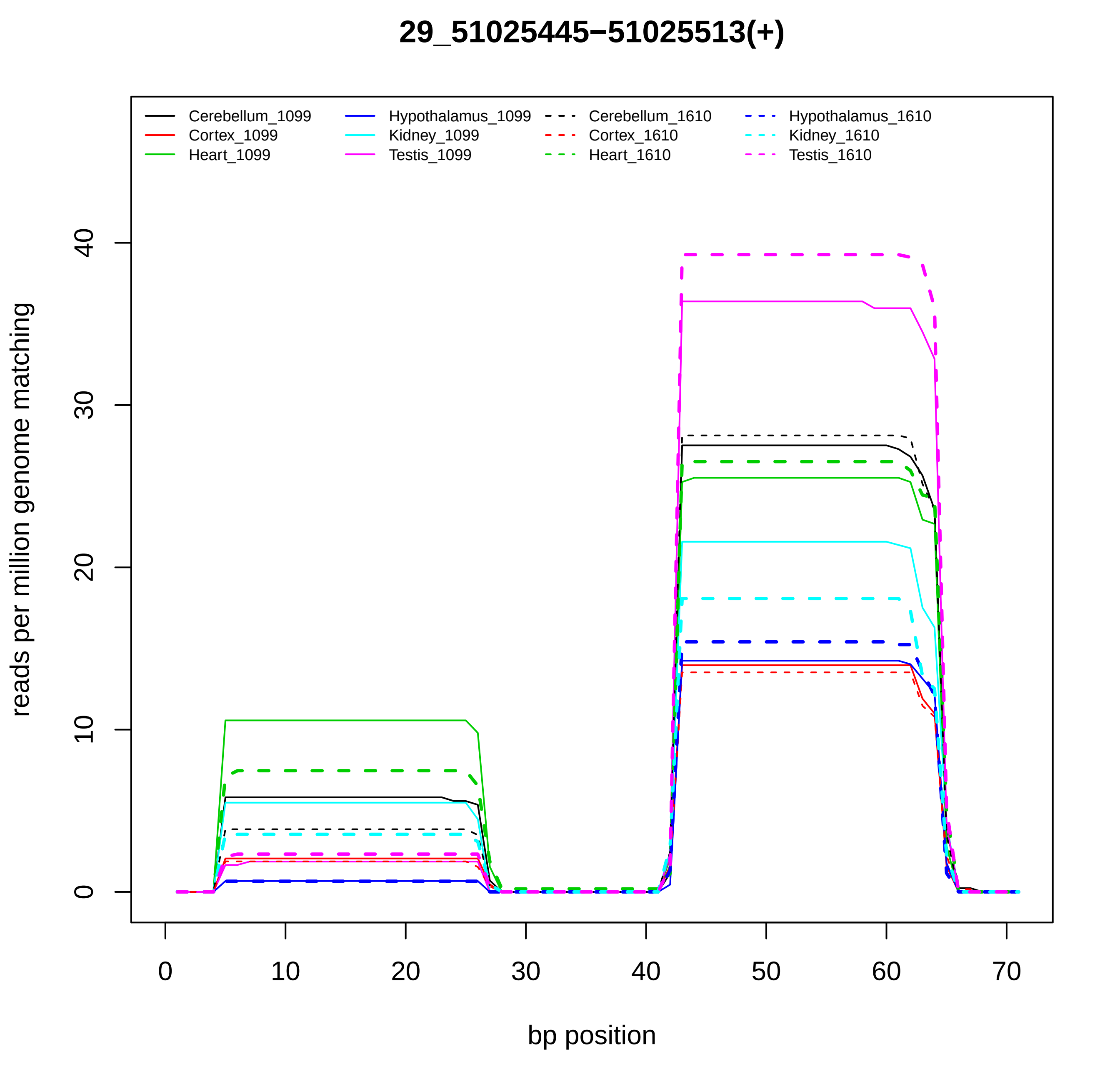

29_51025445−51025513(+)
Cerebellum_1099
Cortex_1099
Heart_1099
Hypothalamus_1099
Kidney_1099
Testis_1099
Cerebellum_1610
Cortex_1610
Heart_1610
Hypothalamus_1610
Kidney_1610
Testis_1610
reads per million genome matching
0
10
20
30
40
bp position
0
10
20
30
40
50
60
70

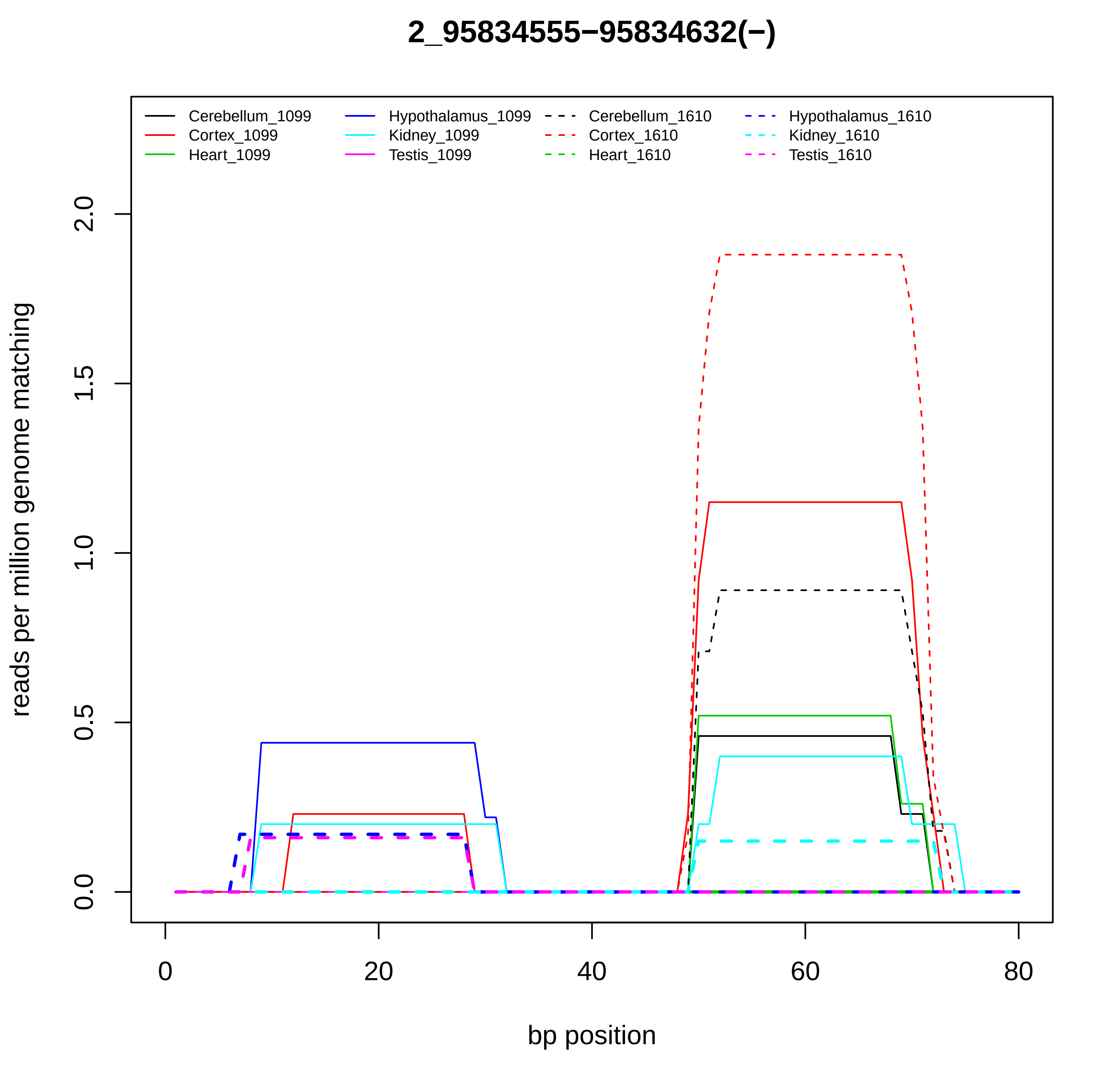

2_95834555−95834632(−)
Cerebellum_1099
Cortex_1099
Heart_1099
Hypothalamus_1099
Kidney_1099
Testis_1099
Cerebellum_1610
Cortex_1610
Heart_1610
Hypothalamus_1610
Kidney_1610
Testis_1610
reads per million genome matching
0.0
0.5
1.0
1.5
2.0
0
20
40
60
80
bp position

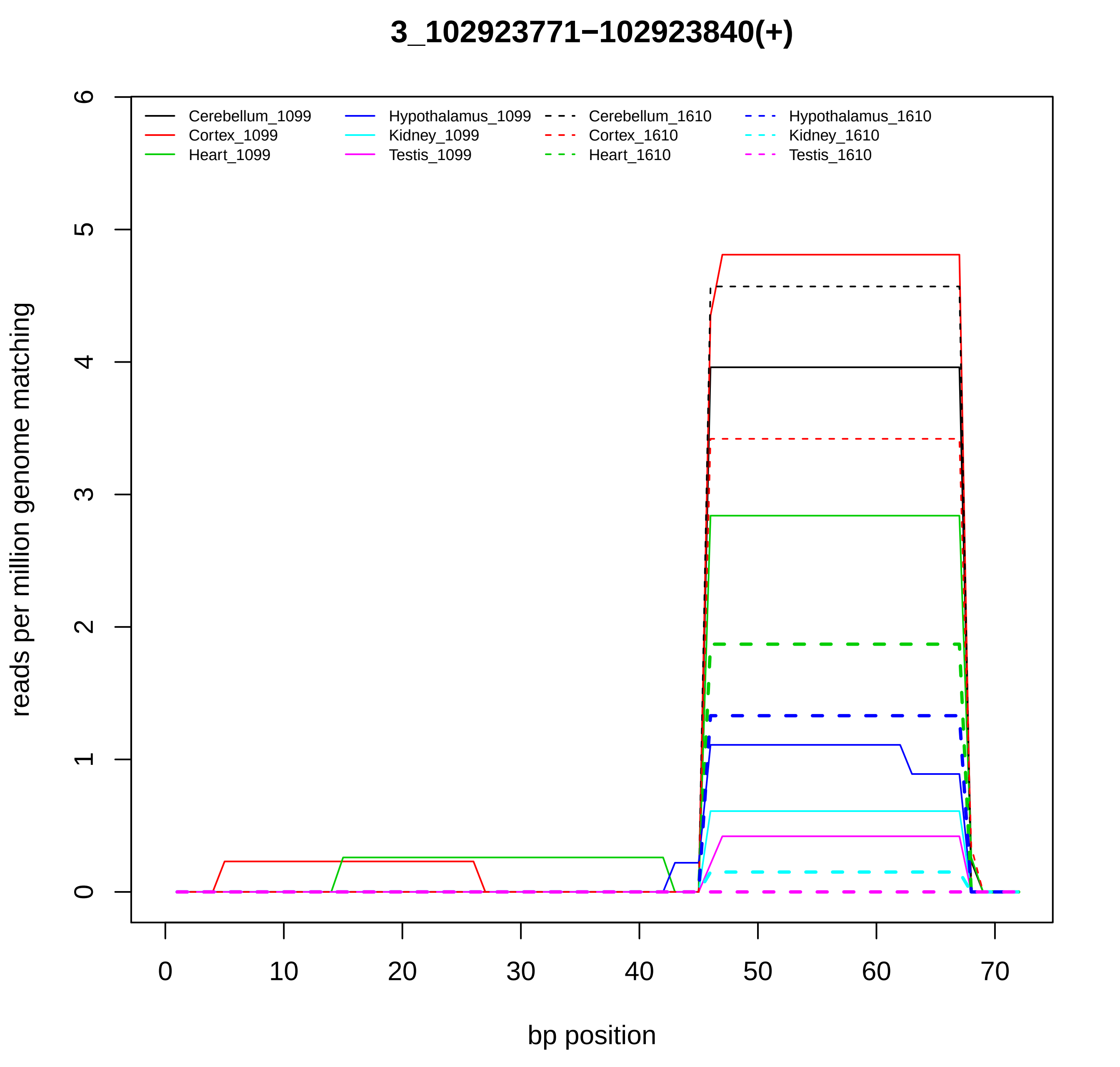
3_102923771−102923840(+)
Cerebellum_1099
Cortex_1099
Heart_1099
Hypothalamus_1099
Kidney_1099
Testis_1099
Cerebellum_1610
Cortex_1610
Heart_1610
Hypothalamus_1610
Kidney_1610
Testis_1610
reads per million genome matching
bp position
0
1
2
3
4
5
6
0
10
20
30
40
50
60
70

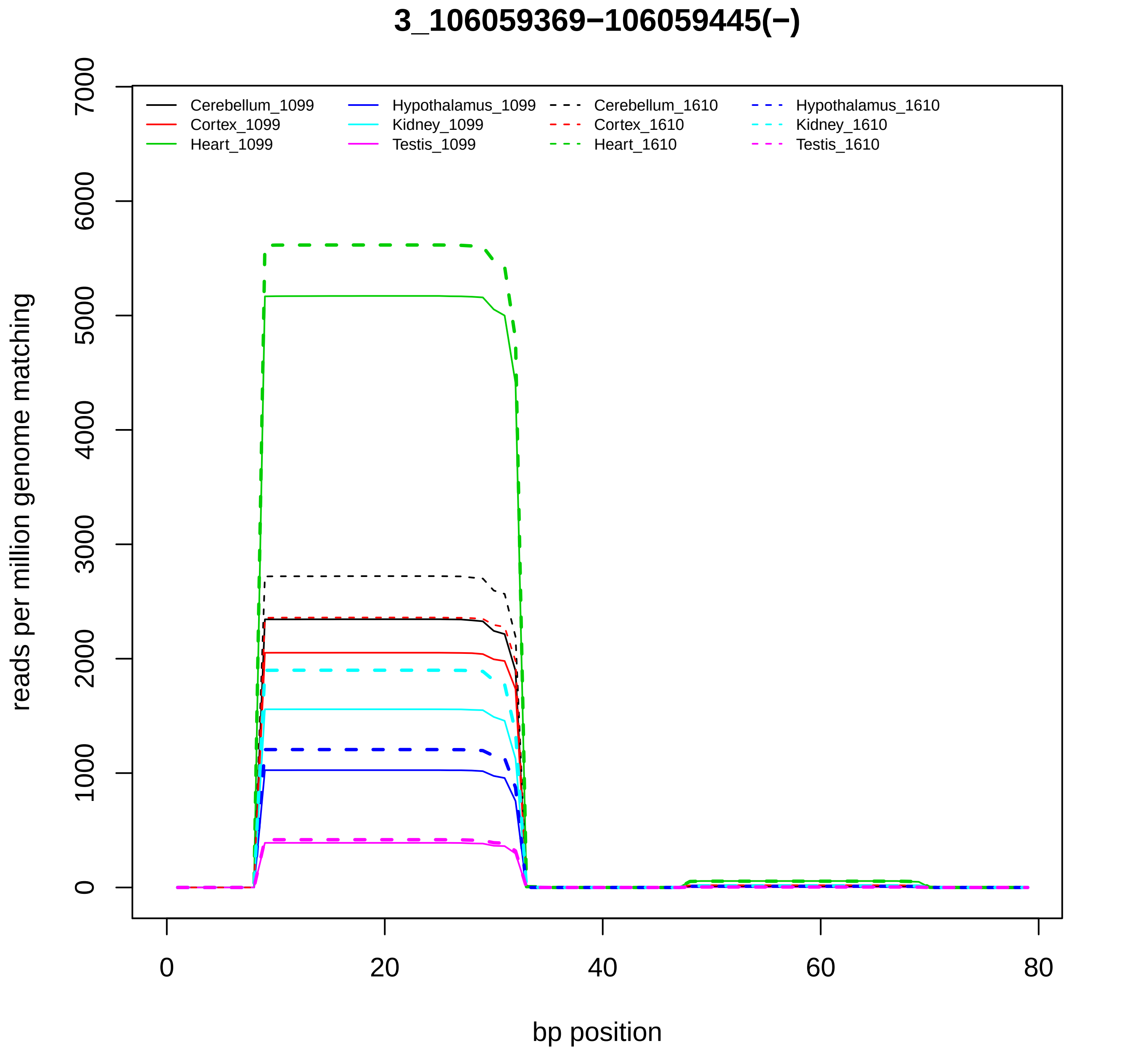
3_106059369−106059445(−)
Cerebellum_1099
Cortex_1099
Heart_1099
Hypothalamus_1099
Kidney_1099
Testis_1099
Cerebellum_1610
Cortex_1610
Heart_1610
Hypothalamus_1610
Kidney_1610
Testis_1610
reads per million genome matching
bp position
0
1000
2000
3000
4000
5000
6000
7000
0
20
40
60
80

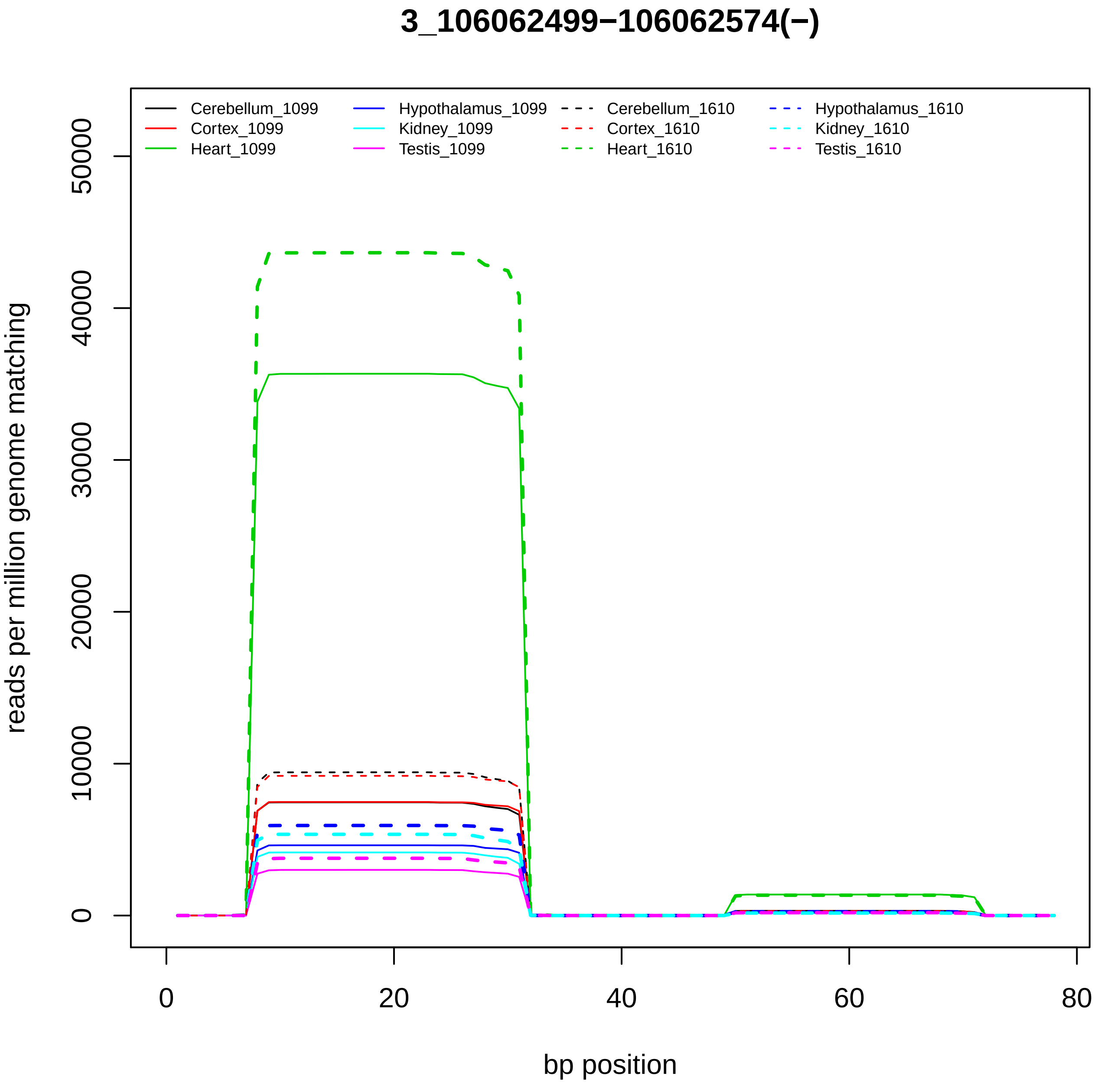

3_106062499−106062574(−)
Cerebellum_1099
Cortex_1099
Heart_1099
Hypothalamus_1099
Kidney_1099
Testis_1099
Cerebellum_1610
Cortex_1610
Heart_1610
Hypothalamus_1610
Kidney_1610
Testis_1610
reads per million genome matching
0
10000
20000
30000
40000
50000
bp position
0
20
40
60
80

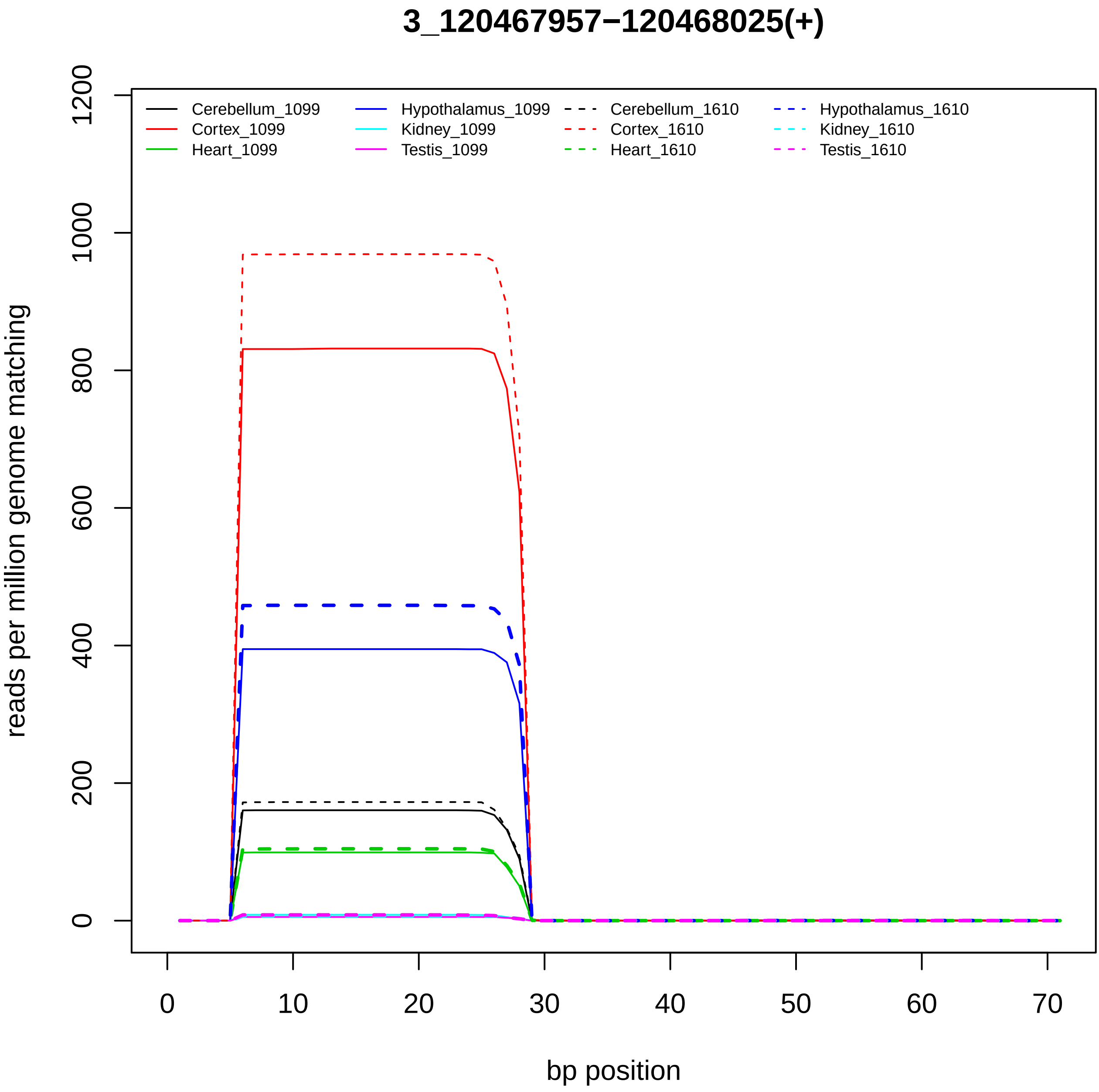

3_120467957−120468025(+)
Cerebellum_1099
Cortex_1099
Heart_1099
Hypothalamus_1099
Kidney_1099
Testis_1099
Cerebellum_1610
Cortex_1610
Heart_1610
Hypothalamus_1610
Kidney_1610
Testis_1610
reads per million genome matching
0
200
400
600
800
1000
1200
0
10
20
30
40
50
60
70
bp position

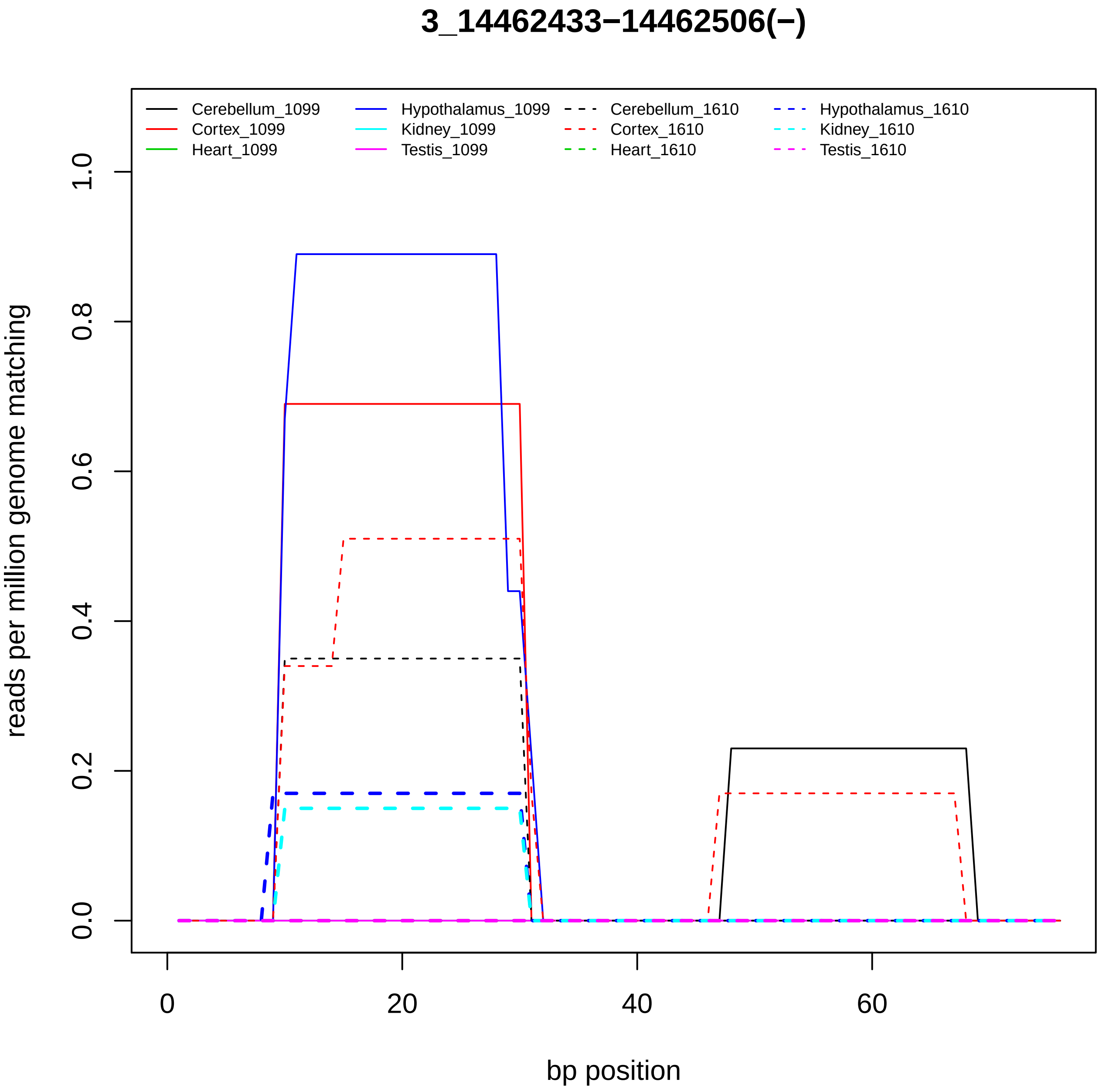

3_14462433−14462506(−)
Cerebellum_1099
Cortex_1099
Heart_1099
Hypothalamus_1099
Kidney_1099
Testis_1099
Cerebellum_1610
Cortex_1610
Heart_1610
Hypothalamus_1610
Kidney_1610
Testis_1610
reads per million genome matching
0.0
0.2
0.4
0.6
0.8
1.0
0
20
40
60
bp position

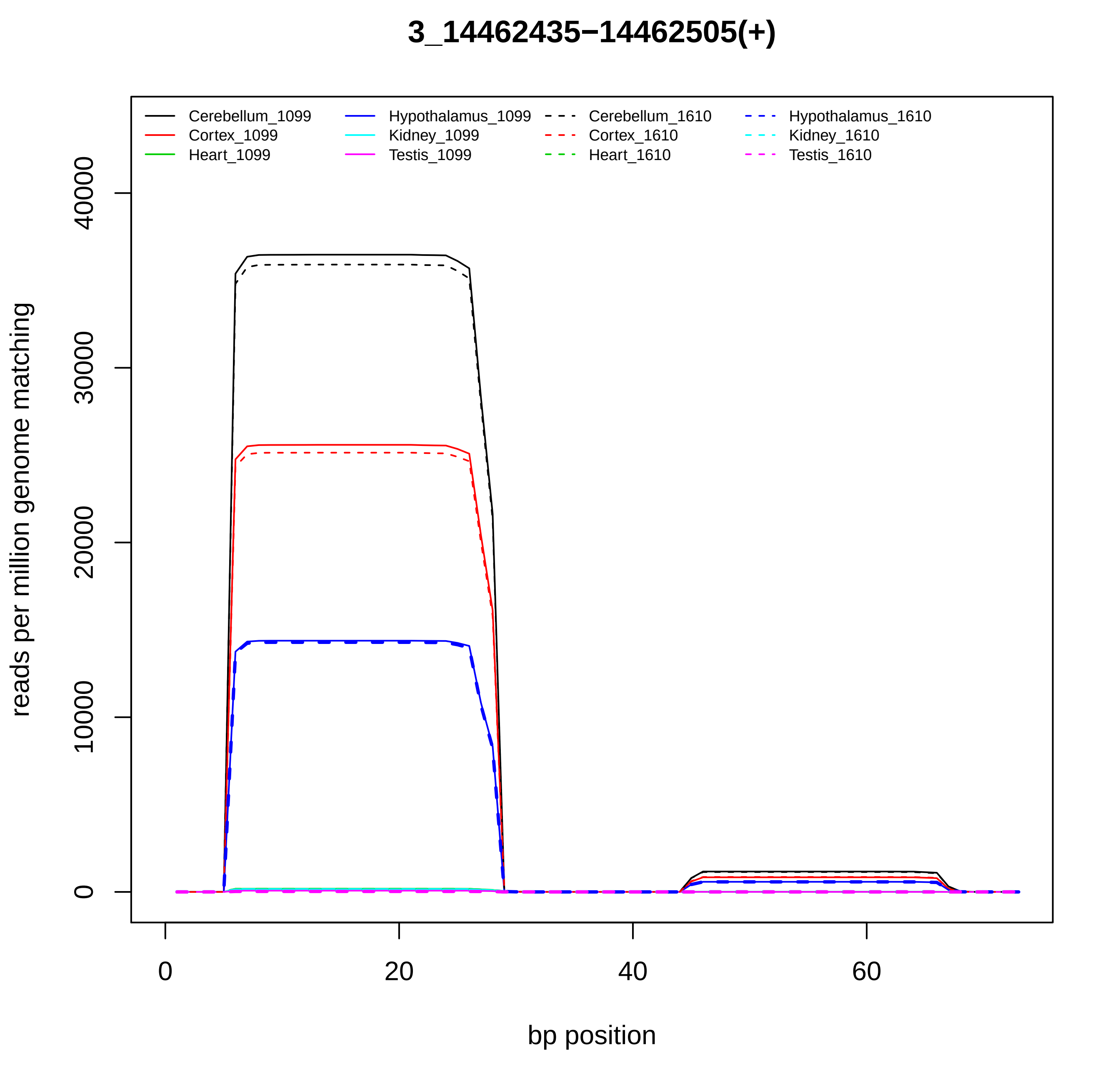

3_14462435−14462505(+)
Cerebellum_1099
Cortex_1099
Heart_1099
Hypothalamus_1099
Kidney_1099
Testis_1099
Cerebellum_1610
Cortex_1610
Heart_1610
Hypothalamus_1610
Kidney_1610
Testis_1610
reads per million genome matching
0
10000
20000
30000
40000
0
20
40
60
bp position

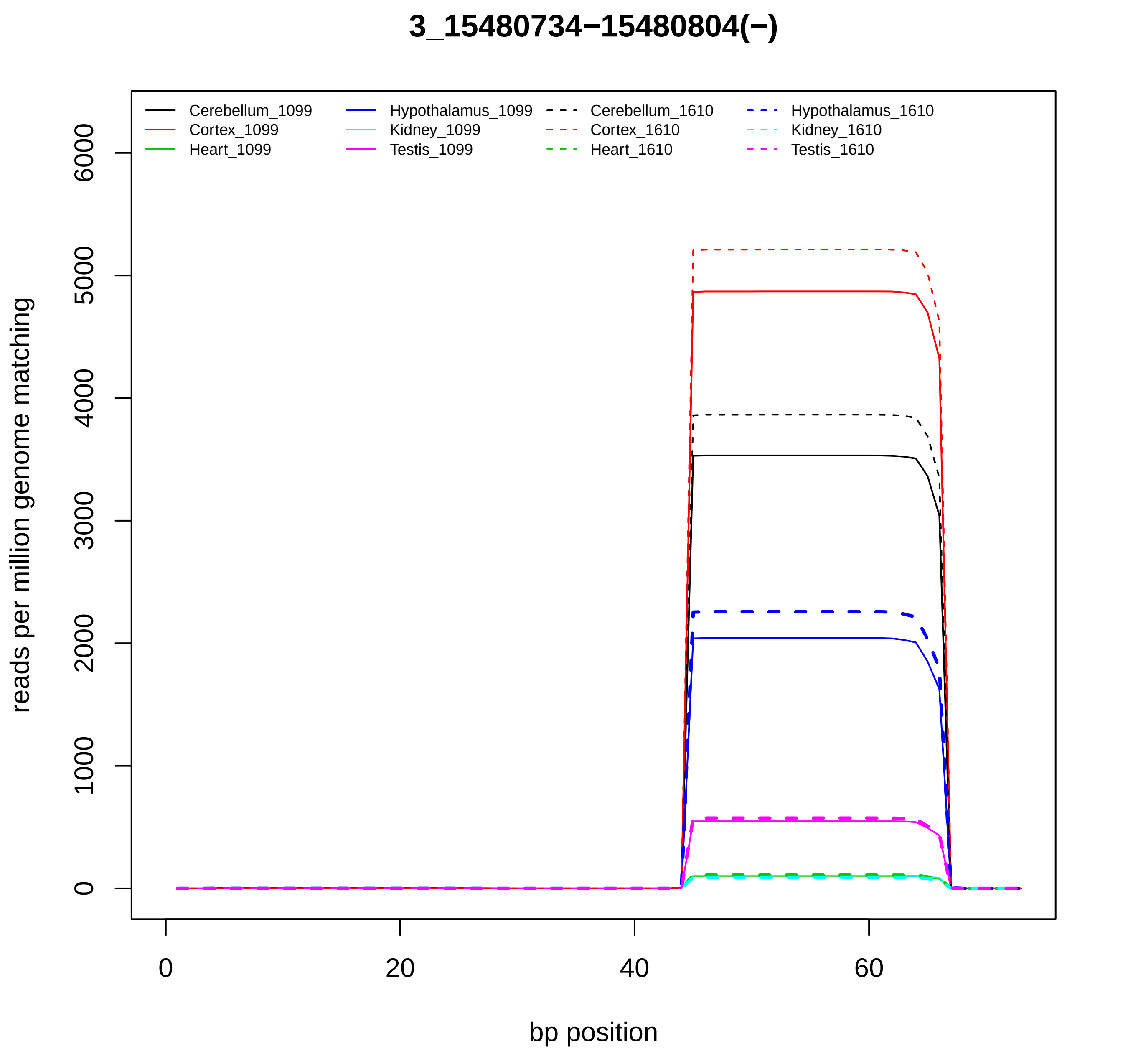

3_15480734−15480804(−)
Cerebellum_1099
Cortex_1099
Heart_1099
Hypothalamus_1099
Kidney_1099
Testis_1099
Cerebellum_1610
Cortex_1610
Heart_1610
Hypothalamus_1610
Kidney_1610
Testis_1610
reads per million genome matching
0
1000
2000
3000
4000
5000
6000
0
20
40
60
bp position

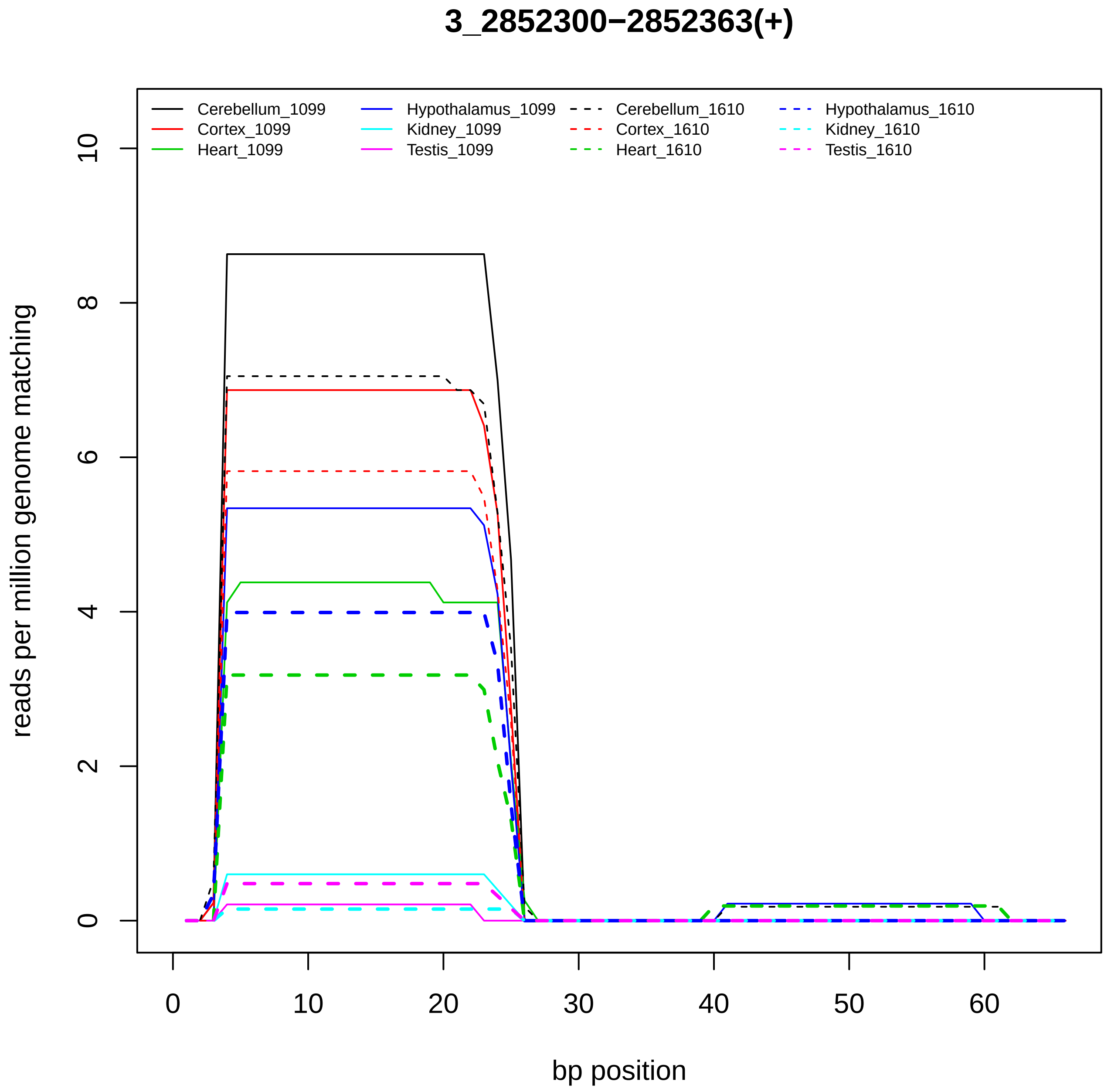

3_2852300−2852363(+)
Cerebellum_1099
Cortex_1099
Heart_1099
Hypothalamus_1099
Kidney_1099
Testis_1099
Cerebellum_1610
Cortex_1610
Heart_1610
Hypothalamus_1610
Kidney_1610
Testis_1610
reads per million genome matching
0
2
4
6
8
10
0
10
20
30
40
50
60
bp position

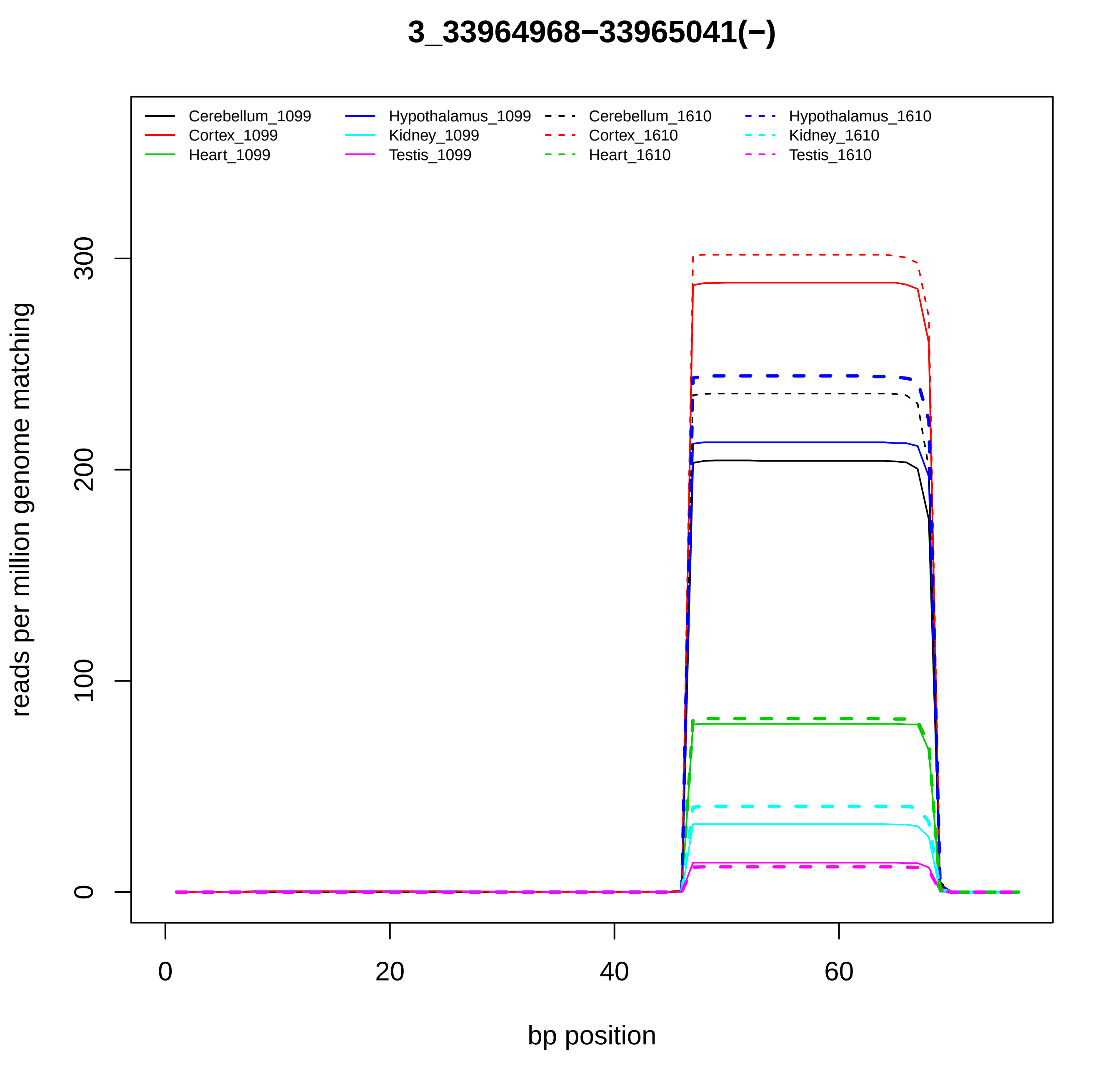

3_33964968−33965041(−)
Cerebellum_1099
Cortex_1099
Heart_1099
Hypothalamus_1099
Kidney_1099
Testis_1099
Cerebellum_1610
Cortex_1610
Heart_1610
Hypothalamus_1610
Kidney_1610
Testis_1610
reads per million genome matching
0
100
200
300
0
20
40
60
bp position

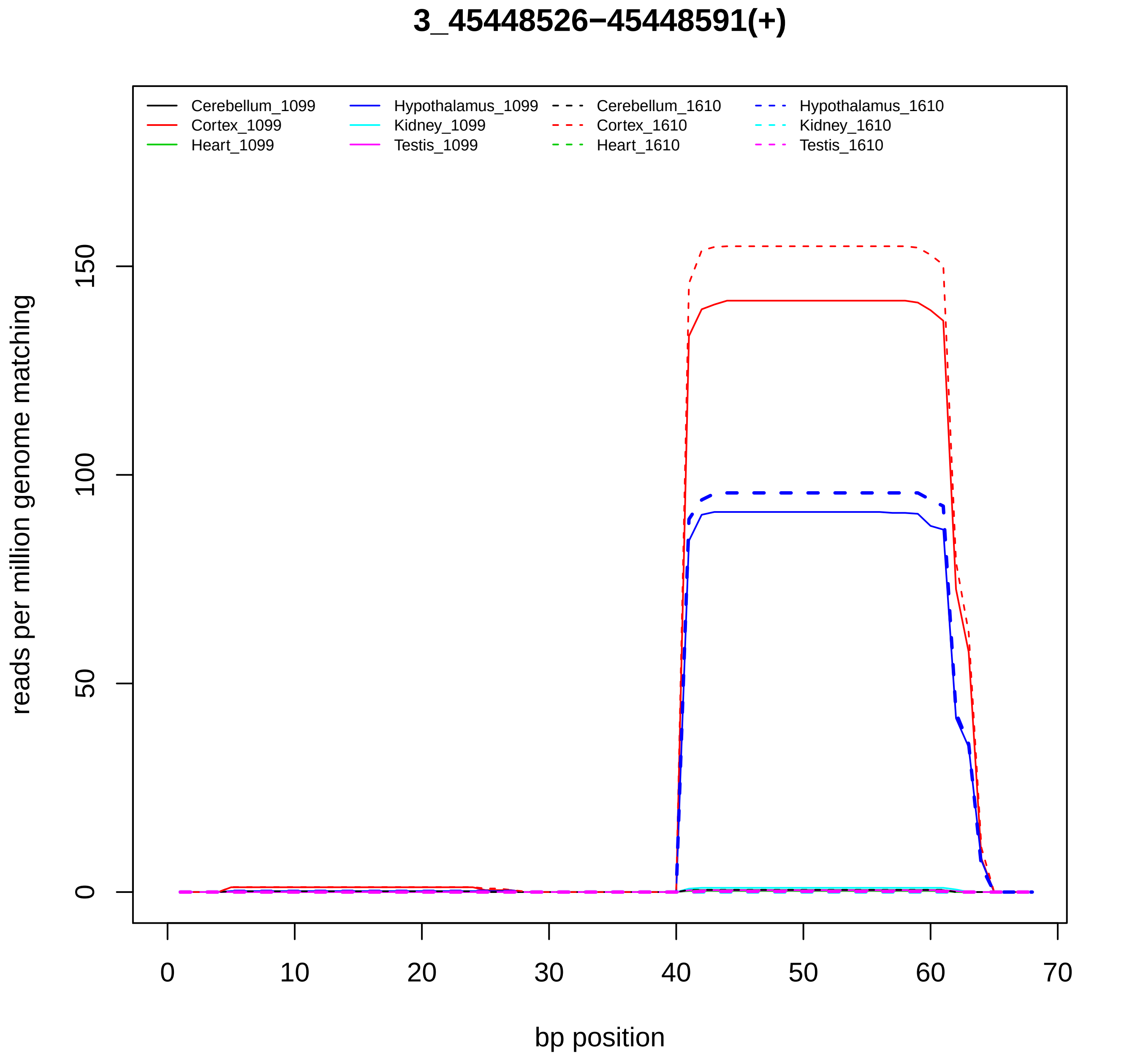

3_45448526−45448591(+)
Cerebellum_1099
Cortex_1099
Heart_1099
Hypothalamus_1099
Kidney_1099
Testis_1099
Cerebellum_1610
Cortex_1610
Heart_1610
Hypothalamus_1610
Kidney_1610
Testis_1610
reads per million genome matching
0
50
100
150
0
10
20
30
40
50
60
70
bp position

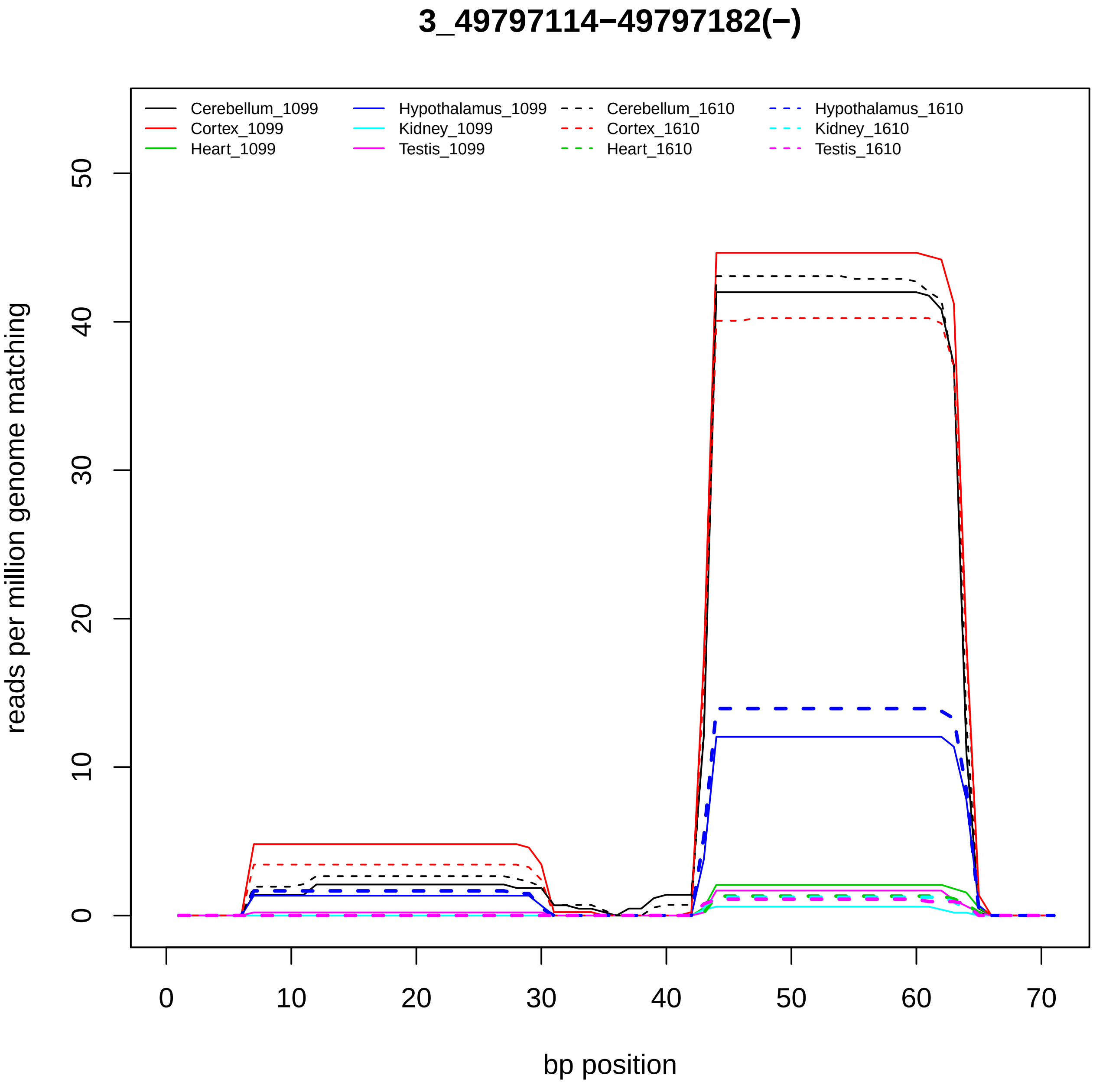

3_49797114−49797182(−)
Cerebellum_1099
Cortex_1099
Heart_1099
Hypothalamus_1099
Kidney_1099
Testis_1099
Cerebellum_1610
Cortex_1610
Heart_1610
Hypothalamus_1610
Kidney_1610
Testis_1610
reads per million genome matching
0
10
20
30
40
50
0
10
20
30
40
50
60
70
bp position

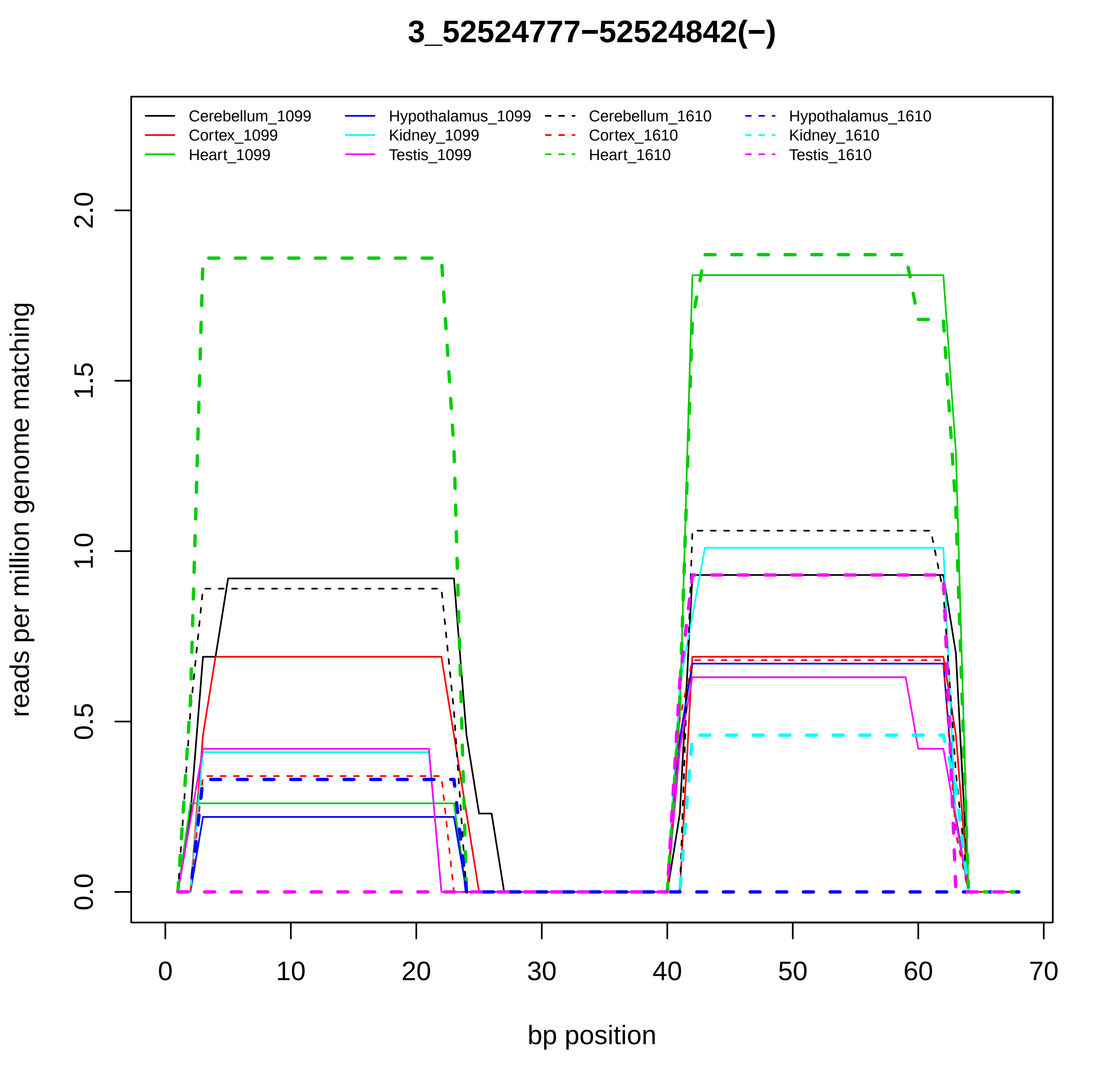

3_52524777−52524842(−)
Cerebellum_1099
Cortex_1099
Heart_1099
Hypothalamus_1099
Kidney_1099
Testis_1099
Cerebellum_1610
Cortex_1610
Heart_1610
Hypothalamus_1610
Kidney_1610
Testis_1610
reads per million genome matching
bp position
0.0
0.5
1.0
1.5
2.0
0
10
20
30
40
50
60
70

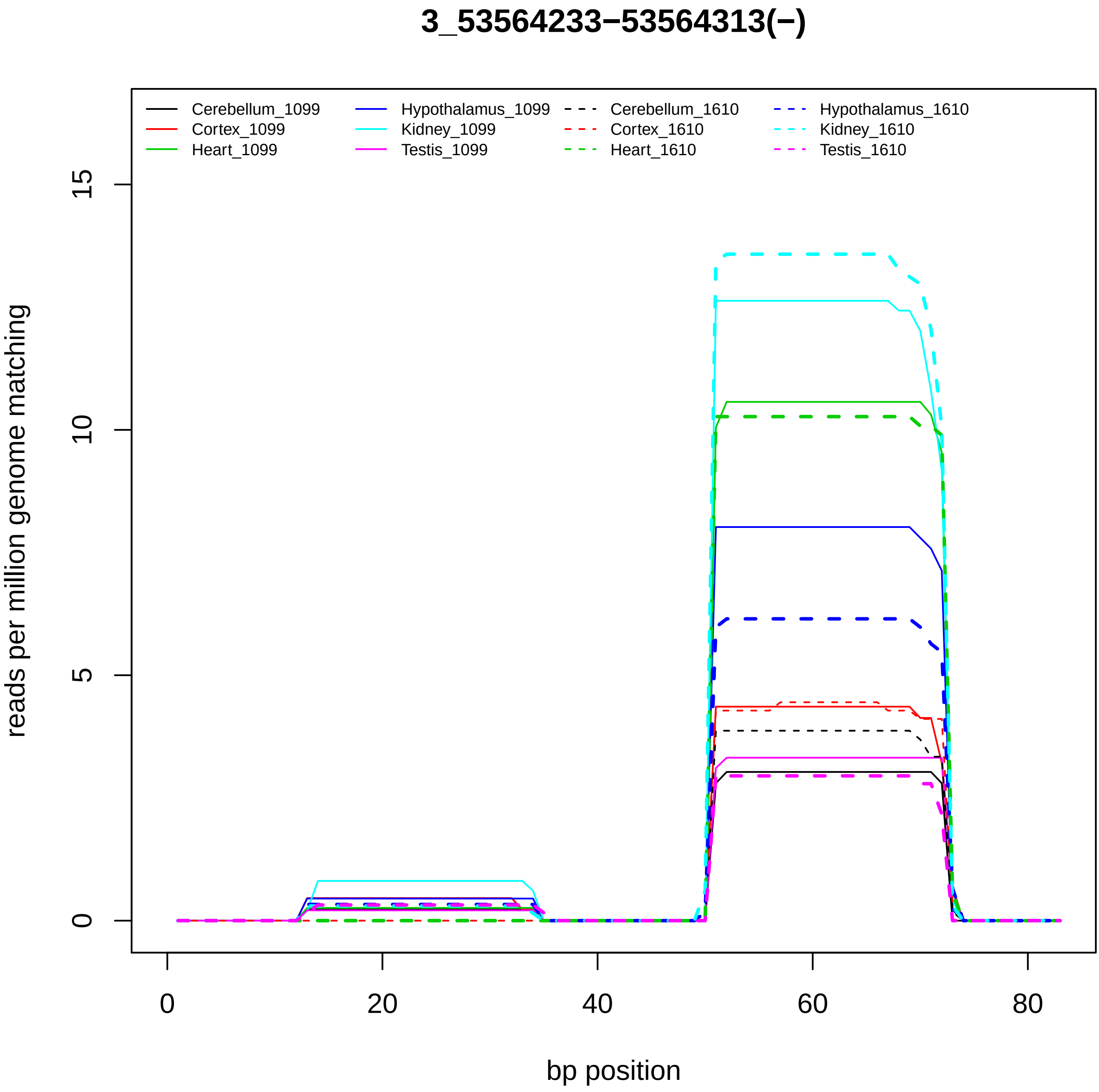

3_53564233−53564313(−)
Cerebellum_1099
Cortex_1099
Heart_1099
Hypothalamus_1099
Kidney_1099
Testis_1099
Cerebellum_1610
Cortex_1610
Heart_1610
Hypothalamus_1610
Kidney_1610
Testis_1610
reads per million genome matching
bp position
0
5
10
15
0
20
40
60
80

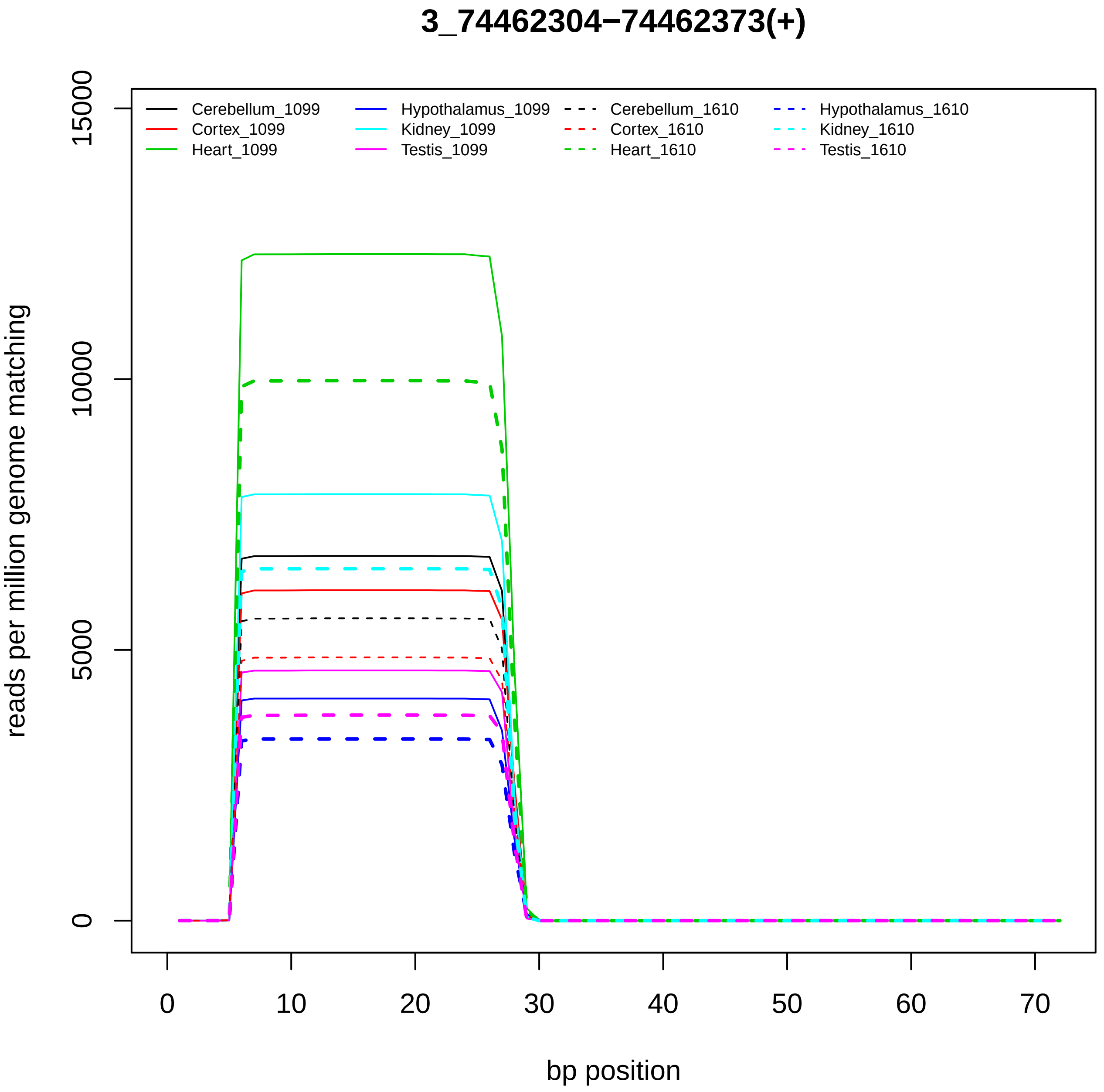

3_74462304−74462373(+)
Cerebellum_1099
Cortex_1099
Heart_1099
Hypothalamus_1099
Kidney_1099
Testis_1099
Cerebellum_1610
Cortex_1610
Heart_1610
Hypothalamus_1610
Kidney_1610
Testis_1610
reads per million genome matching
bp position
0
5000
10000
15000
0
10
20
30
40
50
60
70

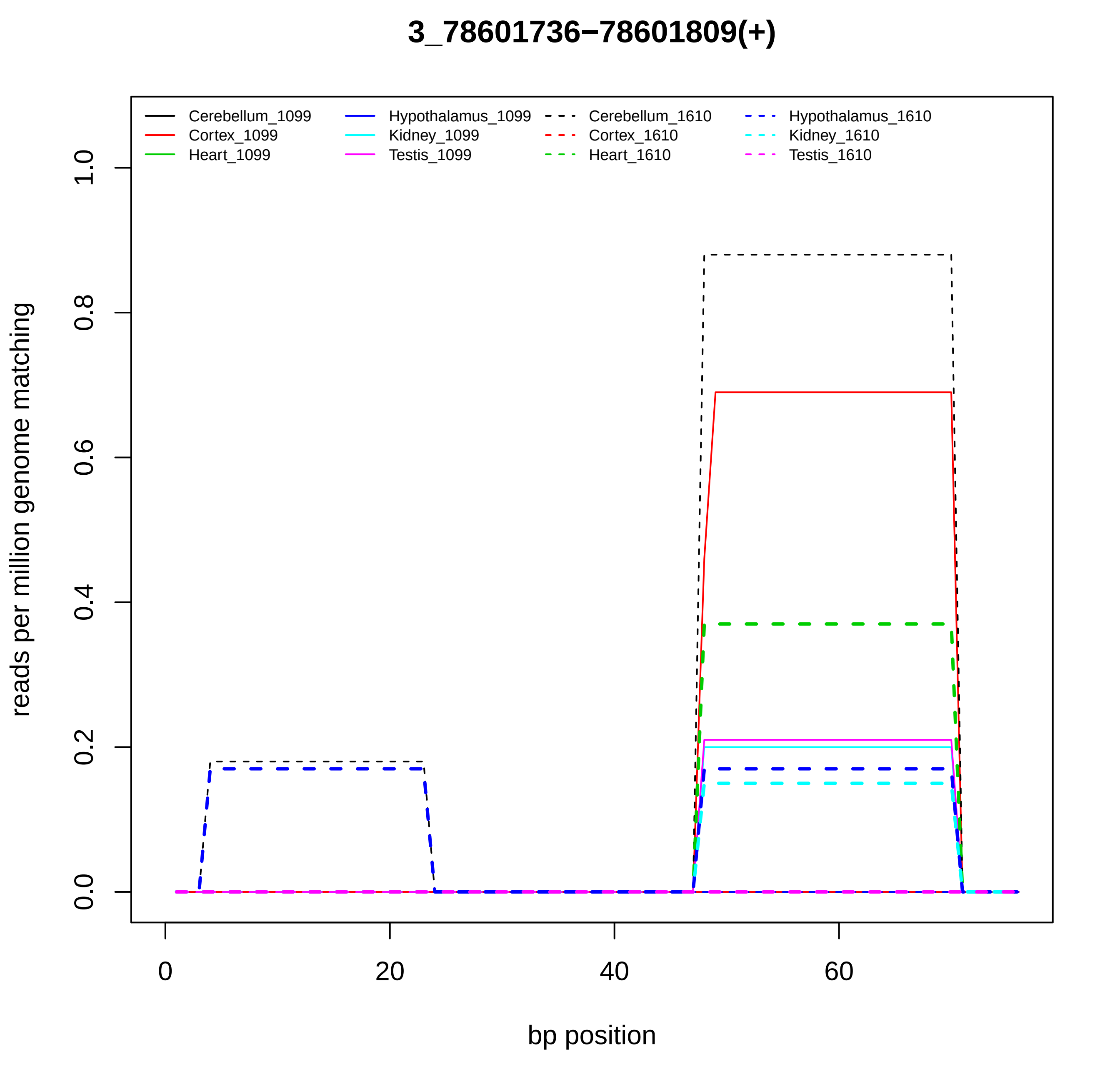

3_78601736−78601809(+)
Cerebellum_1099
Cortex_1099
Heart_1099
Hypothalamus_1099
Kidney_1099
Testis_1099
Cerebellum_1610
Cortex_1610
Heart_1610
Hypothalamus_1610
Kidney_1610
Testis_1610
reads per million genome matching
0.0
0.2
0.4
0.6
0.8
1.0
0
20
40
60
bp position

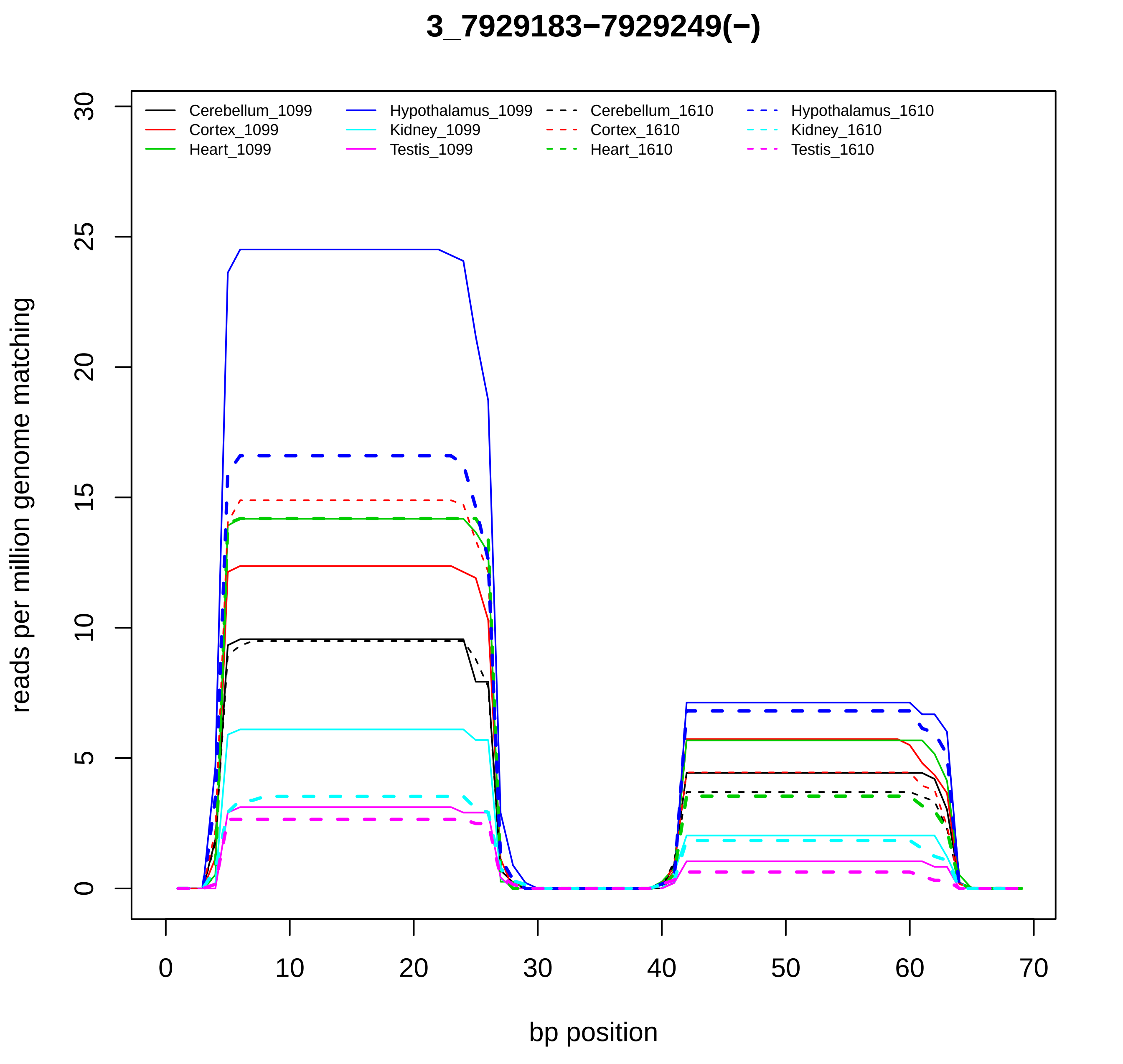

3_7929183−7929249(−)
Cerebellum_1099
Cortex_1099
Heart_1099
Hypothalamus_1099
Kidney_1099
Testis_1099
Cerebellum_1610
Cortex_1610
Heart_1610
Hypothalamus_1610
Kidney_1610
Testis_1610
reads per million genome matching
bp position
0
5
10
15
20
25
30
0
10
20
30
40
50
60
70

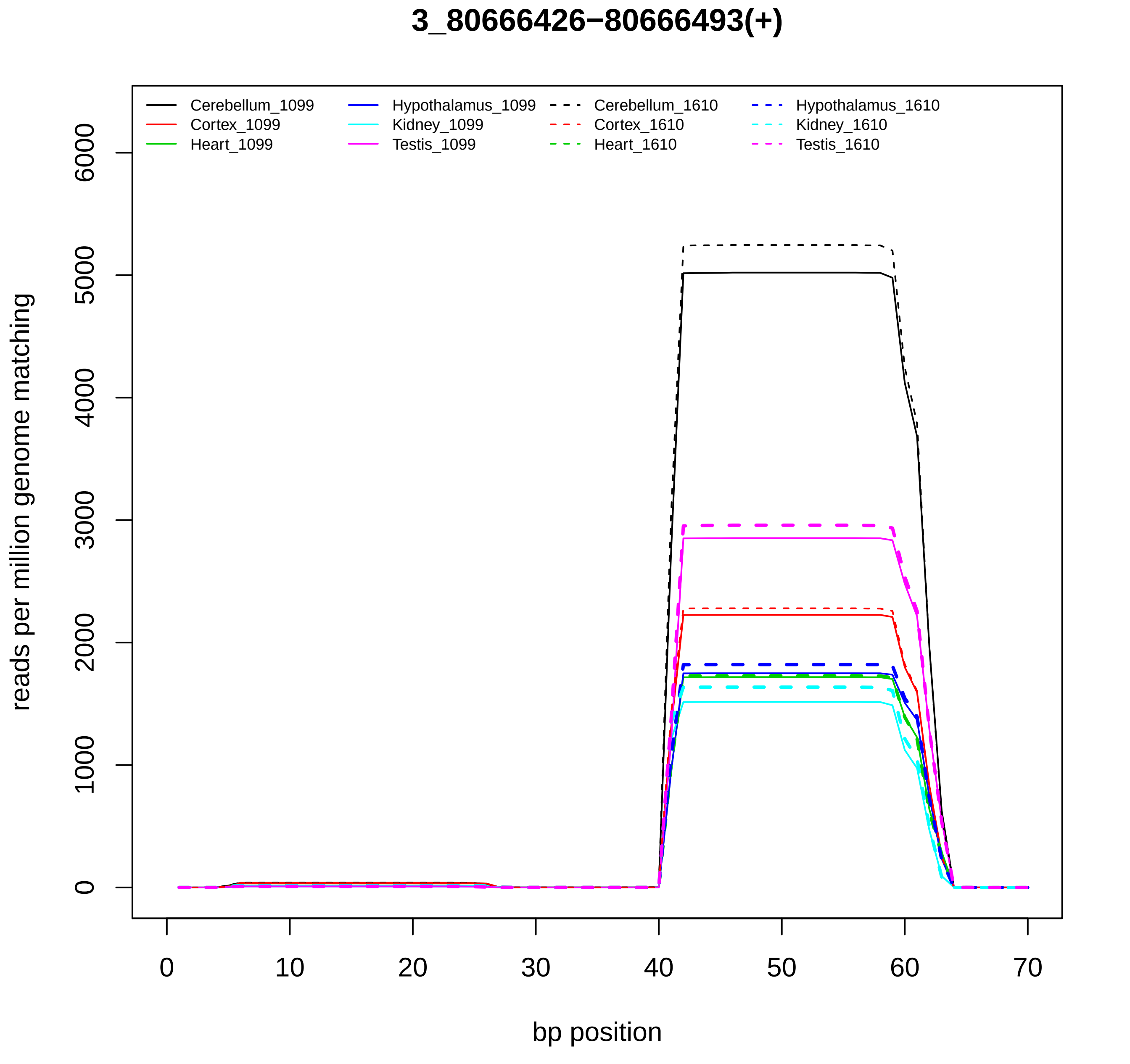
3_80666426−80666493(+)
Cerebellum_1099
Cortex_1099
Heart_1099
Hypothalamus_1099
Kidney_1099
Testis_1099
Cerebellum_1610
Cortex_1610
Heart_1610
Hypothalamus_1610
Kidney_1610
Testis_1610
reads per million genome matching
0
1000
2000
3000
4000
5000
6000
0
10
20
30
40
50
60
70
bp position

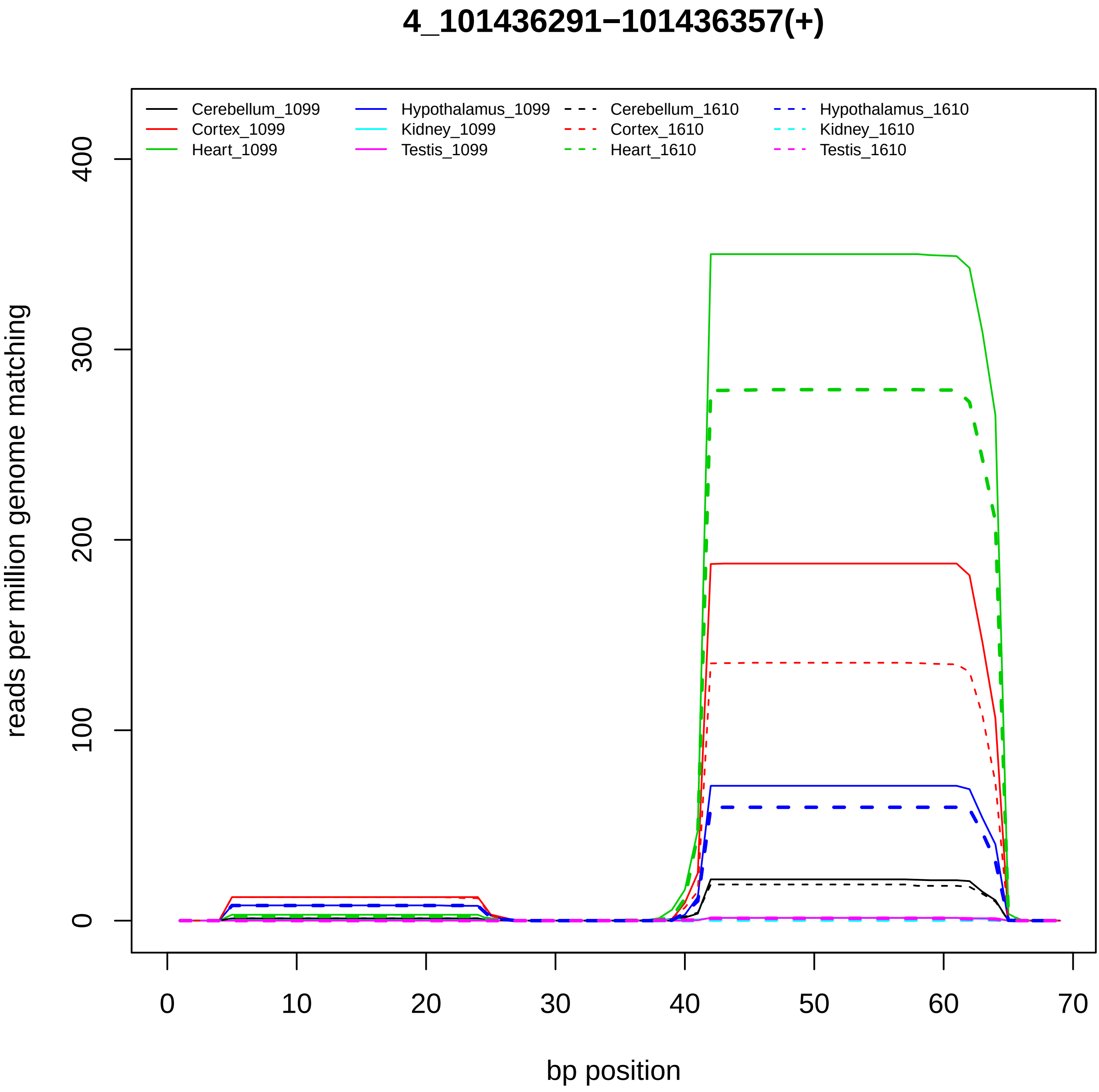
4_101436291−101436357(+)
Cerebellum_1099
Cortex_1099
Heart_1099
Hypothalamus_1099
Kidney_1099
Testis_1099
Cerebellum_1610
Cortex_1610
Heart_1610
Hypothalamus_1610
Kidney_1610
Testis_1610
reads per million genome matching
0
100
200
300
400
0
10
20
30
40
50
60
70
bp position

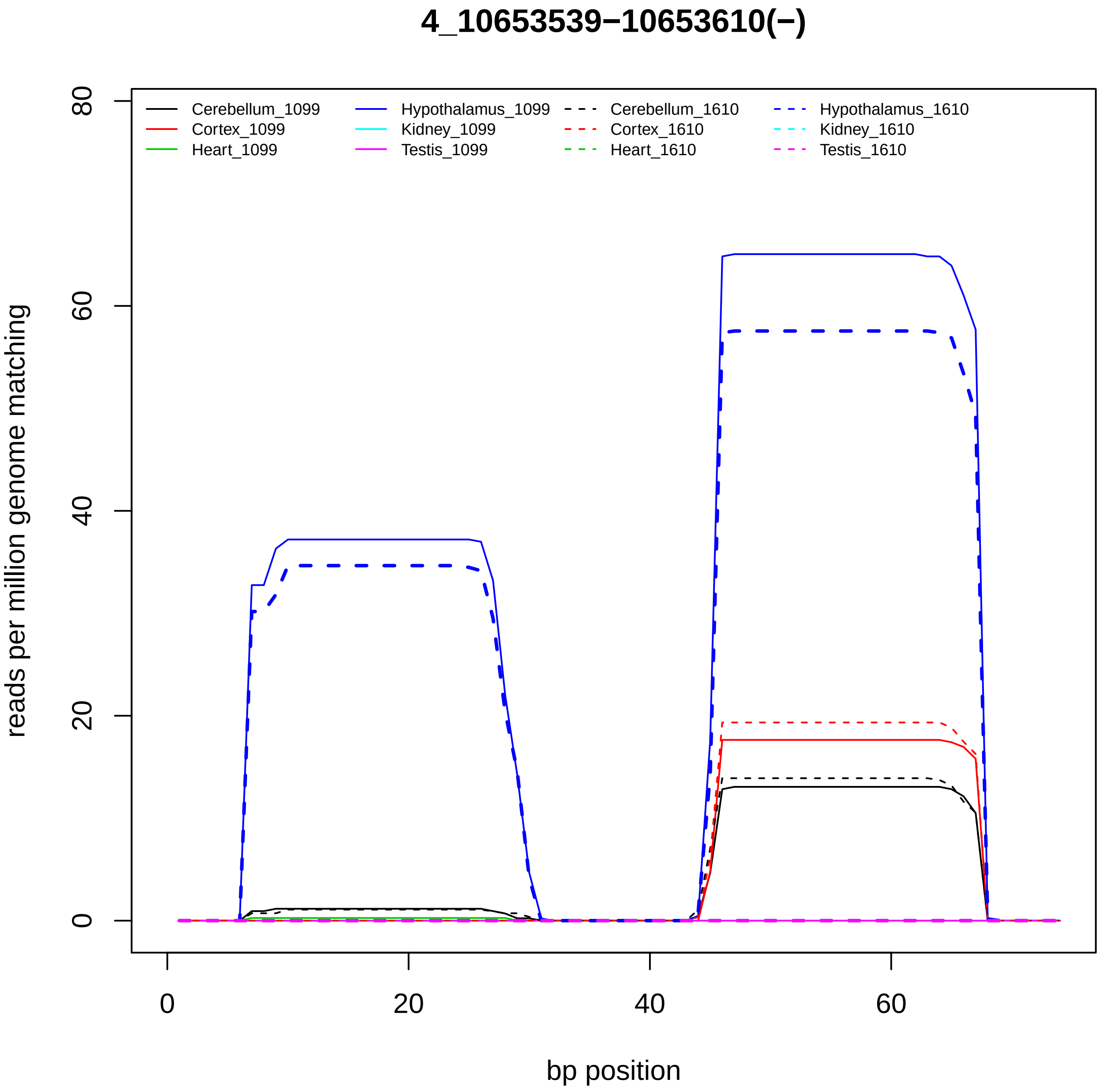

4_10653539−10653610(−)
Cerebellum_1099
Cortex_1099
Heart_1099
Hypothalamus_1099
Kidney_1099
Testis_1099
Cerebellum_1610
Cortex_1610
Heart_1610
Hypothalamus_1610
Kidney_1610
Testis_1610
reads per million genome matching
0
20
40
60
80
0
20
40
60
bp position

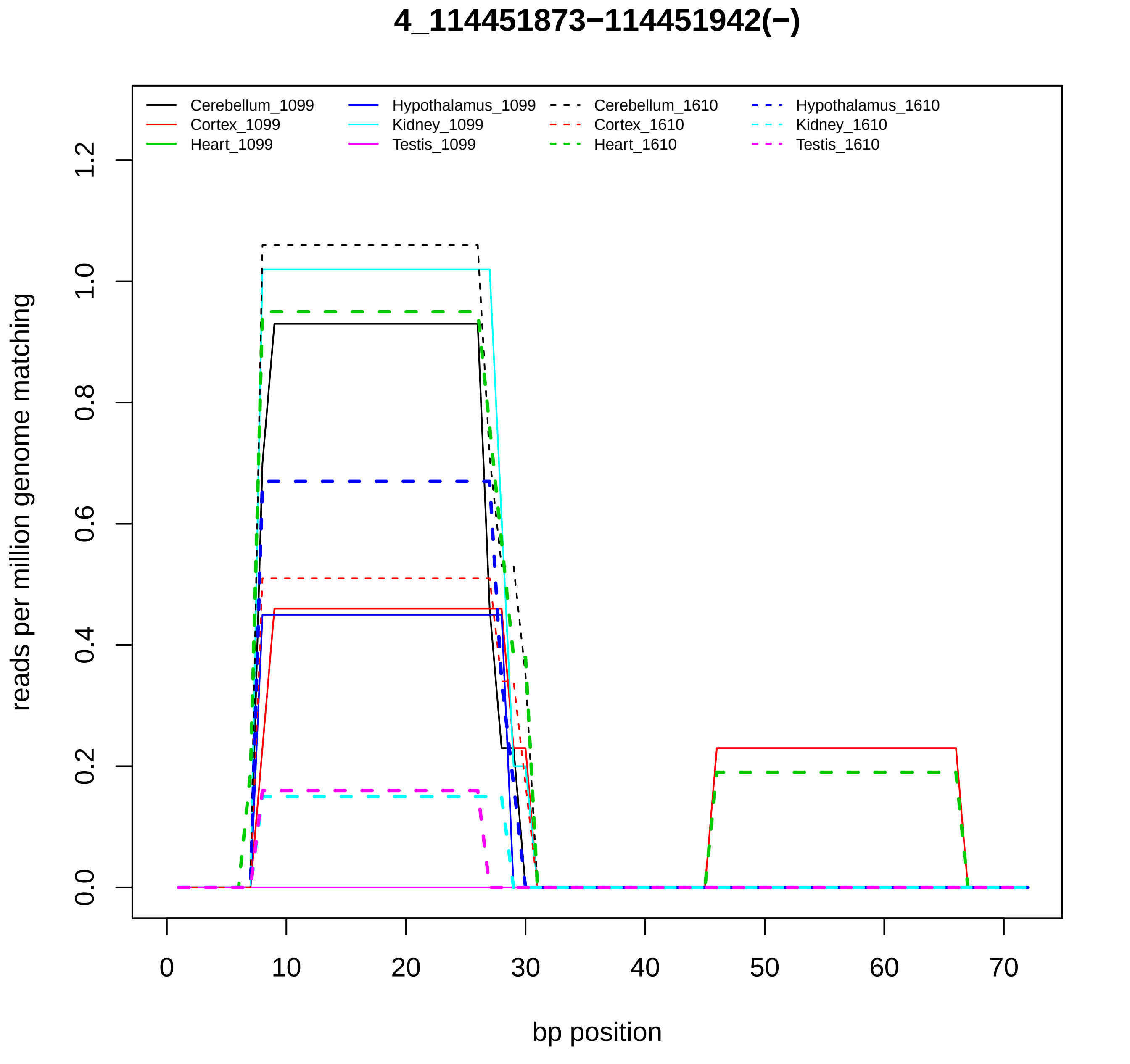

4_114451873−114451942(−)
Cerebellum_1099
Cortex_1099
Heart_1099
Hypothalamus_1099
Kidney_1099
Testis_1099
Cerebellum_1610
Cortex_1610
Heart_1610
Hypothalamus_1610
Kidney_1610
Testis_1610
reads per million genome matching
0.0
0.2
0.4
0.6
0.8
1.0
1.2
0
10
20
30
40
50
60
70
bp position

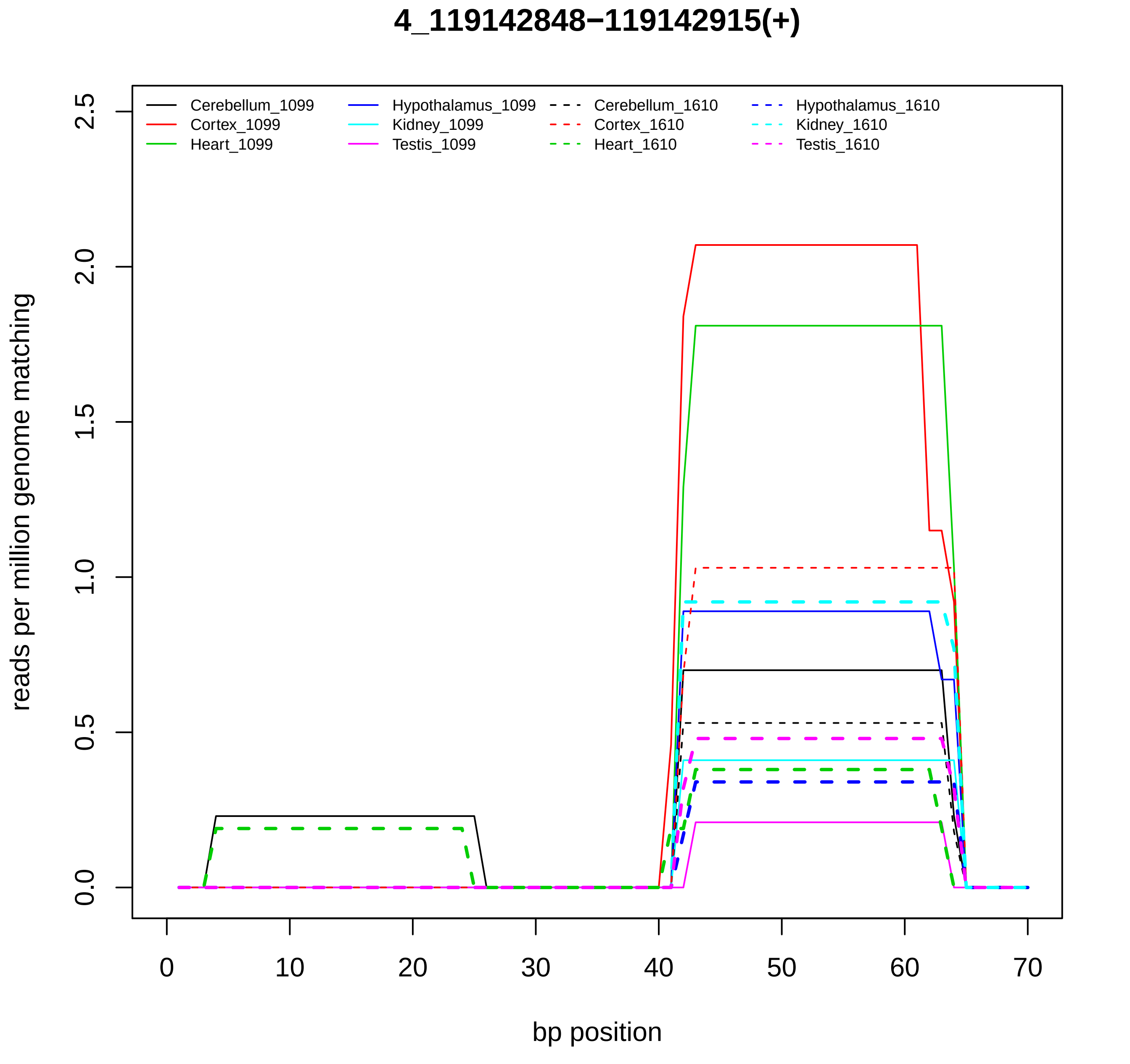

4_119142848−119142915(+)
Cerebellum_1099
Cortex_1099
Heart_1099
Hypothalamus_1099
Kidney_1099
Testis_1099
Cerebellum_1610
Cortex_1610
Heart_1610
Hypothalamus_1610
Kidney_1610
Testis_1610
reads per million genome matching
bp position
0.0
0.5
1.0
1.5
2.0
2.5
0
10
20
30
40
50
60
70

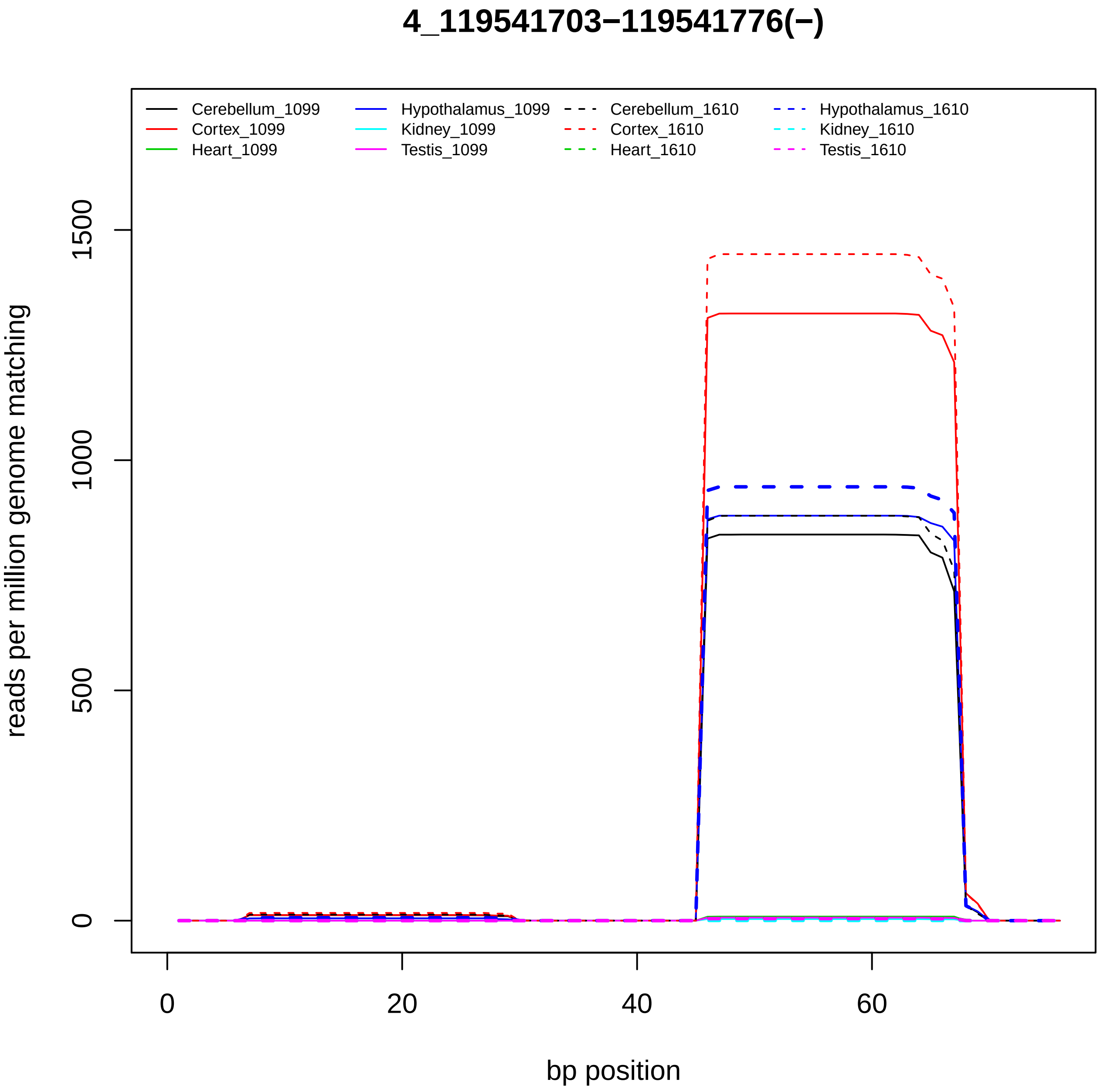
4_119541703−119541776(−)
Cerebellum_1099
Cortex_1099
Heart_1099
Hypothalamus_1099
Kidney_1099
Testis_1099
Cerebellum_1610
Cortex_1610
Heart_1610
Hypothalamus_1610
Kidney_1610
Testis_1610
reads per million genome matching
0
500
1000
1500
0
20
40
60
bp position

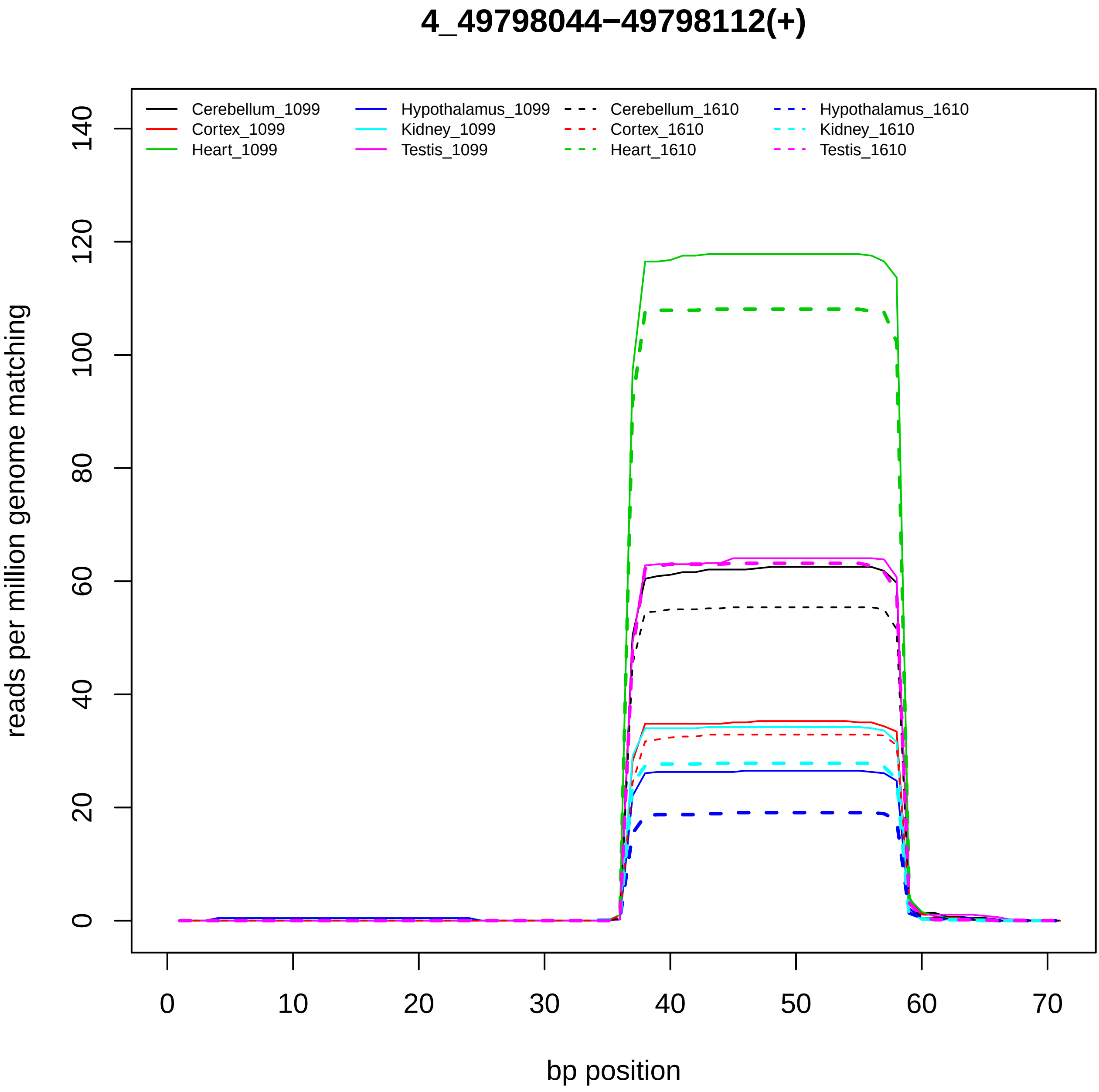
4_49798044−49798112(+)
Cerebellum_1099
Cortex_1099
Heart_1099
Hypothalamus_1099
Kidney_1099
Testis_1099
Cerebellum_1610
Cortex_1610
Heart_1610
Hypothalamus_1610
Kidney_1610
Testis_1610
reads per million genome matching
bp position
0
20
40
60
80
100
120
140
0
10
20
30
40
50
60
70

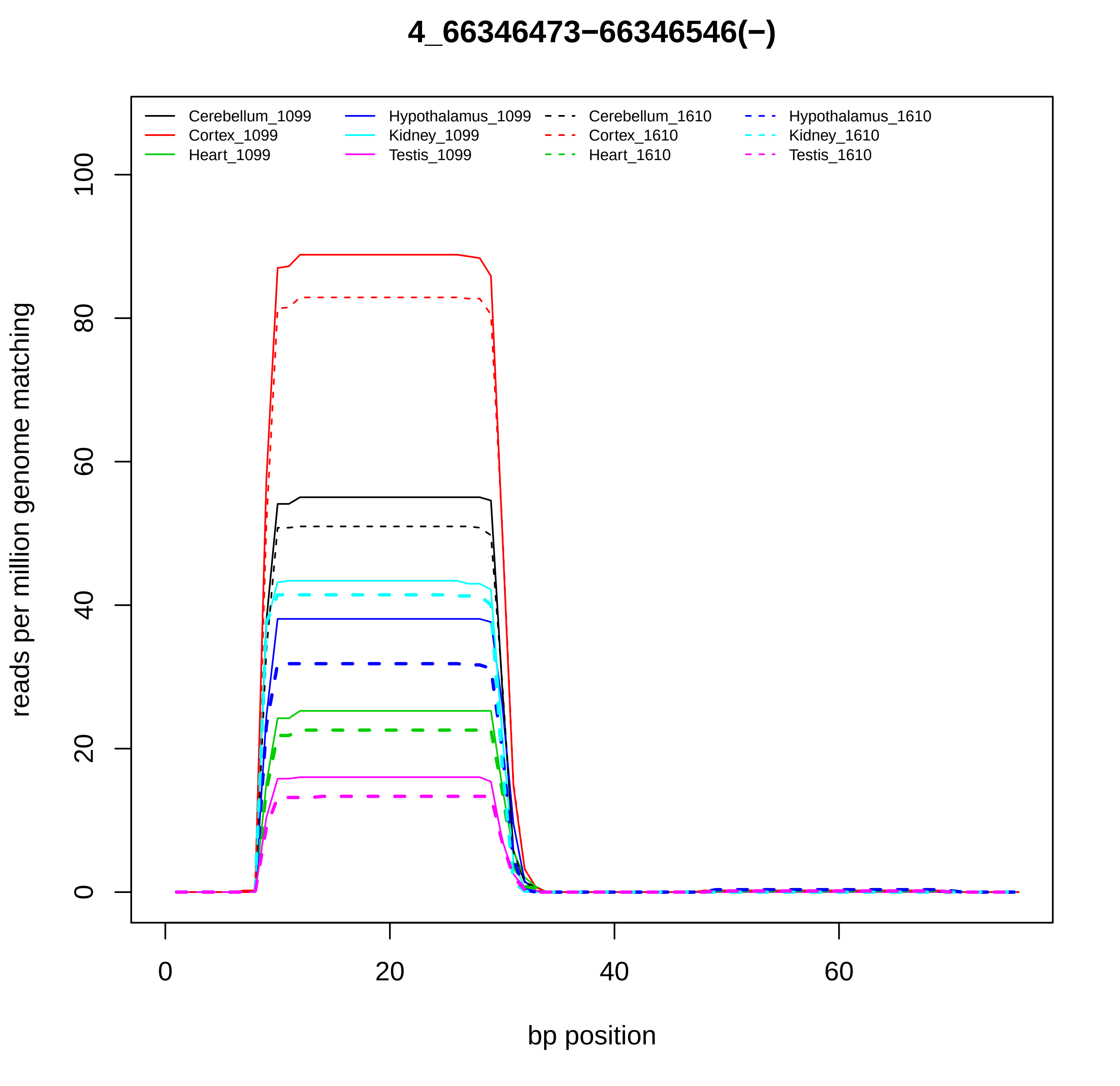

4_66346473−66346546(−)
Cerebellum_1099
Cortex_1099
Heart_1099
Hypothalamus_1099
Kidney_1099
Testis_1099
Cerebellum_1610
Cortex_1610
Heart_1610
Hypothalamus_1610
Kidney_1610
Testis_1610
reads per million genome matching
0
20
40
60
80
100
0
20
40
60
bp position

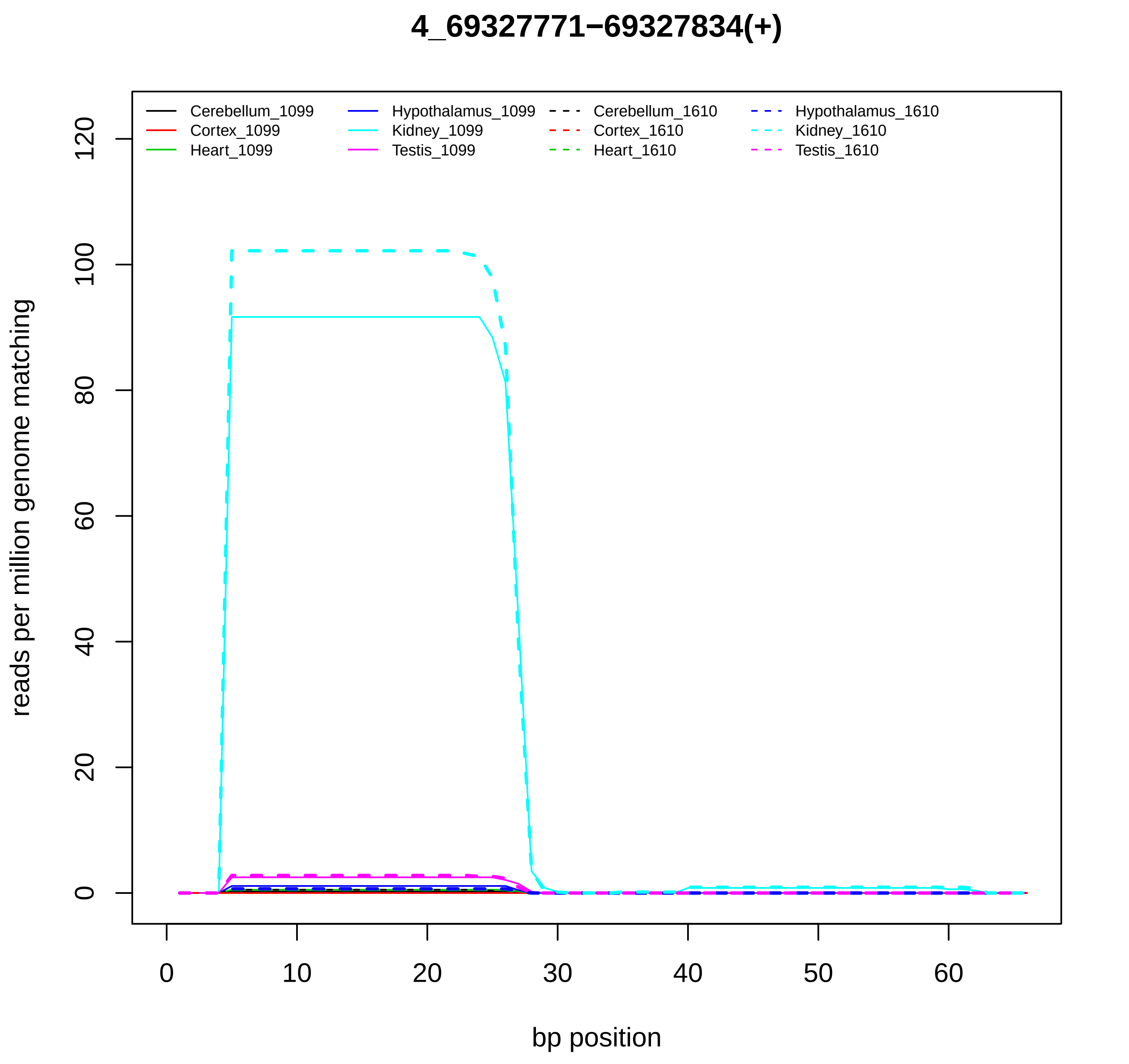

4_69327771−69327834(+)
Cerebellum_1099
Cortex_1099
Heart_1099
Hypothalamus_1099
Kidney_1099
Testis_1099
Cerebellum_1610
Cortex_1610
Heart_1610
Hypothalamus_1610
Kidney_1610
Testis_1610
reads per million genome matching
bp position
0
20
40
60
80
100
120
0
10
20
30
40
50
60

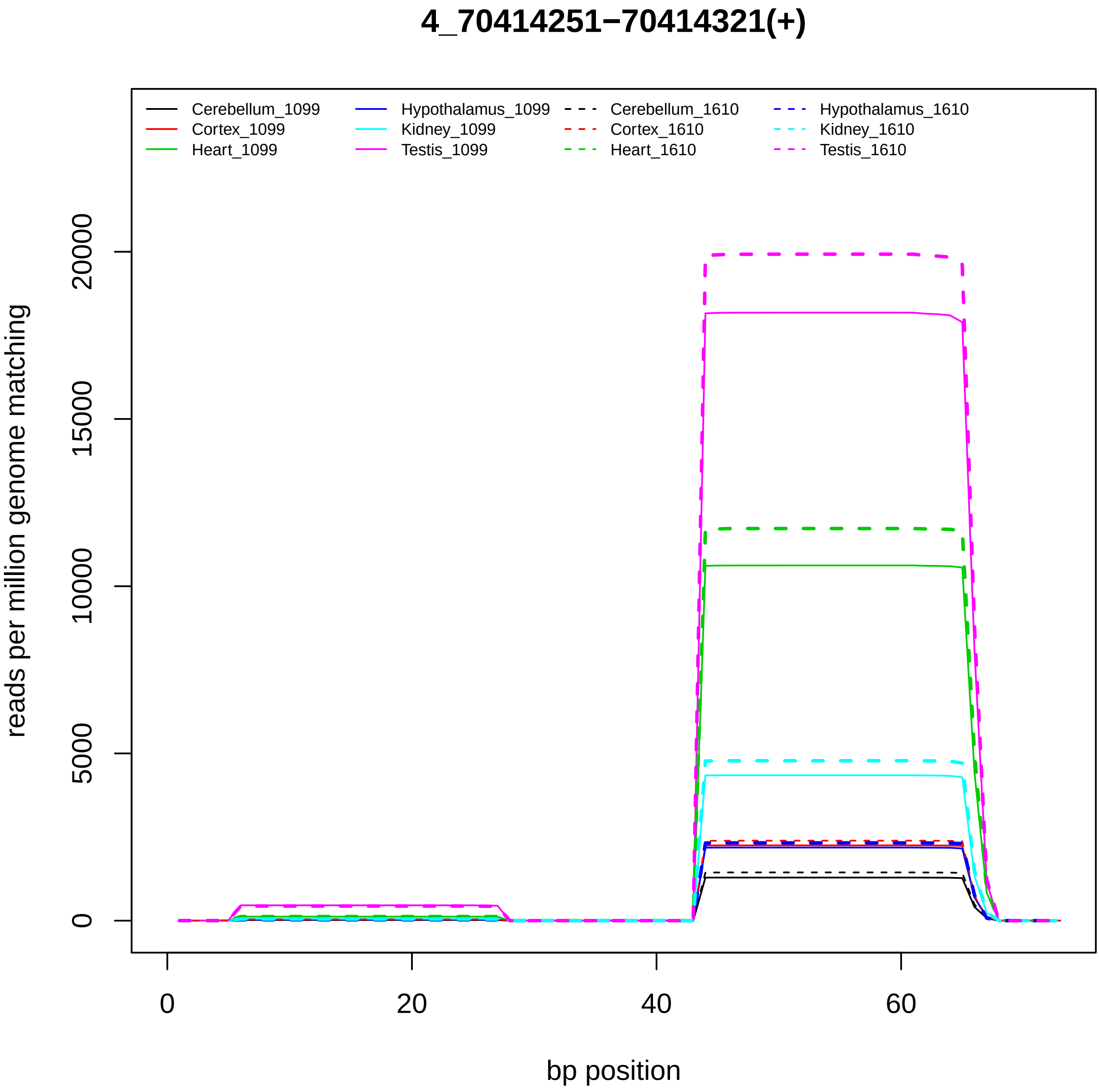

4_70414251−70414321(+)
Cerebellum_1099
Cortex_1099
Heart_1099
Hypothalamus_1099
Kidney_1099
Testis_1099
Cerebellum_1610
Cortex_1610
Heart_1610
Hypothalamus_1610
Kidney_1610
Testis_1610
reads per million genome matching
0
5000
10000
15000
20000
0
20
40
60
bp position

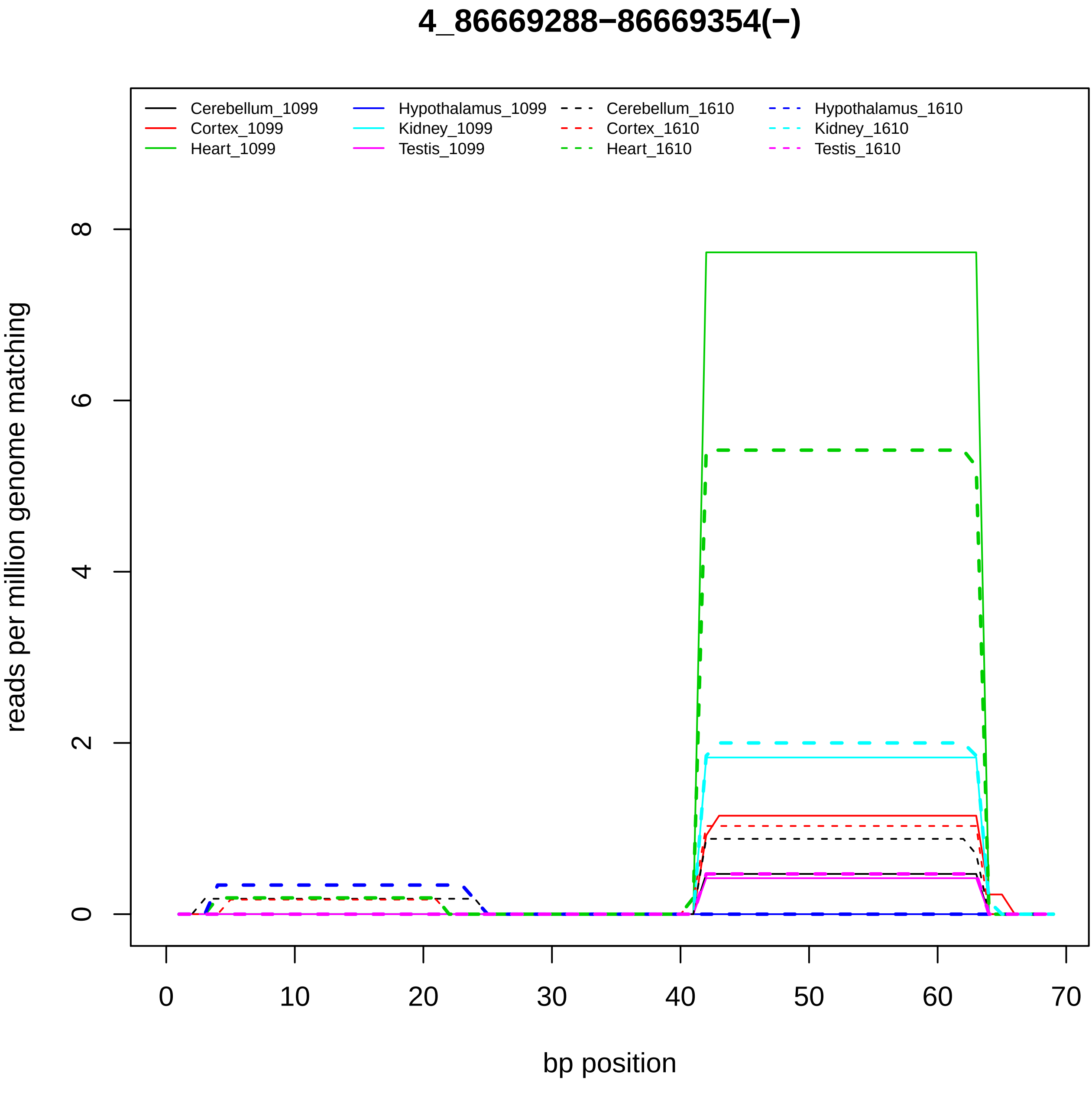

4_86669288−86669354(−)
Cerebellum_1099
Cortex_1099
Heart_1099
Hypothalamus_1099
Kidney_1099
Testis_1099
Cerebellum_1610
Cortex_1610
Heart_1610
Hypothalamus_1610
Kidney_1610
Testis_1610
reads per million genome matching
0
2
4
6
8
0
10
20
30
40
50
60
70
bp position

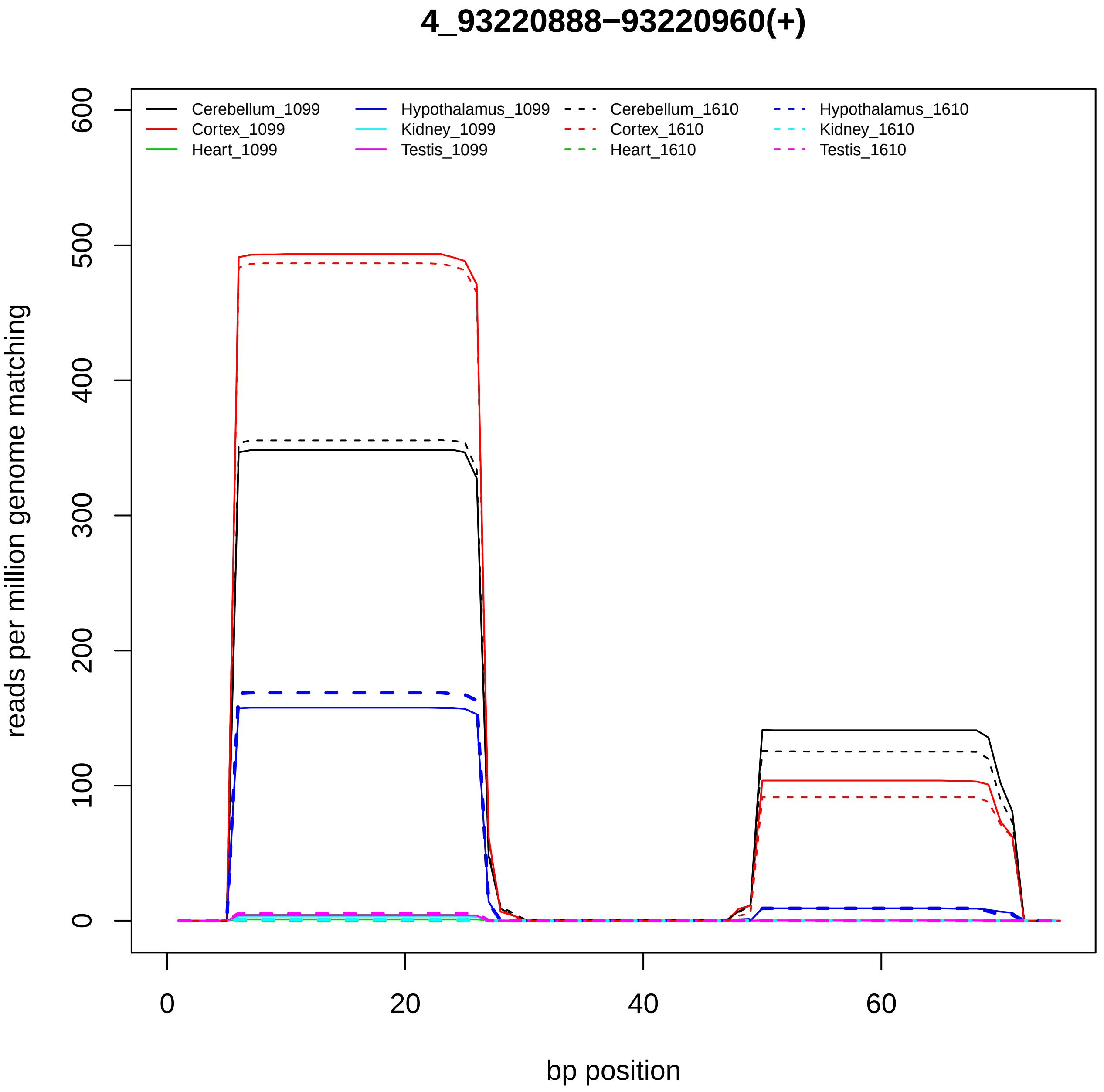

4_93220888−93220960(+)
Cerebellum_1099
Cortex_1099
Heart_1099
Hypothalamus_1099
Kidney_1099
Testis_1099
Cerebellum_1610
Cortex_1610
Heart_1610
Hypothalamus_1610
Kidney_1610
Testis_1610
reads per million genome matching
bp position
0
100
200
300
400
500
600
0
20
40
60

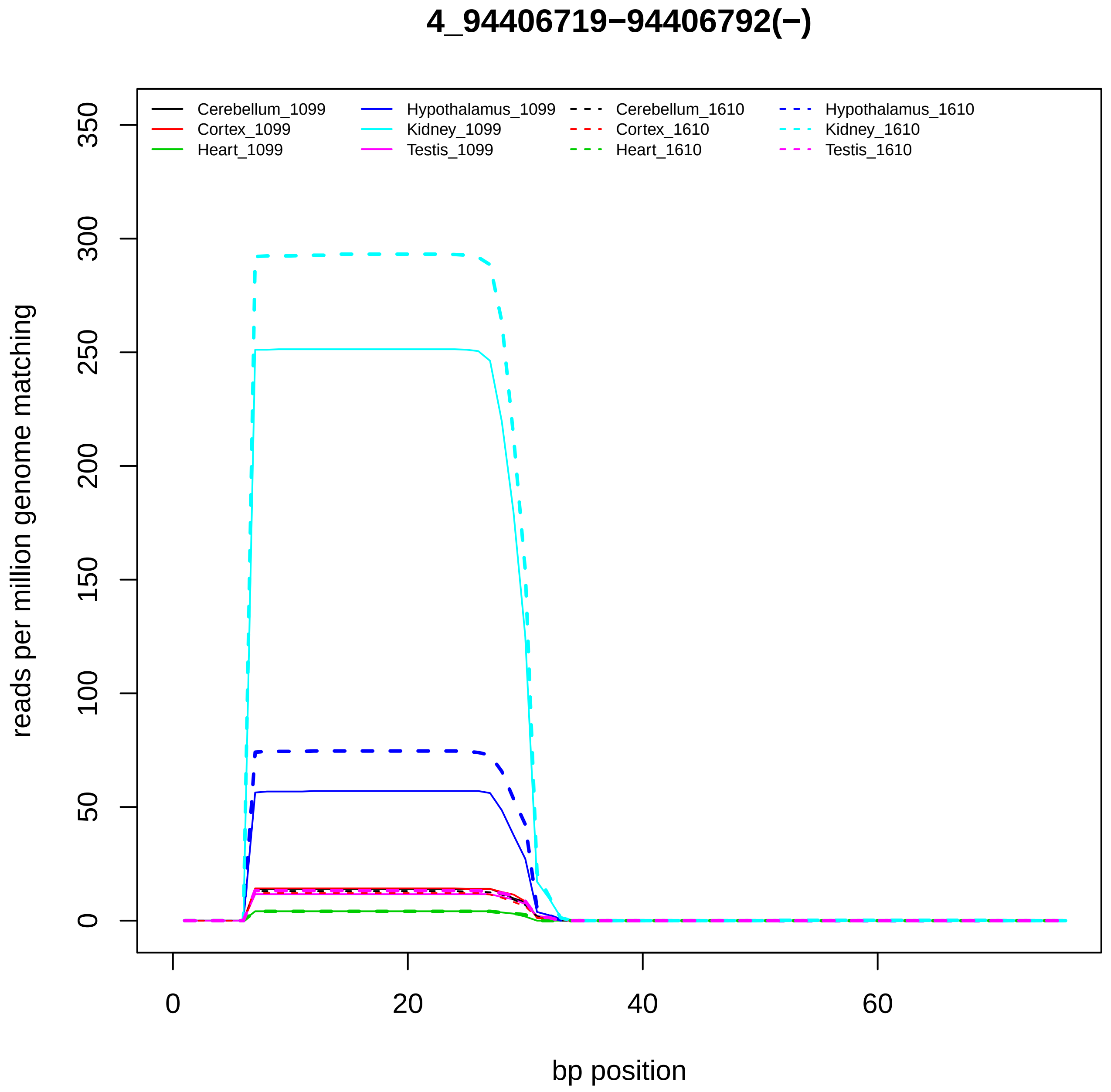

4_94406719−94406792(−)
Cerebellum_1099
Cortex_1099
Heart_1099
Hypothalamus_1099
Kidney_1099
Testis_1099
Cerebellum_1610
Cortex_1610
Heart_1610
Hypothalamus_1610
Kidney_1610
Testis_1610
reads per million genome matching
bp position
0
50
100
150
200
250
300
350
0
20
40
60

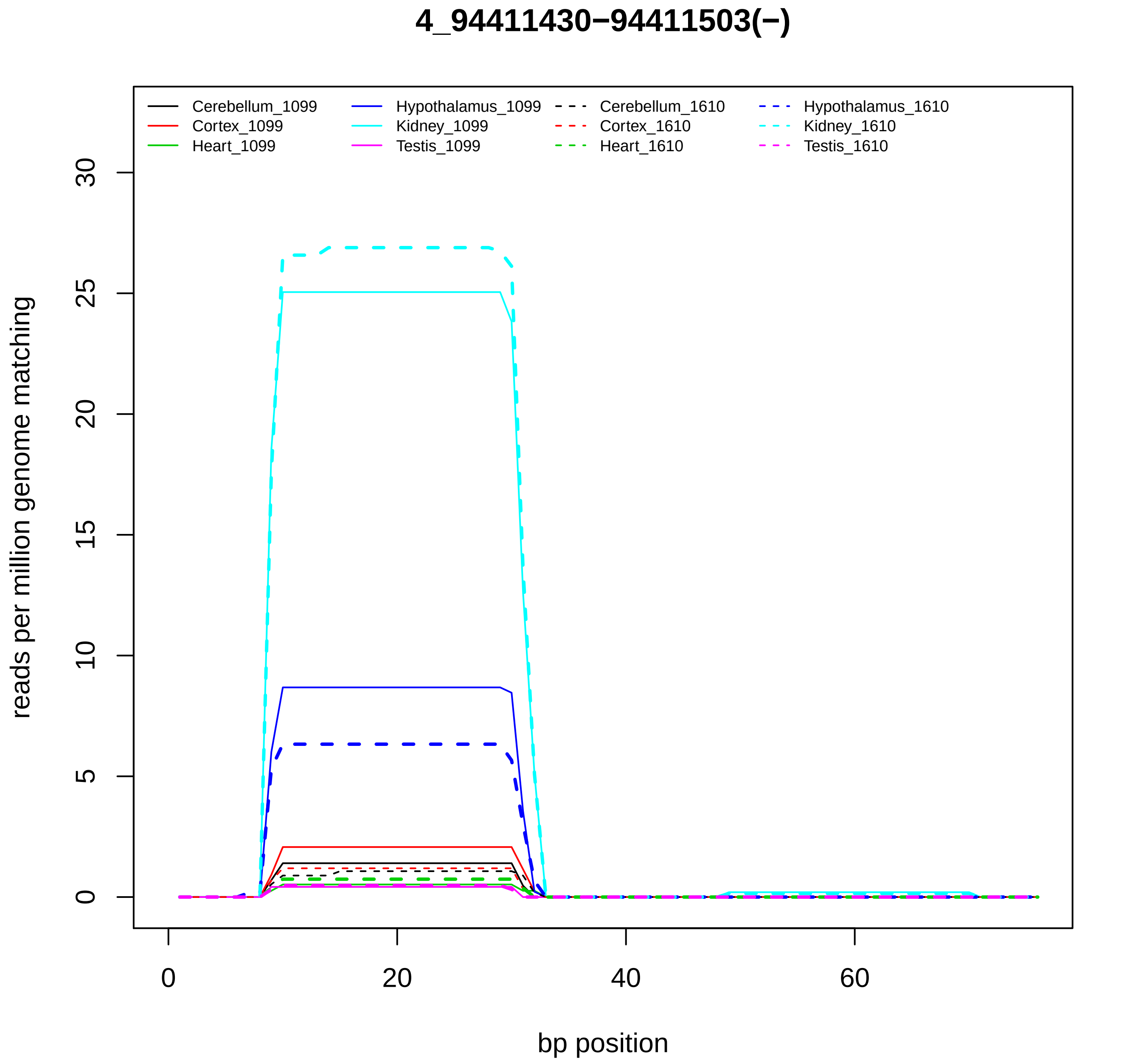

4_94411430−94411503(−)
Cerebellum_1099
Cortex_1099
Heart_1099
Hypothalamus_1099
Kidney_1099
Testis_1099
Cerebellum_1610
Cortex_1610
Heart_1610
Hypothalamus_1610
Kidney_1610
Testis_1610
reads per million genome matching
bp position
0
5
10
15
20
25
30
0
20
40
60

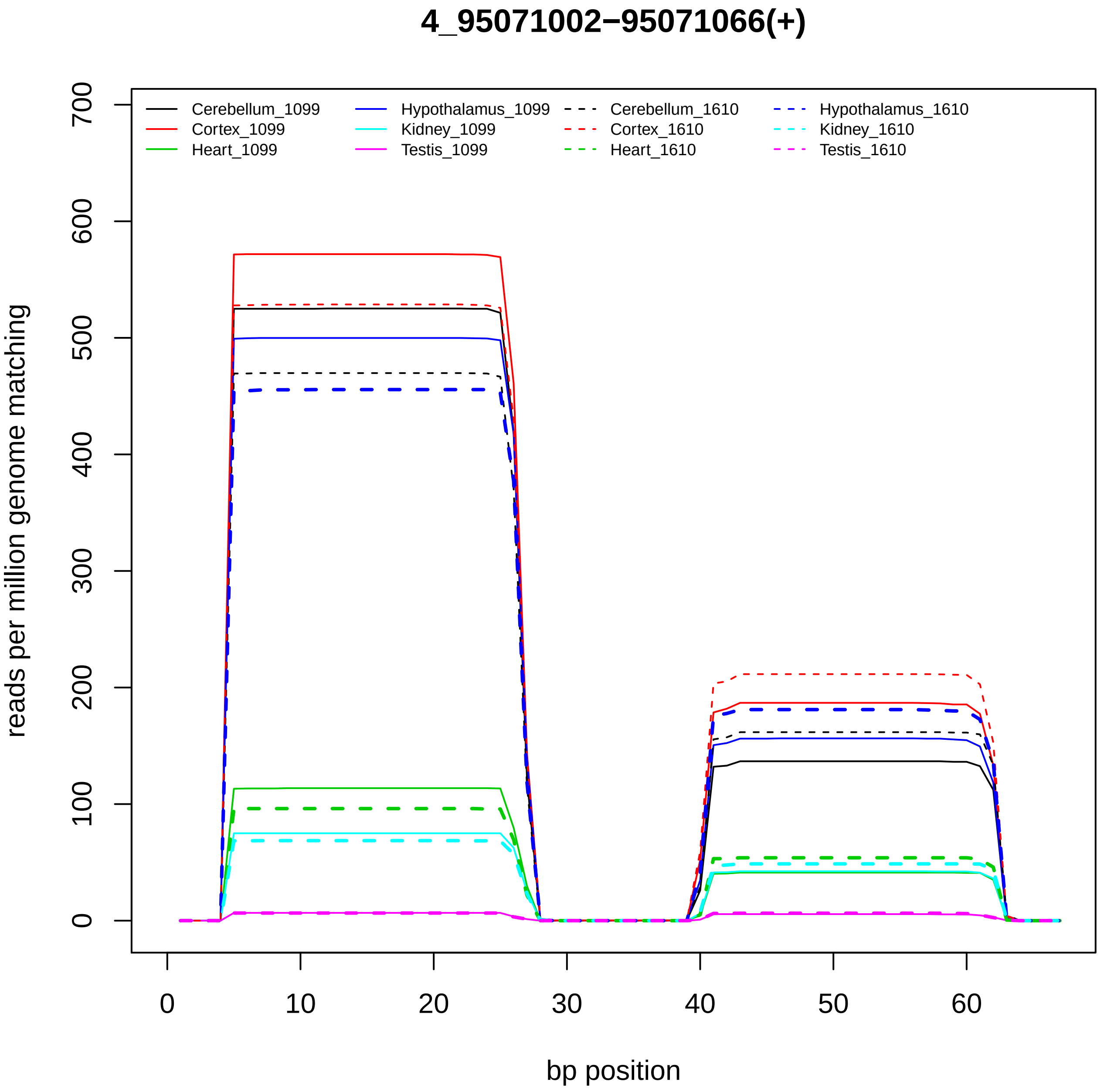

4_95071002−95071066(+)
Cerebellum_1099
Cortex_1099
Heart_1099
Hypothalamus_1099
Kidney_1099
Testis_1099
Cerebellum_1610
Cortex_1610
Heart_1610
Hypothalamus_1610
Kidney_1610
Testis_1610
reads per million genome matching
0
100
200
300
400
500
600
700
bp position
0
10
20
30
40
50
60

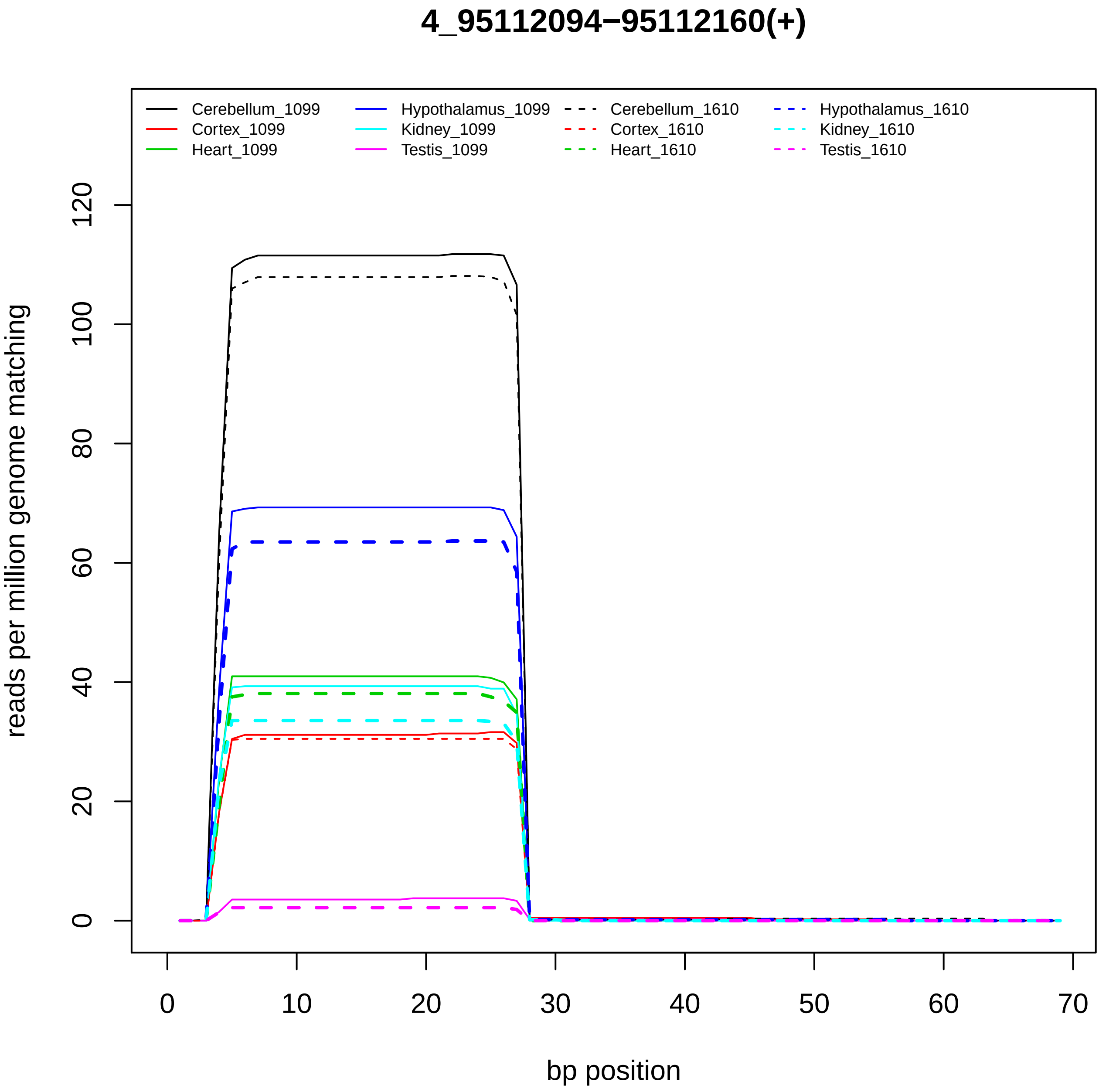

4_95112094−95112160(+)
Cerebellum_1099
Cortex_1099
Heart_1099
Hypothalamus_1099
Kidney_1099
Testis_1099
Cerebellum_1610
Cortex_1610
Heart_1610
Hypothalamus_1610
Kidney_1610
Testis_1610
reads per million genome matching
0
20
40
60
80
100
120
bp position
0
10
20
30
40
50
60
70

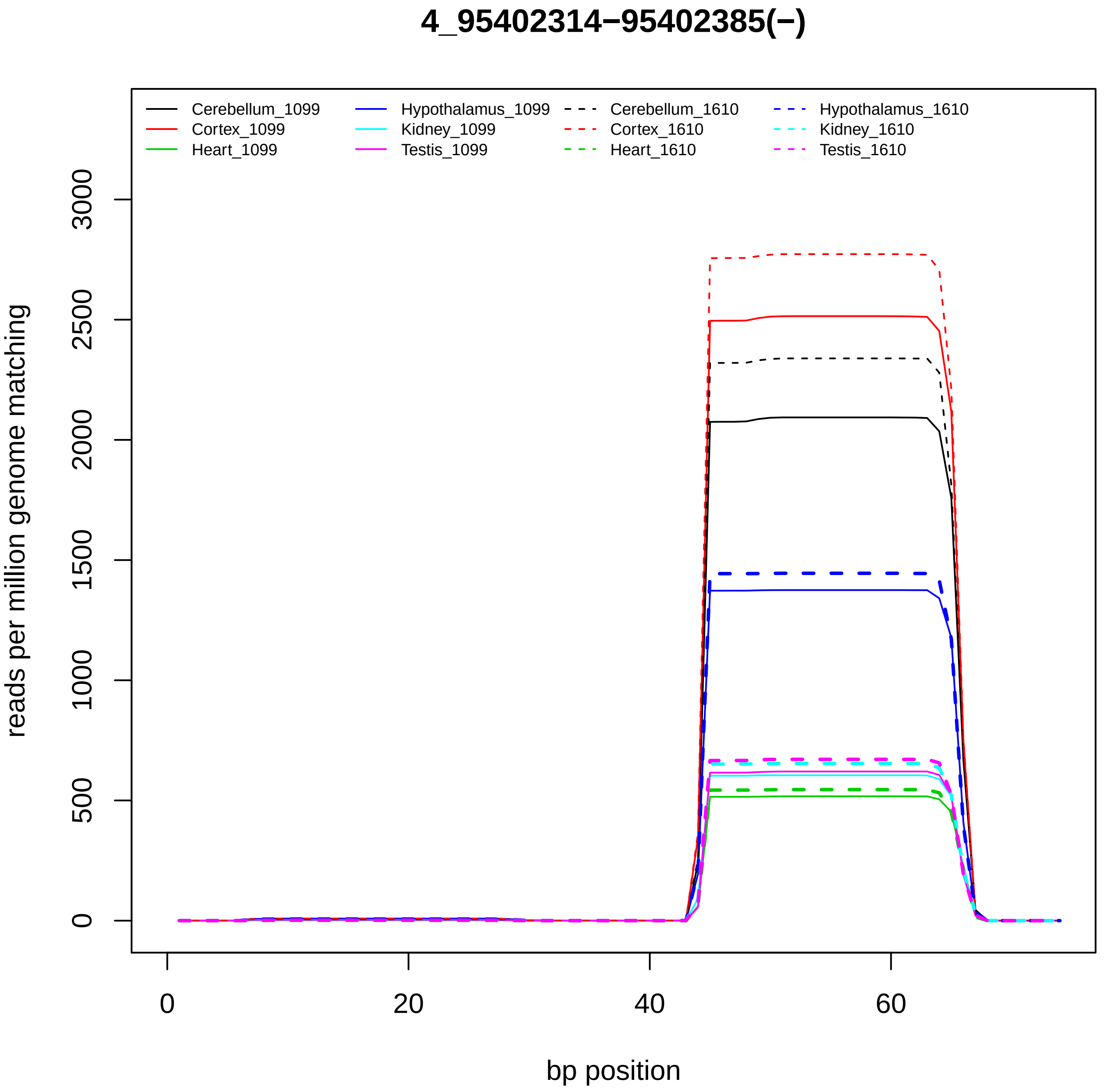

4_95402314−95402385(−)
Cerebellum_1099
Cortex_1099
Heart_1099
Hypothalamus_1099
Kidney_1099
Testis_1099
Cerebellum_1610
Cortex_1610
Heart_1610
Hypothalamus_1610
Kidney_1610
Testis_1610
reads per million genome matching
0
500
1000
1500
2000
2500
3000
bp position
0
20
40
60

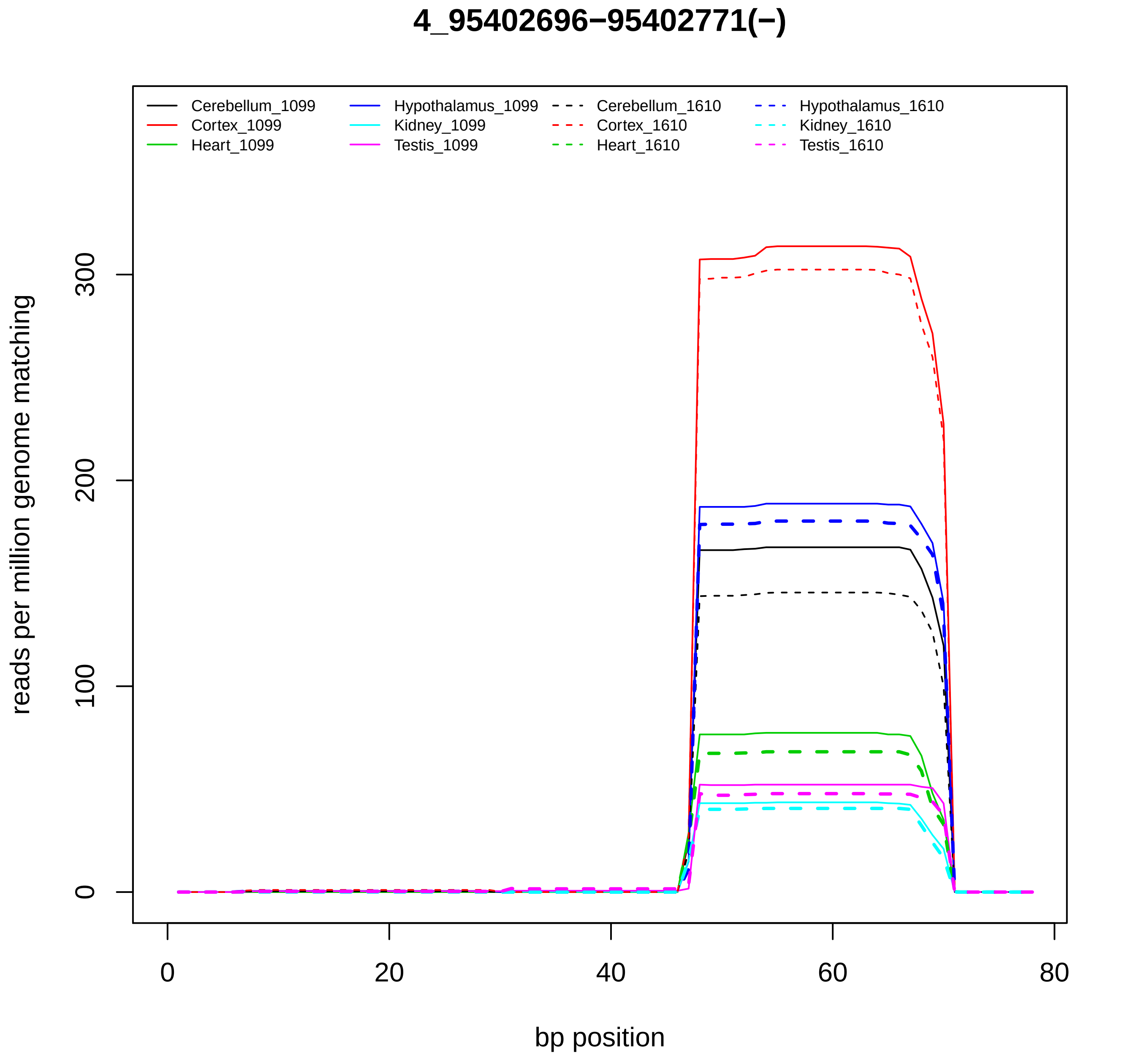
4_95402696−95402771(−)
Cerebellum_1099
Cortex_1099
Heart_1099
Hypothalamus_1099
Kidney_1099
Testis_1099
Cerebellum_1610
Cortex_1610
Heart_1610
Hypothalamus_1610
Kidney_1610
Testis_1610
reads per million genome matching
0
100
200
300
0
20
40
60
80
bp position

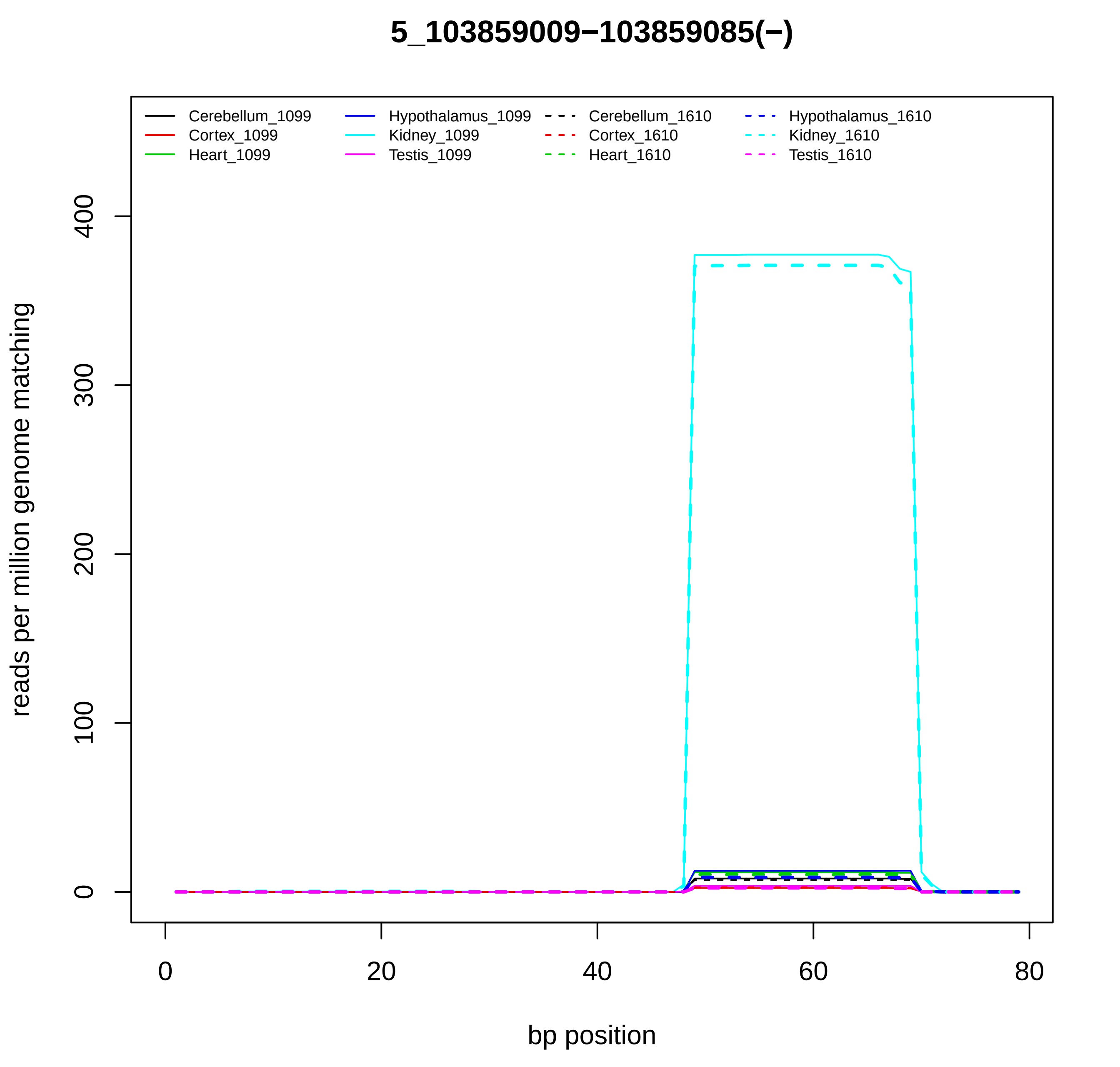

5_103859009−103859085(−)
Cerebellum_1099
Cortex_1099
Heart_1099
Hypothalamus_1099
Kidney_1099
Testis_1099
Cerebellum_1610
Cortex_1610
Heart_1610
Hypothalamus_1610
Kidney_1610
Testis_1610
reads per million genome matching
0
100
200
300
400
0
20
40
60
80
bp position

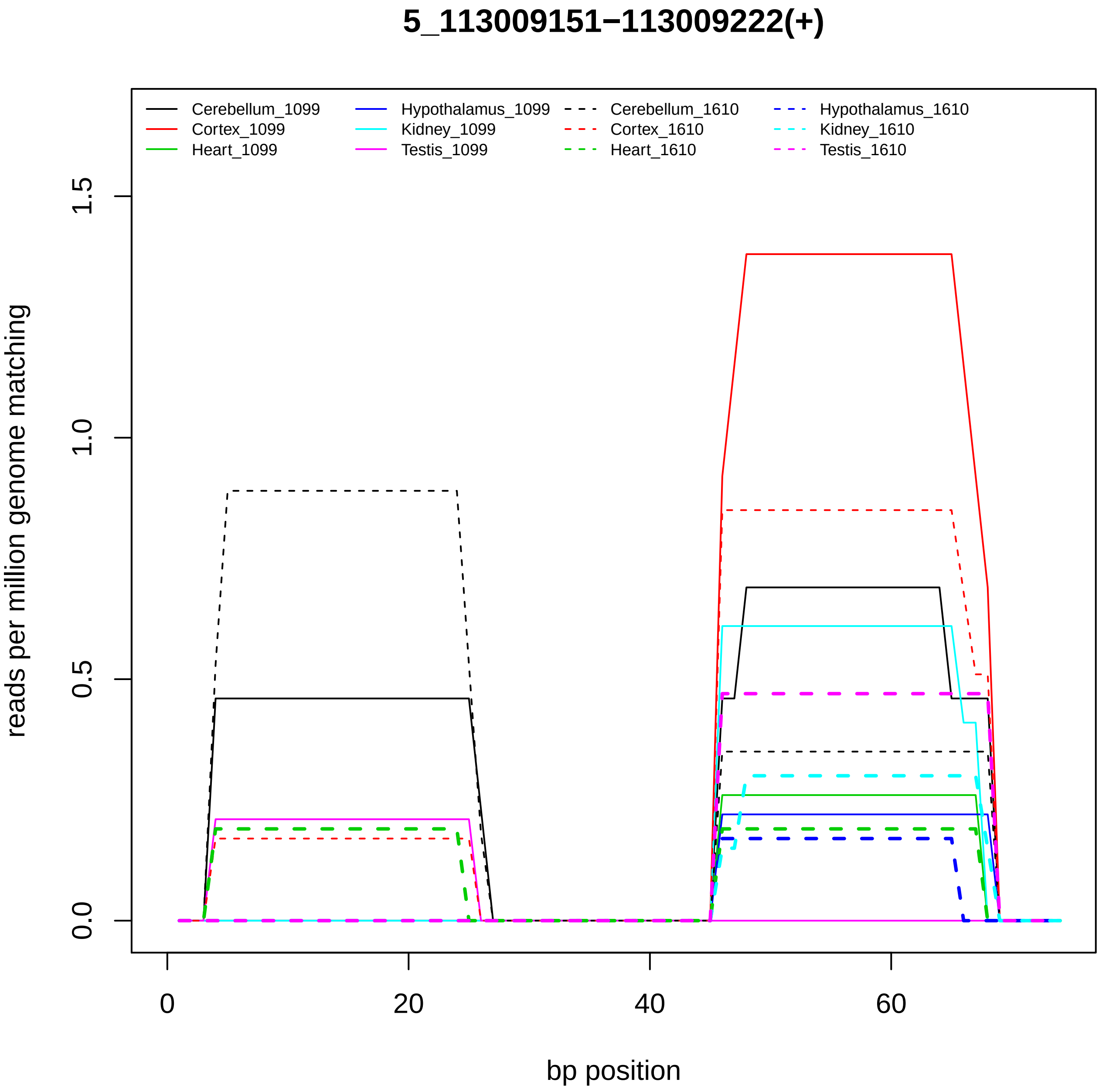

5_113009151−113009222(+)
Cerebellum_1099
Cortex_1099
Heart_1099
Hypothalamus_1099
Kidney_1099
Testis_1099
Cerebellum_1610
Cortex_1610
Heart_1610
Hypothalamus_1610
Kidney_1610
Testis_1610
reads per million genome matching
0.0
0.5
1.0
1.5
0
20
40
60
bp position

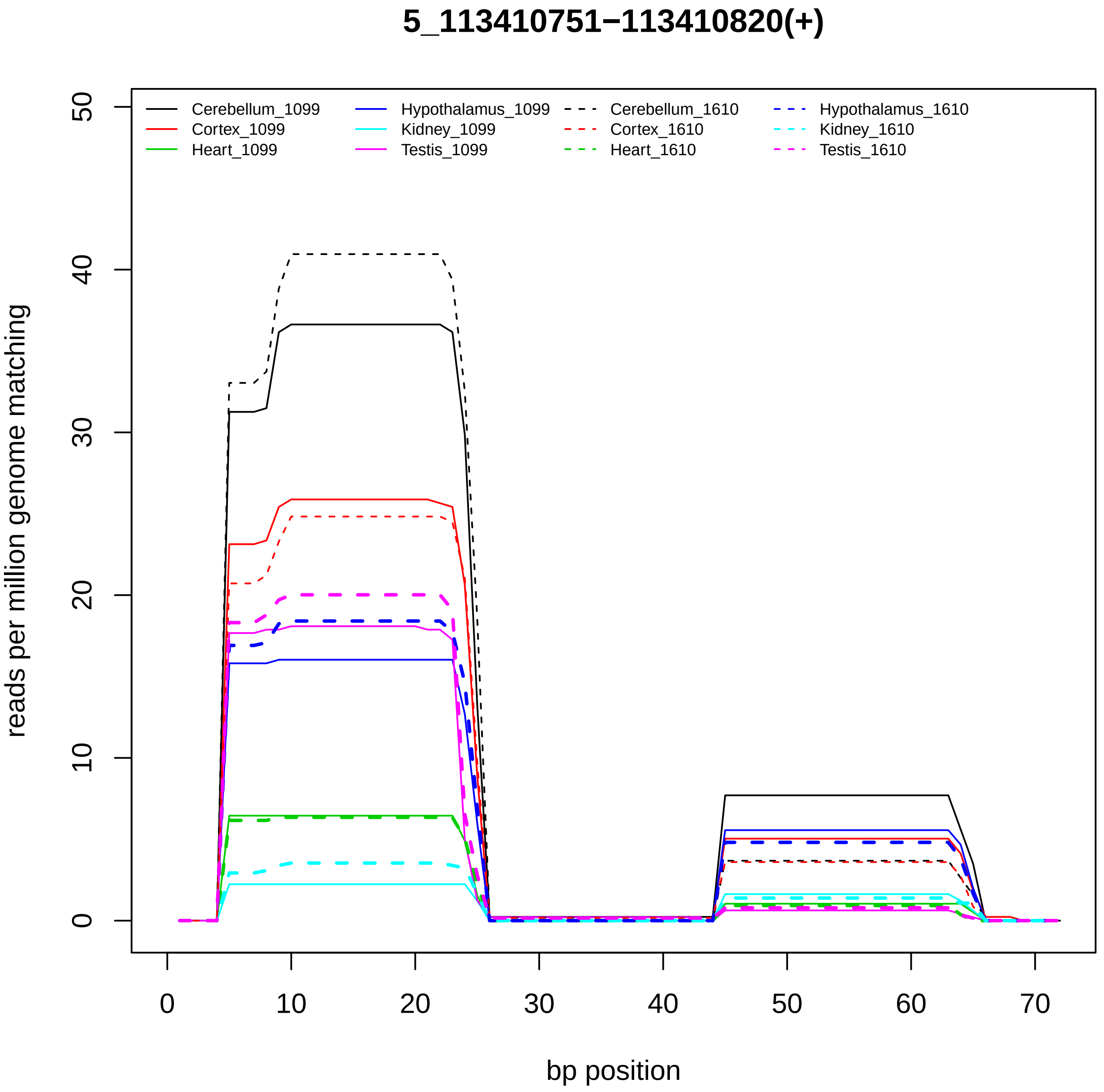

5_113410751−113410820(+)
Cerebellum_1099
Cortex_1099
Heart_1099
Hypothalamus_1099
Kidney_1099
Testis_1099
Cerebellum_1610
Cortex_1610
Heart_1610
Hypothalamus_1610
Kidney_1610
Testis_1610
reads per million genome matching
bp position
0
10
20
30
40
50
60
70

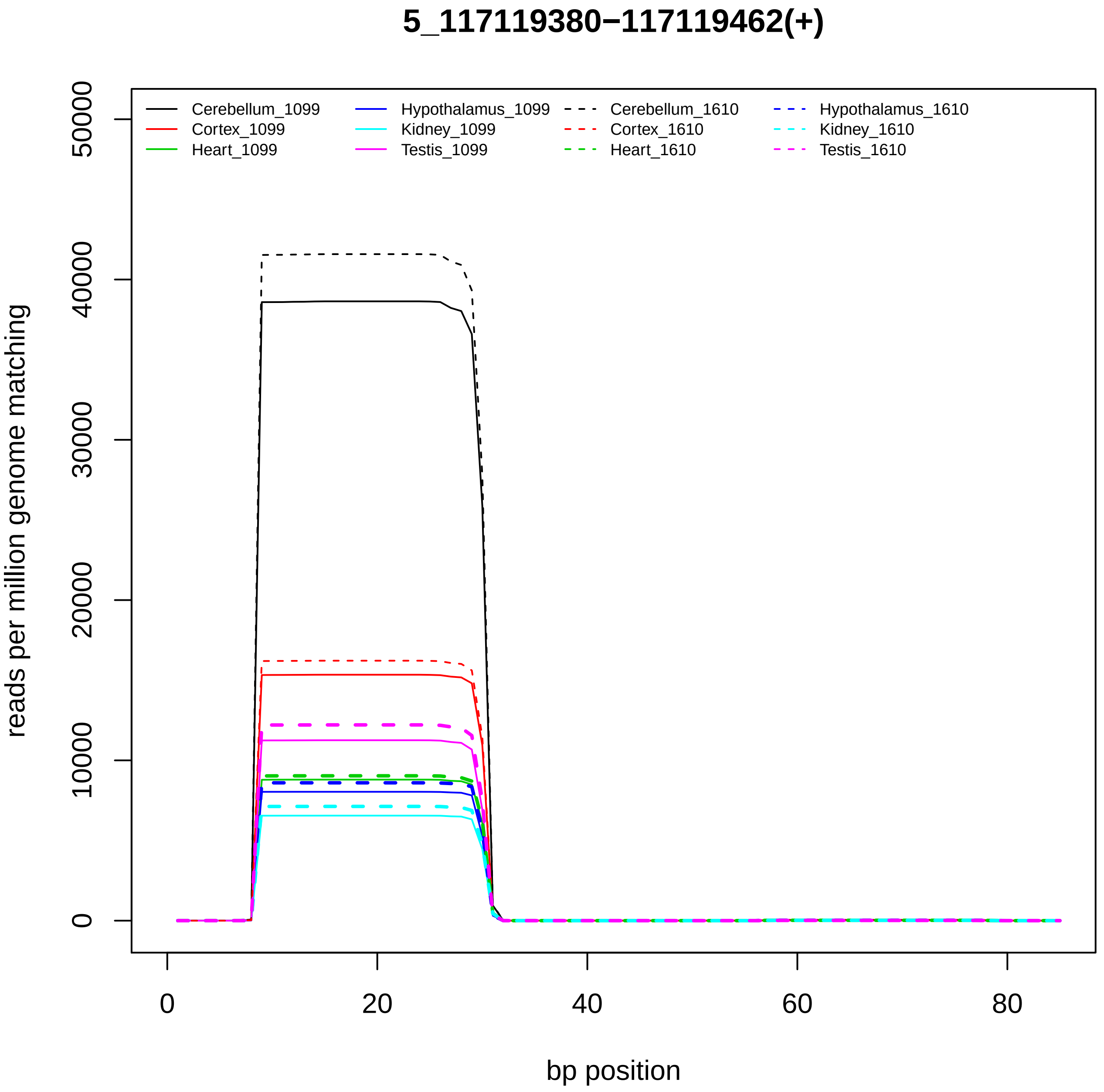

5_117119380−117119462(+)
Cerebellum_1099
Cortex_1099
Heart_1099
Hypothalamus_1099
Kidney_1099
Testis_1099
Cerebellum_1610
Cortex_1610
Heart_1610
Hypothalamus_1610
Kidney_1610
Testis_1610
reads per million genome matching
0
10000
20000
30000
40000
50000
bp position
0
20
40
60
80

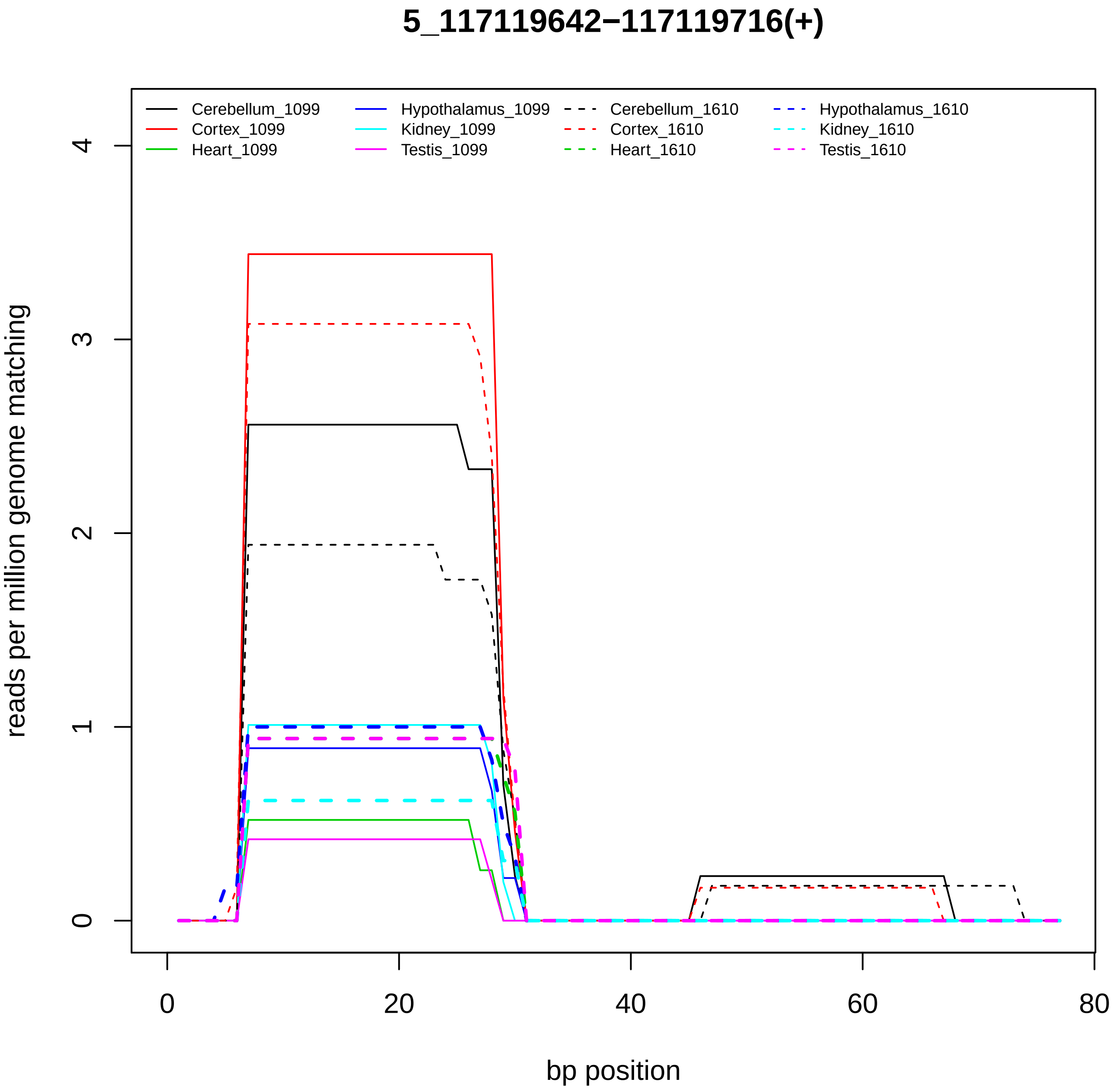

5_117119642−117119716(+)
Cerebellum_1099
Cortex_1099
Heart_1099
Hypothalamus_1099
Kidney_1099
Testis_1099
Cerebellum_1610
Cortex_1610
Heart_1610
Hypothalamus_1610
Kidney_1610
Testis_1610
reads per million genome matching
bp position
0
1
2
3
4
0
20
40
60
80

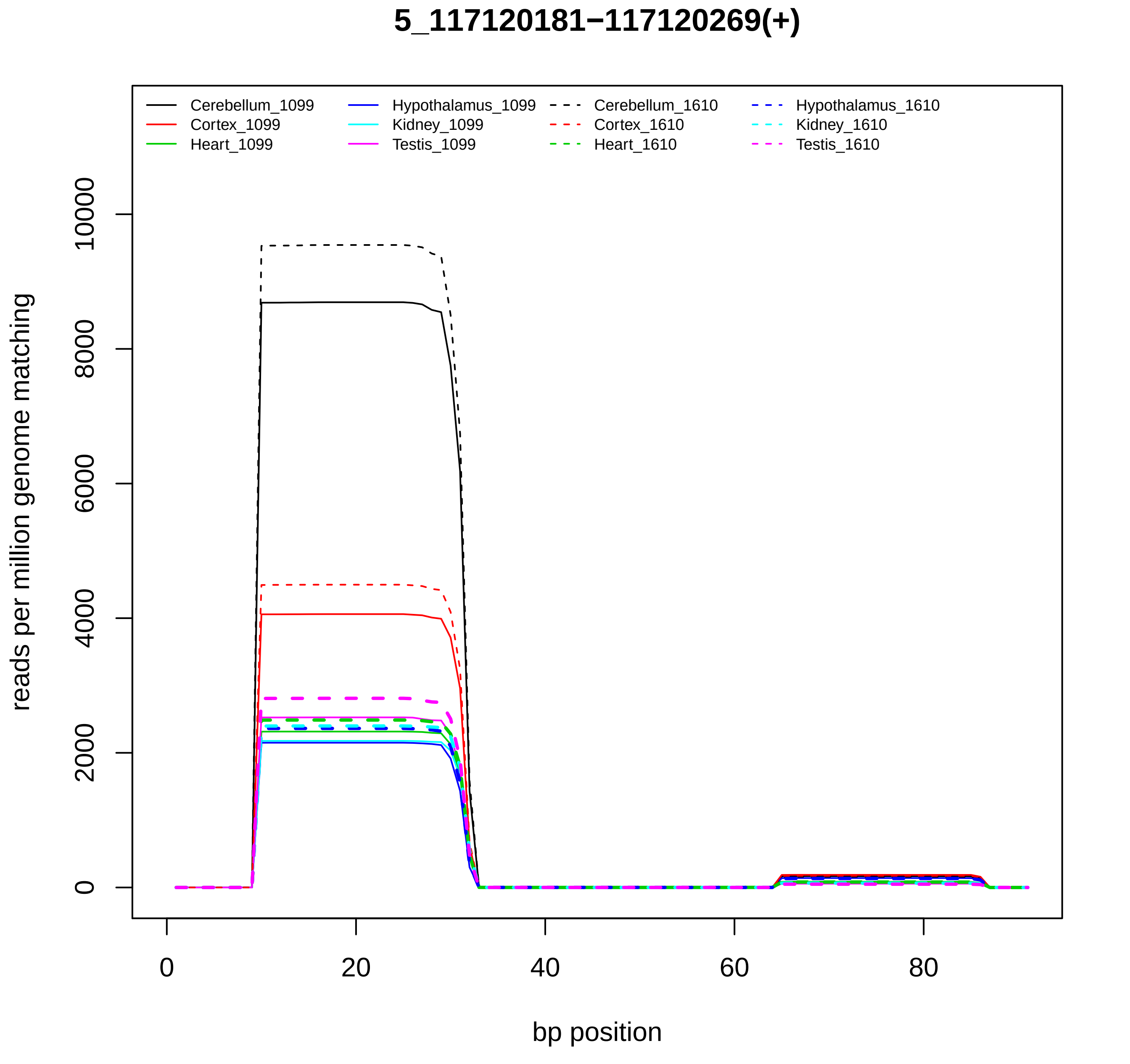

5_117120181−117120269(+)
Cerebellum_1099
Cortex_1099
Heart_1099
Hypothalamus_1099
Kidney_1099
Testis_1099
Cerebellum_1610
Cortex_1610
Heart_1610
Hypothalamus_1610
Kidney_1610
Testis_1610
reads per million genome matching
0
2000
4000
6000
8000
10000
0
20
40
60
80
bp position

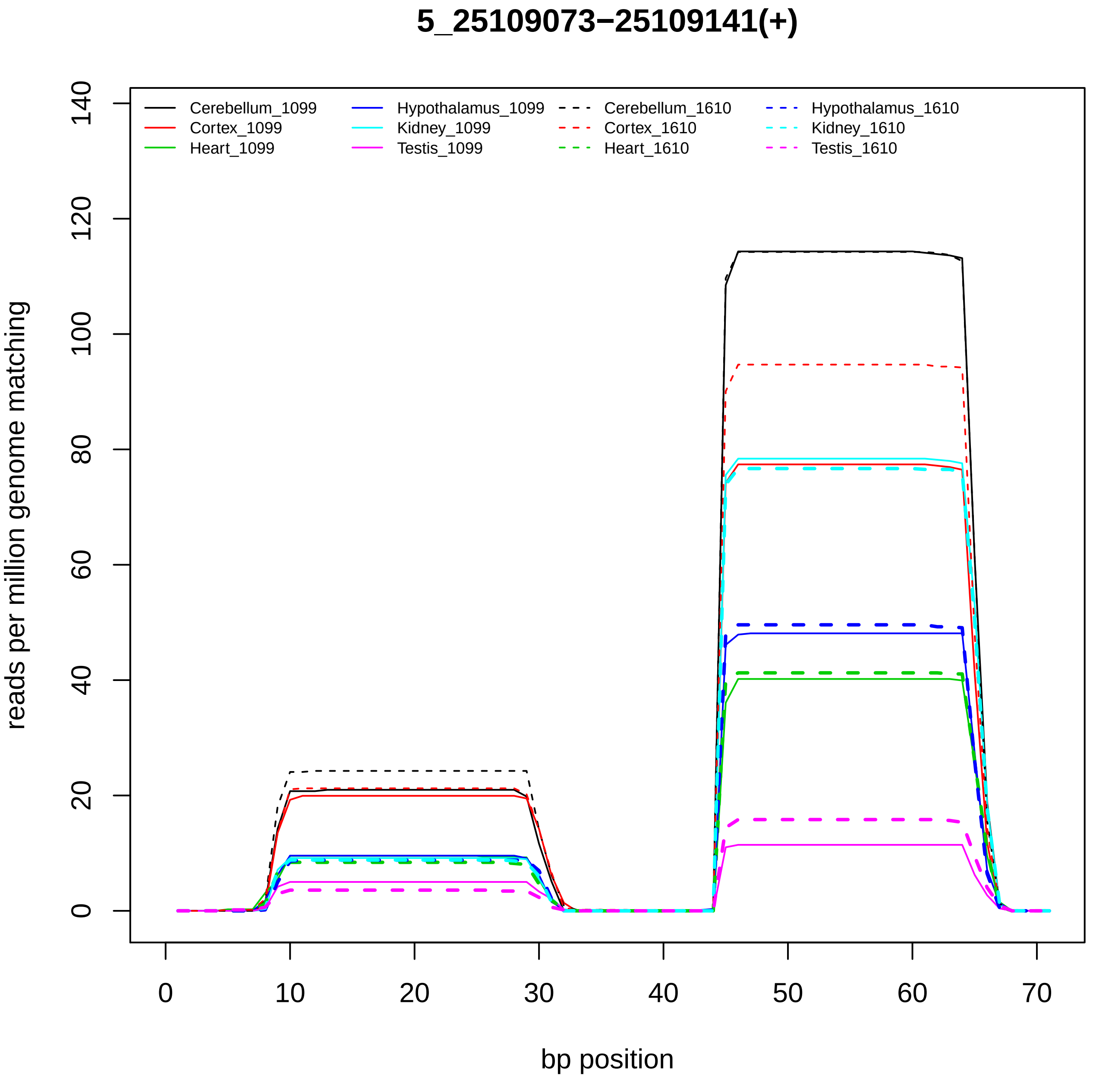

5_25109073−25109141(+)
Cerebellum_1099
Cortex_1099
Heart_1099
Hypothalamus_1099
Kidney_1099
Testis_1099
Cerebellum_1610
Cortex_1610
Heart_1610
Hypothalamus_1610
Kidney_1610
Testis_1610
reads per million genome matching
0
20
40
60
80
100
120
140
0
10
20
30
40
50
60
70
bp position

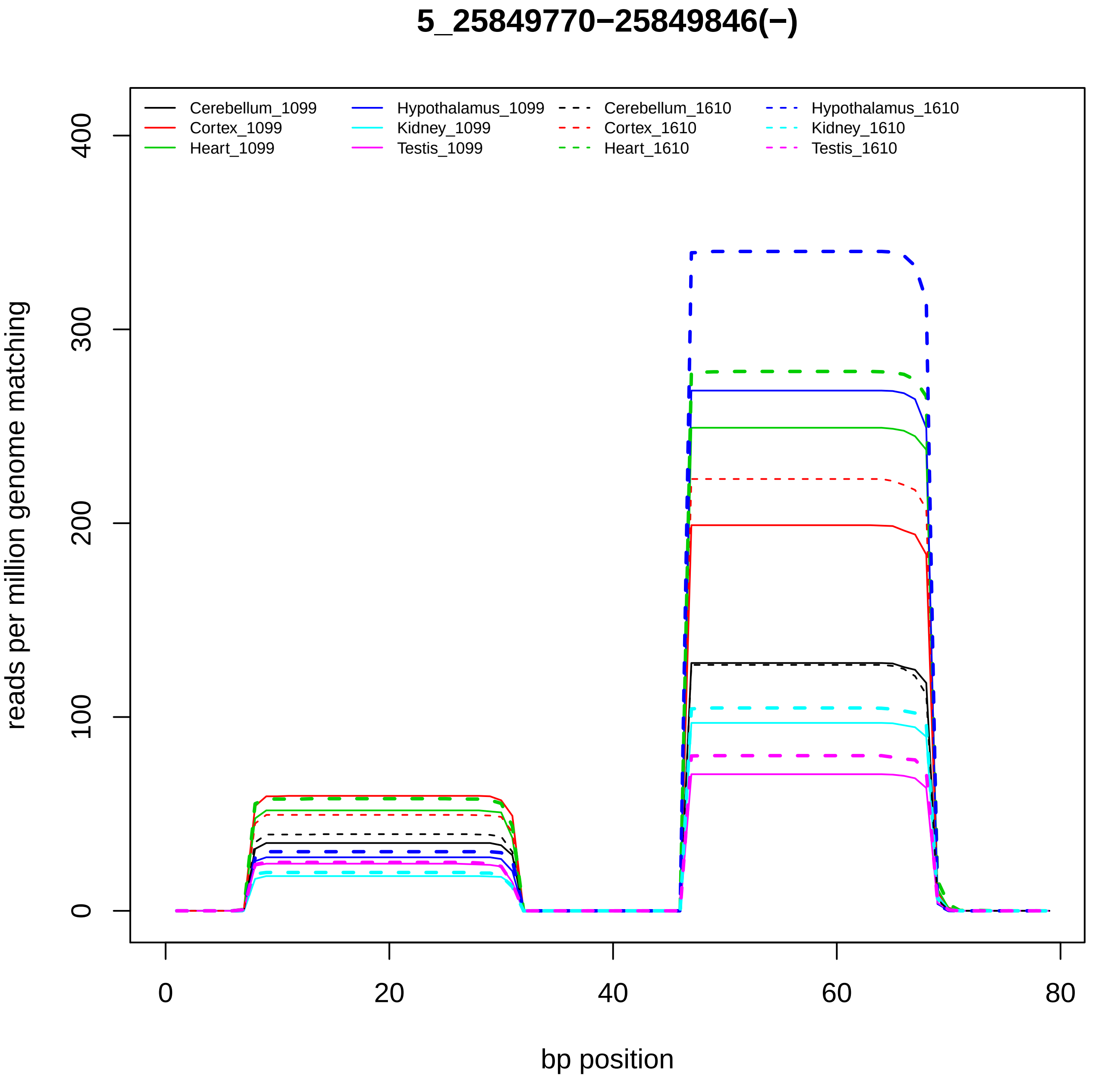

5_25849770−25849846(−)
Cerebellum_1099
Cortex_1099
Heart_1099
Hypothalamus_1099
Kidney_1099
Testis_1099
Cerebellum_1610
Cortex_1610
Heart_1610
Hypothalamus_1610
Kidney_1610
Testis_1610
reads per million genome matching
0
100
200
300
400
0
20
40
60
80
bp position

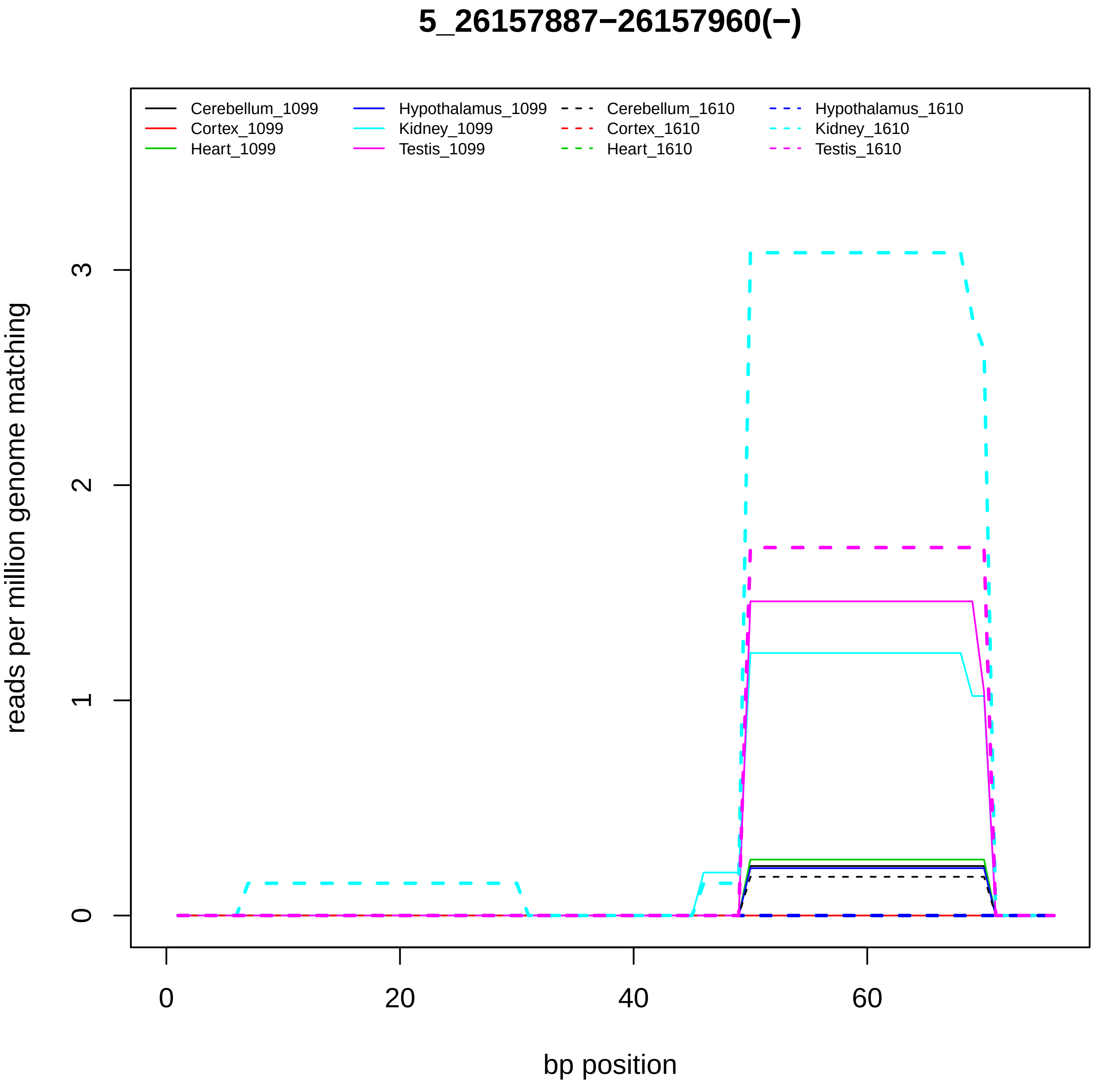
5_26157887−26157960(−)
Cerebellum_1099
Cortex_1099
Heart_1099
Hypothalamus_1099
Kidney_1099
Testis_1099
Cerebellum_1610
Cortex_1610
Heart_1610
Hypothalamus_1610
Kidney_1610
Testis_1610
reads per million genome matching
bp position
0
1
2
3
0
20
40
60

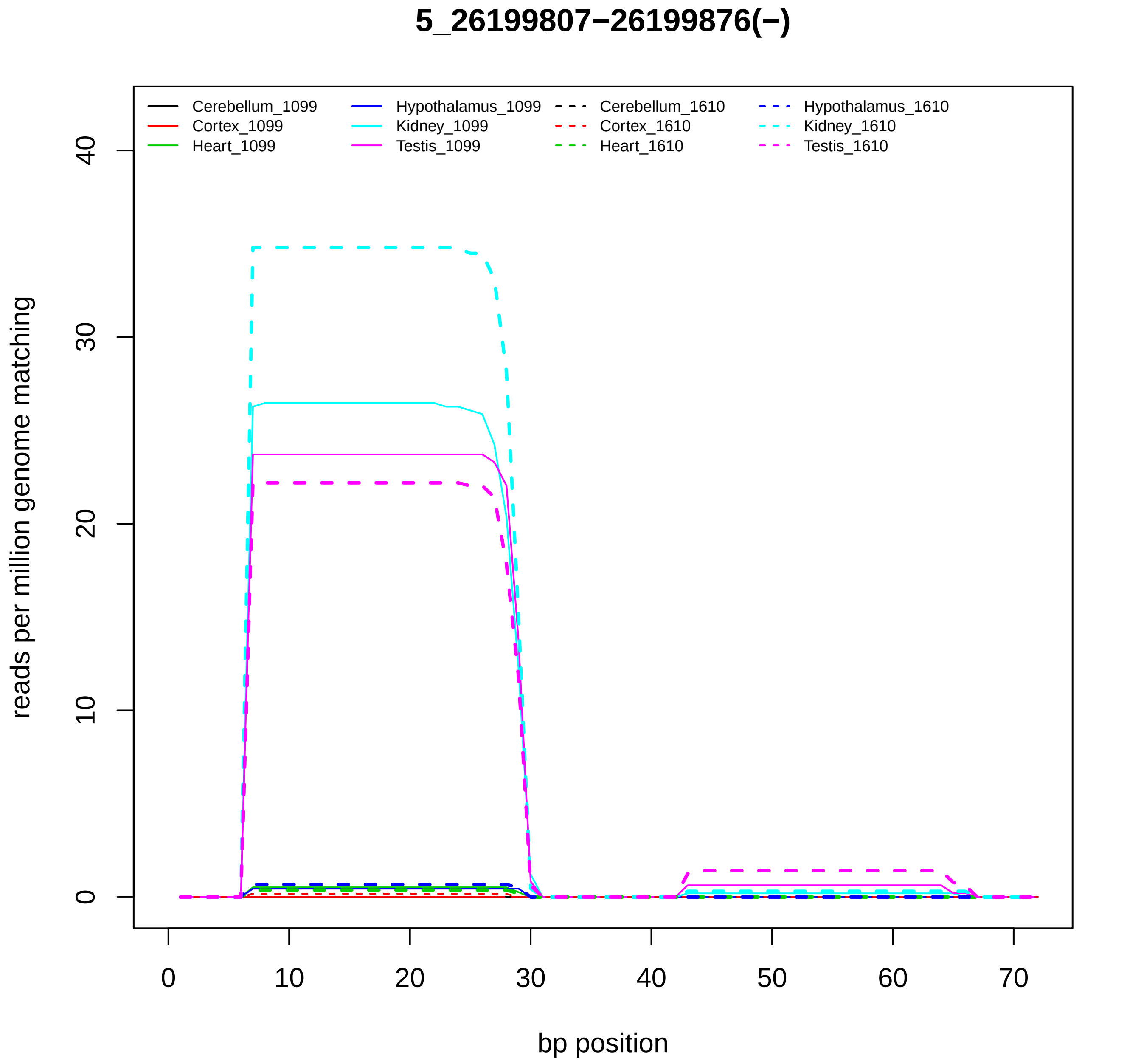

5_26199807−26199876(−)
Cerebellum_1099
Cortex_1099
Heart_1099
Hypothalamus_1099
Kidney_1099
Testis_1099
Cerebellum_1610
Cortex_1610
Heart_1610
Hypothalamus_1610
Kidney_1610
Testis_1610
reads per million genome matching
0
10
20
30
40
0
10
20
30
40
50
60
70
bp position

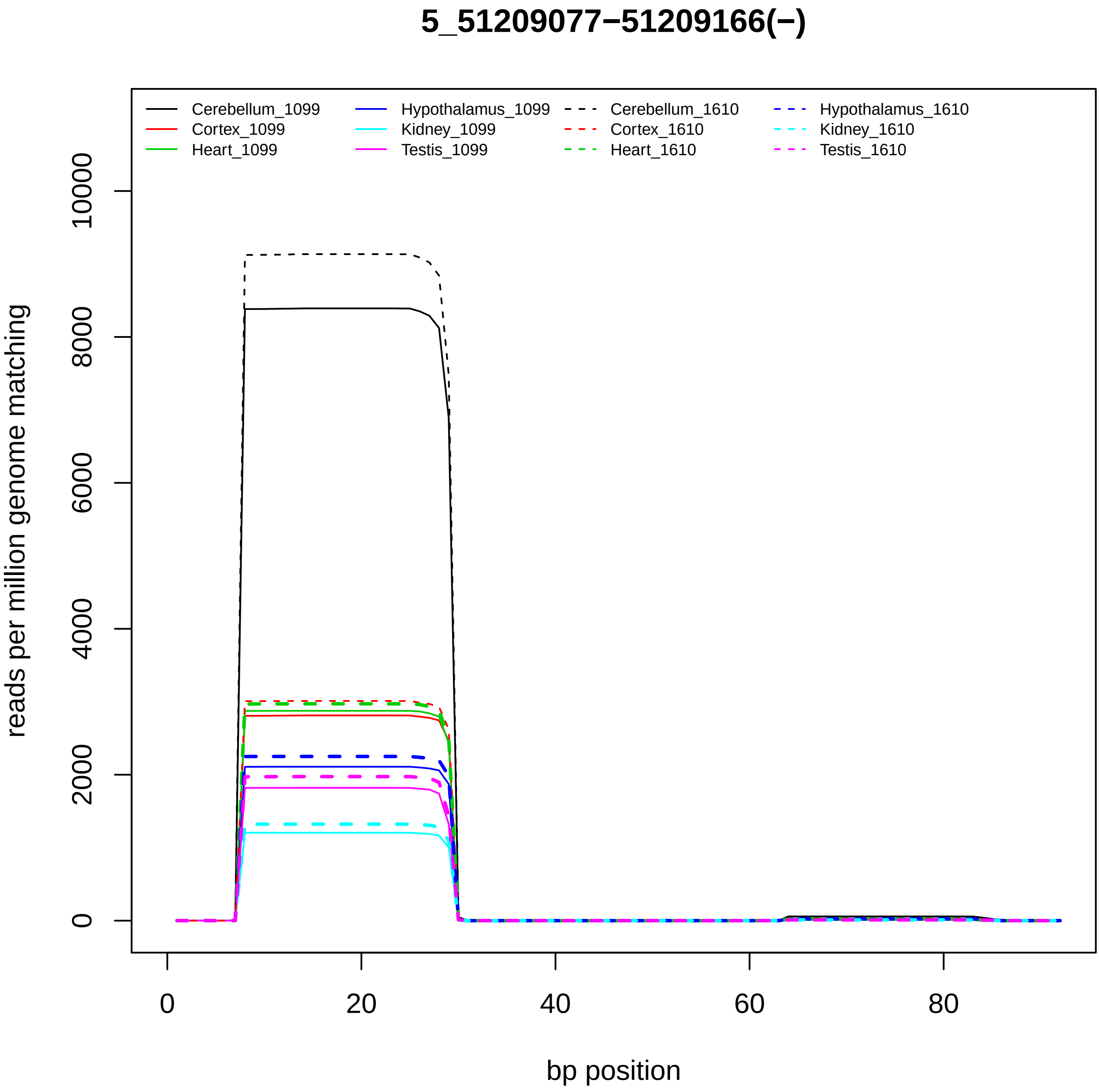

5_51209077−51209166(−)
Cerebellum_1099
Cortex_1099
Heart_1099
Hypothalamus_1099
Kidney_1099
Testis_1099
Cerebellum_1610
Cortex_1610
Heart_1610
Hypothalamus_1610
Kidney_1610
Testis_1610
reads per million genome matching
0
2000
4000
6000
8000
10000
0
20
40
60
80
bp position

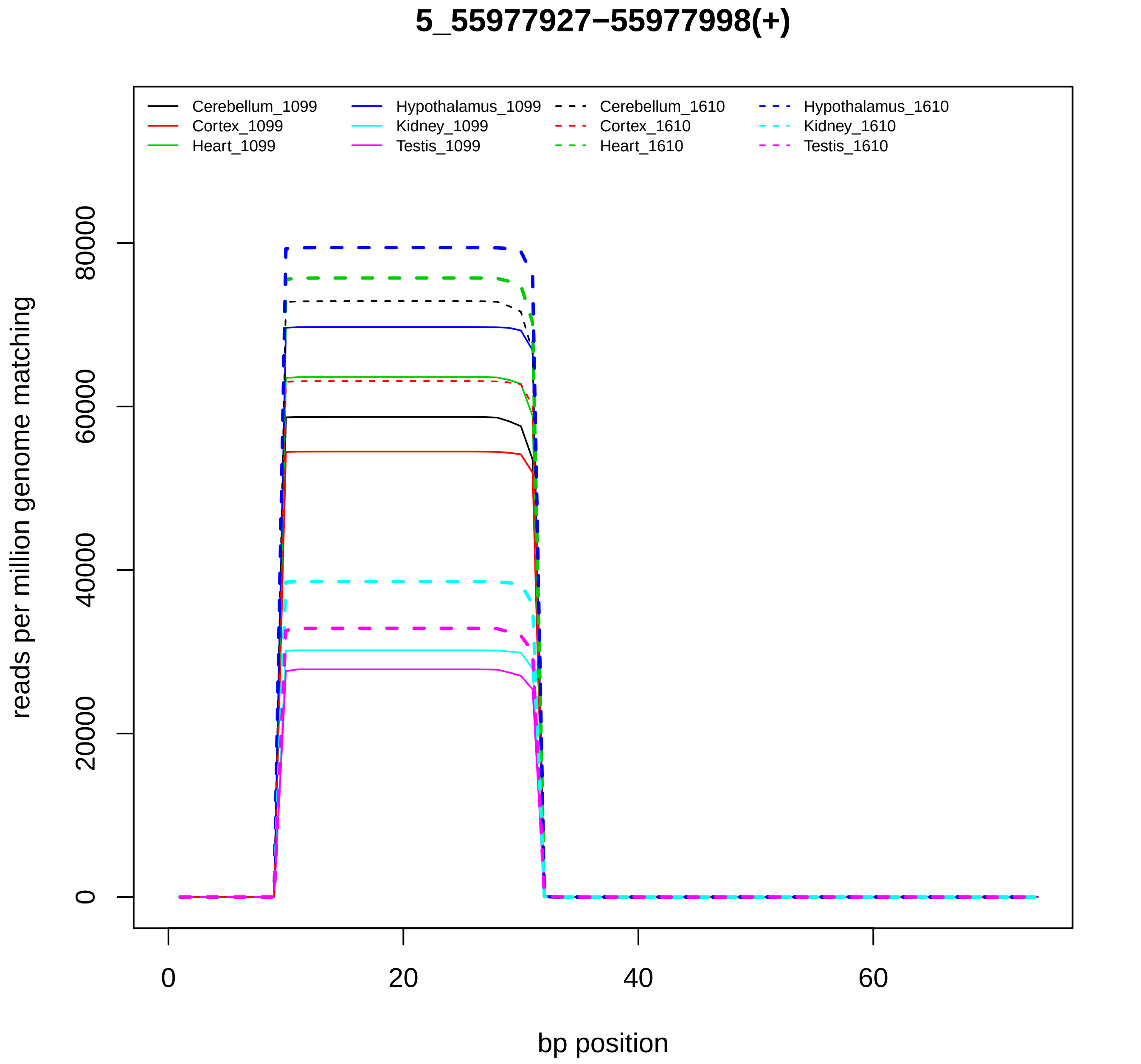
5_55977927−55977998(+)
Cerebellum_1099
Cortex_1099
Heart_1099
Hypothalamus_1099
Kidney_1099
Testis_1099
Cerebellum_1610
Cortex_1610
Heart_1610
Hypothalamus_1610
Kidney_1610
Testis_1610
reads per million genome matching
0
20000
40000
60000
80000
0
20
40
60
bp position

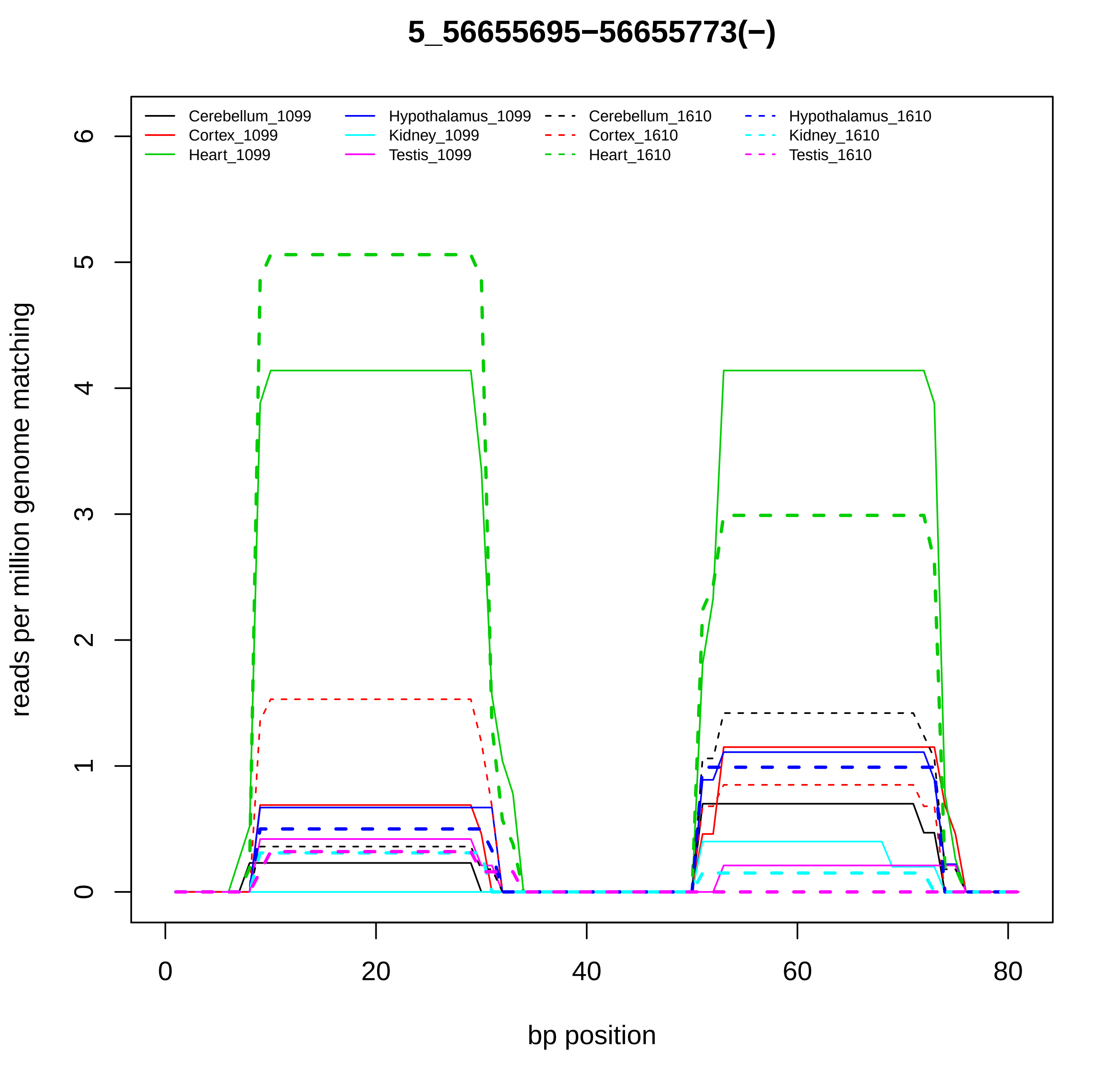

5_56655695−56655773(−)
Cerebellum_1099
Cortex_1099
Heart_1099
Hypothalamus_1099
Kidney_1099
Testis_1099
Cerebellum_1610
Cortex_1610
Heart_1610
Hypothalamus_1610
Kidney_1610
Testis_1610
reads per million genome matching
bp position
0
1
2
3
4
5
6
0
20
40
60
80

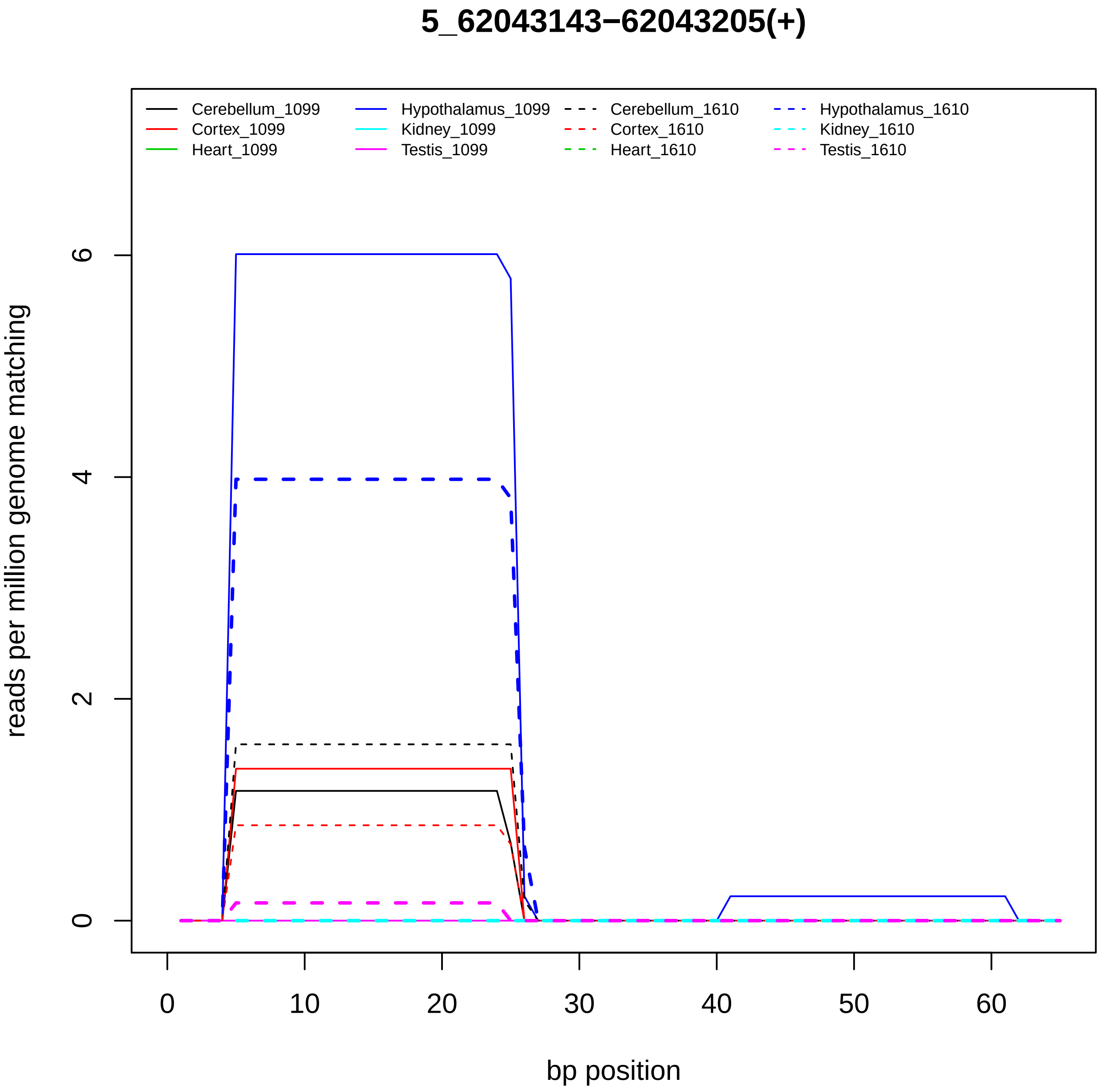

5_62043143−62043205(+)
Cerebellum_1099
Cortex_1099
Heart_1099
Hypothalamus_1099
Kidney_1099
Testis_1099
Cerebellum_1610
Cortex_1610
Heart_1610
Hypothalamus_1610
Kidney_1610
Testis_1610
reads per million genome matching
0
2
4
6
0
10
20
30
40
50
60
bp position

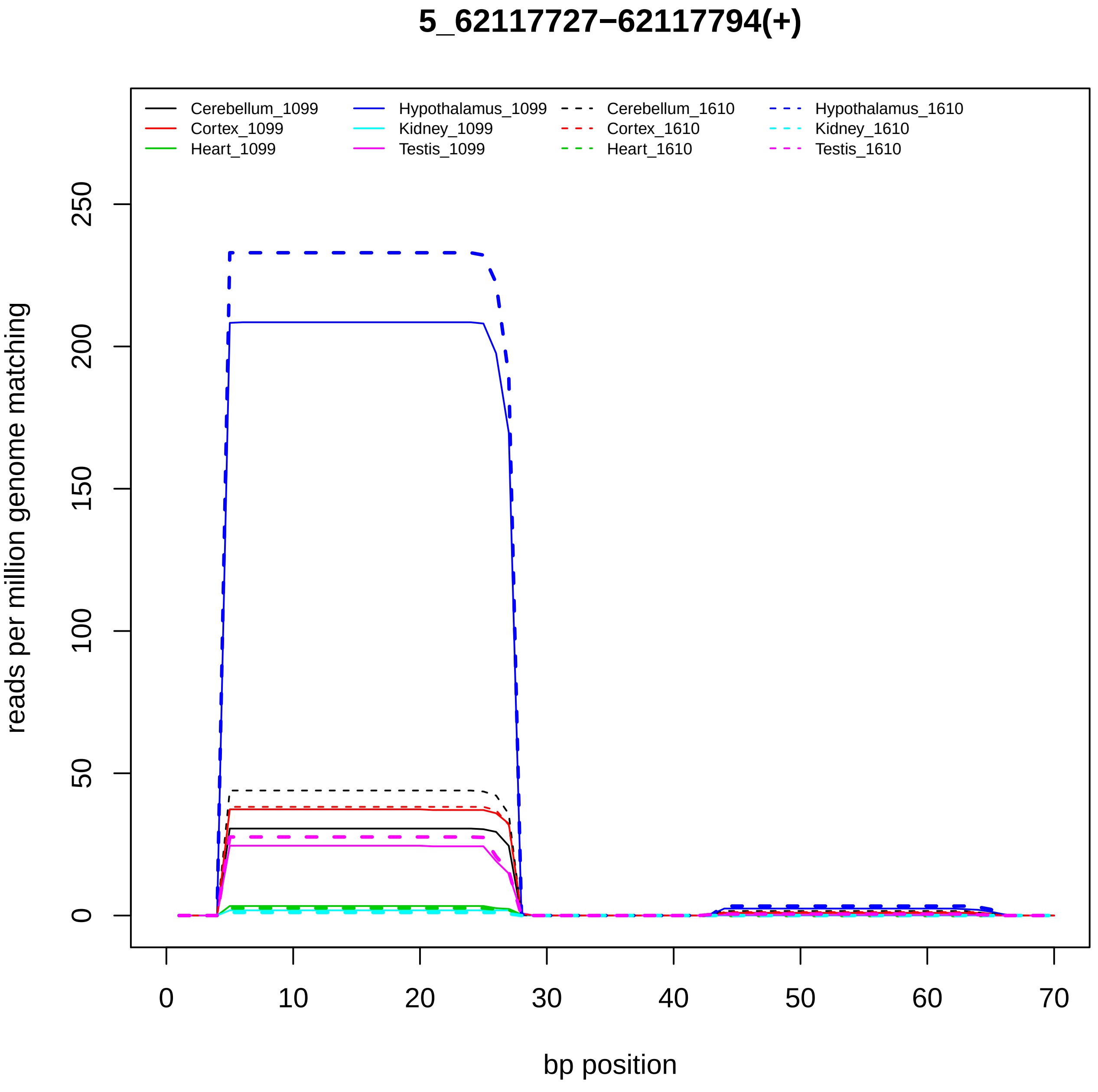

5_62117727−62117794(+)
Cerebellum_1099
Cortex_1099
Heart_1099
Hypothalamus_1099
Kidney_1099
Testis_1099
Cerebellum_1610
Cortex_1610
Heart_1610
Hypothalamus_1610
Kidney_1610
Testis_1610
reads per million genome matching
0
50
100
150
200
250
0
10
20
30
40
50
60
70
bp position

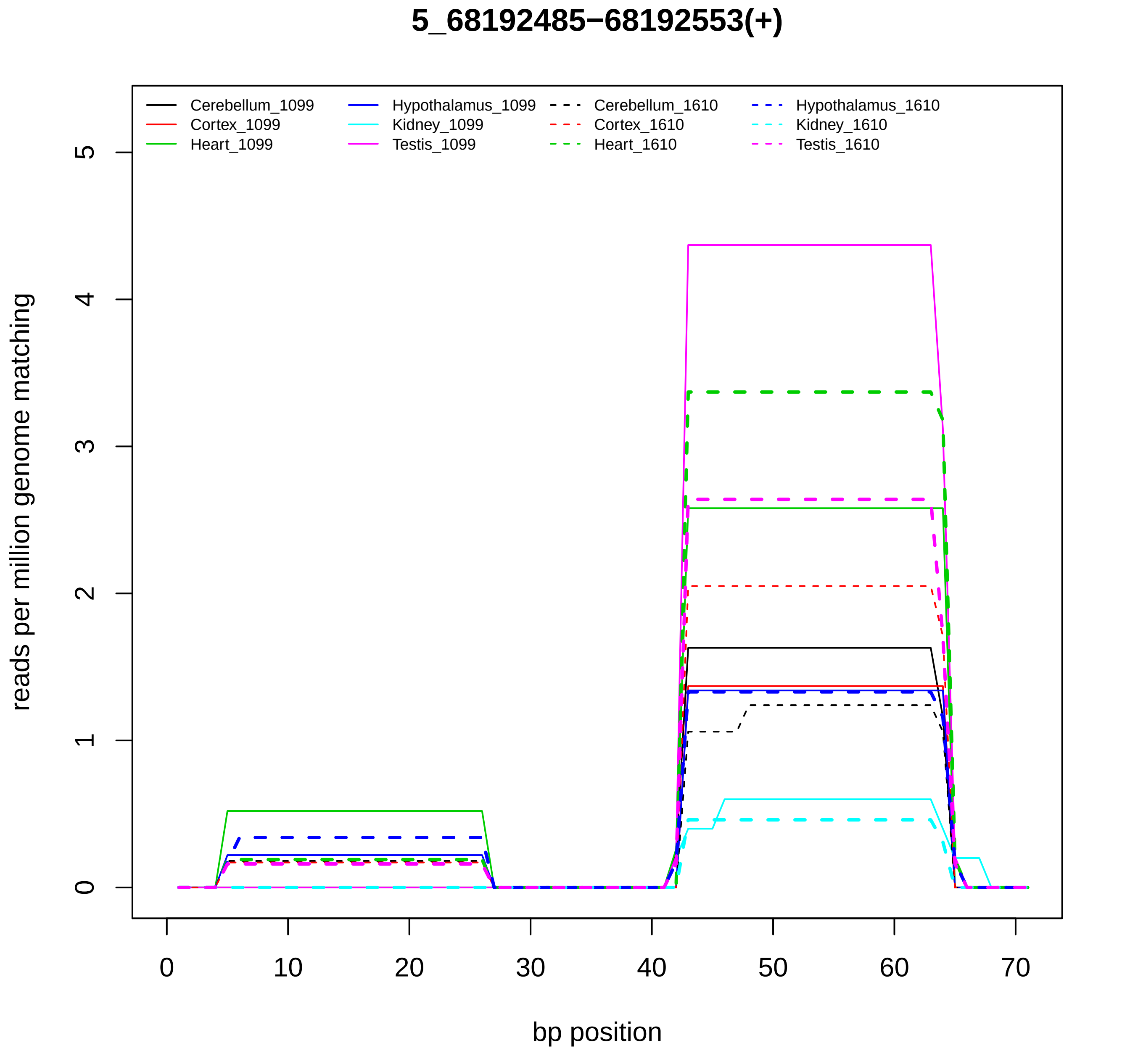

5_68192485−68192553(+)
Cerebellum_1099
Cortex_1099
Heart_1099
Hypothalamus_1099
Kidney_1099
Testis_1099
Cerebellum_1610
Cortex_1610
Heart_1610
Hypothalamus_1610
Kidney_1610
Testis_1610
reads per million genome matching
0
1
2
3
4
5
0
10
20
30
40
50
60
70
bp position

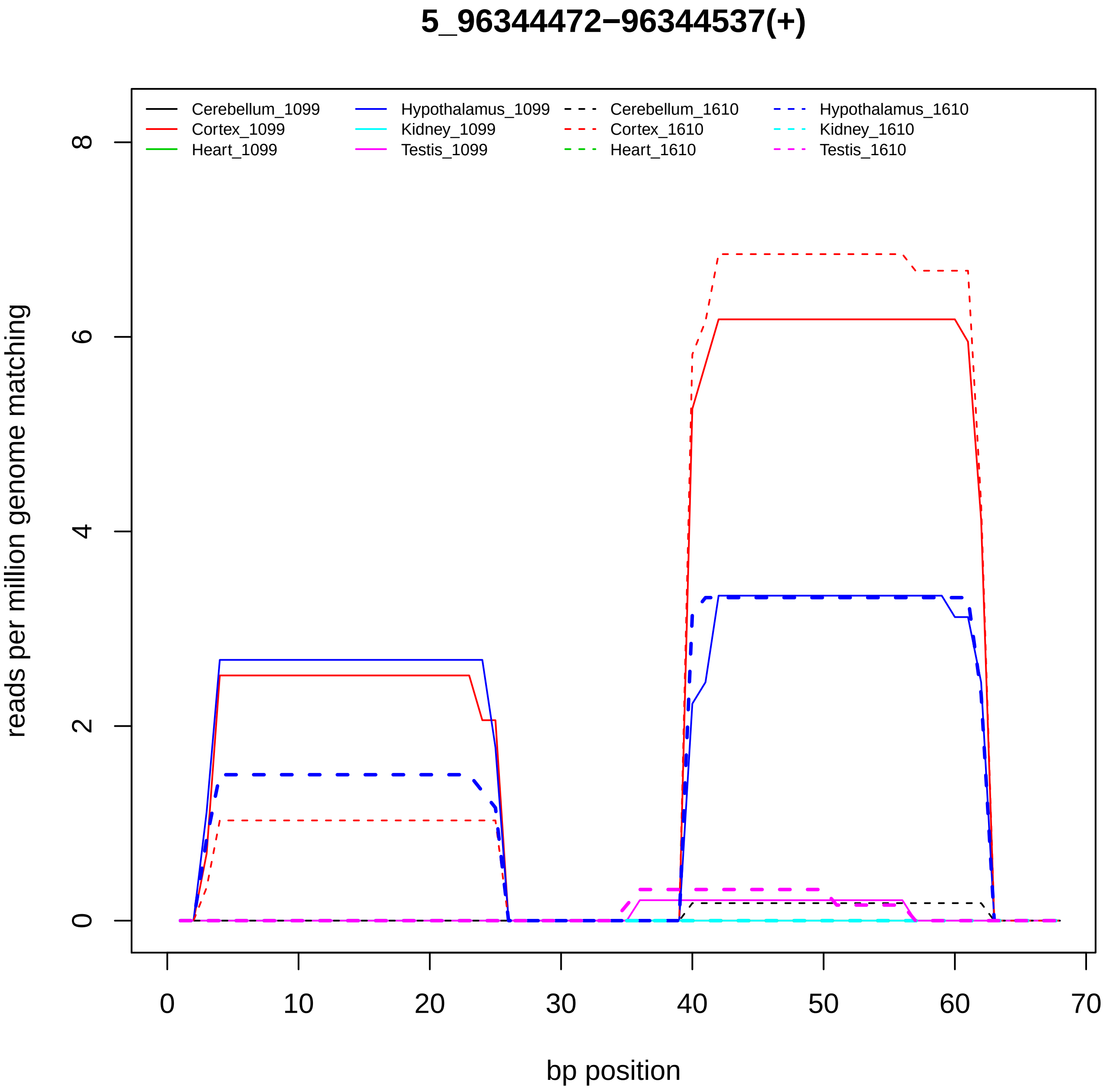

5_96344472−96344537(+)
Cerebellum_1099
Cortex_1099
Heart_1099
Hypothalamus_1099
Kidney_1099
Testis_1099
Cerebellum_1610
Cortex_1610
Heart_1610
Hypothalamus_1610
Kidney_1610
Testis_1610
reads per million genome matching
0
2
4
6
8
bp position
0
10
20
30
40
50
60
70

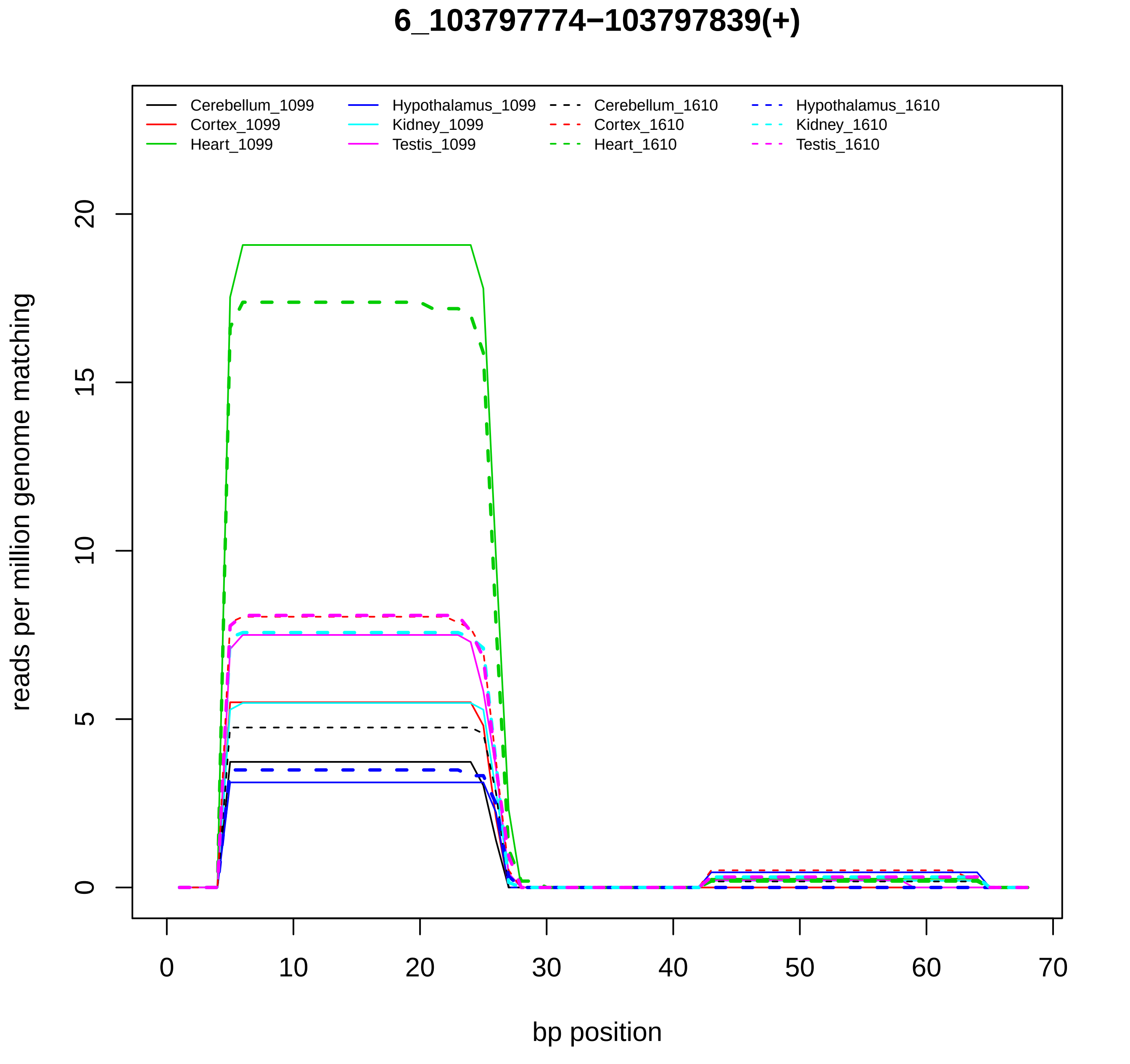
6_103797774−103797839(+)
Cerebellum_1099
Cortex_1099
Heart_1099
Hypothalamus_1099
Kidney_1099
Testis_1099
Cerebellum_1610
Cortex_1610
Heart_1610
Hypothalamus_1610
Kidney_1610
Testis_1610
reads per million genome matching
0
5
10
15
20
bp position
0
10
20
30
40
50
60
70

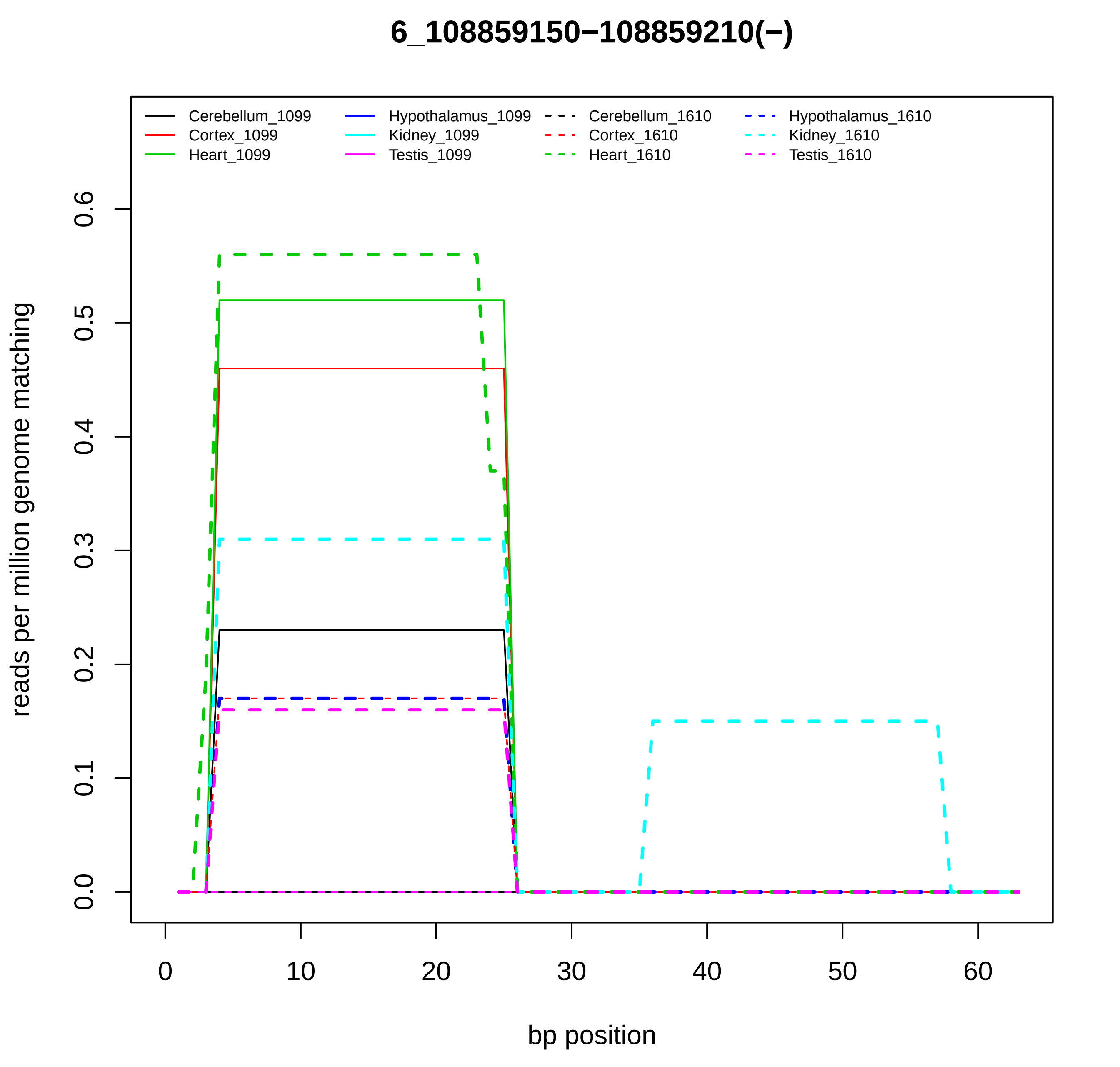
6_108859150−108859210(−)
Cerebellum_1099
Cortex_1099
Heart_1099
Hypothalamus_1099
Kidney_1099
Testis_1099
Cerebellum_1610
Cortex_1610
Heart_1610
Hypothalamus_1610
Kidney_1610
Testis_1610
reads per million genome matching
0.0
0.1
0.2
0.3
0.4
0.5
0.6
0
10
20
30
40
50
60
bp position

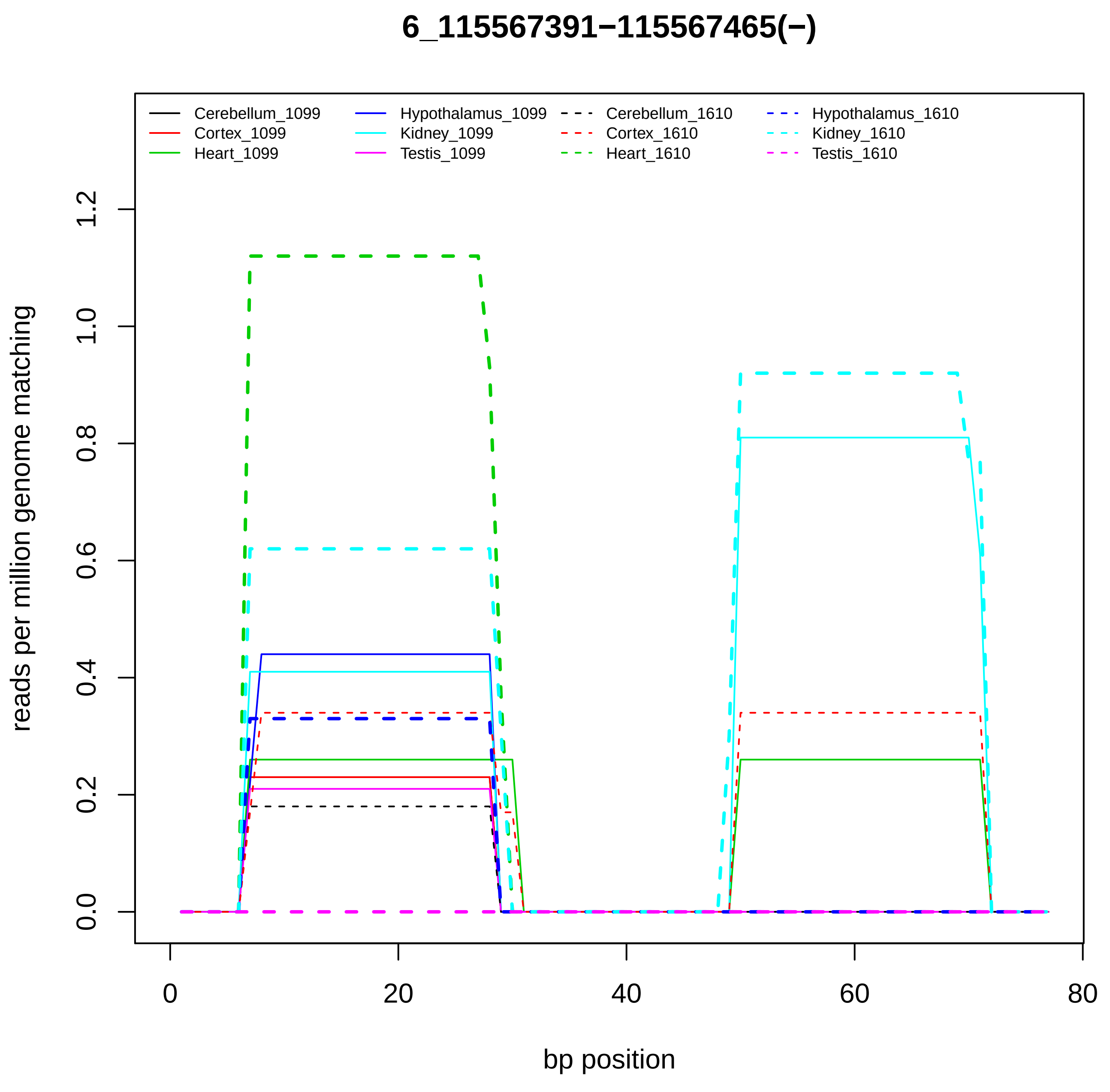

6_115567391−115567465(−)
Cerebellum_1099
Cortex_1099
Heart_1099
Hypothalamus_1099
Kidney_1099
Testis_1099
Cerebellum_1610
Cortex_1610
Heart_1610
Hypothalamus_1610
Kidney_1610
Testis_1610
reads per million genome matching
0.0
0.2
0.4
0.6
0.8
1.0
1.2
0
20
40
60
80
bp position

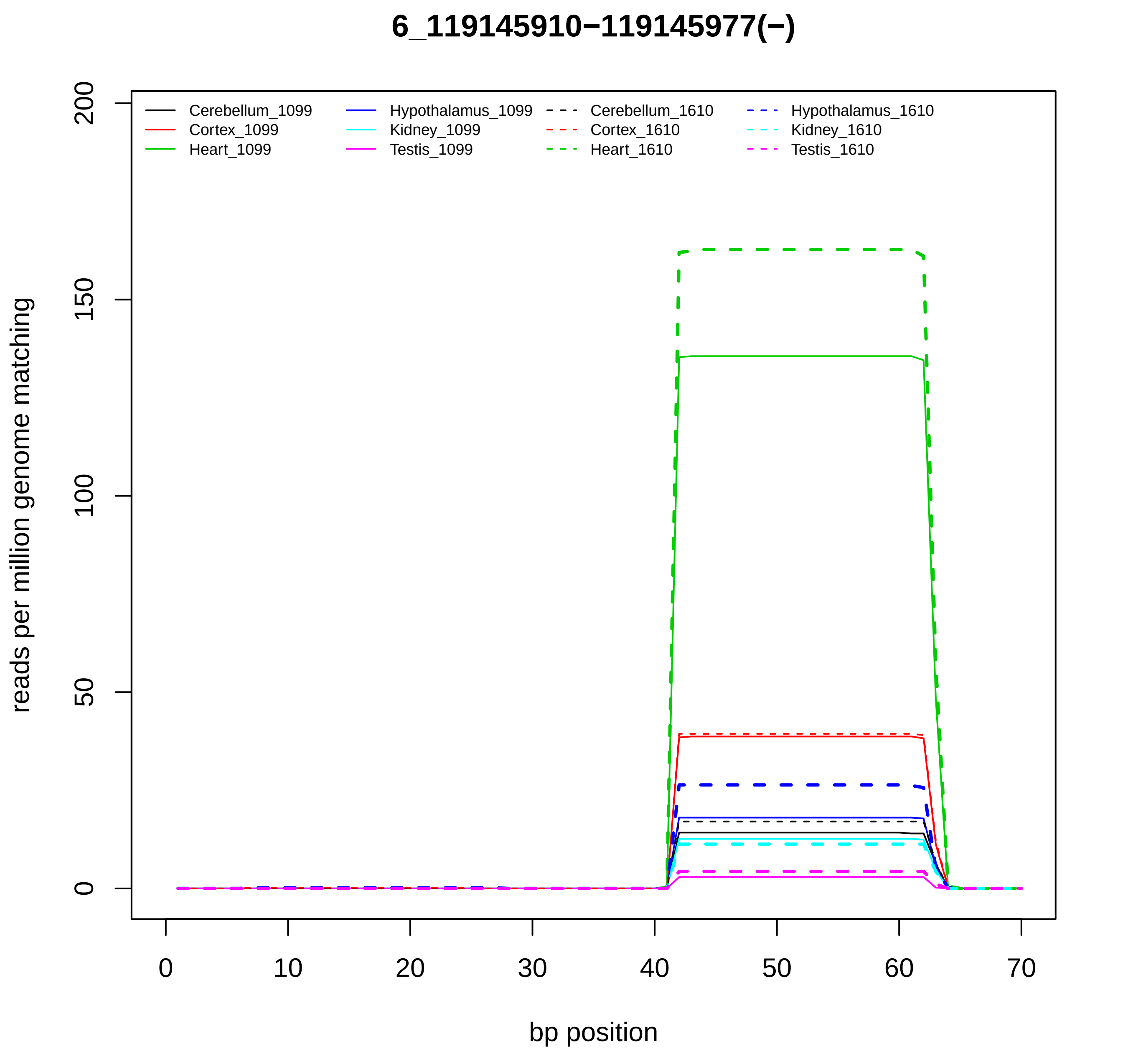
6_119145910−119145977(−)
reads per million genome matching
bp position
Cerebellum_1099
Cortex_1099
Heart_1099
Hypothalamus_1099
Kidney_1099
Testis_1099
Cerebellum_1610
Cortex_1610
Heart_1610
Hypothalamus_1610
Kidney_1610
Testis_1610
0
50
100
150
200
0
10
20
30
40
50
60
70

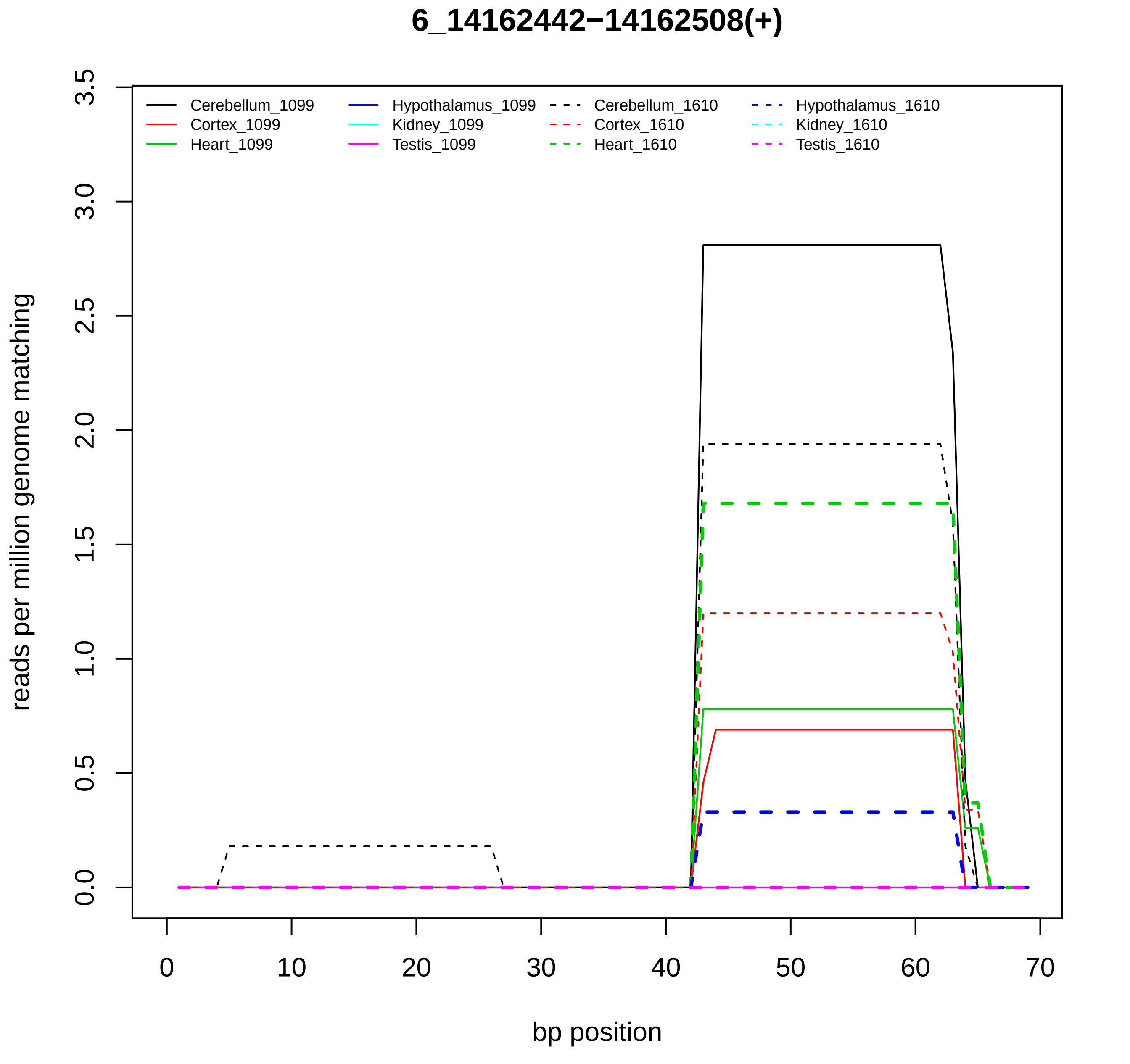

6_14162442−14162508(+)
Cerebellum_1099
Cortex_1099
Heart_1099
Hypothalamus_1099
Kidney_1099
Testis_1099
Cerebellum_1610
Cortex_1610
Heart_1610
Hypothalamus_1610
Kidney_1610
Testis_1610
reads per million genome matching
bp position
0.0
0.5
1.0
1.5
2.0
2.5
3.0
3.5
0
10
20
30
40
50
60
70

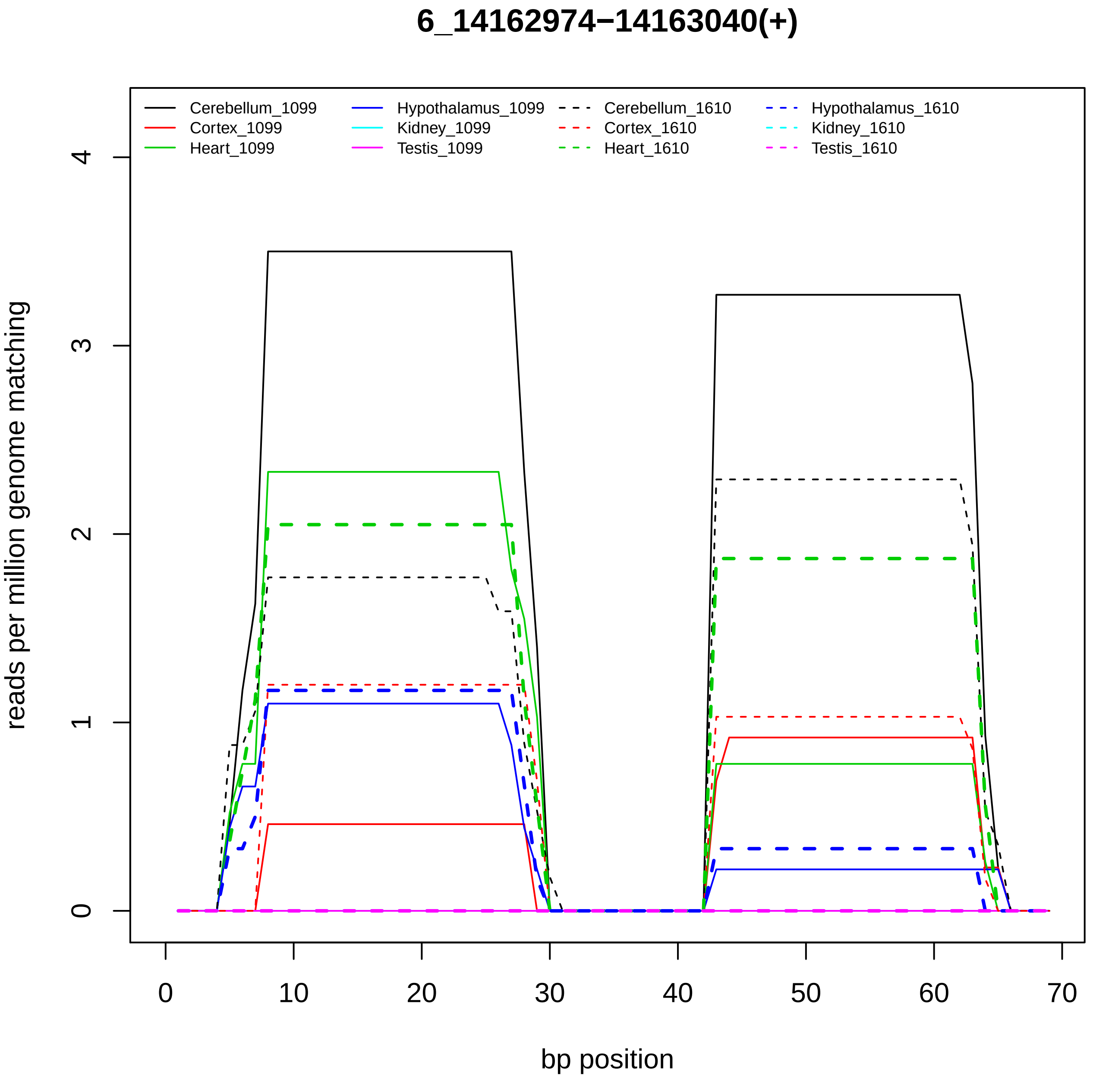

6_14162974−14163040(+)
Cerebellum_1099
Cortex_1099
Heart_1099
Hypothalamus_1099
Kidney_1099
Testis_1099
Cerebellum_1610
Cortex_1610
Heart_1610
Hypothalamus_1610
Kidney_1610
Testis_1610
reads per million genome matching
bp position
0
1
2
3
4
0
10
20
30
40
50
60
70

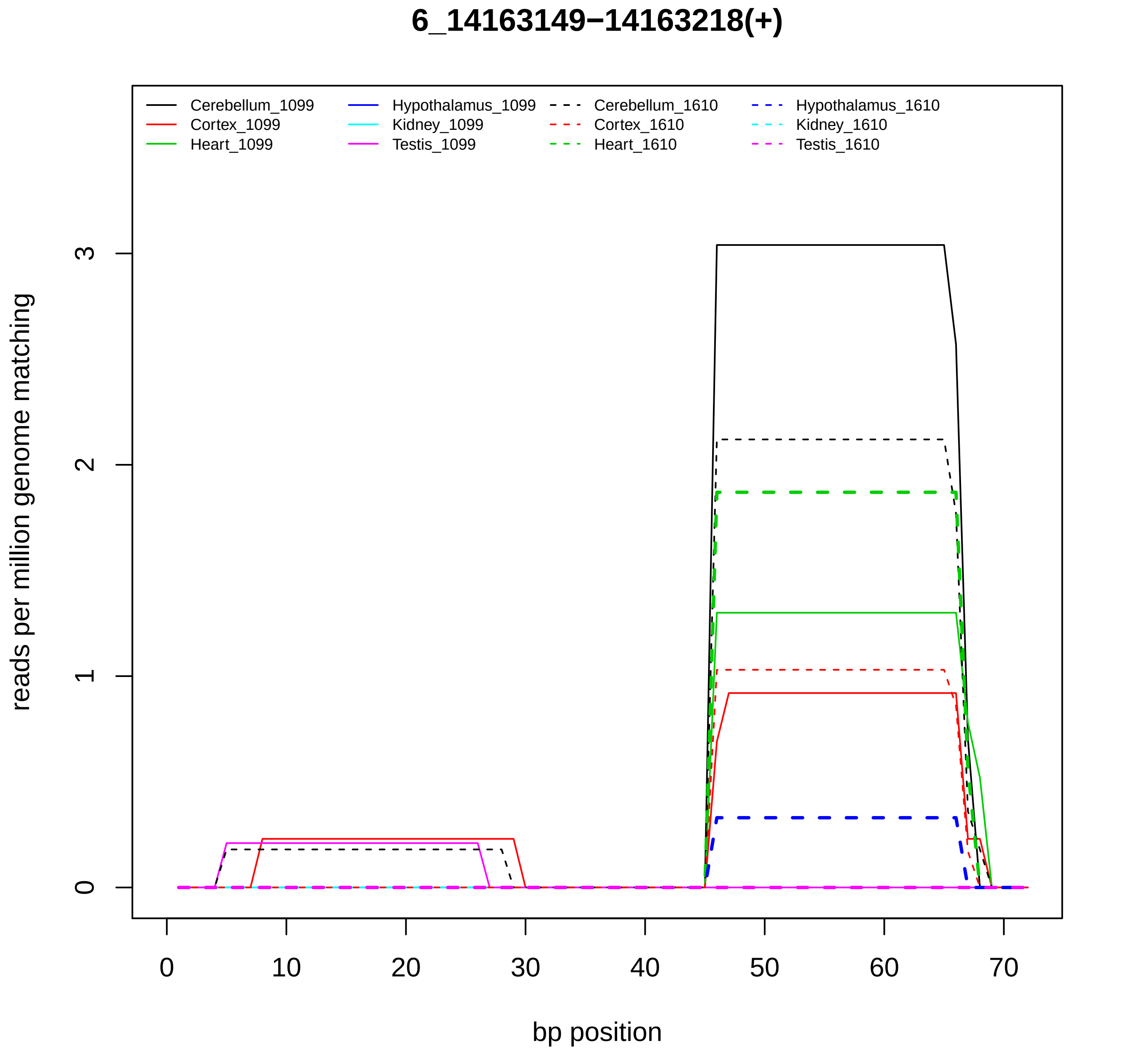

6_14163149−14163218(+)
Cerebellum_1099
Cortex_1099
Heart_1099
Hypothalamus_1099
Kidney_1099
Testis_1099
Cerebellum_1610
Cortex_1610
Heart_1610
Hypothalamus_1610
Kidney_1610
Testis_1610
reads per million genome matching
0
1
2
3
0
10
20
30
40
50
60
70
bp position

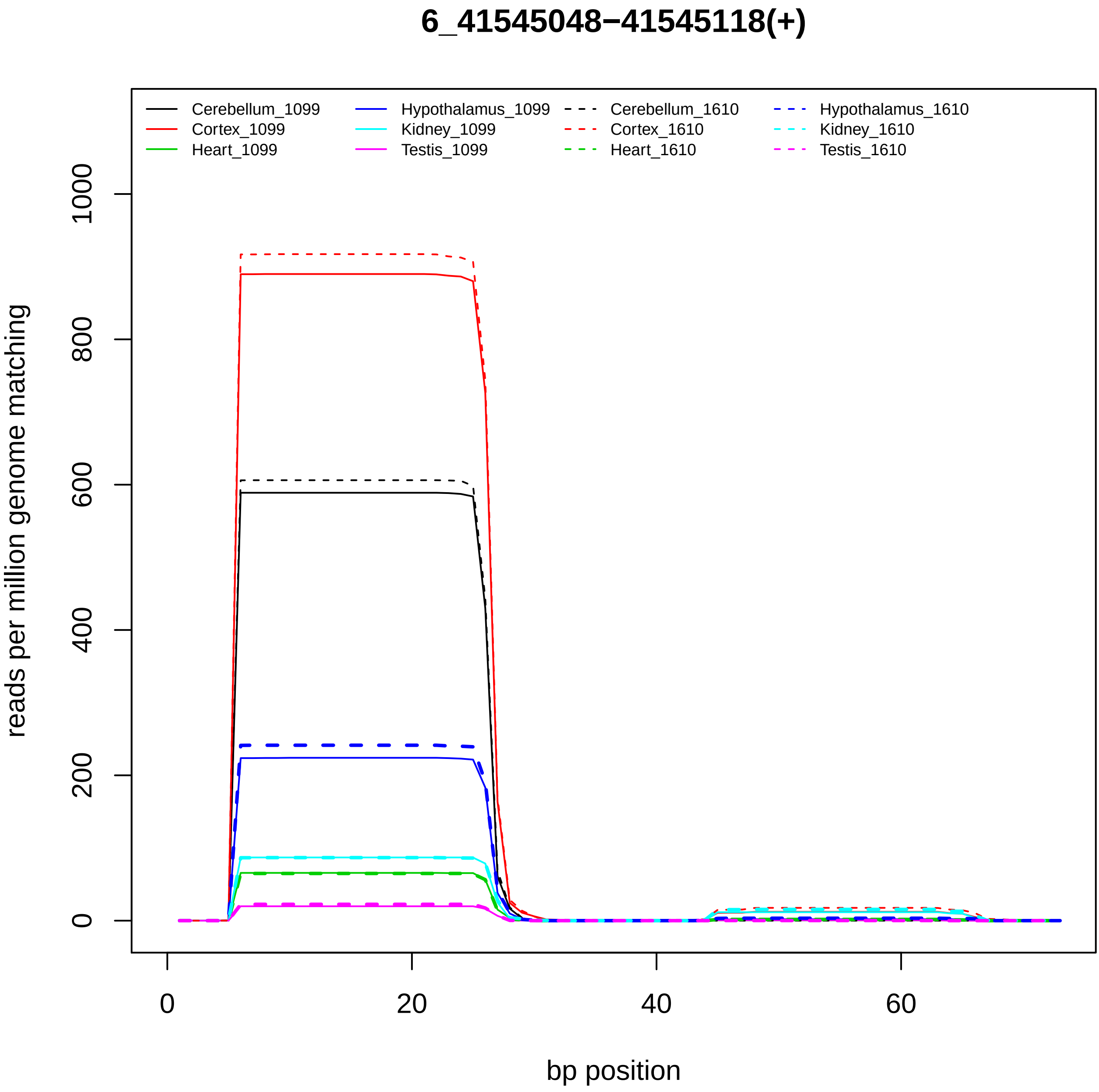
6_41545048−41545118(+)
Cerebellum_1099
Cortex_1099
Heart_1099
Hypothalamus_1099
Kidney_1099
Testis_1099
Cerebellum_1610
Cortex_1610
Heart_1610
Hypothalamus_1610
Kidney_1610
Testis_1610
reads per million genome matching
0
200
400
600
800
1000
0
20
40
60
bp position

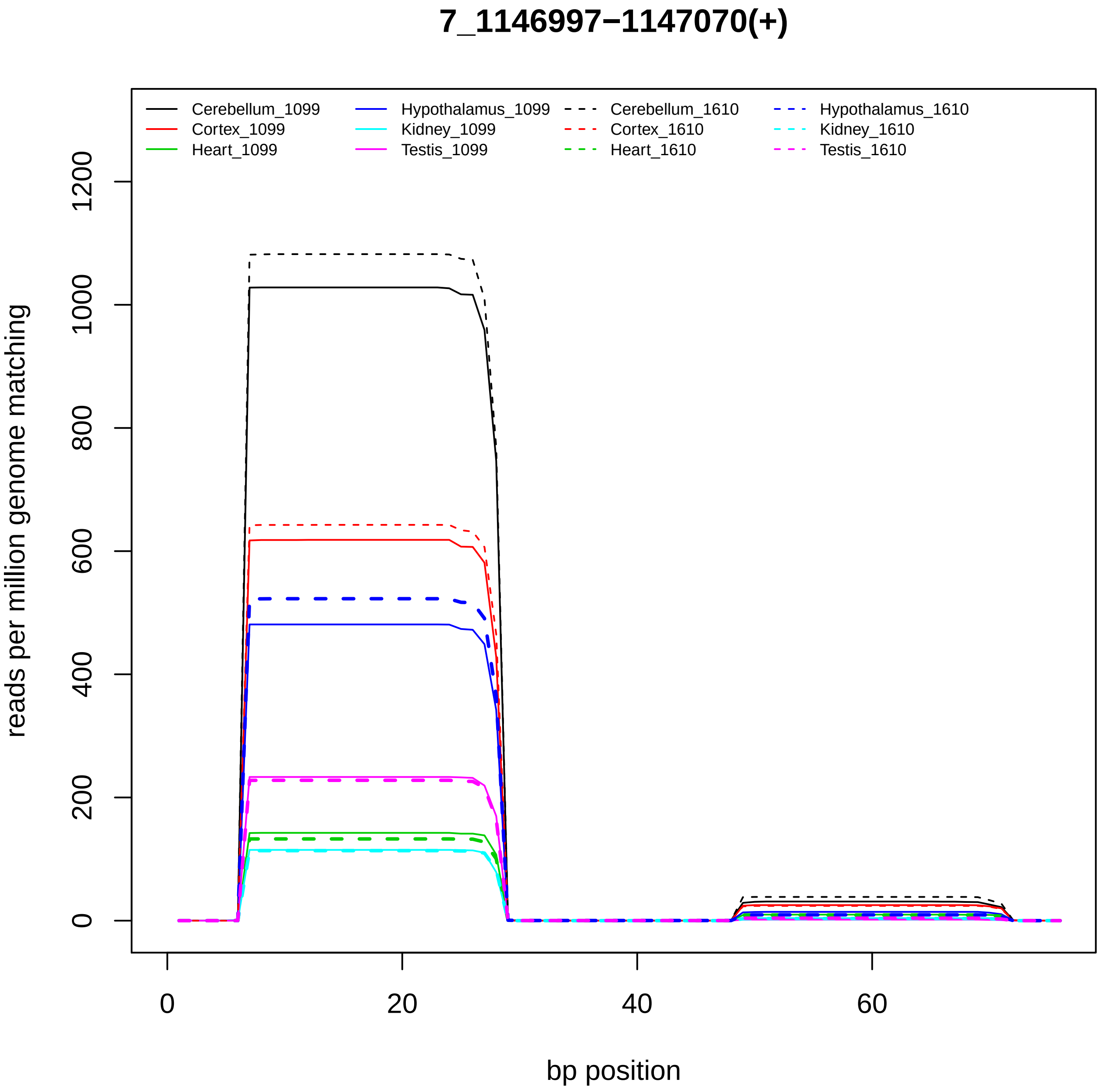

7_1146997−1147070(+)
Cerebellum_1099
Cortex_1099
Heart_1099
Hypothalamus_1099
Kidney_1099
Testis_1099
Cerebellum_1610
Cortex_1610
Heart_1610
Hypothalamus_1610
Kidney_1610
Testis_1610
reads per million genome matching
0
200
400
600
800
1000
1200
0
20
40
60
bp position

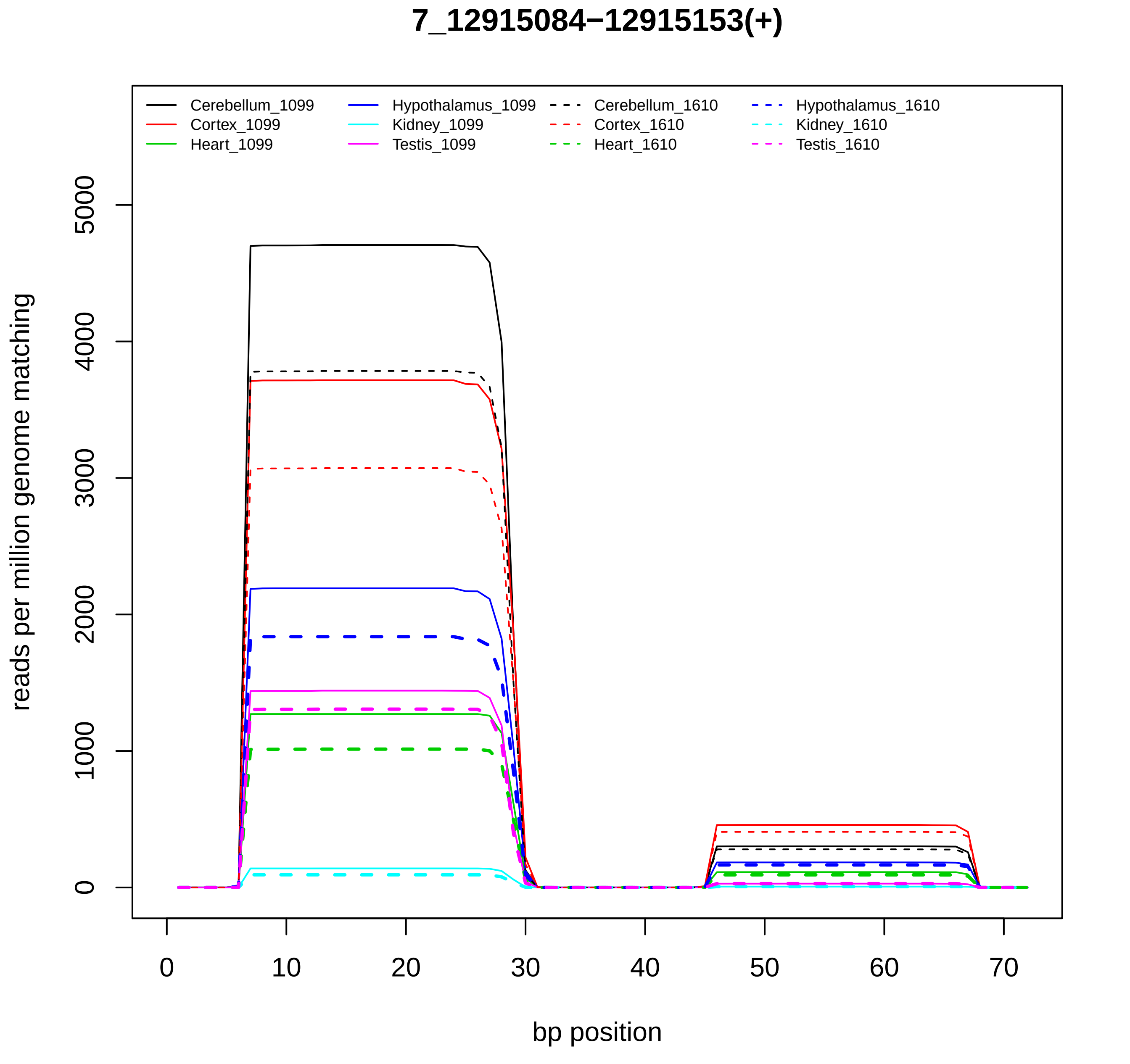
7_12915084−12915153(+)
Cerebellum_1099
Cortex_1099
Heart_1099
Hypothalamus_1099
Kidney_1099
Testis_1099
Cerebellum_1610
Cortex_1610
Heart_1610
Hypothalamus_1610
Kidney_1610
Testis_1610
reads per million genome matching
0
1000
2000
3000
4000
5000
bp position
0
10
20
30
40
50
60
70

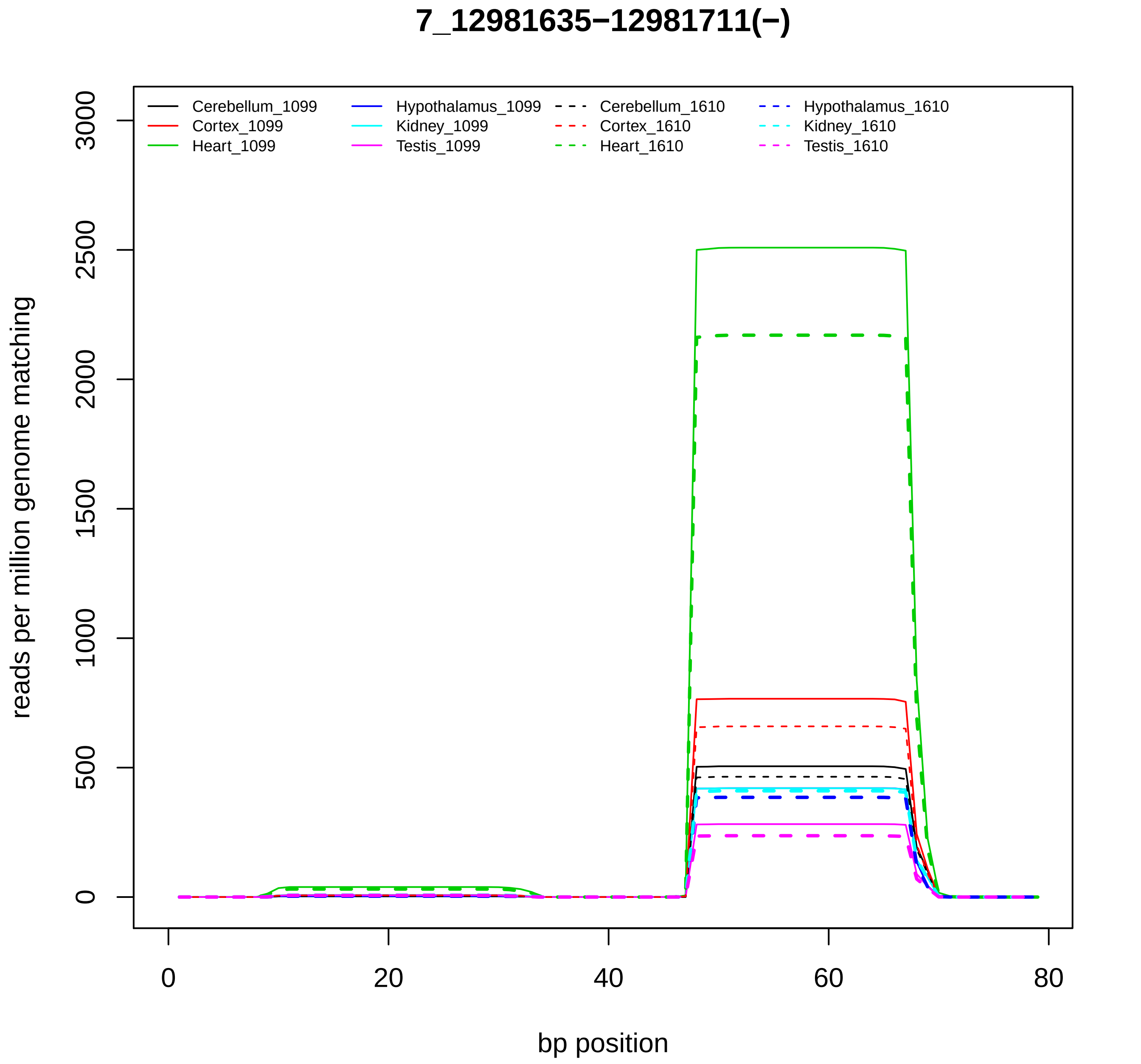

7_12981635−12981711(−)
Cerebellum_1099
Cortex_1099
Heart_1099
Hypothalamus_1099
Kidney_1099
Testis_1099
Cerebellum_1610
Cortex_1610
Heart_1610
Hypothalamus_1610
Kidney_1610
Testis_1610
reads per million genome matching
0
500
1000
1500
2000
2500
3000
0
20
40
60
80
bp position

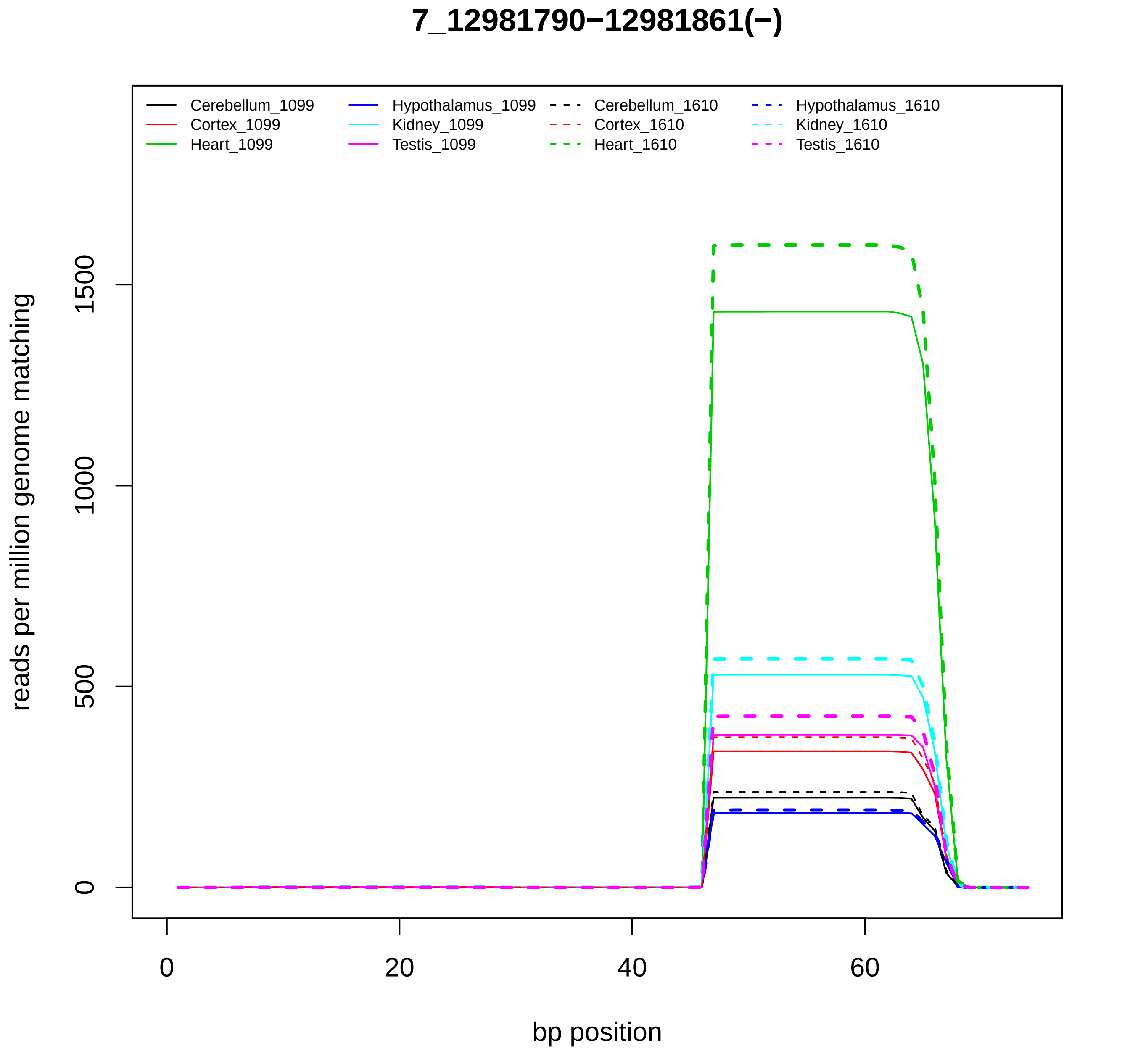

7_12981790−12981861(−)
Cerebellum_1099
Cortex_1099
Heart_1099
Hypothalamus_1099
Kidney_1099
Testis_1099
Cerebellum_1610
Cortex_1610
Heart_1610
Hypothalamus_1610
Kidney_1610
Testis_1610
reads per million genome matching
0
500
1000
1500
0
20
40
60
bp position

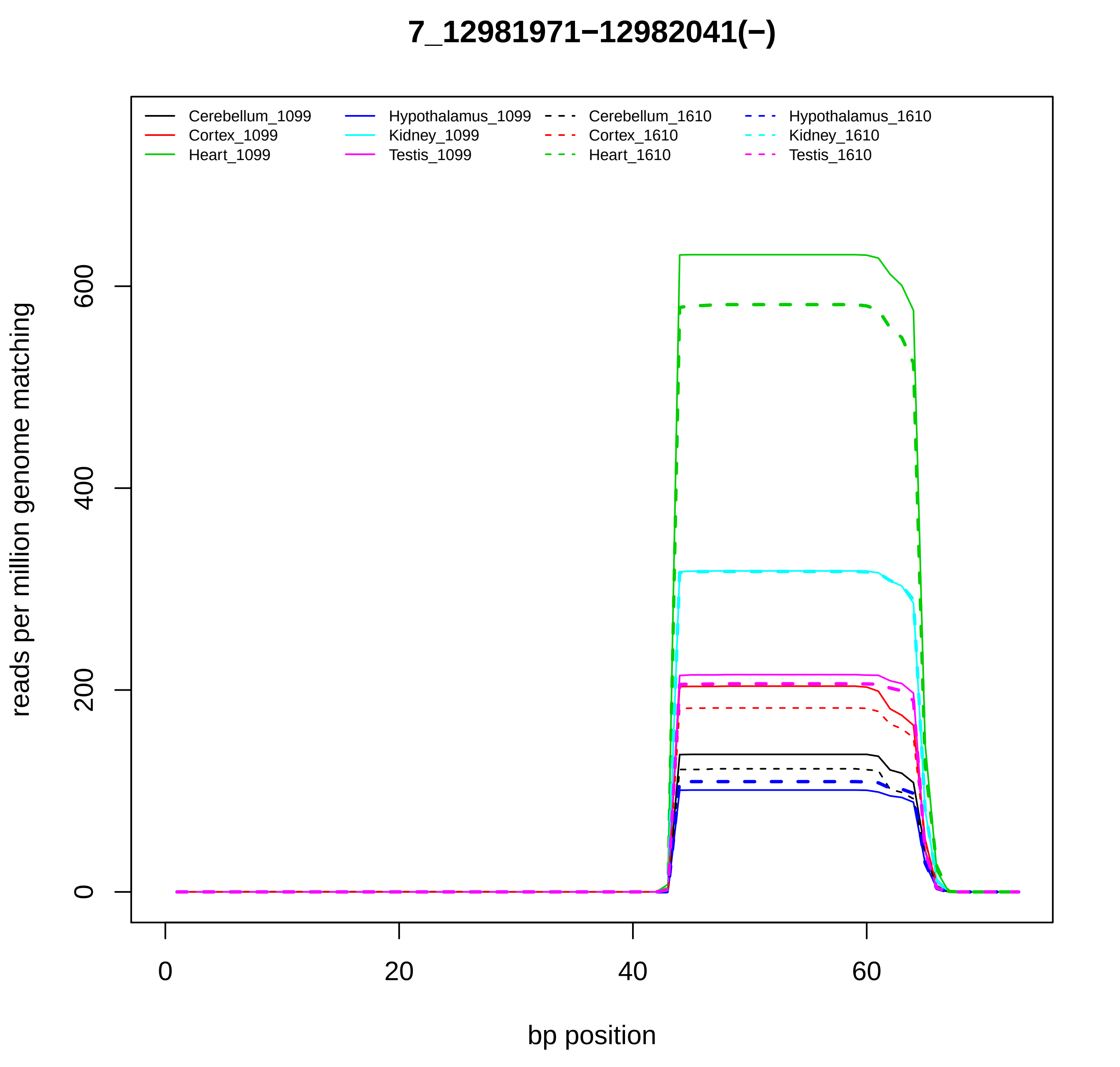
7_12981971−12982041(−)
Cerebellum_1099
Cortex_1099
Heart_1099
Hypothalamus_1099
Kidney_1099
Testis_1099
Cerebellum_1610
Cortex_1610
Heart_1610
Hypothalamus_1610
Kidney_1610
Testis_1610
reads per million genome matching
0
200
400
600
0
20
40
60
bp position

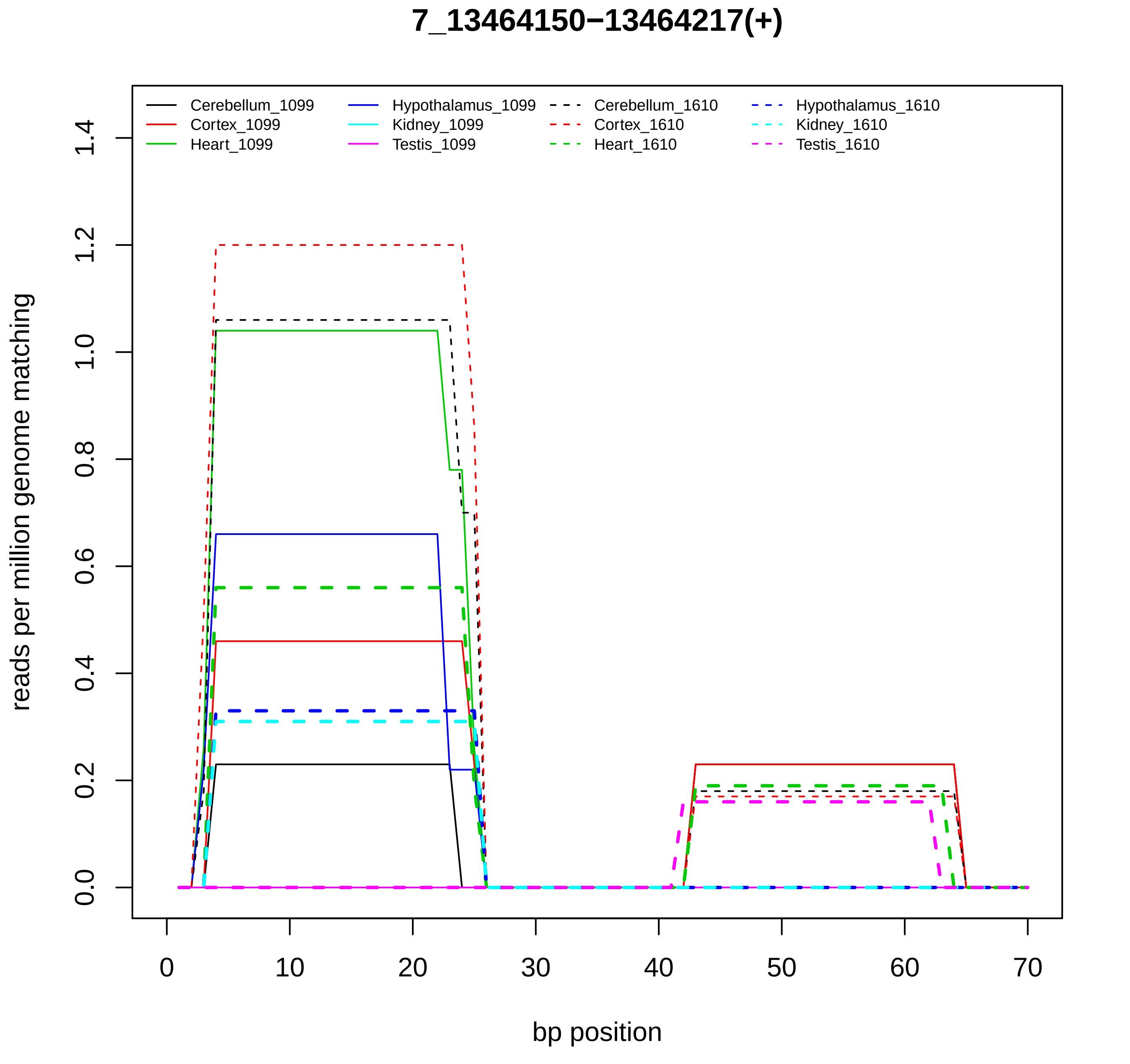
7_13464150−13464217(+)
Cerebellum_1099
Cortex_1099
Heart_1099
Hypothalamus_1099
Kidney_1099
Testis_1099
Cerebellum_1610
Cortex_1610
Heart_1610
Hypothalamus_1610
Kidney_1610
Testis_1610
reads per million genome matching
0.0
0.2
0.4
0.6
0.8
1.0
1.2
1.4
0
10
20
30
40
50
60
70
bp position

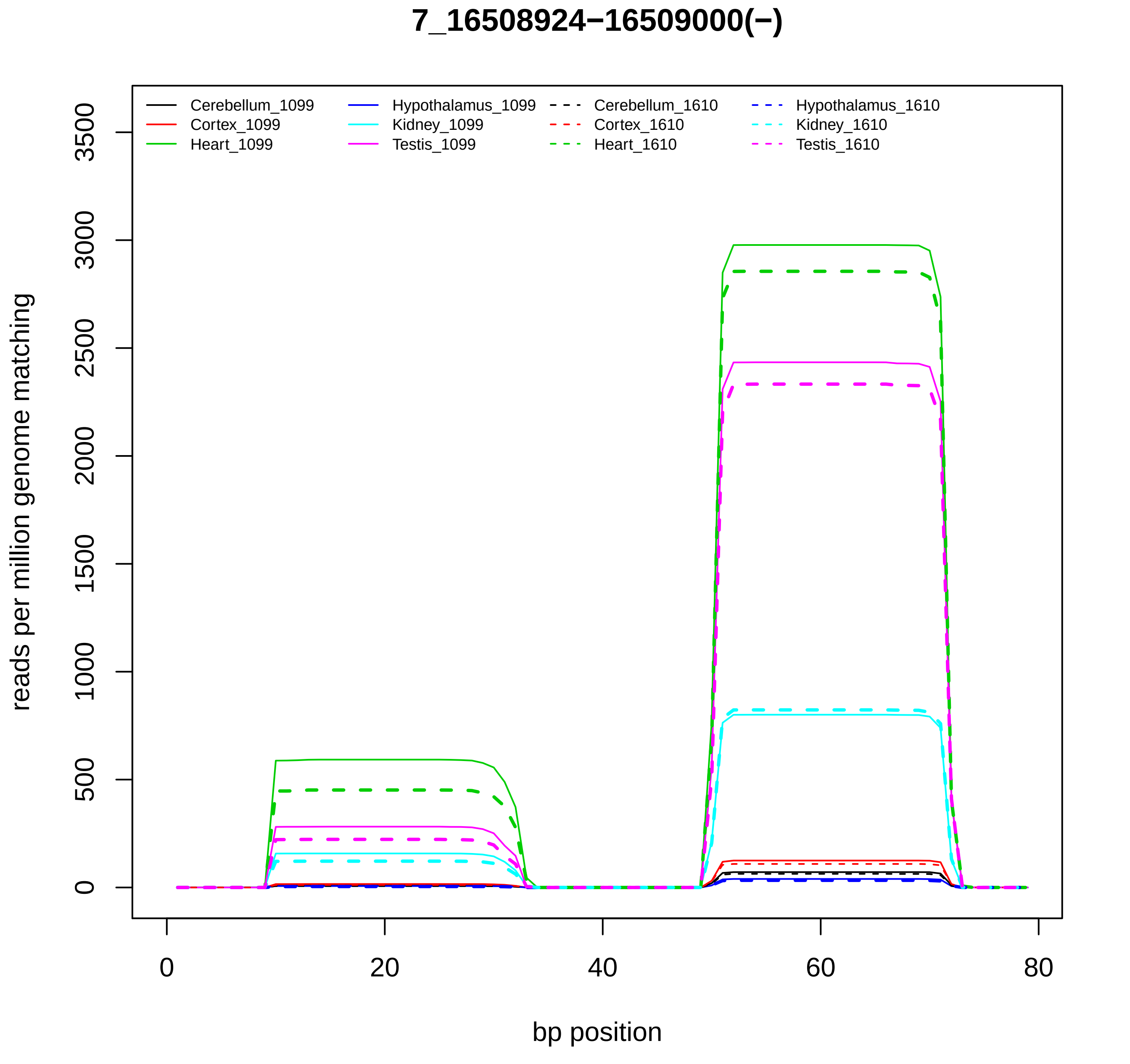

7_16508924−16509000(−)
Cerebellum_1099
Cortex_1099
Heart_1099
Hypothalamus_1099
Kidney_1099
Testis_1099
Cerebellum_1610
Cortex_1610
Heart_1610
Hypothalamus_1610
Kidney_1610
Testis_1610
reads per million genome matching
0
500
1000
1500
2000
2500
3000
3500
0
20
40
60
80
bp position

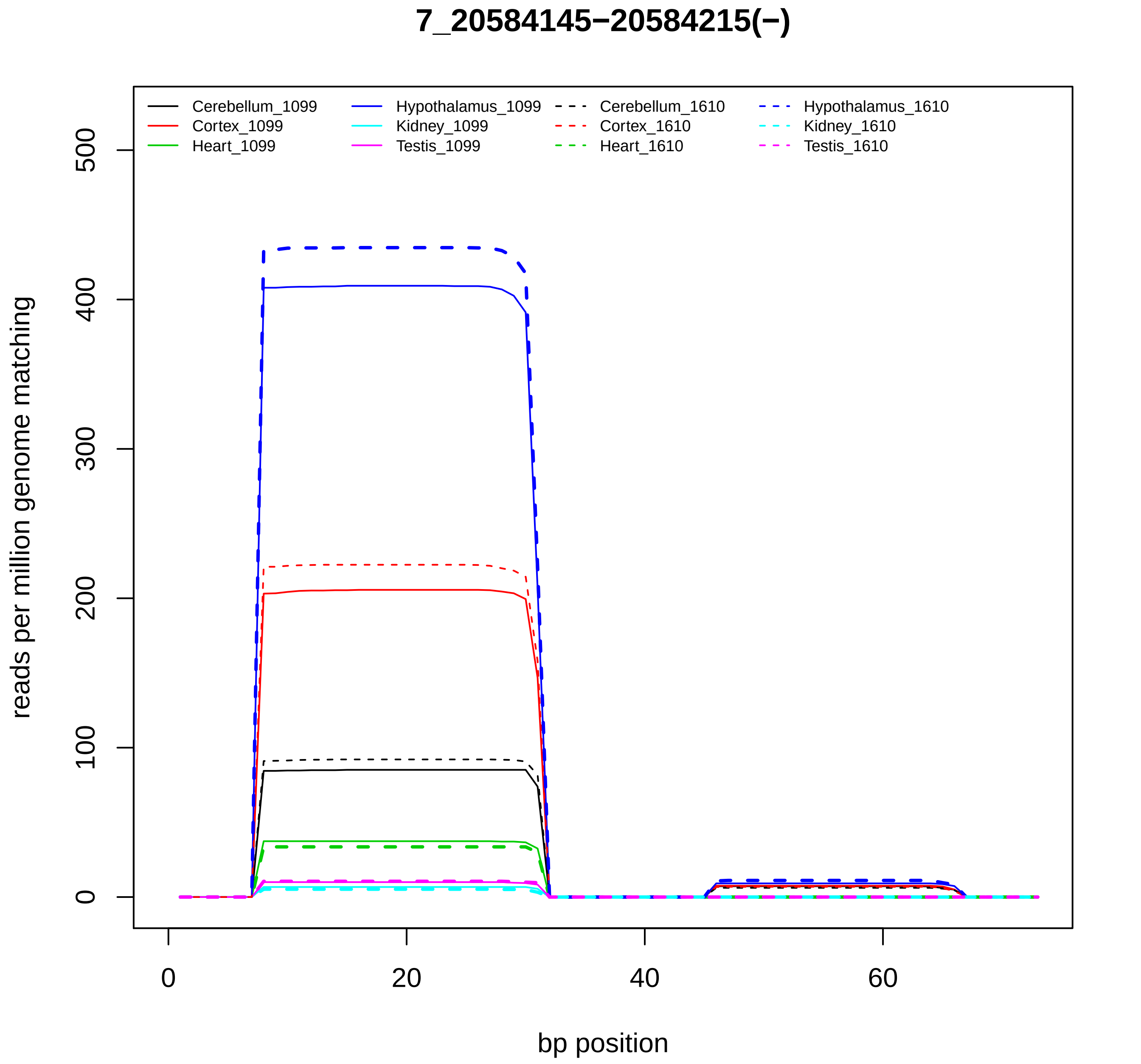

7_20584145−20584215(−)
Cerebellum_1099
Cortex_1099
Heart_1099
Hypothalamus_1099
Kidney_1099
Testis_1099
Cerebellum_1610
Cortex_1610
Heart_1610
Hypothalamus_1610
Kidney_1610
Testis_1610
reads per million genome matching
0
100
200
300
400
500
0
20
40
60
bp position

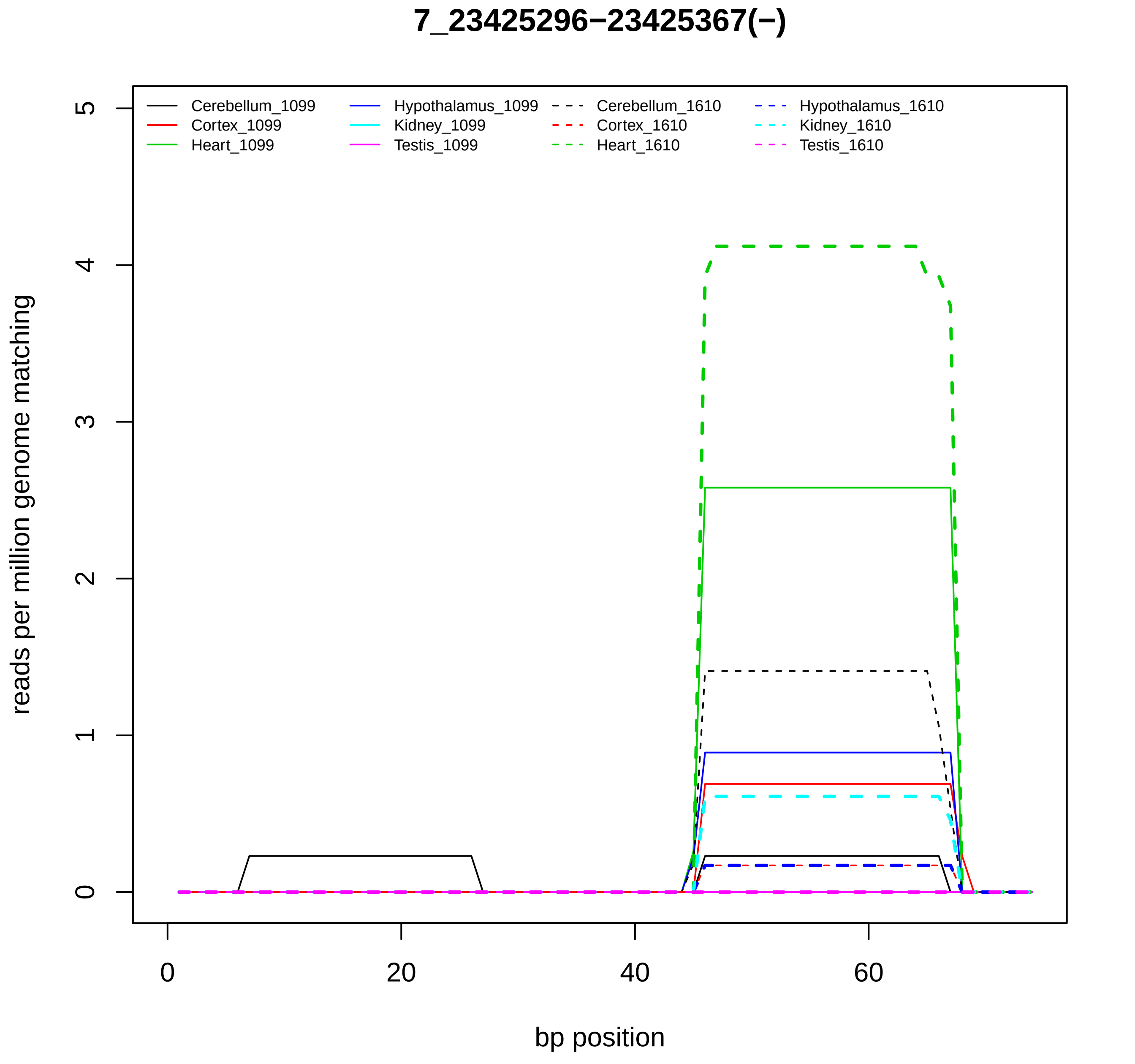

7_23425296−23425367(−)
Cerebellum_1099
Cortex_1099
Heart_1099
Hypothalamus_1099
Kidney_1099
Testis_1099
Cerebellum_1610
Cortex_1610
Heart_1610
Hypothalamus_1610
Kidney_1610
Testis_1610
reads per million genome matching
bp position
0
1
2
3
4
5
0
20
40
60

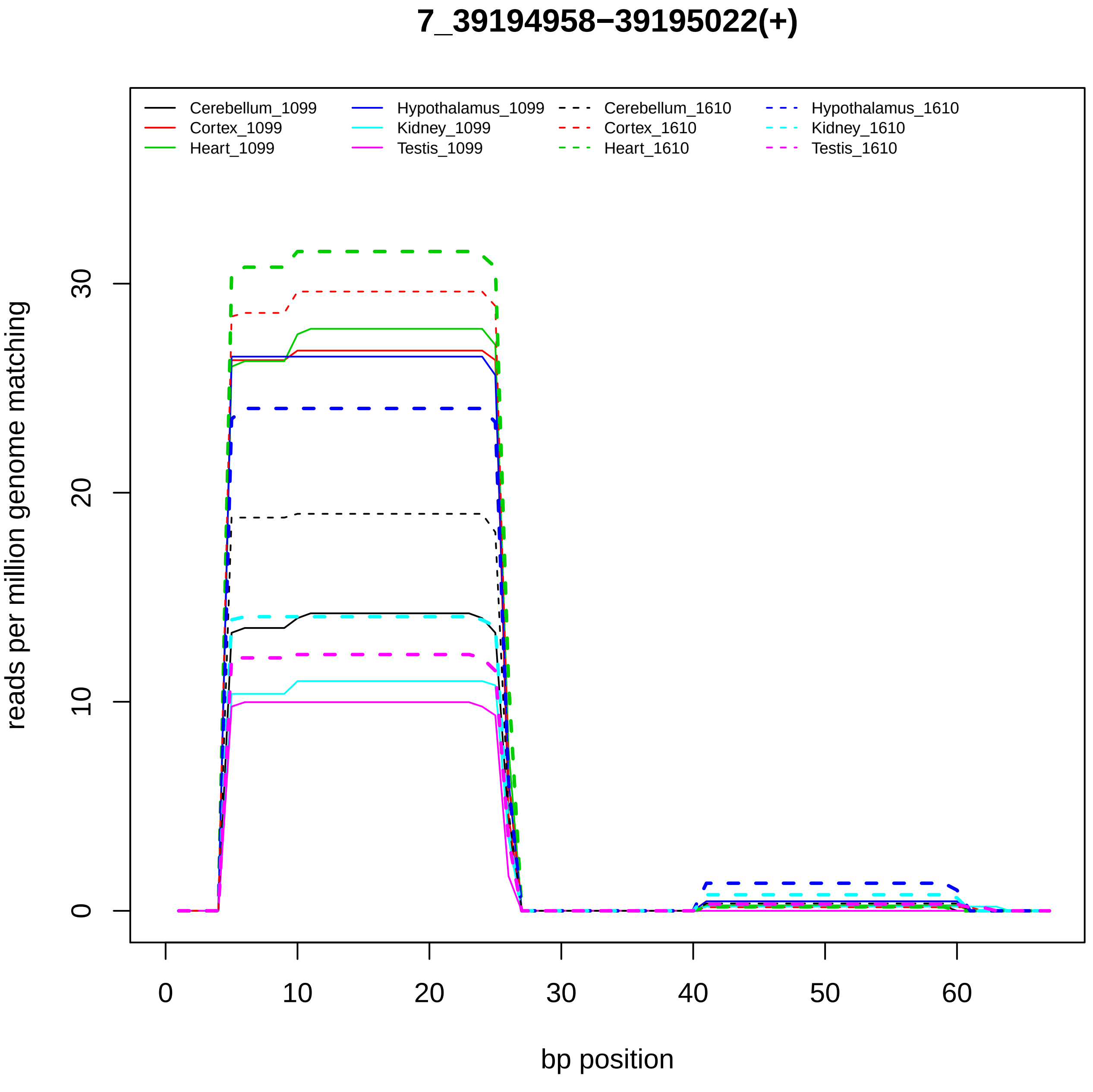

7_39194958−39195022(+)
Cerebellum_1099
Cortex_1099
Heart_1099
Hypothalamus_1099
Kidney_1099
Testis_1099
Cerebellum_1610
Cortex_1610
Heart_1610
Hypothalamus_1610
Kidney_1610
Testis_1610
reads per million genome matching
bp position
0
10
20
30
0
10
20
30
40
50
60

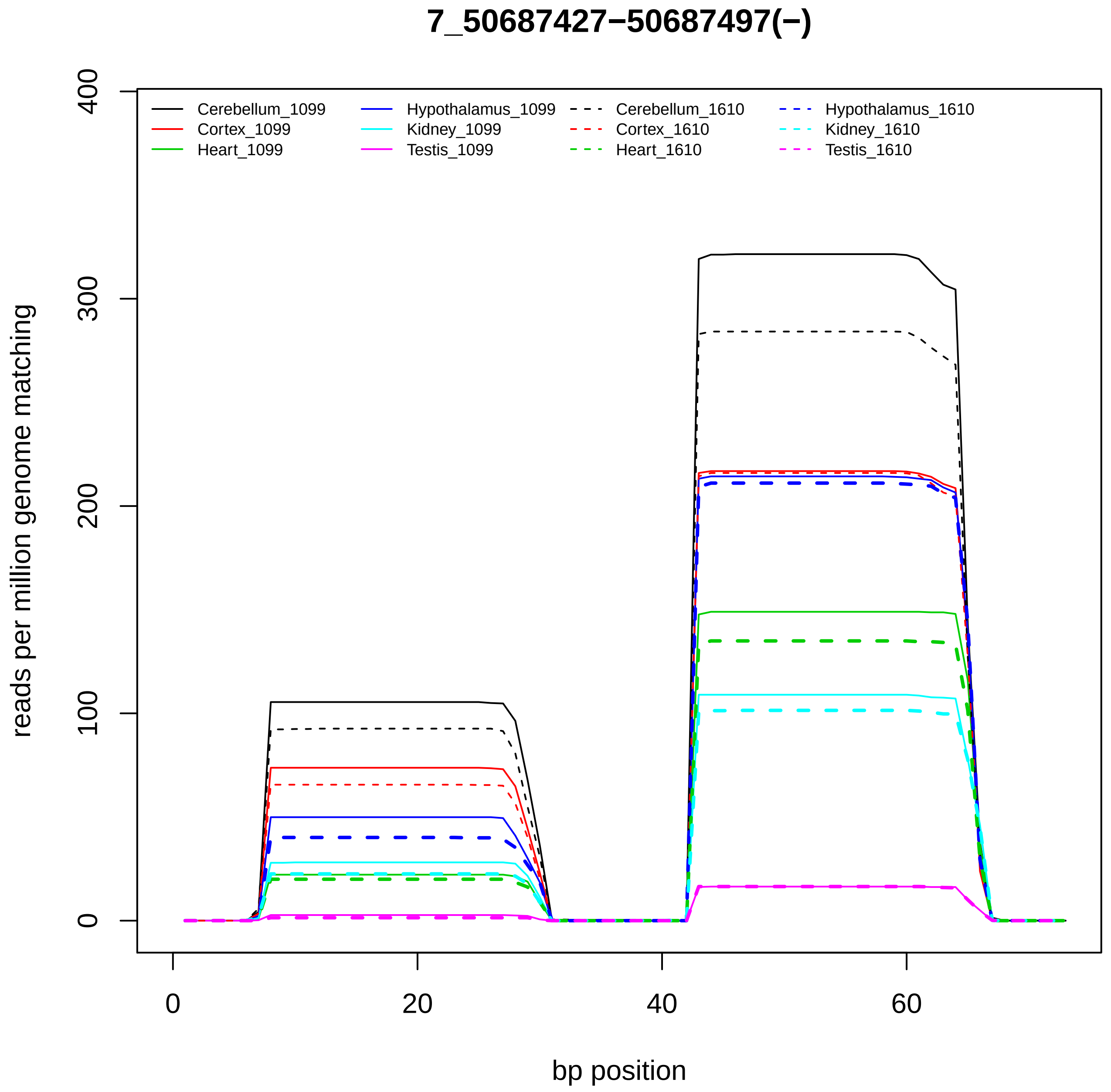
7_50687427−50687497(−)
Cerebellum_1099
Cortex_1099
Heart_1099
Hypothalamus_1099
Kidney_1099
Testis_1099
Cerebellum_1610
Cortex_1610
Heart_1610
Hypothalamus_1610
Kidney_1610
Testis_1610
reads per million genome matching
0
100
200
300
400
bp position
0
20
40
60

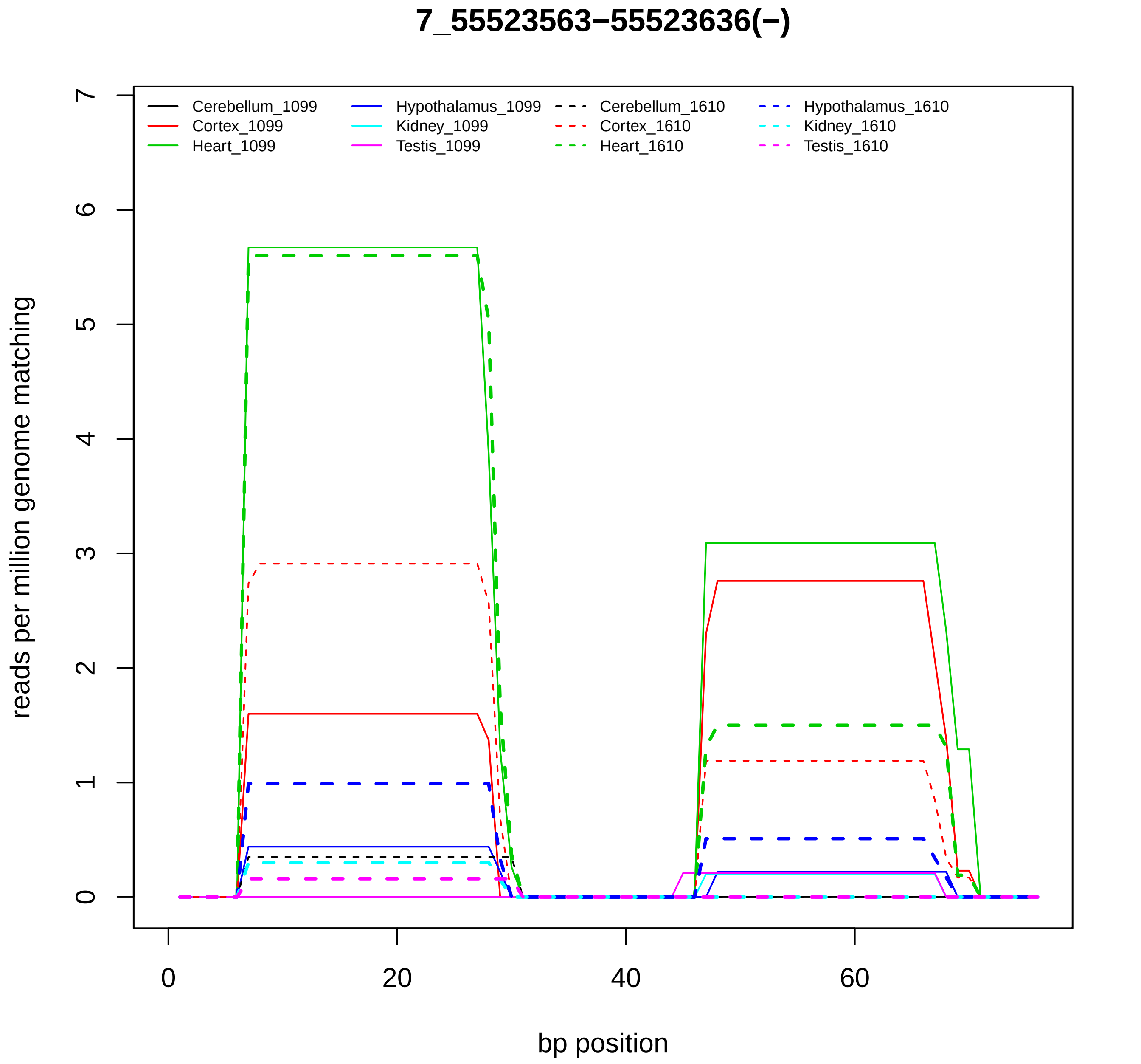

7_55523563−55523636(−)
Cerebellum_1099
Cortex_1099
Heart_1099
Hypothalamus_1099
Kidney_1099
Testis_1099
Cerebellum_1610
Cortex_1610
Heart_1610
Hypothalamus_1610
Kidney_1610
Testis_1610
reads per million genome matching
bp position
0
1
2
3
4
5
6
7
20
40
60

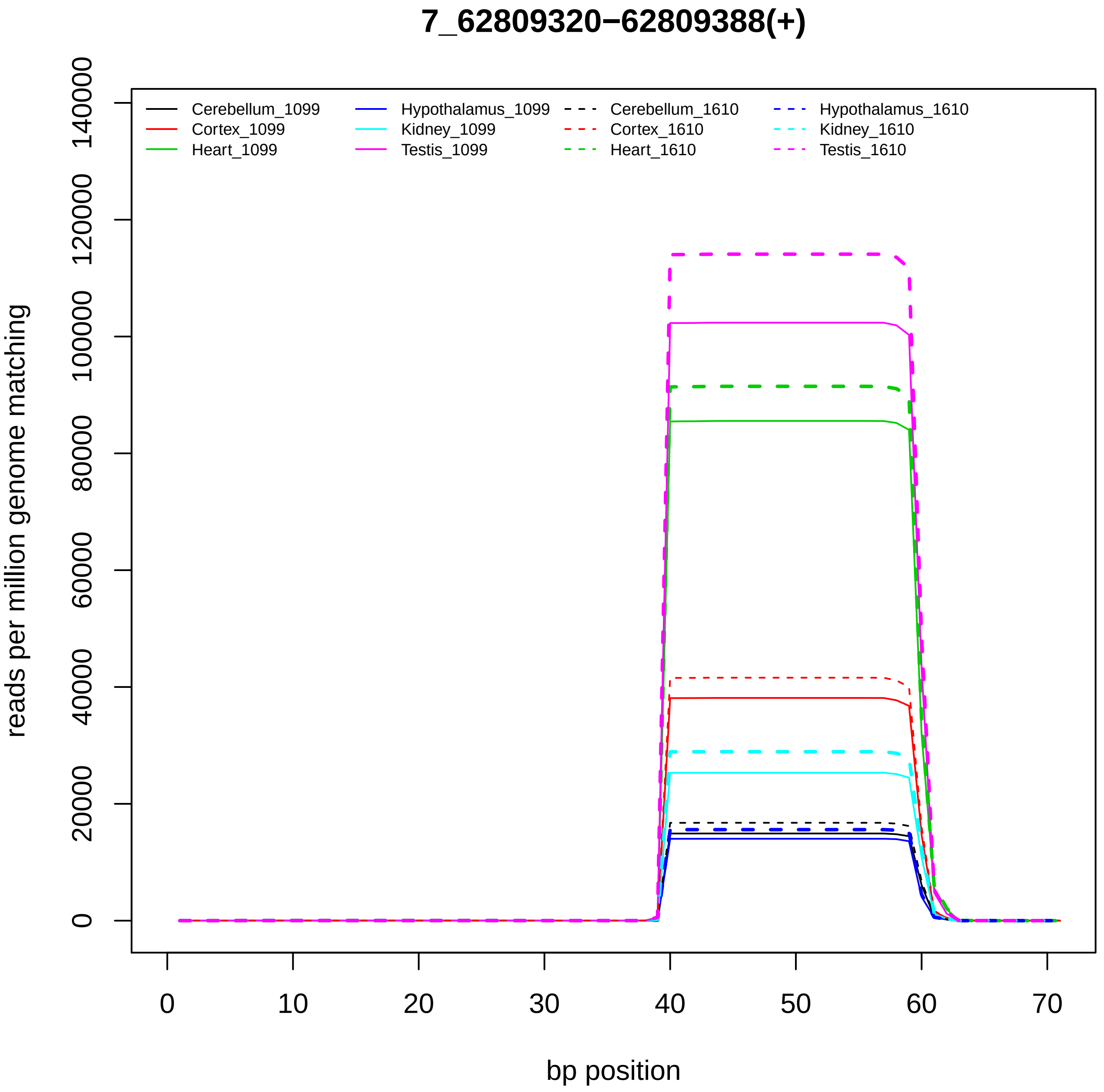

7_62809320−62809388(+)
Cerebellum_1099
Cortex_1099
Heart_1099
Hypothalamus_1099
Kidney_1099
Testis_1099
Cerebellum_1610
Cortex_1610
Heart_1610
Hypothalamus_1610
Kidney_1610
Testis_1610
reads per million genome matching
0
20000
40000
60000
80000
100000
120000
140000
bp position
0
10
20
30
40
50
60
70

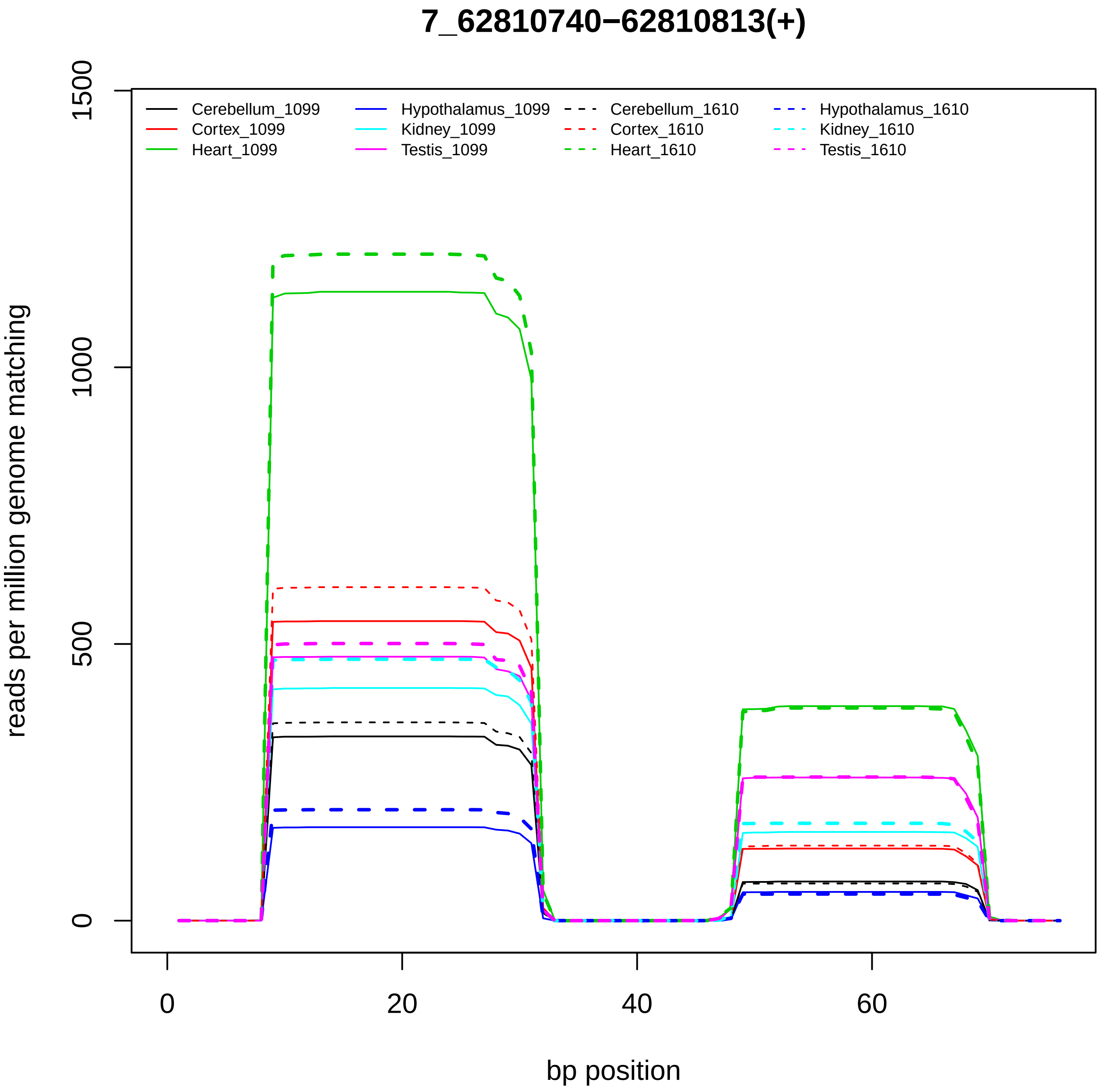

7_62810740−62810813(+)
reads per million genome matching
bp position
0
500
1000
1500
0
20
40
60
Cerebellum_1099
Cortex_1099
Heart_1099
Hypothalamus_1099
Kidney_1099
Testis_1099
Cerebellum_1610
Cortex_1610
Heart_1610
Hypothalamus_1610
Kidney_1610
Testis_1610

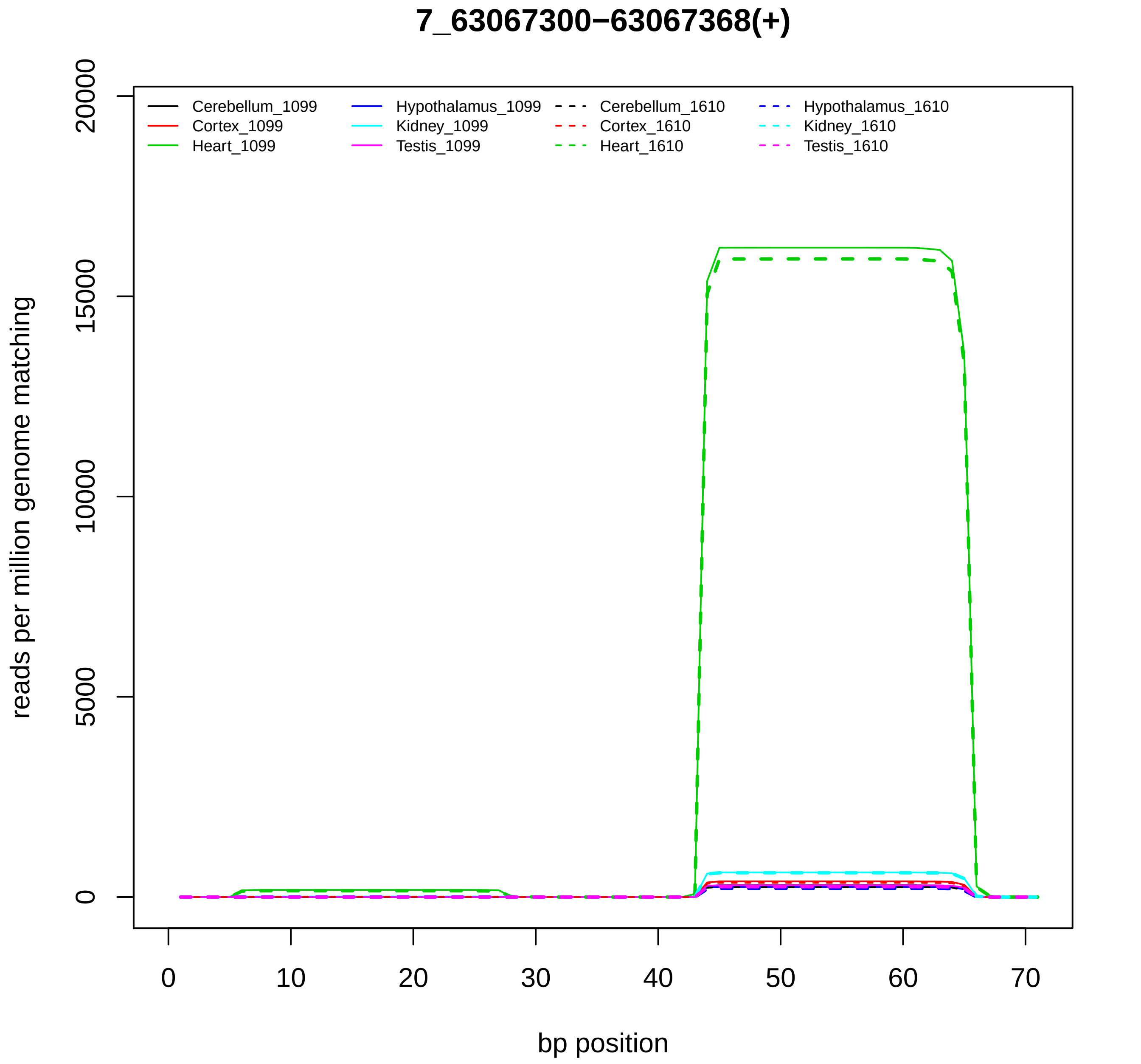
7_63067300−63067368(+)
reads per million genome matching
bp position
0
5000
10000
15000
20000
0
10
20
30
40
50
60
70
Cerebellum_1099
Cortex_1099
Heart_1099
Hypothalamus_1099
Kidney_1099
Testis_1099
Cerebellum_1610
Cortex_1610
Heart_1610
Hypothalamus_1610
Kidney_1610
Testis_1610

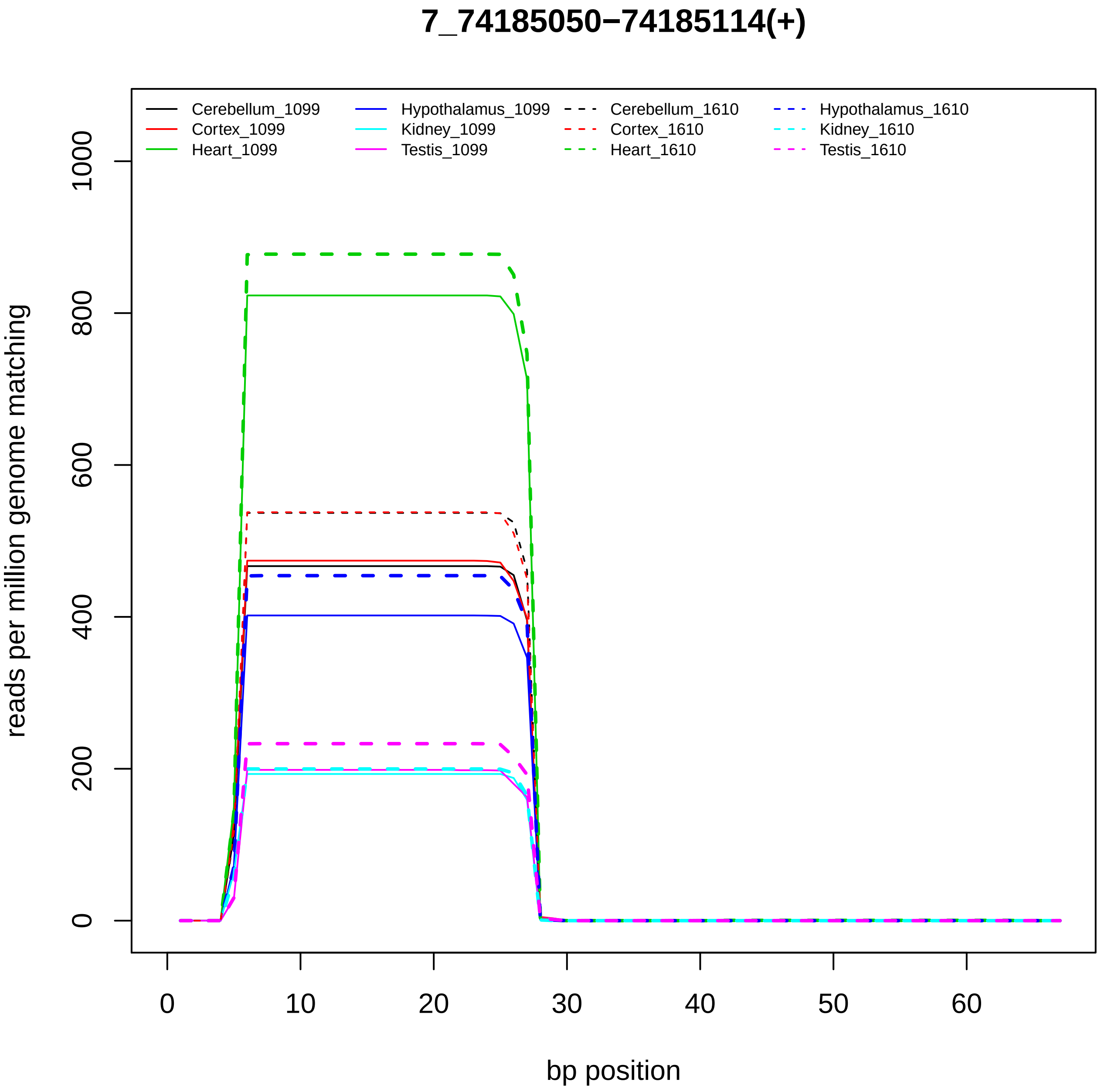
7_74185050−74185114(+)
Cerebellum_1099
Cortex_1099
Heart_1099
Hypothalamus_1099
Kidney_1099
Testis_1099
Cerebellum_1610
Cortex_1610
Heart_1610
Hypothalamus_1610
Kidney_1610
Testis_1610
reads per million genome matching
0
200
400
600
800
1000
0
10
20
30
40
50
60
bp position

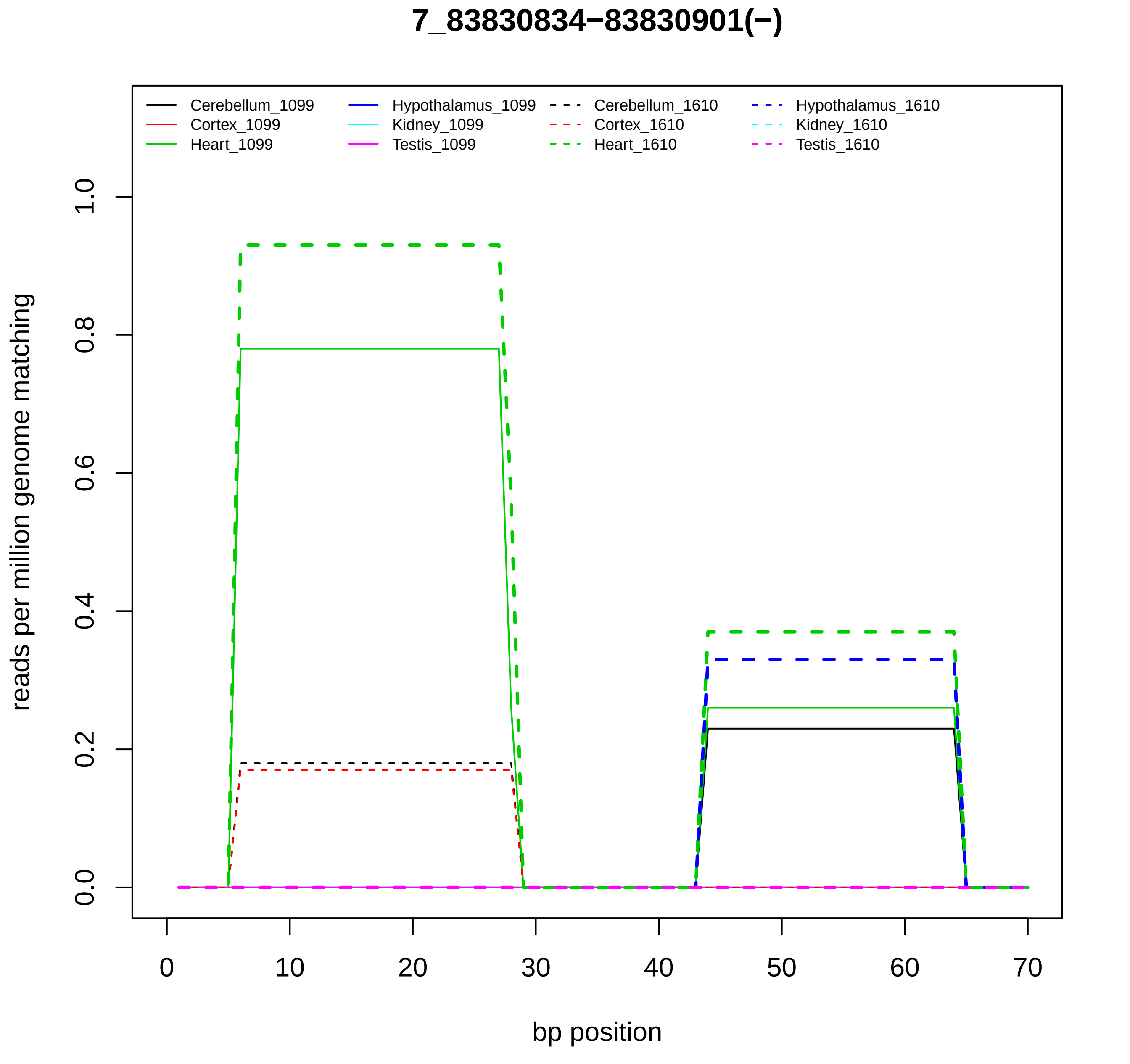

7_83830834−83830901(−)
Cerebellum_1099
Cortex_1099
Heart_1099
Hypothalamus_1099
Kidney_1099
Testis_1099
Cerebellum_1610
Cortex_1610
Heart_1610
Hypothalamus_1610
Kidney_1610
Testis_1610
reads per million genome matching
0.0
0.2
0.4
0.6
0.8
1.0
0
10
20
30
40
50
60
70
bp position

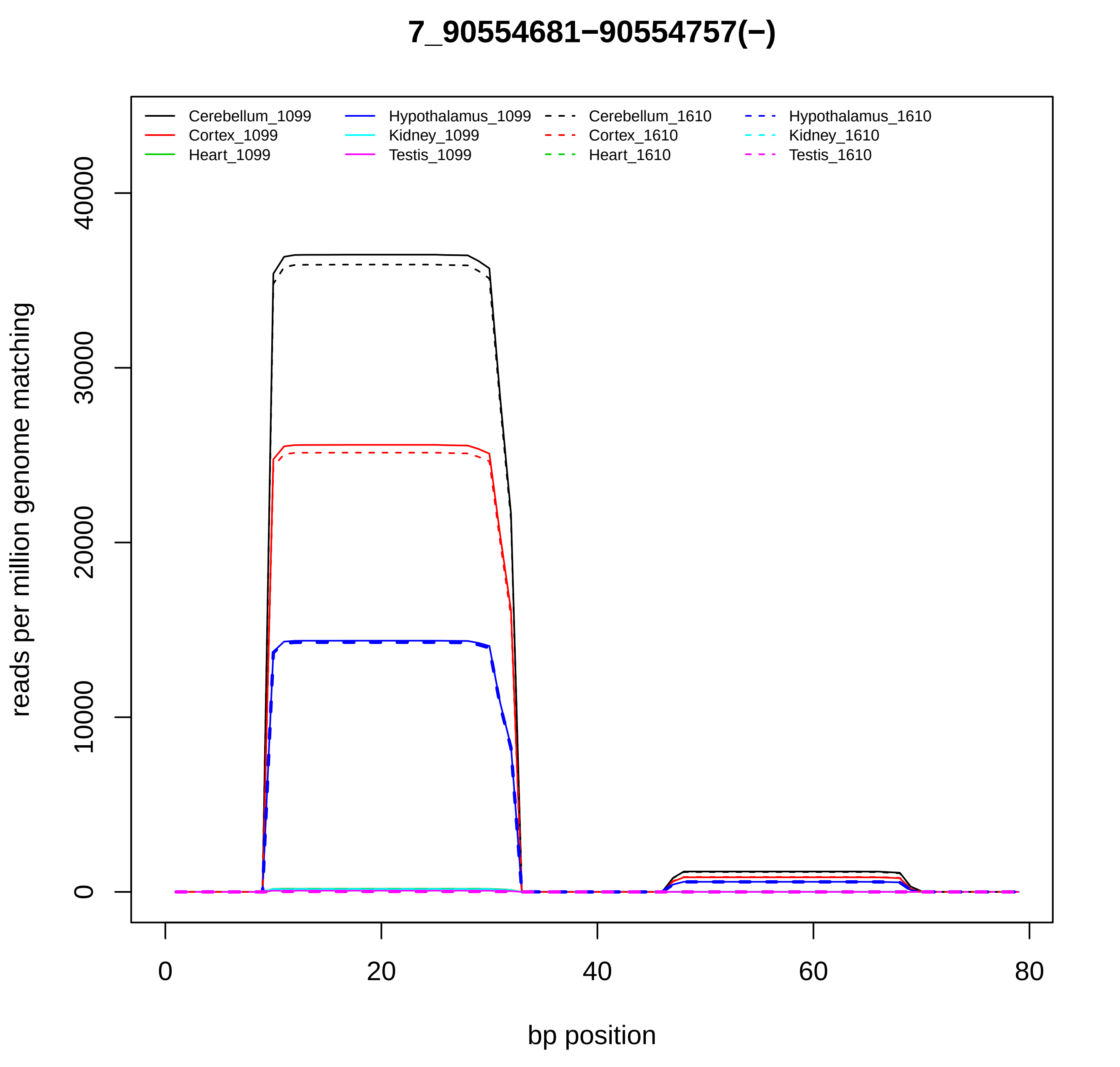

7_90554681−90554757(−)
Cerebellum_1099
Cortex_1099
Heart_1099
Hypothalamus_1099
Kidney_1099
Testis_1099
Cerebellum_1610
Cortex_1610
Heart_1610
Hypothalamus_1610
Kidney_1610
Testis_1610
reads per million genome matching
0
10000
20000
30000
40000
0
20
40
60
80
bp position

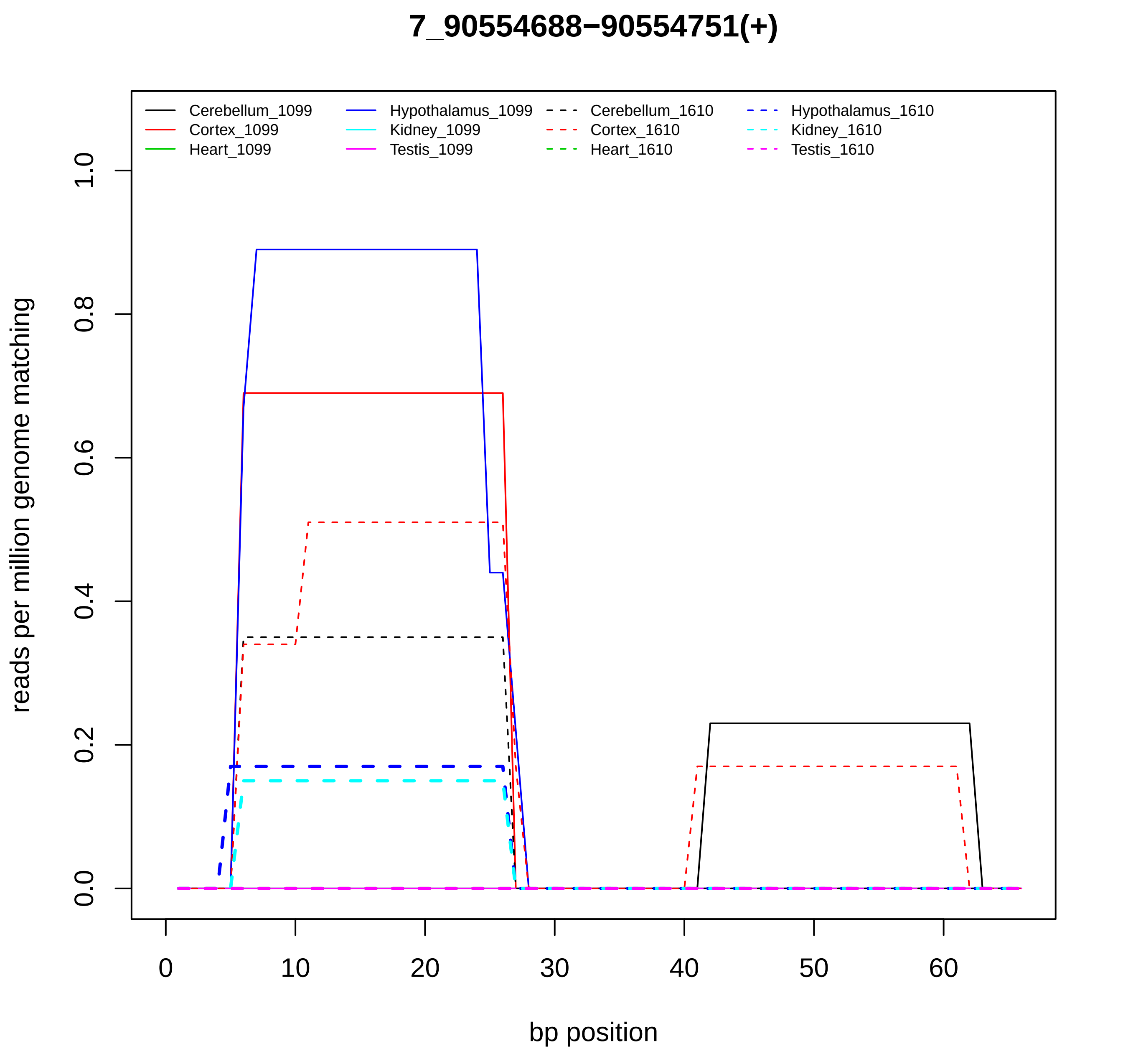

7_90554688−90554751(+)
Cerebellum_1099
Cortex_1099
Heart_1099
Hypothalamus_1099
Kidney_1099
Testis_1099
Cerebellum_1610
Cortex_1610
Heart_1610
Hypothalamus_1610
Kidney_1610
Testis_1610
reads per million genome matching
0.0
0.2
0.4
0.6
0.8
1.0
0
10
20
30
40
50
60
bp position

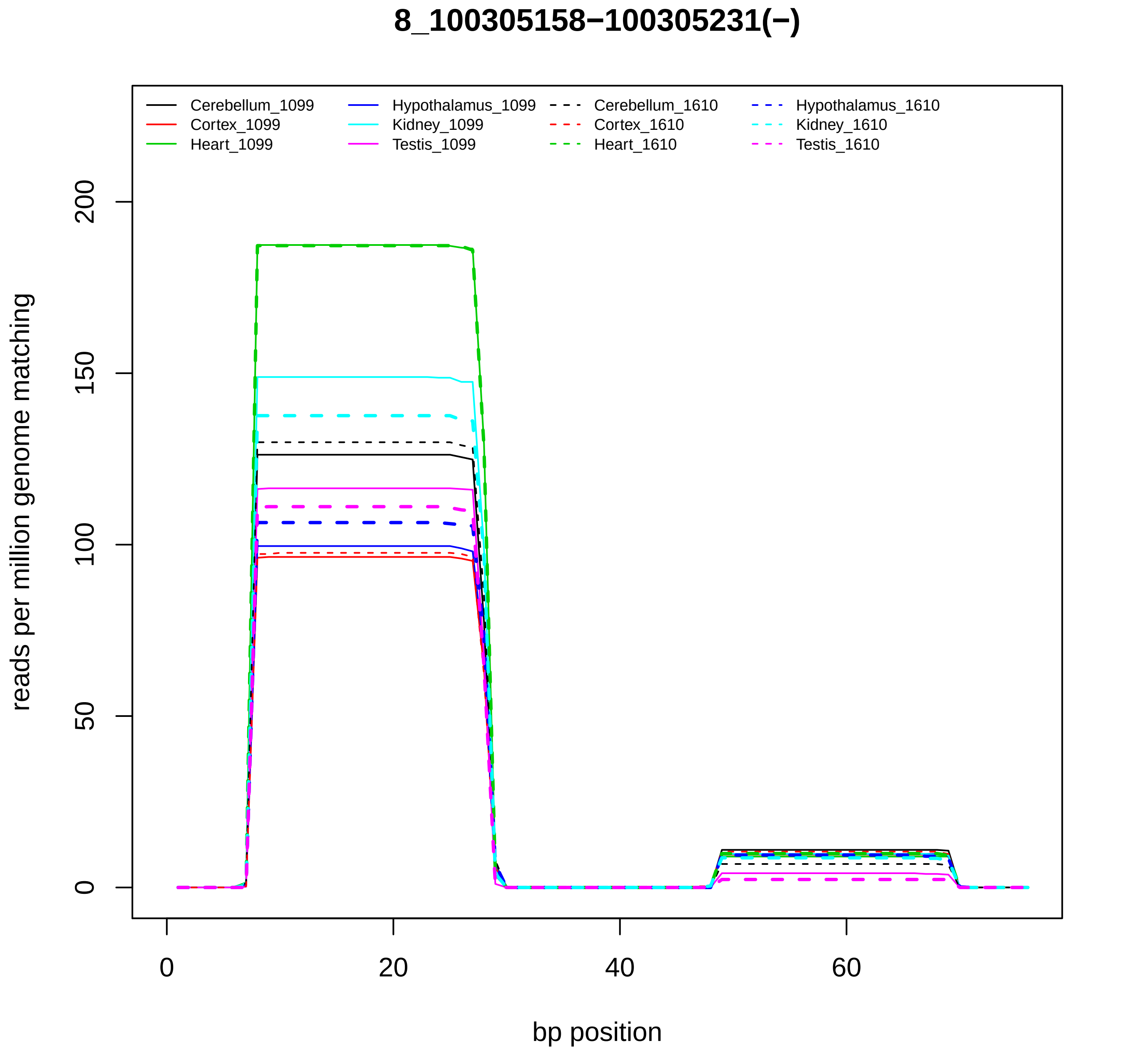

8_100305158−100305231(−)
Cerebellum_1099
Cortex_1099
Heart_1099
Hypothalamus_1099
Kidney_1099
Testis_1099
Cerebellum_1610
Cortex_1610
Heart_1610
Hypothalamus_1610
Kidney_1610
Testis_1610
reads per million genome matching
0
50
100
150
200
0
20
40
60
bp position

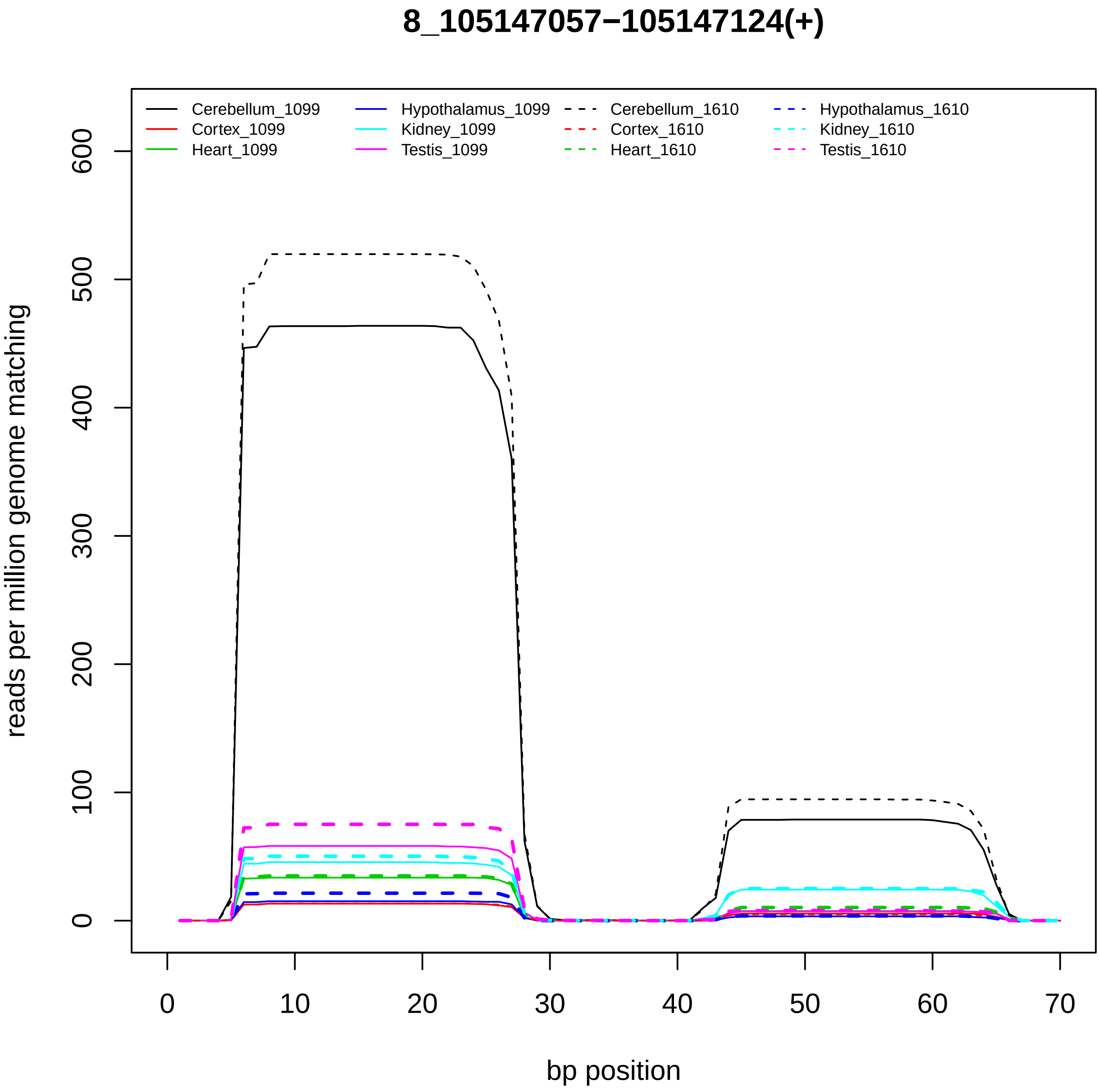
8_105147057−105147124(+)
Cerebellum_1099
Cortex_1099
Heart_1099
Hypothalamus_1099
Kidney_1099
Testis_1099
Cerebellum_1610
Cortex_1610
Heart_1610
Hypothalamus_1610
Kidney_1610
Testis_1610
reads per million genome matching
0
100
200
300
400
500
600
bp position
0
10
20
30
40
50
60
70

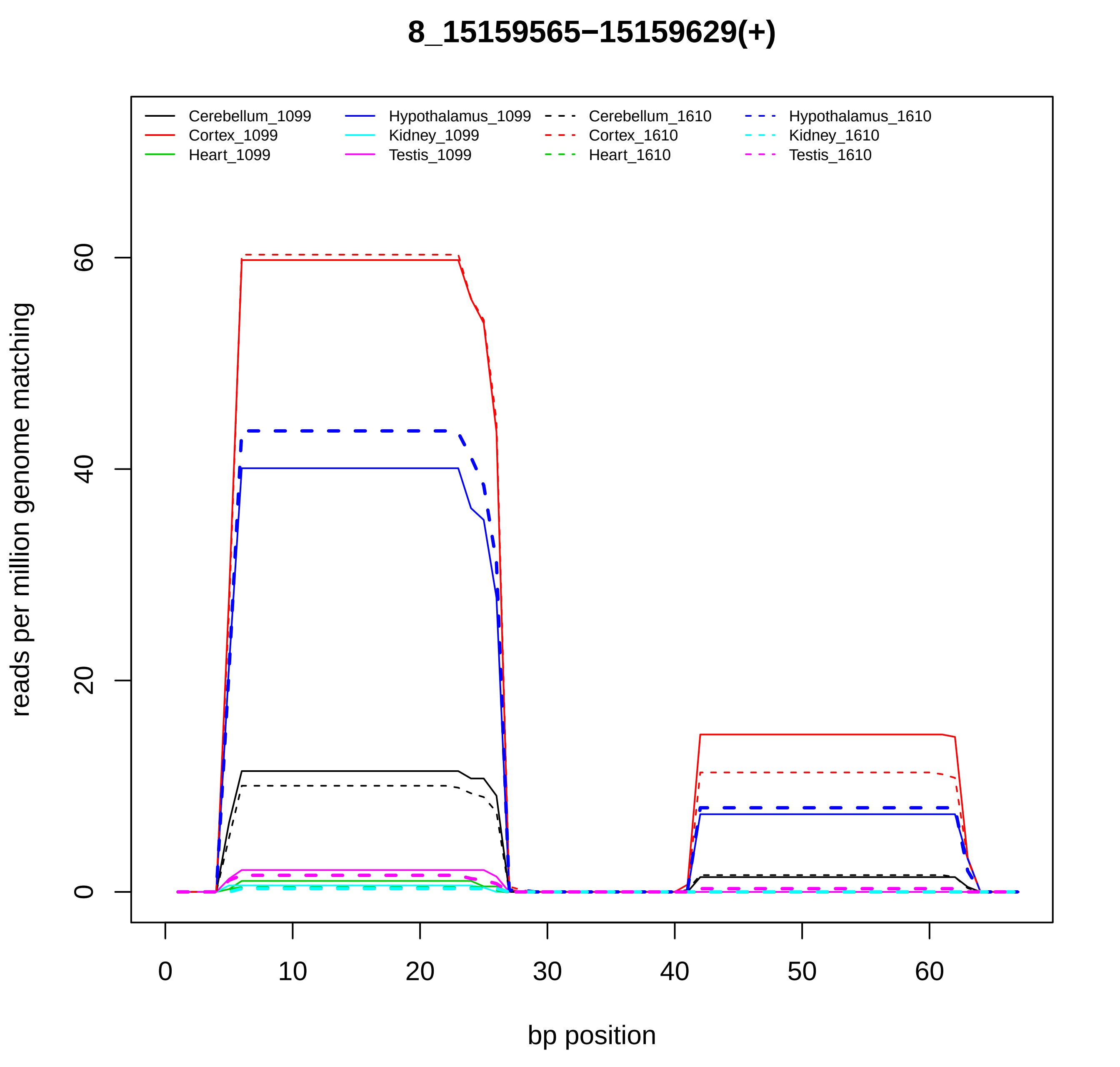

8_15159565−15159629(+)
Cerebellum_1099
Cortex_1099
Heart_1099
Hypothalamus_1099
Kidney_1099
Testis_1099
Cerebellum_1610
Cortex_1610
Heart_1610
Hypothalamus_1610
Kidney_1610
Testis_1610
reads per million genome matching
bp position
0
20
40
60
0
10
20
30
40
50
60

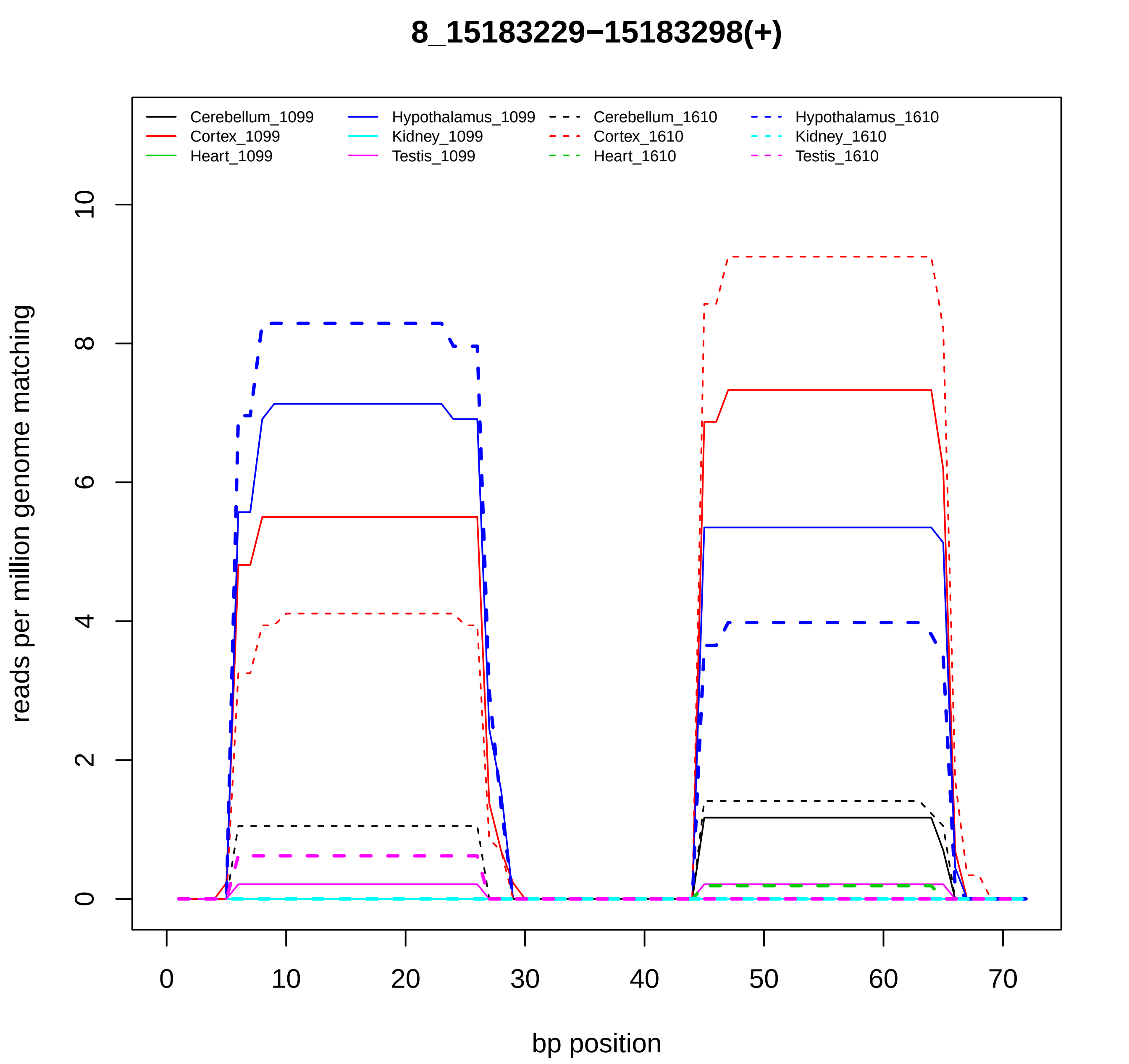
8_15183229−15183298(+)
Cerebellum_1099
Cortex_1099
Heart_1099
Hypothalamus_1099
Kidney_1099
Testis_1099
Cerebellum_1610
Cortex_1610
Heart_1610
Hypothalamus_1610
Kidney_1610
Testis_1610
reads per million genome matching
bp position
0
2
4
6
8
10
0
10
20
30
40
50
60
70

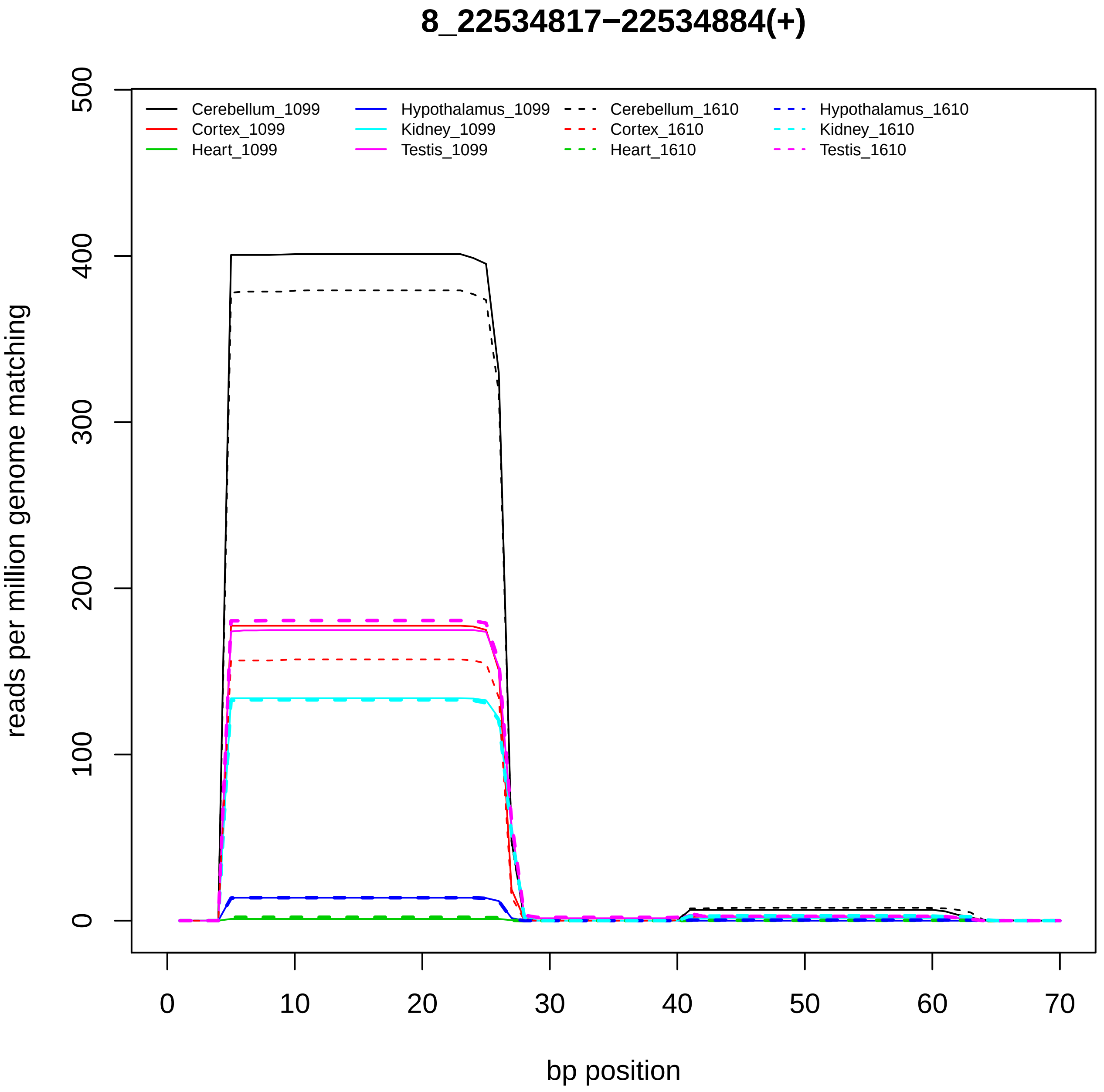

8_22534817−22534884(+)
Cerebellum_1099
Cortex_1099
Heart_1099
Hypothalamus_1099
Kidney_1099
Testis_1099
Cerebellum_1610
Cortex_1610
Heart_1610
Hypothalamus_1610
Kidney_1610
Testis_1610
reads per million genome matching
bp position
0
100
200
300
400
500
0
10
20
30
40
50
60
70

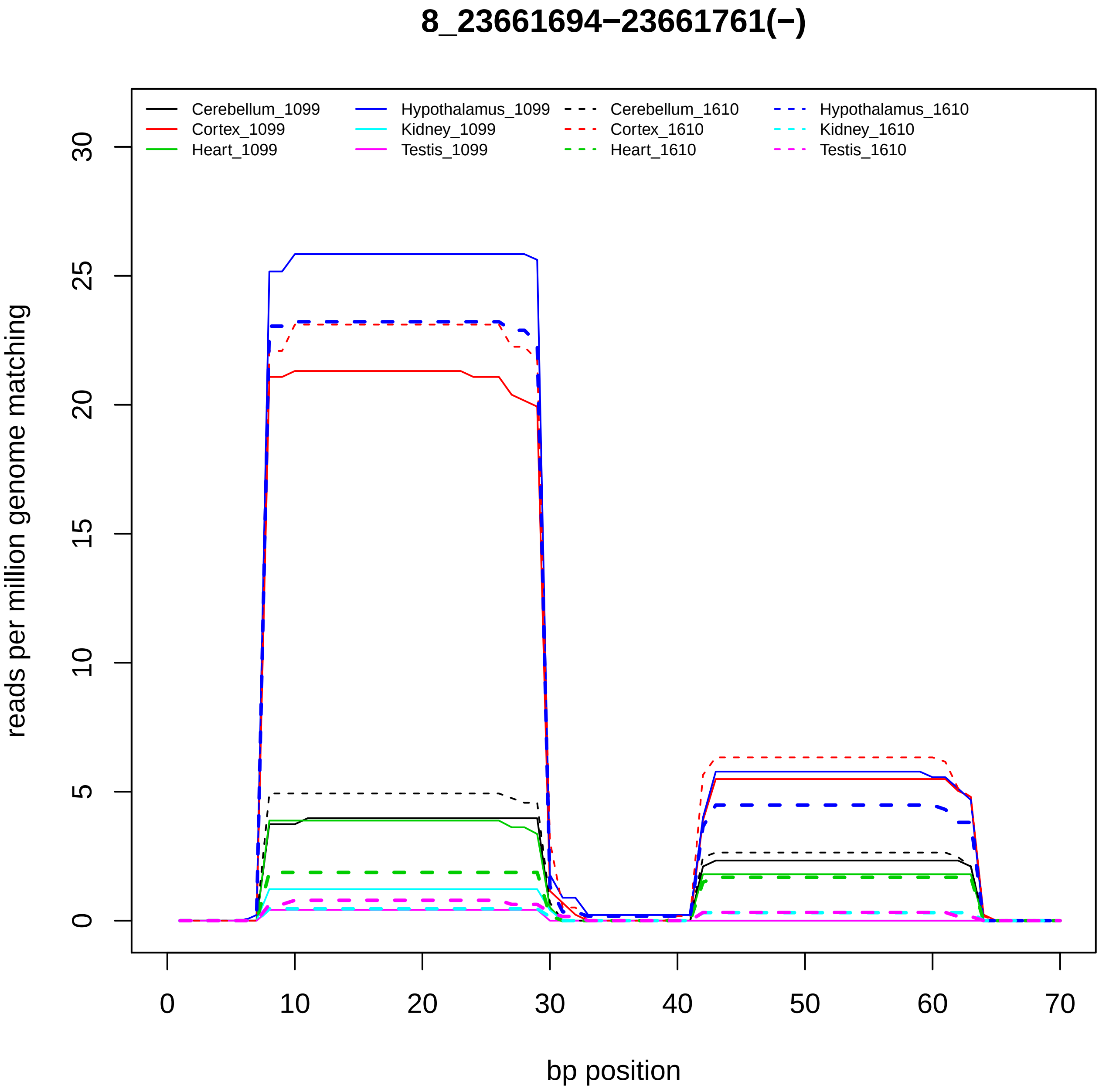

8_23661694−23661761(−)
Cerebellum_1099
Cortex_1099
Heart_1099
Hypothalamus_1099
Kidney_1099
Testis_1099
Cerebellum_1610
Cortex_1610
Heart_1610
Hypothalamus_1610
Kidney_1610
Testis_1610
reads per million genome matching
0
5
10
15
20
25
30
0
10
20
30
40
50
60
70
bp position

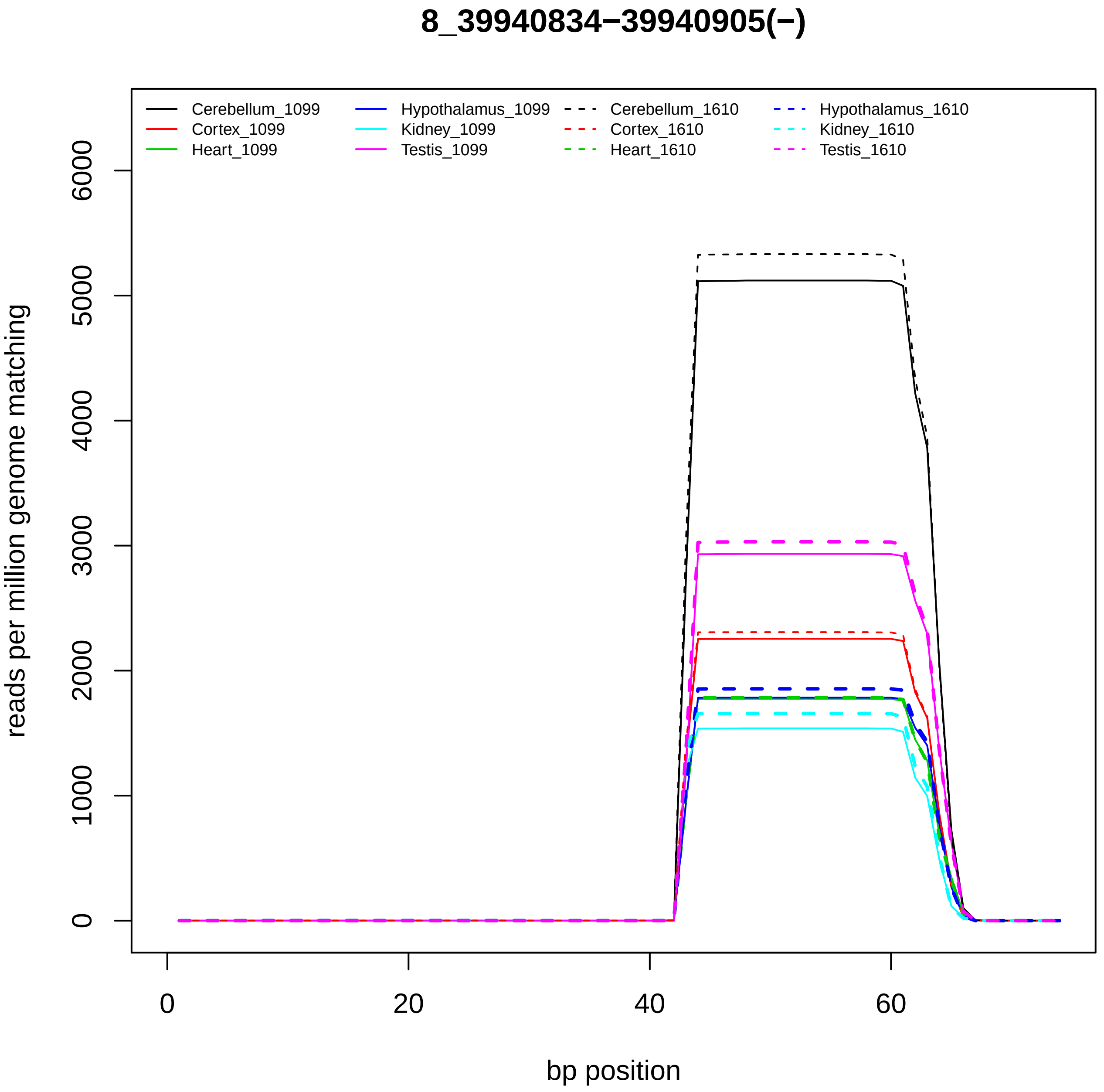
8_39940834−39940905(−)
Cerebellum_1099
Cortex_1099
Heart_1099
Hypothalamus_1099
Kidney_1099
Testis_1099
Cerebellum_1610
Cortex_1610
Heart_1610
Hypothalamus_1610
Kidney_1610
Testis_1610
reads per million genome matching
0
1000
2000
3000
4000
5000
6000
0
20
40
60
bp position

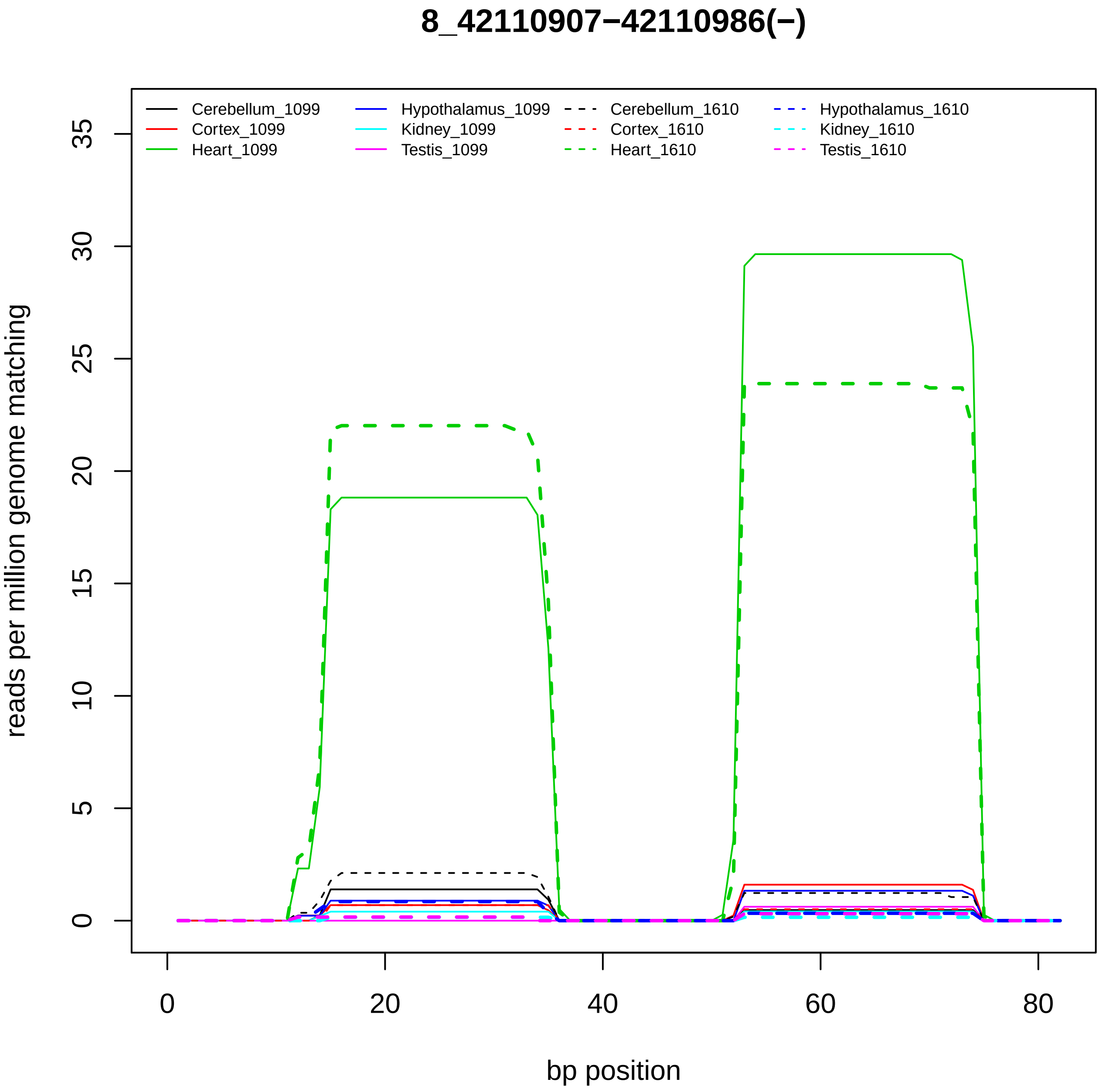

8_42110907−42110986(−)
Cerebellum_1099
Cortex_1099
Heart_1099
Hypothalamus_1099
Kidney_1099
Testis_1099
Cerebellum_1610
Cortex_1610
Heart_1610
Hypothalamus_1610
Kidney_1610
Testis_1610
reads per million genome matching
bp position
0
5
10
15
20
25
30
35
0
20
40
60
80

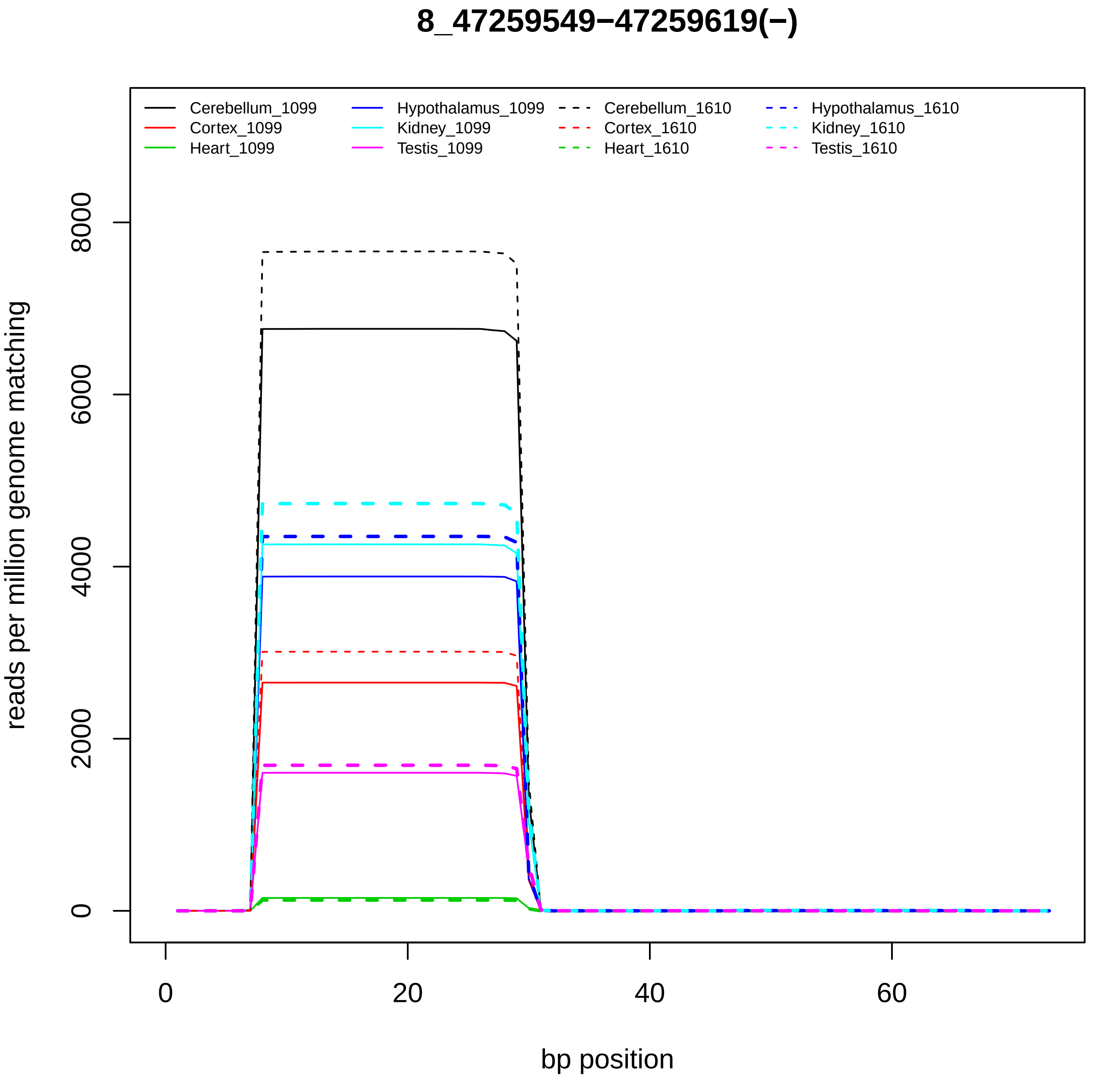

8_47259549−47259619(−)
Cerebellum_1099
Cortex_1099
Heart_1099
Hypothalamus_1099
Kidney_1099
Testis_1099
Cerebellum_1610
Cortex_1610
Heart_1610
Hypothalamus_1610
Kidney_1610
Testis_1610
reads per million genome matching
0
2000
4000
6000
8000
0
20
40
60
bp position

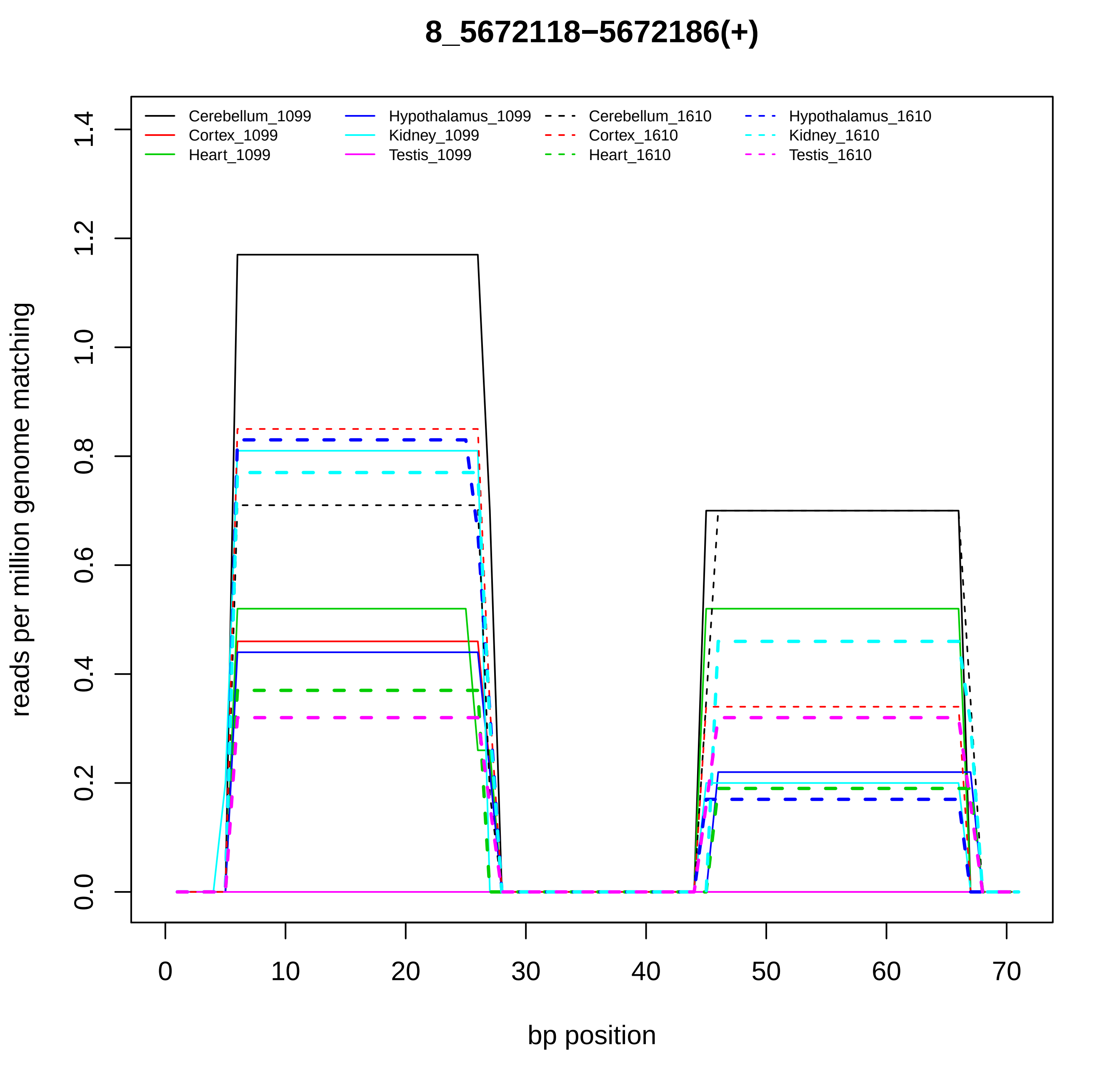

8_5672118−5672186(+)
Cerebellum_1099
Cortex_1099
Heart_1099
Hypothalamus_1099
Kidney_1099
Testis_1099
Cerebellum_1610
Cortex_1610
Heart_1610
Hypothalamus_1610
Kidney_1610
Testis_1610
reads per million genome matching
0.0
0.2
0.4
0.6
0.8
1.0
1.2
1.4
bp position
0
10
20
30
40
50
60
70

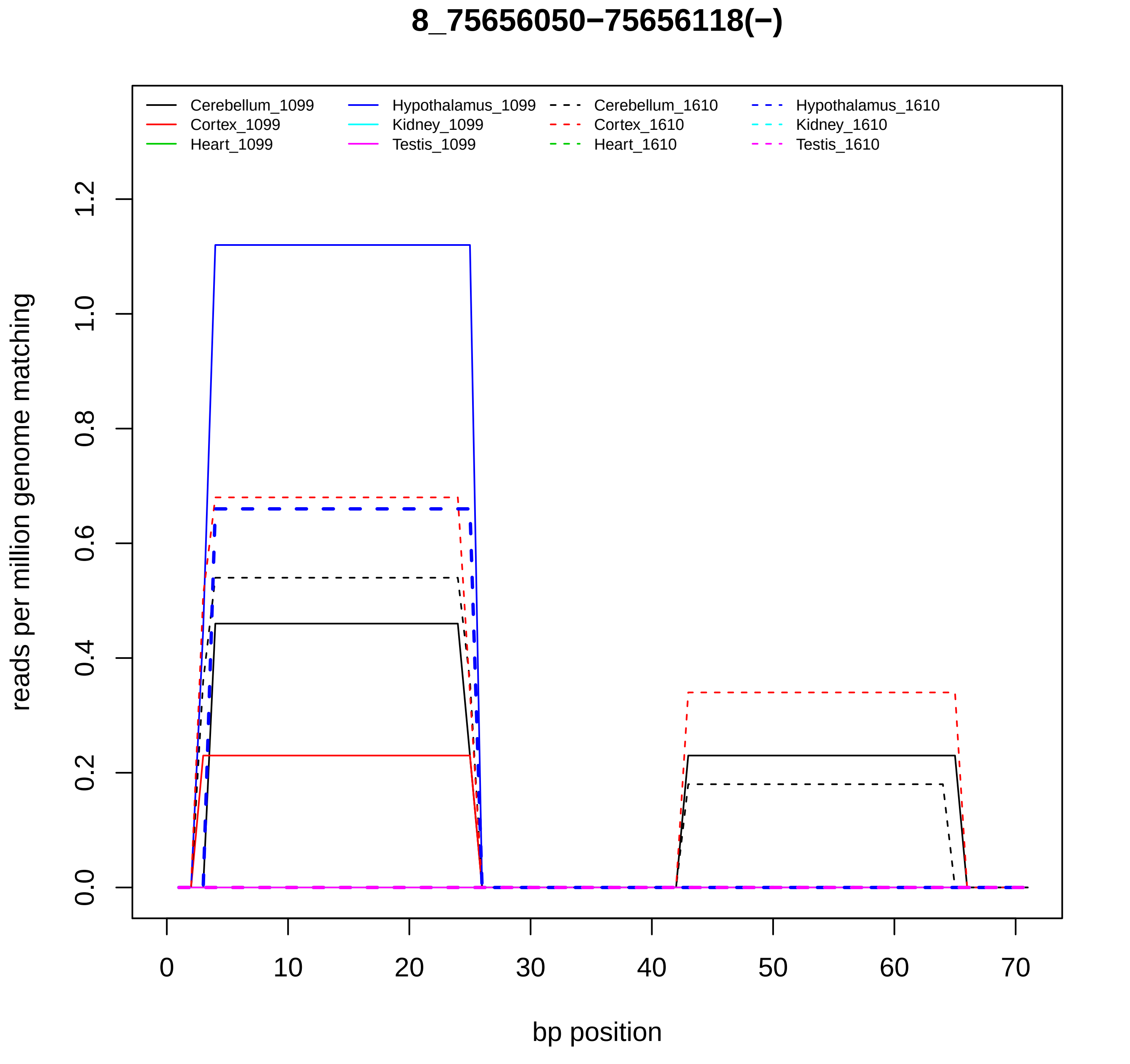

8_75656050−75656118(−)
Cerebellum_1099
Cortex_1099
Heart_1099
Hypothalamus_1099
Kidney_1099
Testis_1099
Cerebellum_1610
Cortex_1610
Heart_1610
Hypothalamus_1610
Kidney_1610
Testis_1610
reads per million genome matching
0.0
0.2
0.4
0.6
0.8
1.0
1.2
0
10
20
30
40
50
60
70
bp position

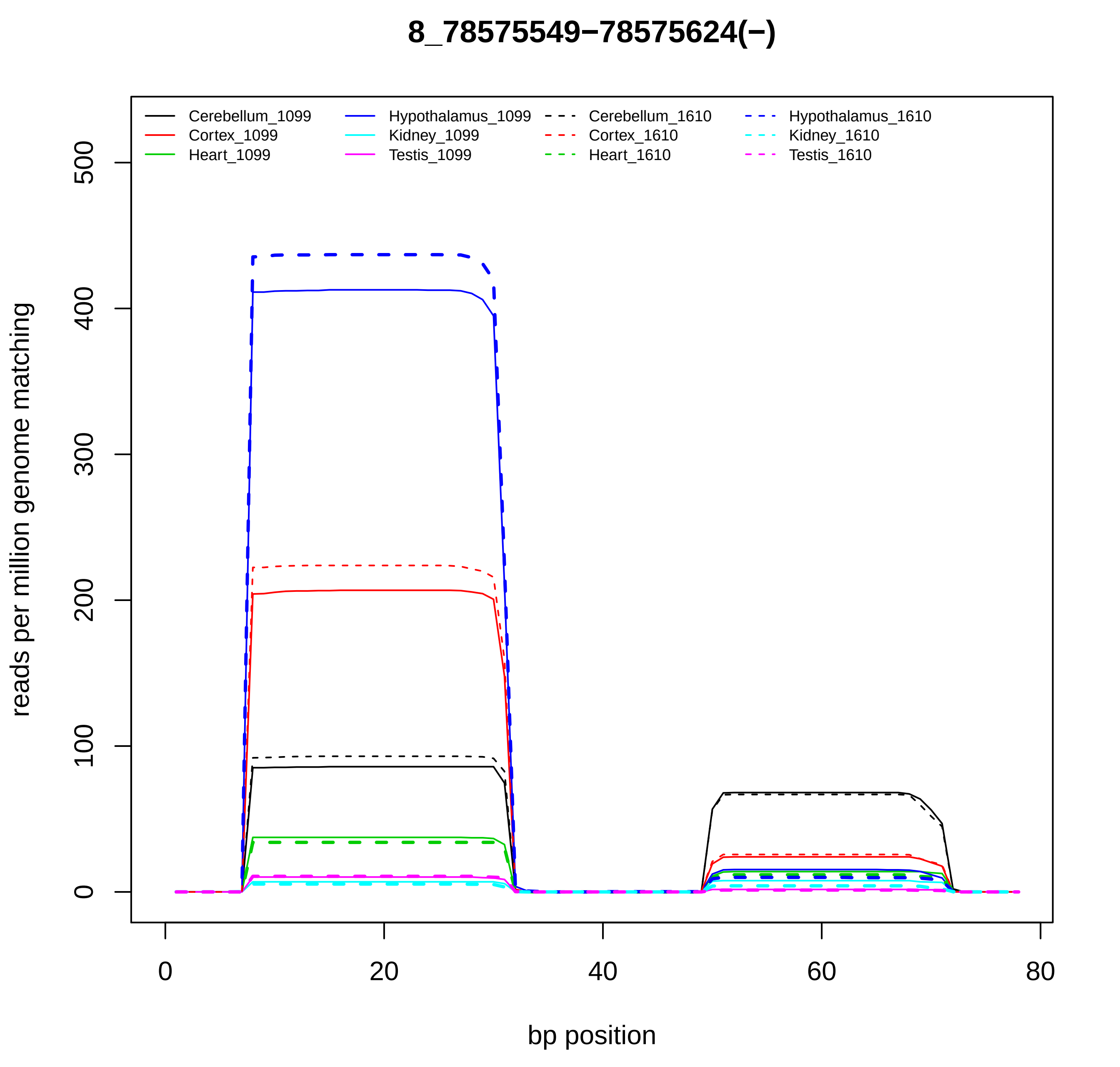
8_78575549−78575624(−)
Cerebellum_1099
Cortex_1099
Heart_1099
Hypothalamus_1099
Kidney_1099
Testis_1099
Cerebellum_1610
Cortex_1610
Heart_1610
Hypothalamus_1610
Kidney_1610
Testis_1610
reads per million genome matching
0
100
200
300
400
500
0
20
40
60
80
bp position

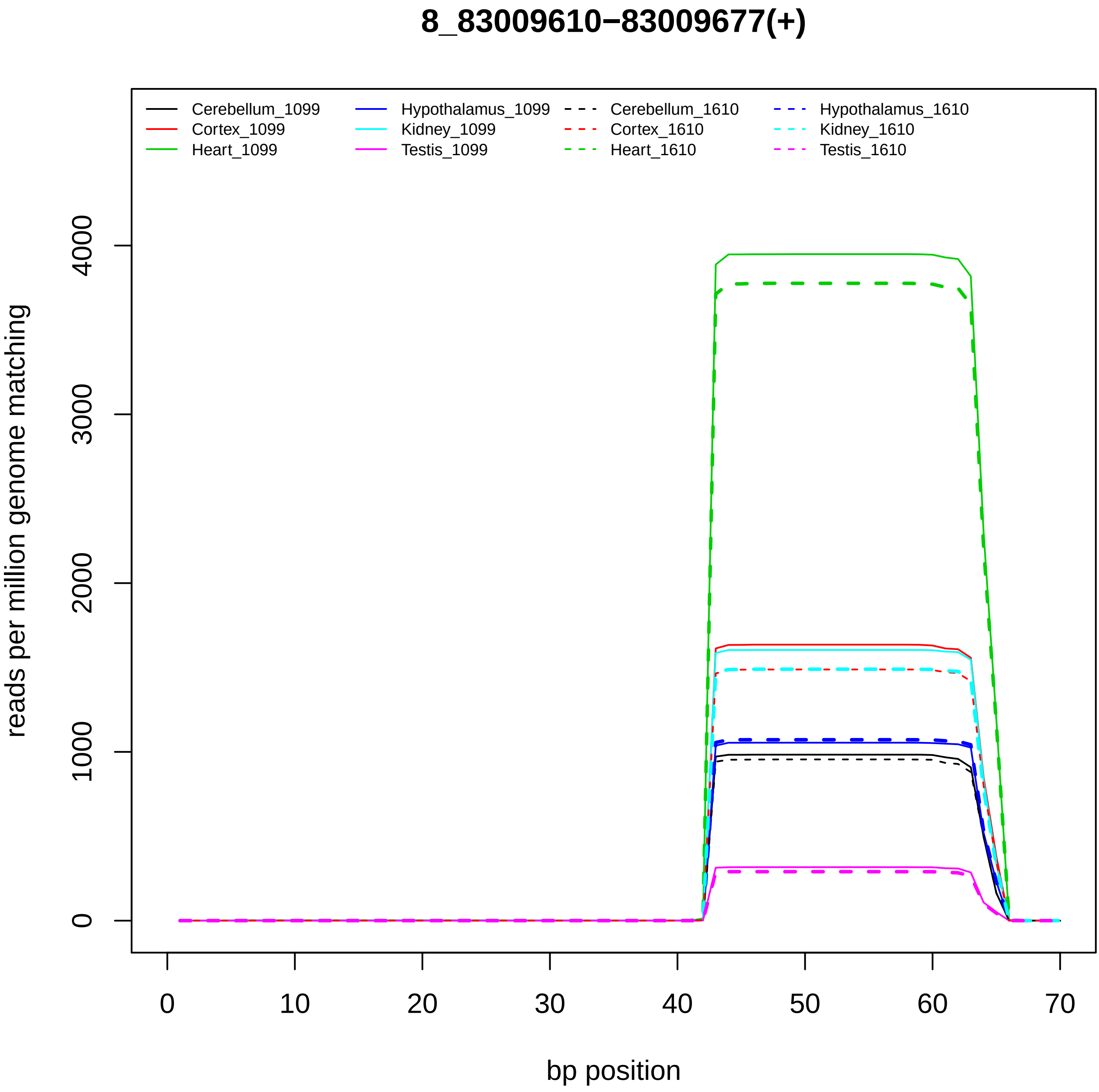

8_83009610−83009677(+)
Cerebellum_1099
Cortex_1099
Heart_1099
Hypothalamus_1099
Kidney_1099
Testis_1099
Cerebellum_1610
Cortex_1610
Heart_1610
Hypothalamus_1610
Kidney_1610
Testis_1610
reads per million genome matching
0
1000
2000
3000
4000
0
10
20
30
40
50
60
70
bp position

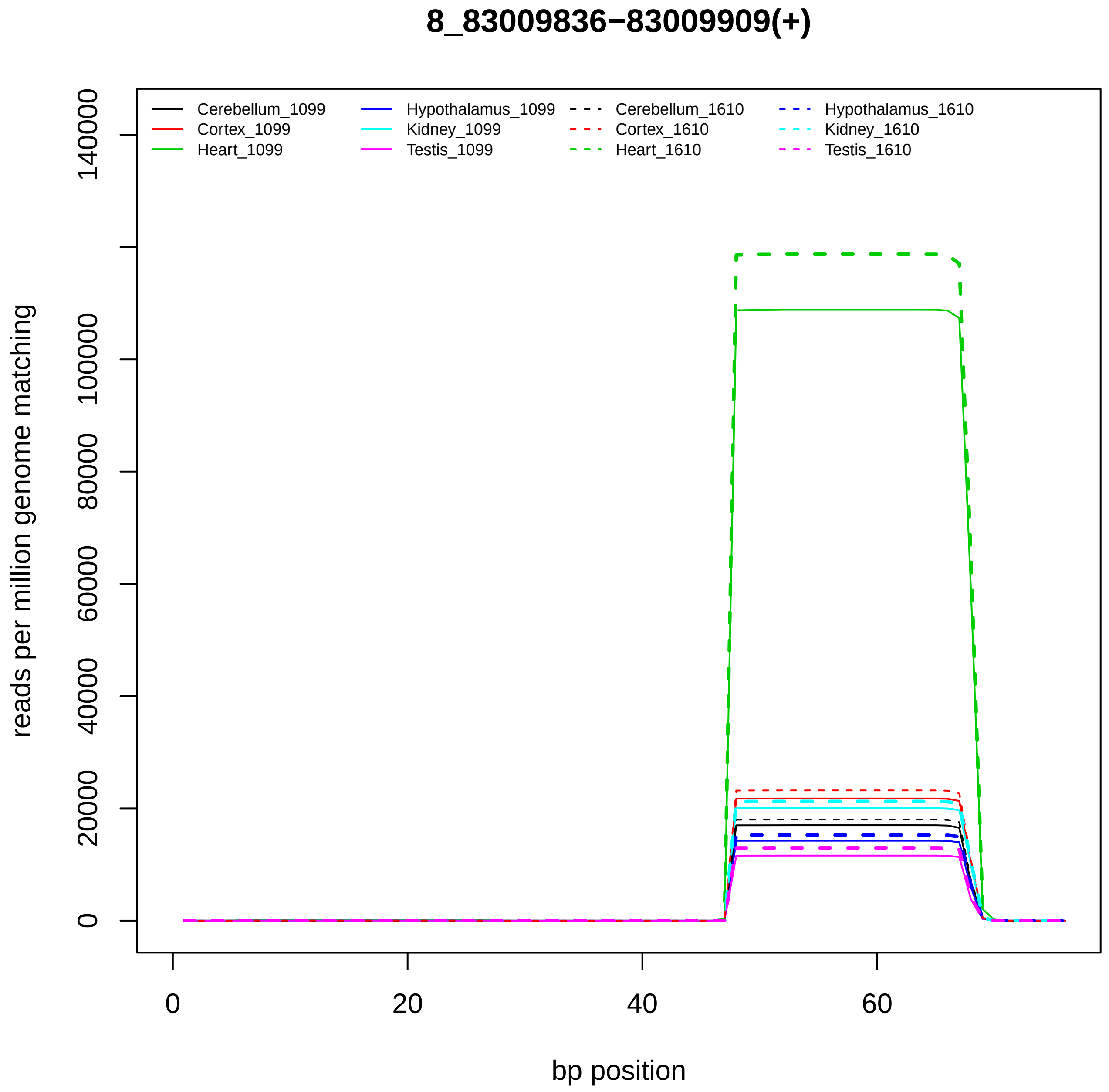

8_83009836−83009909(+)
Cerebellum_1099
Cortex_1099
Heart_1099
Hypothalamus_1099
Kidney_1099
Testis_1099
Cerebellum_1610
Cortex_1610
Heart_1610
Hypothalamus_1610
Kidney_1610
Testis_1610
reads per million genome matching
0
20000
40000
60000
80000
100000
140000
bp position
0
20
40
60

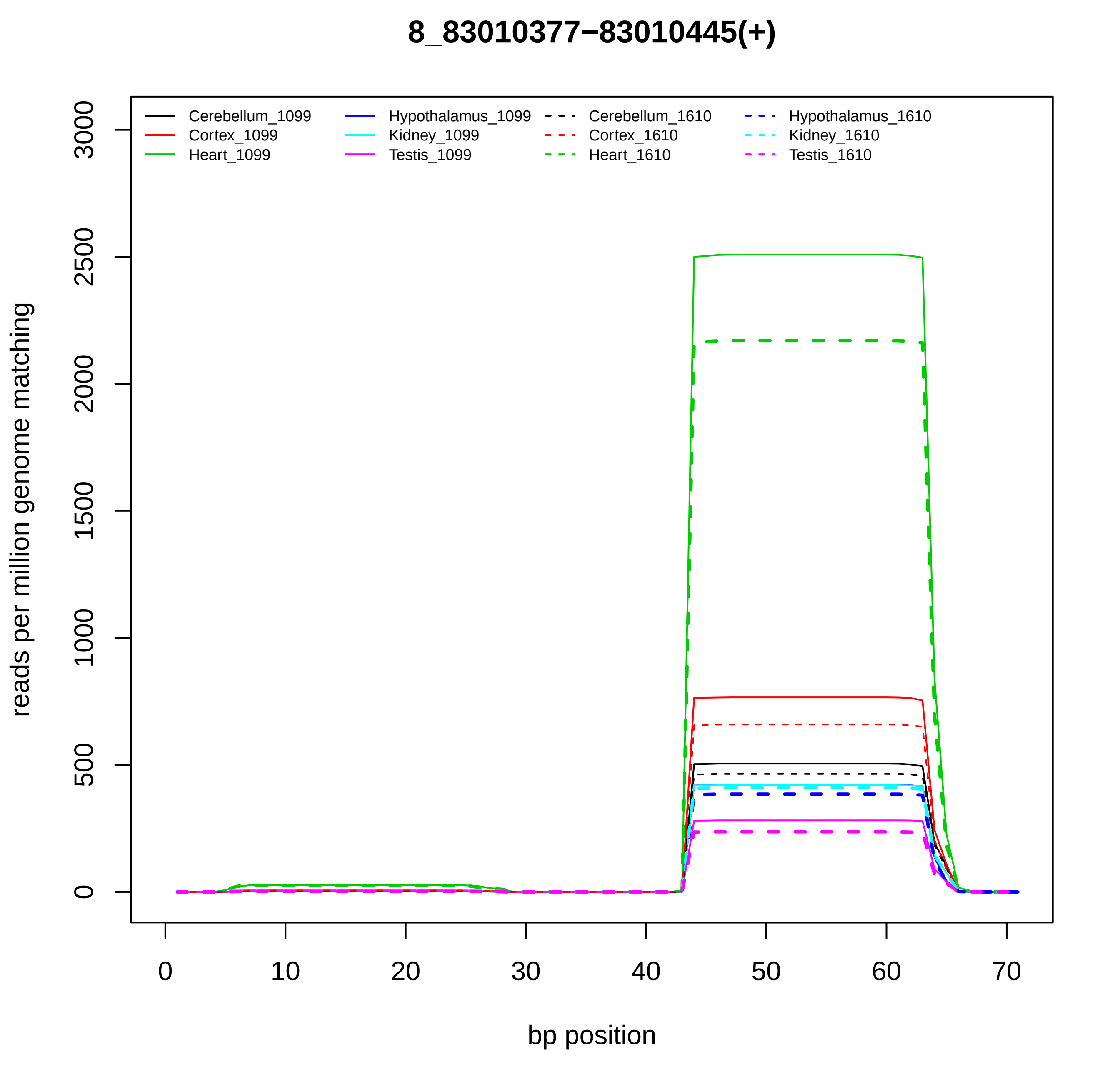

8_83010377−83010445(+)
Cerebellum_1099
Cortex_1099
Heart_1099
Hypothalamus_1099
Kidney_1099
Testis_1099
Cerebellum_1610
Cortex_1610
Heart_1610
Hypothalamus_1610
Kidney_1610
Testis_1610
reads per million genome matching
bp position
0
500
1000
1500
2000
2500
3000
0
10
20
30
40
50
60
70

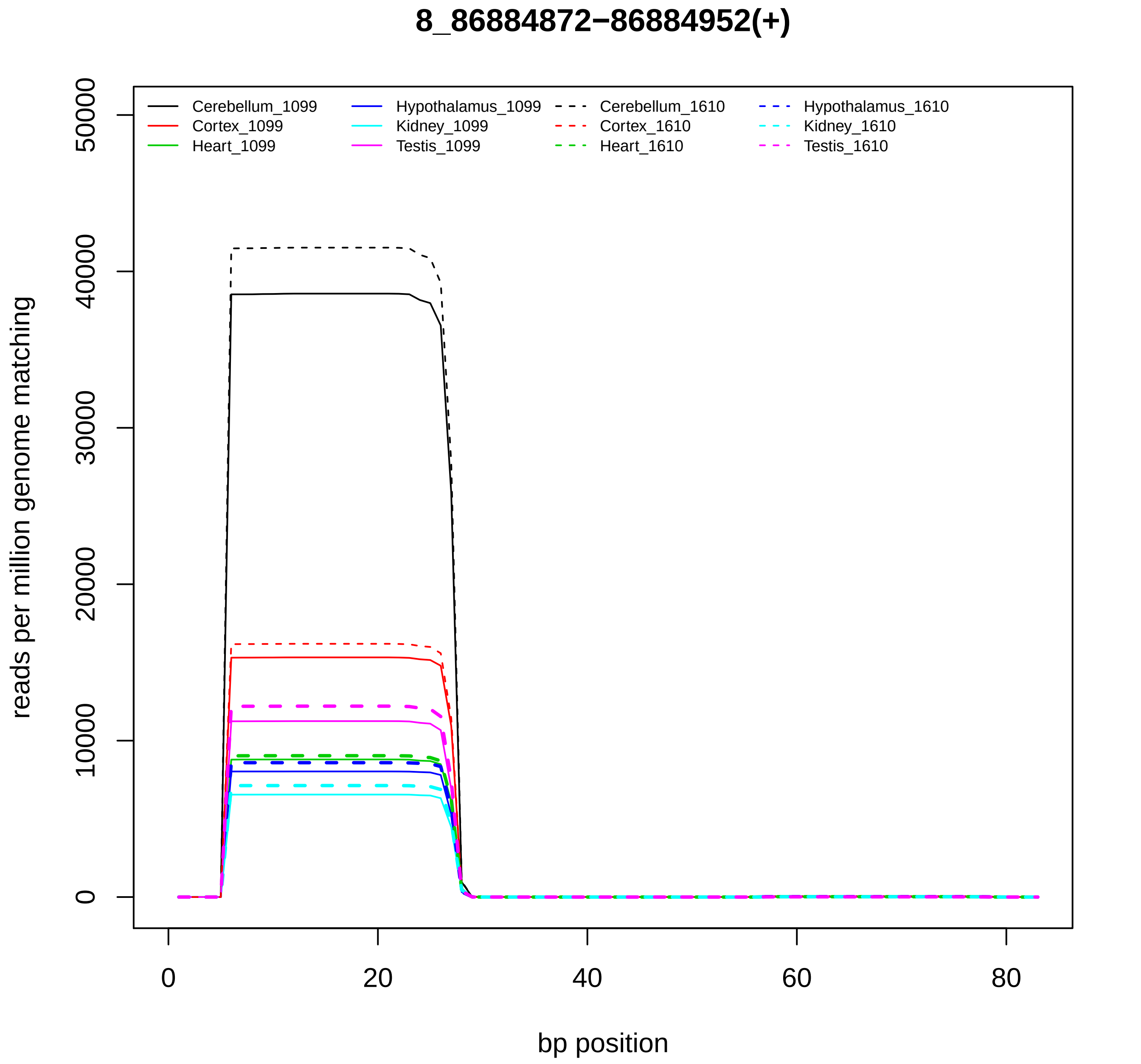

8_86884872−86884952(+)
Cerebellum_1099
Cortex_1099
Heart_1099
Hypothalamus_1099
Kidney_1099
Testis_1099
Cerebellum_1610
Cortex_1610
Heart_1610
Hypothalamus_1610
Kidney_1610
Testis_1610
reads per million genome matching
0
10000
20000
30000
40000
50000
0
20
40
60
80
bp position

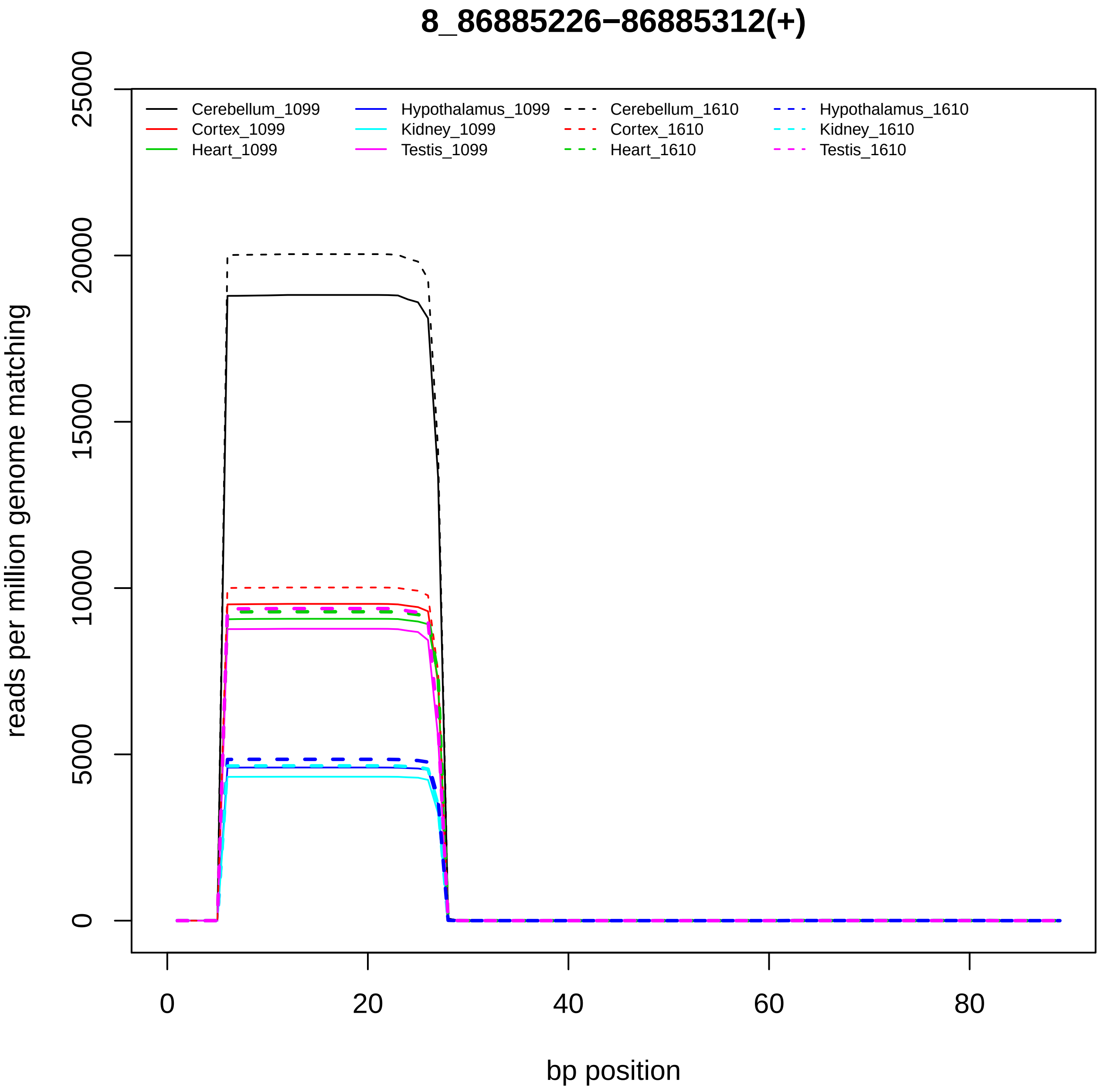
8_86885226−86885312(+)
Cerebellum_1099
Cortex_1099
Heart_1099
Hypothalamus_1099
Kidney_1099
Testis_1099
Cerebellum_1610
Cortex_1610
Heart_1610
Hypothalamus_1610
Kidney_1610
Testis_1610
reads per million genome matching
0
5000
10000
15000
20000
25000
0
20
40
60
80
bp position

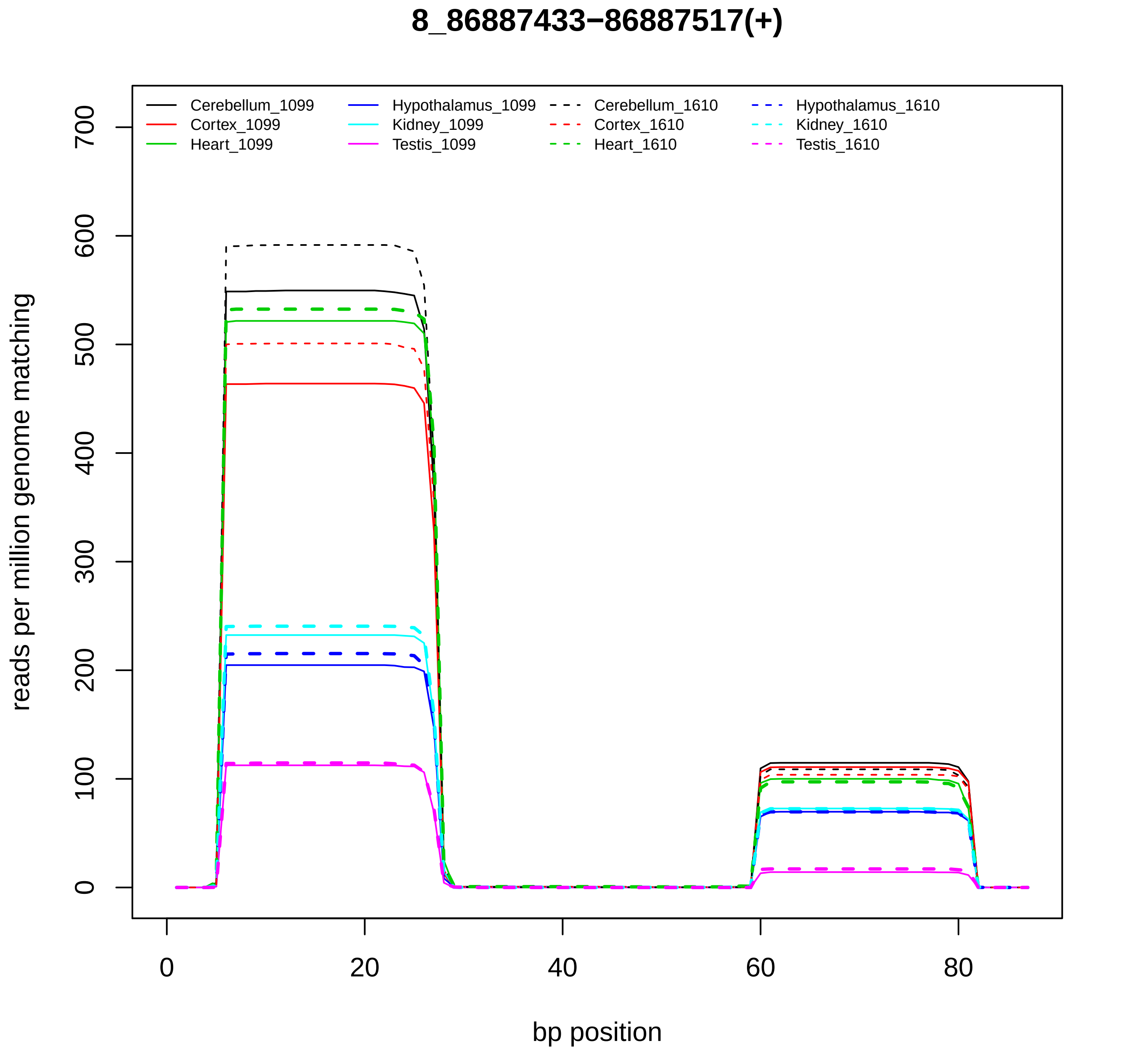

8_86887433−86887517(+)
Cerebellum_1099
Cortex_1099
Heart_1099
Hypothalamus_1099
Kidney_1099
Testis_1099
Cerebellum_1610
Cortex_1610
Heart_1610
Hypothalamus_1610
Kidney_1610
Testis_1610
reads per million genome matching
0
100
200
300
400
500
600
700
bp position
0
20
40
60
80

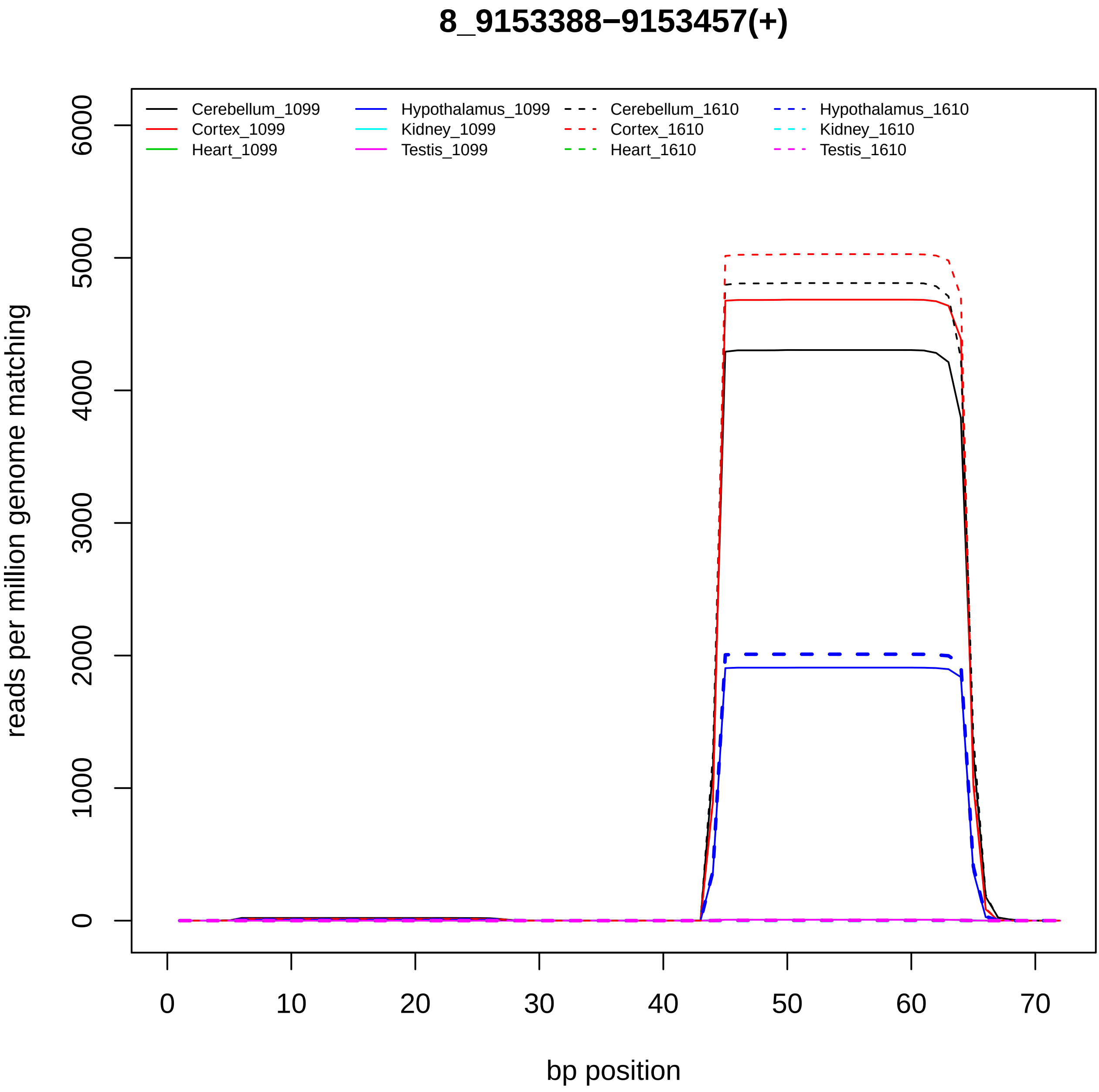

8_9153388−9153457(+)
Cerebellum_1099
Cortex_1099
Heart_1099
Hypothalamus_1099
Kidney_1099
Testis_1099
Cerebellum_1610
Cortex_1610
Heart_1610
Hypothalamus_1610
Kidney_1610
Testis_1610
reads per million genome matching
0
1000
2000
3000
4000
5000
6000
0
10
20
30
40
50
60
70
bp position

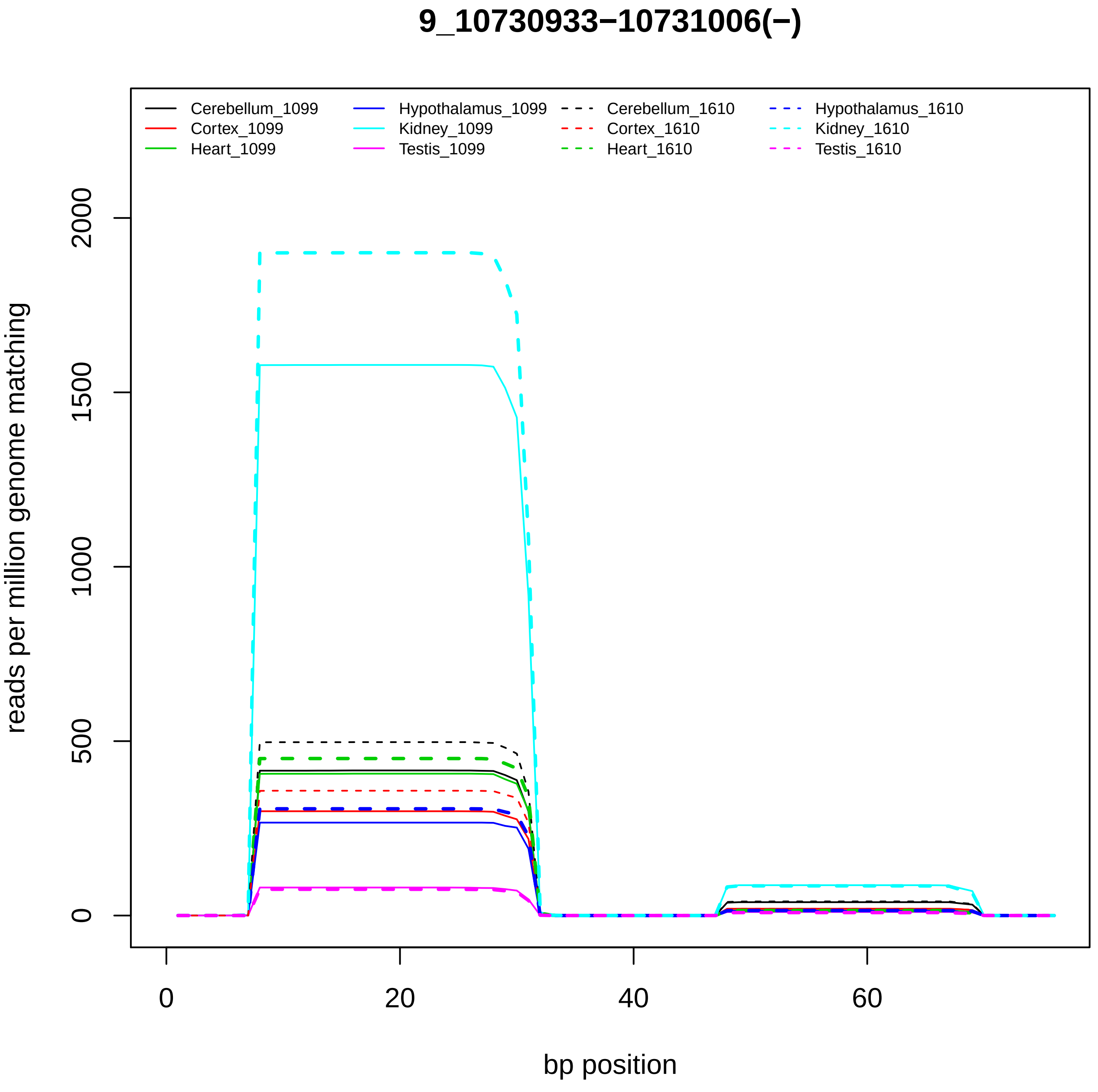

9_10730933−10731006(−)
Cerebellum_1099
Cortex_1099
Heart_1099
Hypothalamus_1099
Kidney_1099
Testis_1099
Cerebellum_1610
Cortex_1610
Heart_1610
Hypothalamus_1610
Kidney_1610
Testis_1610
reads per million genome matching
0
500
1000
1500
2000
0
20
40
60
bp position

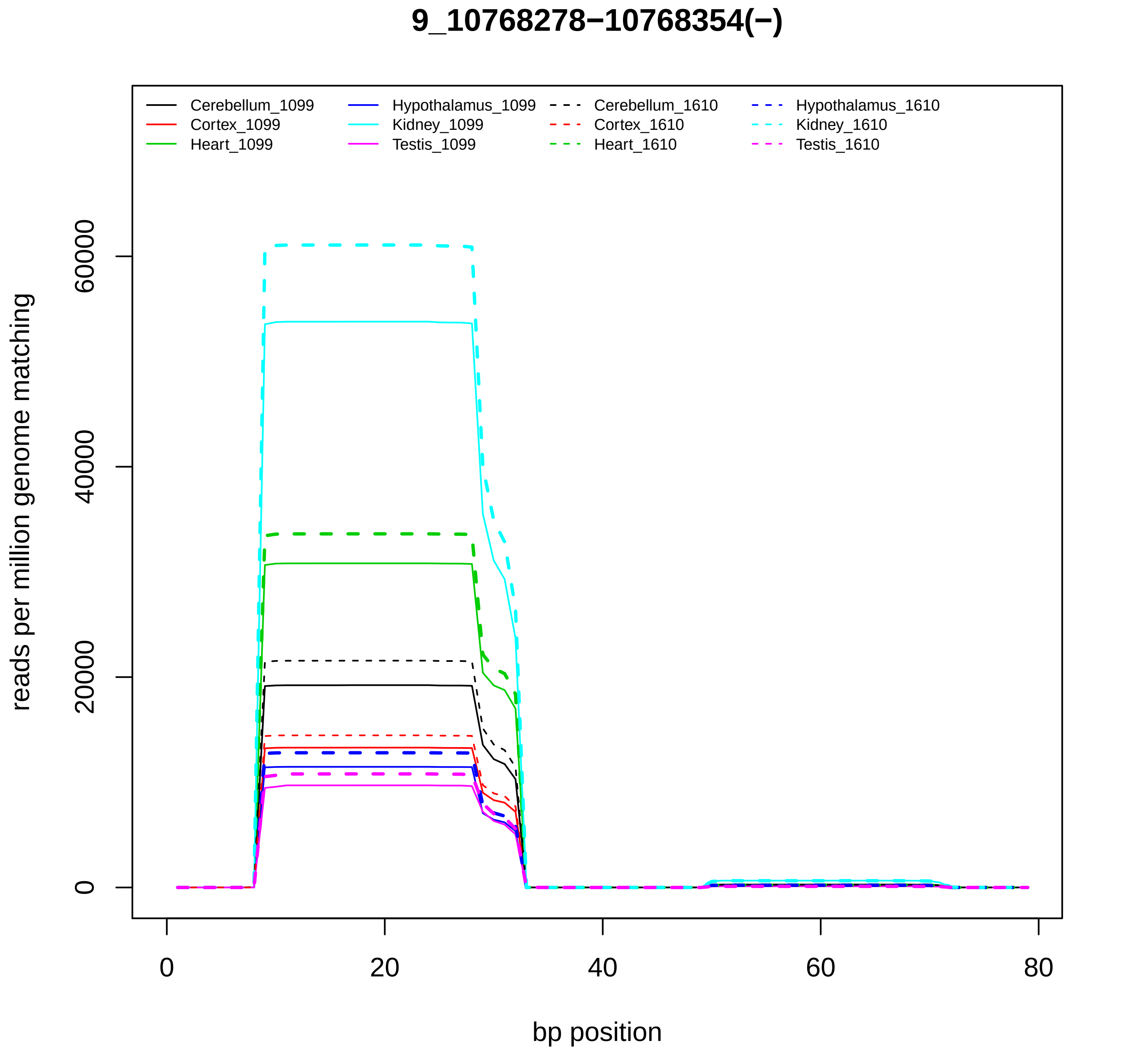

9_10768278−10768354(−)
Cerebellum_1099
Cortex_1099
Heart_1099
Hypothalamus_1099
Kidney_1099
Testis_1099
Cerebellum_1610
Cortex_1610
Heart_1610
Hypothalamus_1610
Kidney_1610
Testis_1610
reads per million genome matching
0
20000
40000
60000
0
20
40
60
80
bp position

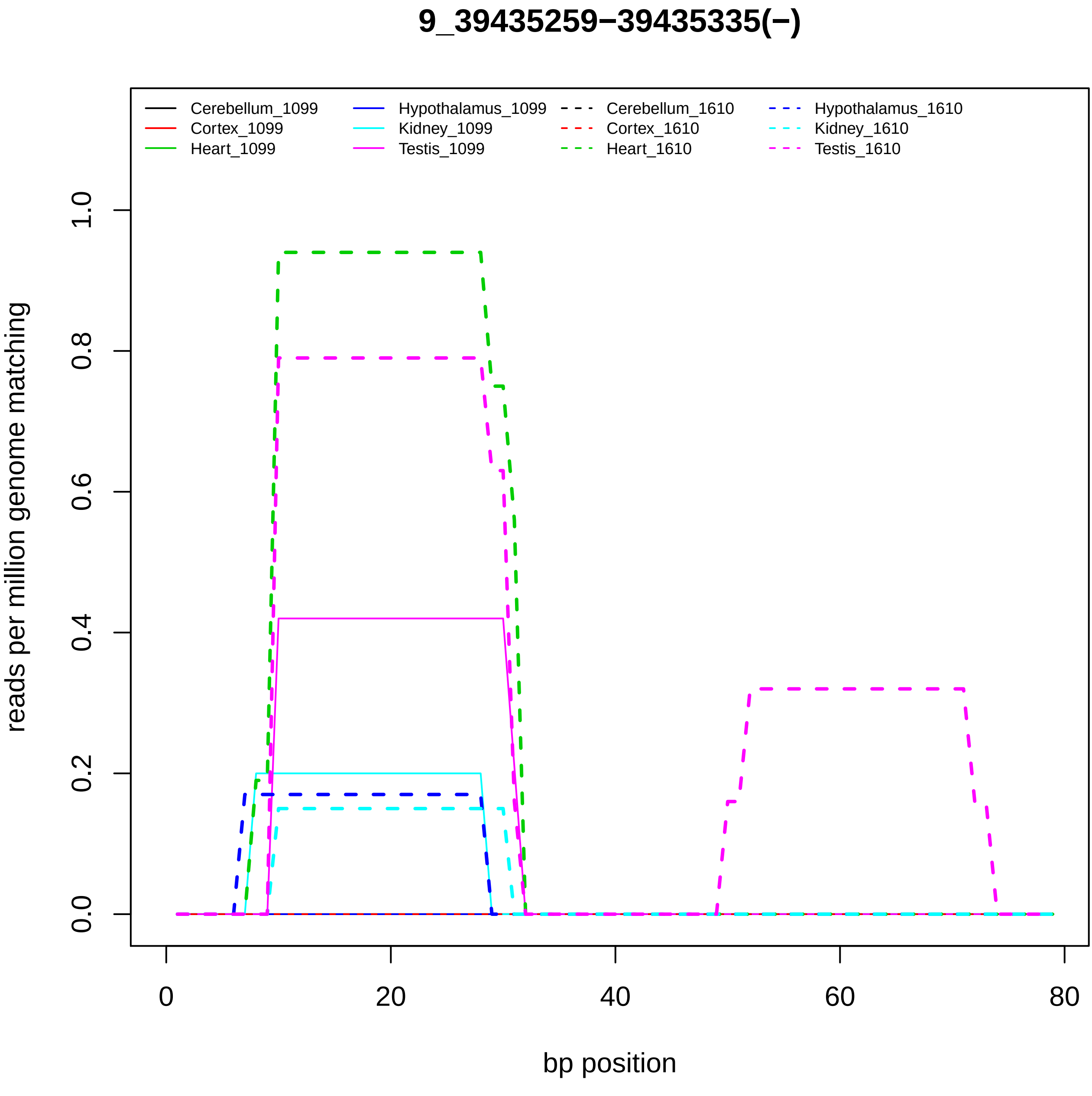
9_39435259−39435335(−)
Cerebellum_1099
Cortex_1099
Heart_1099
Hypothalamus_1099
Kidney_1099
Testis_1099
Cerebellum_1610
Cortex_1610
Heart_1610
Hypothalamus_1610
Kidney_1610
Testis_1610
reads per million genome matching
0.0
0.2
0.4
0.6
0.8
1.0
0
20
40
60
80
bp position

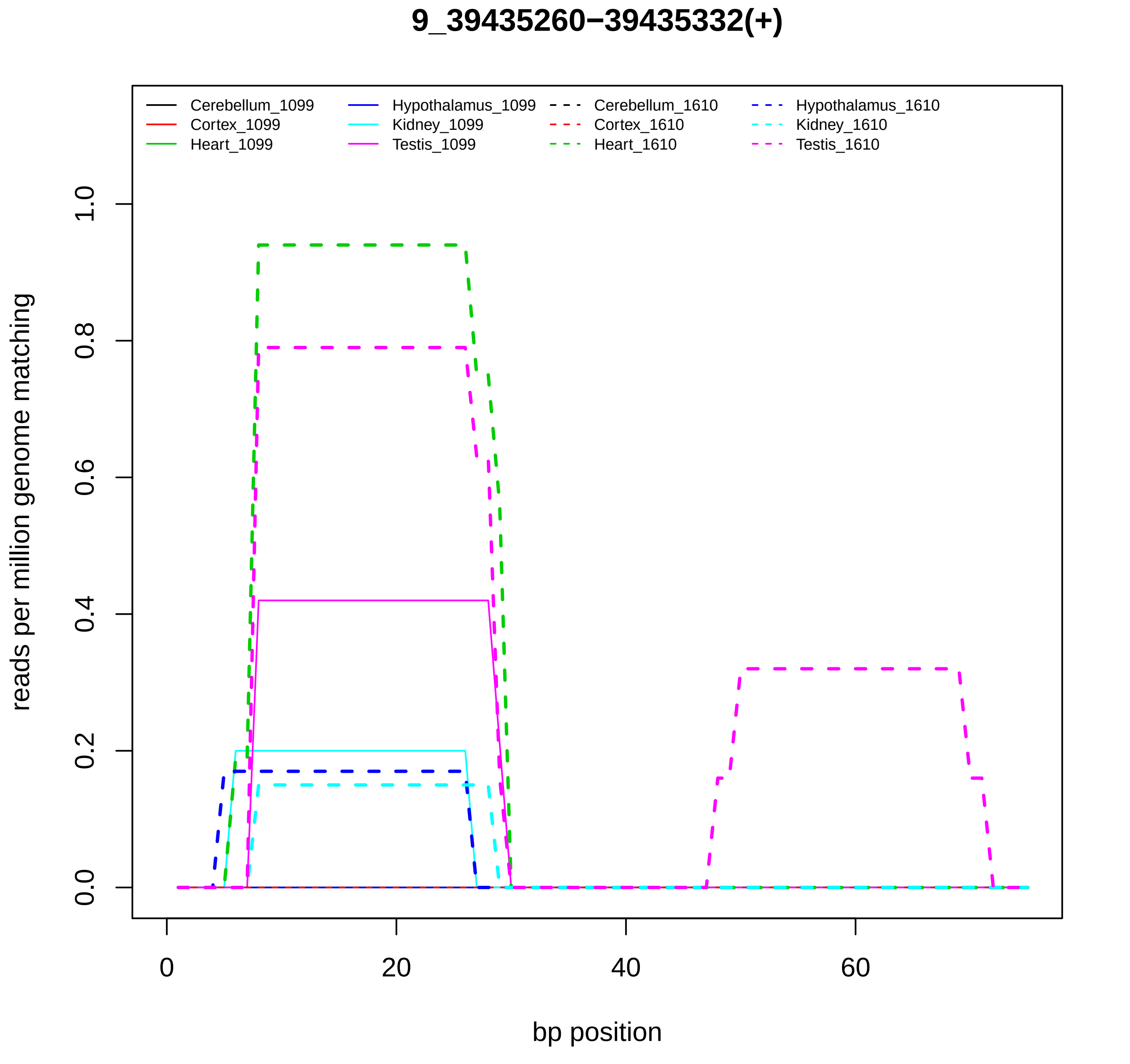
9_39435260−39435332(+)
Cerebellum_1099
Cortex_1099
Heart_1099
Hypothalamus_1099
Kidney_1099
Testis_1099
Cerebellum_1610
Cortex_1610
Heart_1610
Hypothalamus_1610
Kidney_1610
Testis_1610
reads per million genome matching
0.0
0.2
0.4
0.6
0.8
1.0
0
20
40
60
bp position

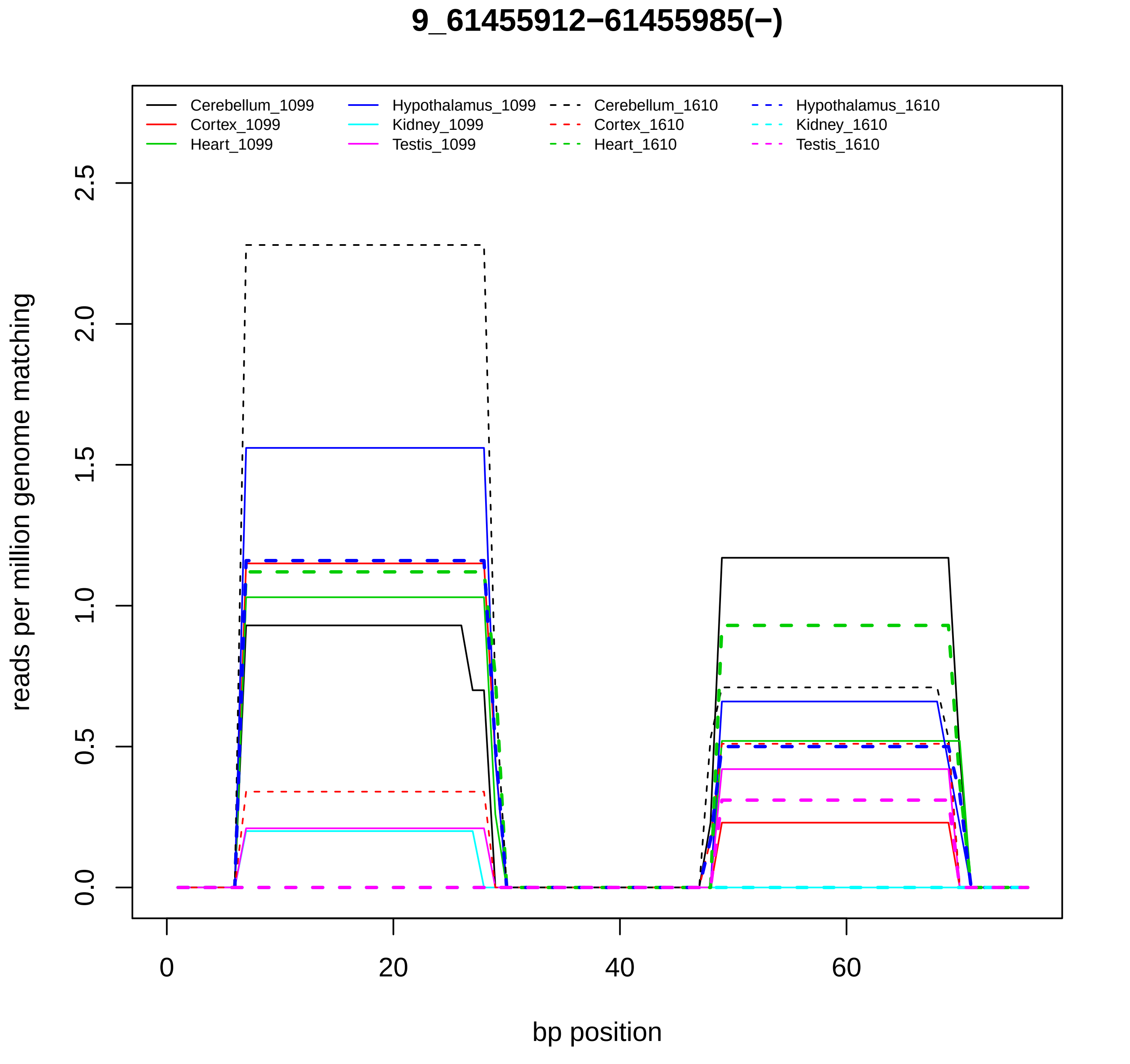

9_61455912−61455985(−)
Cerebellum_1099
Cortex_1099
Heart_1099
Hypothalamus_1099
Kidney_1099
Testis_1099
Cerebellum_1610
Cortex_1610
Heart_1610
Hypothalamus_1610
Kidney_1610
Testis_1610
reads per million genome matching
0.0
0.5
1.0
1.5
2.0
2.5
0
20
40
60
bp position

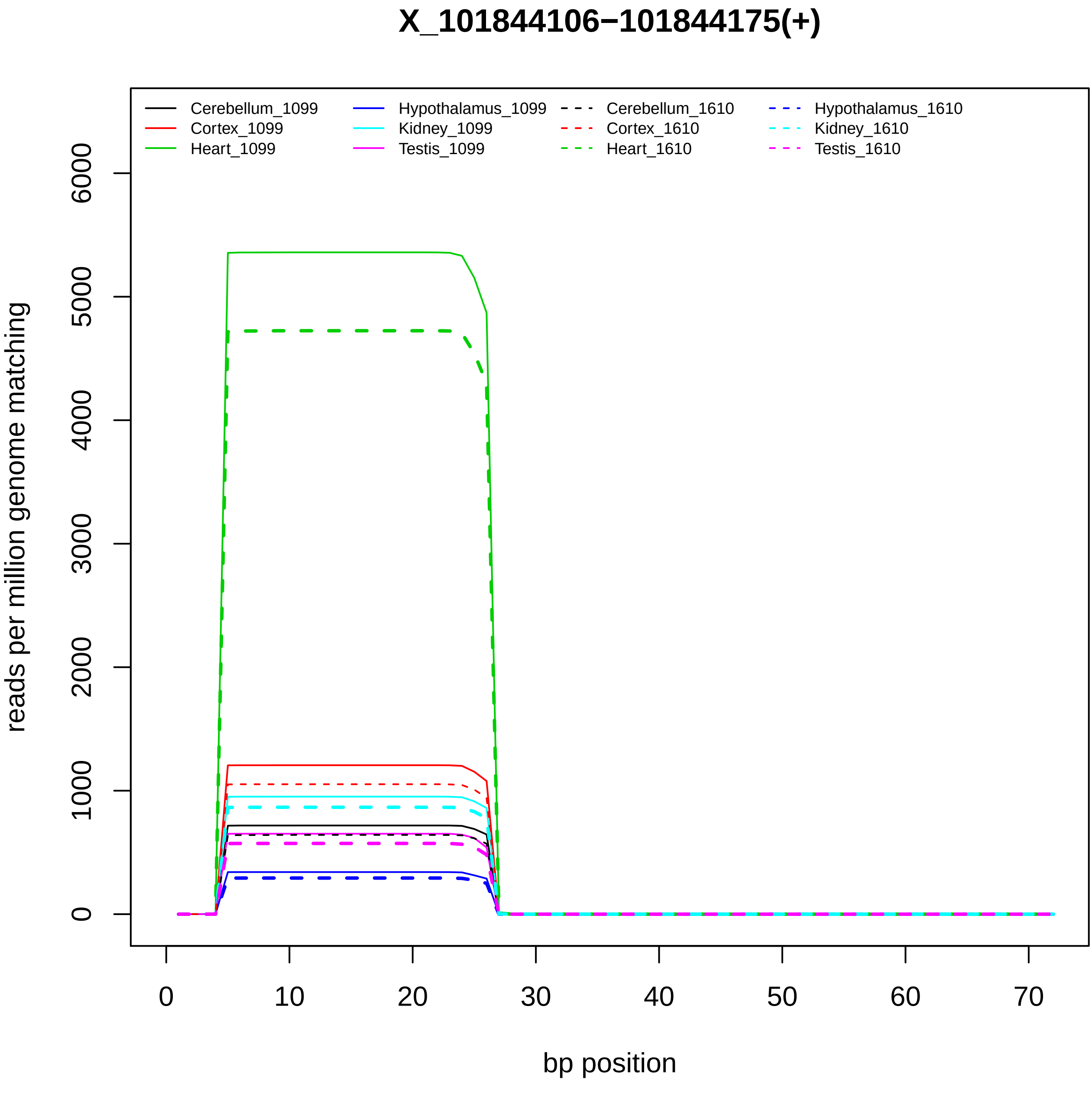

X_101844106−101844175(+)
Cerebellum_1099
Cortex_1099
Heart_1099
Hypothalamus_1099
Kidney_1099
Testis_1099
Cerebellum_1610
Cortex_1610
Heart_1610
Hypothalamus_1610
Kidney_1610
Testis_1610
reads per million genome matching
0
1000
2000
3000
4000
5000
6000
0
10
20
30
40
50
60
70
bp position

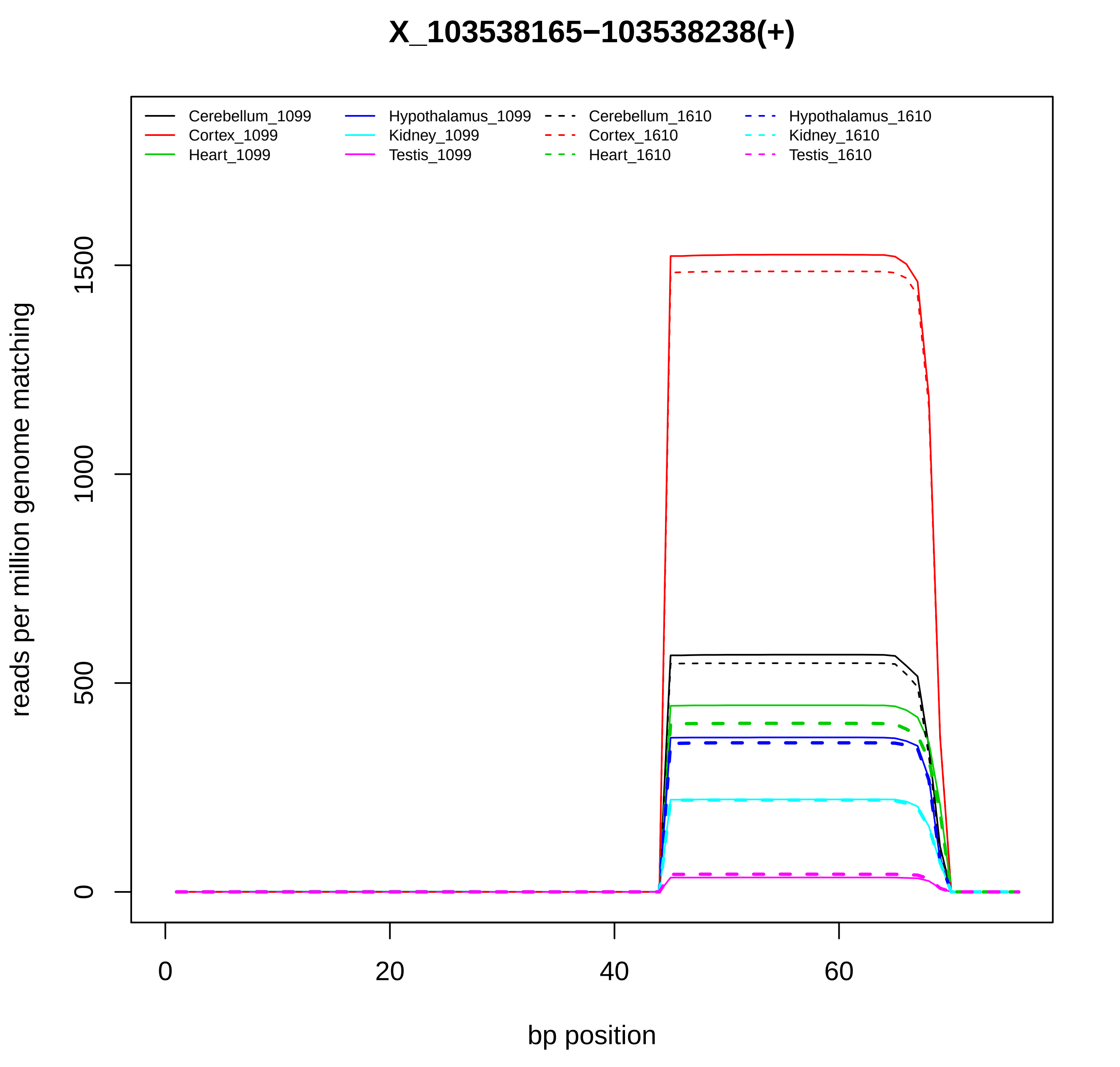

X_103538165−103538238(+)
Cerebellum_1099
Cortex_1099
Heart_1099
Hypothalamus_1099
Kidney_1099
Testis_1099
Cerebellum_1610
Cortex_1610
Heart_1610
Hypothalamus_1610
Kidney_1610
Testis_1610
reads per million genome matching
0
500
1000
1500
0
20
40
60
bp position

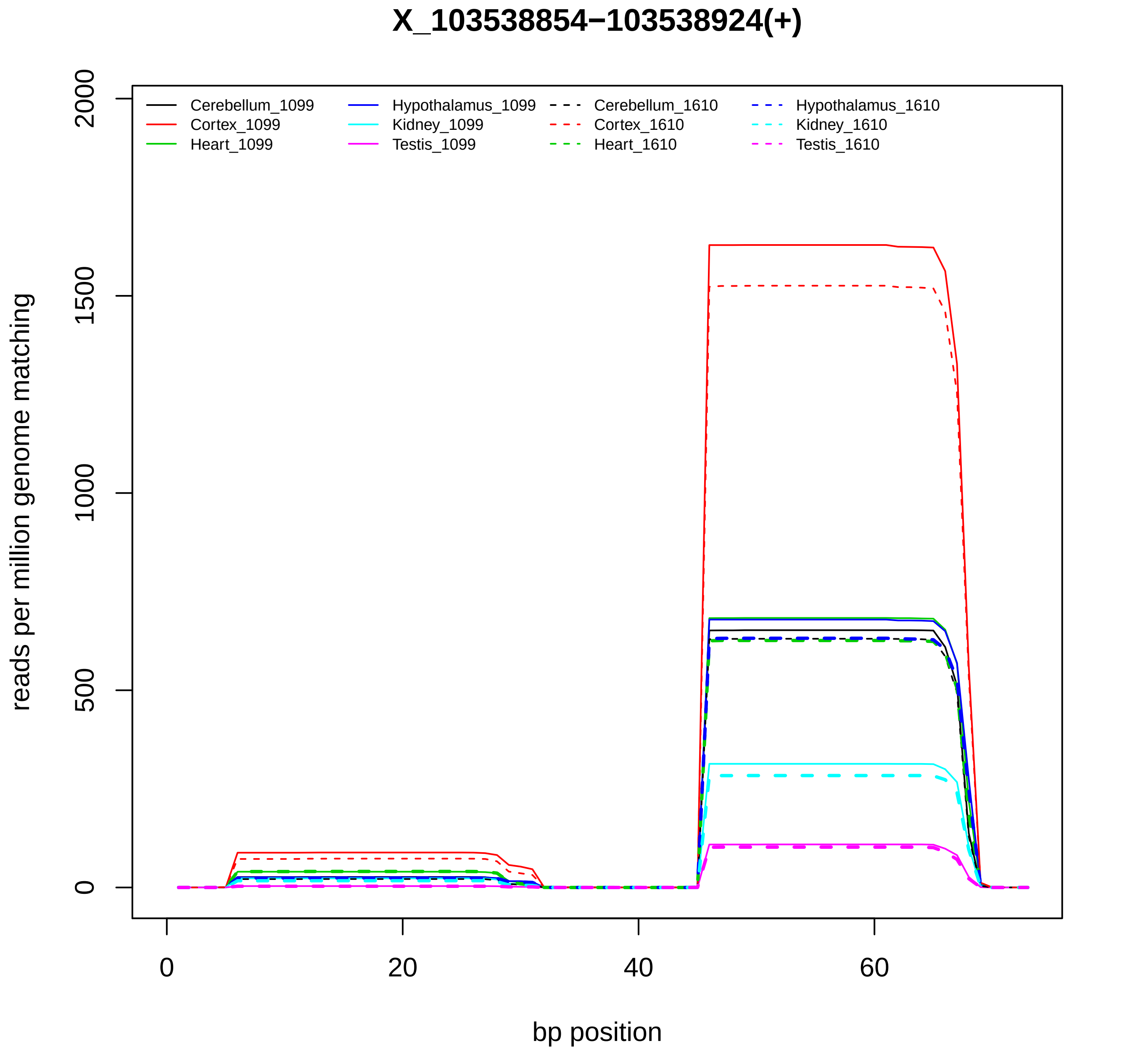

X_103538854−103538924(+)
Cerebellum_1099
Cortex_1099
Heart_1099
Hypothalamus_1099
Kidney_1099
Testis_1099
Cerebellum_1610
Cortex_1610
Heart_1610
Hypothalamus_1610
Kidney_1610
Testis_1610
reads per million genome matching
0
500
1000
1500
2000
0
20
40
60
bp position

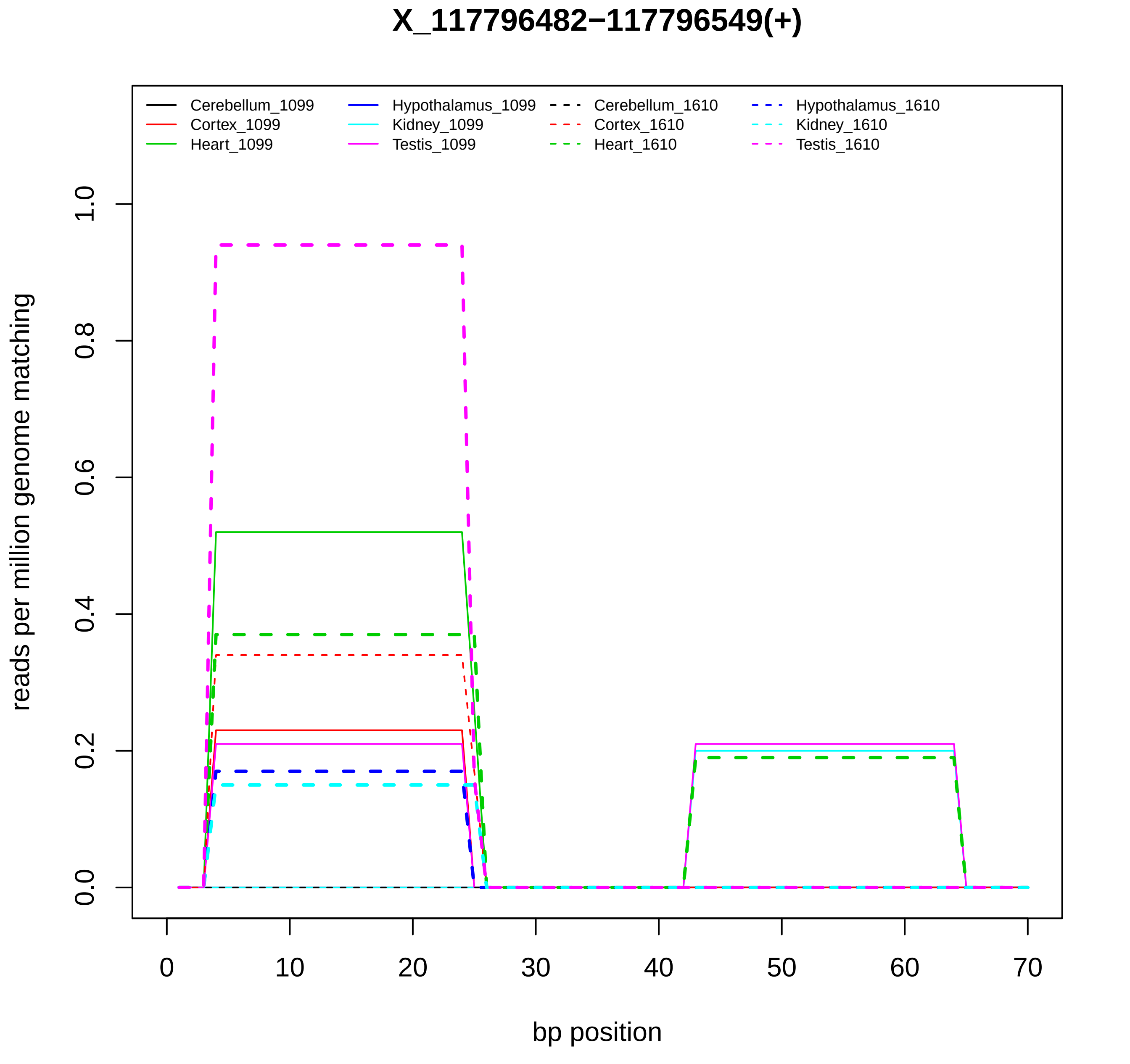

X_117796482−117796549(+)
Cerebellum_1099
Cortex_1099
Heart_1099
Hypothalamus_1099
Kidney_1099
Testis_1099
Cerebellum_1610
Cortex_1610
Heart_1610
Hypothalamus_1610
Kidney_1610
Testis_1610
reads per million genome matching
0.0
0.2
0.4
0.6
0.8
1.0
0
10
20
30
40
50
60
70
bp position

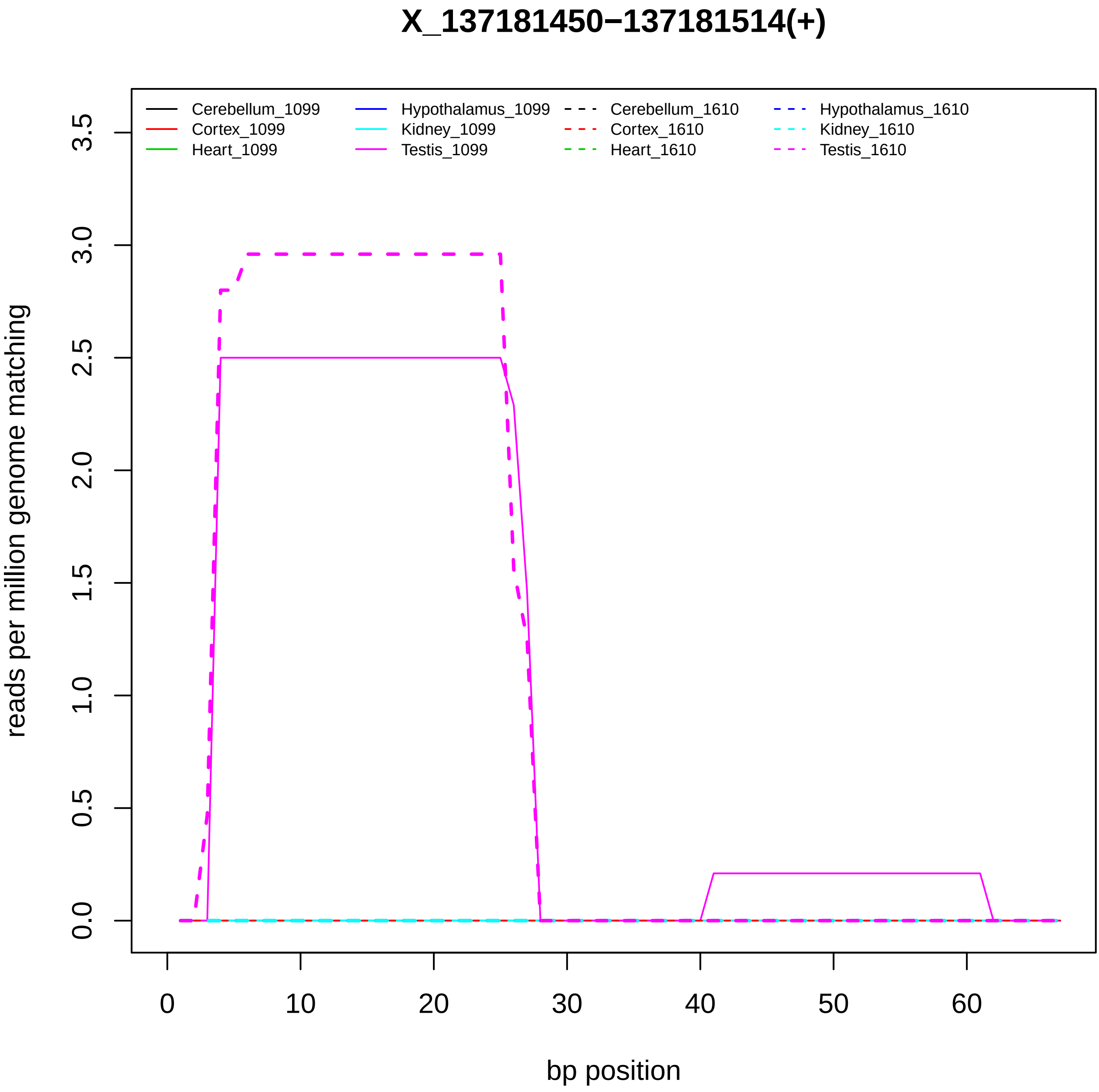

X_137181450−137181514(+)
Cerebellum_1099
Cortex_1099
Heart_1099
Hypothalamus_1099
Kidney_1099
Testis_1099
Cerebellum_1610
Cortex_1610
Heart_1610
Hypothalamus_1610
Kidney_1610
Testis_1610
reads per million genome matching
bp position
0.0
0.5
1.0
1.5
2.0
2.5
3.0
3.5
0
10
20
30
40
50
60

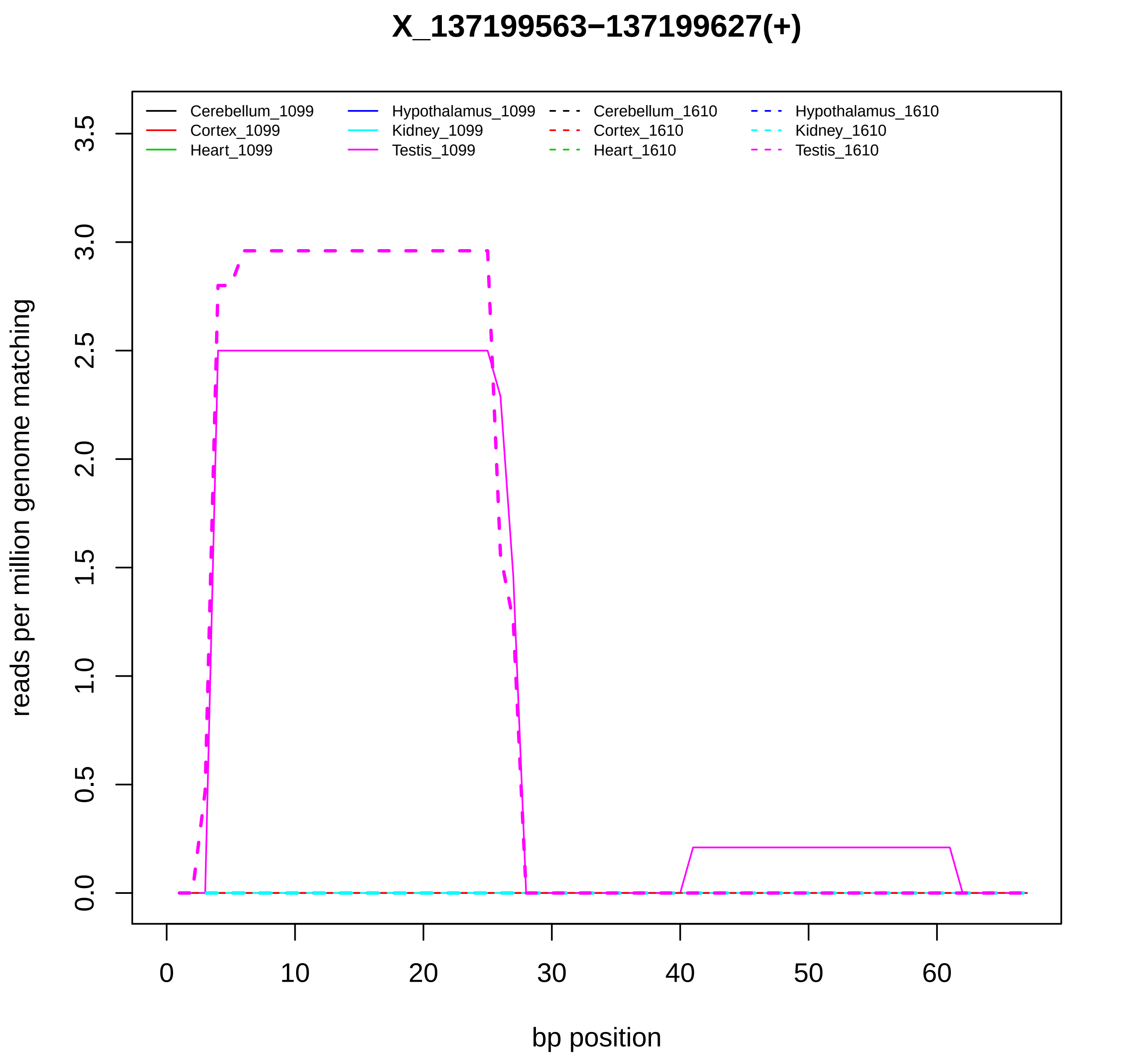

X_137199563−137199627(+)
Cerebellum_1099
Cortex_1099
Heart_1099
Hypothalamus_1099
Kidney_1099
Testis_1099
Cerebellum_1610
Cortex_1610
Heart_1610
Hypothalamus_1610
Kidney_1610
Testis_1610
reads per million genome matching
0.0
0.5
1.0
1.5
2.0
2.5
3.0
3.5
0
10
20
30
40
50
60
bp position

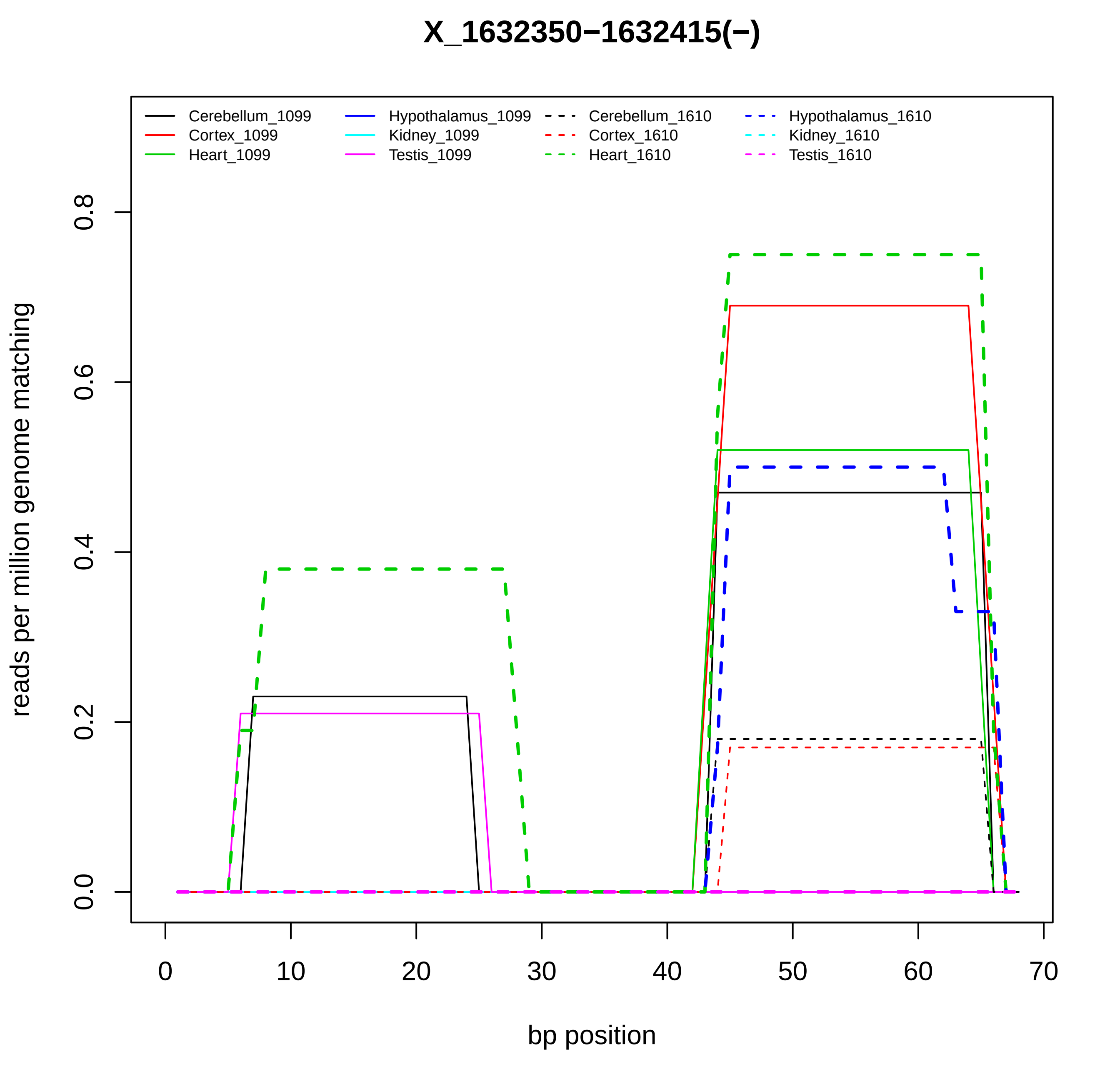

X_1632350−1632415(−)
Cerebellum_1099
Cortex_1099
Heart_1099
Hypothalamus_1099
Kidney_1099
Testis_1099
Cerebellum_1610
Cortex_1610
Heart_1610
Hypothalamus_1610
Kidney_1610
Testis_1610
reads per million genome matching
0.0
0.2
0.4
0.6
0.8
0
10
20
30
40
50
60
70
bp position

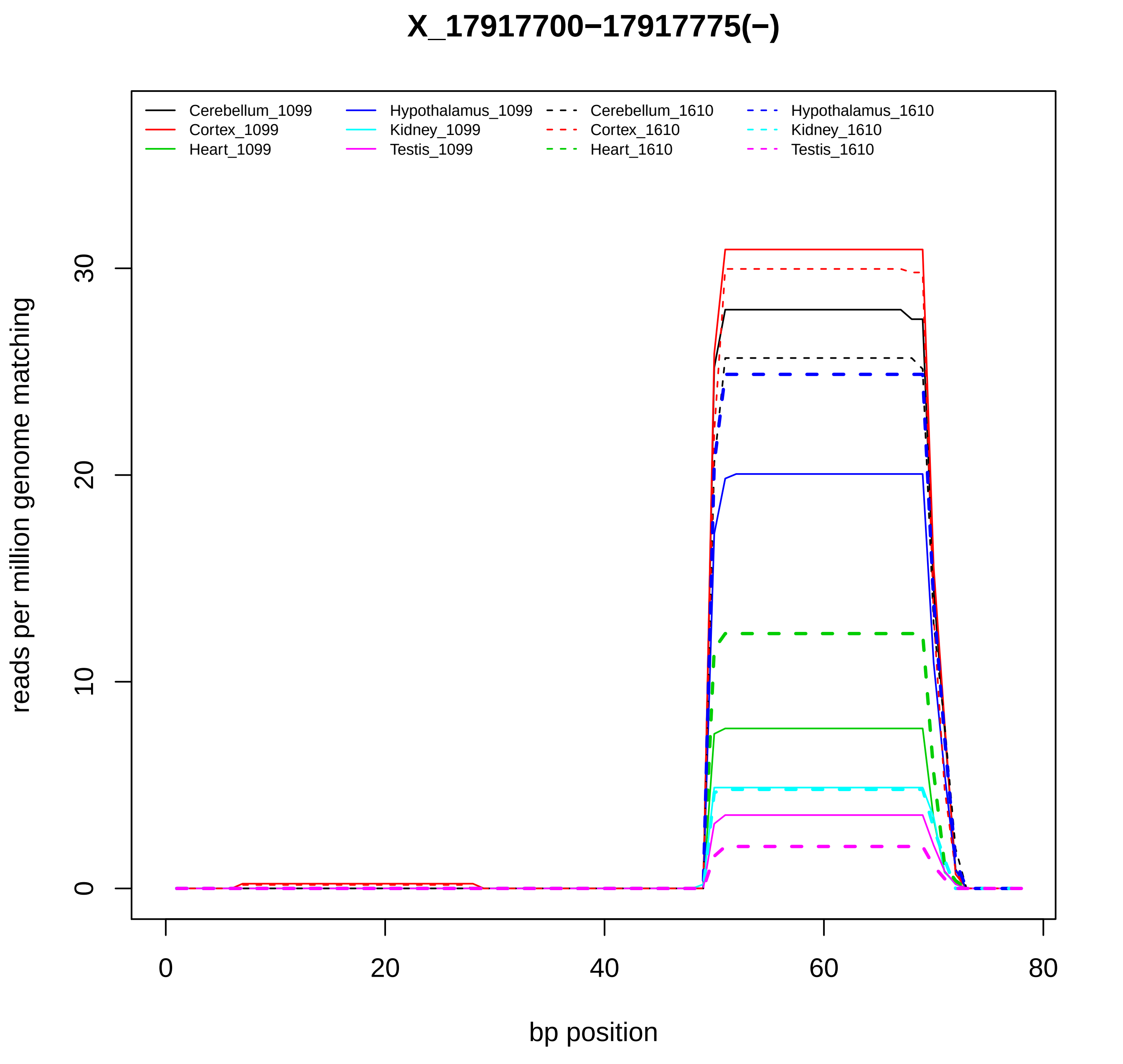
X_17917700−17917775(−)
Cerebellum_1099
Cortex_1099
Heart_1099
Hypothalamus_1099
Kidney_1099
Testis_1099
Cerebellum_1610
Cortex_1610
Heart_1610
Hypothalamus_1610
Kidney_1610
Testis_1610
reads per million genome matching
0
10
20
30
0
20
40
60
80
bp position

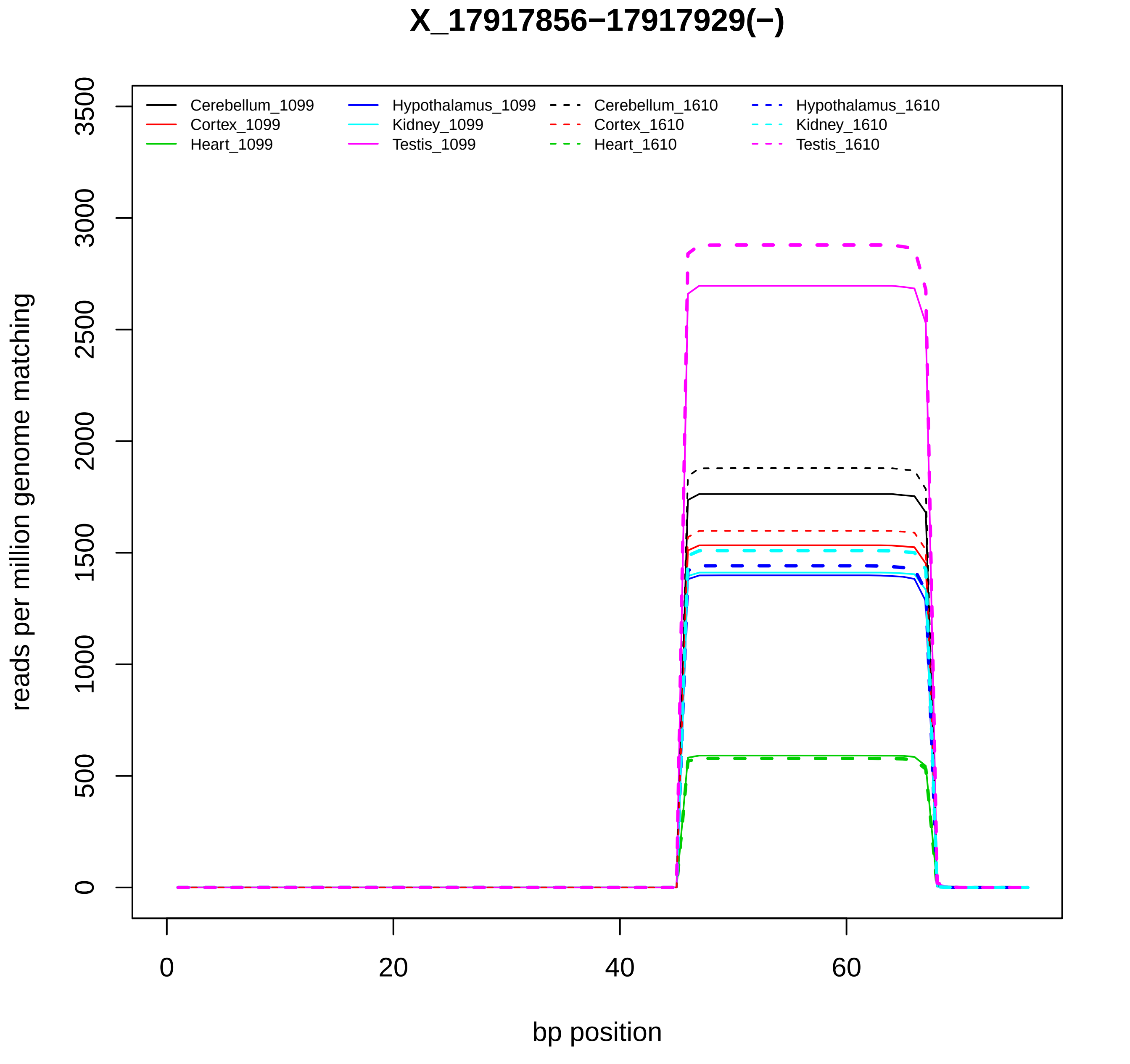

X_17917856−17917929(−)
Cerebellum_1099
Cortex_1099
Heart_1099
Hypothalamus_1099
Kidney_1099
Testis_1099
Cerebellum_1610
Cortex_1610
Heart_1610
Hypothalamus_1610
Kidney_1610
Testis_1610
reads per million genome matching
0
500
1000
1500
2000
2500
3000
3500
0
20
40
60
bp position

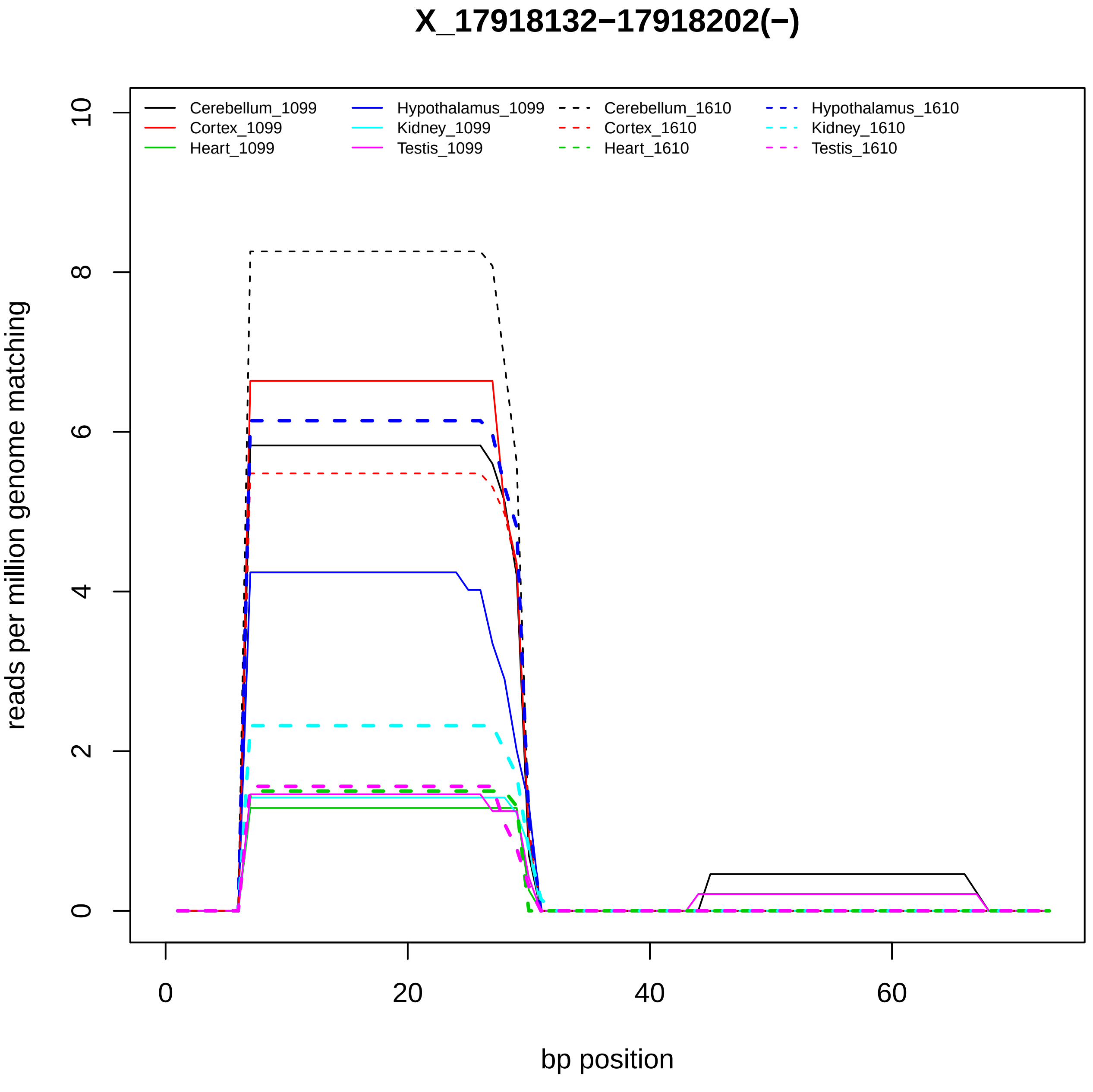
X_17918132−17918202(−)
reads per million genome matching
bp position
0
2
4
6
8
10
0
20
40
60
Cerebellum_1099
Cortex_1099
Heart_1099
Hypothalamus_1099
Kidney_1099
Testis_1099
Cerebellum_1610
Cortex_1610
Heart_1610
Hypothalamus_1610
Kidney_1610
Testis_1610

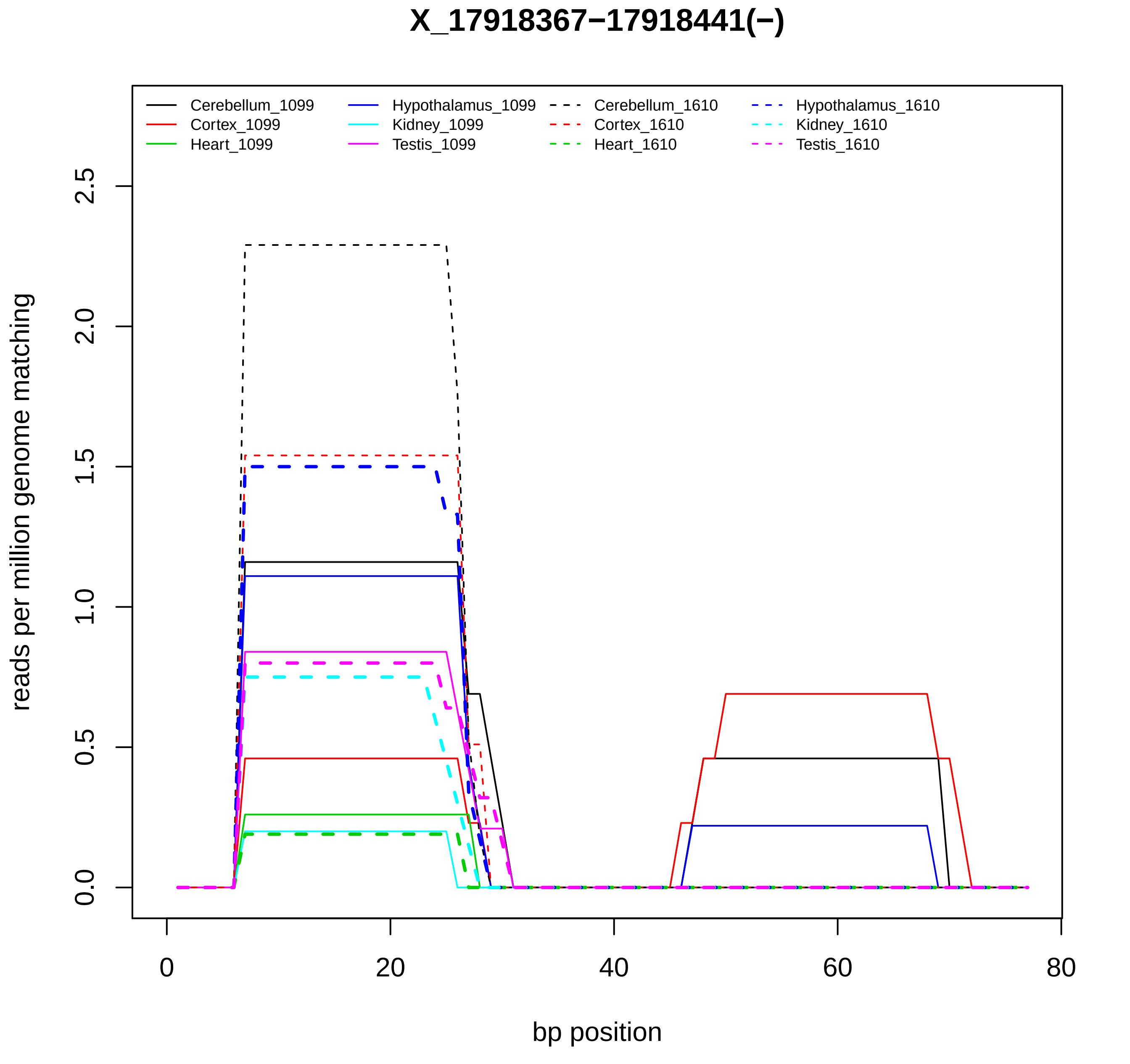

X_17918367−17918441(−)
Cerebellum_1099
Cortex_1099
Heart_1099
Hypothalamus_1099
Kidney_1099
Testis_1099
Cerebellum_1610
Cortex_1610
Heart_1610
Hypothalamus_1610
Kidney_1610
Testis_1610
reads per million genome matching
0.0
0.5
1.0
1.5
2.0
2.5
0
20
40
60
80
bp position

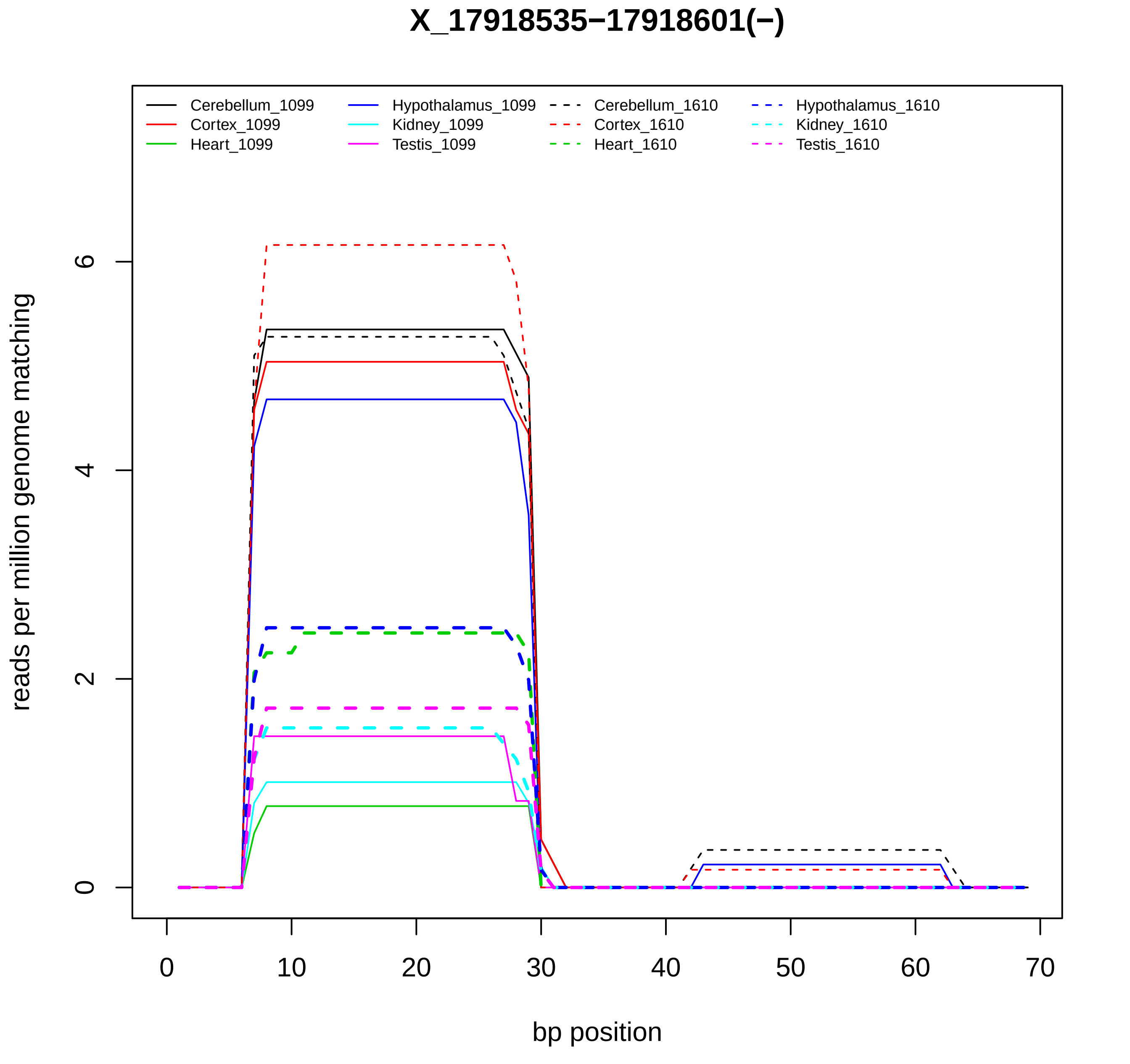

X_17918535−17918601(−)
Cerebellum_1099
Cortex_1099
Heart_1099
Hypothalamus_1099
Kidney_1099
Testis_1099
Cerebellum_1610
Cortex_1610
Heart_1610
Hypothalamus_1610
Kidney_1610
Testis_1610
reads per million genome matching
bp position
0
2
4
6
0
10
20
30
40
50
60
70

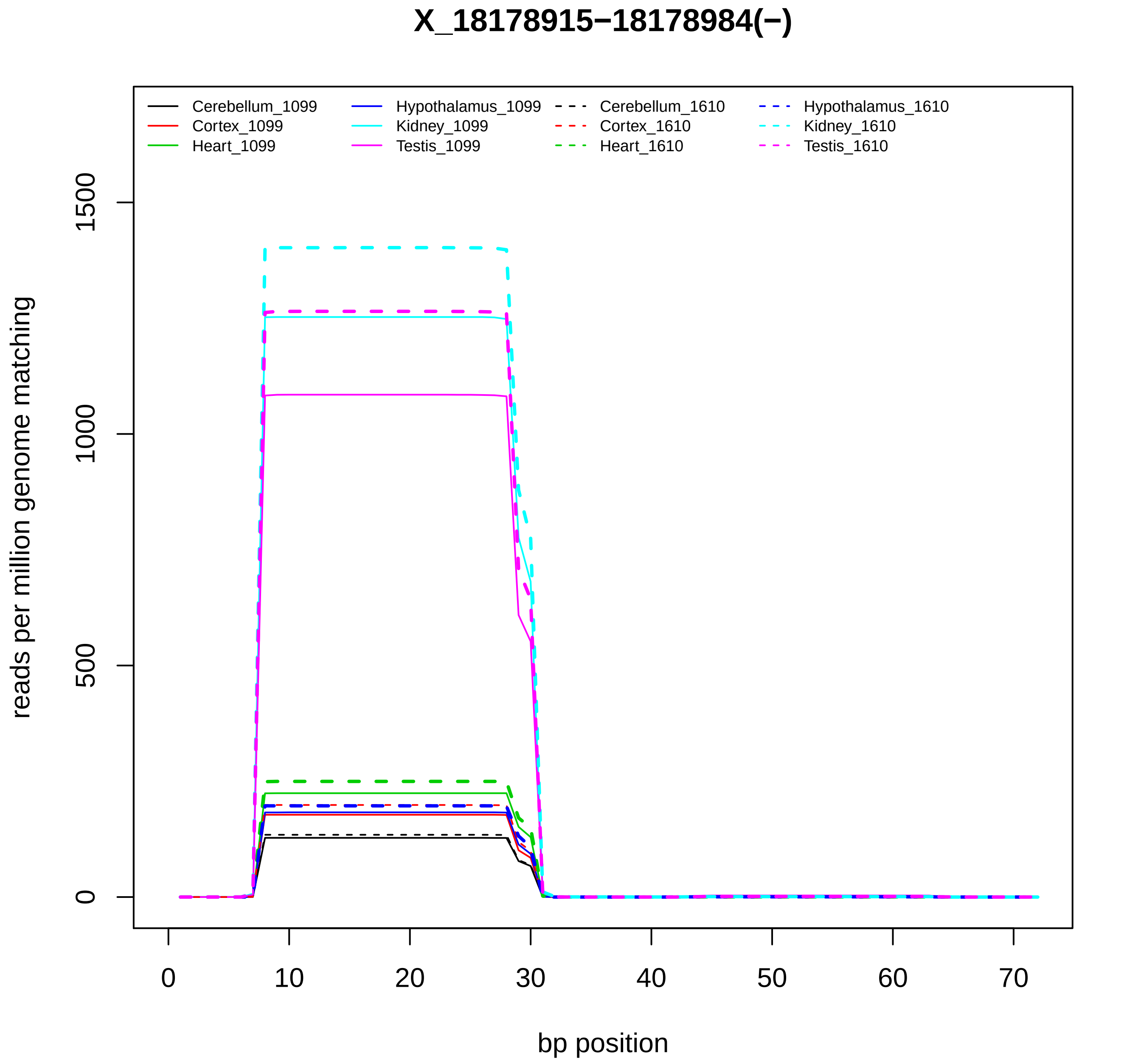

X_18178915−18178984(−)
Cerebellum_1099
Cortex_1099
Heart_1099
Hypothalamus_1099
Kidney_1099
Testis_1099
Cerebellum_1610
Cortex_1610
Heart_1610
Hypothalamus_1610
Kidney_1610
Testis_1610
reads per million genome matching
0
500
1000
1500
0
10
20
30
40
50
60
70
bp position

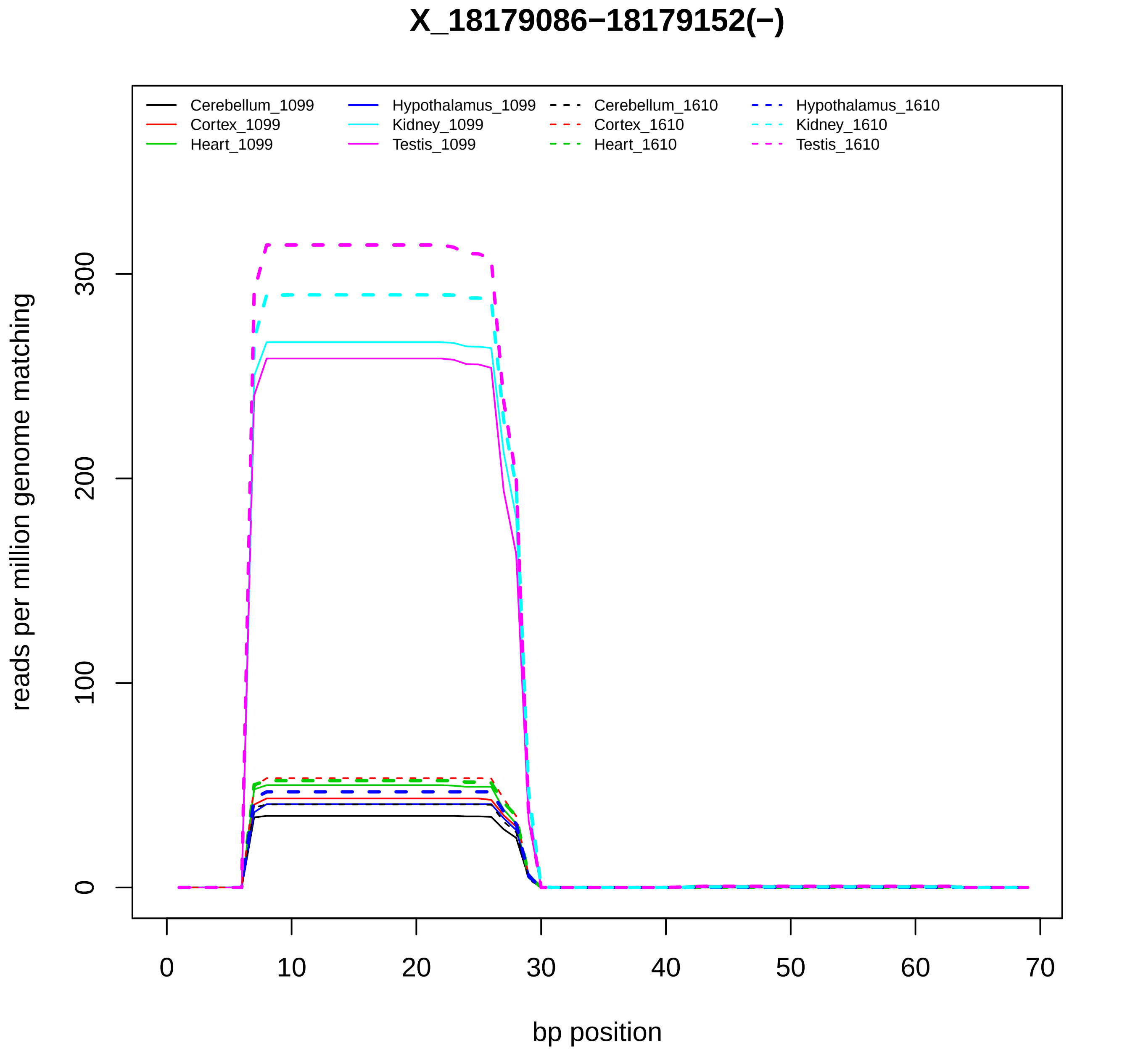

X_18179086−18179152(−)
Cerebellum_1099
Cortex_1099
Heart_1099
Hypothalamus_1099
Kidney_1099
Testis_1099
Cerebellum_1610
Cortex_1610
Heart_1610
Hypothalamus_1610
Kidney_1610
Testis_1610
reads per million genome matching
0
100
200
300
bp position
0
10
20
30
40
50
60
70

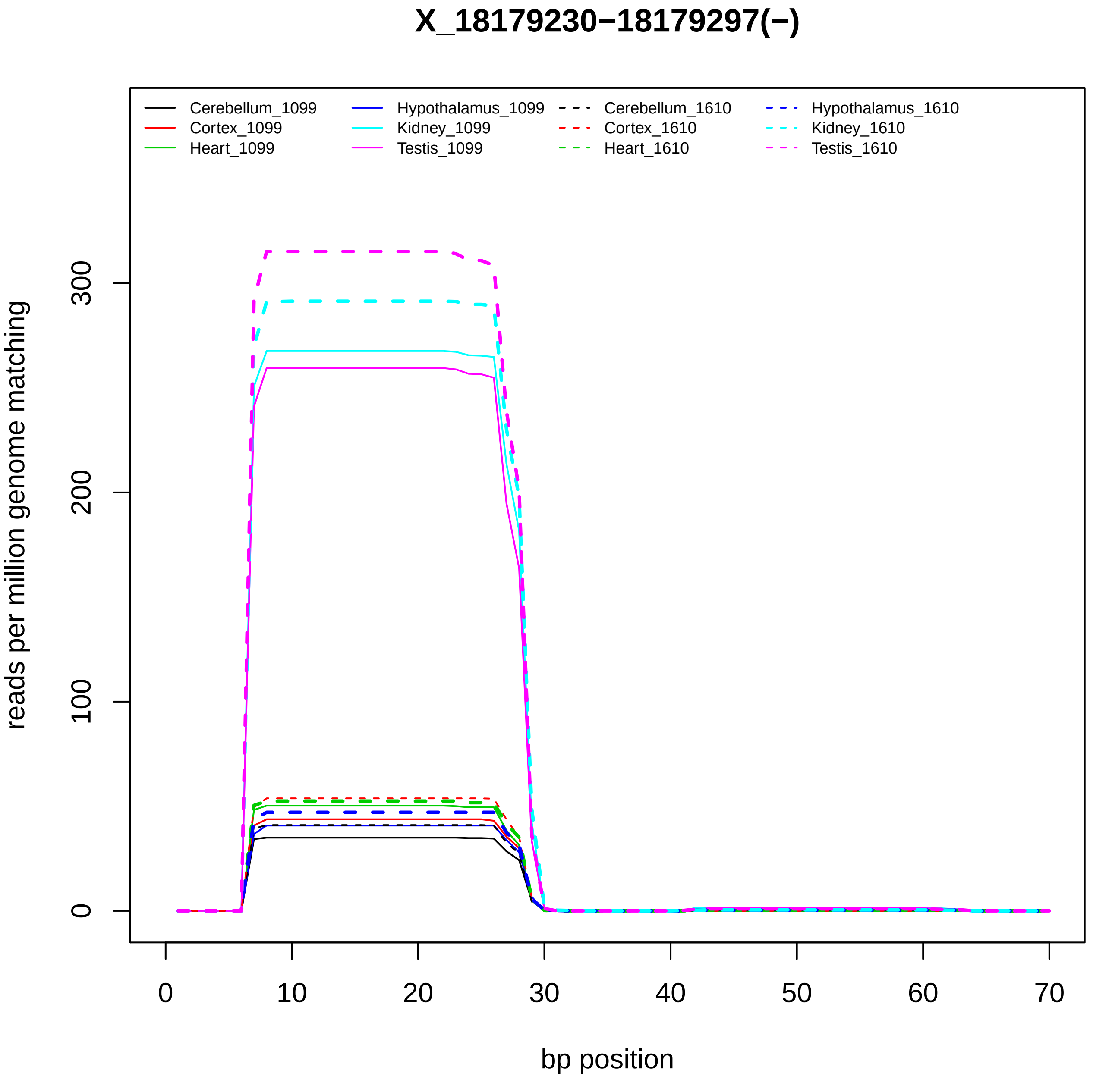

X_18179230−18179297(−)
Cerebellum_1099
Cortex_1099
Heart_1099
Hypothalamus_1099
Kidney_1099
Testis_1099
Cerebellum_1610
Cortex_1610
Heart_1610
Hypothalamus_1610
Kidney_1610
Testis_1610
reads per million genome matching
0
100
200
300
0
10
20
30
40
50
60
70
bp position

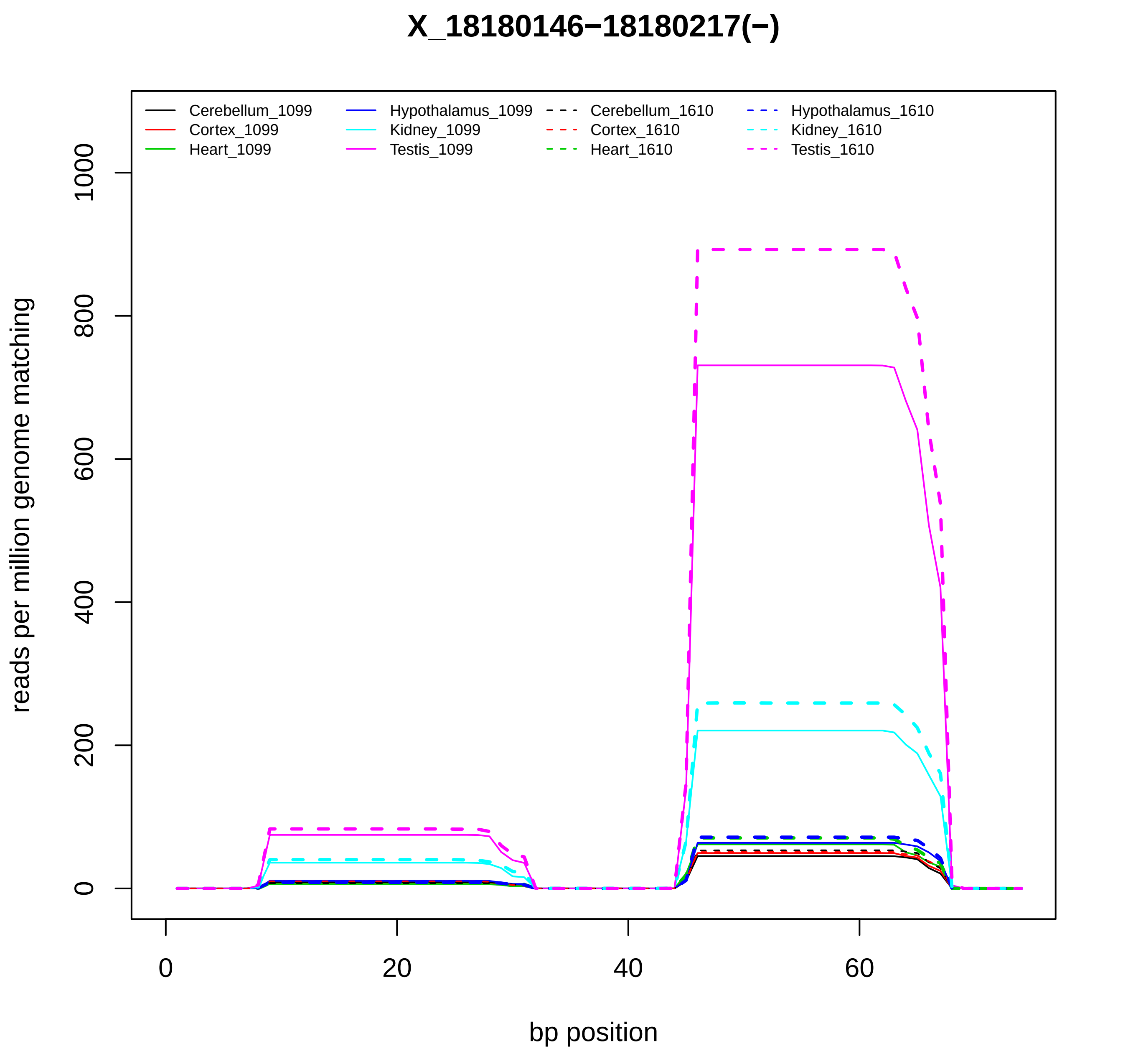

X_18180146−18180217(−)
Cerebellum_1099
Cortex_1099
Heart_1099
Hypothalamus_1099
Kidney_1099
Testis_1099
Cerebellum_1610
Cortex_1610
Heart_1610
Hypothalamus_1610
Kidney_1610
Testis_1610
reads per million genome matching
0
200
400
600
800
1000
0
20
40
60
bp position

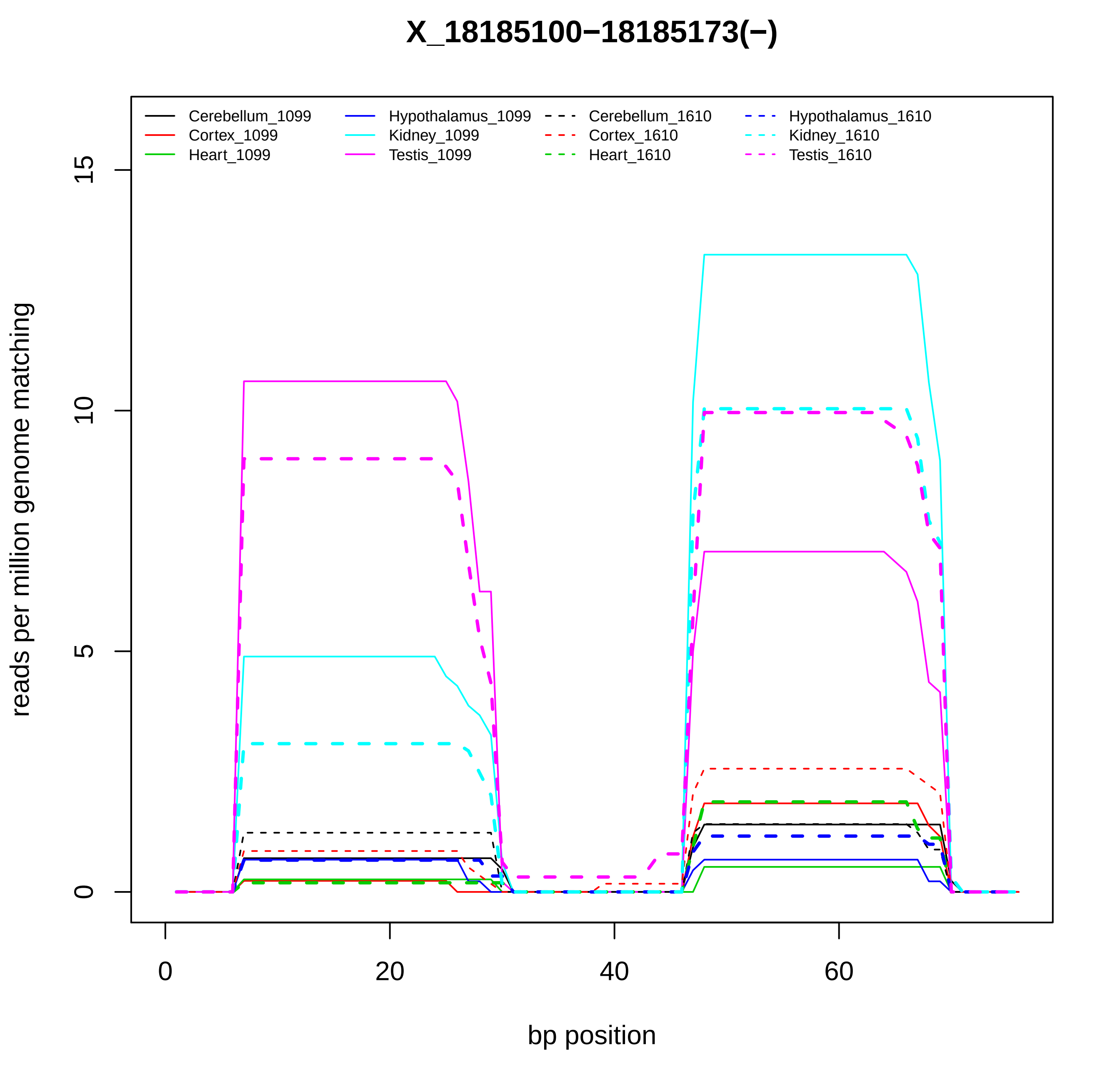

X_18185100−18185173(−)
Cerebellum_1099
Cortex_1099
Heart_1099
Hypothalamus_1099
Kidney_1099
Testis_1099
Cerebellum_1610
Cortex_1610
Heart_1610
Hypothalamus_1610
Kidney_1610
Testis_1610
reads per million genome matching
0
5
10
15
0
20
40
60
bp position

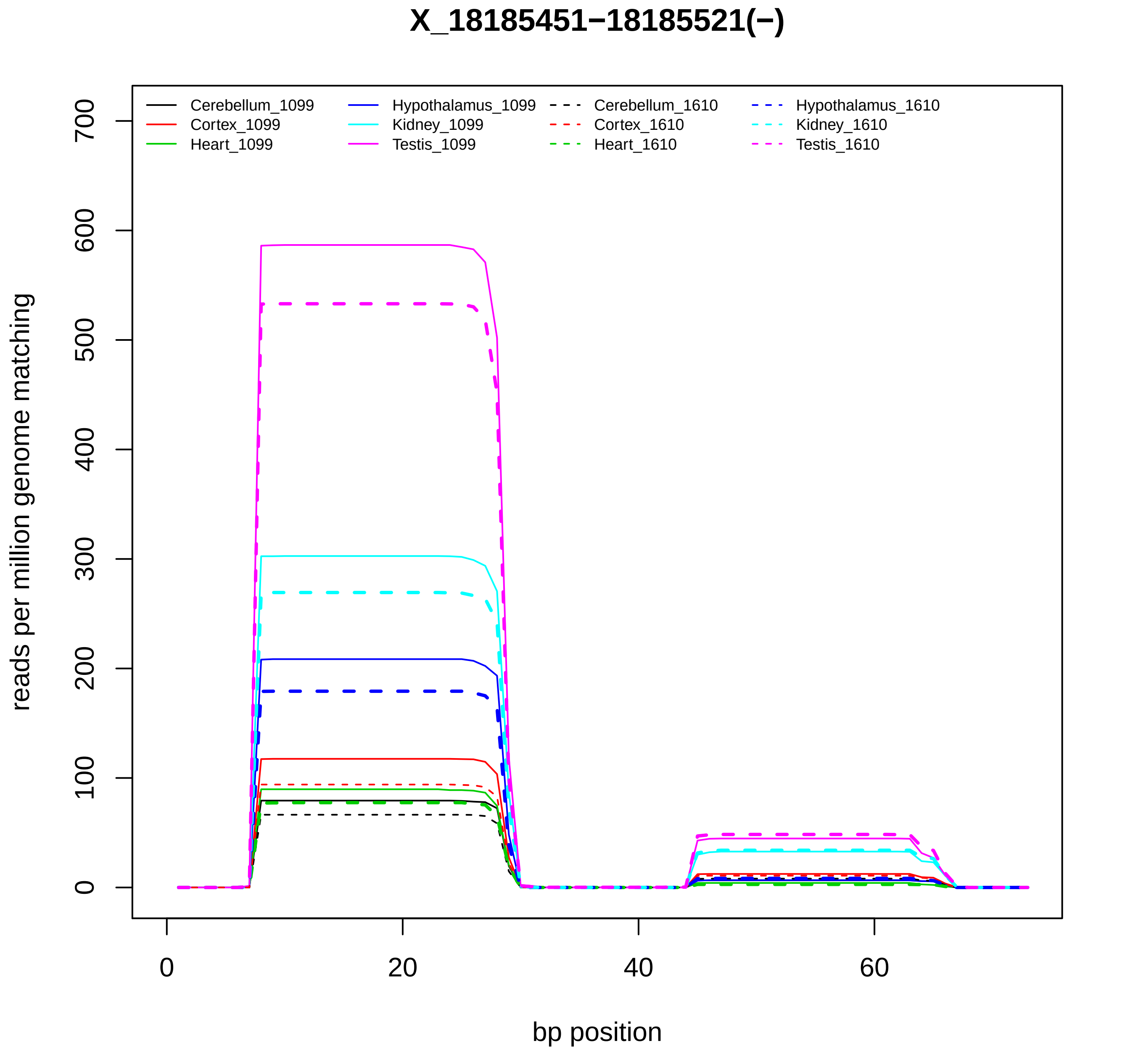
X_18185451−18185521(−)
Cerebellum_1099
Cortex_1099
Heart_1099
Hypothalamus_1099
Kidney_1099
Testis_1099
Cerebellum_1610
Cortex_1610
Heart_1610
Hypothalamus_1610
Kidney_1610
Testis_1610
reads per million genome matching
bp position
0
100
200
300
400
500
600
700
0
20
40
60

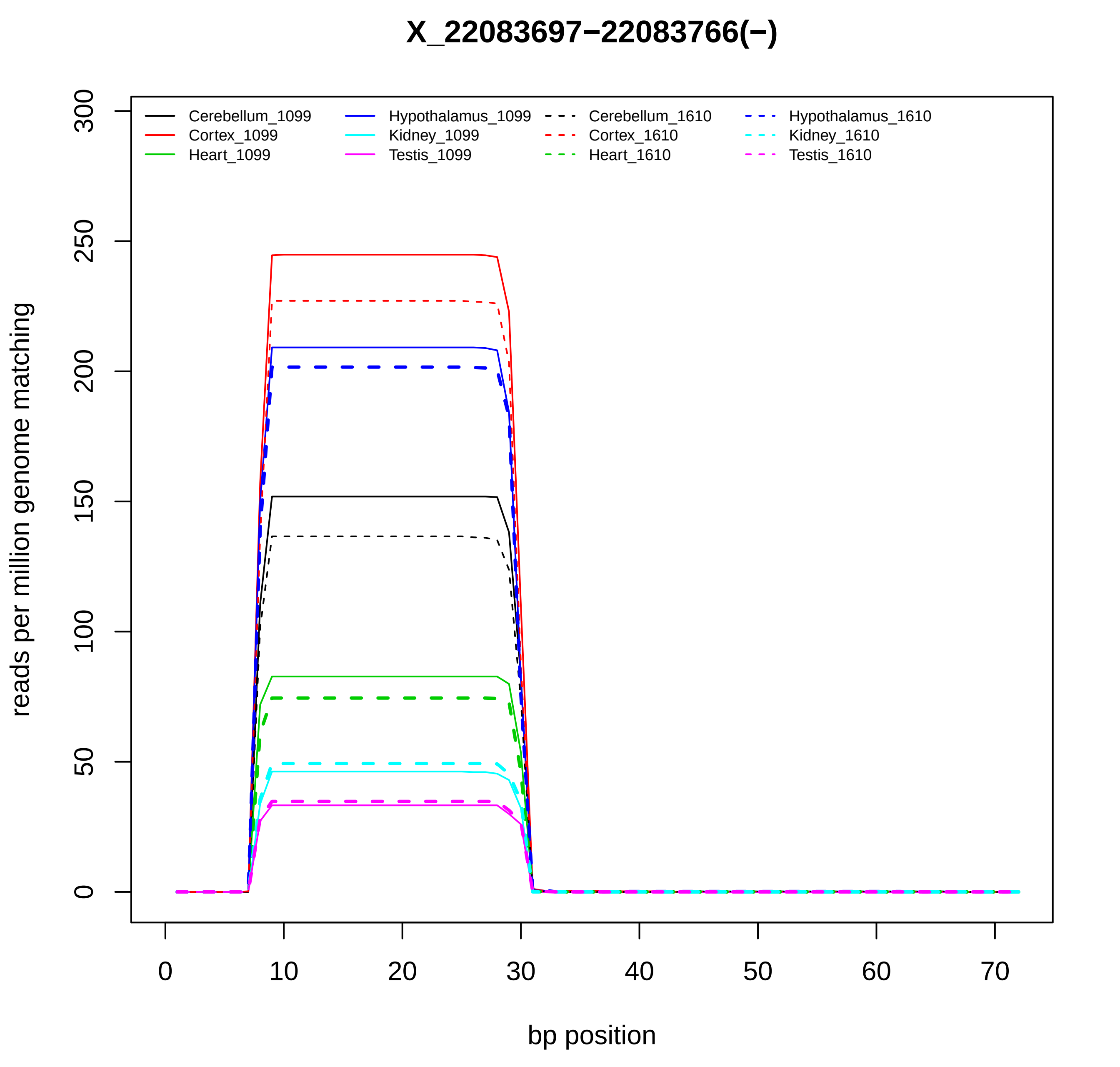
X_22083697−22083766(−)
Cerebellum_1099
Cortex_1099
Heart_1099
Hypothalamus_1099
Kidney_1099
Testis_1099
Cerebellum_1610
Cortex_1610
Heart_1610
Hypothalamus_1610
Kidney_1610
Testis_1610
reads per million genome matching
bp position
0
50
100
150
200
250
300
0
10
20
30
40
50
60
70

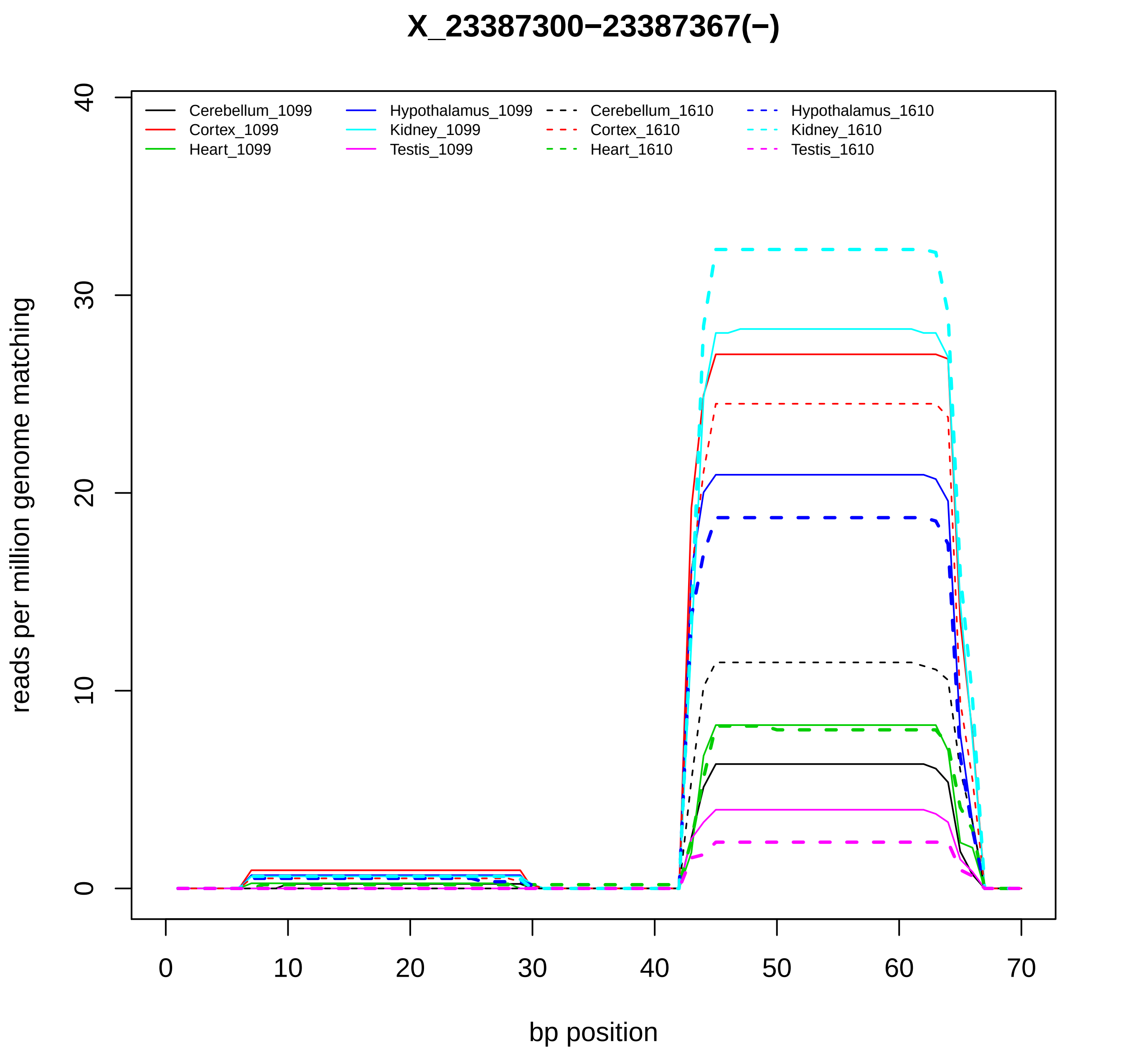

X_23387300−23387367(−)
Cerebellum_1099
Cortex_1099
Heart_1099
Hypothalamus_1099
Kidney_1099
Testis_1099
Cerebellum_1610
Cortex_1610
Heart_1610
Hypothalamus_1610
Kidney_1610
Testis_1610
reads per million genome matching
bp position
0
10
20
30
40
50
60
70

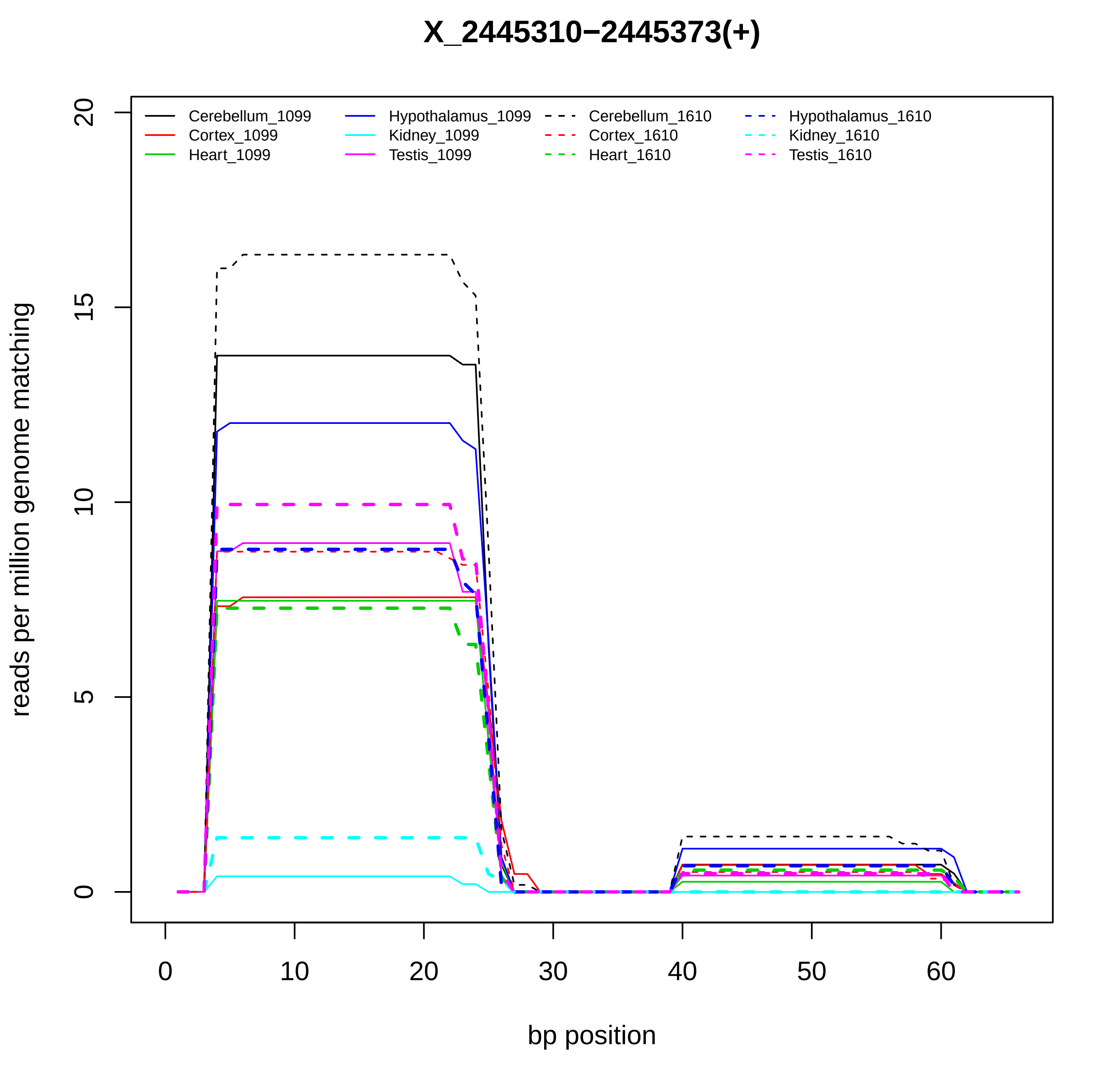

X_2445310−2445373(+)
Cerebellum_1099
Cortex_1099
Heart_1099
Hypothalamus_1099
Kidney_1099
Testis_1099
Cerebellum_1610
Cortex_1610
Heart_1610
Hypothalamus_1610
Kidney_1610
Testis_1610
reads per million genome matching
bp position
0
5
10
15
20
0
10
20
30
40
50
60

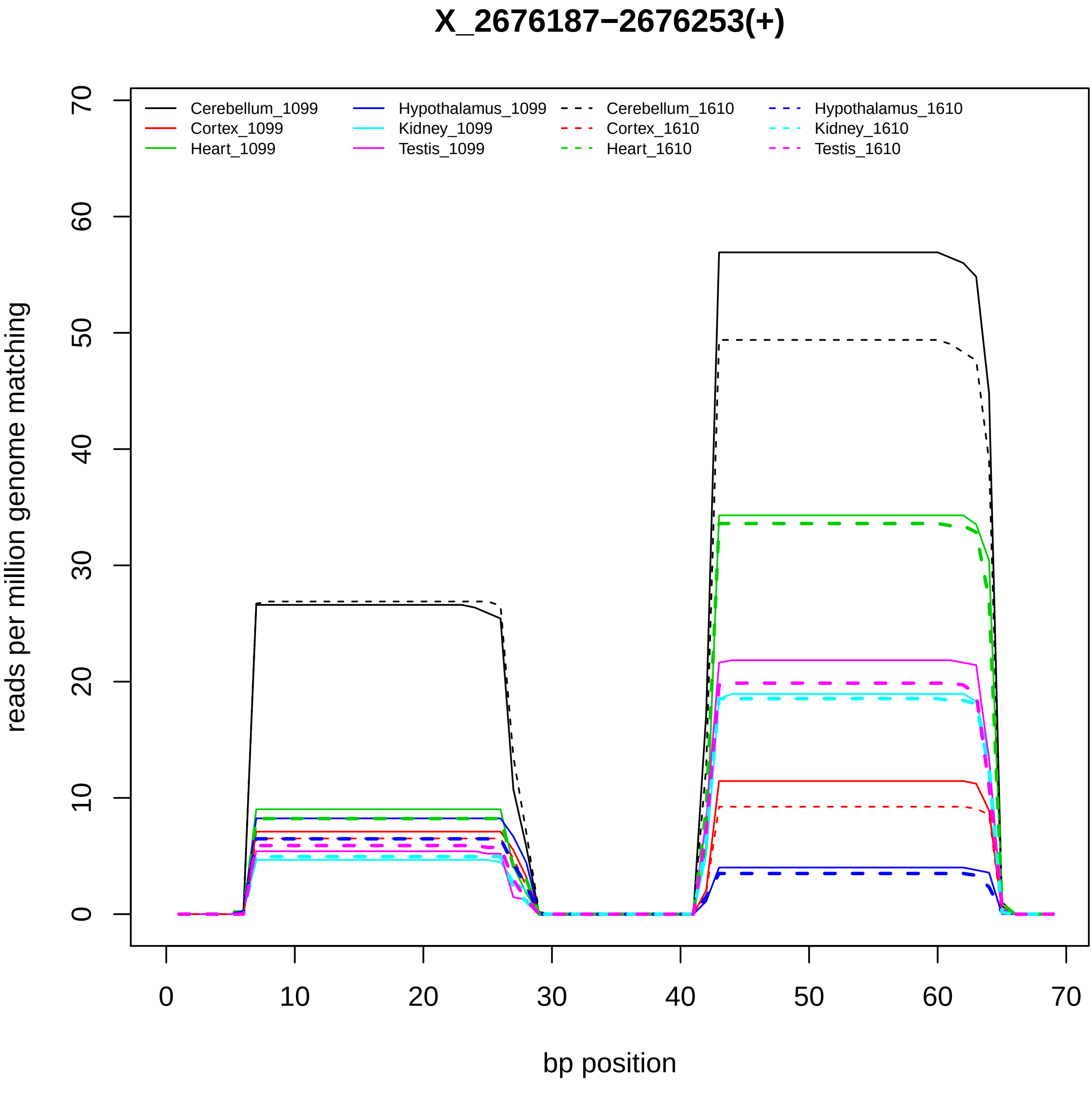
X_2676187−2676253(+)
Cerebellum_1099
Cortex_1099
Heart_1099
Hypothalamus_1099
Kidney_1099
Testis_1099
Cerebellum_1610
Cortex_1610
Heart_1610
Hypothalamus_1610
Kidney_1610
Testis_1610
reads per million genome matching
bp position
0
10
20
30
40
50
60
70

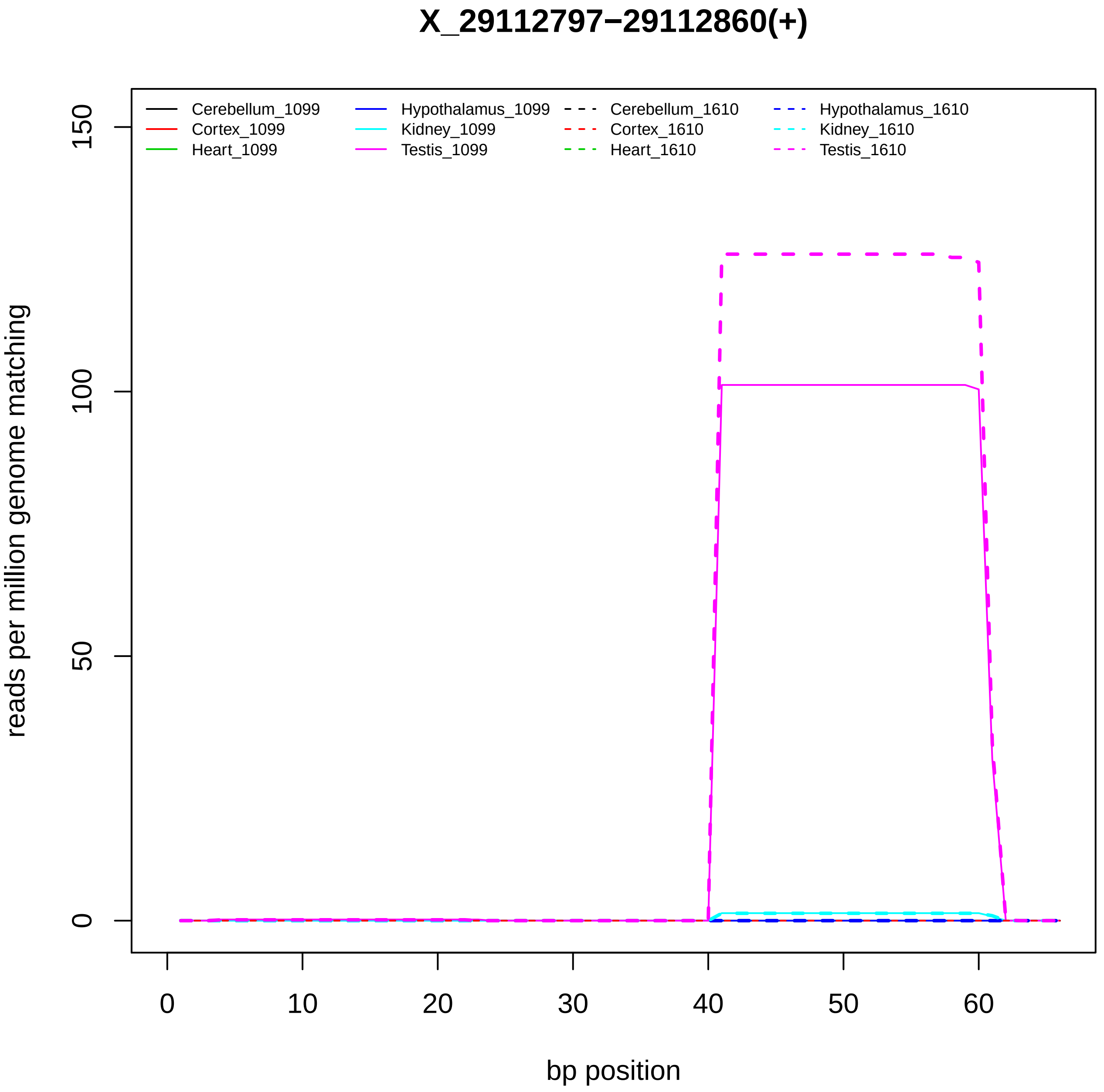

X_29112797−29112860(+)
Cerebellum_1099
Cortex_1099
Heart_1099
Hypothalamus_1099
Kidney_1099
Testis_1099
Cerebellum_1610
Cortex_1610
Heart_1610
Hypothalamus_1610
Kidney_1610
Testis_1610
reads per million genome matching
0
50
100
150
0
10
20
30
40
50
60
bp position

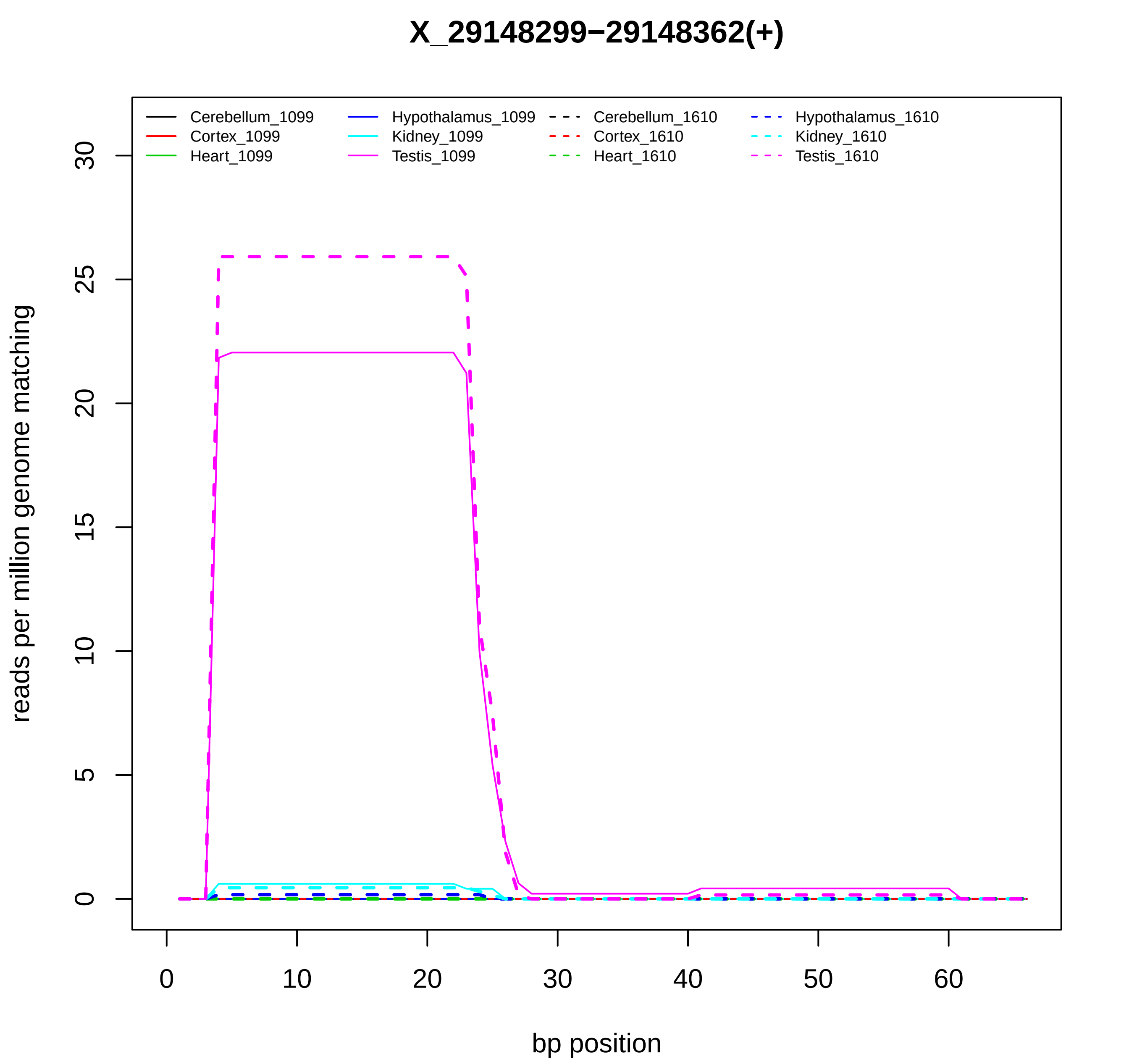

X_29148299−29148362(+)
Cerebellum_1099
Cortex_1099
Heart_1099
Hypothalamus_1099
Kidney_1099
Testis_1099
Cerebellum_1610
Cortex_1610
Heart_1610
Hypothalamus_1610
Kidney_1610
Testis_1610
reads per million genome matching
0
5
10
15
20
25
30
0
10
20
30
40
50
60
bp position

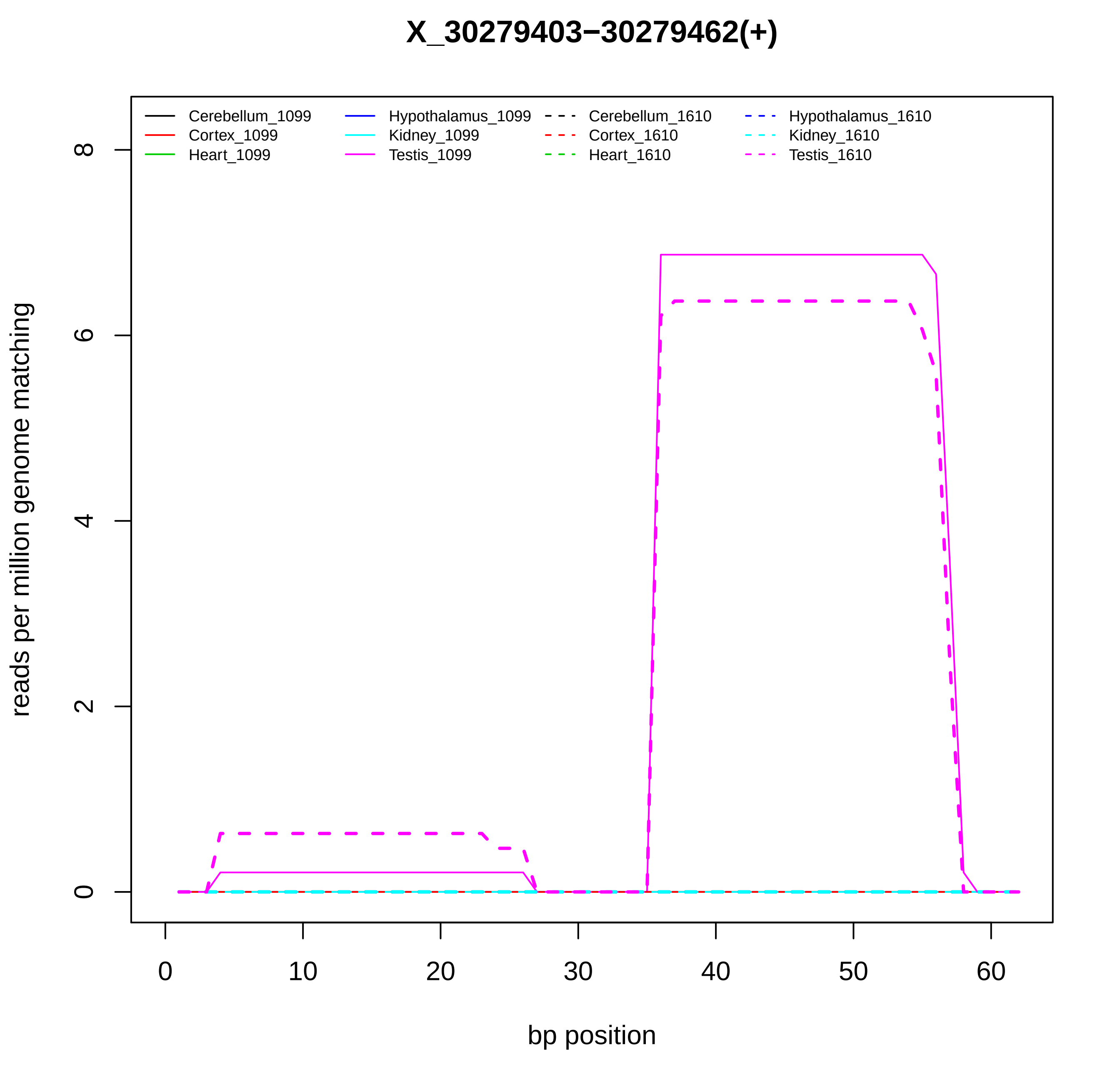

X_30279403−30279462(+)
Cerebellum_1099
Cortex_1099
Heart_1099
Hypothalamus_1099
Kidney_1099
Testis_1099
Cerebellum_1610
Cortex_1610
Heart_1610
Hypothalamus_1610
Kidney_1610
Testis_1610
reads per million genome matching
0
2
4
6
8
0
10
20
30
40
50
60
bp position

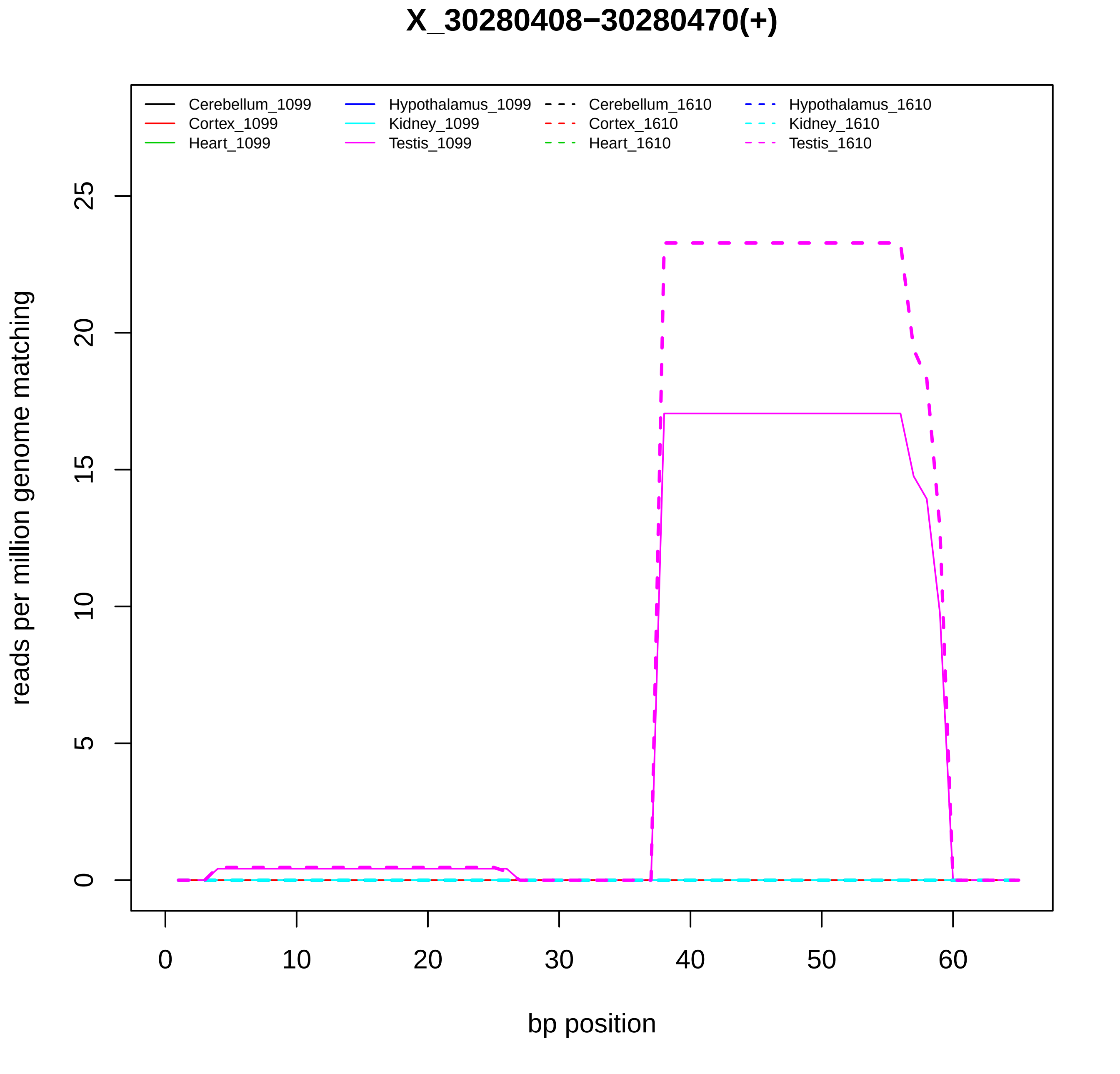

X_30280408−30280470(+)
Cerebellum_1099
Cortex_1099
Heart_1099
Hypothalamus_1099
Kidney_1099
Testis_1099
Cerebellum_1610
Cortex_1610
Heart_1610
Hypothalamus_1610
Kidney_1610
Testis_1610
reads per million genome matching
0
5
10
15
20
25
0
10
20
30
40
50
60
bp position

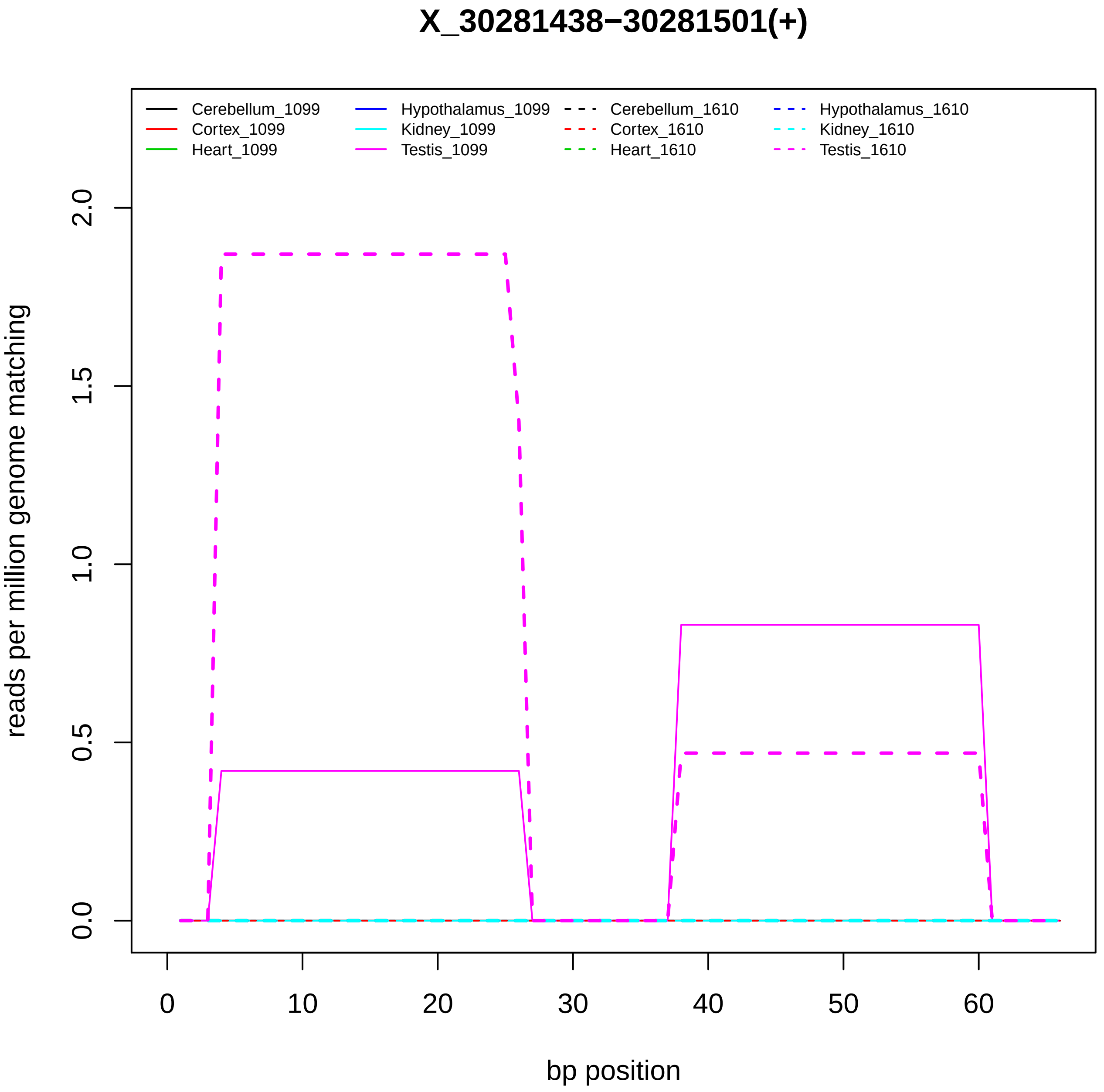

X_30281438−30281501(+)
Cerebellum_1099
Cortex_1099
Heart_1099
Hypothalamus_1099
Kidney_1099
Testis_1099
Cerebellum_1610
Cortex_1610
Heart_1610
Hypothalamus_1610
Kidney_1610
Testis_1610
reads per million genome matching
0.0
0.5
1.0
1.5
2.0
0
10
20
30
40
50
60
bp position

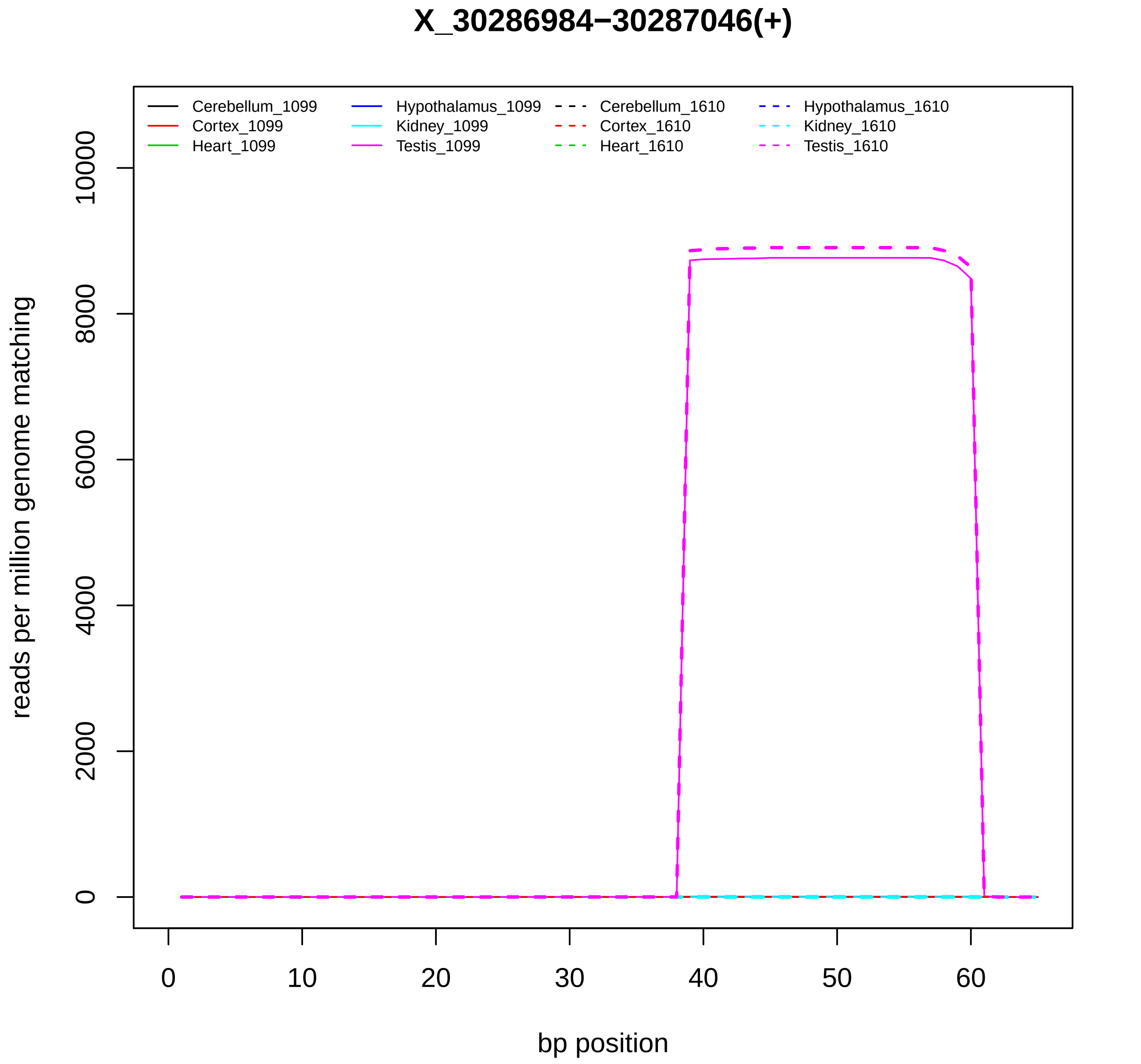

X_30286984−30287046(+)
Cerebellum_1099
Cortex_1099
Heart_1099
Hypothalamus_1099
Kidney_1099
Testis_1099
Cerebellum_1610
Cortex_1610
Heart_1610
Hypothalamus_1610
Kidney_1610
Testis_1610
reads per million genome matching
0
2000
4000
6000
8000
10000
0
10
20
30
40
50
60
bp position

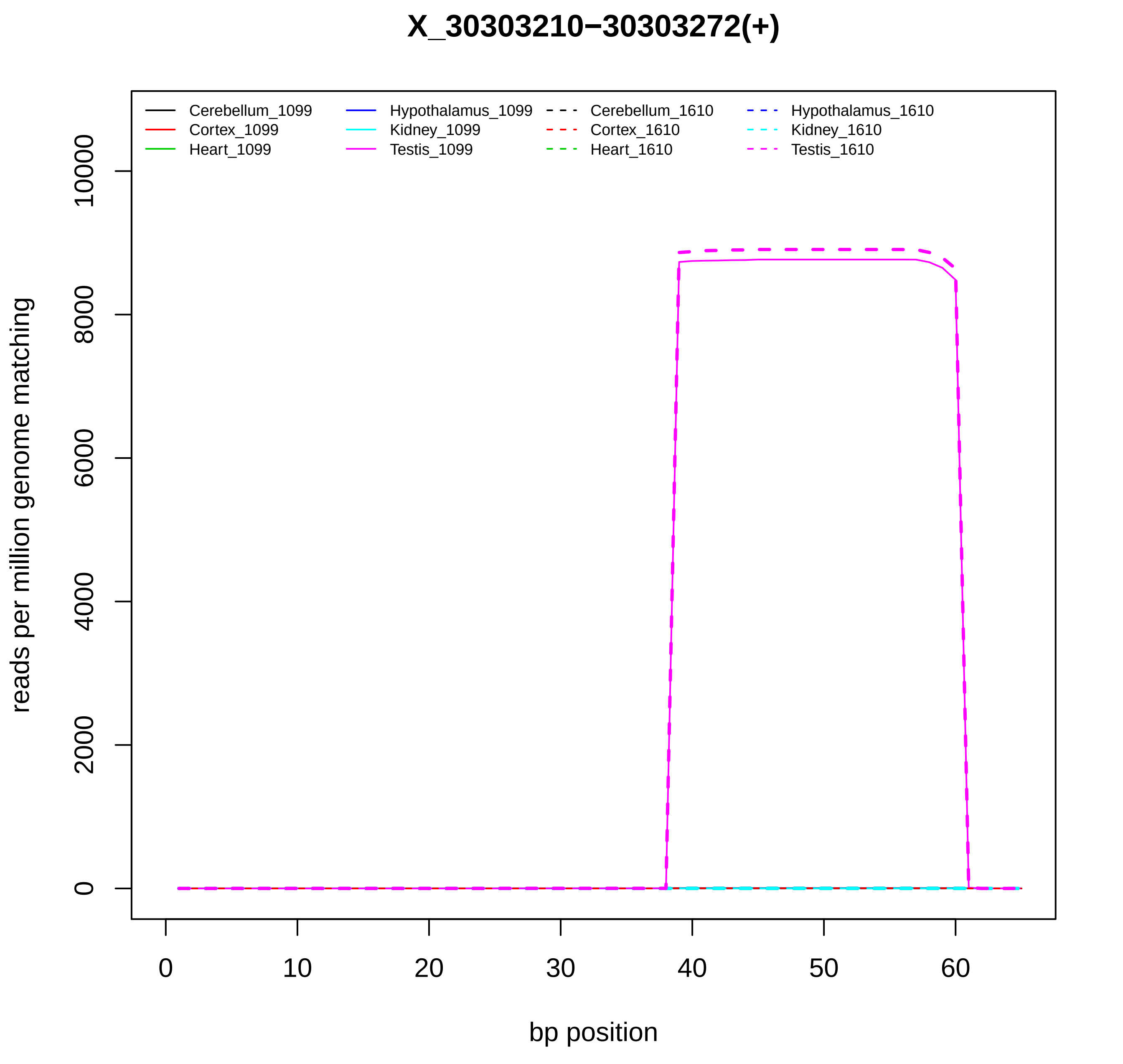

X_30303210−30303272(+)
Cerebellum_1099
Cortex_1099
Heart_1099
Hypothalamus_1099
Kidney_1099
Testis_1099
Cerebellum_1610
Cortex_1610
Heart_1610
Hypothalamus_1610
Kidney_1610
Testis_1610
reads per million genome matching
0
2000
4000
6000
8000
10000
0
10
20
30
40
50
60
bp position

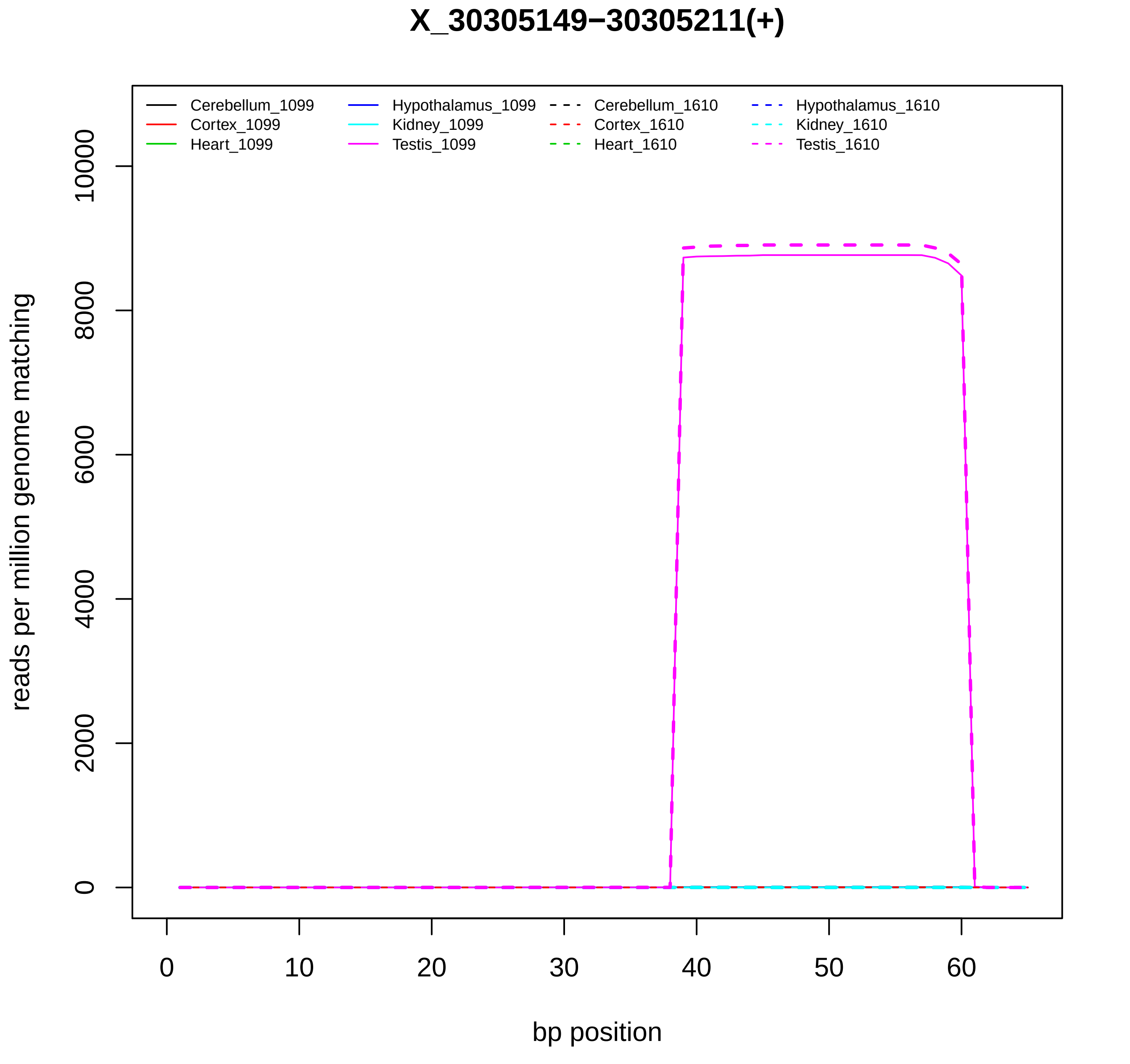
X_30305149−30305211(+)
Cerebellum_1099
Cortex_1099
Heart_1099
Hypothalamus_1099
Kidney_1099
Testis_1099
Cerebellum_1610
Cortex_1610
Heart_1610
Hypothalamus_1610
Kidney_1610
Testis_1610
reads per million genome matching
0
2000
4000
6000
8000
10000
0
10
20
30
40
50
60
bp position

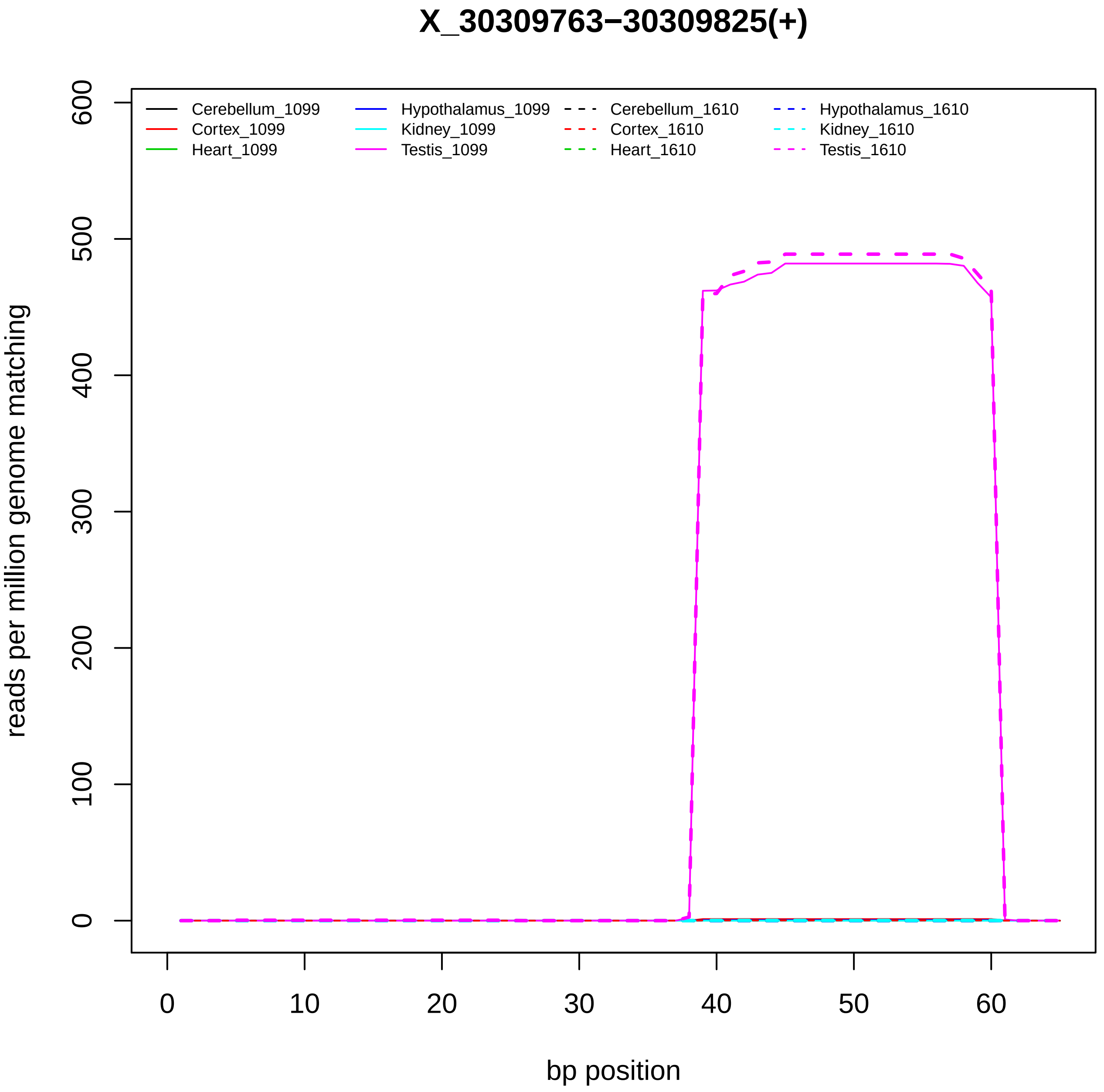

X_30309763−30309825(+)
Cerebellum_1099
Cortex_1099
Heart_1099
Hypothalamus_1099
Kidney_1099
Testis_1099
Cerebellum_1610
Cortex_1610
Heart_1610
Hypothalamus_1610
Kidney_1610
Testis_1610
reads per million genome matching
bp position
0
100
200
300
400
500
600
0
10
20
30
40
50
60

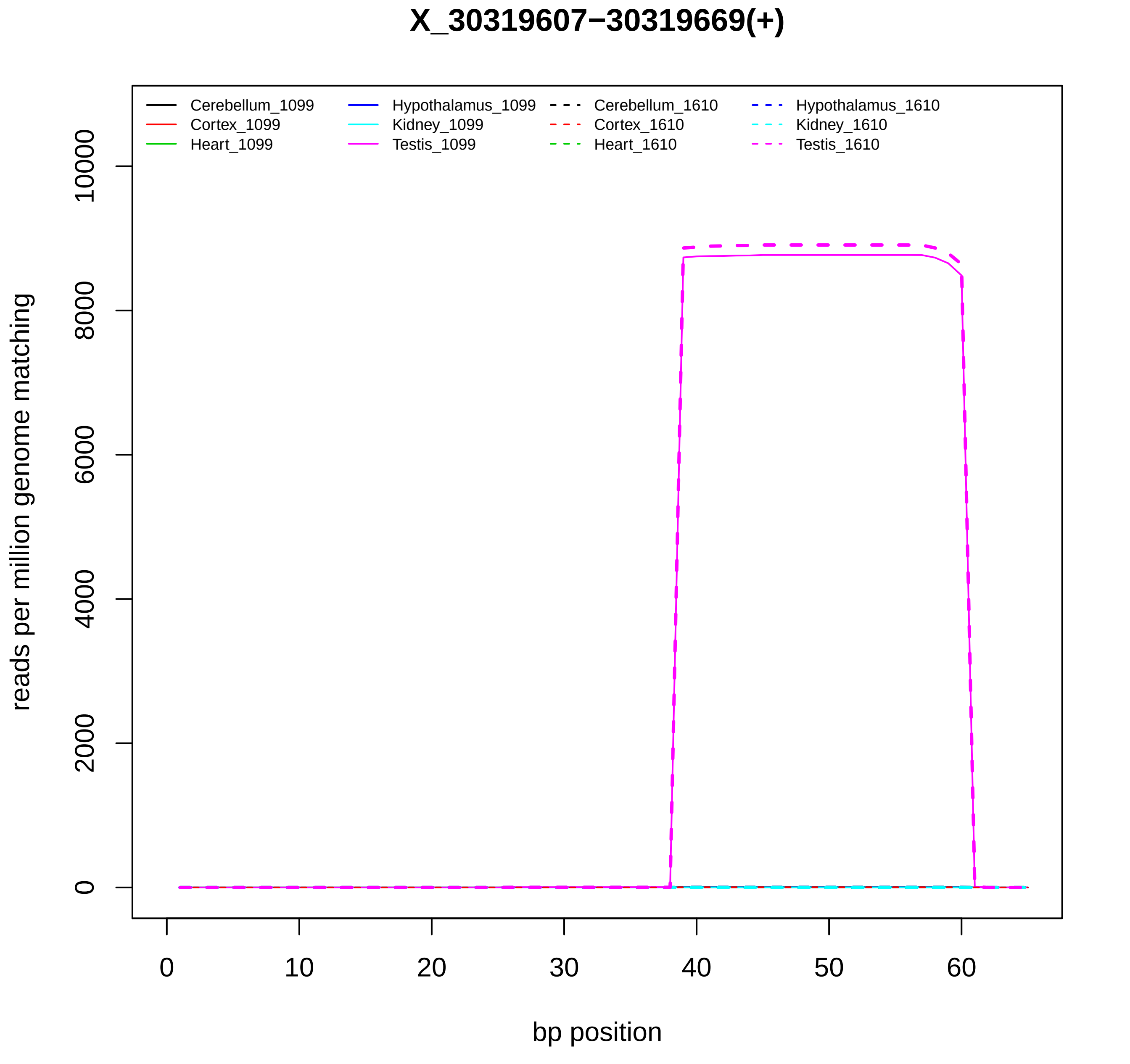

X_30319607−30319669(+)
Cerebellum_1099
Cortex_1099
Heart_1099
Hypothalamus_1099
Kidney_1099
Testis_1099
Cerebellum_1610
Cortex_1610
Heart_1610
Hypothalamus_1610
Kidney_1610
Testis_1610
reads per million genome matching
0
2000
4000
6000
8000
10000
0
10
20
30
40
50
60
bp position

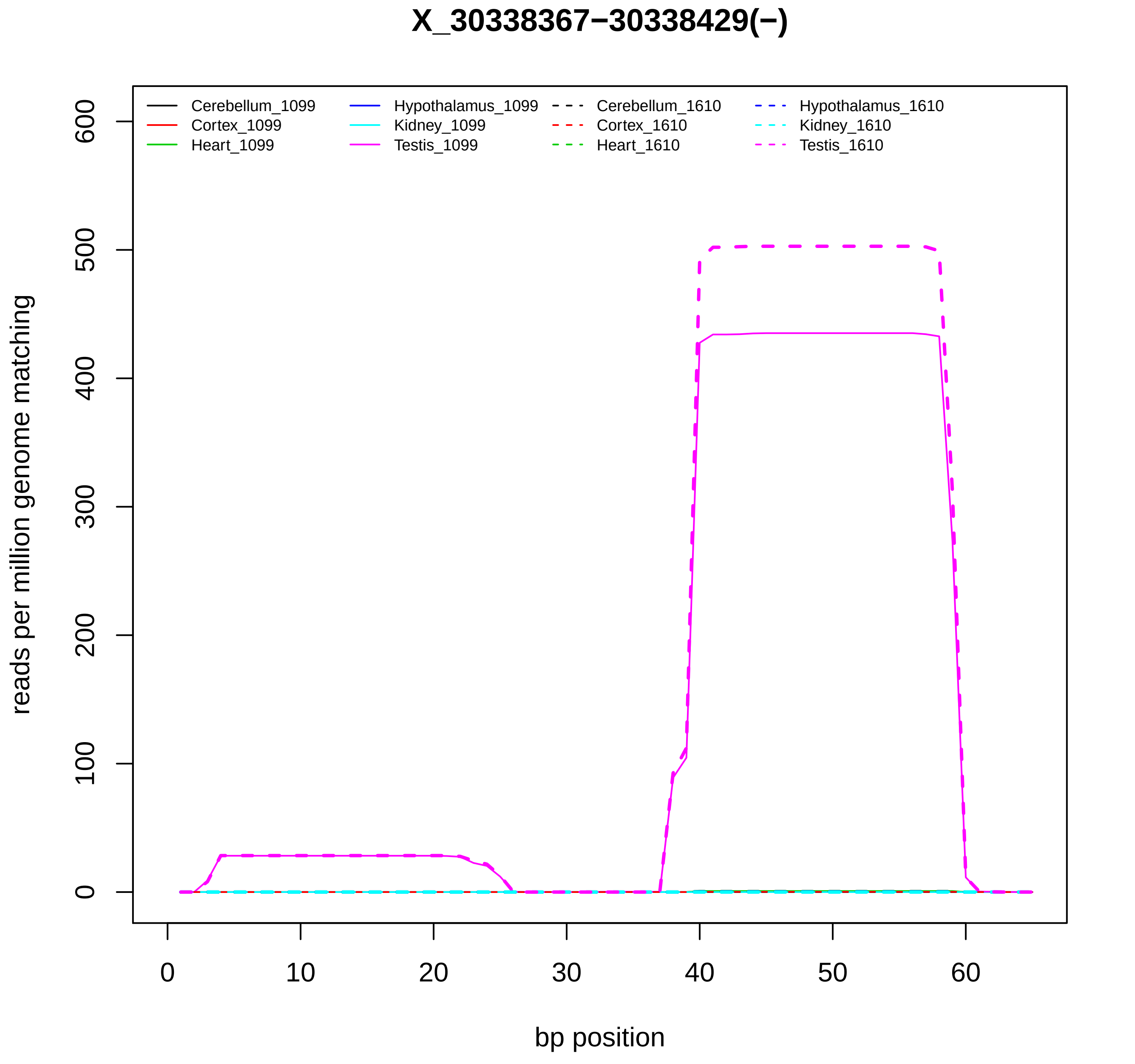

X_30338367−30338429(−)
Cerebellum_1099
Cortex_1099
Heart_1099
Hypothalamus_1099
Kidney_1099
Testis_1099
Cerebellum_1610
Cortex_1610
Heart_1610
Hypothalamus_1610
Kidney_1610
Testis_1610
reads per million genome matching
0
100
200
300
400
500
600
0
10
20
30
40
50
60
bp position

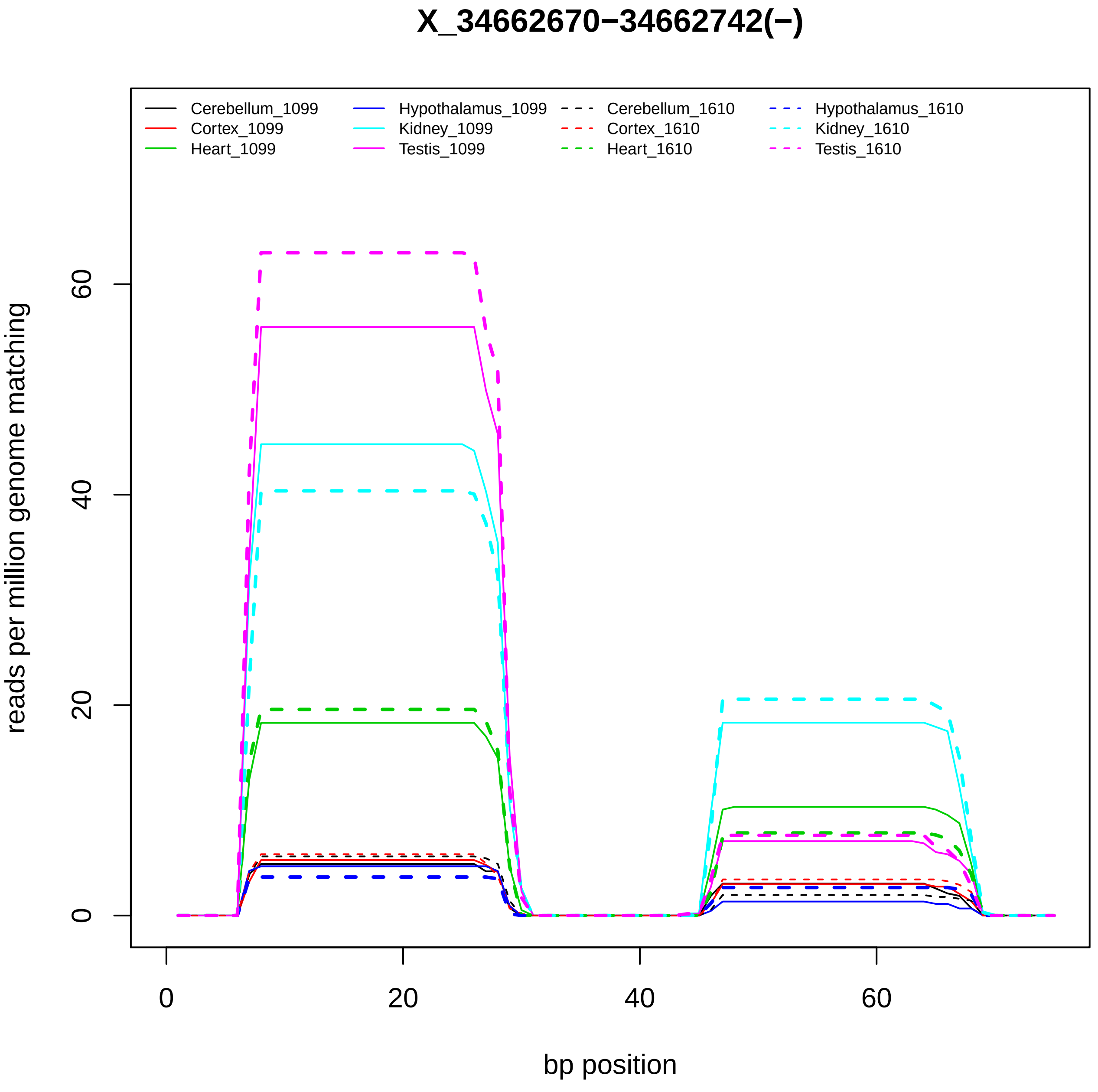

X_34662670−34662742(−)
Cerebellum_1099
Cortex_1099
Heart_1099
Hypothalamus_1099
Kidney_1099
Testis_1099
Cerebellum_1610
Cortex_1610
Heart_1610
Hypothalamus_1610
Kidney_1610
Testis_1610
reads per million genome matching
bp position
0
20
40
60
0
20
40
60

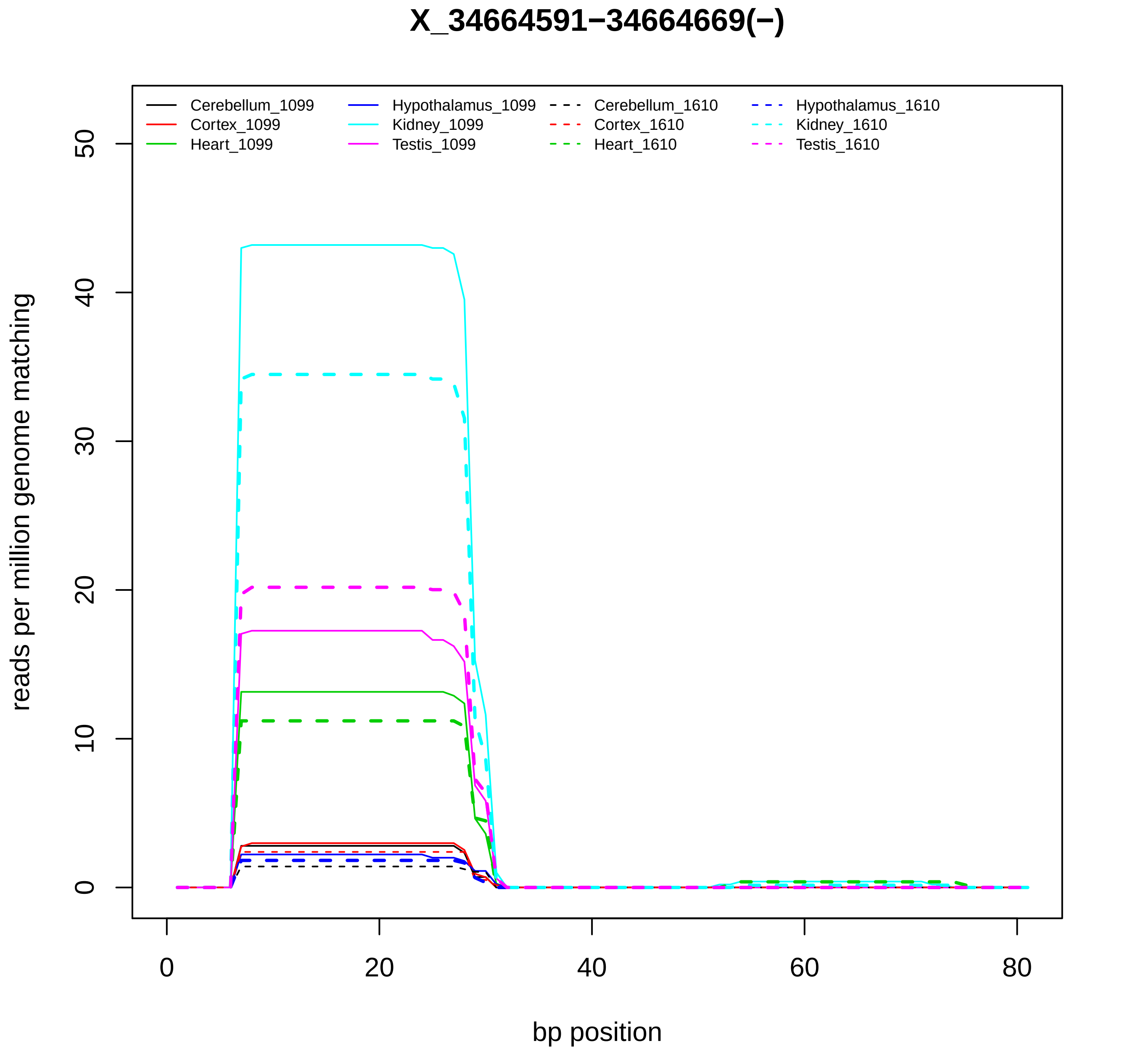

X_34664591−34664669(−)
Cerebellum_1099
Cortex_1099
Heart_1099
Hypothalamus_1099
Kidney_1099
Testis_1099
Cerebellum_1610
Cortex_1610
Heart_1610
Hypothalamus_1610
Kidney_1610
Testis_1610
reads per million genome matching
bp position
0
10
20
30
40
50
0
20
40
60
80

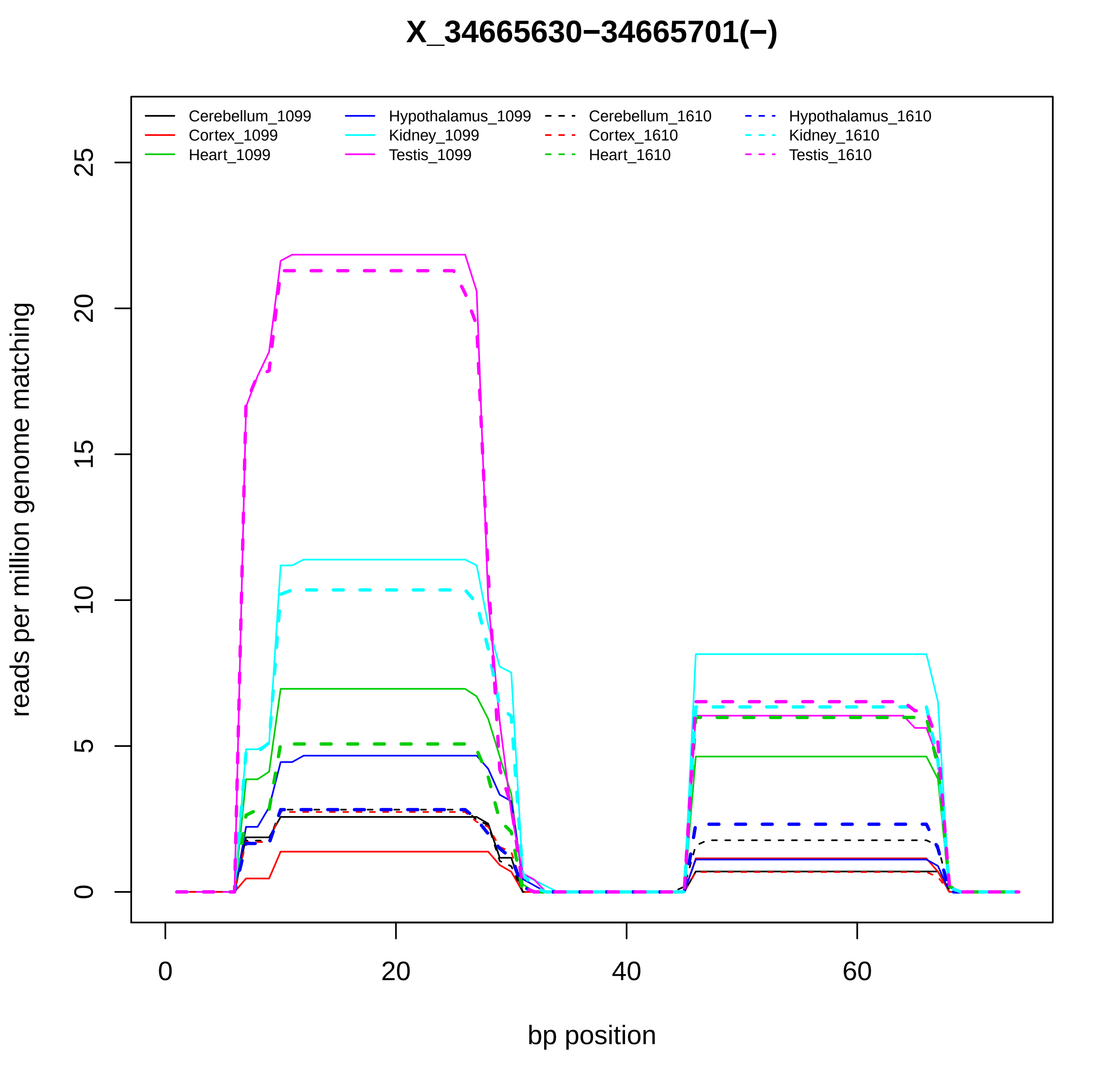

X_34665630−34665701(−)
Cerebellum_1099
Cortex_1099
Heart_1099
Hypothalamus_1099
Kidney_1099
Testis_1099
Cerebellum_1610
Cortex_1610
Heart_1610
Hypothalamus_1610
Kidney_1610
Testis_1610
reads per million genome matching
bp position
0
5
10
15
20
25
0
20
40
60

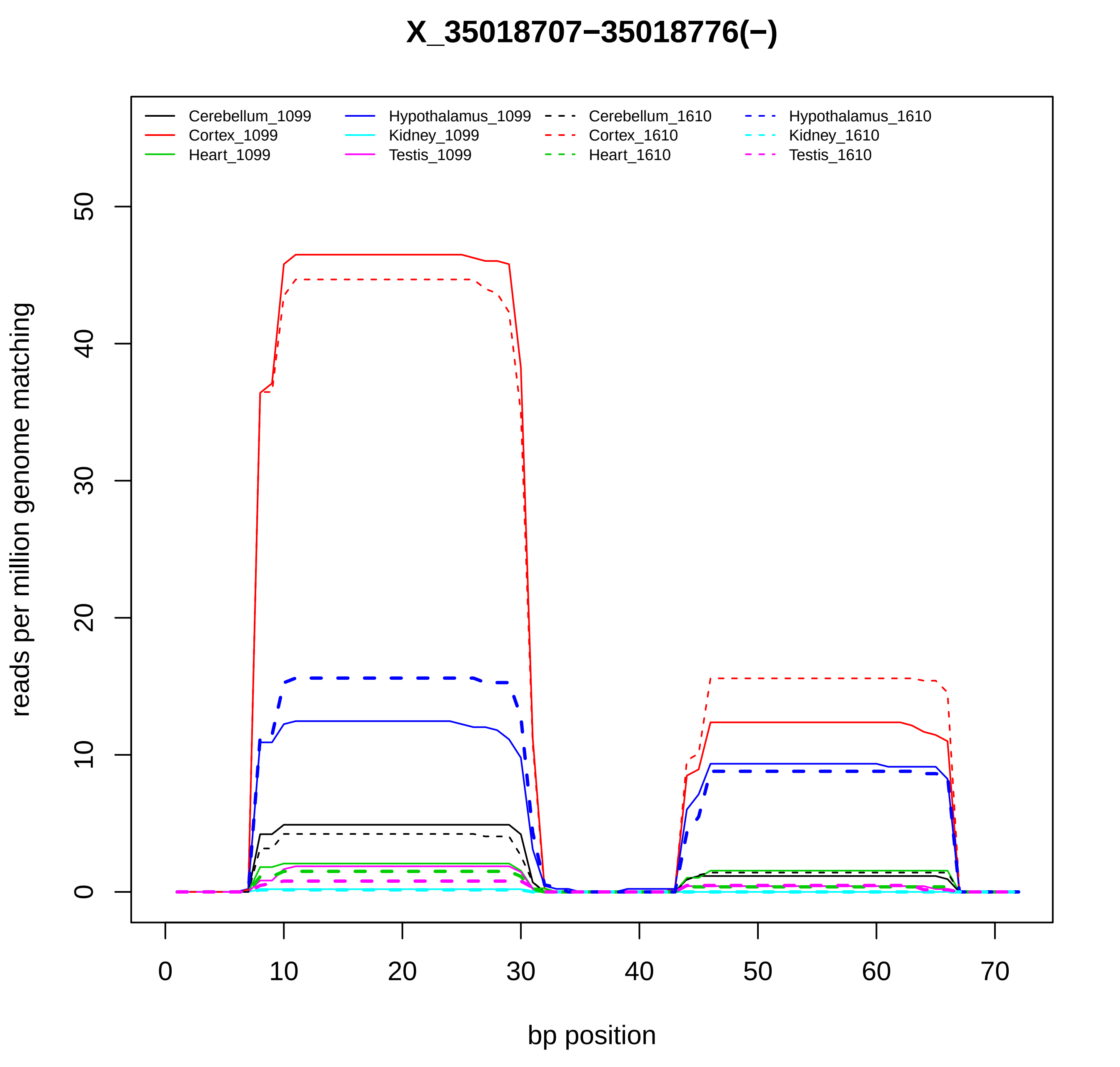

X_35018707−35018776(−)
Cerebellum_1099
Cortex_1099
Heart_1099
Hypothalamus_1099
Kidney_1099
Testis_1099
Cerebellum_1610
Cortex_1610
Heart_1610
Hypothalamus_1610
Kidney_1610
Testis_1610
reads per million genome matching
bp position
0
10
20
30
40
50
60
70

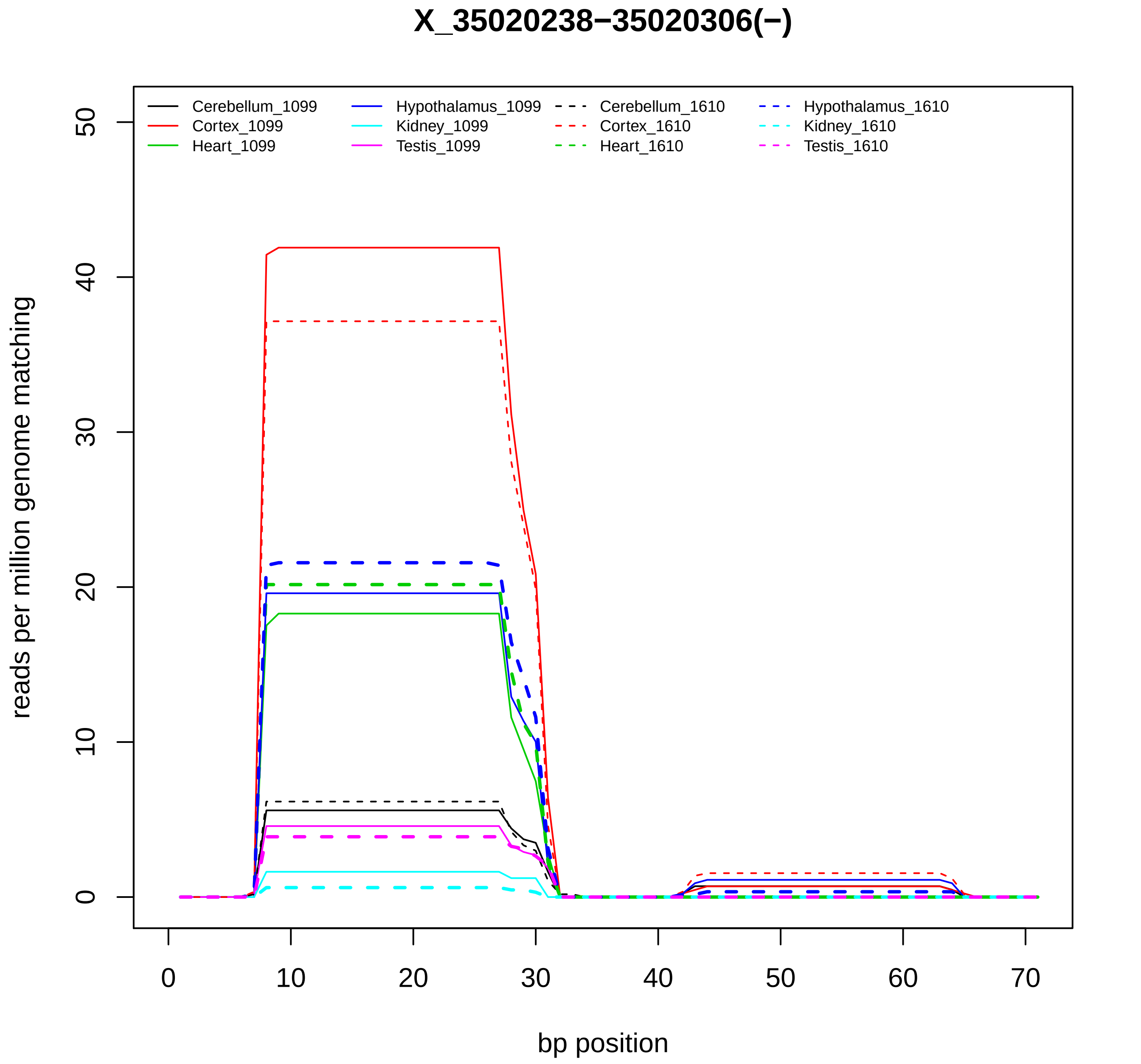
X_35020238−35020306(−)
Cerebellum_1099
Cortex_1099
Heart_1099
Hypothalamus_1099
Kidney_1099
Testis_1099
Cerebellum_1610
Cortex_1610
Heart_1610
Hypothalamus_1610
Kidney_1610
Testis_1610
reads per million genome matching
bp position
0
10
20
30
40
50
60
70

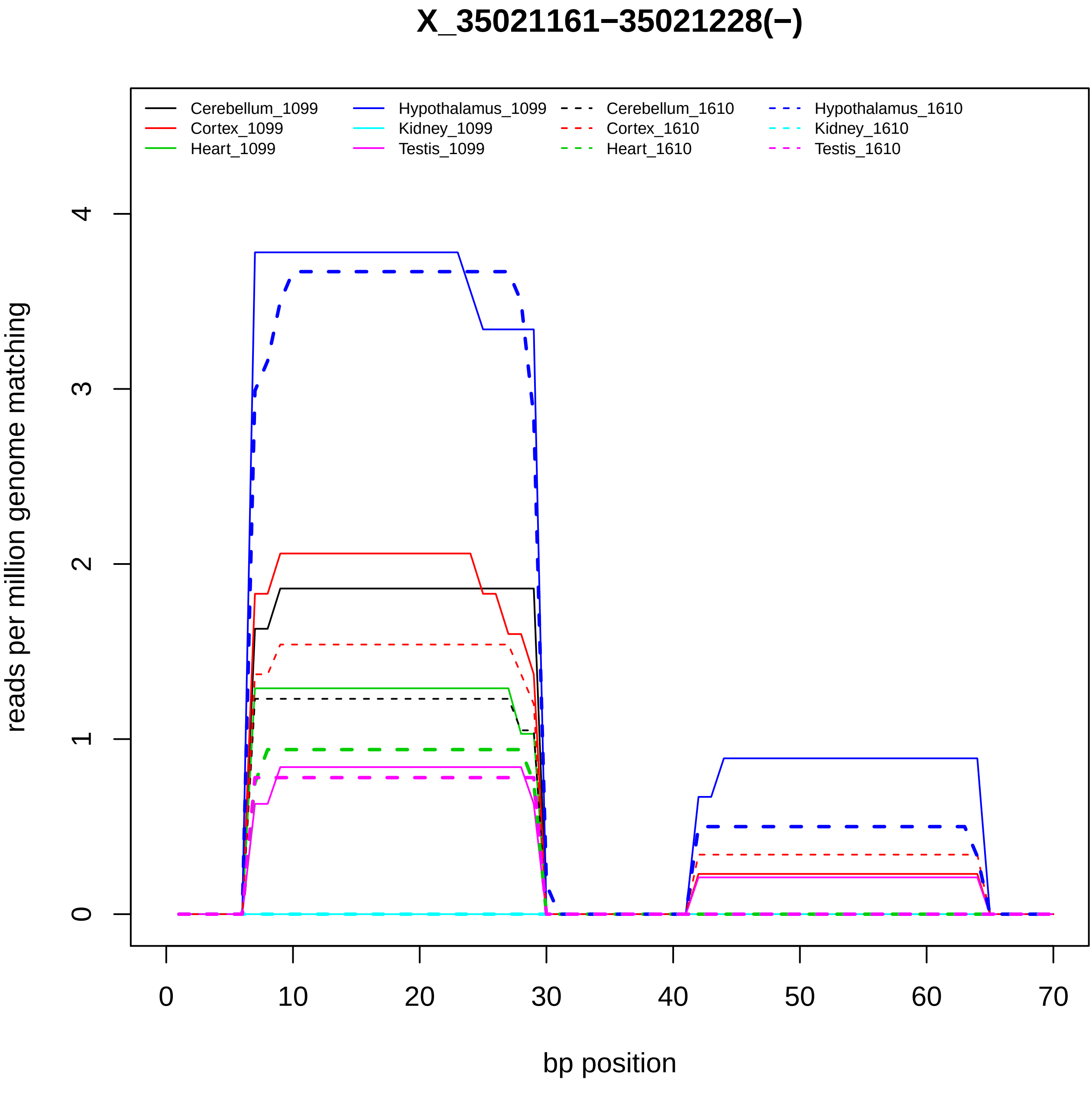

X_35021161−35021228(−)
Cerebellum_1099
Cortex_1099
Heart_1099
Hypothalamus_1099
Kidney_1099
Testis_1099
Cerebellum_1610
Cortex_1610
Heart_1610
Hypothalamus_1610
Kidney_1610
Testis_1610
reads per million genome matching
0
1
2
3
4
0
10
20
30
40
50
60
70
bp position

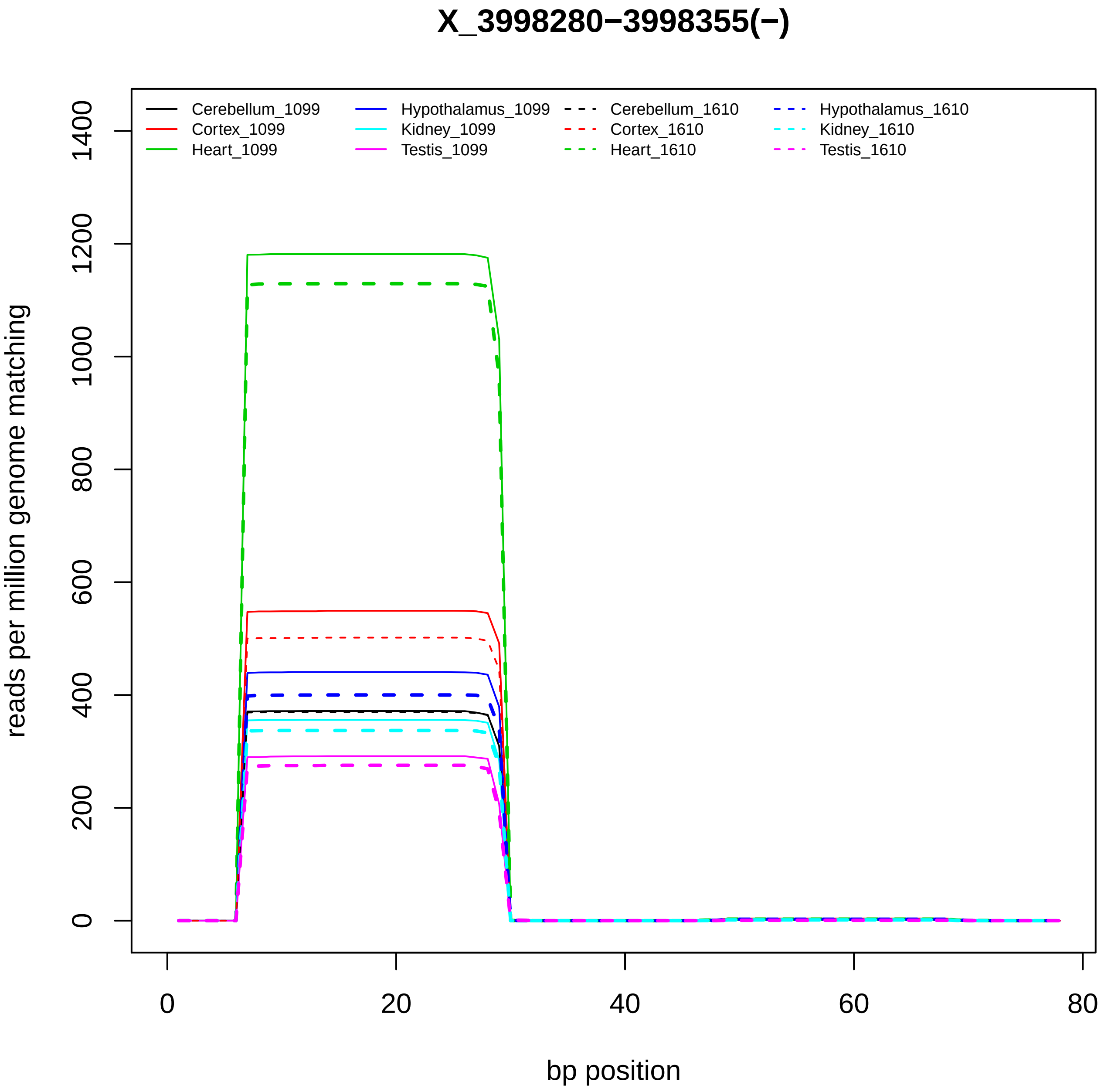

X_3998280−3998355(−)
Cerebellum_1099
Cortex_1099
Heart_1099
Hypothalamus_1099
Kidney_1099
Testis_1099
Cerebellum_1610
Cortex_1610
Heart_1610
Hypothalamus_1610
Kidney_1610
Testis_1610
reads per million genome matching
0
200
400
600
800
1000
1200
1400
bp position
0
20
40
60
80

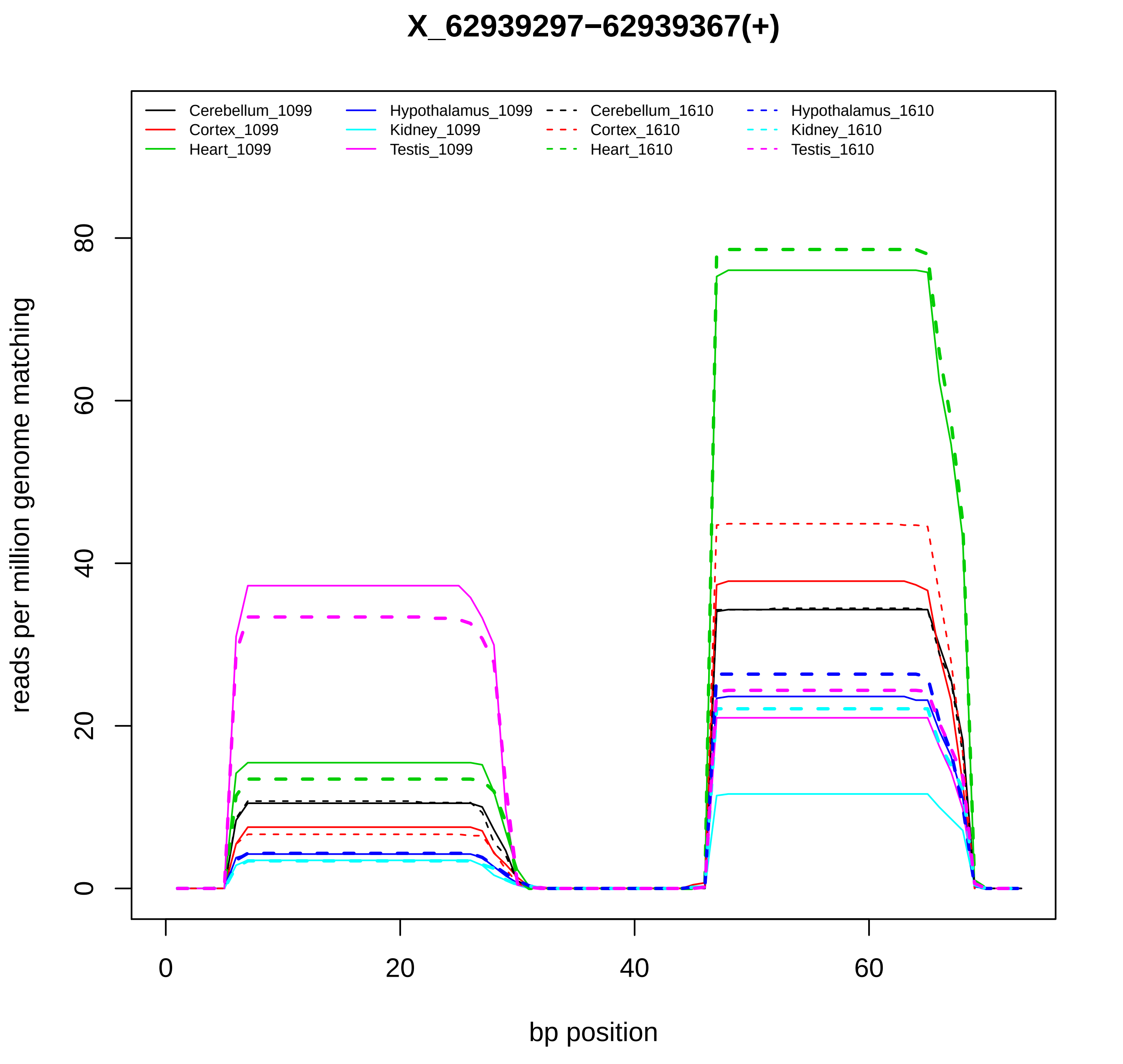

X_62939297−62939367(+)
Cerebellum_1099
Cortex_1099
Heart_1099
Hypothalamus_1099
Kidney_1099
Testis_1099
Cerebellum_1610
Cortex_1610
Heart_1610
Hypothalamus_1610
Kidney_1610
Testis_1610
reads per million genome matching
bp position
0
20
40
60
80

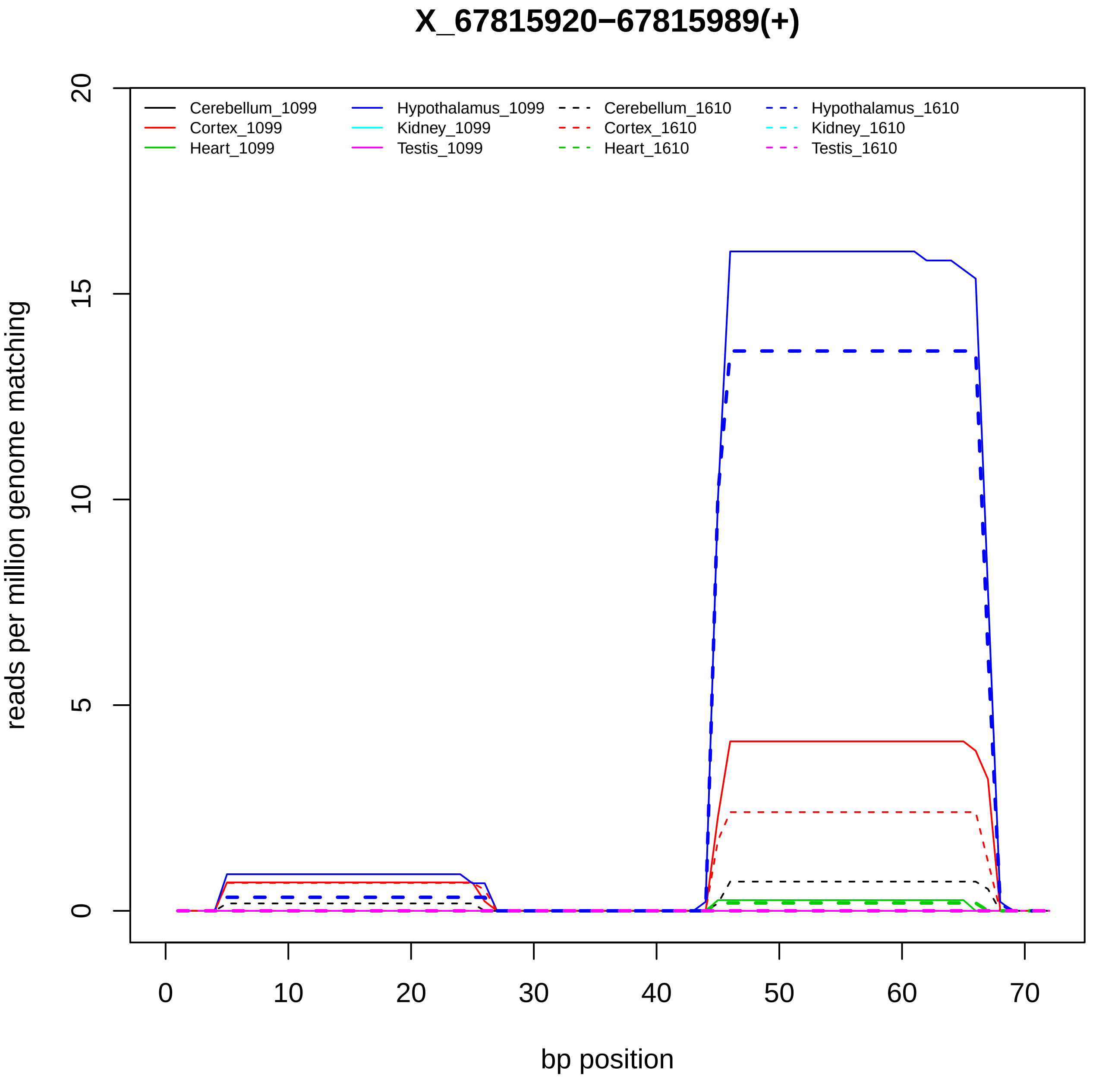

X_67815920−67815989(+)
Cerebellum_1099
Cortex_1099
Heart_1099
Hypothalamus_1099
Kidney_1099
Testis_1099
Cerebellum_1610
Cortex_1610
Heart_1610
Hypothalamus_1610
Kidney_1610
Testis_1610
reads per million genome matching
0
5
10
15
20
bp position
0
10
20
30
40
50
60
70

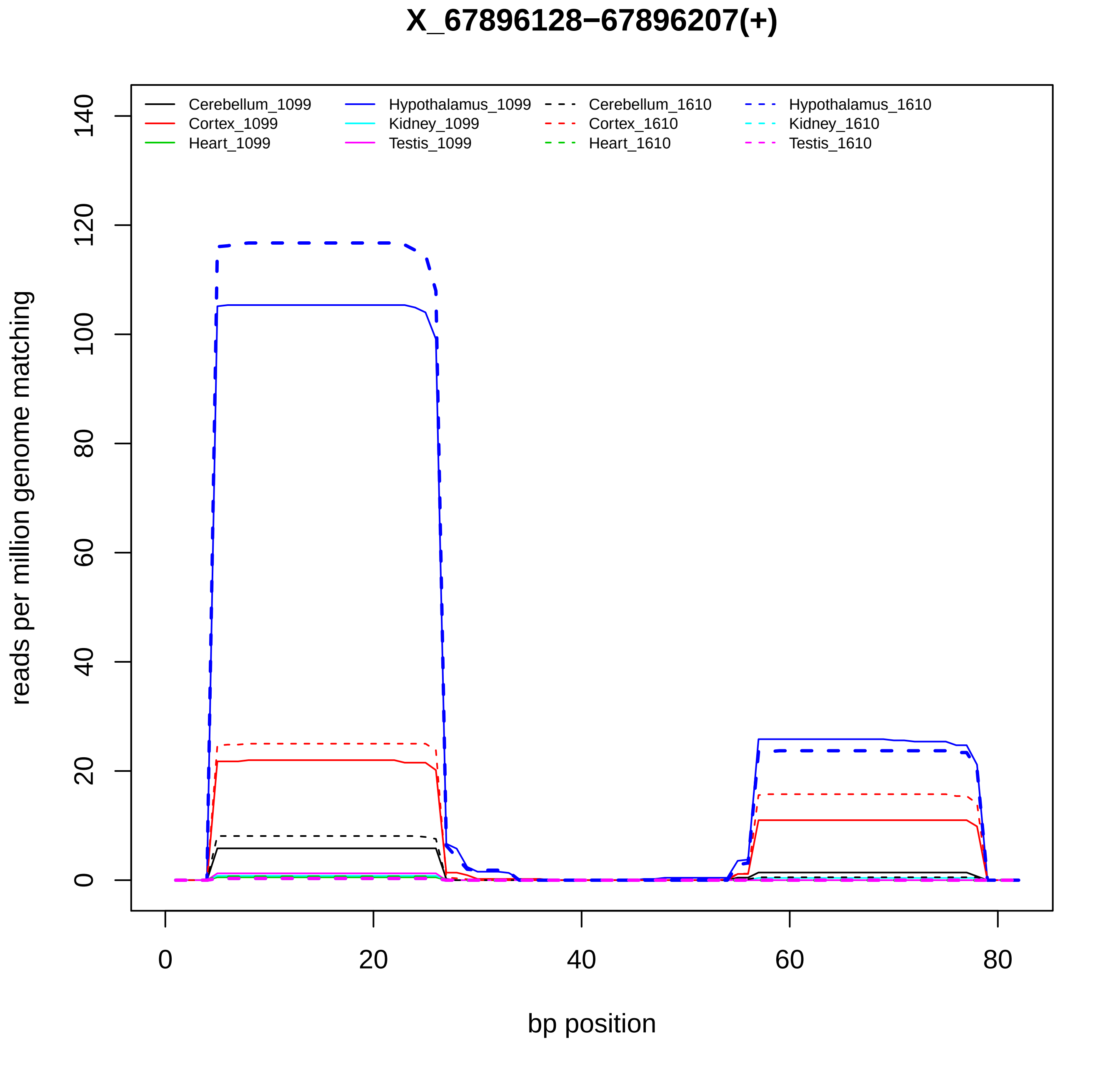

X_67896128−67896207(+)
Cerebellum_1099
Cortex_1099
Heart_1099
Hypothalamus_1099
Kidney_1099
Testis_1099
Cerebellum_1610
Cortex_1610
Heart_1610
Hypothalamus_1610
Kidney_1610
Testis_1610
reads per million genome matching
bp position
0
20
40
60
80
100
120
140

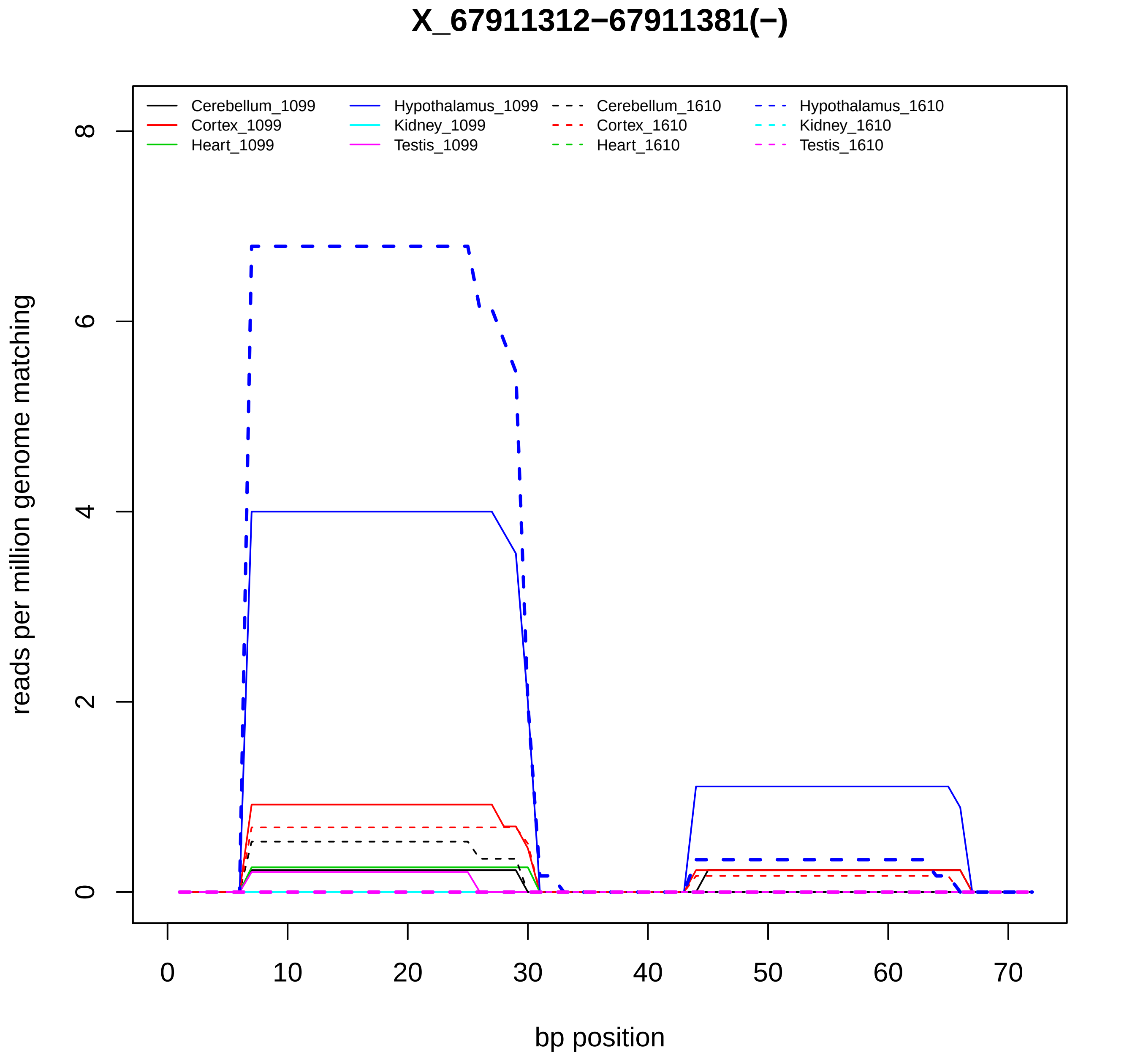

X_67911312−67911381(−)
Cerebellum_1099
Cortex_1099
Heart_1099
Hypothalamus_1099
Kidney_1099
Testis_1099
Cerebellum_1610
Cortex_1610
Heart_1610
Hypothalamus_1610
Kidney_1610
Testis_1610
reads per million genome matching
0
2
4
6
8
0
10
20
30
40
50
60
70
bp position

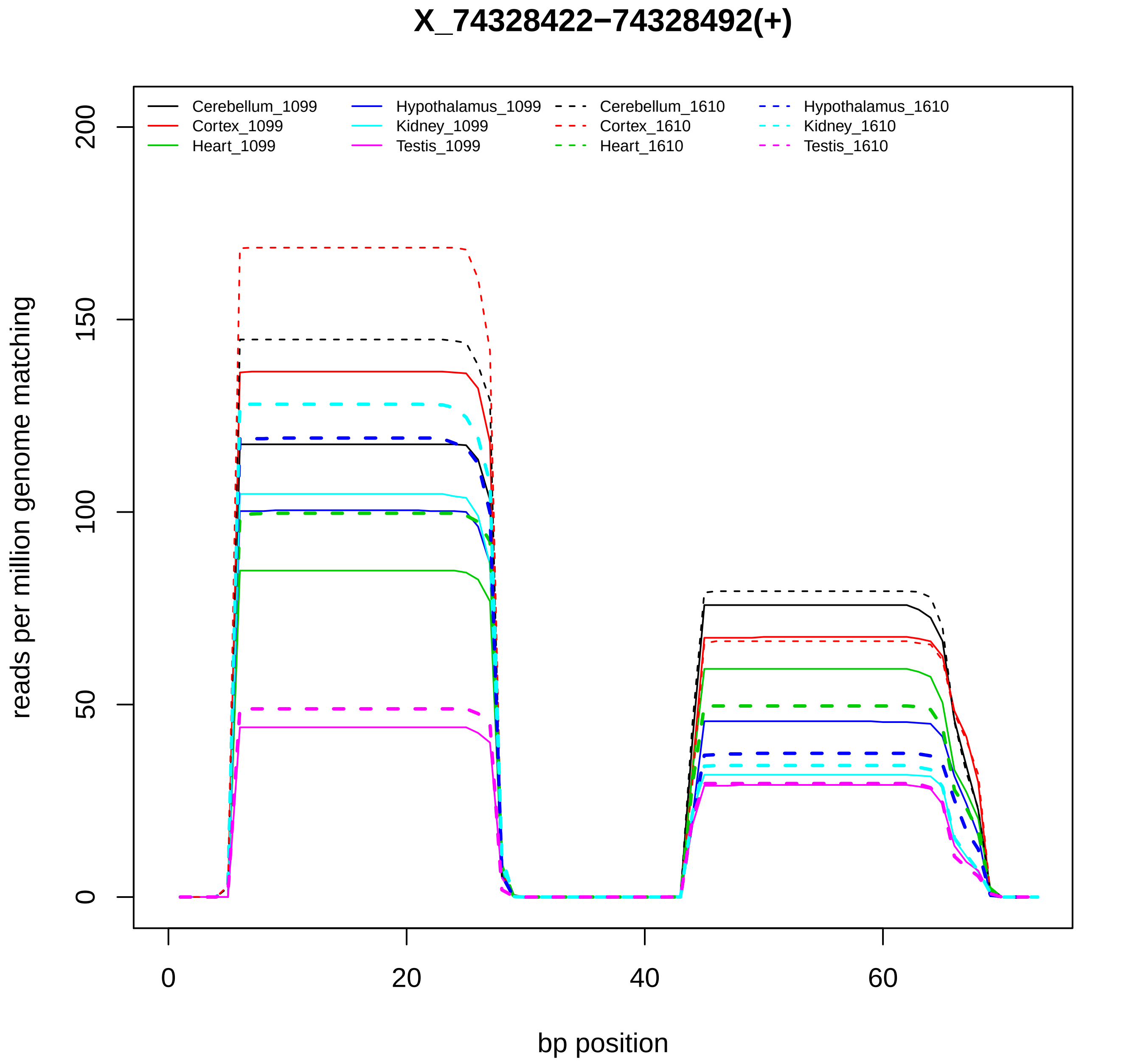
X_74328422−74328492(+)
Cerebellum_1099
Cortex_1099
Heart_1099
Hypothalamus_1099
Kidney_1099
Testis_1099
Cerebellum_1610
Cortex_1610
Heart_1610
Hypothalamus_1610
Kidney_1610
Testis_1610
reads per million genome matching
0
50
100
150
200
0
20
40
60
bp position

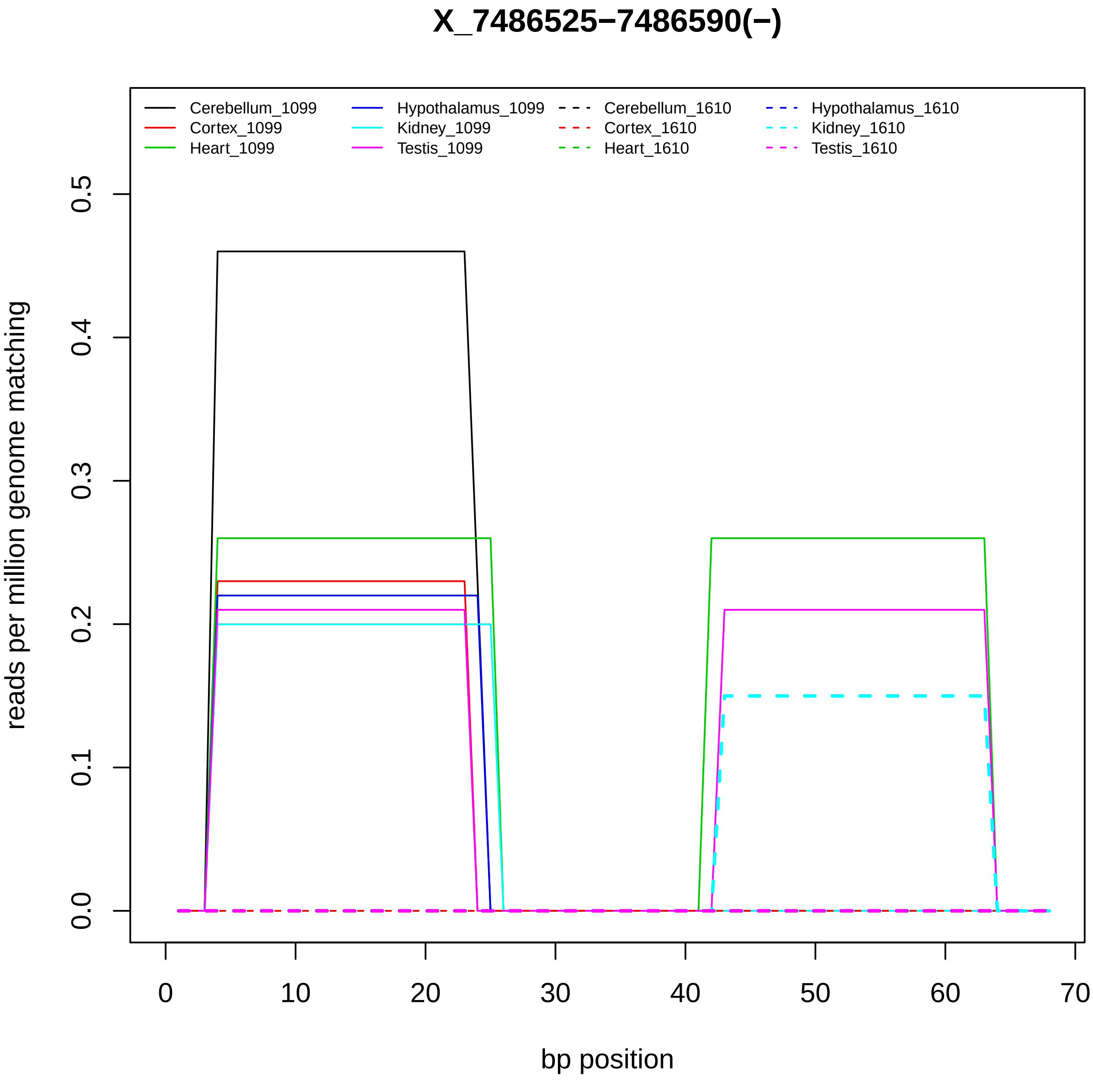

X_7486525−7486590(−)
Cerebellum_1099
Cortex_1099
Heart_1099
Hypothalamus_1099
Kidney_1099
Testis_1099
Cerebellum_1610
Cortex_1610
Heart_1610
Hypothalamus_1610
Kidney_1610
Testis_1610
reads per million genome matching
0.0
0.1
0.2
0.3
0.4
0.5
0
10
20
30
40
50
60
70
bp position

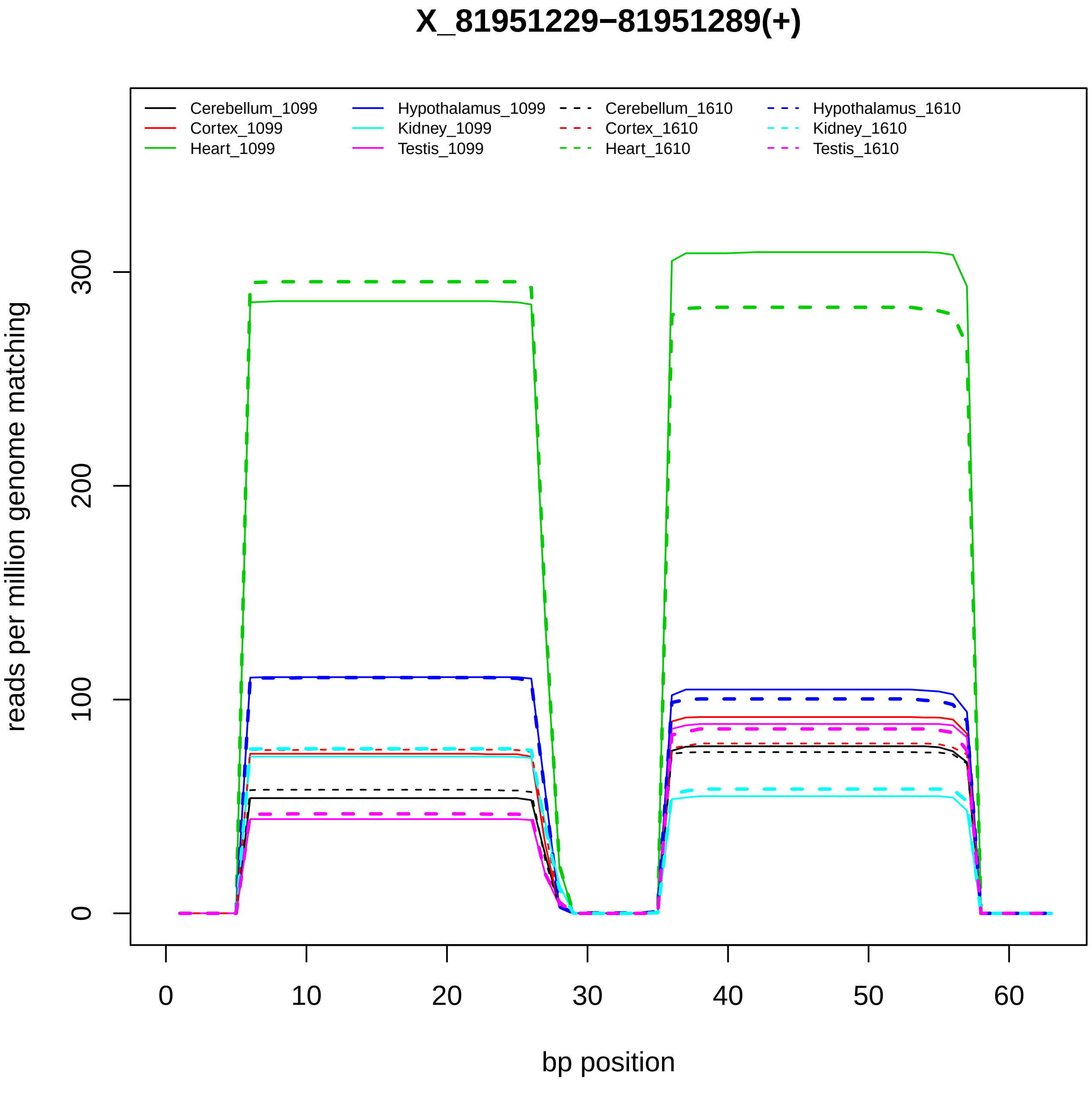
X_81951229−81951289(+)
Cerebellum_1099
Cortex_1099
Heart_1099
Hypothalamus_1099
Kidney_1099
Testis_1099
Cerebellum_1610
Cortex_1610
Heart_1610
Hypothalamus_1610
Kidney_1610
Testis_1610
reads per million genome matching
0
100
200
300
0
10
20
30
40
50
60
bp position

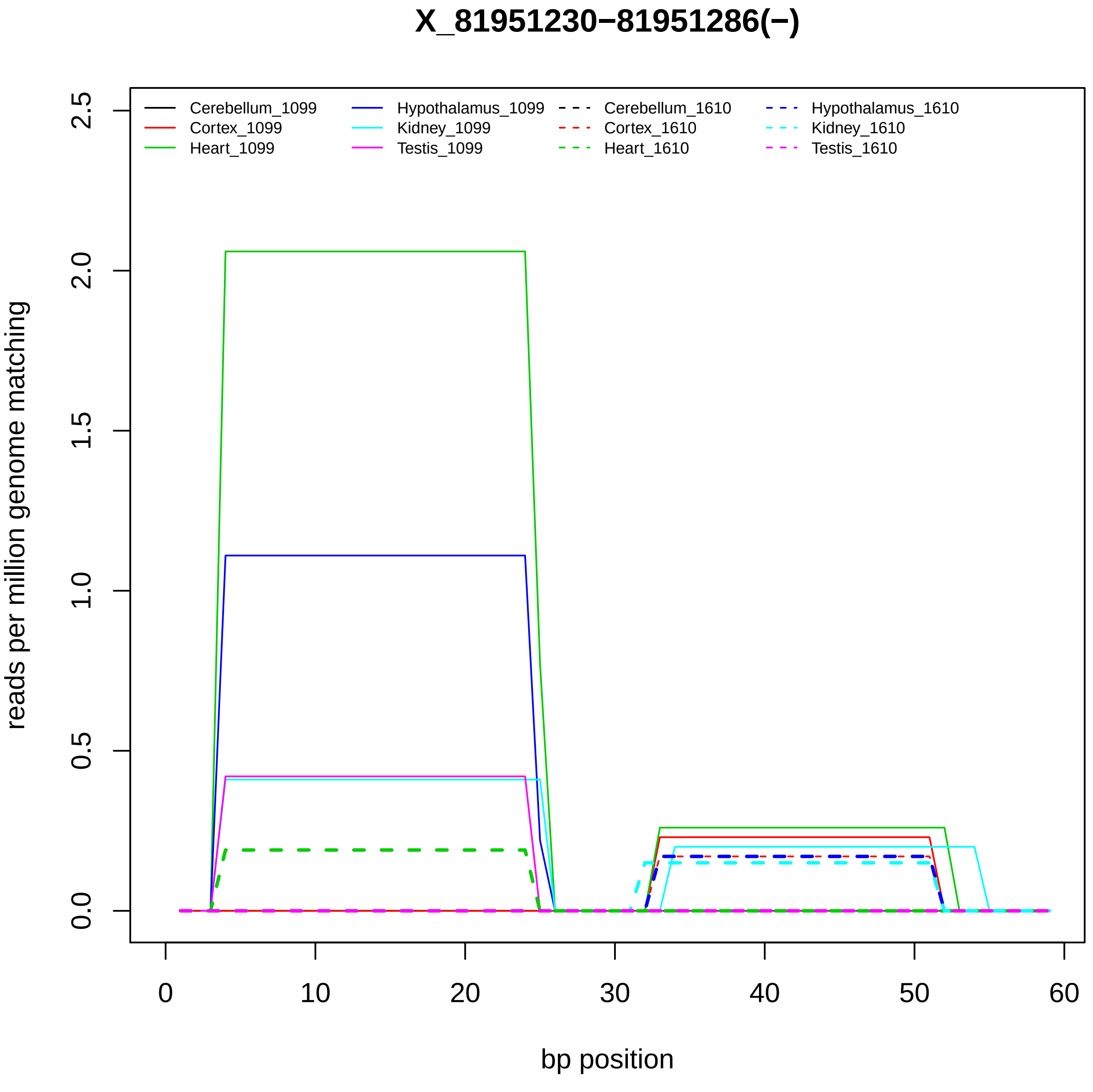

X_81951230−81951286(−)
Cerebellum_1099
Cortex_1099
Heart_1099
Hypothalamus_1099
Kidney_1099
Testis_1099
Cerebellum_1610
Cortex_1610
Heart_1610
Hypothalamus_1610
Kidney_1610
Testis_1610
reads per million genome matching
bp position
0.0
0.5
1.0
1.5
2.0
2.5
0
10
20
30
40
50
60

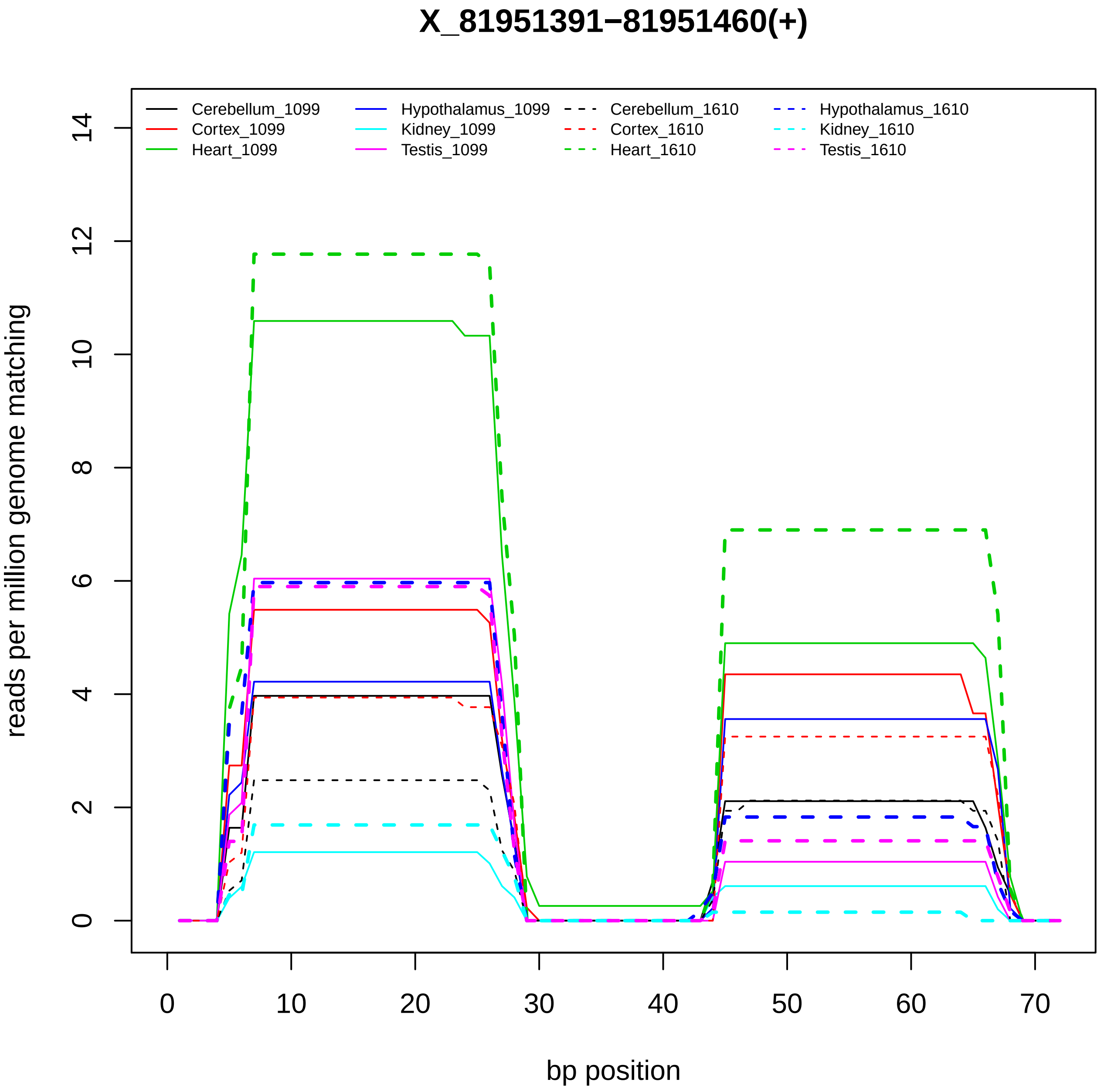

X_81951391−81951460(+)
Cerebellum_1099
Cortex_1099
Heart_1099
Hypothalamus_1099
Kidney_1099
Testis_1099
Cerebellum_1610
Cortex_1610
Heart_1610
Hypothalamus_1610
Kidney_1610
Testis_1610
reads per million genome matching
bp position
0
2
4
6
8
10
12
14
0
10
20
30
40
50
60
70

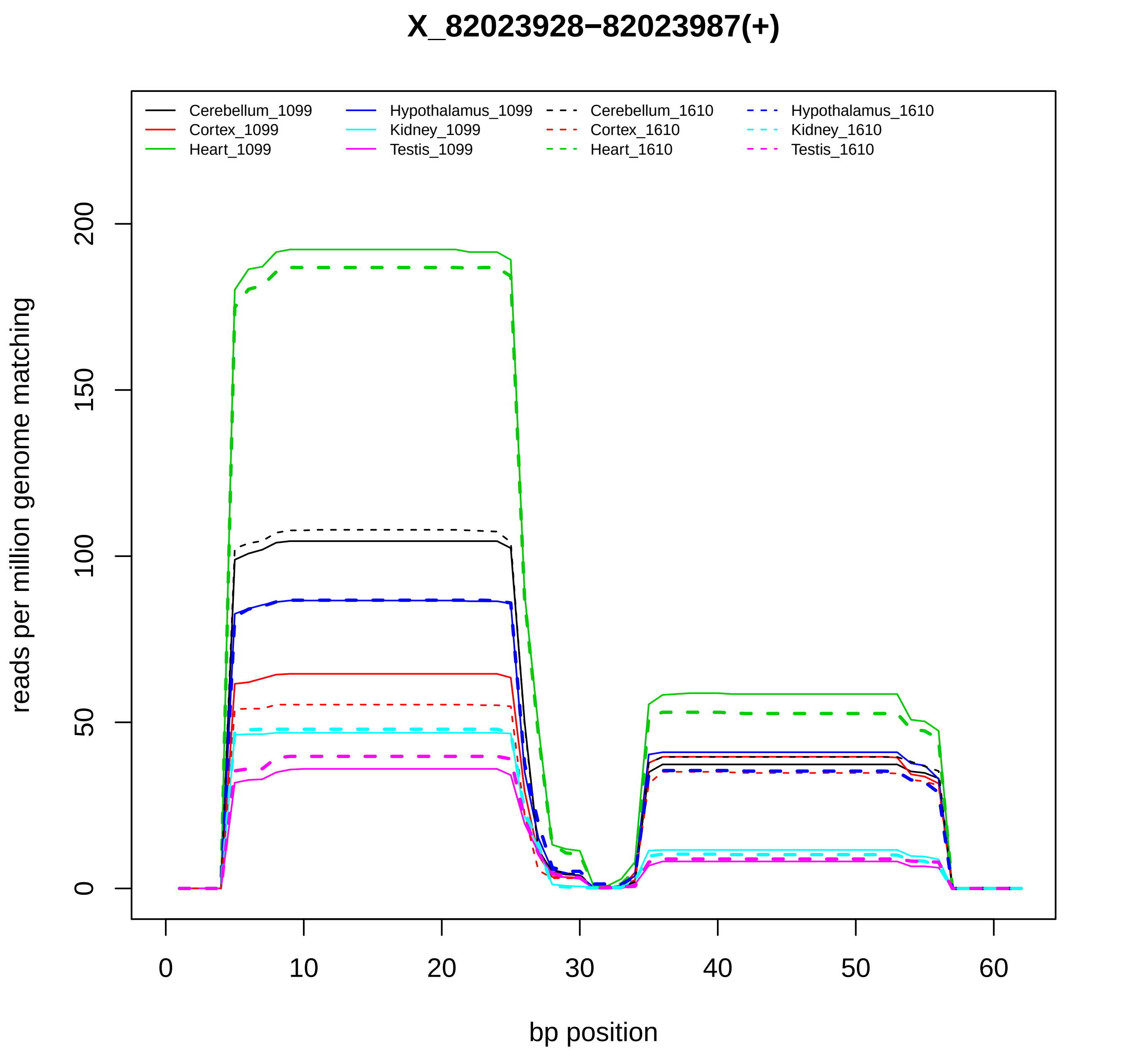

X_82023928−82023987(+)
Cerebellum_1099
Cortex_1099
Heart_1099
Hypothalamus_1099
Kidney_1099
Testis_1099
Cerebellum_1610
Cortex_1610
Heart_1610
Hypothalamus_1610
Kidney_1610
Testis_1610
reads per million genome matching
bp position
0
50
100
150
200
0
10
20
30
40
50
60

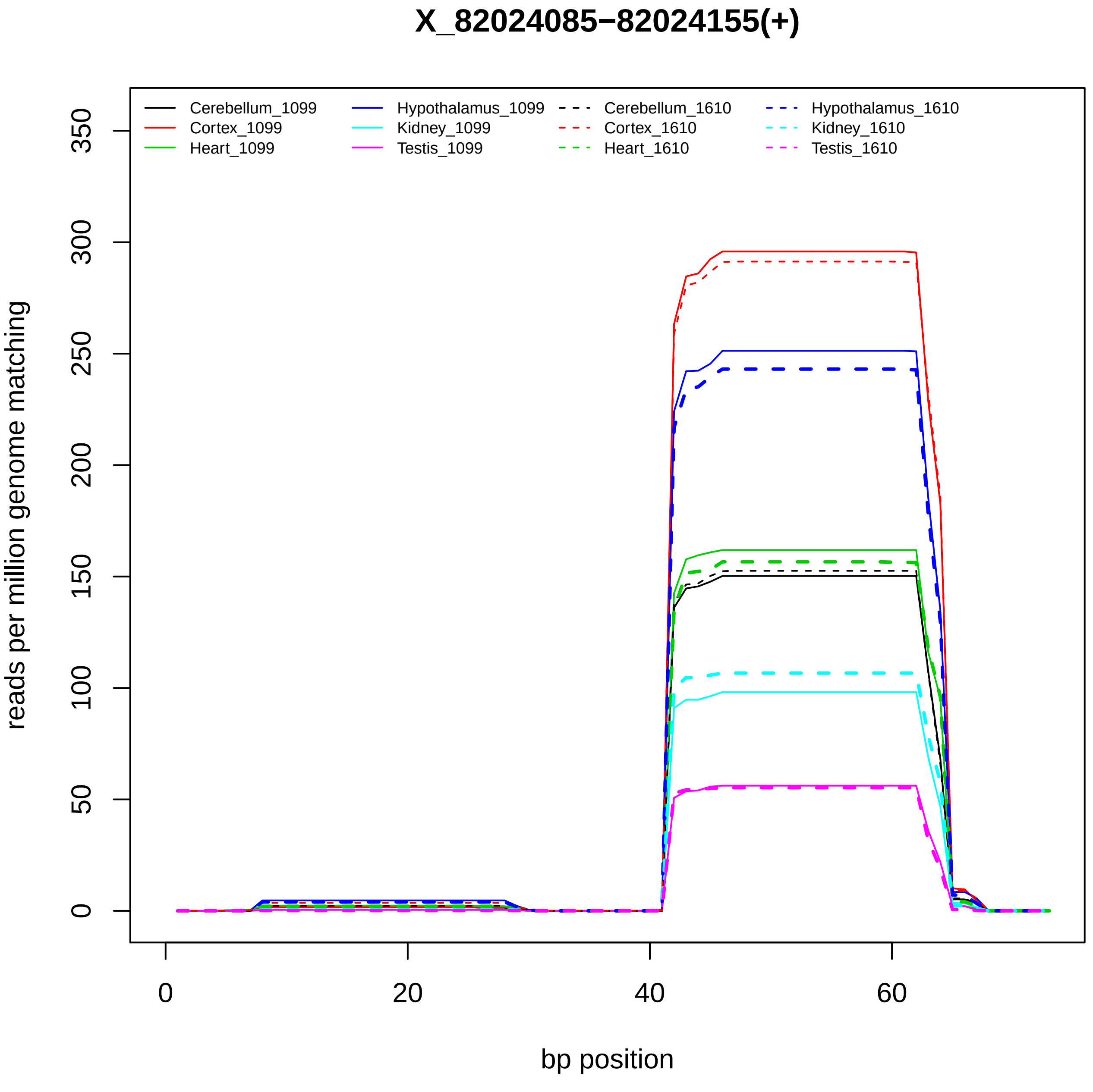

X_82024085−82024155(+)
Cerebellum_1099
Cortex_1099
Heart_1099
Hypothalamus_1099
Kidney_1099
Testis_1099
Cerebellum_1610
Cortex_1610
Heart_1610
Hypothalamus_1610
Kidney_1610
Testis_1610
reads per million genome matching
0
50
100
150
200
250
300
350
0
20
40
60
bp position

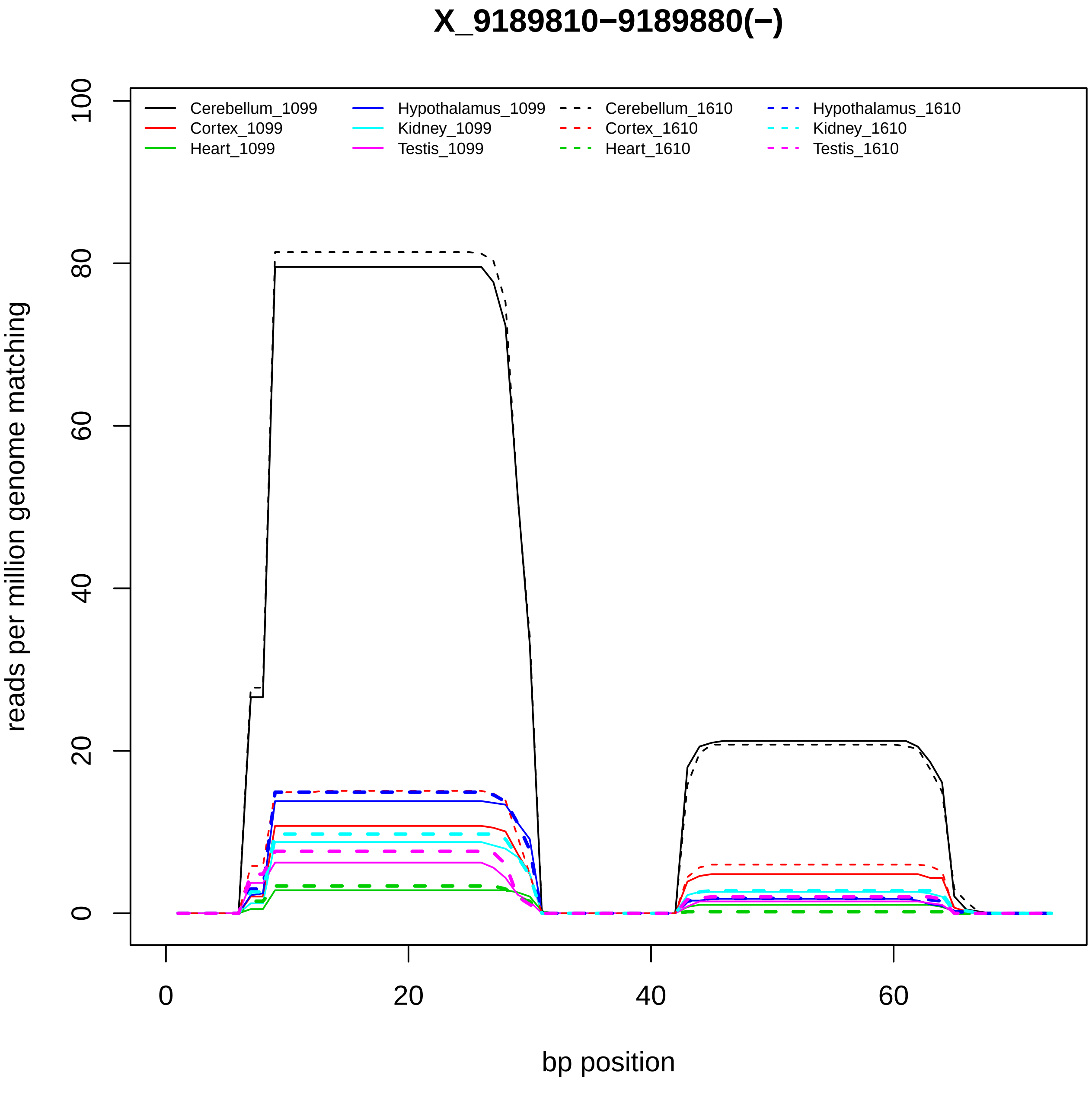

X_9189810−9189880(−)
Cerebellum_1099
Cortex_1099
Heart_1099
Hypothalamus_1099
Kidney_1099
Testis_1099
Cerebellum_1610
Cortex_1610
Heart_1610
Hypothalamus_1610
Kidney_1610
Testis_1610
reads per million genome matching
bp position
0
20
40
60
80
100

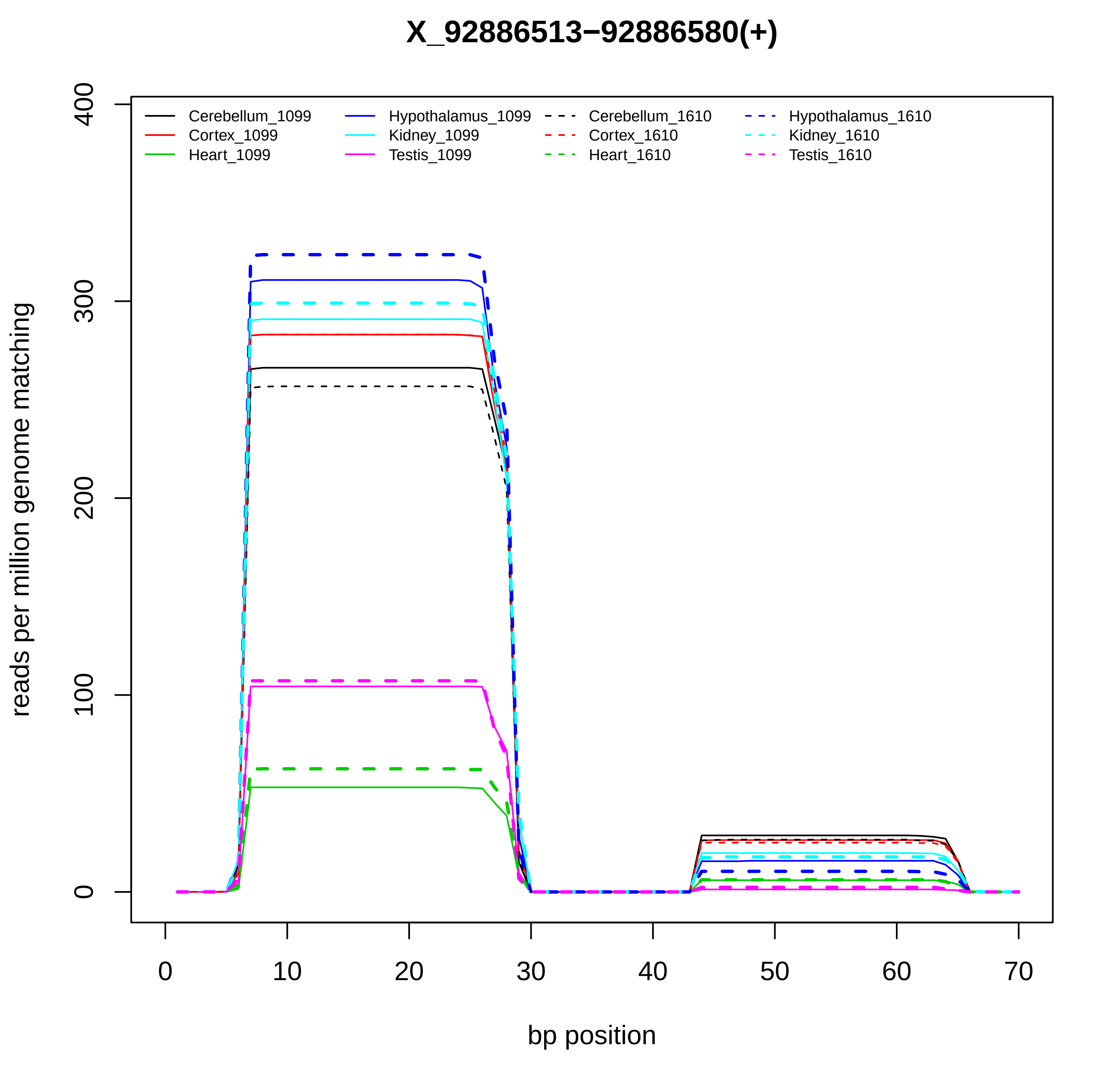
X_92886513−92886580(+)
reads per million genome matching
bp position
0
100
200
300
400
0
10
20
30
40
50
60
70
Cerebellum_1099
Cortex_1099
Heart_1099
Hypothalamus_1099
Kidney_1099
Testis_1099
Cerebellum_1610
Cortex_1610
Heart_1610
Hypothalamus_1610
Kidney_1610
Testis_1610

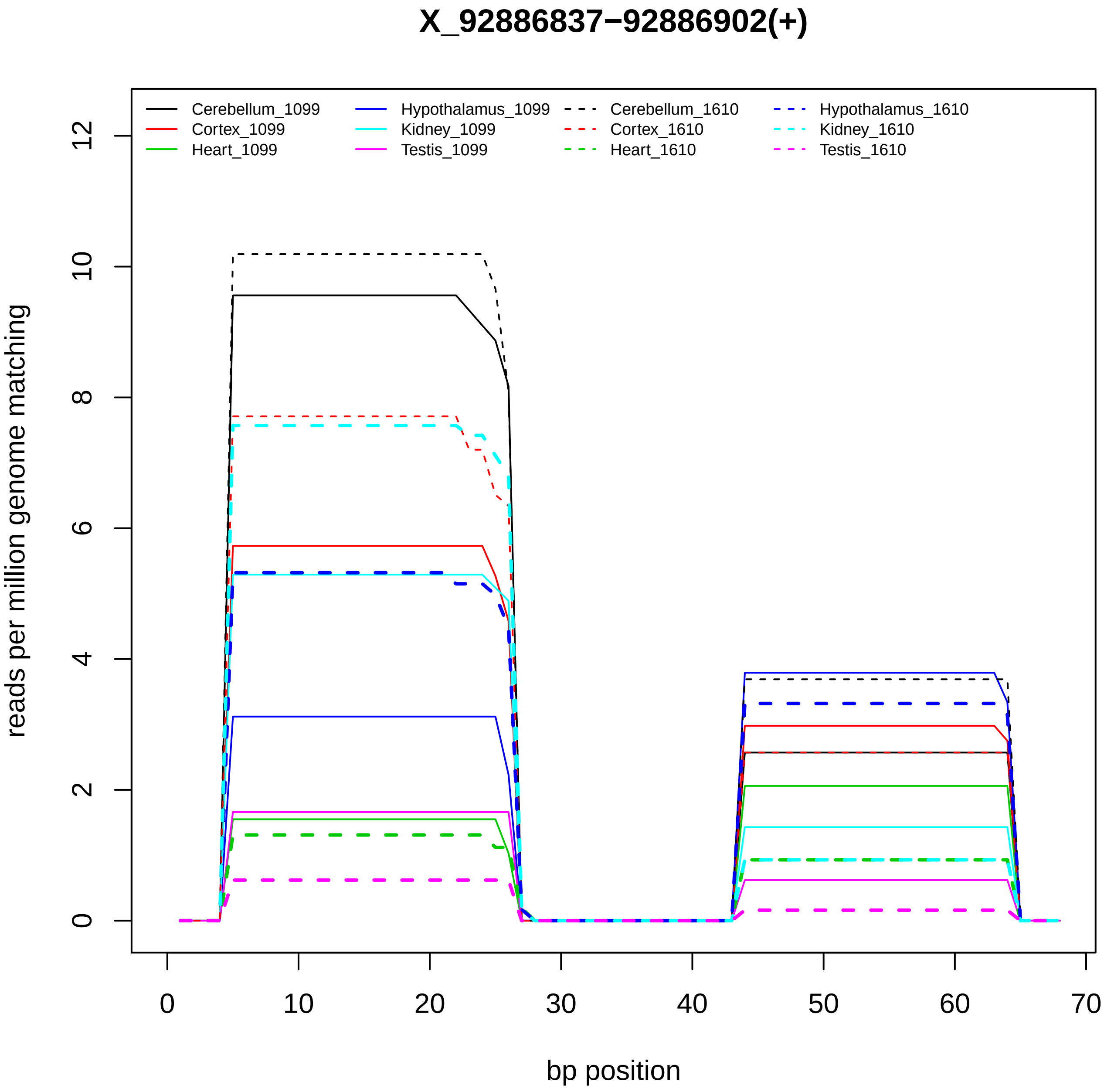

X_92886837−92886902(+)
Cerebellum_1099
Cortex_1099
Heart_1099
Hypothalamus_1099
Kidney_1099
Testis_1099
Cerebellum_1610
Cortex_1610
Heart_1610
Hypothalamus_1610
Kidney_1610
Testis_1610
reads per million genome matching
bp position
0
2
4
6
8
10
12
0
10
20
30
40
50
60
70

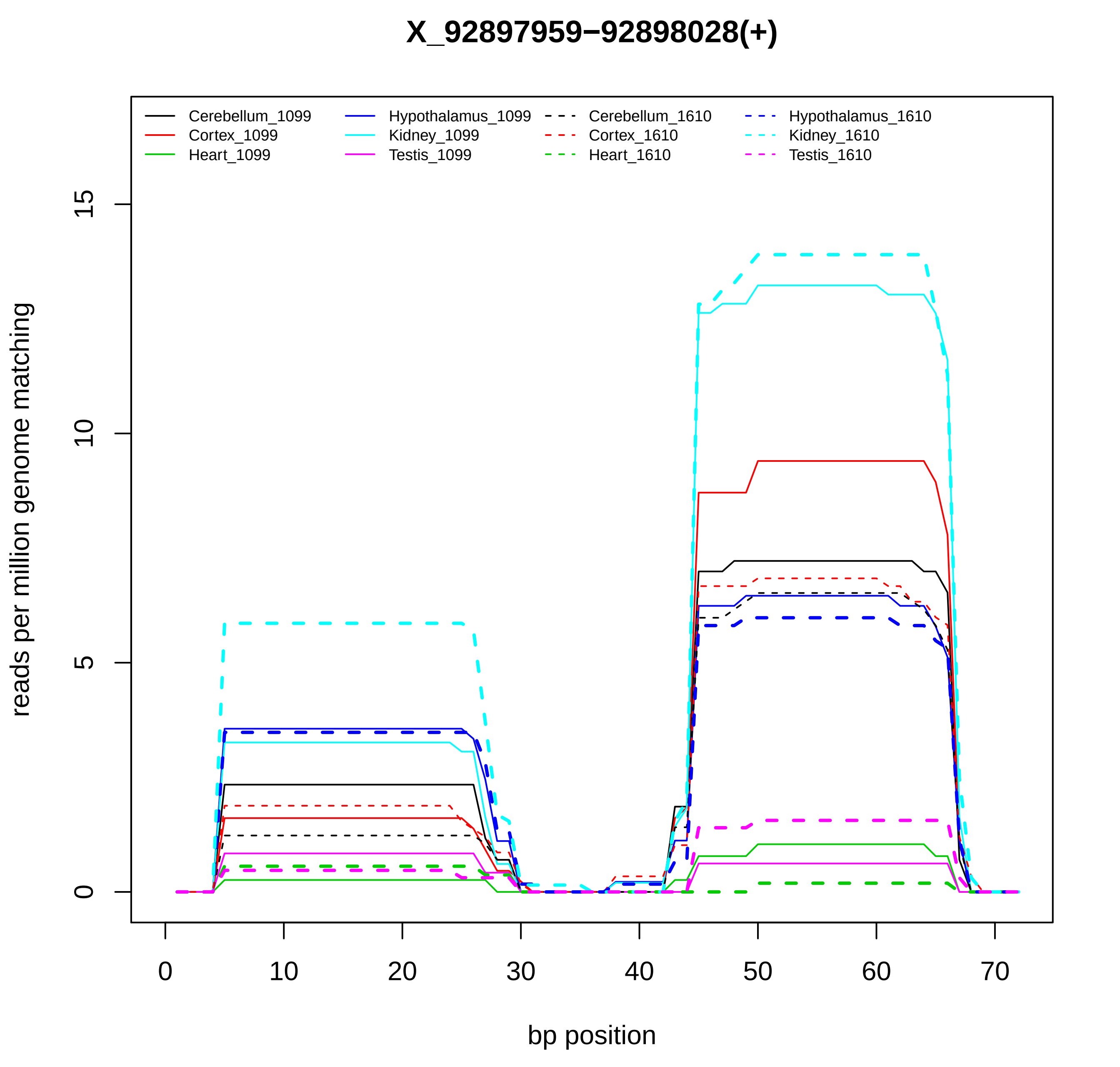

X_92897959−92898028(+)
Cerebellum_1099
Cortex_1099
Heart_1099
Hypothalamus_1099
Kidney_1099
Testis_1099
Cerebellum_1610
Cortex_1610
Heart_1610
Hypothalamus_1610
Kidney_1610
Testis_1610
reads per million genome matching
0
5
10
15
0
10
20
30
40
50
60
70
bp position

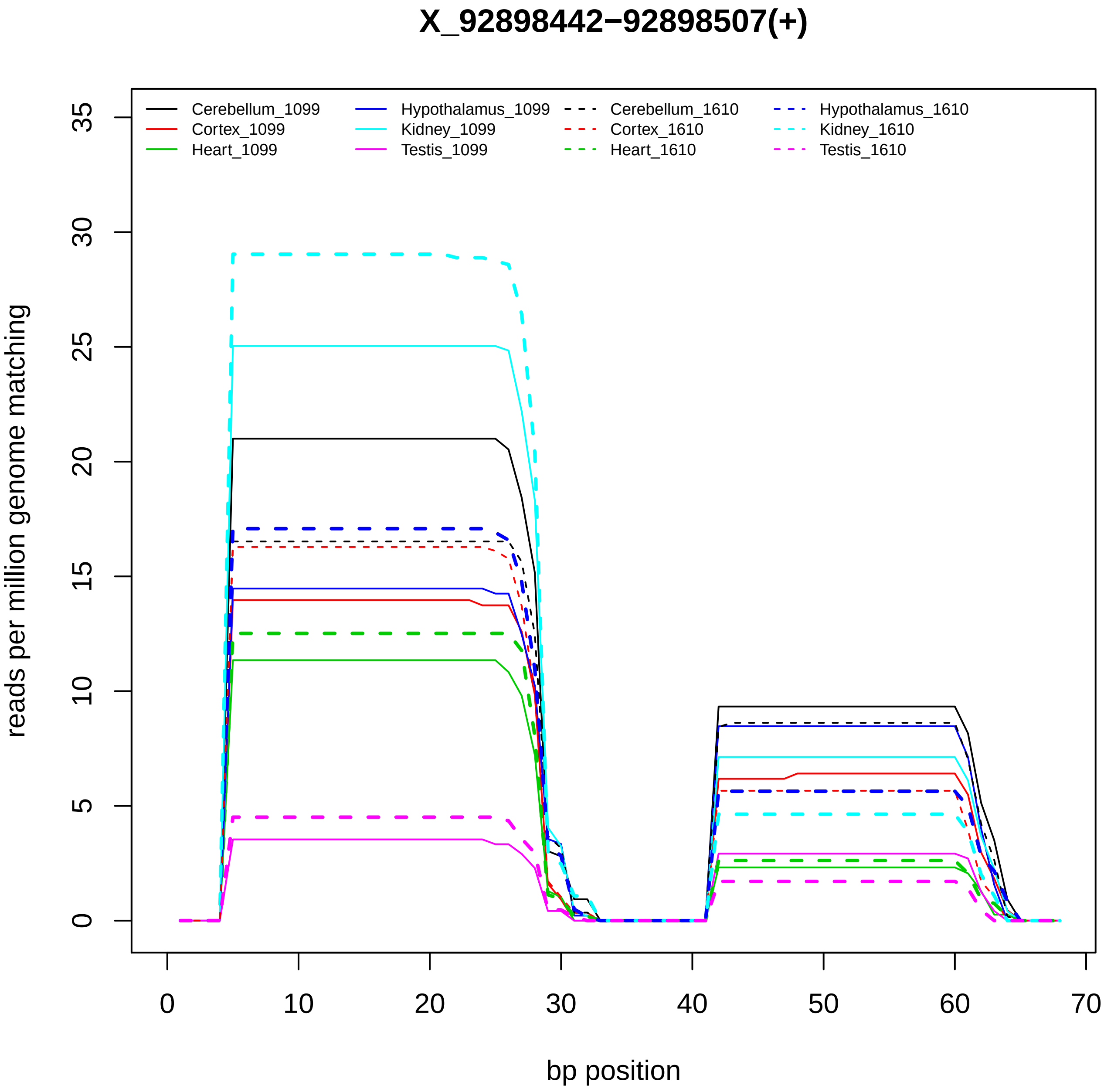

X_92898442−92898507(+)
Cerebellum_1099
Cortex_1099
Heart_1099
Hypothalamus_1099
Kidney_1099
Testis_1099
Cerebellum_1610
Cortex_1610
Heart_1610
Hypothalamus_1610
Kidney_1610
Testis_1610
reads per million genome matching
bp position
0
5
10
15
20
25
30
35
0
10
20
30
40
50
60
70

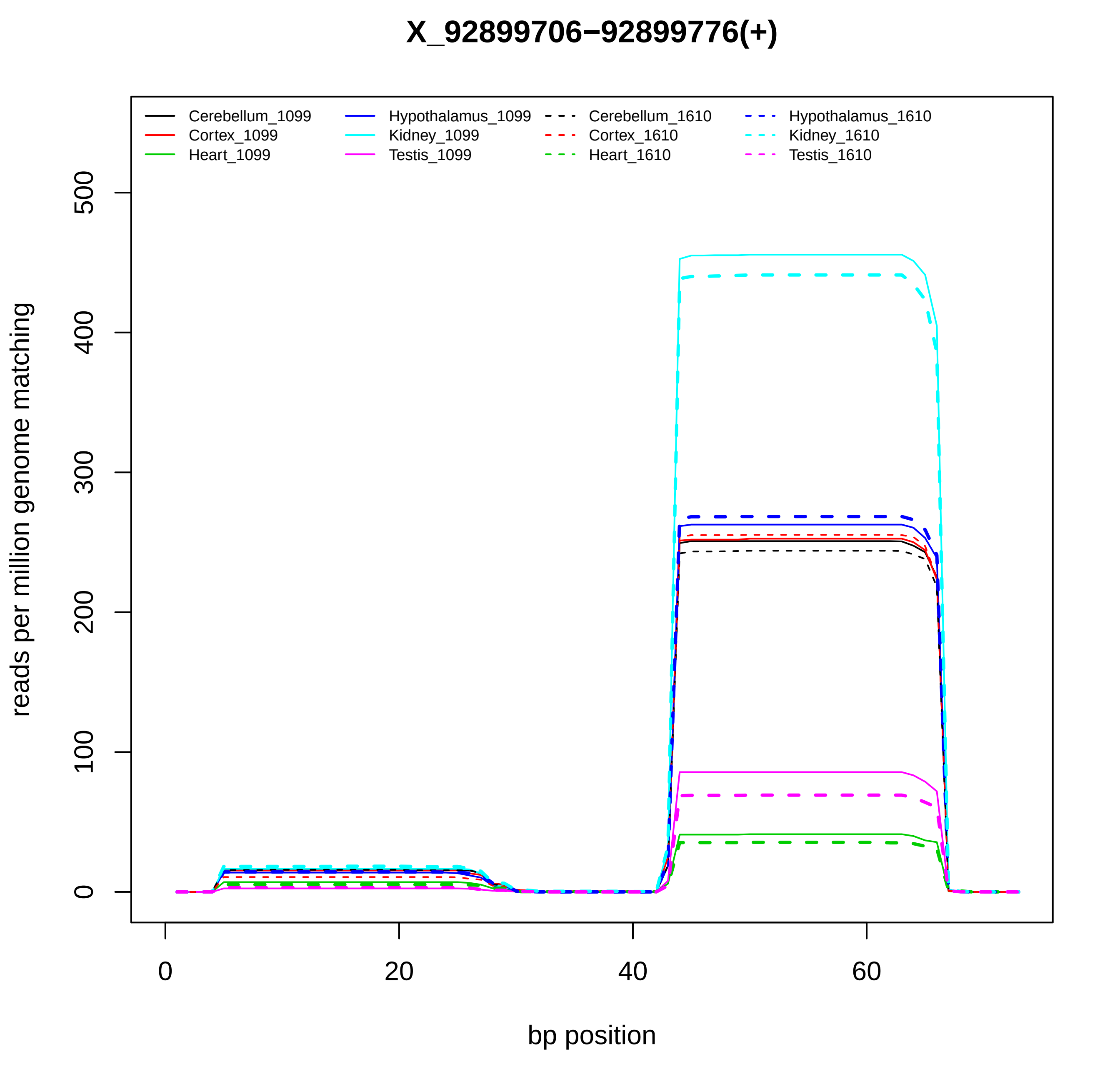

X_92899706−92899776(+)
Cerebellum_1099
Cortex_1099
Heart_1099
Hypothalamus_1099
Kidney_1099
Testis_1099
Cerebellum_1610
Cortex_1610
Heart_1610
Hypothalamus_1610
Kidney_1610
Testis_1610
reads per million genome matching
bp position
0
100
200
300
400
500
0
20
40
60

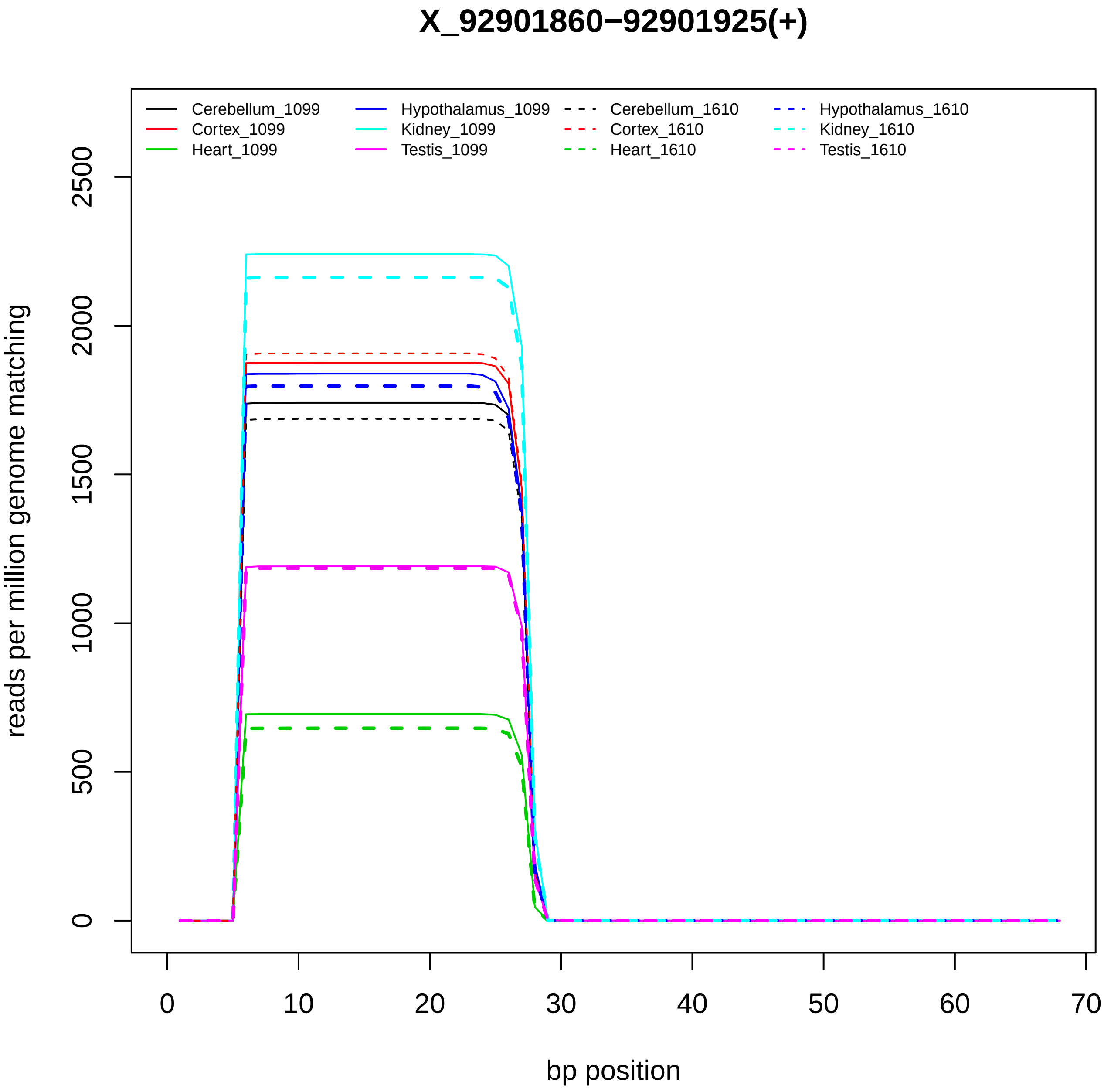

X_92901860−92901925(+)
Cerebellum_1099
Cortex_1099
Heart_1099
Hypothalamus_1099
Kidney_1099
Testis_1099
Cerebellum_1610
Cortex_1610
Heart_1610
Hypothalamus_1610
Kidney_1610
Testis_1610
reads per million genome matching
0
500
1000
1500
2000
2500
bp position
0
10
20
30
40
50
60
70

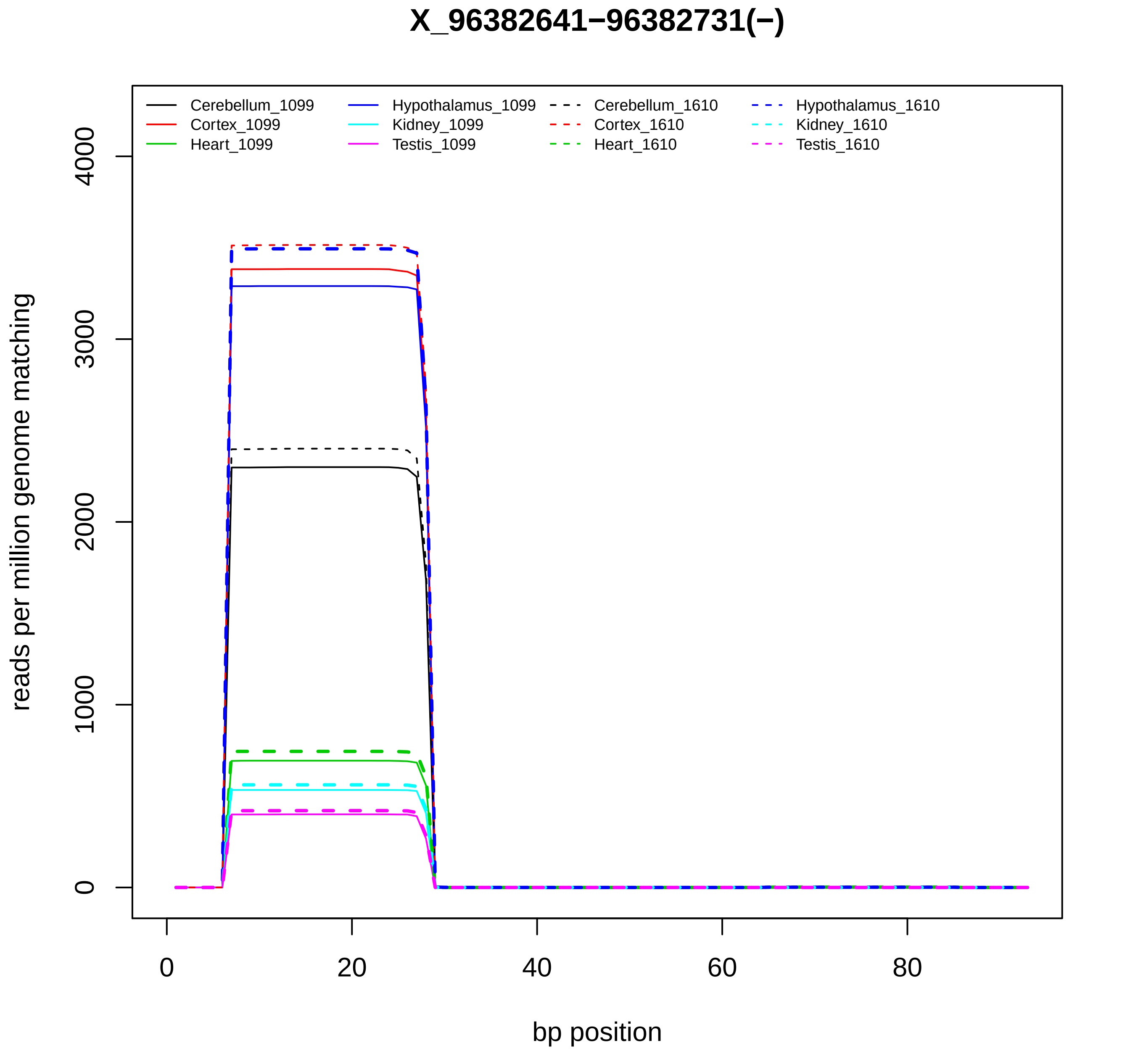

X_96382641−96382731(−)
Cerebellum_1099
Cortex_1099
Heart_1099
Hypothalamus_1099
Kidney_1099
Testis_1099
Cerebellum_1610
Cortex_1610
Heart_1610
Hypothalamus_1610
Kidney_1610
Testis_1610
reads per million genome matching
0
1000
2000
3000
4000
0
20
40
60
80
bp position

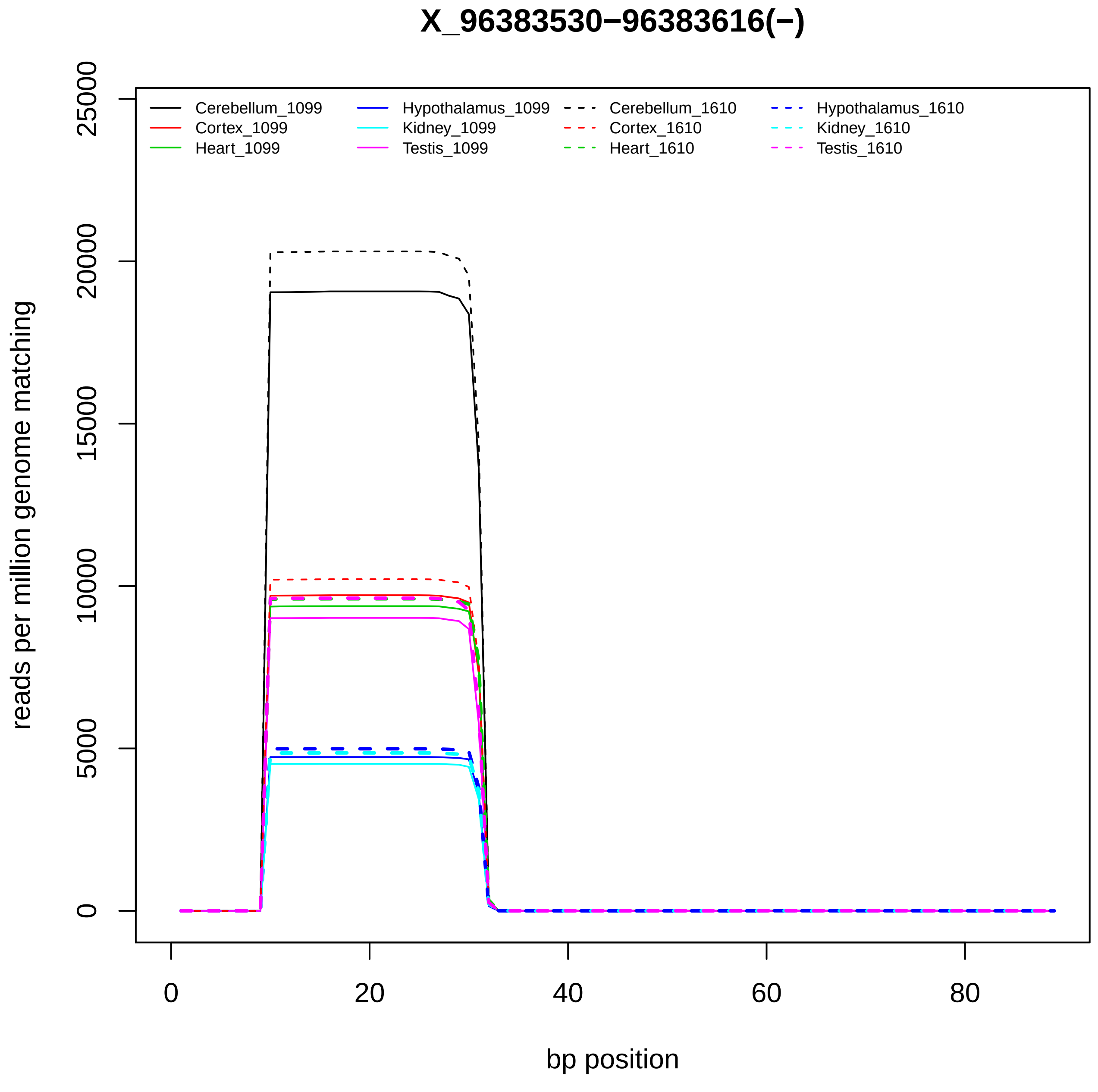

X_96383530−96383616(−)
Cerebellum_1099
Cortex_1099
Heart_1099
Hypothalamus_1099
Kidney_1099
Testis_1099
Cerebellum_1610
Cortex_1610
Heart_1610
Hypothalamus_1610
Kidney_1610
Testis_1610
reads per million genome matching
0
5000
10000
15000
20000
25000
0
20
40
60
80
bp position

Fig. S5

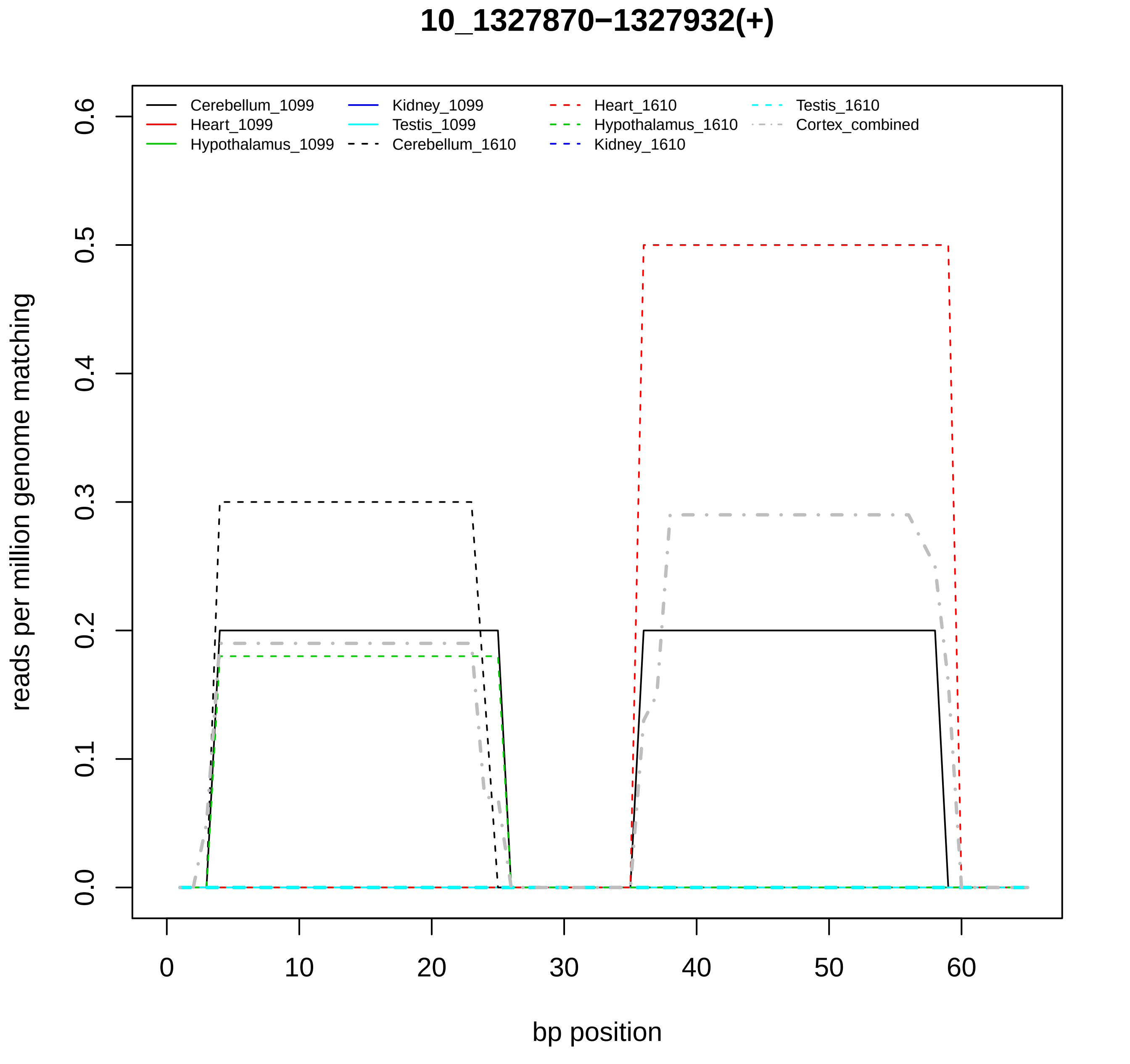

10_1327870−1327932(+)
Cerebellum_1099
Heart_1099
Hypothalamus_1099
Kidney_1099
Testis_1099
Cerebellum_1610
Heart_1610
Hypothalamus_1610
Kidney_1610
Testis_1610
Cortex_combined
reads per million genome matching
0.0
0.1
0.2
0.3
0.4
0.5
0.6
0
10
20
30
40
50
60
bp position

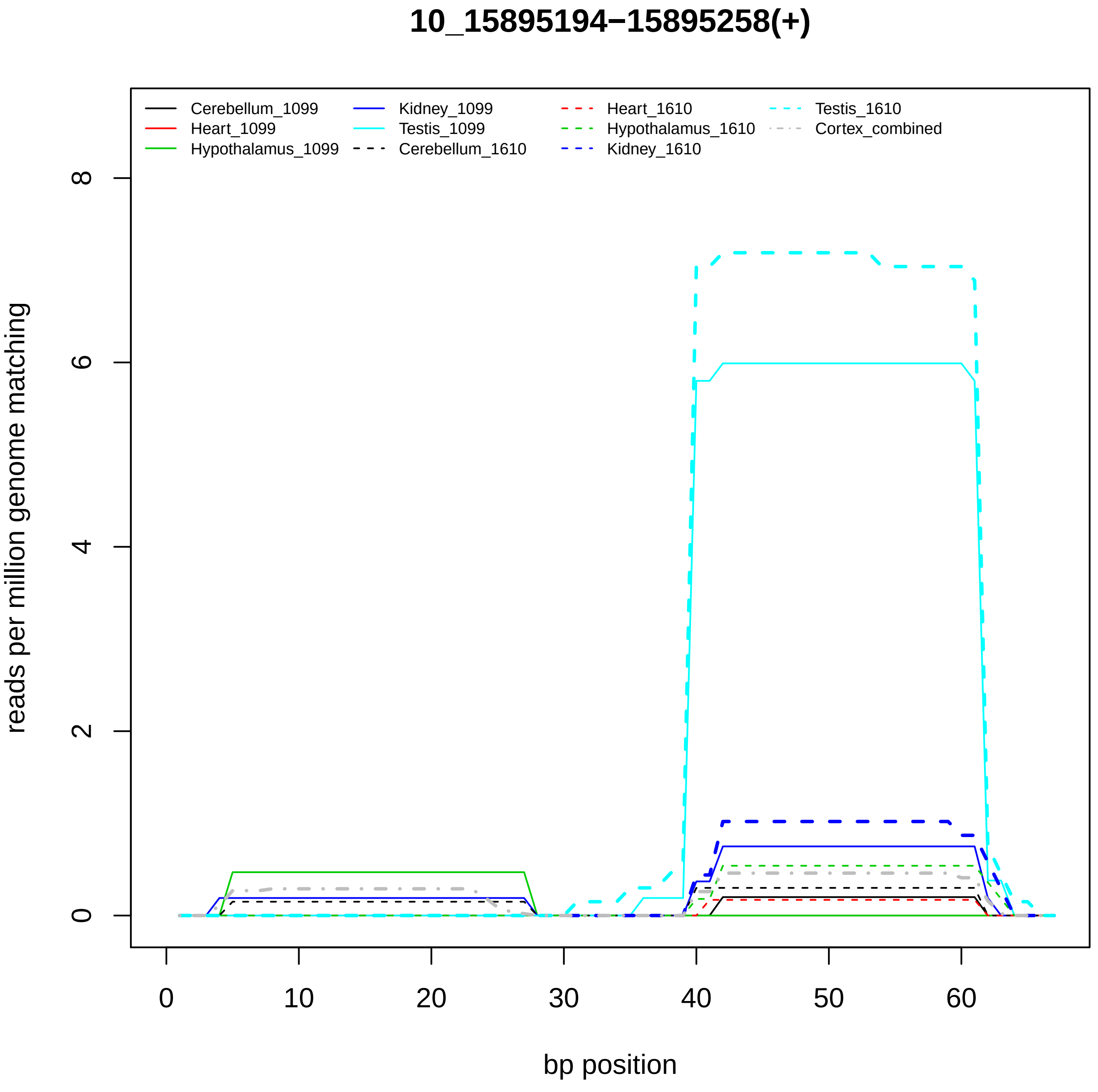
10_15895194−15895258(+)
Cerebellum_1099
Heart_1099
Hypothalamus_1099
Kidney_1099
Testis_1099
Cerebellum_1610
Heart_1610
Hypothalamus_1610
Kidney_1610
Testis_1610
Cortex_combined
reads per million genome matching
bp position
0
2
4
6
8
0
10
20
30
40
50
60

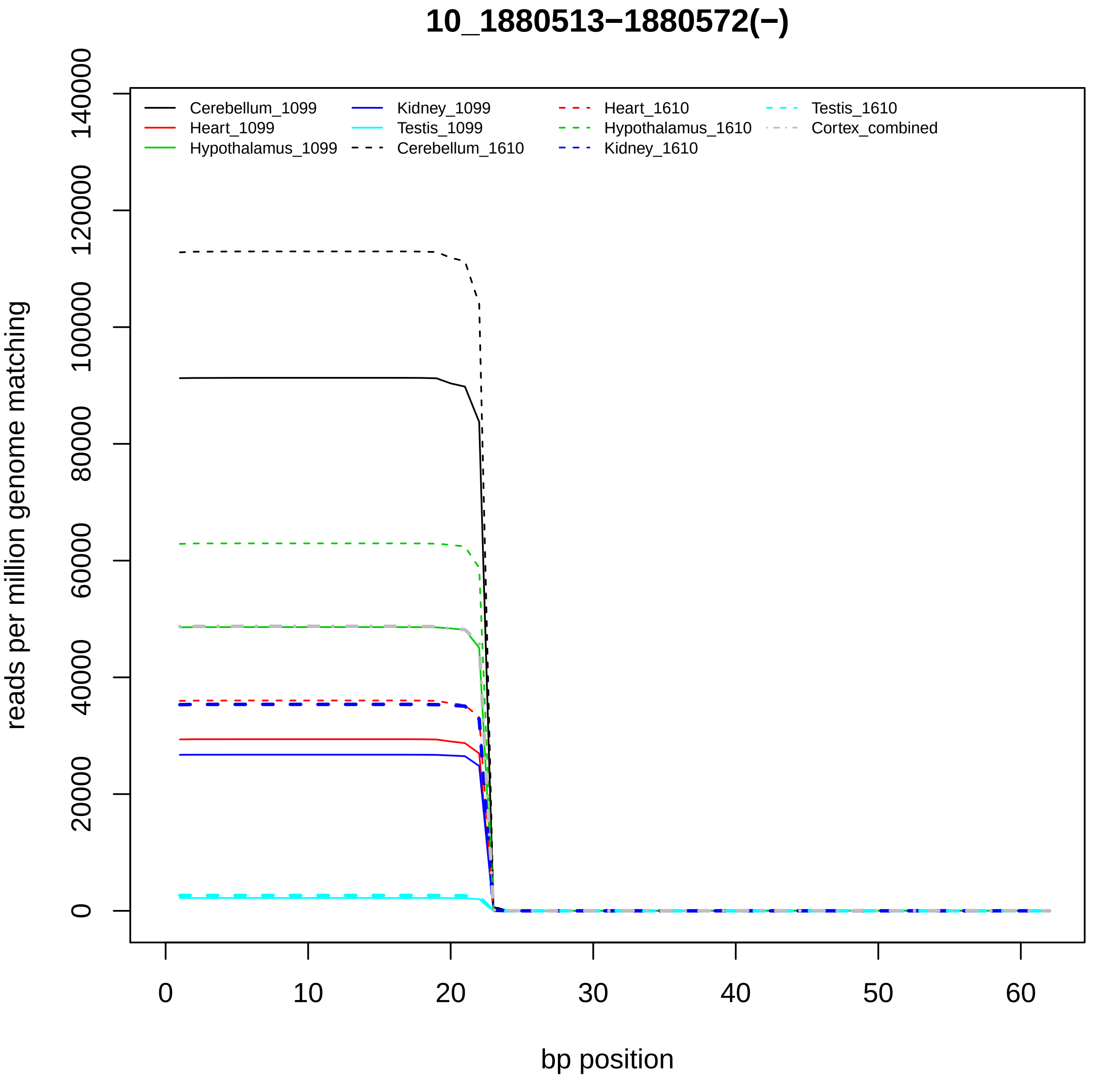

10_1880513−1880572(−)
Cerebellum_1099
Heart_1099
Hypothalamus_1099
Kidney_1099
Testis_1099
Cerebellum_1610
Heart_1610
Hypothalamus_1610
Kidney_1610
Testis_1610
Cortex_combined
reads per million genome matching
bp position
0
20000
40000
60000
80000
100000
120000
140000
0
10
20
30
40
50
60

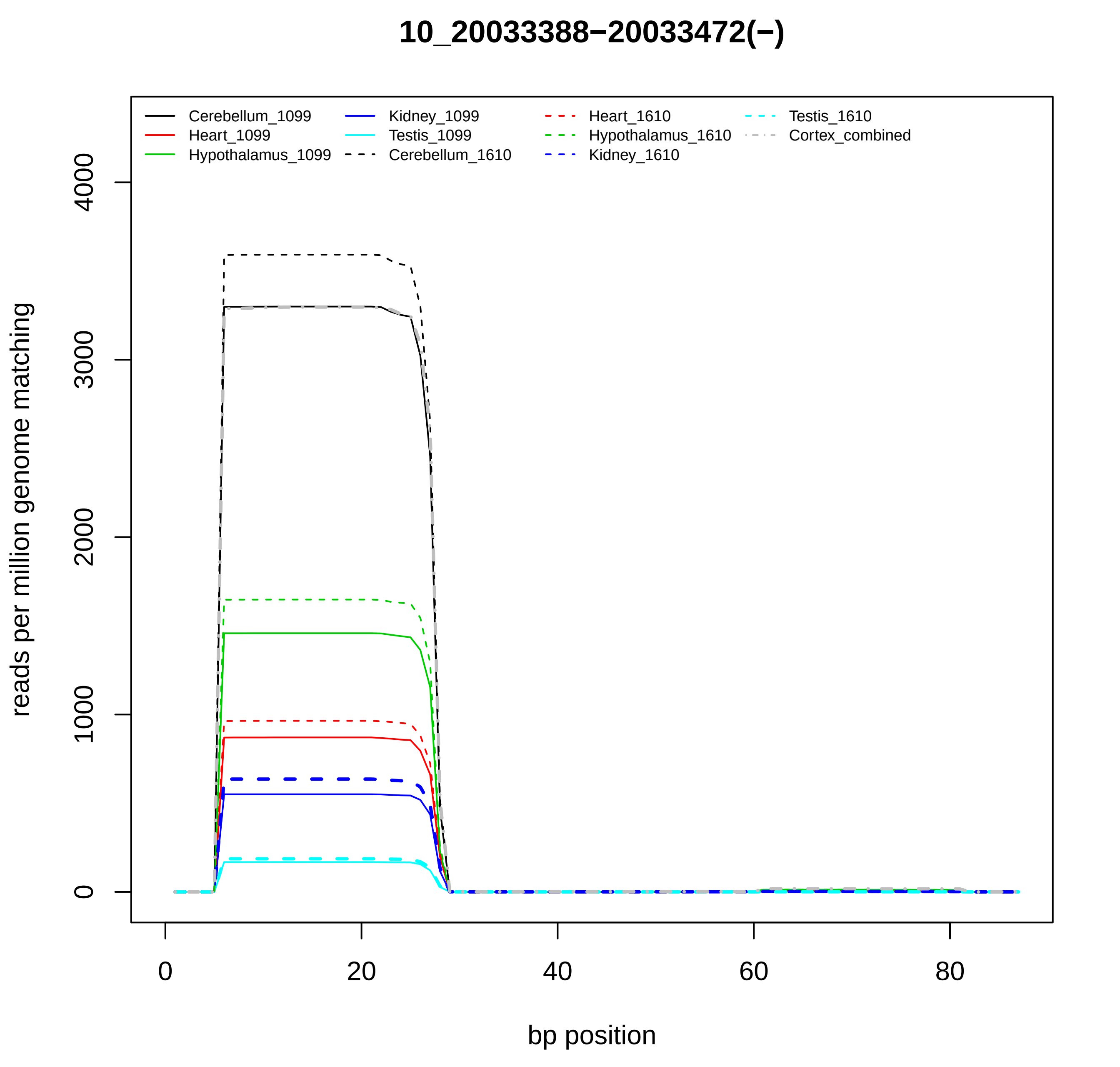
10_20033388−20033472(−)
Cerebellum_1099
Heart_1099
Hypothalamus_1099
Kidney_1099
Testis_1099
Cerebellum_1610
Heart_1610
Hypothalamus_1610
Kidney_1610
Testis_1610
Cortex_combined
reads per million genome matching
0
1000
2000
3000
4000
0
20
40
60
80
bp position

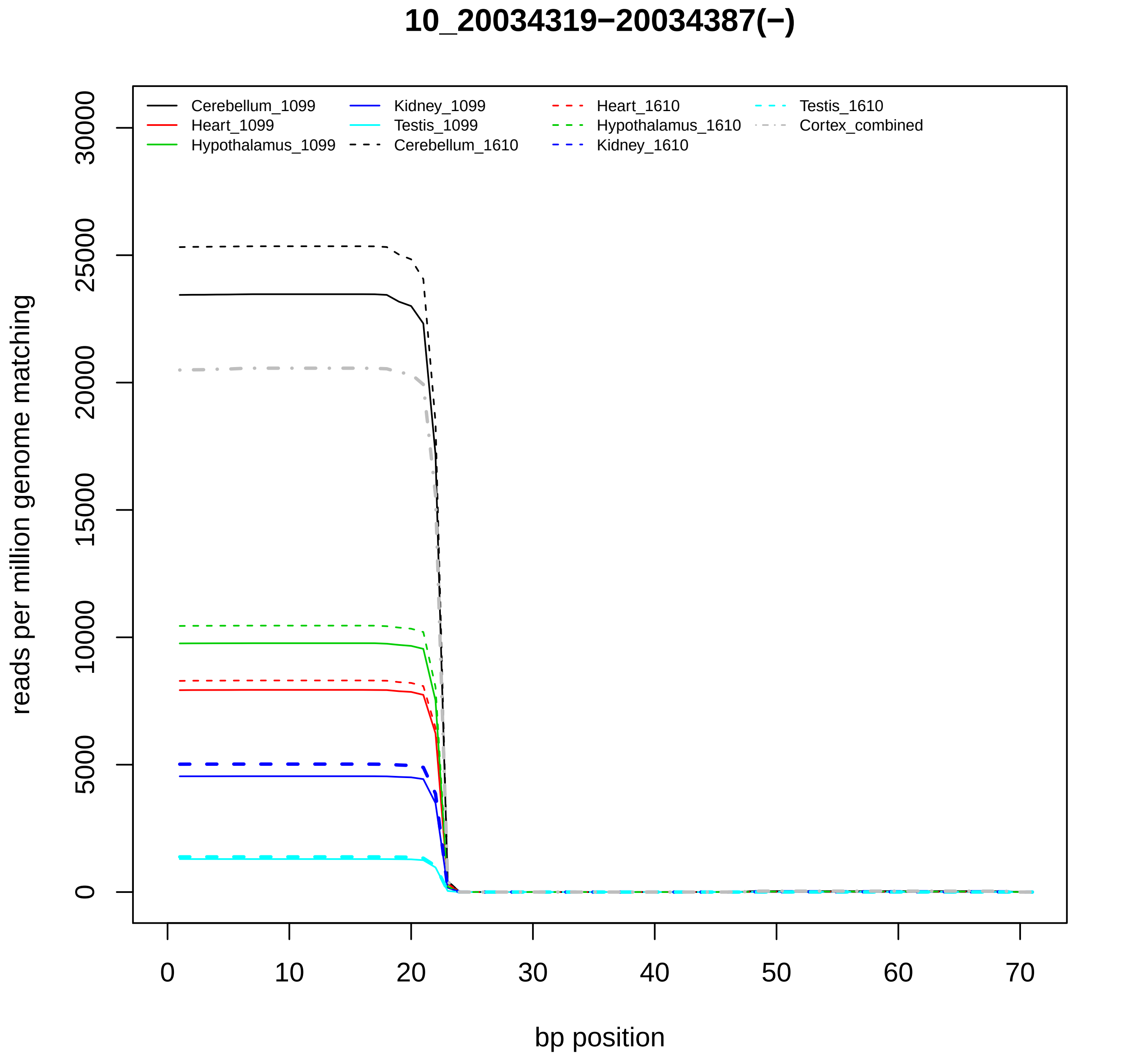

10_20034319−20034387(−)
Cerebellum_1099
Heart_1099
Hypothalamus_1099
Kidney_1099
Testis_1099
Cerebellum_1610
Heart_1610
Hypothalamus_1610
Kidney_1610
Testis_1610
Cortex_combined
reads per million genome matching
0
5000
10000
15000
20000
25000
30000
0
10
20
30
40
50
60
70
bp position

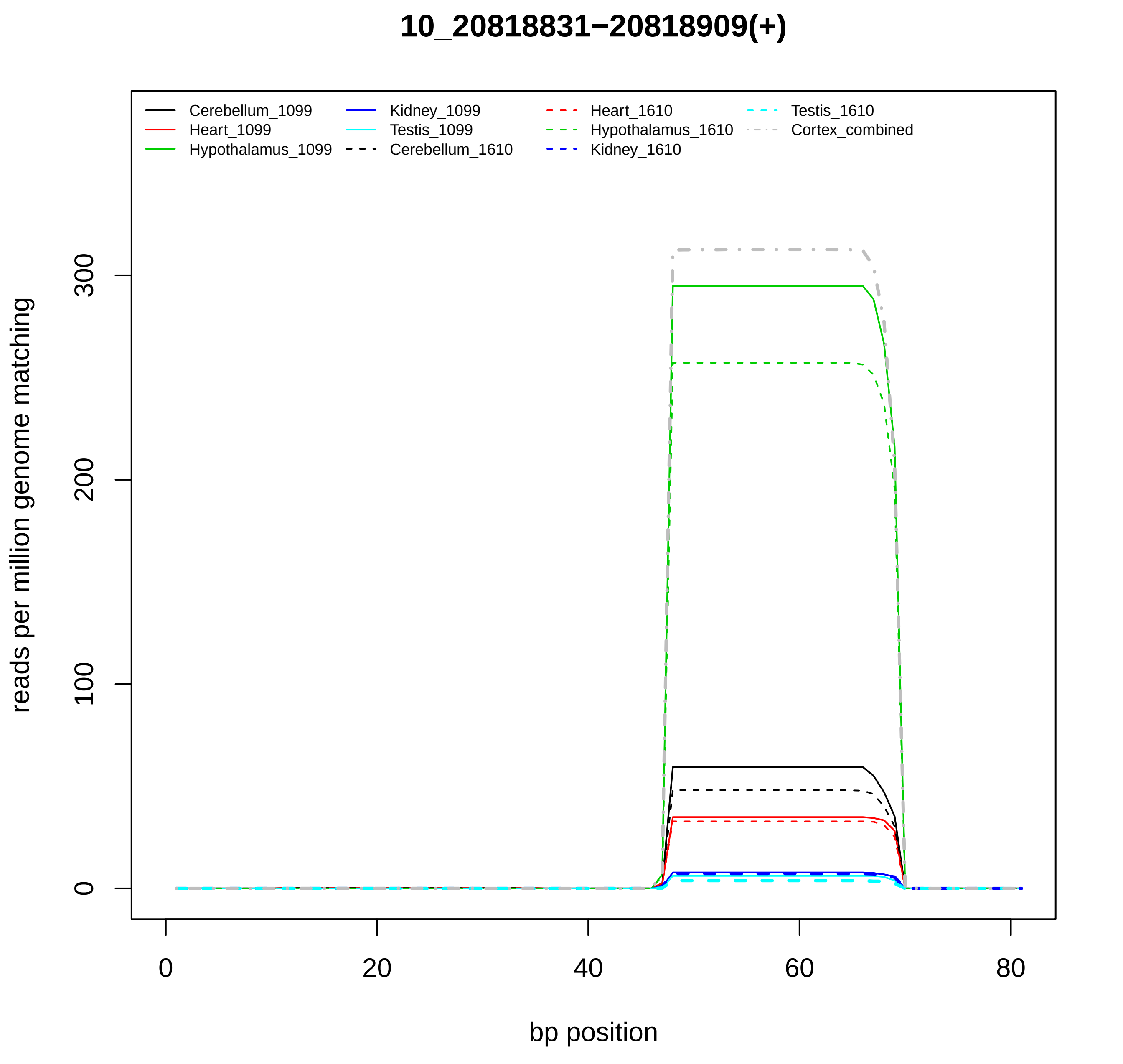

10_20818831−20818909(+)
Cerebellum_1099
Heart_1099
Hypothalamus_1099
Kidney_1099
Testis_1099
Cerebellum_1610
Heart_1610
Hypothalamus_1610
Kidney_1610
Testis_1610
Cortex_combined
reads per million genome matching
0
100
200
300
0
20
40
60
80
bp position

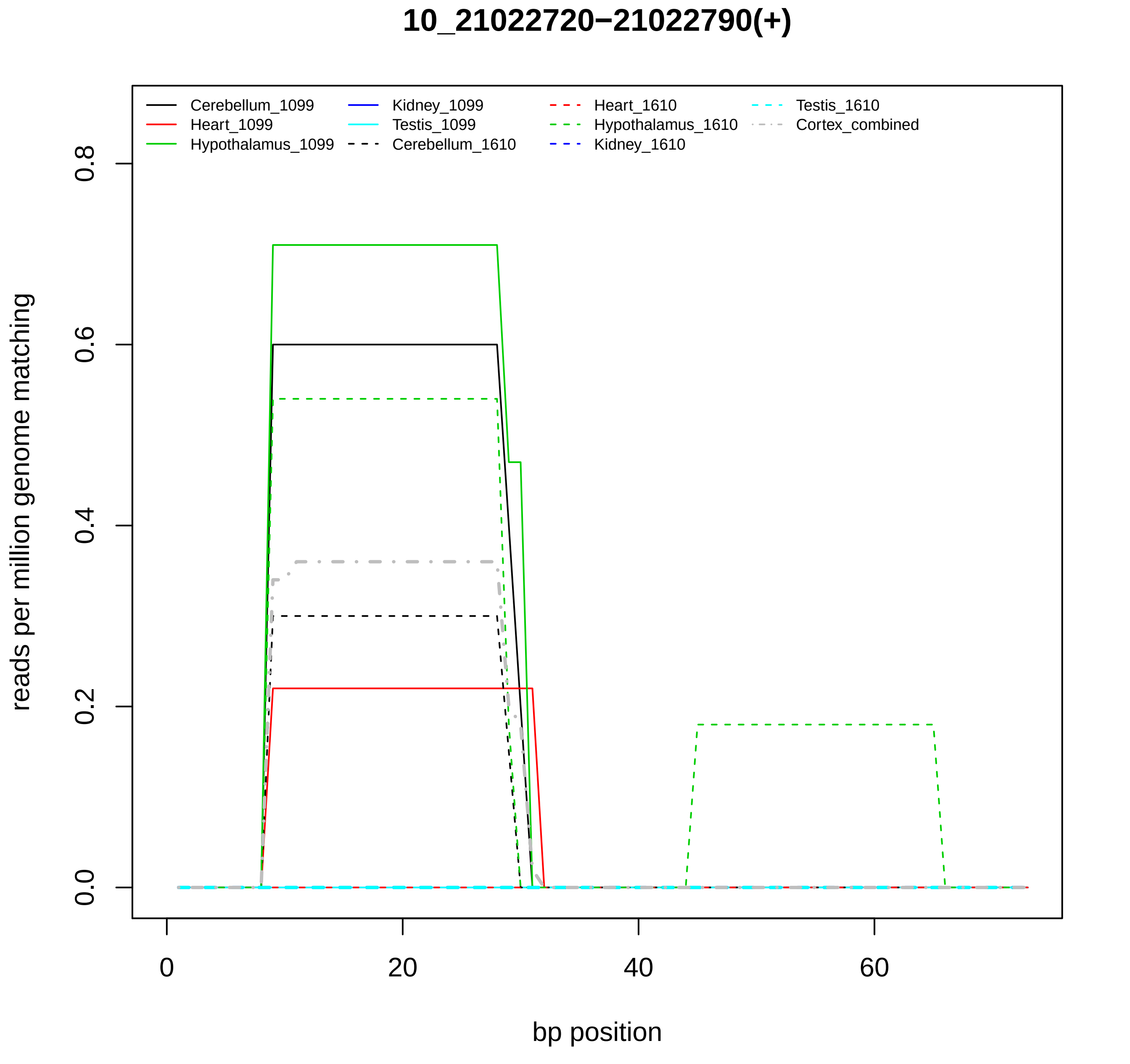

10_21022720−21022790(+)
Cerebellum_1099
Heart_1099
Hypothalamus_1099
Kidney_1099
Testis_1099
Cerebellum_1610
Heart_1610
Hypothalamus_1610
Kidney_1610
Testis_1610
Cortex_combined
reads per million genome matching
0.0
0.2
0.4
0.6
0.8
0
20
40
60
bp position

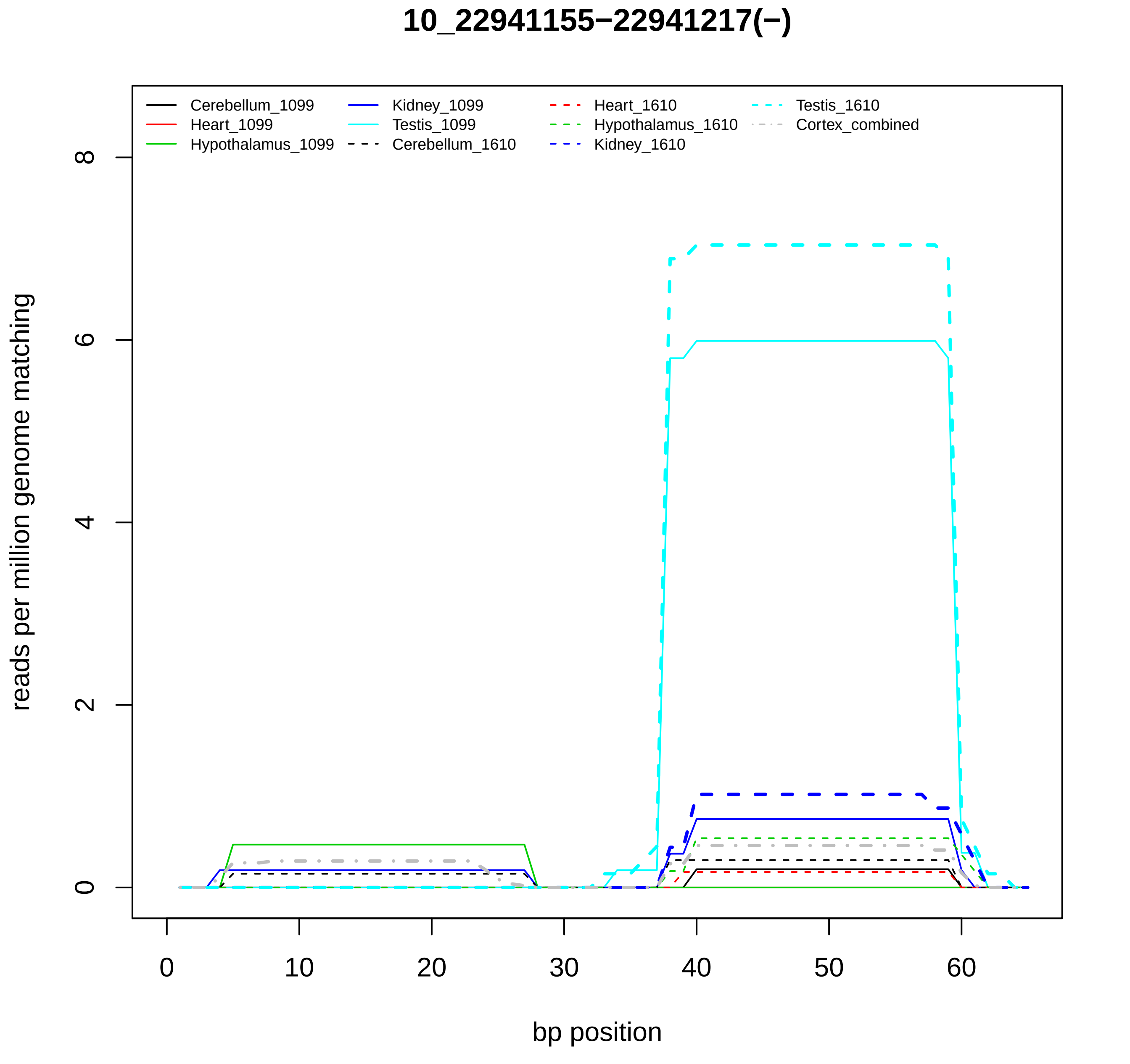
10_22941155−22941217(−)
Cerebellum_1099
Heart_1099
Hypothalamus_1099
Kidney_1099
Testis_1099
Cerebellum_1610
Heart_1610
Hypothalamus_1610
Kidney_1610
Testis_1610
Cortex_combined
reads per million genome matching
0
2
4
6
8
0
10
20
30
40
50
60
bp position

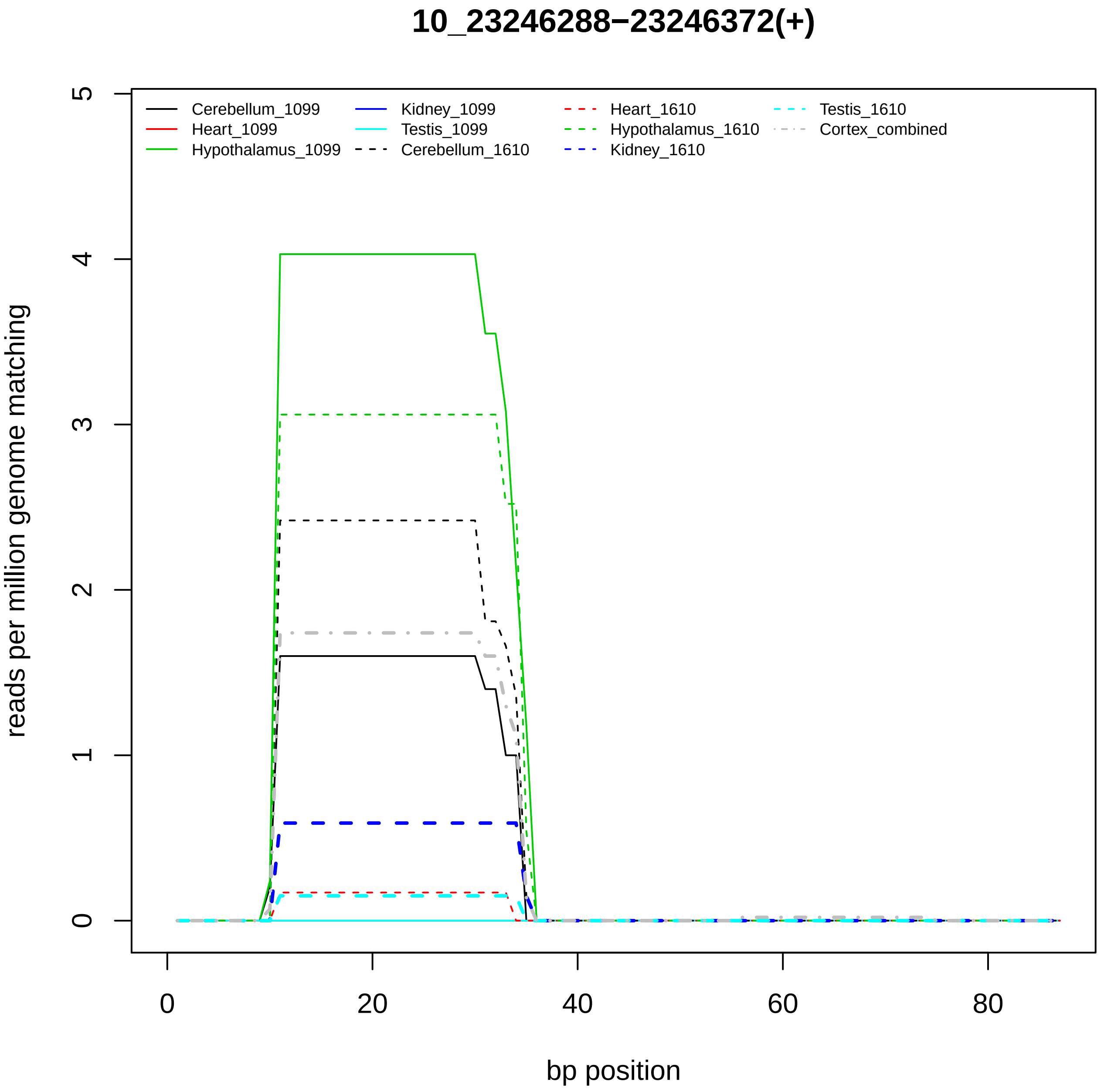

10_23246288−23246372(+)
Cerebellum_1099
Heart_1099
Hypothalamus_1099
Kidney_1099
Testis_1099
Cerebellum_1610
Heart_1610
Hypothalamus_1610
Kidney_1610
Testis_1610
Cortex_combined
reads per million genome matching
bp position
0
1
2
3
4
5
0
20
40
60
80

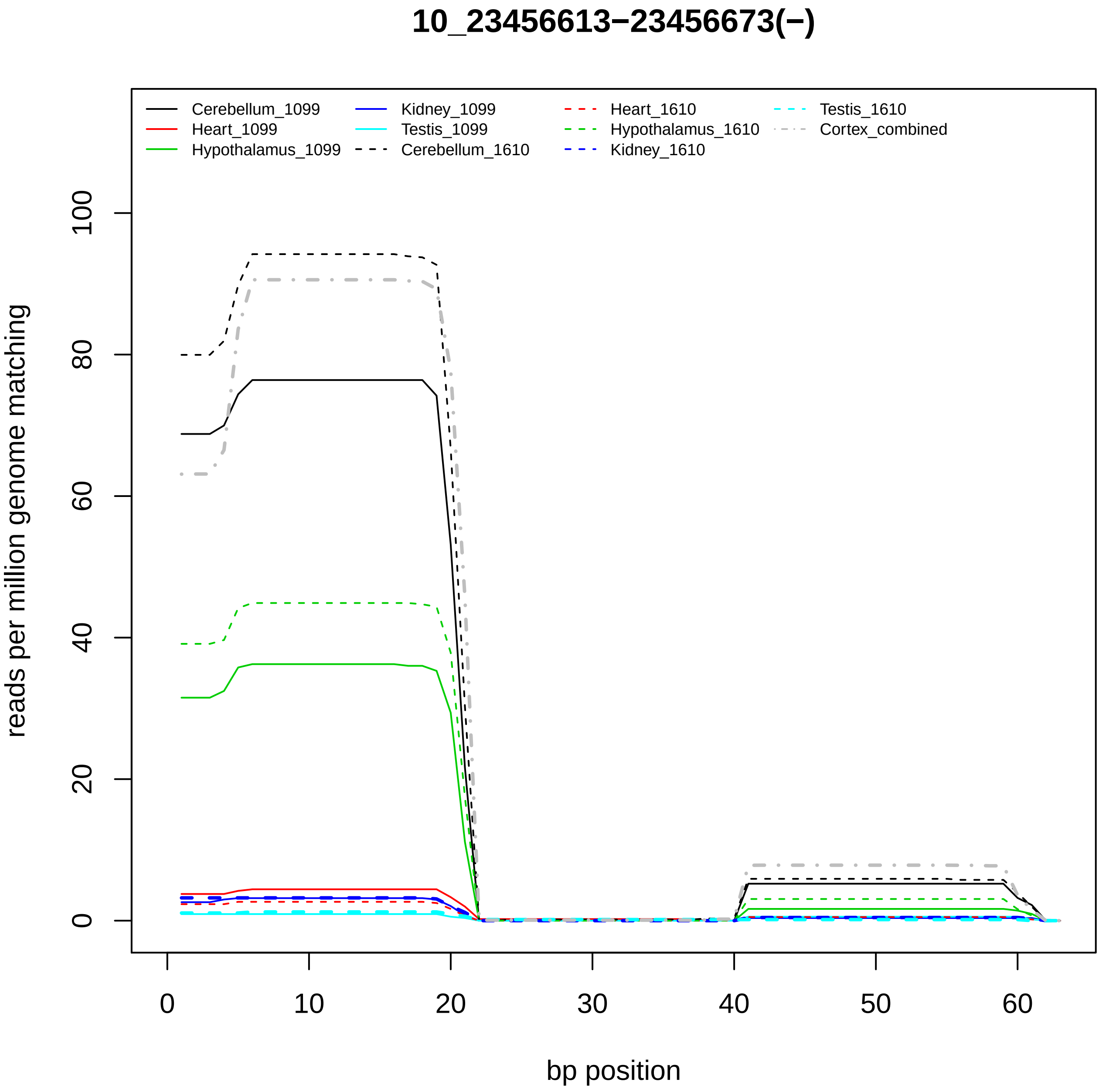

10_23456613−23456673(−)
Cerebellum_1099
Heart_1099
Hypothalamus_1099
Kidney_1099
Testis_1099
Cerebellum_1610
Heart_1610
Hypothalamus_1610
Kidney_1610
Testis_1610
Cortex_combined
reads per million genome matching
bp position
0
20
40
60
80
100
0
10
20
30
40
50
60

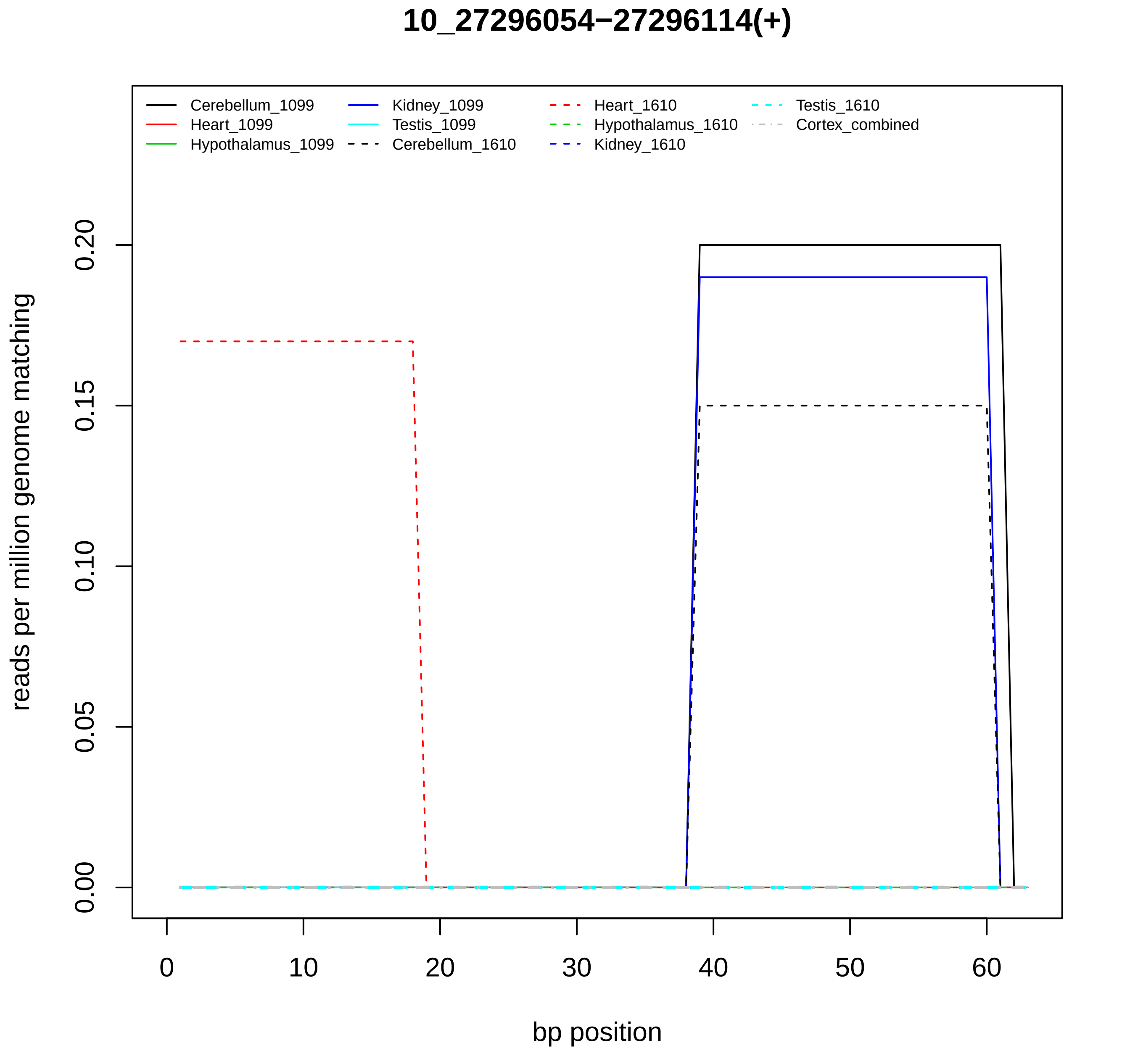

10_27296054−27296114(+)
Cerebellum_1099
Heart_1099
Hypothalamus_1099
Kidney_1099
Testis_1099
Cerebellum_1610
Heart_1610
Hypothalamus_1610
Kidney_1610
Testis_1610
Cortex_combined
reads per million genome matching
0.00
0.05
0.10
0.15
0.20
0
10
20
30
40
50
60
bp position

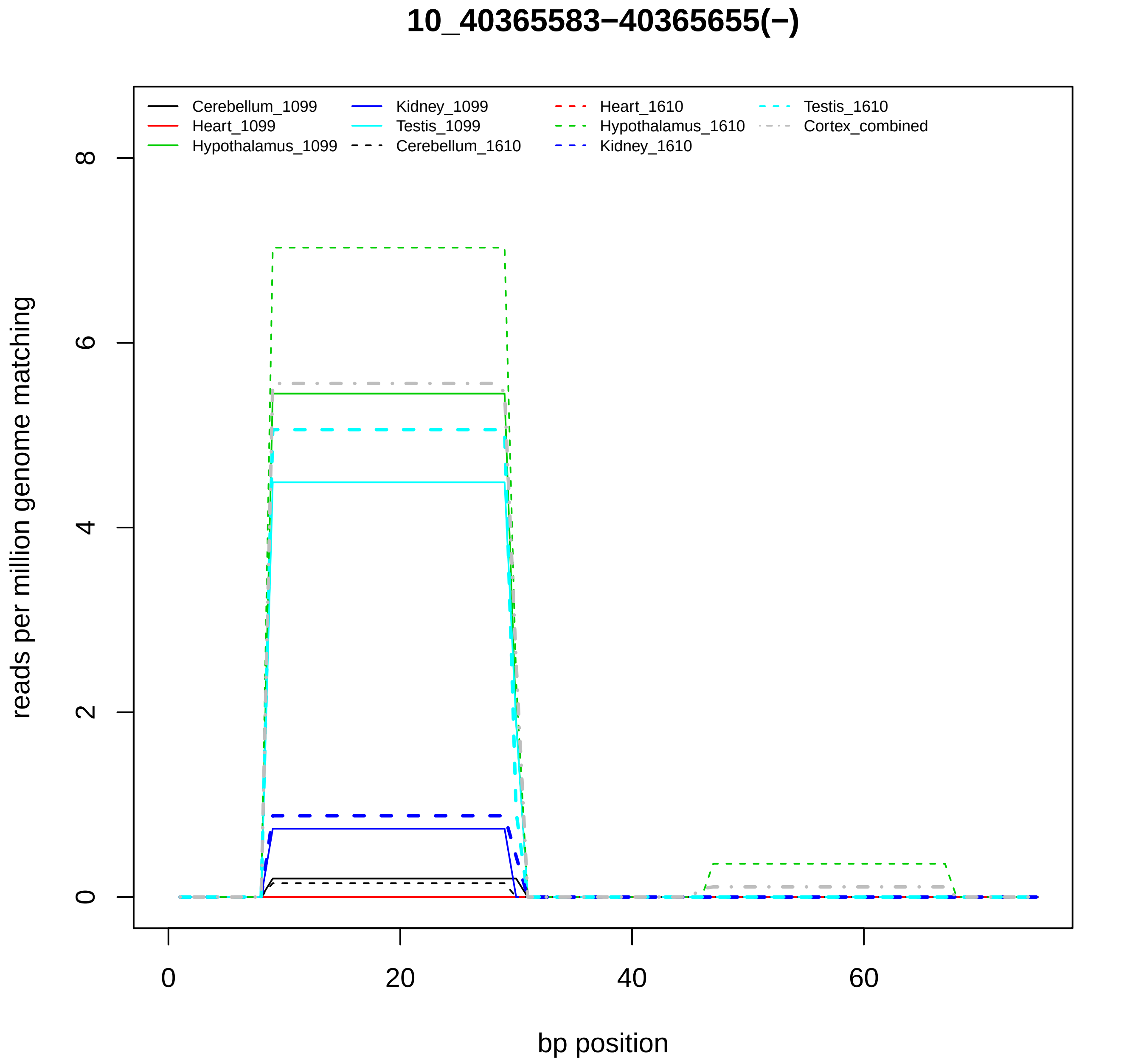

10_40365583−40365655(−)
Cerebellum_1099
Heart_1099
Hypothalamus_1099
Kidney_1099
Testis_1099
Cerebellum_1610
Heart_1610
Hypothalamus_1610
Kidney_1610
Testis_1610
Cortex_combined
reads per million genome matching
bp position
0
2
4
6
8
0
20
40
60

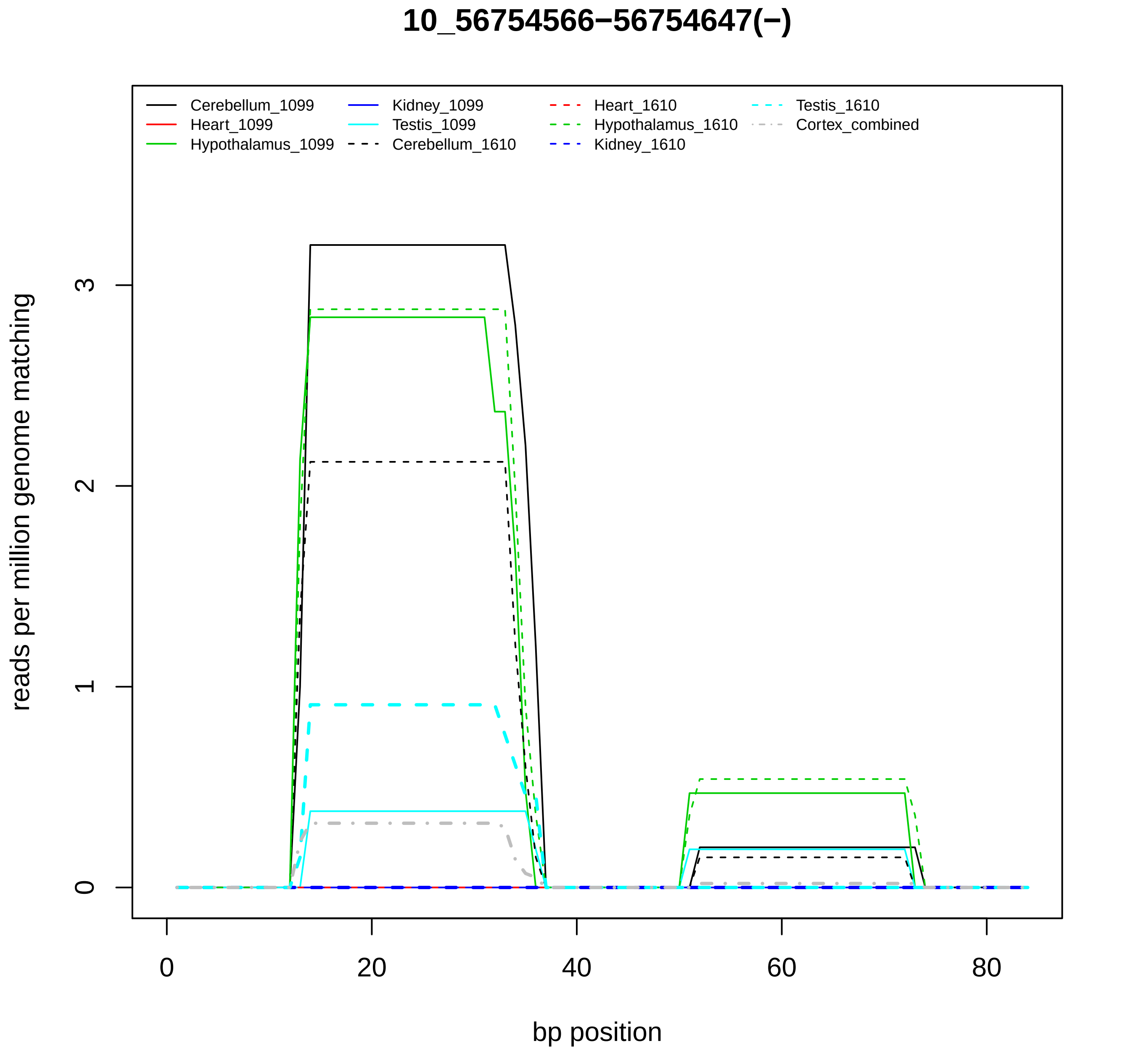

10_56754566−56754647(−)
Cerebellum_1099
Heart_1099
Hypothalamus_1099
Kidney_1099
Testis_1099
Cerebellum_1610
Heart_1610
Hypothalamus_1610
Kidney_1610
Testis_1610
Cortex_combined
reads per million genome matching
0
1
2
3
0
20
40
60
80
bp position

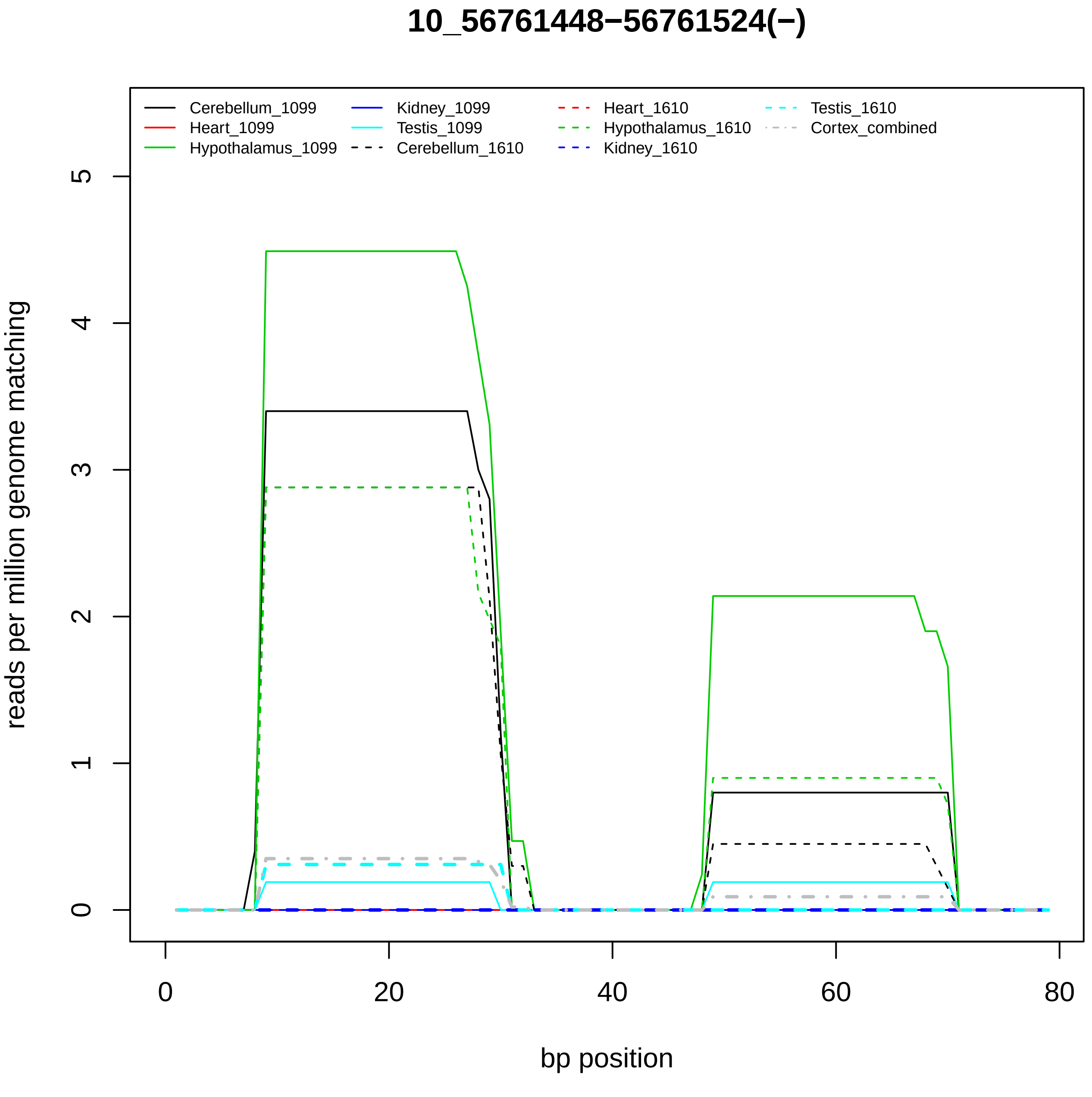

10_56761448−56761524(−)
Cerebellum_1099
Heart_1099
Hypothalamus_1099
Kidney_1099
Testis_1099
Cerebellum_1610
Heart_1610
Hypothalamus_1610
Kidney_1610
Testis_1610
Cortex_combined
reads per million genome matching
bp position
0
1
2
3
4
5
0
20
40
60
80

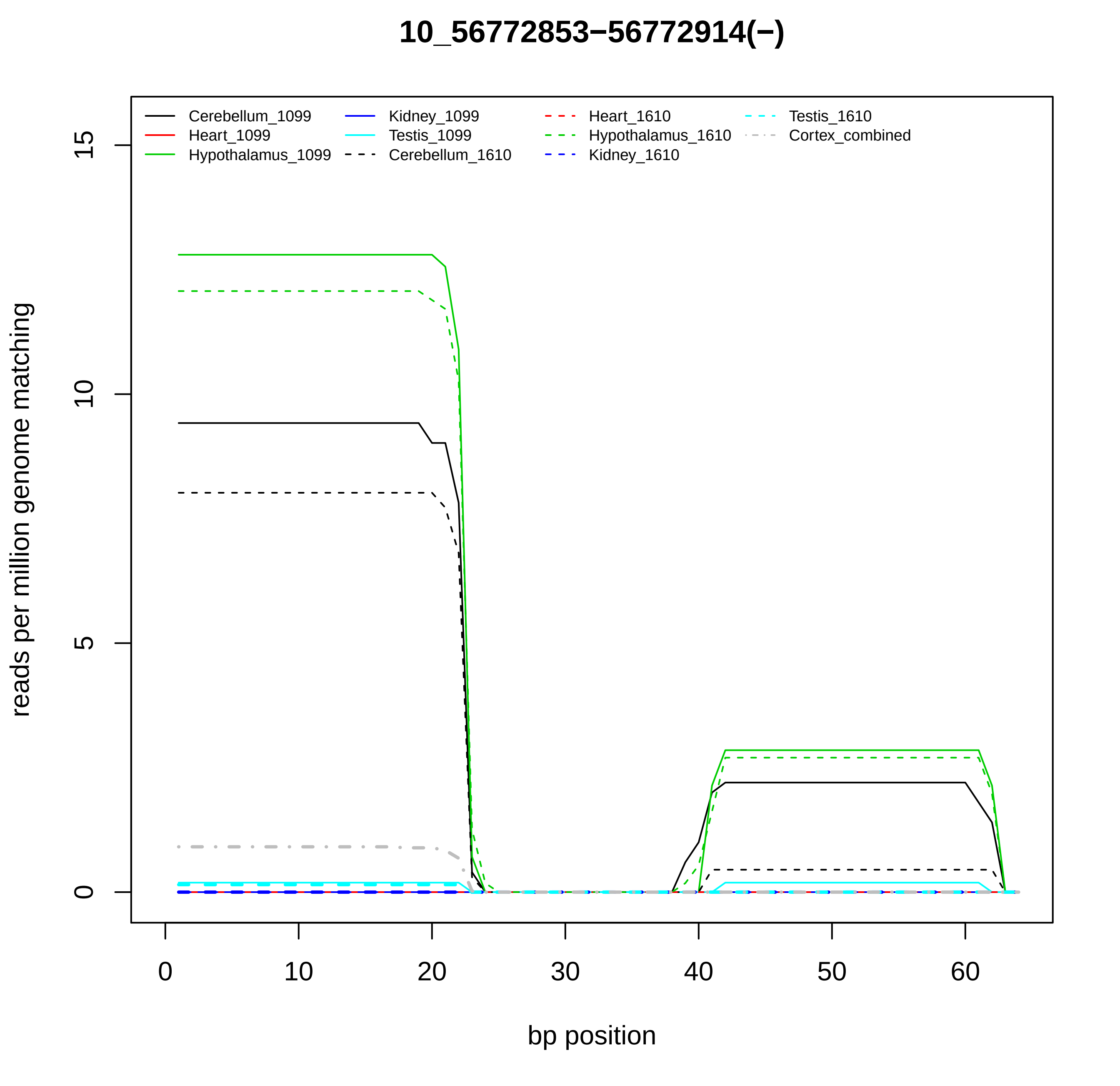

10_56772853−56772914(−)
Cerebellum_1099
Heart_1099
Hypothalamus_1099
Kidney_1099
Testis_1099
Cerebellum_1610
Heart_1610
Hypothalamus_1610
Kidney_1610
Testis_1610
Cortex_combined
reads per million genome matching
bp position
0
5
10
15
0
10
20
30
40
50
60

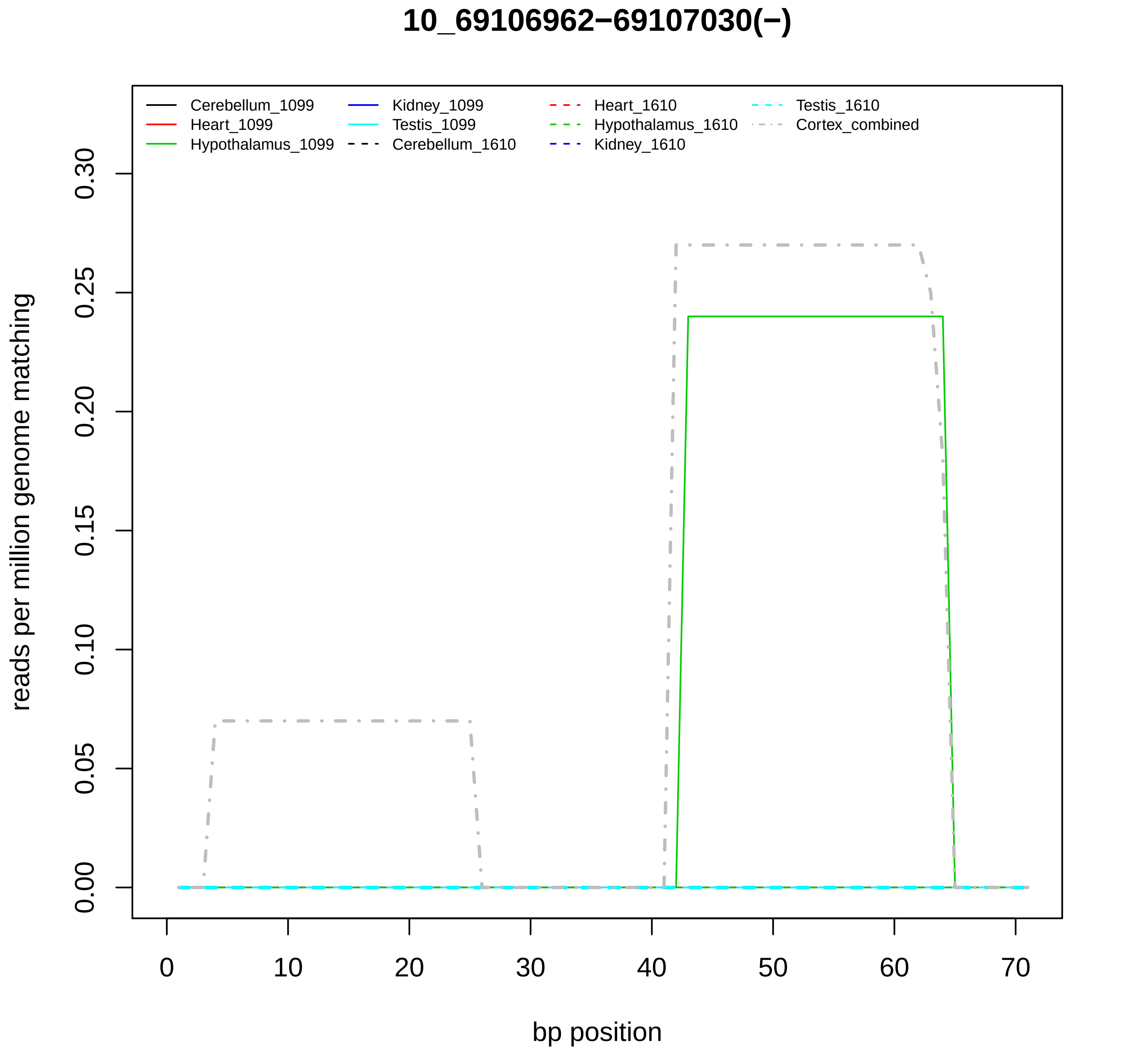
10_69106962−69107030(−)
Cerebellum_1099
Heart_1099
Hypothalamus_1099
Kidney_1099
Testis_1099
Cerebellum_1610
Heart_1610
Hypothalamus_1610
Kidney_1610
Testis_1610
Cortex_combined
reads per million genome matching
0.00
0.05
0.10
0.15
0.20
0.25
0.30
0
10
20
30
40
50
60
70
bp position

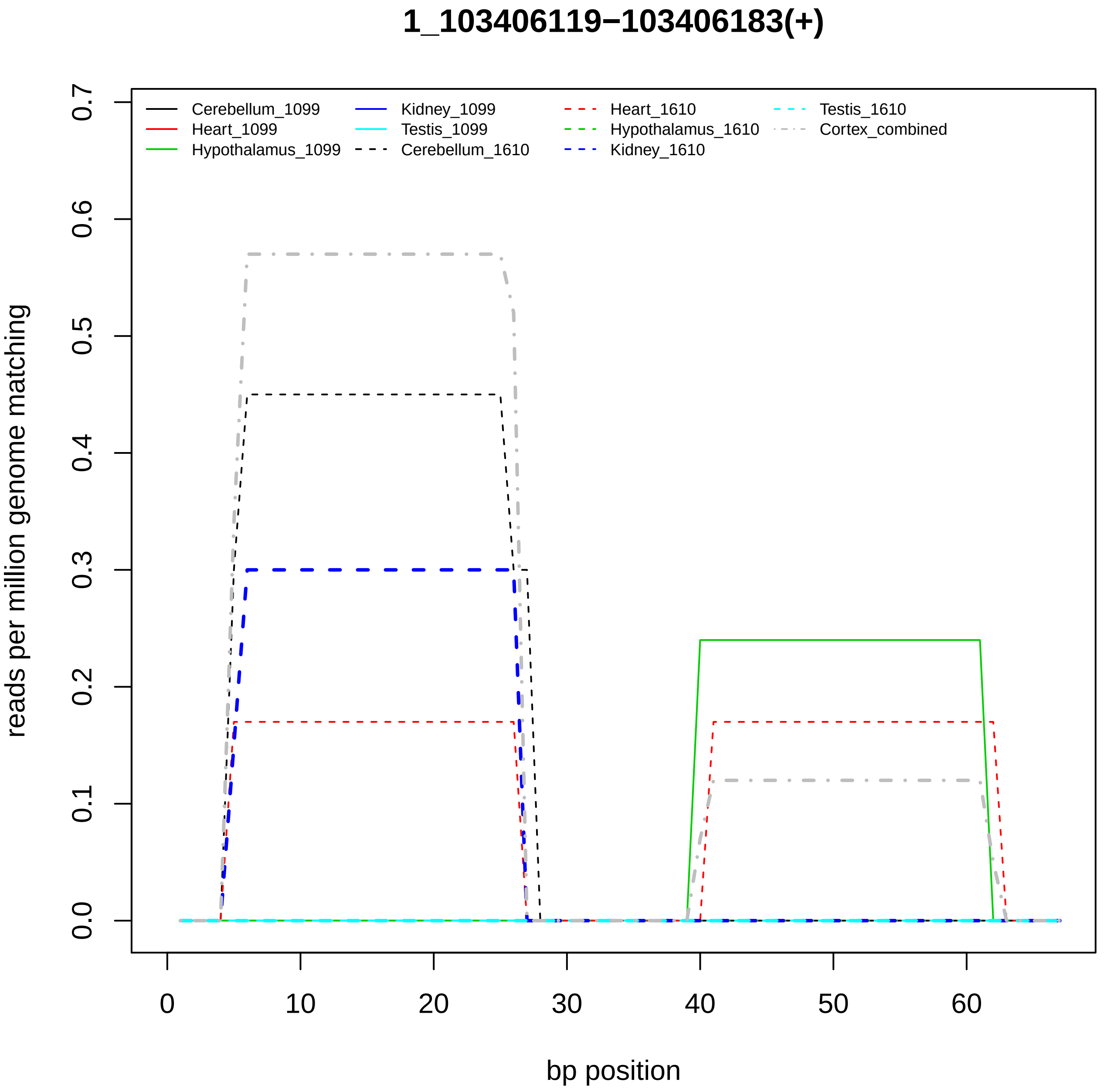

1_103406119−103406183(+)
Cerebellum_1099
Heart_1099
Hypothalamus_1099
Kidney_1099
Testis_1099
Cerebellum_1610
Heart_1610
Hypothalamus_1610
Kidney_1610
Testis_1610
Cortex_combined
reads per million genome matching
bp position
0.0
0.1
0.2
0.3
0.4
0.5
0.6
0.7
0
10
20
30
40
50
60

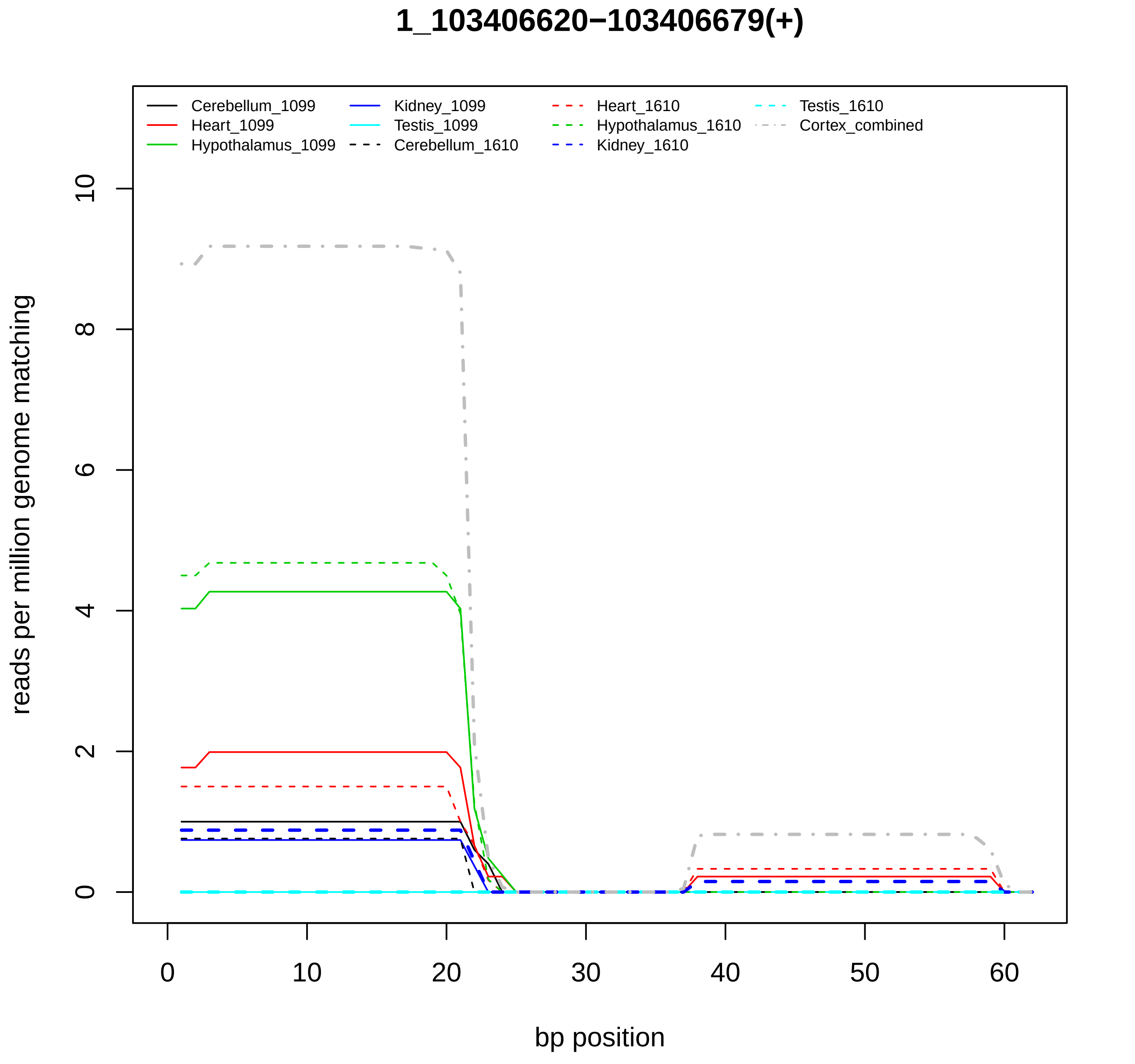

1_103406620−103406679(+)
Cerebellum_1099
Heart_1099
Hypothalamus_1099
Kidney_1099
Testis_1099
Cerebellum_1610
Heart_1610
Hypothalamus_1610
Kidney_1610
Testis_1610
Cortex_combined
reads per million genome matching
0
2
4
6
8
10
0
10
20
30
40
50
60
bp position

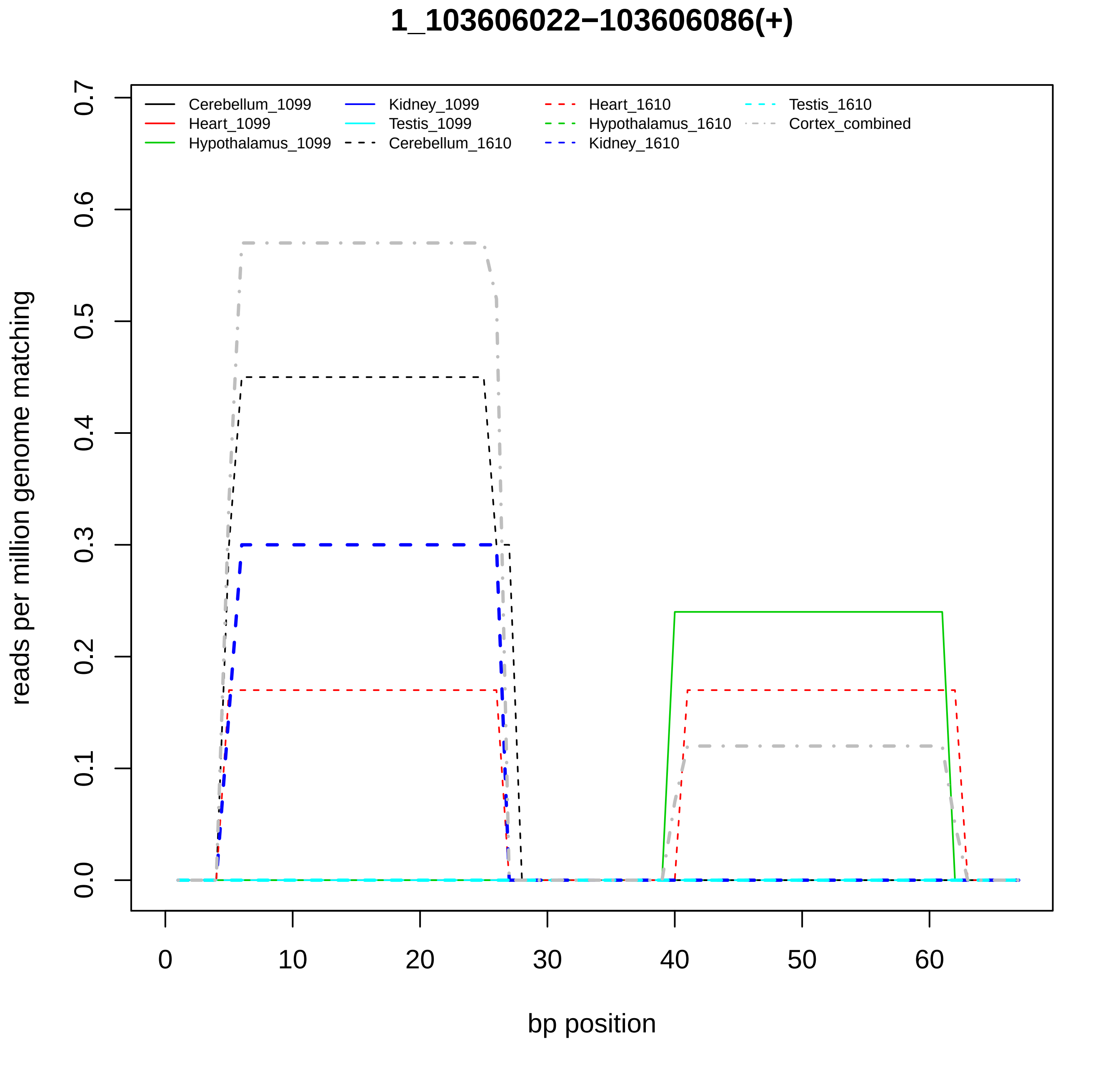

1_103606022−103606086(+)
Cerebellum_1099
Heart_1099
Hypothalamus_1099
Kidney_1099
Testis_1099
Cerebellum_1610
Heart_1610
Hypothalamus_1610
Kidney_1610
Testis_1610
Cortex_combined
reads per million genome matching
bp position
0.0
0.1
0.2
0.3
0.4
0.5
0.6
0.7
0
10
20
30
40
50
60

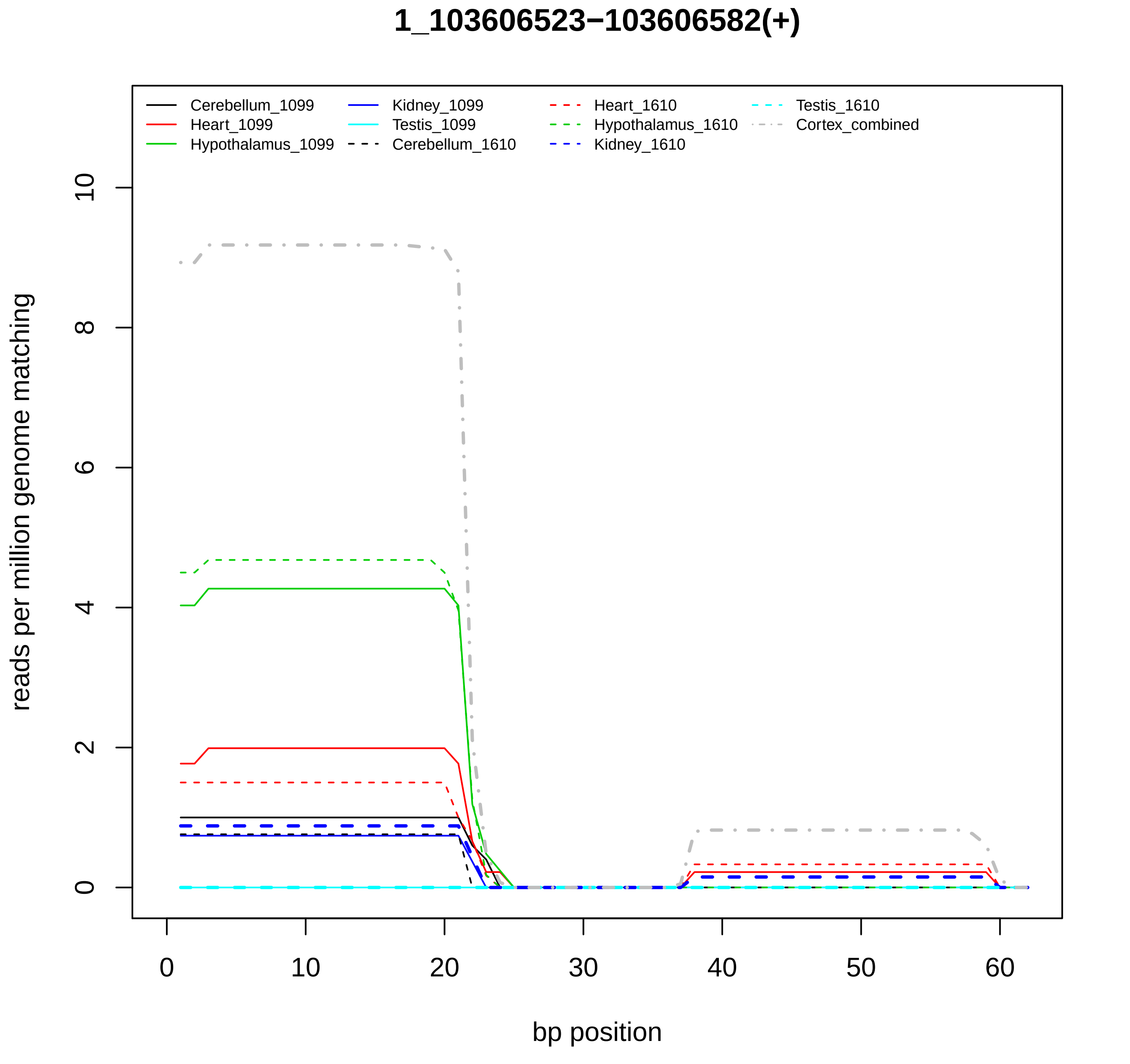

1_103606523−103606582(+)
Cerebellum_1099
Heart_1099
Hypothalamus_1099
Kidney_1099
Testis_1099
Cerebellum_1610
Heart_1610
Hypothalamus_1610
Kidney_1610
Testis_1610
Cortex_combined
reads per million genome matching
bp position
0
2
4
6
8
10
0
10
20
30
40
50
60

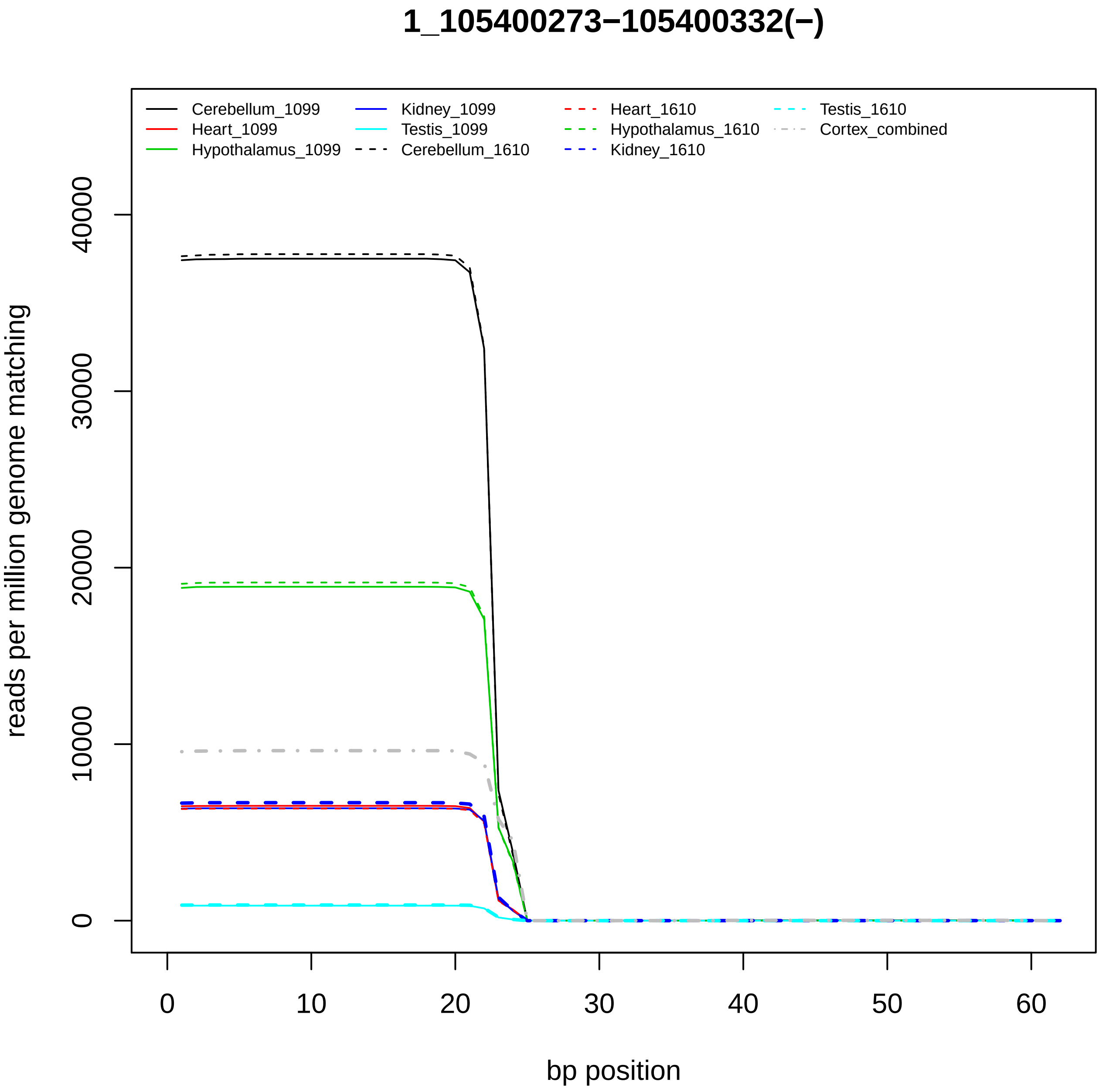
1_105400273−105400332(−)
Cerebellum_1099
Heart_1099
Hypothalamus_1099
Kidney_1099
Testis_1099
Cerebellum_1610
Heart_1610
Hypothalamus_1610
Kidney_1610
Testis_1610
Cortex_combined
reads per million genome matching
0
10000
20000
30000
40000
bp position
0
10
20
30
40
50
60

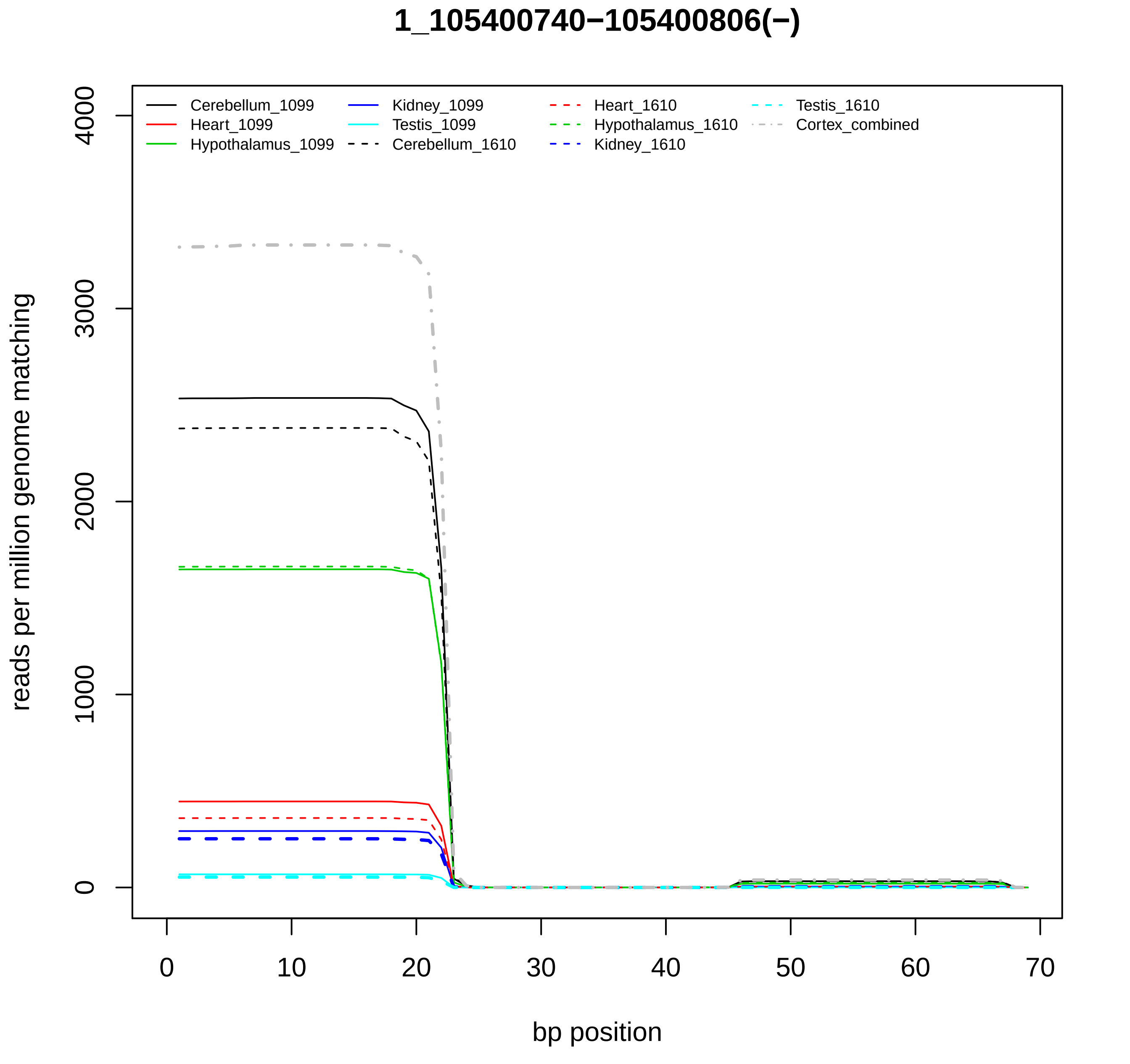

1_105400740−105400806(−)
Cerebellum_1099
Heart_1099
Hypothalamus_1099
Kidney_1099
Testis_1099
Cerebellum_1610
Heart_1610
Hypothalamus_1610
Kidney_1610
Testis_1610
Cortex_combined
reads per million genome matching
0
1000
2000
3000
4000
0
10
20
30
40
50
60
70
bp position

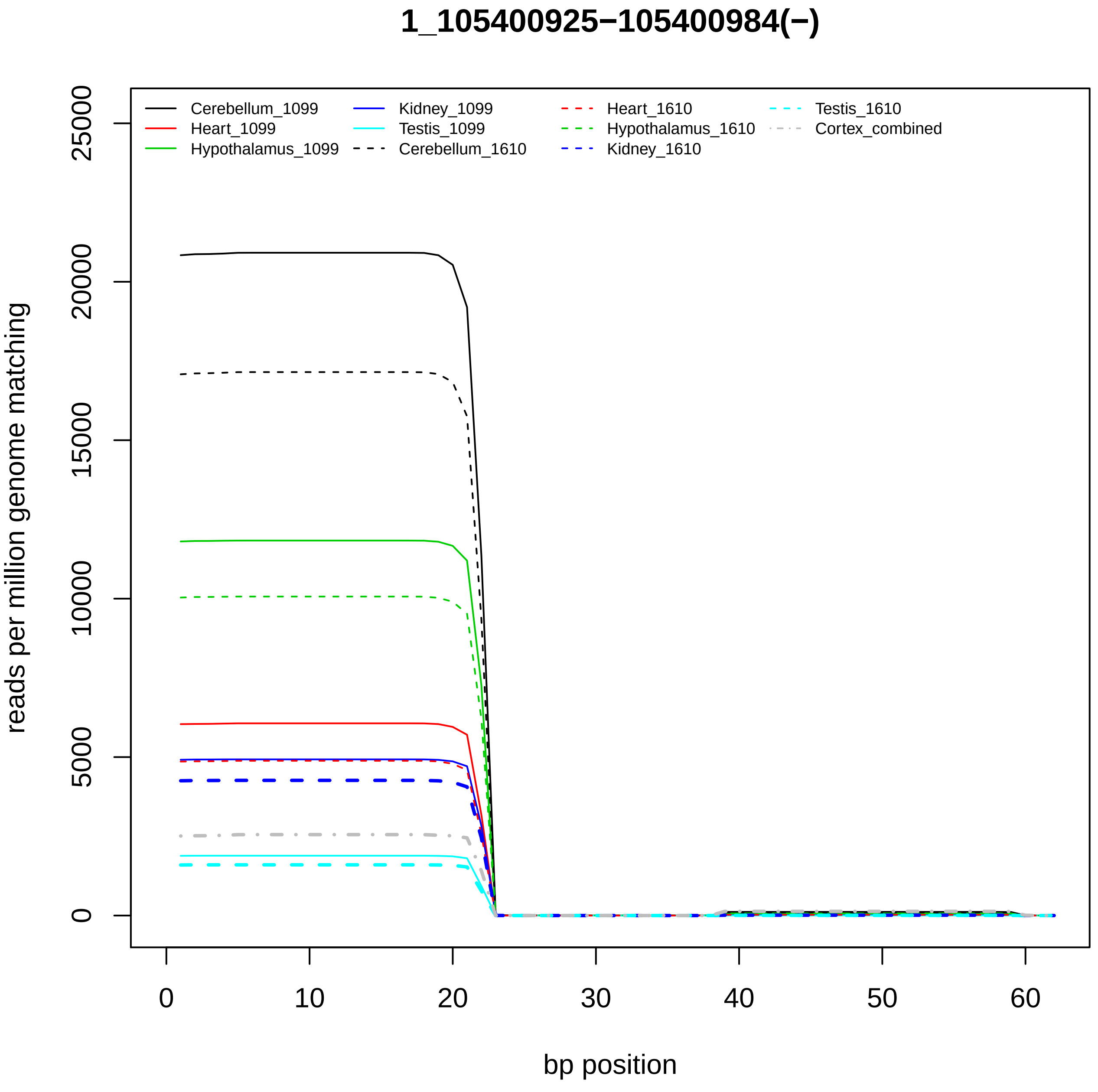
1_105400925−105400984(−)
Cerebellum_1099
Heart_1099
Hypothalamus_1099
Kidney_1099
Testis_1099
Cerebellum_1610
Heart_1610
Hypothalamus_1610
Kidney_1610
Testis_1610
Cortex_combined
reads per million genome matching
0
5000
10000
15000
20000
25000
0
10
20
30
40
50
60
bp position

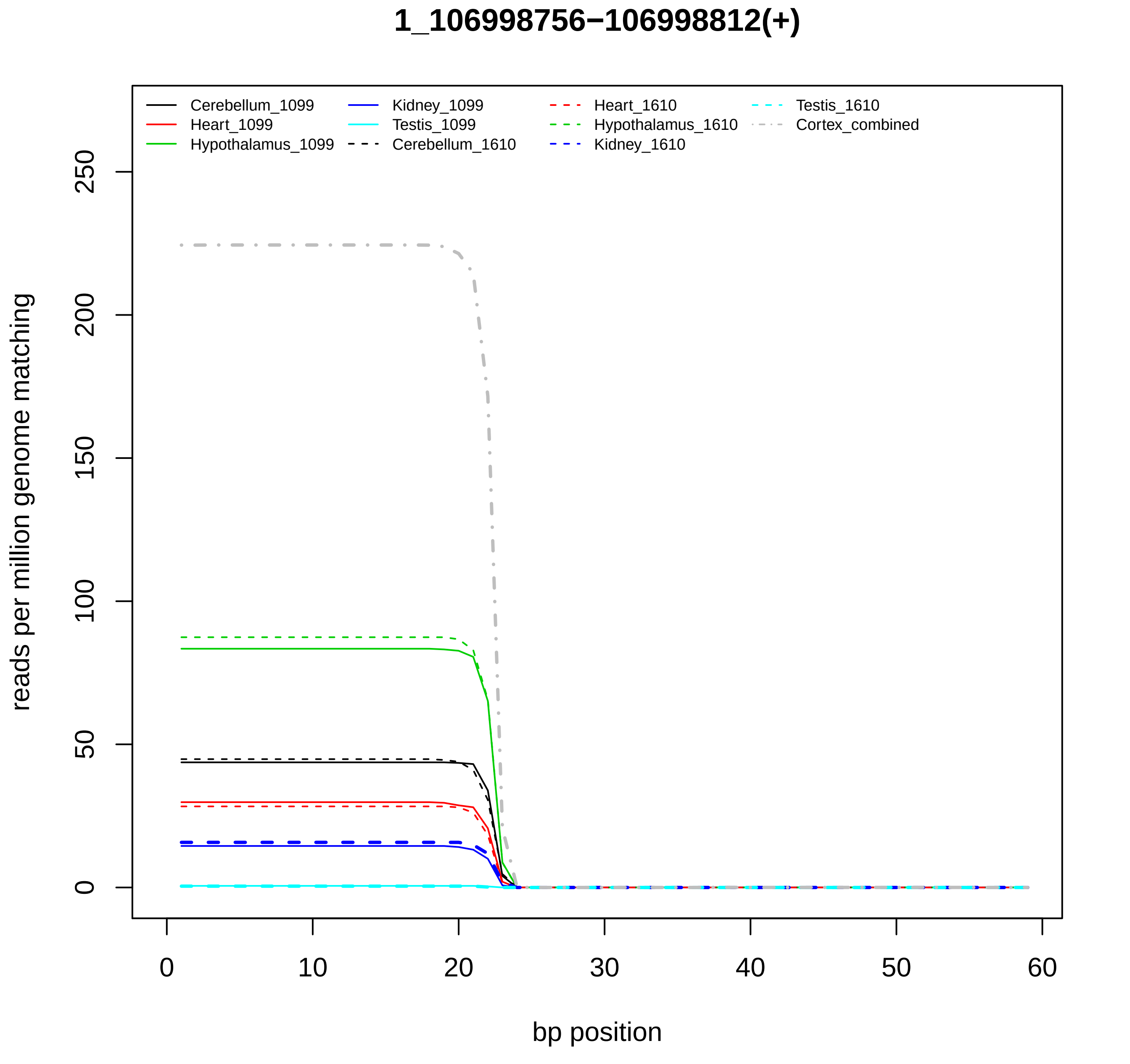
1_106998756−106998812(+)
Cerebellum_1099
Heart_1099
Hypothalamus_1099
Kidney_1099
Testis_1099
Cerebellum_1610
Heart_1610
Hypothalamus_1610
Kidney_1610
Testis_1610
Cortex_combined
reads per million genome matching
bp position
0
50
100
150
200
250
0
10
20
30
40
50
60

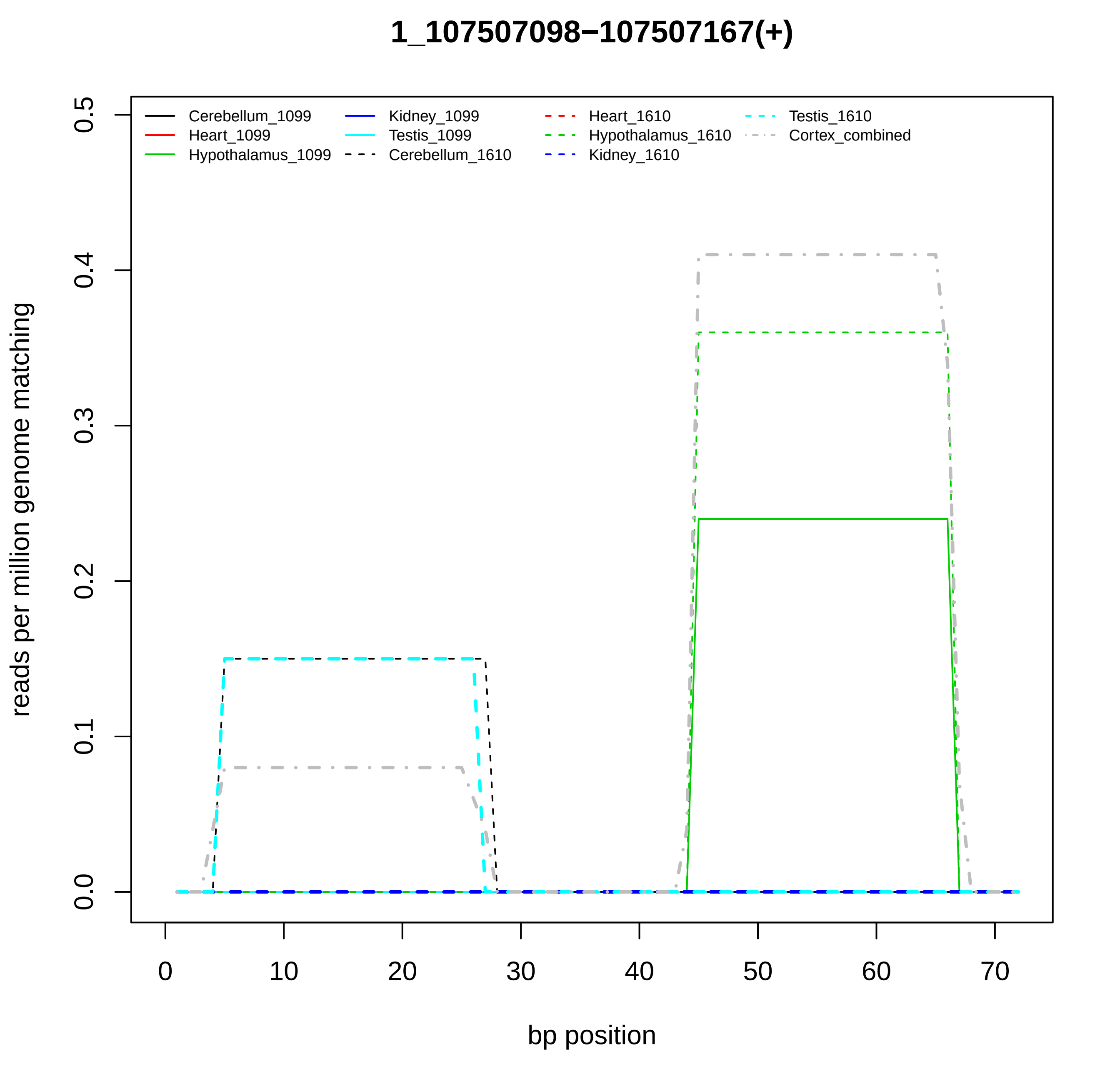

1_107507098−107507167(+)
Cerebellum_1099
Heart_1099
Hypothalamus_1099
Kidney_1099
Testis_1099
Cerebellum_1610
Heart_1610
Hypothalamus_1610
Kidney_1610
Testis_1610
Cortex_combined
reads per million genome matching
bp position
0.0
0.1
0.2
0.3
0.4
0.5
0
10
20
30
40
50
60
70

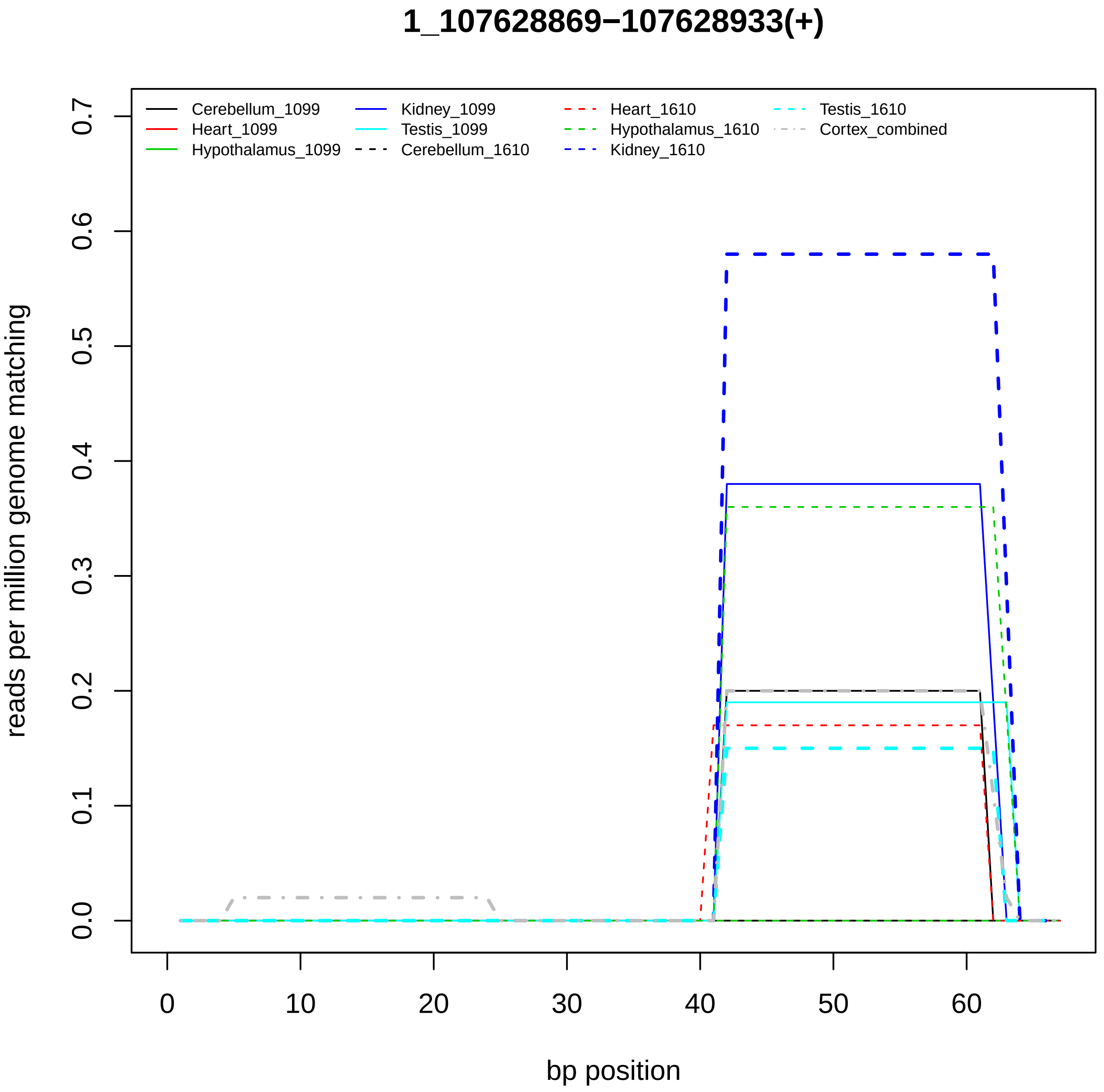
1_107628869−107628933(+)
Cerebellum_1099
Heart_1099
Hypothalamus_1099
Kidney_1099
Testis_1099
Cerebellum_1610
Heart_1610
Hypothalamus_1610
Kidney_1610
Testis_1610
Cortex_combined
reads per million genome matching
bp position
0.0
0.1
0.2
0.3
0.4
0.5
0.6
0.7
0
10
20
30
40
50
60

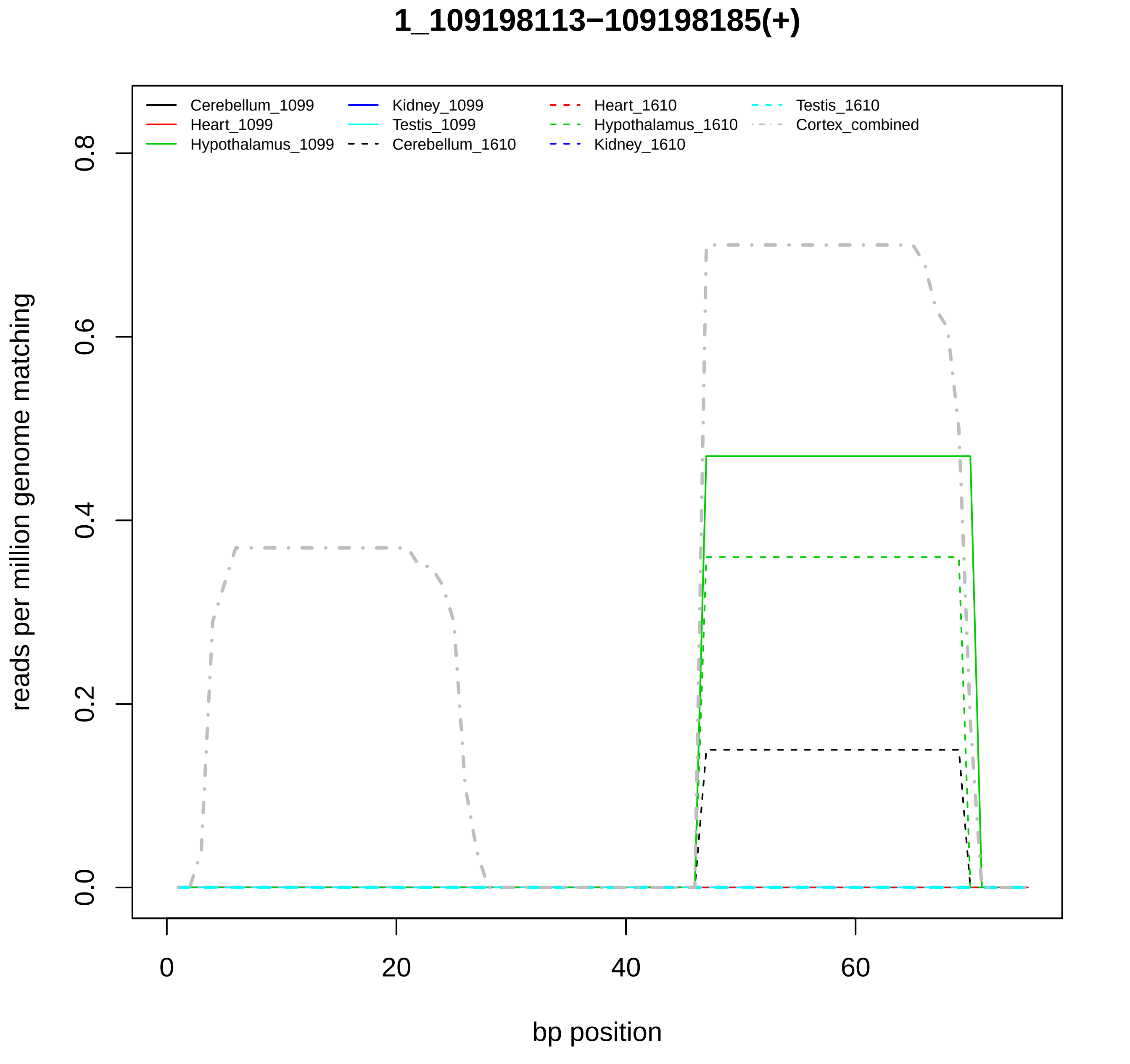

1_109198113−109198185(+)
Cerebellum_1099
Heart_1099
Hypothalamus_1099
Kidney_1099
Testis_1099
Cerebellum_1610
Heart_1610
Hypothalamus_1610
Kidney_1610
Testis_1610
Cortex_combined
reads per million genome matching
0.0
0.2
0.4
0.6
0.8
0
20
40
60
bp position

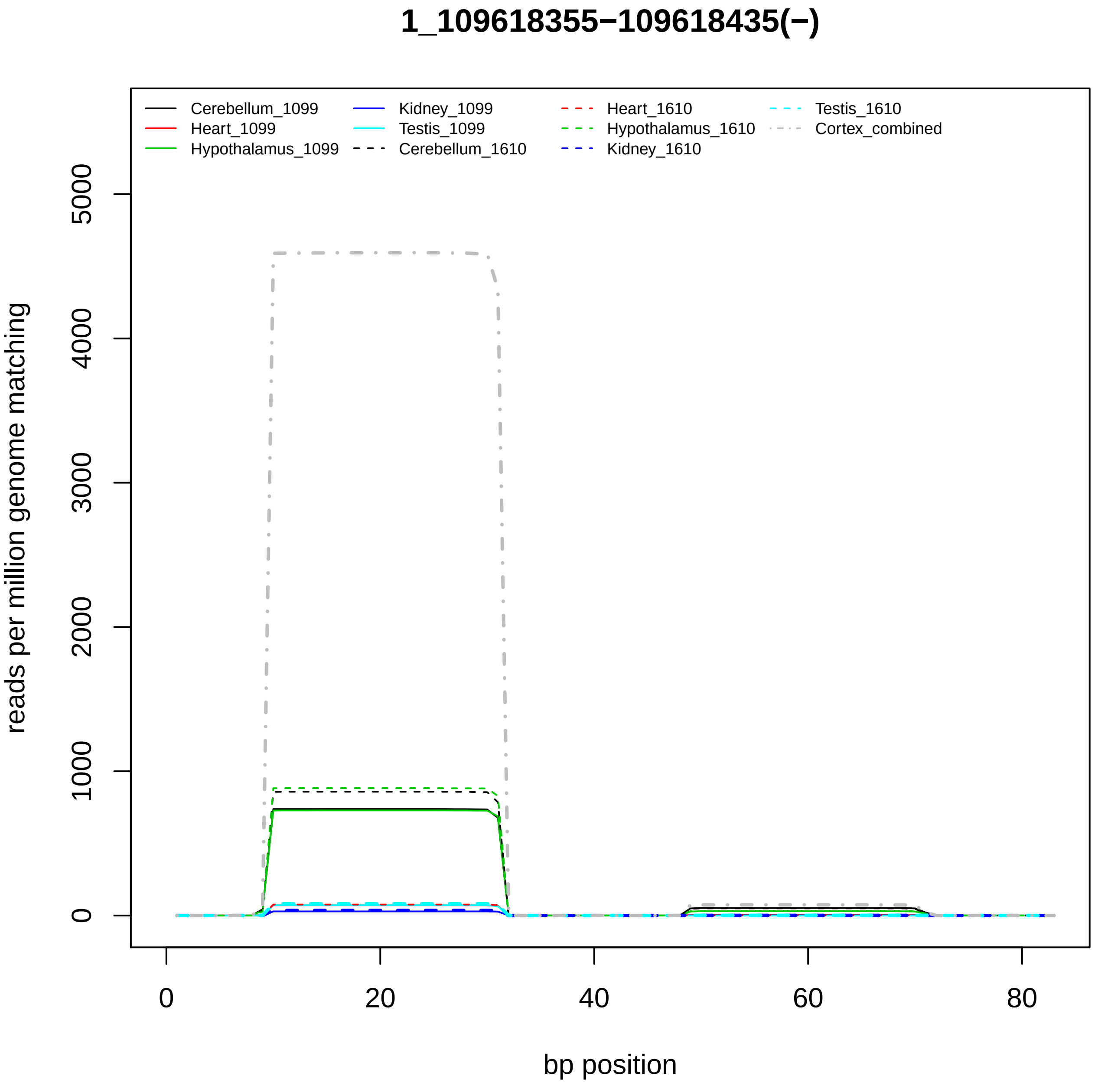

1_109618355−109618435(−)
Cerebellum_1099
Heart_1099
Hypothalamus_1099
Kidney_1099
Testis_1099
Cerebellum_1610
Heart_1610
Hypothalamus_1610
Kidney_1610
Testis_1610
Cortex_combined
reads per million genome matching
0
1000
2000
3000
4000
5000
0
20
40
60
80
bp position

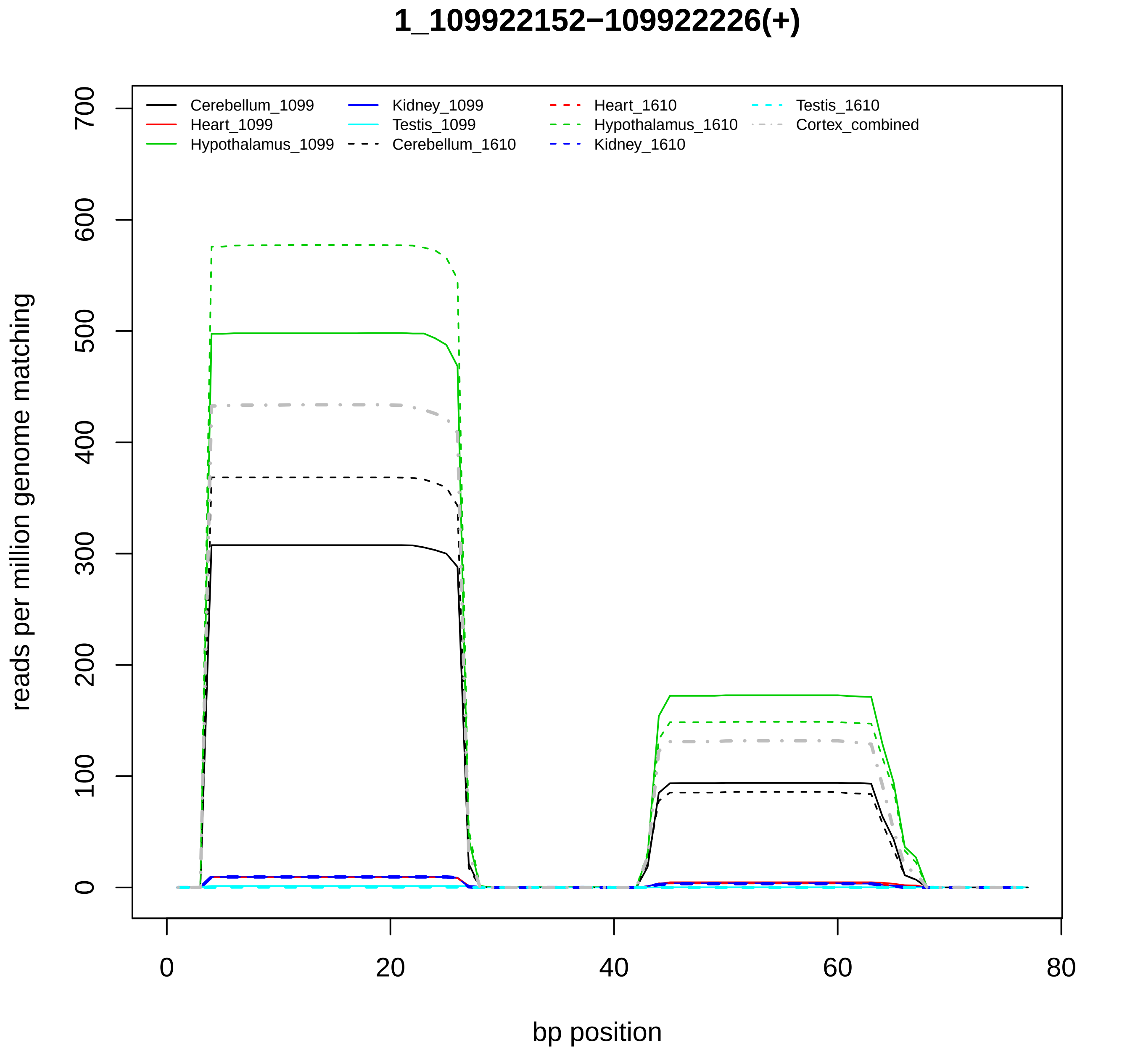

1_109922152−109922226(+)
Cerebellum_1099
Heart_1099
Hypothalamus_1099
Kidney_1099
Testis_1099
Cerebellum_1610
Heart_1610
Hypothalamus_1610
Kidney_1610
Testis_1610
Cortex_combined
reads per million genome matching
0
100
200
300
400
500
600
700
0
20
40
60
80
bp position

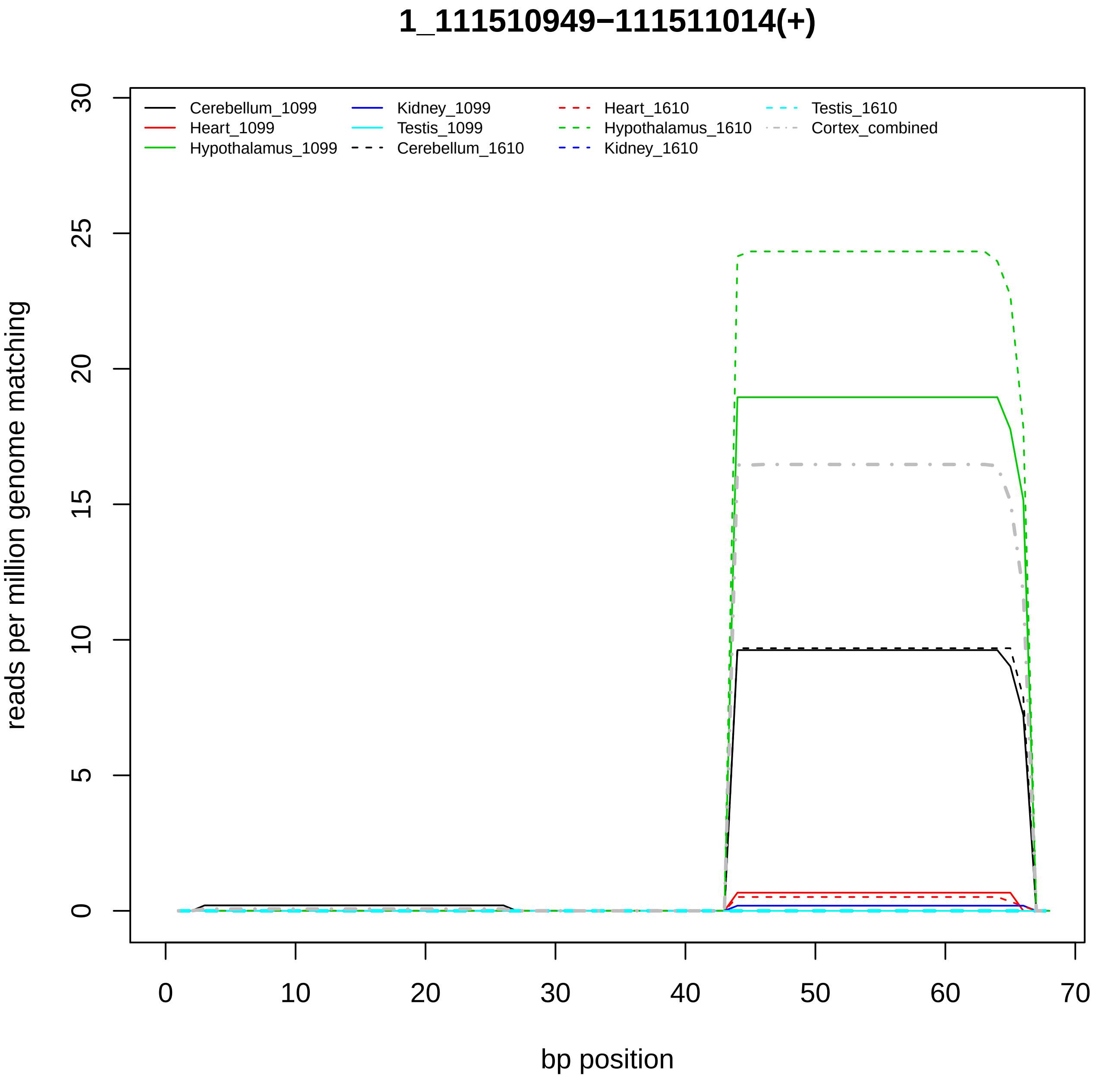

1_111510949−111511014(+)
Cerebellum_1099
Heart_1099
Hypothalamus_1099
Kidney_1099
Testis_1099
Cerebellum_1610
Heart_1610
Hypothalamus_1610
Kidney_1610
Testis_1610
Cortex_combined
reads per million genome matching
bp position
0
5
10
15
20
25
30
0
10
20
30
40
50
60
70

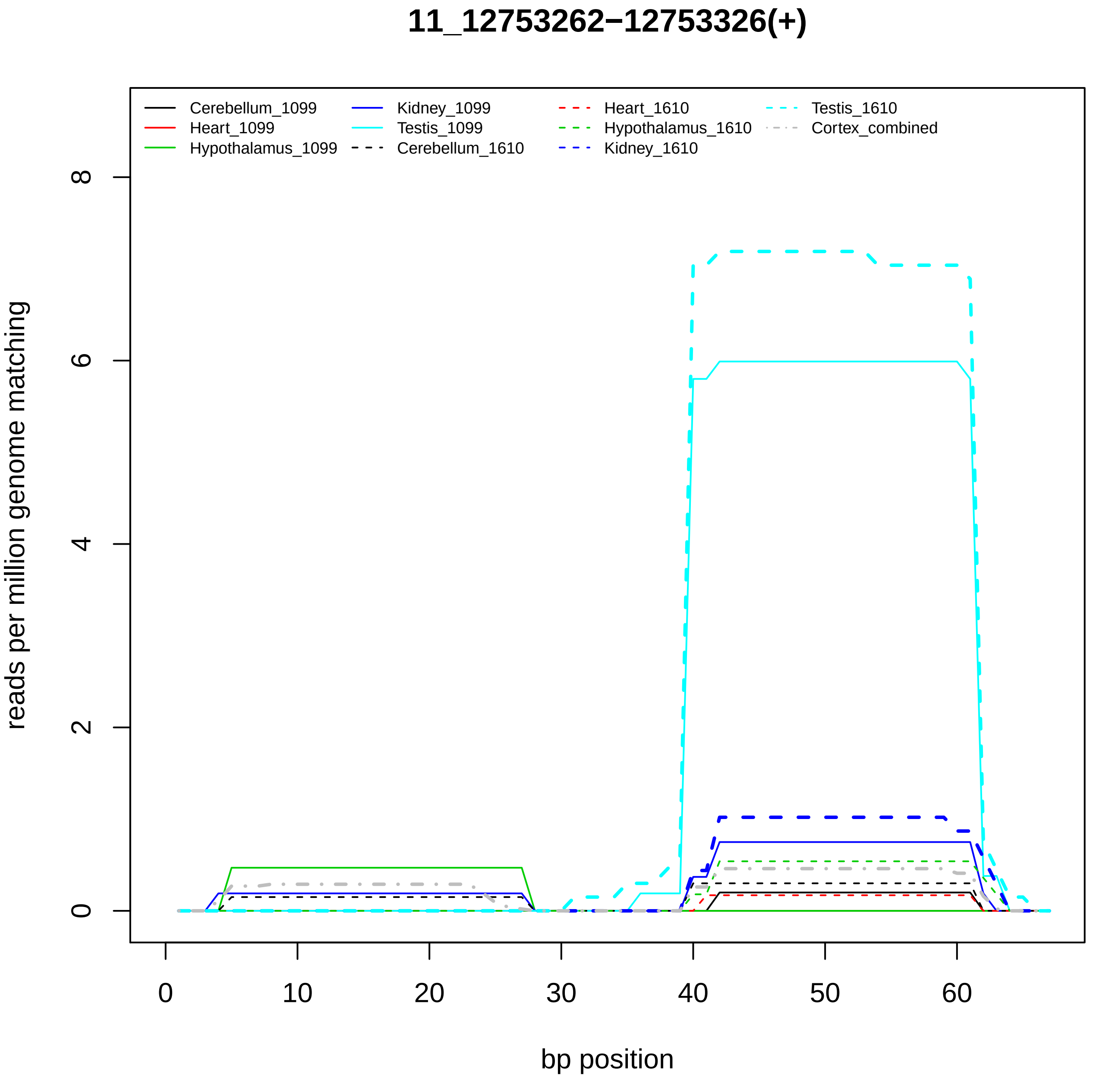

11_12753262−12753326(+)
Cerebellum_1099
Heart_1099
Hypothalamus_1099
Kidney_1099
Testis_1099
Cerebellum_1610
Heart_1610
Hypothalamus_1610
Kidney_1610
Testis_1610
Cortex_combined
reads per million genome matching
bp position
0
2
4
6
8
0
10
20
30
40
50
60

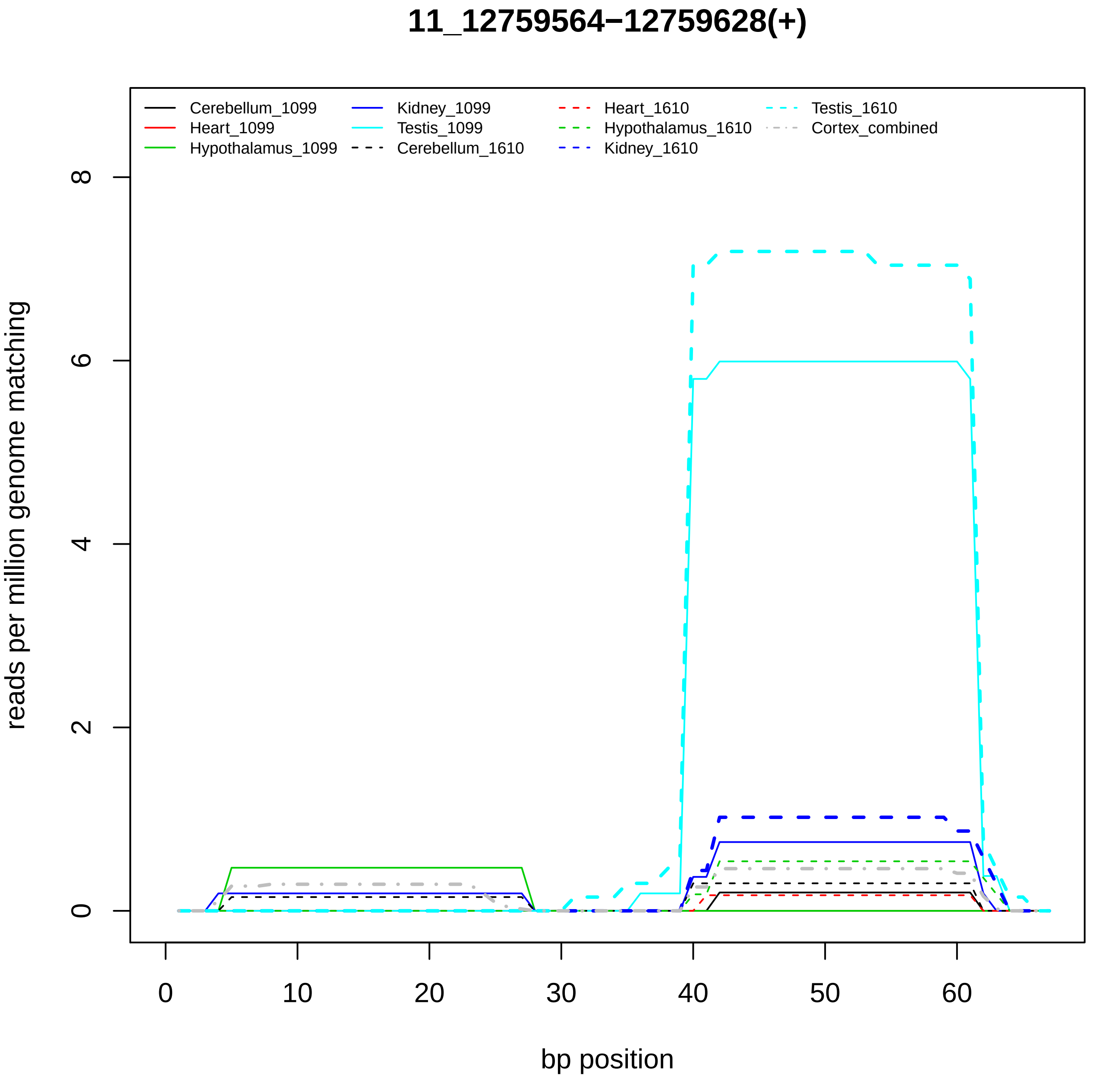
11_12759564−12759628(+)
Cerebellum_1099
Heart_1099
Hypothalamus_1099
Kidney_1099
Testis_1099
Cerebellum_1610
Heart_1610
Hypothalamus_1610
Kidney_1610
Testis_1610
Cortex_combined
reads per million genome matching
bp position
0
2
4
6
8
0
10
20
30
40
50
60

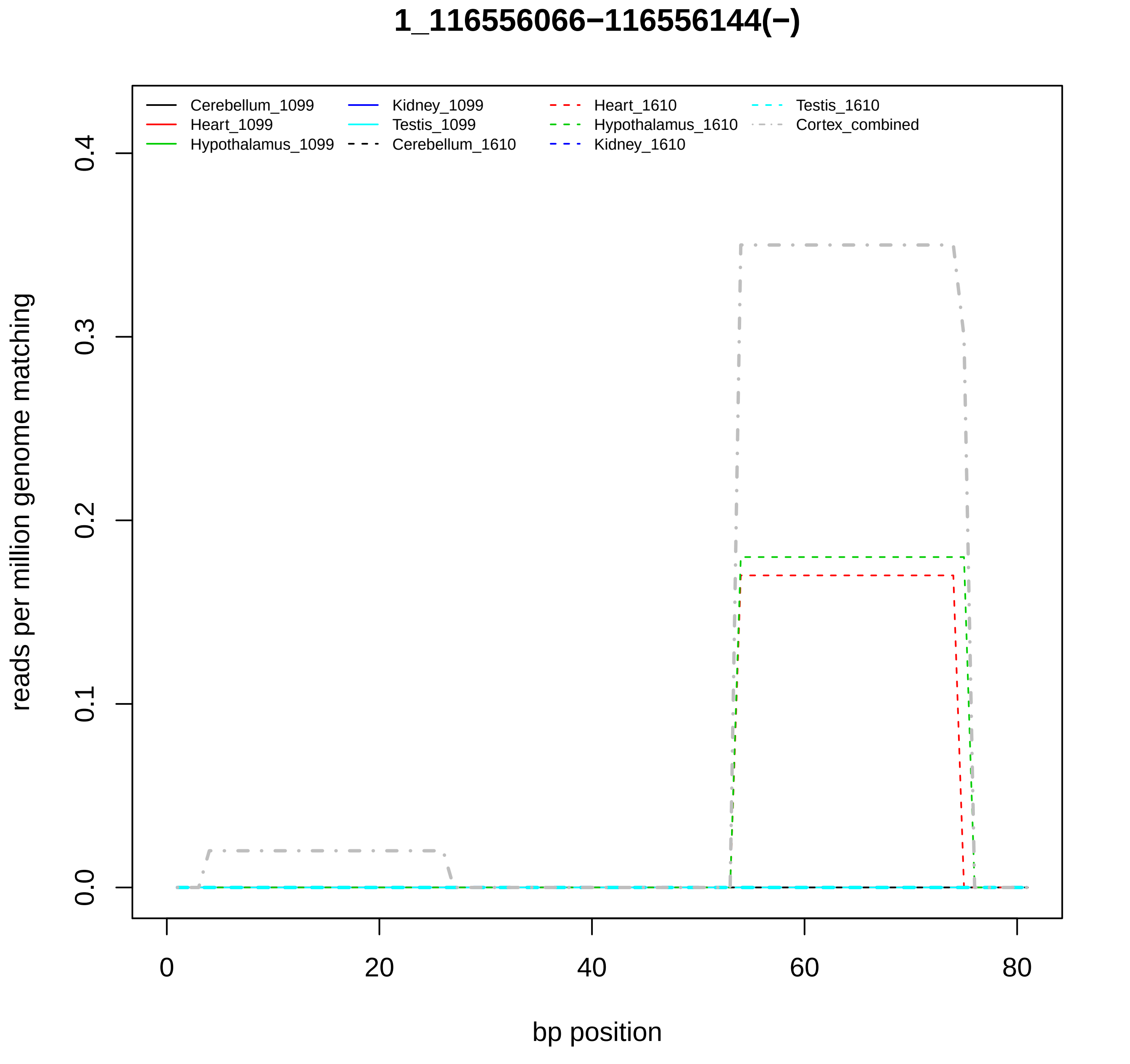

1_116556066−116556144(−)
Cerebellum_1099
Heart_1099
Hypothalamus_1099
Kidney_1099
Testis_1099
Cerebellum_1610
Heart_1610
Hypothalamus_1610
Kidney_1610
Testis_1610
Cortex_combined
reads per million genome matching
0.0
0.1
0.2
0.3
0.4
0
20
40
60
80
bp position

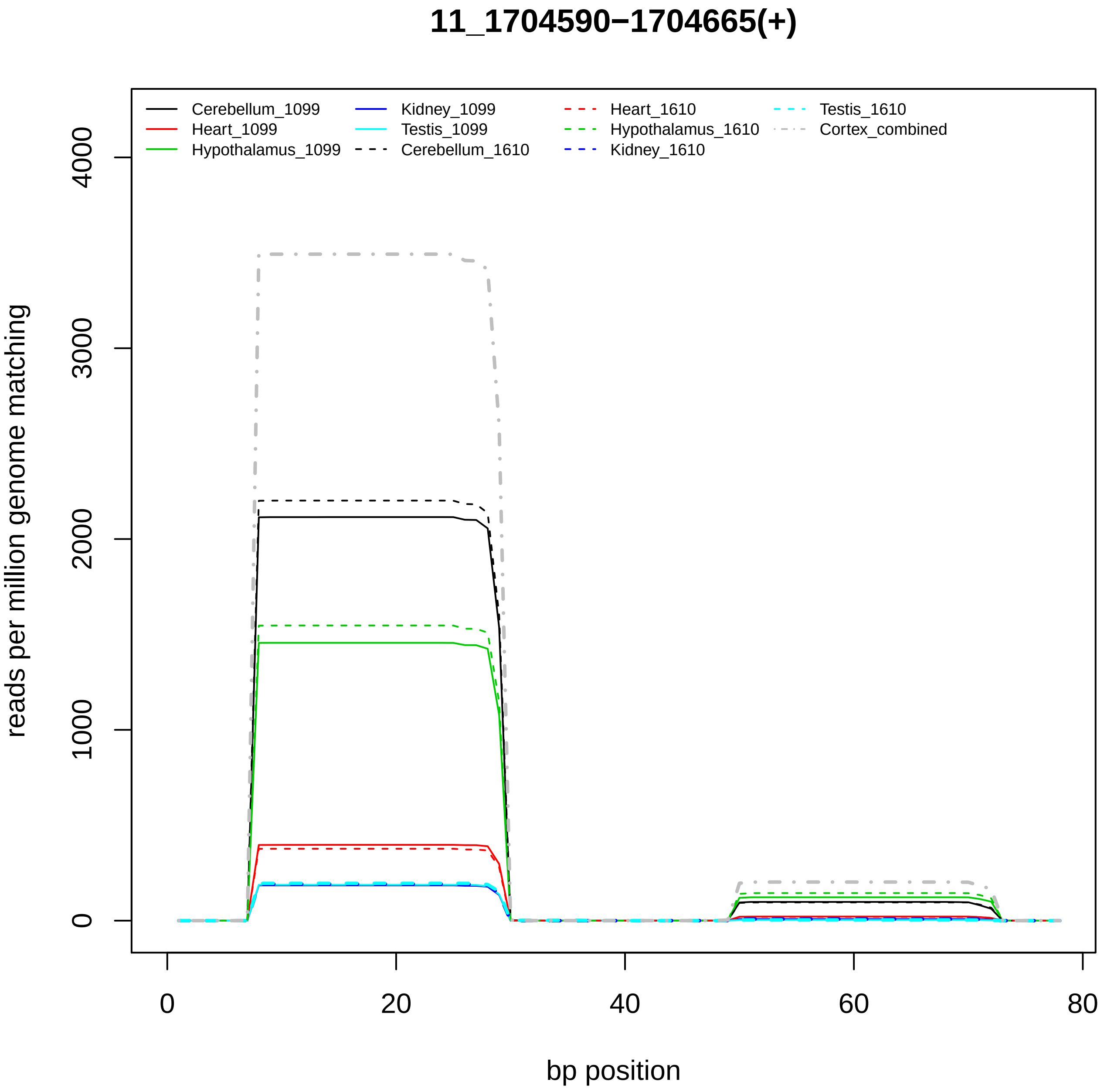

11_1704590−1704665(+)
Cerebellum_1099
Heart_1099
Hypothalamus_1099
Kidney_1099
Testis_1099
Cerebellum_1610
Heart_1610
Hypothalamus_1610
Kidney_1610
Testis_1610
Cortex_combined
reads per million genome matching
0
1000
2000
3000
4000
bp position
0
20
40
60
80

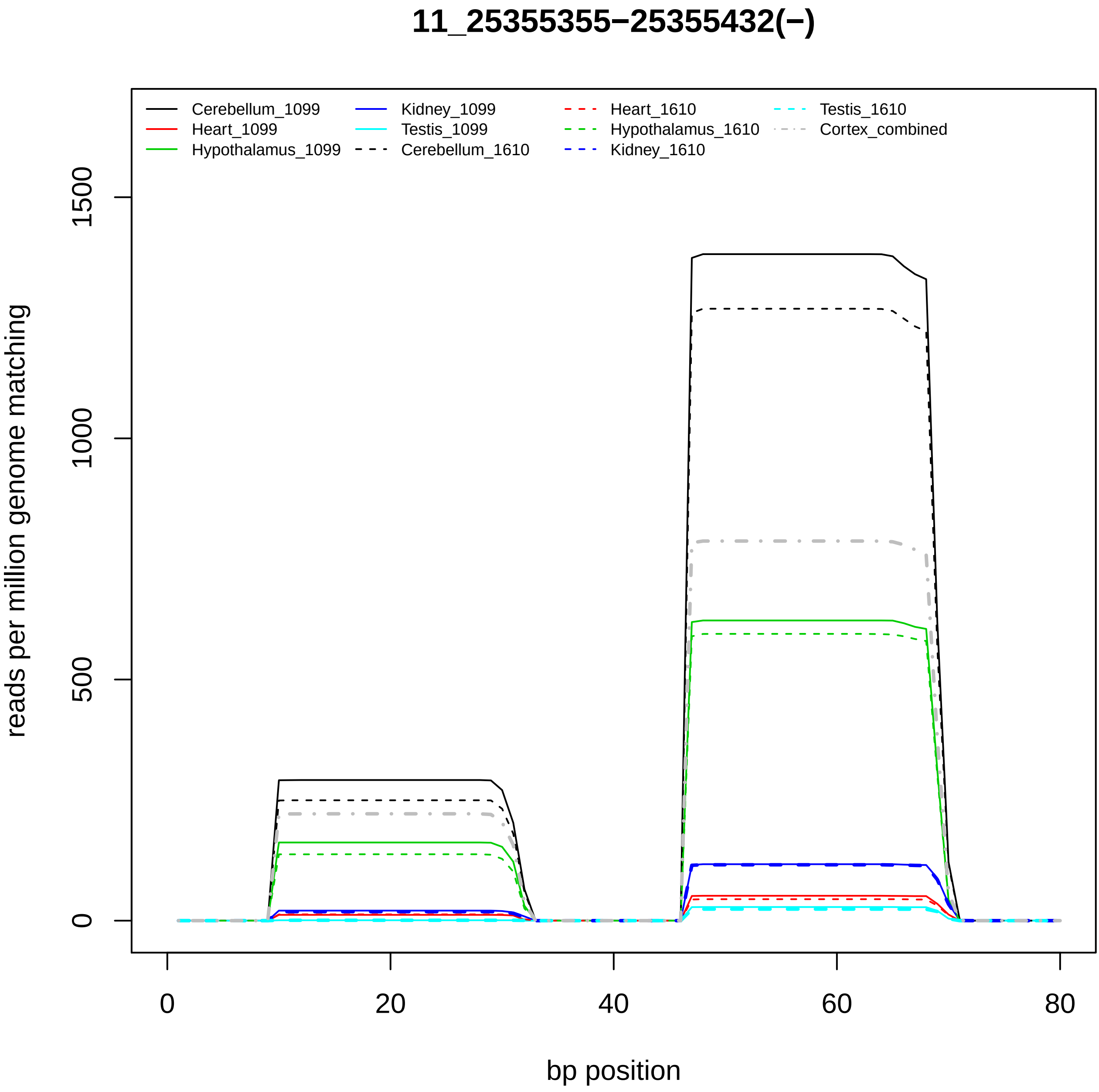

11_25355355−25355432(−)
Cerebellum_1099
Heart_1099
Hypothalamus_1099
Kidney_1099
Testis_1099
Cerebellum_1610
Heart_1610
Hypothalamus_1610
Kidney_1610
Testis_1610
Cortex_combined
reads per million genome matching
0
500
1000
1500
0
20
40
60
80
bp position

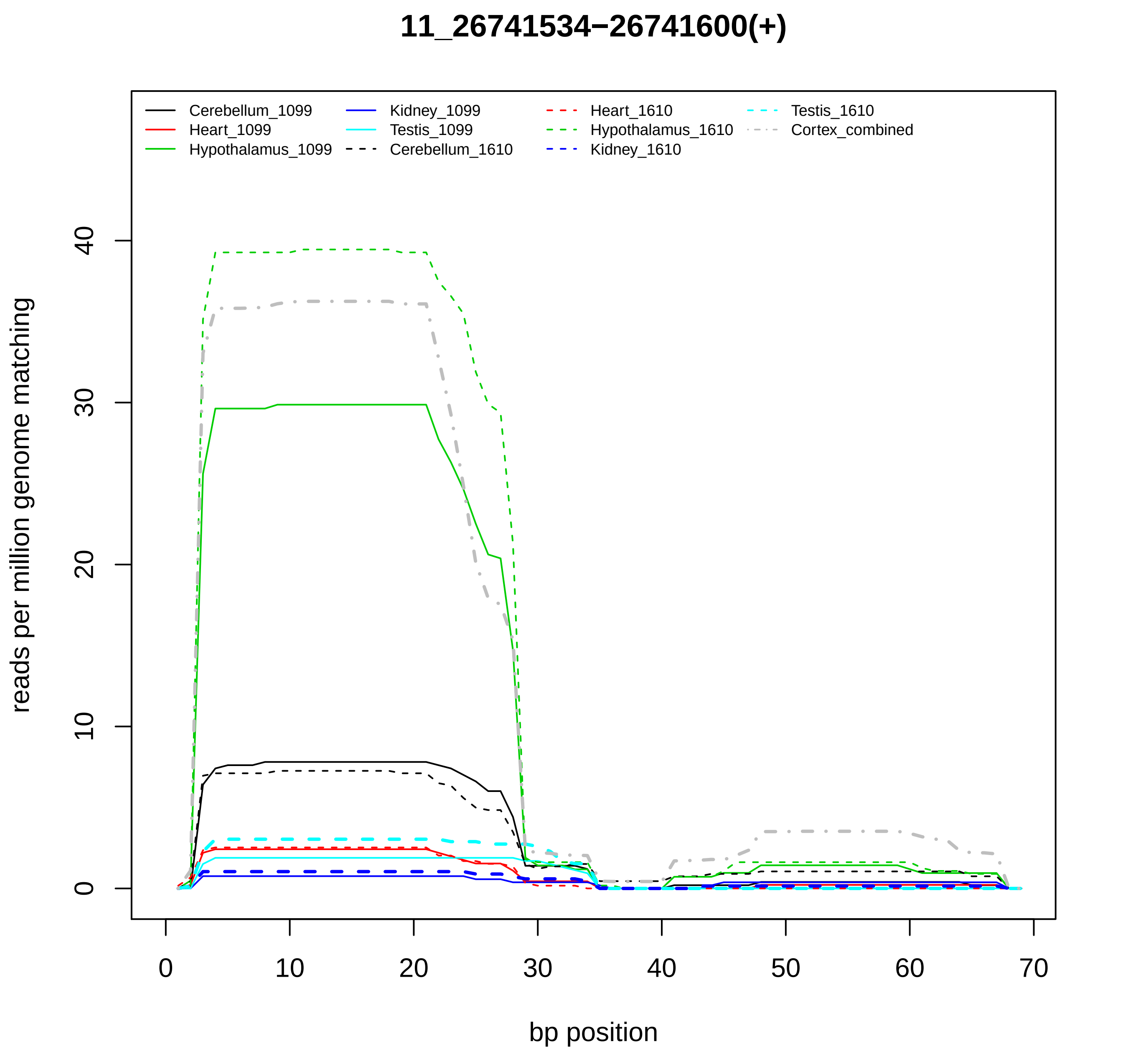

11_26741534−26741600(+)
Cerebellum_1099
Heart_1099
Hypothalamus_1099
Kidney_1099
Testis_1099
Cerebellum_1610
Heart_1610
Hypothalamus_1610
Kidney_1610
Testis_1610
Cortex_combined
reads per million genome matching
0
10
20
30
40
0
10
20
30
40
50
60
70
bp position

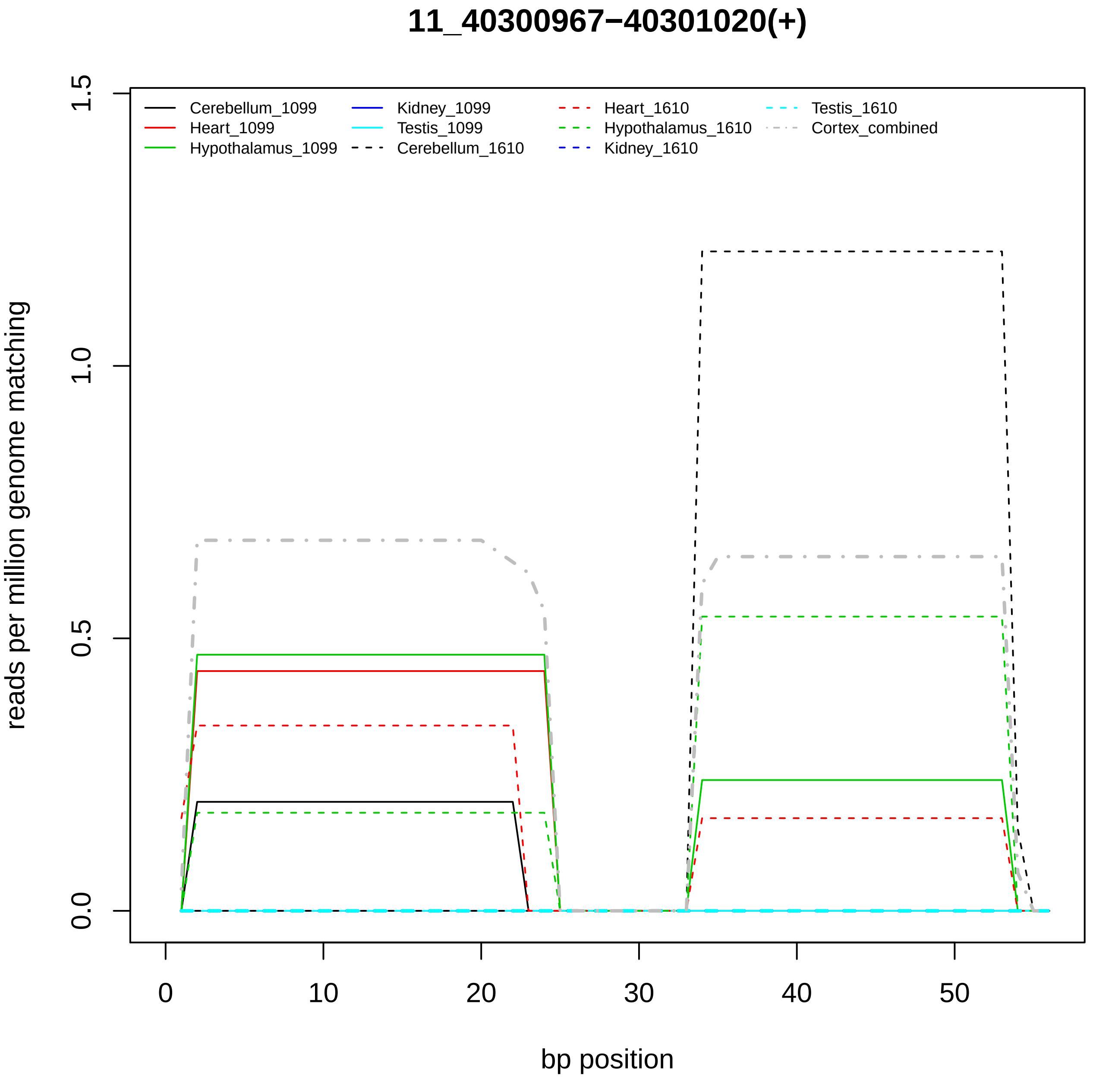

11_40300967−40301020(+)
Cerebellum_1099
Heart_1099
Hypothalamus_1099
Kidney_1099
Testis_1099
Cerebellum_1610
Heart_1610
Hypothalamus_1610
Kidney_1610
Testis_1610
Cortex_combined
reads per million genome matching
0.0
0.5
1.0
1.5
0
10
20
30
40
50
bp position

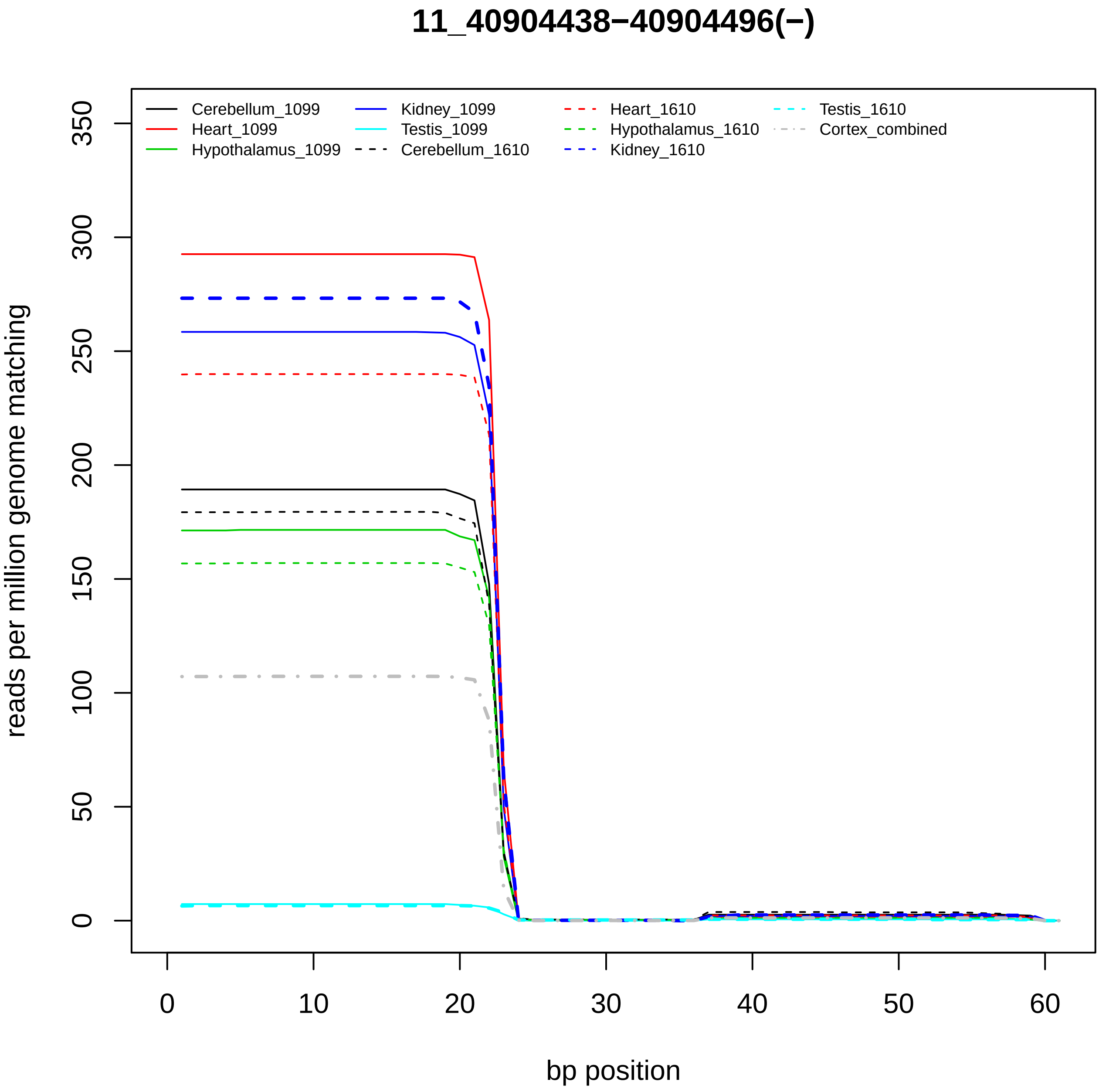

11_40904438−40904496(−)
Cerebellum_1099
Heart_1099
Hypothalamus_1099
Kidney_1099
Testis_1099
Cerebellum_1610
Heart_1610
Hypothalamus_1610
Kidney_1610
Testis_1610
Cortex_combined
reads per million genome matching
bp position
0
50
100
150
200
250
300
350
0
10
20
30
40
50
60

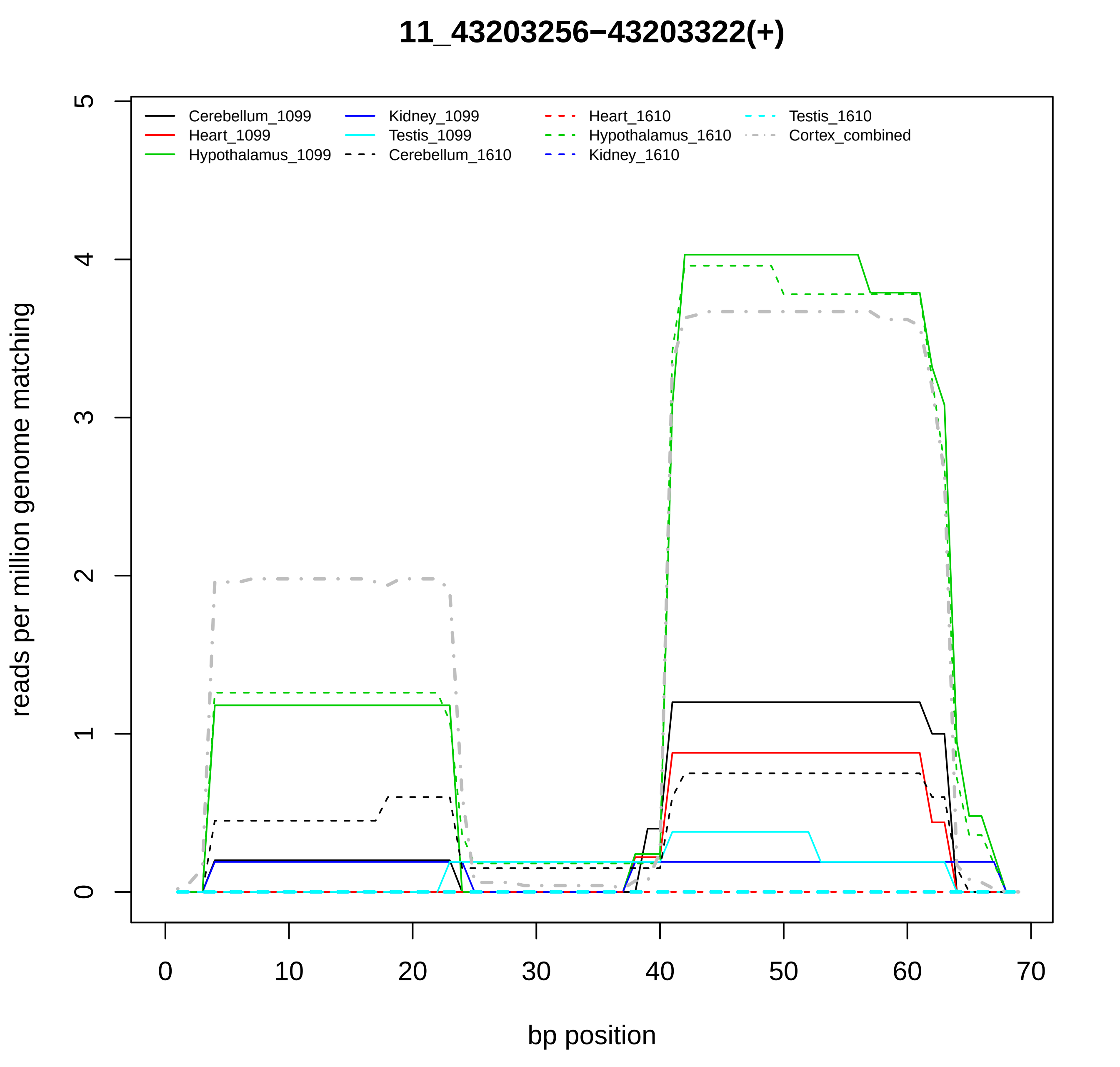

11_43203256−43203322(+)
Cerebellum_1099
Heart_1099
Hypothalamus_1099
Kidney_1099
Testis_1099
Cerebellum_1610
Heart_1610
Hypothalamus_1610
Kidney_1610
Testis_1610
Cortex_combined
reads per million genome matching
bp position
0
1
2
3
4
5
0
10
20
30
40
50
60
70

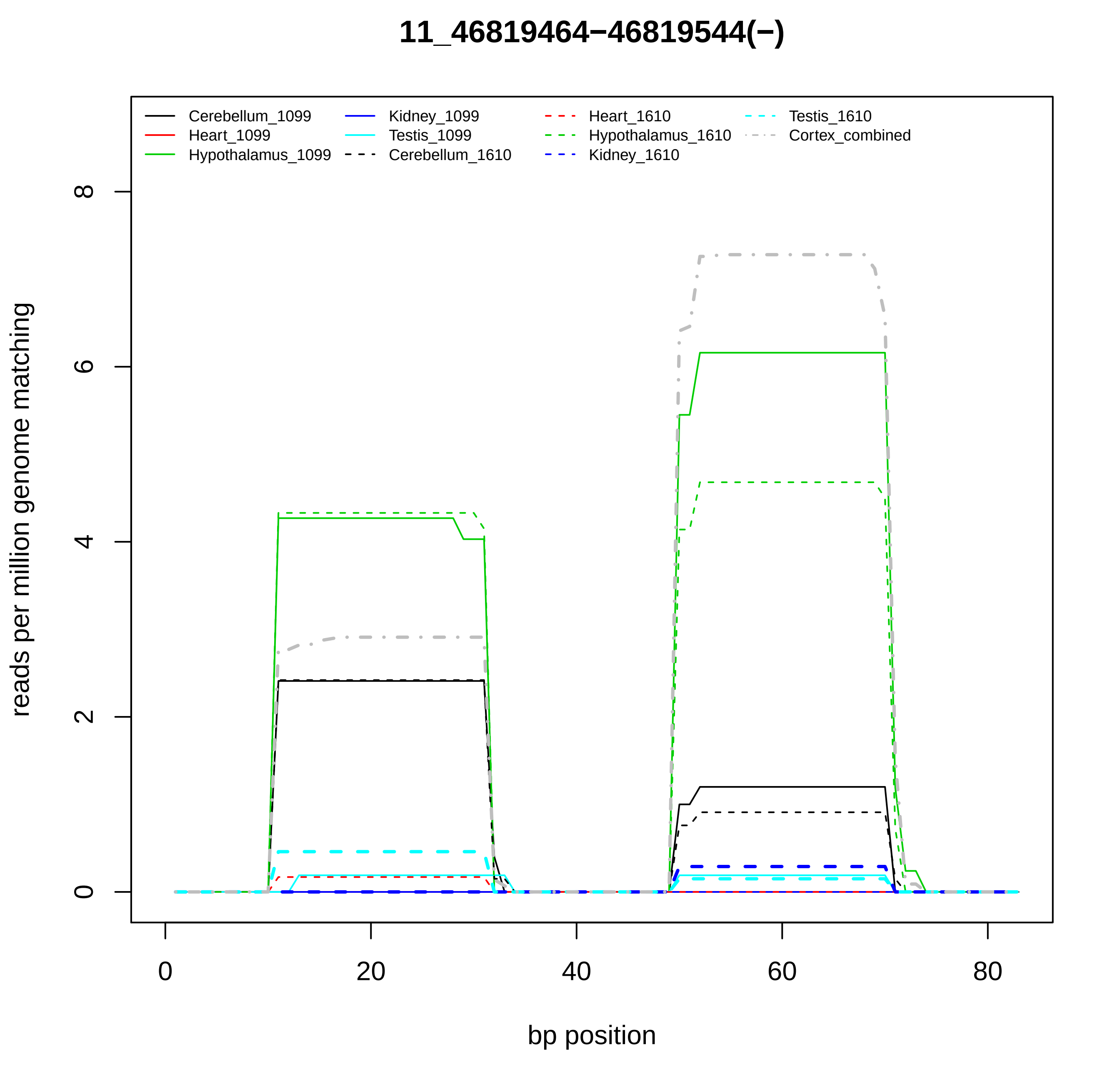

11_46819464−46819544(−)
Cerebellum_1099
Heart_1099
Hypothalamus_1099
Kidney_1099
Testis_1099
Cerebellum_1610
Heart_1610
Hypothalamus_1610
Kidney_1610
Testis_1610
Cortex_combined
reads per million genome matching
bp position
0
2
4
6
8
0
20
40
60
80

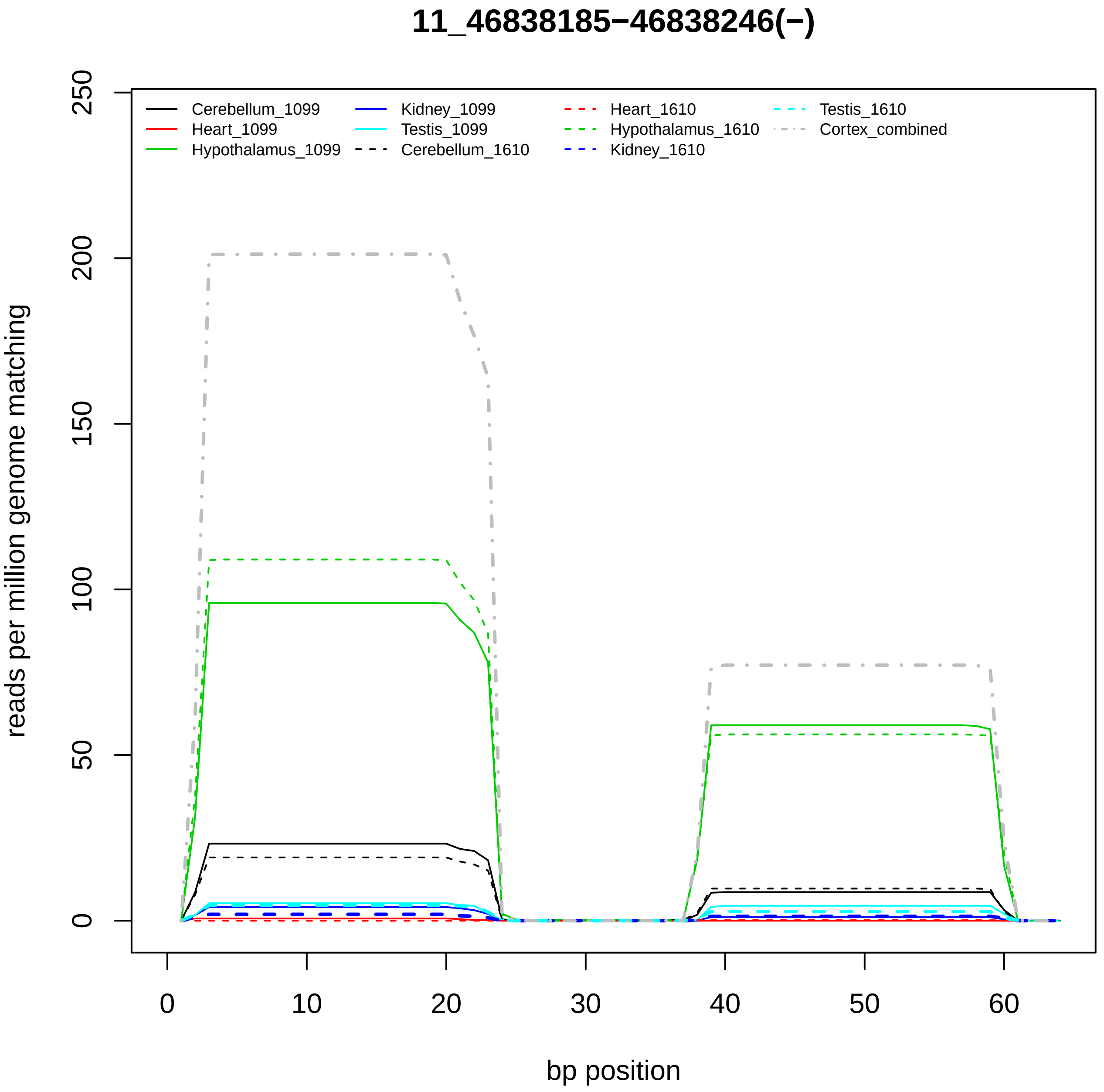

11_46838185−46838246(−)
Cerebellum_1099
Heart_1099
Hypothalamus_1099
Kidney_1099
Testis_1099
Cerebellum_1610
Heart_1610
Hypothalamus_1610
Kidney_1610
Testis_1610
Cortex_combined
reads per million genome matching
bp position
0
50
100
150
200
250
0
10
20
30
40
50
60

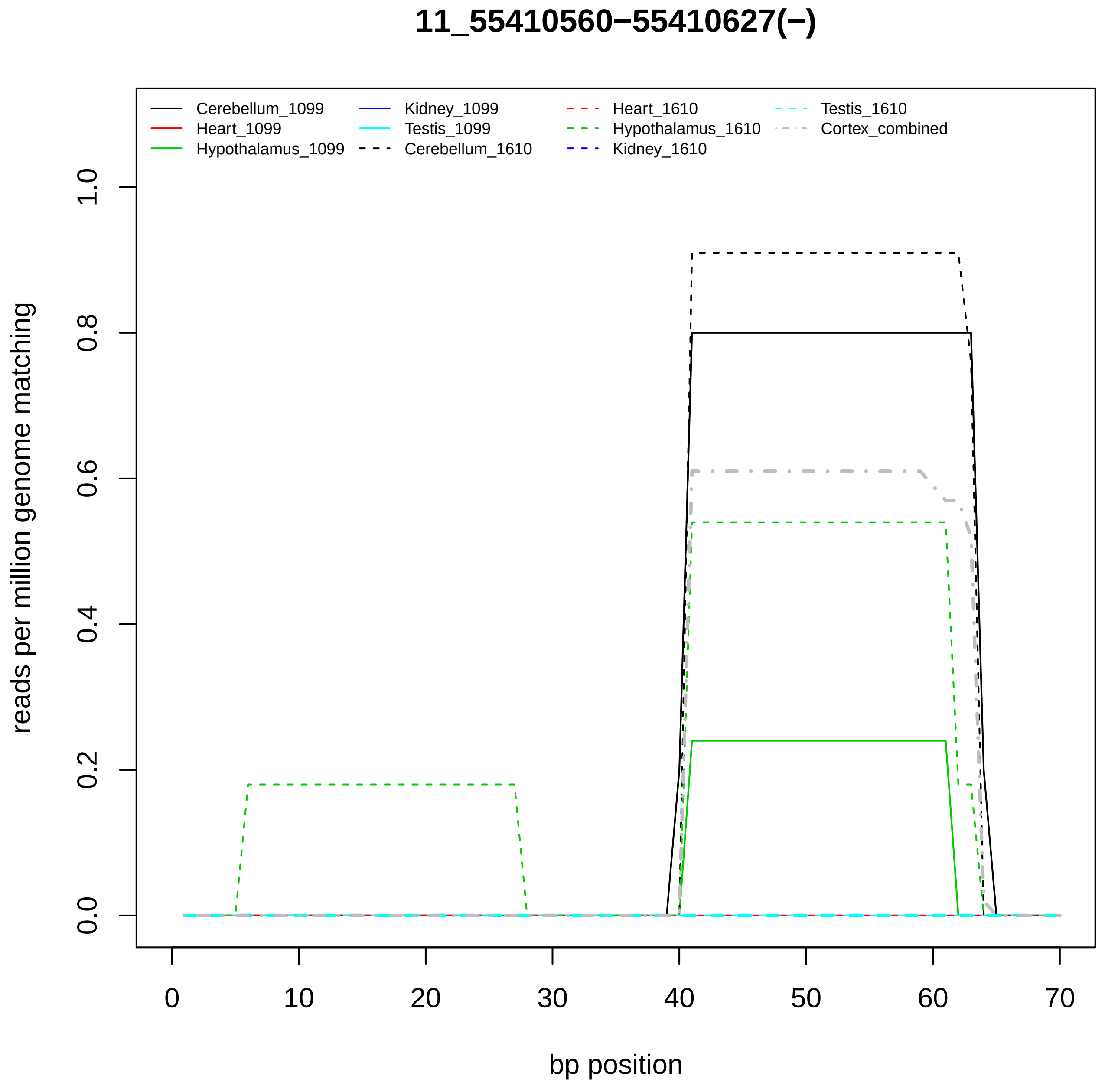

11_55410560−55410627(−)
Cerebellum_1099
Heart_1099
Hypothalamus_1099
Kidney_1099
Testis_1099
Cerebellum_1610
Heart_1610
Hypothalamus_1610
Kidney_1610
Testis_1610
Cortex_combined
reads per million genome matching
0.0
0.2
0.4
0.6
0.8
1.0
0
10
20
30
40
50
60
70
bp position

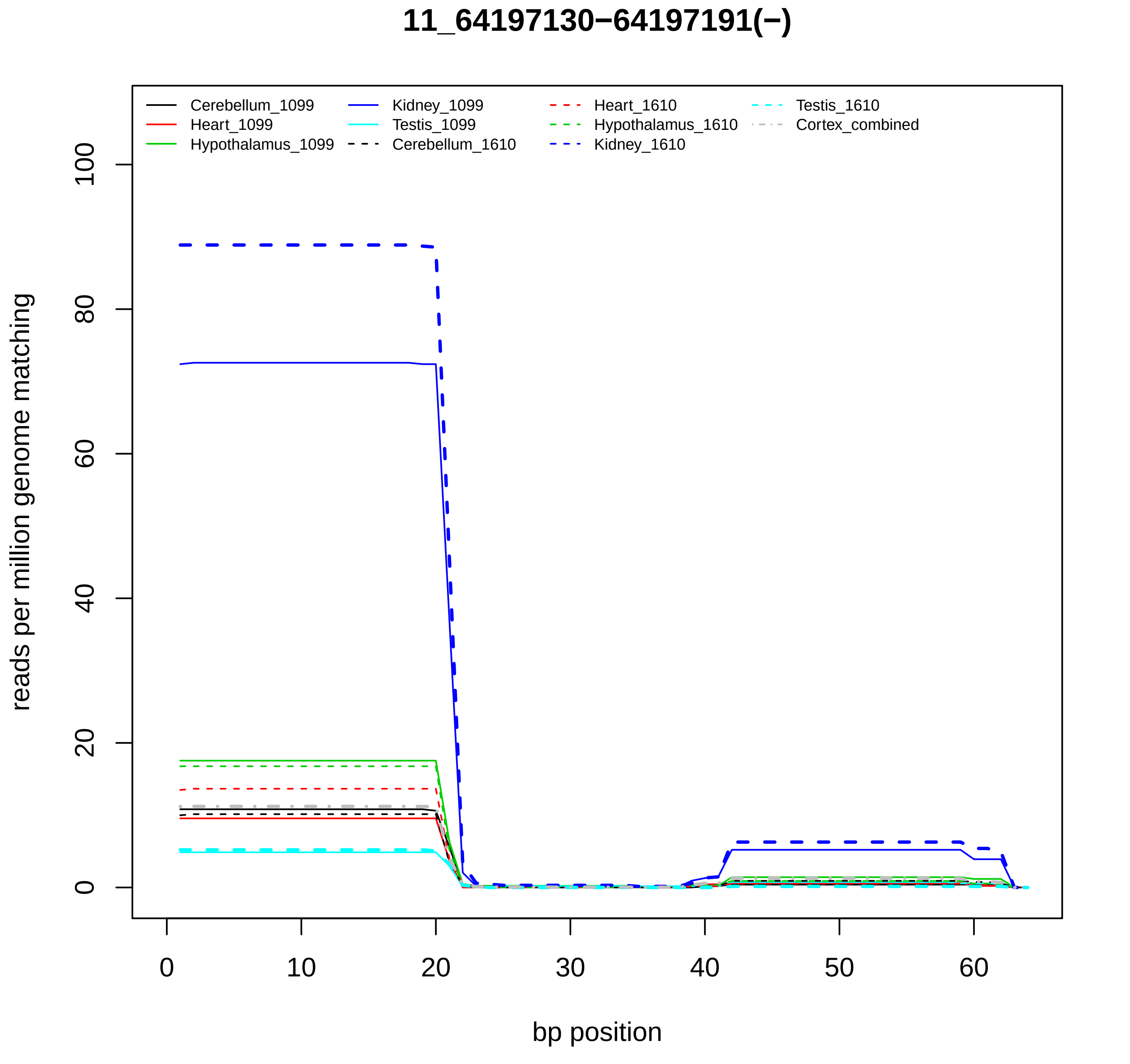

11_64197130−64197191(−)
Cerebellum_1099
Heart_1099
Hypothalamus_1099
Kidney_1099
Testis_1099
Cerebellum_1610
Heart_1610
Hypothalamus_1610
Kidney_1610
Testis_1610
Cortex_combined
reads per million genome matching
bp position
0
20
40
60
80
100
0
10
20
30
40
50
60

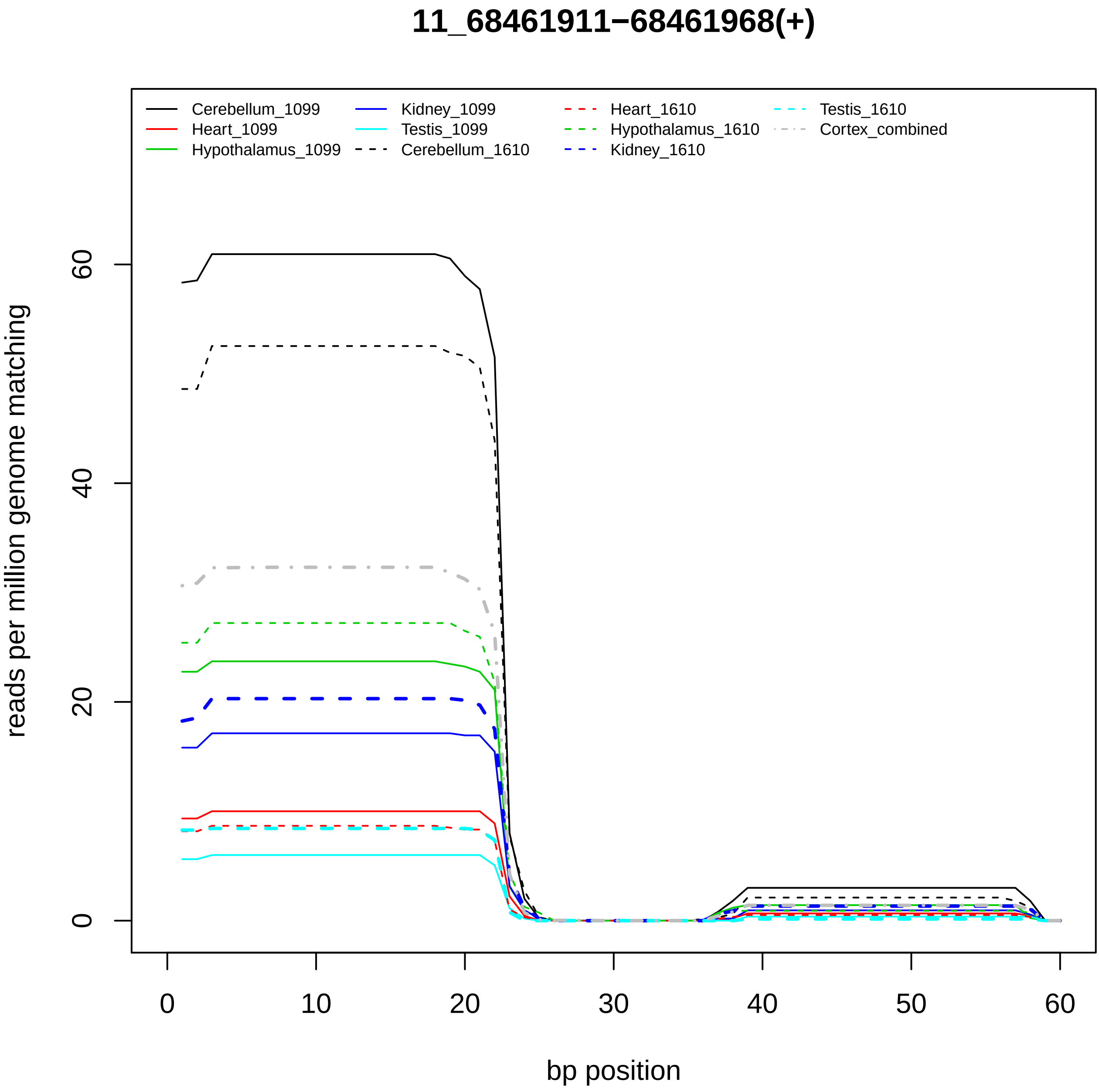
11_68461911−68461968(+)
Cerebellum_1099
Heart_1099
Hypothalamus_1099
Kidney_1099
Testis_1099
Cerebellum_1610
Heart_1610
Hypothalamus_1610
Kidney_1610
Testis_1610
Cortex_combined
reads per million genome matching
0
20
40
60
0
10
20
30
40
50
60
bp position

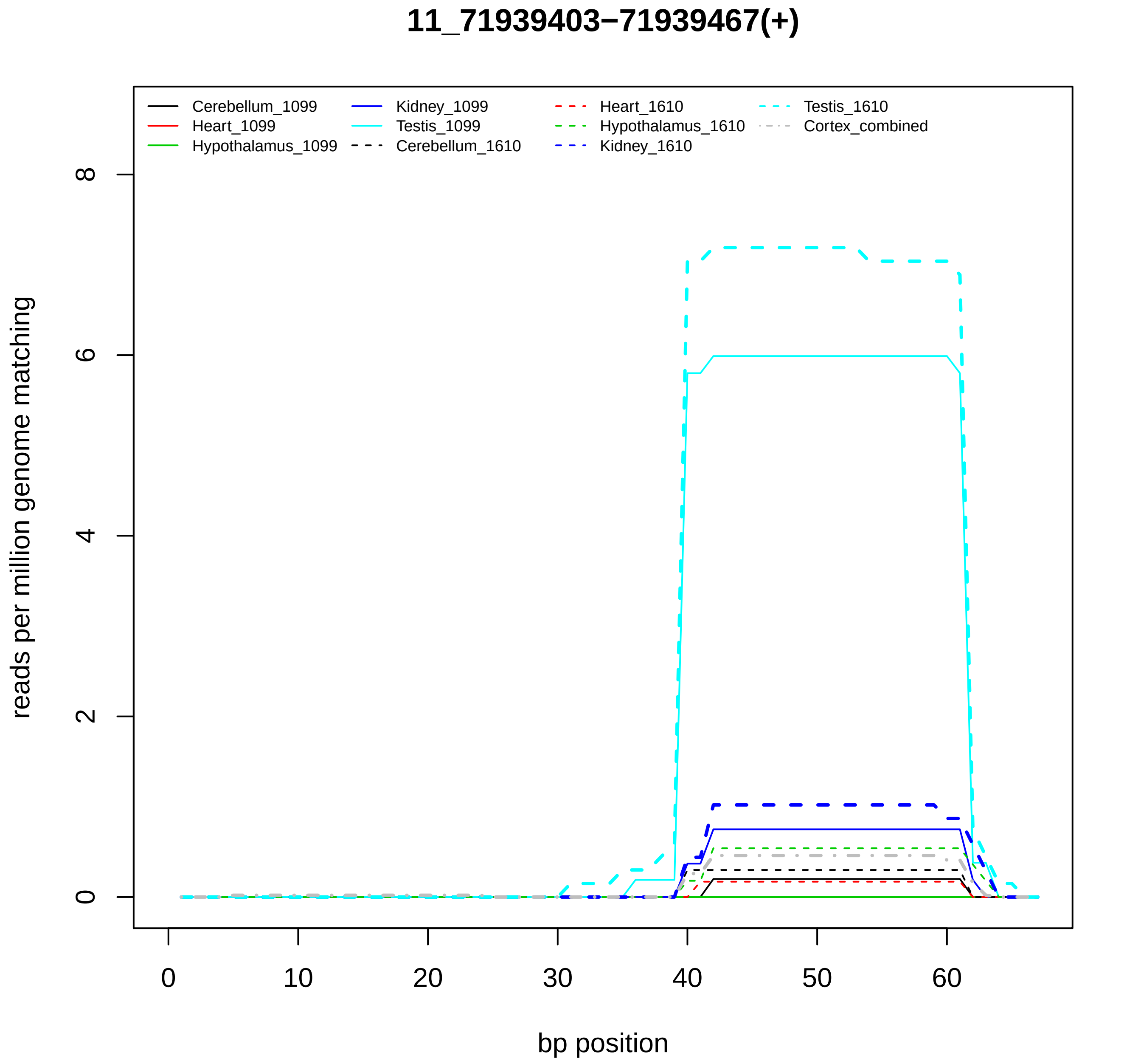

11_71939403−71939467(+)
Cerebellum_1099
Heart_1099
Hypothalamus_1099
Kidney_1099
Testis_1099
Cerebellum_1610
Heart_1610
Hypothalamus_1610
Kidney_1610
Testis_1610
Cortex_combined
reads per million genome matching
bp position
0
2
4
6
8
0
10
20
30
40
50
60

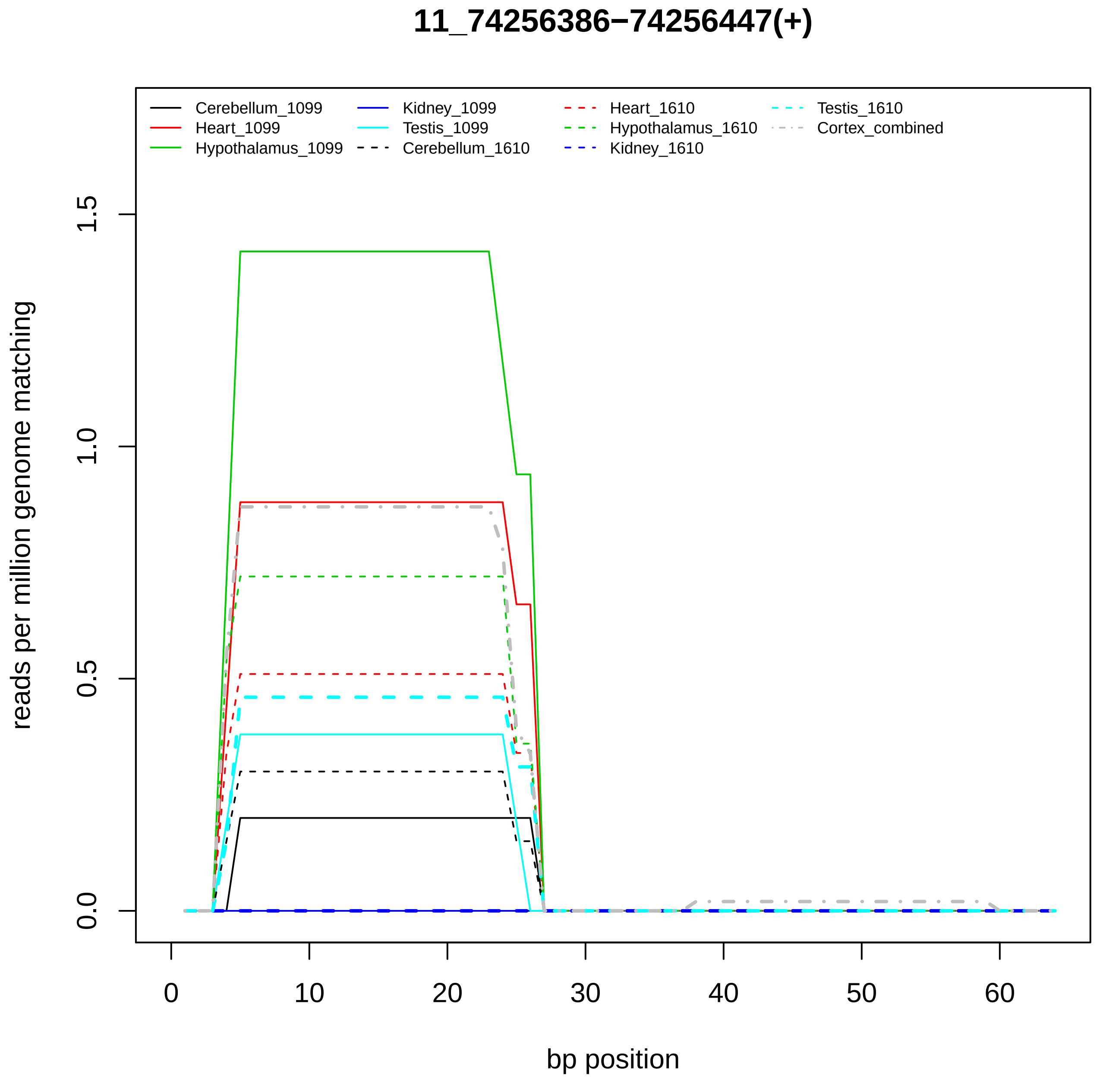

11_74256386−74256447(+)
Cerebellum_1099
Heart_1099
Hypothalamus_1099
Kidney_1099
Testis_1099
Cerebellum_1610
Heart_1610
Hypothalamus_1610
Kidney_1610
Testis_1610
Cortex_combined
reads per million genome matching
0.0
0.5
1.0
1.5
0
10
20
30
40
50
60
bp position

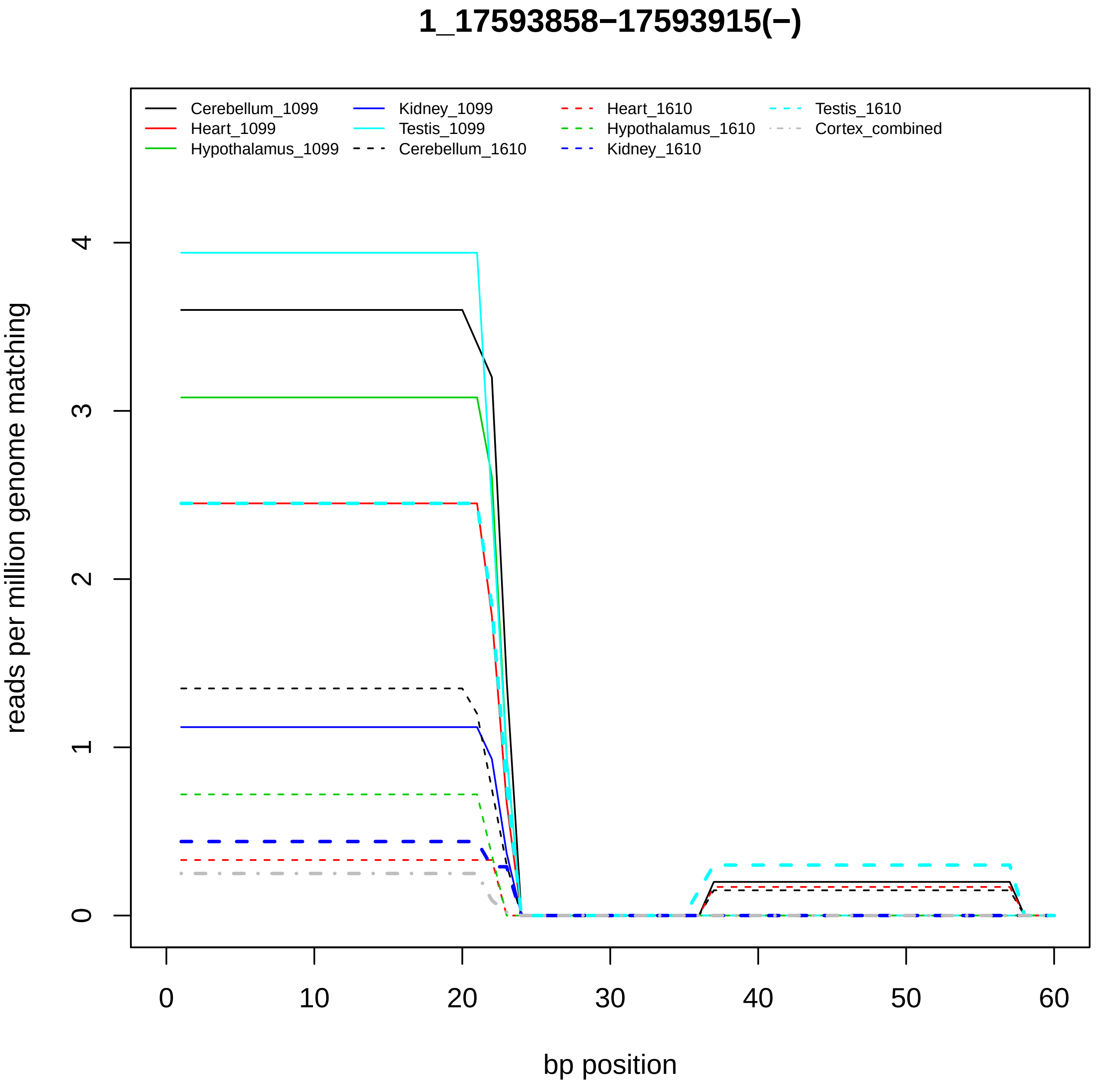
1_17593858−17593915(−)
Cerebellum_1099
Heart_1099
Hypothalamus_1099
Kidney_1099
Testis_1099
Cerebellum_1610
Heart_1610
Hypothalamus_1610
Kidney_1610
Testis_1610
Cortex_combined
reads per million genome matching
0
1
2
3
4
0
10
20
30
40
50
60
bp position

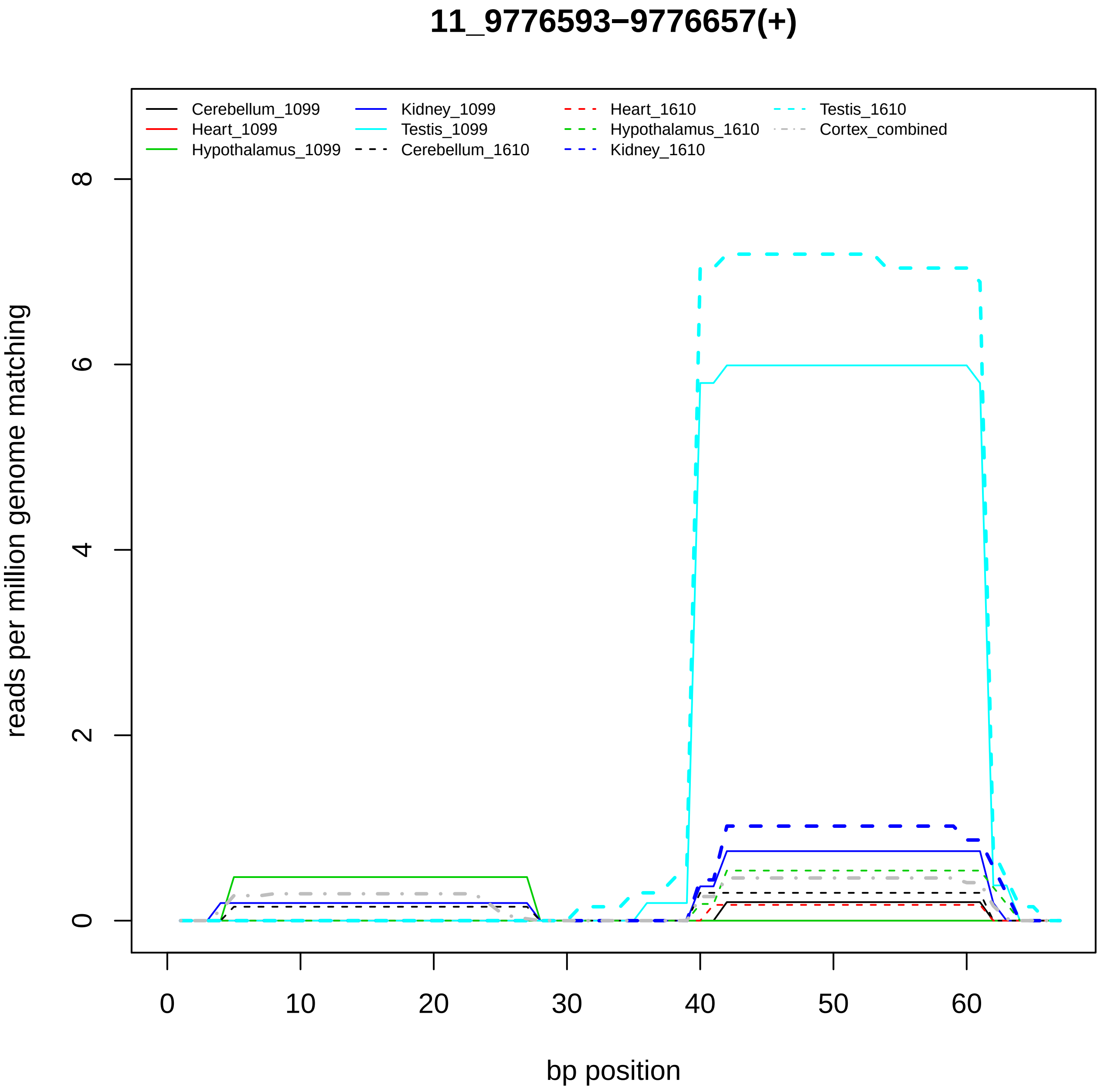

11_9776593−9776657(+)
Cerebellum_1099
Heart_1099
Hypothalamus_1099
Kidney_1099
Testis_1099
Cerebellum_1610
Heart_1610
Hypothalamus_1610
Kidney_1610
Testis_1610
Cortex_combined
reads per million genome matching
bp position
0
2
4
6
8
0
10
20
30
40
50
60

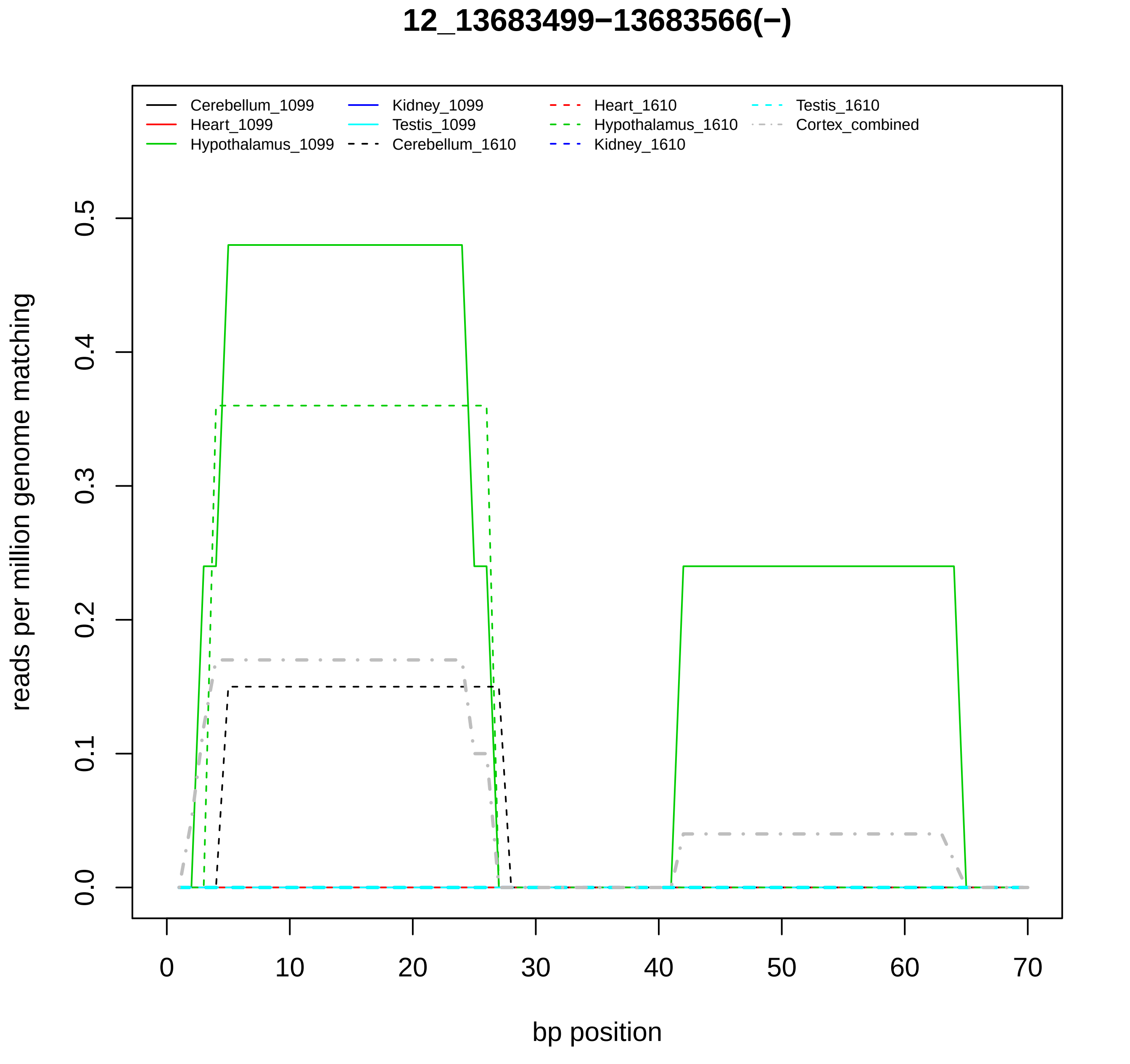

12_13683499−13683566(−)
Cerebellum_1099
Heart_1099
Hypothalamus_1099
Kidney_1099
Testis_1099
Cerebellum_1610
Heart_1610
Hypothalamus_1610
Kidney_1610
Testis_1610
Cortex_combined
reads per million genome matching
0.0
0.1
0.2
0.3
0.4
0.5
0
10
20
30
40
50
60
70
bp position

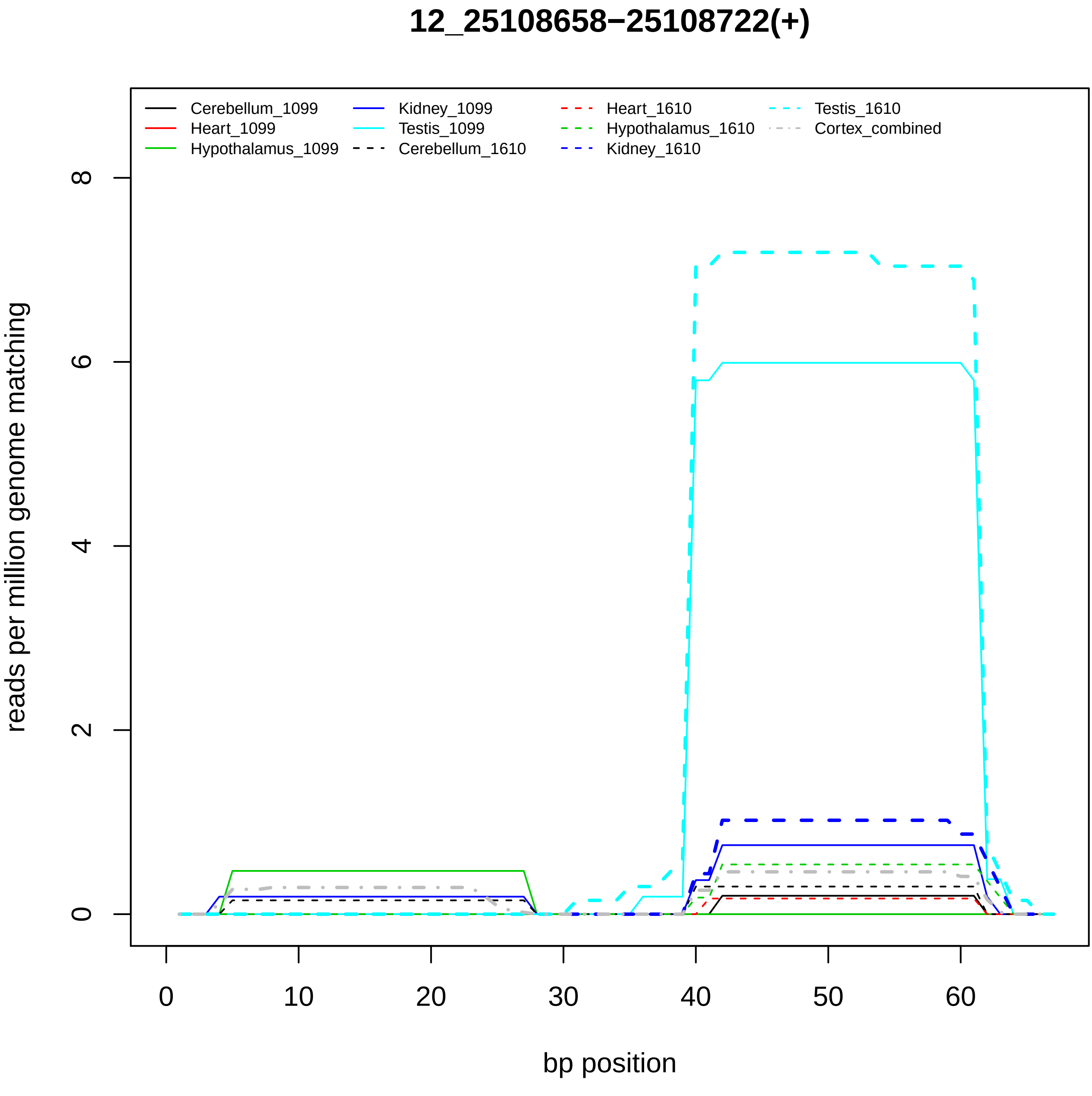

12_25108658−25108722(+)
Cerebellum_1099
Heart_1099
Hypothalamus_1099
Kidney_1099
Testis_1099
Cerebellum_1610
Heart_1610
Hypothalamus_1610
Kidney_1610
Testis_1610
Cortex_combined
reads per million genome matching
bp position
0
2
4
6
8
0
10
20
30
40
50
60

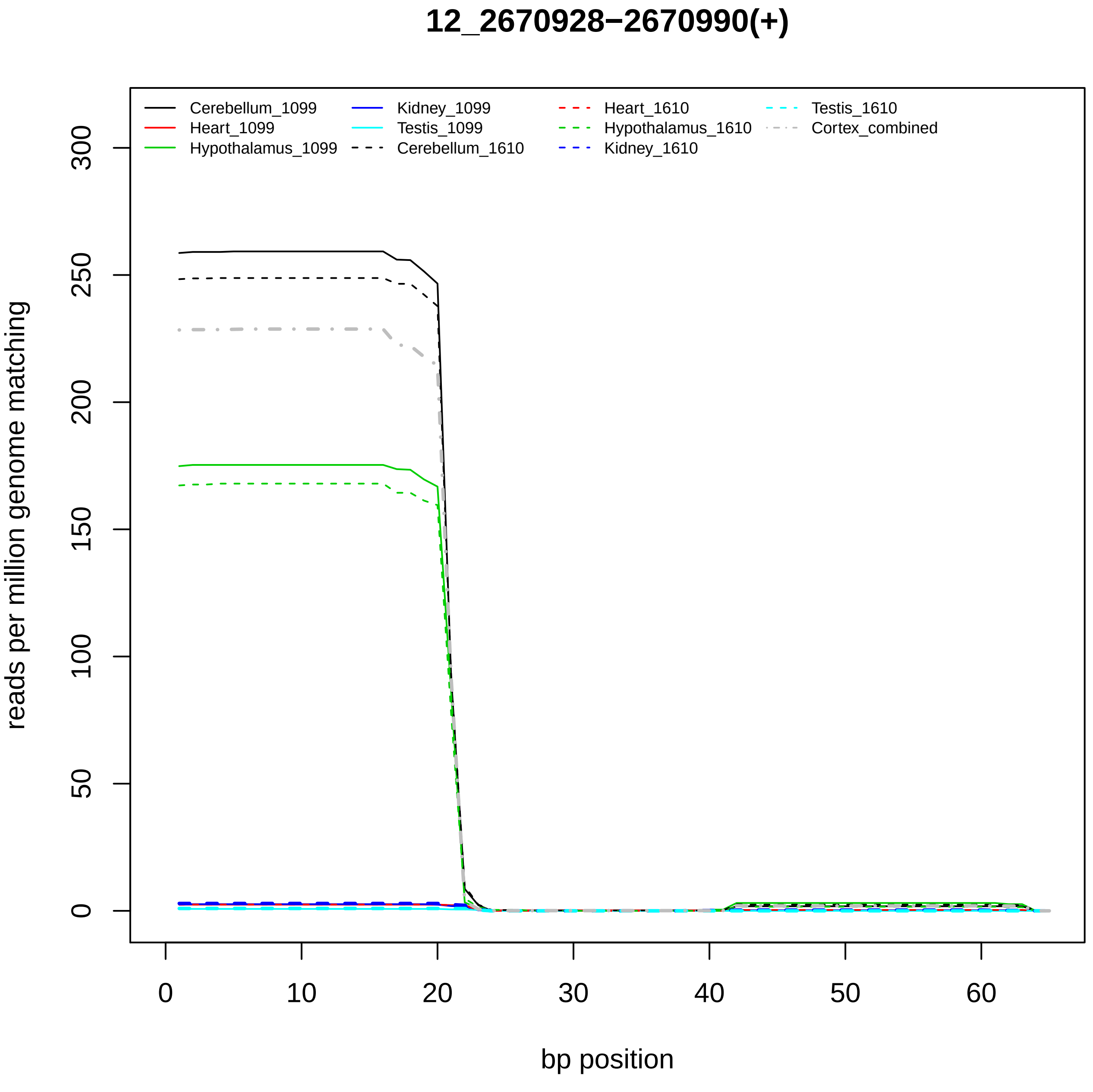
12_2670928−2670990(+)
Cerebellum_1099
Heart_1099
Hypothalamus_1099
Kidney_1099
Testis_1099
Cerebellum_1610
Heart_1610
Hypothalamus_1610
Kidney_1610
Testis_1610
Cortex_combined
reads per million genome matching
bp position
0
50
100
150
200
250
300
0
10
20
30
40
50
60

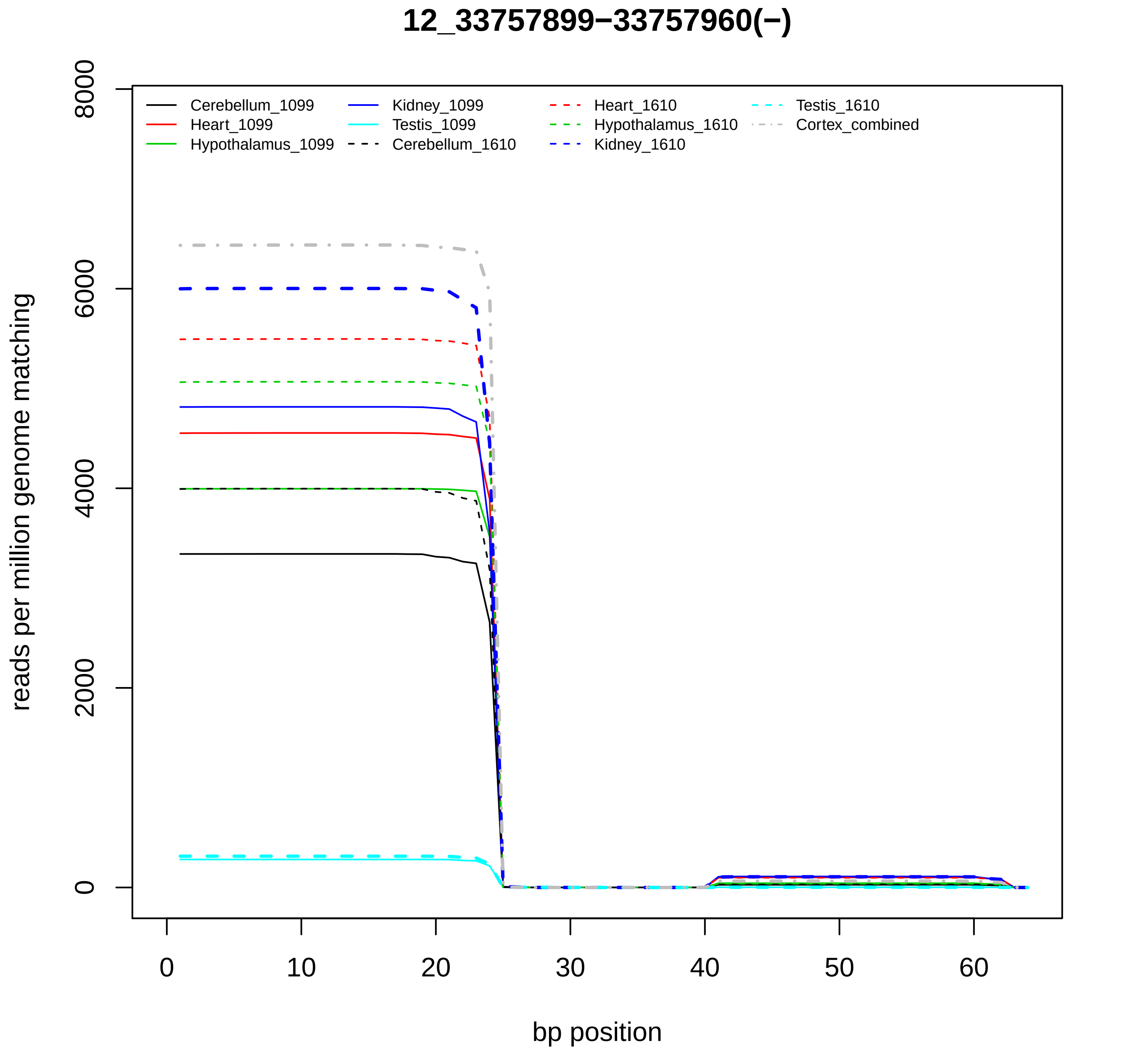

12_33757899−33757960(−)
Cerebellum_1099
Heart_1099
Hypothalamus_1099
Kidney_1099
Testis_1099
Cerebellum_1610
Heart_1610
Hypothalamus_1610
Kidney_1610
Testis_1610
Cortex_combined
reads per million genome matching
bp position
0
2000
4000
6000
8000
0
10
20
30
40
50
60

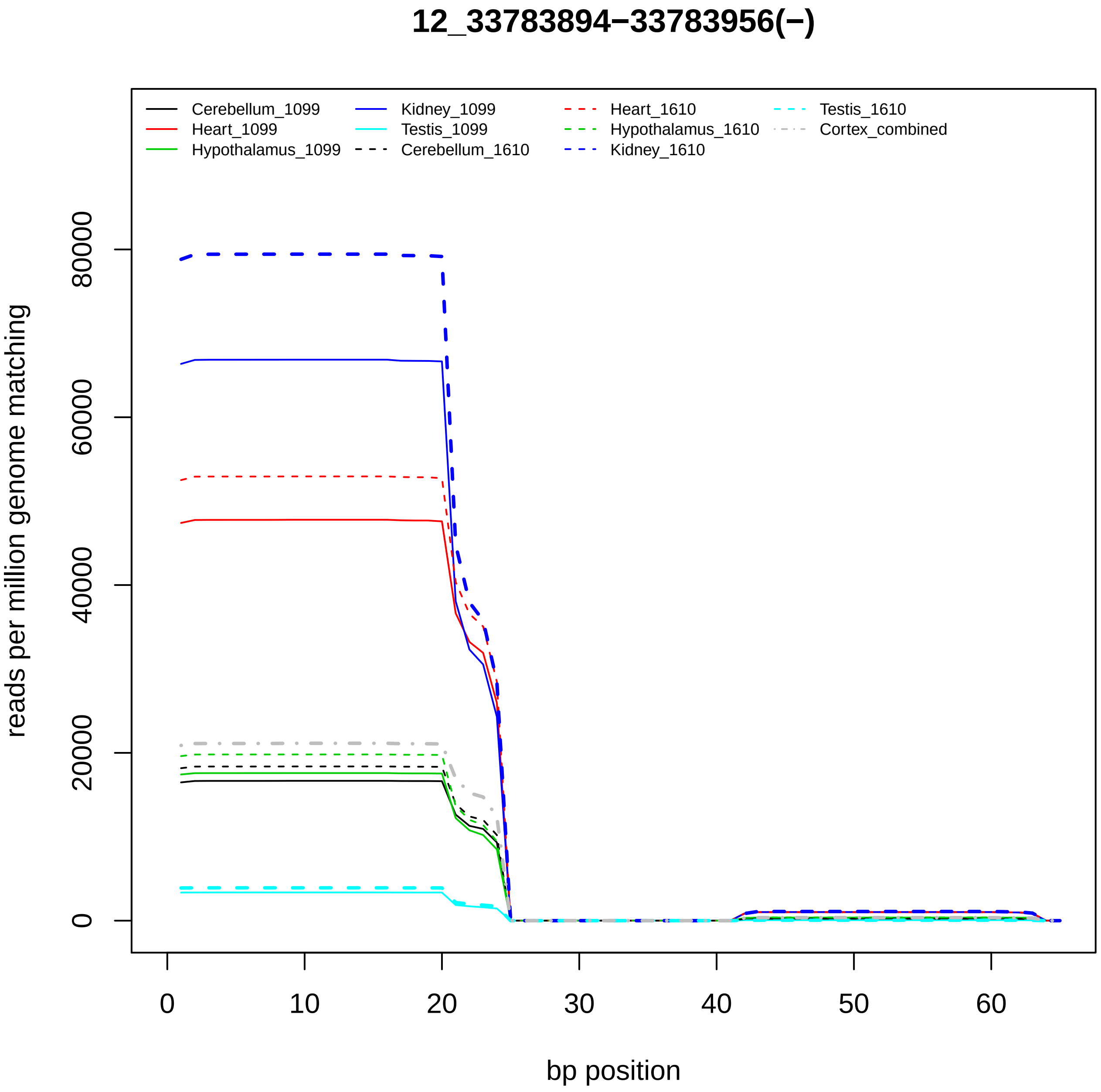

12_33783894−33783956(−)
Cerebellum_1099
Heart_1099
Hypothalamus_1099
Kidney_1099
Testis_1099
Cerebellum_1610
Heart_1610
Hypothalamus_1610
Kidney_1610
Testis_1610
Cortex_combined
reads per million genome matching
0
20000
40000
60000
80000
bp position
0
10
20
30
40
50
60

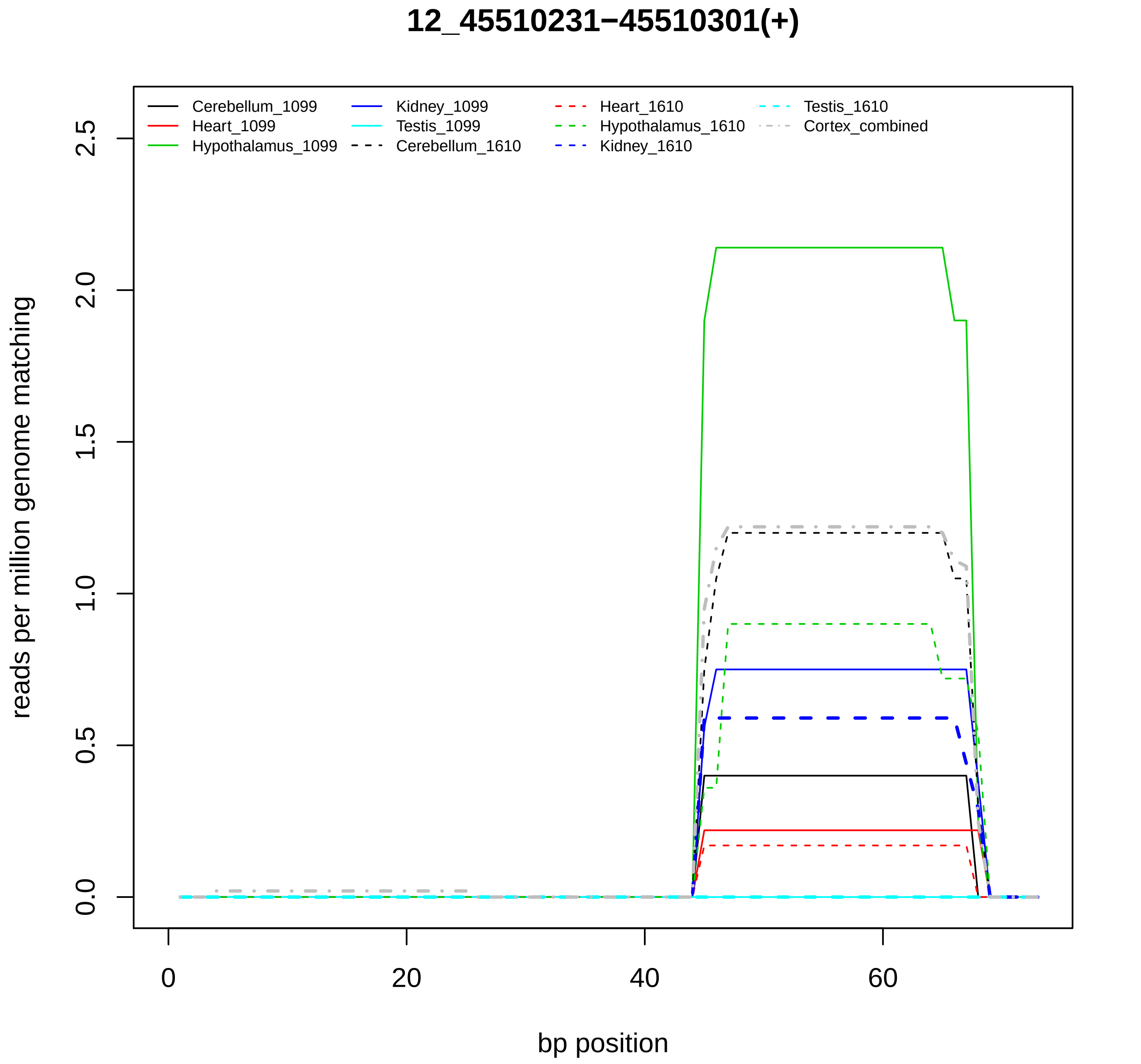

12_45510231−45510301(+)
Cerebellum_1099
Heart_1099
Hypothalamus_1099
Kidney_1099
Testis_1099
Cerebellum_1610
Heart_1610
Hypothalamus_1610
Kidney_1610
Testis_1610
Cortex_combined
reads per million genome matching
bp position
0.0
0.5
1.0
1.5
2.0
2.5
0
20
40
60

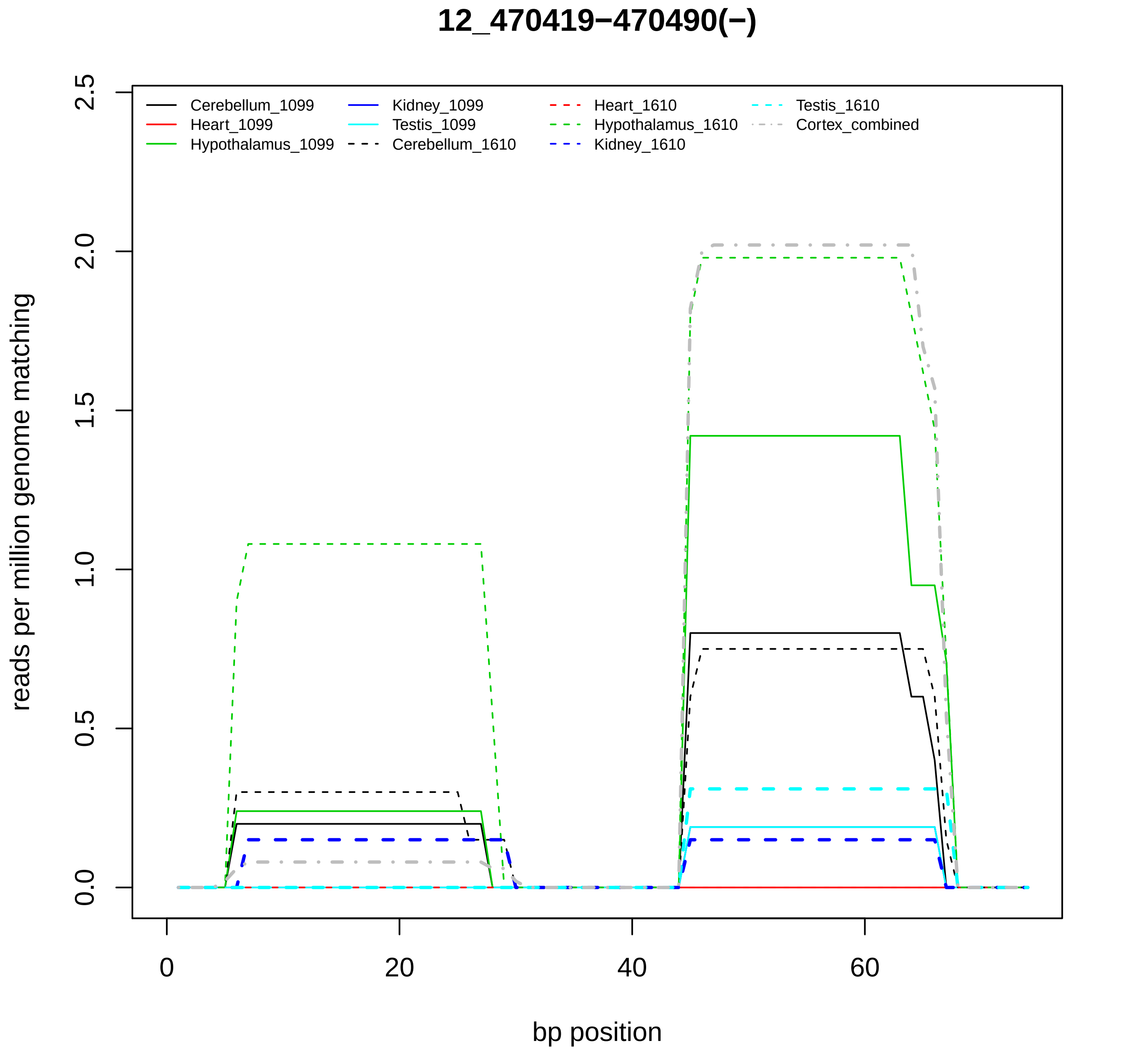

12_470419−470490(−)
Cerebellum_1099
Heart_1099
Hypothalamus_1099
Kidney_1099
Testis_1099
Cerebellum_1610
Heart_1610
Hypothalamus_1610
Kidney_1610
Testis_1610
Cortex_combined
reads per million genome matching
bp position
0.0
0.5
1.0
1.5
2.0
2.5
0
20
40
60

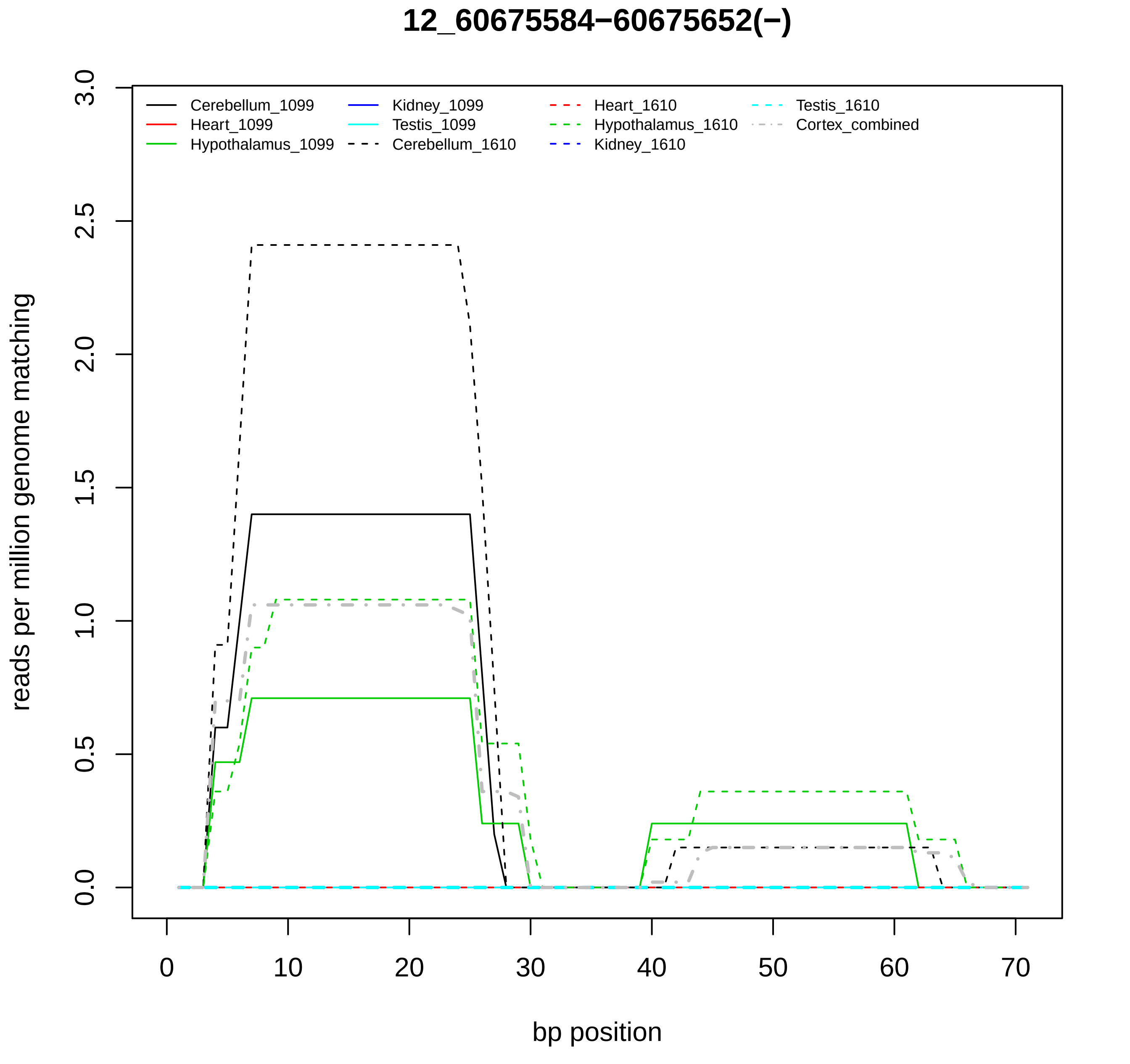

12_60675584−60675652(−)
Cerebellum_1099
Heart_1099
Hypothalamus_1099
Kidney_1099
Testis_1099
Cerebellum_1610
Heart_1610
Hypothalamus_1610
Kidney_1610
Testis_1610
Cortex_combined
reads per million genome matching
bp position
0.0
0.5
1.0
1.5
2.0
2.5
3.0
0
10
20
30
40
50
60
70

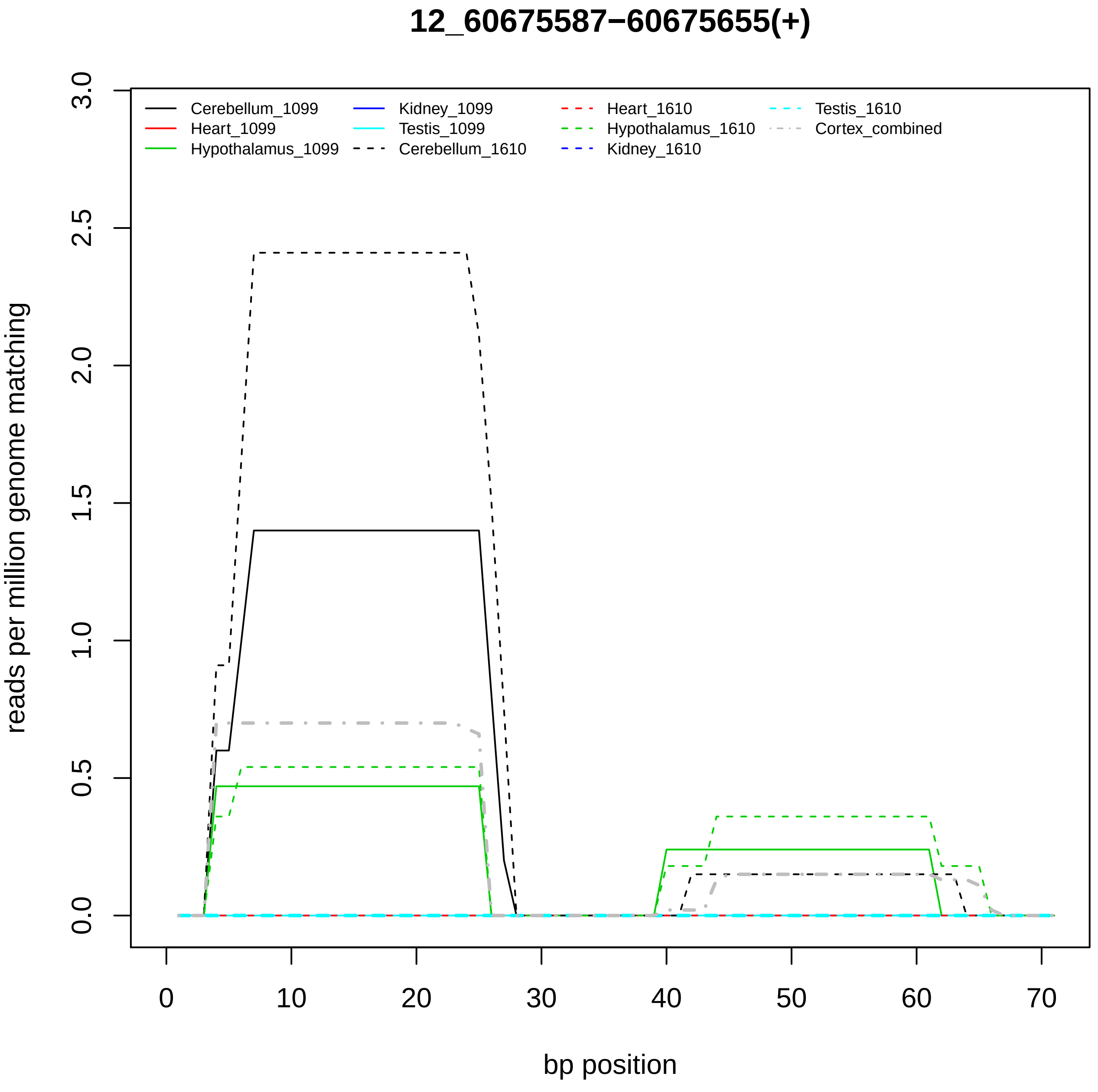

12_60675587−60675655(+)
Cerebellum_1099
Heart_1099
Hypothalamus_1099
Kidney_1099
Testis_1099
Cerebellum_1610
Heart_1610
Hypothalamus_1610
Kidney_1610
Testis_1610
Cortex_combined
reads per million genome matching
bp position
0.0
0.5
1.0
1.5
2.0
2.5
3.0
0
10
20
30
40
50
60
70

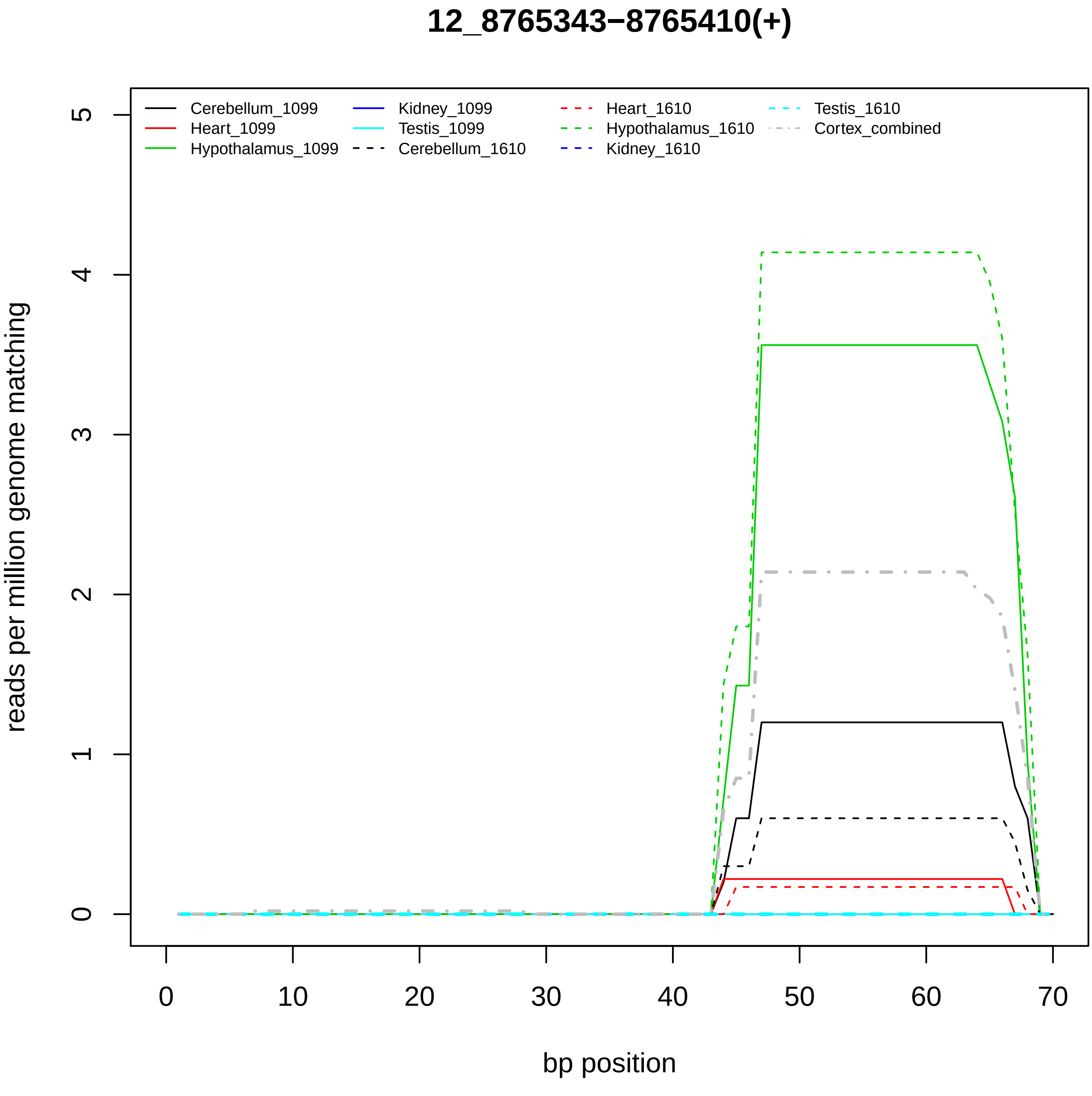
12_8765343−8765410(+)
Cerebellum_1099
Heart_1099
Hypothalamus_1099
Kidney_1099
Testis_1099
Cerebellum_1610
Heart_1610
Hypothalamus_1610
Kidney_1610
Testis_1610
Cortex_combined
reads per million genome matching
bp position
0
1
2
3
4
5
0
10
20
30
40
50
60
70

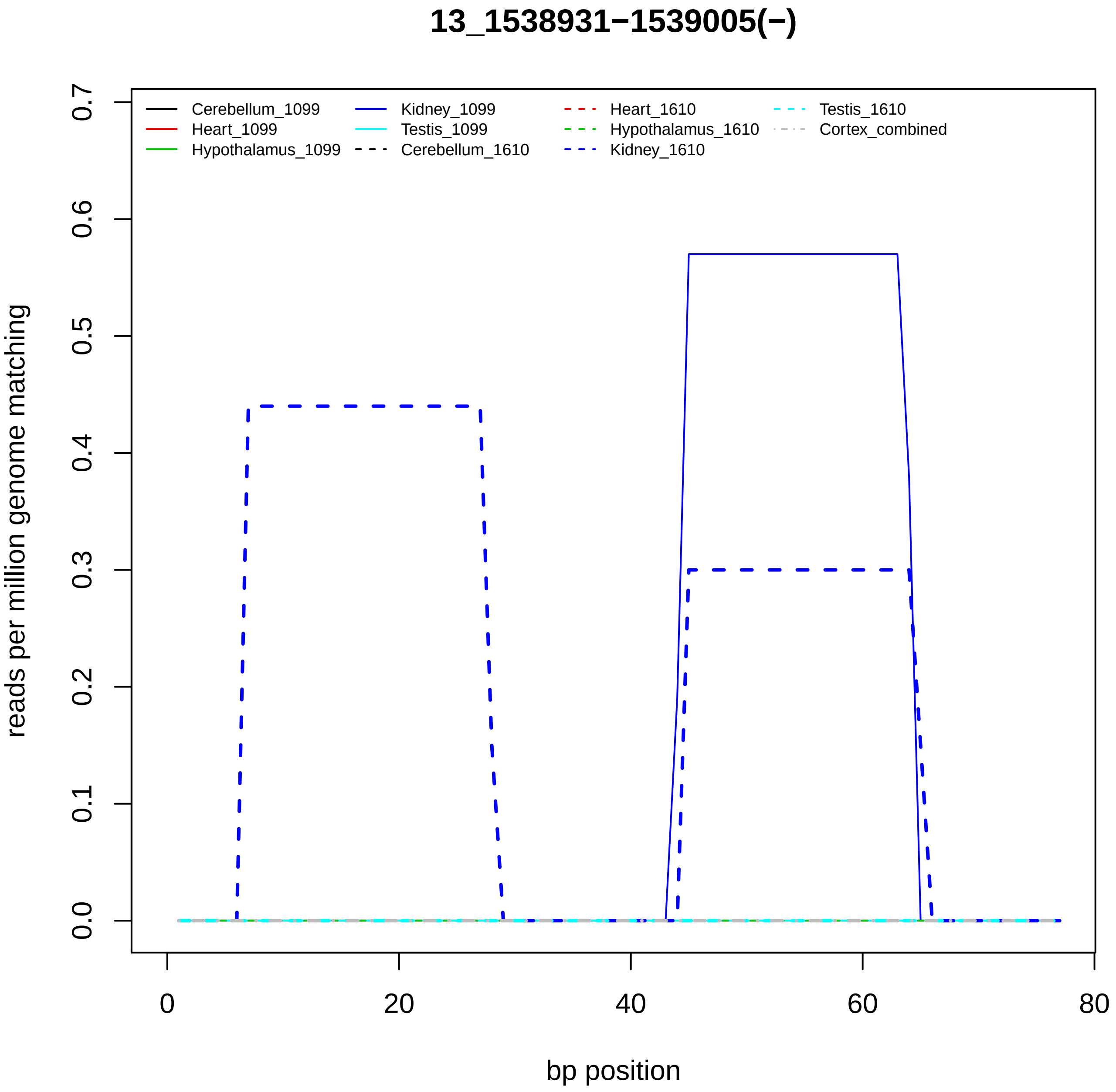
13_1538931−1539005(−)
Cerebellum_1099
Heart_1099
Hypothalamus_1099
Kidney_1099
Testis_1099
Cerebellum_1610
Heart_1610
Hypothalamus_1610
Kidney_1610
Testis_1610
Cortex_combined
reads per million genome matching
0.0
0.1
0.2
0.3
0.4
0.5
0.6
0.7
bp position
0
20
40
60
80

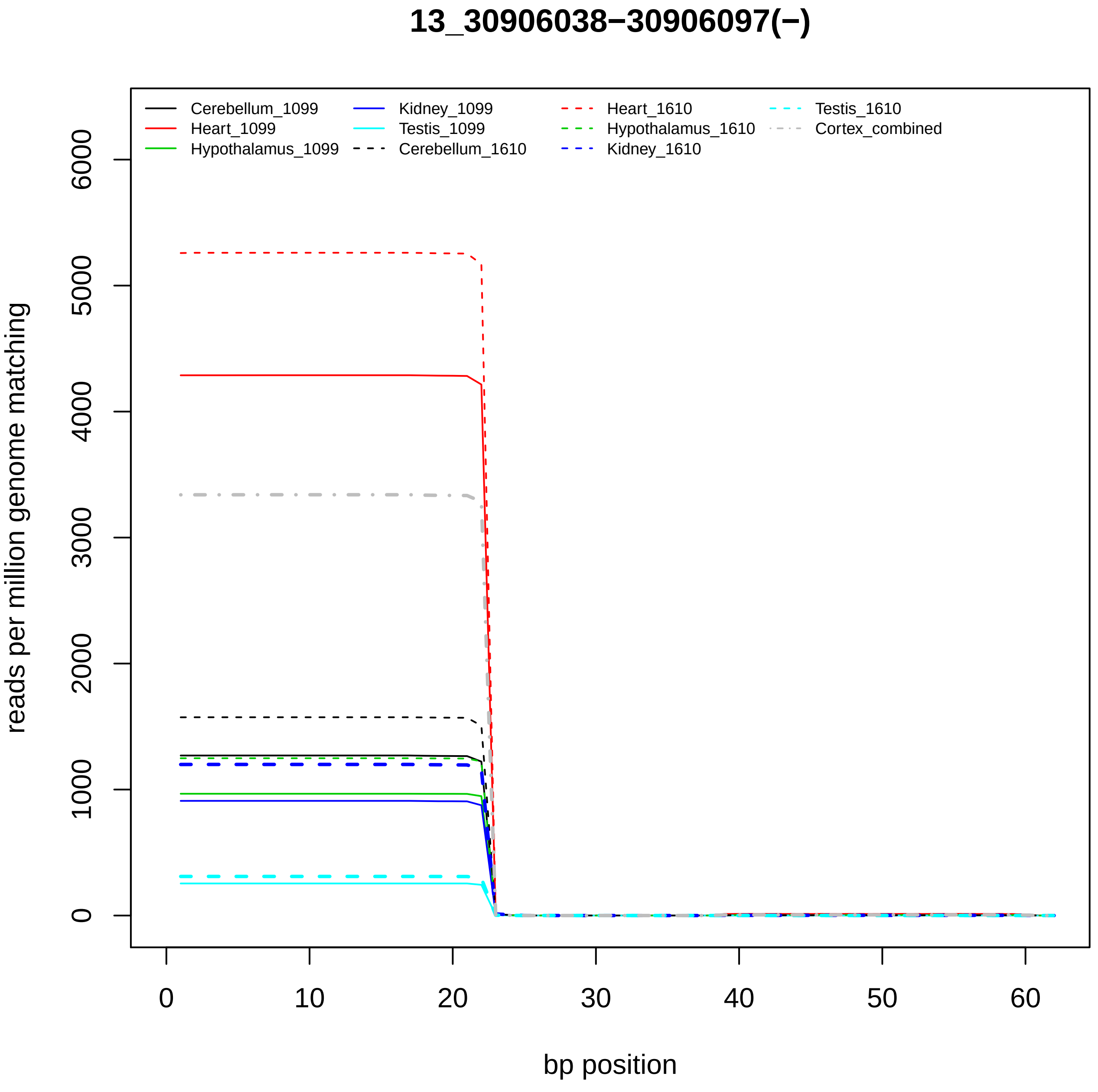
13_30906038−30906097(−)
Cerebellum_1099
Heart_1099
Hypothalamus_1099
Kidney_1099
Testis_1099
Cerebellum_1610
Heart_1610
Hypothalamus_1610
Kidney_1610
Testis_1610
Cortex_combined
reads per million genome matching
0
1000
2000
3000
4000
5000
6000
0
10
20
30
40
50
60
bp position

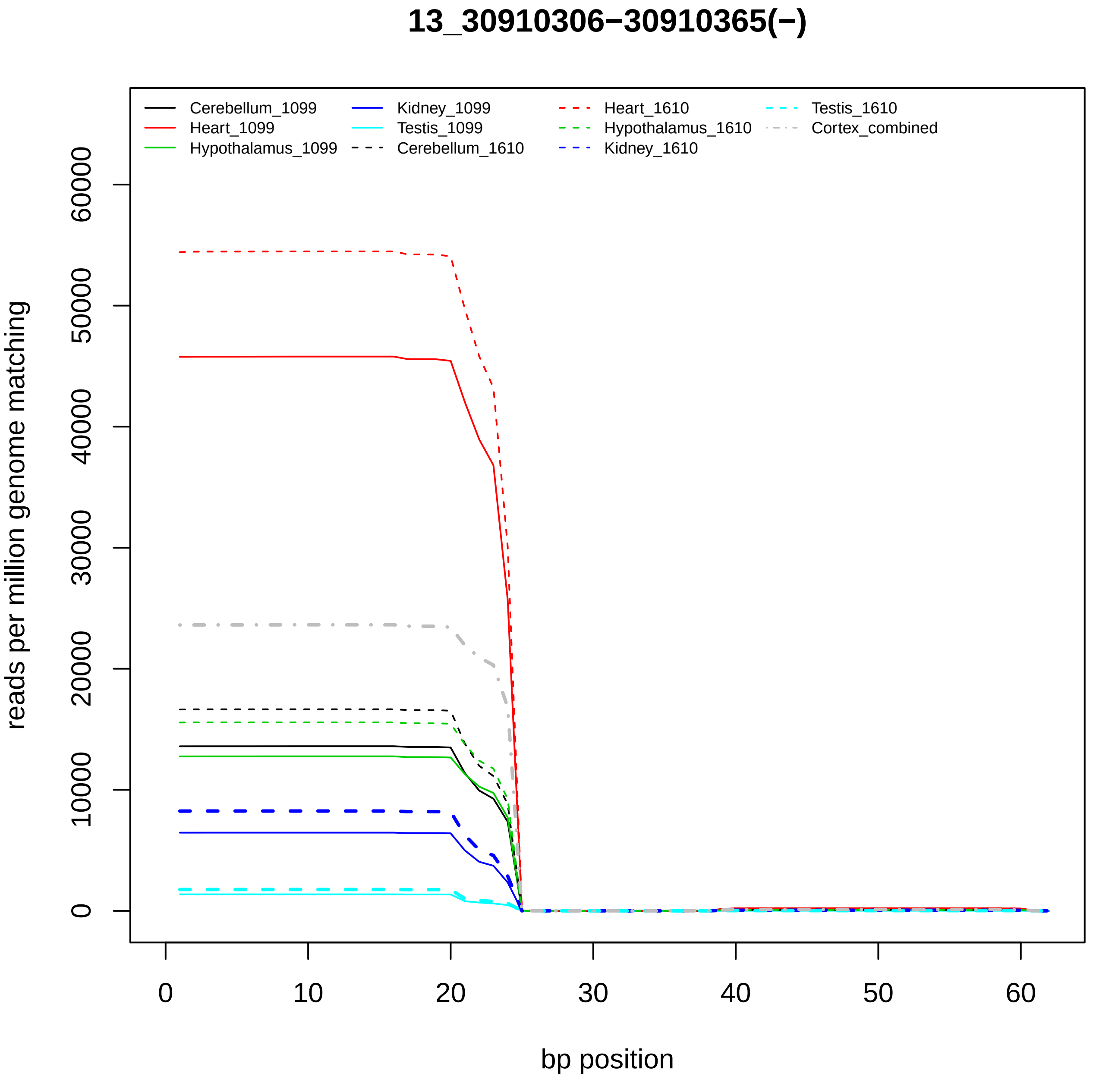

13_30910306−30910365(−)
Cerebellum_1099
Heart_1099
Hypothalamus_1099
Kidney_1099
Testis_1099
Cerebellum_1610
Heart_1610
Hypothalamus_1610
Kidney_1610
Testis_1610
Cortex_combined
reads per million genome matching
0
10000
20000
30000
40000
50000
60000
0
10
20
30
40
50
60
bp position

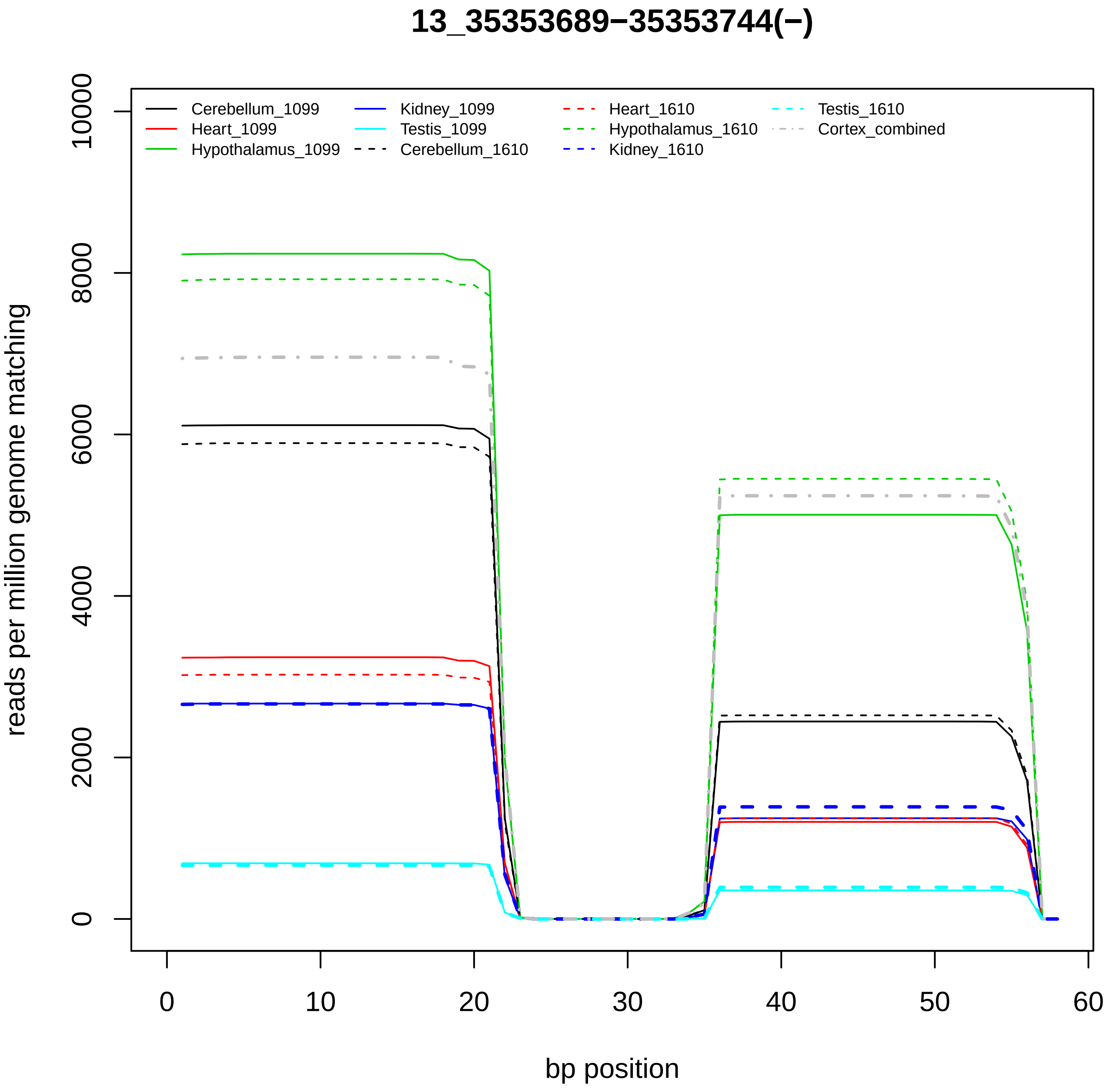

13_35353689−35353744(−)
Cerebellum_1099
Heart_1099
Hypothalamus_1099
Kidney_1099
Testis_1099
Cerebellum_1610
Heart_1610
Hypothalamus_1610
Kidney_1610
Testis_1610
Cortex_combined
reads per million genome matching
0
2000
4000
6000
8000
10000
bp position
0
10
20
30
40
50
60

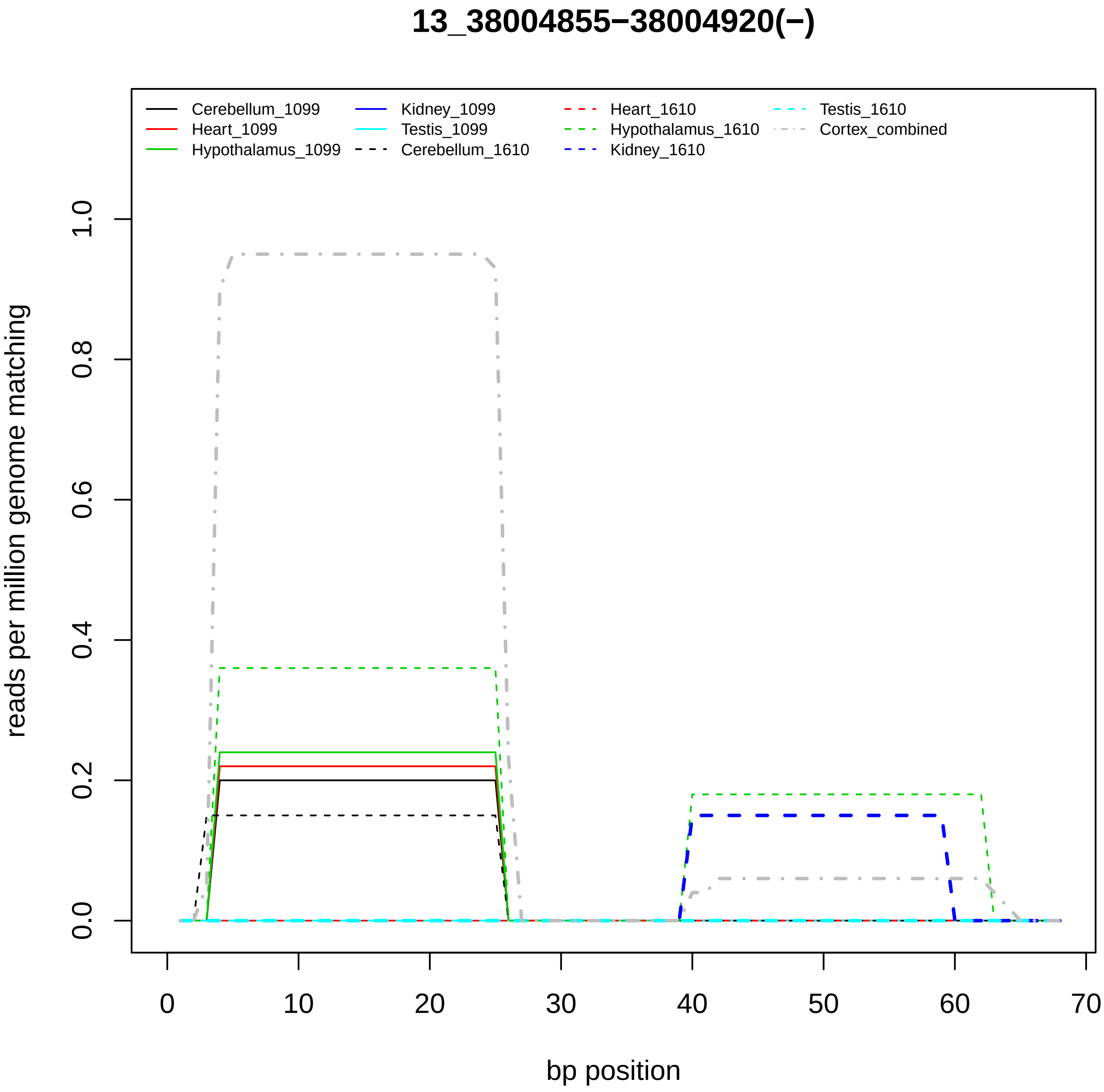
13_38004855−38004920(−)
Cerebellum_1099
Heart_1099
Hypothalamus_1099
Kidney_1099
Testis_1099
Cerebellum_1610
Heart_1610
Hypothalamus_1610
Kidney_1610
Testis_1610
Cortex_combined
reads per million genome matching
0.0
0.2
0.4
0.6
0.8
1.0
0
10
20
30
40
50
60
70
bp position

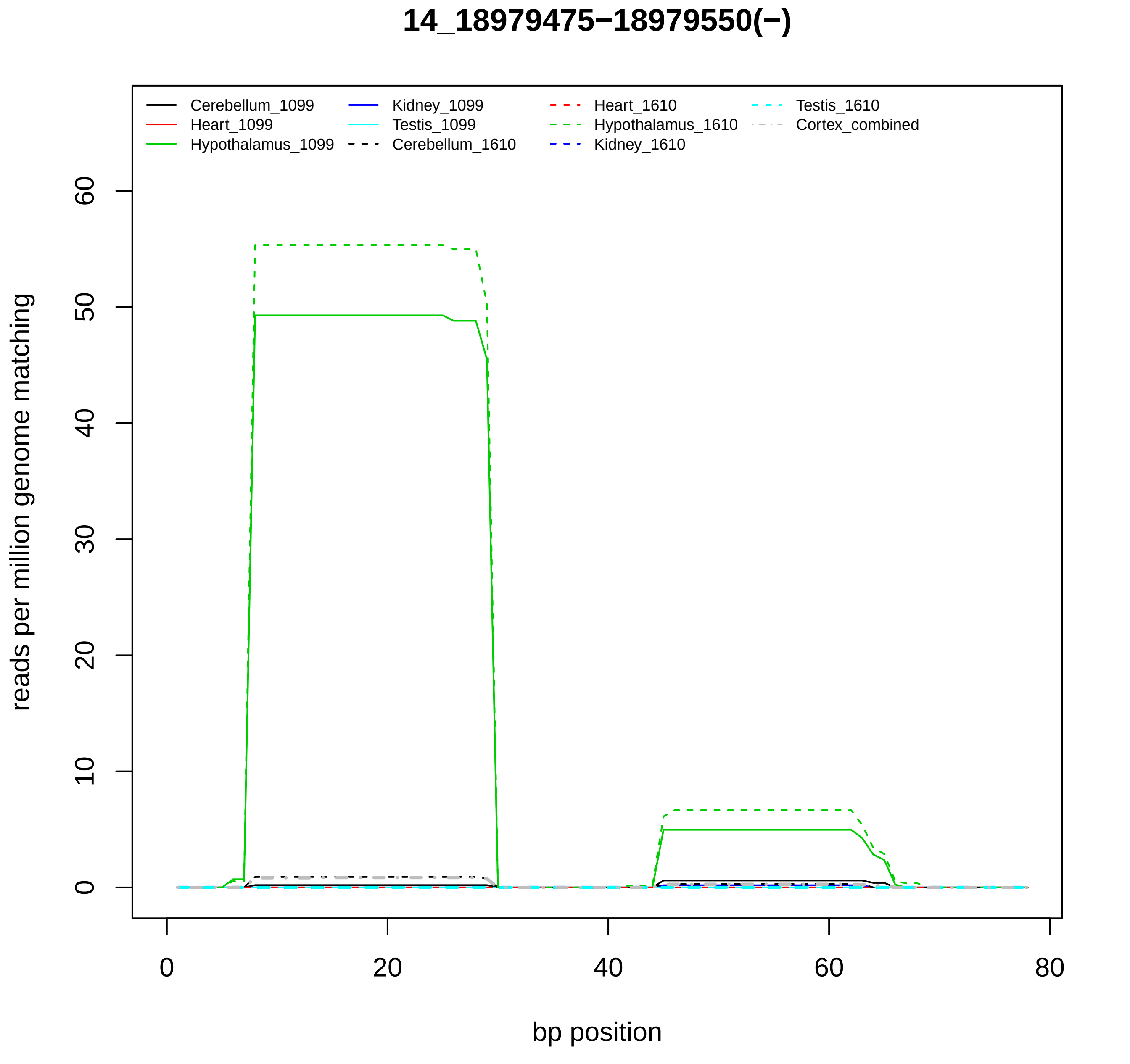

14_18979475−18979550(−)
Cerebellum_1099
Heart_1099
Hypothalamus_1099
Kidney_1099
Testis_1099
Cerebellum_1610
Heart_1610
Hypothalamus_1610
Kidney_1610
Testis_1610
Cortex_combined
reads per million genome matching
bp position
0
10
20
30
40
50
60
0
20
40
60
80

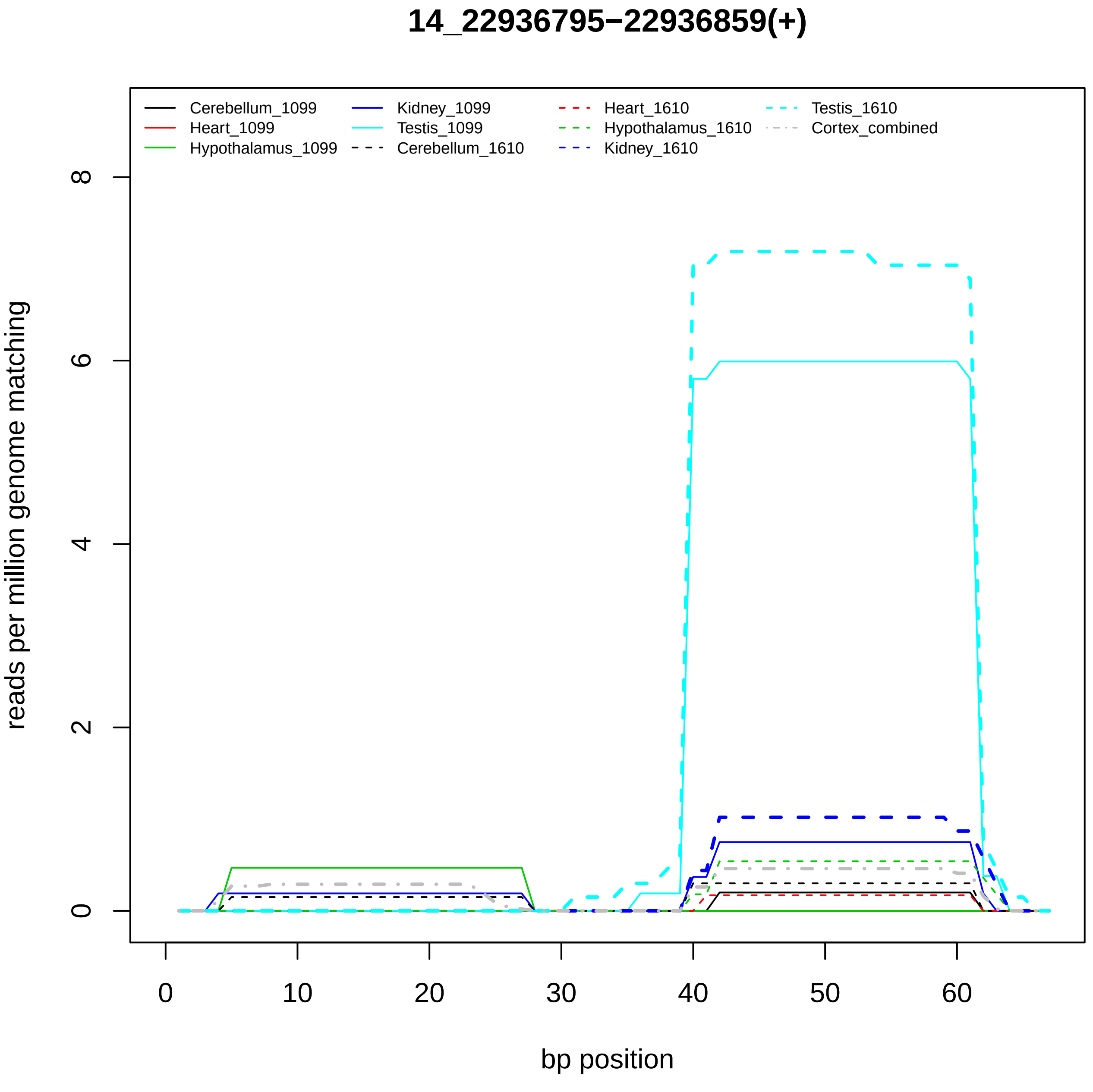

14_22936795−22936859(+)
Cerebellum_1099
Heart_1099
Hypothalamus_1099
Kidney_1099
Testis_1099
Cerebellum_1610
Heart_1610
Hypothalamus_1610
Kidney_1610
Testis_1610
Cortex_combined
reads per million genome matching
0
2
4
6
8
0
10
20
30
40
50
60
bp position

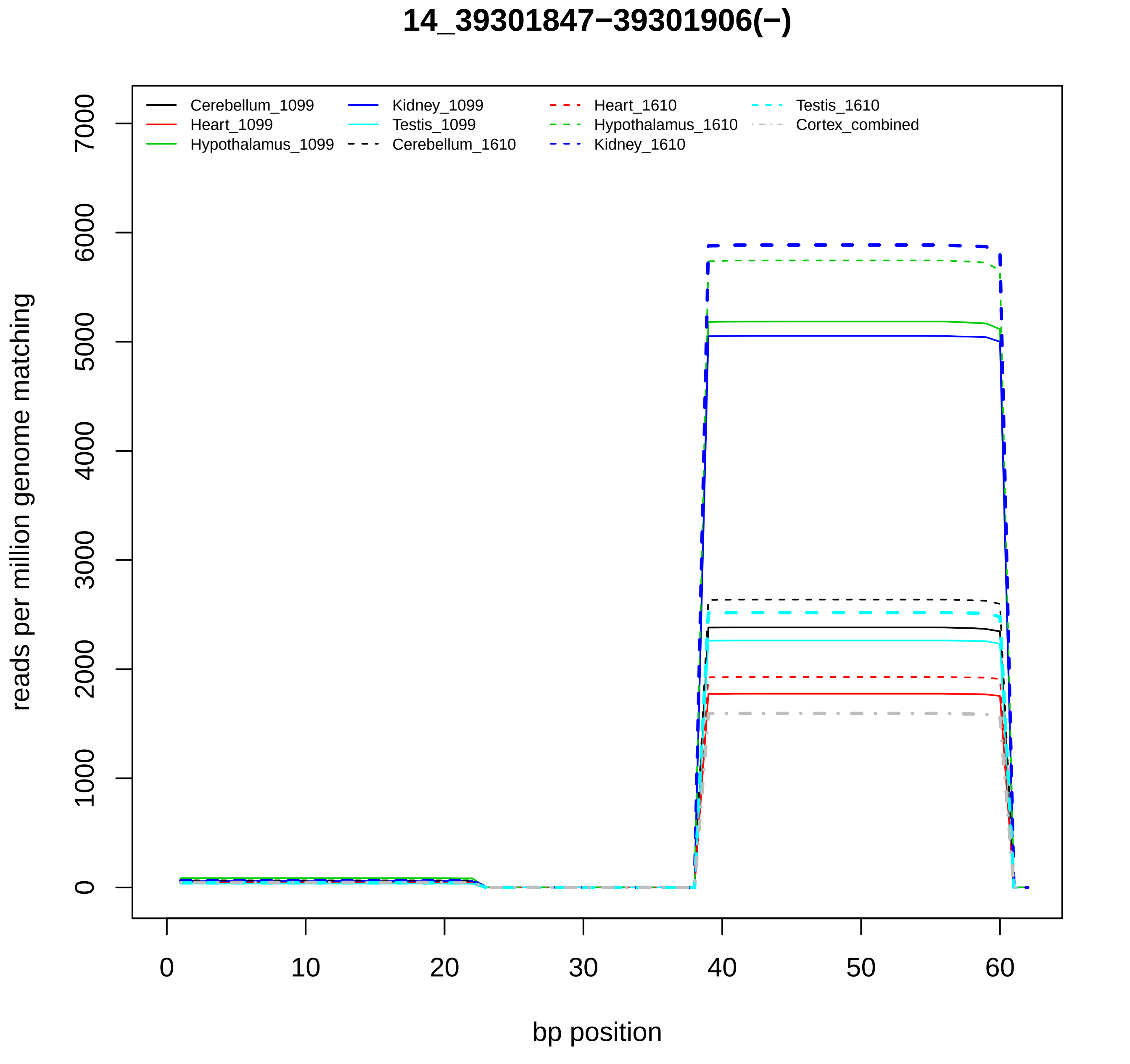

14_39301847−39301906(−)
reads per million genome matching
bp position
0
1000
2000
3000
4000
5000
6000
7000
0
10
20
30
40
50
60
Cerebellum_1099
Heart_1099
Hypothalamus_1099
Kidney_1099
Testis_1099
Cerebellum_1610
Heart_1610
Hypothalamus_1610
Kidney_1610
Testis_1610
Cortex_combined

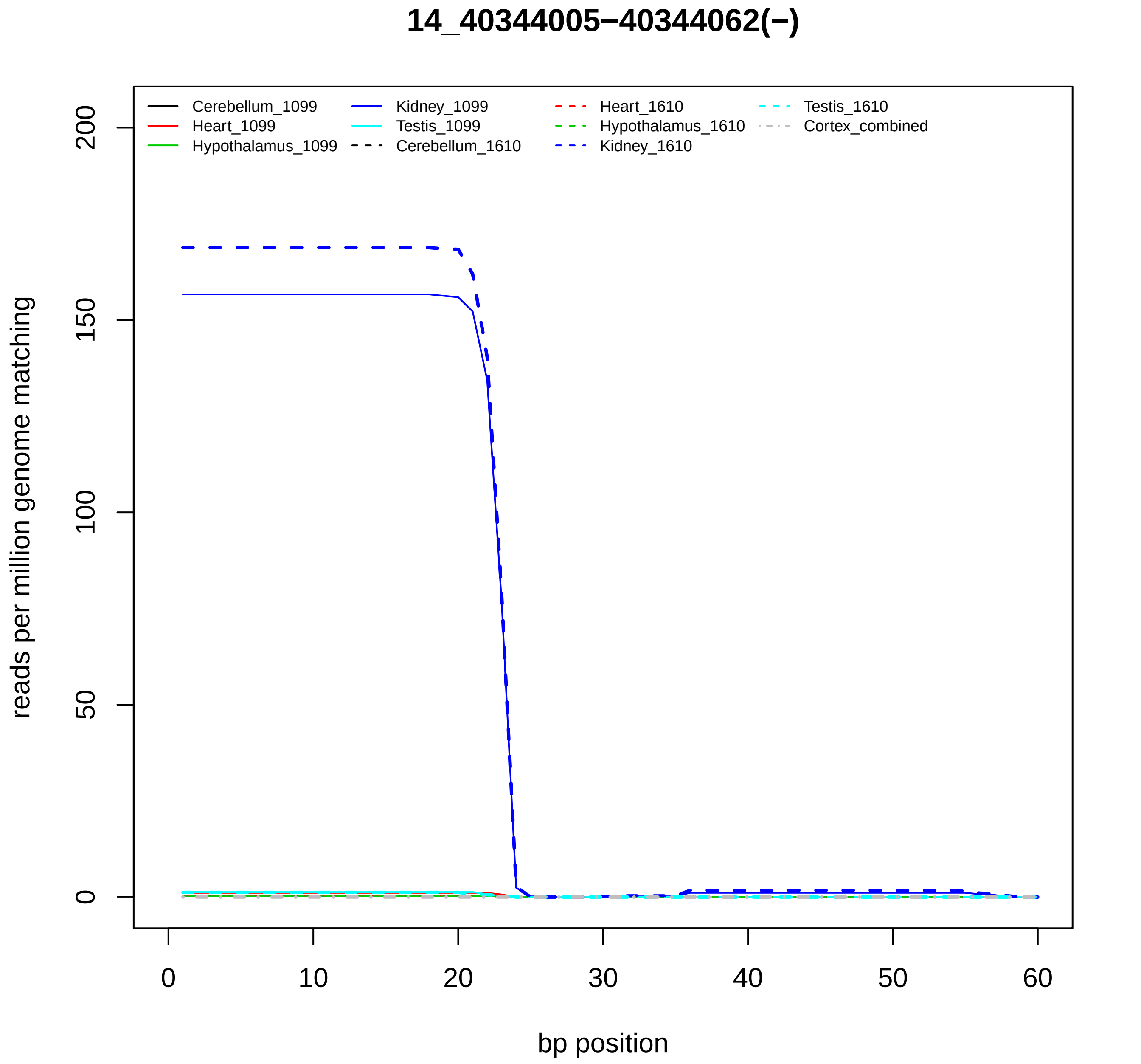

14_40344005−40344062(−)
Cerebellum_1099
Heart_1099
Hypothalamus_1099
Kidney_1099
Testis_1099
Cerebellum_1610
Heart_1610
Hypothalamus_1610
Kidney_1610
Testis_1610
Cortex_combined
reads per million genome matching
bp position
0
50
100
150
200
0
10
20
30
40
50
60

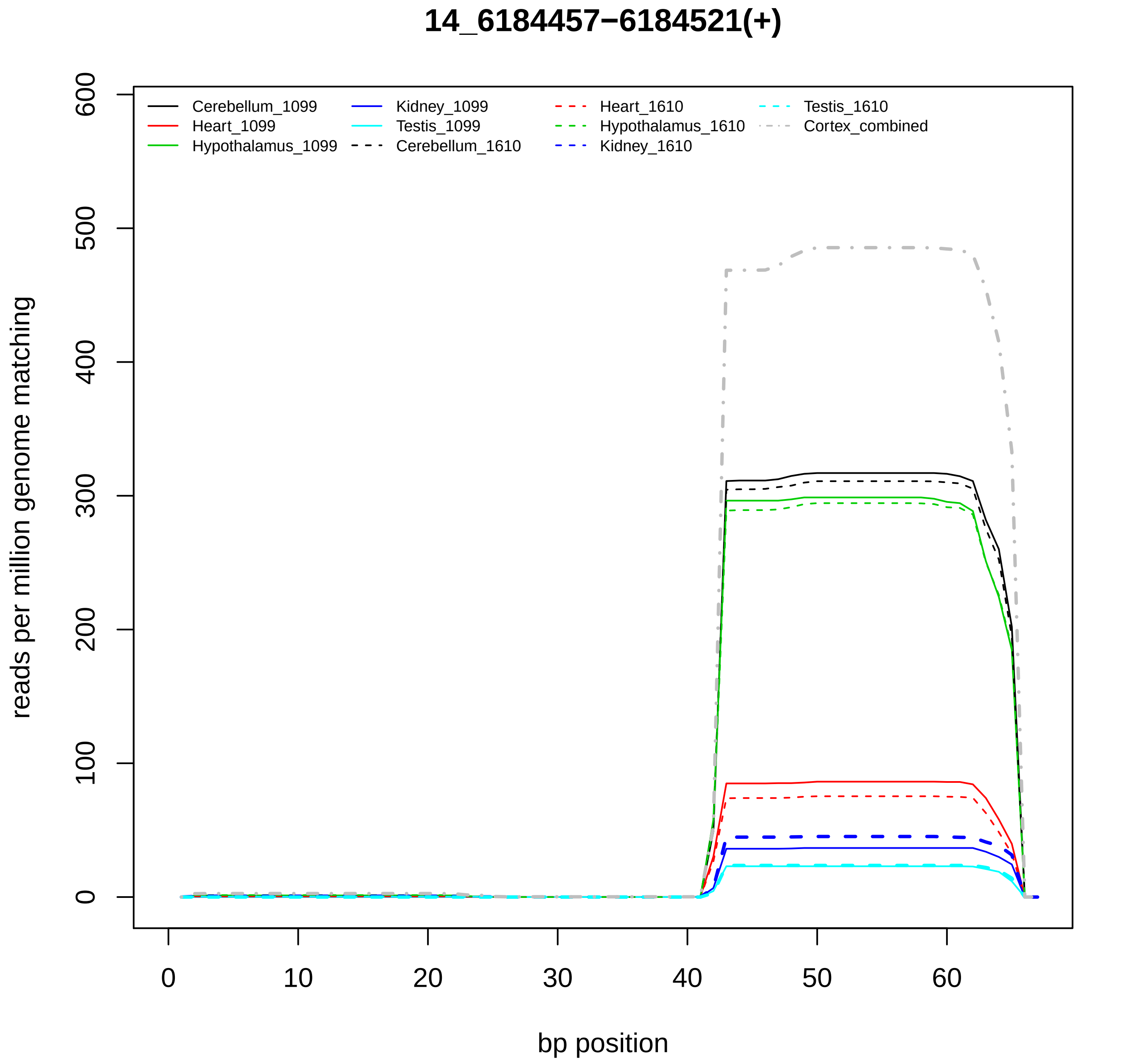

14_6184457−6184521(+)
Cerebellum_1099
Heart_1099
Hypothalamus_1099
Kidney_1099
Testis_1099
Cerebellum_1610
Heart_1610
Hypothalamus_1610
Kidney_1610
Testis_1610
Cortex_combined
reads per million genome matching
bp position
0
100
200
300
400
500
600
0
10
20
30
40
50
60

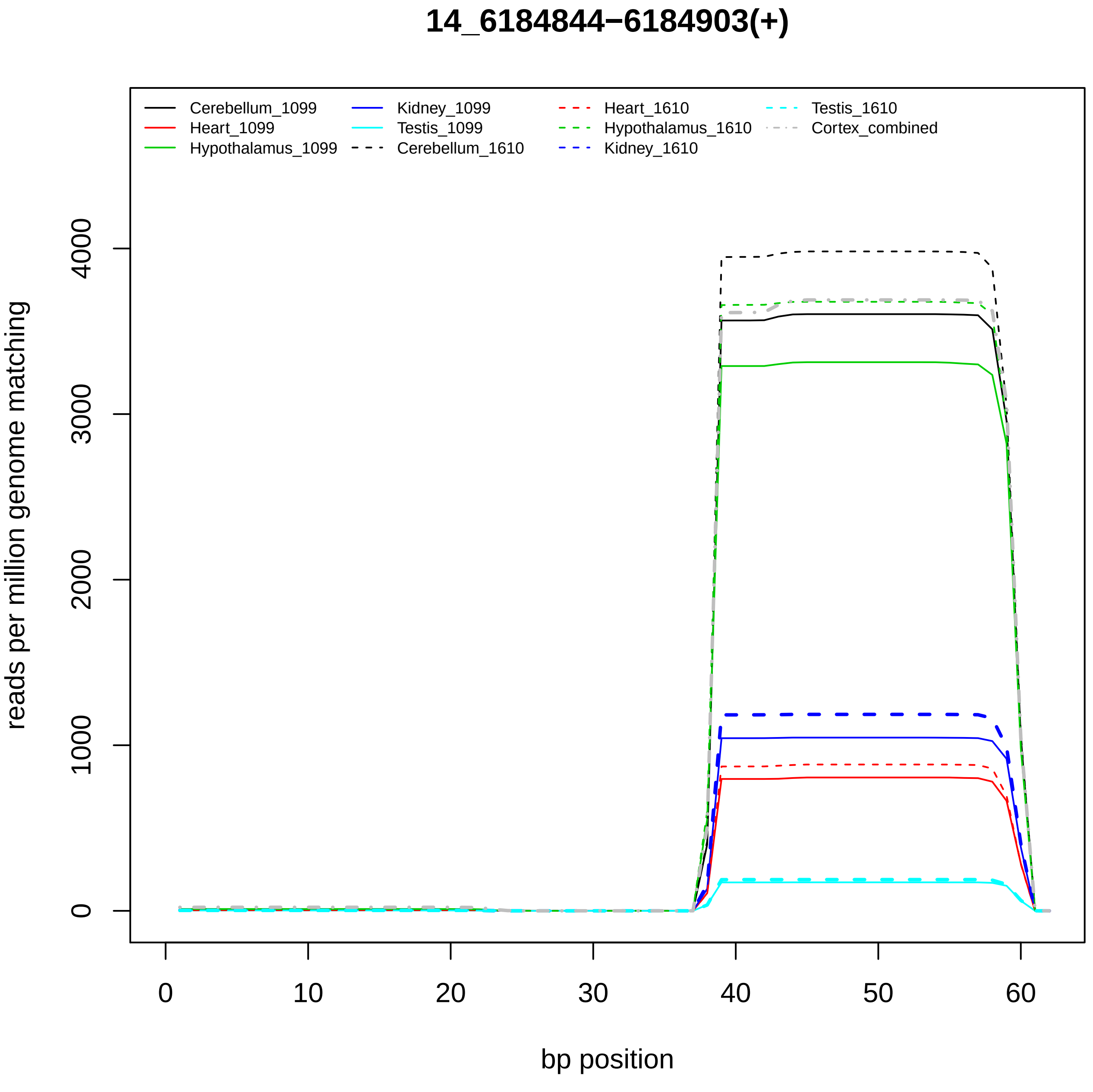
14_6184844−6184903(+)
Cerebellum_1099
Heart_1099
Hypothalamus_1099
Kidney_1099
Testis_1099
Cerebellum_1610
Heart_1610
Hypothalamus_1610
Kidney_1610
Testis_1610
Cortex_combined
reads per million genome matching
0
1000
2000
3000
4000
0
10
20
30
40
50
60
bp position

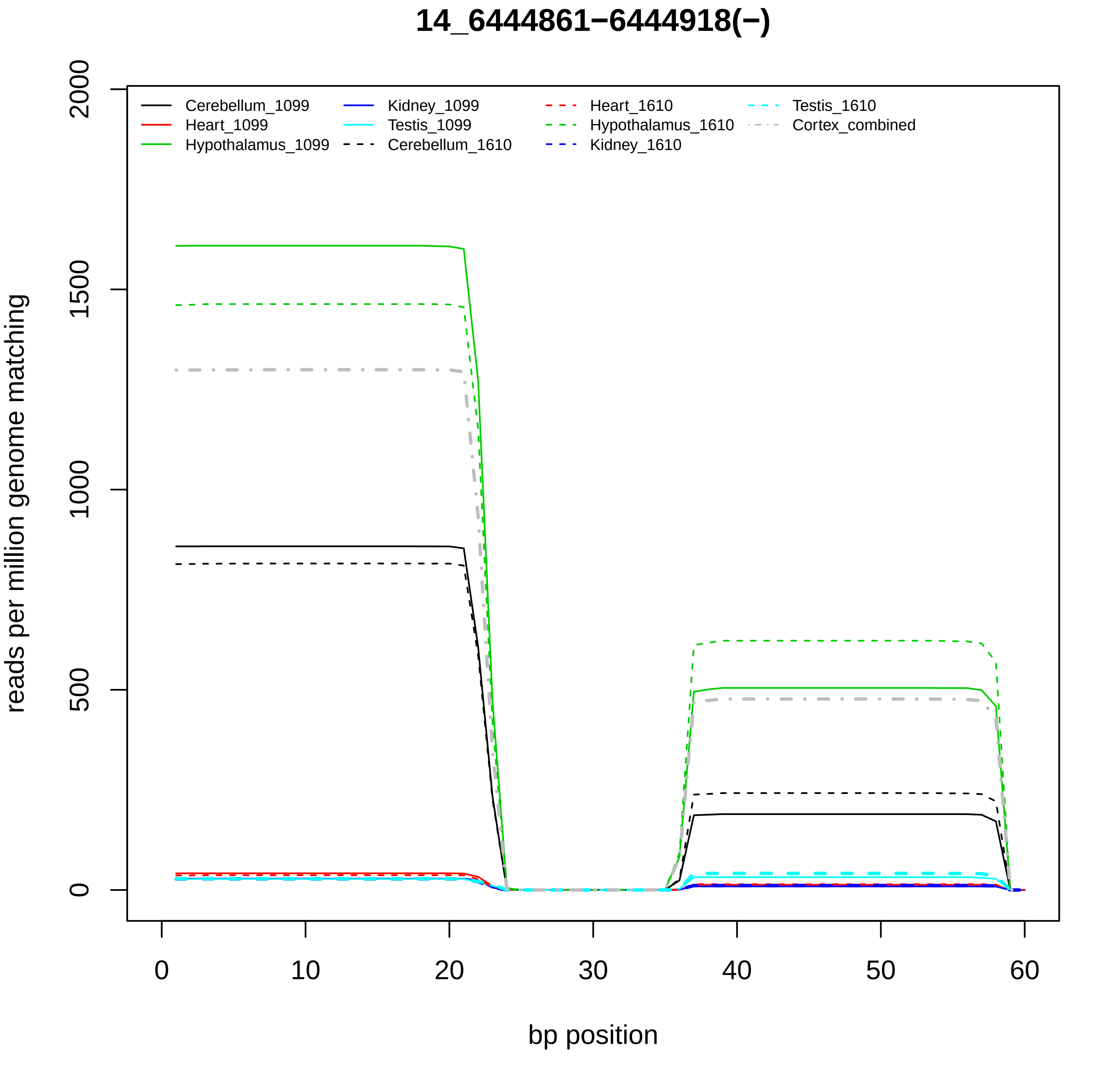
14_6444861−6444918(−)
Cerebellum_1099
Heart_1099
Hypothalamus_1099
Kidney_1099
Testis_1099
Cerebellum_1610
Heart_1610
Hypothalamus_1610
Kidney_1610
Testis_1610
Cortex_combined
reads per million genome matching
bp position
0
500
1000
1500
2000
0
10
20
30
40
50
60

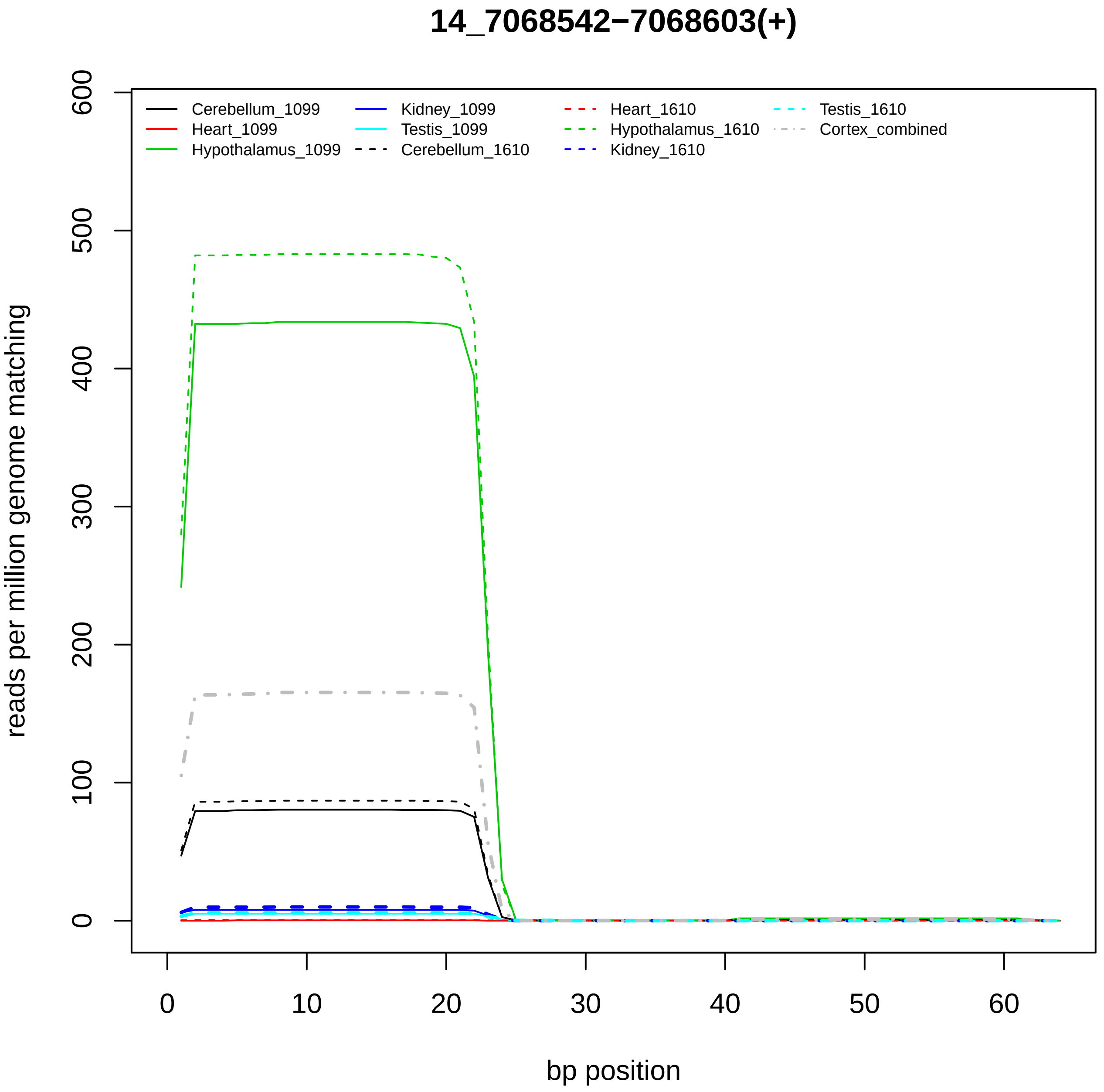

14_7068542−7068603(+)
Cerebellum_1099
Heart_1099
Hypothalamus_1099
Kidney_1099
Testis_1099
Cerebellum_1610
Heart_1610
Hypothalamus_1610
Kidney_1610
Testis_1610
Cortex_combined
reads per million genome matching
bp position
0
100
200
300
400
500
600
0
10
20
30
40
50
60

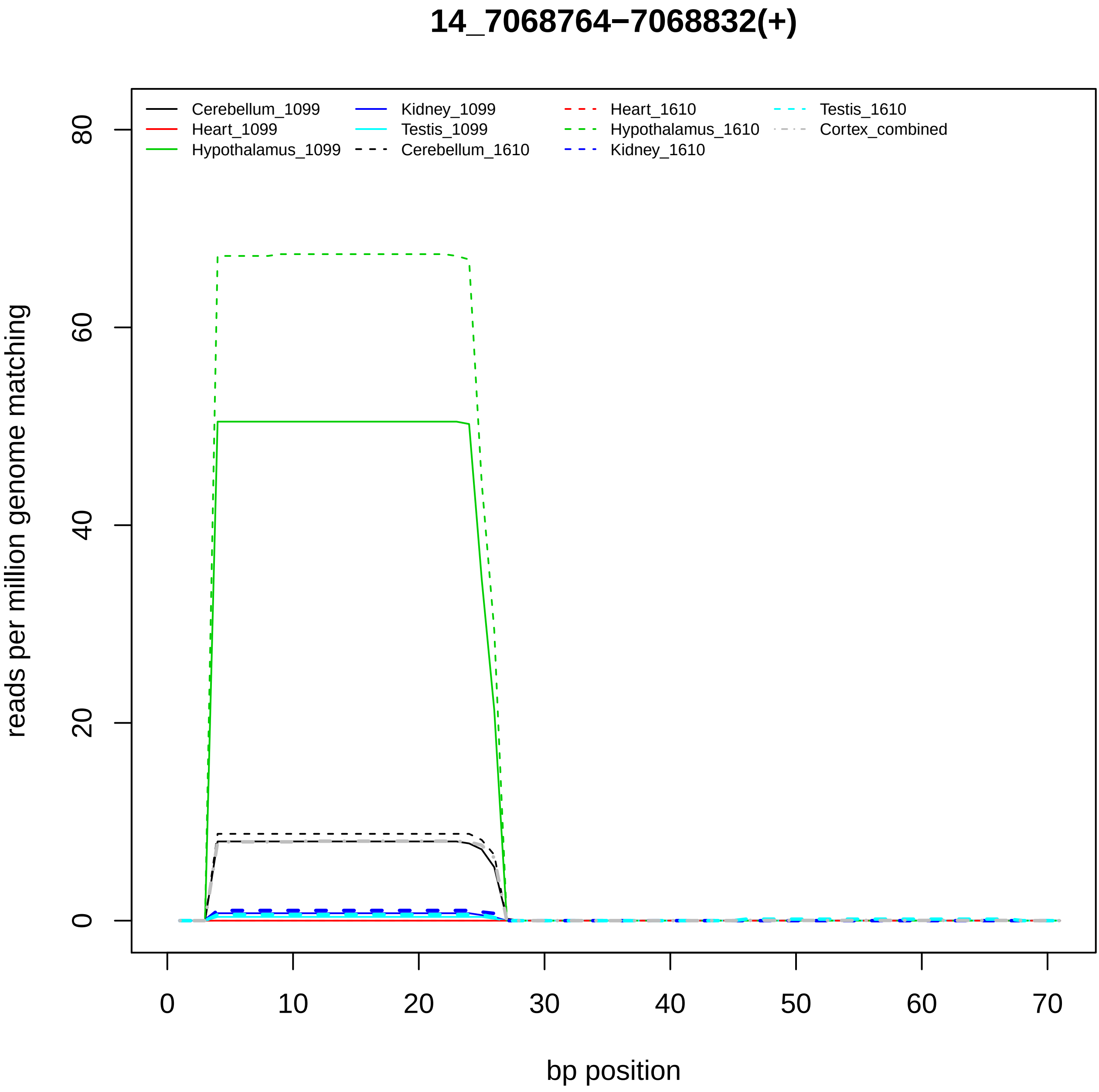
14_7068764−7068832(+)
Cerebellum_1099
Heart_1099
Hypothalamus_1099
Kidney_1099
Testis_1099
Cerebellum_1610
Heart_1610
Hypothalamus_1610
Kidney_1610
Testis_1610
Cortex_combined
reads per million genome matching
0
20
40
60
80
0
10
20
30
40
50
60
70
bp position

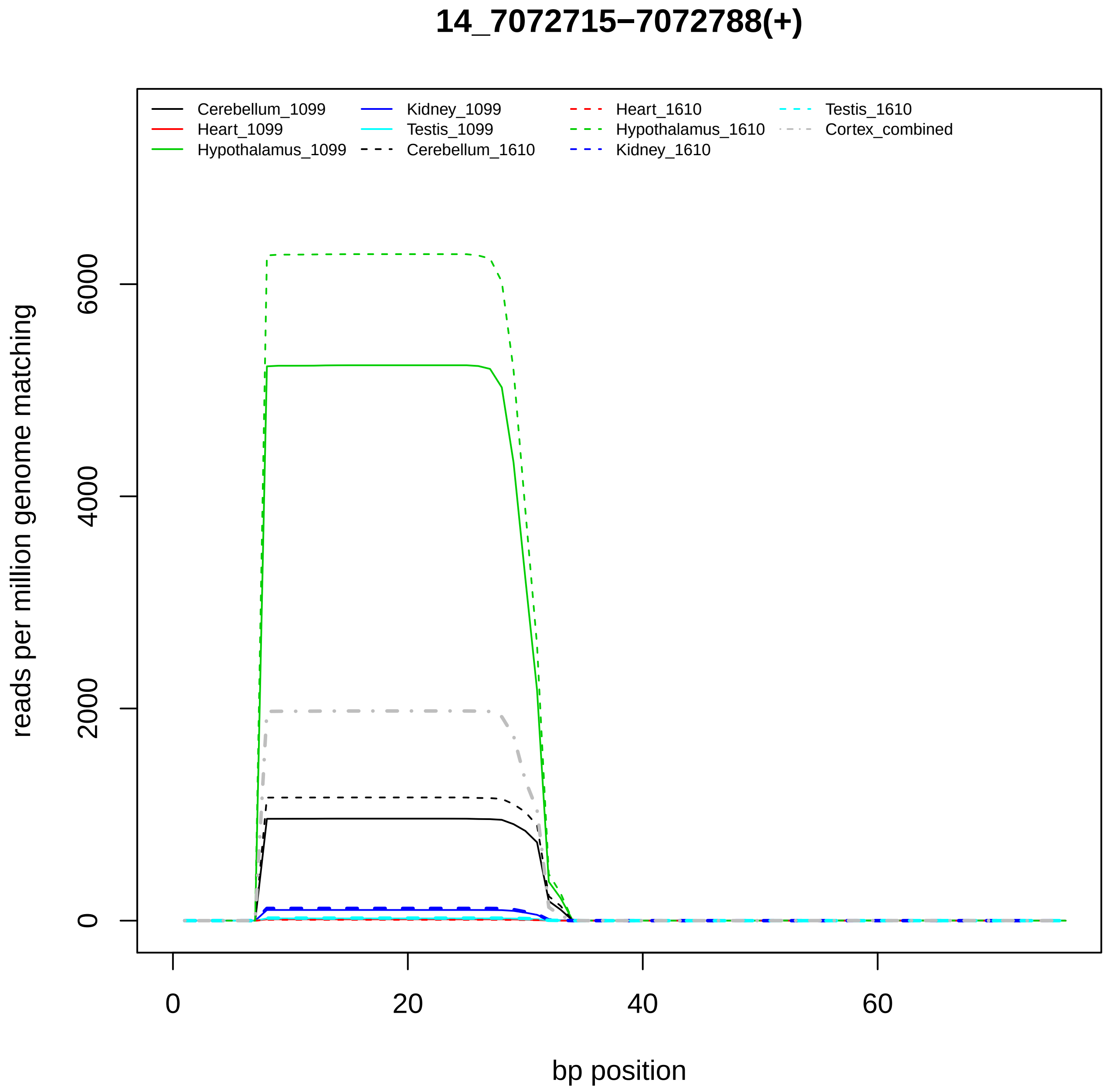

14_7072715−7072788(+)
Cerebellum_1099
Heart_1099
Hypothalamus_1099
Kidney_1099
Testis_1099
Cerebellum_1610
Heart_1610
Hypothalamus_1610
Kidney_1610
Testis_1610
Cortex_combined
reads per million genome matching
0
2000
4000
6000
0
20
40
60
bp position

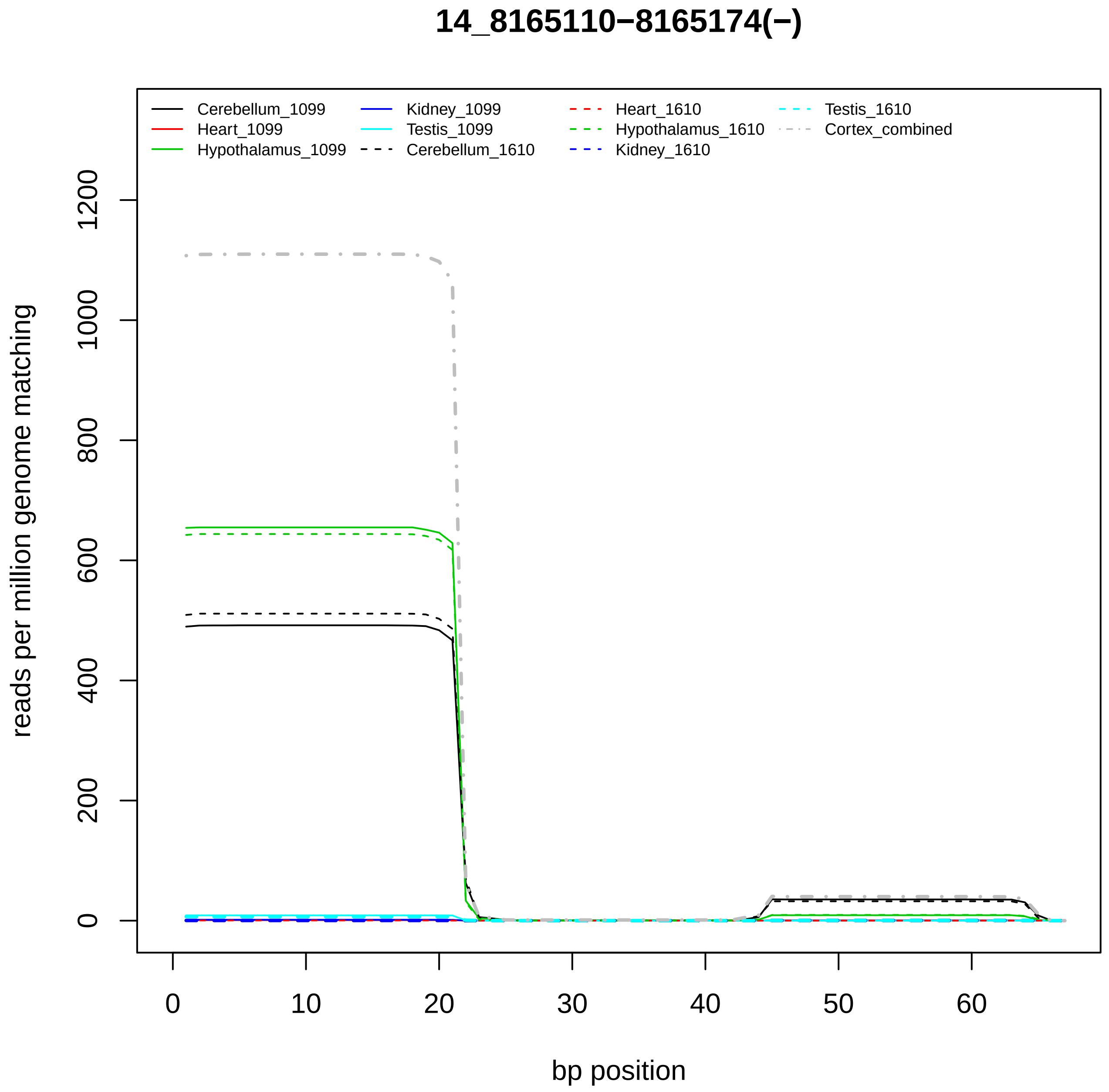
14_8165110−8165174(−)
Cerebellum_1099
Heart_1099
Hypothalamus_1099
Kidney_1099
Testis_1099
Cerebellum_1610
Heart_1610
Hypothalamus_1610
Kidney_1610
Testis_1610
Cortex_combined
reads per million genome matching
0
200
400
600
800
1000
1200
0
10
20
30
40
50
60
bp position

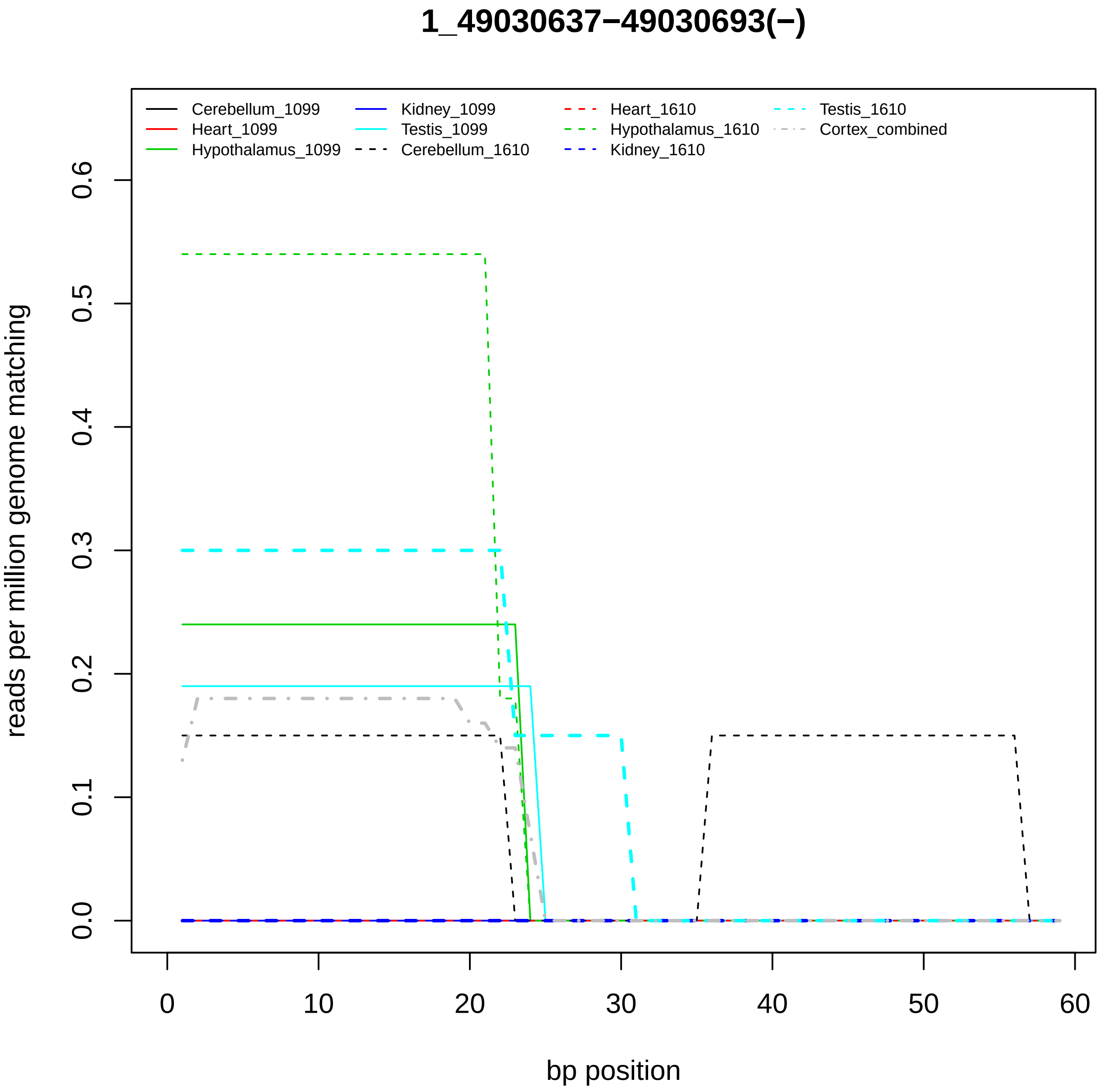

1_49030637−49030693(−)
Cerebellum_1099
Heart_1099
Hypothalamus_1099
Kidney_1099
Testis_1099
Cerebellum_1610
Heart_1610
Hypothalamus_1610
Kidney_1610
Testis_1610
Cortex_combined
reads per million genome matching
0.0
0.1
0.2
0.3
0.4
0.5
0.6
0
10
20
30
40
50
60
bp position

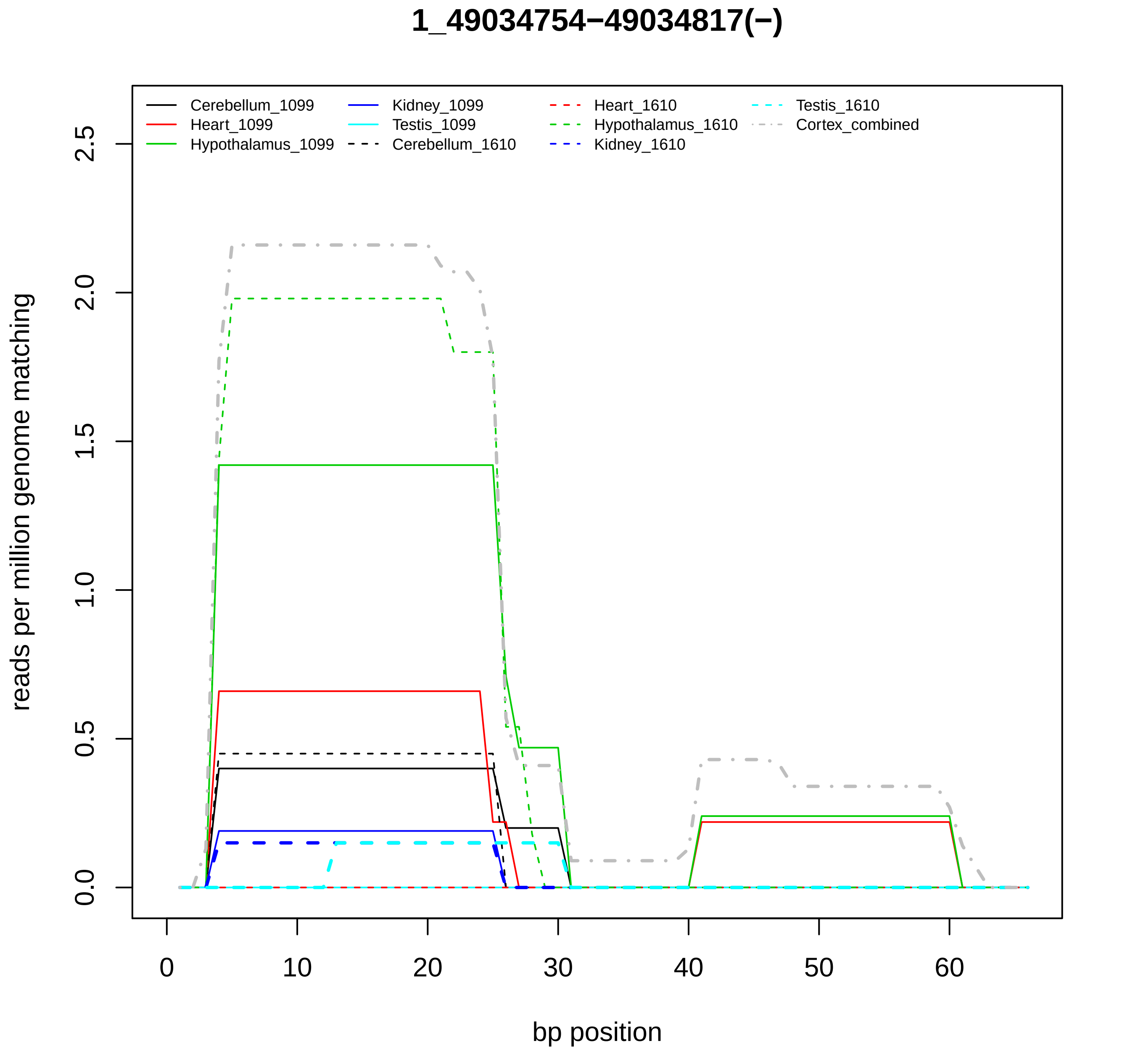

1_49034754−49034817(−)
Cerebellum_1099
Heart_1099
Hypothalamus_1099
Kidney_1099
Testis_1099
Cerebellum_1610
Heart_1610
Hypothalamus_1610
Kidney_1610
Testis_1610
Cortex_combined
reads per million genome matching
0.0
0.5
1.0
1.5
2.0
2.5
0
10
20
30
40
50
60
bp position

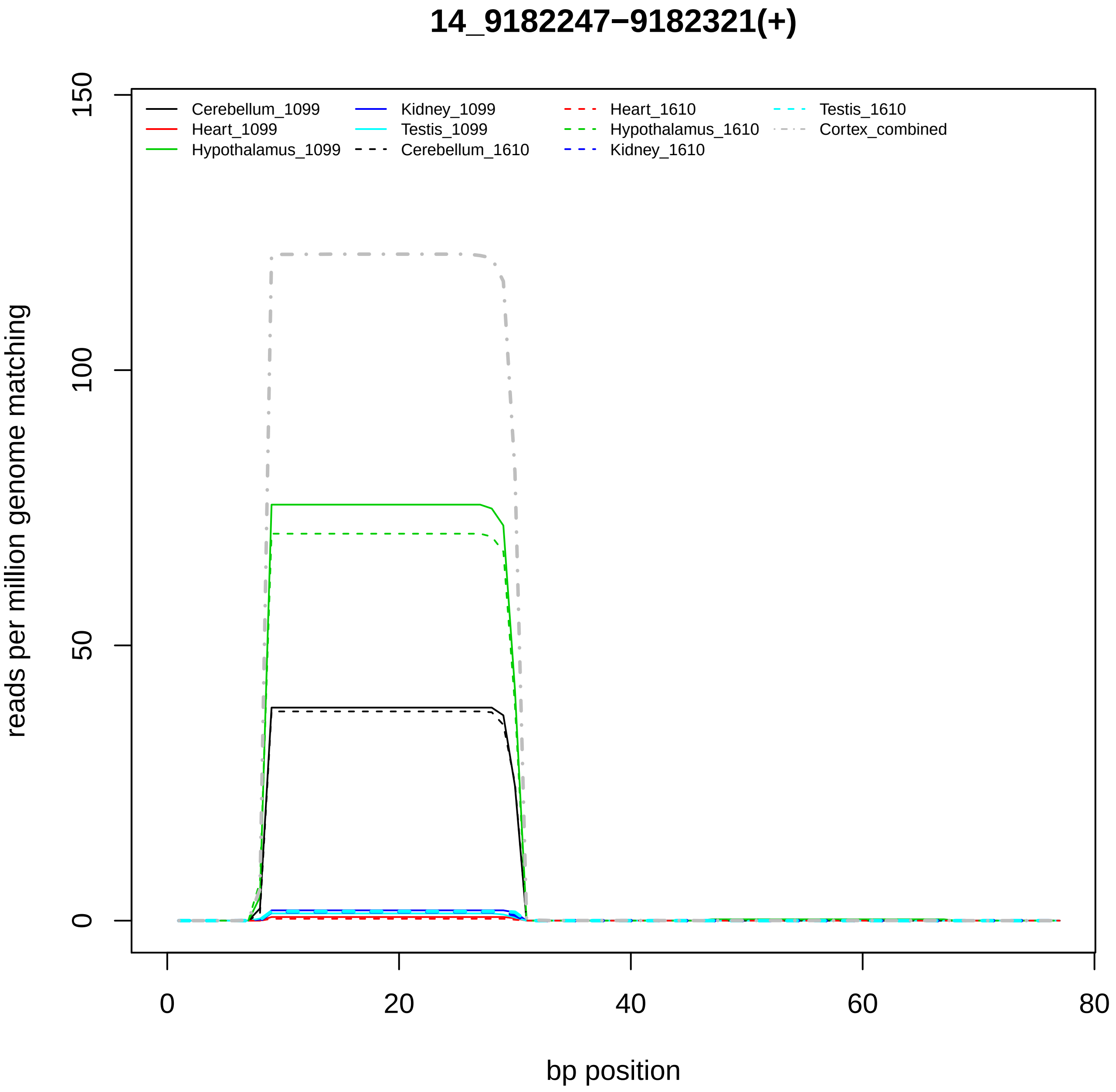

14_9182247−9182321(+)
Cerebellum_1099
Heart_1099
Hypothalamus_1099
Kidney_1099
Testis_1099
Cerebellum_1610
Heart_1610
Hypothalamus_1610
Kidney_1610
Testis_1610
Cortex_combined
reads per million genome matching
bp position
0
50
100
150
0
20
40
60
80

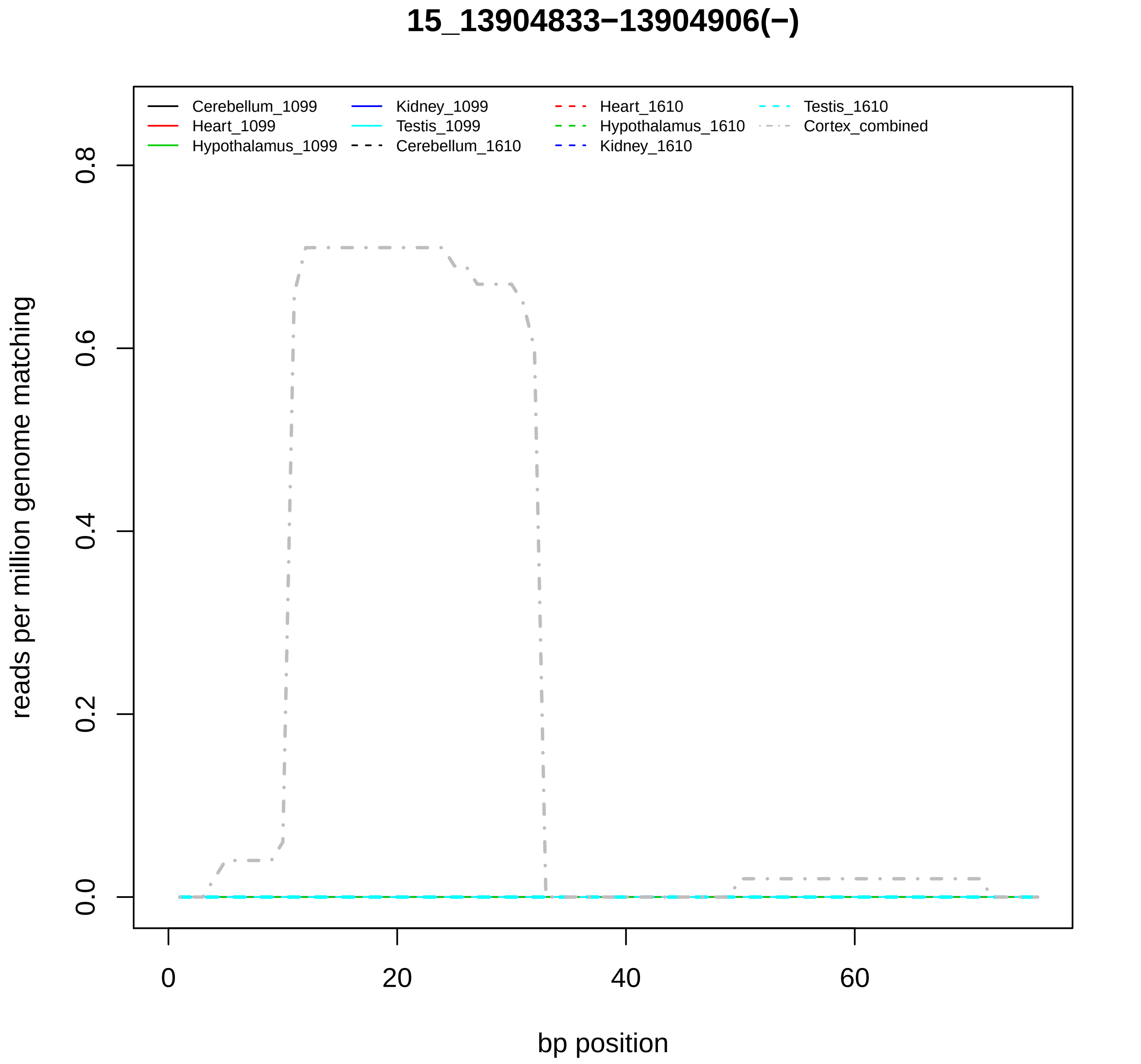

15_13904833−13904906(−)
Cerebellum_1099
Heart_1099
Hypothalamus_1099
Kidney_1099
Testis_1099
Cerebellum_1610
Heart_1610
Hypothalamus_1610
Kidney_1610
Testis_1610
Cortex_combined
reads per million genome matching
0.0
0.2
0.4
0.6
0.8
0
20
40
60
bp position

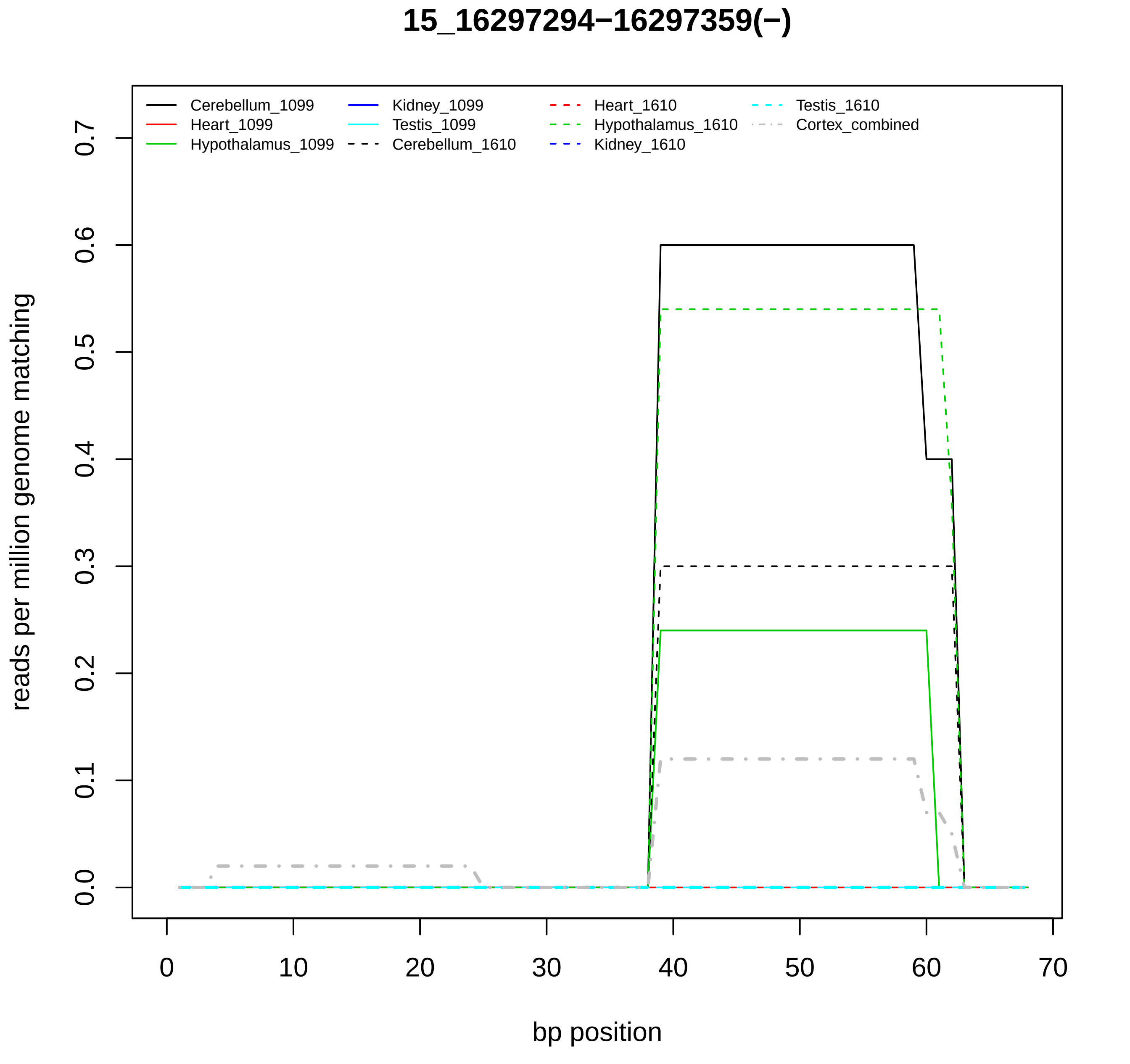

15_16297294−16297359(−)
Cerebellum_1099
Heart_1099
Hypothalamus_1099
Kidney_1099
Testis_1099
Cerebellum_1610
Heart_1610
Hypothalamus_1610
Kidney_1610
Testis_1610
Cortex_combined
reads per million genome matching
bp position
0.0
0.1
0.2
0.3
0.4
0.5
0.6
0.7
0
10
20
30
40
50
60
70

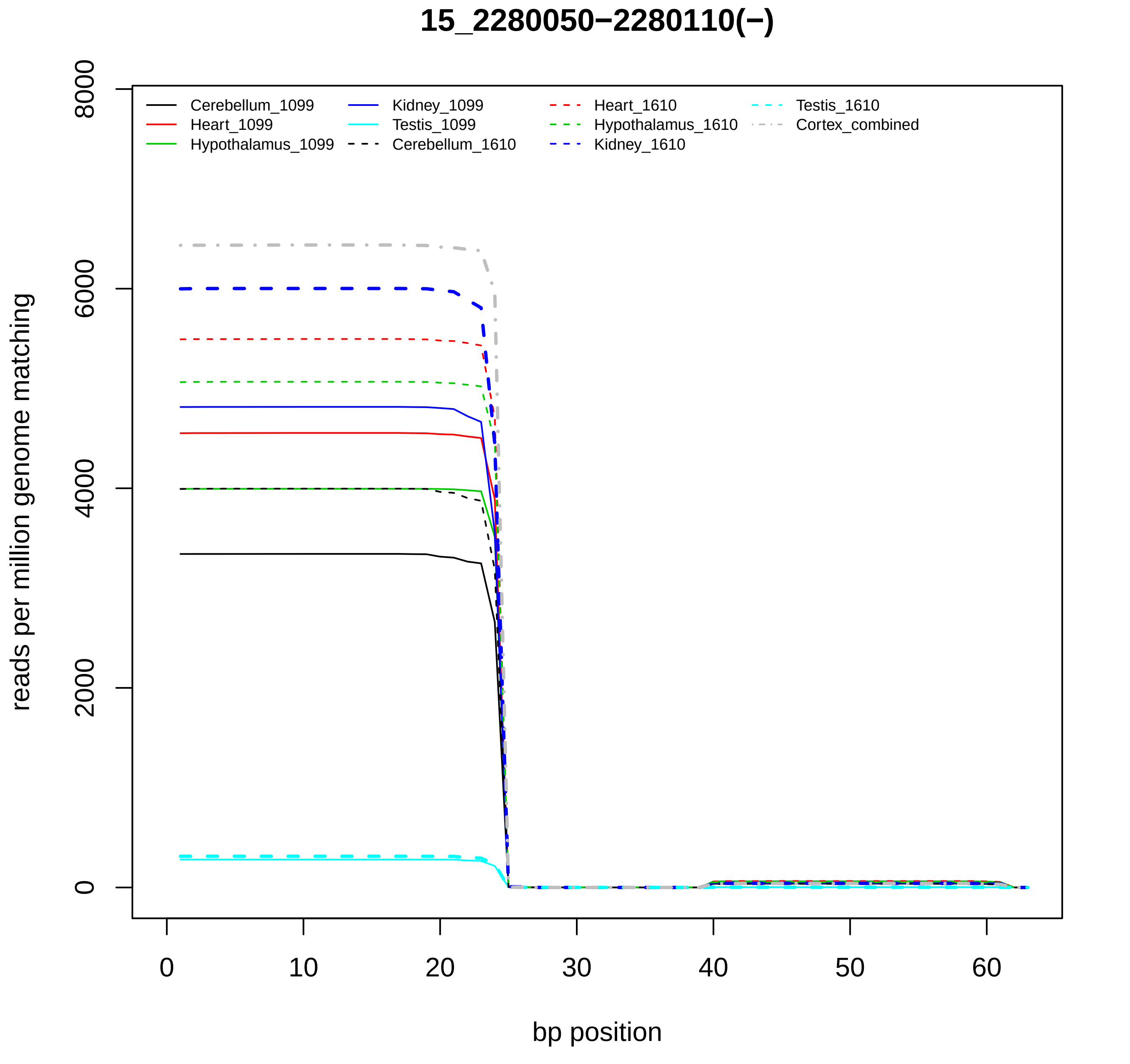
15_2280050−2280110(−)
Cerebellum_1099
Heart_1099
Hypothalamus_1099
Kidney_1099
Testis_1099
Cerebellum_1610
Heart_1610
Hypothalamus_1610
Kidney_1610
Testis_1610
Cortex_combined
reads per million genome matching
bp position
0
2000
4000
6000
8000
0
10
20
30
40
50
60

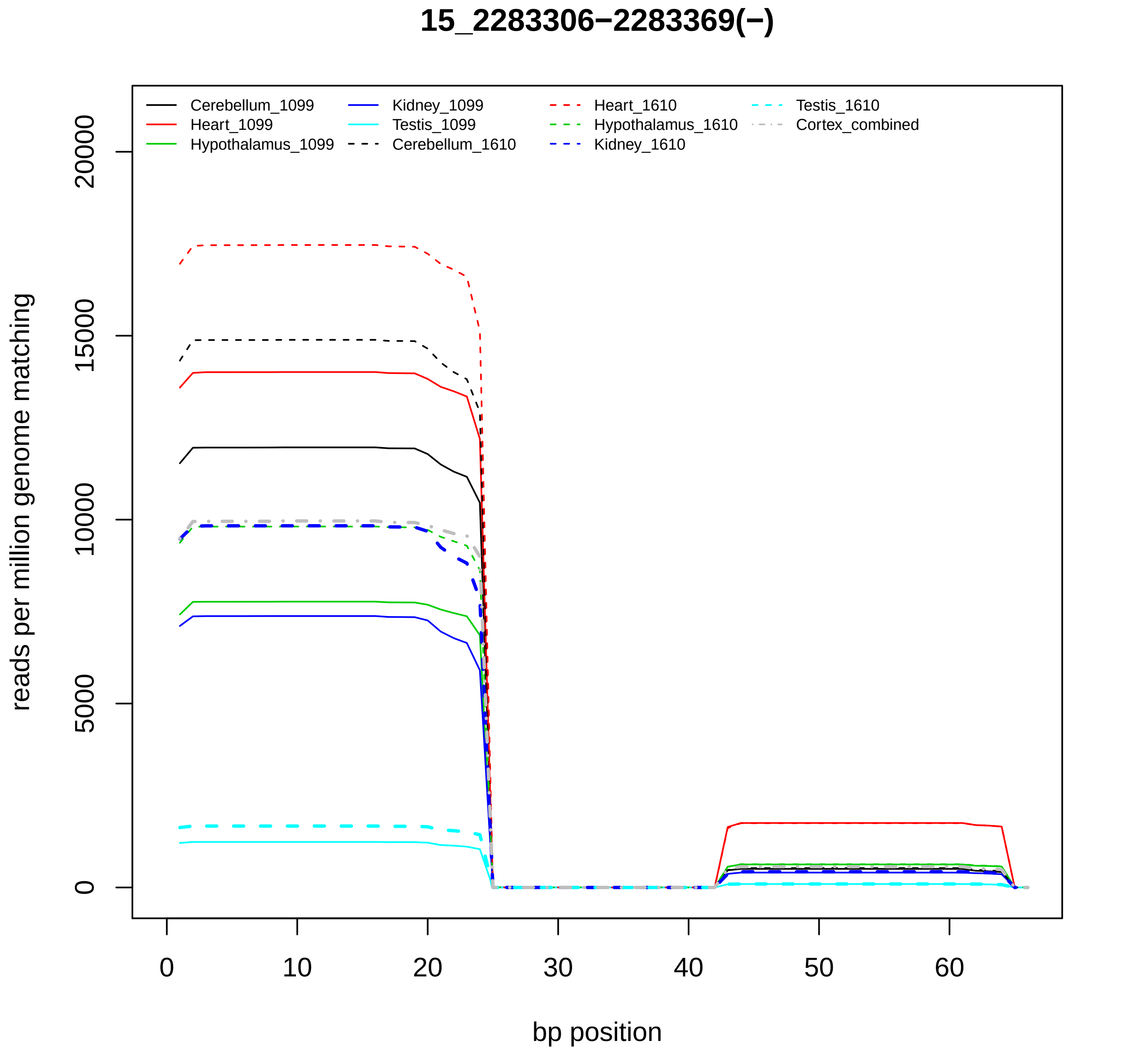

15_2283306−2283369(−)
Cerebellum_1099
Heart_1099
Hypothalamus_1099
Kidney_1099
Testis_1099
Cerebellum_1610
Heart_1610
Hypothalamus_1610
Kidney_1610
Testis_1610
Cortex_combined
reads per million genome matching
bp position
0
5000
10000
15000
20000
0
10
20
30
40
50
60

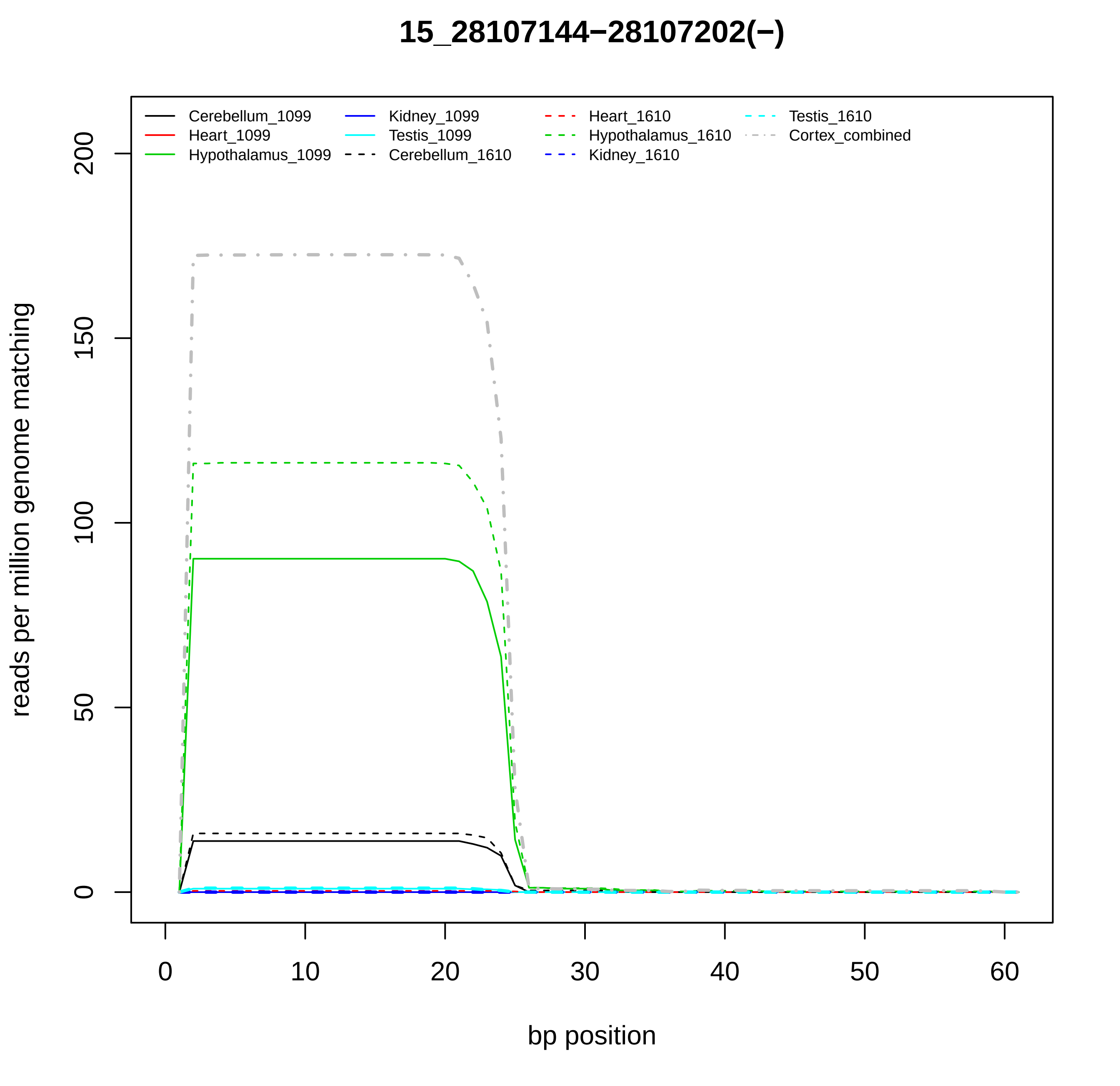

15_28107144−28107202(−)
Cerebellum_1099
Heart_1099
Hypothalamus_1099
Kidney_1099
Testis_1099
Cerebellum_1610
Heart_1610
Hypothalamus_1610
Kidney_1610
Testis_1610
Cortex_combined
reads per million genome matching
0
50
100
150
200
0
10
20
30
40
50
60
bp position

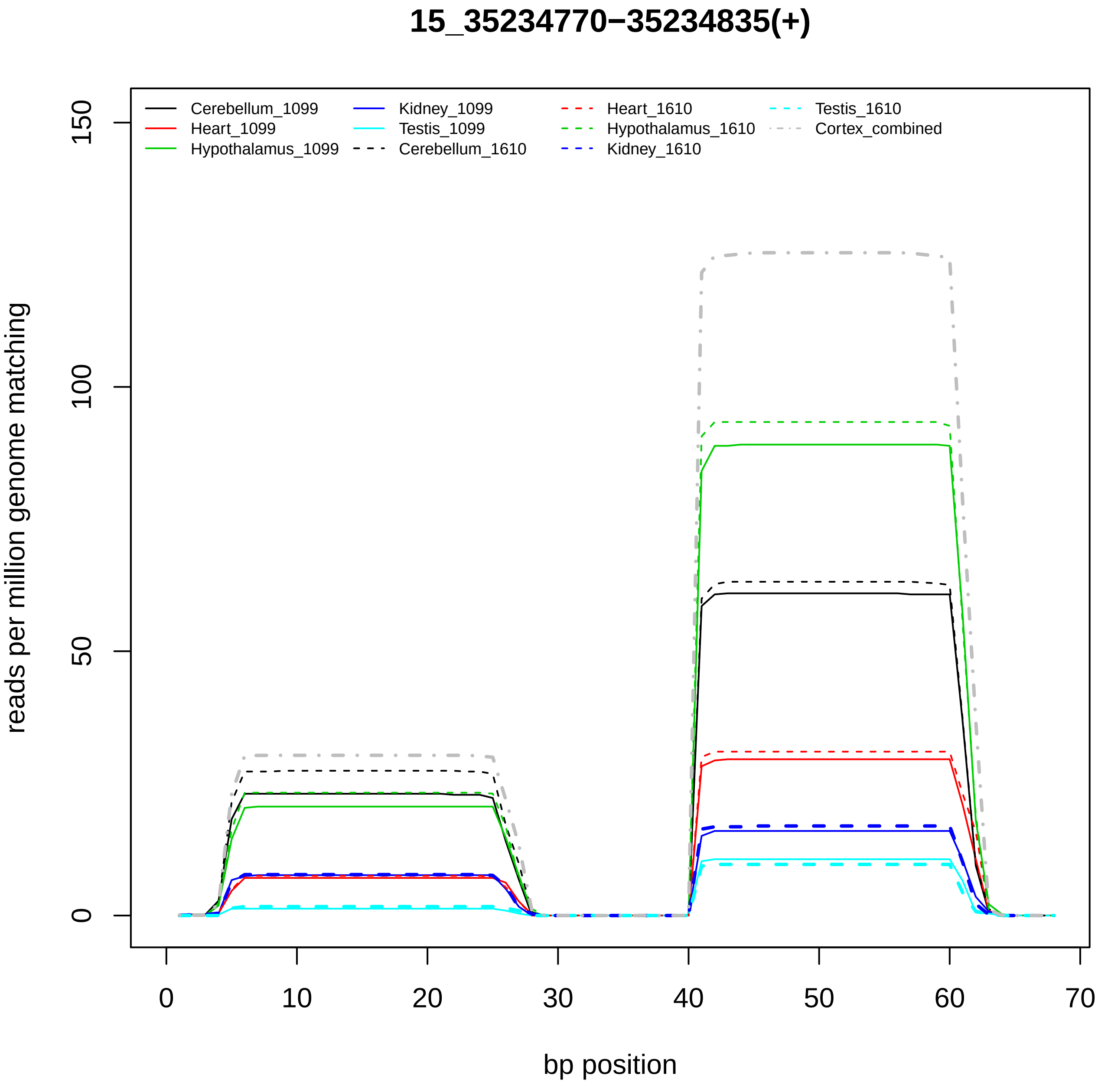

15_35234770−35234835(+)
Cerebellum_1099
Heart_1099
Hypothalamus_1099
Kidney_1099
Testis_1099
Cerebellum_1610
Heart_1610
Hypothalamus_1610
Kidney_1610
Testis_1610
Cortex_combined
reads per million genome matching
0
50
100
150
0
10
20
30
40
50
60
70
bp position

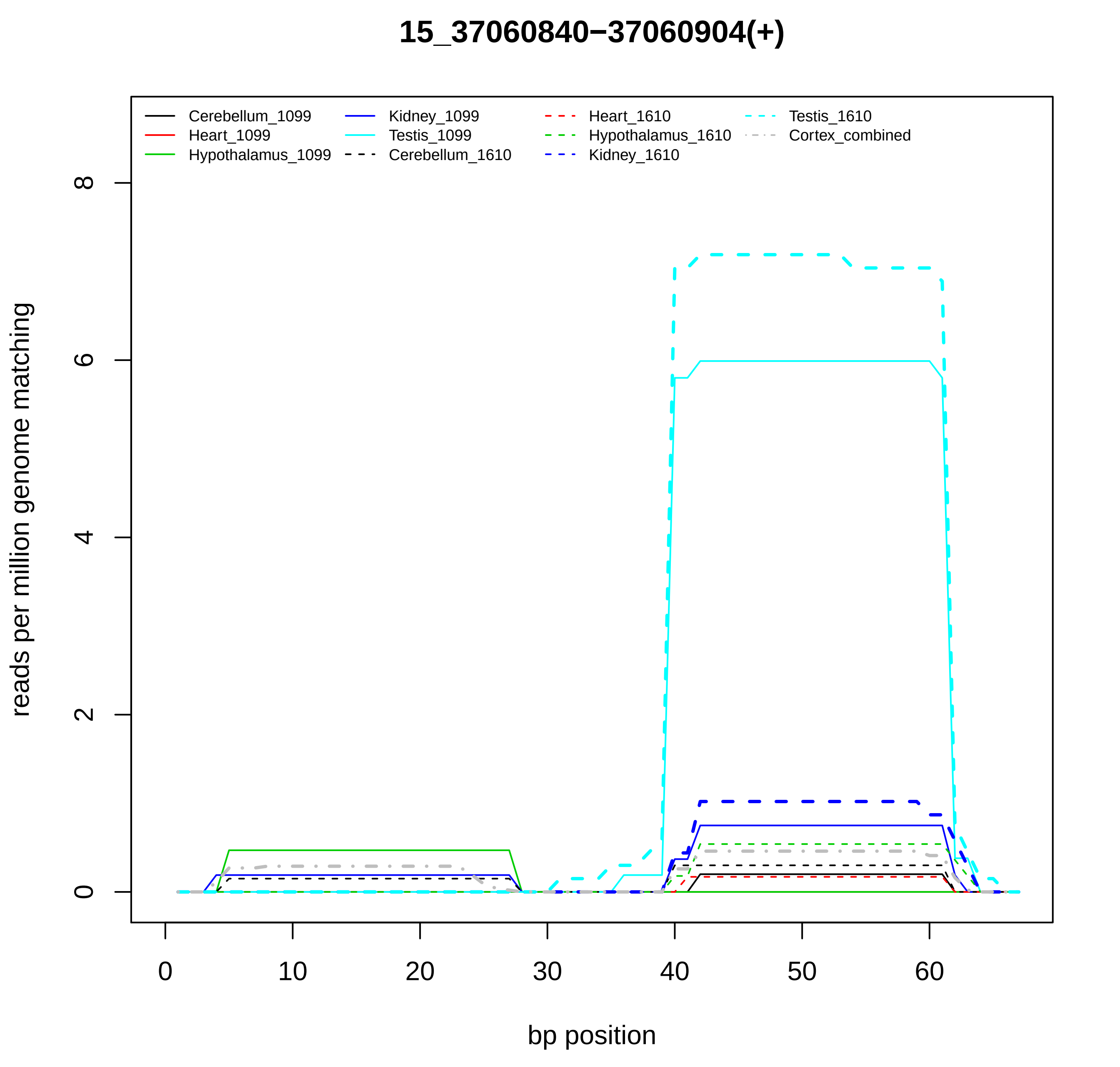
15_37060840−37060904(+)
Cerebellum_1099
Heart_1099
Hypothalamus_1099
Kidney_1099
Testis_1099
Cerebellum_1610
Heart_1610
Hypothalamus_1610
Kidney_1610
Testis_1610
Cortex_combined
reads per million genome matching
bp position
0
2
4
6
8
0
10
20
30
40
50
60

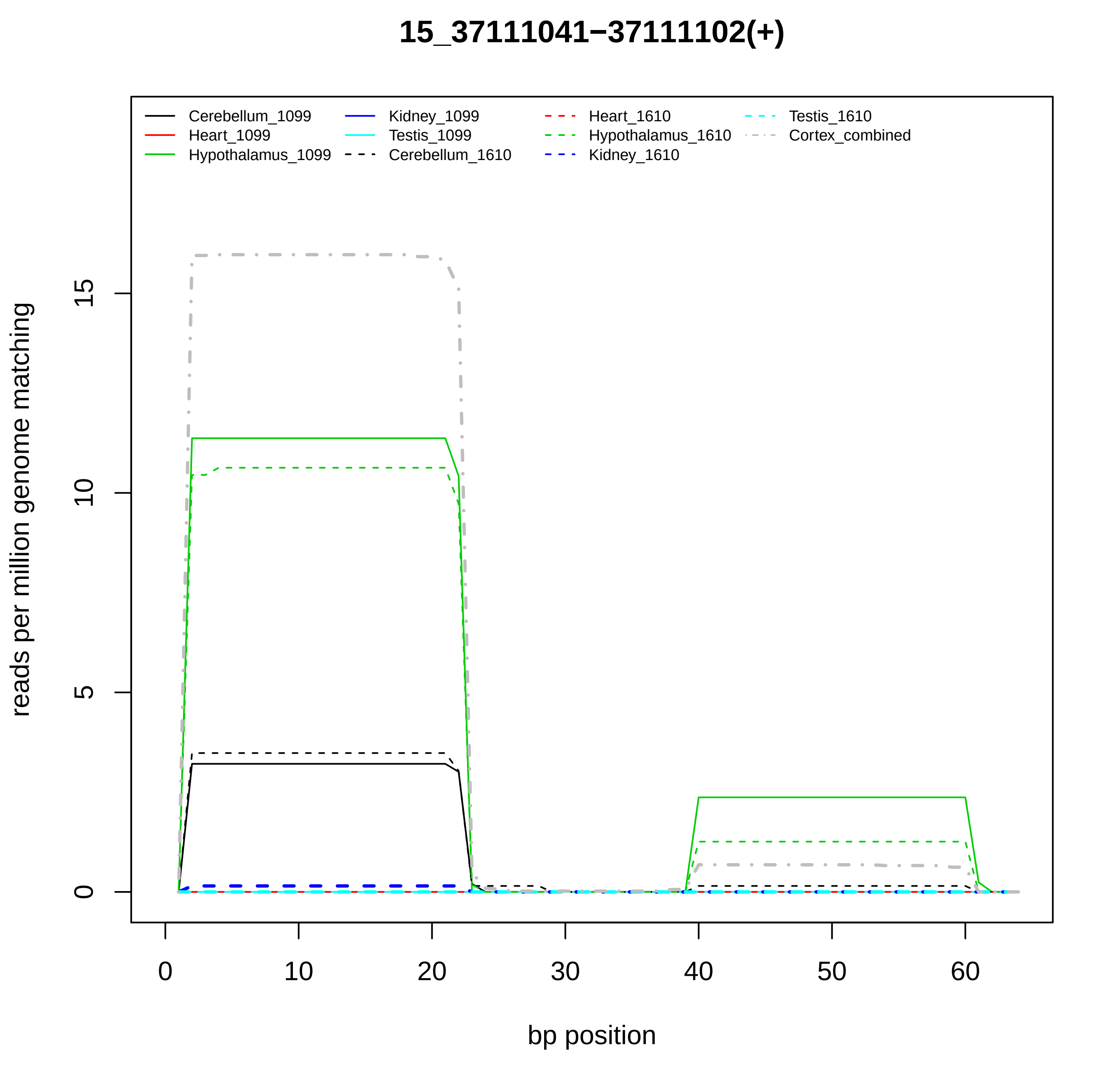

15_37111041−37111102(+)
Cerebellum_1099
Heart_1099
Hypothalamus_1099
Kidney_1099
Testis_1099
Cerebellum_1610
Heart_1610
Hypothalamus_1610
Kidney_1610
Testis_1610
Cortex_combined
reads per million genome matching
bp position
0
5
10
15
0
10
20
30
40
50
60

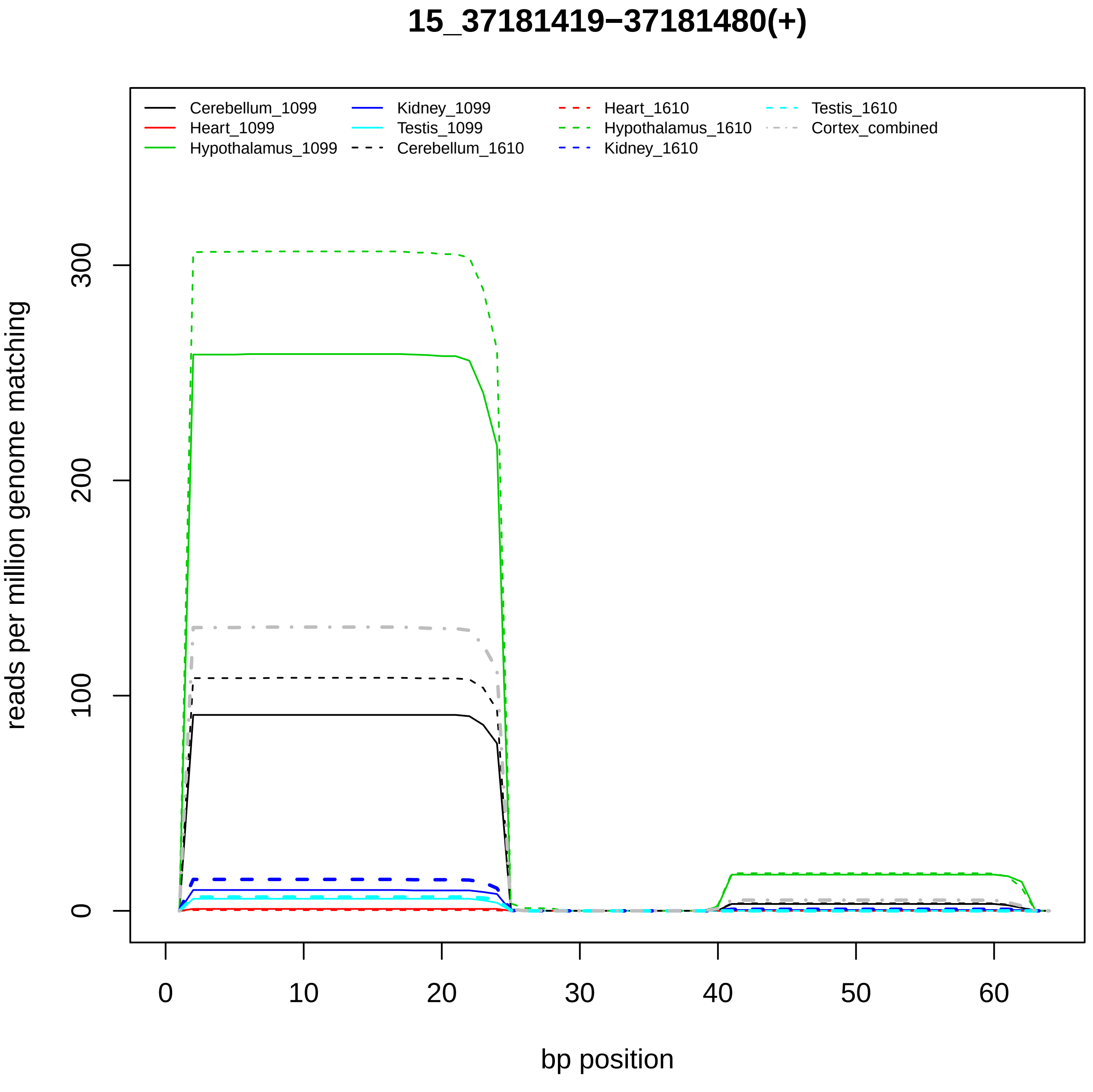

15_37181419−37181480(+)
Cerebellum_1099
Heart_1099
Hypothalamus_1099
Kidney_1099
Testis_1099
Cerebellum_1610
Heart_1610
Hypothalamus_1610
Kidney_1610
Testis_1610
Cortex_combined
reads per million genome matching
0
100
200
300
0
10
20
30
40
50
60
bp position

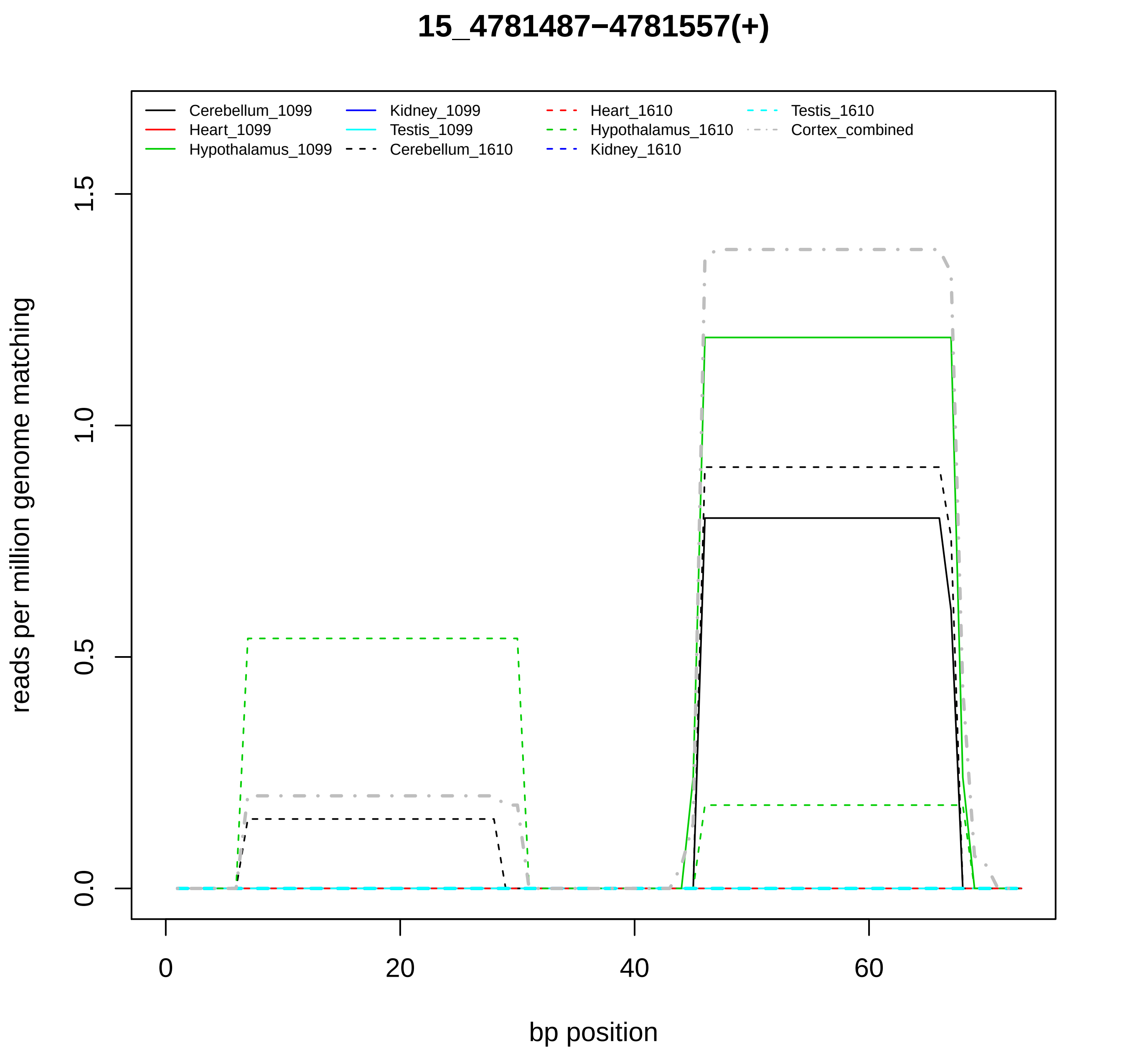

15_4781487−4781557(+)
Cerebellum_1099
Heart_1099
Hypothalamus_1099
Kidney_1099
Testis_1099
Cerebellum_1610
Heart_1610
Hypothalamus_1610
Kidney_1610
Testis_1610
Cortex_combined
reads per million genome matching
0.0
0.5
1.0
1.5
0
20
40
60
bp position

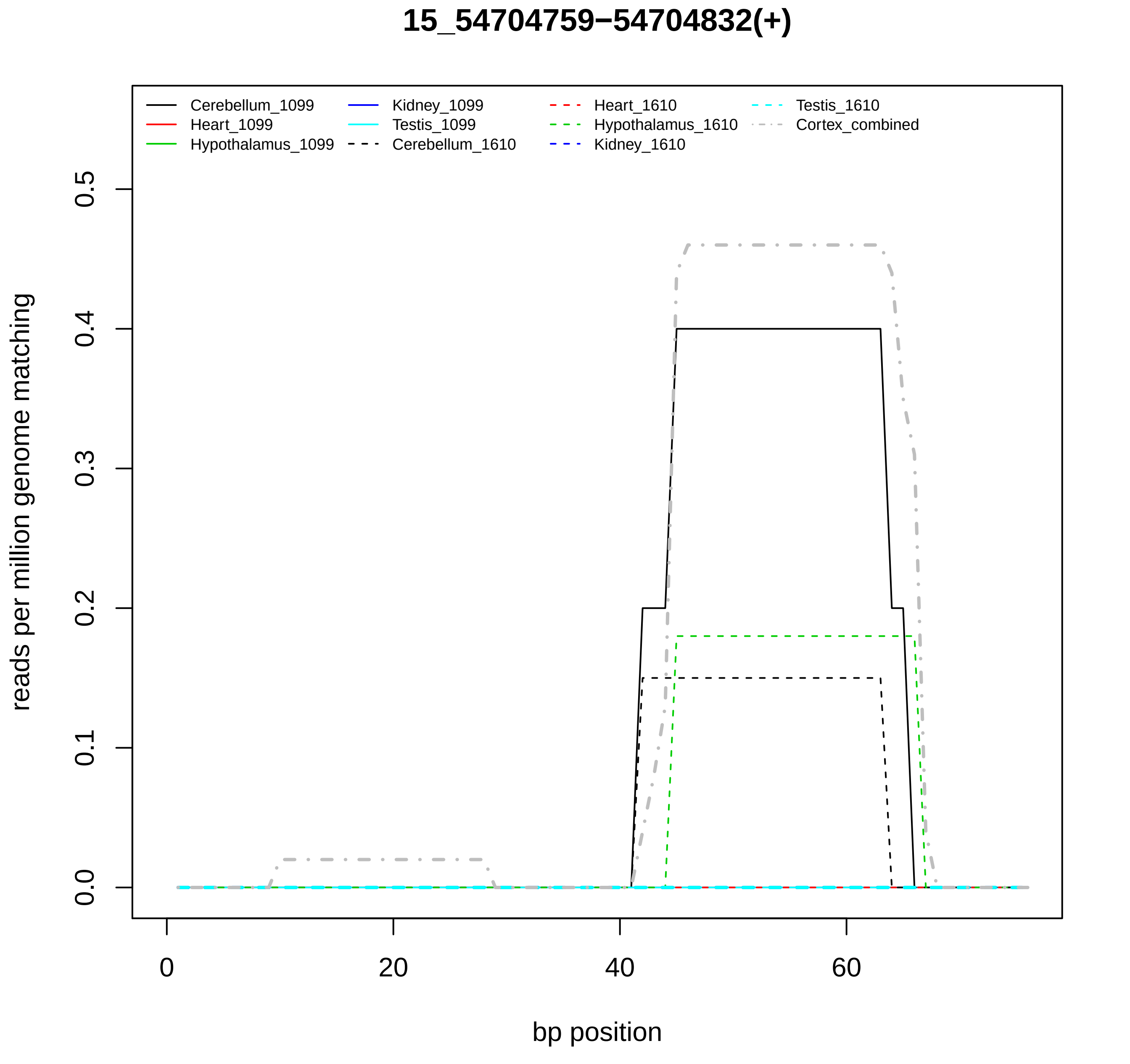
15_54704759−54704832(+)
Cerebellum_1099
Heart_1099
Hypothalamus_1099
Kidney_1099
Testis_1099
Cerebellum_1610
Heart_1610
Hypothalamus_1610
Kidney_1610
Testis_1610
Cortex_combined
reads per million genome matching
bp position
0.0
0.1
0.2
0.3
0.4
0.5
0
20
40
60

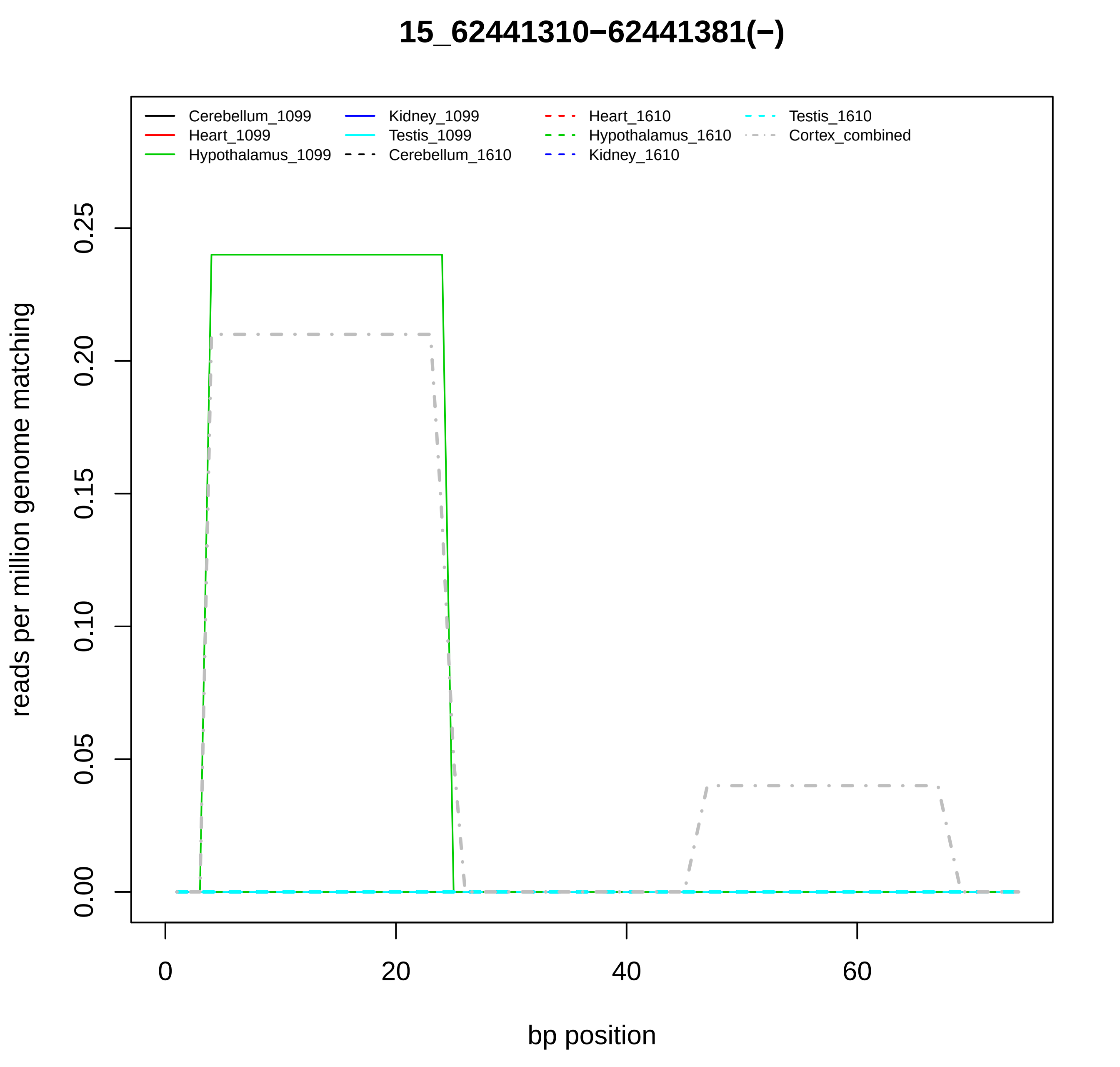

15_62441310−62441381(−)
Cerebellum_1099
Heart_1099
Hypothalamus_1099
Kidney_1099
Testis_1099
Cerebellum_1610
Heart_1610
Hypothalamus_1610
Kidney_1610
Testis_1610
Cortex_combined
reads per million genome matching
0.00
0.05
0.10
0.15
0.20
0.25
0
20
40
60
bp position

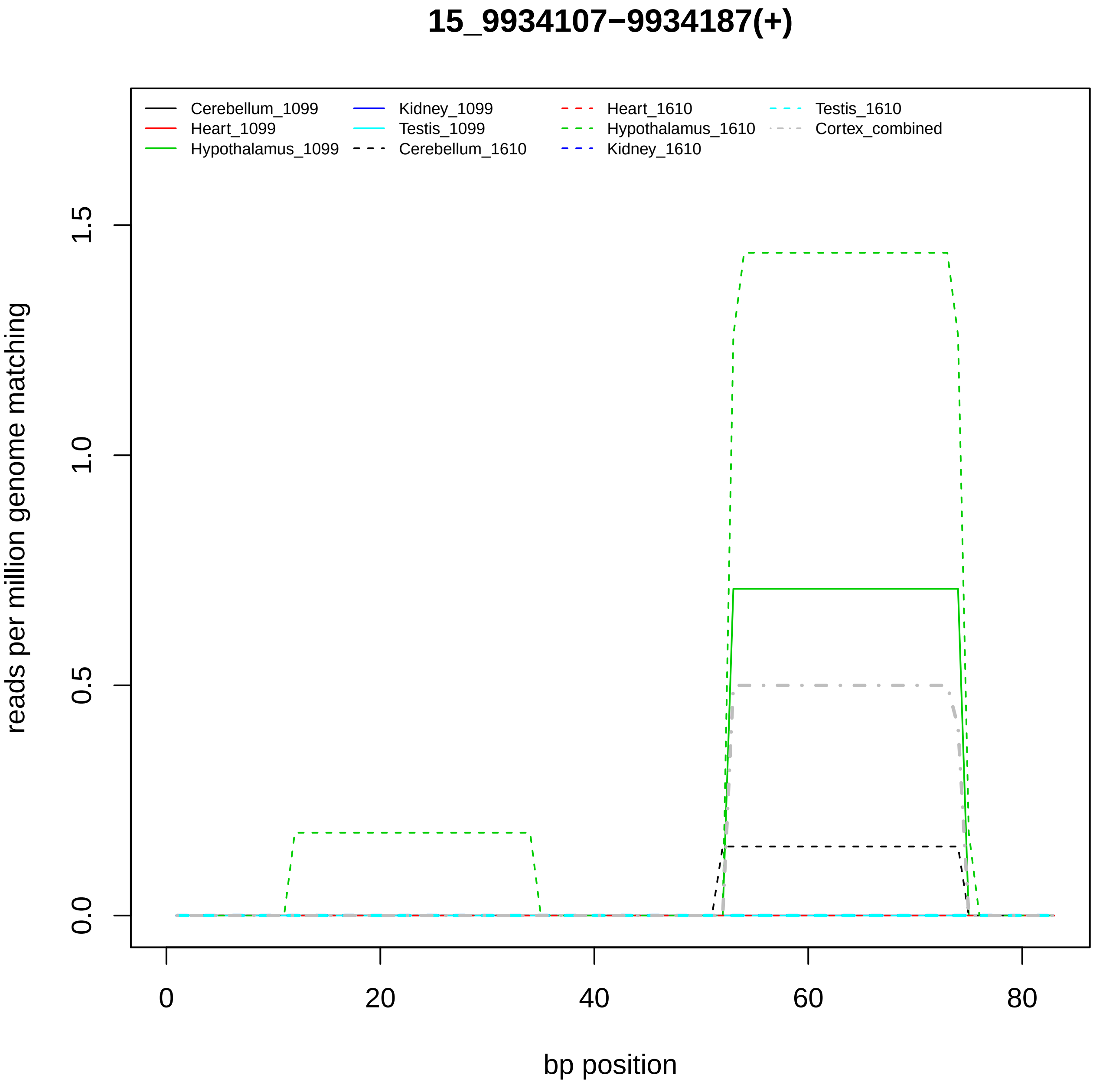

15_9934107−9934187(+)
Cerebellum_1099
Heart_1099
Hypothalamus_1099
Kidney_1099
Testis_1099
Cerebellum_1610
Heart_1610
Hypothalamus_1610
Kidney_1610
Testis_1610
Cortex_combined
reads per million genome matching
bp position
0.0
0.5
1.0
1.5
0
20
40
60
80

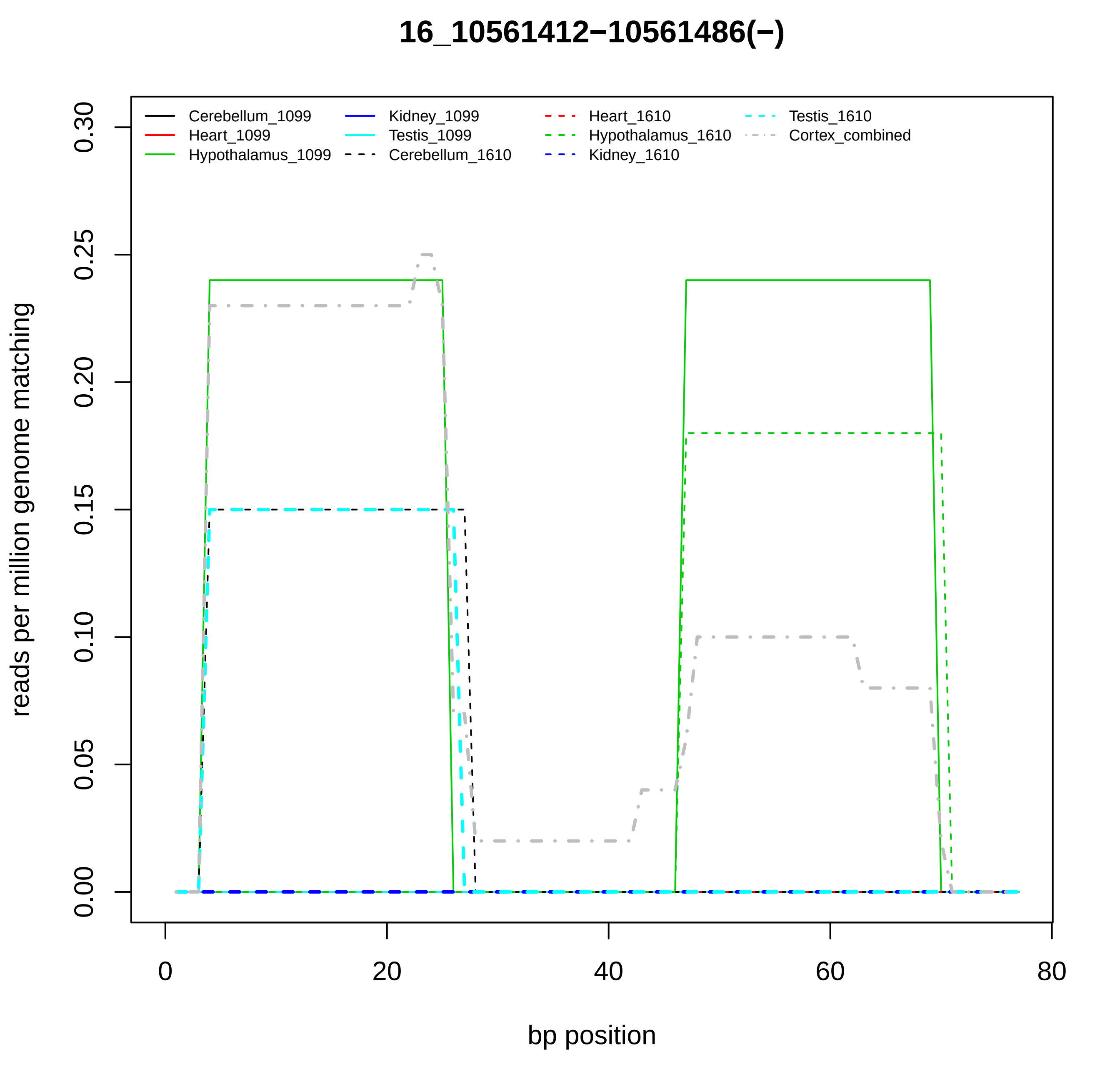

16_10561412−10561486(−)
Cerebellum_1099
Heart_1099
Hypothalamus_1099
Kidney_1099
Testis_1099
Cerebellum_1610
Heart_1610
Hypothalamus_1610
Kidney_1610
Testis_1610
Cortex_combined
reads per million genome matching
0.00
0.05
0.10
0.15
0.20
0.25
0.30
0
20
40
60
80
bp position

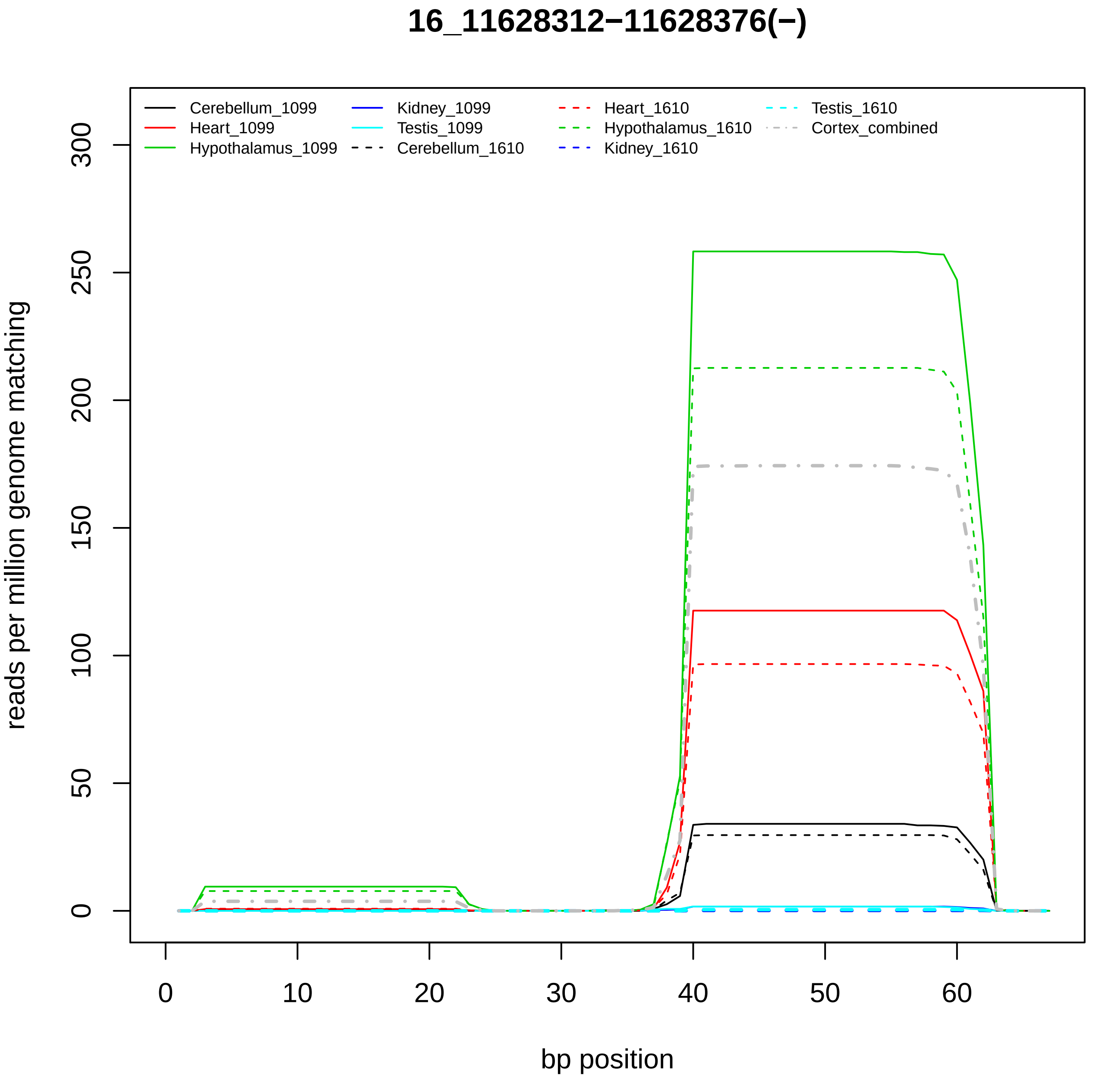

16_11628312−11628376(−)
Cerebellum_1099
Heart_1099
Hypothalamus_1099
Kidney_1099
Testis_1099
Cerebellum_1610
Heart_1610
Hypothalamus_1610
Kidney_1610
Testis_1610
Cortex_combined
reads per million genome matching
bp position
0
50
100
150
200
250
300
0
10
20
30
40
50
60

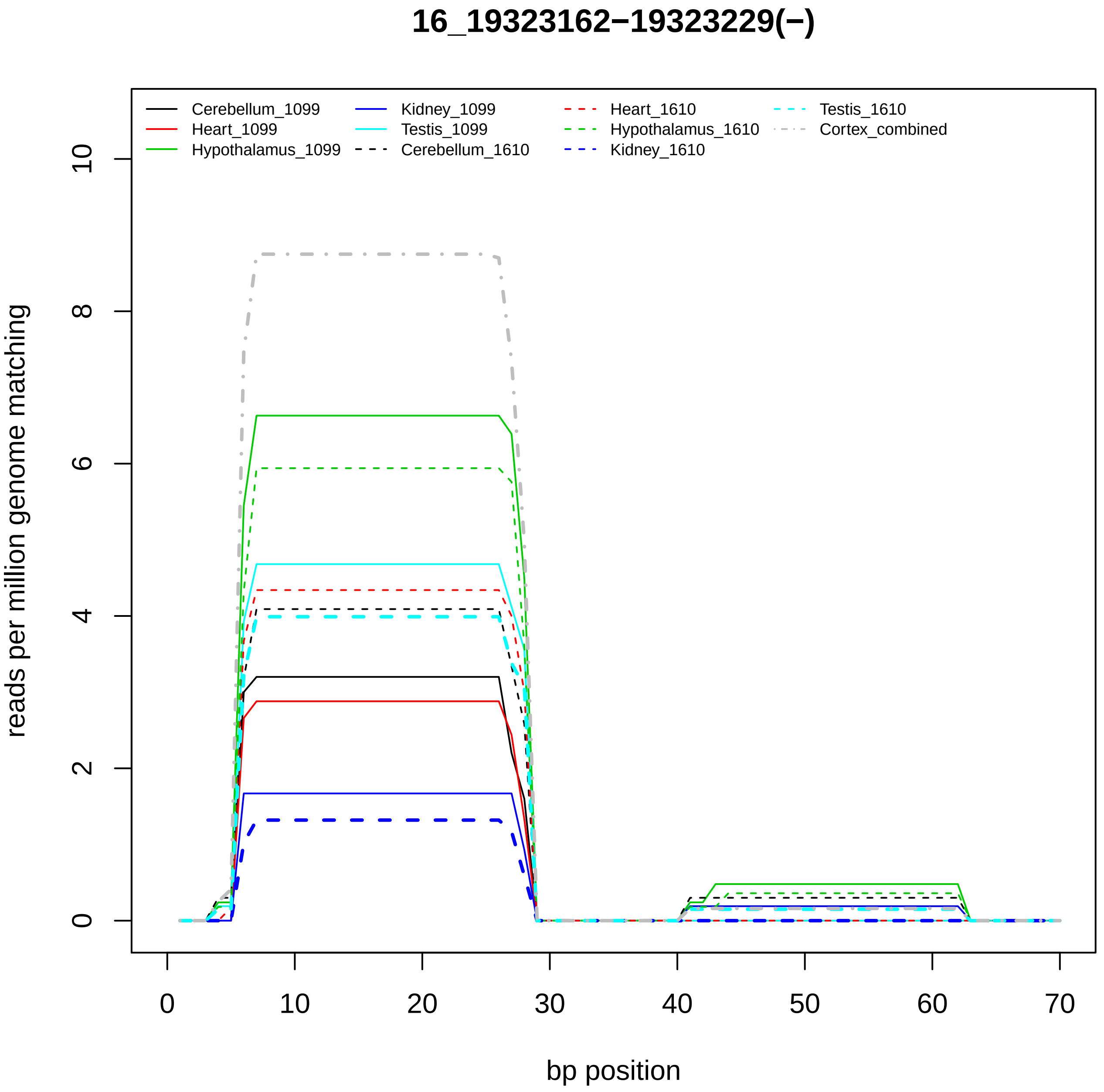

16_19323162−19323229(−)
Cerebellum_1099
Heart_1099
Hypothalamus_1099
Kidney_1099
Testis_1099
Cerebellum_1610
Heart_1610
Hypothalamus_1610
Kidney_1610
Testis_1610
Cortex_combined
reads per million genome matching
0
2
4
6
8
10
0
10
20
30
40
50
60
70
bp position

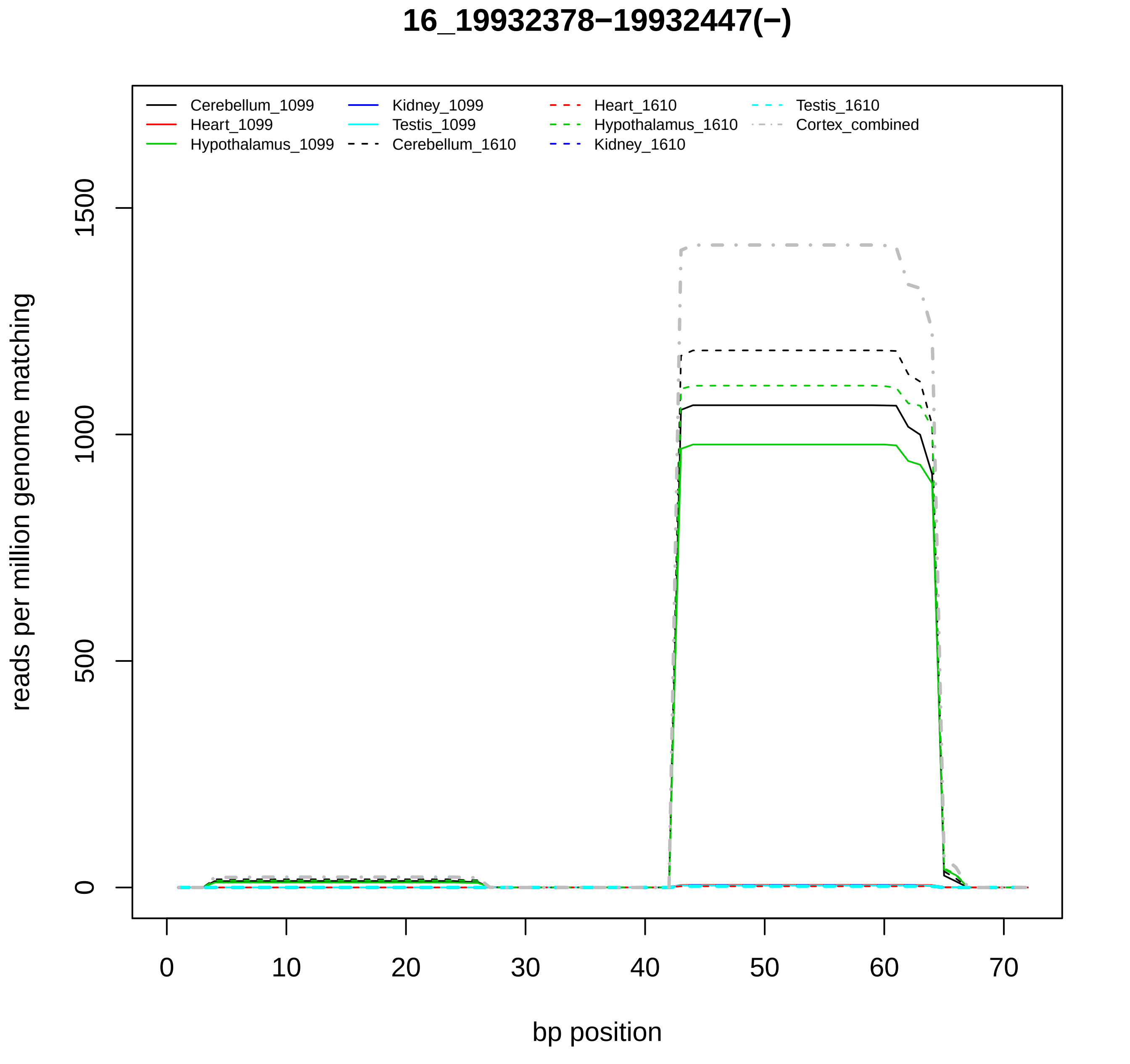
16_19932378−19932447(−)
Cerebellum_1099
Heart_1099
Hypothalamus_1099
Kidney_1099
Testis_1099
Cerebellum_1610
Heart_1610
Hypothalamus_1610
Kidney_1610
Testis_1610
Cortex_combined
reads per million genome matching
0
500
1000
1500
0
10
20
30
40
50
60
70
bp position

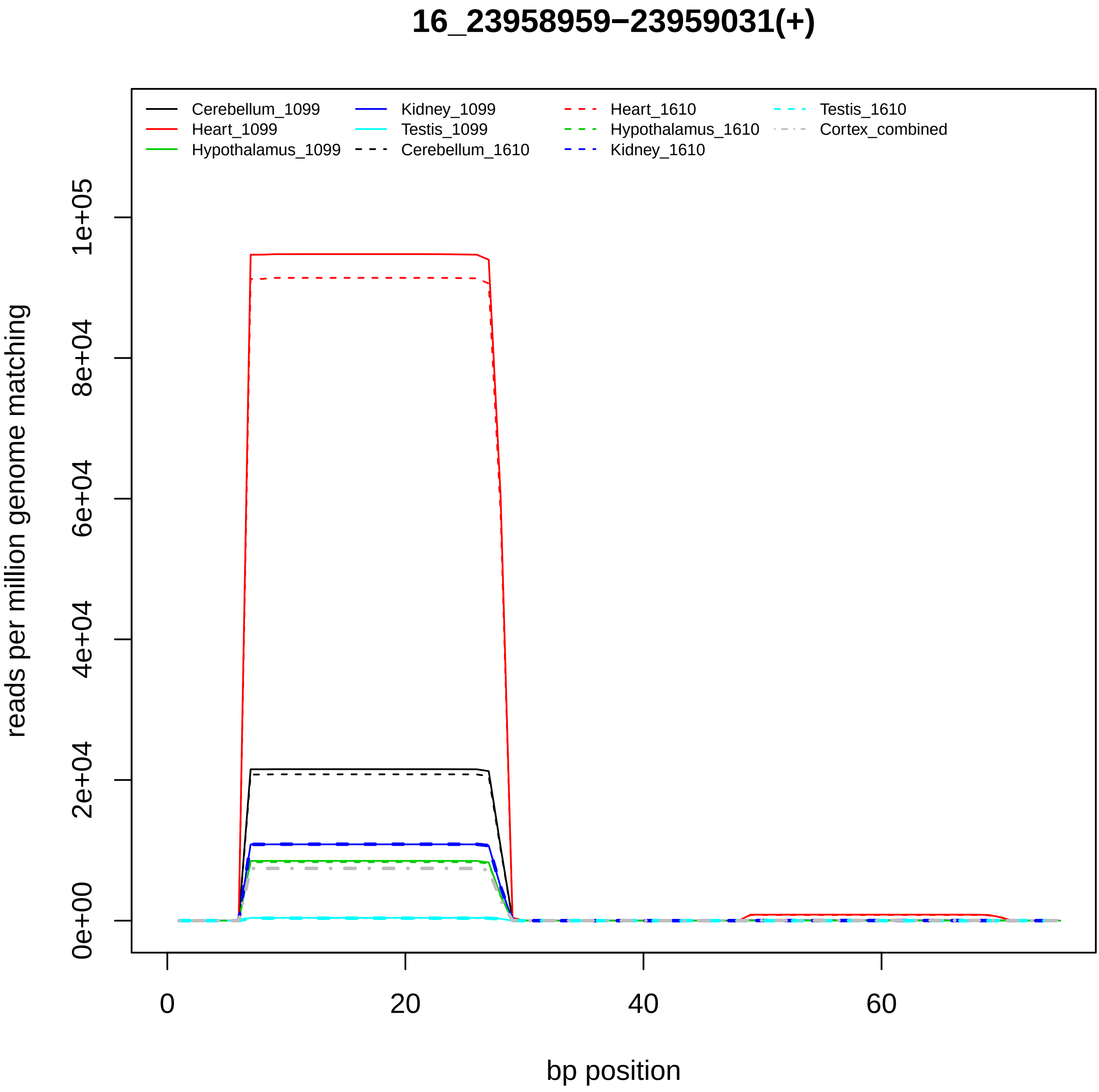
16_23958959−23959031(+)
Cerebellum_1099
Heart_1099
Hypothalamus_1099
Kidney_1099
Testis_1099
Cerebellum_1610
Heart_1610
Hypothalamus_1610
Kidney_1610
Testis_1610
Cortex_combined
reads per million genome matching
0e+00
2e+04
4e+04
6e+04
8e+04
1e+05
0
20
40
60
bp position

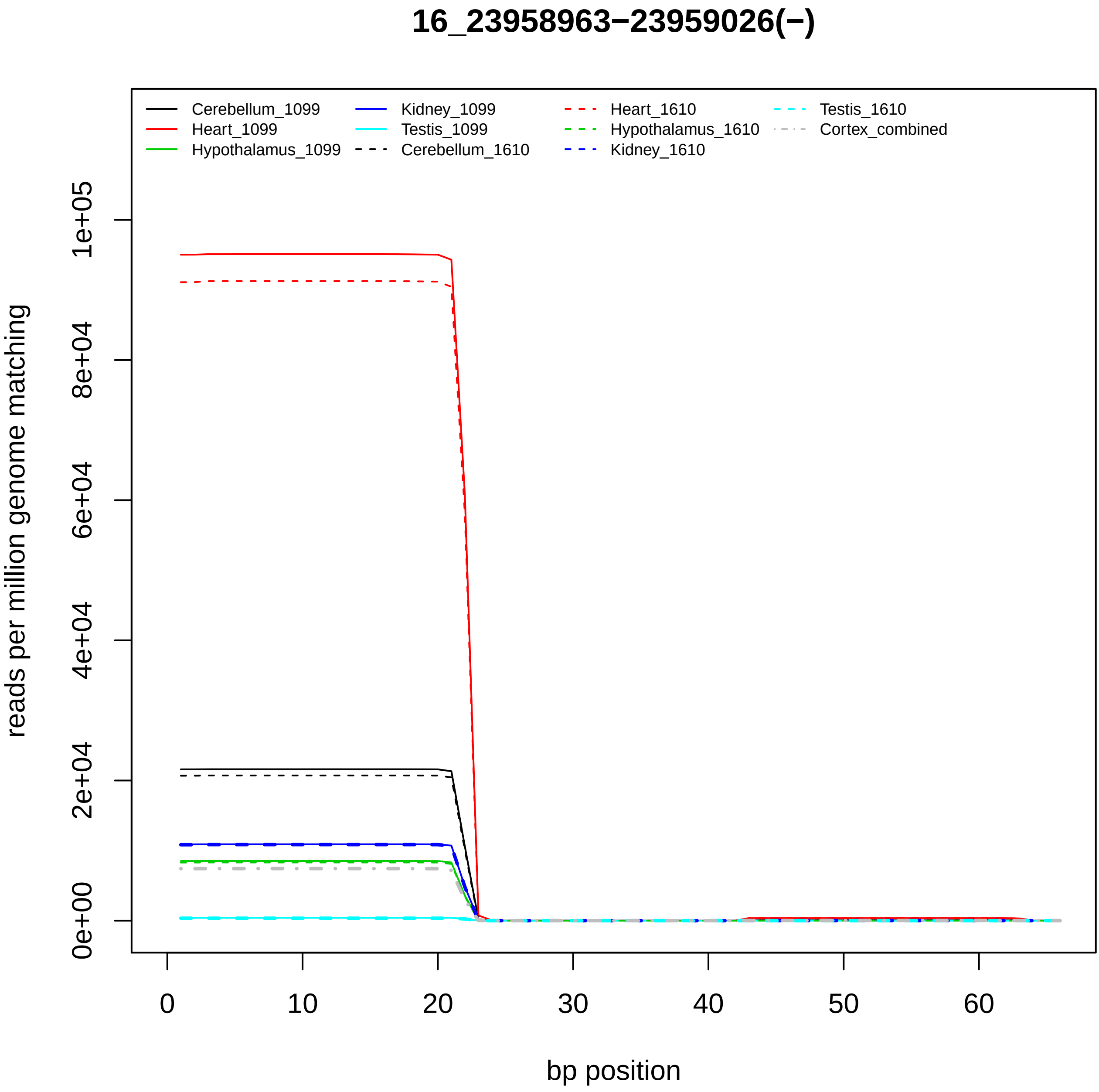

16_23958963−23959026(−)
Cerebellum_1099
Heart_1099
Hypothalamus_1099
Kidney_1099
Testis_1099
Cerebellum_1610
Heart_1610
Hypothalamus_1610
Kidney_1610
Testis_1610
Cortex_combined
reads per million genome matching
0e+00
2e+04
4e+04
6e+04
8e+04
1e+05
0
10
20
30
40
50
60
bp position

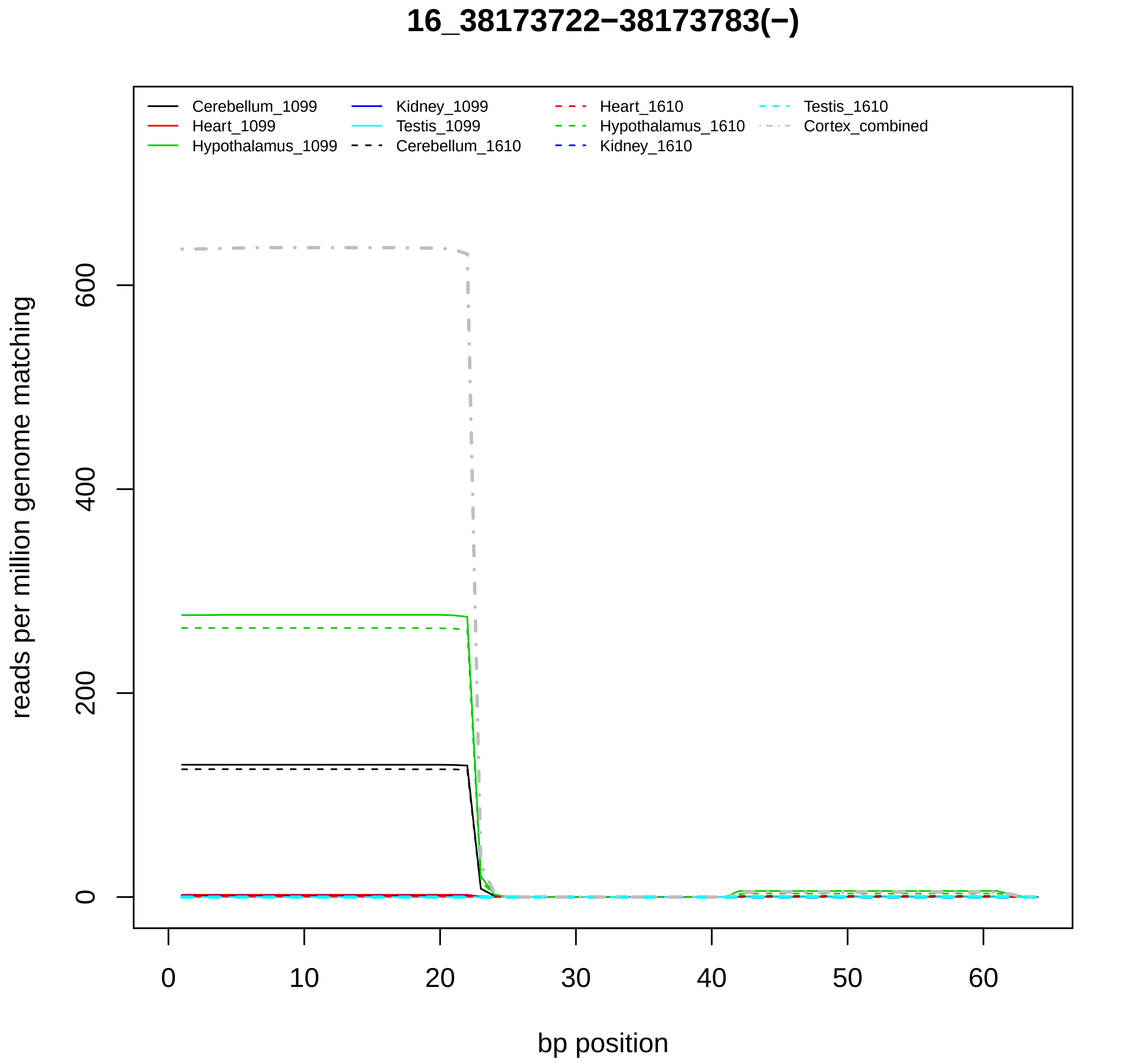

16_38173722−38173783(−)
Cerebellum_1099
Heart_1099
Hypothalamus_1099
Kidney_1099
Testis_1099
Cerebellum_1610
Heart_1610
Hypothalamus_1610
Kidney_1610
Testis_1610
Cortex_combined
reads per million genome matching
0
200
400
600
0
10
20
30
40
50
60
bp position

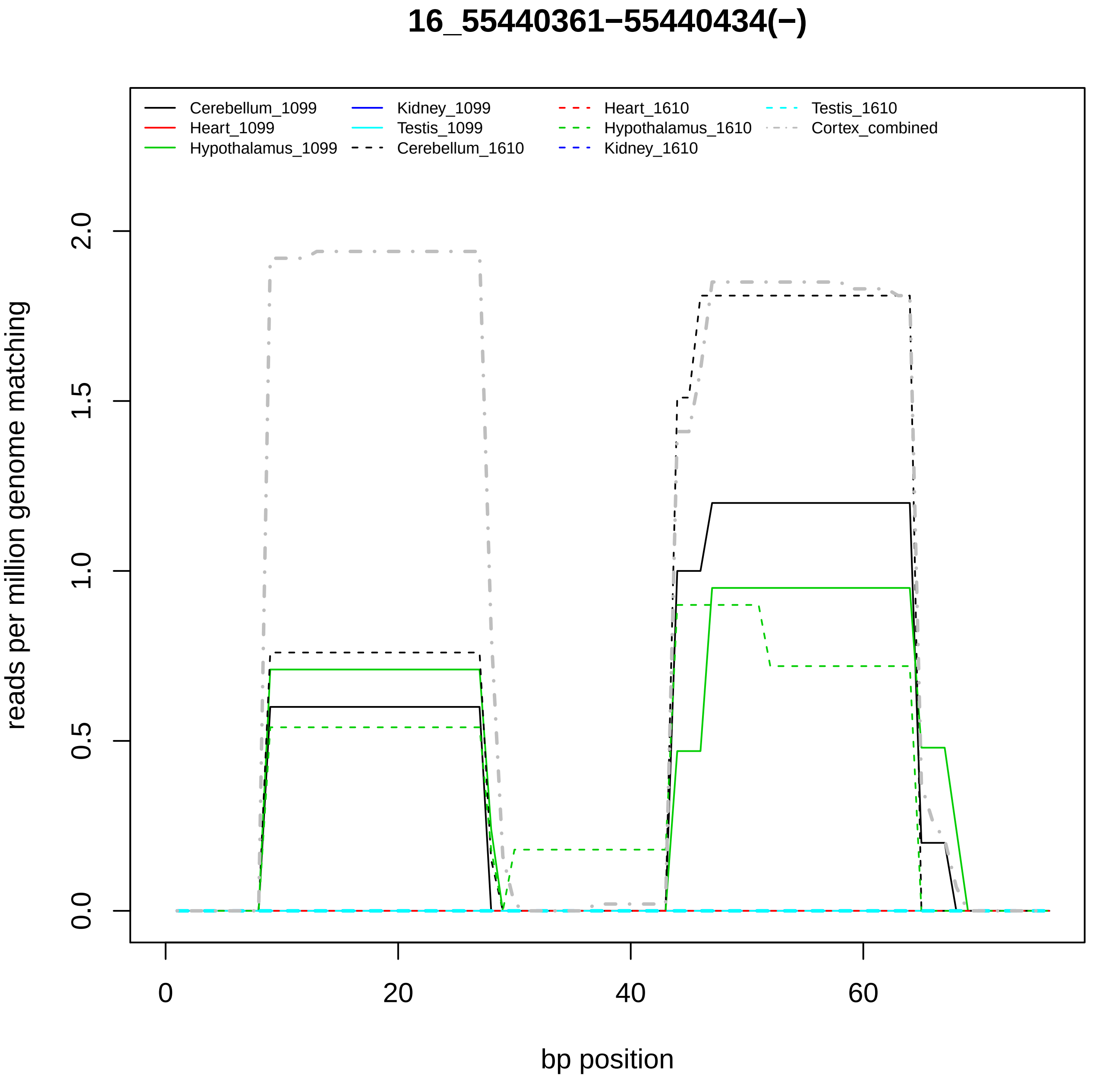

16_55440361−55440434(−)
Cerebellum_1099
Heart_1099
Hypothalamus_1099
Kidney_1099
Testis_1099
Cerebellum_1610
Heart_1610
Hypothalamus_1610
Kidney_1610
Testis_1610
Cortex_combined
reads per million genome matching
0.0
0.5
1.0
1.5
2.0
0
20
40
60
bp position

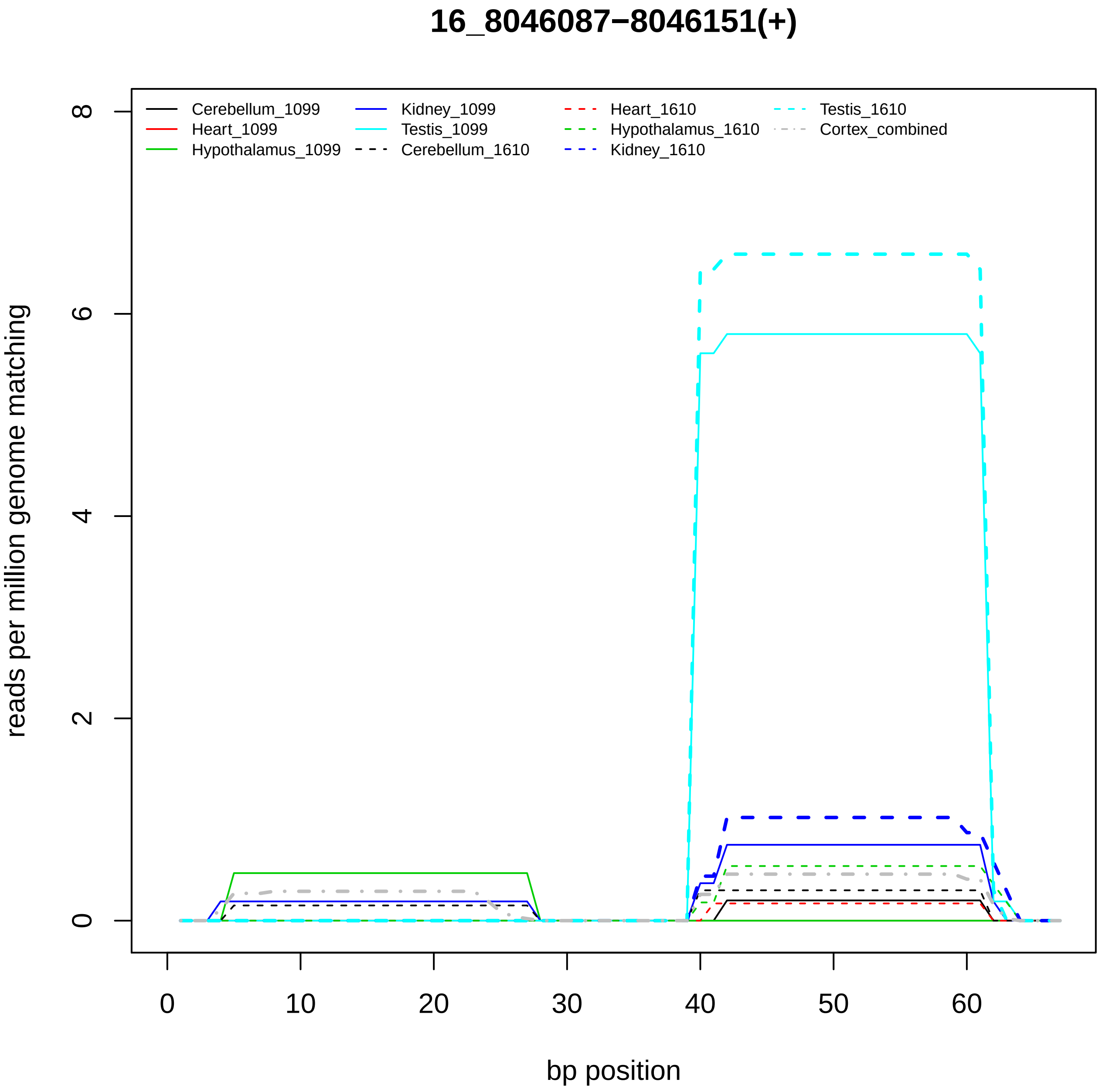

16_8046087−8046151(+)
Cerebellum_1099
Heart_1099
Hypothalamus_1099
Kidney_1099
Testis_1099
Cerebellum_1610
Heart_1610
Hypothalamus_1610
Kidney_1610
Testis_1610
Cortex_combined
reads per million genome matching
bp position
0
2
4
6
8
0
10
20
30
40
50
60

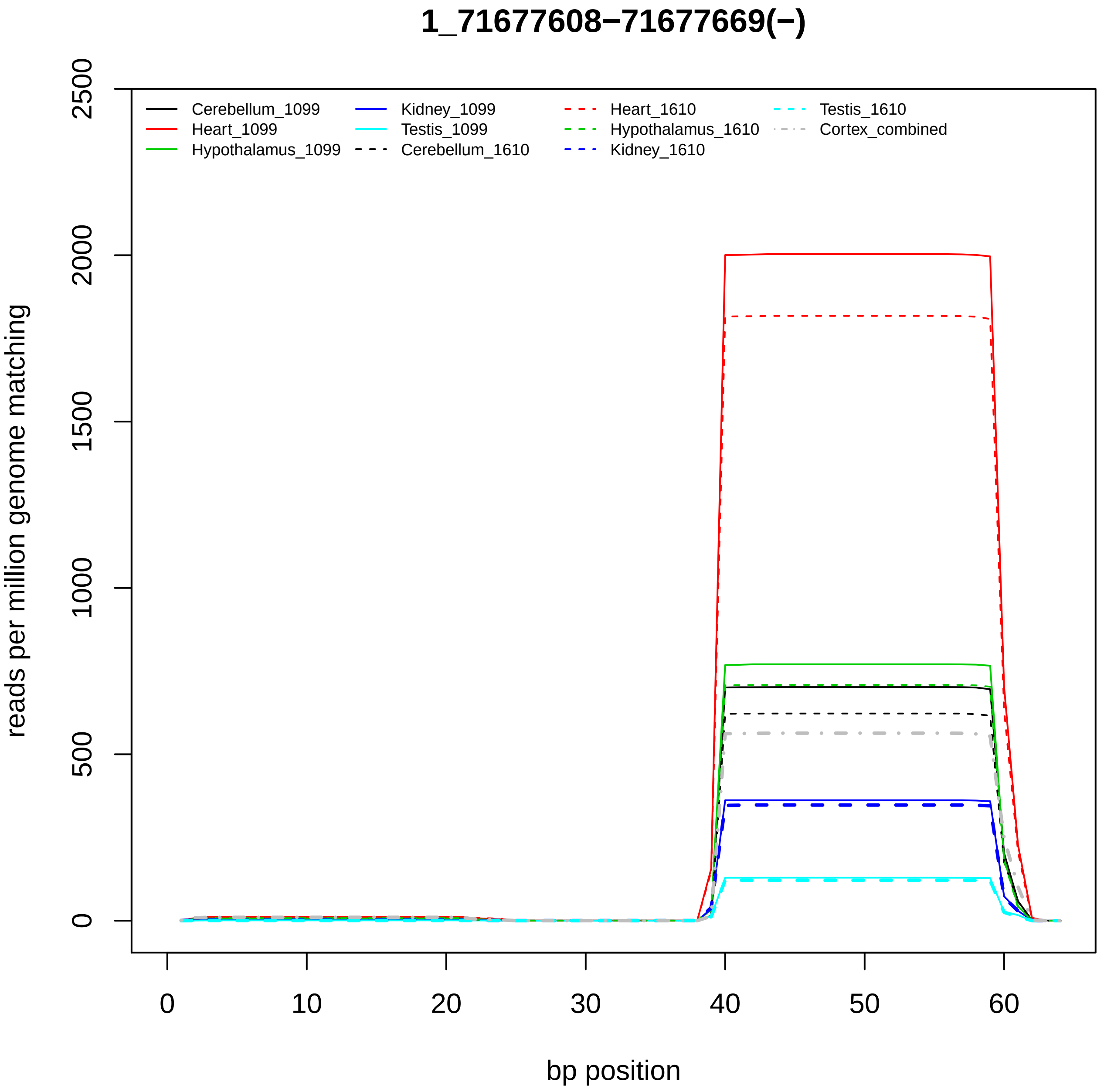

1_71677608−71677669(−)
Cerebellum_1099
Heart_1099
Hypothalamus_1099
Kidney_1099
Testis_1099
Cerebellum_1610
Heart_1610
Hypothalamus_1610
Kidney_1610
Testis_1610
Cortex_combined
reads per million genome matching
bp position
0
500
1000
1500
2000
2500
0
10
20
30
40
50
60

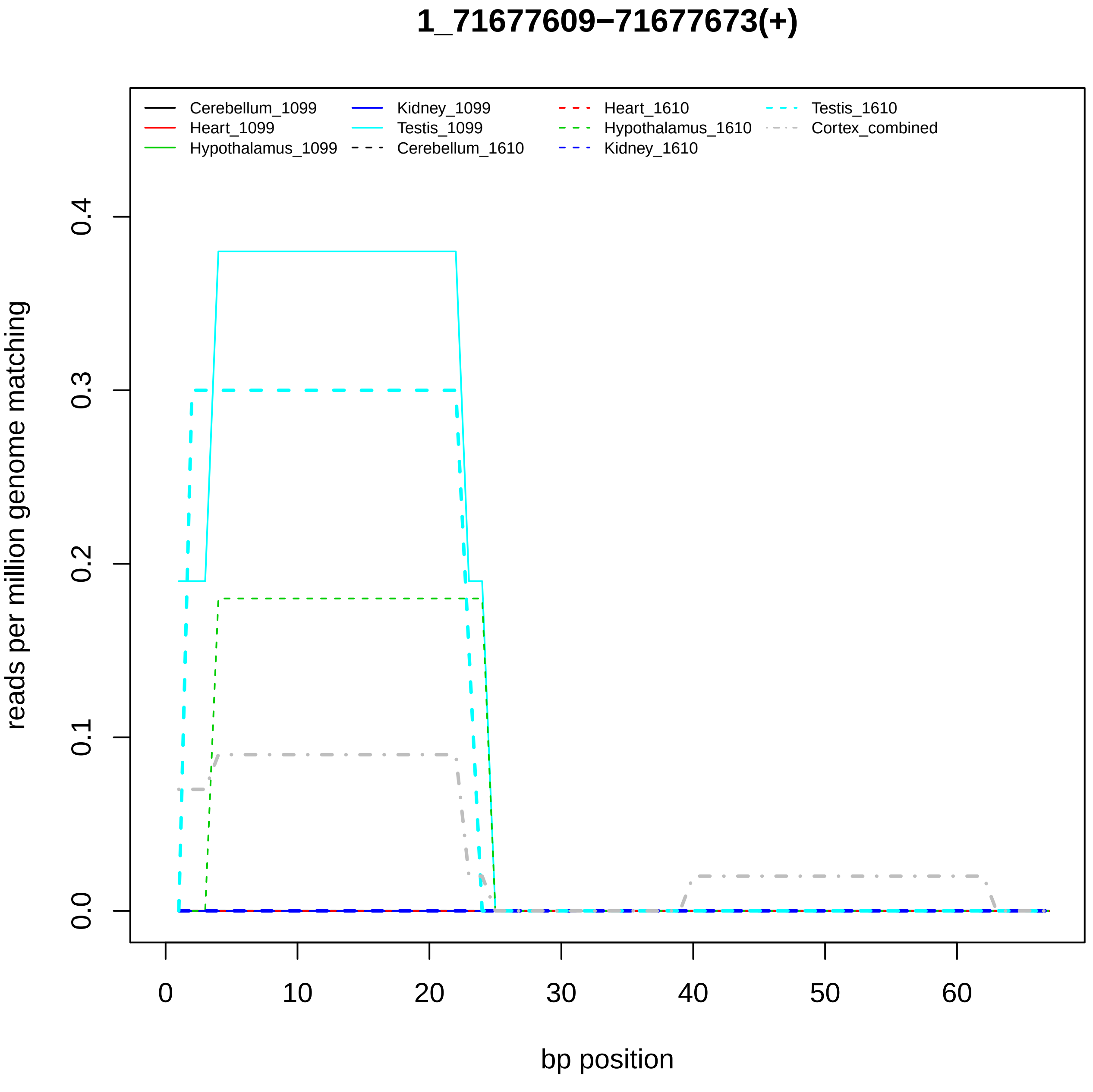

1_71677609−71677673(+)
Cerebellum_1099
Heart_1099
Hypothalamus_1099
Kidney_1099
Testis_1099
Cerebellum_1610
Heart_1610
Hypothalamus_1610
Kidney_1610
Testis_1610
Cortex_combined
reads per million genome matching
0.0
0.1
0.2
0.3
0.4
0
10
20
30
40
50
60
bp position

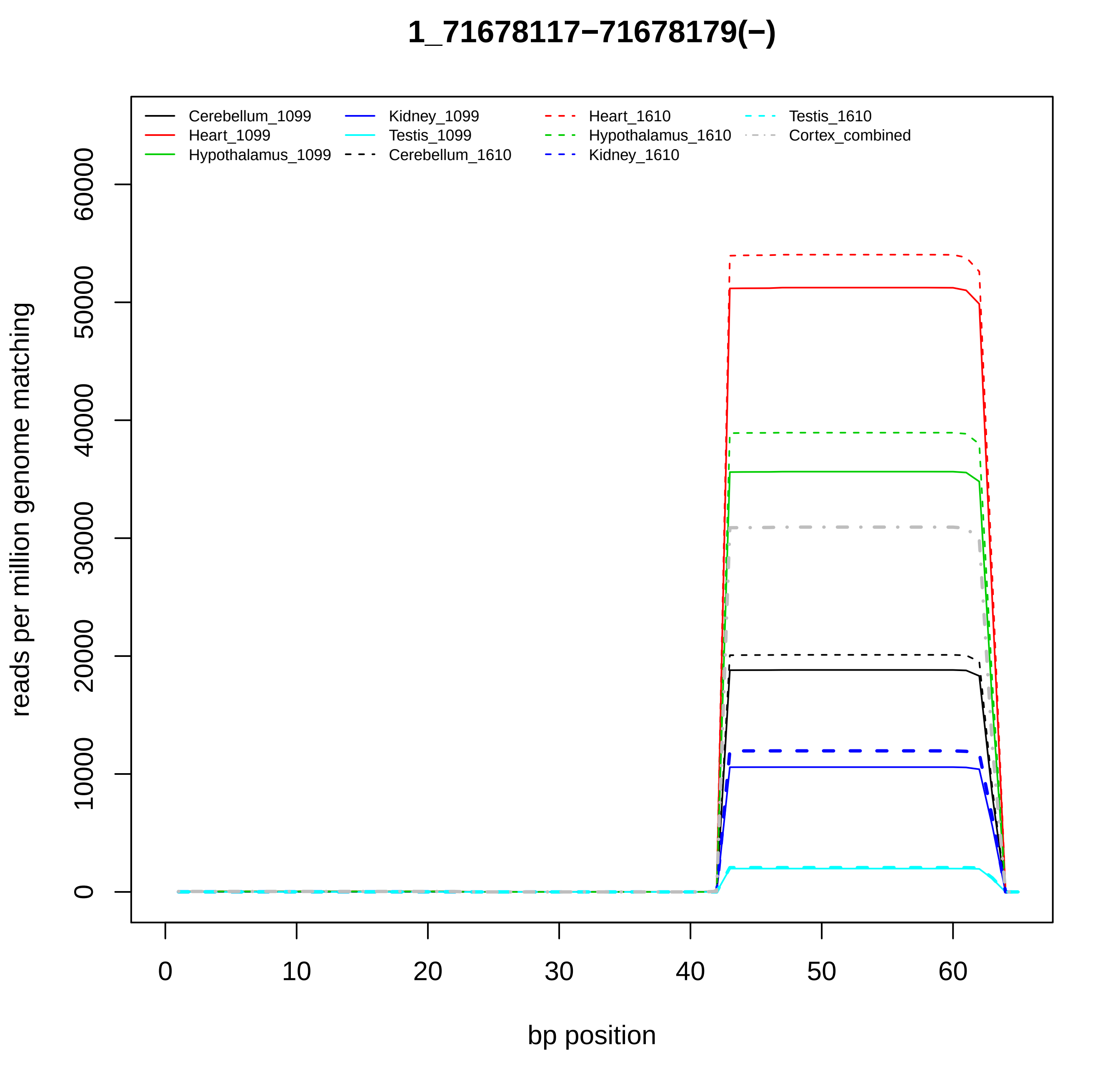
1_71678117−71678179(−)
Cerebellum_1099
Heart_1099
Hypothalamus_1099
Kidney_1099
Testis_1099
Cerebellum_1610
Heart_1610
Hypothalamus_1610
Kidney_1610
Testis_1610
Cortex_combined
reads per million genome matching
0
10000
20000
30000
40000
50000
60000
bp position
0
10
20
30
40
50
60

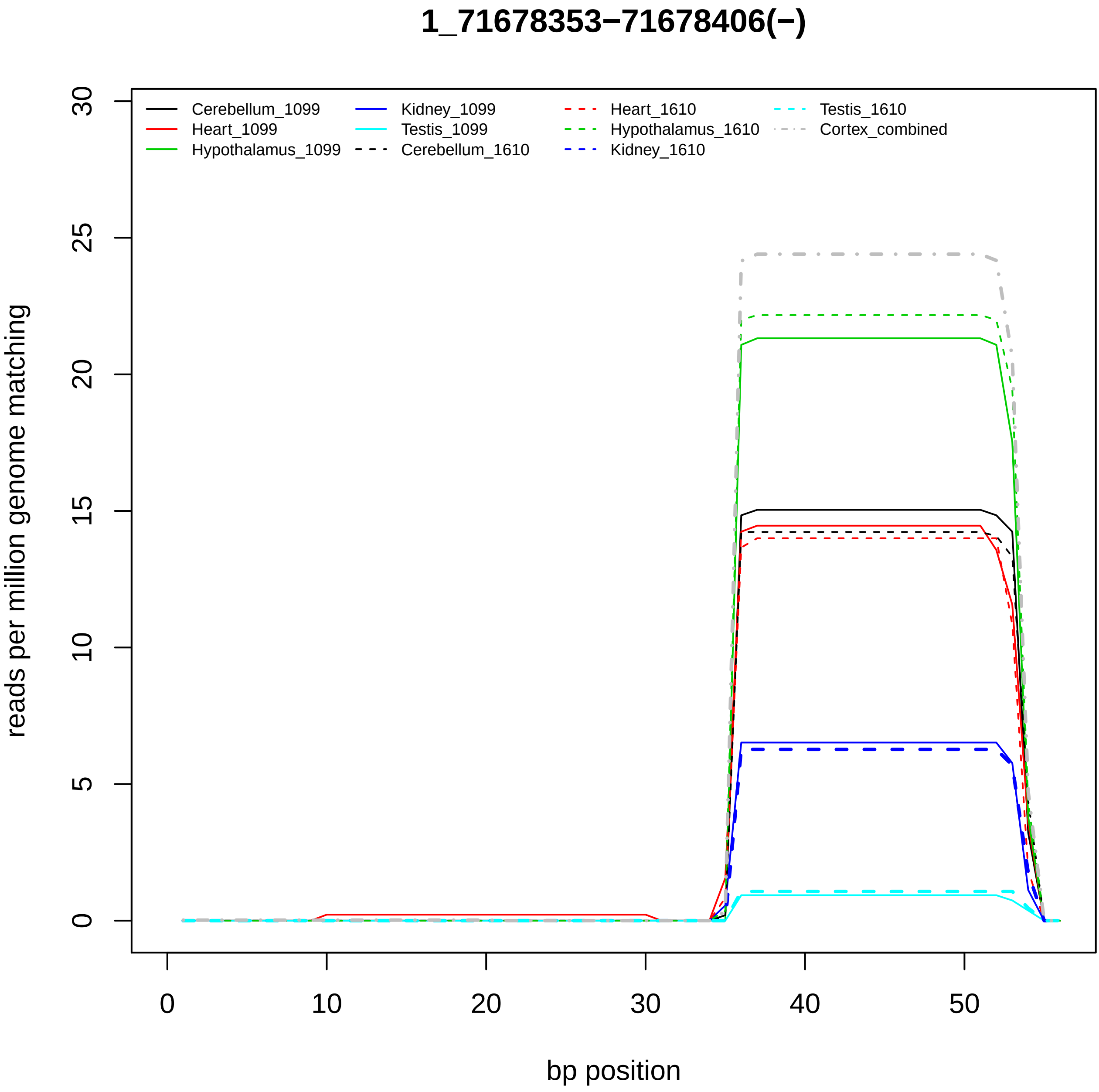
1_71678353−71678406(−)
Cerebellum_1099
Heart_1099
Hypothalamus_1099
Kidney_1099
Testis_1099
Cerebellum_1610
Heart_1610
Hypothalamus_1610
Kidney_1610
Testis_1610
Cortex_combined
reads per million genome matching
bp position
0
5
10
15
20
25
30
40
50

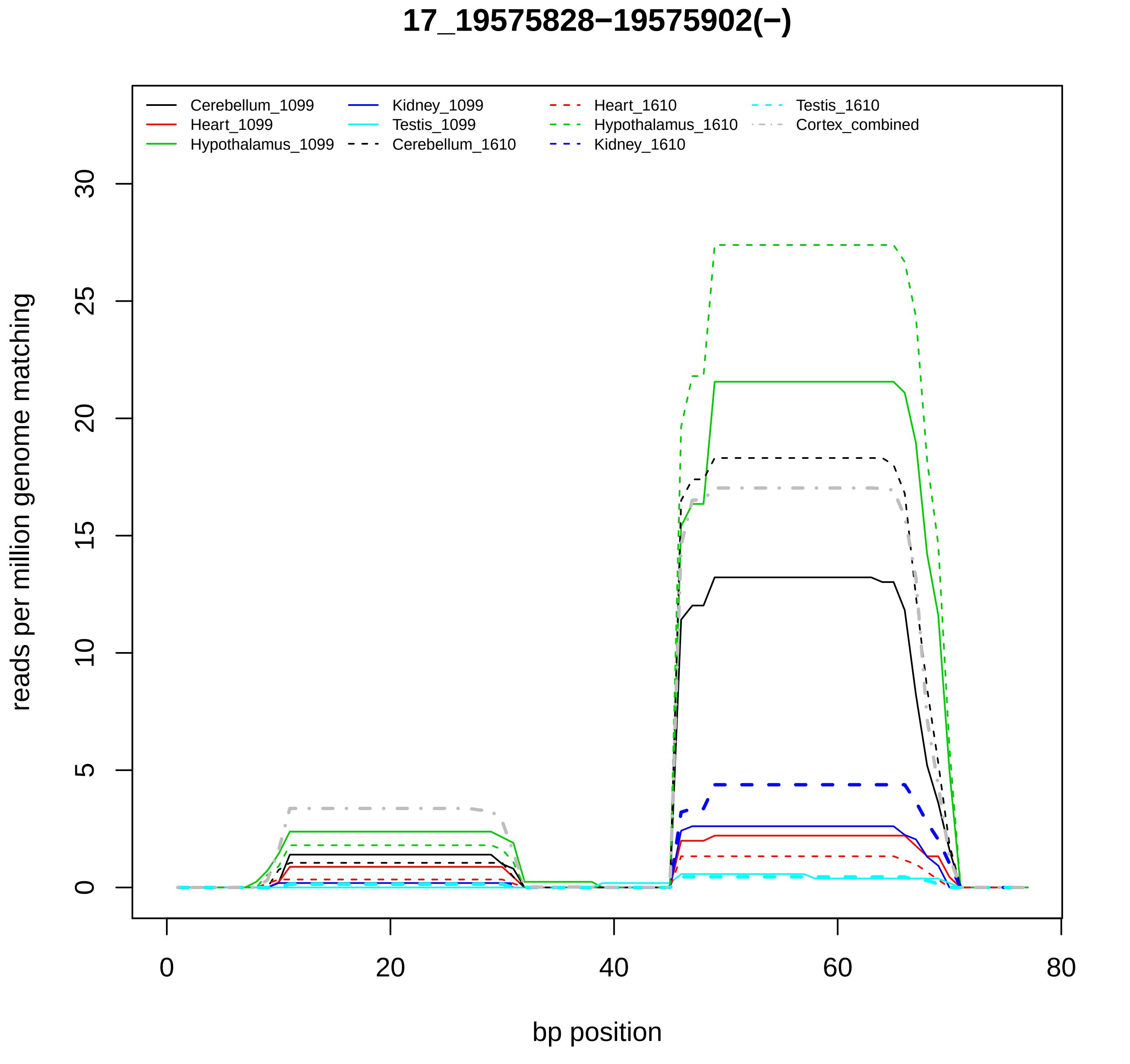

17_19575828−19575902(−)
Cerebellum_1099
Heart_1099
Hypothalamus_1099
Kidney_1099
Testis_1099
Cerebellum_1610
Heart_1610
Hypothalamus_1610
Kidney_1610
Testis_1610
Cortex_combined
reads per million genome matching
0
5
10
15
20
25
30
0
20
40
60
80
bp position

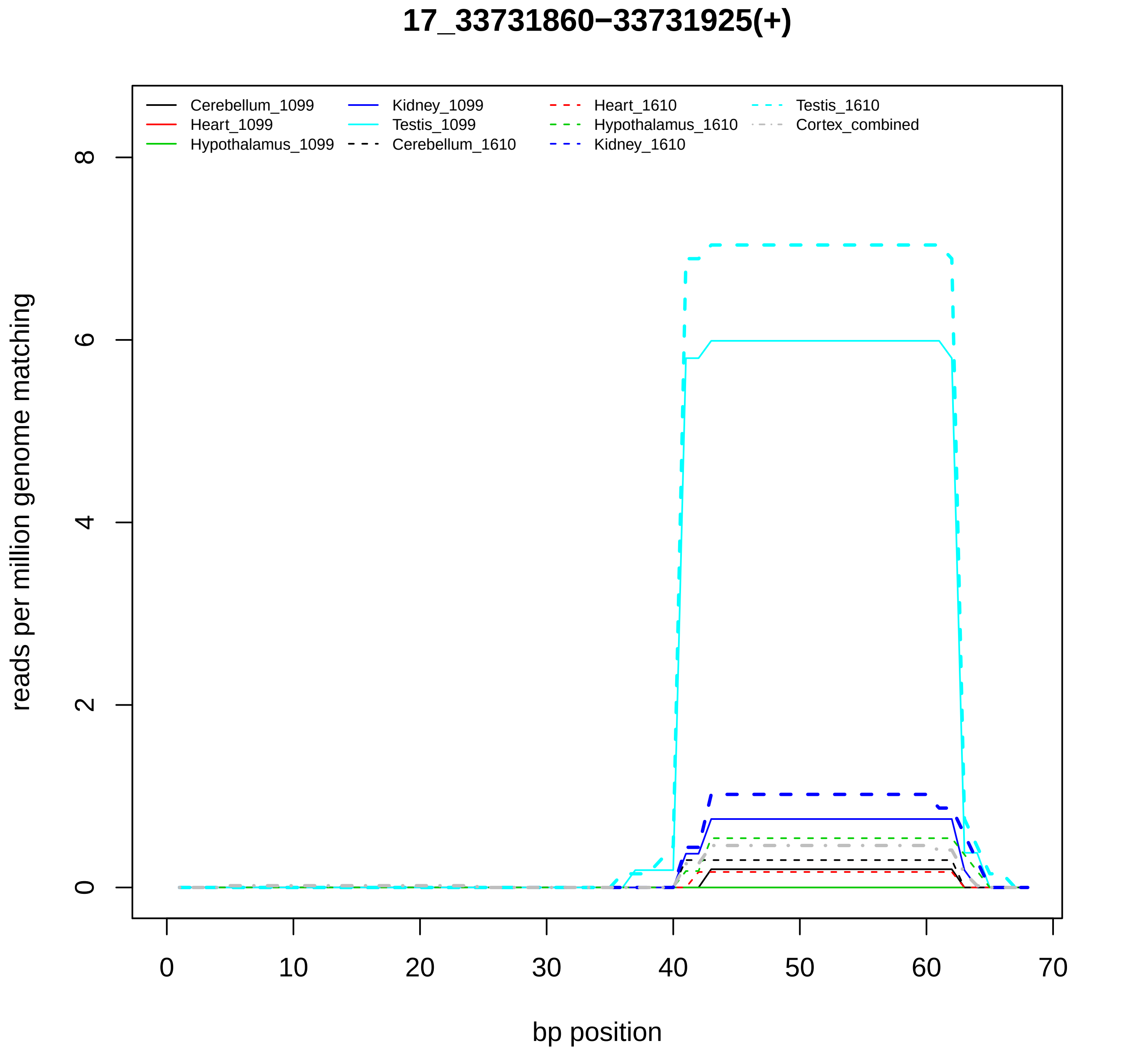
17_33731860−33731925(+)
Cerebellum_1099
Heart_1099
Hypothalamus_1099
Kidney_1099
Testis_1099
Cerebellum_1610
Heart_1610
Hypothalamus_1610
Kidney_1610
Testis_1610
Cortex_combined
reads per million genome matching
bp position
0
2
4
6
8
0
10
20
30
40
50
60
70

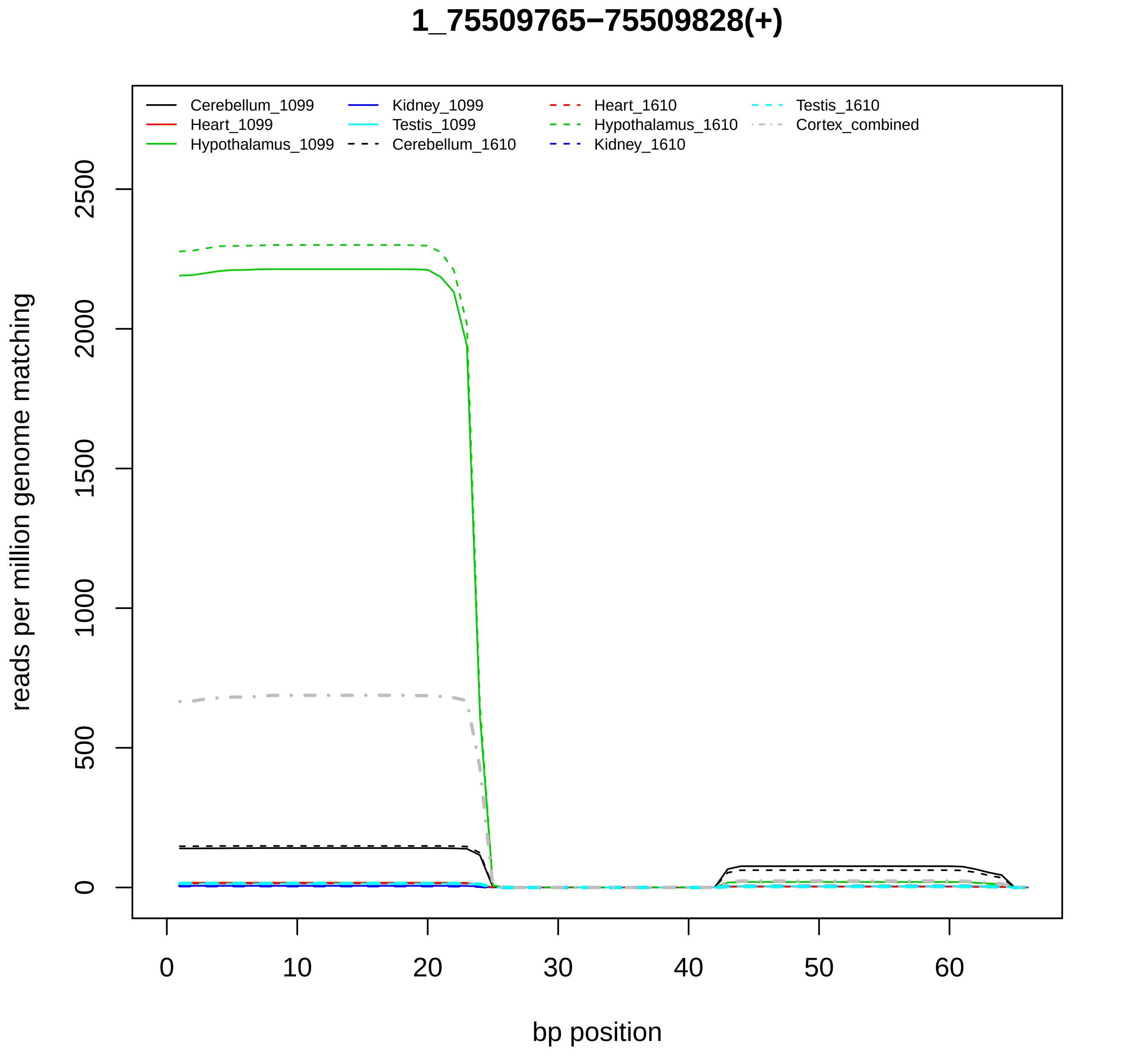

1_75509765−75509828(+)
Cerebellum_1099
Heart_1099
Hypothalamus_1099
Kidney_1099
Testis_1099
Cerebellum_1610
Heart_1610
Hypothalamus_1610
Kidney_1610
Testis_1610
Cortex_combined
reads per million genome matching
0
500
1000
1500
2000
2500
0
10
20
30
40
50
60
bp position

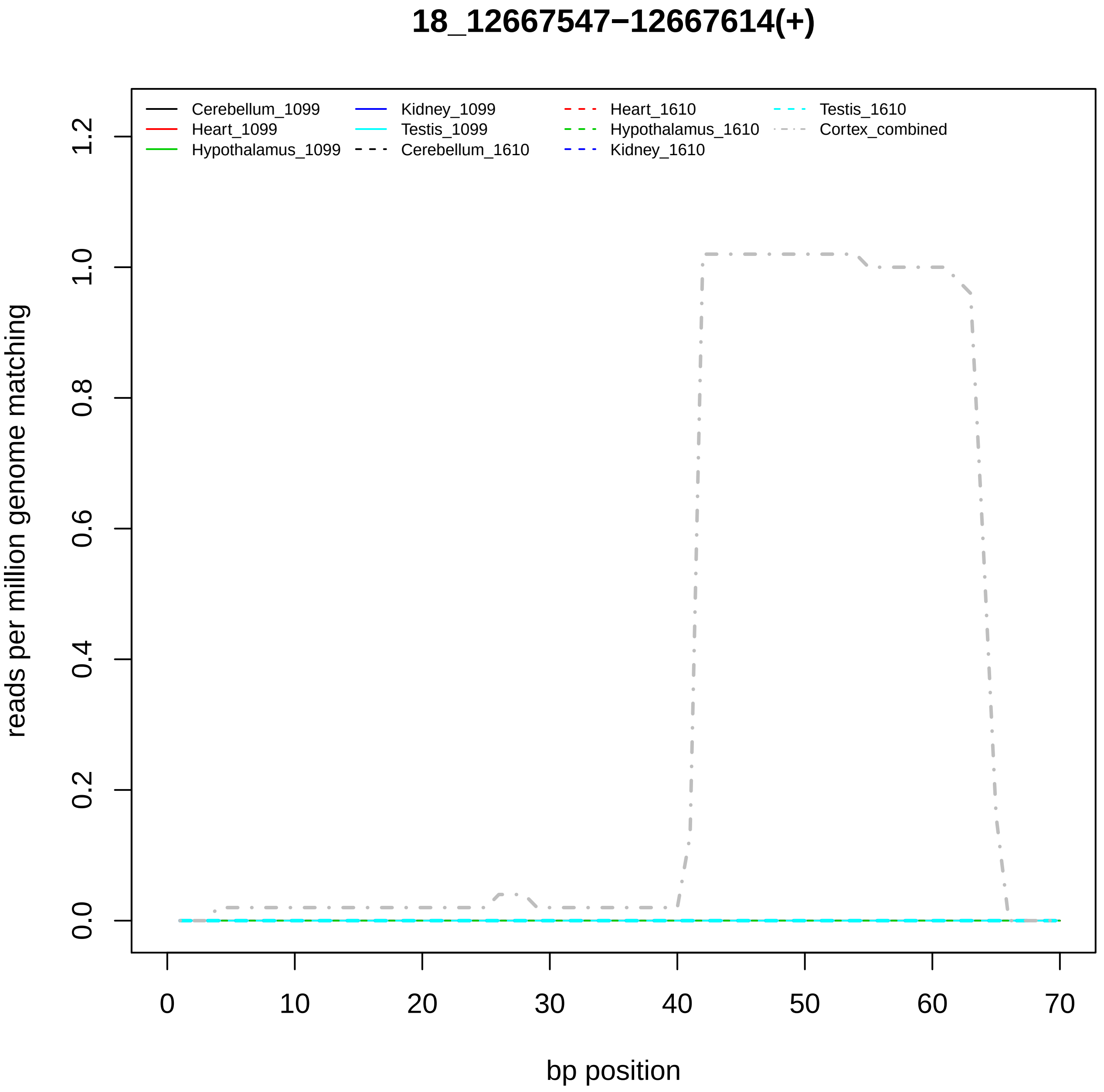

18_12667547−12667614(+)
Cerebellum_1099
Heart_1099
Hypothalamus_1099
Kidney_1099
Testis_1099
Cerebellum_1610
Heart_1610
Hypothalamus_1610
Kidney_1610
Testis_1610
Cortex_combined
reads per million genome matching
bp position
0.0
0.2
0.4
0.6
0.8
1.0
1.2
0
10
20
30
40
50
60
70

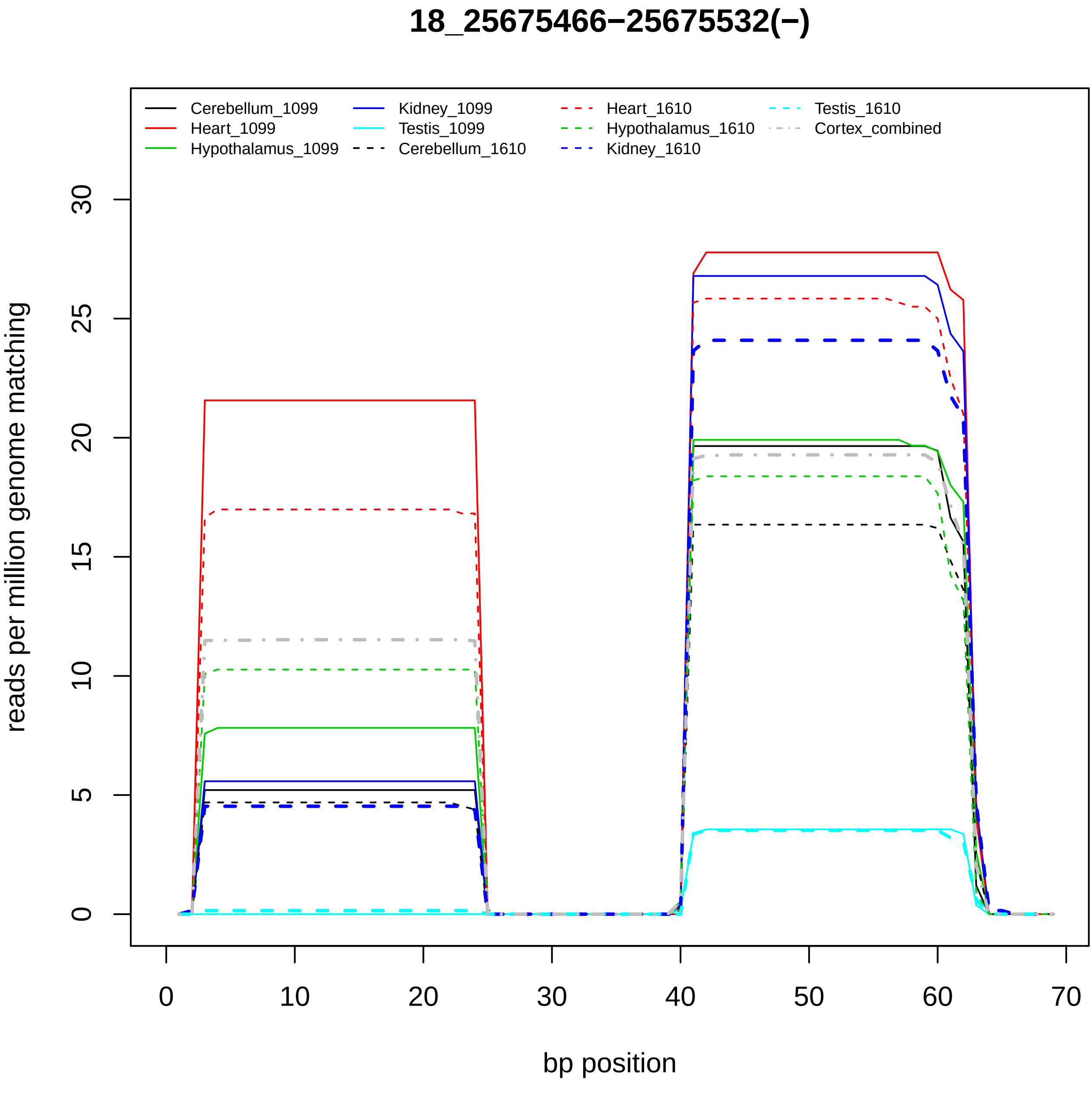

18_25675466−25675532(−)
Cerebellum_1099
Heart_1099
Hypothalamus_1099
Kidney_1099
Testis_1099
Cerebellum_1610
Heart_1610
Hypothalamus_1610
Kidney_1610
Testis_1610
Cortex_combined
reads per million genome matching
bp position
0
5
10
15
20
25
30
0
10
20
30
40
50
60
70

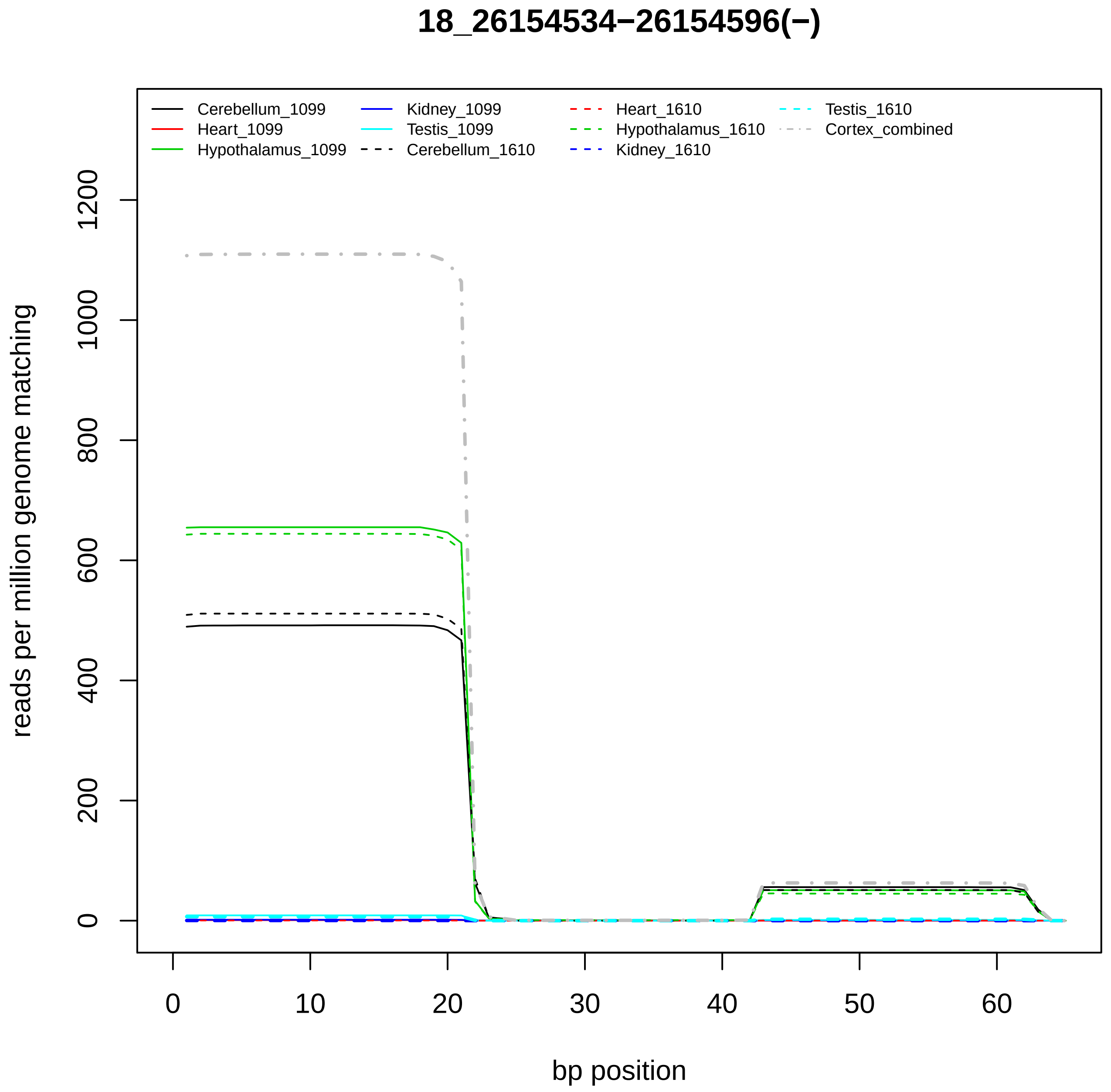
18_26154534−26154596(−)
Cerebellum_1099
Heart_1099
Hypothalamus_1099
Kidney_1099
Testis_1099
Cerebellum_1610
Heart_1610
Hypothalamus_1610
Kidney_1610
Testis_1610
Cortex_combined
reads per million genome matching
0
200
400
600
800
1000
1200
bp position
0
10
20
30
40
50
60

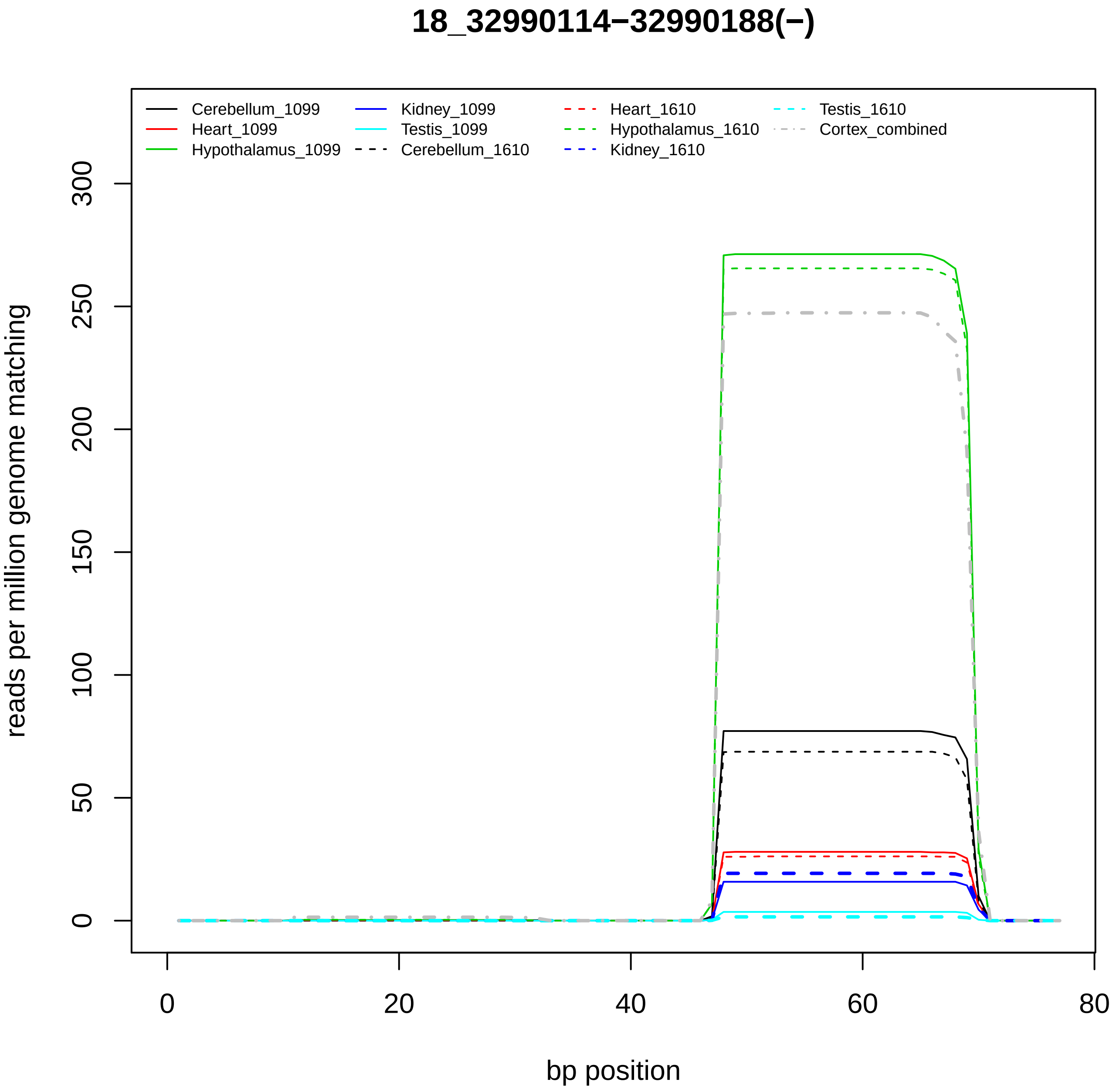

18_32990114−32990188(−)
Cerebellum_1099
Heart_1099
Hypothalamus_1099
Kidney_1099
Testis_1099
Cerebellum_1610
Heart_1610
Hypothalamus_1610
Kidney_1610
Testis_1610
Cortex_combined
reads per million genome matching
0
50
100
150
200
250
300
0
20
40
60
80
bp position

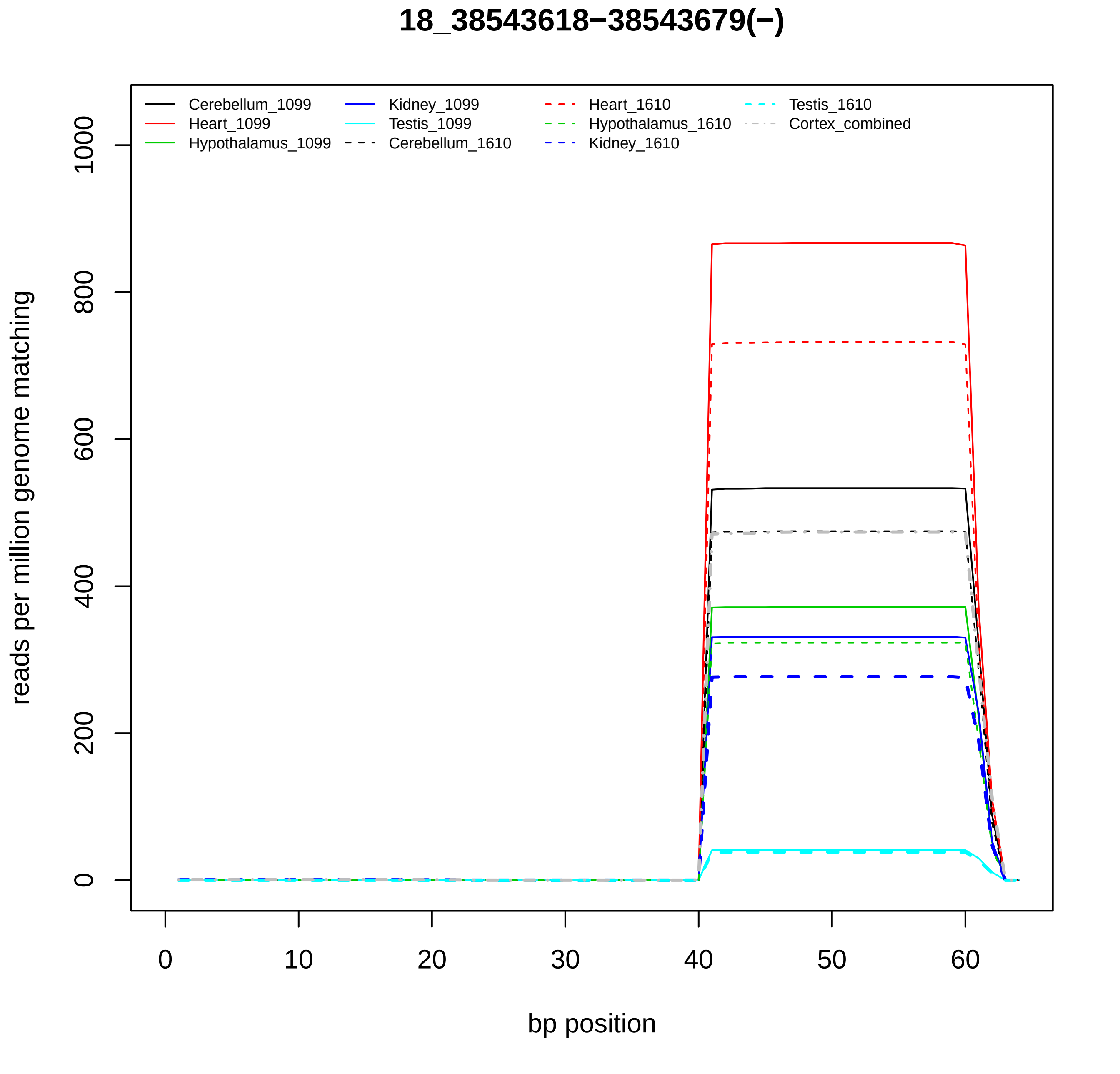

18_38543618−38543679(−)
Cerebellum_1099
Heart_1099
Hypothalamus_1099
Kidney_1099
Testis_1099
Cerebellum_1610
Heart_1610
Hypothalamus_1610
Kidney_1610
Testis_1610
Cortex_combined
reads per million genome matching
0
200
400
600
800
1000
0
10
20
30
40
50
60
bp position

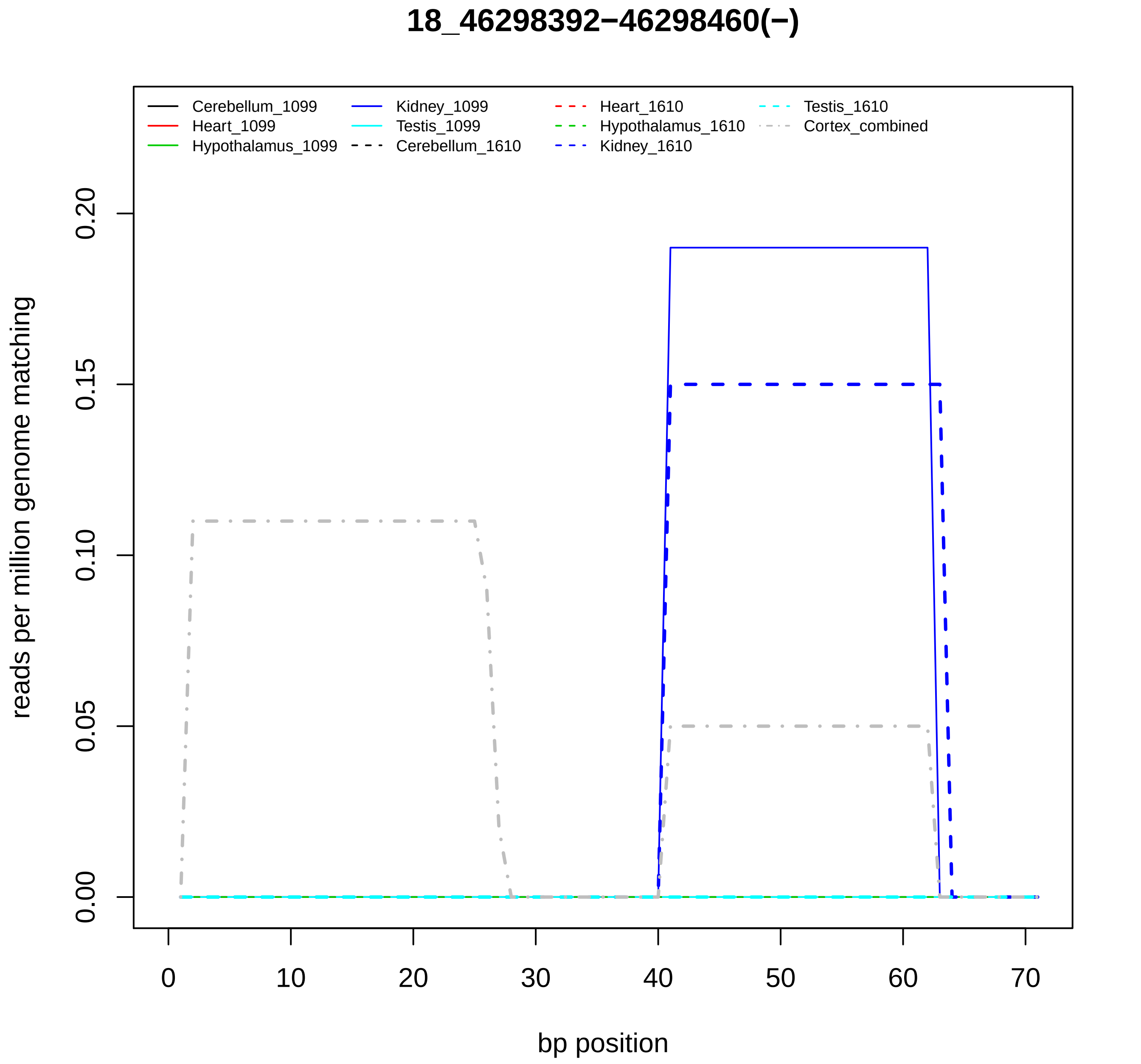

18_46298392−46298460(−)
Cerebellum_1099
Heart_1099
Hypothalamus_1099
Kidney_1099
Testis_1099
Cerebellum_1610
Heart_1610
Hypothalamus_1610
Kidney_1610
Testis_1610
Cortex_combined
reads per million genome matching
0.00
0.05
0.10
0.15
0.20
0
10
20
30
40
50
60
70
bp position

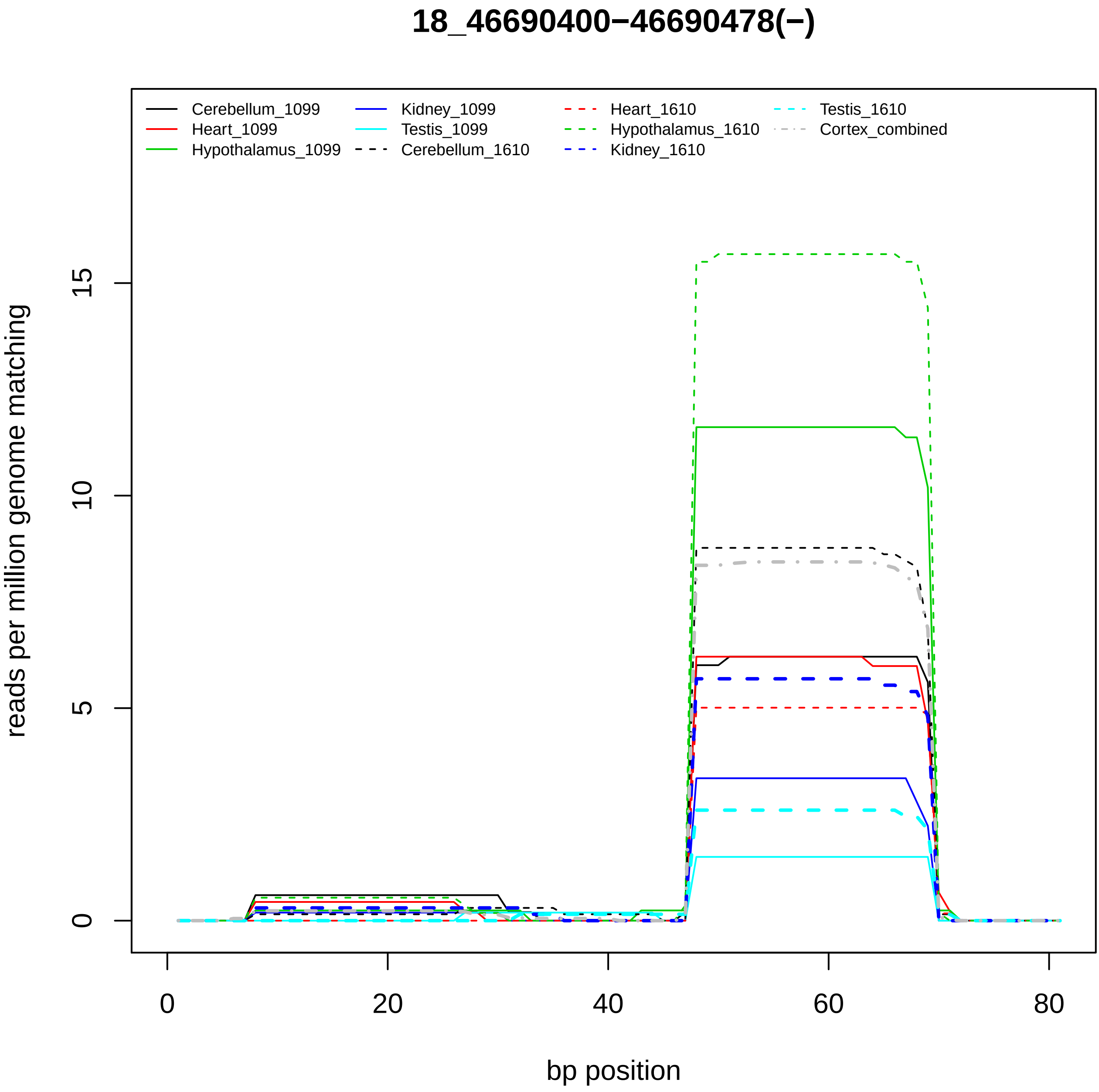
18_46690400−46690478(−)
Cerebellum_1099
Heart_1099
Hypothalamus_1099
Kidney_1099
Testis_1099
Cerebellum_1610
Heart_1610
Hypothalamus_1610
Kidney_1610
Testis_1610
Cortex_combined
reads per million genome matching
bp position
0
5
10
15
0
20
40
60
80

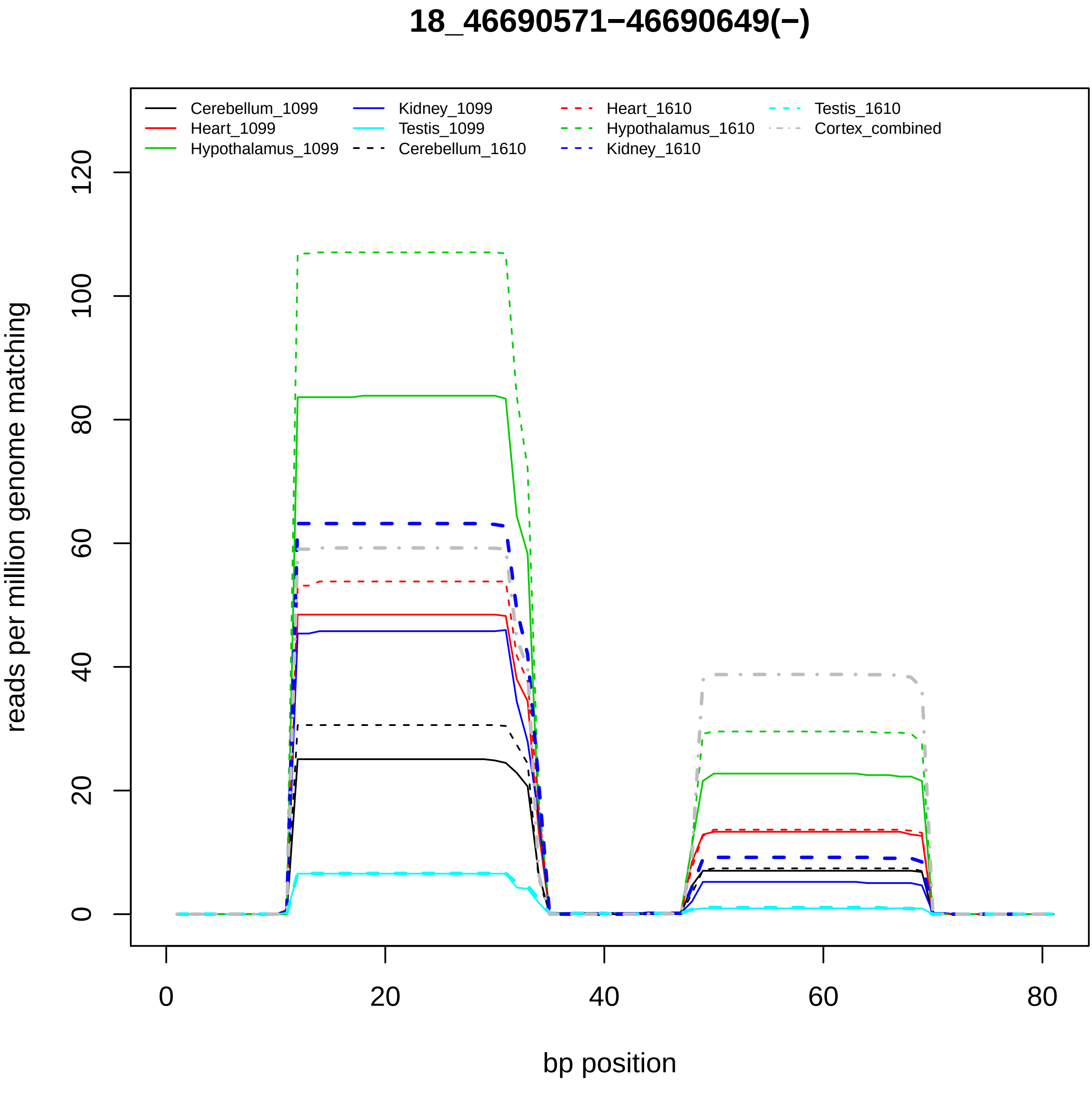
18_46690571−46690649(−)
Cerebellum_1099
Heart_1099
Hypothalamus_1099
Kidney_1099
Testis_1099
Cerebellum_1610
Heart_1610
Hypothalamus_1610
Kidney_1610
Testis_1610
Cortex_combined
reads per million genome matching
0
20
40
60
80
100
120
0
20
40
60
80
bp position

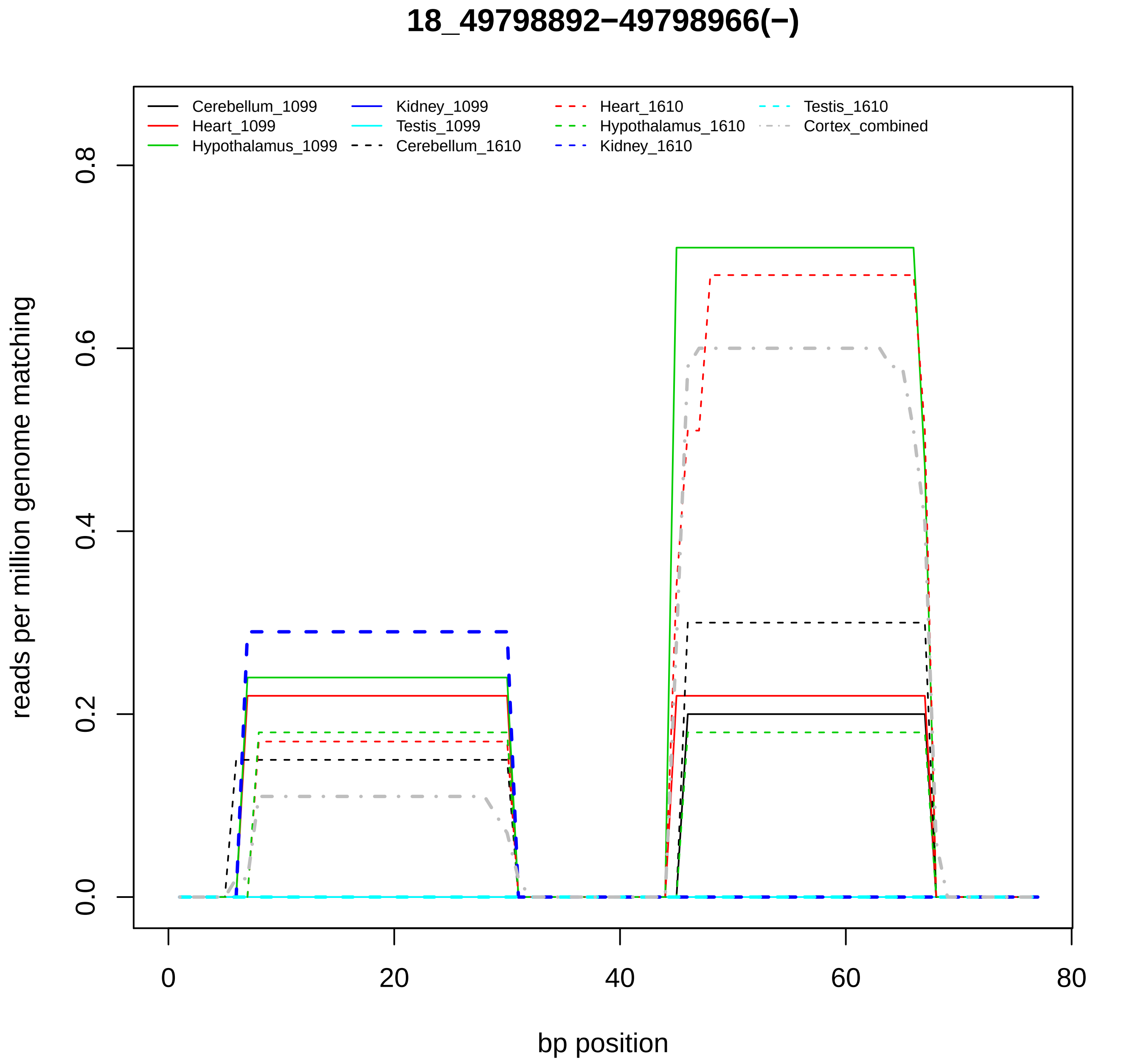

18_49798892−49798966(−)
Cerebellum_1099
Heart_1099
Hypothalamus_1099
Kidney_1099
Testis_1099
Cerebellum_1610
Heart_1610
Hypothalamus_1610
Kidney_1610
Testis_1610
Cortex_combined
reads per million genome matching
bp position
0.0
0.2
0.4
0.6
0.8
0
20
40
60
80

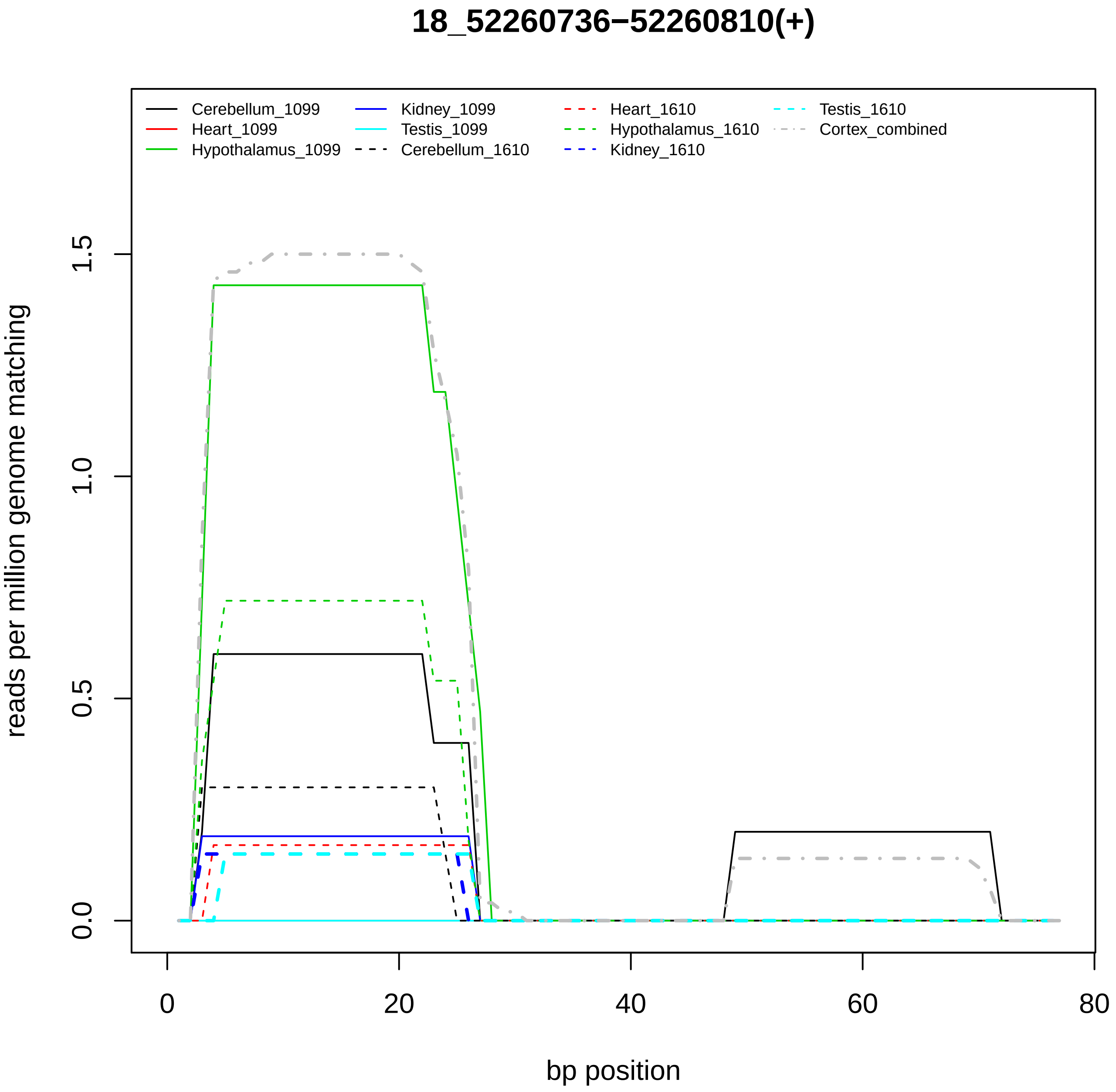

18_52260736−52260810(+)
Cerebellum_1099
Heart_1099
Hypothalamus_1099
Kidney_1099
Testis_1099
Cerebellum_1610
Heart_1610
Hypothalamus_1610
Kidney_1610
Testis_1610
Cortex_combined
reads per million genome matching
bp position
0.0
0.5
1.0
1.5
0
20
40
60
80

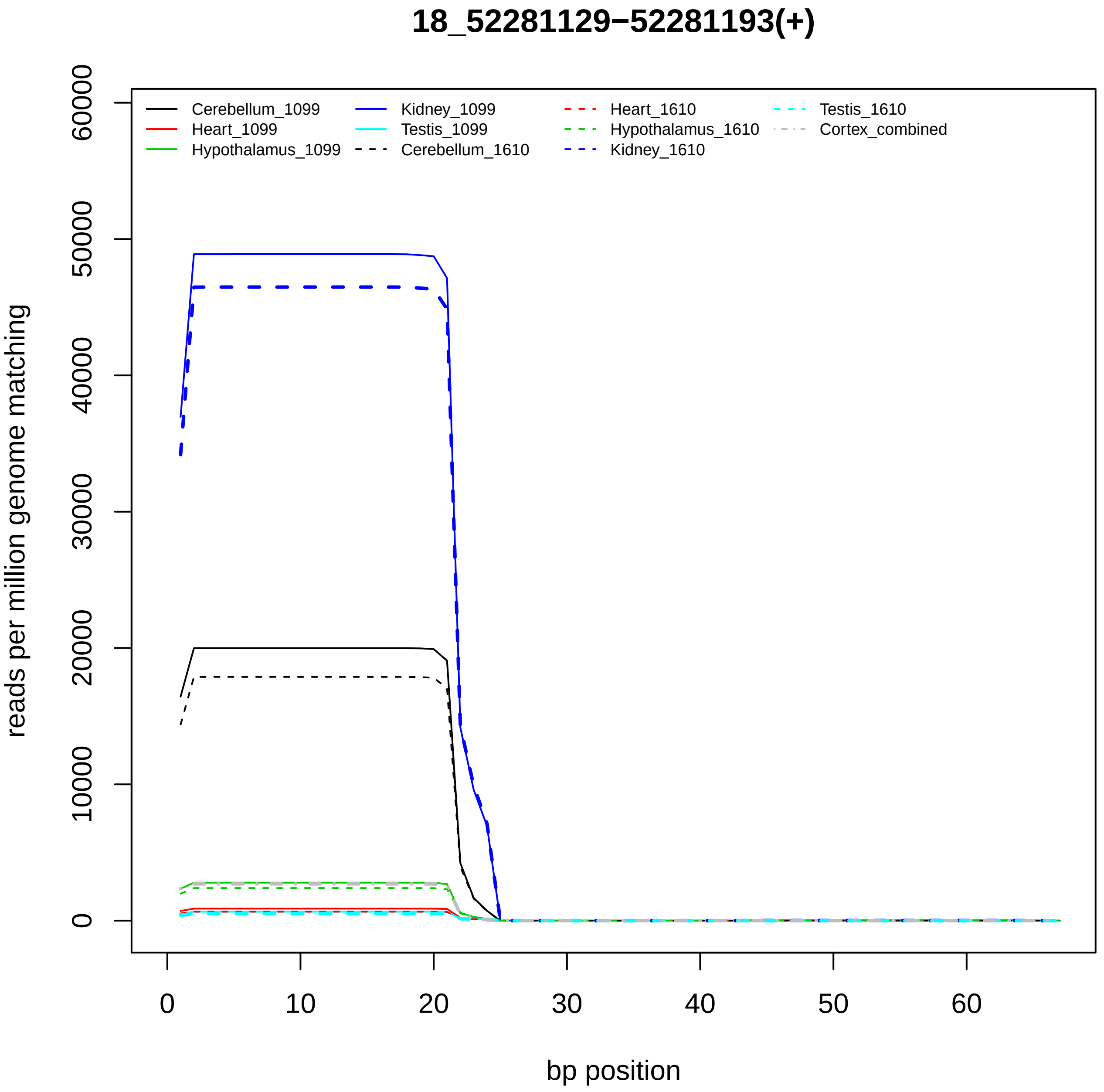
18_52281129−52281193(+)
Cerebellum_1099
Heart_1099
Hypothalamus_1099
Kidney_1099
Testis_1099
Cerebellum_1610
Heart_1610
Hypothalamus_1610
Kidney_1610
Testis_1610
Cortex_combined
reads per million genome matching
bp position
0
10000
20000
30000
40000
50000
60000
0
10
20
30
40
50
60

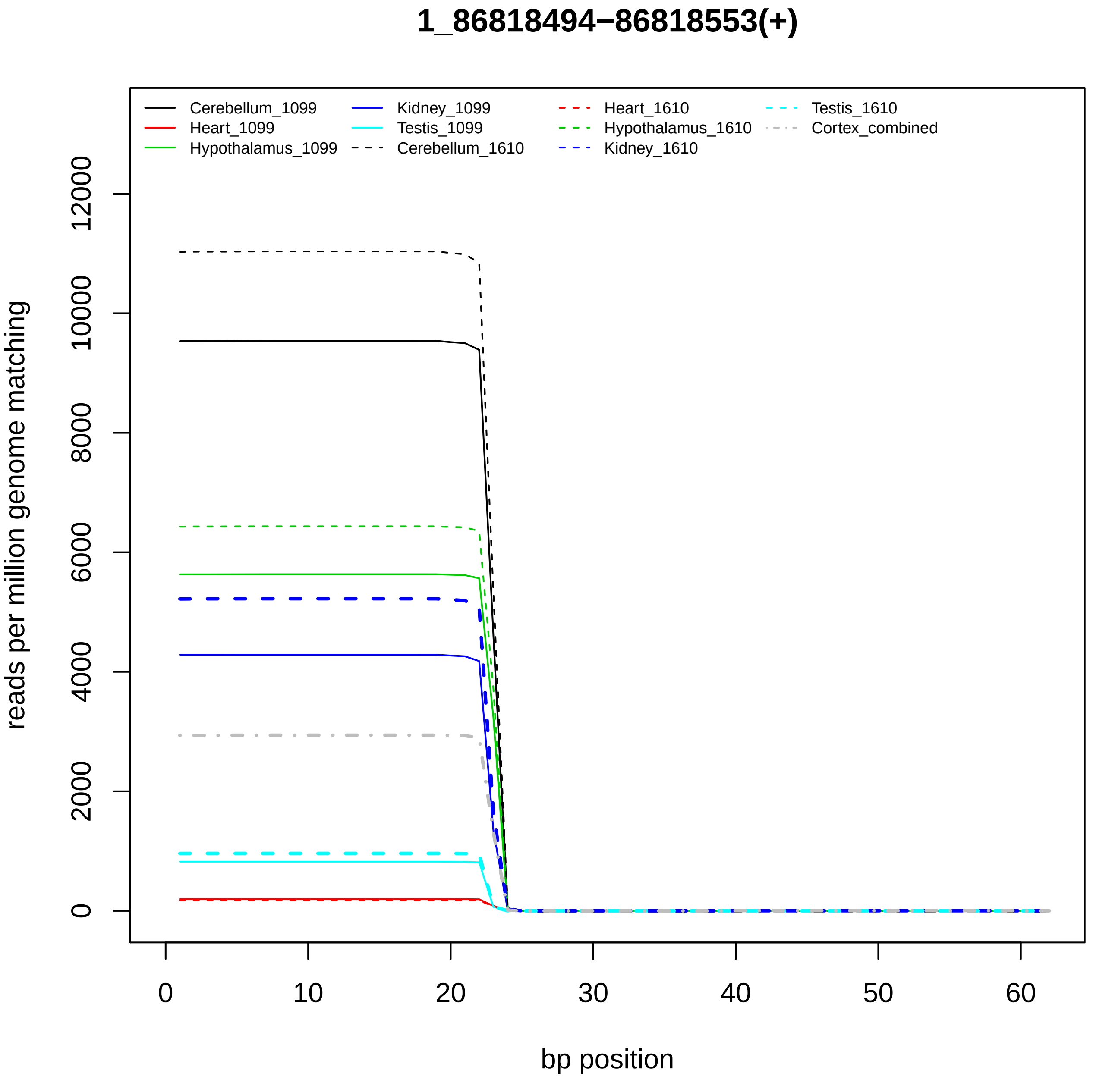

1_86818494−86818553(+)
Cerebellum_1099
Heart_1099
Hypothalamus_1099
Kidney_1099
Testis_1099
Cerebellum_1610
Heart_1610
Hypothalamus_1610
Kidney_1610
Testis_1610
Cortex_combined
reads per million genome matching
bp position
0
2000
4000
6000
8000
10000
12000
10
20
30
40
50
60

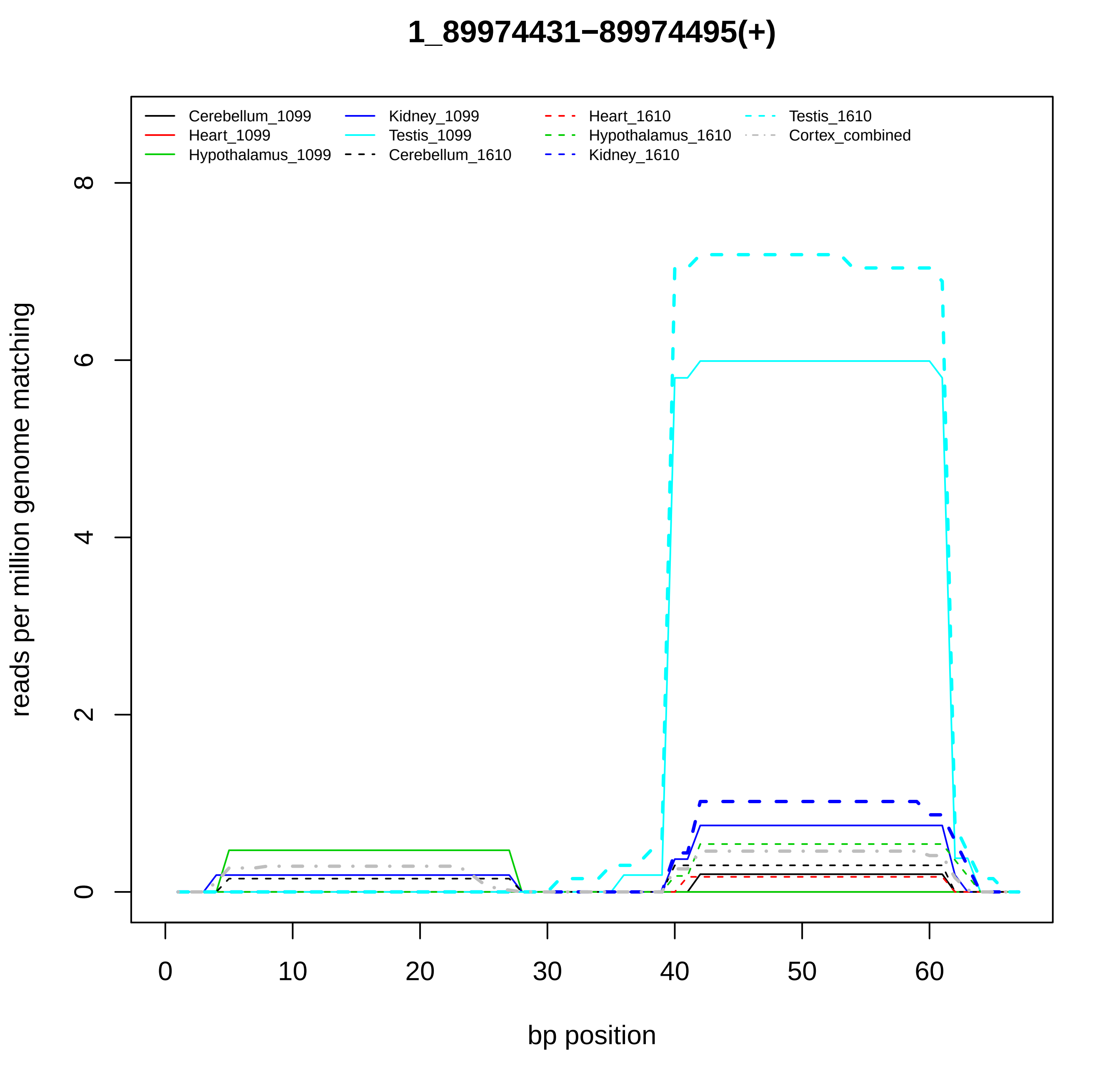

1_89974431−89974495(+)
Cerebellum_1099
Heart_1099
Hypothalamus_1099
Kidney_1099
Testis_1099
Cerebellum_1610
Heart_1610
Hypothalamus_1610
Kidney_1610
Testis_1610
Cortex_combined
reads per million genome matching
bp position
0
2
4
6
8
0
10
20
30
40
50
60

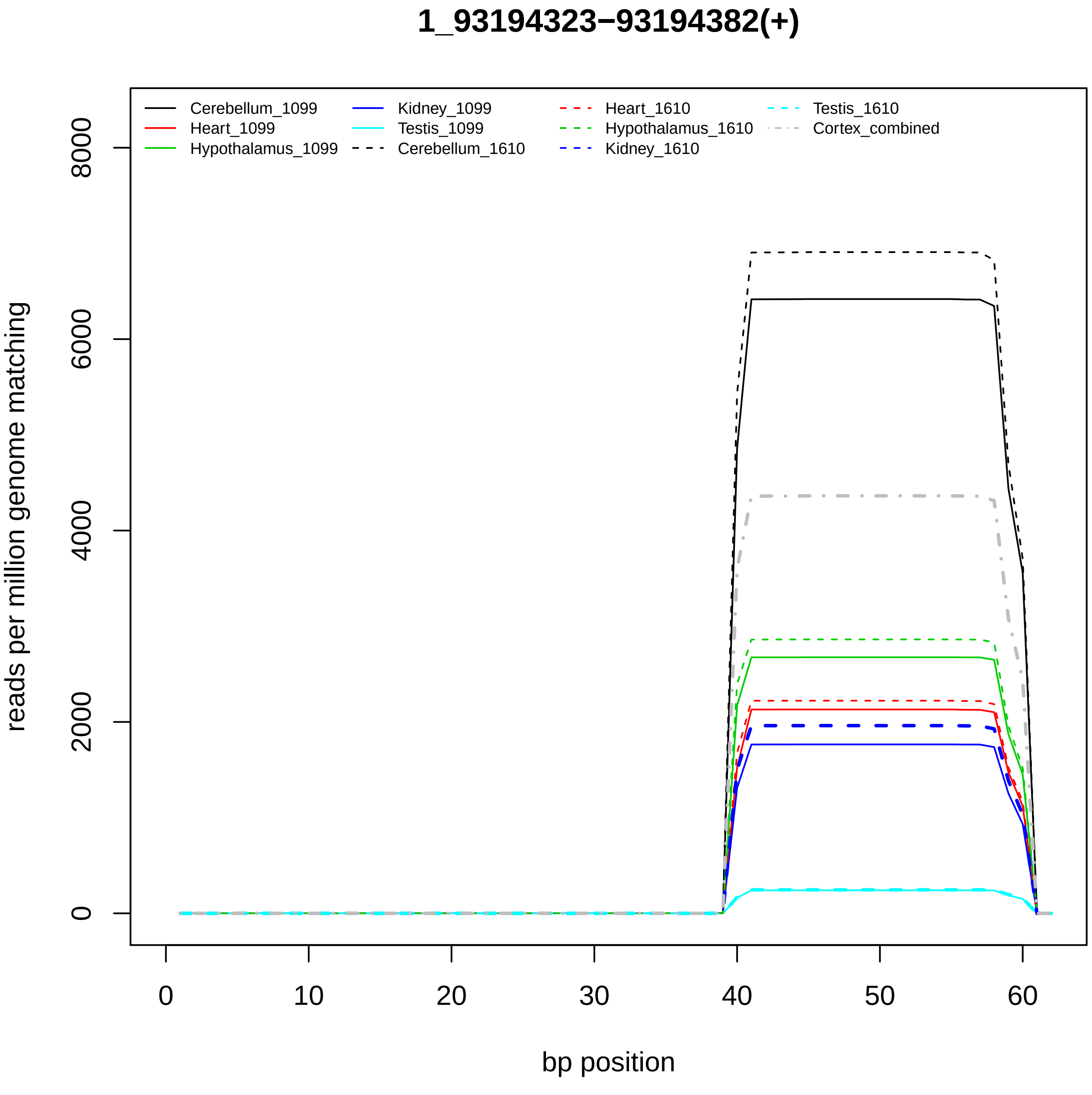

1_93194323−93194382(+)
Cerebellum_1099
Heart_1099
Hypothalamus_1099
Kidney_1099
Testis_1099
Cerebellum_1610
Heart_1610
Hypothalamus_1610
Kidney_1610
Testis_1610
Cortex_combined
reads per million genome matching
bp position
0
2000
4000
6000
8000
0
10
20
30
40
50
60

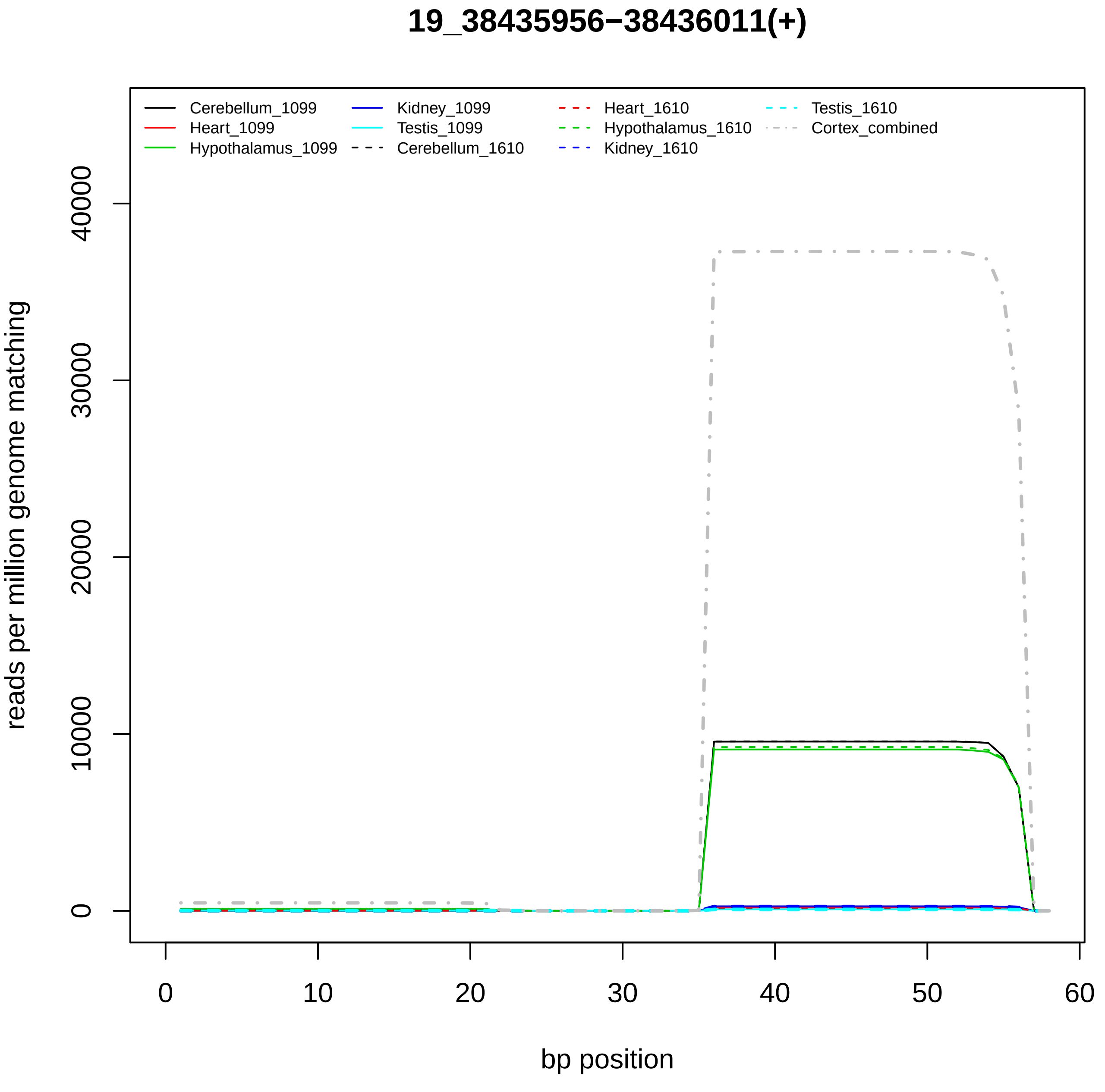

19_38435956−38436011(+)
Cerebellum_1099
Heart_1099
Hypothalamus_1099
Kidney_1099
Testis_1099
Cerebellum_1610
Heart_1610
Hypothalamus_1610
Kidney_1610
Testis_1610
Cortex_combined
reads per million genome matching
0
10000
20000
30000
40000
0
10
20
30
40
50
60
bp position

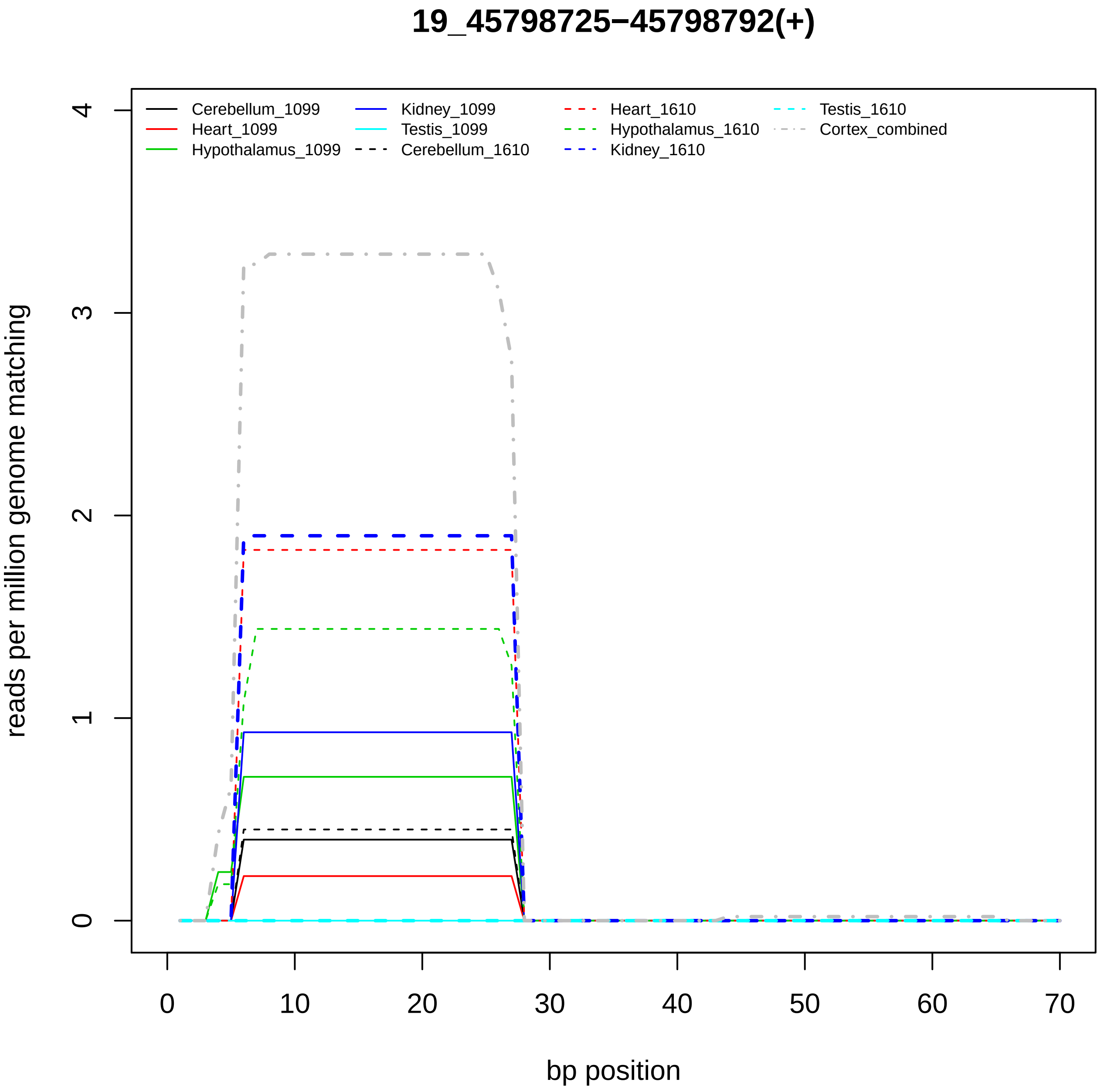

19_45798725−45798792(+)
Cerebellum_1099
Heart_1099
Hypothalamus_1099
Kidney_1099
Testis_1099
Cerebellum_1610
Heart_1610
Hypothalamus_1610
Kidney_1610
Testis_1610
Cortex_combined
reads per million genome matching
bp position
0
1
2
3
4
0
10
20
30
40
50
60
70

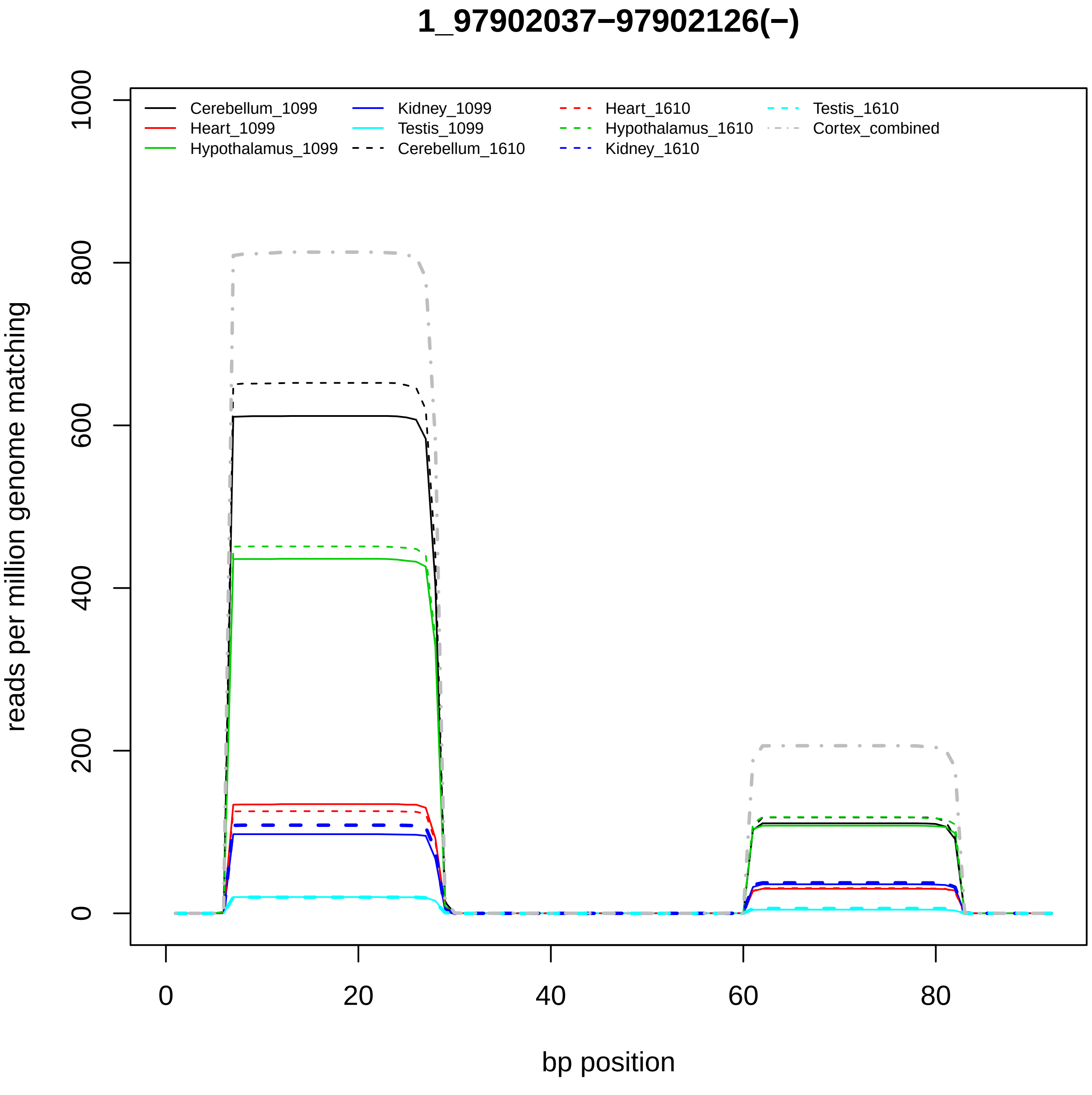
1_97902037−97902126(−)
Cerebellum_1099
Heart_1099
Hypothalamus_1099
Kidney_1099
Testis_1099
Cerebellum_1610
Heart_1610
Hypothalamus_1610
Kidney_1610
Testis_1610
Cortex_combined
reads per million genome matching
bp position
0
200
400
600
800
1000
0
20
40
60
80

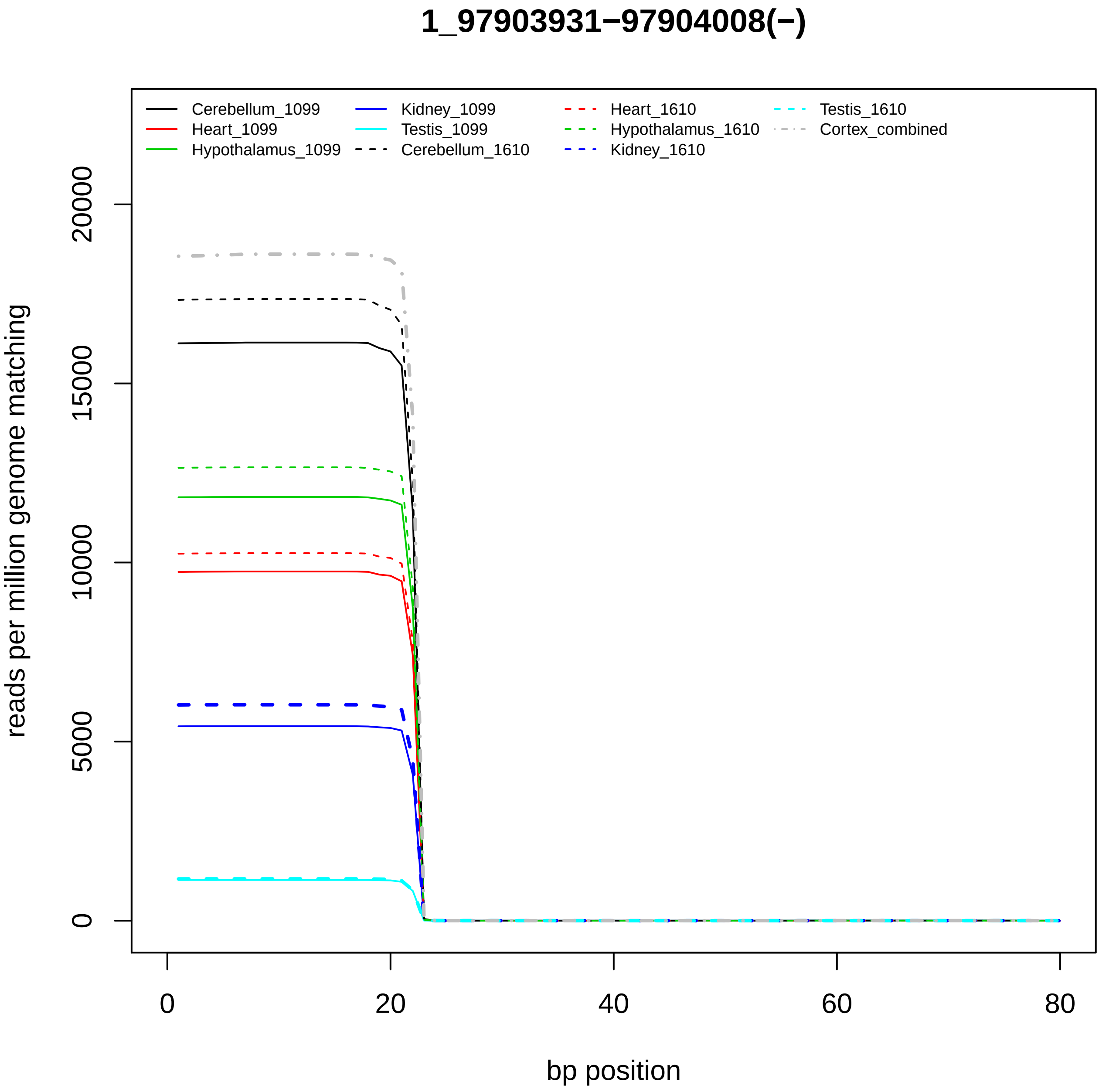

1_97903931−97904008(−)
Cerebellum_1099
Heart_1099
Hypothalamus_1099
Kidney_1099
Testis_1099
Cerebellum_1610
Heart_1610
Hypothalamus_1610
Kidney_1610
Testis_1610
Cortex_combined
reads per million genome matching
0
5000
10000
15000
20000
0
20
40
60
80
bp position

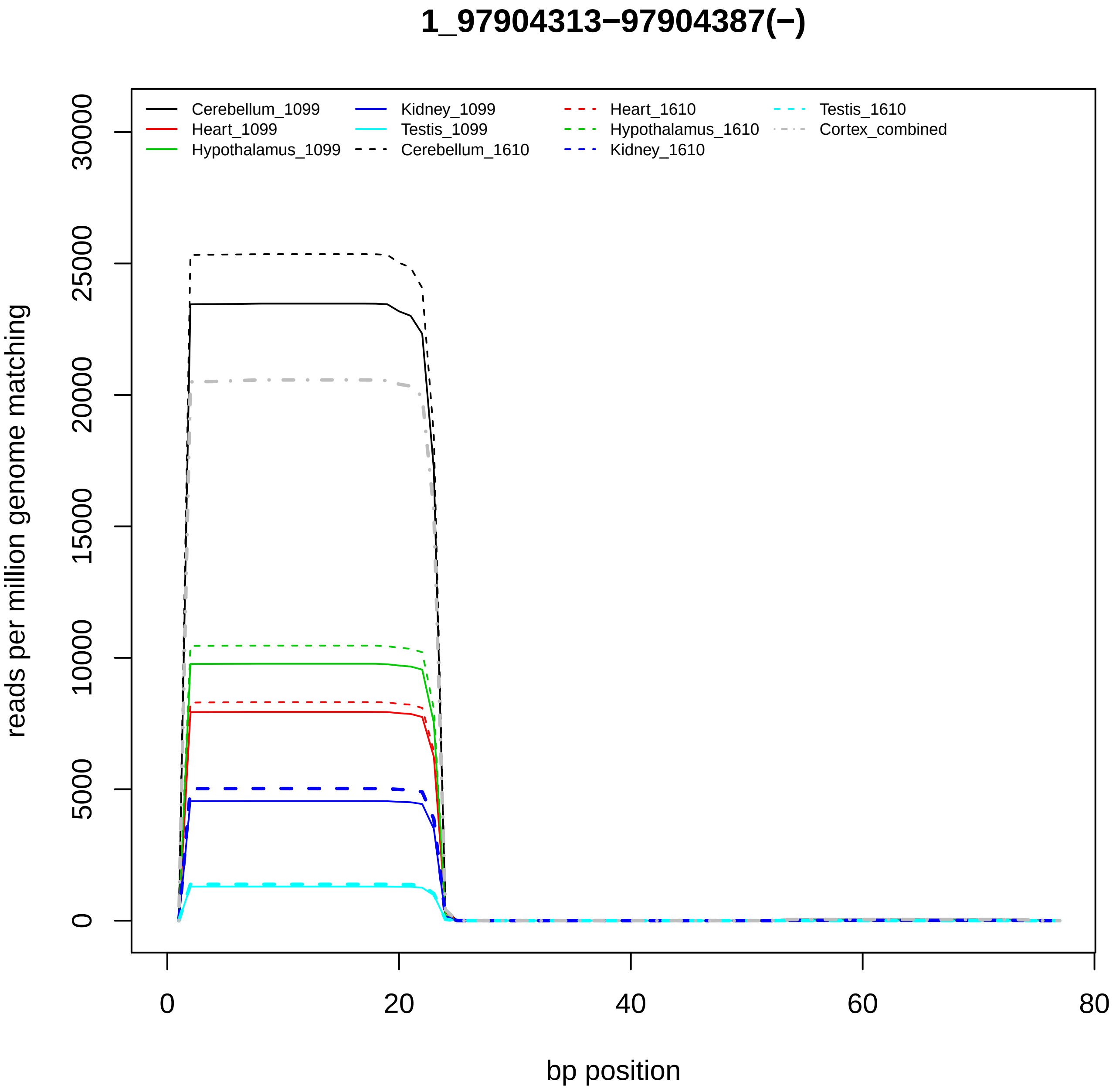

1_97904313−97904387(−)
Cerebellum_1099
Heart_1099
Hypothalamus_1099
Kidney_1099
Testis_1099
Cerebellum_1610
Heart_1610
Hypothalamus_1610
Kidney_1610
Testis_1610
Cortex_combined
reads per million genome matching
0
5000
10000
15000
20000
25000
30000
bp position
0
20
40
60
80

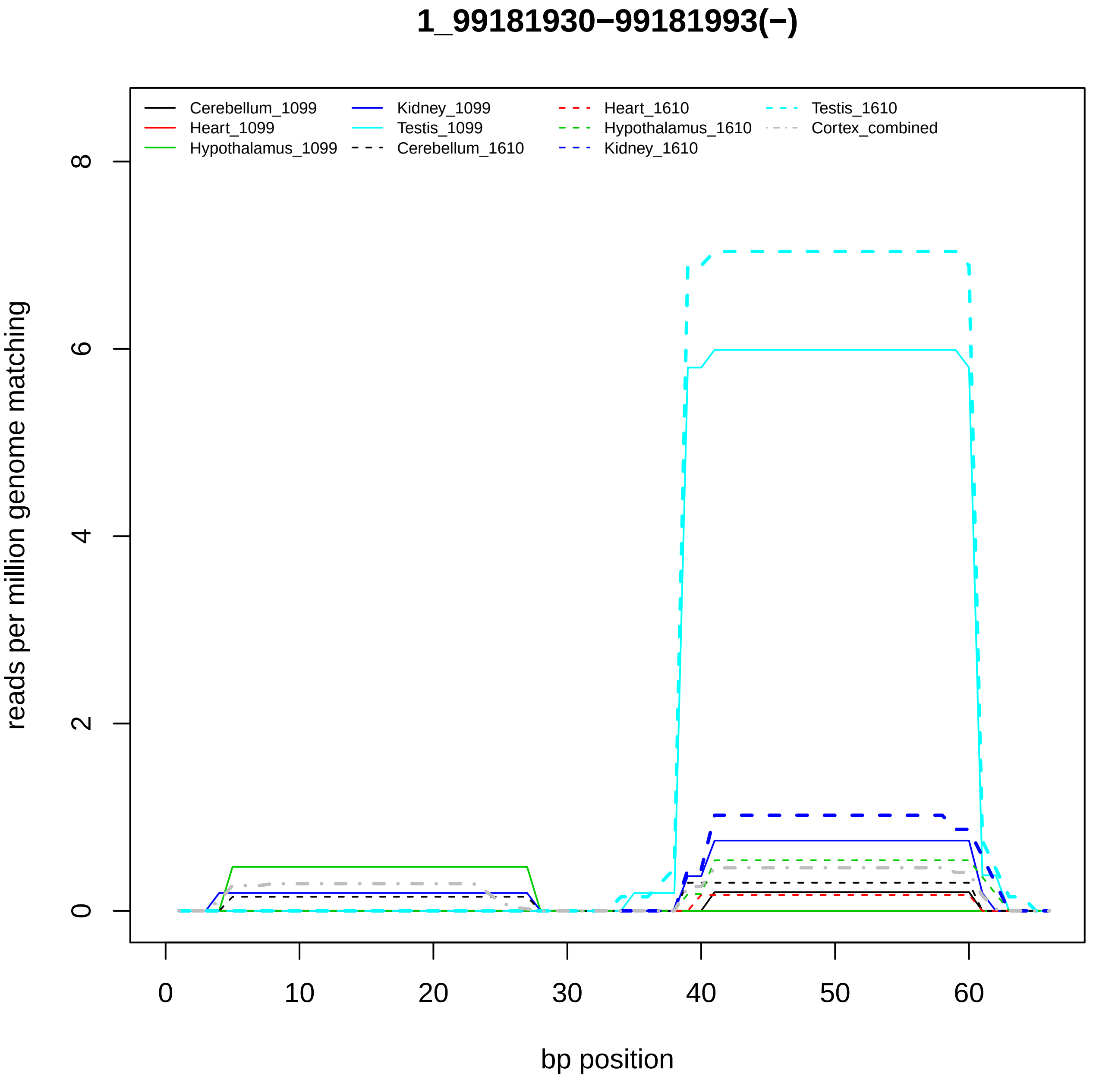

1_99181930−99181993(−)
Cerebellum_1099
Heart_1099
Hypothalamus_1099
Kidney_1099
Testis_1099
Cerebellum_1610
Heart_1610
Hypothalamus_1610
Kidney_1610
Testis_1610
Cortex_combined
reads per million genome matching
bp position
0
2
4
6
8
0
10
20
30
40
50
60

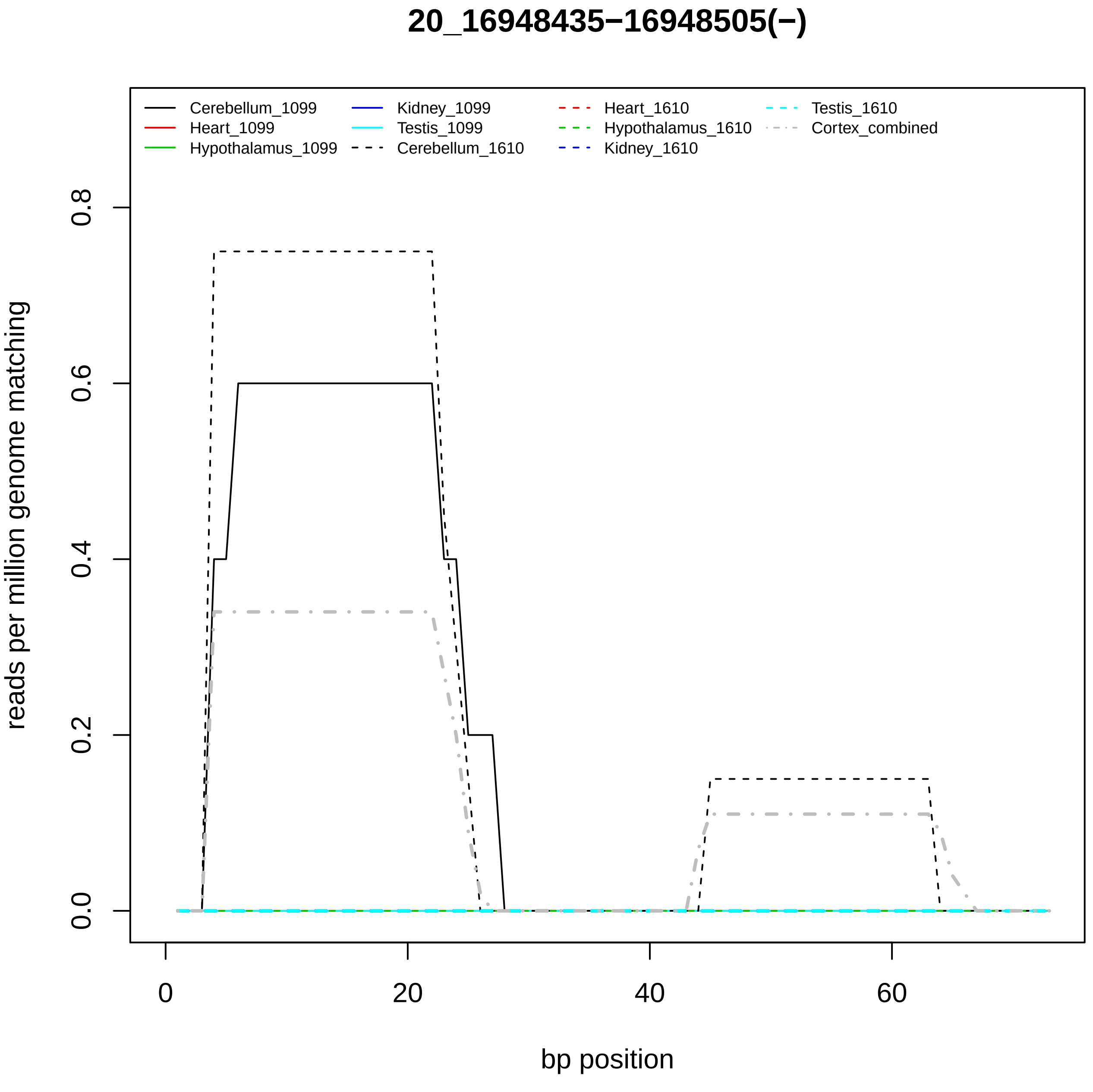

20_16948435−16948505(−)
Cerebellum_1099
Heart_1099
Hypothalamus_1099
Kidney_1099
Testis_1099
Cerebellum_1610
Heart_1610
Hypothalamus_1610
Kidney_1610
Testis_1610
Cortex_combined
reads per million genome matching
0.0
0.2
0.4
0.6
0.8
0
20
40
60
bp position

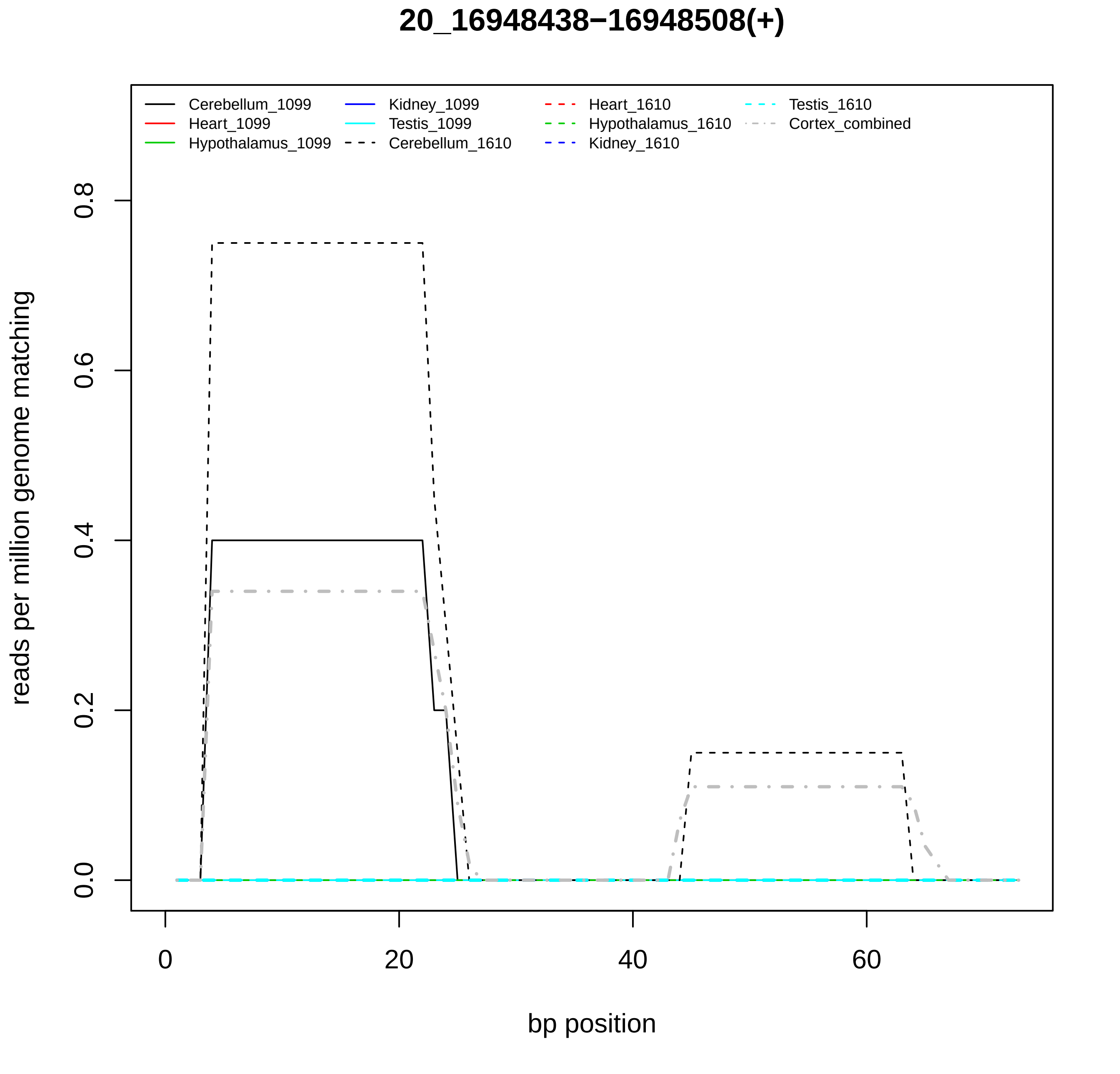
20_16948438−16948508(+)
Cerebellum_1099
Heart_1099
Hypothalamus_1099
Kidney_1099
Testis_1099
Cerebellum_1610
Heart_1610
Hypothalamus_1610
Kidney_1610
Testis_1610
Cortex_combined
reads per million genome matching
0.0
0.2
0.4
0.6
0.8
0
20
40
60
bp position

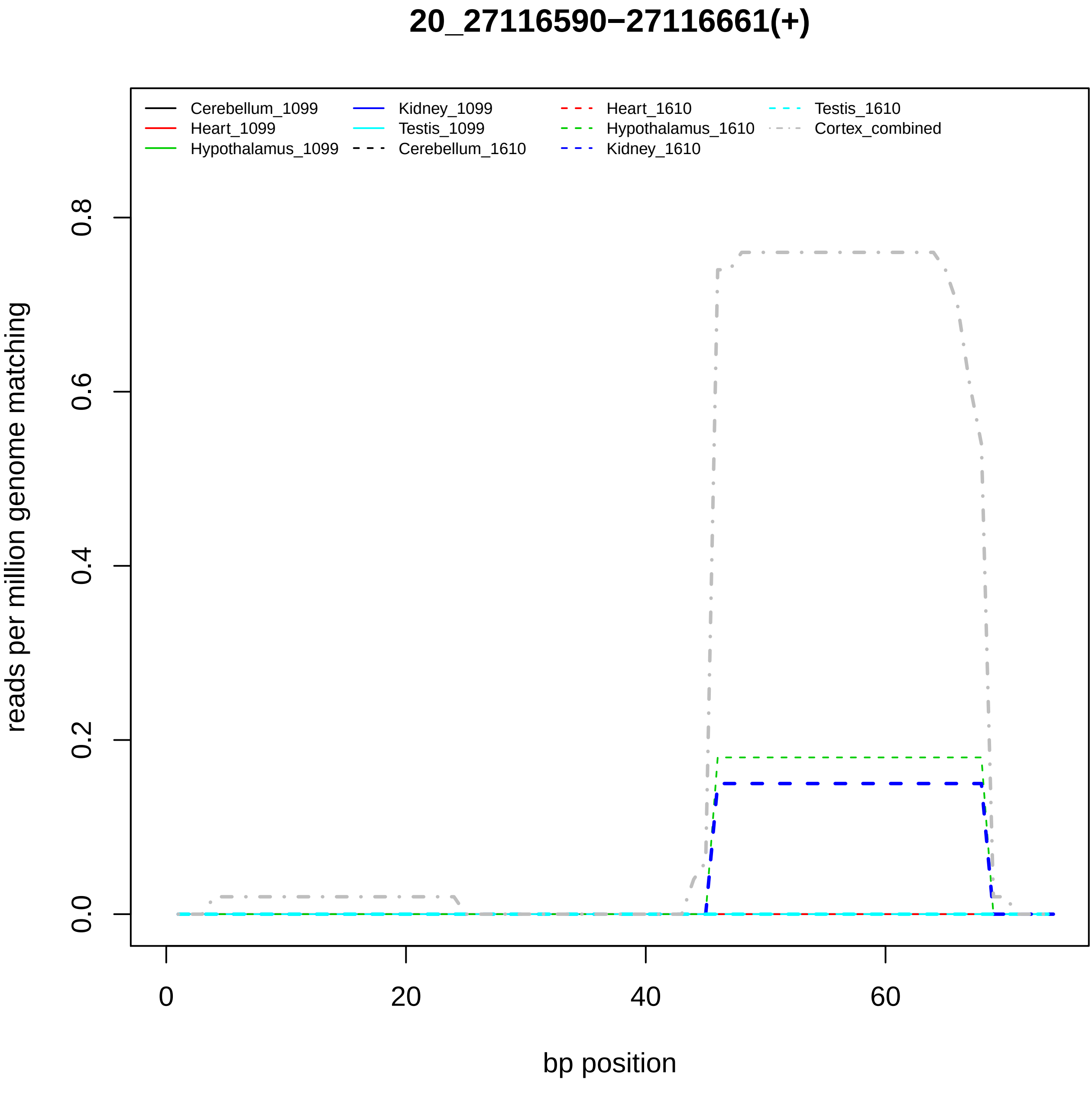

20_27116590−27116661(+)
Cerebellum_1099
Heart_1099
Hypothalamus_1099
Kidney_1099
Testis_1099
Cerebellum_1610
Heart_1610
Hypothalamus_1610
Kidney_1610
Testis_1610
Cortex_combined
reads per million genome matching
0.0
0.2
0.4
0.6
0.8
0
20
40
60
bp position

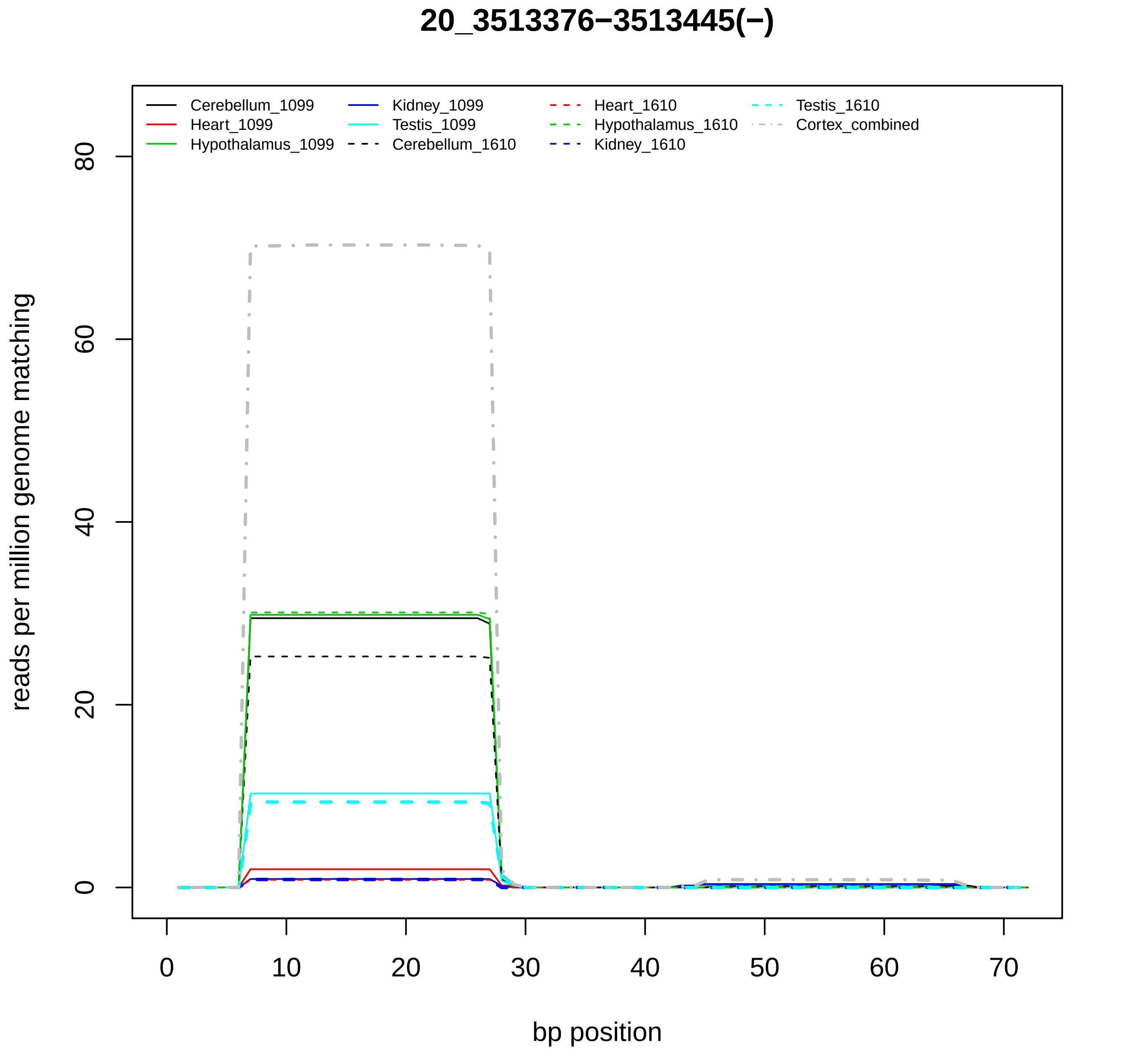
20_3513376−3513445(−)
Cerebellum_1099
Heart_1099
Hypothalamus_1099
Kidney_1099
Testis_1099
Cerebellum_1610
Heart_1610
Hypothalamus_1610
Kidney_1610
Testis_1610
Cortex_combined
reads per million genome matching
0
20
40
60
80
0
10
20
30
40
50
60
70
bp position

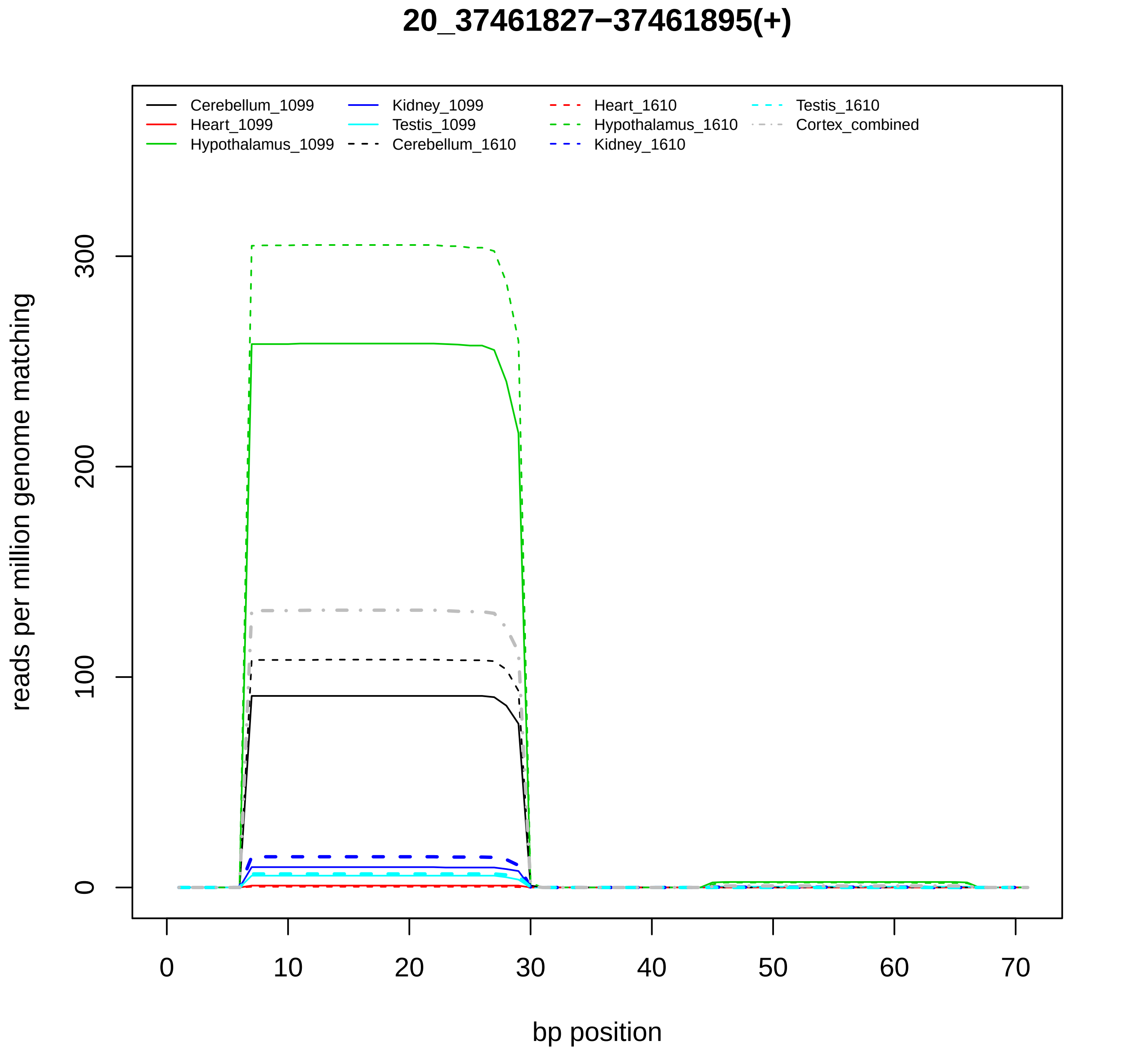

20_37461827−37461895(+)
Cerebellum_1099
Heart_1099
Hypothalamus_1099
Kidney_1099
Testis_1099
Cerebellum_1610
Heart_1610
Hypothalamus_1610
Kidney_1610
Testis_1610
Cortex_combined
reads per million genome matching
0
100
200
300
0
10
20
30
40
50
60
70
bp position

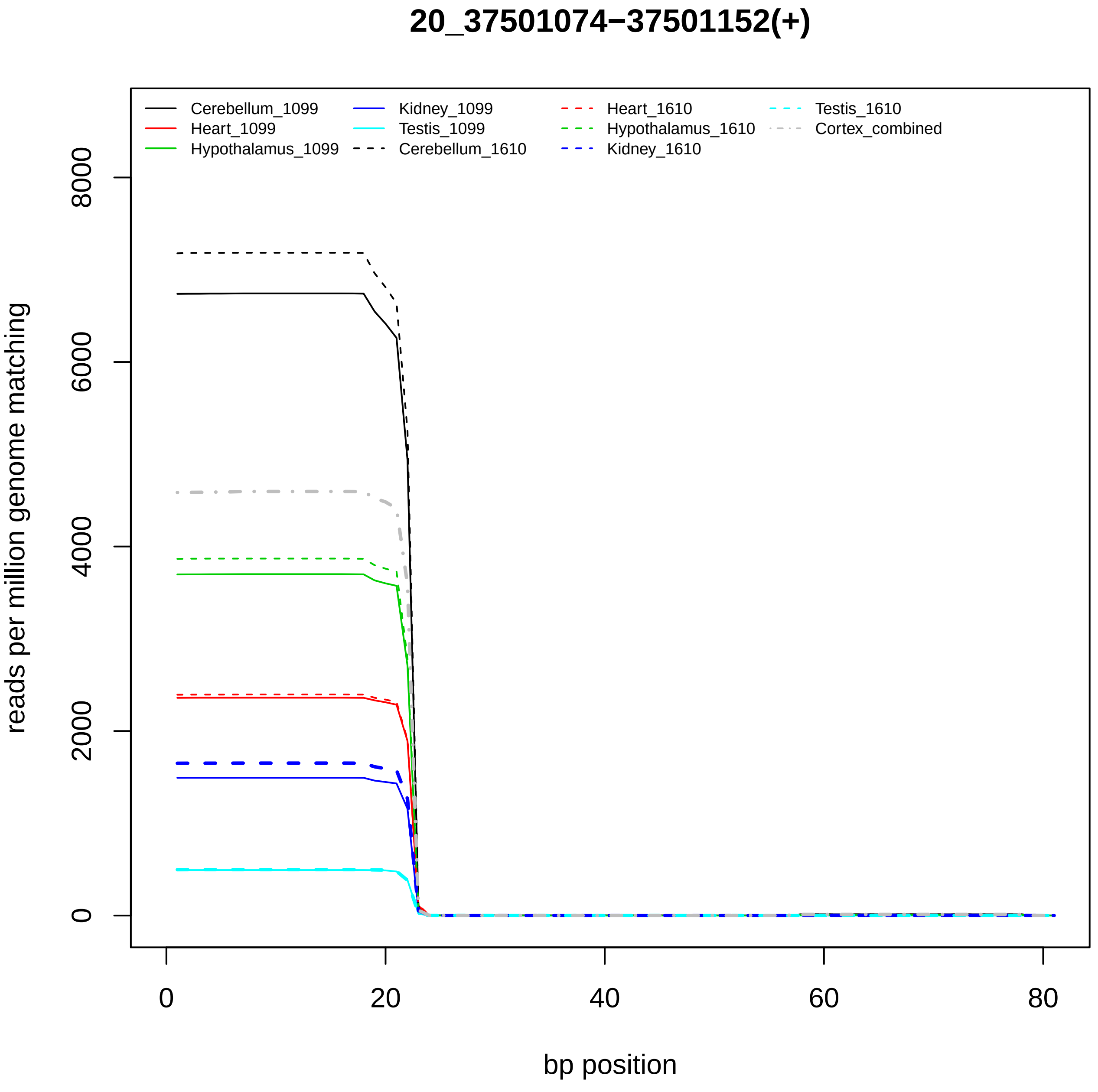

20_37501074−37501152(+)
Cerebellum_1099
Heart_1099
Hypothalamus_1099
Kidney_1099
Testis_1099
Cerebellum_1610
Heart_1610
Hypothalamus_1610
Kidney_1610
Testis_1610
Cortex_combined
reads per million genome matching
0
2000
4000
6000
8000
0
20
40
60
80
bp position

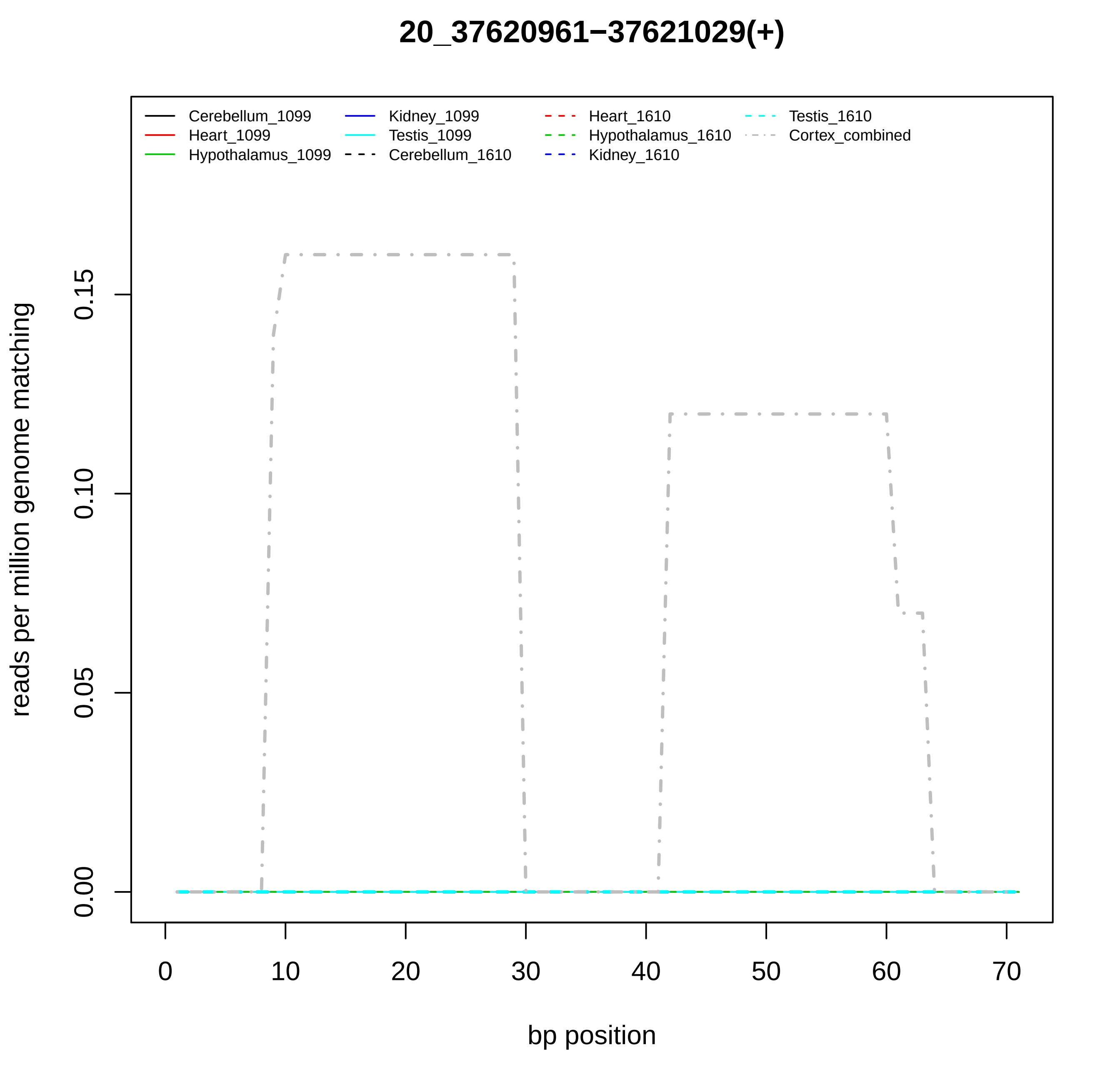

20_37620961−37621029(+)
Cerebellum_1099
Heart_1099
Hypothalamus_1099
Kidney_1099
Testis_1099
Cerebellum_1610
Heart_1610
Hypothalamus_1610
Kidney_1610
Testis_1610
Cortex_combined
reads per million genome matching
0.00
0.05
0.10
0.15
0
10
20
30
40
50
60
70
bp position

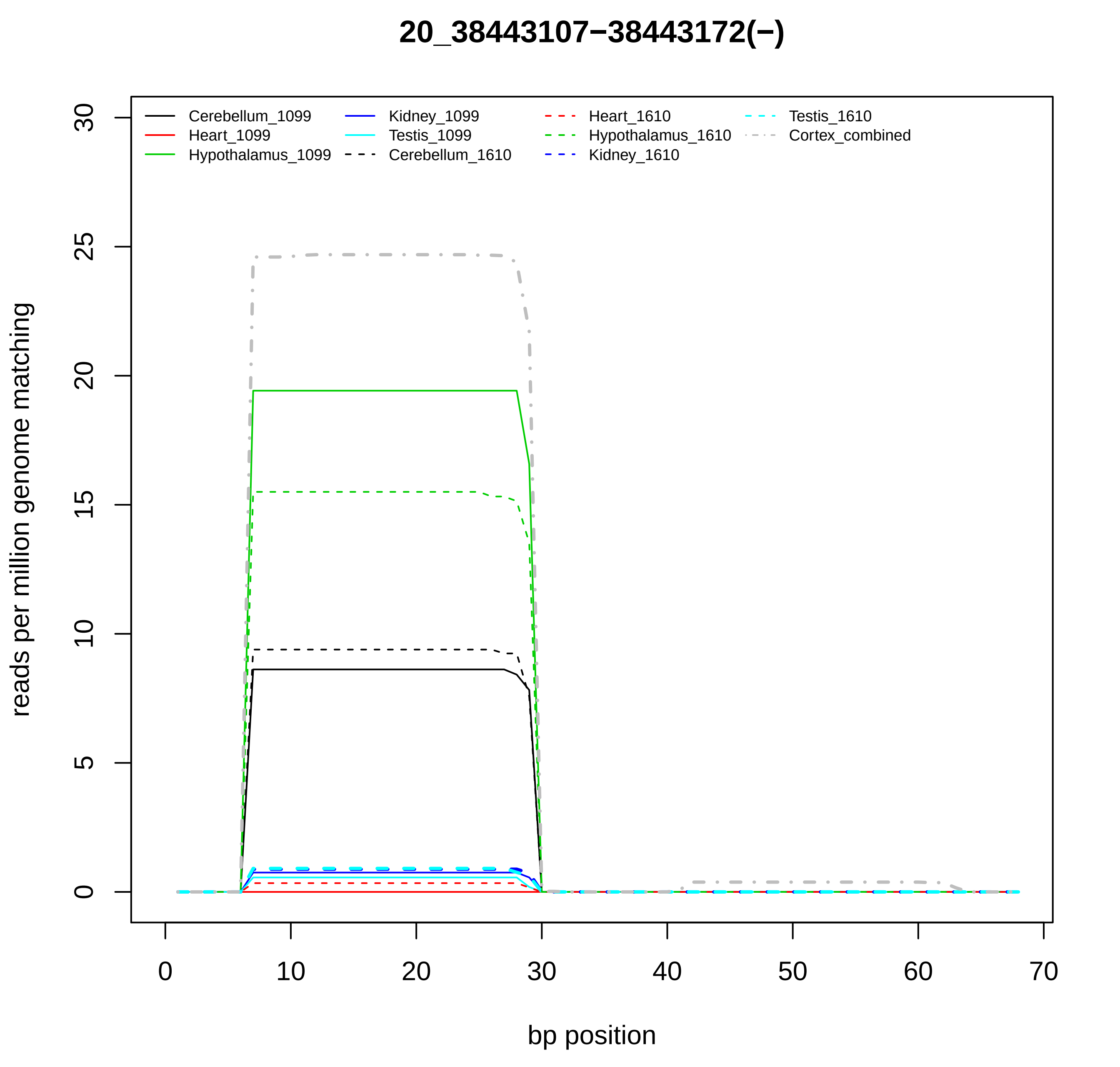
20_38443107−38443172(−)
Cerebellum_1099
Heart_1099
Hypothalamus_1099
Kidney_1099
Testis_1099
Cerebellum_1610
Heart_1610
Hypothalamus_1610
Kidney_1610
Testis_1610
Cortex_combined
reads per million genome matching
bp position
0
5
10
15
20
25
30
0
10
20
30
40
50
60
70

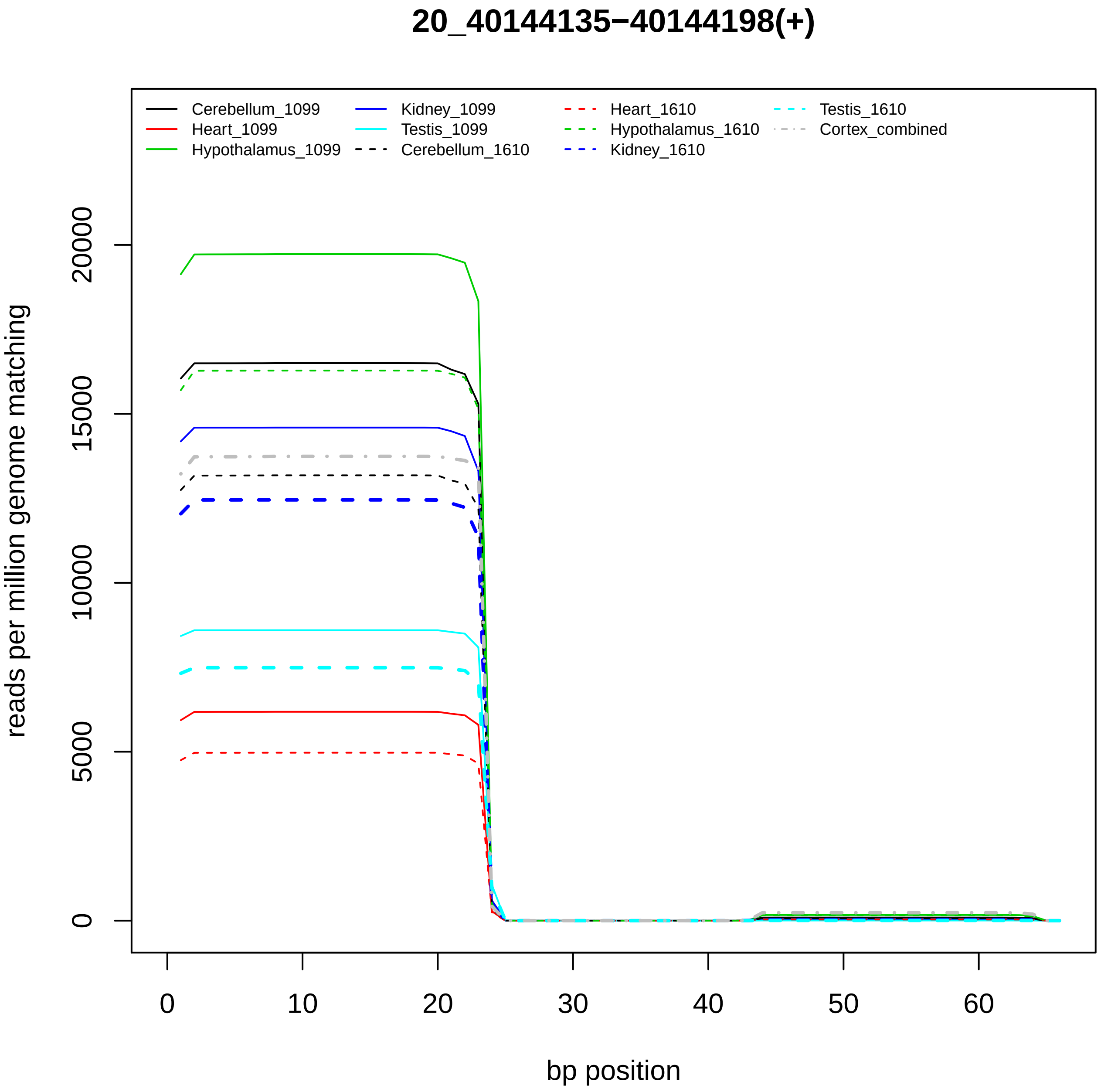

20_40144135−40144198(+)
Cerebellum_1099
Heart_1099
Hypothalamus_1099
Kidney_1099
Testis_1099
Cerebellum_1610
Heart_1610
Hypothalamus_1610
Kidney_1610
Testis_1610
Cortex_combined
reads per million genome matching
0
5000
10000
15000
20000
bp position
0
10
20
30
40
50
60

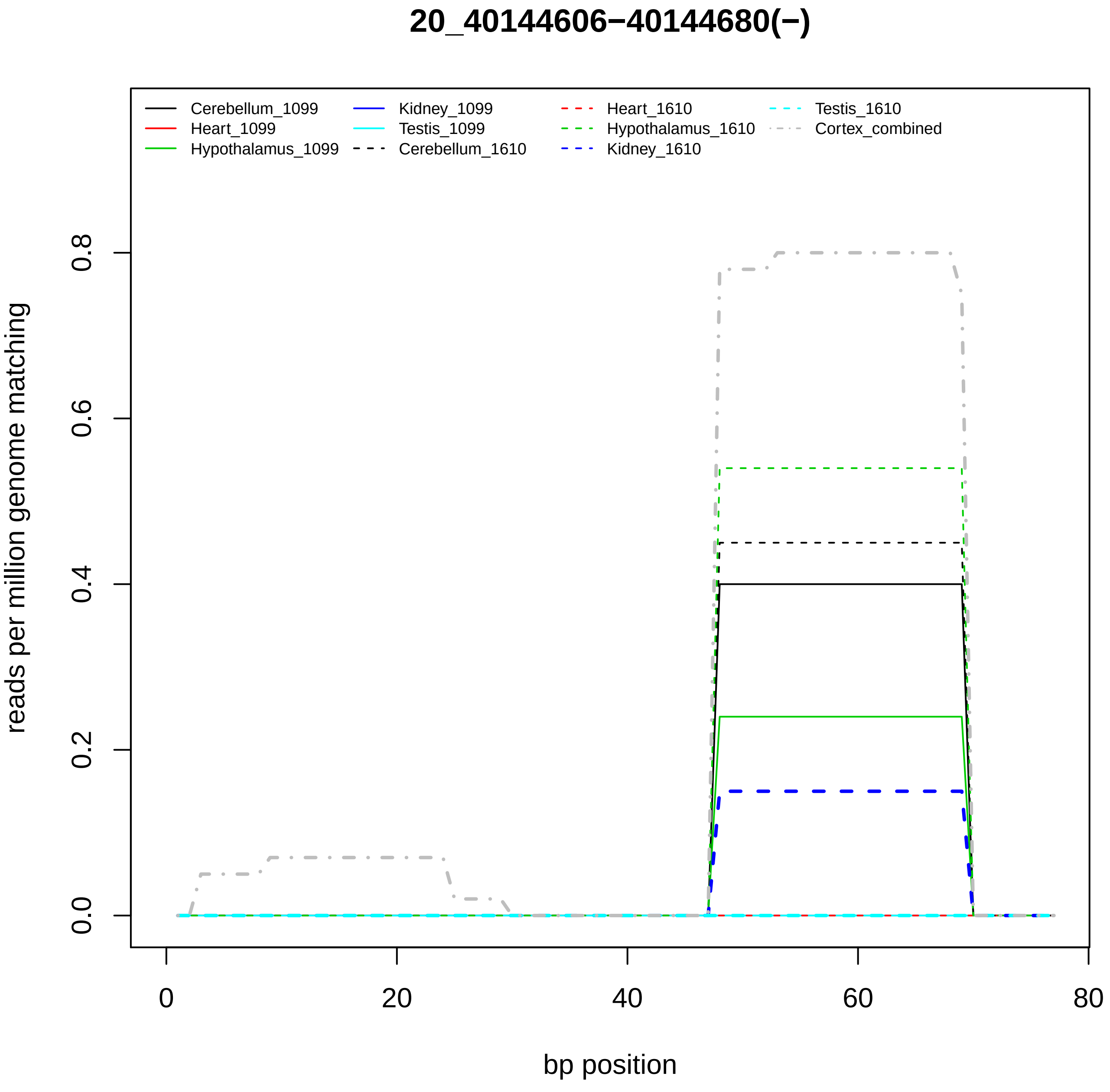
20_40144606−40144680(−)
Cerebellum_1099
Heart_1099
Hypothalamus_1099
Kidney_1099
Testis_1099
Cerebellum_1610
Heart_1610
Hypothalamus_1610
Kidney_1610
Testis_1610
Cortex_combined
reads per million genome matching
0.0
0.2
0.4
0.6
0.8
0
20
40
60
80
bp position

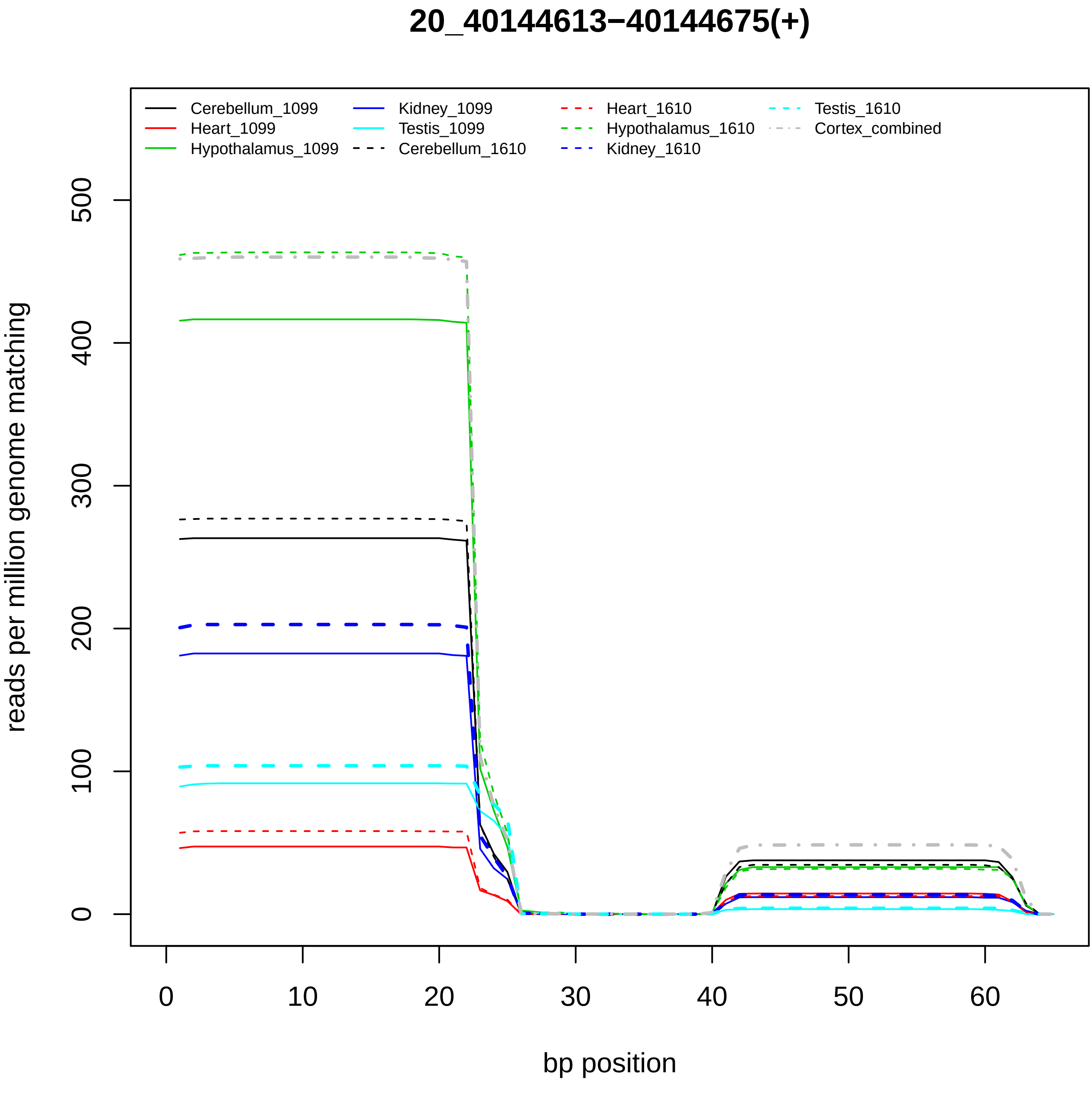

20_40144613−40144675(+)
Cerebellum_1099
Heart_1099
Hypothalamus_1099
Kidney_1099
Testis_1099
Cerebellum_1610
Heart_1610
Hypothalamus_1610
Kidney_1610
Testis_1610
Cortex_combined
reads per million genome matching
0
100
200
300
400
500
0
10
20
30
40
50
60
bp position

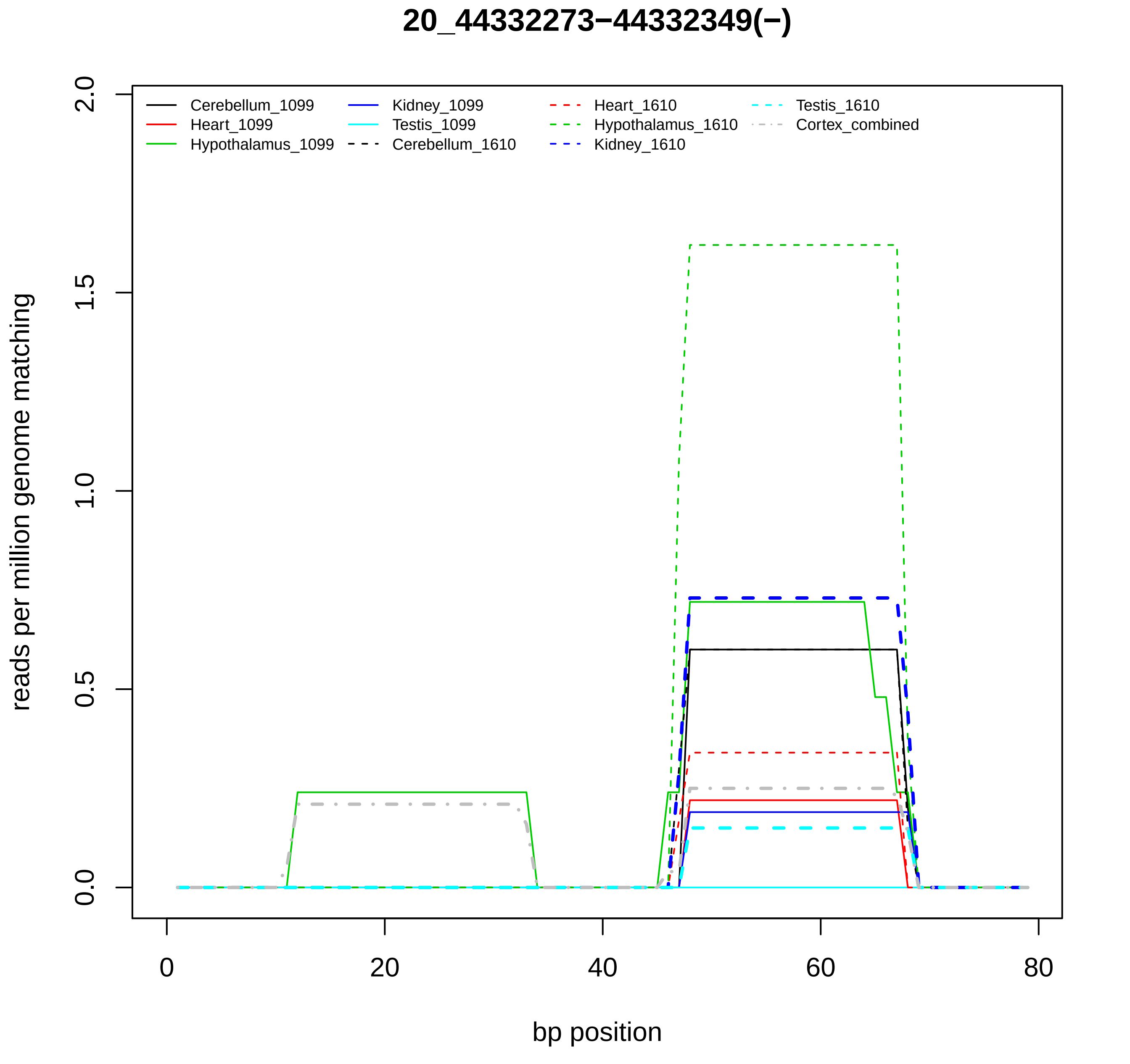
20_44332273−44332349(−)
Cerebellum_1099
Heart_1099
Hypothalamus_1099
Kidney_1099
Testis_1099
Cerebellum_1610
Heart_1610
Hypothalamus_1610
Kidney_1610
Testis_1610
Cortex_combined
reads per million genome matching
bp position
0.0
0.5
1.0
1.5
2.0
0
20
40
60
80

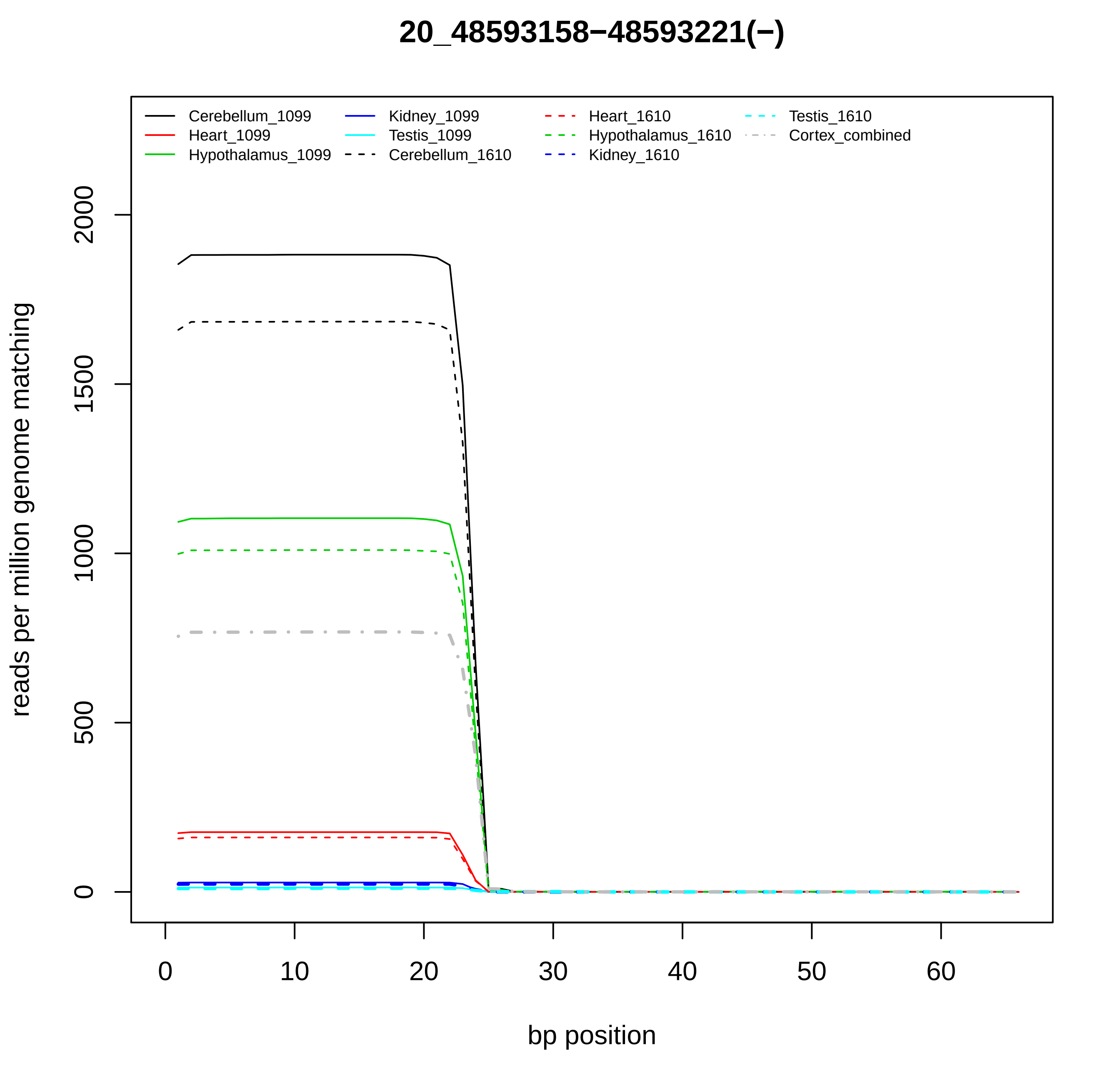
20_48593158−48593221(−)
Cerebellum_1099
Heart_1099
Hypothalamus_1099
Kidney_1099
Testis_1099
Cerebellum_1610
Heart_1610
Hypothalamus_1610
Kidney_1610
Testis_1610
Cortex_combined
reads per million genome matching
0
500
1000
1500
2000
0
10
20
30
40
50
60
bp position

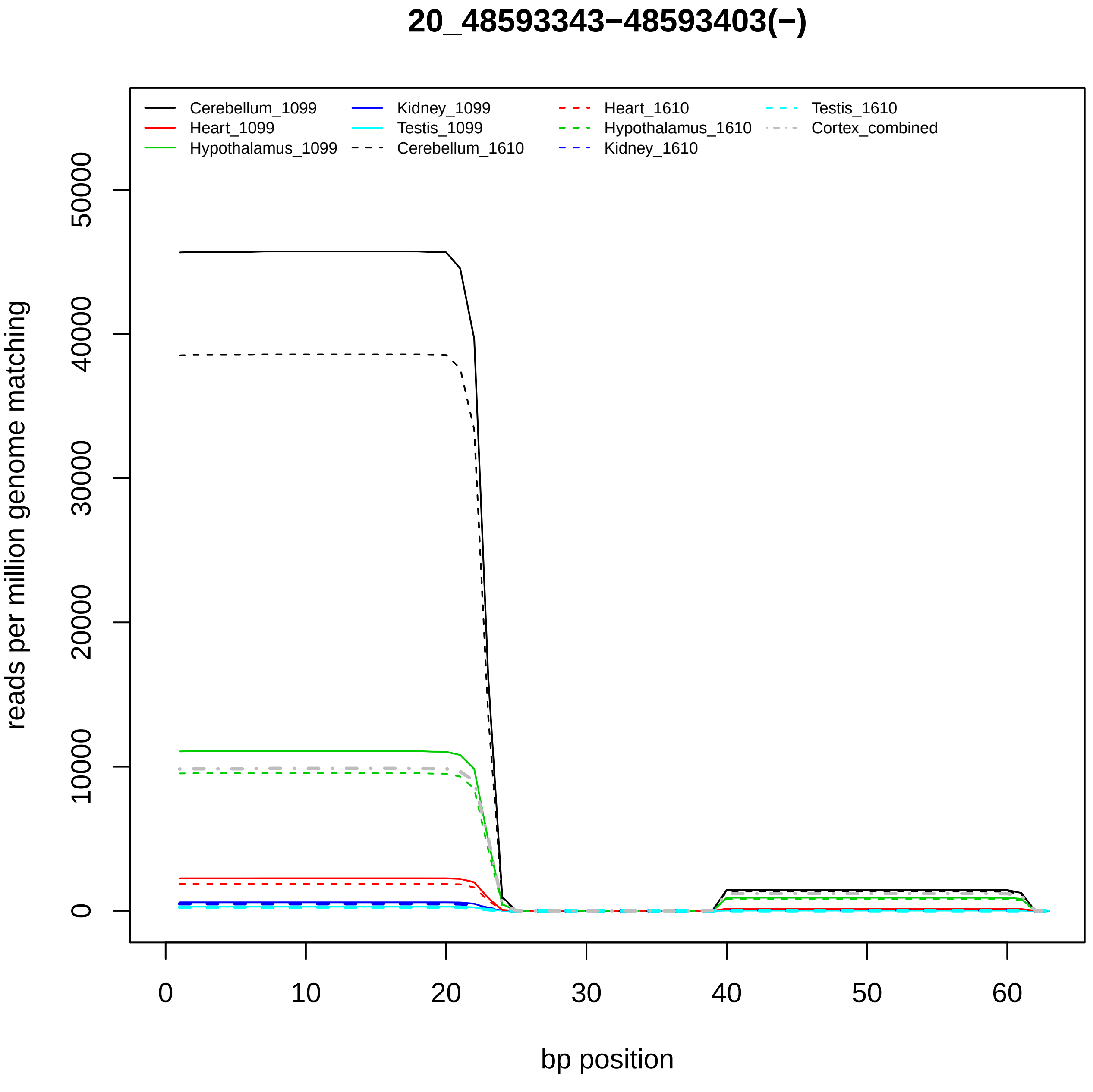

20_48593343−48593403(−)
Cerebellum_1099
Heart_1099
Hypothalamus_1099
Kidney_1099
Testis_1099
Cerebellum_1610
Heart_1610
Hypothalamus_1610
Kidney_1610
Testis_1610
Cortex_combined
reads per million genome matching
0
10000
20000
30000
40000
50000
bp position
0
10
20
30
40
50
60

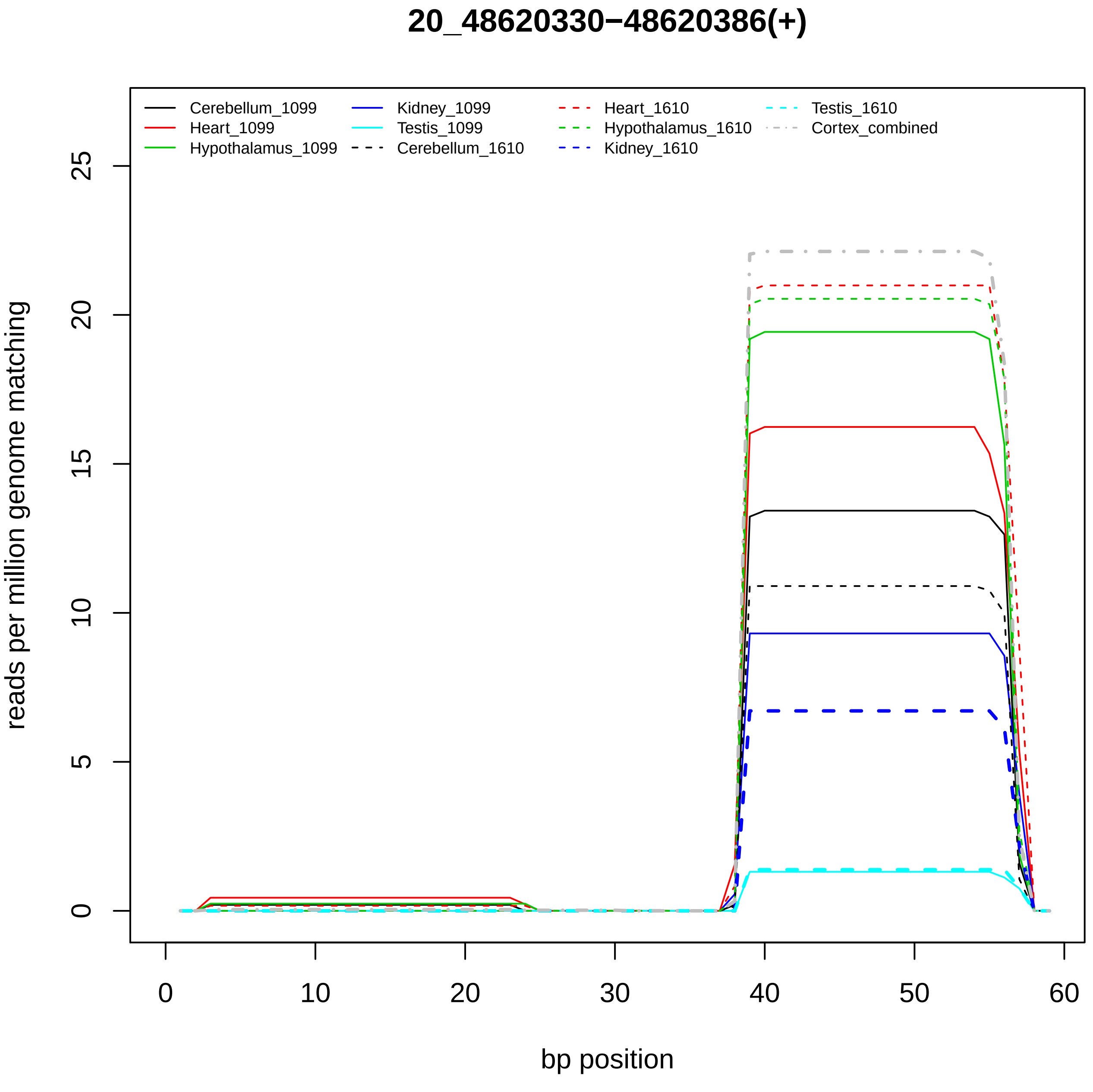
20_48620330−48620386(+)
Cerebellum_1099
Heart_1099
Hypothalamus_1099
Kidney_1099
Testis_1099
Cerebellum_1610
Heart_1610
Hypothalamus_1610
Kidney_1610
Testis_1610
Cortex_combined
reads per million genome matching
0
5
10
15
20
25
bp position
0
10
20
30
40
50
60

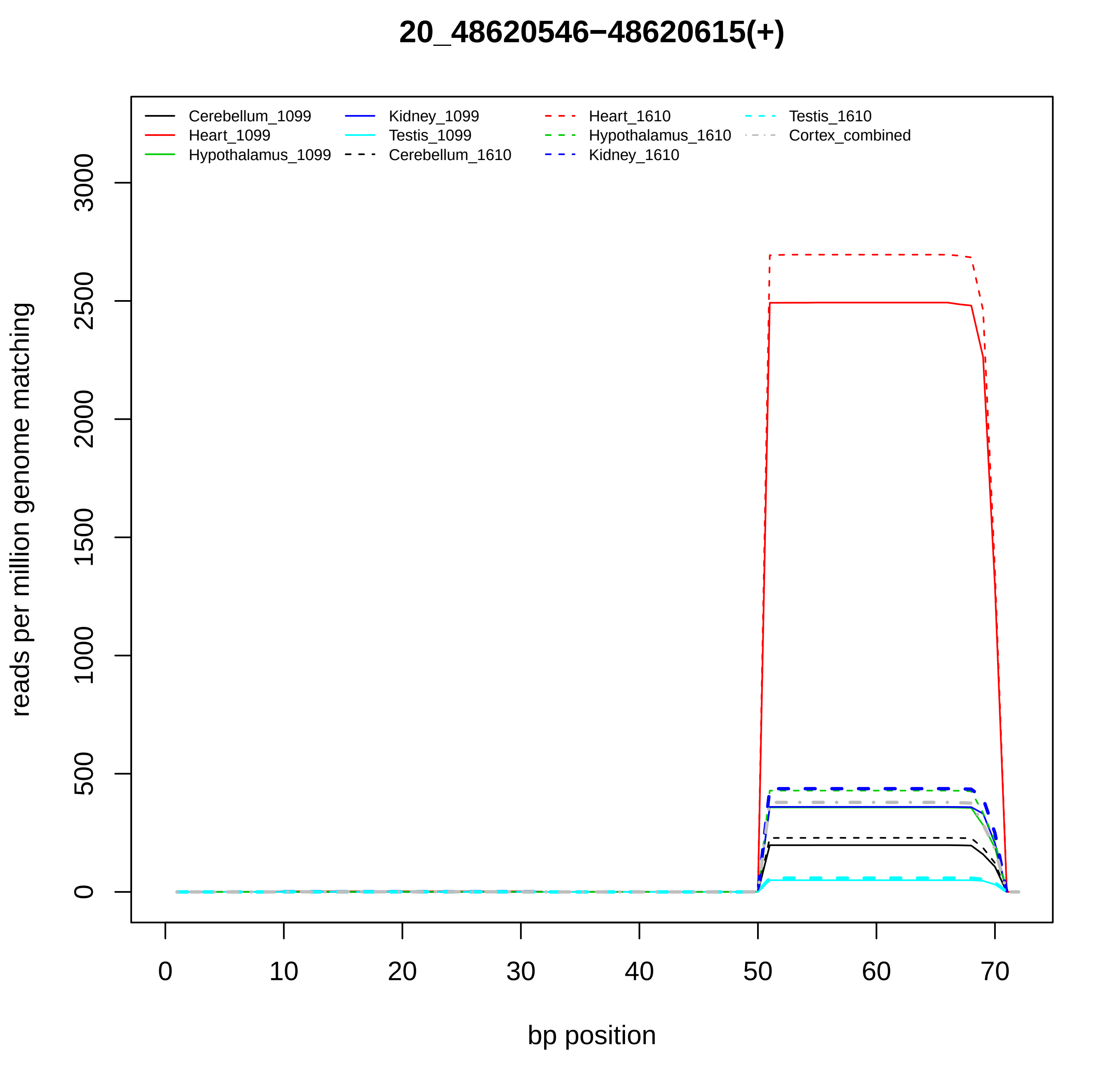

20_48620546−48620615(+)
Cerebellum_1099
Heart_1099
Hypothalamus_1099
Kidney_1099
Testis_1099
Cerebellum_1610
Heart_1610
Hypothalamus_1610
Kidney_1610
Testis_1610
Cortex_combined
reads per million genome matching
0
500
1000
1500
2000
2500
3000
0
10
20
30
40
50
60
70
bp position

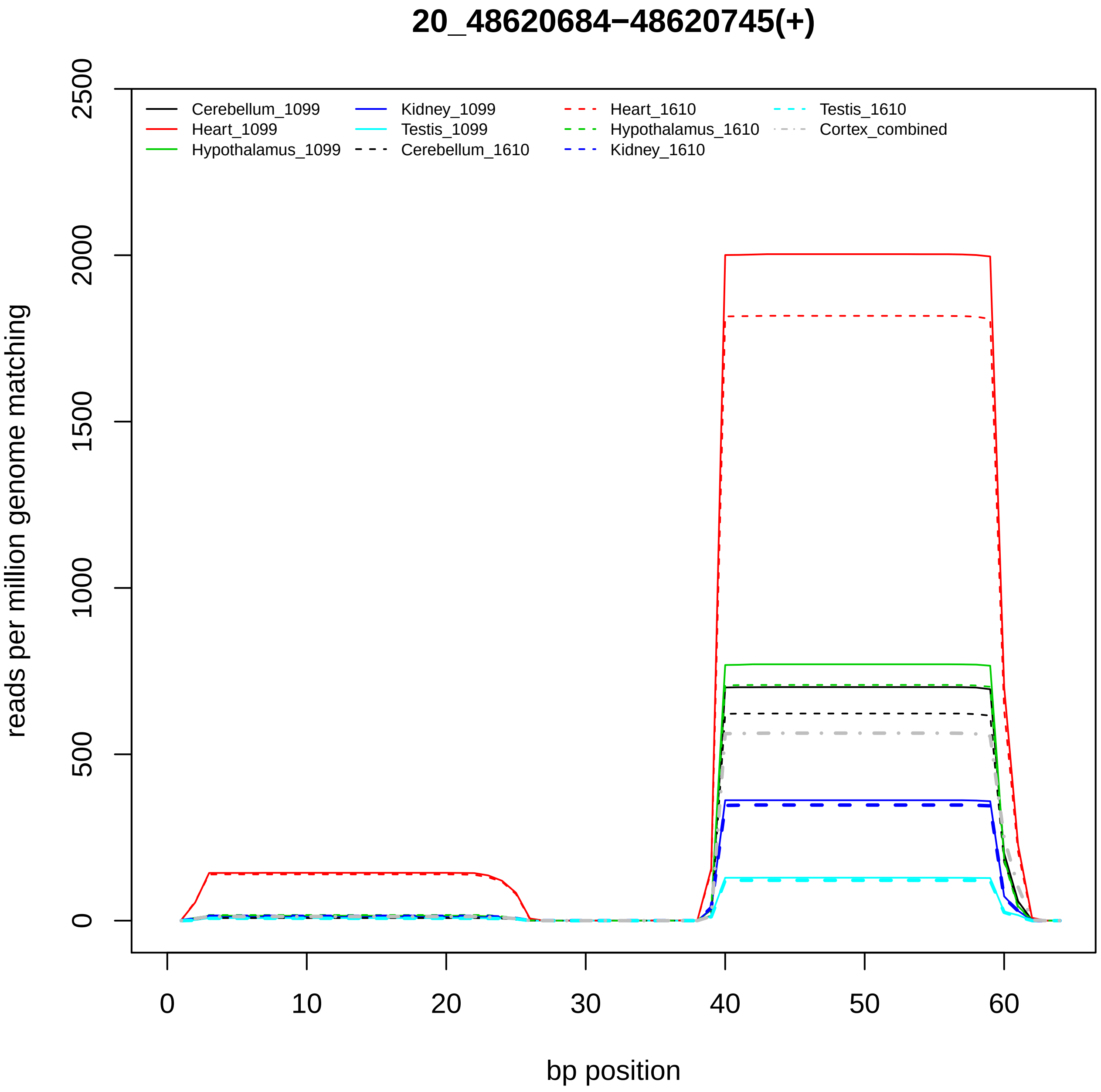
20_48620684−48620745(+)
Cerebellum_1099
Heart_1099
Hypothalamus_1099
Kidney_1099
Testis_1099
Cerebellum_1610
Heart_1610
Hypothalamus_1610
Kidney_1610
Testis_1610
Cortex_combined
reads per million genome matching
bp position
0
500
1000
1500
2000
2500
0
10
20
30
40
50
60

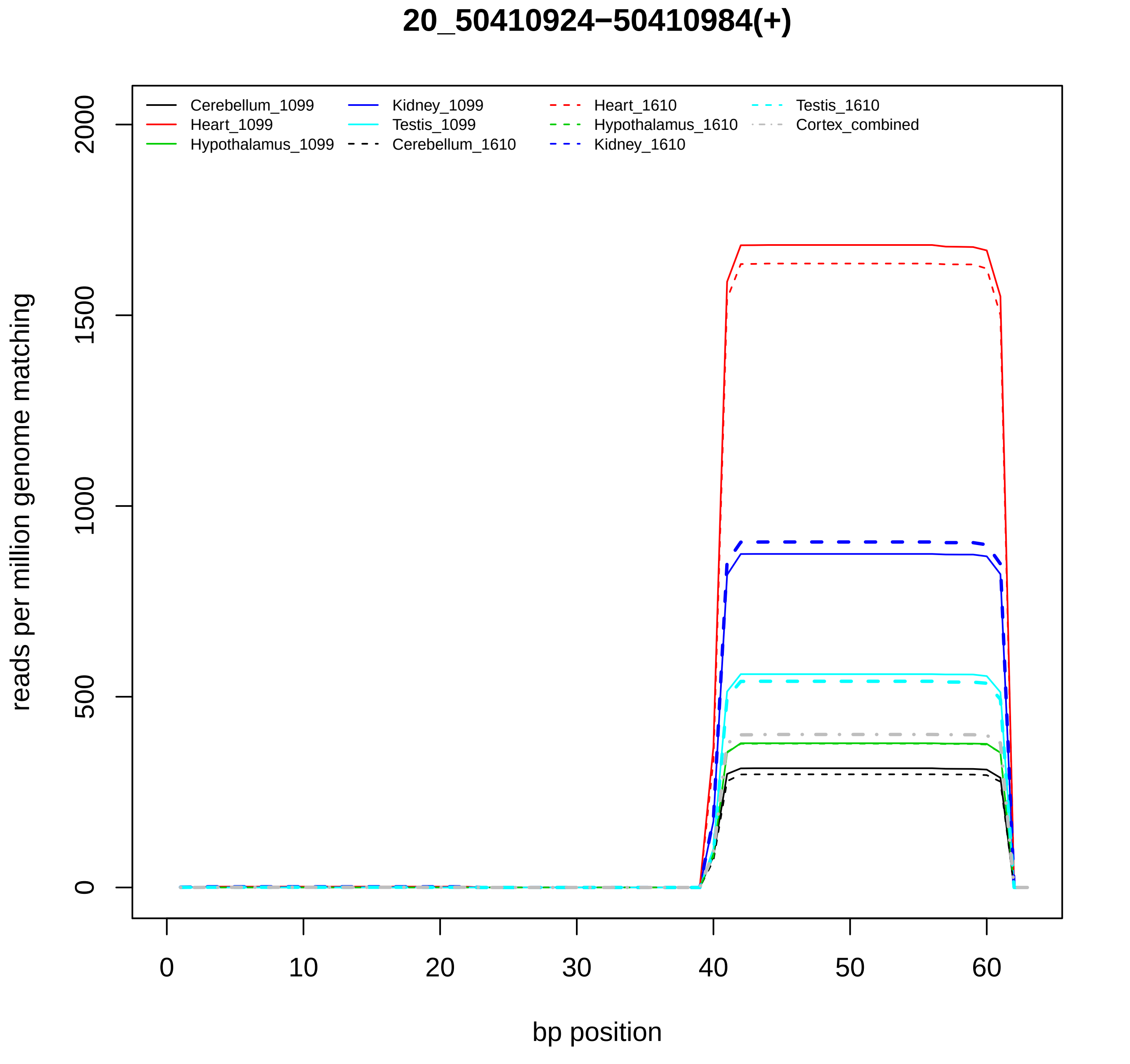

20_50410924−50410984(+)
Cerebellum_1099
Heart_1099
Hypothalamus_1099
Kidney_1099
Testis_1099
Cerebellum_1610
Heart_1610
Hypothalamus_1610
Kidney_1610
Testis_1610
Cortex_combined
reads per million genome matching
0
500
1000
1500
2000
0
10
20
30
40
50
60
bp position

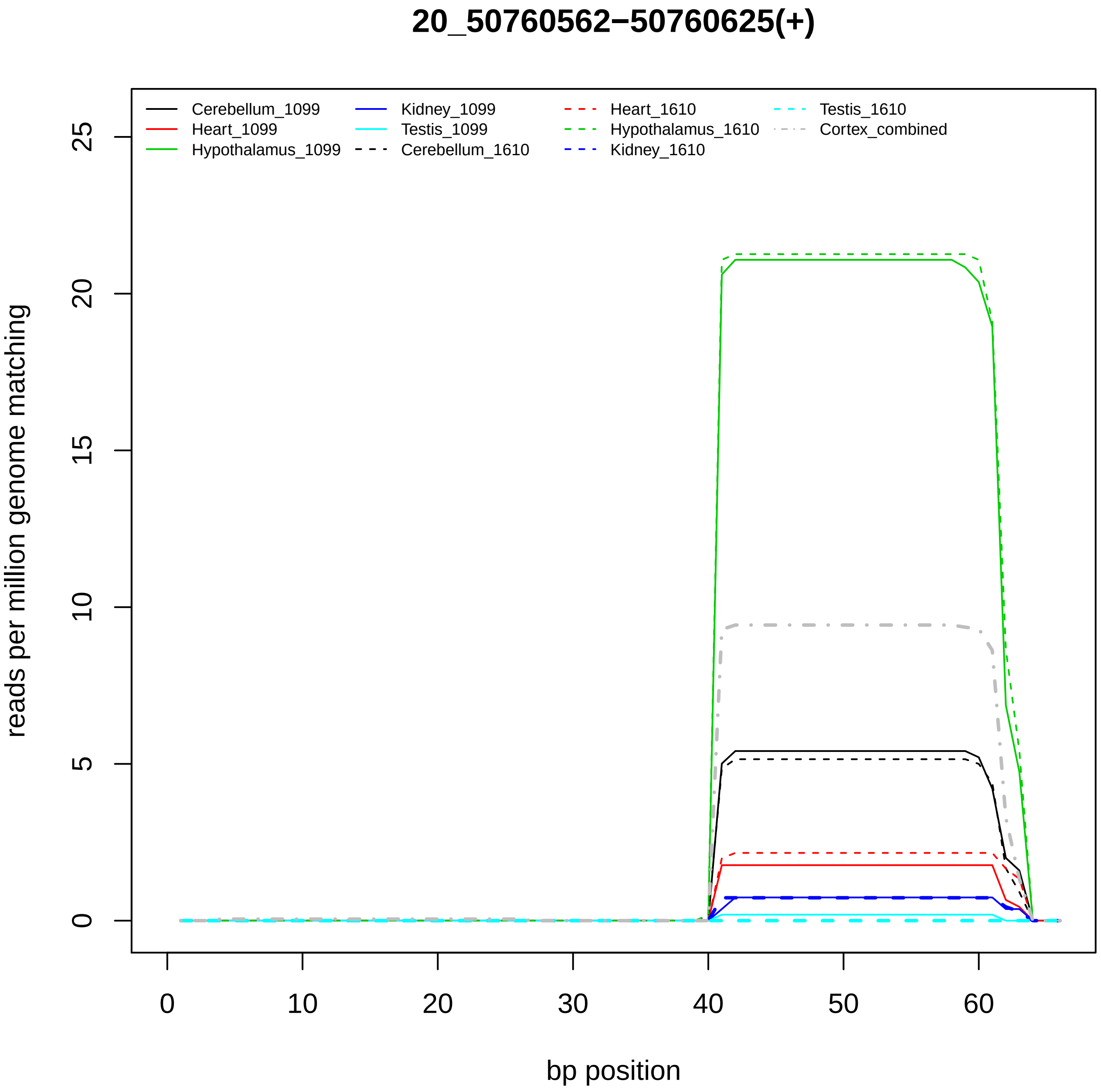

20_50760562−50760625(+)
Cerebellum_1099
Heart_1099
Hypothalamus_1099
Kidney_1099
Testis_1099
Cerebellum_1610
Heart_1610
Hypothalamus_1610
Kidney_1610
Testis_1610
Cortex_combined
reads per million genome matching
bp position
0
5
10
15
20
25
0
10
20
30
40
50
60

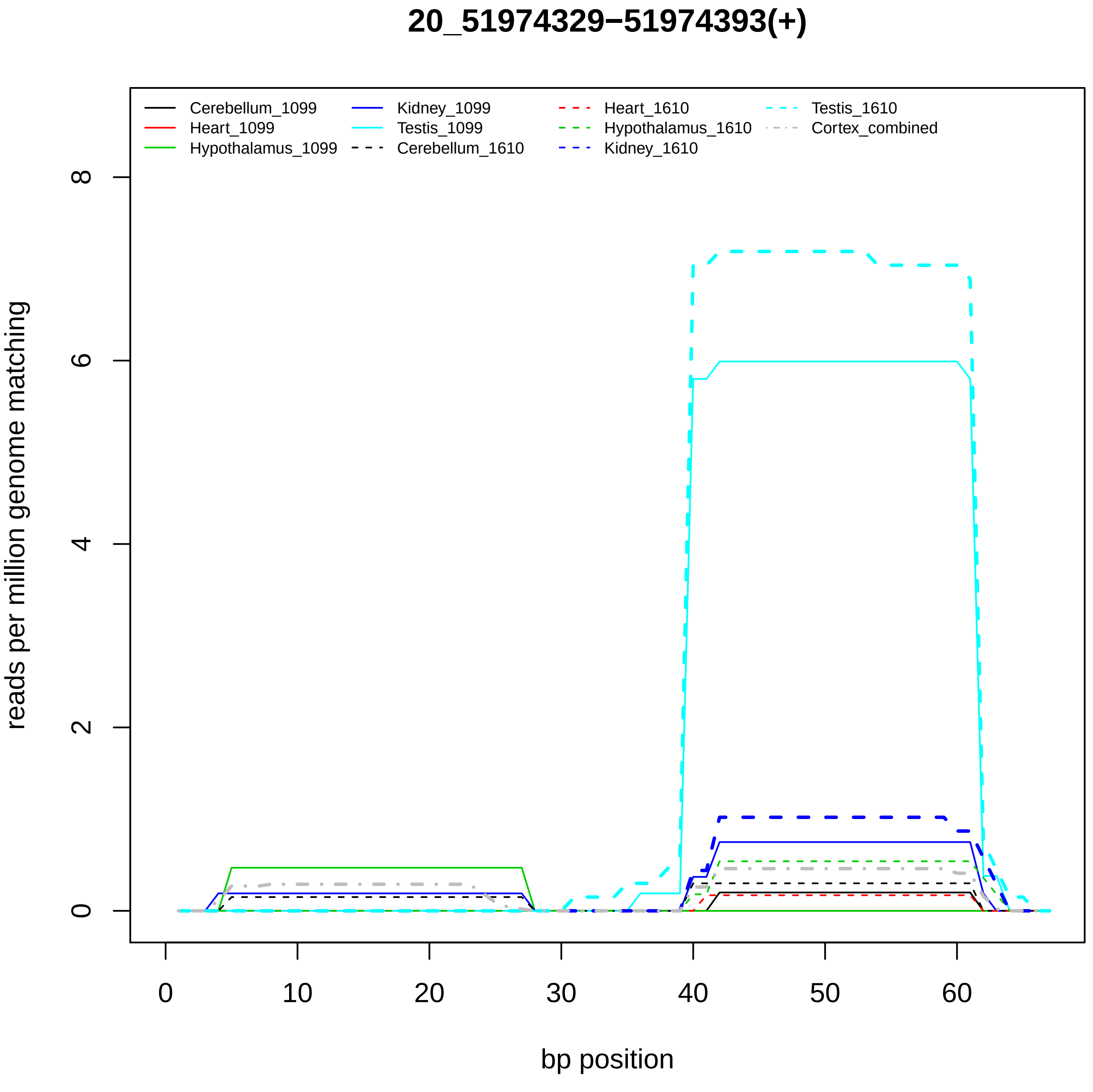

20_51974329−51974393(+)
Cerebellum_1099
Heart_1099
Hypothalamus_1099
Kidney_1099
Testis_1099
Cerebellum_1610
Heart_1610
Hypothalamus_1610
Kidney_1610
Testis_1610
Cortex_combined
reads per million genome matching
0
2
4
6
8
0
10
20
30
40
50
60
bp position

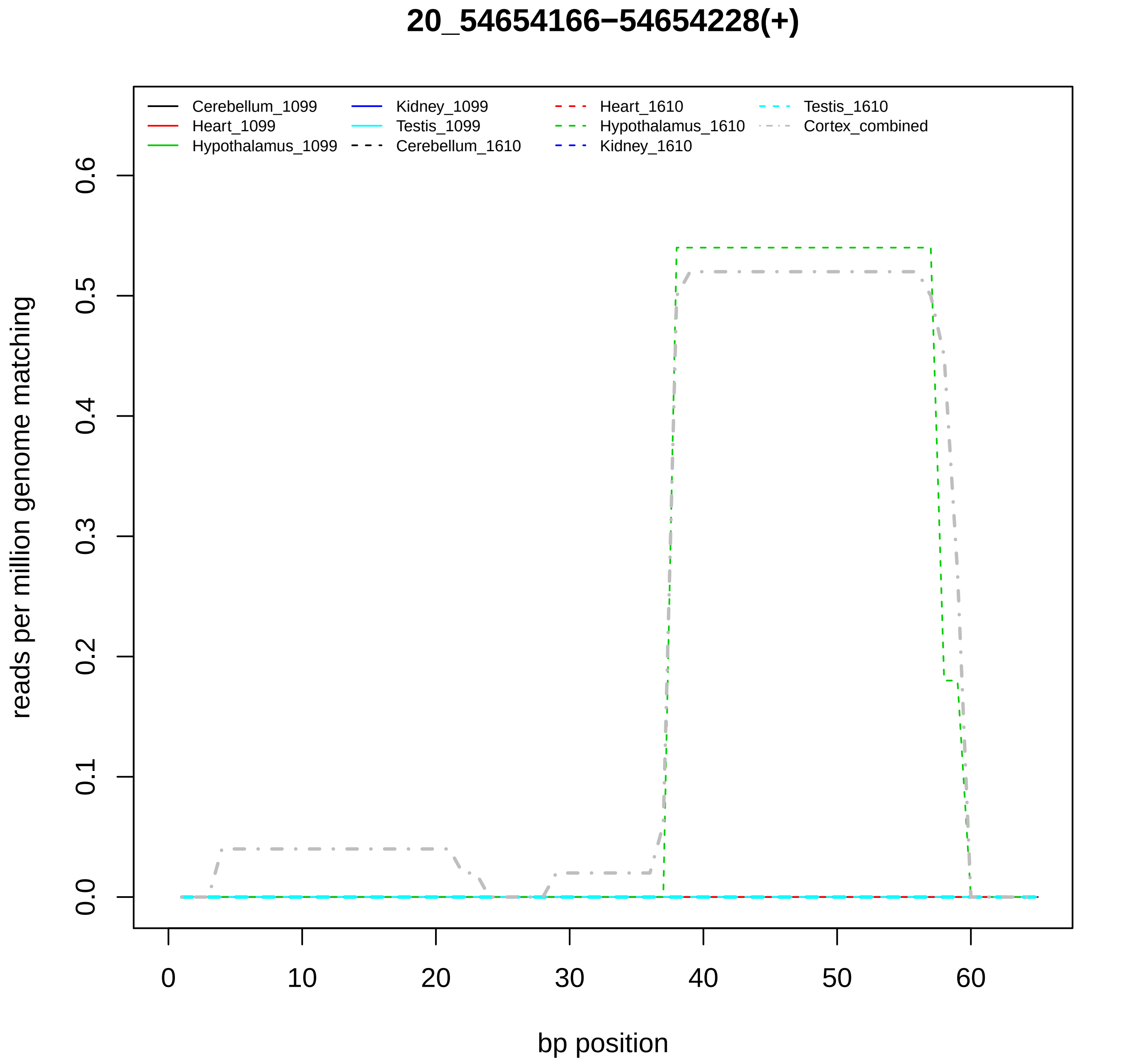
20_54654166−54654228(+)
Cerebellum_1099
Heart_1099
Hypothalamus_1099
Kidney_1099
Testis_1099
Cerebellum_1610
Heart_1610
Hypothalamus_1610
Kidney_1610
Testis_1610
Cortex_combined
reads per million genome matching
0.0
0.1
0.2
0.3
0.4
0.5
0.6
0
10
20
30
40
50
60
bp position

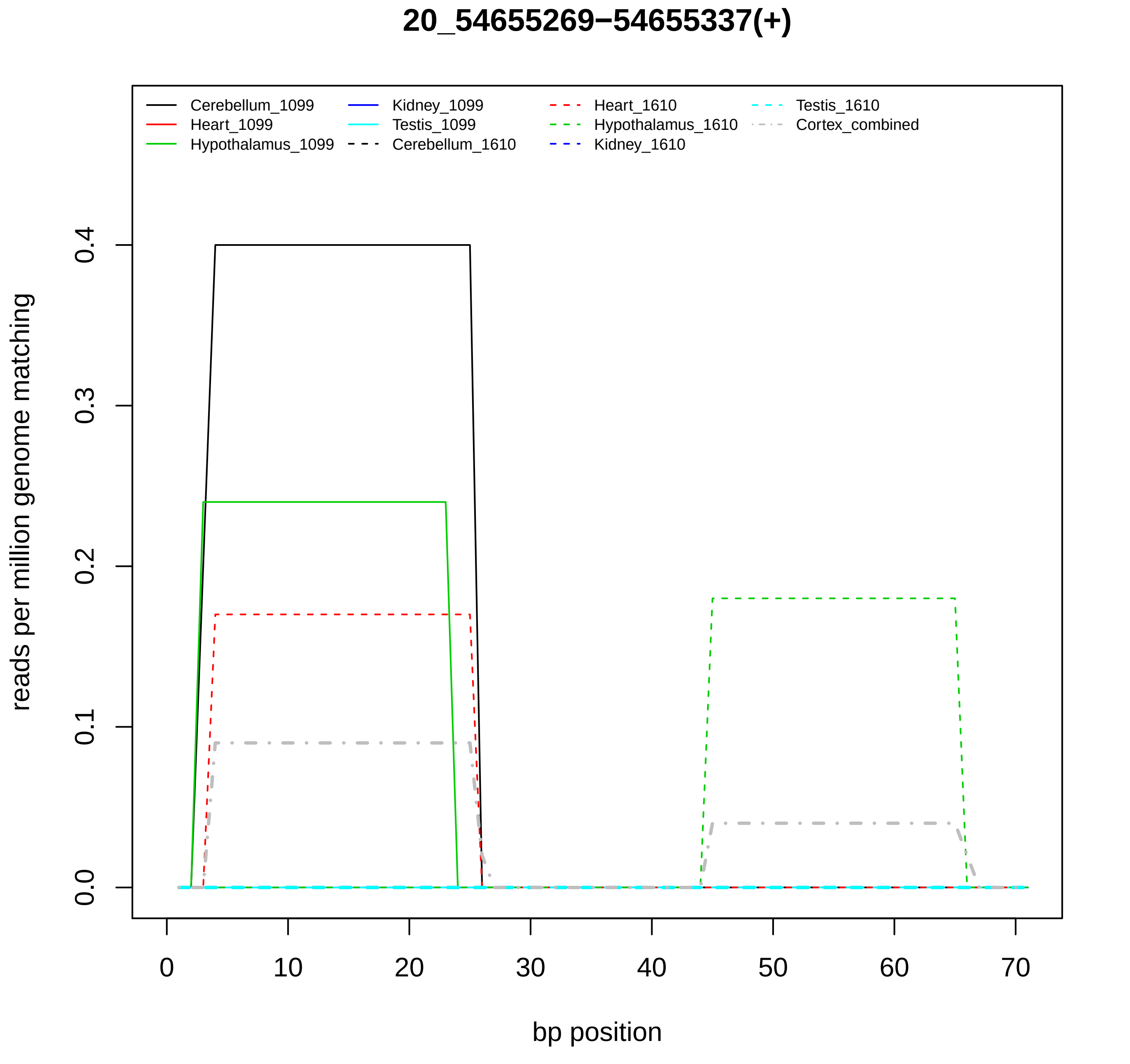

20_54655269−54655337(+)
Cerebellum_1099
Heart_1099
Hypothalamus_1099
Kidney_1099
Testis_1099
Cerebellum_1610
Heart_1610
Hypothalamus_1610
Kidney_1610
Testis_1610
Cortex_combined
reads per million genome matching
0.0
0.1
0.2
0.3
0.4
0
10
20
30
40
50
60
70
bp position

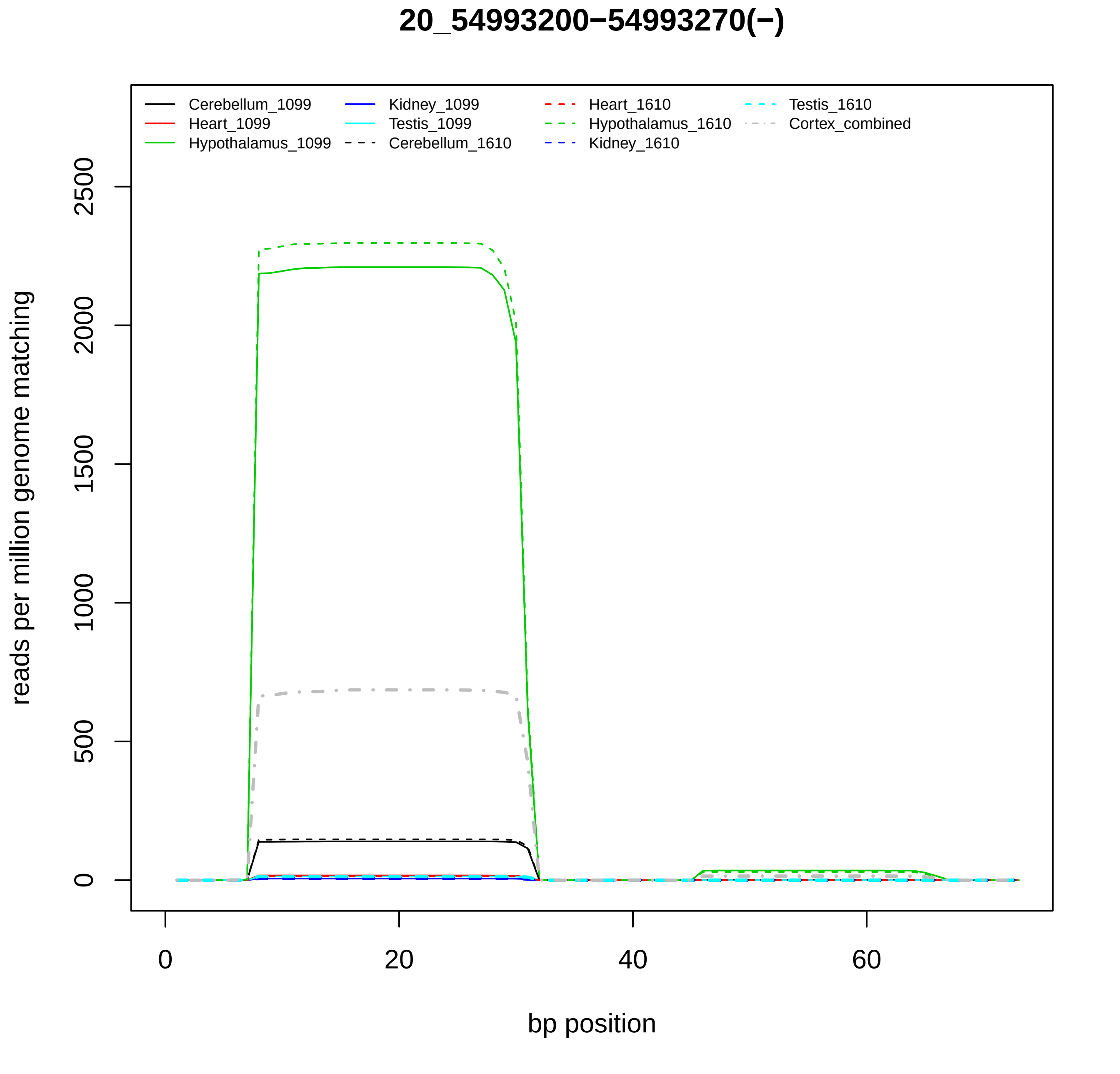

20_54993200−54993270(−)
Cerebellum_1099
Heart_1099
Hypothalamus_1099
Kidney_1099
Testis_1099
Cerebellum_1610
Heart_1610
Hypothalamus_1610
Kidney_1610
Testis_1610
Cortex_combined
reads per million genome matching
0
500
1000
1500
2000
2500
0
20
40
60
bp position

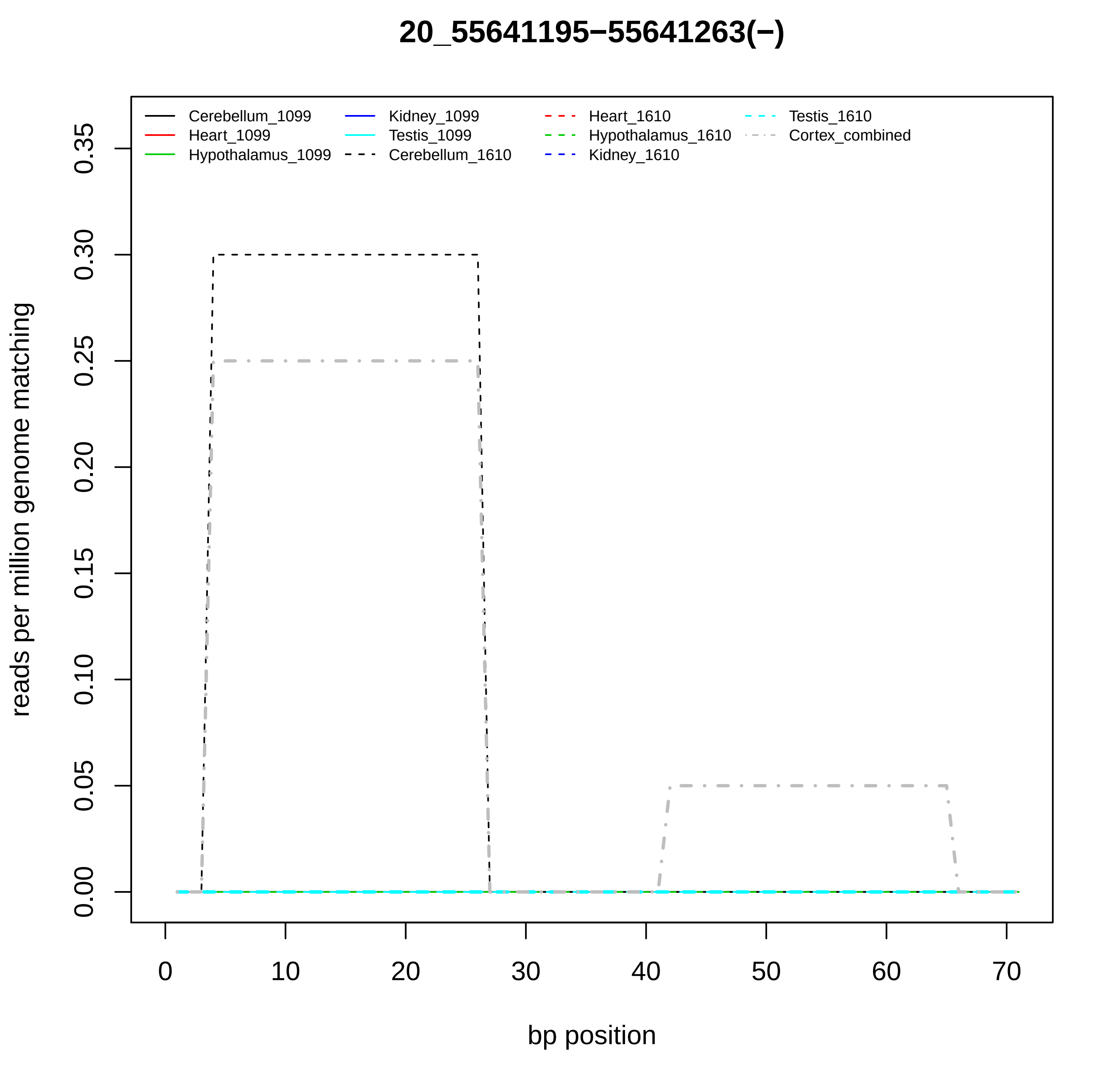

20_55641195−55641263(−)
Cerebellum_1099
Heart_1099
Hypothalamus_1099
Kidney_1099
Testis_1099
Cerebellum_1610
Heart_1610
Hypothalamus_1610
Kidney_1610
Testis_1610
Cortex_combined
reads per million genome matching
0.00
0.05
0.10
0.15
0.20
0.25
0.30
0.35
bp position
0
10
20
30
40
50
60
70

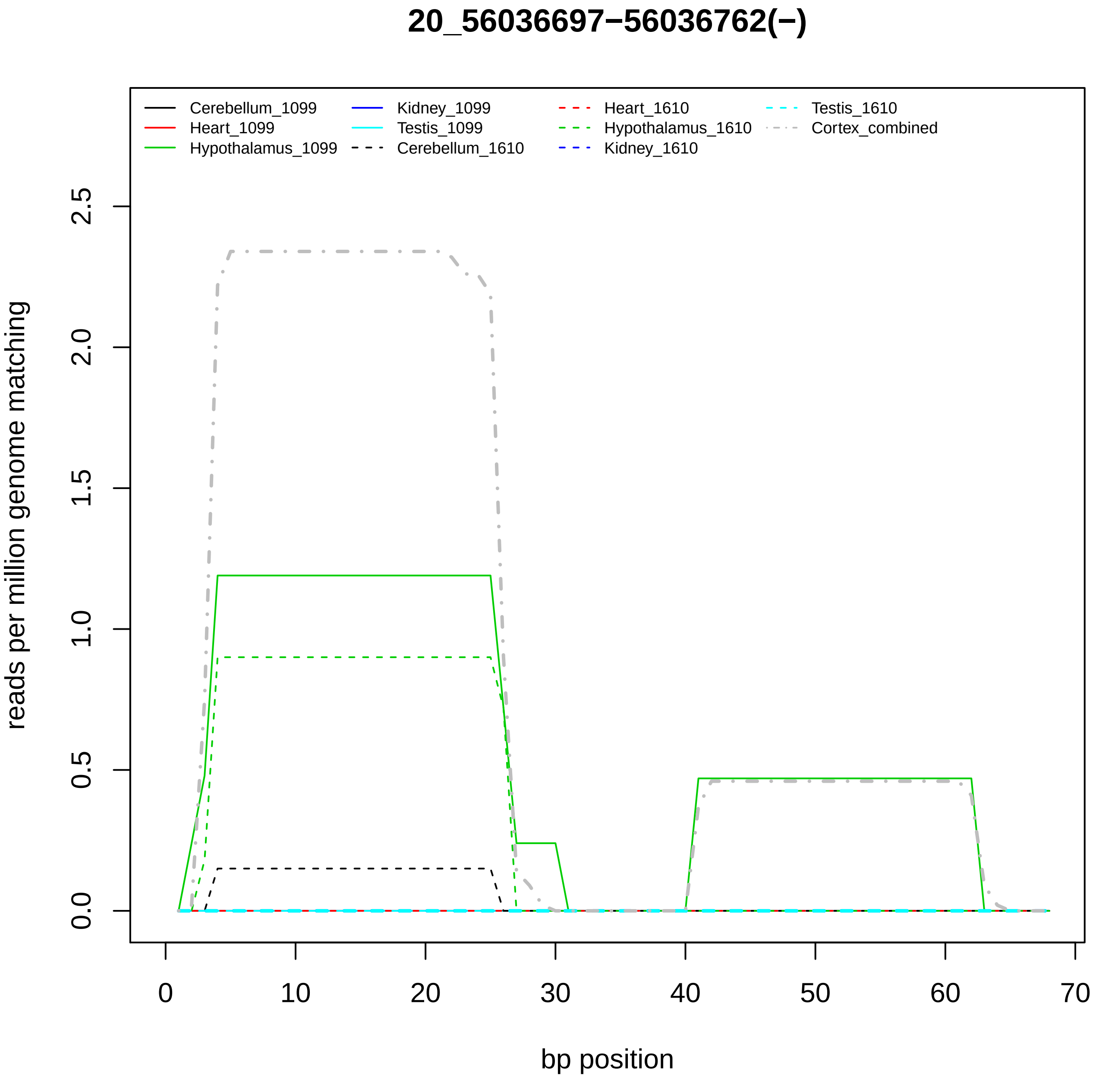

20_56036697−56036762(−)
Cerebellum_1099
Heart_1099
Hypothalamus_1099
Kidney_1099
Testis_1099
Cerebellum_1610
Heart_1610
Hypothalamus_1610
Kidney_1610
Testis_1610
Cortex_combined
reads per million genome matching
0.0
0.5
1.0
1.5
2.0
2.5
0
10
20
30
40
50
60
70
bp position

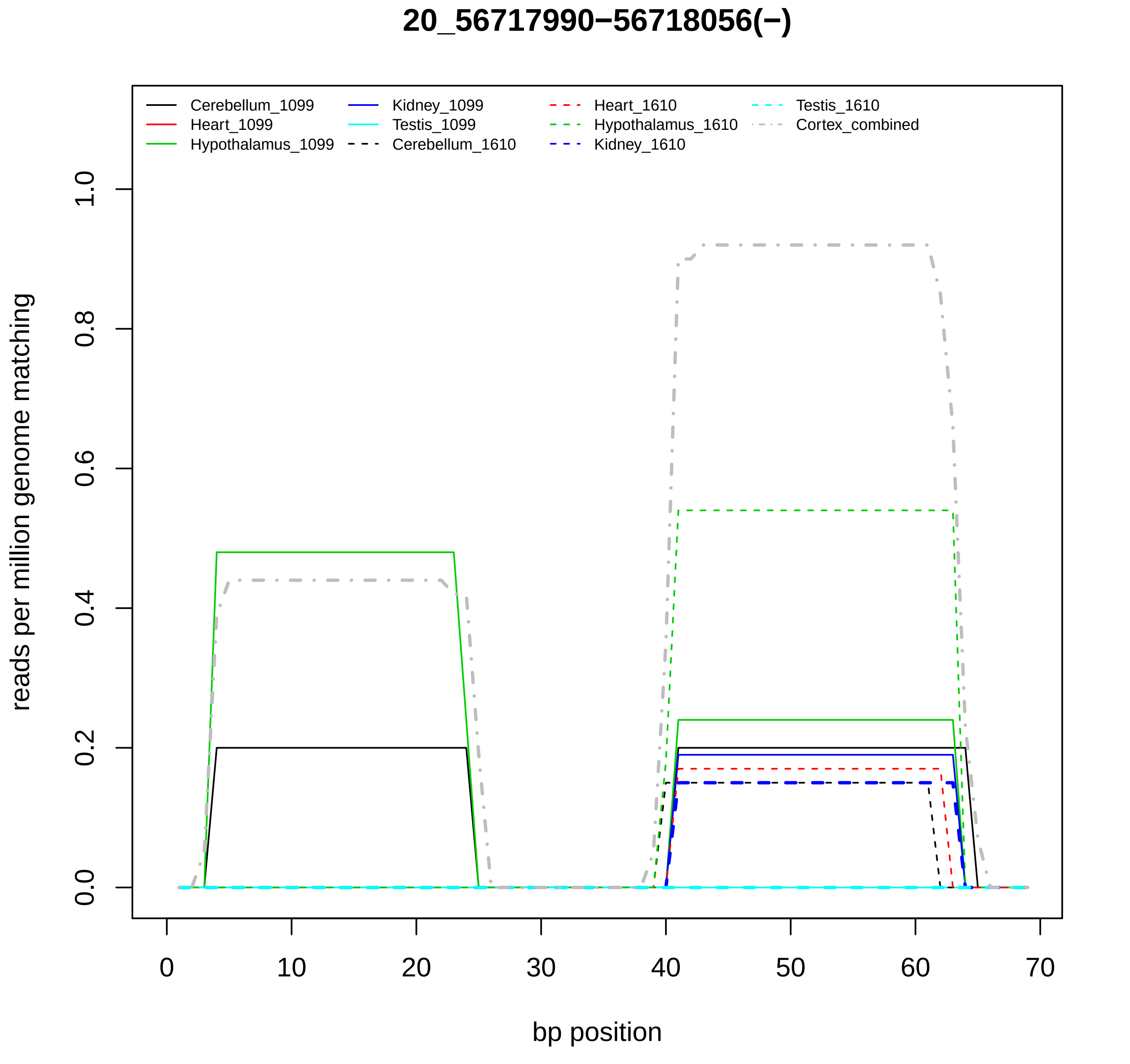

20_56717990−56718056(−)
Cerebellum_1099
Heart_1099
Hypothalamus_1099
Kidney_1099
Testis_1099
Cerebellum_1610
Heart_1610
Hypothalamus_1610
Kidney_1610
Testis_1610
Cortex_combined
reads per million genome matching
0.0
0.2
0.4
0.6
0.8
1.0
0
10
20
30
40
50
60
70
bp position

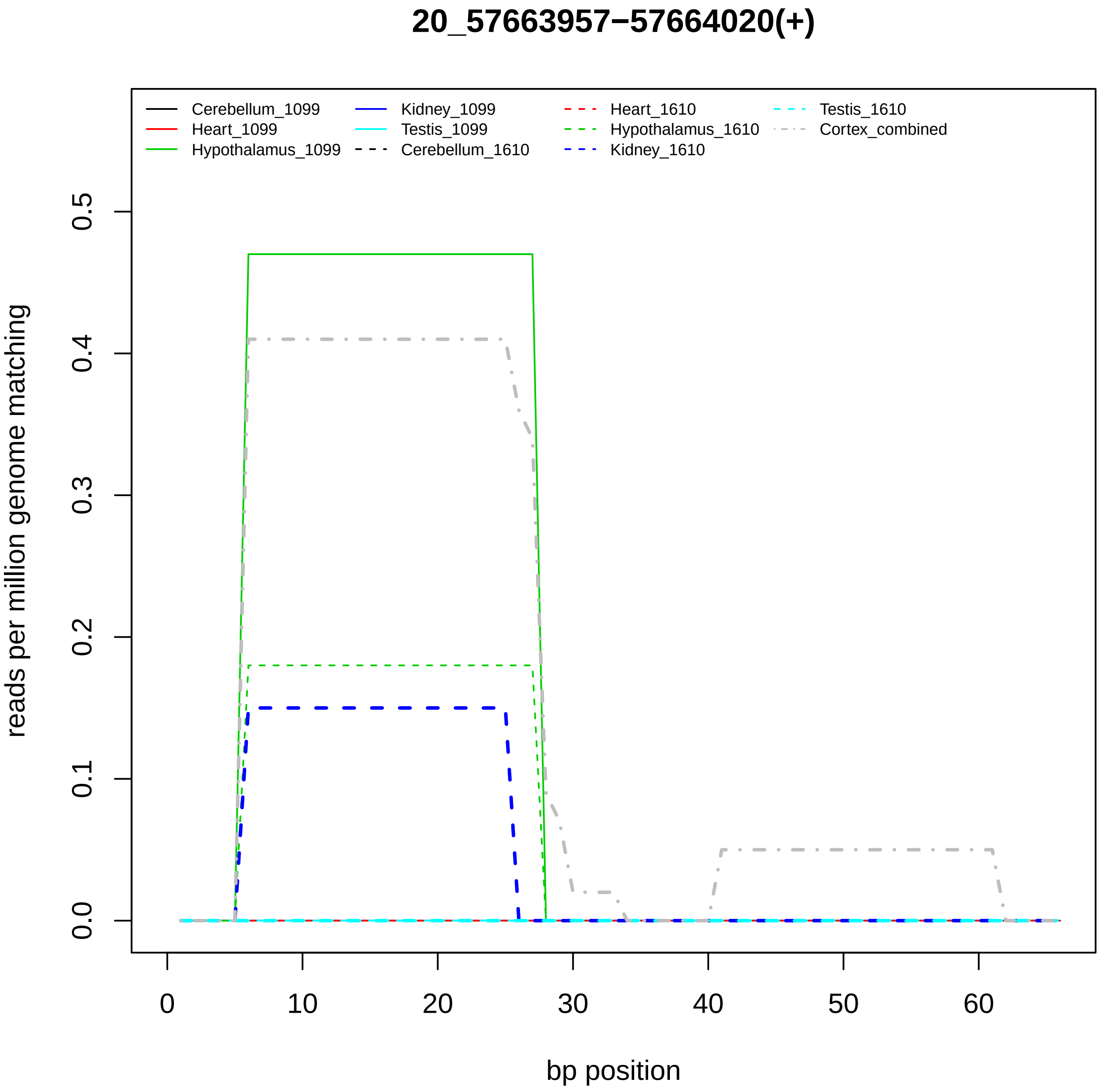

20_57663957−57664020(+)
Cerebellum_1099
Heart_1099
Hypothalamus_1099
Kidney_1099
Testis_1099
Cerebellum_1610
Heart_1610
Hypothalamus_1610
Kidney_1610
Testis_1610
Cortex_combined
reads per million genome matching
bp position
0.0
0.1
0.2
0.3
0.4
0.5
0
10
20
30
40
50
60

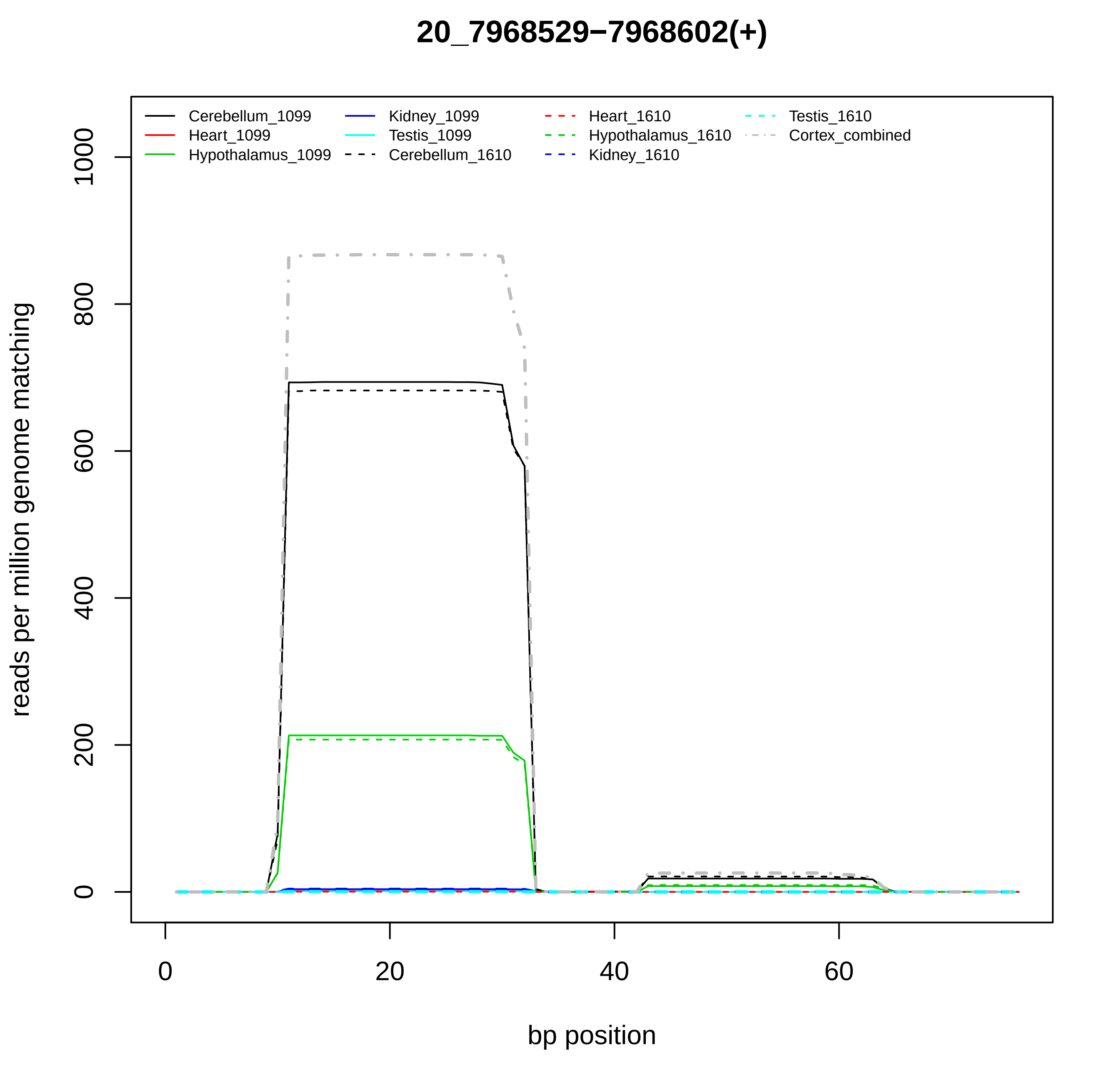

20_7968529−7968602(+)
Cerebellum_1099
Heart_1099
Hypothalamus_1099
Kidney_1099
Testis_1099
Cerebellum_1610
Heart_1610
Hypothalamus_1610
Kidney_1610
Testis_1610
Cortex_combined
reads per million genome matching
0
200
400
600
800
1000
0
20
40
60
bp position

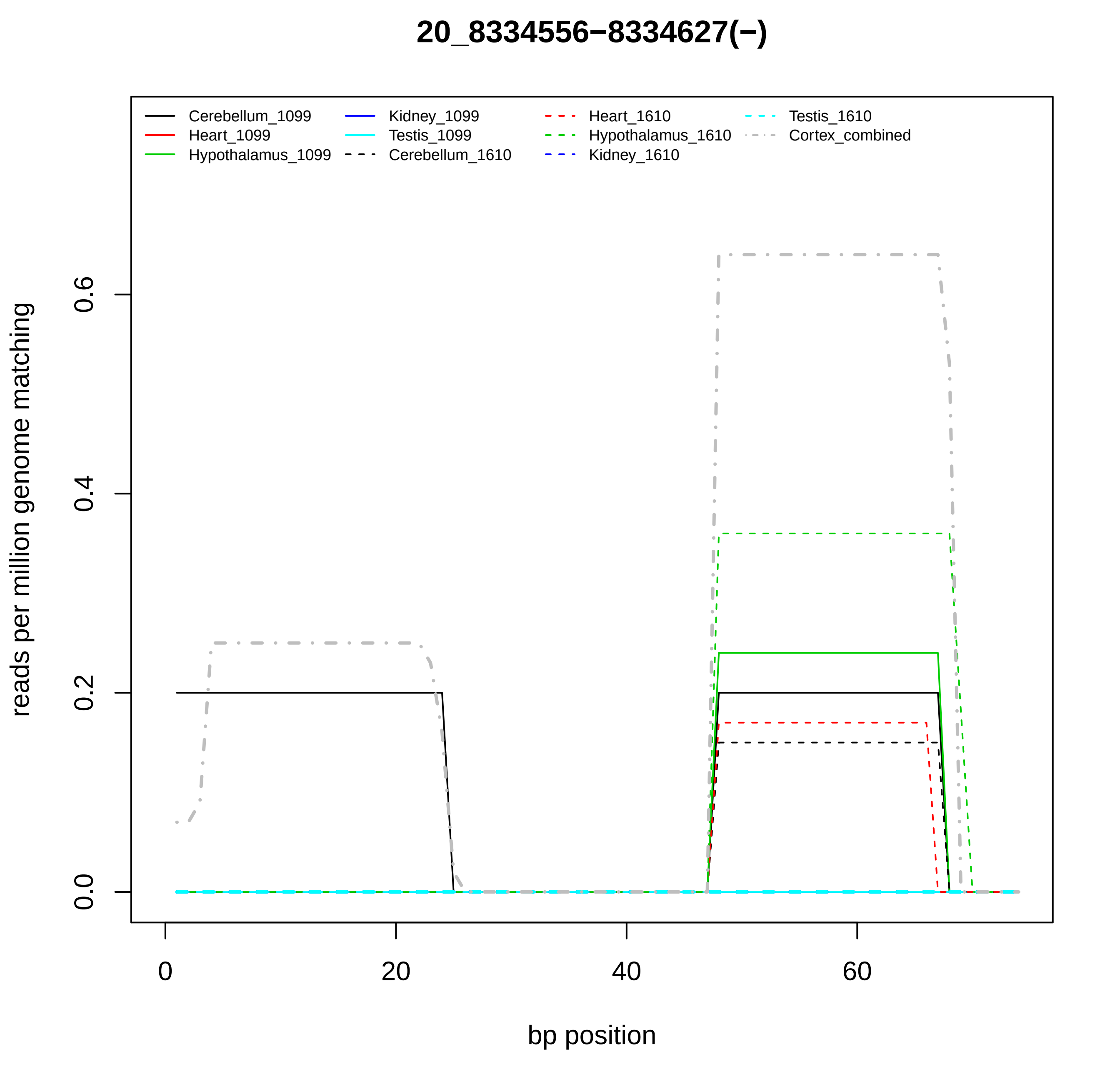

20_8334556−8334627(−)
Cerebellum_1099
Heart_1099
Hypothalamus_1099
Kidney_1099
Testis_1099
Cerebellum_1610
Heart_1610
Hypothalamus_1610
Kidney_1610
Testis_1610
Cortex_combined
reads per million genome matching
0.0
0.2
0.4
0.6
0
20
40
60
bp position

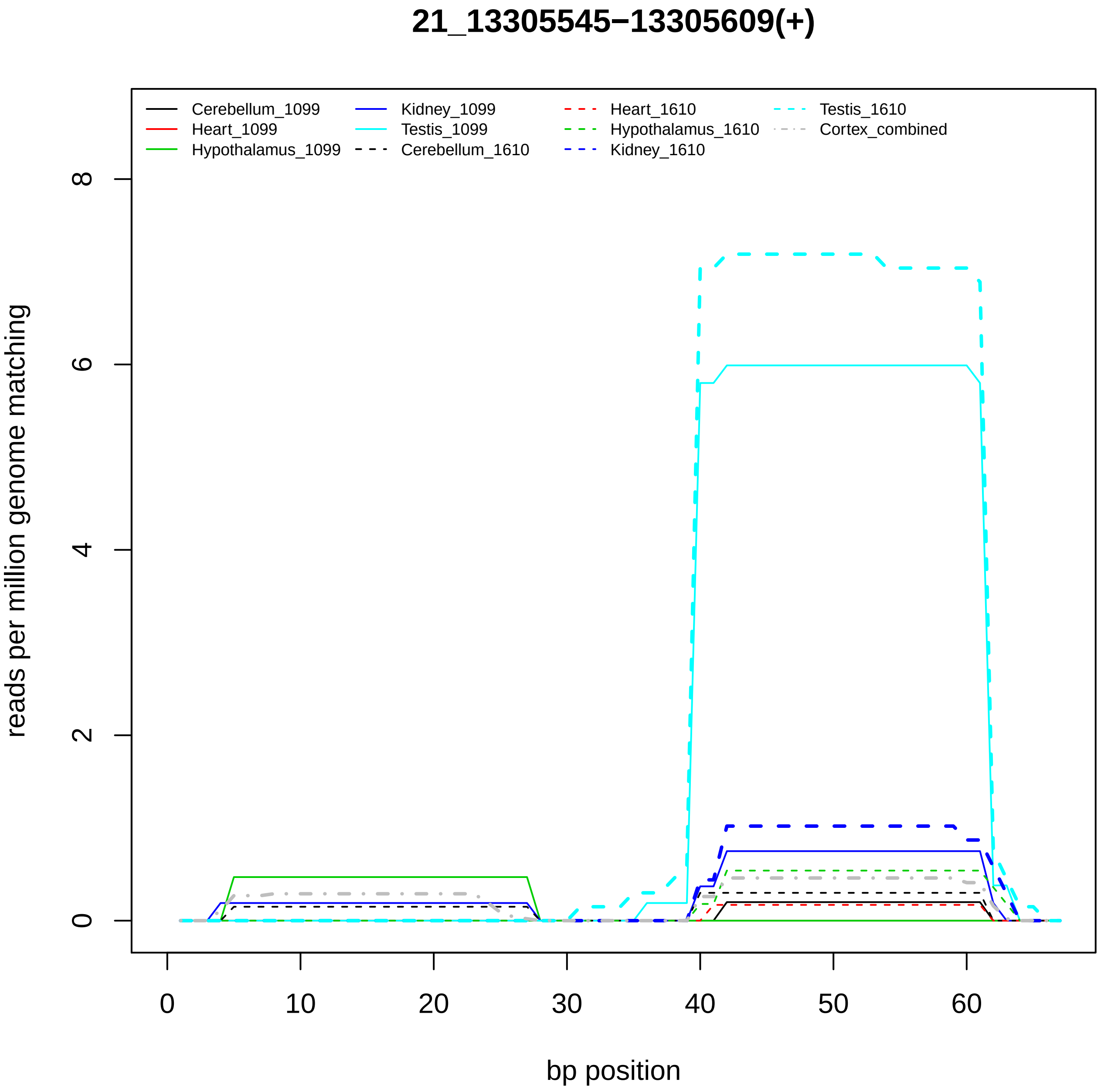

21_13305545−13305609(+)
Cerebellum_1099
Heart_1099
Hypothalamus_1099
Kidney_1099
Testis_1099
Cerebellum_1610
Heart_1610
Hypothalamus_1610
Kidney_1610
Testis_1610
Cortex_combined
reads per million genome matching
0
2
4
6
8
0
10
20
30
40
50
60
bp position

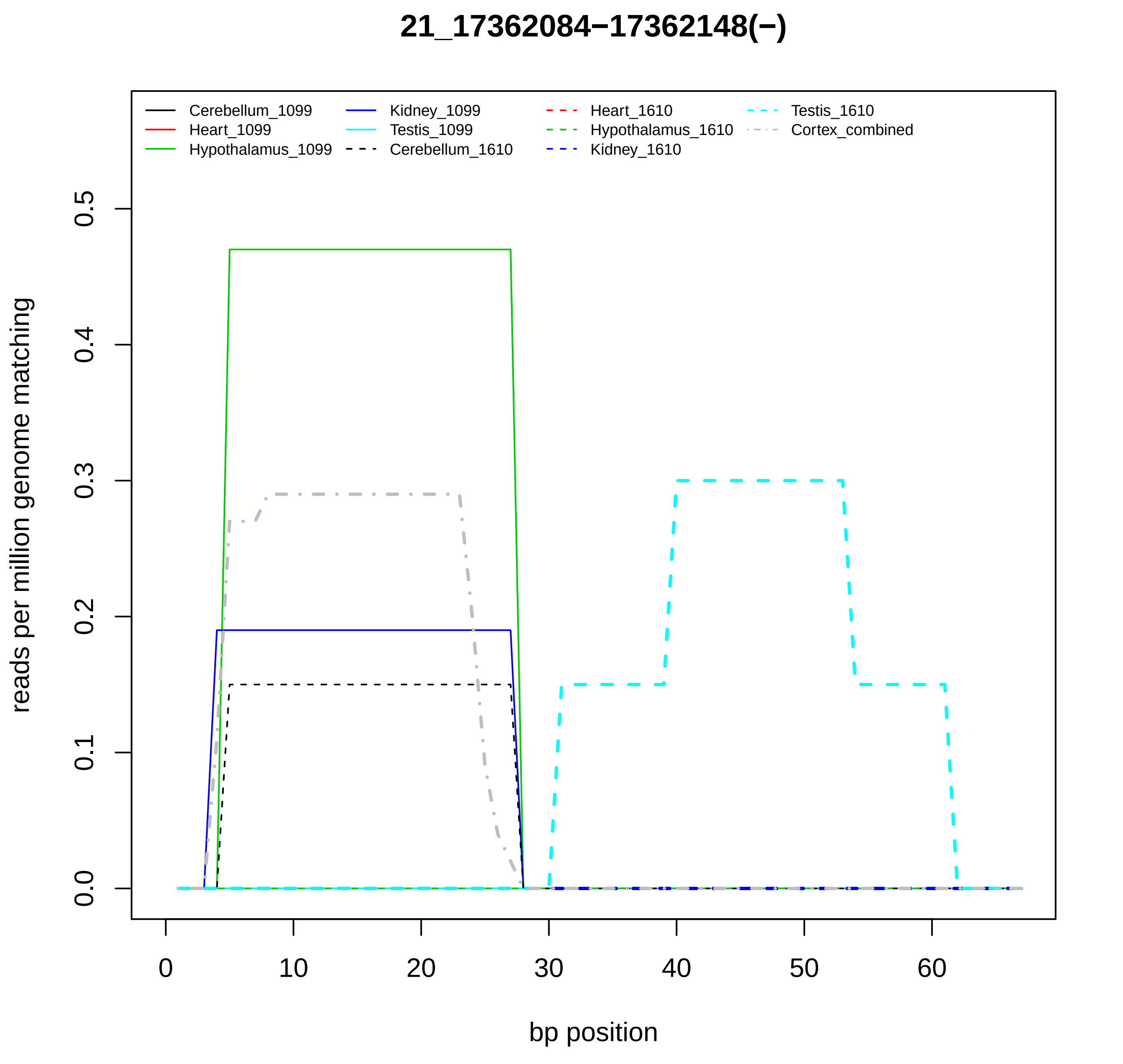
21_17362084−17362148(−)
Cerebellum_1099
Heart_1099
Hypothalamus_1099
Kidney_1099
Testis_1099
Cerebellum_1610
Heart_1610
Hypothalamus_1610
Kidney_1610
Testis_1610
Cortex_combined
reads per million genome matching
0.0
0.1
0.2
0.3
0.4
0.5
0
10
20
30
40
50
60
bp position

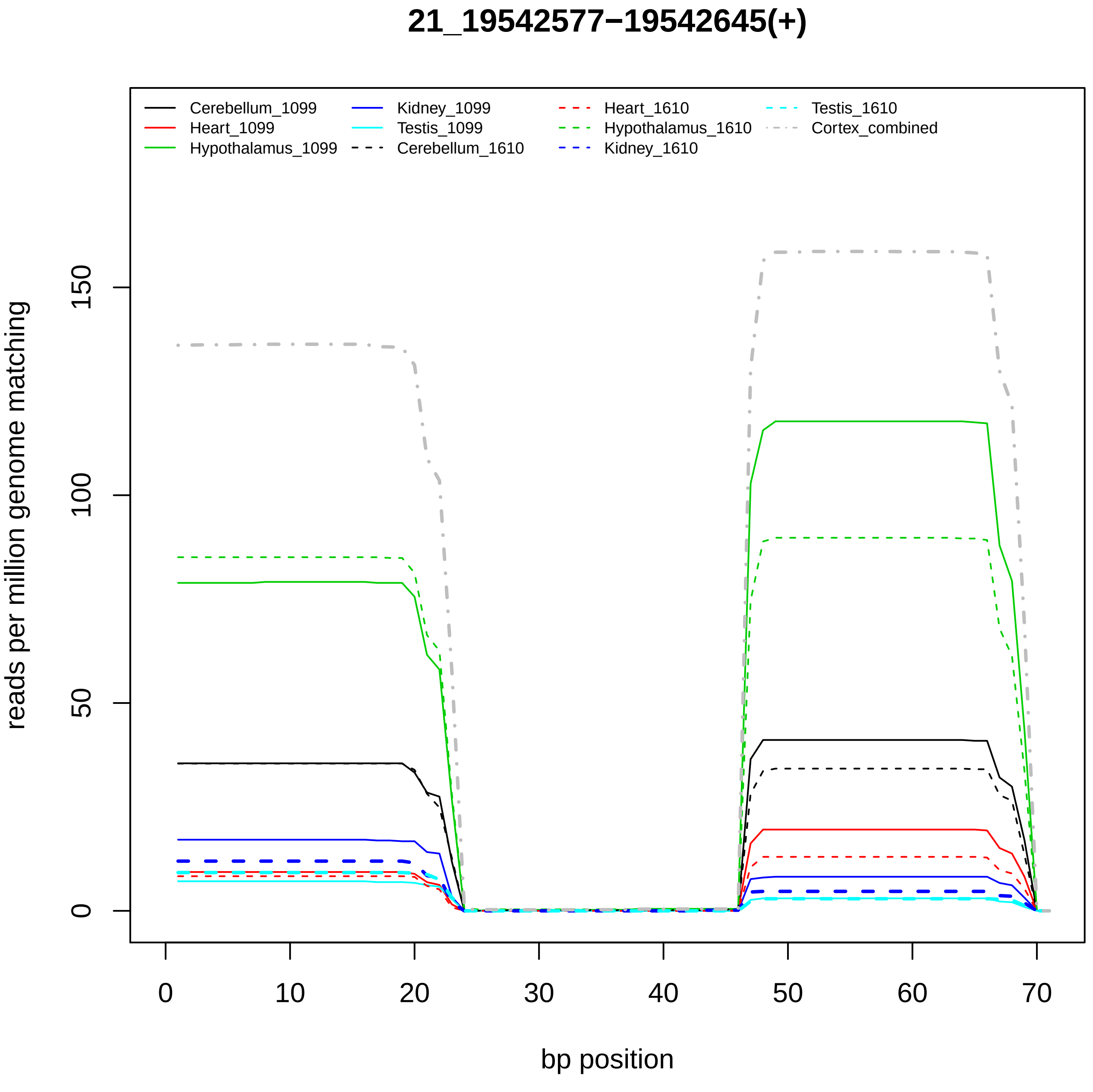
21_19542577−19542645(+)
Cerebellum_1099
Heart_1099
Hypothalamus_1099
Kidney_1099
Testis_1099
Cerebellum_1610
Heart_1610
Hypothalamus_1610
Kidney_1610
Testis_1610
Cortex_combined
reads per million genome matching
bp position
0
50
100
150
0
10
20
30
40
50
60
70

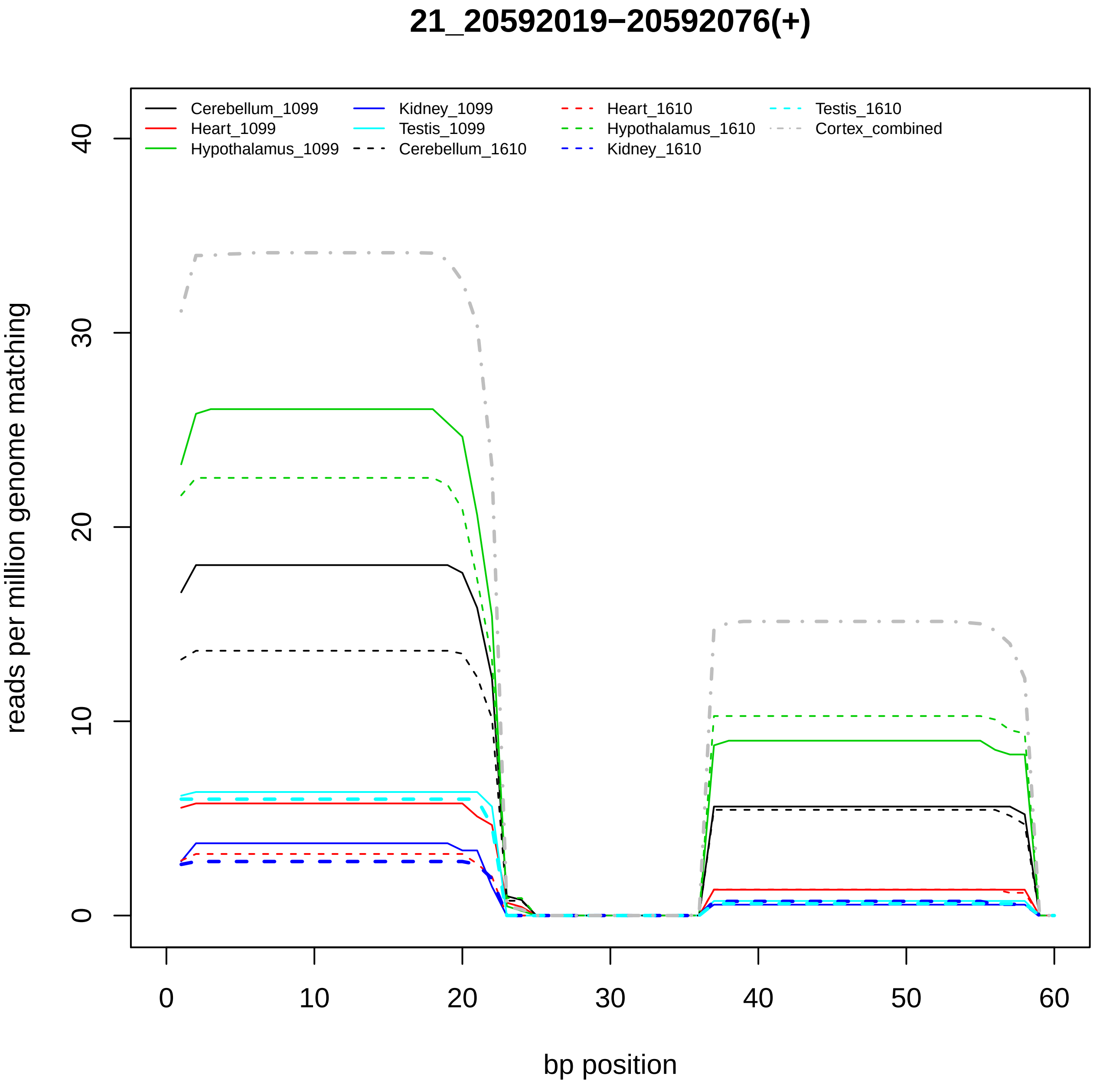
21_20592019−20592076(+)
Cerebellum_1099
Heart_1099
Hypothalamus_1099
Kidney_1099
Testis_1099
Cerebellum_1610
Heart_1610
Hypothalamus_1610
Kidney_1610
Testis_1610
Cortex_combined
reads per million genome matching
bp position
0
10
20
30
40
50
60

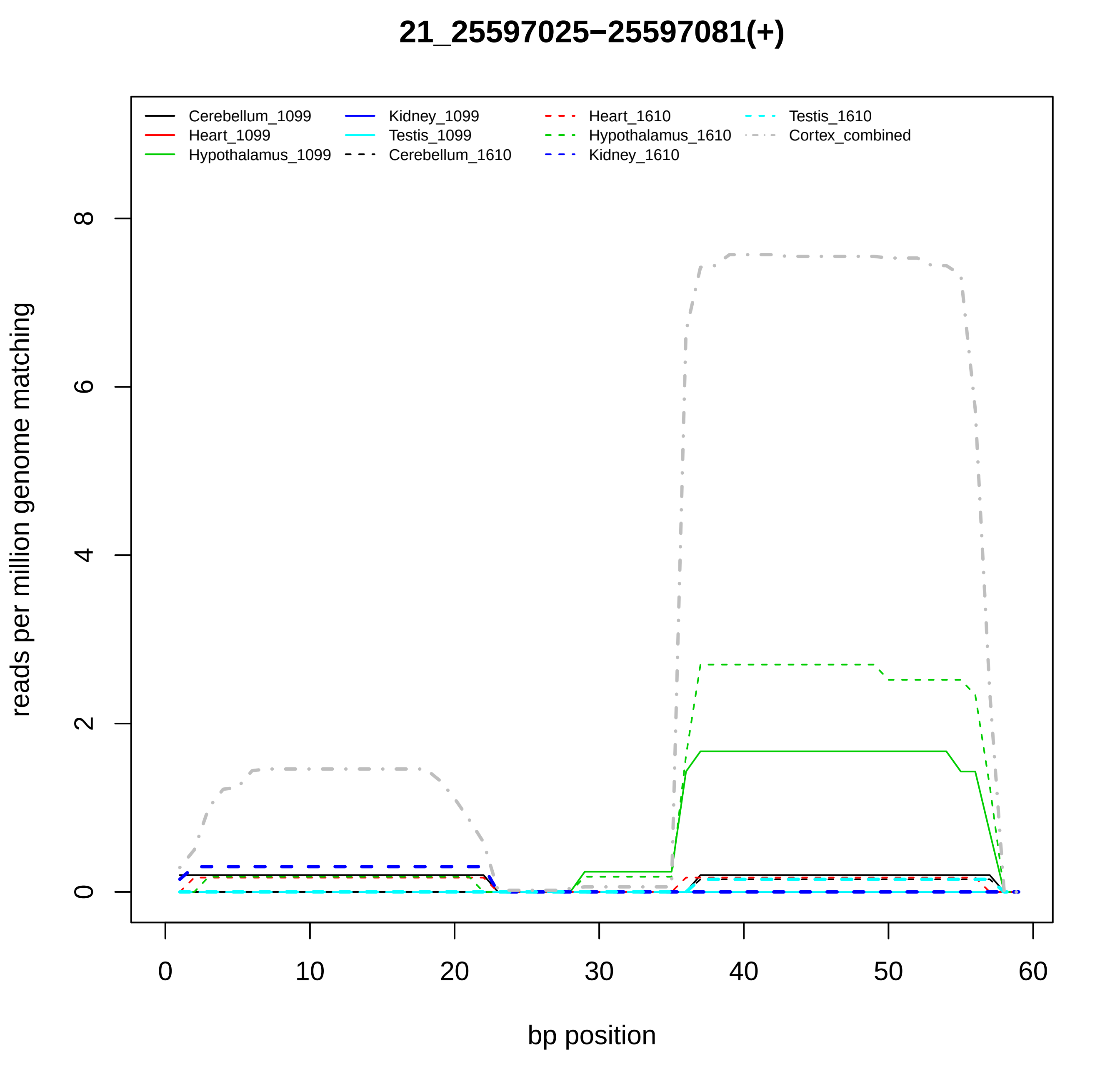

21_25597025−25597081(+)
Cerebellum_1099
Heart_1099
Hypothalamus_1099
Kidney_1099
Testis_1099
Cerebellum_1610
Heart_1610
Hypothalamus_1610
Kidney_1610
Testis_1610
Cortex_combined
reads per million genome matching
0
2
4
6
8
0
10
20
30
40
50
60
bp position

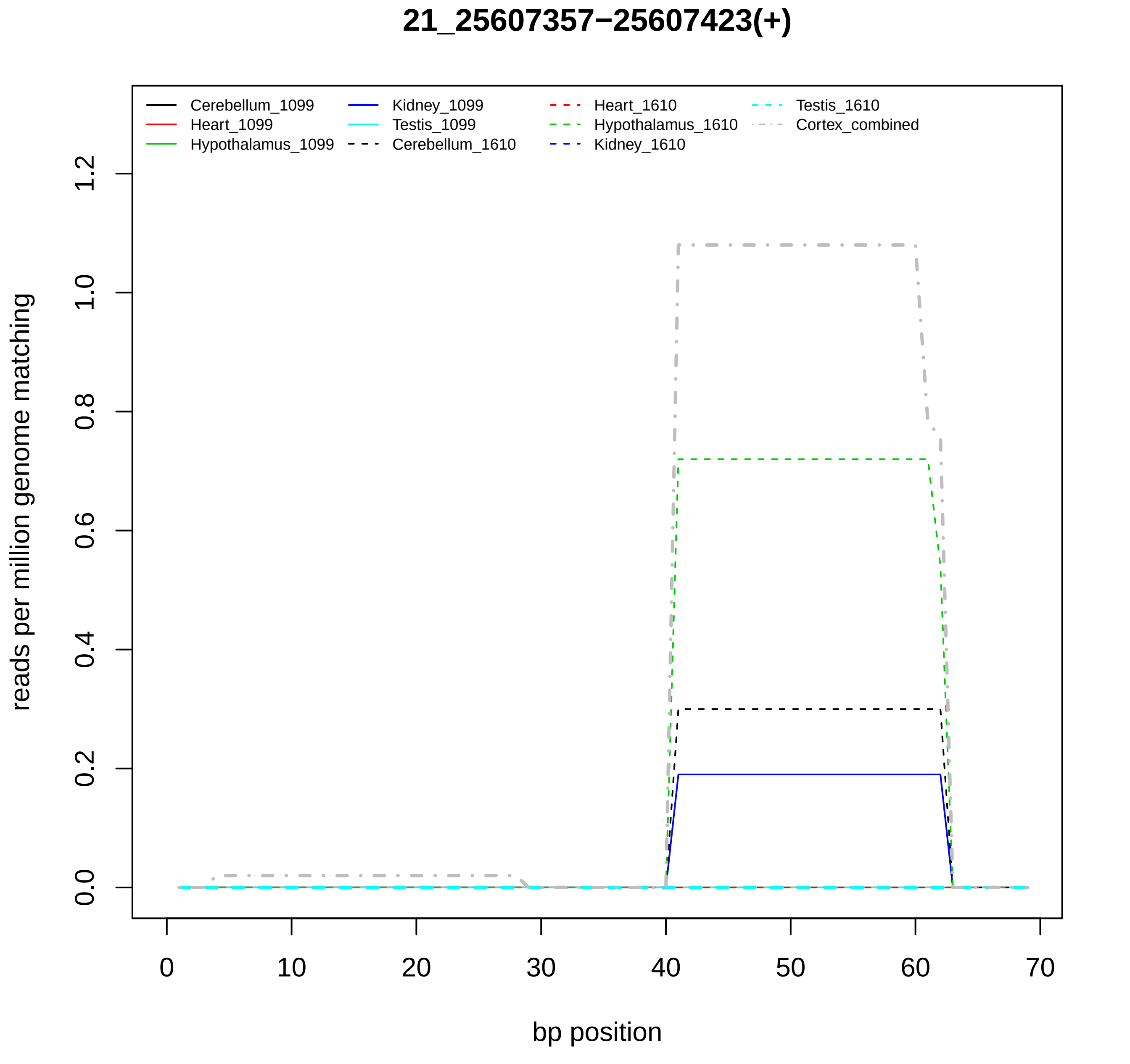

21_25607357−25607423(+)
Cerebellum_1099
Heart_1099
Hypothalamus_1099
Kidney_1099
Testis_1099
Cerebellum_1610
Heart_1610
Hypothalamus_1610
Kidney_1610
Testis_1610
Cortex_combined
reads per million genome matching
bp position
0.0
0.2
0.4
0.6
0.8
1.0
1.2
0
10
20
30
40
50
60
70

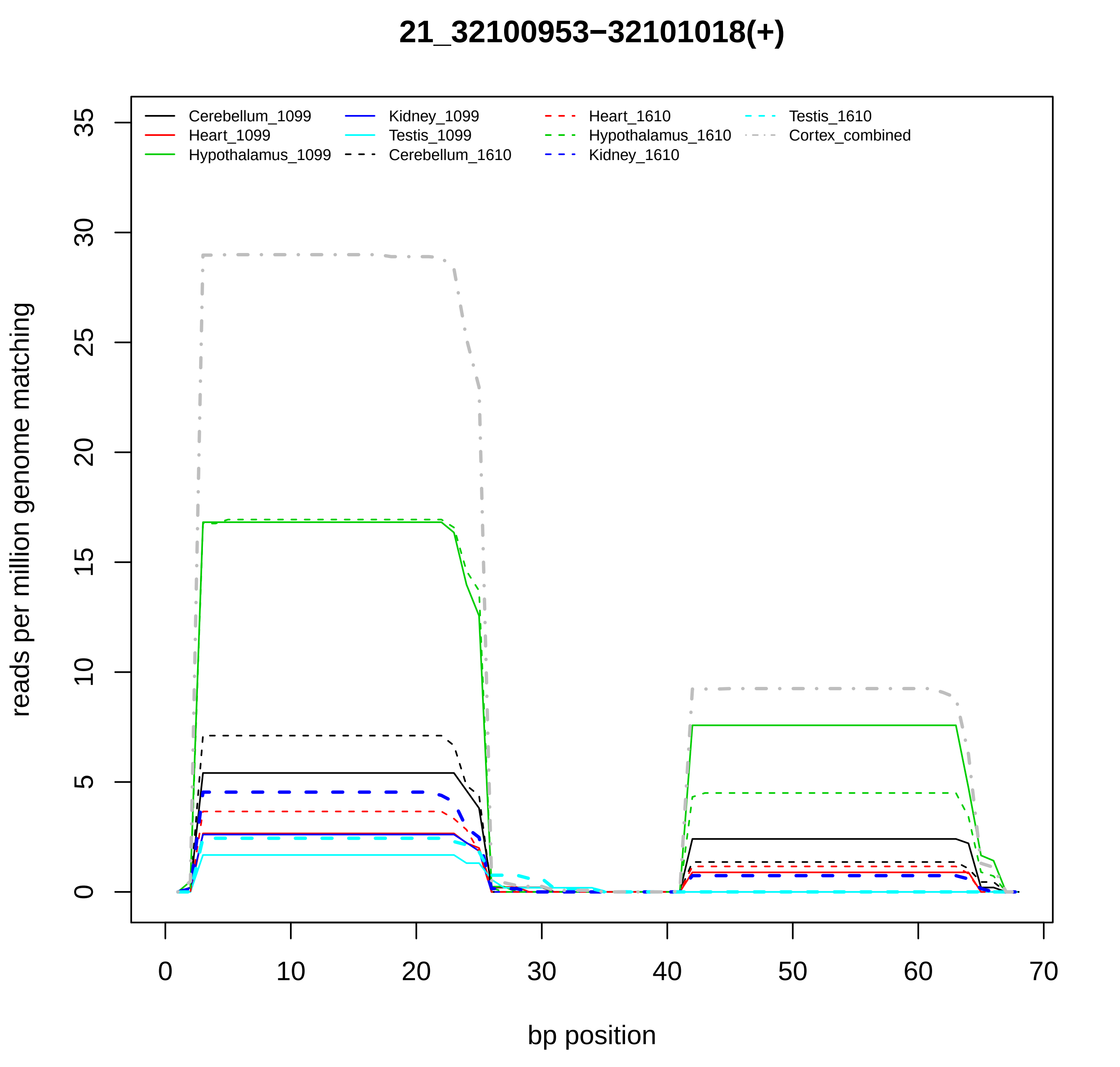

21_32100953−32101018(+)
Cerebellum_1099
Heart_1099
Hypothalamus_1099
Kidney_1099
Testis_1099
Cerebellum_1610
Heart_1610
Hypothalamus_1610
Kidney_1610
Testis_1610
Cortex_combined
reads per million genome matching
bp position
0
5
10
15
20
25
30
35
0
10
20
30
40
50
60
70

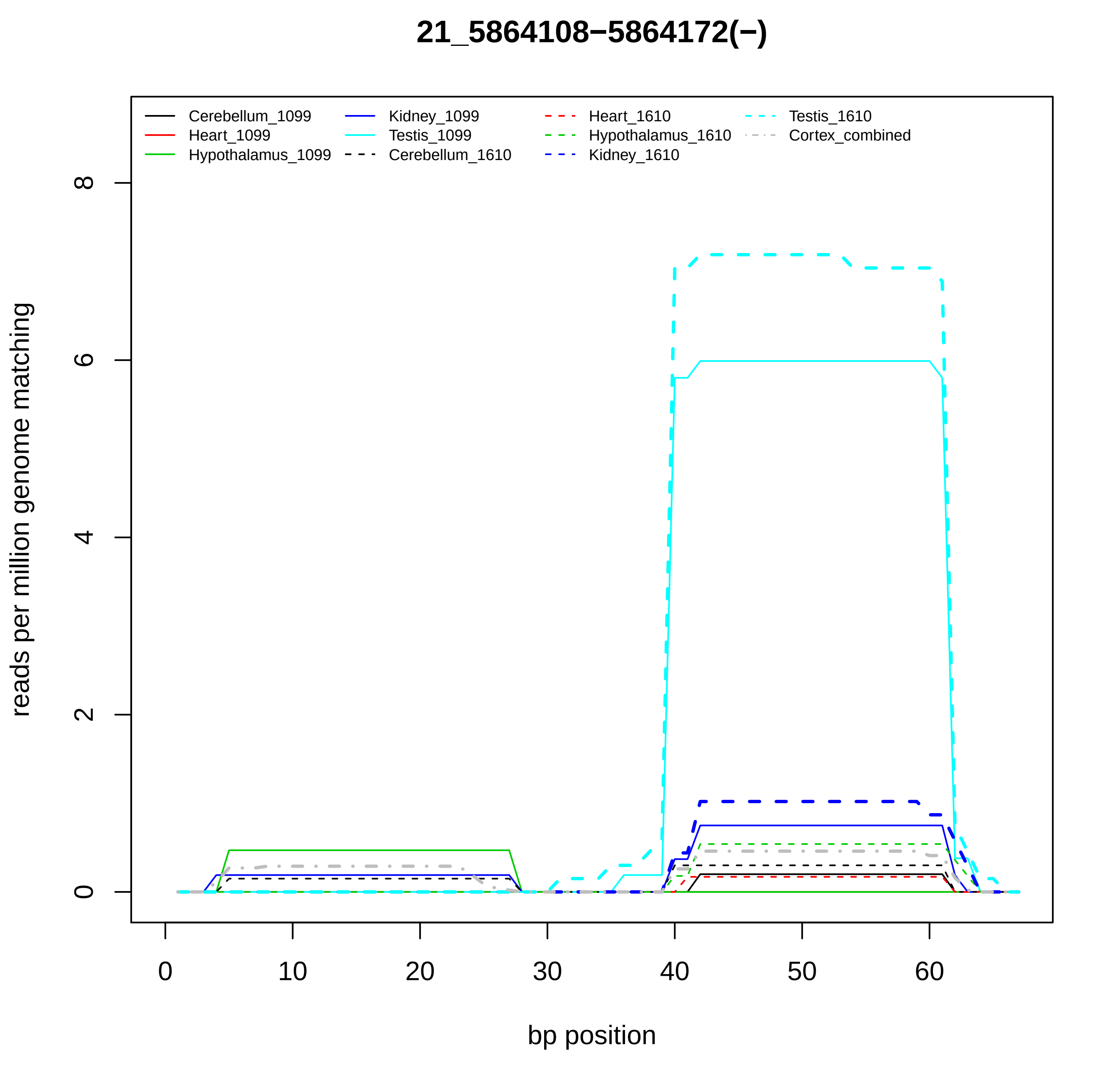

21_5864108−5864172(−)
Cerebellum_1099
Heart_1099
Hypothalamus_1099
Kidney_1099
Testis_1099
Cerebellum_1610
Heart_1610
Hypothalamus_1610
Kidney_1610
Testis_1610
Cortex_combined
reads per million genome matching
0
2
4
6
8
0
10
20
30
40
50
60
bp position

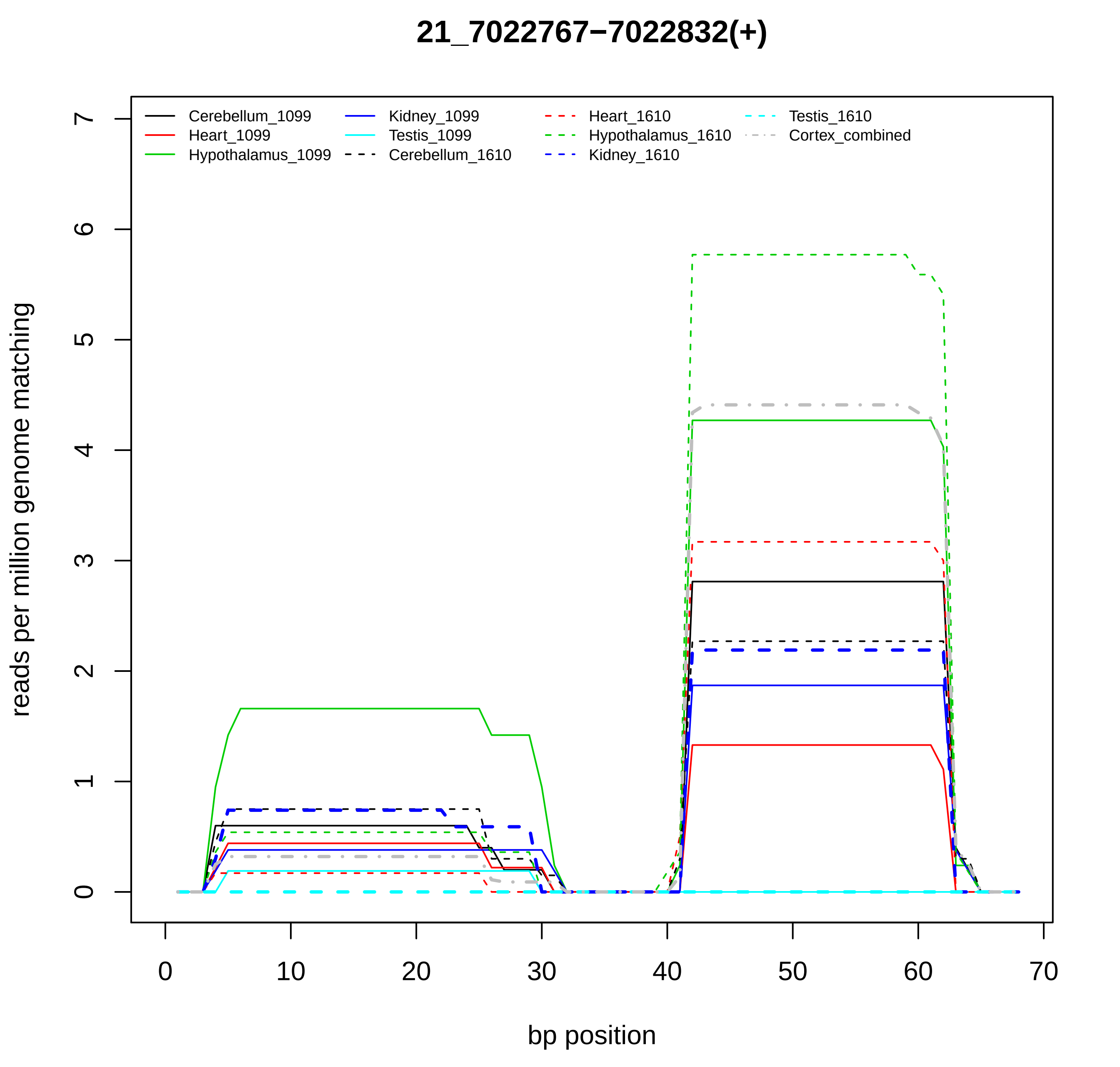

21_7022767−7022832(+)
Cerebellum_1099
Heart_1099
Hypothalamus_1099
Kidney_1099
Testis_1099
Cerebellum_1610
Heart_1610
Hypothalamus_1610
Kidney_1610
Testis_1610
Cortex_combined
reads per million genome matching
bp position
0
1
2
3
4
5
6
7
0
10
20
30
40
50
60
70

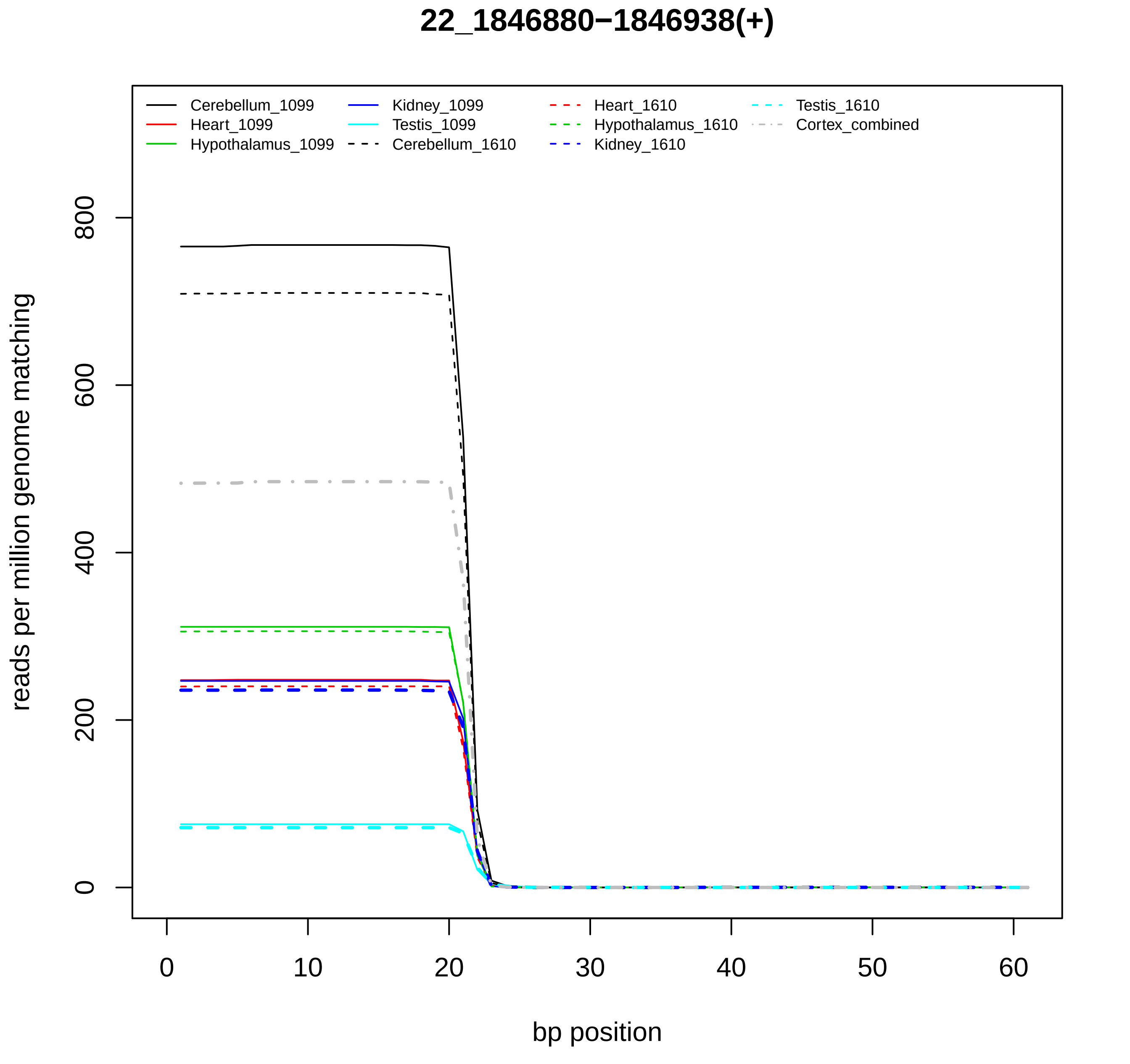
22_1846880−1846938(+)
Cerebellum_1099
Heart_1099
Hypothalamus_1099
Kidney_1099
Testis_1099
Cerebellum_1610
Heart_1610
Hypothalamus_1610
Kidney_1610
Testis_1610
Cortex_combined
reads per million genome matching
0
200
400
600
800
0
10
20
30
40
50
60
bp position

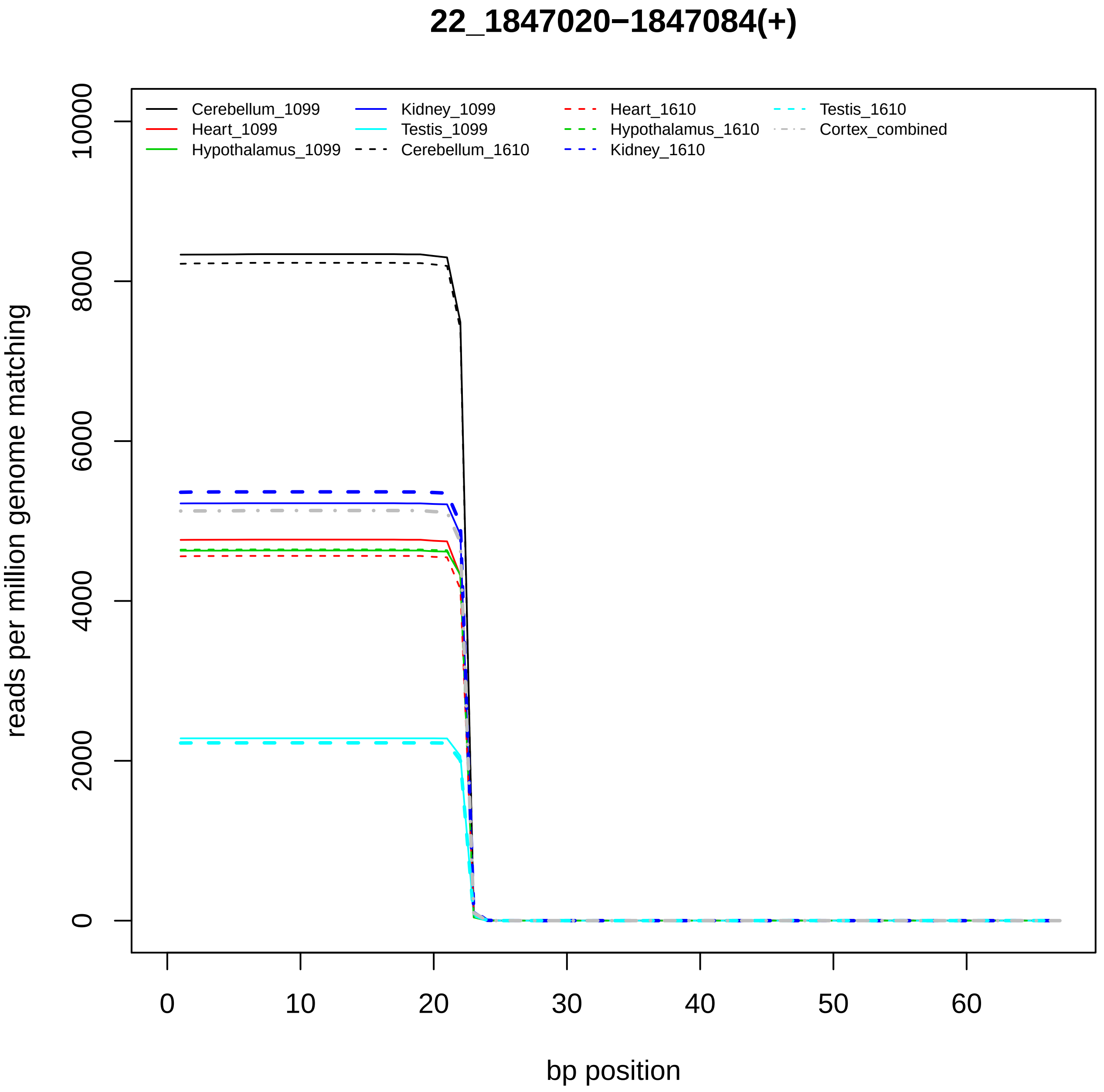

22_1847020−1847084(+)
Cerebellum_1099
Heart_1099
Hypothalamus_1099
Kidney_1099
Testis_1099
Cerebellum_1610
Heart_1610
Hypothalamus_1610
Kidney_1610
Testis_1610
Cortex_combined
reads per million genome matching
0
2000
4000
6000
8000
10000
0
10
20
30
40
50
60
bp position

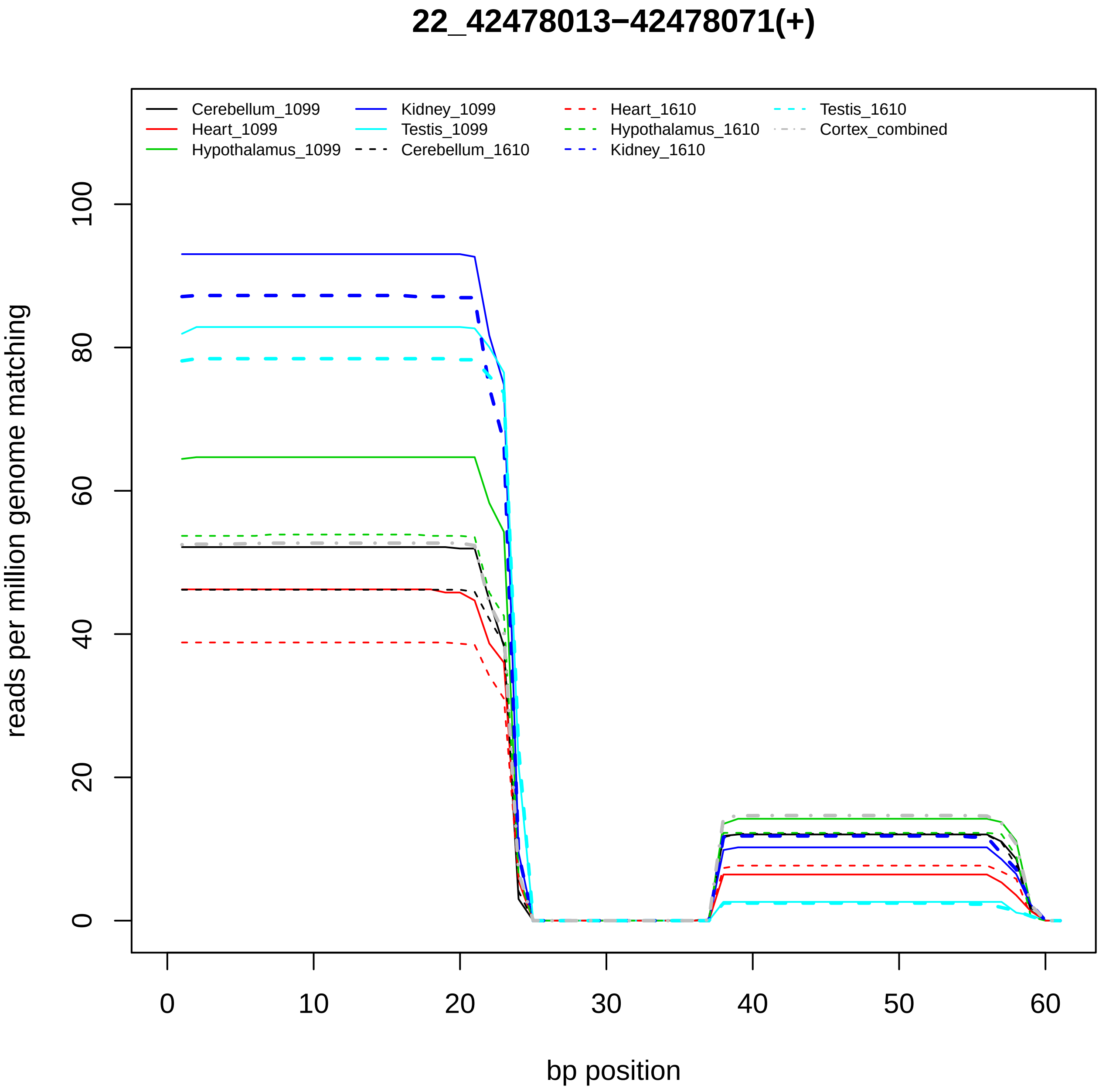
22_42478013−42478071(+)
Cerebellum_1099
Heart_1099
Hypothalamus_1099
Kidney_1099
Testis_1099
Cerebellum_1610
Heart_1610
Hypothalamus_1610
Kidney_1610
Testis_1610
Cortex_combined
reads per million genome matching
0
20
40
60
80
100
0
10
20
30
40
50
60
bp position

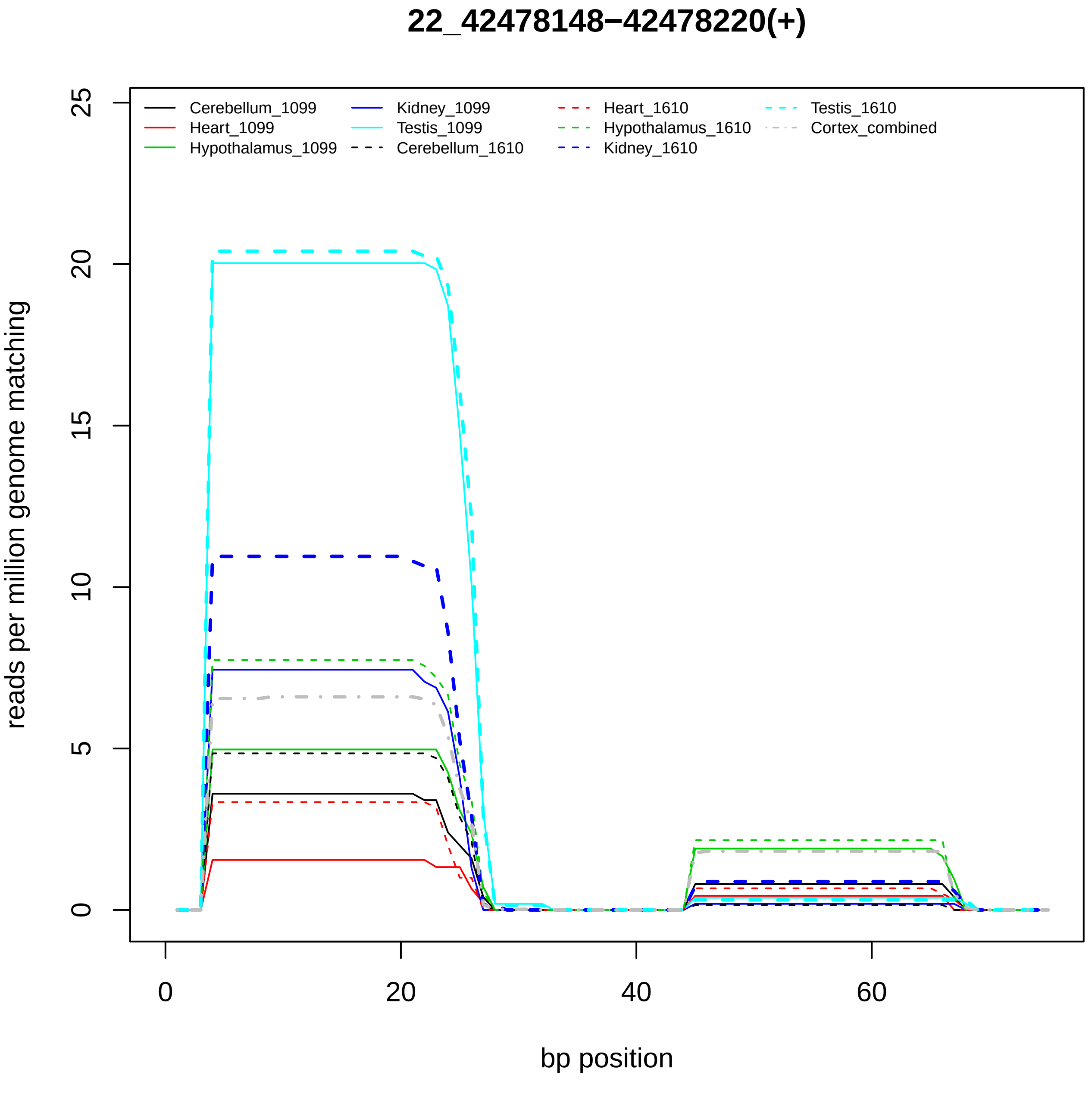

22_42478148−42478220(+)
Cerebellum_1099
Heart_1099
Hypothalamus_1099
Kidney_1099
Testis_1099
Cerebellum_1610
Heart_1610
Hypothalamus_1610
Kidney_1610
Testis_1610
Cortex_combined
reads per million genome matching
bp position
0
5
10
15
20
25
0
20
40
60

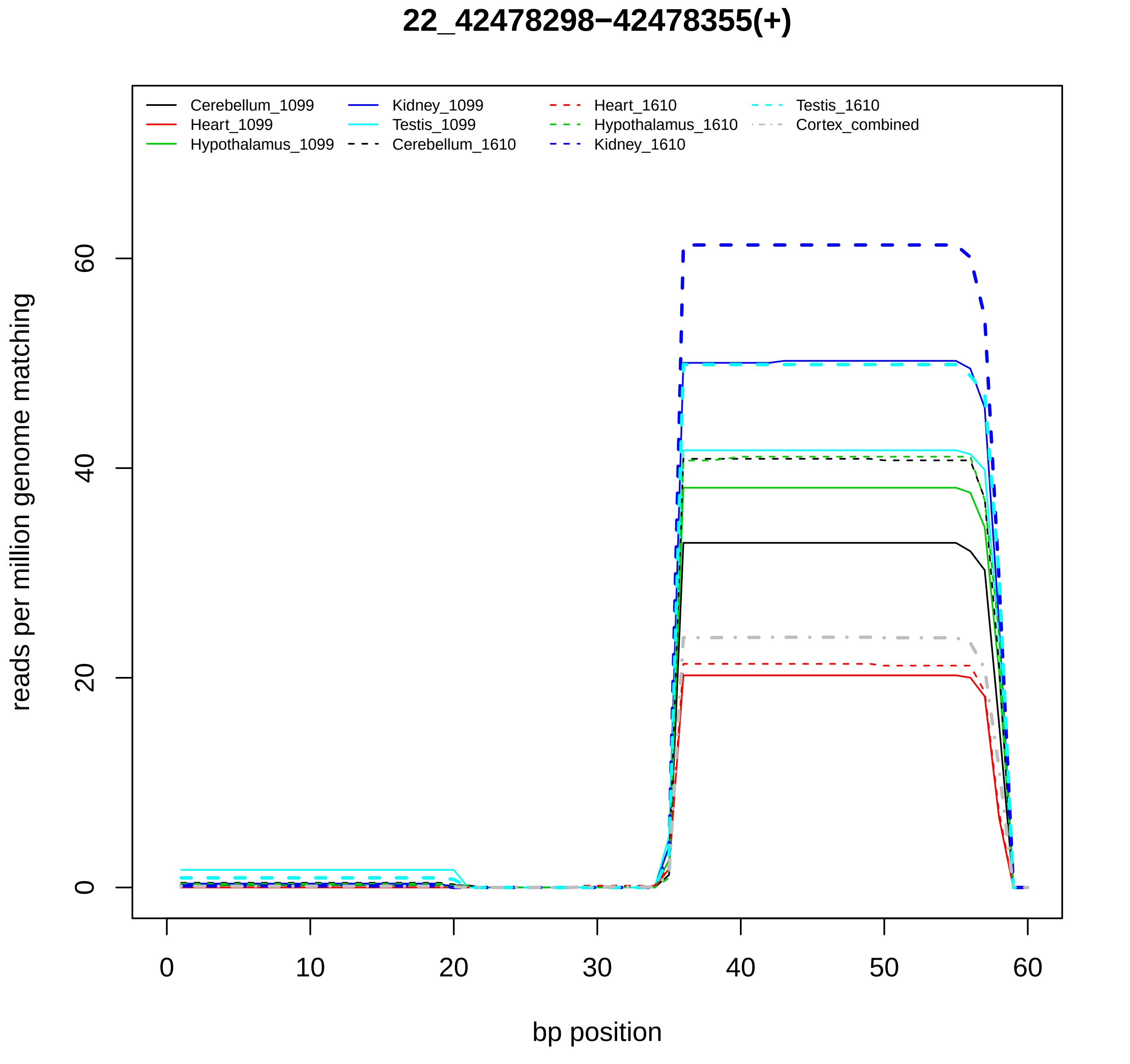
22_42478298−42478355(+)
Cerebellum_1099
Heart_1099
Hypothalamus_1099
Kidney_1099
Testis_1099
Cerebellum_1610
Heart_1610
Hypothalamus_1610
Kidney_1610
Testis_1610
Cortex_combined
reads per million genome matching
0
20
40
60
0
10
20
30
40
50
60
bp position

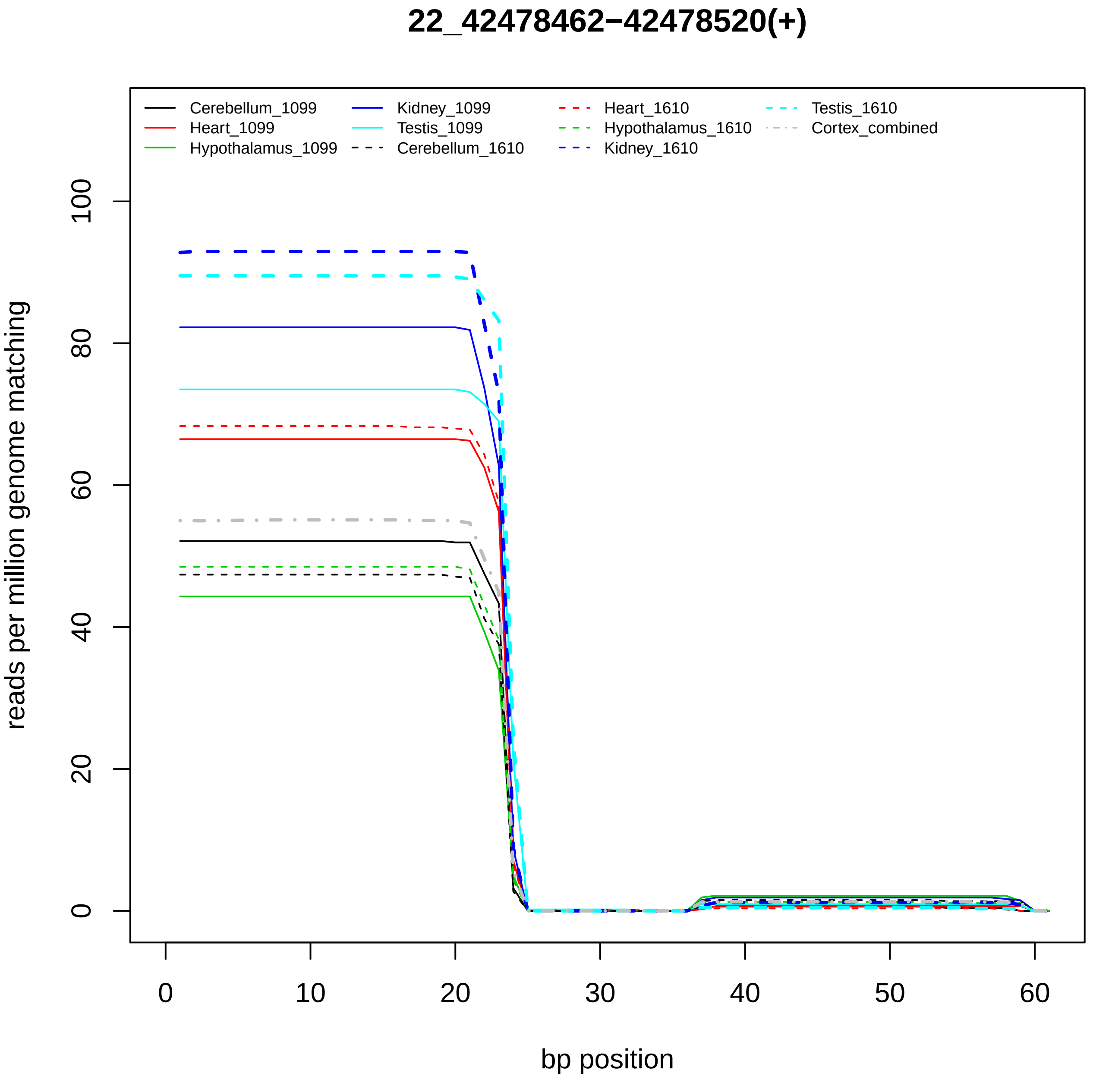
22_42478462−42478520(+)
Cerebellum_1099
Heart_1099
Hypothalamus_1099
Kidney_1099
Testis_1099
Cerebellum_1610
Heart_1610
Hypothalamus_1610
Kidney_1610
Testis_1610
Cortex_combined
reads per million genome matching
0
20
40
60
80
100
0
10
20
30
40
50
60
bp position

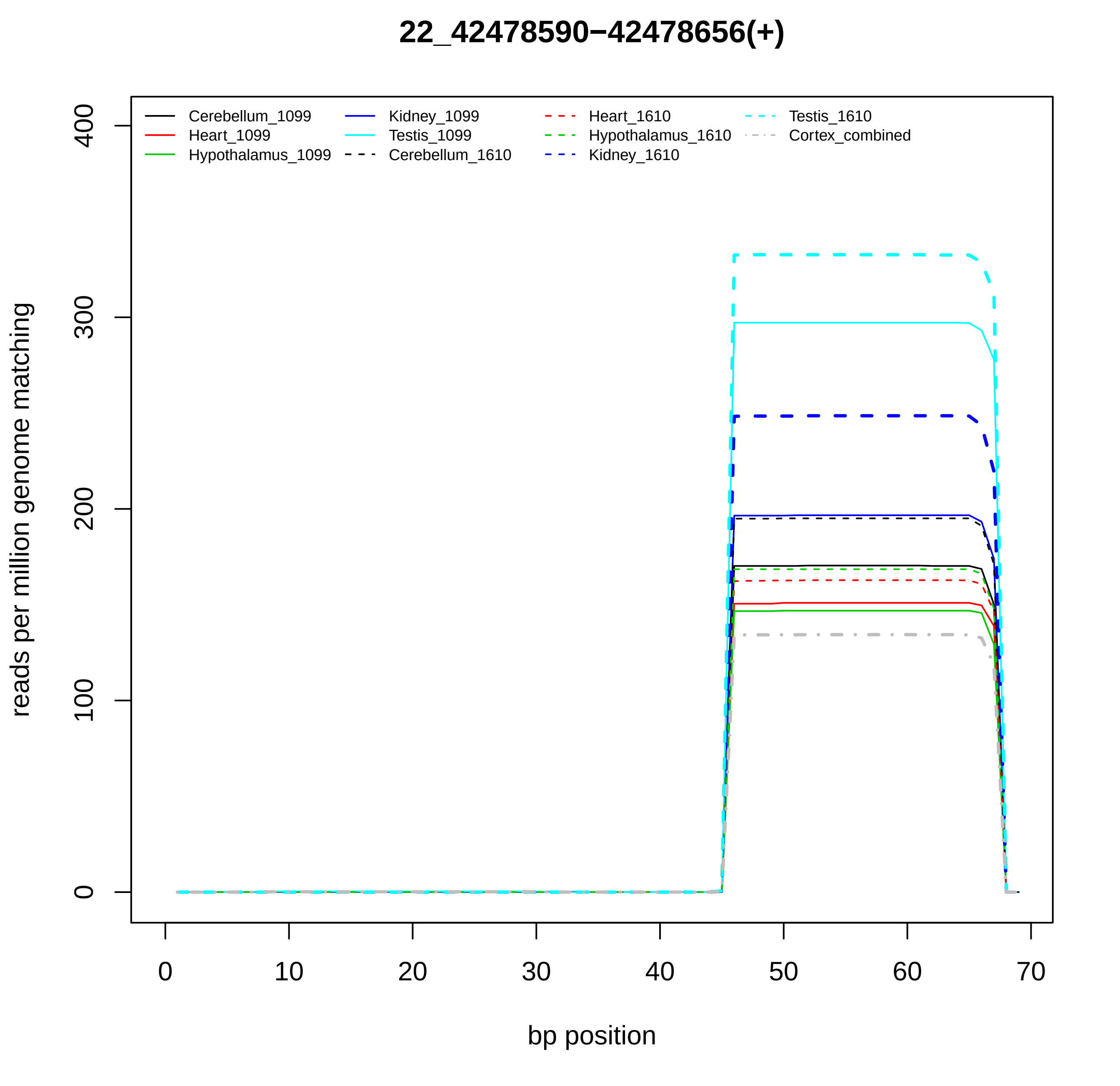

22_42478590−42478656(+)
Cerebellum_1099
Heart_1099
Hypothalamus_1099
Kidney_1099
Testis_1099
Cerebellum_1610
Heart_1610
Hypothalamus_1610
Kidney_1610
Testis_1610
Cortex_combined
reads per million genome matching
bp position
0
100
200
300
400
0
10
20
30
40
50
60
70

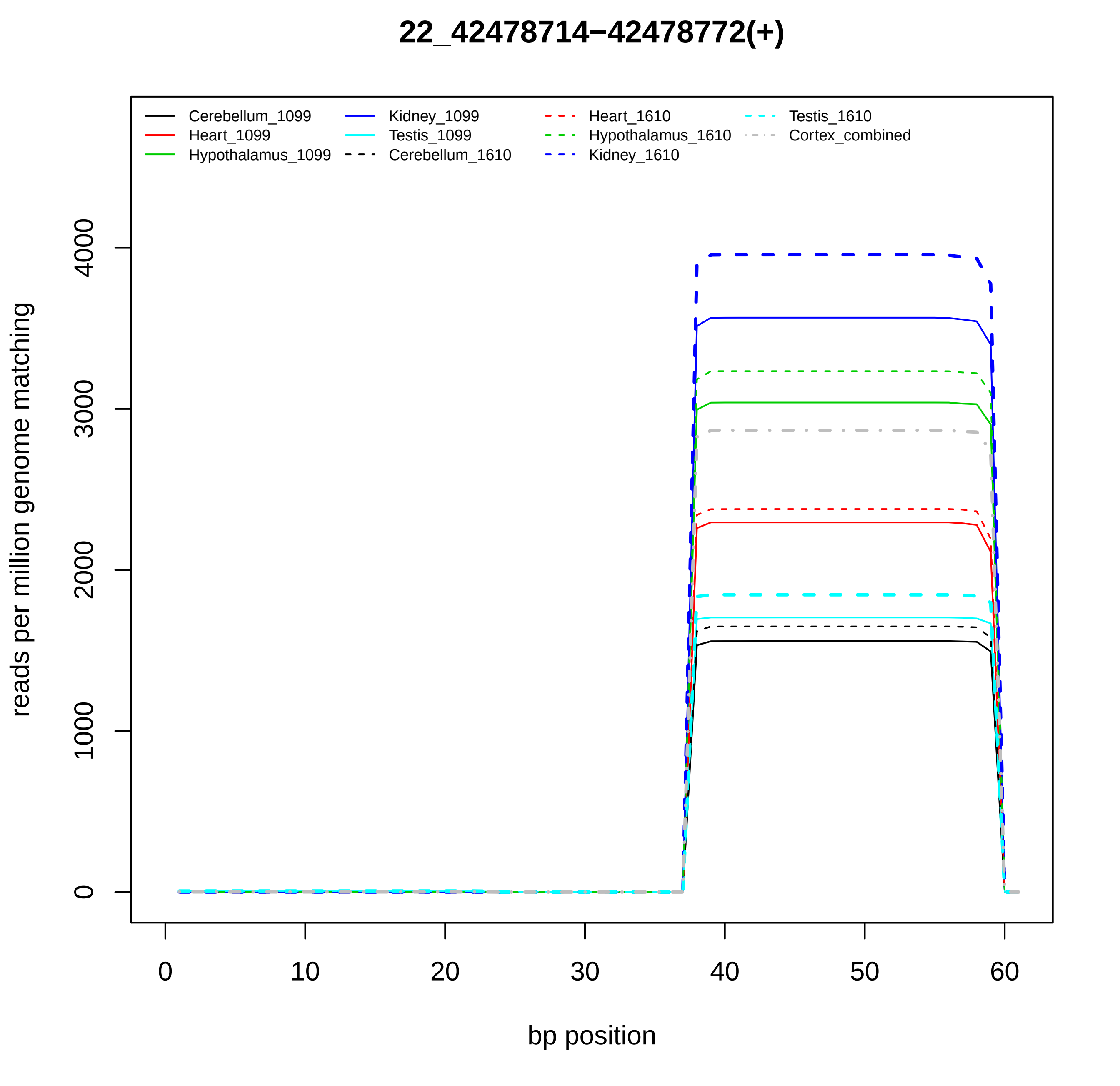

22_42478714−42478772(+)
Cerebellum_1099
Heart_1099
Hypothalamus_1099
Kidney_1099
Testis_1099
Cerebellum_1610
Heart_1610
Hypothalamus_1610
Kidney_1610
Testis_1610
Cortex_combined
reads per million genome matching
0
1000
2000
3000
4000
0
10
20
30
40
50
60
bp position

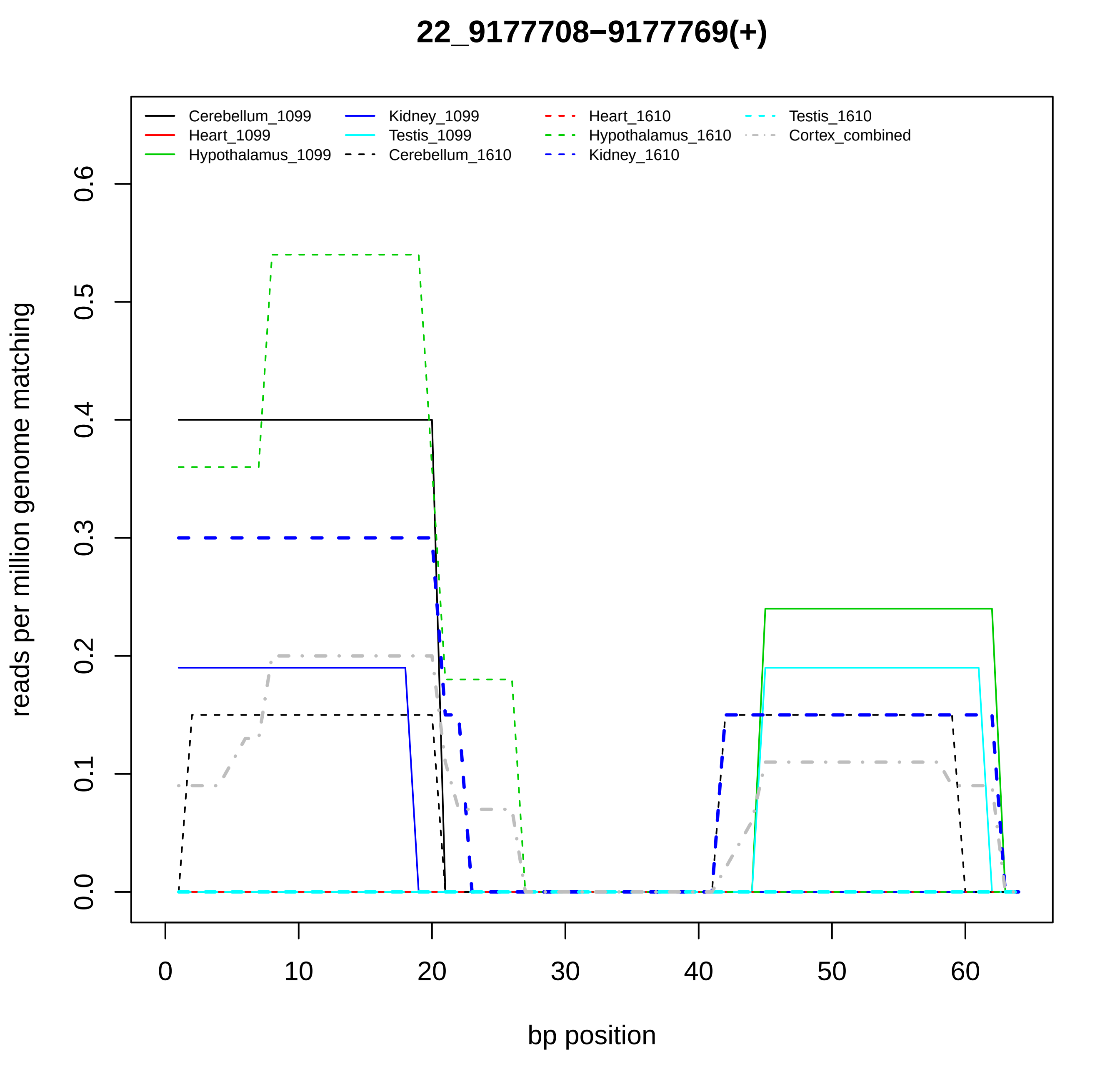

22_9177708−9177769(+)
Cerebellum_1099
Heart_1099
Hypothalamus_1099
Kidney_1099
Testis_1099
Cerebellum_1610
Heart_1610
Hypothalamus_1610
Kidney_1610
Testis_1610
Cortex_combined
reads per million genome matching
0.0
0.1
0.2
0.3
0.4
0.5
0.6
0
10
20
30
40
50
60
bp position

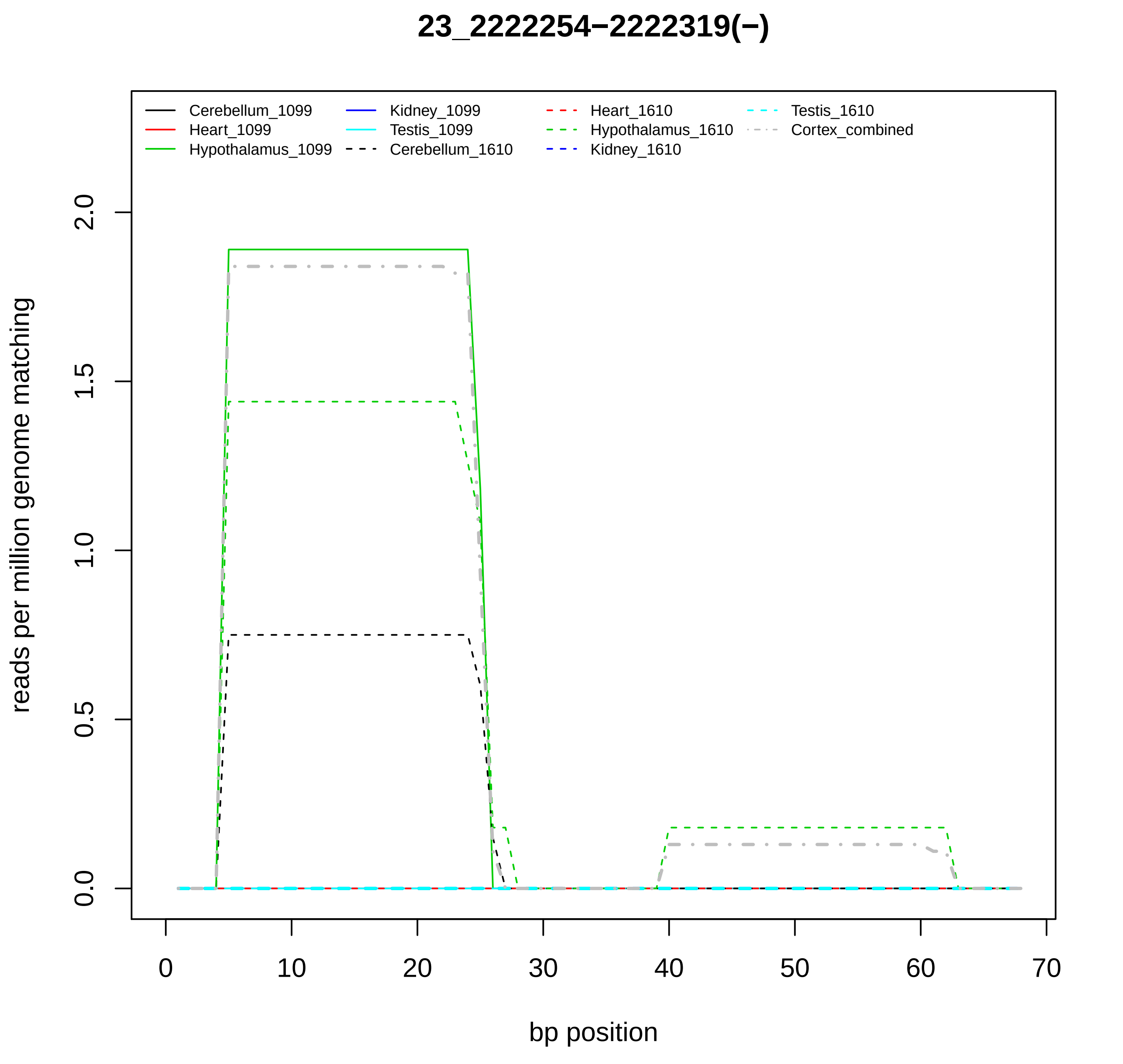

23_2222254−2222319(−)
Cerebellum_1099
Heart_1099
Hypothalamus_1099
Kidney_1099
Testis_1099
Cerebellum_1610
Heart_1610
Hypothalamus_1610
Kidney_1610
Testis_1610
Cortex_combined
reads per million genome matching
0.0
0.5
1.0
1.5
2.0
0
10
20
30
40
50
60
70
bp position

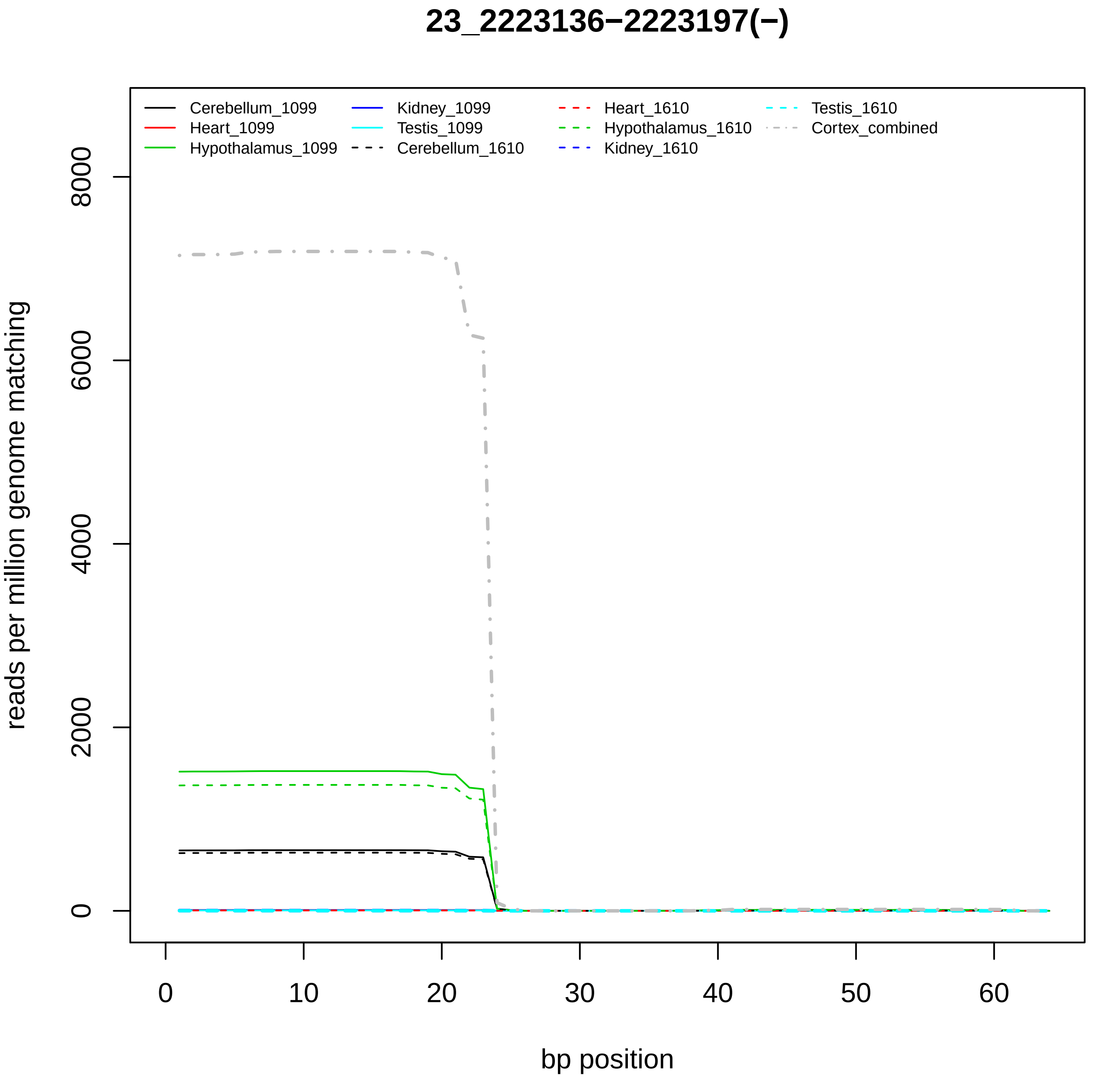

23_2223136−2223197(−)
Cerebellum_1099
Heart_1099
Hypothalamus_1099
Kidney_1099
Testis_1099
Cerebellum_1610
Heart_1610
Hypothalamus_1610
Kidney_1610
Testis_1610
Cortex_combined
reads per million genome matching
0
2000
4000
6000
8000
0
10
20
30
40
50
60
bp position

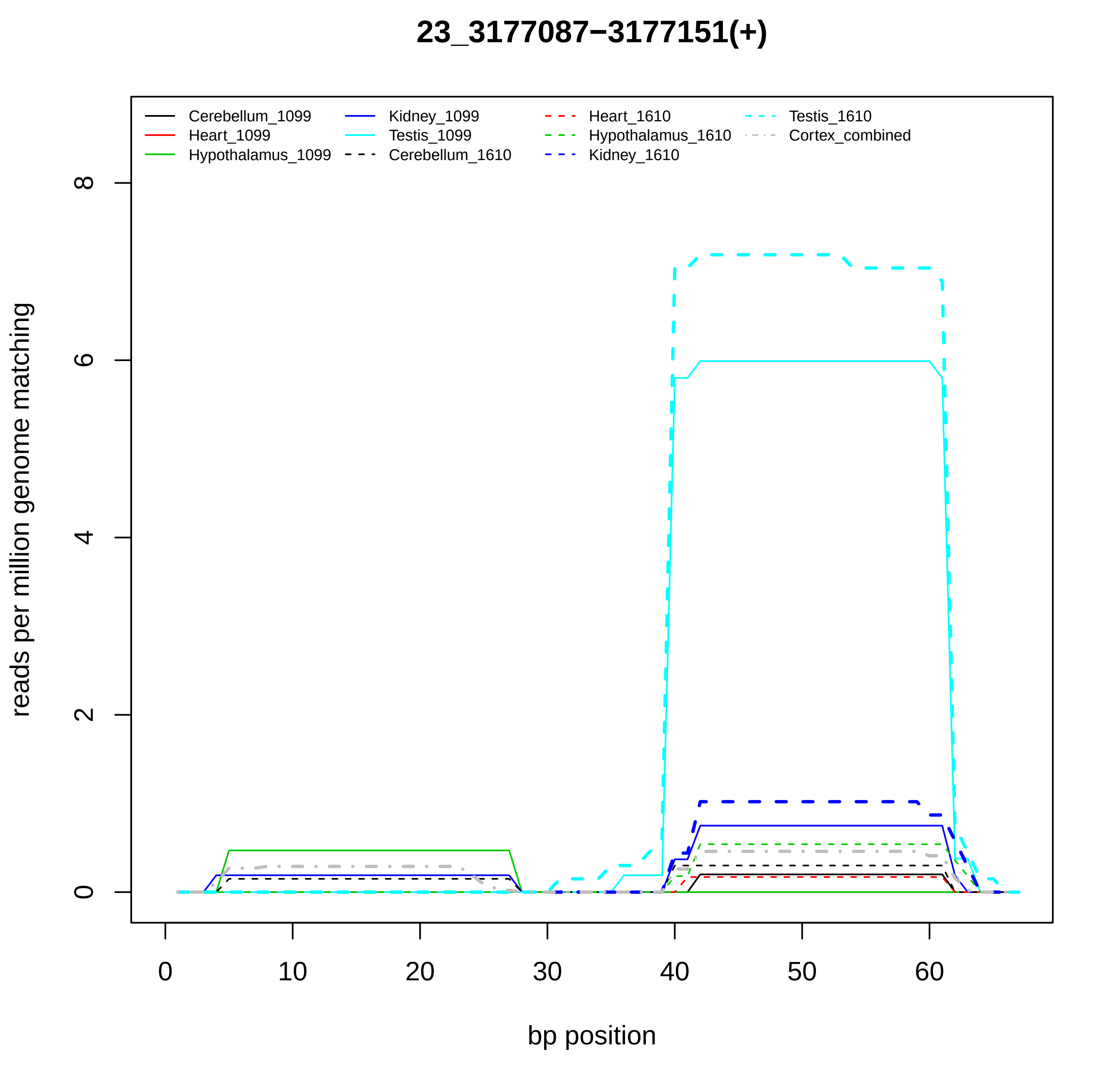
23_3177087−3177151(+)
Cerebellum_1099
Heart_1099
Hypothalamus_1099
Kidney_1099
Testis_1099
Cerebellum_1610
Heart_1610
Hypothalamus_1610
Kidney_1610
Testis_1610
Cortex_combined
reads per million genome matching
bp position
0
2
4
6
8
0
10
20
30
40
50
60

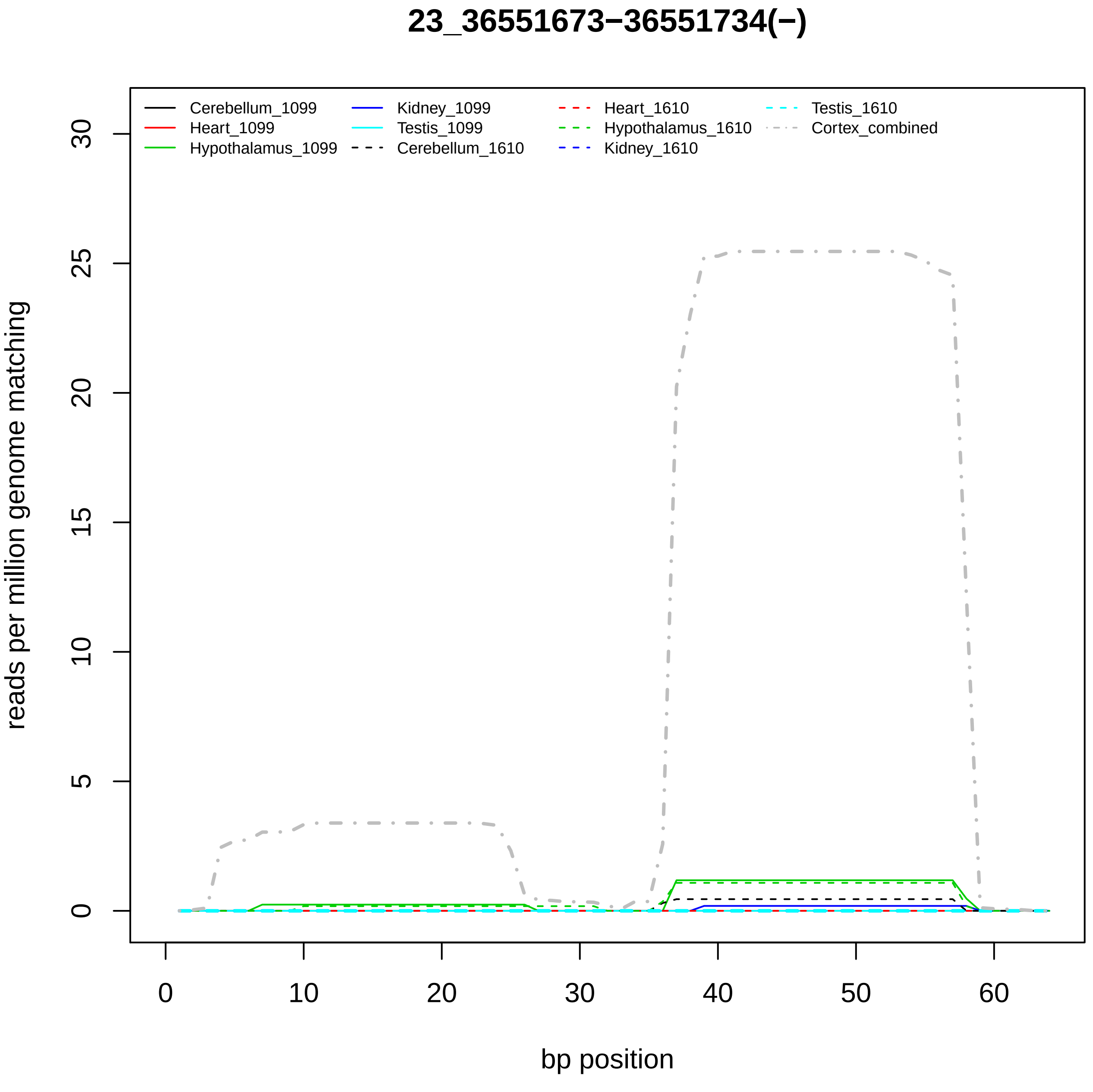
23_36551673−36551734(−)
Cerebellum_1099
Heart_1099
Hypothalamus_1099
Kidney_1099
Testis_1099
Cerebellum_1610
Heart_1610
Hypothalamus_1610
Kidney_1610
Testis_1610
Cortex_combined
reads per million genome matching
bp position
0
5
10
15
20
25
30
0
10
20
30
40
50
60

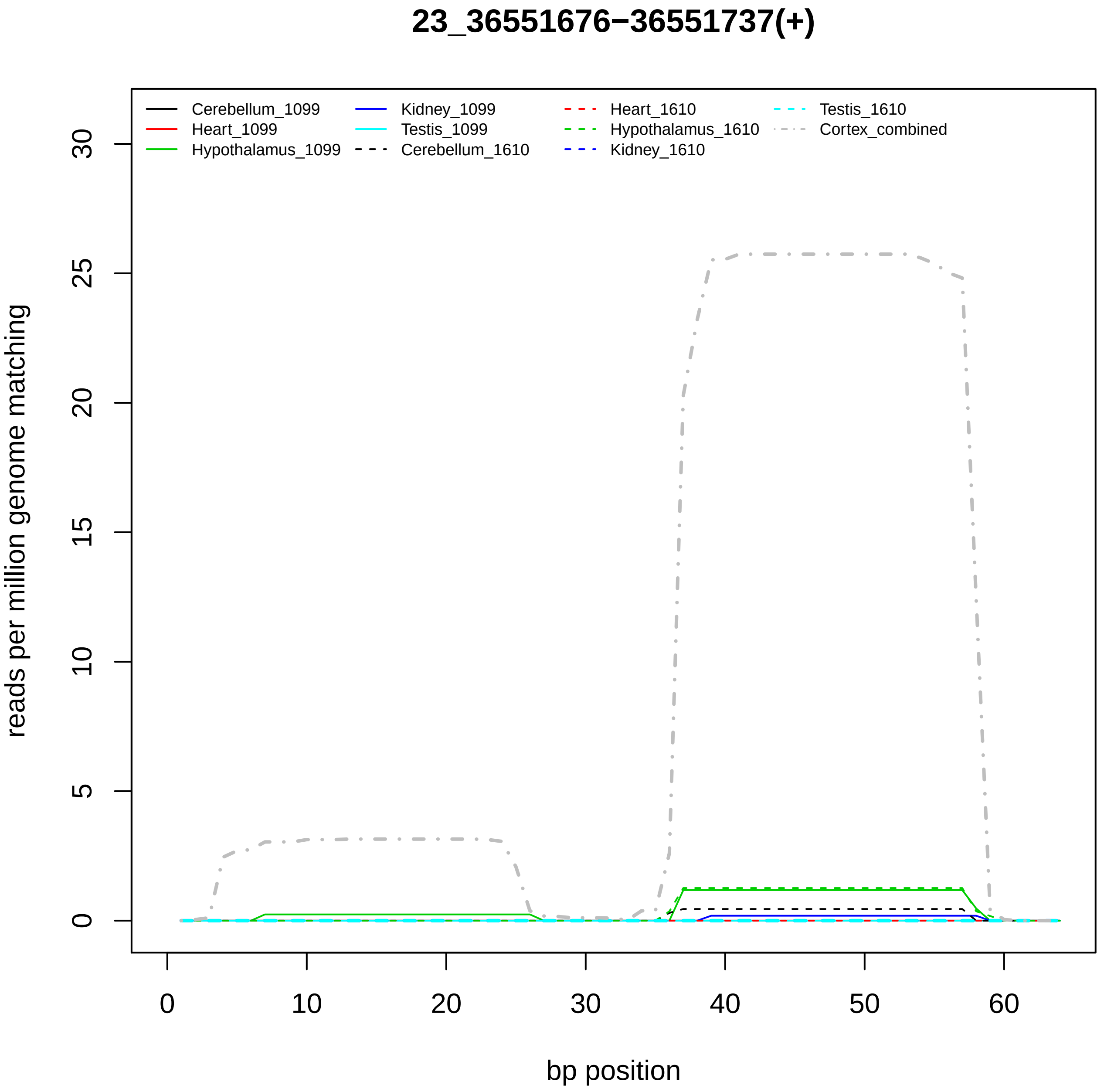
23_36551676−36551737(+)
Cerebellum_1099
Heart_1099
Hypothalamus_1099
Kidney_1099
Testis_1099
Cerebellum_1610
Heart_1610
Hypothalamus_1610
Kidney_1610
Testis_1610
Cortex_combined
reads per million genome matching
bp position
0
5
10
15
20
25
30
0
10
20
30
40
50
60

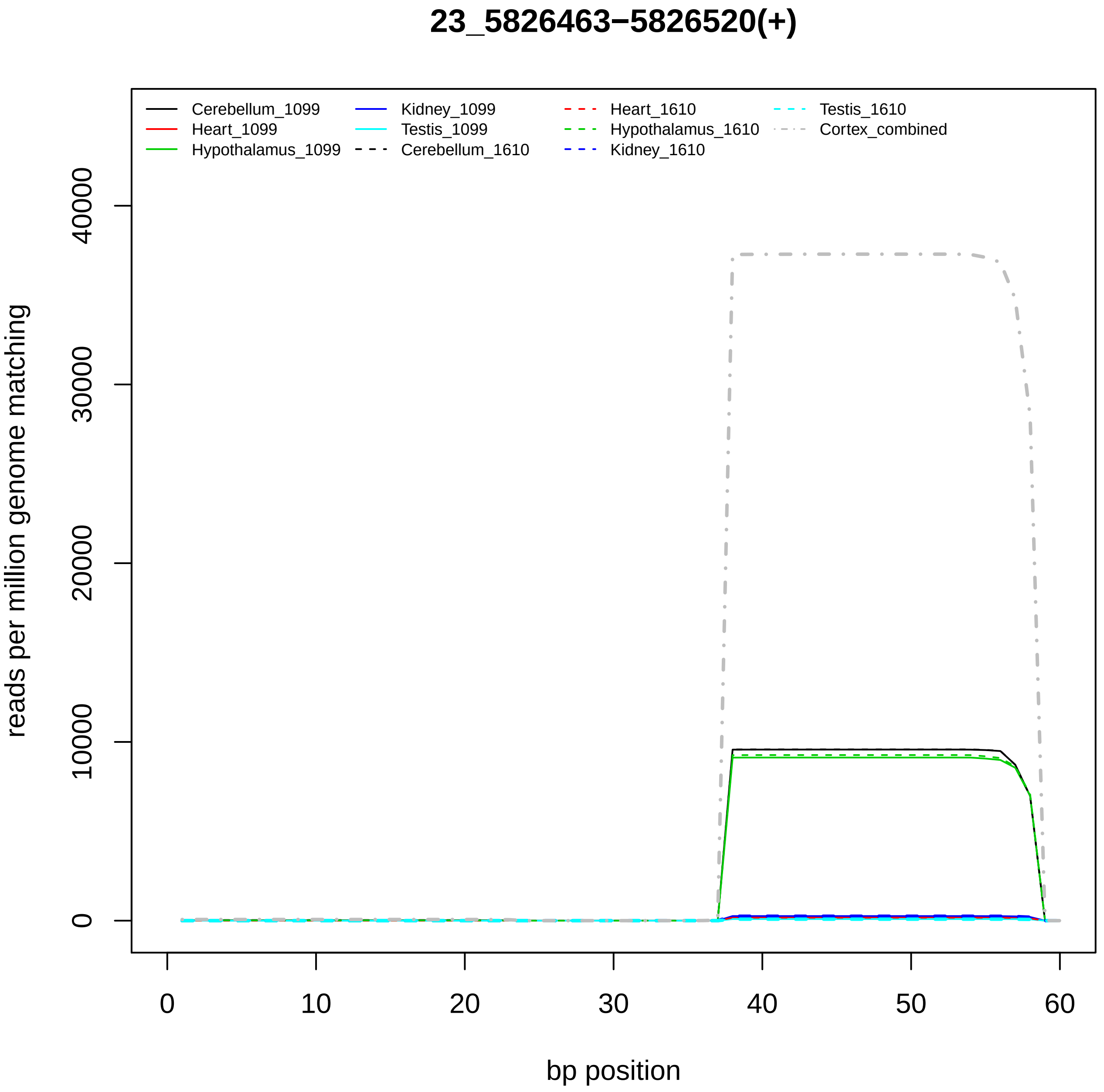
23_5826463−5826520(+)
Cerebellum_1099
Heart_1099
Hypothalamus_1099
Kidney_1099
Testis_1099
Cerebellum_1610
Heart_1610
Hypothalamus_1610
Kidney_1610
Testis_1610
Cortex_combined
reads per million genome matching
0
10000
20000
30000
40000
0
10
20
30
40
50
60
bp position

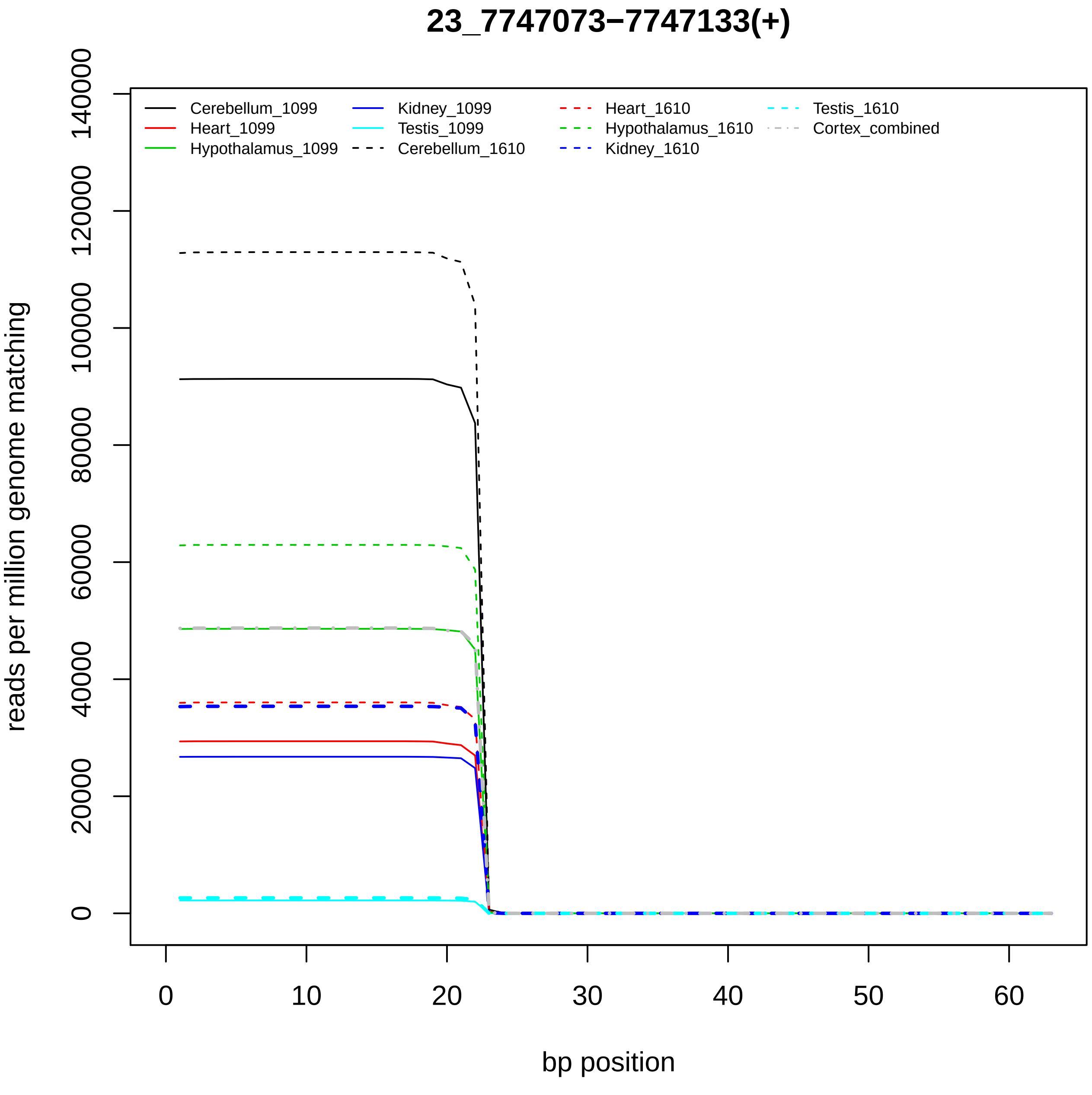

23_7747073−7747133(+)
Cerebellum_1099
Heart_1099
Hypothalamus_1099
Kidney_1099
Testis_1099
Cerebellum_1610
Heart_1610
Hypothalamus_1610
Kidney_1610
Testis_1610
Cortex_combined
reads per million genome matching
bp position
0
20000
40000
60000
80000
100000
120000
140000
0
10
20
30
40
50
60

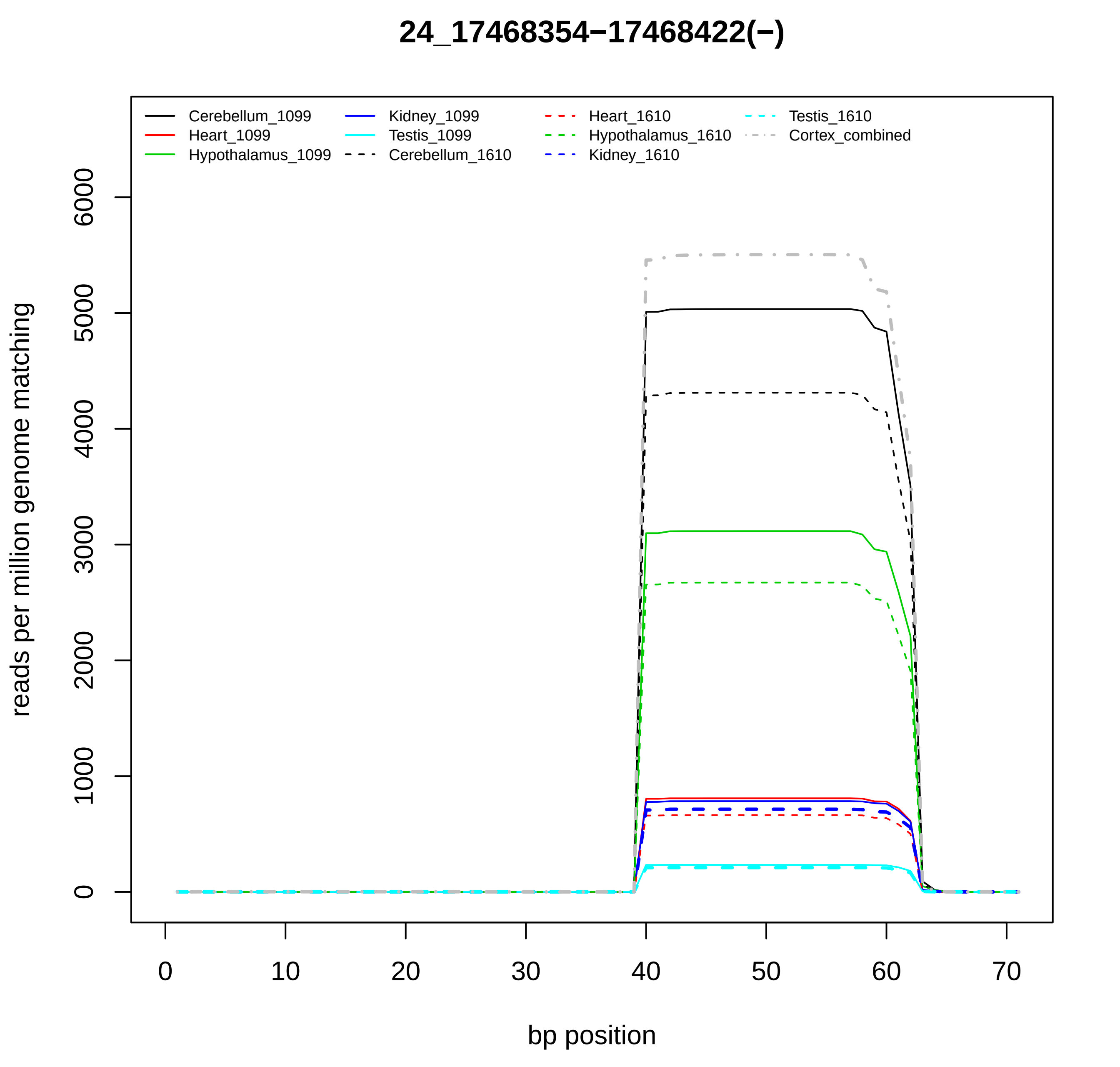

24_17468354−17468422(−)
Cerebellum_1099
Heart_1099
Hypothalamus_1099
Kidney_1099
Testis_1099
Cerebellum_1610
Heart_1610
Hypothalamus_1610
Kidney_1610
Testis_1610
Cortex_combined
reads per million genome matching
0
1000
2000
3000
4000
5000
6000
bp position
0
10
20
30
40
50
60
70

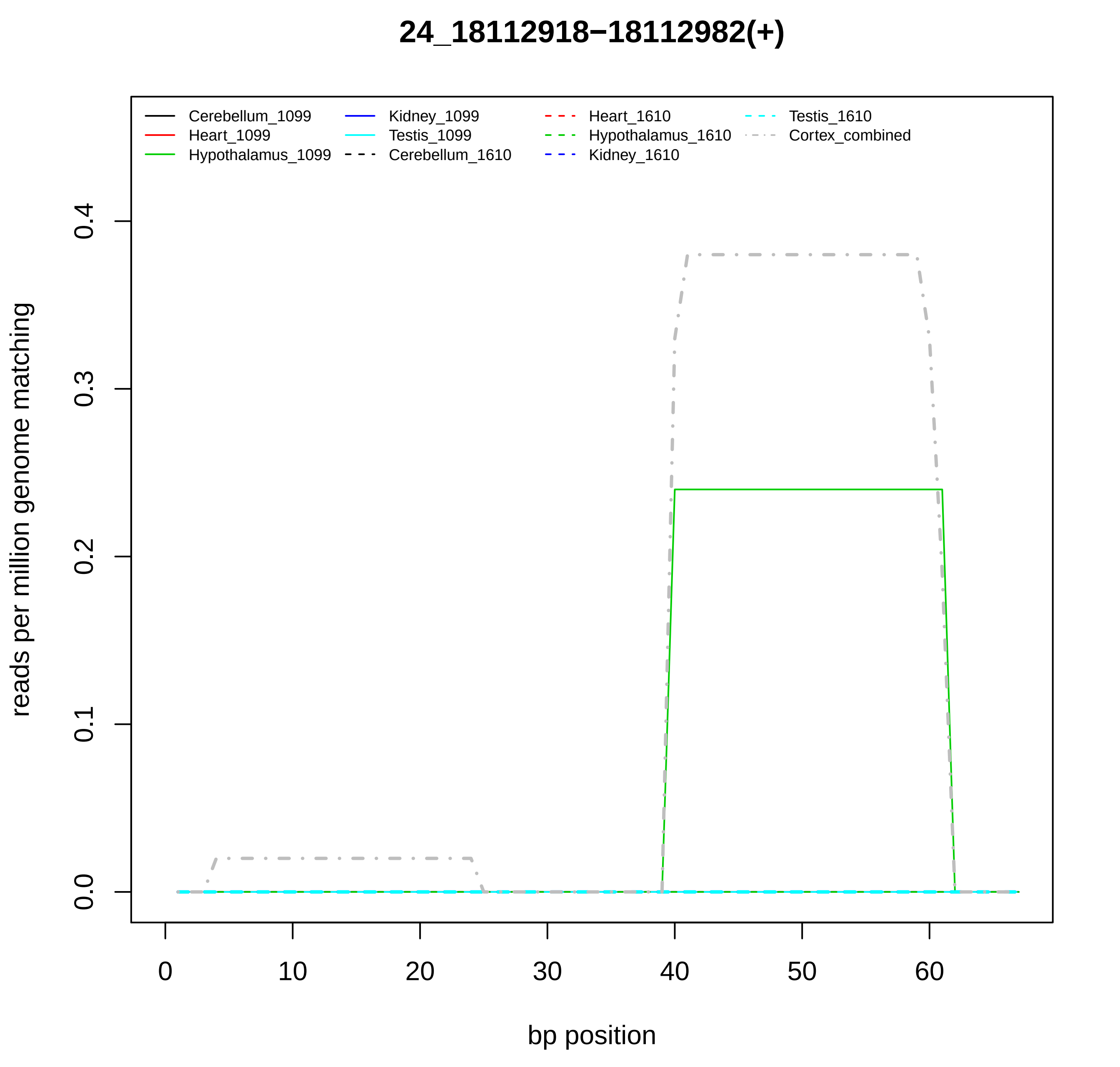

24_18112918−18112982(+)
Cerebellum_1099
Heart_1099
Hypothalamus_1099
Kidney_1099
Testis_1099
Cerebellum_1610
Heart_1610
Hypothalamus_1610
Kidney_1610
Testis_1610
Cortex_combined
reads per million genome matching
0.0
0.1
0.2
0.3
0.4
0
10
20
30
40
50
60
bp position

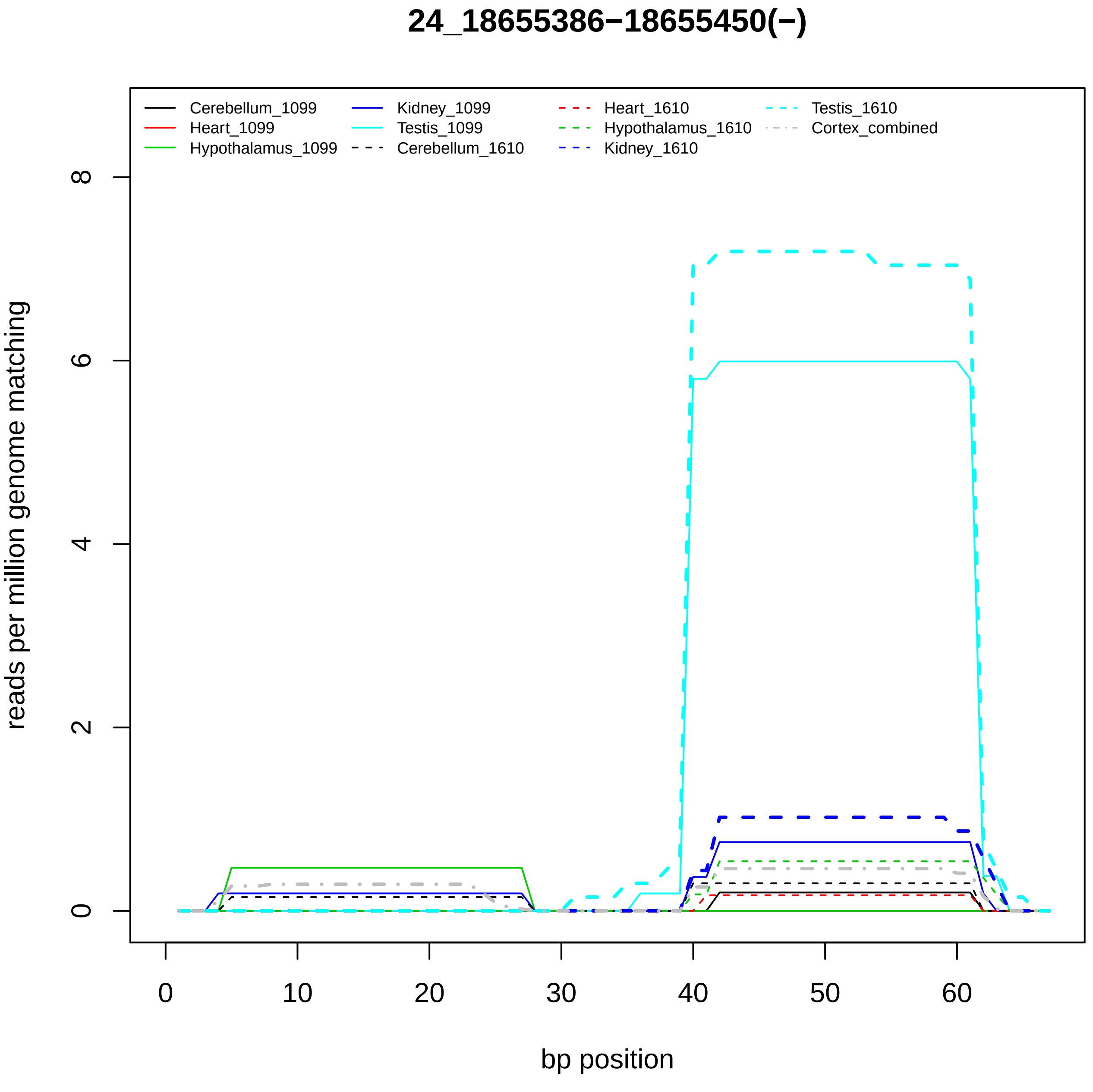

24_18655386−18655450(−)
Cerebellum_1099
Heart_1099
Hypothalamus_1099
Kidney_1099
Testis_1099
Cerebellum_1610
Heart_1610
Hypothalamus_1610
Kidney_1610
Testis_1610
Cortex_combined
reads per million genome matching
0
2
4
6
8
0
10
20
30
40
50
60
bp position

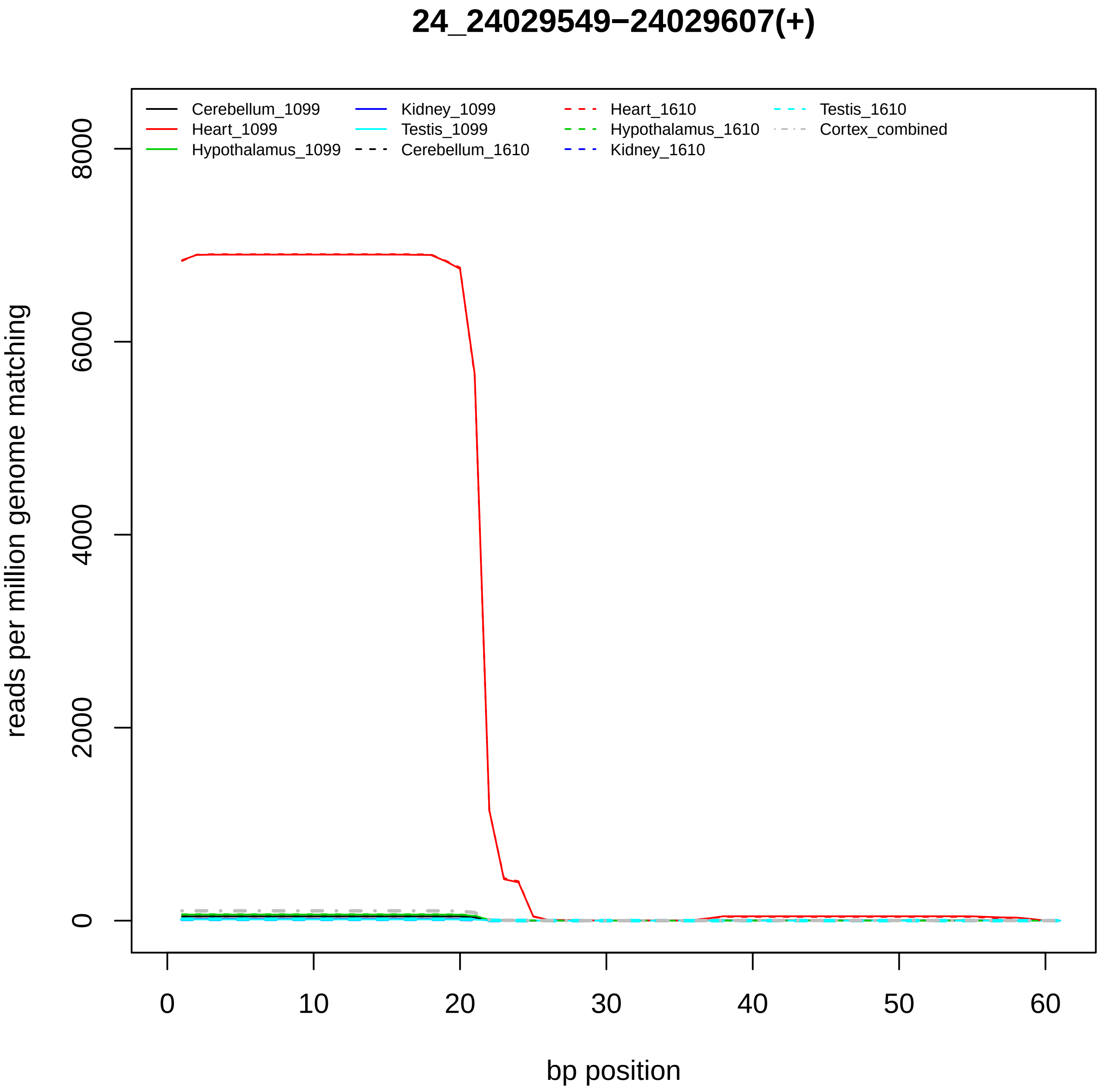
24_24029549−24029607(+)
Cerebellum_1099
Heart_1099
Hypothalamus_1099
Kidney_1099
Testis_1099
Cerebellum_1610
Heart_1610
Hypothalamus_1610
Kidney_1610
Testis_1610
Cortex_combined
reads per million genome matching
0
2000
4000
6000
8000
bp position
0
10
20
30
40
50
60

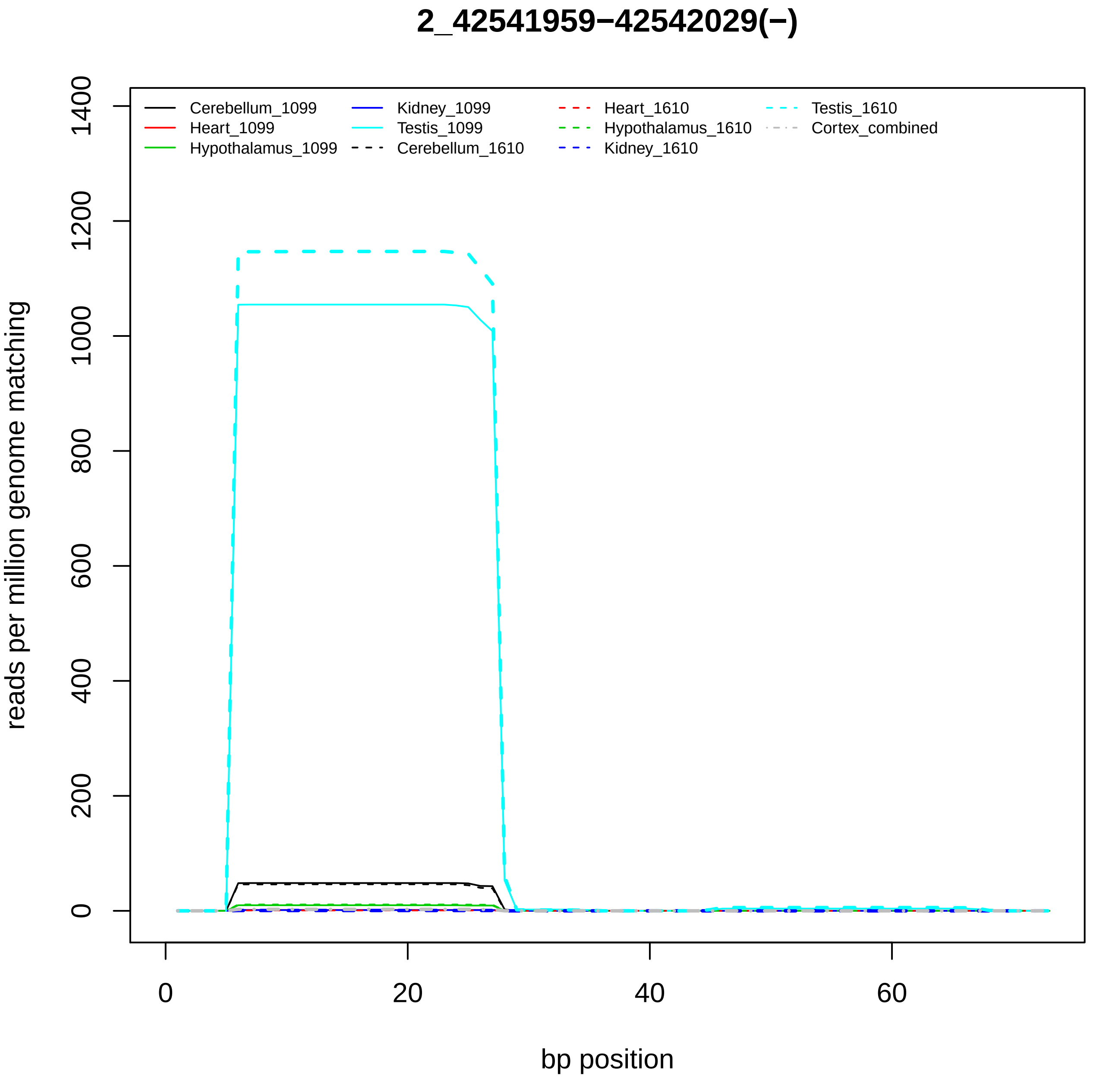

2_42541959−42542029(−)
Cerebellum_1099
Heart_1099
Hypothalamus_1099
Kidney_1099
Testis_1099
Cerebellum_1610
Heart_1610
Hypothalamus_1610
Kidney_1610
Testis_1610
Cortex_combined
reads per million genome matching
0
200
400
600
800
1000
1200
1400
0
20
40
60
bp position

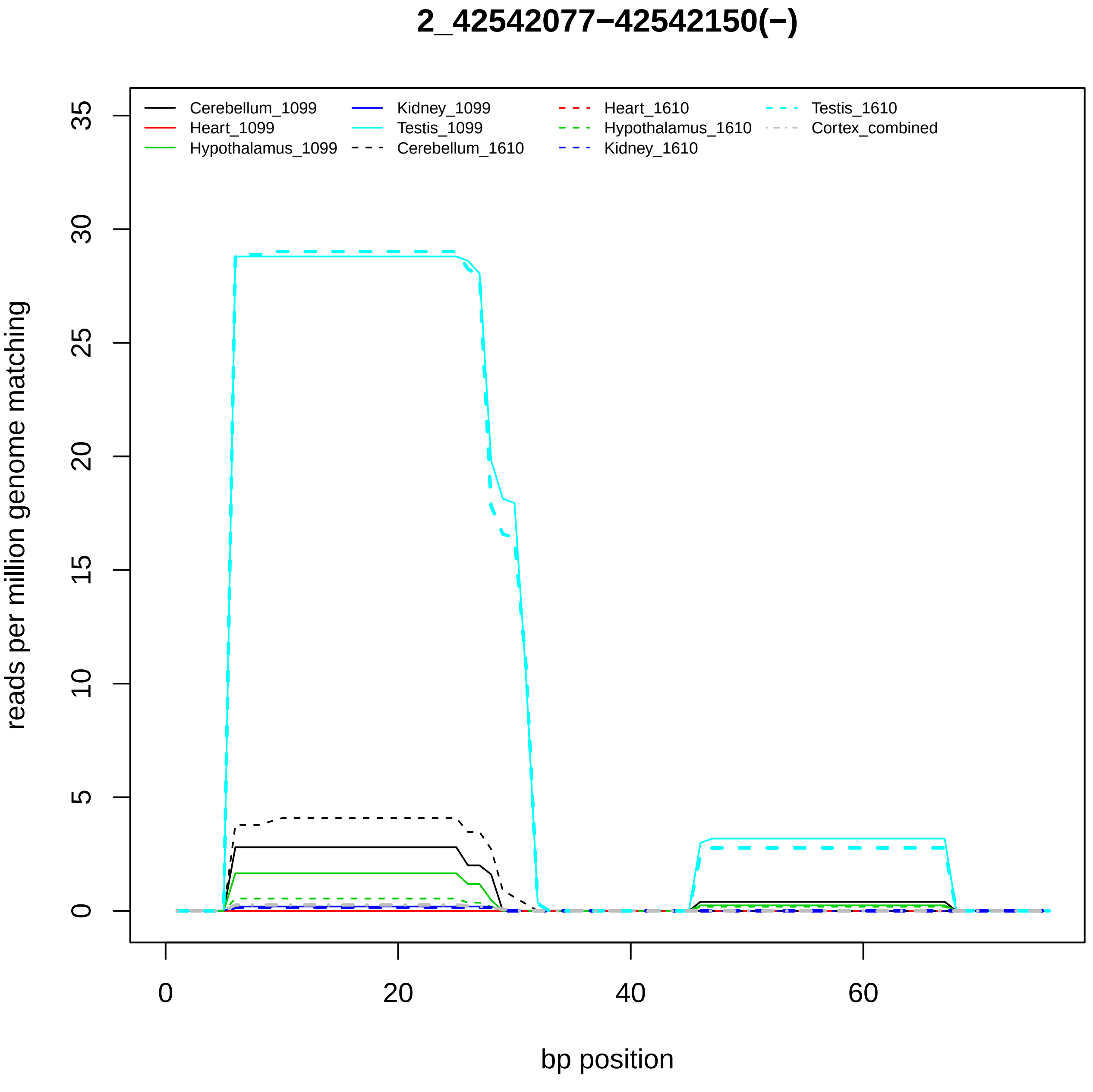

2_42542077−42542150(−)
Cerebellum_1099
Heart_1099
Hypothalamus_1099
Kidney_1099
Testis_1099
Cerebellum_1610
Heart_1610
Hypothalamus_1610
Kidney_1610
Testis_1610
Cortex_combined
reads per million genome matching
bp position
0
5
10
15
20
25
30
35
0
20
40
60

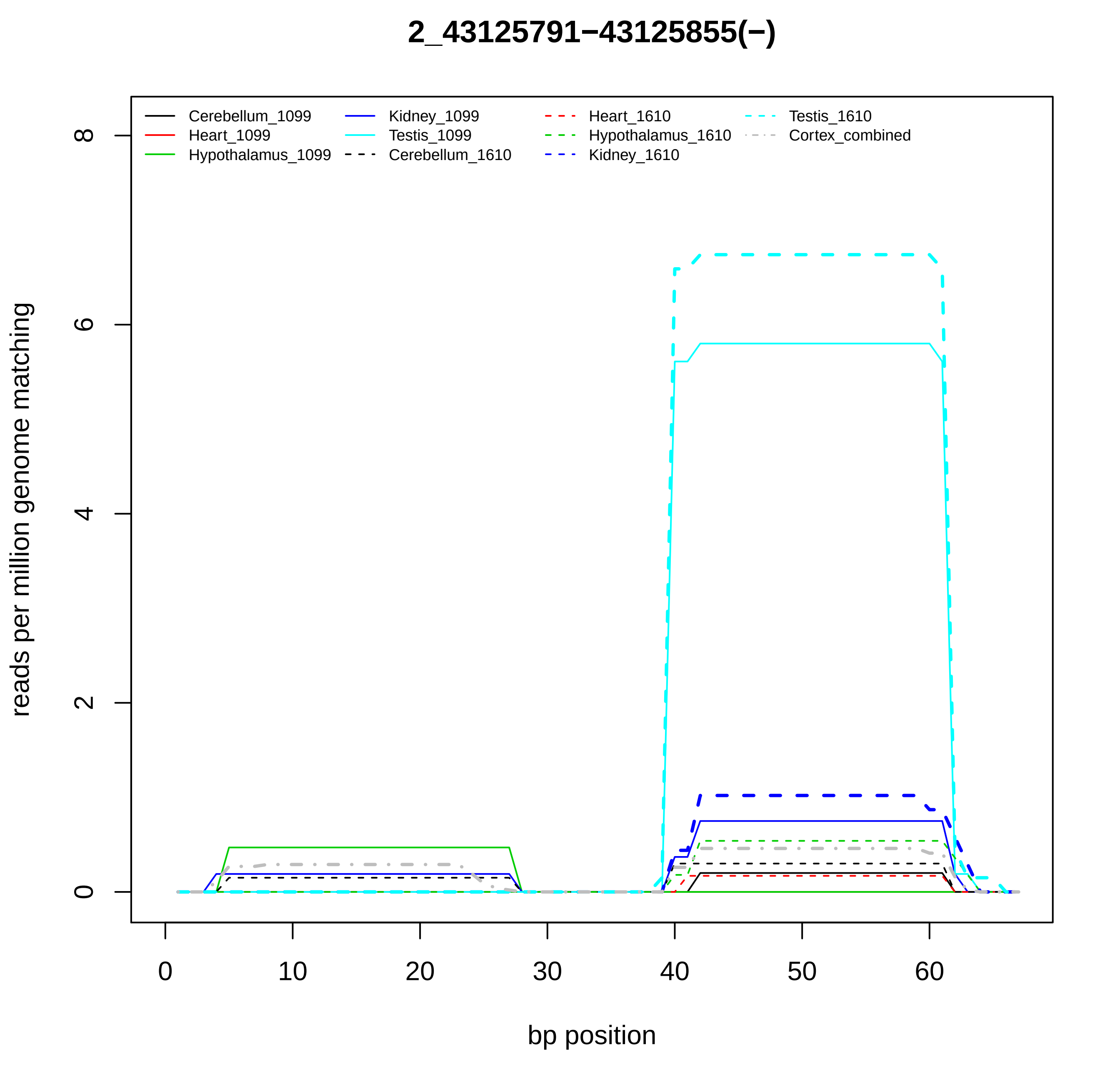

2_43125791−43125855(−)
Cerebellum_1099
Heart_1099
Hypothalamus_1099
Kidney_1099
Testis_1099
Cerebellum_1610
Heart_1610
Hypothalamus_1610
Kidney_1610
Testis_1610
Cortex_combined
reads per million genome matching
bp position
0
2
4
6
8
0
10
20
30
40
50
60

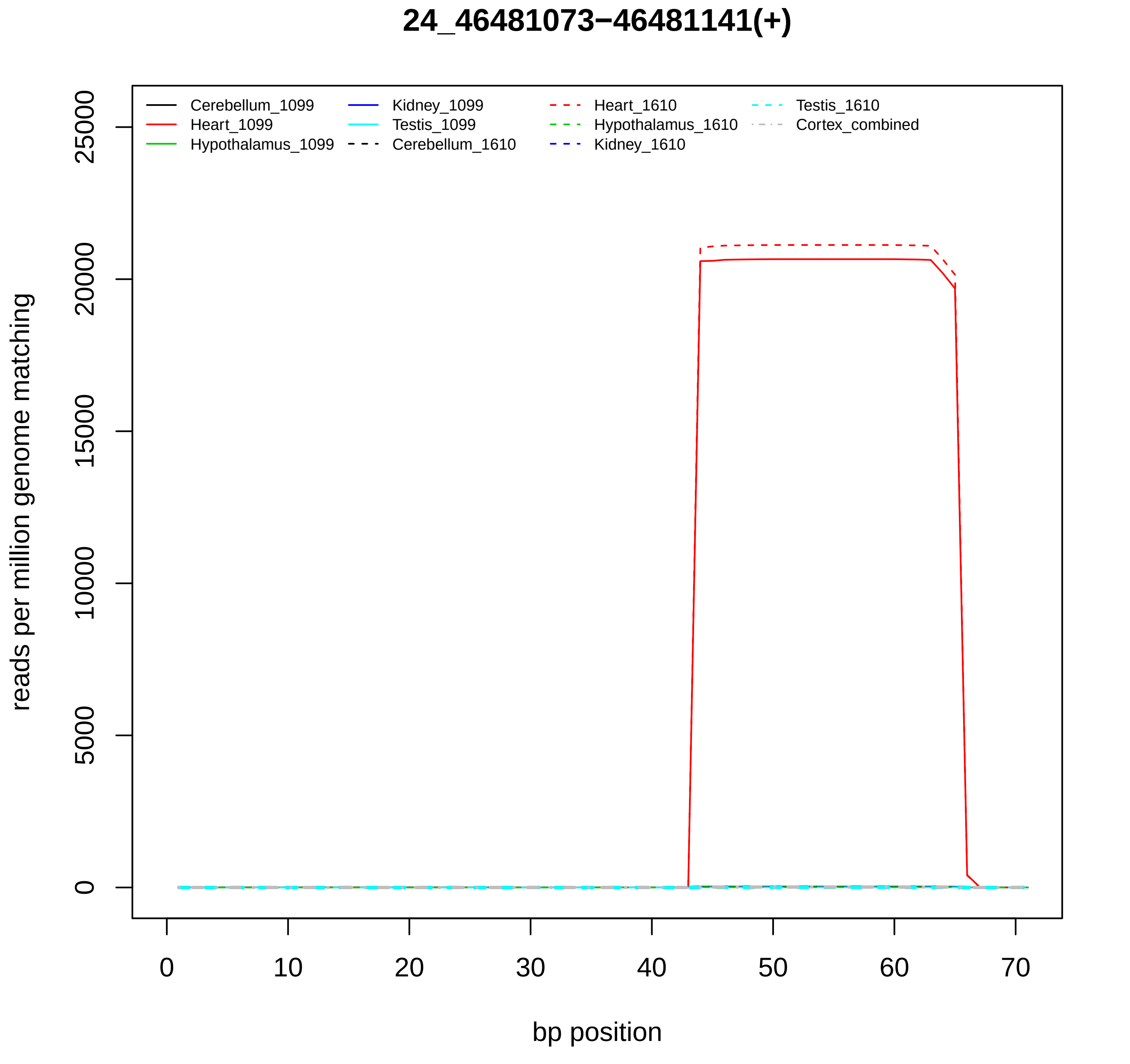

24_46481073−46481141(+)
Cerebellum_1099
Heart_1099
Hypothalamus_1099
Kidney_1099
Testis_1099
Cerebellum_1610
Heart_1610
Hypothalamus_1610
Kidney_1610
Testis_1610
Cortex_combined
reads per million genome matching
0
5000
10000
15000
20000
25000
0
10
20
30
40
50
60
70
bp position

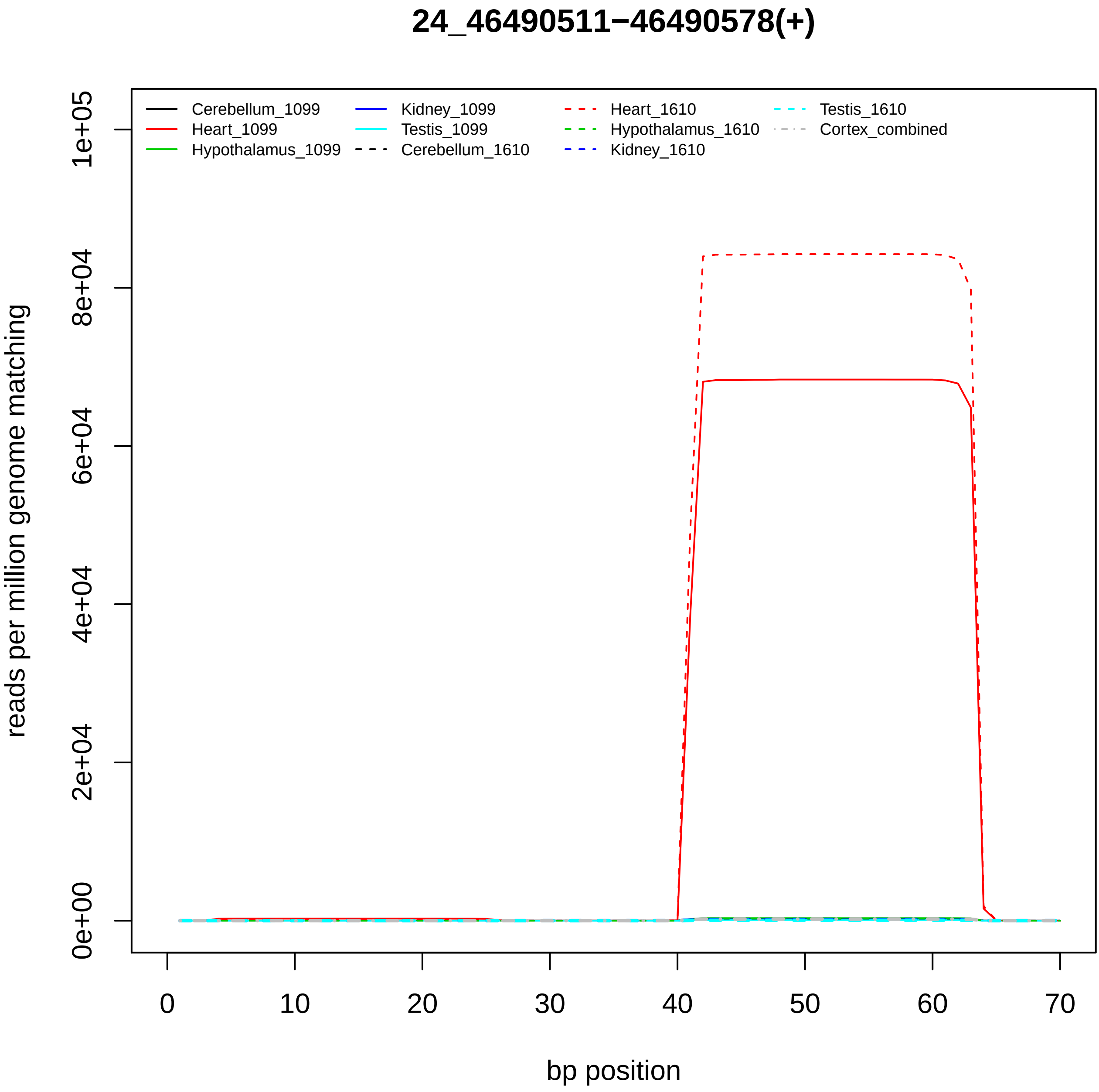

24_46490511−46490578(+)
Cerebellum_1099
Heart_1099
Hypothalamus_1099
Kidney_1099
Testis_1099
Cerebellum_1610
Heart_1610
Hypothalamus_1610
Kidney_1610
Testis_1610
Cortex_combined
reads per million genome matching
0e+00
2e+04
4e+04
6e+04
8e+04
1e+05
0
10
20
30
40
50
60
70
bp position

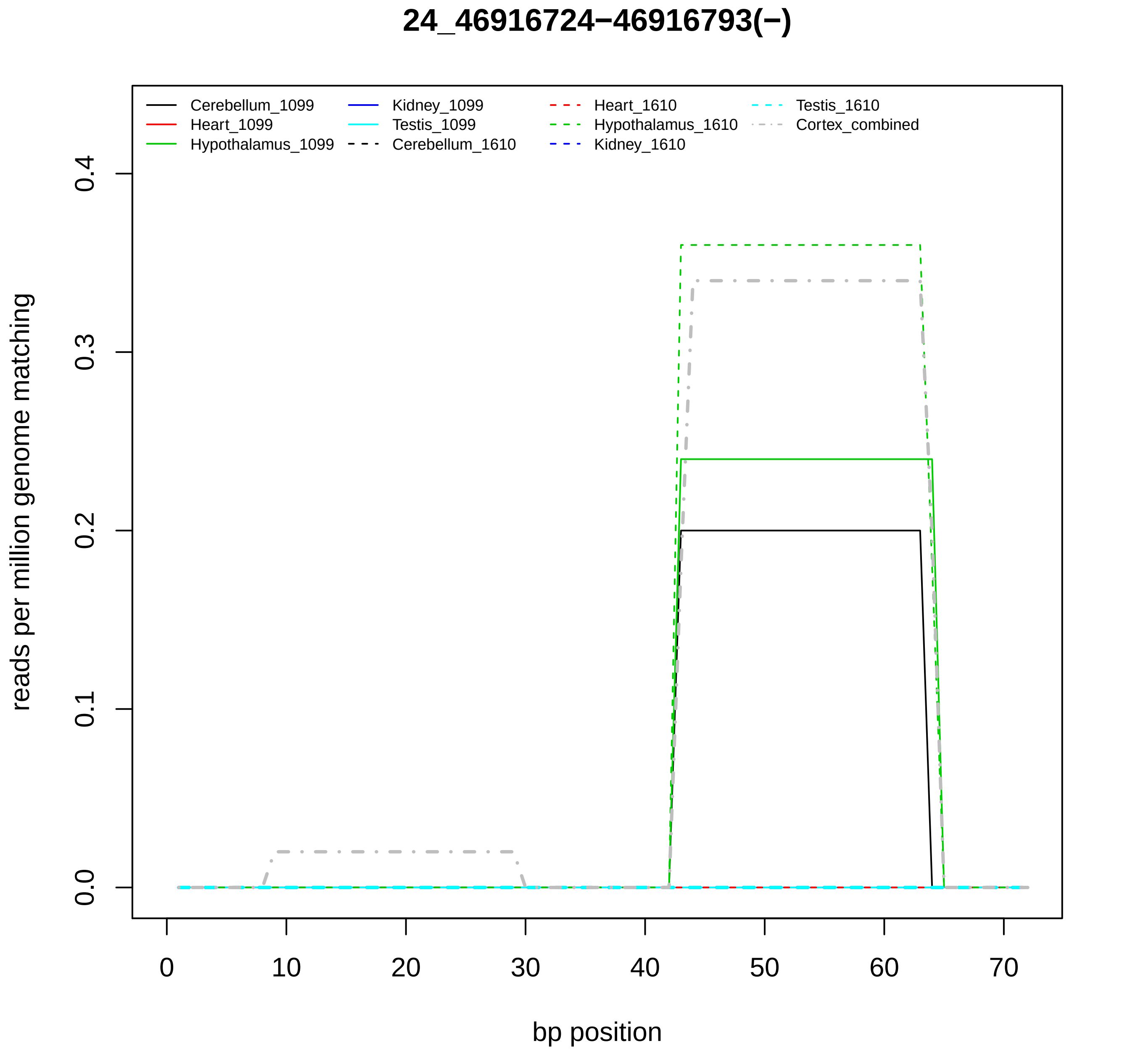
24_46916724−46916793(−)
Cerebellum_1099
Heart_1099
Hypothalamus_1099
Kidney_1099
Testis_1099
Cerebellum_1610
Heart_1610
Hypothalamus_1610
Kidney_1610
Testis_1610
Cortex_combined
reads per million genome matching
0.0
0.1
0.2
0.3
0.4
0
10
20
30
40
50
60
70
bp position

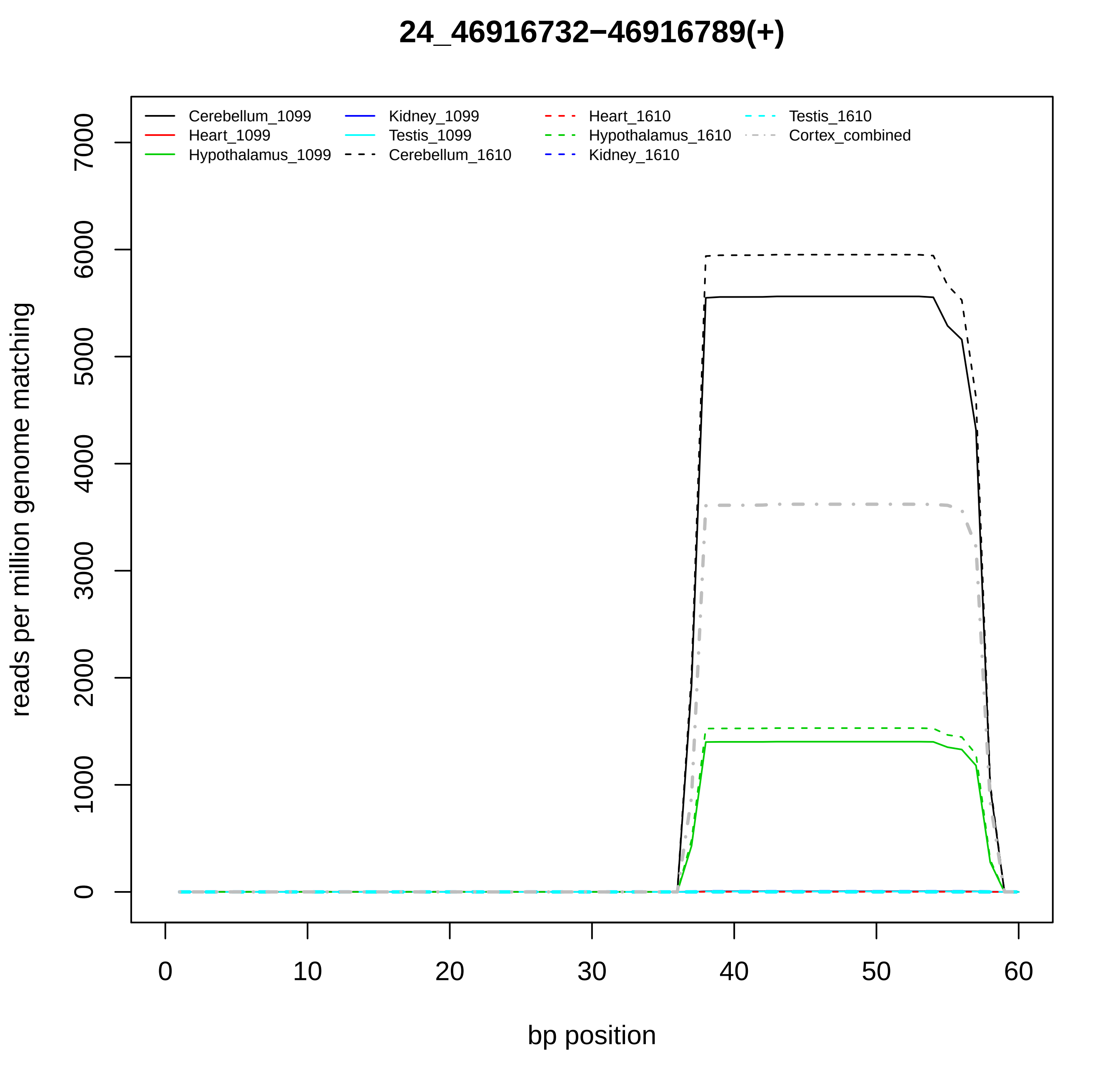

24_46916732−46916789(+)
Cerebellum_1099
Heart_1099
Hypothalamus_1099
Kidney_1099
Testis_1099
Cerebellum_1610
Heart_1610
Hypothalamus_1610
Kidney_1610
Testis_1610
Cortex_combined
reads per million genome matching
bp position
0
1000
2000
3000
4000
5000
6000
7000
0
10
20
30
40
50
60

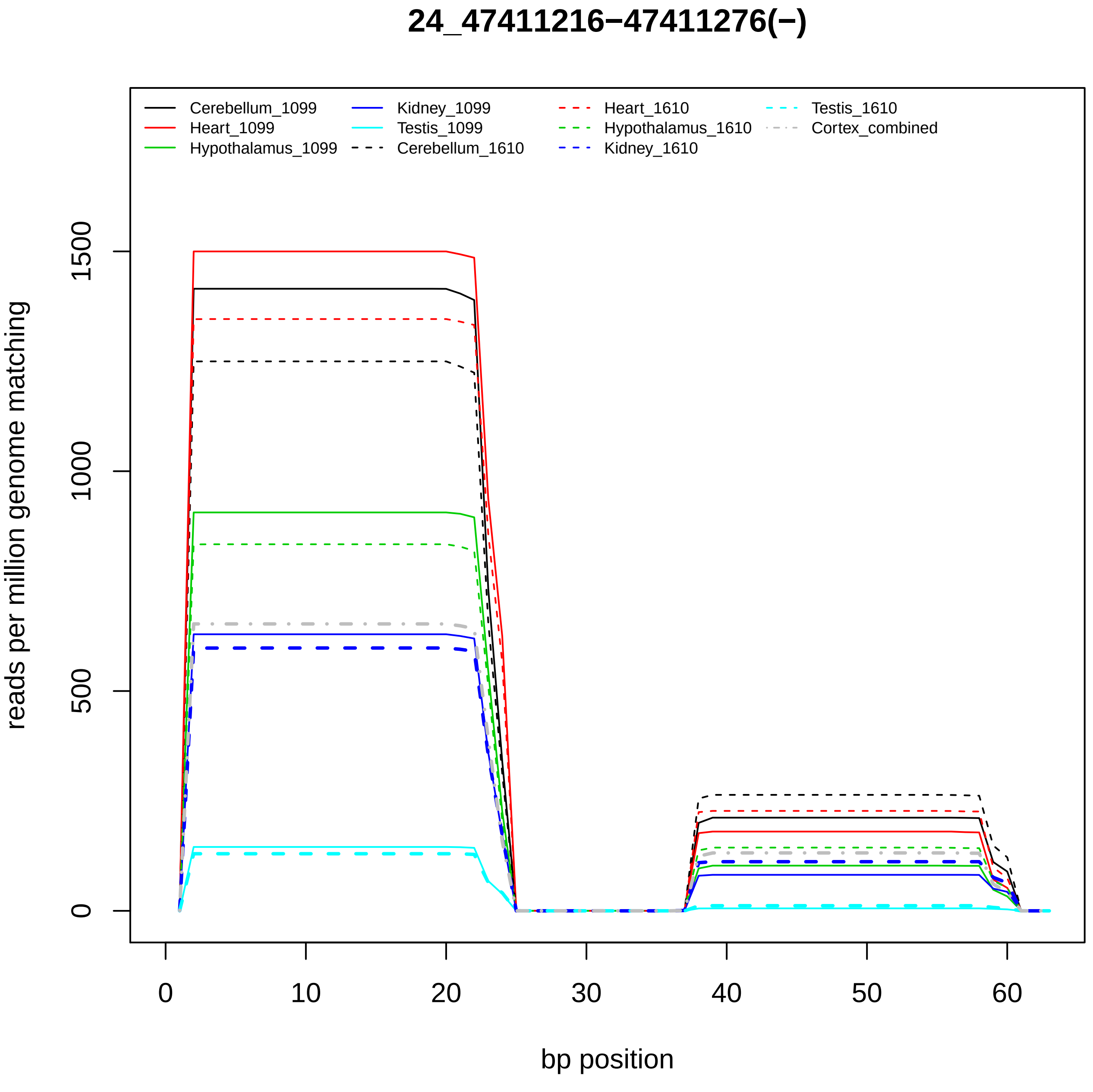
24_47411216−47411276(−)
Cerebellum_1099
Heart_1099
Hypothalamus_1099
Kidney_1099
Testis_1099
Cerebellum_1610
Heart_1610
Hypothalamus_1610
Kidney_1610
Testis_1610
Cortex_combined
reads per million genome matching
0
500
1000
1500
0
10
20
30
40
50
60
bp position

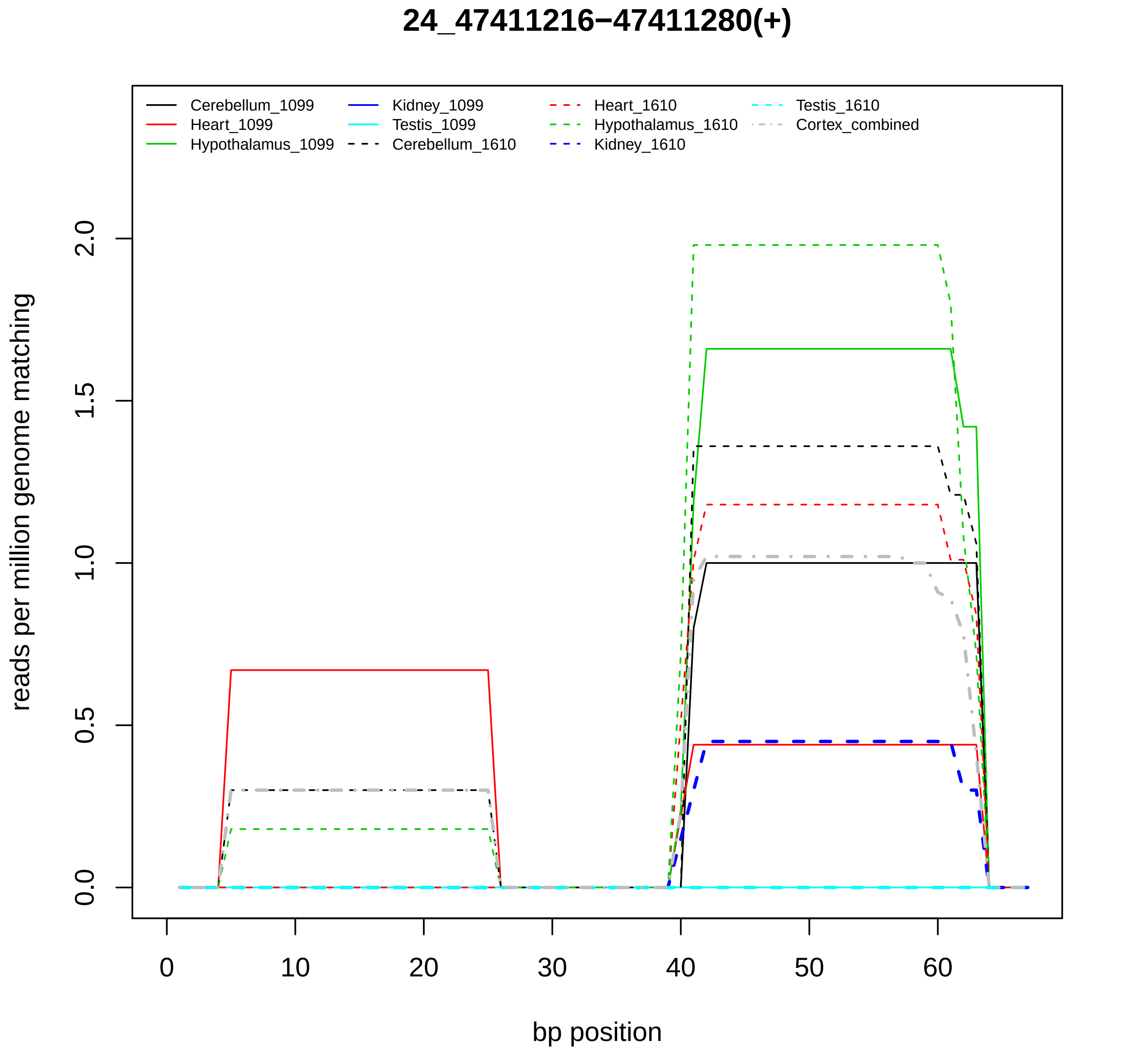

24_47411216−47411280(+)
Cerebellum_1099
Heart_1099
Hypothalamus_1099
Kidney_1099
Testis_1099
Cerebellum_1610
Heart_1610
Hypothalamus_1610
Kidney_1610
Testis_1610
Cortex_combined
reads per million genome matching
0.0
0.5
1.0
1.5
2.0
0
10
20
30
40
50
60
bp position

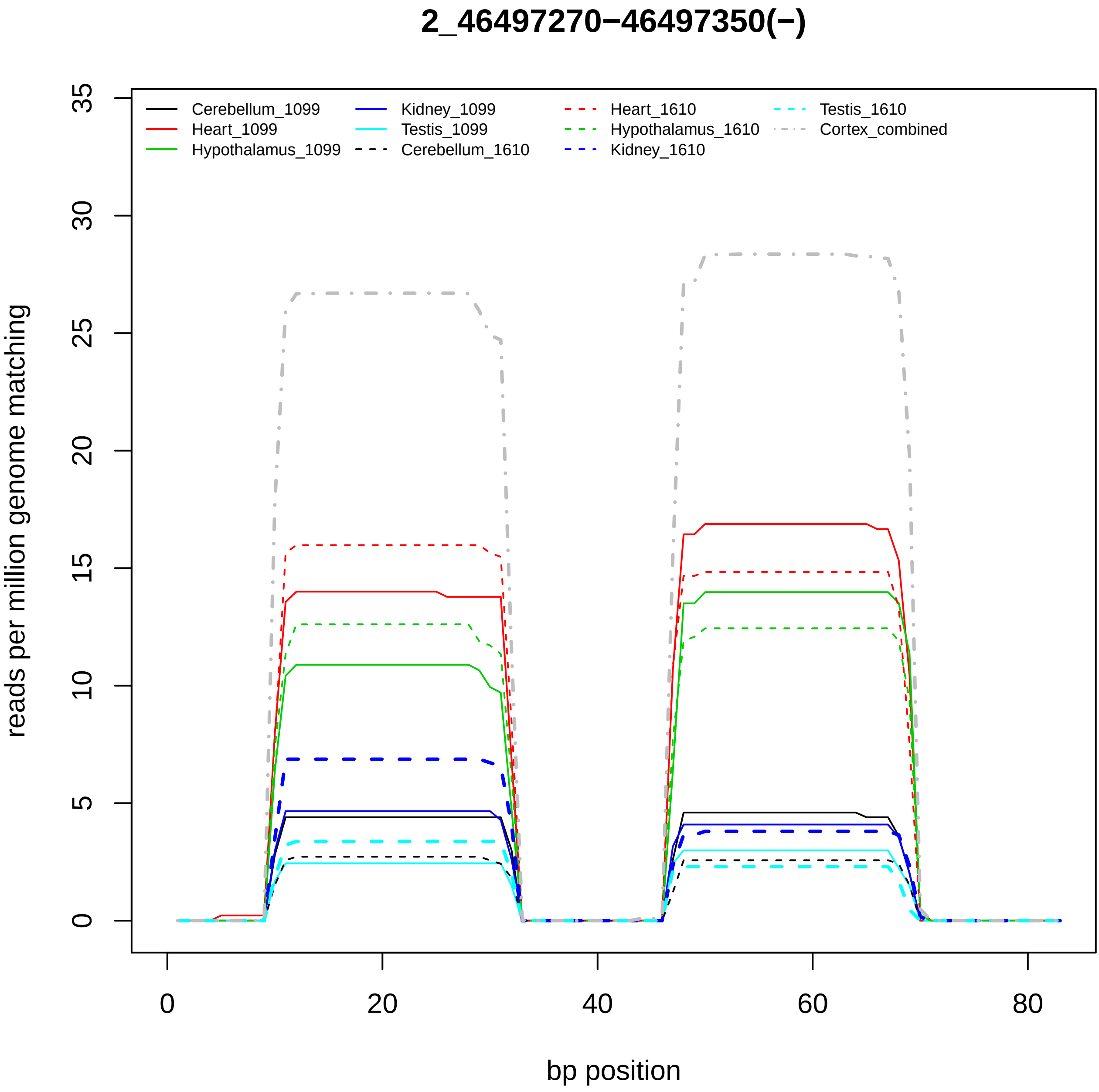
2_46497270−46497350(−)
Cerebellum_1099
Heart_1099
Hypothalamus_1099
Kidney_1099
Testis_1099
Cerebellum_1610
Heart_1610
Hypothalamus_1610
Kidney_1610
Testis_1610
Cortex_combined
reads per million genome matching
bp position
0
5
10
15
20
25
30
35
0
20
40
60
80

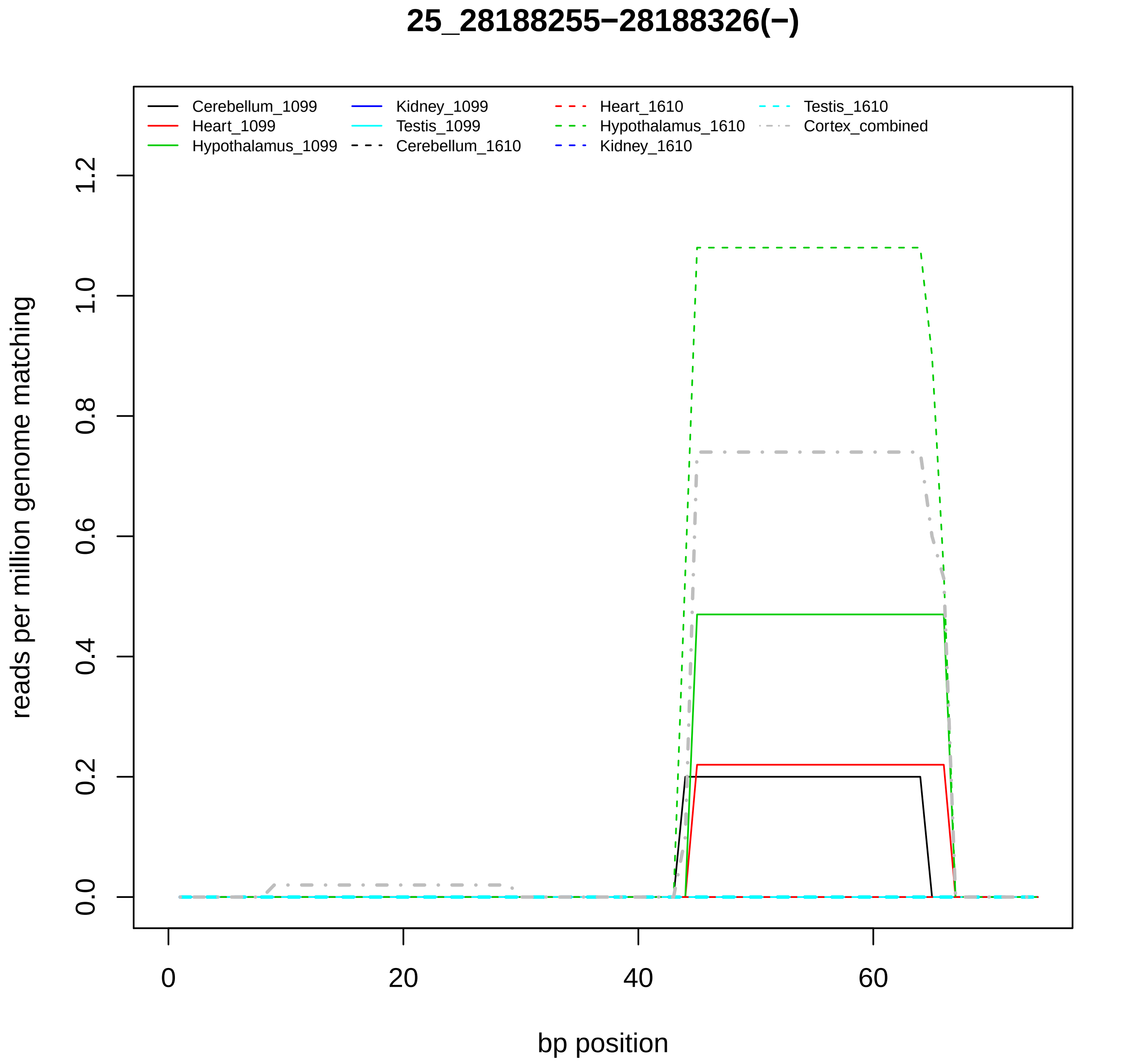

25_28188255−28188326(−)
Cerebellum_1099
Heart_1099
Hypothalamus_1099
Kidney_1099
Testis_1099
Cerebellum_1610
Heart_1610
Hypothalamus_1610
Kidney_1610
Testis_1610
Cortex_combined
reads per million genome matching
0.0
0.2
0.4
0.6
0.8
1.0
1.2
0
20
40
60
bp position

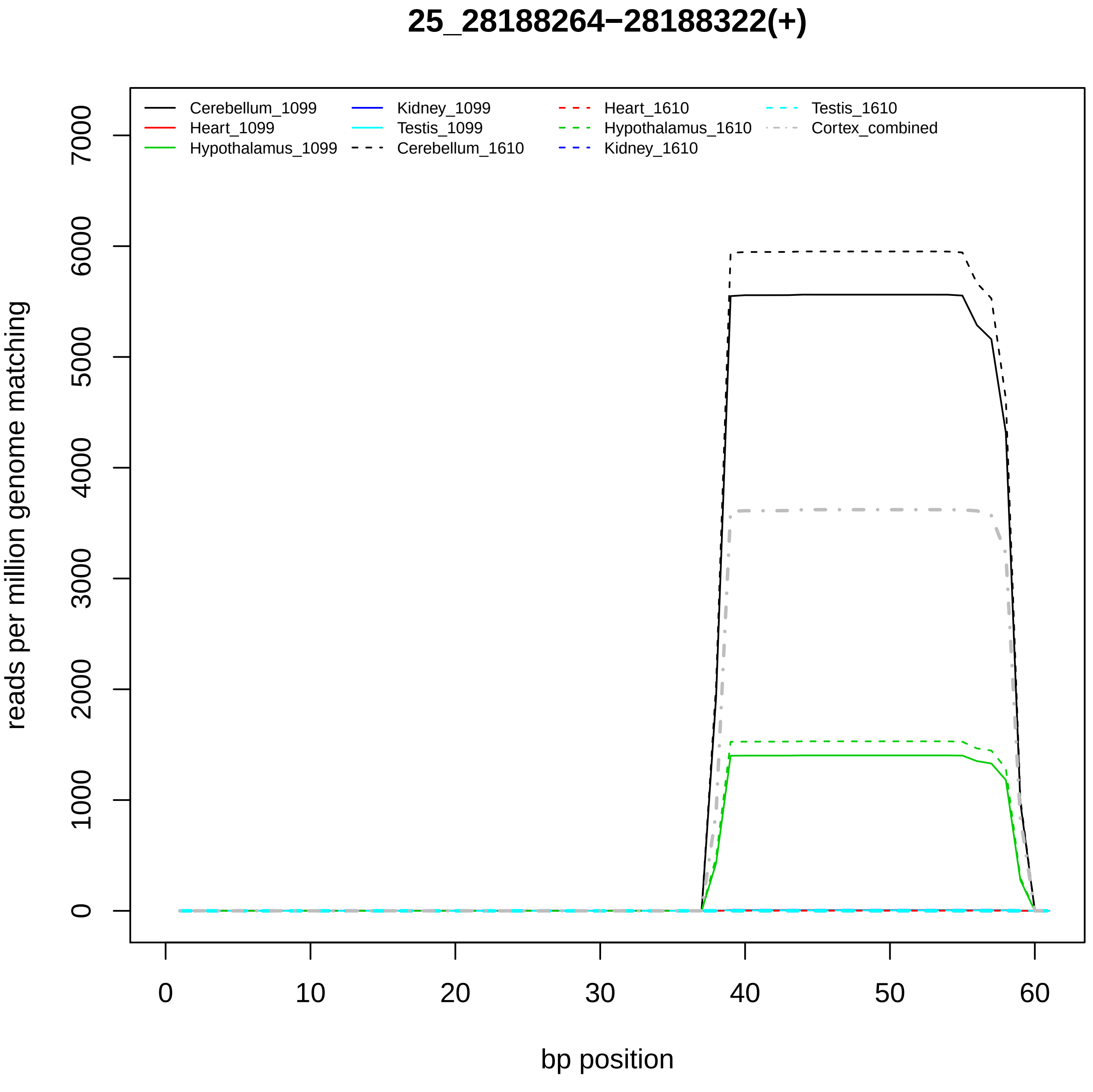

25_28188264−28188322(+)
Cerebellum_1099
Heart_1099
Hypothalamus_1099
Kidney_1099
Testis_1099
Cerebellum_1610
Heart_1610
Hypothalamus_1610
Kidney_1610
Testis_1610
Cortex_combined
reads per million genome matching
bp position
0
1000
2000
3000
4000
5000
6000
7000
0
10
20
30
40
50
60

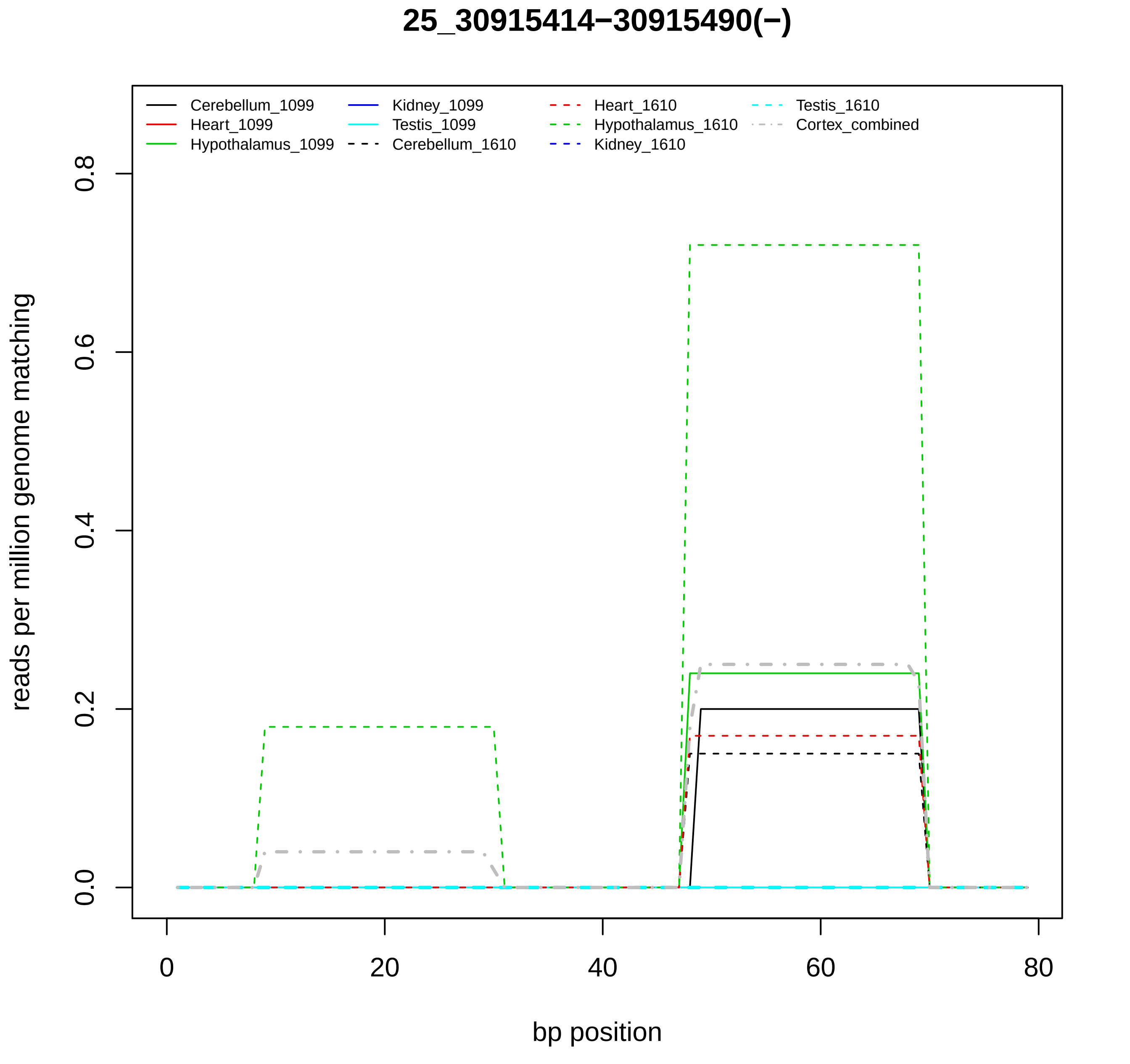

25_30915414−30915490(−)
Cerebellum_1099
Heart_1099
Hypothalamus_1099
Kidney_1099
Testis_1099
Cerebellum_1610
Heart_1610
Hypothalamus_1610
Kidney_1610
Testis_1610
Cortex_combined
reads per million genome matching
0.0
0.2
0.4
0.6
0.8
0
20
40
60
80
bp position

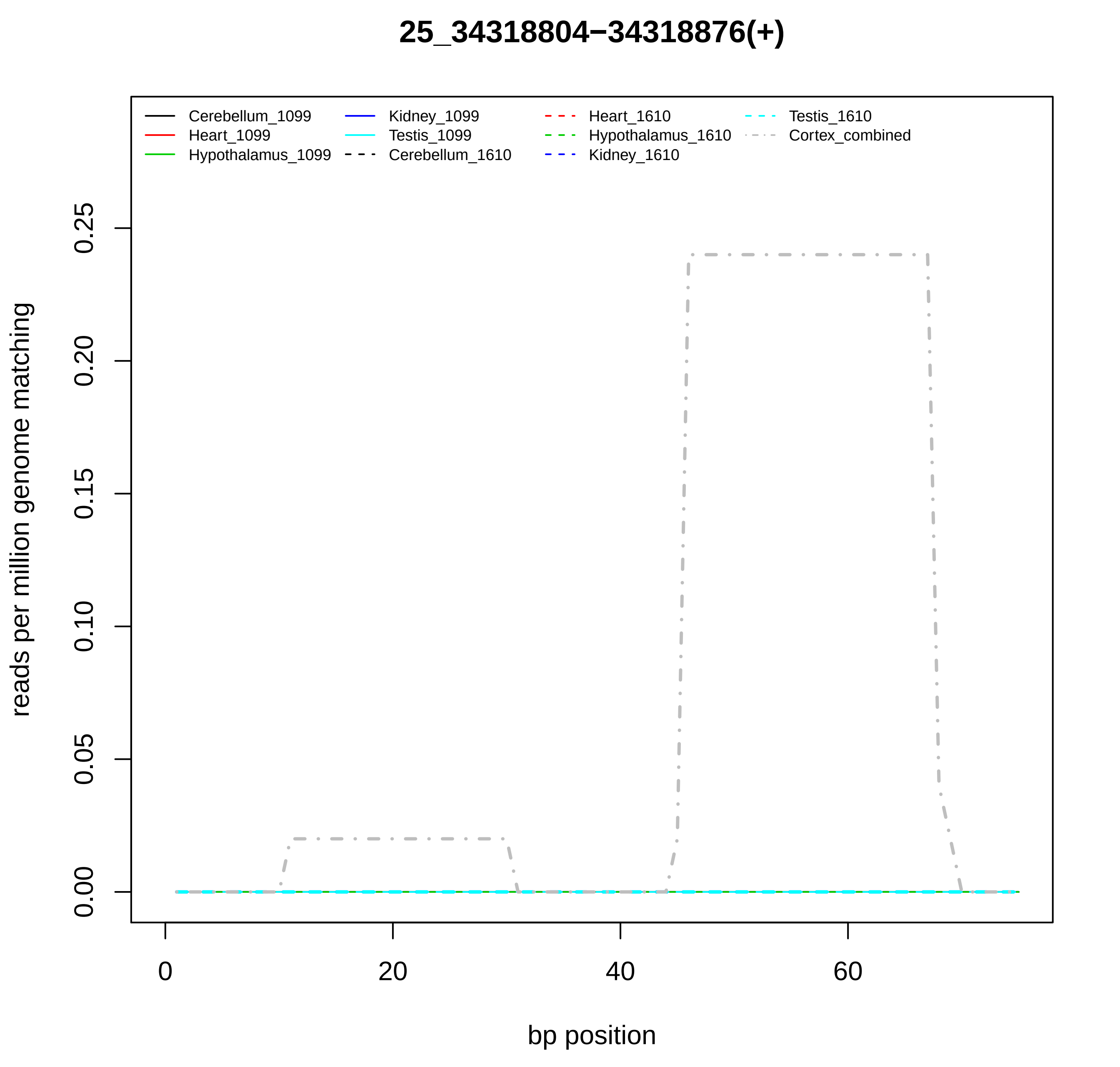

25_34318804−34318876(+)
Cerebellum_1099
Heart_1099
Hypothalamus_1099
Kidney_1099
Testis_1099
Cerebellum_1610
Heart_1610
Hypothalamus_1610
Kidney_1610
Testis_1610
Cortex_combined
reads per million genome matching
0.00
0.05
0.10
0.15
0.20
0.25
0
20
40
60
bp position

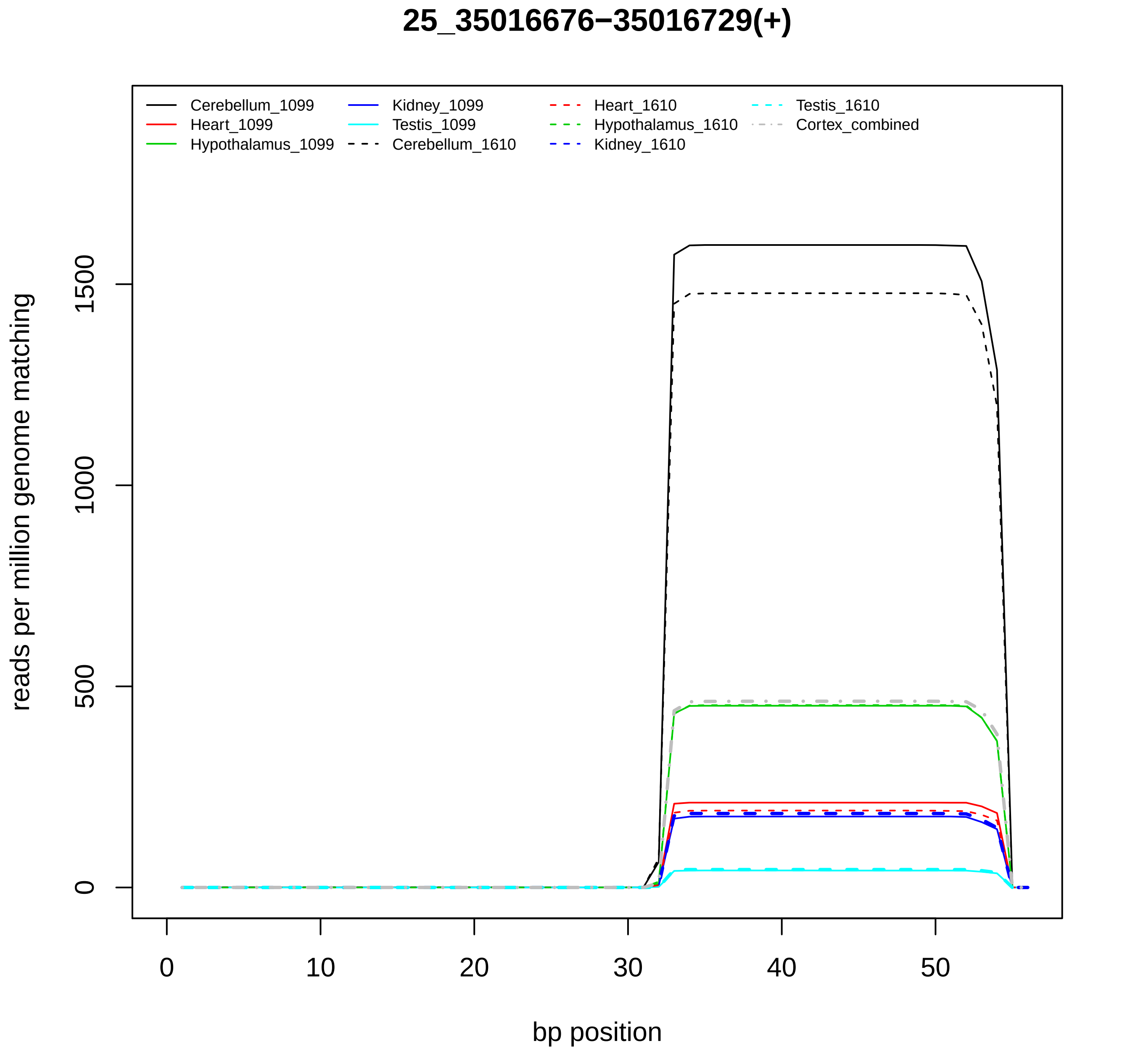

25_35016676−35016729(+)
Cerebellum_1099
Heart_1099
Hypothalamus_1099
Kidney_1099
Testis_1099
Cerebellum_1610
Heart_1610
Hypothalamus_1610
Kidney_1610
Testis_1610
Cortex_combined
reads per million genome matching
0
500
1000
1500
0
10
20
30
40
50
bp position

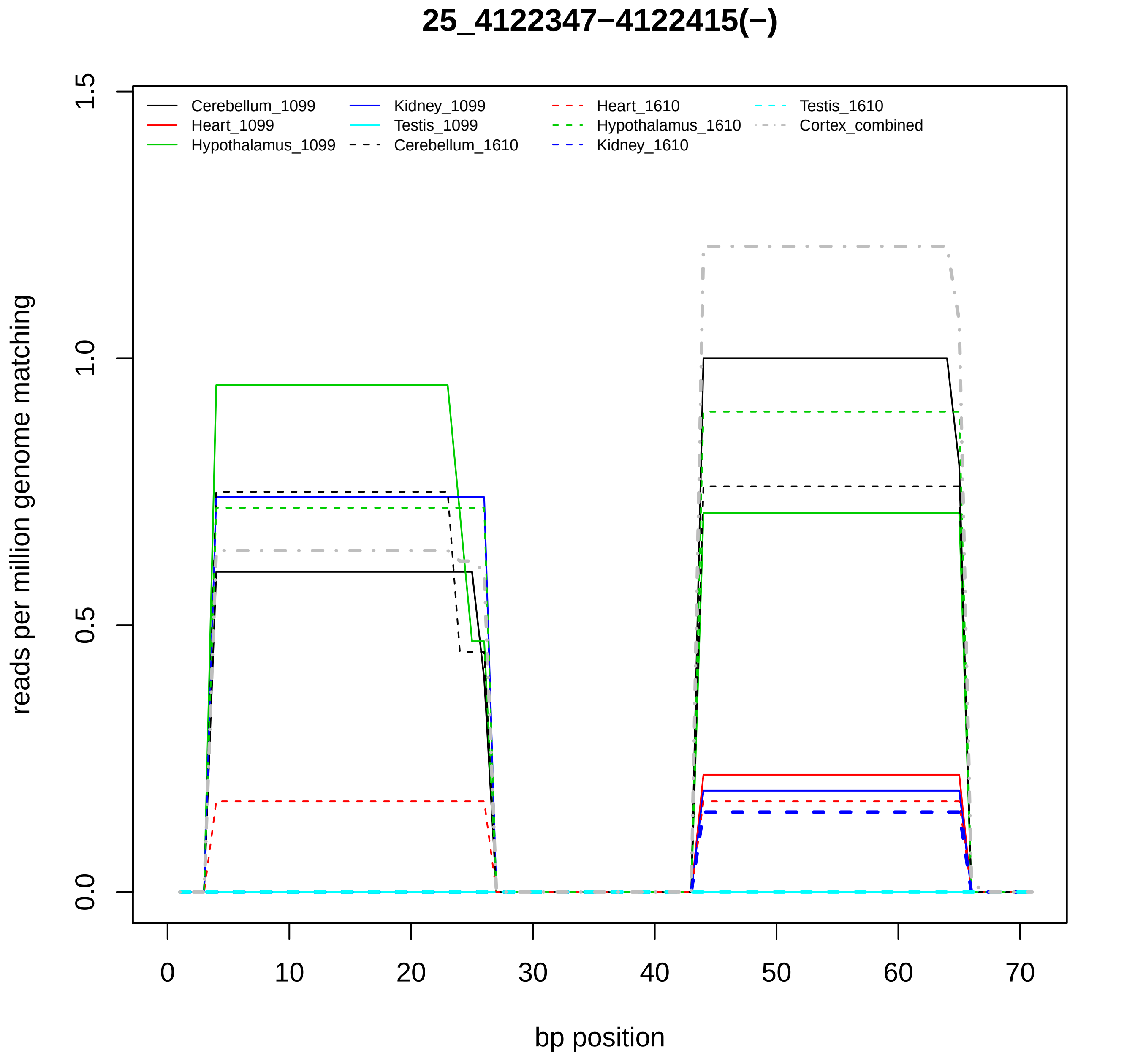

25_4122347−4122415(−)
Cerebellum_1099
Heart_1099
Hypothalamus_1099
Kidney_1099
Testis_1099
Cerebellum_1610
Heart_1610
Hypothalamus_1610
Kidney_1610
Testis_1610
Cortex_combined
reads per million genome matching
bp position
0.0
0.5
1.0
1.5
0
10
20
30
40
50
60
70

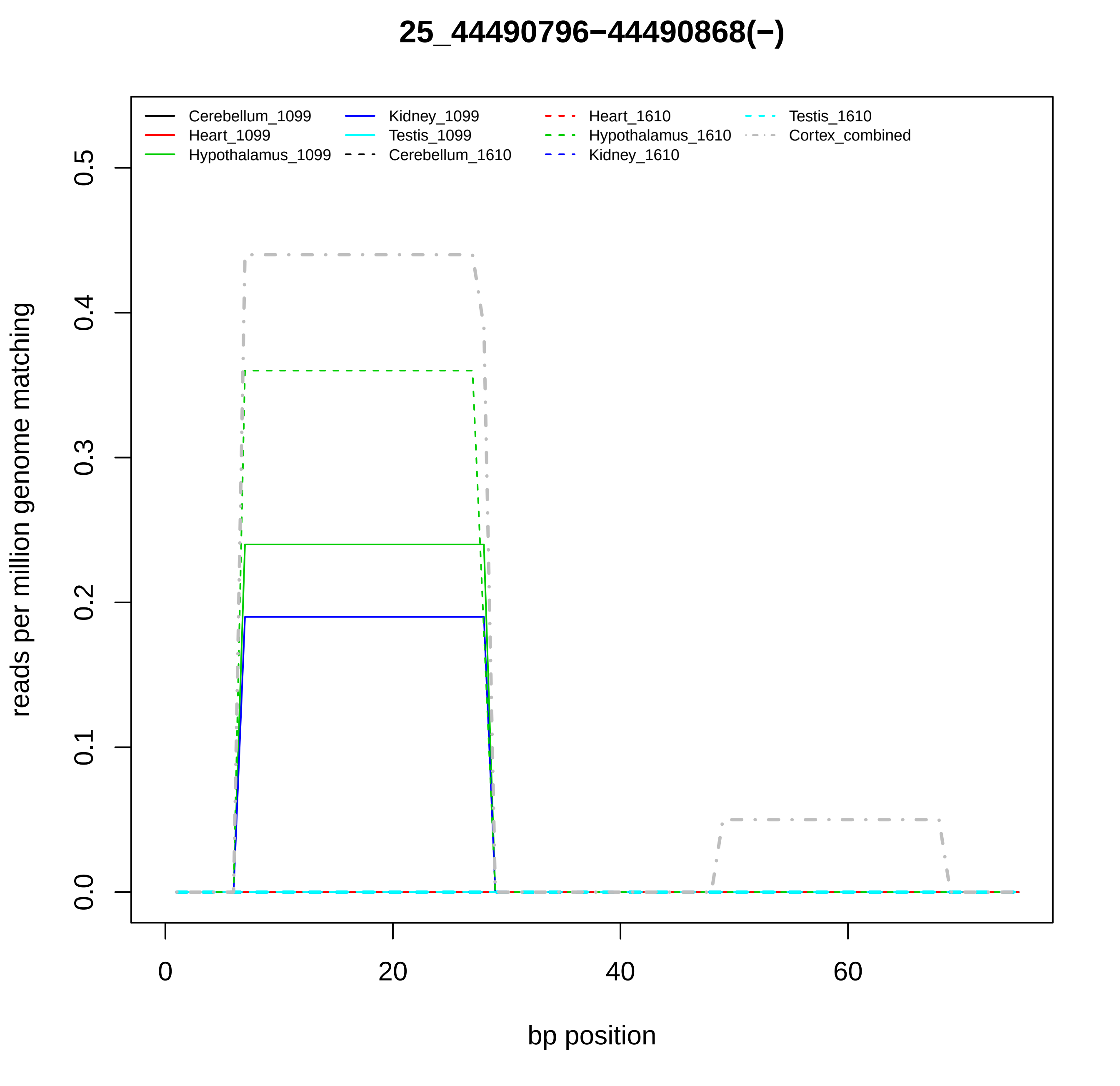

25_44490796−44490868(−)
Cerebellum_1099
Heart_1099
Hypothalamus_1099
Kidney_1099
Testis_1099
Cerebellum_1610
Heart_1610
Hypothalamus_1610
Kidney_1610
Testis_1610
Cortex_combined
reads per million genome matching
bp position
0.0
0.1
0.2
0.3
0.4
0.5
0
20
40
60

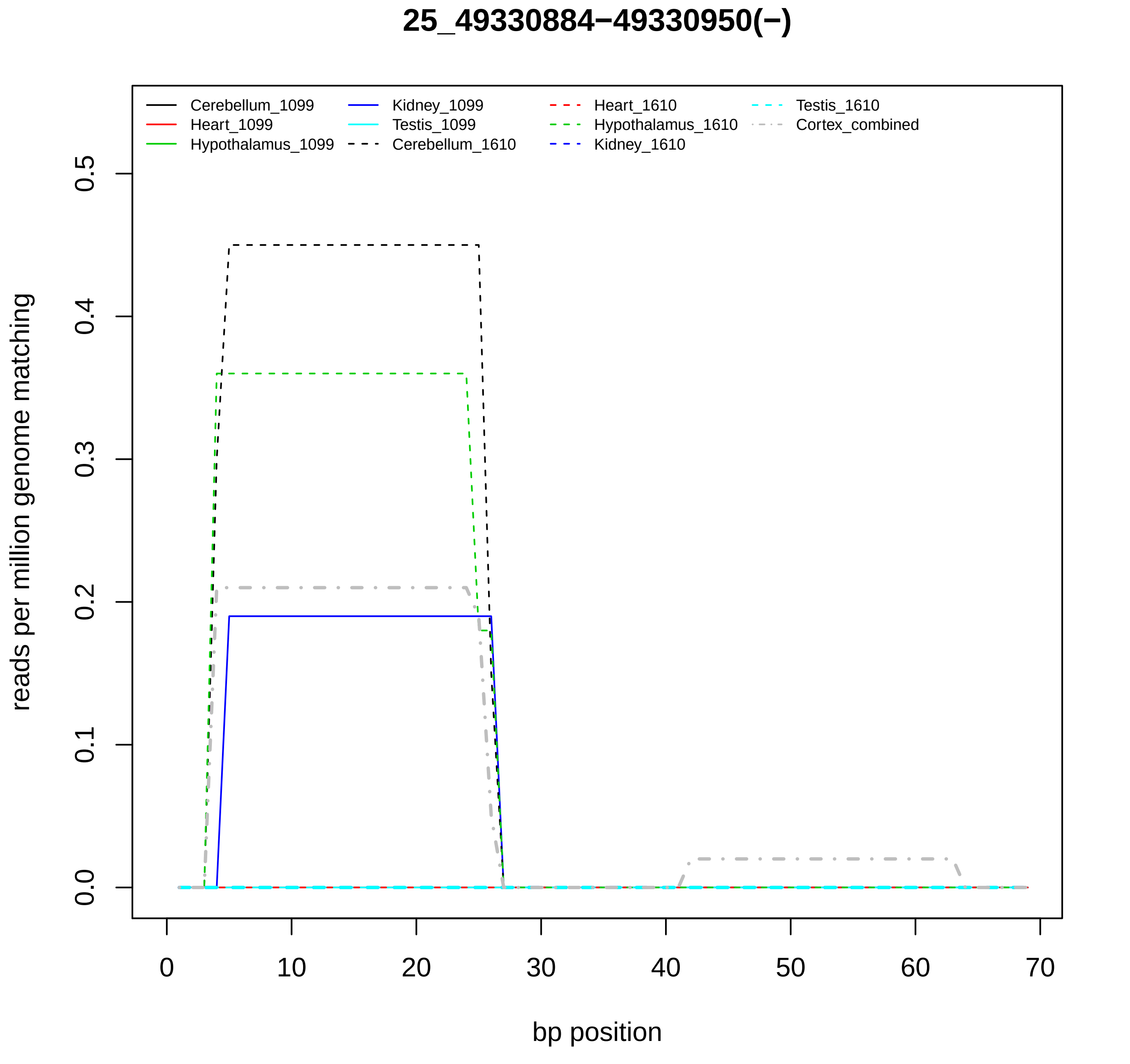

25_49330884−49330950(−)
Cerebellum_1099
Heart_1099
Hypothalamus_1099
Kidney_1099
Testis_1099
Cerebellum_1610
Heart_1610
Hypothalamus_1610
Kidney_1610
Testis_1610
Cortex_combined
reads per million genome matching
0.0
0.1
0.2
0.3
0.4
0.5
0
10
20
30
40
50
60
70
bp position

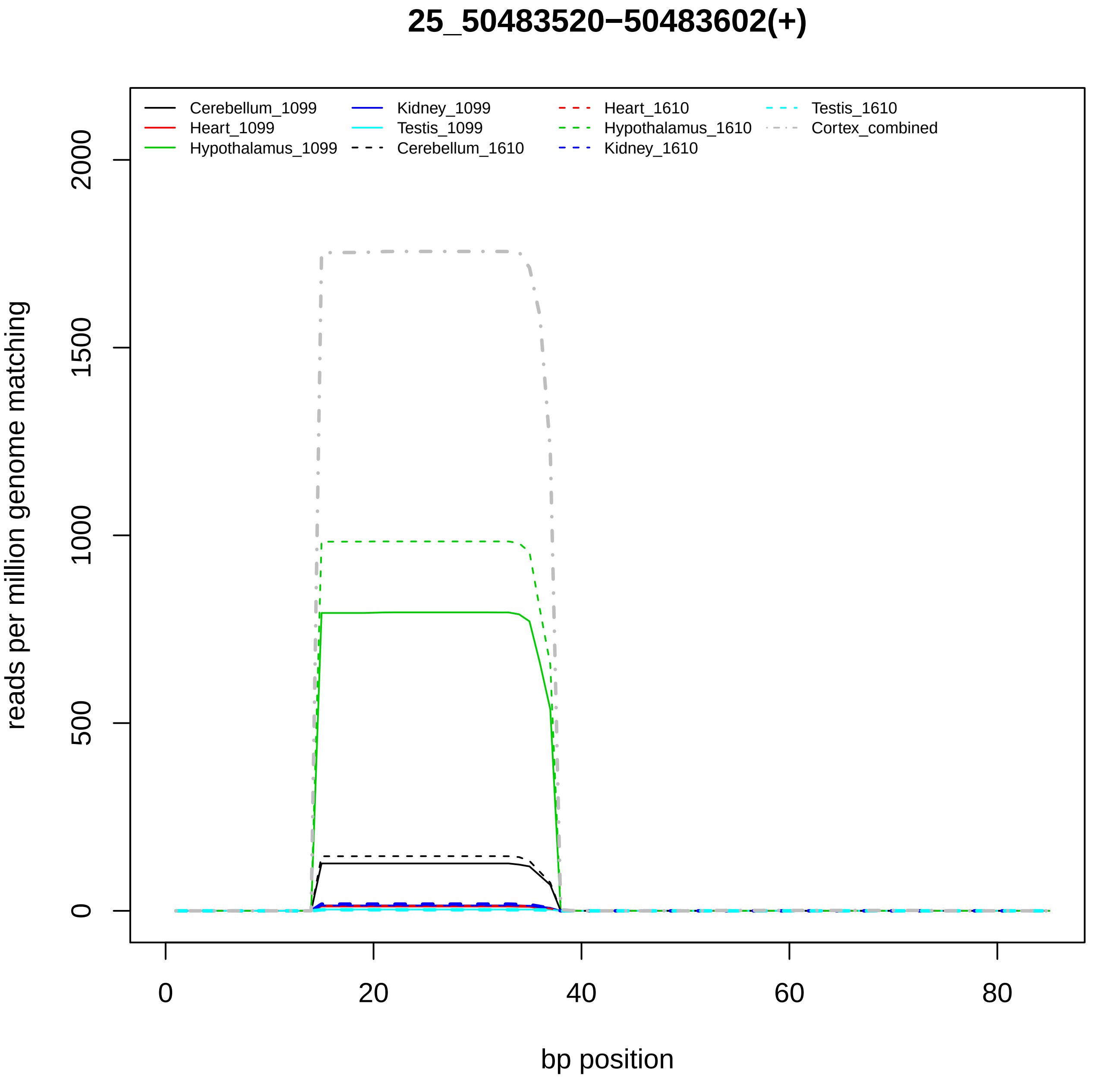

25_50483520−50483602(+)
Cerebellum_1099
Heart_1099
Hypothalamus_1099
Kidney_1099
Testis_1099
Cerebellum_1610
Heart_1610
Hypothalamus_1610
Kidney_1610
Testis_1610
Cortex_combined
reads per million genome matching
0
500
1000
1500
2000
0
20
40
60
80
bp position

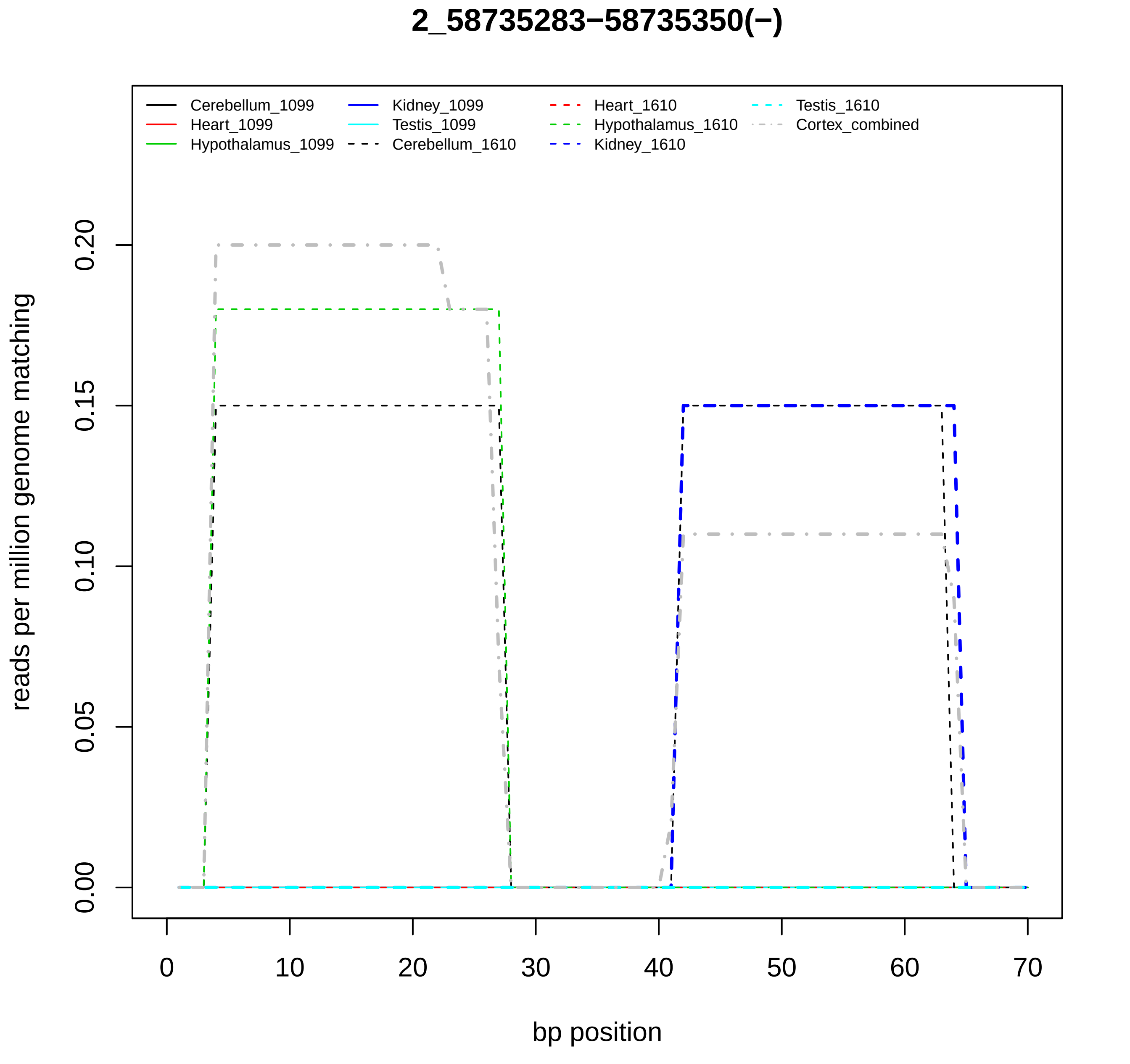

2_58735283−58735350(−)
Cerebellum_1099
Heart_1099
Hypothalamus_1099
Kidney_1099
Testis_1099
Cerebellum_1610
Heart_1610
Hypothalamus_1610
Kidney_1610
Testis_1610
Cortex_combined
reads per million genome matching
0.00
0.05
0.10
0.15
0.20
0
10
20
30
40
50
60
70
bp position

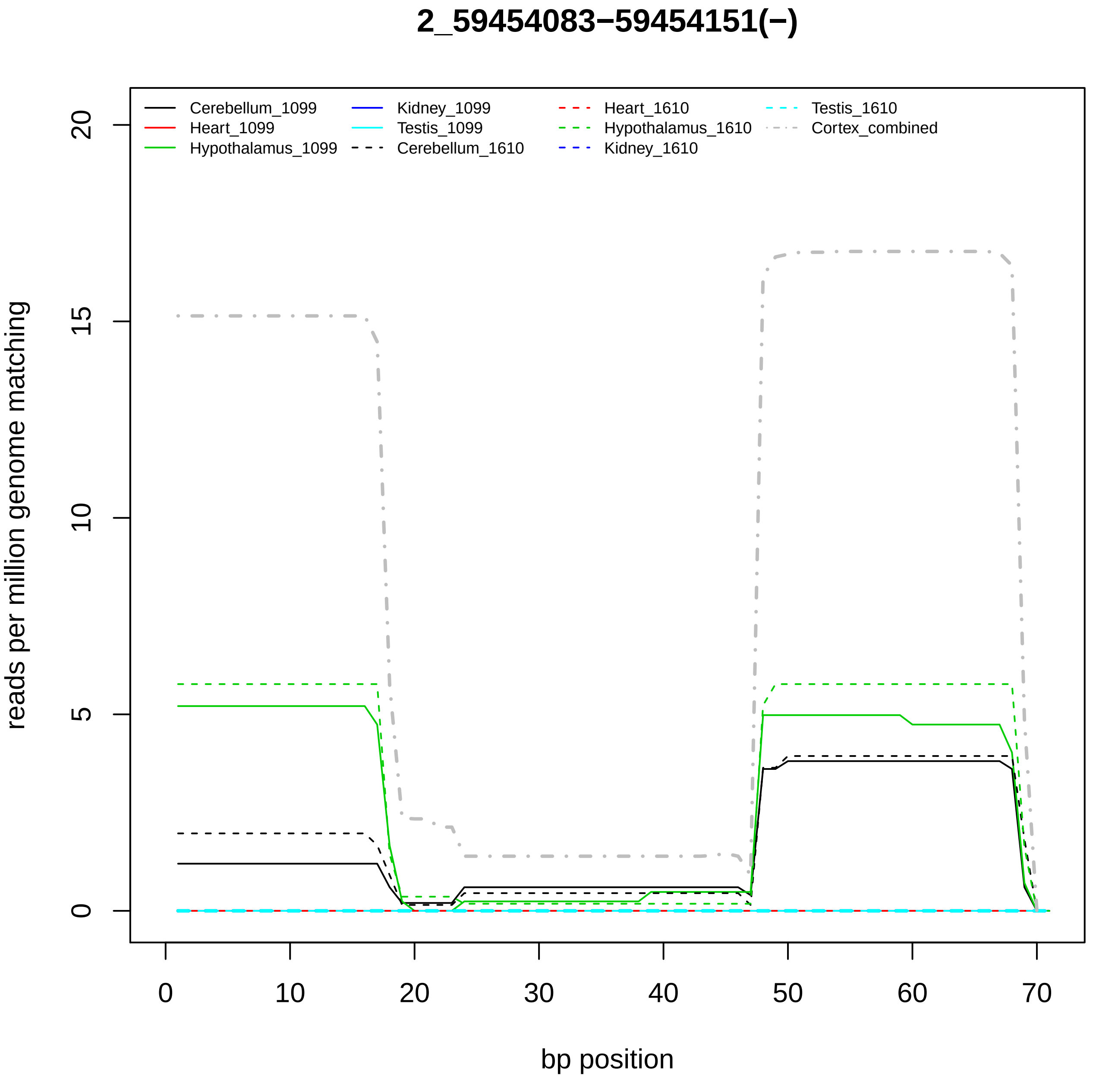

2_59454083−59454151(−)
Cerebellum_1099
Heart_1099
Hypothalamus_1099
Kidney_1099
Testis_1099
Cerebellum_1610
Heart_1610
Hypothalamus_1610
Kidney_1610
Testis_1610
Cortex_combined
reads per million genome matching
0
5
10
15
20
0
10
20
30
40
50
60
70
bp position

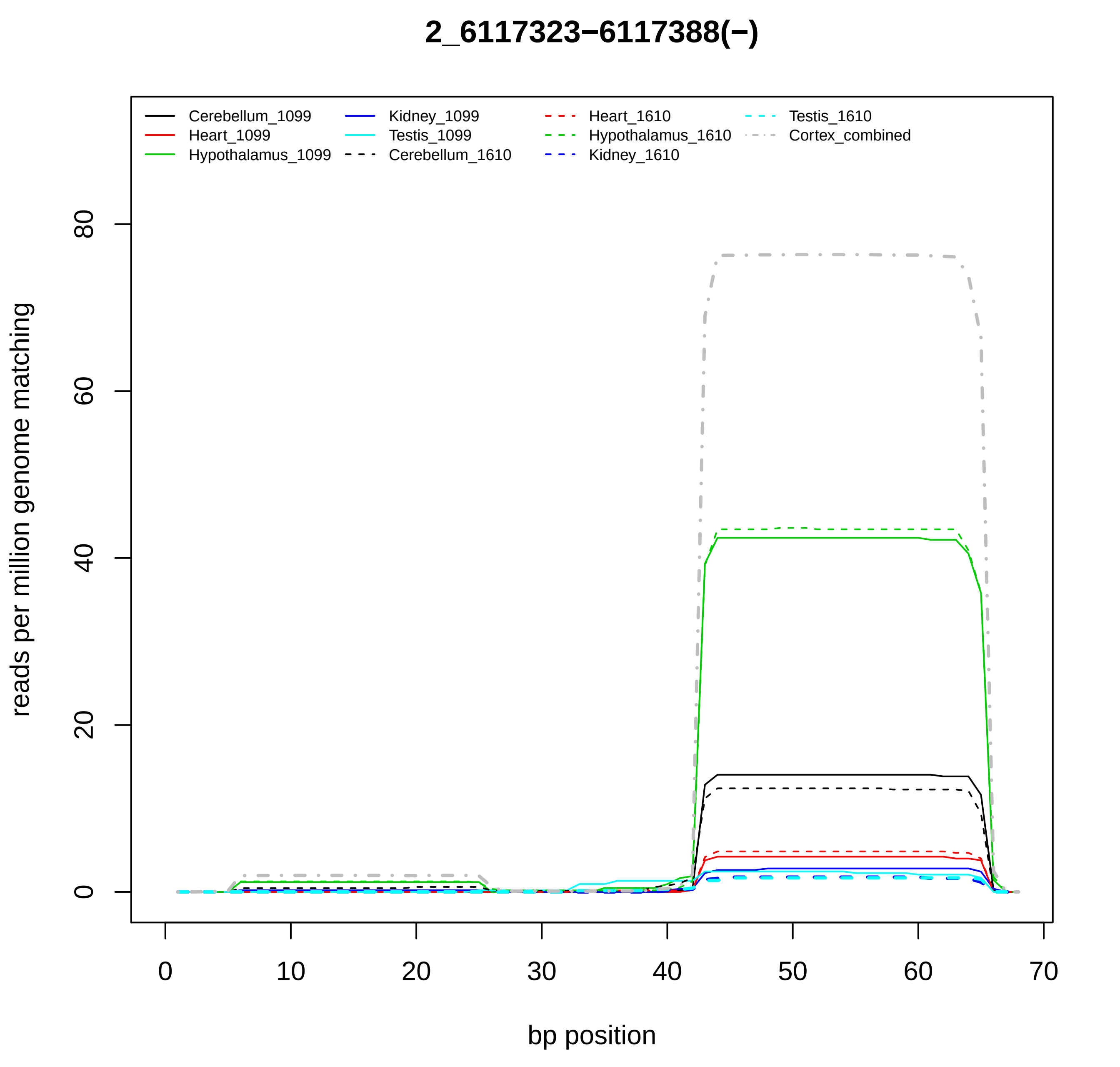

2_6117323−6117388(−)
Cerebellum_1099
Heart_1099
Hypothalamus_1099
Kidney_1099
Testis_1099
Cerebellum_1610
Heart_1610
Hypothalamus_1610
Kidney_1610
Testis_1610
Cortex_combined
reads per million genome matching
0
20
40
60
80
bp position
0
10
20
30
40
50
60
70

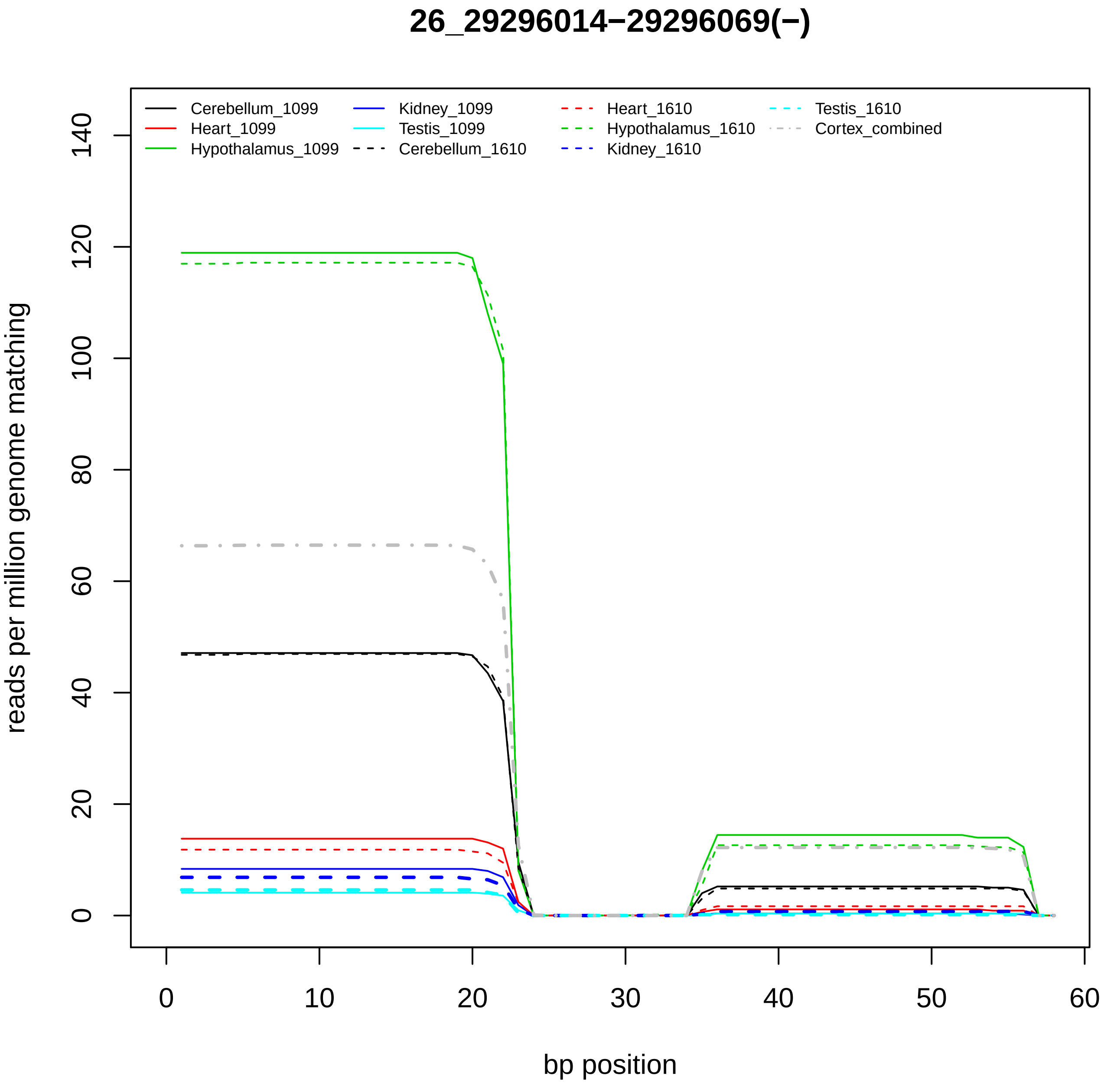

26_29296014−29296069(−)
Cerebellum_1099
Heart_1099
Hypothalamus_1099
Kidney_1099
Testis_1099
Cerebellum_1610
Heart_1610
Hypothalamus_1610
Kidney_1610
Testis_1610
Cortex_combined
reads per million genome matching
0
20
40
60
80
100
120
140
bp position
0
10
20
30
40
50
60

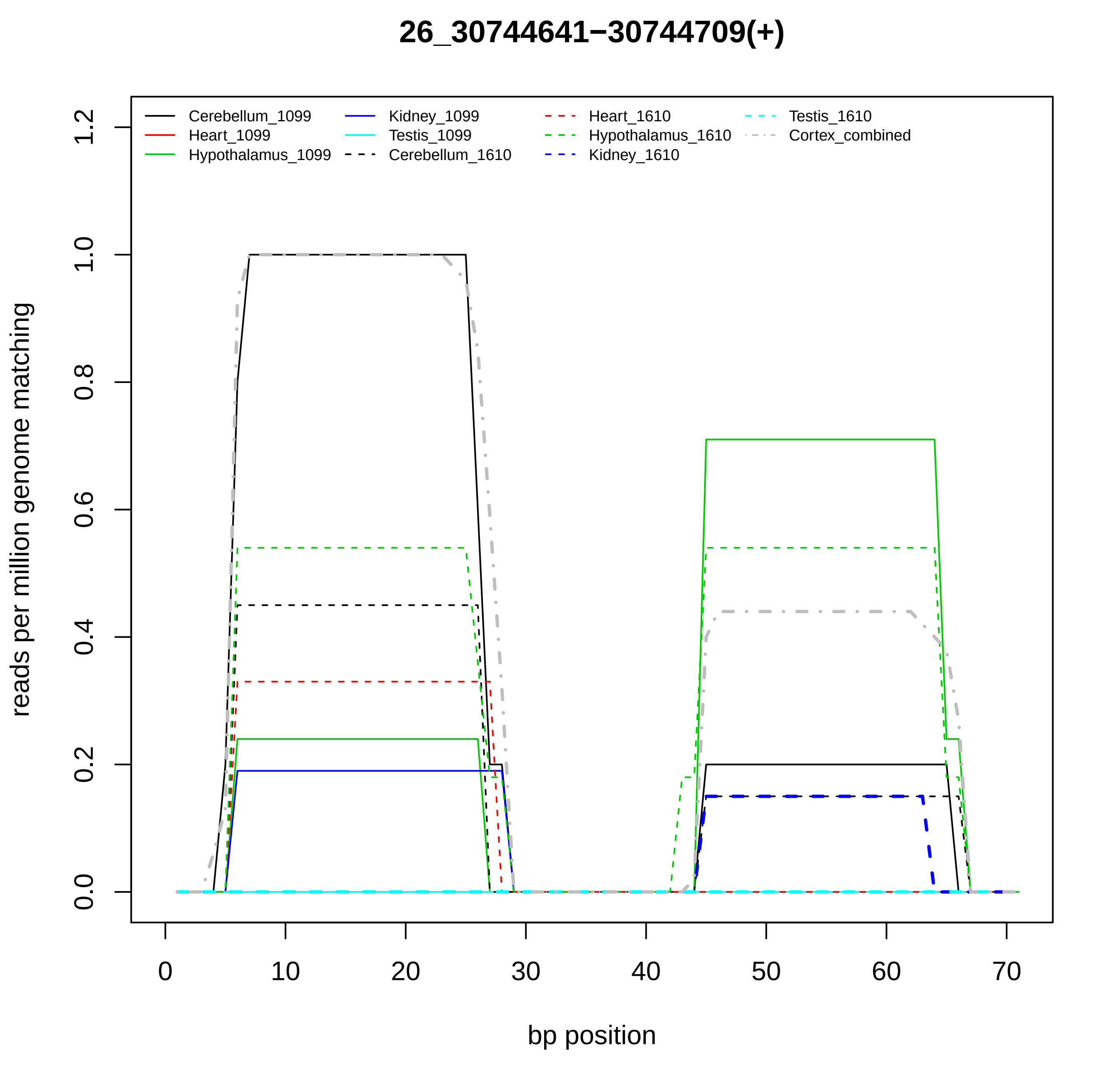
26_30744641−30744709(+)
Cerebellum_1099
Heart_1099
Hypothalamus_1099
Kidney_1099
Testis_1099
Cerebellum_1610
Heart_1610
Hypothalamus_1610
Kidney_1610
Testis_1610
Cortex_combined
reads per million genome matching
bp position
0.0
0.2
0.4
0.6
0.8
1.0
1.2
0
10
20
30
40
50
60
70

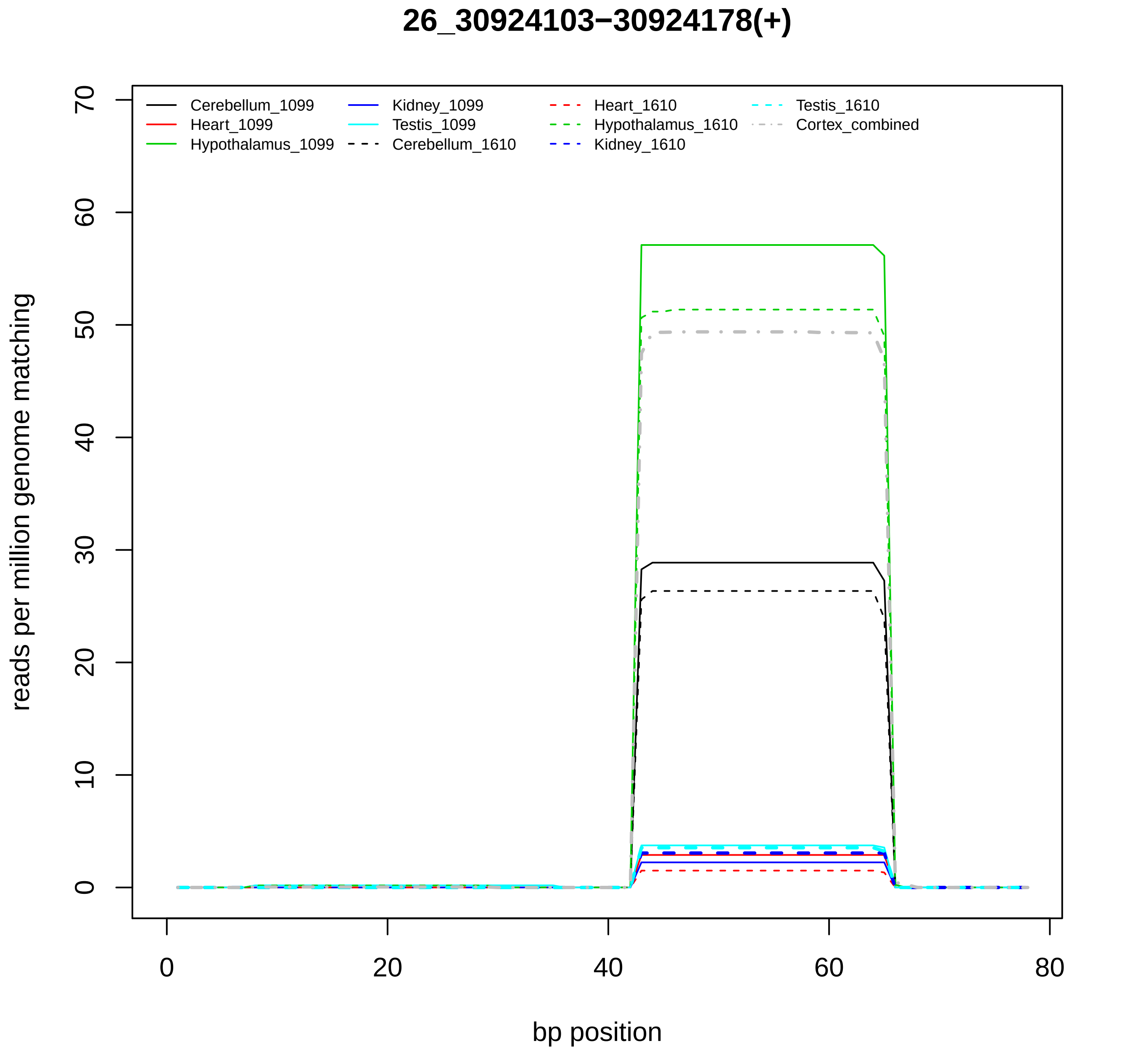
26_30924103−30924178(+)
Cerebellum_1099
Heart_1099
Hypothalamus_1099
Kidney_1099
Testis_1099
Cerebellum_1610
Heart_1610
Hypothalamus_1610
Kidney_1610
Testis_1610
Cortex_combined
reads per million genome matching
bp position
0
10
20
30
40
50
60
70
80

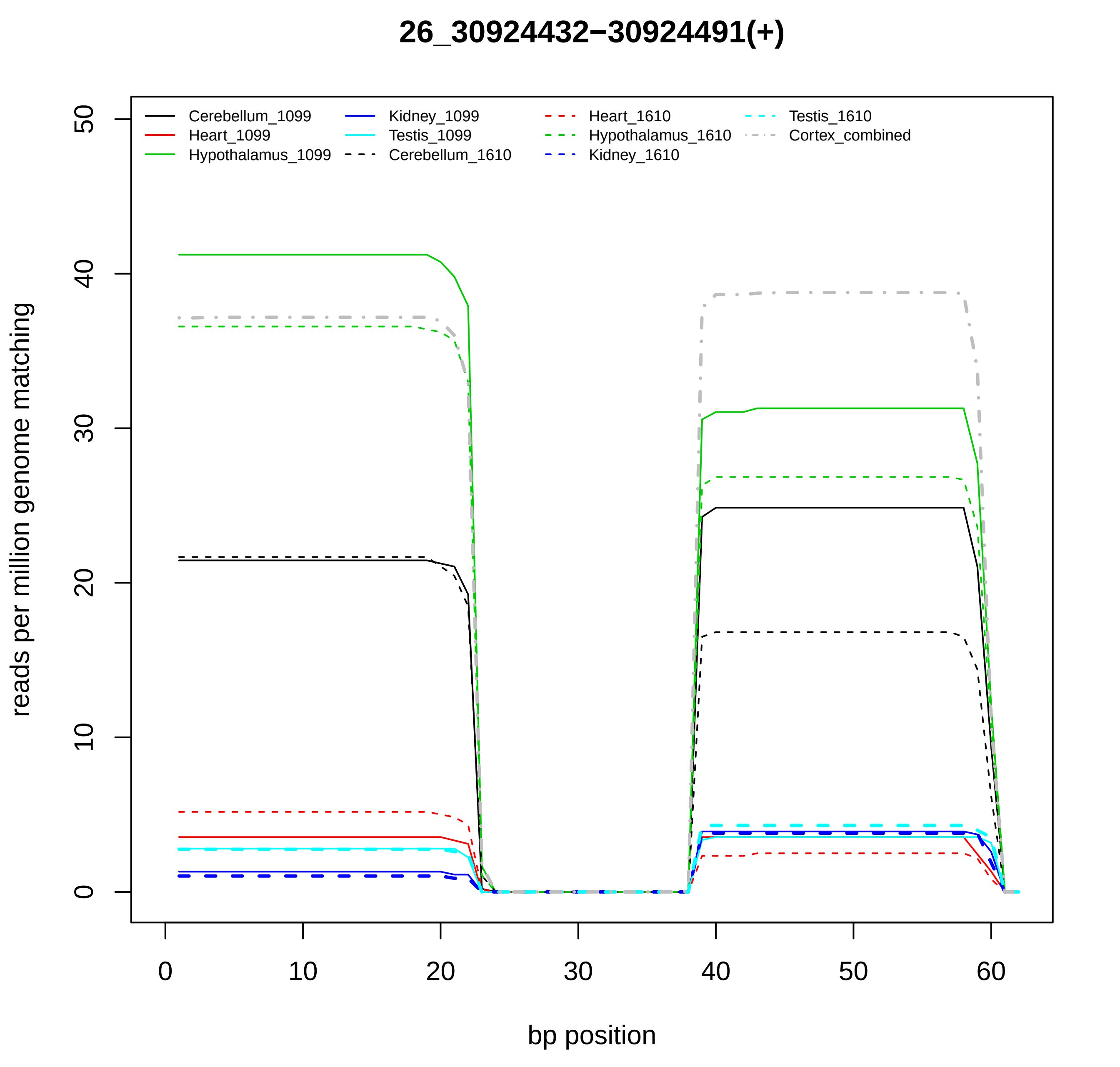

26_30924432−30924491(+)
Cerebellum_1099
Heart_1099
Hypothalamus_1099
Kidney_1099
Testis_1099
Cerebellum_1610
Heart_1610
Hypothalamus_1610
Kidney_1610
Testis_1610
Cortex_combined
reads per million genome matching
bp position
0
10
20
30
40
50
60

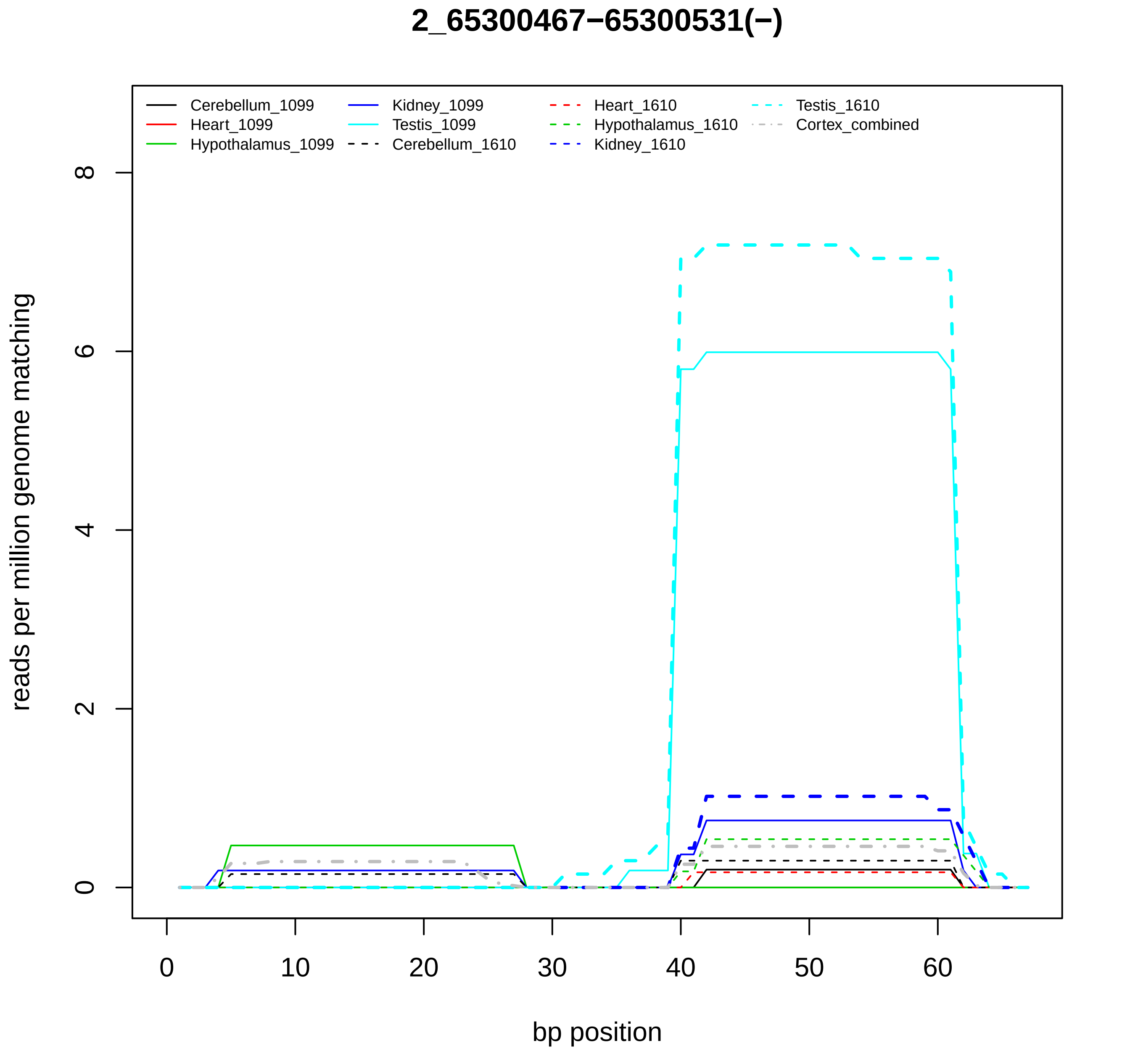

2_65300467−65300531(−)
Cerebellum_1099
Heart_1099
Hypothalamus_1099
Kidney_1099
Testis_1099
Cerebellum_1610
Heart_1610
Hypothalamus_1610
Kidney_1610
Testis_1610
Cortex_combined
reads per million genome matching
bp position
0
2
4
6
8
0
10
20
30
40
50
60

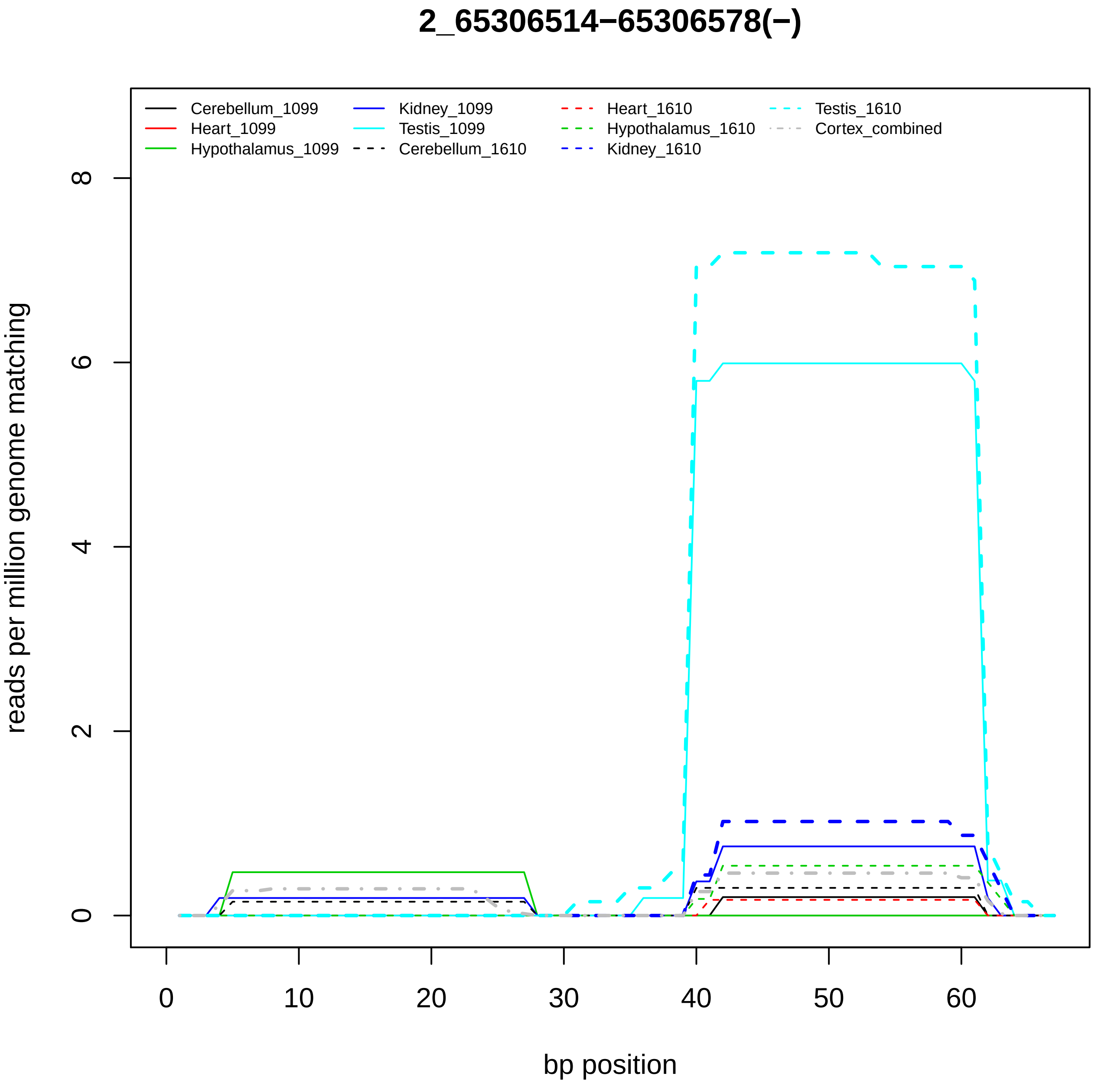

2_65306514−65306578(−)
Cerebellum_1099
Heart_1099
Hypothalamus_1099
Kidney_1099
Testis_1099
Cerebellum_1610
Heart_1610
Hypothalamus_1610
Kidney_1610
Testis_1610
Cortex_combined
reads per million genome matching
bp position
0
2
4
6
8
0
10
20
30
40
50
60

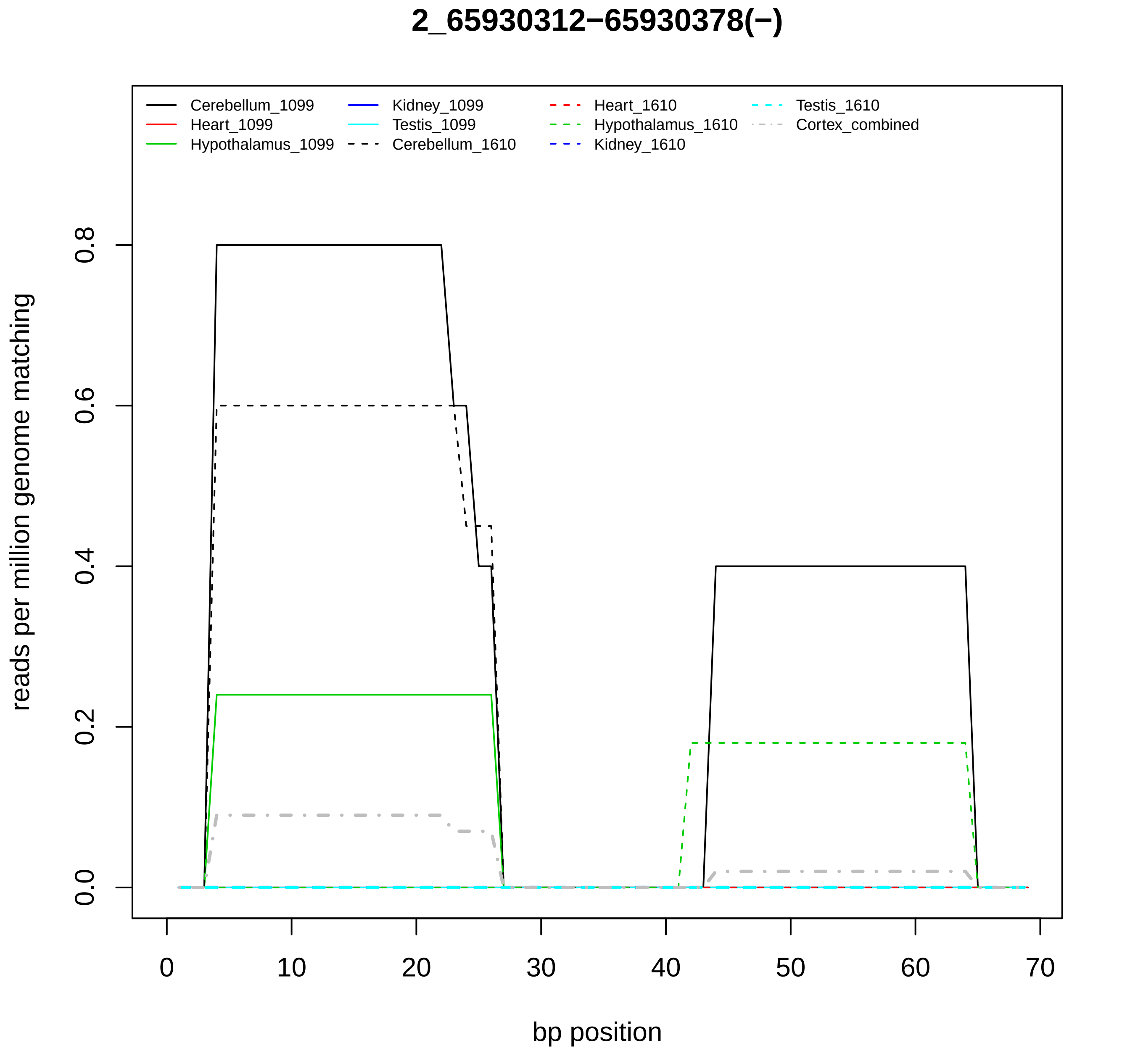

2_65930312−65930378(−)
Cerebellum_1099
Heart_1099
Hypothalamus_1099
Kidney_1099
Testis_1099
Cerebellum_1610
Heart_1610
Hypothalamus_1610
Kidney_1610
Testis_1610
Cortex_combined
reads per million genome matching
0.0
0.2
0.4
0.6
0.8
0
10
20
30
40
50
60
70
bp position

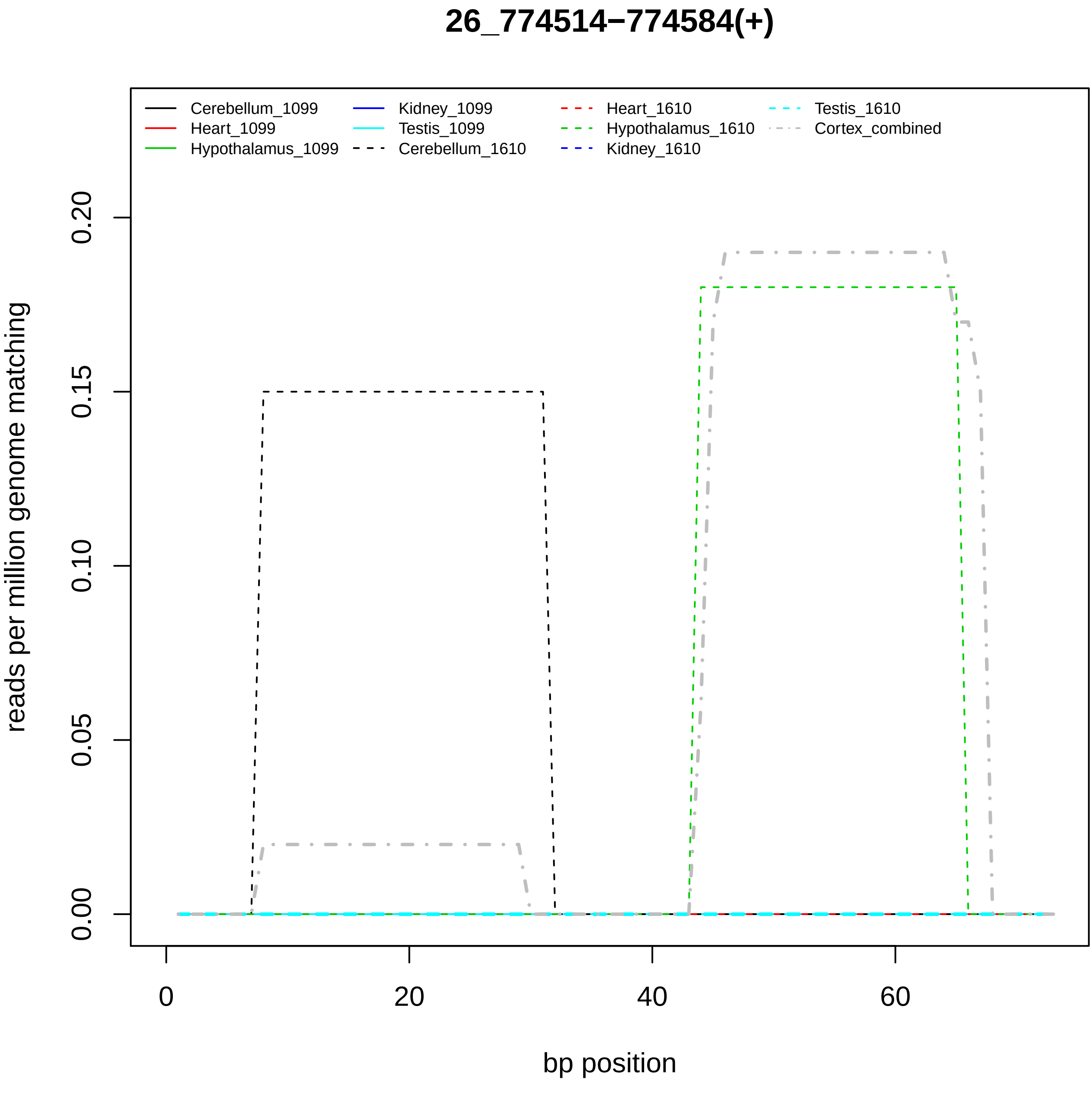

26_774514−774584(+)
Cerebellum_1099
Heart_1099
Hypothalamus_1099
Kidney_1099
Testis_1099
Cerebellum_1610
Heart_1610
Hypothalamus_1610
Kidney_1610
Testis_1610
Cortex_combined
reads per million genome matching
0.00
0.05
0.10
0.15
0.20
0
20
40
60
bp position

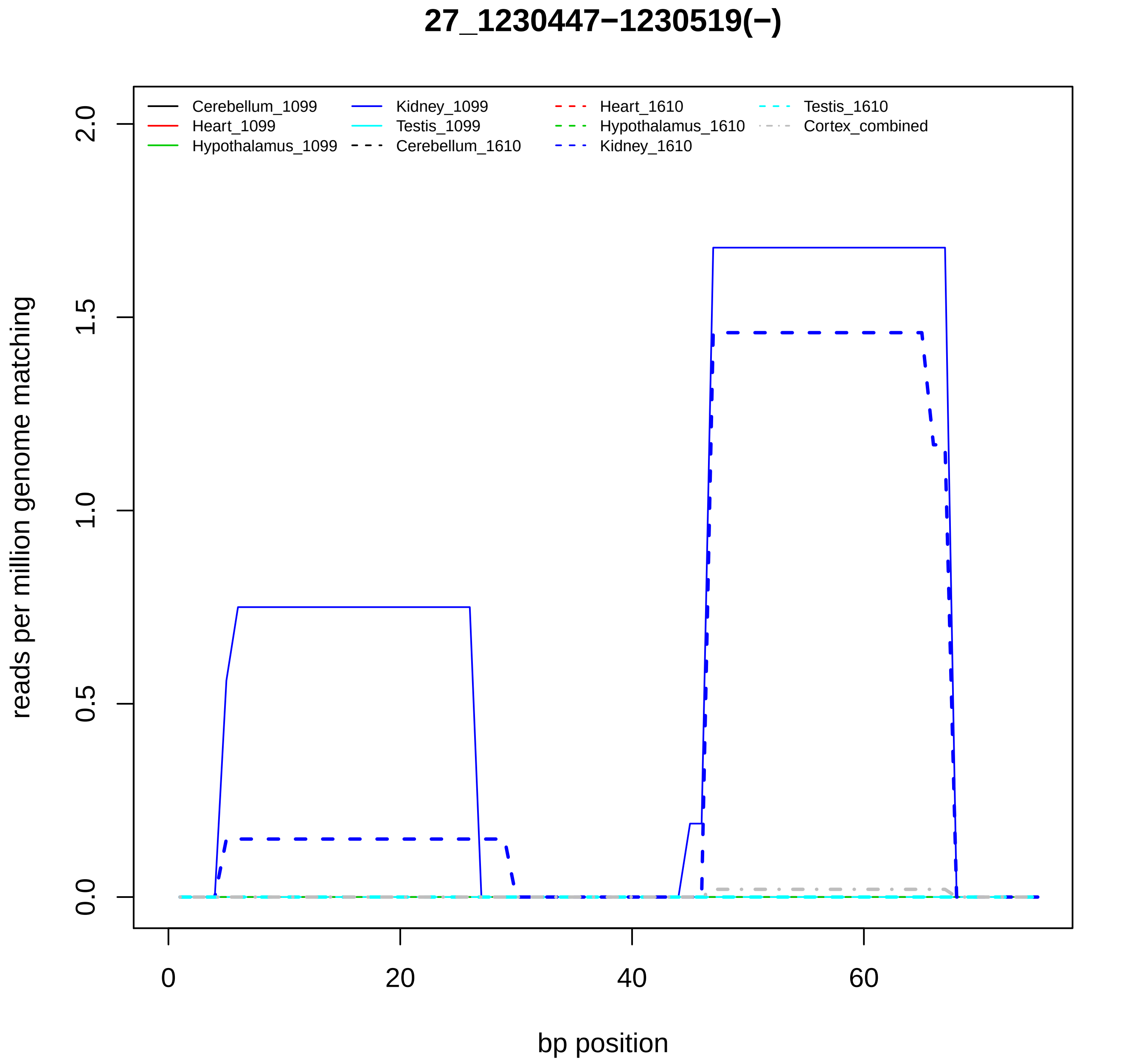

27_1230447−1230519(−)
Cerebellum_1099
Heart_1099
Hypothalamus_1099
Kidney_1099
Testis_1099
Cerebellum_1610
Heart_1610
Hypothalamus_1610
Kidney_1610
Testis_1610
Cortex_combined
reads per million genome matching
bp position
0.0
0.5
1.0
1.5
2.0
0
20
40
60

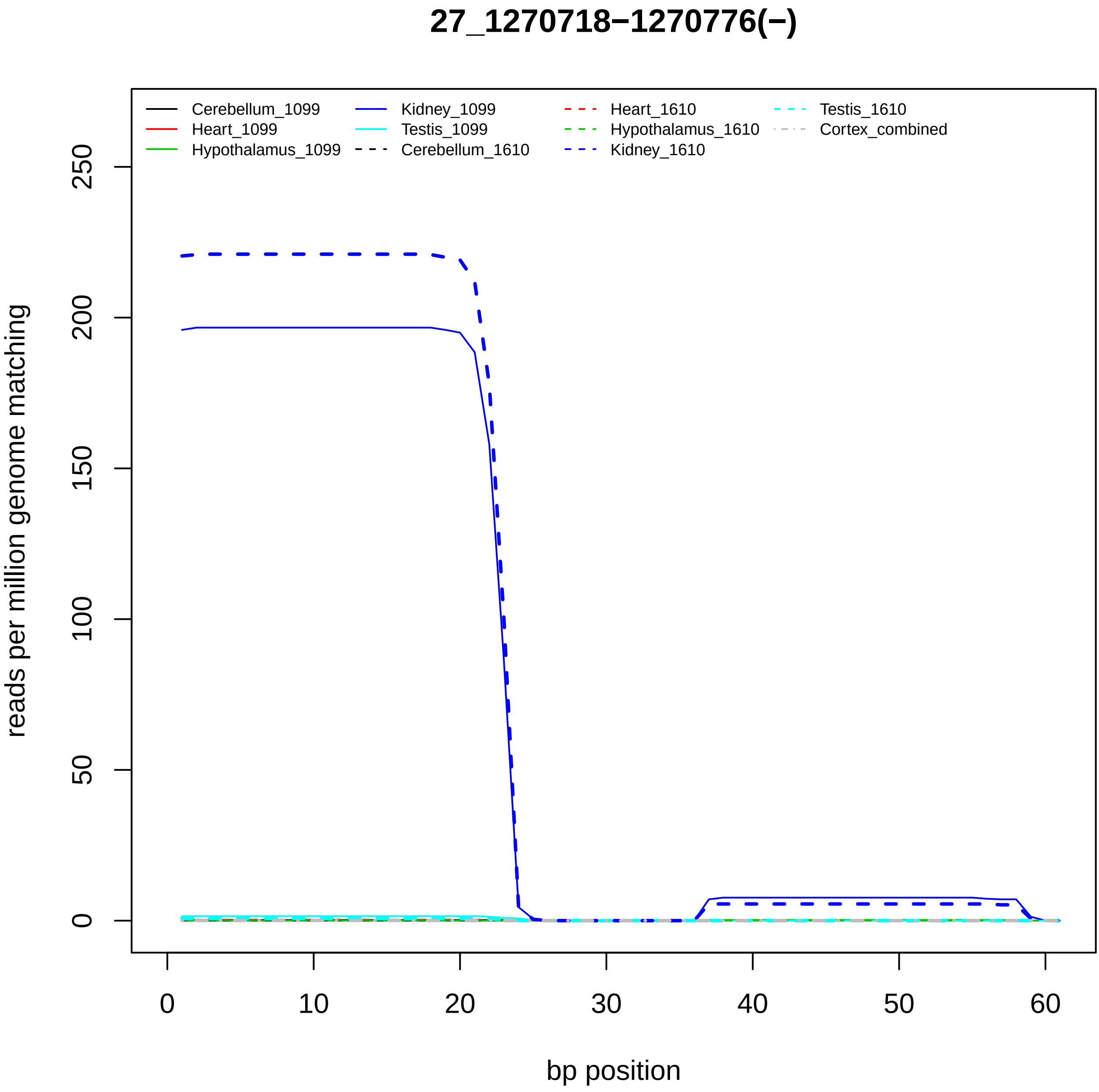

27_1270718−1270776(−)
Cerebellum_1099
Heart_1099
Hypothalamus_1099
Kidney_1099
Testis_1099
Cerebellum_1610
Heart_1610
Hypothalamus_1610
Kidney_1610
Testis_1610
Cortex_combined
reads per million genome matching
bp position
0
50
100
150
200
250
0
10
20
30
40
50
60

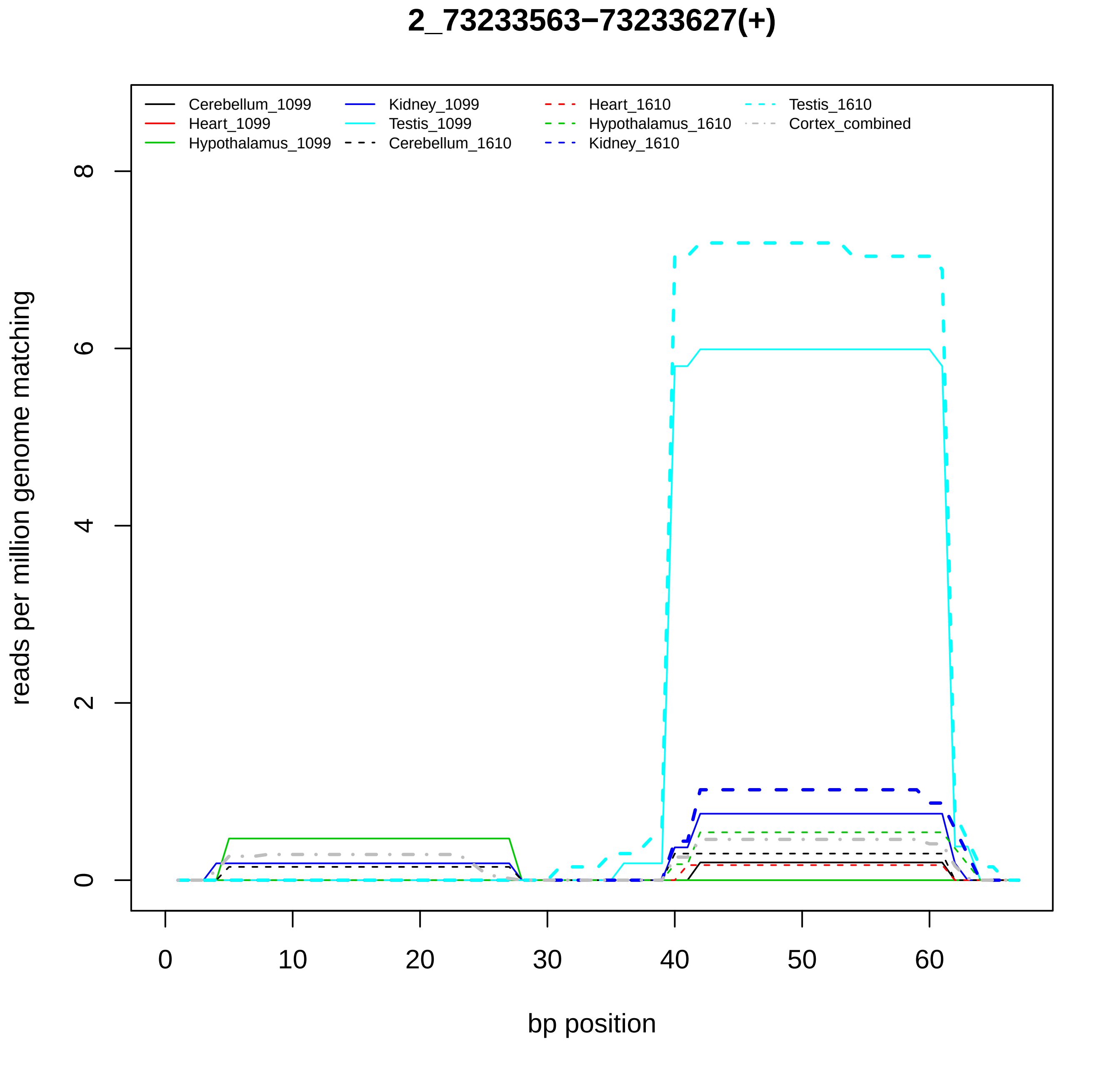

2_73233563−73233627(+)
Cerebellum_1099
Heart_1099
Hypothalamus_1099
Kidney_1099
Testis_1099
Cerebellum_1610
Heart_1610
Hypothalamus_1610
Kidney_1610
Testis_1610
Cortex_combined
reads per million genome matching
bp position
0
2
4
6
8
0
10
20
30
40
50
60

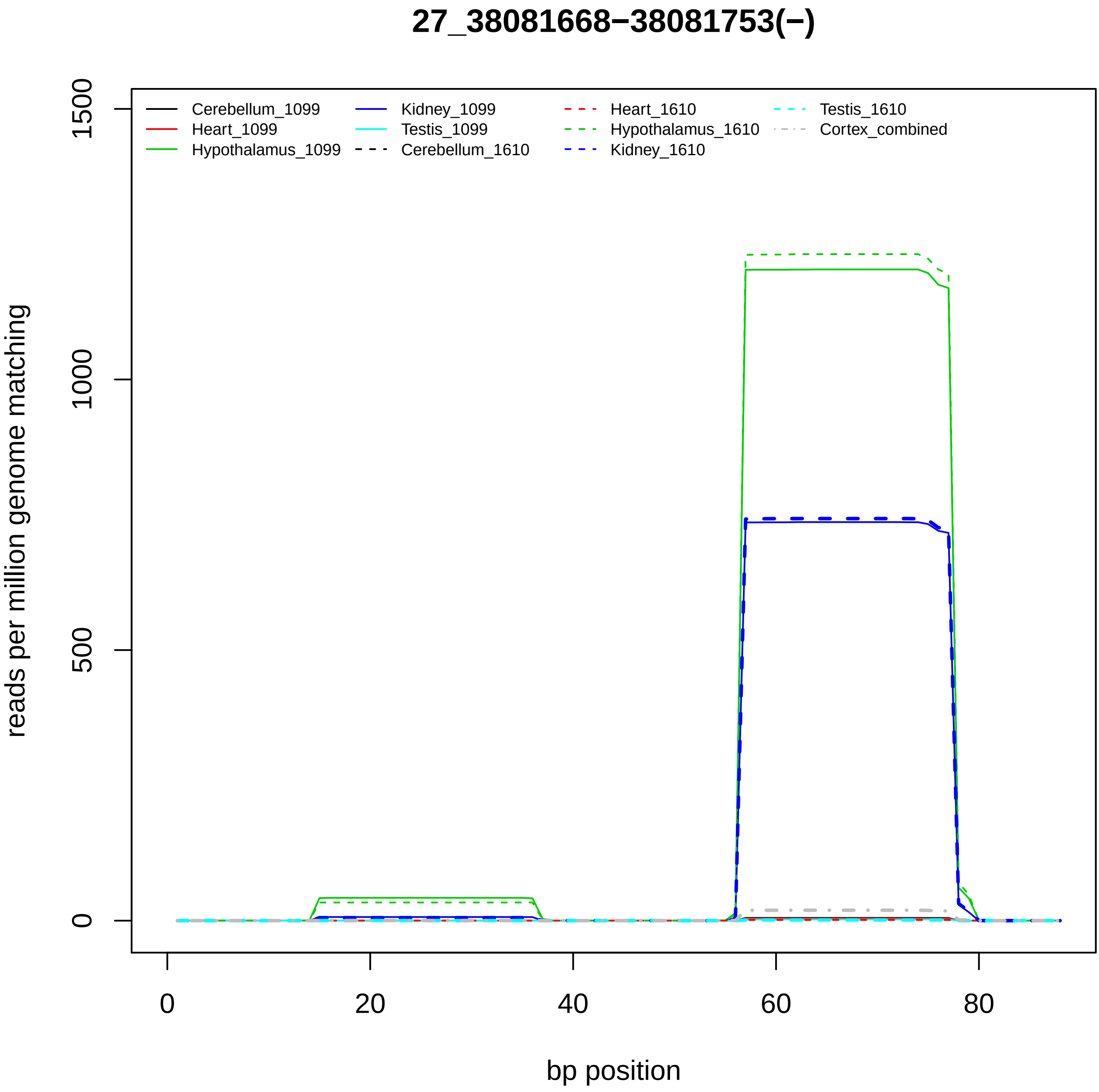
27_38081668−38081753(−)
Cerebellum_1099
Heart_1099
Hypothalamus_1099
Kidney_1099
Testis_1099
Cerebellum_1610
Heart_1610
Hypothalamus_1610
Kidney_1610
Testis_1610
Cortex_combined
reads per million genome matching
bp position
0
500
1000
1500
0
20
40
60
80

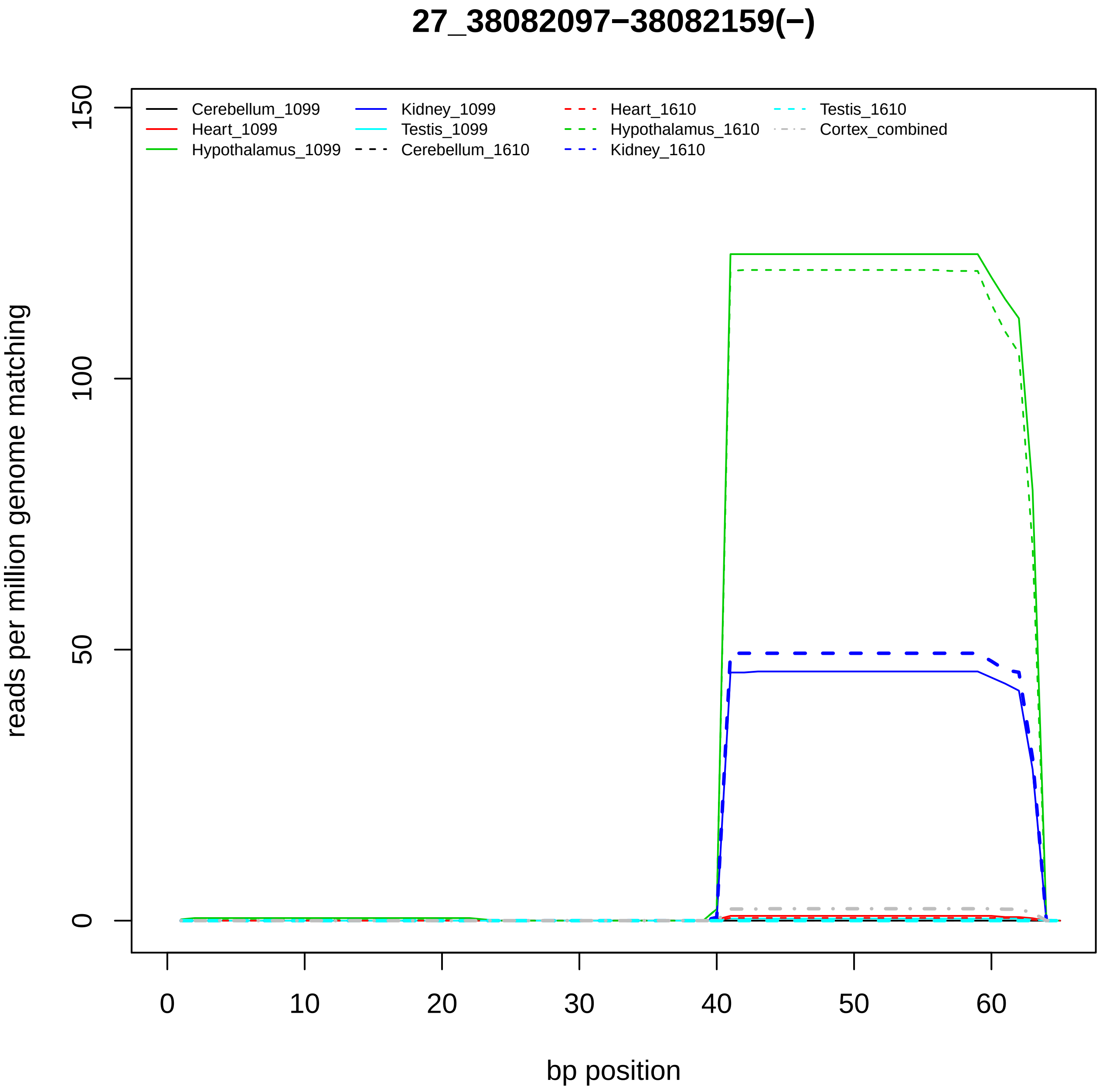
27_38082097−38082159(−)
Cerebellum_1099
Heart_1099
Hypothalamus_1099
Kidney_1099
Testis_1099
Cerebellum_1610
Heart_1610
Hypothalamus_1610
Kidney_1610
Testis_1610
Cortex_combined
reads per million genome matching
bp position
0
50
100
150
0
10
20
30
40
50
60

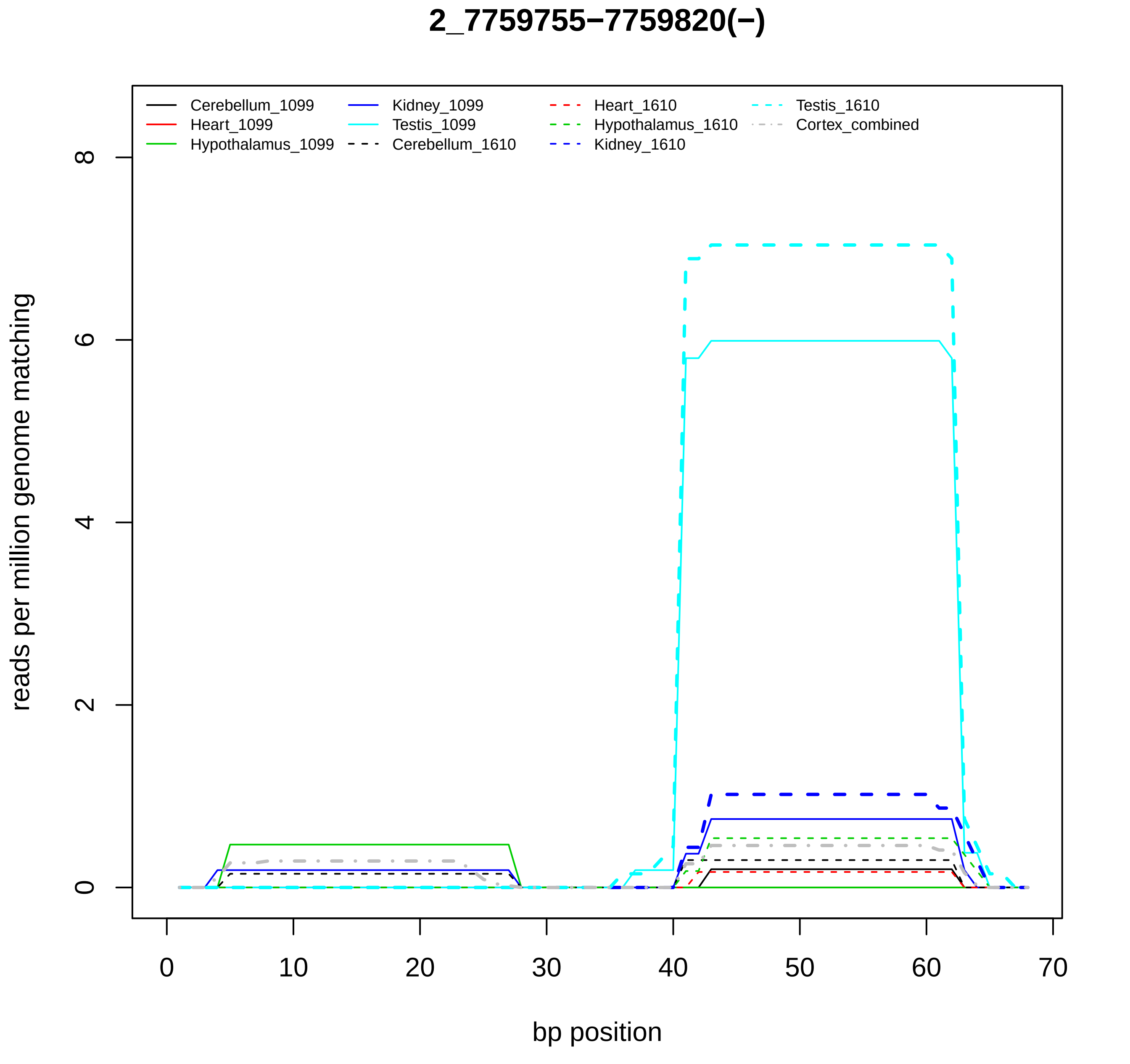

2_7759755−7759820(−)
Cerebellum_1099
Heart_1099
Hypothalamus_1099
Kidney_1099
Testis_1099
Cerebellum_1610
Heart_1610
Hypothalamus_1610
Kidney_1610
Testis_1610
Cortex_combined
reads per million genome matching
0
2
4
6
8
0
10
20
30
40
50
60
70
bp position

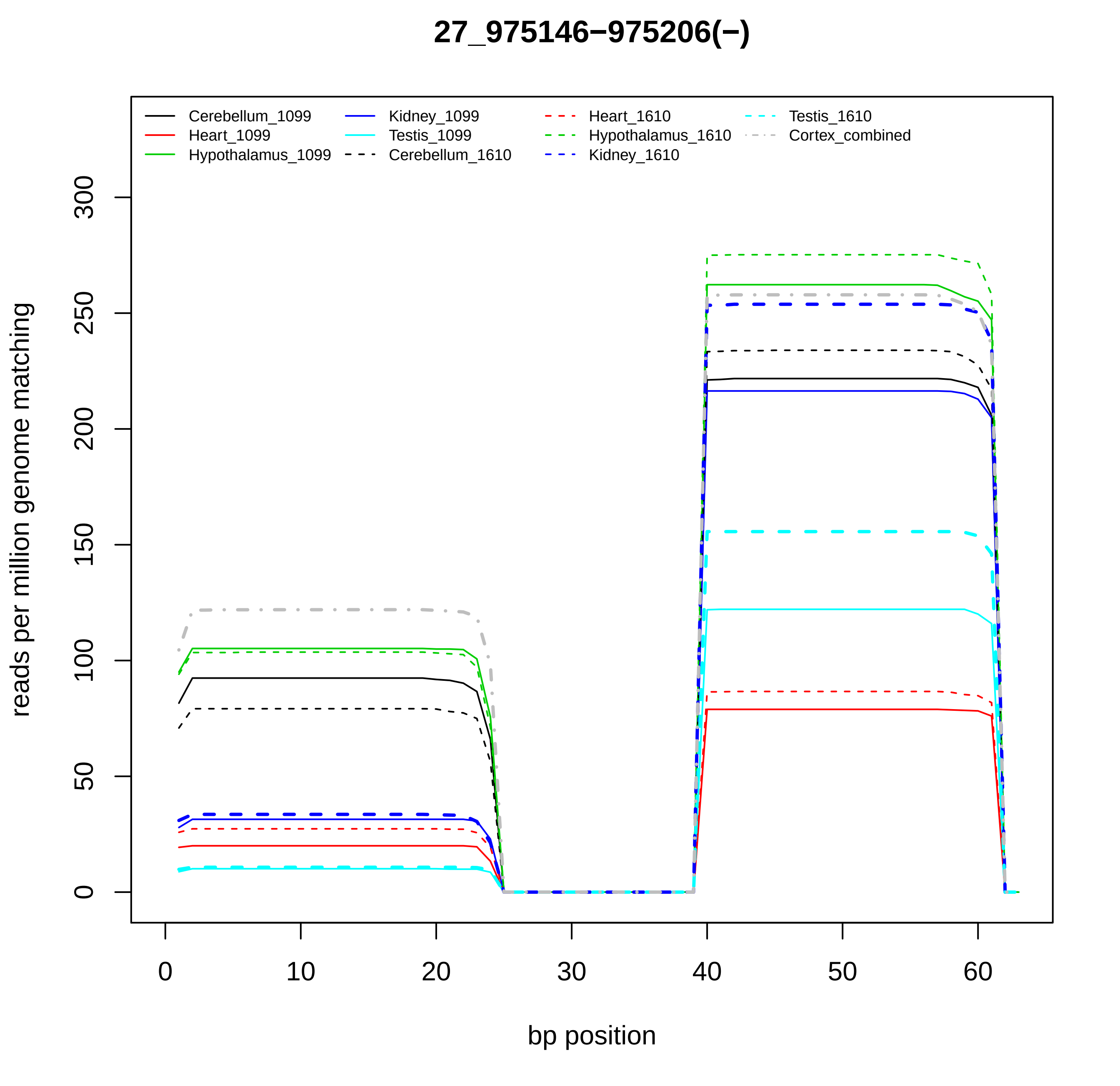
27_975146−975206(−)
Cerebellum_1099
Heart_1099
Hypothalamus_1099
Kidney_1099
Testis_1099
Cerebellum_1610
Heart_1610
Hypothalamus_1610
Kidney_1610
Testis_1610
Cortex_combined
reads per million genome matching
0
50
100
150
200
250
300
bp position
0
10
20
30
40
50
60

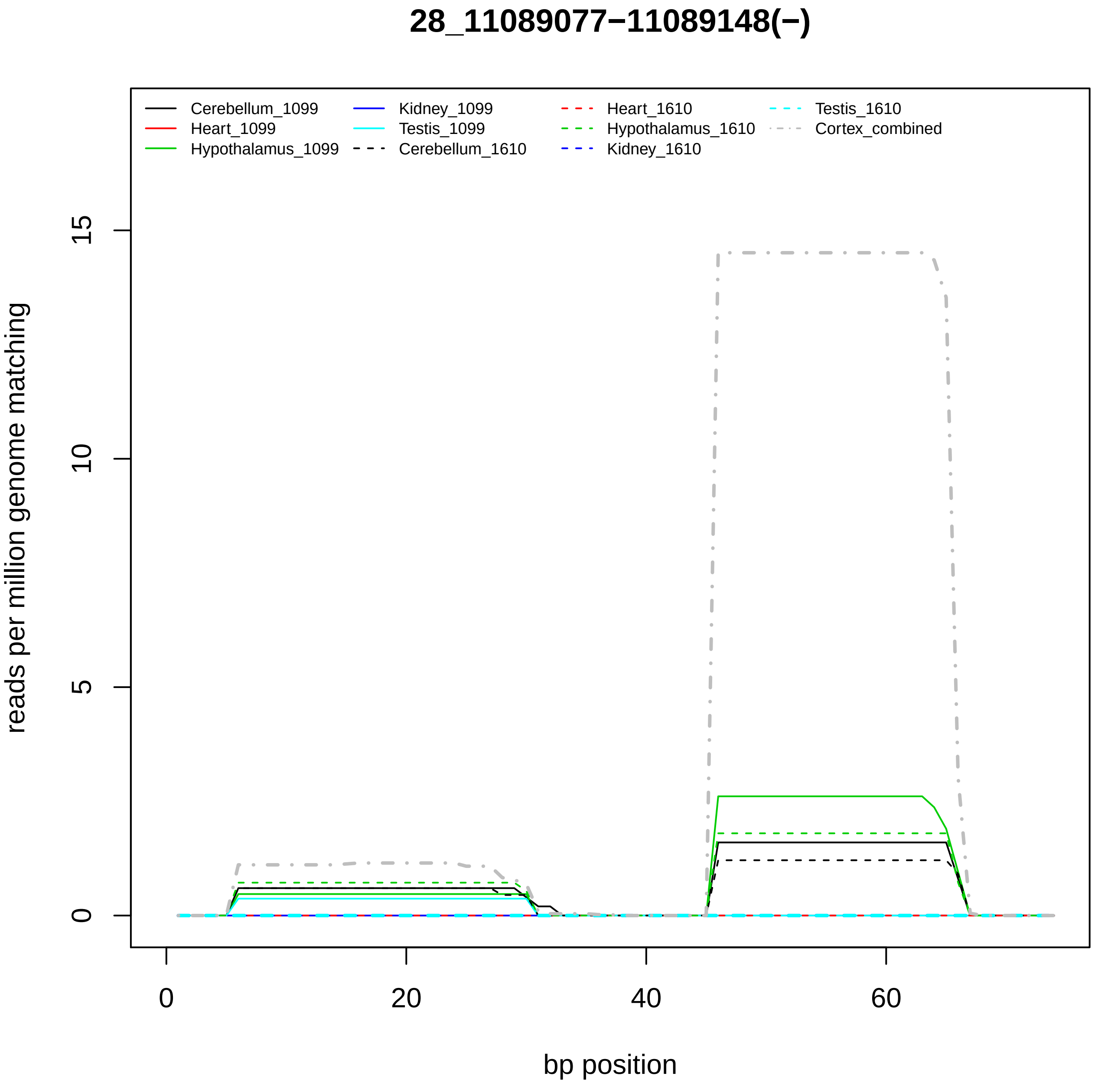

28_11089077−11089148(−)
Cerebellum_1099
Heart_1099
Hypothalamus_1099
Kidney_1099
Testis_1099
Cerebellum_1610
Heart_1610
Hypothalamus_1610
Kidney_1610
Testis_1610
Cortex_combined
reads per million genome matching
bp position
0
5
10
15
0
20
40
60

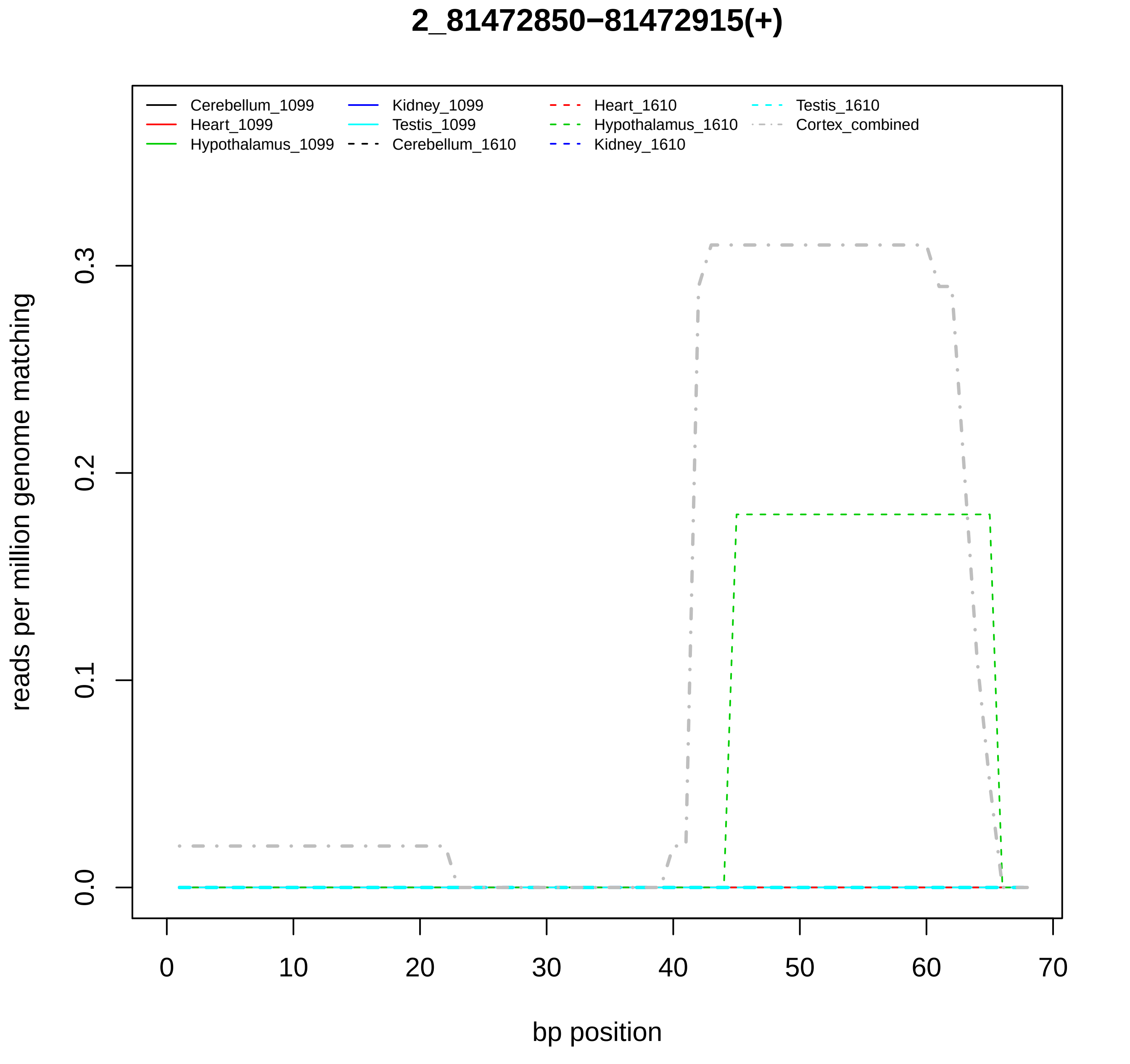

2_81472850−81472915(+)
Cerebellum_1099
Heart_1099
Hypothalamus_1099
Kidney_1099
Testis_1099
Cerebellum_1610
Heart_1610
Hypothalamus_1610
Kidney_1610
Testis_1610
Cortex_combined
reads per million genome matching
0.0
0.1
0.2
0.3
0
10
20
30
40
50
60
70
bp position

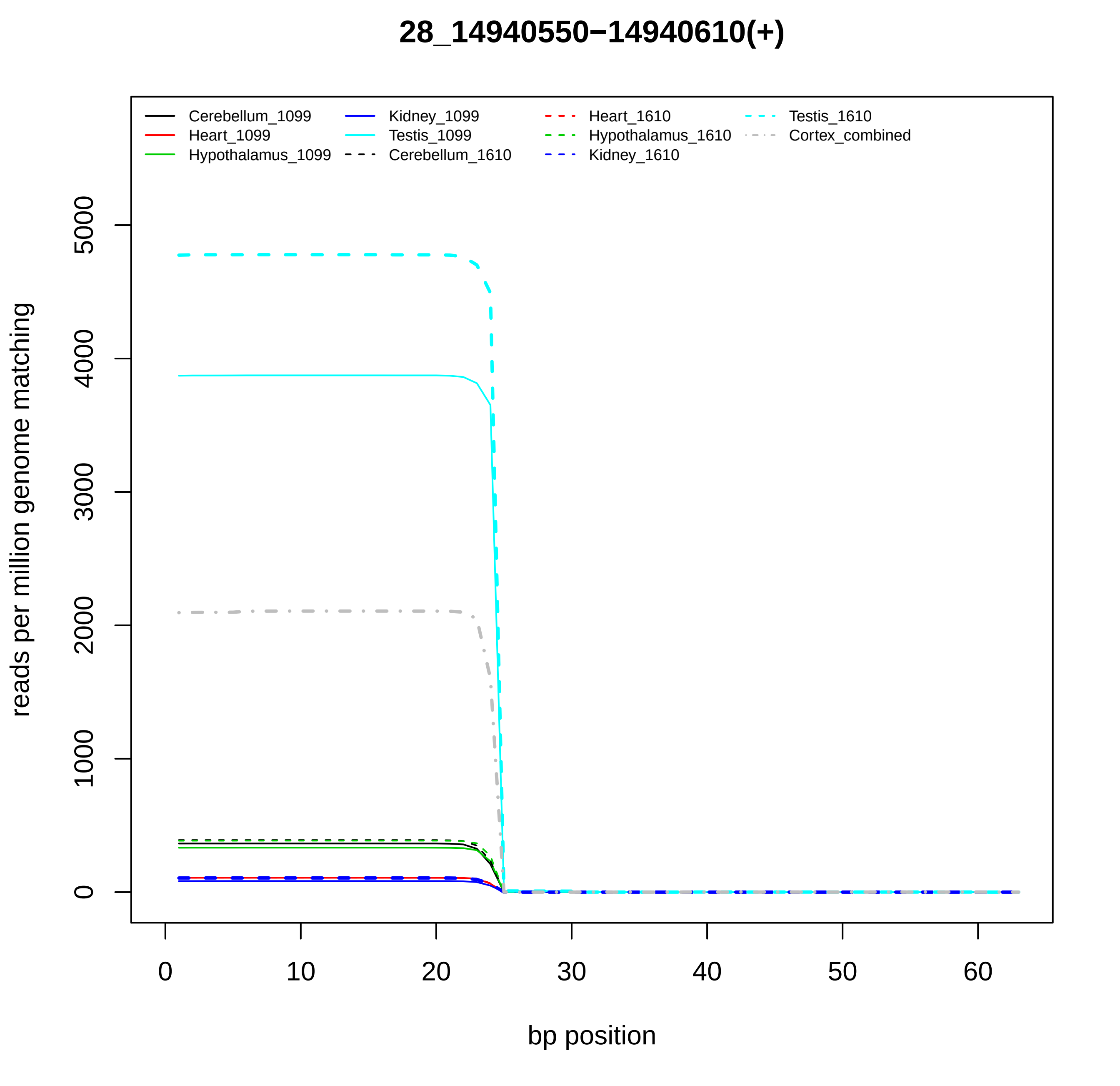

28_14940550−14940610(+)
Cerebellum_1099
Heart_1099
Hypothalamus_1099
Kidney_1099
Testis_1099
Cerebellum_1610
Heart_1610
Hypothalamus_1610
Kidney_1610
Testis_1610
Cortex_combined
reads per million genome matching
0
1000
2000
3000
4000
5000
0
10
20
30
40
50
60
bp position

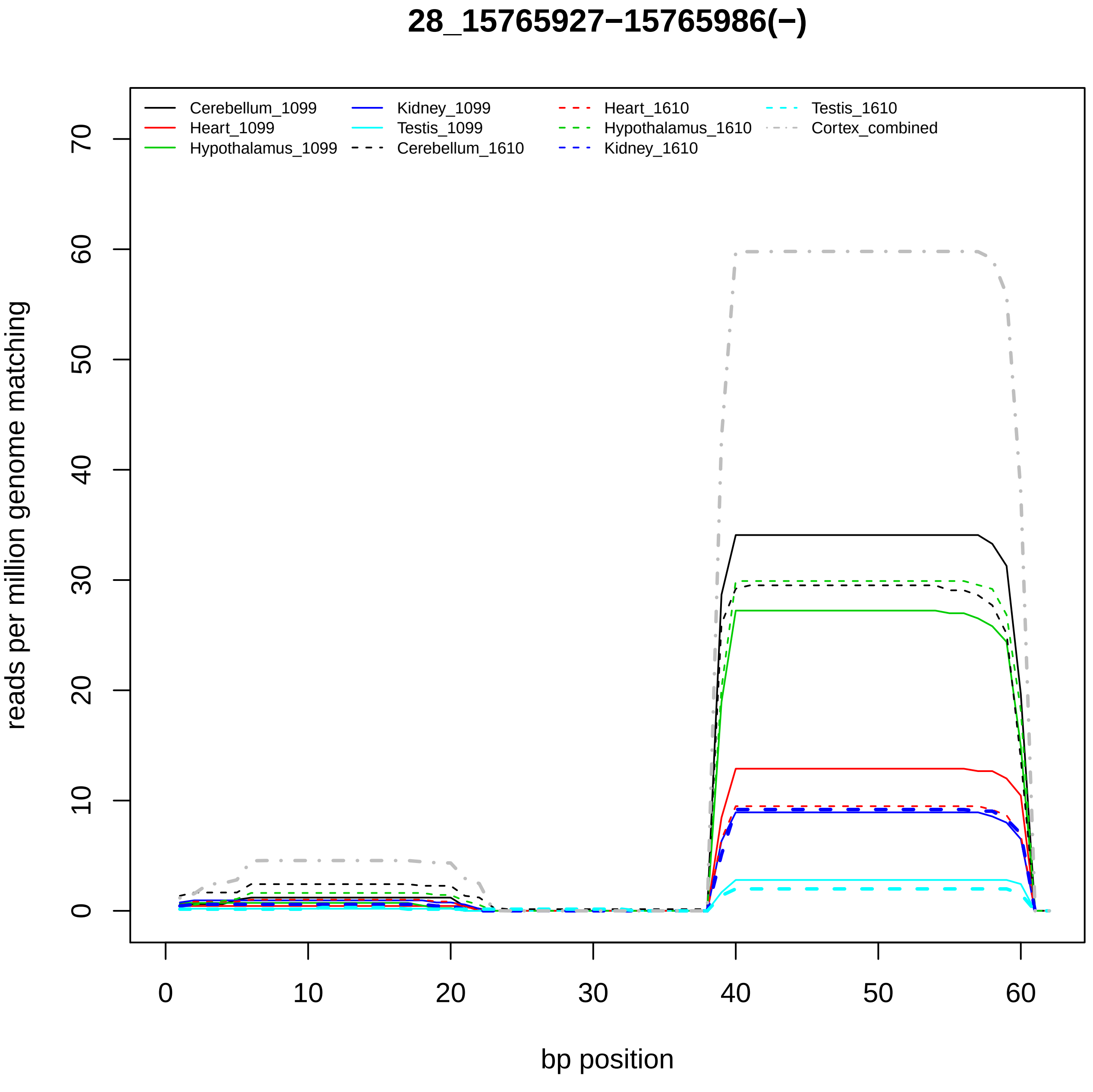
28_15765927−15765986(−)
Cerebellum_1099
Heart_1099
Hypothalamus_1099
Kidney_1099
Testis_1099
Cerebellum_1610
Heart_1610
Hypothalamus_1610
Kidney_1610
Testis_1610
Cortex_combined
reads per million genome matching
bp position
0
10
20
30
40
50
60
70

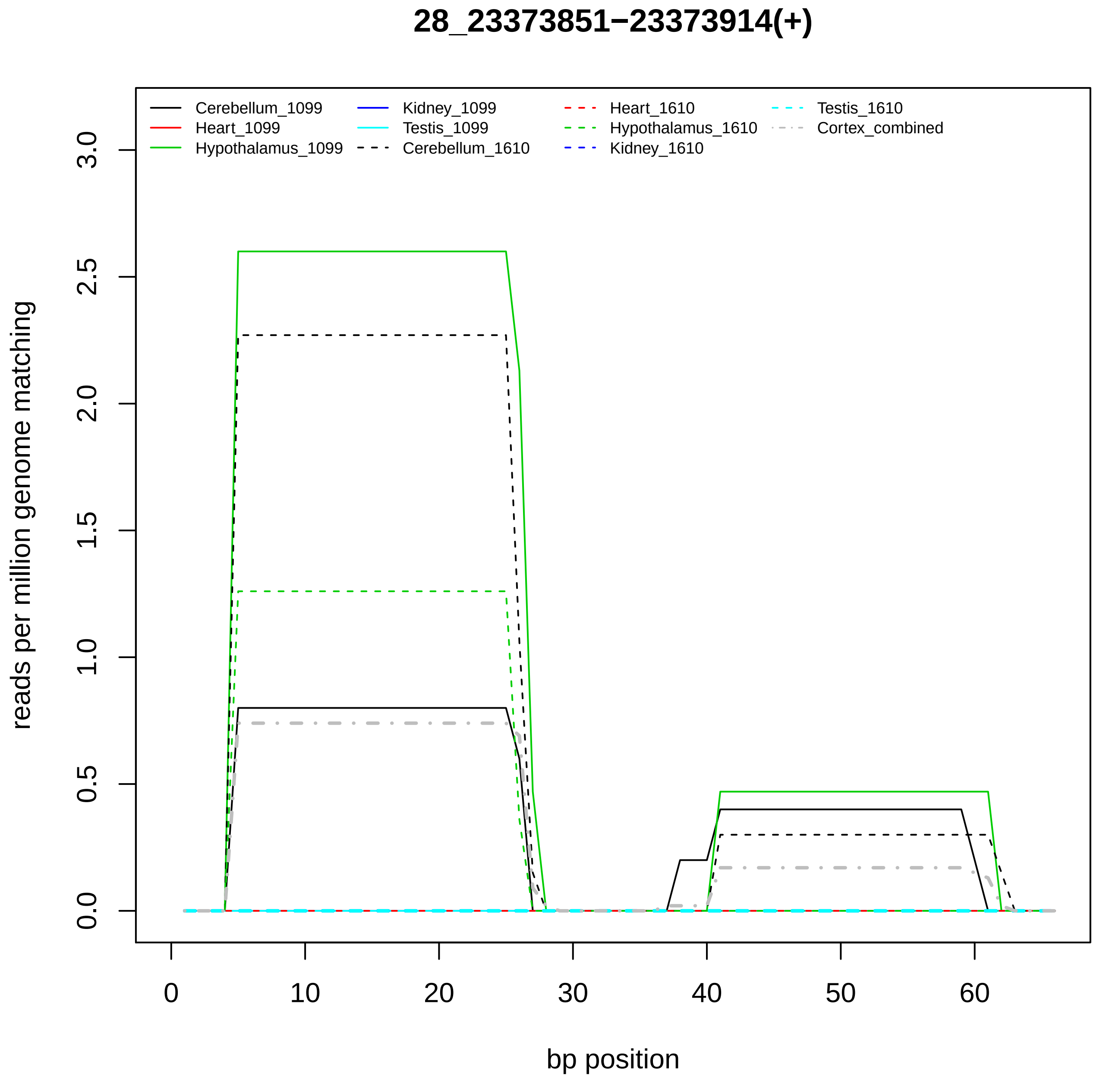
28_23373851−23373914(+)
Cerebellum_1099
Heart_1099
Hypothalamus_1099
Kidney_1099
Testis_1099
Cerebellum_1610
Heart_1610
Hypothalamus_1610
Kidney_1610
Testis_1610
Cortex_combined
reads per million genome matching
0.0
0.5
1.0
1.5
2.0
2.5
3.0
0
10
20
30
40
50
60
bp position

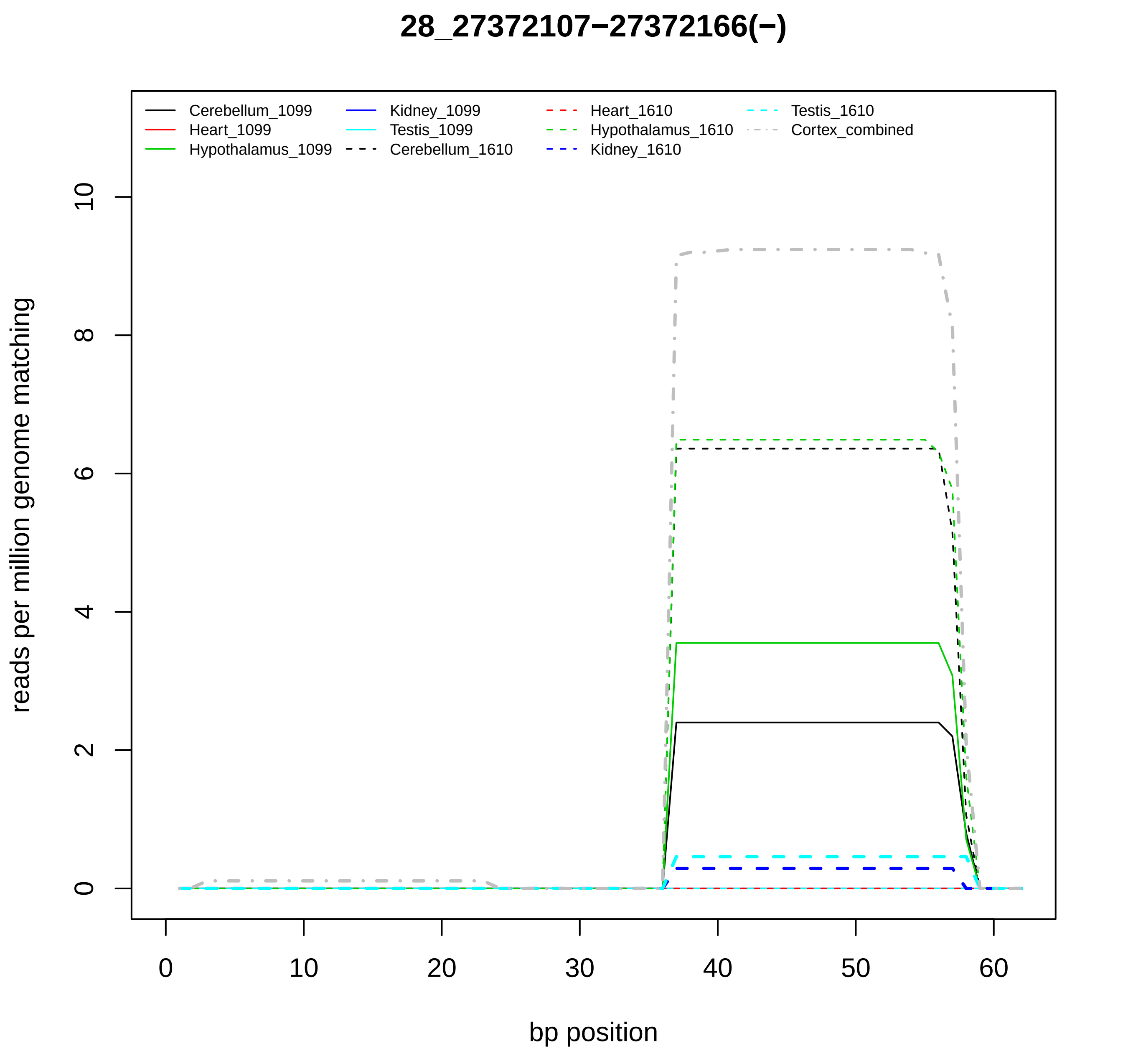

28_27372107−27372166(−)
Cerebellum_1099
Heart_1099
Hypothalamus_1099
Kidney_1099
Testis_1099
Cerebellum_1610
Heart_1610
Hypothalamus_1610
Kidney_1610
Testis_1610
Cortex_combined
reads per million genome matching
bp position
0
2
4
6
8
10
0
10
20
30
40
50
60

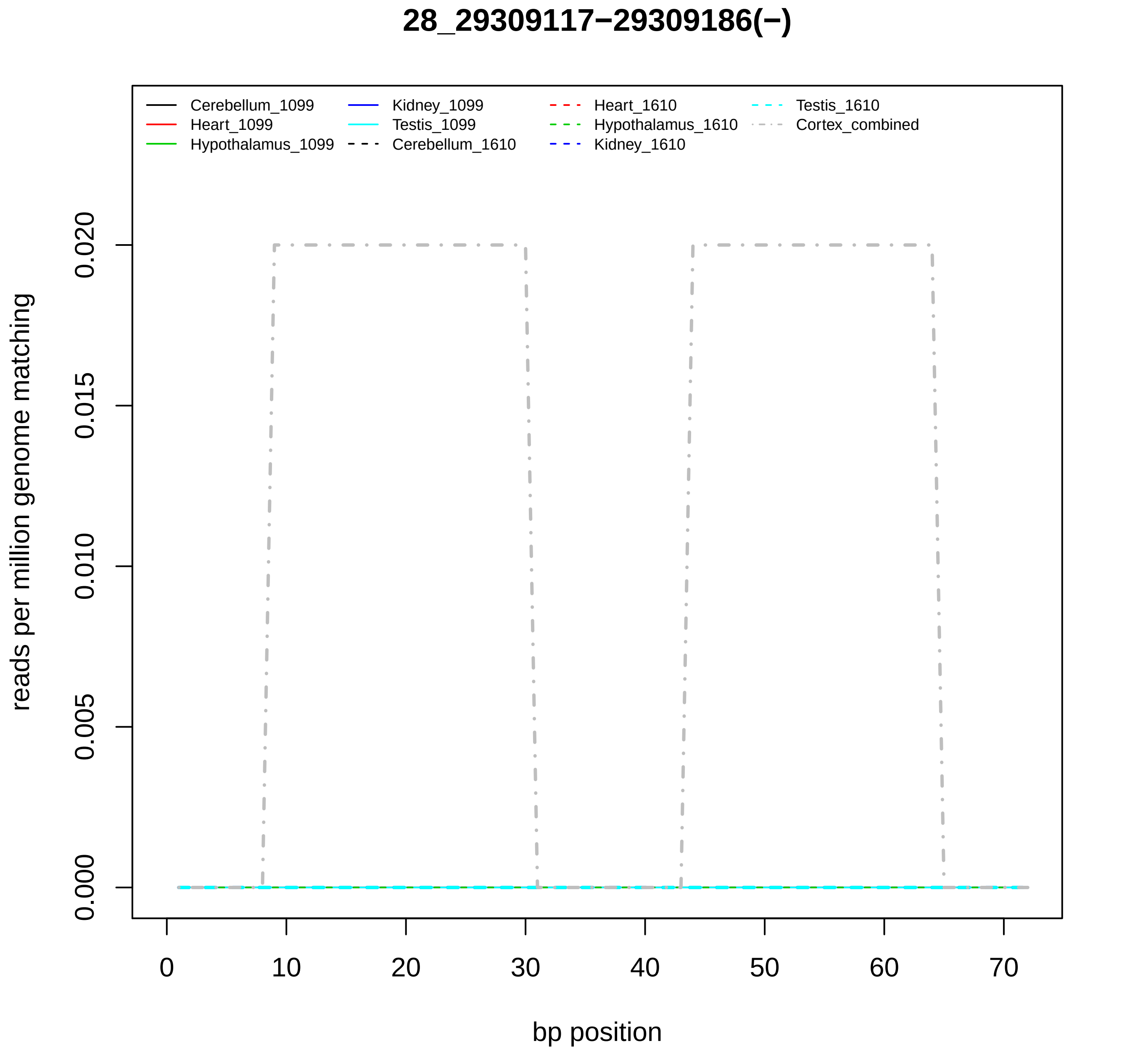

28_29309117−29309186(−)
Cerebellum_1099
Heart_1099
Hypothalamus_1099
Kidney_1099
Testis_1099
Cerebellum_1610
Heart_1610
Hypothalamus_1610
Kidney_1610
Testis_1610
Cortex_combined
reads per million genome matching
0.000
0.005
0.010
0.015
0.020
0
10
20
30
40
50
60
70
bp position

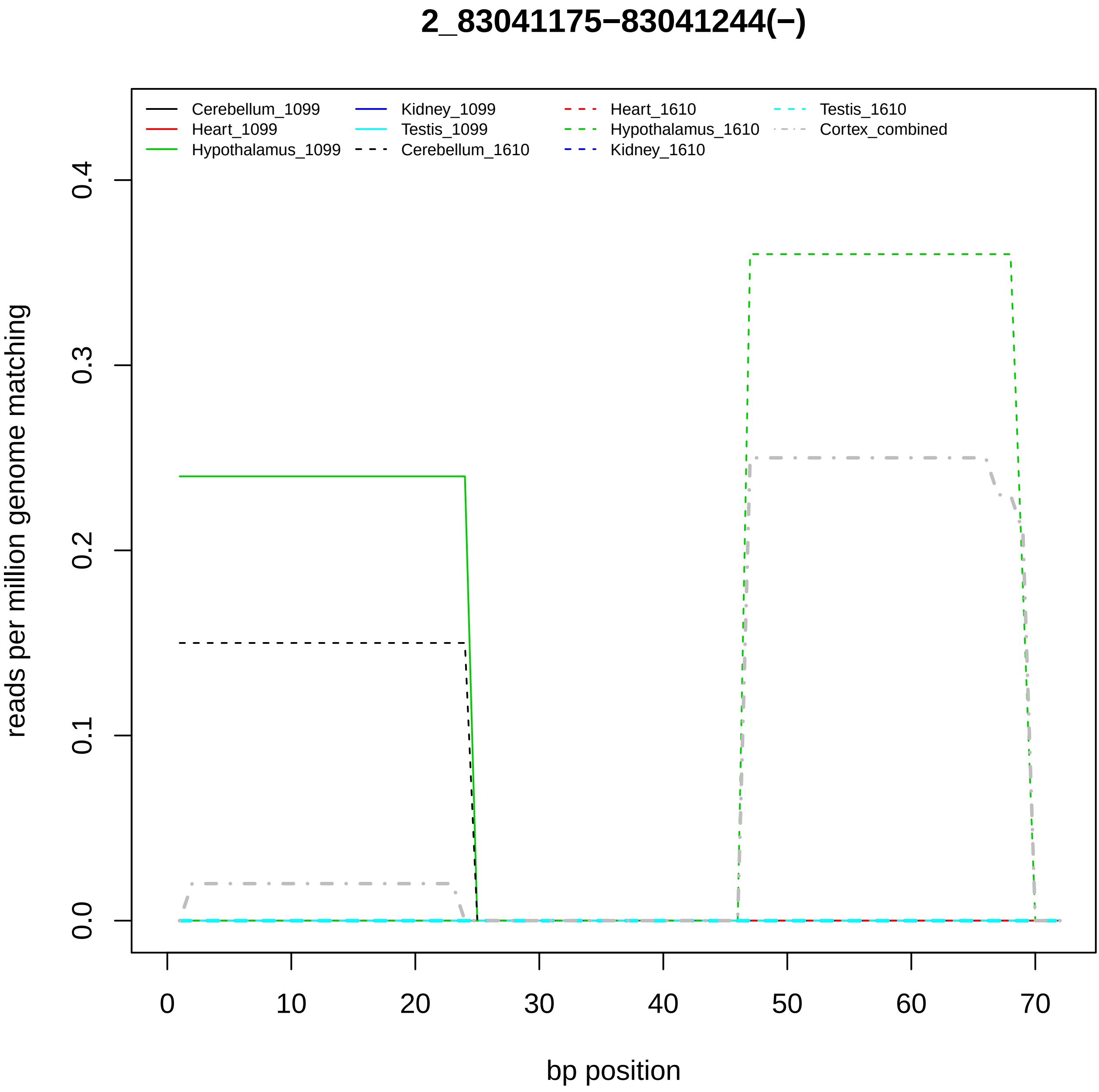

2_83041175−83041244(−)
Cerebellum_1099
Heart_1099
Hypothalamus_1099
Kidney_1099
Testis_1099
Cerebellum_1610
Heart_1610
Hypothalamus_1610
Kidney_1610
Testis_1610
Cortex_combined
reads per million genome matching
0.0
0.1
0.2
0.3
0.4
0
10
20
30
40
50
60
70
bp position

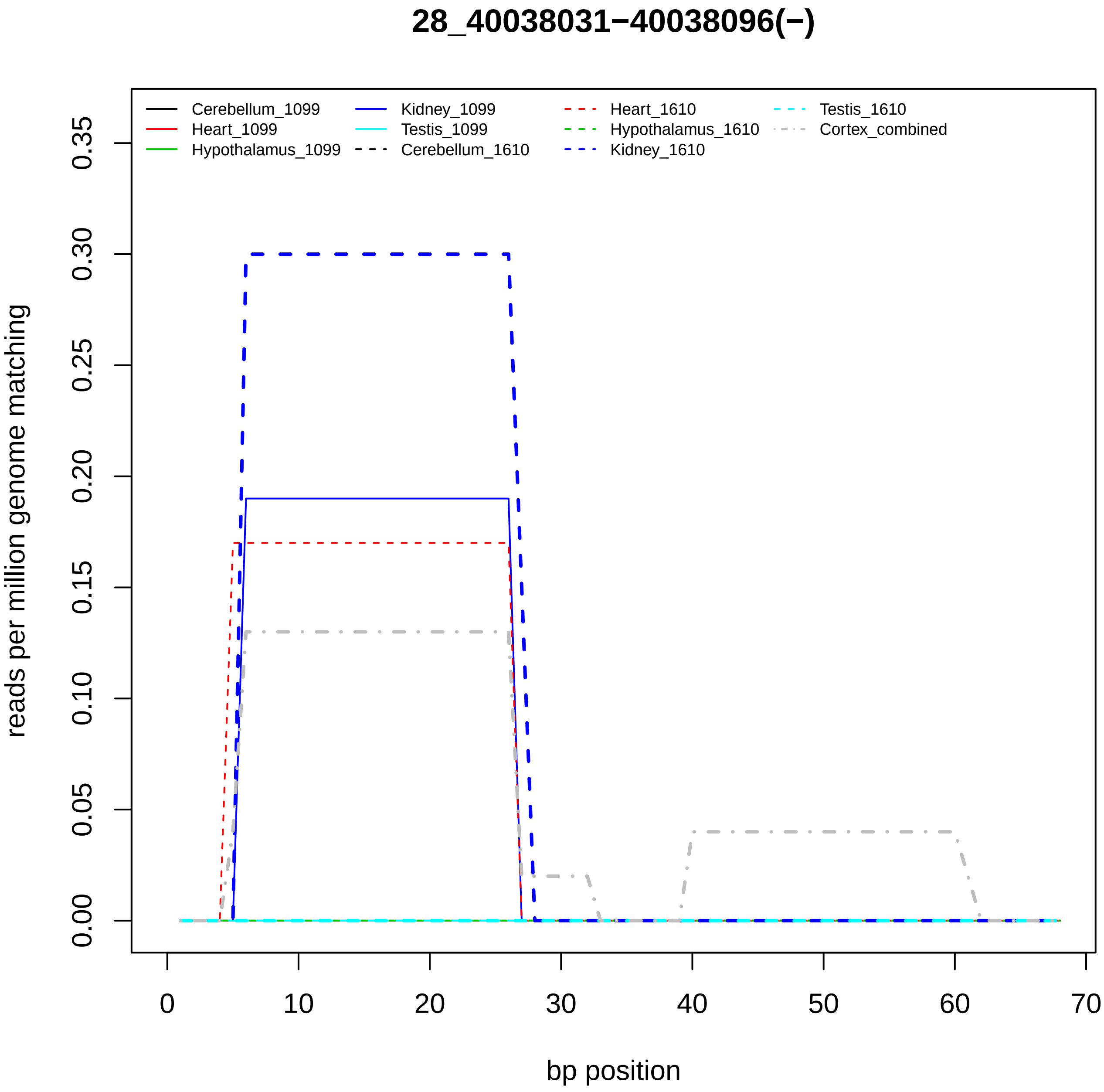
28_40038031−40038096(−)
Cerebellum_1099
Heart_1099
Hypothalamus_1099
Kidney_1099
Testis_1099
Cerebellum_1610
Heart_1610
Hypothalamus_1610
Kidney_1610
Testis_1610
Cortex_combined
reads per million genome matching
0.00
0.05
0.10
0.15
0.20
0.25
0.30
0.35
0
10
20
30
40
50
60
70
bp position

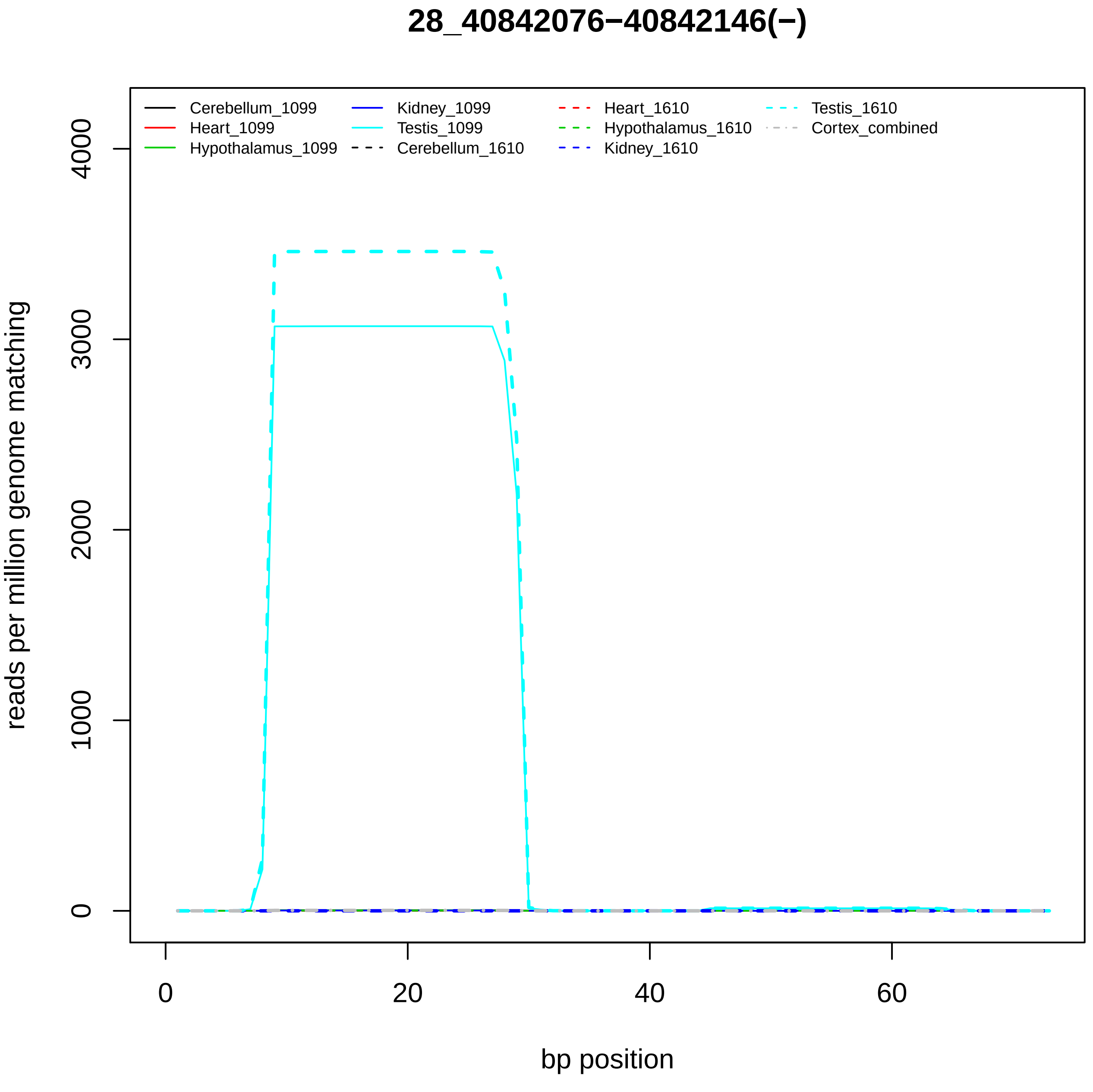

28_40842076−40842146(−)
Cerebellum_1099
Heart_1099
Hypothalamus_1099
Kidney_1099
Testis_1099
Cerebellum_1610
Heart_1610
Hypothalamus_1610
Kidney_1610
Testis_1610
Cortex_combined
reads per million genome matching
0
1000
2000
3000
4000
0
20
40
60
bp position

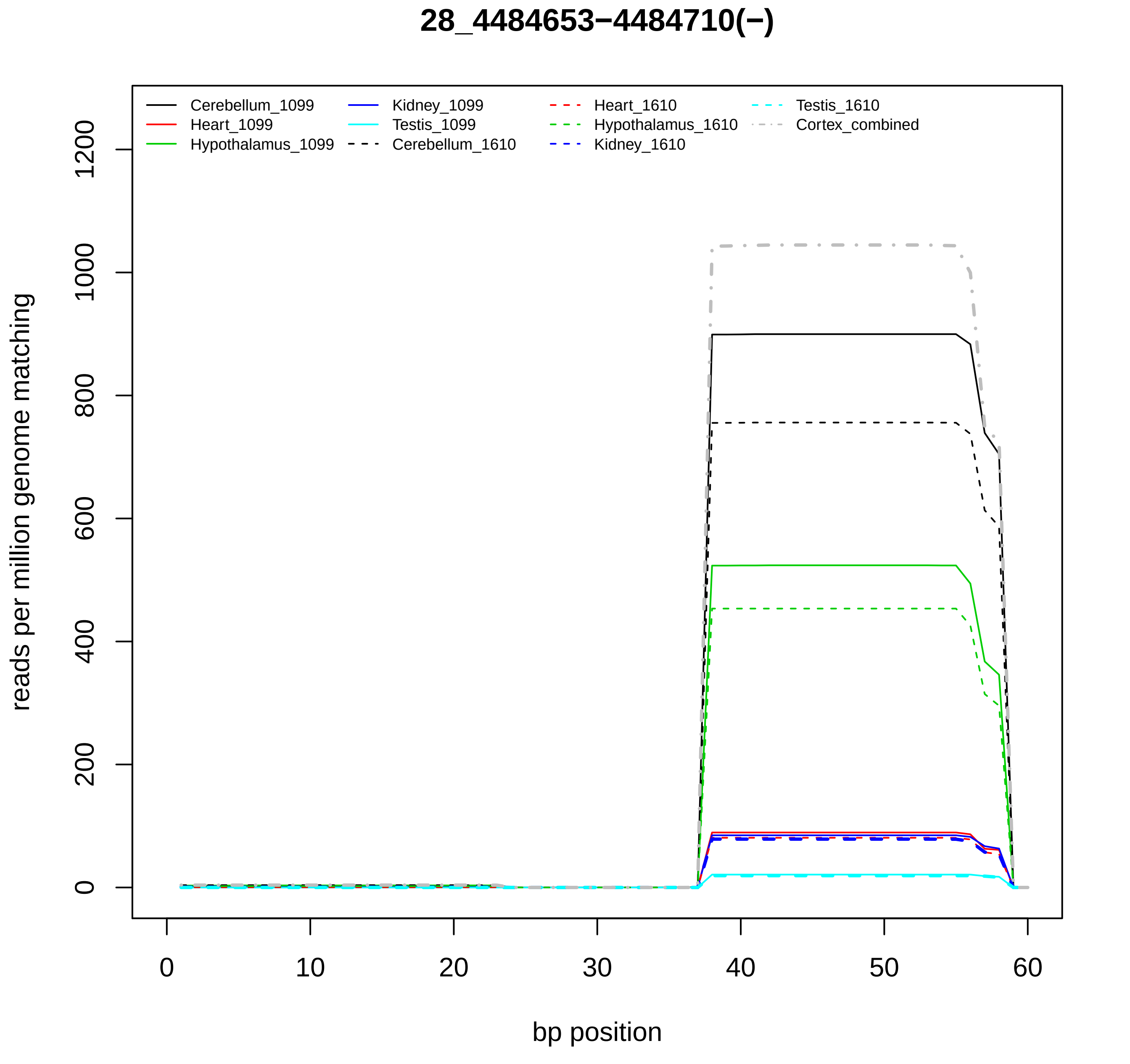

28_4484653−4484710(−)
Cerebellum_1099
Heart_1099
Hypothalamus_1099
Kidney_1099
Testis_1099
Cerebellum_1610
Heart_1610
Hypothalamus_1610
Kidney_1610
Testis_1610
Cortex_combined
reads per million genome matching
bp position
0
200
400
600
800
1000
1200
0
10
20
30
40
50
60

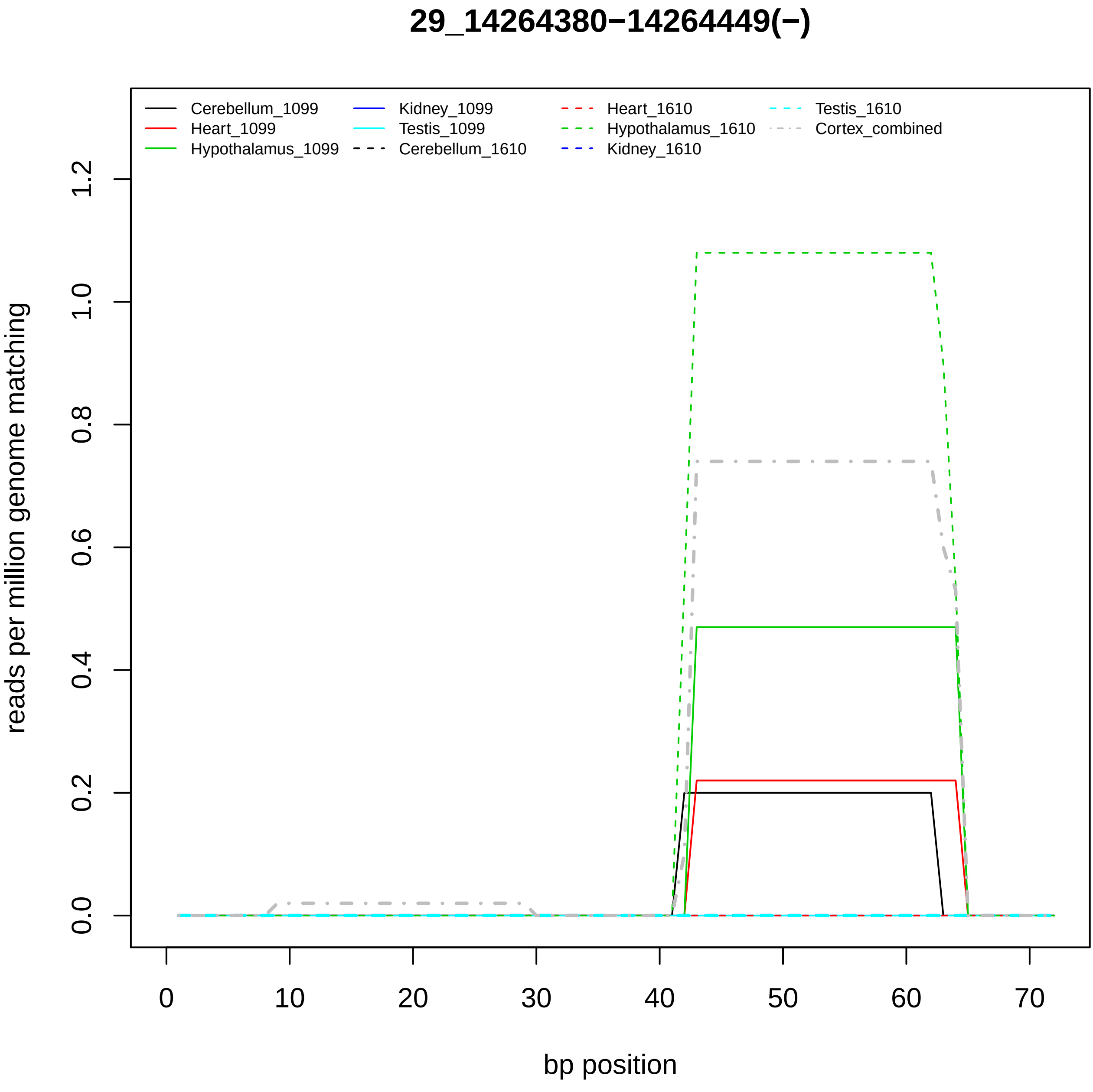

29_14264380−14264449(−)
Cerebellum_1099
Heart_1099
Hypothalamus_1099
Kidney_1099
Testis_1099
Cerebellum_1610
Heart_1610
Hypothalamus_1610
Kidney_1610
Testis_1610
Cortex_combined
reads per million genome matching
bp position
0.0
0.2
0.4
0.6
0.8
1.0
1.2
0
10
20
30
40
50
60
70

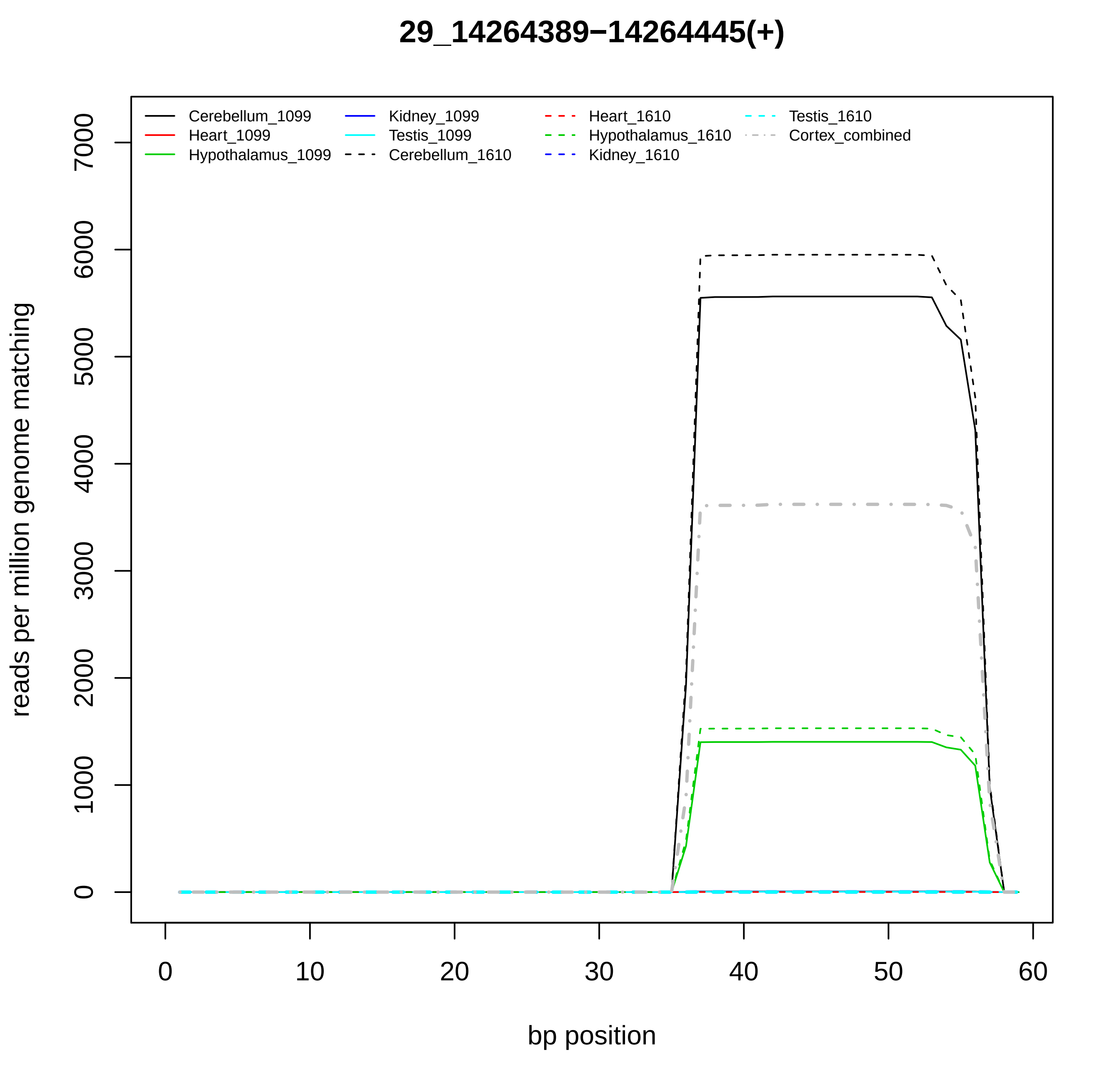
29_14264389−14264445(+)
Cerebellum_1099
Heart_1099
Hypothalamus_1099
Kidney_1099
Testis_1099
Cerebellum_1610
Heart_1610
Hypothalamus_1610
Kidney_1610
Testis_1610
Cortex_combined
reads per million genome matching
bp position
0
1000
2000
3000
4000
5000
6000
7000
0
10
20
30
40
50
60

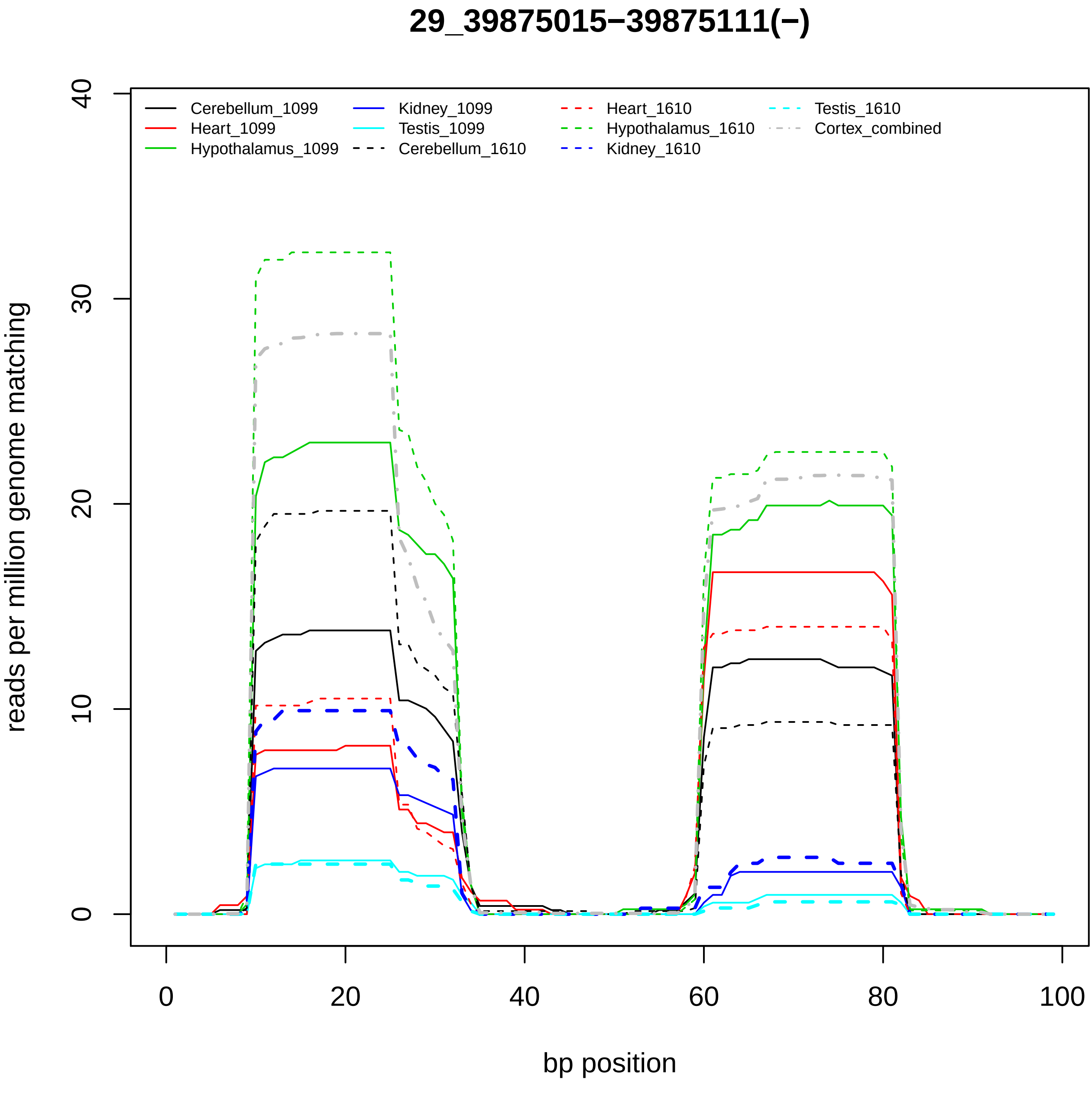

29_39875015−39875111(−)
Cerebellum_1099
Heart_1099
Hypothalamus_1099
Kidney_1099
Testis_1099
Cerebellum_1610
Heart_1610
Hypothalamus_1610
Kidney_1610
Testis_1610
Cortex_combined
reads per million genome matching
bp position
0
10
20
30
40
0
20
40
60
80
100

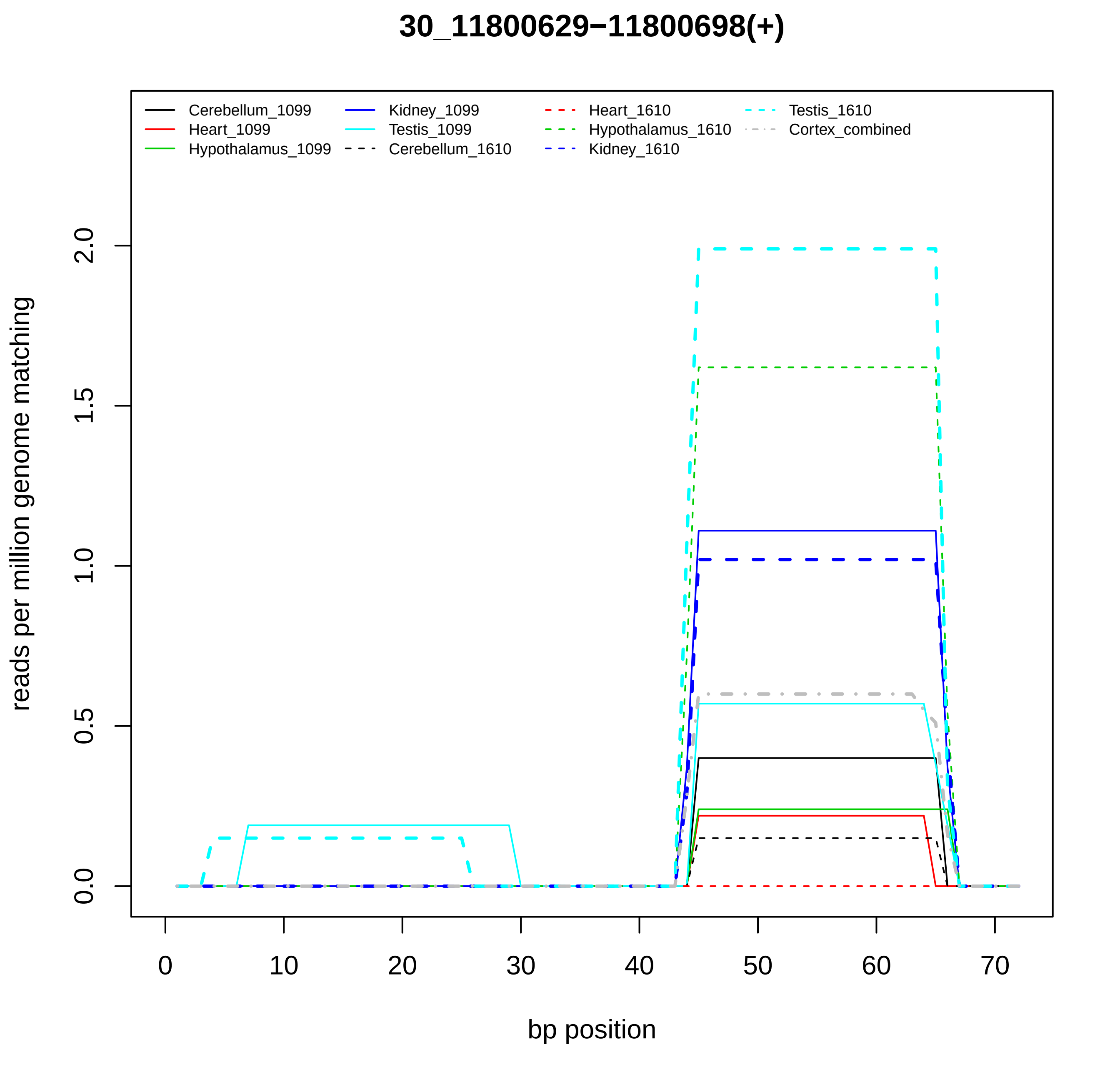

30_11800629−11800698(+)
Cerebellum_1099
Heart_1099
Hypothalamus_1099
Kidney_1099
Testis_1099
Cerebellum_1610
Heart_1610
Hypothalamus_1610
Kidney_1610
Testis_1610
Cortex_combined
reads per million genome matching
0.0
0.5
1.0
1.5
2.0
0
10
20
30
40
50
60
70
bp position

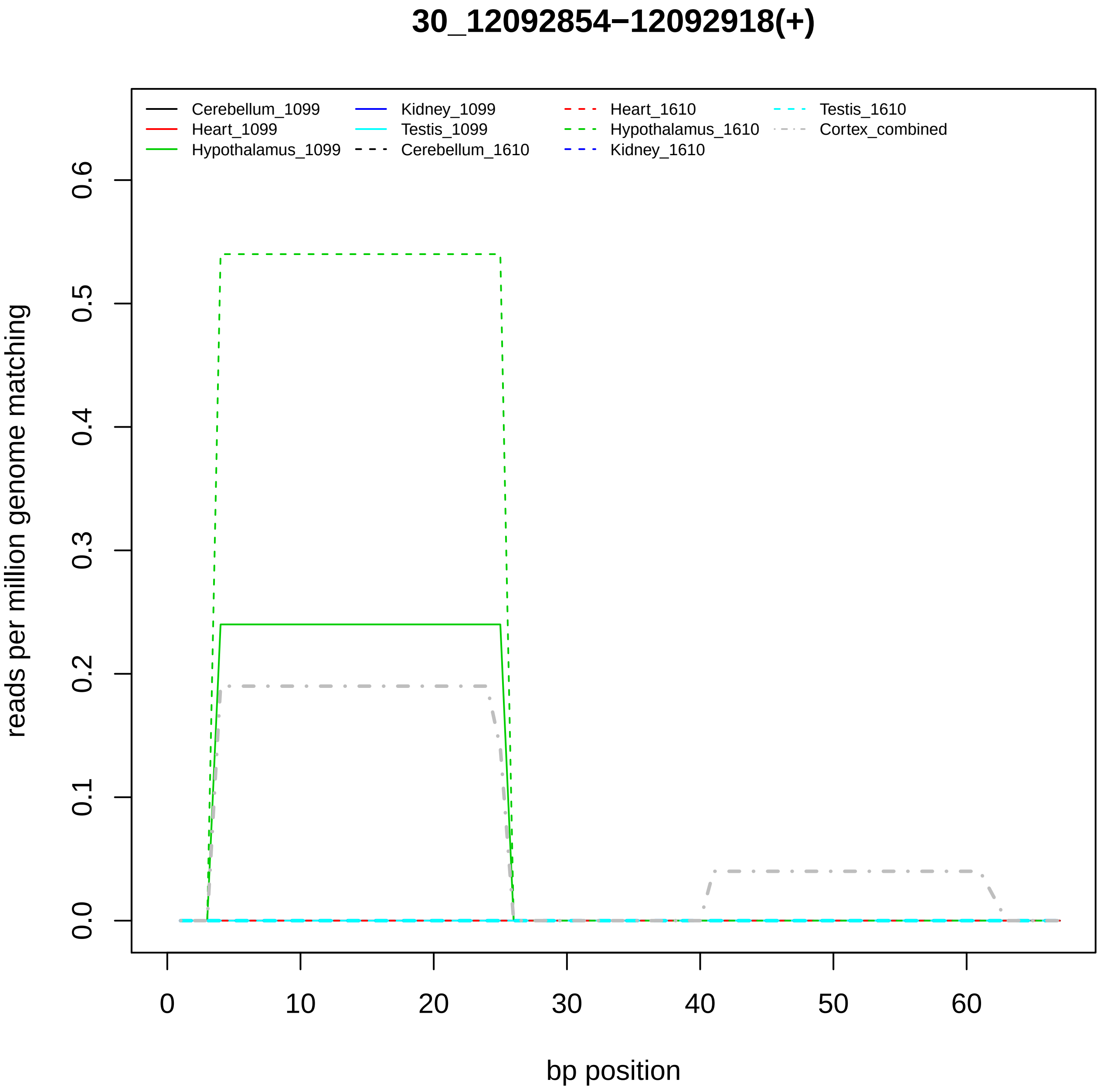
30_12092854−12092918(+)
Cerebellum_1099
Heart_1099
Hypothalamus_1099
Kidney_1099
Testis_1099
Cerebellum_1610
Heart_1610
Hypothalamus_1610
Kidney_1610
Testis_1610
Cortex_combined
reads per million genome matching
0.0
0.1
0.2
0.3
0.4
0.5
0.6
0
10
20
30
40
50
60
bp position

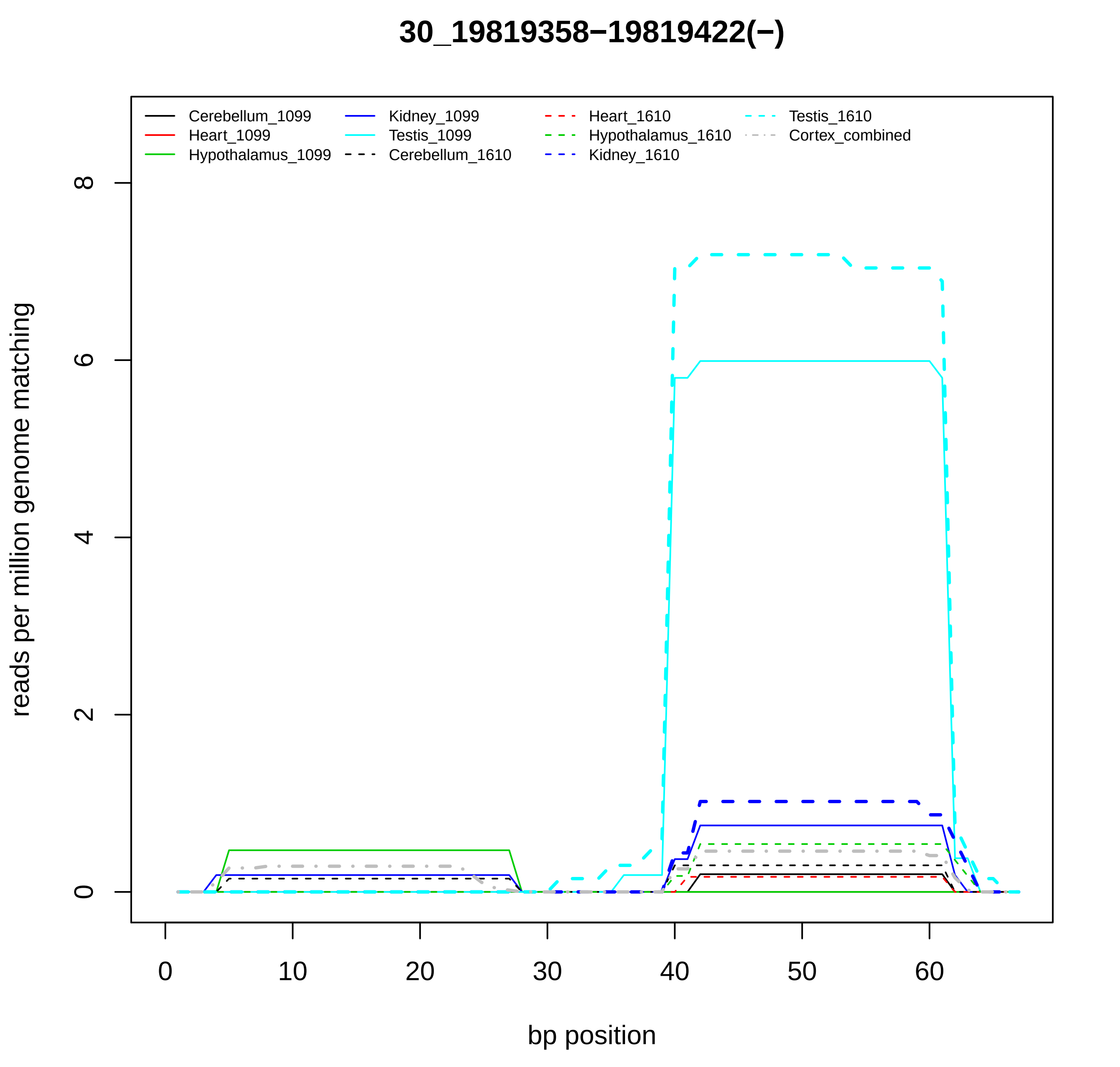

30_19819358−19819422(−)
Cerebellum_1099
Heart_1099
Hypothalamus_1099
Kidney_1099
Testis_1099
Cerebellum_1610
Heart_1610
Hypothalamus_1610
Kidney_1610
Testis_1610
Cortex_combined
reads per million genome matching
bp position
0
2
4
6
8
0
10
20
30
40
50
60

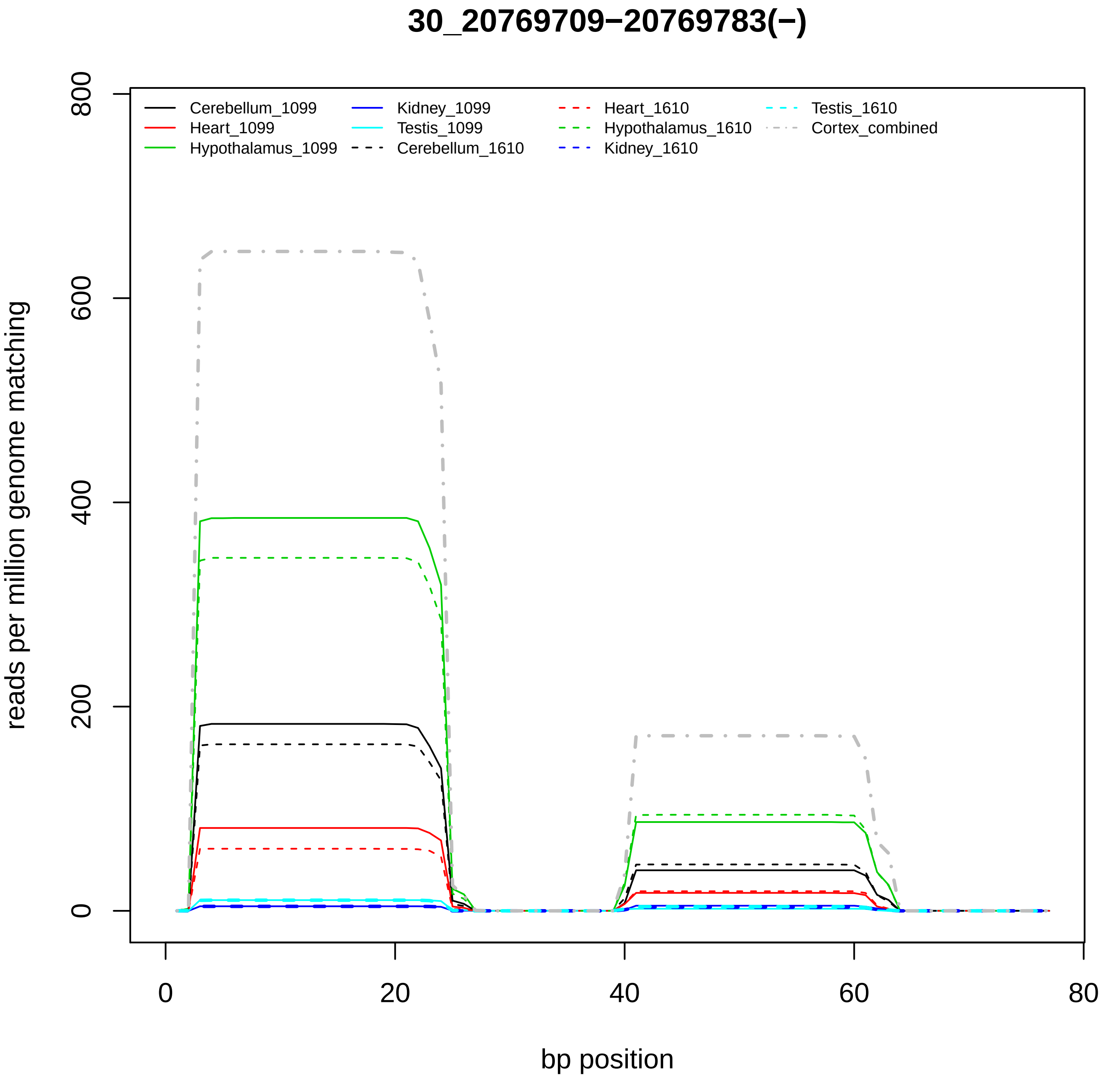
30_20769709−20769783(−)
reads per million genome matching
bp position
0
200
400
600
800
0
20
40
60
80
Cerebellum_1099
Heart_1099
Hypothalamus_1099
Kidney_1099
Testis_1099
Cerebellum_1610
Heart_1610
Hypothalamus_1610
Kidney_1610
Testis_1610
Cortex_combined

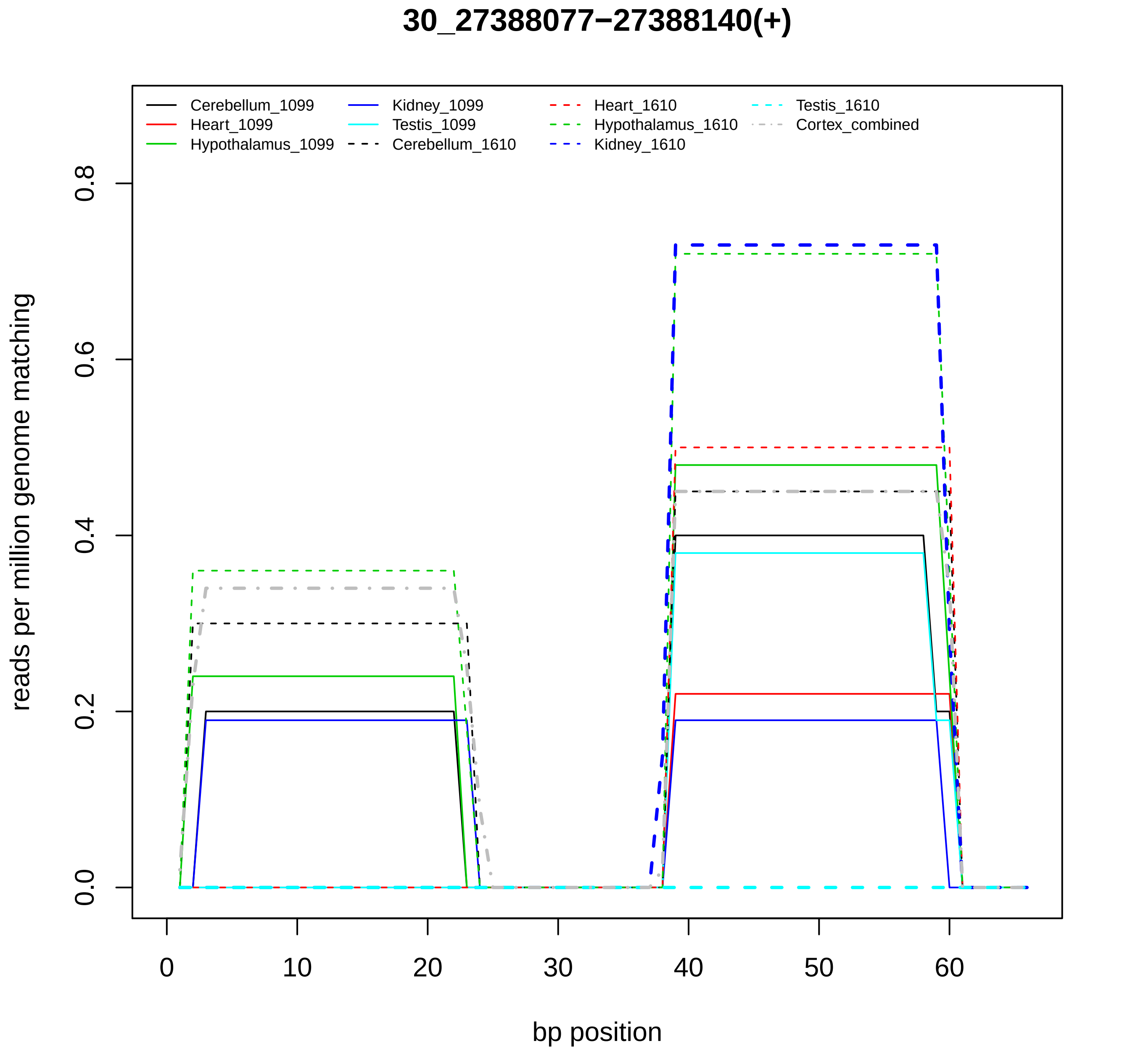

30_27388077−27388140(+)
Cerebellum_1099
Heart_1099
Hypothalamus_1099
Kidney_1099
Testis_1099
Cerebellum_1610
Heart_1610
Hypothalamus_1610
Kidney_1610
Testis_1610
Cortex_combined
reads per million genome matching
0.0
0.2
0.4
0.6
0.8
0
10
20
30
40
50
60
bp position

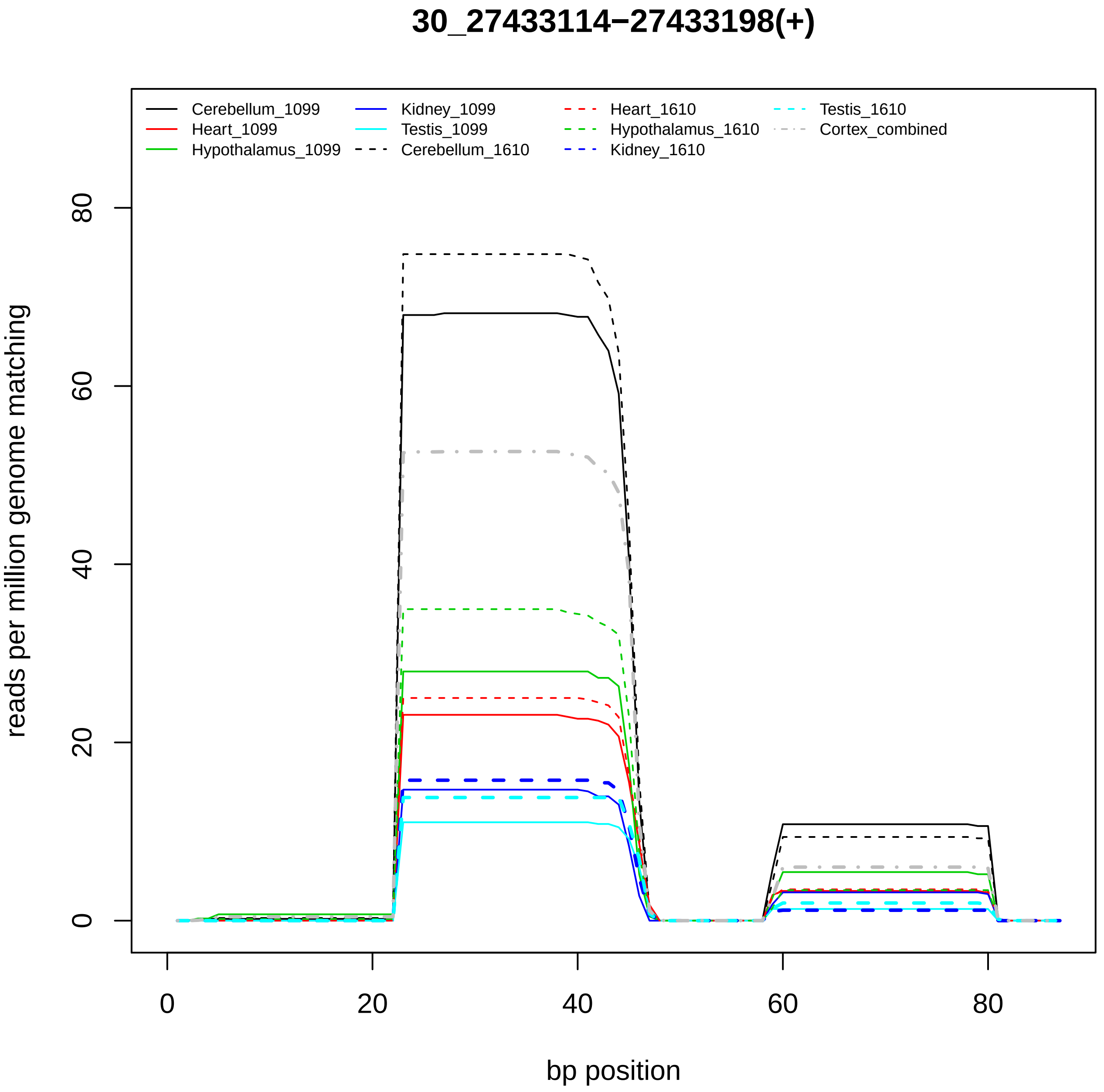
30_27433114−27433198(+)
Cerebellum_1099
Heart_1099
Hypothalamus_1099
Kidney_1099
Testis_1099
Cerebellum_1610
Heart_1610
Hypothalamus_1610
Kidney_1610
Testis_1610
Cortex_combined
reads per million genome matching
0
20
40
60
80
bp position
0
20
40
60
80

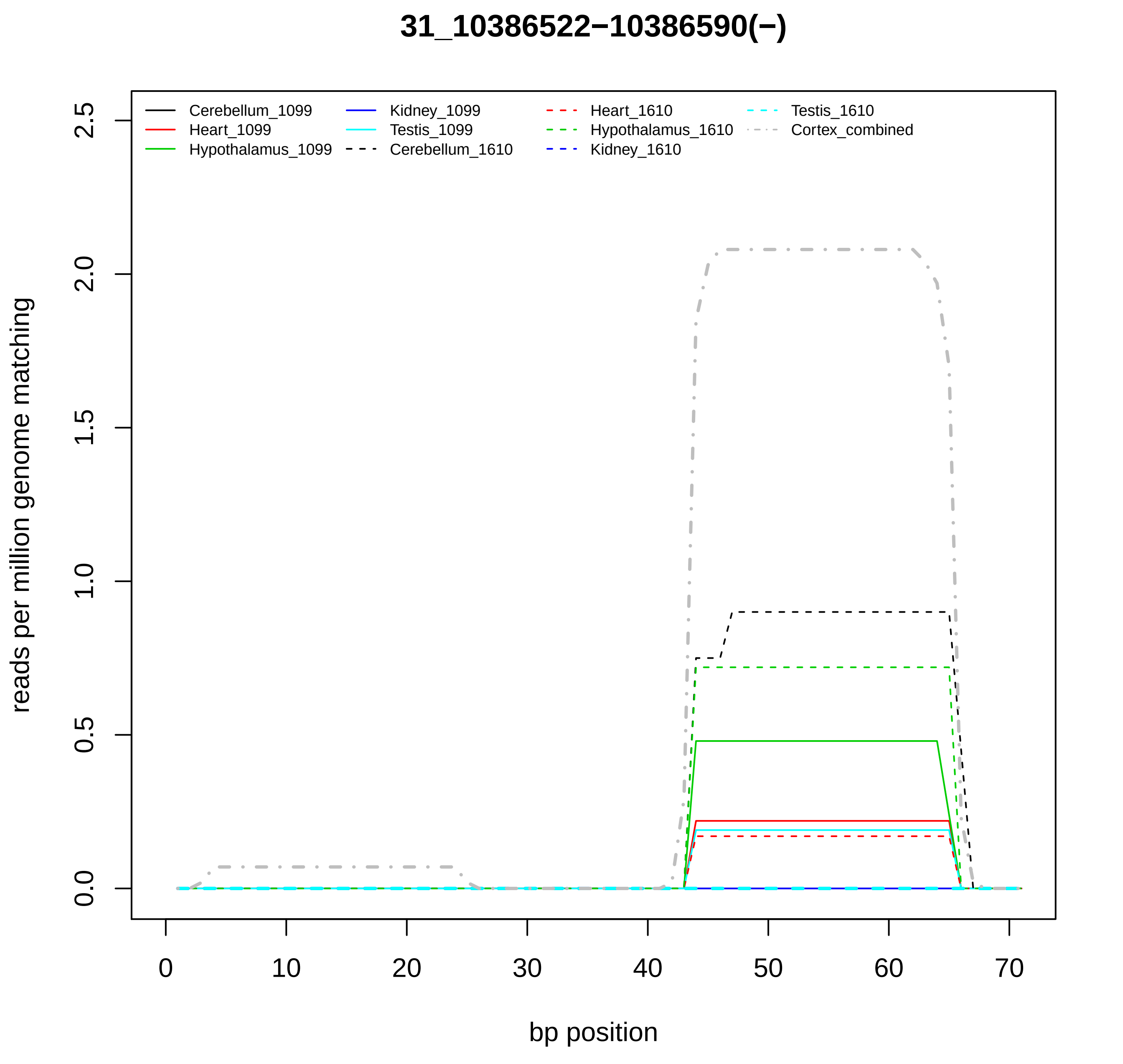
31_10386522−10386590(−)
Cerebellum_1099
Heart_1099
Hypothalamus_1099
Kidney_1099
Testis_1099
Cerebellum_1610
Heart_1610
Hypothalamus_1610
Kidney_1610
Testis_1610
Cortex_combined
reads per million genome matching
0.0
0.5
1.0
1.5
2.0
2.5
0
10
20
30
40
50
60
70
bp position

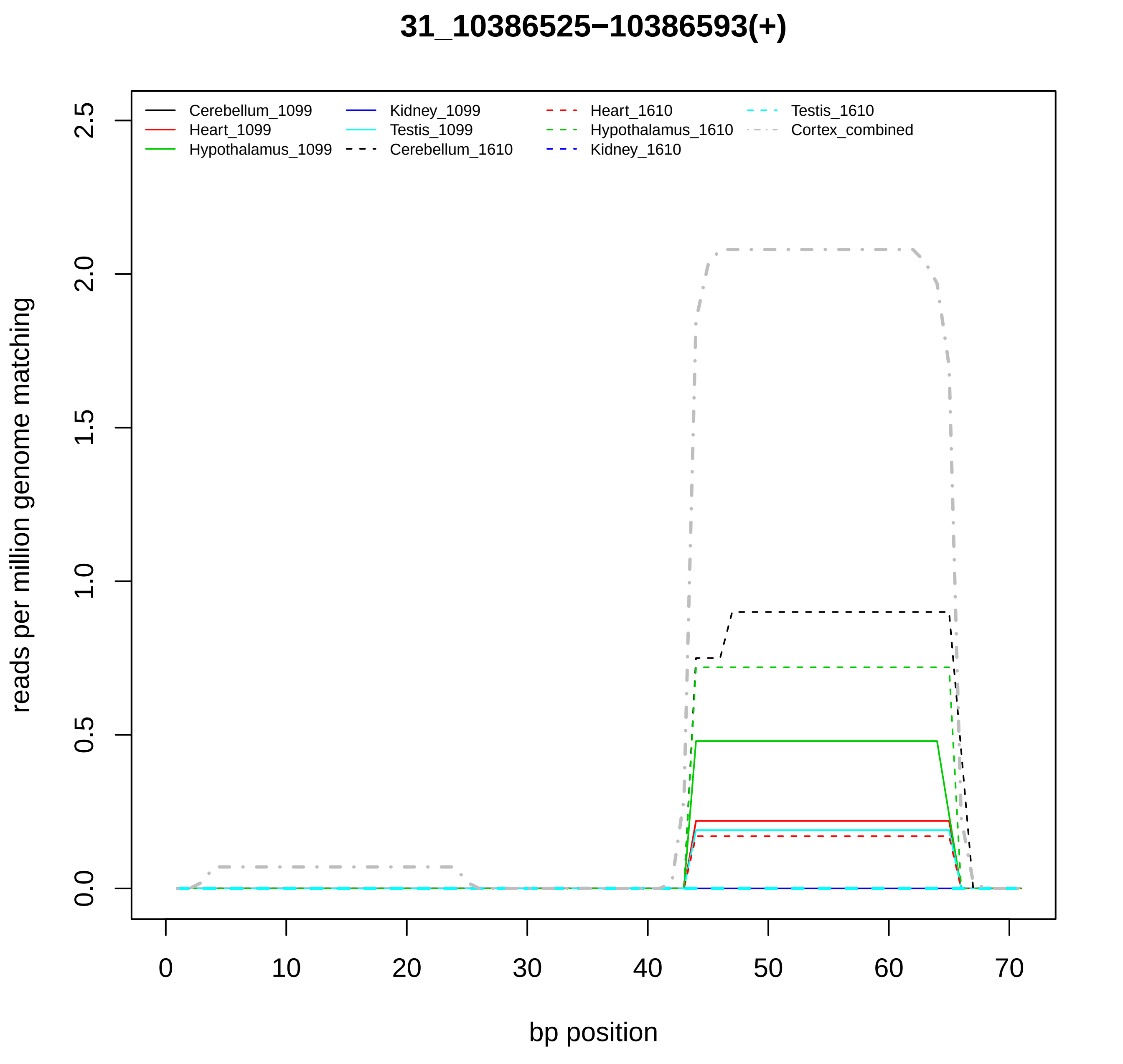

31_10386525−10386593(+)
Cerebellum_1099
Heart_1099
Hypothalamus_1099
Kidney_1099
Testis_1099
Cerebellum_1610
Heart_1610
Hypothalamus_1610
Kidney_1610
Testis_1610
Cortex_combined
reads per million genome matching
bp position
0.0
0.5
1.0
1.5
2.0
2.5
0
10
20
30
40
50
60
70

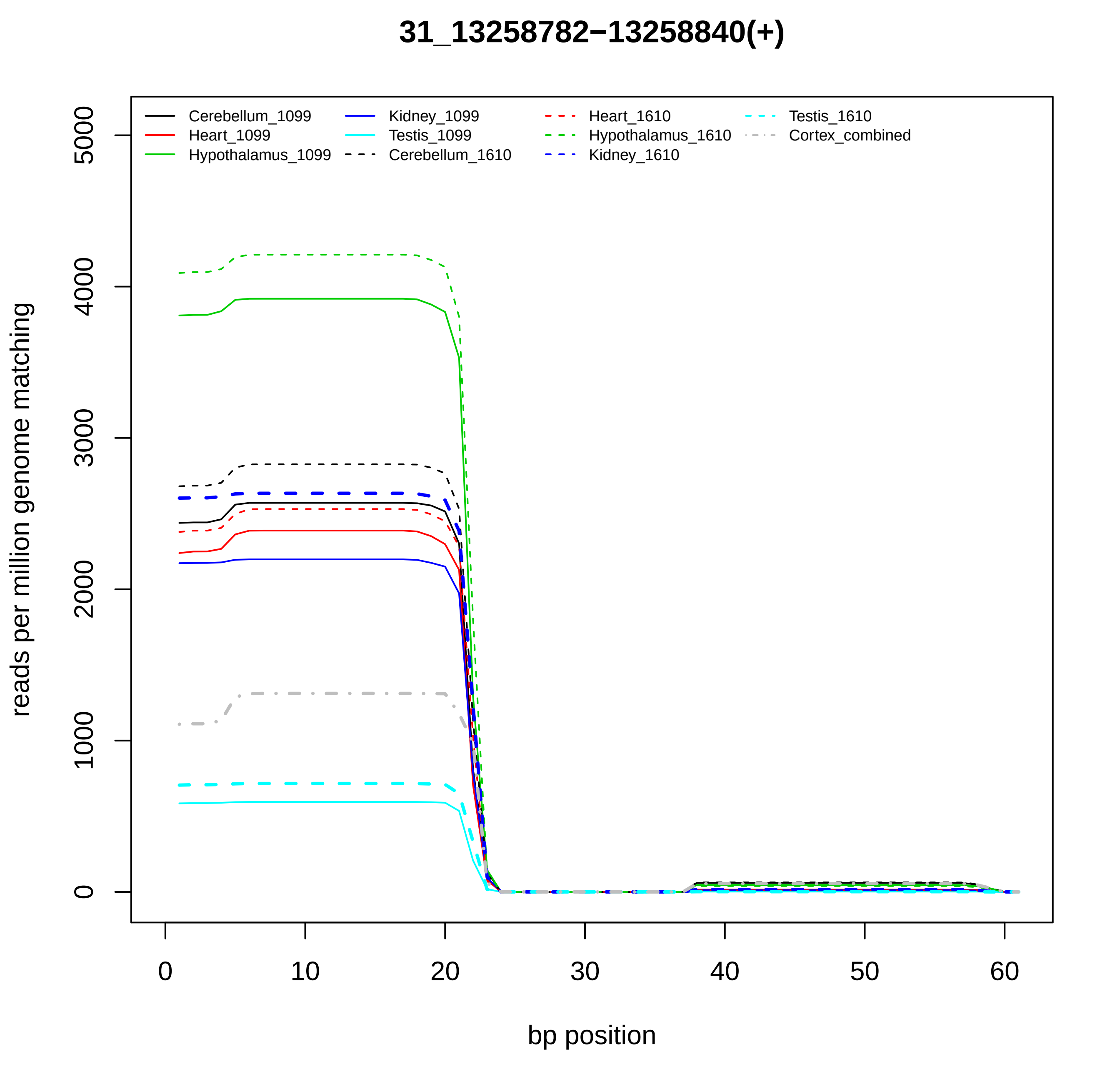
31_13258782−13258840(+)
Cerebellum_1099
Heart_1099
Hypothalamus_1099
Kidney_1099
Testis_1099
Cerebellum_1610
Heart_1610
Hypothalamus_1610
Kidney_1610
Testis_1610
Cortex_combined
reads per million genome matching
0
1000
2000
3000
4000
5000
0
10
20
30
40
50
60
bp position

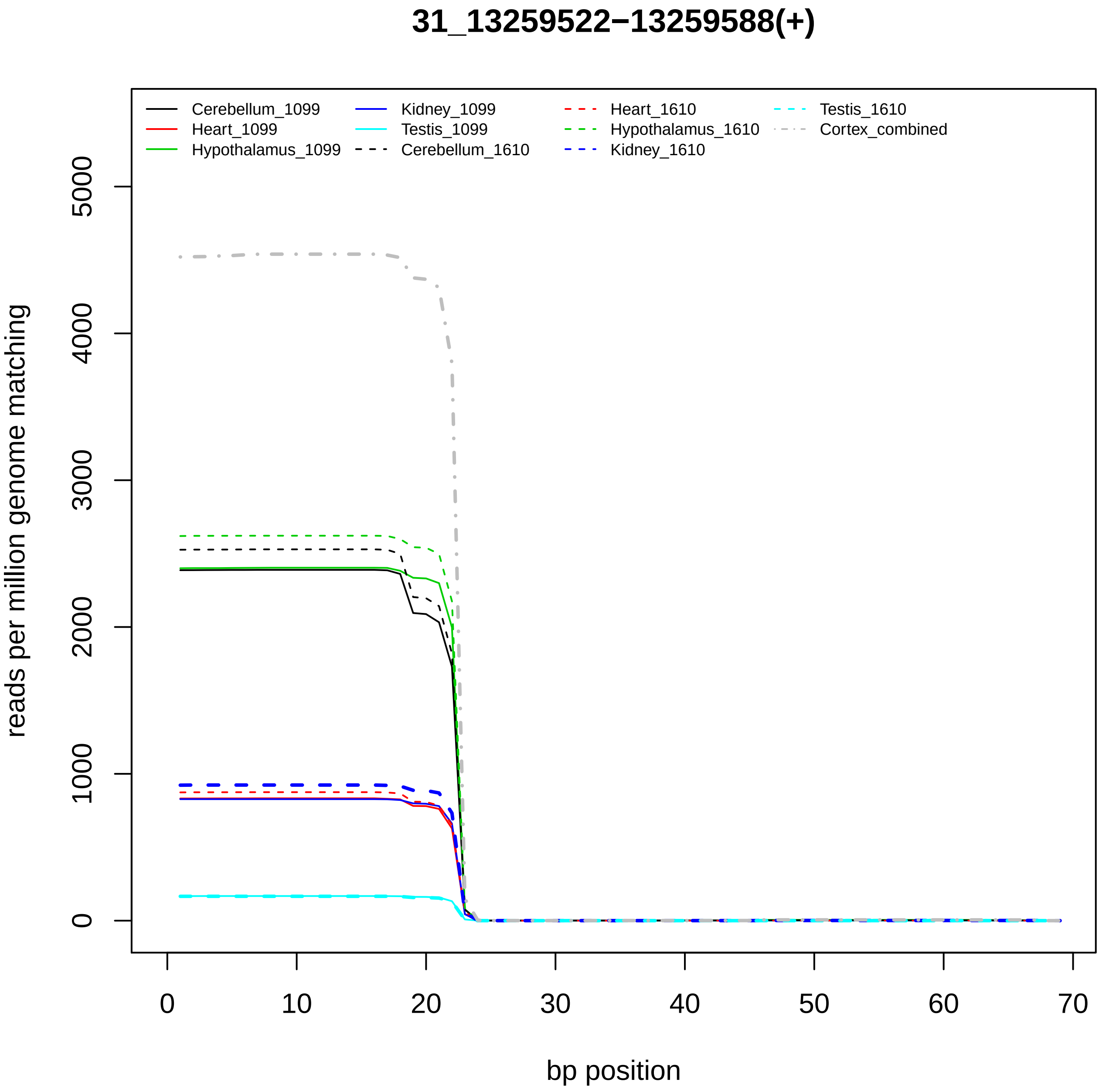

31_13259522−13259588(+)
Cerebellum_1099
Heart_1099
Hypothalamus_1099
Kidney_1099
Testis_1099
Cerebellum_1610
Heart_1610
Hypothalamus_1610
Kidney_1610
Testis_1610
Cortex_combined
reads per million genome matching
0
1000
2000
3000
4000
5000
0
10
20
30
40
50
60
70
bp position

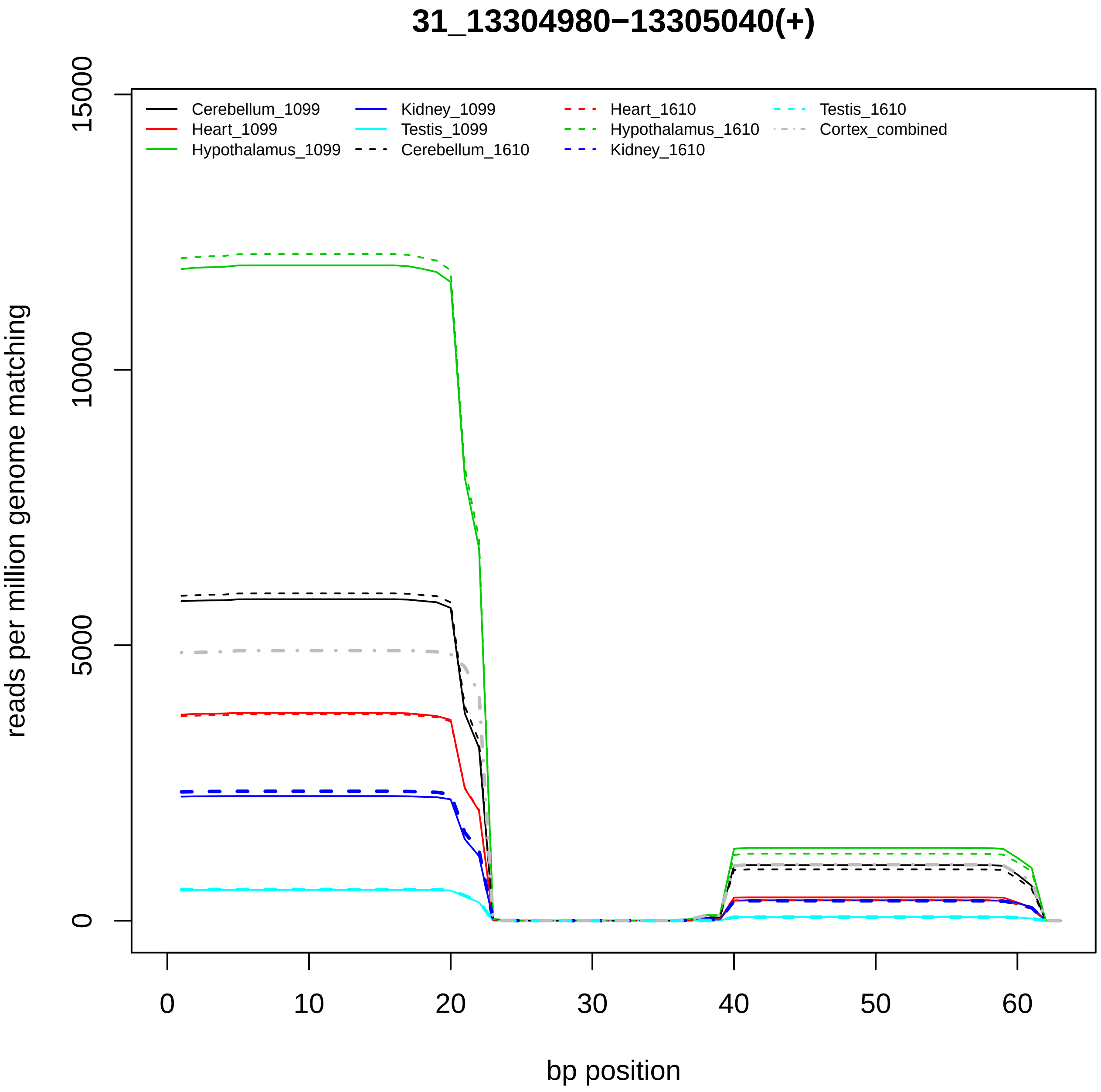

31_13304980−13305040(+)
Cerebellum_1099
Heart_1099
Hypothalamus_1099
Kidney_1099
Testis_1099
Cerebellum_1610
Heart_1610
Hypothalamus_1610
Kidney_1610
Testis_1610
Cortex_combined
reads per million genome matching
15000
10000
5000
0
0
10
20
30
40
50
60
bp position

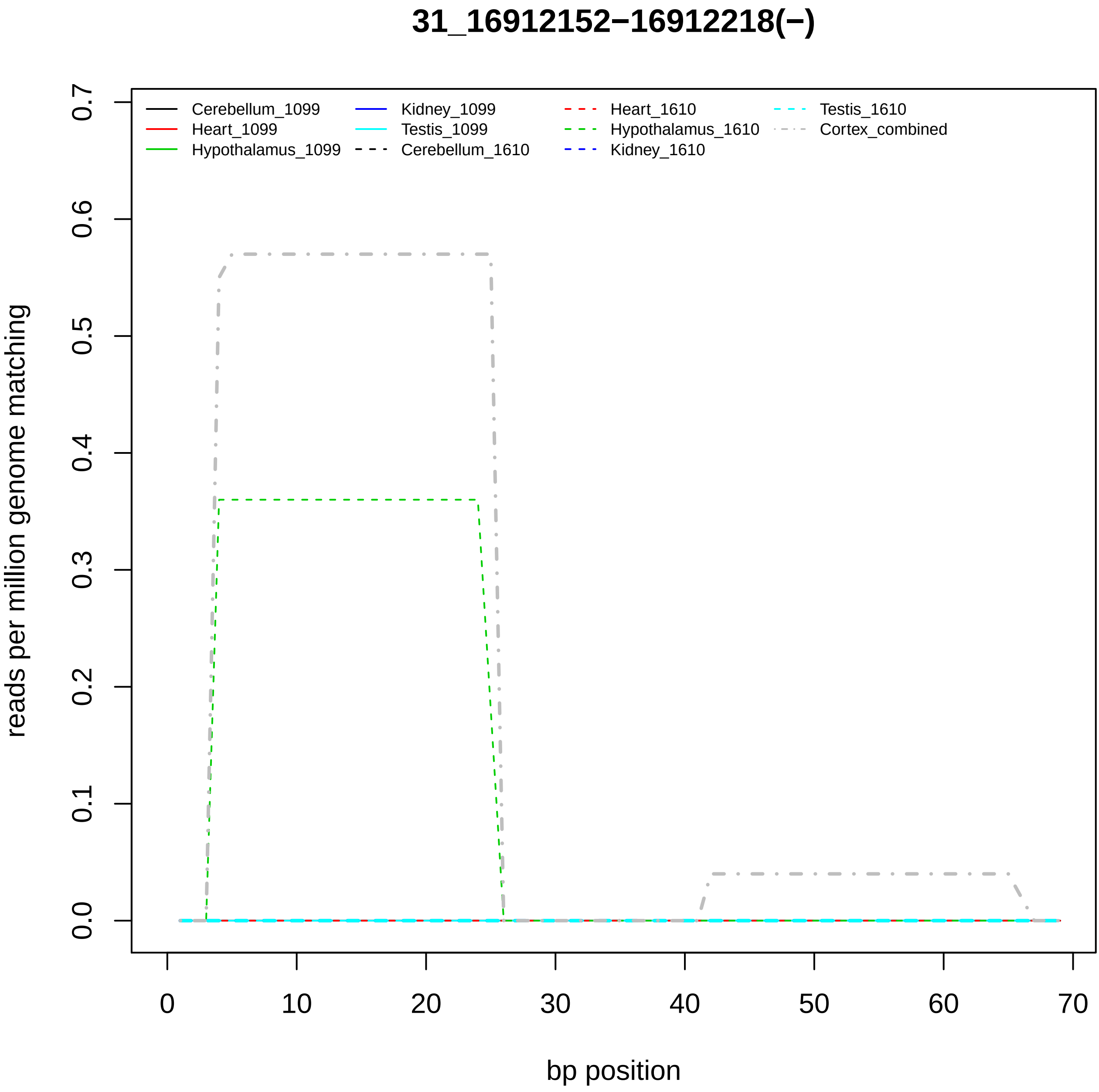

31_16912152−16912218(−)
Cerebellum_1099
Heart_1099
Hypothalamus_1099
Kidney_1099
Testis_1099
Cerebellum_1610
Heart_1610
Hypothalamus_1610
Kidney_1610
Testis_1610
Cortex_combined
reads per million genome matching
bp position
0.0
0.1
0.2
0.3
0.4
0.5
0.6
0.7
0
10
20
30
40
50
60
70

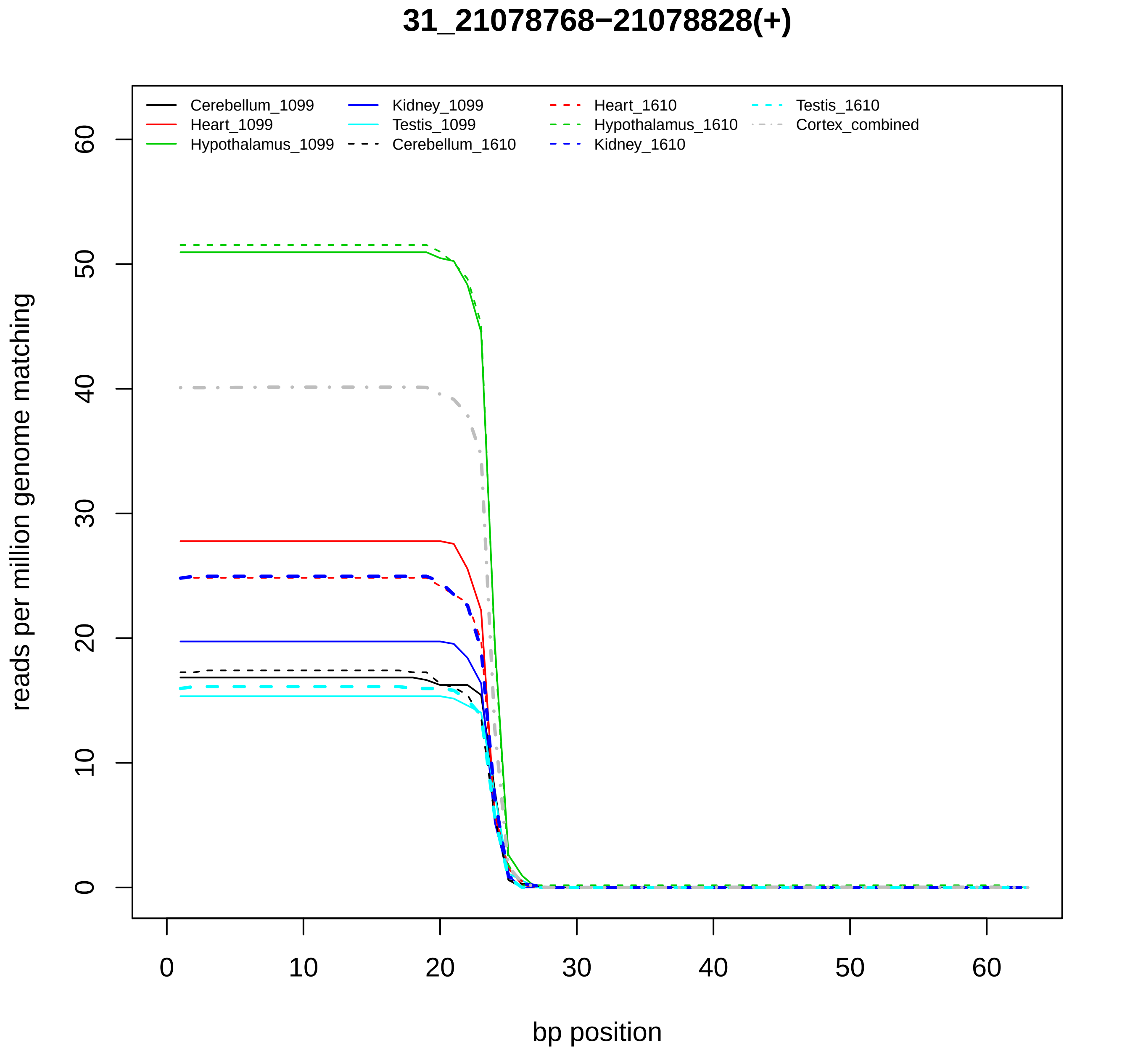

31_21078768−21078828(+)
Cerebellum_1099
Heart_1099
Hypothalamus_1099
Kidney_1099
Testis_1099
Cerebellum_1610
Heart_1610
Hypothalamus_1610
Kidney_1610
Testis_1610
Cortex_combined
reads per million genome matching
bp position
0
10
20
30
40
50
60

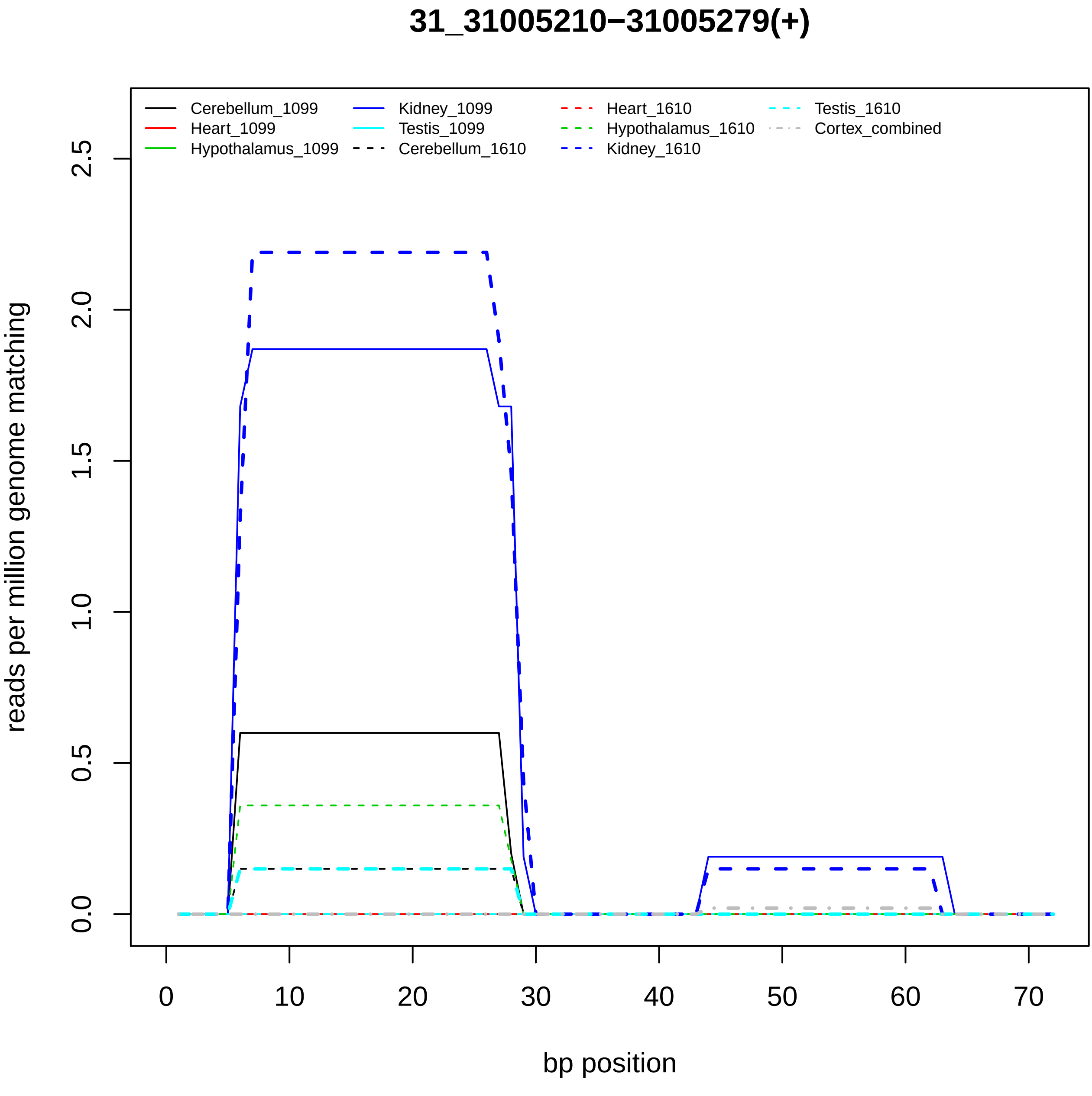

31_31005210−31005279(+)
Cerebellum_1099
Heart_1099
Hypothalamus_1099
Kidney_1099
Testis_1099
Cerebellum_1610
Heart_1610
Hypothalamus_1610
Kidney_1610
Testis_1610
Cortex_combined
reads per million genome matching
0.0
0.5
1.0
1.5
2.0
2.5
0
10
20
30
40
50
60
70
bp position

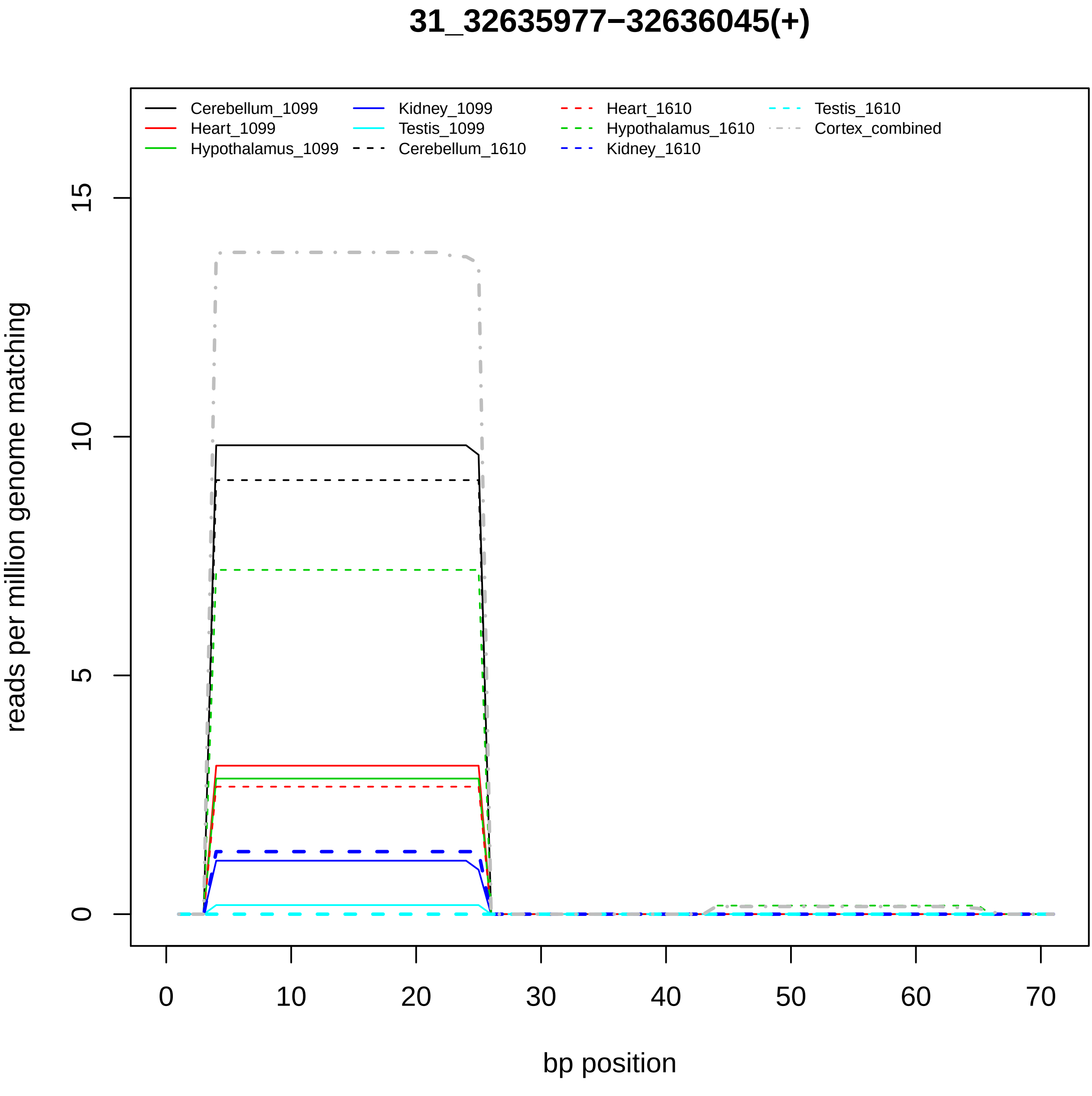
31_32635977−32636045(+)
Cerebellum_1099
Heart_1099
Hypothalamus_1099
Kidney_1099
Testis_1099
Cerebellum_1610
Heart_1610
Hypothalamus_1610
Kidney_1610
Testis_1610
Cortex_combined
reads per million genome matching
bp position
0
5
10
15
0
10
20
30
40
50
60
70

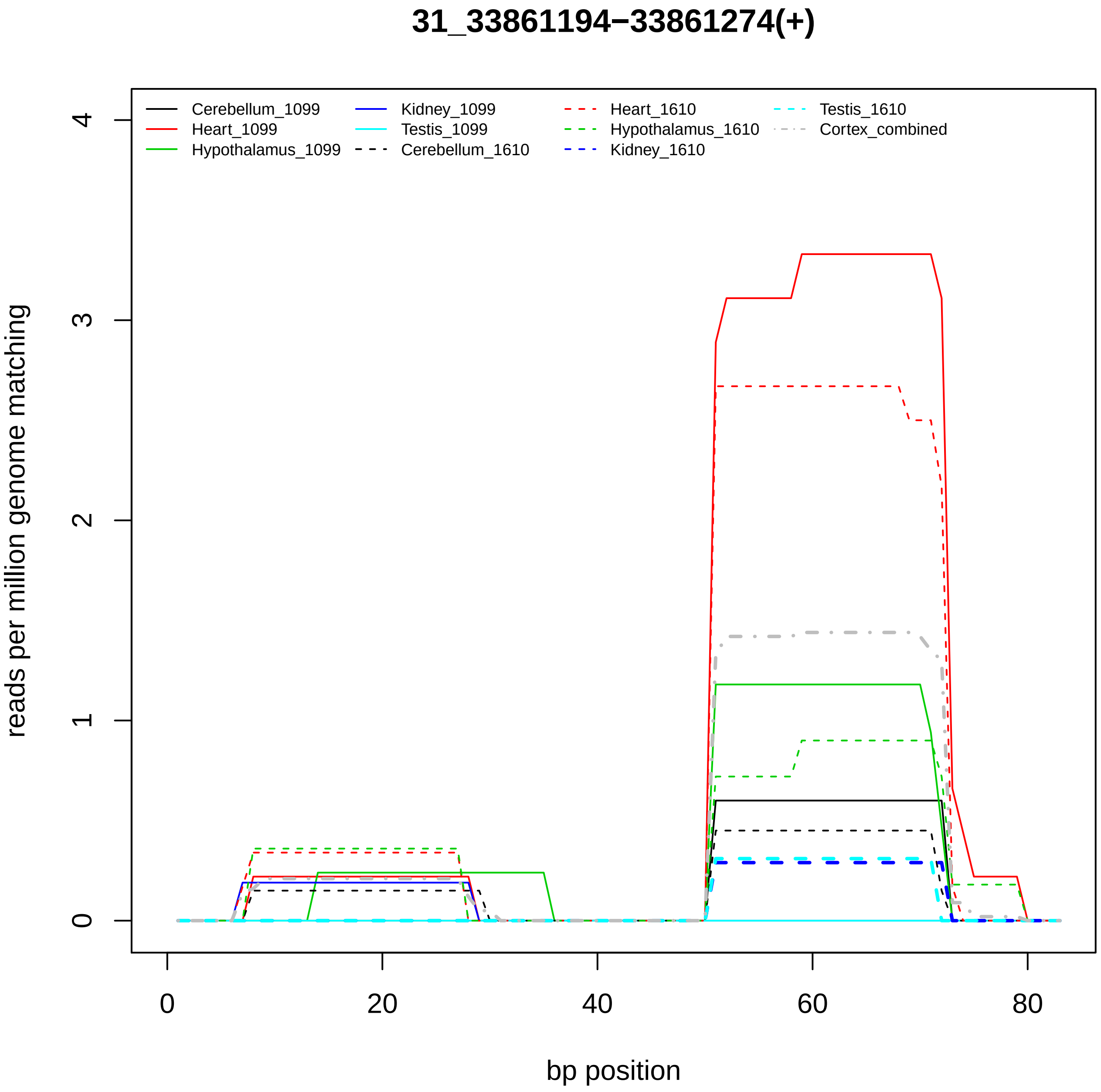

31_33861194−33861274(+)
Cerebellum_1099
Heart_1099
Hypothalamus_1099
Kidney_1099
Testis_1099
Cerebellum_1610
Heart_1610
Hypothalamus_1610
Kidney_1610
Testis_1610
Cortex_combined
reads per million genome matching
bp position
0
1
2
3
4
0
20
40
60
80

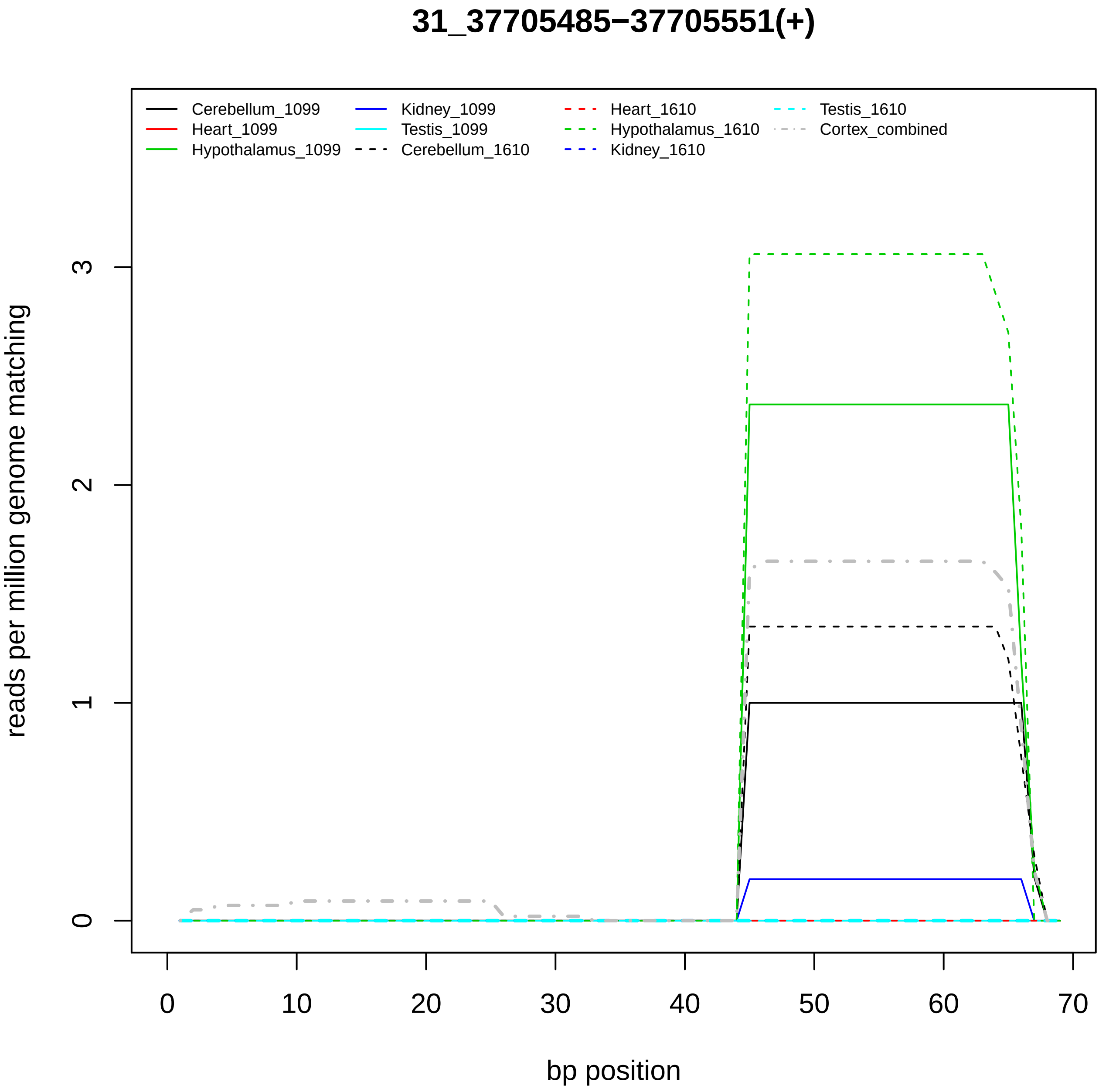

31_37705485−37705551(+)
Cerebellum_1099
Heart_1099
Hypothalamus_1099
Kidney_1099
Testis_1099
Cerebellum_1610
Heart_1610
Hypothalamus_1610
Kidney_1610
Testis_1610
Cortex_combined
reads per million genome matching
bp position
0
1
2
3
0
10
20
30
40
50
60
70

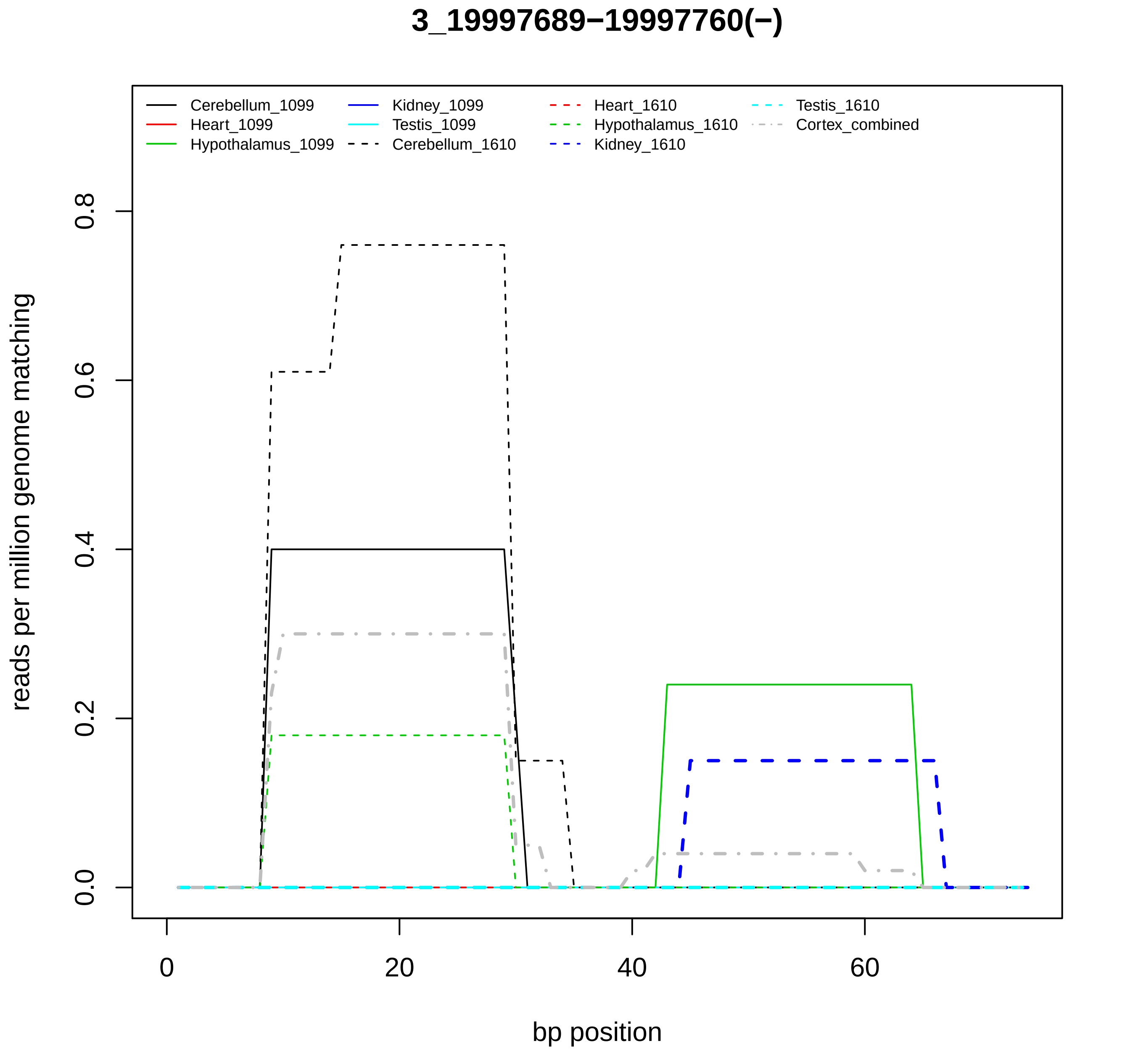

3_19997689−19997760(−)
Cerebellum_1099
Heart_1099
Hypothalamus_1099
Kidney_1099
Testis_1099
Cerebellum_1610
Heart_1610
Hypothalamus_1610
Kidney_1610
Testis_1610
Cortex_combined
reads per million genome matching
0.0
0.2
0.4
0.6
0.8
0
20
40
60
bp position

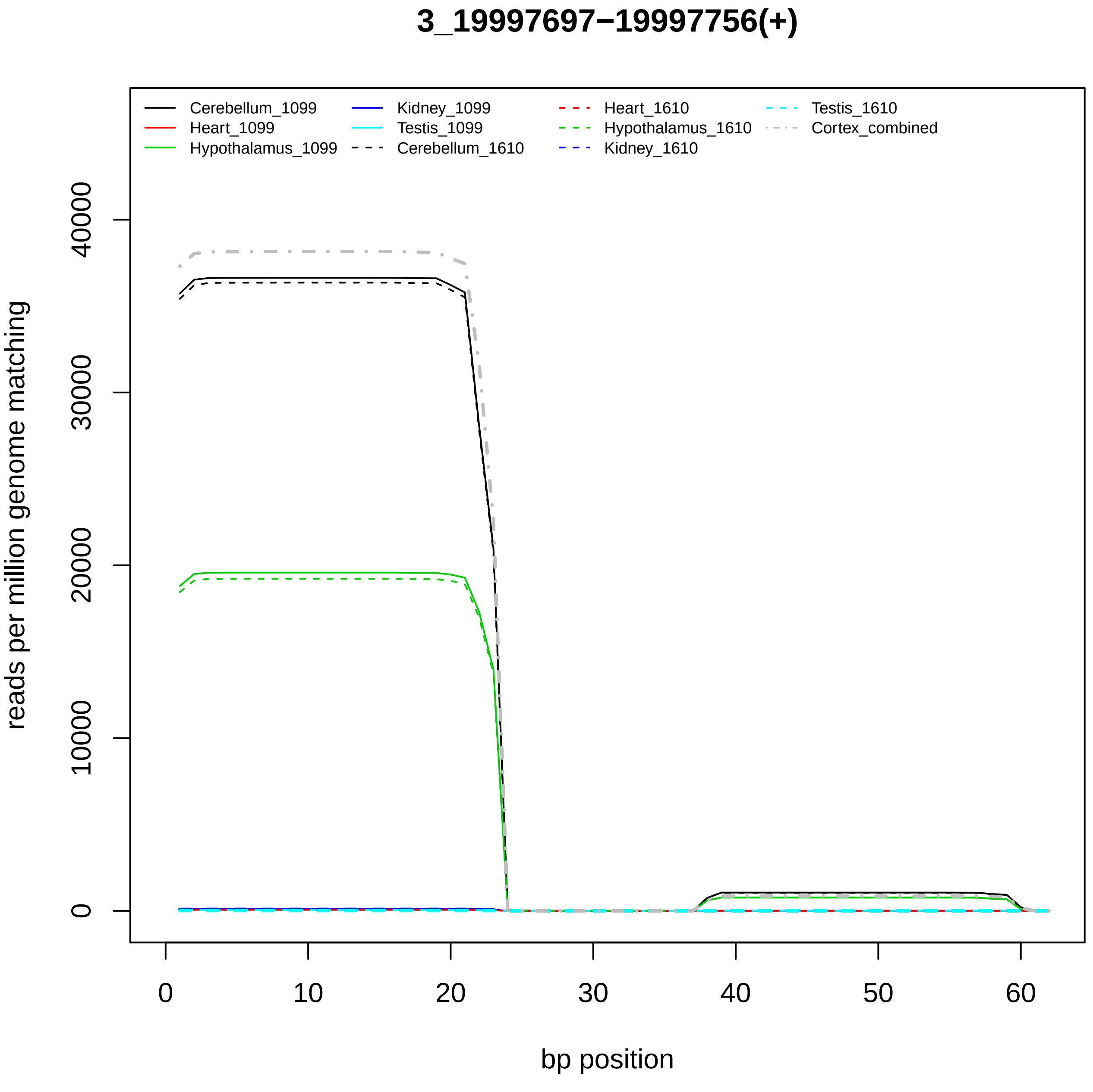

3_19997697−19997756(+)
Cerebellum_1099
Heart_1099
Hypothalamus_1099
Kidney_1099
Testis_1099
Cerebellum_1610
Heart_1610
Hypothalamus_1610
Kidney_1610
Testis_1610
Cortex_combined
reads per million genome matching
0
10000
20000
30000
40000
bp position
0
10
20
30
40
50
60

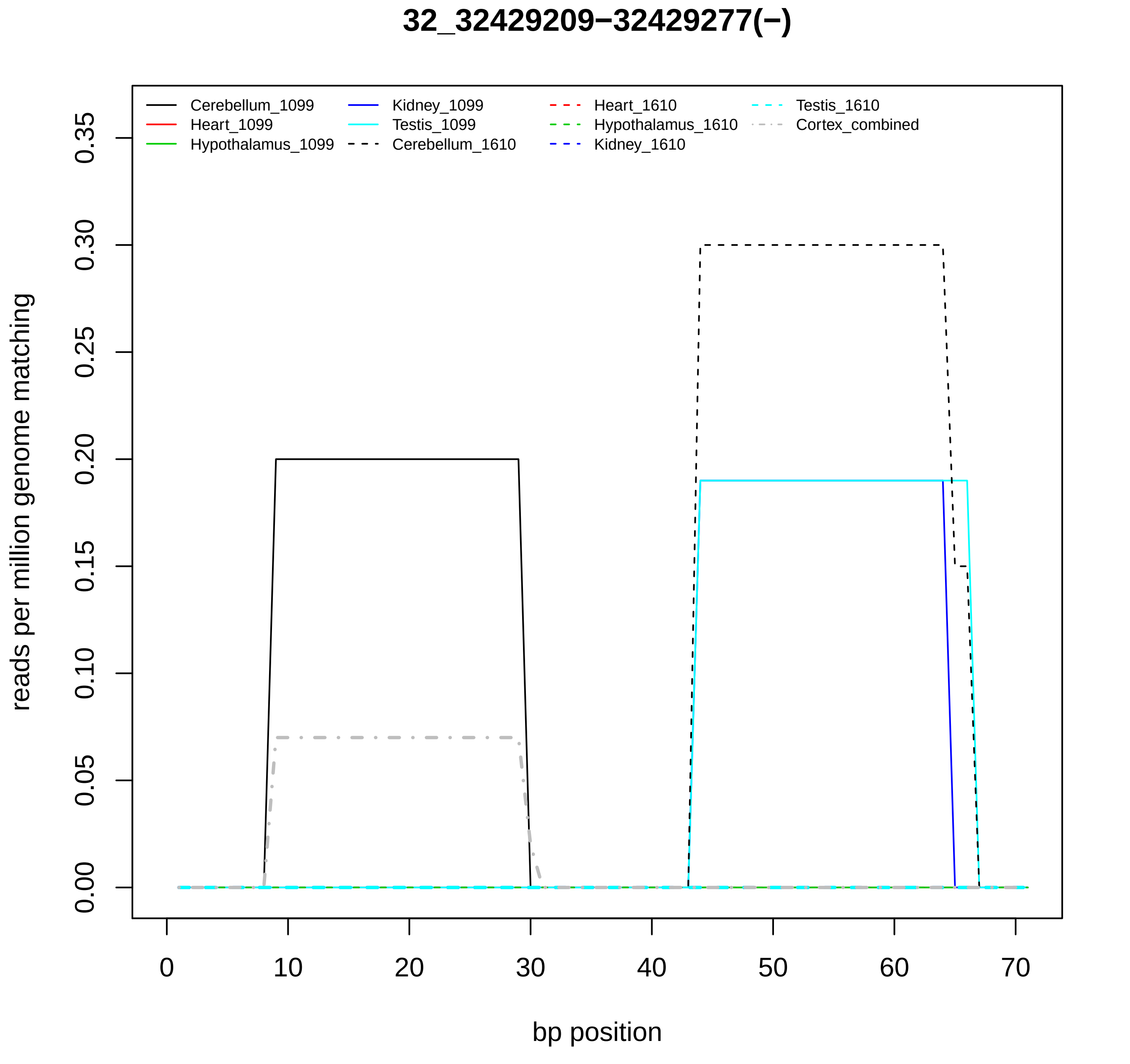

32_32429209−32429277(−)
Cerebellum_1099
Heart_1099
Hypothalamus_1099
Kidney_1099
Testis_1099
Cerebellum_1610
Heart_1610
Hypothalamus_1610
Kidney_1610
Testis_1610
Cortex_combined
reads per million genome matching
0.00
0.05
0.10
0.15
0.20
0.25
0.30
0.35
0
10
20
30
40
50
60
70
bp position

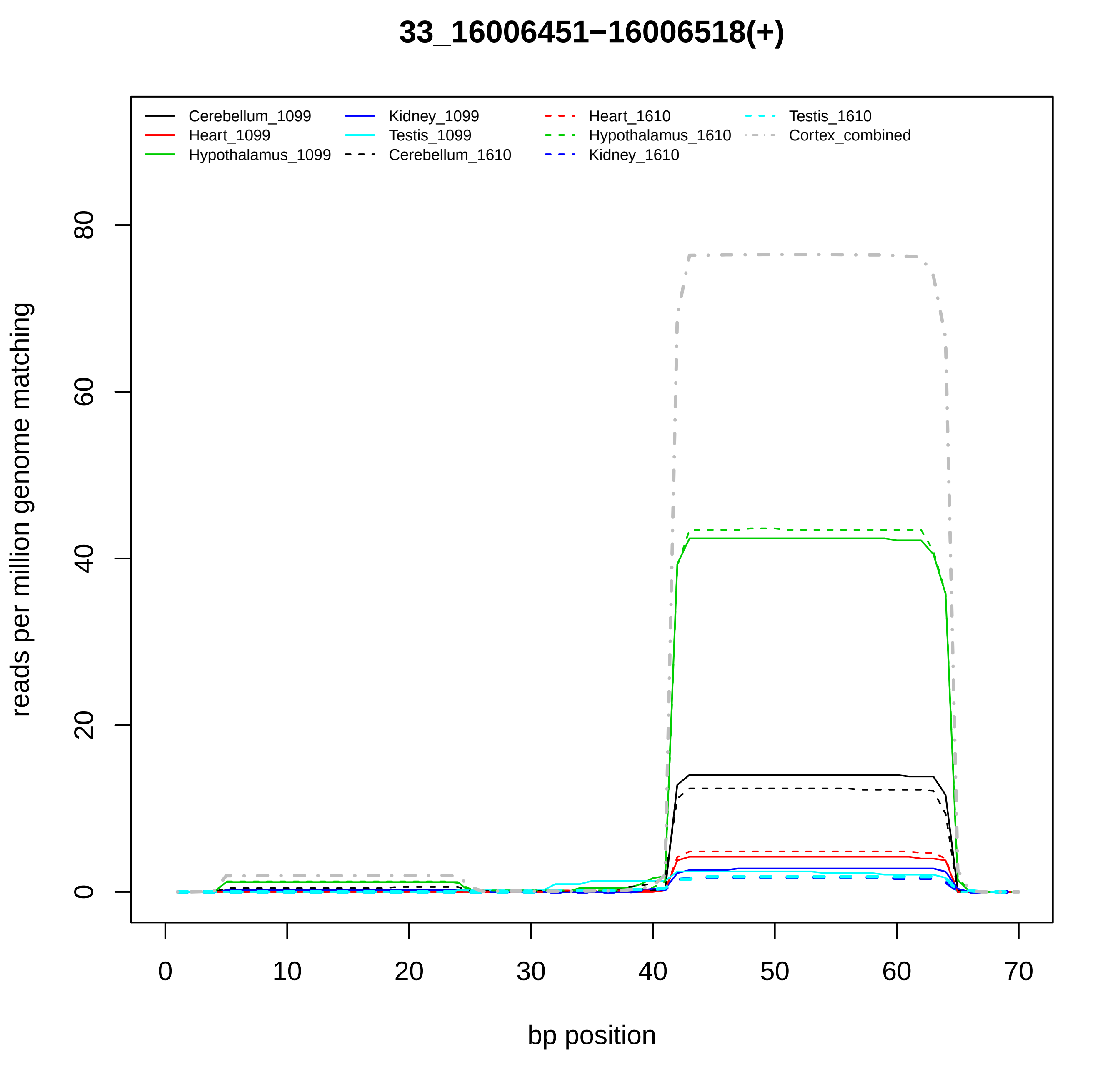

33_16006451−16006518(+)
Cerebellum_1099
Heart_1099
Hypothalamus_1099
Kidney_1099
Testis_1099
Cerebellum_1610
Heart_1610
Hypothalamus_1610
Kidney_1610
Testis_1610
Cortex_combined
reads per million genome matching
0
20
40
60
80
0
10
20
30
40
50
60
70
bp position

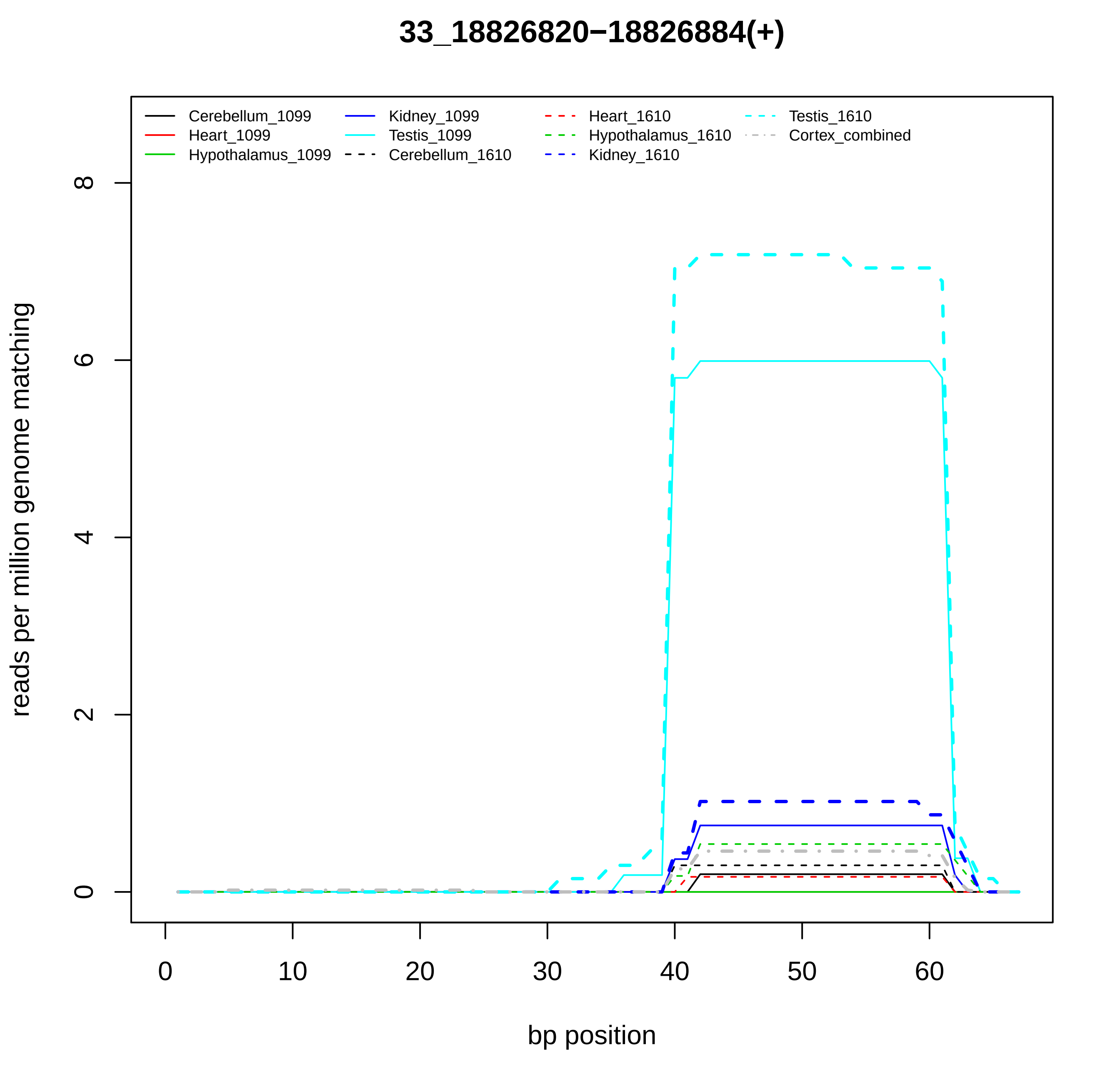

33_18826820−18826884(+)
Cerebellum_1099
Heart_1099
Hypothalamus_1099
Kidney_1099
Testis_1099
Cerebellum_1610
Heart_1610
Hypothalamus_1610
Kidney_1610
Testis_1610
Cortex_combined
reads per million genome matching
bp position
0
2
4
6
8
0
10
20
30
40
50
60

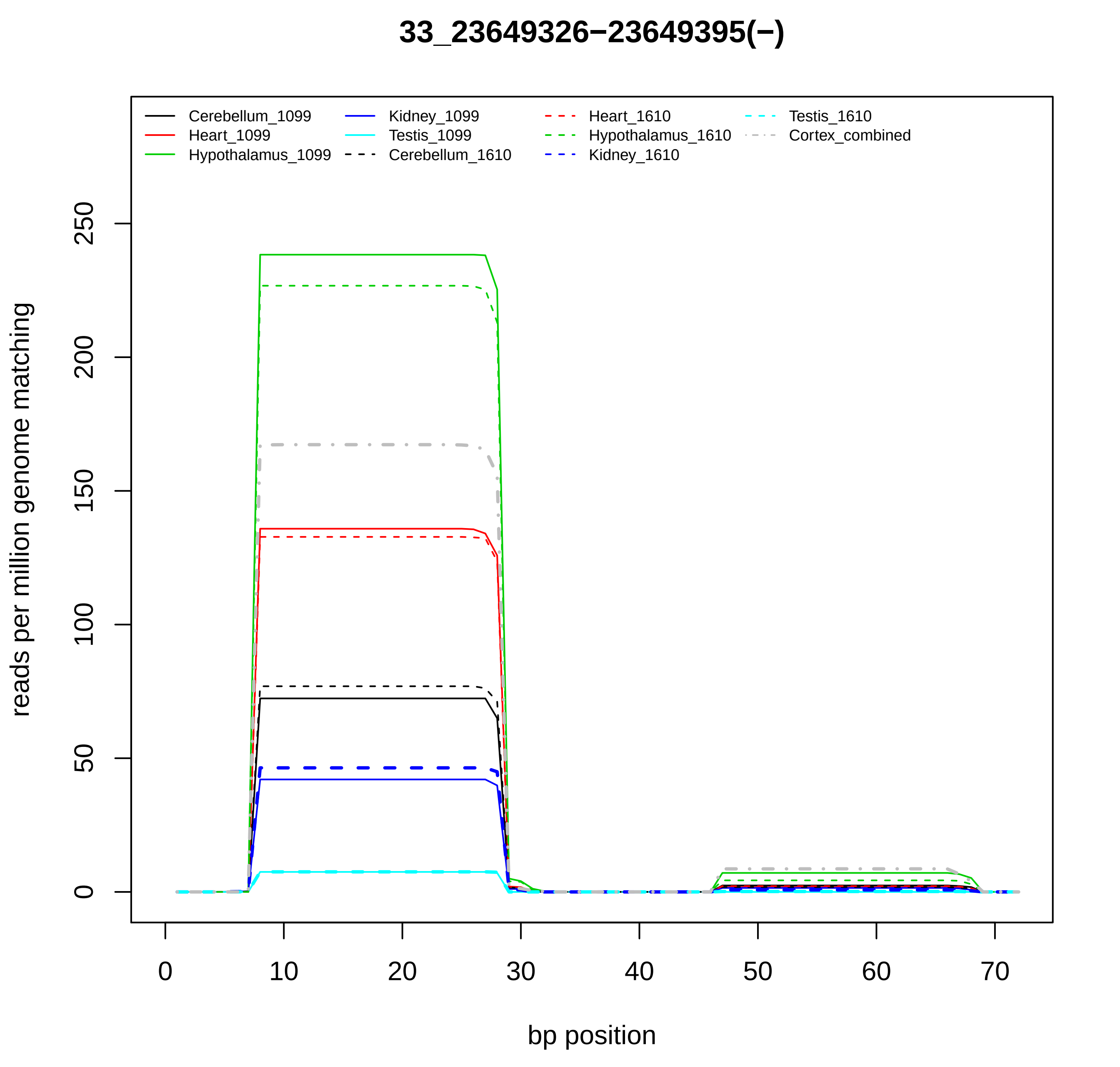
33_23649326−23649395(−)
Cerebellum_1099
Heart_1099
Hypothalamus_1099
Kidney_1099
Testis_1099
Cerebellum_1610
Heart_1610
Hypothalamus_1610
Kidney_1610
Testis_1610
Cortex_combined
reads per million genome matching
0
50
100
150
200
250
0
10
20
30
40
50
60
70
bp position

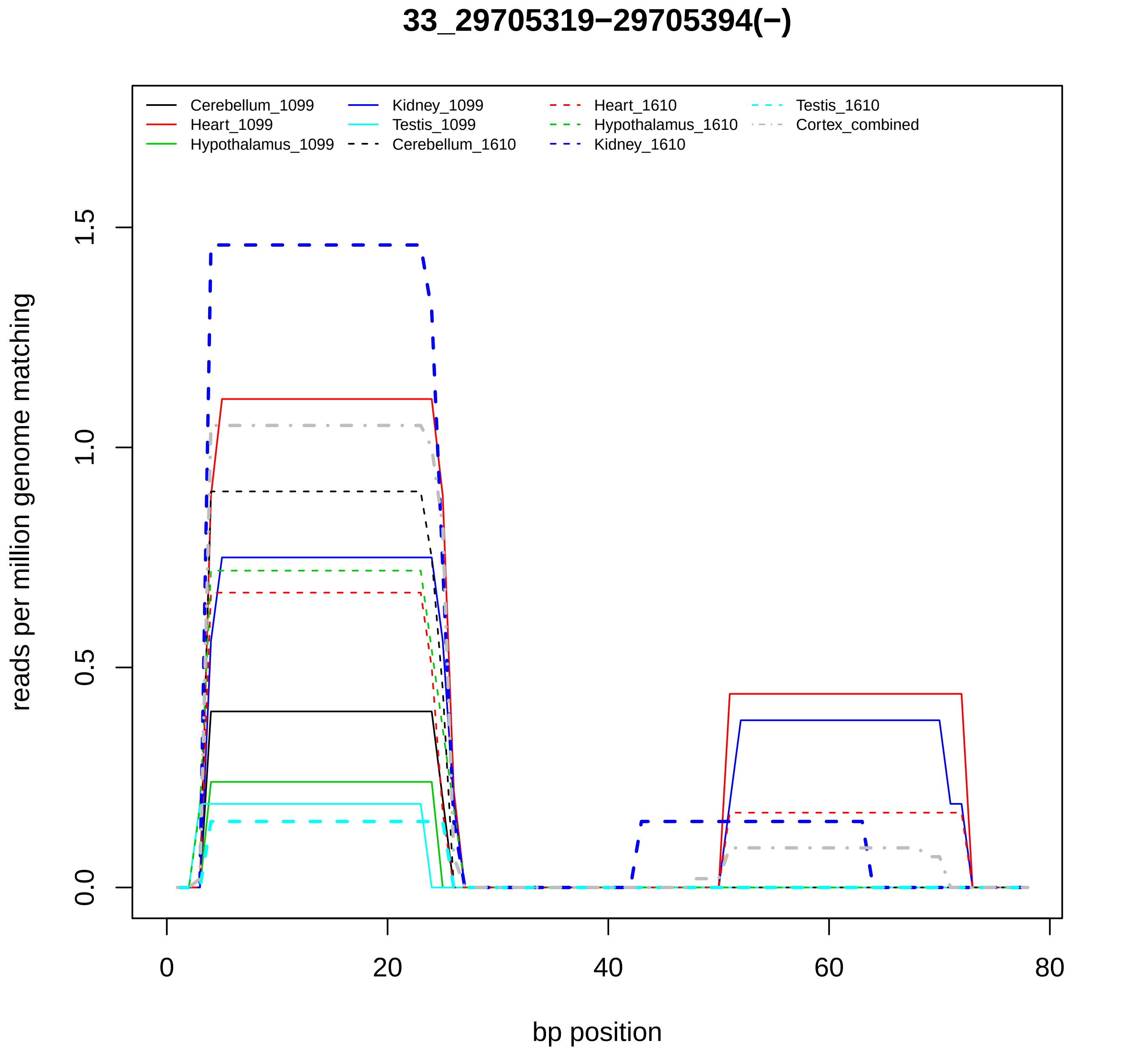

33_29705319−29705394(−)
Cerebellum_1099
Heart_1099
Hypothalamus_1099
Kidney_1099
Testis_1099
Cerebellum_1610
Heart_1610
Hypothalamus_1610
Kidney_1610
Testis_1610
Cortex_combined
reads per million genome matching
0.0
0.5
1.0
1.5
0
20
40
60
80
bp position

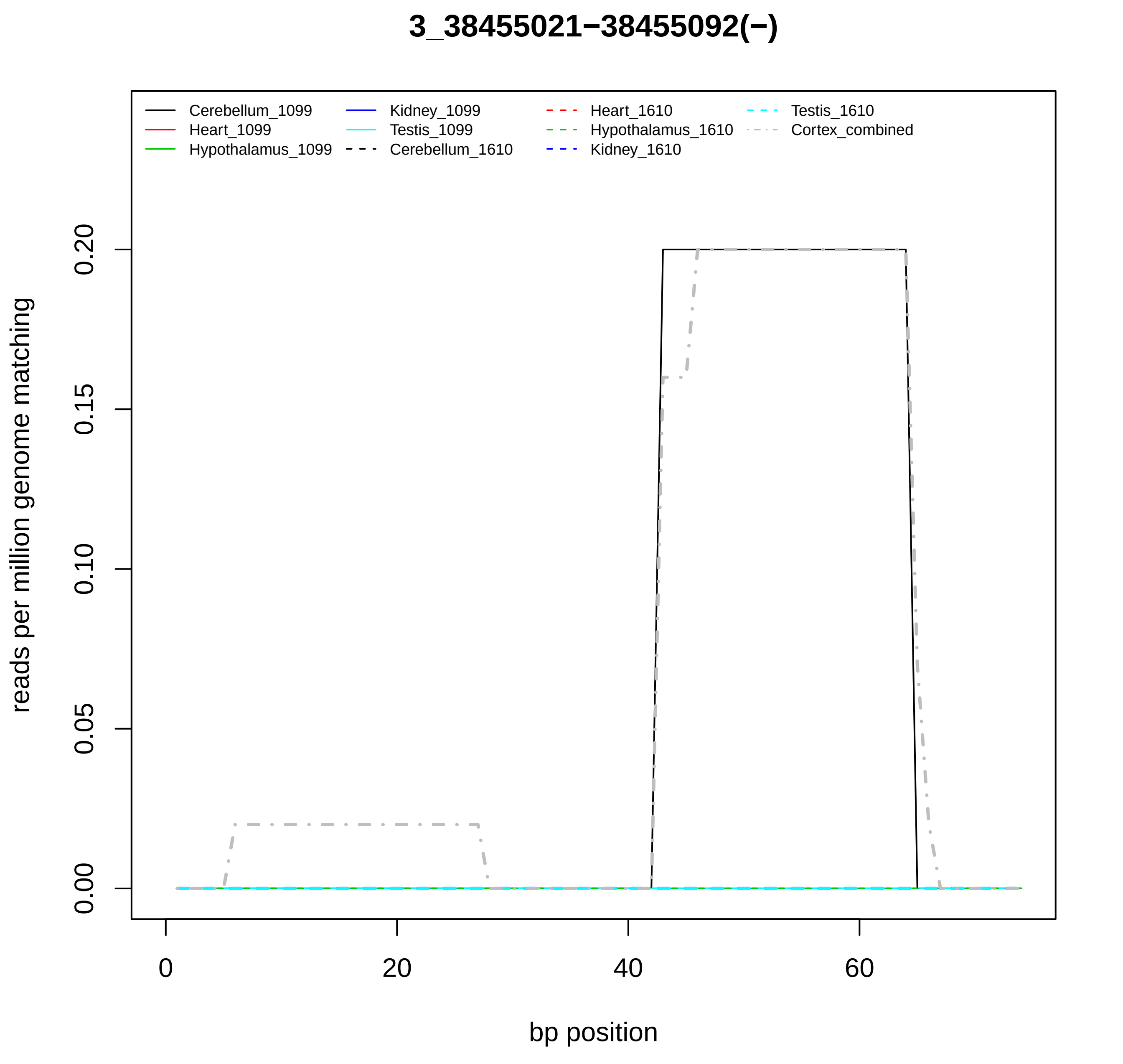

3_38455021−38455092(−)
Cerebellum_1099
Heart_1099
Hypothalamus_1099
Kidney_1099
Testis_1099
Cerebellum_1610
Heart_1610
Hypothalamus_1610
Kidney_1610
Testis_1610
Cortex_combined
reads per million genome matching
0.00
0.05
0.10
0.15
0.20
0
20
40
60
bp position

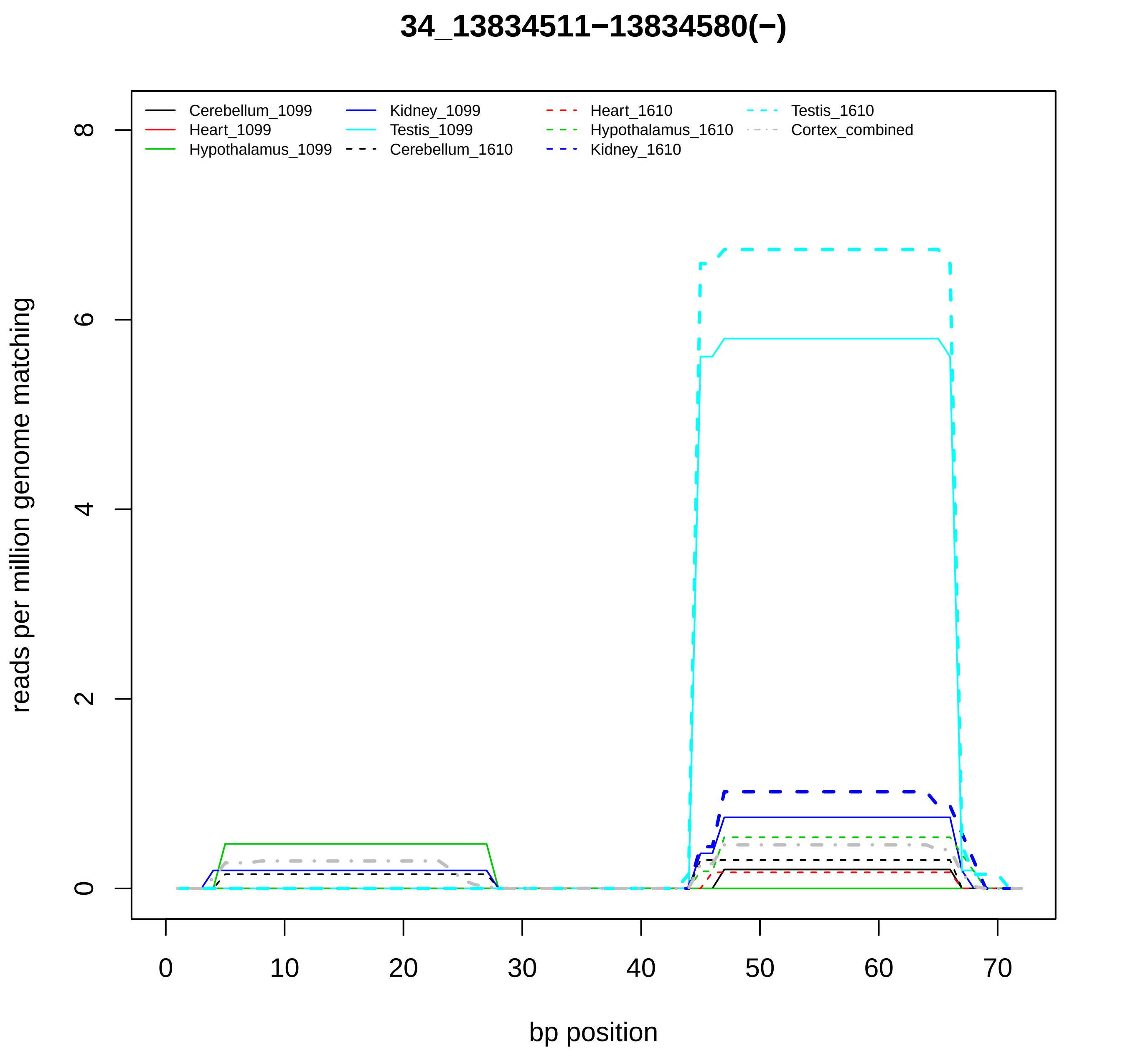

34_13834511−13834580(−)
Cerebellum_1099
Heart_1099
Hypothalamus_1099
Kidney_1099
Testis_1099
Cerebellum_1610
Heart_1610
Hypothalamus_1610
Kidney_1610
Testis_1610
Cortex_combined
reads per million genome matching
bp position
0
2
4
6
8
0
10
20
30
40
50
60
70

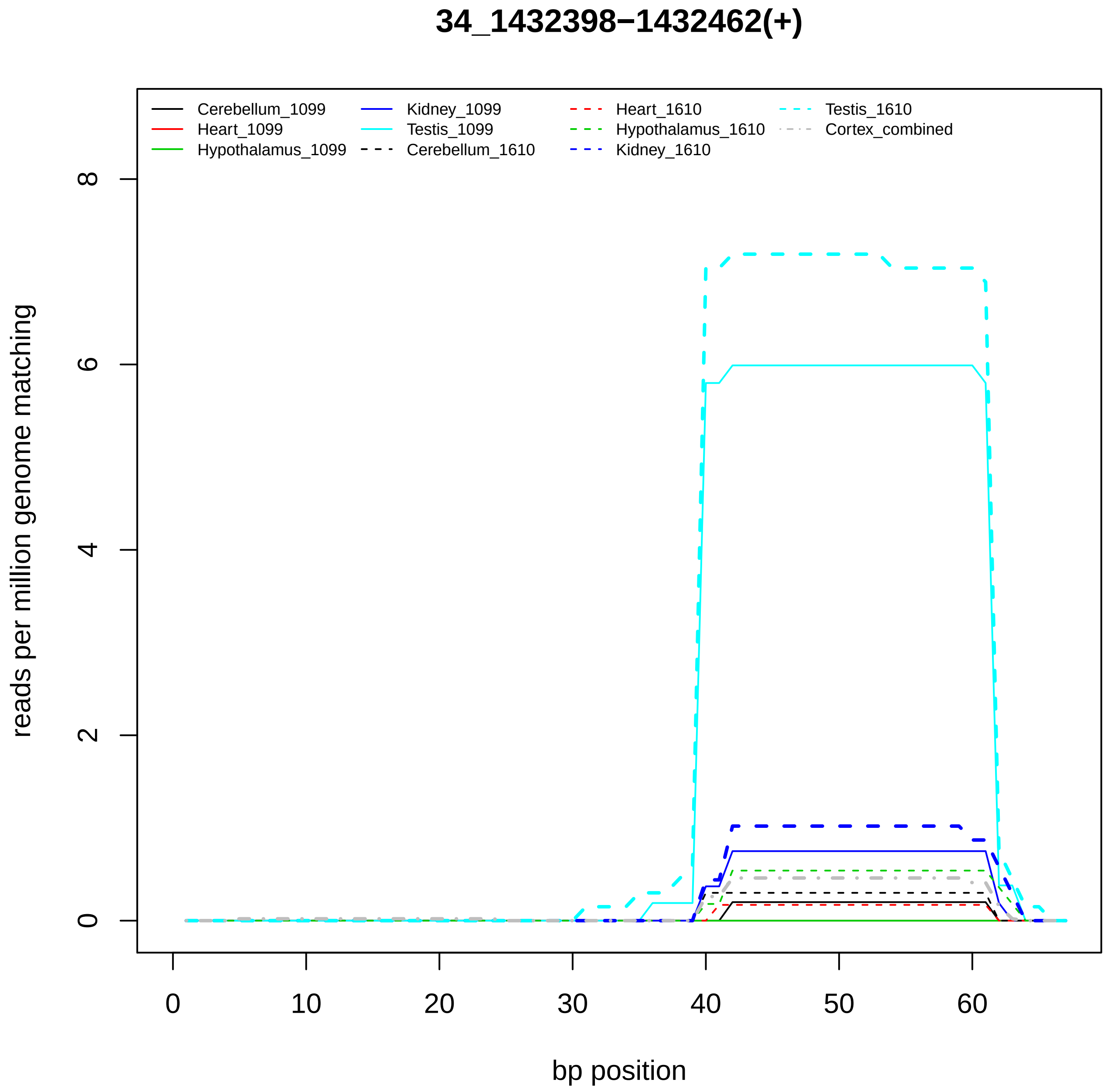

34_1432398−1432462(+)
Cerebellum_1099
Heart_1099
Hypothalamus_1099
Kidney_1099
Testis_1099
Cerebellum_1610
Heart_1610
Hypothalamus_1610
Kidney_1610
Testis_1610
Cortex_combined
reads per million genome matching
0
2
4
6
8
0
10
20
30
40
50
60
bp position

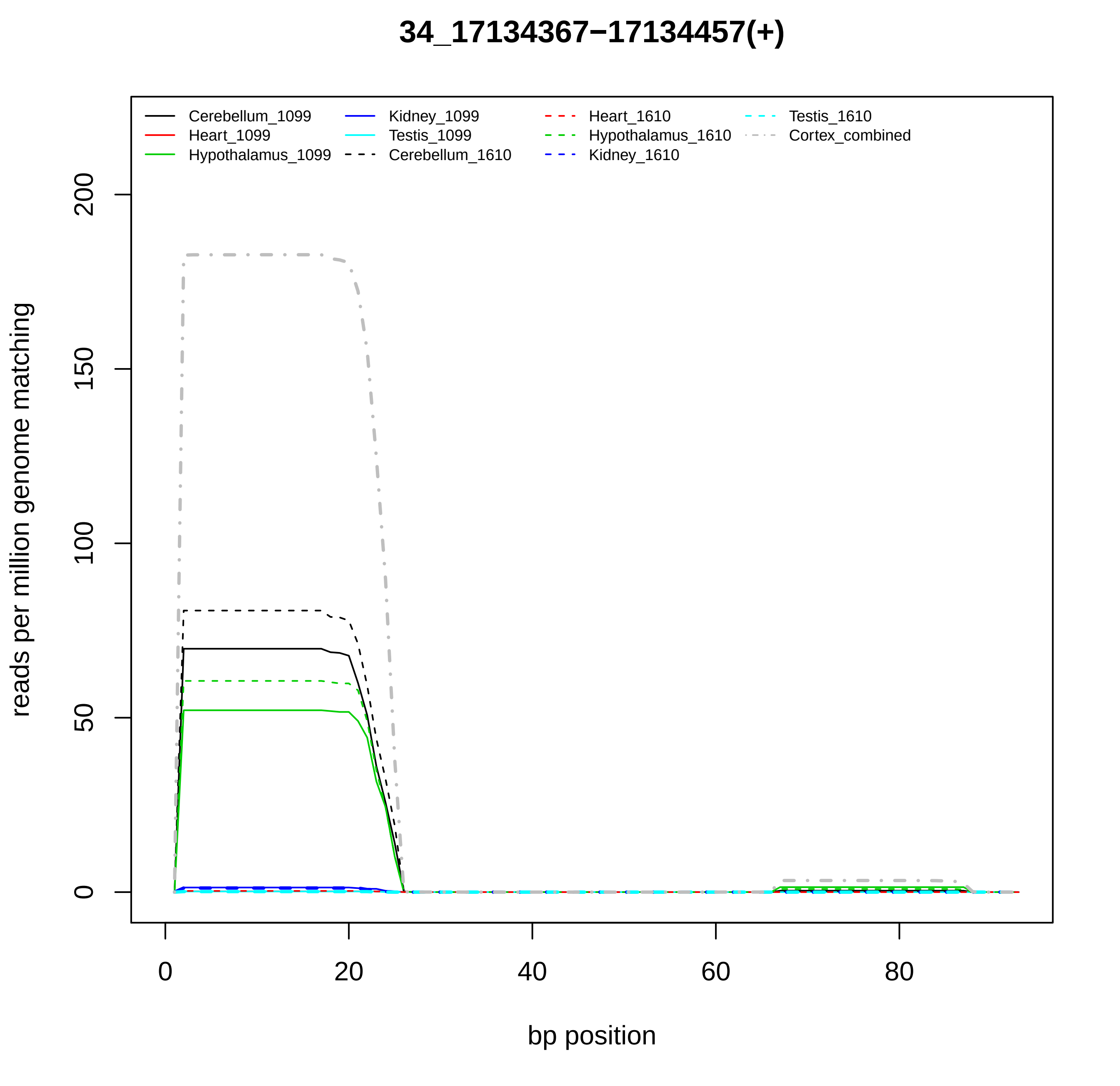

34_17134367−17134457(+)
Cerebellum_1099
Heart_1099
Hypothalamus_1099
Kidney_1099
Testis_1099
Cerebellum_1610
Heart_1610
Hypothalamus_1610
Kidney_1610
Testis_1610
Cortex_combined
reads per million genome matching
bp position
0
50
100
150
200
0
20
40
60
80

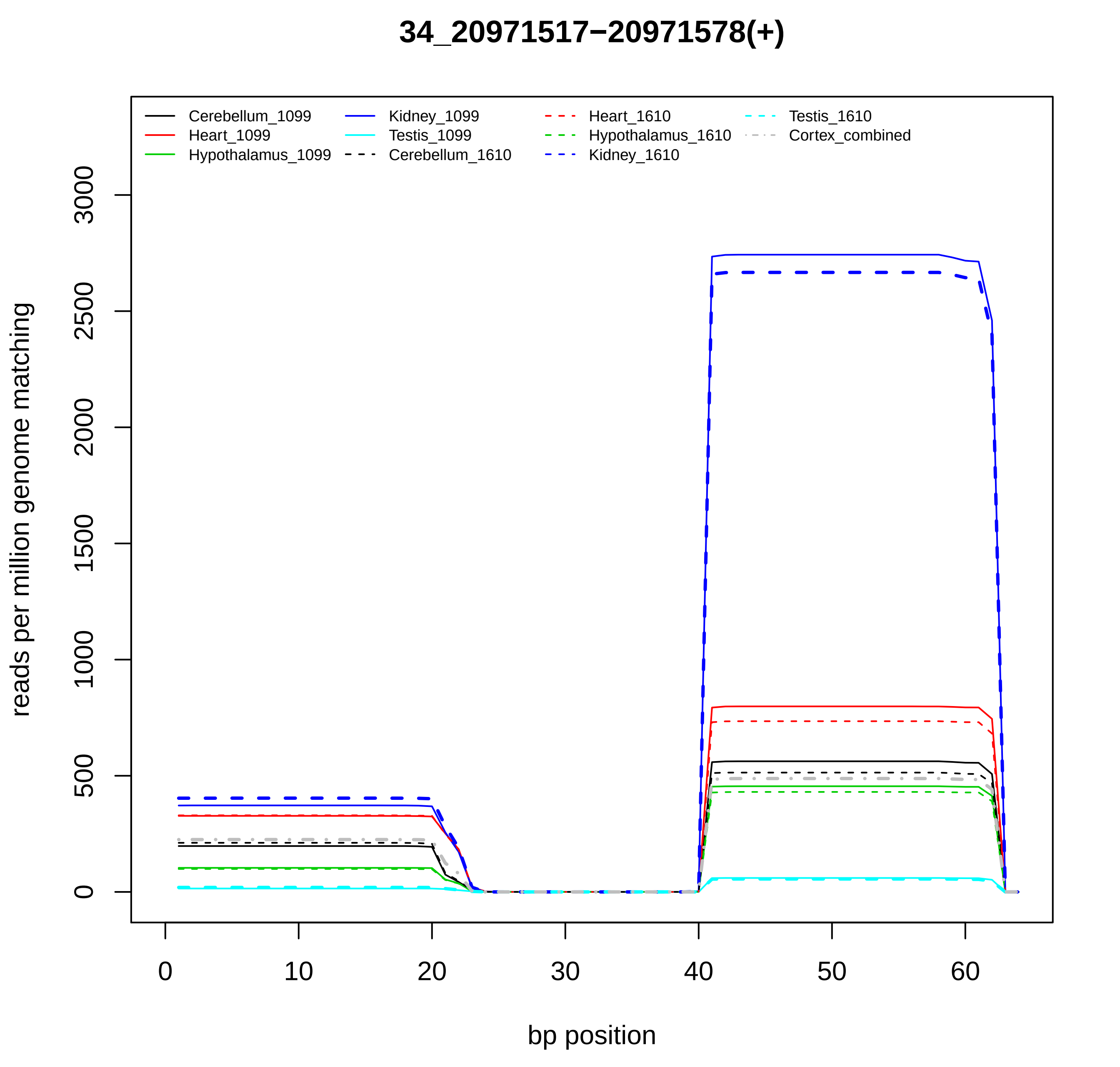
34_20971517−20971578(+)
Cerebellum_1099
Heart_1099
Hypothalamus_1099
Kidney_1099
Testis_1099
Cerebellum_1610
Heart_1610
Hypothalamus_1610
Kidney_1610
Testis_1610
Cortex_combined
reads per million genome matching
bp position
0
500
1000
1500
2000
2500
3000
0
10
20
30
40
50
60

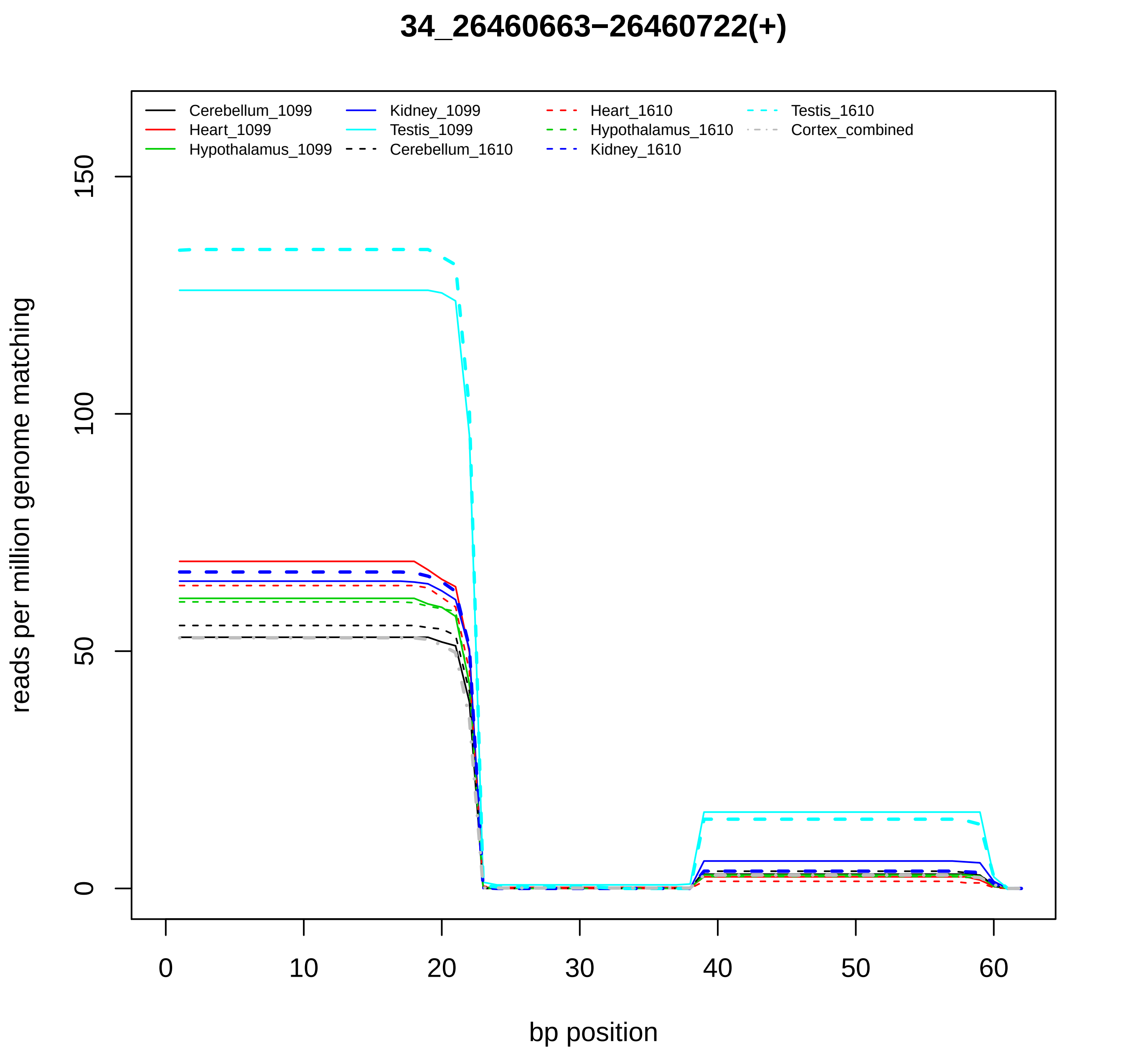

34_26460663−26460722(+)
Cerebellum_1099
Heart_1099
Hypothalamus_1099
Kidney_1099
Testis_1099
Cerebellum_1610
Heart_1610
Hypothalamus_1610
Kidney_1610
Testis_1610
Cortex_combined
reads per million genome matching
0
50
100
150
0
10
20
30
40
50
60
bp position

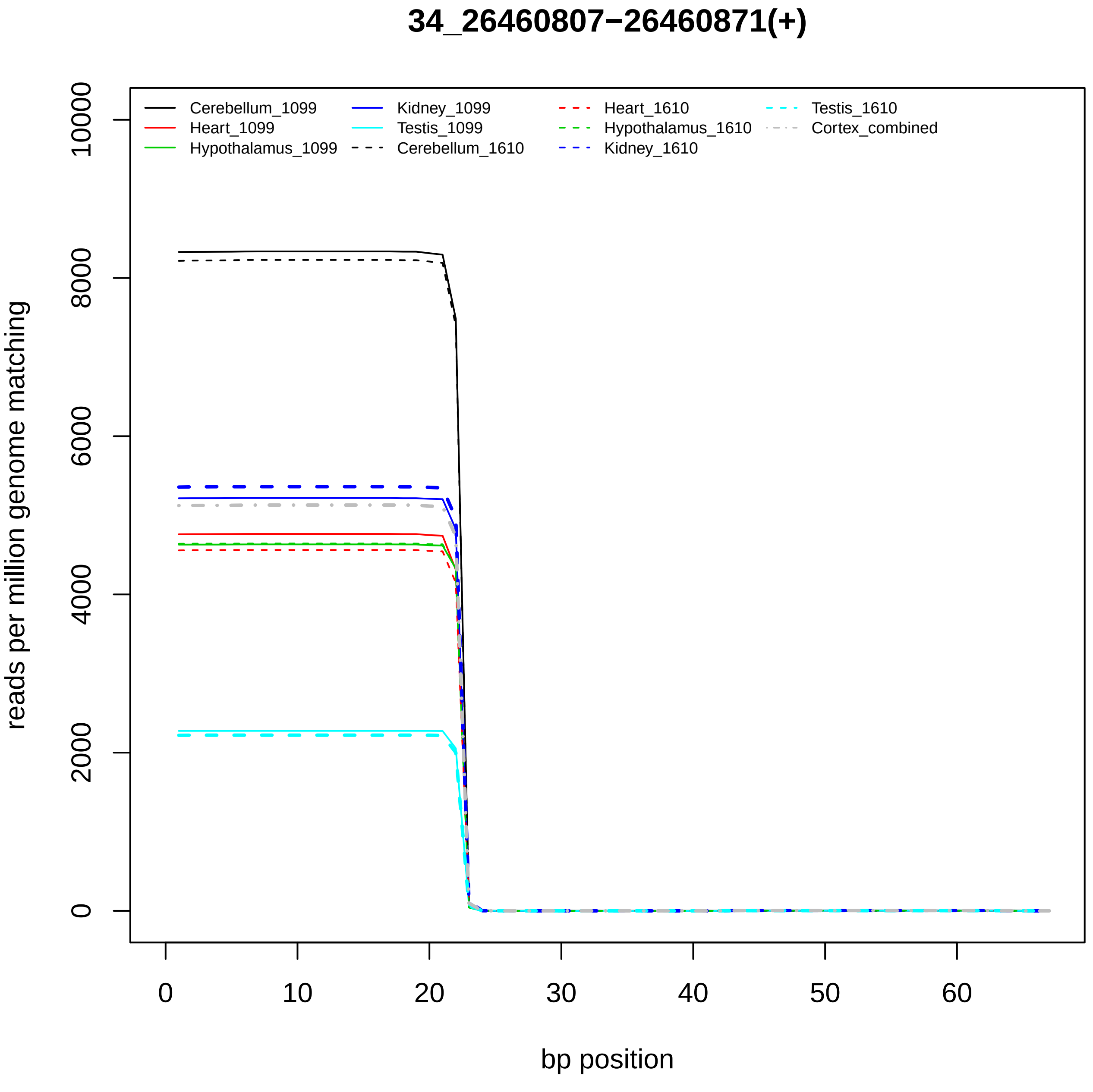

34_26460807−26460871(+)
Cerebellum_1099
Heart_1099
Hypothalamus_1099
Kidney_1099
Testis_1099
Cerebellum_1610
Heart_1610
Hypothalamus_1610
Kidney_1610
Testis_1610
Cortex_combined
reads per million genome matching
0
2000
4000
6000
8000
10000
0
10
20
30
40
50
60
bp position

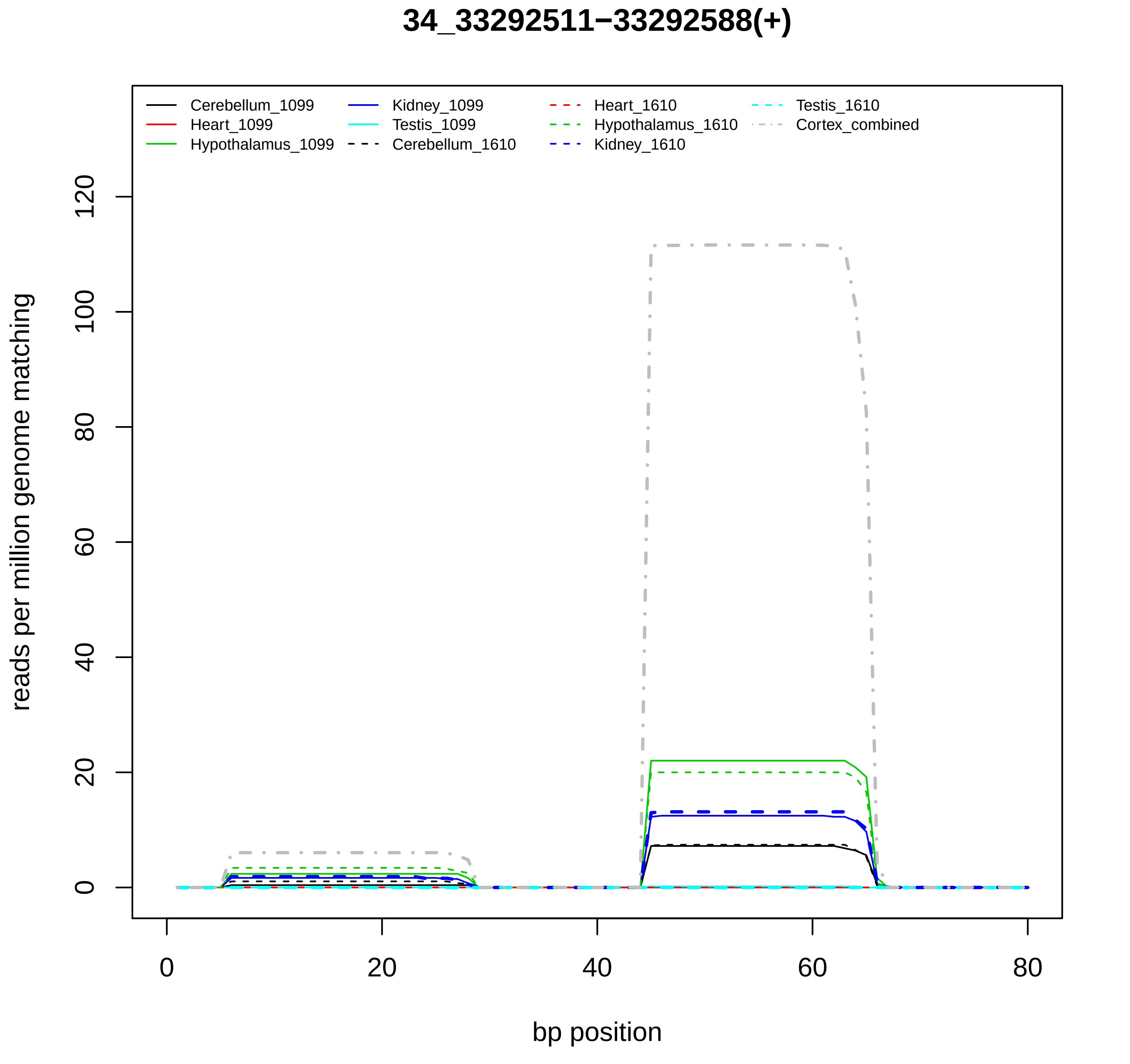

34_33292511−33292588(+)
Cerebellum_1099
Heart_1099
Hypothalamus_1099
Kidney_1099
Testis_1099
Cerebellum_1610
Heart_1610
Hypothalamus_1610
Kidney_1610
Testis_1610
Cortex_combined
reads per million genome matching
0
20
40
60
80
100
120
bp position
0
20
40
60
80

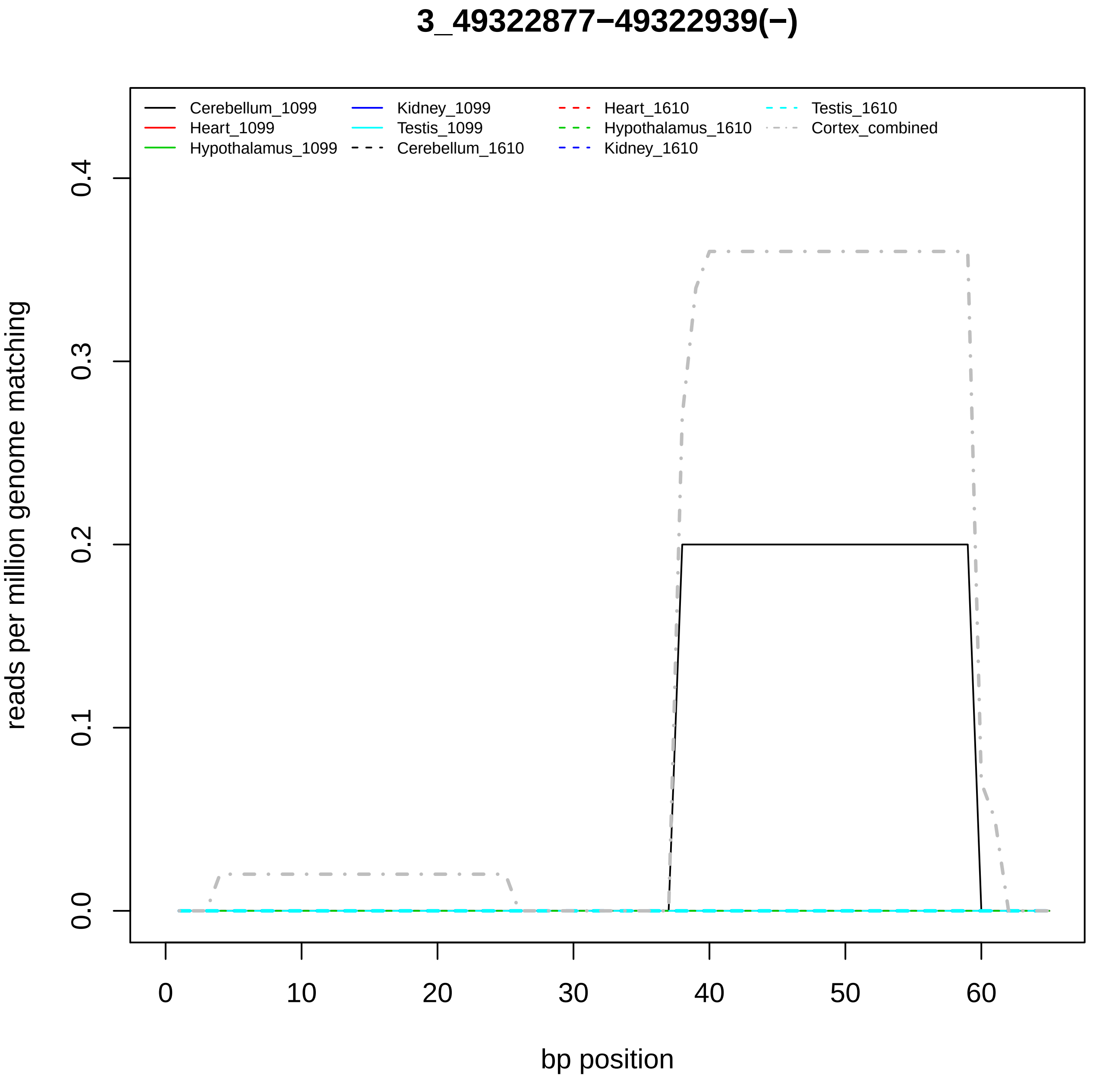
3_49322877−49322939(−)
Cerebellum_1099
Heart_1099
Hypothalamus_1099
Kidney_1099
Testis_1099
Cerebellum_1610
Heart_1610
Hypothalamus_1610
Kidney_1610
Testis_1610
Cortex_combined
0.0
0.1
0.2
0.3
0.4
reads per million genome matching
0
10
20
30
40
50
60
bp position

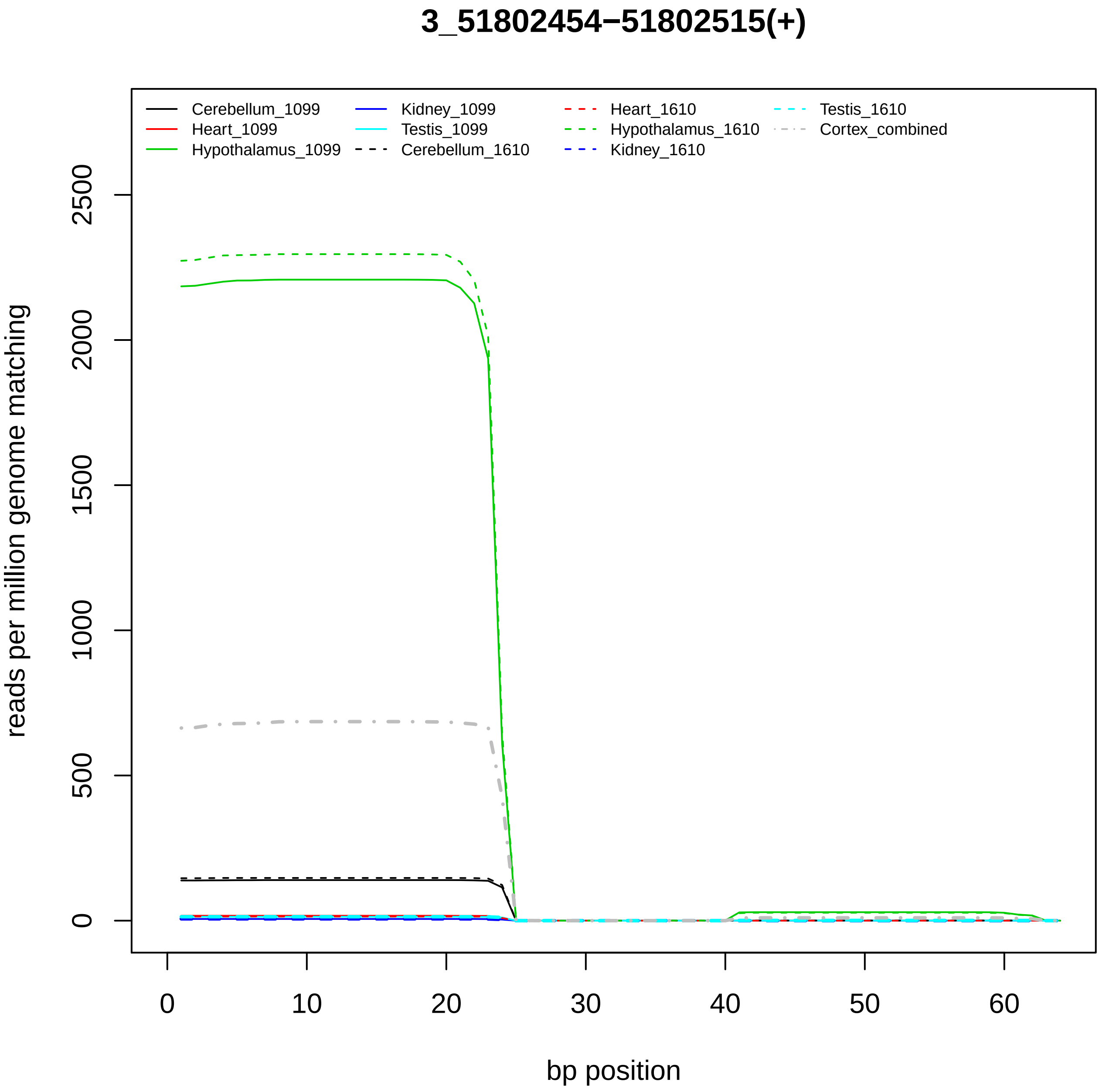

3_51802454−51802515(+)
Cerebellum_1099
Heart_1099
Hypothalamus_1099
Kidney_1099
Testis_1099
Cerebellum_1610
Heart_1610
Hypothalamus_1610
Kidney_1610
Testis_1610
Cortex_combined
reads per million genome matching
0
500
1000
1500
2000
2500
0
10
20
30
40
50
60
bp position

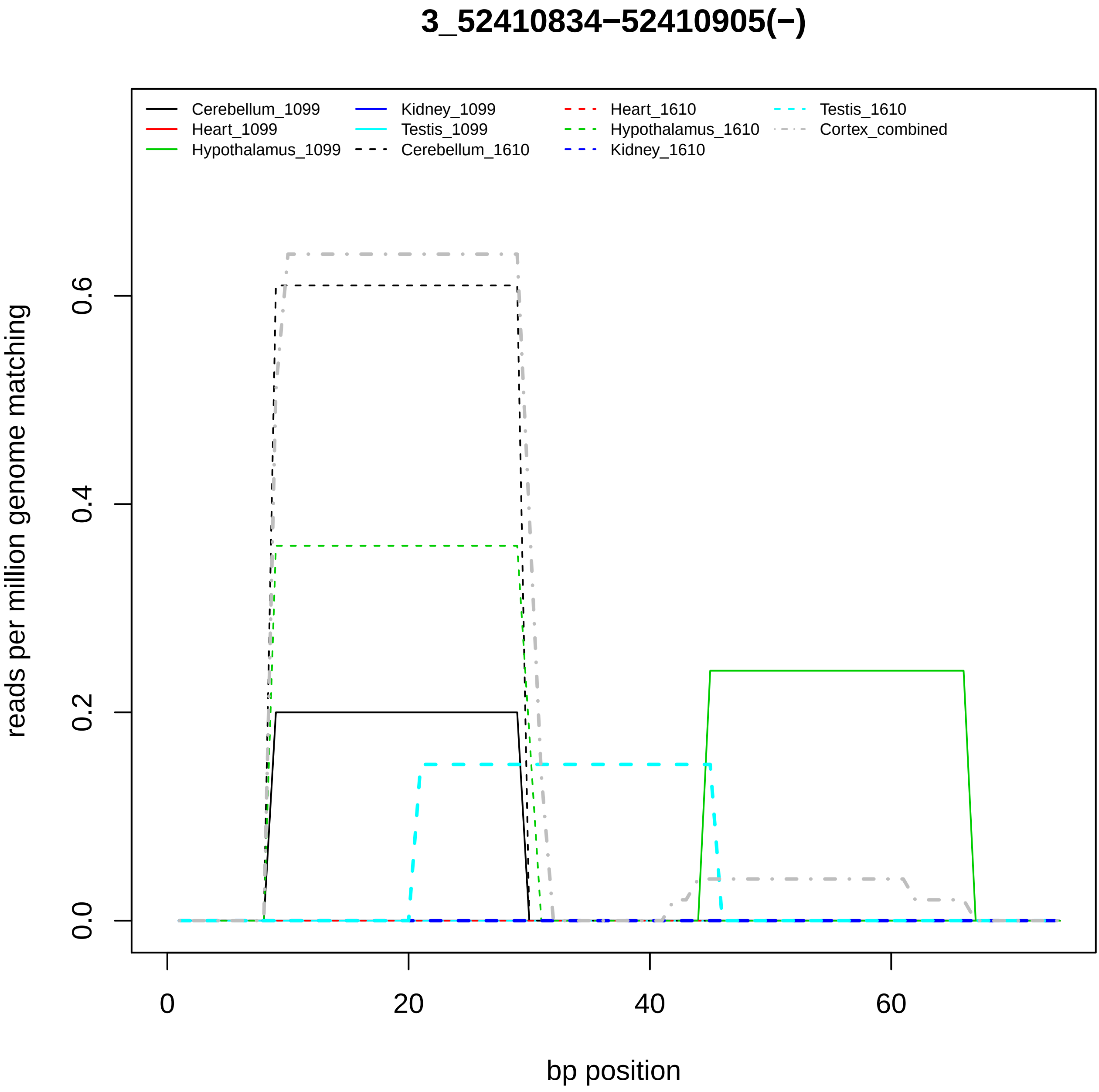
3_52410834−52410905(−)
Cerebellum_1099
Heart_1099
Hypothalamus_1099
Kidney_1099
Testis_1099
Cerebellum_1610
Heart_1610
Hypothalamus_1610
Kidney_1610
Testis_1610
Cortex_combined
reads per million genome matching
0.0
0.2
0.4
0.6
0
20
40
60
bp position

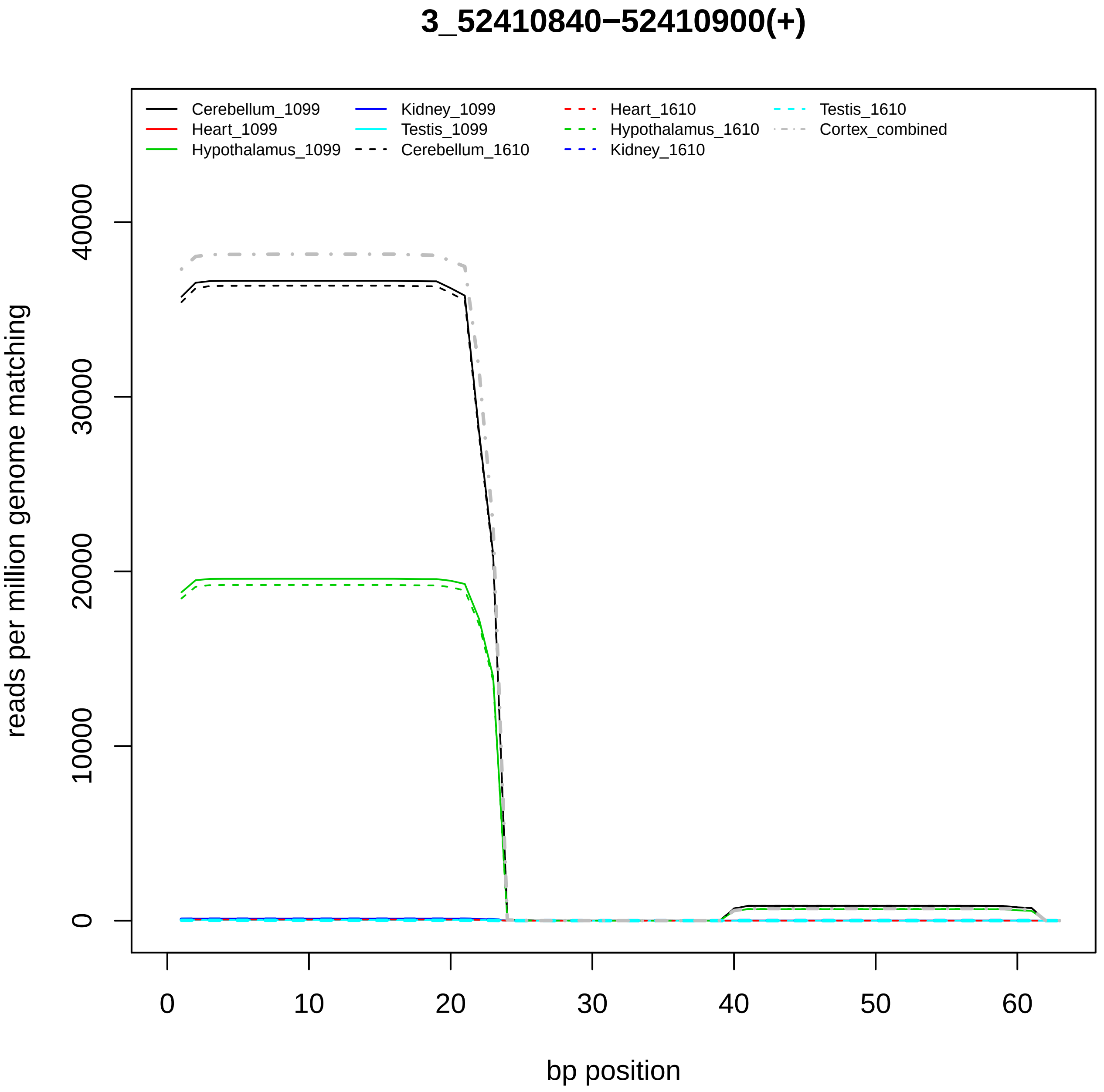

3_52410840−52410900(+)
Cerebellum_1099
Heart_1099
Hypothalamus_1099
Kidney_1099
Testis_1099
Cerebellum_1610
Heart_1610
Hypothalamus_1610
Kidney_1610
Testis_1610
Cortex_combined
reads per million genome matching
0
10000
20000
30000
40000
0
10
20
30
40
50
60
bp position

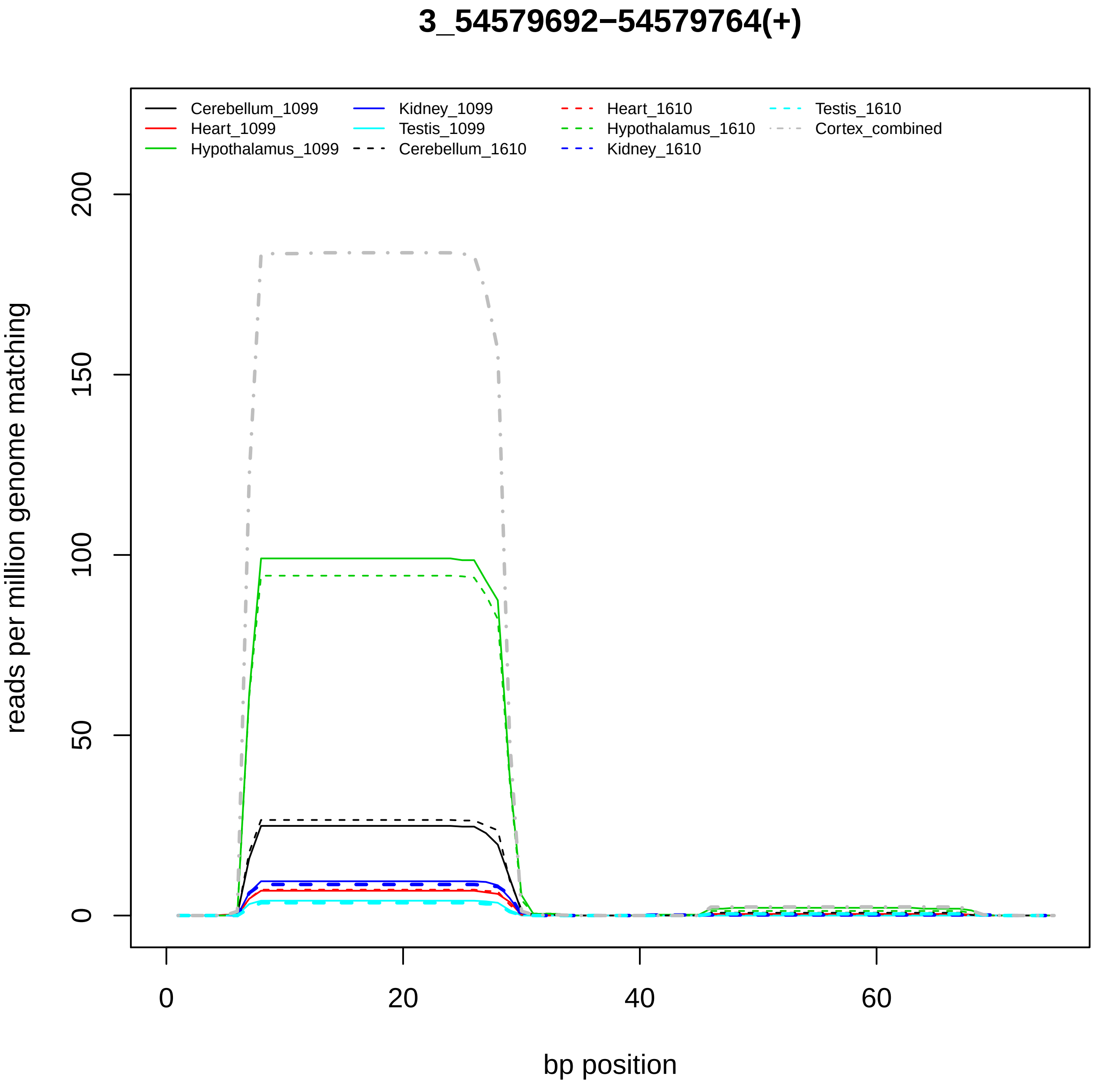

3_54579692−54579764(+)
Cerebellum_1099
Heart_1099
Hypothalamus_1099
Kidney_1099
Testis_1099
Cerebellum_1610
Heart_1610
Hypothalamus_1610
Kidney_1610
Testis_1610
Cortex_combined
reads per million genome matching
bp position
0
50
100
150
200
0
20
40
60

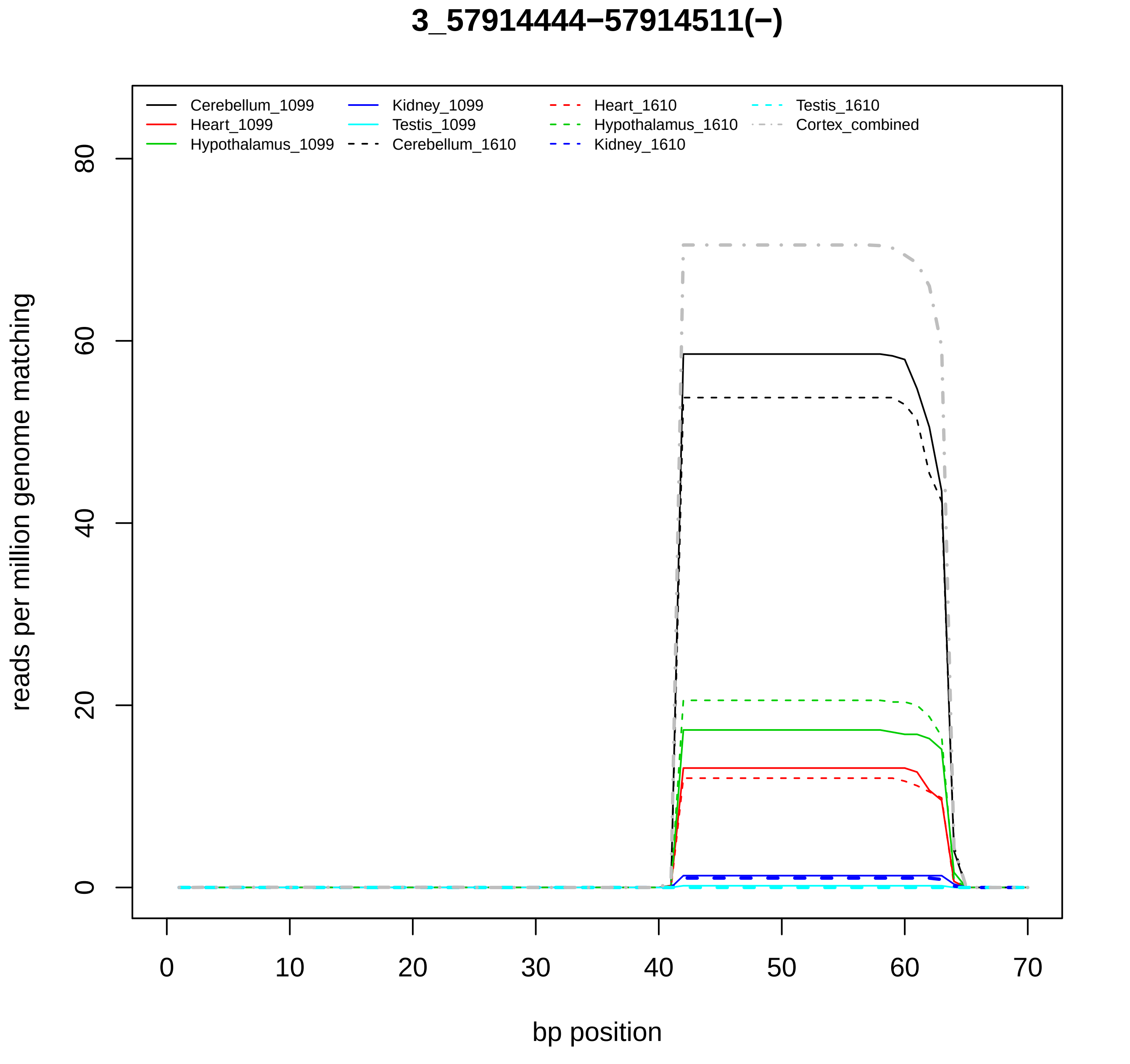
3_57914444−57914511(−)
Cerebellum_1099
Heart_1099
Hypothalamus_1099
Kidney_1099
Testis_1099
Cerebellum_1610
Heart_1610
Hypothalamus_1610
Kidney_1610
Testis_1610
Cortex_combined
reads per million genome matching
0
20
40
60
80
0
10
20
30
40
50
60
70
bp position

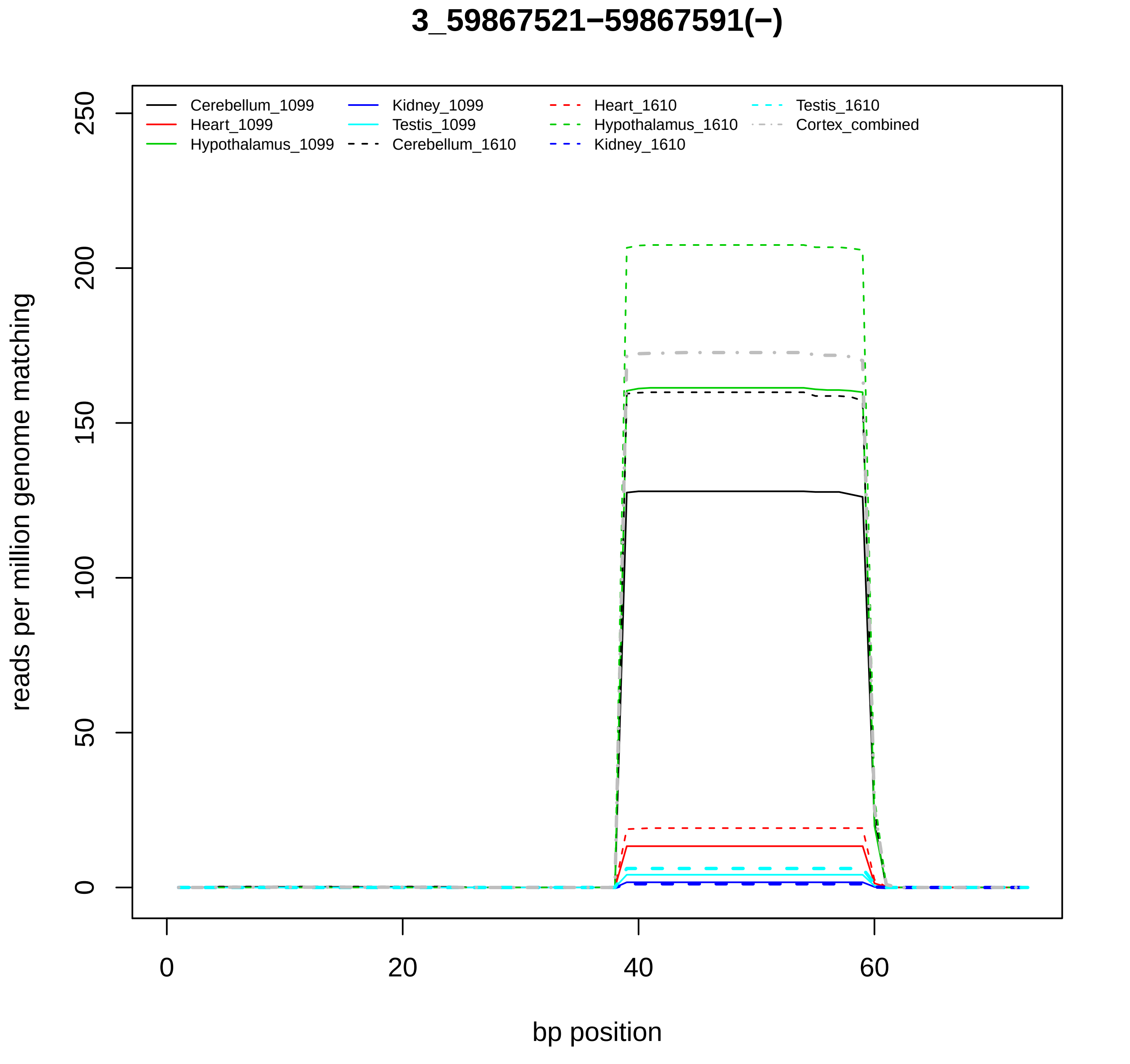

3_59867521−59867591(−)
Cerebellum_1099
Heart_1099
Hypothalamus_1099
Kidney_1099
Testis_1099
Cerebellum_1610
Heart_1610
Hypothalamus_1610
Kidney_1610
Testis_1610
Cortex_combined
reads per million genome matching
bp position
0
50
100
150
200
250
0
20
40
60

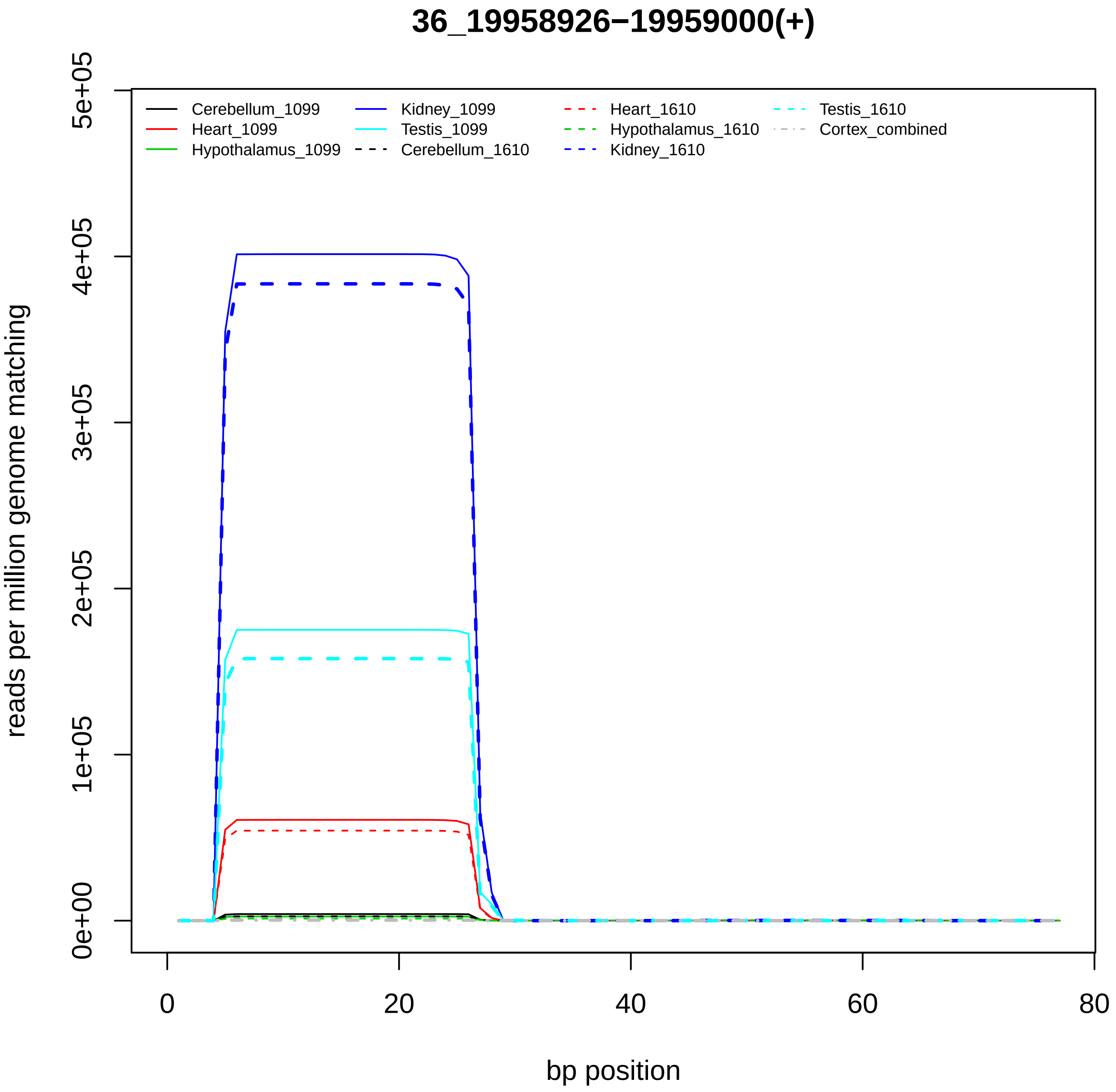
36_19958926−19959000(+)
Cerebellum_1099
Heart_1099
Hypothalamus_1099
Kidney_1099
Testis_1099
Cerebellum_1610
Heart_1610
Hypothalamus_1610
Kidney_1610
Testis_1610
Cortex_combined
reads per million genome matching
0e+00
1e+05
2e+05
3e+05
4e+05
5e+05
0
20
40
60
80
bp position

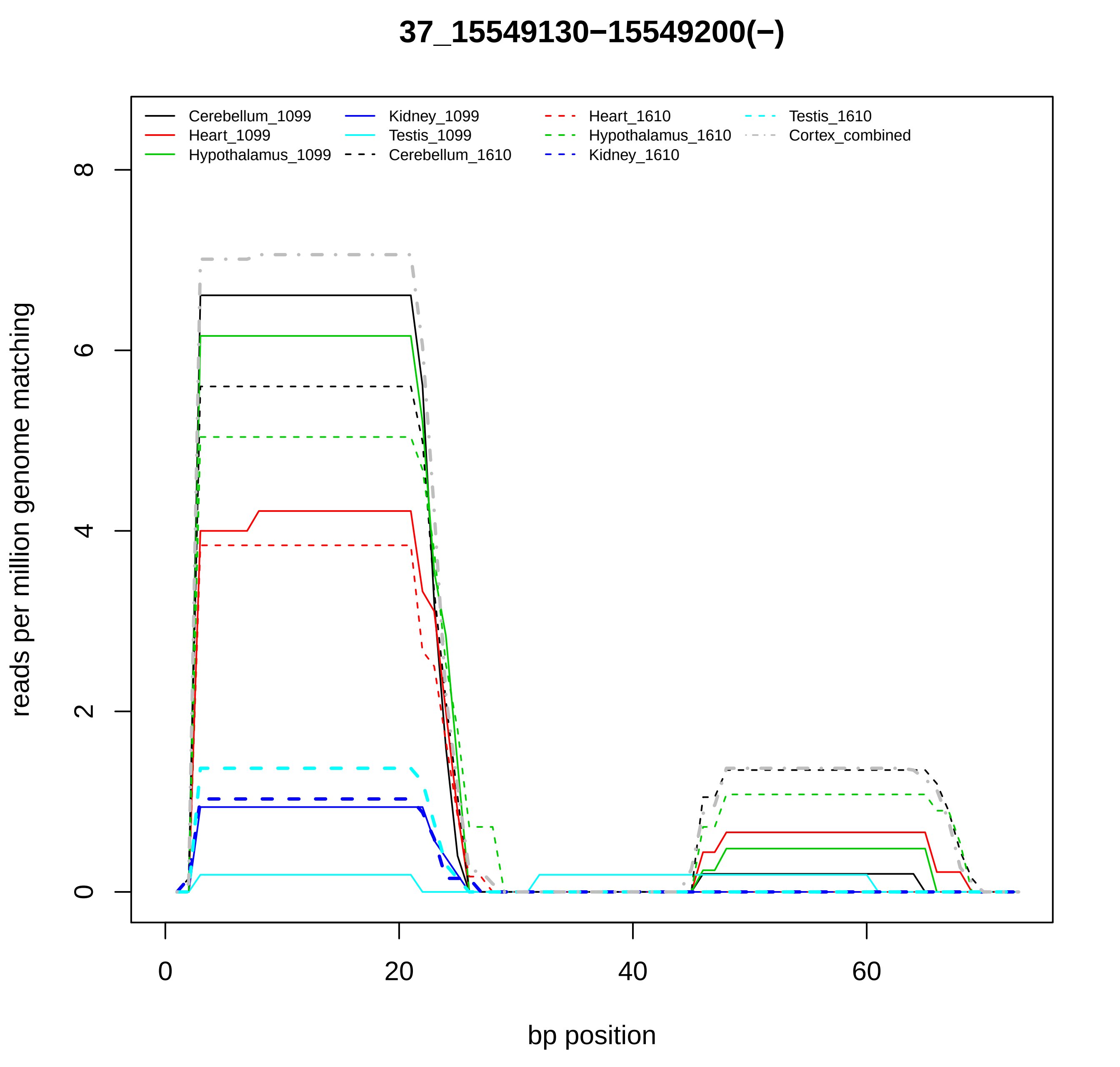
37_15549130−15549200(−)
Cerebellum_1099
Heart_1099
Hypothalamus_1099
Kidney_1099
Testis_1099
Cerebellum_1610
Heart_1610
Hypothalamus_1610
Kidney_1610
Testis_1610
Cortex_combined
reads per million genome matching
bp position
0
2
4
6
8
0
20
40
60

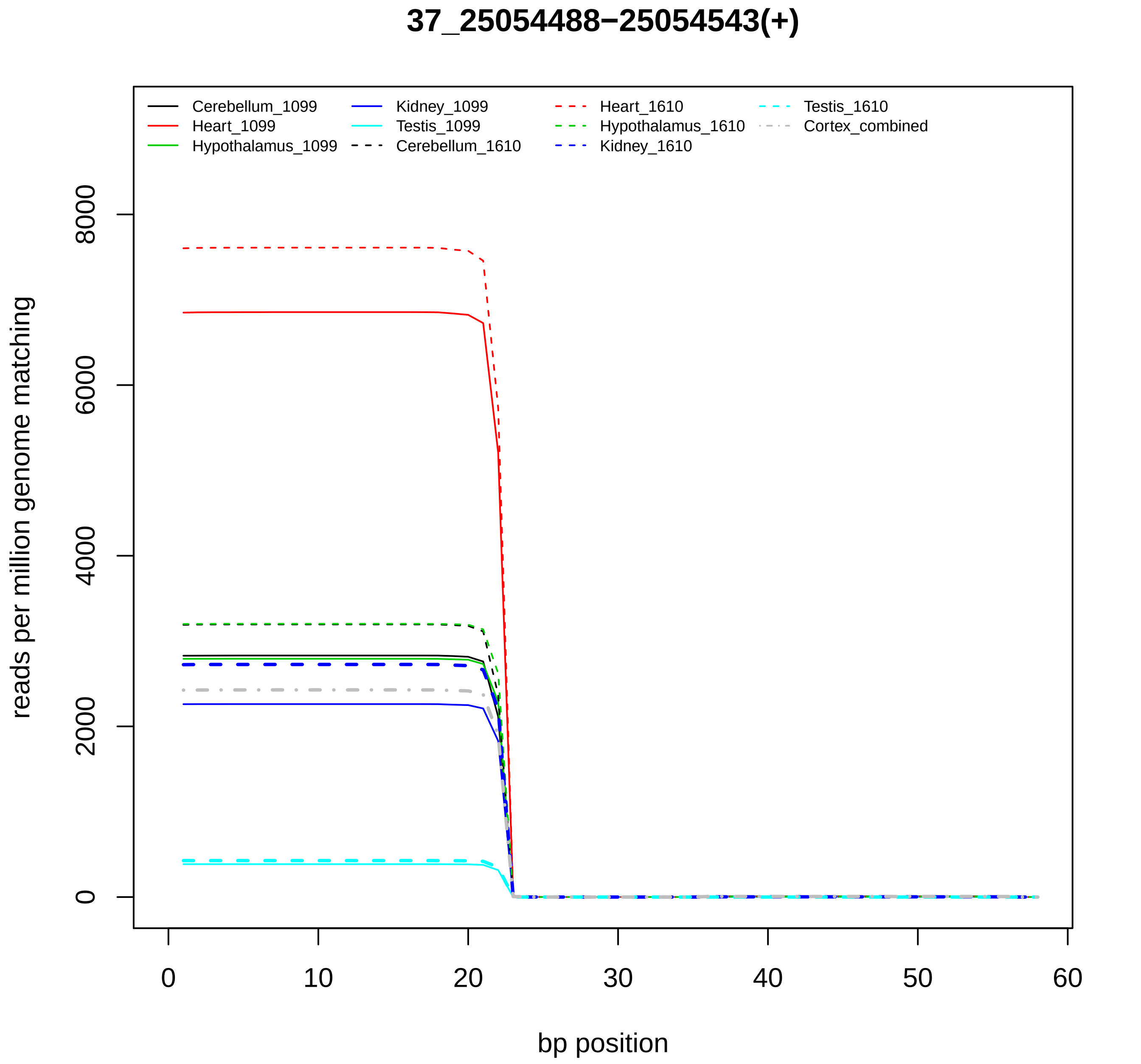
37_25054488−25054543(+)
Cerebellum_1099
Heart_1099
Hypothalamus_1099
Kidney_1099
Testis_1099
Cerebellum_1610
Heart_1610
Hypothalamus_1610
Kidney_1610
Testis_1610
Cortex_combined
reads per million genome matching
0
2000
4000
6000
8000
bp position
0
10
20
30
40
50
60

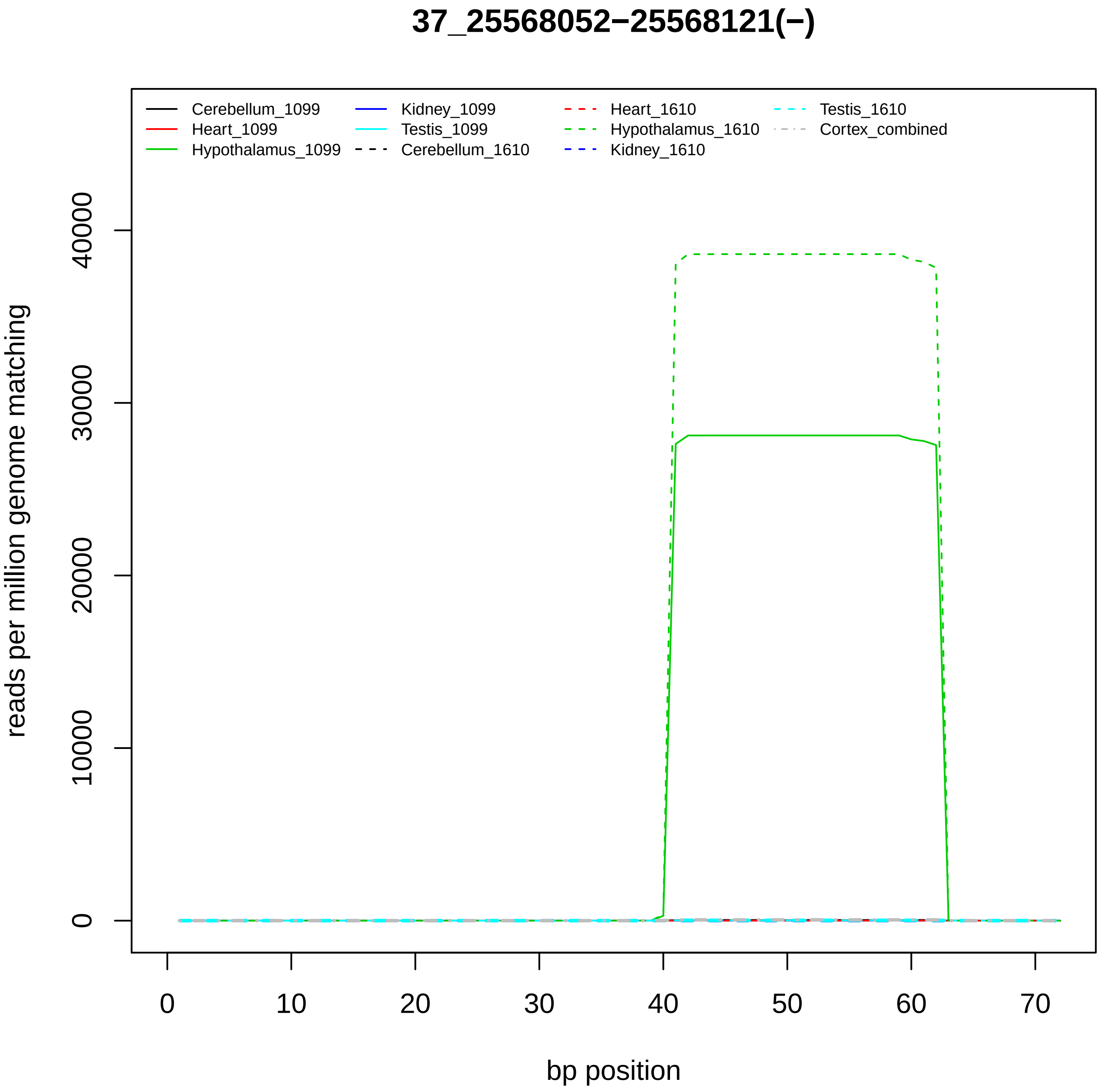

37_25568052−25568121(−)
Cerebellum_1099
Heart_1099
Hypothalamus_1099
Kidney_1099
Testis_1099
Cerebellum_1610
Heart_1610
Hypothalamus_1610
Kidney_1610
Testis_1610
Cortex_combined
reads per million genome matching
0
10000
20000
30000
40000
0
10
20
30
40
50
60
70
bp position

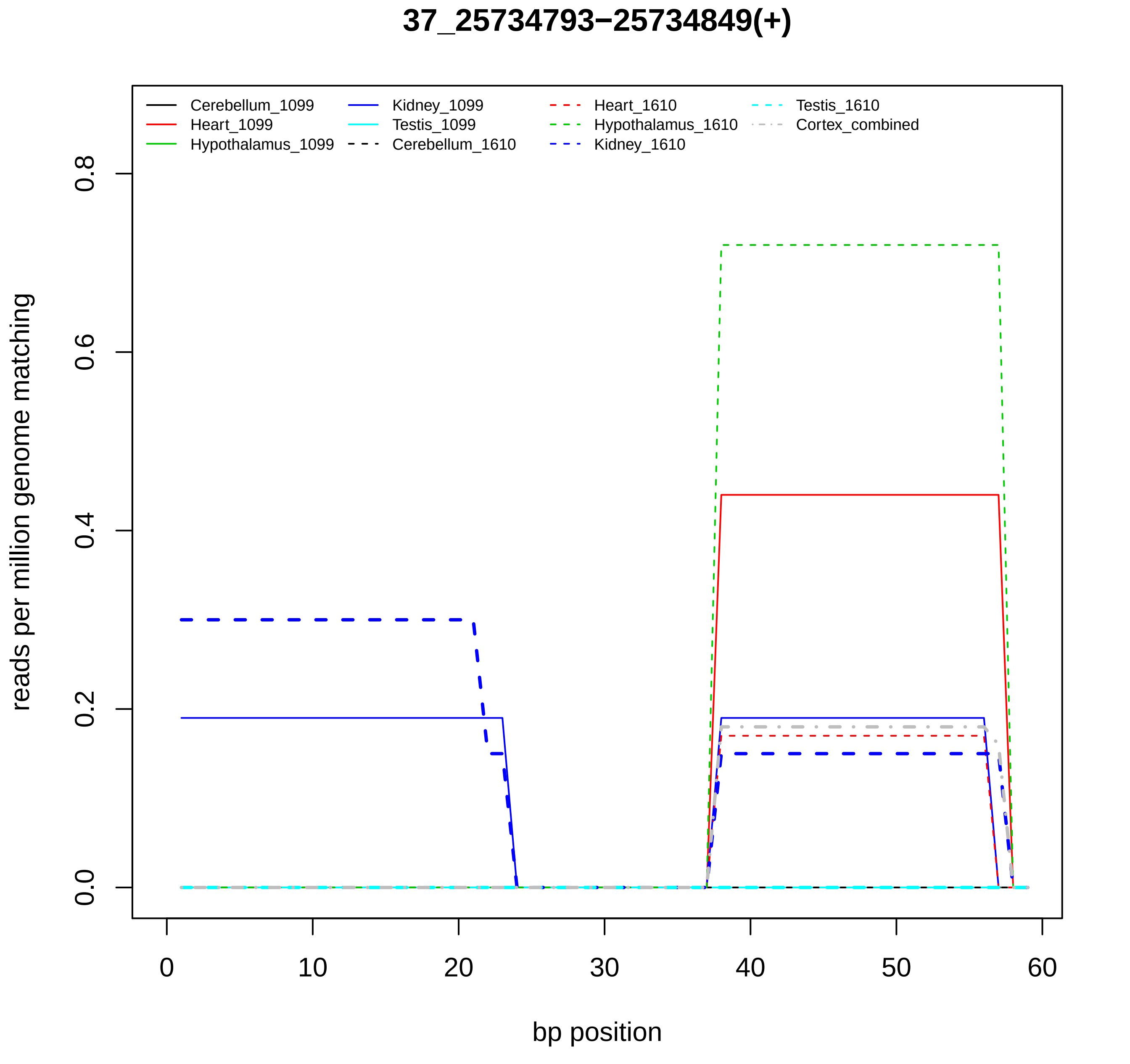

37_25734793−25734849(+)
Cerebellum_1099
Heart_1099
Hypothalamus_1099
Kidney_1099
Testis_1099
Cerebellum_1610
Heart_1610
Hypothalamus_1610
Kidney_1610
Testis_1610
Cortex_combined
reads per million genome matching
0.0
0.2
0.4
0.6
0.8
0
10
20
30
40
50
60
bp position

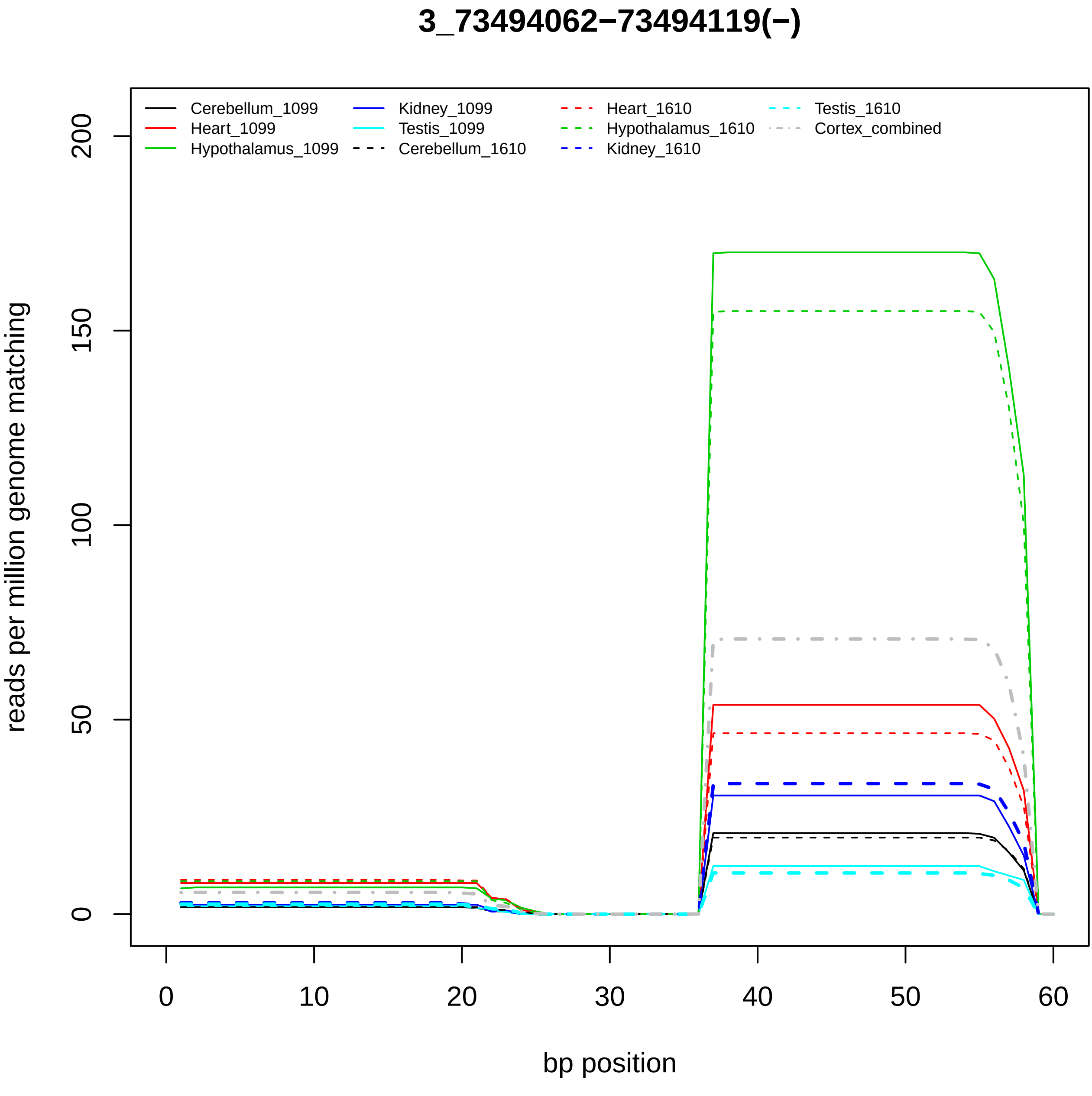
3_73494062−73494119(−)
Cerebellum_1099
Heart_1099
Hypothalamus_1099
Kidney_1099
Testis_1099
Cerebellum_1610
Heart_1610
Hypothalamus_1610
Kidney_1610
Testis_1610
Cortex_combined
reads per million genome matching
bp position
0
50
100
150
200
0
10
20
30
40
50
60

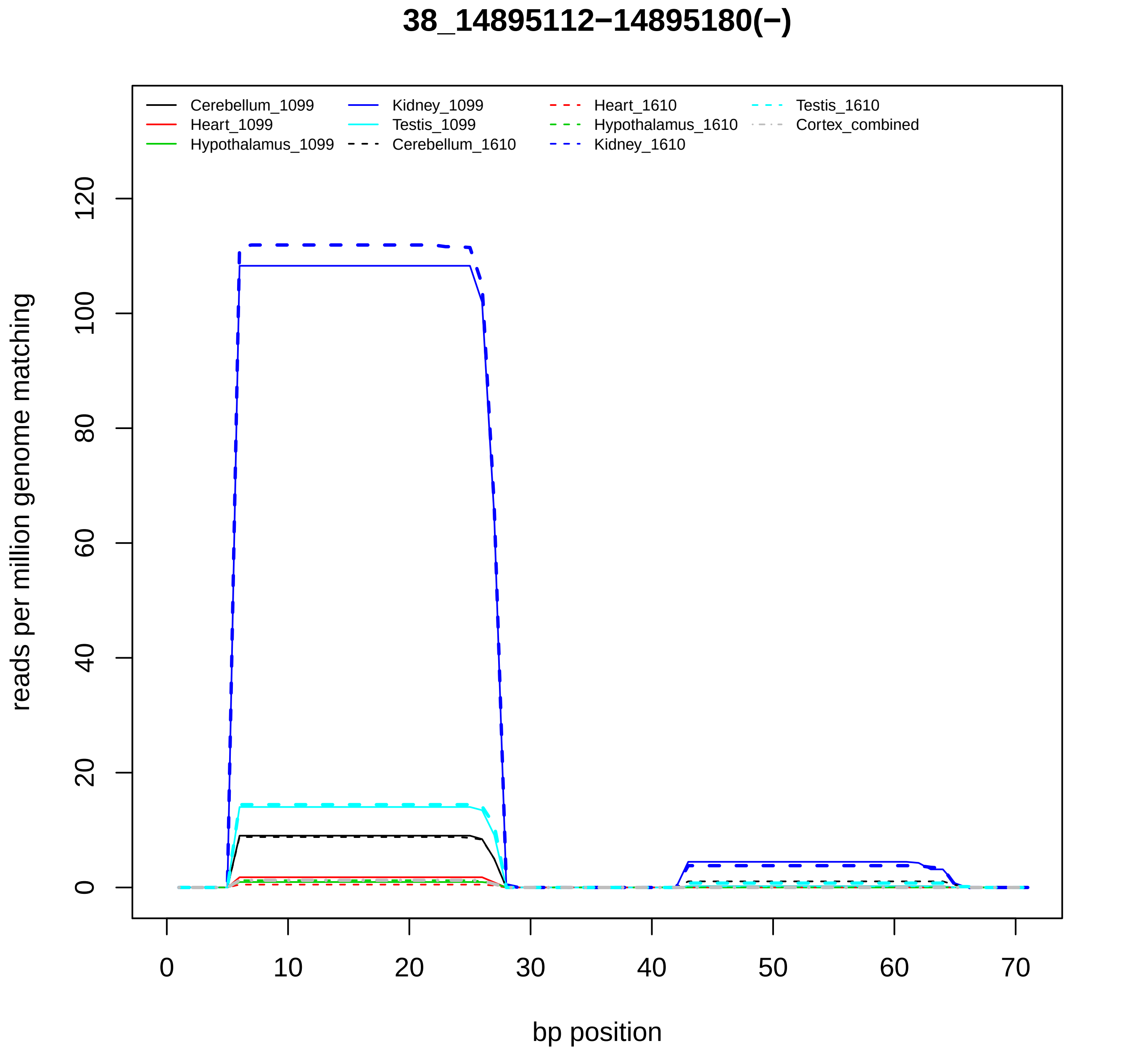
38_14895112−14895180(−)
Cerebellum_1099
Heart_1099
Hypothalamus_1099
Kidney_1099
Testis_1099
Cerebellum_1610
Heart_1610
Hypothalamus_1610
Kidney_1610
Testis_1610
Cortex_combined
reads per million genome matching
0
20
40
60
80
100
120
0
10
20
30
40
50
60
70
bp position

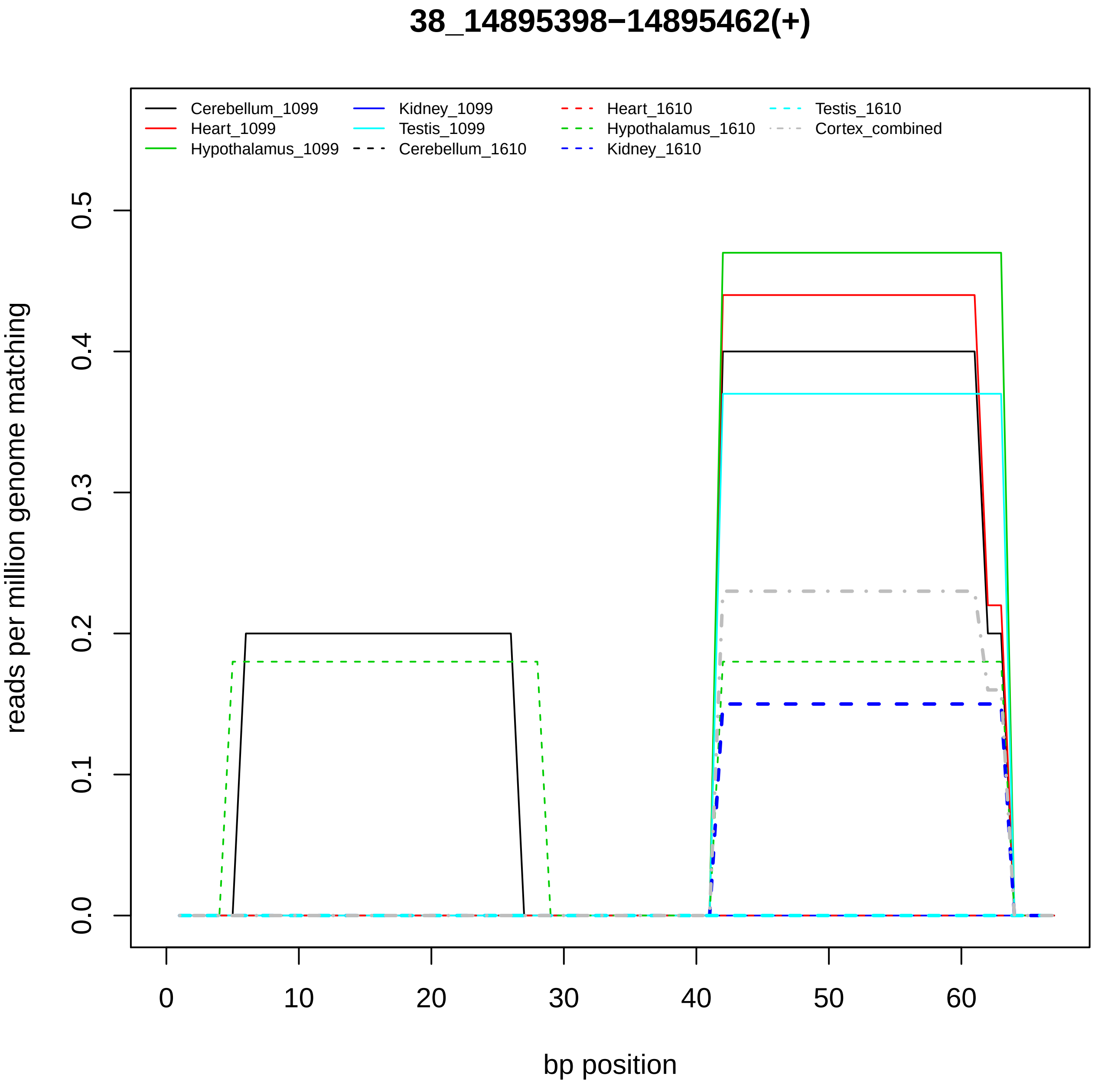

38_14895398−14895462(+)
Cerebellum_1099
Heart_1099
Hypothalamus_1099
Kidney_1099
Testis_1099
Cerebellum_1610
Heart_1610
Hypothalamus_1610
Kidney_1610
Testis_1610
Cortex_combined
reads per million genome matching
bp position
0.0
0.1
0.2
0.3
0.4
0.5
0
10
20
30
40
50
60

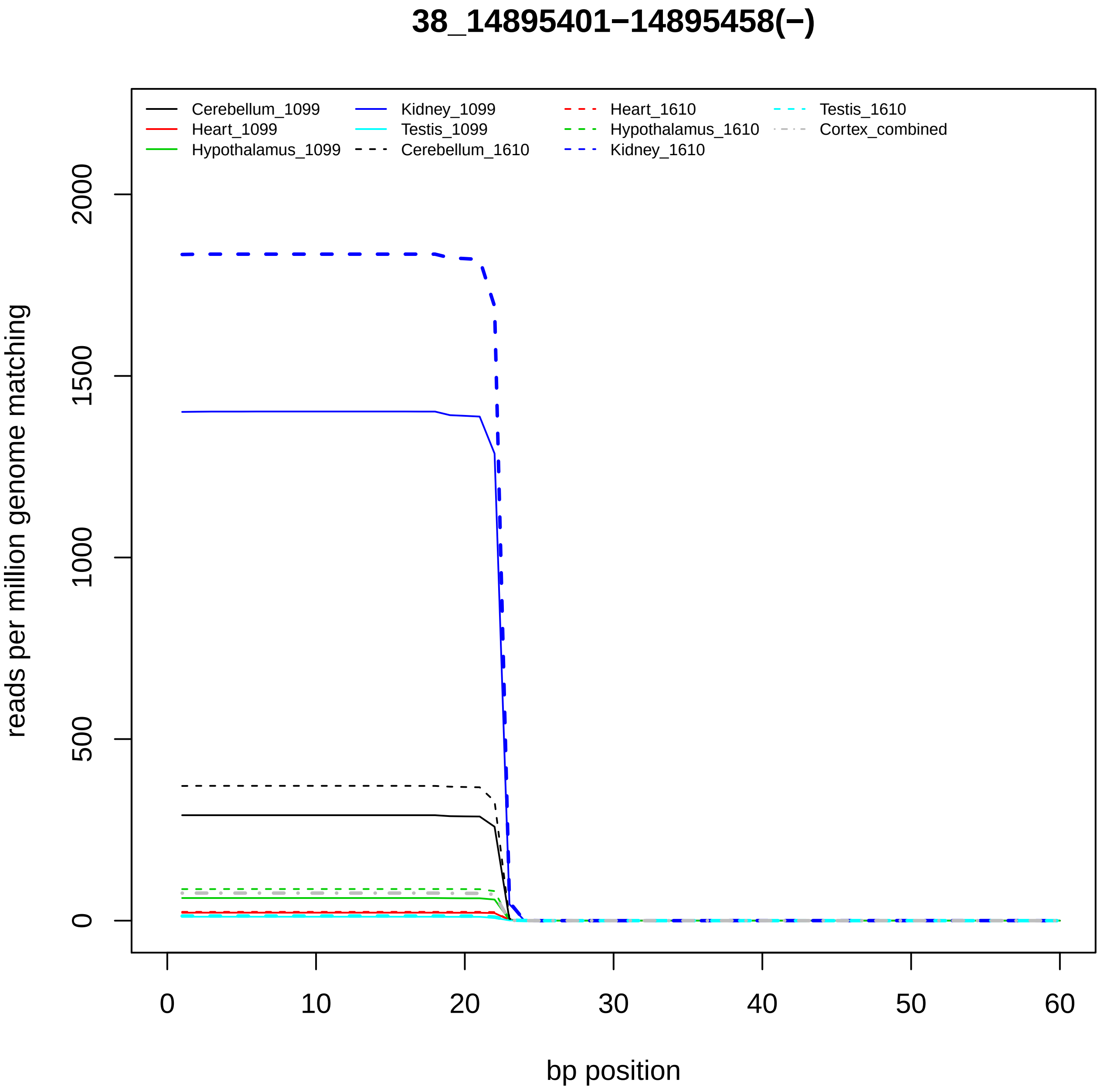

38_14895401−14895458(−)
Cerebellum_1099
Heart_1099
Hypothalamus_1099
Kidney_1099
Testis_1099
Cerebellum_1610
Heart_1610
Hypothalamus_1610
Kidney_1610
Testis_1610
Cortex_combined
reads per million genome matching
0
500
1000
1500
2000
0
10
20
30
40
50
60
bp position

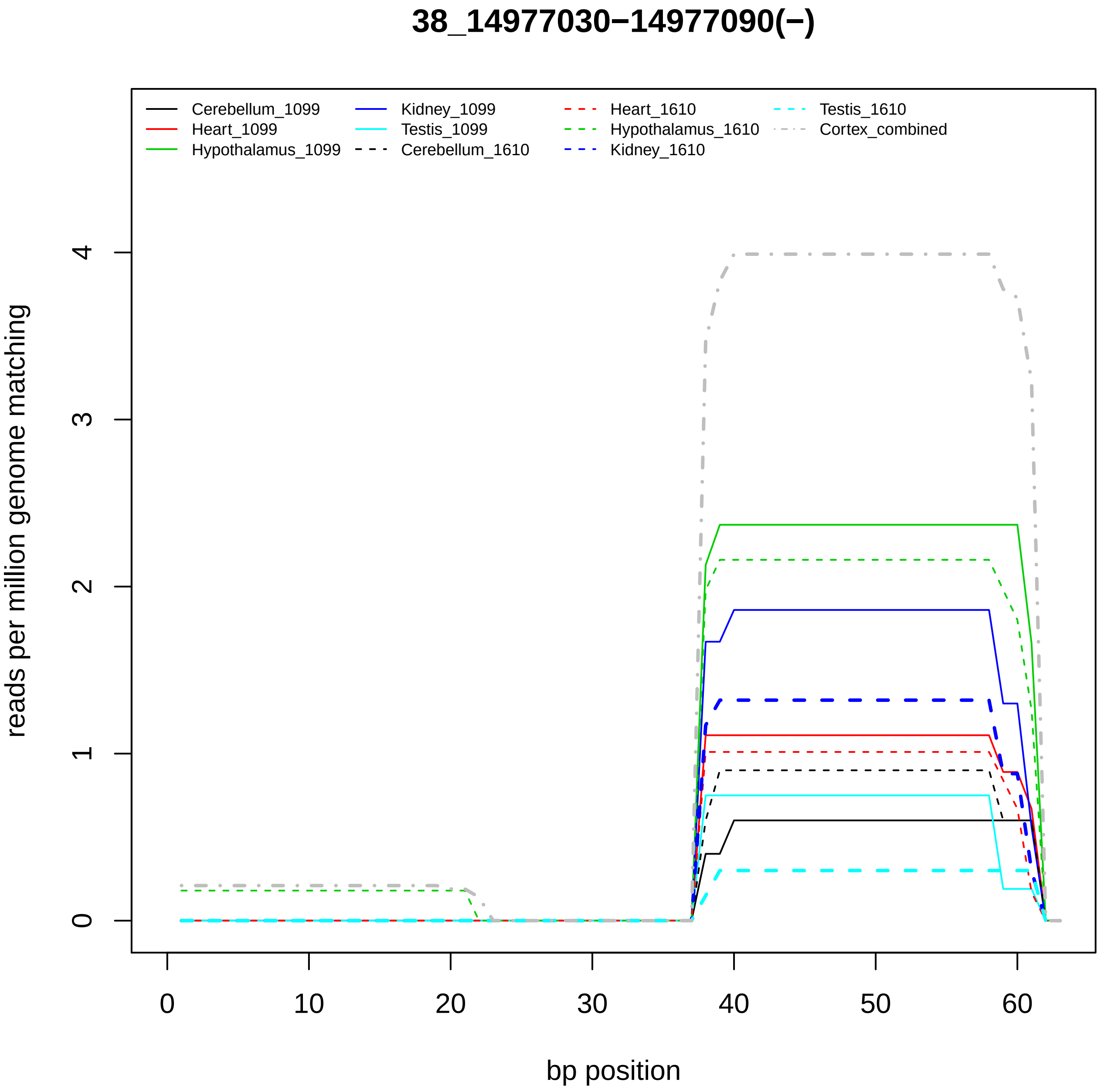
38_14977030−14977090(−)
Cerebellum_1099
Heart_1099
Hypothalamus_1099
Kidney_1099
Testis_1099
Cerebellum_1610
Heart_1610
Hypothalamus_1610
Kidney_1610
Testis_1610
Cortex_combined
reads per million genome matching
bp position
0
1
2
3
4
0
10
20
30
40
50
60

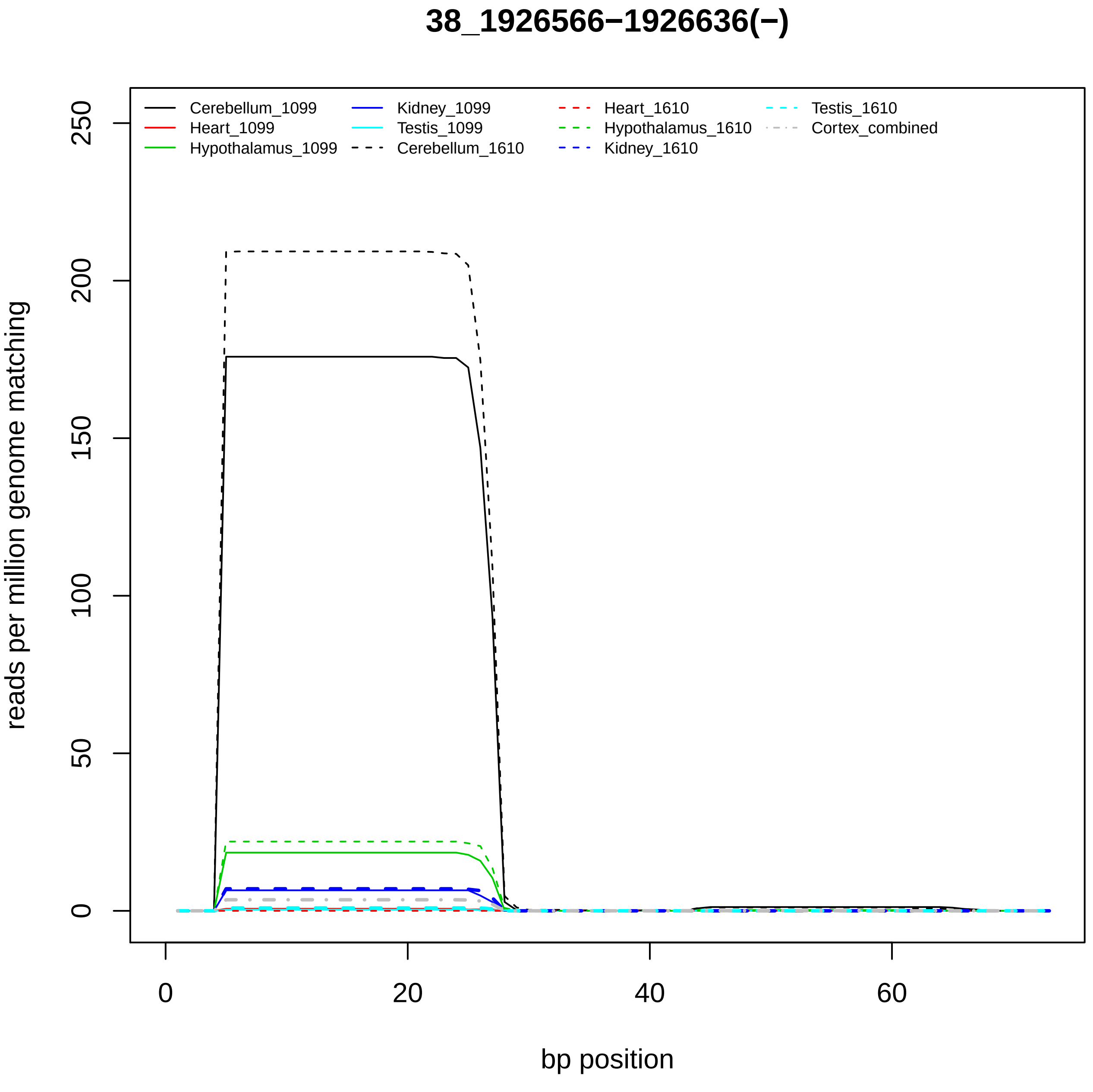

38_1926566−1926636(−)
Cerebellum_1099
Heart_1099
Hypothalamus_1099
Kidney_1099
Testis_1099
Cerebellum_1610
Heart_1610
Hypothalamus_1610
Kidney_1610
Testis_1610
Cortex_combined
reads per million genome matching
bp position
0
50
100
150
200
250
0
20
40
60

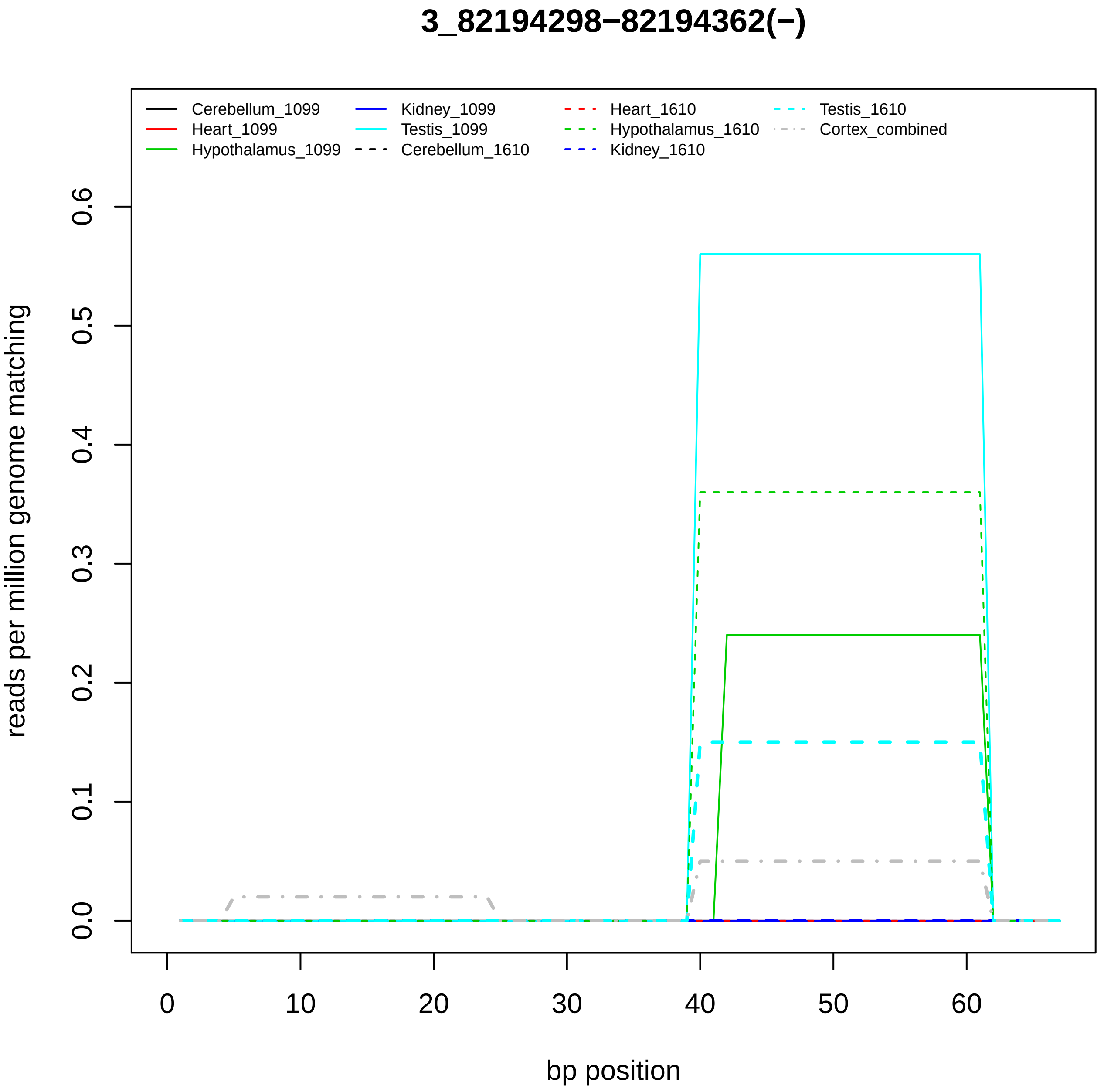
3_82194298−82194362(−)
Cerebellum_1099
Heart_1099
Hypothalamus_1099
Kidney_1099
Testis_1099
Cerebellum_1610
Heart_1610
Hypothalamus_1610
Kidney_1610
Testis_1610
Cortex_combined
reads per million genome matching
0.0
0.1
0.2
0.3
0.4
0.5
0.6
0
10
20
30
40
50
60
bp position

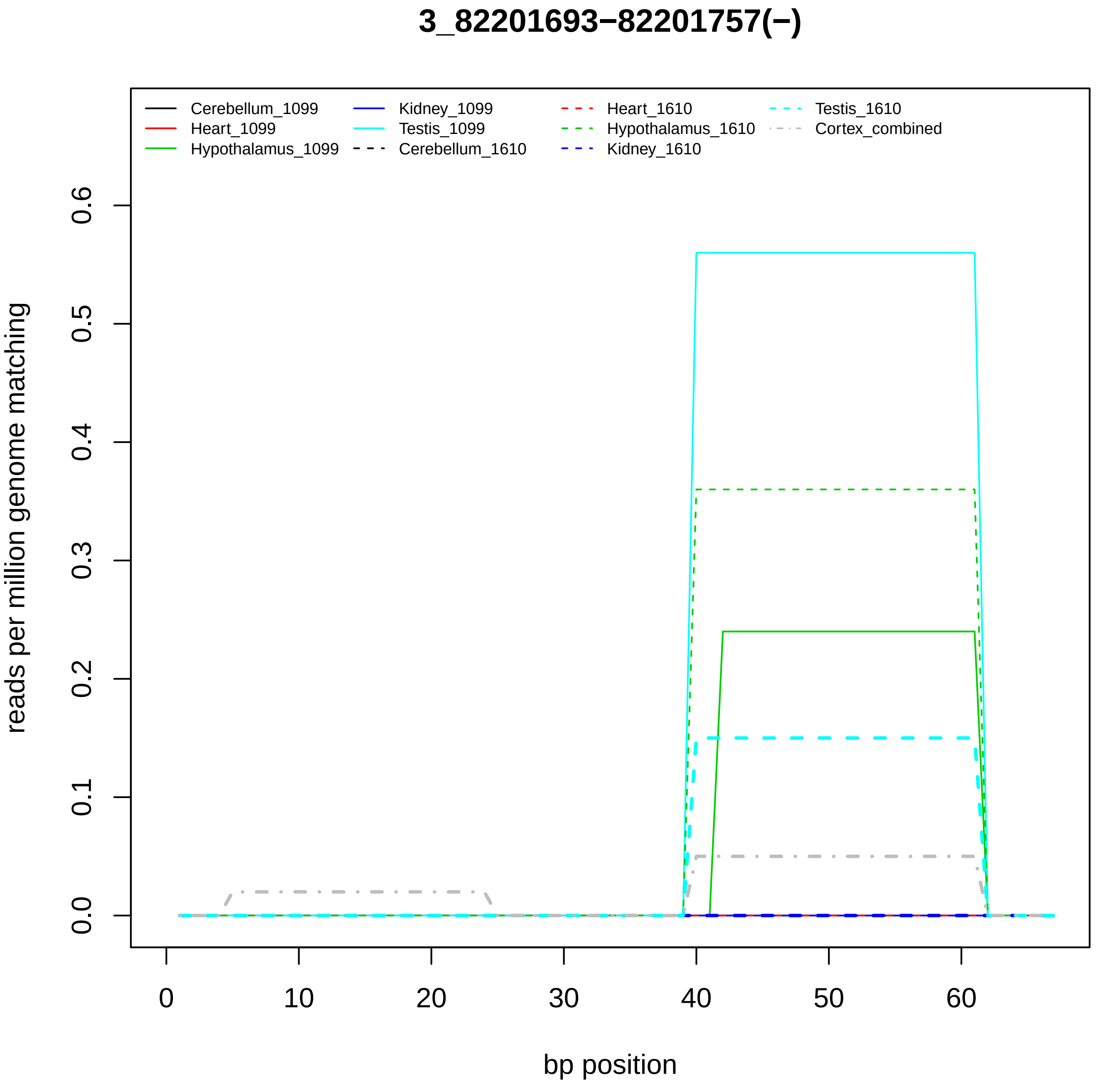

3_82201693−82201757(−)
Cerebellum_1099
Heart_1099
Hypothalamus_1099
Kidney_1099
Testis_1099
Cerebellum_1610
Heart_1610
Hypothalamus_1610
Kidney_1610
Testis_1610
Cortex_combined
reads per million genome matching
0.0
0.1
0.2
0.3
0.4
0.5
0.6
0
10
20
30
40
50
60
bp position

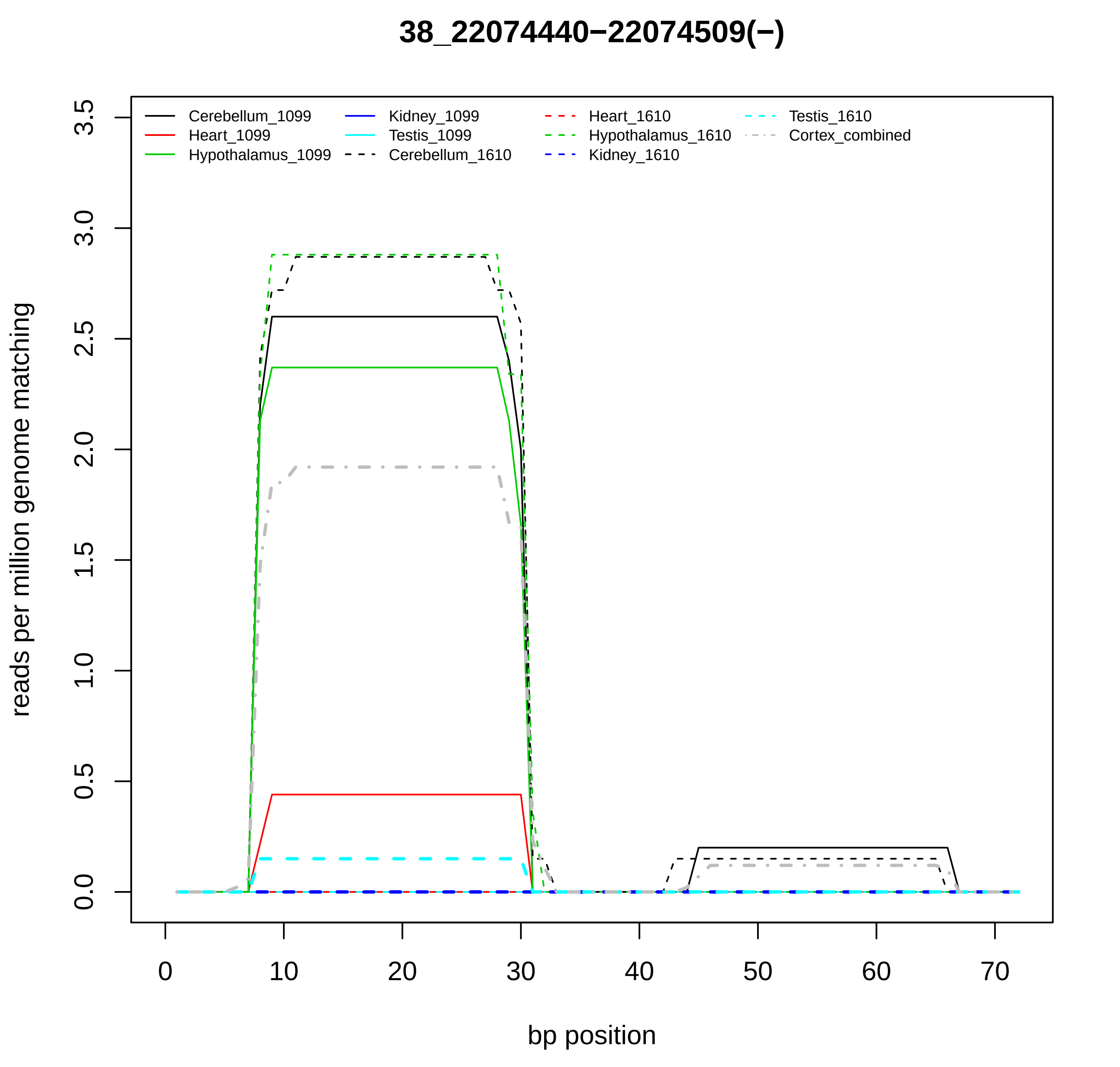

38_22074440−22074509(−)
Cerebellum_1099
Heart_1099
Hypothalamus_1099
Kidney_1099
Testis_1099
Cerebellum_1610
Heart_1610
Hypothalamus_1610
Kidney_1610
Testis_1610
Cortex_combined
reads per million genome matching
bp position
0.0
0.5
1.0
1.5
2.0
2.5
3.0
3.5
0
10
20
30
40
50
60
70

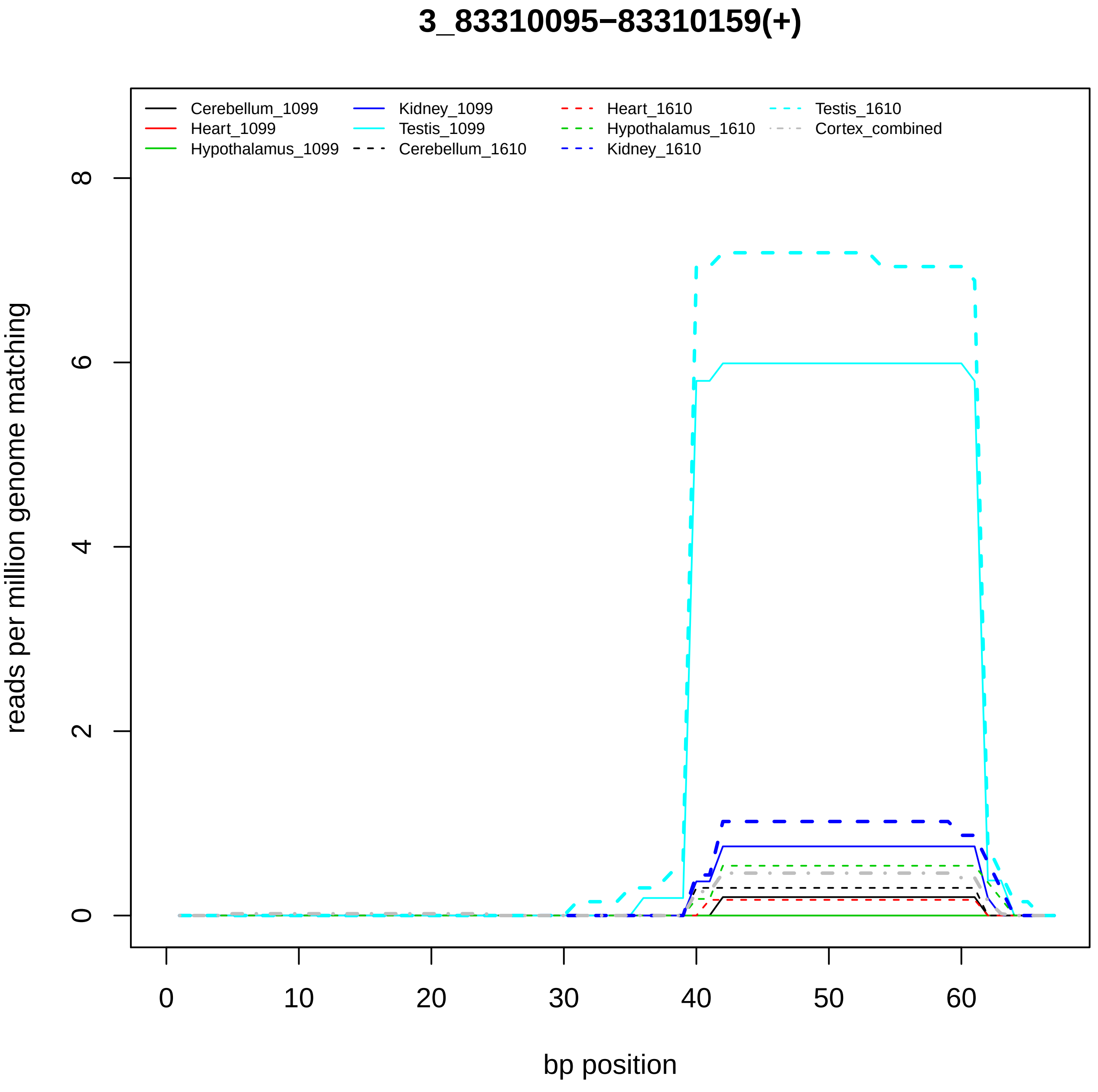

3_83310095−83310159(+)
Cerebellum_1099
Heart_1099
Hypothalamus_1099
Kidney_1099
Testis_1099
Cerebellum_1610
Heart_1610
Hypothalamus_1610
Kidney_1610
Testis_1610
Cortex_combined
reads per million genome matching
bp position
0
2
4
6
8
0
10
20
30
40
50
60

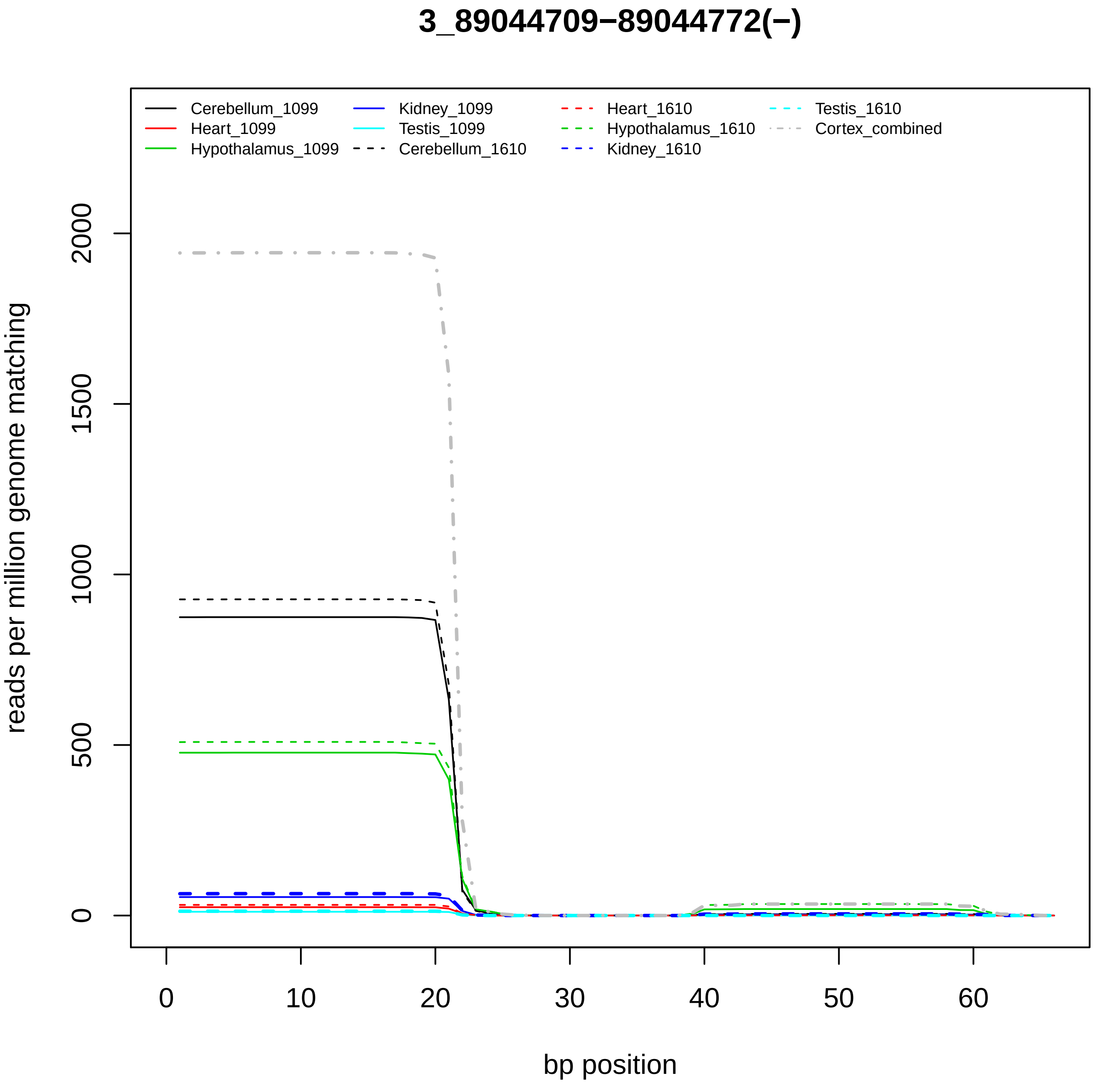

3_89044709−89044772(−)
Cerebellum_1099
Heart_1099
Hypothalamus_1099
Kidney_1099
Testis_1099
Cerebellum_1610
Heart_1610
Hypothalamus_1610
Kidney_1610
Testis_1610
Cortex_combined
reads per million genome matching
0
500
1000
1500
2000
0
10
20
30
40
50
60
bp position

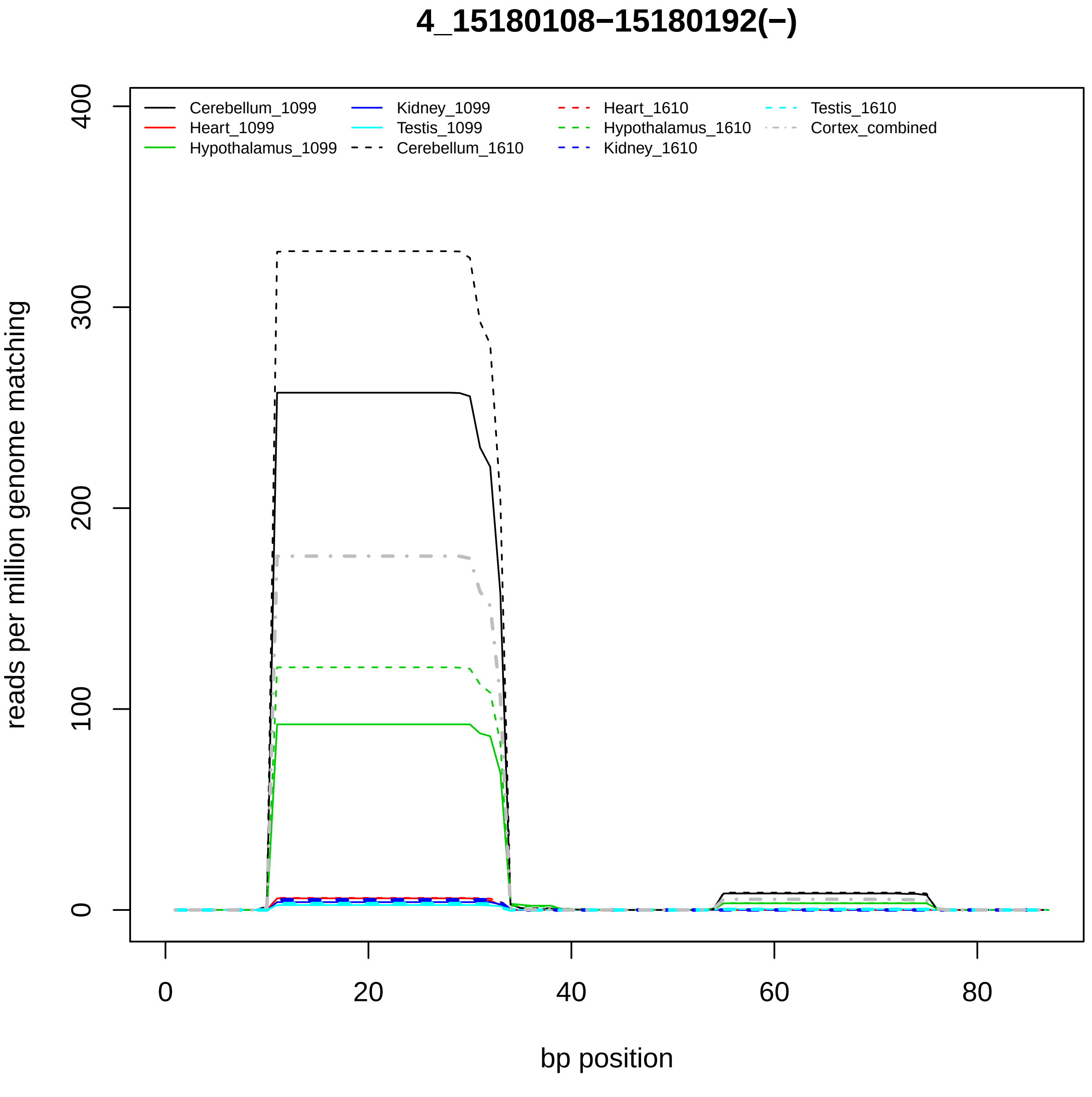

4_15180108−15180192(−)
Cerebellum_1099
Heart_1099
Hypothalamus_1099
Kidney_1099
Testis_1099
Cerebellum_1610
Heart_1610
Hypothalamus_1610
Kidney_1610
Testis_1610
Cortex_combined
reads per million genome matching
bp position
0
100
200
300
400
0
20
40
60
80

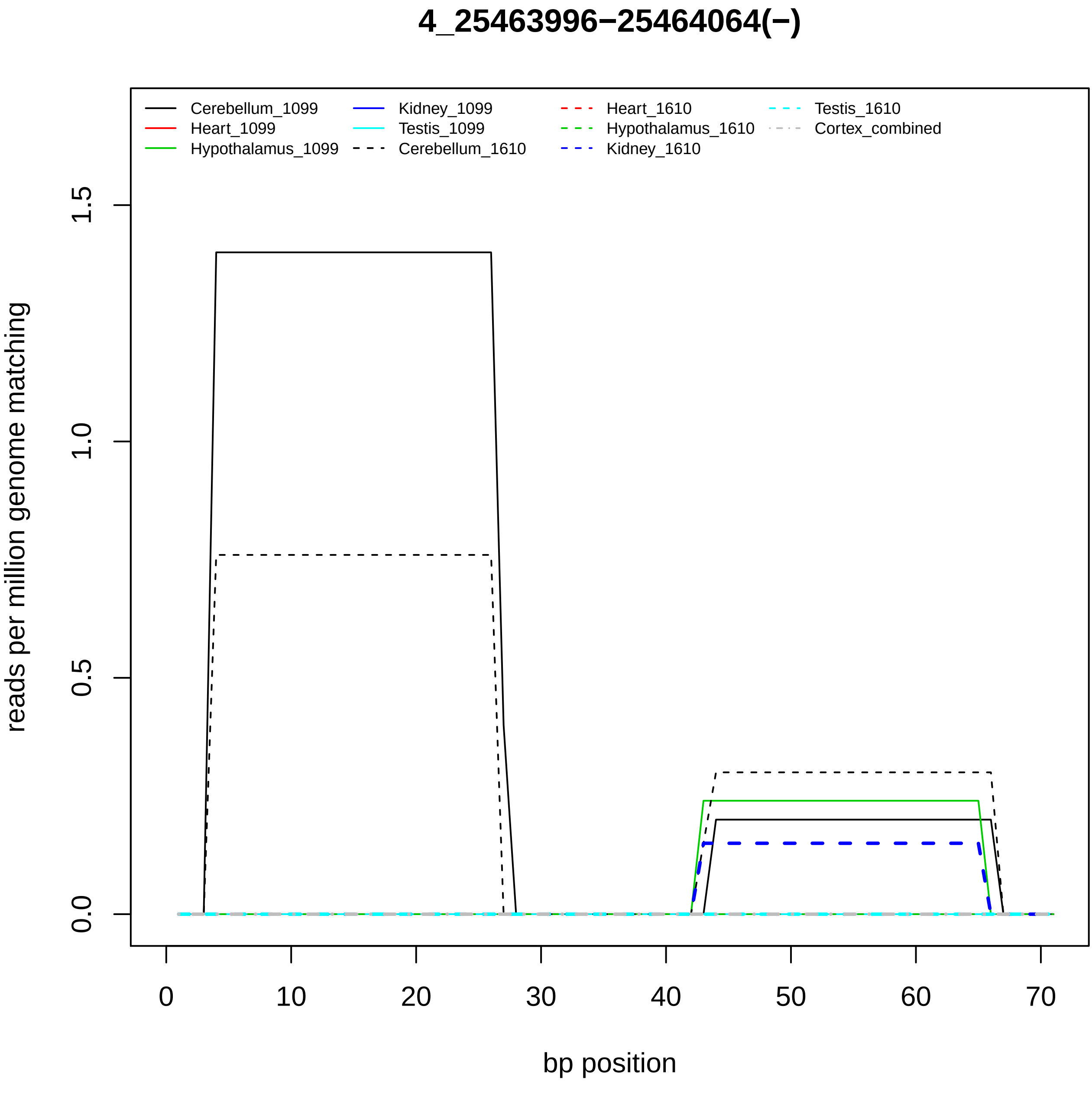
4_25463996−25464064(−)
Cerebellum_1099
Heart_1099
Hypothalamus_1099
Kidney_1099
Testis_1099
Cerebellum_1610
Heart_1610
Hypothalamus_1610
Kidney_1610
Testis_1610
Cortex_combined
reads per million genome matching
0.0
0.5
1.0
1.5
0
10
20
30
40
50
60
70
bp position

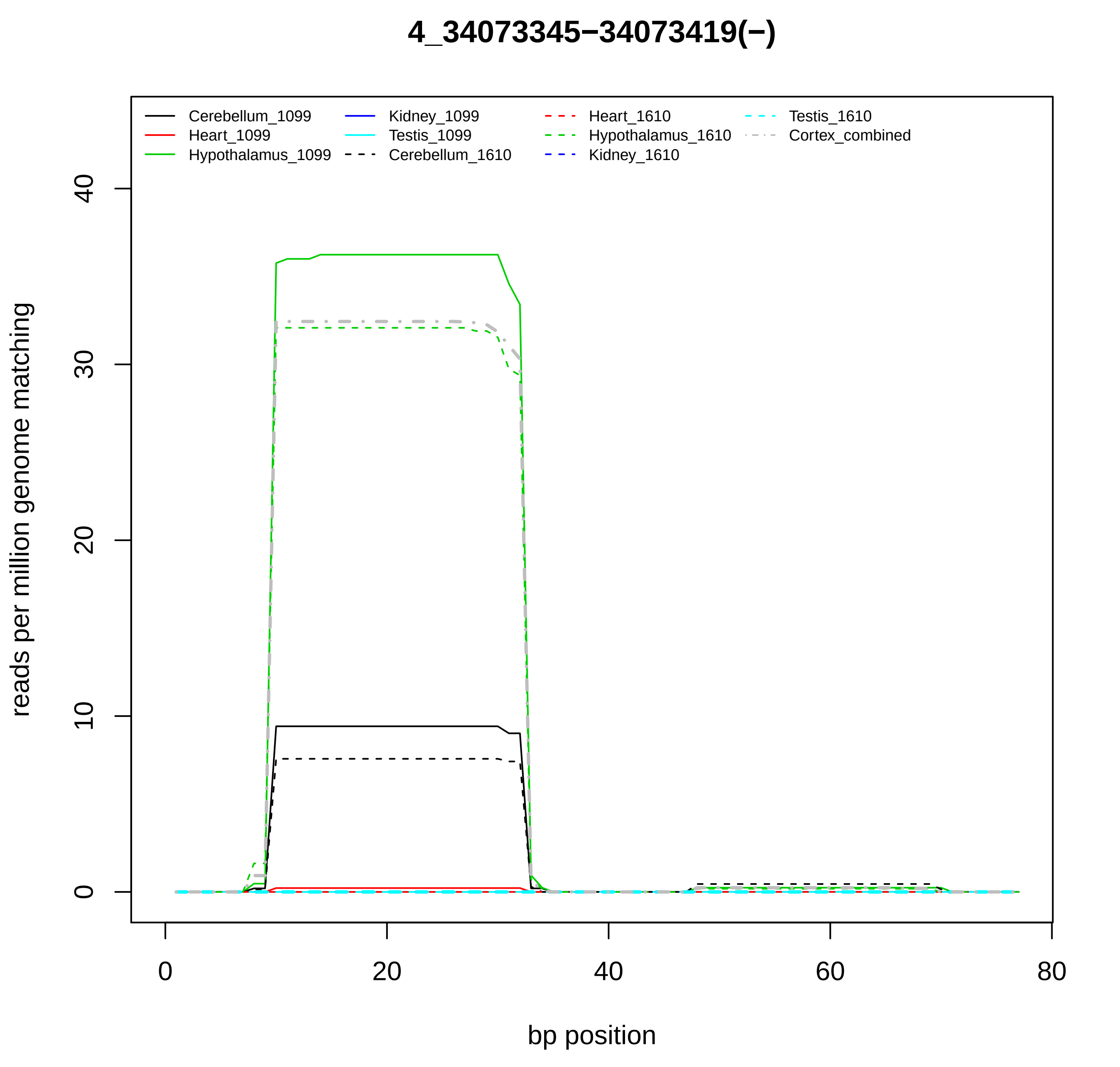

4_34073345−34073419(−)
Cerebellum_1099
Heart_1099
Hypothalamus_1099
Kidney_1099
Testis_1099
Cerebellum_1610
Heart_1610
Hypothalamus_1610
Kidney_1610
Testis_1610
Cortex_combined
reads per million genome matching
bp position
0
10
20
30
40
0
20
40
60
80

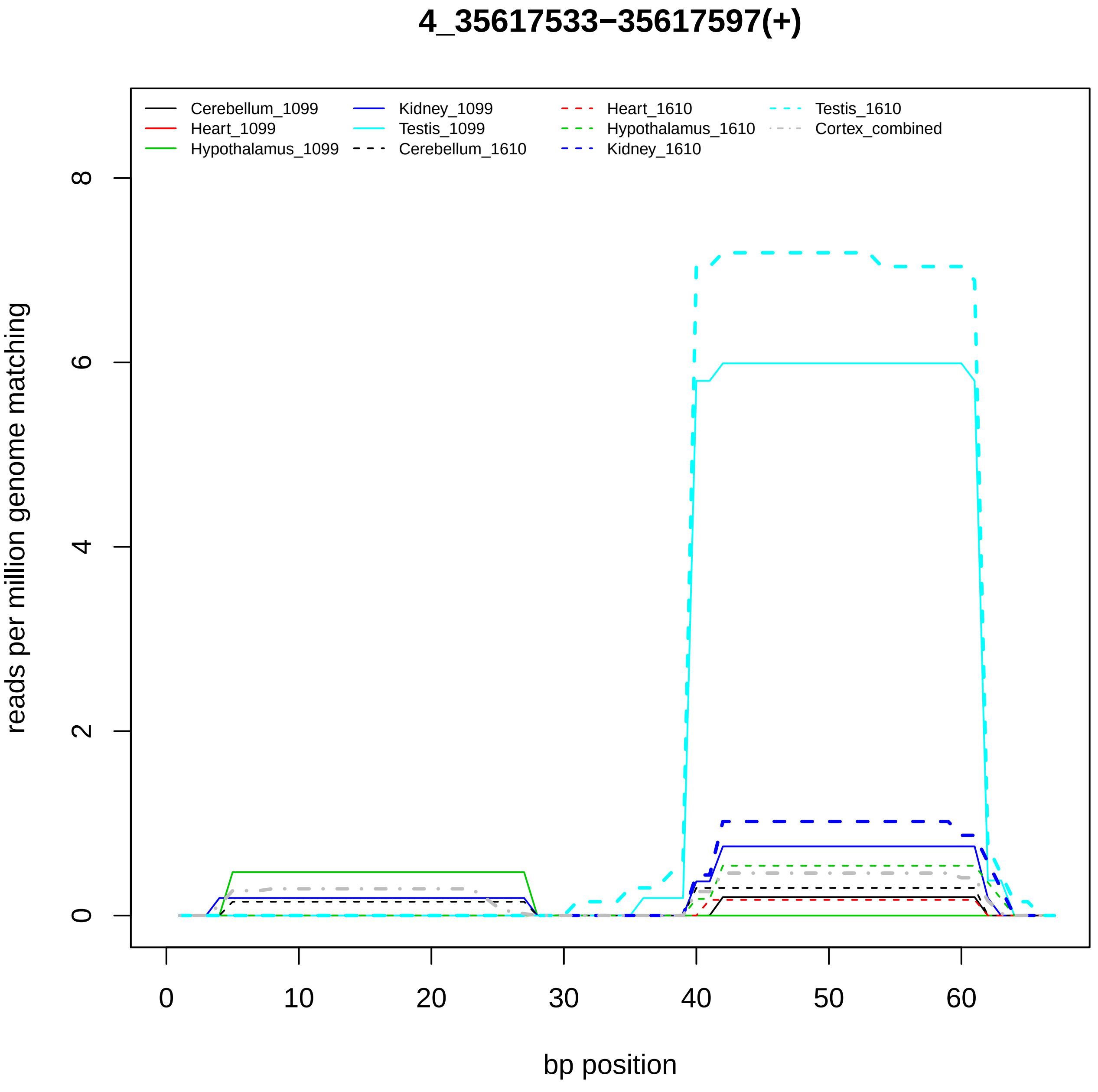

4_35617533−35617597(+)
Cerebellum_1099
Heart_1099
Hypothalamus_1099
Kidney_1099
Testis_1099
Cerebellum_1610
Heart_1610
Hypothalamus_1610
Kidney_1610
Testis_1610
Cortex_combined
reads per million genome matching
bp position
0
2
4
6
8
0
10
20
30
40
50
60

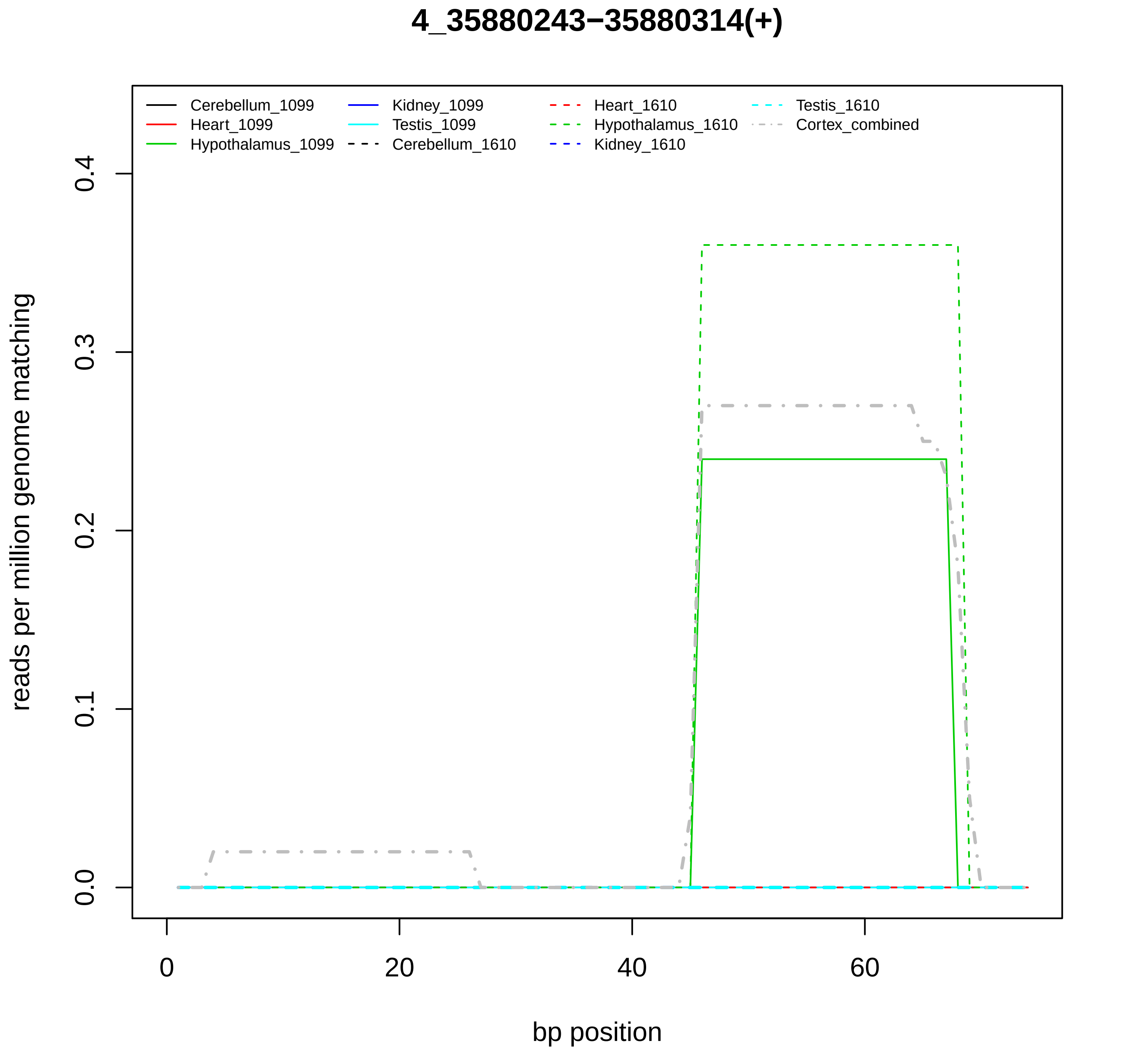

4_35880243−35880314(+)
Cerebellum_1099
Heart_1099
Hypothalamus_1099
Kidney_1099
Testis_1099
Cerebellum_1610
Heart_1610
Hypothalamus_1610
Kidney_1610
Testis_1610
Cortex_combined
reads per million genome matching
0.0
0.1
0.2
0.3
0.4
0
20
40
60
bp position

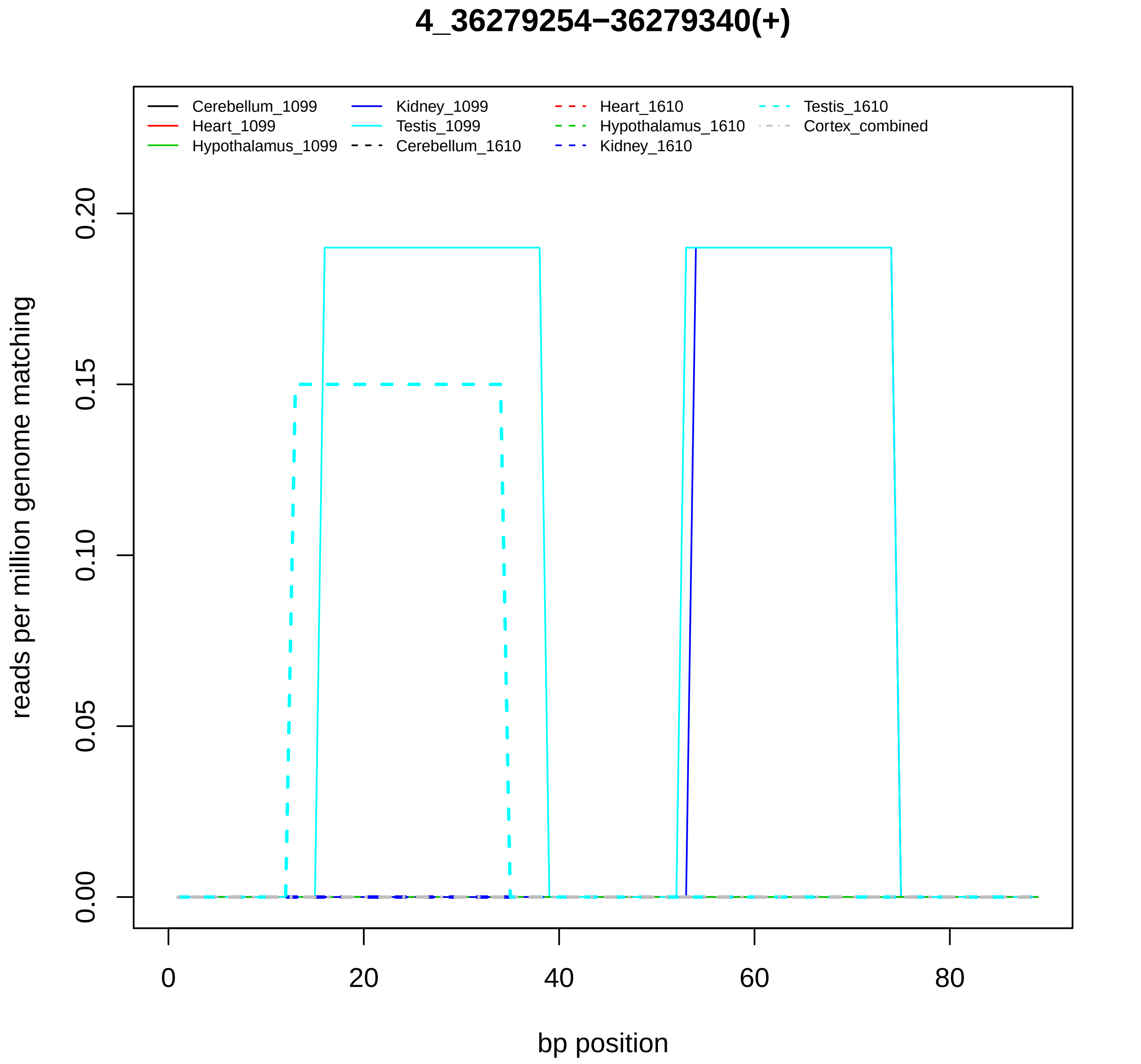

4_36279254−36279340(+)
Cerebellum_1099
Heart_1099
Hypothalamus_1099
Kidney_1099
Testis_1099
Cerebellum_1610
Heart_1610
Hypothalamus_1610
Kidney_1610
Testis_1610
Cortex_combined
reads per million genome matching
0.00
0.05
0.10
0.15
0.20
0
20
40
60
80
bp position

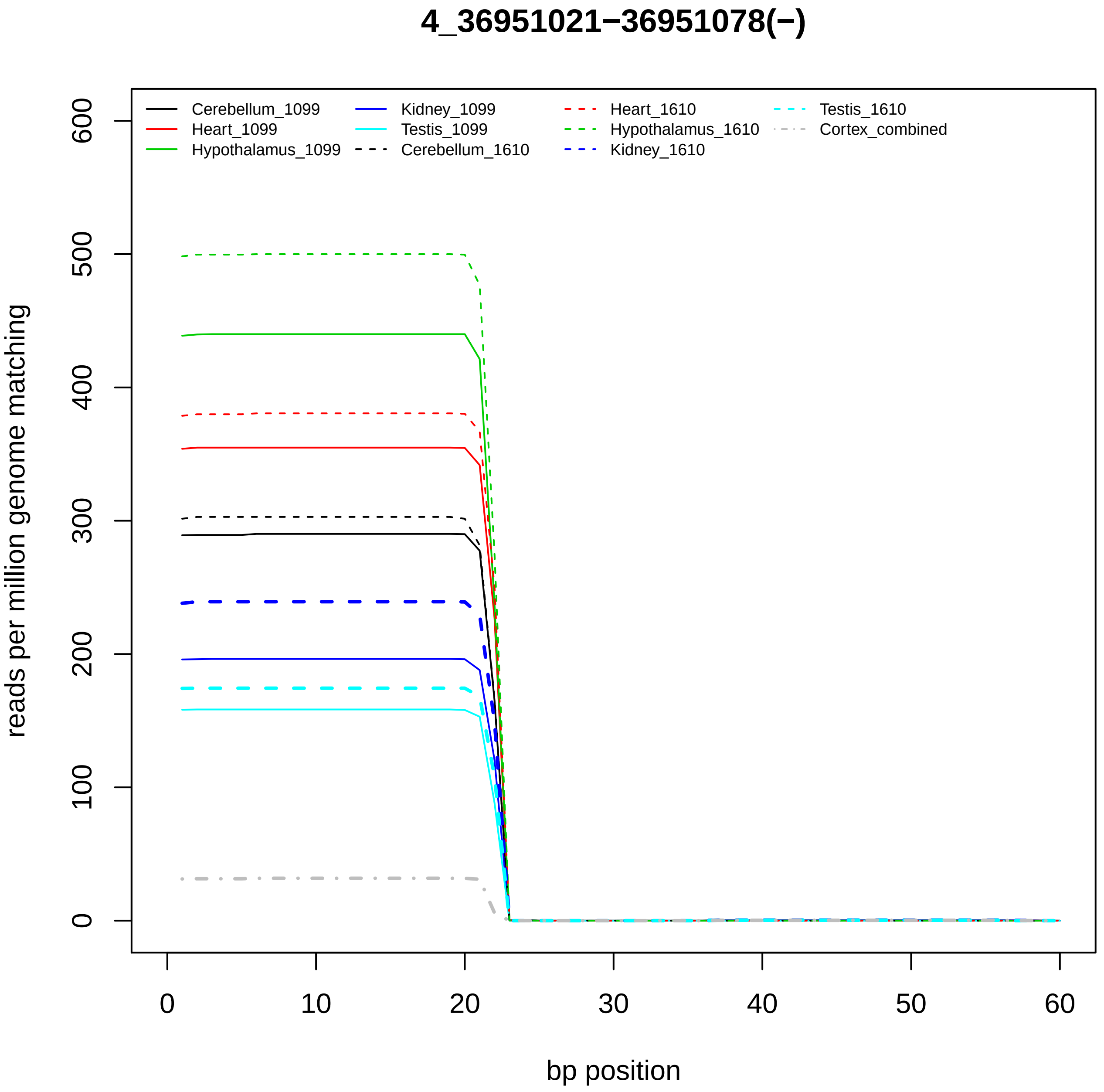
4_36951021−36951078(−)
Cerebellum_1099
Heart_1099
Hypothalamus_1099
Kidney_1099
Testis_1099
Cerebellum_1610
Heart_1610
Hypothalamus_1610
Kidney_1610
Testis_1610
Cortex_combined
reads per million genome matching
bp position
0
100
200
300
400
500
600
0
10
20
30
40
50
60

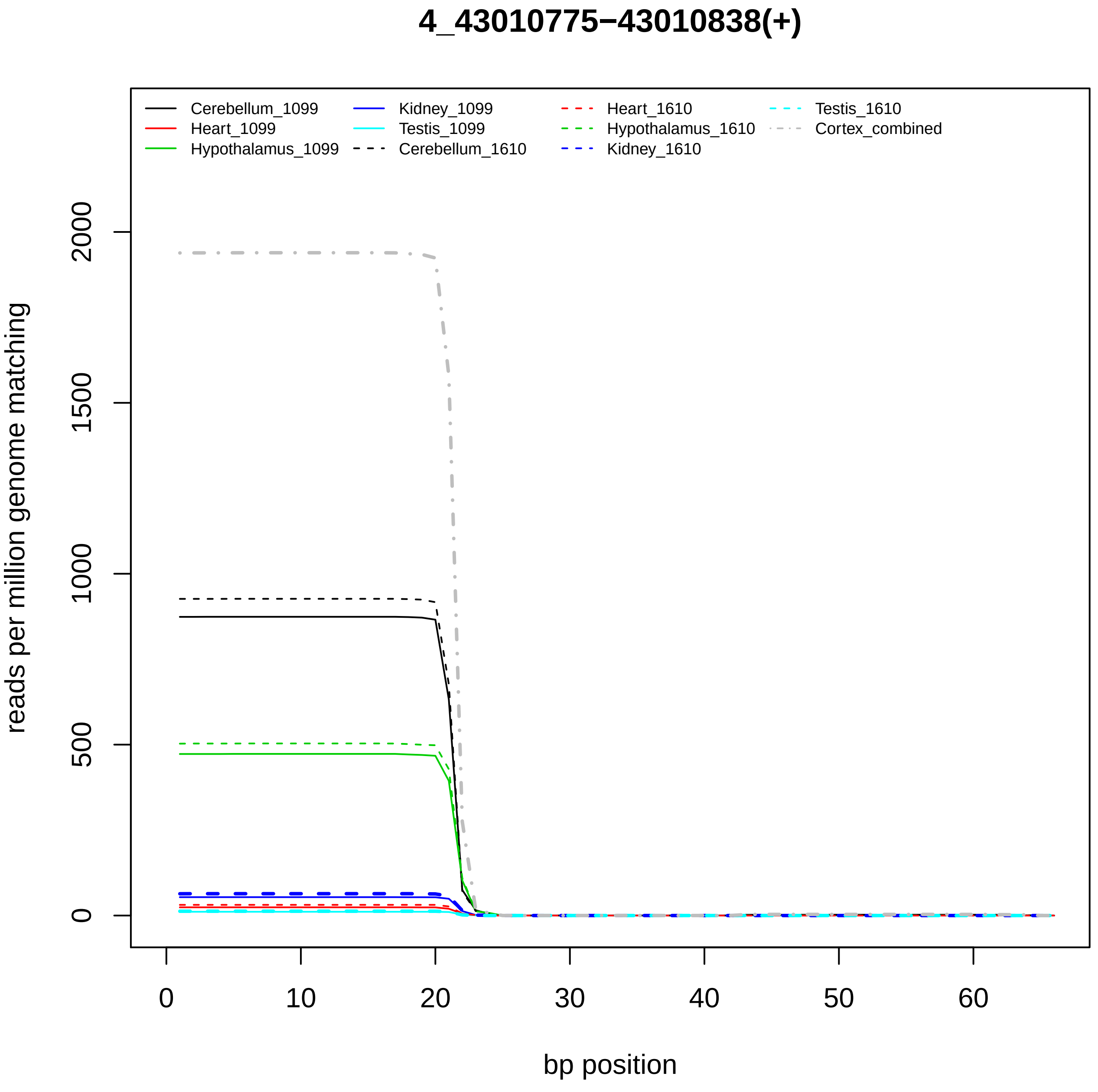
4_43010775−43010838(+)
Cerebellum_1099
Heart_1099
Hypothalamus_1099
Kidney_1099
Testis_1099
Cerebellum_1610
Heart_1610
Hypothalamus_1610
Kidney_1610
Testis_1610
Cortex_combined
reads per million genome matching
0
500
1000
1500
2000
bp position
0
10
20
30
40
50
60

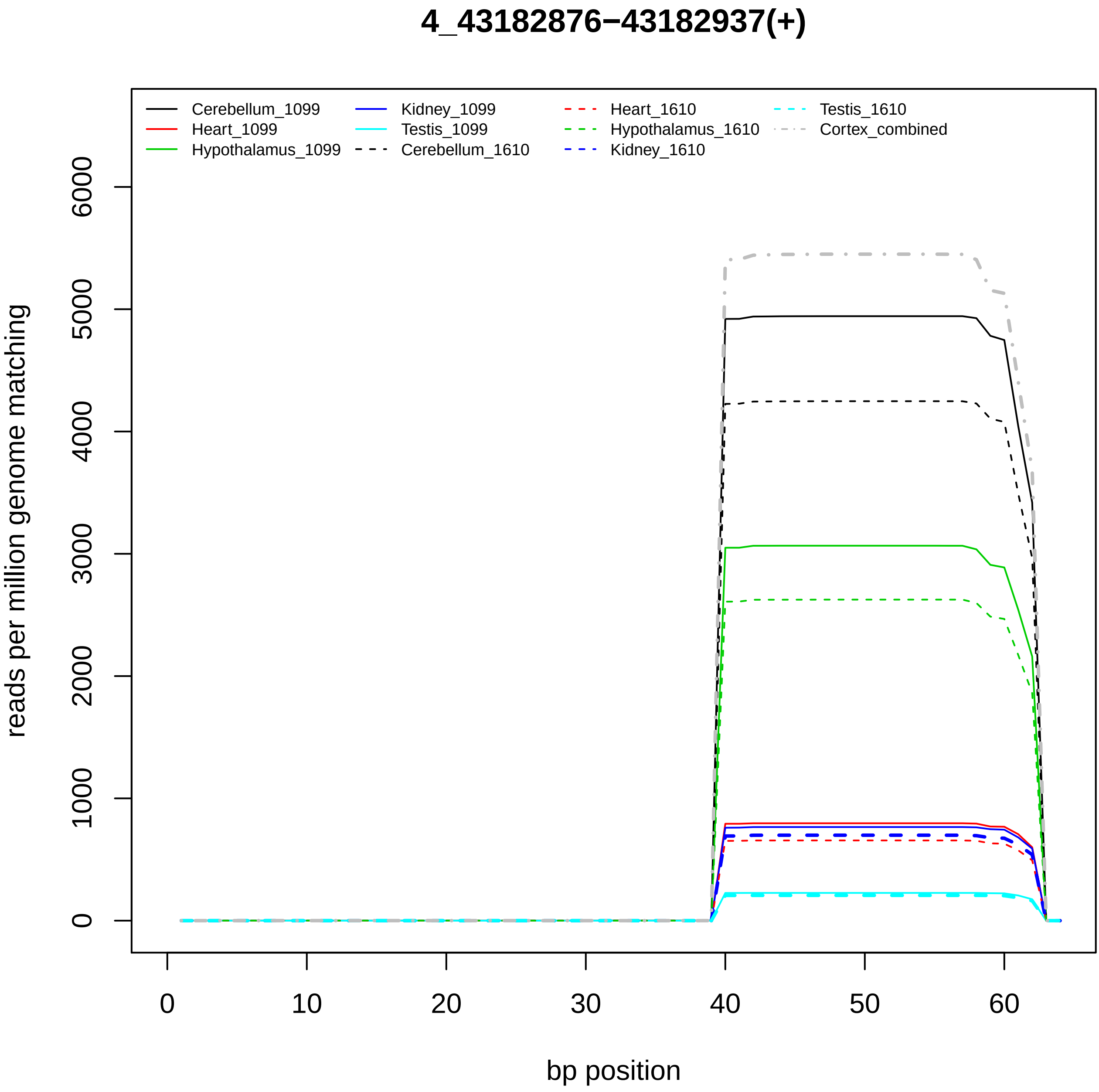

4_43182876−43182937(+)
Cerebellum_1099
Heart_1099
Hypothalamus_1099
Kidney_1099
Testis_1099
Cerebellum_1610
Heart_1610
Hypothalamus_1610
Kidney_1610
Testis_1610
Cortex_combined
reads per million genome matching
bp position
0
1000
2000
3000
4000
5000
6000
0
10
20
30
40
50
60

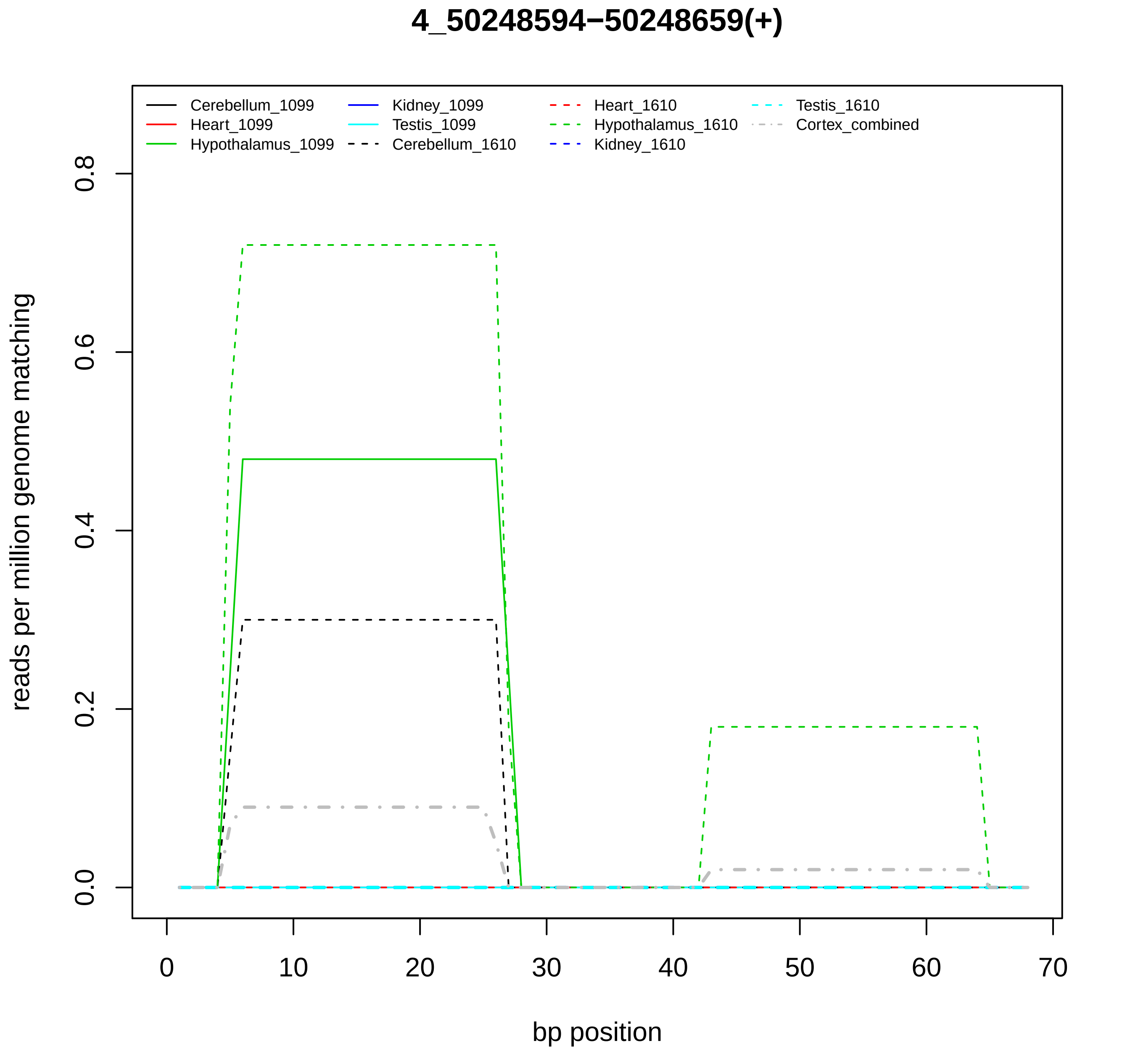

4_50248594−50248659(+)
Cerebellum_1099
Heart_1099
Hypothalamus_1099
Kidney_1099
Testis_1099
Cerebellum_1610
Heart_1610
Hypothalamus_1610
Kidney_1610
Testis_1610
Cortex_combined
reads per million genome matching
0.0
0.2
0.4
0.6
0.8
0
10
20
30
40
50
60
70
bp position

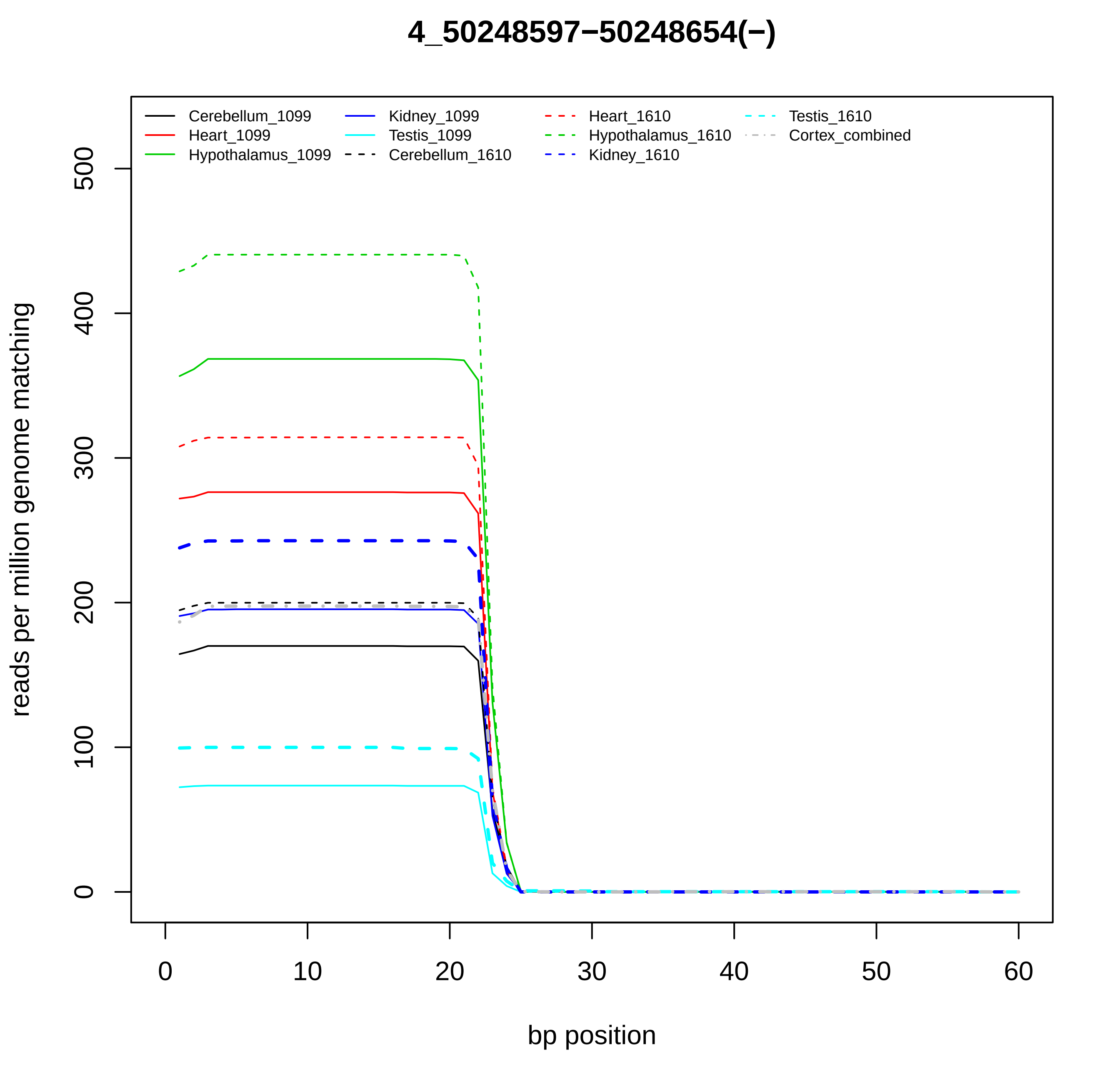

4_50248597−50248654(−)
Cerebellum_1099
Heart_1099
Hypothalamus_1099
Kidney_1099
Testis_1099
Cerebellum_1610
Heart_1610
Hypothalamus_1610
Kidney_1610
Testis_1610
Cortex_combined
reads per million genome matching
bp position
0
100
200
300
400
500
0
10
20
30
40
50
60

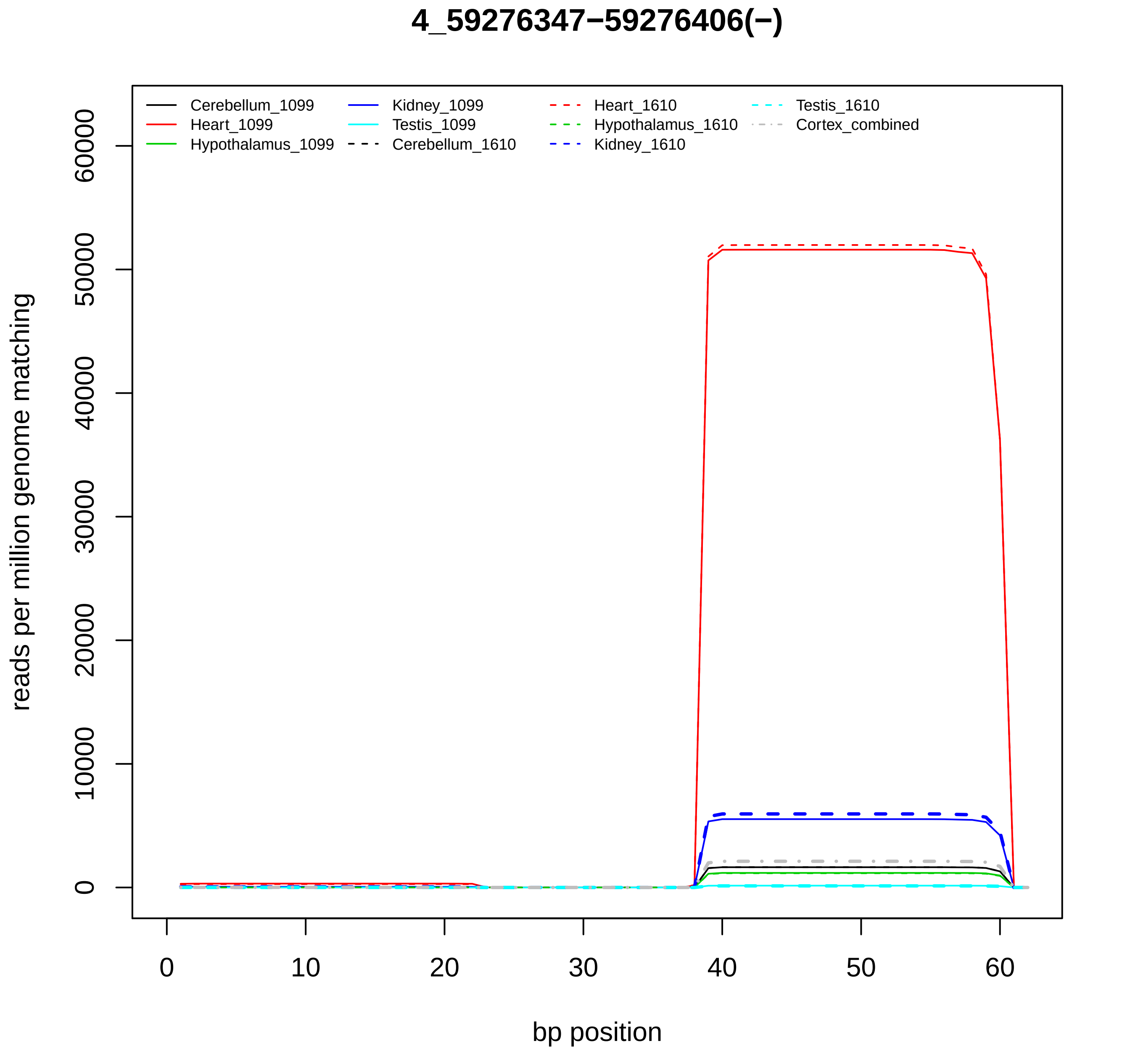
4_59276347−59276406(−)
Cerebellum_1099
Heart_1099
Hypothalamus_1099
Kidney_1099
Testis_1099
Cerebellum_1610
Heart_1610
Hypothalamus_1610
Kidney_1610
Testis_1610
Cortex_combined
reads per million genome matching
bp position
0
10000
20000
30000
40000
50000
60000
0
10
20
30
40
50
60

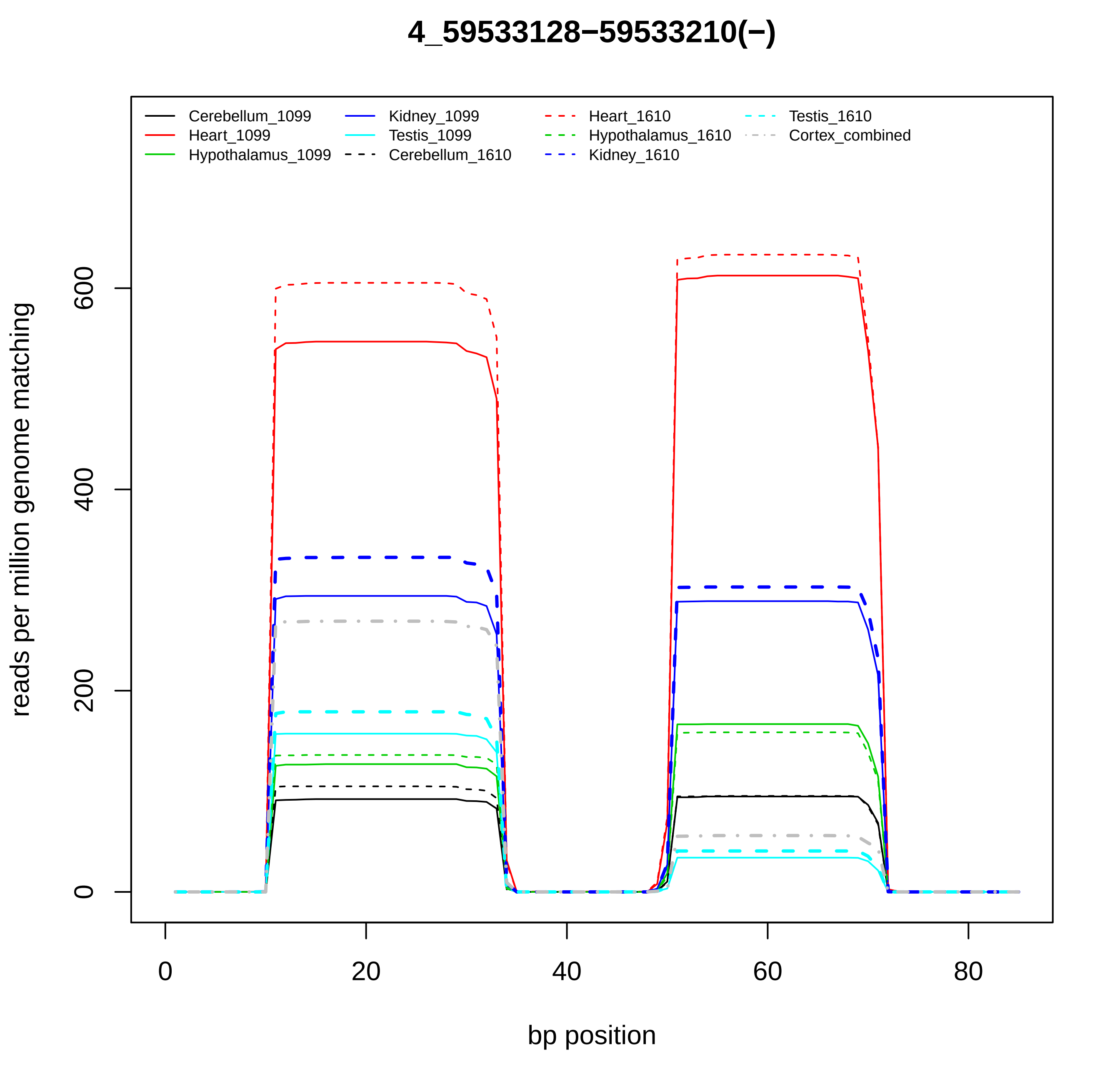
4_59533128−59533210(−)
Cerebellum_1099
Heart_1099
Hypothalamus_1099
Kidney_1099
Testis_1099
Cerebellum_1610
Heart_1610
Hypothalamus_1610
Kidney_1610
Testis_1610
Cortex_combined
reads per million genome matching
0
200
400
600
0
20
40
60
80
bp position

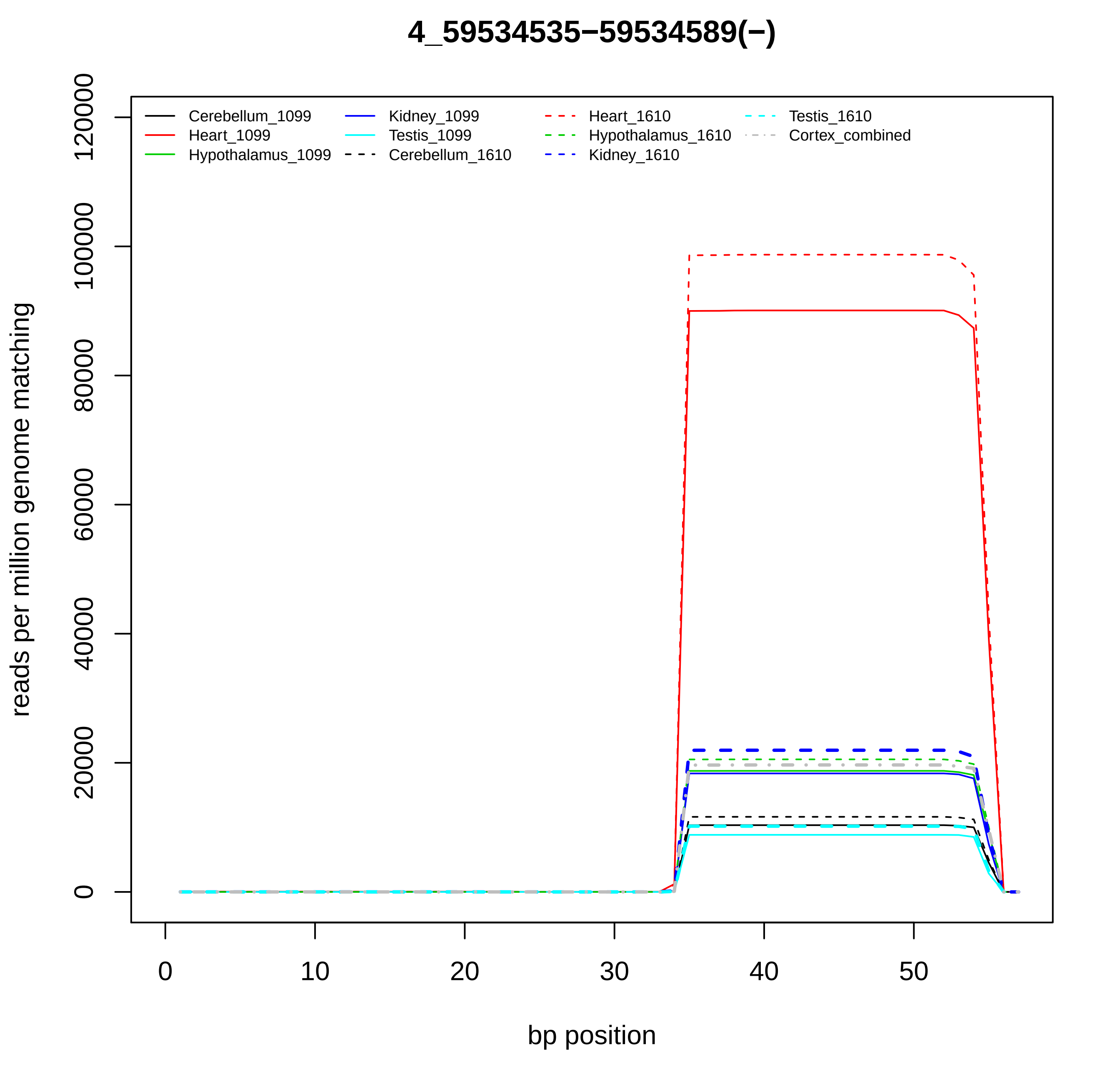

4_59534535−59534589(−)
Cerebellum_1099
Heart_1099
Hypothalamus_1099
Kidney_1099
Testis_1099
Cerebellum_1610
Heart_1610
Hypothalamus_1610
Kidney_1610
Testis_1610
Cortex_combined
reads per million genome matching
0
20000
40000
60000
80000
100000
120000
0
10
20
30
40
50
bp position

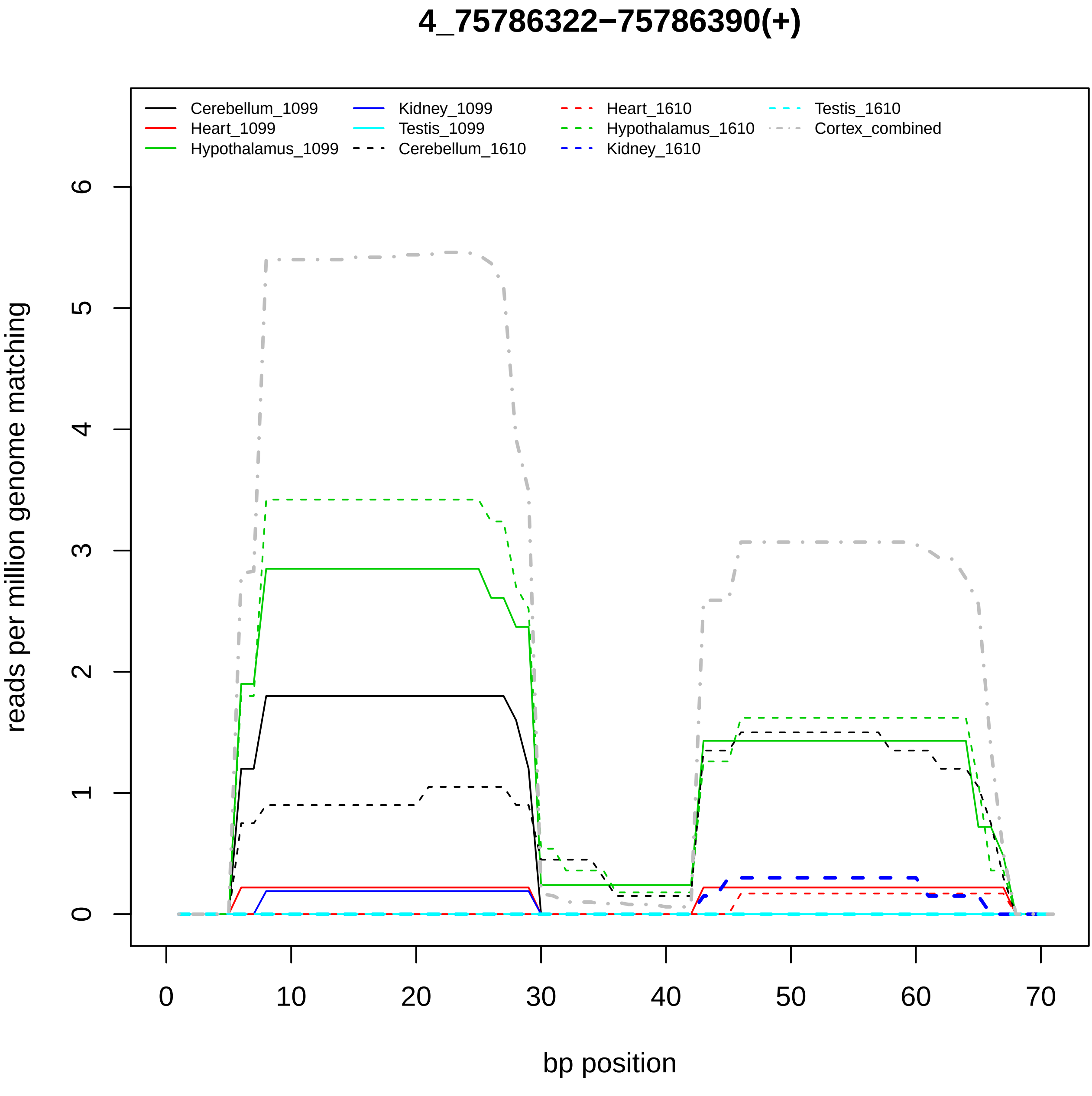

4_75786322−75786390(+)
Cerebellum_1099
Heart_1099
Hypothalamus_1099
Kidney_1099
Testis_1099
Cerebellum_1610
Heart_1610
Hypothalamus_1610
Kidney_1610
Testis_1610
Cortex_combined
reads per million genome matching
bp position
0
1
2
3
4
5
6
0
10
20
30
40
50
60
70

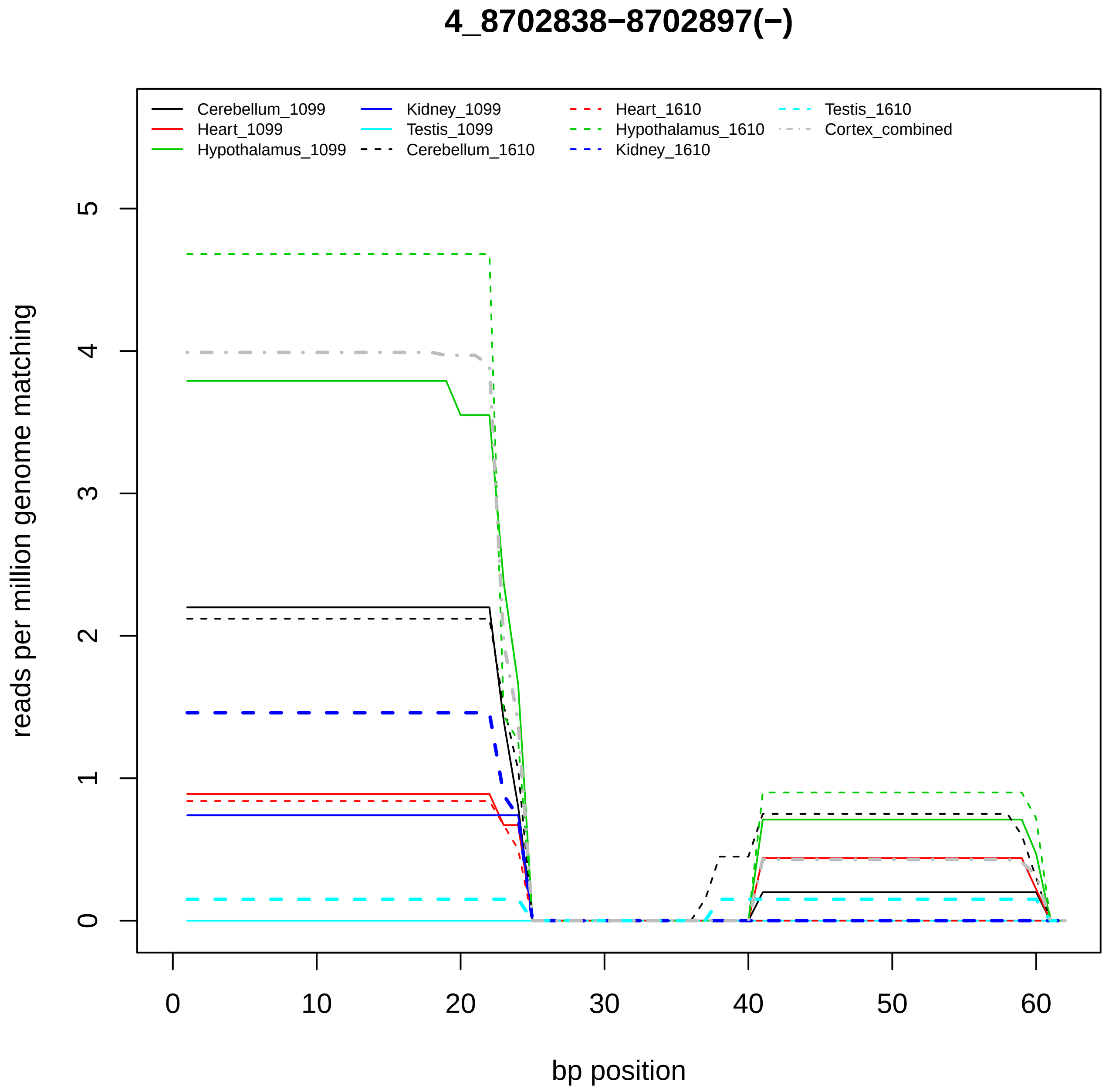

4_8702838−8702897(−)
Cerebellum_1099
Heart_1099
Hypothalamus_1099
Kidney_1099
Testis_1099
Cerebellum_1610
Heart_1610
Hypothalamus_1610
Kidney_1610
Testis_1610
Cortex_combined
reads per million genome matching
bp position
0
1
2
3
4
5
10
20
30
40
50
60

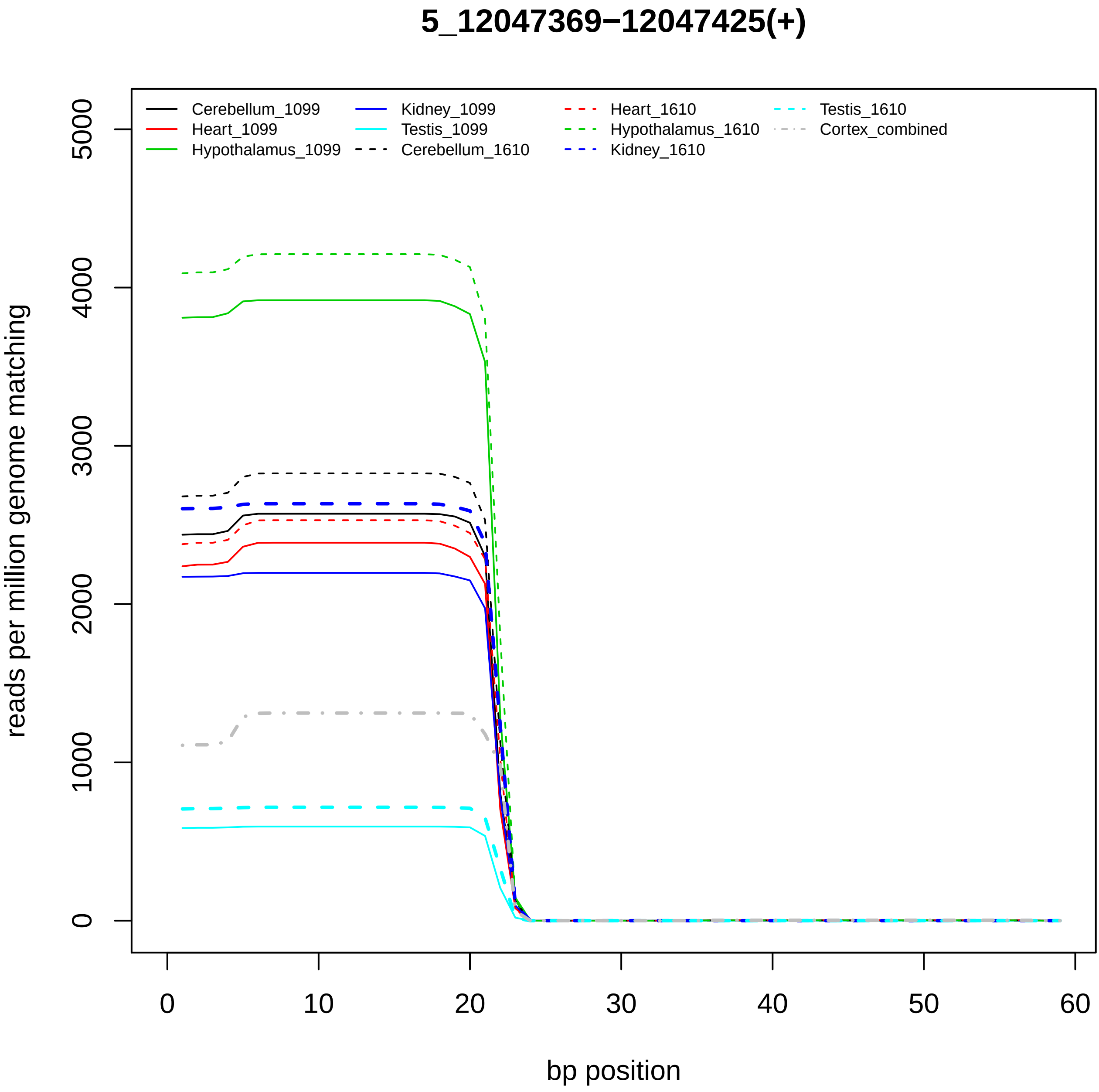

5_12047369−12047425(+)
Cerebellum_1099
Heart_1099
Hypothalamus_1099
Kidney_1099
Testis_1099
Cerebellum_1610
Heart_1610
Hypothalamus_1610
Kidney_1610
Testis_1610
Cortex_combined
reads per million genome matching
0
1000
2000
3000
4000
5000
bp position
0
10
20
30
40
50
60

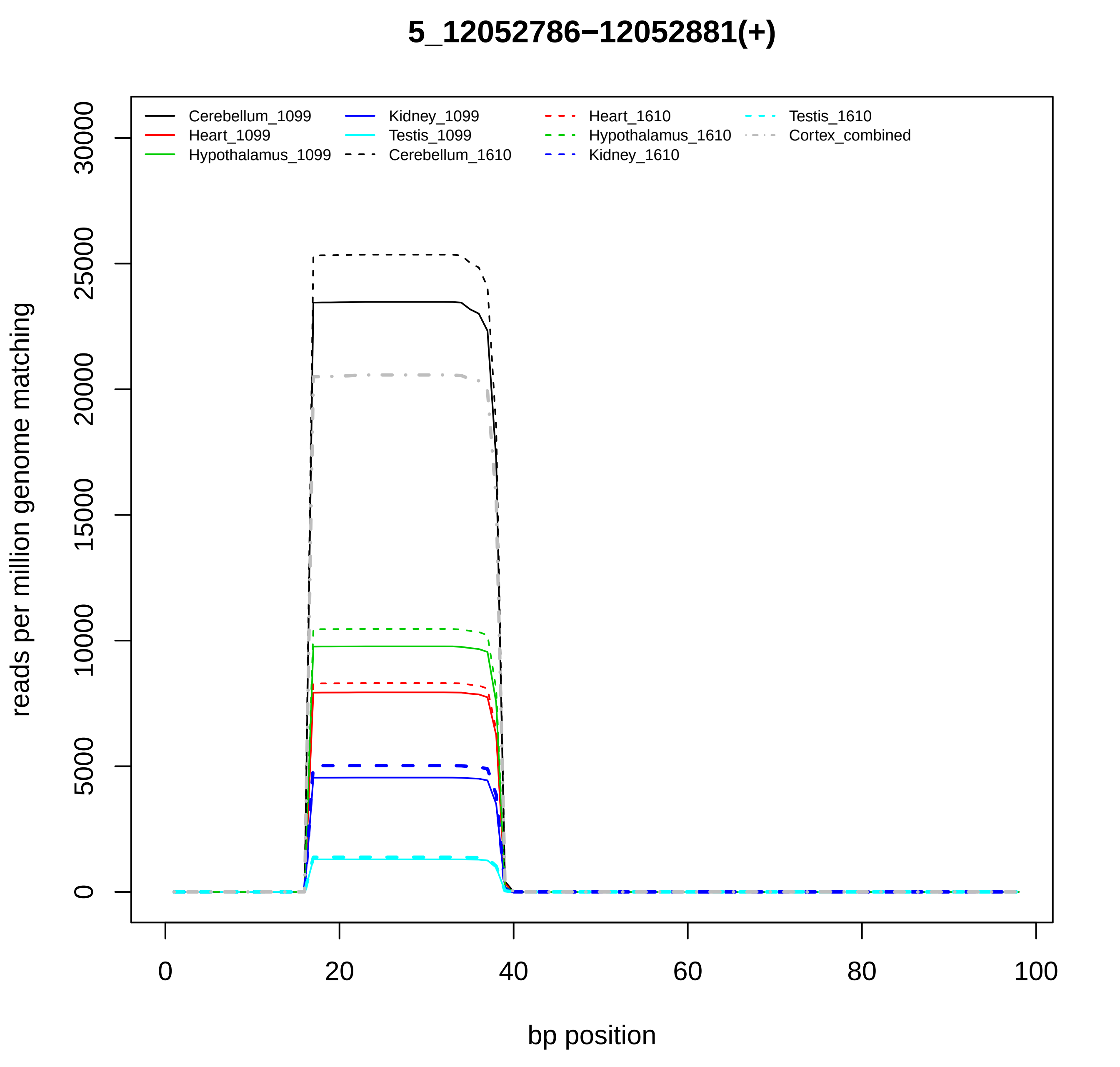

5_12052786−12052881(+)
Cerebellum_1099
Heart_1099
Hypothalamus_1099
Kidney_1099
Testis_1099
Cerebellum_1610
Heart_1610
Hypothalamus_1610
Kidney_1610
Testis_1610
Cortex_combined
reads per million genome matching
0
5000
10000
15000
20000
25000
30000
0
20
40
60
80
100
bp position

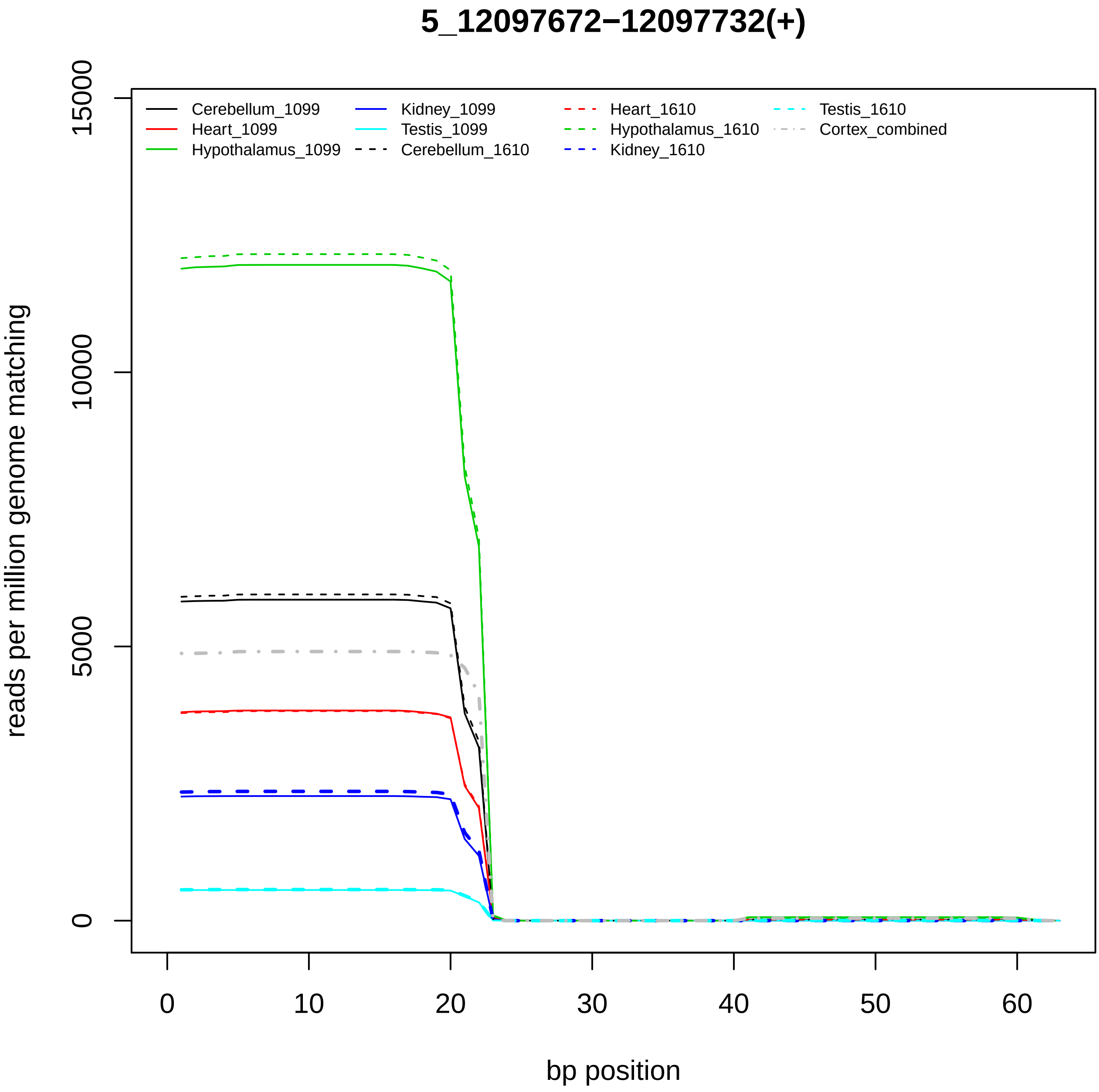

5_12097672−12097732(+)
reads per million genome matching
bp position
Cerebellum_1099
Heart_1099
Hypothalamus_1099
Kidney_1099
Testis_1099
Cerebellum_1610
Heart_1610
Hypothalamus_1610
Kidney_1610
Testis_1610
Cortex_combined
0
5000
10000
15000
0
10
20
30
40
50
60

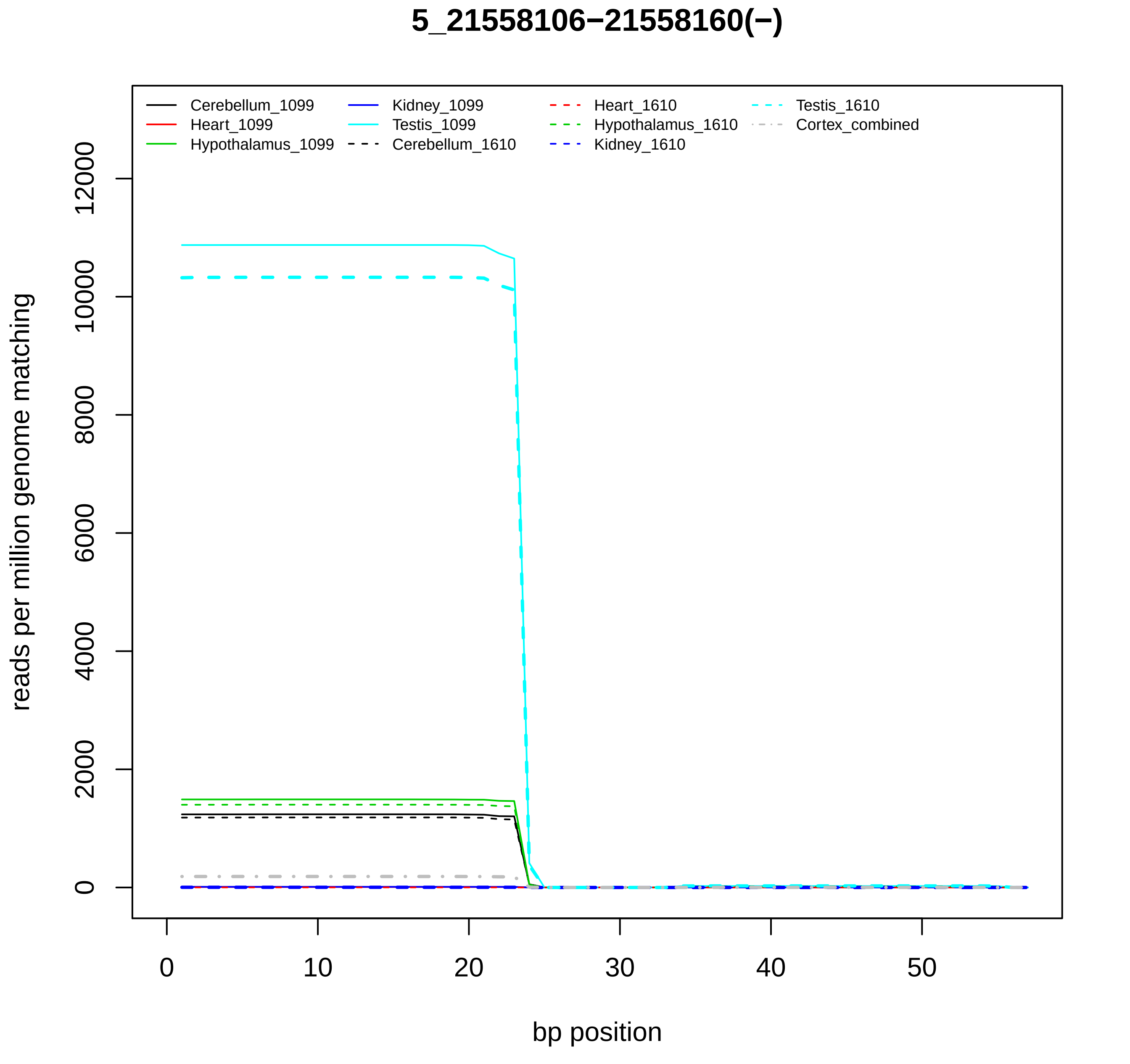

5_21558106−21558160(−)
Cerebellum_1099
Heart_1099
Hypothalamus_1099
Kidney_1099
Testis_1099
Cerebellum_1610
Heart_1610
Hypothalamus_1610
Kidney_1610
Testis_1610
Cortex_combined
reads per million genome matching
0
2000
4000
6000
8000
10000
12000
0
10
20
30
40
50
bp position

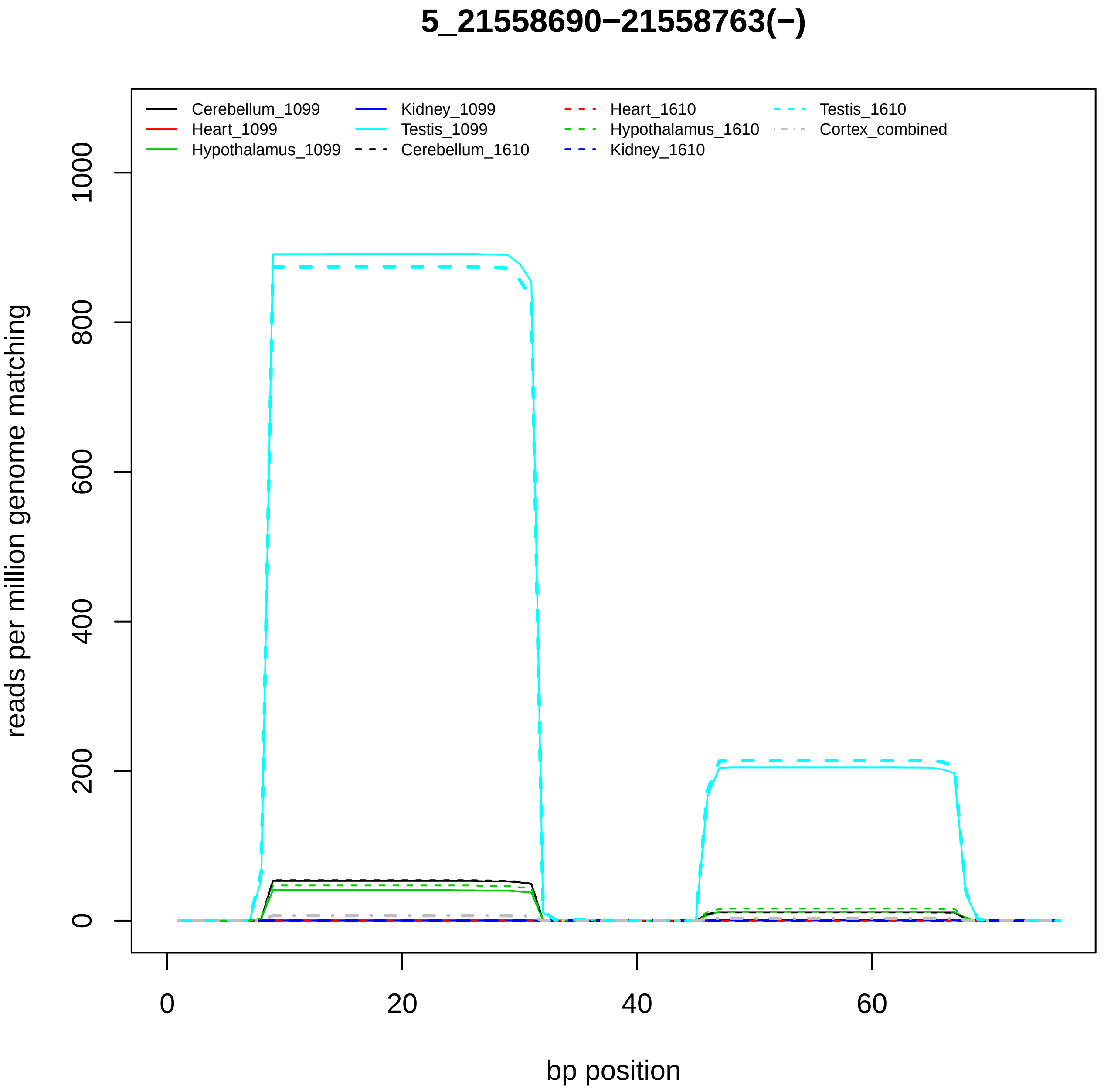

5_21558690−21558763(−)
Cerebellum_1099
Heart_1099
Hypothalamus_1099
Kidney_1099
Testis_1099
Cerebellum_1610
Heart_1610
Hypothalamus_1610
Kidney_1610
Testis_1610
Cortex_combined
reads per million genome matching
bp position
0
200
400
600
800
1000
0
20
40
60

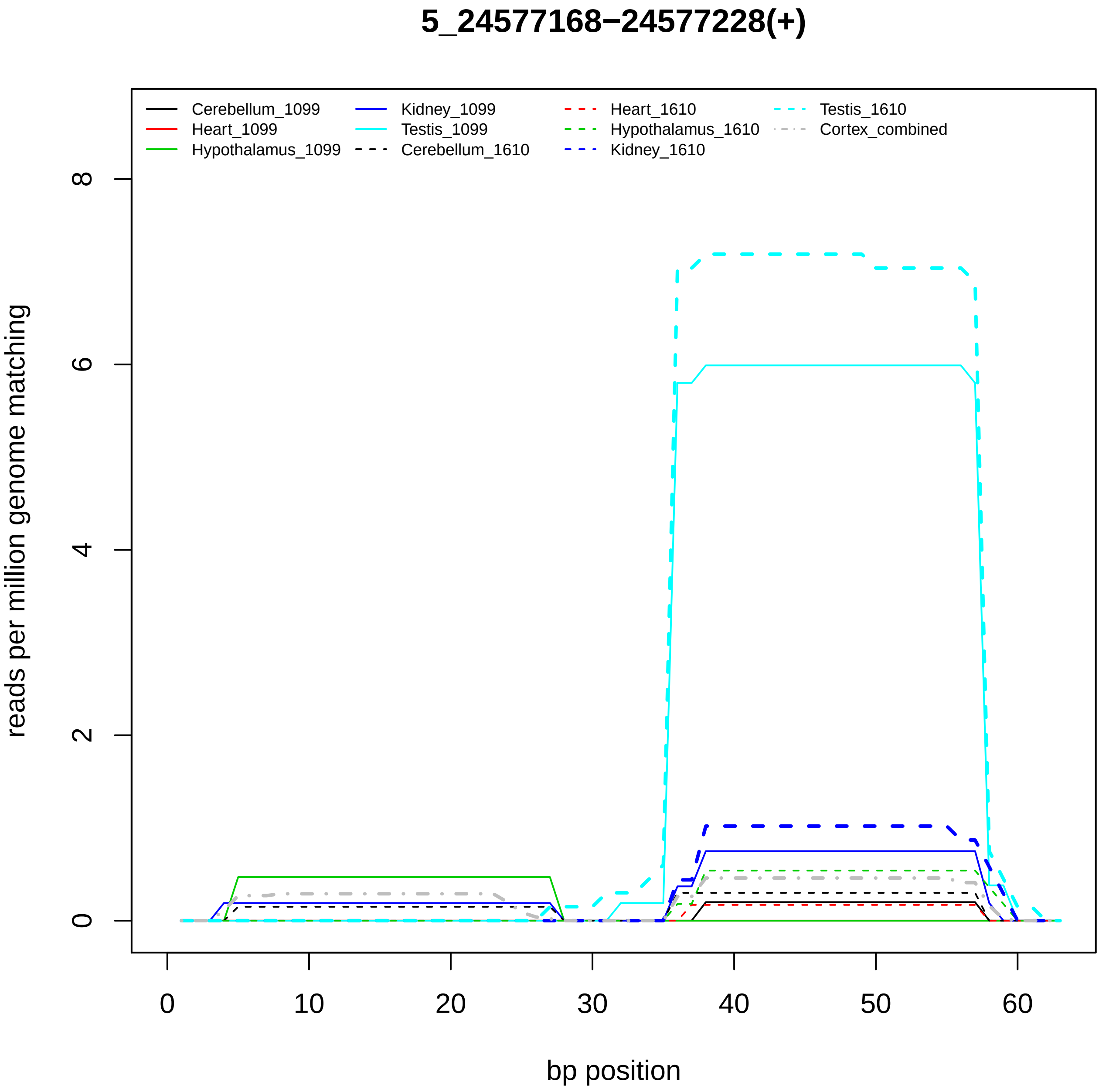

5_24577168−24577228(+)
Cerebellum_1099
Heart_1099
Hypothalamus_1099
Kidney_1099
Testis_1099
Cerebellum_1610
Heart_1610
Hypothalamus_1610
Kidney_1610
Testis_1610
Cortex_combined
reads per million genome matching
0
2
4
6
8
0
10
20
30
40
50
60
bp position

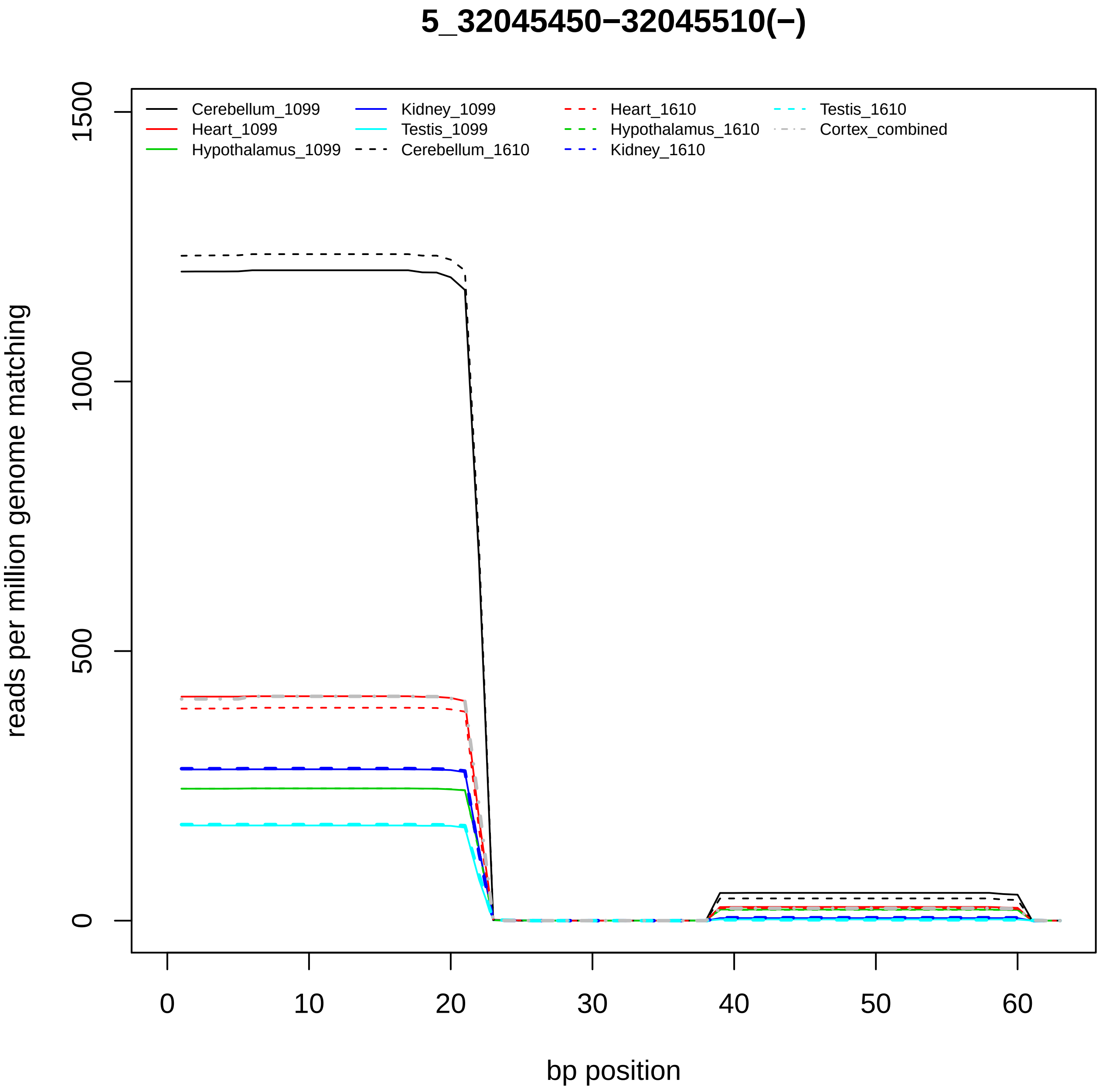

5_32045450−32045510(−)
Cerebellum_1099
Heart_1099
Hypothalamus_1099
Kidney_1099
Testis_1099
Cerebellum_1610
Heart_1610
Hypothalamus_1610
Kidney_1610
Testis_1610
Cortex_combined
reads per million genome matching
bp position
0
500
1000
1500
0
10
20
30
40
50
60

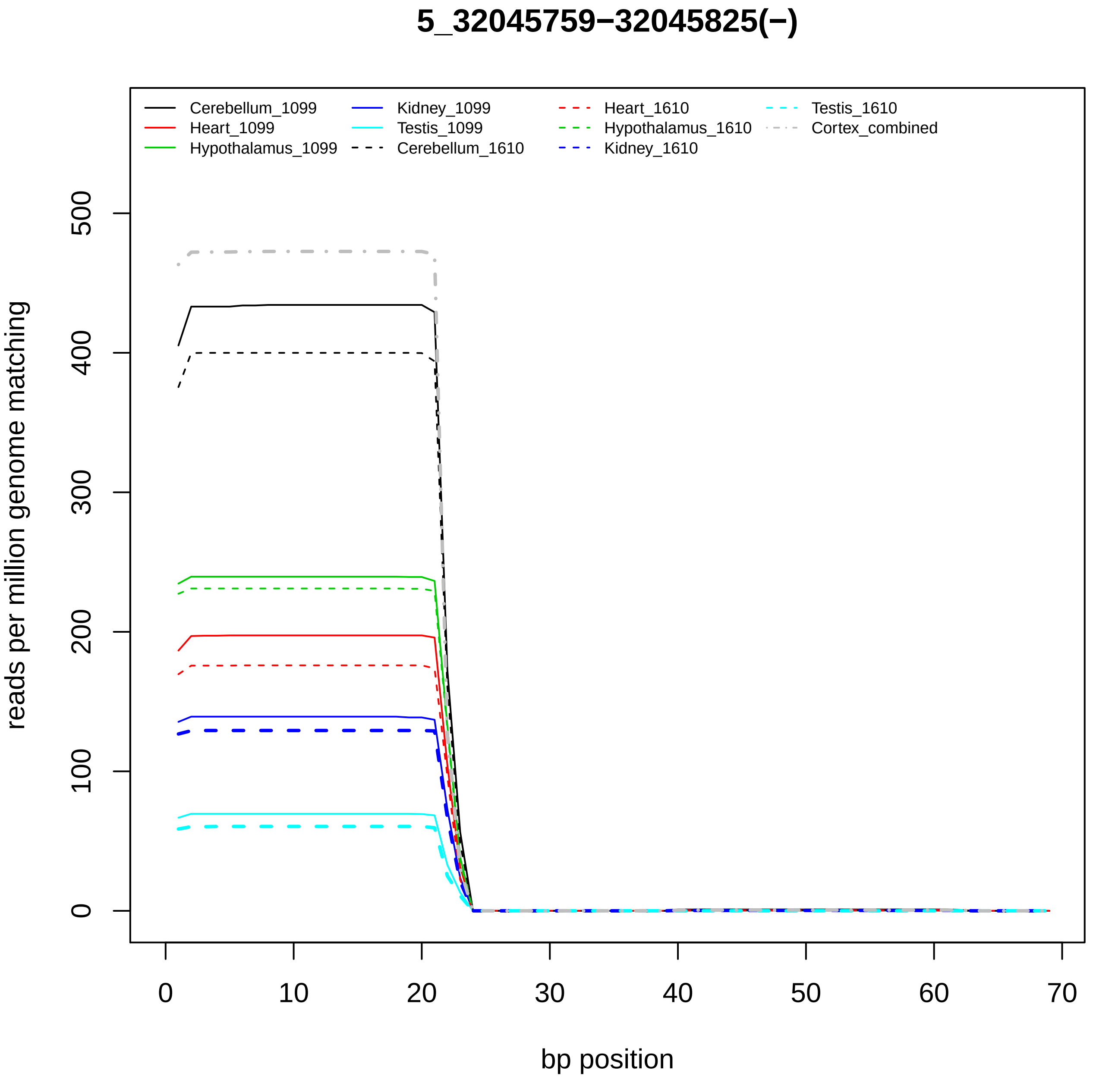

5_32045759−32045825(−)
Cerebellum_1099
Heart_1099
Hypothalamus_1099
Kidney_1099
Testis_1099
Cerebellum_1610
Heart_1610
Hypothalamus_1610
Kidney_1610
Testis_1610
Cortex_combined
reads per million genome matching
bp position
0
100
200
300
400
500
0
10
20
30
40
50
60
70

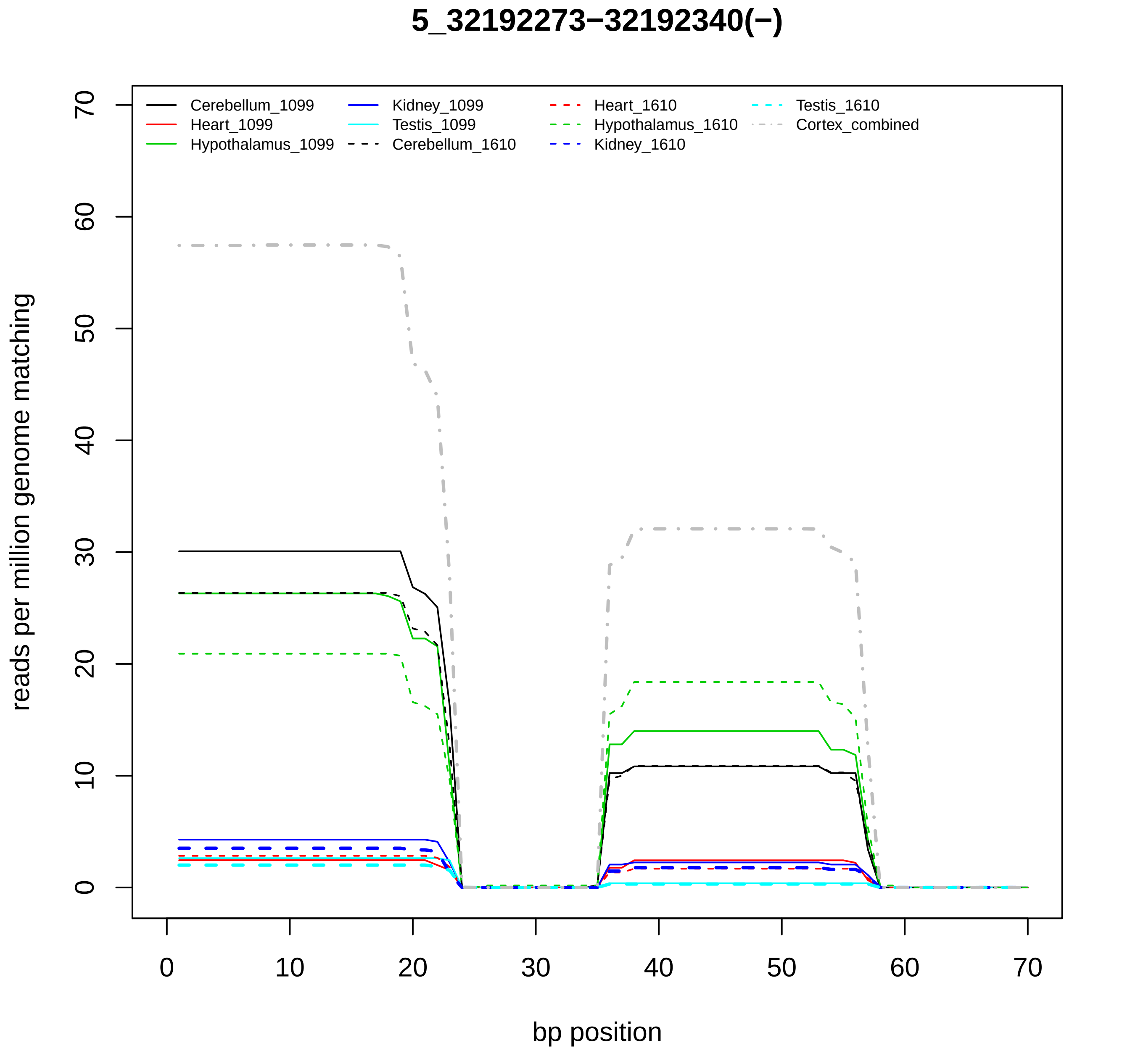
5_32192273−32192340(−)
Cerebellum_1099
Heart_1099
Hypothalamus_1099
Kidney_1099
Testis_1099
Cerebellum_1610
Heart_1610
Hypothalamus_1610
Kidney_1610
Testis_1610
Cortex_combined
reads per million genome matching
bp position
0
10
20
30
40
50
60
70

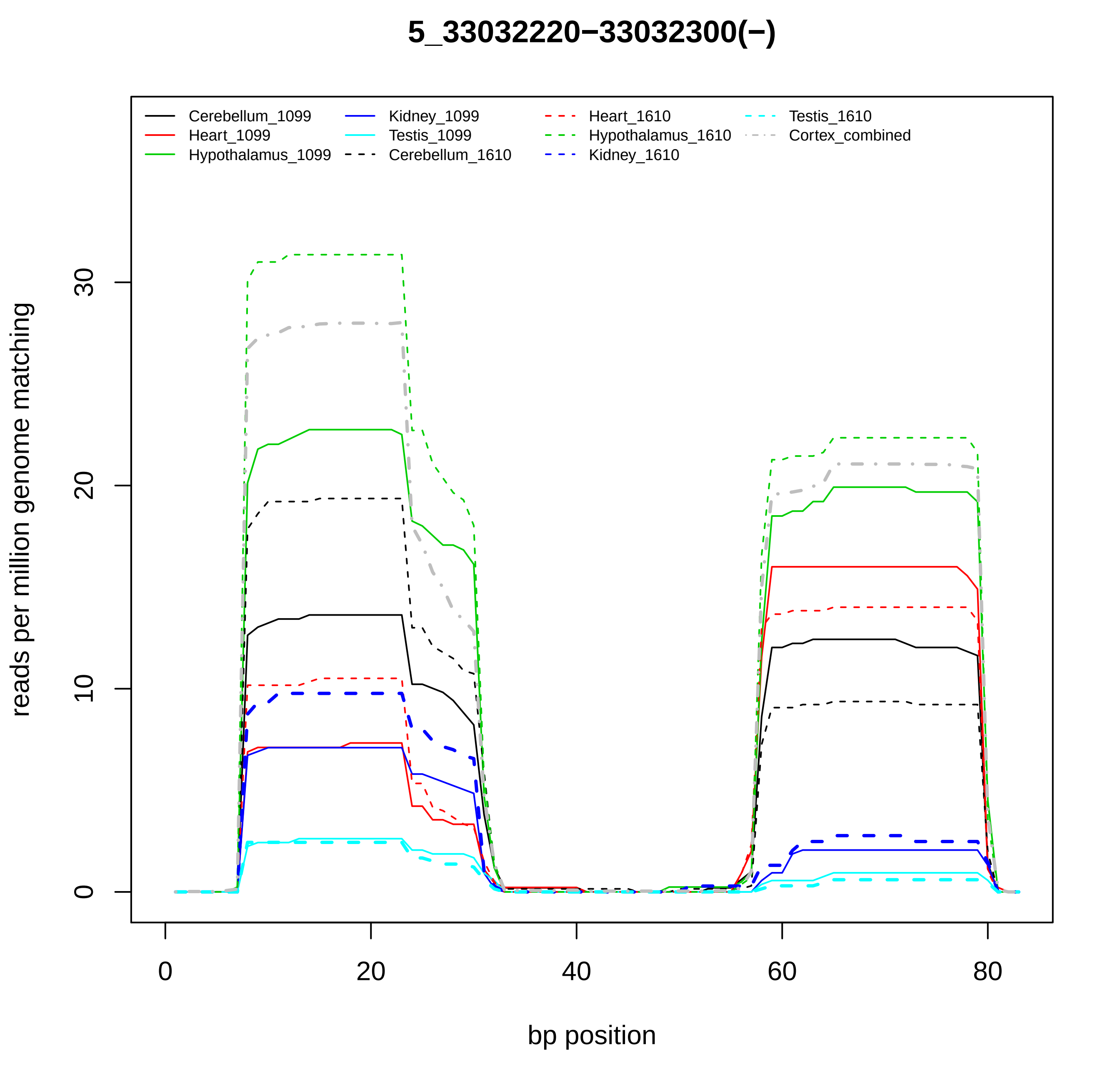

5_33032220−33032300(−)
Cerebellum_1099
Heart_1099
Hypothalamus_1099
Kidney_1099
Testis_1099
Cerebellum_1610
Heart_1610
Hypothalamus_1610
Kidney_1610
Testis_1610
Cortex_combined
reads per million genome matching
0
10
20
30
0
20
40
60
80
bp position

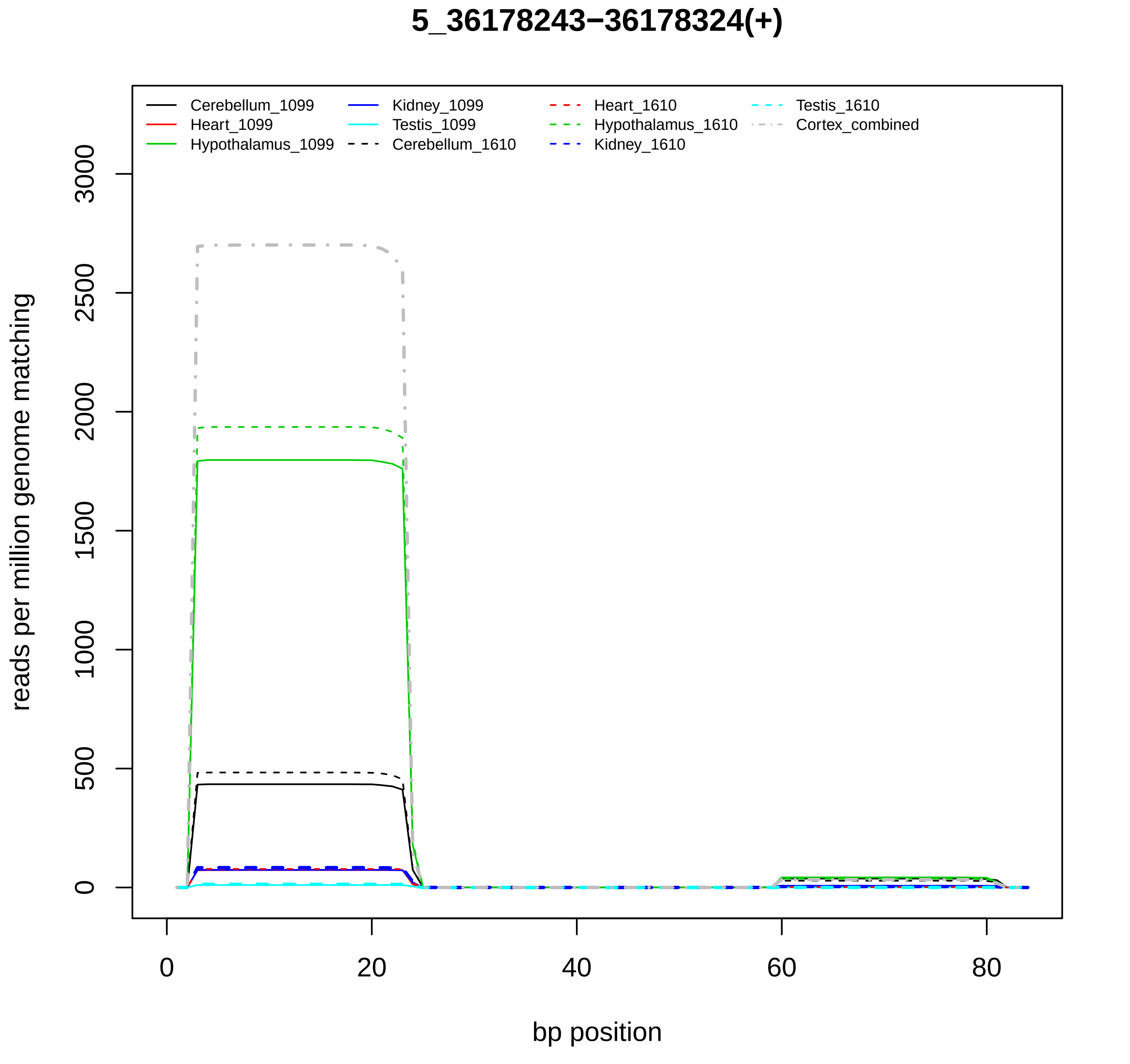

5_36178243−36178324(+)
Cerebellum_1099
Heart_1099
Hypothalamus_1099
Kidney_1099
Testis_1099
Cerebellum_1610
Heart_1610
Hypothalamus_1610
Kidney_1610
Testis_1610
Cortex_combined
reads per million genome matching
0
500
1000
1500
2000
2500
3000
0
20
40
60
80
bp position

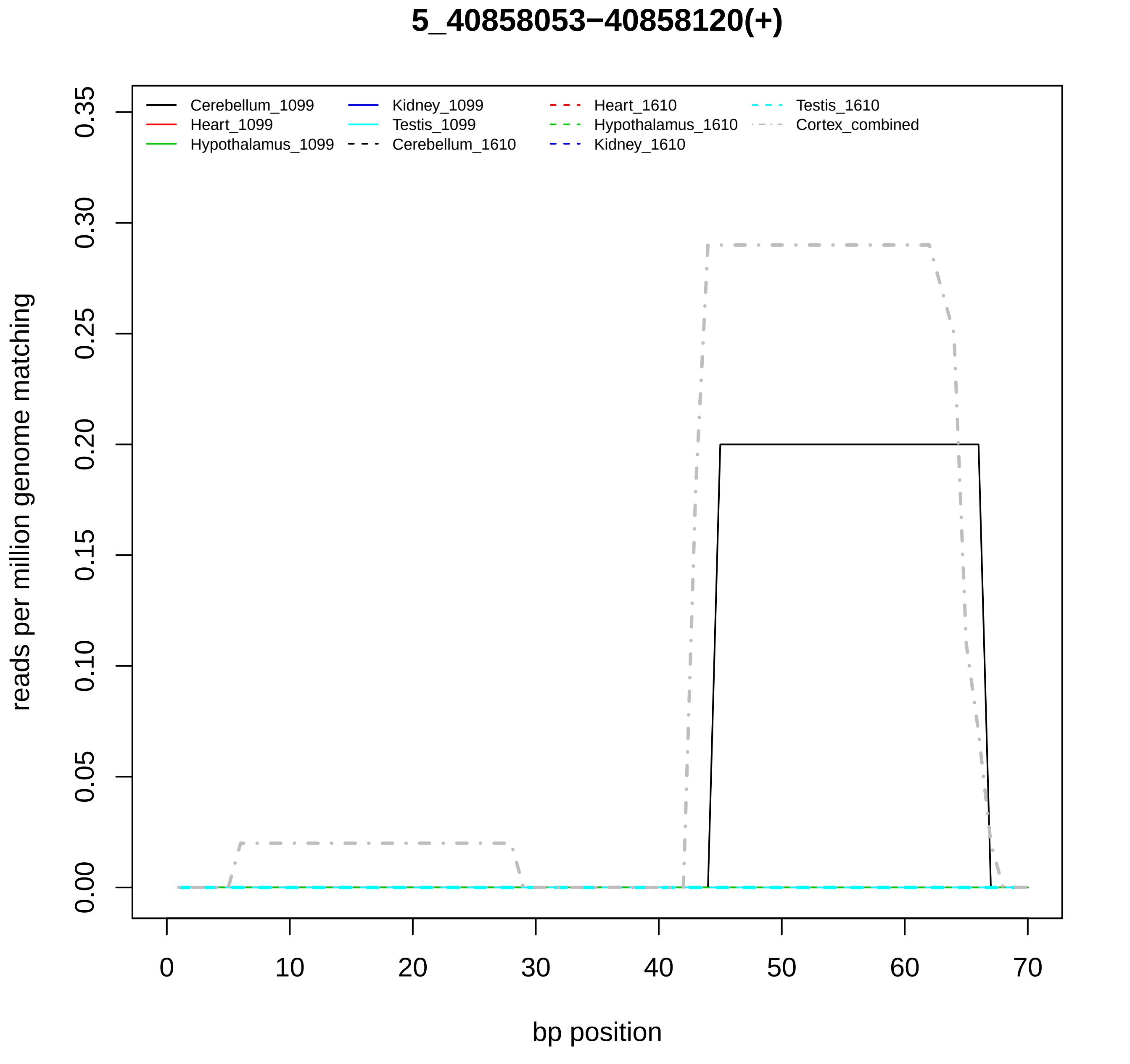

5_40858053−40858120(+)
Cerebellum_1099
Heart_1099
Hypothalamus_1099
Kidney_1099
Testis_1099
Cerebellum_1610
Heart_1610
Hypothalamus_1610
Kidney_1610
Testis_1610
Cortex_combined
reads per million genome matching
0.00
0.05
0.10
0.15
0.20
0.25
0.30
0.35
0
10
20
30
40
50
60
70
bp position

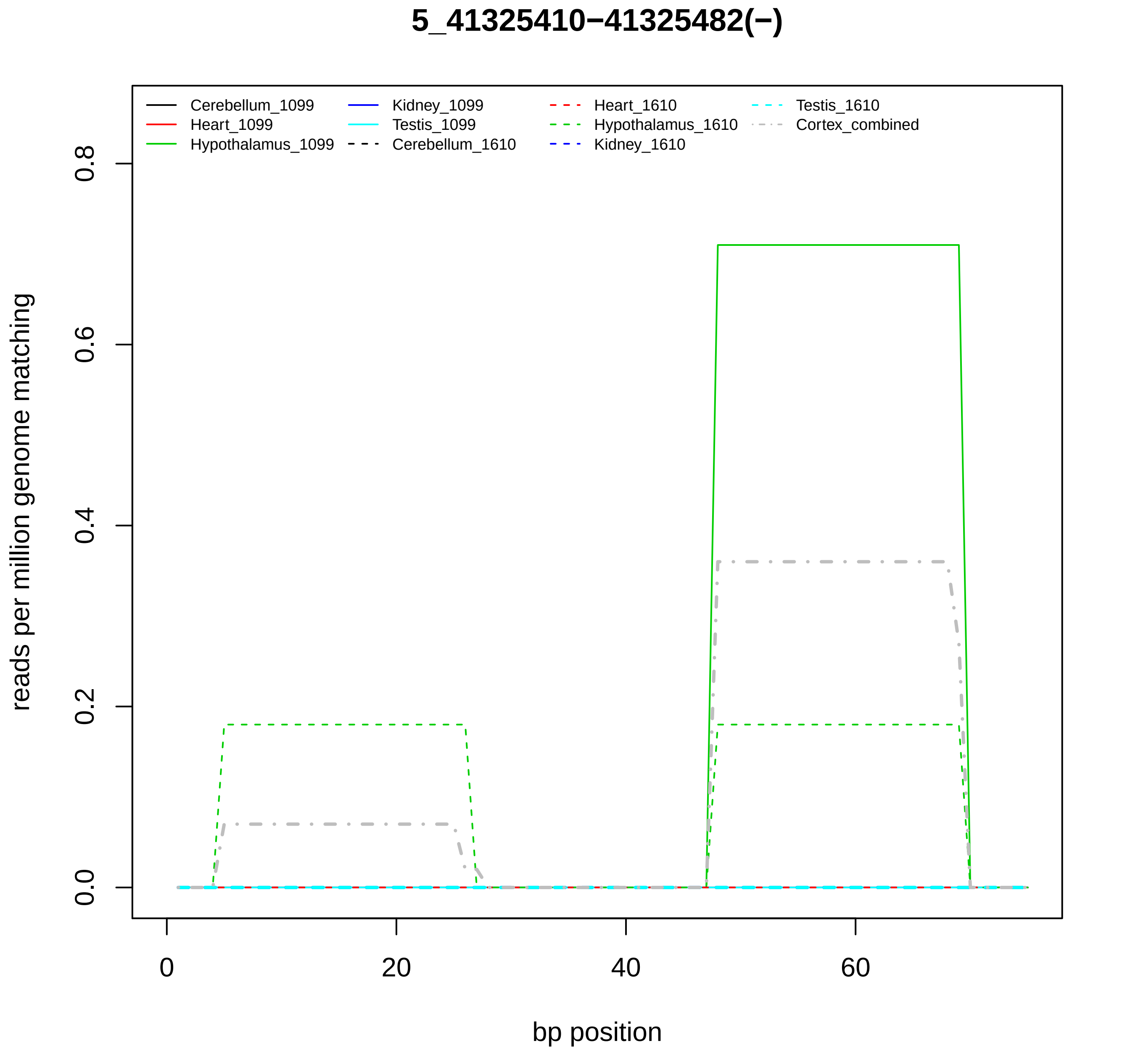

5_41325410−41325482(−)
Cerebellum_1099
Heart_1099
Hypothalamus_1099
Kidney_1099
Testis_1099
Cerebellum_1610
Heart_1610
Hypothalamus_1610
Kidney_1610
Testis_1610
Cortex_combined
reads per million genome matching
0.0
0.2
0.4
0.6
0.8
0
20
40
60
bp position

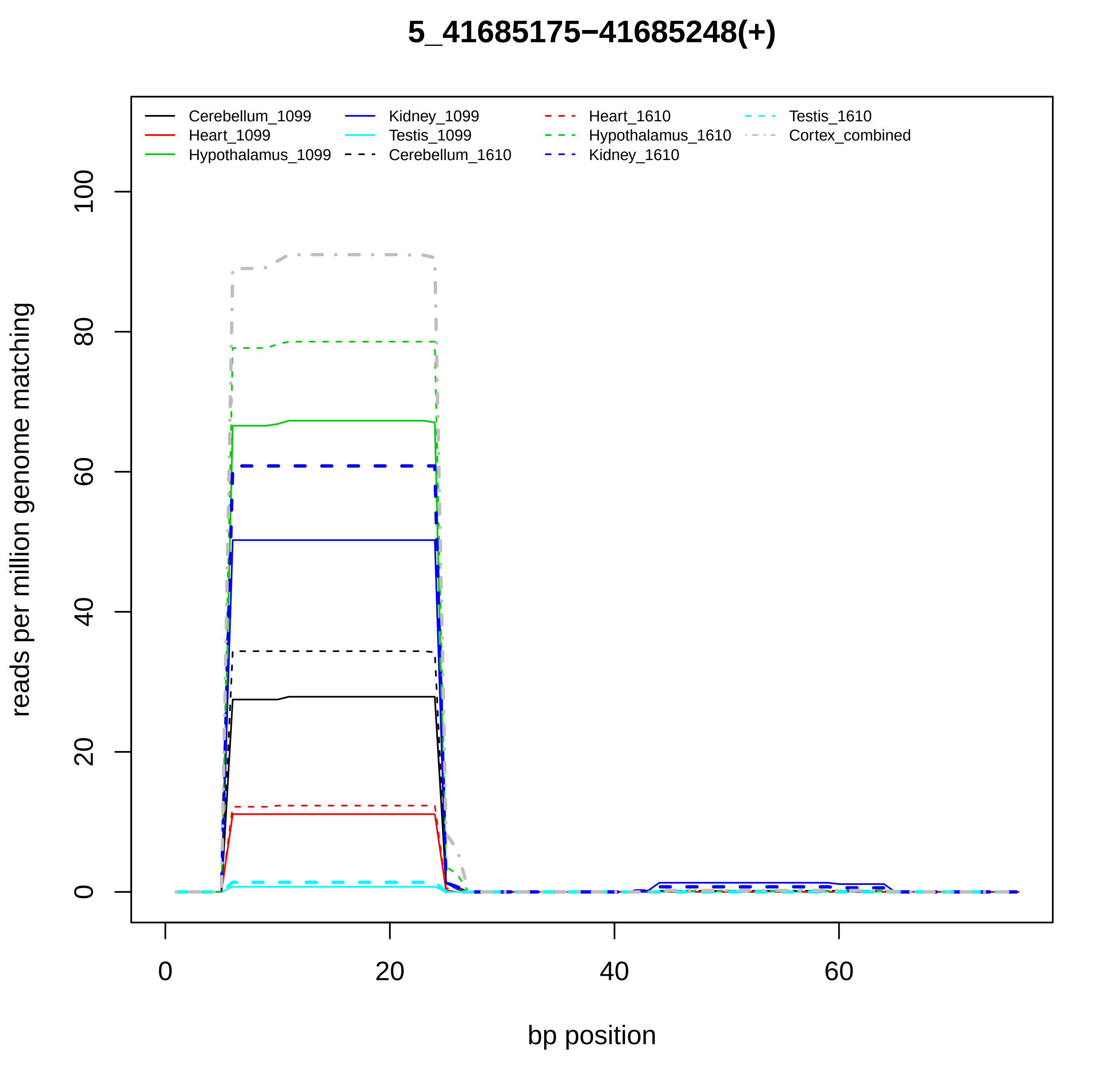

5_41685175−41685248(+)
Cerebellum_1099
Heart_1099
Hypothalamus_1099
Kidney_1099
Testis_1099
Cerebellum_1610
Heart_1610
Hypothalamus_1610
Kidney_1610
Testis_1610
Cortex_combined
reads per million genome matching
bp position
0
20
40
60
80
100

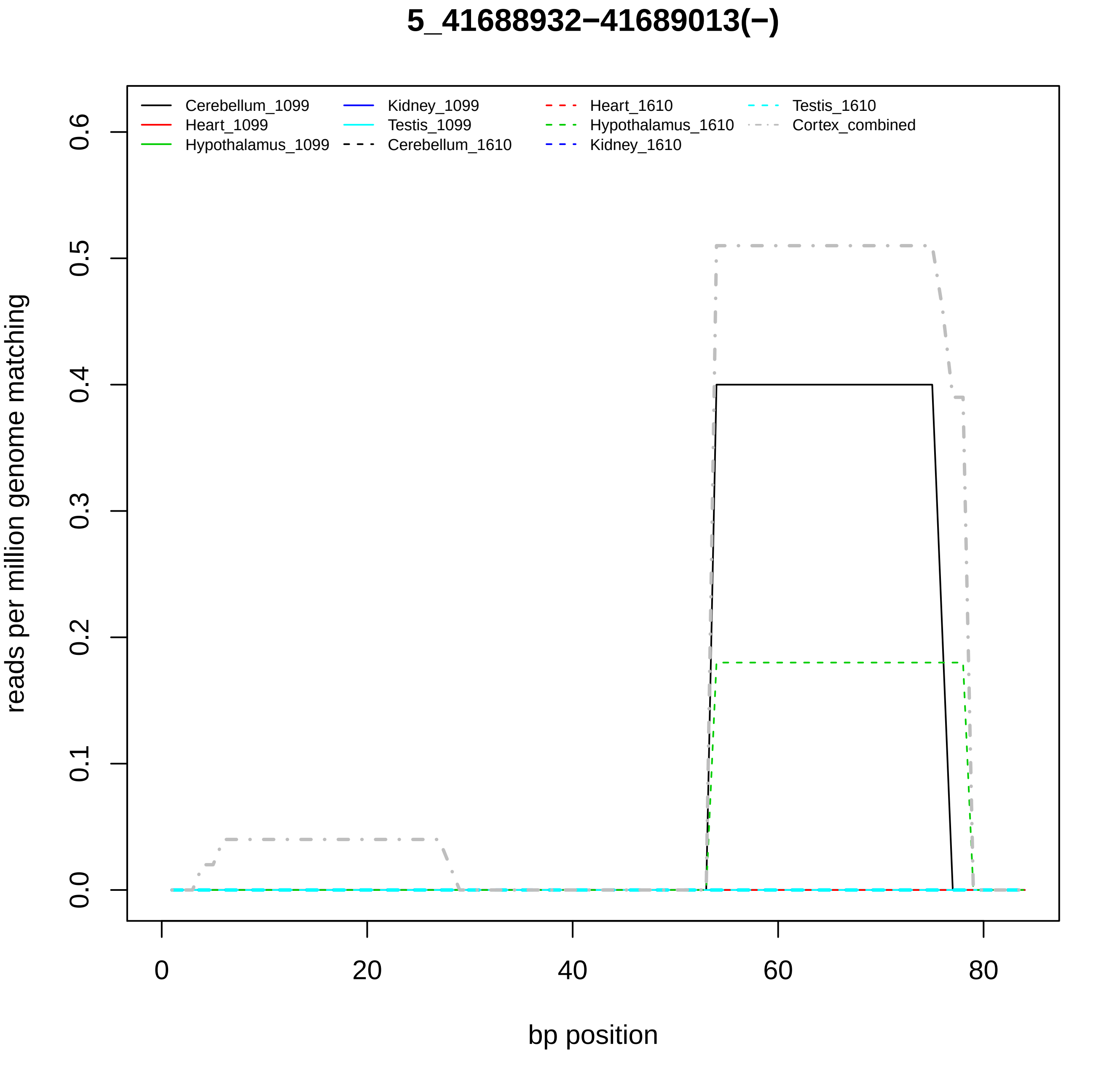

5_41688932−41689013(−)
Cerebellum_1099
Heart_1099
Hypothalamus_1099
Kidney_1099
Testis_1099
Cerebellum_1610
Heart_1610
Hypothalamus_1610
Kidney_1610
Testis_1610
Cortex_combined
reads per million genome matching
bp position
0.0
0.1
0.2
0.3
0.4
0.5
0.6
0
20
40
60
80

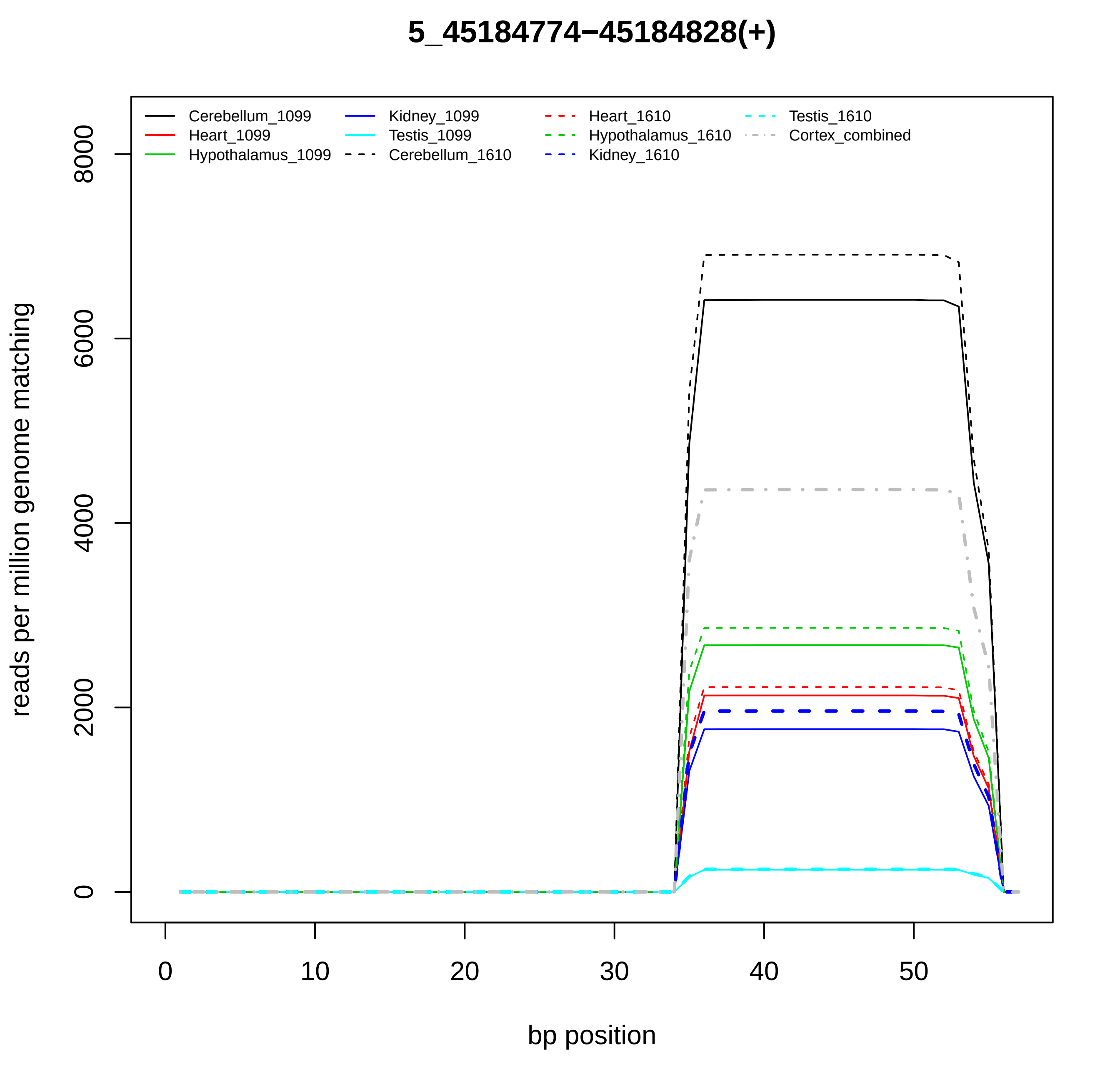

5_45184774−45184828(+)
Cerebellum_1099
Heart_1099
Hypothalamus_1099
Kidney_1099
Testis_1099
Cerebellum_1610
Heart_1610
Hypothalamus_1610
Kidney_1610
Testis_1610
Cortex_combined
reads per million genome matching
0
2000
4000
6000
8000
0
10
20
30
40
50
bp position

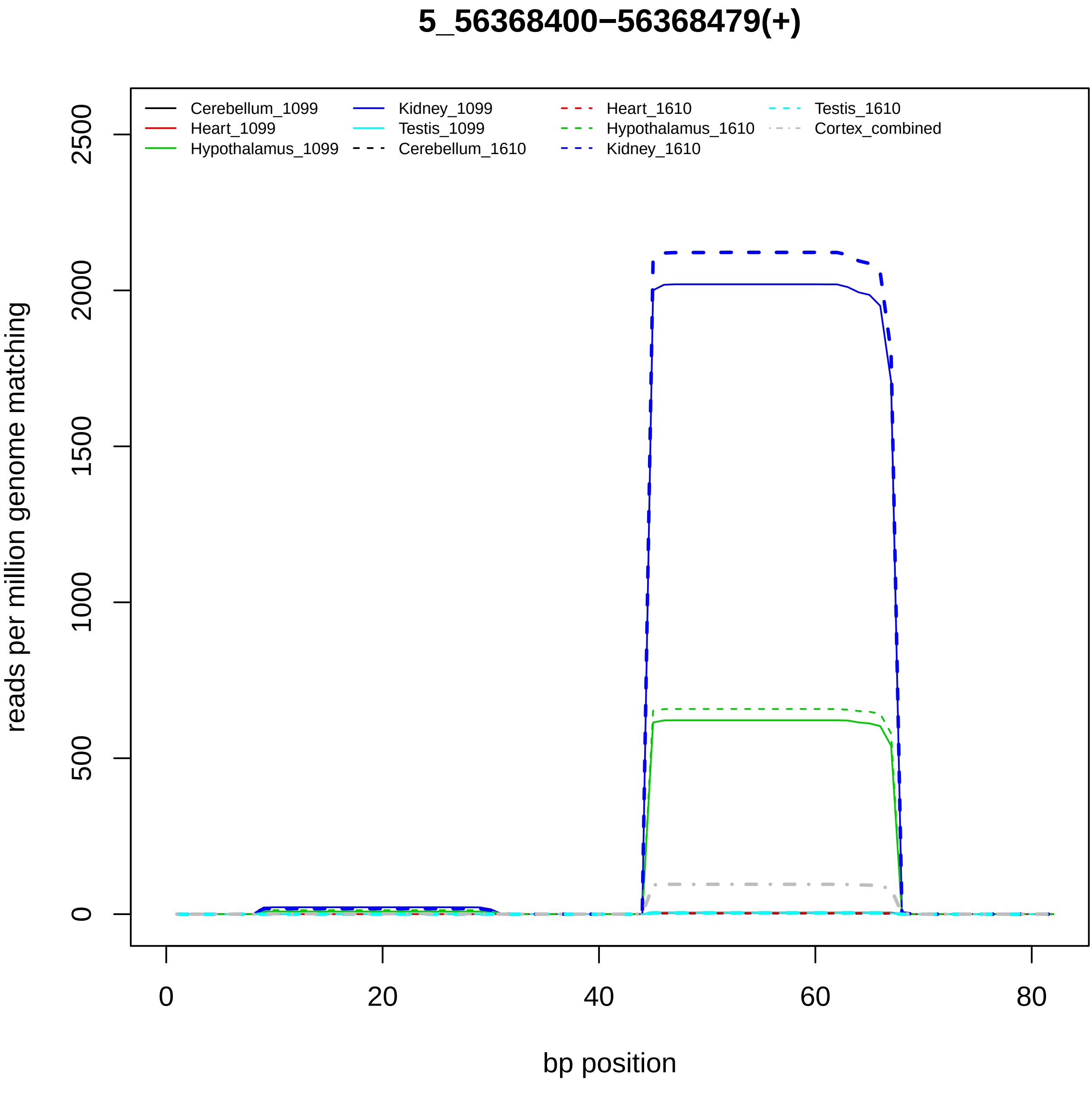

5_56368400−56368479(+)
Cerebellum_1099
Heart_1099
Hypothalamus_1099
Kidney_1099
Testis_1099
Cerebellum_1610
Heart_1610
Hypothalamus_1610
Kidney_1610
Testis_1610
Cortex_combined
reads per million genome matching
bp position
0
500
1000
1500
2000
2500
0
20
40
60
80

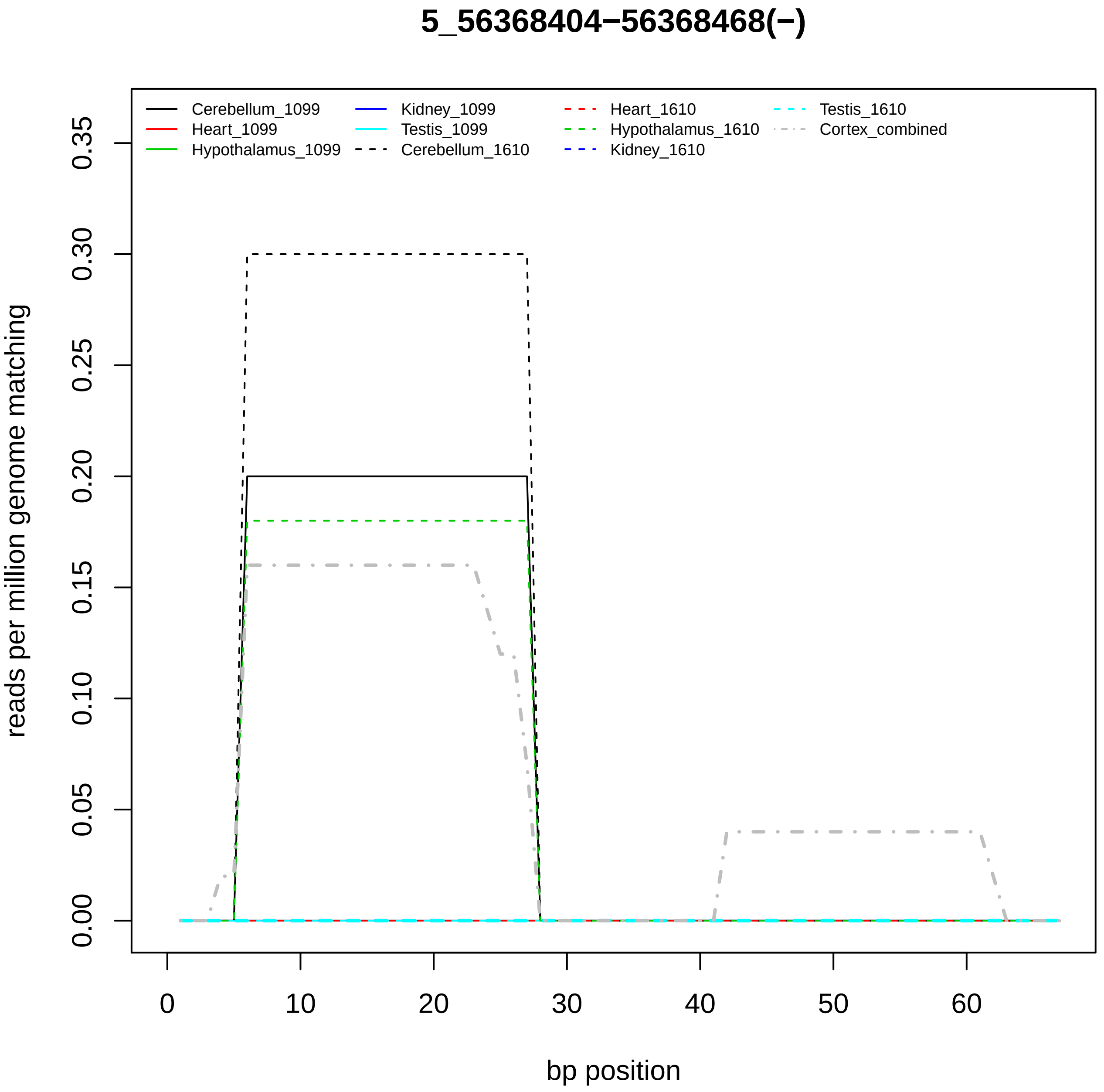

5_56368404−56368468(−)
Cerebellum_1099
Heart_1099
Hypothalamus_1099
Kidney_1099
Testis_1099
Cerebellum_1610
Heart_1610
Hypothalamus_1610
Kidney_1610
Testis_1610
Cortex_combined
reads per million genome matching
0.00
0.05
0.10
0.15
0.20
0.25
0.30
0.35
bp position
0
10
20
30
40
50
60

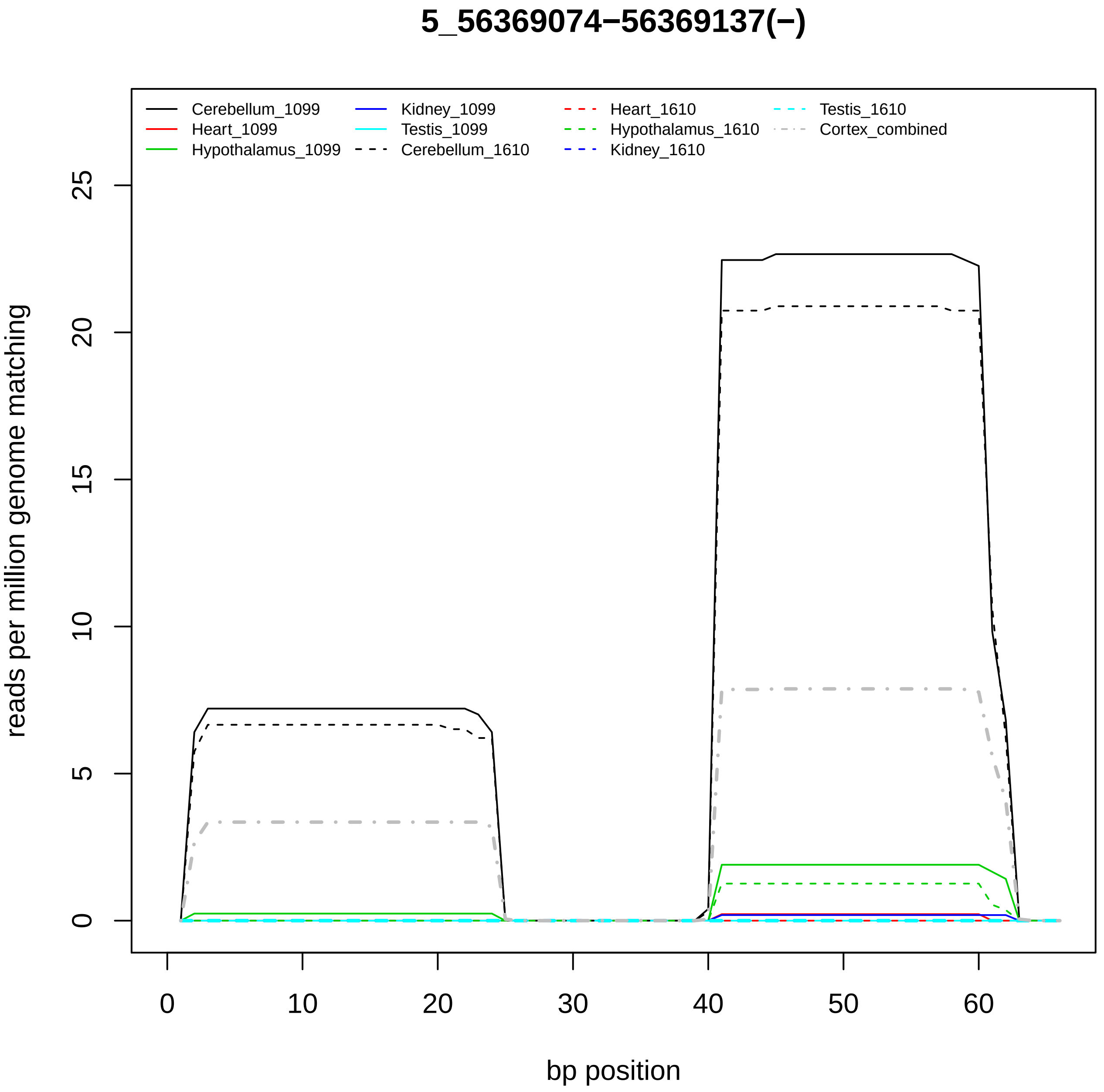

5_56369074−56369137(−)
Cerebellum_1099
Heart_1099
Hypothalamus_1099
Kidney_1099
Testis_1099
Cerebellum_1610
Heart_1610
Hypothalamus_1610
Kidney_1610
Testis_1610
Cortex_combined
reads per million genome matching
bp position
0
5
10
15
20
25
0
10
20
30
40
50
60

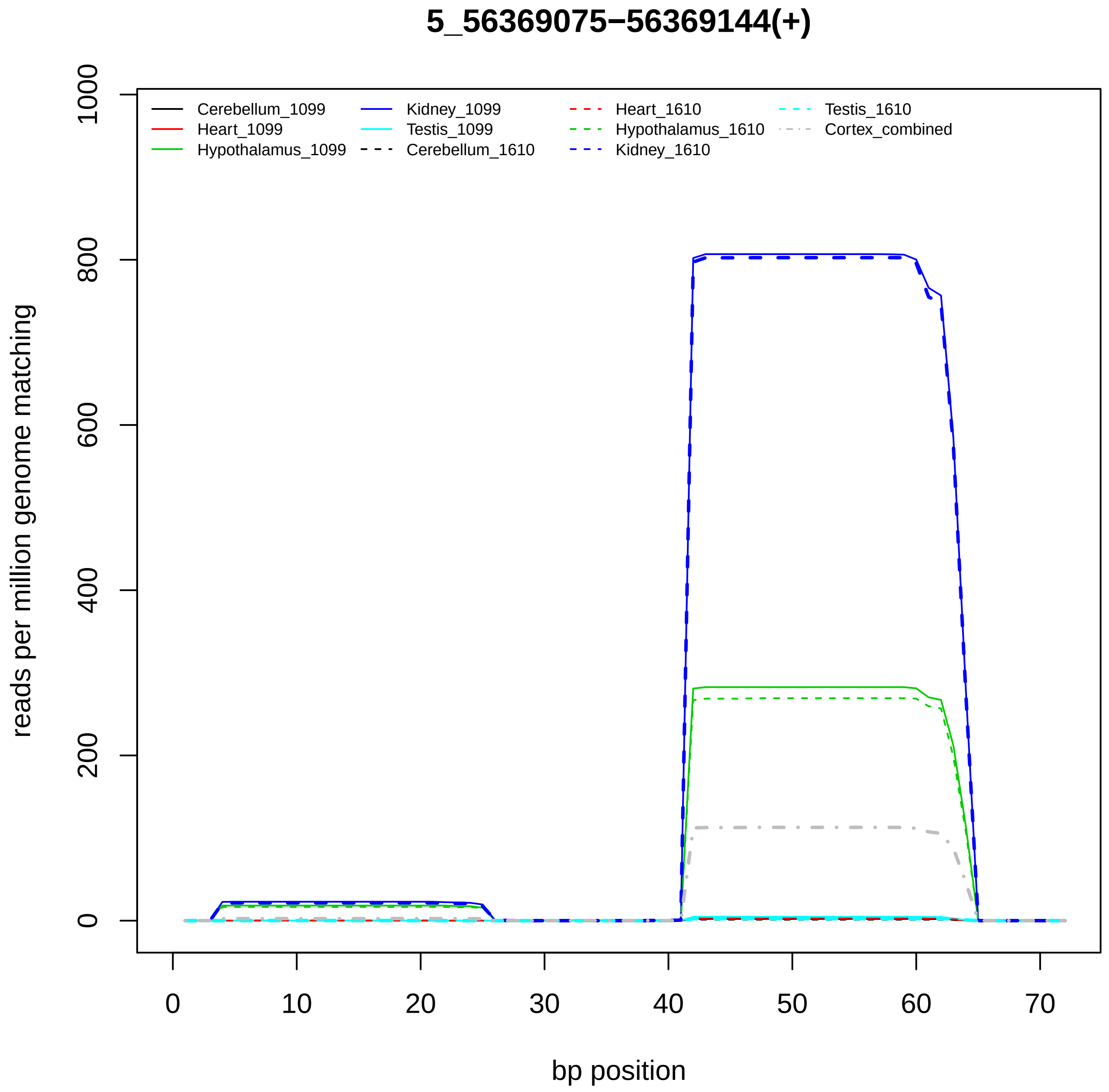

5_56369075−56369144(+)
reads per million genome matching
bp position
0
200
400
600
800
1000
0
10
20
30
40
50
60
70
Cerebellum_1099
Heart_1099
Hypothalamus_1099
Kidney_1099
Testis_1099
Cerebellum_1610
Heart_1610
Hypothalamus_1610
Kidney_1610
Testis_1610
Cortex_combined

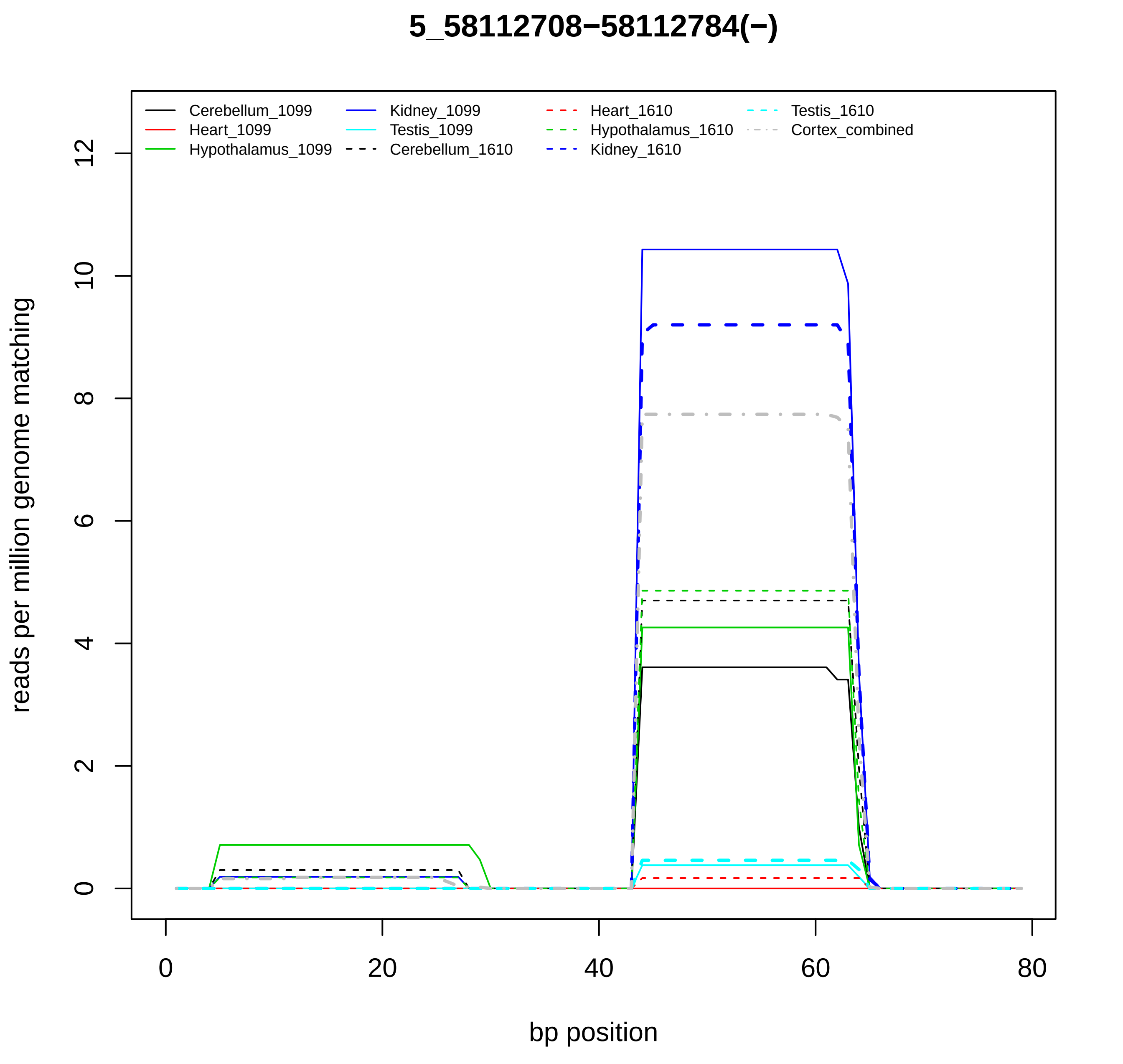

5_58112708−58112784(−)
Cerebellum_1099
Heart_1099
Hypothalamus_1099
Kidney_1099
Testis_1099
Cerebellum_1610
Heart_1610
Hypothalamus_1610
Kidney_1610
Testis_1610
Cortex_combined
reads per million genome matching
0
2
4
6
8
10
12
0
20
40
60
80
bp position

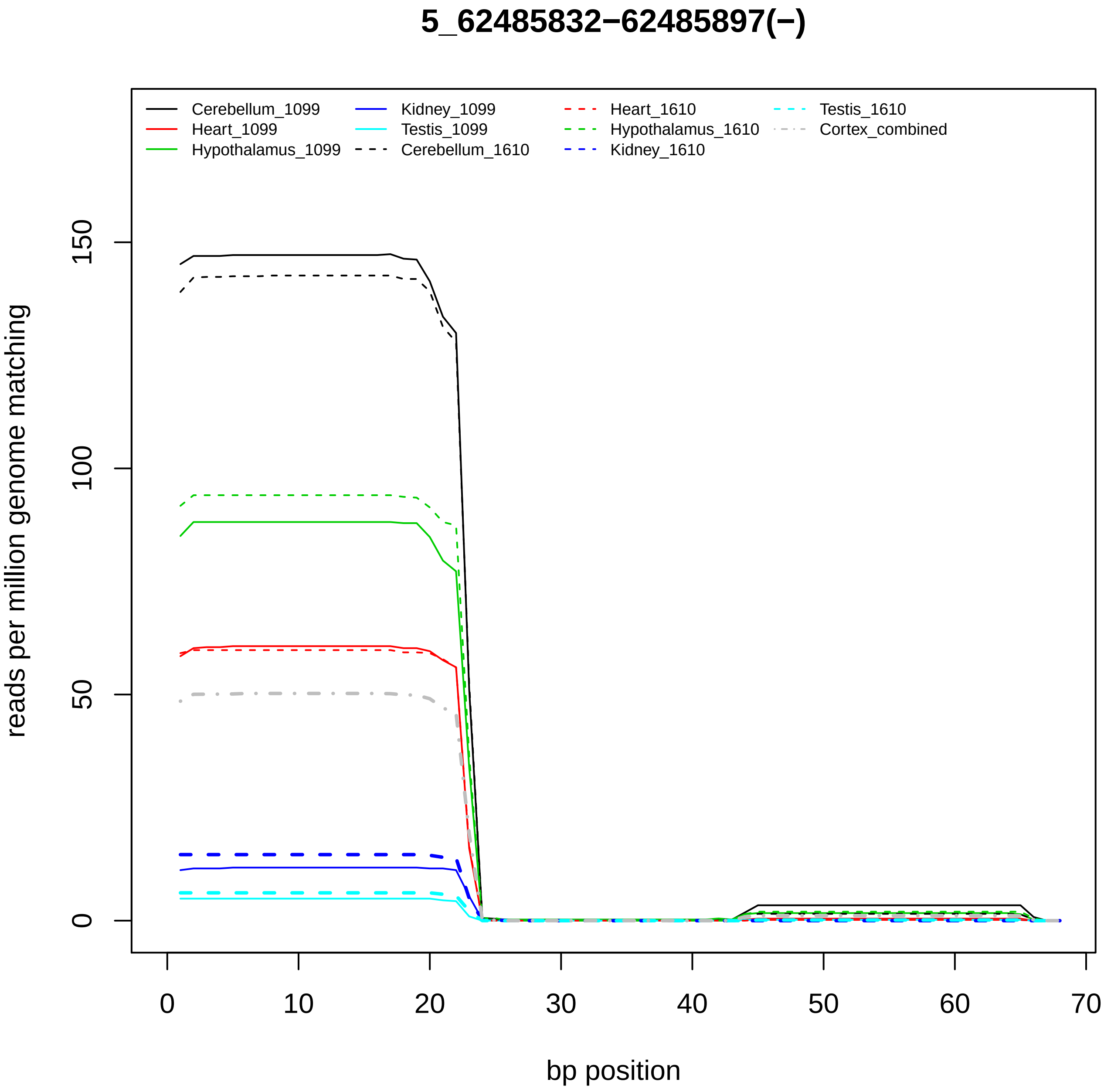

5_62485832−62485897(−)
Cerebellum_1099
Heart_1099
Hypothalamus_1099
Kidney_1099
Testis_1099
Cerebellum_1610
Heart_1610
Hypothalamus_1610
Kidney_1610
Testis_1610
Cortex_combined
reads per million genome matching
bp position
0
50
100
150
0
10
20
30
40
50
60
70

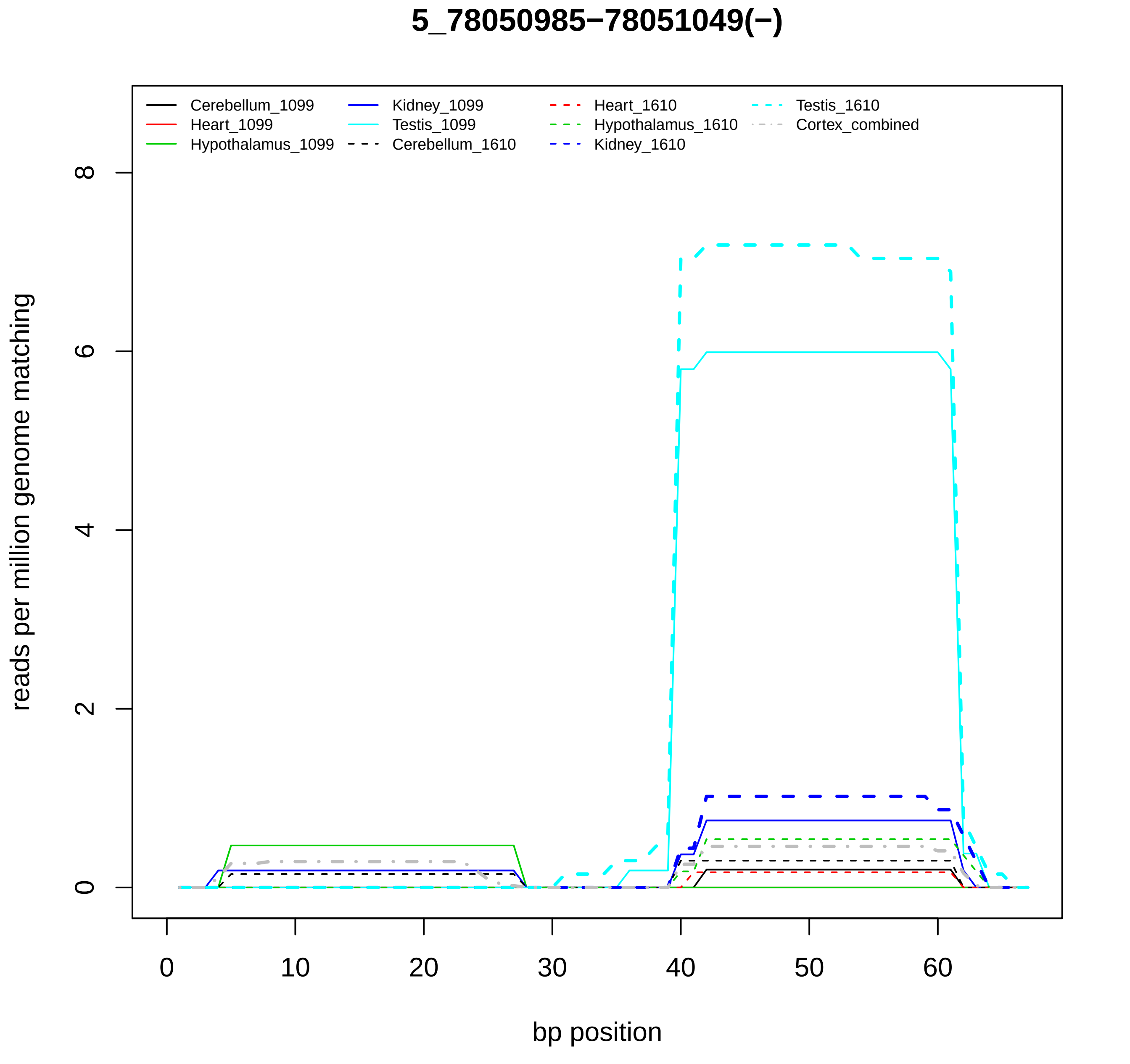
5_78050985−78051049(−)
Cerebellum_1099
Heart_1099
Hypothalamus_1099
Kidney_1099
Testis_1099
Cerebellum_1610
Heart_1610
Hypothalamus_1610
Kidney_1610
Testis_1610
Cortex_combined
reads per million genome matching
0
2
4
6
8
bp position
0
10
20
30
40
50
60

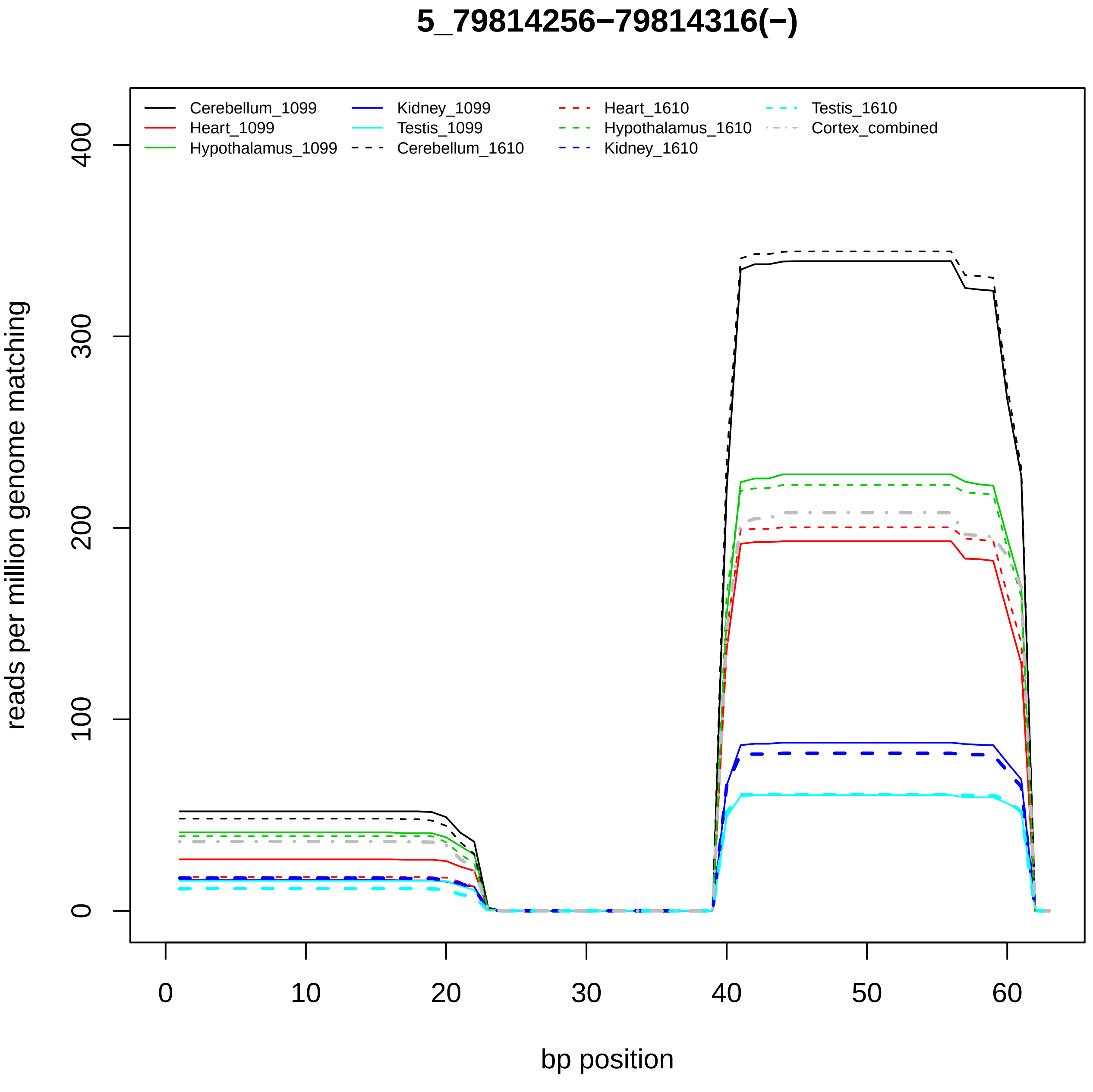
5_79814256−79814316(−)
Cerebellum_1099
Heart_1099
Hypothalamus_1099
Kidney_1099
Testis_1099
Cerebellum_1610
Heart_1610
Hypothalamus_1610
Kidney_1610
Testis_1610
Cortex_combined
reads per million genome matching
bp position
0
100
200
300
400
0
10
20
30
40
50
60

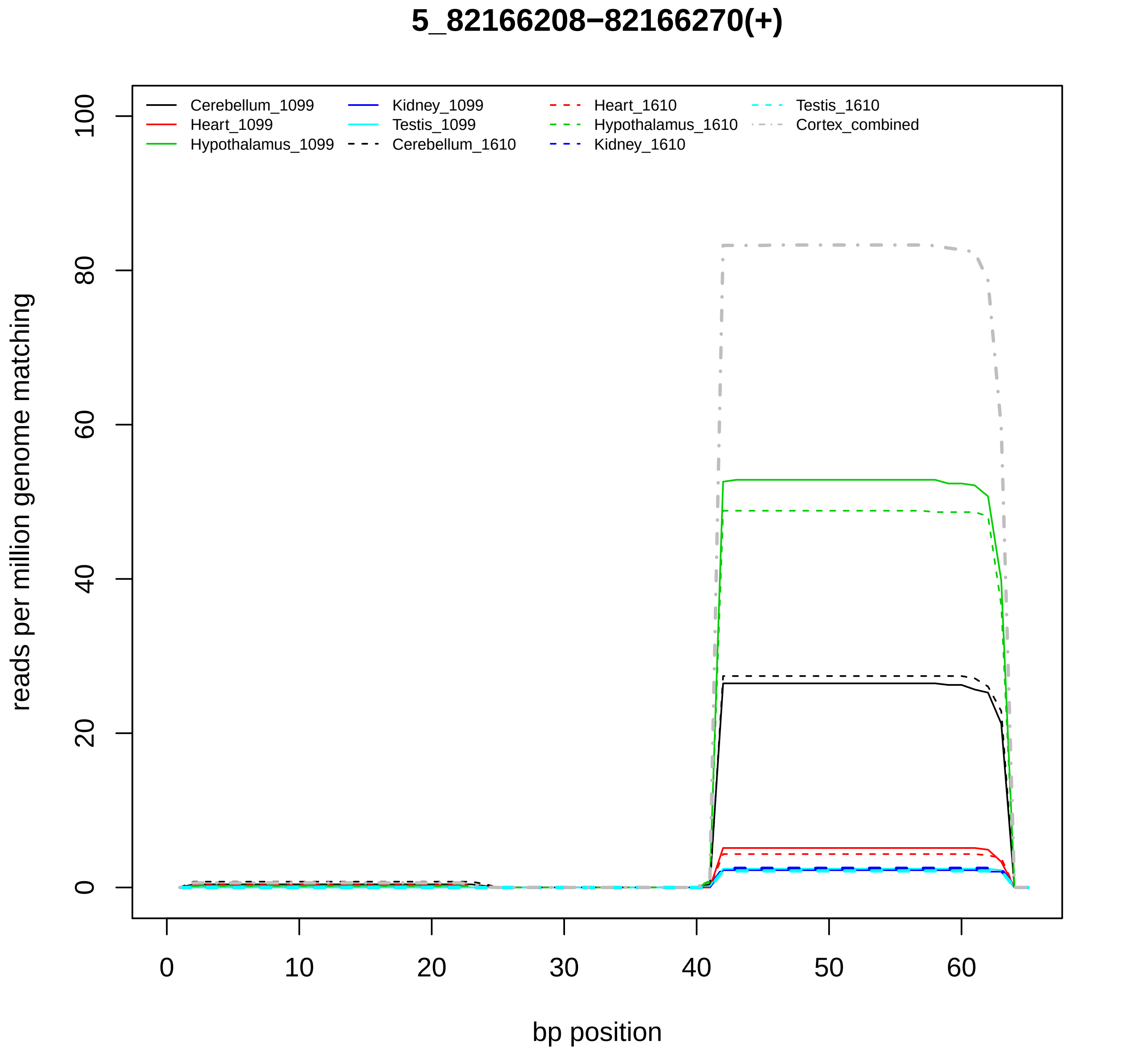

5_82166208−82166270(+)
Cerebellum_1099
Heart_1099
Hypothalamus_1099
Kidney_1099
Testis_1099
Cerebellum_1610
Heart_1610
Hypothalamus_1610
Kidney_1610
Testis_1610
Cortex_combined
reads per million genome matching
0
20
40
60
80
100
0
10
20
30
40
50
60
bp position

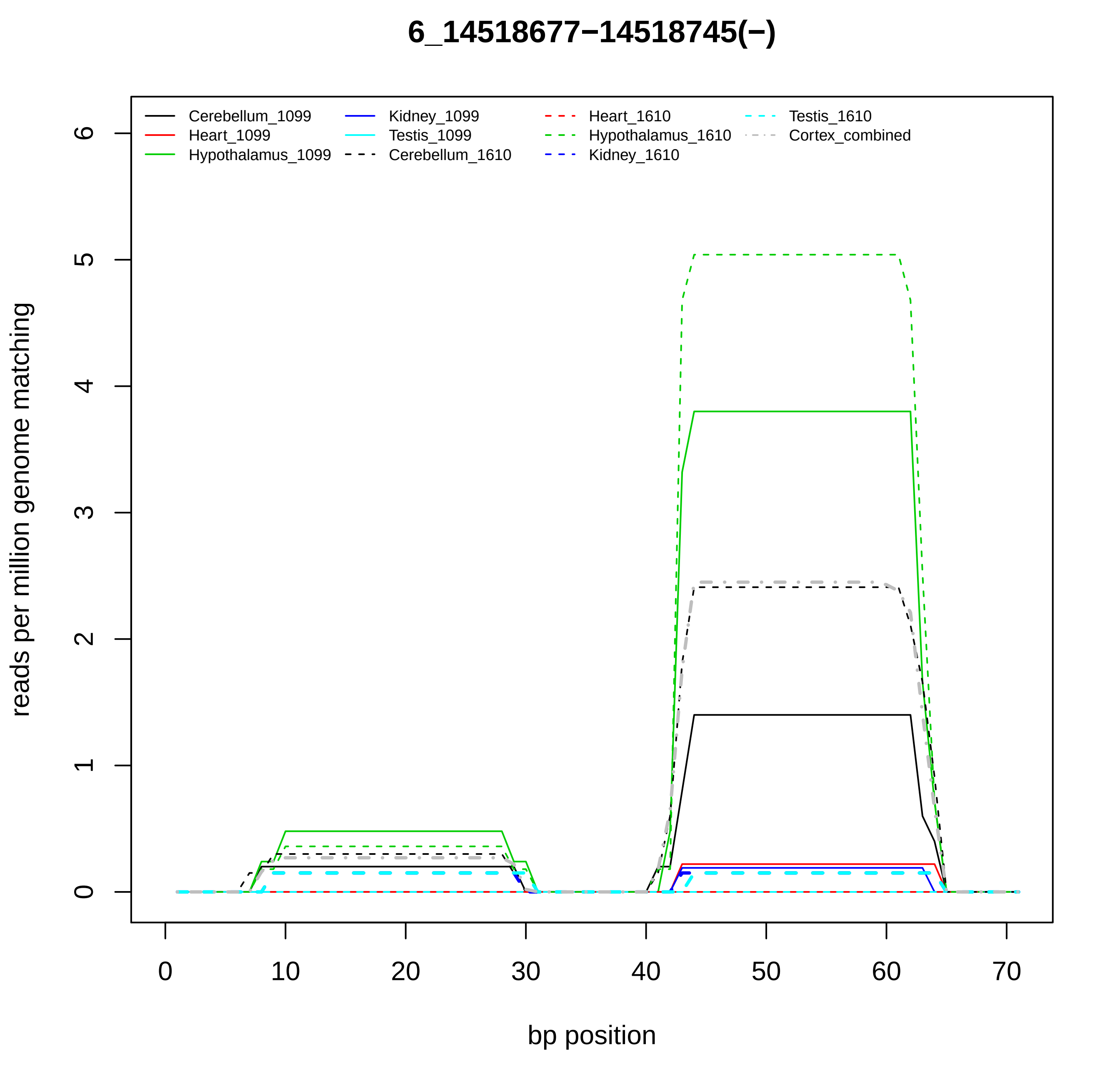
6_14518677−14518745(−)
Cerebellum_1099
Heart_1099
Hypothalamus_1099
Kidney_1099
Testis_1099
Cerebellum_1610
Heart_1610
Hypothalamus_1610
Kidney_1610
Testis_1610
Cortex_combined
reads per million genome matching
bp position
0
1
2
3
4
5
6
0
10
20
30
40
50
60
70

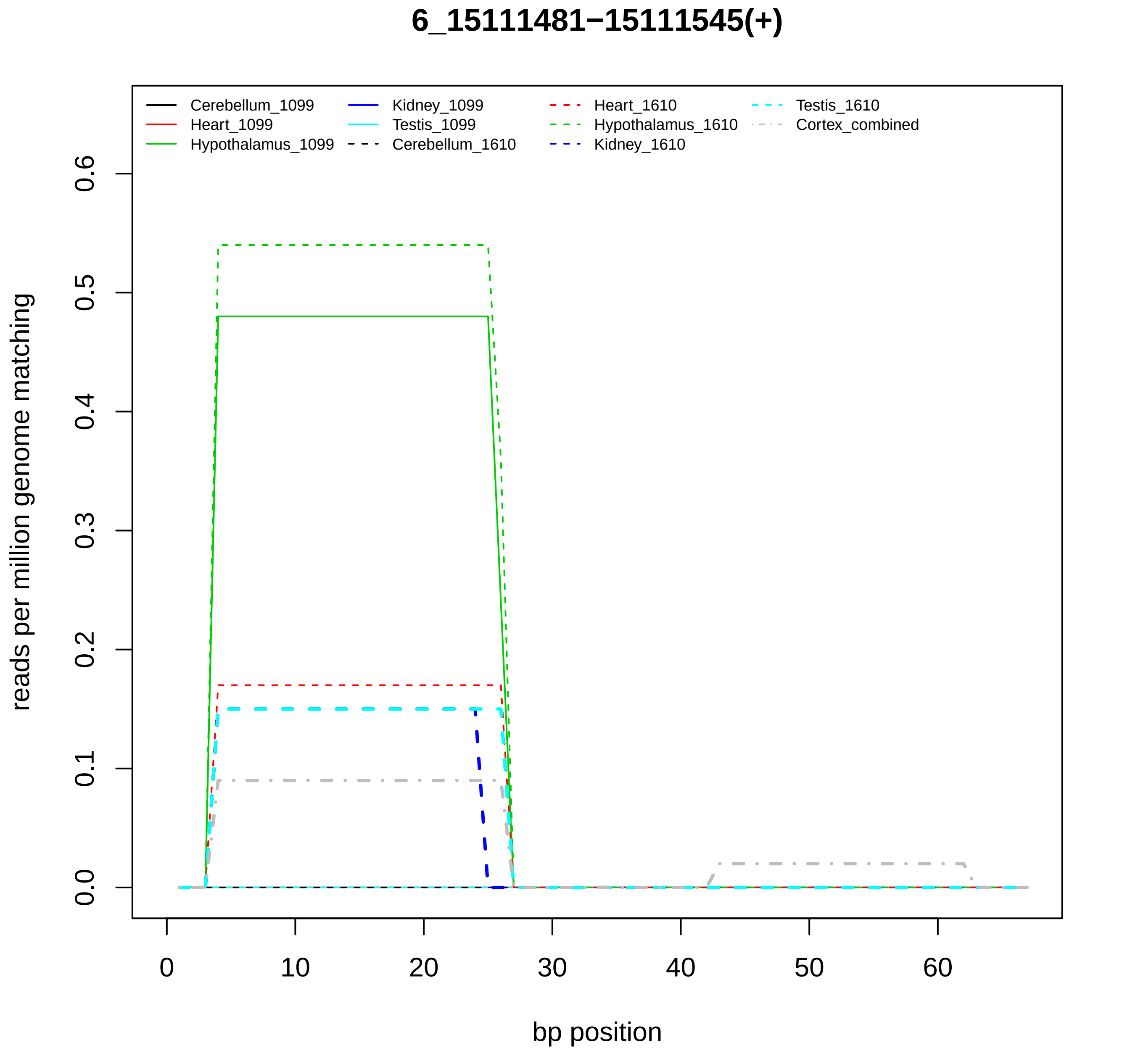

6_15111481−15111545(+)
Cerebellum_1099
Heart_1099
Hypothalamus_1099
Kidney_1099
Testis_1099
Cerebellum_1610
Heart_1610
Hypothalamus_1610
Kidney_1610
Testis_1610
Cortex_combined
reads per million genome matching
0.0
0.1
0.2
0.3
0.4
0.5
0.6
0
10
20
30
40
50
60
bp position

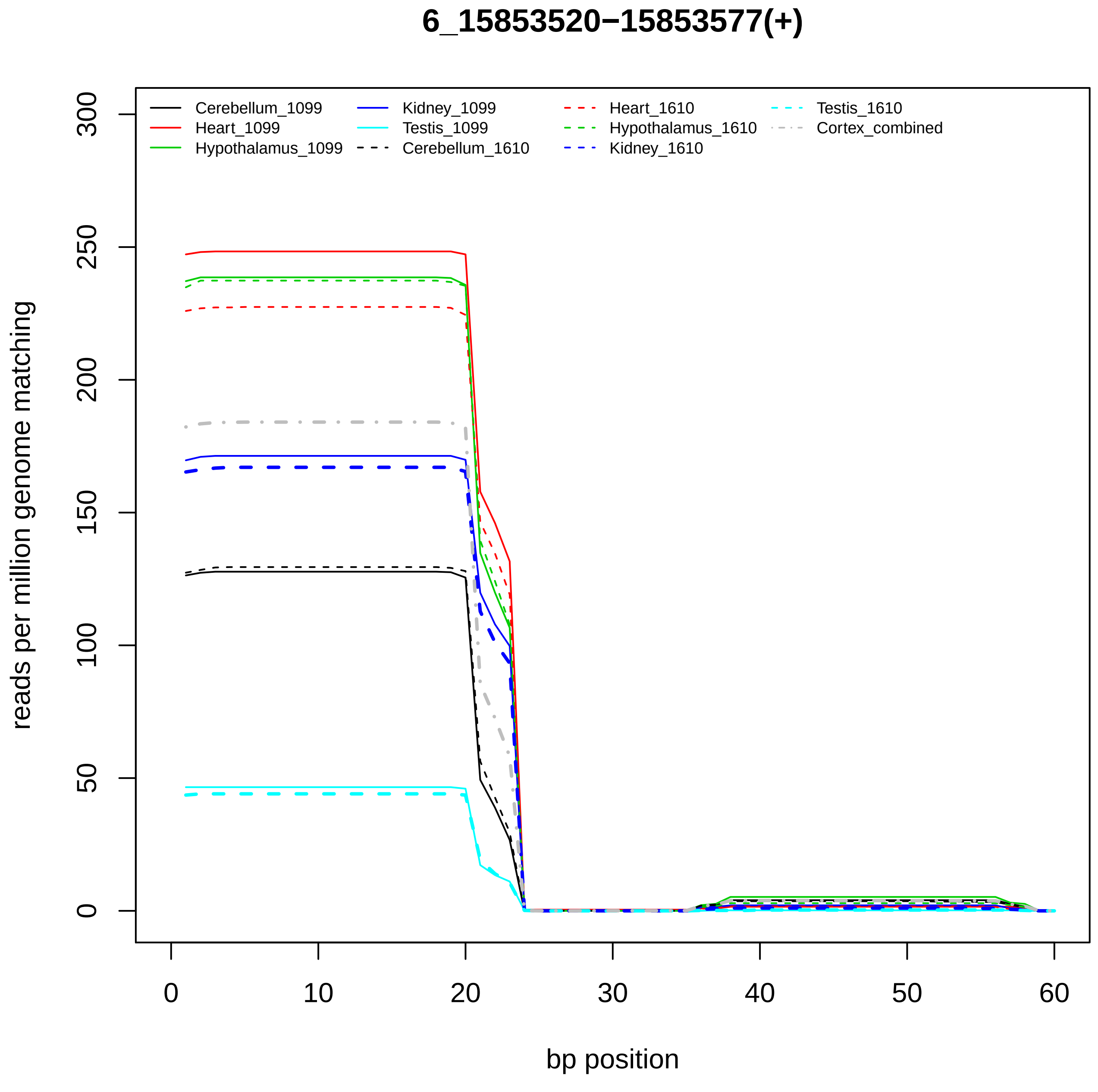

6_15853520−15853577(+)
Cerebellum_1099
Heart_1099
Hypothalamus_1099
Kidney_1099
Testis_1099
Cerebellum_1610
Heart_1610
Hypothalamus_1610
Kidney_1610
Testis_1610
Cortex_combined
reads per million genome matching
bp position
0
50
100
150
200
250
300
0
10
20
30
40
50
60

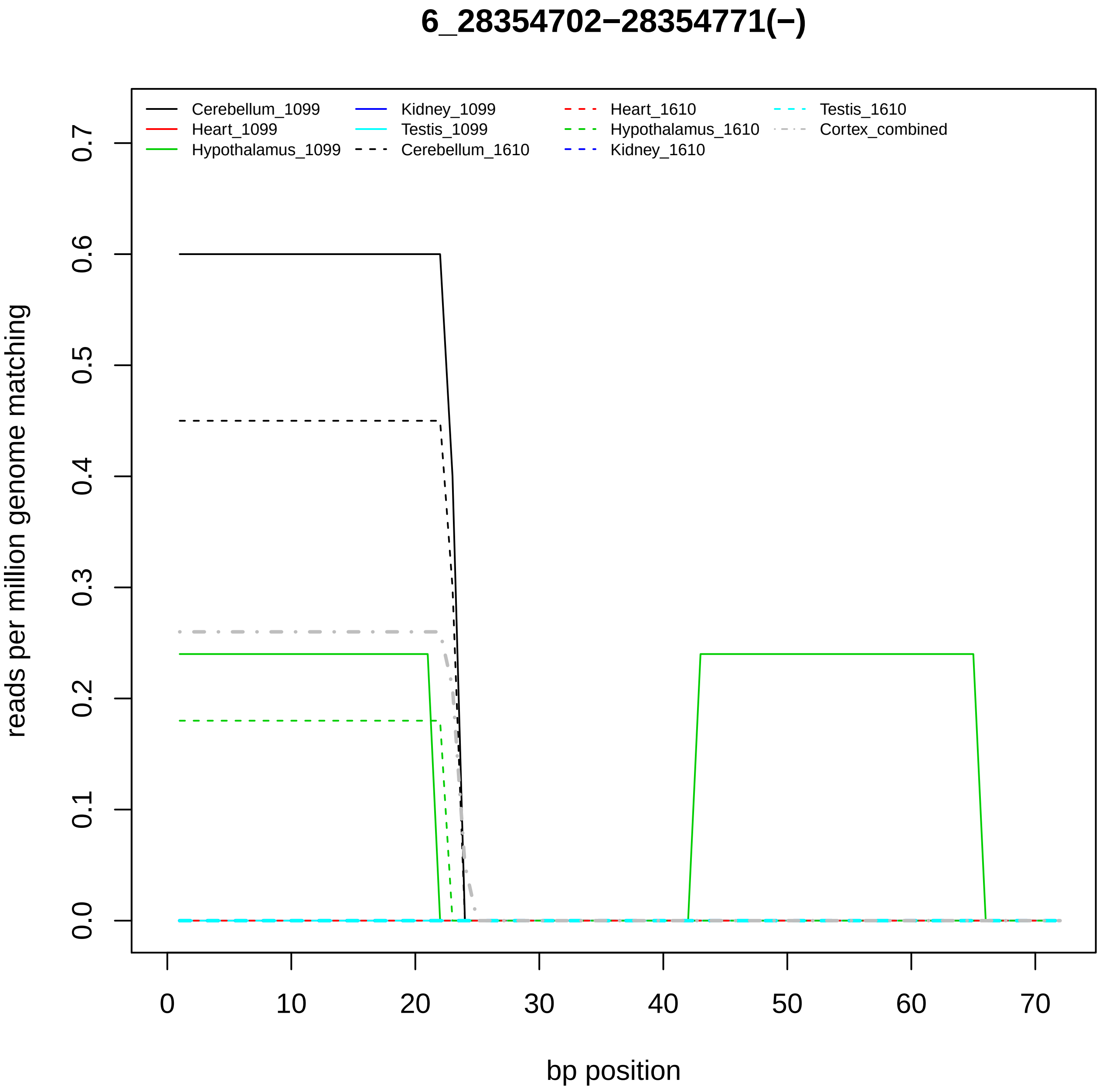

6_28354702−28354771(−)
Cerebellum_1099
Heart_1099
Hypothalamus_1099
Kidney_1099
Testis_1099
Cerebellum_1610
Heart_1610
Hypothalamus_1610
Kidney_1610
Testis_1610
Cortex_combined
reads per million genome matching
0.0
0.1
0.2
0.3
0.4
0.5
0.6
0.7
0
10
20
30
40
50
60
70
bp position

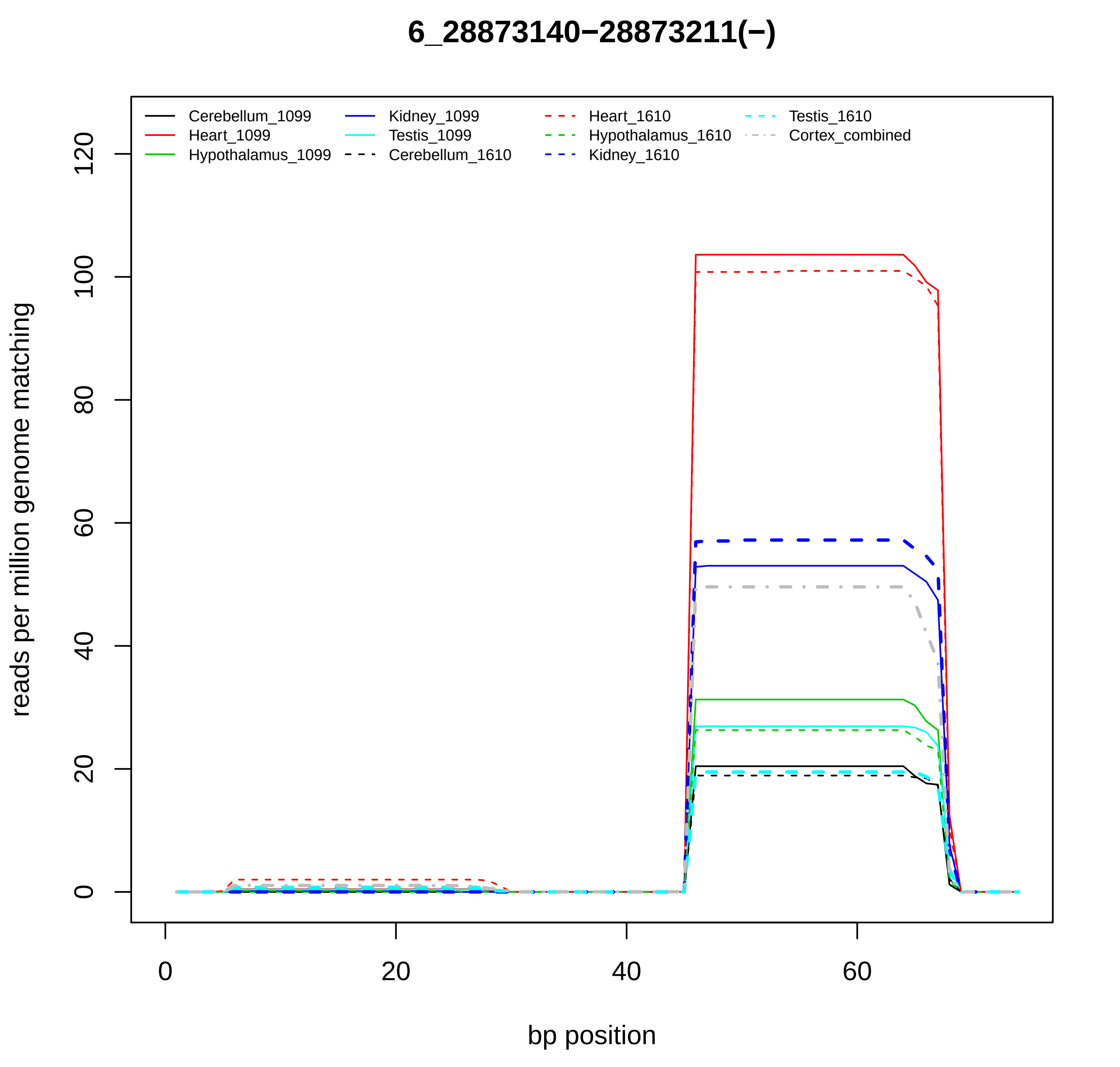

6_28873140−28873211(−)
Cerebellum_1099
Heart_1099
Hypothalamus_1099
Kidney_1099
Testis_1099
Cerebellum_1610
Heart_1610
Hypothalamus_1610
Kidney_1610
Testis_1610
Cortex_combined
reads per million genome matching
bp position
0
20
40
60
80
100
120

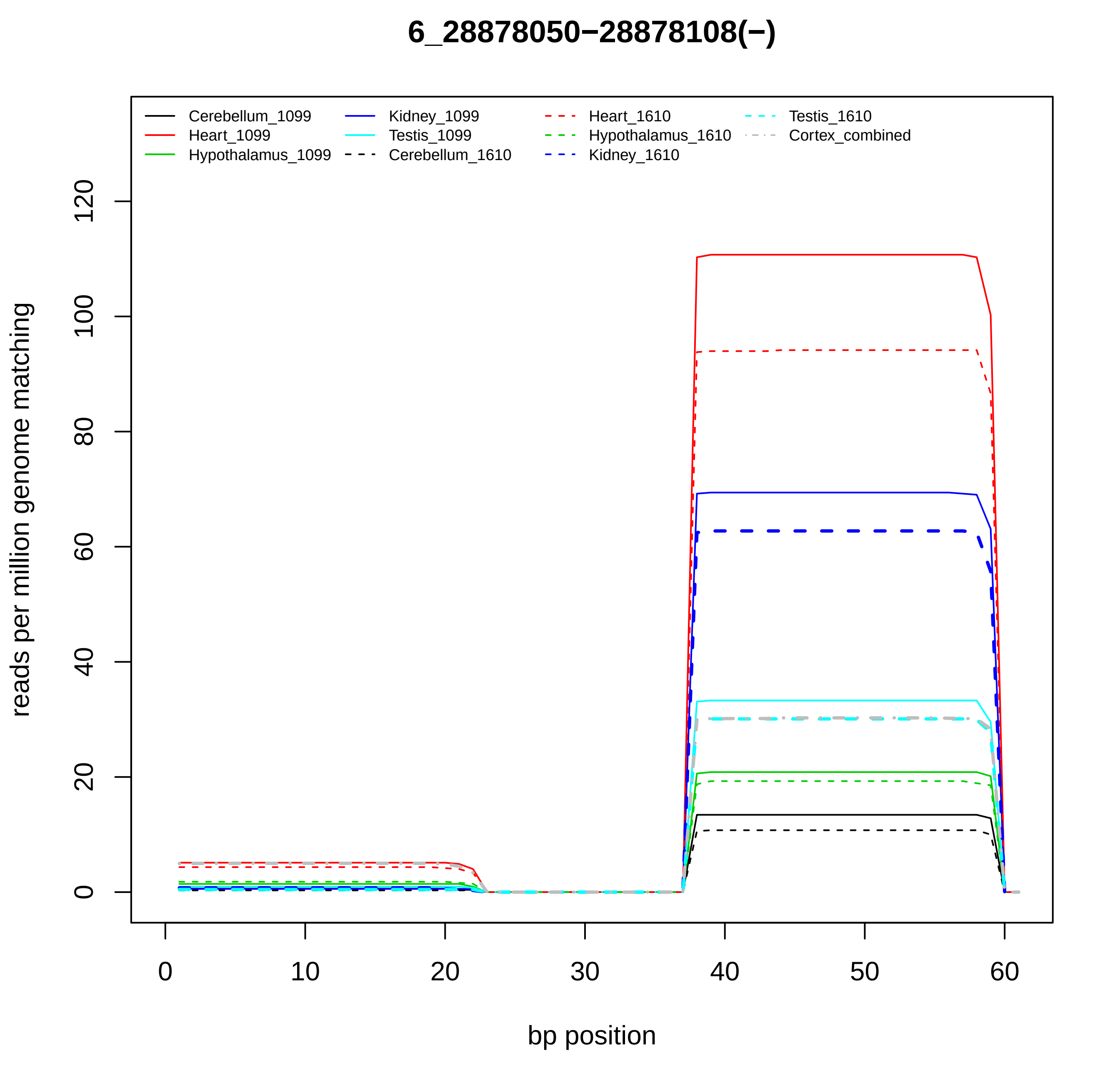
6_28878050−28878108(−)
Cerebellum_1099
Heart_1099
Hypothalamus_1099
Kidney_1099
Testis_1099
Cerebellum_1610
Heart_1610
Hypothalamus_1610
Kidney_1610
Testis_1610
Cortex_combined
reads per million genome matching
0
20
40
60
80
100
120
bp position
0
10
20
30
40
50
60

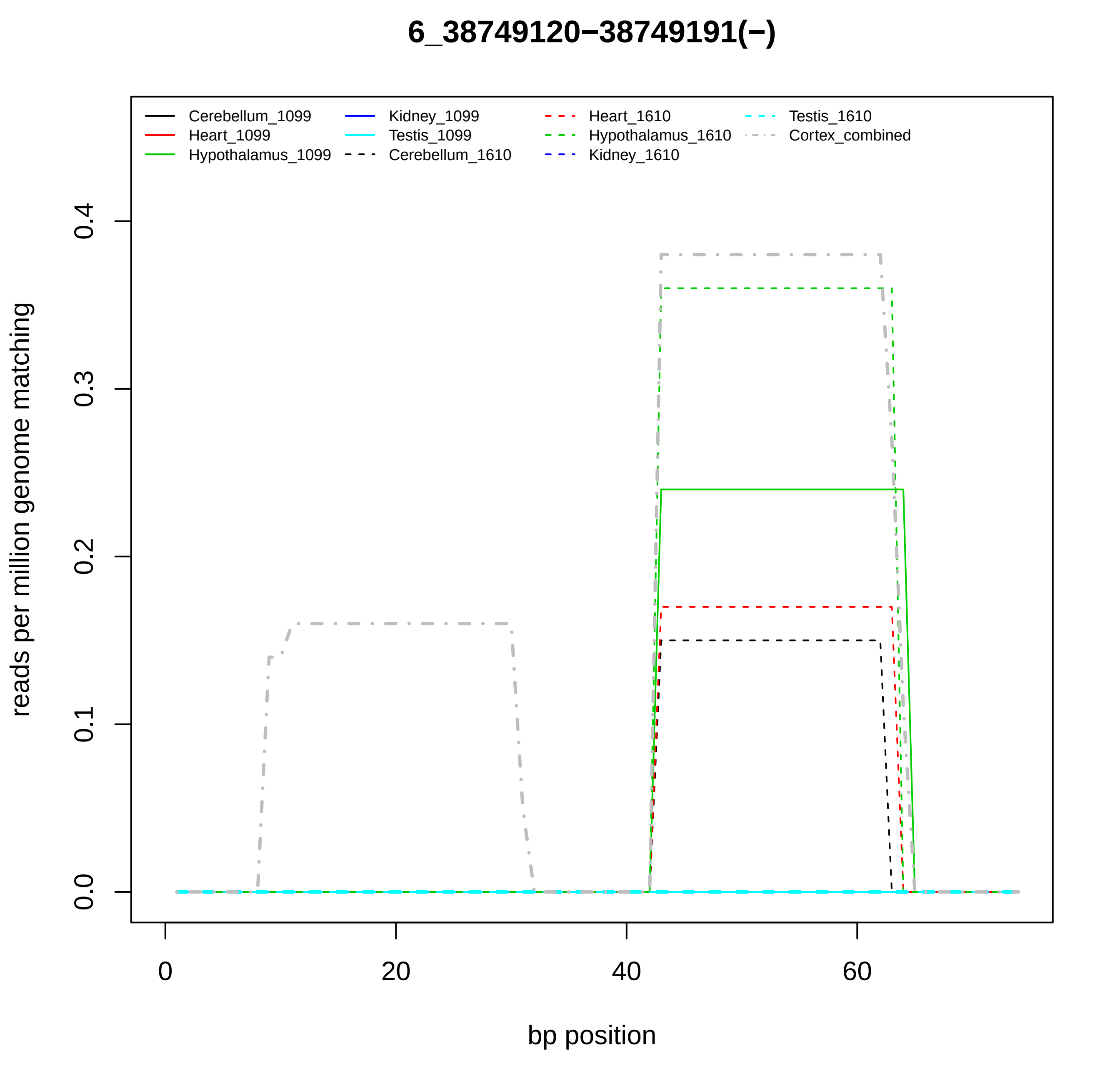

6_38749120−38749191(−)
Cerebellum_1099
Heart_1099
Hypothalamus_1099
Kidney_1099
Testis_1099
Cerebellum_1610
Heart_1610
Hypothalamus_1610
Kidney_1610
Testis_1610
Cortex_combined
reads per million genome matching
0.0
0.1
0.2
0.3
0.4
0
20
40
60
bp position

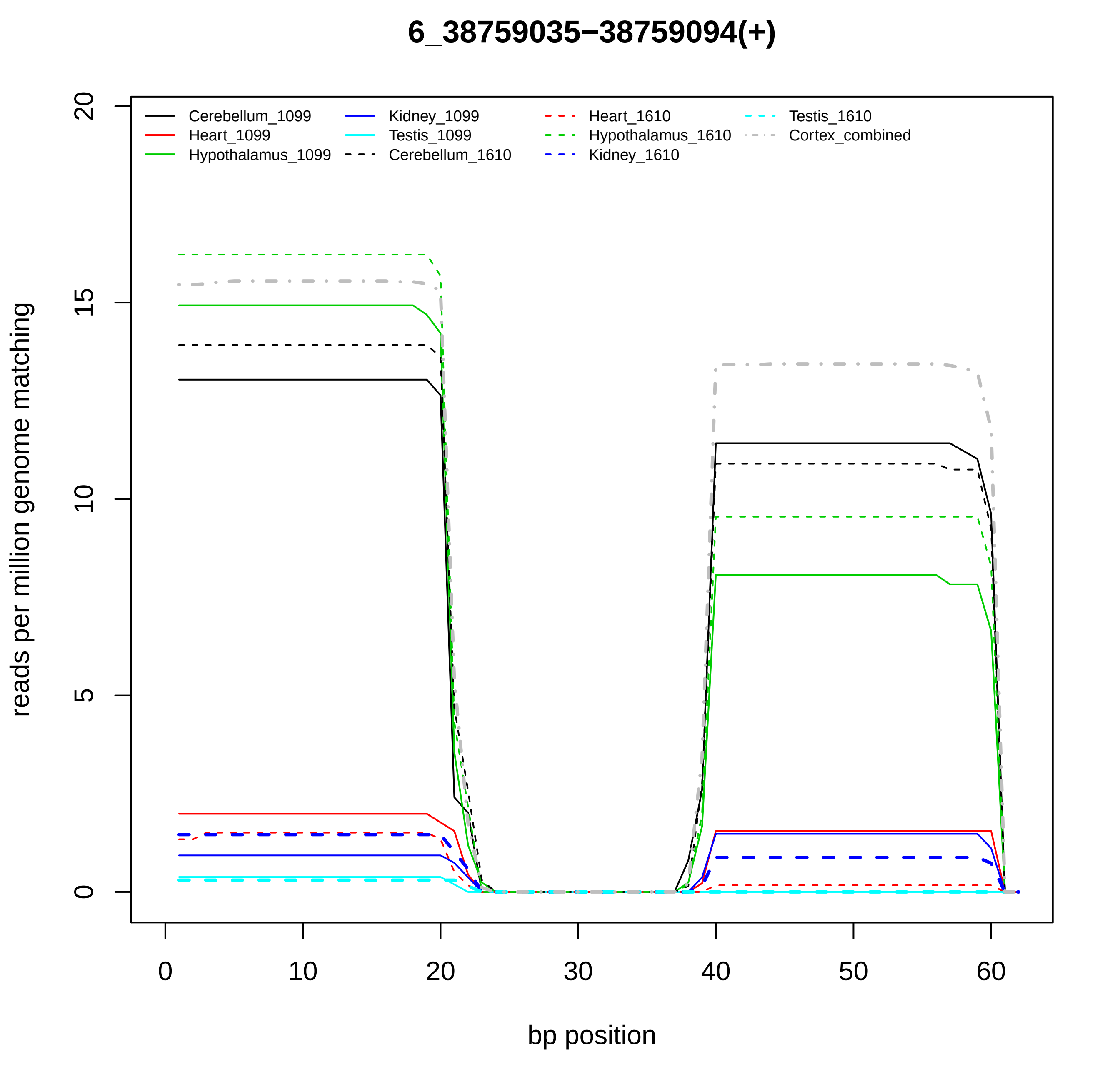

6_38759035−38759094(+)
Cerebellum_1099
Heart_1099
Hypothalamus_1099
Kidney_1099
Testis_1099
Cerebellum_1610
Heart_1610
Hypothalamus_1610
Kidney_1610
Testis_1610
Cortex_combined
reads per million genome matching
bp position
0
5
10
15
20
0
10
20
30
40
50
60

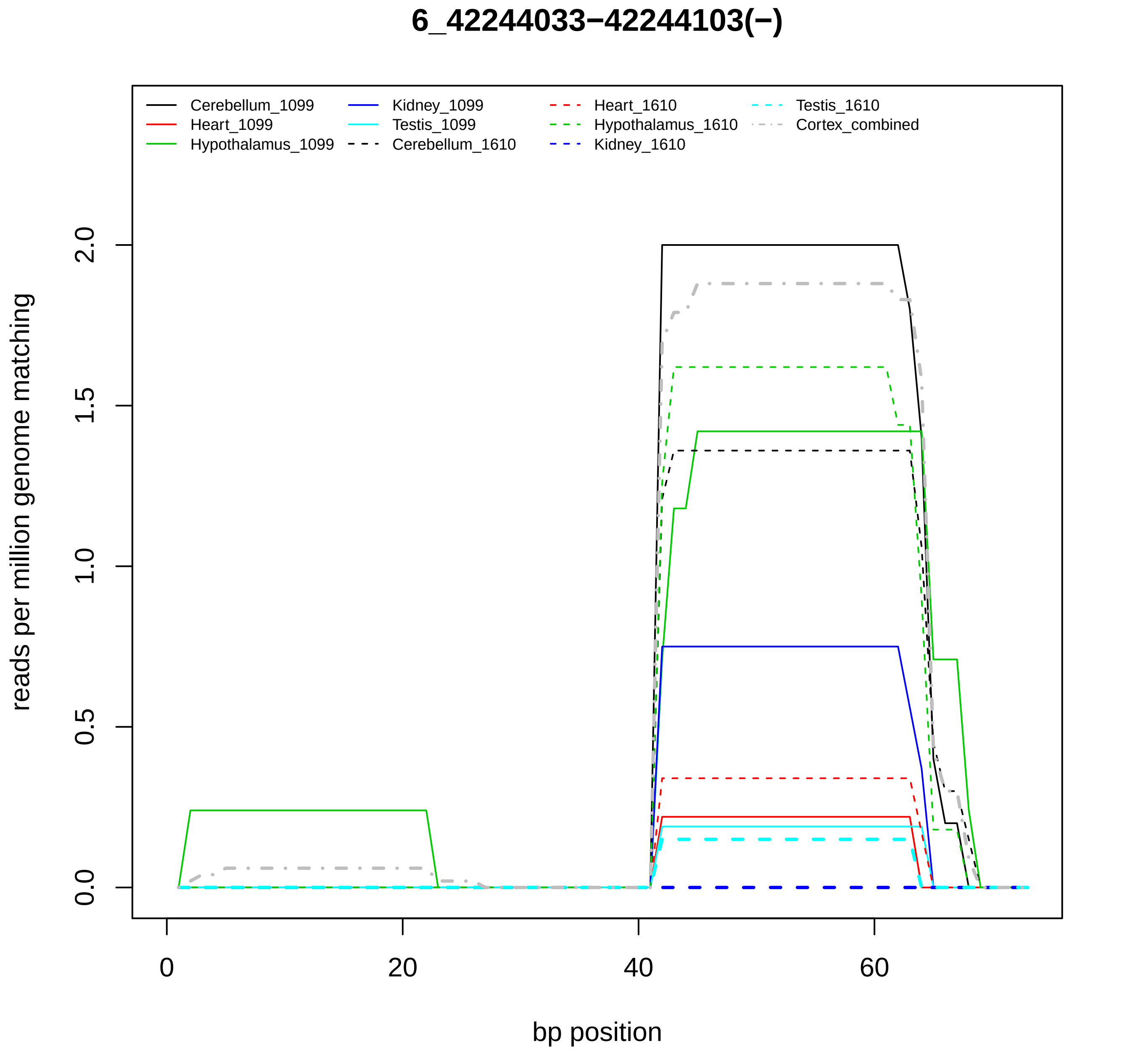
6_42244033−42244103(−)
Cerebellum_1099
Heart_1099
Hypothalamus_1099
Kidney_1099
Testis_1099
Cerebellum_1610
Heart_1610
Hypothalamus_1610
Kidney_1610
Testis_1610
Cortex_combined
reads per million genome matching
0.0
0.5
1.0
1.5
2.0
0
20
40
60
bp position

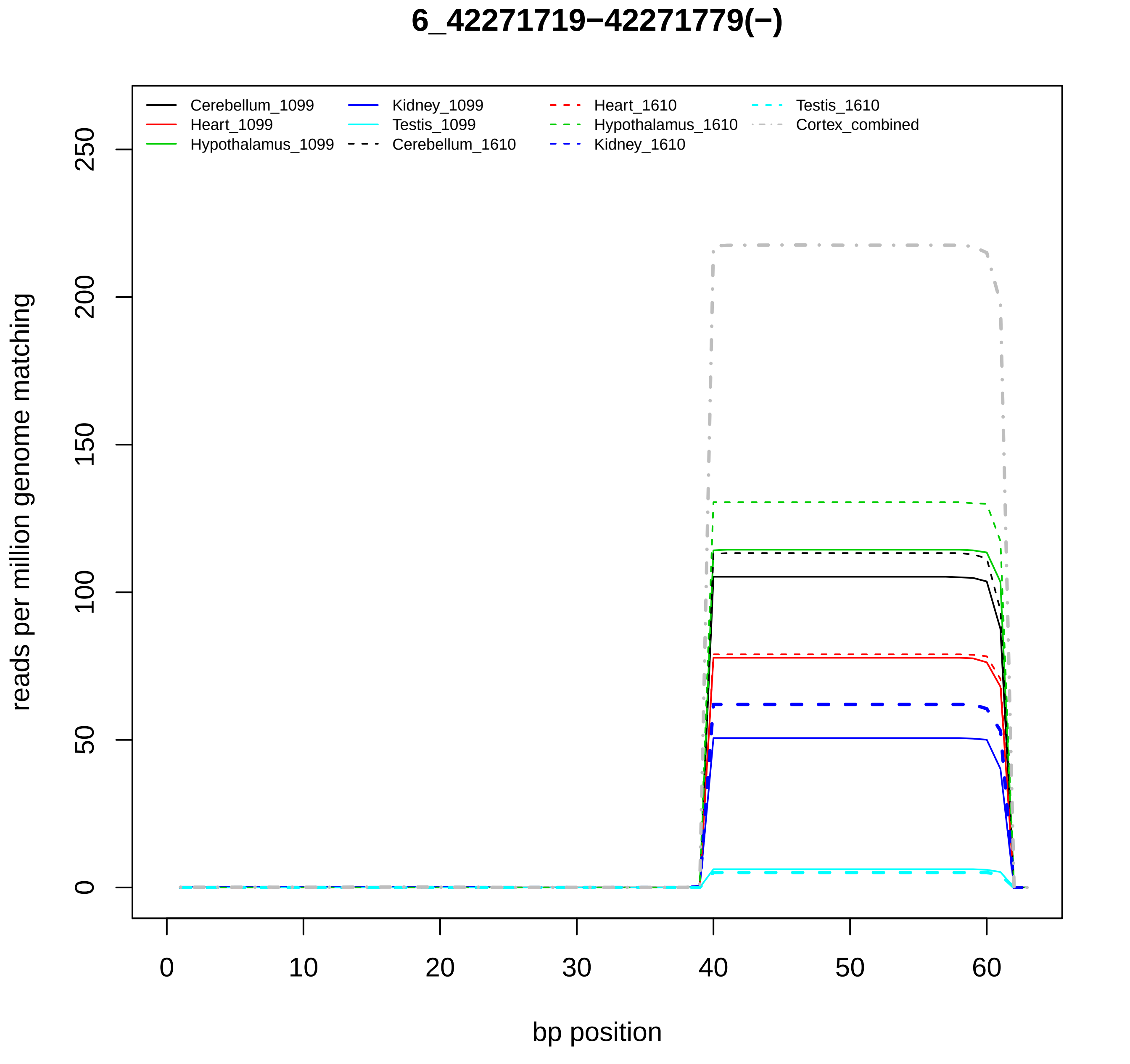

6_42271719−42271779(−)
Cerebellum_1099
Heart_1099
Hypothalamus_1099
Kidney_1099
Testis_1099
Cerebellum_1610
Heart_1610
Hypothalamus_1610
Kidney_1610
Testis_1610
Cortex_combined
reads per million genome matching
bp position
0
50
100
150
200
250
0
10
20
30
40
50
60

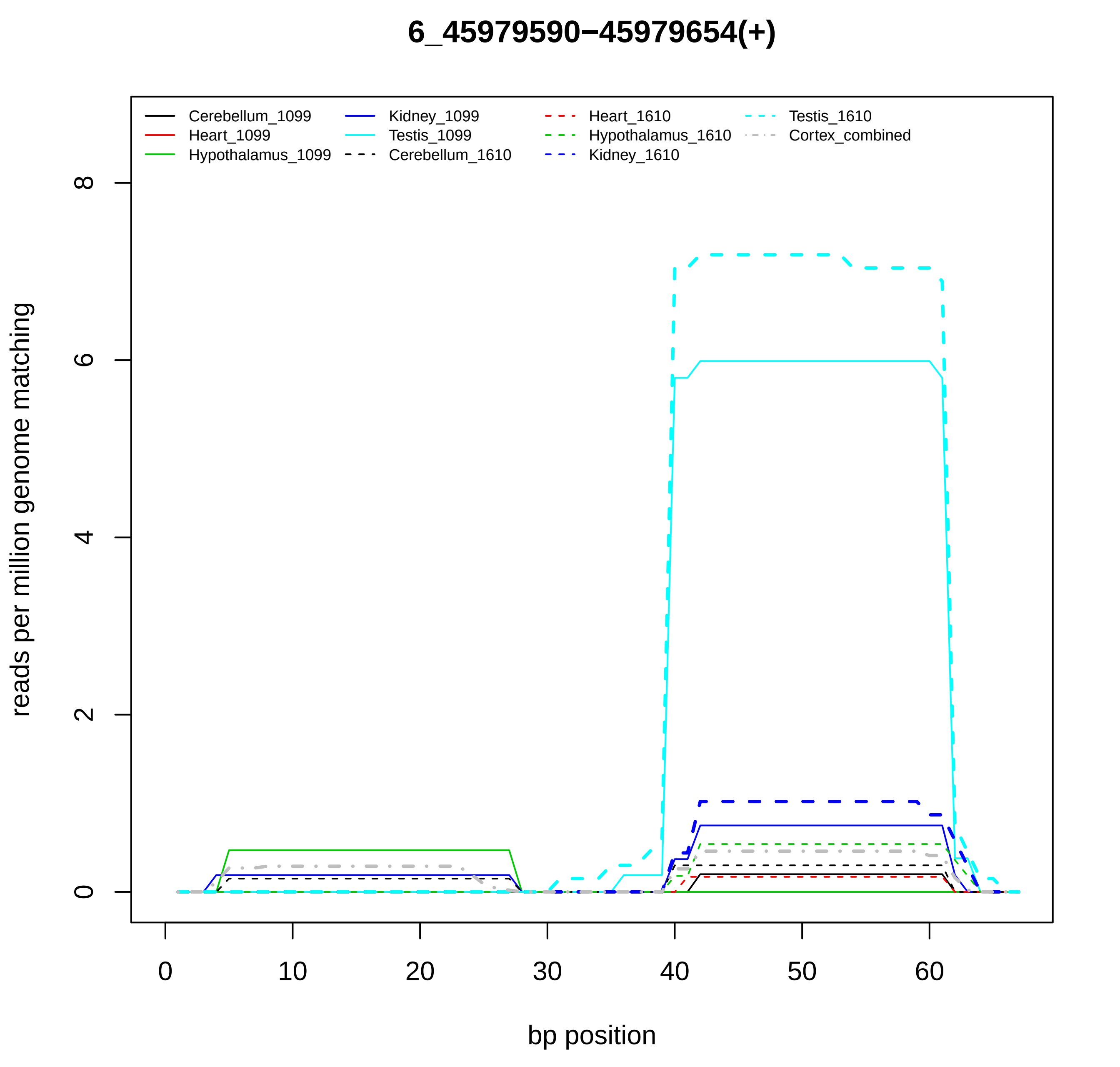

6_45979590−45979654(+)
Cerebellum_1099
Heart_1099
Hypothalamus_1099
Kidney_1099
Testis_1099
Cerebellum_1610
Heart_1610
Hypothalamus_1610
Kidney_1610
Testis_1610
Cortex_combined
reads per million genome matching
bp position
0
2
4
6
8
0
10
20
30
40
50
60

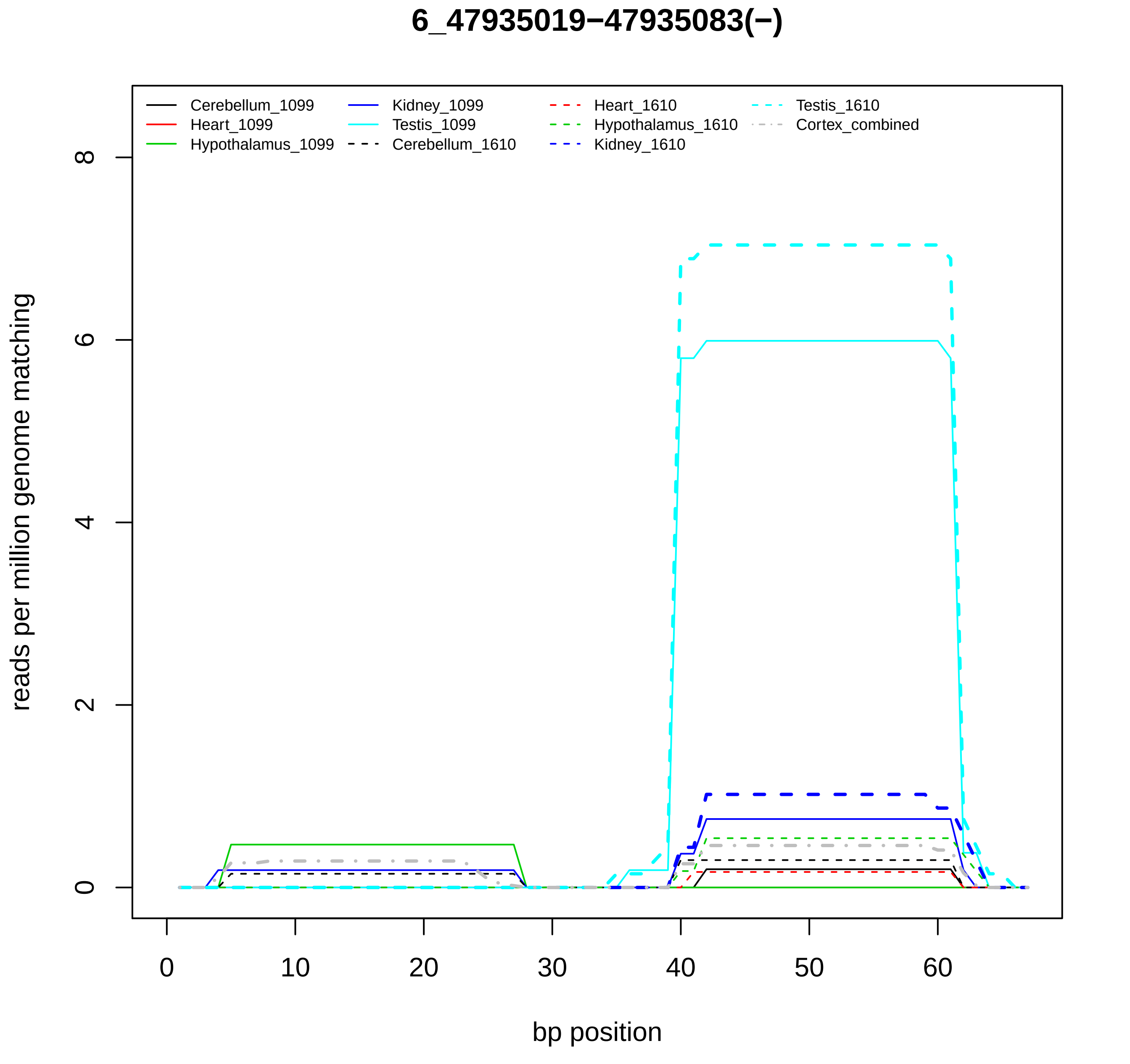
6_47935019−47935083(−)
Cerebellum_1099
Heart_1099
Hypothalamus_1099
Kidney_1099
Testis_1099
Cerebellum_1610
Heart_1610
Hypothalamus_1610
Kidney_1610
Testis_1610
Cortex_combined
reads per million genome matching
0
2
4
6
8
0
10
20
30
40
50
60
bp position

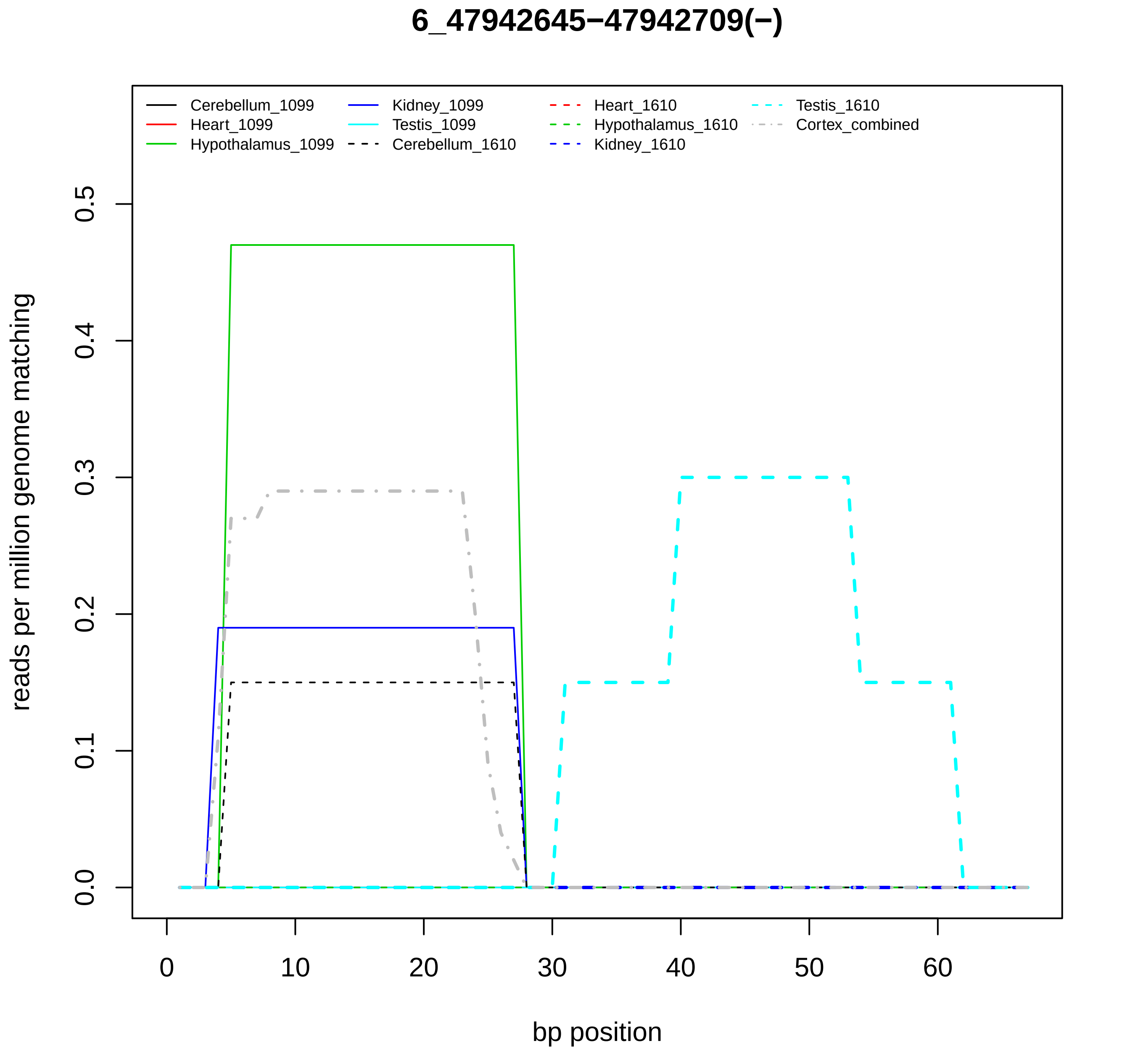

6_47942645−47942709(−)
Cerebellum_1099
Heart_1099
Hypothalamus_1099
Kidney_1099
Testis_1099
Cerebellum_1610
Heart_1610
Hypothalamus_1610
Kidney_1610
Testis_1610
Cortex_combined
reads per million genome matching
0.0
0.1
0.2
0.3
0.4
0.5
0
10
20
30
40
50
60
bp position

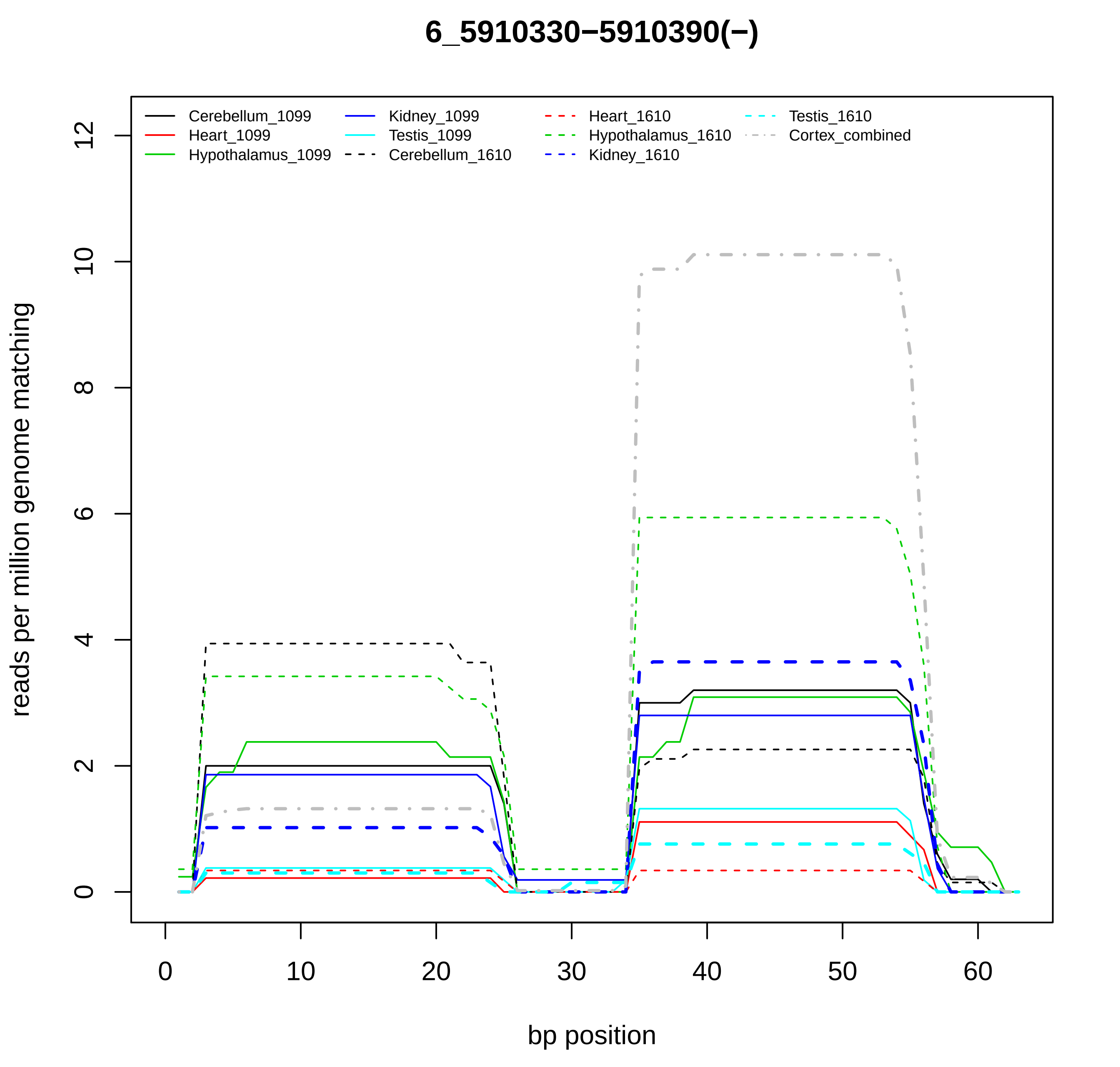

6_5910330−5910390(−)
Cerebellum_1099
Heart_1099
Hypothalamus_1099
Kidney_1099
Testis_1099
Cerebellum_1610
Heart_1610
Hypothalamus_1610
Kidney_1610
Testis_1610
Cortex_combined
reads per million genome matching
bp position
0
2
4
6
8
10
12
0
10
20
30
40
50
60

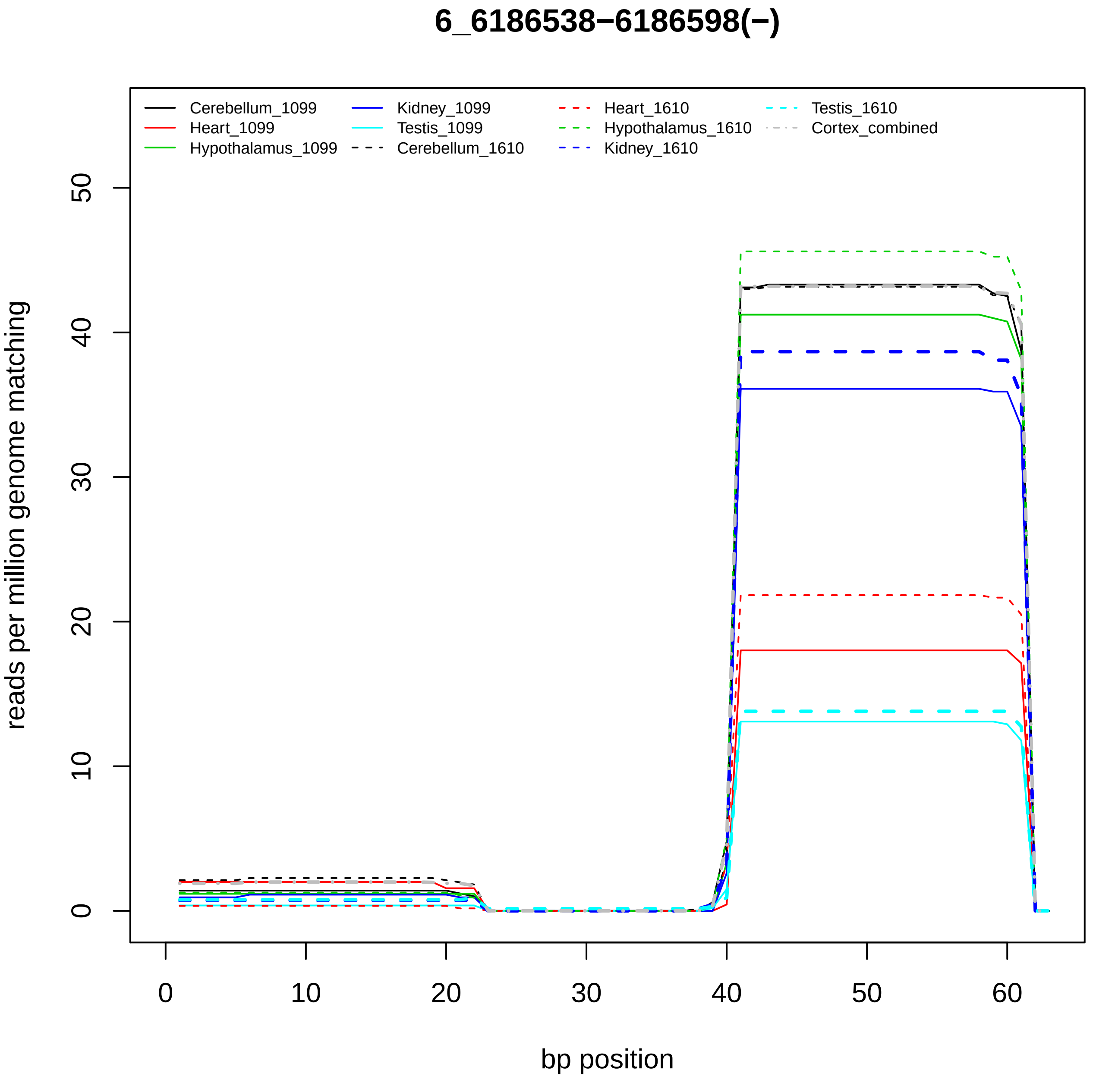

6_6186538−6186598(−)
Cerebellum_1099
Heart_1099
Hypothalamus_1099
Kidney_1099
Testis_1099
Cerebellum_1610
Heart_1610
Hypothalamus_1610
Kidney_1610
Testis_1610
Cortex_combined
reads per million genome matching
bp position
0
10
20
30
40
50
60

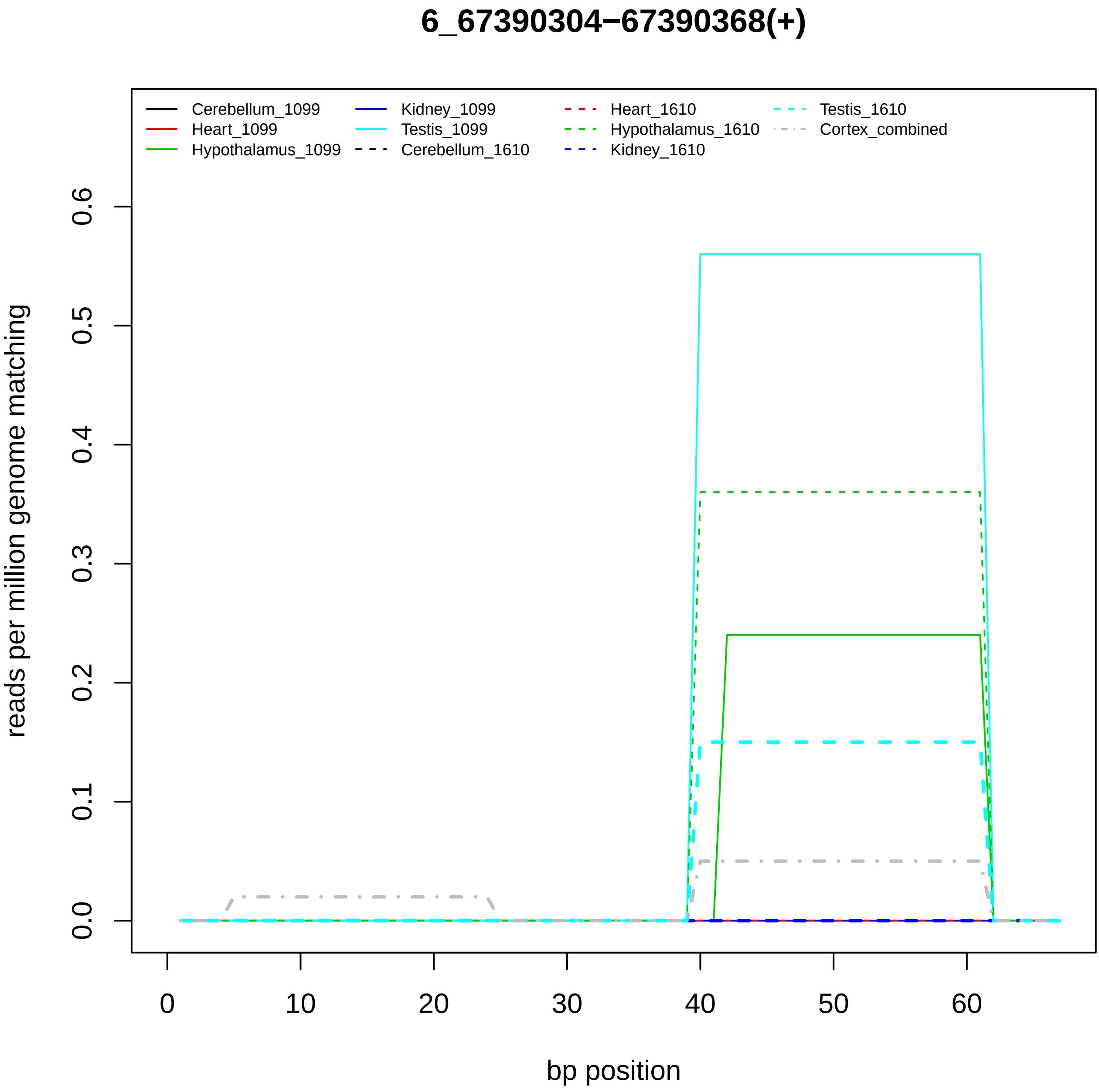

6_67390304−67390368(+)
Cerebellum_1099
Heart_1099
Hypothalamus_1099
Kidney_1099
Testis_1099
Cerebellum_1610
Heart_1610
Hypothalamus_1610
Kidney_1610
Testis_1610
Cortex_combined
reads per million genome matching
0.0
0.1
0.2
0.3
0.4
0.5
0.6
0
10
20
30
40
50
60
bp position

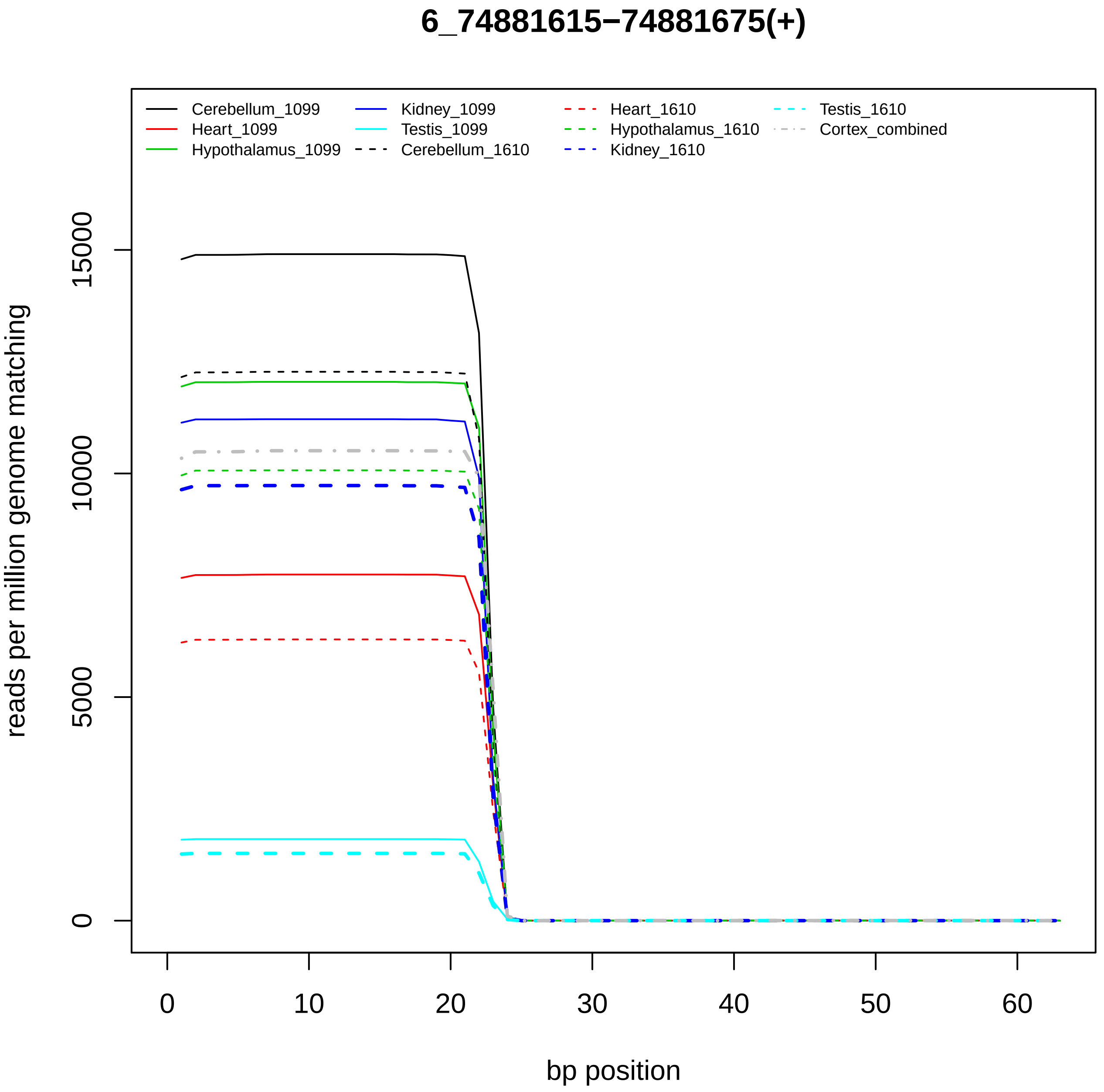

6_74881615−74881675(+)
Cerebellum_1099
Heart_1099
Hypothalamus_1099
Kidney_1099
Testis_1099
Cerebellum_1610
Heart_1610
Hypothalamus_1610
Kidney_1610
Testis_1610
Cortex_combined
reads per million genome matching
0
5000
10000
15000
0
10
20
30
40
50
60
bp position

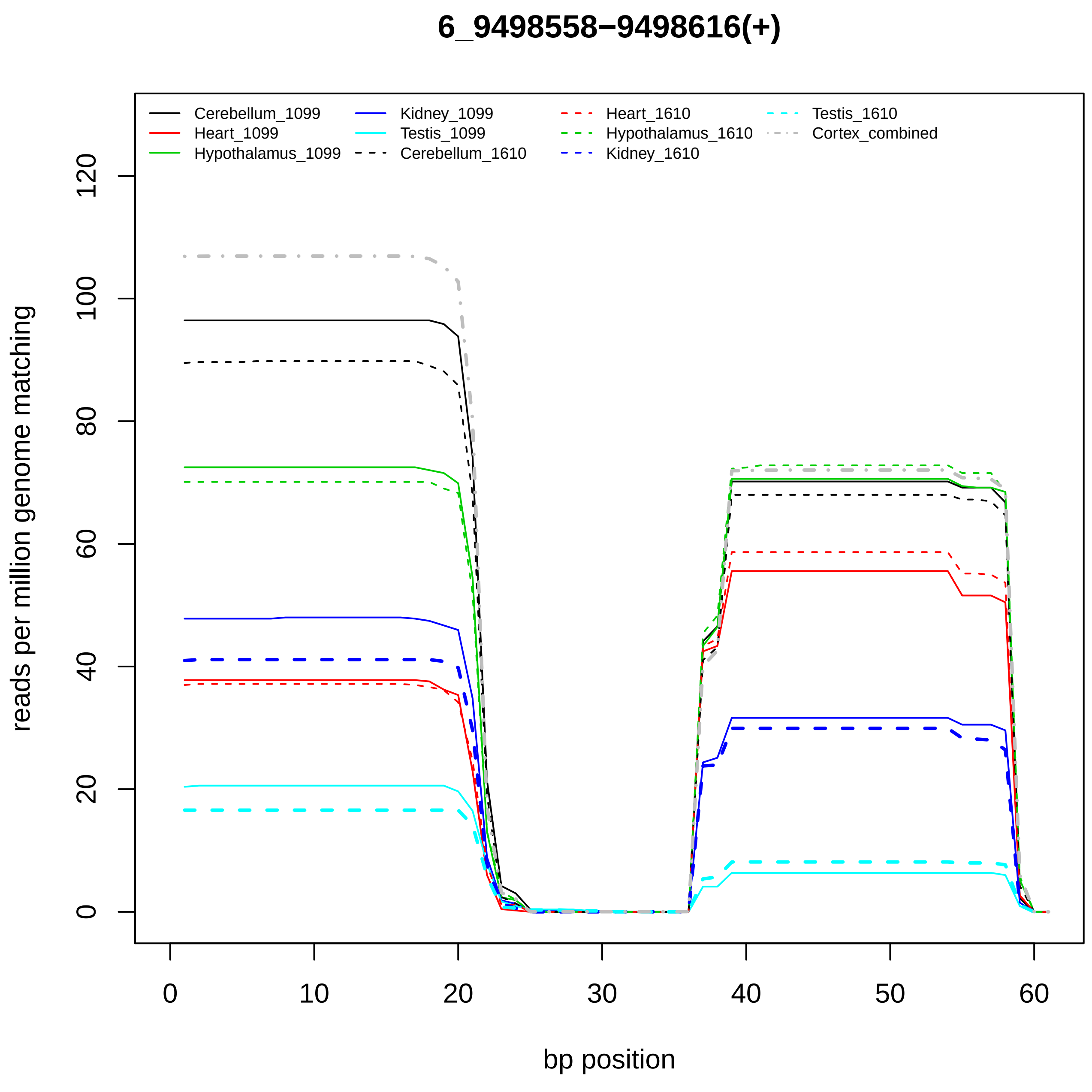

6_9498558−9498616(+)
Cerebellum_1099
Heart_1099
Hypothalamus_1099
Kidney_1099
Testis_1099
Cerebellum_1610
Heart_1610
Hypothalamus_1610
Kidney_1610
Testis_1610
Cortex_combined
reads per million genome matching
bp position
0
20
40
60
80
100
120
0
10
20
30
40
50
60

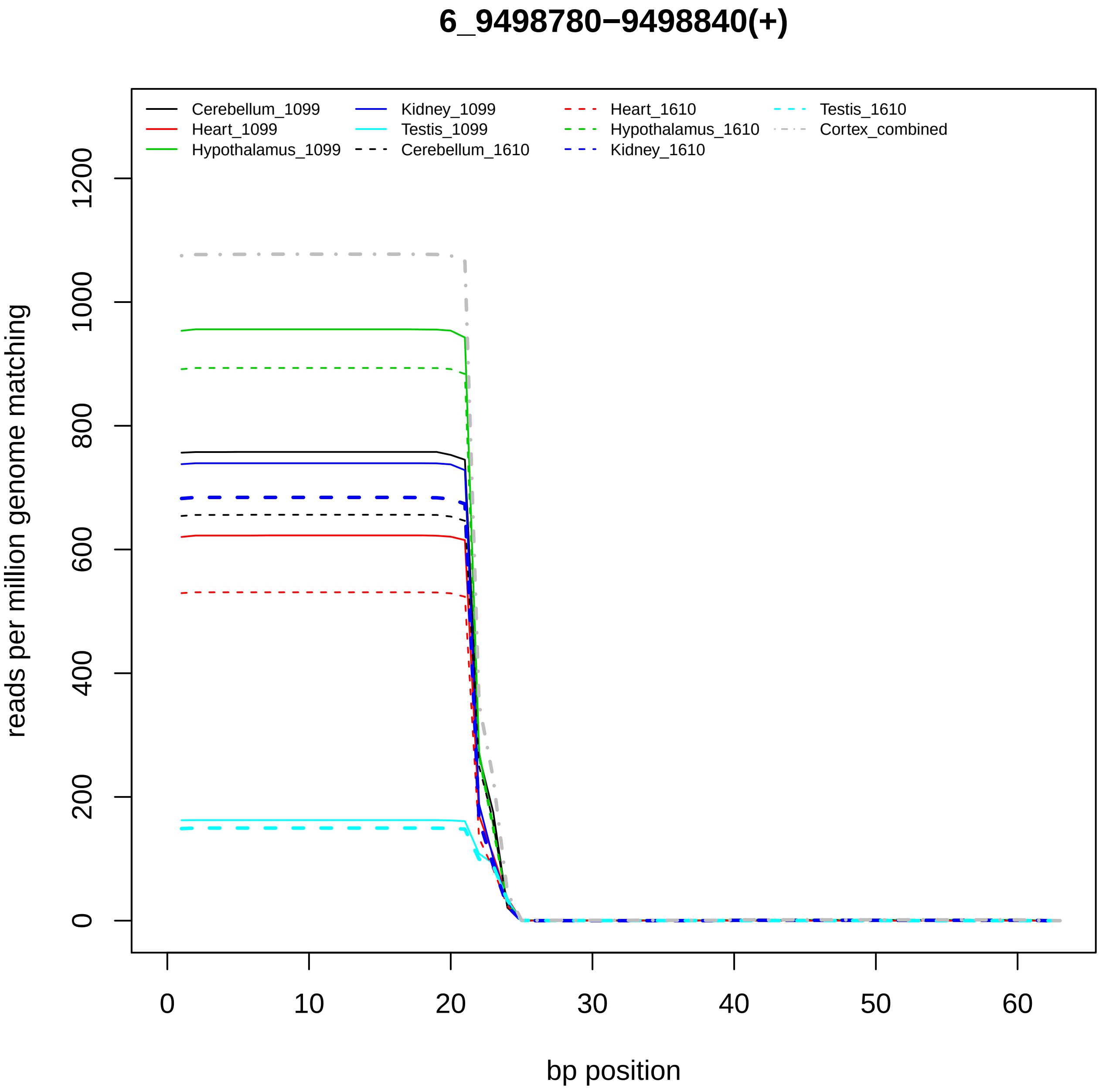

6_9498780−9498840(+)
Cerebellum_1099
Heart_1099
Hypothalamus_1099
Kidney_1099
Testis_1099
Cerebellum_1610
Heart_1610
Hypothalamus_1610
Kidney_1610
Testis_1610
Cortex_combined
reads per million genome matching
0
200
400
600
800
1000
1200
bp position
0
10
20
30
40
50
60

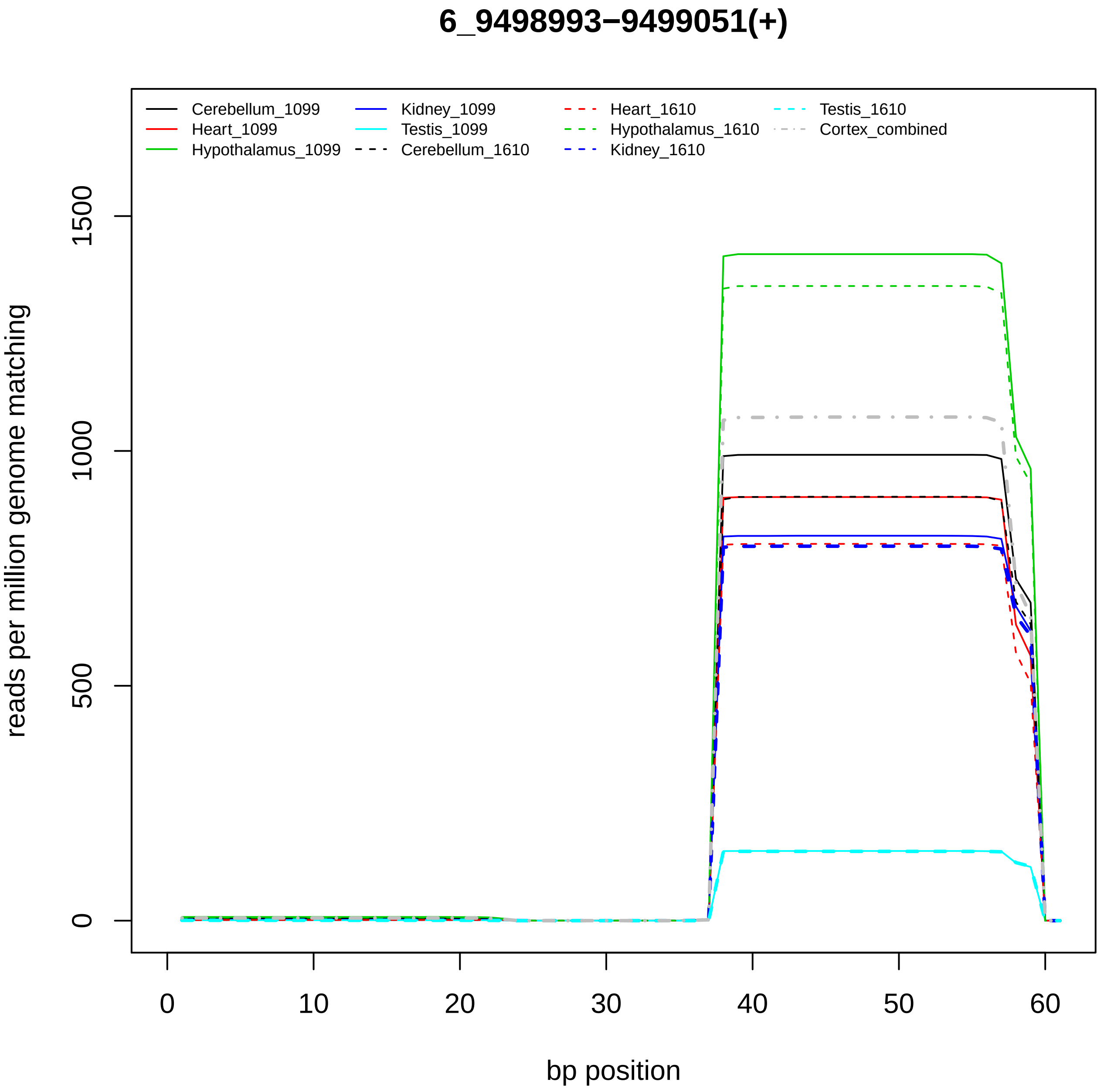

6_9498993−9499051(+)
Cerebellum_1099
Heart_1099
Hypothalamus_1099
Kidney_1099
Testis_1099
Cerebellum_1610
Heart_1610
Hypothalamus_1610
Kidney_1610
Testis_1610
Cortex_combined
reads per million genome matching
0
500
1000
1500
0
10
20
30
40
50
60
bp position

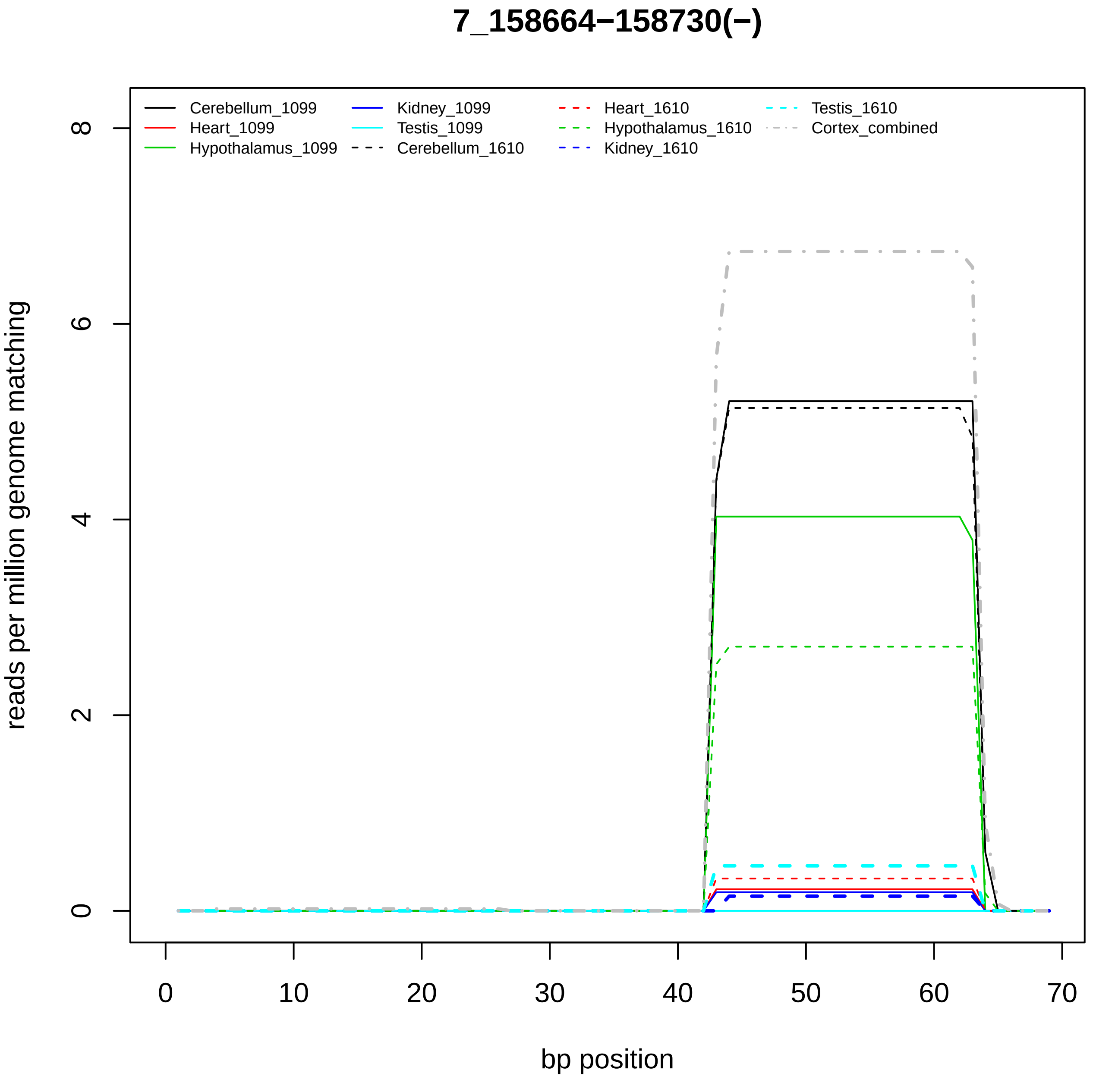

7_158664−158730(−)
Cerebellum_1099
Heart_1099
Hypothalamus_1099
Kidney_1099
Testis_1099
Cerebellum_1610
Heart_1610
Hypothalamus_1610
Kidney_1610
Testis_1610
Cortex_combined
reads per million genome matching
bp position
0
2
4
6
8
0
10
20
30
40
50
60
70

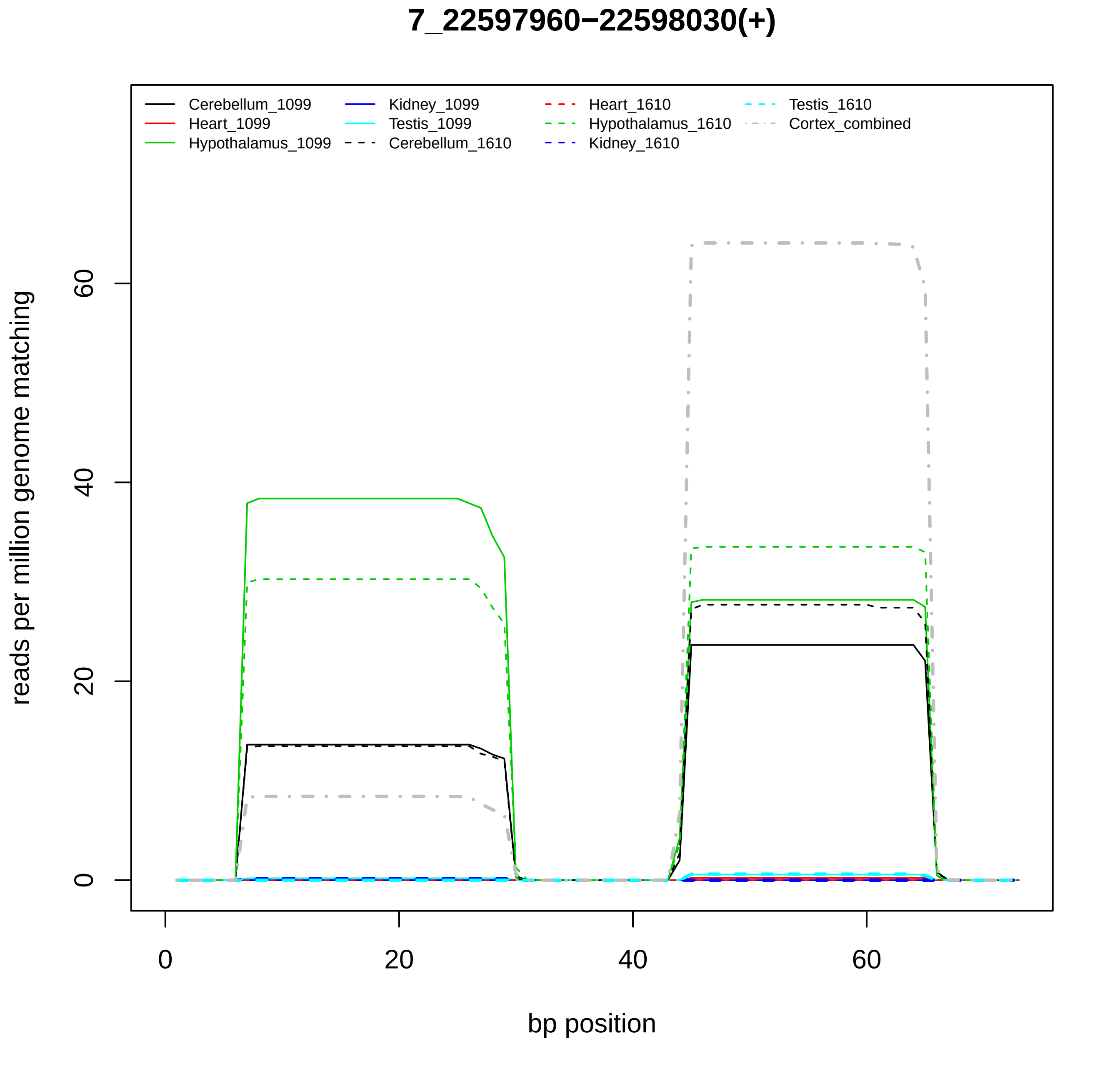

7_22597960−22598030(+)
Cerebellum_1099
Heart_1099
Hypothalamus_1099
Kidney_1099
Testis_1099
Cerebellum_1610
Heart_1610
Hypothalamus_1610
Kidney_1610
Testis_1610
Cortex_combined
reads per million genome matching
bp position
0
20
40
60
0
20
40
60

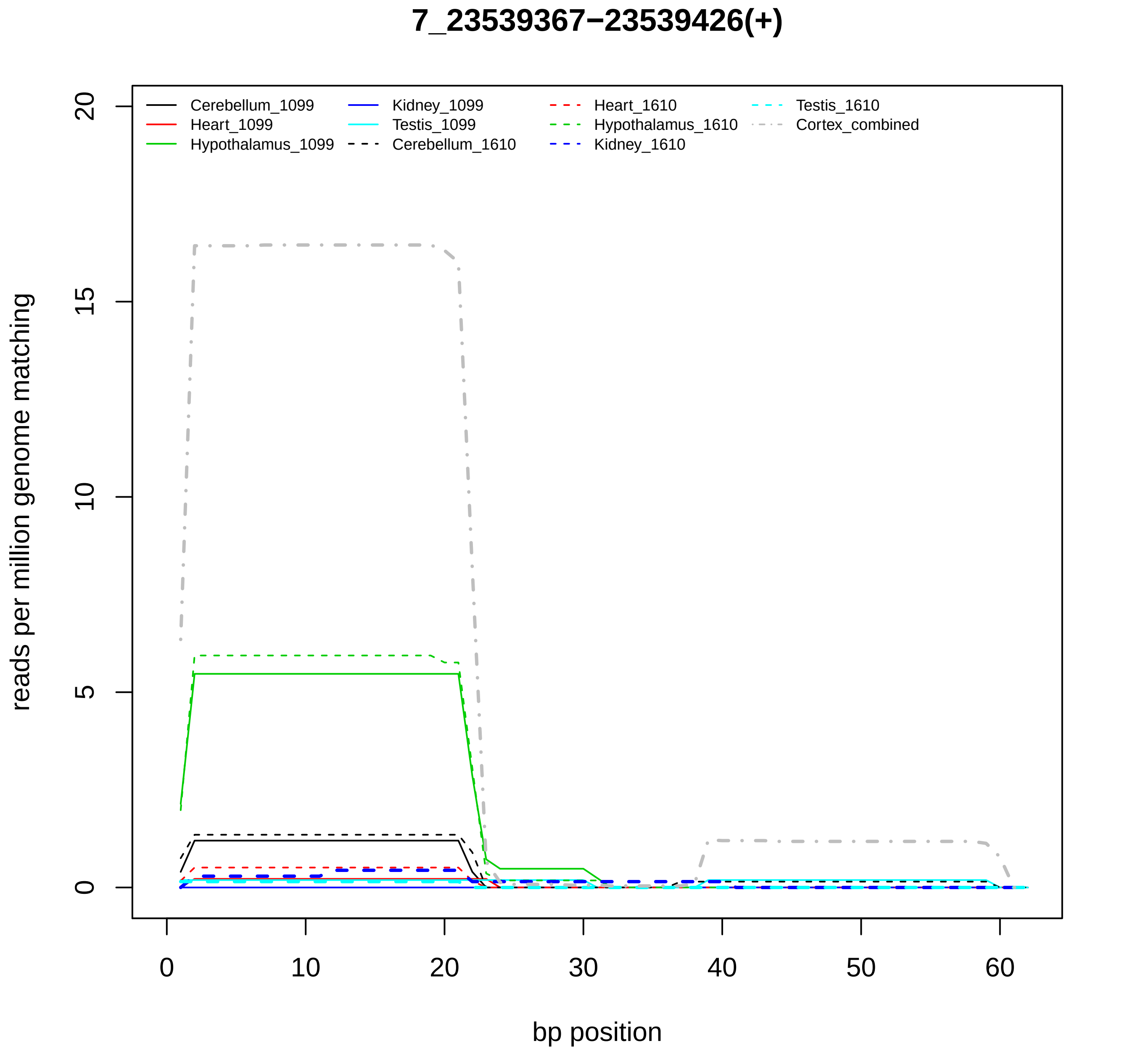

7_23539367−23539426(+)
Cerebellum_1099
Heart_1099
Hypothalamus_1099
Kidney_1099
Testis_1099
Cerebellum_1610
Heart_1610
Hypothalamus_1610
Kidney_1610
Testis_1610
Cortex_combined
reads per million genome matching
bp position
0
5
10
15
20
0
10
20
30
40
50
60

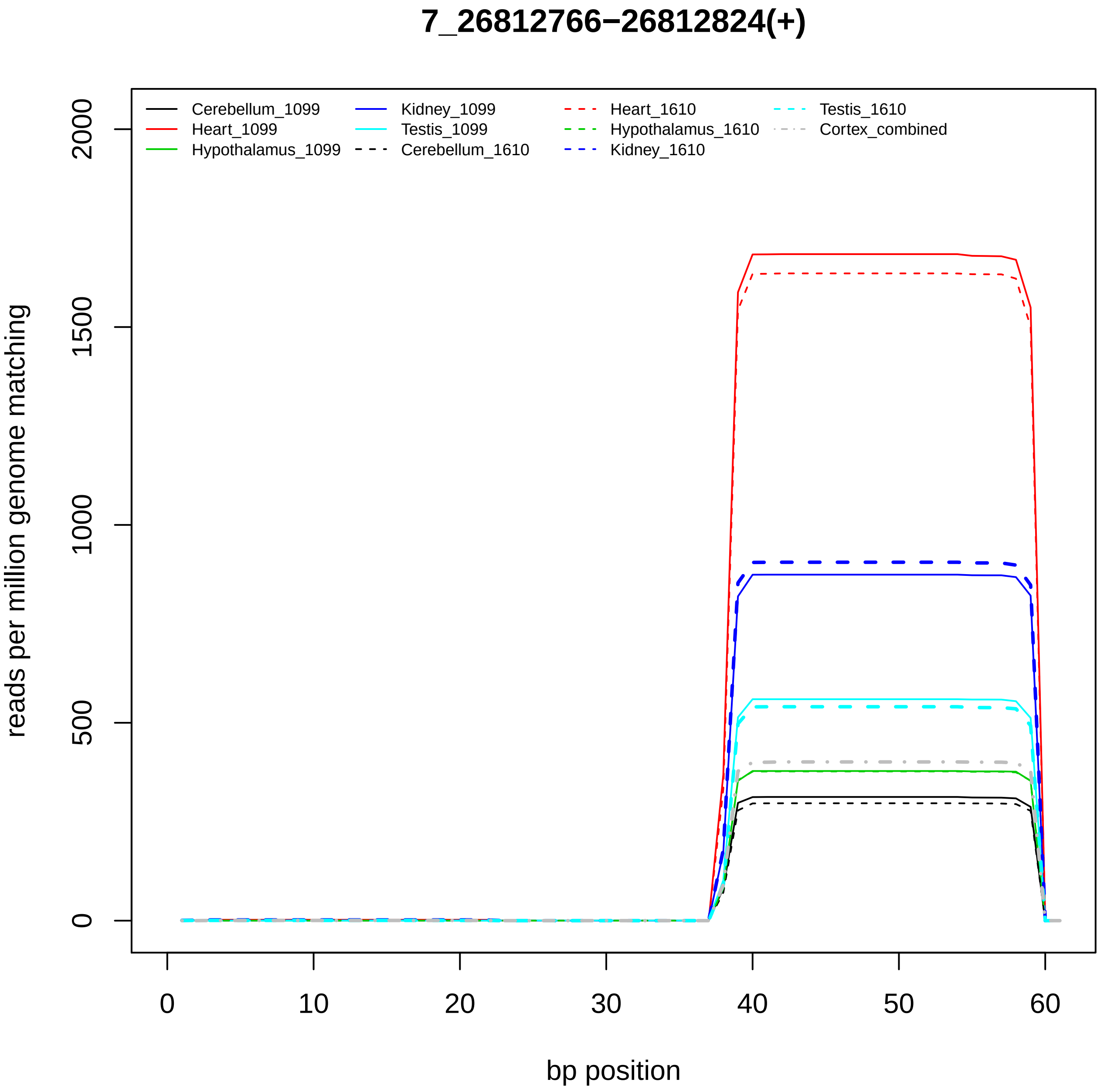

7_26812766−26812824(+)
Cerebellum_1099
Heart_1099
Hypothalamus_1099
Kidney_1099
Testis_1099
Cerebellum_1610
Heart_1610
Hypothalamus_1610
Kidney_1610
Testis_1610
Cortex_combined
reads per million genome matching
0
500
1000
1500
2000
bp position
0
10
20
30
40
50
60

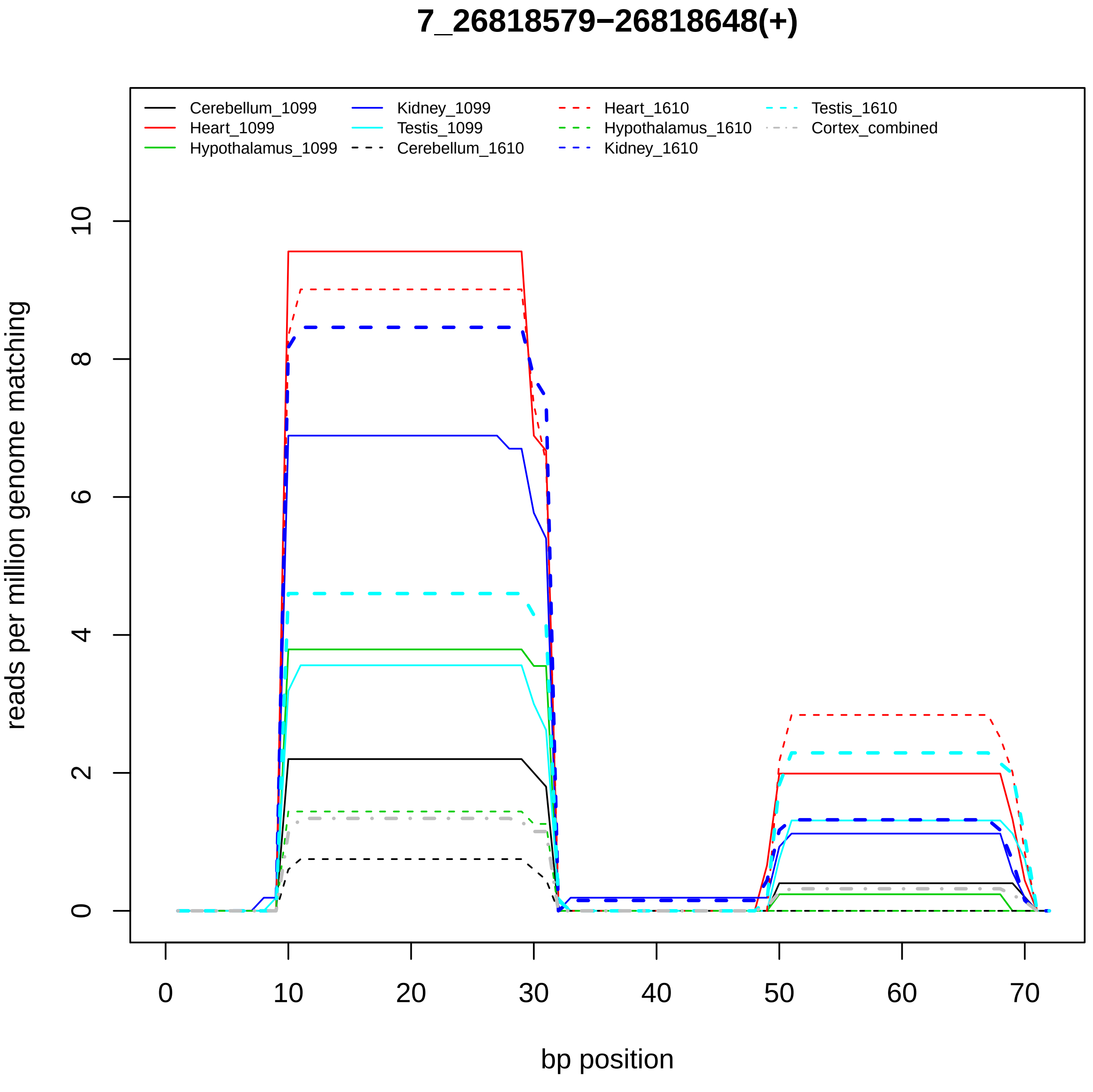
7_26818579−26818648(+)
Cerebellum_1099
Heart_1099
Hypothalamus_1099
Kidney_1099
Testis_1099
Cerebellum_1610
Heart_1610
Hypothalamus_1610
Kidney_1610
Testis_1610
Cortex_combined
reads per million genome matching
bp position
0
2
4
6
8
10
0
10
20
30
40
50
60
70

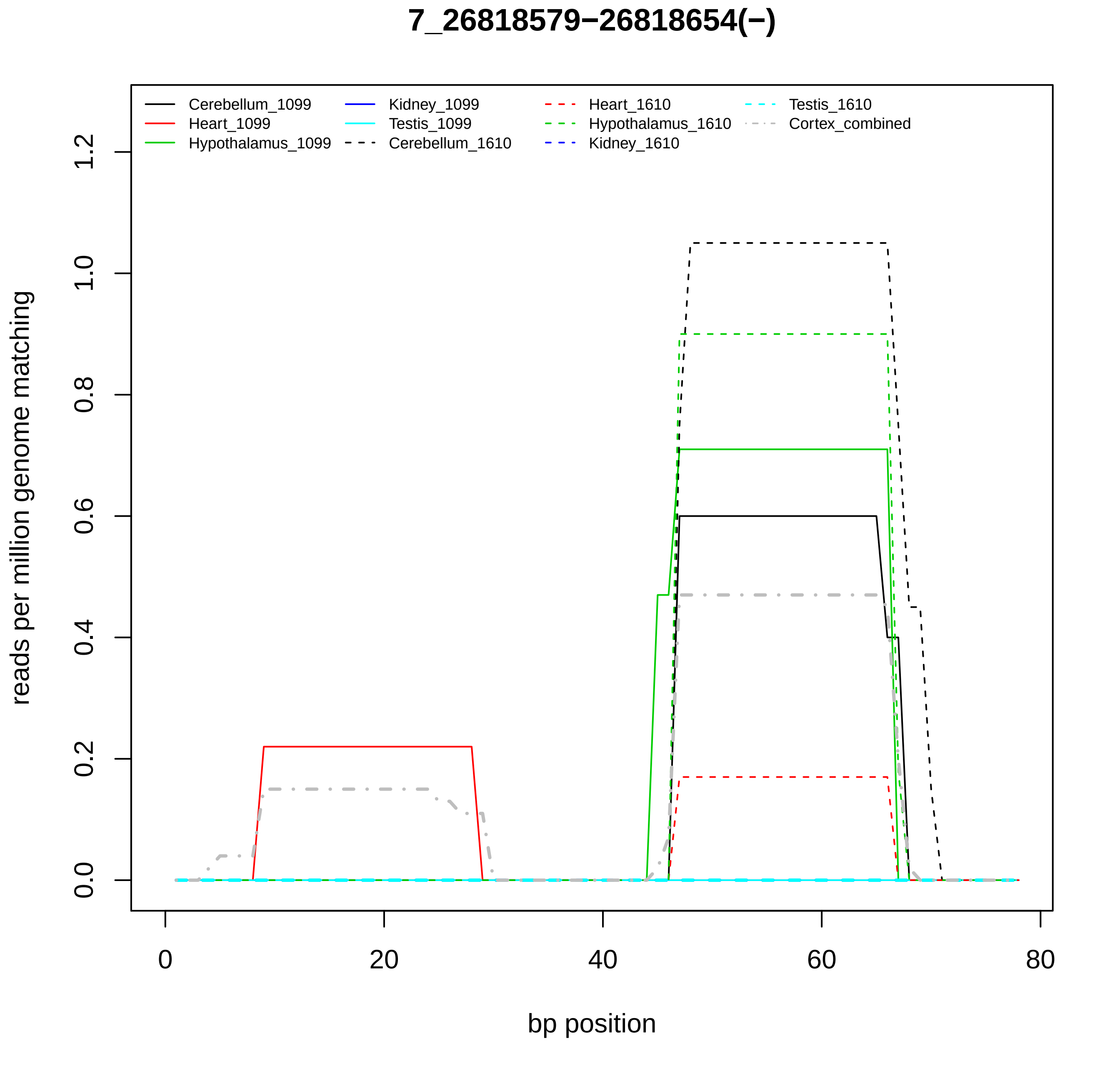

7_26818579−26818654(−)
Cerebellum_1099
Heart_1099
Hypothalamus_1099
Kidney_1099
Testis_1099
Cerebellum_1610
Heart_1610
Hypothalamus_1610
Kidney_1610
Testis_1610
Cortex_combined
reads per million genome matching
bp position
0.0
0.2
0.4
0.6
0.8
1.0
1.2
0
20
40
60
80

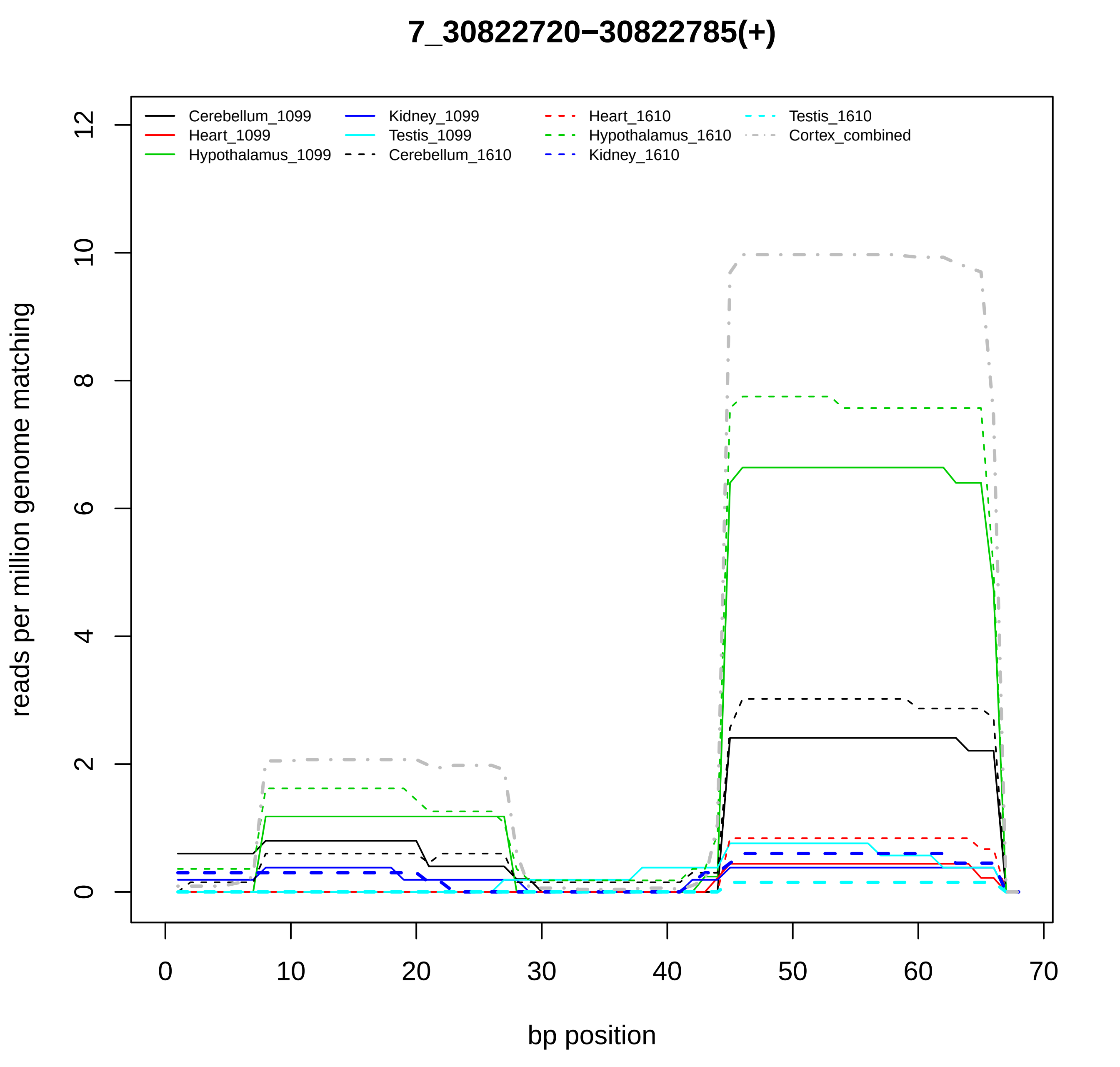
7_30822720−30822785(+)
Cerebellum_1099
Heart_1099
Hypothalamus_1099
Kidney_1099
Testis_1099
Cerebellum_1610
Heart_1610
Hypothalamus_1610
Kidney_1610
Testis_1610
Cortex_combined
reads per million genome matching
bp position
0
2
4
6
8
10
12
0
10
20
30
40
50
60
70

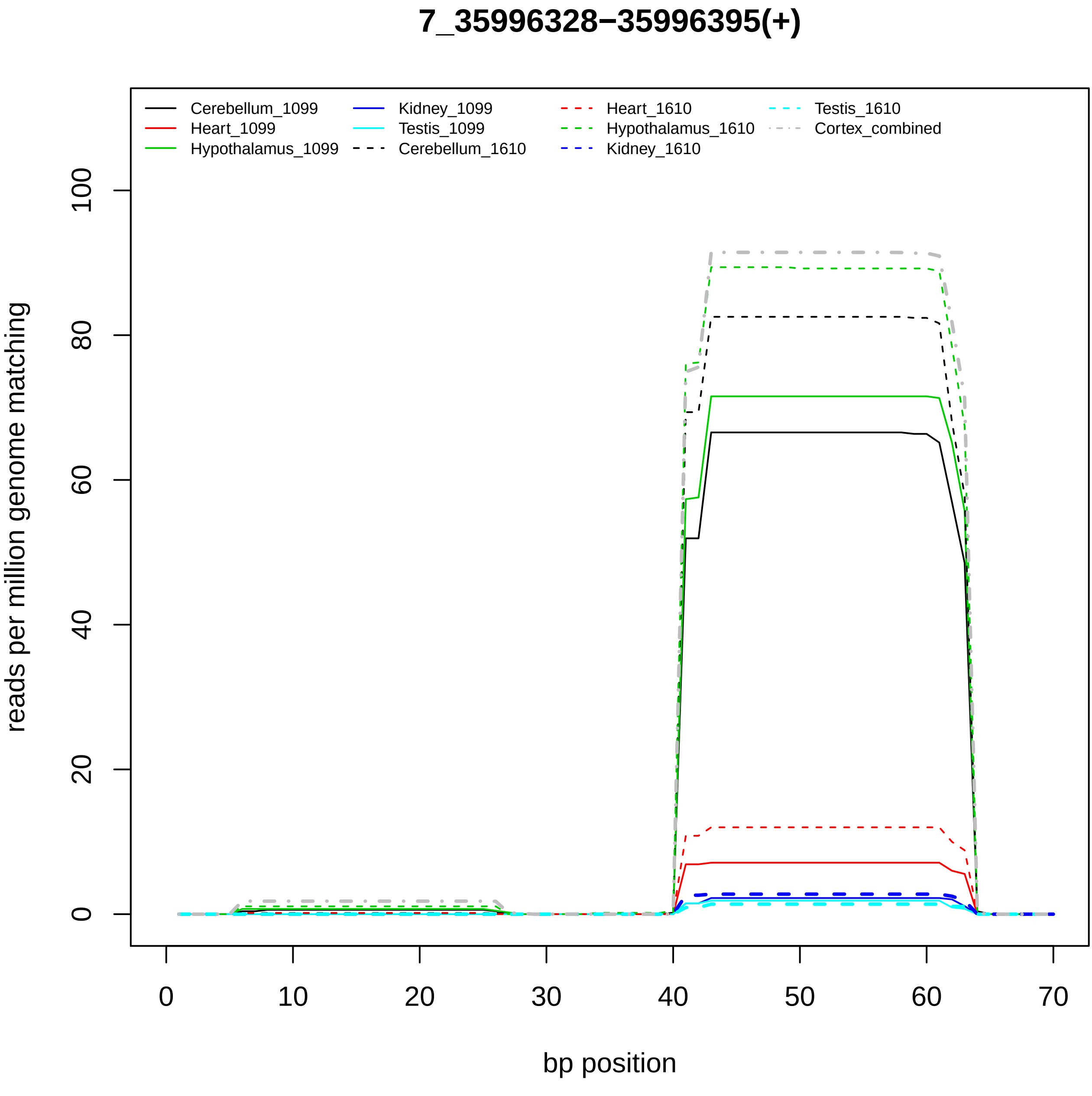

7_35996328−35996395(+)
Cerebellum_1099
Heart_1099
Hypothalamus_1099
Kidney_1099
Testis_1099
Cerebellum_1610
Heart_1610
Hypothalamus_1610
Kidney_1610
Testis_1610
Cortex_combined
reads per million genome matching
bp position
0
20
40
60
80
100
0
10
20
30
40
50
60
70

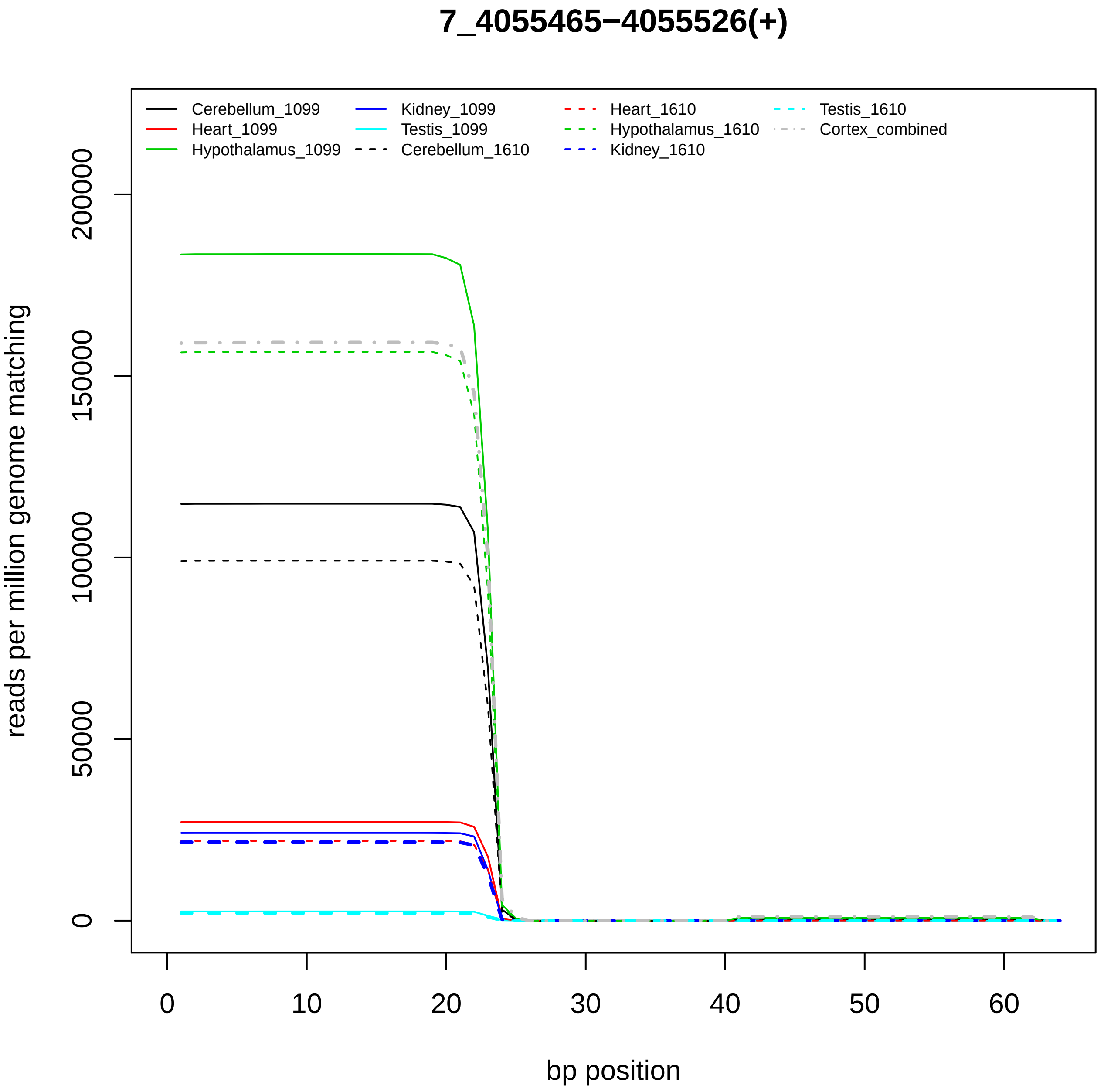

7_4055465−4055526(+)
Cerebellum_1099
Heart_1099
Hypothalamus_1099
Kidney_1099
Testis_1099
Cerebellum_1610
Heart_1610
Hypothalamus_1610
Kidney_1610
Testis_1610
Cortex_combined
reads per million genome matching
0
50000
100000
150000
200000
0
10
20
30
40
50
60
bp position

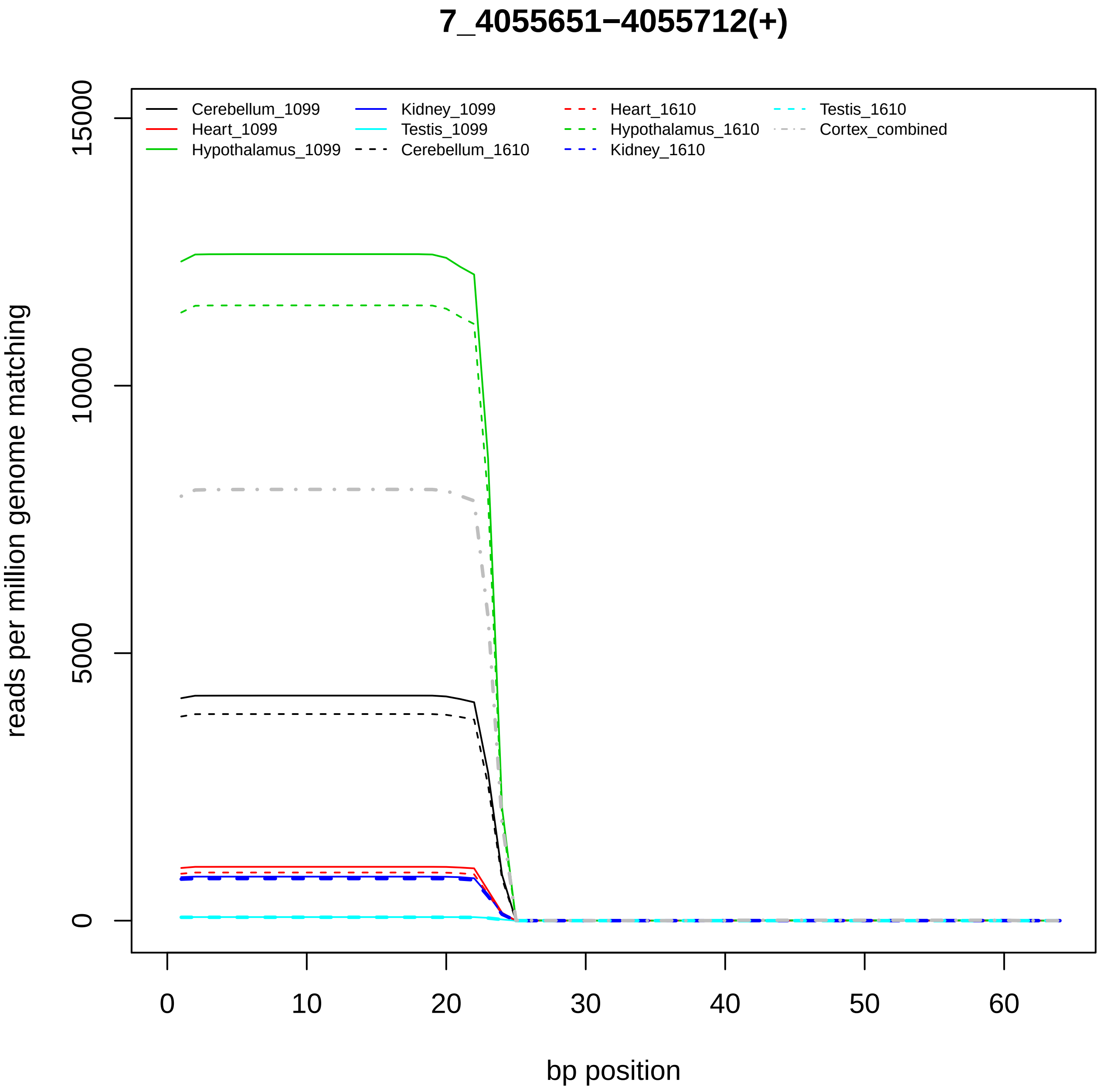

7_4055651−4055712(+)
Cerebellum_1099
Heart_1099
Hypothalamus_1099
Kidney_1099
Testis_1099
Cerebellum_1610
Heart_1610
Hypothalamus_1610
Kidney_1610
Testis_1610
Cortex_combined
reads per million genome matching
bp position
0
5000
10000
15000
0
10
20
30
40
50
60

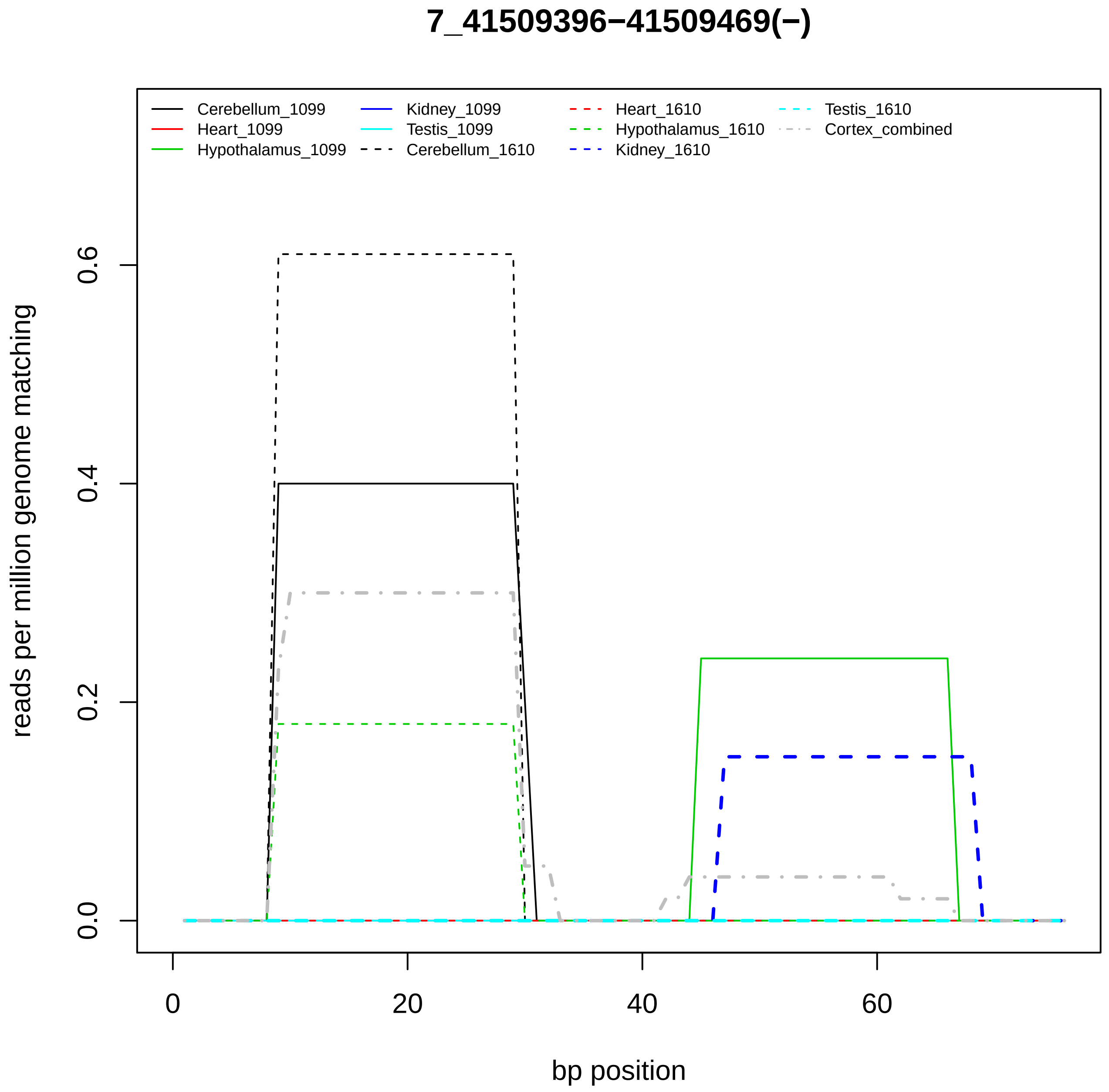

7_41509396−41509469(−)
Cerebellum_1099
Heart_1099
Hypothalamus_1099
Kidney_1099
Testis_1099
Cerebellum_1610
Heart_1610
Hypothalamus_1610
Kidney_1610
Testis_1610
Cortex_combined
reads per million genome matching
0.0
0.2
0.4
0.6
0
20
40
60
bp position

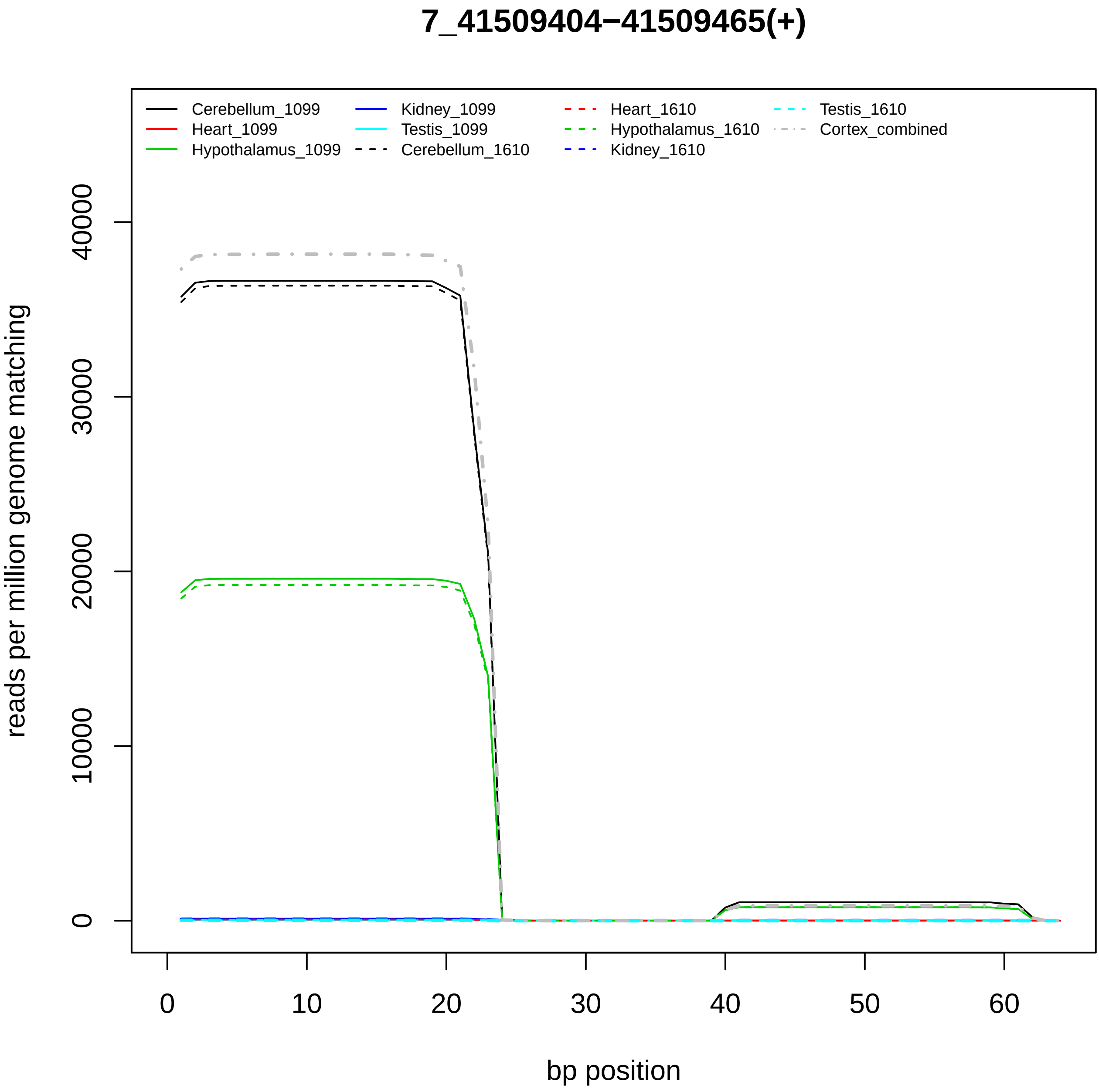

7_41509404−41509465(+)
Cerebellum_1099
Heart_1099
Hypothalamus_1099
Kidney_1099
Testis_1099
Cerebellum_1610
Heart_1610
Hypothalamus_1610
Kidney_1610
Testis_1610
Cortex_combined
reads per million genome matching
0
10000
20000
30000
40000
bp position
0
10
20
30
40
50
60

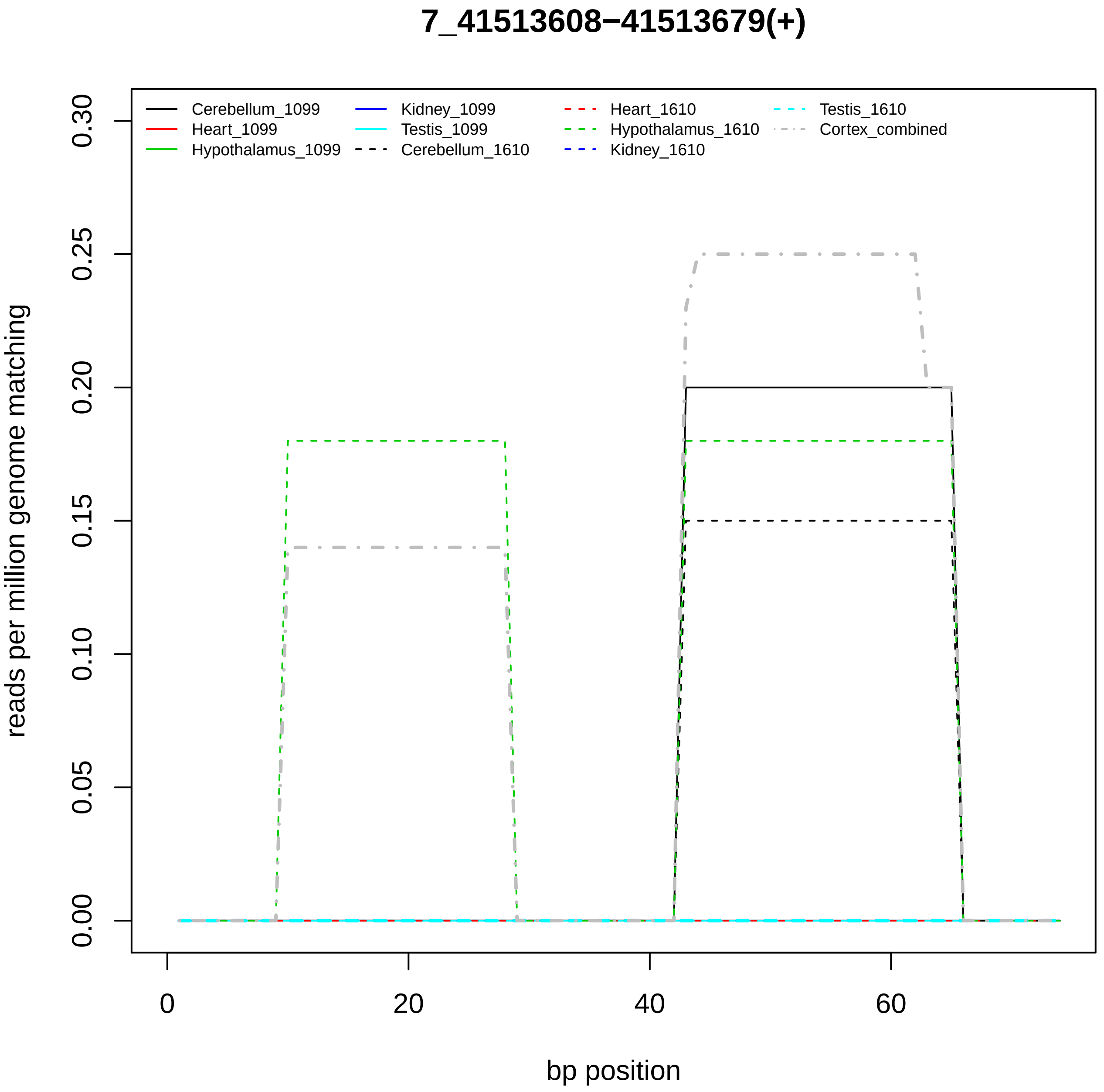

7_41513608−41513679(+)
Cerebellum_1099
Heart_1099
Hypothalamus_1099
Kidney_1099
Testis_1099
Cerebellum_1610
Heart_1610
Hypothalamus_1610
Kidney_1610
Testis_1610
Cortex_combined
reads per million genome matching
0.00
0.05
0.10
0.15
0.20
0.25
0.30
0
20
40
60
bp position

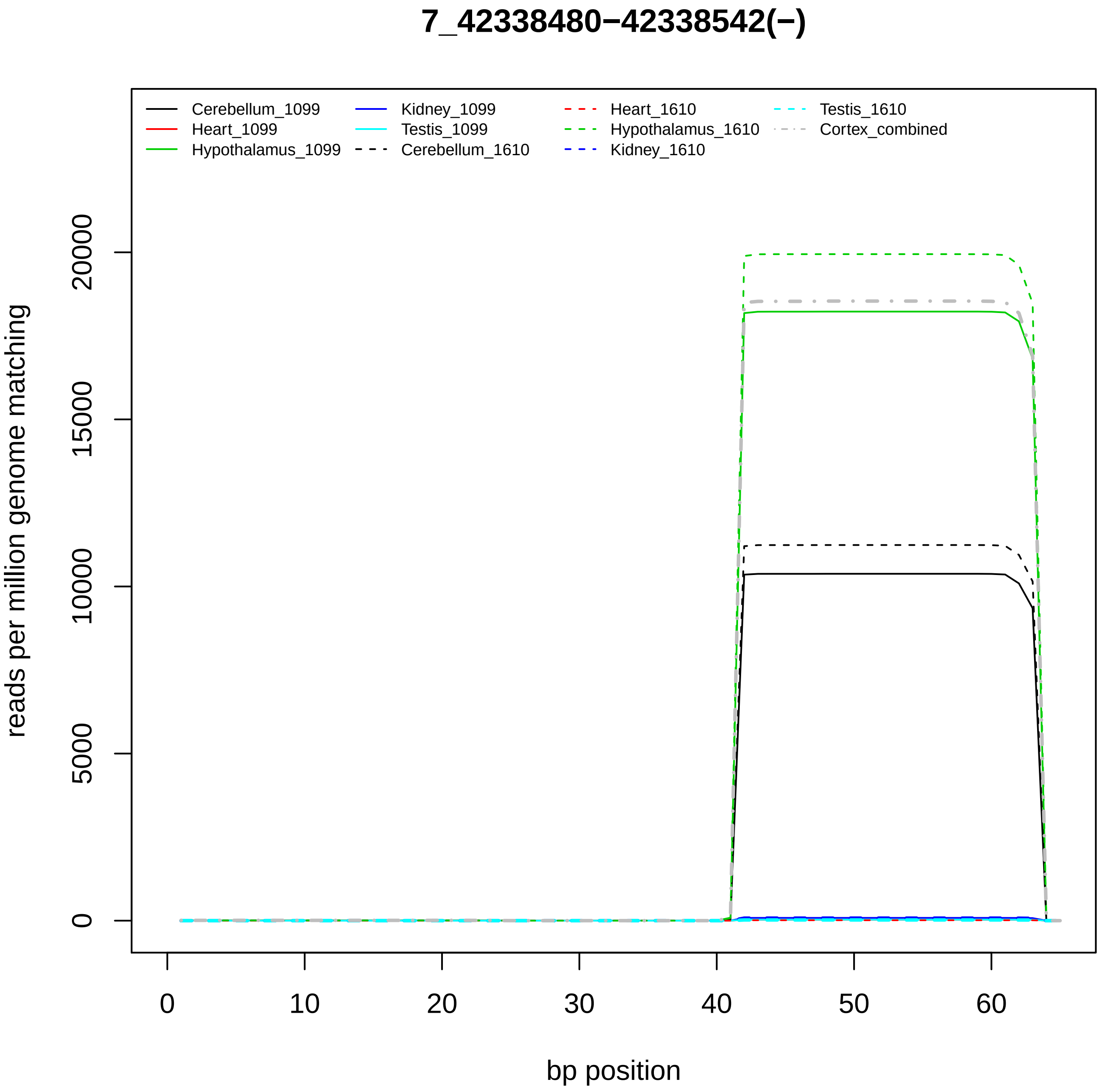
7_42338480−42338542(−)
Cerebellum_1099
Heart_1099
Hypothalamus_1099
Kidney_1099
Testis_1099
Cerebellum_1610
Heart_1610
Hypothalamus_1610
Kidney_1610
Testis_1610
Cortex_combined
reads per million genome matching
bp position
0
5000
10000
15000
20000
0
10
20
30
40
50
60

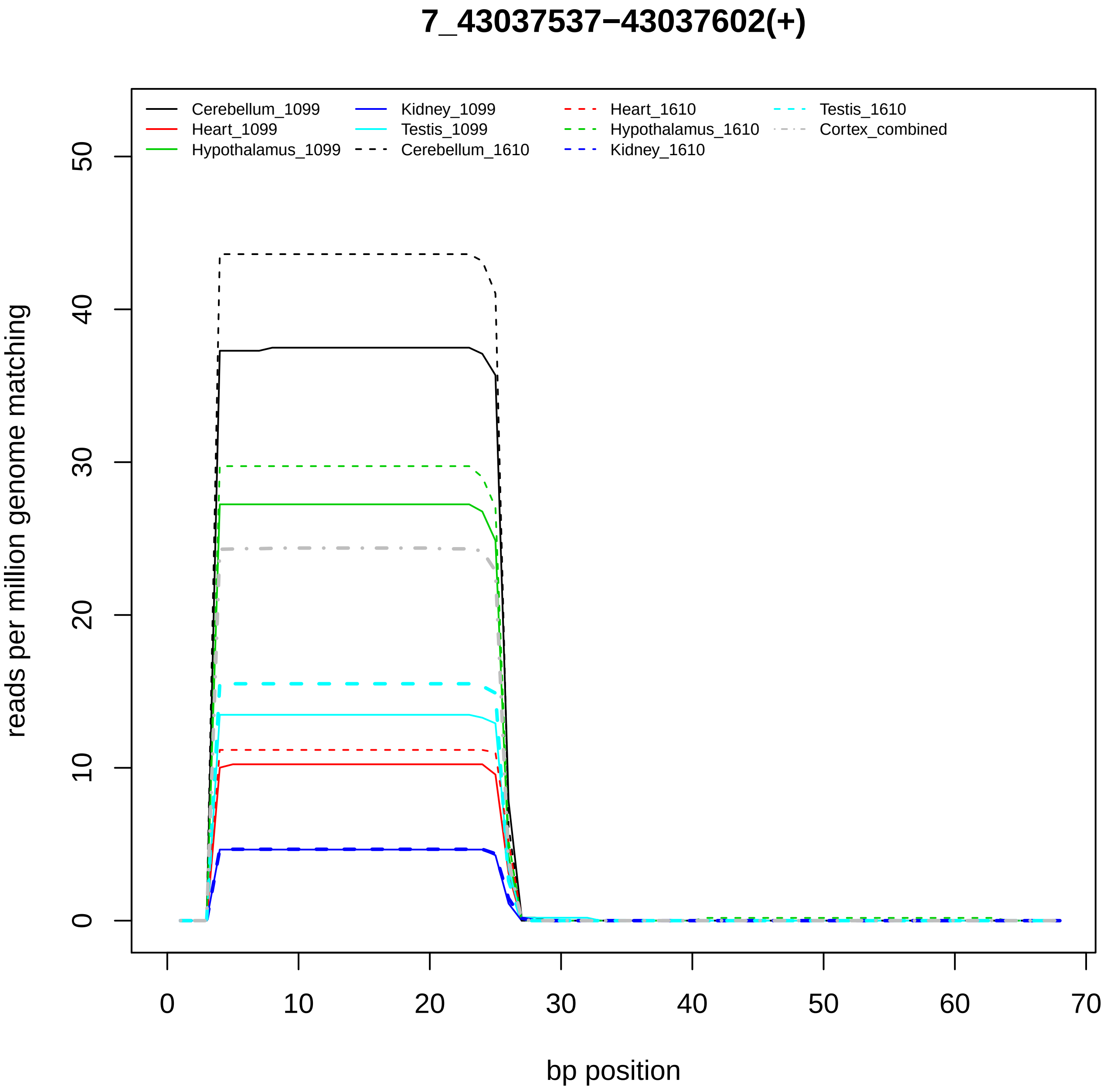
7_43037537−43037602(+)
Cerebellum_1099
Heart_1099
Hypothalamus_1099
Kidney_1099
Testis_1099
Cerebellum_1610
Heart_1610
Hypothalamus_1610
Kidney_1610
Testis_1610
Cortex_combined
reads per million genome matching
bp position
0
10
20
30
40
50
60
70

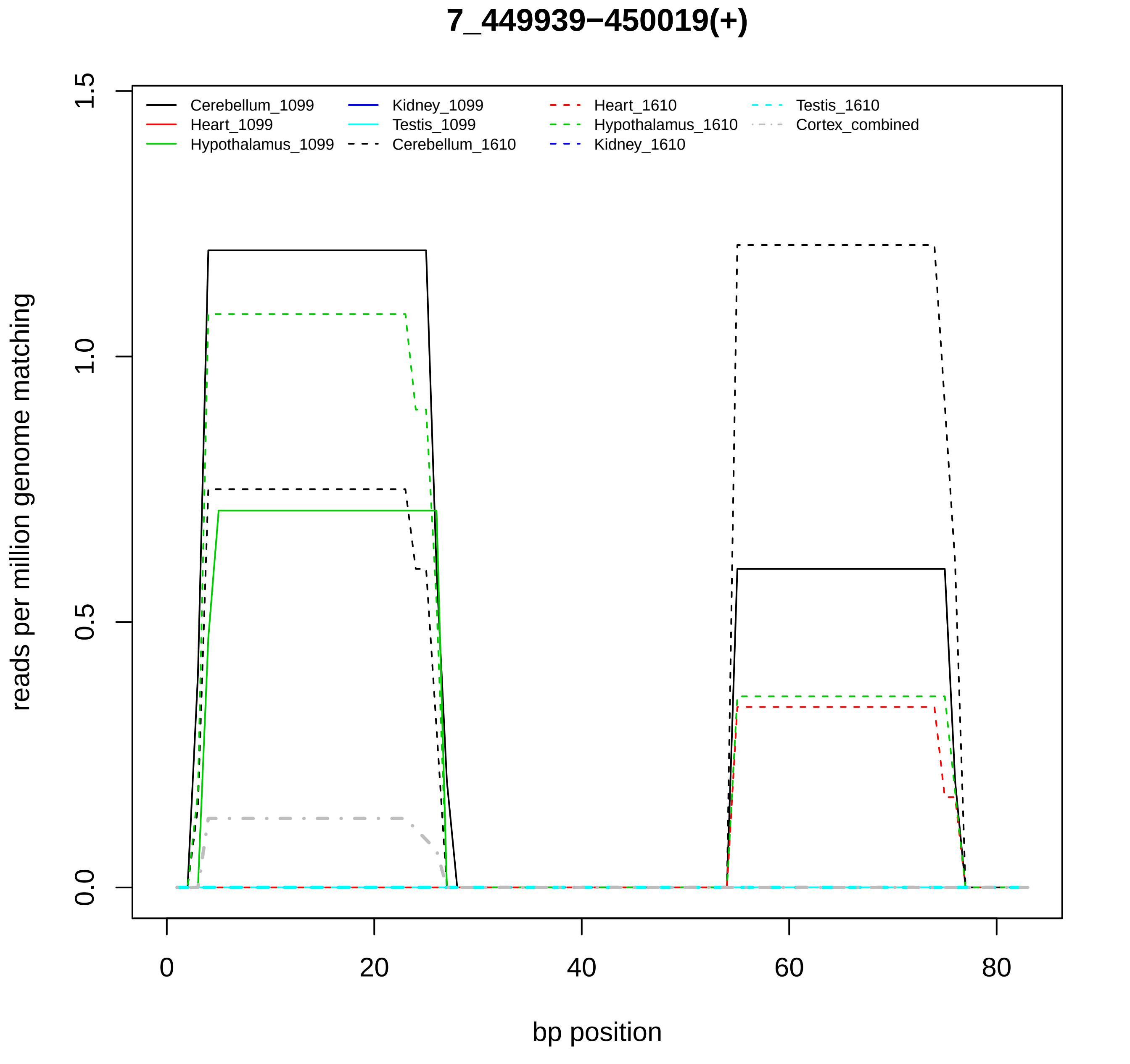
7_449939−450019(+)
Cerebellum_1099
Heart_1099
Hypothalamus_1099
Kidney_1099
Testis_1099
Cerebellum_1610
Heart_1610
Hypothalamus_1610
Kidney_1610
Testis_1610
Cortex_combined
reads per million genome matching
bp position
0.0
0.5
1.0
1.5
0
20
40
60
80

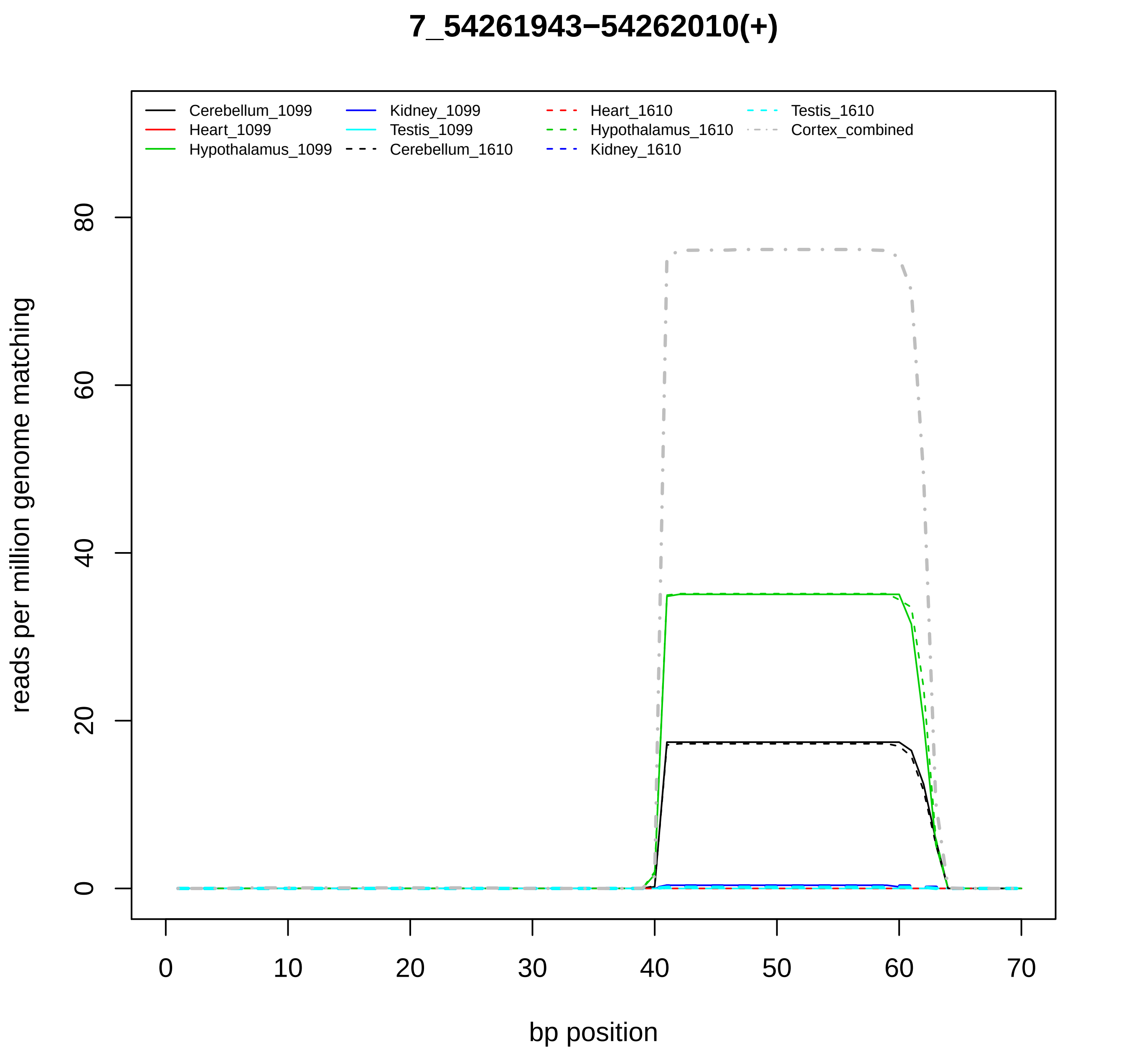

7_54261943−54262010(+)
Cerebellum_1099
Heart_1099
Hypothalamus_1099
Kidney_1099
Testis_1099
Cerebellum_1610
Heart_1610
Hypothalamus_1610
Kidney_1610
Testis_1610
Cortex_combined
reads per million genome matching
bp position
0
20
40
60
80
0
10
20
30
40
50
60
70

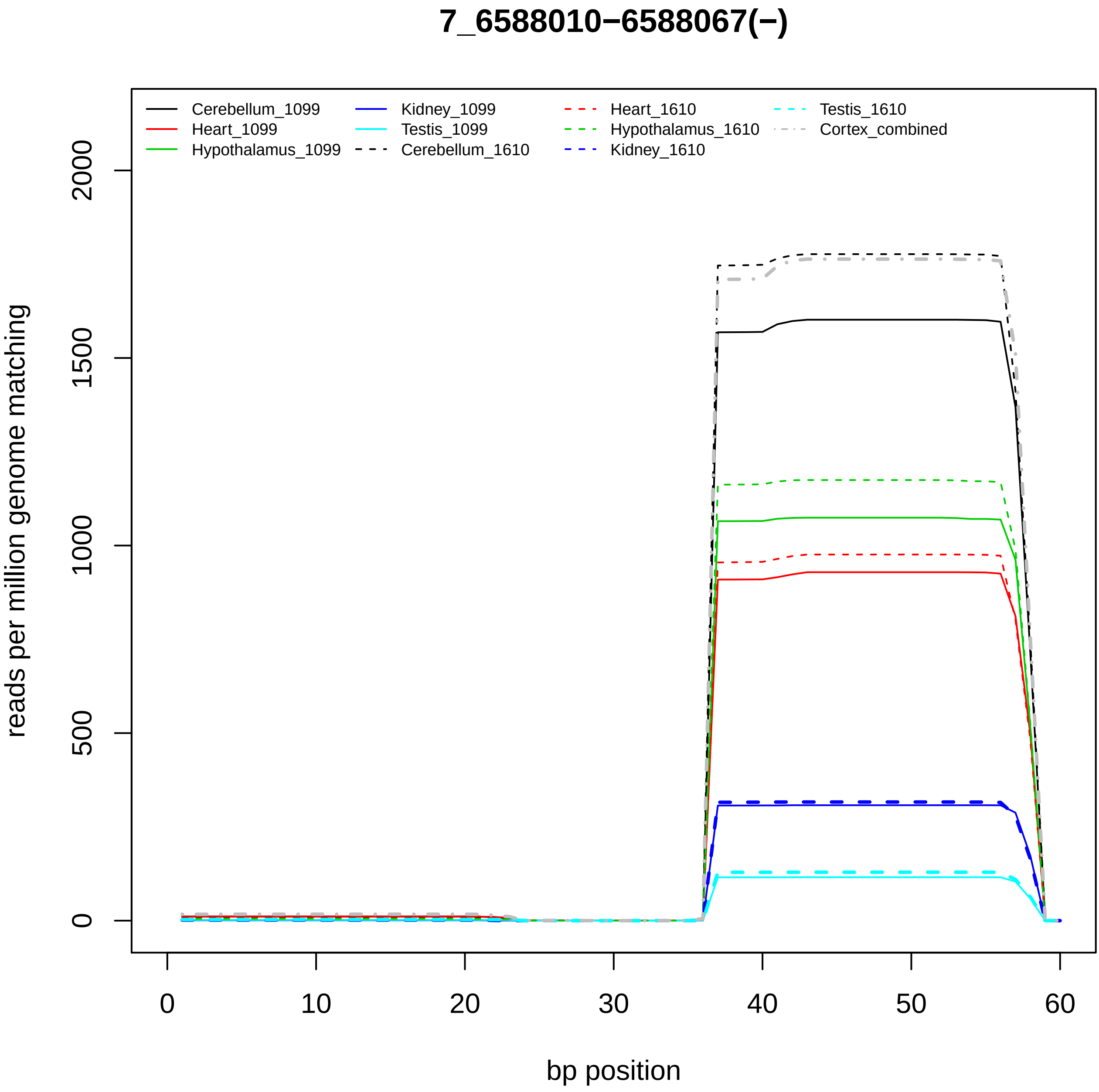

7_6588010−6588067(−)
Cerebellum_1099
Heart_1099
Hypothalamus_1099
Kidney_1099
Testis_1099
Cerebellum_1610
Heart_1610
Hypothalamus_1610
Kidney_1610
Testis_1610
Cortex_combined
reads per million genome matching
0
500
1000
1500
2000
bp position
0
10
20
30
40
50
60

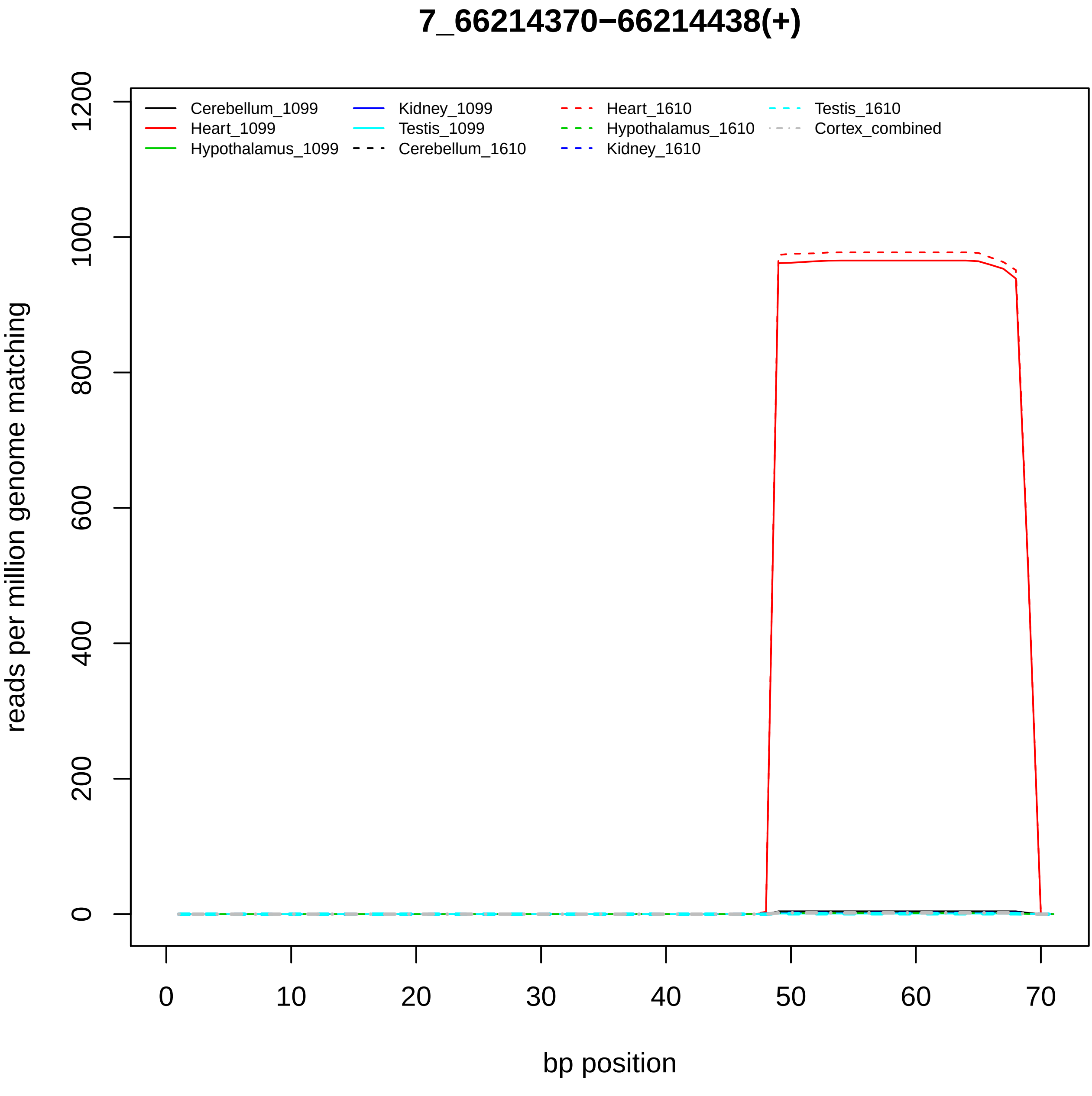

7_66214370−66214438(+)
Cerebellum_1099
Heart_1099
Hypothalamus_1099
Kidney_1099
Testis_1099
Cerebellum_1610
Heart_1610
Hypothalamus_1610
Kidney_1610
Testis_1610
Cortex_combined
reads per million genome matching
0
200
400
600
800
1000
1200
bp position
0
10
20
30
40
50
60
70

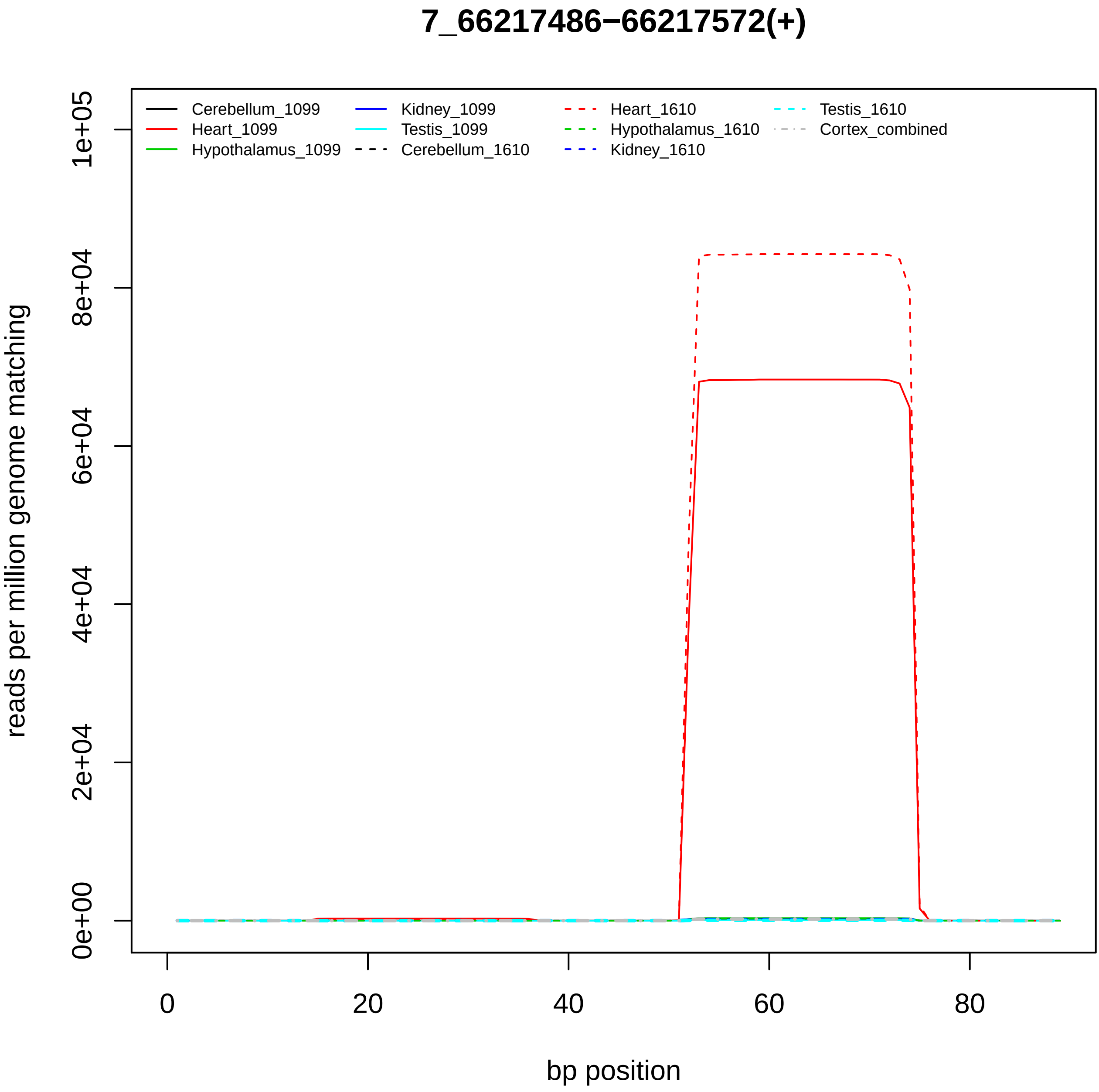

7_66217486−66217572(+)
Cerebellum_1099
Heart_1099
Hypothalamus_1099
Kidney_1099
Testis_1099
Cerebellum_1610
Heart_1610
Hypothalamus_1610
Kidney_1610
Testis_1610
Cortex_combined
reads per million genome matching
0e+00
2e+04
4e+04
6e+04
8e+04
1e+05
0
20
40
60
80
bp position

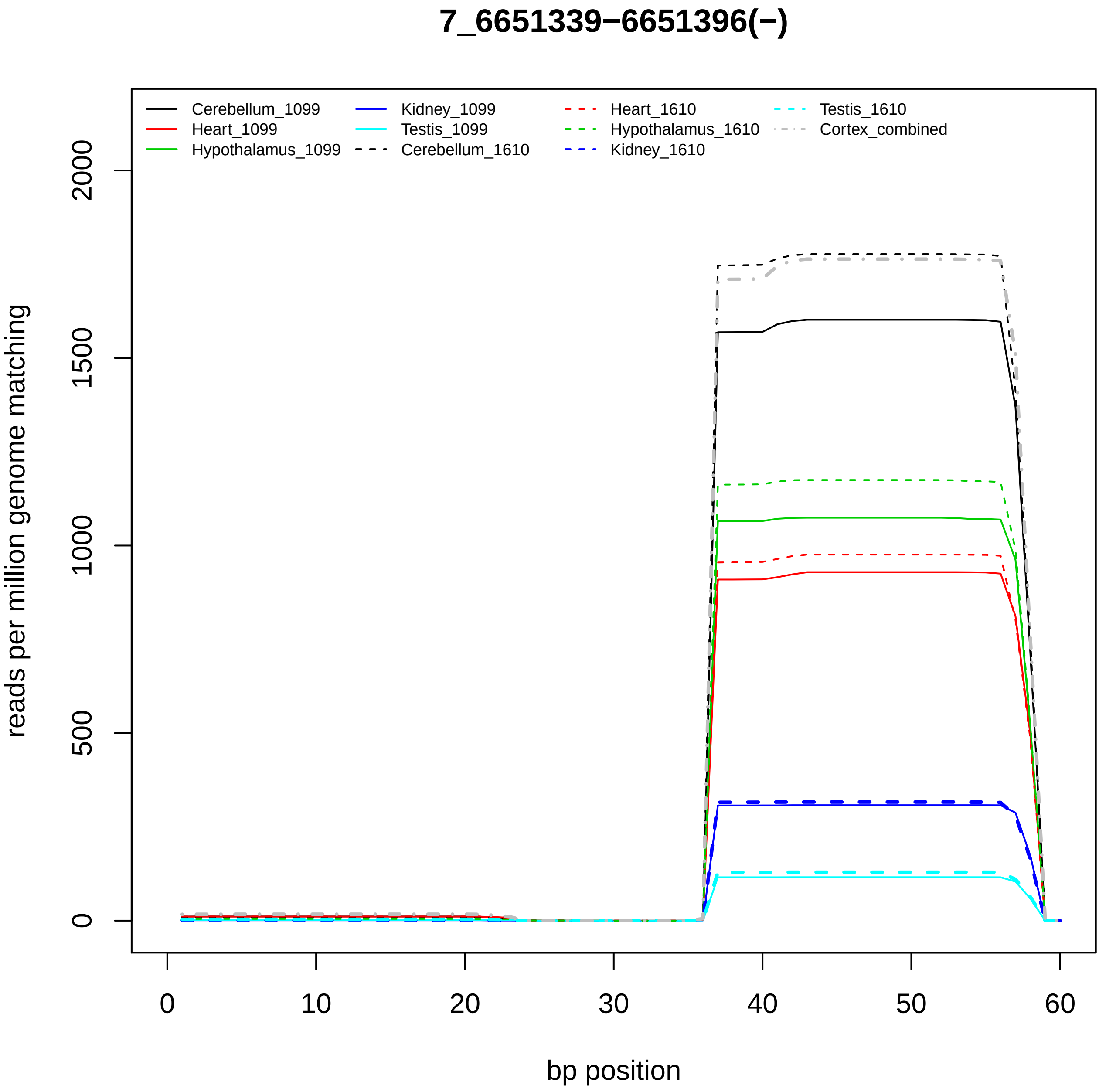

7_6651339−6651396(−)
Cerebellum_1099
Heart_1099
Hypothalamus_1099
Kidney_1099
Testis_1099
Cerebellum_1610
Heart_1610
Hypothalamus_1610
Kidney_1610
Testis_1610
Cortex_combined
reads per million genome matching
0
500
1000
1500
2000
bp position
0
10
20
30
40
50
60

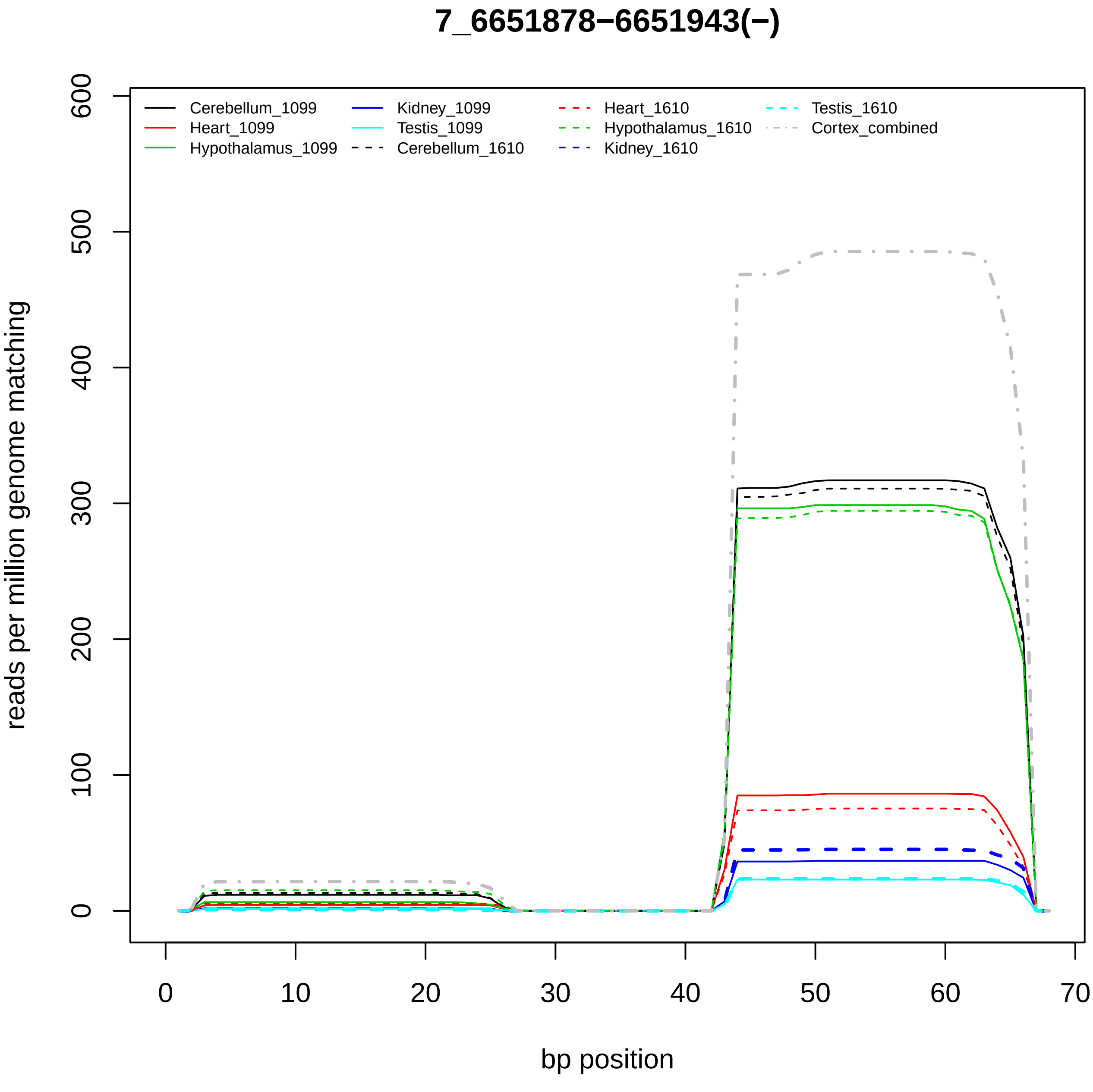

7_6651878−6651943(−)
Cerebellum_1099
Heart_1099
Hypothalamus_1099
Kidney_1099
Testis_1099
Cerebellum_1610
Heart_1610
Hypothalamus_1610
Kidney_1610
Testis_1610
Cortex_combined
reads per million genome matching
bp position
0
100
200
300
400
500
600
0
10
20
30
40
50
60
70

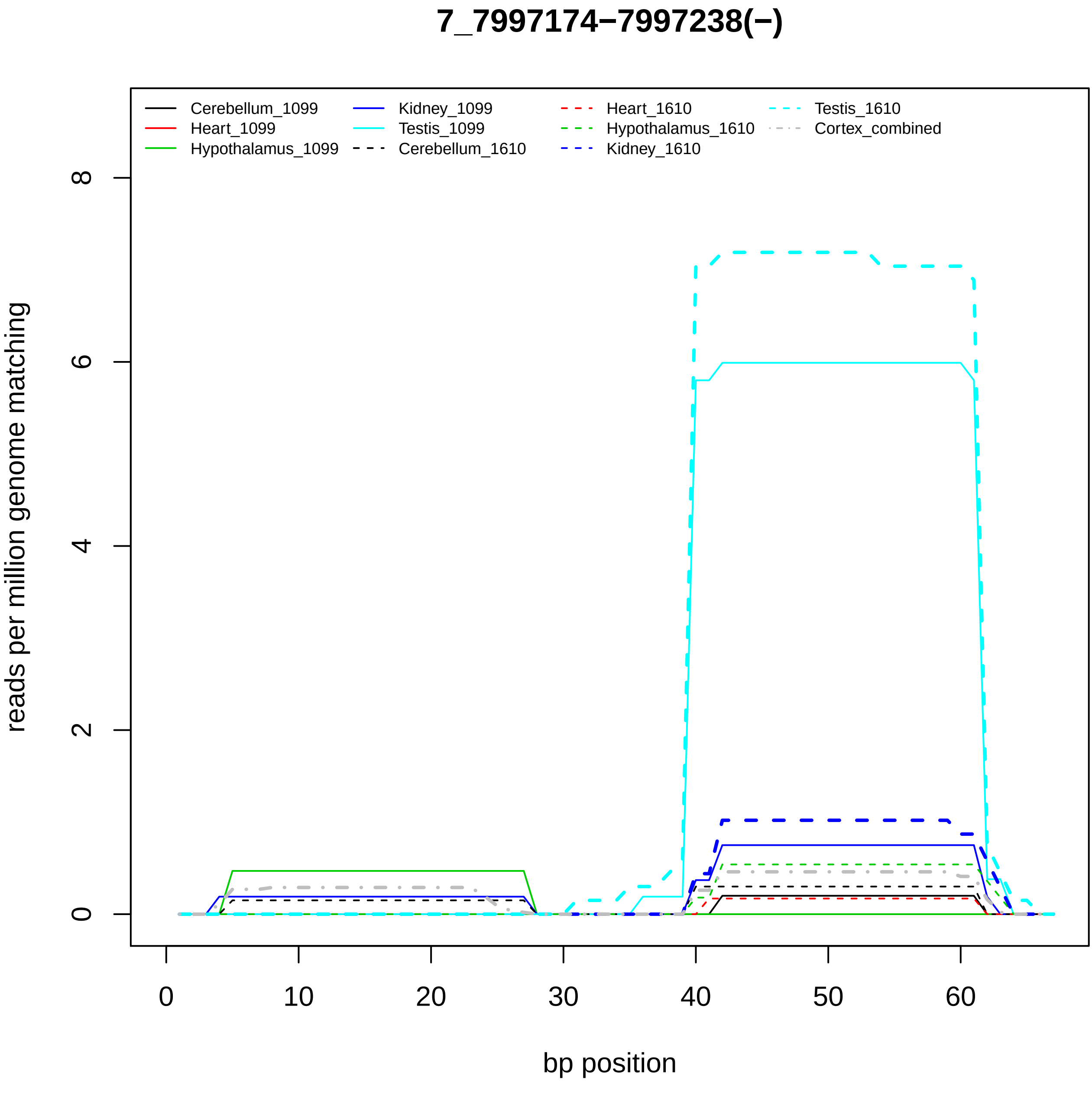

7_7997174−7997238(−)
Cerebellum_1099
Heart_1099
Hypothalamus_1099
Kidney_1099
Testis_1099
Cerebellum_1610
Heart_1610
Hypothalamus_1610
Kidney_1610
Testis_1610
Cortex_combined
reads per million genome matching
0
2
4
6
8
0
10
20
30
40
50
60
bp position

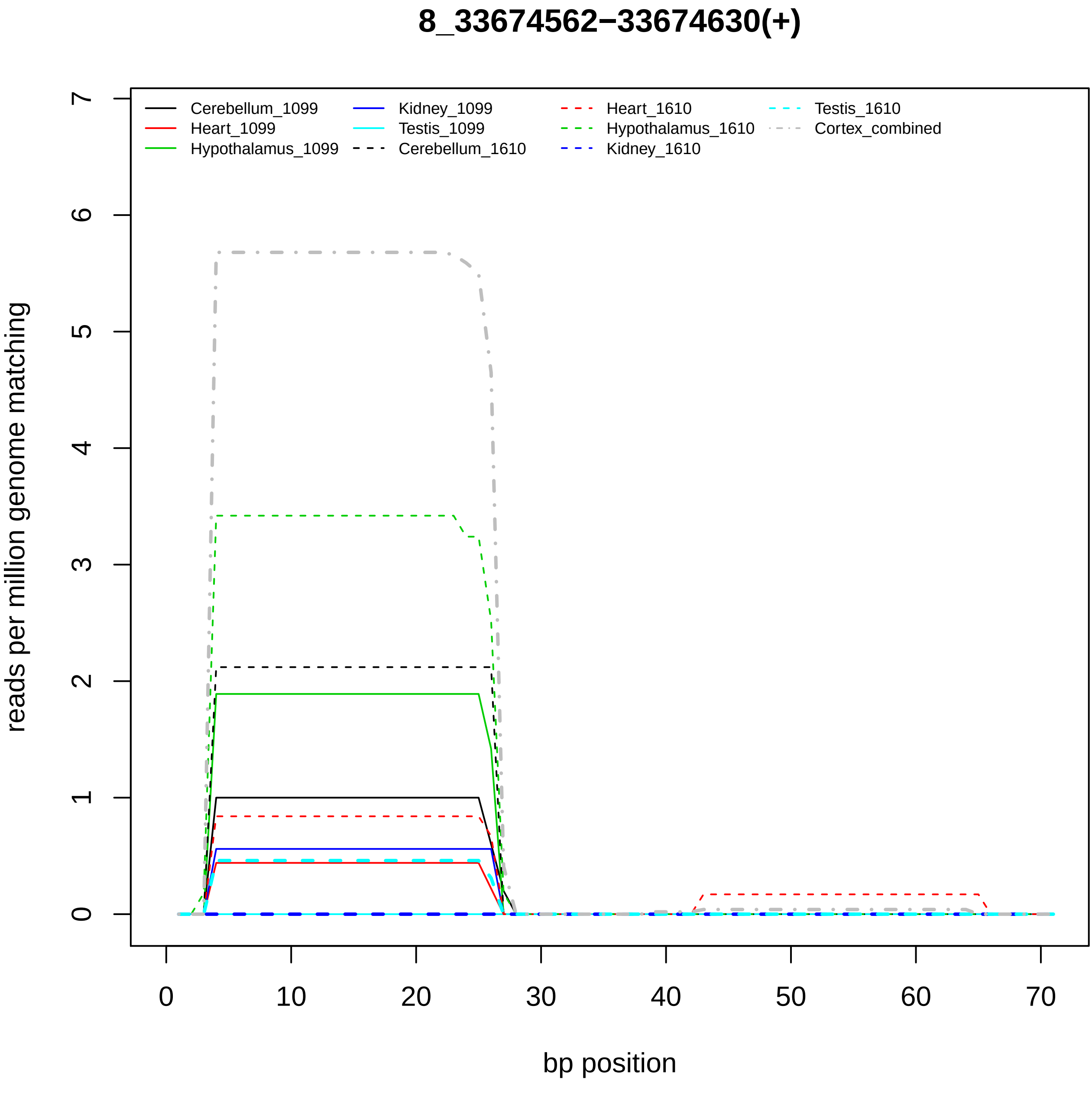

8_33674562−33674630(+)
Cerebellum_1099
Heart_1099
Hypothalamus_1099
Kidney_1099
Testis_1099
Cerebellum_1610
Heart_1610
Hypothalamus_1610
Kidney_1610
Testis_1610
Cortex_combined
reads per million genome matching
bp position
0
1
2
3
4
5
6
7
0
10
20
30
40
50
60
70

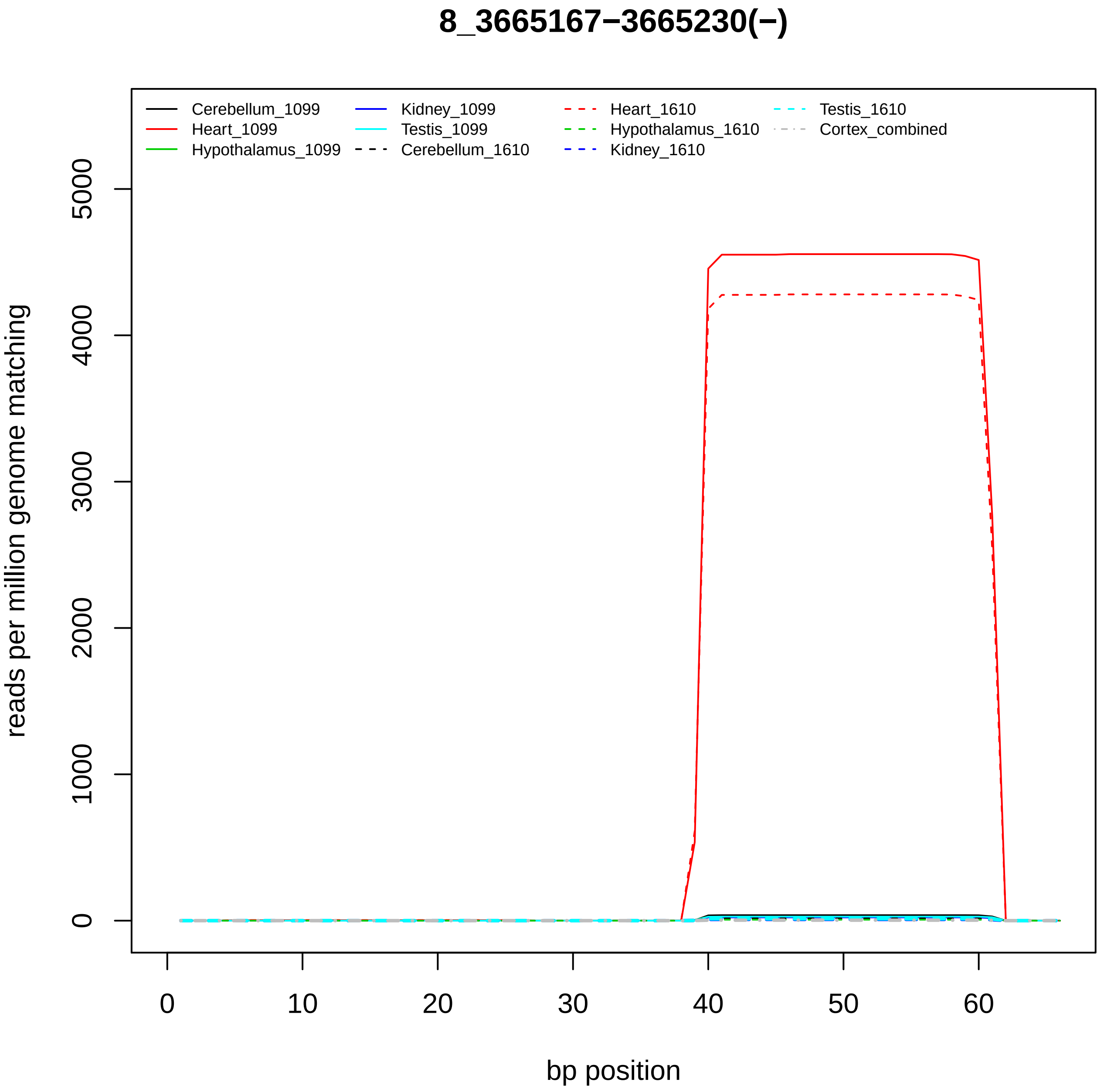

8_3665167−3665230(−)
Cerebellum_1099
Heart_1099
Hypothalamus_1099
Kidney_1099
Testis_1099
Cerebellum_1610
Heart_1610
Hypothalamus_1610
Kidney_1610
Testis_1610
Cortex_combined
reads per million genome matching
0
1000
2000
3000
4000
5000
0
10
20
30
40
50
60
bp position

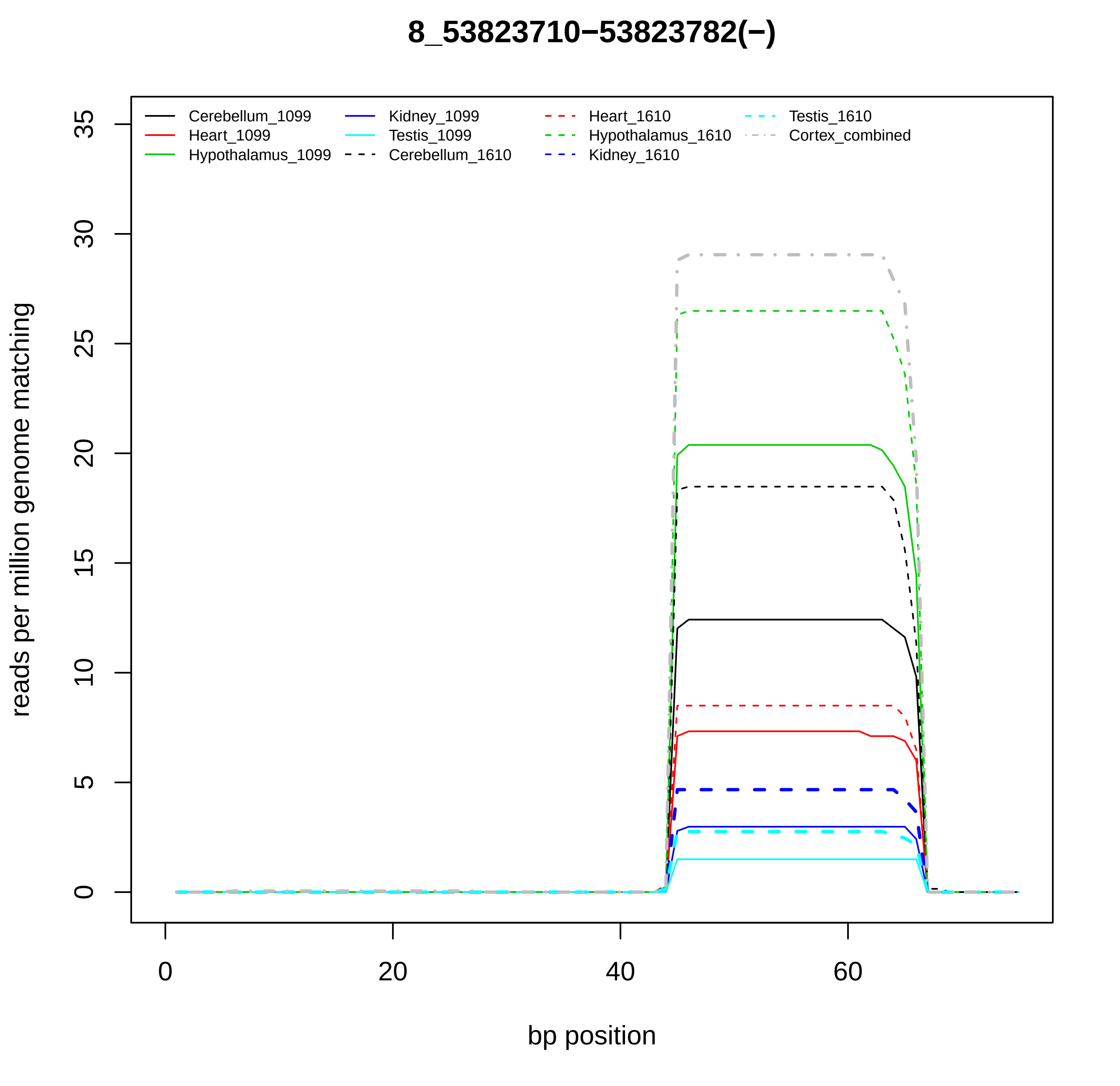

8_53823710−53823782(−)
Cerebellum_1099
Heart_1099
Hypothalamus_1099
Kidney_1099
Testis_1099
Cerebellum_1610
Heart_1610
Hypothalamus_1610
Kidney_1610
Testis_1610
Cortex_combined
reads per million genome matching
bp position
0
5
10
15
20
25
30
35
0
20
40
60

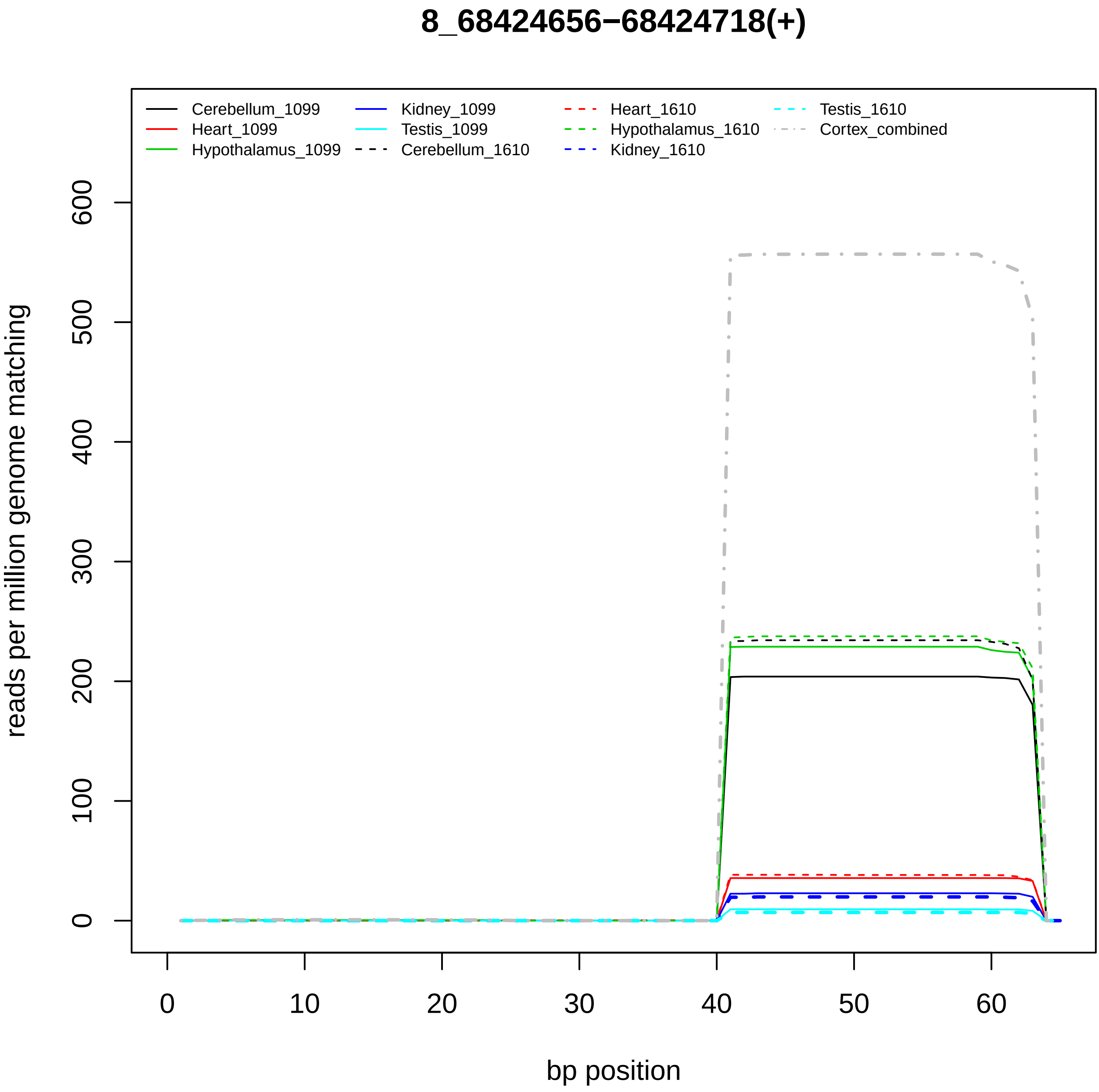
8_68424656−68424718(+)
Cerebellum_1099
Heart_1099
Hypothalamus_1099
Kidney_1099
Testis_1099
Cerebellum_1610
Heart_1610
Hypothalamus_1610
Kidney_1610
Testis_1610
Cortex_combined
reads per million genome matching
bp position
0
100
200
300
400
500
600
10
20
30
40
50
60

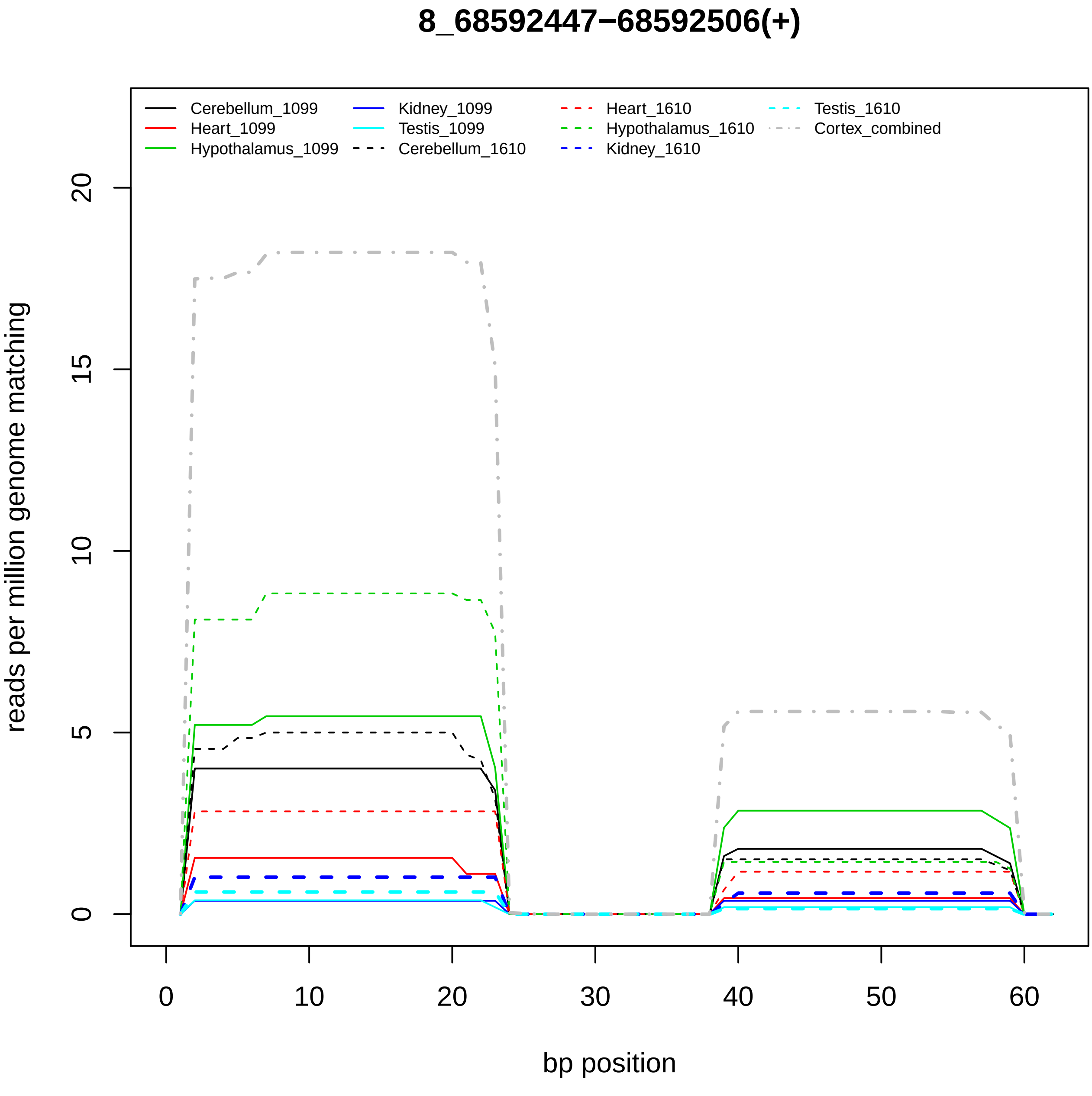

8_68592447−68592506(+)
Cerebellum_1099
Heart_1099
Hypothalamus_1099
Kidney_1099
Testis_1099
Cerebellum_1610
Heart_1610
Hypothalamus_1610
Kidney_1610
Testis_1610
Cortex_combined
reads per million genome matching
0
5
10
15
20
bp position
0
10
20
30
40
50
60

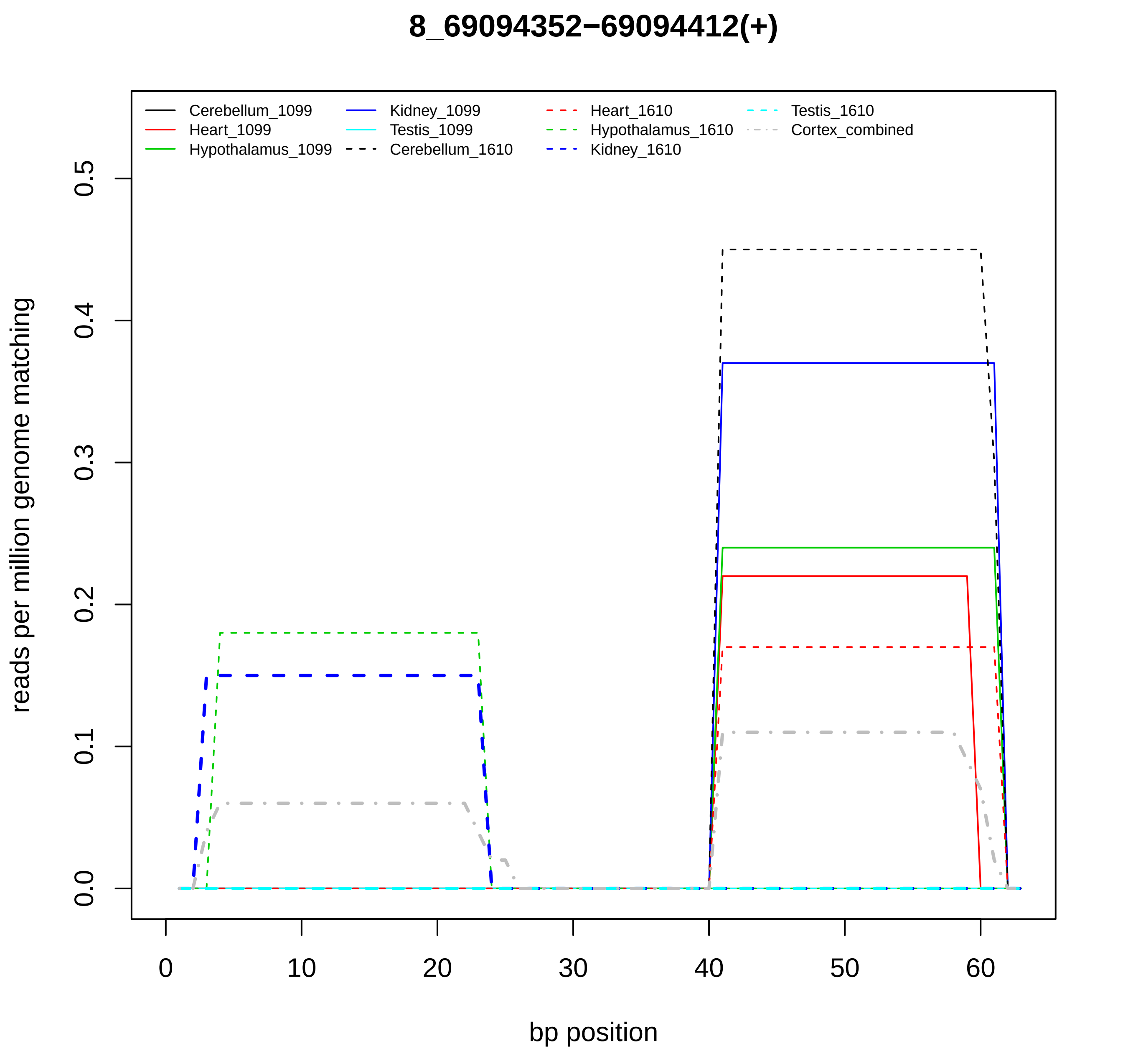

8_69094352−69094412(+)
Cerebellum_1099
Heart_1099
Hypothalamus_1099
Kidney_1099
Testis_1099
Cerebellum_1610
Heart_1610
Hypothalamus_1610
Kidney_1610
Testis_1610
Cortex_combined
reads per million genome matching
0.0
0.1
0.2
0.3
0.4
0.5
bp position
0
10
20
30
40
50
60

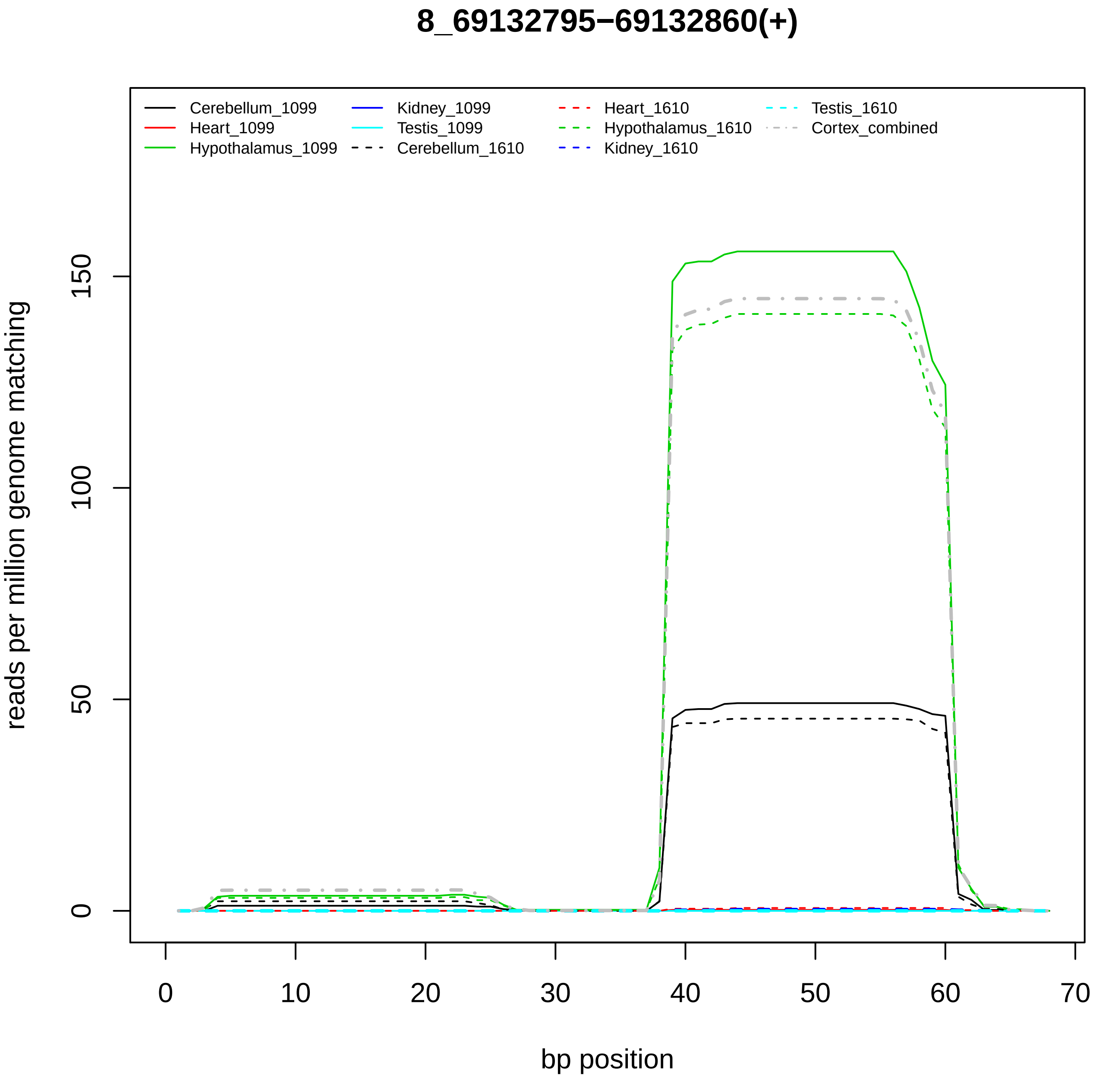
8_69132795−69132860(+)
Cerebellum_1099
Heart_1099
Hypothalamus_1099
Kidney_1099
Testis_1099
Cerebellum_1610
Heart_1610
Hypothalamus_1610
Kidney_1610
Testis_1610
Cortex_combined
reads per million genome matching
bp position
0
50
100
150
0
10
20
30
40
50
60
70

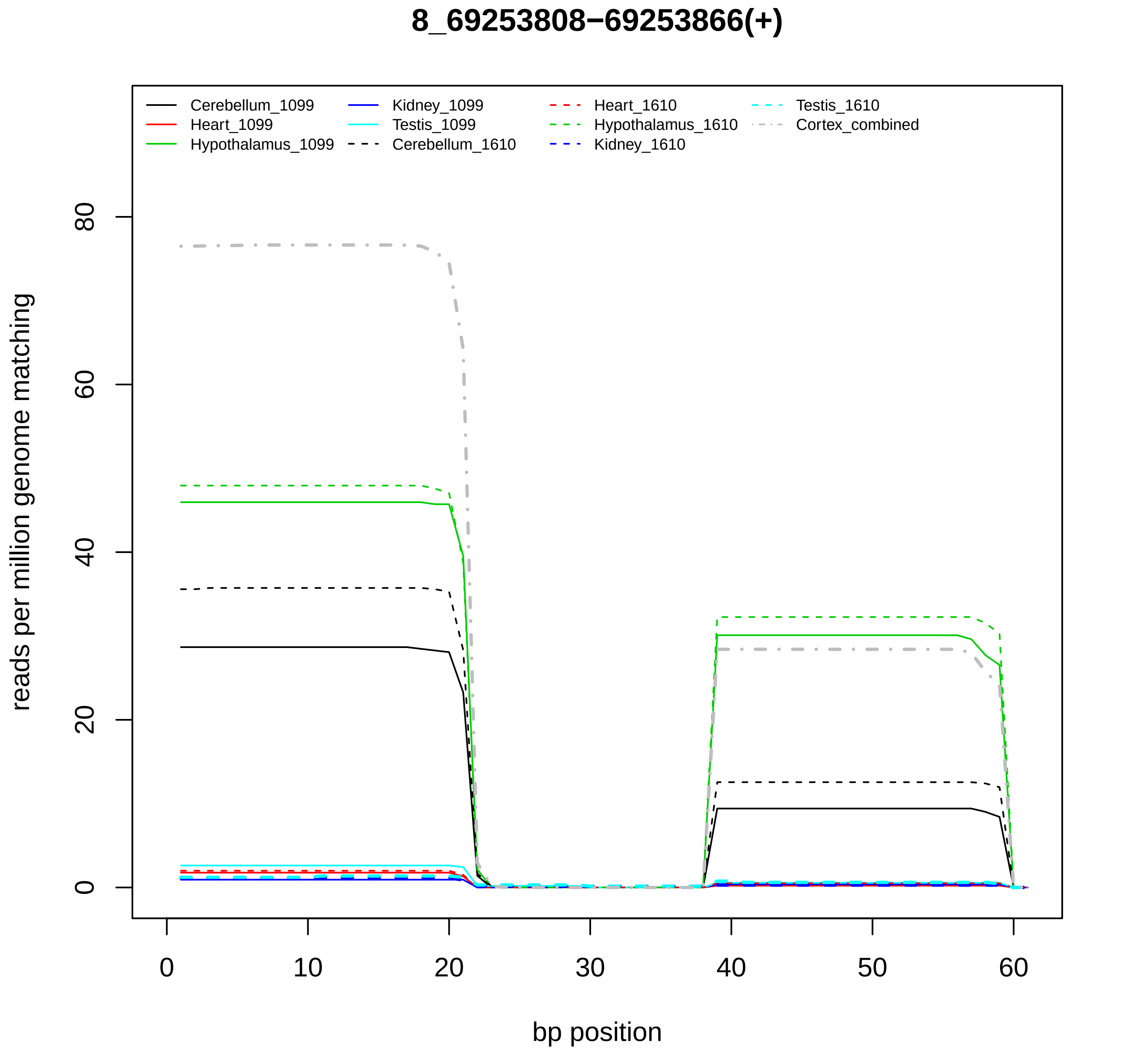

8_69253808−69253866(+)
Cerebellum_1099
Heart_1099
Hypothalamus_1099
Kidney_1099
Testis_1099
Cerebellum_1610
Heart_1610
Hypothalamus_1610
Kidney_1610
Testis_1610
Cortex_combined
reads per million genome matching
0
20
40
60
80
0
10
20
30
40
50
60
bp position

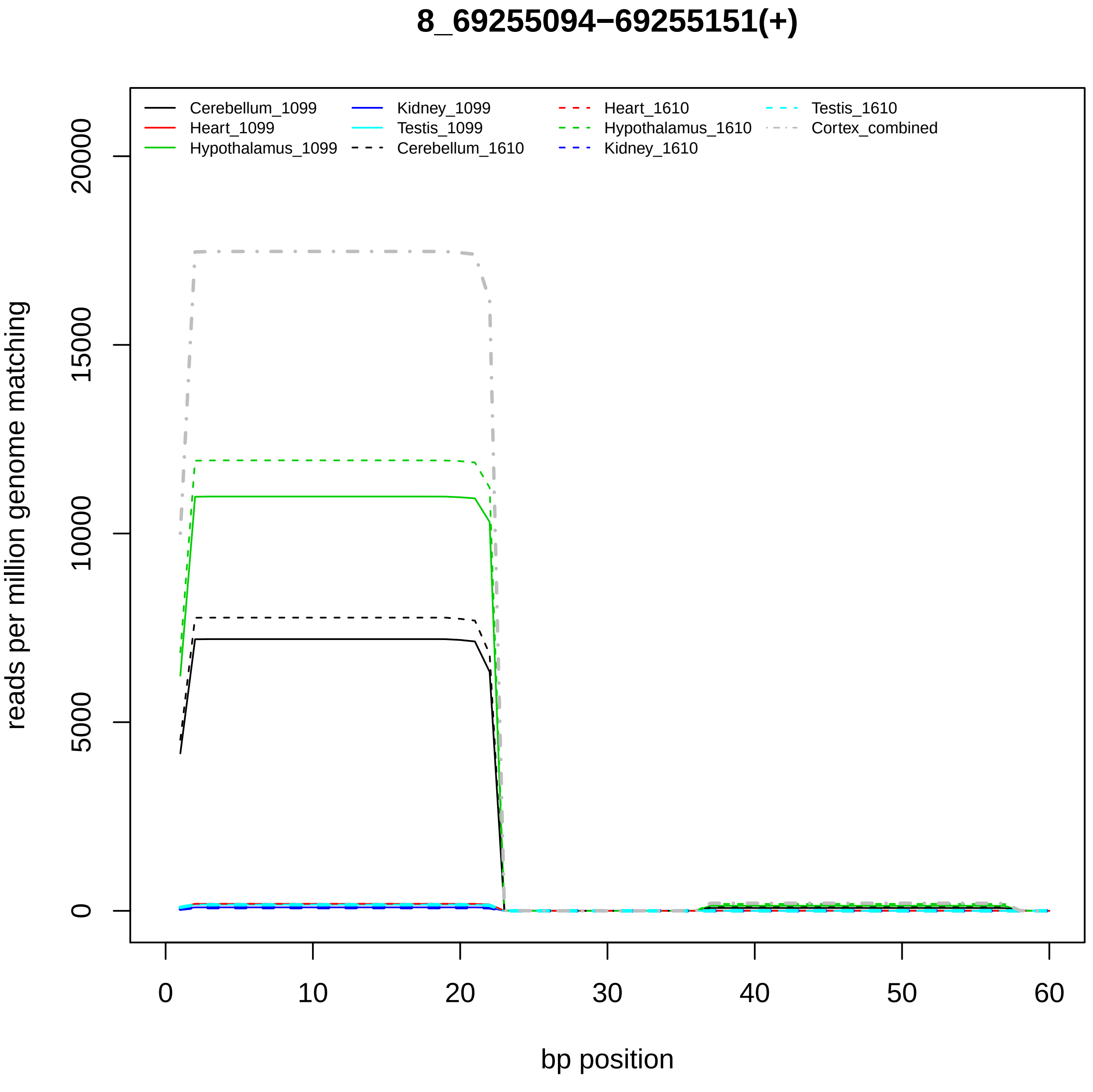
8_69255094−69255151(+)
Cerebellum_1099
Heart_1099
Hypothalamus_1099
Kidney_1099
Testis_1099
Cerebellum_1610
Heart_1610
Hypothalamus_1610
Kidney_1610
Testis_1610
Cortex_combined
reads per million genome matching
bp position
0
5000
10000
15000
20000
0
10
20
30
40
50
60

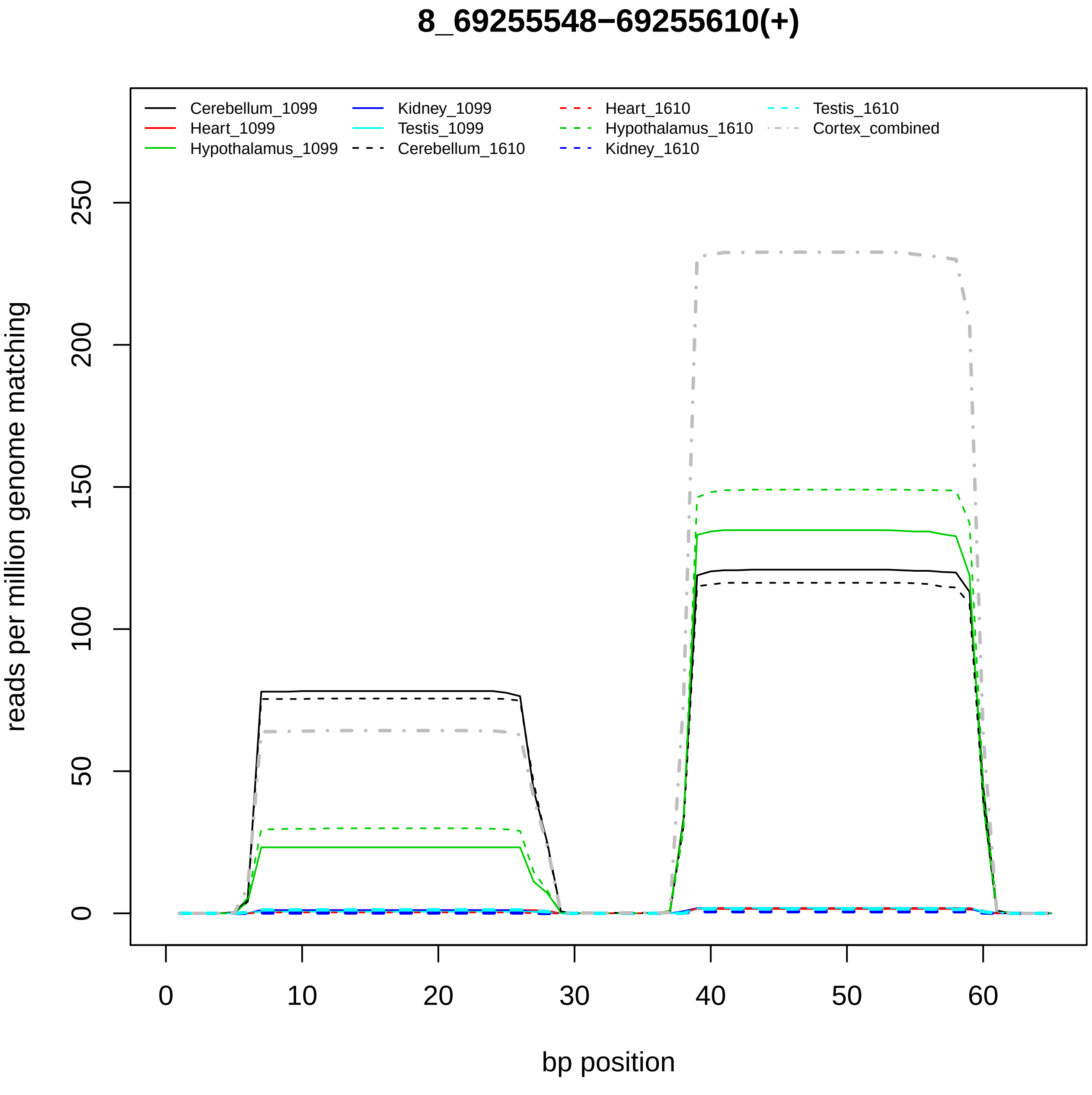

8_69255548−69255610(+)
Cerebellum_1099
Heart_1099
Hypothalamus_1099
Kidney_1099
Testis_1099
Cerebellum_1610
Heart_1610
Hypothalamus_1610
Kidney_1610
Testis_1610
Cortex_combined
reads per million genome matching
0
50
100
150
200
250
0
10
20
30
40
50
60
bp position

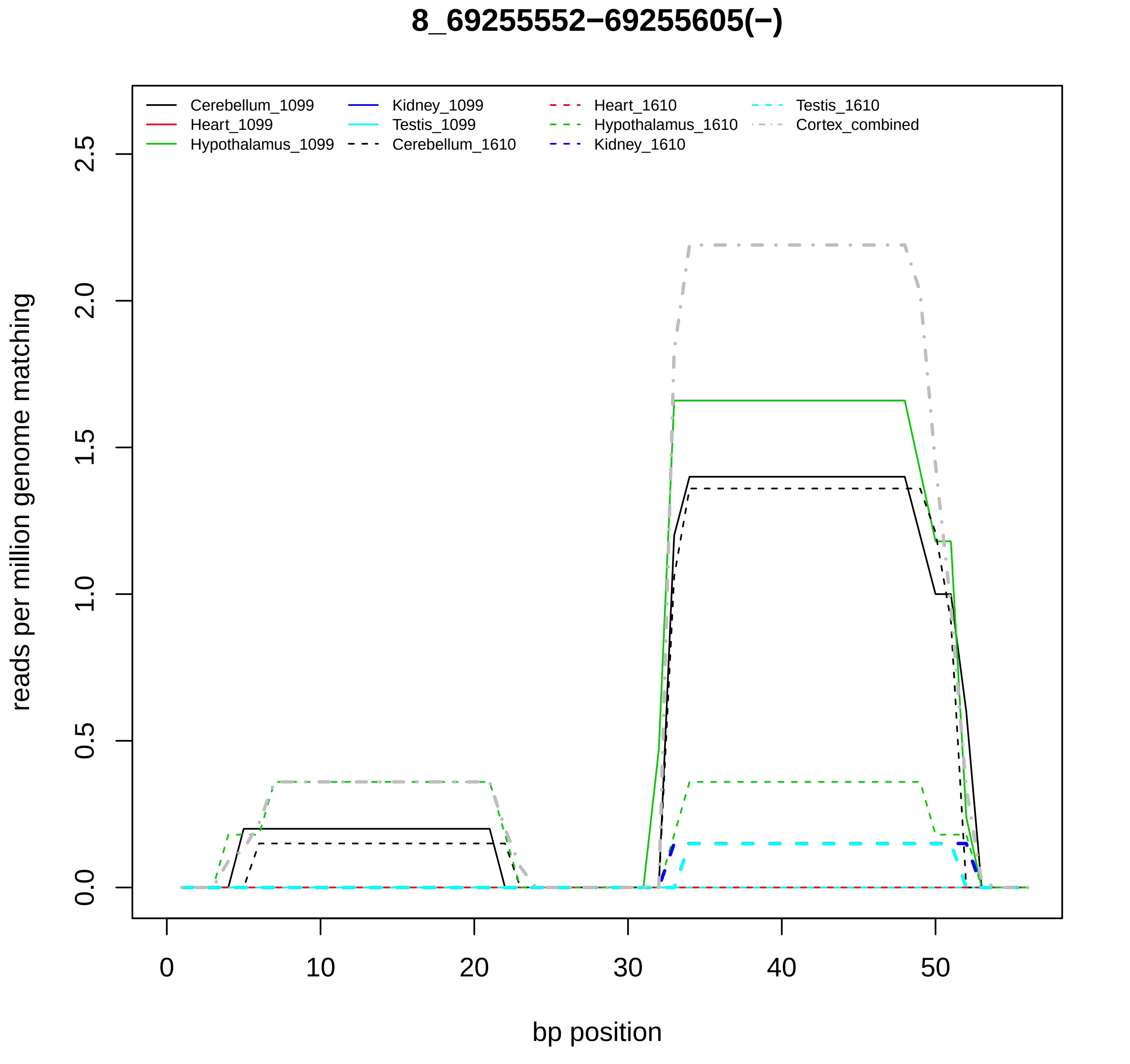
8_69255552−69255605(−)
Cerebellum_1099
Heart_1099
Hypothalamus_1099
Kidney_1099
Testis_1099
Cerebellum_1610
Heart_1610
Hypothalamus_1610
Kidney_1610
Testis_1610
Cortex_combined
reads per million genome matching
bp position
0.0
0.5
1.0
1.5
2.0
2.5
0
10
20
30
40
50

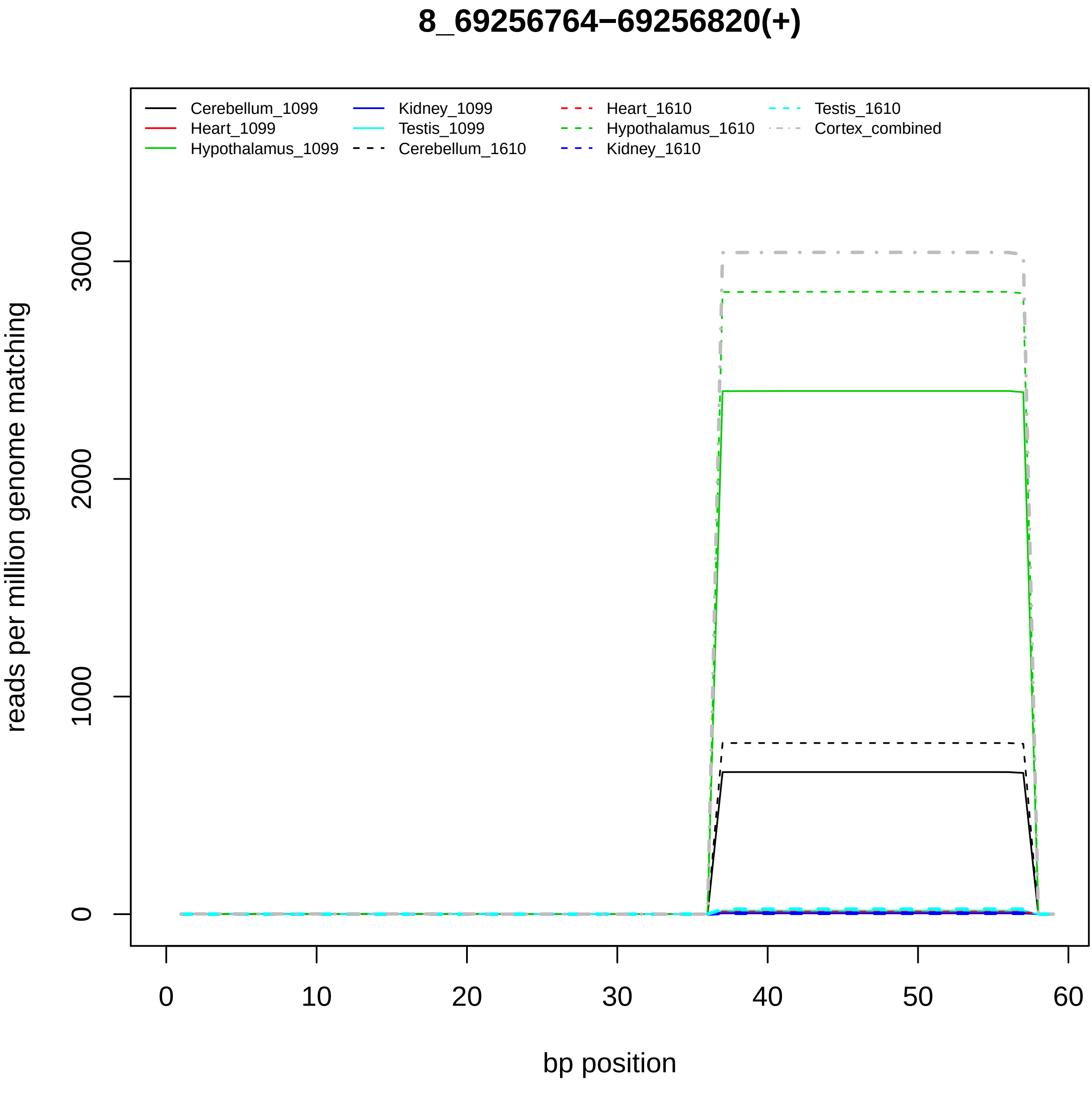

8_69256764−69256820(+)
Cerebellum_1099
Heart_1099
Hypothalamus_1099
Kidney_1099
Testis_1099
Cerebellum_1610
Heart_1610
Hypothalamus_1610
Kidney_1610
Testis_1610
Cortex_combined
reads per million genome matching
0
1000
2000
3000
0
10
20
30
40
50
60
bp position

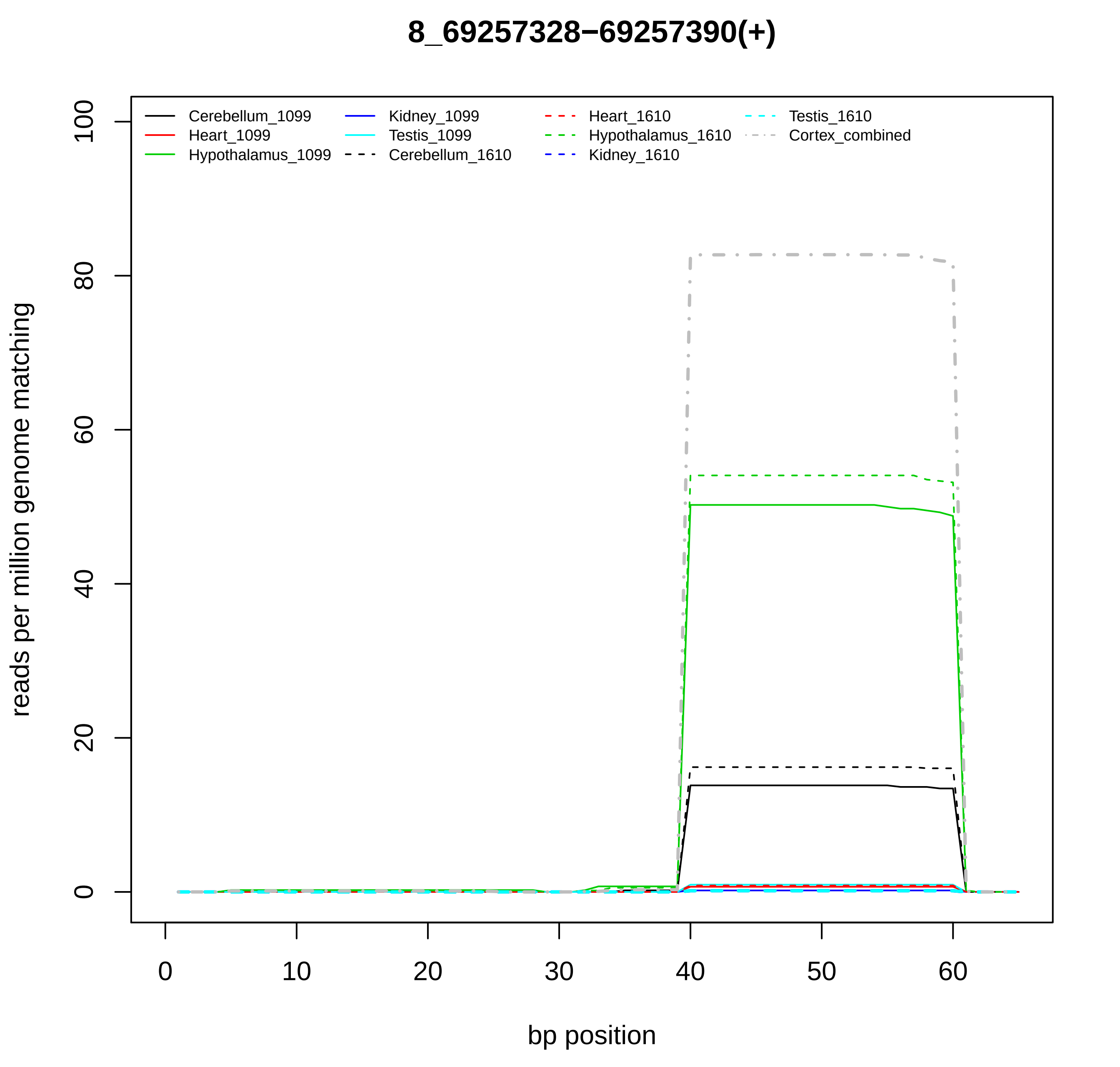
8_69257328−69257390(+)
Cerebellum_1099
Heart_1099
Hypothalamus_1099
Kidney_1099
Testis_1099
Cerebellum_1610
Heart_1610
Hypothalamus_1610
Kidney_1610
Testis_1610
Cortex_combined
reads per million genome matching
0
20
40
60
80
100
bp position
0
10
20
30
40
50
60

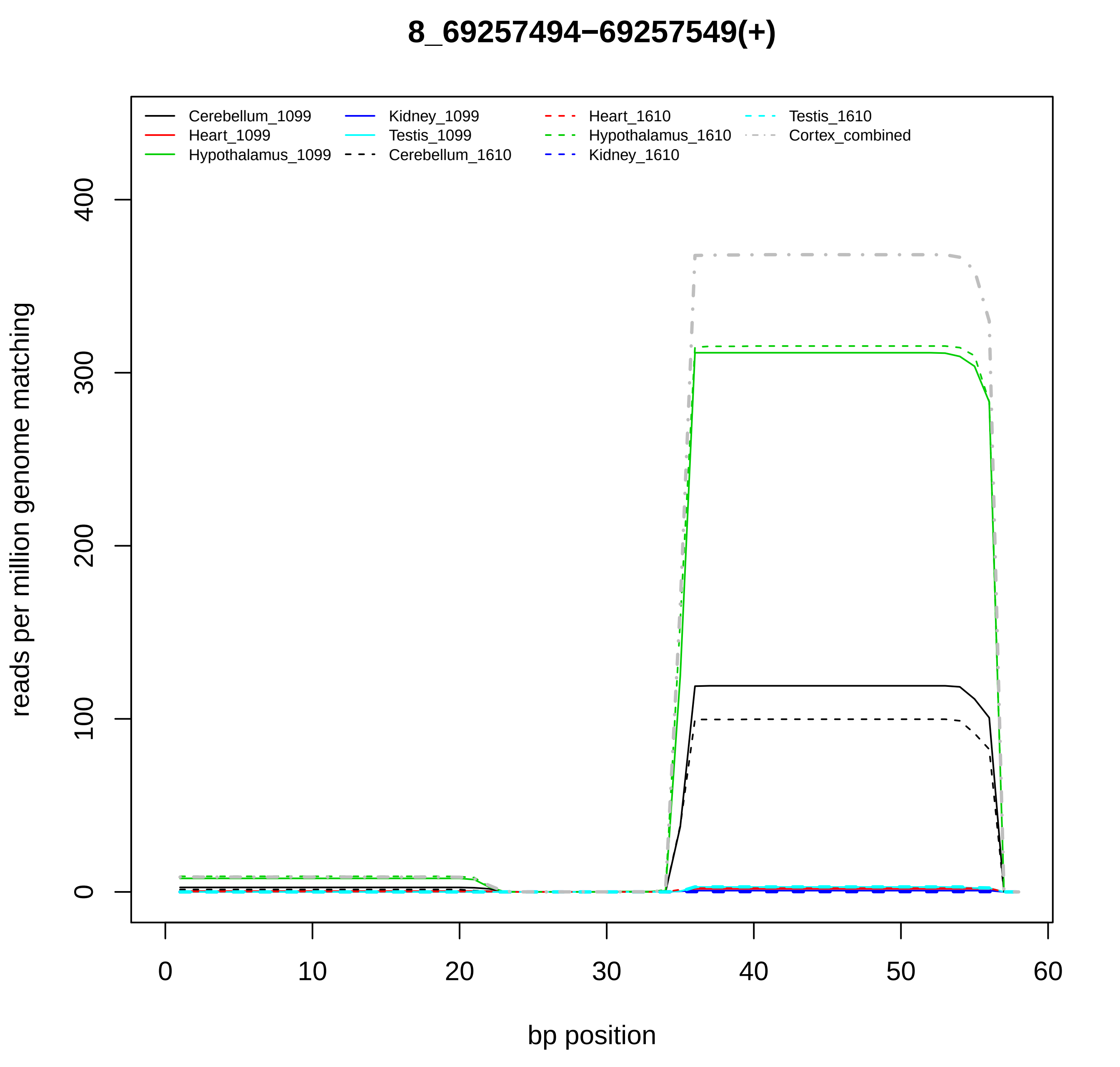

8_69257494−69257549(+)
Cerebellum_1099
Heart_1099
Hypothalamus_1099
Kidney_1099
Testis_1099
Cerebellum_1610
Heart_1610
Hypothalamus_1610
Kidney_1610
Testis_1610
Cortex_combined
reads per million genome matching
bp position
0
100
200
300
400
10
20
30
40
50
60

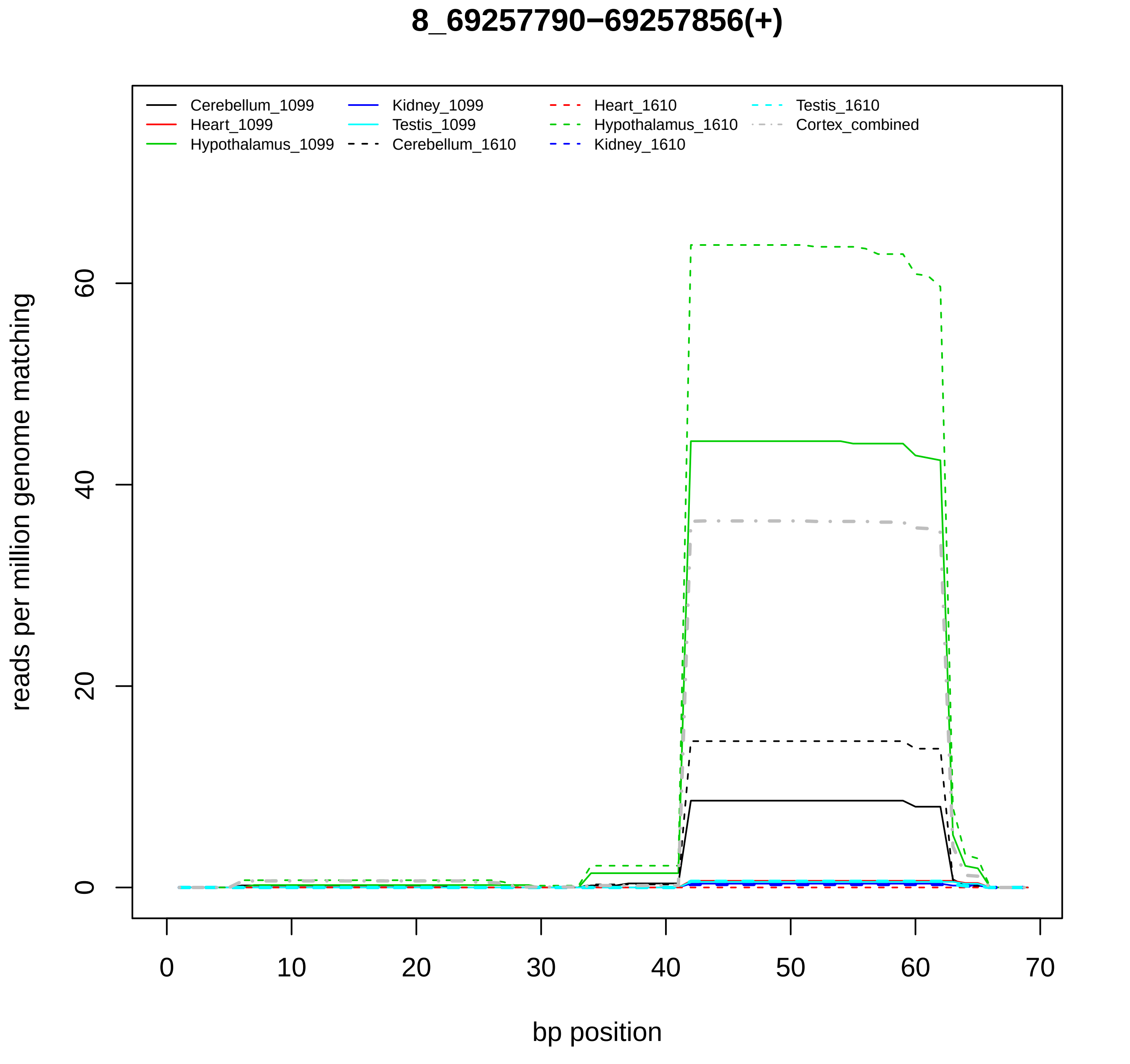
8_69257790−69257856(+)
Cerebellum_1099
Heart_1099
Hypothalamus_1099
Kidney_1099
Testis_1099
Cerebellum_1610
Heart_1610
Hypothalamus_1610
Kidney_1610
Testis_1610
Cortex_combined
reads per million genome matching
0
20
40
60
0
10
20
30
40
50
60
70
bp position

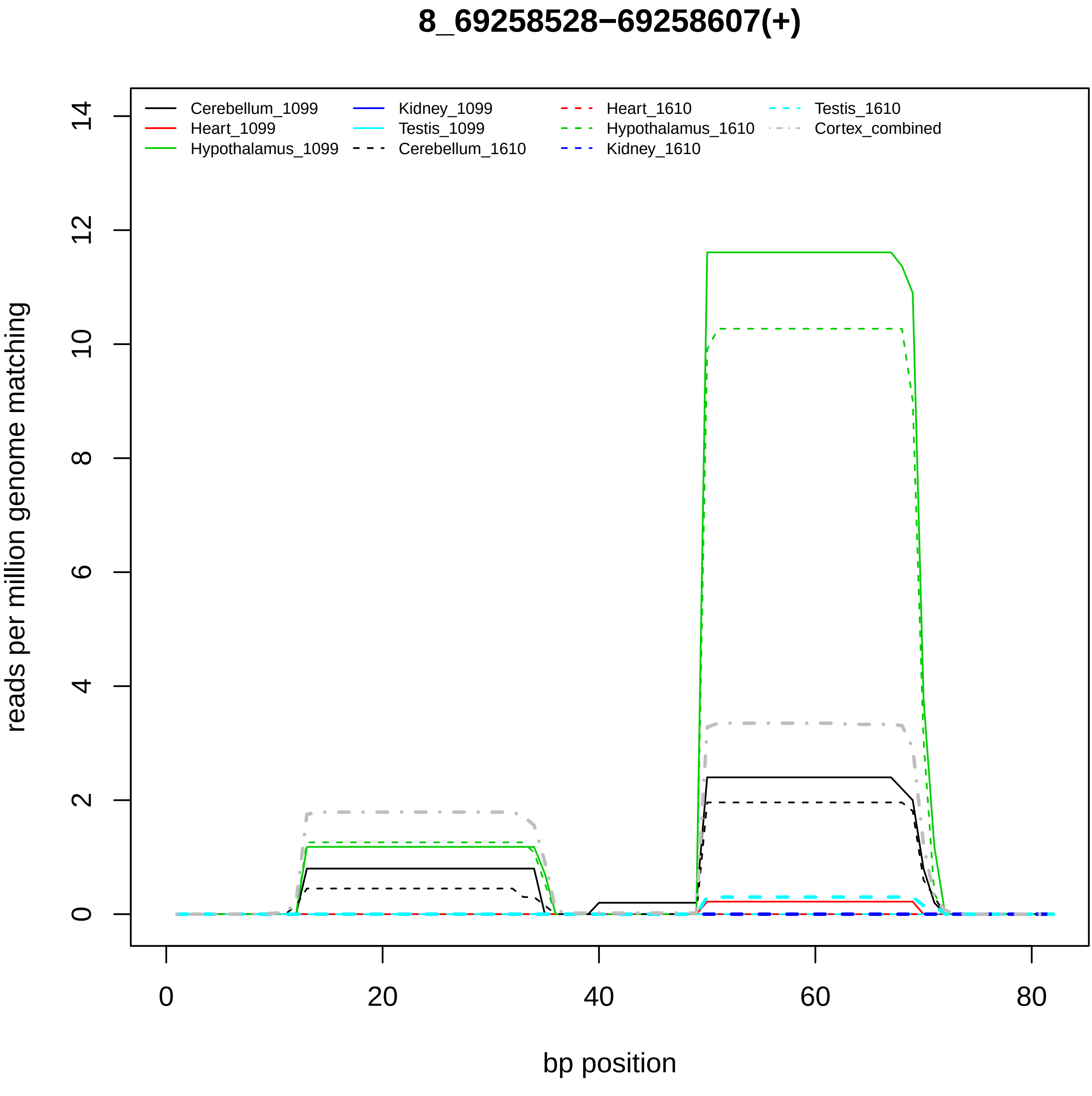
8_69258528−69258607(+)
Cerebellum_1099
Heart_1099
Hypothalamus_1099
Kidney_1099
Testis_1099
Cerebellum_1610
Heart_1610
Hypothalamus_1610
Kidney_1610
Testis_1610
Cortex_combined
reads per million genome matching
bp position
0
2
4
6
8
10
12
14
0
20
40
60
80

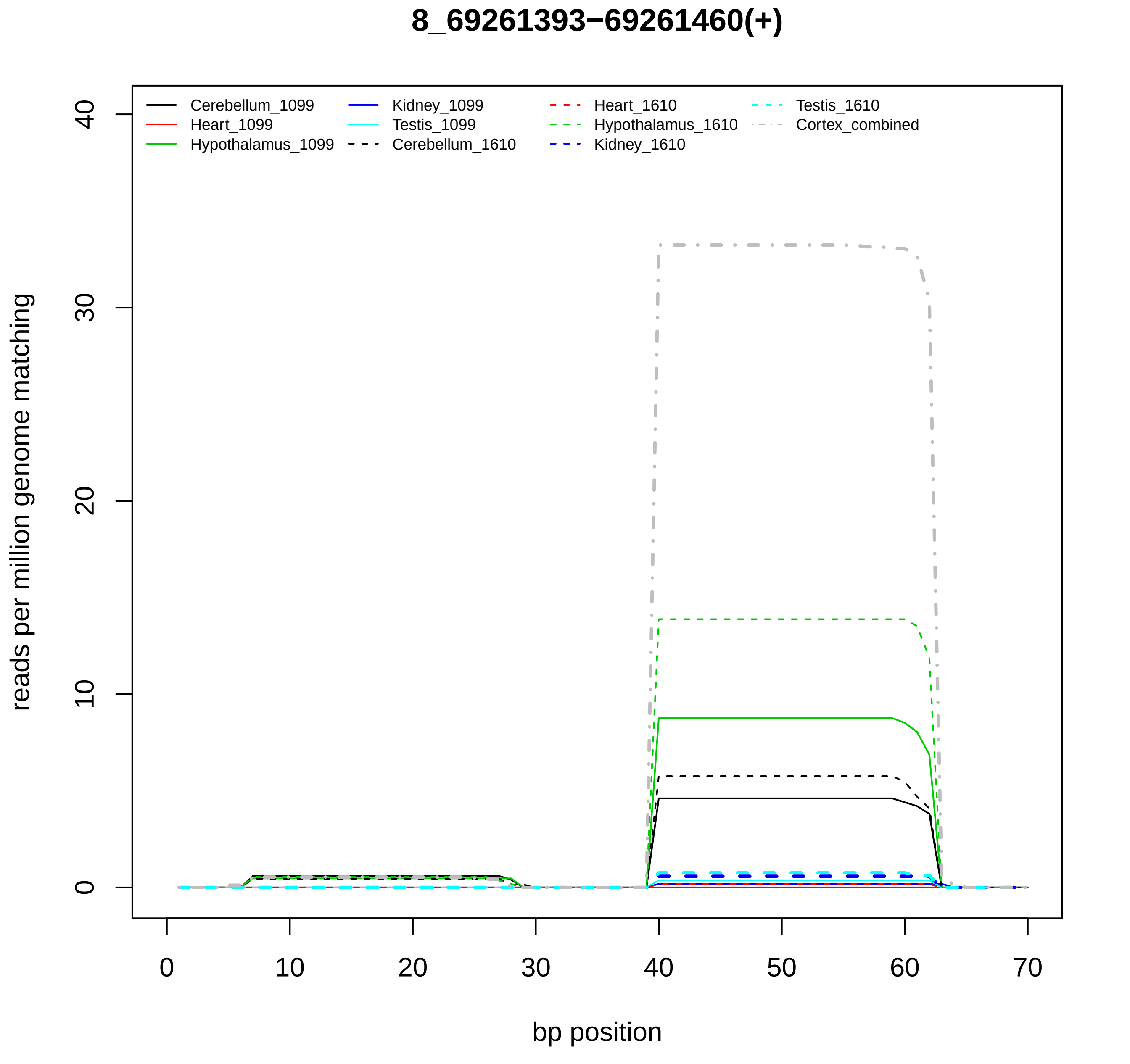

8_69261393−69261460(+)
Cerebellum_1099
Heart_1099
Hypothalamus_1099
Kidney_1099
Testis_1099
Cerebellum_1610
Heart_1610
Hypothalamus_1610
Kidney_1610
Testis_1610
Cortex_combined
reads per million genome matching
bp position
0
10
20
30
40
50
60
70

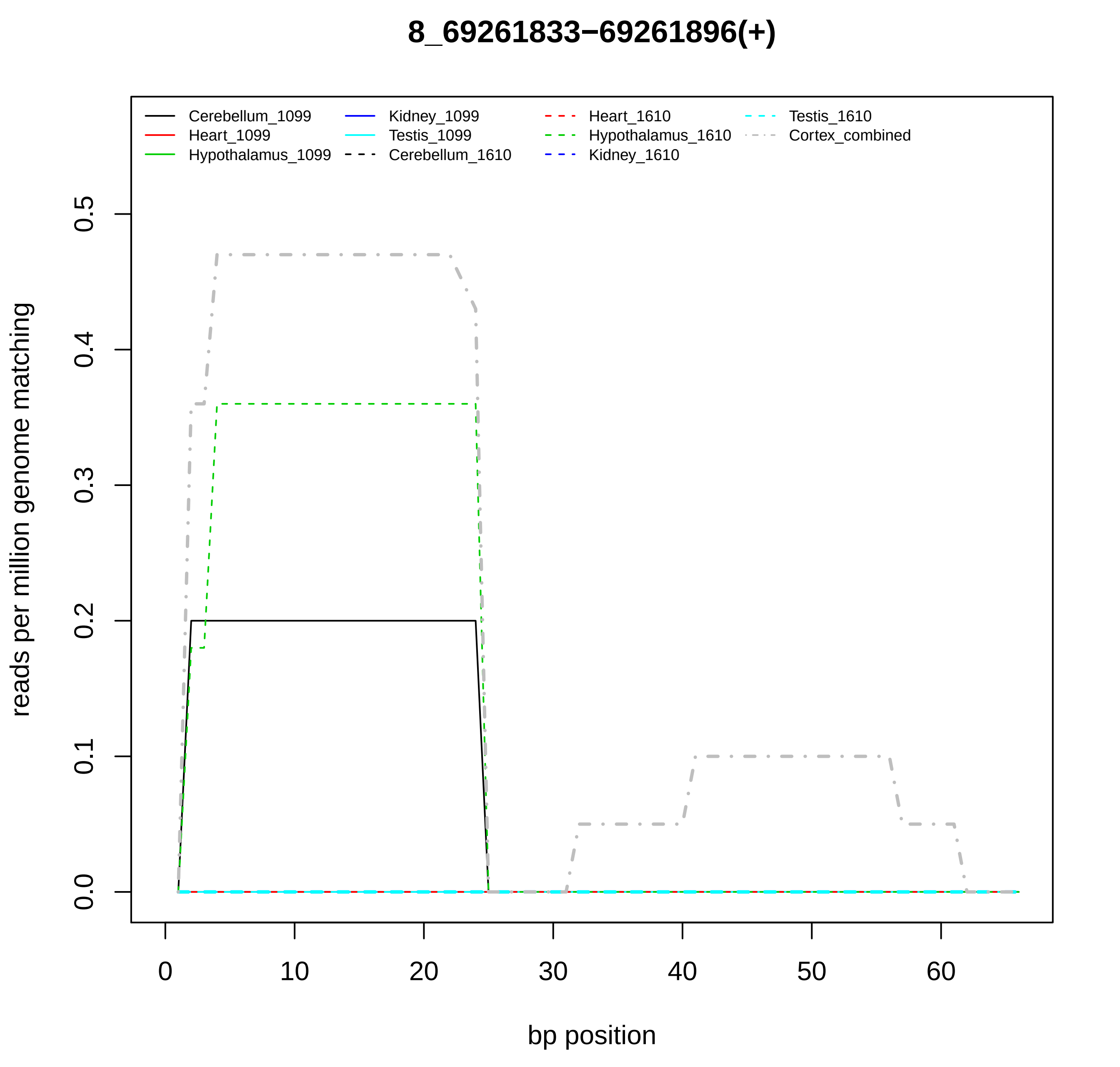

8_69261833−69261896(+)
Cerebellum_1099
Heart_1099
Hypothalamus_1099
Kidney_1099
Testis_1099
Cerebellum_1610
Heart_1610
Hypothalamus_1610
Kidney_1610
Testis_1610
Cortex_combined
reads per million genome matching
0.0
0.1
0.2
0.3
0.4
0.5
0
10
20
30
40
50
60
bp position

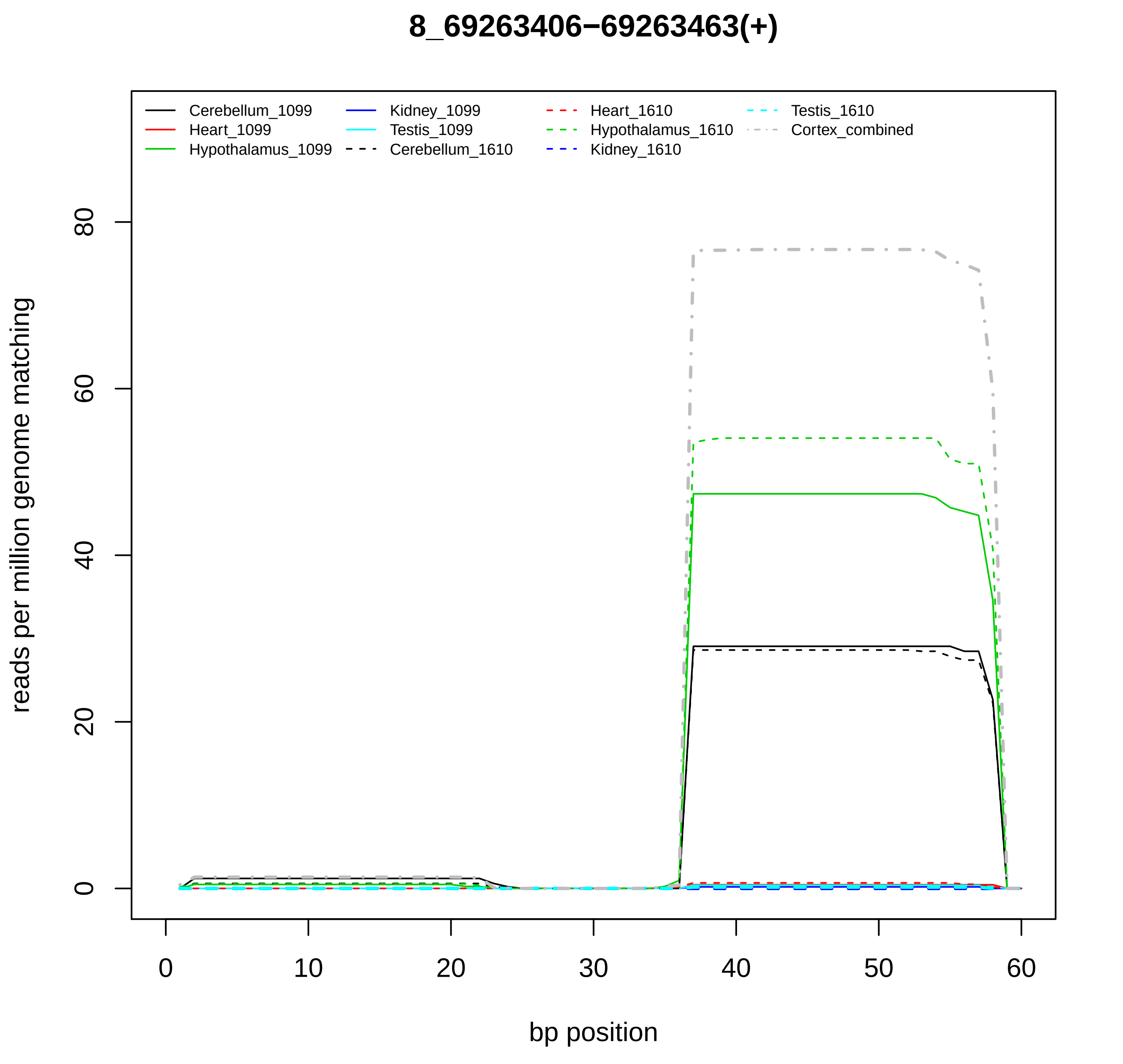

8_69263406−69263463(+)
Cerebellum_1099
Heart_1099
Hypothalamus_1099
Kidney_1099
Testis_1099
Cerebellum_1610
Heart_1610
Hypothalamus_1610
Kidney_1610
Testis_1610
Cortex_combined
reads per million genome matching
0
20
40
60
80
0
10
20
30
40
50
60
bp position

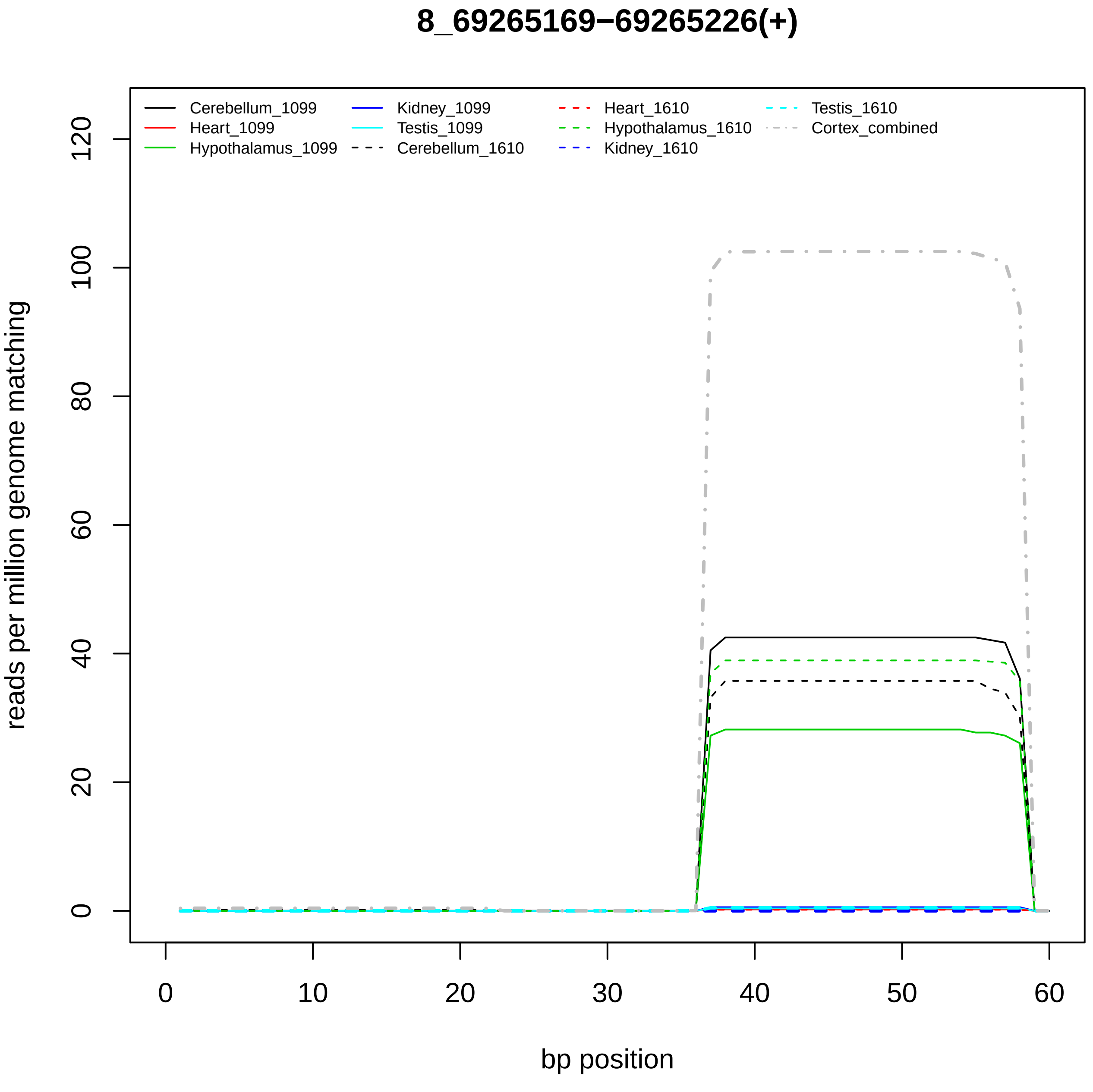

8_69265169−69265226(+)
Cerebellum_1099
Heart_1099
Hypothalamus_1099
Kidney_1099
Testis_1099
Cerebellum_1610
Heart_1610
Hypothalamus_1610
Kidney_1610
Testis_1610
Cortex_combined
reads per million genome matching
bp position
0
20
40
60
80
100
120
0
10
20
30
40
50
60

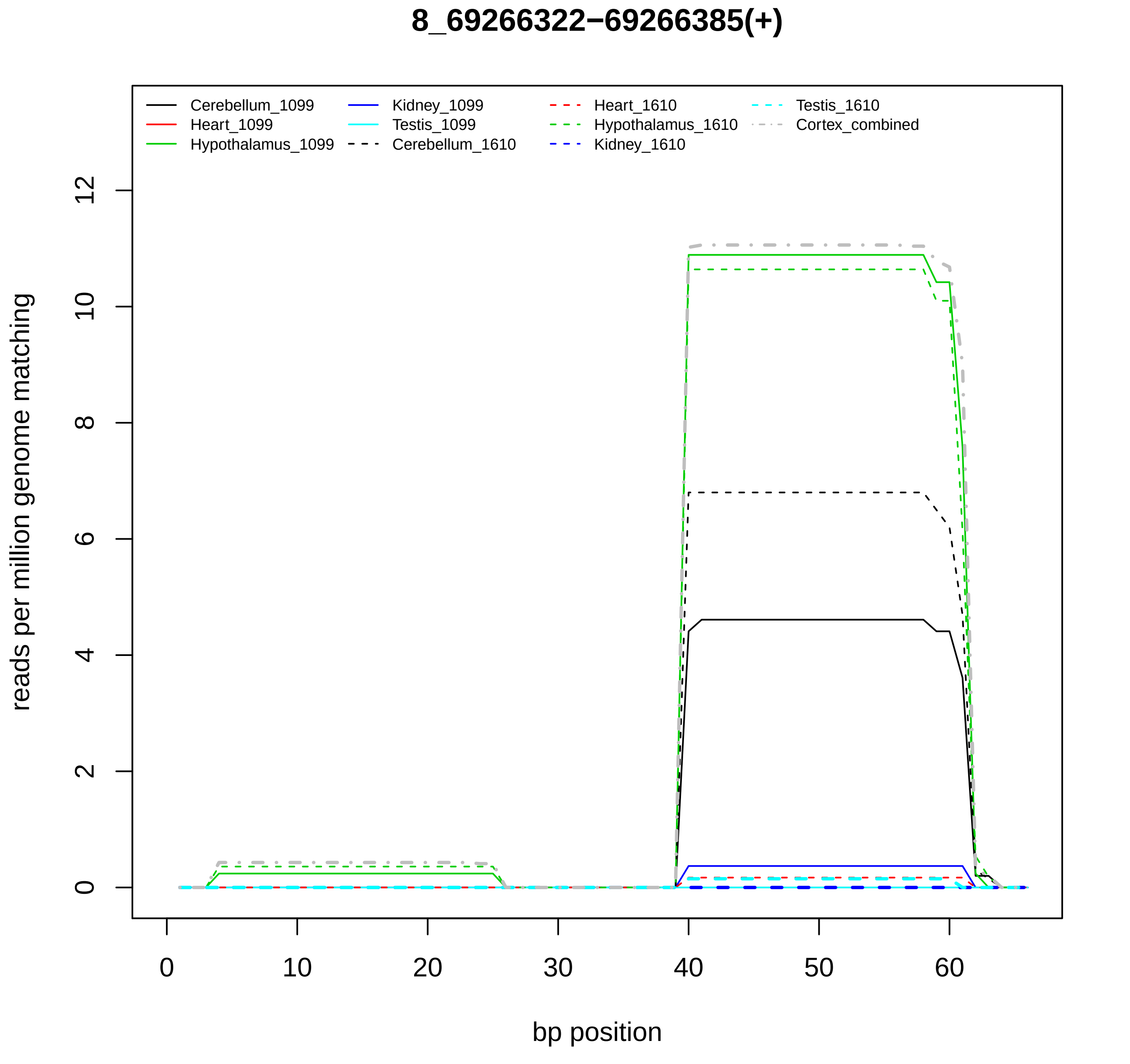
8_69266322−69266385(+)
Cerebellum_1099
Heart_1099
Hypothalamus_1099
Kidney_1099
Testis_1099
Cerebellum_1610
Heart_1610
Hypothalamus_1610
Kidney_1610
Testis_1610
Cortex_combined
reads per million genome matching
0
2
4
6
8
10
12
0
10
20
30
40
50
60
bp position

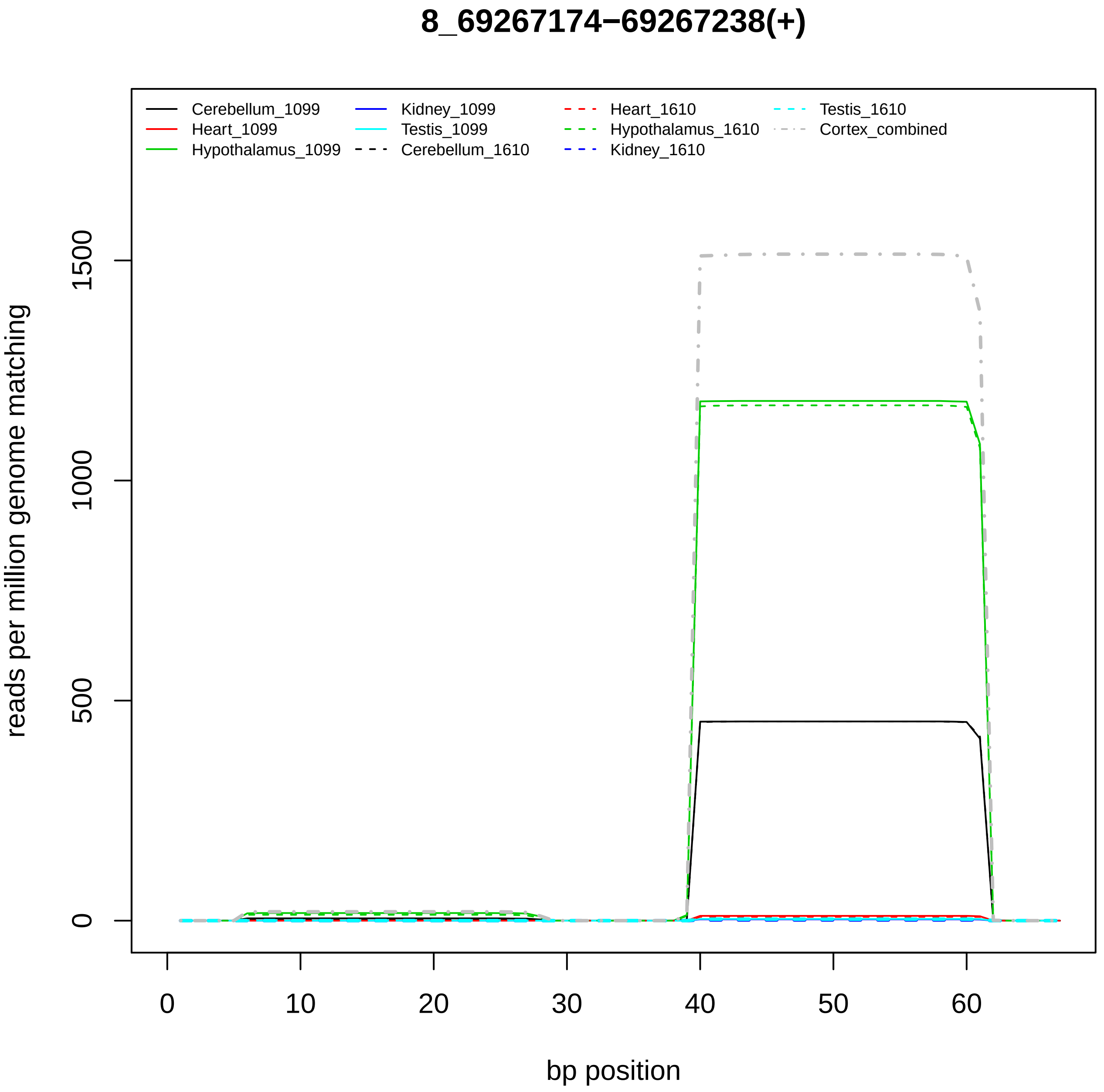
8_69267174−69267238(+)
Cerebellum_1099
Heart_1099
Hypothalamus_1099
Kidney_1099
Testis_1099
Cerebellum_1610
Heart_1610
Hypothalamus_1610
Kidney_1610
Testis_1610
Cortex_combined
reads per million genome matching
0
500
1000
1500
0
10
20
30
40
50
60
bp position

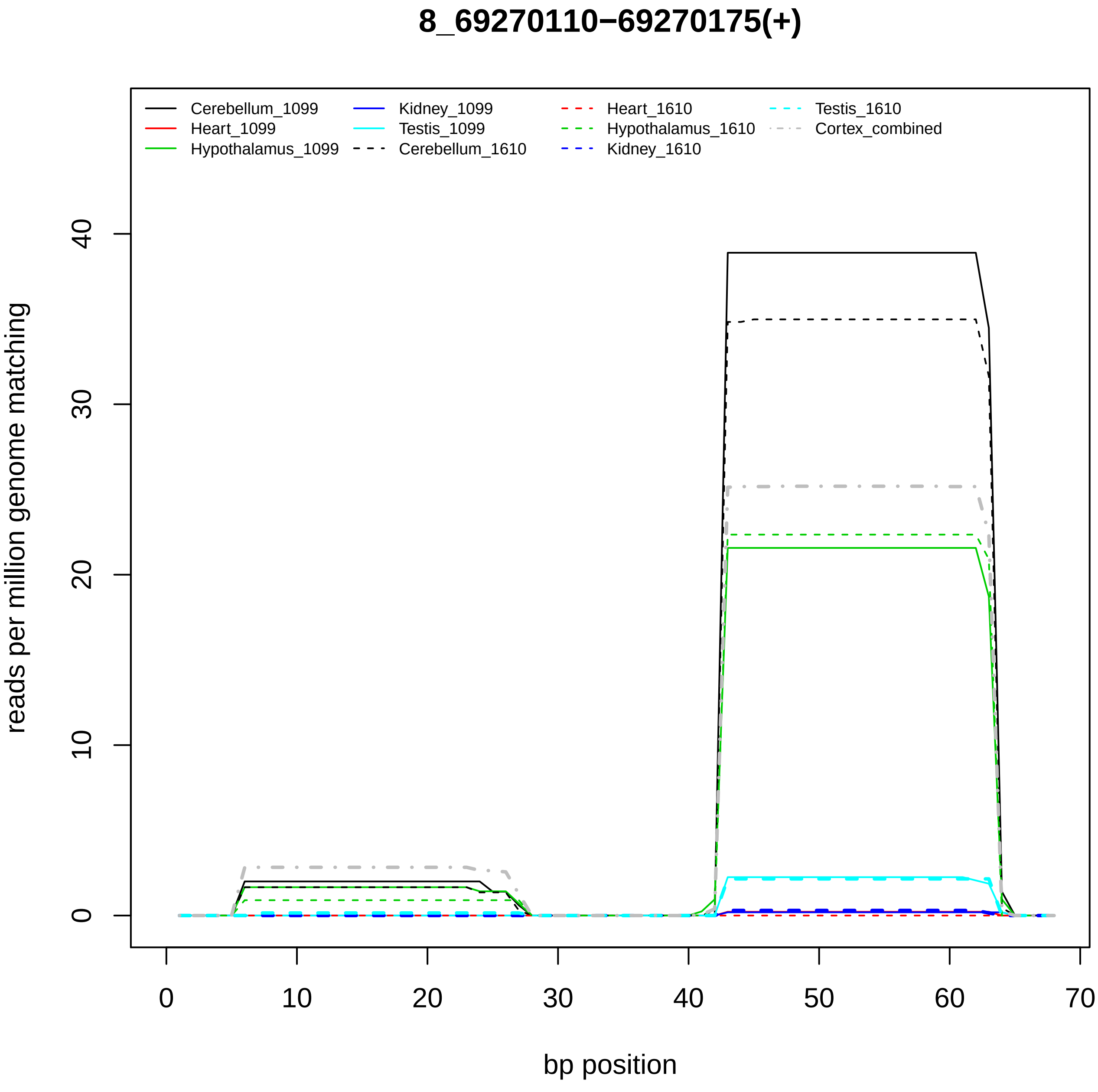
8_69270110−69270175(+)
Cerebellum_1099
Heart_1099
Hypothalamus_1099
Kidney_1099
Testis_1099
Cerebellum_1610
Heart_1610
Hypothalamus_1610
Kidney_1610
Testis_1610
Cortex_combined
reads per million genome matching
bp position
0
10
20
30
40
0
10
20
30
40
50
60
70

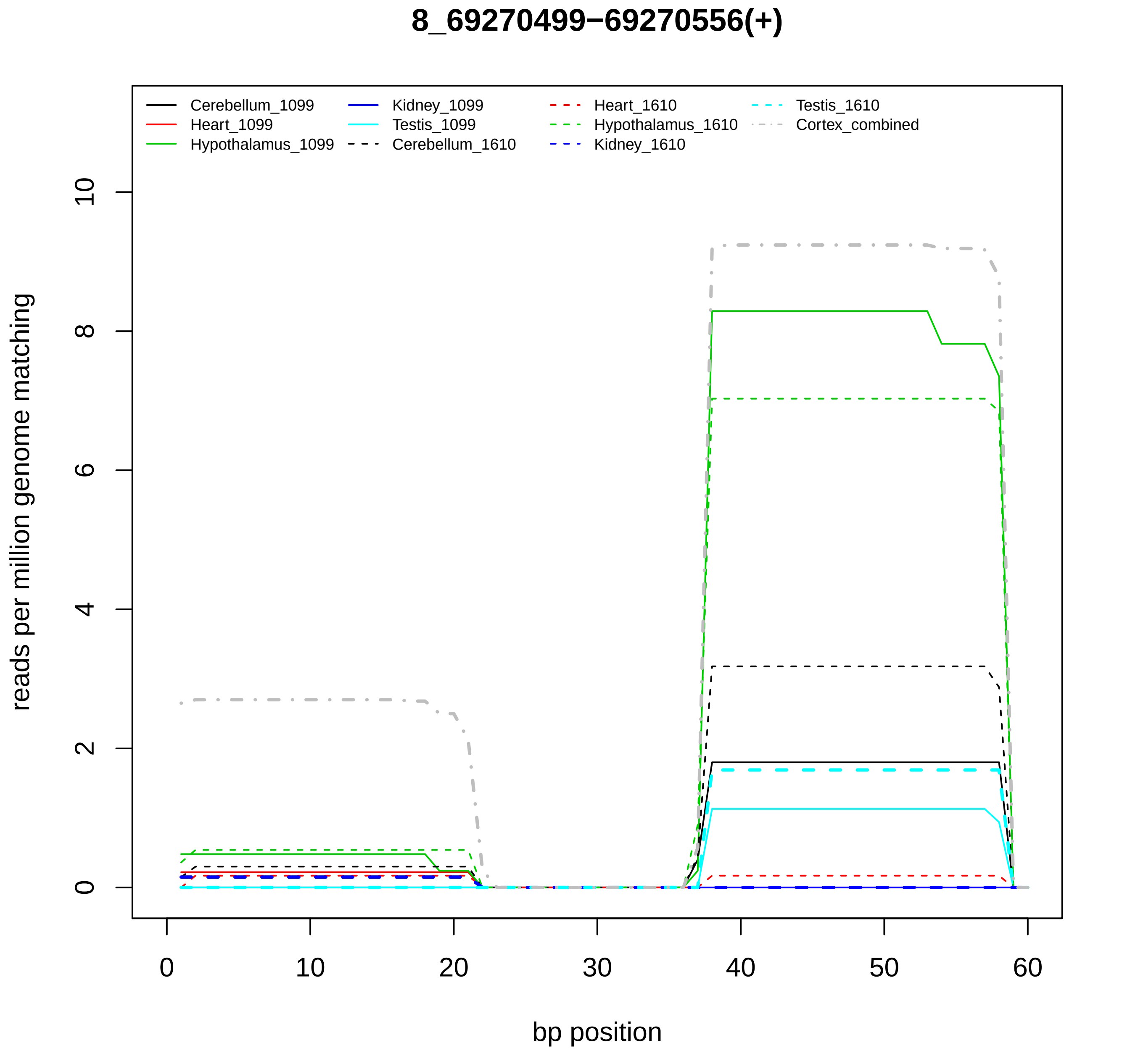

8_69270499−69270556(+)
Cerebellum_1099
Heart_1099
Hypothalamus_1099
Kidney_1099
Testis_1099
Cerebellum_1610
Heart_1610
Hypothalamus_1610
Kidney_1610
Testis_1610
Cortex_combined
reads per million genome matching
0
2
4
6
8
10
0
10
20
30
40
50
60
bp position

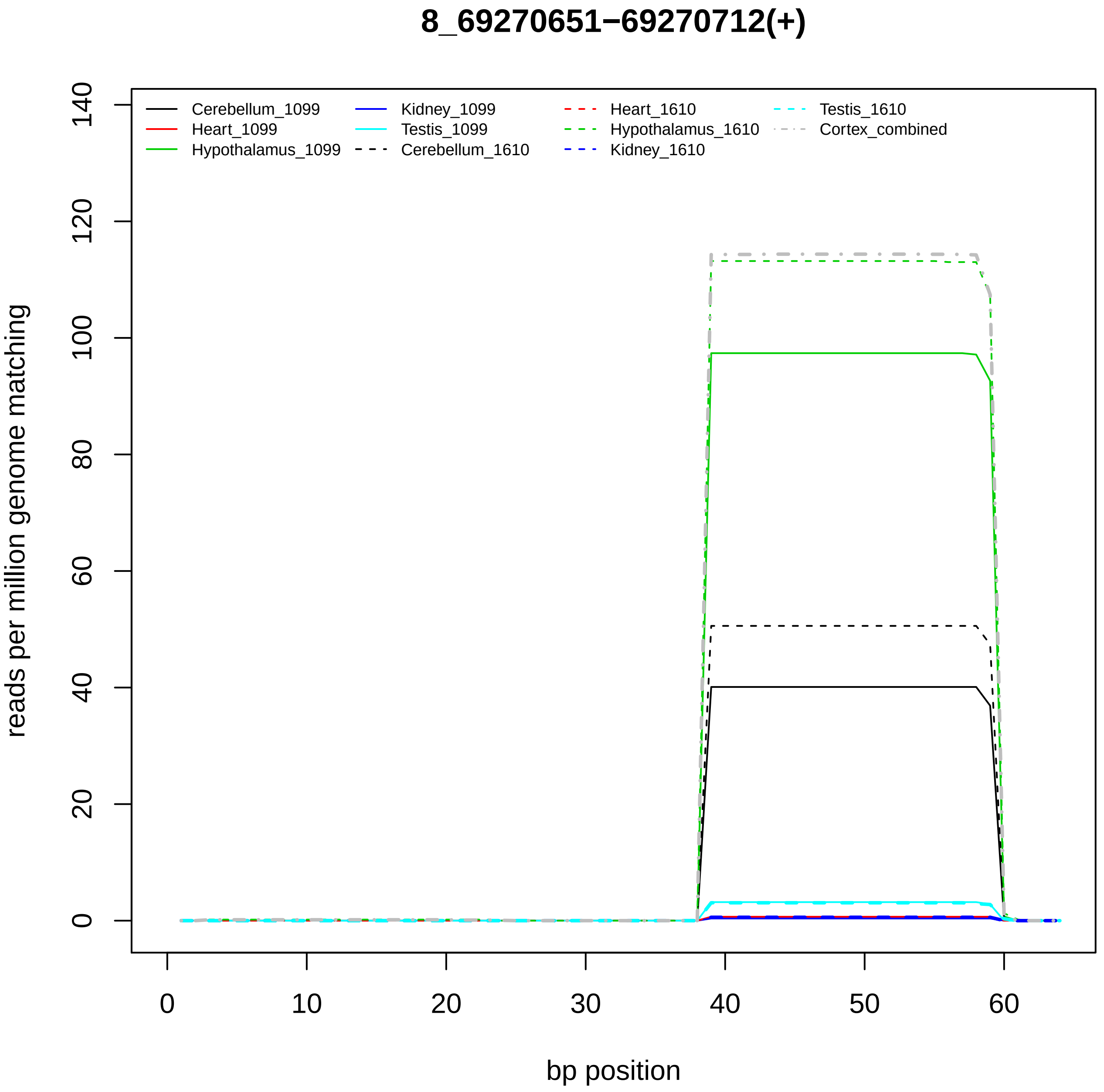

8_69270651−69270712(+)
Cerebellum_1099
Heart_1099
Hypothalamus_1099
Kidney_1099
Testis_1099
Cerebellum_1610
Heart_1610
Hypothalamus_1610
Kidney_1610
Testis_1610
Cortex_combined
reads per million genome matching
bp position
0
20
40
60
80
100
120
140
0
10
20
30
40
50
60

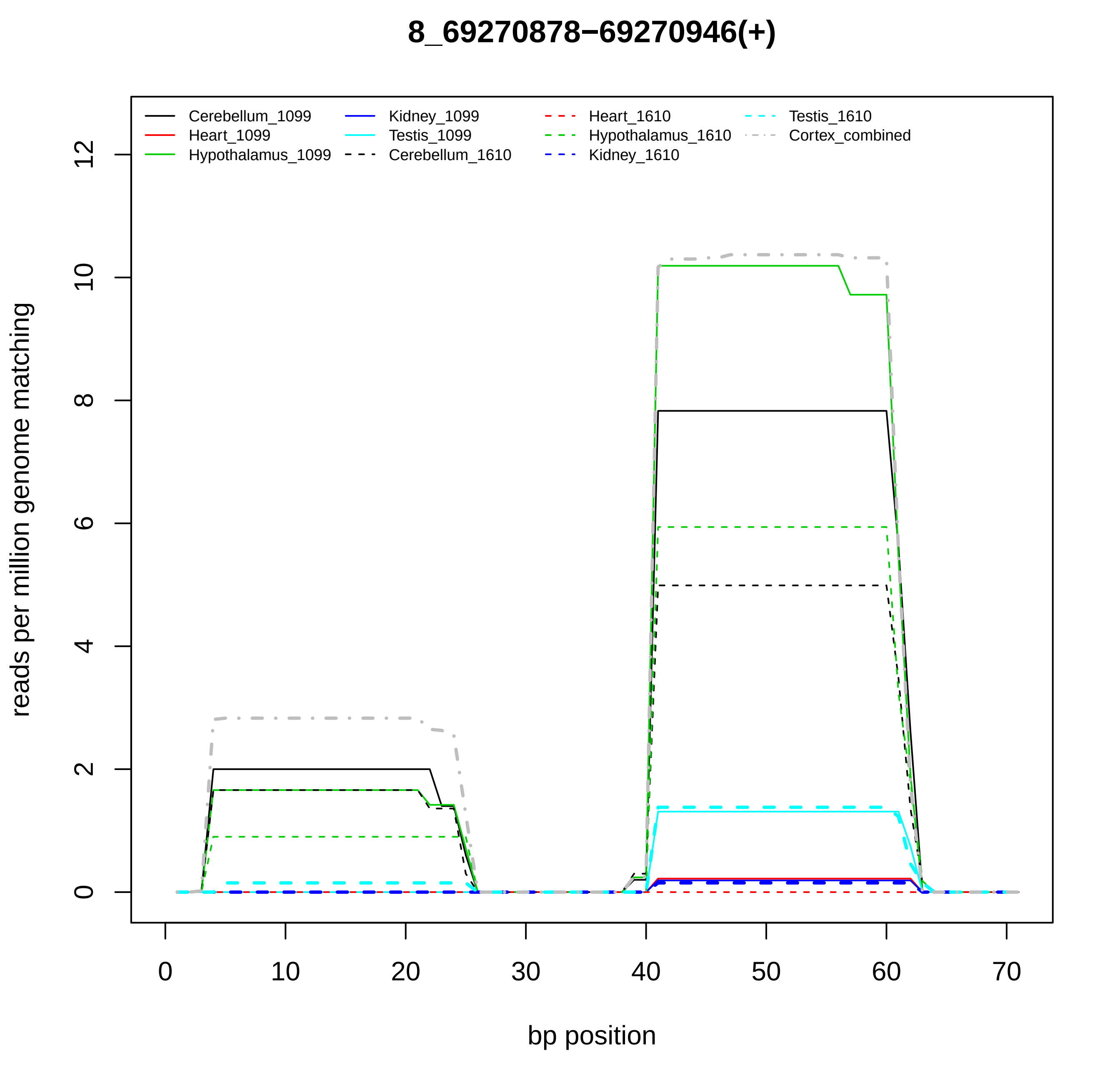

8_69270878−69270946(+)
Cerebellum_1099
Heart_1099
Hypothalamus_1099
Kidney_1099
Testis_1099
Cerebellum_1610
Heart_1610
Hypothalamus_1610
Kidney_1610
Testis_1610
Cortex_combined
reads per million genome matching
bp position
0
2
4
6
8
10
12
0
10
20
30
40
50
60
70

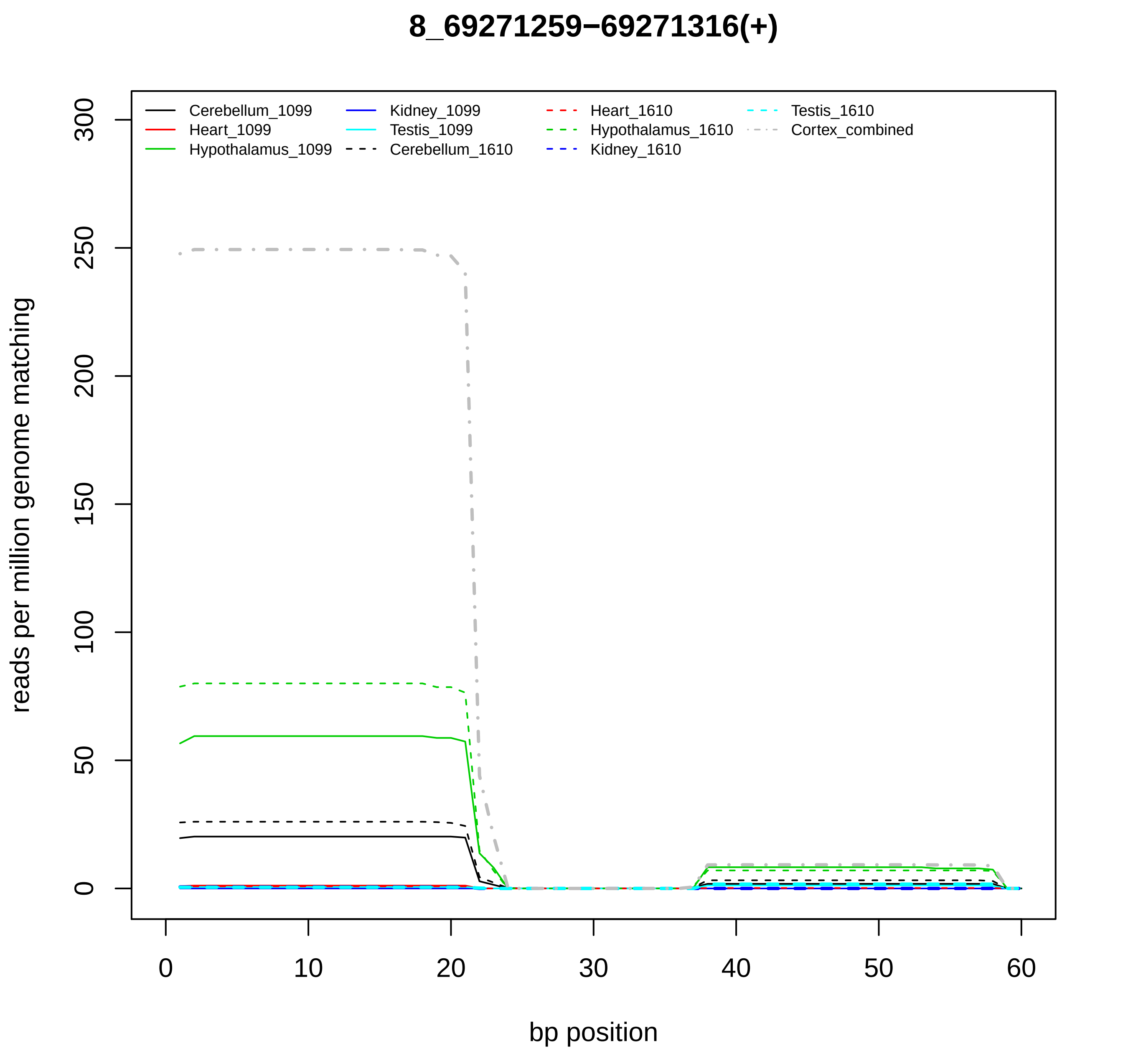

8_69271259−69271316(+)
Cerebellum_1099
Heart_1099
Hypothalamus_1099
Kidney_1099
Testis_1099
Cerebellum_1610
Heart_1610
Hypothalamus_1610
Kidney_1610
Testis_1610
Cortex_combined
reads per million genome matching
bp position
0
50
100
150
200
250
300
0
10
20
30
40
50
60

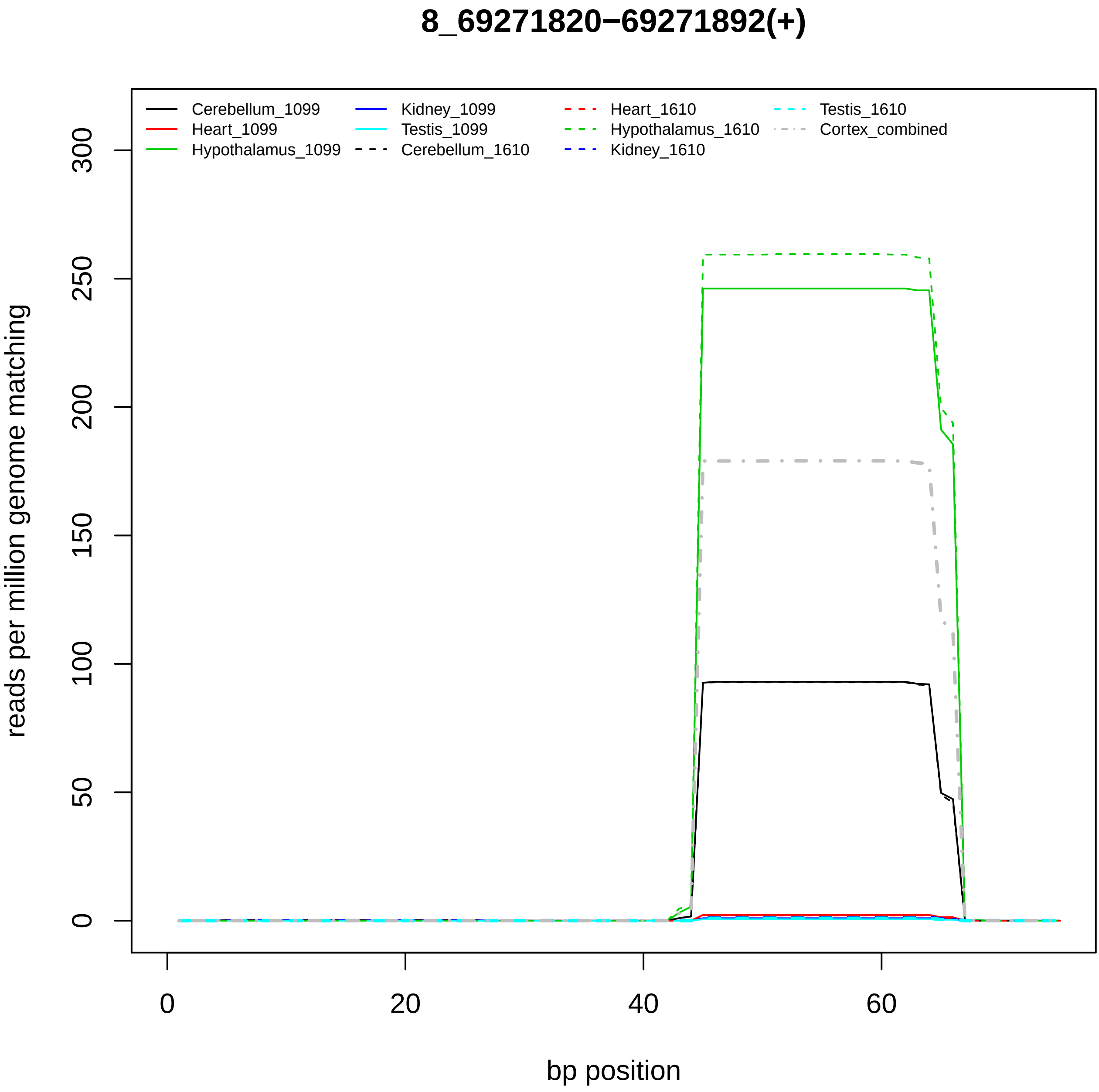

8_69271820−69271892(+)
Cerebellum_1099
Heart_1099
Hypothalamus_1099
Kidney_1099
Testis_1099
Cerebellum_1610
Heart_1610
Hypothalamus_1610
Kidney_1610
Testis_1610
Cortex_combined
reads per million genome matching
0
50
100
150
200
250
300
bp position
0
20
40
60

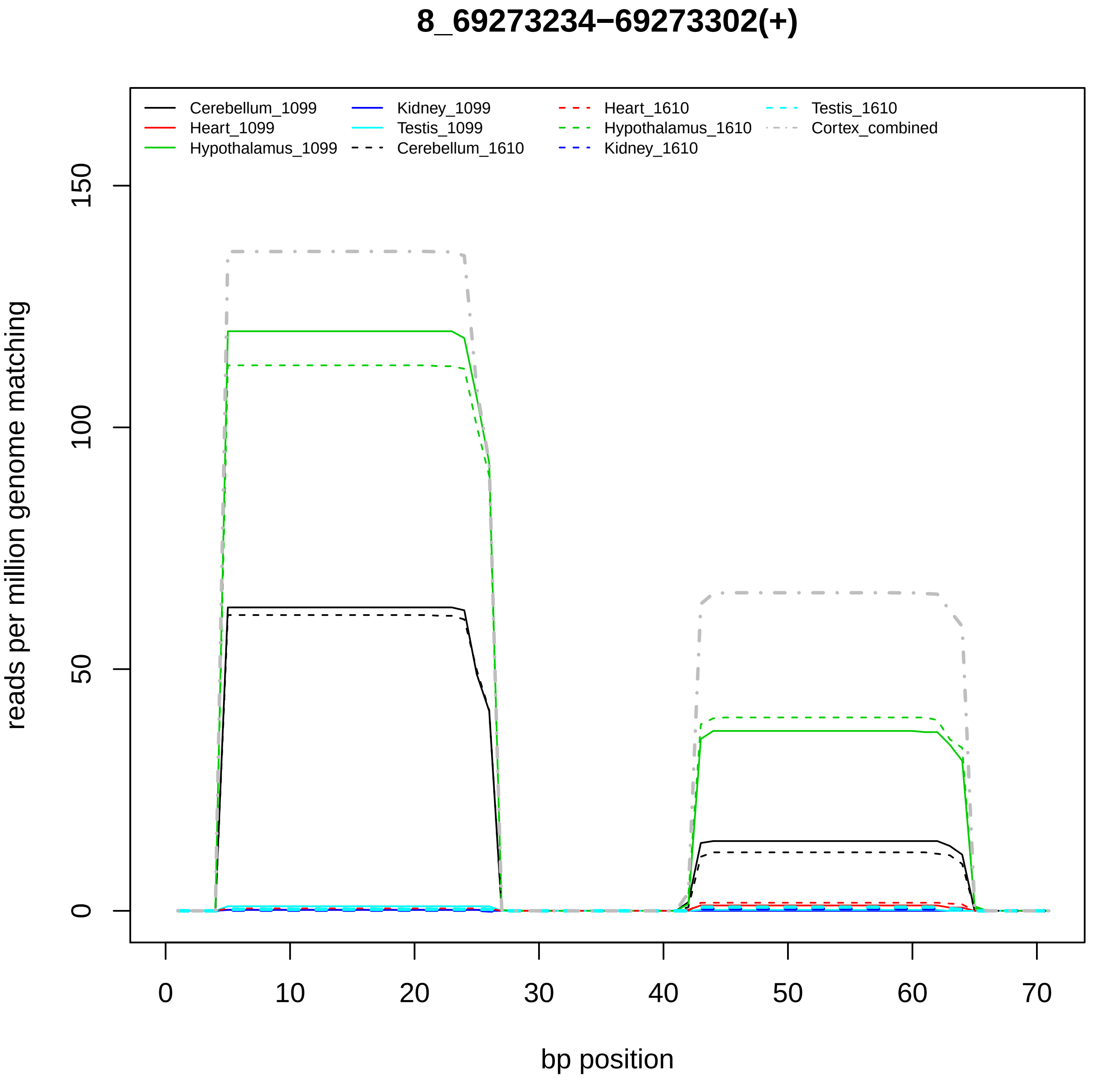

8_69273234−69273302(+)
Cerebellum_1099
Heart_1099
Hypothalamus_1099
Kidney_1099
Testis_1099
Cerebellum_1610
Heart_1610
Hypothalamus_1610
Kidney_1610
Testis_1610
Cortex_combined
reads per million genome matching
0
50
100
150
bp position
0
10
20
30
40
50
60
70

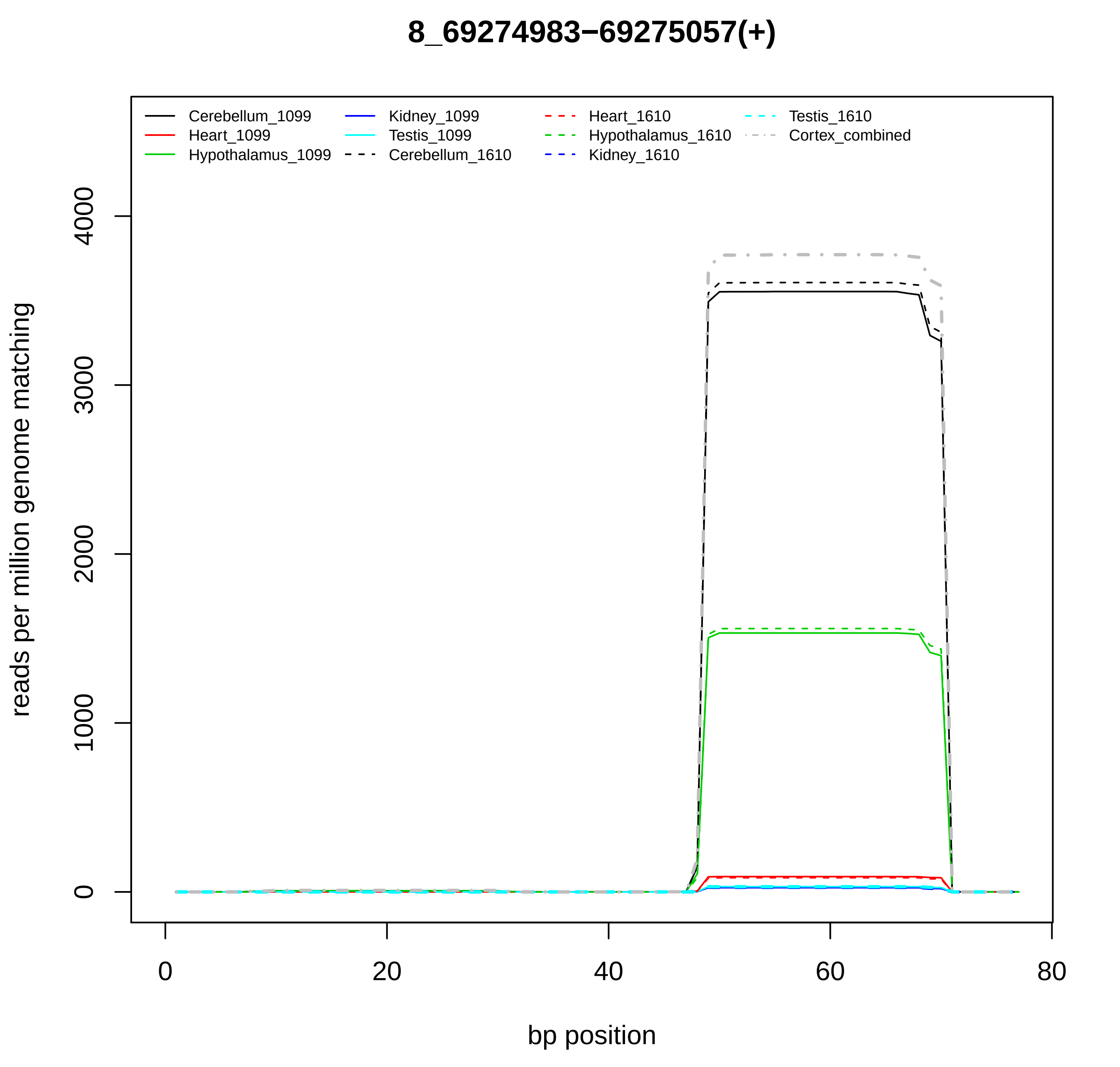

8_69274983−69275057(+)
Cerebellum_1099
Heart_1099
Hypothalamus_1099
Kidney_1099
Testis_1099
Cerebellum_1610
Heart_1610
Hypothalamus_1610
Kidney_1610
Testis_1610
Cortex_combined
reads per million genome matching
bp position
0
1000
2000
3000
4000
0
20
40
60
80

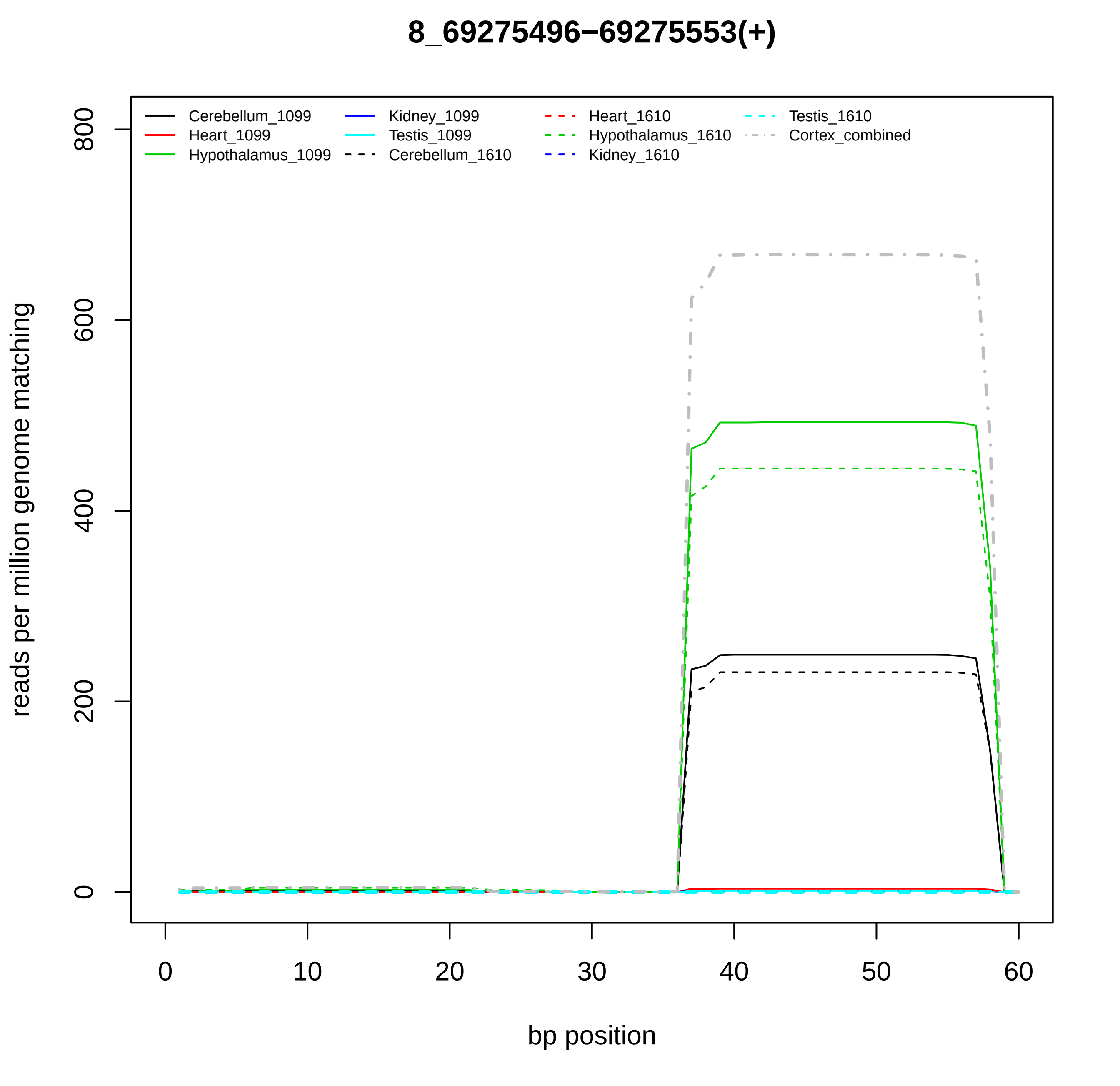
8_69275496−69275553(+)
Cerebellum_1099
Heart_1099
Hypothalamus_1099
Kidney_1099
Testis_1099
Cerebellum_1610
Heart_1610
Hypothalamus_1610
Kidney_1610
Testis_1610
Cortex_combined
reads per million genome matching
bp position
0
200
400
600
800
0
10
20
30
40
50
60

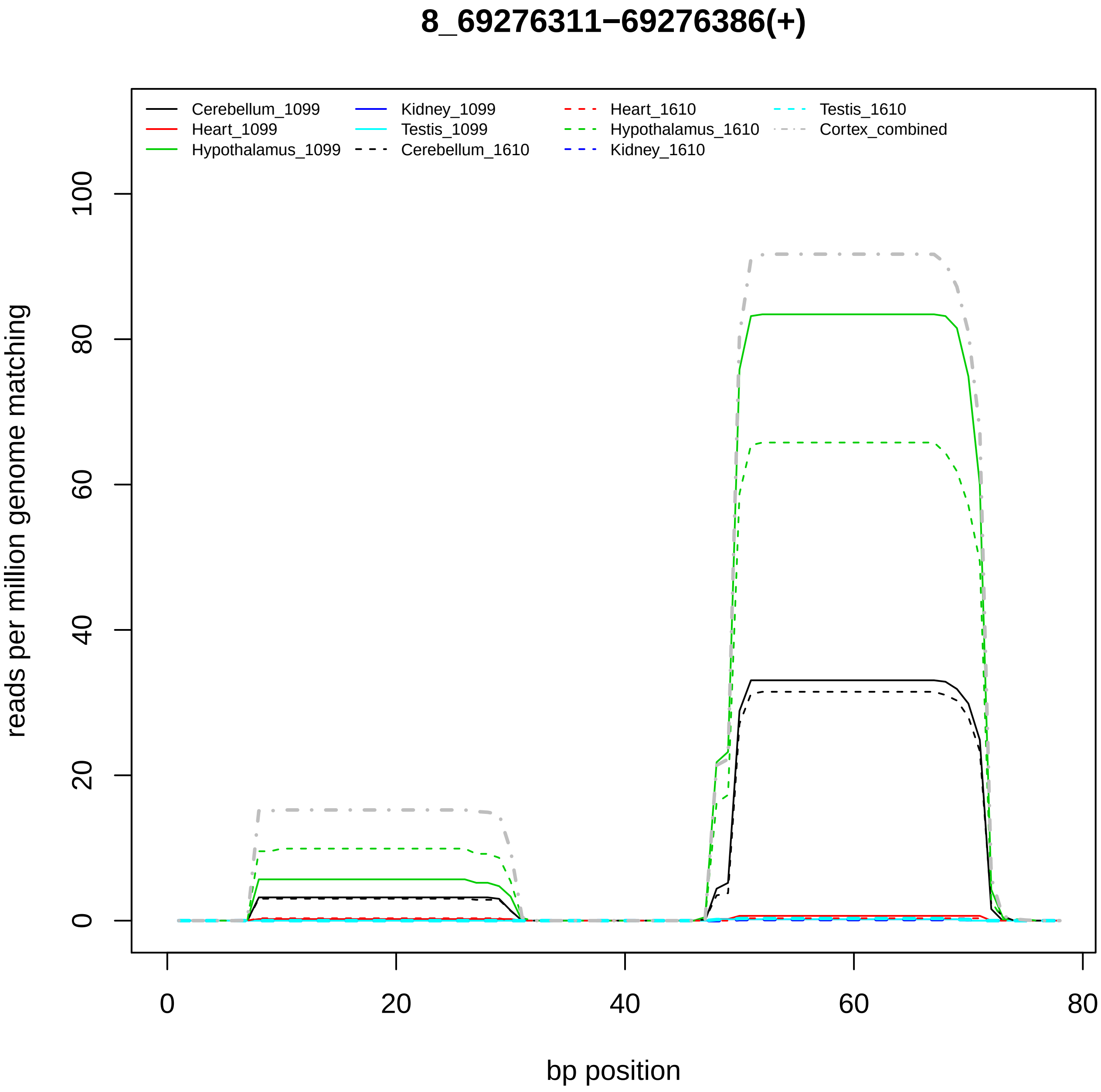

8_69276311−69276386(+)
Cerebellum_1099
Heart_1099
Hypothalamus_1099
Kidney_1099
Testis_1099
Cerebellum_1610
Heart_1610
Hypothalamus_1610
Kidney_1610
Testis_1610
Cortex_combined
reads per million genome matching
0
20
40
60
80
100
bp position
0
20
40
60
80

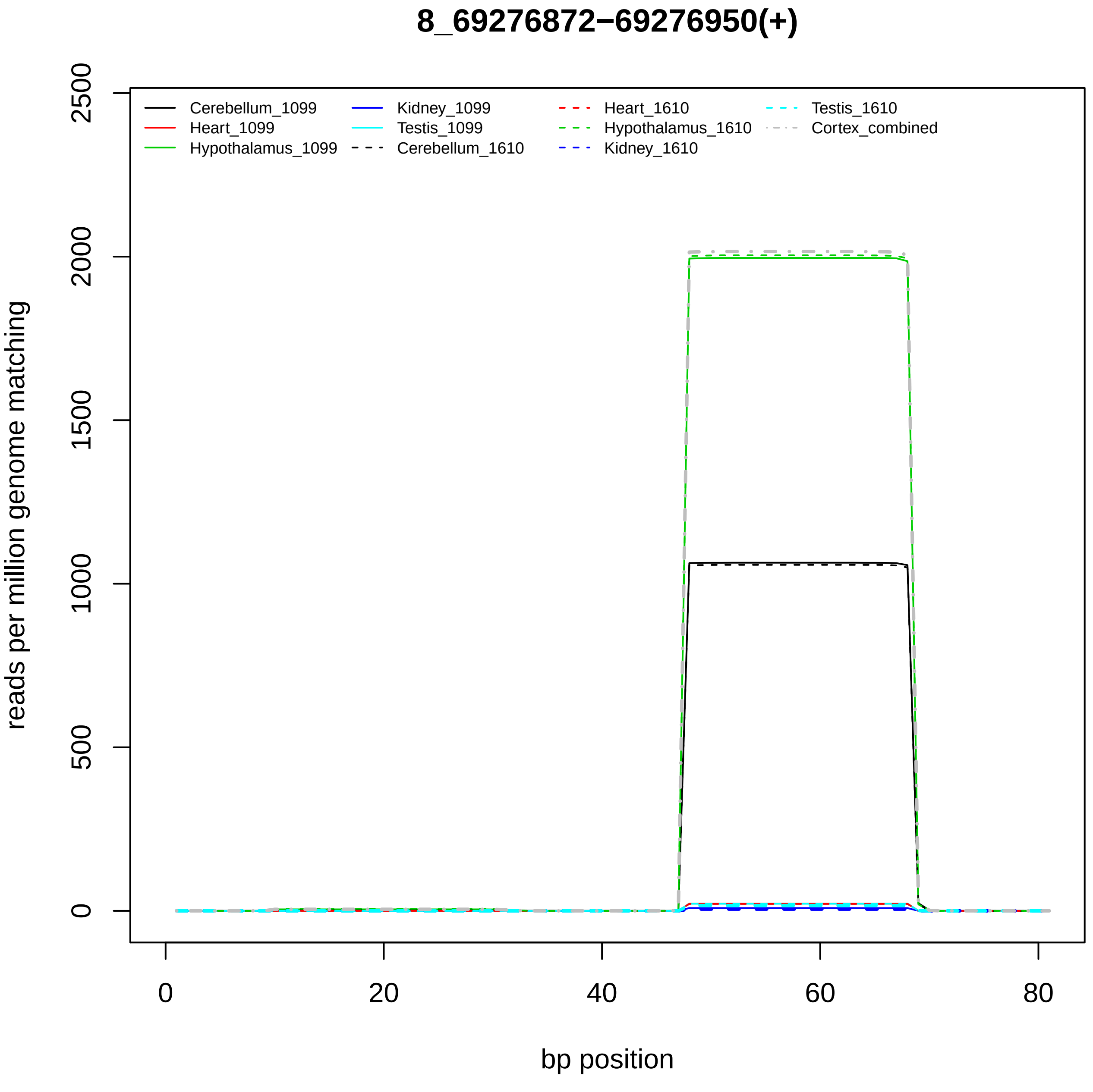

8_69276872−69276950(+)
Cerebellum_1099
Heart_1099
Hypothalamus_1099
Kidney_1099
Testis_1099
Cerebellum_1610
Heart_1610
Hypothalamus_1610
Kidney_1610
Testis_1610
Cortex_combined
reads per million genome matching
bp position
0
500
1000
1500
2000
2500
0
20
40
60
80

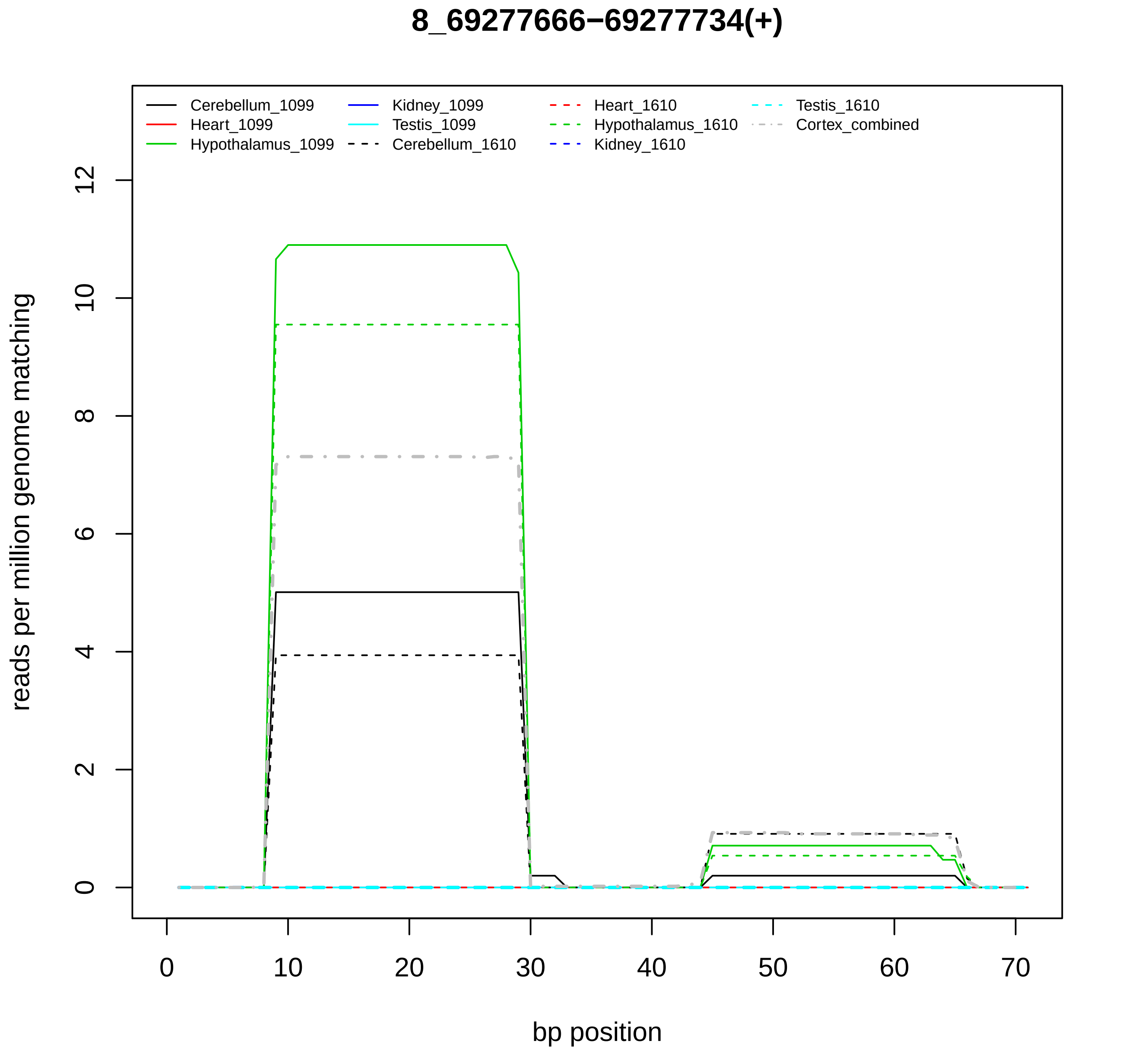

8_69277666−69277734(+)
Cerebellum_1099
Heart_1099
Hypothalamus_1099
Kidney_1099
Testis_1099
Cerebellum_1610
Heart_1610
Hypothalamus_1610
Kidney_1610
Testis_1610
Cortex_combined
reads per million genome matching
bp position
0
2
4
6
8
10
12
0
10
20
30
40
50
60
70

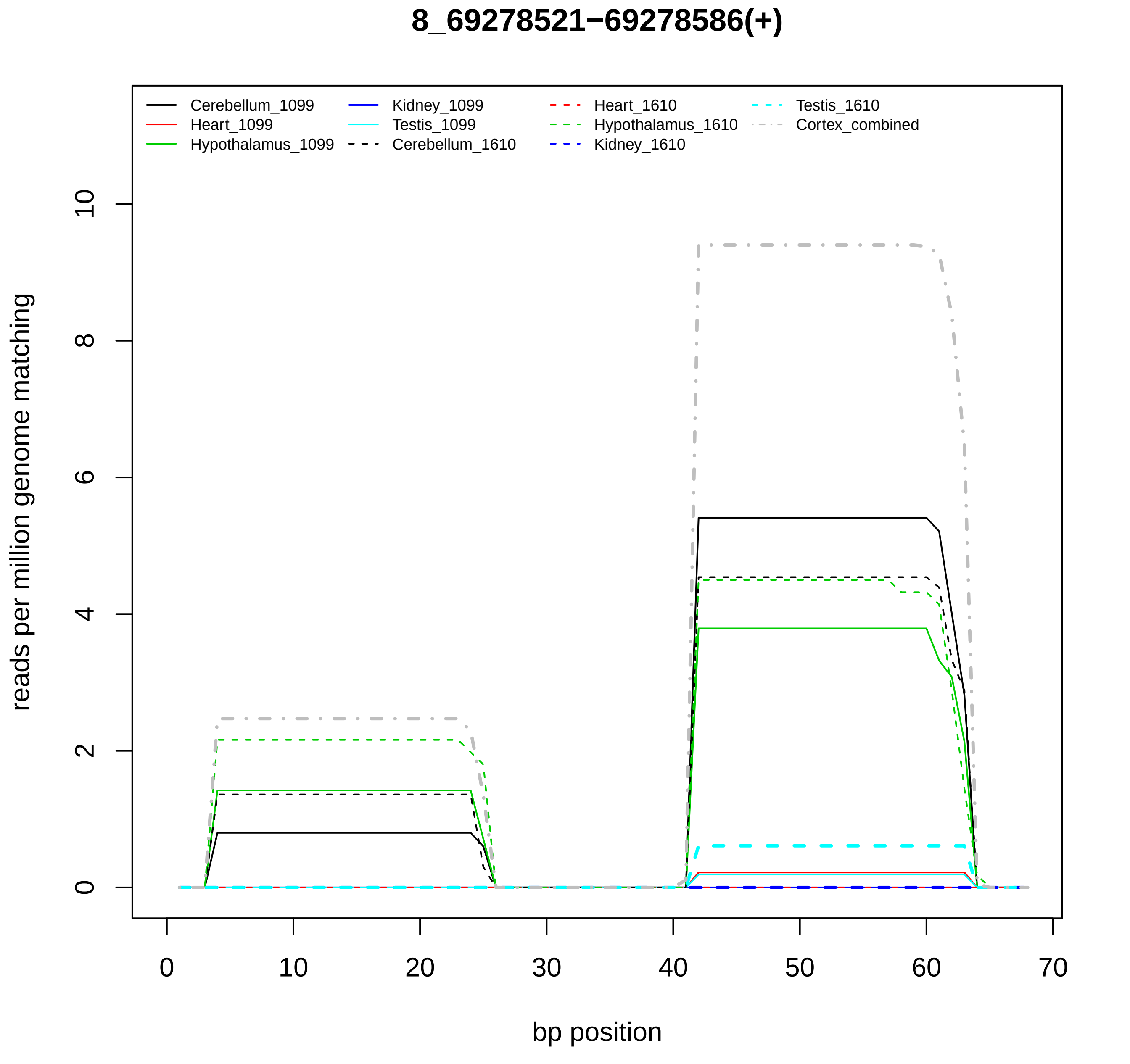

8_69278521−69278586(+)
Cerebellum_1099
Heart_1099
Hypothalamus_1099
Kidney_1099
Testis_1099
Cerebellum_1610
Heart_1610
Hypothalamus_1610
Kidney_1610
Testis_1610
Cortex_combined
reads per million genome matching
bp position
0
2
4
6
8
10
0
10
20
30
40
50
60
70

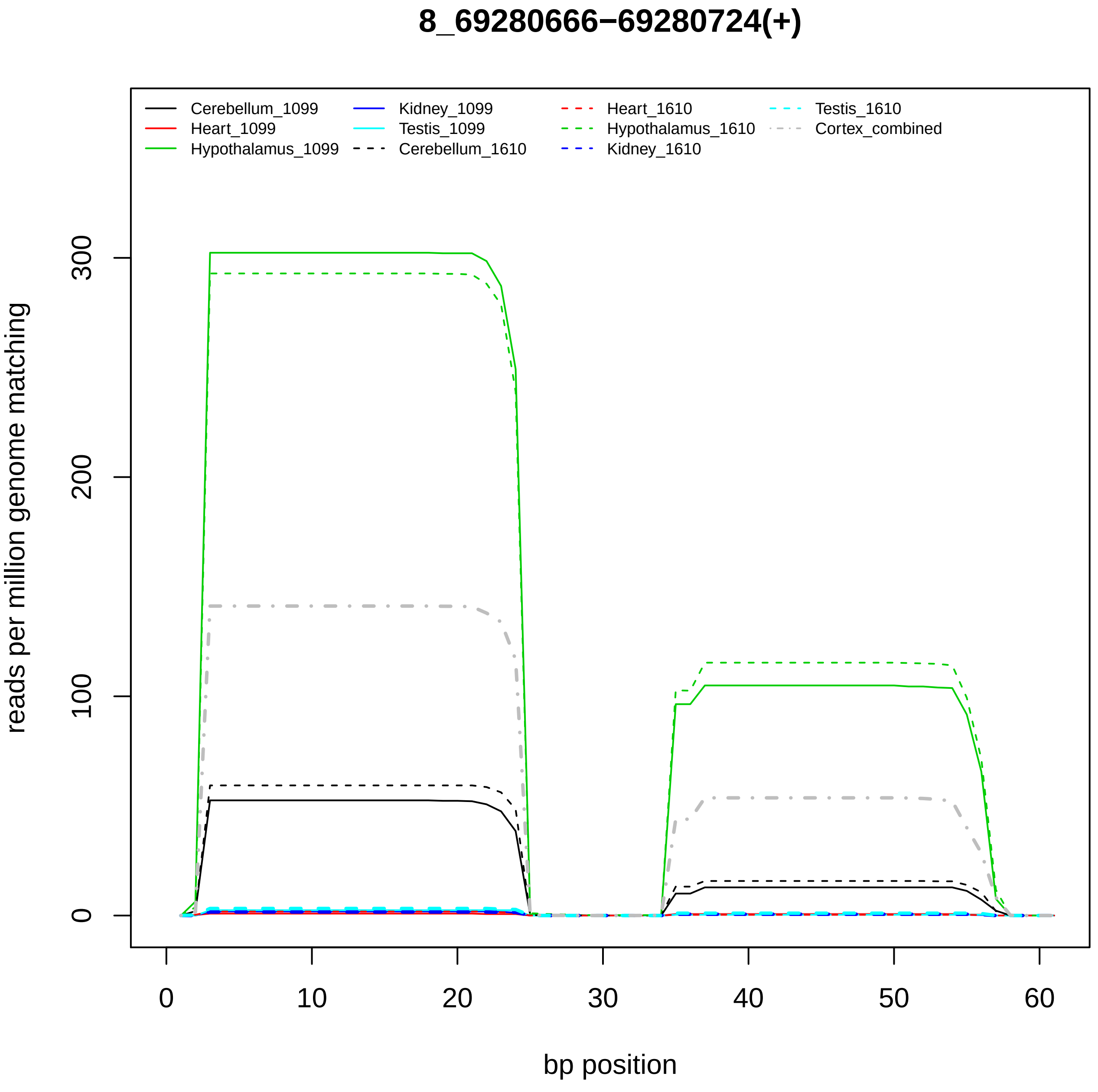
8_69280666−69280724(+)
Cerebellum_1099
Heart_1099
Hypothalamus_1099
Kidney_1099
Testis_1099
Cerebellum_1610
Heart_1610
Hypothalamus_1610
Kidney_1610
Testis_1610
Cortex_combined
reads per million genome matching
bp position
0
100
200
300
0
10
20
30
40
50
60

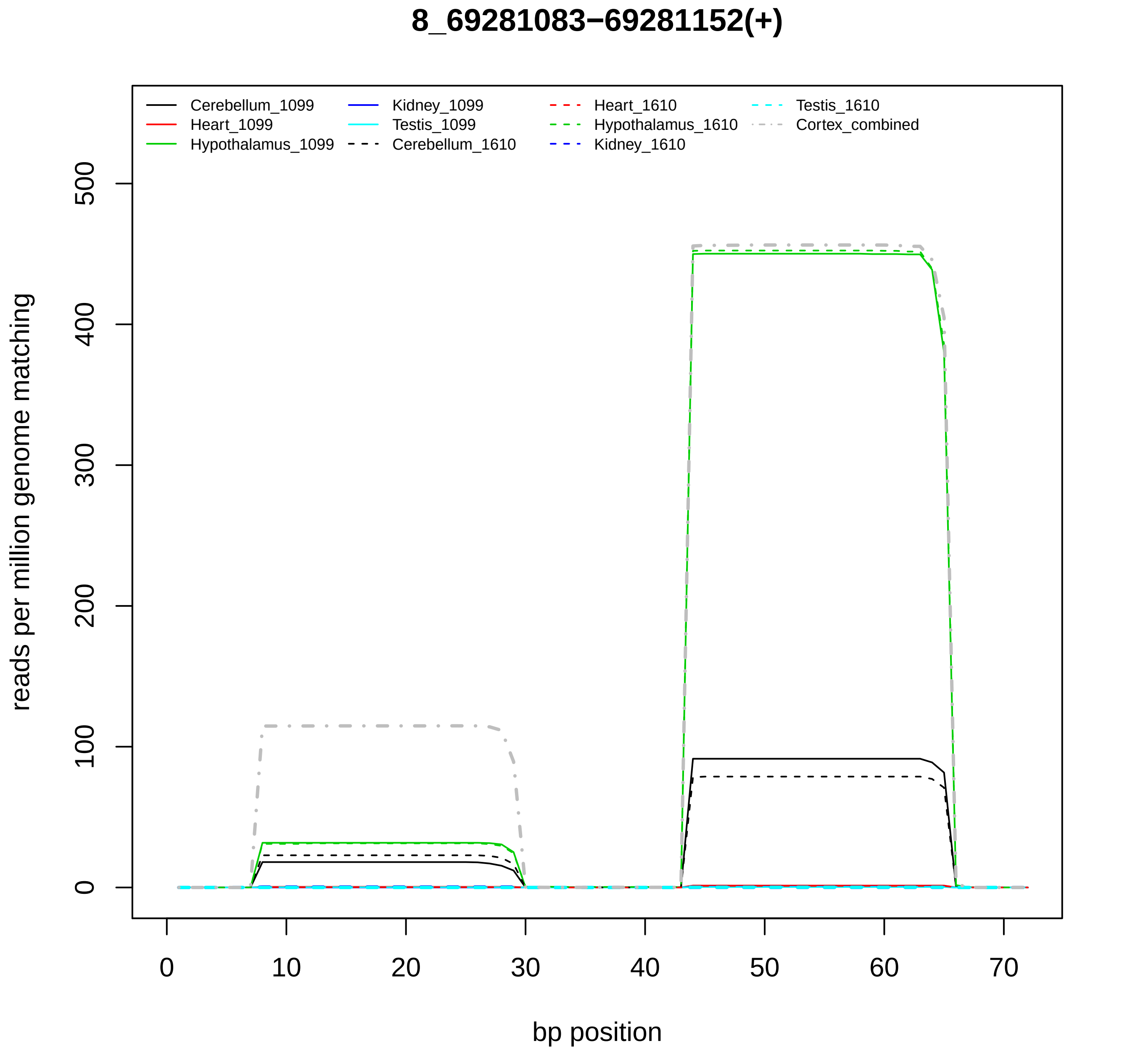

8_69281083−69281152(+)
Cerebellum_1099
Heart_1099
Hypothalamus_1099
Kidney_1099
Testis_1099
Cerebellum_1610
Heart_1610
Hypothalamus_1610
Kidney_1610
Testis_1610
Cortex_combined
reads per million genome matching
0
100
200
300
400
500
0
10
20
30
40
50
60
70
bp position

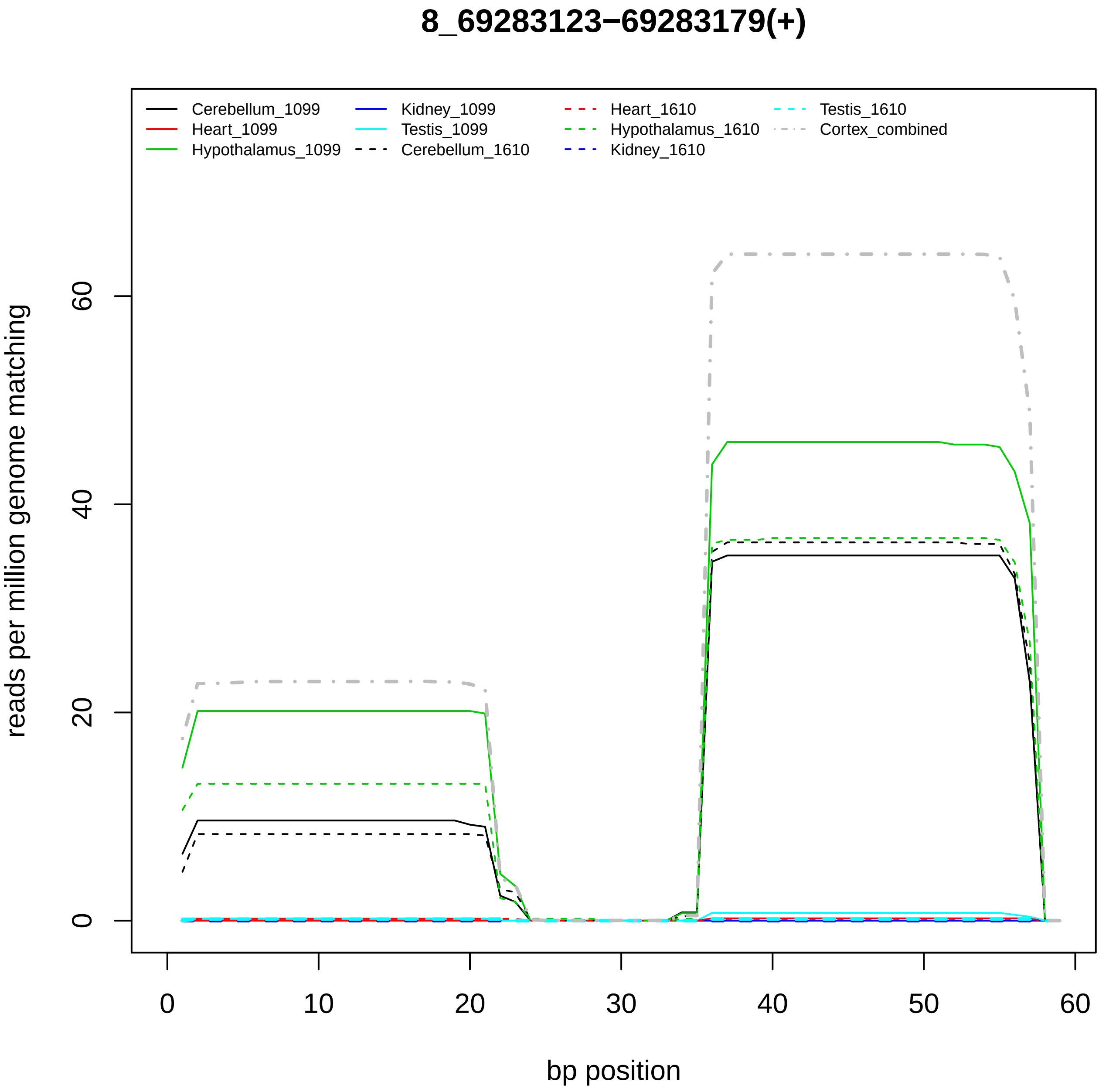
8_69283123−69283179(+)
Cerebellum_1099
Heart_1099
Hypothalamus_1099
Kidney_1099
Testis_1099
Cerebellum_1610
Heart_1610
Hypothalamus_1610
Kidney_1610
Testis_1610
Cortex_combined
reads per million genome matching
bp position
0
20
40
60
0
10
20
30
40
50
60

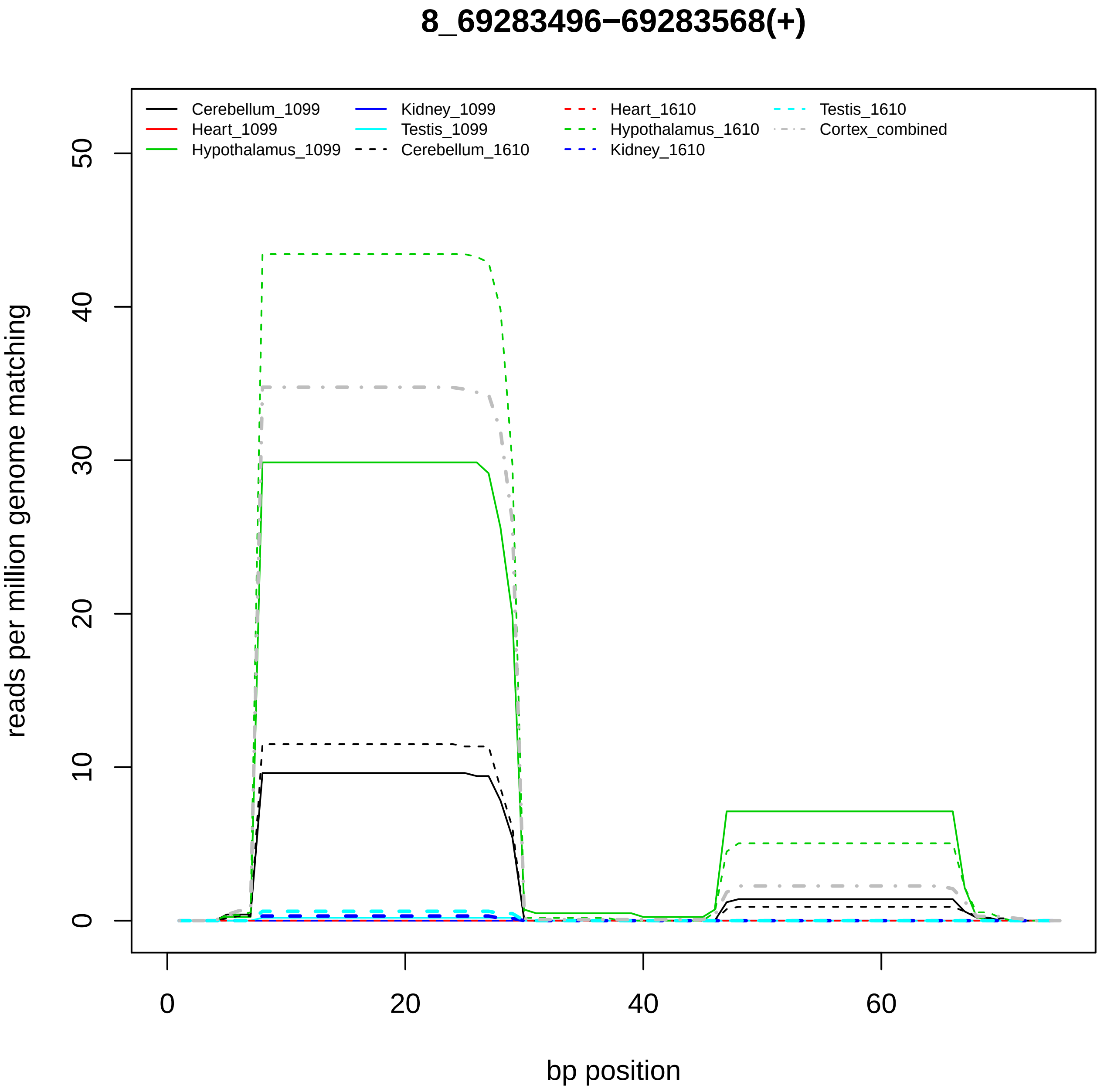
8_69283496−69283568(+)
Cerebellum_1099
Heart_1099
Hypothalamus_1099
Kidney_1099
Testis_1099
Cerebellum_1610
Heart_1610
Hypothalamus_1610
Kidney_1610
Testis_1610
Cortex_combined
reads per million genome matching
bp position
0
10
20
30
40
50
60

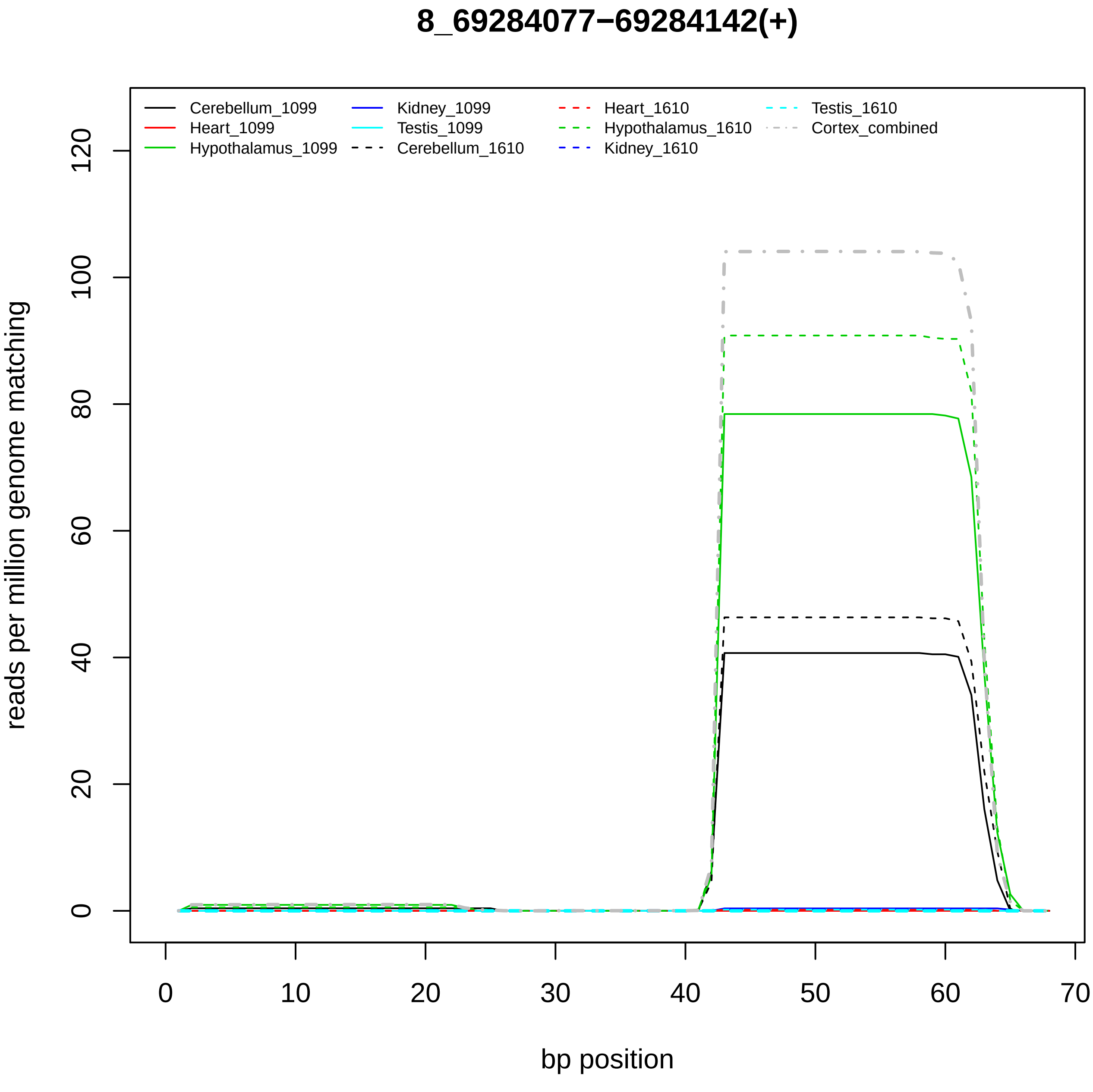

8_69284077−69284142(+)
Cerebellum_1099
Heart_1099
Hypothalamus_1099
Kidney_1099
Testis_1099
Cerebellum_1610
Heart_1610
Hypothalamus_1610
Kidney_1610
Testis_1610
Cortex_combined
reads per million genome matching
bp position
0
20
40
60
80
100
120
0
10
20
30
40
50
60
70

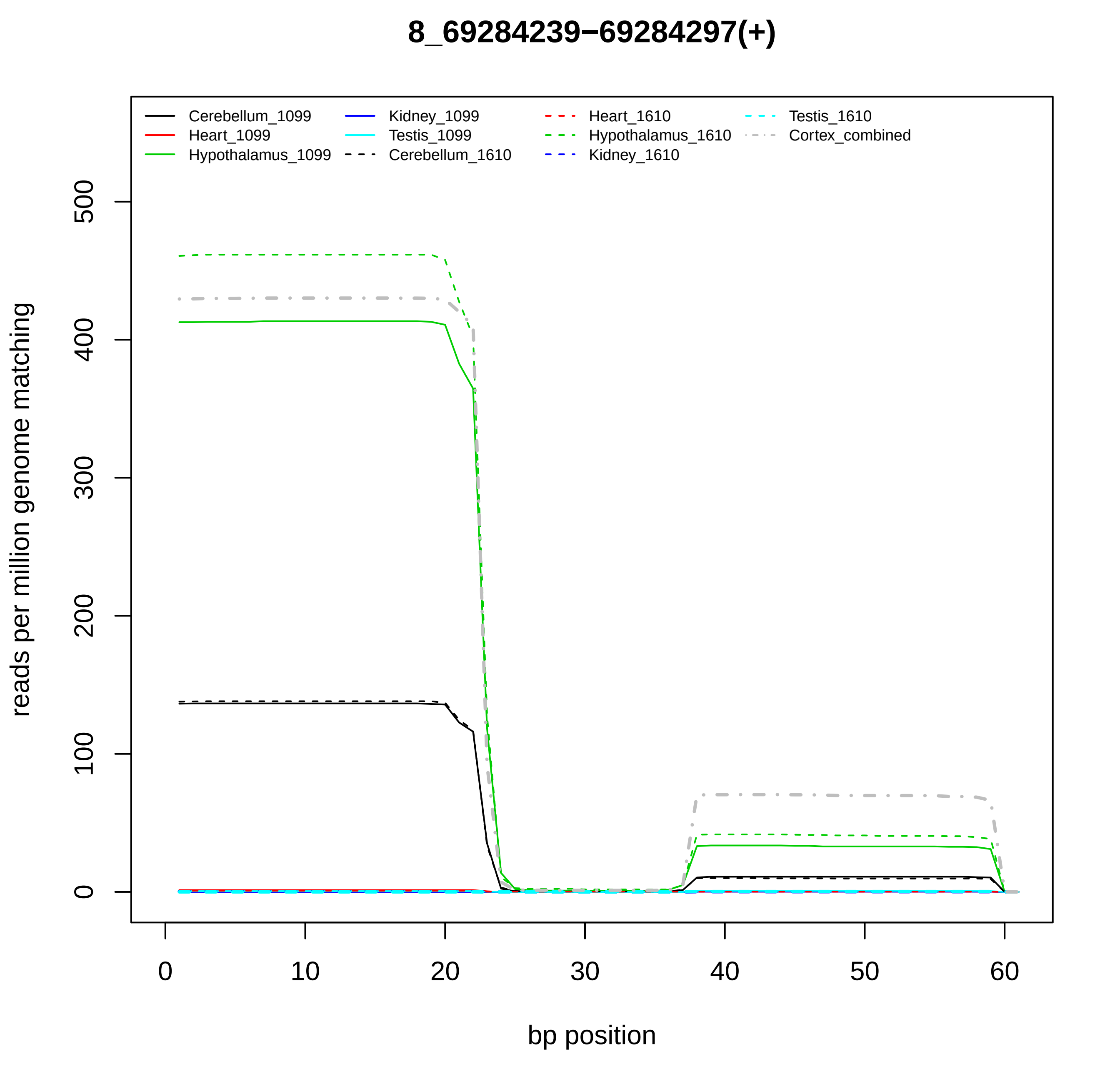

8_69284239−69284297(+)
Cerebellum_1099
Heart_1099
Hypothalamus_1099
Kidney_1099
Testis_1099
Cerebellum_1610
Heart_1610
Hypothalamus_1610
Kidney_1610
Testis_1610
Cortex_combined
reads per million genome matching
0
100
200
300
400
500
0
10
20
30
40
50
60
bp position

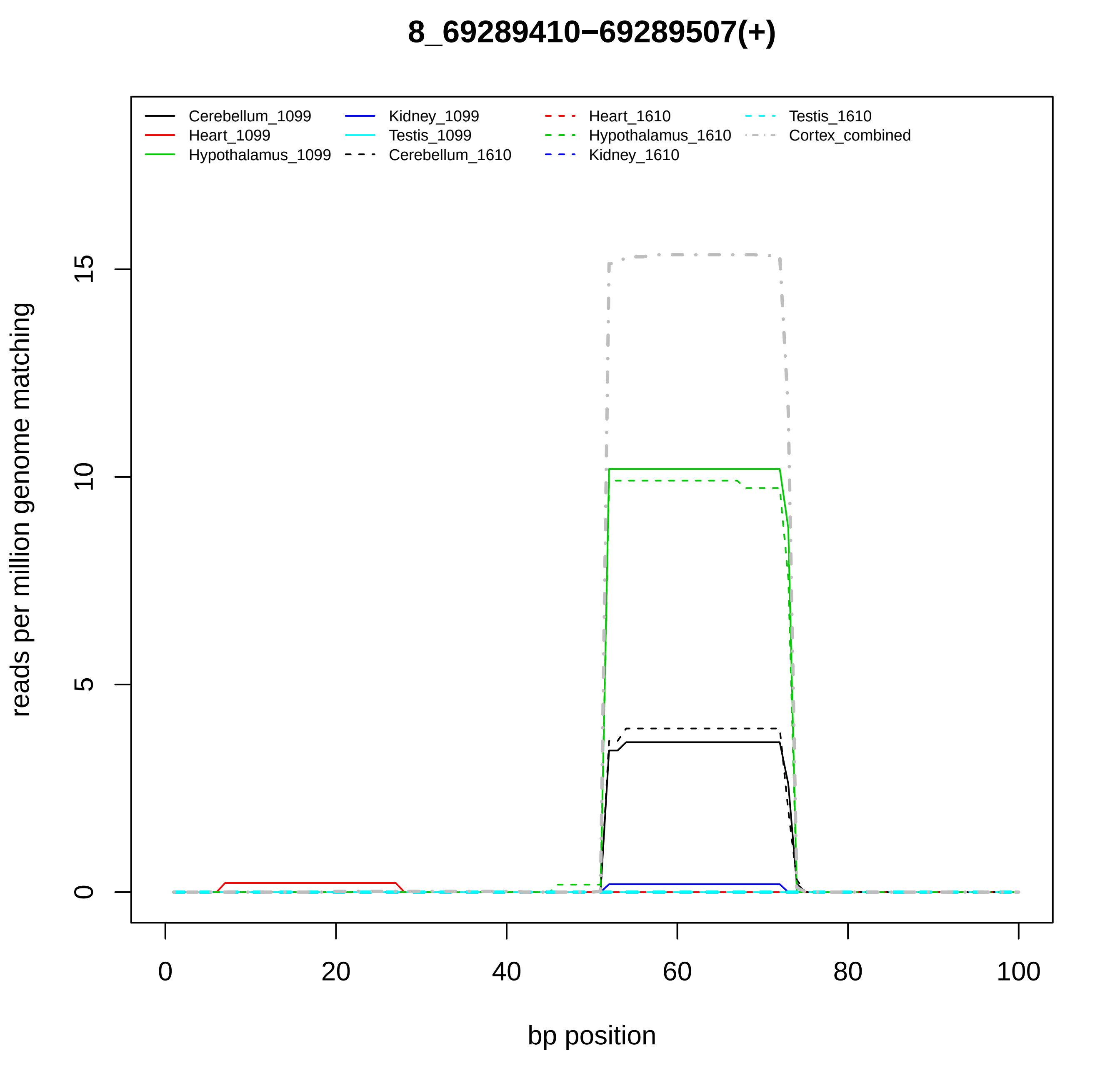
8_69289410−69289507(+)
Cerebellum_1099
Heart_1099
Hypothalamus_1099
Kidney_1099
Testis_1099
Cerebellum_1610
Heart_1610
Hypothalamus_1610
Kidney_1610
Testis_1610
Cortex_combined
reads per million genome matching
bp position
0
5
10
15
0
20
40
60
80
100

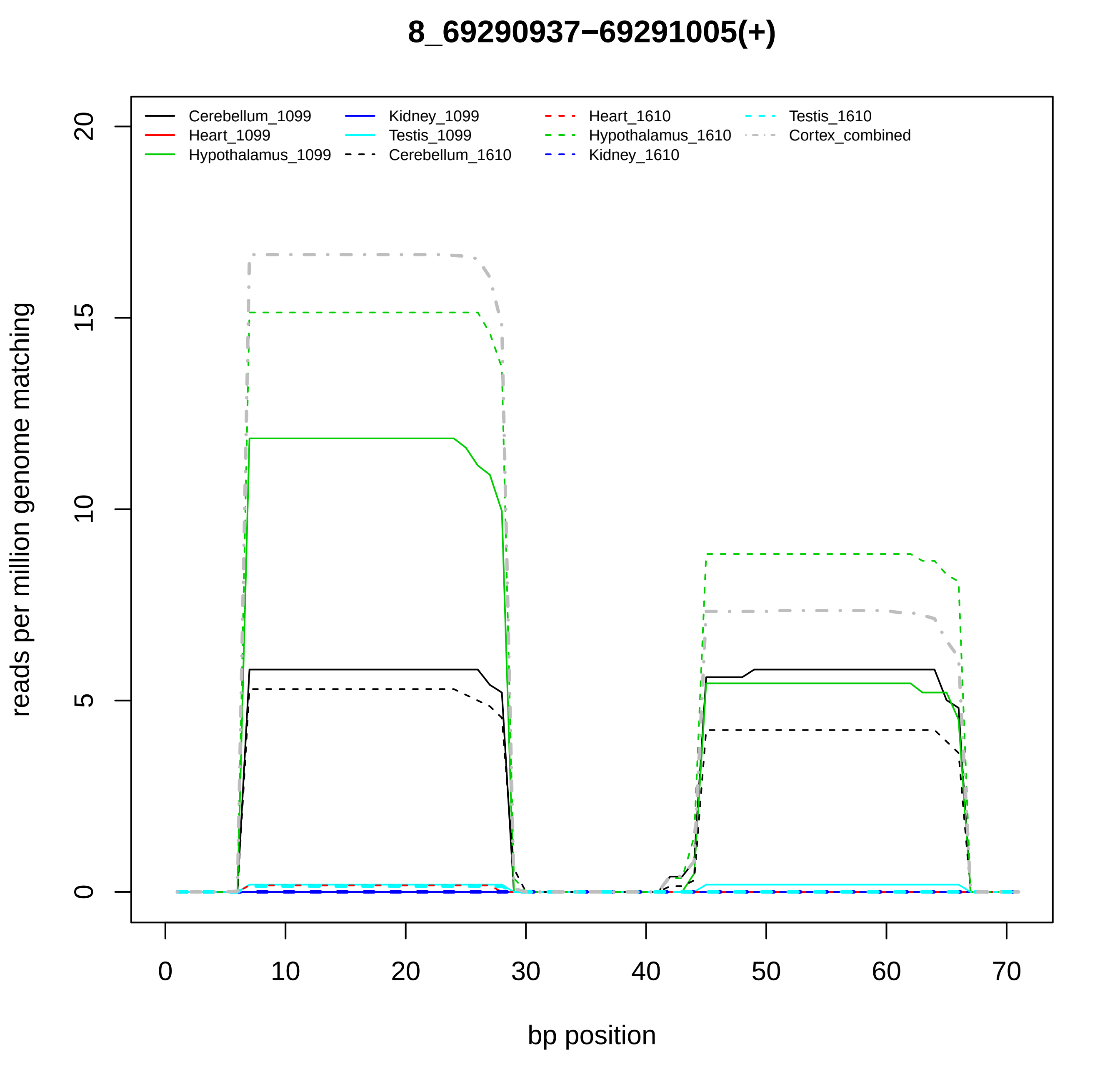

8_69290937−69291005(+)
Cerebellum_1099
Heart_1099
Hypothalamus_1099
Kidney_1099
Testis_1099
Cerebellum_1610
Heart_1610
Hypothalamus_1610
Kidney_1610
Testis_1610
Cortex_combined
reads per million genome matching
0
5
10
15
20
0
10
20
30
40
50
60
70
bp position

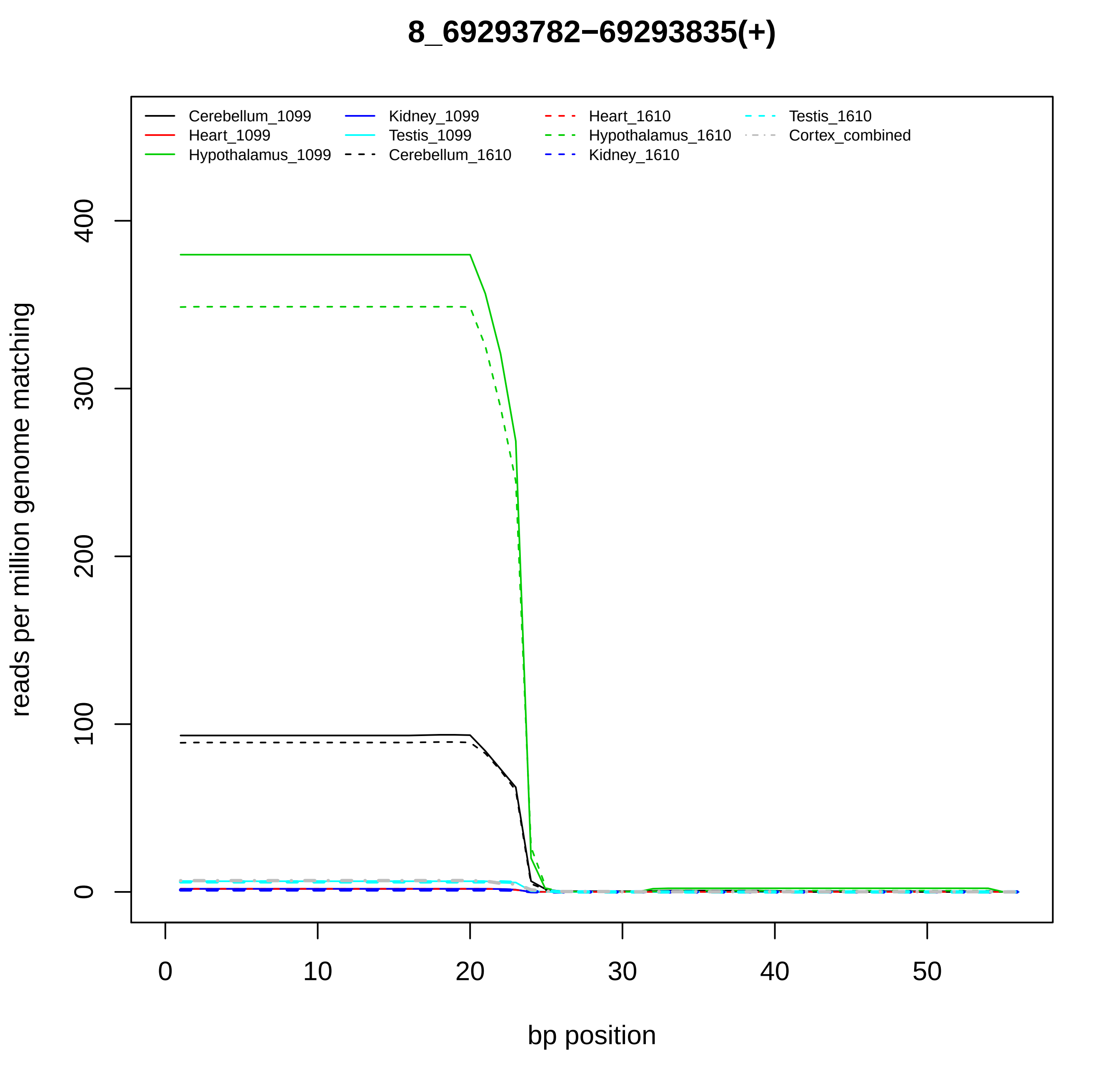

8_69293782−69293835(+)
Cerebellum_1099
Heart_1099
Hypothalamus_1099
Kidney_1099
Testis_1099
Cerebellum_1610
Heart_1610
Hypothalamus_1610
Kidney_1610
Testis_1610
Cortex_combined
reads per million genome matching
0
100
200
300
400
bp position
0
10
20
30
40
50

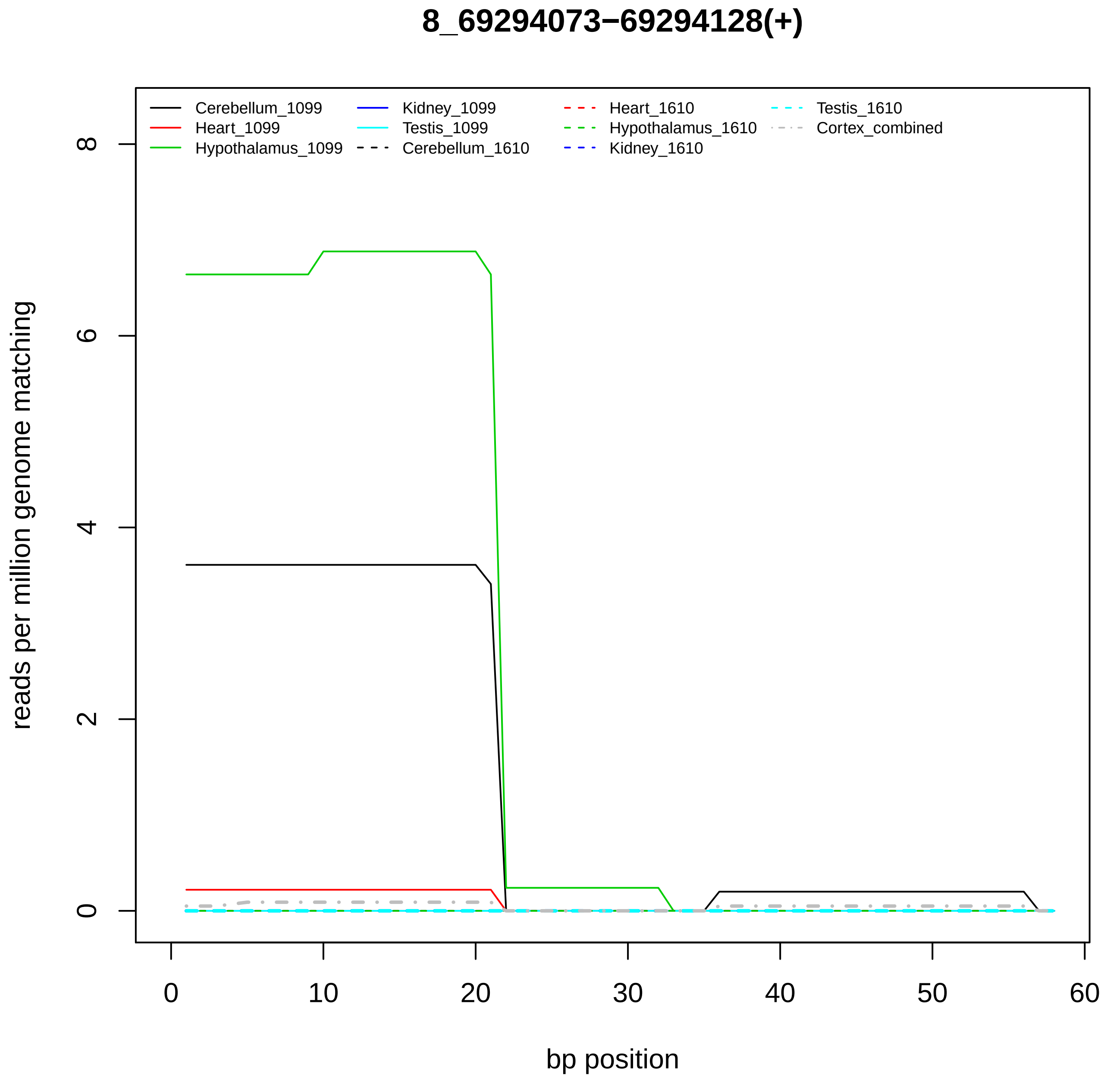
8_69294073−69294128(+)
Cerebellum_1099
Heart_1099
Hypothalamus_1099
Kidney_1099
Testis_1099
Cerebellum_1610
Heart_1610
Hypothalamus_1610
Kidney_1610
Testis_1610
Cortex_combined
reads per million genome matching
bp position
0
2
4
6
8
0
10
20
30
40
50
60

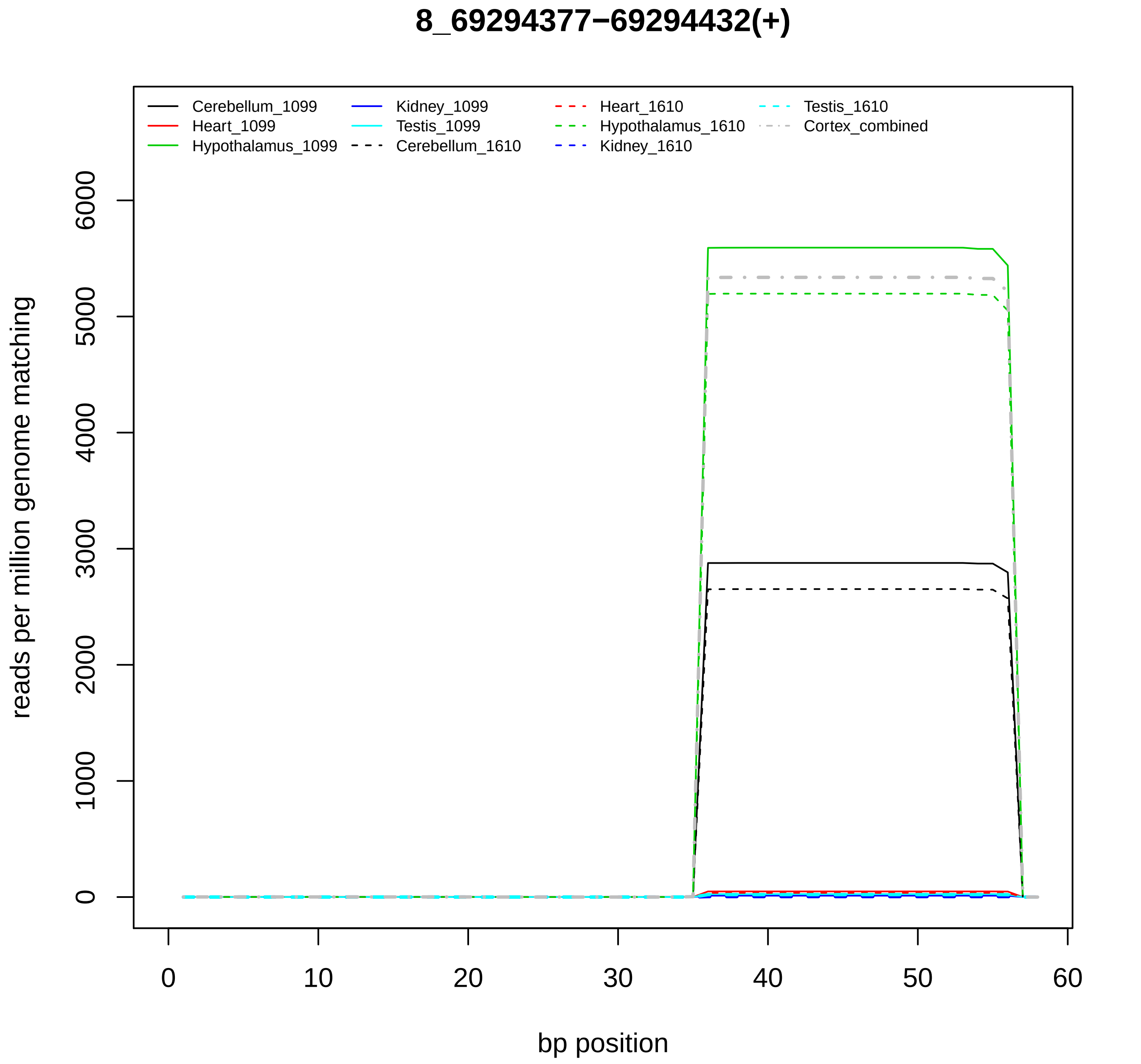
8_69294377−69294432(+)
Cerebellum_1099
Heart_1099
Hypothalamus_1099
Kidney_1099
Testis_1099
Cerebellum_1610
Heart_1610
Hypothalamus_1610
Kidney_1610
Testis_1610
Cortex_combined
reads per million genome matching
0
1000
2000
3000
4000
5000
6000
bp position
0
10
20
30
40
50
60

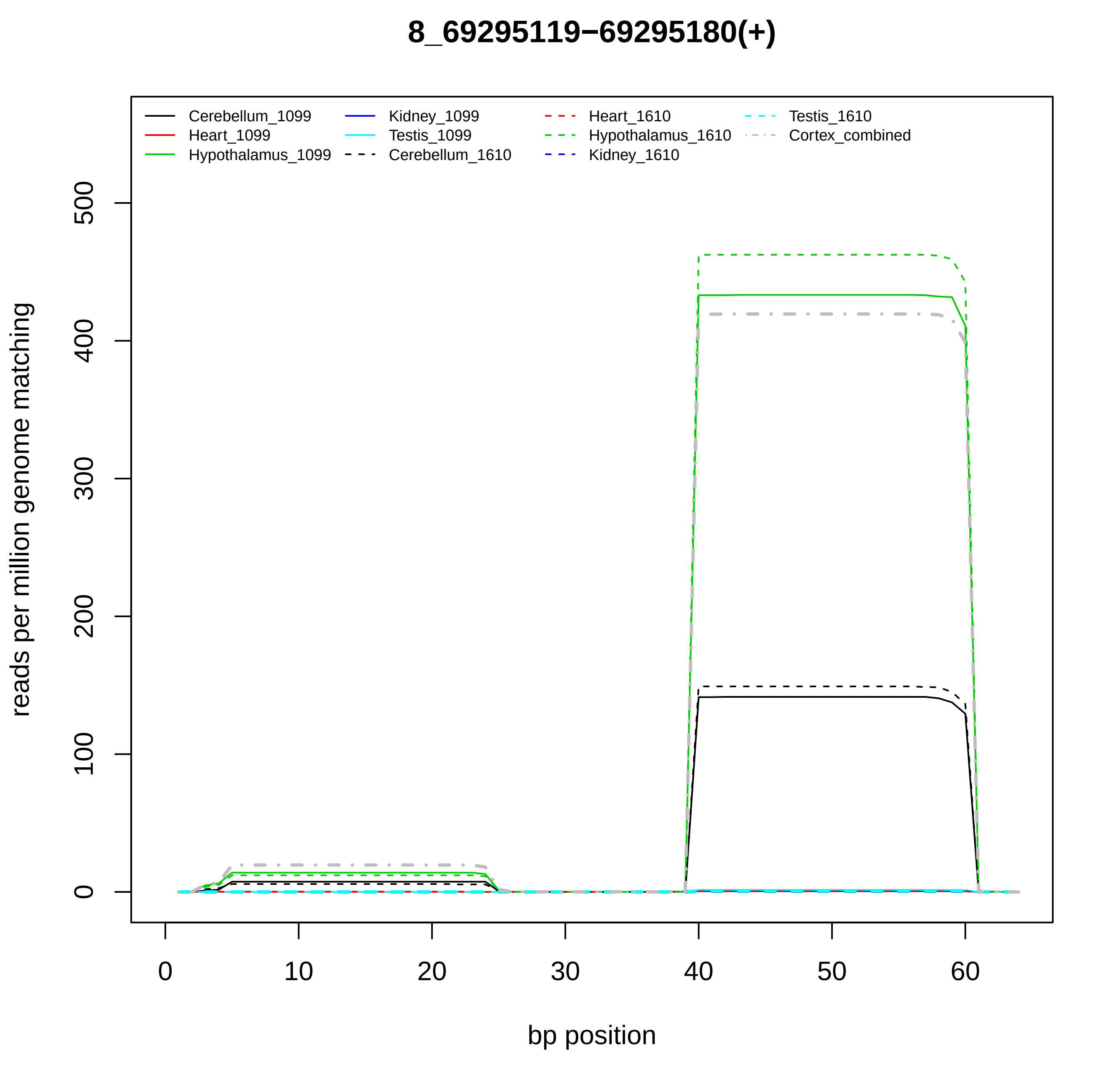

8_69295119−69295180(+)
Cerebellum_1099
Heart_1099
Hypothalamus_1099
Kidney_1099
Testis_1099
Cerebellum_1610
Heart_1610
Hypothalamus_1610
Kidney_1610
Testis_1610
Cortex_combined
reads per million genome matching
0
100
200
300
400
500
0
10
20
30
40
50
60
bp position

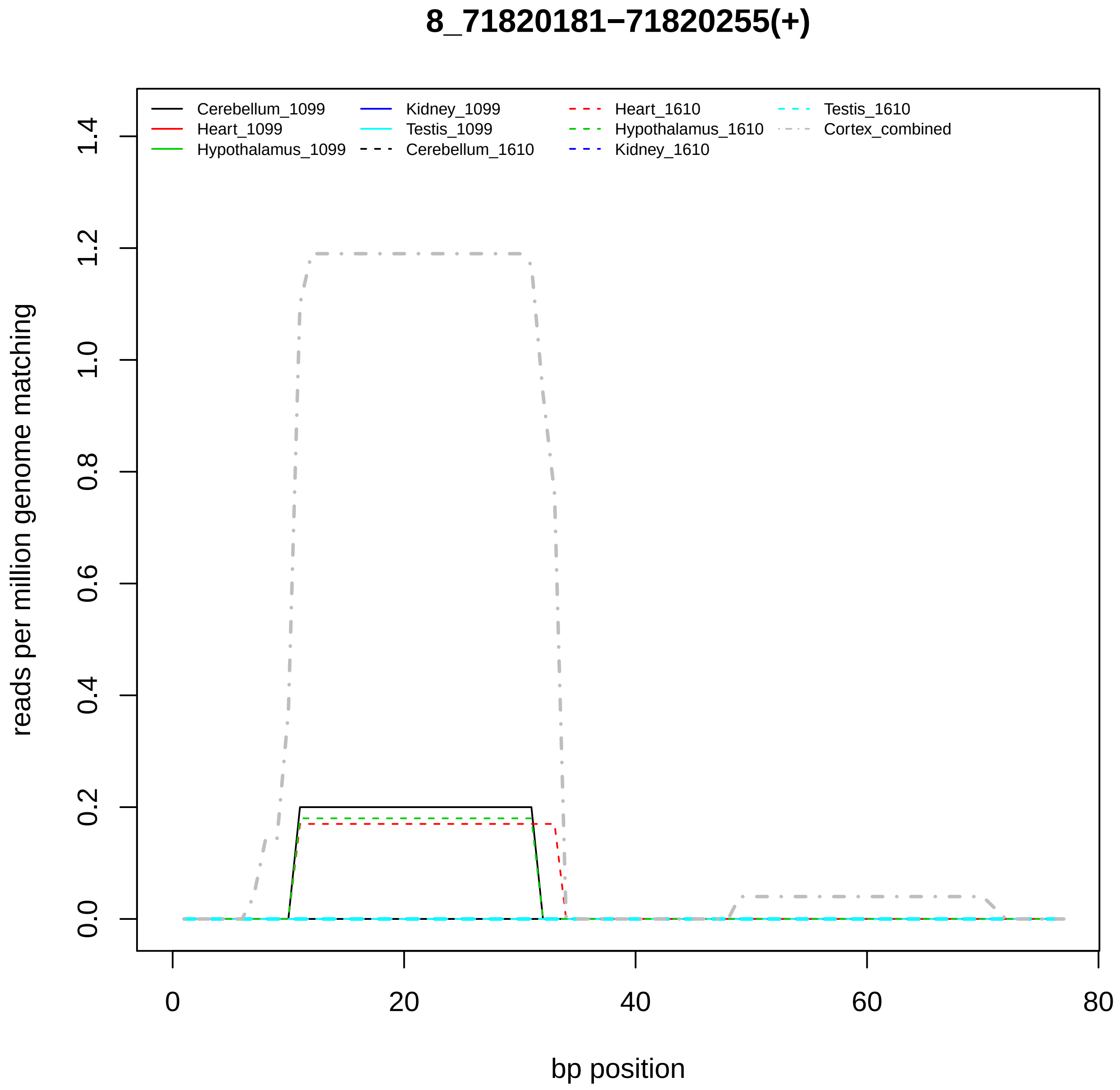

8_71820181−71820255(+)
Cerebellum_1099
Heart_1099
Hypothalamus_1099
Kidney_1099
Testis_1099
Cerebellum_1610
Heart_1610
Hypothalamus_1610
Kidney_1610
Testis_1610
Cortex_combined
reads per million genome matching
bp position
0.0
0.2
0.4
0.6
0.8
1.0
1.2
1.4
0
20
40
60
80

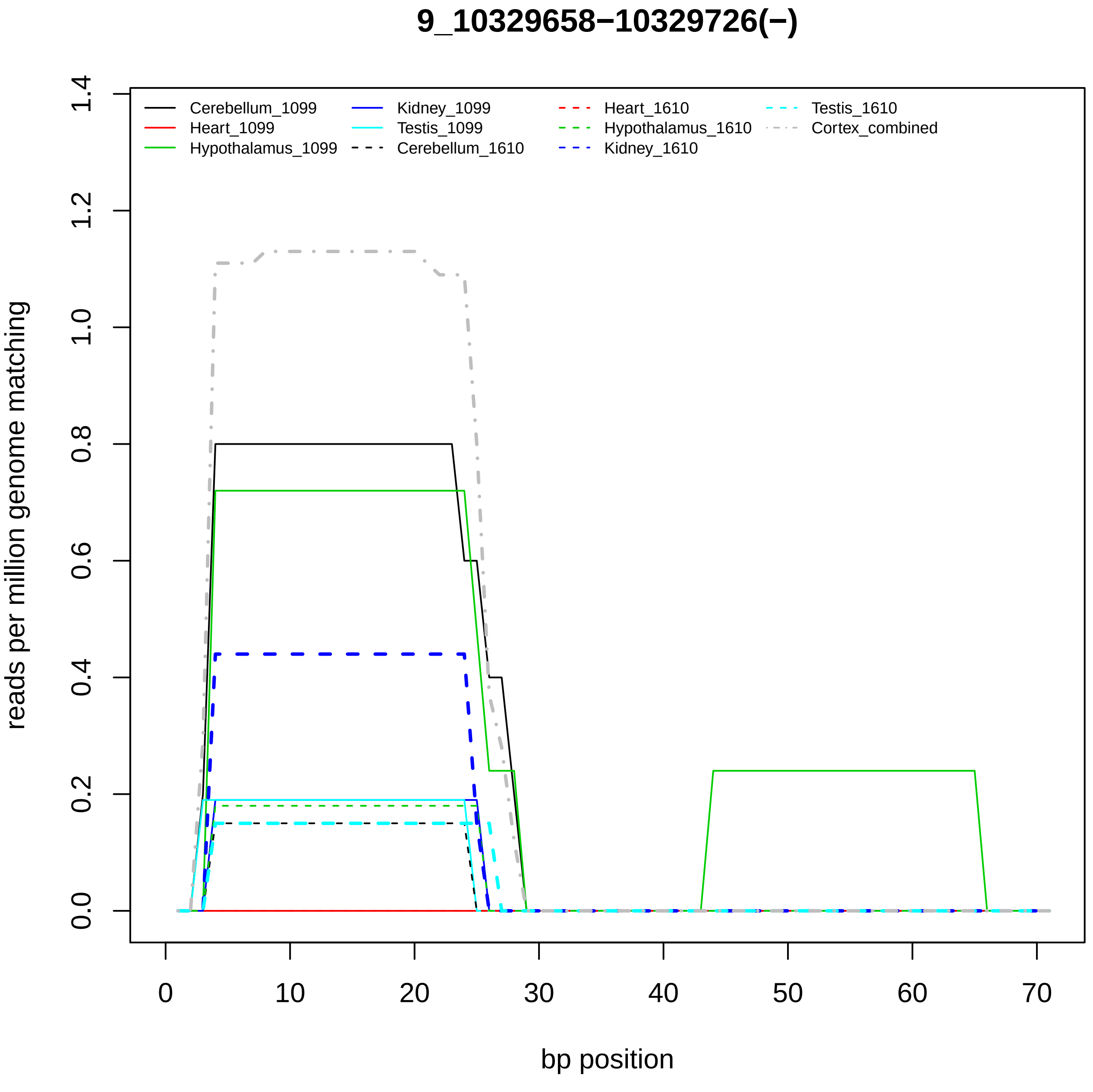

9_10329658−10329726(−)
Cerebellum_1099
Heart_1099
Hypothalamus_1099
Kidney_1099
Testis_1099
Cerebellum_1610
Heart_1610
Hypothalamus_1610
Kidney_1610
Testis_1610
Cortex_combined
reads per million genome matching
bp position
0.0
0.2
0.4
0.6
0.8
1.0
1.2
1.4
0
10
20
30
40
50
60
70

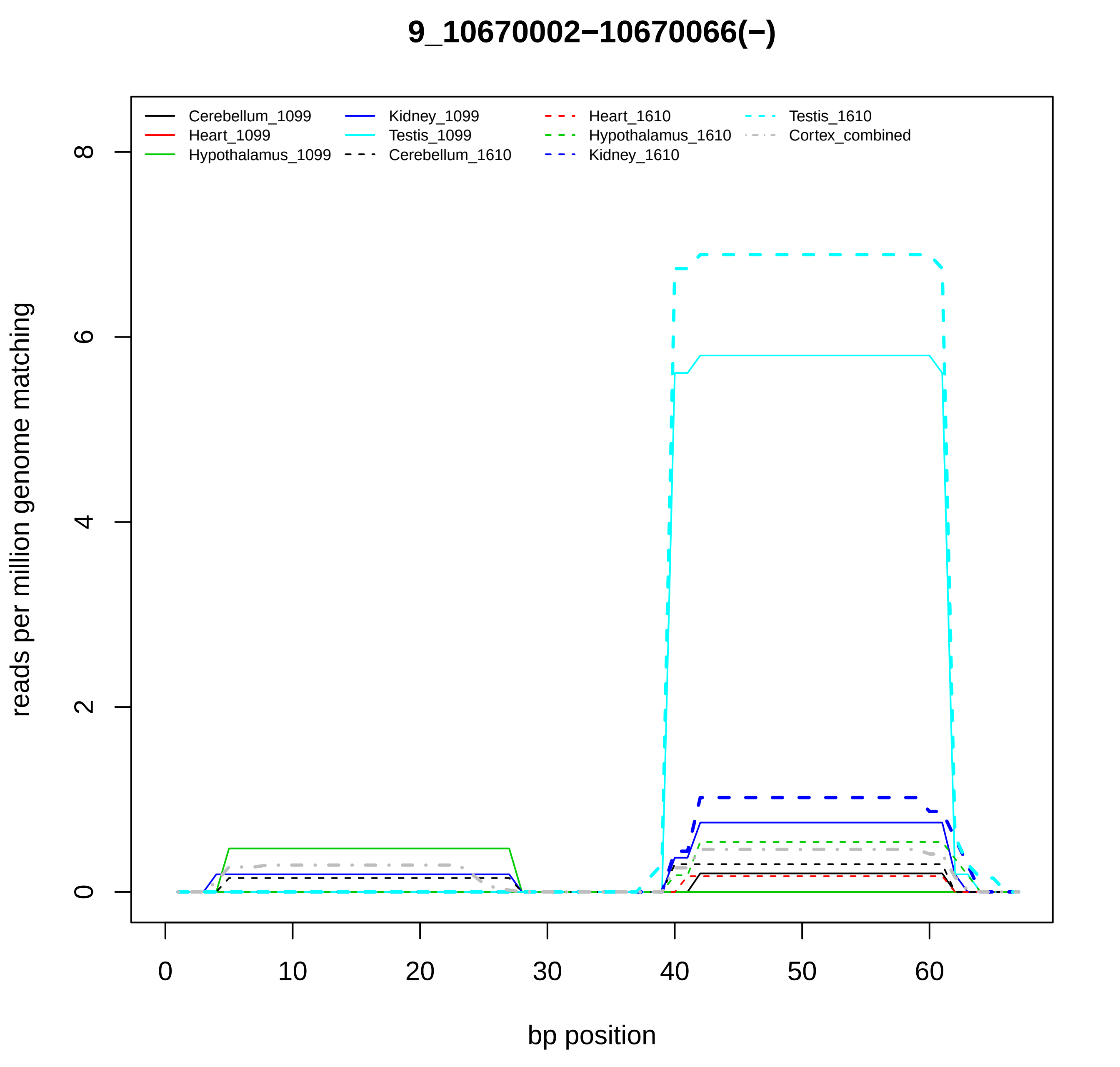

9_10670002−10670066(−)
Cerebellum_1099
Heart_1099
Hypothalamus_1099
Kidney_1099
Testis_1099
Cerebellum_1610
Heart_1610
Hypothalamus_1610
Kidney_1610
Testis_1610
Cortex_combined
reads per million genome matching
0
2
4
6
8
0
10
20
30
40
50
60
bp position

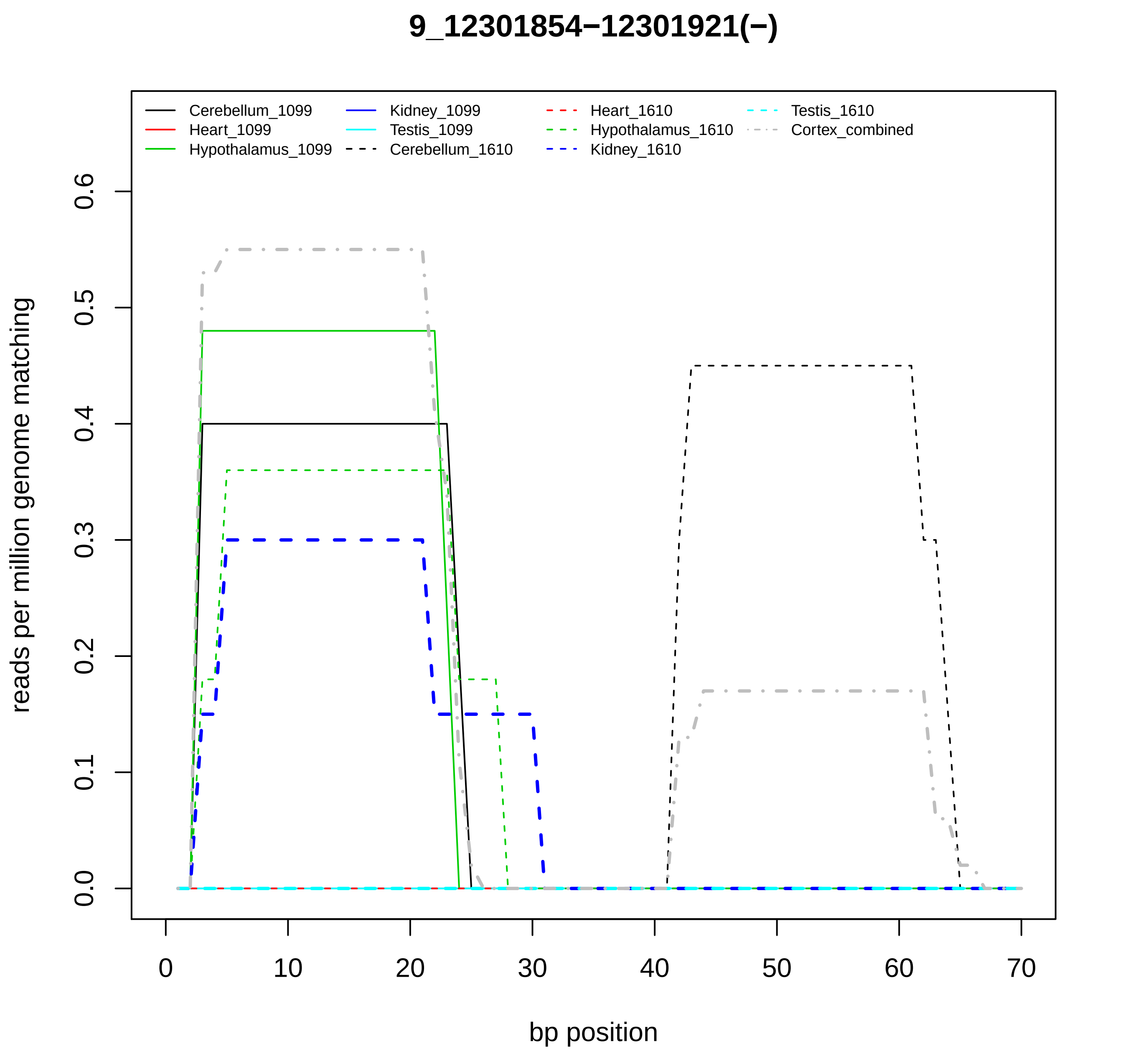

9_12301854−12301921(−)
Cerebellum_1099
Heart_1099
Hypothalamus_1099
Kidney_1099
Testis_1099
Cerebellum_1610
Heart_1610
Hypothalamus_1610
Kidney_1610
Testis_1610
Cortex_combined
reads per million genome matching
0.0
0.1
0.2
0.3
0.4
0.5
0.6
0
10
20
30
40
50
60
70
bp position

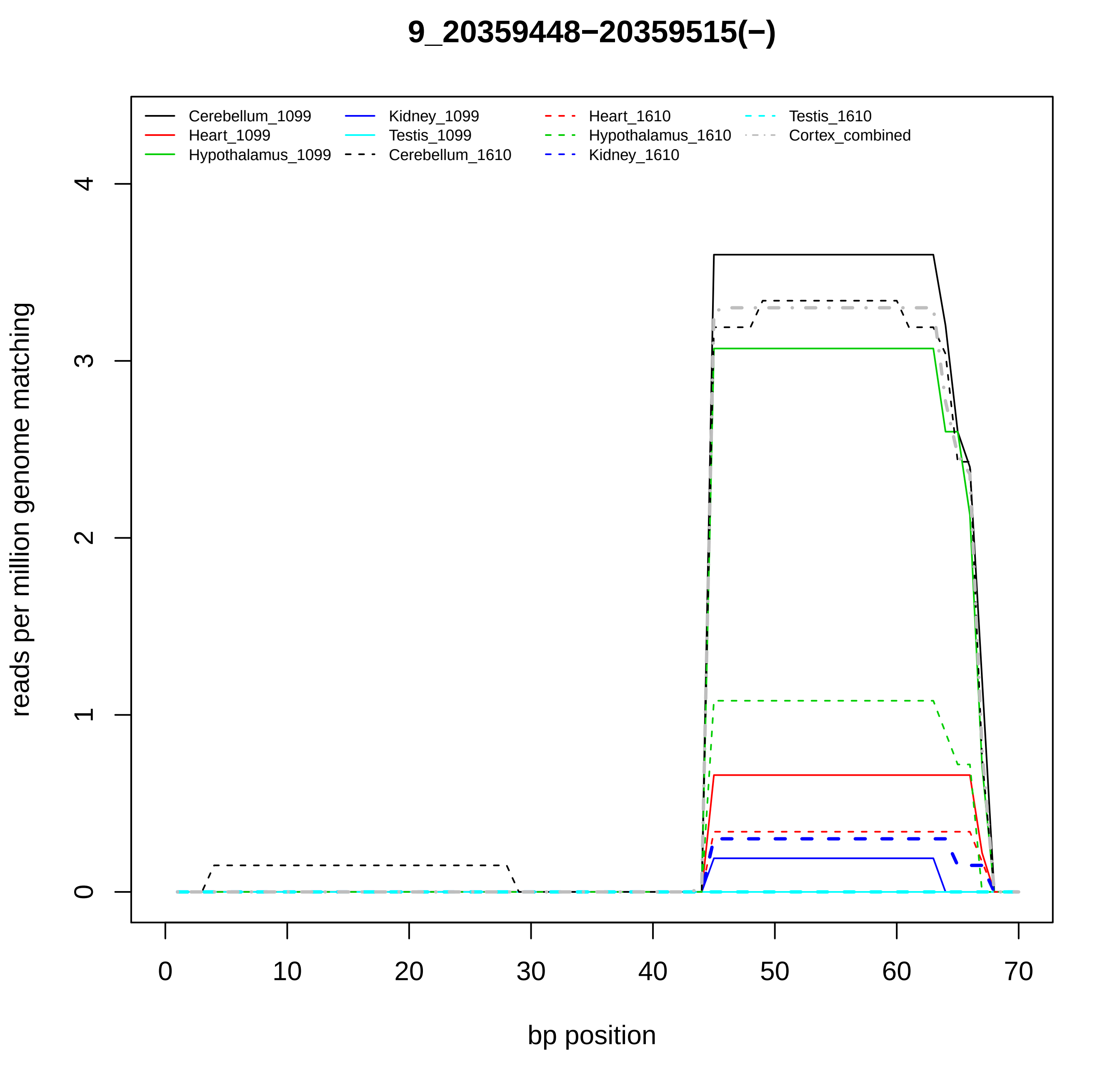
9_20359448−20359515(−)
Cerebellum_1099
Heart_1099
Hypothalamus_1099
Kidney_1099
Testis_1099
Cerebellum_1610
Heart_1610
Hypothalamus_1610
Kidney_1610
Testis_1610
Cortex_combined
reads per million genome matching
0
1
2
3
4
0
10
20
30
40
50
60
70
bp position

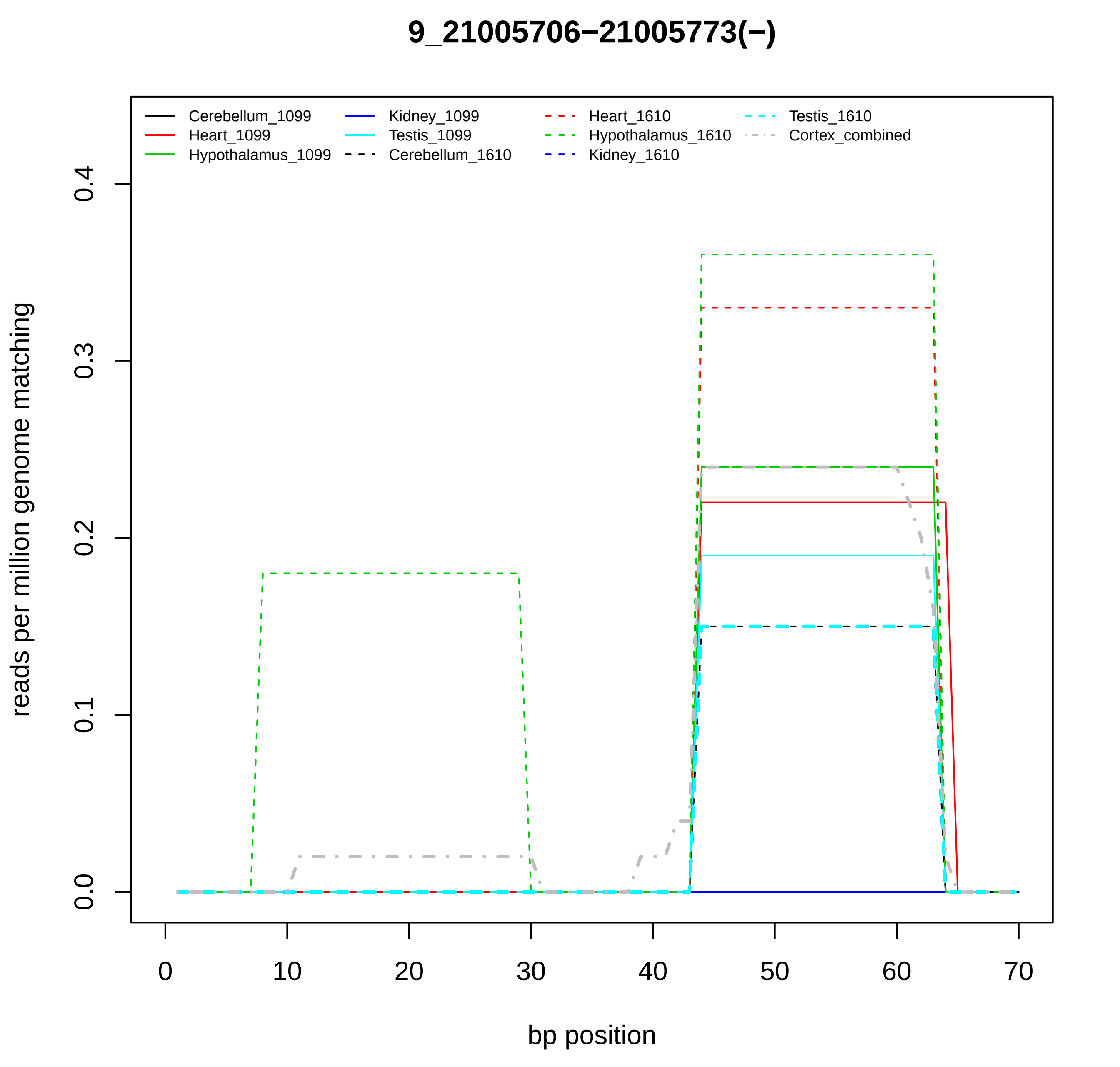

9_21005706−21005773(−)
Cerebellum_1099
Heart_1099
Hypothalamus_1099
Kidney_1099
Testis_1099
Cerebellum_1610
Heart_1610
Hypothalamus_1610
Kidney_1610
Testis_1610
Cortex_combined
reads per million genome matching
0.0
0.1
0.2
0.3
0.4
0
10
20
30
40
50
60
70
bp position

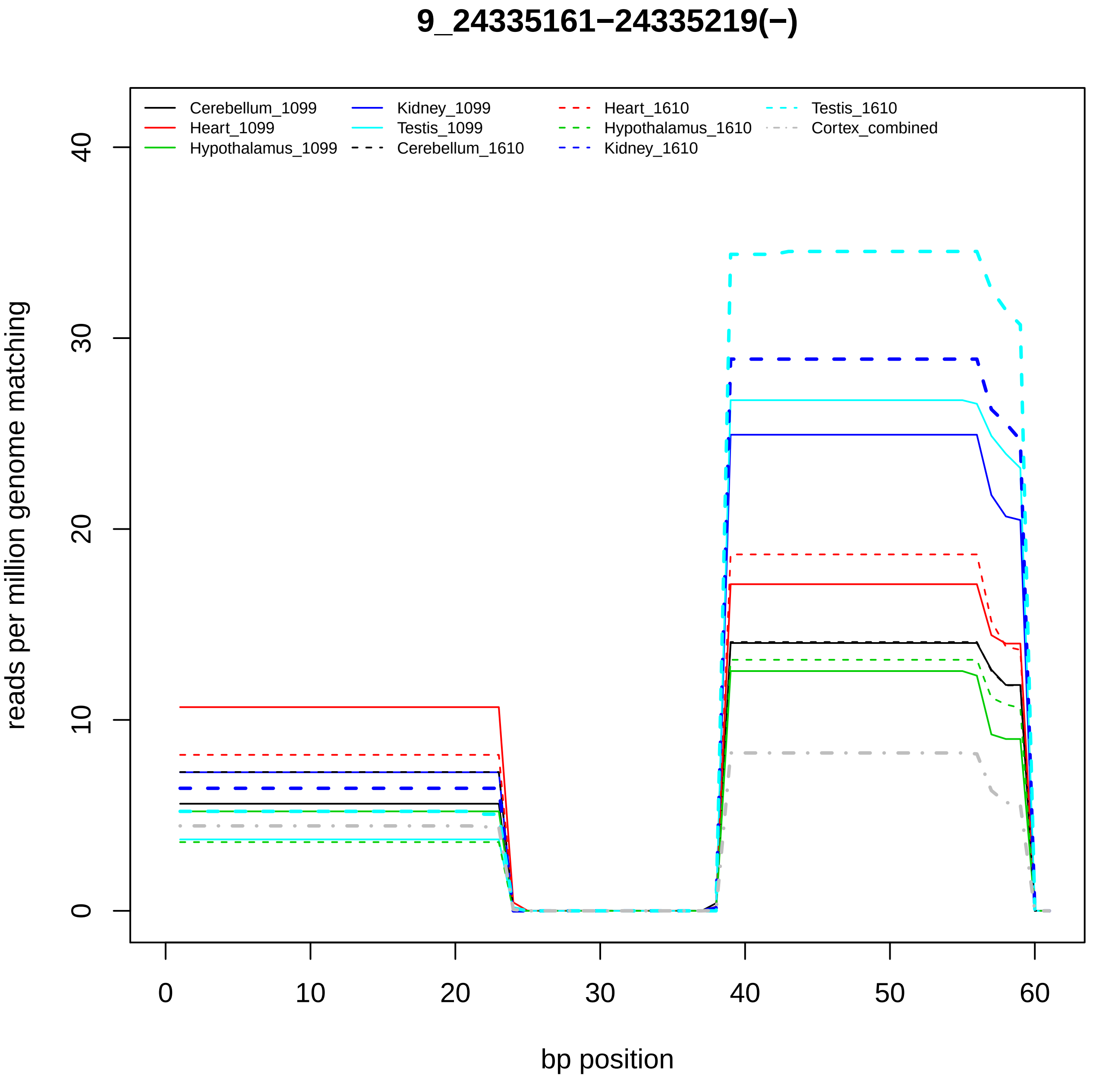
9_24335161−24335219(−)
Cerebellum_1099
Heart_1099
Hypothalamus_1099
Kidney_1099
Testis_1099
Cerebellum_1610
Heart_1610
Hypothalamus_1610
Kidney_1610
Testis_1610
Cortex_combined
reads per million genome matching
0
10
20
30
40
bp position
0
10
20
30
40
50
60

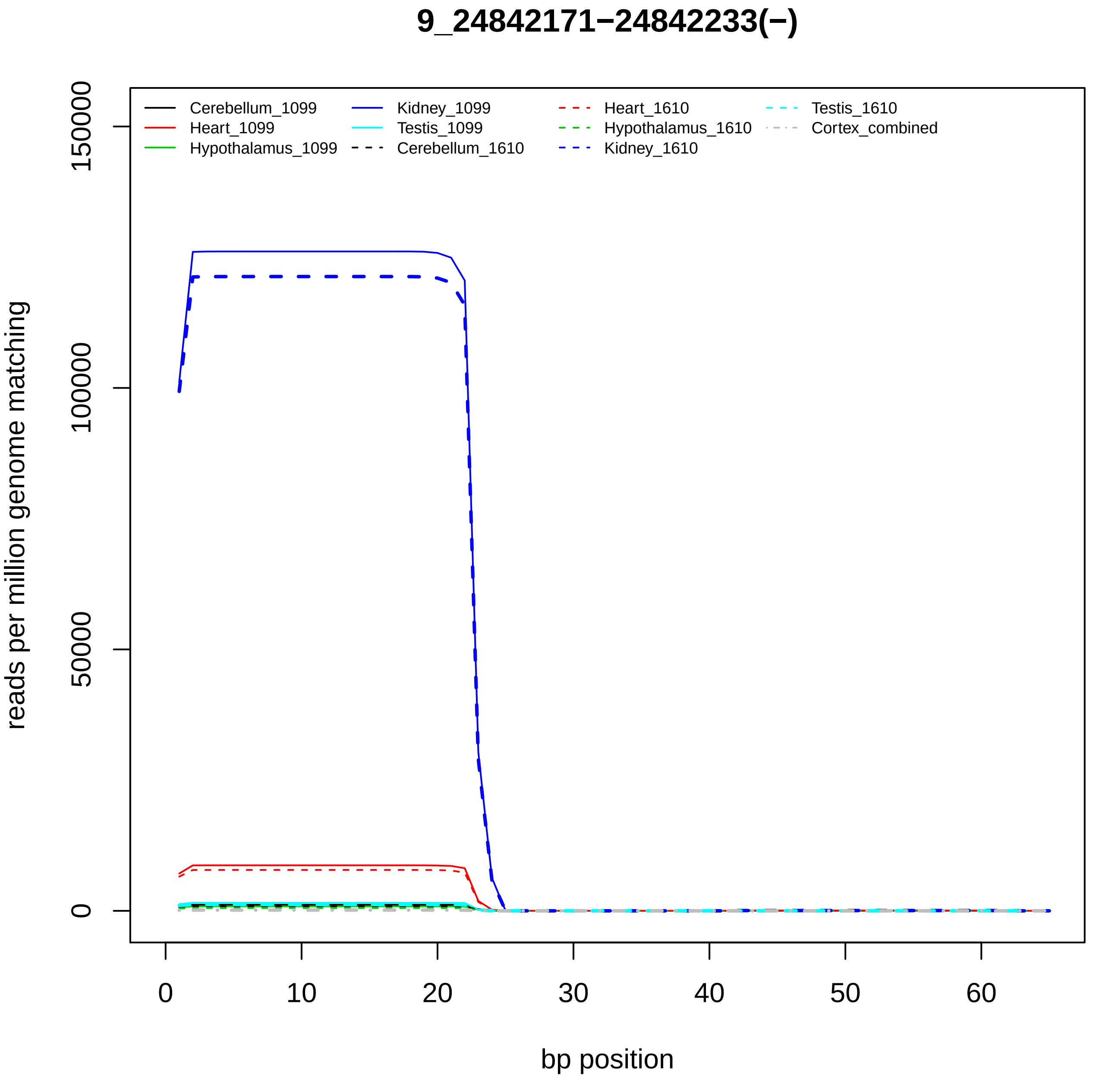

9_24842171−24842233(−)
Cerebellum_1099
Heart_1099
Hypothalamus_1099
Kidney_1099
Testis_1099
Cerebellum_1610
Heart_1610
Hypothalamus_1610
Kidney_1610
Testis_1610
Cortex_combined
reads per million genome matching
bp position
0
50000
100000
150000
0
10
20
30
40
50
60

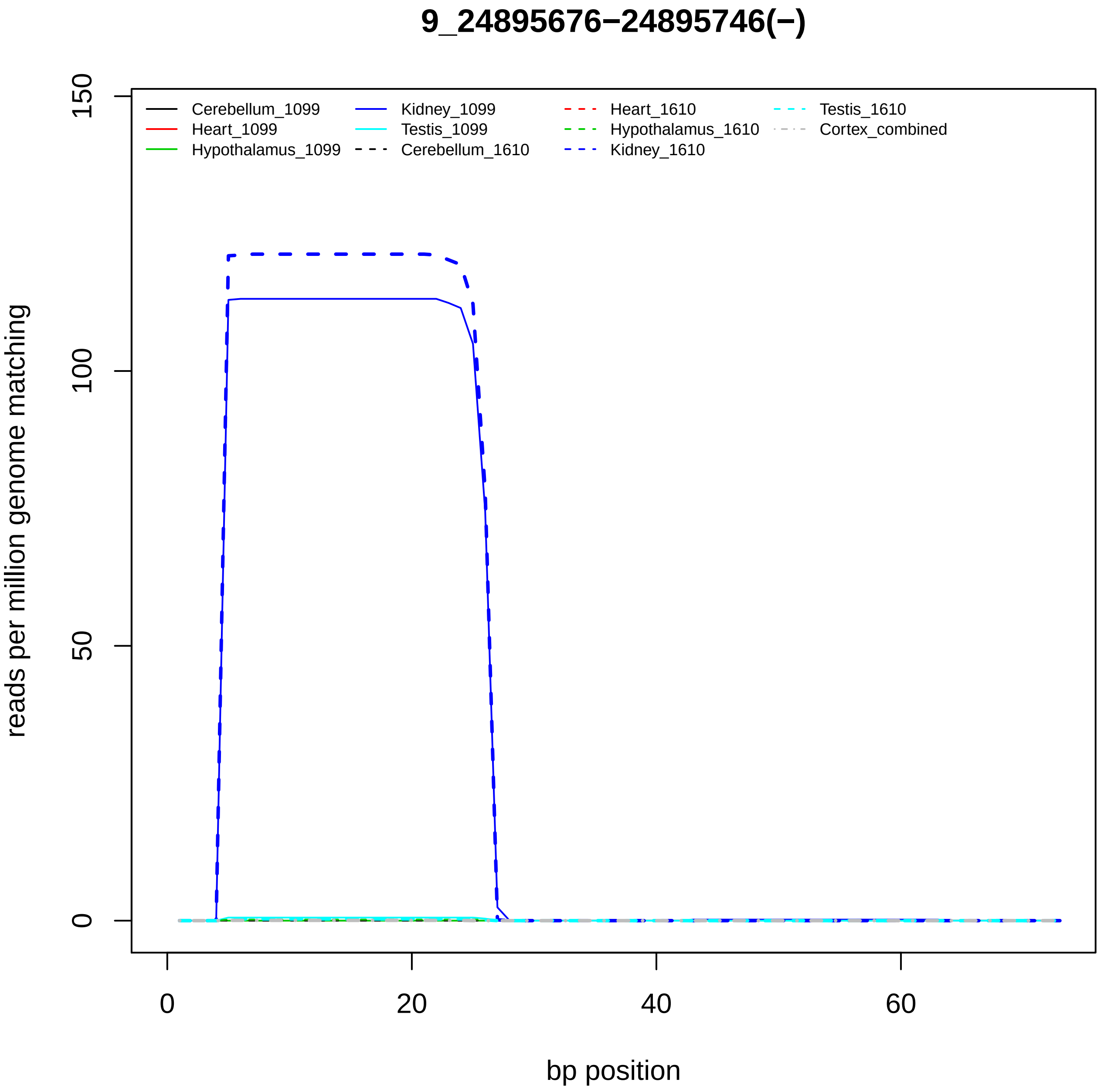

9_24895676−24895746(−)
Cerebellum_1099
Heart_1099
Hypothalamus_1099
Kidney_1099
Testis_1099
Cerebellum_1610
Heart_1610
Hypothalamus_1610
Kidney_1610
Testis_1610
Cortex_combined
reads per million genome matching
bp position
0
50
100
150
0
20
40
60

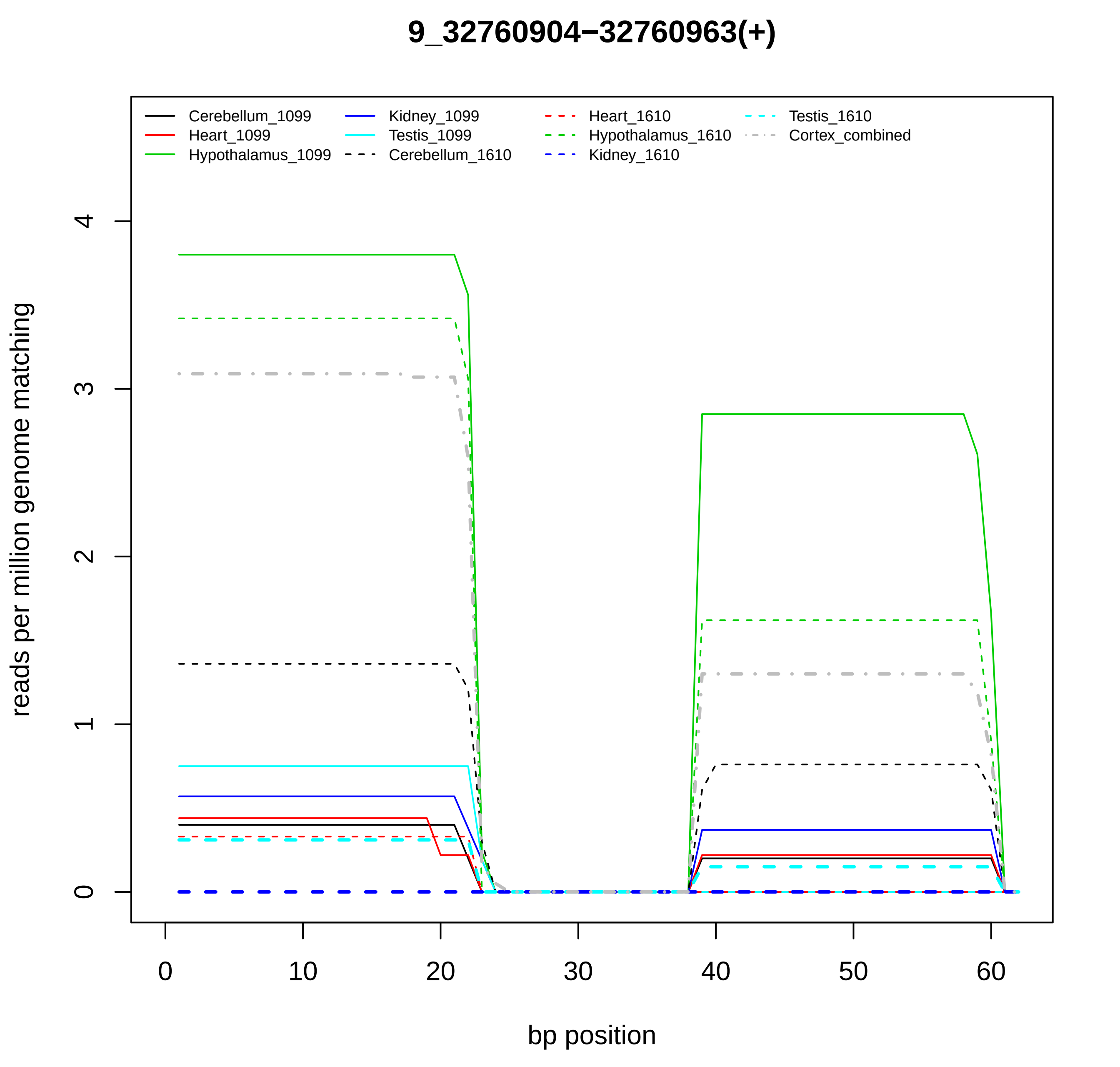

9_32760904−32760963(+)
Cerebellum_1099
Heart_1099
Hypothalamus_1099
Kidney_1099
Testis_1099
Cerebellum_1610
Heart_1610
Hypothalamus_1610
Kidney_1610
Testis_1610
Cortex_combined
reads per million genome matching
bp position
0
1
2
3
4
0
10
20
30
40
50
60

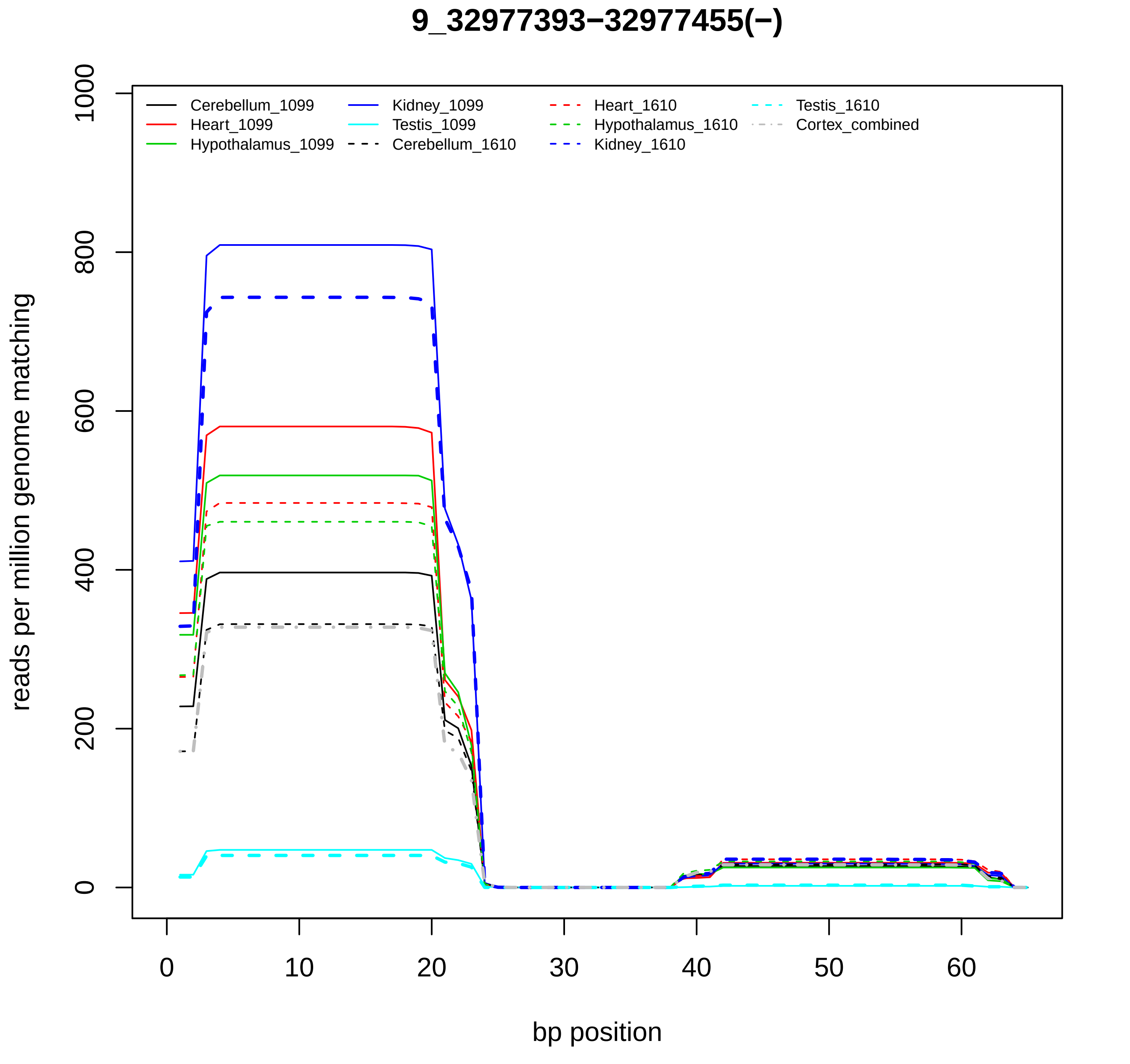

9_32977393−32977455(−)
Cerebellum_1099
Heart_1099
Hypothalamus_1099
Kidney_1099
Testis_1099
Cerebellum_1610
Heart_1610
Hypothalamus_1610
Kidney_1610
Testis_1610
Cortex_combined
reads per million genome matching
bp position
0
200
400
600
800
1000
0
10
20
30
40
50
60

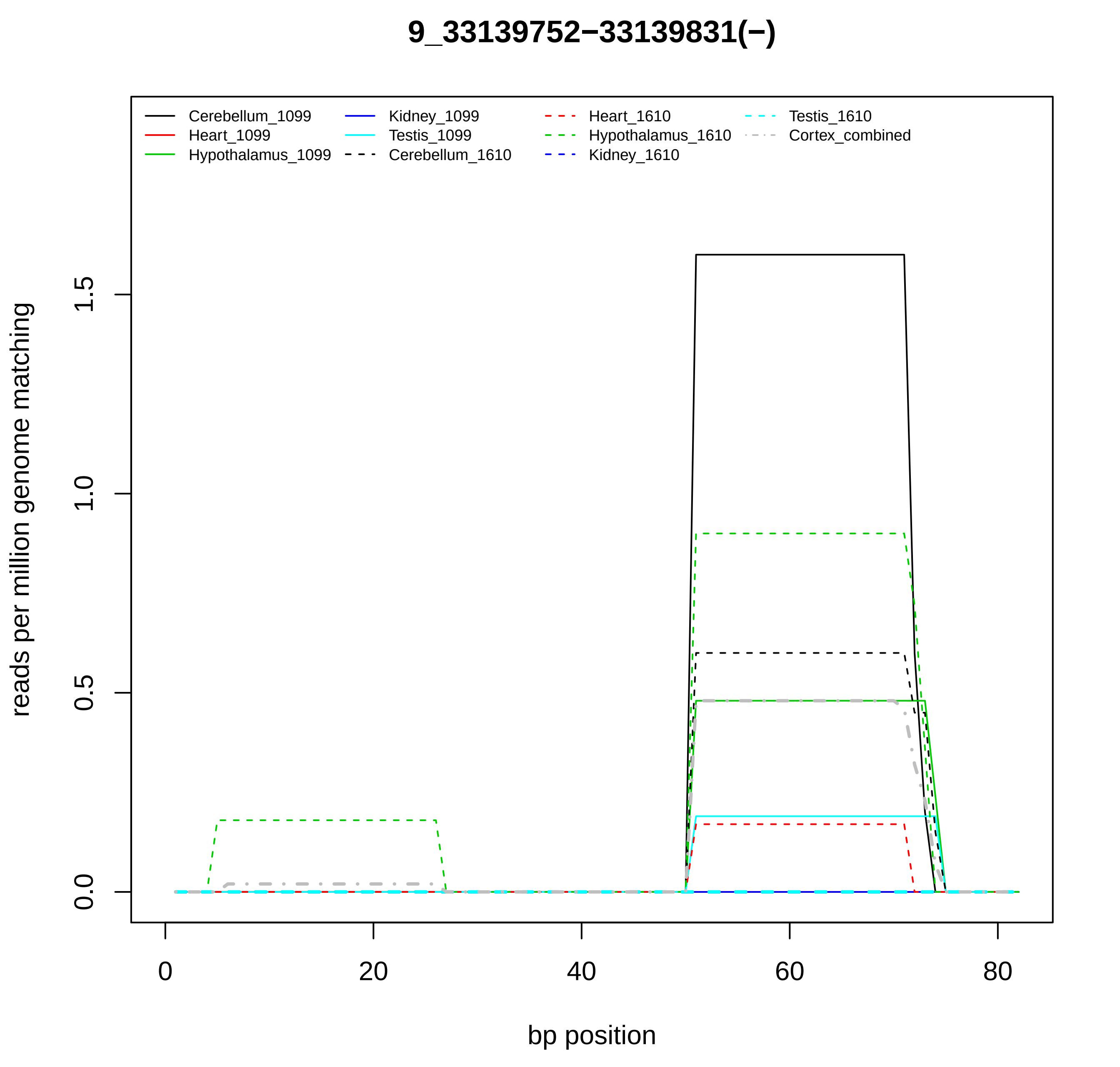

9_33139752−33139831(−)
Cerebellum_1099
Heart_1099
Hypothalamus_1099
Kidney_1099
Testis_1099
Cerebellum_1610
Heart_1610
Hypothalamus_1610
Kidney_1610
Testis_1610
Cortex_combined
reads per million genome matching
0.0
0.5
1.0
1.5
0
20
40
60
80
bp position

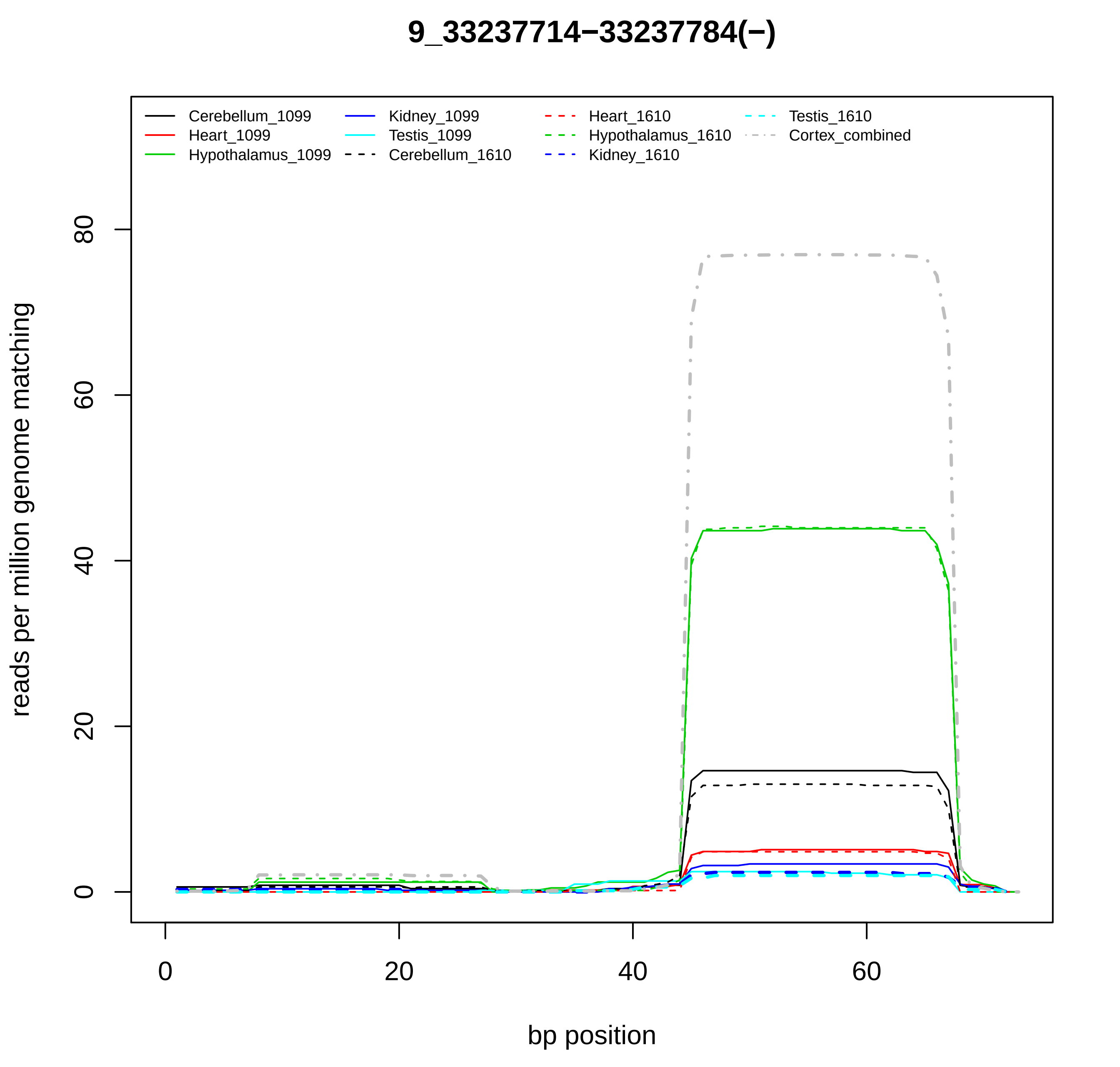

9_33237714−33237784(−)
Cerebellum_1099
Heart_1099
Hypothalamus_1099
Kidney_1099
Testis_1099
Cerebellum_1610
Heart_1610
Hypothalamus_1610
Kidney_1610
Testis_1610
Cortex_combined
reads per million genome matching
0
20
40
60
80
0
20
40
60
bp position

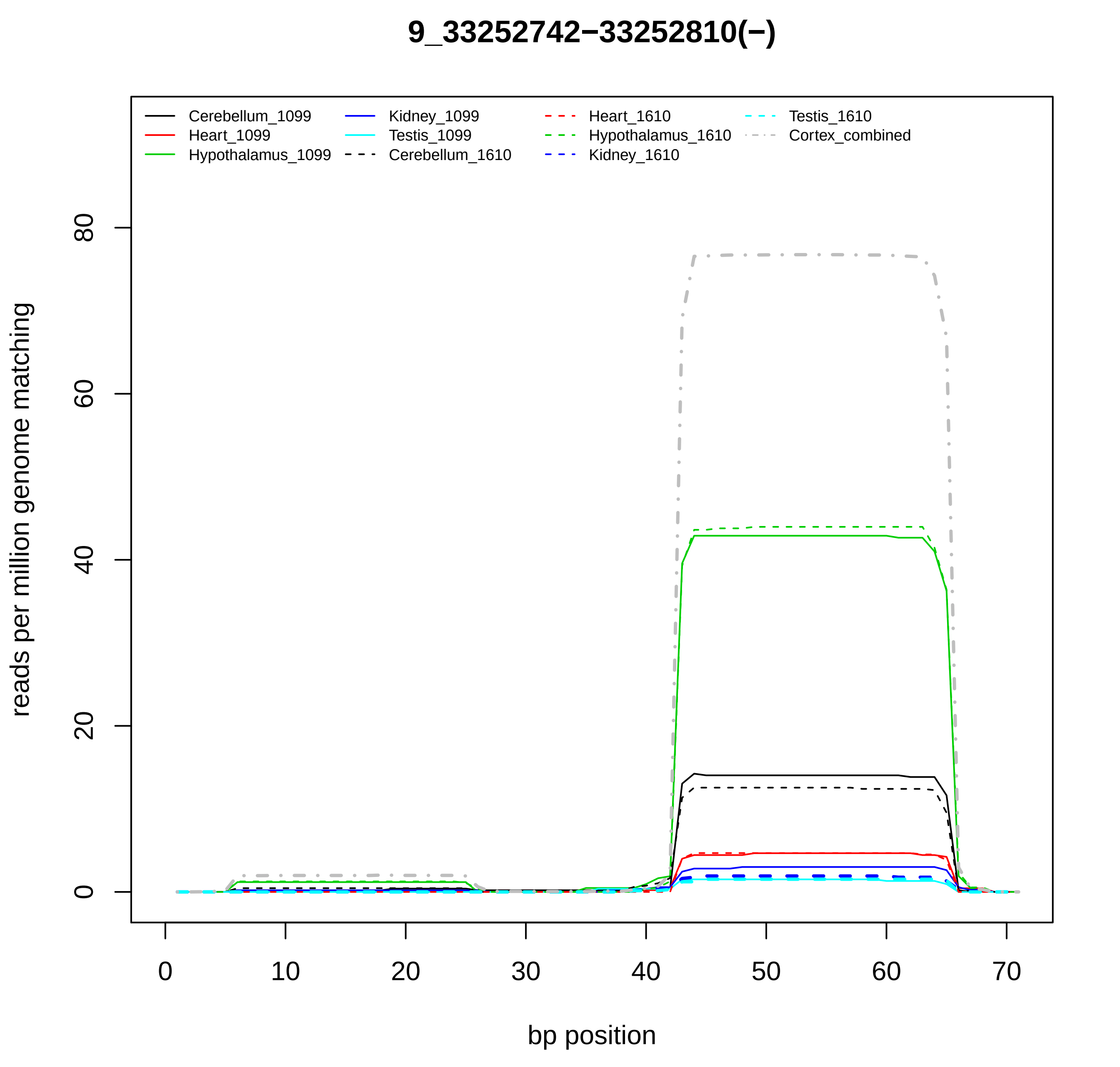

9_33252742−33252810(−)
Cerebellum_1099
Heart_1099
Hypothalamus_1099
Kidney_1099
Testis_1099
Cerebellum_1610
Heart_1610
Hypothalamus_1610
Kidney_1610
Testis_1610
Cortex_combined
reads per million genome matching
bp position
0
20
40
60
80
0
10
20
30
40
50
60
70

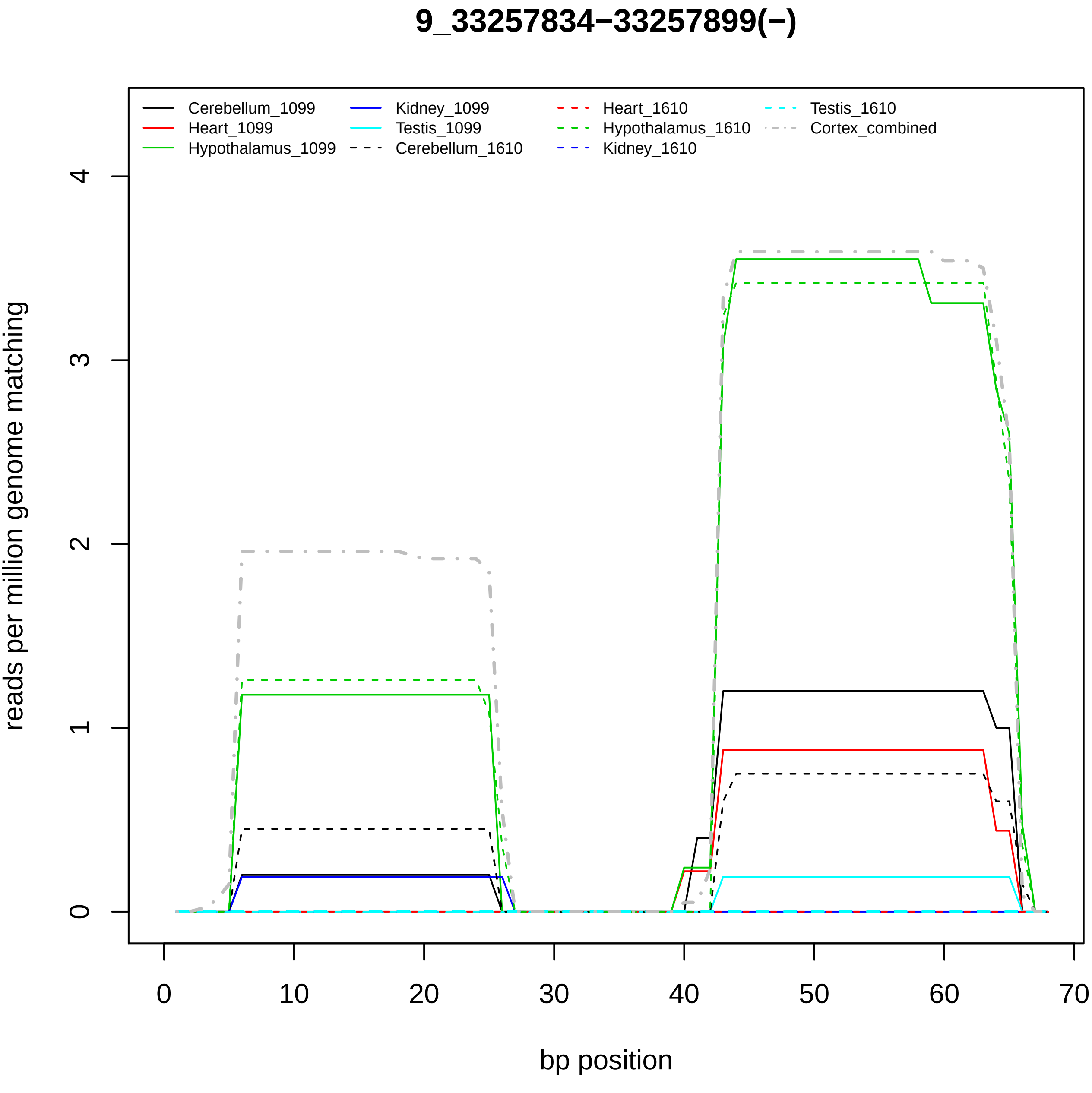
9_33257834−33257899(−)
Cerebellum_1099
Heart_1099
Hypothalamus_1099
Kidney_1099
Testis_1099
Cerebellum_1610
Heart_1610
Hypothalamus_1610
Kidney_1610
Testis_1610
Cortex_combined
reads per million genome matching
bp position
0
1
2
3
4
0
10
20
30
40
50
60
70

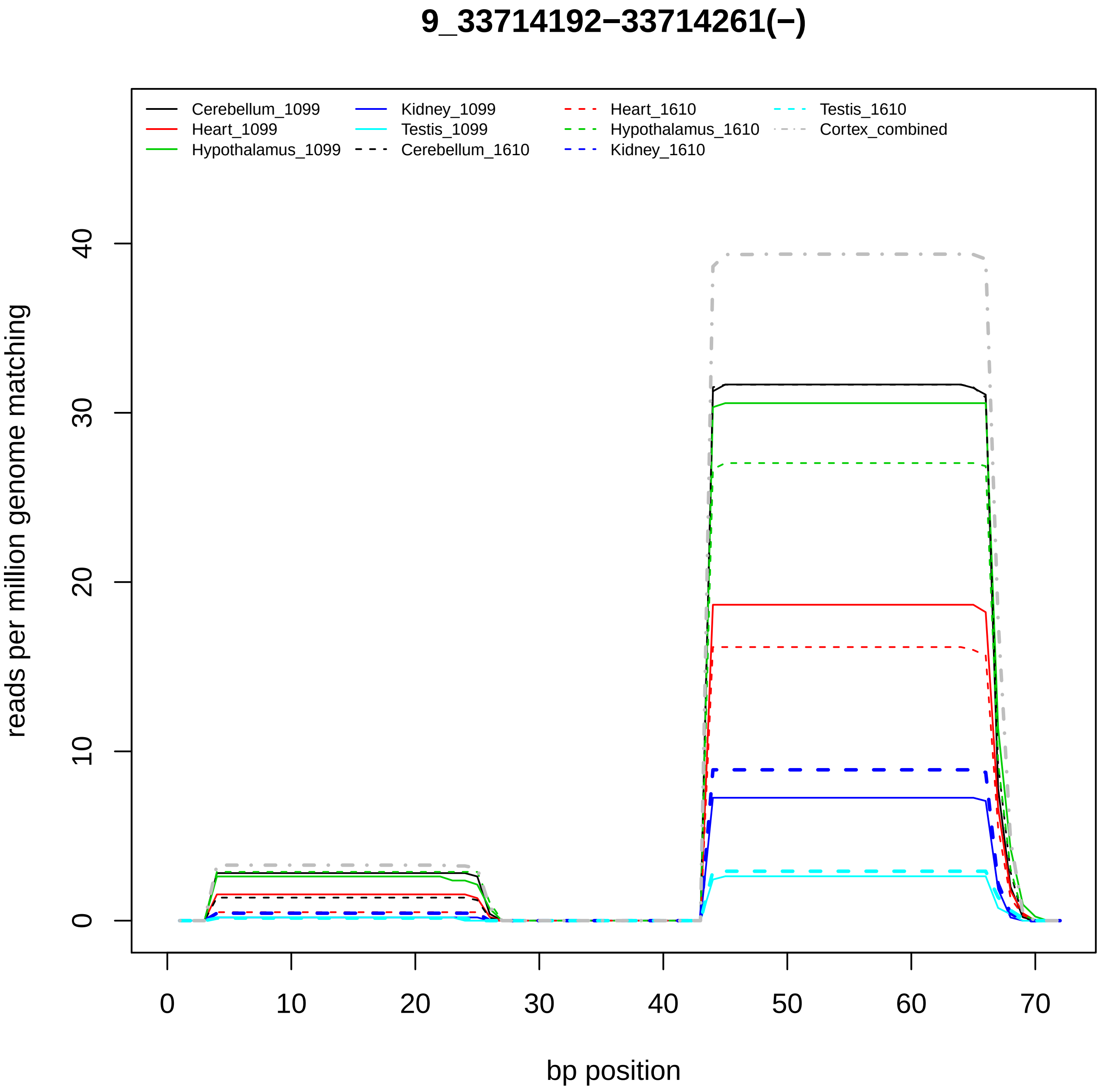

9_33714192−33714261(−)
Cerebellum_1099
Heart_1099
Hypothalamus_1099
Kidney_1099
Testis_1099
Cerebellum_1610
Heart_1610
Hypothalamus_1610
Kidney_1610
Testis_1610
Cortex_combined
reads per million genome matching
bp position
0
10
20
30
40
0
10
20
30
40
50
60
70

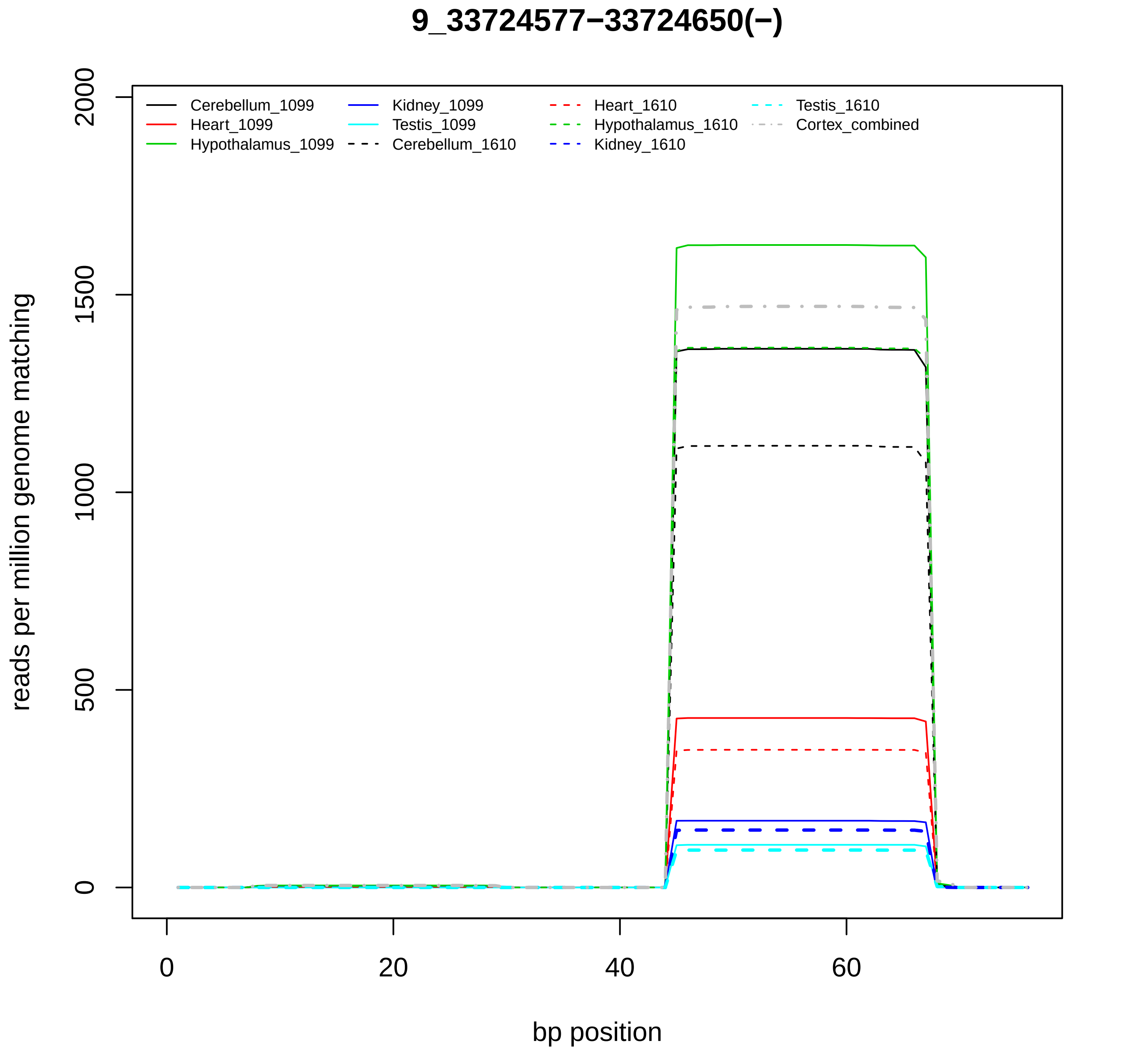

9_33724577−33724650(−)
Cerebellum_1099
Heart_1099
Hypothalamus_1099
Kidney_1099
Testis_1099
Cerebellum_1610
Heart_1610
Hypothalamus_1610
Kidney_1610
Testis_1610
Cortex_combined
reads per million genome matching
bp position
0
500
1000
1500
2000
0
20
40
60

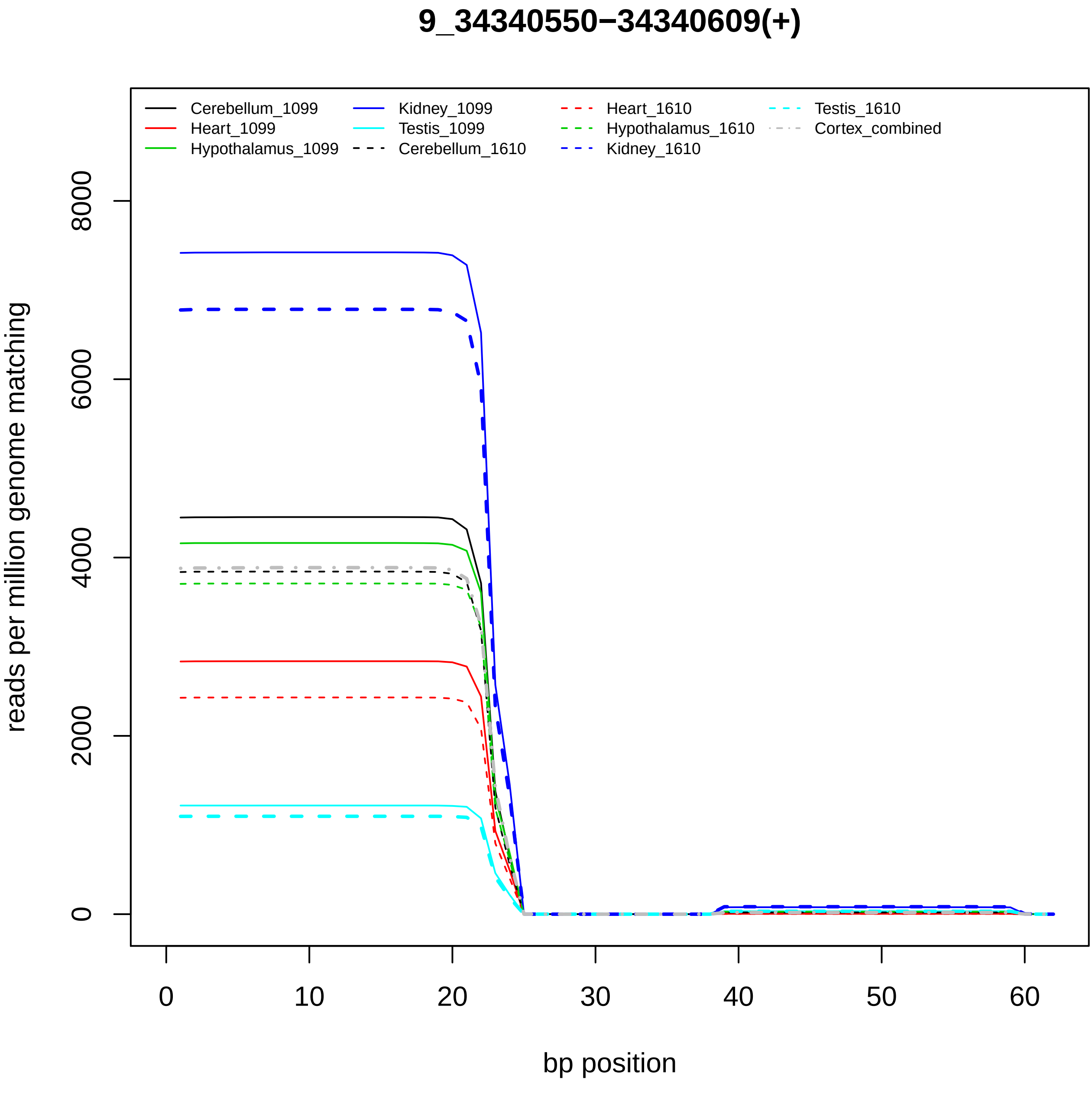

9_34340550−34340609(+)
Cerebellum_1099
Heart_1099
Hypothalamus_1099
Kidney_1099
Testis_1099
Cerebellum_1610
Heart_1610
Hypothalamus_1610
Kidney_1610
Testis_1610
Cortex_combined
reads per million genome matching
0
2000
4000
6000
8000
0
10
20
30
40
50
60
bp position

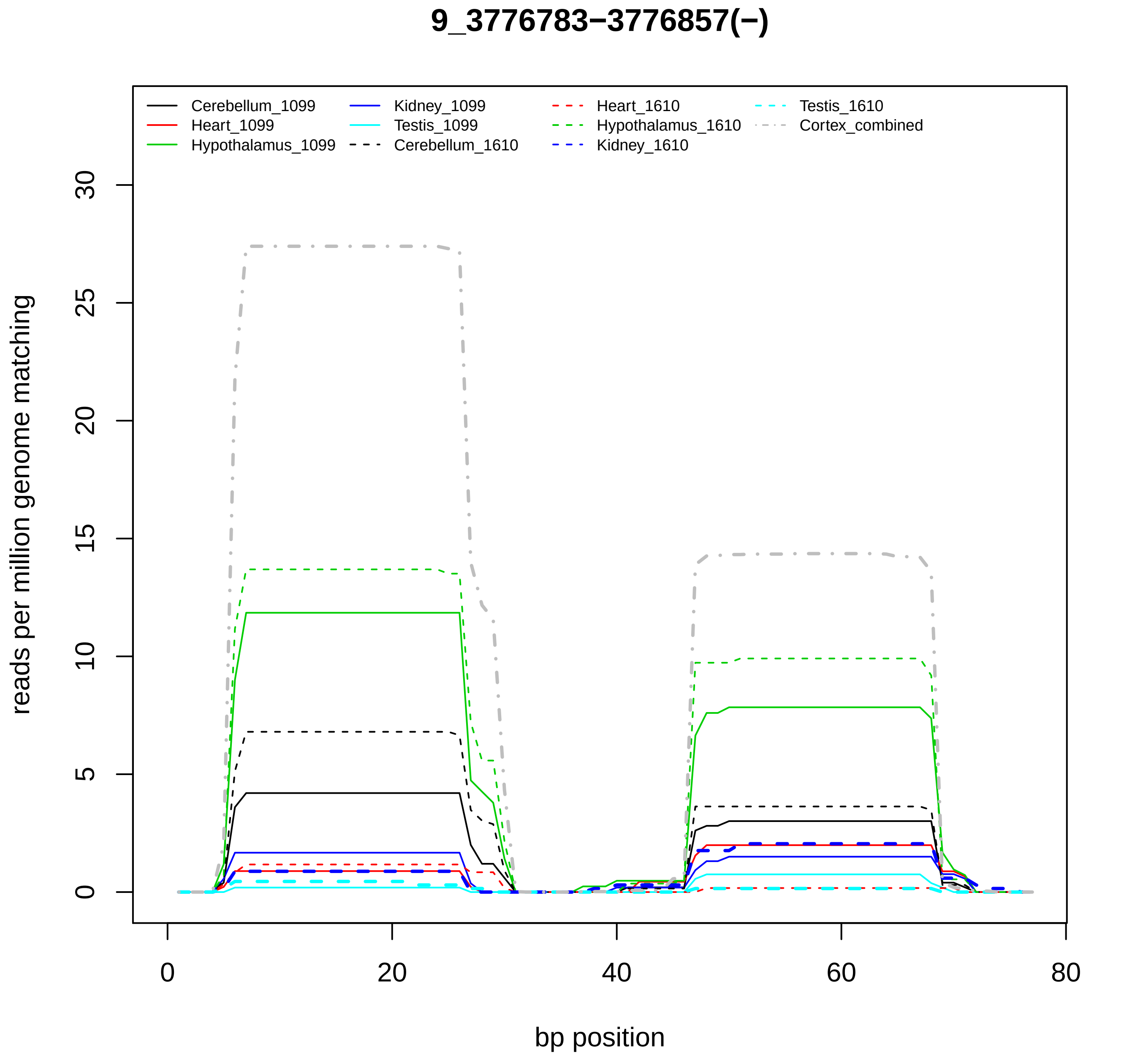
9_3776783−3776857(−)
Cerebellum_1099
Heart_1099
Hypothalamus_1099
Kidney_1099
Testis_1099
Cerebellum_1610
Heart_1610
Hypothalamus_1610
Kidney_1610
Testis_1610
Cortex_combined
reads per million genome matching
bp position
0
5
10
15
20
25
30
0
20
40
60
80

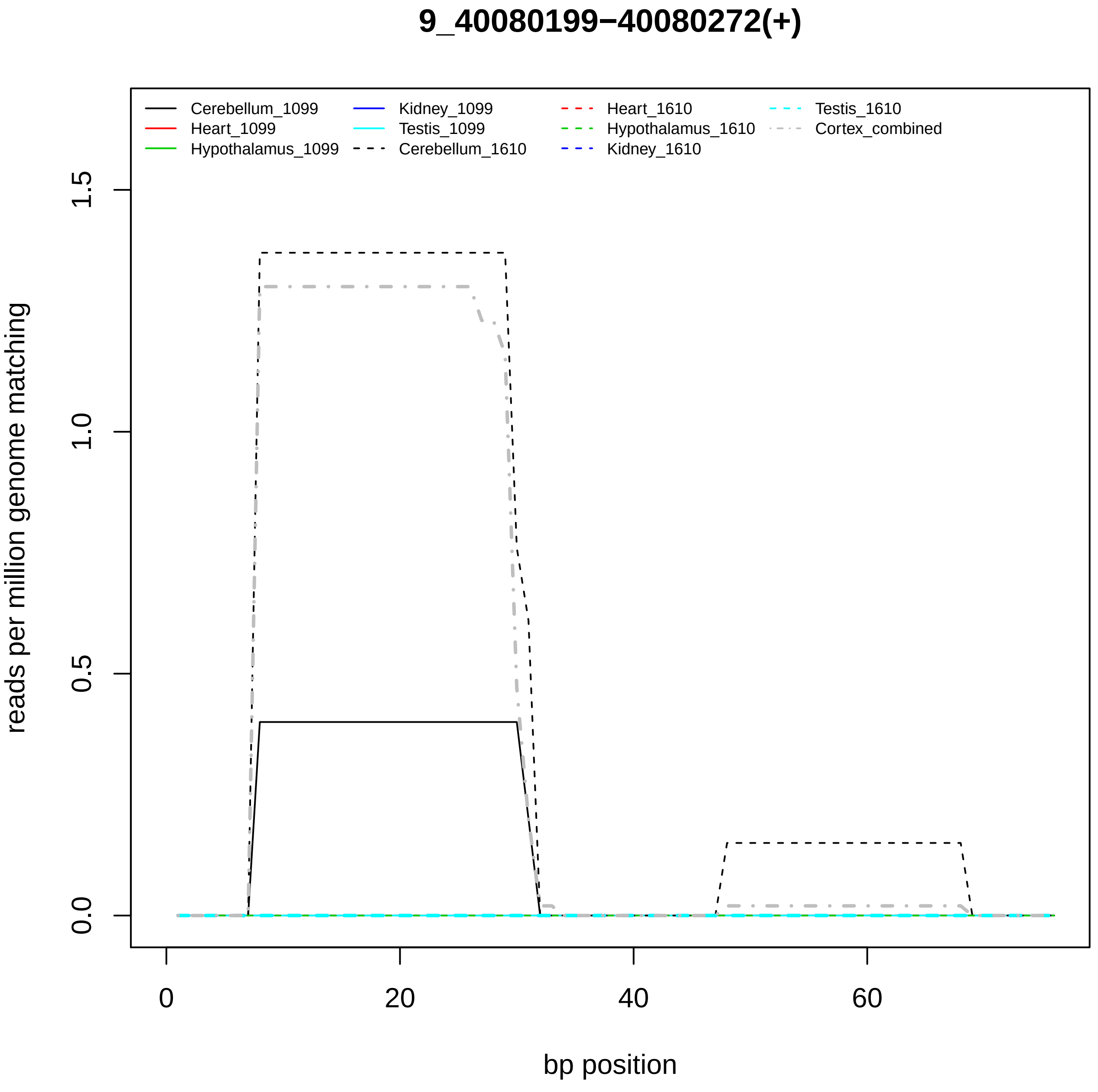

9_40080199−40080272(+)
Cerebellum_1099
Heart_1099
Hypothalamus_1099
Kidney_1099
Testis_1099
Cerebellum_1610
Heart_1610
Hypothalamus_1610
Kidney_1610
Testis_1610
Cortex_combined
reads per million genome matching
0.0
0.5
1.0
1.5
0
20
40
60
bp position

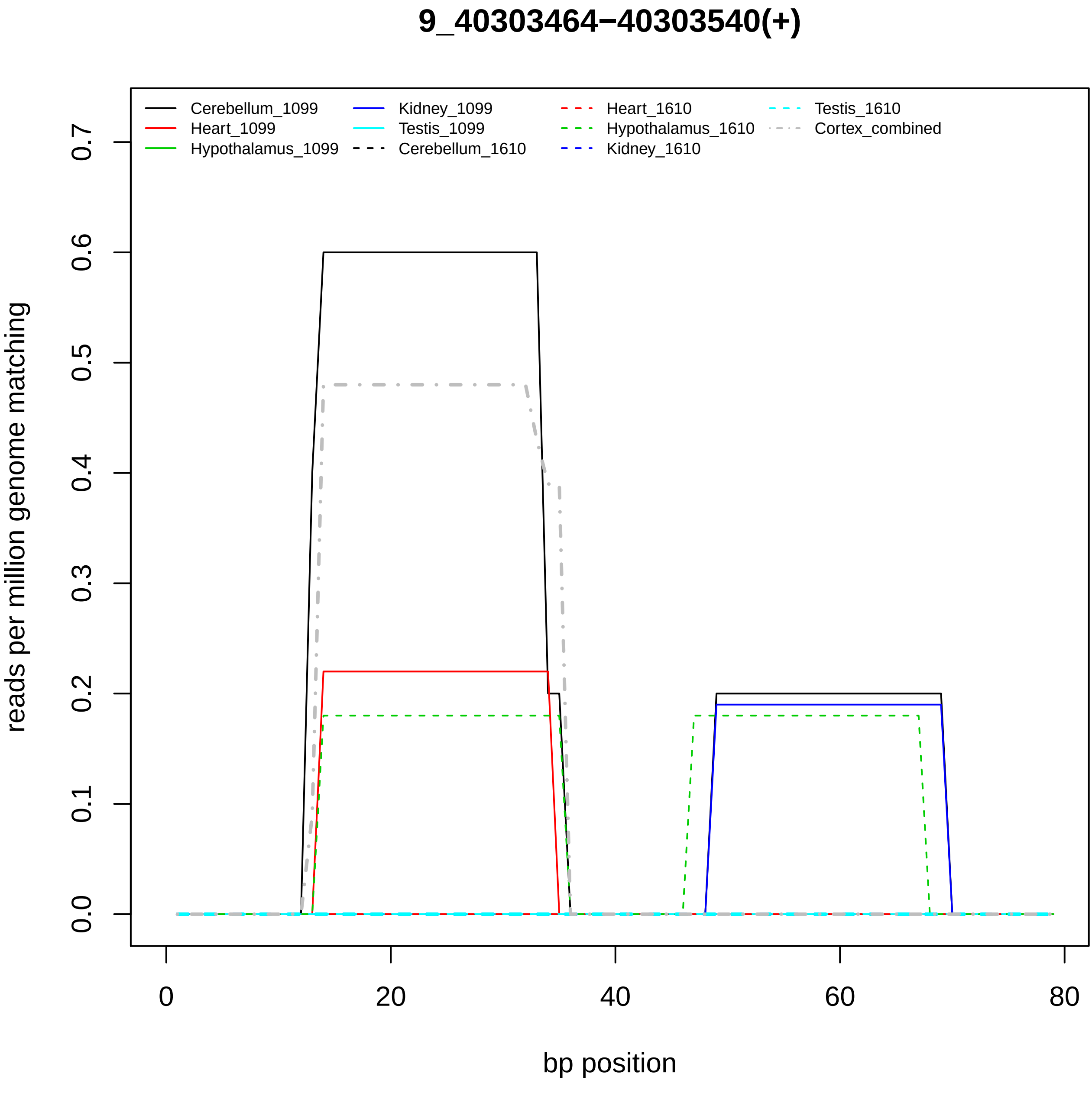

9_40303464−40303540(+)
Cerebellum_1099
Heart_1099
Hypothalamus_1099
Kidney_1099
Testis_1099
Cerebellum_1610
Heart_1610
Hypothalamus_1610
Kidney_1610
Testis_1610
Cortex_combined
reads per million genome matching
0.0
0.1
0.2
0.3
0.4
0.5
0.6
0.7
0
20
40
60
80
bp position

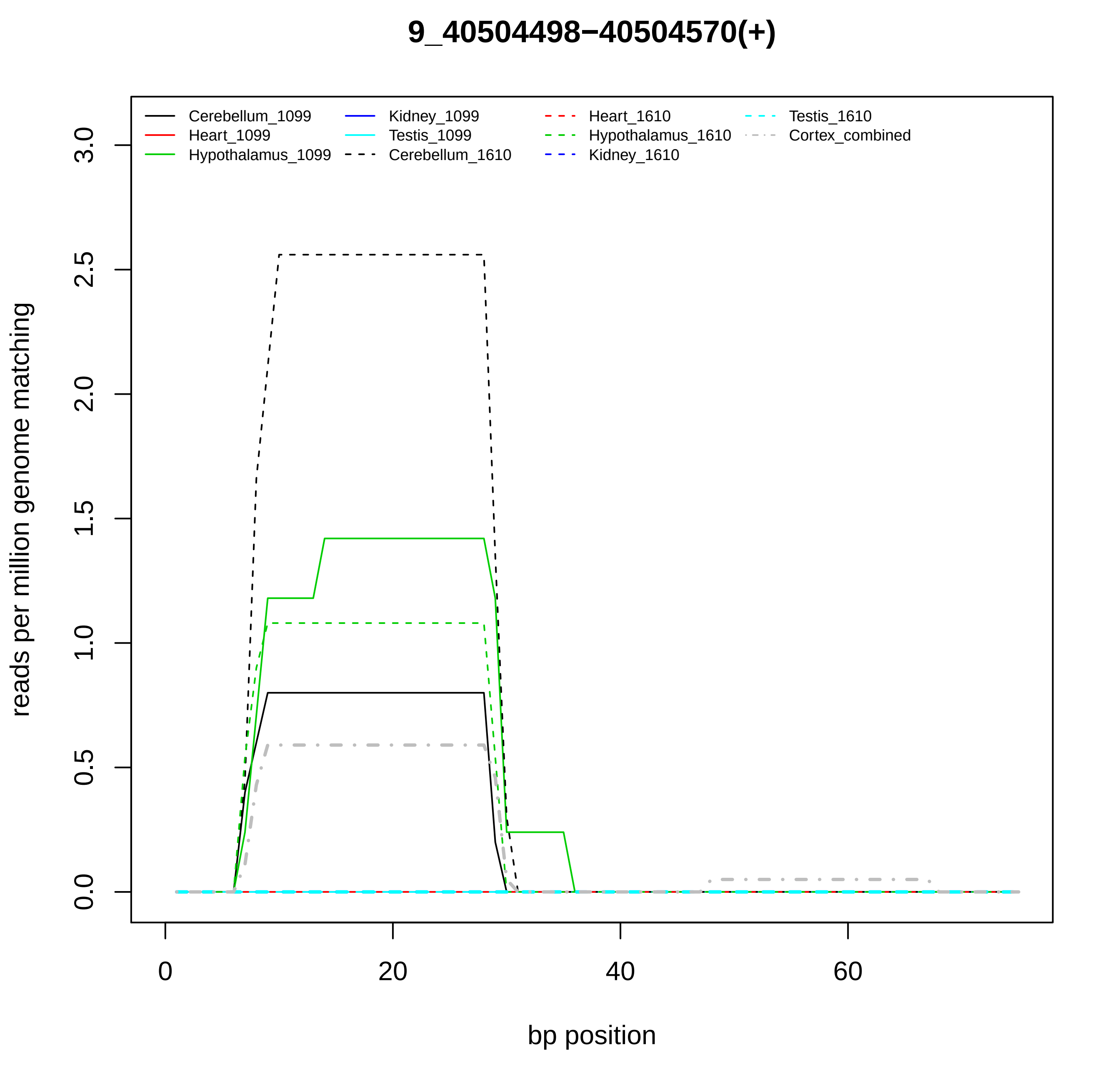
9_40504498−40504570(+)
Cerebellum_1099
Heart_1099
Hypothalamus_1099
Kidney_1099
Testis_1099
Cerebellum_1610
Heart_1610
Hypothalamus_1610
Kidney_1610
Testis_1610
Cortex_combined
reads per million genome matching
bp position
0.0
0.5
1.0
1.5
2.0
2.5
3.0
0
20
40
60

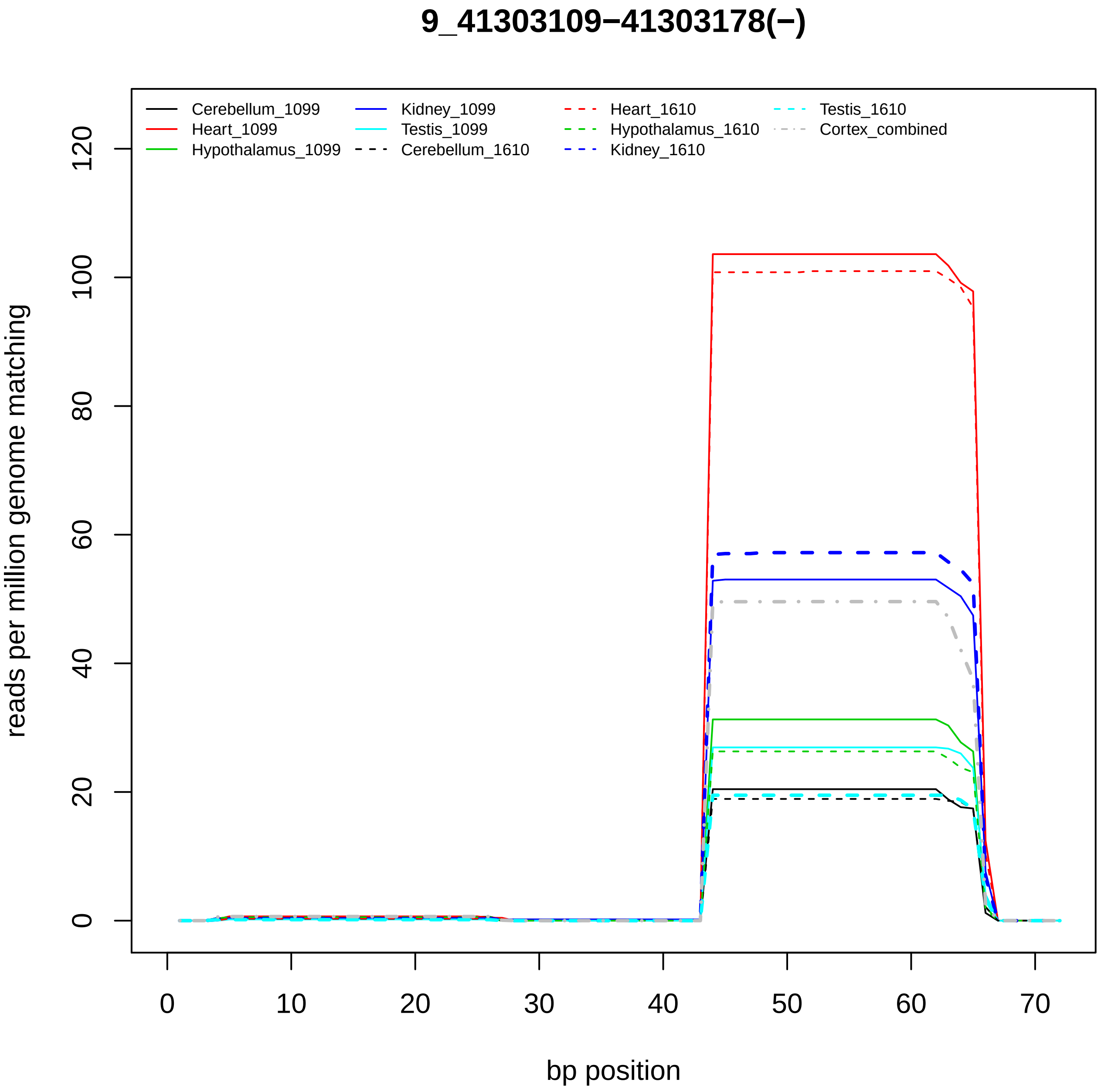

9_41303109−41303178(−)
Cerebellum_1099
Heart_1099
Hypothalamus_1099
Kidney_1099
Testis_1099
Cerebellum_1610
Heart_1610
Hypothalamus_1610
Kidney_1610
Testis_1610
Cortex_combined
reads per million genome matching
bp position
0
20
40
60
80
100
120
0
10
20
30
40
50
60
70

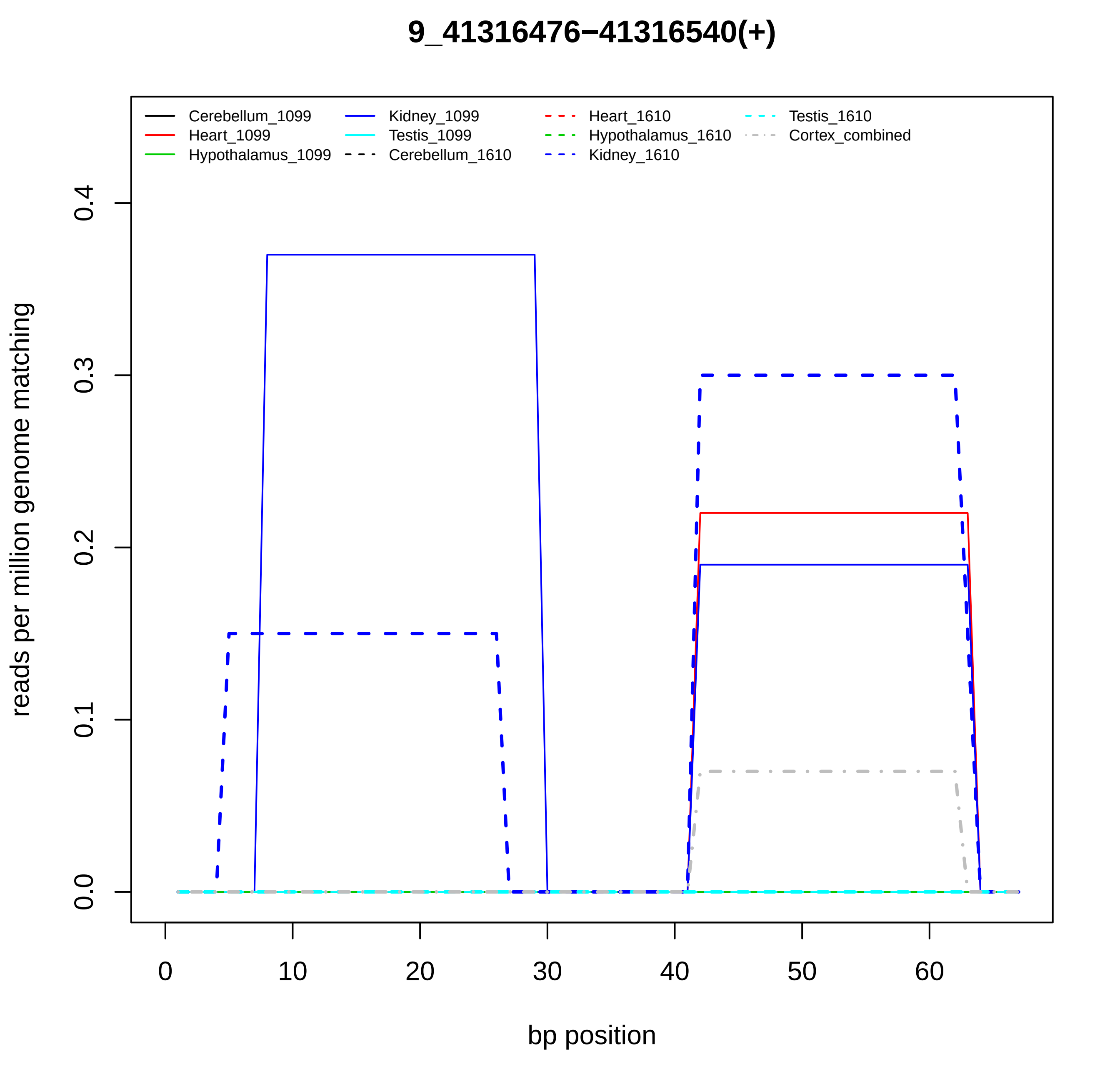

9_41316476−41316540(+)
Cerebellum_1099
Heart_1099
Hypothalamus_1099
Kidney_1099
Testis_1099
Cerebellum_1610
Heart_1610
Hypothalamus_1610
Kidney_1610
Testis_1610
Cortex_combined
reads per million genome matching
0.0
0.1
0.2
0.3
0.4
0
10
20
30
40
50
60
bp position

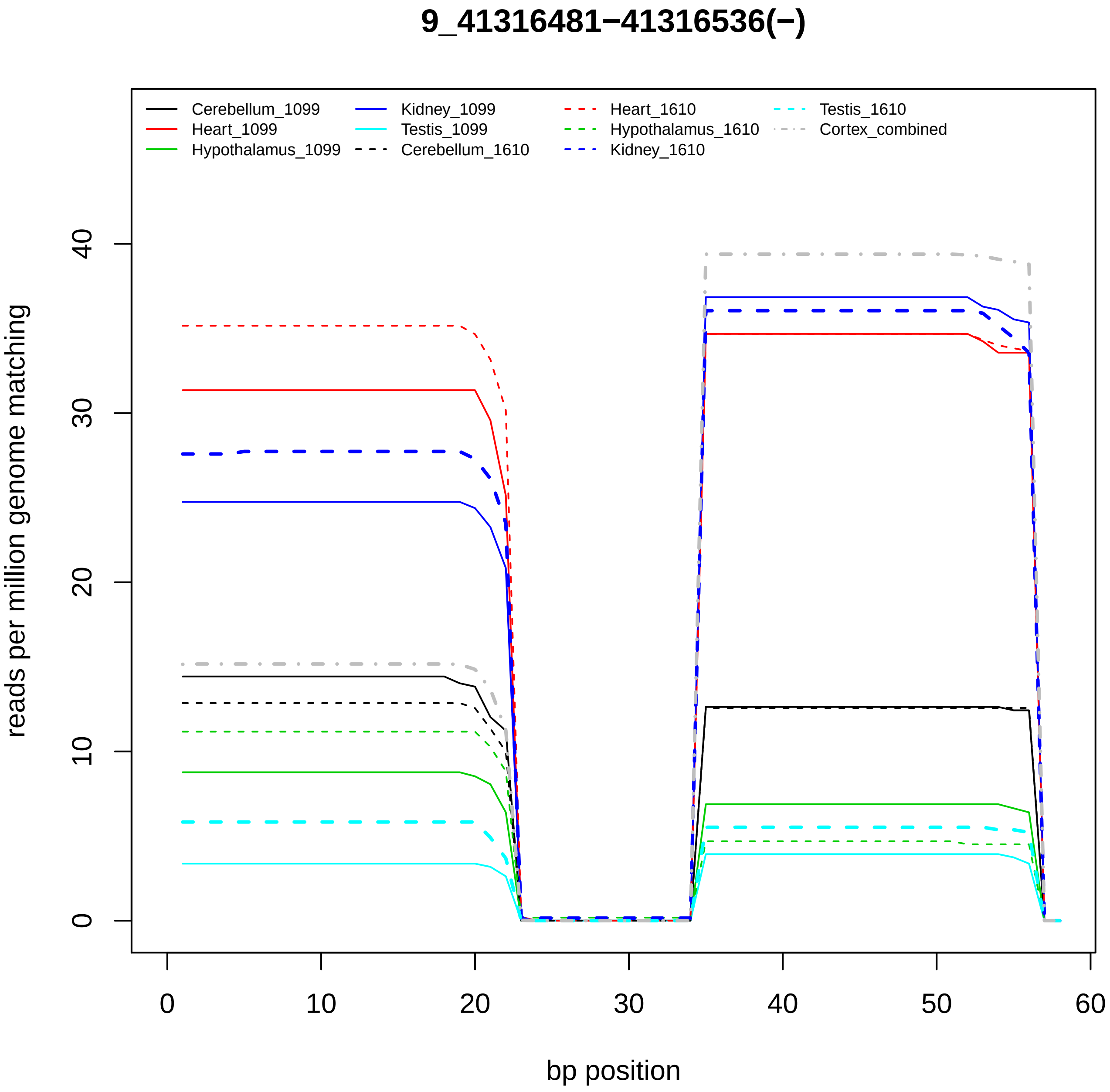

9_41316481−41316536(−)
Cerebellum_1099
Heart_1099
Hypothalamus_1099
Kidney_1099
Testis_1099
Cerebellum_1610
Heart_1610
Hypothalamus_1610
Kidney_1610
Testis_1610
Cortex_combined
reads per million genome matching
bp position
0
10
20
30
40
0
10
20
30
40
50
60

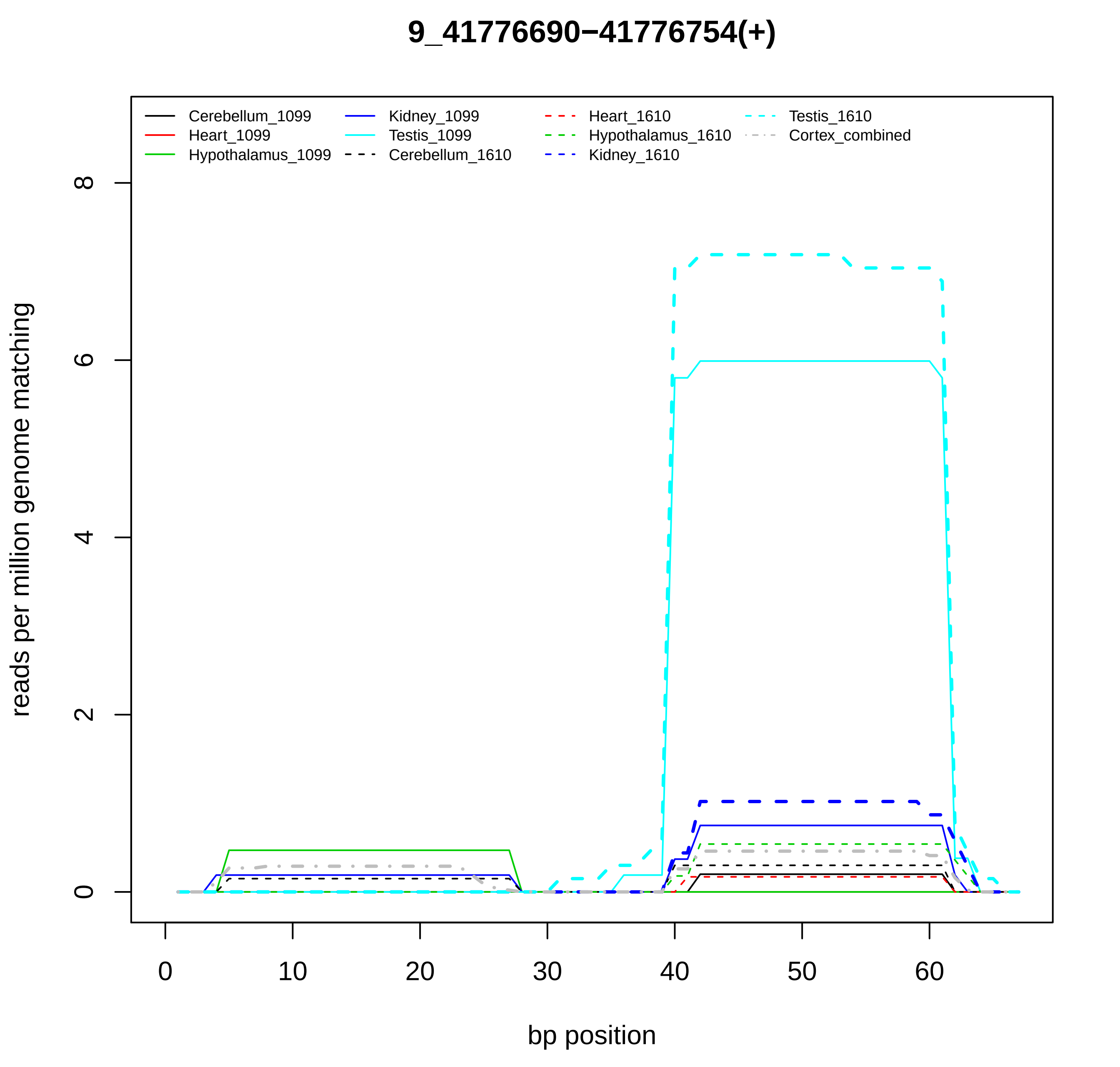

9_41776690−41776754(+)
Cerebellum_1099
Heart_1099
Hypothalamus_1099
Kidney_1099
Testis_1099
Cerebellum_1610
Heart_1610
Hypothalamus_1610
Kidney_1610
Testis_1610
Cortex_combined
reads per million genome matching
bp position
0
2
4
6
8
0
10
20
30
40
50
60

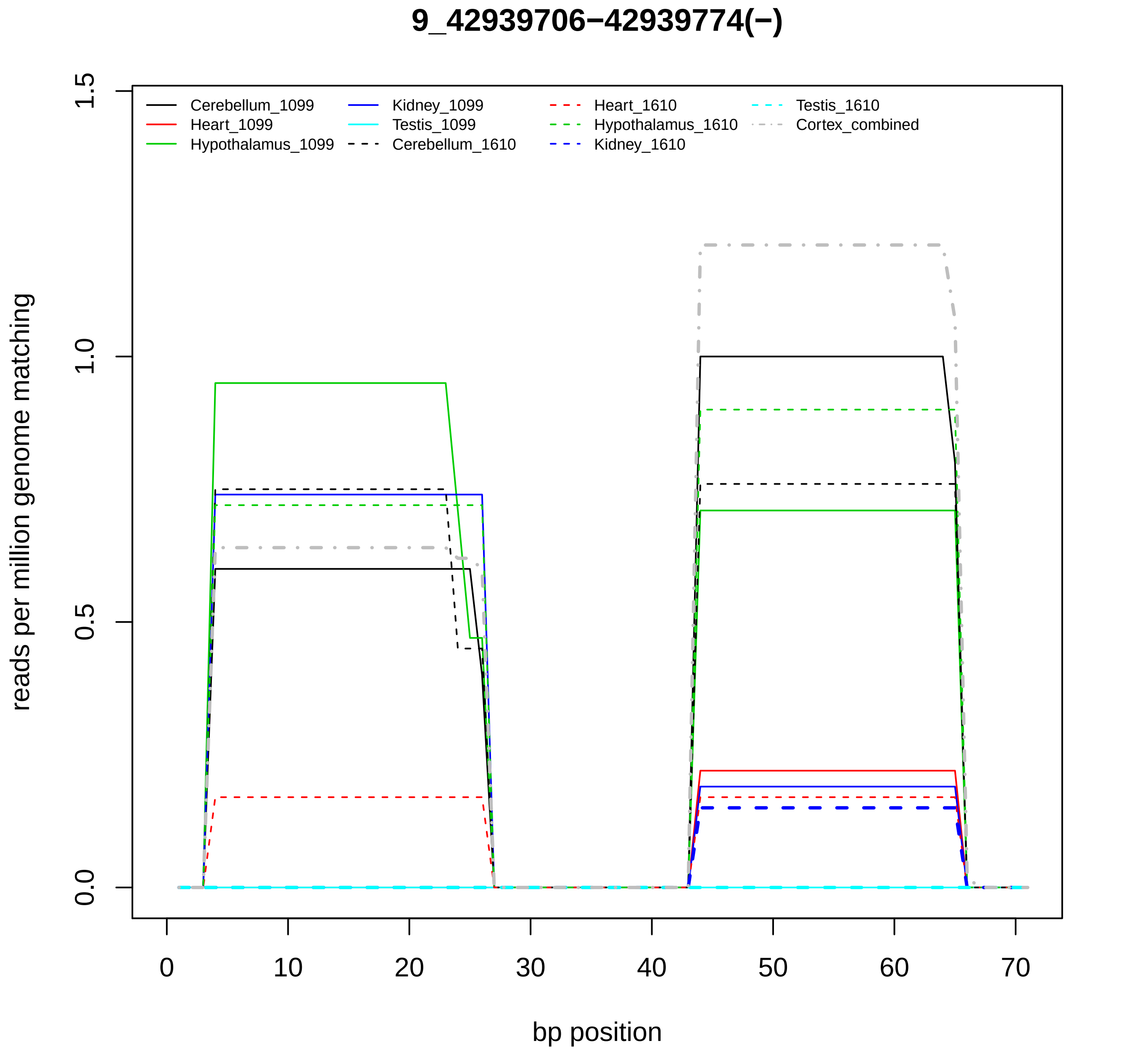

9_42939706−42939774(−)
Cerebellum_1099
Heart_1099
Hypothalamus_1099
Kidney_1099
Testis_1099
Cerebellum_1610
Heart_1610
Hypothalamus_1610
Kidney_1610
Testis_1610
Cortex_combined
reads per million genome matching
bp position
0.0
0.5
1.0
1.5
0
10
20
30
40
50
60
70

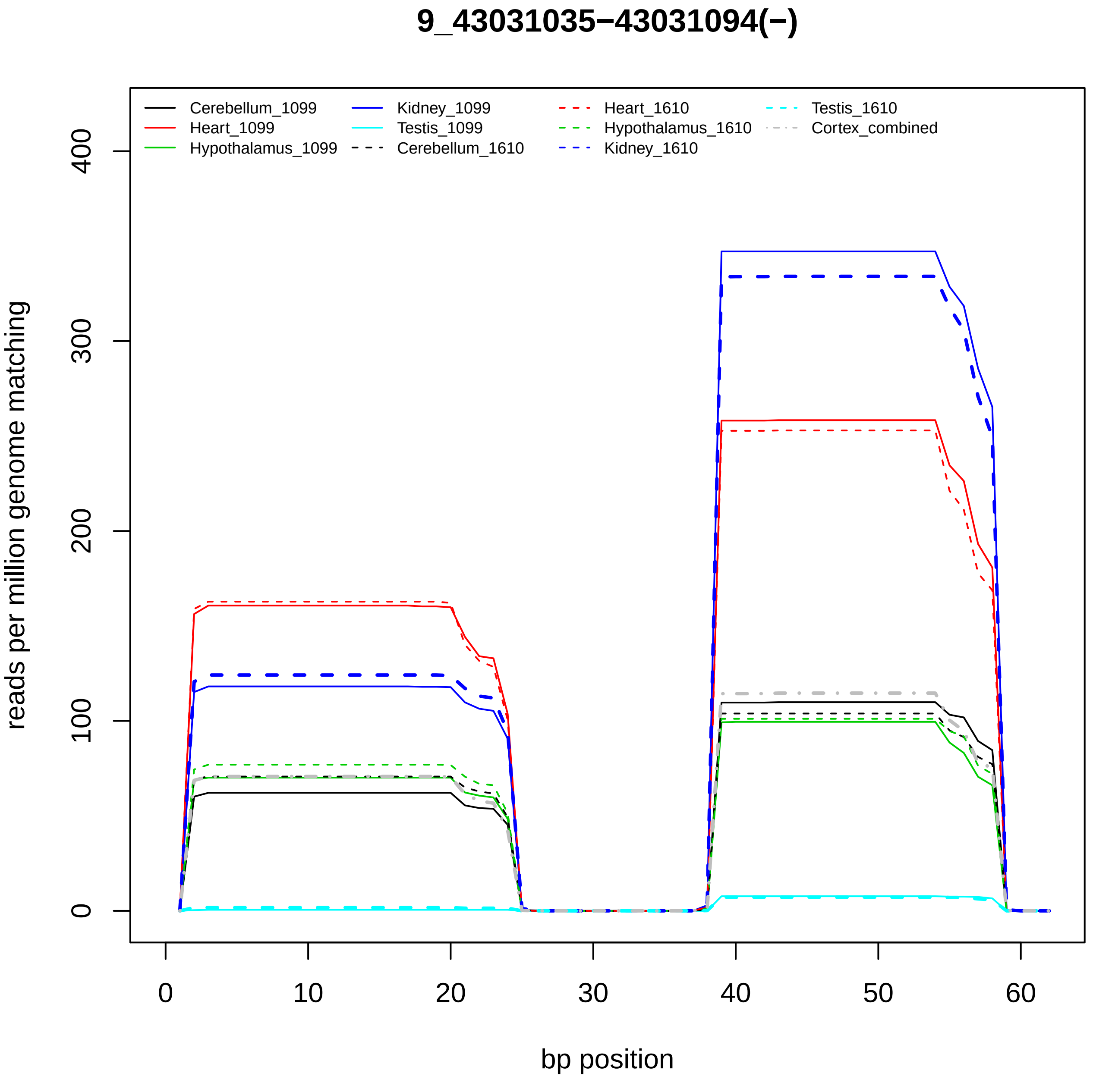

9_43031035−43031094(−)
Cerebellum_1099
Heart_1099
Hypothalamus_1099
Kidney_1099
Testis_1099
Cerebellum_1610
Heart_1610
Hypothalamus_1610
Kidney_1610
Testis_1610
Cortex_combined
reads per million genome matching
0
100
200
300
400
0
10
20
30
40
50
60
bp position

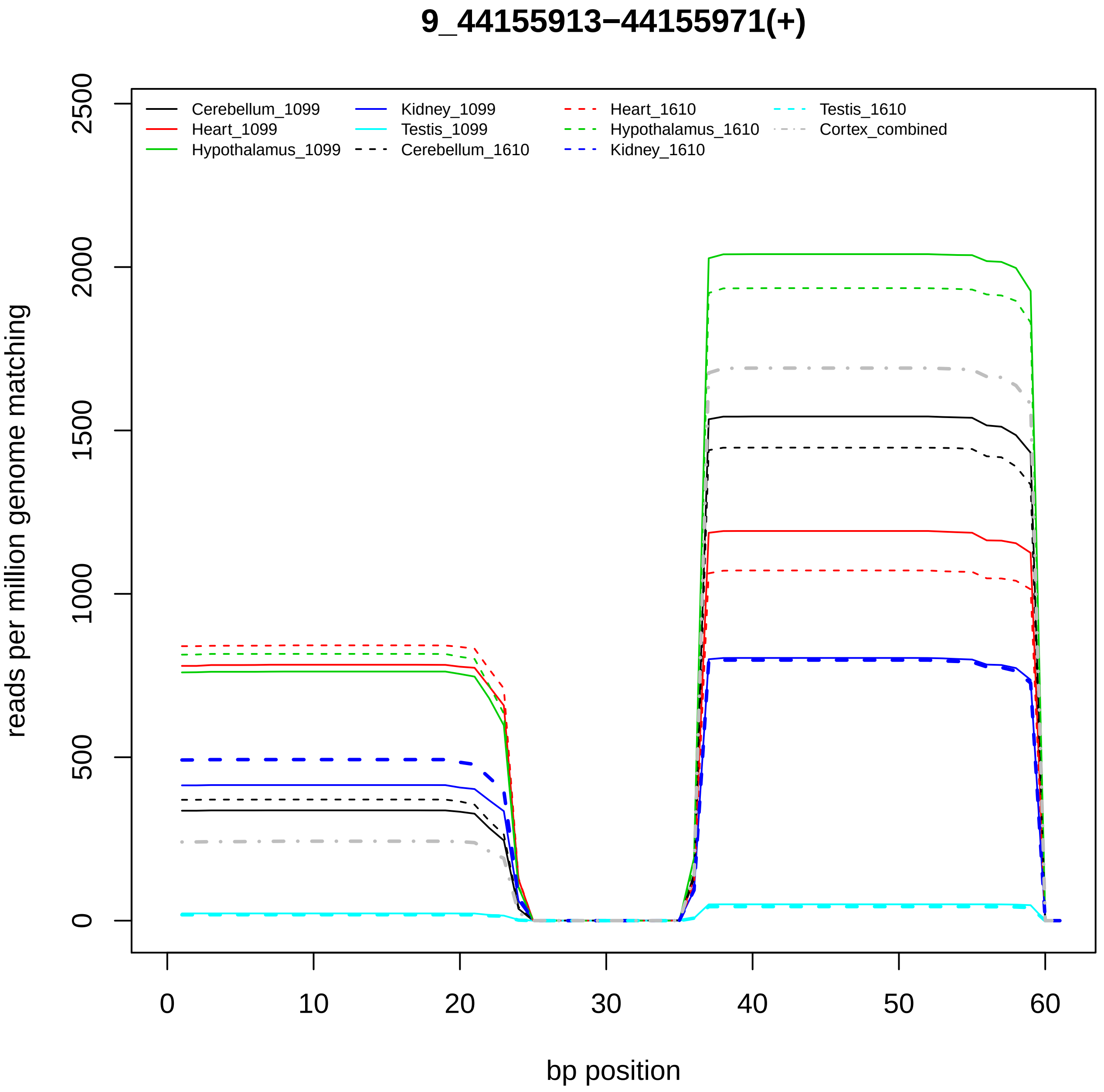

9_44155913−44155971(+)
Cerebellum_1099
Heart_1099
Hypothalamus_1099
Kidney_1099
Testis_1099
Cerebellum_1610
Heart_1610
Hypothalamus_1610
Kidney_1610
Testis_1610
Cortex_combined
reads per million genome matching
0
500
1000
1500
2000
2500
bp position
0
10
20
30
40
50
60

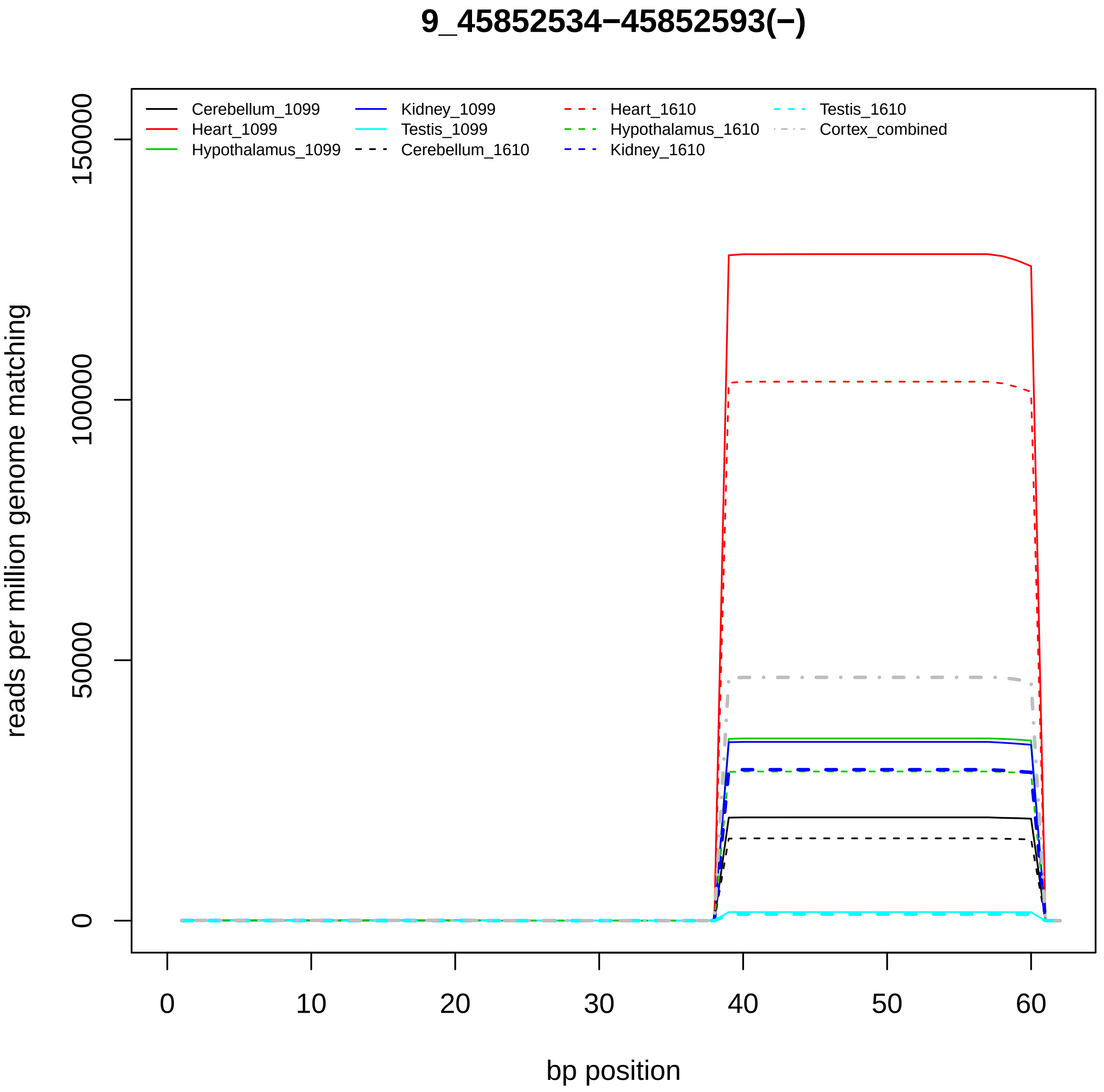

9_45852534−45852593(−)
Cerebellum_1099
Heart_1099
Hypothalamus_1099
Kidney_1099
Testis_1099
Cerebellum_1610
Heart_1610
Hypothalamus_1610
Kidney_1610
Testis_1610
Cortex_combined
reads per million genome matching
0
50000
100000
150000
0
10
20
30
40
50
60
bp position

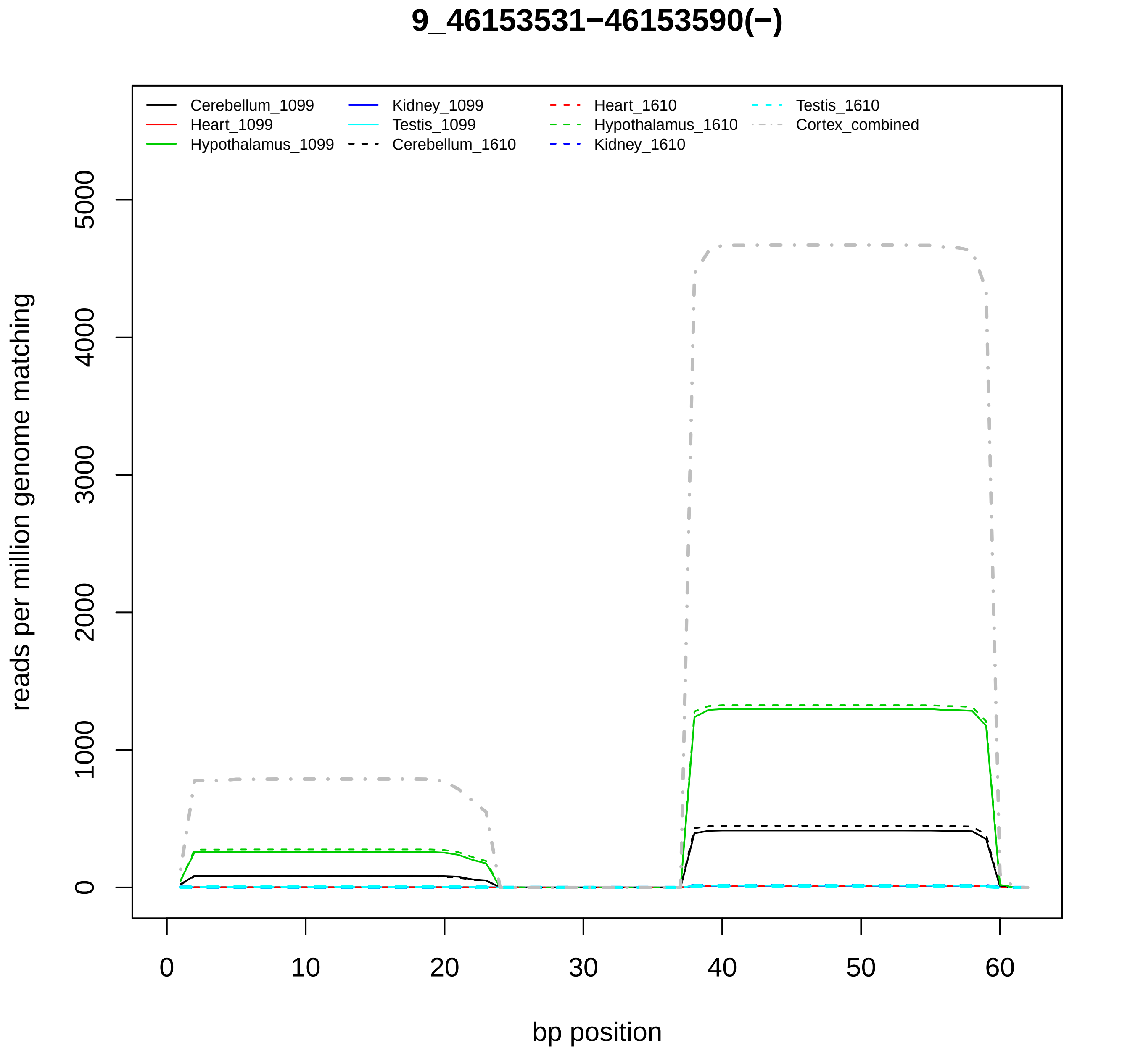

9_46153531−46153590(−)
Cerebellum_1099
Heart_1099
Hypothalamus_1099
Kidney_1099
Testis_1099
Cerebellum_1610
Heart_1610
Hypothalamus_1610
Kidney_1610
Testis_1610
Cortex_combined
reads per million genome matching
bp position
0
1000
2000
3000
4000
5000
0
10
20
30
40
50
60

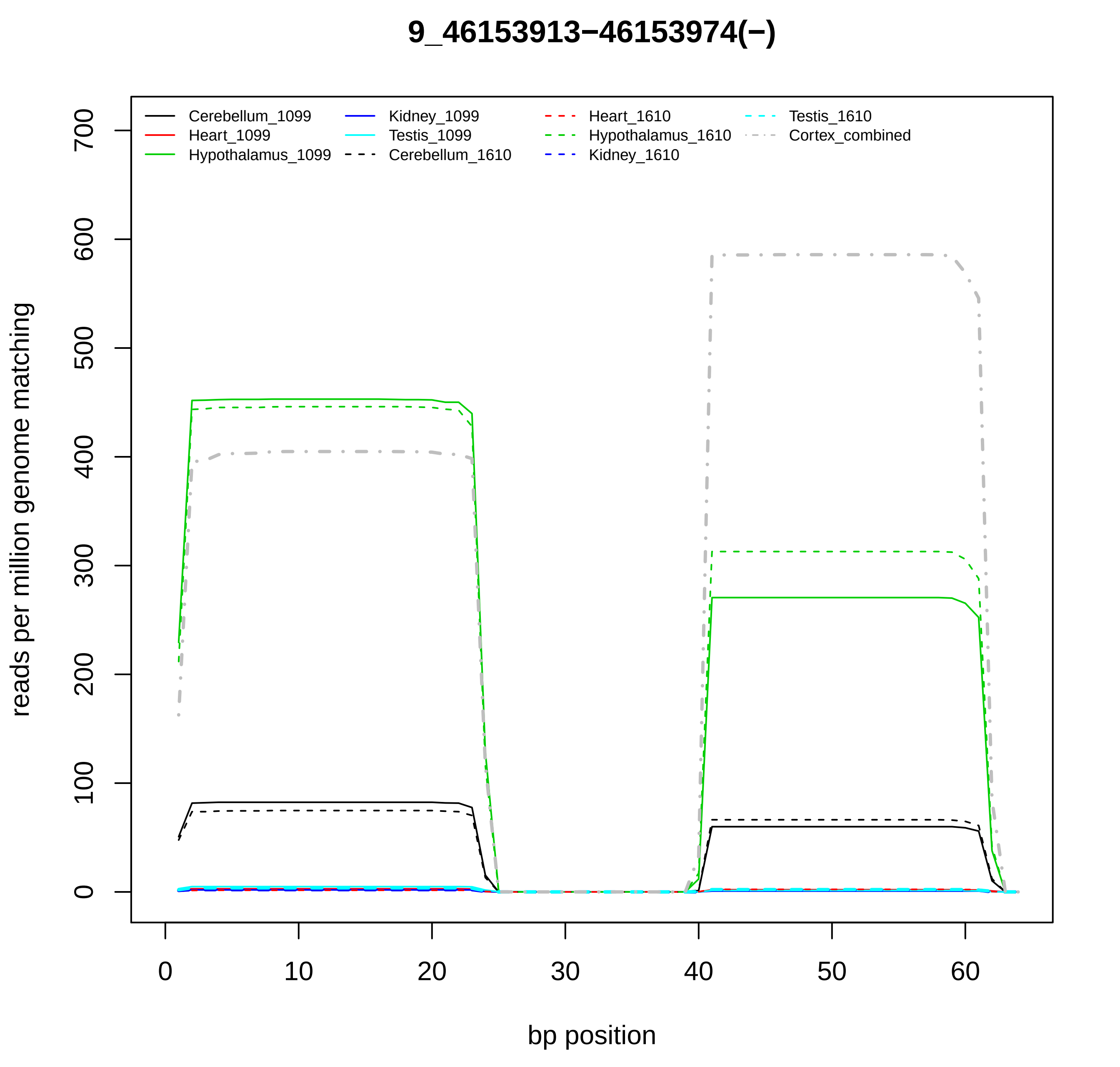
9_46153913−46153974(−)
Cerebellum_1099
Heart_1099
Hypothalamus_1099
Kidney_1099
Testis_1099
Cerebellum_1610
Heart_1610
Hypothalamus_1610
Kidney_1610
Testis_1610
Cortex_combined
reads per million genome matching
bp position
0
100
200
300
400
500
600
700
0
10
20
30
40
50
60

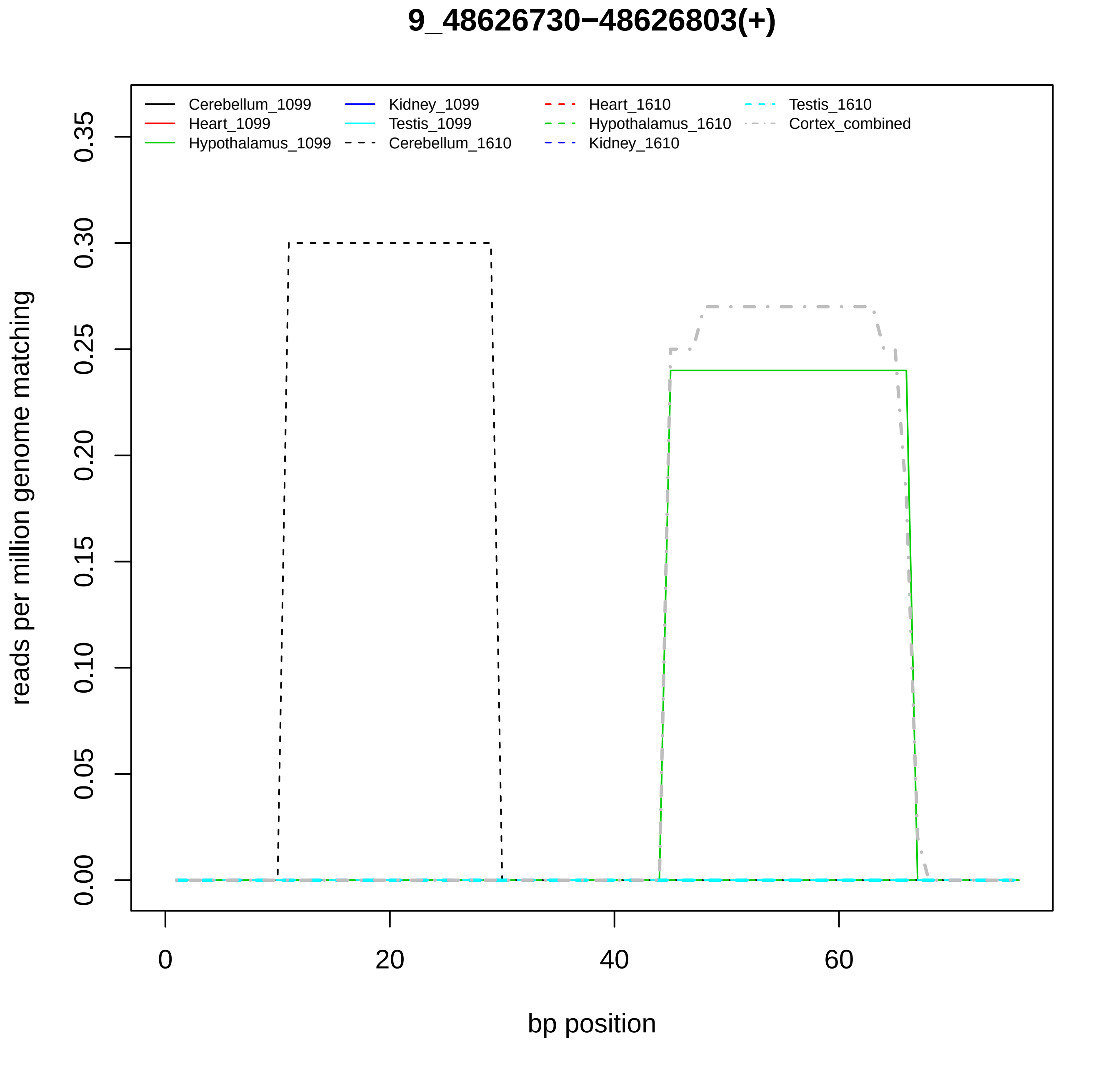
9_48626730−48626803(+)
Cerebellum_1099
Heart_1099
Hypothalamus_1099
Kidney_1099
Testis_1099
Cerebellum_1610
Heart_1610
Hypothalamus_1610
Kidney_1610
Testis_1610
Cortex_combined
reads per million genome matching
0.00
0.05
0.10
0.15
0.20
0.25
0.30
0.35
0
20
40
60
bp position

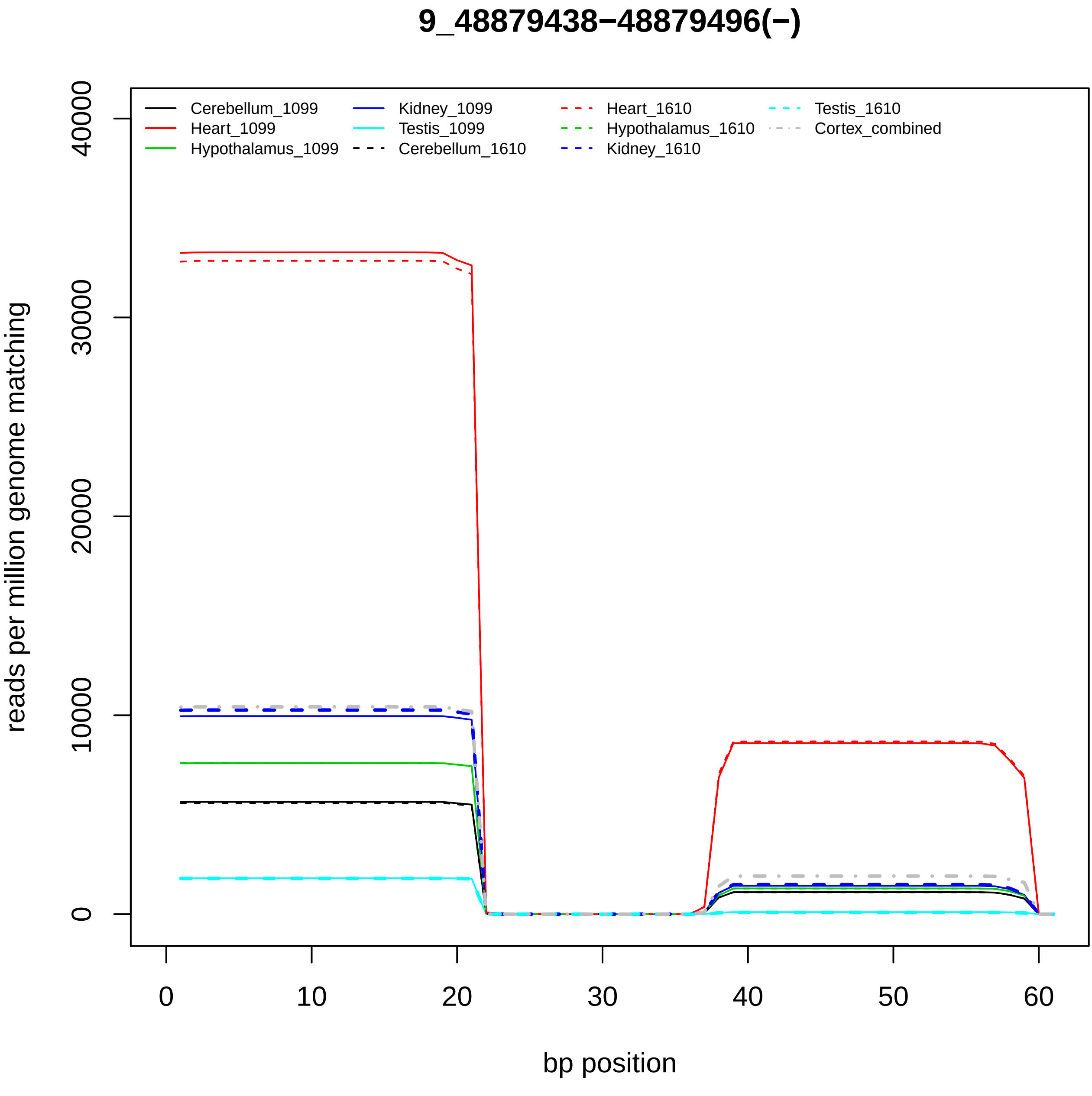

9_48879438−48879496(−)
Cerebellum_1099
Heart_1099
Hypothalamus_1099
Kidney_1099
Testis_1099
Cerebellum_1610
Heart_1610
Hypothalamus_1610
Kidney_1610
Testis_1610
Cortex_combined
reads per million genome matching
0
10000
20000
30000
40000
bp position
0
10
20
30
40
50
60

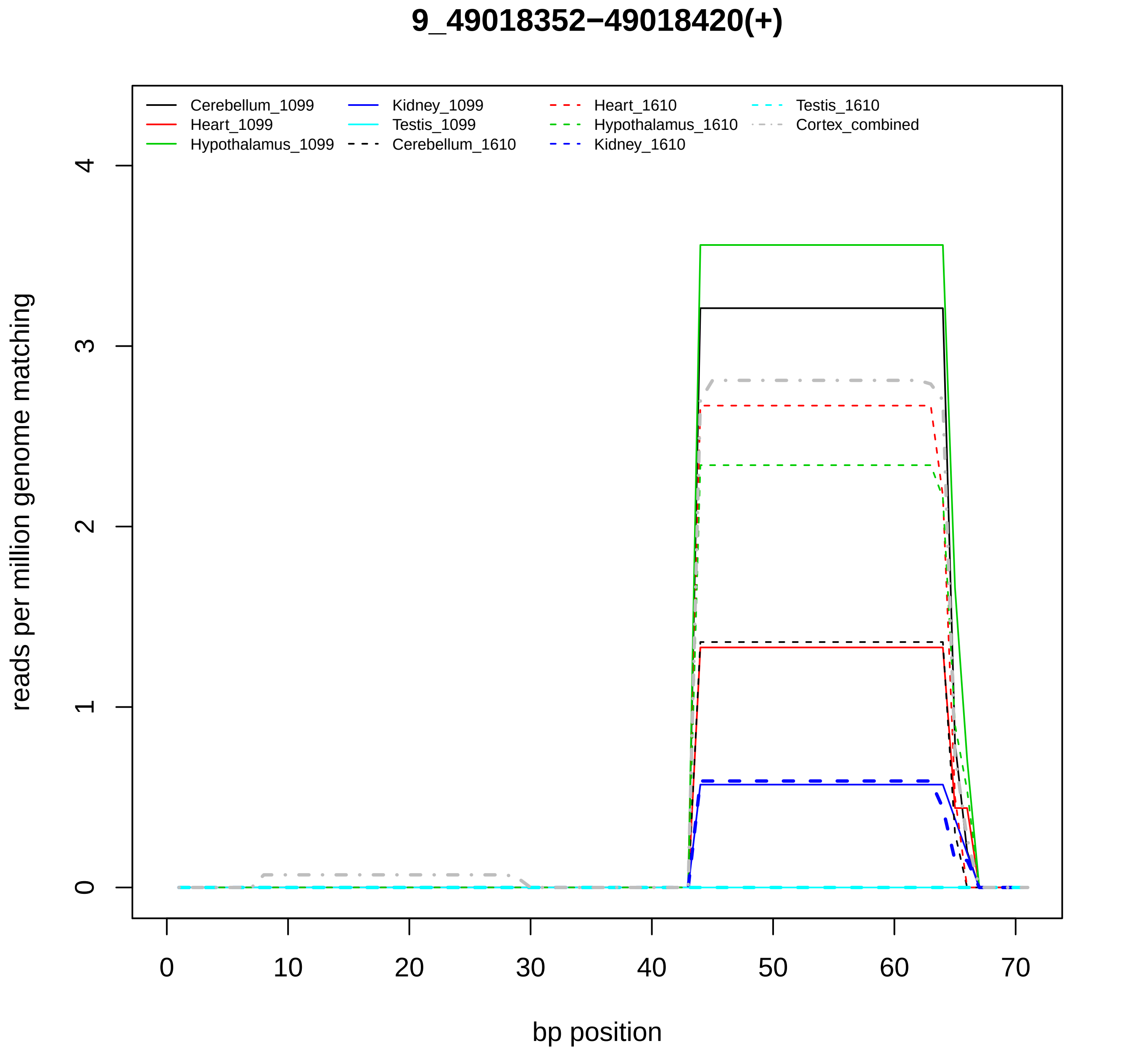
9_49018352−49018420(+)
Cerebellum_1099
Heart_1099
Hypothalamus_1099
Kidney_1099
Testis_1099
Cerebellum_1610
Heart_1610
Hypothalamus_1610
Kidney_1610
Testis_1610
Cortex_combined
reads per million genome matching
bp position
0
1
2
3
4
0
10
20
30
40
50
60
70

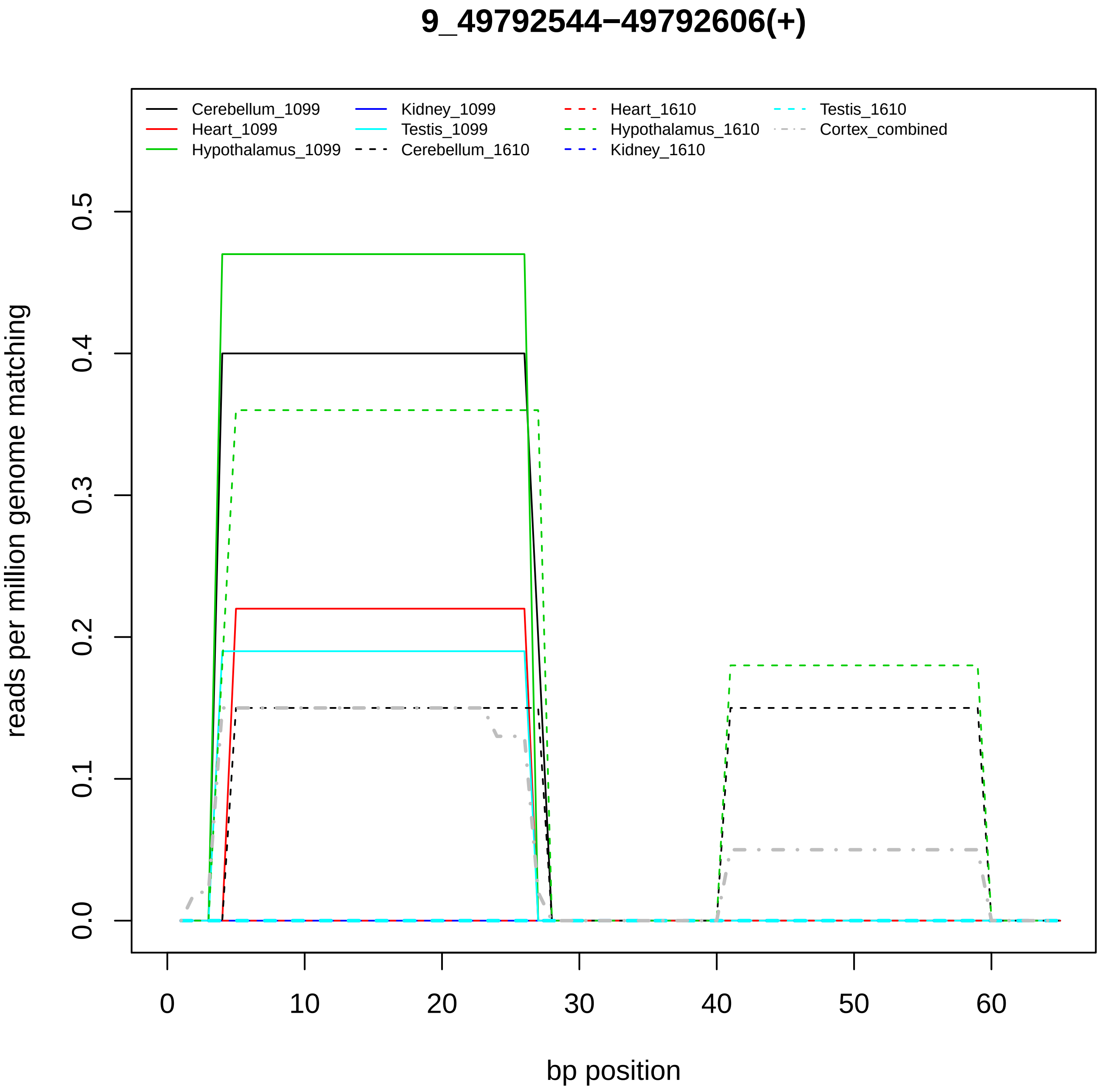

9_49792544−49792606(+)
Cerebellum_1099
Heart_1099
Hypothalamus_1099
Kidney_1099
Testis_1099
Cerebellum_1610
Heart_1610
Hypothalamus_1610
Kidney_1610
Testis_1610
Cortex_combined
reads per million genome matching
0.0
0.1
0.2
0.3
0.4
0.5
0
10
20
30
40
50
60
bp position

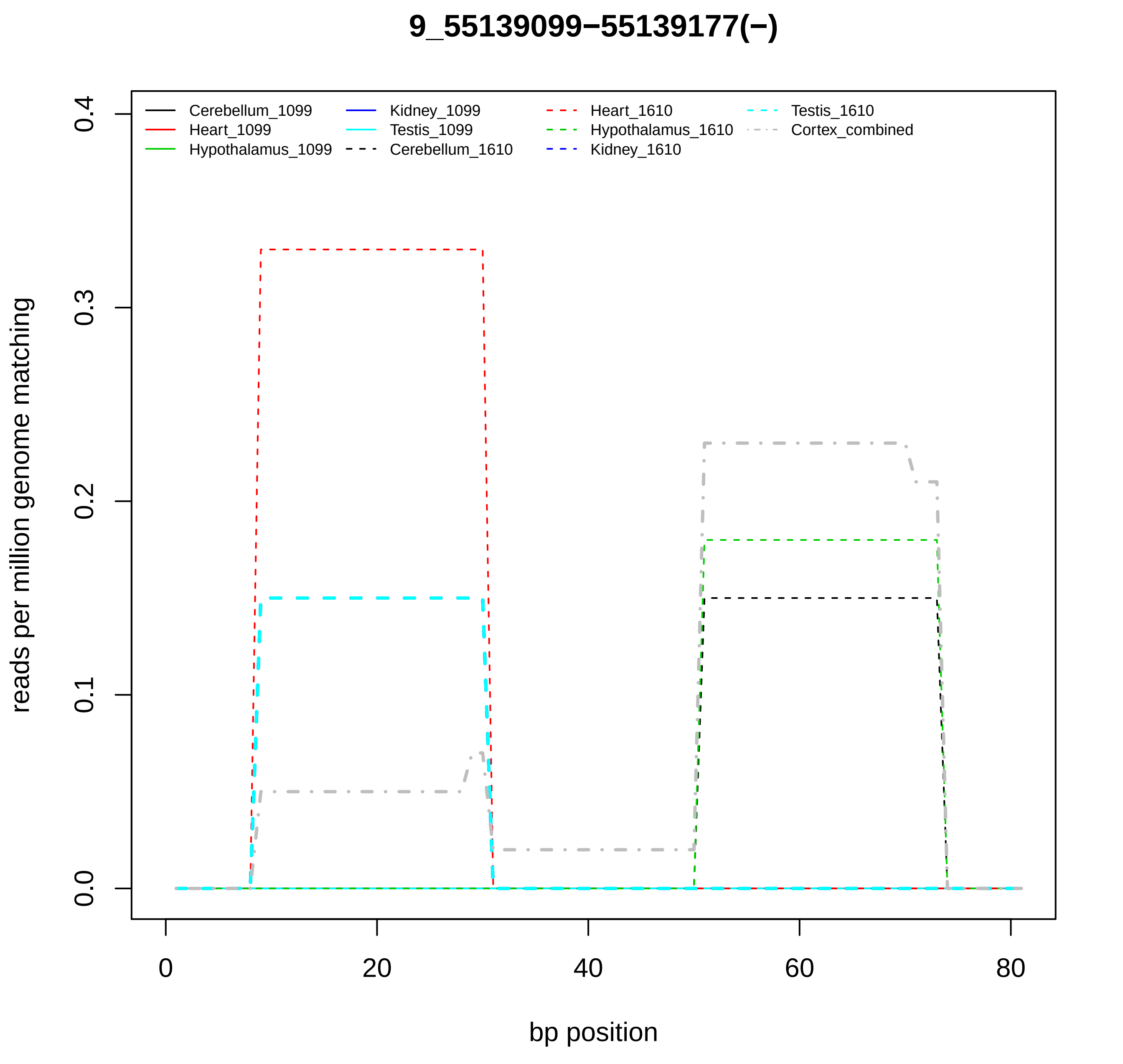
9_55139099−55139177(−)
Cerebellum_1099
Heart_1099
Hypothalamus_1099
Kidney_1099
Testis_1099
Cerebellum_1610
Heart_1610
Hypothalamus_1610
Kidney_1610
Testis_1610
Cortex_combined
reads per million genome matching
bp position
0.0
0.1
0.2
0.3
0.4
0
20
40
60
80

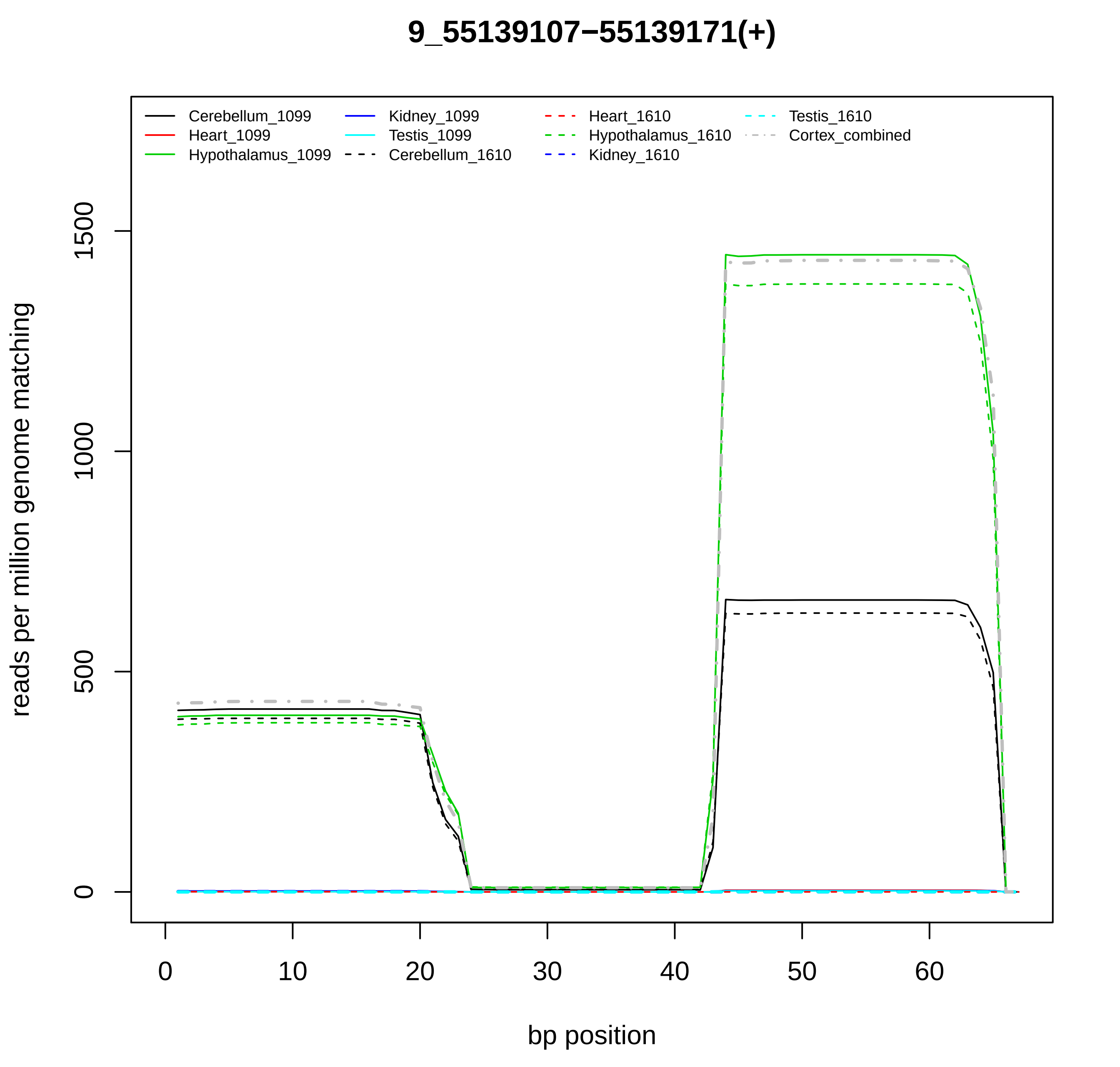

9_55139107−55139171(+)
Cerebellum_1099
Heart_1099
Hypothalamus_1099
Kidney_1099
Testis_1099
Cerebellum_1610
Heart_1610
Hypothalamus_1610
Kidney_1610
Testis_1610
Cortex_combined
reads per million genome matching
0
500
1000
1500
0
10
20
30
40
50
60
bp position

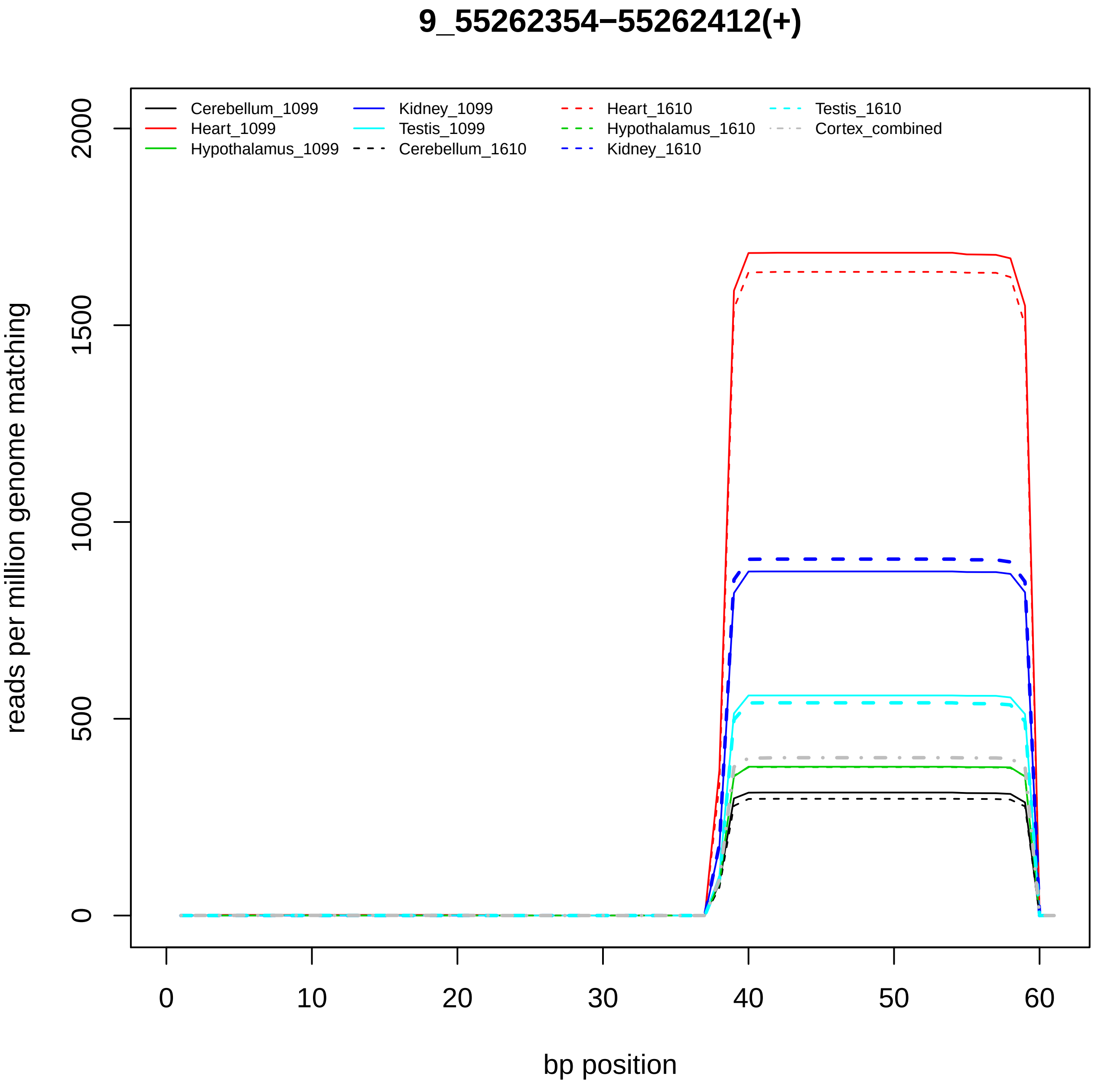
9_55262354−55262412(+)
Cerebellum_1099
Heart_1099
Hypothalamus_1099
Kidney_1099
Testis_1099
Cerebellum_1610
Heart_1610
Hypothalamus_1610
Kidney_1610
Testis_1610
Cortex_combined
reads per million genome matching
bp position
0
500
1000
1500
2000
0
10
20
30
40
50
60

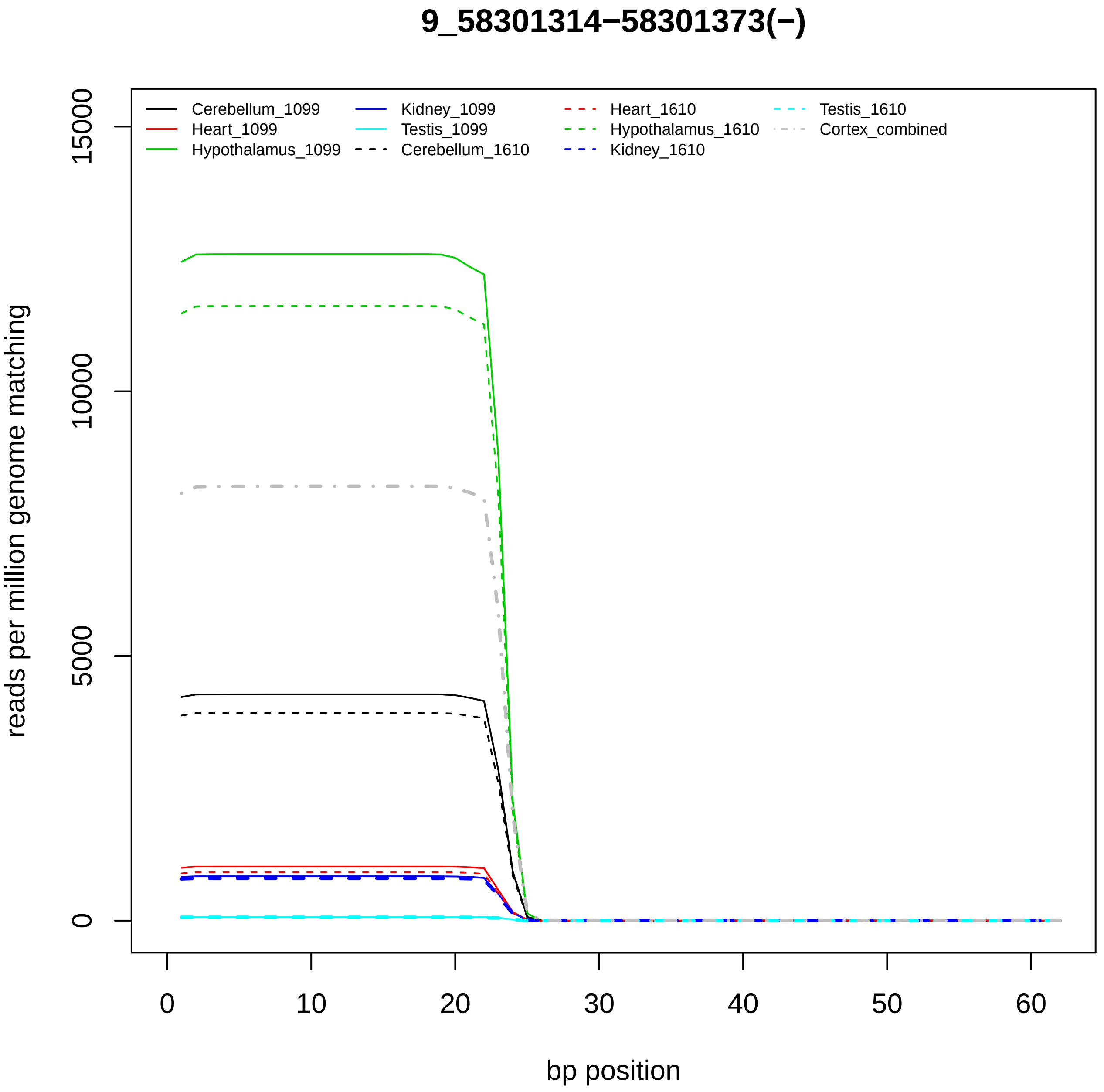

9_58301314−58301373(−)
Cerebellum_1099
Heart_1099
Hypothalamus_1099
Kidney_1099
Testis_1099
Cerebellum_1610
Heart_1610
Hypothalamus_1610
Kidney_1610
Testis_1610
Cortex_combined
reads per million genome matching
bp position
0
5000
10000
15000
0
10
20
30
40
50
60

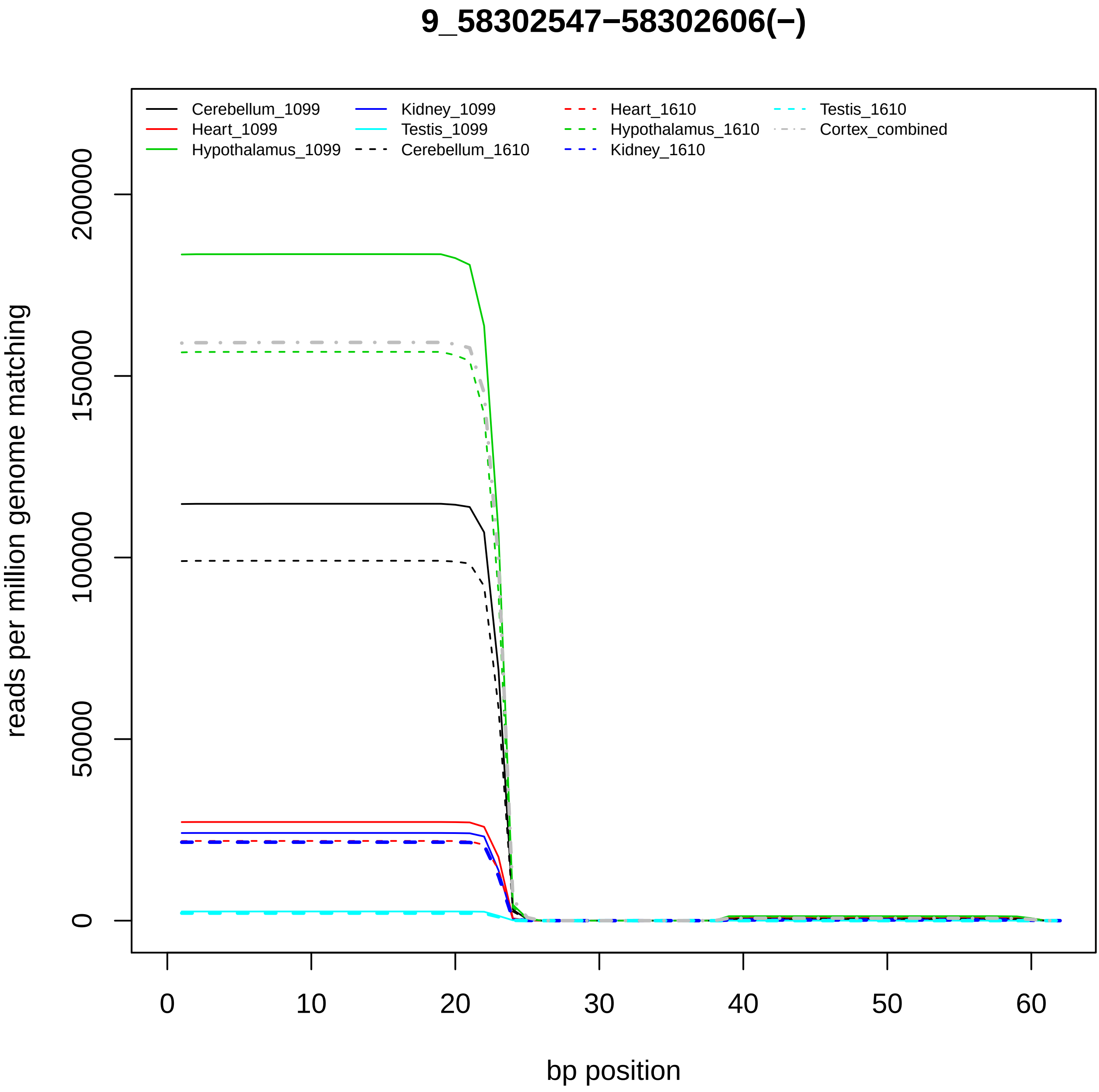

9_58302547−58302606(−)
Cerebellum_1099
Heart_1099
Hypothalamus_1099
Kidney_1099
Testis_1099
Cerebellum_1610
Heart_1610
Hypothalamus_1610
Kidney_1610
Testis_1610
Cortex_combined
reads per million genome matching
bp position
0
50000
100000
150000
200000
0
10
20
30
40
50
60

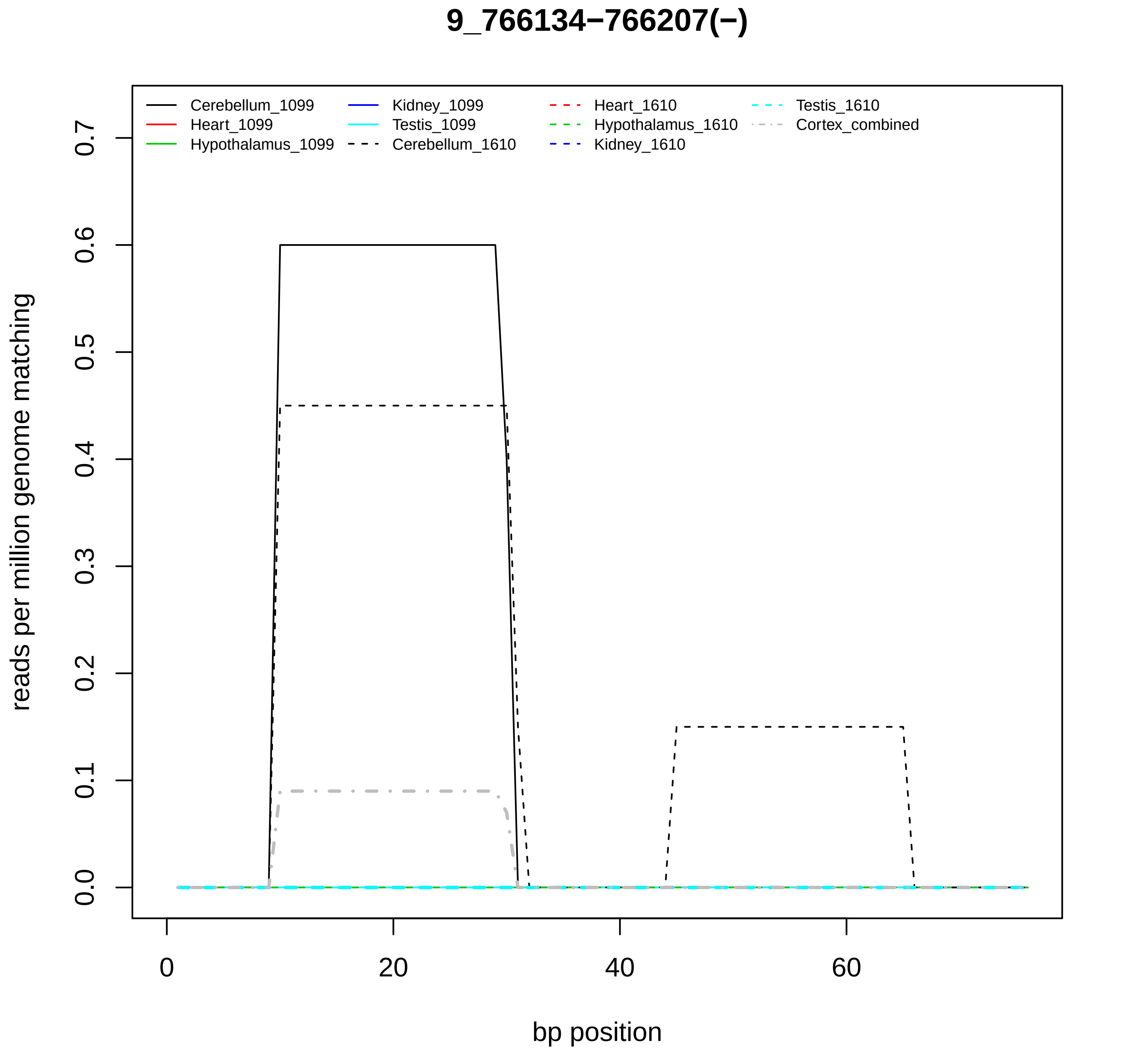

9_766134−766207(−)
Cerebellum_1099
Heart_1099
Hypothalamus_1099
Kidney_1099
Testis_1099
Cerebellum_1610
Heart_1610
Hypothalamus_1610
Kidney_1610
Testis_1610
Cortex_combined
reads per million genome matching
0.0
0.1
0.2
0.3
0.4
0.5
0.6
0.7
0
20
40
60
bp position

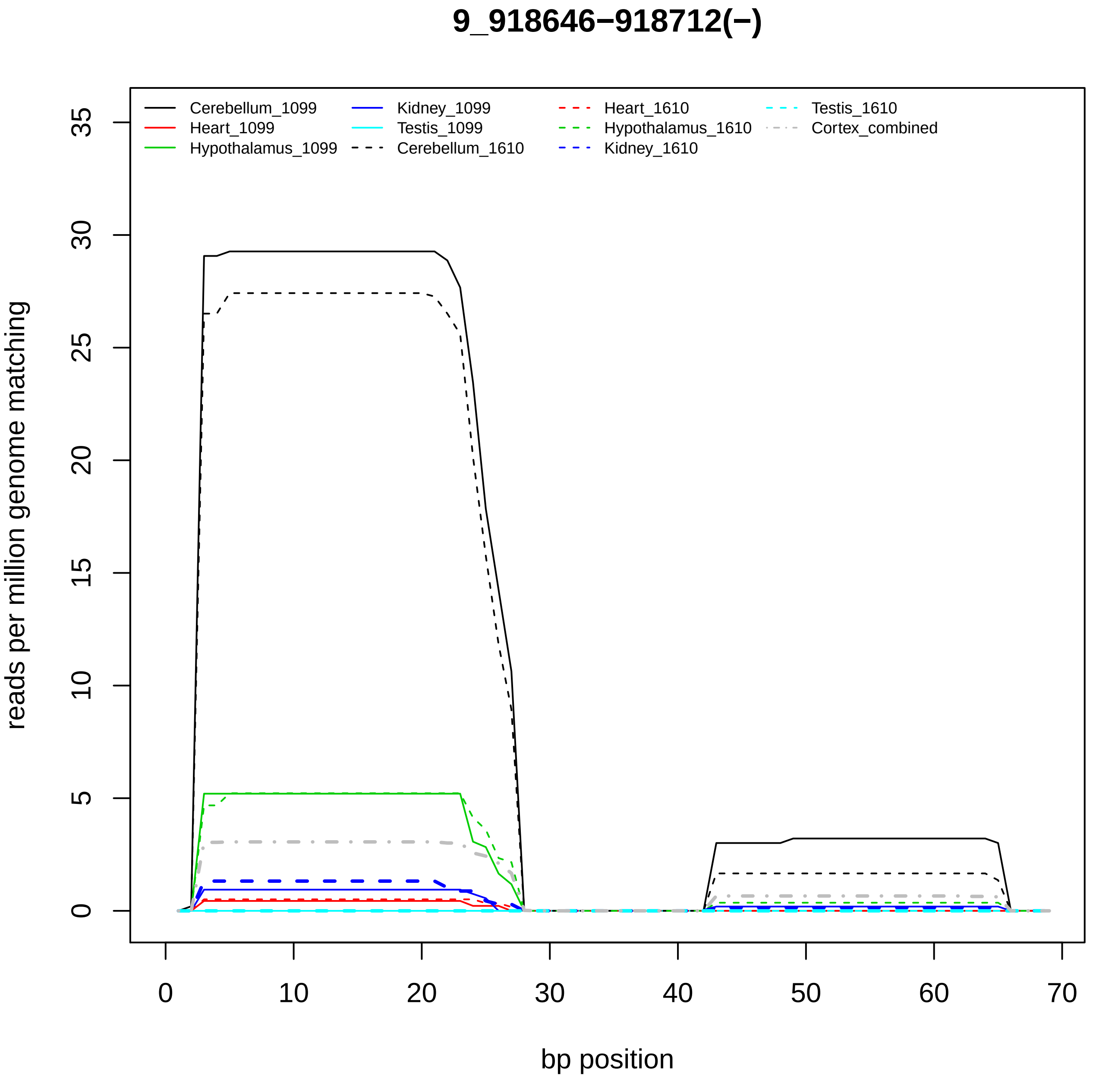
9_918646−918712(−)
Cerebellum_1099
Heart_1099
Hypothalamus_1099
Kidney_1099
Testis_1099
Cerebellum_1610
Heart_1610
Hypothalamus_1610
Kidney_1610
Testis_1610
Cortex_combined
reads per million genome matching
bp position
0
5
10
15
20
25
30
35
0
10
20
30
40
50
60
70

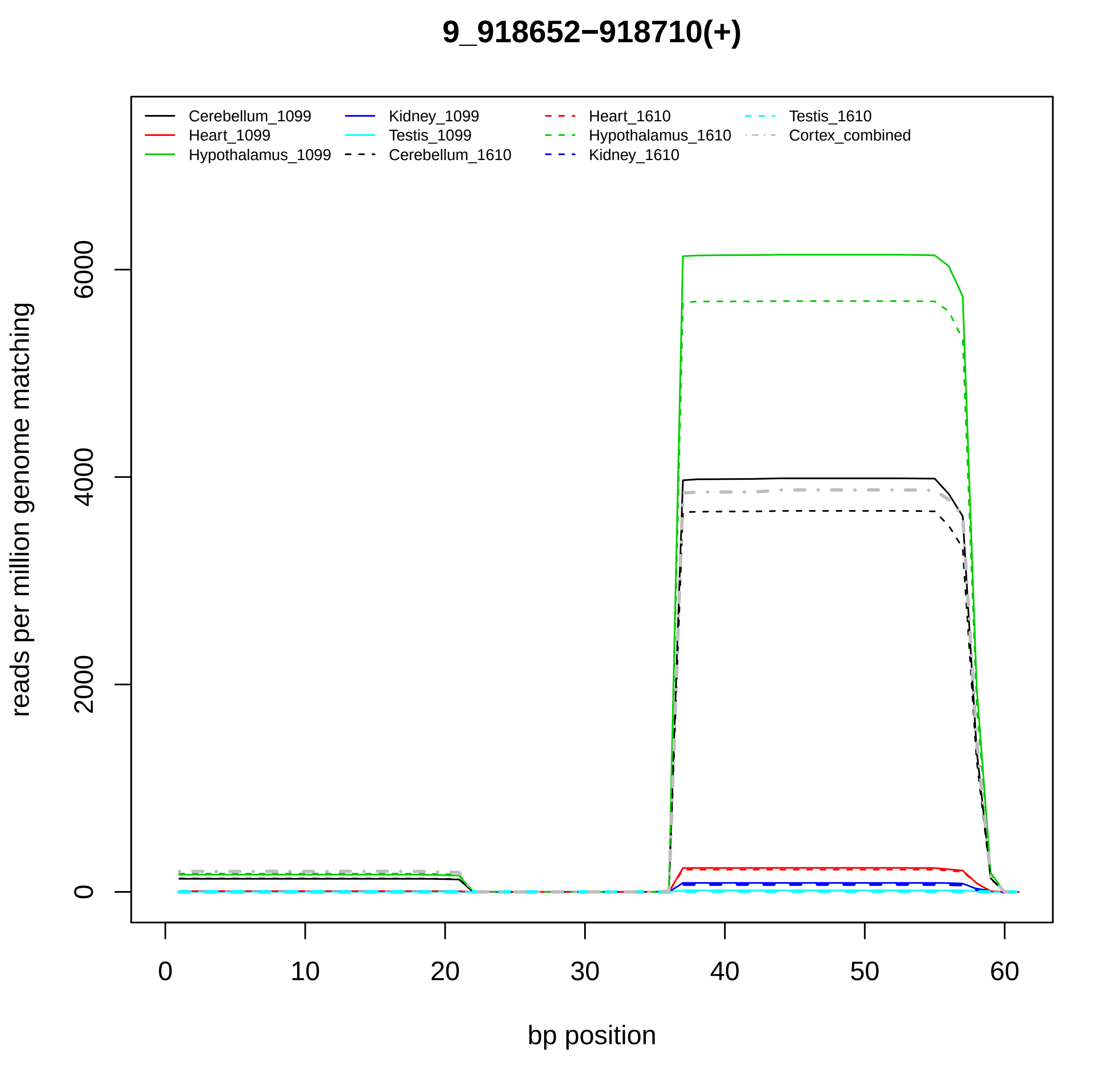

9_918652−918710(+)
Cerebellum_1099
Heart_1099
Hypothalamus_1099
Kidney_1099
Testis_1099
Cerebellum_1610
Heart_1610
Hypothalamus_1610
Kidney_1610
Testis_1610
Cortex_combined
reads per million genome matching
0
2000
4000
6000
bp position
0
10
20
30
40
50
60

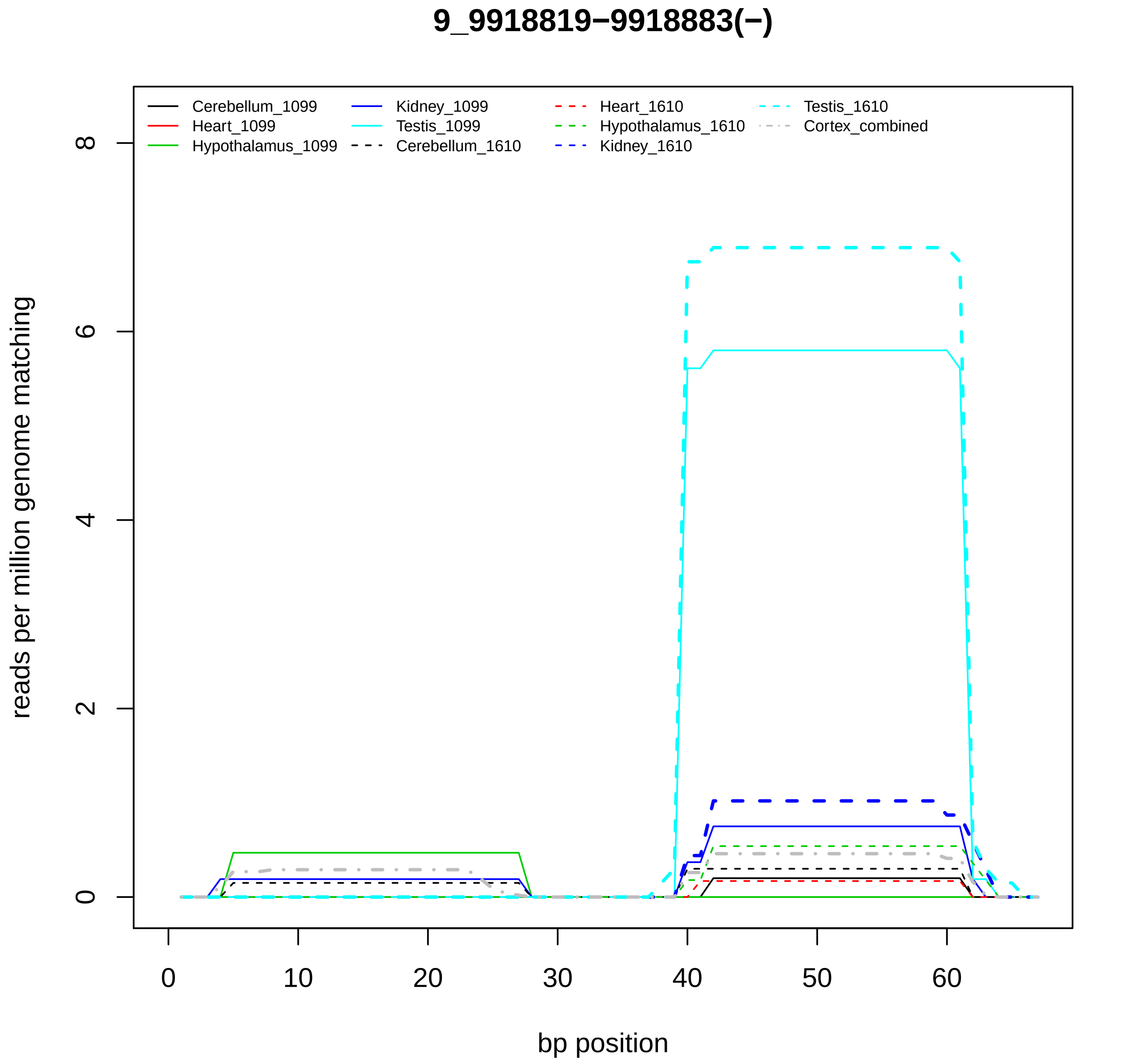
9_9918819−9918883(−)
Cerebellum_1099
Heart_1099
Hypothalamus_1099
Kidney_1099
Testis_1099
Cerebellum_1610
Heart_1610
Hypothalamus_1610
Kidney_1610
Testis_1610
Cortex_combined
reads per million genome matching
bp position
0
2
4
6
8
0
10
20
30
40
50
60

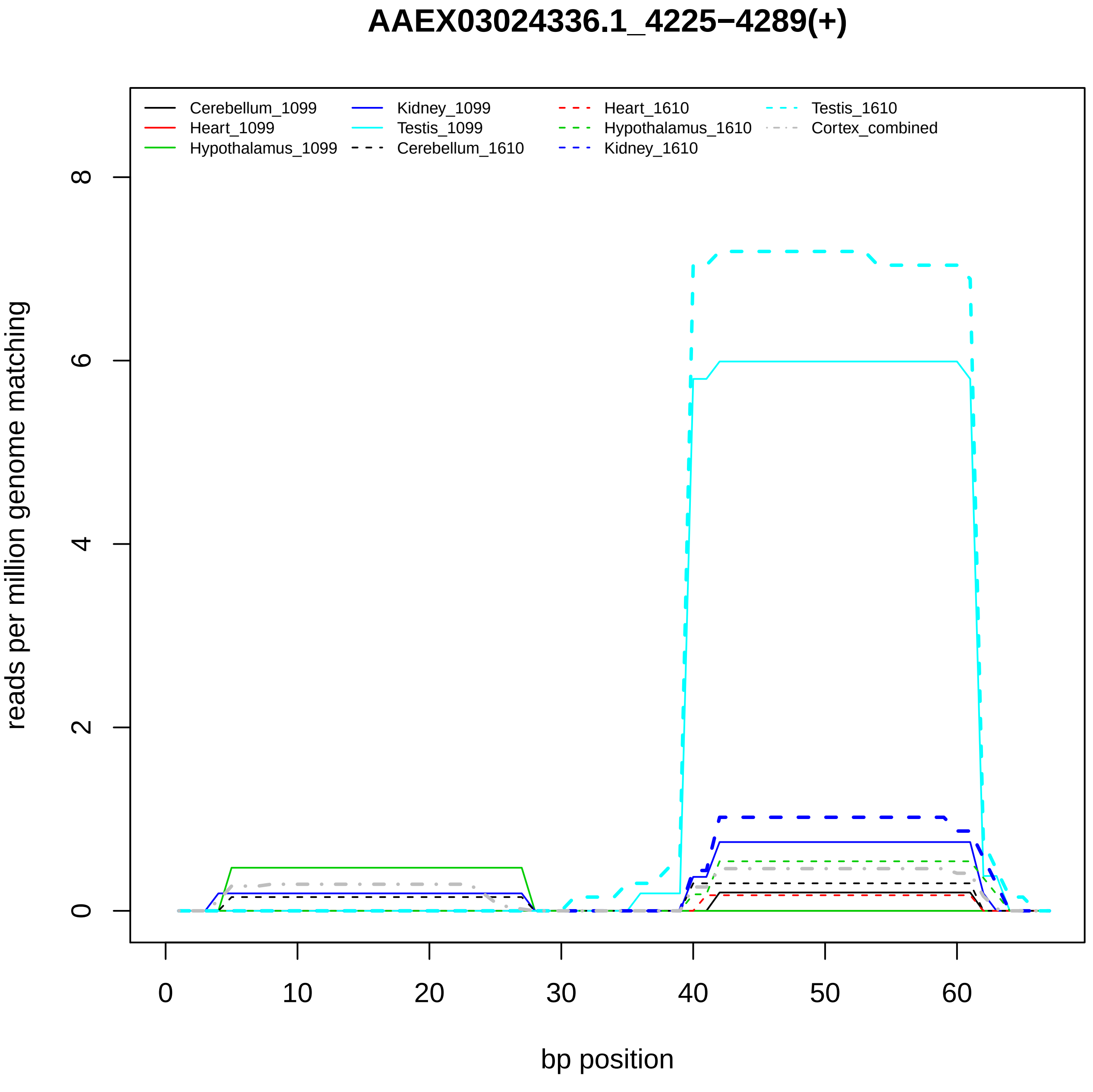
AAEX03024336.1_4225−4289(+)
Cerebellum_1099
Heart_1099
Hypothalamus_1099
Kidney_1099
Testis_1099
Cerebellum_1610
Heart_1610
Hypothalamus_1610
Kidney_1610
Testis_1610
Cortex_combined
reads per million genome matching
0
2
4
6
8
0
10
20
30
40
50
60
bp position

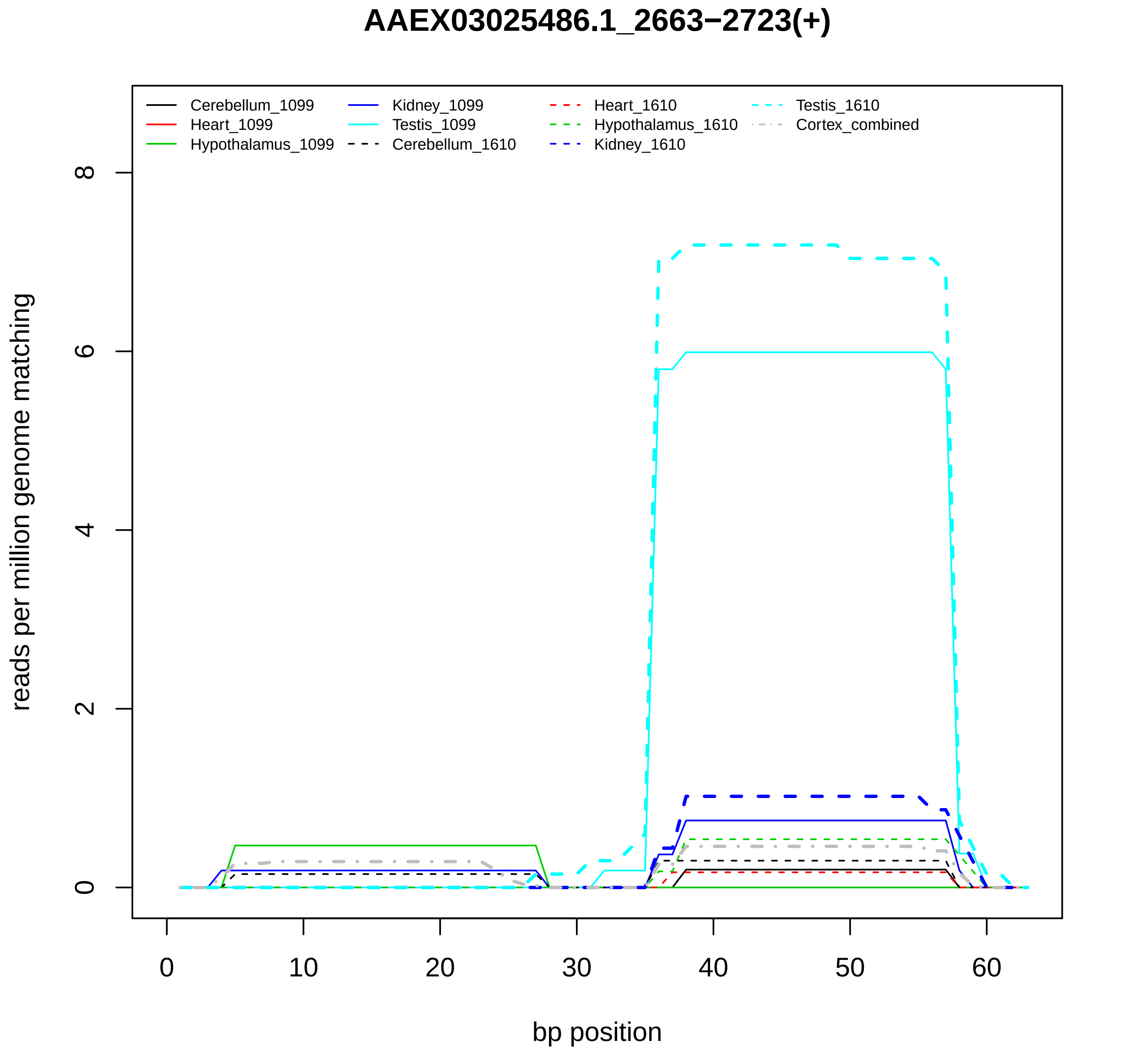

AAEX03025486.1_2663−2723(+)
Cerebellum_1099
Heart_1099
Hypothalamus_1099
Kidney_1099
Testis_1099
Cerebellum_1610
Heart_1610
Hypothalamus_1610
Kidney_1610
Testis_1610
Cortex_combined
reads per million genome matching
bp position
0
2
4
6
8
0
10
20
30
40
50
60

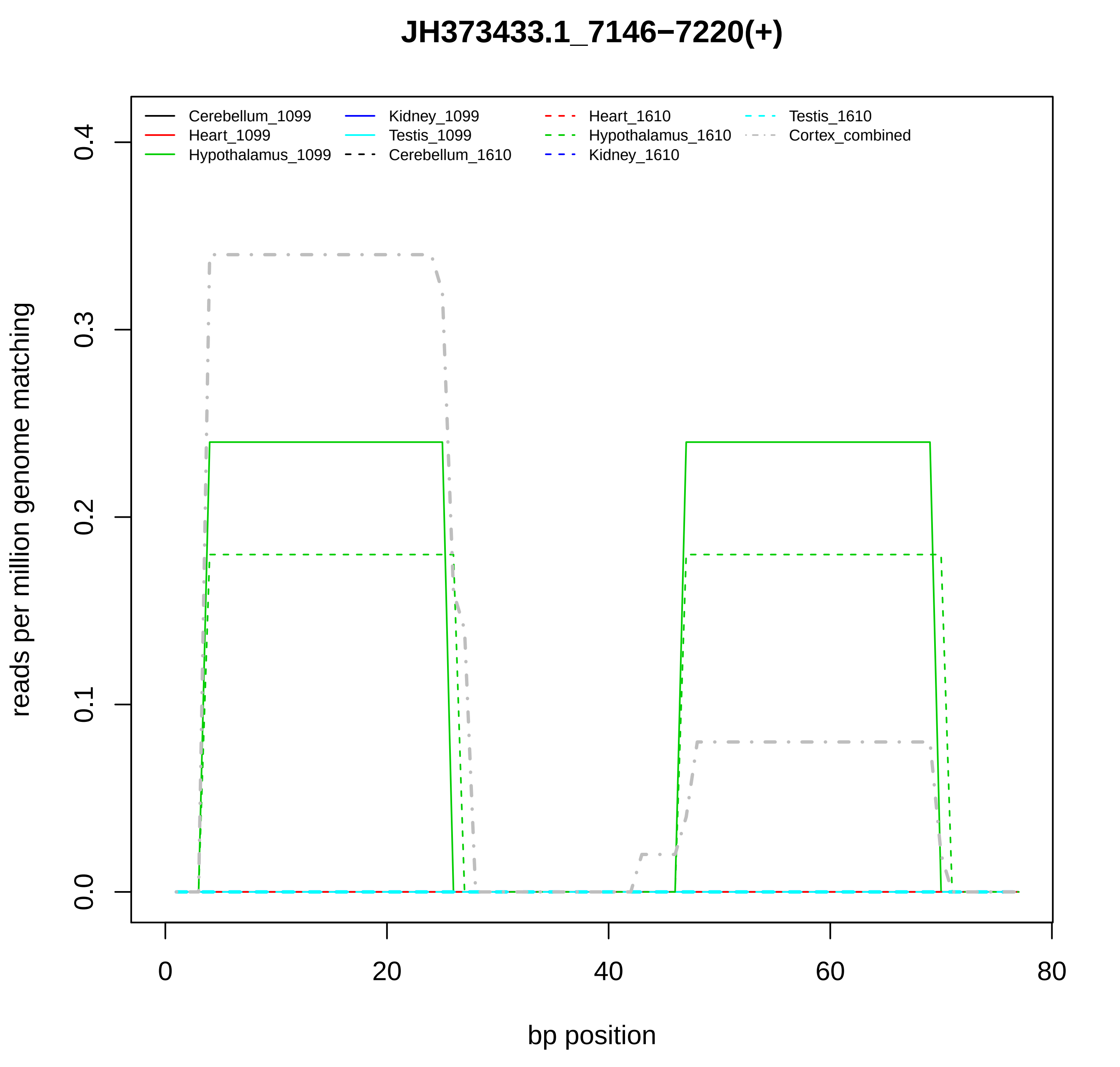

JH373433.1_7146−7220(+)
Cerebellum_1099
Heart_1099
Hypothalamus_1099
Kidney_1099
Testis_1099
Cerebellum_1610
Heart_1610
Hypothalamus_1610
Kidney_1610
Testis_1610
Cortex_combined
reads per million genome matching
0.0
0.1
0.2
0.3
0.4
0
20
40
60
80
bp position

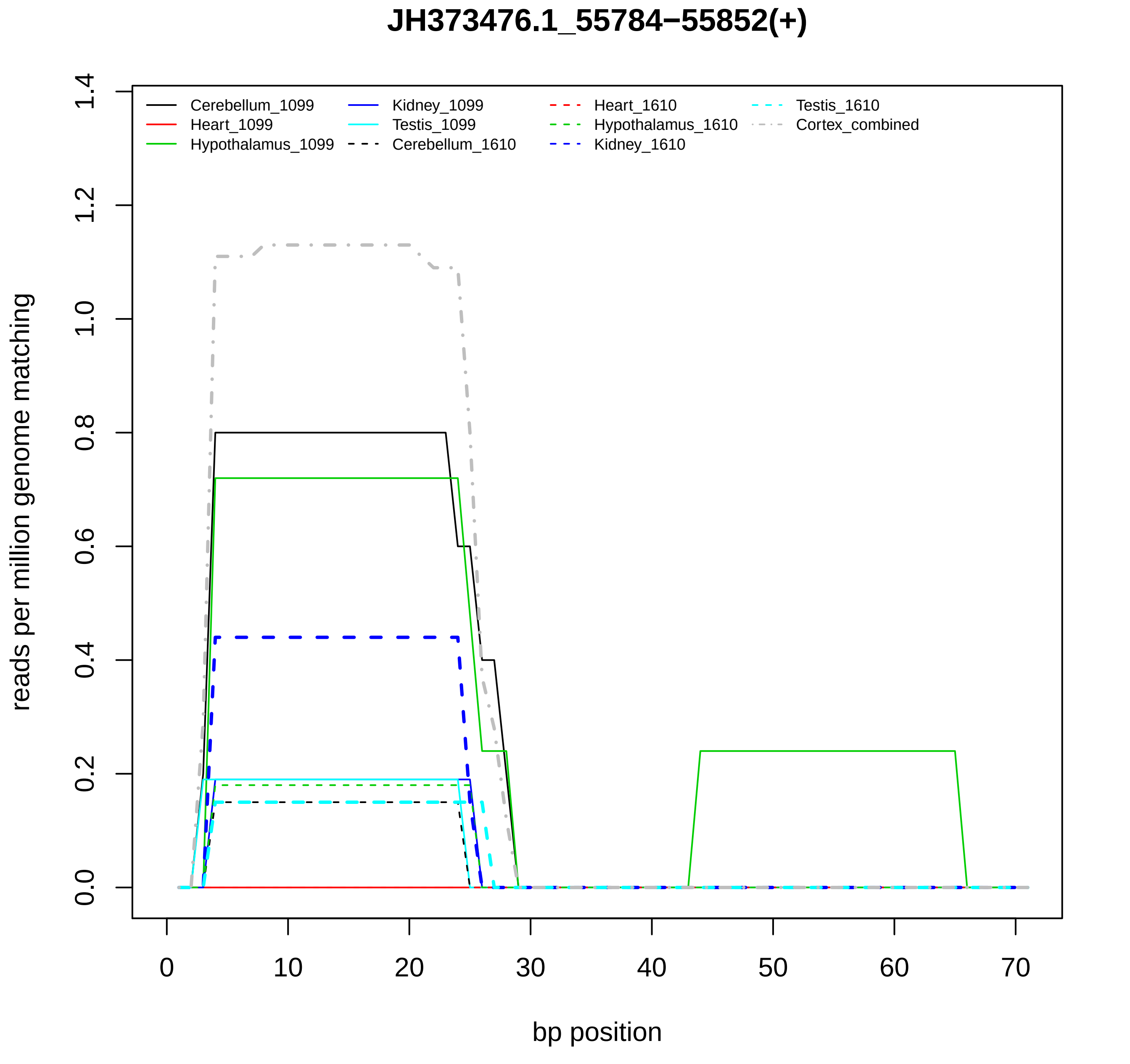
JH373476.1_55784−55852(+)
Cerebellum_1099
Heart_1099
Hypothalamus_1099
Kidney_1099
Testis_1099
Cerebellum_1610
Heart_1610
Hypothalamus_1610
Kidney_1610
Testis_1610
Cortex_combined
reads per million genome matching
bp position
0.0
0.2
0.4
0.6
0.8
1.0
1.2
1.4
0
10
20
30
40
50
60
70

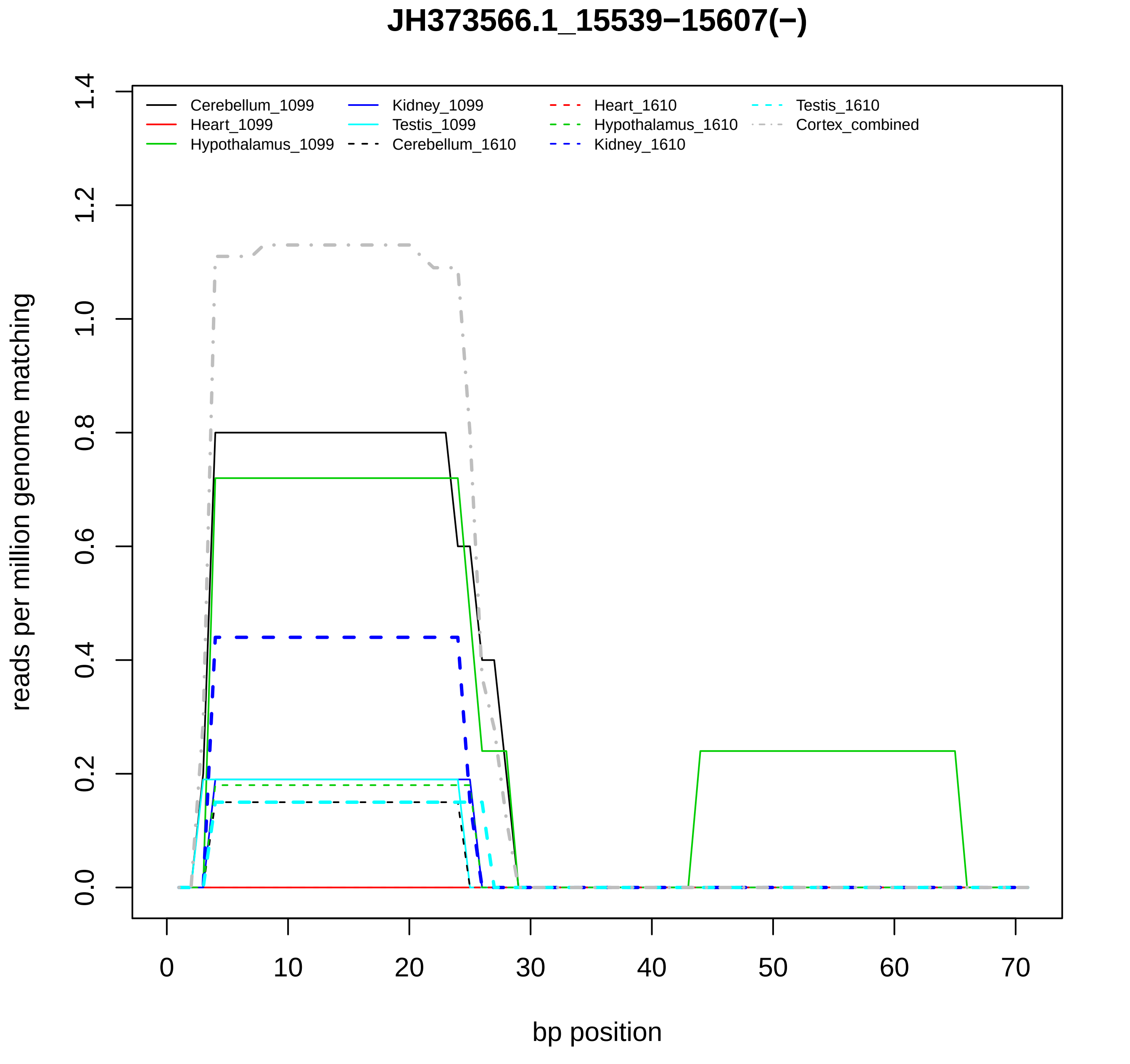

JH373566.1_15539−15607(−)
Cerebellum_1099
Heart_1099
Hypothalamus_1099
Kidney_1099
Testis_1099
Cerebellum_1610
Heart_1610
Hypothalamus_1610
Kidney_1610
Testis_1610
Cortex_combined
reads per million genome matching
bp position
0.0
0.2
0.4
0.6
0.8
1.0
1.2
1.4
0
10
20
30
40
50
60
70

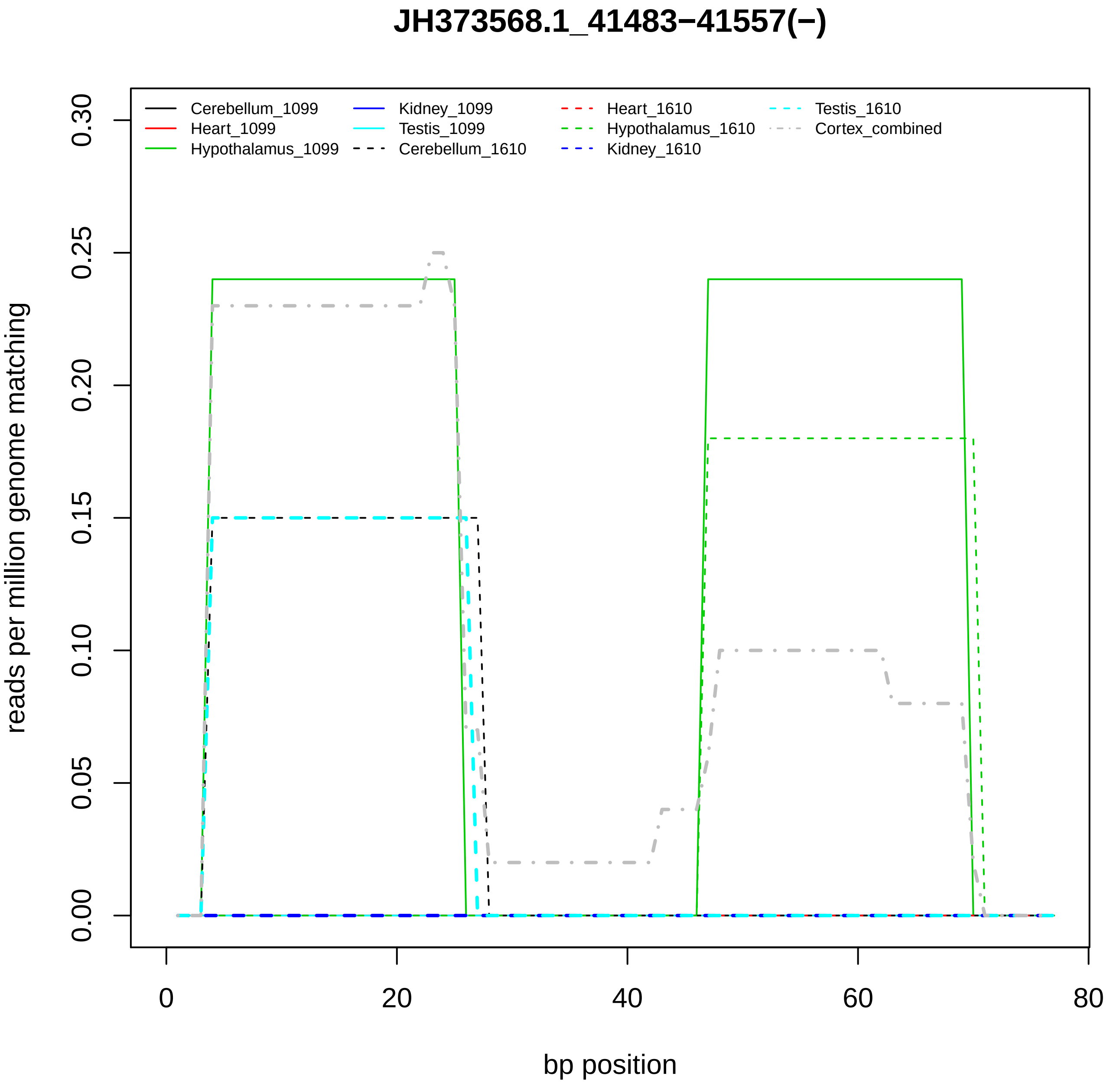

JH373568.1_41483−41557(−)
Cerebellum_1099
Heart_1099
Hypothalamus_1099
Kidney_1099
Testis_1099
Cerebellum_1610
Heart_1610
Hypothalamus_1610
Kidney_1610
Testis_1610
Cortex_combined
reads per million genome matching
bp position
0.00
0.05
0.10
0.15
0.20
0.25
0.30
0
20
40
60
80

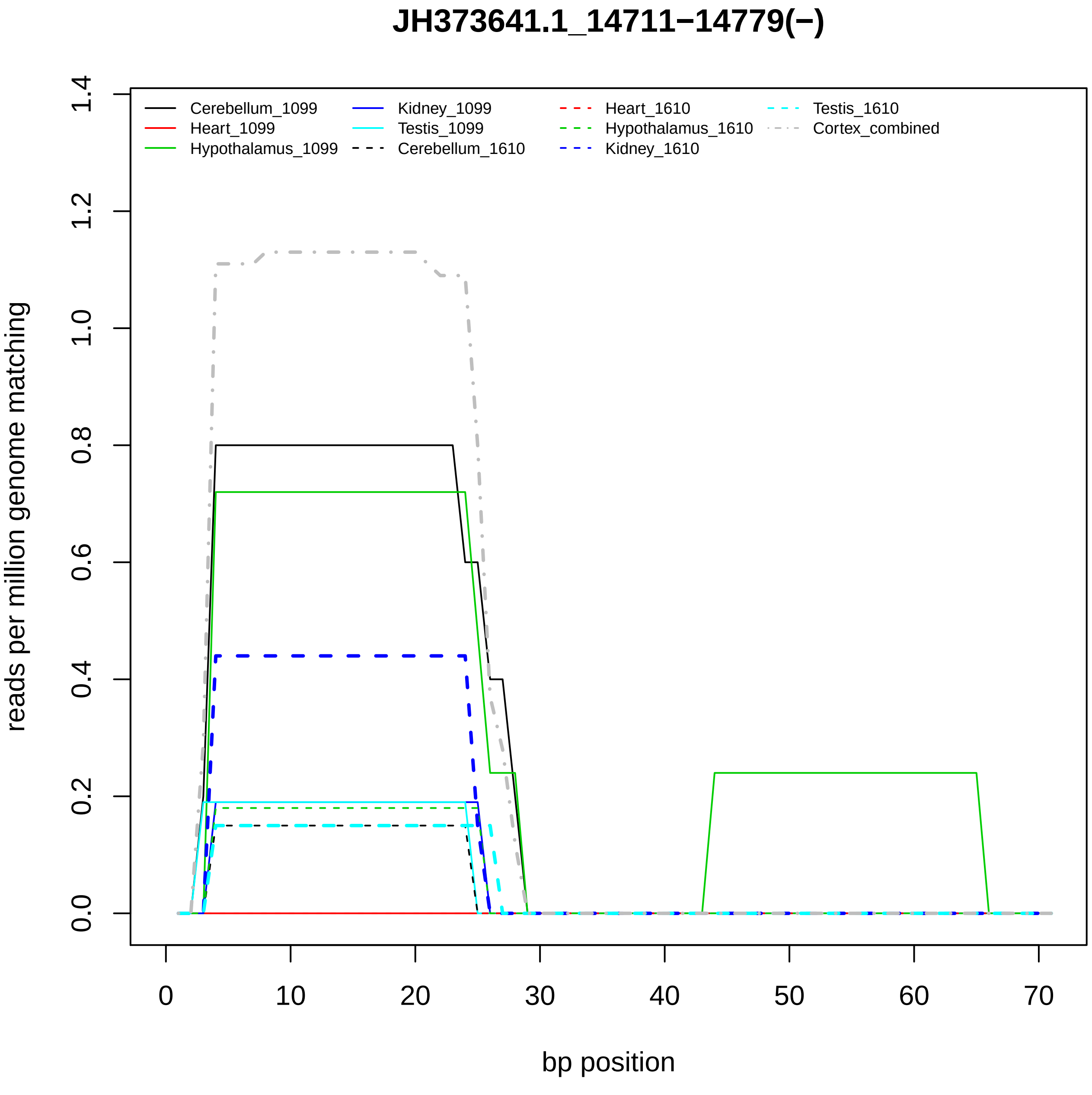

JH373641.1_14711−14779(−)
Cerebellum_1099
Heart_1099
Hypothalamus_1099
Kidney_1099
Testis_1099
Cerebellum_1610
Heart_1610
Hypothalamus_1610
Kidney_1610
Testis_1610
Cortex_combined
reads per million genome matching
bp position
0.0
0.2
0.4
0.6
0.8
1.0
1.2
1.4
0
10
20
30
40
50
60
70

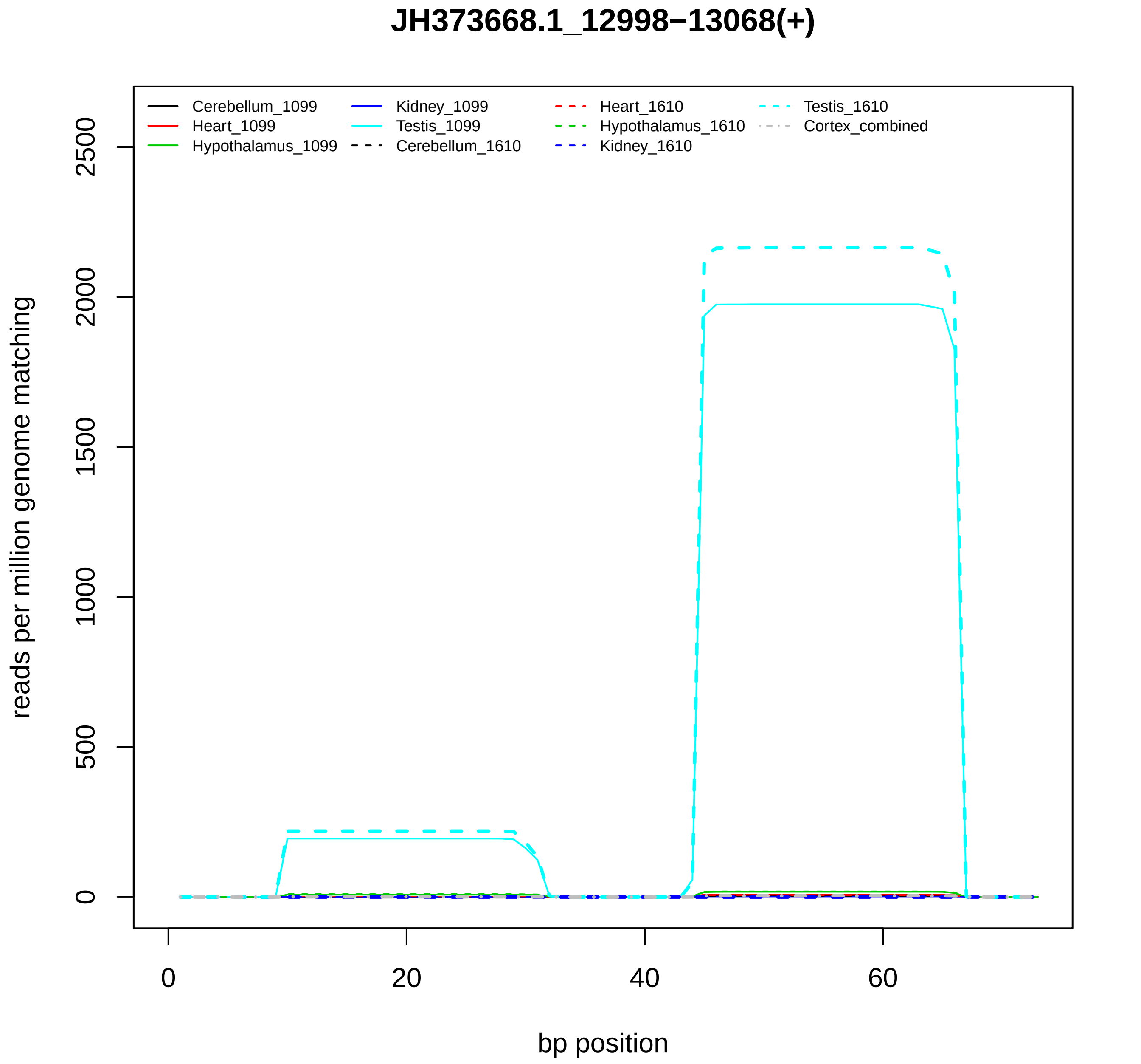
JH373668.1_12998−13068(+)
Cerebellum_1099
Heart_1099
Hypothalamus_1099
Kidney_1099
Testis_1099
Cerebellum_1610
Heart_1610
Hypothalamus_1610
Kidney_1610
Testis_1610
Cortex_combined
reads per million genome matching
0
500
1000
1500
2000
2500
0
20
40
60
bp position

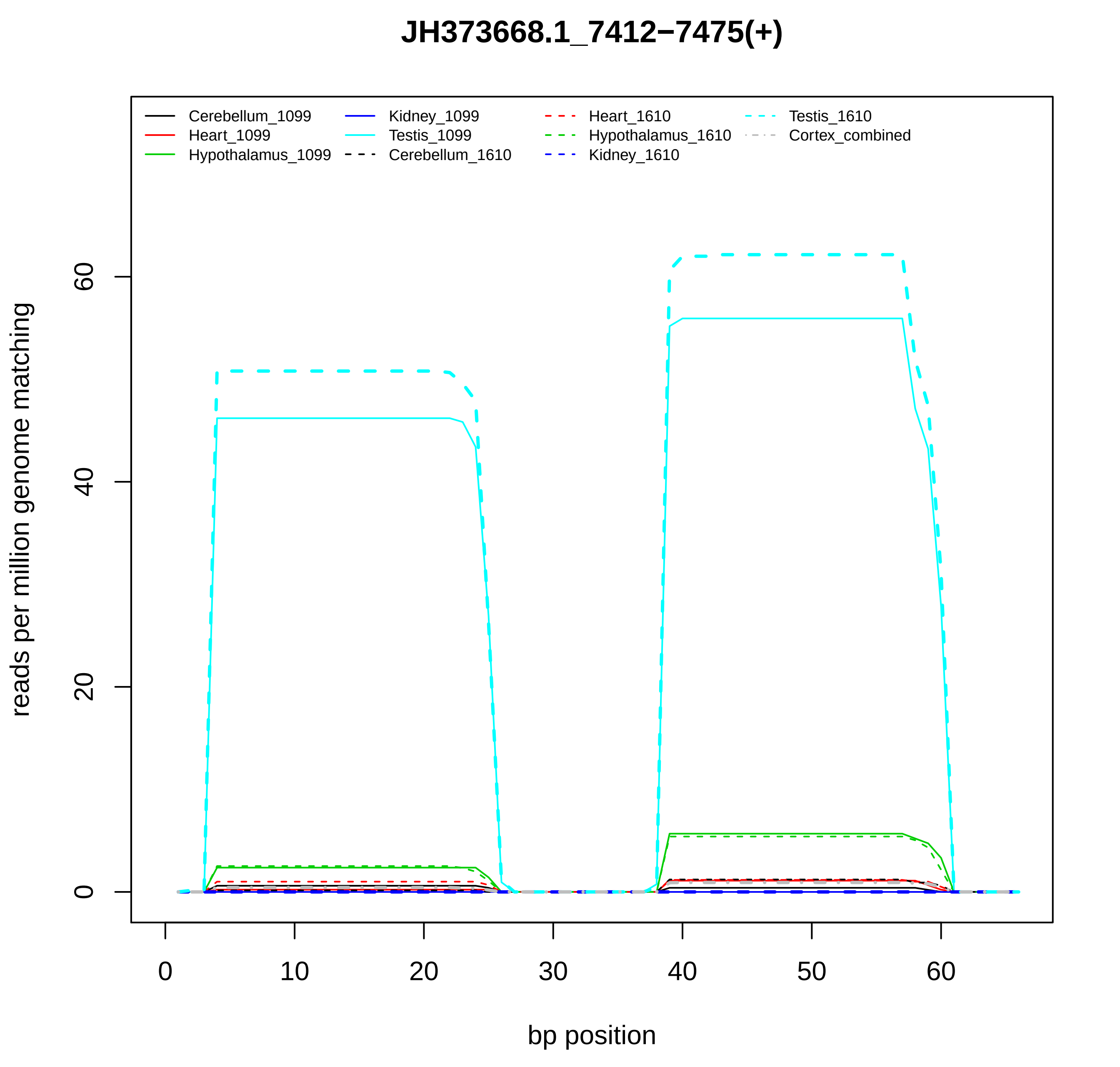

JH373668.1_7412−7475(+)
Cerebellum_1099
Heart_1099
Hypothalamus_1099
Kidney_1099
Testis_1099
Cerebellum_1610
Heart_1610
Hypothalamus_1610
Kidney_1610
Testis_1610
Cortex_combined
reads per million genome matching
0
20
40
60
0
10
20
30
40
50
60
bp position

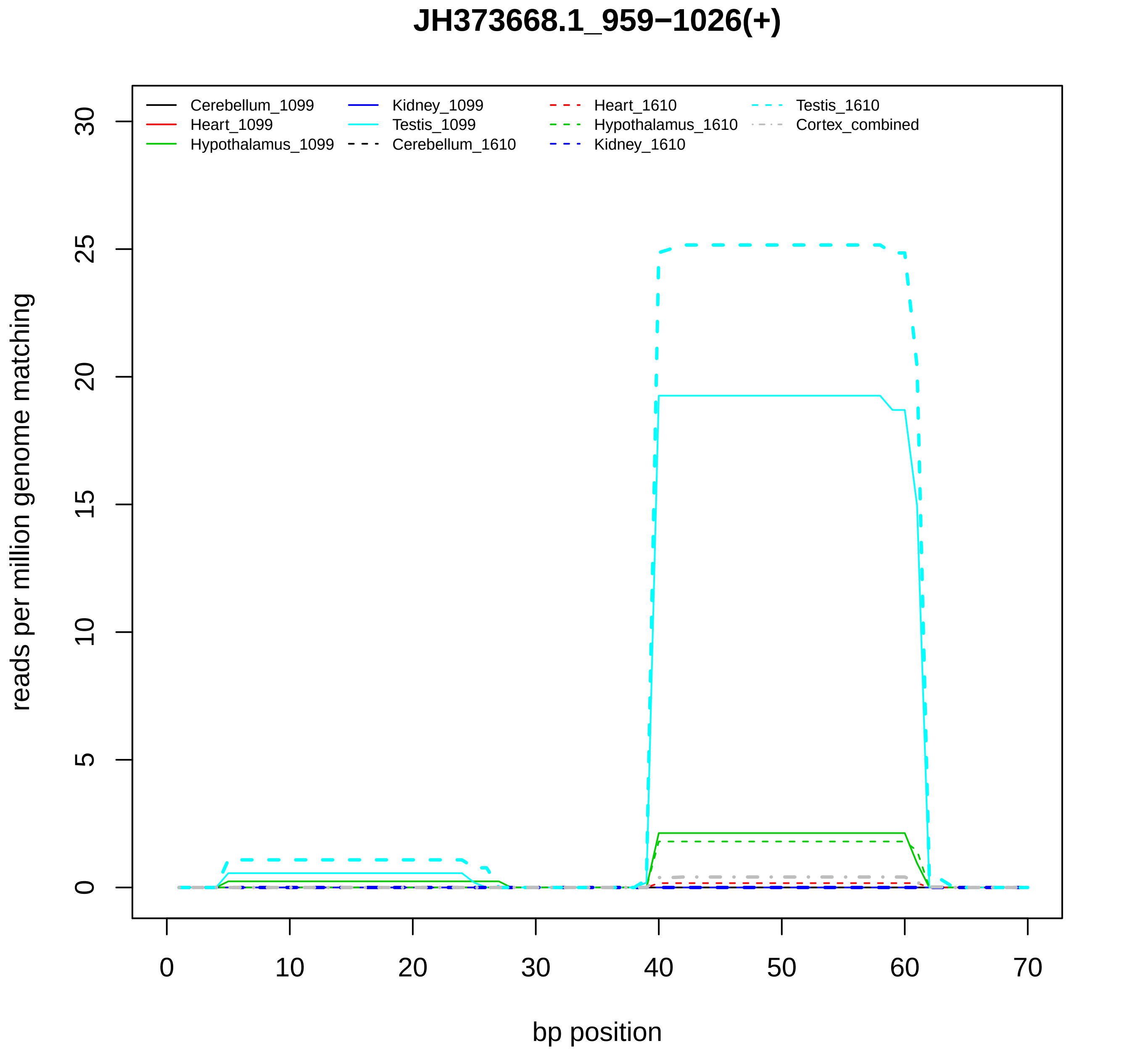

JH373668.1_959−1026(+)
Cerebellum_1099
Heart_1099
Hypothalamus_1099
Kidney_1099
Testis_1099
Cerebellum_1610
Heart_1610
Hypothalamus_1610
Kidney_1610
Testis_1610
Cortex_combined
reads per million genome matching
bp position
0
5
10
15
20
25
30
0
10
20
30
40
50
60
70

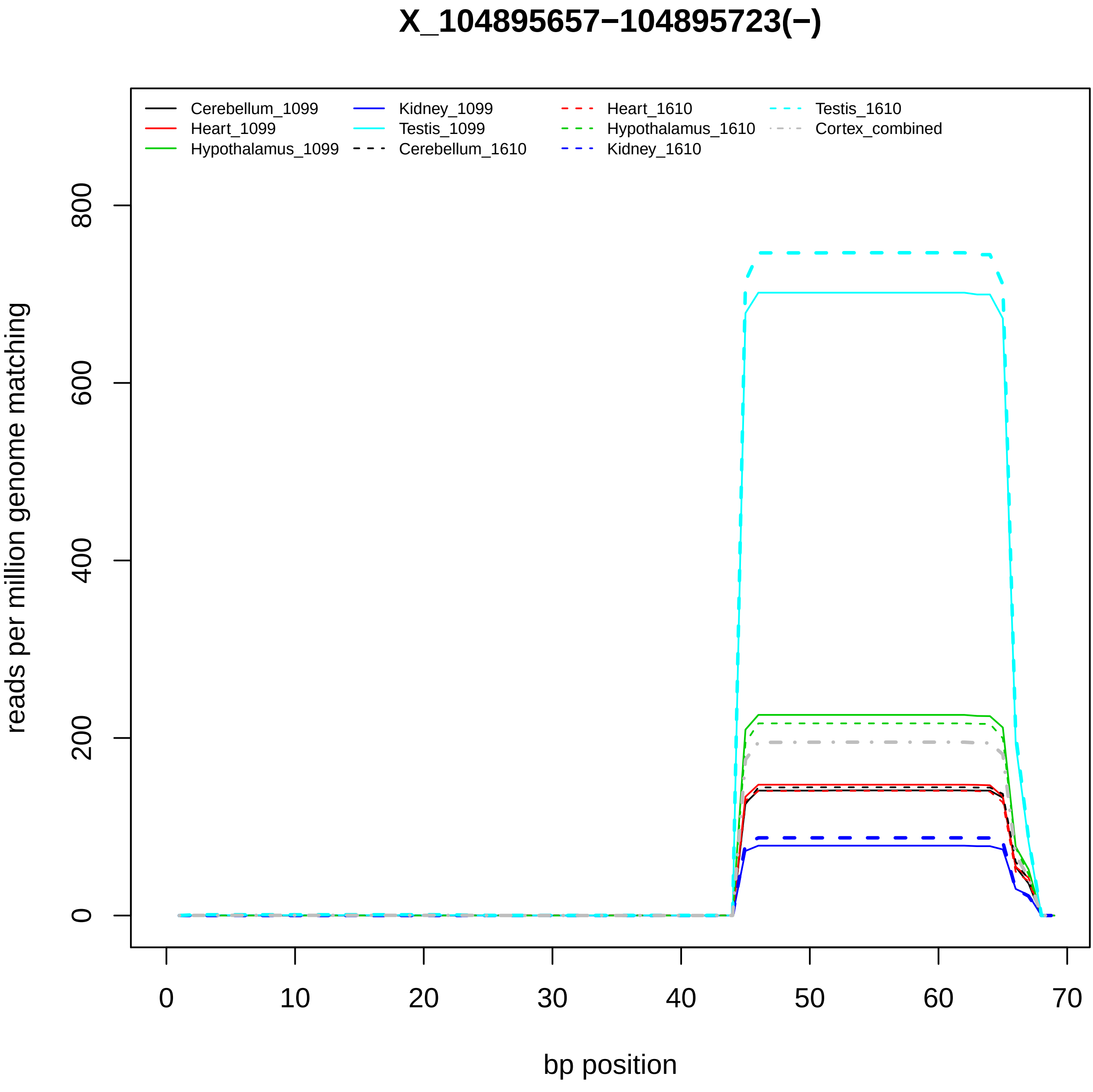
X_104895657−104895723(−)
Cerebellum_1099
Heart_1099
Hypothalamus_1099
Kidney_1099
Testis_1099
Cerebellum_1610
Heart_1610
Hypothalamus_1610
Kidney_1610
Testis_1610
Cortex_combined
reads per million genome matching
bp position
0
200
400
600
800
0
10
20
30
40
50
60
70

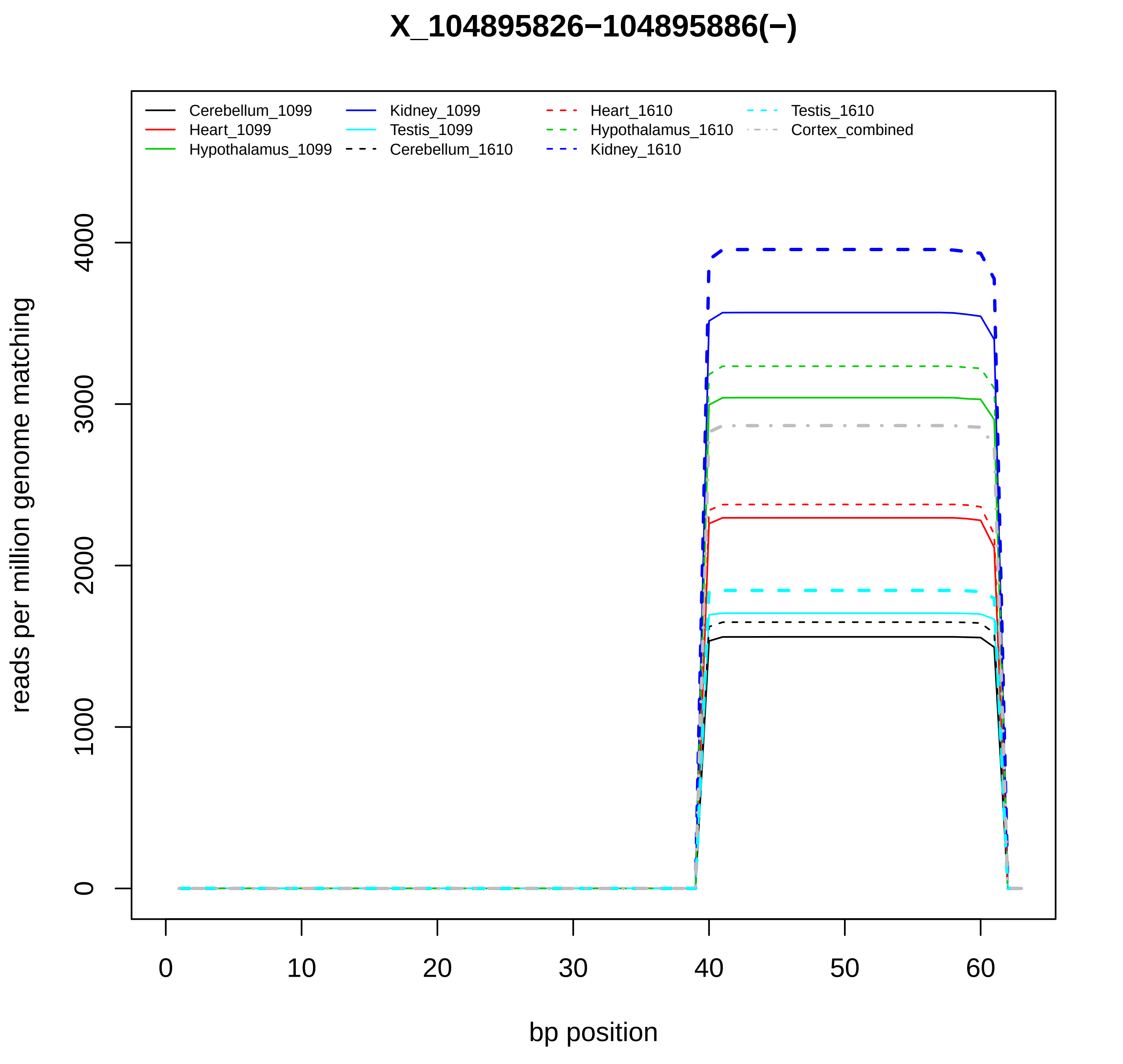

X_104895826−104895886(−)
Cerebellum_1099
Heart_1099
Hypothalamus_1099
Kidney_1099
Testis_1099
Cerebellum_1610
Heart_1610
Hypothalamus_1610
Kidney_1610
Testis_1610
Cortex_combined
reads per million genome matching
0
1000
2000
3000
4000
bp position
0
10
20
30
40
50
60

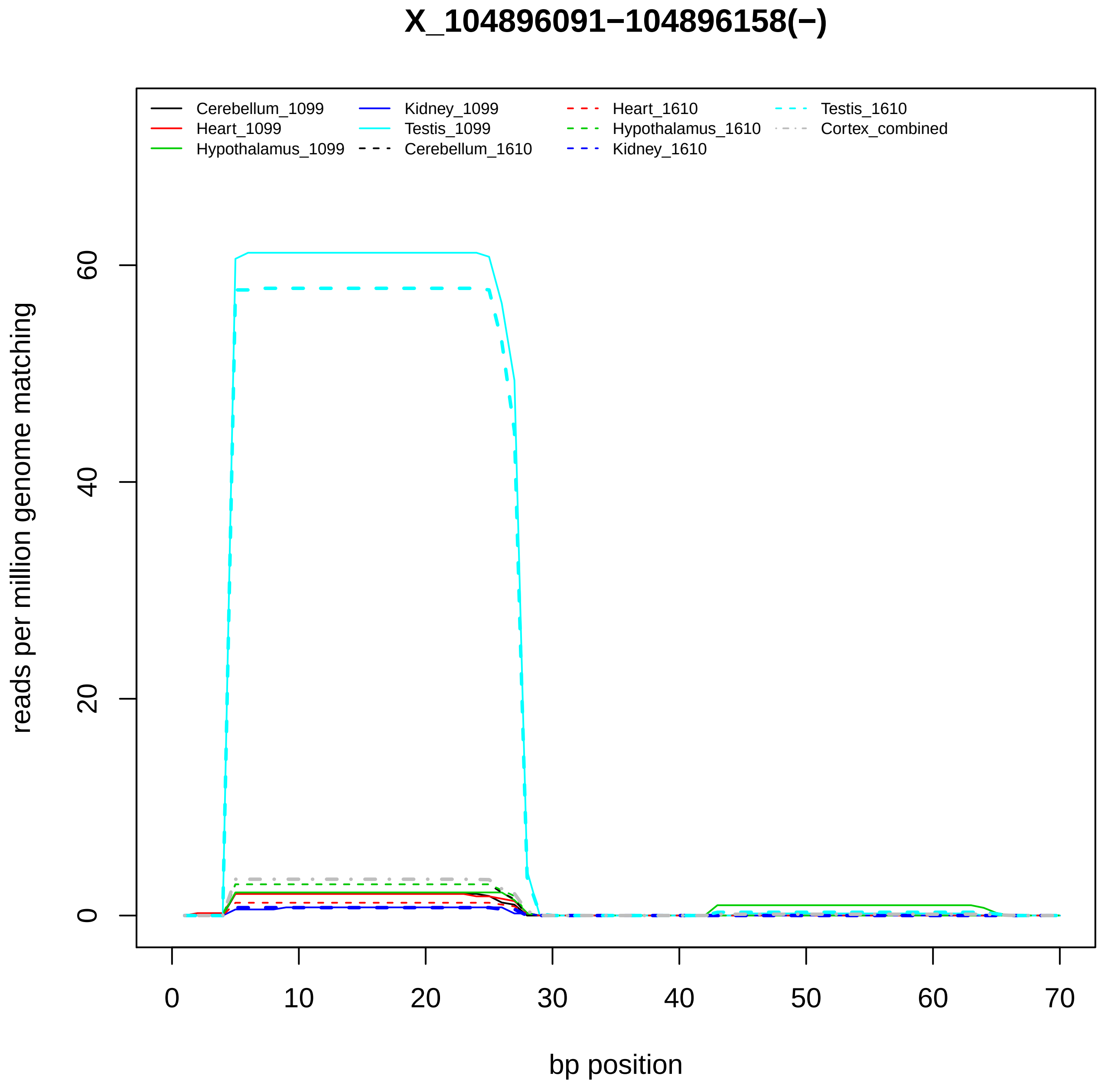

X_104896091−104896158(−)
Cerebellum_1099
Heart_1099
Hypothalamus_1099
Kidney_1099
Testis_1099
Cerebellum_1610
Heart_1610
Hypothalamus_1610
Kidney_1610
Testis_1610
Cortex_combined
reads per million genome matching
bp position
0
20
40
60
0
10
20
30
40
50
60
70

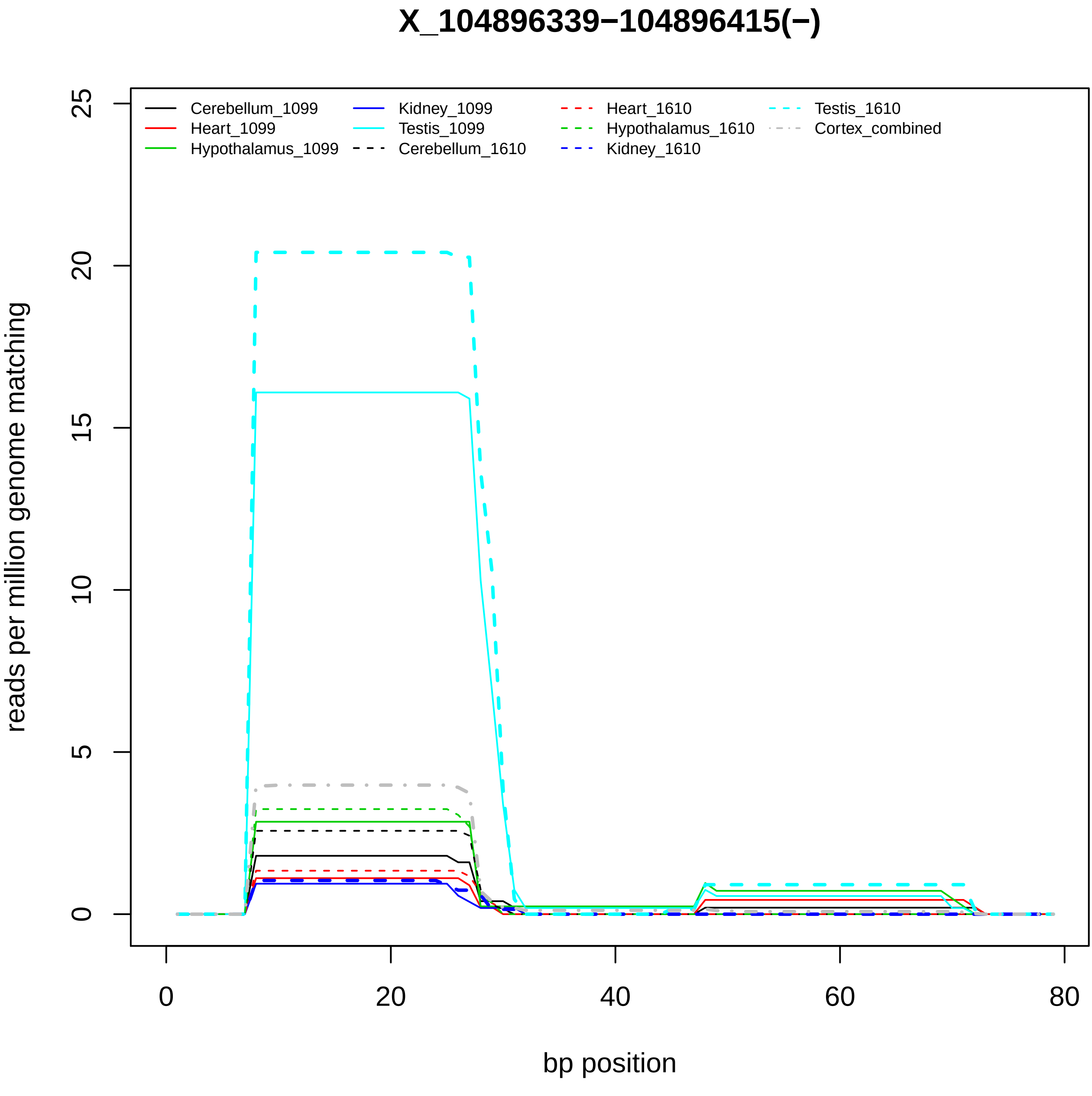
X_104896339−104896415(−)
Cerebellum_1099
Heart_1099
Hypothalamus_1099
Kidney_1099
Testis_1099
Cerebellum_1610
Heart_1610
Hypothalamus_1610
Kidney_1610
Testis_1610
Cortex_combined
reads per million genome matching
bp position
0
5
10
15
20
25
0
20
40
60
80

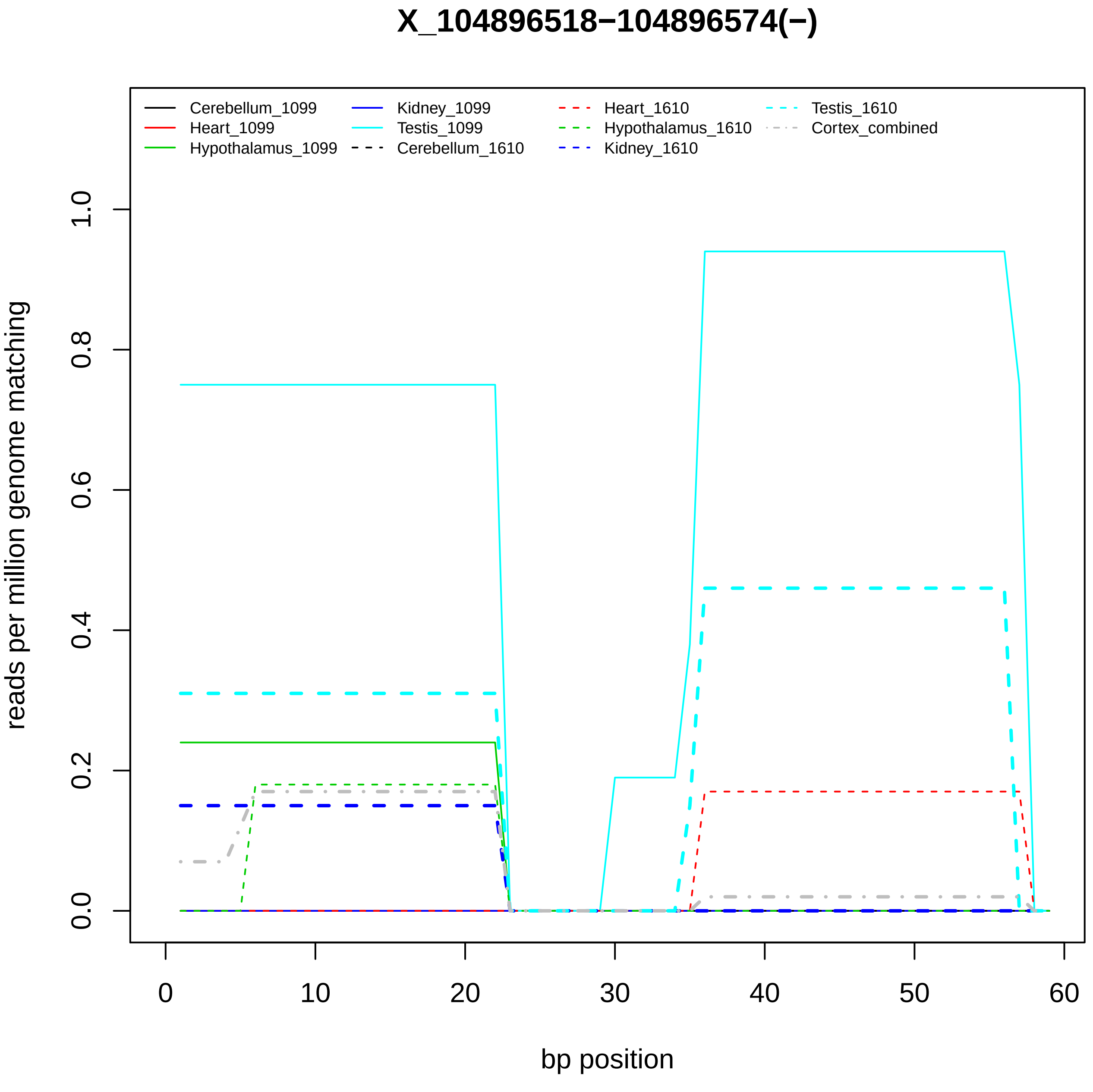
X_104896518−104896574(−)
Cerebellum_1099
Heart_1099
Hypothalamus_1099
Kidney_1099
Testis_1099
Cerebellum_1610
Heart_1610
Hypothalamus_1610
Kidney_1610
Testis_1610
Cortex_combined
reads per million genome matching
0.0
0.2
0.4
0.6
0.8
1.0
bp position
0
10
20
30
40
50
60

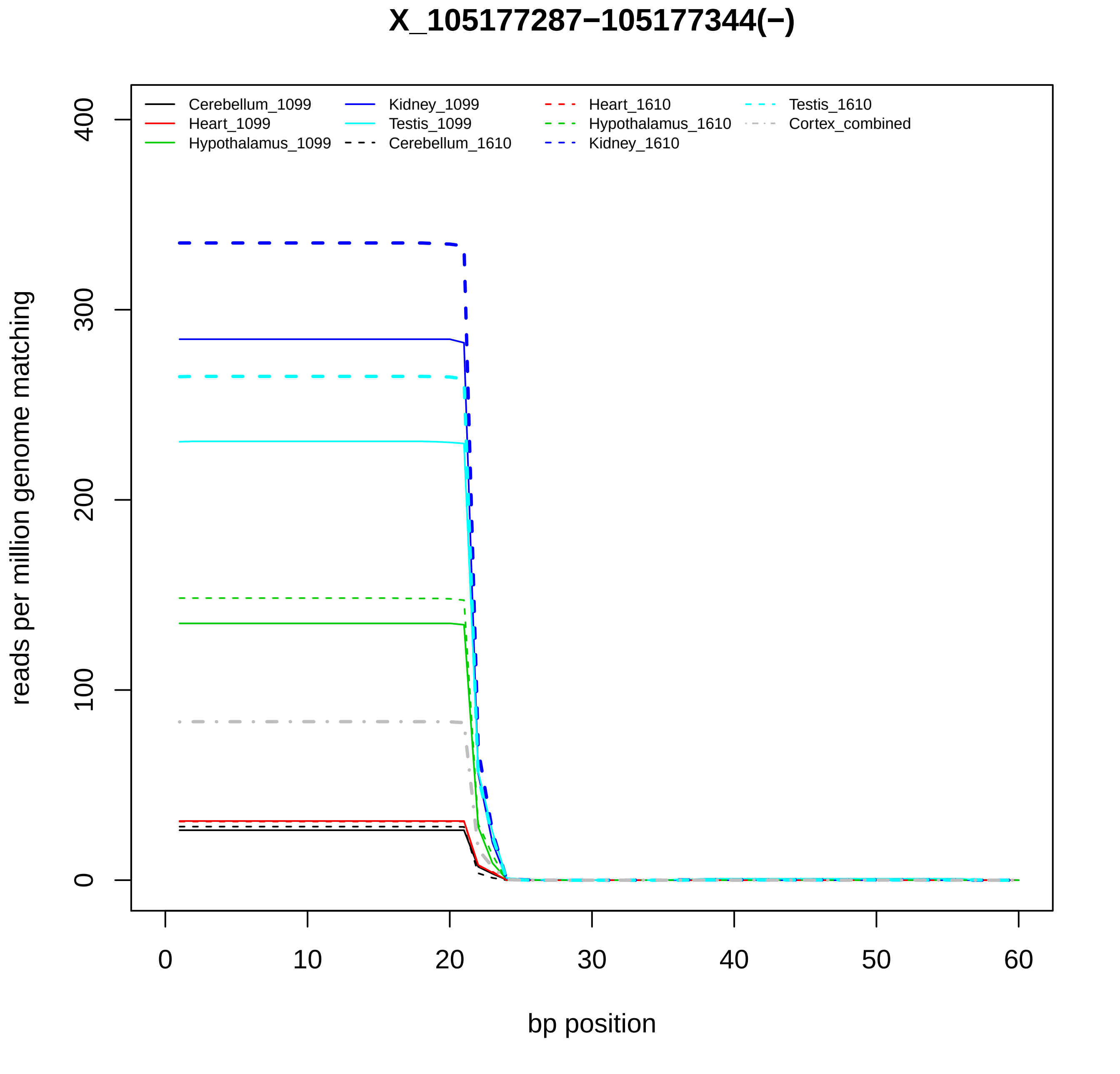

X_105177287−105177344(−)
Cerebellum_1099
Heart_1099
Hypothalamus_1099
Kidney_1099
Testis_1099
Cerebellum_1610
Heart_1610
Hypothalamus_1610
Kidney_1610
Testis_1610
Cortex_combined
reads per million genome matching
bp position
0
100
200
300
400
0
10
20
30
40
50
60

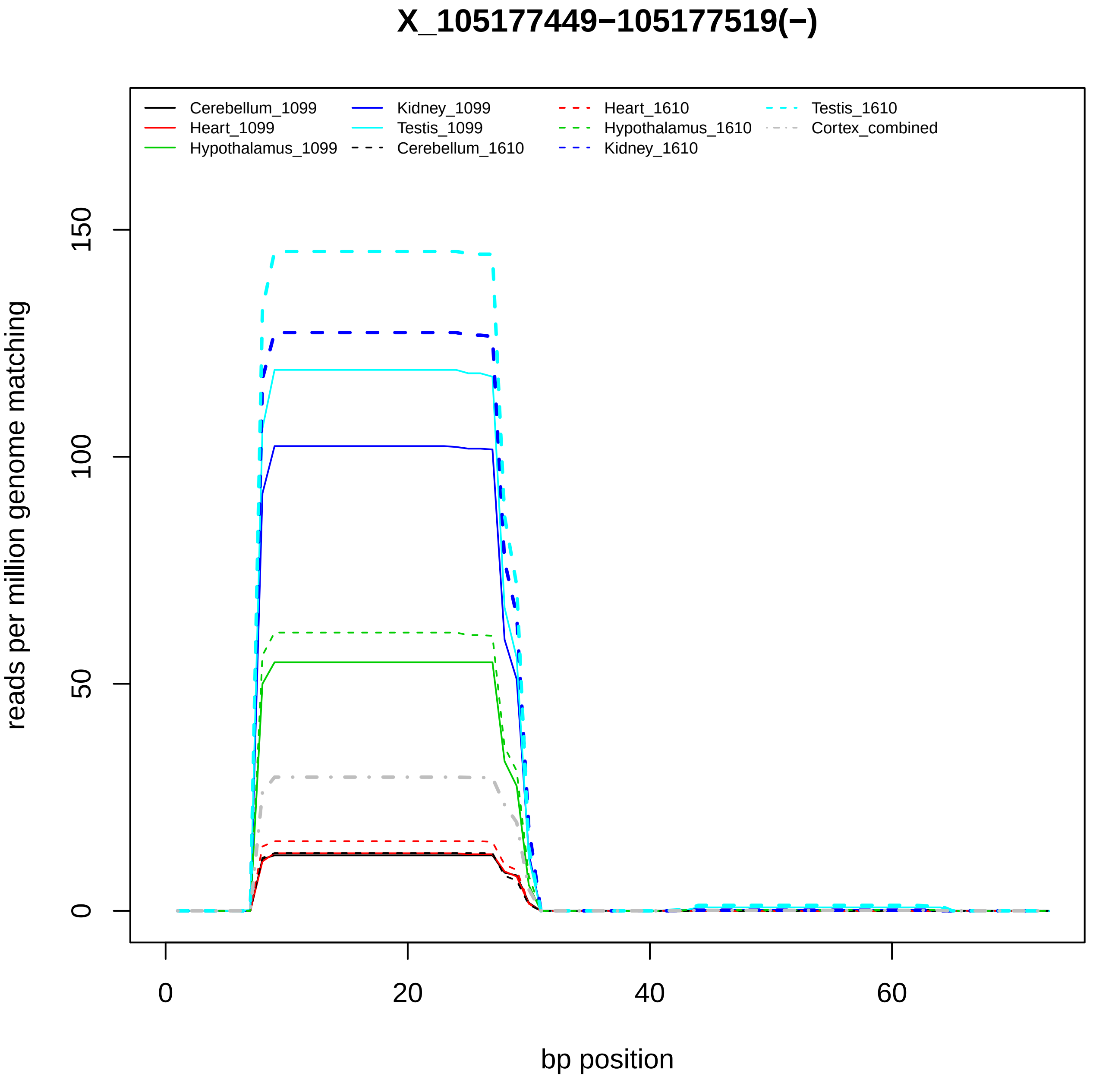

X_105177449−105177519(−)
Cerebellum_1099
Heart_1099
Hypothalamus_1099
Kidney_1099
Testis_1099
Cerebellum_1610
Heart_1610
Hypothalamus_1610
Kidney_1610
Testis_1610
Cortex_combined
reads per million genome matching
0
50
100
150
0
20
40
60
bp position

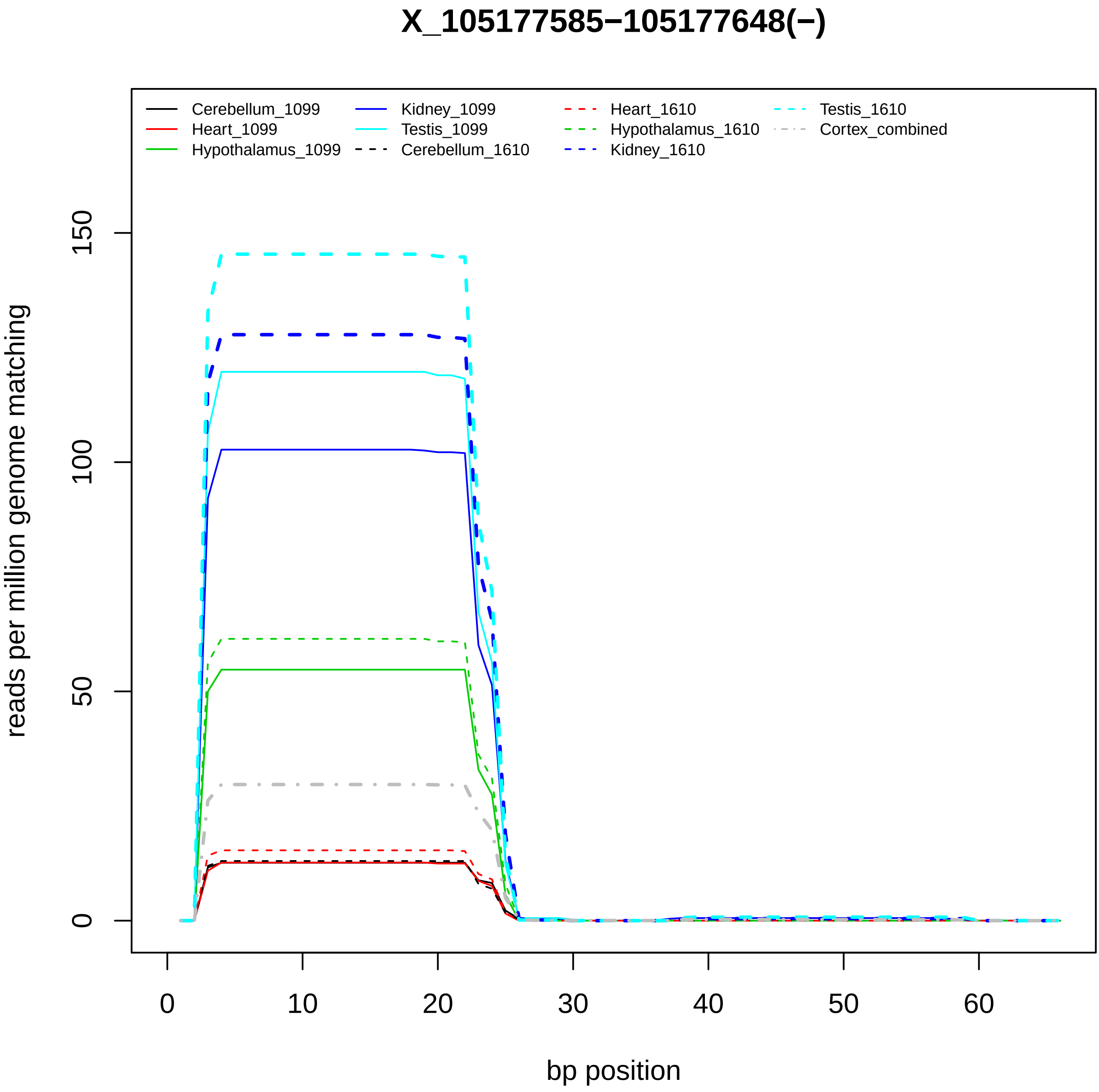
X_105177585−105177648(−)
Cerebellum_1099
Heart_1099
Hypothalamus_1099
Kidney_1099
Testis_1099
Cerebellum_1610
Heart_1610
Hypothalamus_1610
Kidney_1610
Testis_1610
Cortex_combined
reads per million genome matching
0
50
100
150
0
10
20
30
40
50
60
bp position

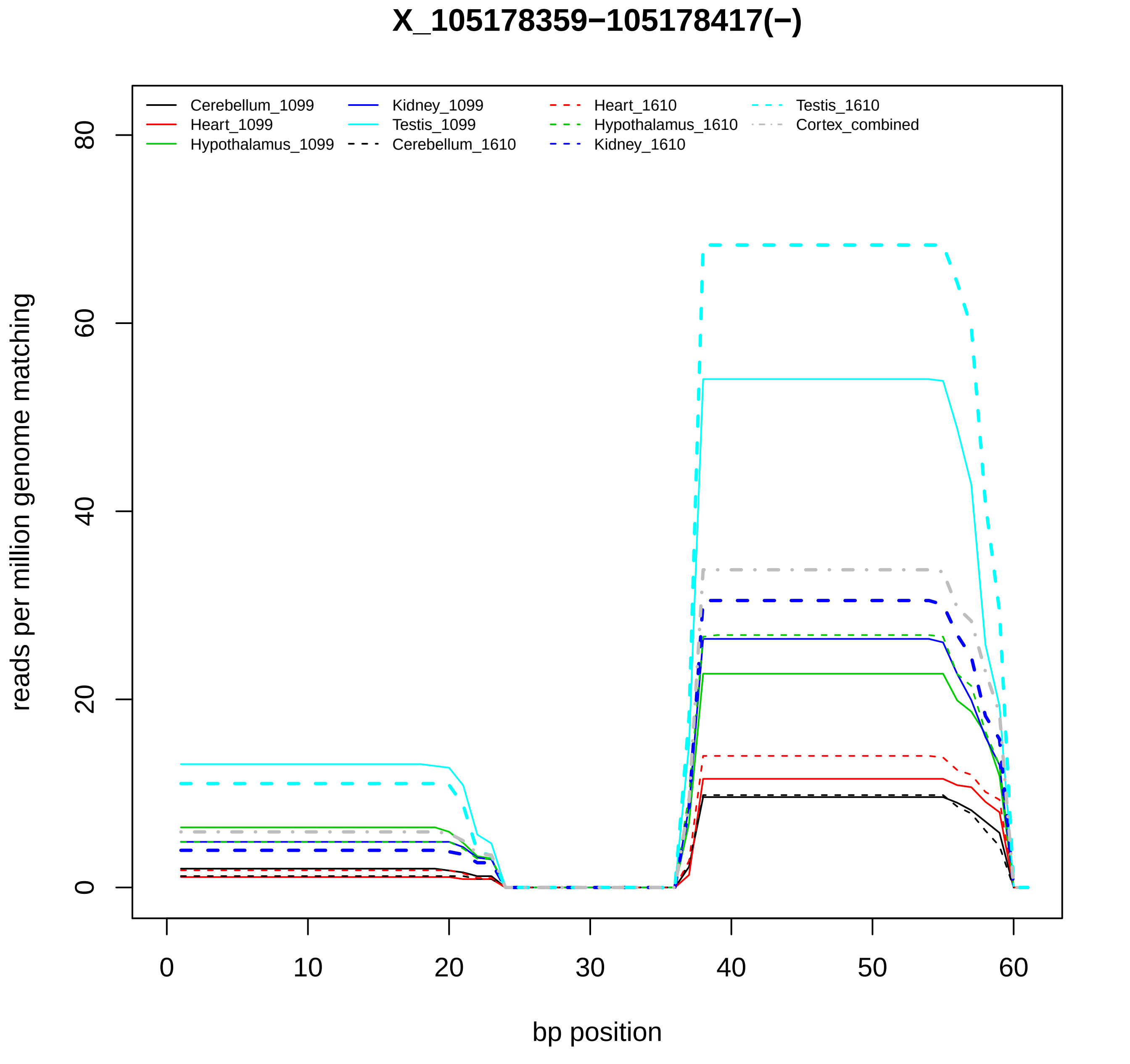

X_105178359−105178417(−)
Cerebellum_1099
Heart_1099
Hypothalamus_1099
Kidney_1099
Testis_1099
Cerebellum_1610
Heart_1610
Hypothalamus_1610
Kidney_1610
Testis_1610
Cortex_combined
reads per million genome matching
0
20
40
60
80
0
10
20
30
40
50
60
bp position

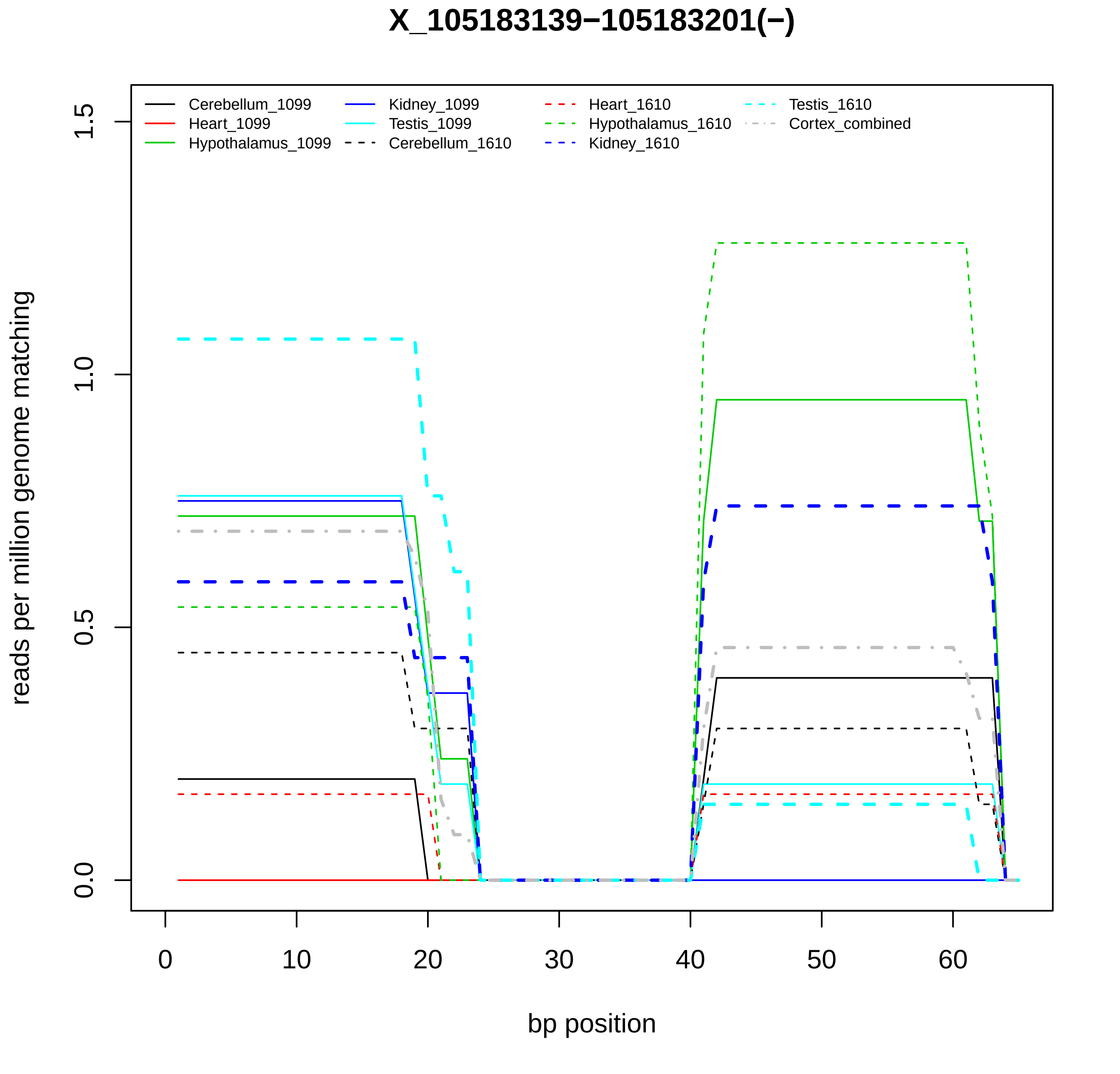

X_105183139−105183201(−)
Cerebellum_1099
Heart_1099
Hypothalamus_1099
Kidney_1099
Testis_1099
Cerebellum_1610
Heart_1610
Hypothalamus_1610
Kidney_1610
Testis_1610
Cortex_combined
reads per million genome matching
bp position
0.0
0.5
1.0
1.5
0
10
20
30
40
50
60

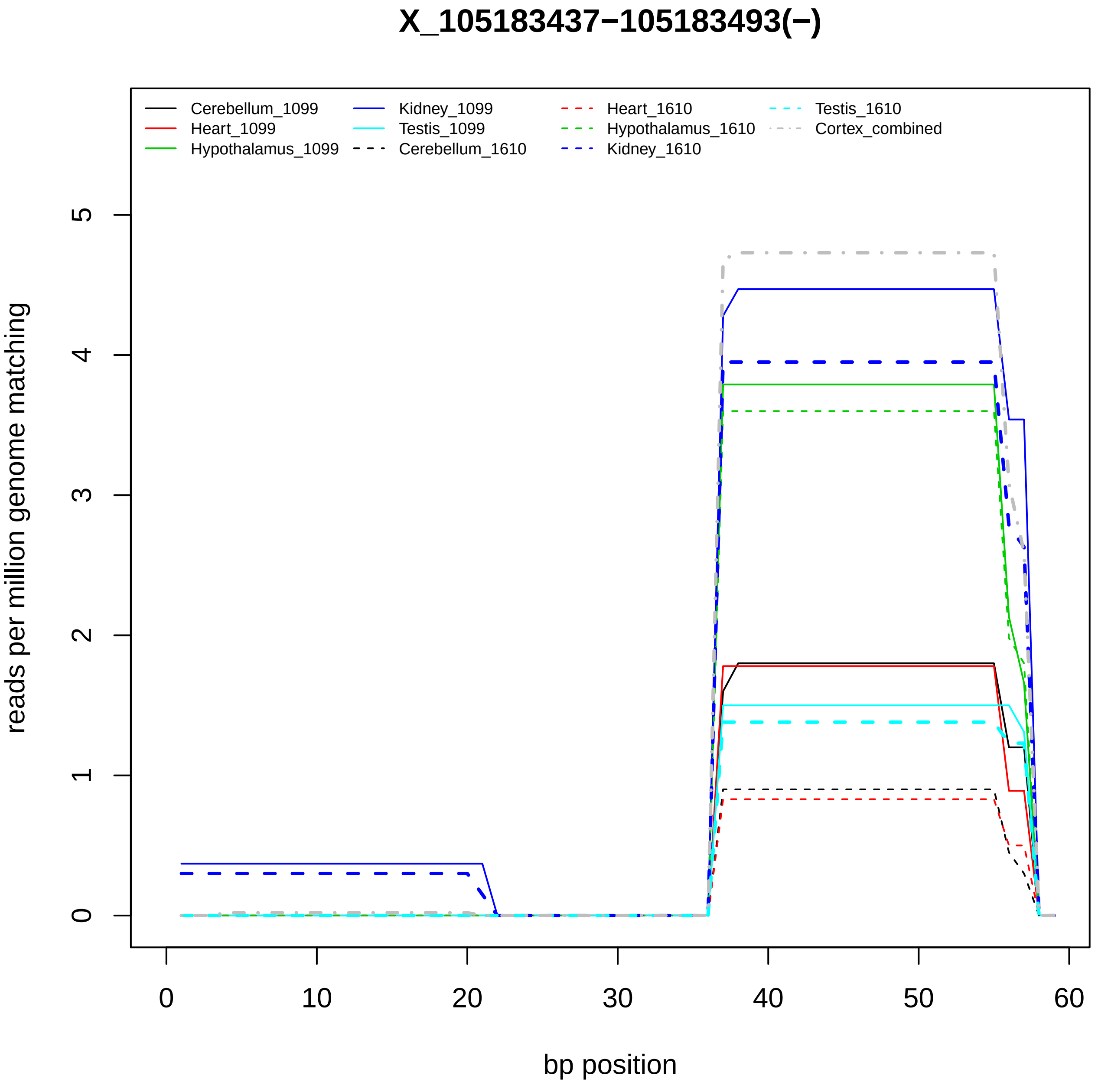
X_105183437−105183493(−)
Cerebellum_1099
Heart_1099
Hypothalamus_1099
Kidney_1099
Testis_1099
Cerebellum_1610
Heart_1610
Hypothalamus_1610
Kidney_1610
Testis_1610
Cortex_combined
reads per million genome matching
bp position
0
1
2
3
4
5
0
10
20
30
40
50
60

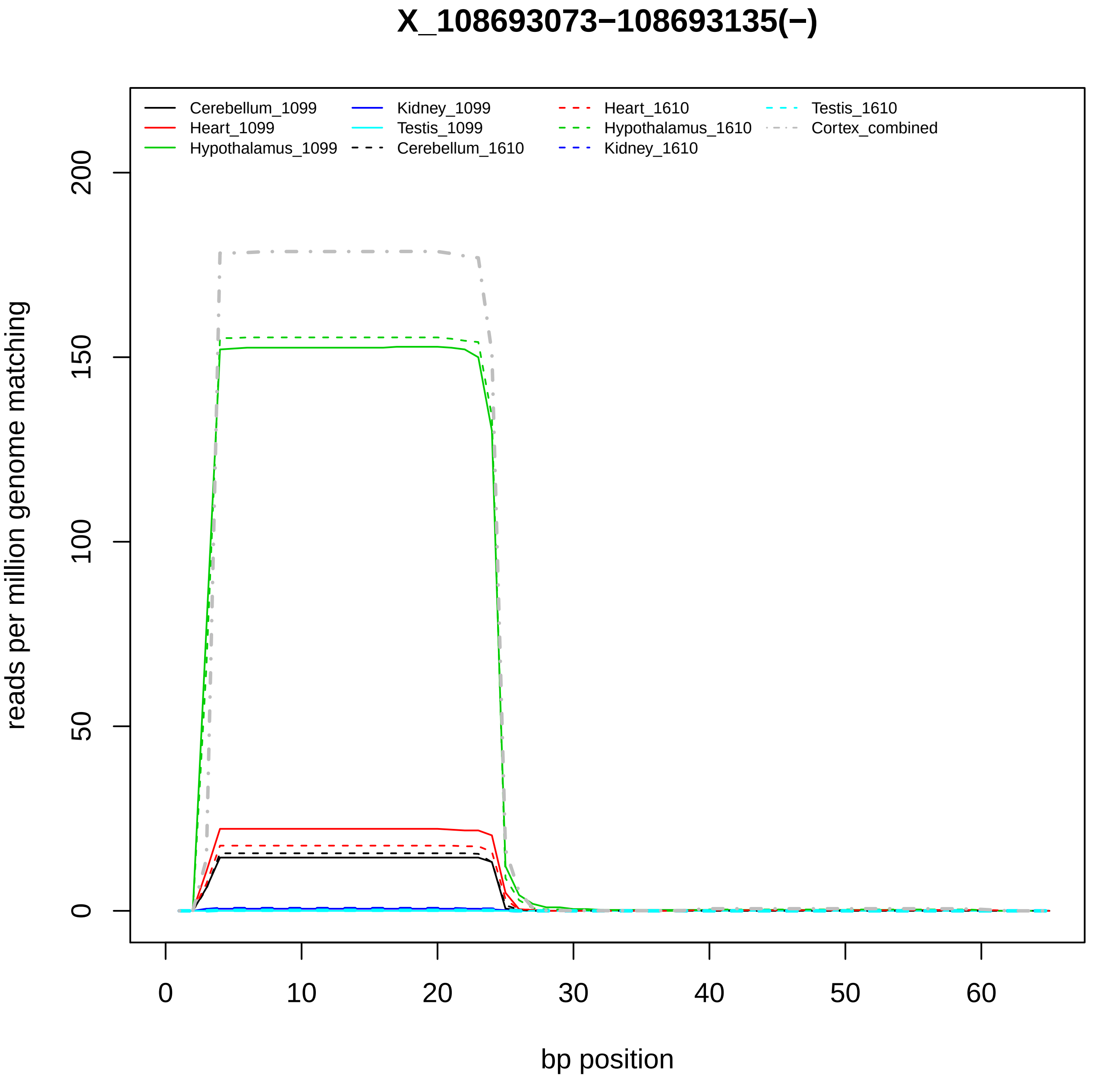

X_108693073−108693135(−)
Cerebellum_1099
Heart_1099
Hypothalamus_1099
Kidney_1099
Testis_1099
Cerebellum_1610
Heart_1610
Hypothalamus_1610
Kidney_1610
Testis_1610
Cortex_combined
reads per million genome matching
bp position
0
50
100
150
200
0
10
20
30
40
50
60

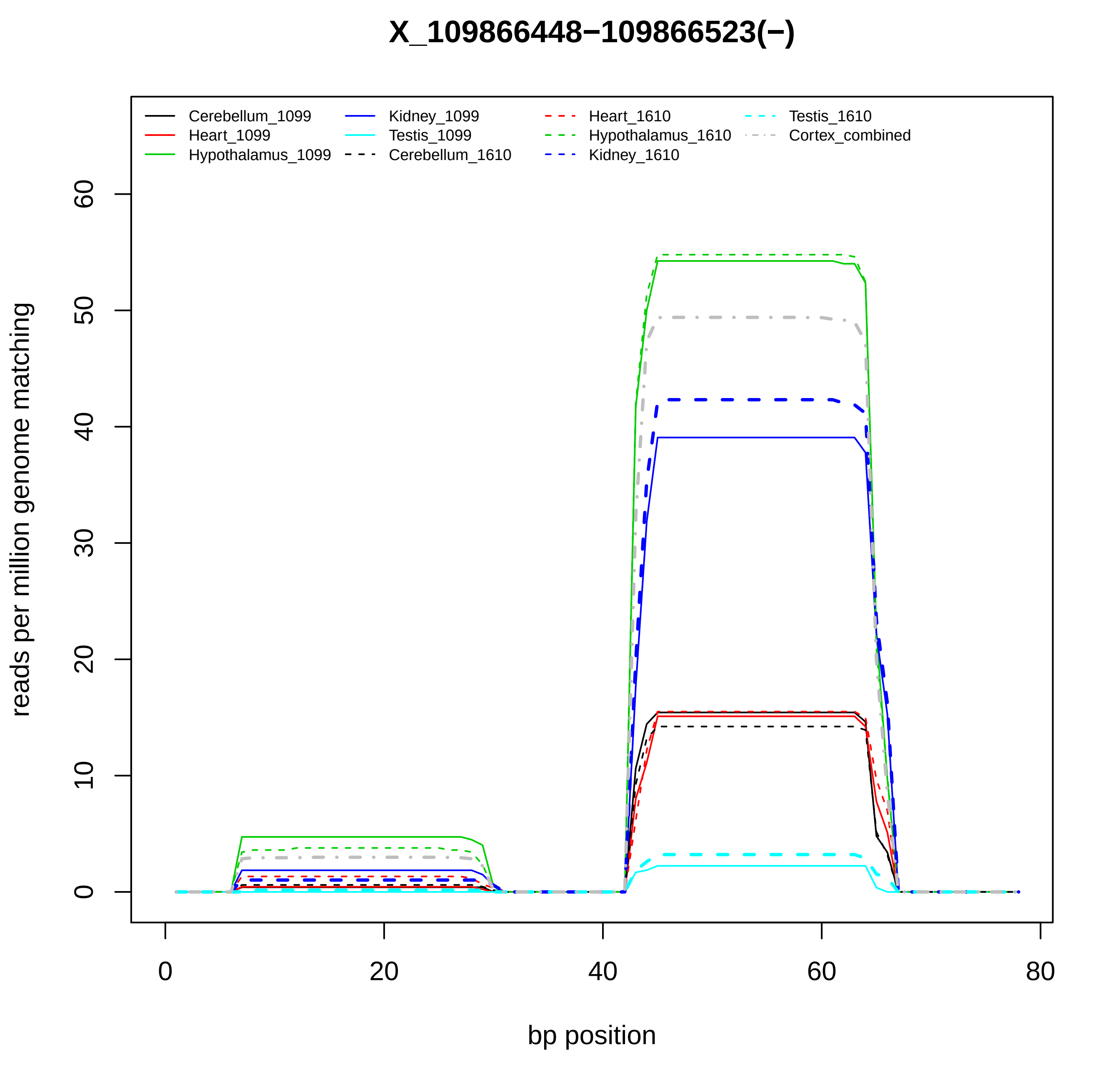

X_109866448−109866523(−)
Cerebellum_1099
Heart_1099
Hypothalamus_1099
Kidney_1099
Testis_1099
Cerebellum_1610
Heart_1610
Hypothalamus_1610
Kidney_1610
Testis_1610
Cortex_combined
reads per million genome matching
0
10
20
30
40
50
60
0
20
40
60
80
bp position

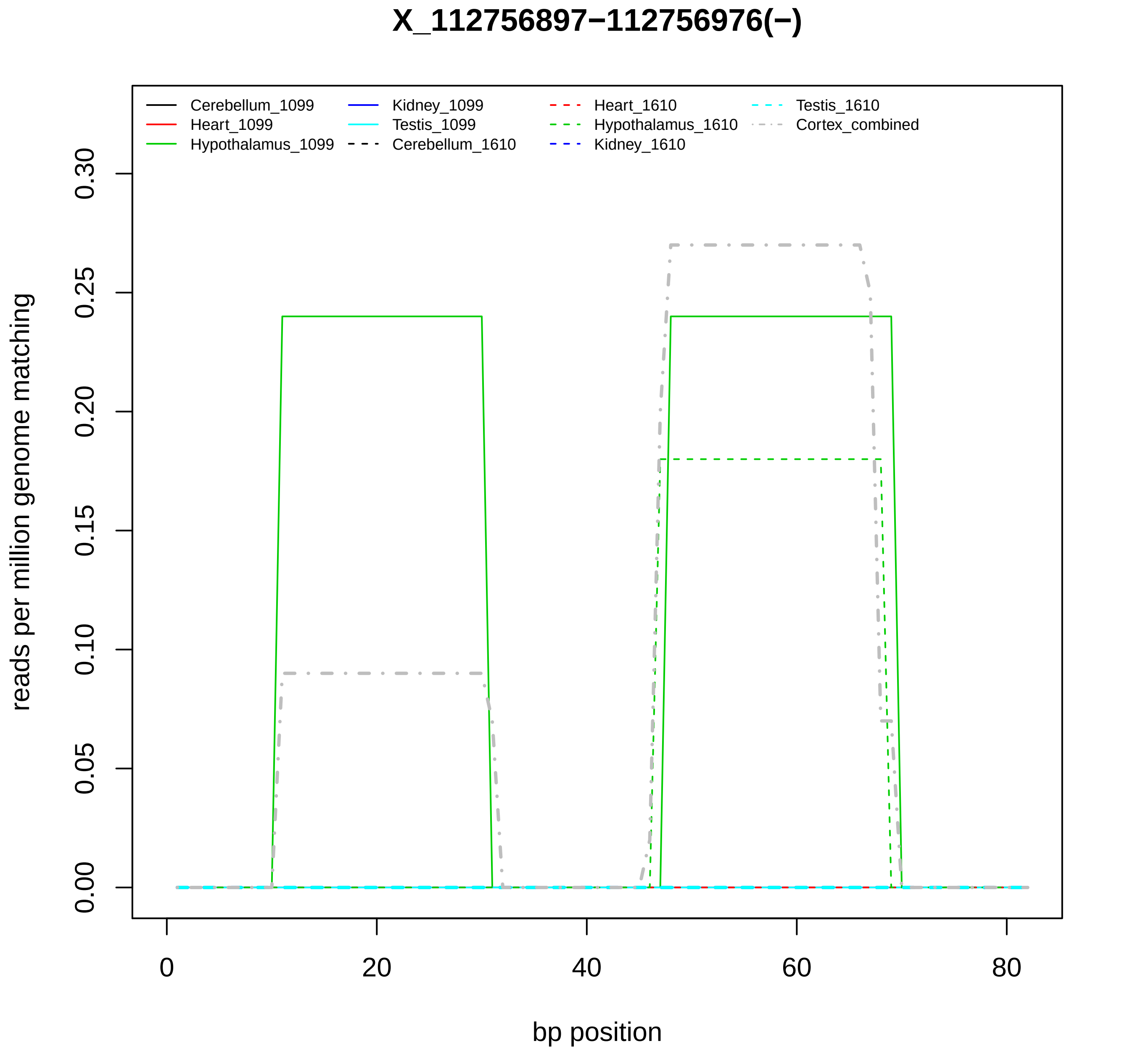
X_112756897−112756976(−)
Cerebellum_1099
Heart_1099
Hypothalamus_1099
Kidney_1099
Testis_1099
Cerebellum_1610
Heart_1610
Hypothalamus_1610
Kidney_1610
Testis_1610
Cortex_combined
reads per million genome matching
0.00
0.05
0.10
0.15
0.20
0.25
0.30
0
20
40
60
80
bp position

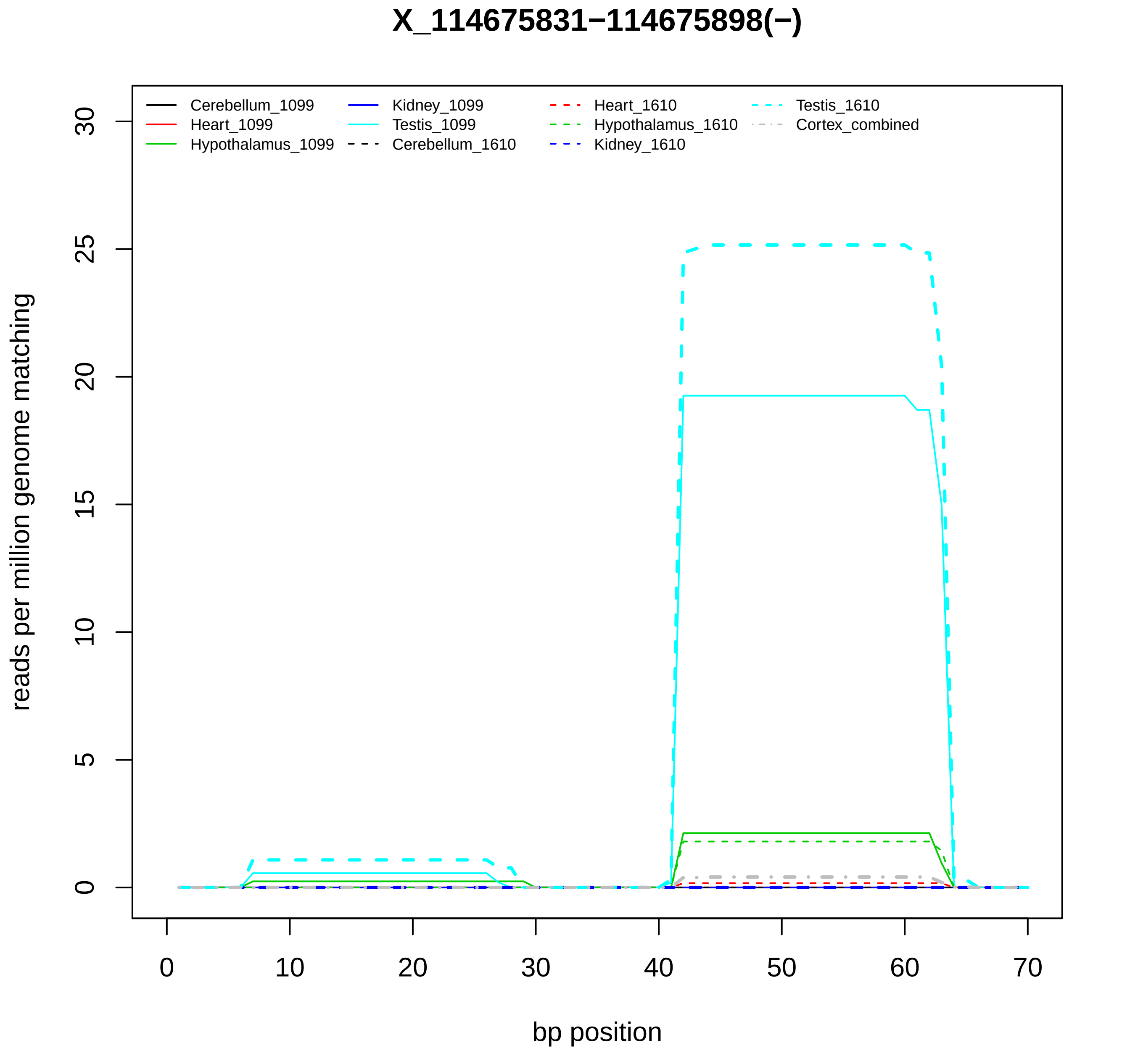

X_114675831−114675898(−)
Cerebellum_1099
Heart_1099
Hypothalamus_1099
Kidney_1099
Testis_1099
Cerebellum_1610
Heart_1610
Hypothalamus_1610
Kidney_1610
Testis_1610
Cortex_combined
reads per million genome matching
bp position
0
5
10
15
20
25
30
0
10
20
30
40
50
60
70

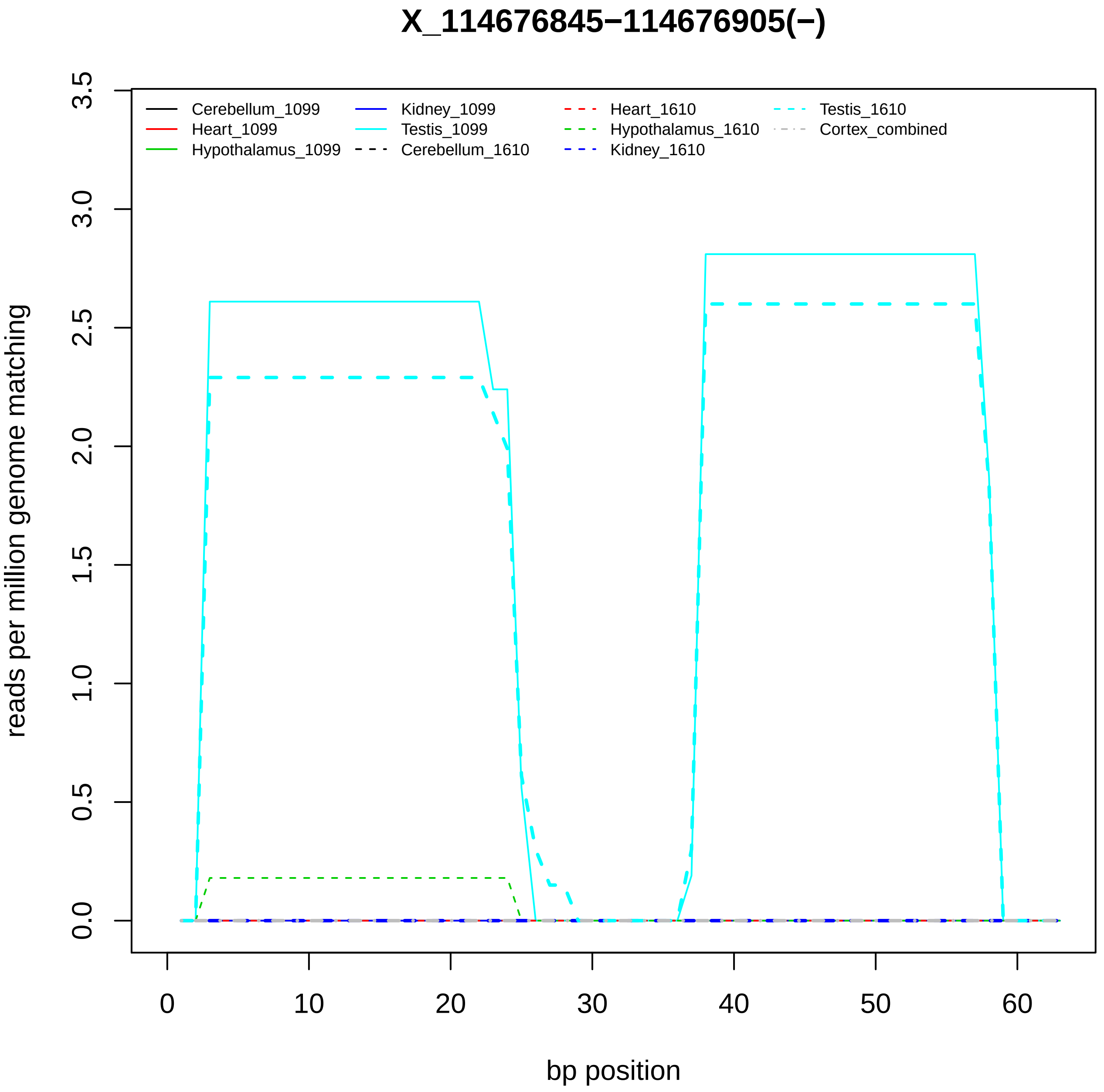
X_114676845−114676905(−)
Cerebellum_1099
Heart_1099
Hypothalamus_1099
Kidney_1099
Testis_1099
Cerebellum_1610
Heart_1610
Hypothalamus_1610
Kidney_1610
Testis_1610
Cortex_combined
reads per million genome matching
bp position
0.0
0.5
1.0
1.5
2.0
2.5
3.0
3.5
0
10
20
30
40
50
60

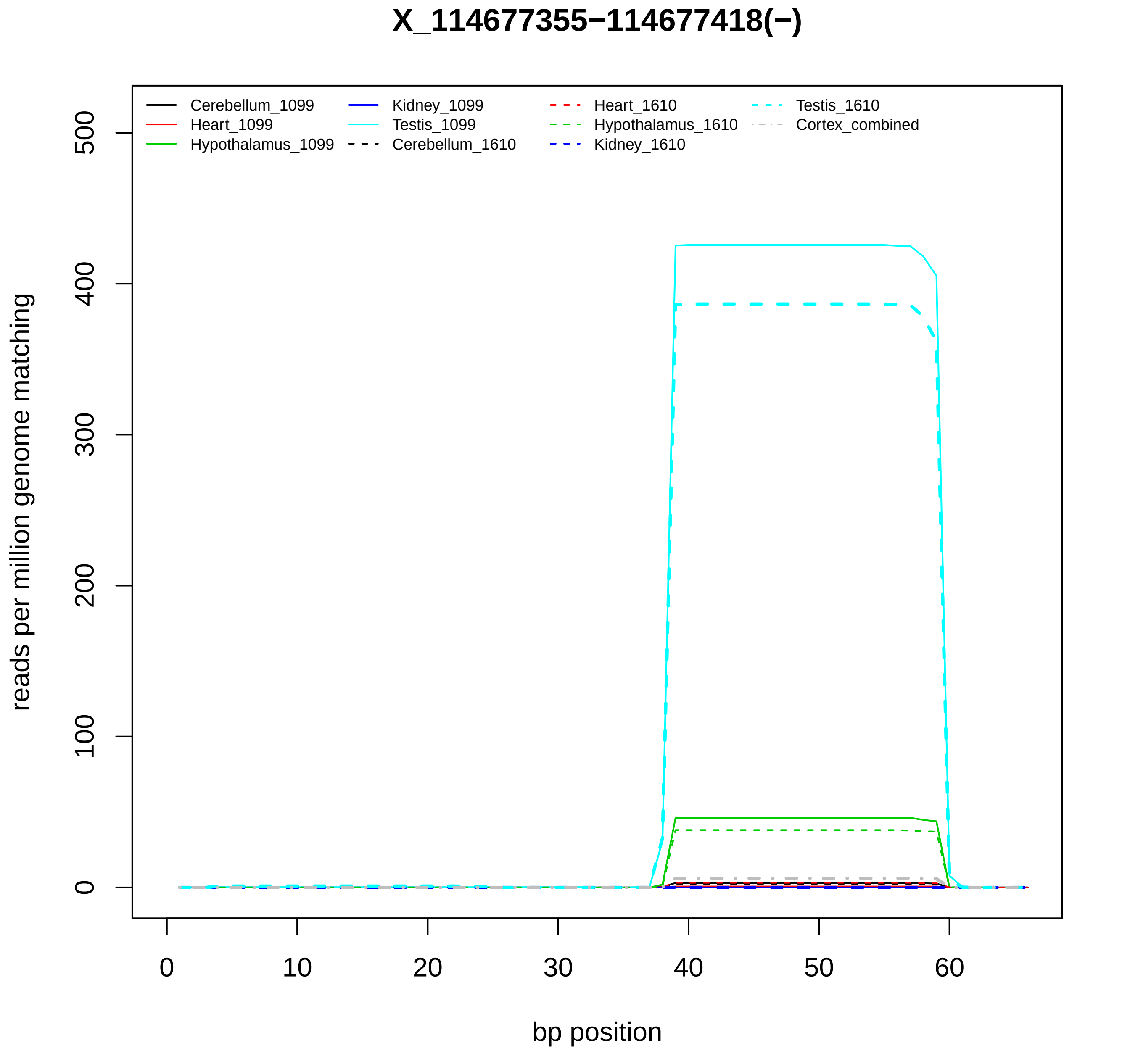
X_114677355−114677418(−)
Cerebellum_1099
Heart_1099
Hypothalamus_1099
Kidney_1099
Testis_1099
Cerebellum_1610
Heart_1610
Hypothalamus_1610
Kidney_1610
Testis_1610
Cortex_combined
reads per million genome matching
bp position
0
100
200
300
400
500
0
10
20
30
40
50
60

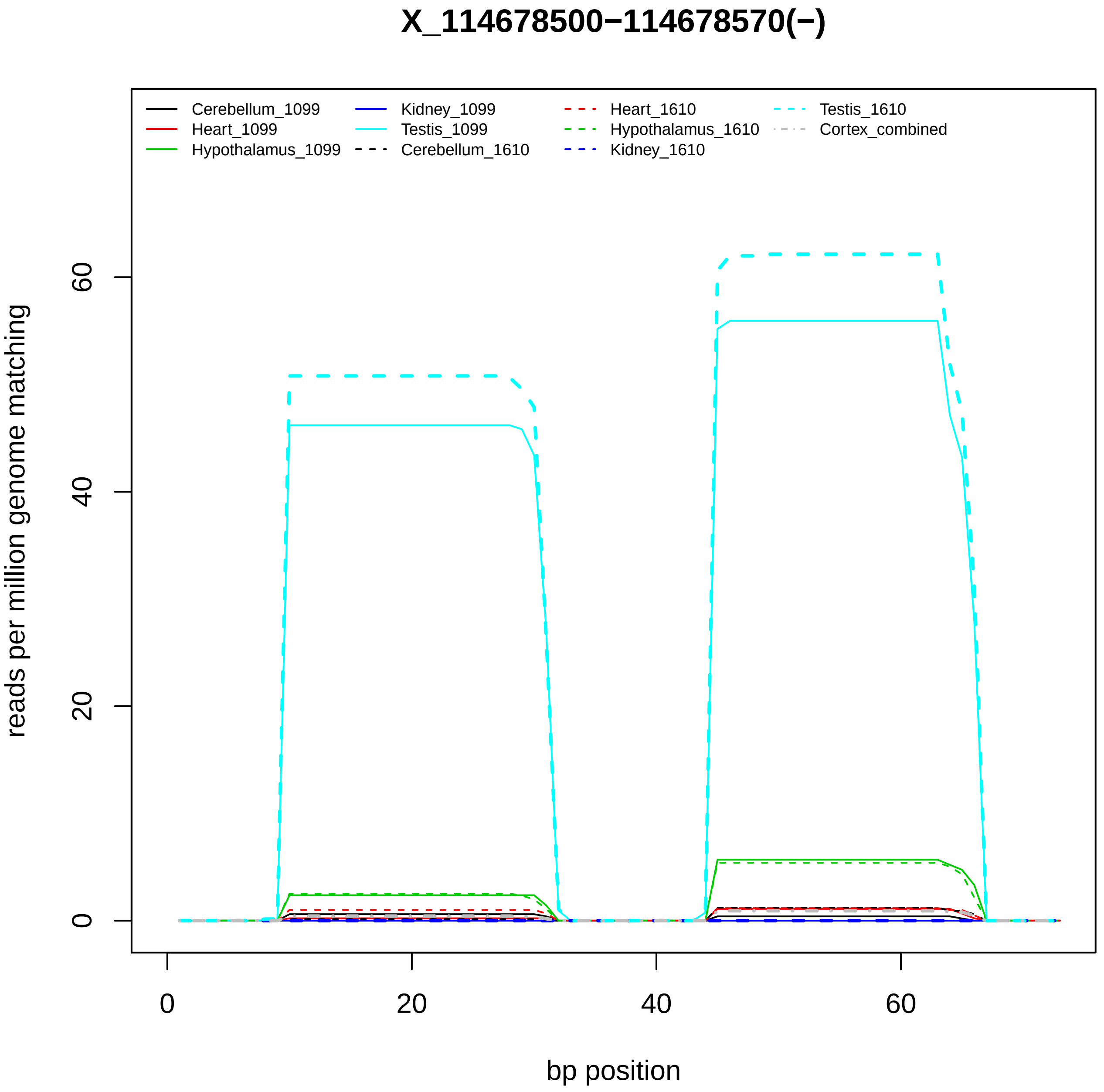

X_114678500−114678570(−)
Cerebellum_1099
Heart_1099
Hypothalamus_1099
Kidney_1099
Testis_1099
Cerebellum_1610
Heart_1610
Hypothalamus_1610
Kidney_1610
Testis_1610
Cortex_combined
reads per million genome matching
0
20
40
60
bp position

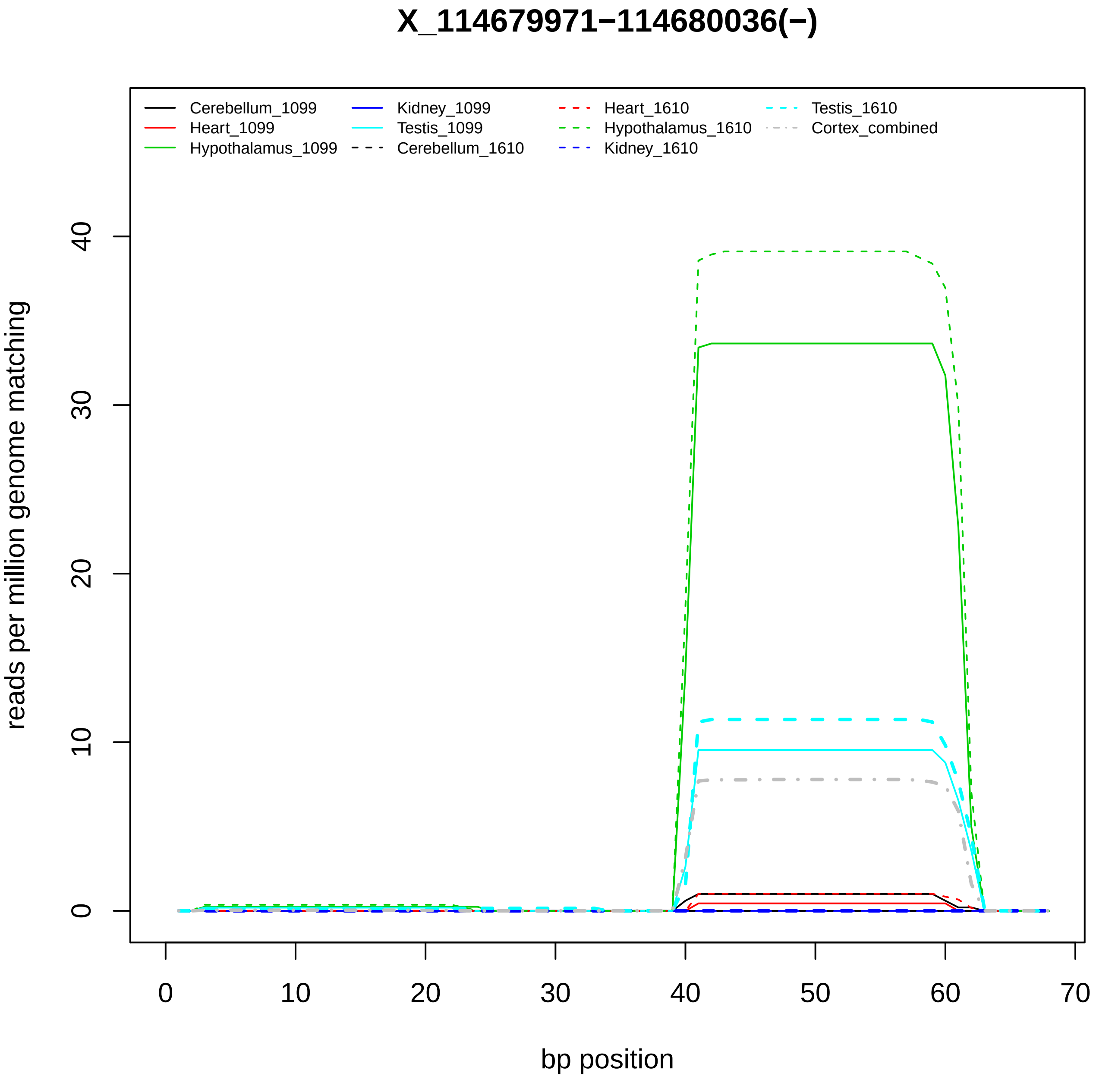

X_114679971−114680036(−)
Cerebellum_1099
Heart_1099
Hypothalamus_1099
Kidney_1099
Testis_1099
Cerebellum_1610
Heart_1610
Hypothalamus_1610
Kidney_1610
Testis_1610
Cortex_combined
reads per million genome matching
0
10
20
30
40
0
10
20
30
40
50
60
70
bp position

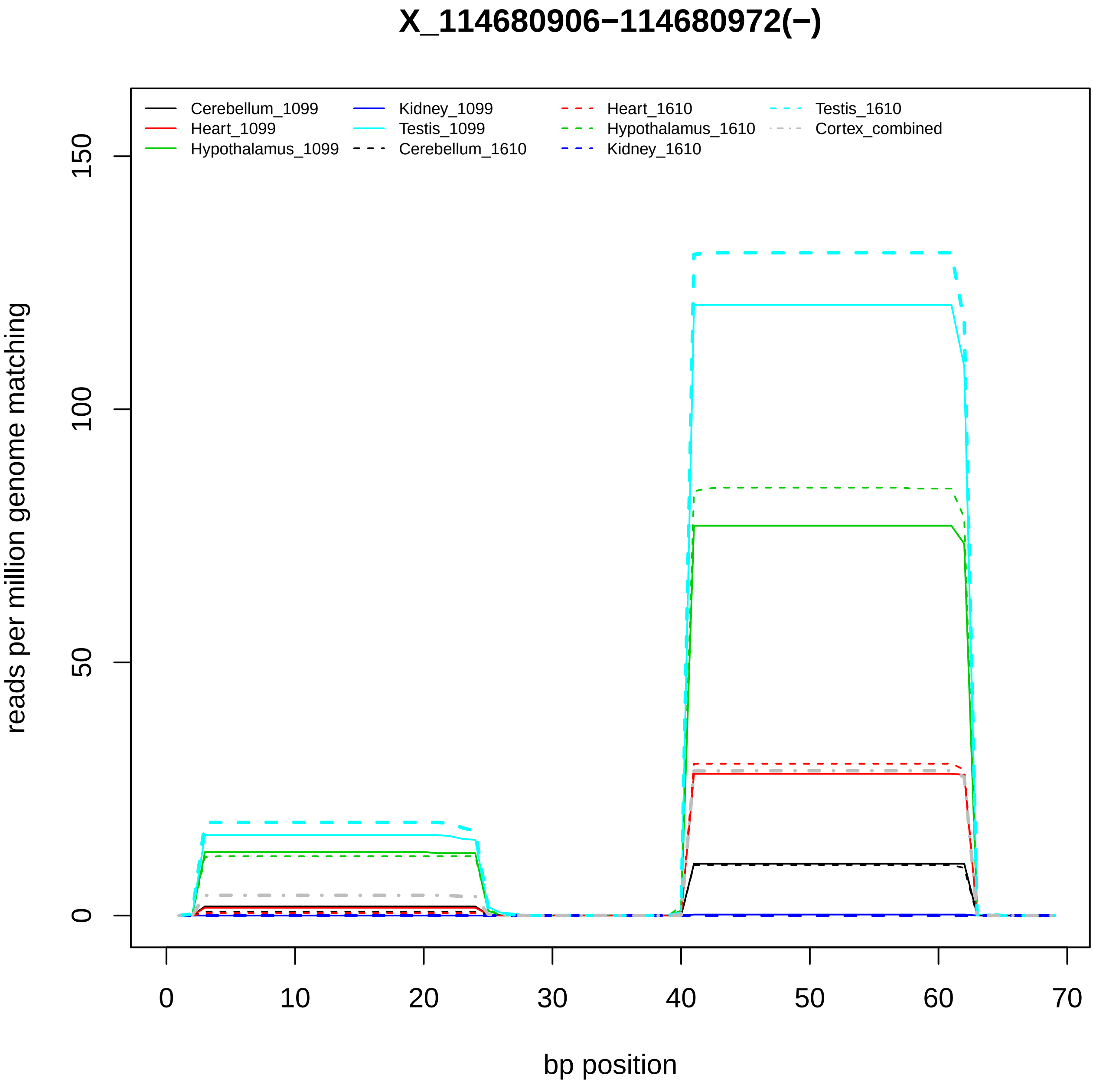
X_114680906−114680972(−)
Cerebellum_1099
Heart_1099
Hypothalamus_1099
Kidney_1099
Testis_1099
Cerebellum_1610
Heart_1610
Hypothalamus_1610
Kidney_1610
Testis_1610
Cortex_combined
reads per million genome matching
bp position
0
50
100
150
0
10
20
30
40
50
60
70

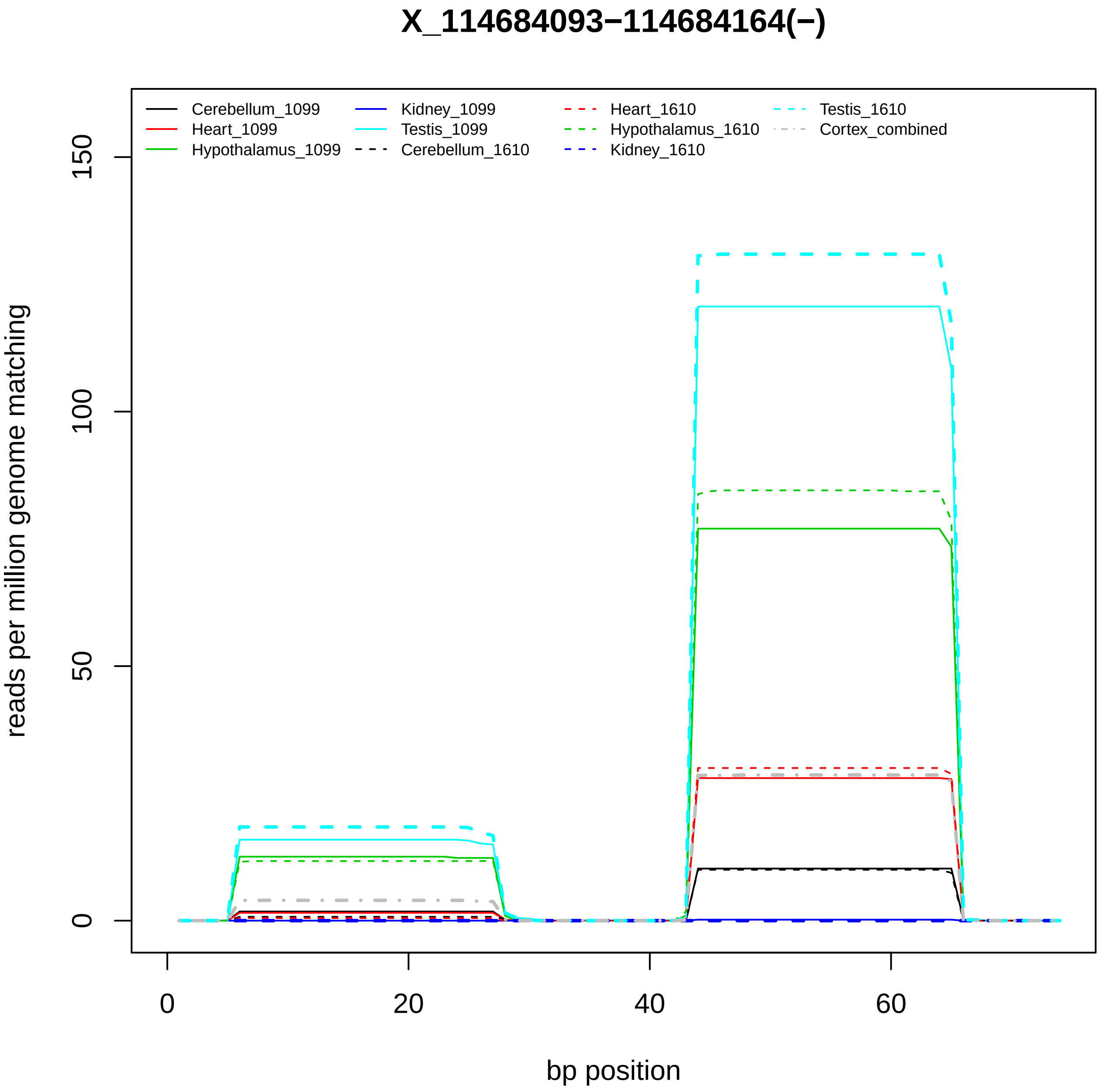
X_114684093−114684164(−)
Cerebellum_1099
Heart_1099
Hypothalamus_1099
Kidney_1099
Testis_1099
Cerebellum_1610
Heart_1610
Hypothalamus_1610
Kidney_1610
Testis_1610
Cortex_combined
reads per million genome matching
0
50
100
150
0
20
40
60
bp position

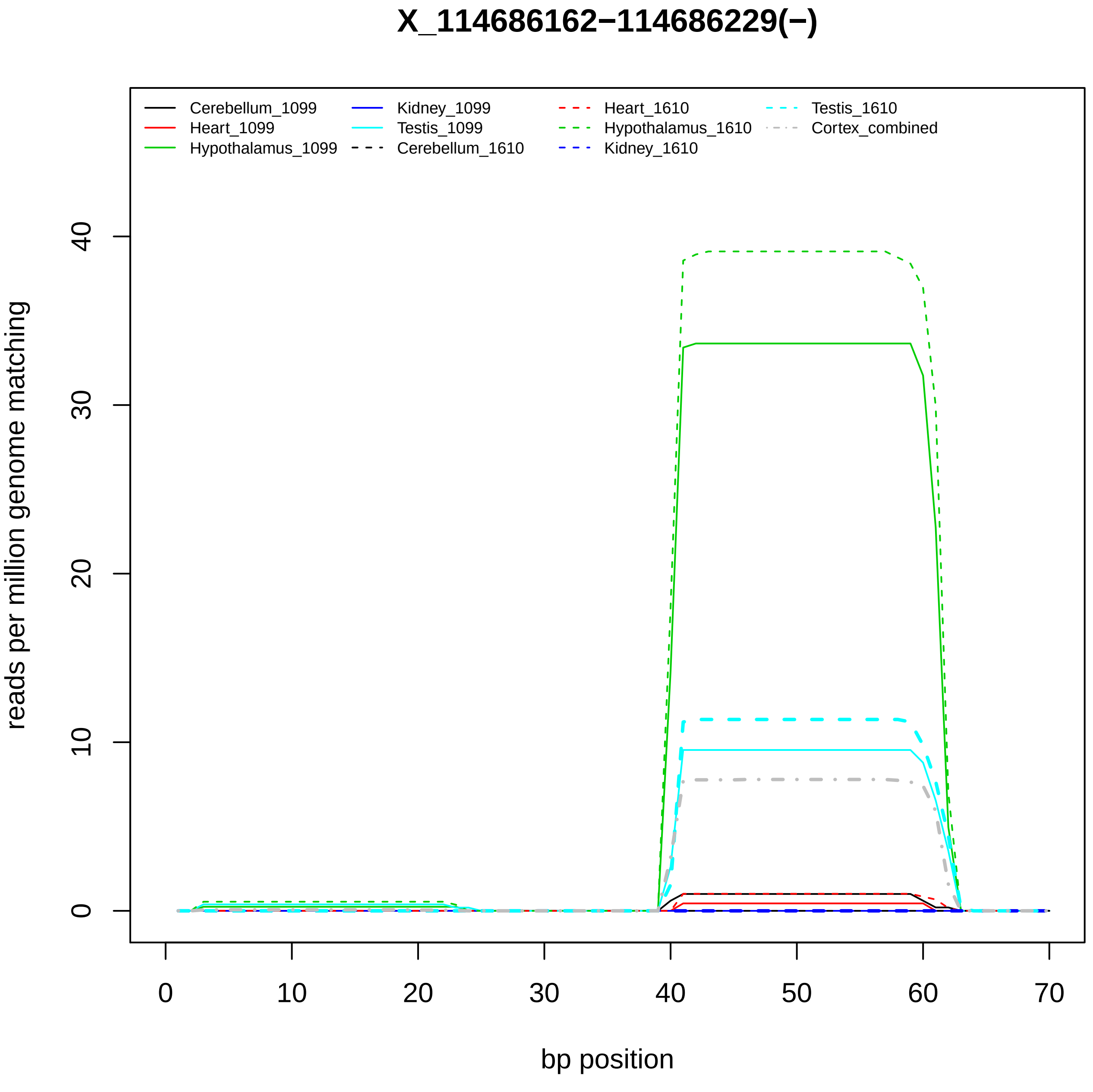
X_114686162−114686229(−)
Cerebellum_1099
Heart_1099
Hypothalamus_1099
Kidney_1099
Testis_1099
Cerebellum_1610
Heart_1610
Hypothalamus_1610
Kidney_1610
Testis_1610
Cortex_combined
reads per million genome matching
bp position
0
10
20
30
40
0
10
20
30
40
50
60
70

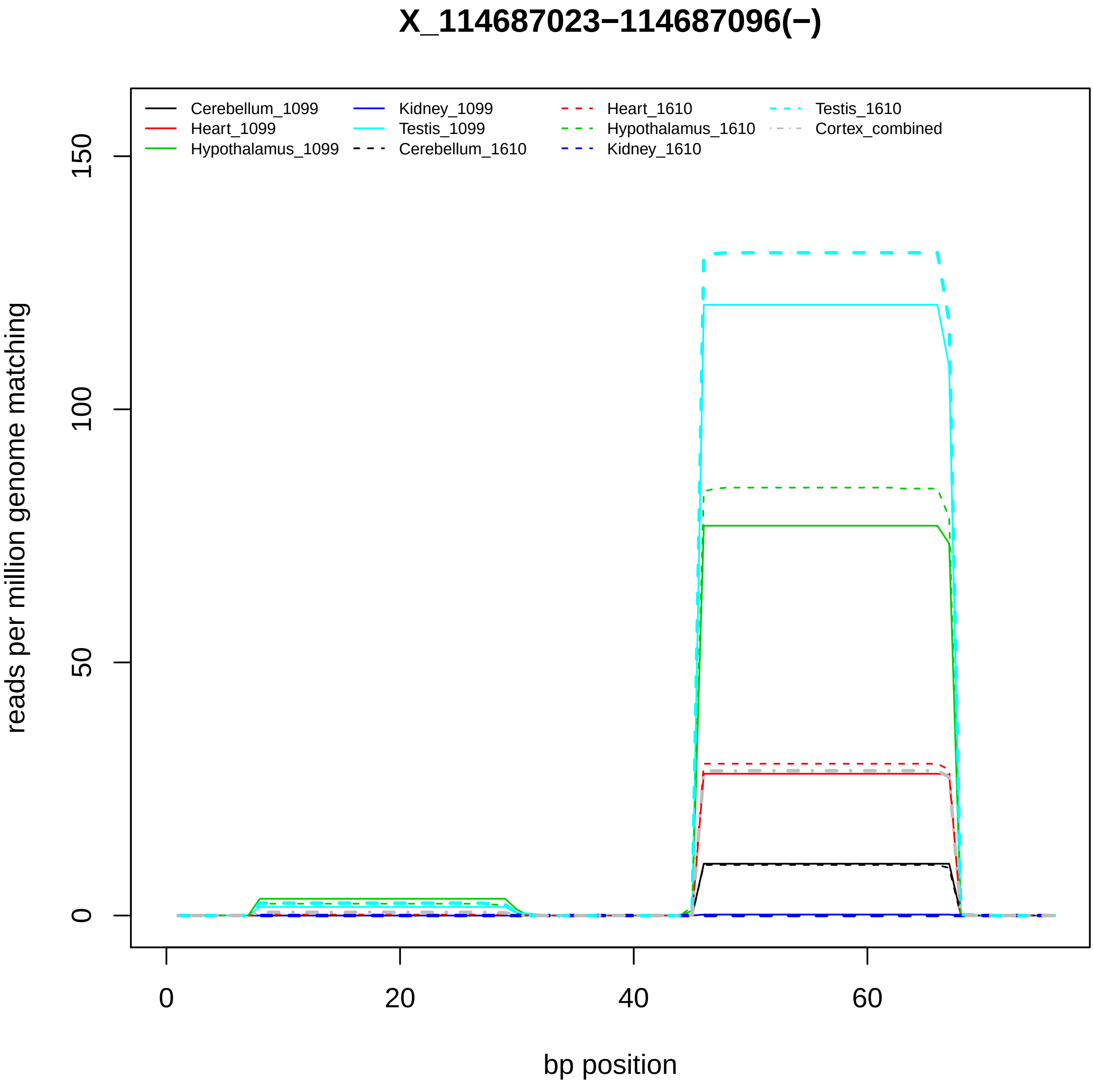
X_114687023−114687096(−)
Cerebellum_1099
Heart_1099
Hypothalamus_1099
Kidney_1099
Testis_1099
Cerebellum_1610
Heart_1610
Hypothalamus_1610
Kidney_1610
Testis_1610
Cortex_combined
reads per million genome matching
bp position
0
50
100
150
0
20
40
60

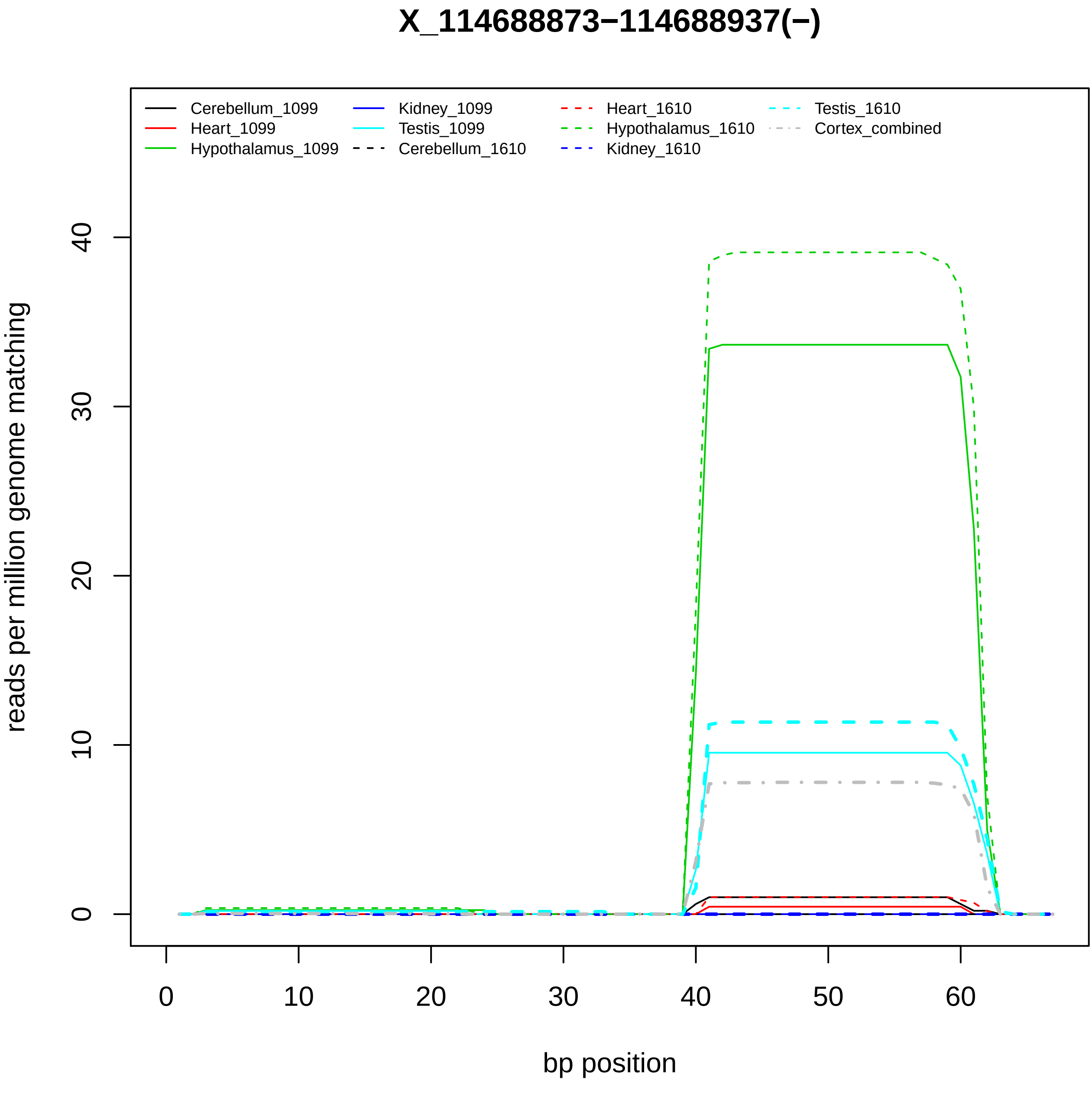

X_114688873−114688937(−)
Cerebellum_1099
Heart_1099
Hypothalamus_1099
Kidney_1099
Testis_1099
Cerebellum_1610
Heart_1610
Hypothalamus_1610
Kidney_1610
Testis_1610
Cortex_combined
reads per million genome matching
0
10
20
30
40
0
10
20
30
40
50
60
bp position

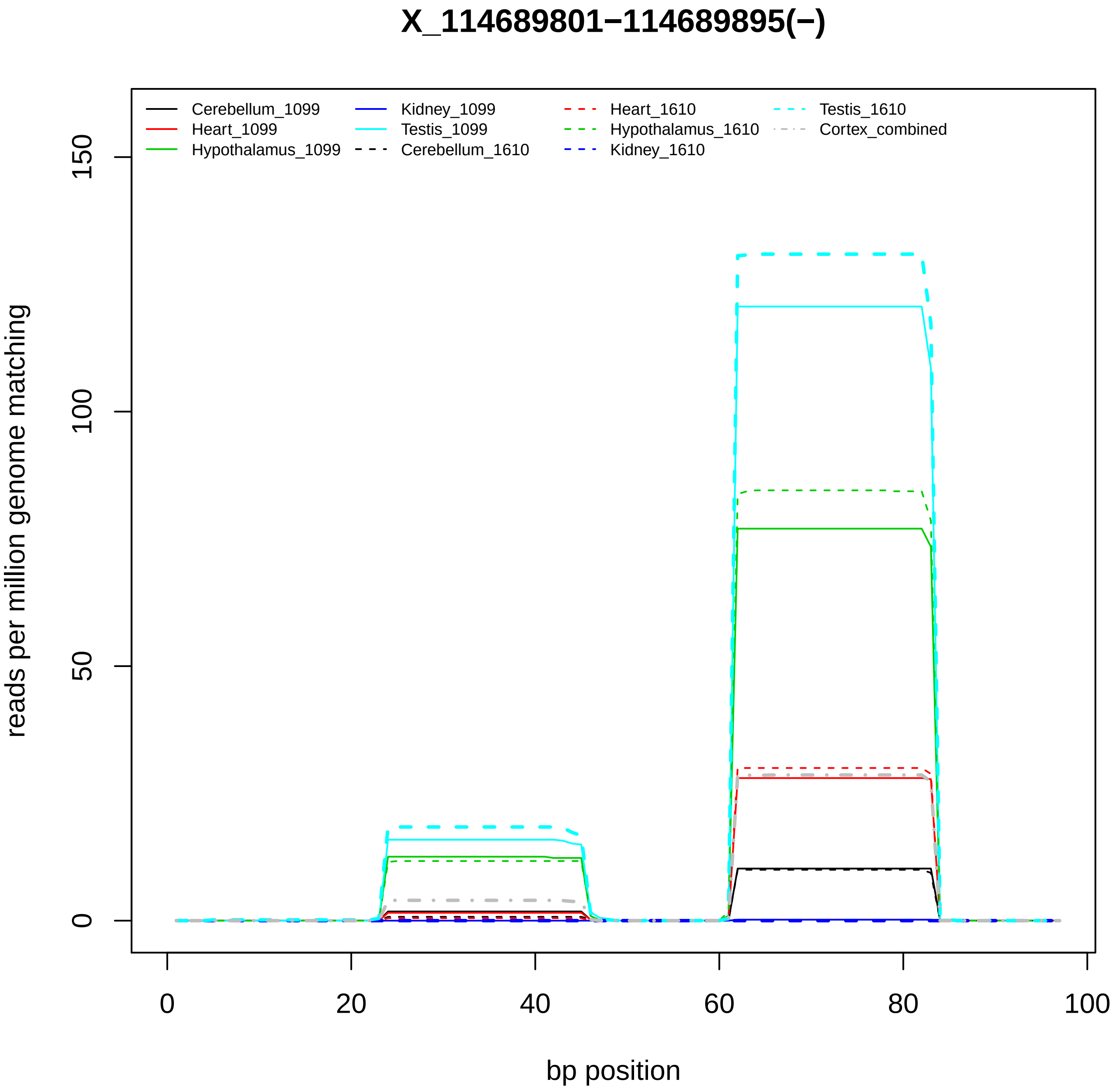
X_114689801−114689895(−)
Cerebellum_1099
Heart_1099
Hypothalamus_1099
Kidney_1099
Testis_1099
Cerebellum_1610
Heart_1610
Hypothalamus_1610
Kidney_1610
Testis_1610
Cortex_combined
reads per million genome matching
bp position
0
50
100
150
0
20
40
60
80
100

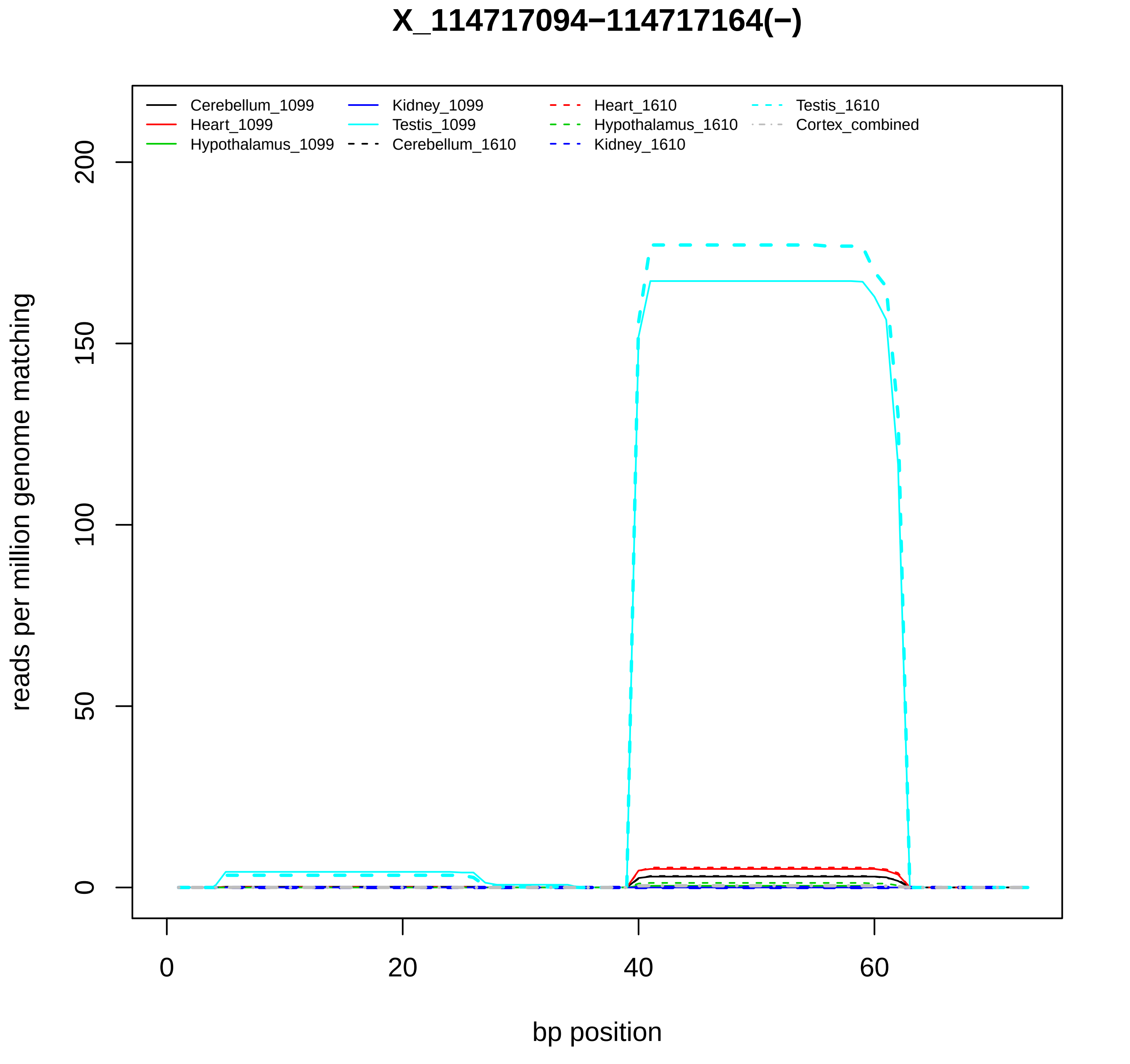

X_114717094−114717164(−)
Cerebellum_1099
Heart_1099
Hypothalamus_1099
Kidney_1099
Testis_1099
Cerebellum_1610
Heart_1610
Hypothalamus_1610
Kidney_1610
Testis_1610
Cortex_combined
reads per million genome matching
bp position
0
50
100
150
200
0
20
40
60

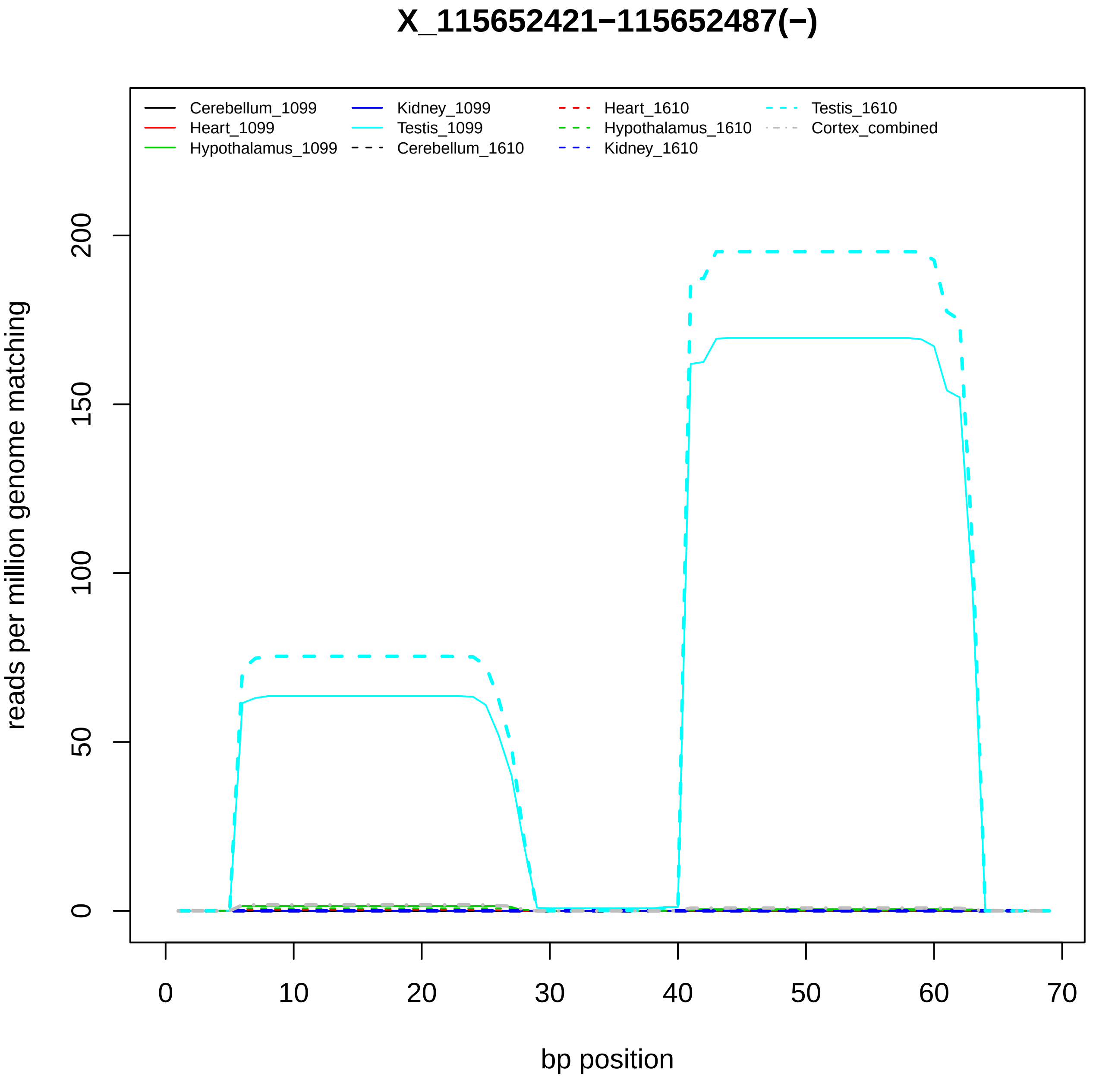
X_115652421−115652487(−)
Cerebellum_1099
Heart_1099
Hypothalamus_1099
Kidney_1099
Testis_1099
Cerebellum_1610
Heart_1610
Hypothalamus_1610
Kidney_1610
Testis_1610
Cortex_combined
reads per million genome matching
bp position
0
50
100
150
200
0
10
20
30
40
50
60
70

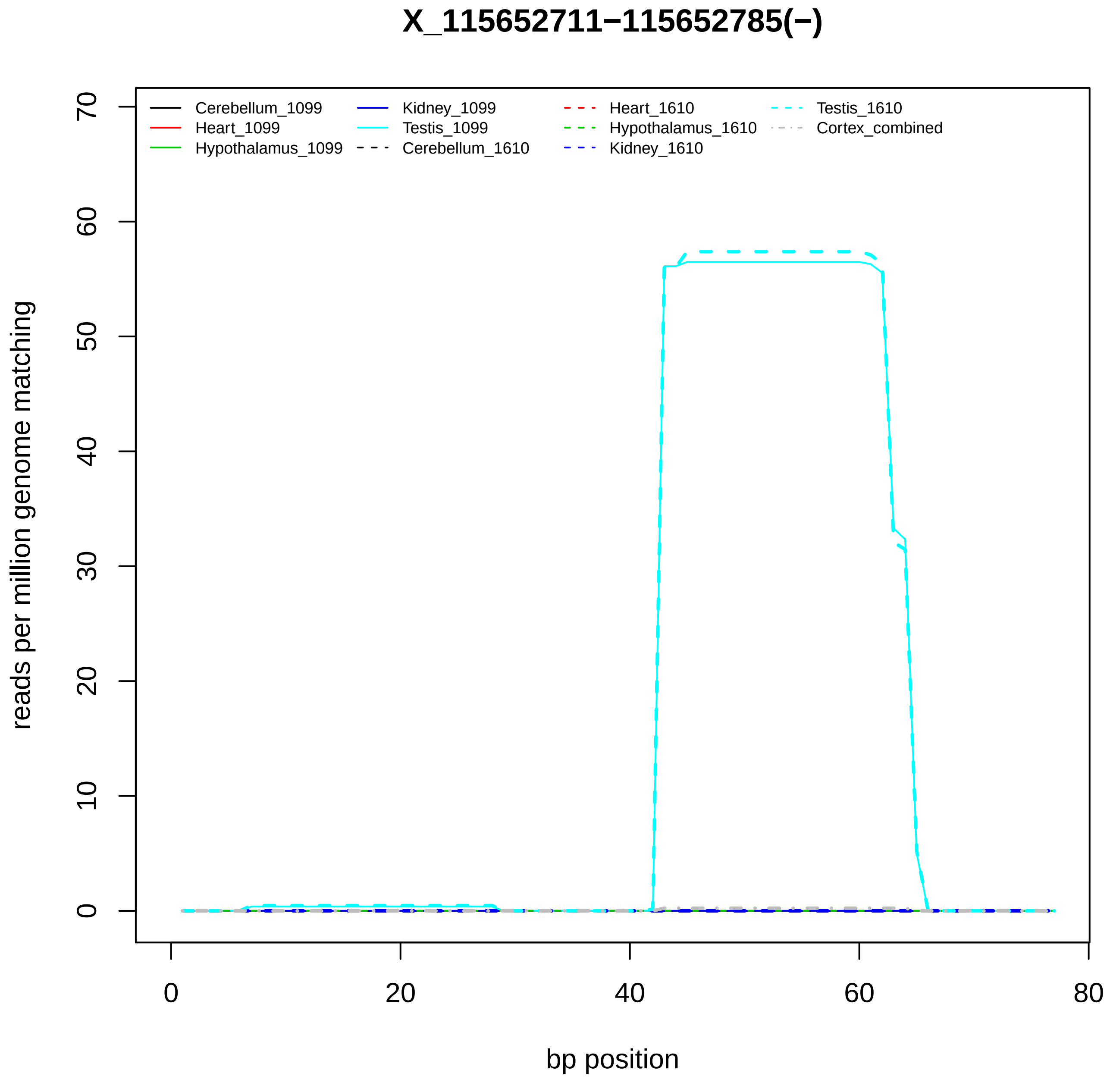

X_115652711−115652785(−)
reads per million genome matching
bp position
0
10
20
30
40
50
60
70
0
20
40
60
80
Cerebellum_1099
Heart_1099
Hypothalamus_1099
Kidney_1099
Testis_1099
Cerebellum_1610
Heart_1610
Hypothalamus_1610
Kidney_1610
Testis_1610
Cortex_combined

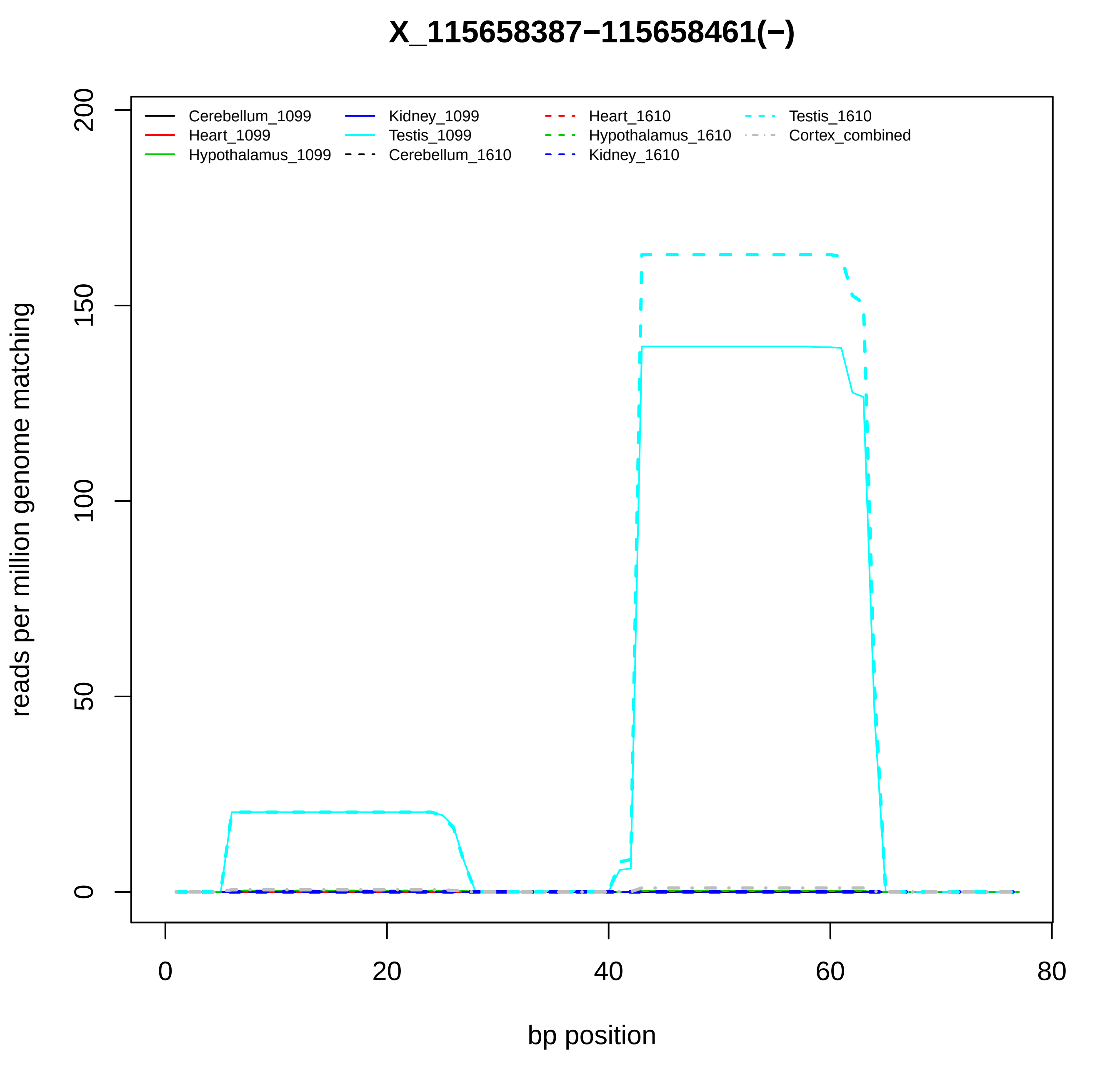
X_115658387−115658461(−)
Cerebellum_1099
Heart_1099
Hypothalamus_1099
Kidney_1099
Testis_1099
Cerebellum_1610
Heart_1610
Hypothalamus_1610
Kidney_1610
Testis_1610
Cortex_combined
reads per million genome matching
bp position
0
50
100
150
200
0
20
40
60
80

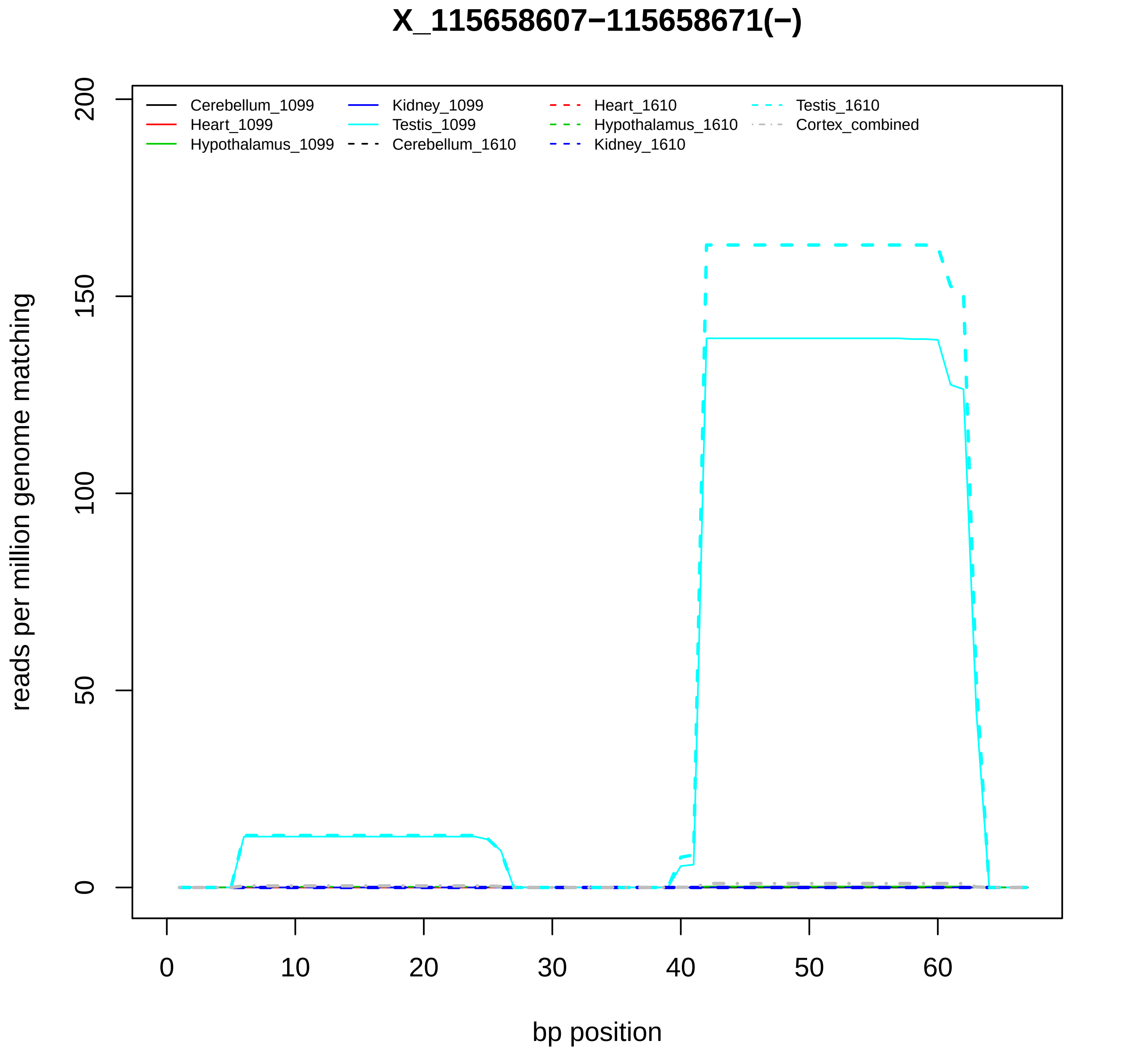
X_115658607−115658671(−)
Cerebellum_1099
Heart_1099
Hypothalamus_1099
Kidney_1099
Testis_1099
Cerebellum_1610
Heart_1610
Hypothalamus_1610
Kidney_1610
Testis_1610
Cortex_combined
reads per million genome matching
bp position
0
50
100
150
200
0
10
20
30
40
50
60

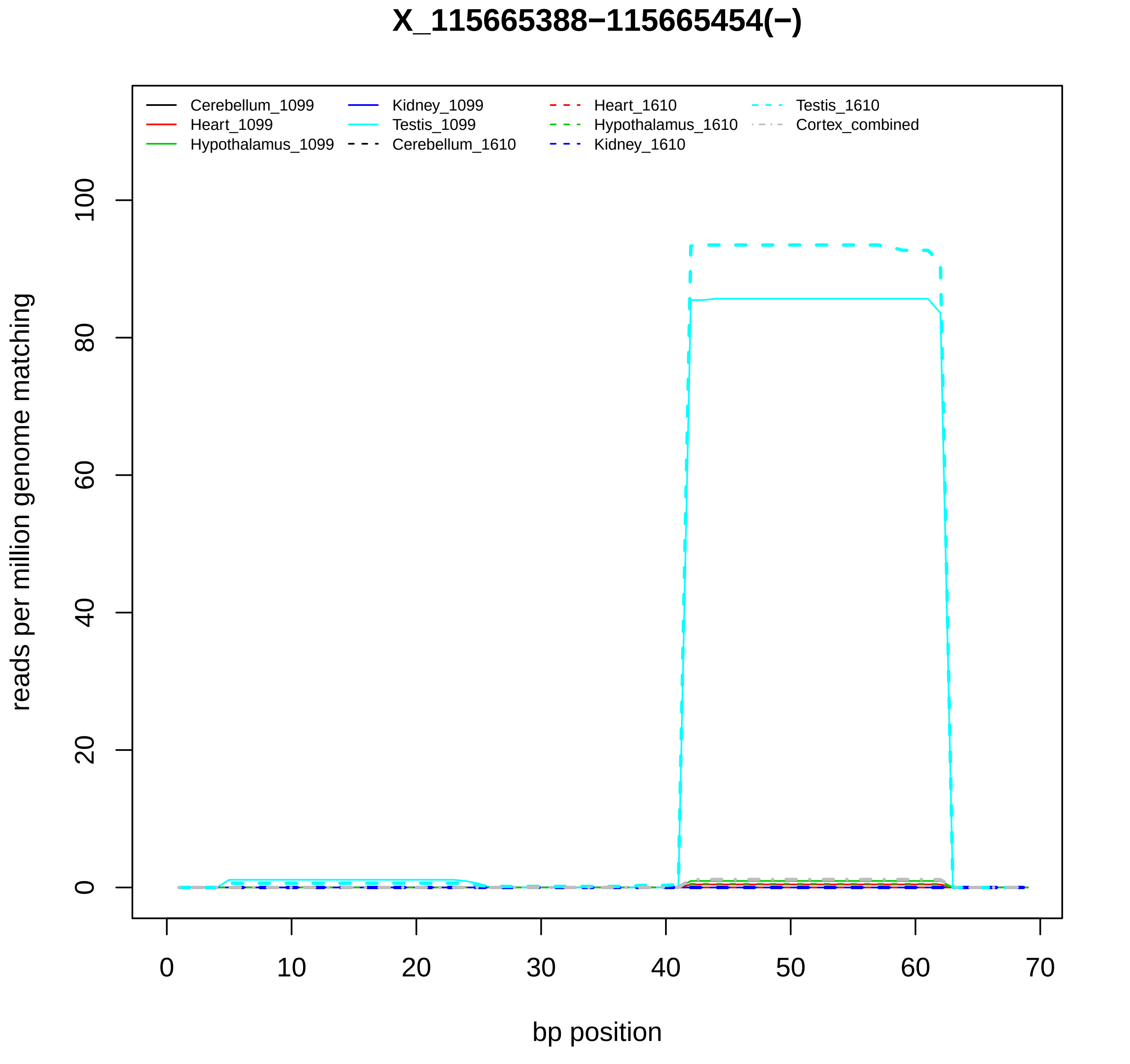

X_115665388−115665454(−)
Cerebellum_1099
Heart_1099
Hypothalamus_1099
Kidney_1099
Testis_1099
Cerebellum_1610
Heart_1610
Hypothalamus_1610
Kidney_1610
Testis_1610
Cortex_combined
reads per million genome matching
bp position
0
20
40
60
80
100
0
10
20
30
40
50
60
70

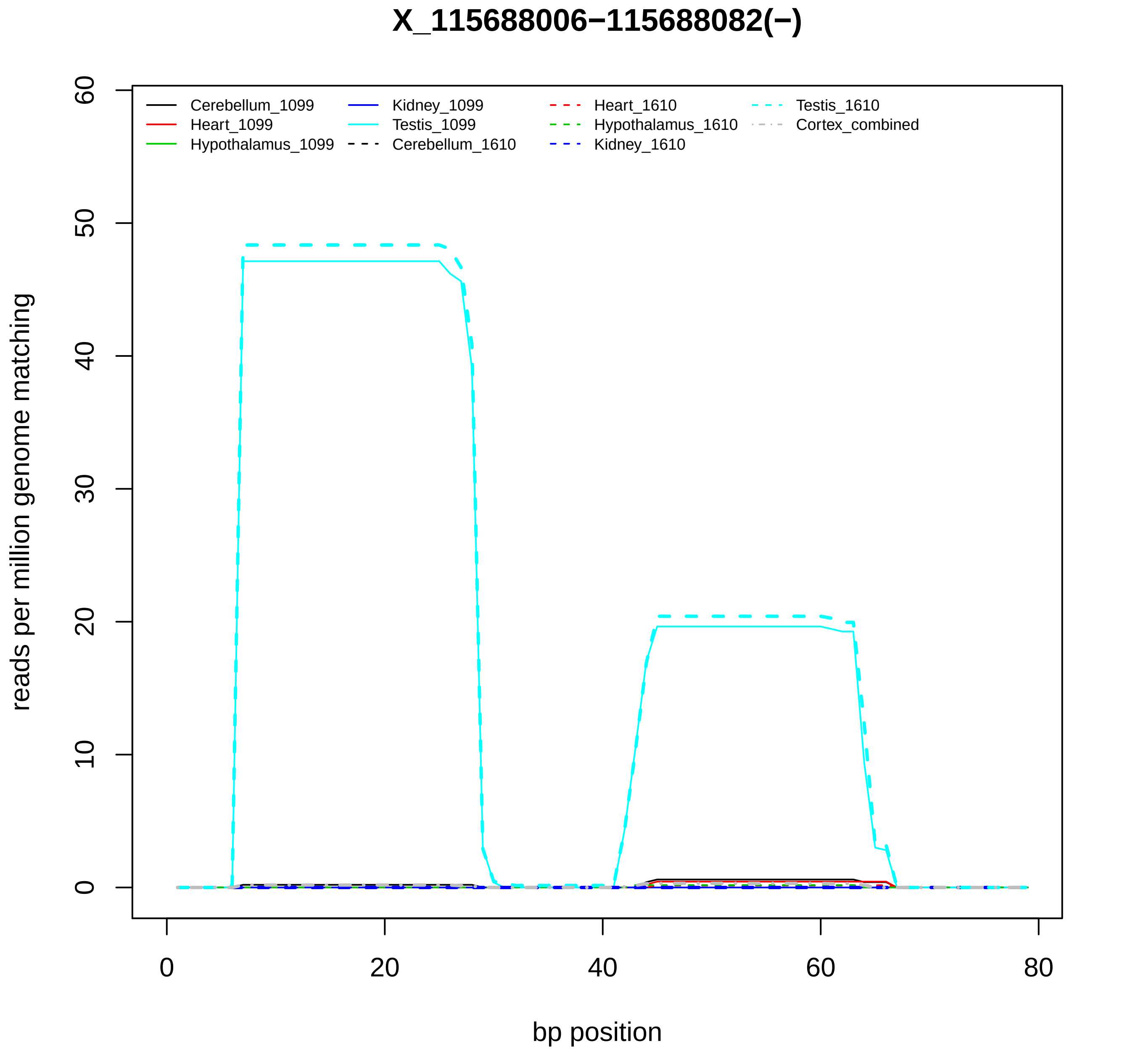

X_115688006−115688082(−)
Cerebellum_1099
Heart_1099
Hypothalamus_1099
Kidney_1099
Testis_1099
Cerebellum_1610
Heart_1610
Hypothalamus_1610
Kidney_1610
Testis_1610
Cortex_combined
reads per million genome matching
bp position
0
10
20
30
40
50
60
0
20
40
60
80

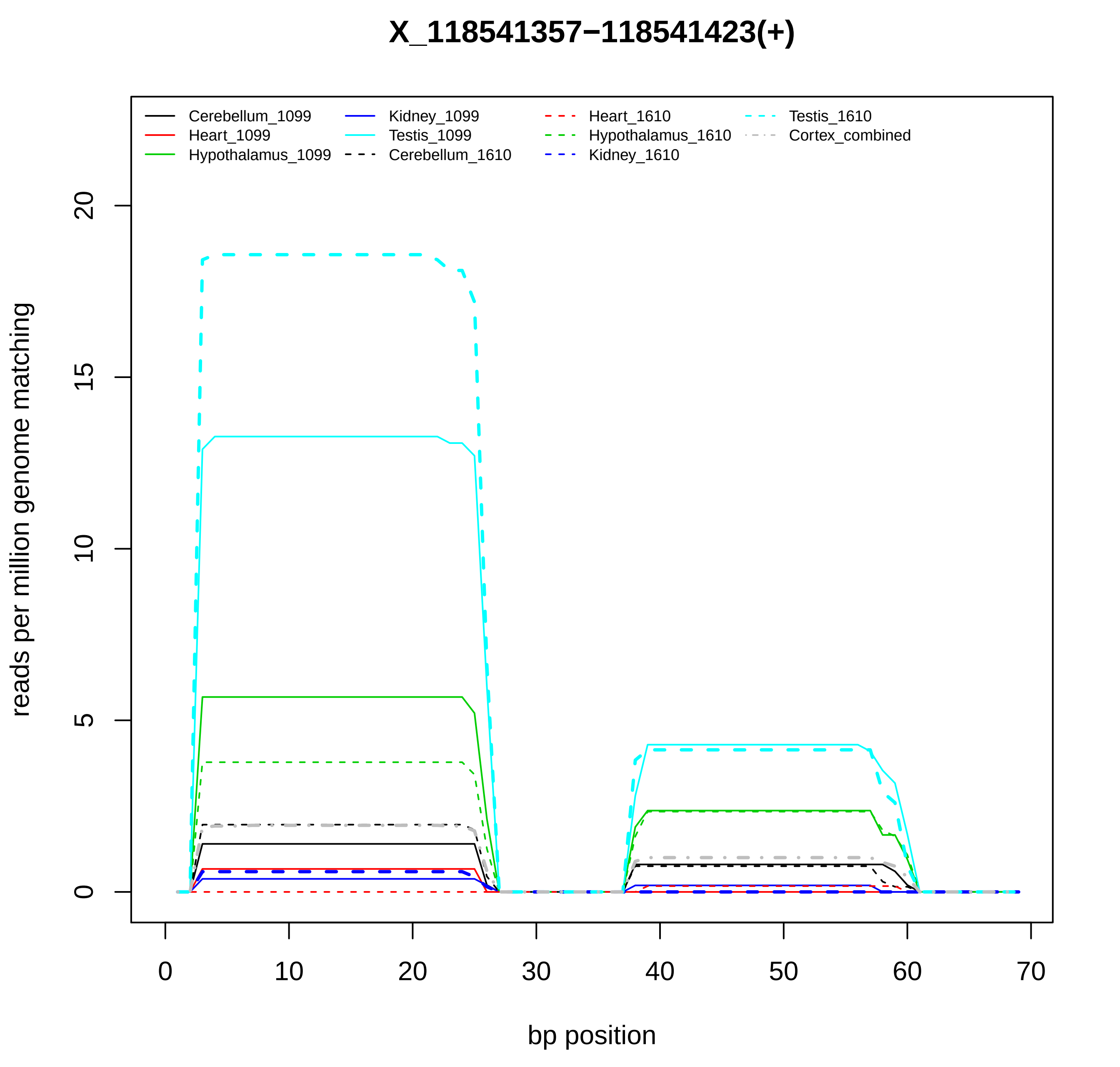
X_118541357−118541423(+)
Cerebellum_1099
Heart_1099
Hypothalamus_1099
Kidney_1099
Testis_1099
Cerebellum_1610
Heart_1610
Hypothalamus_1610
Kidney_1610
Testis_1610
Cortex_combined
reads per million genome matching
0
5
10
15
20
0
10
20
30
40
50
60
70
bp position

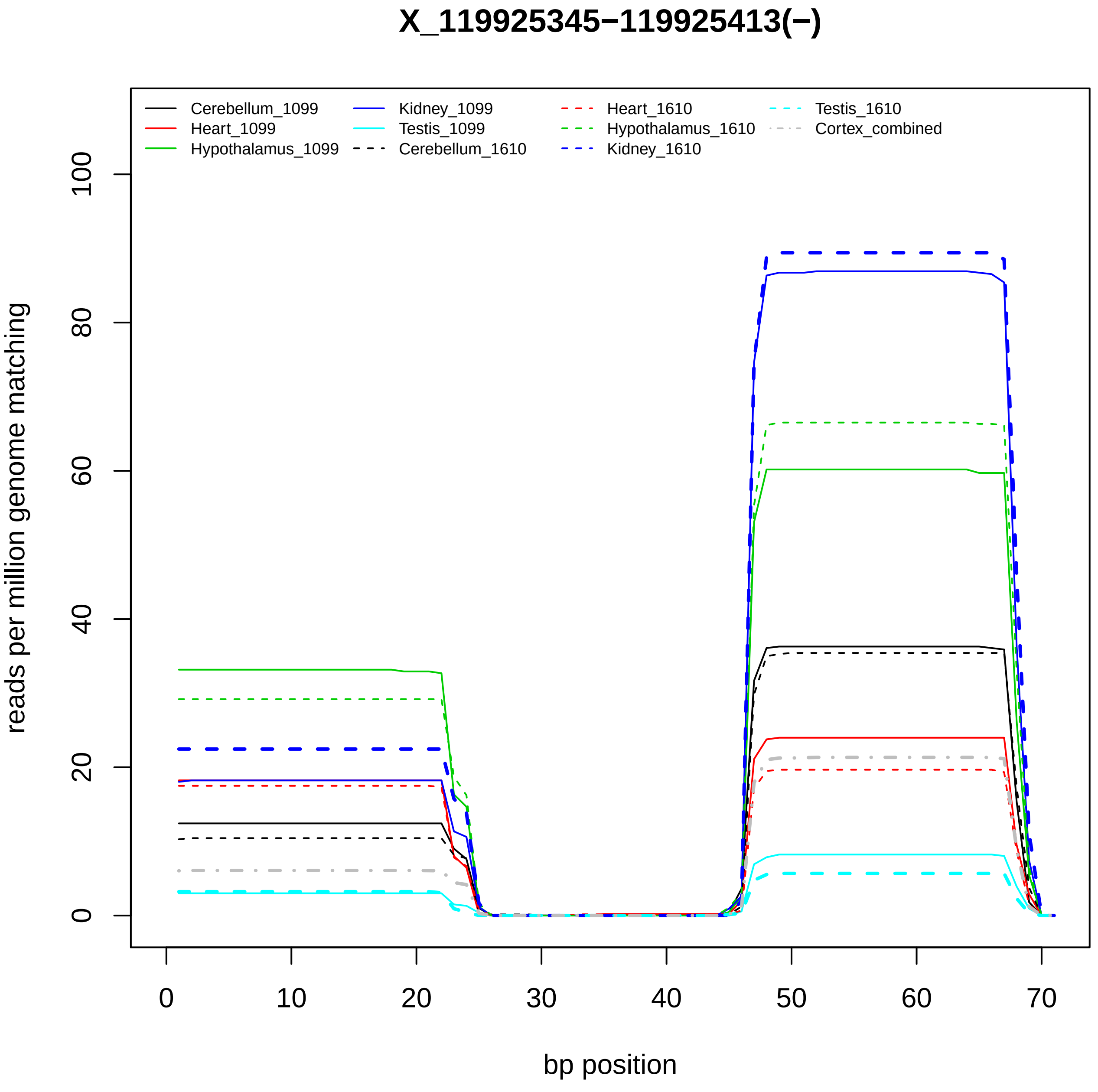

X_119925345−119925413(−)
Cerebellum_1099
Heart_1099
Hypothalamus_1099
Kidney_1099
Testis_1099
Cerebellum_1610
Heart_1610
Hypothalamus_1610
Kidney_1610
Testis_1610
Cortex_combined
reads per million genome matching
0
20
40
60
80
100
bp position
0
10
20
30
40
50
60
70

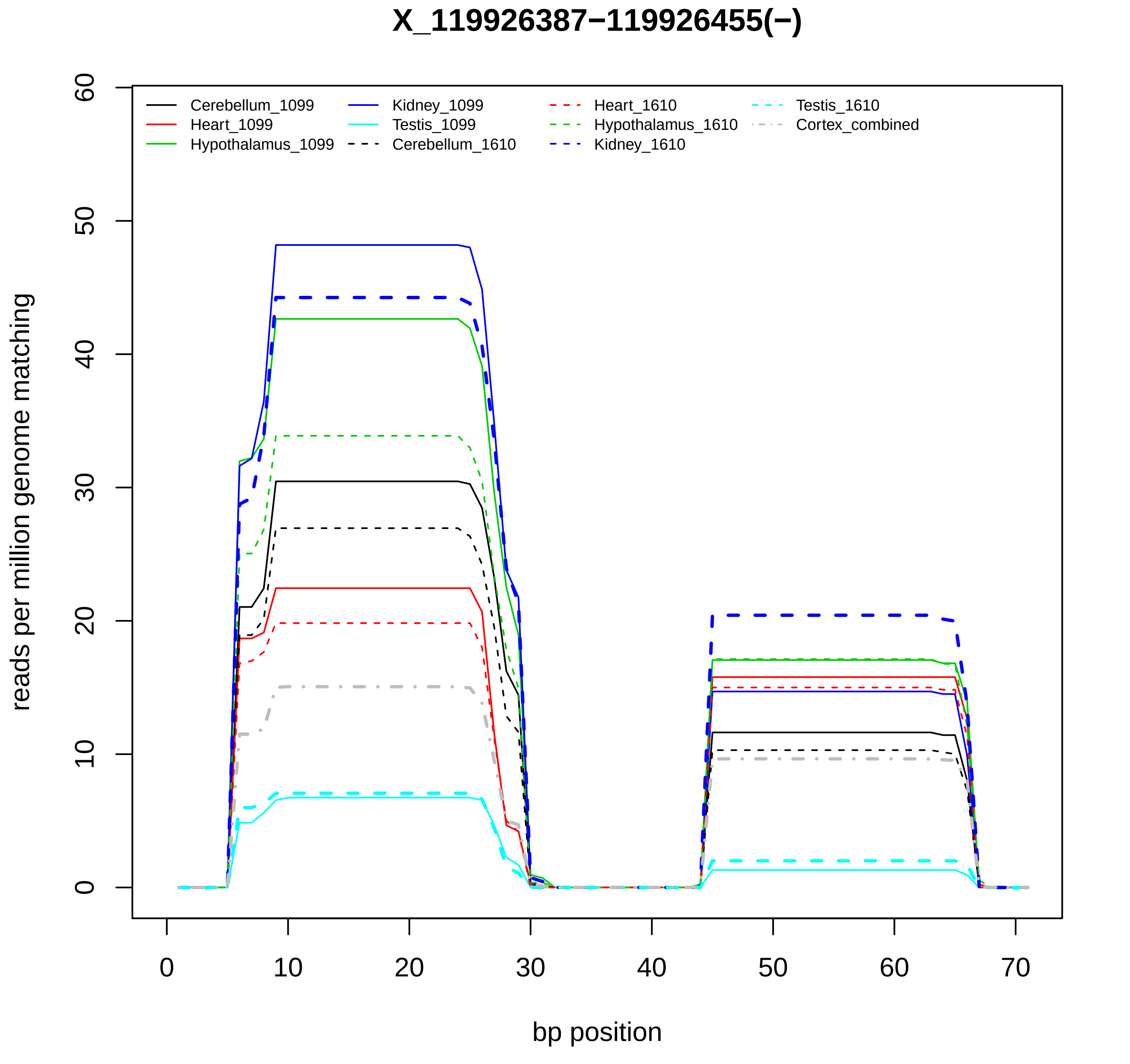
X_119926387−119926455(−)
Cerebellum_1099
Heart_1099
Hypothalamus_1099
Kidney_1099
Testis_1099
Cerebellum_1610
Heart_1610
Hypothalamus_1610
Kidney_1610
Testis_1610
Cortex_combined
reads per million genome matching
bp position
0
10
20
30
40
50
60
70

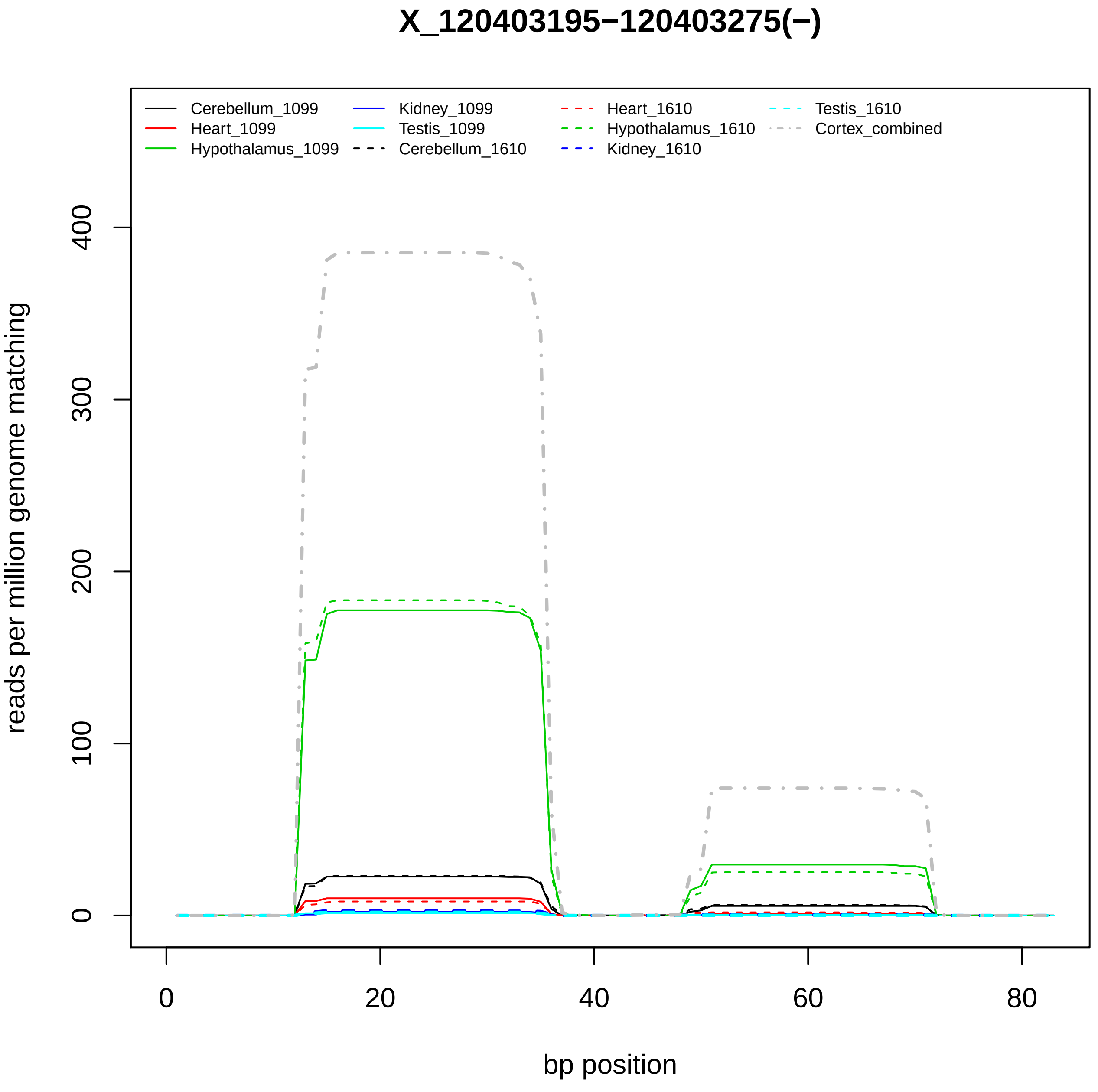

X_120403195−120403275(−)
Cerebellum_1099
Heart_1099
Hypothalamus_1099
Kidney_1099
Testis_1099
Cerebellum_1610
Heart_1610
Hypothalamus_1610
Kidney_1610
Testis_1610
Cortex_combined
reads per million genome matching
bp position
0
100
200
300
400
0
20
40
60
80

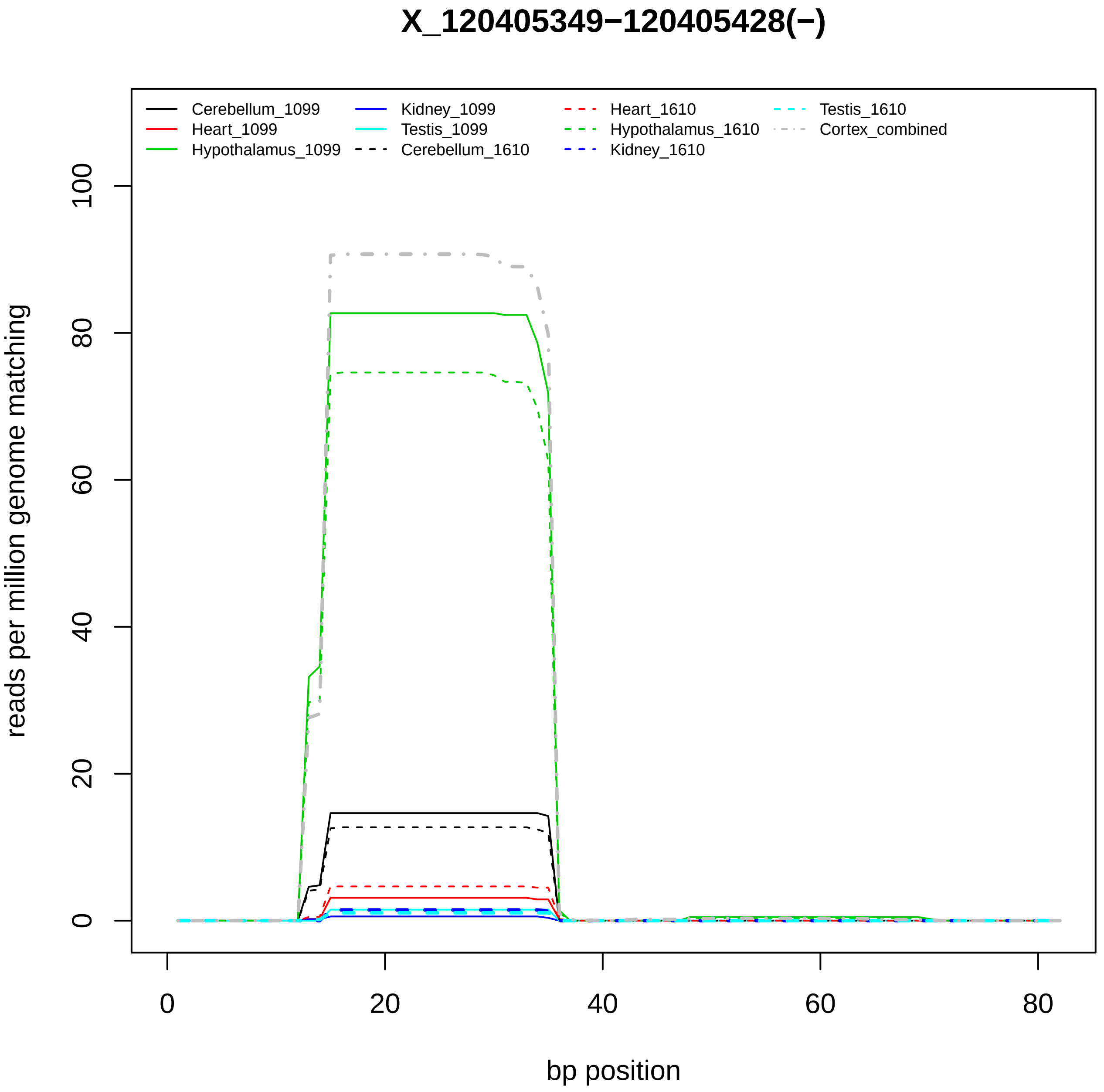

X_120405349−120405428(−)
Cerebellum_1099
Heart_1099
Hypothalamus_1099
Kidney_1099
Testis_1099
Cerebellum_1610
Heart_1610
Hypothalamus_1610
Kidney_1610
Testis_1610
Cortex_combined
reads per million genome matching
0
20
40
60
80
100
0
20
40
60
80
bp position

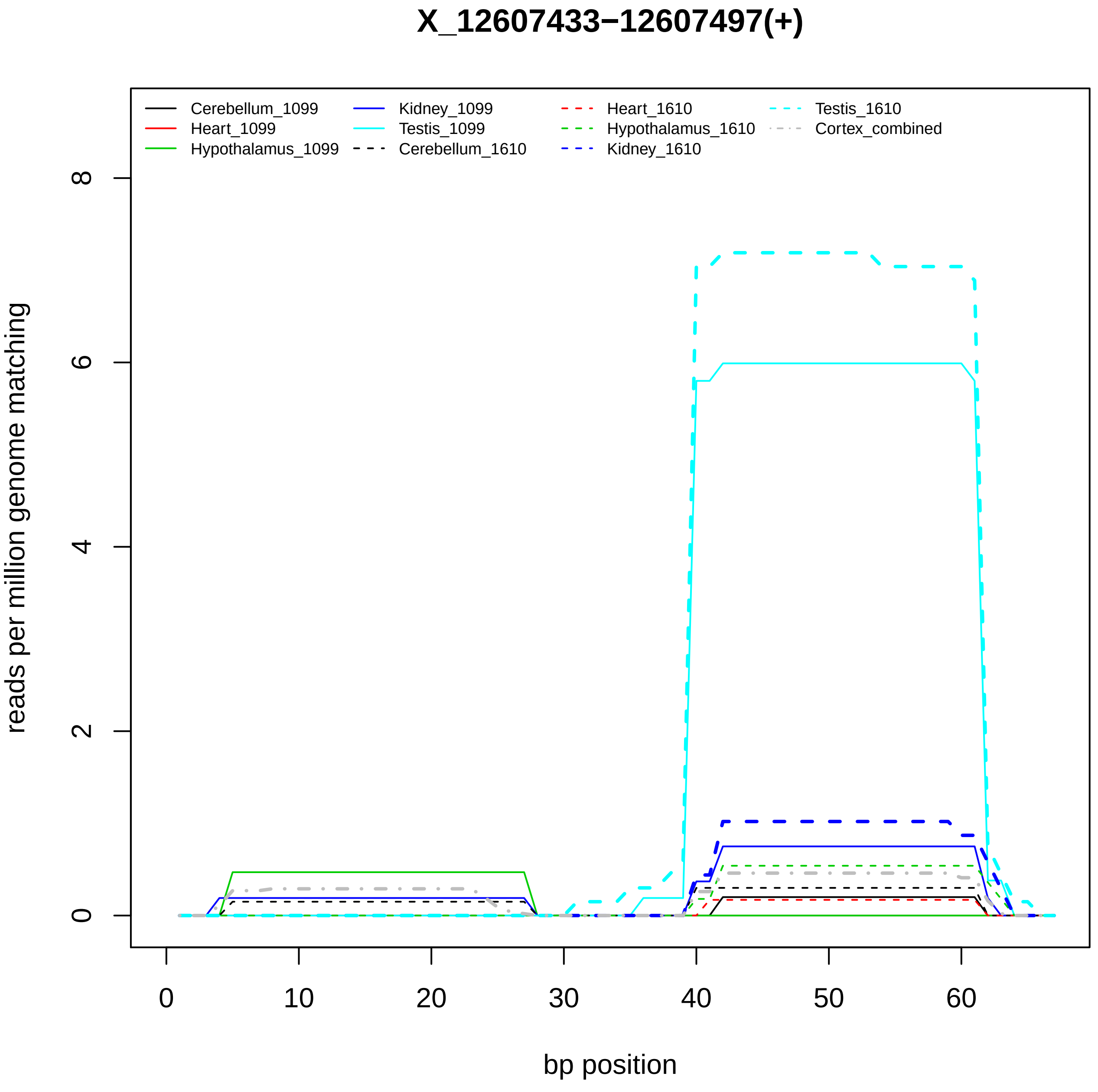

X_12607433−12607497(+)
Cerebellum_1099
Heart_1099
Hypothalamus_1099
Kidney_1099
Testis_1099
Cerebellum_1610
Heart_1610
Hypothalamus_1610
Kidney_1610
Testis_1610
Cortex_combined
reads per million genome matching
0
2
4
6
8
0
10
20
30
40
50
60
bp position

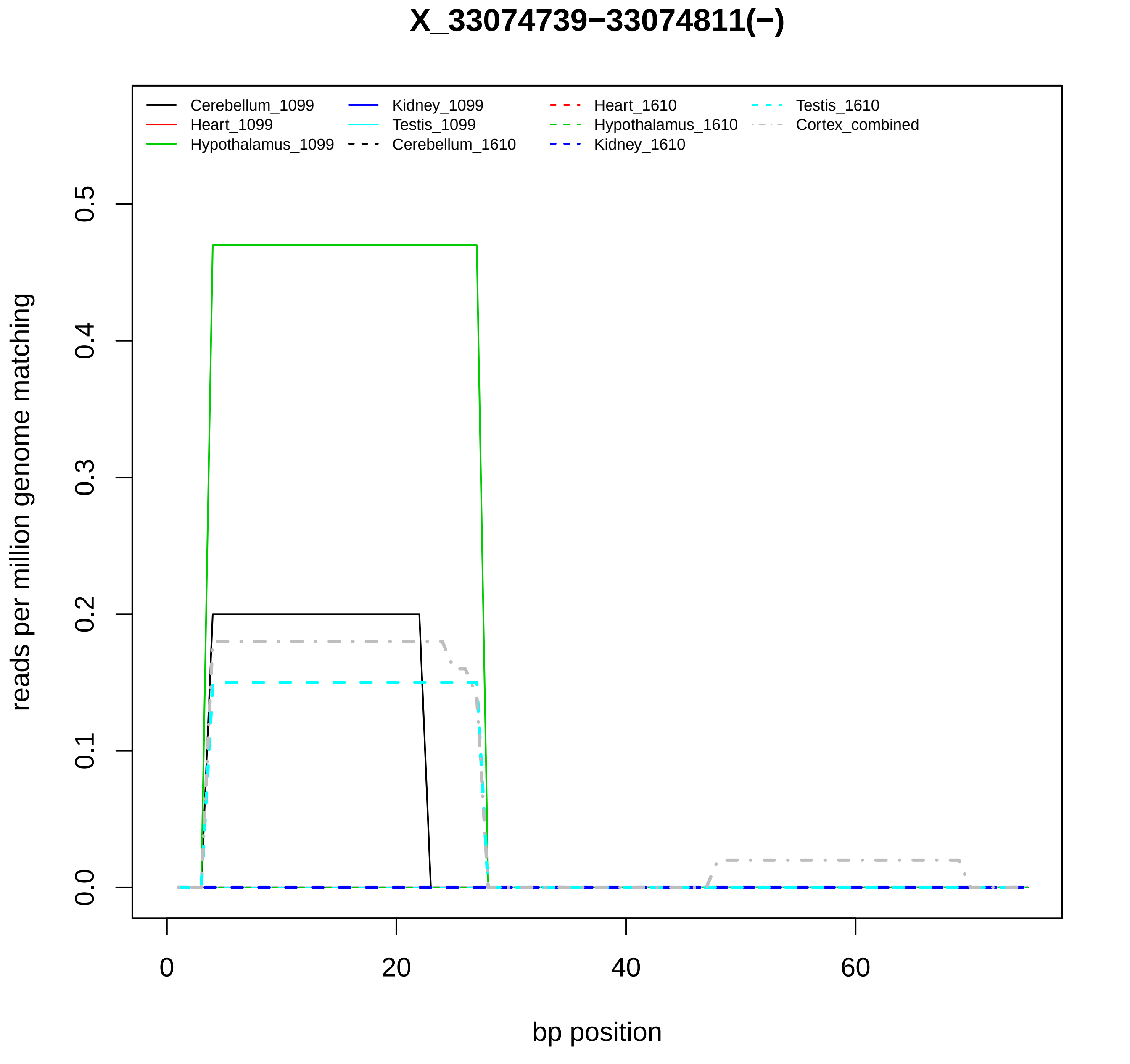

X_33074739−33074811(−)
Cerebellum_1099
Heart_1099
Hypothalamus_1099
Kidney_1099
Testis_1099
Cerebellum_1610
Heart_1610
Hypothalamus_1610
Kidney_1610
Testis_1610
Cortex_combined
reads per million genome matching
0.0
0.1
0.2
0.3
0.4
0.5
0
20
40
60
bp position

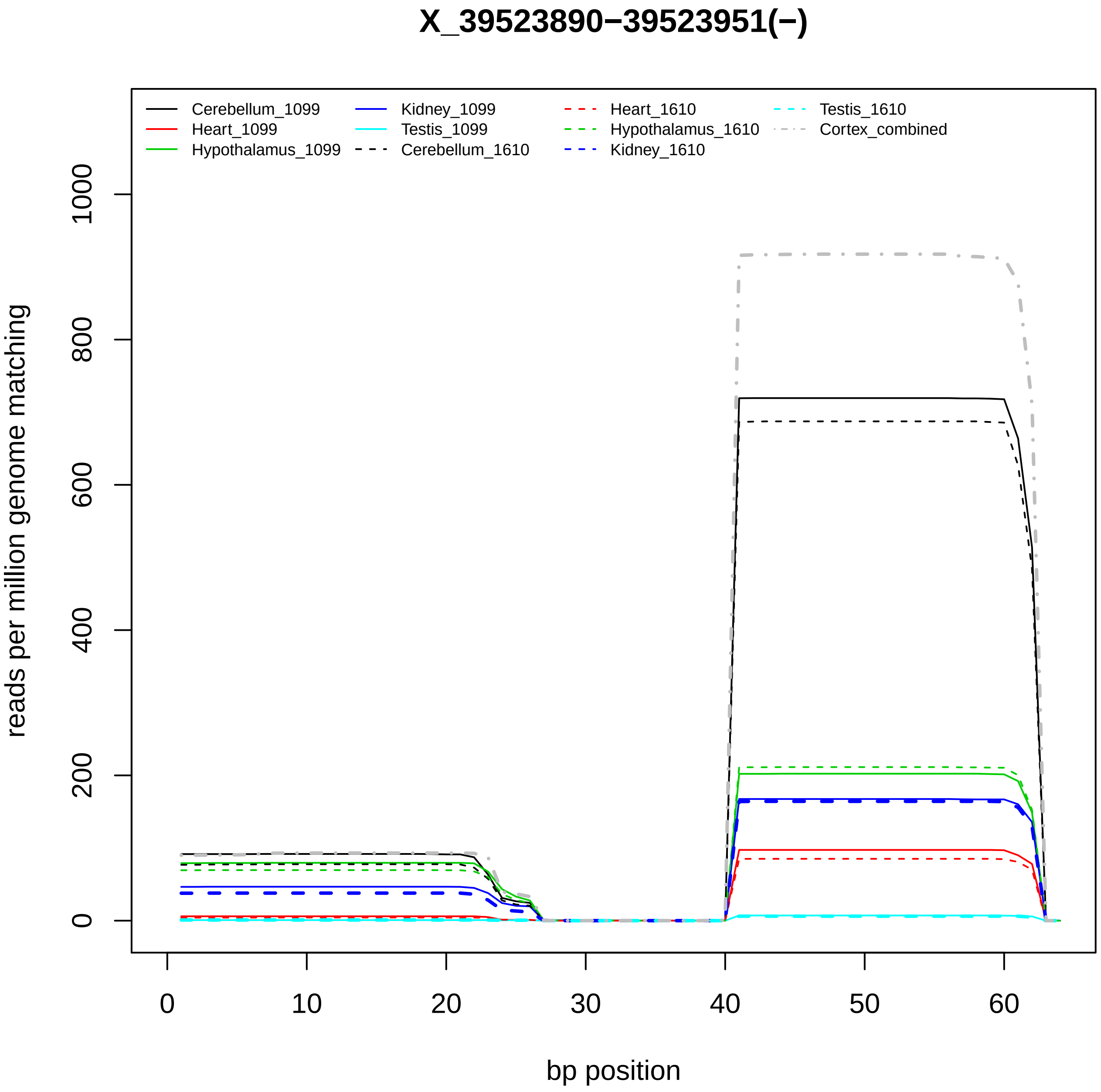

X_39523890−39523951(−)
Cerebellum_1099
Heart_1099
Hypothalamus_1099
Kidney_1099
Testis_1099
Cerebellum_1610
Heart_1610
Hypothalamus_1610
Kidney_1610
Testis_1610
Cortex_combined
reads per million genome matching
0
200
400
600
800
1000
0
10
20
30
40
50
60
bp position

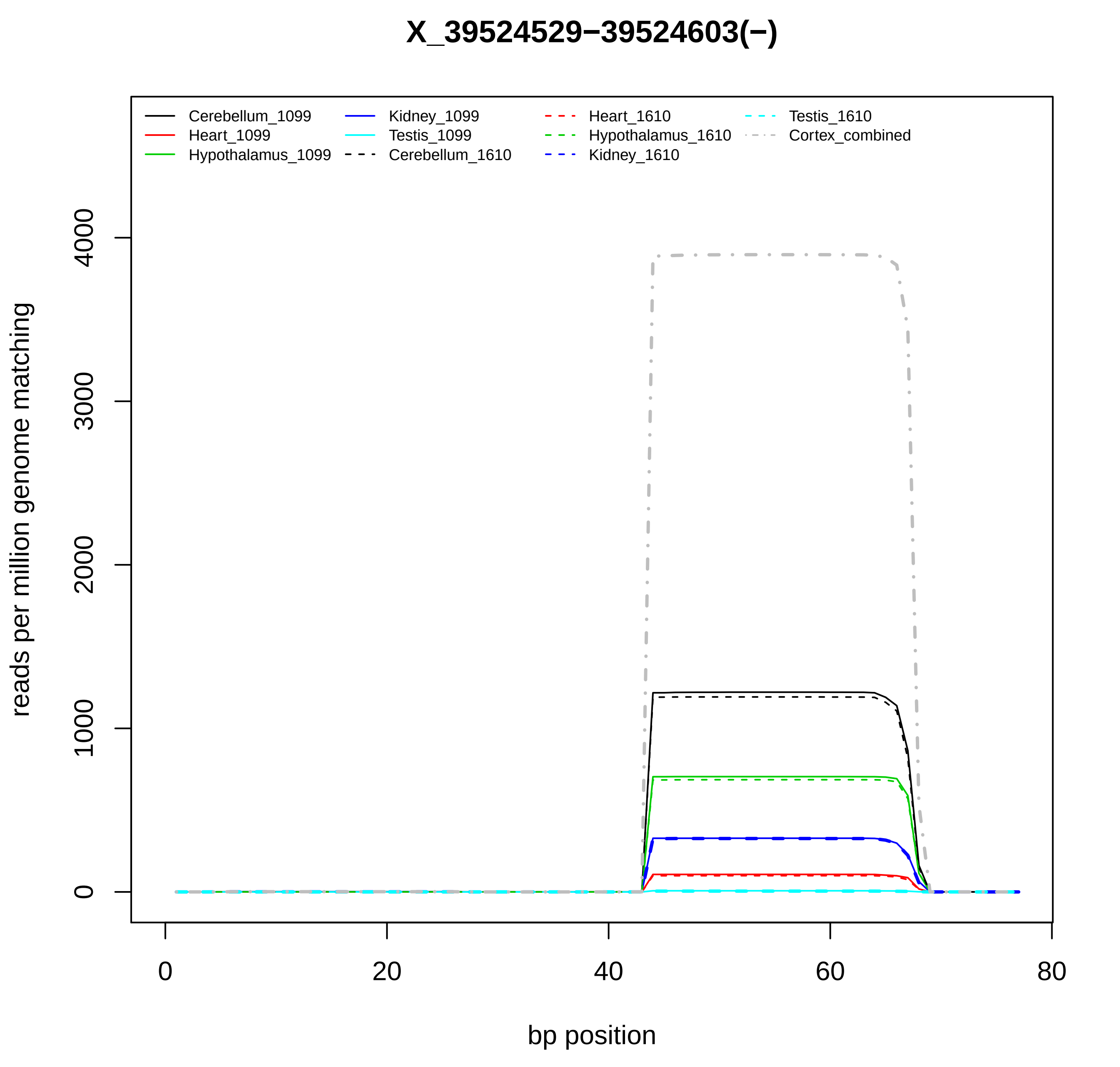

X_39524529−39524603(−)
Cerebellum_1099
Heart_1099
Hypothalamus_1099
Kidney_1099
Testis_1099
Cerebellum_1610
Heart_1610
Hypothalamus_1610
Kidney_1610
Testis_1610
Cortex_combined
reads per million genome matching
0
1000
2000
3000
4000
0
20
40
60
80
bp position

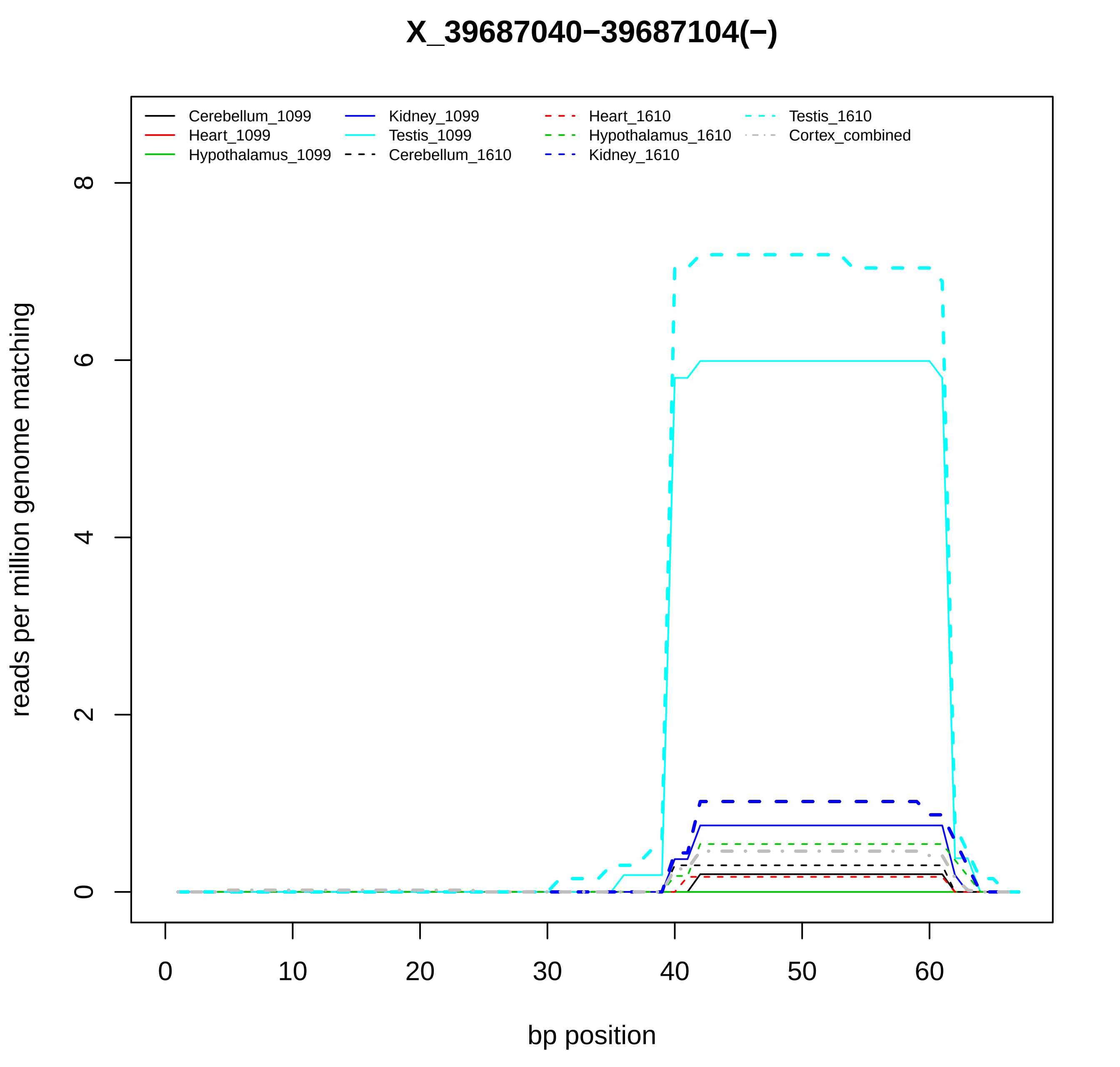

X_39687040−39687104(−)
Cerebellum_1099
Heart_1099
Hypothalamus_1099
Kidney_1099
Testis_1099
Cerebellum_1610
Heart_1610
Hypothalamus_1610
Kidney_1610
Testis_1610
Cortex_combined
reads per million genome matching
0
2
4
6
8
0
10
20
30
40
50
60
bp position

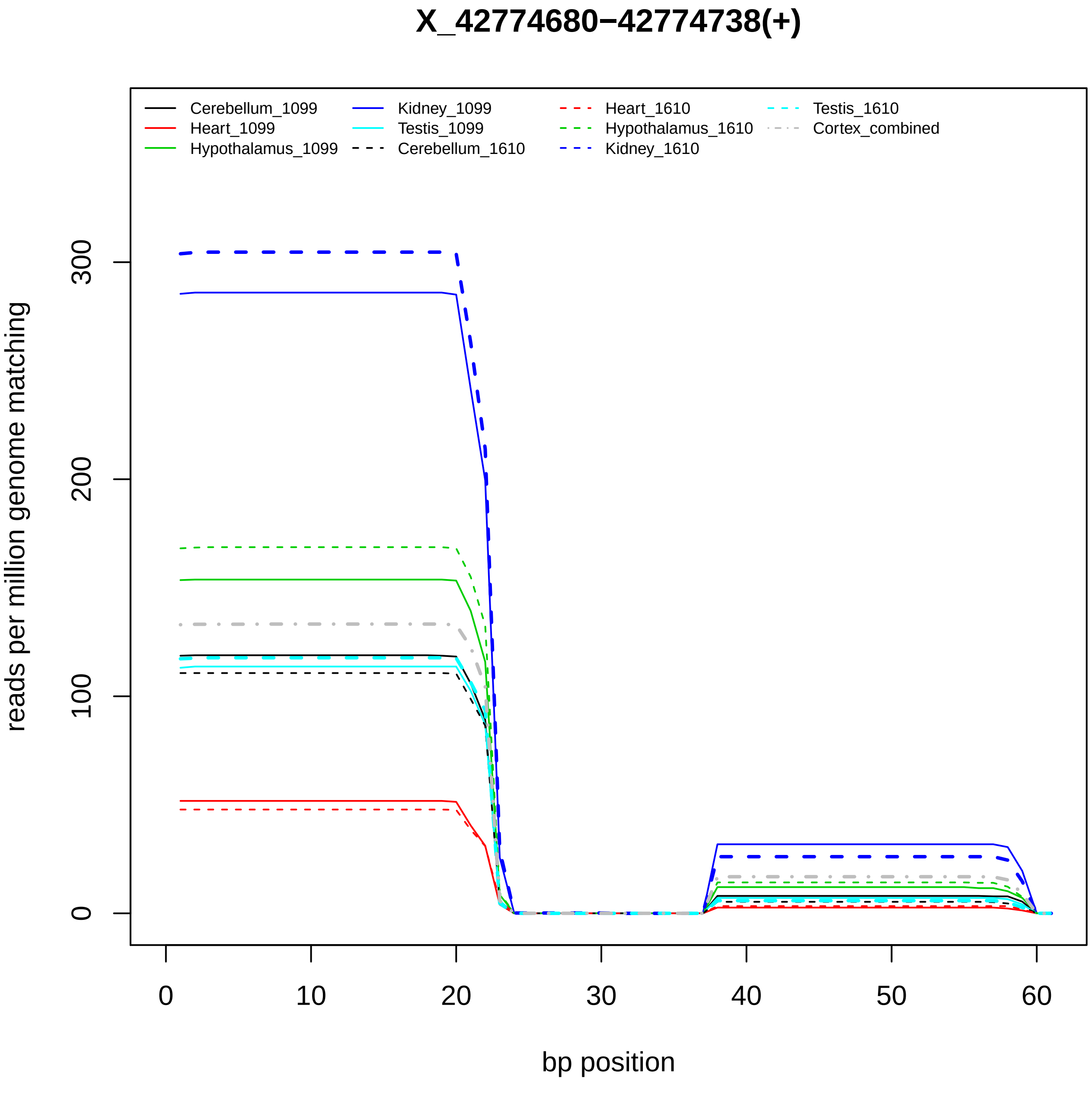

X_42774680−42774738(+)
Cerebellum_1099
Heart_1099
Hypothalamus_1099
Kidney_1099
Testis_1099
Cerebellum_1610
Heart_1610
Hypothalamus_1610
Kidney_1610
Testis_1610
Cortex_combined
reads per million genome matching
0
100
200
300
0
10
20
30
40
50
60
bp position

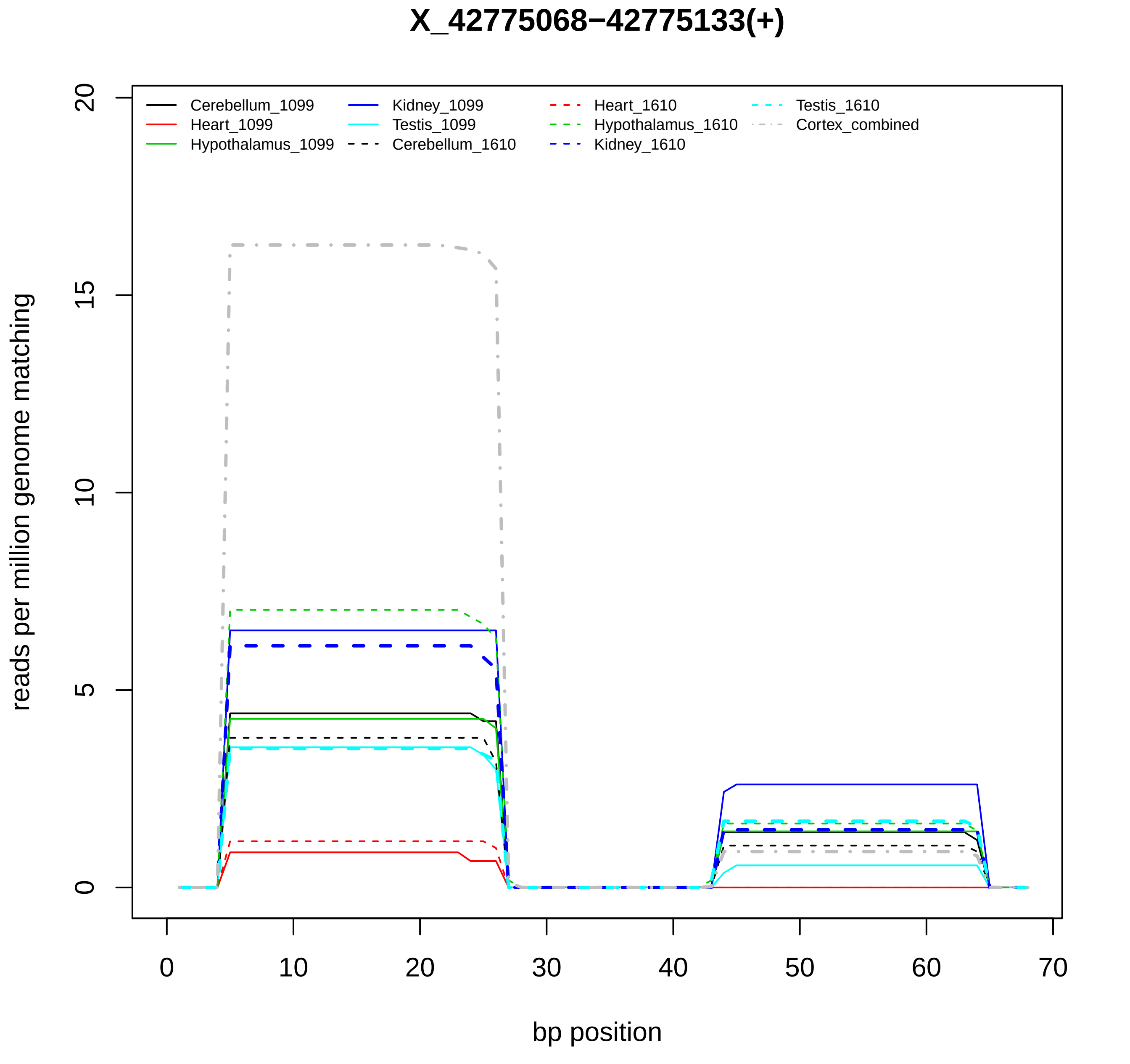

X_42775068−42775133(+)
Cerebellum_1099
Heart_1099
Hypothalamus_1099
Kidney_1099
Testis_1099
Cerebellum_1610
Heart_1610
Hypothalamus_1610
Kidney_1610
Testis_1610
Cortex_combined
reads per million genome matching
bp position
0
5
10
15
20
0
10
20
30
40
50
60
70

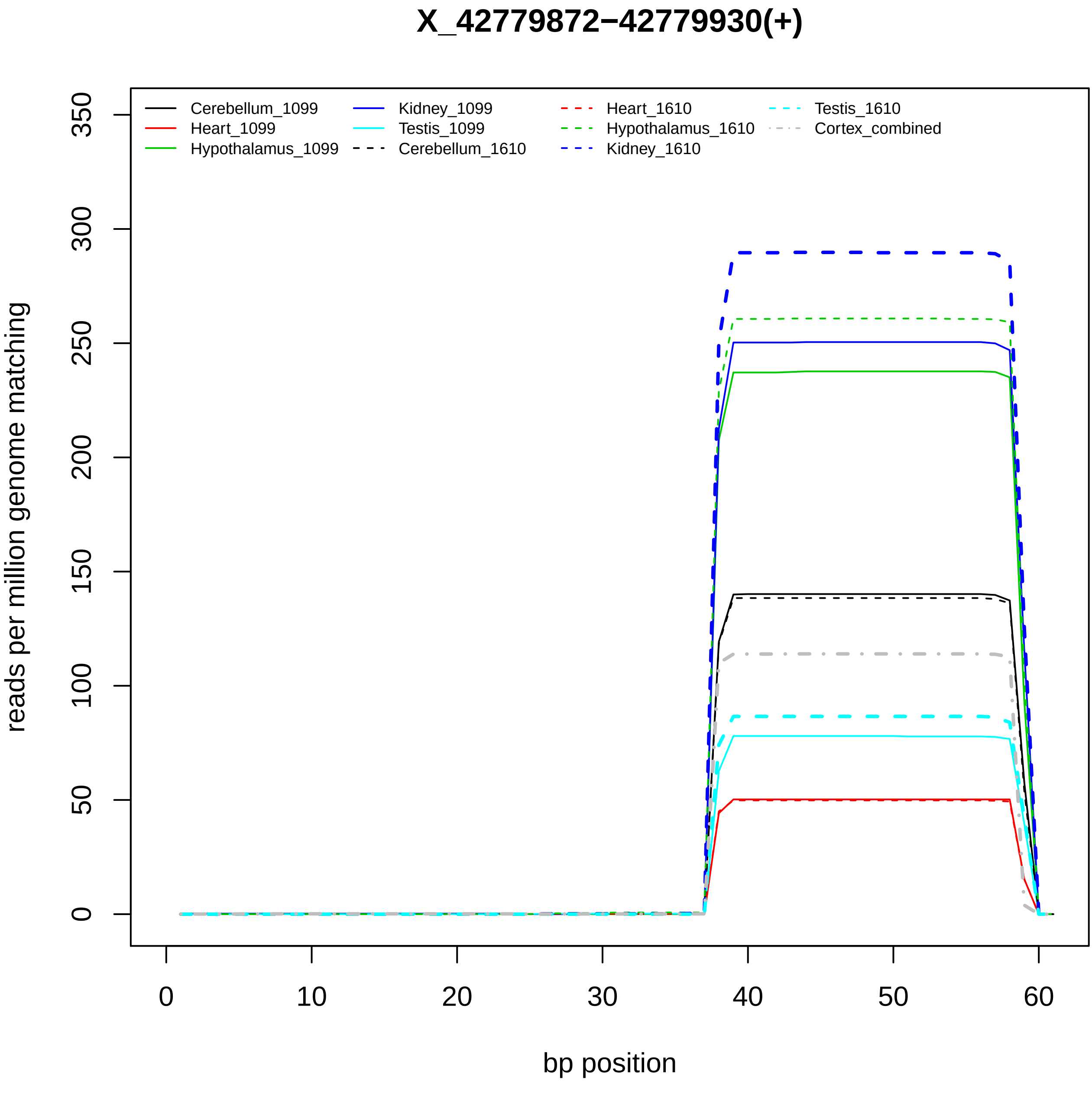

X_42779872−42779930(+)
Cerebellum_1099
Heart_1099
Hypothalamus_1099
Kidney_1099
Testis_1099
Cerebellum_1610
Heart_1610
Hypothalamus_1610
Kidney_1610
Testis_1610
Cortex_combined
reads per million genome matching
bp position
0
50
100
150
200
250
300
350
0
10
20
30
40
50
60

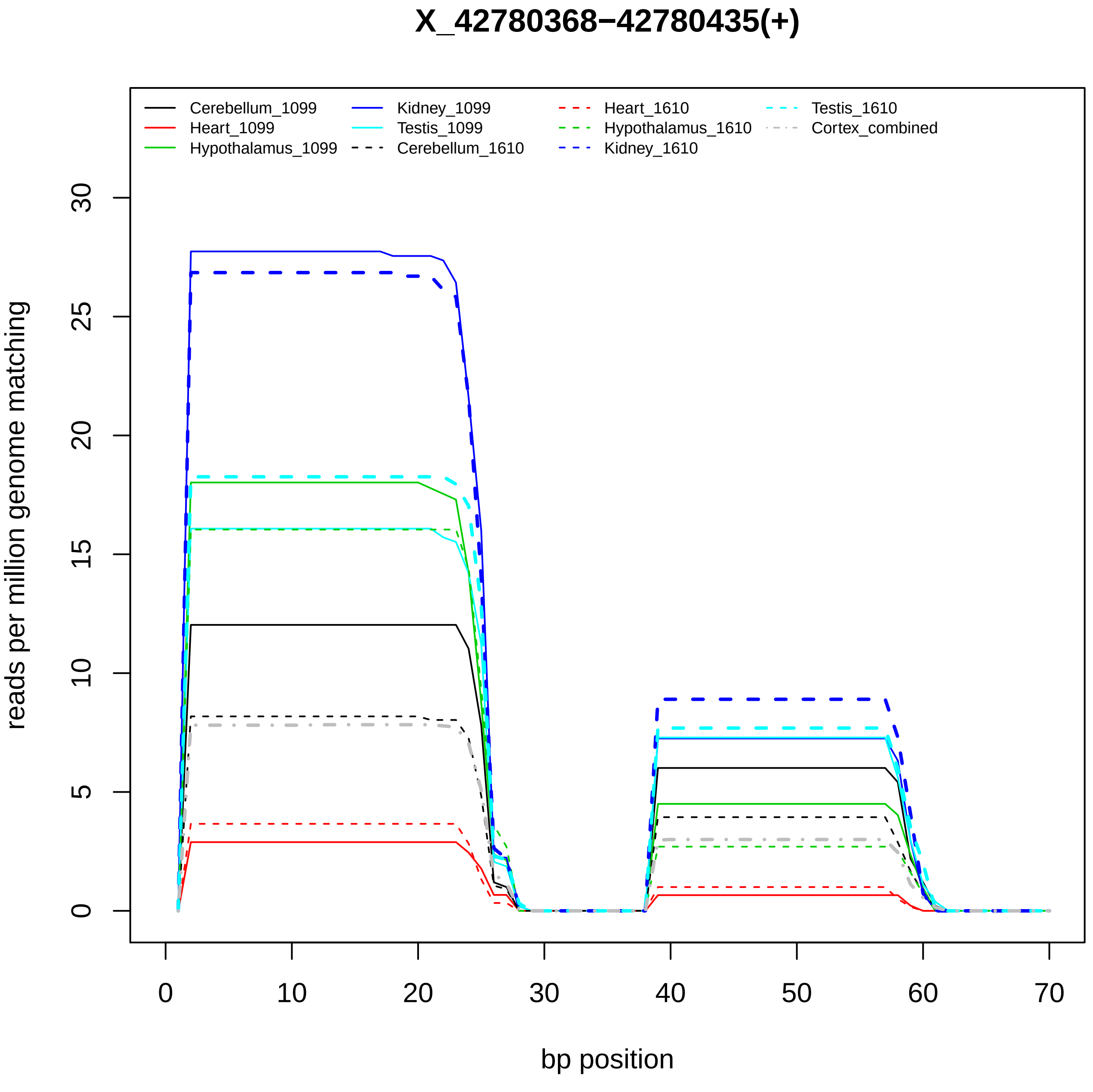
X_42780368−42780435(+)
Cerebellum_1099
Heart_1099
Hypothalamus_1099
Kidney_1099
Testis_1099
Cerebellum_1610
Heart_1610
Hypothalamus_1610
Kidney_1610
Testis_1610
Cortex_combined
reads per million genome matching
bp position
0
5
10
15
20
25
30
40
50
60
70

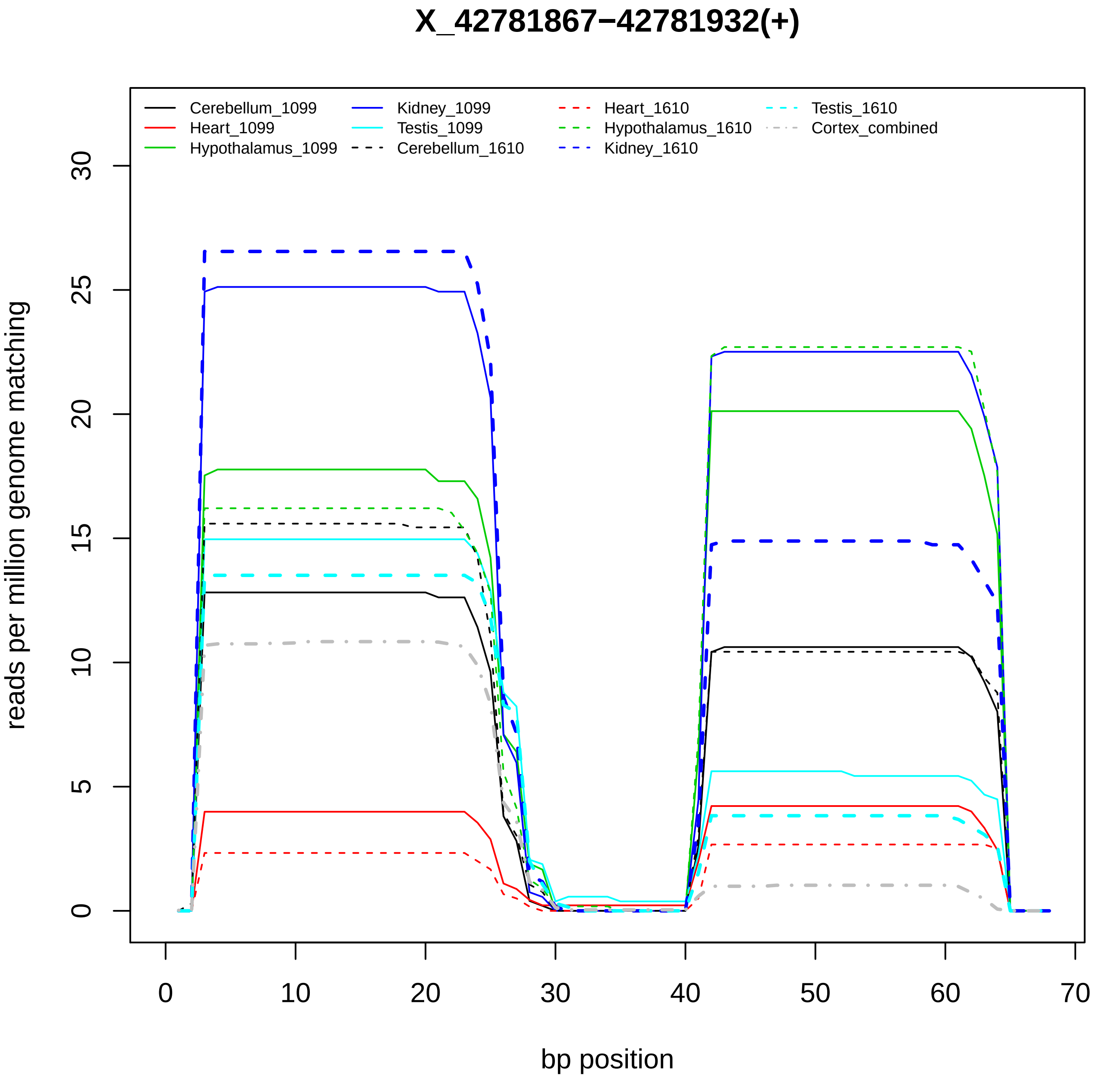

X_42781867−42781932(+)
Cerebellum_1099
Heart_1099
Hypothalamus_1099
Kidney_1099
Testis_1099
Cerebellum_1610
Heart_1610
Hypothalamus_1610
Kidney_1610
Testis_1610
Cortex_combined
reads per million genome matching
0
5
10
15
20
25
30
0
10
20
30
40
50
60
70
bp position

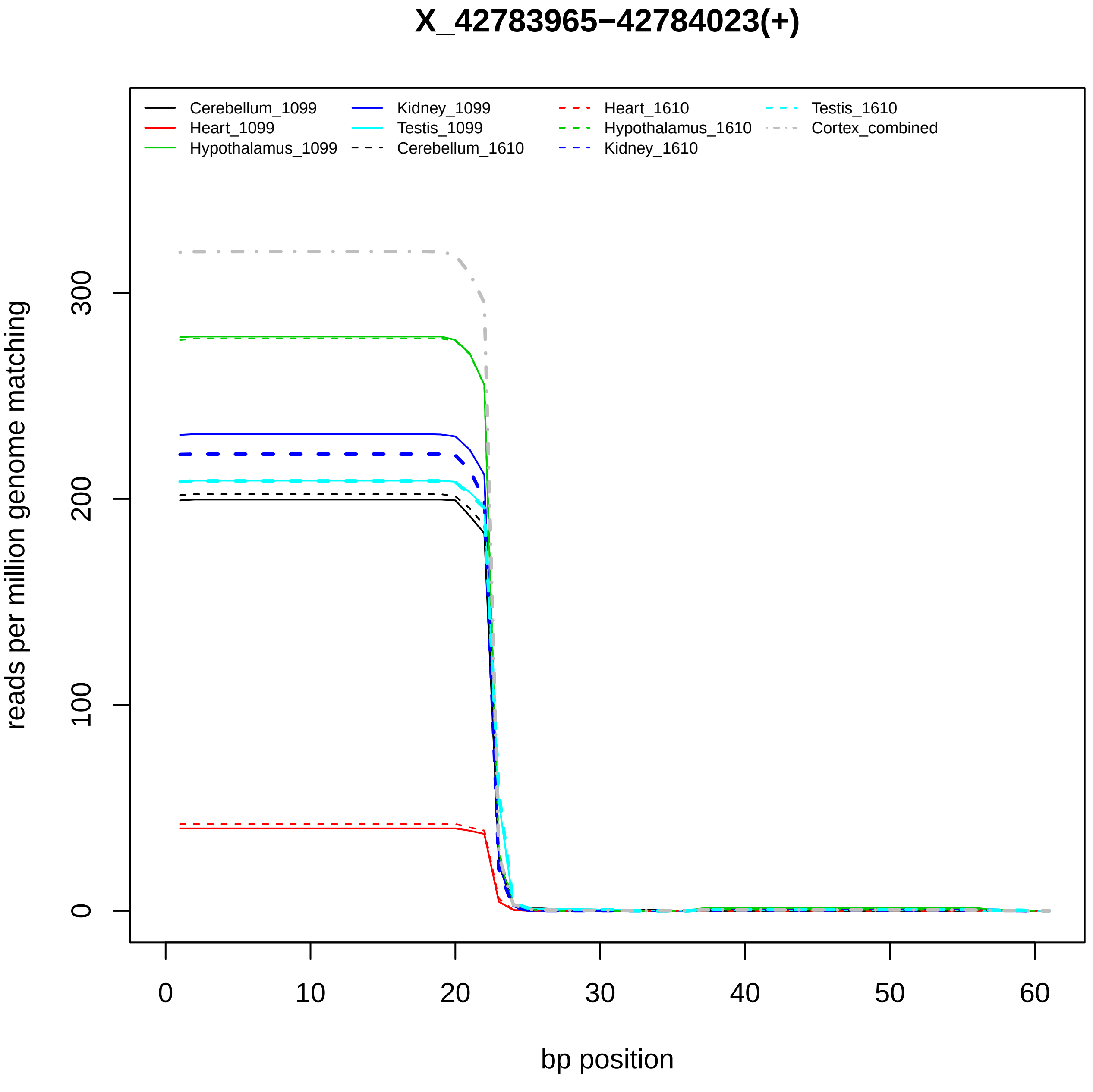

X_42783965−42784023(+)
Cerebellum_1099
Heart_1099
Hypothalamus_1099
Kidney_1099
Testis_1099
Cerebellum_1610
Heart_1610
Hypothalamus_1610
Kidney_1610
Testis_1610
Cortex_combined
reads per million genome matching
0
100
200
300
0
10
20
30
40
50
60
bp position

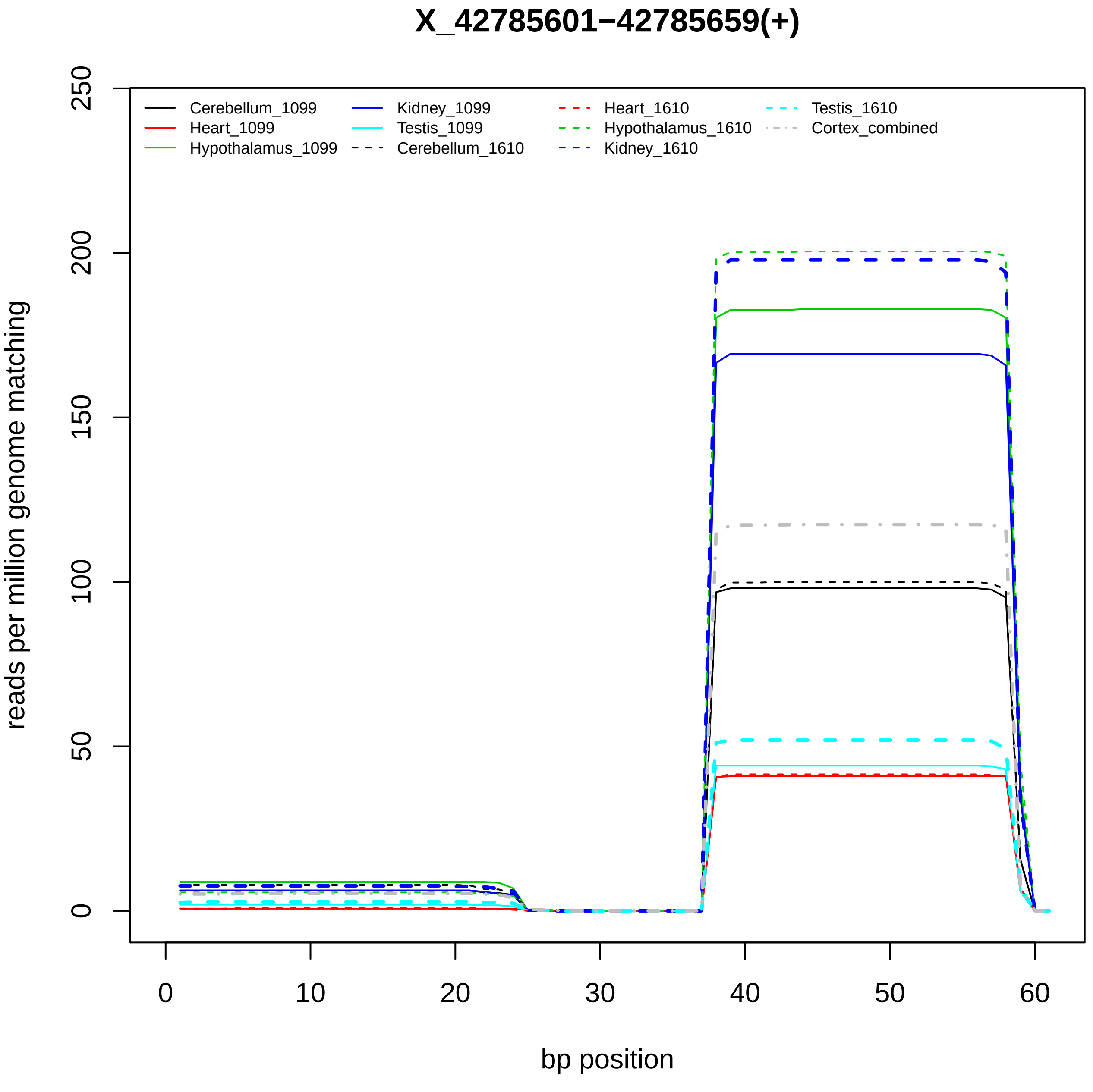
X_42785601−42785659(+)
Cerebellum_1099
Heart_1099
Hypothalamus_1099
Kidney_1099
Testis_1099
Cerebellum_1610
Heart_1610
Hypothalamus_1610
Kidney_1610
Testis_1610
Cortex_combined
reads per million genome matching
bp position
0
50
100
150
200
250
0
10
20
30
40
50
60

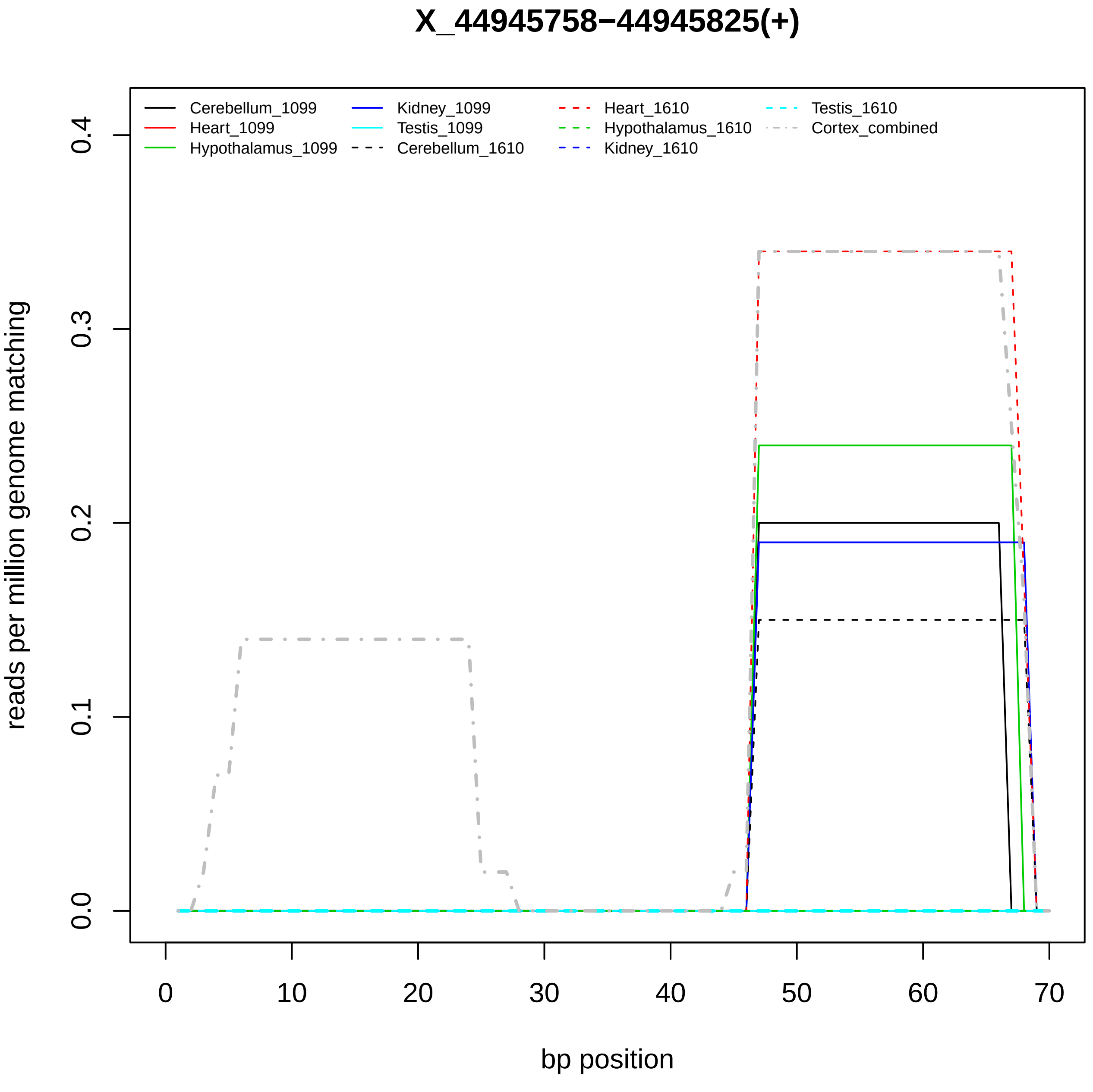

X_44945758−44945825(+)
Cerebellum_1099
Heart_1099
Hypothalamus_1099
Kidney_1099
Testis_1099
Cerebellum_1610
Heart_1610
Hypothalamus_1610
Kidney_1610
Testis_1610
Cortex_combined
reads per million genome matching
0.0
0.1
0.2
0.3
0.4
0
10
20
30
40
50
60
70
bp position

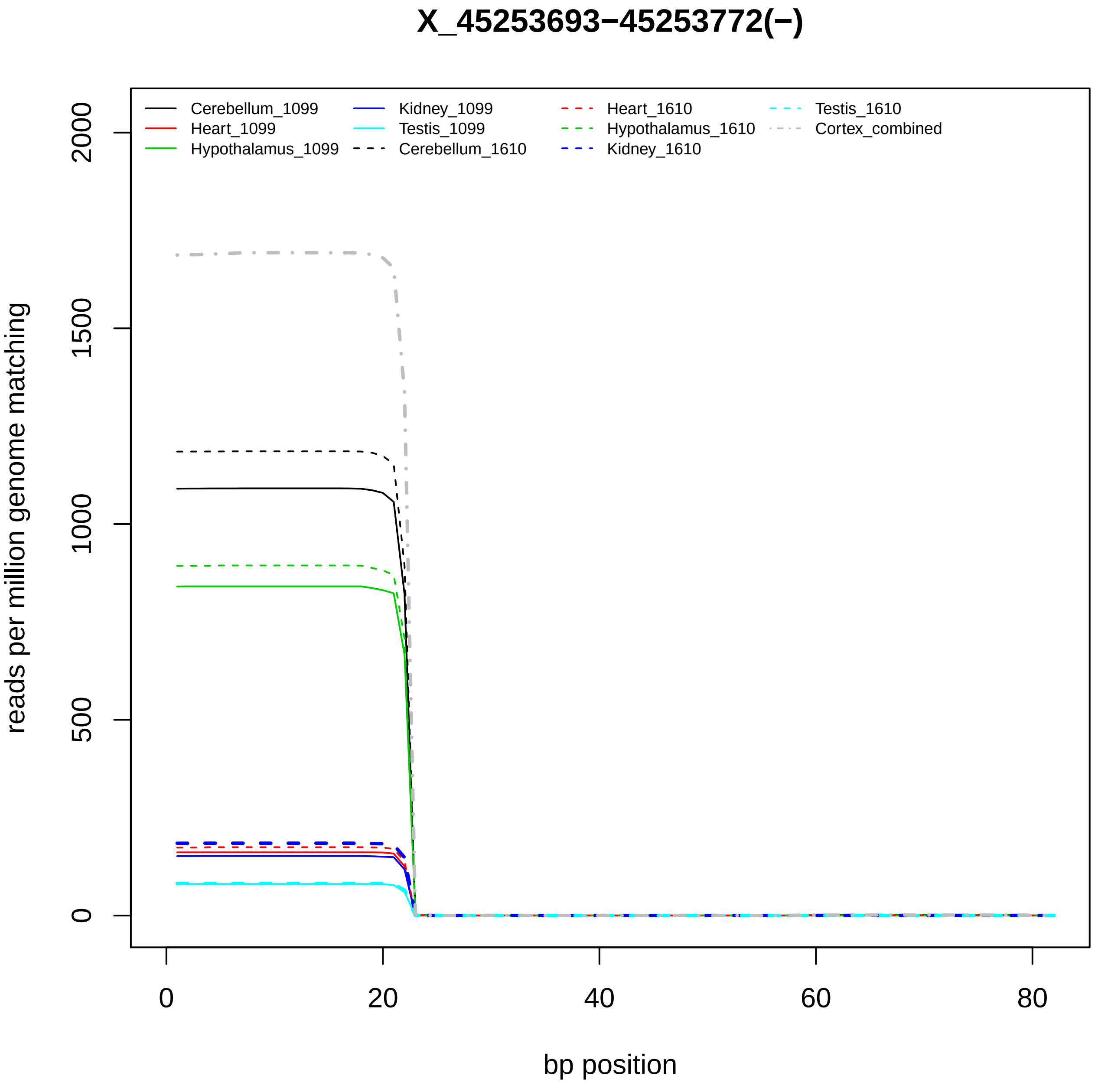

X_45253693−45253772(−)
Cerebellum_1099
Heart_1099
Hypothalamus_1099
Kidney_1099
Testis_1099
Cerebellum_1610
Heart_1610
Hypothalamus_1610
Kidney_1610
Testis_1610
Cortex_combined
reads per million genome matching
bp position
0
500
1000
1500
2000
0
20
40
60
80

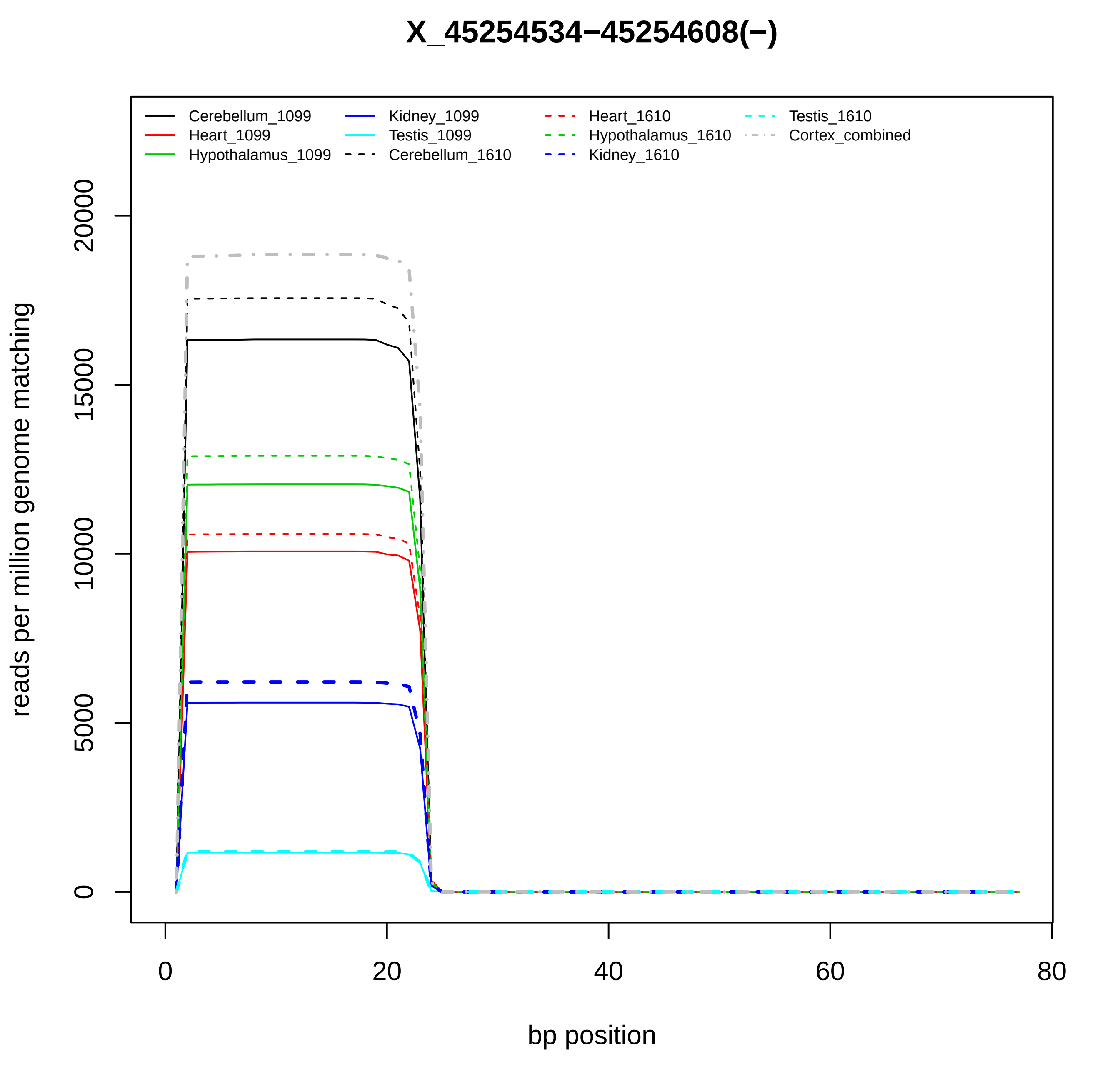

X_45254534−45254608(−)
Cerebellum_1099
Heart_1099
Hypothalamus_1099
Kidney_1099
Testis_1099
Cerebellum_1610
Heart_1610
Hypothalamus_1610
Kidney_1610
Testis_1610
Cortex_combined
reads per million genome matching
0
5000
10000
15000
20000
0
20
40
60
80
bp position

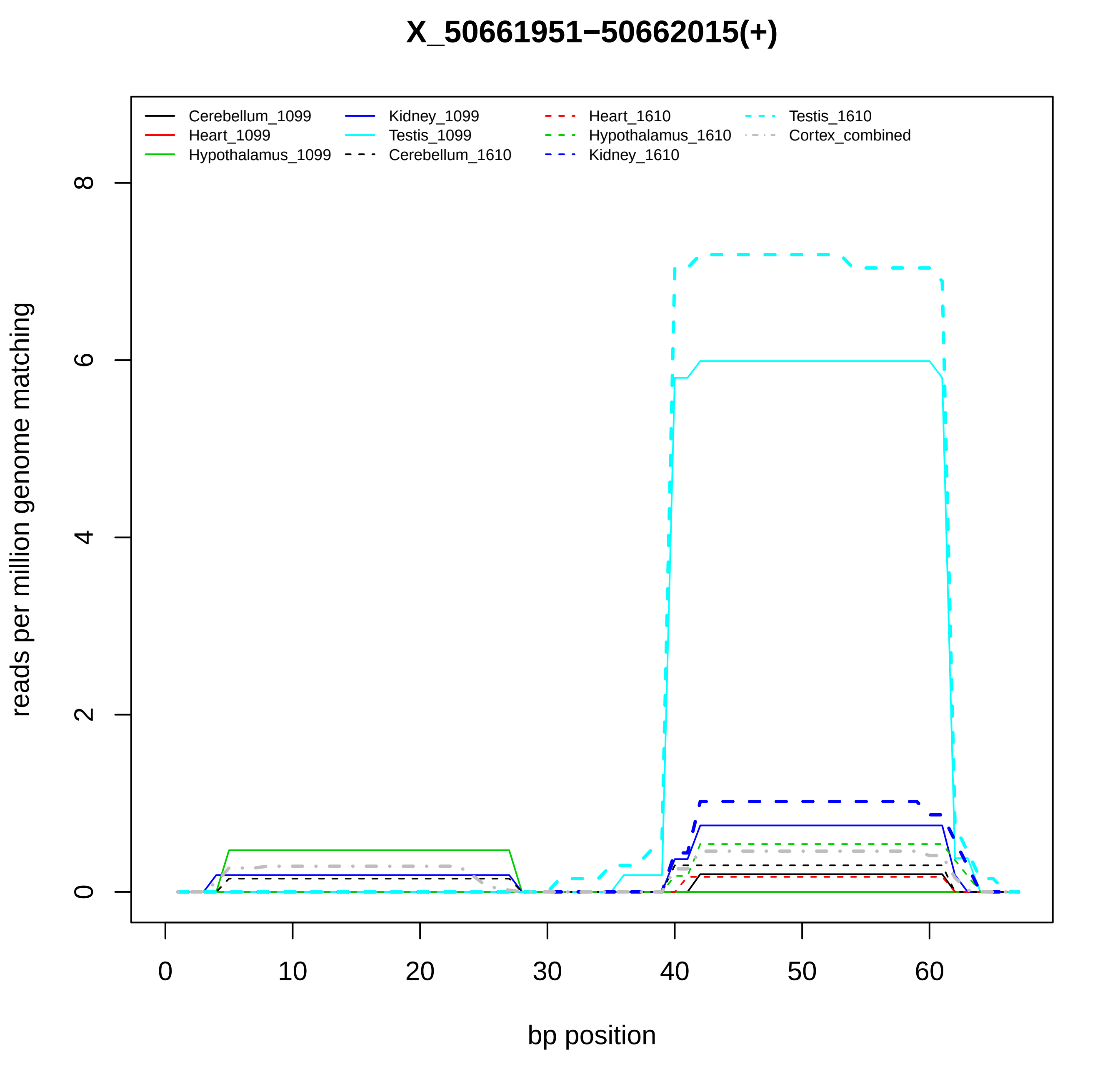

X_50661951−50662015(+)
Cerebellum_1099
Heart_1099
Hypothalamus_1099
Kidney_1099
Testis_1099
Cerebellum_1610
Heart_1610
Hypothalamus_1610
Kidney_1610
Testis_1610
Cortex_combined
reads per million genome matching
bp position
0
2
4
6
8
0
10
20
30
40
50
60

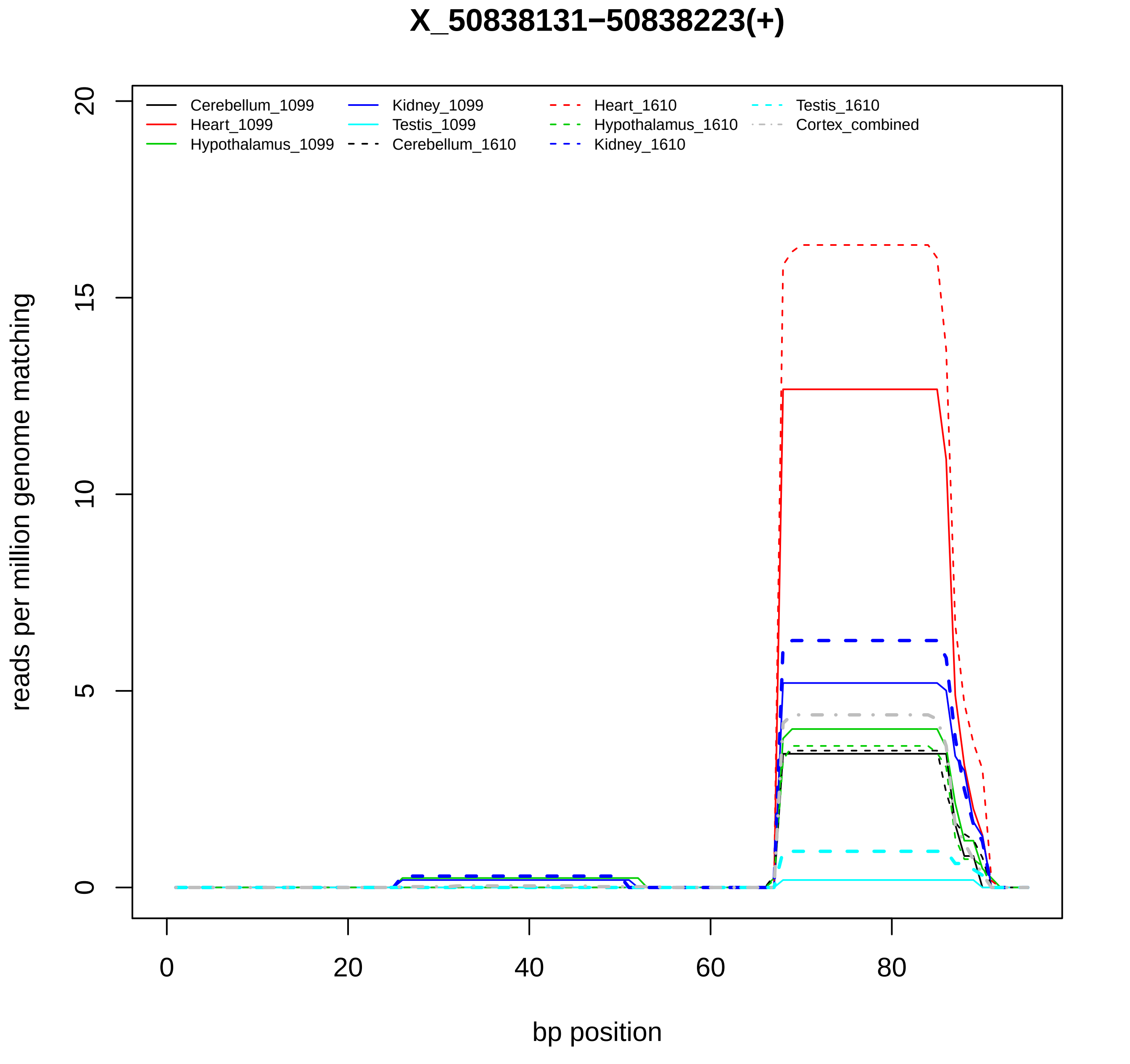

X_50838131−50838223(+)
Cerebellum_1099
Heart_1099
Hypothalamus_1099
Kidney_1099
Testis_1099
Cerebellum_1610
Heart_1610
Hypothalamus_1610
Kidney_1610
Testis_1610
Cortex_combined
reads per million genome matching
bp position
0
5
10
15
20
0
20
40
60
80

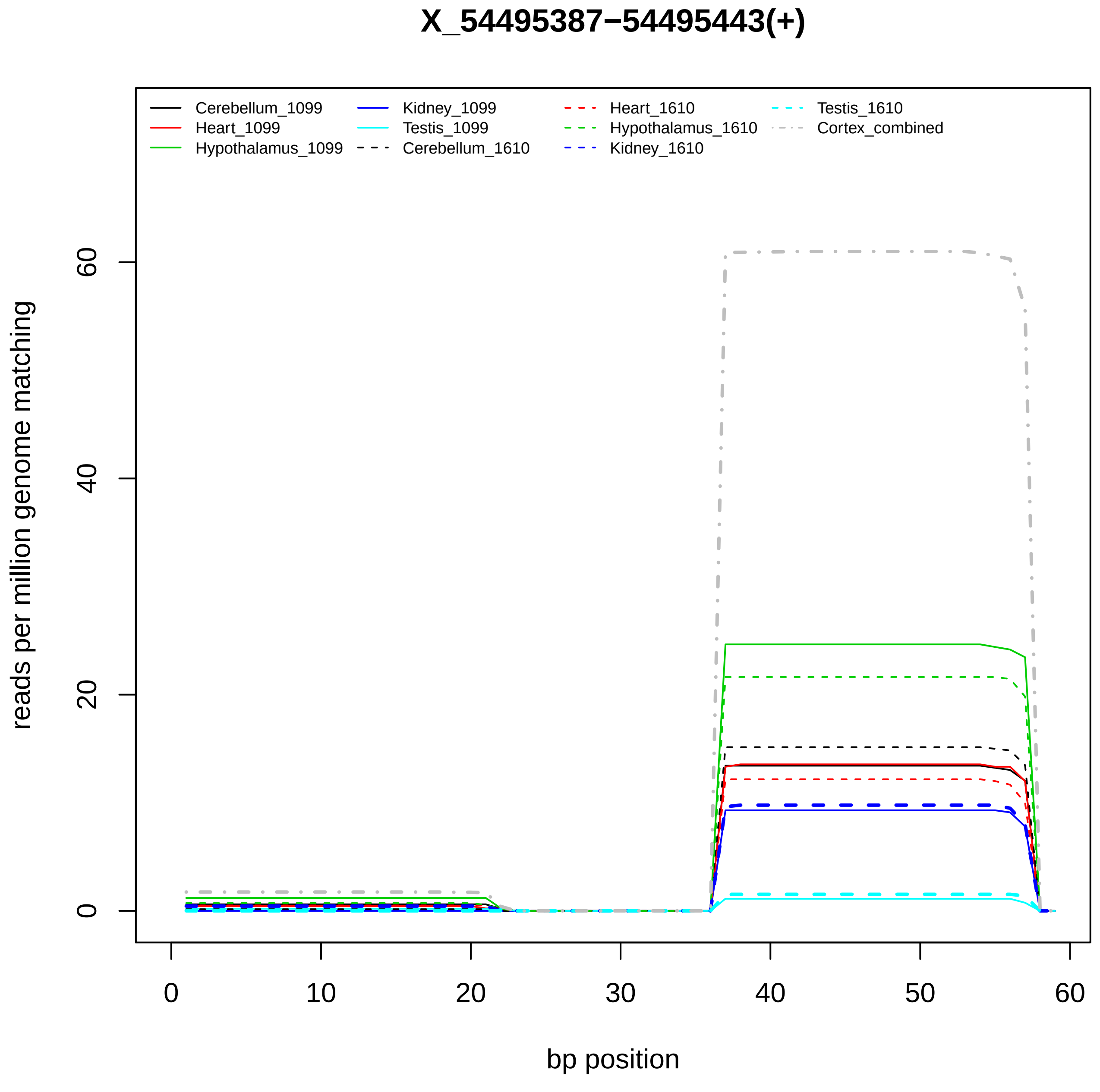

X_54495387−54495443(+)
Cerebellum_1099
Heart_1099
Hypothalamus_1099
Kidney_1099
Testis_1099
Cerebellum_1610
Heart_1610
Hypothalamus_1610
Kidney_1610
Testis_1610
Cortex_combined
reads per million genome matching
0
20
40
60
0
10
20
30
40
50
60
bp position

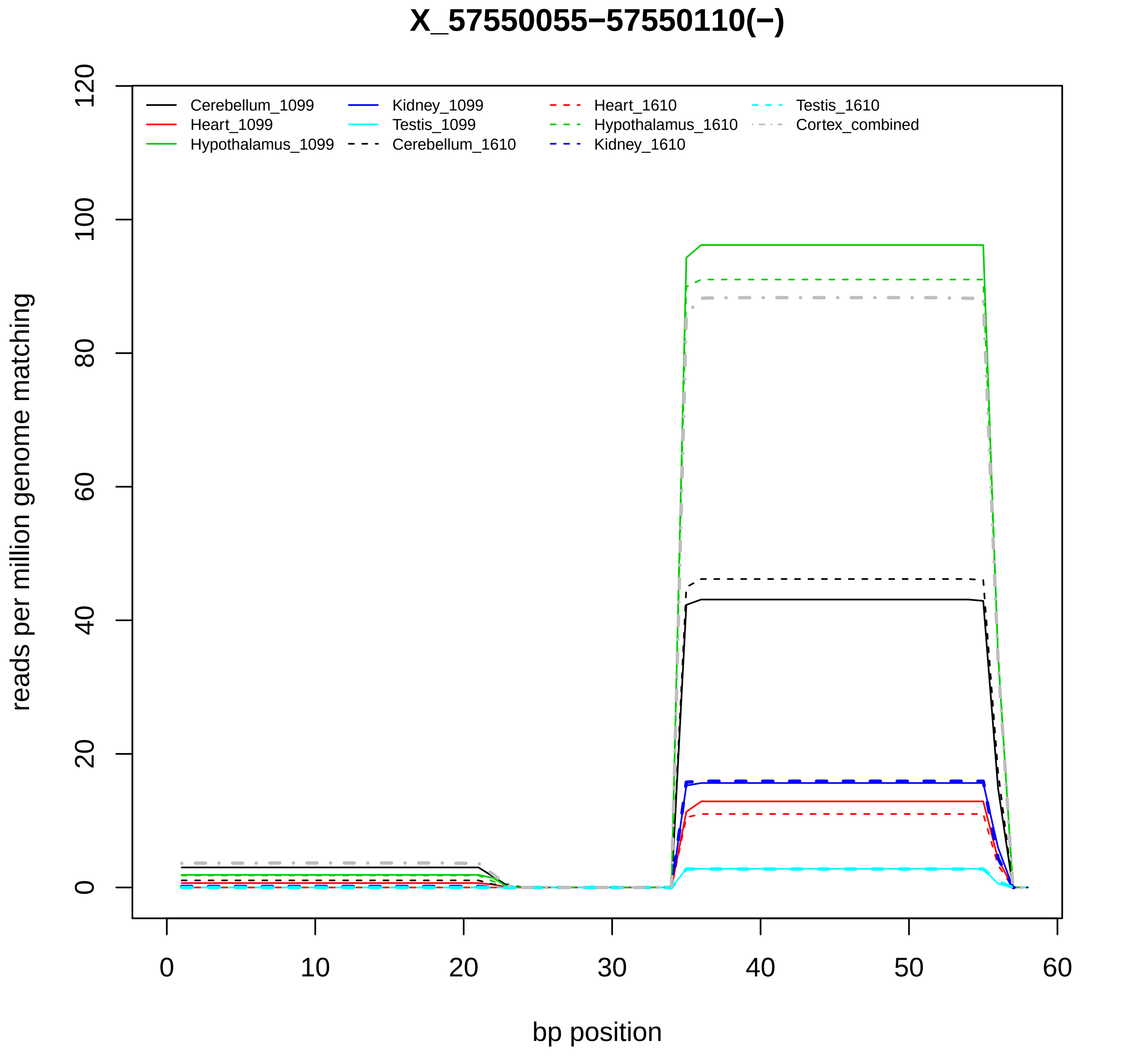

X_57550055−57550110(−)
Cerebellum_1099
Heart_1099
Hypothalamus_1099
Kidney_1099
Testis_1099
Cerebellum_1610
Heart_1610
Hypothalamus_1610
Kidney_1610
Testis_1610
Cortex_combined
reads per million genome matching
bp position
0
20
40
60
80
100
120
0
10
20
30
40
50
60

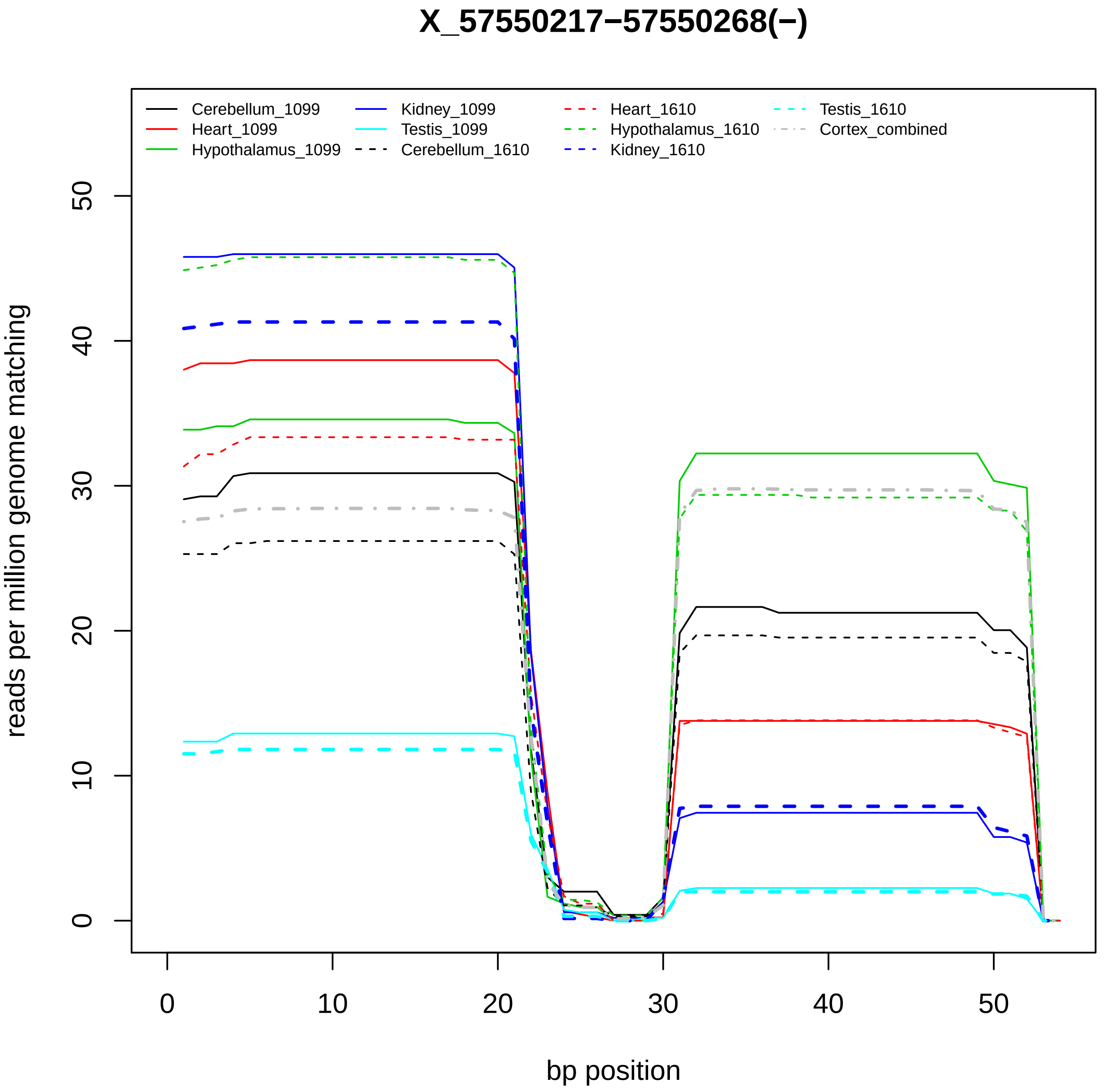

X_57550217−57550268(−)
Cerebellum_1099
Heart_1099
Hypothalamus_1099
Kidney_1099
Testis_1099
Cerebellum_1610
Heart_1610
Hypothalamus_1610
Kidney_1610
Testis_1610
Cortex_combined
reads per million genome matching
0
10
20
30
40
50
bp position

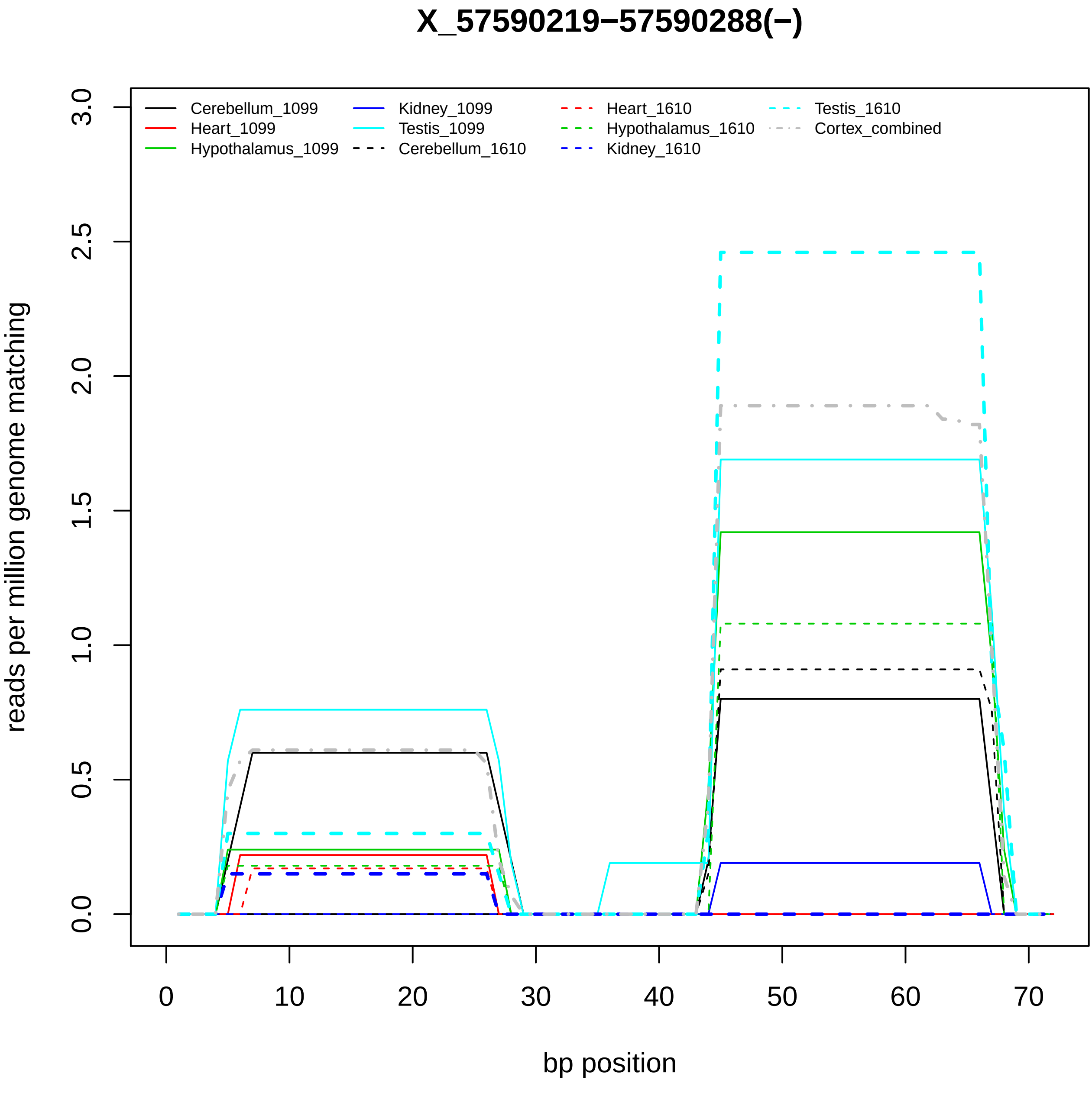

X_57590219−57590288(−)
Cerebellum_1099
Heart_1099
Hypothalamus_1099
Kidney_1099
Testis_1099
Cerebellum_1610
Heart_1610
Hypothalamus_1610
Kidney_1610
Testis_1610
Cortex_combined
reads per million genome matching
bp position
0.0
0.5
1.0
1.5
2.0
2.5
3.0
0
10
20
30
40
50
60
70

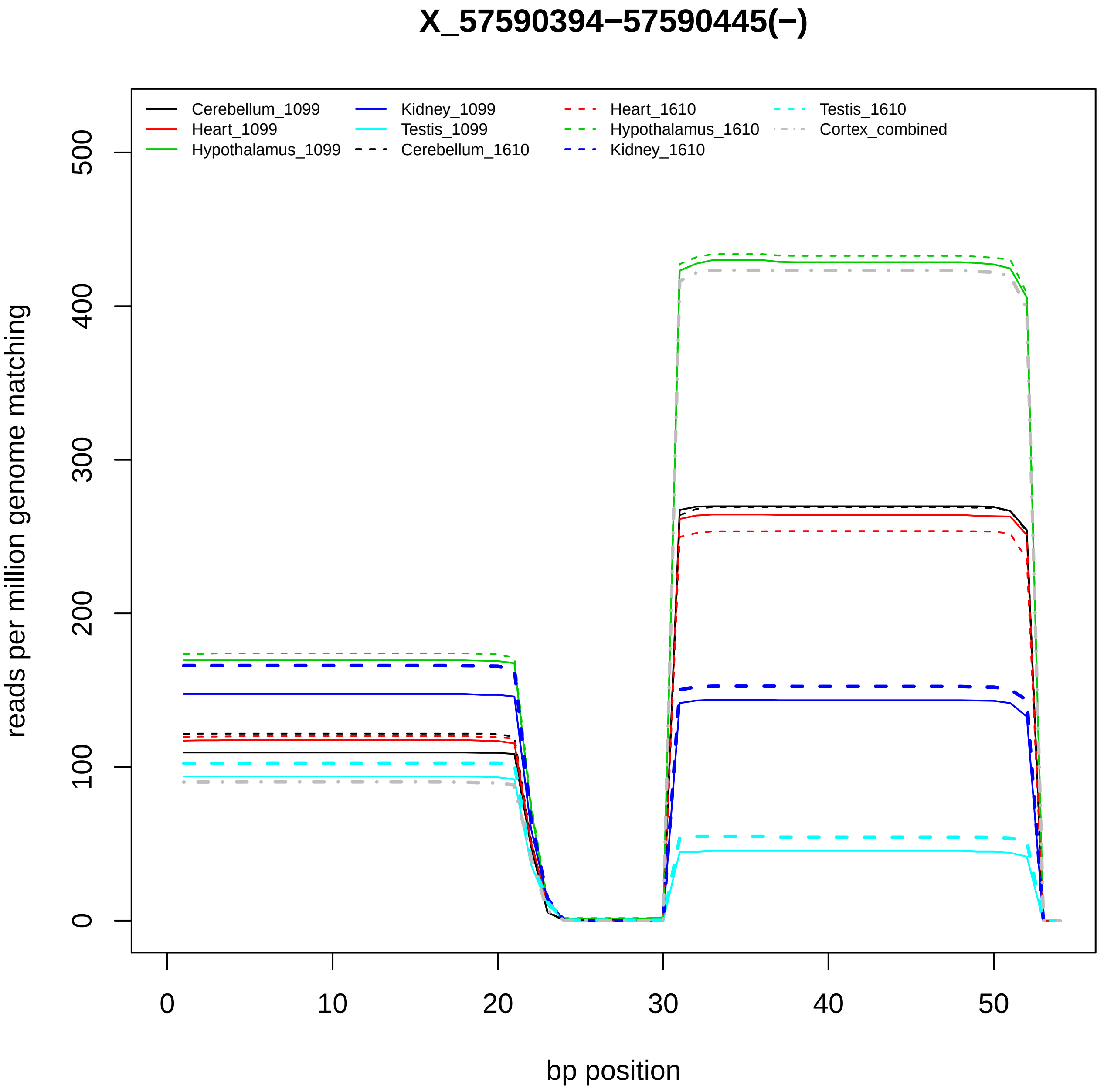
X_57590394−57590445(−)
Cerebellum_1099
Heart_1099
Hypothalamus_1099
Kidney_1099
Testis_1099
Cerebellum_1610
Heart_1610
Hypothalamus_1610
Kidney_1610
Testis_1610
Cortex_combined
reads per million genome matching
bp position
0
100
200
300
400
500
0
10
20
30
40
50

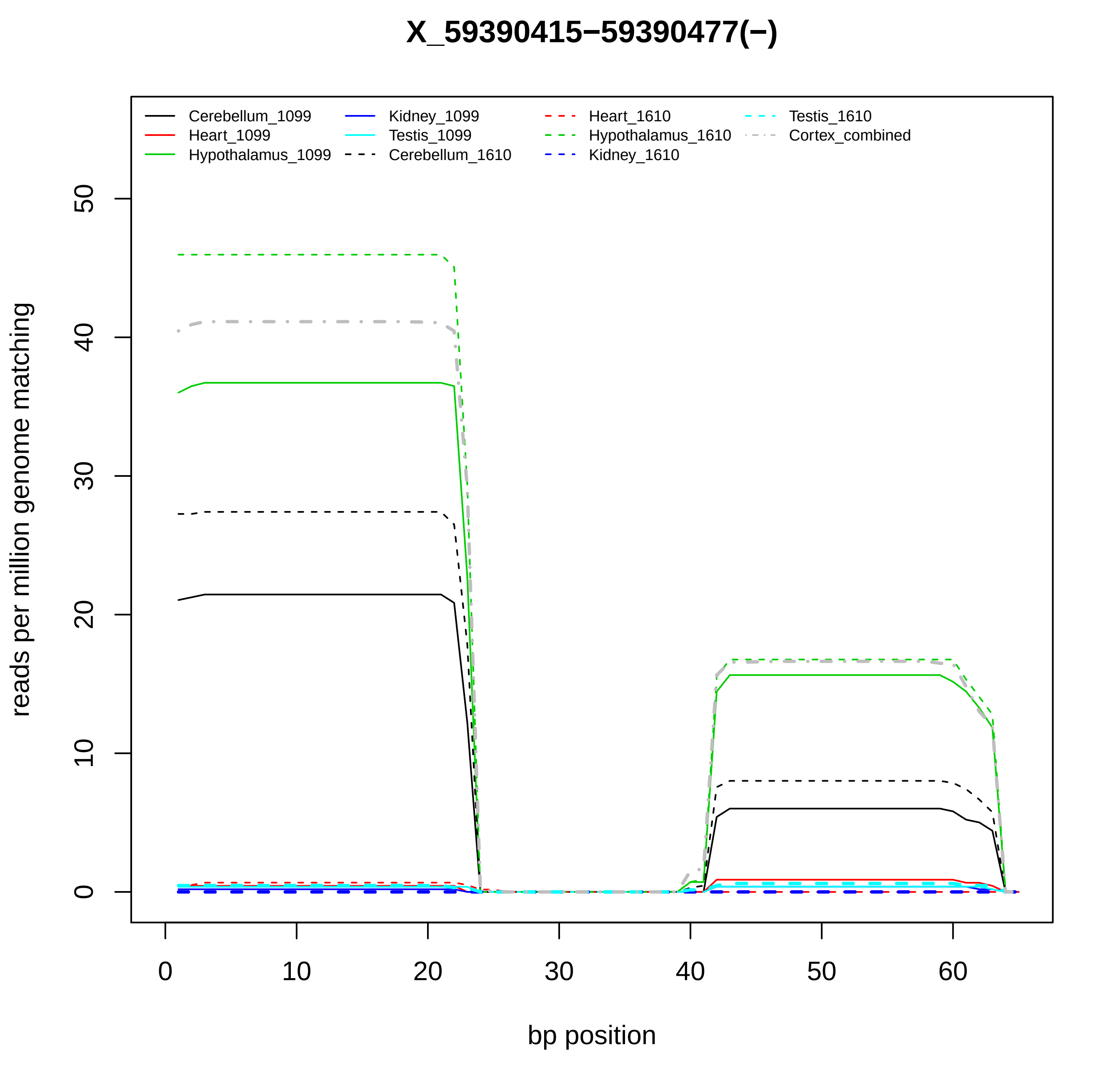

X_59390415−59390477(−)
Cerebellum_1099
Heart_1099
Hypothalamus_1099
Kidney_1099
Testis_1099
Cerebellum_1610
Heart_1610
Hypothalamus_1610
Kidney_1610
Testis_1610
Cortex_combined
reads per million genome matching
bp position
0
10
20
30
40
50
60

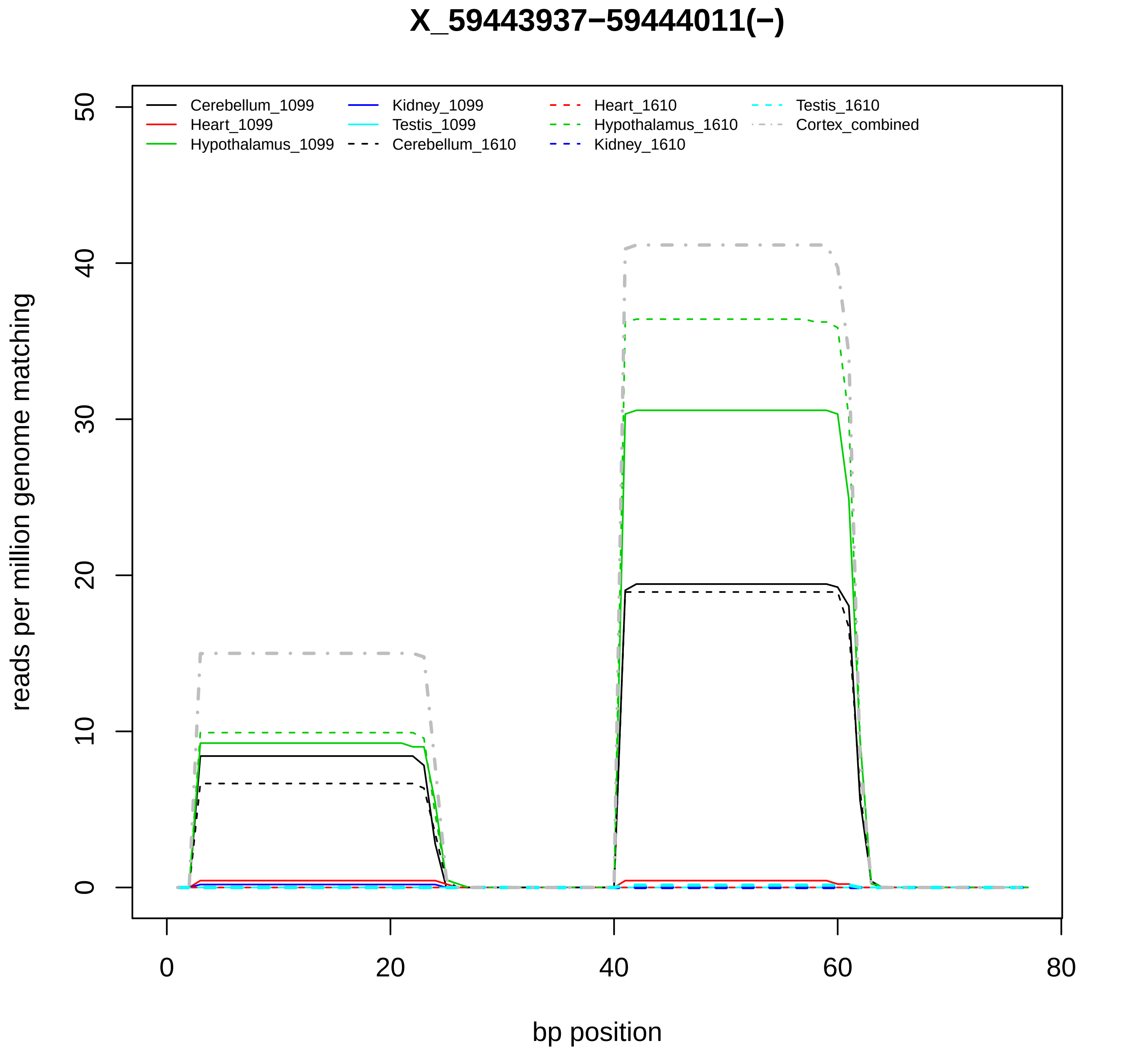
X_59443937−59444011(−)
Cerebellum_1099
Heart_1099
Hypothalamus_1099
Kidney_1099
Testis_1099
Cerebellum_1610
Heart_1610
Hypothalamus_1610
Kidney_1610
Testis_1610
Cortex_combined
reads per million genome matching
bp position
0
10
20
30
40
50
0
20
40
60
80

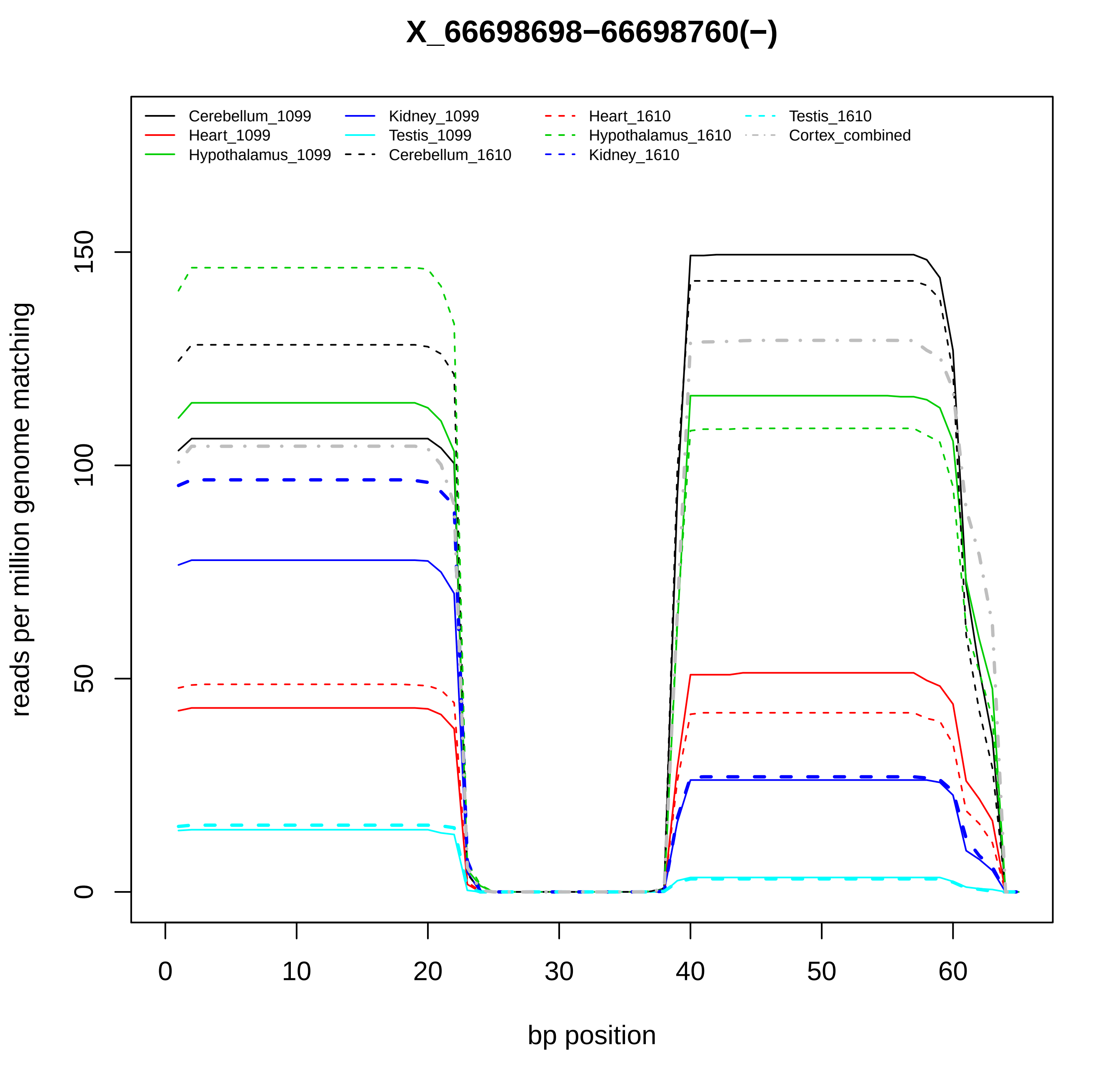
X_66698698−66698760(−)
Cerebellum_1099
Heart_1099
Hypothalamus_1099
Kidney_1099
Testis_1099
Cerebellum_1610
Heart_1610
Hypothalamus_1610
Kidney_1610
Testis_1610
Cortex_combined
reads per million genome matching
0
50
100
150
0
10
20
30
40
50
60
bp position

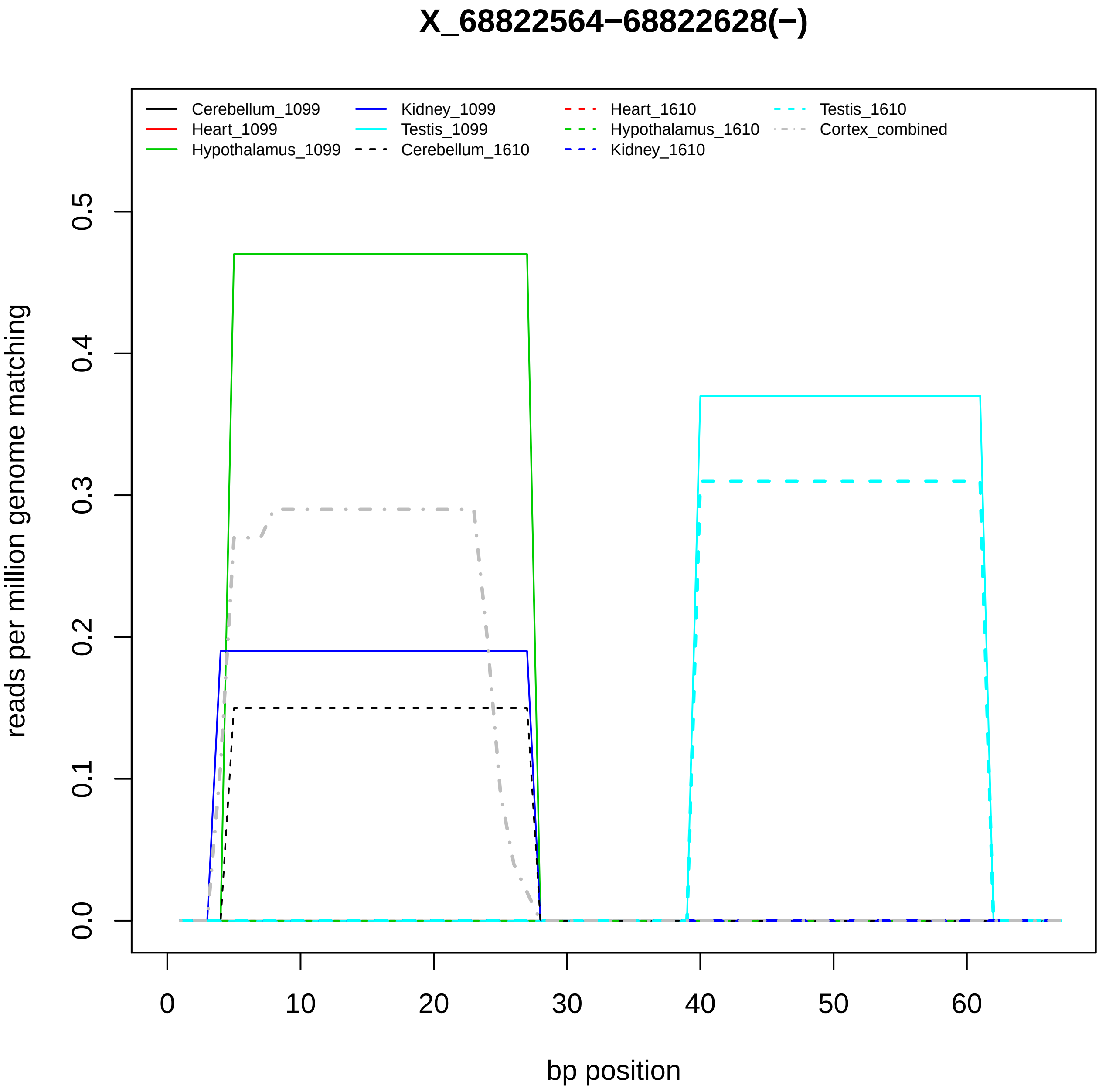
X_68822564−68822628(−)
Cerebellum_1099
Heart_1099
Hypothalamus_1099
Kidney_1099
Testis_1099
Cerebellum_1610
Heart_1610
Hypothalamus_1610
Kidney_1610
Testis_1610
Cortex_combined
reads per million genome matching
0.0
0.1
0.2
0.3
0.4
0.5
0
10
20
30
40
50
60
bp position

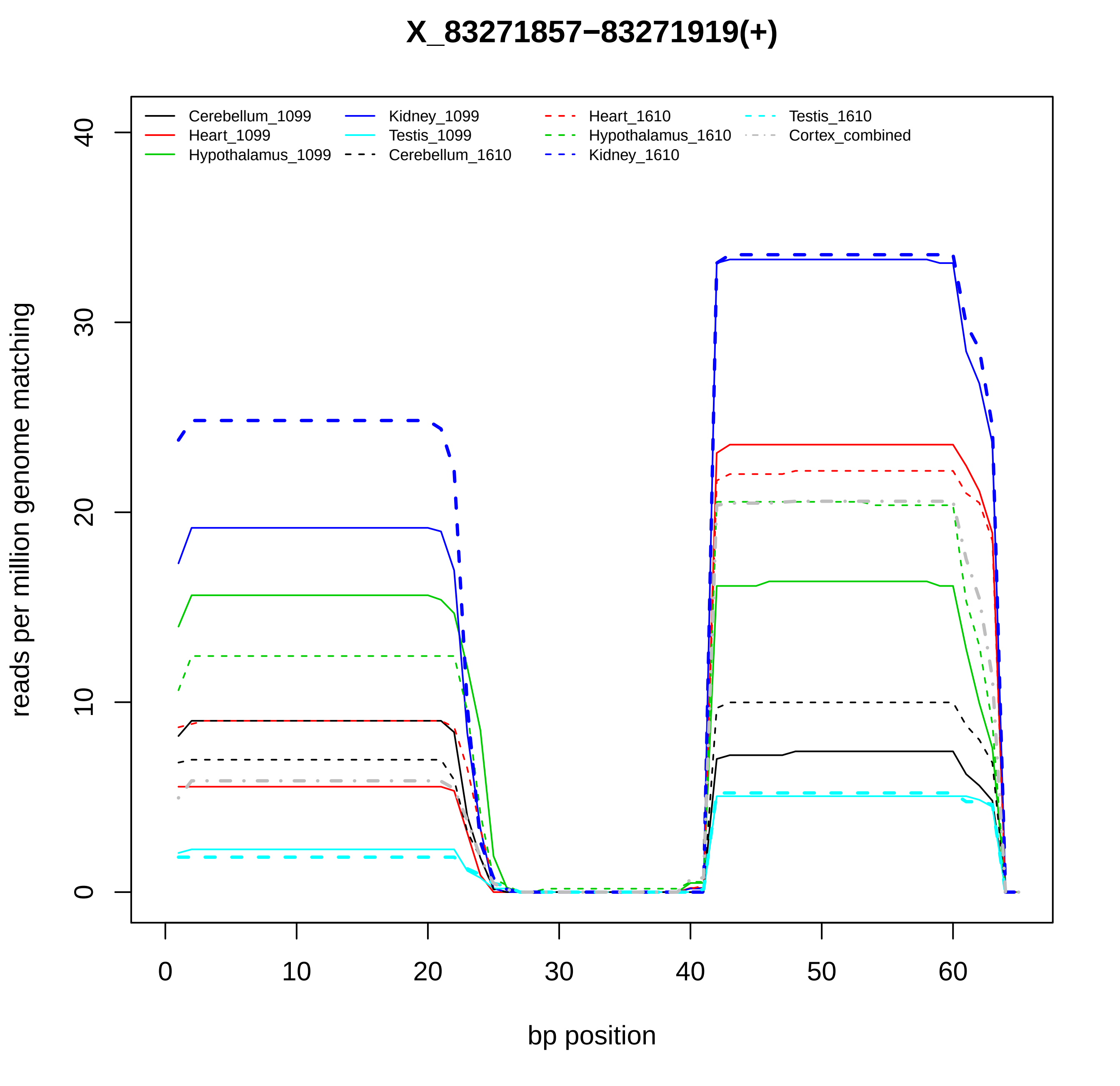
X_83271857−83271919(+)
Cerebellum_1099
Heart_1099
Hypothalamus_1099
Kidney_1099
Testis_1099
Cerebellum_1610
Heart_1610
Hypothalamus_1610
Kidney_1610
Testis_1610
Cortex_combined
reads per million genome matching
bp position
0
10
20
30
40
50
60

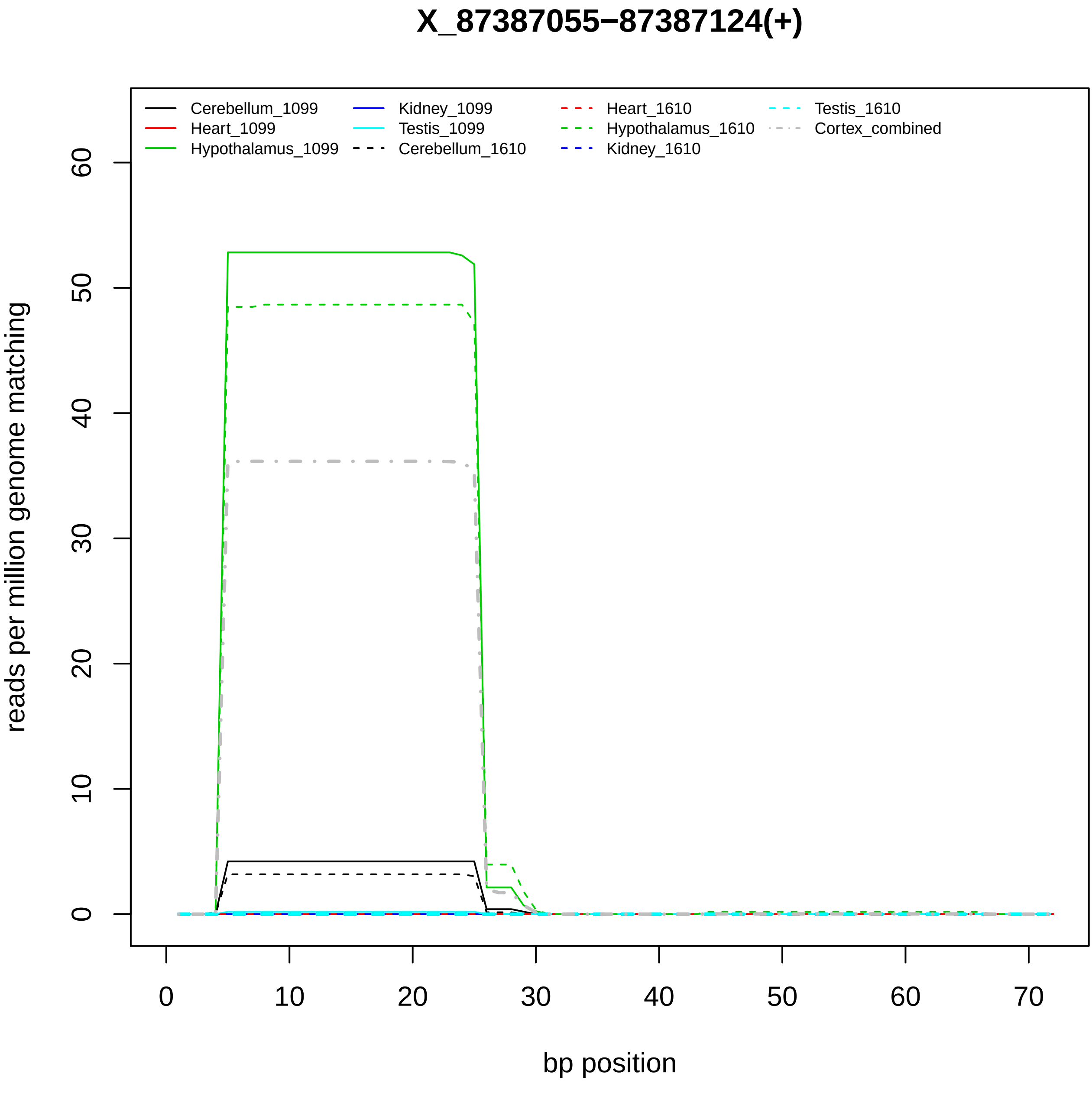

X_87387055−87387124(+)
Cerebellum_1099
Heart_1099
Hypothalamus_1099
Kidney_1099
Testis_1099
Cerebellum_1610
Heart_1610
Hypothalamus_1610
Kidney_1610
Testis_1610
Cortex_combined
reads per million genome matching
bp position
0
10
20
30
40
50
60
70

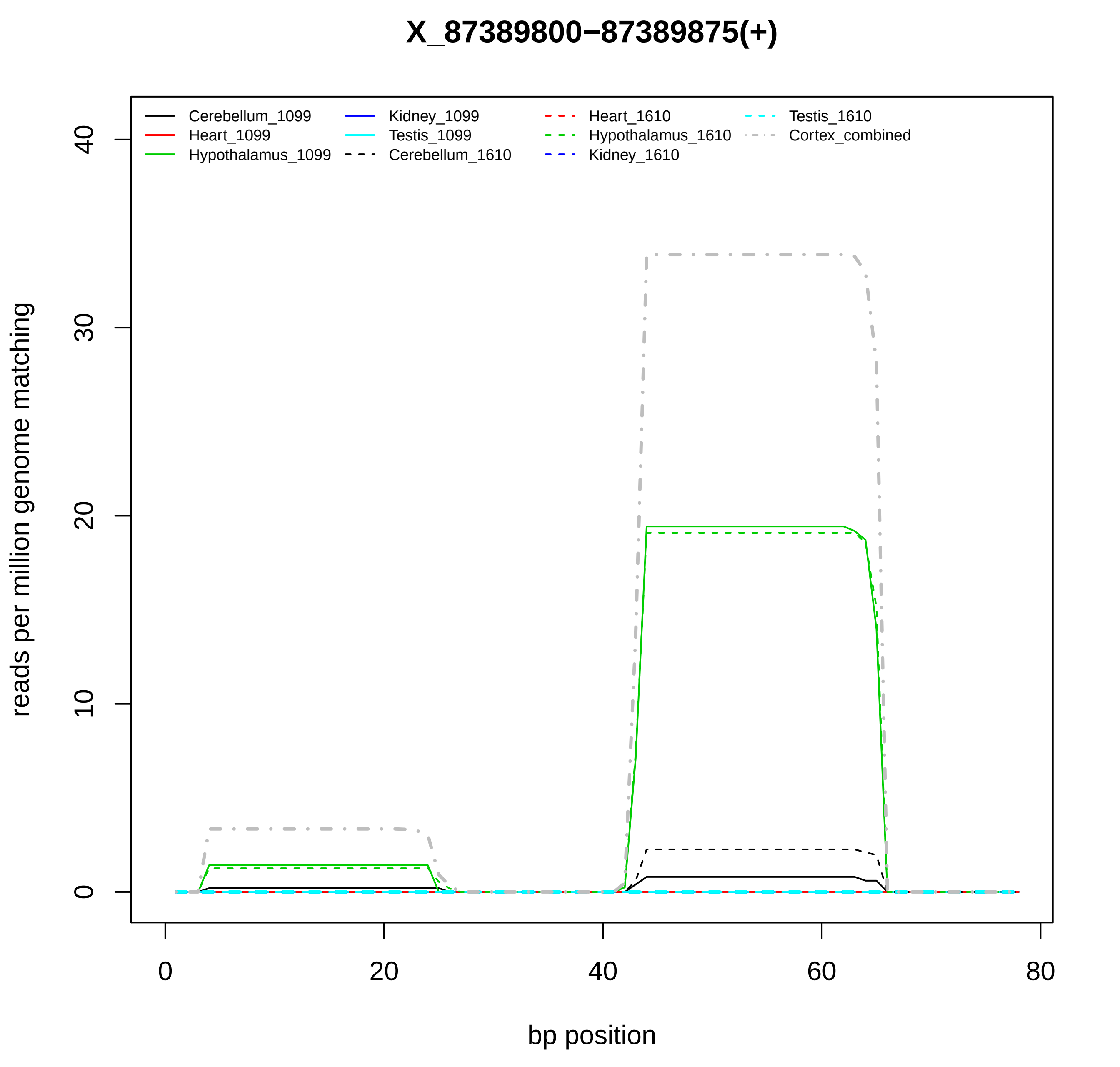

X_87389800−87389875(+)
Cerebellum_1099
Heart_1099
Hypothalamus_1099
Kidney_1099
Testis_1099
Cerebellum_1610
Heart_1610
Hypothalamus_1610
Kidney_1610
Testis_1610
Cortex_combined
reads per million genome matching
0
10
20
30
40
0
20
40
60
80
bp position

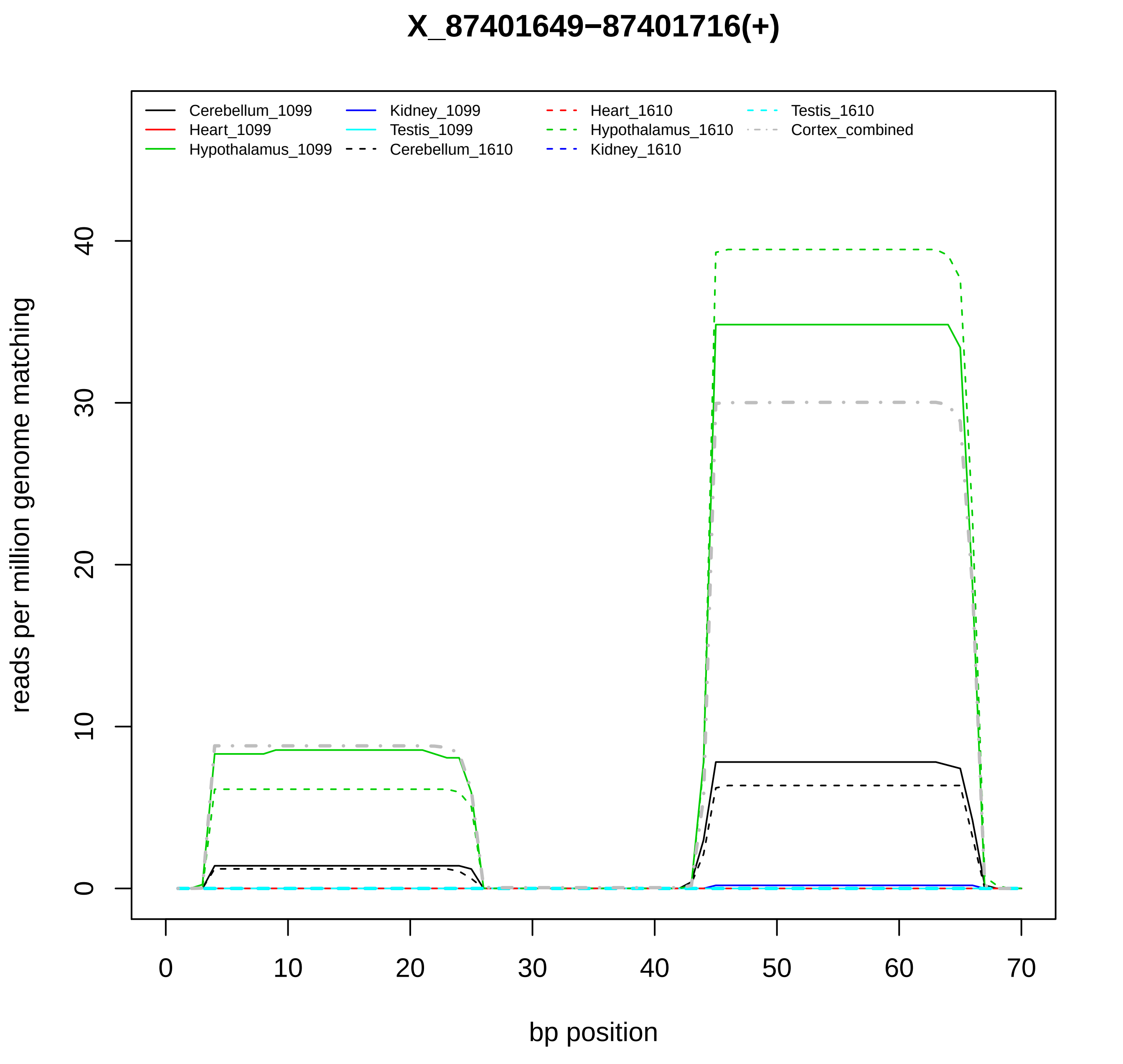
X_87401649−87401716(+)
Cerebellum_1099
Heart_1099
Hypothalamus_1099
Kidney_1099
Testis_1099
Cerebellum_1610
Heart_1610
Hypothalamus_1610
Kidney_1610
Testis_1610
Cortex_combined
reads per million genome matching
0
10
20
30
40
0
10
20
30
40
50
60
70
bp position

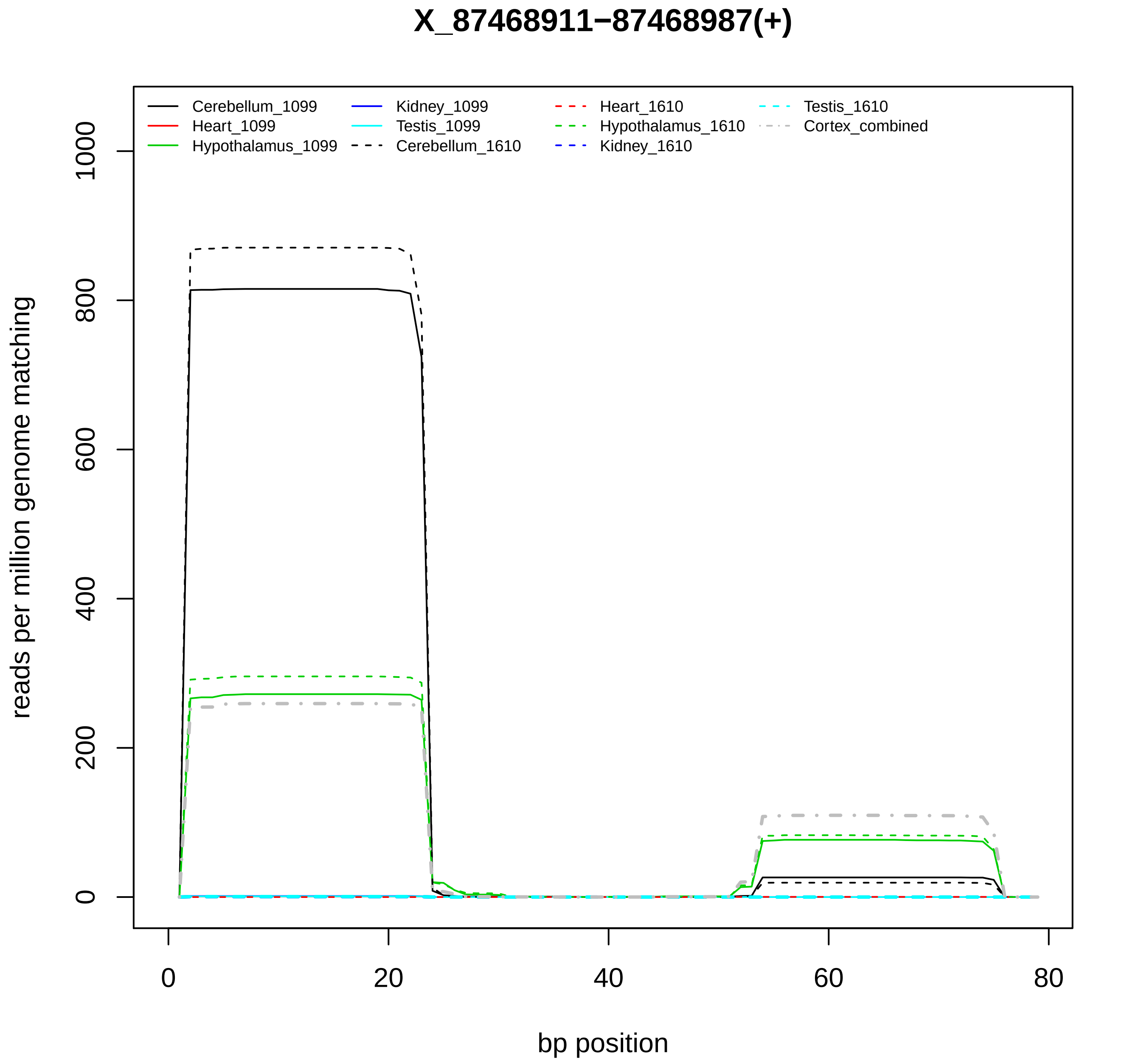
X_87468911−87468987(+)
Cerebellum_1099
Heart_1099
Hypothalamus_1099
Kidney_1099
Testis_1099
Cerebellum_1610
Heart_1610
Hypothalamus_1610
Kidney_1610
Testis_1610
Cortex_combined
reads per million genome matching
0
200
400
600
800
1000
0
20
40
60
80
bp position

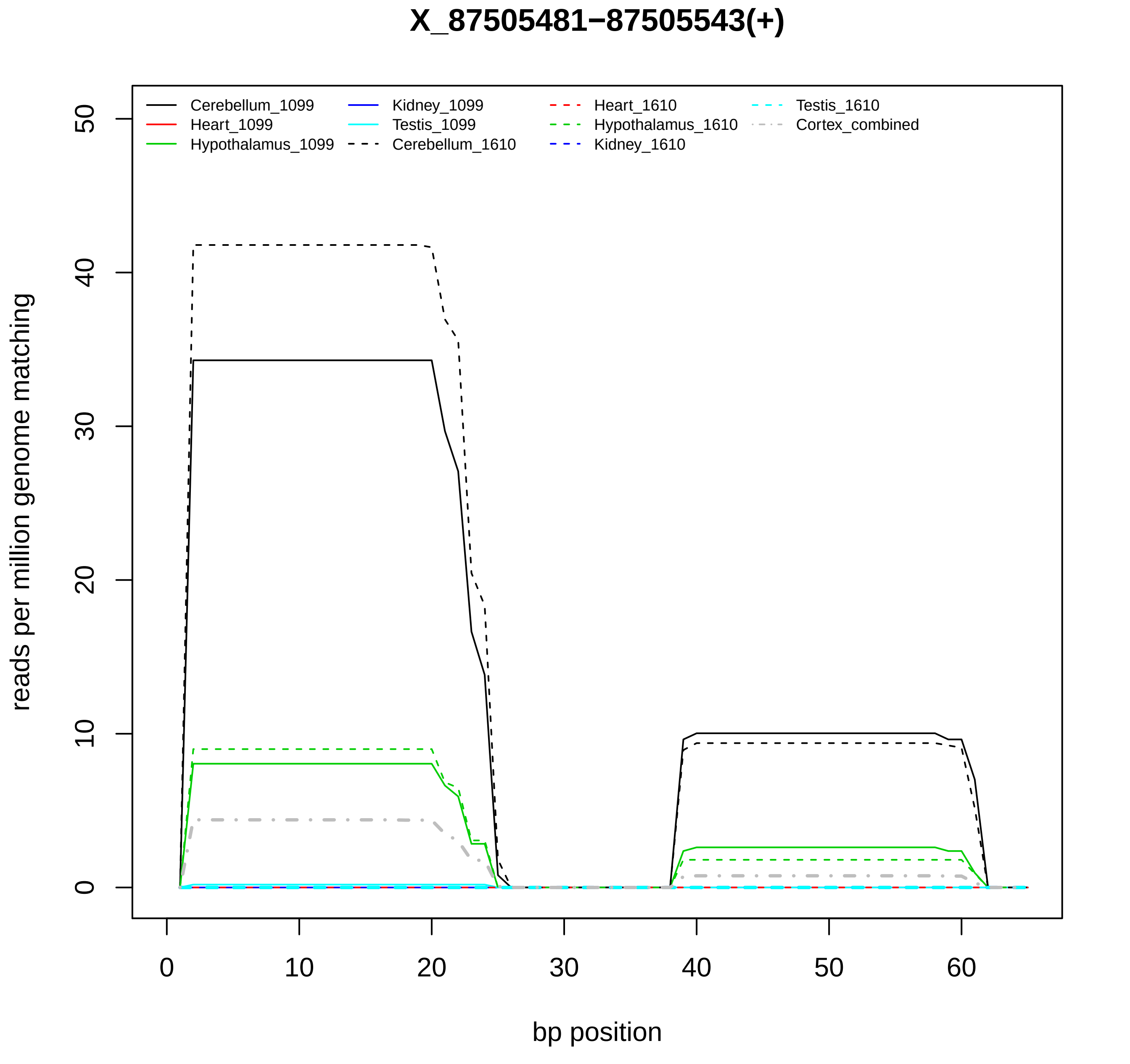

X_87505481−87505543(+)
Cerebellum_1099
Heart_1099
Hypothalamus_1099
Kidney_1099
Testis_1099
Cerebellum_1610
Heart_1610
Hypothalamus_1610
Kidney_1610
Testis_1610
Cortex_combined
reads per million genome matching
bp position
0
10
20
30
40
50
60

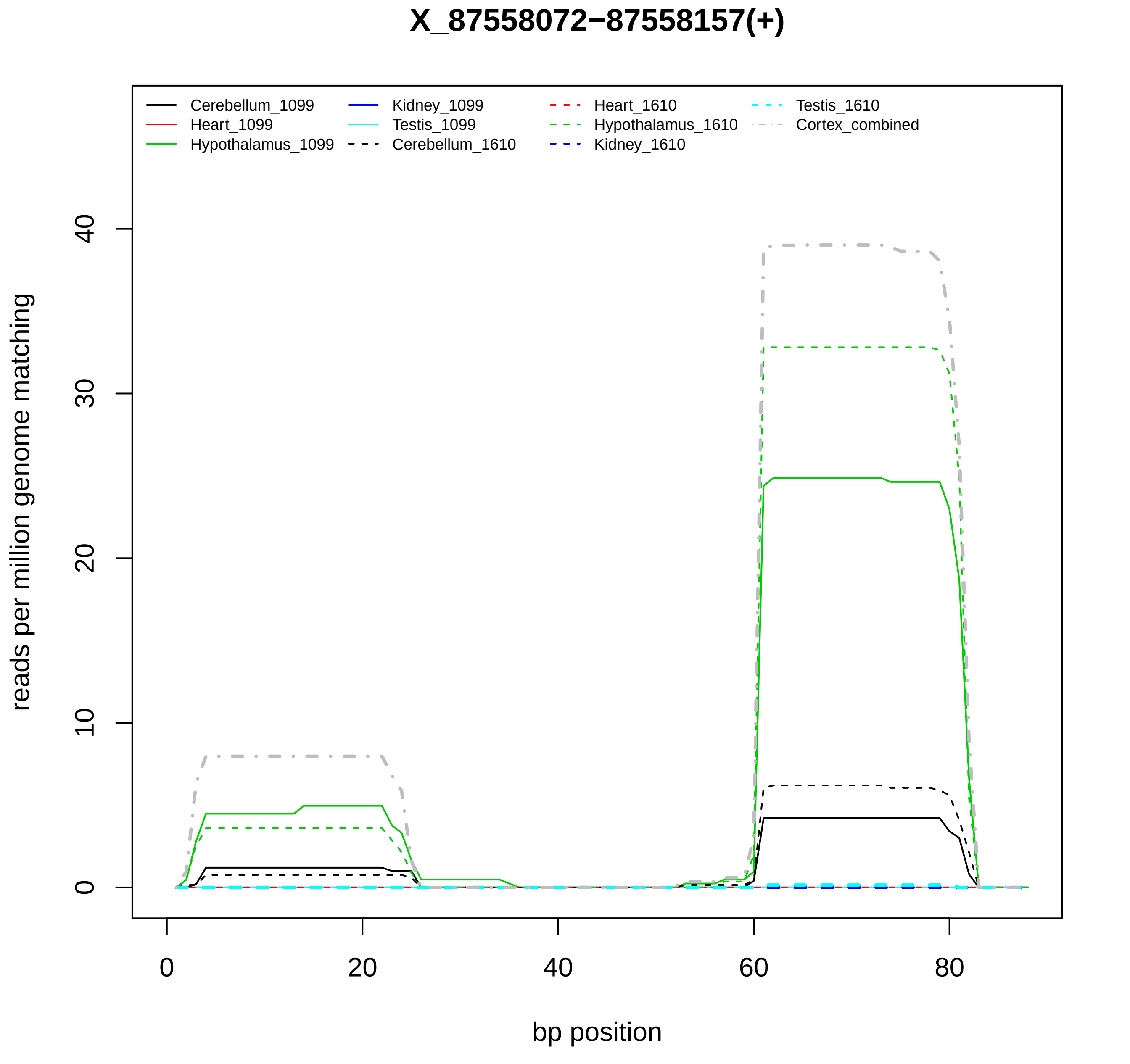
X_87558072−87558157(+)
Cerebellum_1099
Heart_1099
Hypothalamus_1099
Kidney_1099
Testis_1099
Cerebellum_1610
Heart_1610
Hypothalamus_1610
Kidney_1610
Testis_1610
Cortex_combined
reads per million genome matching
bp position
0
10
20
30
40
0
20
40
60
80

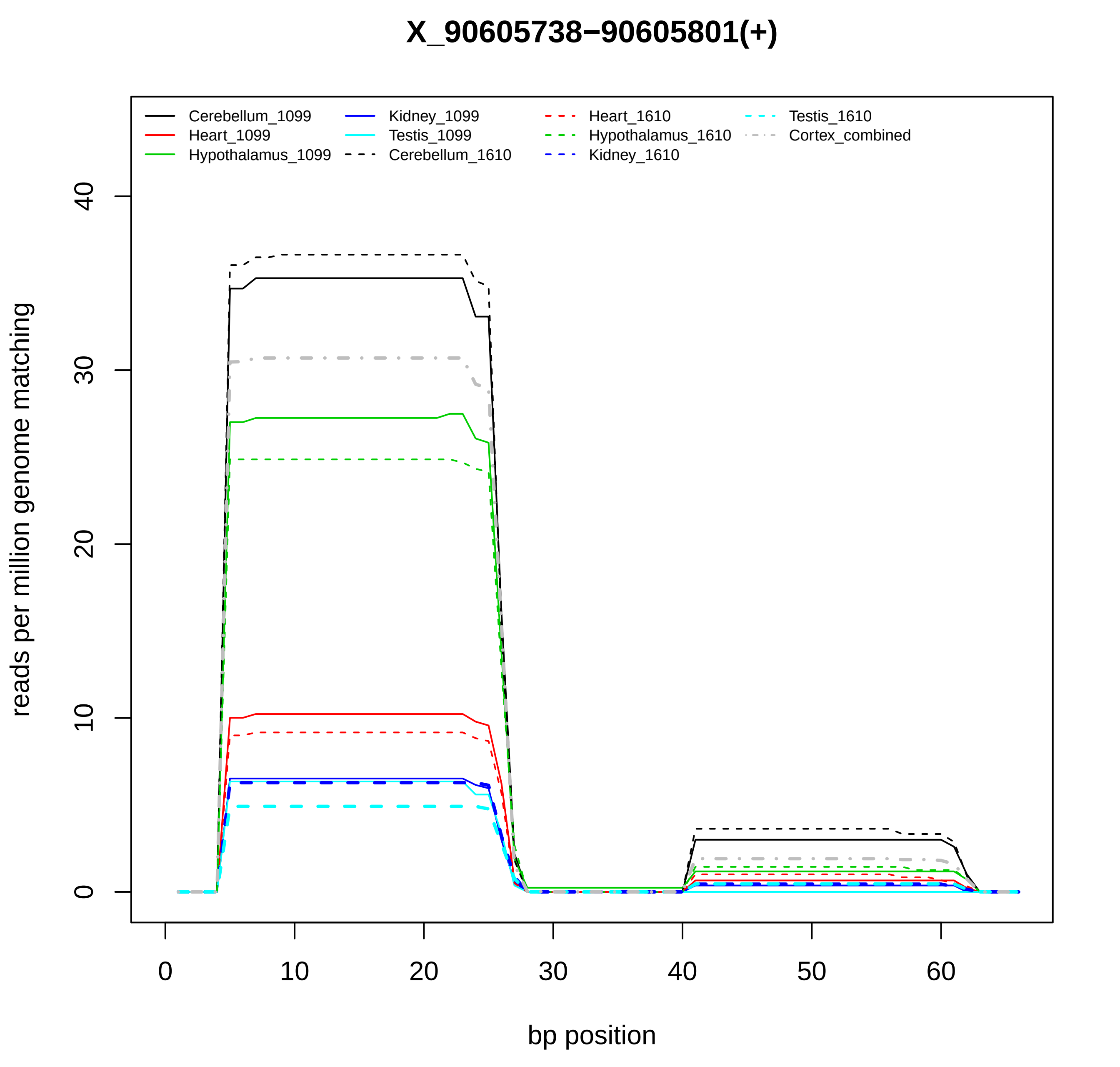

X_90605738−90605801(+)
Cerebellum_1099
Heart_1099
Hypothalamus_1099
Kidney_1099
Testis_1099
Cerebellum_1610
Heart_1610
Hypothalamus_1610
Kidney_1610
Testis_1610
Cortex_combined
reads per million genome matching
bp position
0
10
20
30
40
0
10
20
30
40
50
60

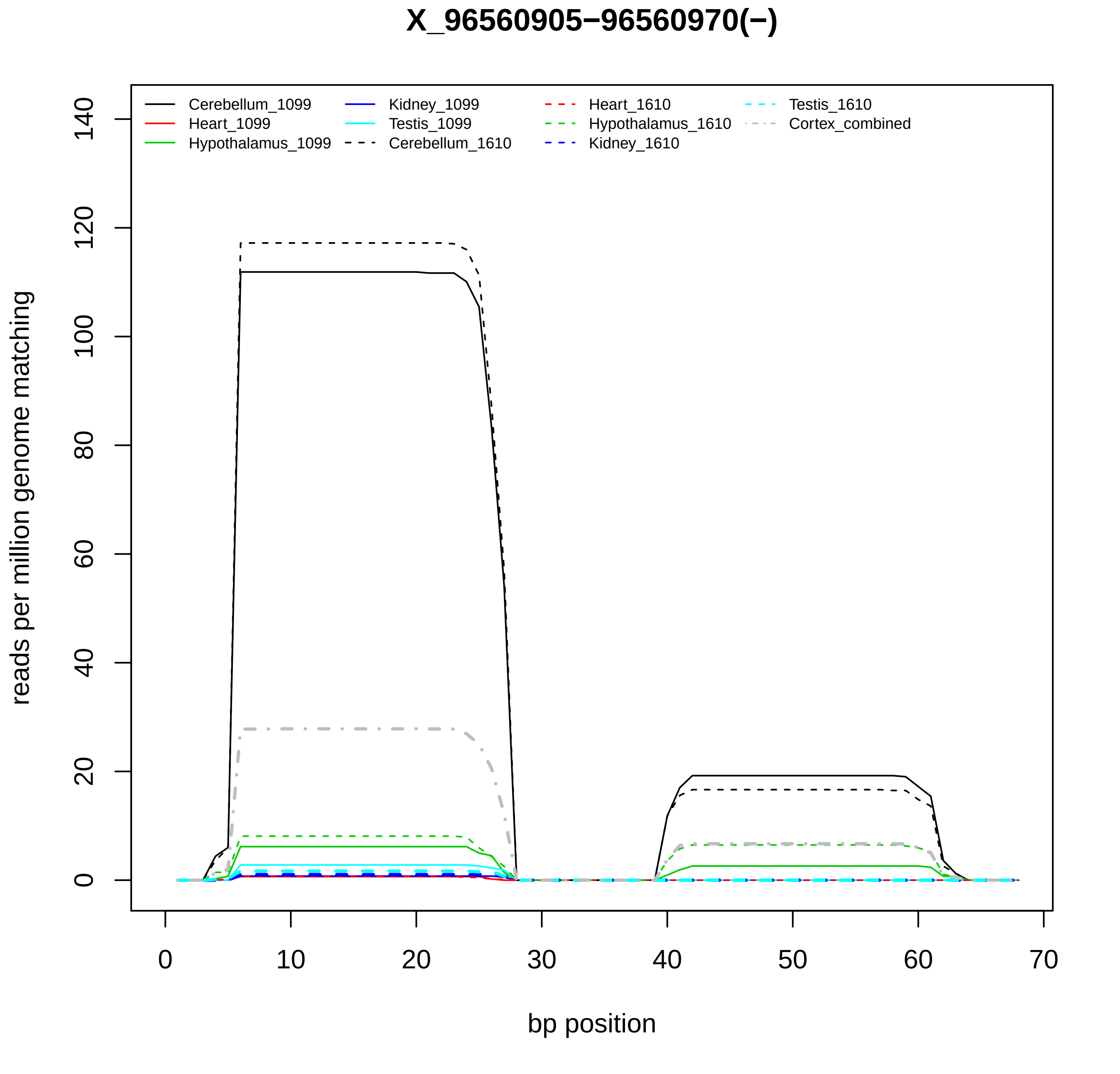
X_96560905−96560970(−)
Cerebellum_1099
Heart_1099
Hypothalamus_1099
Kidney_1099
Testis_1099
Cerebellum_1610
Heart_1610
Hypothalamus_1610
Kidney_1610
Testis_1610
Cortex_combined
reads per million genome matching
bp position
0
20
40
60
80
100
120
140
0
10
20
30
40
50
60
70

Fig. S6

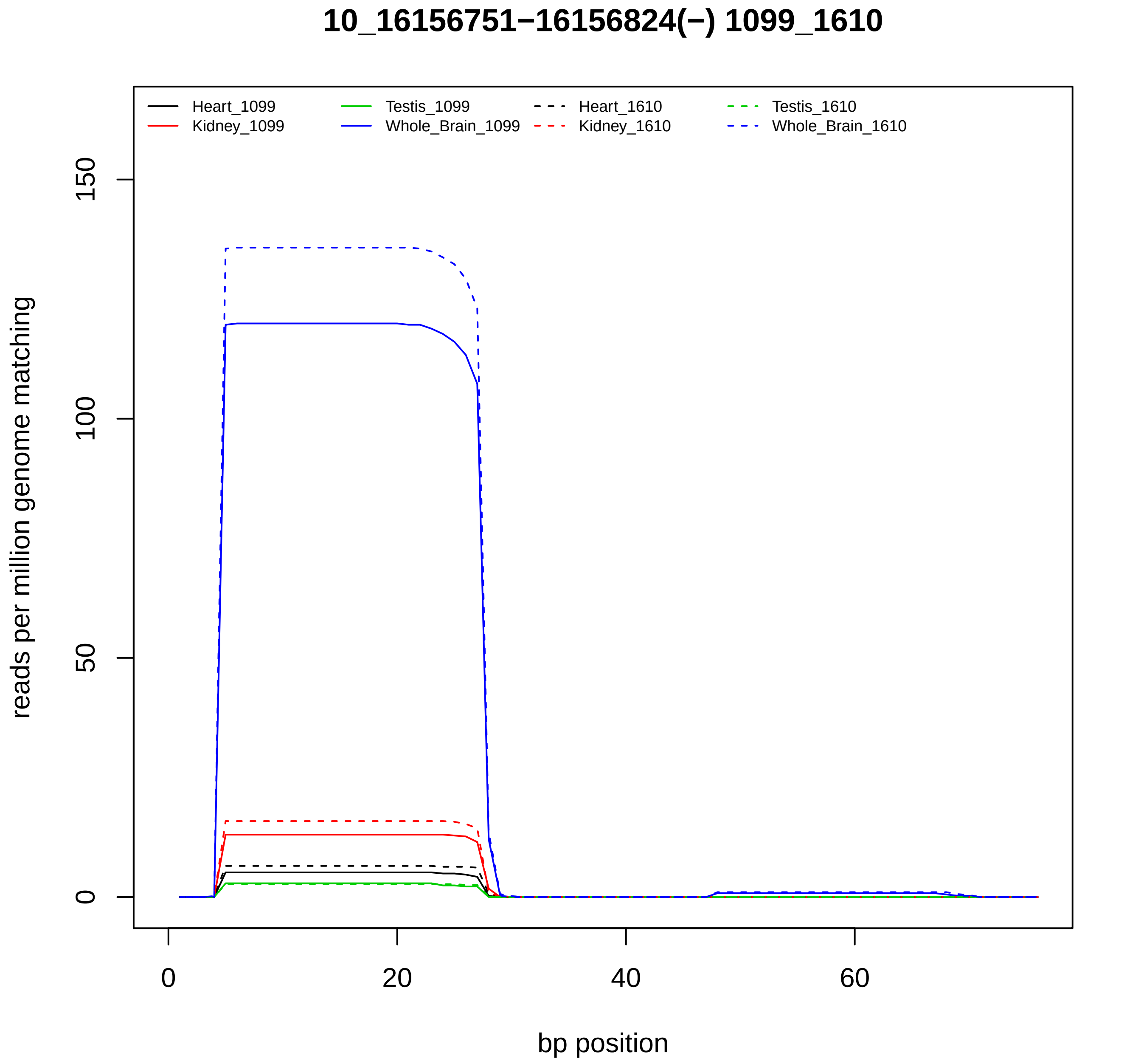

10_16156751−16156824(−) 1099_1610
Heart_1099
Kidney_1099
Testis_1099
Whole_Brain_1099
Heart_1610
Kidney_1610
Testis_1610
Whole_Brain_1610
reads per million genome matching
bp position
0
50
100
150
0
20
40
60

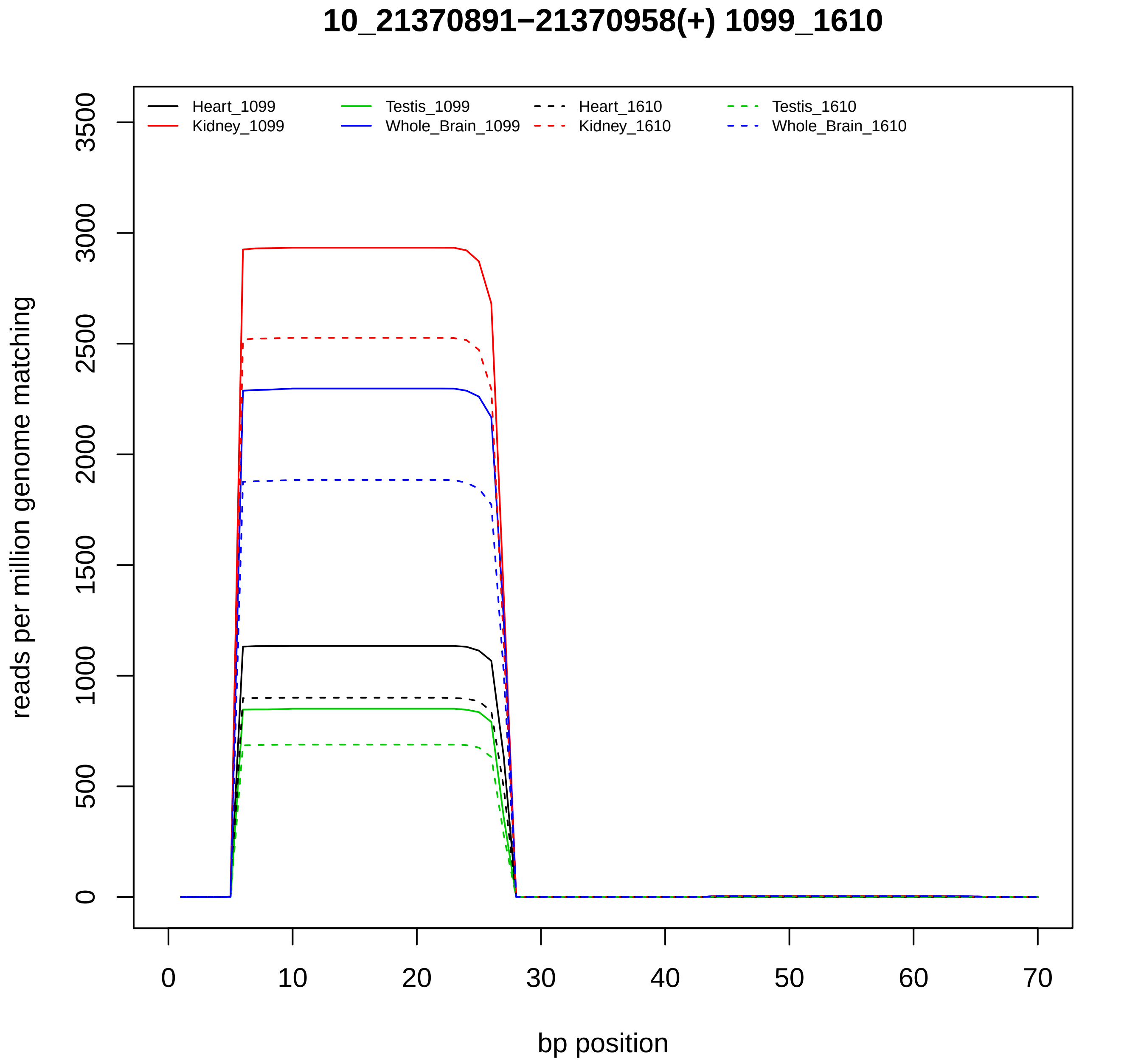

10_21370891−21370958(+) 1099_1610
Heart_1099
Kidney_1099
Testis_1099
Whole_Brain_1099
Heart_1610
Kidney_1610
Testis_1610
Whole_Brain_1610
reads per million genome matching
0
500
1000
1500
2000
2500
3000
3500
0
10
20
30
40
50
60
70
bp position

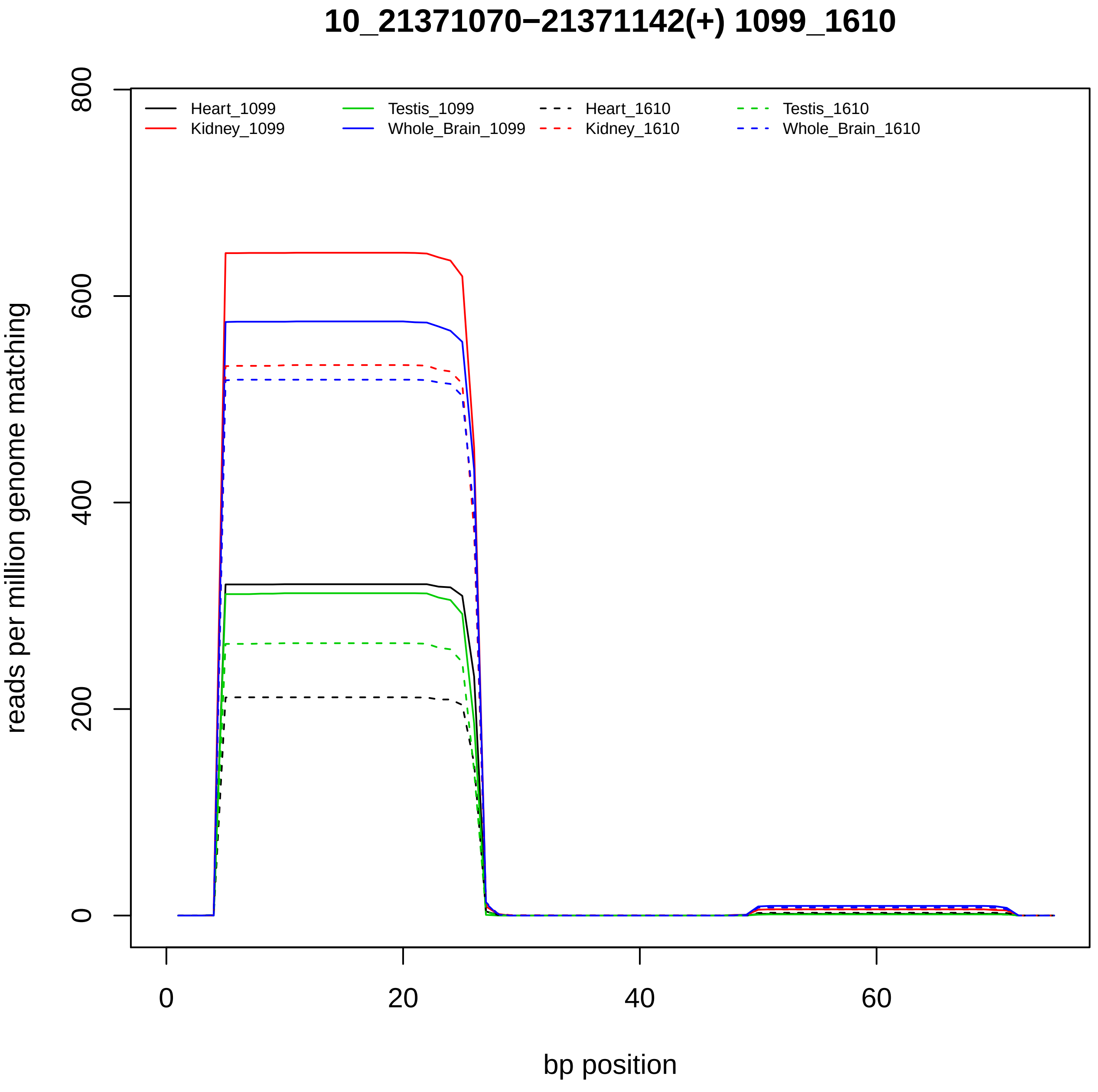
10_21371070−21371142(+) 1099_1610
Heart_1099
Kidney_1099
Testis_1099
Whole_Brain_1099
Heart_1610
Kidney_1610
Testis_1610
Whole_Brain_1610
reads per million genome matching
bp position
0
200
400
600
800
0
20
40
60

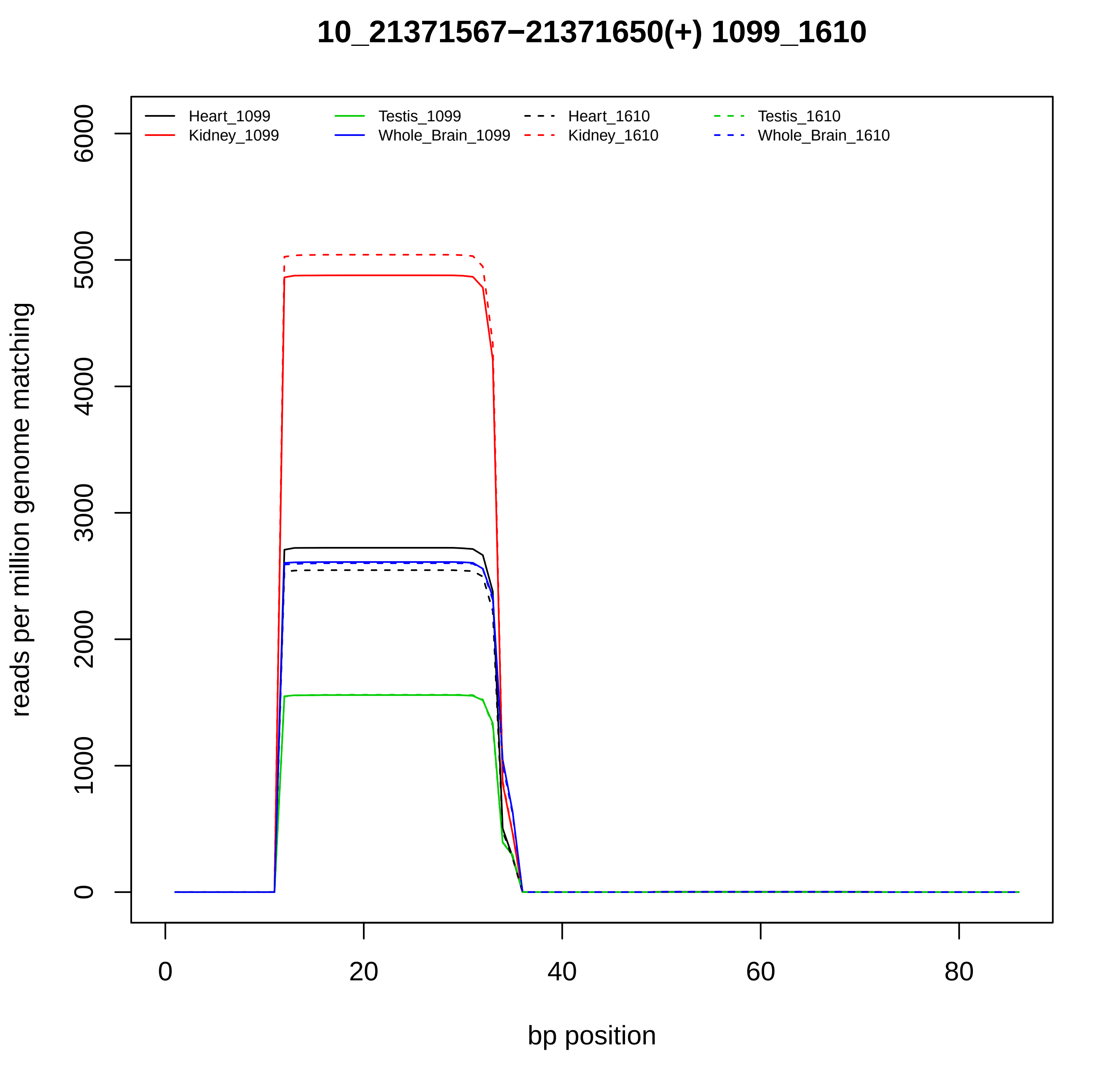

10_21371567−21371650(+) 1099_1610
Heart_1099
Kidney_1099
Testis_1099
Whole_Brain_1099
Heart_1610
Kidney_1610
Testis_1610
Whole_Brain_1610
reads per million genome matching
0
1000
2000
3000
4000
5000
6000
bp position
0
20
40
60
80

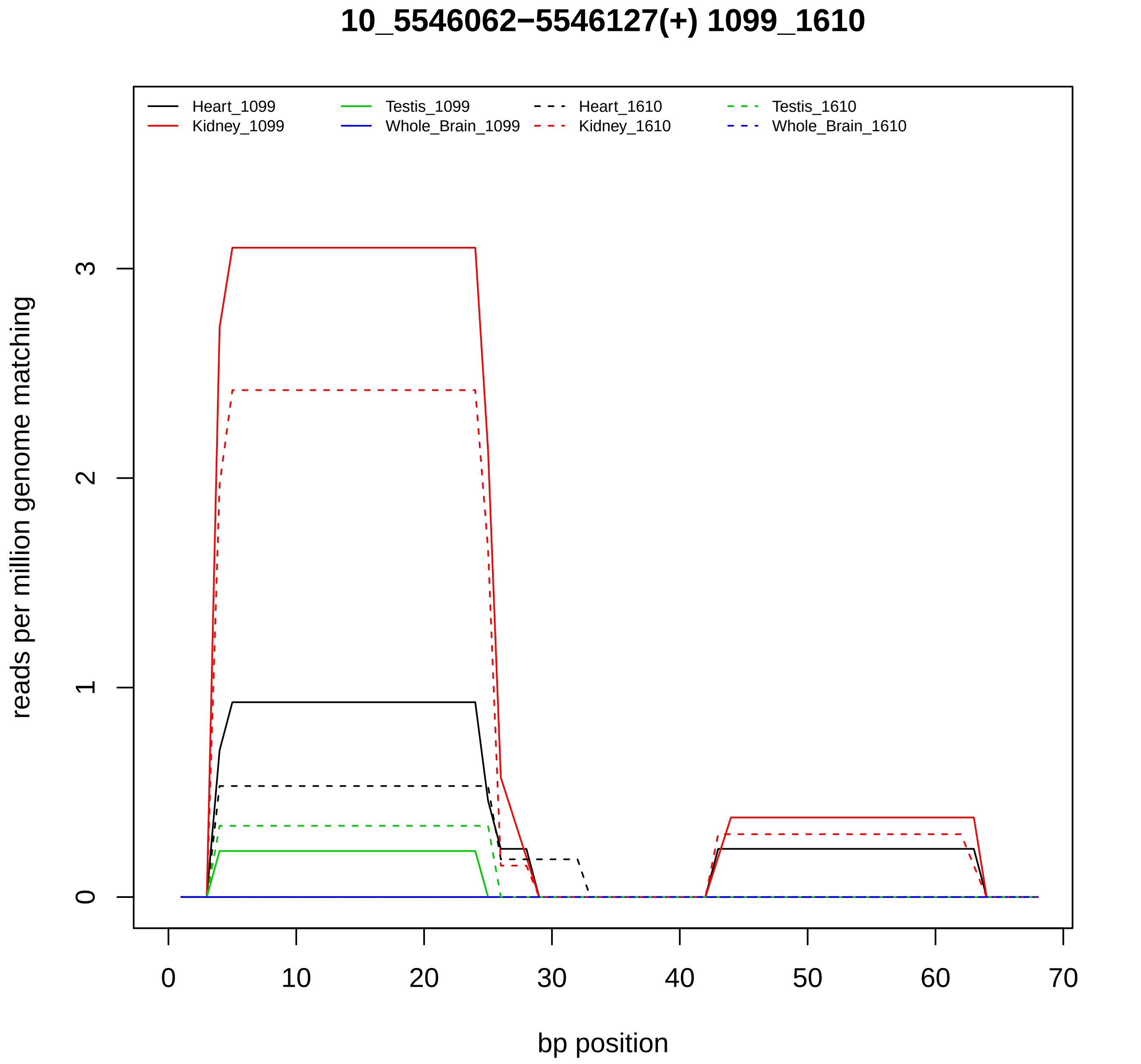

10_5546062−5546127(+) 1099_1610
Heart_1099
Kidney_1099
Testis_1099
Whole_Brain_1099
Heart_1610
Kidney_1610
Testis_1610
Whole_Brain_1610
reads per million genome matching
0
1
2
3
bp position
0
10
20
30
40
50
60
70

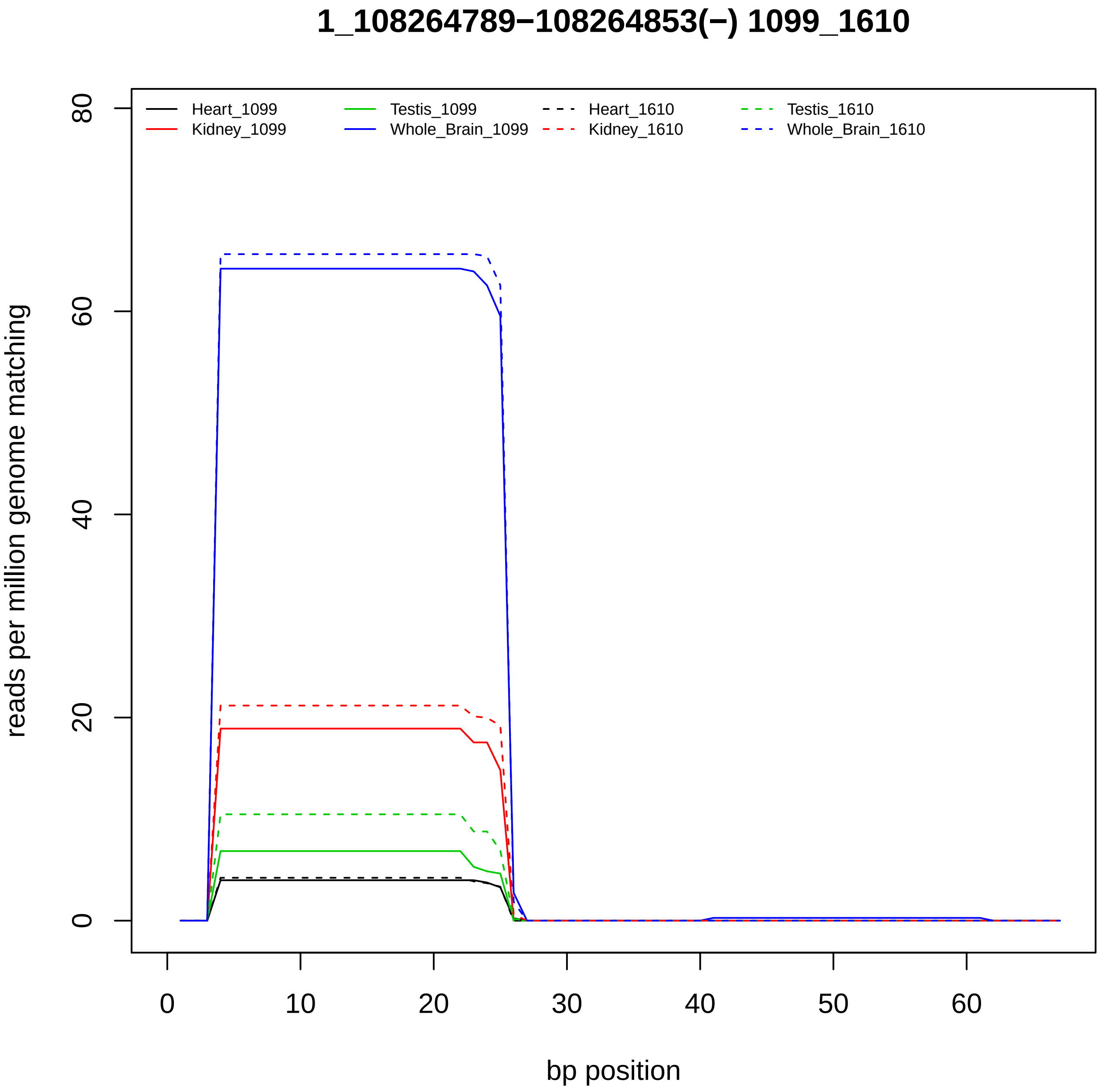

1_108264789−108264853(−) 1099_1610
Heart_1099
Kidney_1099
Testis_1099
Whole_Brain_1099
Heart_1610
Kidney_1610
Testis_1610
Whole_Brain_1610
reads per million genome matching
bp position
0
20
40
60
80
0
10
20
30
40
50
60

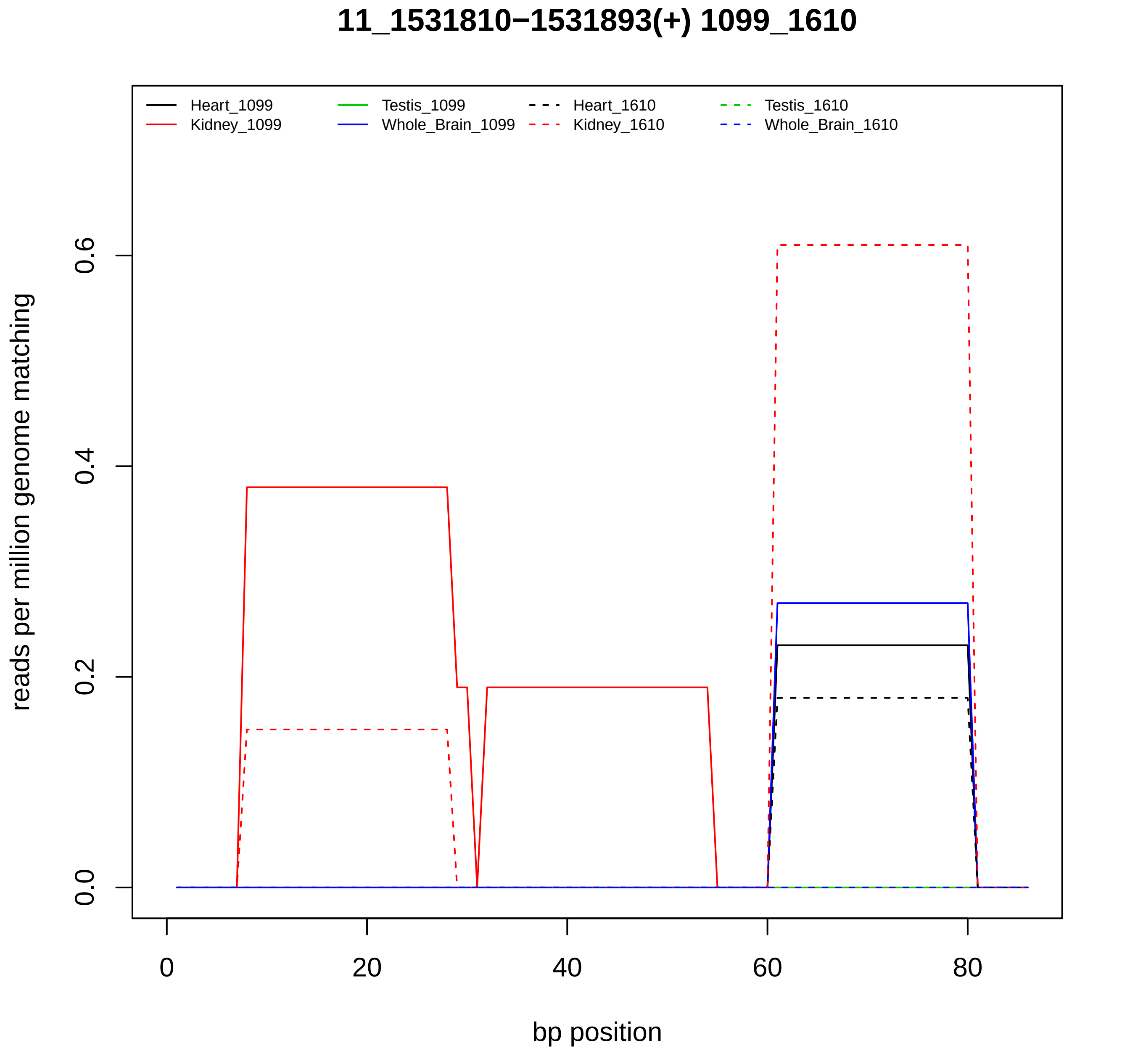

11_1531810−1531893(+) 1099_1610
Heart_1099
Kidney_1099
Testis_1099
Whole_Brain_1099
Heart_1610
Kidney_1610
Testis_1610
Whole_Brain_1610
reads per million genome matching
0.0
0.2
0.4
0.6
0
20
40
60
80
bp position

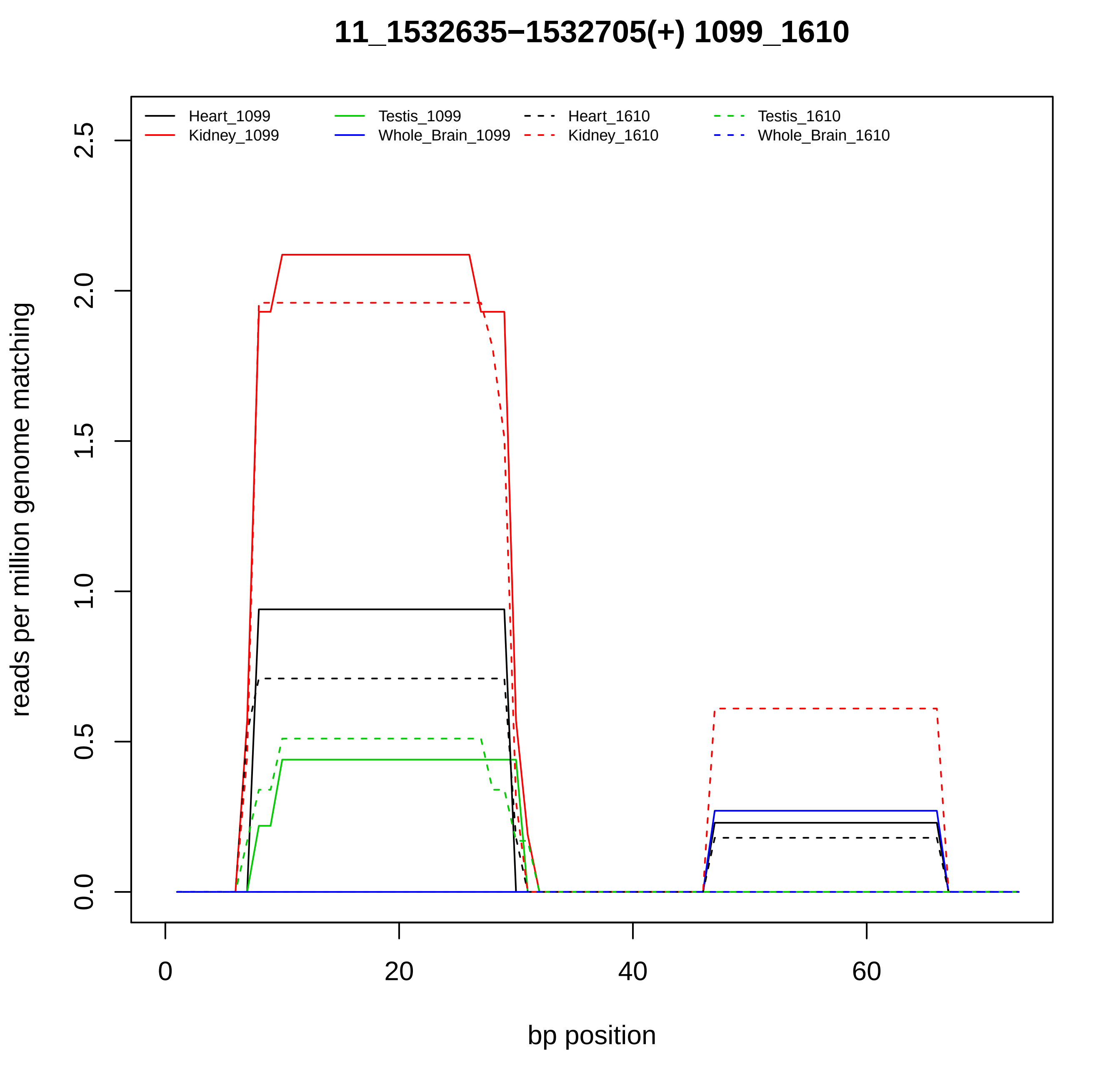

11_1532635−1532705(+) 1099_1610
Heart_1099
Kidney_1099
Testis_1099
Whole_Brain_1099
Heart_1610
Kidney_1610
Testis_1610
Whole_Brain_1610
reads per million genome matching
0.0
0.5
1.0
1.5
2.0
2.5
0
20
40
60
bp position

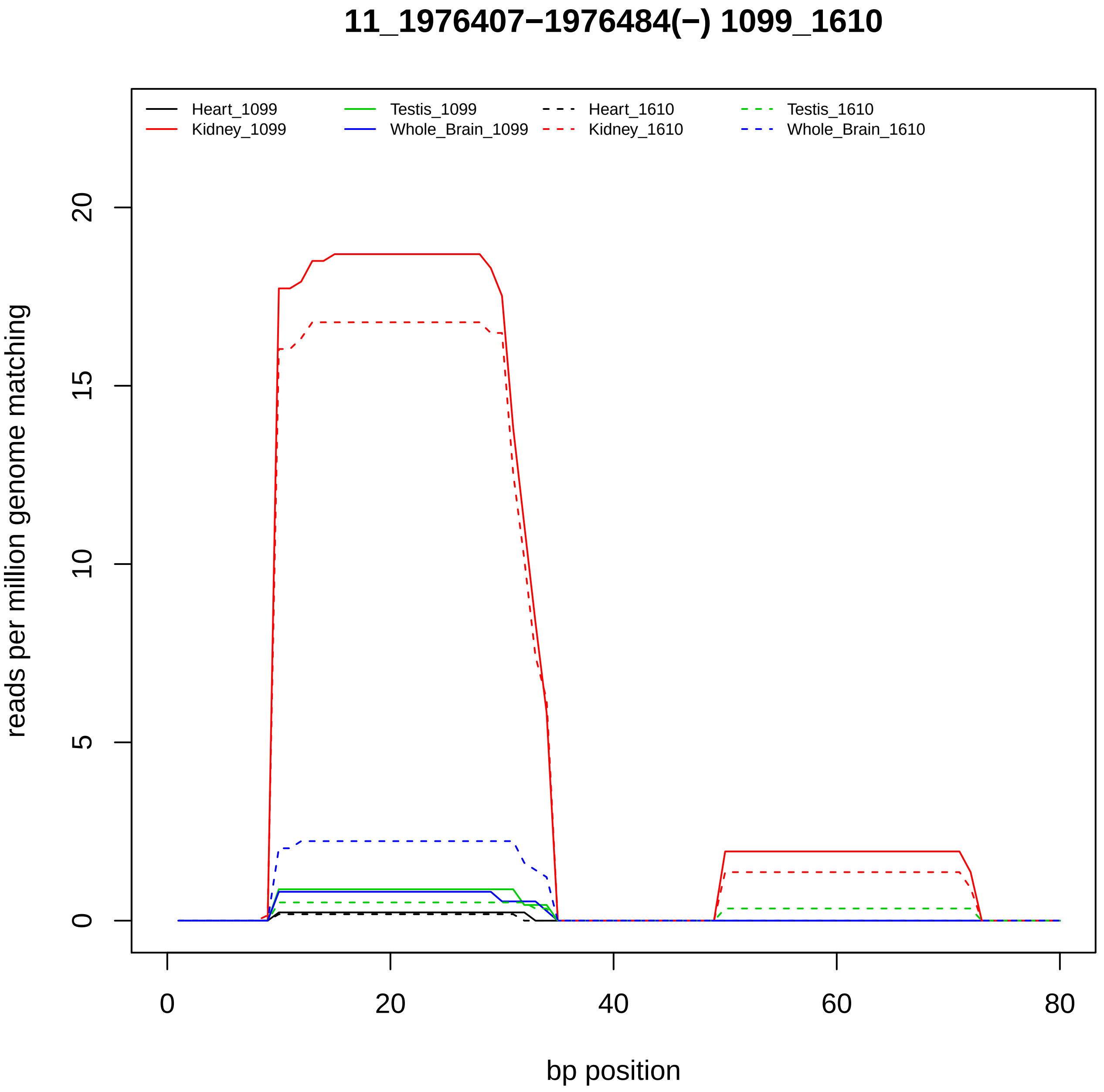

11_1976407−1976484(−) 1099_1610
Heart_1099
Kidney_1099
Testis_1099
Whole_Brain_1099
Heart_1610
Kidney_1610
Testis_1610
Whole_Brain_1610
reads per million genome matching
bp position
0
5
10
15
20
0
20
40
60
80

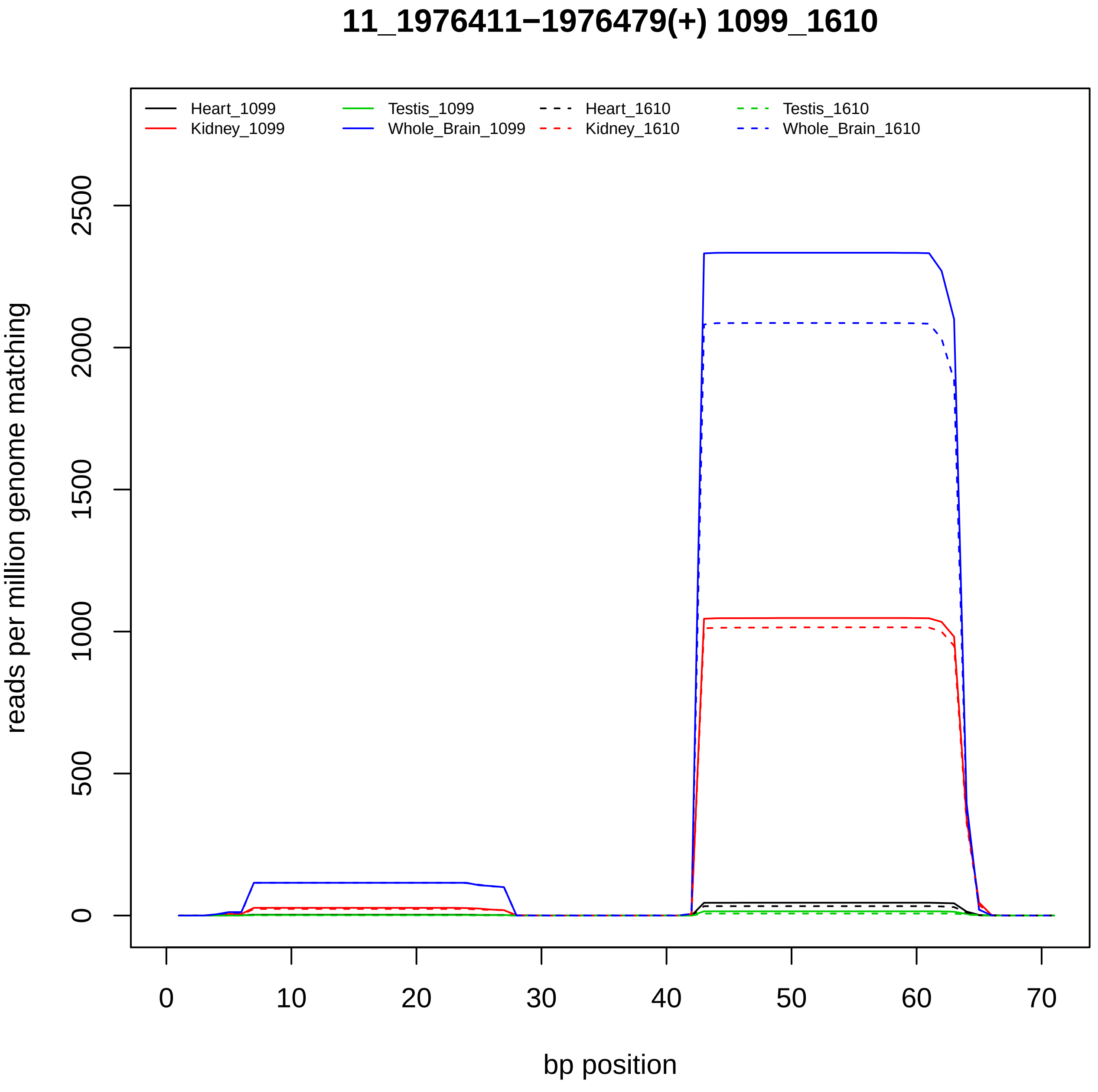

11_1976411−1976479(+) 1099_1610
Heart_1099
Kidney_1099
Testis_1099
Whole_Brain_1099
Heart_1610
Kidney_1610
Testis_1610
Whole_Brain_1610
reads per million genome matching
0
500
1000
1500
2000
2500
bp position
0
10
20
30
40
50
60
70

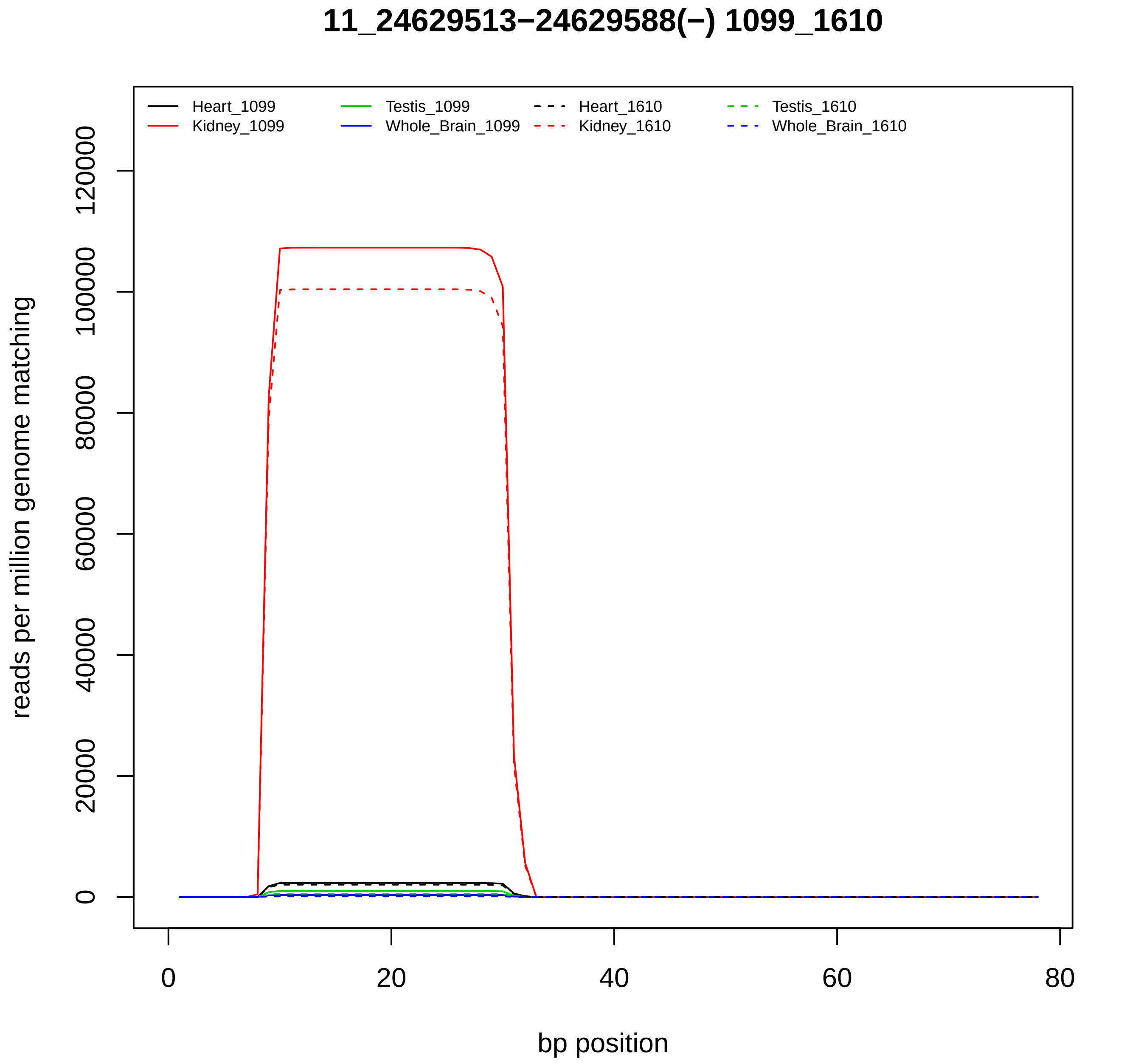

11_24629513−24629588(−) 1099_1610
Heart_1099
Kidney_1099
Testis_1099
Whole_Brain_1099
Heart_1610
Kidney_1610
Testis_1610
Whole_Brain_1610
reads per million genome matching
0
20000
40000
60000
80000
100000
120000
0
20
40
60
80
bp position

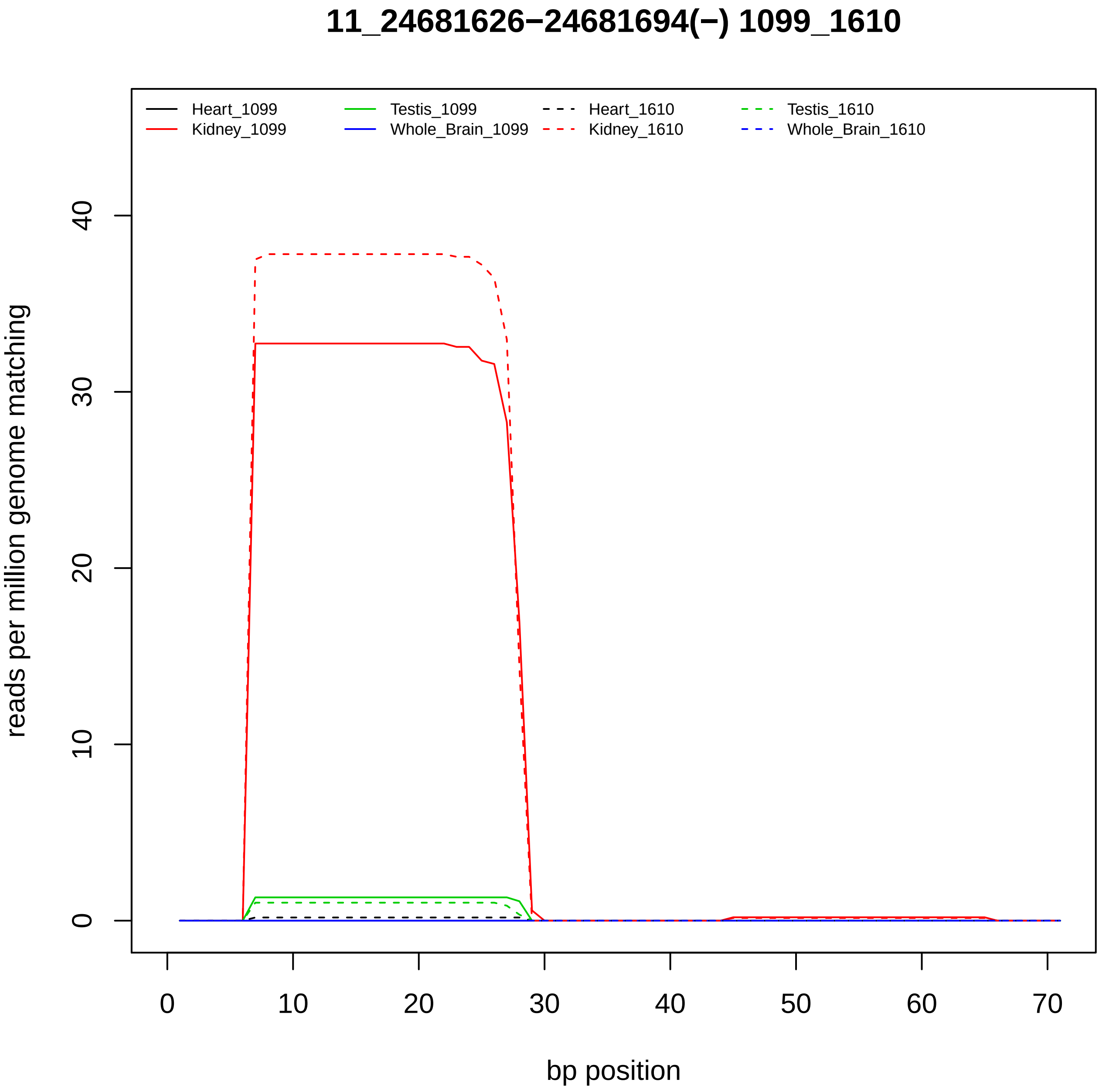
11_24681626−24681694(−) 1099_1610
Heart_1099
Kidney_1099
Testis_1099
Whole_Brain_1099
Heart_1610
Kidney_1610
Testis_1610
Whole_Brain_1610
reads per million genome matching
bp position
0
10
20
30
40
0
10
20
30
40
50
60
70

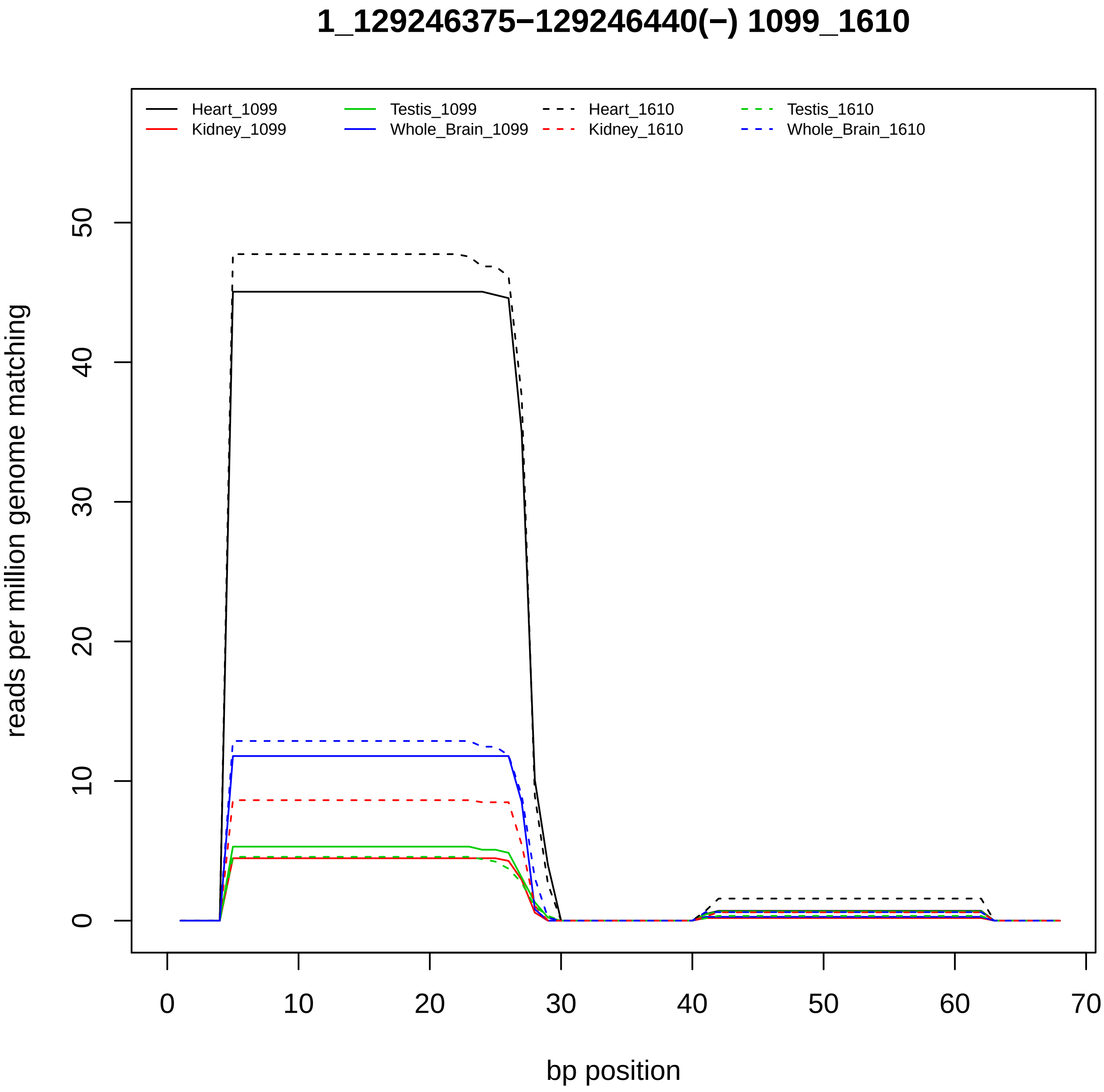

1_129246375−129246440(−) 1099_1610
Heart_1099
Kidney_1099
Testis_1099
Whole_Brain_1099
Heart_1610
Kidney_1610
Testis_1610
Whole_Brain_1610
reads per million genome matching
bp position
0
10
20
30
40
50
60
70

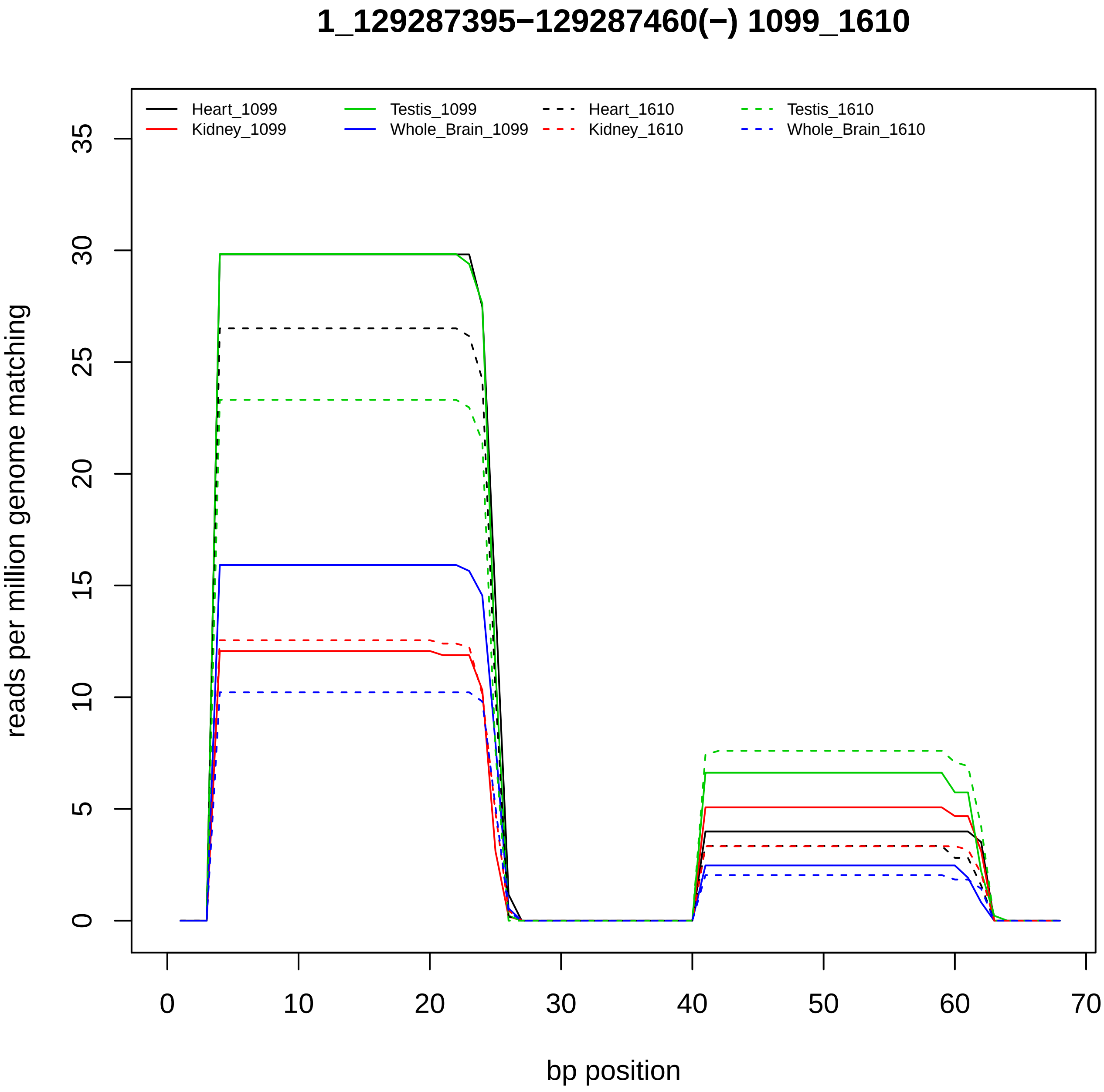
1_129287395−129287460(−) 1099_1610
Heart_1099
Kidney_1099
Testis_1099
Whole_Brain_1099
Heart_1610
Kidney_1610
Testis_1610
Whole_Brain_1610
reads per million genome matching
bp position
0
5
10
15
20
25
30
35
0
10
20
30
40
50
60
70

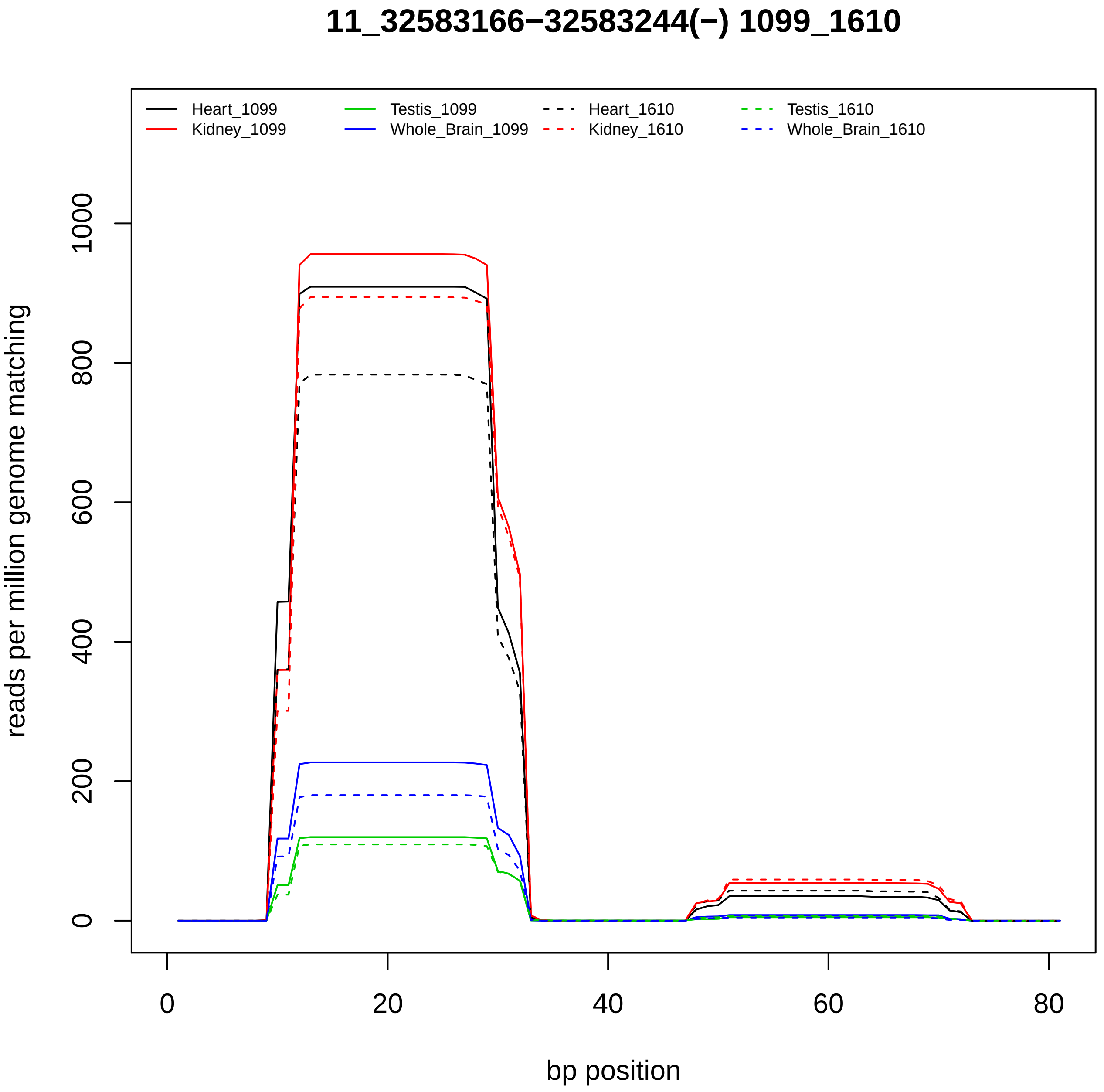

11_32583166−32583244(−) 1099_1610
Heart_1099
Kidney_1099
Testis_1099
Whole_Brain_1099
Heart_1610
Kidney_1610
Testis_1610
Whole_Brain_1610
reads per million genome matching
0
200
400
600
800
1000
bp position
0
20
40
60
80

11_33283288−33283363(−) 1099_1610

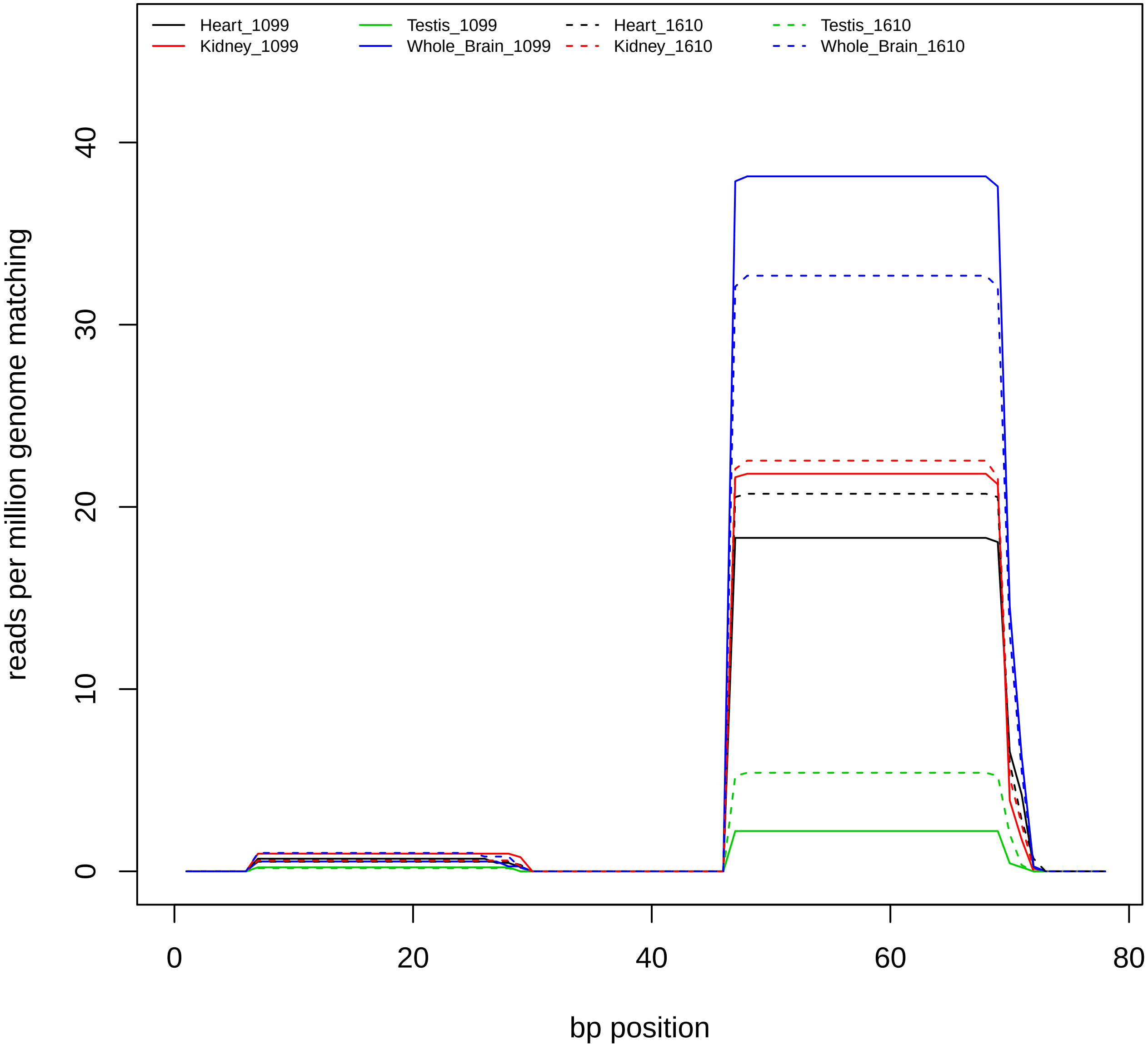

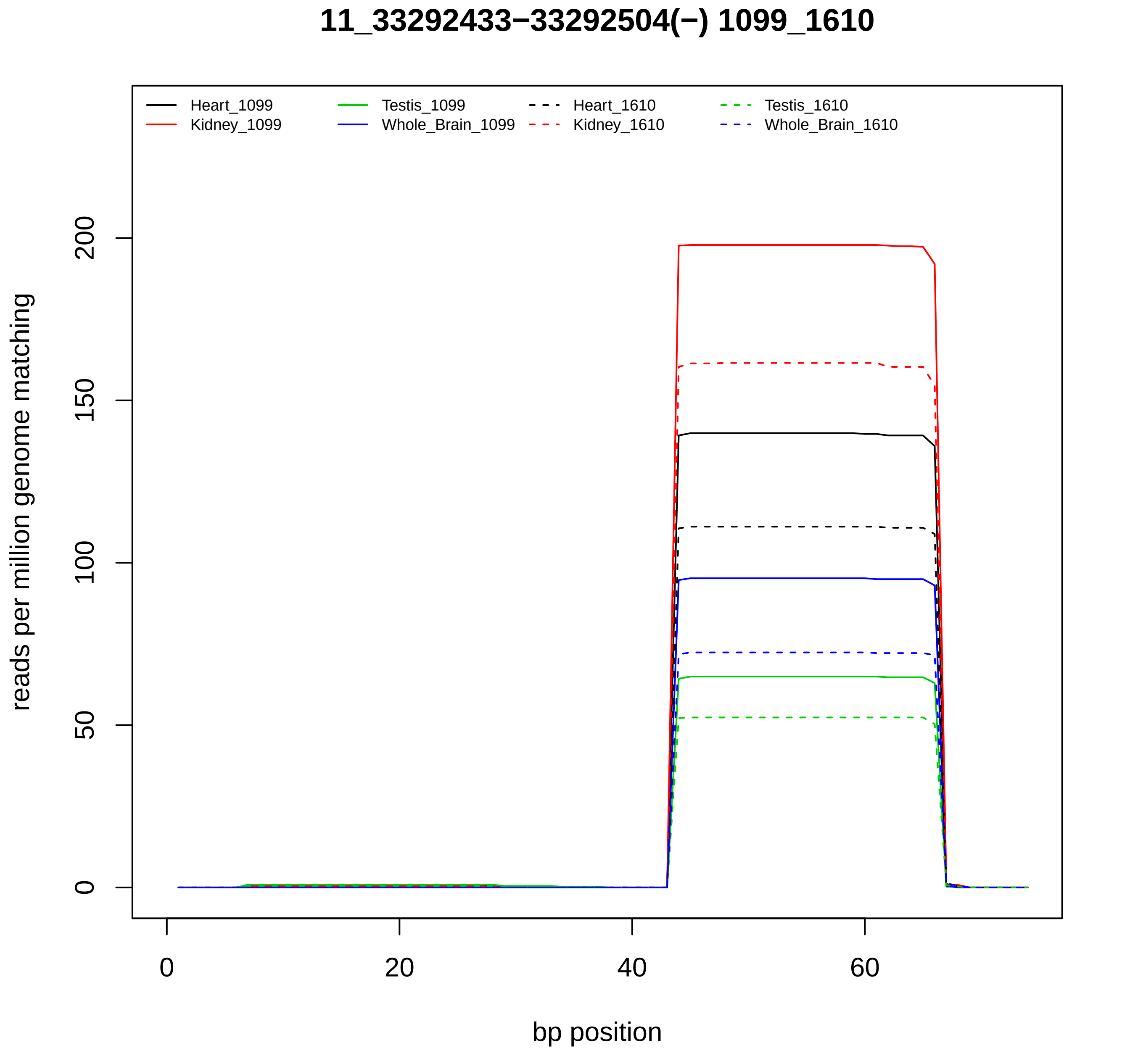

11_33292433−33292504(−) 1099_1610
Heart_1099
Kidney_1099
Testis_1099
Whole_Brain_1099
Heart_1610
Kidney_1610
Testis_1610
Whole_Brain_1610
reads per million genome matching
0
50
100
150
200
0
20
40
60
bp position

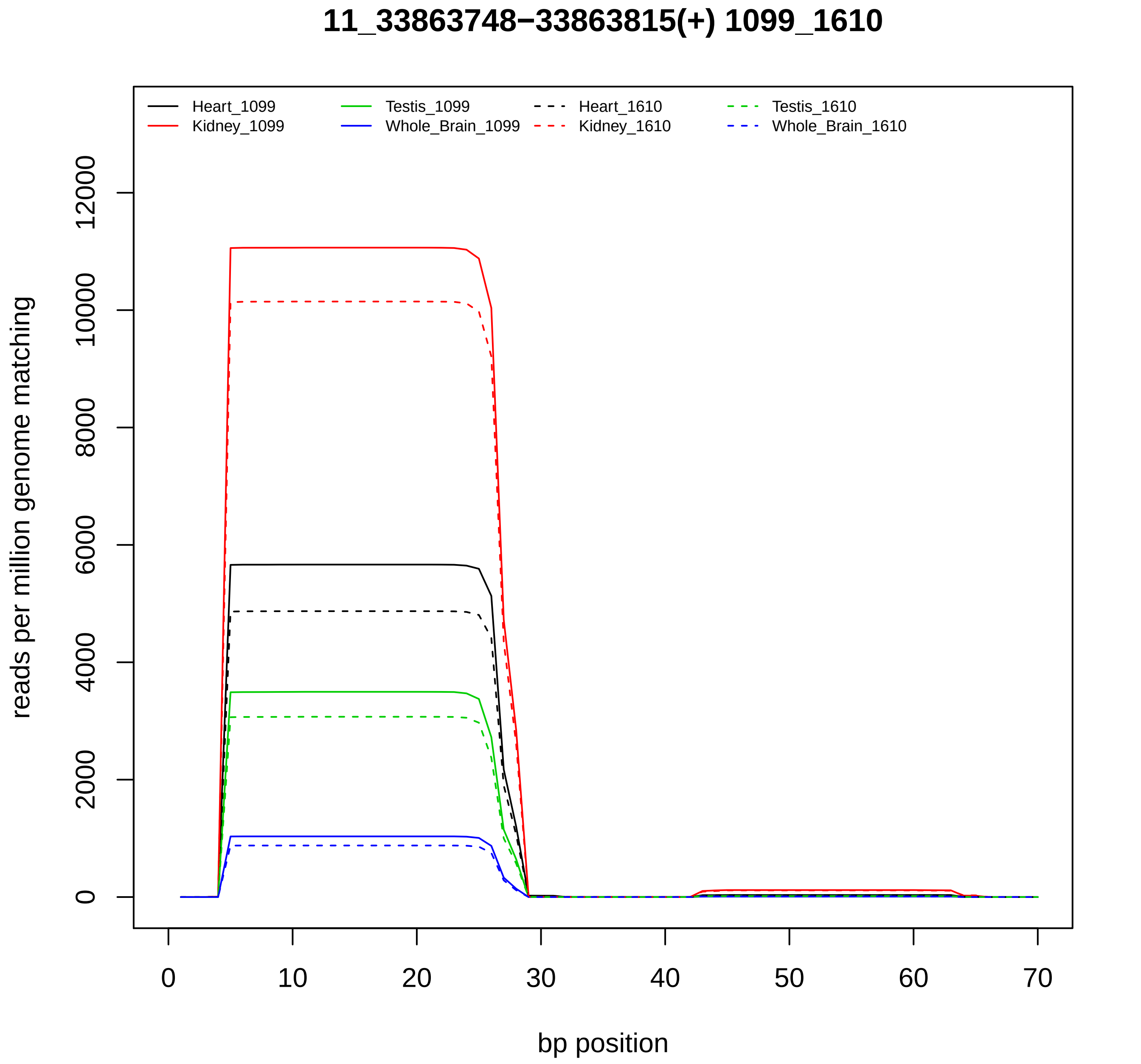

11_33863748−33863815(+) 1099_1610
Heart_1099
Kidney_1099
Testis_1099
Whole_Brain_1099
Heart_1610
Kidney_1610
Testis_1610
Whole_Brain_1610
reads per million genome matching
0
2000
4000
6000
8000
10000
12000
0
10
20
30
40
50
60
70
bp position

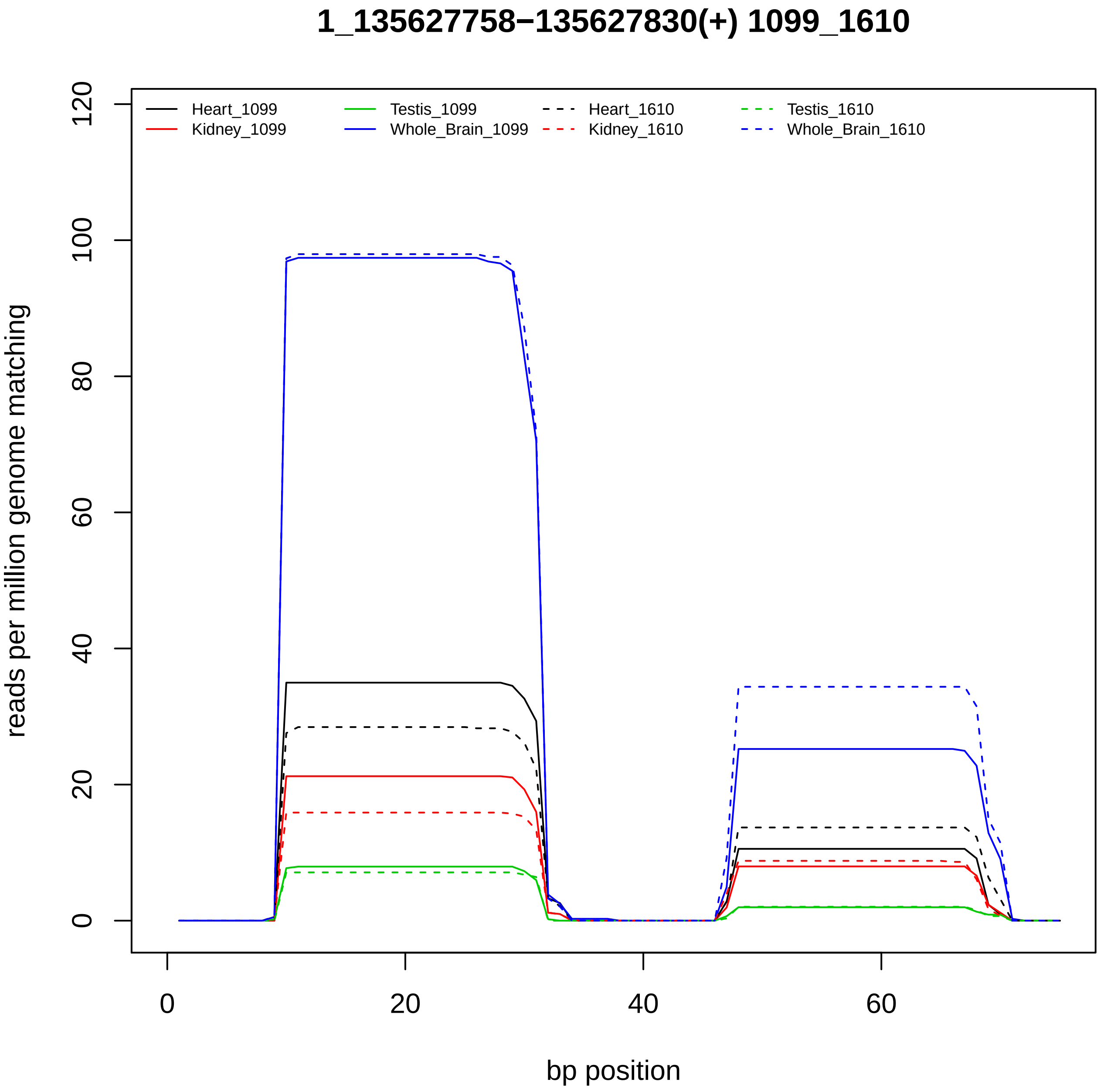

1_135627758−135627830(+) 1099_1610
Heart_1099
Kidney_1099
Testis_1099
Whole_Brain_1099
Heart_1610
Kidney_1610
Testis_1610
Whole_Brain_1610
reads per million genome matching
0
20
40
60
80
100
120
bp position
0
20
40
60

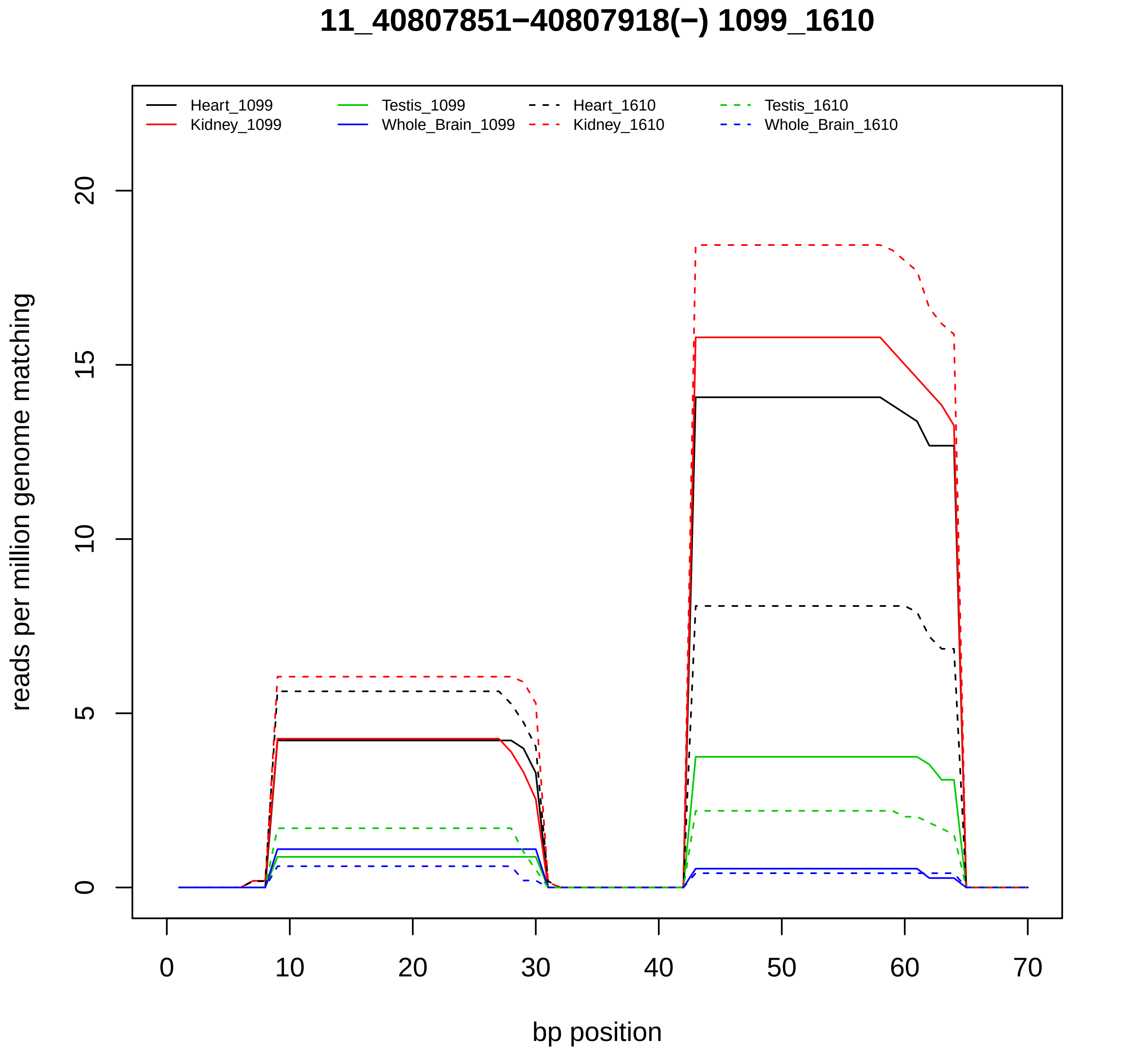

11_40807851−40807918(−) 1099_1610
Heart_1099
Kidney_1099
Testis_1099
Whole_Brain_1099
Heart_1610
Kidney_1610
Testis_1610
Whole_Brain_1610
reads per million genome matching
bp position
0
5
10
15
20
0
10
20
30
40
50
60
70

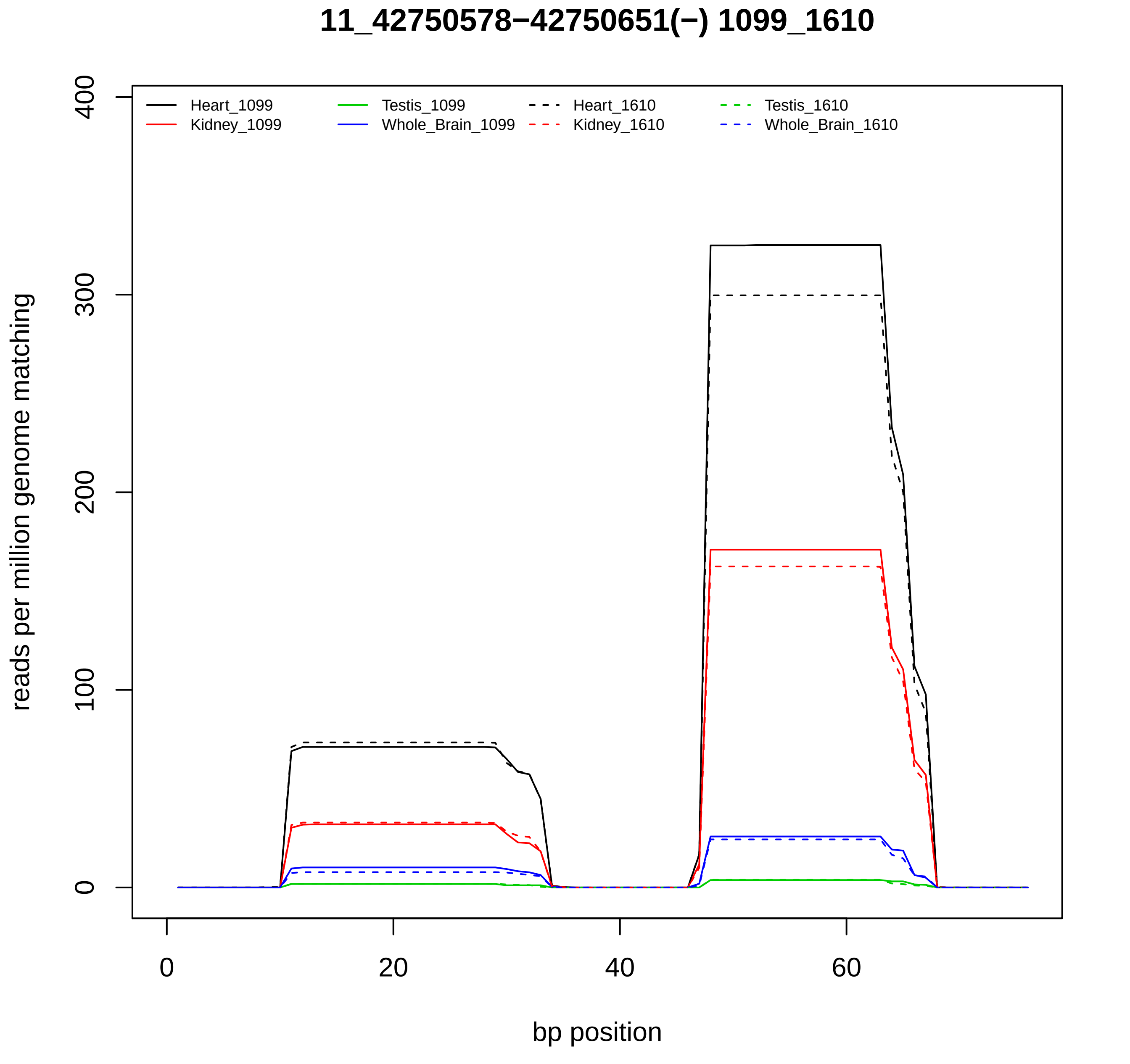

11_42750578−42750651(−) 1099_1610
Heart_1099
Kidney_1099
Testis_1099
Whole_Brain_1099
Heart_1610
Kidney_1610
Testis_1610
Whole_Brain_1610
reads per million genome matching
bp position
0
100
200
300
400
0
20
40
60

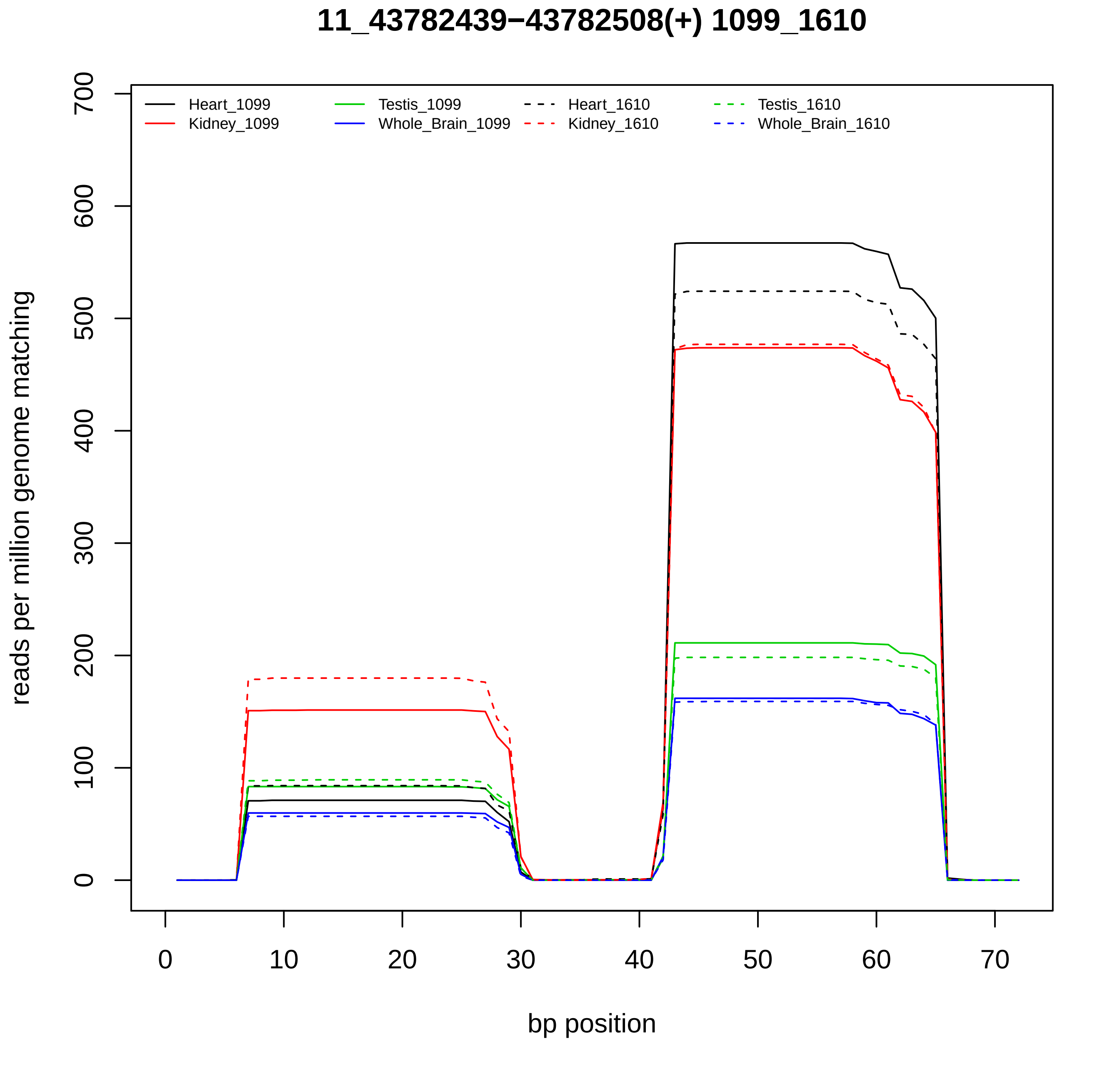

11_43782439−43782508(+) 1099_1610
Heart_1099
Kidney_1099
Testis_1099
Whole_Brain_1099
Heart_1610
Kidney_1610
Testis_1610
Whole_Brain_1610
reads per million genome matching
bp position
0
100
200
300
400
500
600
700
0
10
20
30
40
50
60
70

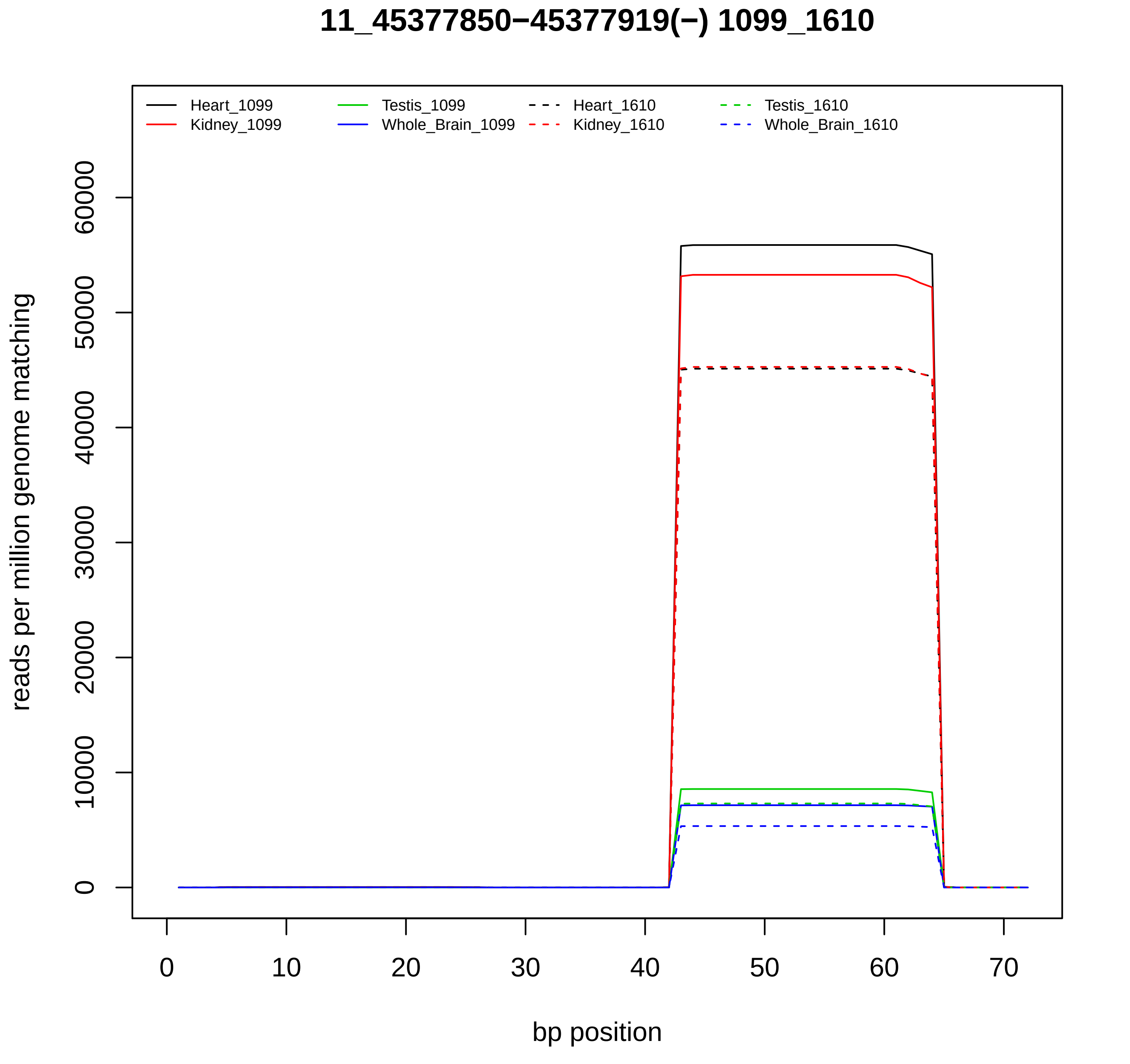

11_45377850−45377919(−) 1099_1610
Heart_1099
Kidney_1099
Testis_1099
Whole_Brain_1099
Heart_1610
Kidney_1610
Testis_1610
Whole_Brain_1610
reads per million genome matching
0
10000
20000
30000
40000
50000
60000
bp position
0
10
20
30
40
50
60
70

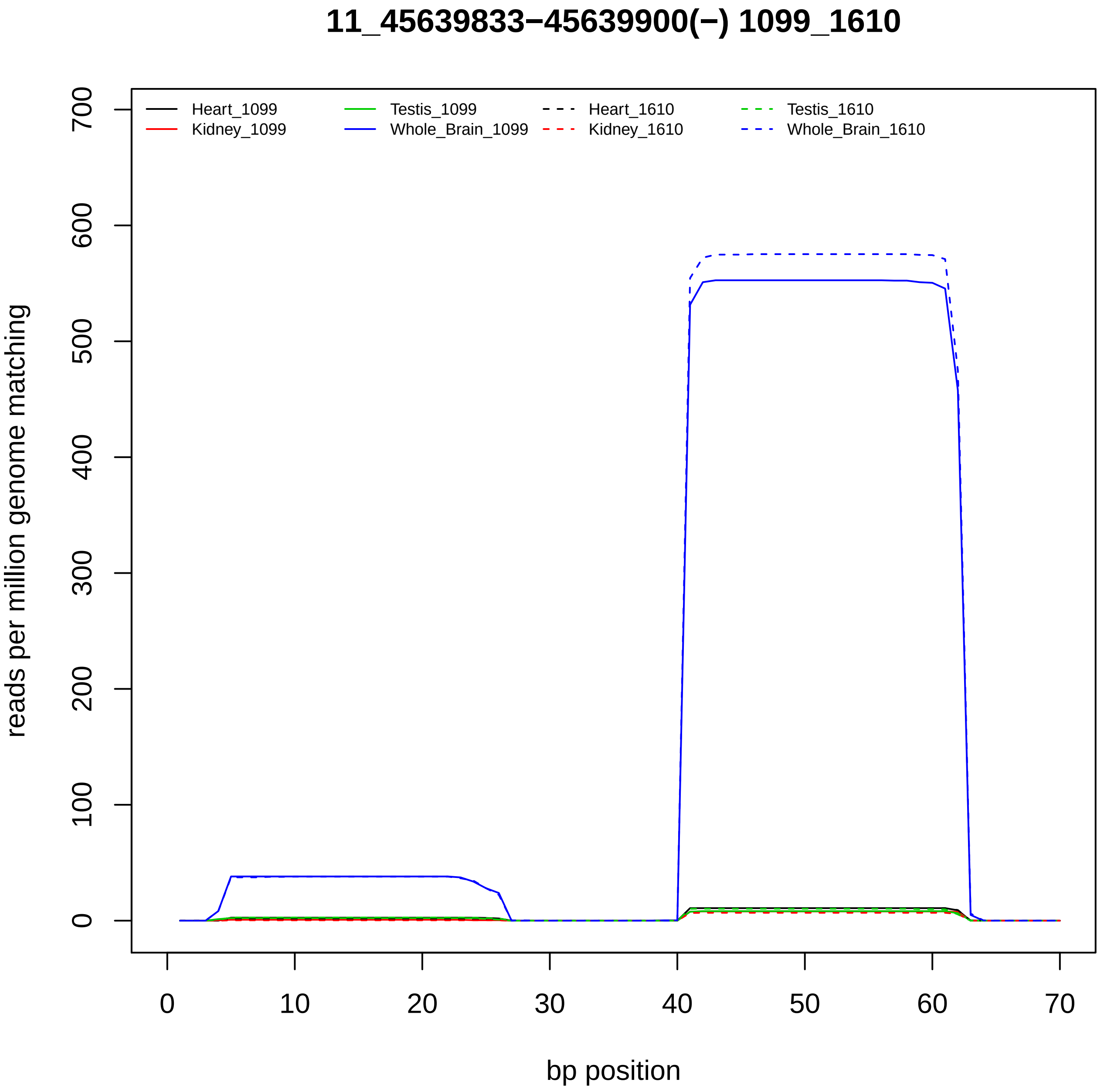

11_45639833−45639900(−) 1099_1610
Heart_1099
Kidney_1099
Testis_1099
Whole_Brain_1099
Heart_1610
Kidney_1610
Testis_1610
Whole_Brain_1610
reads per million genome matching
0
100
200
300
400
500
600
700
bp position
0
10
20
30
40
50
60
70

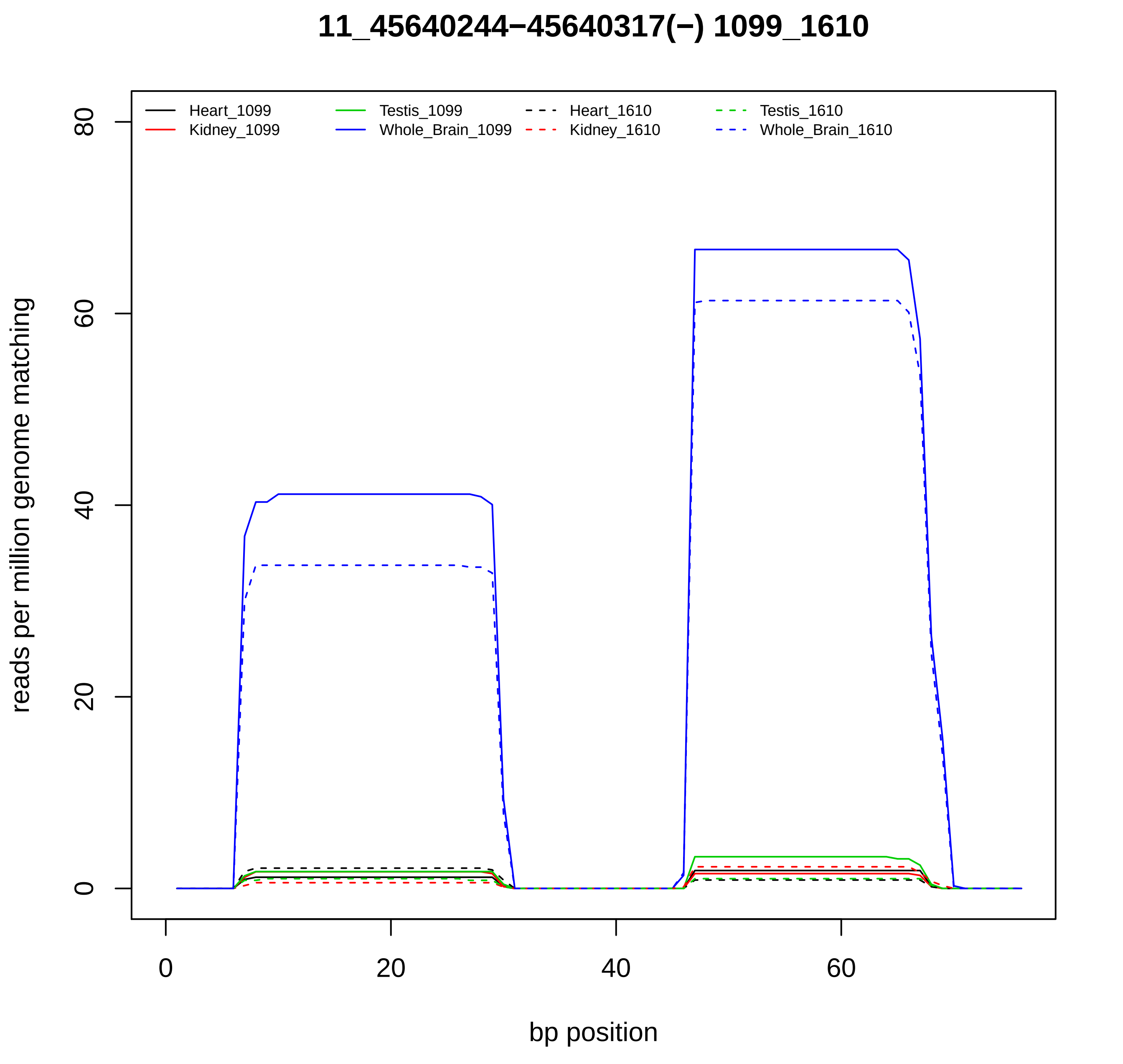

11_45640244−45640317(−) 1099_1610
Heart_1099
Kidney_1099
Testis_1099
Whole_Brain_1099
Heart_1610
Kidney_1610
Testis_1610
Whole_Brain_1610
reads per million genome matching
0
20
40
60
80
bp position
0
20
40
60

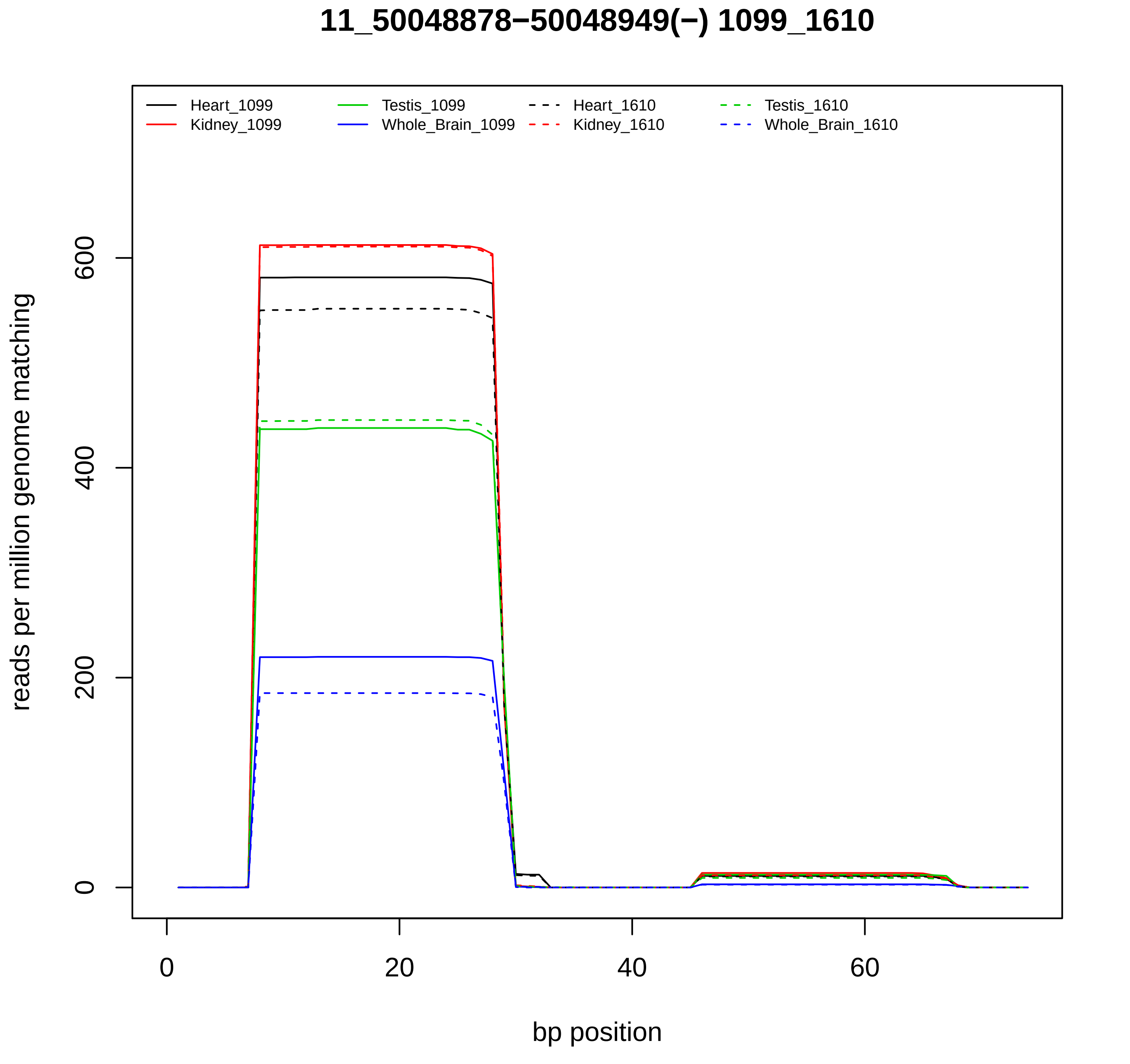

11_50048878−50048949(−) 1099_1610
Heart_1099
Kidney_1099
Testis_1099
Whole_Brain_1099
Heart_1610
Kidney_1610
Testis_1610
Whole_Brain_1610
reads per million genome matching
0
200
400
600
0
20
40
60
bp position

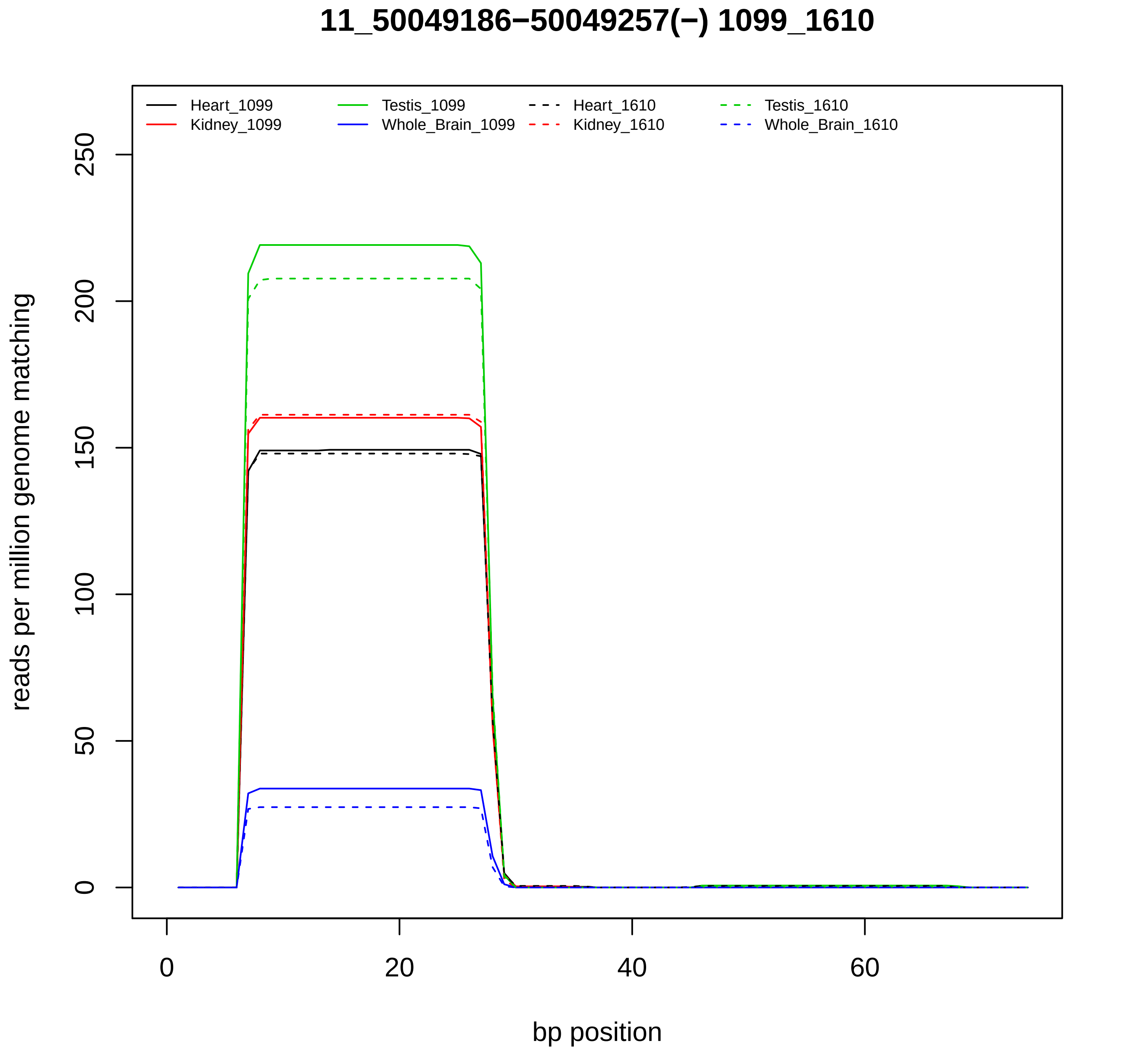
11_50049186−50049257(−) 1099_1610
Heart_1099
Kidney_1099
Testis_1099
Whole_Brain_1099
Heart_1610
Kidney_1610
Testis_1610
Whole_Brain_1610
reads per million genome matching
0
50
100
150
200
250
0
20
40
60
bp position

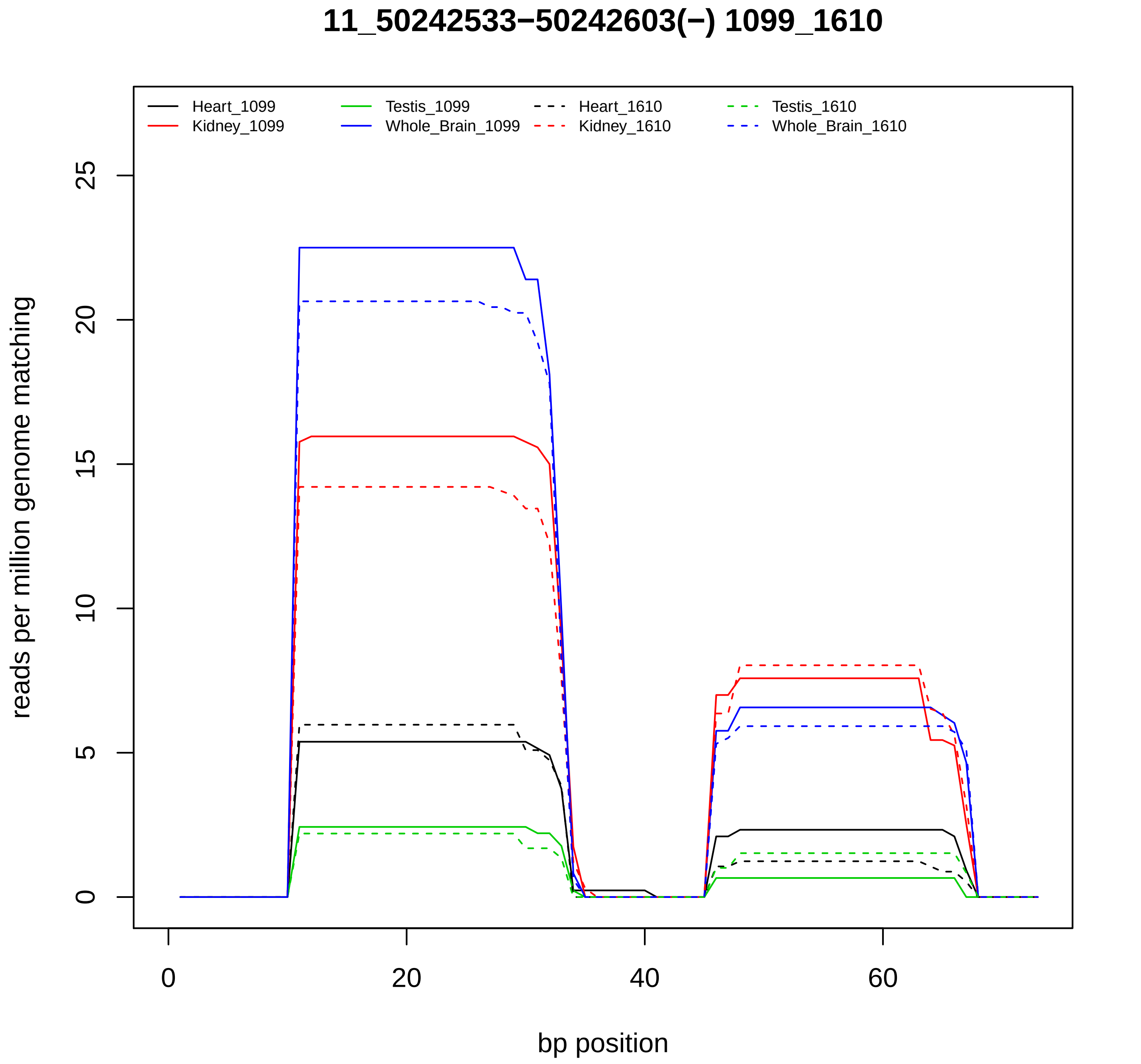
11_50242533−50242603(−) 1099_1610
Heart_1099
Kidney_1099
Testis_1099
Whole_Brain_1099
Heart_1610
Kidney_1610
Testis_1610
Whole_Brain_1610
reads per million genome matching
0
5
10
15
20
25
0
20
40
60
bp position

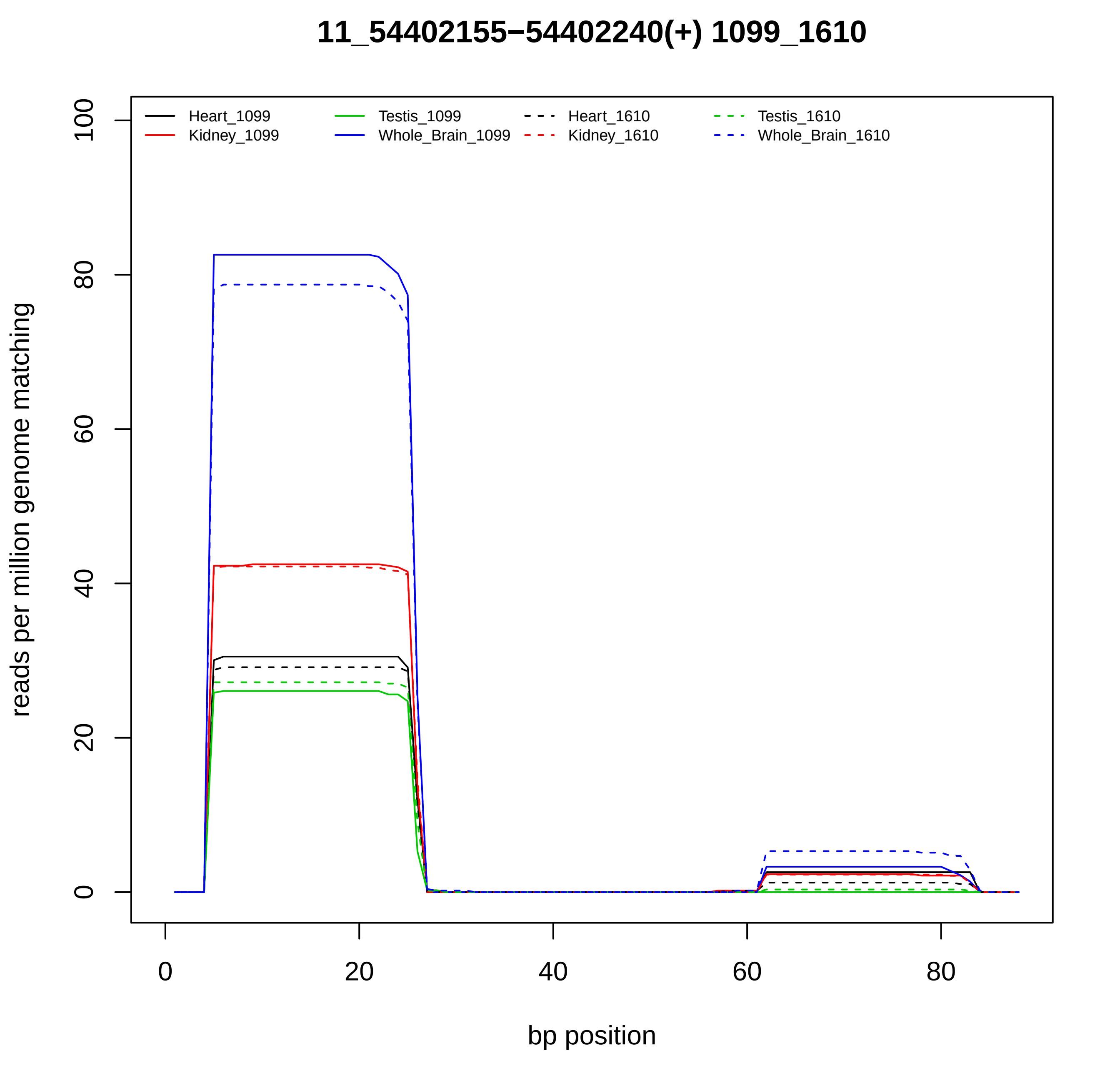
11_54402155−54402240(+) 1099_1610
Heart_1099
Kidney_1099
Testis_1099
Whole_Brain_1099
Heart_1610
Kidney_1610
Testis_1610
Whole_Brain_1610
reads per million genome matching
bp position
0
20
40
60
80
100

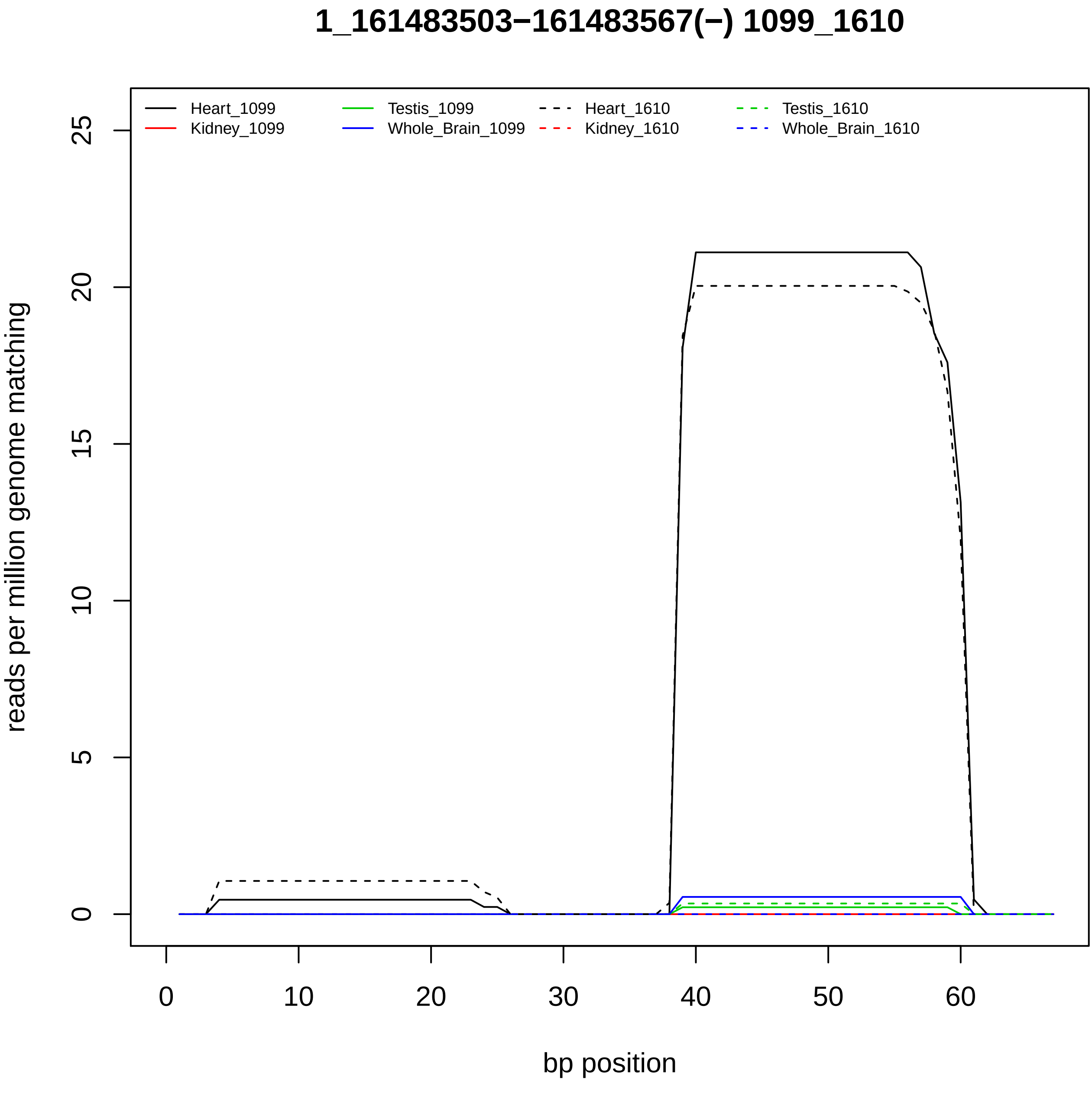

1_161483503−161483567(−) 1099_1610
Heart_1099
Kidney_1099
Testis_1099
Whole_Brain_1099
Heart_1610
Kidney_1610
Testis_1610
Whole_Brain_1610
reads per million genome matching
bp position
0
5
10
15
20
25
0
10
20
30
40
50
60

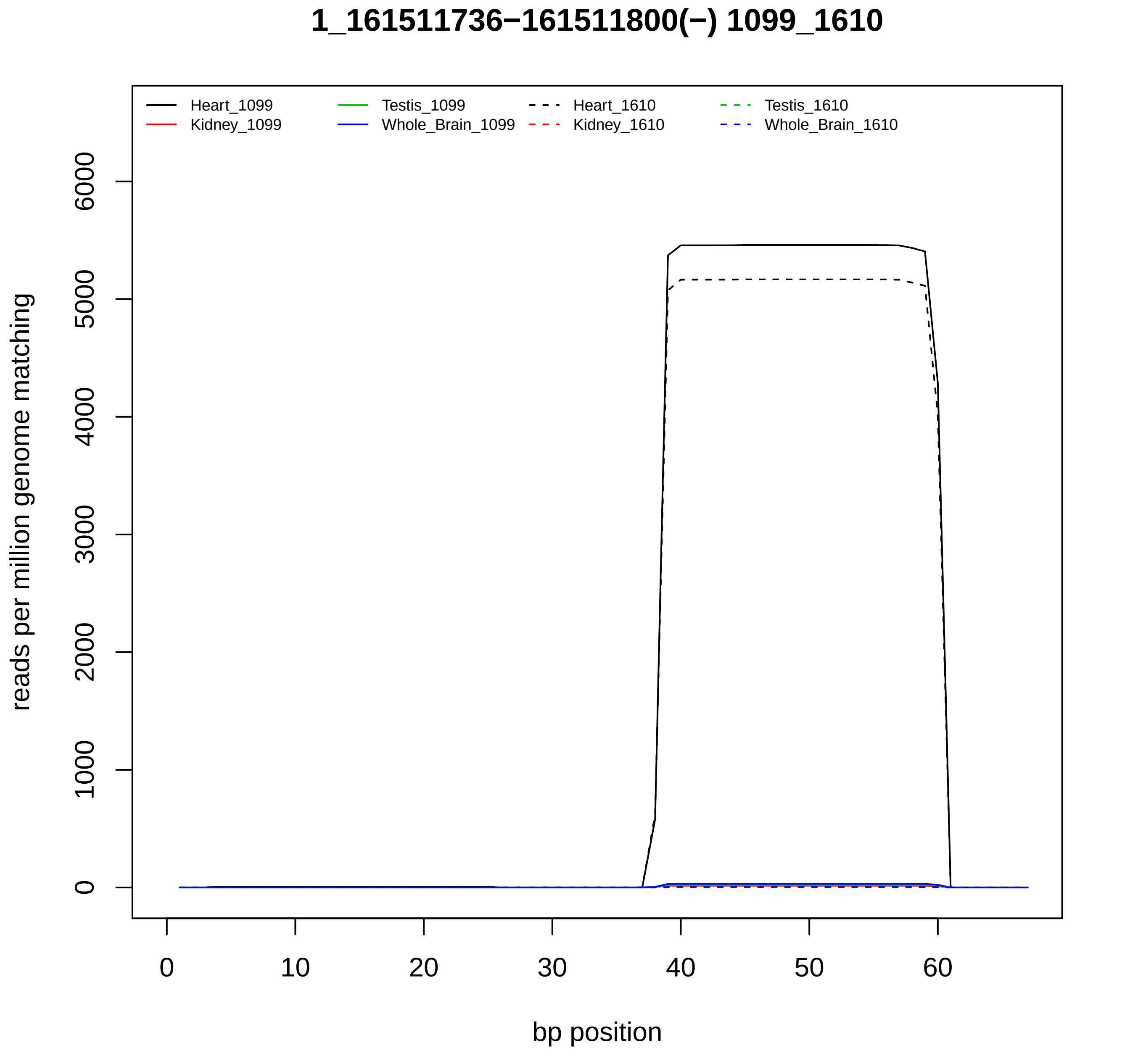

1_161511736−161511800(−) 1099_1610
Heart_1099
Kidney_1099
Testis_1099
Whole_Brain_1099
Heart_1610
Kidney_1610
Testis_1610
Whole_Brain_1610
reads per million genome matching
0
1000
2000
3000
4000
5000
6000
bp position
0
10
20
30
40
50
60

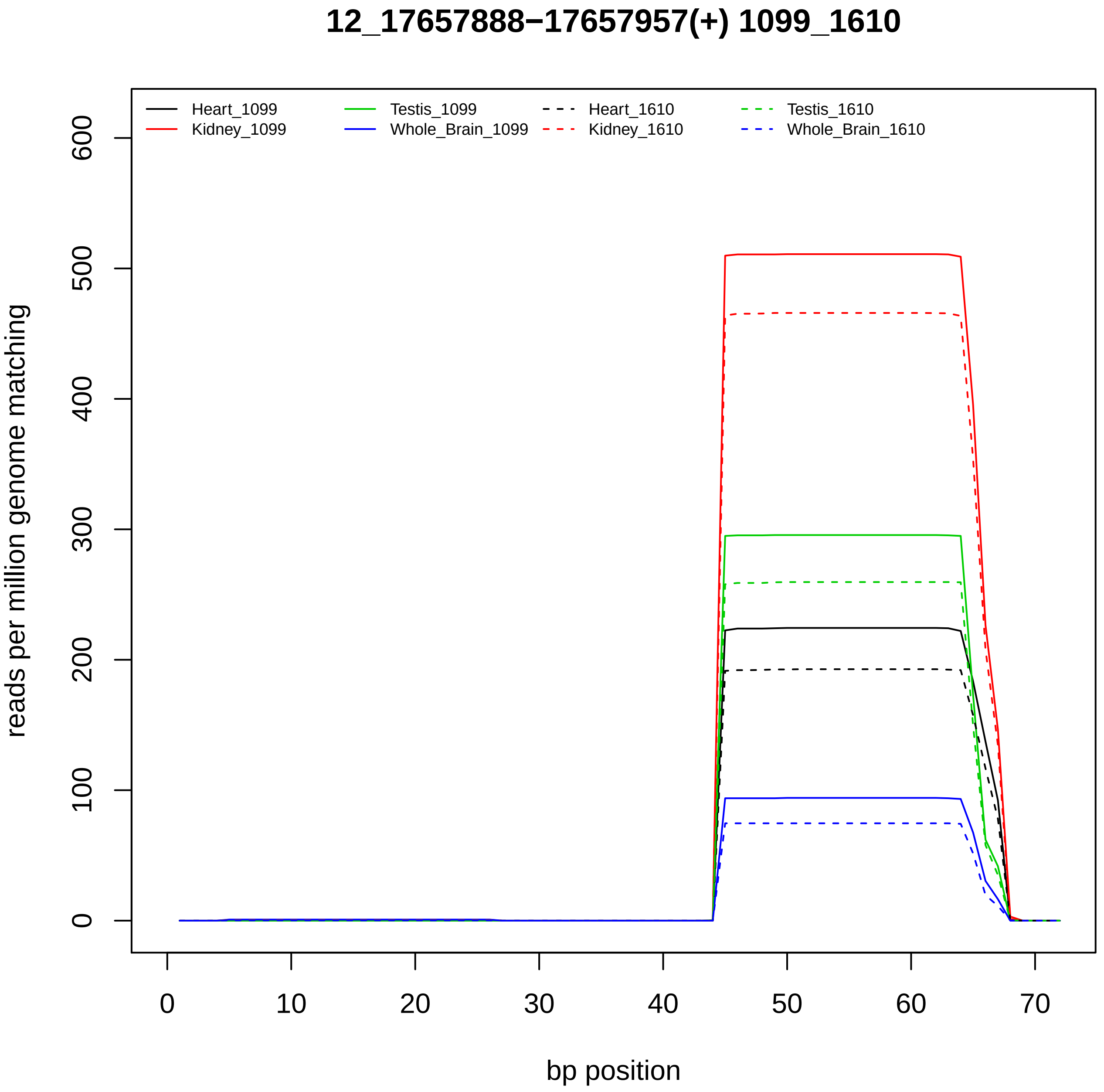

12_17657888−17657957(+) 1099_1610
Heart_1099
Kidney_1099
Testis_1099
Whole_Brain_1099
Heart_1610
Kidney_1610
Testis_1610
Whole_Brain_1610
reads per million genome matching
0
100
200
300
400
500
600
0
10
20
30
40
50
60
70
bp position

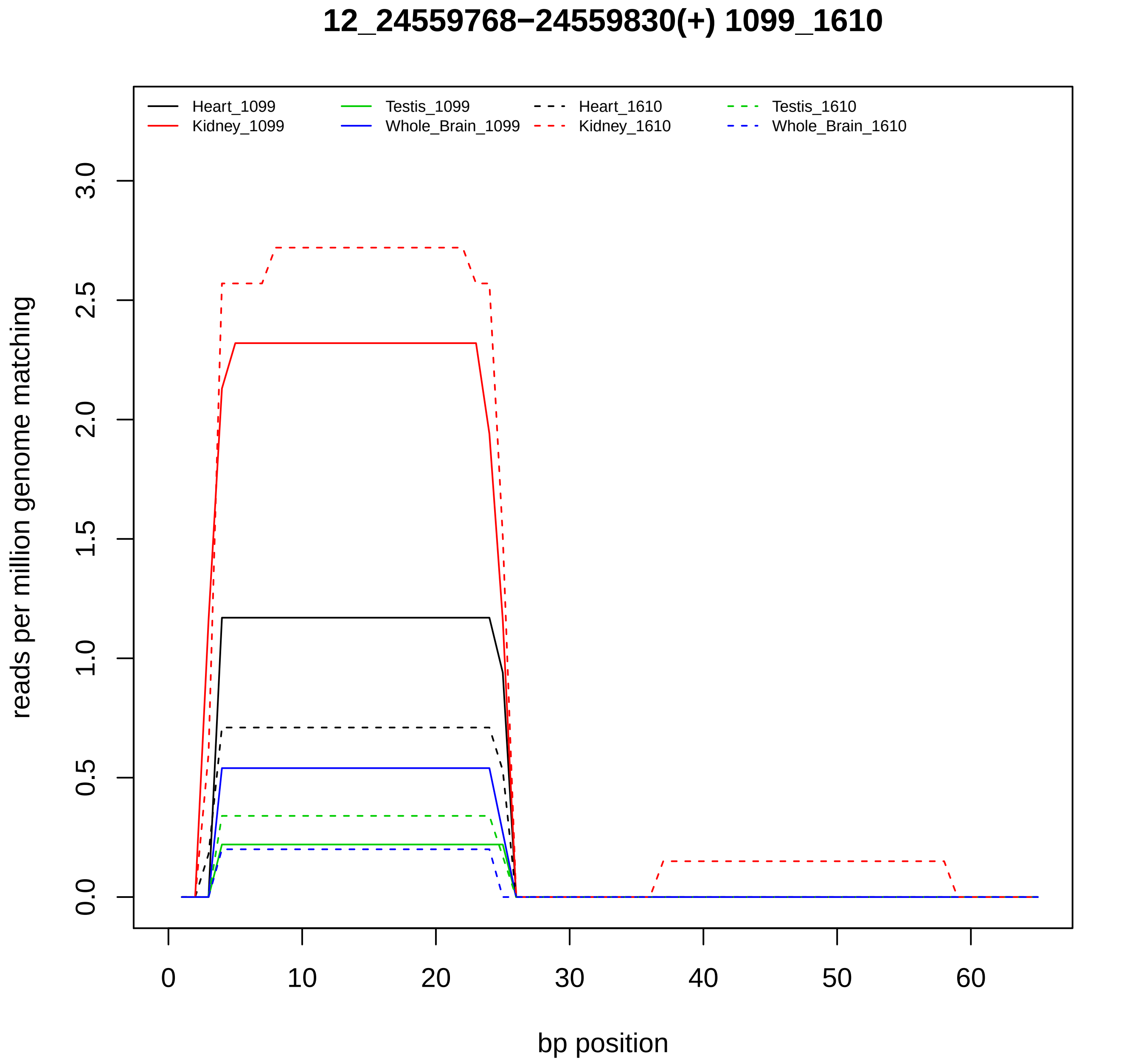

12_24559768−24559830(+) 1099_1610
Heart_1099
Kidney_1099
Testis_1099
Whole_Brain_1099
Heart_1610
Kidney_1610
Testis_1610
Whole_Brain_1610
reads per million genome matching
0.0
0.5
1.0
1.5
2.0
2.5
3.0
0
10
20
30
40
50
60
bp position

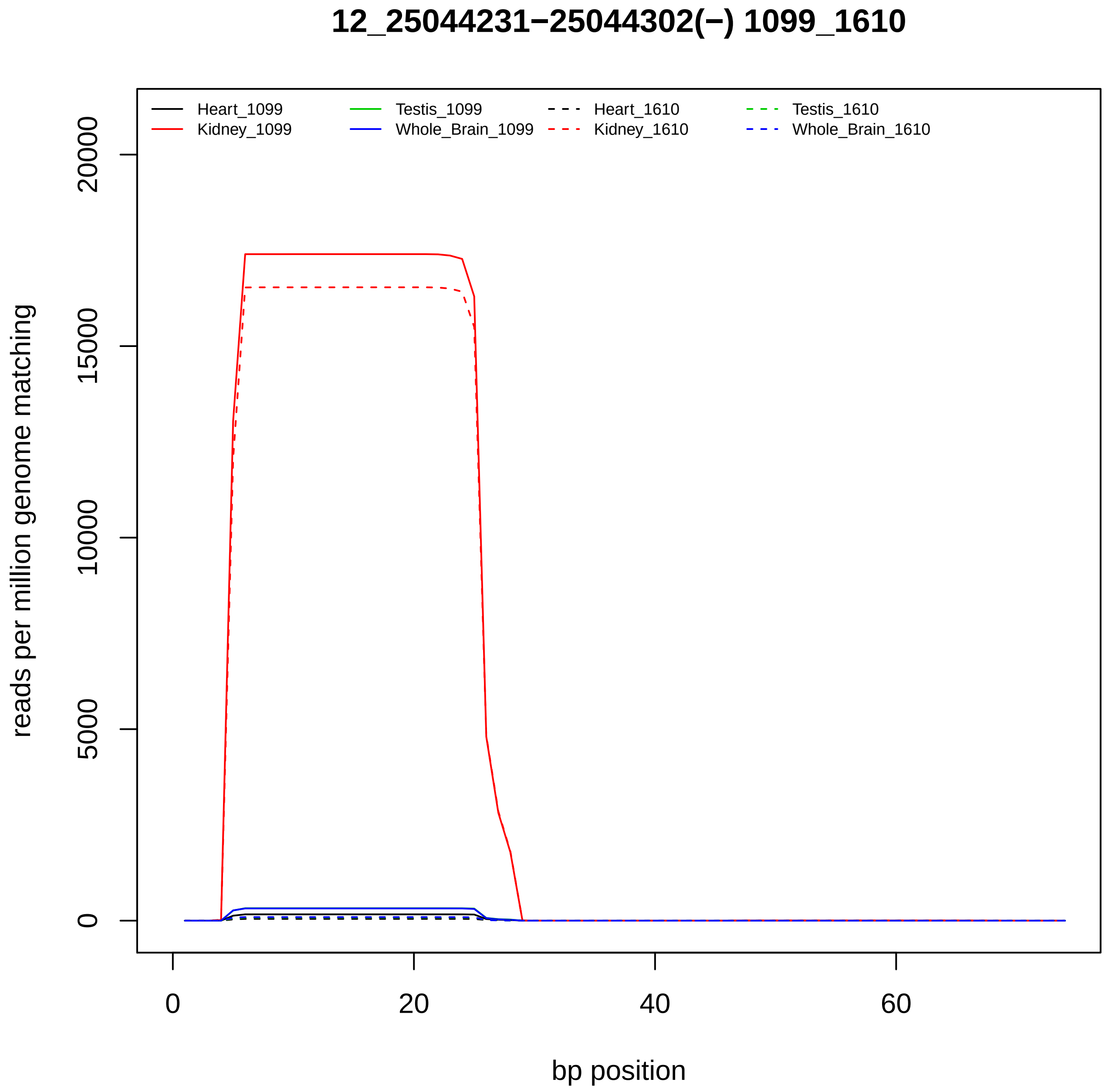
12_25044231−25044302(−) 1099_1610
Heart_1099
Kidney_1099
Testis_1099
Whole_Brain_1099
Heart_1610
Kidney_1610
Testis_1610
Whole_Brain_1610
reads per million genome matching
0
5000
10000
15000
20000
bp position
0
20
40
60

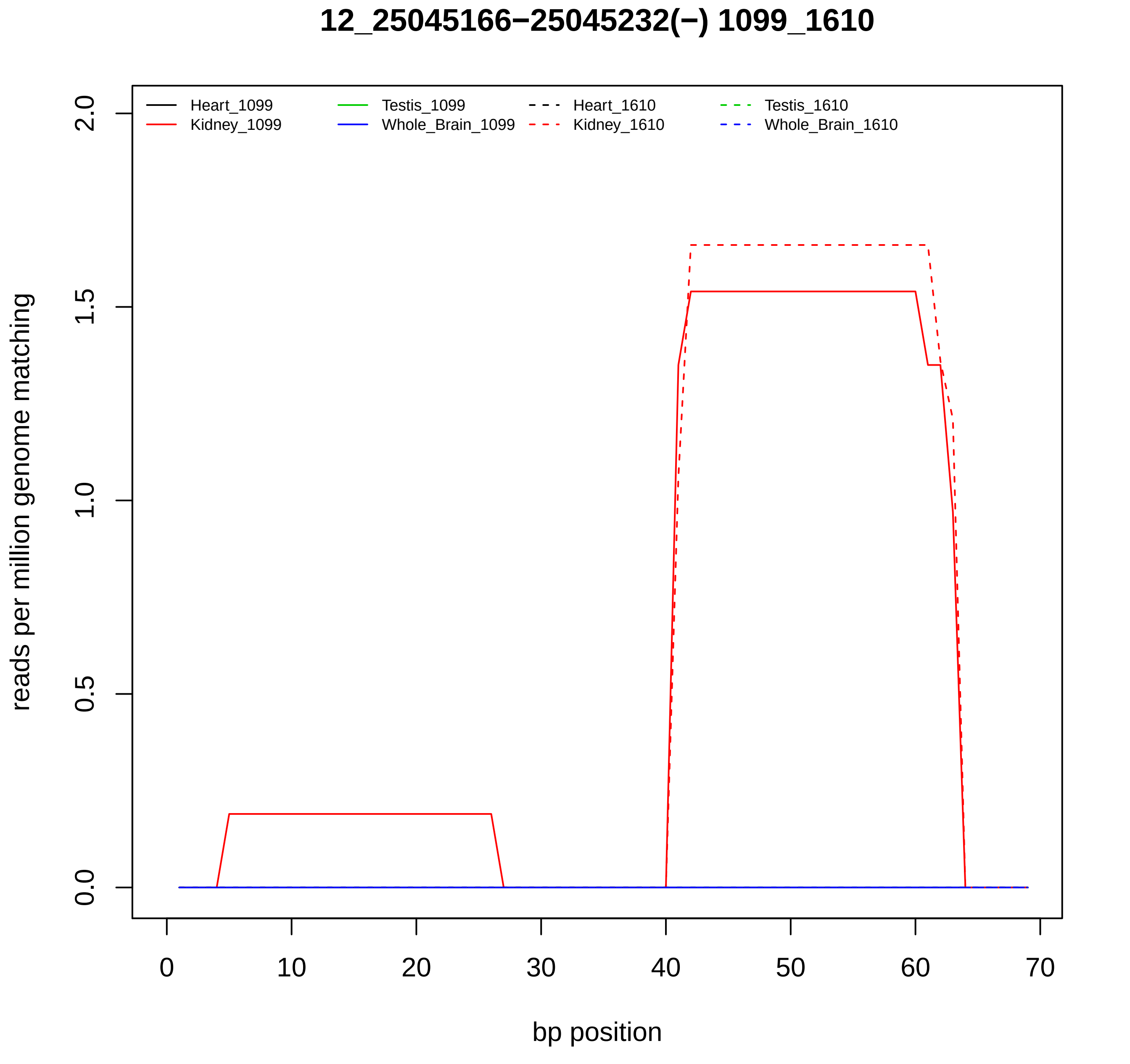

12_25045166−25045232(−) 1099_1610
Heart_1099
Kidney_1099
Testis_1099
Whole_Brain_1099
Heart_1610
Kidney_1610
Testis_1610
Whole_Brain_1610
reads per million genome matching
0.0
0.5
1.0
1.5
2.0
0
10
20
30
40
50
60
70
bp position

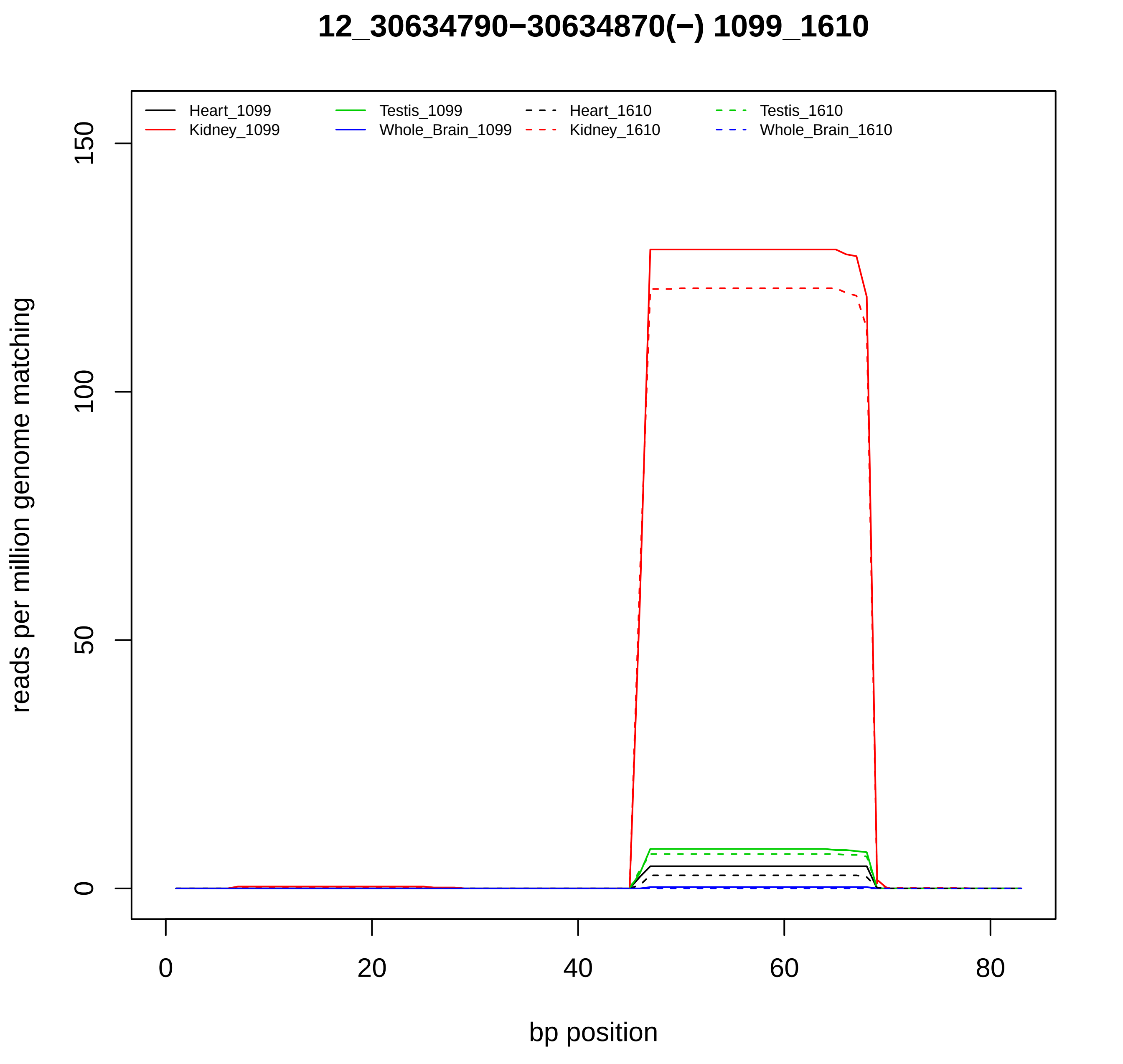
12_30634790−30634870(−) 1099_1610
Heart_1099
Kidney_1099
Testis_1099
Whole_Brain_1099
Heart_1610
Kidney_1610
Testis_1610
Whole_Brain_1610
150
100
50
0
reads per million genome matching
0
20
40
60
80
bp position

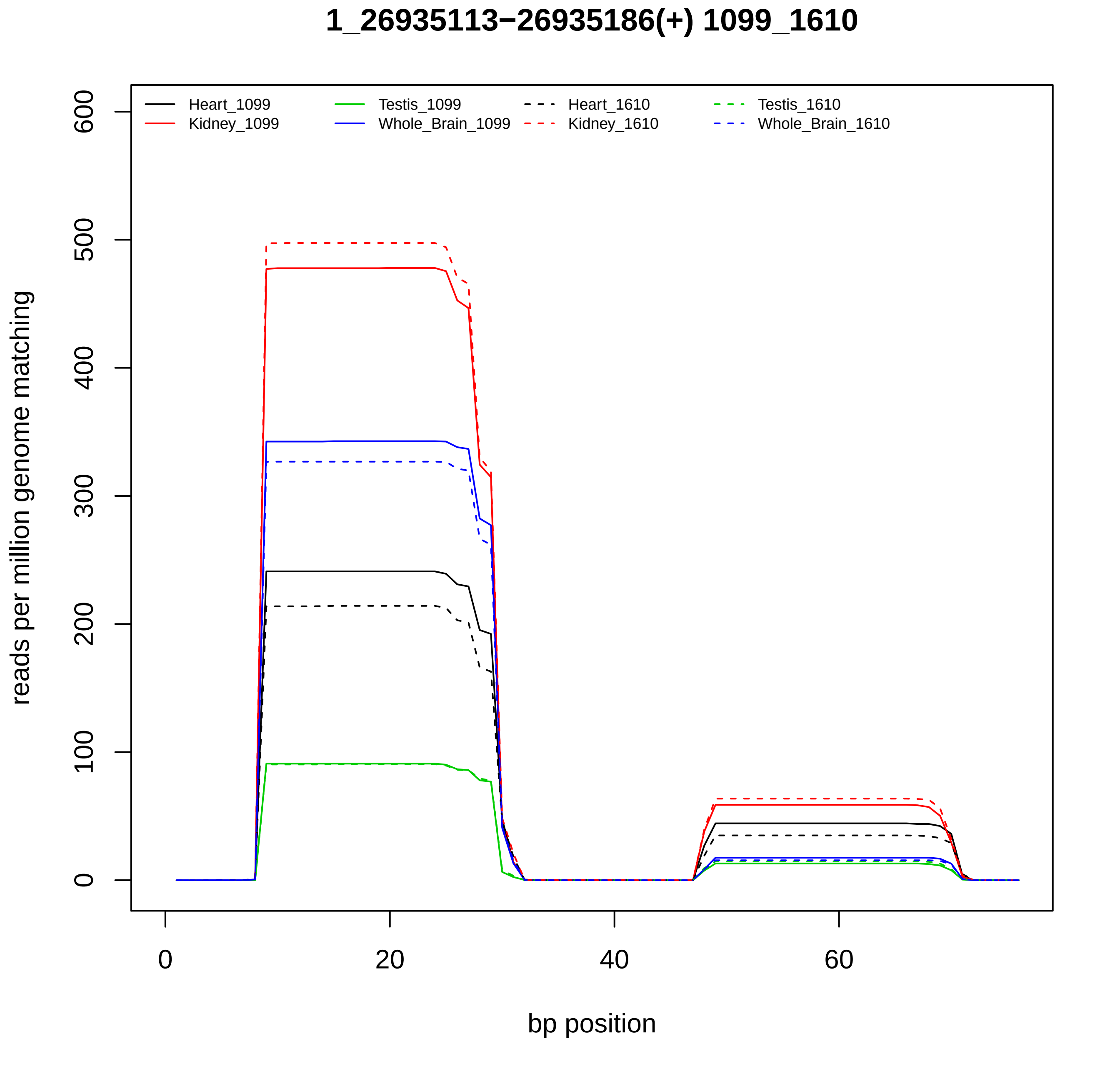
1_26935113−26935186(+) 1099_1610
Heart_1099
Kidney_1099
Testis_1099
Whole_Brain_1099
Heart_1610
Kidney_1610
Testis_1610
Whole_Brain_1610
reads per million genome matching
bp position
0
100
200
300
400
500
600
0
20
40
60

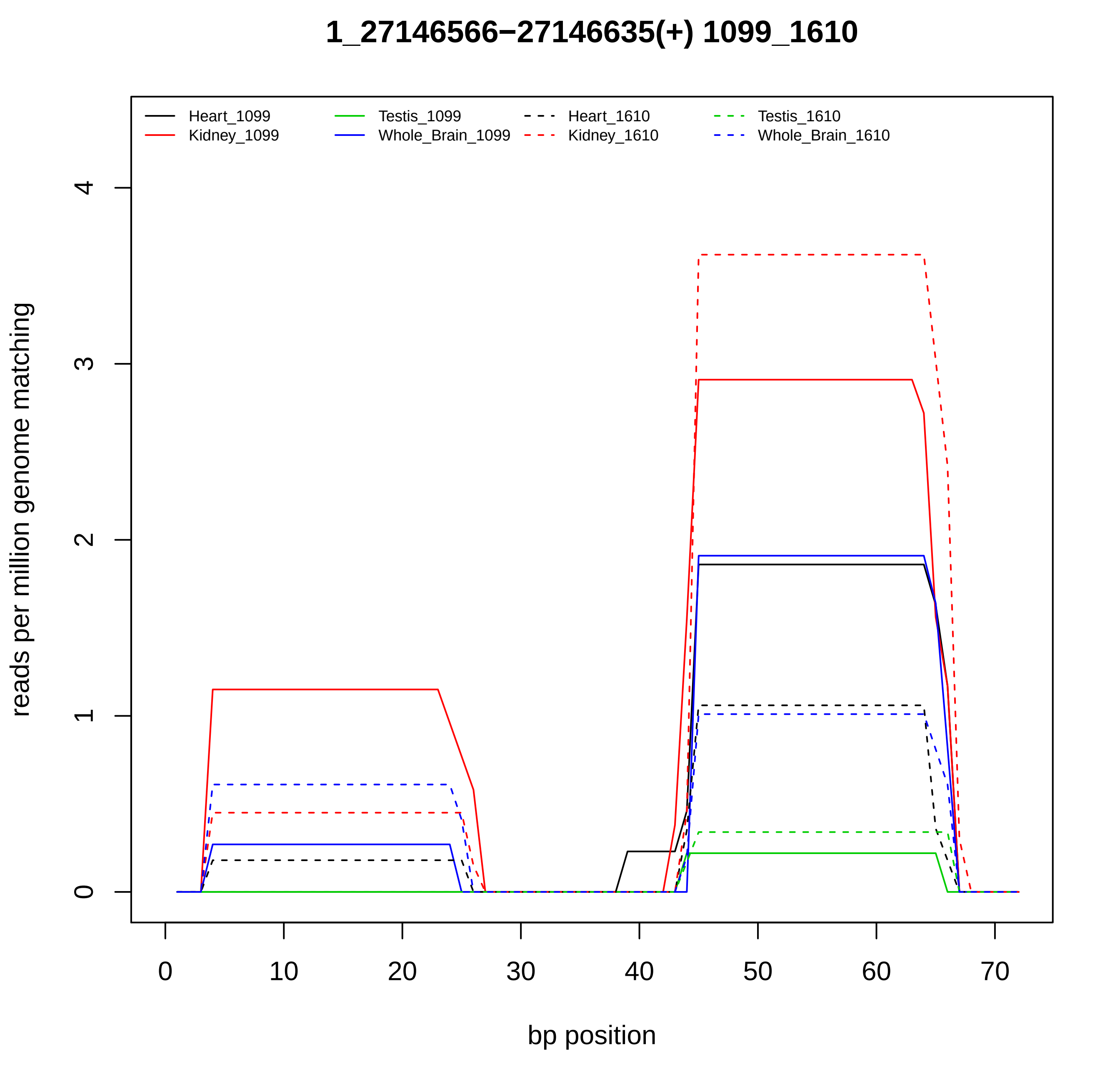

1_27146566−27146635(+) 1099_1610
Heart_1099
Kidney_1099
Testis_1099
Whole_Brain_1099
Heart_1610
Kidney_1610
Testis_1610
Whole_Brain_1610
reads per million genome matching
bp position
0
1
2
3
4
0
10
20
30
40
50
60
70

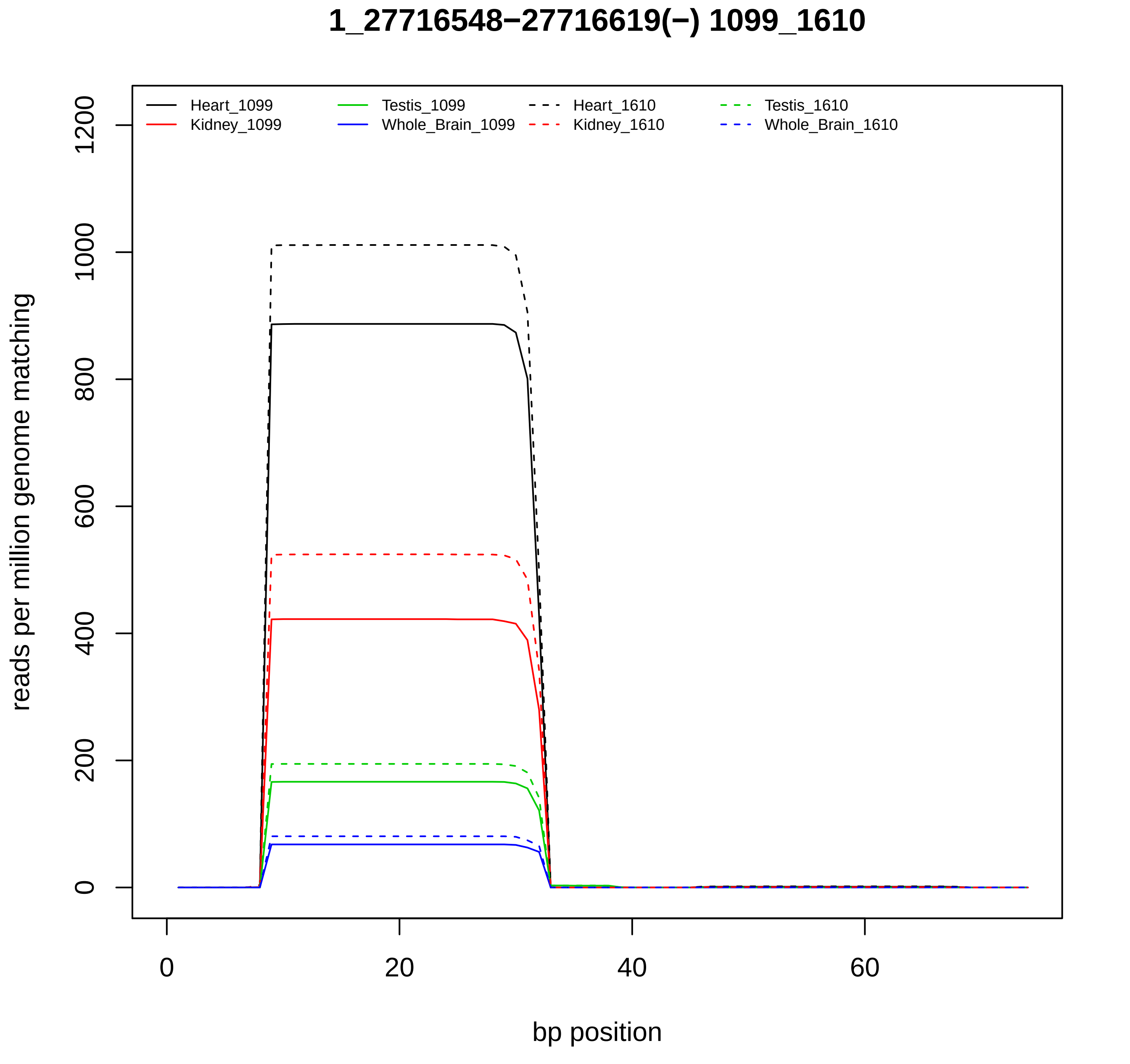

1_27716548−27716619(−) 1099_1610
Heart_1099
Kidney_1099
Testis_1099
Whole_Brain_1099
Heart_1610
Kidney_1610
Testis_1610
Whole_Brain_1610
reads per million genome matching
0
200
400
600
800
1000
1200
0
20
40
60
bp position

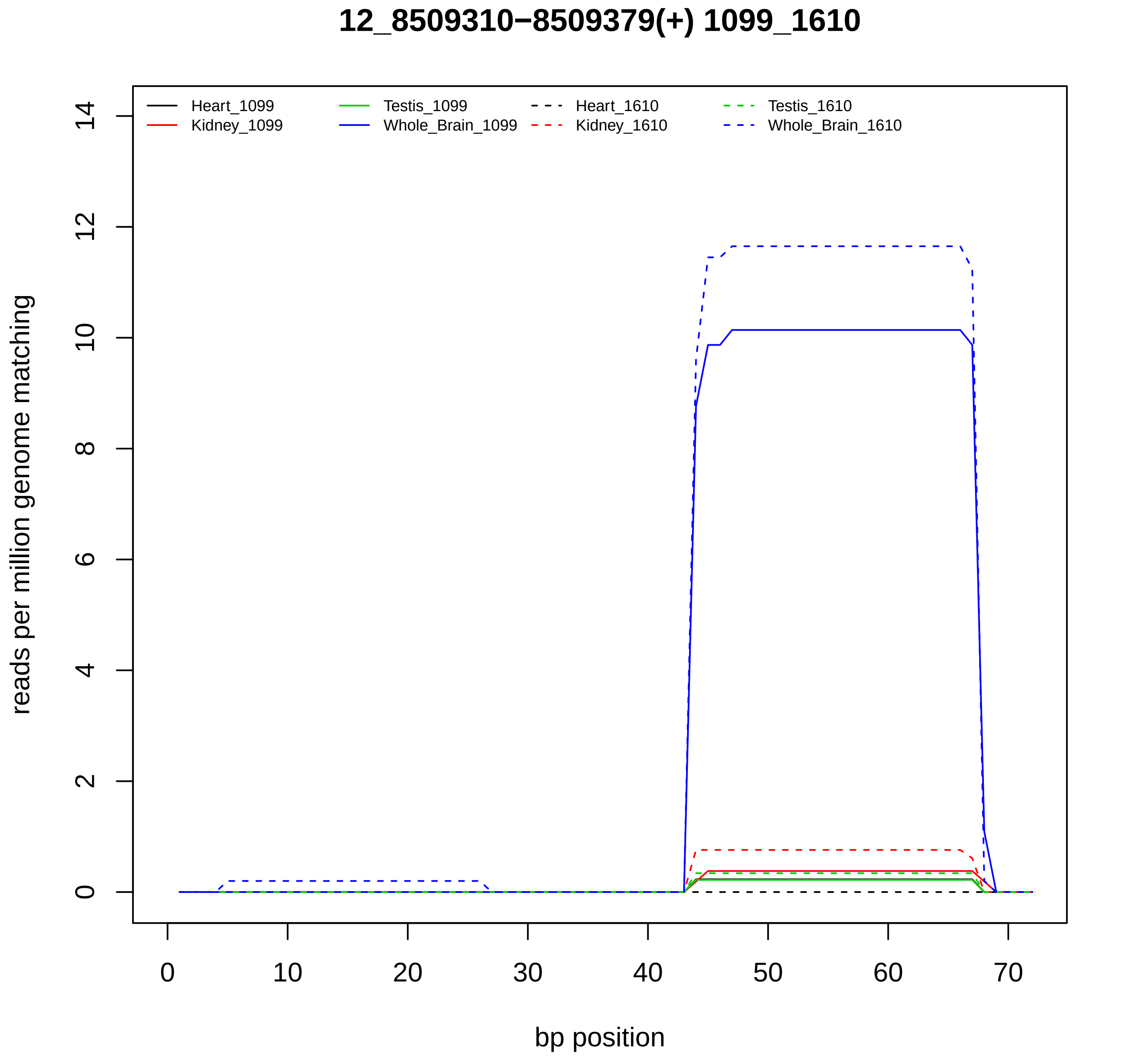

12_8509310−8509379(+) 1099_1610
Heart_1099
Kidney_1099
Testis_1099
Whole_Brain_1099
Heart_1610
Kidney_1610
Testis_1610
Whole_Brain_1610
reads per million genome matching
bp position
0
2
4
6
8
10
12
14
0
10
20
30
40
50
60
70

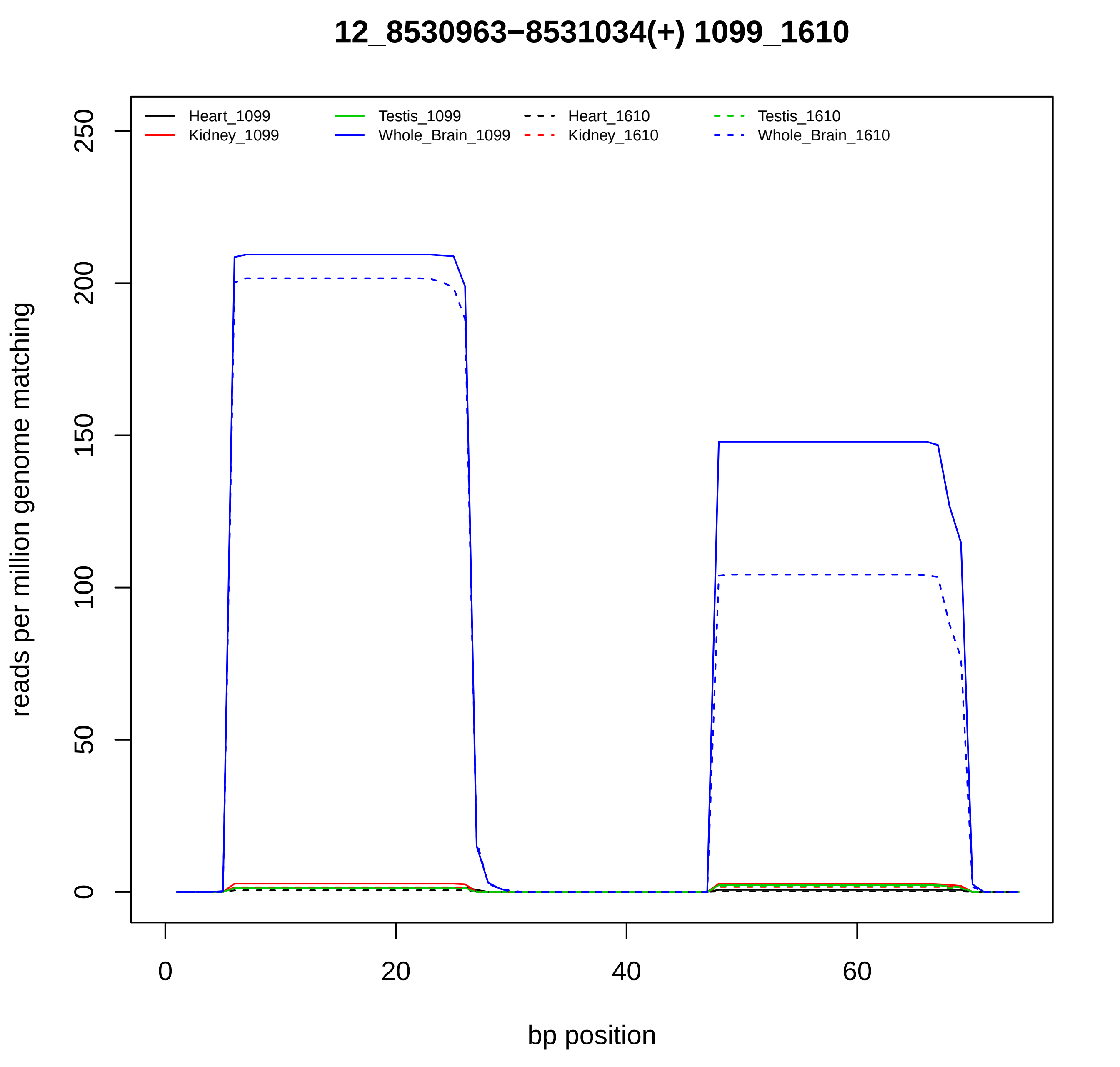
12_8530963−8531034(+) 1099_1610
Heart_1099
Kidney_1099
Testis_1099
Whole_Brain_1099
Heart_1610
Kidney_1610
Testis_1610
Whole_Brain_1610
reads per million genome matching
bp position
0
50
100
150
200
250
0
20
40
60

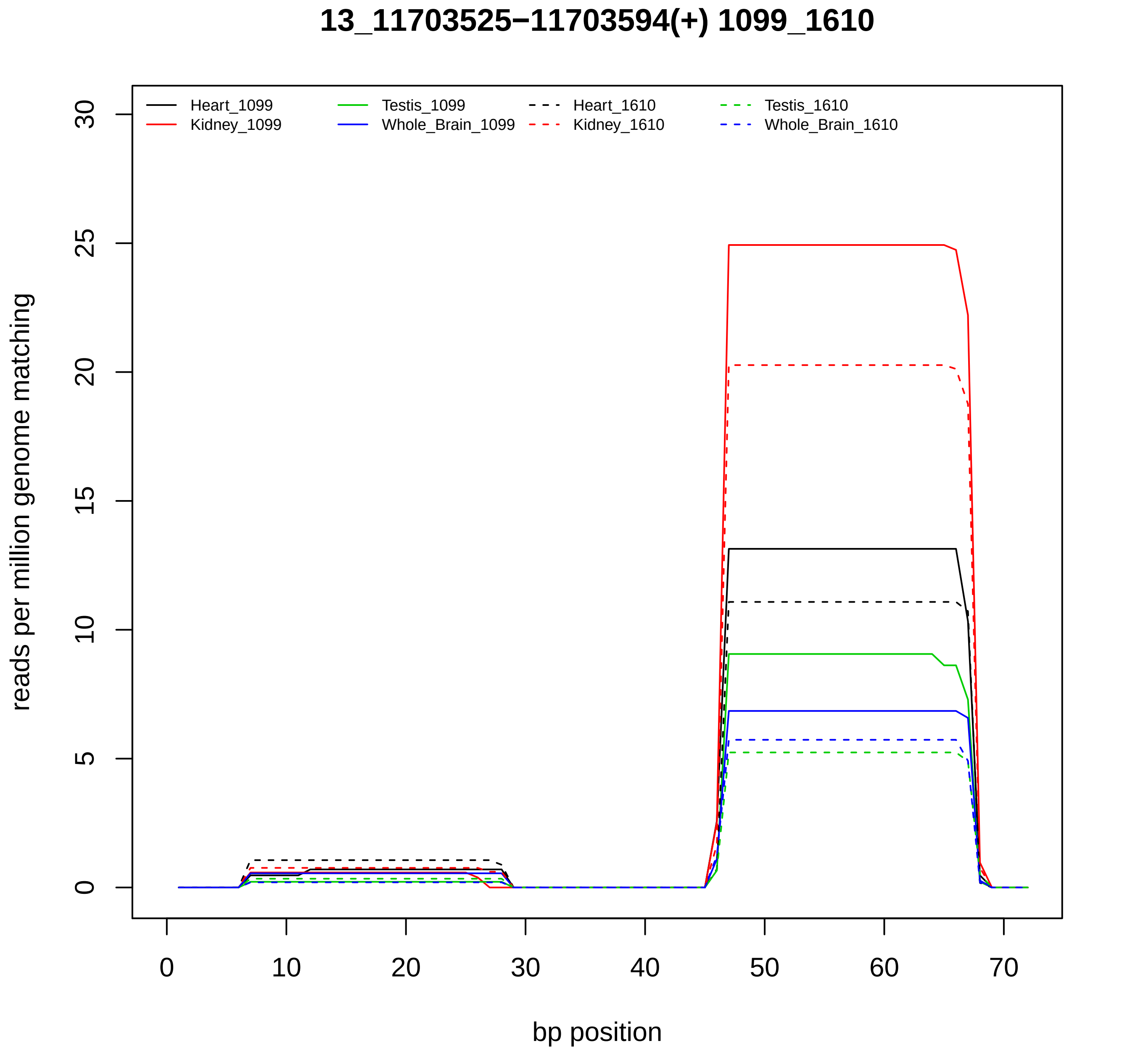
13_11703525−11703594(+) 1099_1610
Heart_1099
Kidney_1099
Testis_1099
Whole_Brain_1099
Heart_1610
Kidney_1610
Testis_1610
Whole_Brain_1610
reads per million genome matching
bp position
0
5
10
15
20
25
30
0
10
20
30
40
50
60
70

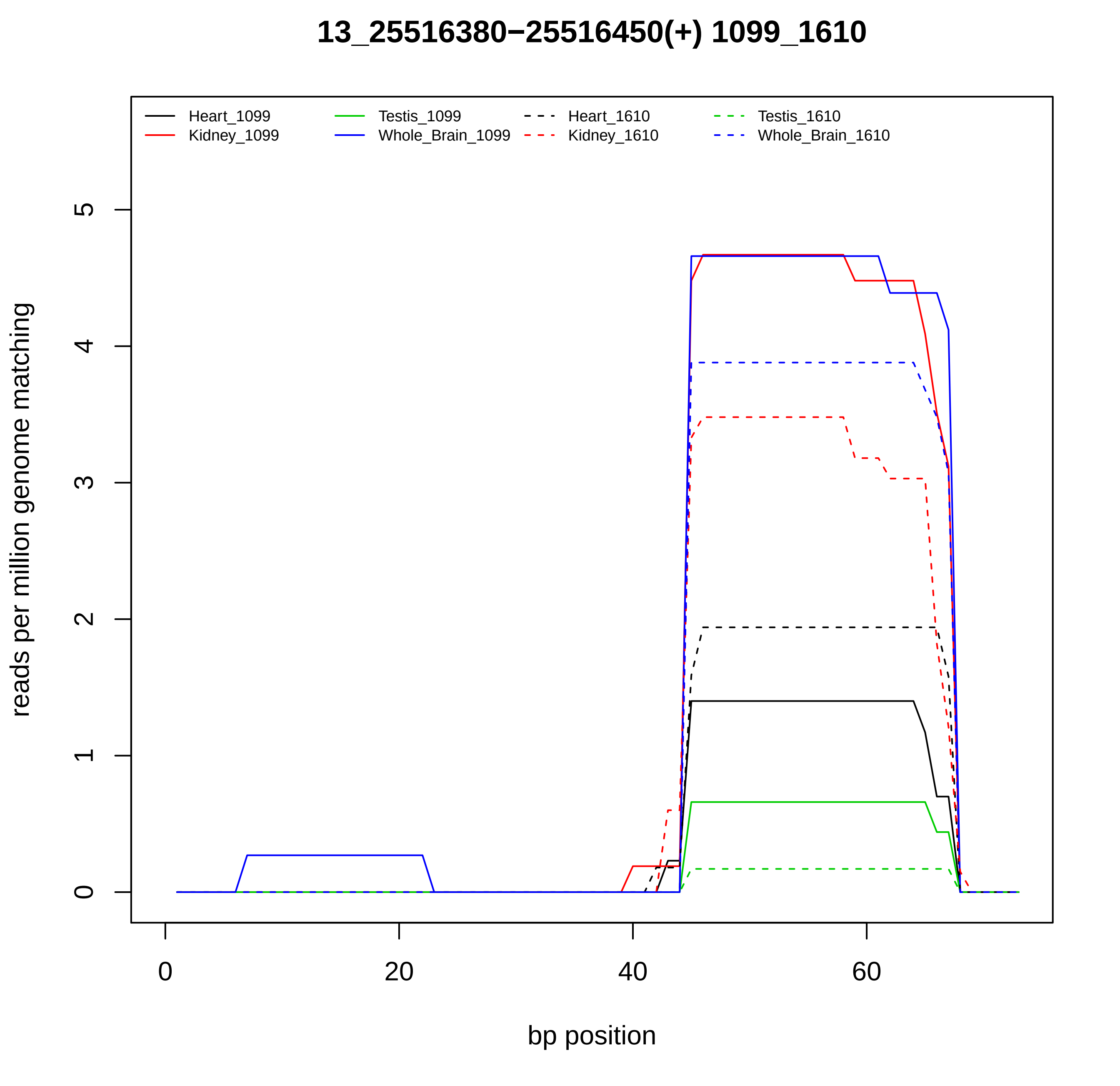
13_25516380−25516450(+) 1099_1610
Heart_1099
Kidney_1099
Testis_1099
Whole_Brain_1099
Heart_1610
Kidney_1610
Testis_1610
Whole_Brain_1610
reads per million genome matching
0
1
2
3
4
5
0
20
40
60
bp position

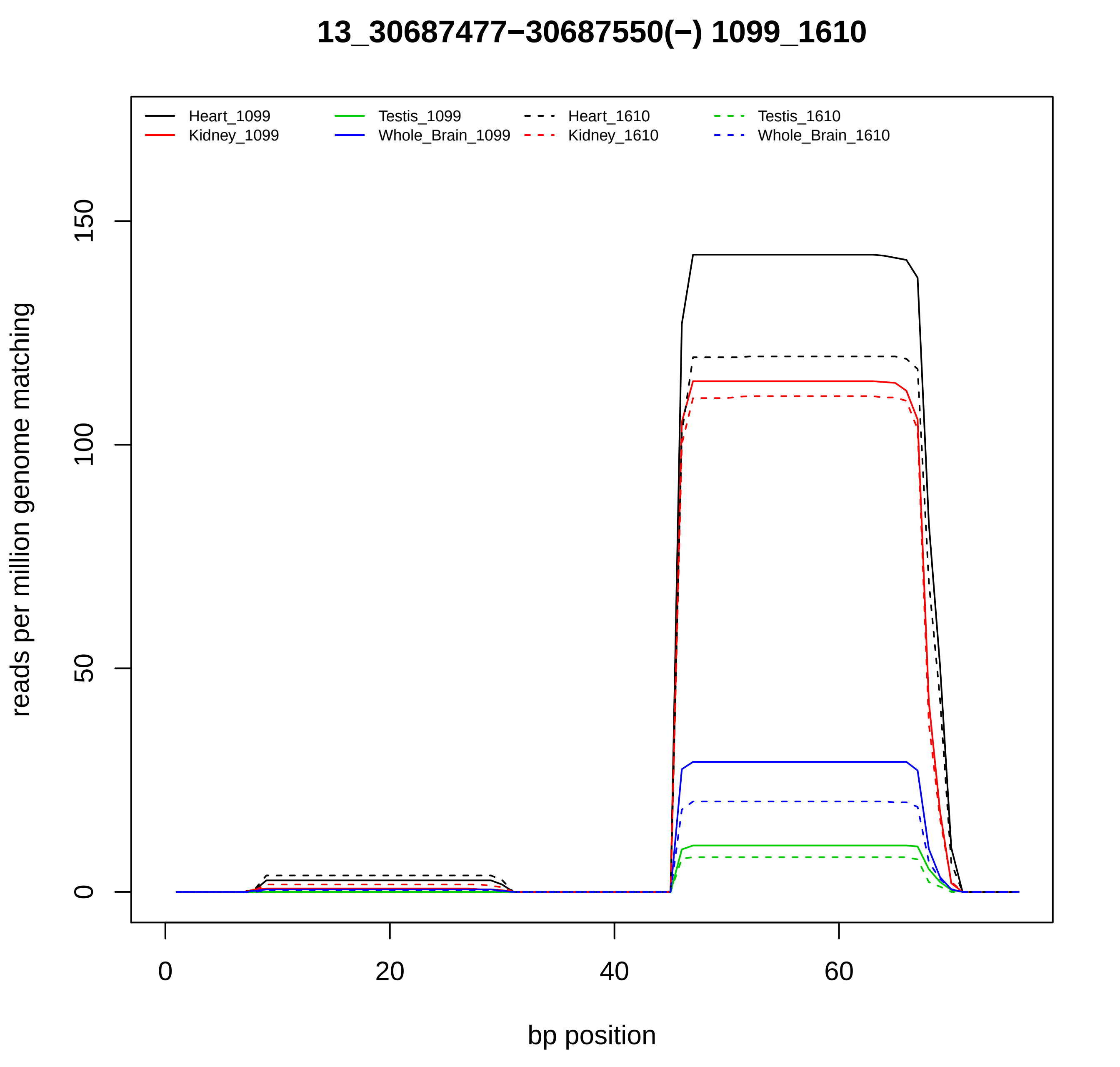
13_30687477−30687550(−) 1099_1610
Heart_1099
Kidney_1099
Testis_1099
Whole_Brain_1099
Heart_1610
Kidney_1610
Testis_1610
Whole_Brain_1610
reads per million genome matching
150
100
50
0
0
20
40
60
bp position

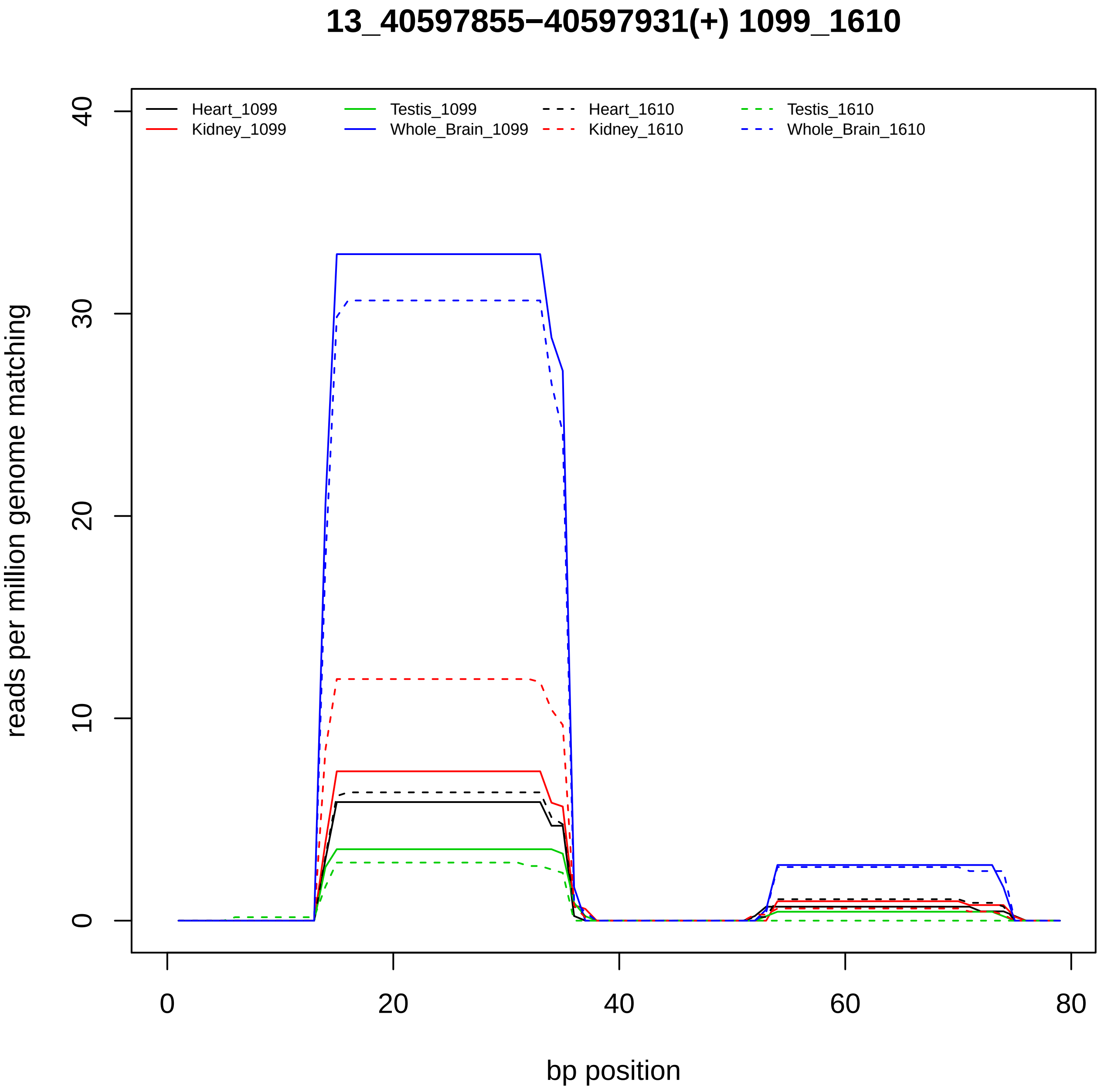

13_40597855−40597931(+) 1099_1610
Heart_1099
Kidney_1099
Testis_1099
Whole_Brain_1099
Heart_1610
Kidney_1610
Testis_1610
Whole_Brain_1610
reads per million genome matching
bp position
0
10
20
30
40
0
20
40
60
80

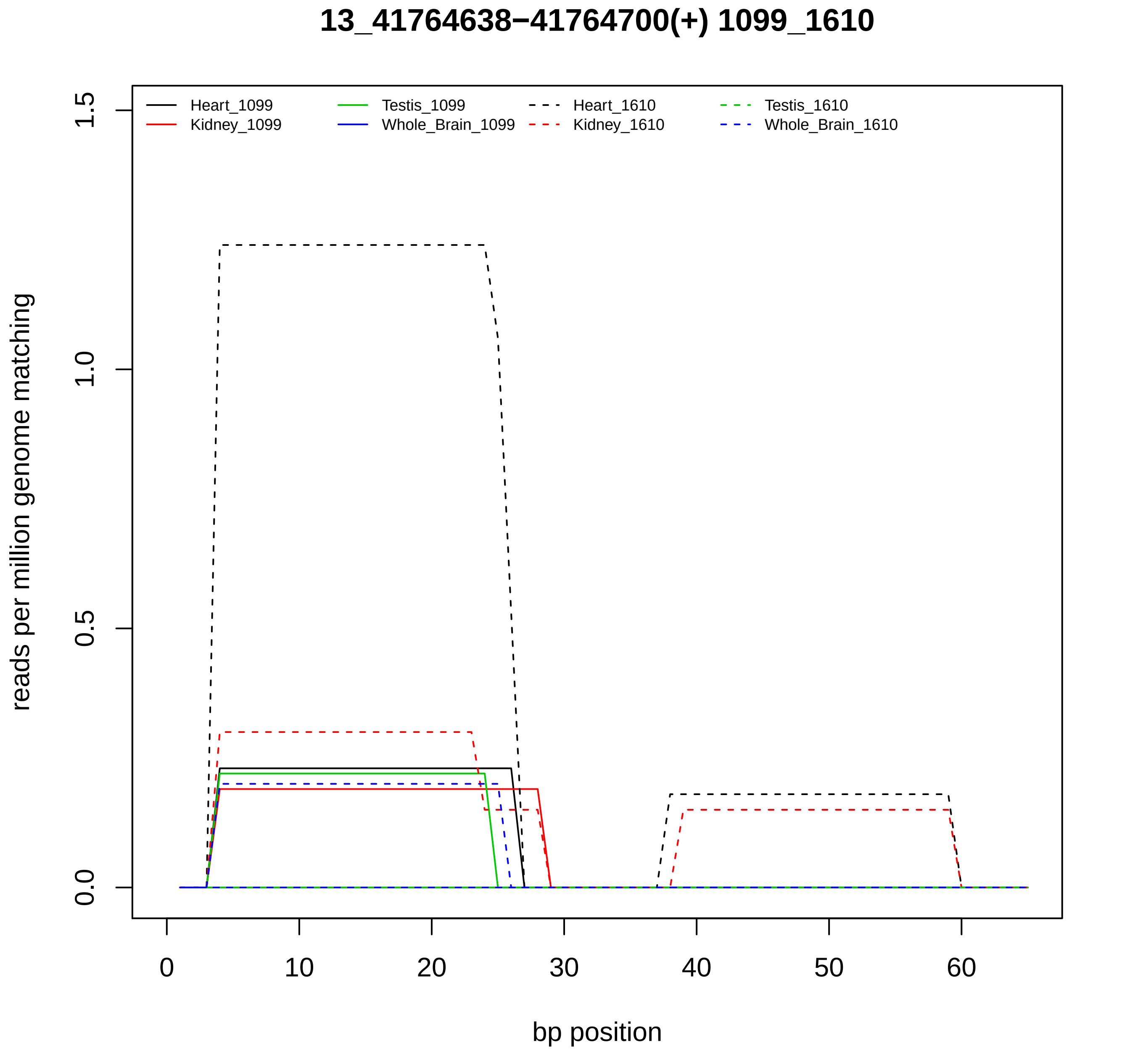

13_41764638−41764700(+) 1099_1610
Heart_1099
Kidney_1099
Testis_1099
Whole_Brain_1099
Heart_1610
Kidney_1610
Testis_1610
Whole_Brain_1610
reads per million genome matching
0.0
0.5
1.0
1.5
0
10
20
30
40
50
60
bp position

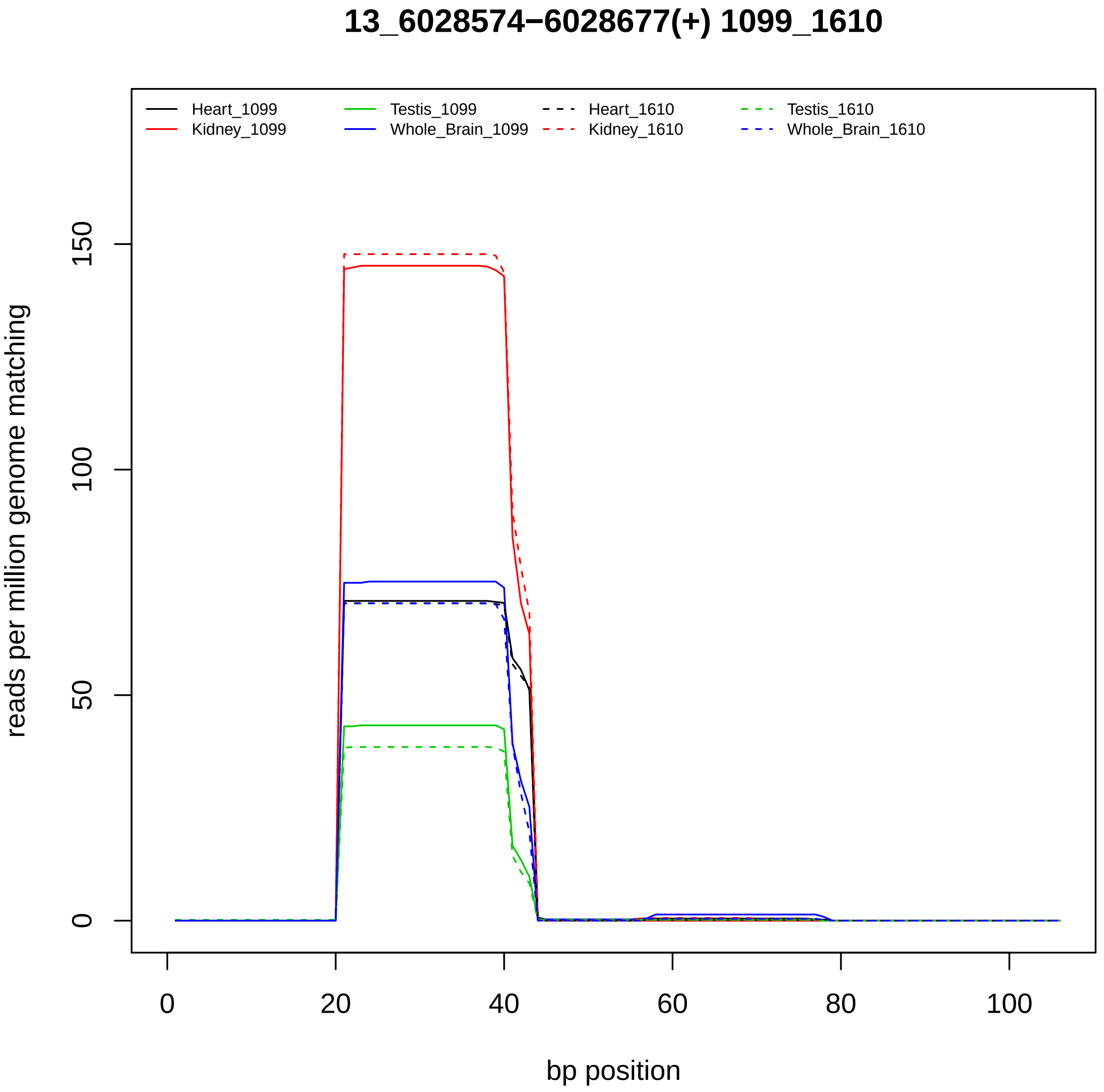
13_6028574−6028677(+) 1099_1610
Heart_1099
Kidney_1099
Testis_1099
Whole_Brain_1099
Heart_1610
Kidney_1610
Testis_1610
Whole_Brain_1610
reads per million genome matching
0
50
100
150
0
20
40
60
80
100
bp position

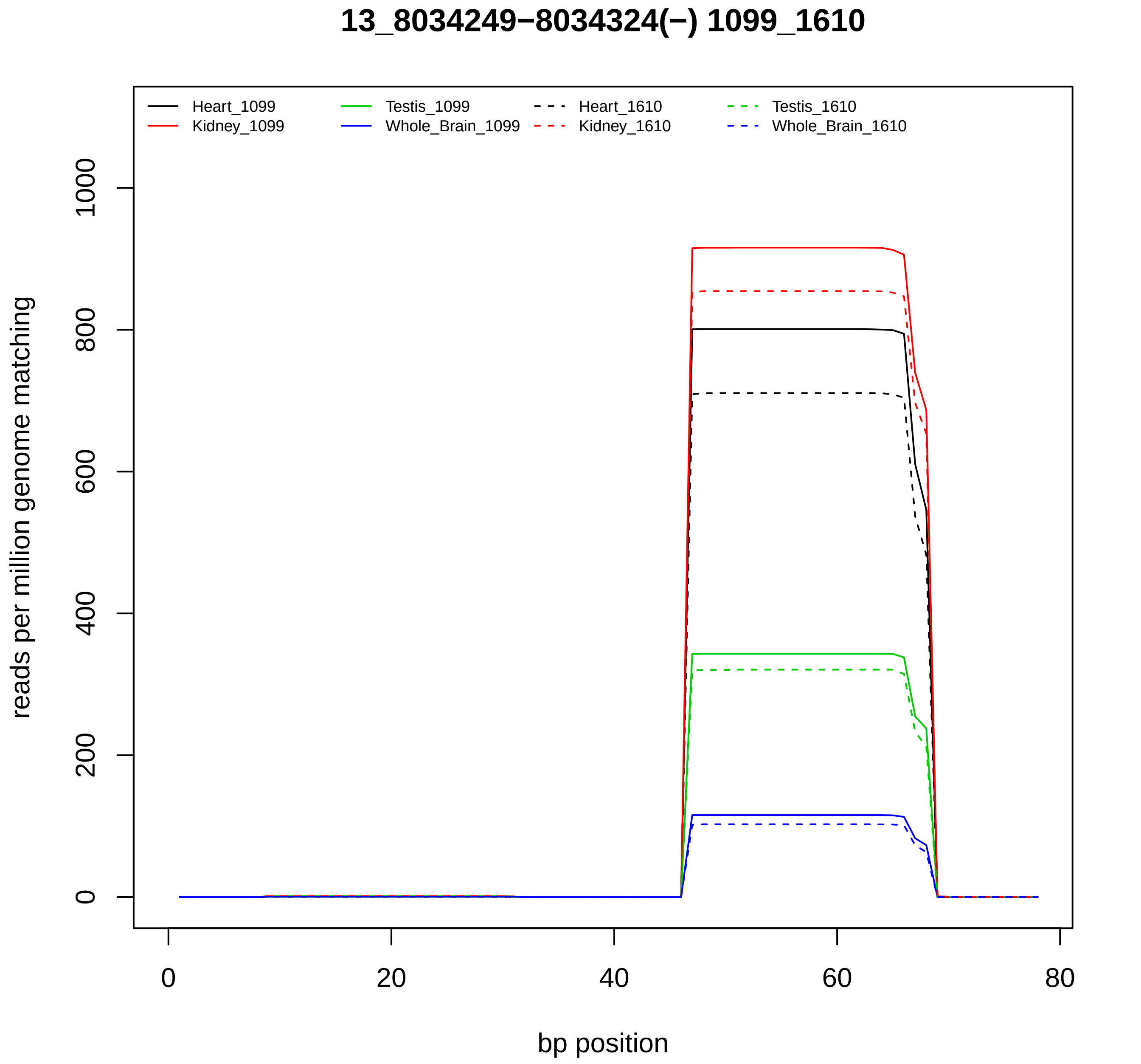

13_8034249−8034324(−) 1099_1610
Heart_1099
Kidney_1099
Testis_1099
Whole_Brain_1099
Heart_1610
Kidney_1610
Testis_1610
Whole_Brain_1610
reads per million genome matching
0
200
400
600
800
1000
bp position
0
20
40
60
80

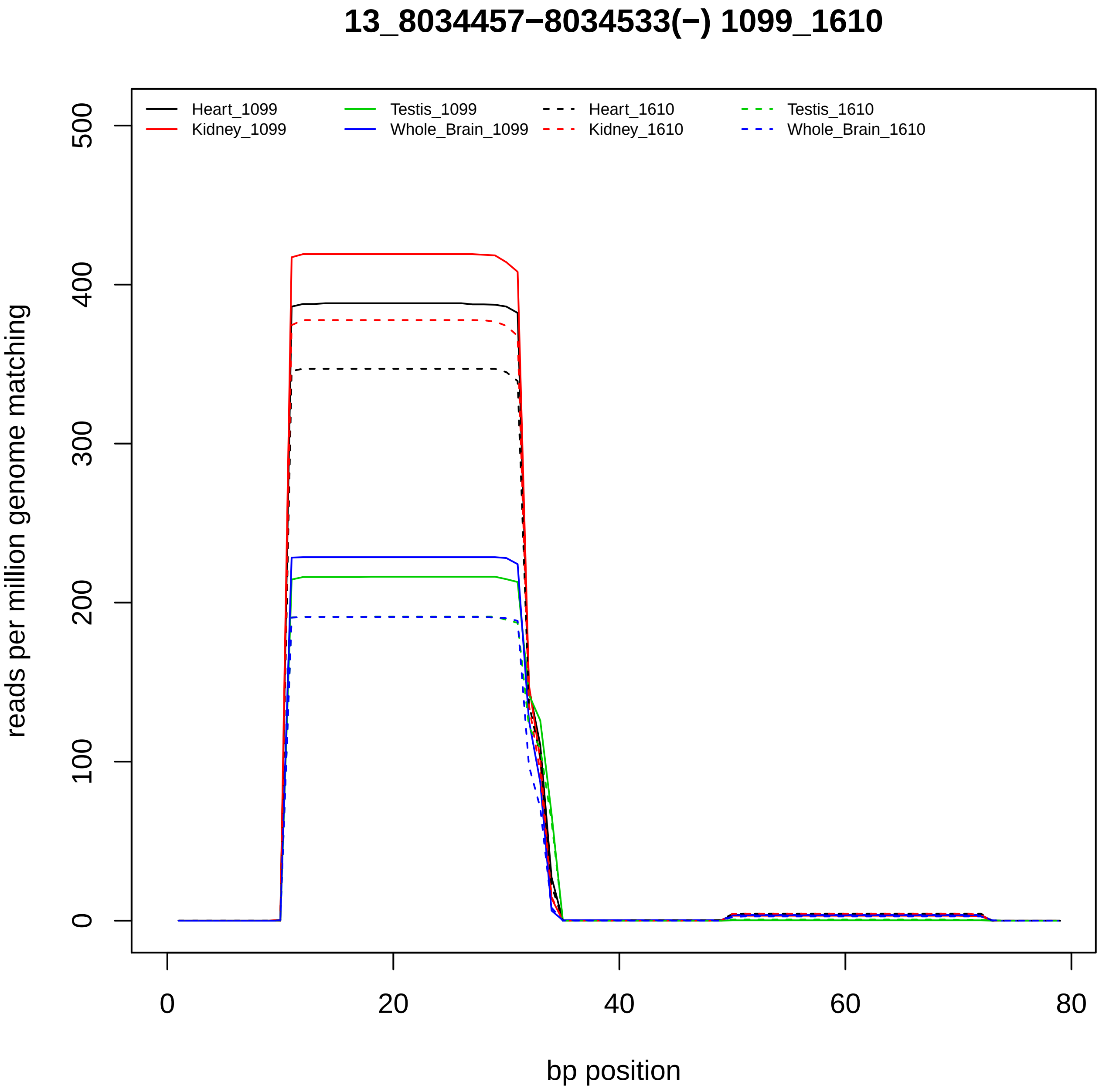

13_8034457−8034533(−) 1099_1610
Heart_1099
Kidney_1099
Testis_1099
Whole_Brain_1099
Heart_1610
Kidney_1610
Testis_1610
Whole_Brain_1610
reads per million genome matching
bp position
0
100
200
300
400
500
0
20
40
60
80

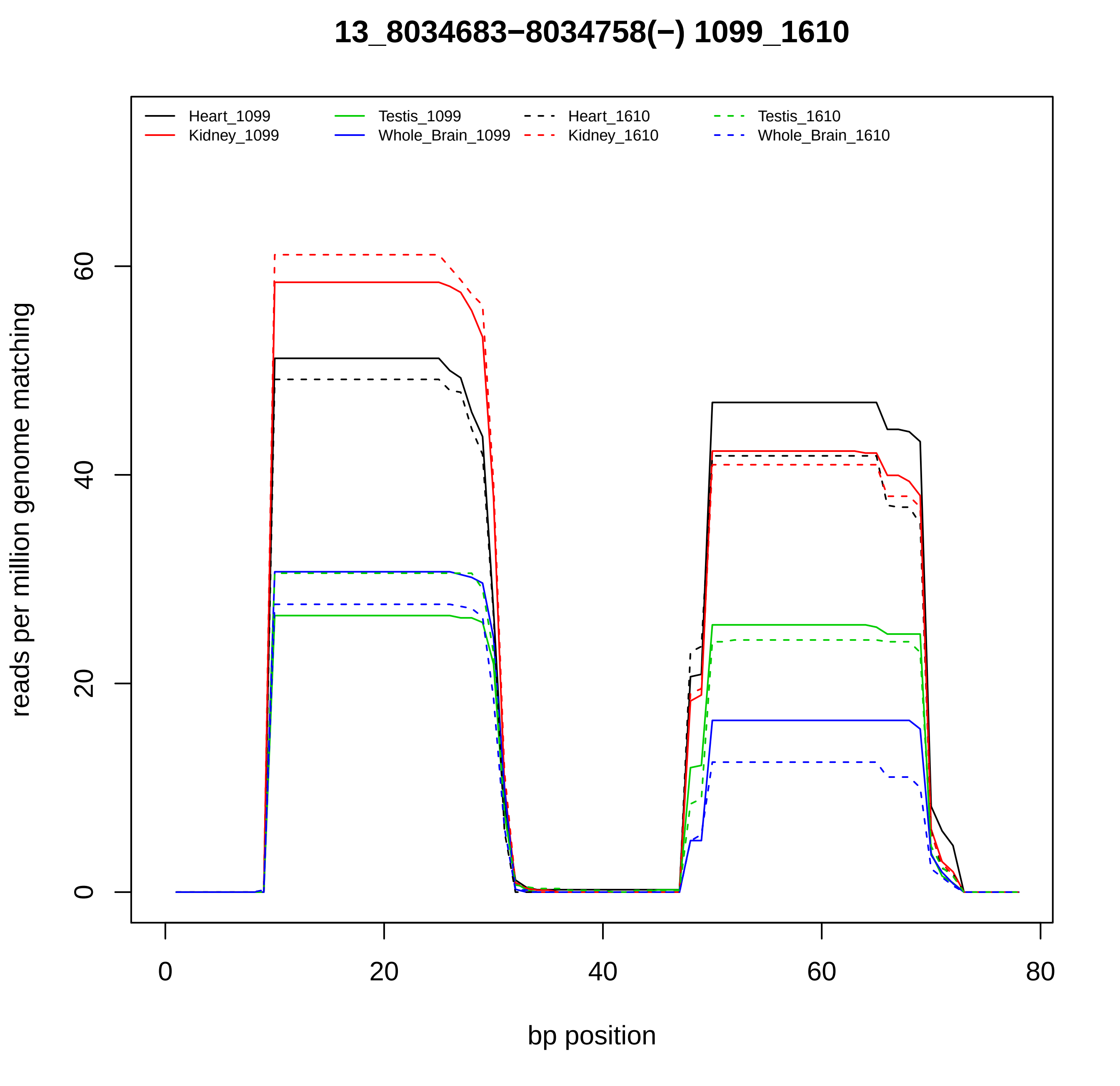
13_8034683−8034758(−) 1099_1610
Heart_1099
Kidney_1099
Testis_1099
Whole_Brain_1099
Heart_1610
Kidney_1610
Testis_1610
Whole_Brain_1610
reads per million genome matching
bp position
0
20
40
60
80
0
20
40
60

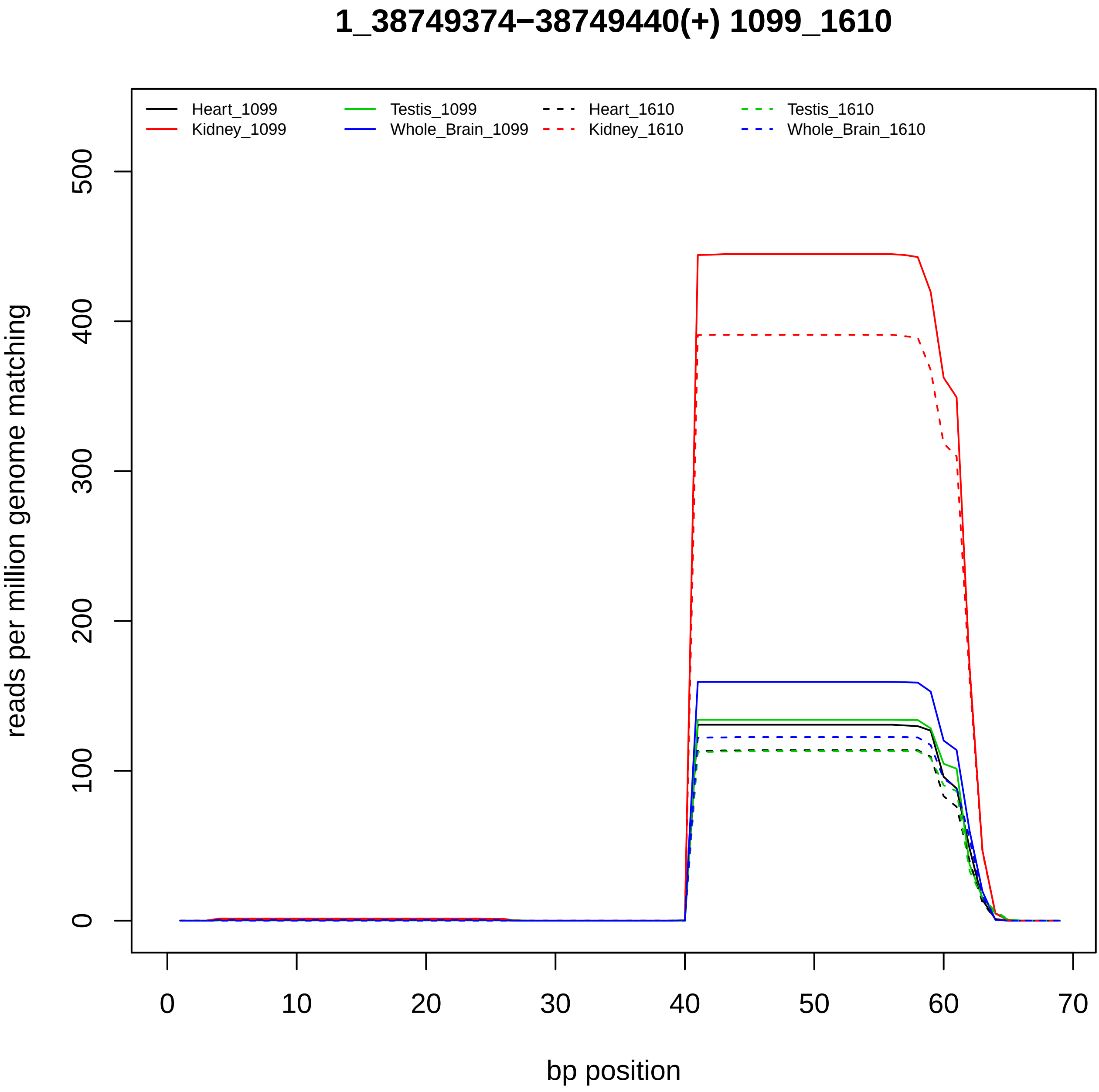
1_38749374−38749440(+) 1099_1610
Heart_1099
Kidney_1099
Testis_1099
Whole_Brain_1099
Heart_1610
Kidney_1610
Testis_1610
Whole_Brain_1610
reads per million genome matching
bp position
0
100
200
300
400
500
0
10
20
30
40
50
60
70

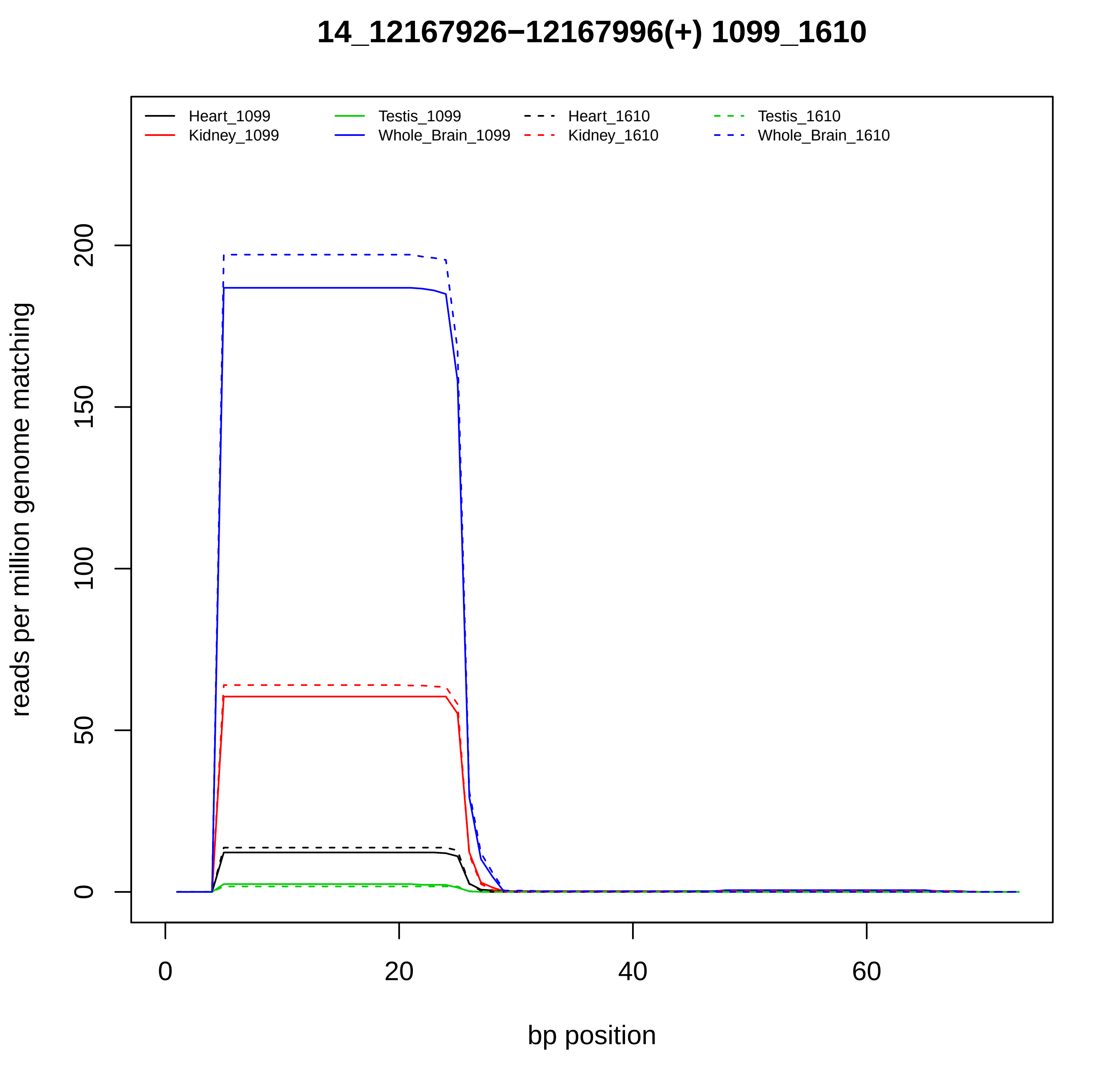
14_12167926−12167996(+) 1099_1610
Heart_1099
Kidney_1099
Testis_1099
Whole_Brain_1099
Heart_1610
Kidney_1610
Testis_1610
Whole_Brain_1610
reads per million genome matching
0
50
100
150
200
bp position
0
20
40
60

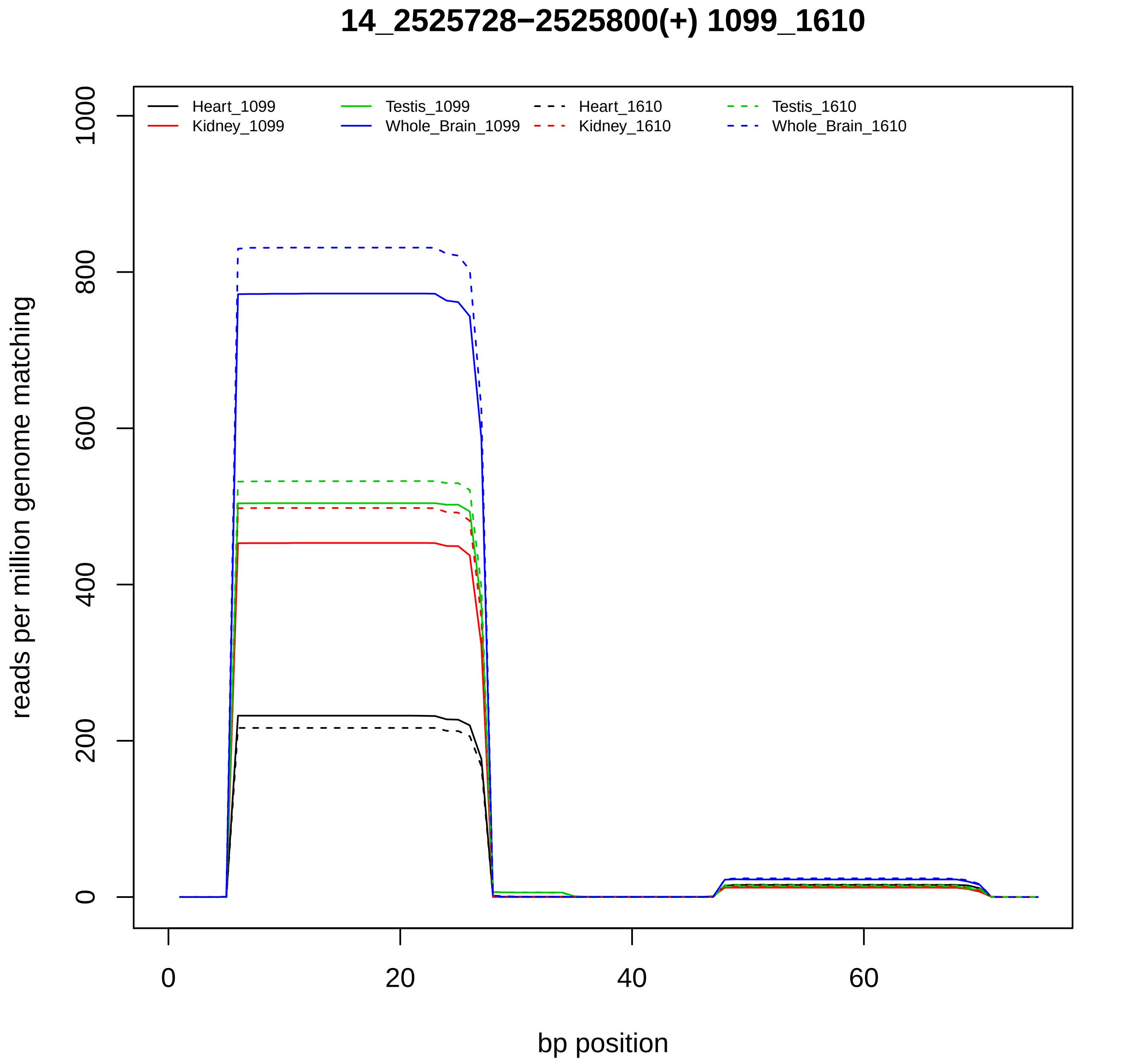

14_2525728−2525800(+) 1099_1610
Heart_1099
Kidney_1099
Testis_1099
Whole_Brain_1099
Heart_1610
Kidney_1610
Testis_1610
Whole_Brain_1610
reads per million genome matching
0
200
400
600
800
1000
0
20
40
60
bp position

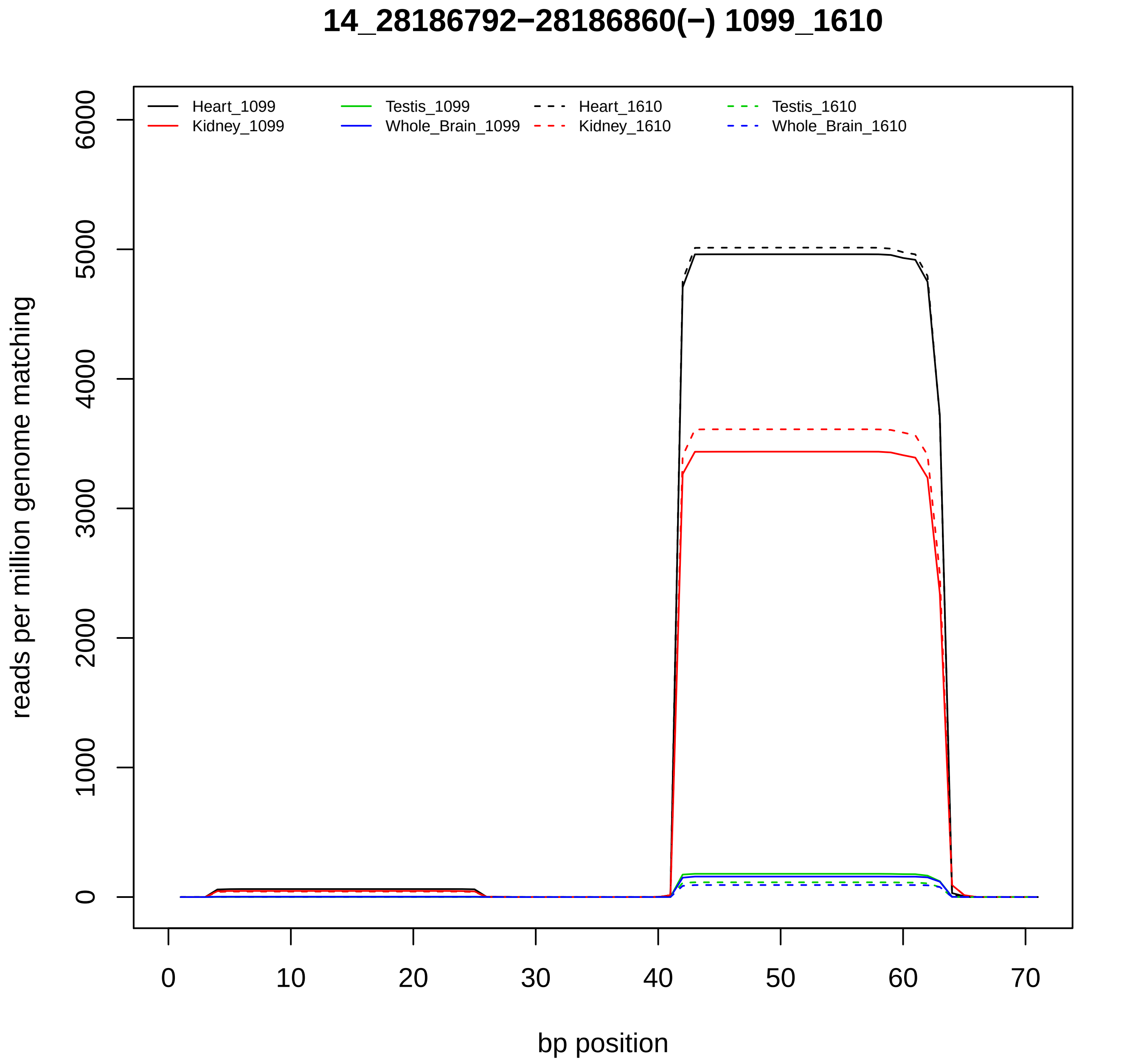

14_28186792−28186860(−) 1099_1610
Heart_1099
Kidney_1099
Testis_1099
Whole_Brain_1099
Heart_1610
Kidney_1610
Testis_1610
Whole_Brain_1610
reads per million genome matching
bp position
0
1000
2000
3000
4000
5000
6000
0
10
20
30
40
50
60
70

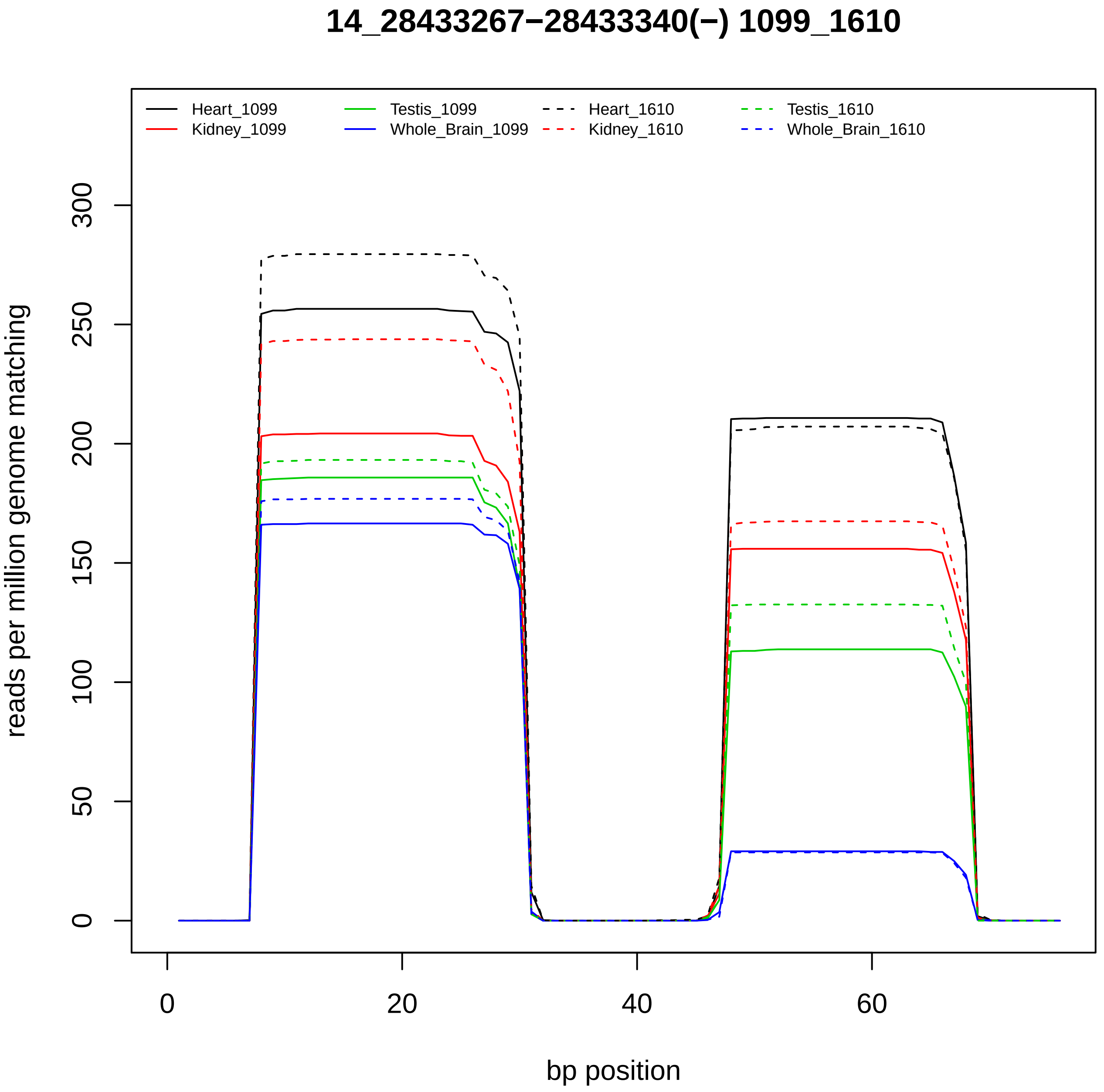

14_28433267−28433340(−) 1099_1610
Heart_1099
Kidney_1099
Testis_1099
Whole_Brain_1099
Heart_1610
Kidney_1610
Testis_1610
Whole_Brain_1610
reads per million genome matching
0
50
100
150
200
250
300
0
20
40
60
bp position

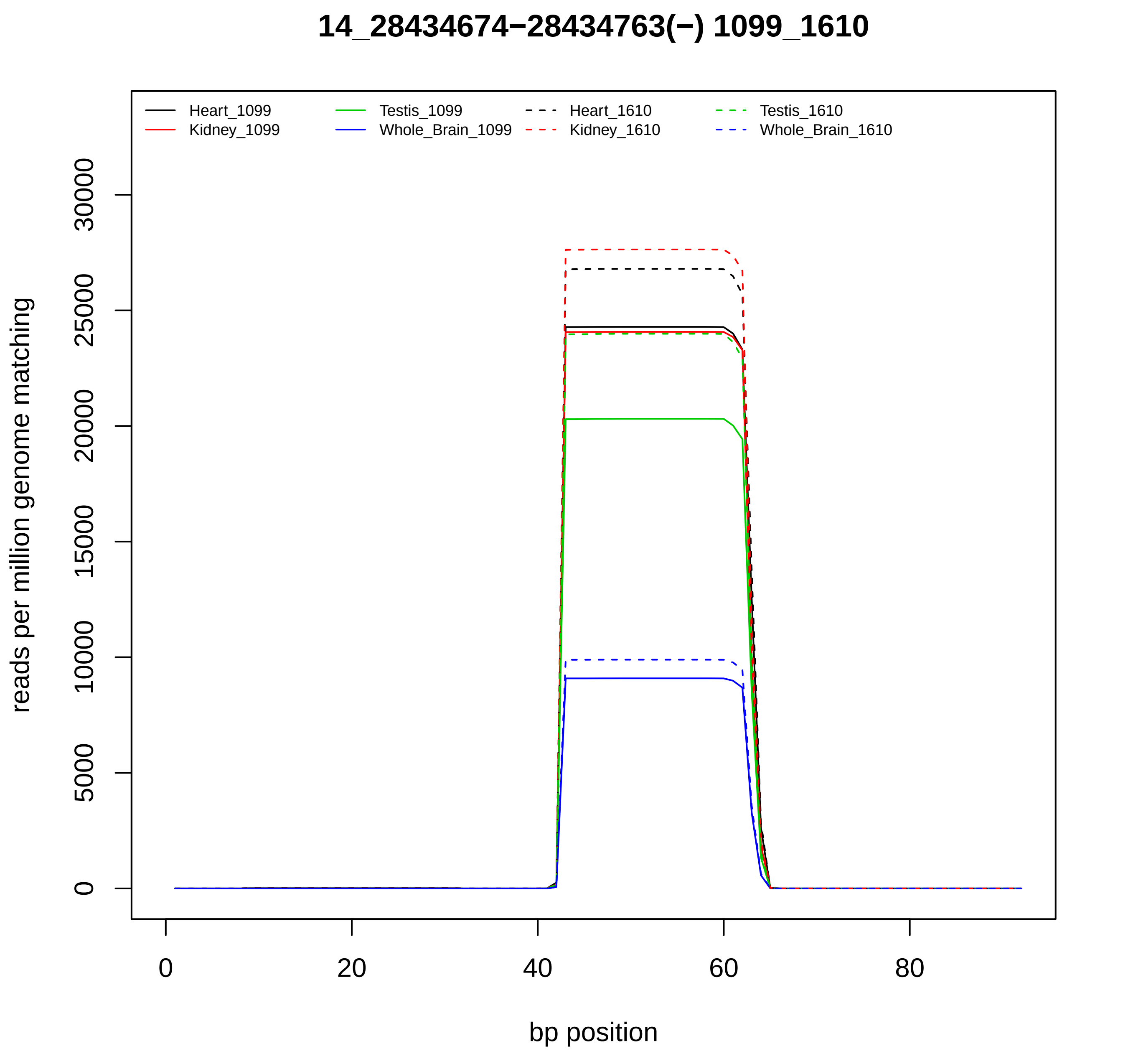

14_28434674−28434763(−) 1099_1610
Heart_1099
Kidney_1099
Testis_1099
Whole_Brain_1099
Heart_1610
Kidney_1610
Testis_1610
Whole_Brain_1610
reads per million genome matching
0
5000
10000
15000
20000
25000
30000
0
20
40
60
80
bp position

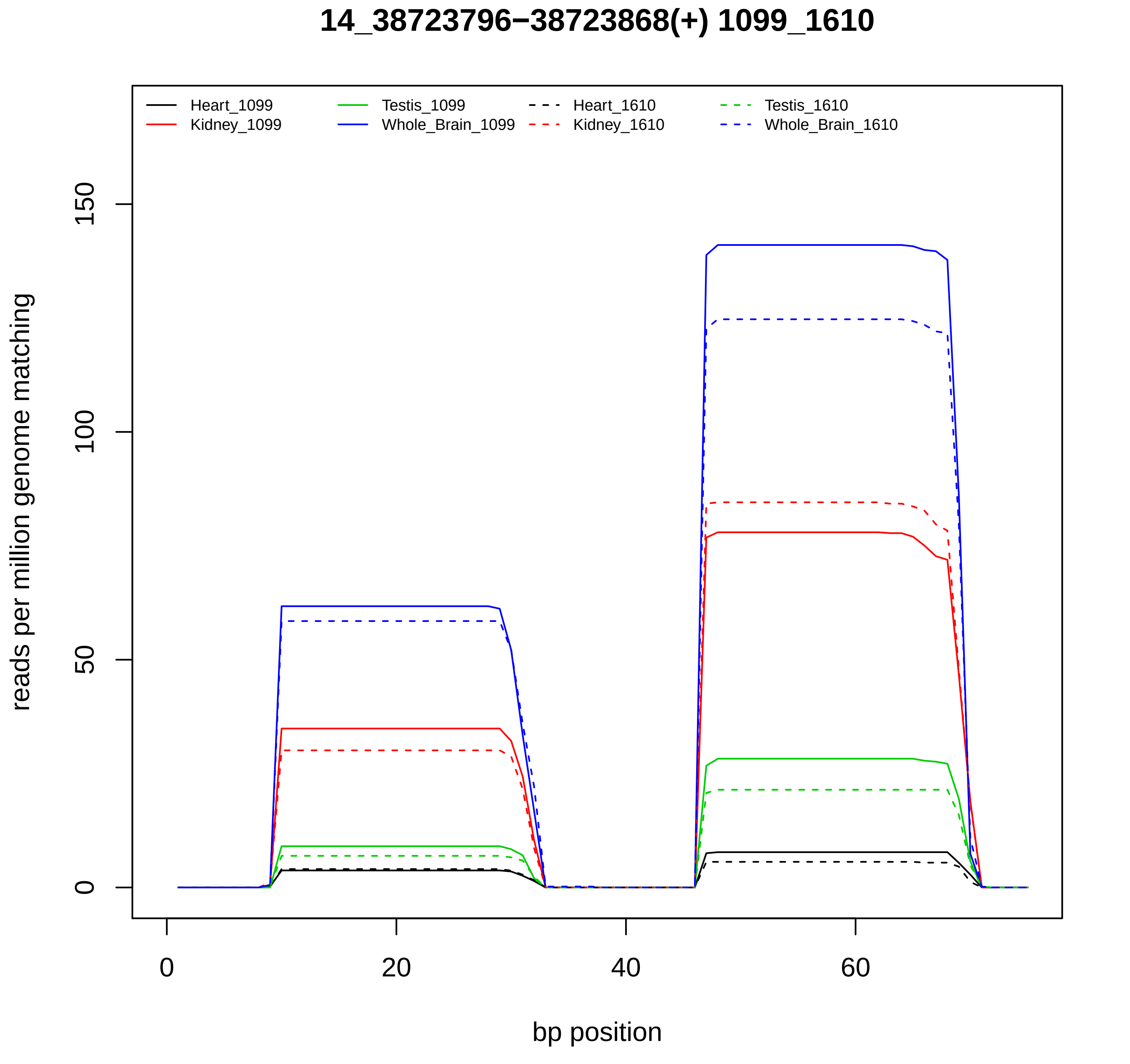

14_38723796−38723868(+) 1099_1610
Heart_1099
Kidney_1099
Testis_1099
Whole_Brain_1099
Heart_1610
Kidney_1610
Testis_1610
Whole_Brain_1610
reads per million genome matching
0
50
100
150
0
20
40
60
bp position

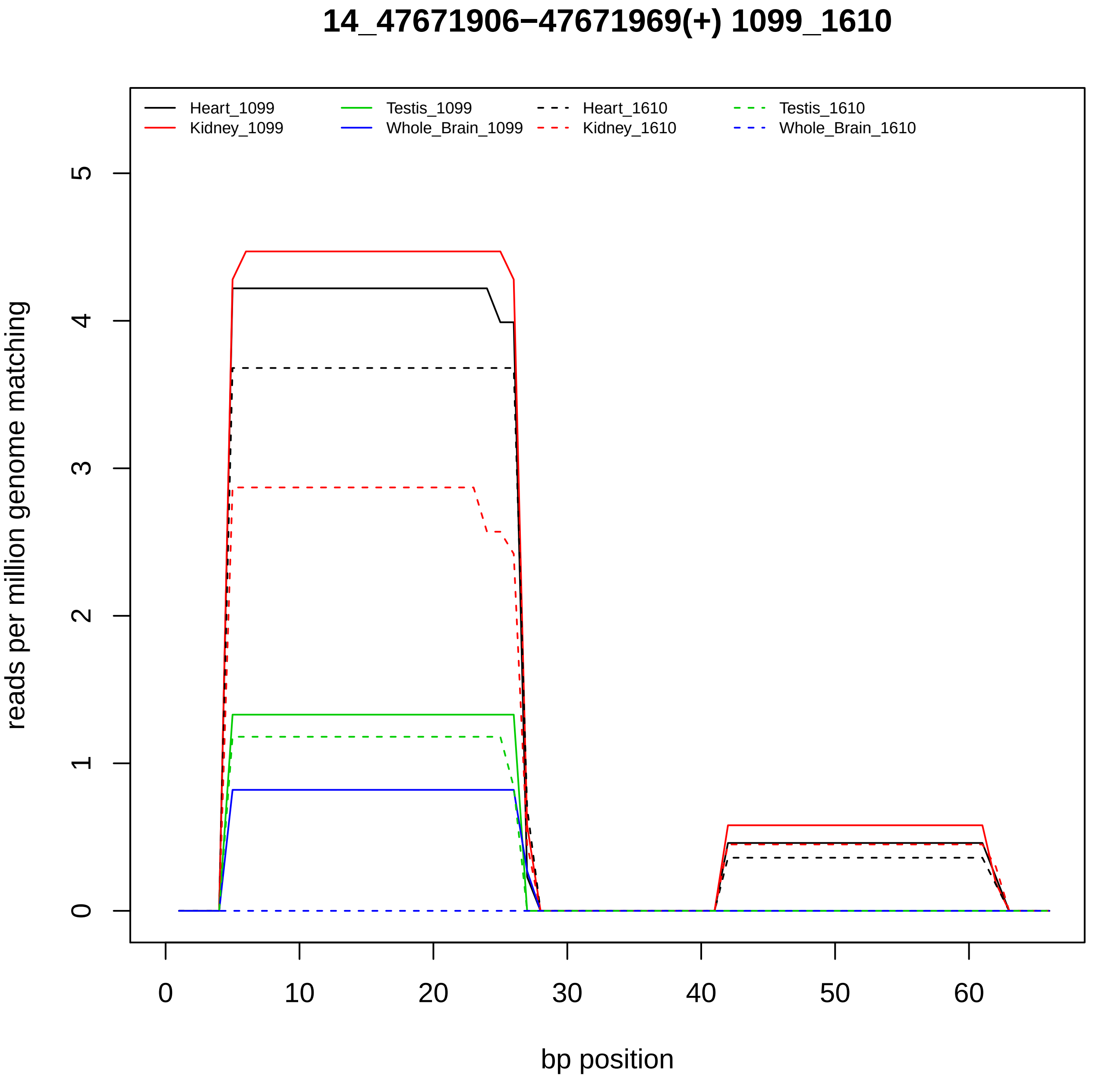

14_47671906−47671969(+) 1099_1610
Heart_1099
Kidney_1099
Testis_1099
Whole_Brain_1099
Heart_1610
Kidney_1610
Testis_1610
Whole_Brain_1610
reads per million genome matching
0
1
2
3
4
5
0
10
20
30
40
50
60
bp position

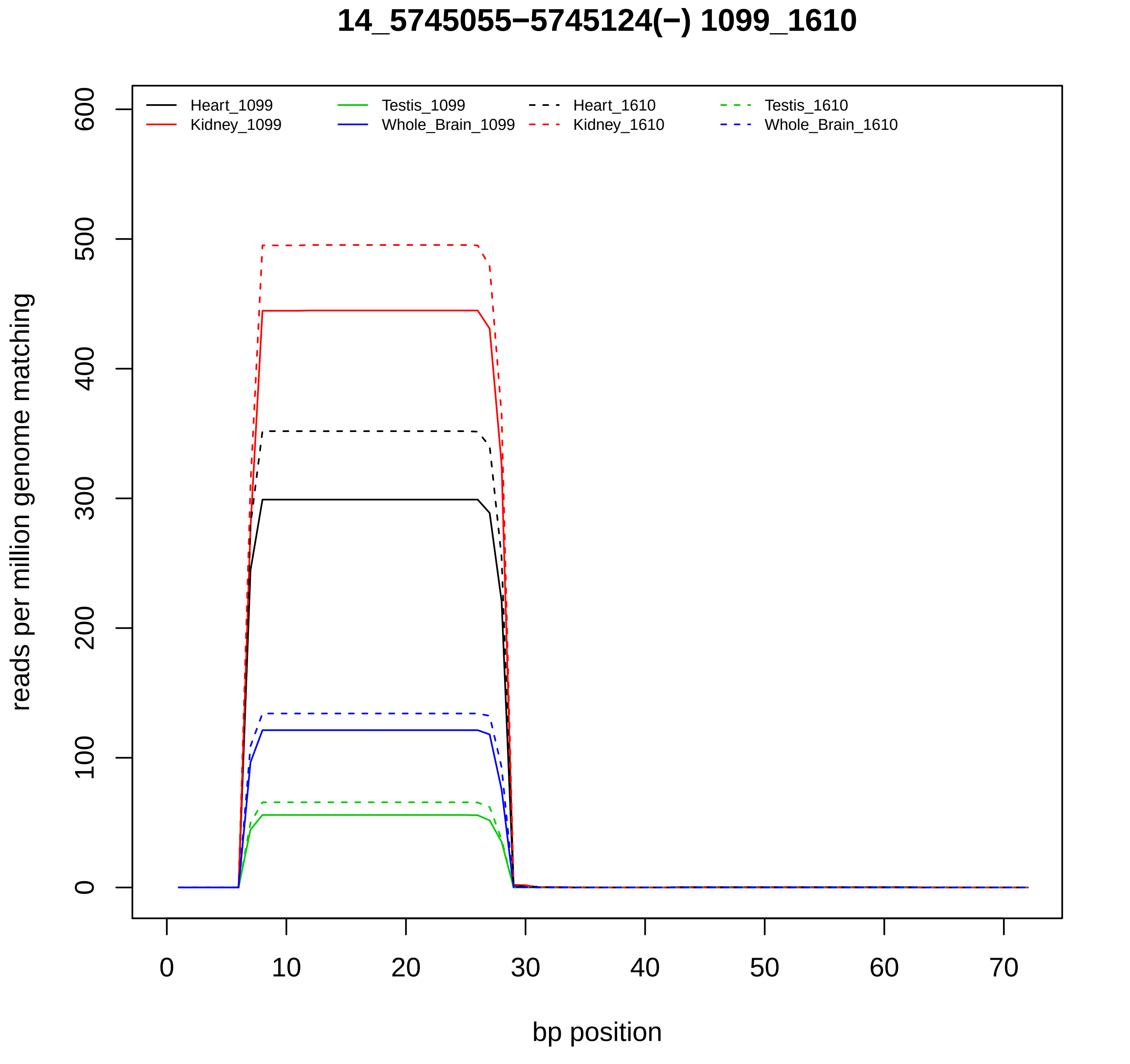

14_5745055−5745124(−) 1099_1610
Heart_1099
Kidney_1099
Testis_1099
Whole_Brain_1099
Heart_1610
Kidney_1610
Testis_1610
Whole_Brain_1610
reads per million genome matching
bp position
0
100
200
300
400
500
600
0
10
20
30
40
50
60
70

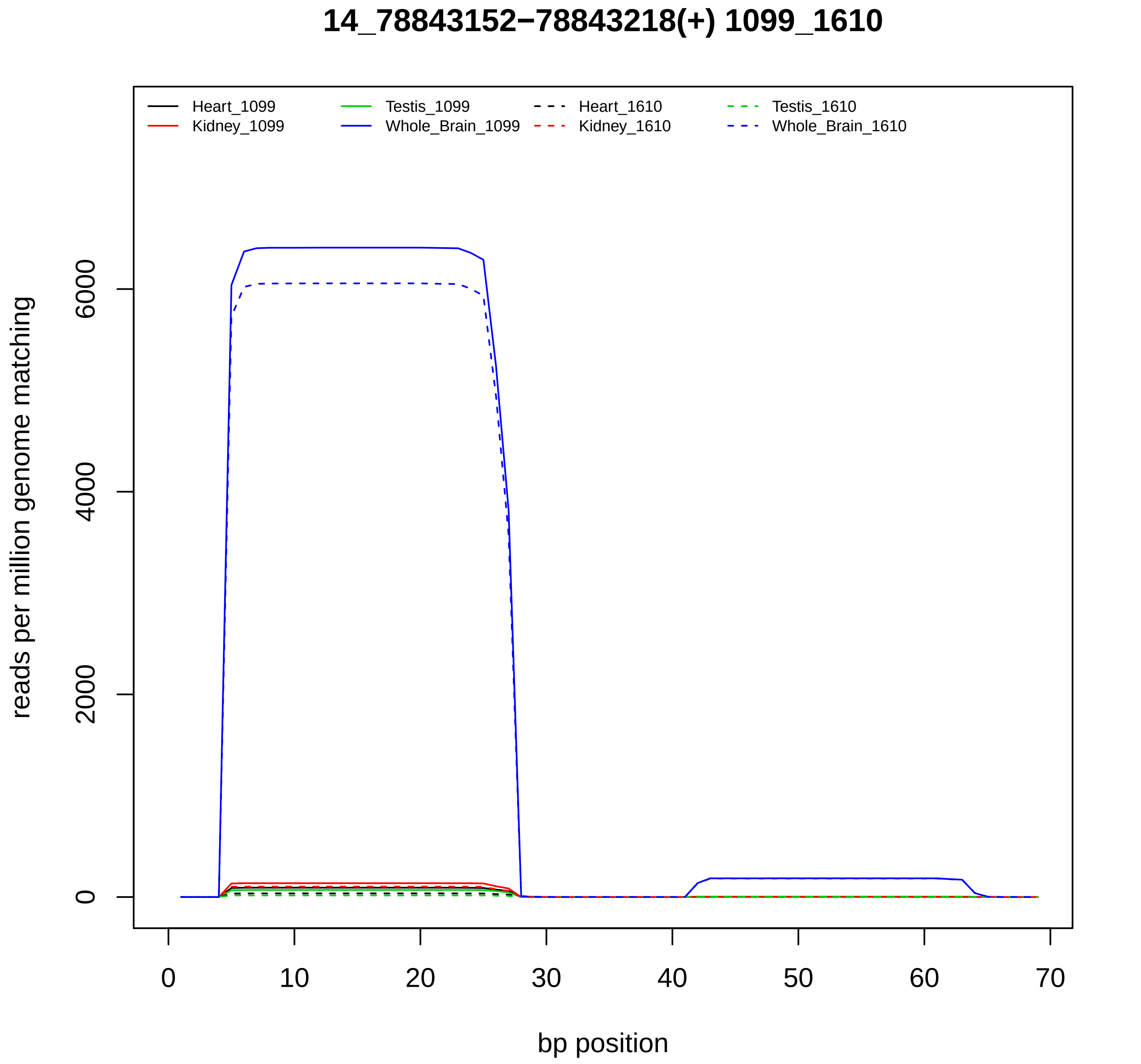

14_78843152−78843218(+) 1099_1610
Heart_1099
Kidney_1099
Testis_1099
Whole_Brain_1099
Heart_1610
Kidney_1610
Testis_1610
Whole_Brain_1610
reads per million genome matching
0
2000
4000
6000
0
10
20
30
40
50
60
70
bp position

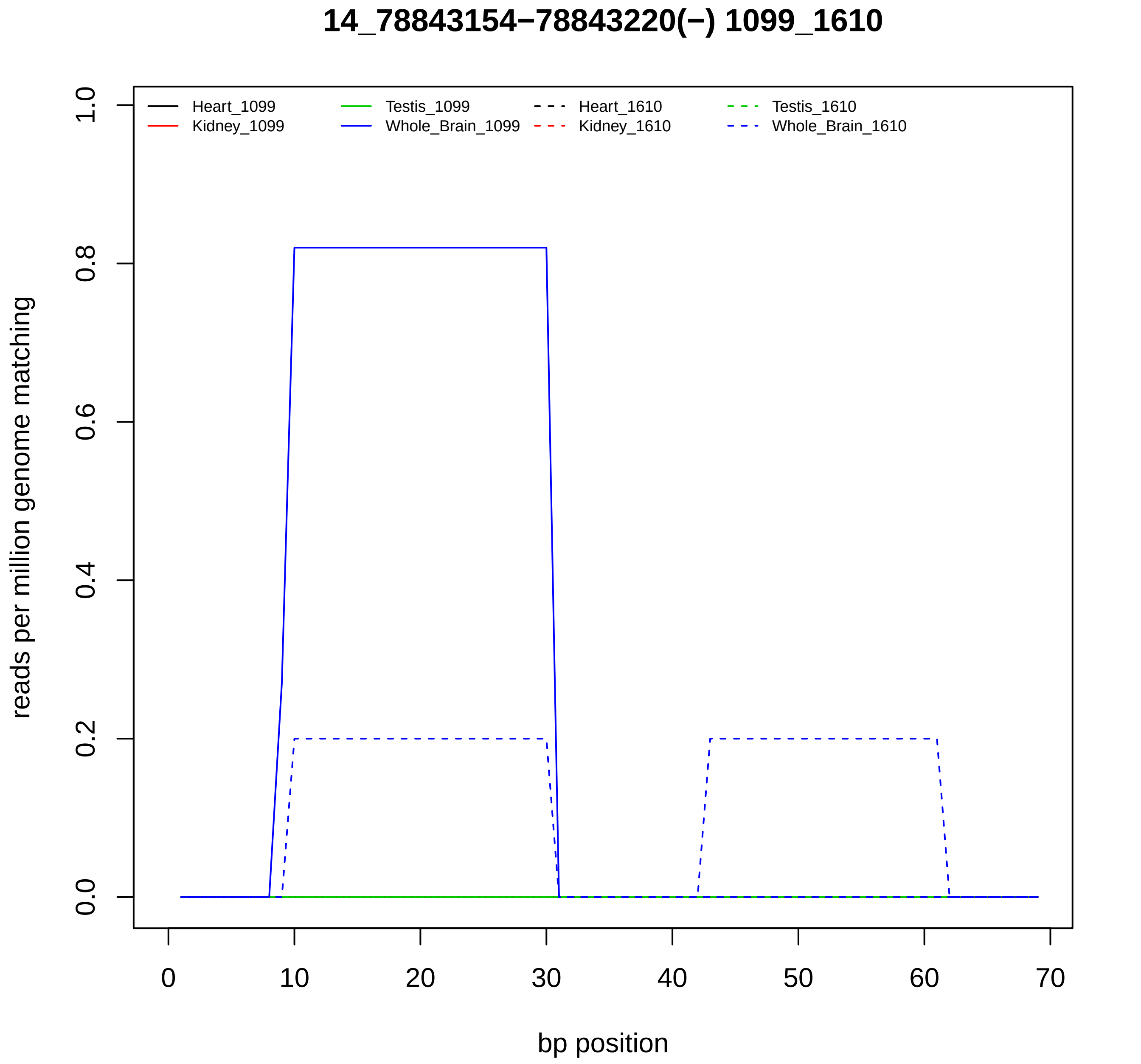

14_78843154−78843220(−) 1099_1610
Heart_1099
Kidney_1099
Testis_1099
Whole_Brain_1099
Heart_1610
Kidney_1610
Testis_1610
Whole_Brain_1610
reads per million genome matching
0.0
0.2
0.4
0.6
0.8
1.0
0
10
20
30
40
50
60
70
bp position

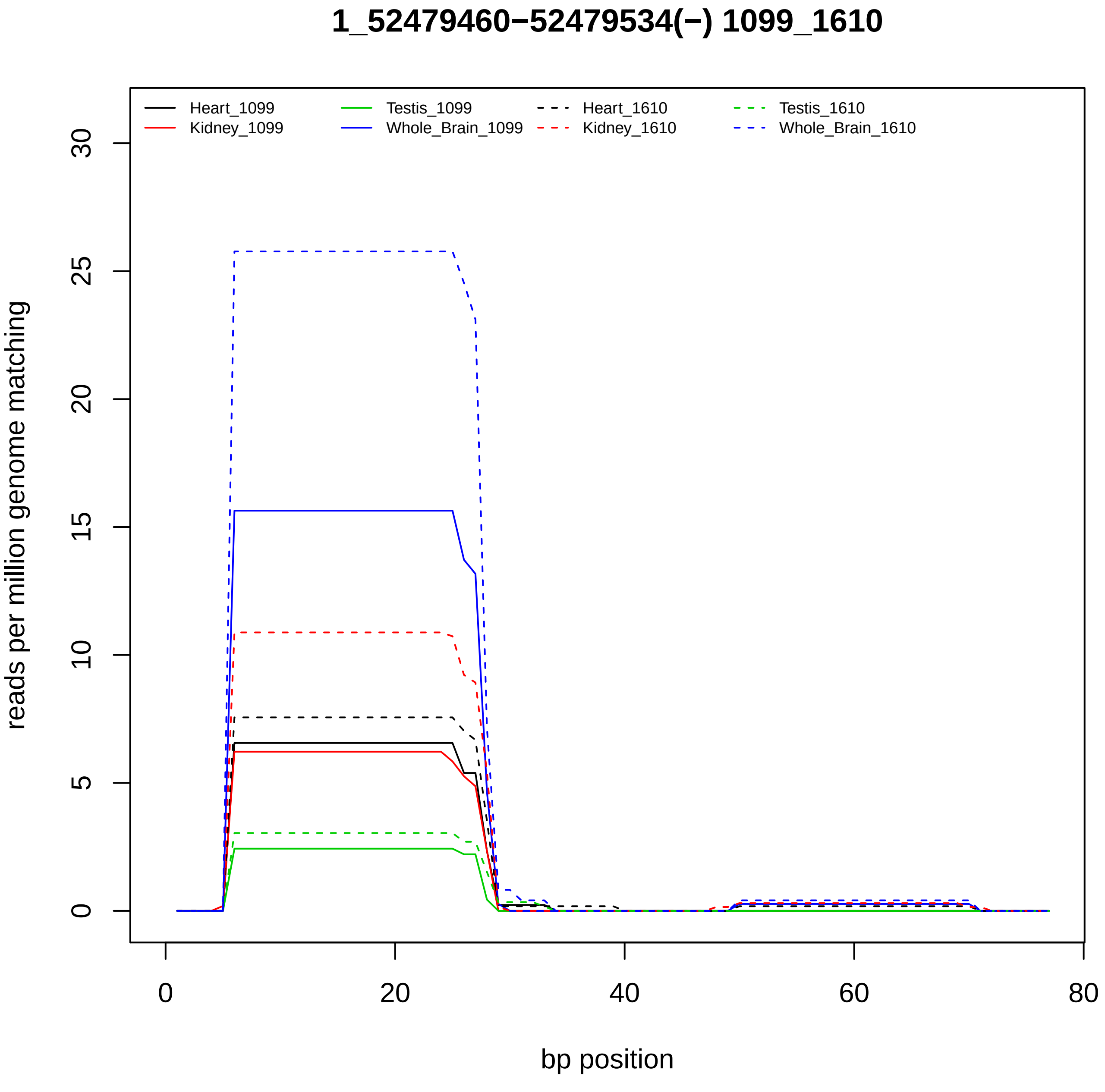
1_52479460−52479534(−) 1099_1610
Heart_1099
Kidney_1099
Testis_1099
Whole_Brain_1099
Heart_1610
Kidney_1610
Testis_1610
Whole_Brain_1610
reads per million genome matching
0
5
10
15
20
25
30
0
20
40
60
80
bp position

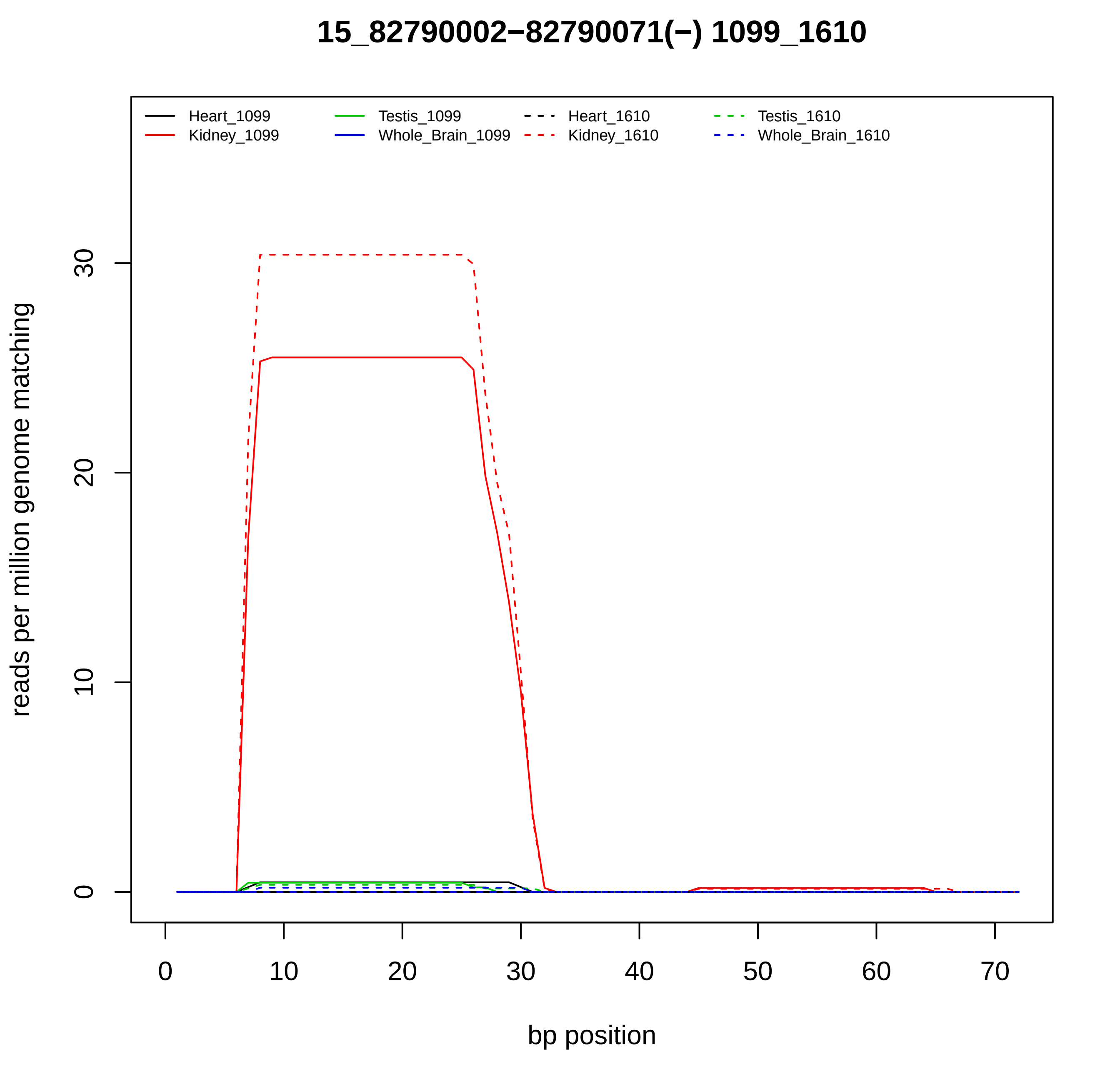

15_82790002−82790071(−) 1099_1610
Heart_1099
Kidney_1099
Testis_1099
Whole_Brain_1099
Heart_1610
Kidney_1610
Testis_1610
Whole_Brain_1610
reads per million genome matching
0
10
20
30
bp position
0
10
20
30
40
50
60
70

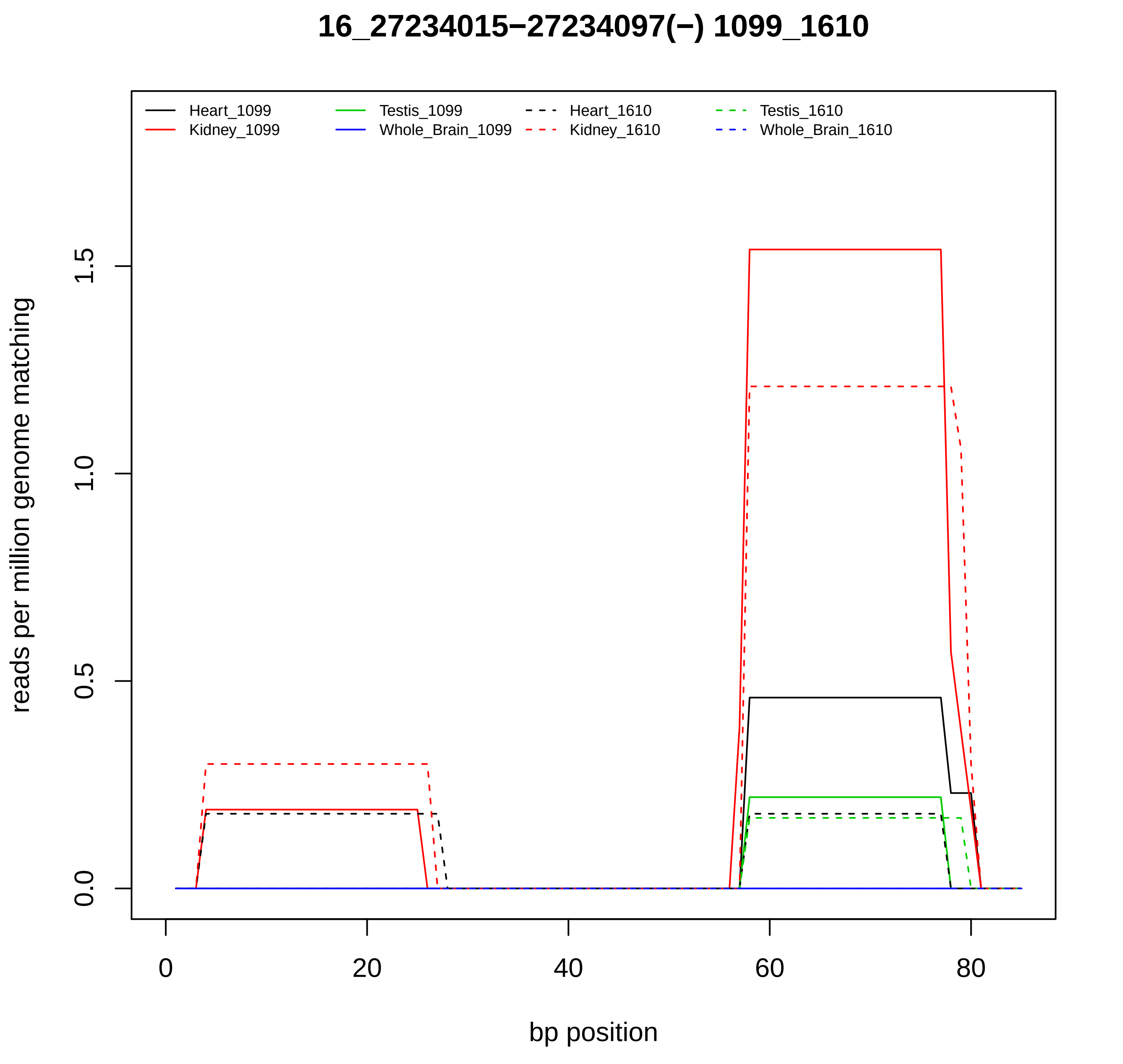
16_27234015−27234097(−) 1099_1610
Heart_1099
Kidney_1099
Testis_1099
Whole_Brain_1099
Heart_1610
Kidney_1610
Testis_1610
Whole_Brain_1610
reads per million genome matching
0.0
0.5
1.0
1.5
0
20
40
60
80
bp position

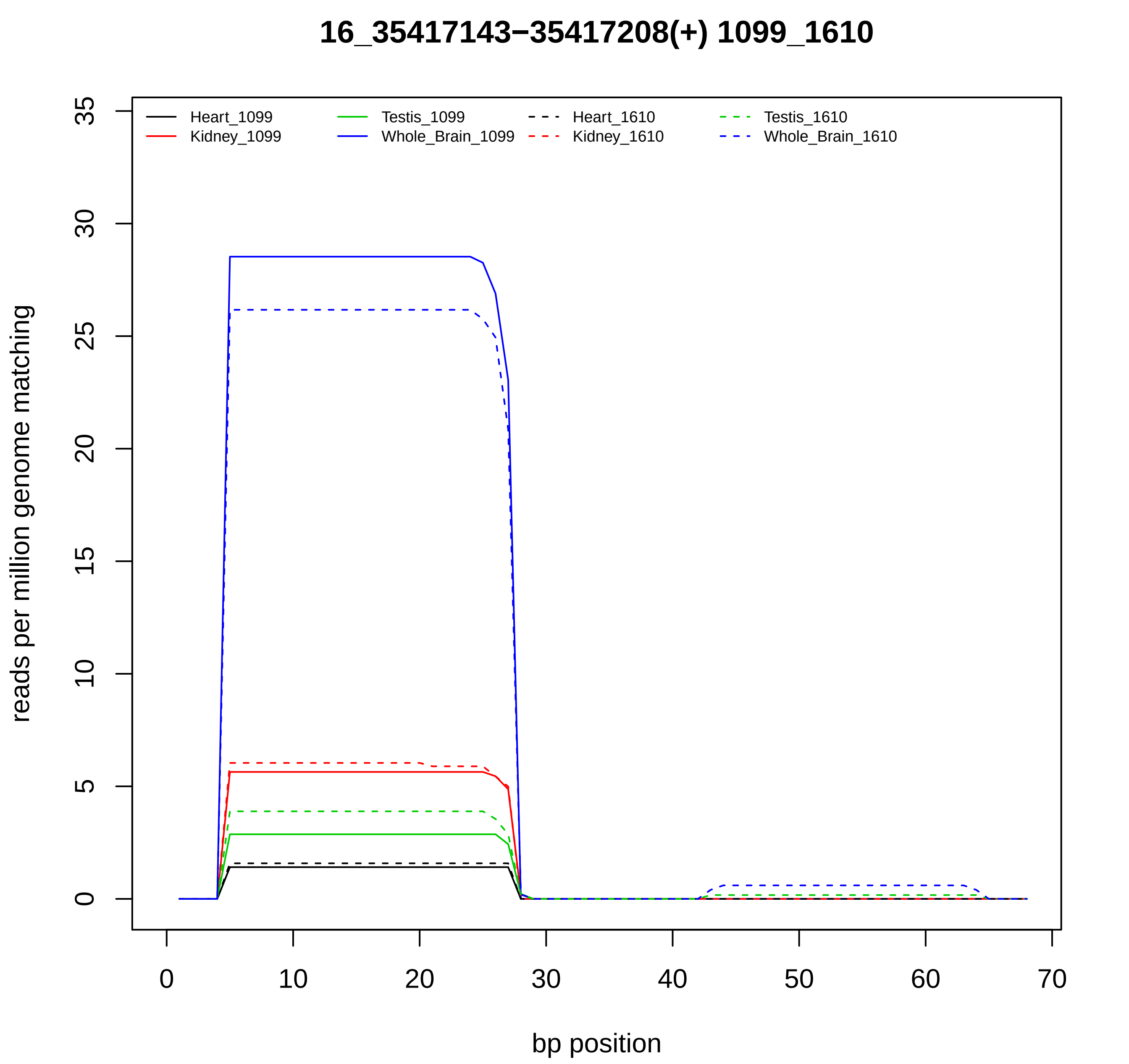

16_35417143−35417208(+) 1099_1610
Heart_1099
Kidney_1099
Testis_1099
Whole_Brain_1099
Heart_1610
Kidney_1610
Testis_1610
Whole_Brain_1610
reads per million genome matching
bp position
0
5
10
15
20
25
30
35
0
10
20
30
40
50
60
70

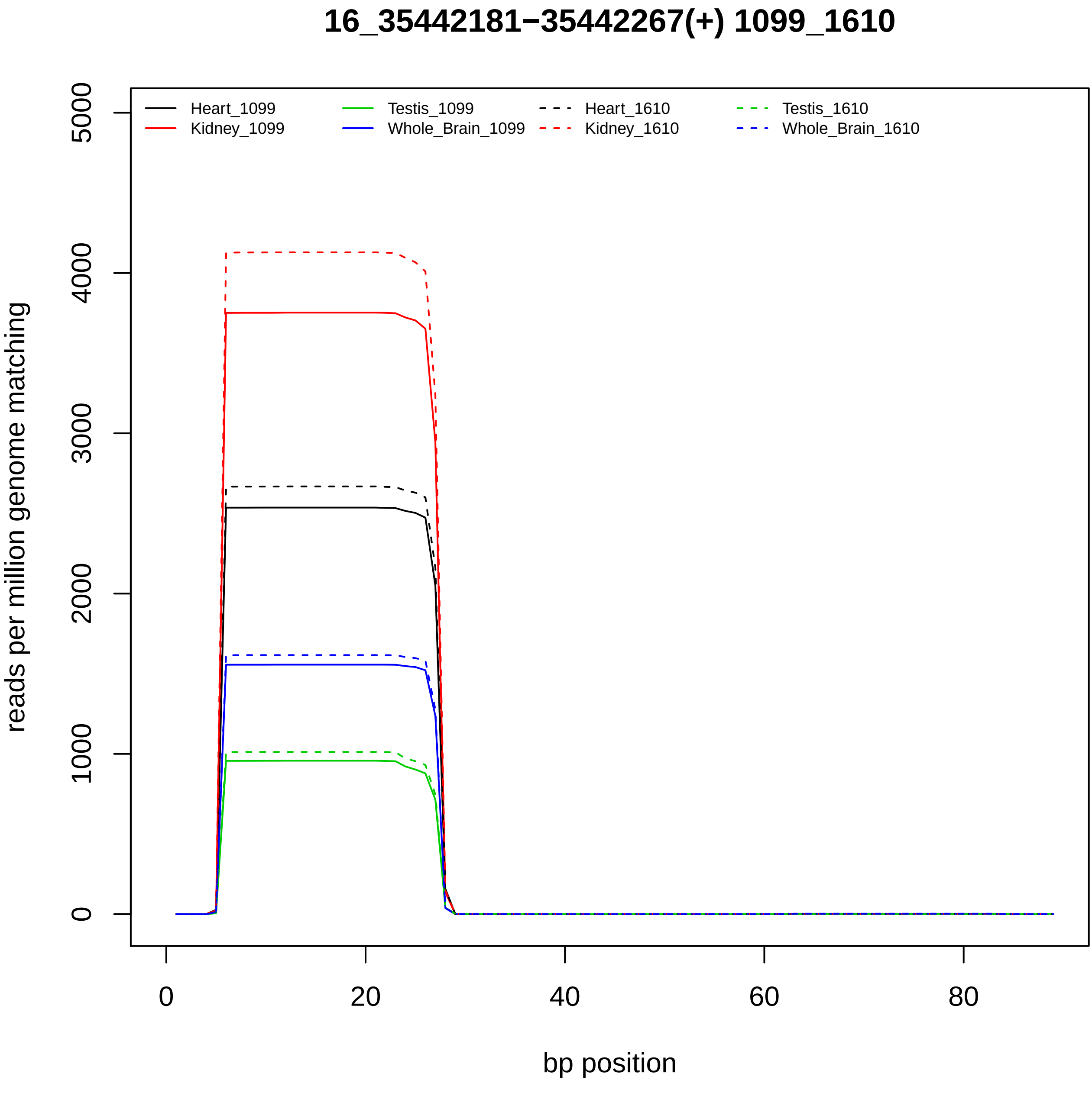

16_35442181−35442267(+) 1099_1610
Heart_1099
Kidney_1099
Testis_1099
Whole_Brain_1099
Heart_1610
Kidney_1610
Testis_1610
Whole_Brain_1610
reads per million genome matching
0
1000
2000
3000
4000
5000
bp position
0
20
40
60
80

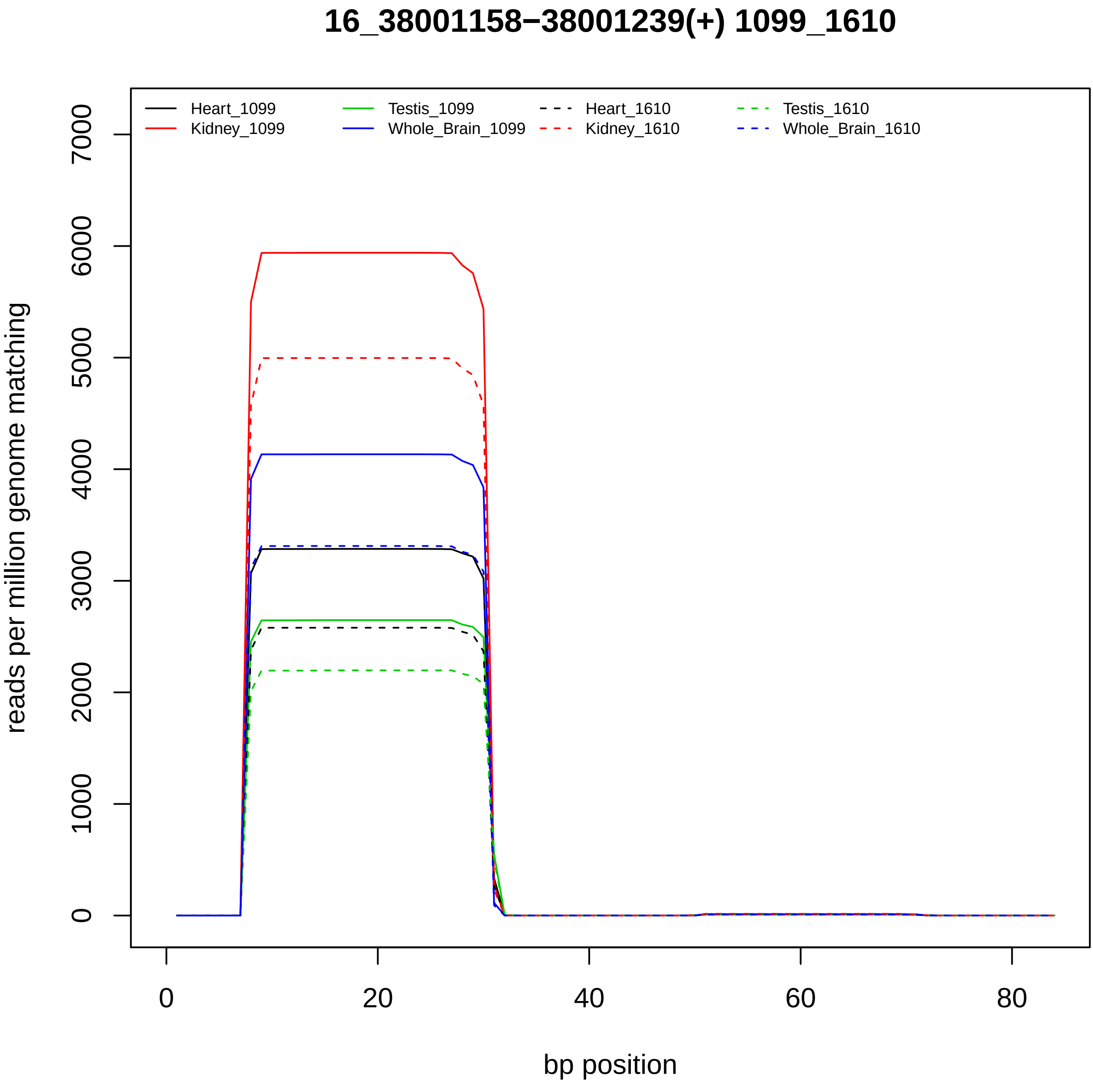
16_38001158−38001239(+) 1099_1610
Heart_1099
Kidney_1099
Testis_1099
Whole_Brain_1099
Heart_1610
Kidney_1610
Testis_1610
Whole_Brain_1610
reads per million genome matching
0
1000
2000
3000
4000
5000
6000
7000
bp position
0
20
40
60
80

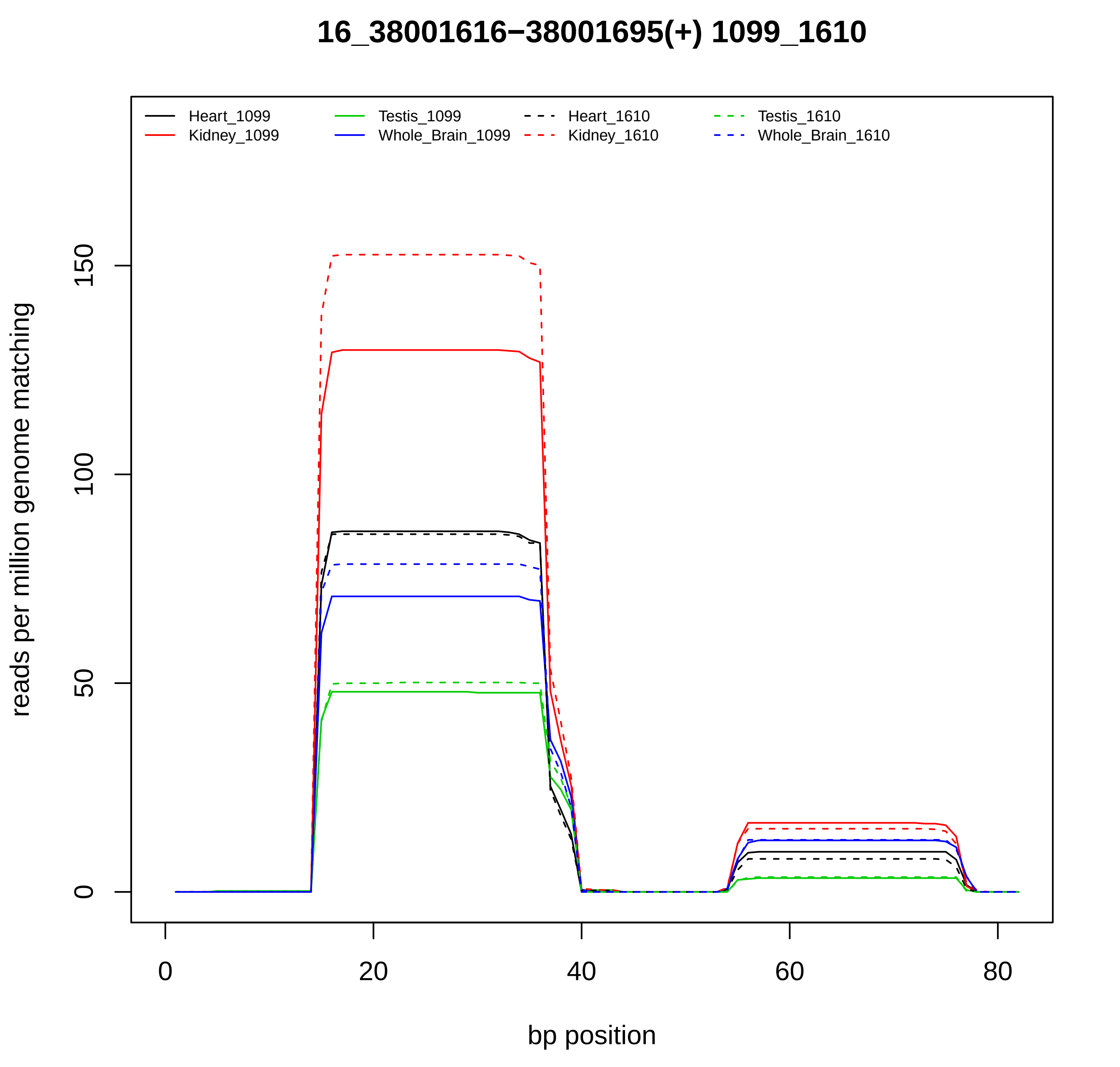

16_38001616−38001695(+) 1099_1610
Heart_1099
Kidney_1099
Testis_1099
Whole_Brain_1099
Heart_1610
Kidney_1610
Testis_1610
Whole_Brain_1610
reads per million genome matching
0
50
100
150
0
20
40
60
80
bp position

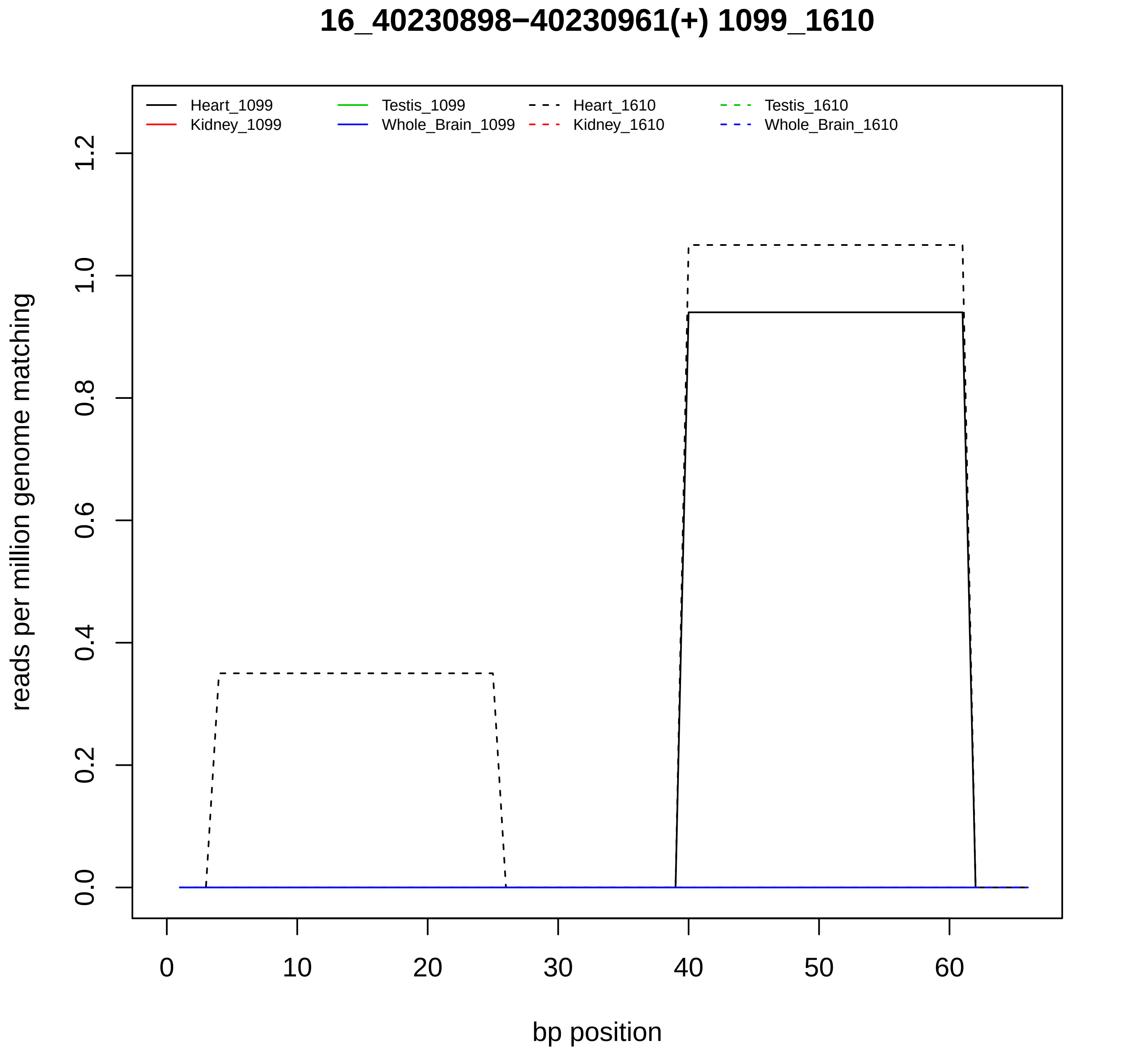
16_40230898−40230961(+) 1099_1610
Heart_1099
Kidney_1099
Testis_1099
Whole_Brain_1099
Heart_1610
Kidney_1610
Testis_1610
Whole_Brain_1610
reads per million genome matching
0.0
0.2
0.4
0.6
0.8
1.0
1.2
0
10
20
30
40
50
60
bp position

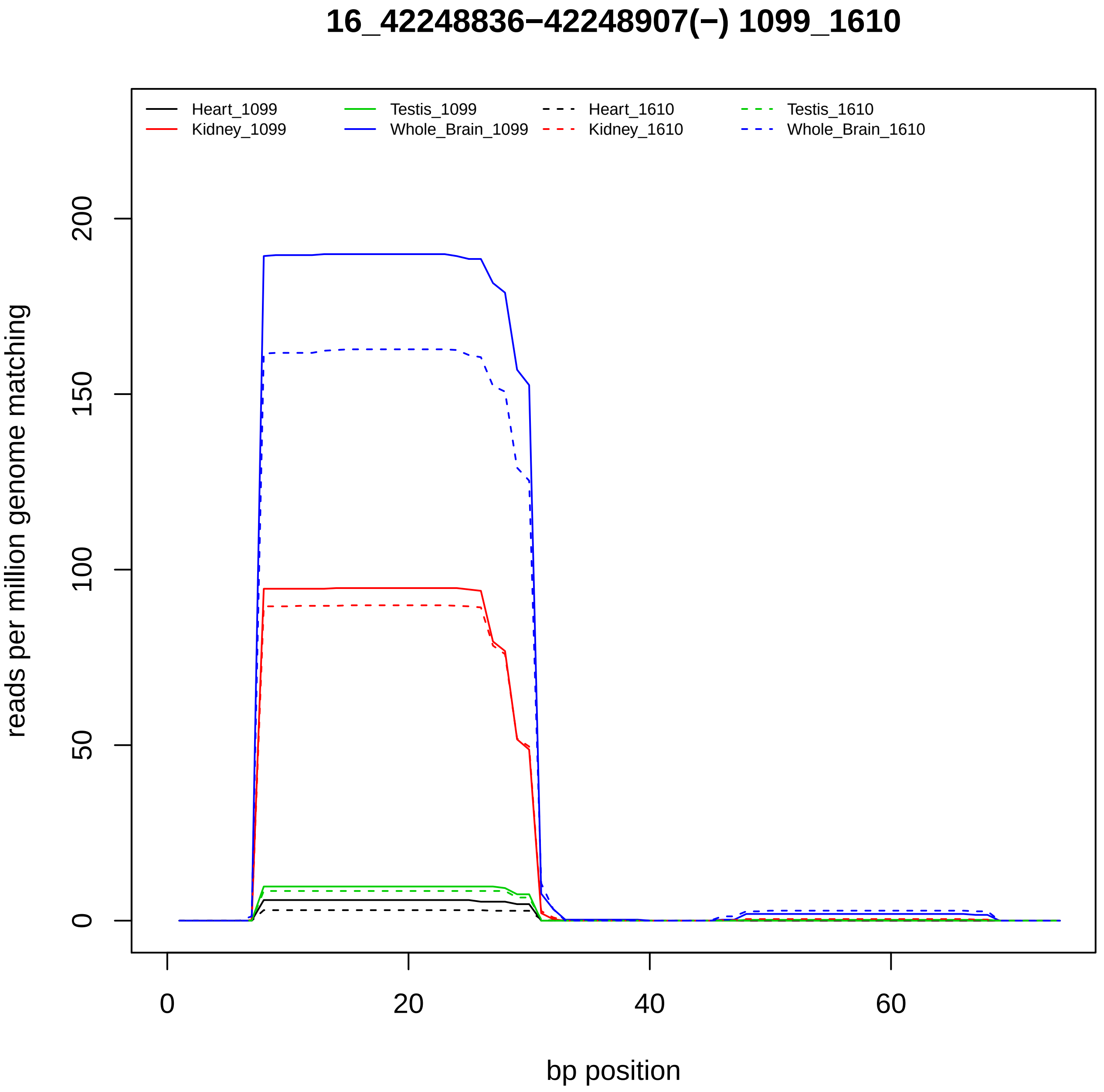
16_42248836−42248907(−) 1099_1610
Heart_1099
Kidney_1099
Testis_1099
Whole_Brain_1099
Heart_1610
Kidney_1610
Testis_1610
Whole_Brain_1610
reads per million genome matching
0
50
100
150
200
bp position
0
20
40
60

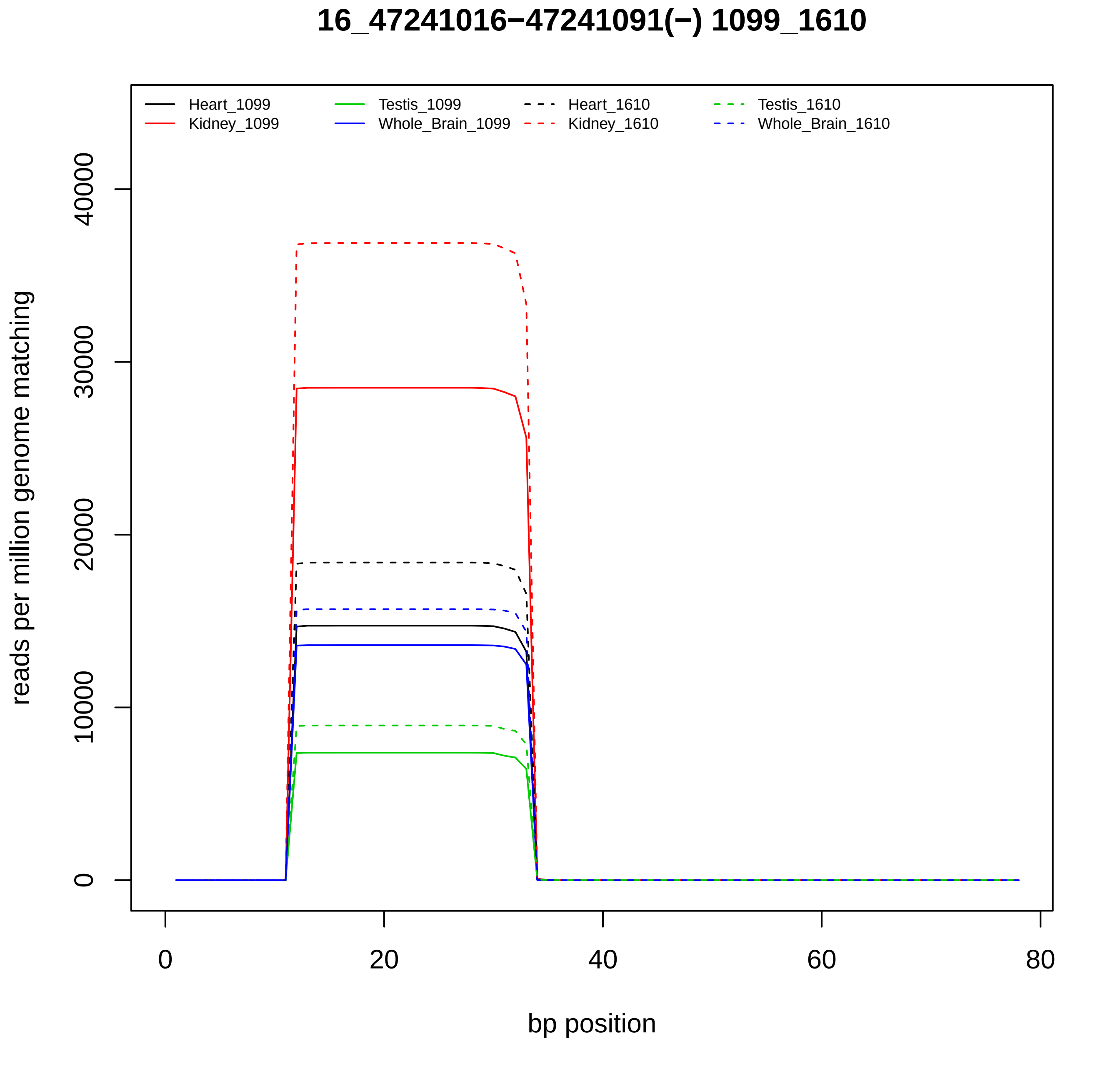
16_47241016−47241091(−) 1099_1610
Heart_1099
Kidney_1099
Testis_1099
Whole_Brain_1099
Heart_1610
Kidney_1610
Testis_1610
Whole_Brain_1610
reads per million genome matching
0
10000
20000
30000
40000
0
20
40
60
80
bp position

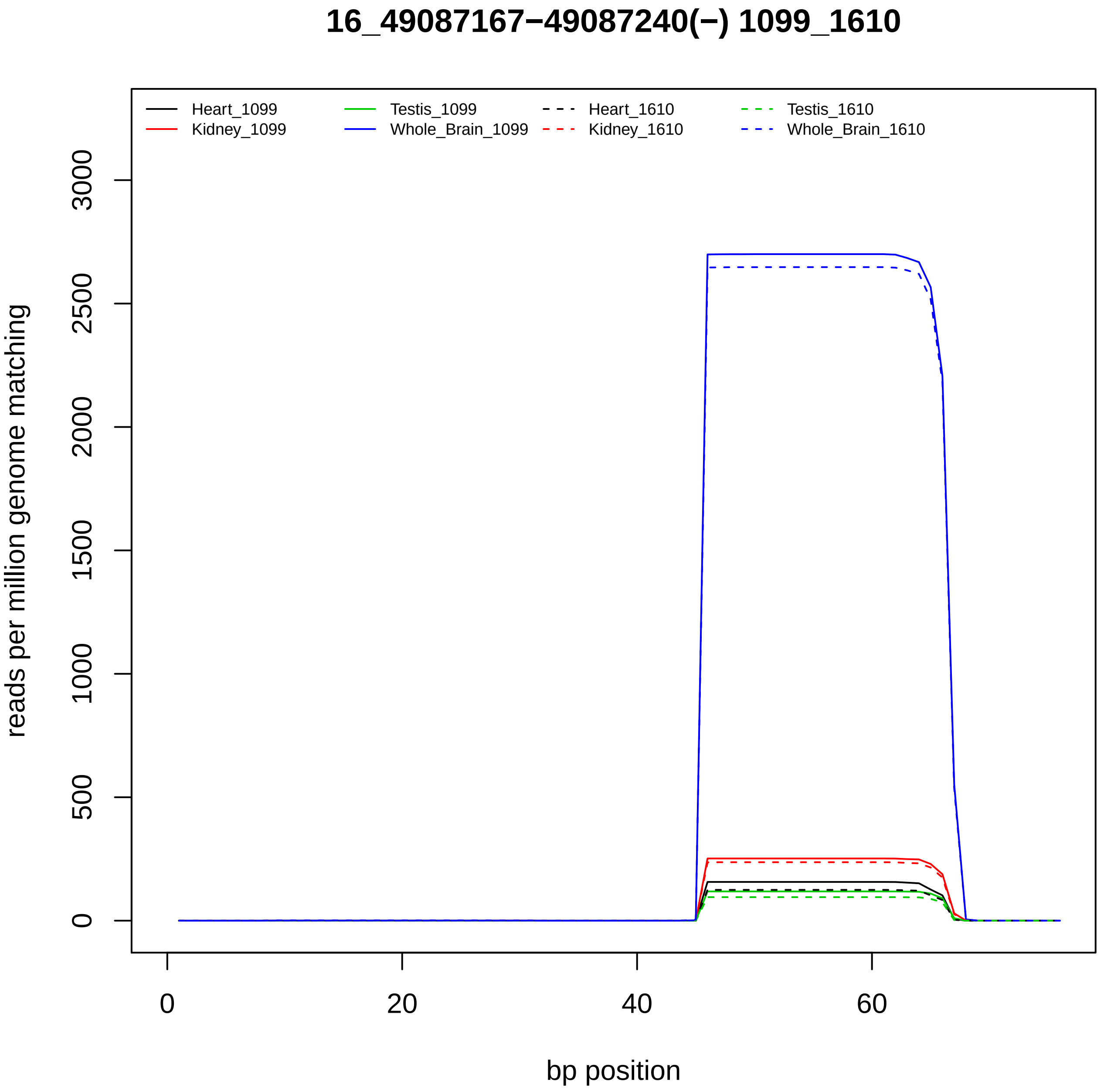

16_49087167−49087240(−) 1099_1610
Heart_1099
Kidney_1099
Testis_1099
Whole_Brain_1099
Heart_1610
Kidney_1610
Testis_1610
Whole_Brain_1610
reads per million genome matching
0
500
1000
1500
2000
2500
3000
0
20
40
60
bp position

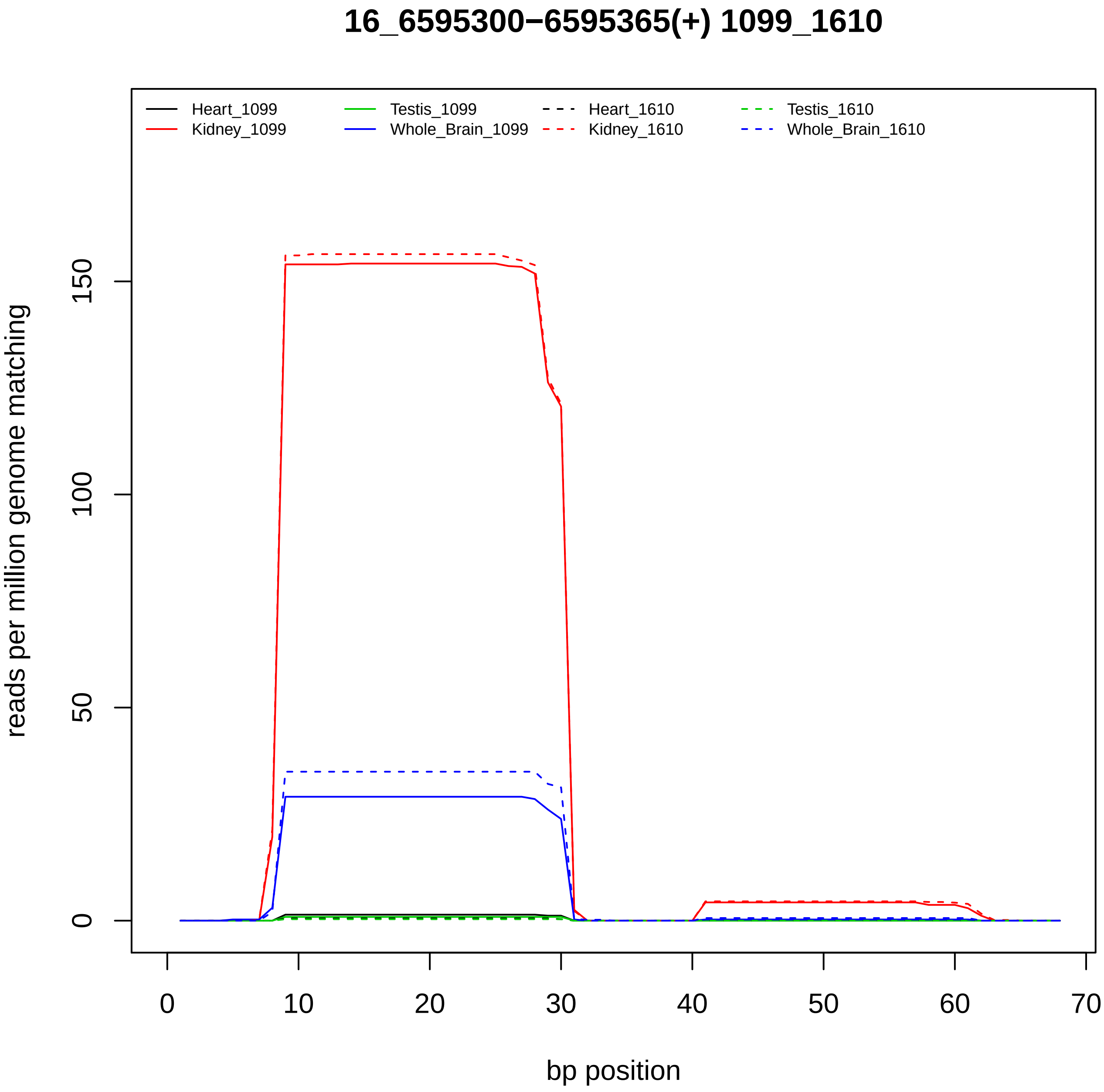
16_6595300−6595365(+) 1099_1610
Heart_1099
Kidney_1099
Testis_1099
Whole_Brain_1099
Heart_1610
Kidney_1610
Testis_1610
Whole_Brain_1610
reads per million genome matching
0
50
100
150
bp position
0
10
20
30
40
50
60
70

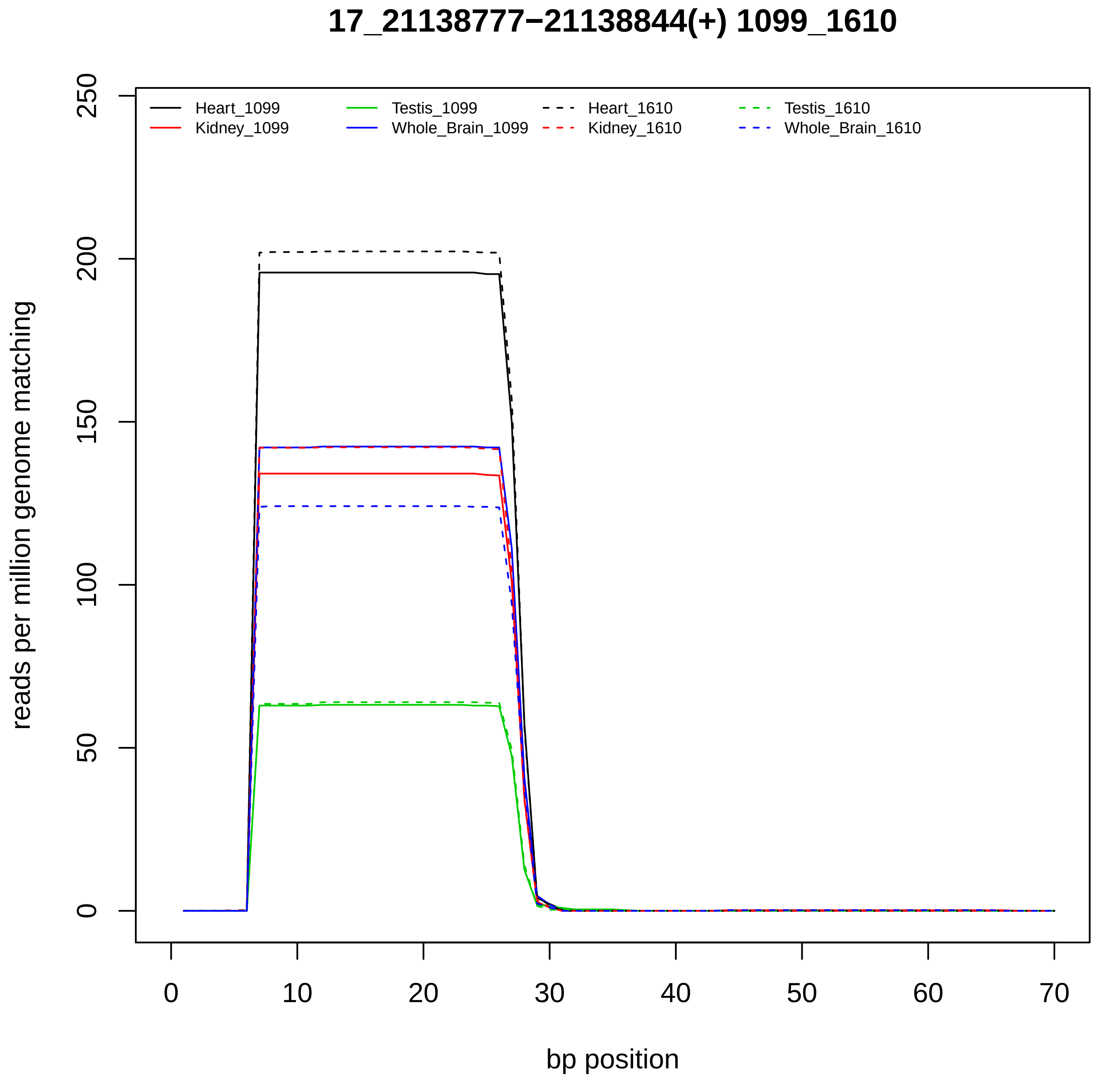

17_21138777−21138844(+) 1099_1610
Heart_1099
Kidney_1099
Testis_1099
Whole_Brain_1099
Heart_1610
Kidney_1610
Testis_1610
Whole_Brain_1610
reads per million genome matching
bp position
0
50
100
150
200
250
0
10
20
30
40
50
60
70

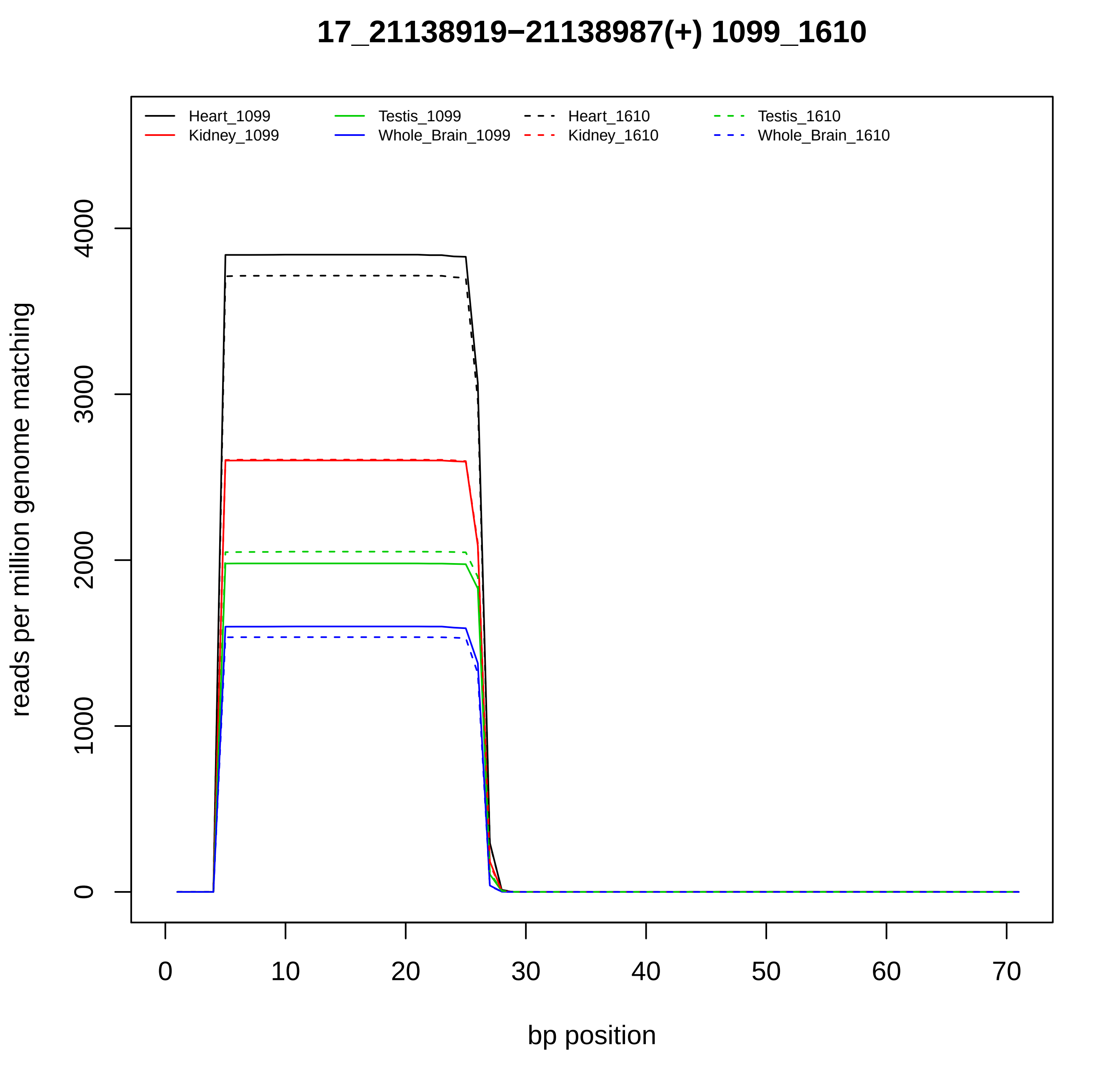

17_21138919−21138987(+) 1099_1610
Heart_1099
Kidney_1099
Testis_1099
Whole_Brain_1099
Heart_1610
Kidney_1610
Testis_1610
Whole_Brain_1610
reads per million genome matching
0
1000
2000
3000
4000
bp position
0
10
20
30
40
50
60
70

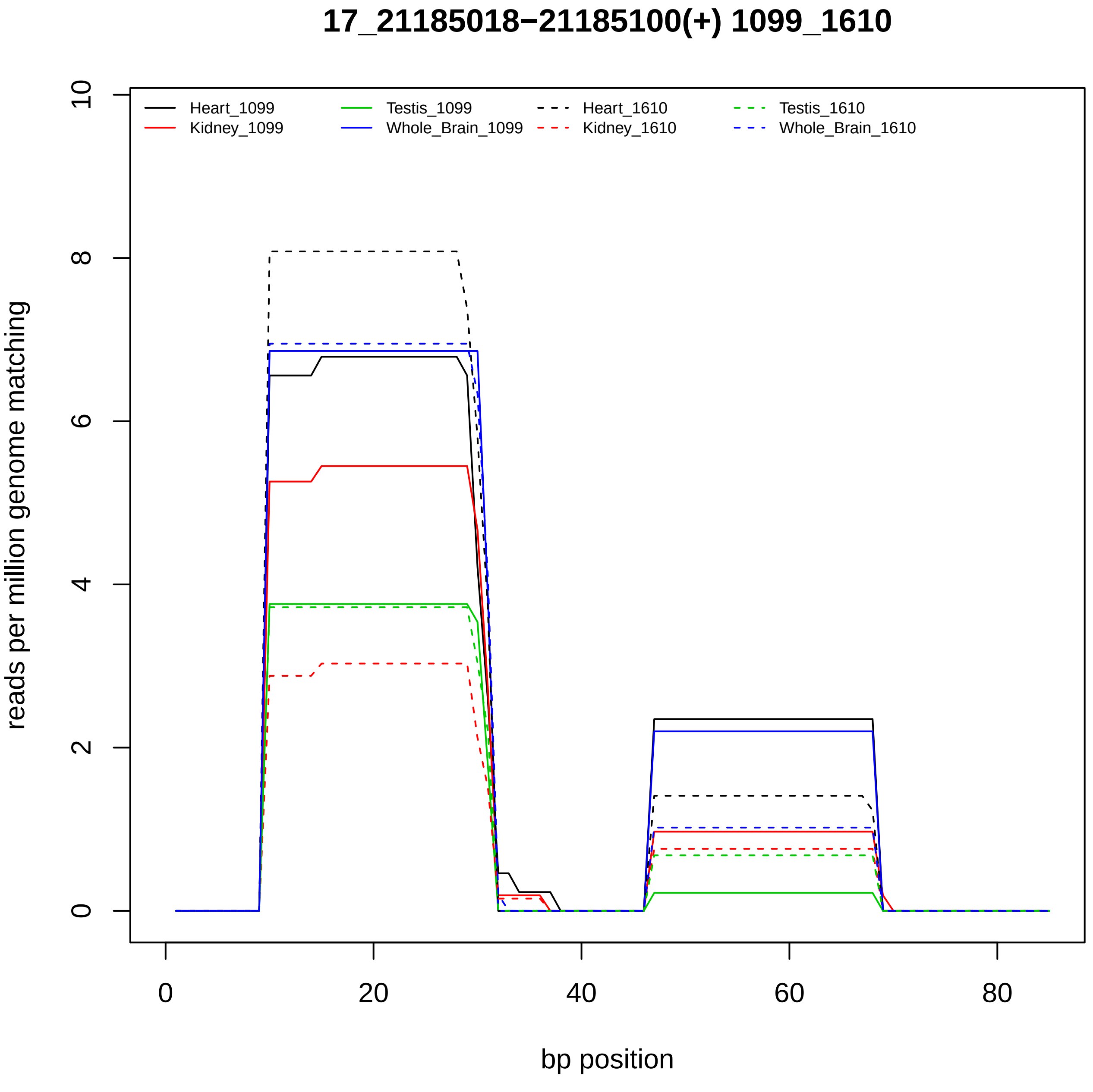
17_21185018−21185100(+) 1099_1610
Heart_1099
Kidney_1099
Testis_1099
Whole_Brain_1099
Heart_1610
Kidney_1610
Testis_1610
Whole_Brain_1610
reads per million genome matching
bp position
0
2
4
6
8
10
0
20
40
60
80

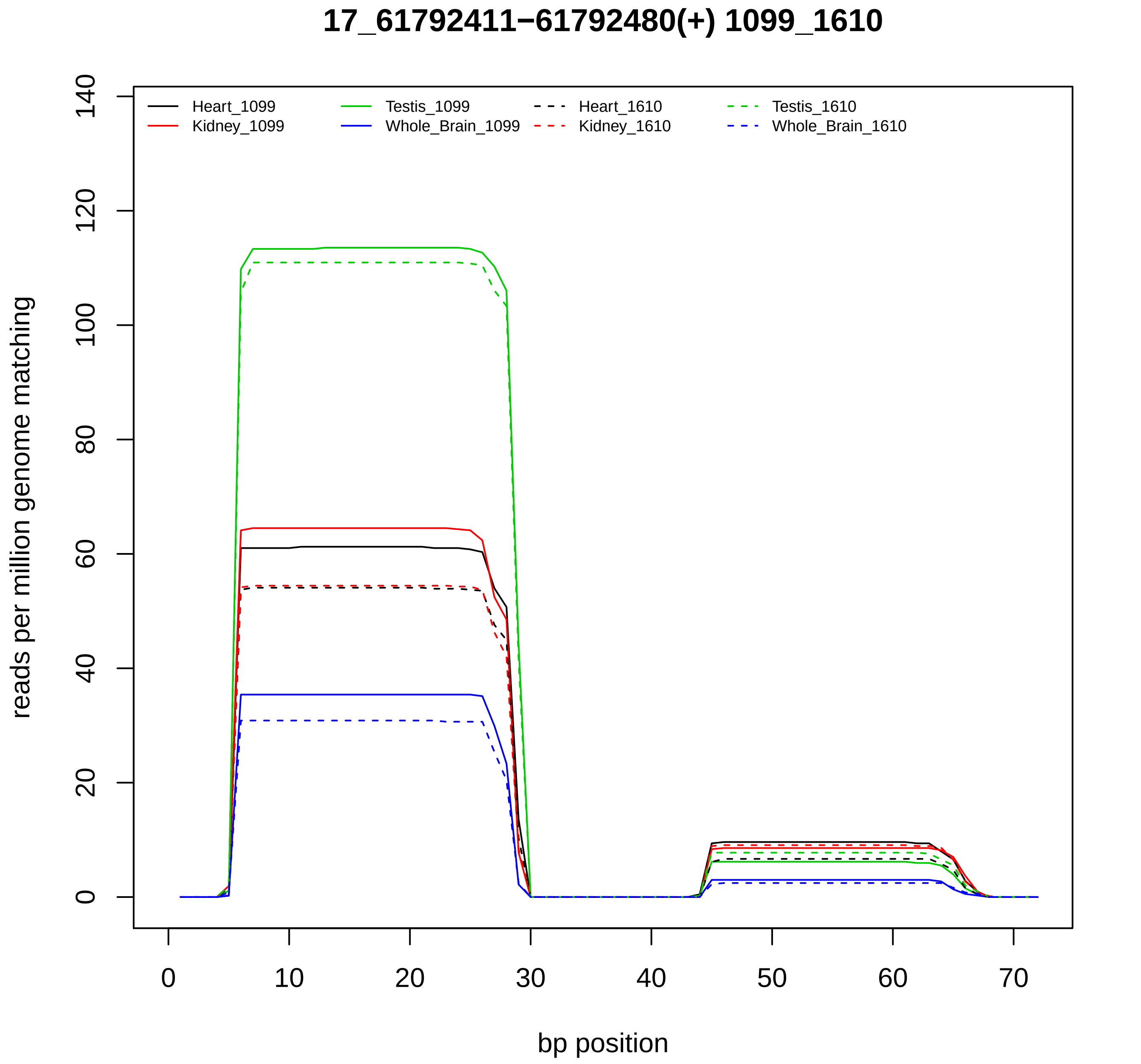
17_61792411−61792480(+) 1099_1610
Heart_1099
Kidney_1099
Testis_1099
Whole_Brain_1099
Heart_1610
Kidney_1610
Testis_1610
Whole_Brain_1610
reads per million genome matching
bp position
0
20
40
60
80
100
120
140
0
10
20
30
40
50
60
70

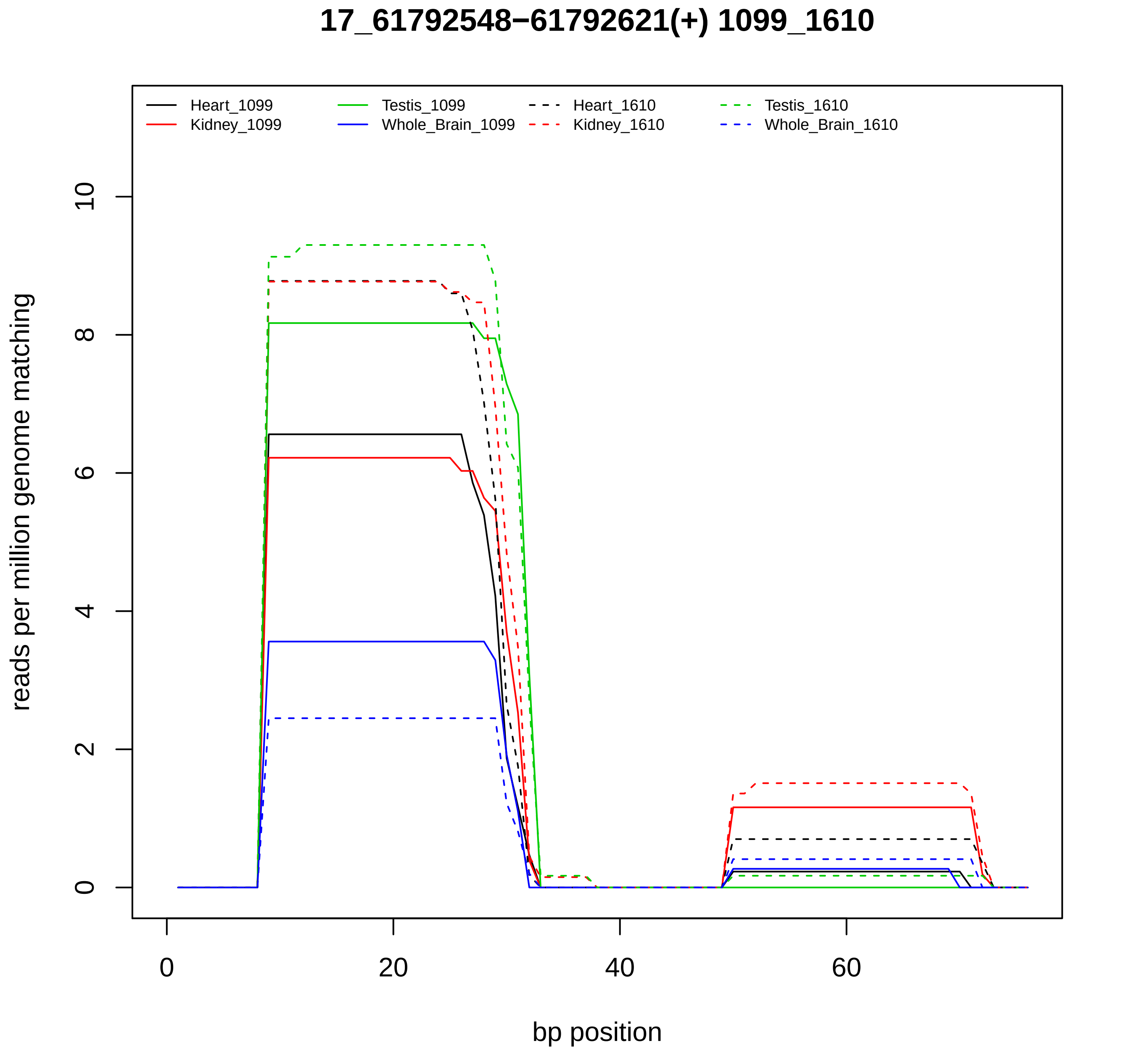

17_61792548−61792621(+) 1099_1610
Heart_1099
Kidney_1099
Testis_1099
Whole_Brain_1099
Heart_1610
Kidney_1610
Testis_1610
Whole_Brain_1610
reads per million genome matching
bp position
0
2
4
6
8
10
0
20
40
60

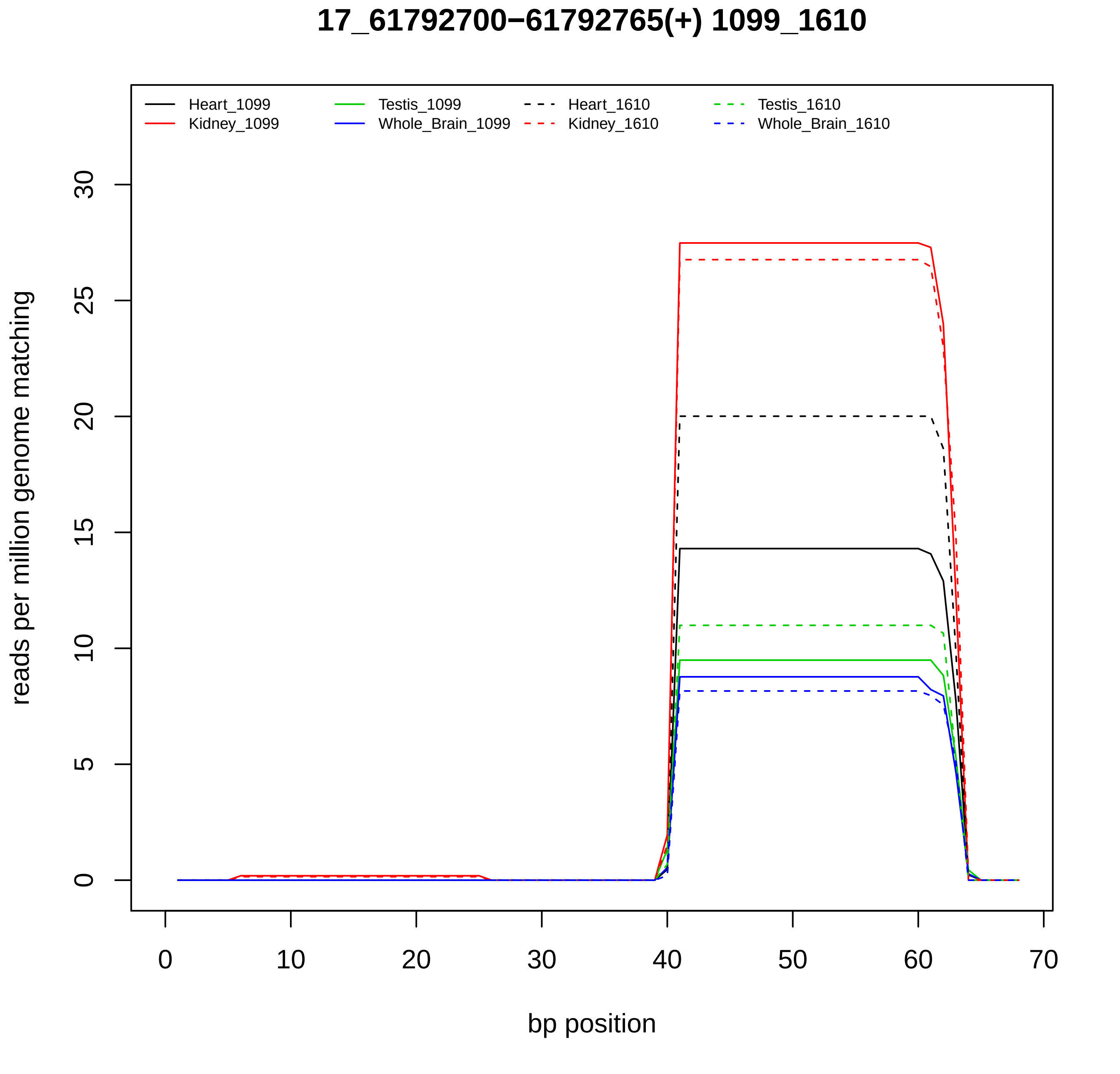

17_61792700−61792765(+) 1099_1610
Heart_1099
Kidney_1099
Testis_1099
Whole_Brain_1099
Heart_1610
Kidney_1610
Testis_1610
Whole_Brain_1610
reads per million genome matching
bp position
0
5
10
15
20
25
30
0
10
20
30
40
50
60
70

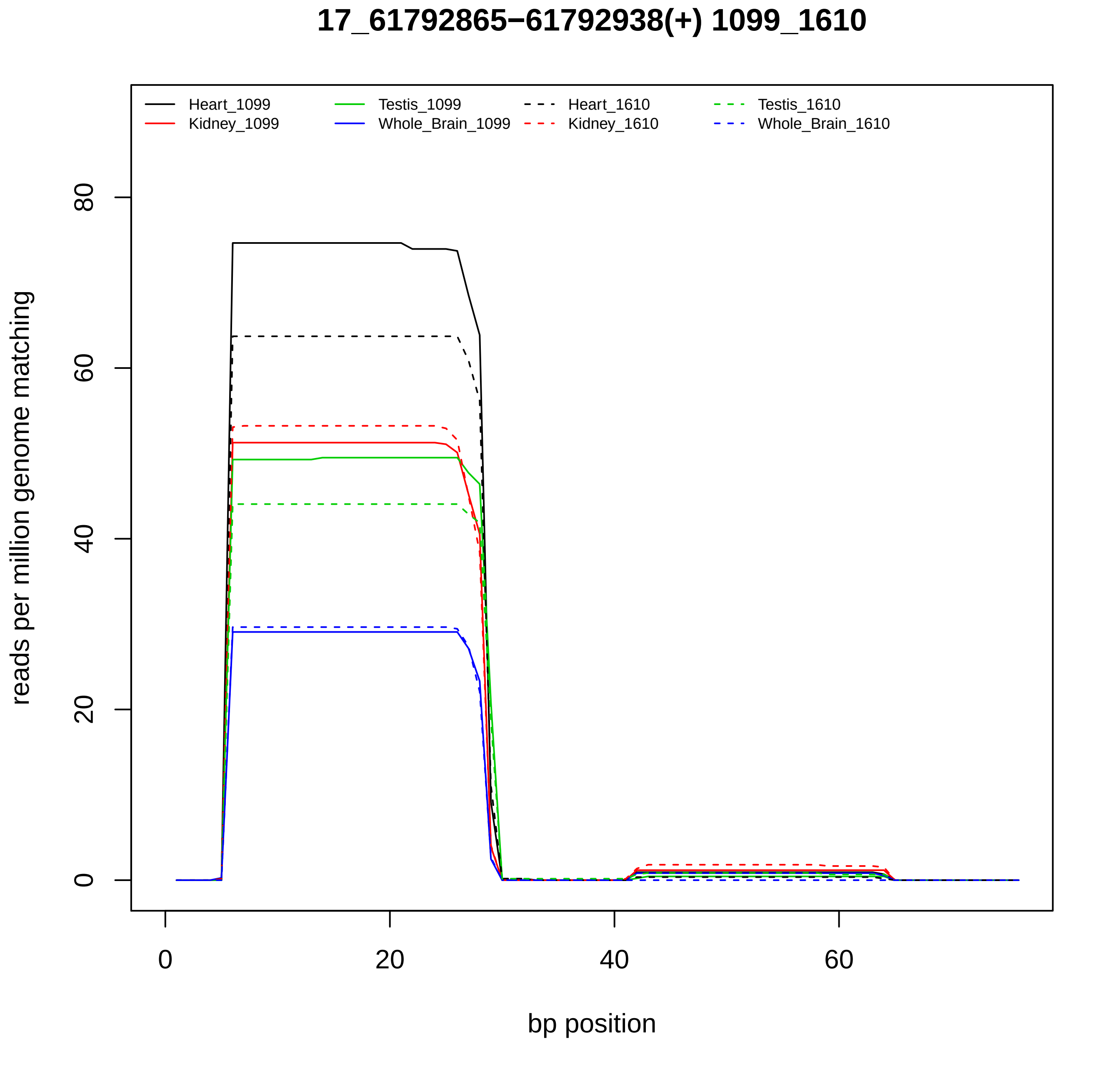

17_61792865−61792938(+) 1099_1610
Heart_1099
Kidney_1099
Testis_1099
Whole_Brain_1099
Heart_1610
Kidney_1610
Testis_1610
Whole_Brain_1610
reads per million genome matching
bp position
0
20
40
60
80

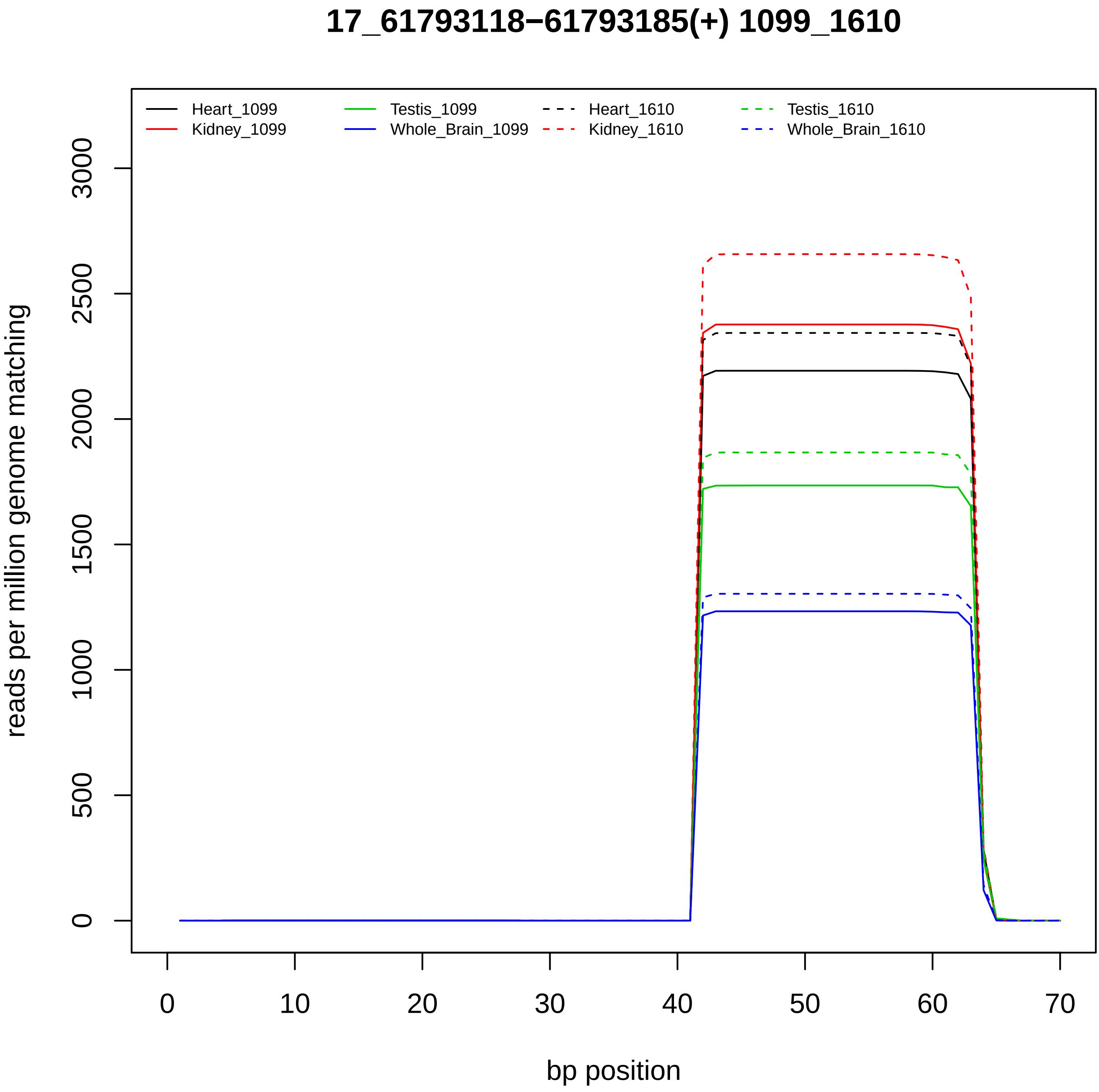

17_61793118−61793185(+) 1099_1610
Heart_1099
Kidney_1099
Testis_1099
Whole_Brain_1099
Heart_1610
Kidney_1610
Testis_1610
Whole_Brain_1610
reads per million genome matching
0
500
1000
1500
2000
2500
3000
bp position
0
10
20
30
40
50
60
70

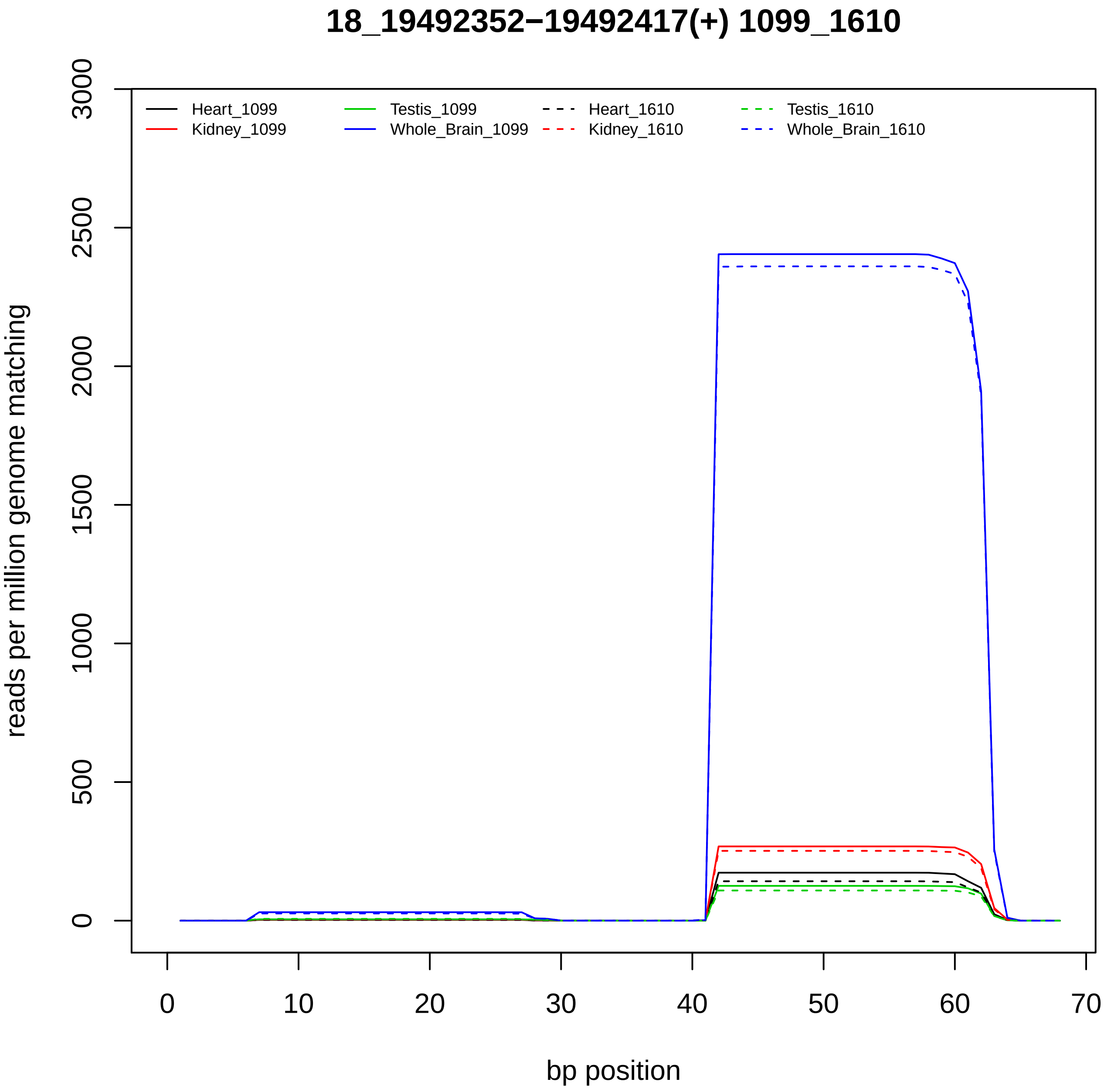

18_19492352−19492417(+) 1099_1610
Heart_1099
Kidney_1099
Testis_1099
Whole_Brain_1099
Heart_1610
Kidney_1610
Testis_1610
Whole_Brain_1610
reads per million genome matching
0
500
1000
1500
2000
2500
3000
bp position
0
10
20
30
40
50
60
70

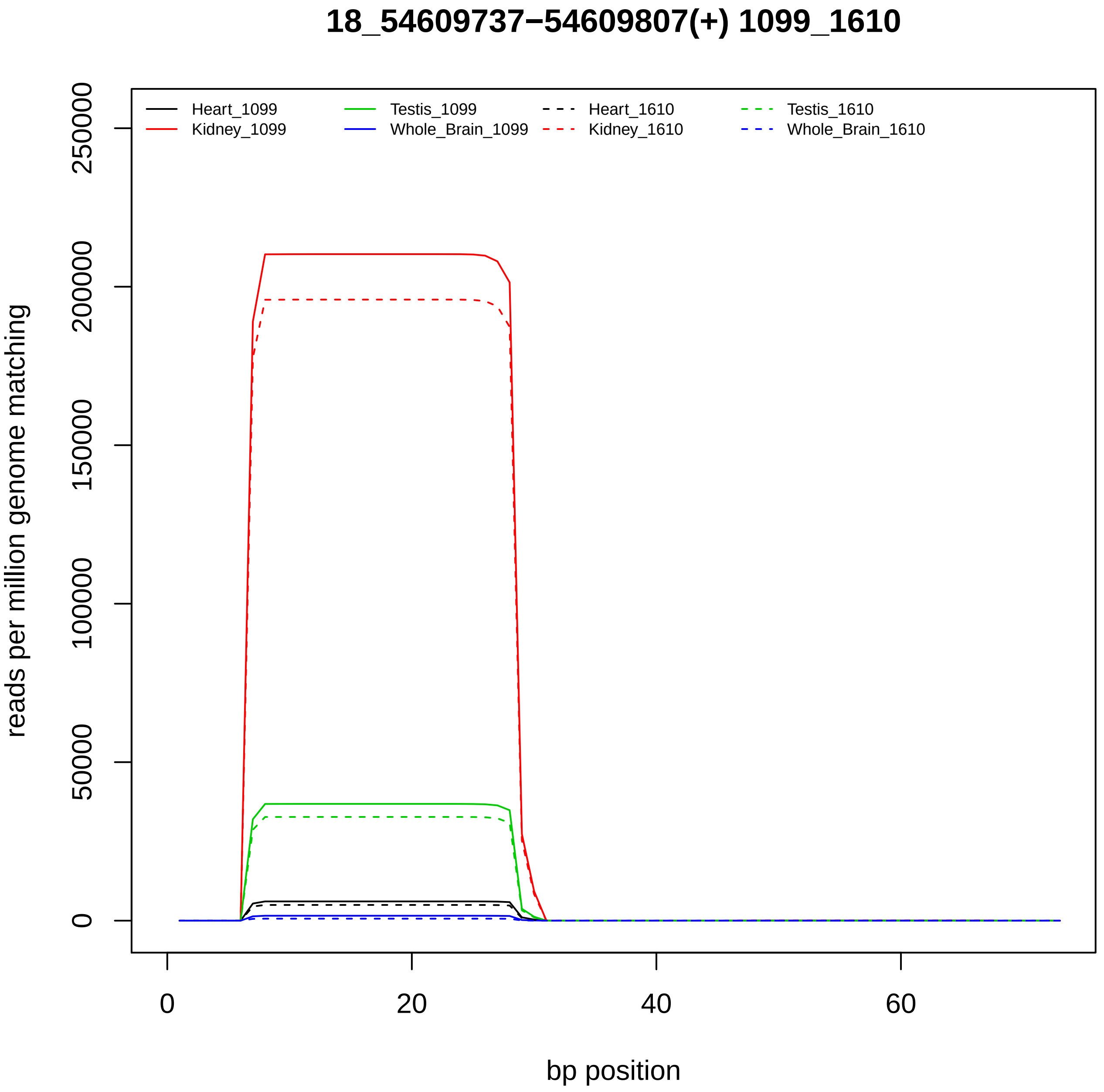

18_54609737−54609807(+) 1099_1610
Heart_1099
Kidney_1099
Testis_1099
Whole_Brain_1099
Heart_1610
Kidney_1610
Testis_1610
Whole_Brain_1610
reads per million genome matching
0
50000
100000
150000
200000
250000
0
20
40
60
bp position

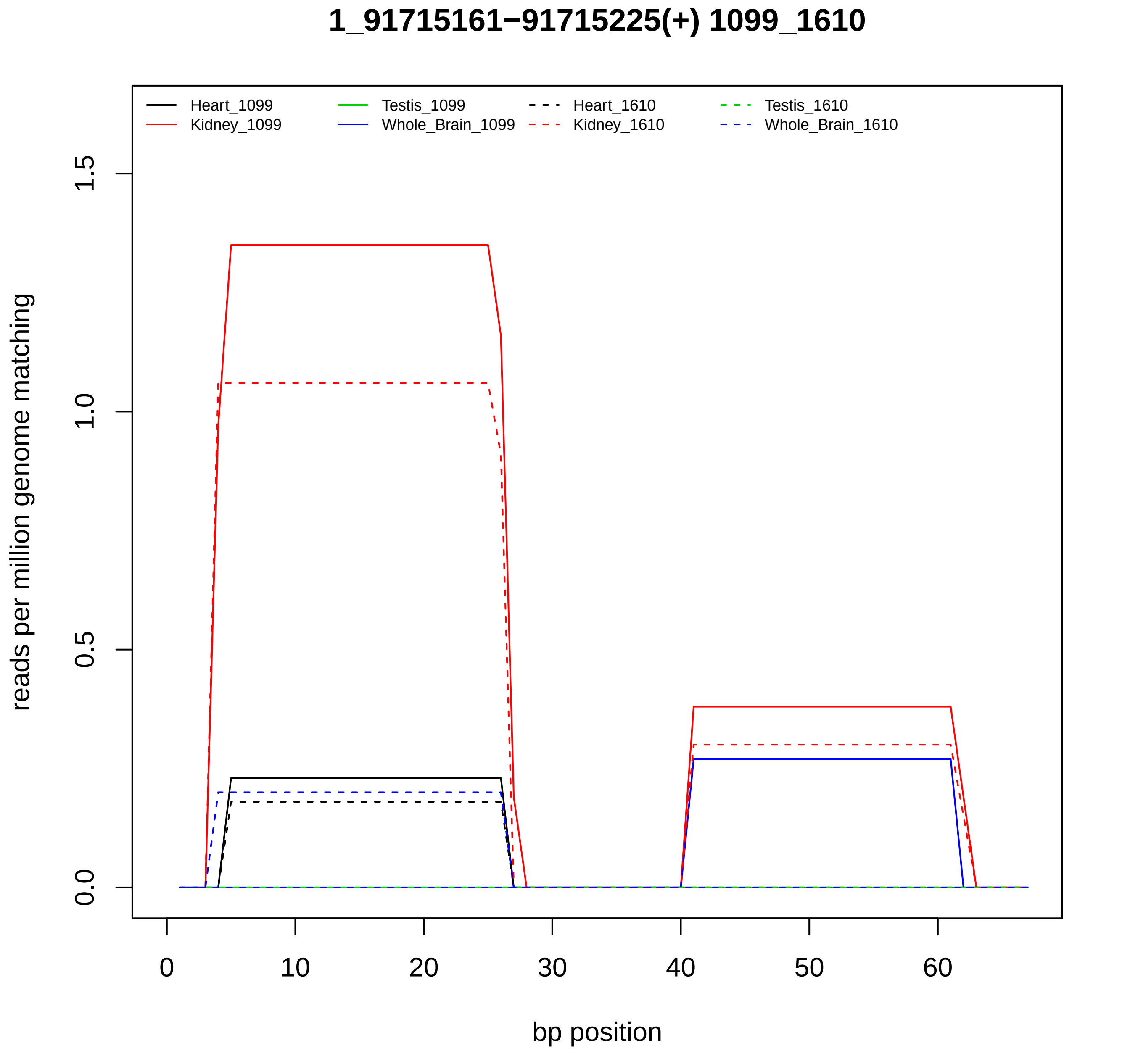

1_91715161−91715225(+) 1099_1610
Heart_1099
Kidney_1099
Testis_1099
Whole_Brain_1099
Heart_1610
Kidney_1610
Testis_1610
Whole_Brain_1610
reads per million genome matching
0.0
0.5
1.0
1.5
0
10
20
30
40
50
60
bp position

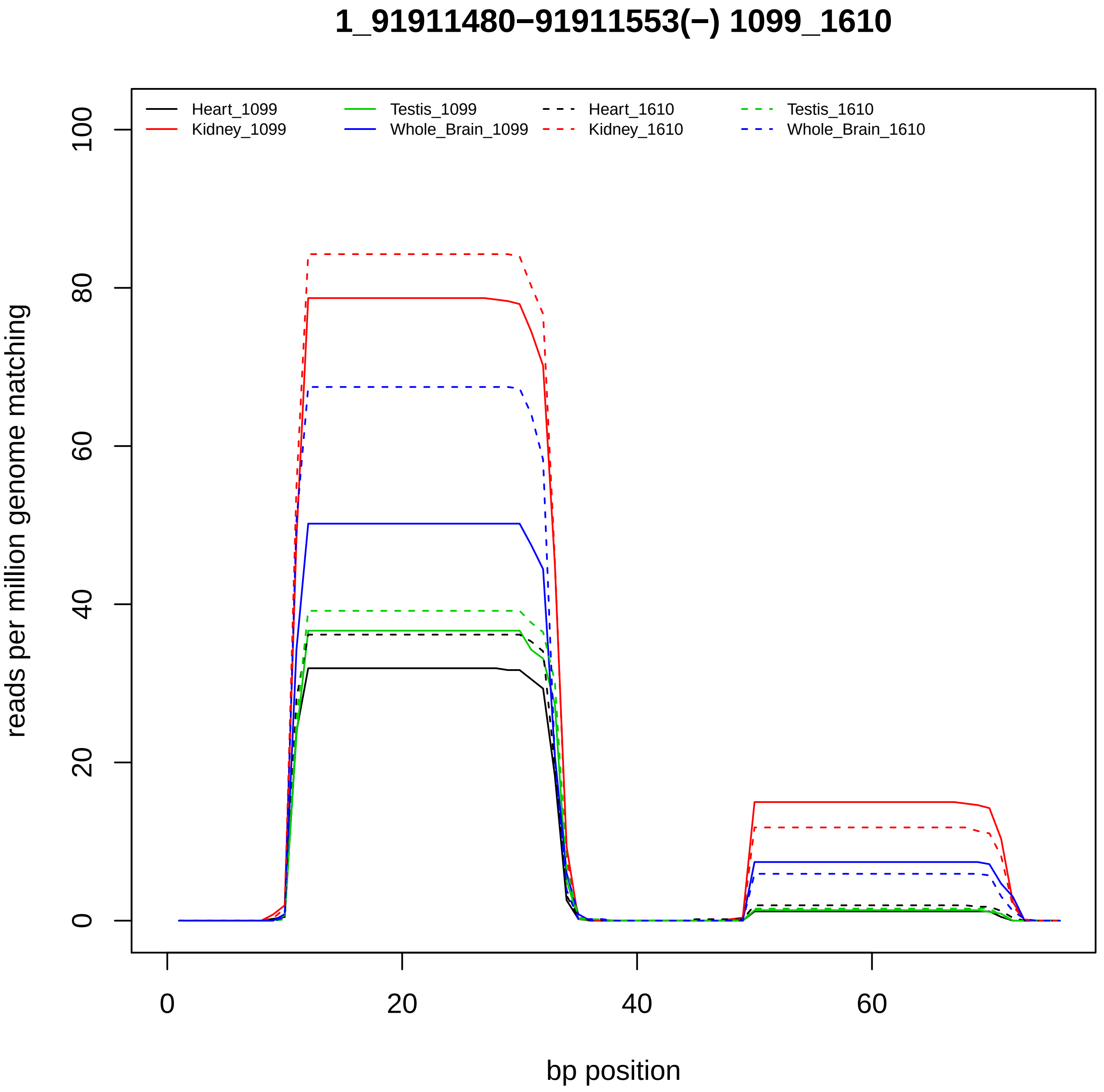
1_91911480−91911553(−) 1099_1610
Heart_1099
Kidney_1099
Testis_1099
Whole_Brain_1099
Heart_1610
Kidney_1610
Testis_1610
Whole_Brain_1610
reads per million genome matching
bp position
0
20
40
60
80
100
0
20
40
60

19_26408754−26408825(+) 1099_1610

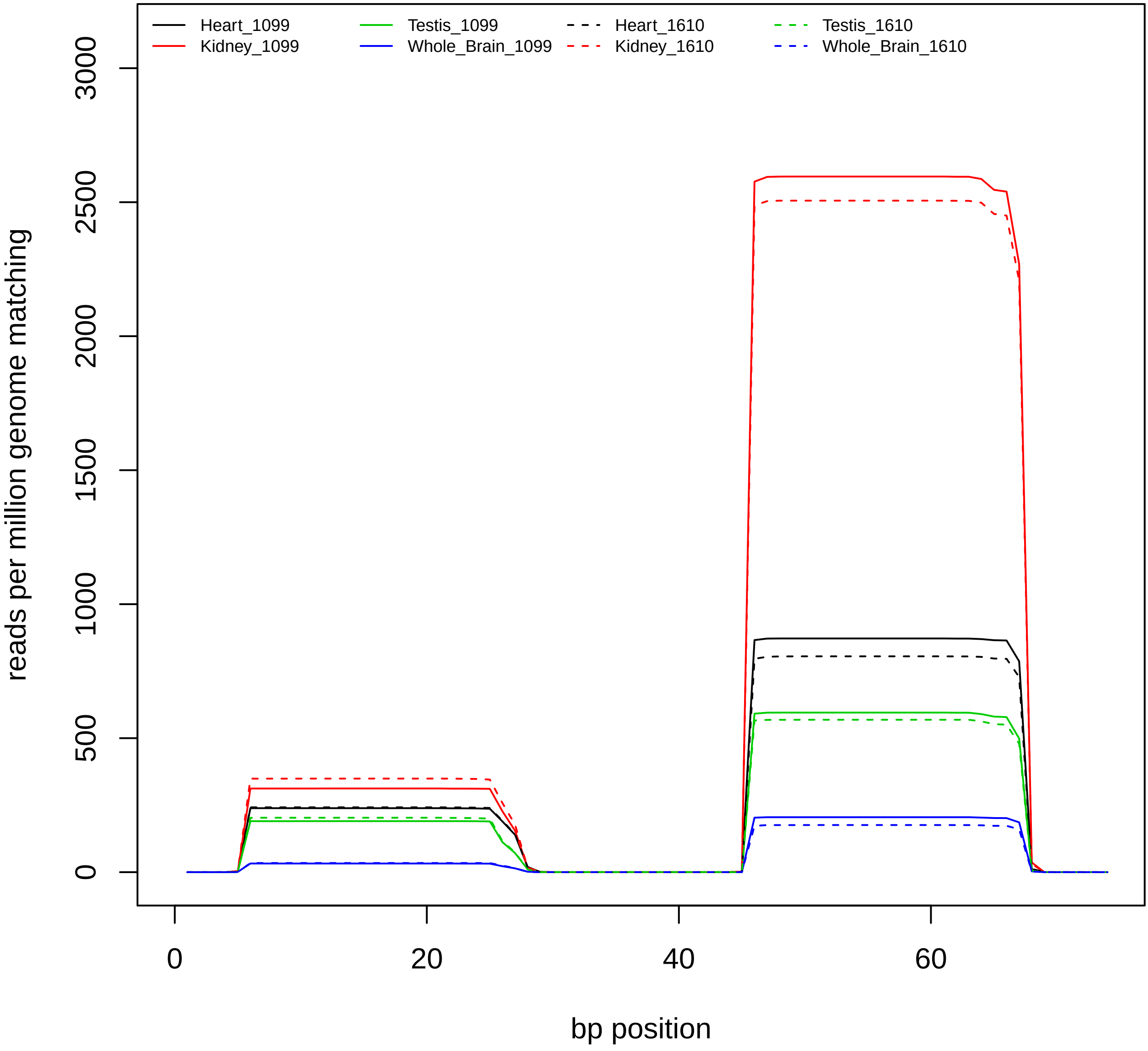

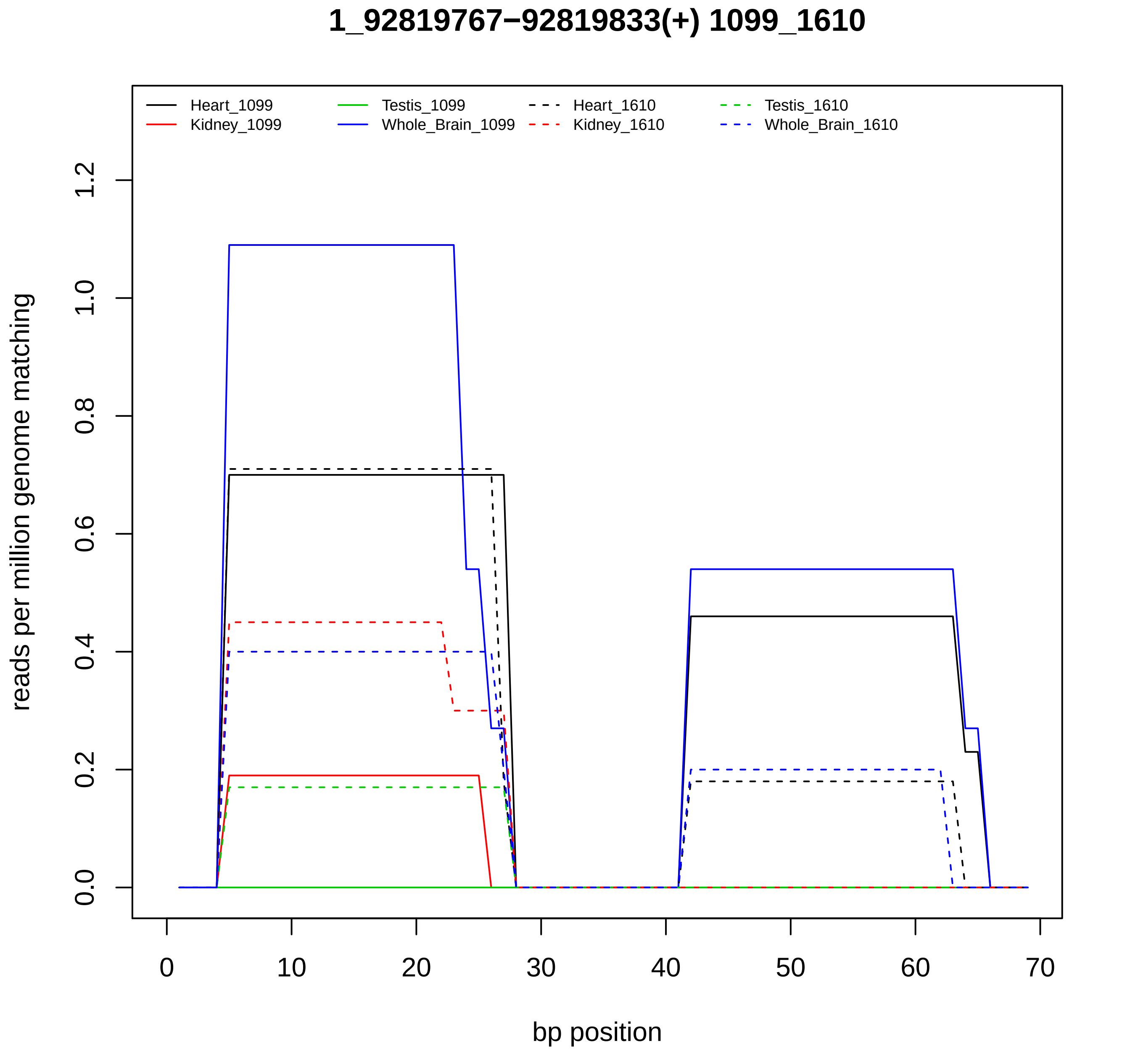
1_92819767−92819833(+) 1099_1610
Heart_1099
Kidney_1099
Testis_1099
Whole_Brain_1099
Heart_1610
Kidney_1610
Testis_1610
Whole_Brain_1610
reads per million genome matching
0.0
0.2
0.4
0.6
0.8
1.0
1.2
0
10
20
30
40
50
60
70
bp position

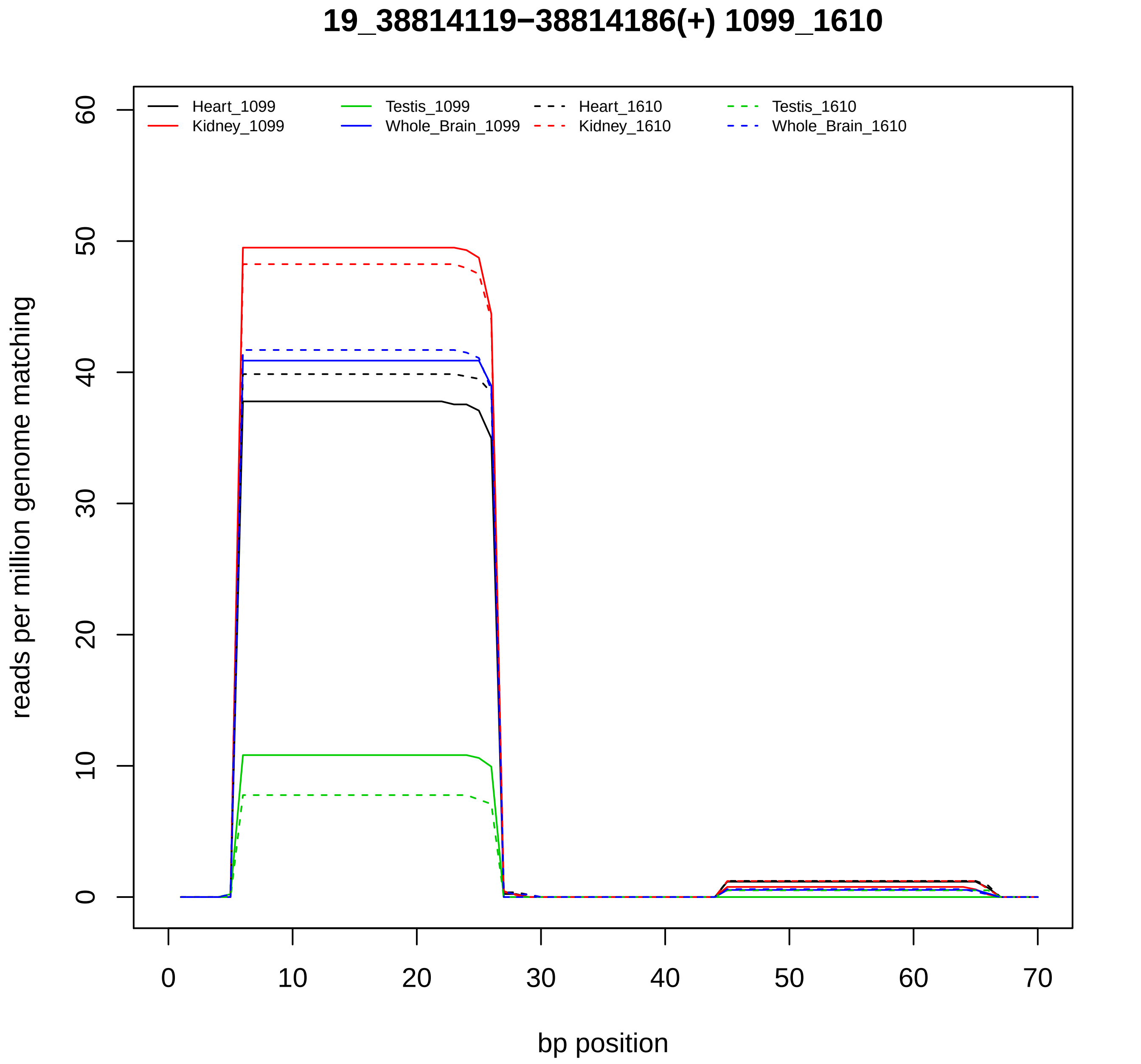

19_38814119−38814186(+) 1099_1610
Heart_1099
Kidney_1099
Testis_1099
Whole_Brain_1099
Heart_1610
Kidney_1610
Testis_1610
Whole_Brain_1610
reads per million genome matching
bp position
0
10
20
30
40
50
60
70

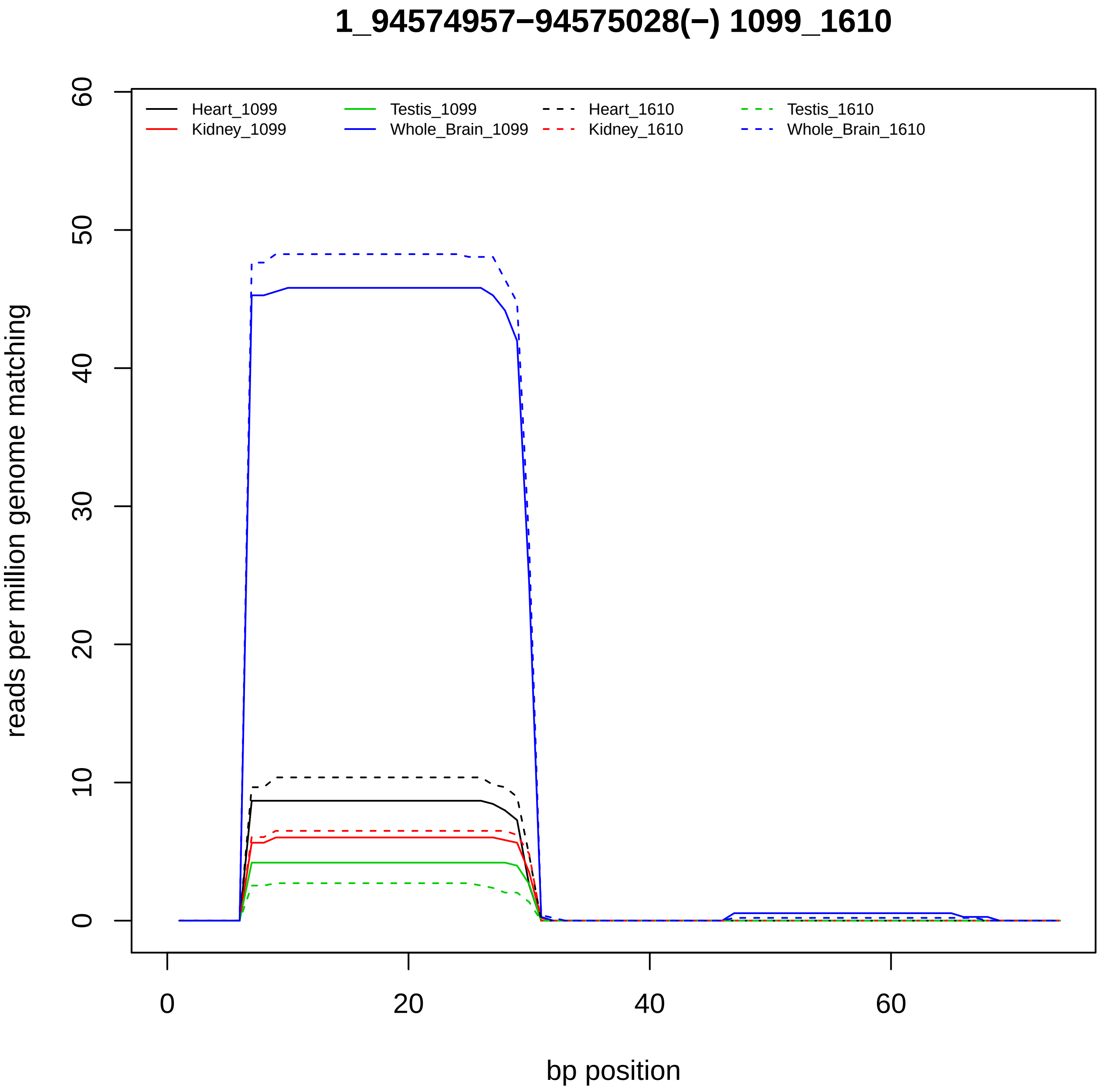

1_94574957−94575028(−) 1099_1610
Heart_1099
Kidney_1099
Testis_1099
Whole_Brain_1099
Heart_1610
Kidney_1610
Testis_1610
Whole_Brain_1610
reads per million genome matching
bp position
0
10
20
30
40
50
60
0
20
40
60

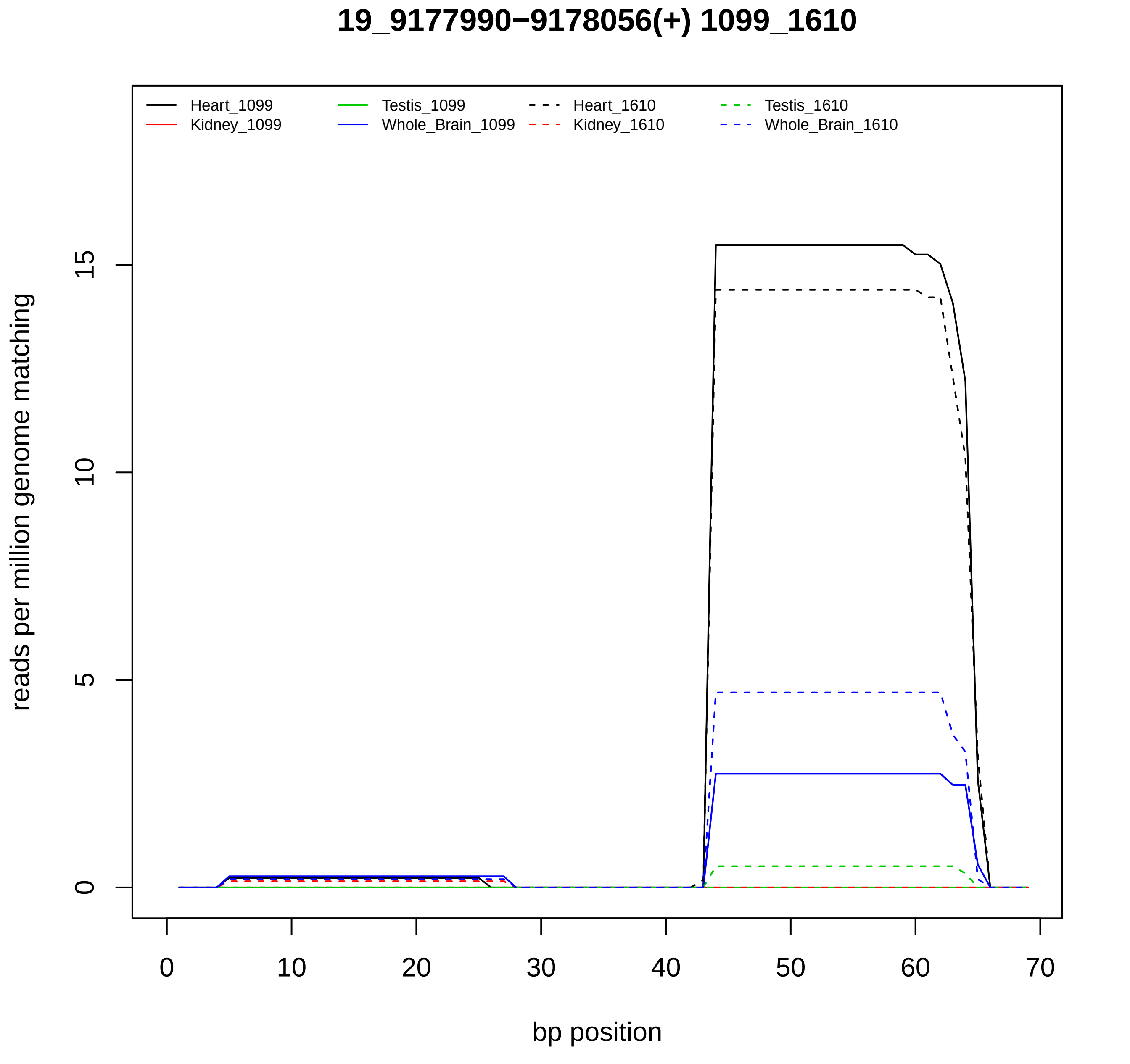
19_9177990−9178056(+) 1099_1610
Heart_1099
Kidney_1099
Testis_1099
Whole_Brain_1099
Heart_1610
Kidney_1610
Testis_1610
Whole_Brain_1610
reads per million genome matching
0
5
10
15
0
10
20
30
40
50
60
70
bp position

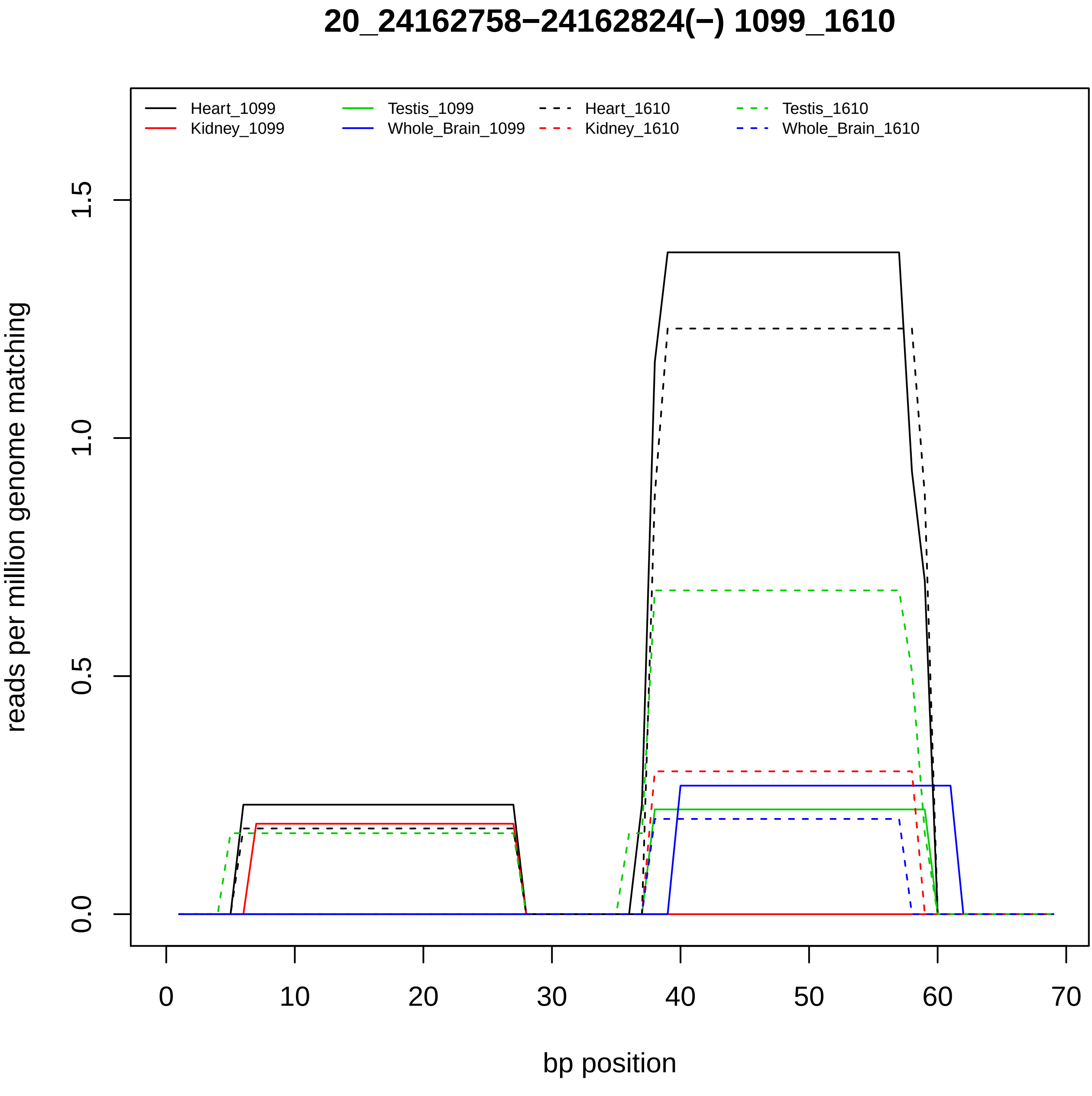

20_24162758−24162824(−) 1099_1610
Heart_1099
Kidney_1099
Testis_1099
Whole_Brain_1099
Heart_1610
Kidney_1610
Testis_1610
Whole_Brain_1610
reads per million genome matching
0.0
0.5
1.0
1.5
0
10
20
30
40
50
60
70
bp position

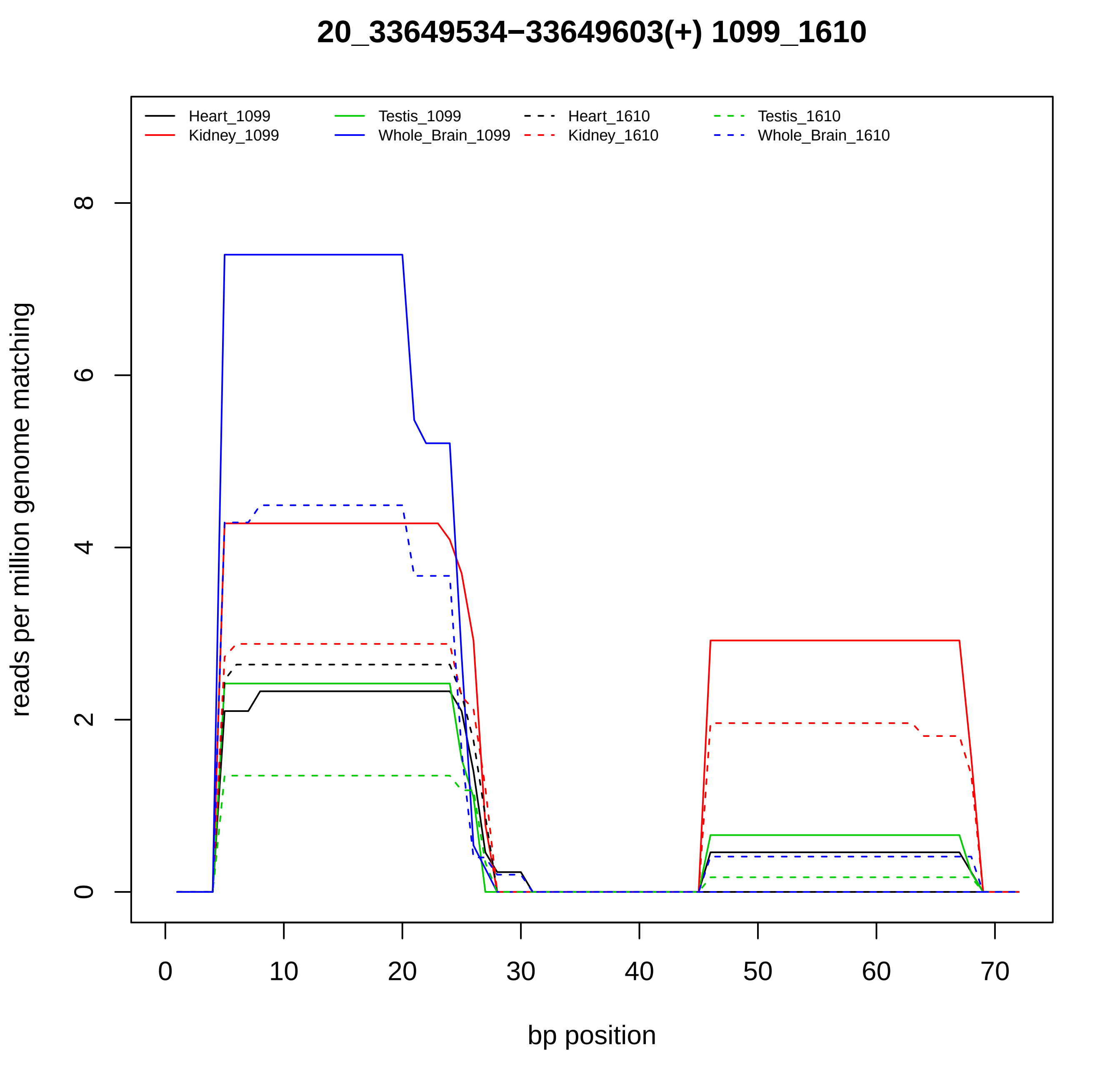

20_33649534−33649603(+) 1099_1610
Heart_1099
Kidney_1099
Testis_1099
Whole_Brain_1099
Heart_1610
Kidney_1610
Testis_1610
Whole_Brain_1610
reads per million genome matching
0
2
4
6
8
bp position
0
10
20
30
40
50
60
70

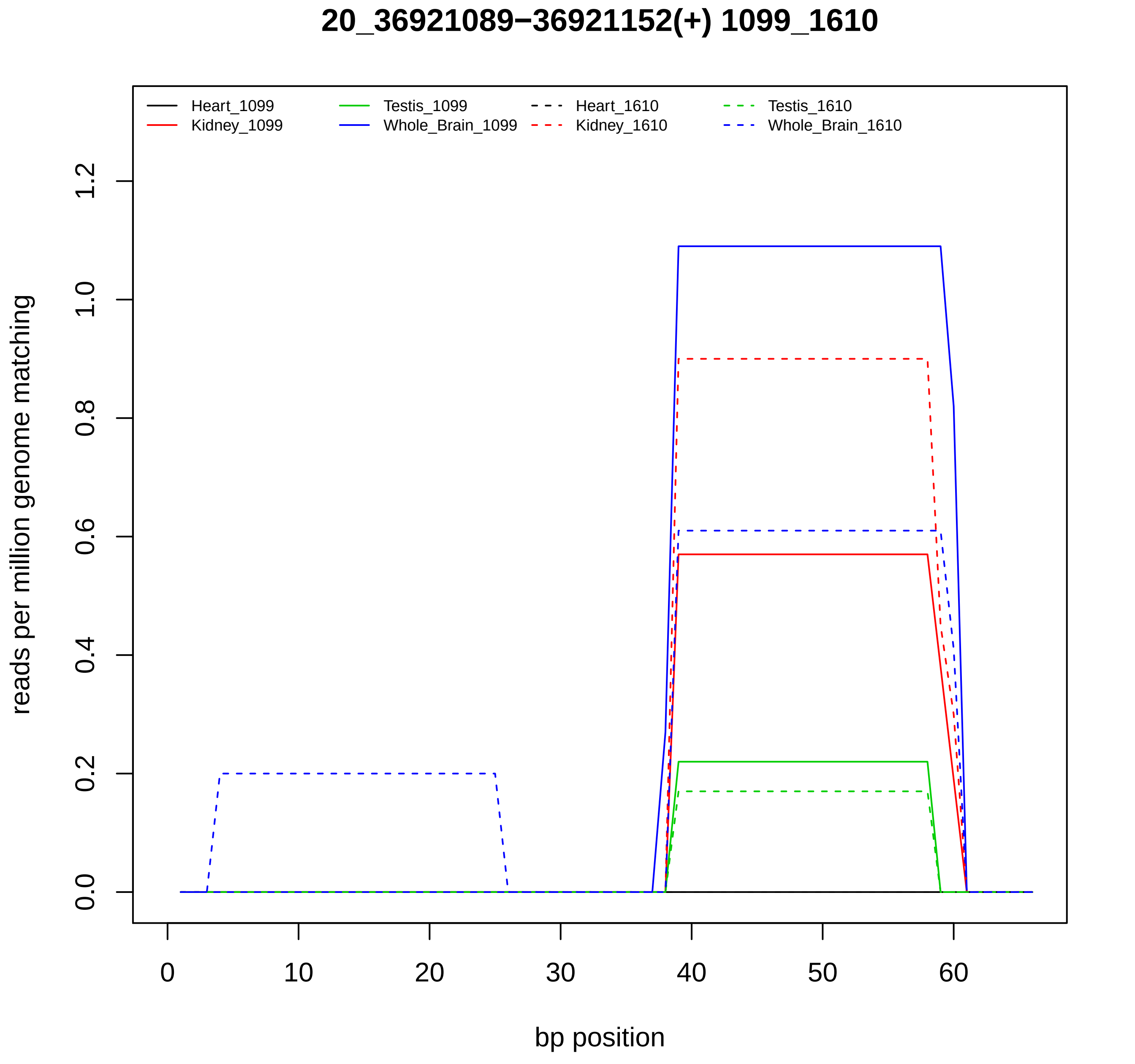

20_36921089−36921152(+) 1099_1610
Heart_1099
Kidney_1099
Testis_1099
Whole_Brain_1099
Heart_1610
Kidney_1610
Testis_1610
Whole_Brain_1610
reads per million genome matching
0.0
0.2
0.4
0.6
0.8
1.0
1.2
bp position
0
10
20
30
40
50
60

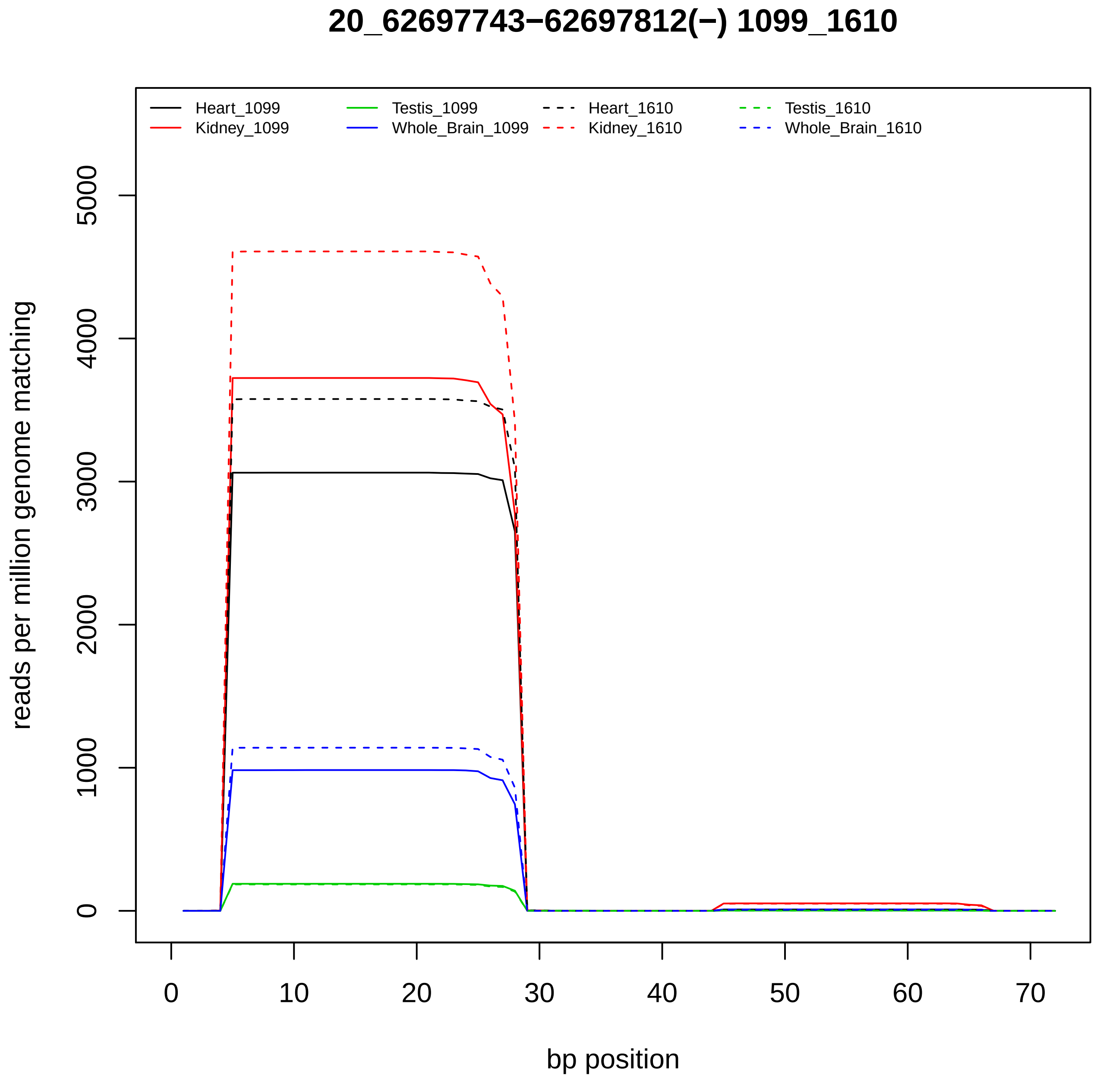

20_62697743−62697812(−) 1099_1610
Heart_1099
Kidney_1099
Testis_1099
Whole_Brain_1099
Heart_1610
Kidney_1610
Testis_1610
Whole_Brain_1610
reads per million genome matching
0
1000
2000
3000
4000
5000
0
10
20
30
40
50
60
70
bp position

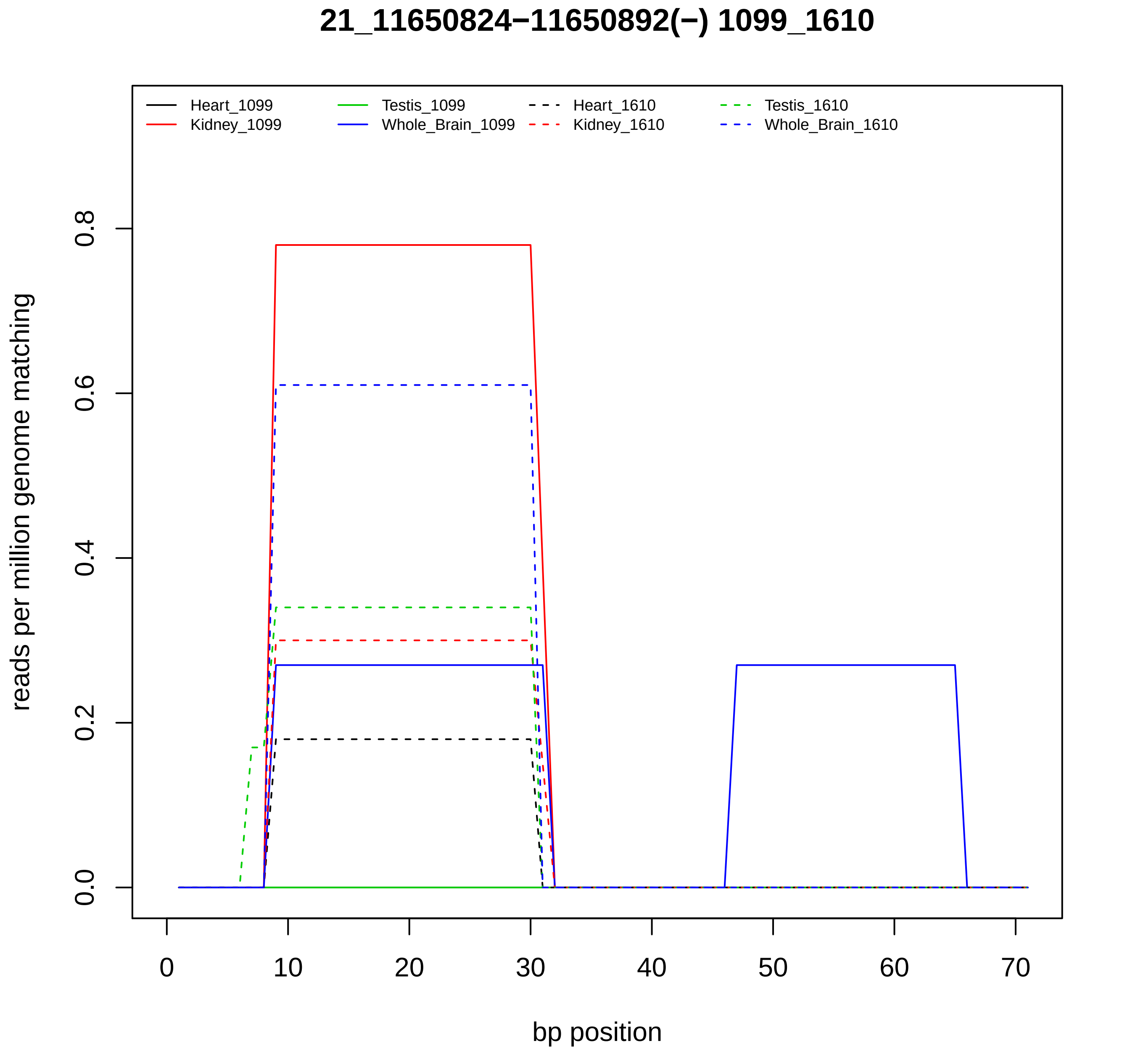

21_11650824−11650892(−) 1099_1610
Heart_1099
Kidney_1099
Testis_1099
Whole_Brain_1099
Heart_1610
Kidney_1610
Testis_1610
Whole_Brain_1610
reads per million genome matching
0.0
0.2
0.4
0.6
0.8
0
10
20
30
40
50
60
70
bp position

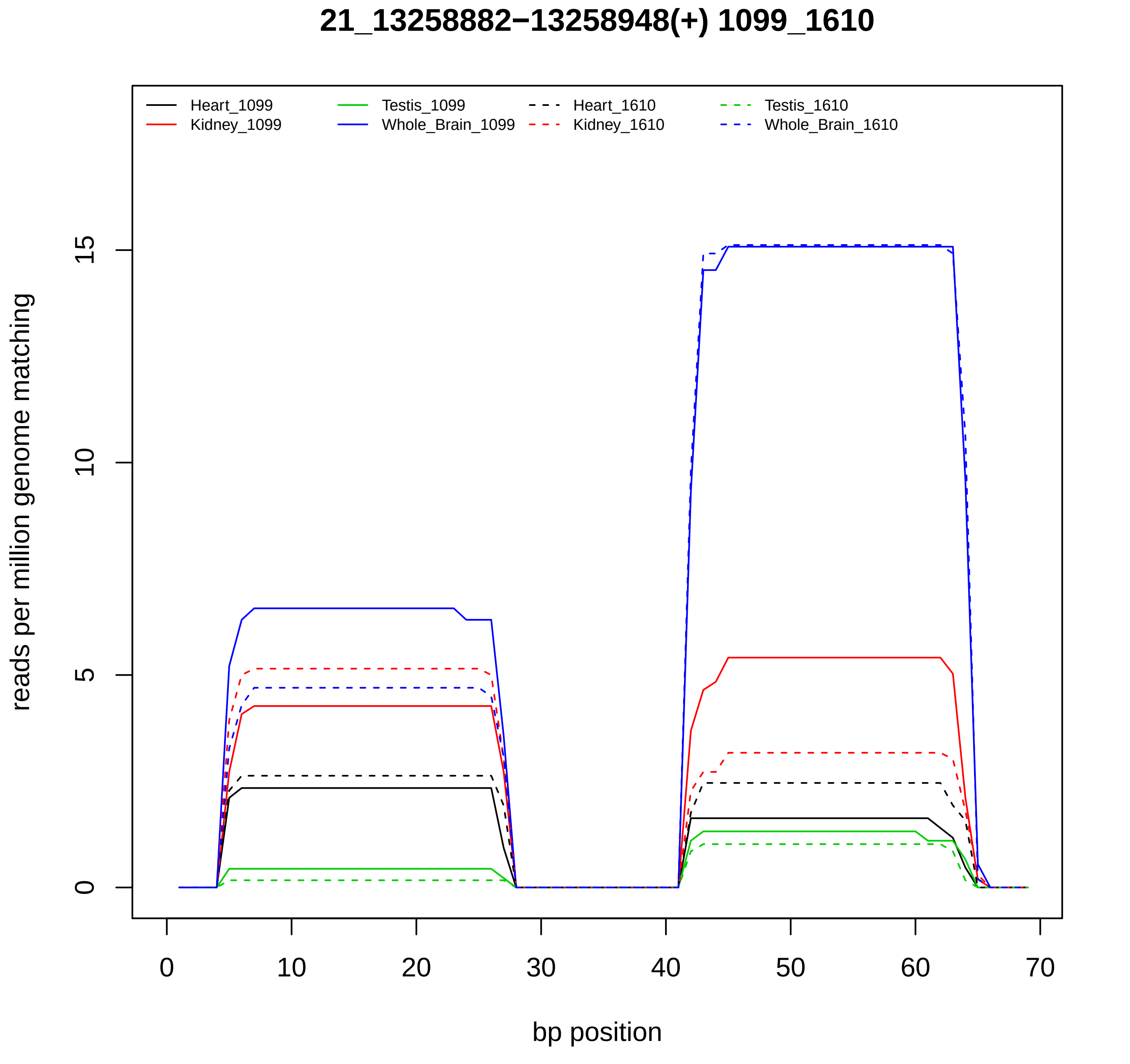
21_13258882−13258948(+) 1099_1610
Heart_1099
Kidney_1099
Testis_1099
Whole_Brain_1099
Heart_1610
Kidney_1610
Testis_1610
Whole_Brain_1610
reads per million genome matching
bp position
0
5
10
15
0
10
20
30
40
50
60
70

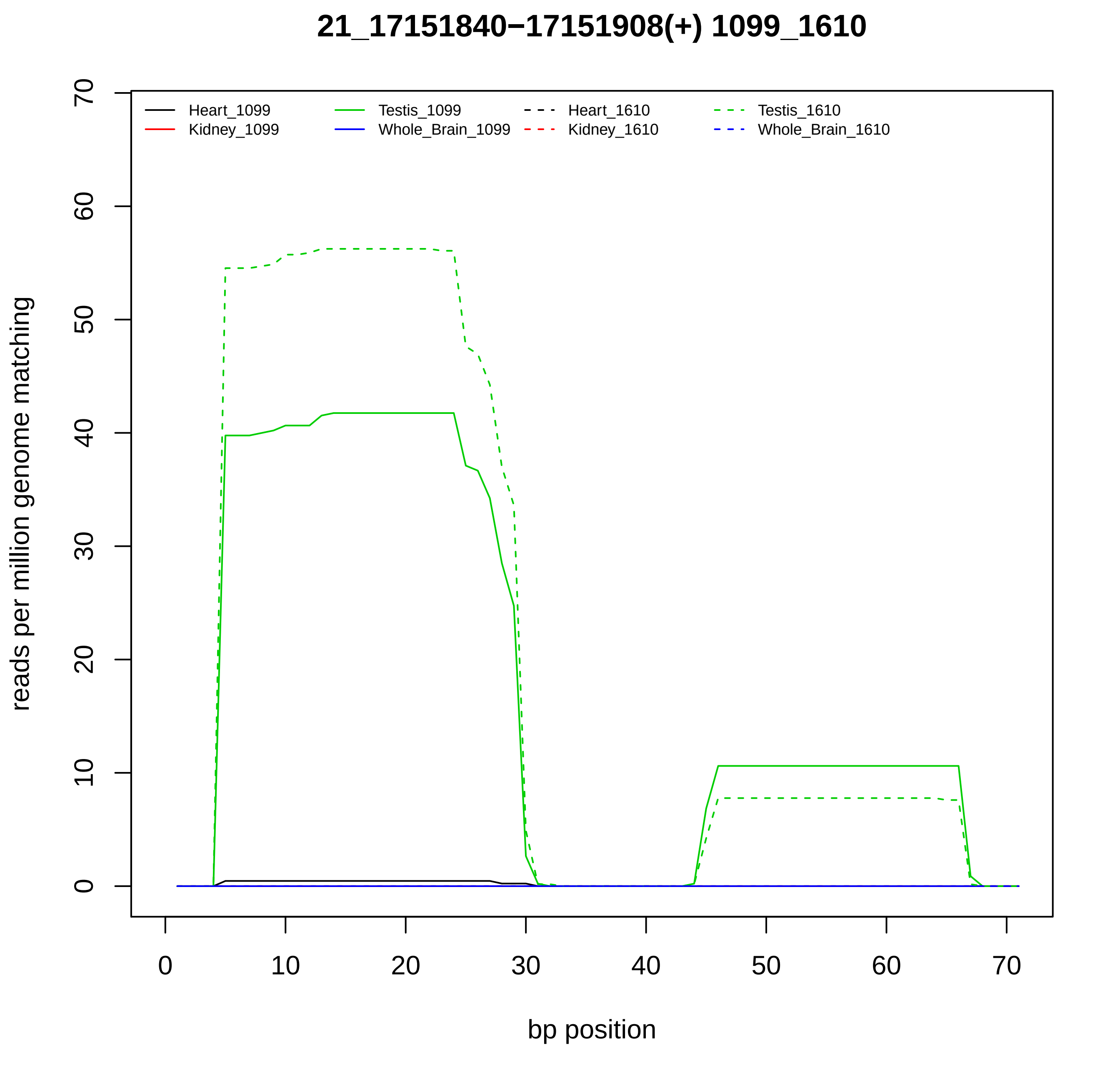
21_17151840−17151908(+) 1099_1610
Heart_1099
Kidney_1099
Testis_1099
Whole_Brain_1099
Heart_1610
Kidney_1610
Testis_1610
Whole_Brain_1610
reads per million genome matching
bp position
0
10
20
30
40
50
60
70

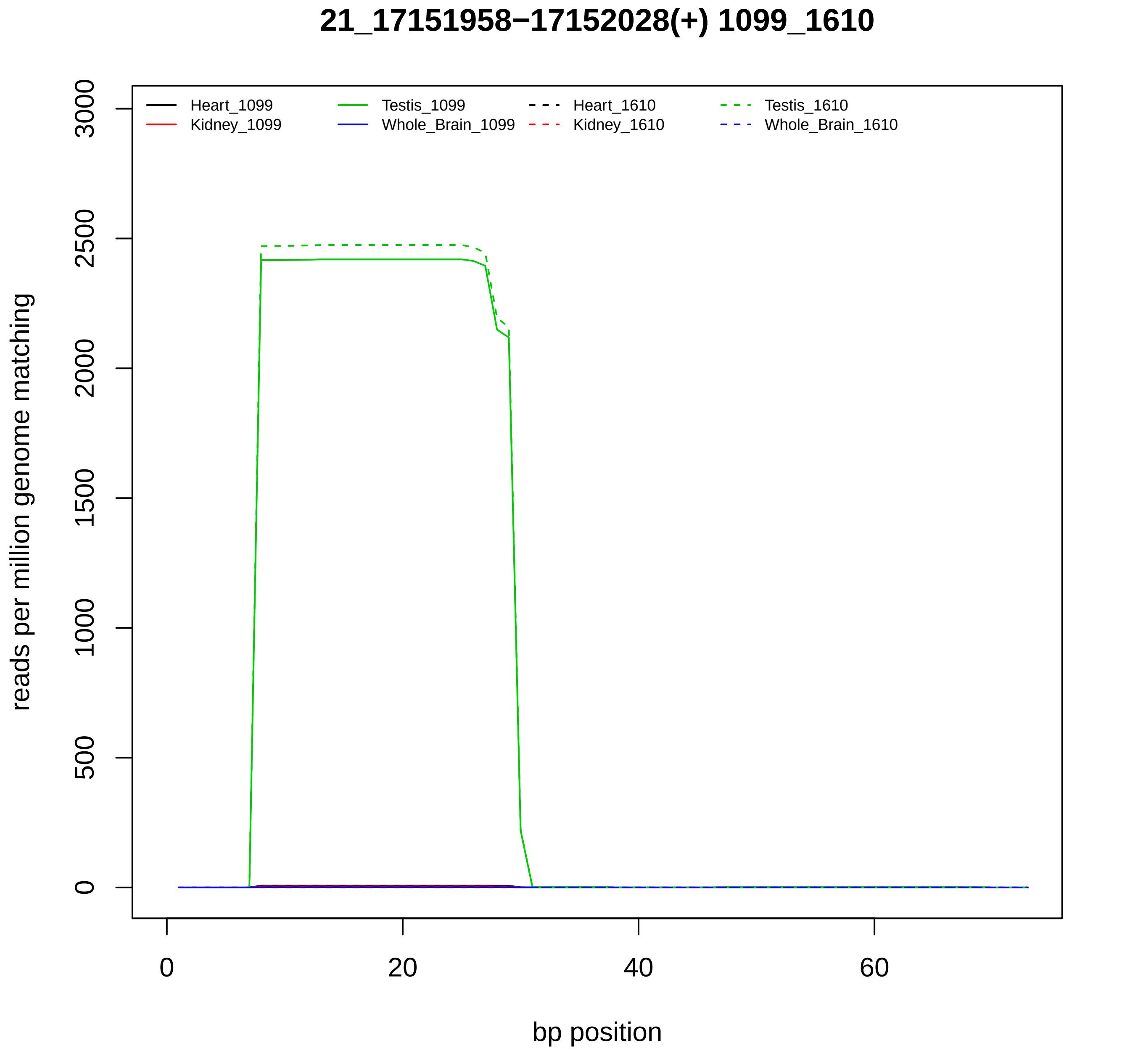

21_17151958−17152028(+) 1099_1610
Heart_1099
Kidney_1099
Testis_1099
Whole_Brain_1099
Heart_1610
Kidney_1610
Testis_1610
Whole_Brain_1610
reads per million genome matching
0
500
1000
1500
2000
2500
3000
0
20
40
60
bp position

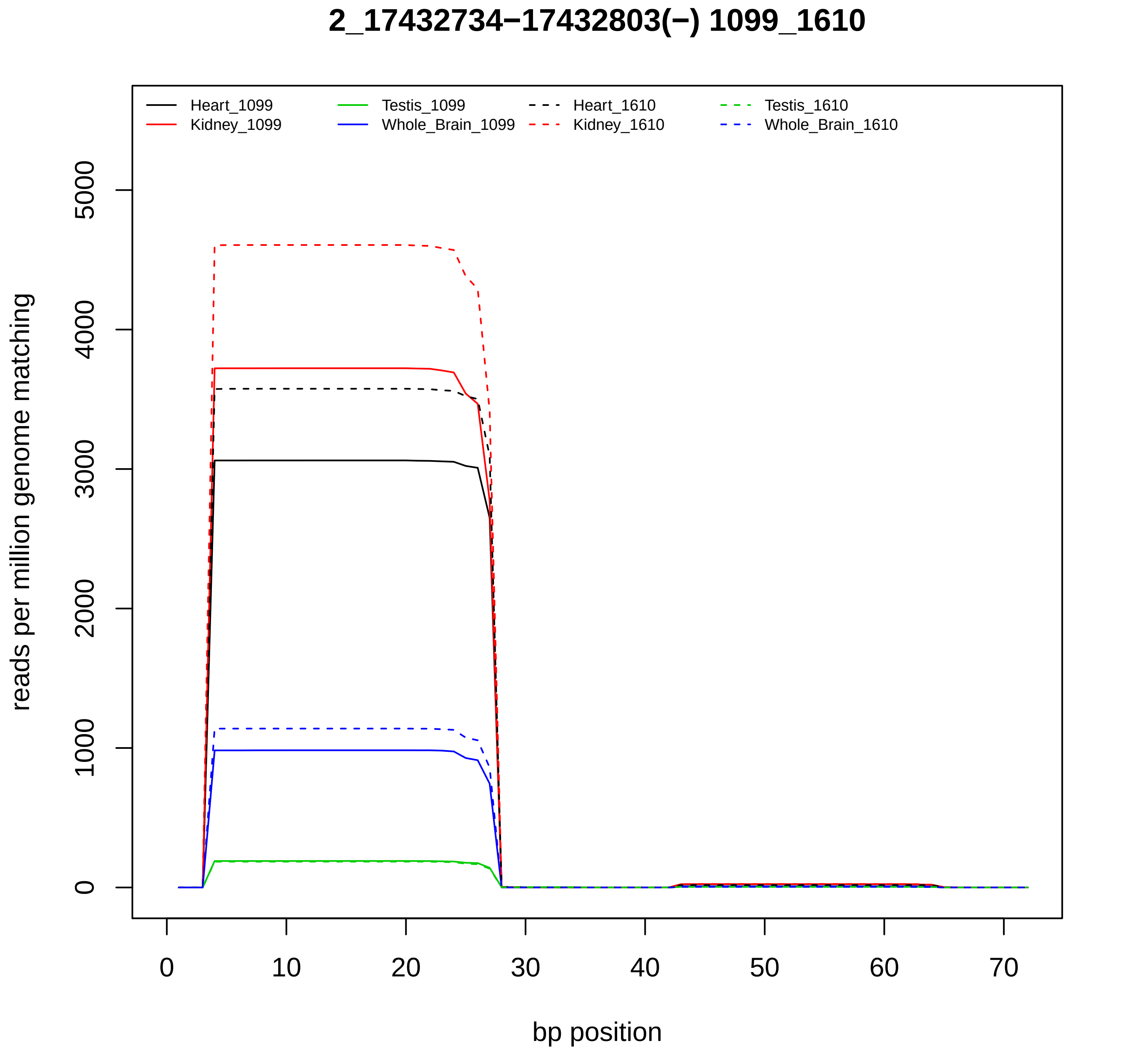

2_17432734−17432803(−) 1099_1610
Heart_1099
Kidney_1099
Testis_1099
Whole_Brain_1099
Heart_1610
Kidney_1610
Testis_1610
Whole_Brain_1610
reads per million genome matching
0
1000
2000
3000
4000
5000
0
10
20
30
40
50
60
70
bp position

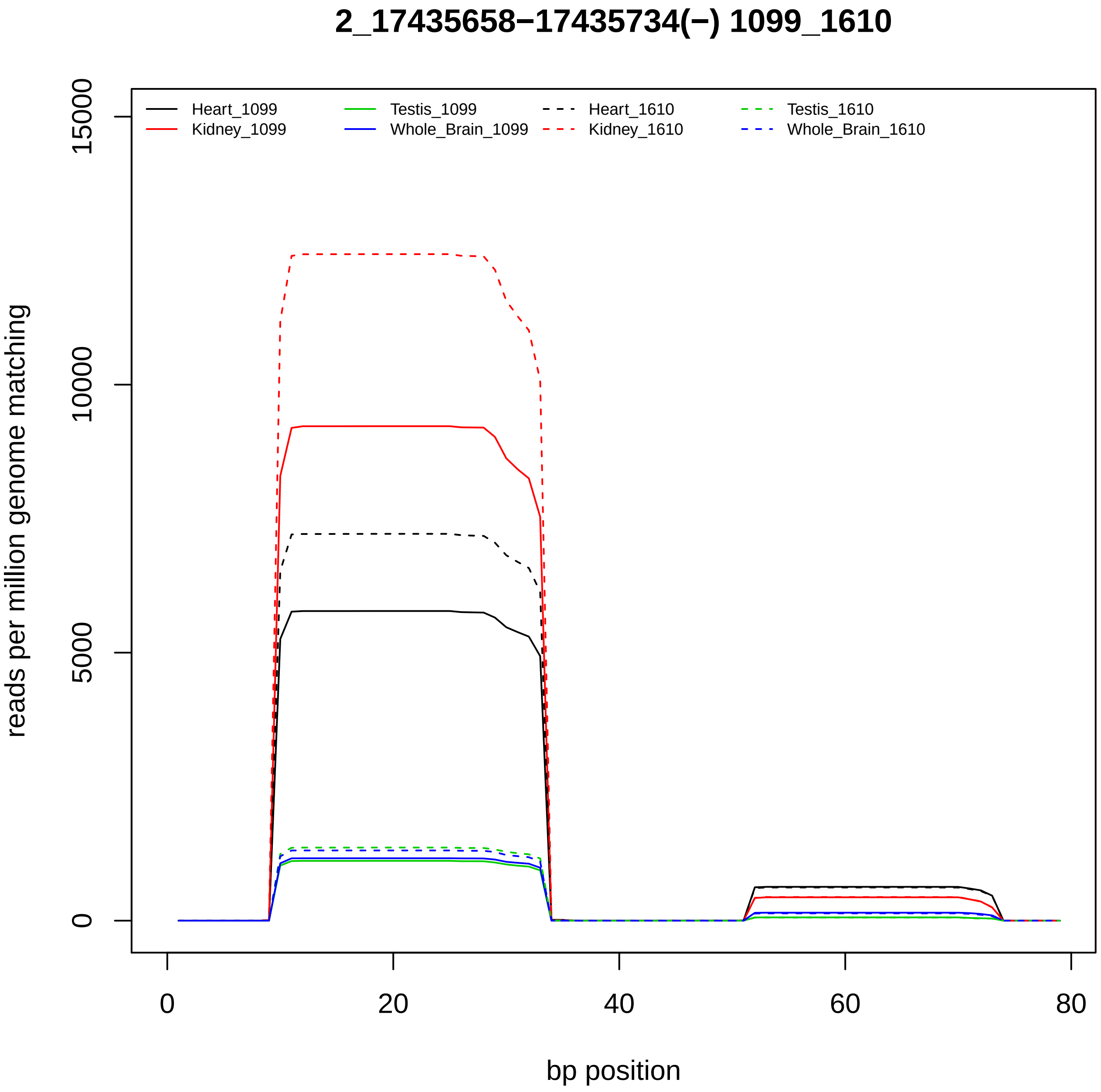
2_17435658−17435734(−) 1099_1610
Heart_1099
Kidney_1099
Testis_1099
Whole_Brain_1099
Heart_1610
Kidney_1610
Testis_1610
Whole_Brain_1610
reads per million genome matching
0
5000
10000
15000
bp position
0
20
40
60
80

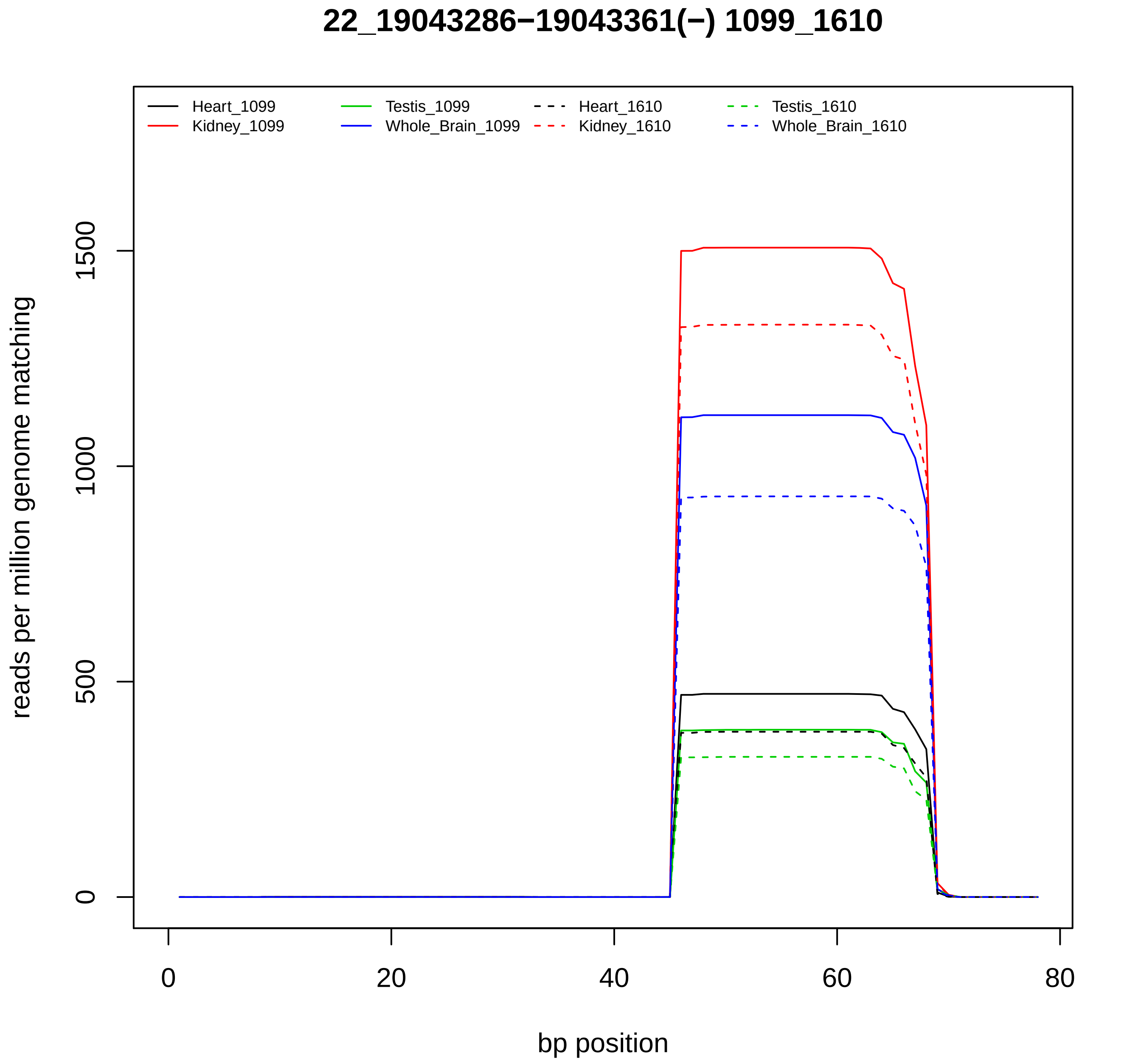

22_19043286−19043361(−) 1099_1610
Heart_1099
Kidney_1099
Testis_1099
Whole_Brain_1099
Heart_1610
Kidney_1610
Testis_1610
Whole_Brain_1610
reads per million genome matching
bp position
0
500
1000
1500
0
20
40
60
80

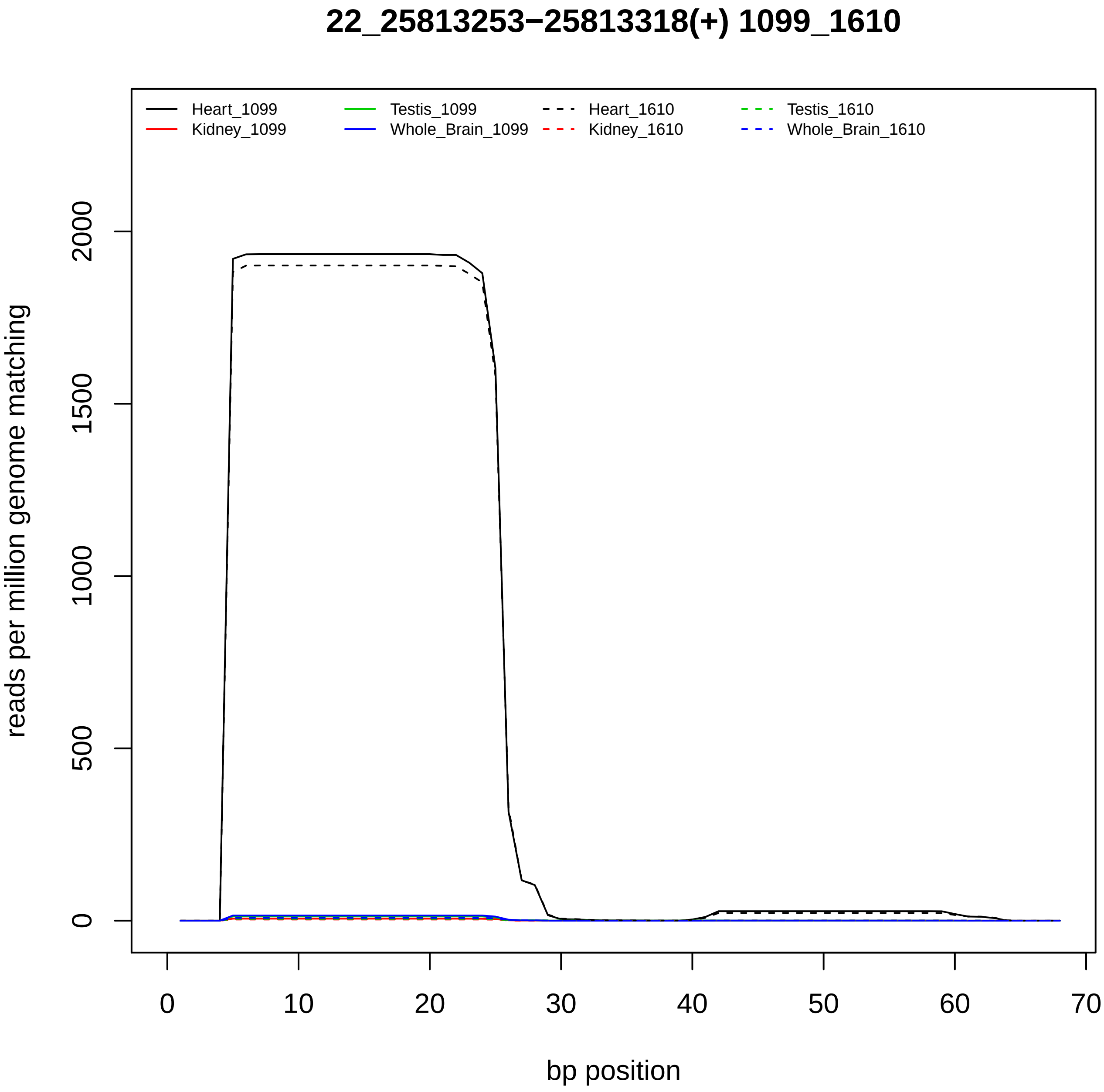

22_25813253−25813318(+) 1099_1610
Heart_1099
Kidney_1099
Testis_1099
Whole_Brain_1099
Heart_1610
Kidney_1610
Testis_1610
Whole_Brain_1610
reads per million genome matching
0
500
1000
1500
2000
bp position
0
10
20
30
40
50
60
70

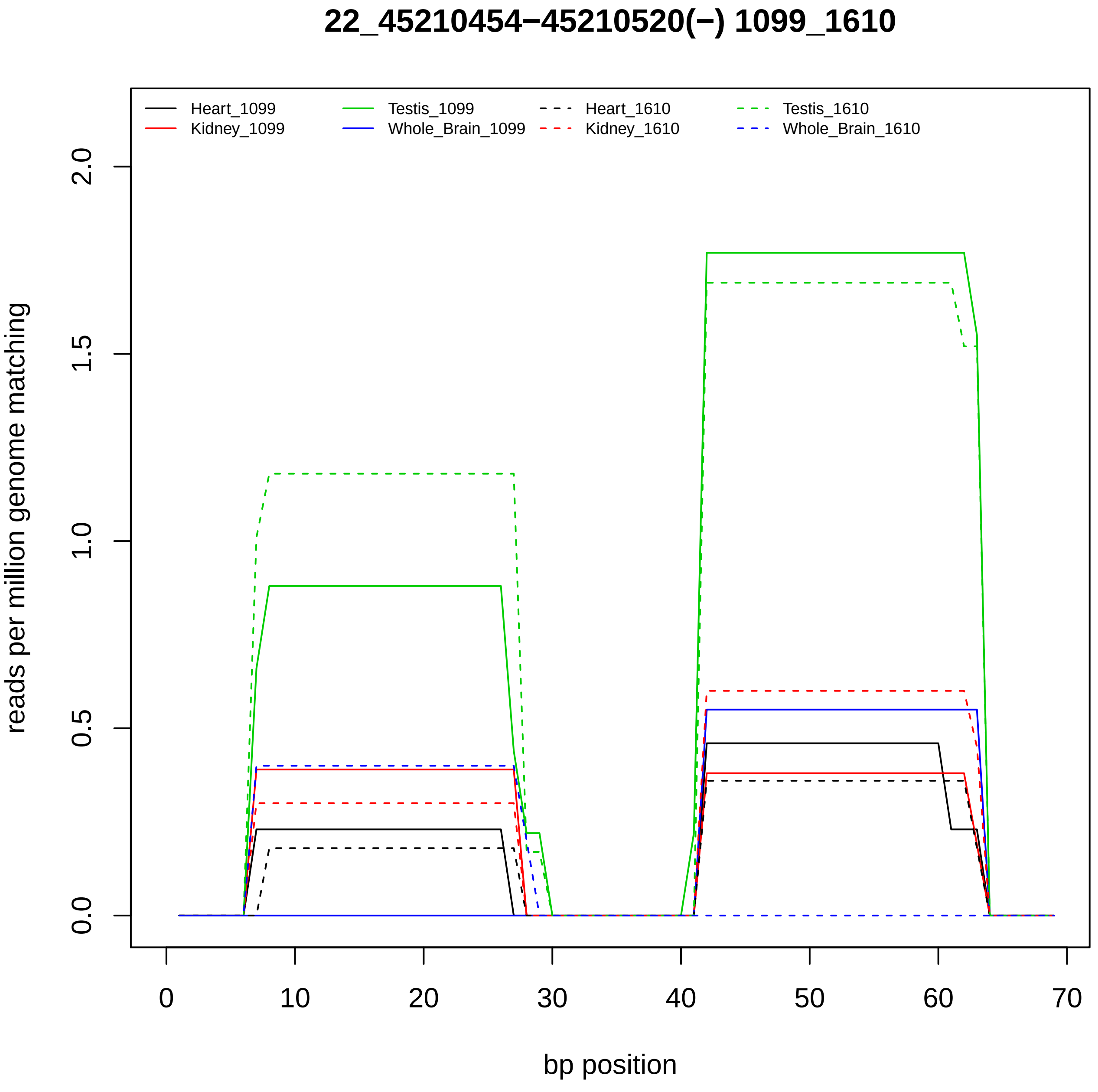
22_45210454−45210520(−) 1099_1610
Heart_1099
Kidney_1099
Testis_1099
Whole_Brain_1099
Heart_1610
Kidney_1610
Testis_1610
Whole_Brain_1610
reads per million genome matching
0.0
0.5
1.0
1.5
2.0
bp position
0
10
20
30
40
50
60
70

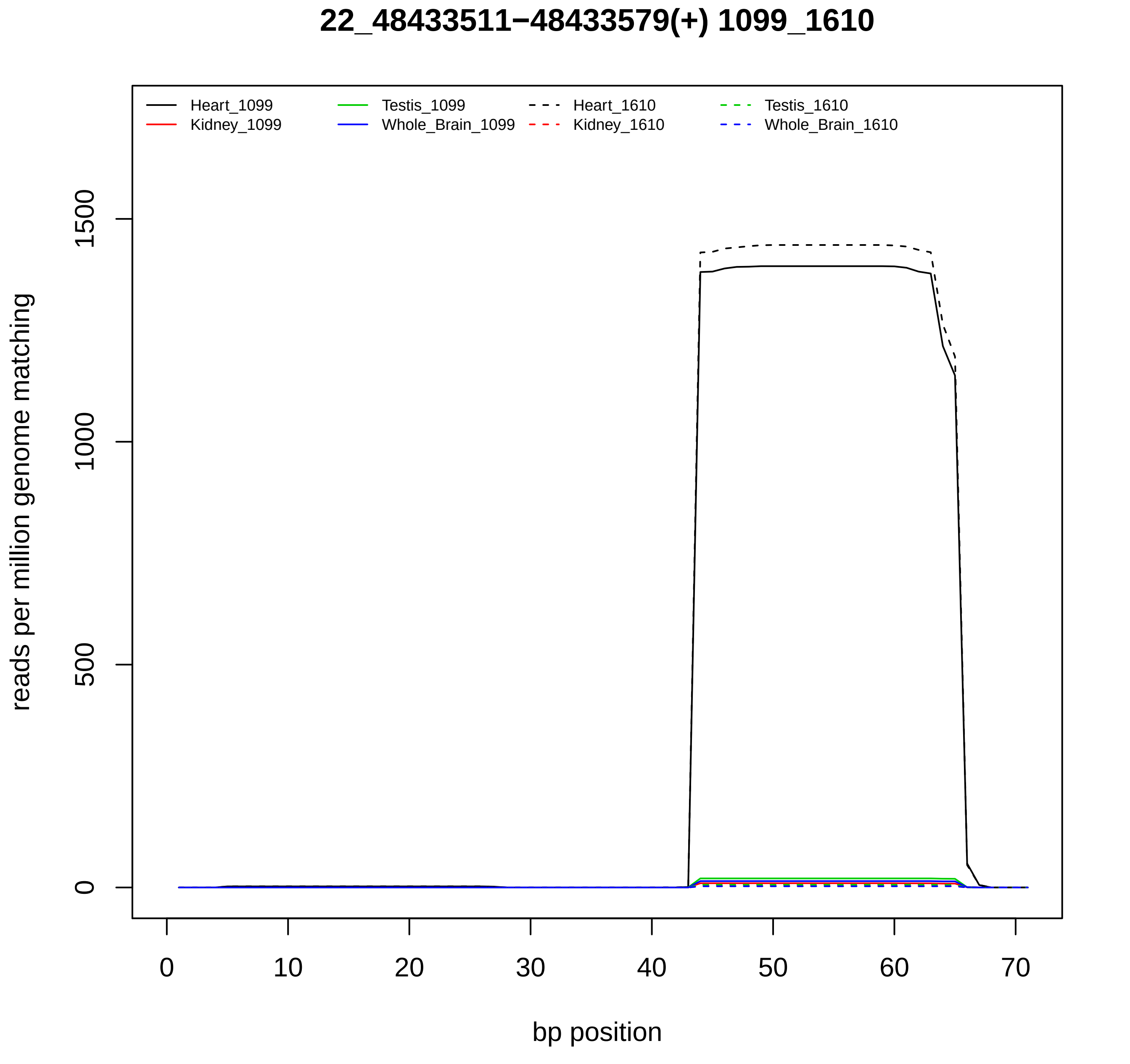

22_48433511−48433579(+) 1099_1610
Heart_1099
Kidney_1099
Testis_1099
Whole_Brain_1099
Heart_1610
Kidney_1610
Testis_1610
Whole_Brain_1610
reads per million genome matching
bp position
0
500
1000
1500
0
10
20
30
40
50
60
70

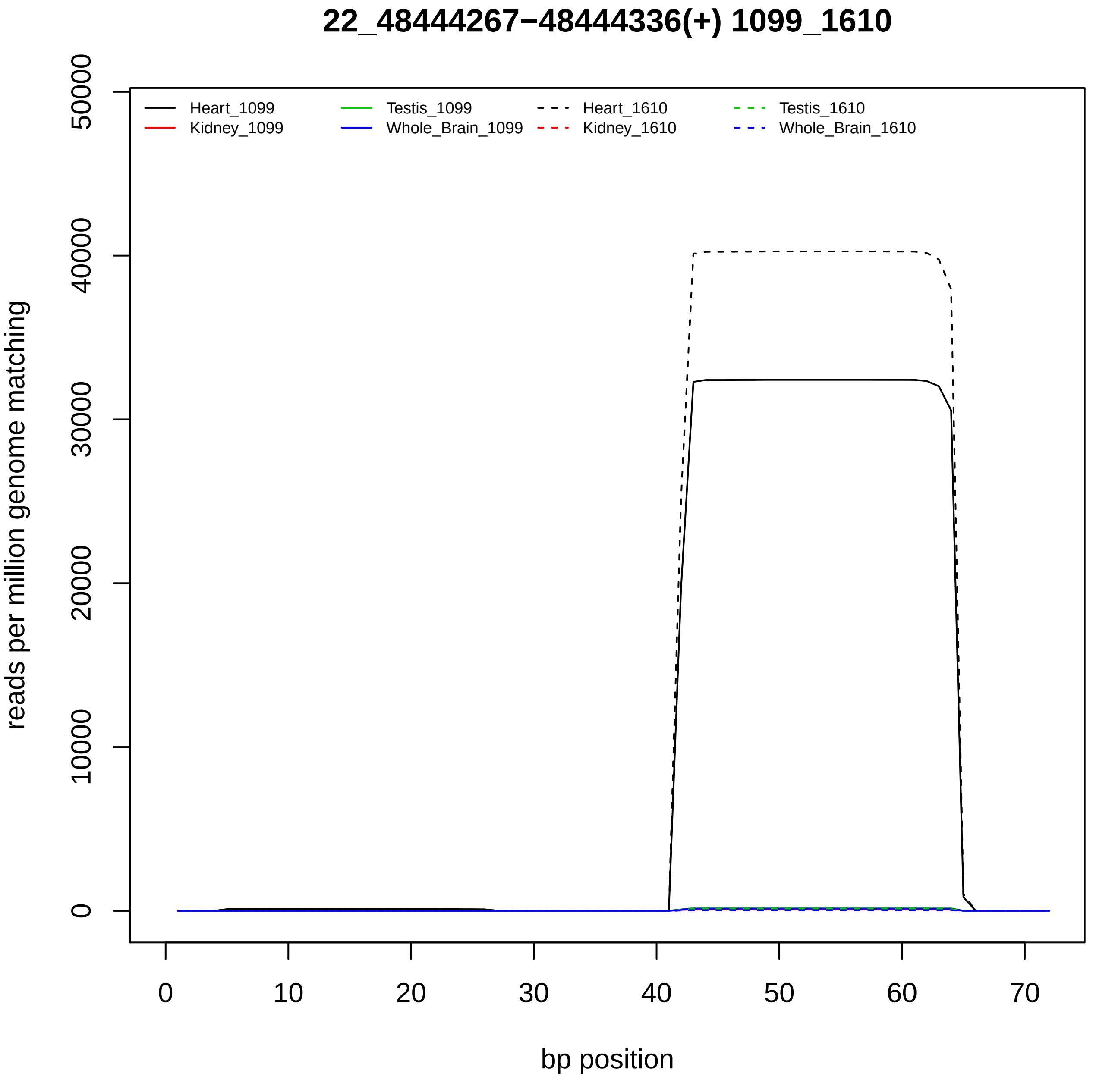
22_48444267−48444336(+) 1099_1610
Heart_1099
Kidney_1099
Testis_1099
Whole_Brain_1099
Heart_1610
Kidney_1610
Testis_1610
Whole_Brain_1610
reads per million genome matching
0
10000
20000
30000
40000
50000
bp position
0
10
20
30
40
50
60
70

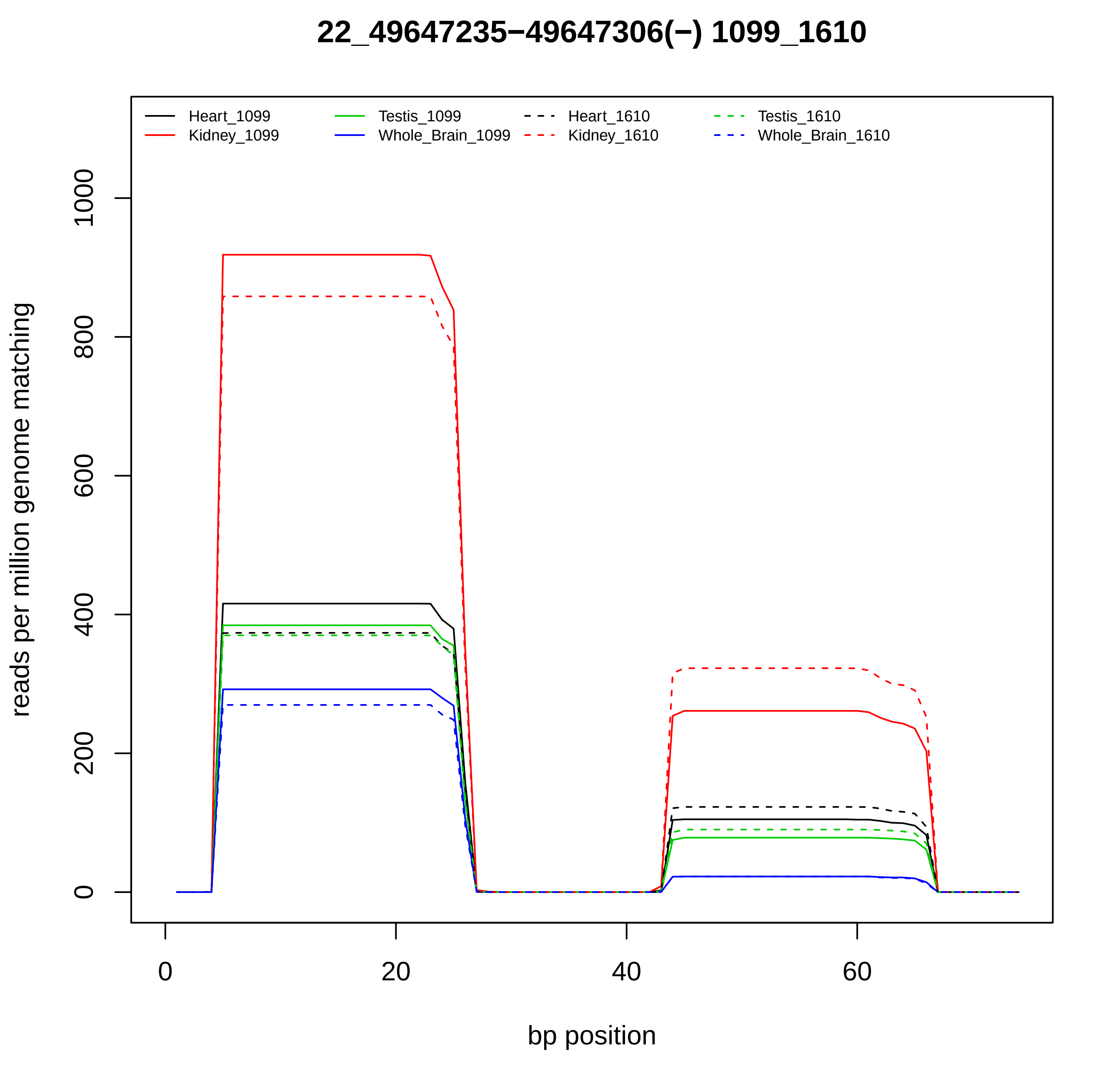

22_49647235−49647306(−) 1099_1610
Heart_1099
Kidney_1099
Testis_1099
Whole_Brain_1099
Heart_1610
Kidney_1610
Testis_1610
Whole_Brain_1610
reads per million genome matching
0
200
400
600
800
1000
0
20
40
60
bp position

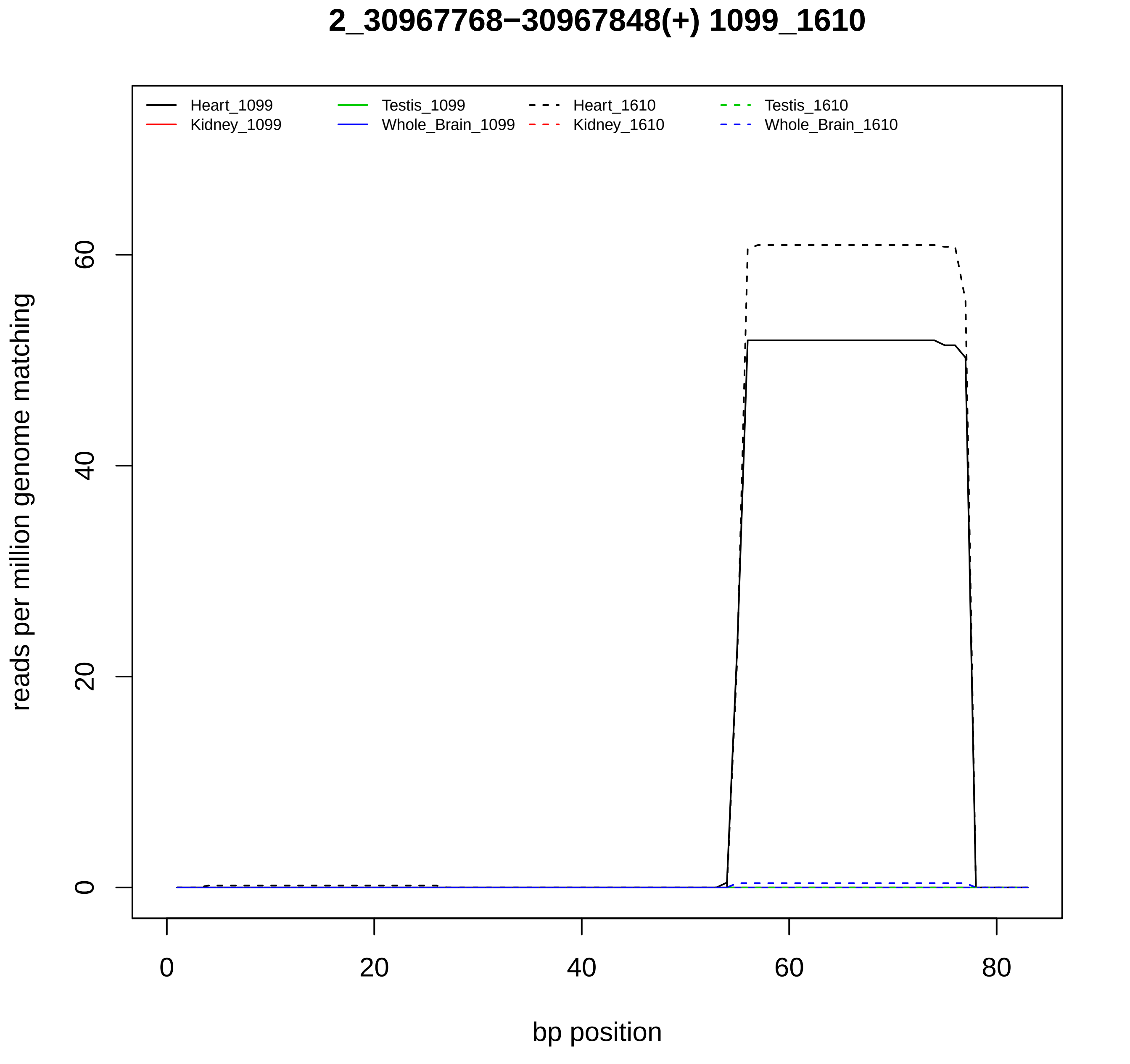

2_30967768−30967848(+) 1099_1610
Heart_1099
Kidney_1099
Testis_1099
Whole_Brain_1099
Heart_1610
Kidney_1610
Testis_1610
Whole_Brain_1610
reads per million genome matching
bp position
0
20
40
60
80

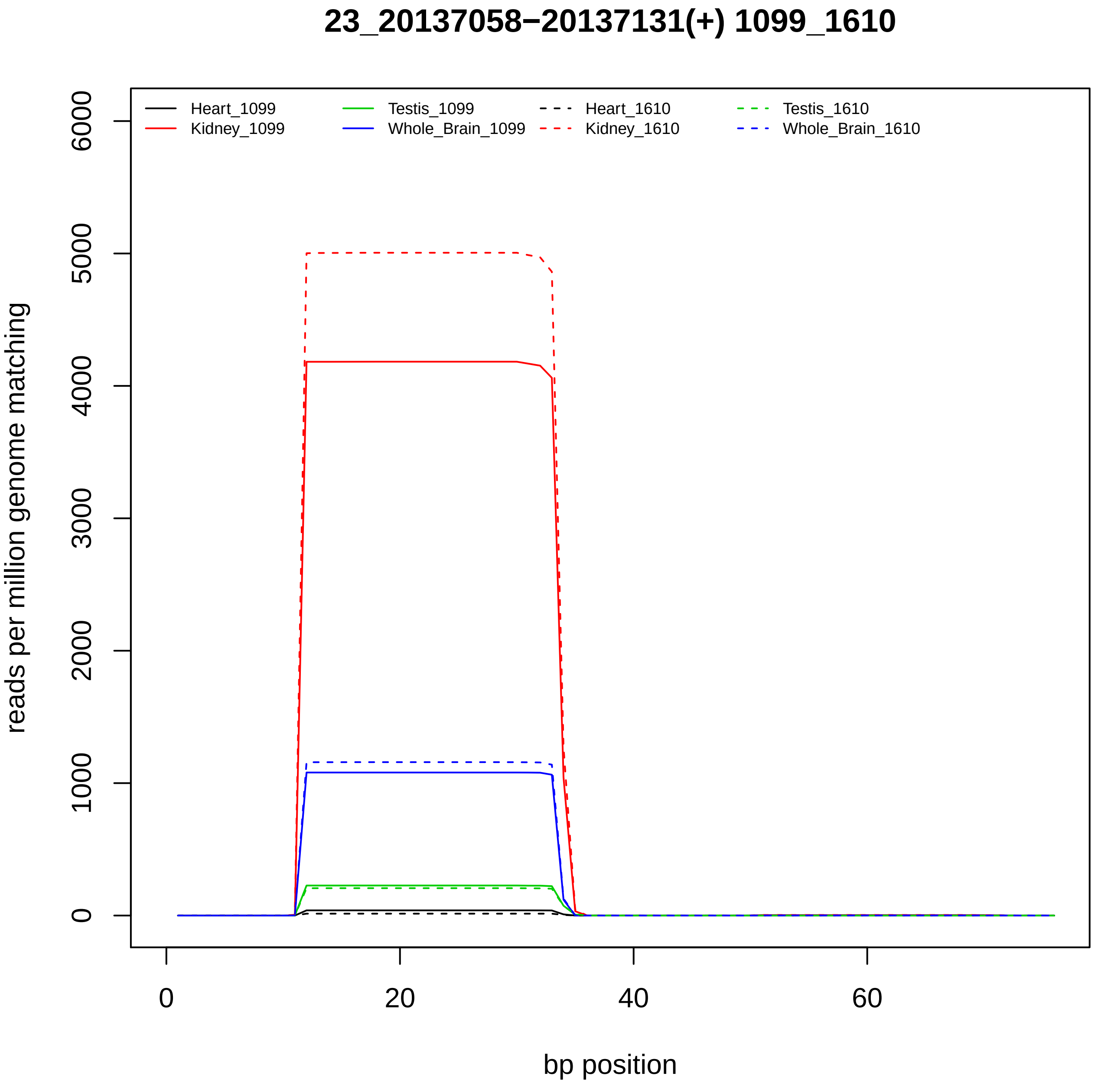
23_20137058−20137131(+) 1099_1610
Heart_1099
Kidney_1099
Testis_1099
Whole_Brain_1099
Heart_1610
Kidney_1610
Testis_1610
Whole_Brain_1610
reads per million genome matching
0
1000
2000
3000
4000
5000
6000
bp position
0
20
40
60

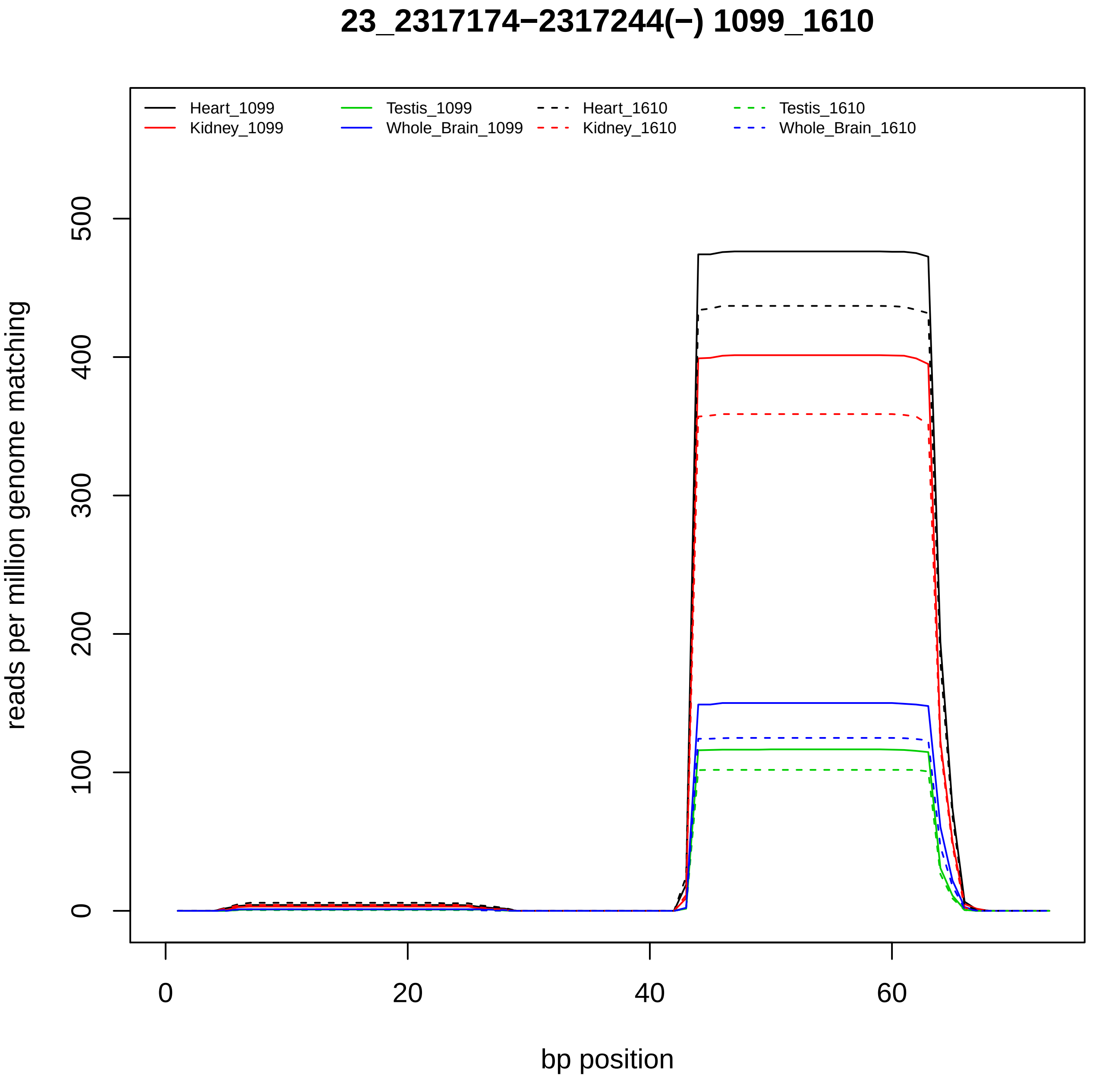

23_2317174−2317244(−) 1099_1610
Heart_1099
Kidney_1099
Testis_1099
Whole_Brain_1099
Heart_1610
Kidney_1610
Testis_1610
Whole_Brain_1610
reads per million genome matching
0
100
200
300
400
500
0
20
40
60
bp position

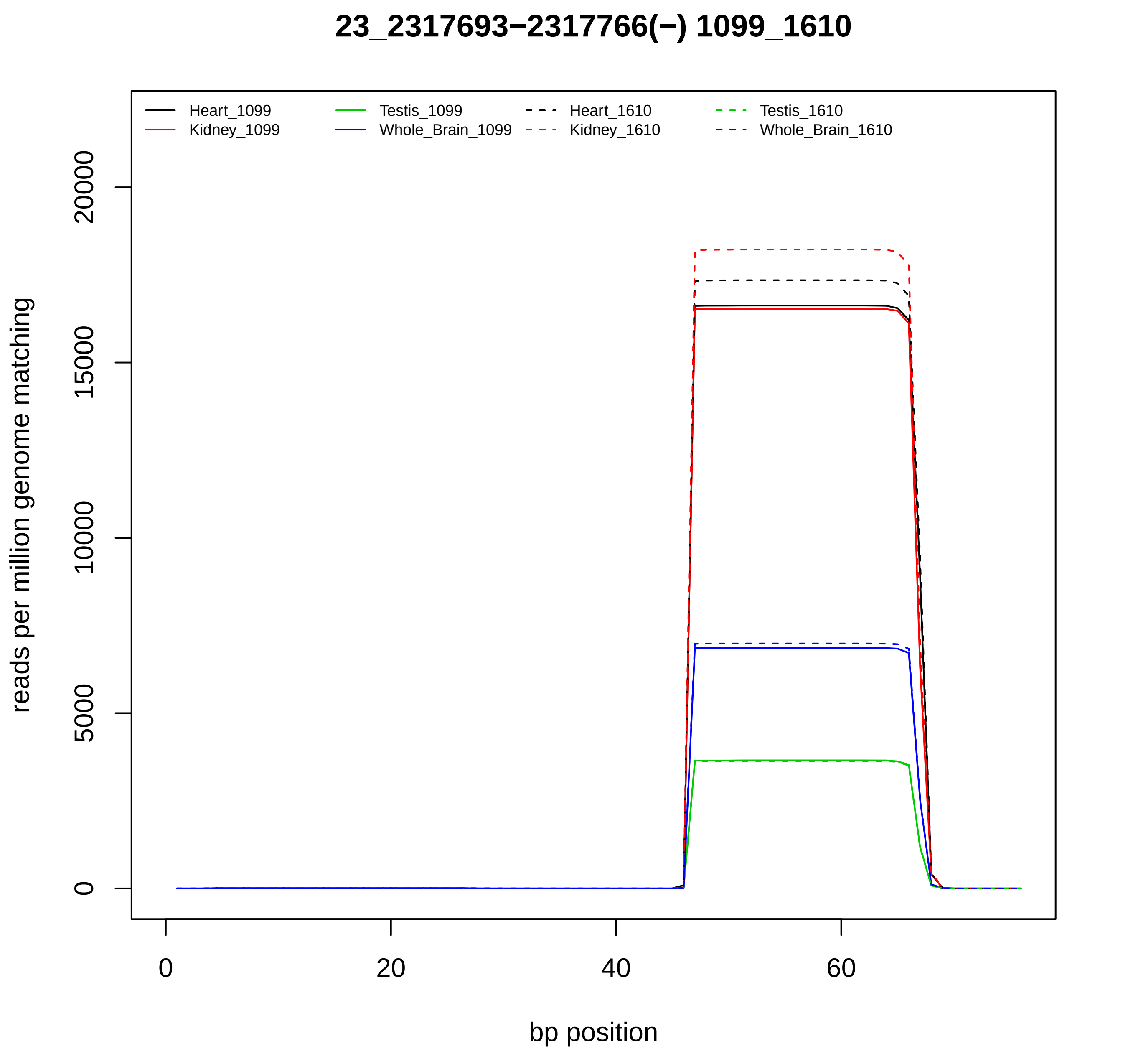
23_2317693−2317766(−) 1099_1610
Heart_1099
Kidney_1099
Testis_1099
Whole_Brain_1099
Heart_1610
Kidney_1610
Testis_1610
Whole_Brain_1610
reads per million genome matching
0
5000
10000
15000
20000
0
20
40
60
bp position

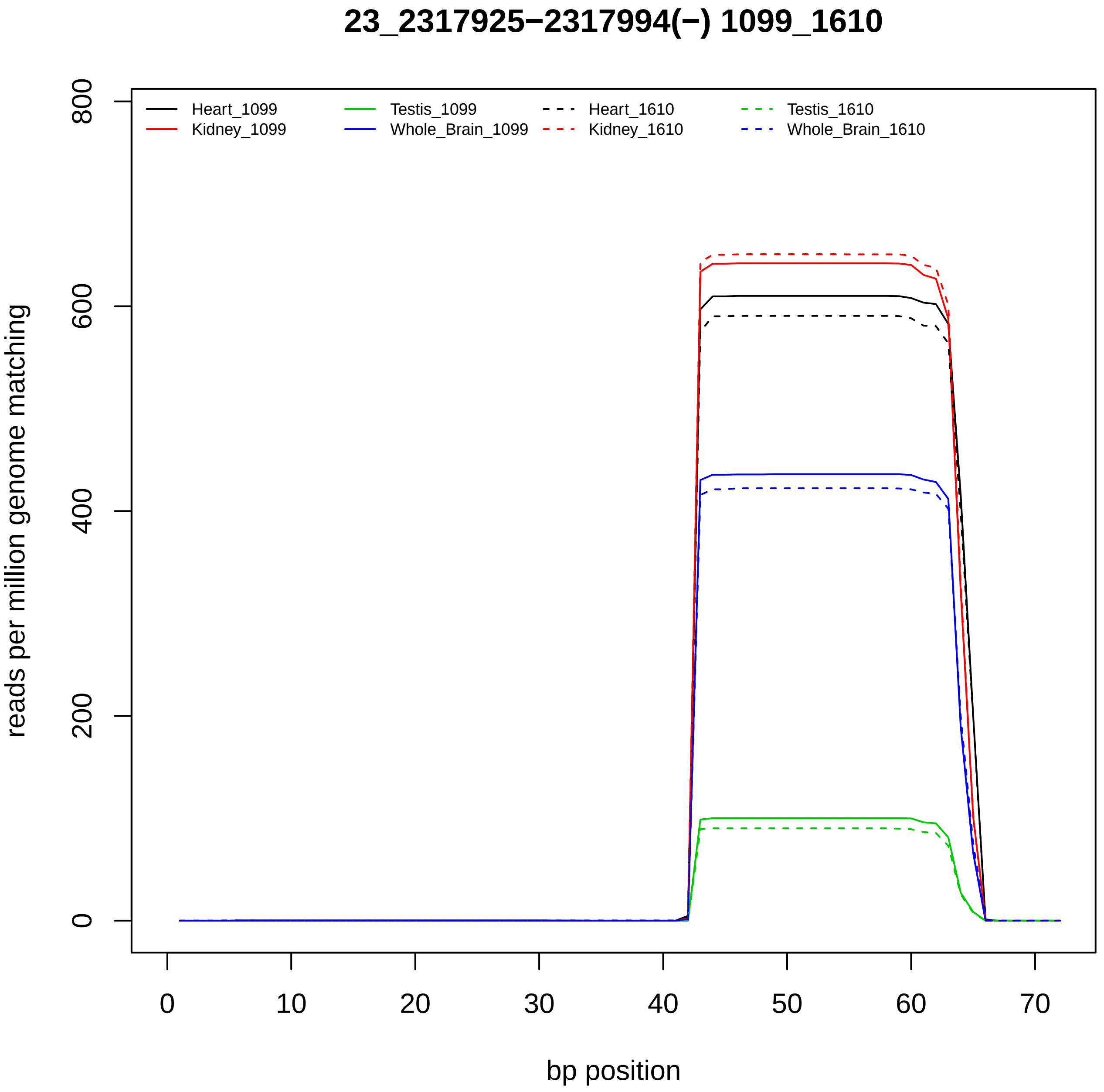

23_2317925−2317994(−) 1099_1610
Heart_1099
Kidney_1099
Testis_1099
Whole_Brain_1099
Heart_1610
Kidney_1610
Testis_1610
Whole_Brain_1610
reads per million genome matching
0
200
400
600
800
0
10
20
30
40
50
60
70
bp position

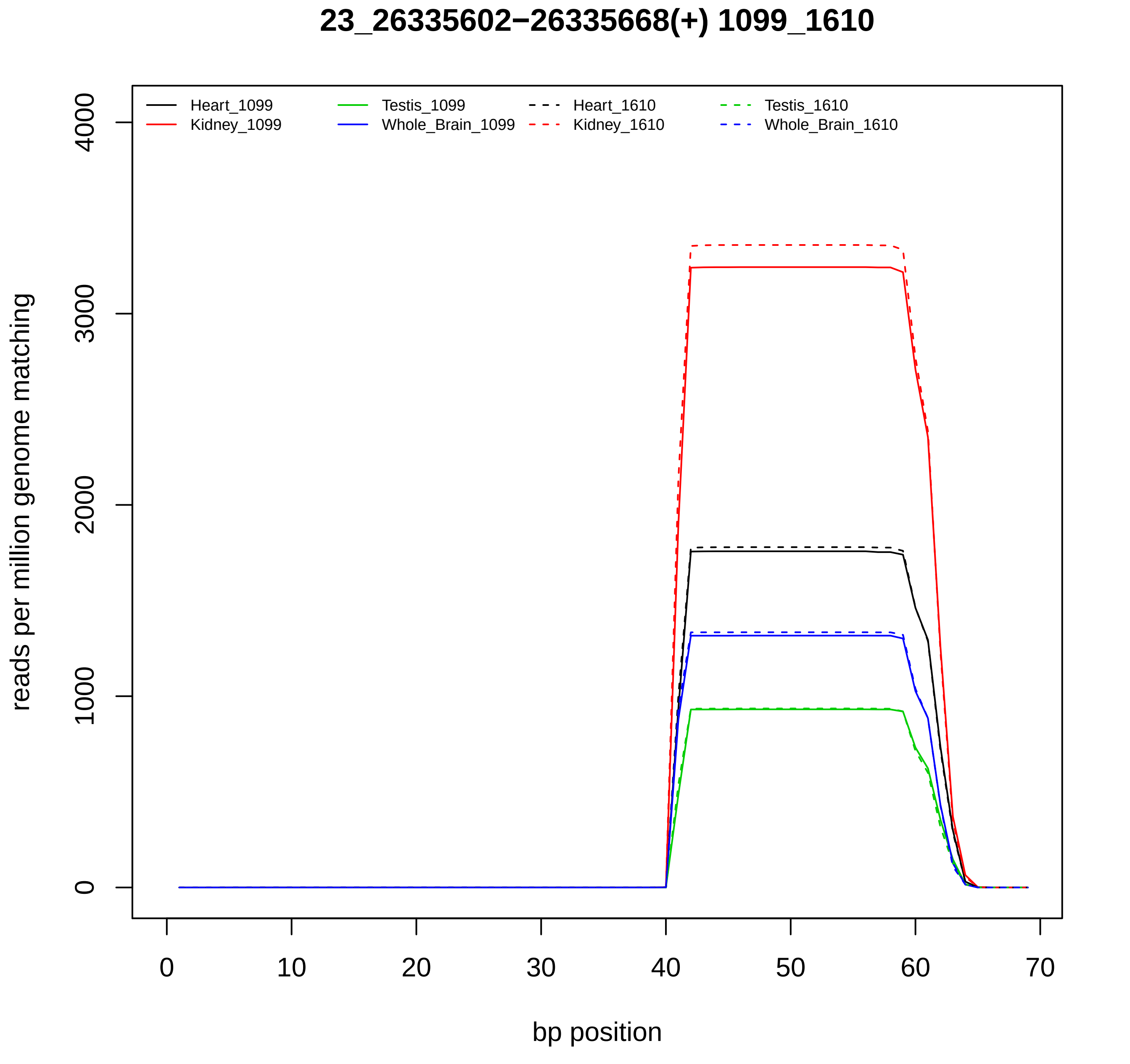

23_26335602−26335668(+) 1099_1610
Heart_1099
Kidney_1099
Testis_1099
Whole_Brain_1099
Heart_1610
Kidney_1610
Testis_1610
Whole_Brain_1610
reads per million genome matching
0
1000
2000
3000
4000
0
10
20
30
40
50
60
70
bp position

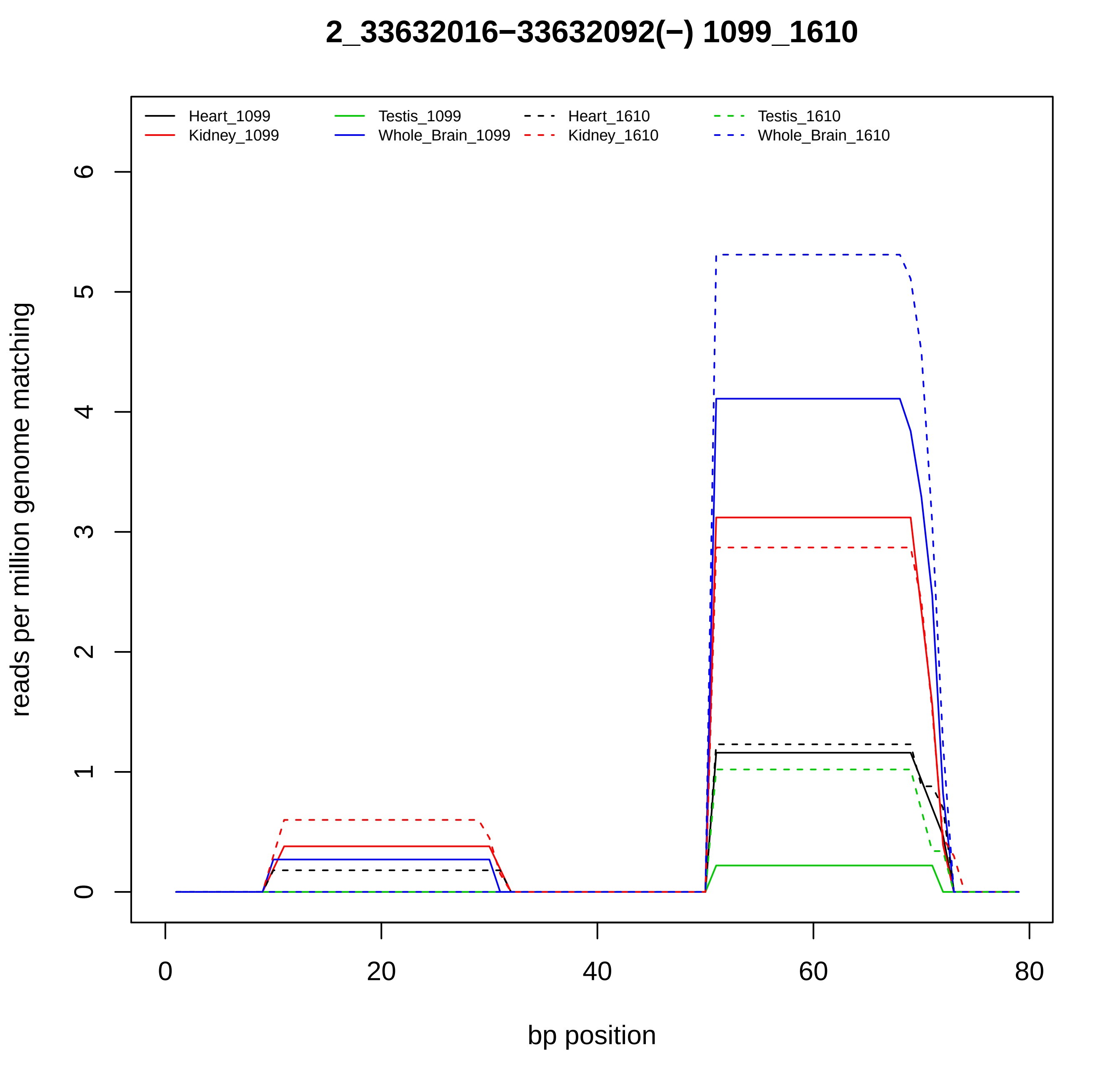
2_33632016−33632092(−) 1099_1610
Heart_1099
Kidney_1099
Testis_1099
Whole_Brain_1099
Heart_1610
Kidney_1610
Testis_1610
Whole_Brain_1610
reads per million genome matching
bp position
0
1
2
3
4
5
6
20
40
60
80

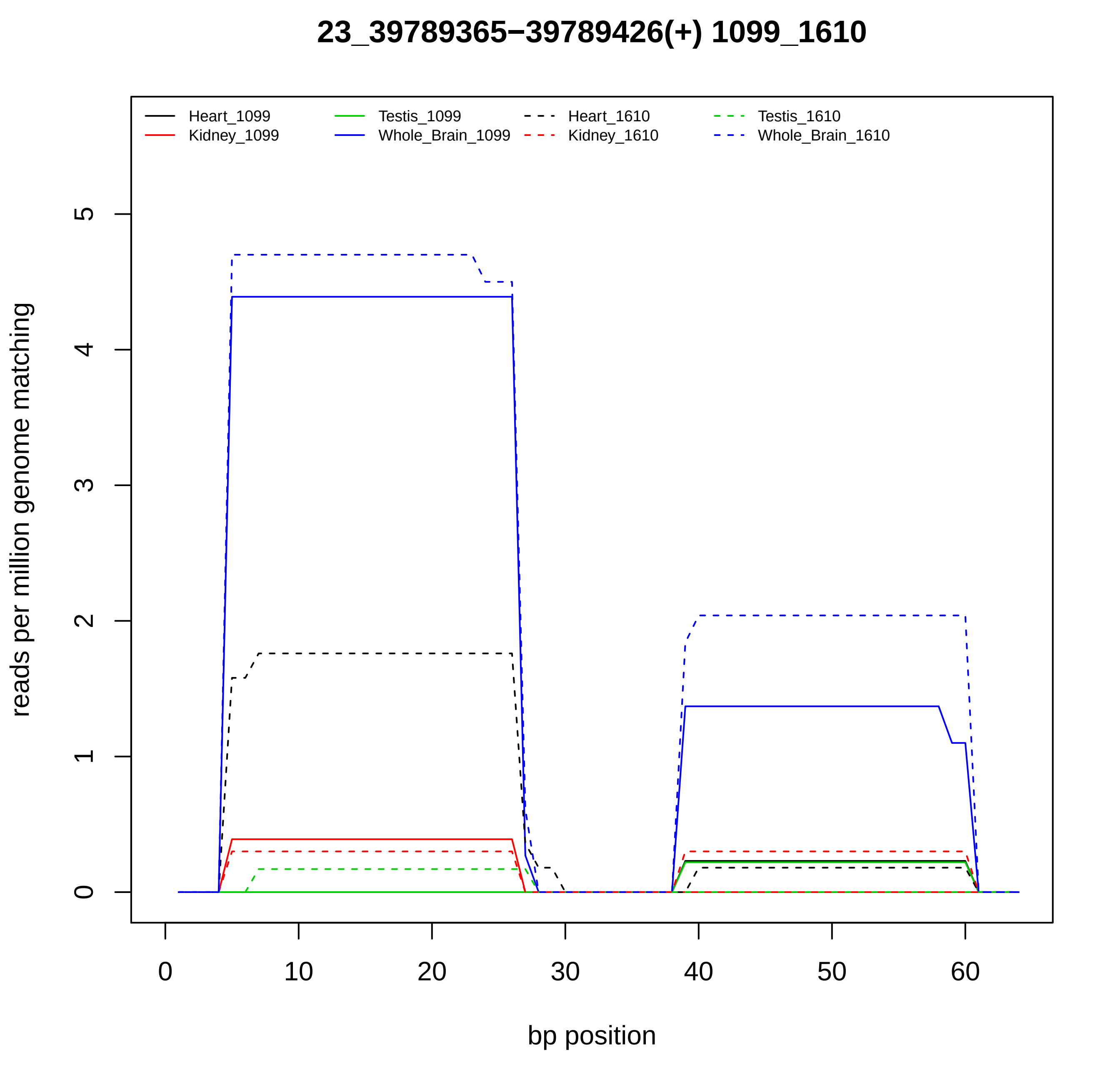

23_39789365−39789426(+) 1099_1610
Heart_1099
Kidney_1099
Testis_1099
Whole_Brain_1099
Heart_1610
Kidney_1610
Testis_1610
Whole_Brain_1610
reads per million genome matching
bp position
0
1
2
3
4
5
0
10
20
30
40
50
60

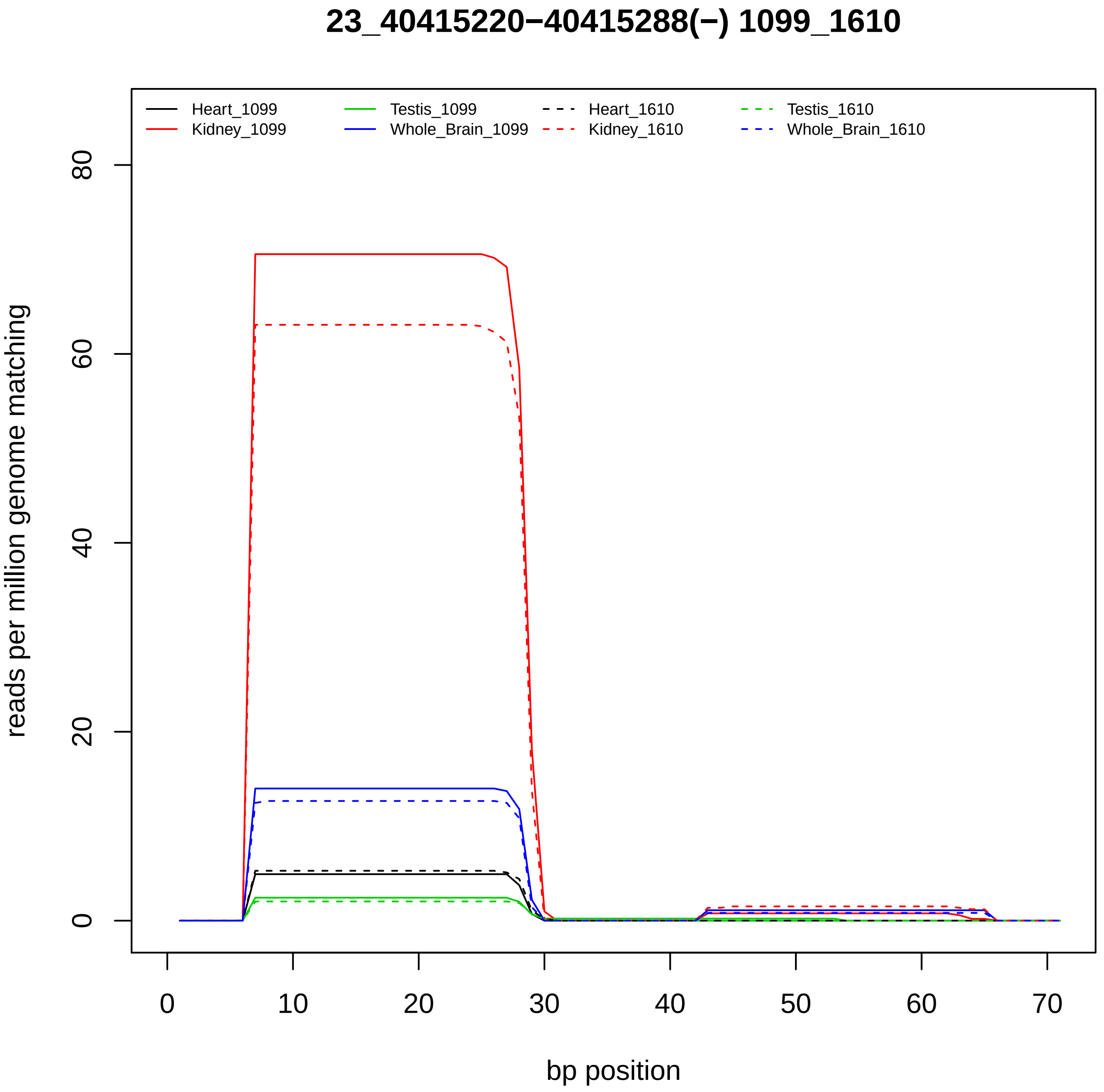

23_40415220−40415288(−) 1099_1610
Heart_1099
Kidney_1099
Testis_1099
Whole_Brain_1099
Heart_1610
Kidney_1610
Testis_1610
Whole_Brain_1610
reads per million genome matching
0
20
40
60
80
bp position
0
10
20
30
40
50
60
70

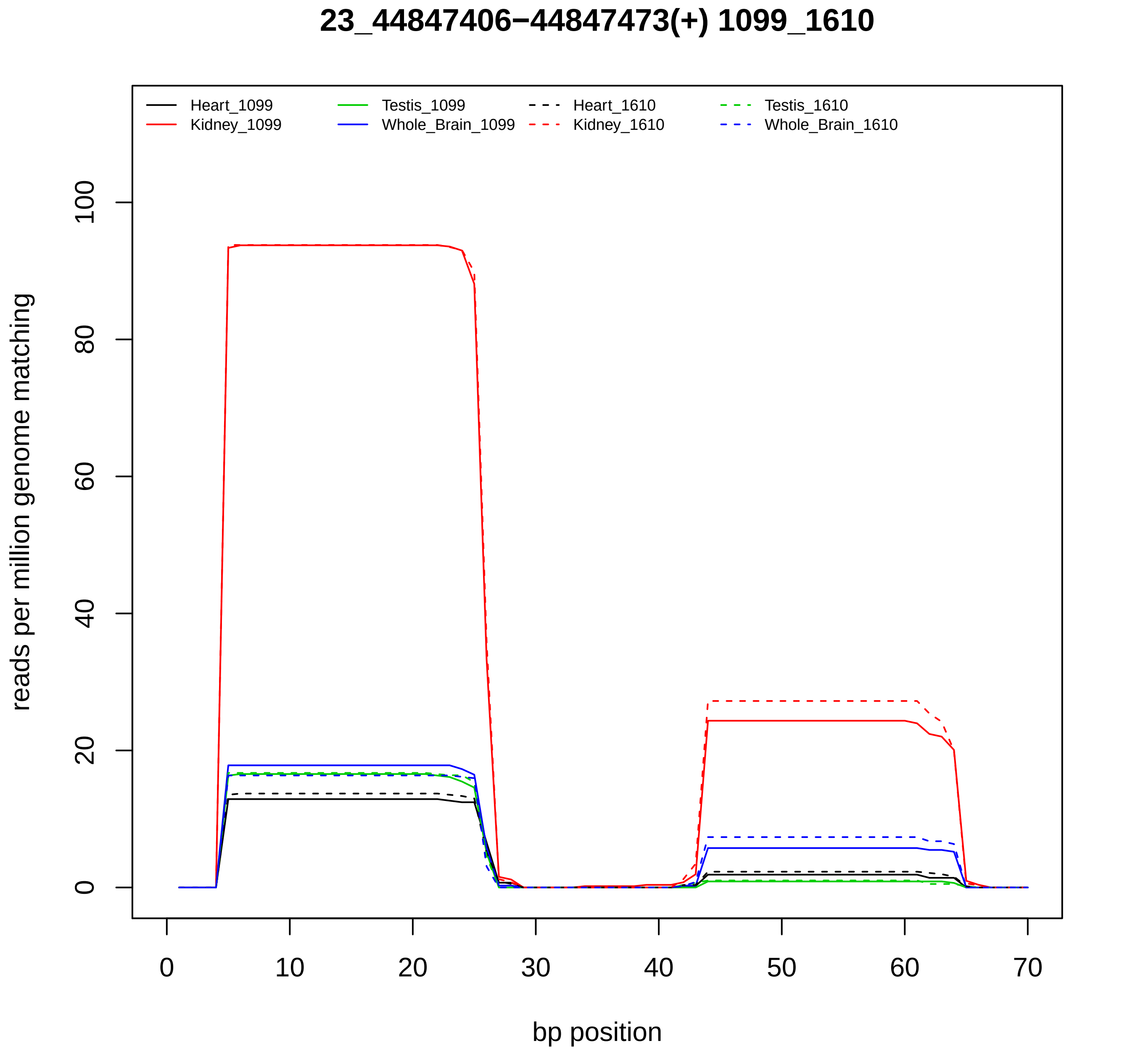

23_44847406−44847473(+) 1099_1610
Heart_1099
Kidney_1099
Testis_1099
Whole_Brain_1099
Heart_1610
Kidney_1610
Testis_1610
Whole_Brain_1610
reads per million genome matching
0
20
40
60
80
100
0
10
20
30
40
50
60
70
bp position

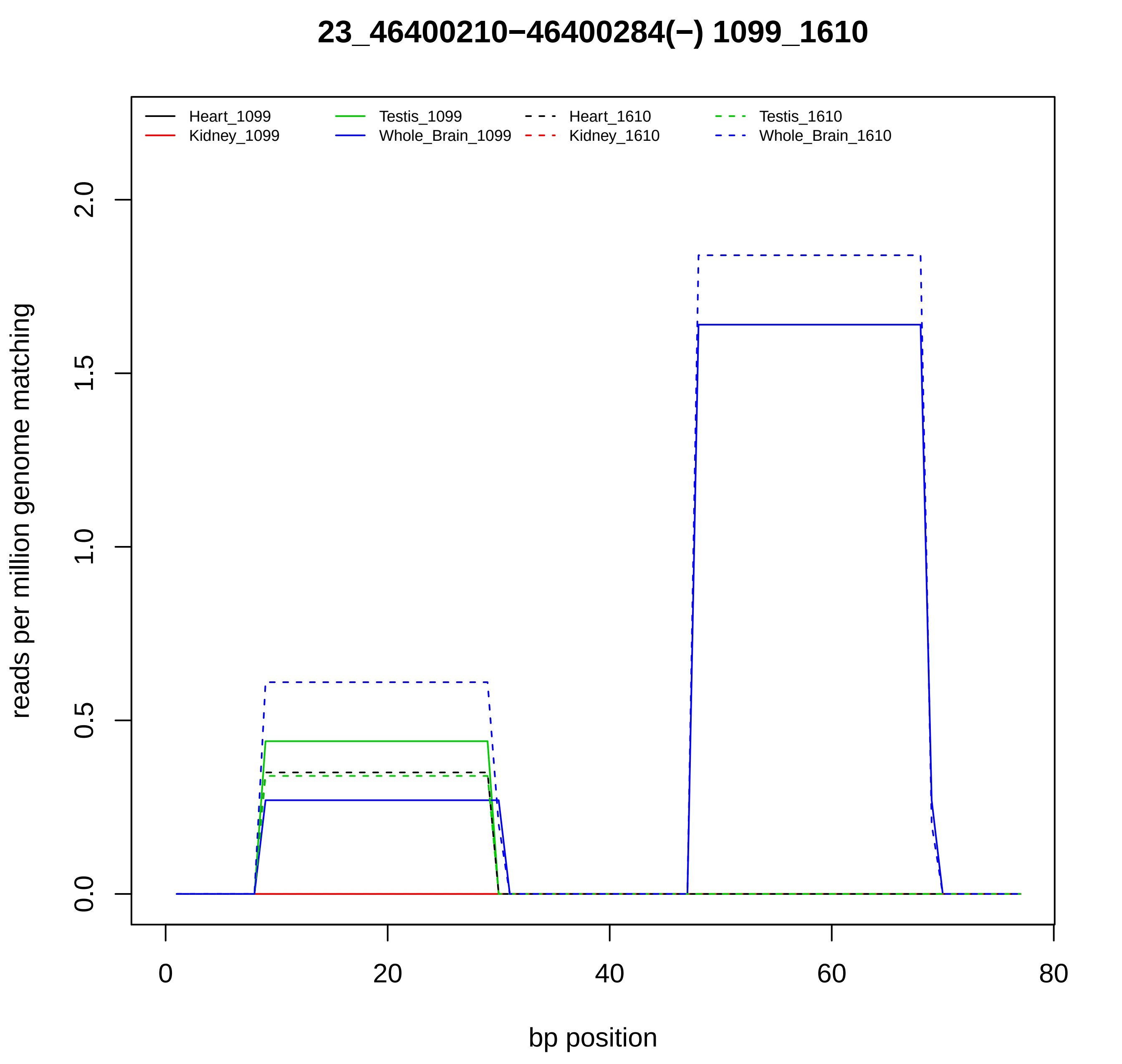

23_46400210−46400284(−) 1099_1610
Heart_1099
Kidney_1099
Testis_1099
Whole_Brain_1099
Heart_1610
Kidney_1610
Testis_1610
Whole_Brain_1610
reads per million genome matching
0.0
0.5
1.0
1.5
2.0
0
20
40
60
80
bp position

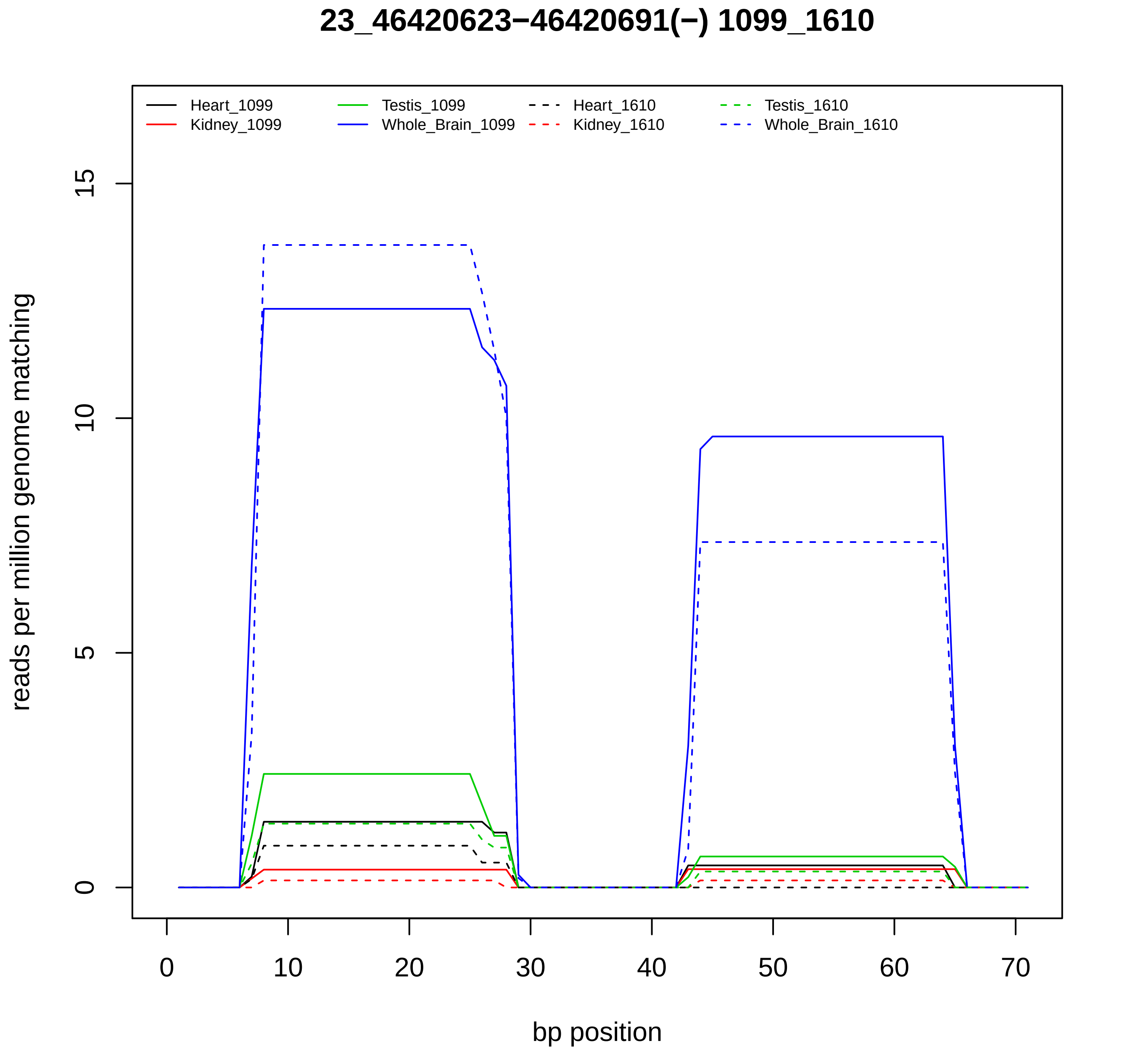

23_46420623−46420691(−) 1099_1610
Heart_1099
Kidney_1099
Testis_1099
Whole_Brain_1099
Heart_1610
Kidney_1610
Testis_1610
Whole_Brain_1610
reads per million genome matching
0
5
10
15
0
10
20
30
40
50
60
70
bp position

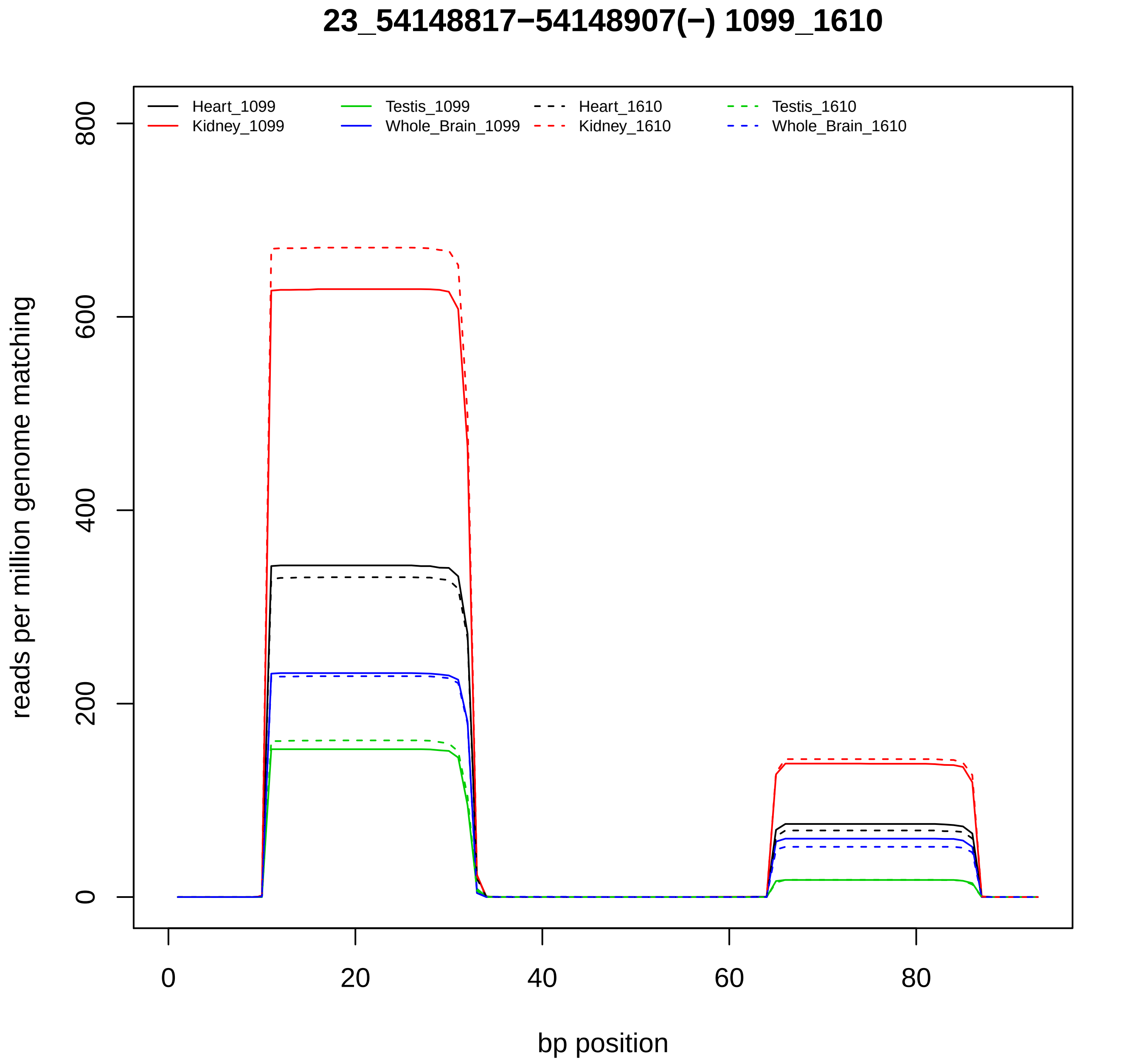

23_54148817−54148907(−) 1099_1610
Heart_1099
Kidney_1099
Testis_1099
Whole_Brain_1099
Heart_1610
Kidney_1610
Testis_1610
Whole_Brain_1610
reads per million genome matching
0
200
400
600
800
0
20
40
60
80
bp position

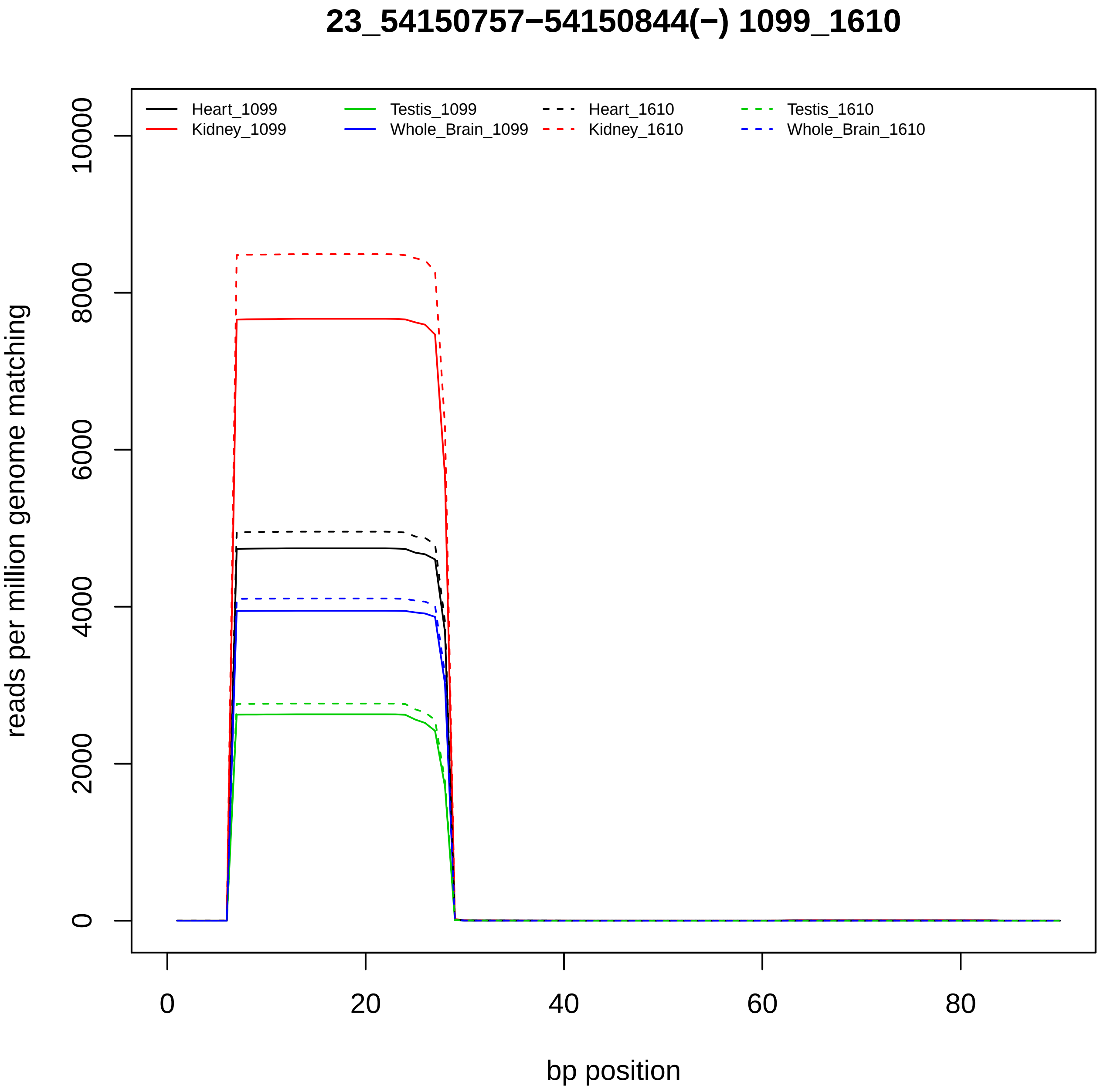

23_54150757−54150844(−) 1099_1610
Heart_1099
Kidney_1099
Testis_1099
Whole_Brain_1099
Heart_1610
Kidney_1610
Testis_1610
Whole_Brain_1610
reads per million genome matching
0
2000
4000
6000
8000
10000
bp position
0
20
40
60
80

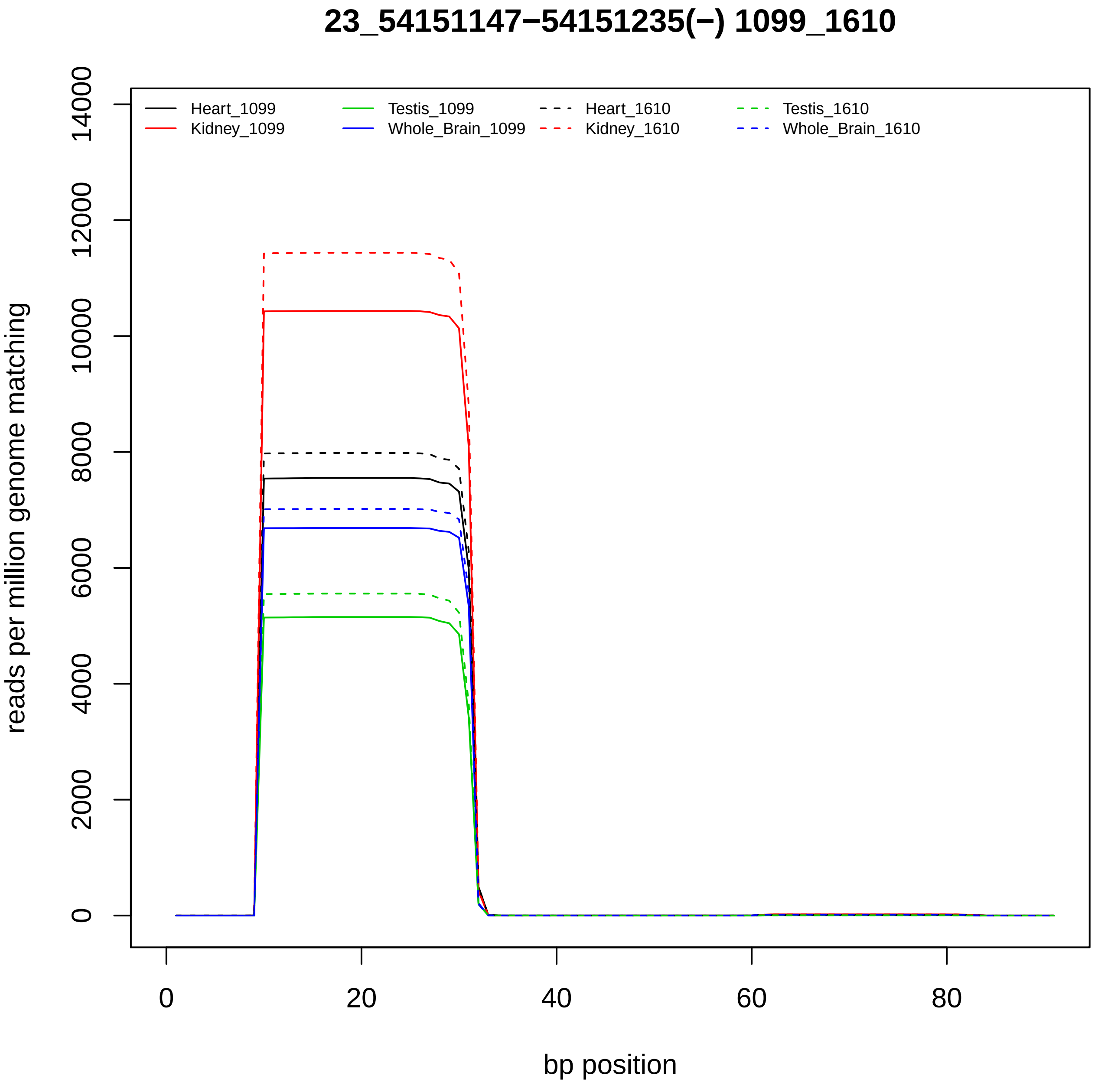
23_54151147−54151235(−) 1099_1610
Heart_1099
Kidney_1099
Testis_1099
Whole_Brain_1099
Heart_1610
Kidney_1610
Testis_1610
Whole_Brain_1610
reads per million genome matching
0
2000
4000
6000
8000
10000
12000
14000
0
20
40
60
80
bp position

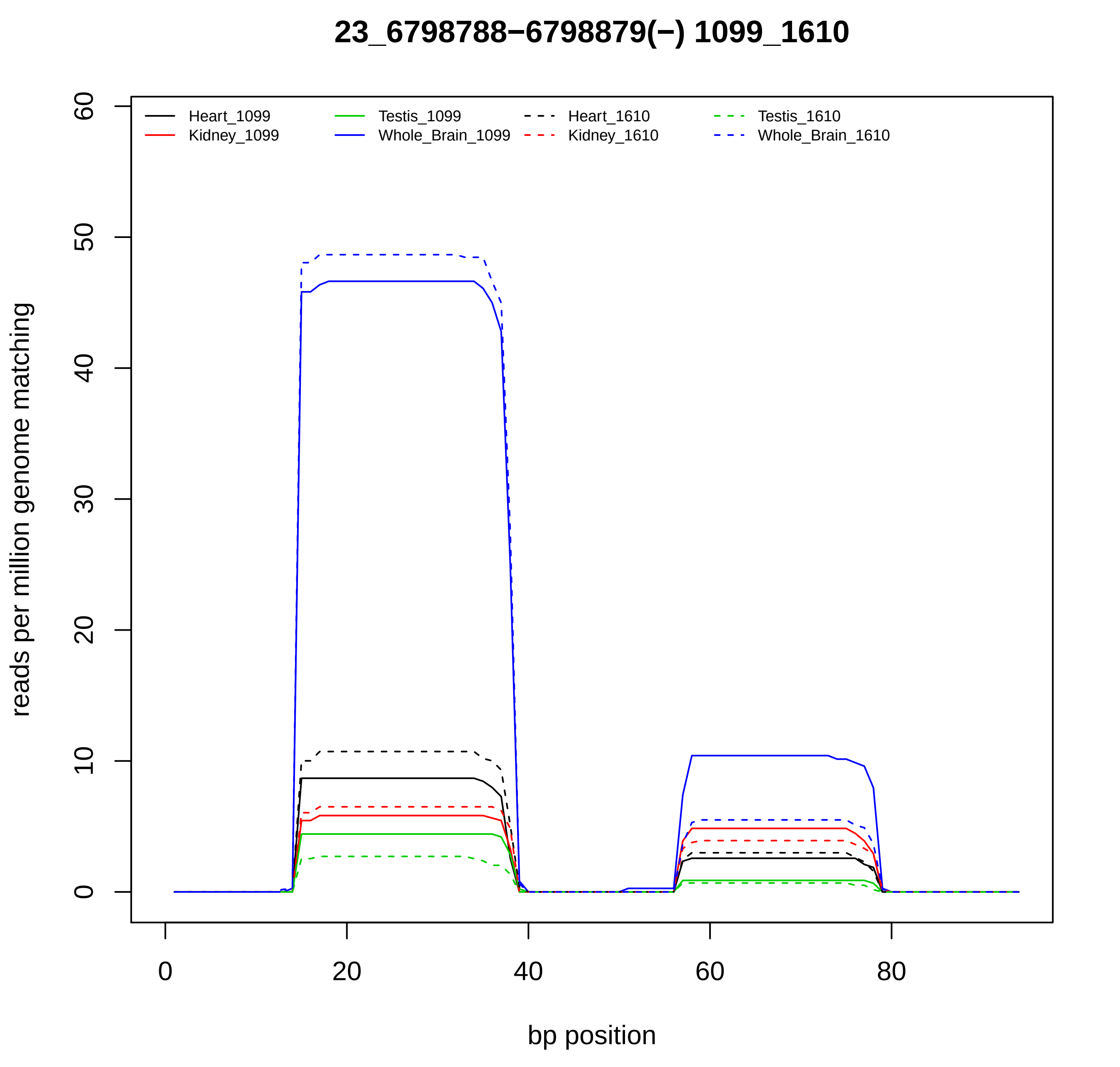

23_6798788−6798879(−) 1099_1610
Heart_1099
Kidney_1099
Testis_1099
Whole_Brain_1099
Heart_1610
Kidney_1610
Testis_1610
Whole_Brain_1610
reads per million genome matching
bp position
0
10
20
30
40
50
60
0
20
40
60
80

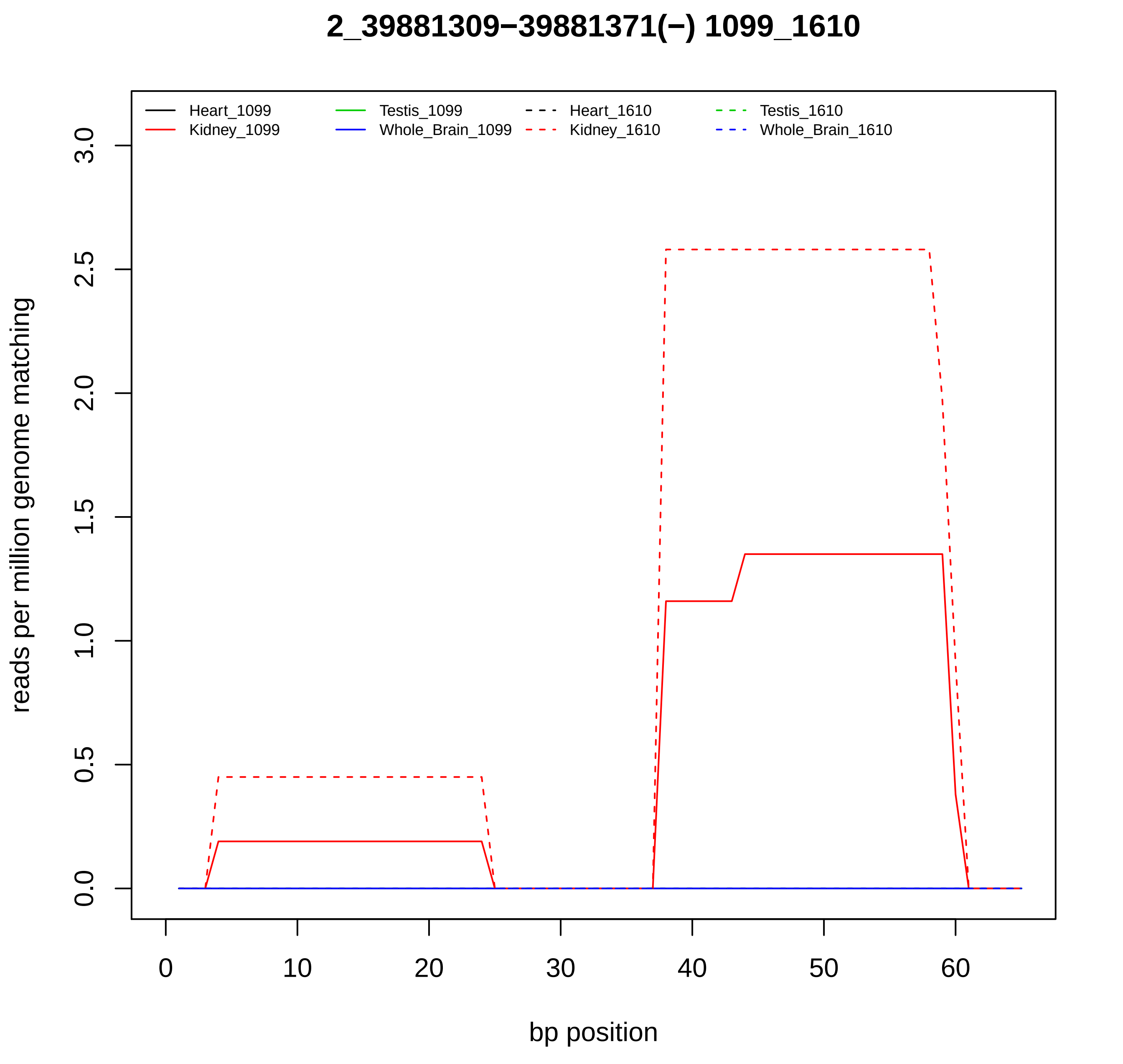
2_39881309−39881371(−) 1099_1610
Heart_1099
Kidney_1099
Testis_1099
Whole_Brain_1099
Heart_1610
Kidney_1610
Testis_1610
Whole_Brain_1610
reads per million genome matching
0.0
0.5
1.0
1.5
2.0
2.5
3.0
bp position
0
10
20
30
40
50
60

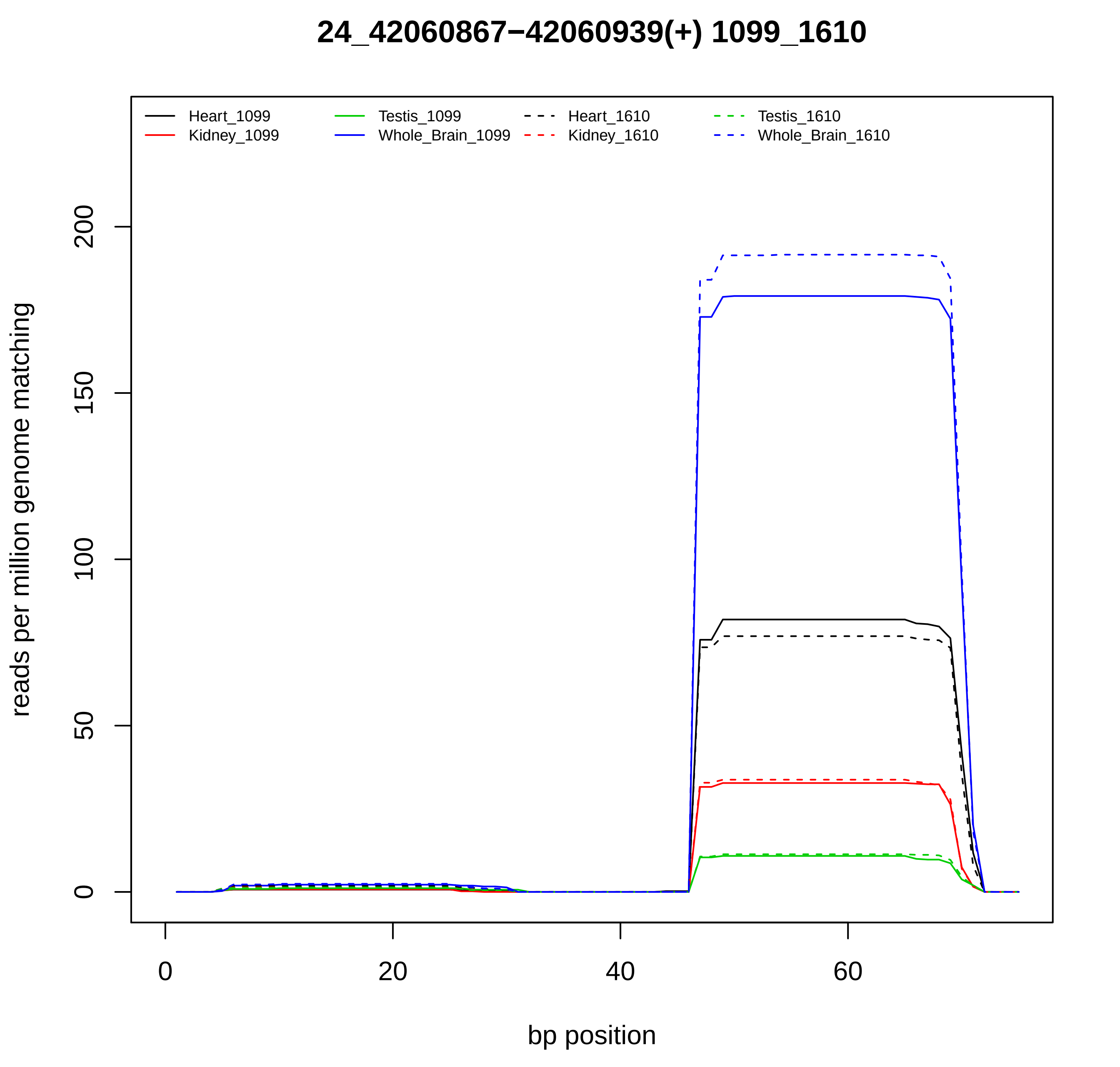

24_42060867−42060939(+) 1099_1610
Heart_1099
Kidney_1099
Testis_1099
Whole_Brain_1099
Heart_1610
Kidney_1610
Testis_1610
Whole_Brain_1610
reads per million genome matching
bp position
0
50
100
150
200
0
20
40
60

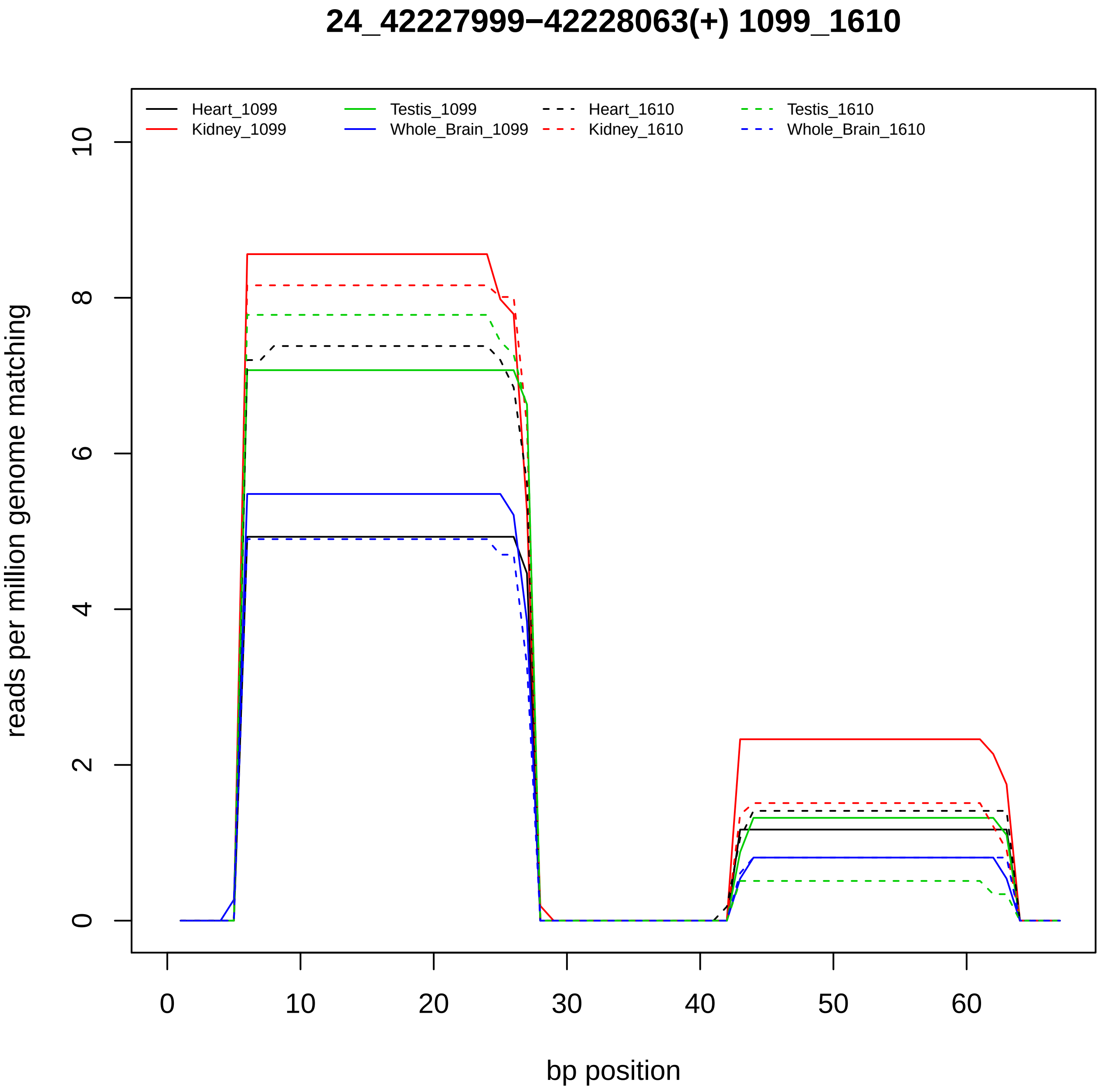

24_42227999−42228063(+) 1099_1610
Heart_1099
Kidney_1099
Testis_1099
Whole_Brain_1099
Heart_1610
Kidney_1610
Testis_1610
Whole_Brain_1610
reads per million genome matching
bp position
0
2
4
6
8
10
0
10
20
30
40
50
60

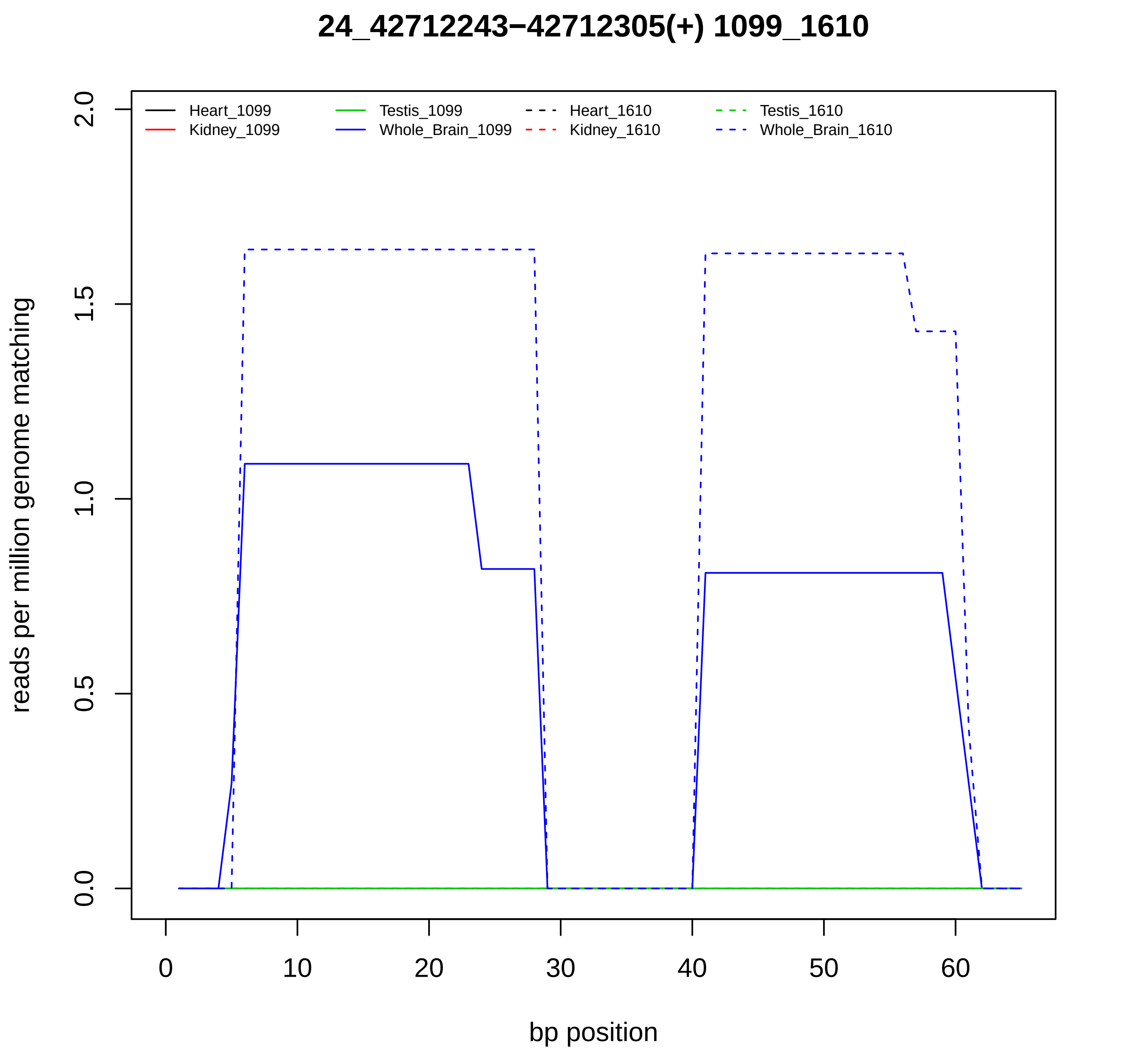

24_42712243−42712305(+) 1099_1610
Heart_1099
Kidney_1099
Testis_1099
Whole_Brain_1099
Heart_1610
Kidney_1610
Testis_1610
Whole_Brain_1610
reads per million genome matching
bp position
0.0
0.5
1.0
1.5
2.0
0
10
20
30
40
50
60

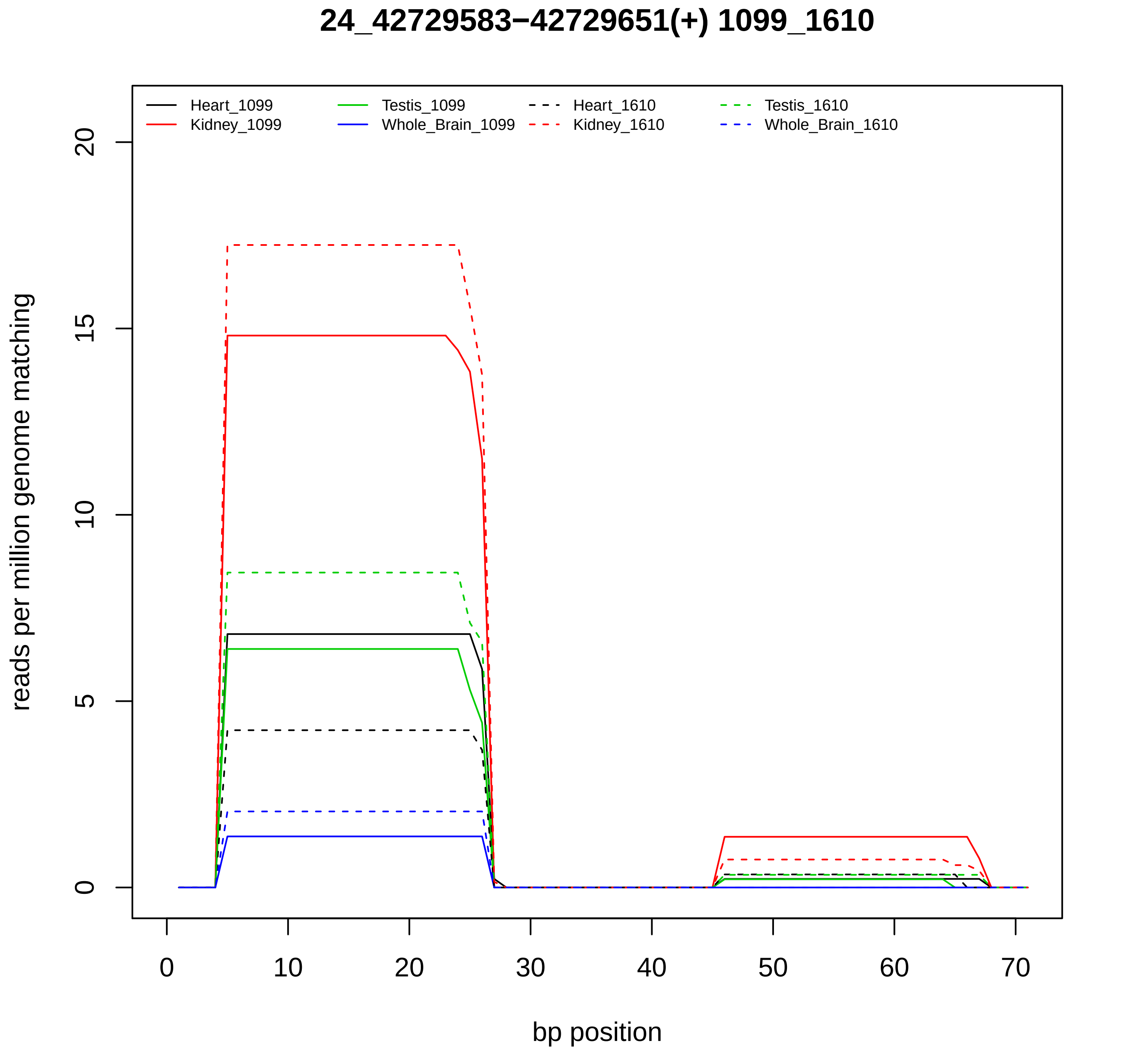

24_42729583−42729651(+) 1099_1610
Heart_1099
Kidney_1099
Testis_1099
Whole_Brain_1099
Heart_1610
Kidney_1610
Testis_1610
Whole_Brain_1610
reads per million genome matching
0
5
10
15
20
0
10
20
30
40
50
60
70
bp position

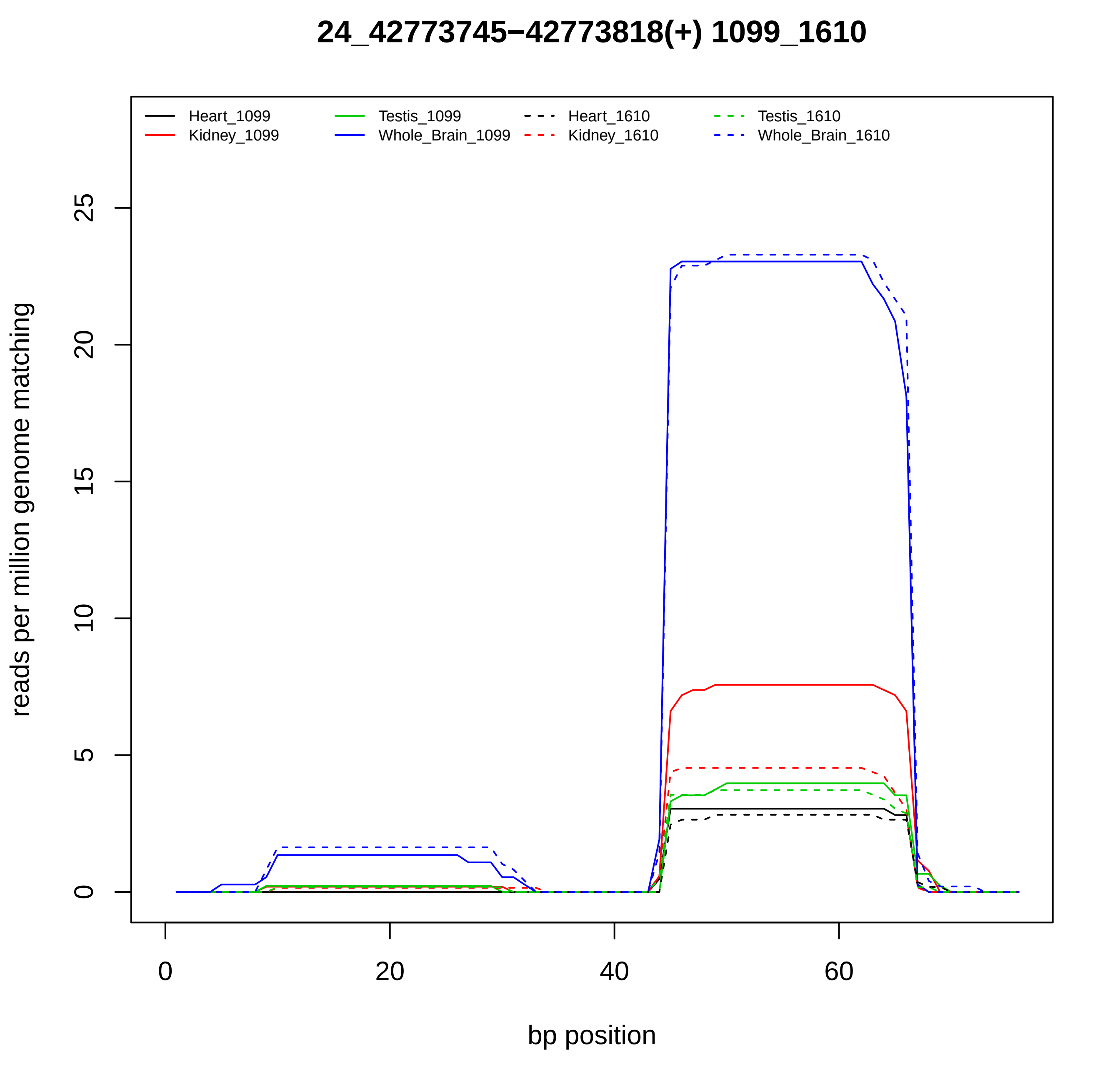
24_42773745−42773818(+) 1099_1610
Heart_1099
Kidney_1099
Testis_1099
Whole_Brain_1099
Heart_1610
Kidney_1610
Testis_1610
Whole_Brain_1610
reads per million genome matching
bp position
0
5
10
15
20
25
0
20
40
60

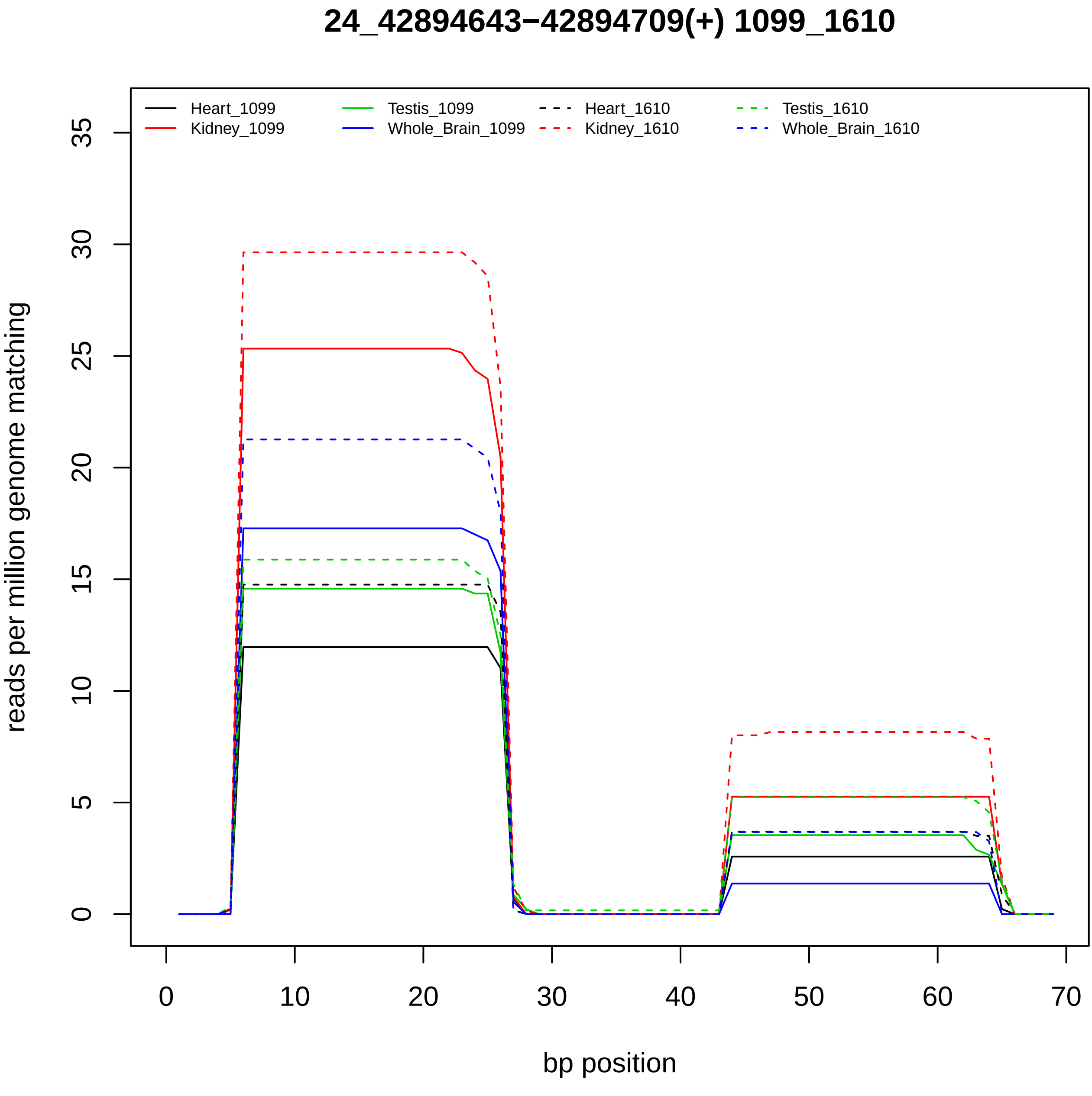
24_42894643−42894709(+) 1099_1610
Heart_1099
Kidney_1099
Testis_1099
Whole_Brain_1099
Heart_1610
Kidney_1610
Testis_1610
Whole_Brain_1610
reads per million genome matching
bp position
0
5
10
15
20
25
30
35
0
10
20
30
40
50
60
70

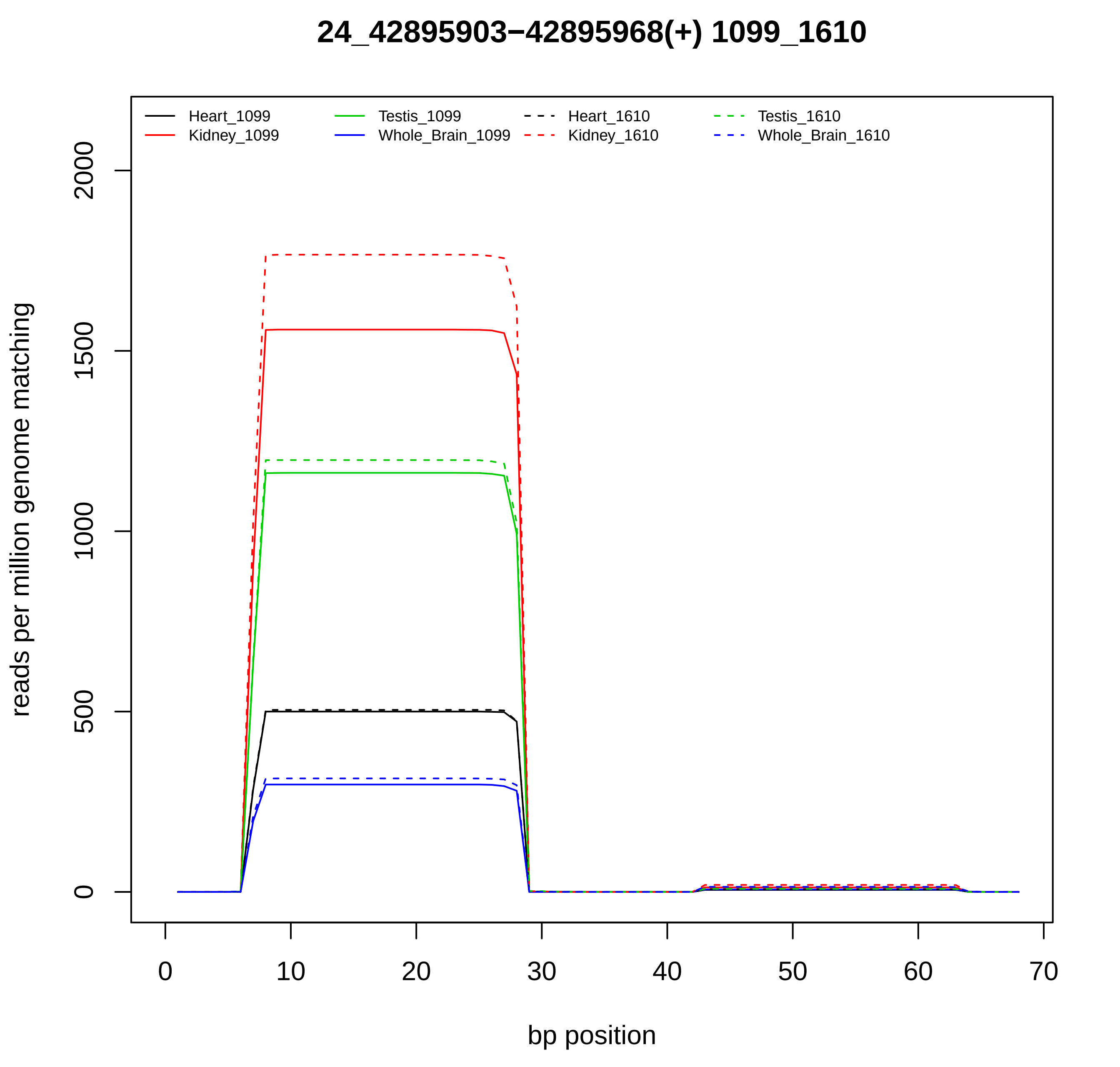
24_42895903−42895968(+) 1099_1610
Heart_1099
Kidney_1099
Testis_1099
Whole_Brain_1099
Heart_1610
Kidney_1610
Testis_1610
Whole_Brain_1610
reads per million genome matching
0
500
1000
1500
2000
0
10
20
30
40
50
60
70
bp position

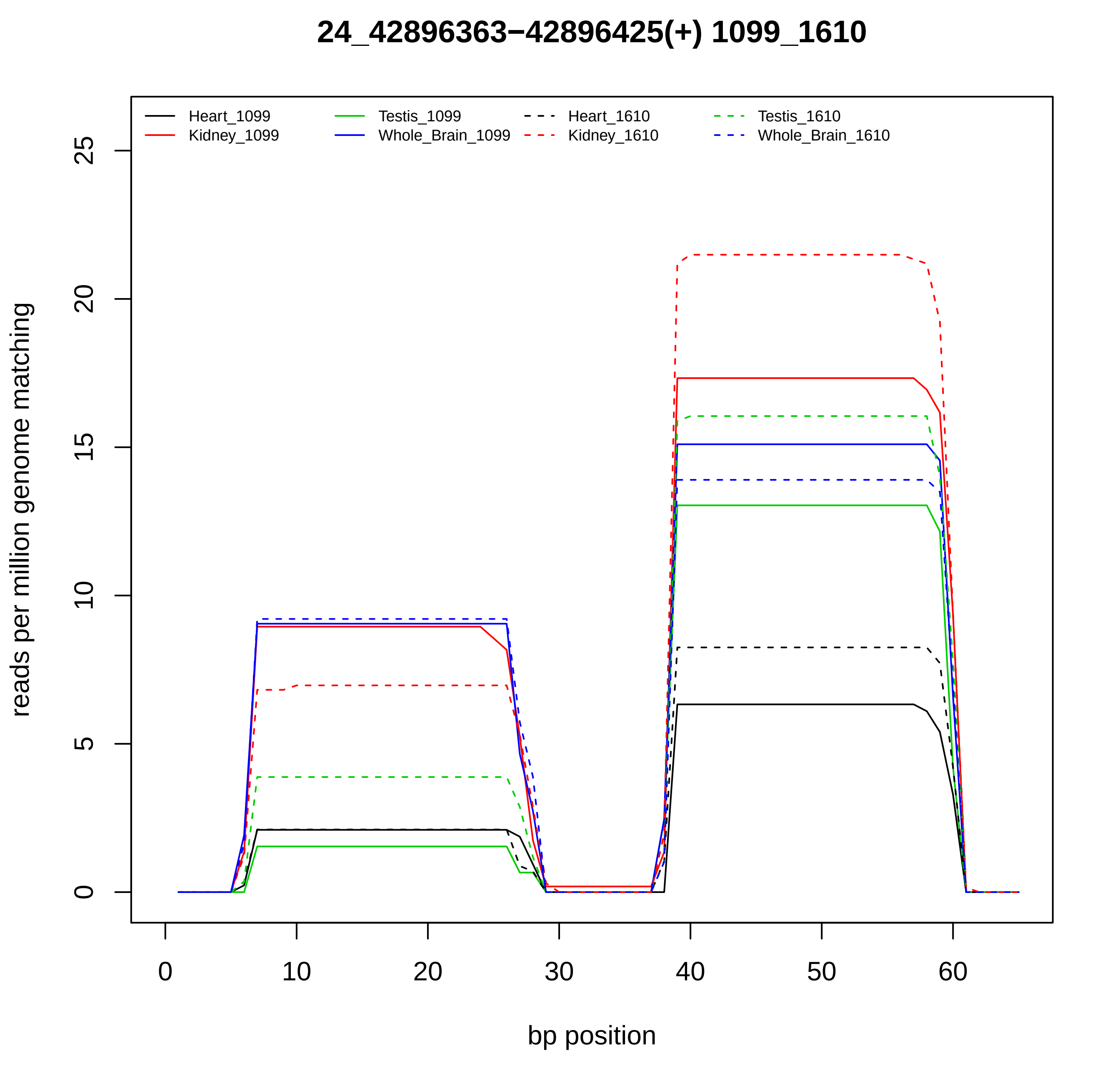

24_42896363−42896425(+) 1099_1610
Heart_1099
Kidney_1099
Testis_1099
Whole_Brain_1099
Heart_1610
Kidney_1610
Testis_1610
Whole_Brain_1610
reads per million genome matching
bp position
0
5
10
15
20
25
0
10
20
30
40
50
60

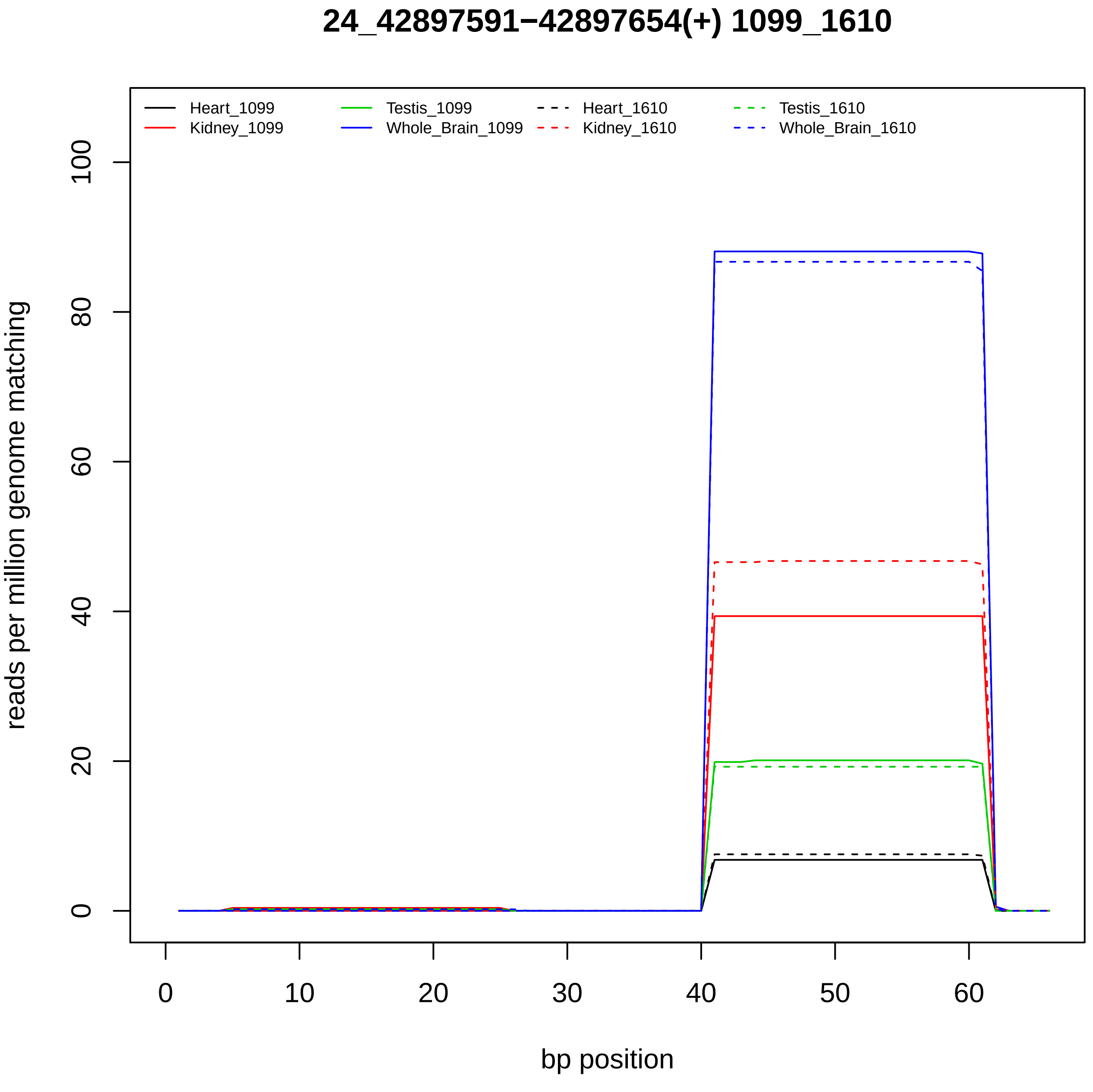

24_42897591−42897654(+) 1099_1610
Heart_1099
Kidney_1099
Testis_1099
Whole_Brain_1099
Heart_1610
Kidney_1610
Testis_1610
Whole_Brain_1610
reads per million genome matching
bp position
0
20
40
60
80
100
0
10
20
30
40
50
60

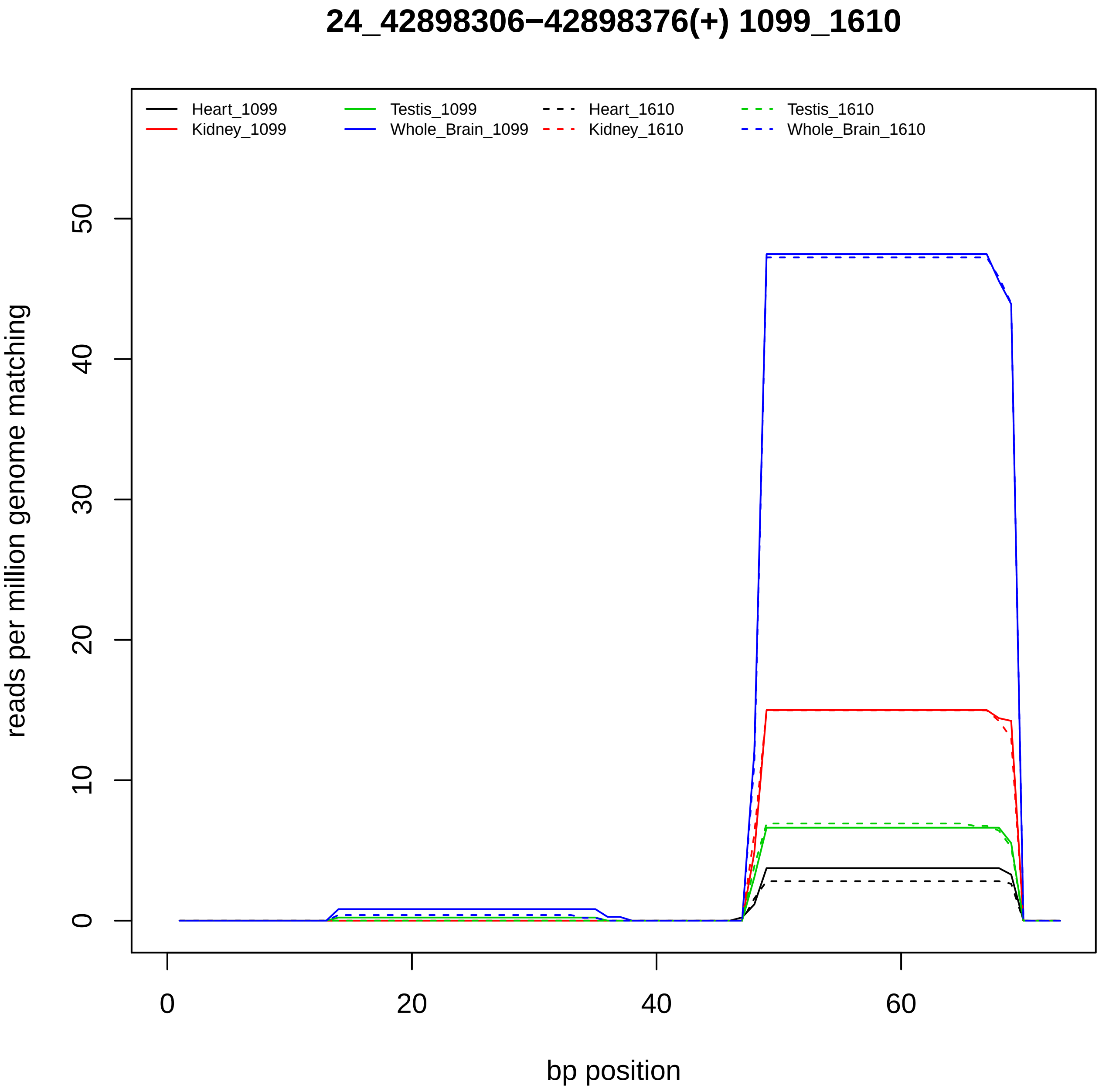

24_42898306−42898376(+) 1099_1610
Heart_1099
Kidney_1099
Testis_1099
Whole_Brain_1099
Heart_1610
Kidney_1610
Testis_1610
Whole_Brain_1610
reads per million genome matching
0
10
20
30
40
50
0
20
40
60
bp position

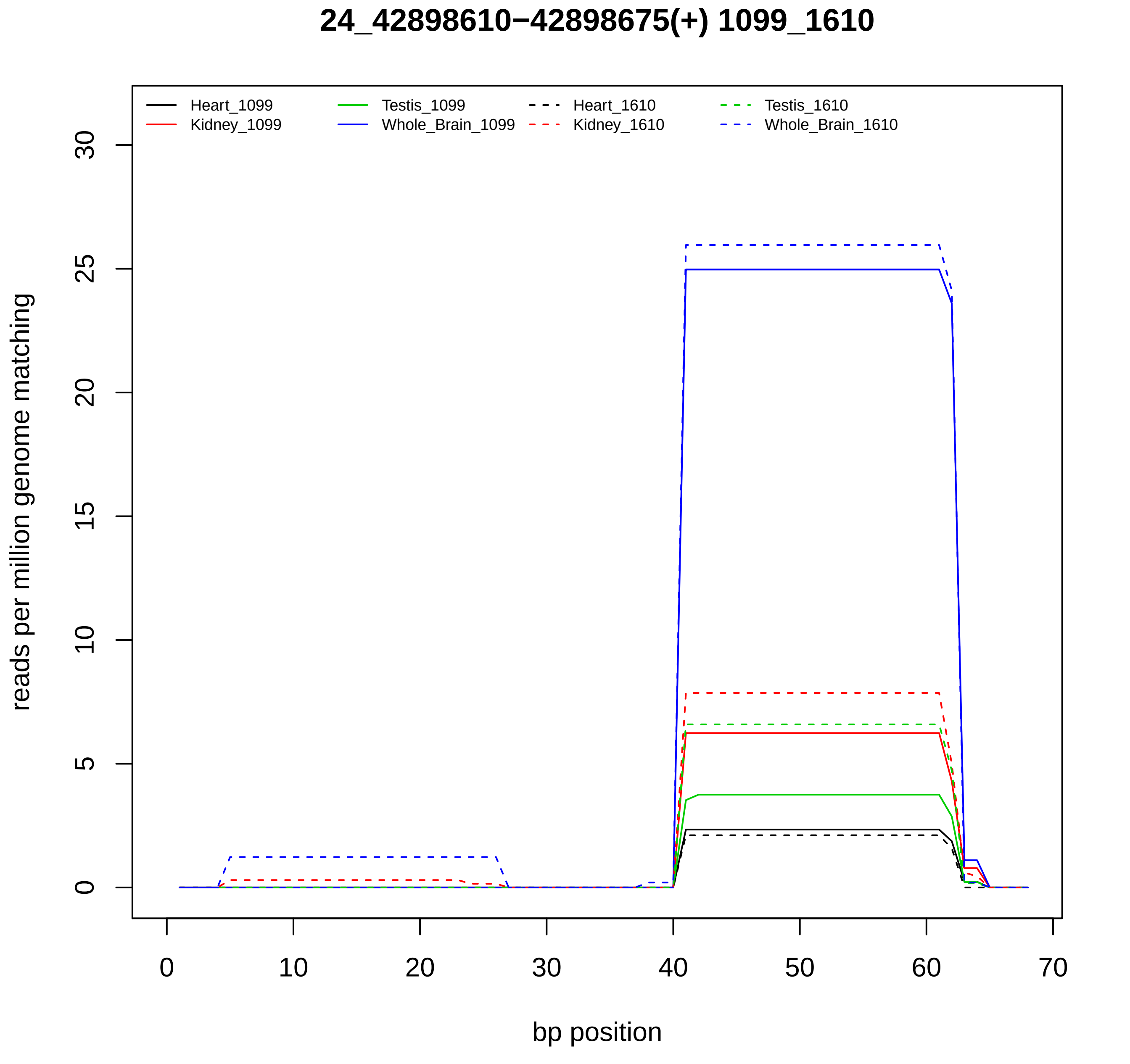

24_42898610−42898675(+) 1099_1610
Heart_1099
Kidney_1099
Testis_1099
Whole_Brain_1099
Heart_1610
Kidney_1610
Testis_1610
Whole_Brain_1610
reads per million genome matching
bp position
0
5
10
15
20
25
30
0
10
20
30
40
50
60
70

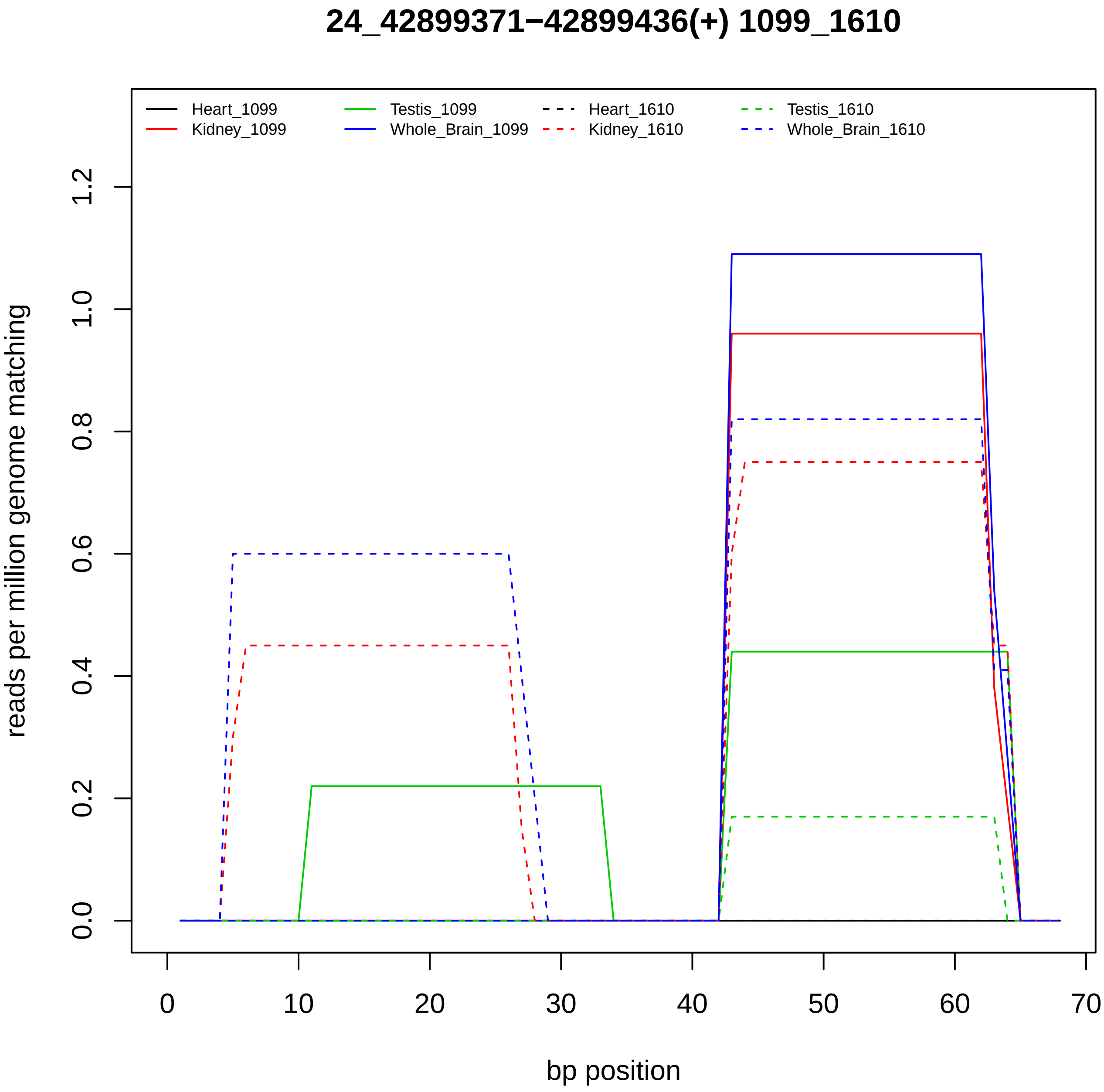
24_42899371−42899436(+) 1099_1610
Heart_1099
Kidney_1099
Testis_1099
Whole_Brain_1099
Heart_1610
Kidney_1610
Testis_1610
Whole_Brain_1610
reads per million genome matching
0.0
0.2
0.4
0.6
0.8
1.0
1.2
0
10
20
30
40
50
60
70
bp position

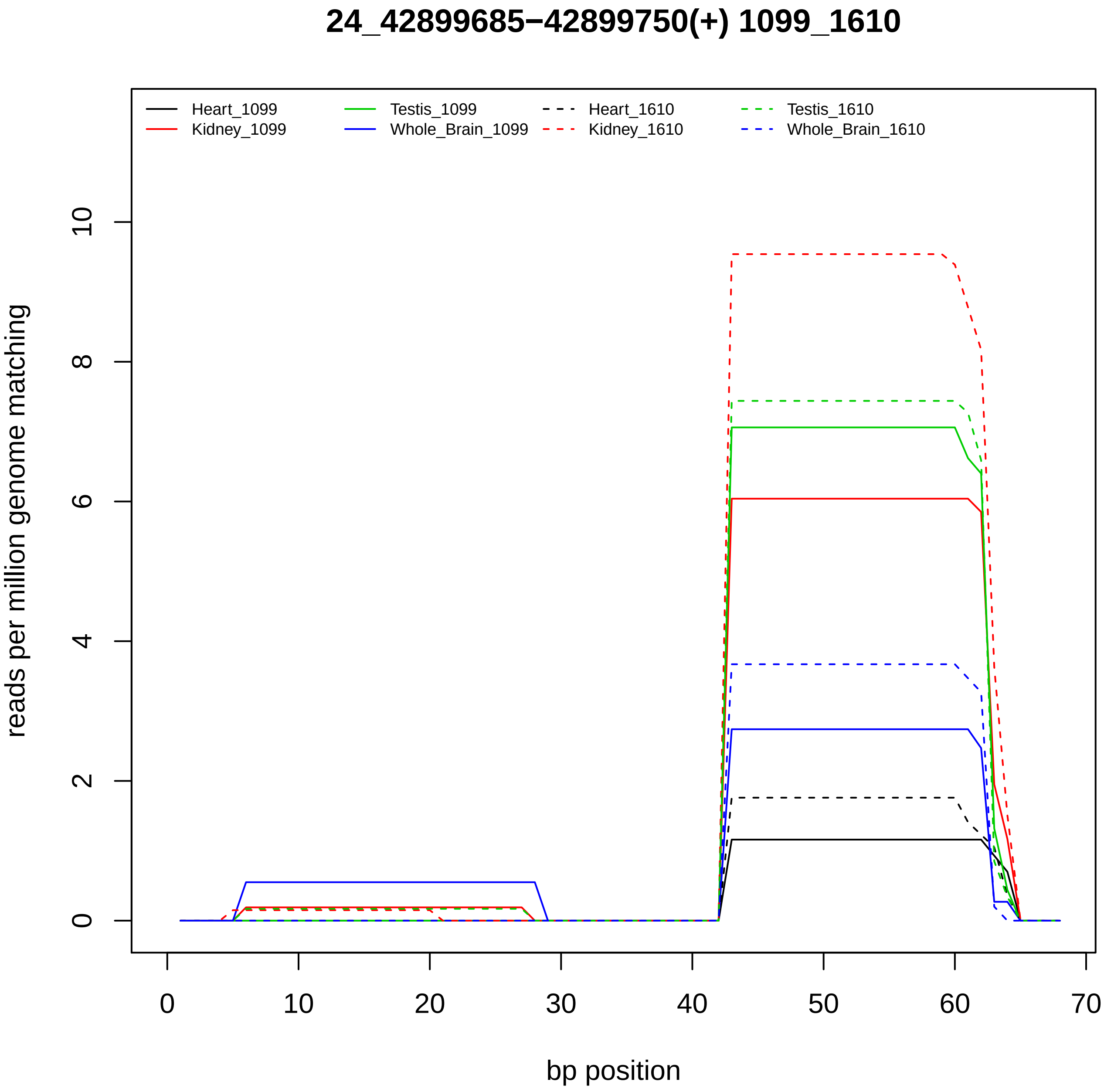
24_42899685−42899750(+) 1099_1610
Heart_1099
Kidney_1099
Testis_1099
Whole_Brain_1099
Heart_1610
Kidney_1610
Testis_1610
Whole_Brain_1610
reads per million genome matching
bp position
0
2
4
6
8
10
0
10
20
30
40
50
60
70

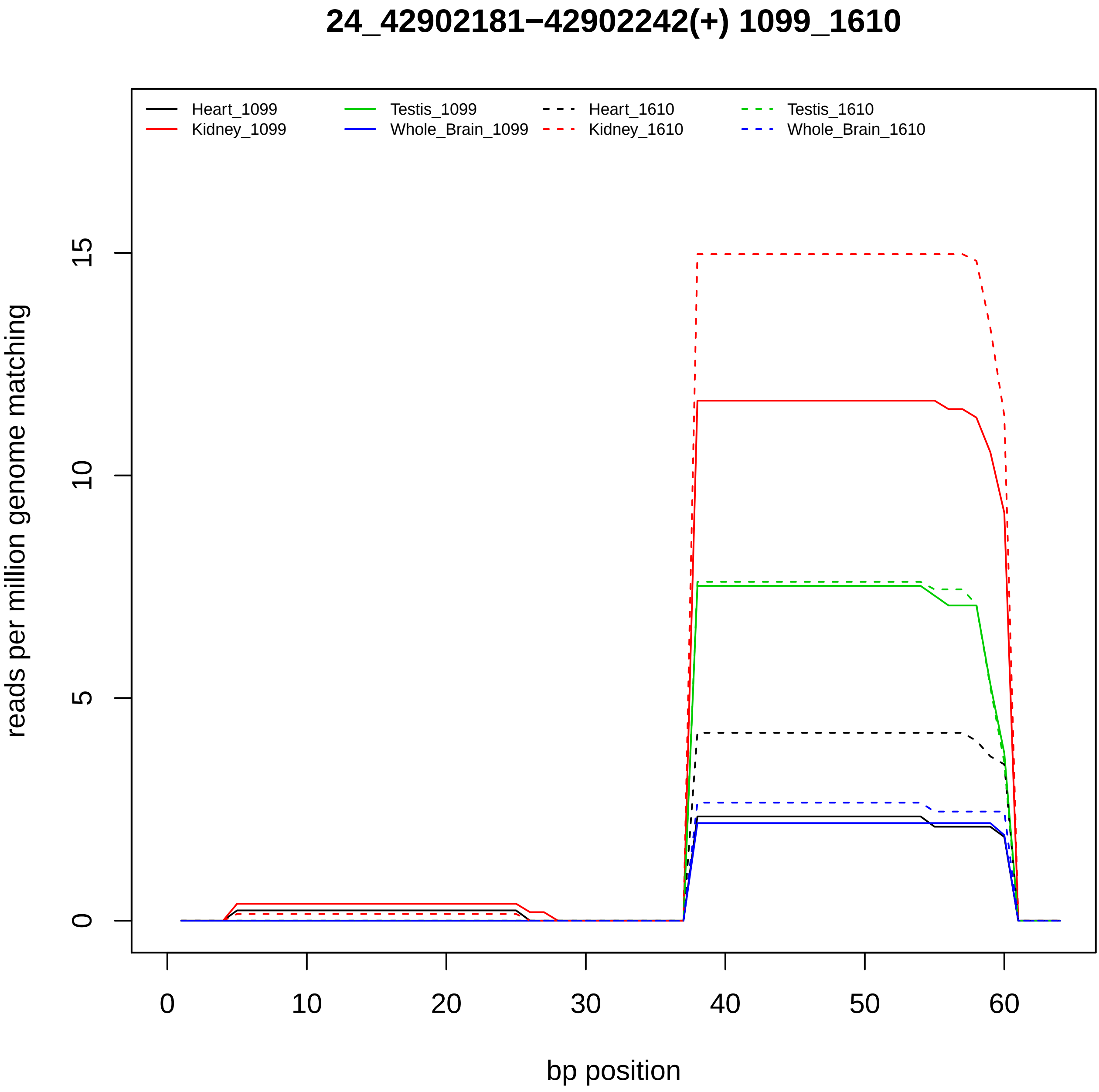
24_42902181−42902242(+) 1099_1610
Heart_1099
Kidney_1099
Testis_1099
Whole_Brain_1099
Heart_1610
Kidney_1610
Testis_1610
Whole_Brain_1610
reads per million genome matching
bp position
0
5
10
15
0
10
20
30
40
50
60

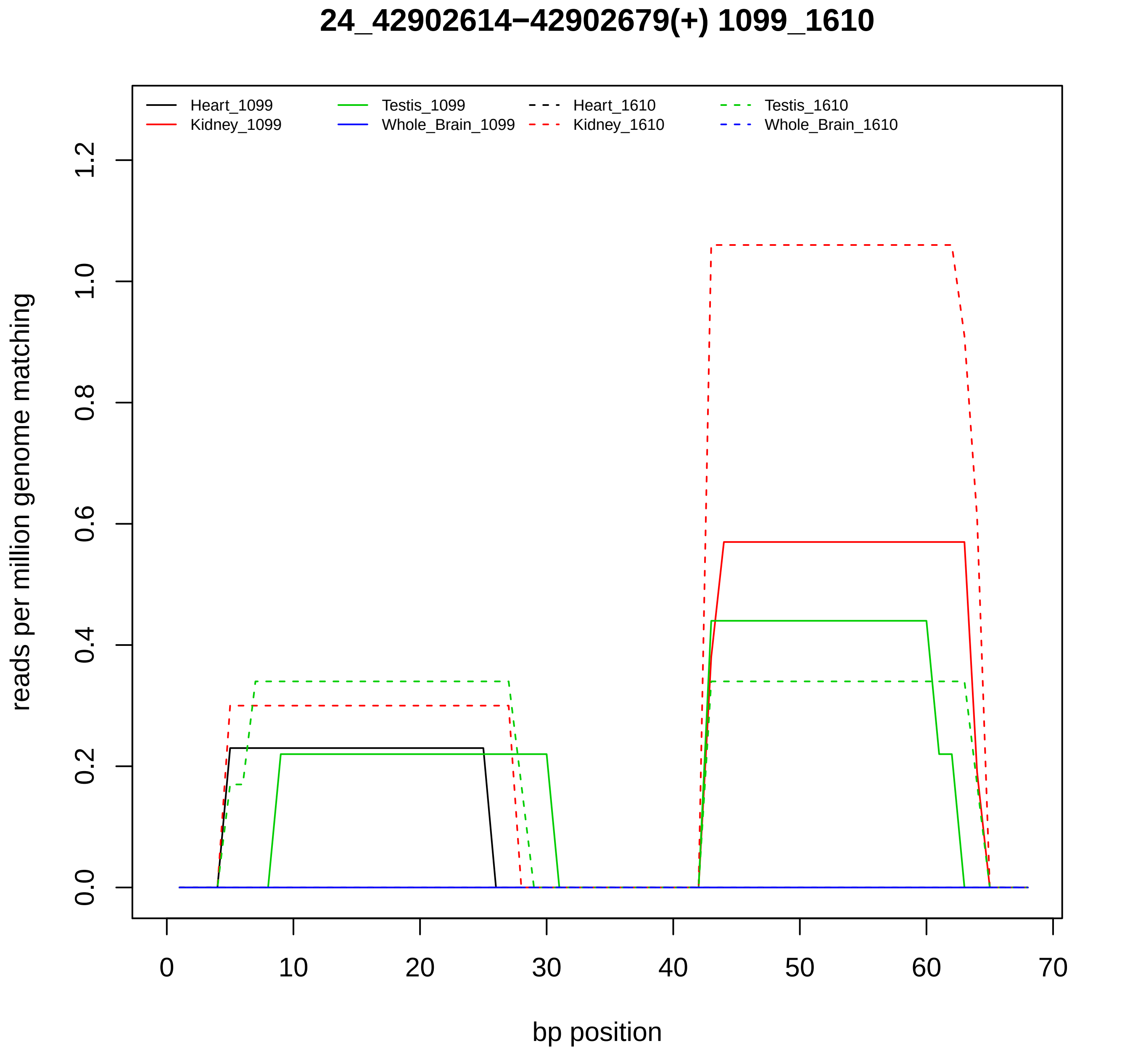
24_42902614−42902679(+) 1099_1610
Heart_1099
Kidney_1099
Testis_1099
Whole_Brain_1099
Heart_1610
Kidney_1610
Testis_1610
Whole_Brain_1610
reads per million genome matching
0.0
0.2
0.4
0.6
0.8
1.0
1.2
bp position
0
10
20
30
40
50
60
70

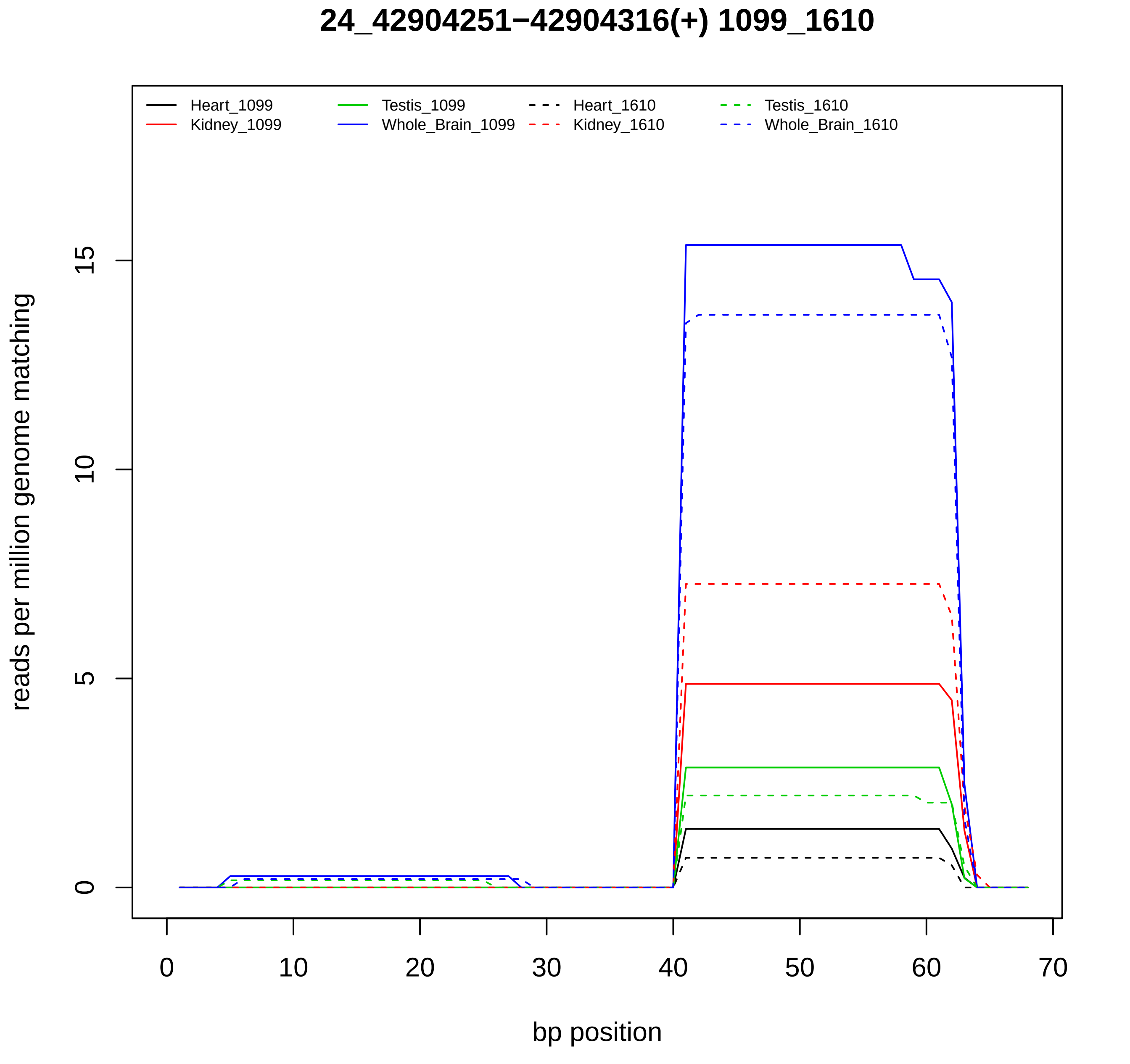

24_42904251−42904316(+) 1099_1610
Heart_1099
Kidney_1099
Testis_1099
Whole_Brain_1099
Heart_1610
Kidney_1610
Testis_1610
Whole_Brain_1610
reads per million genome matching
0
5
10
15
bp position
0
10
20
30
40
50
60
70

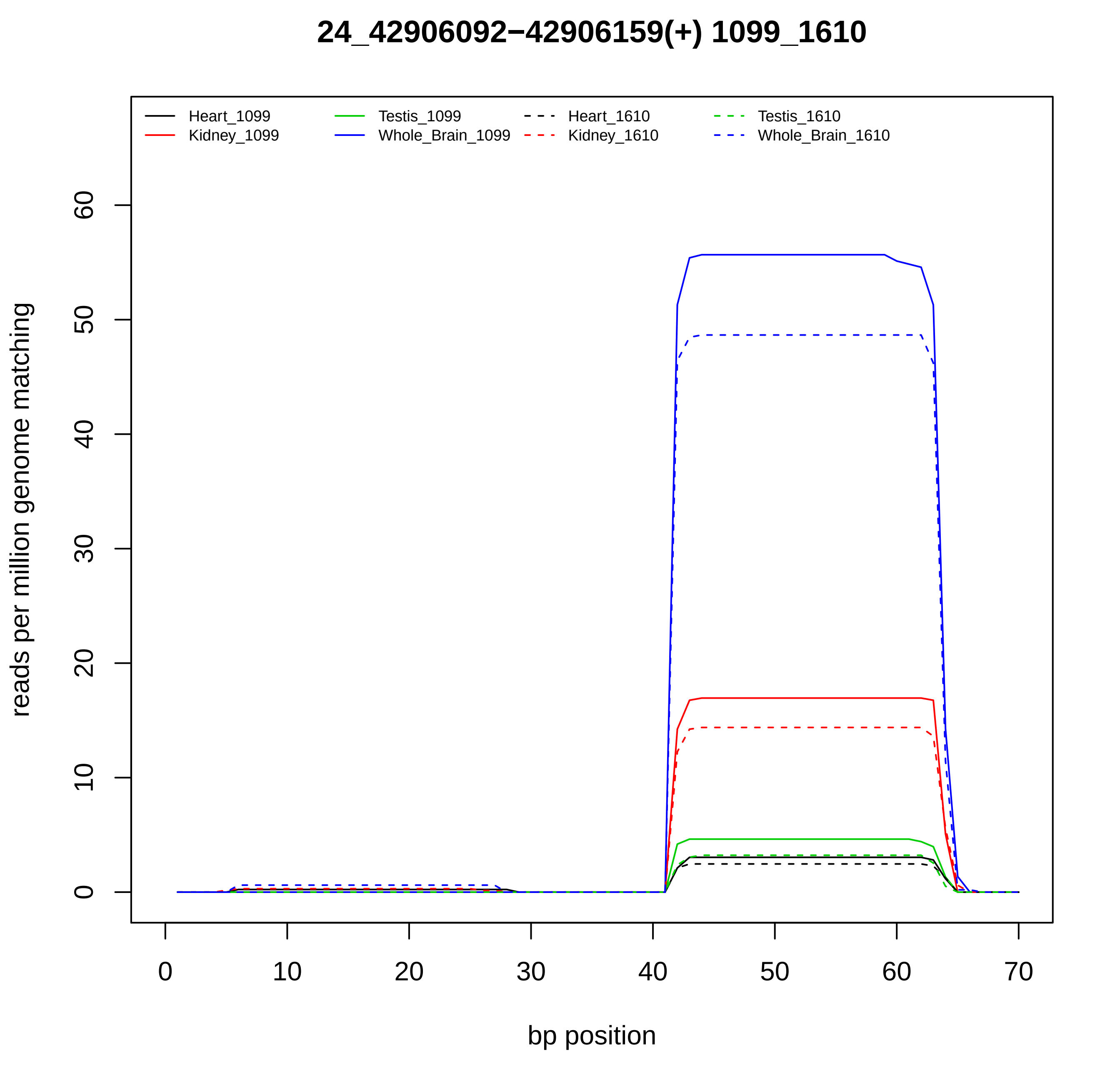

24_42906092−42906159(+) 1099_1610
Heart_1099
Kidney_1099
Testis_1099
Whole_Brain_1099
Heart_1610
Kidney_1610
Testis_1610
Whole_Brain_1610
reads per million genome matching
bp position
0
10
20
30
40
50
60
70

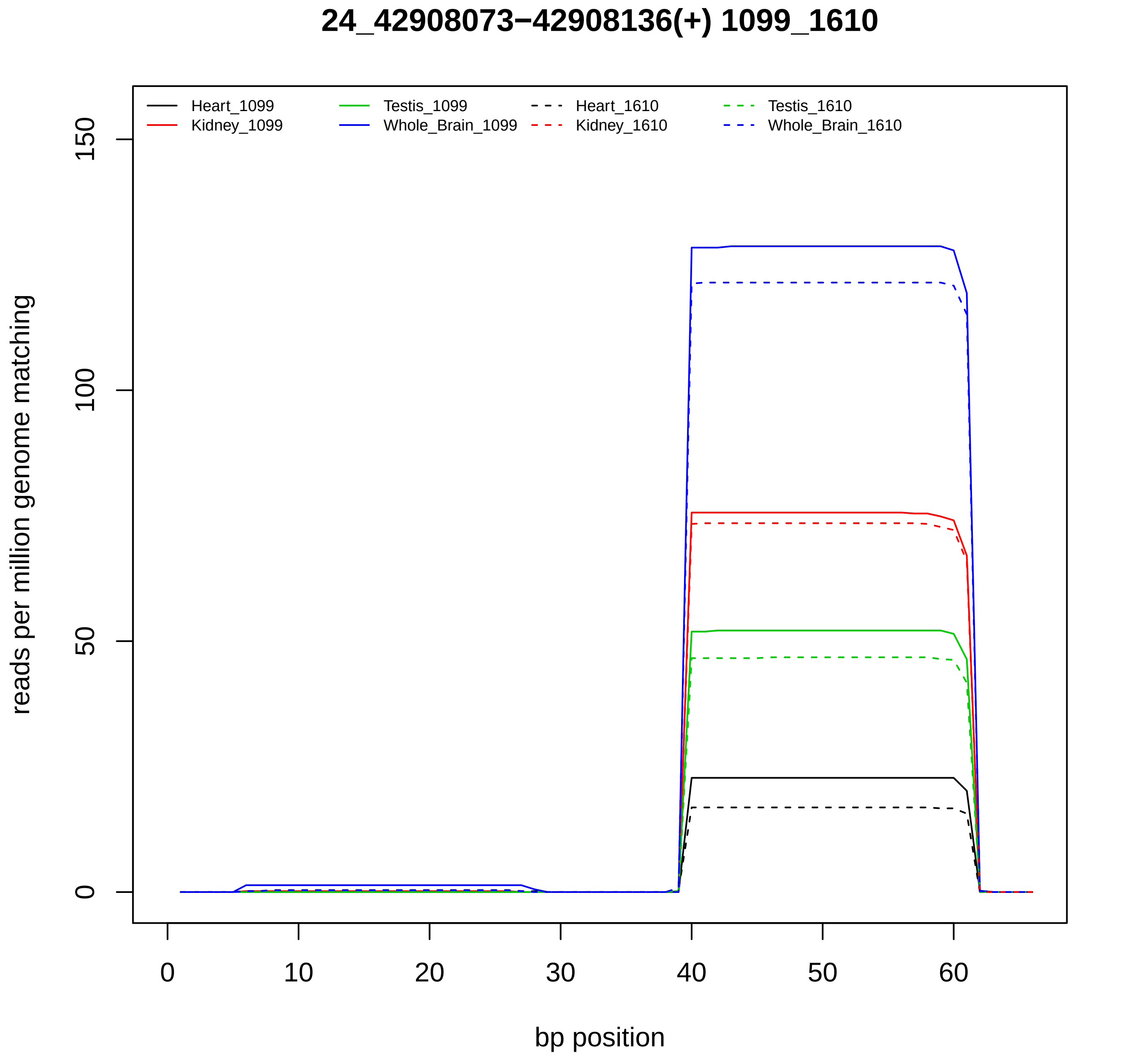
24_42908073−42908136(+) 1099_1610
Heart_1099
Kidney_1099
Testis_1099
Whole_Brain_1099
Heart_1610
Kidney_1610
Testis_1610
Whole_Brain_1610
reads per million genome matching
0
50
100
150
0
10
20
30
40
50
60
bp position

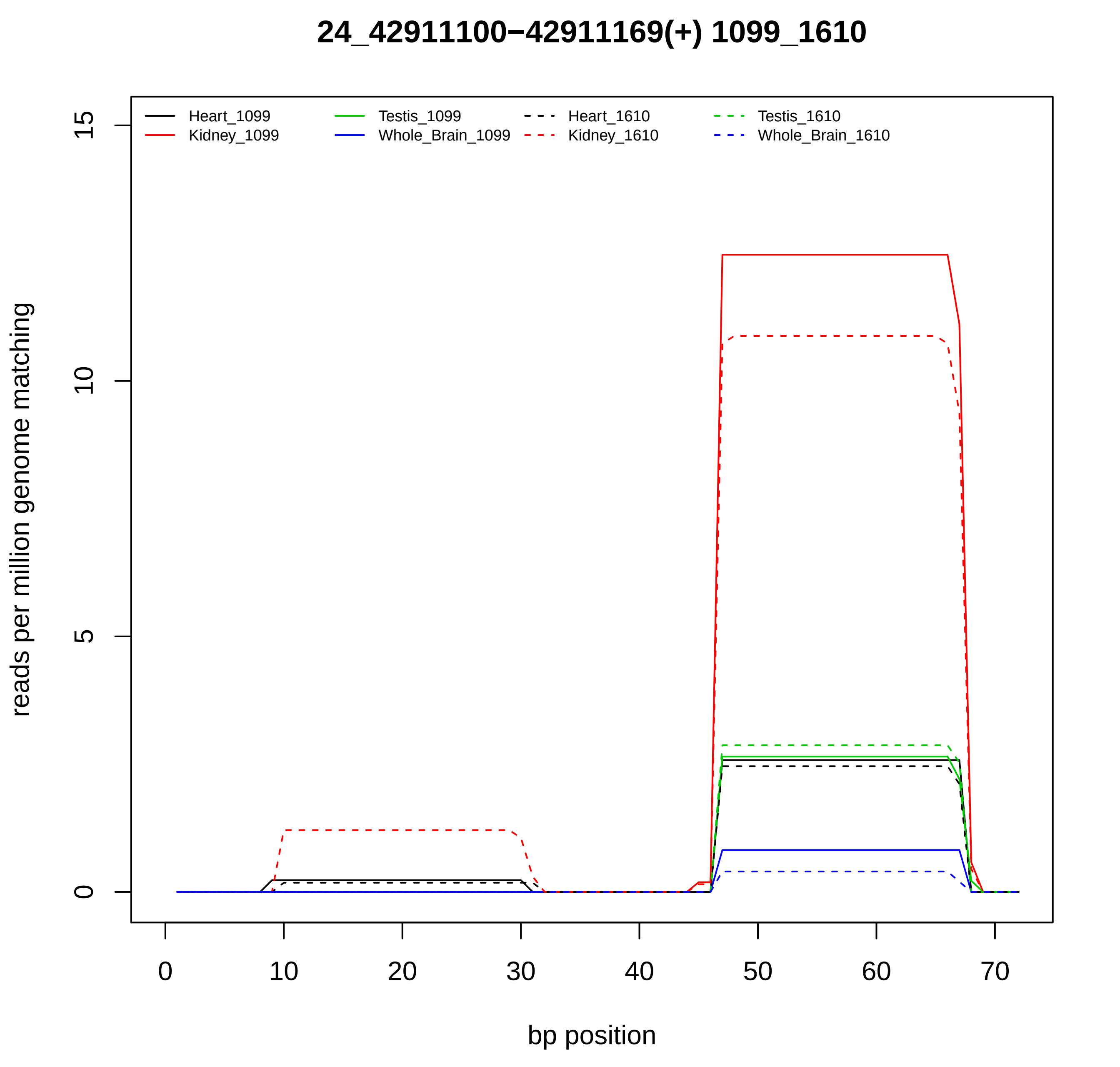

24_42911100−42911169(+) 1099_1610
Heart_1099
Kidney_1099
Testis_1099
Whole_Brain_1099
Heart_1610
Kidney_1610
Testis_1610
Whole_Brain_1610
reads per million genome matching
0
5
10
15
0
10
20
30
40
50
60
70
bp position

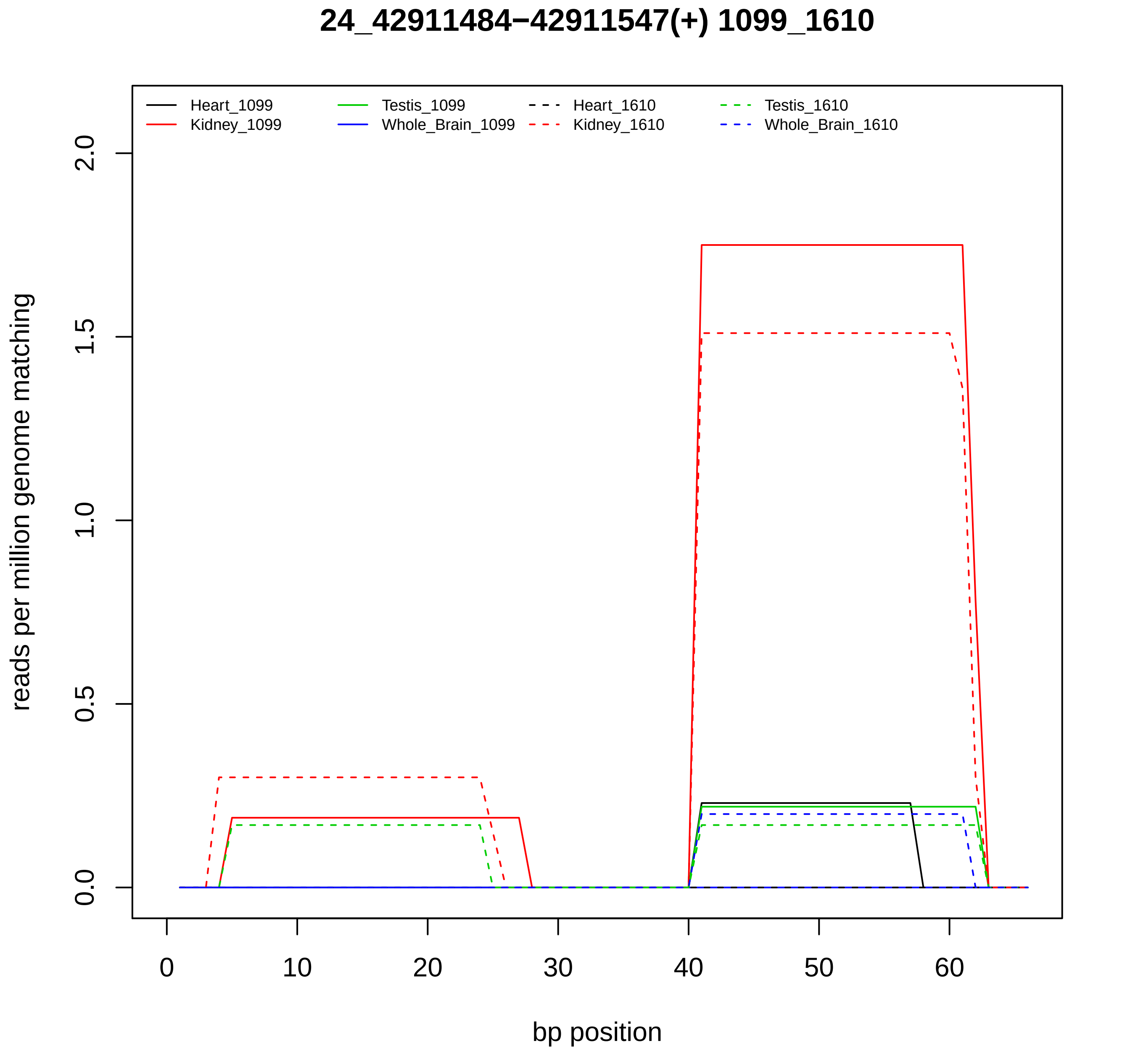

24_42911484−42911547(+) 1099_1610
Heart_1099
Kidney_1099
Testis_1099
Whole_Brain_1099
Heart_1610
Kidney_1610
Testis_1610
Whole_Brain_1610
reads per million genome matching
bp position
0.0
0.5
1.0
1.5
2.0
0
10
20
30
40
50
60

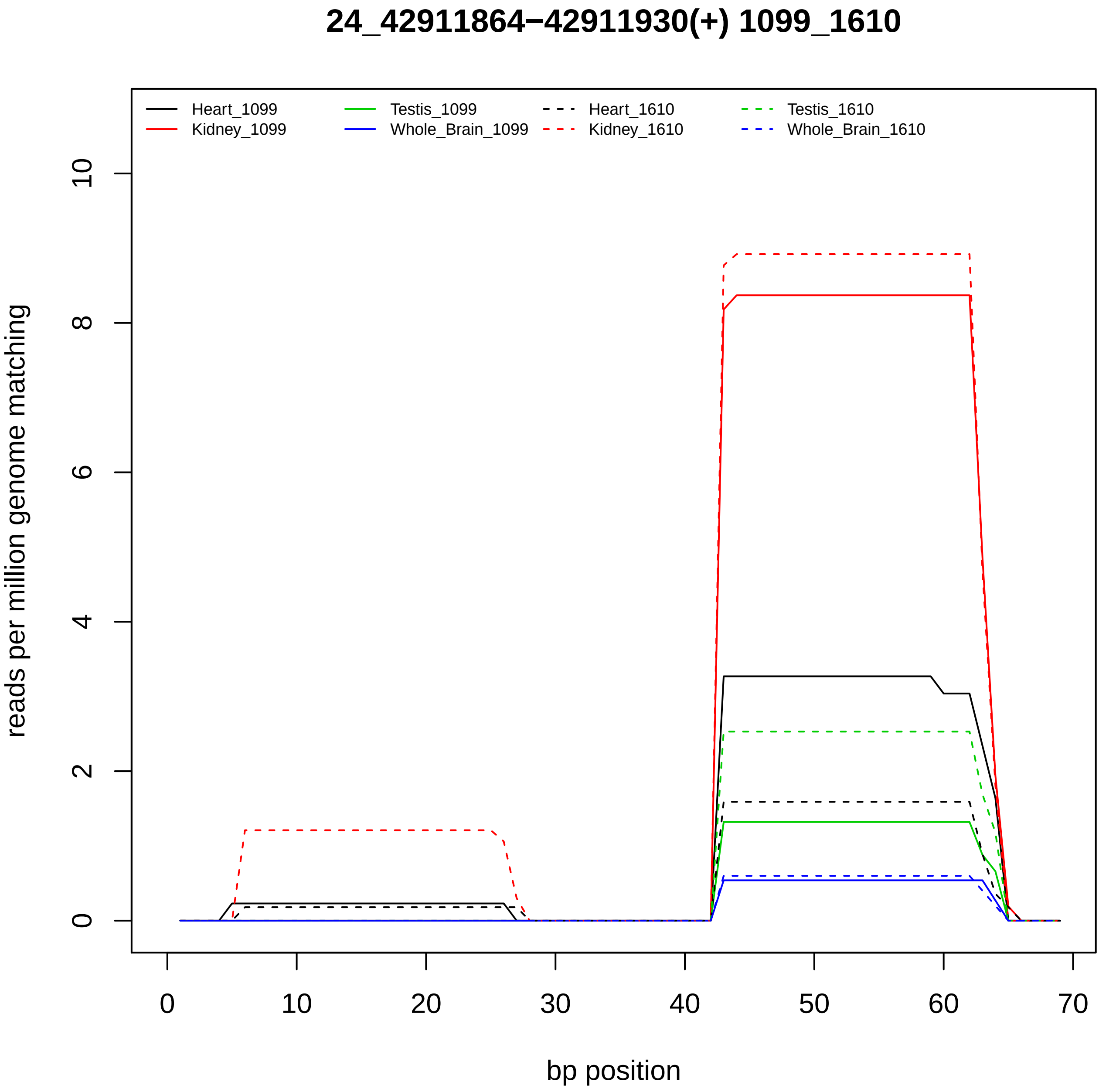
24_42911864−42911930(+) 1099_1610
Heart_1099
Kidney_1099
Testis_1099
Whole_Brain_1099
Heart_1610
Kidney_1610
Testis_1610
Whole_Brain_1610
reads per million genome matching
bp position
0
2
4
6
8
10
0
10
20
30
40
50
60
70

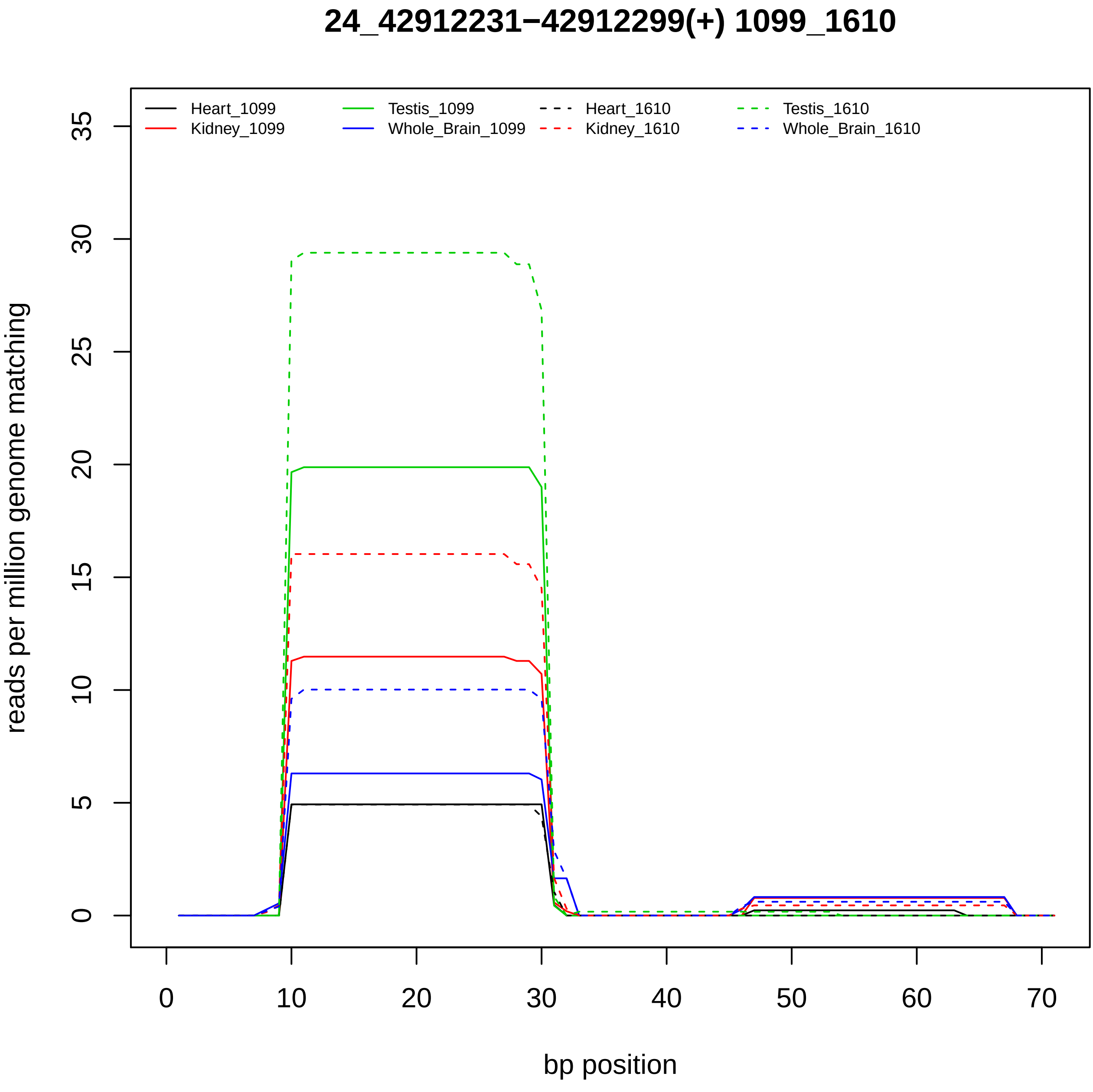

24_42912231−42912299(+) 1099_1610
Heart_1099
Kidney_1099
Testis_1099
Whole_Brain_1099
Heart_1610
Kidney_1610
Testis_1610
Whole_Brain_1610
reads per million genome matching
0
5
10
15
20
25
30
35
0
10
20
30
40
50
60
70
bp position

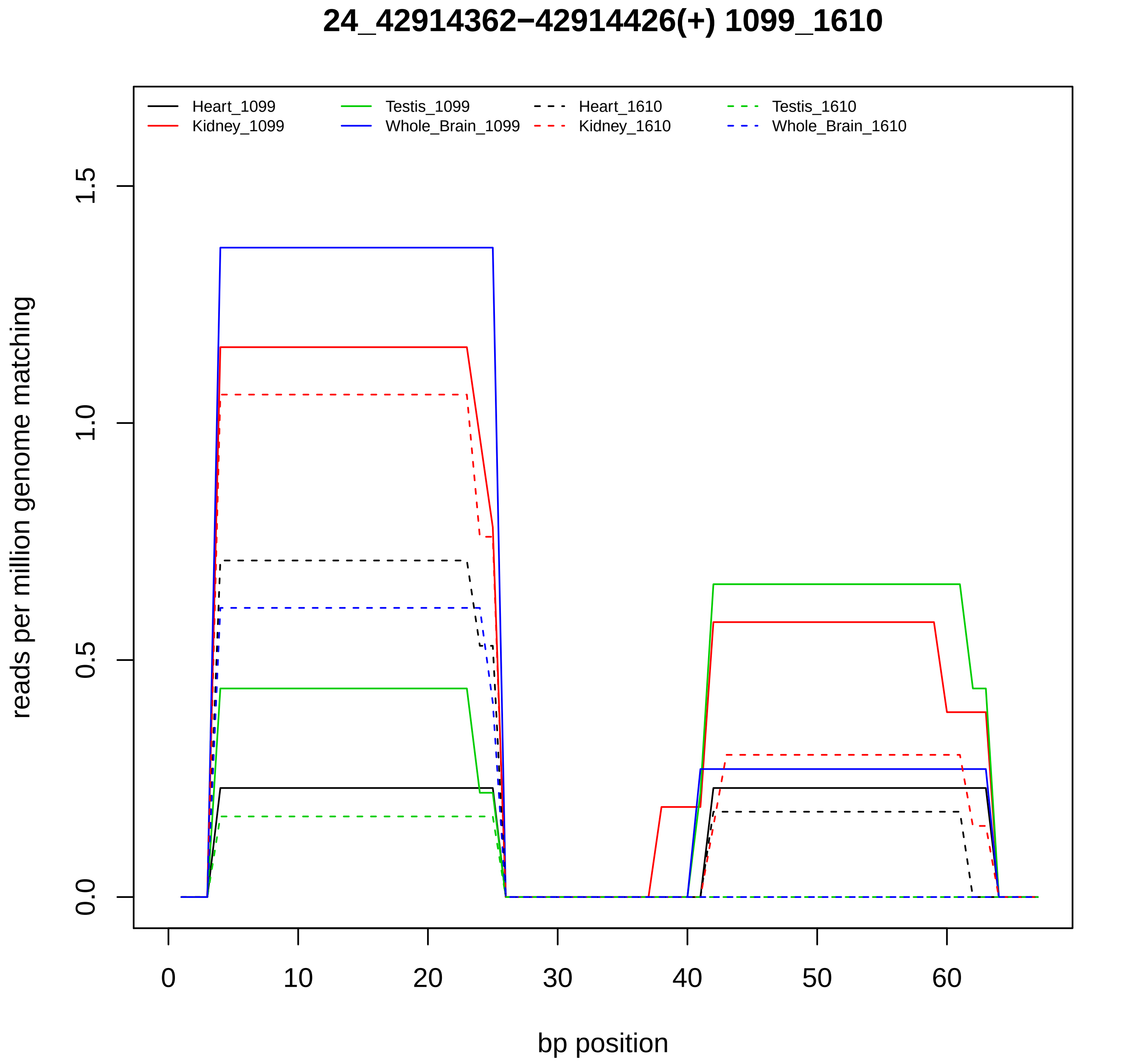

24_42914362−42914426(+) 1099_1610
Heart_1099
Kidney_1099
Testis_1099
Whole_Brain_1099
Heart_1610
Kidney_1610
Testis_1610
Whole_Brain_1610
reads per million genome matching
0.0
0.5
1.0
1.5
bp position
0
10
20
30
40
50
60

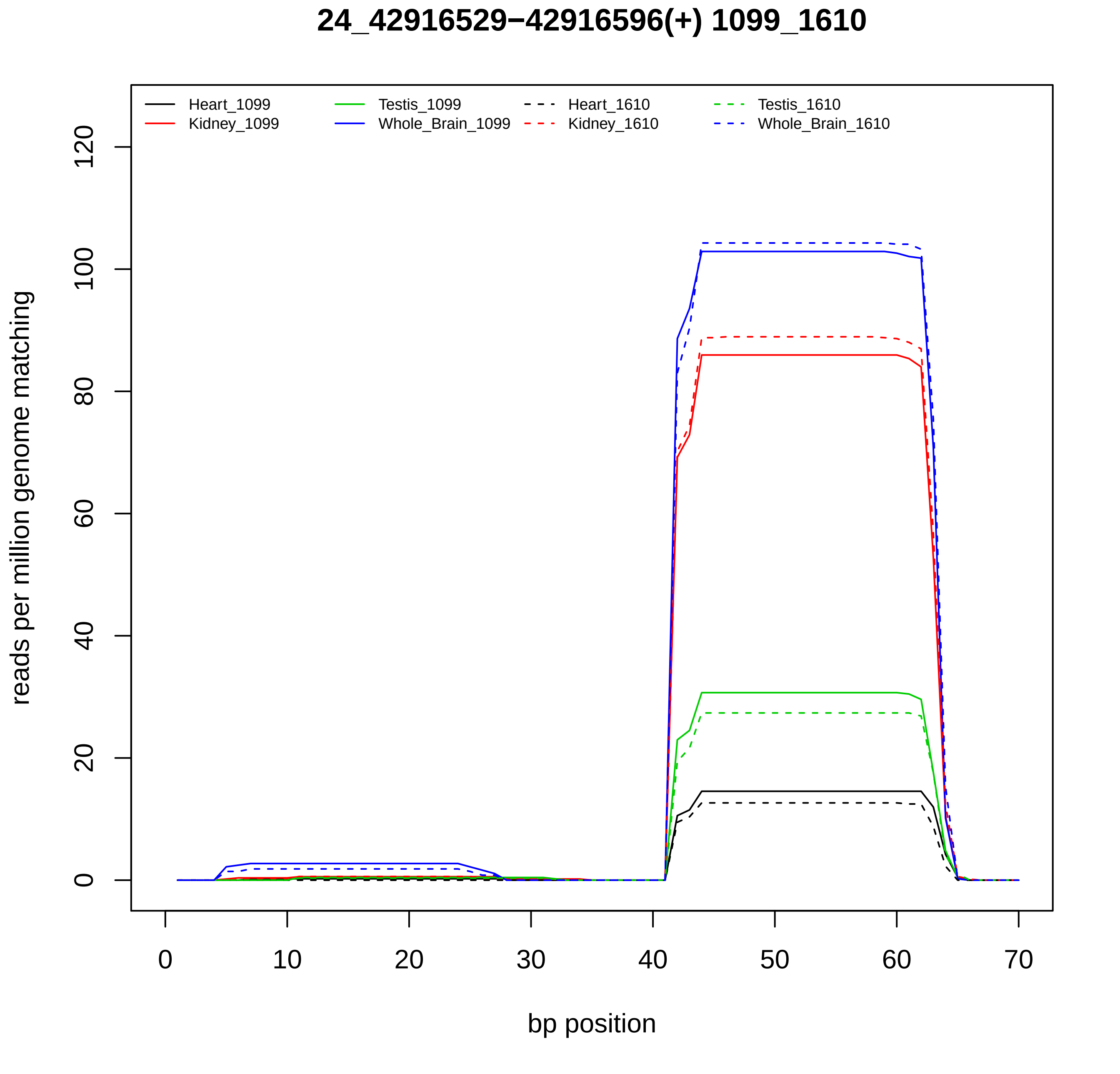
24_42916529−42916596(+) 1099_1610
Heart_1099
Kidney_1099
Testis_1099
Whole_Brain_1099
Heart_1610
Kidney_1610
Testis_1610
Whole_Brain_1610
reads per million genome matching
0
20
40
60
80
100
120
bp position
0
10
20
30
40
50
60
70

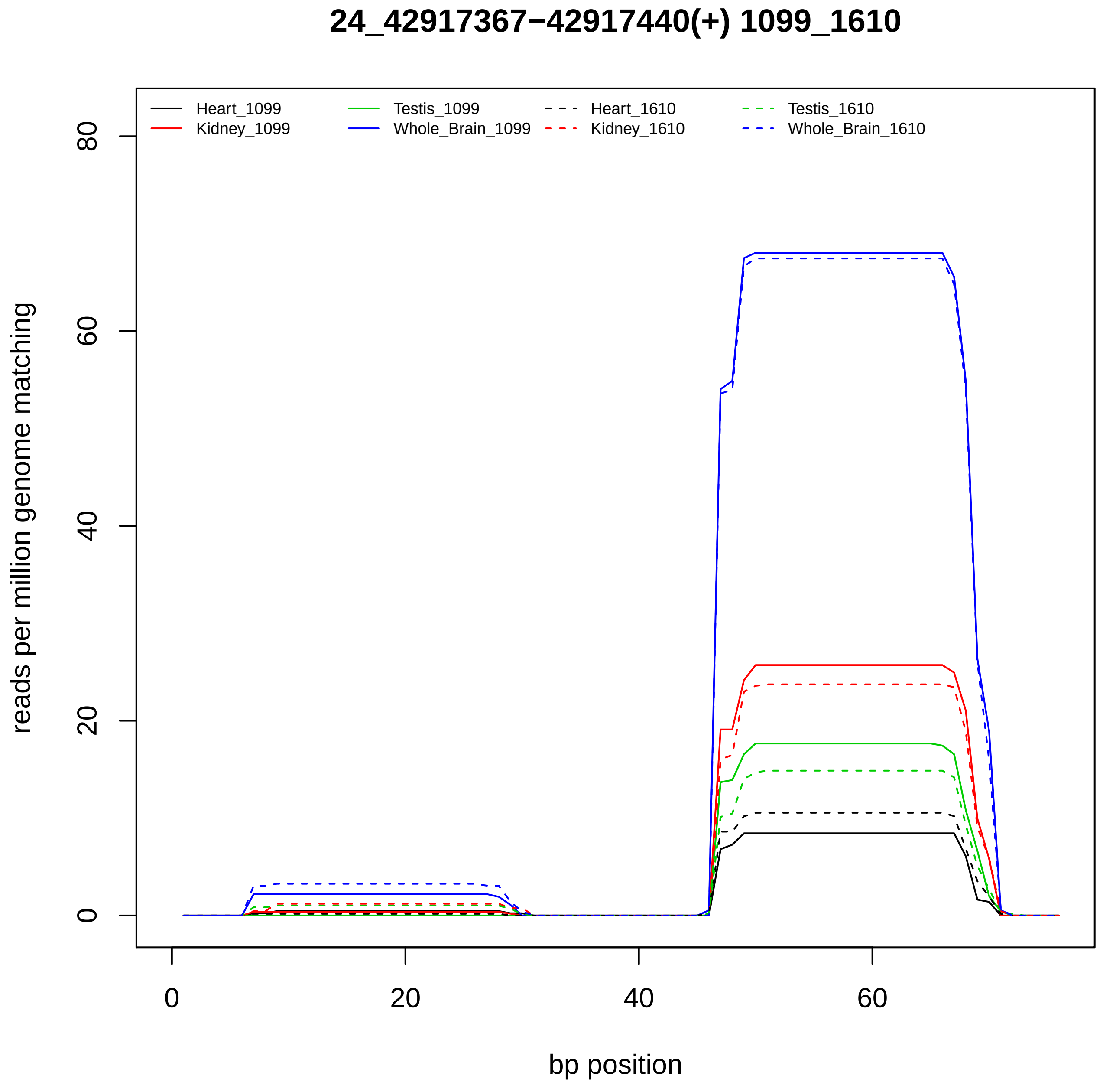

24_42917367−42917440(+) 1099_1610
Heart_1099
Kidney_1099
Testis_1099
Whole_Brain_1099
Heart_1610
Kidney_1610
Testis_1610
Whole_Brain_1610
reads per million genome matching
bp position
0
20
40
60
80

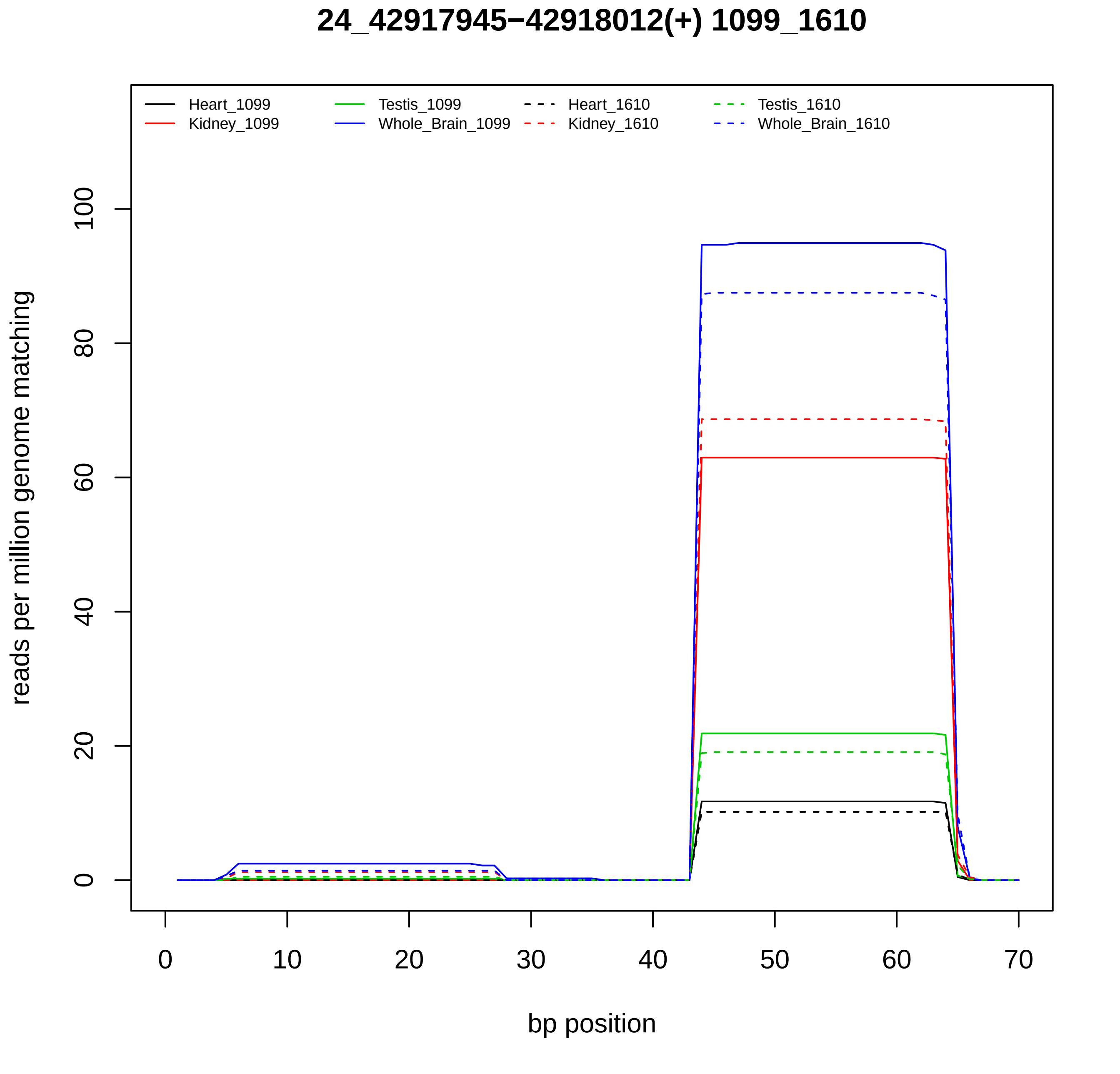

24_42917945−42918012(+) 1099_1610
Heart_1099
Kidney_1099
Testis_1099
Whole_Brain_1099
Heart_1610
Kidney_1610
Testis_1610
Whole_Brain_1610
reads per million genome matching
bp position
0
20
40
60
80
100
0
10
20
30
40
50
60
70

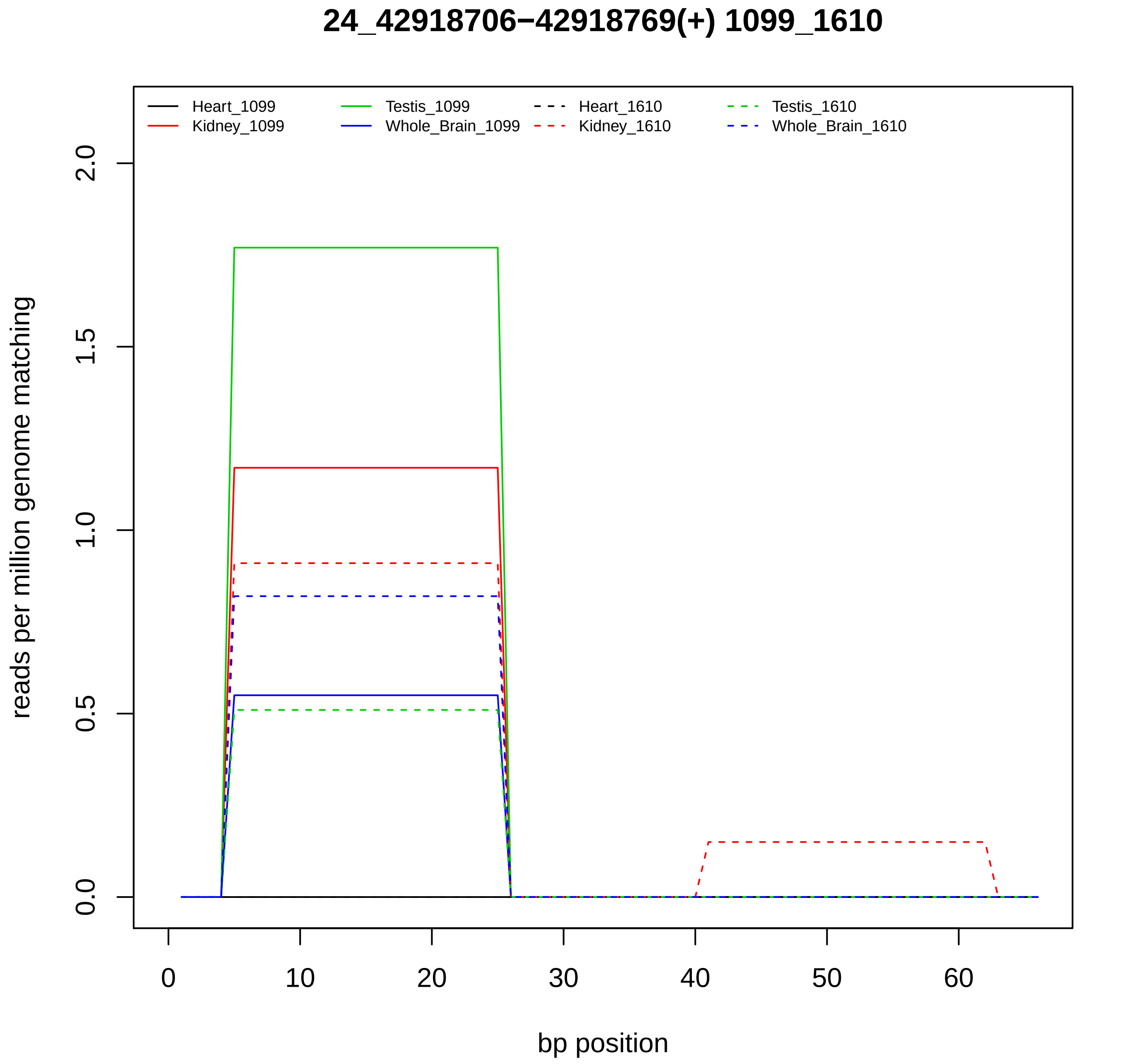

24_42918706−42918769(+) 1099_1610
Heart_1099
Kidney_1099
Testis_1099
Whole_Brain_1099
Heart_1610
Kidney_1610
Testis_1610
Whole_Brain_1610
reads per million genome matching
0.0
0.5
1.0
1.5
2.0
0
10
20
30
40
50
60
bp position

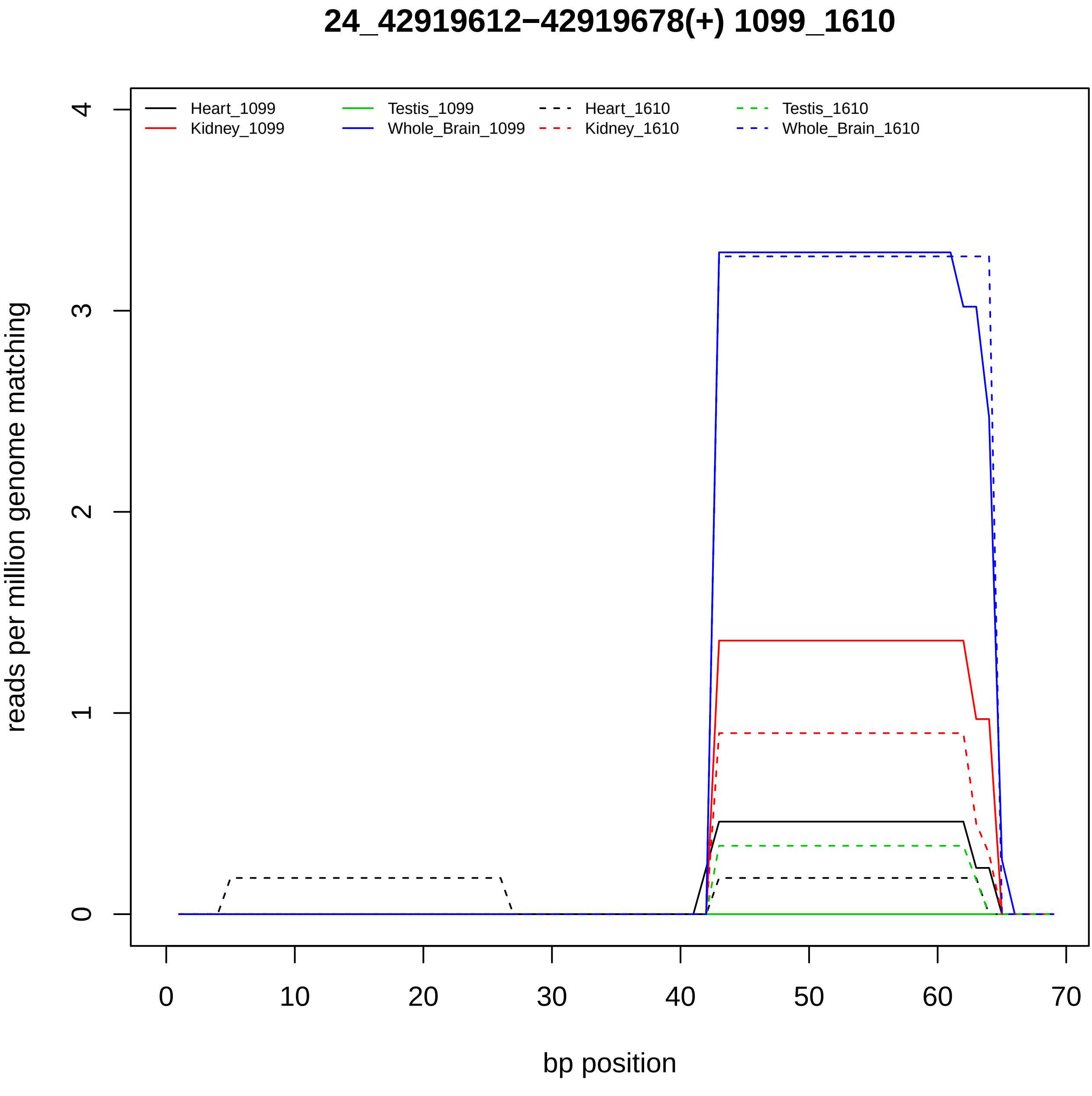

24_42919612−42919678(+) 1099_1610
Heart_1099
Kidney_1099
Testis_1099
Whole_Brain_1099
Heart_1610
Kidney_1610
Testis_1610
Whole_Brain_1610
reads per million genome matching
bp position
0
1
2
3
4
0
10
20
30
40
50
60
70

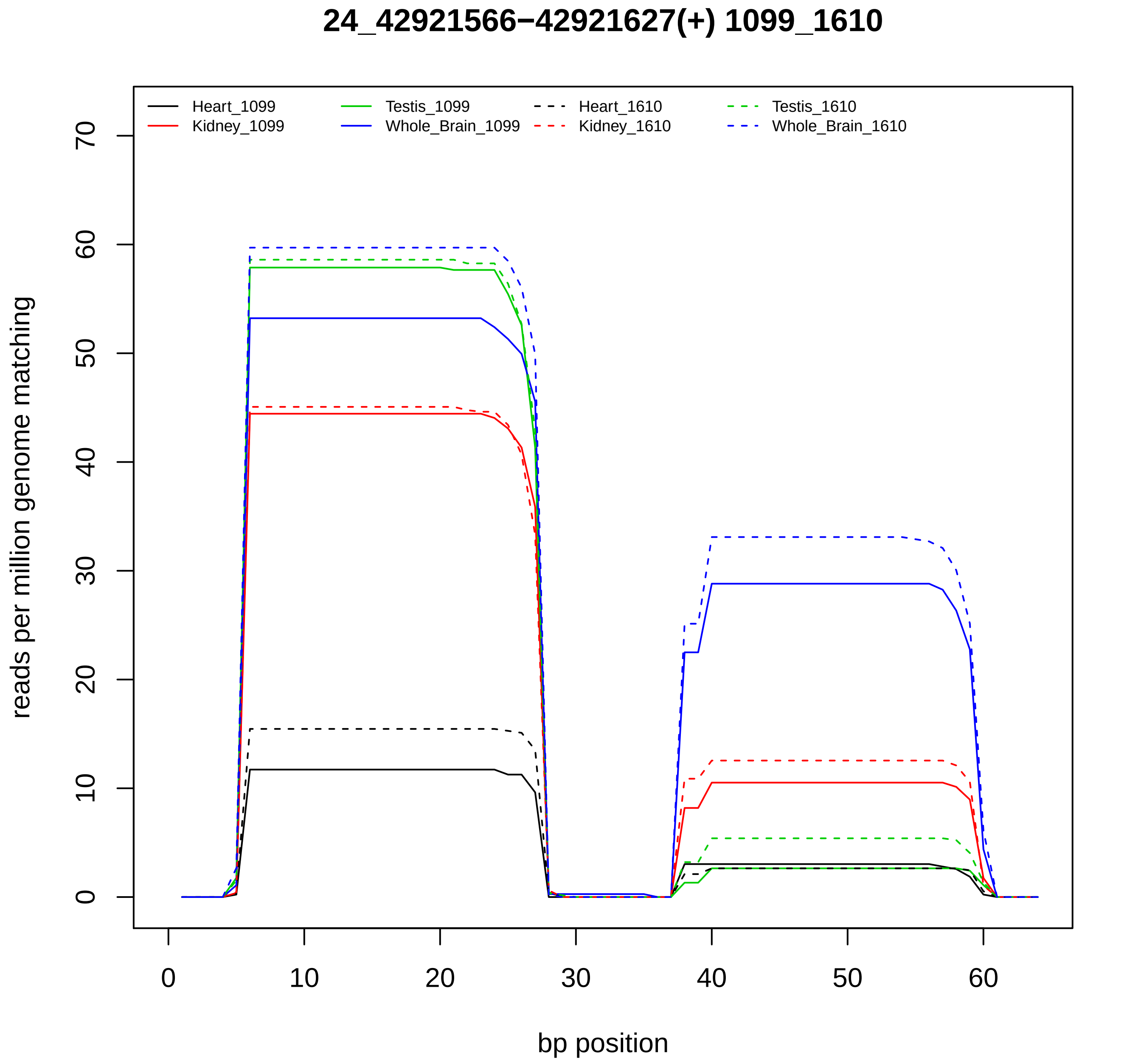
24_42921566−42921627(+) 1099_1610
Heart_1099
Kidney_1099
Testis_1099
Whole_Brain_1099
Heart_1610
Kidney_1610
Testis_1610
Whole_Brain_1610
reads per million genome matching
0
10
20
30
40
50
60
70
bp position
0
10
20
30
40
50
60

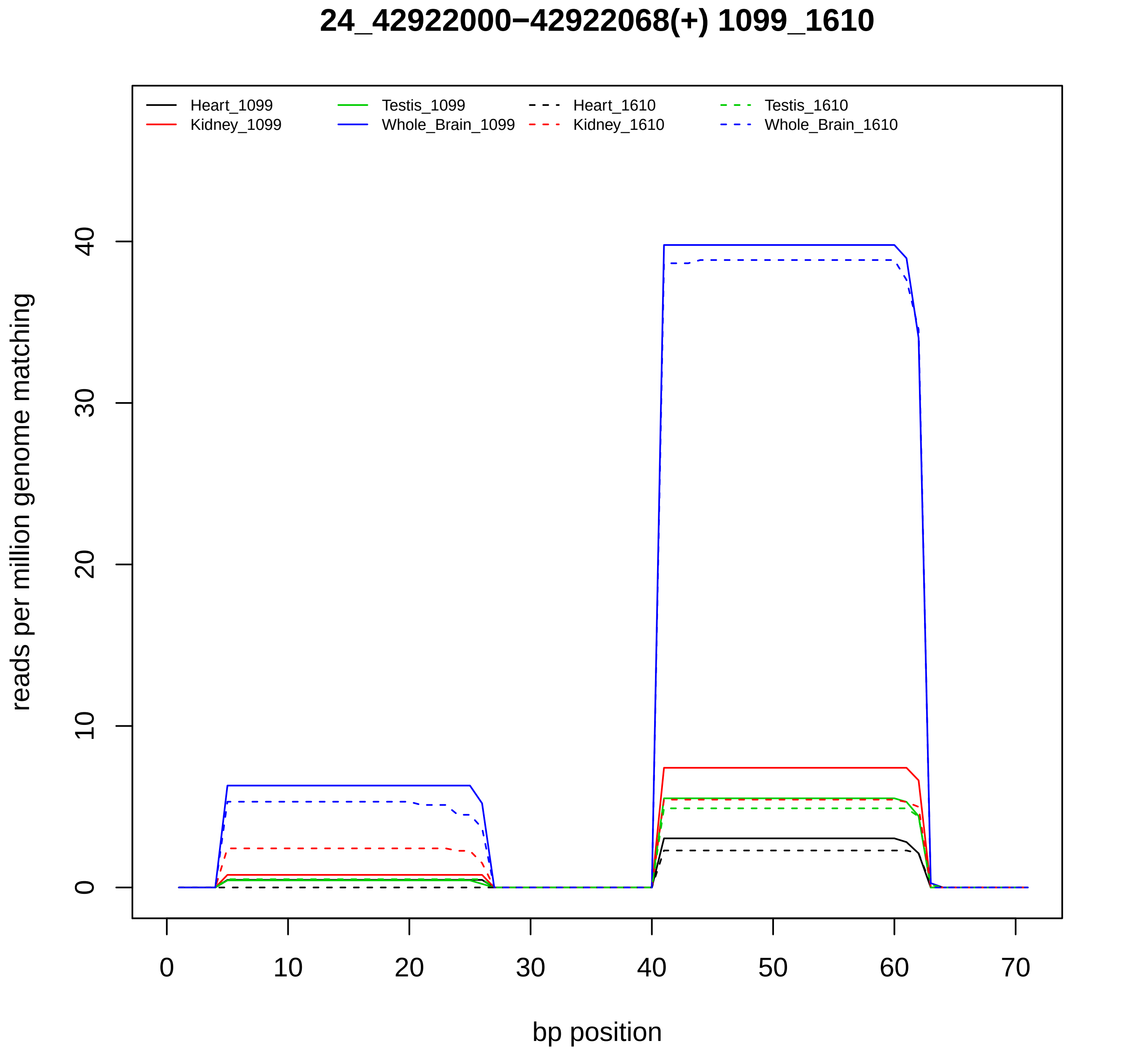

24_42922000−42922068(+) 1099_1610
Heart_1099
Kidney_1099
Testis_1099
Whole_Brain_1099
Heart_1610
Kidney_1610
Testis_1610
Whole_Brain_1610
reads per million genome matching
0
10
20
30
40
0
10
20
30
40
50
60
70
bp position

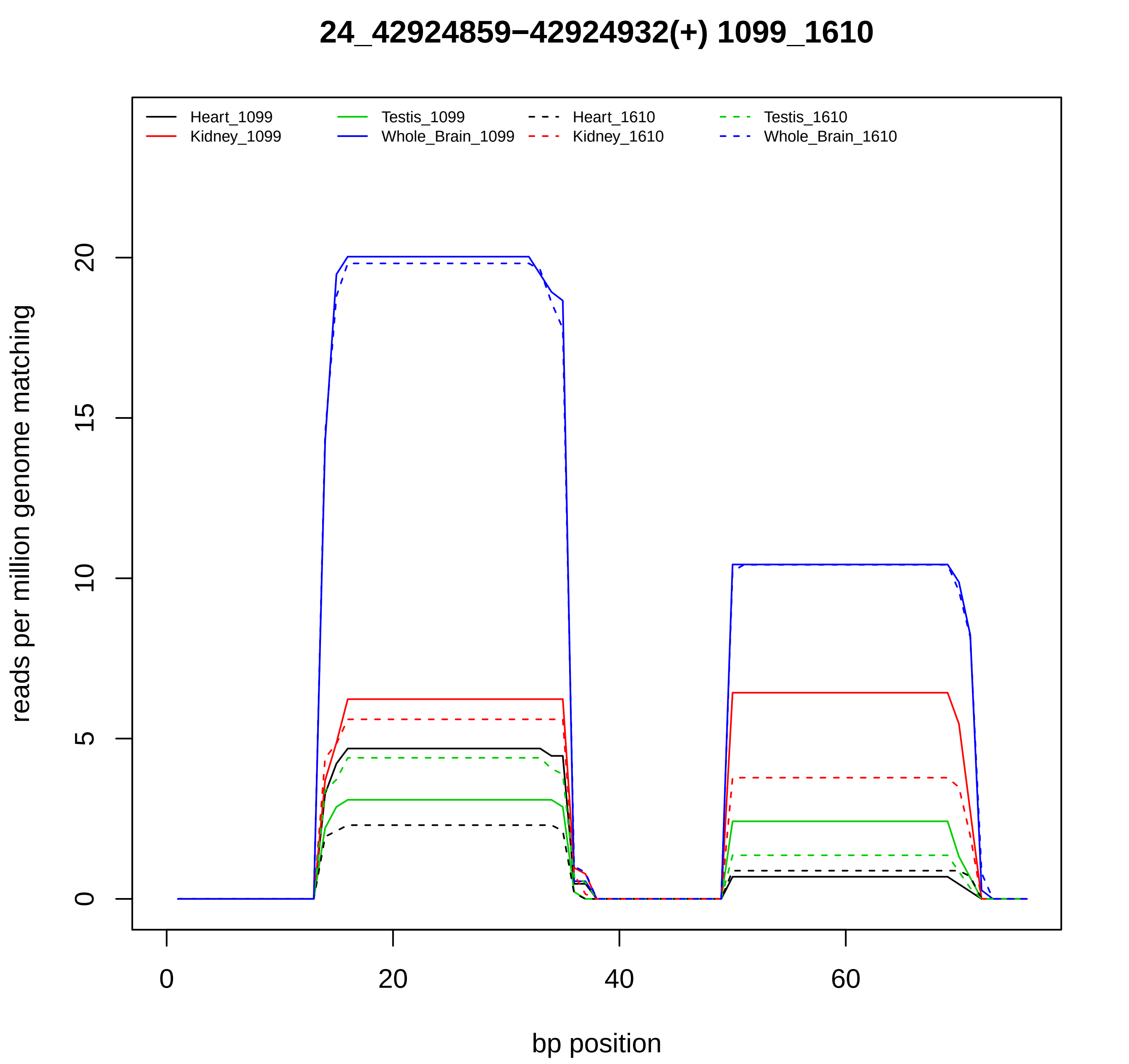
24_42924859−42924932(+) 1099_1610
Heart_1099
Kidney_1099
Testis_1099
Whole_Brain_1099
Heart_1610
Kidney_1610
Testis_1610
Whole_Brain_1610
reads per million genome matching
0
5
10
15
20
0
20
40
60
bp position

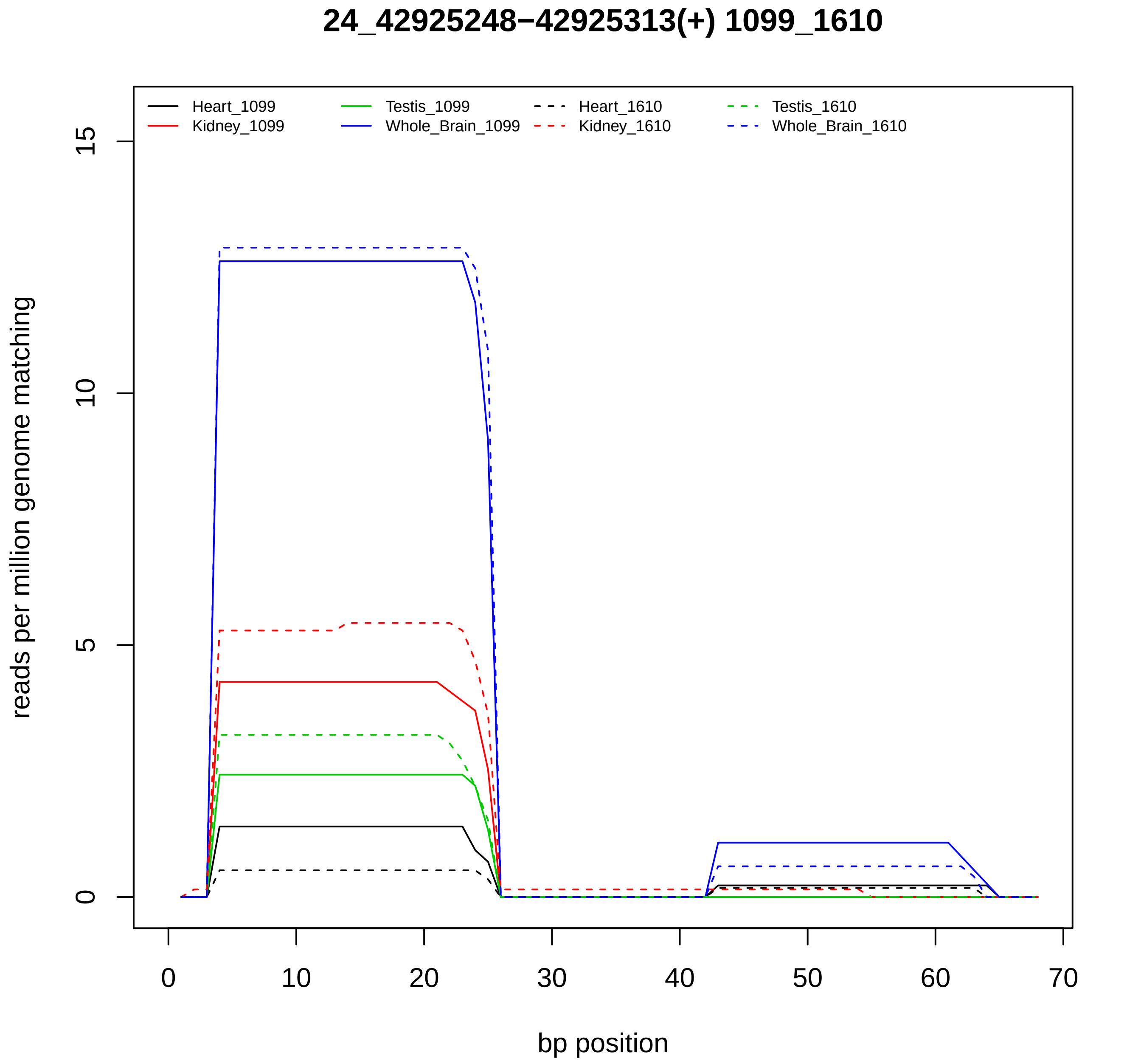

24_42925248−42925313(+) 1099_1610
Heart_1099
Kidney_1099
Testis_1099
Whole_Brain_1099
Heart_1610
Kidney_1610
Testis_1610
Whole_Brain_1610
reads per million genome matching
0
5
10
15
0
10
20
30
40
50
60
70
bp position

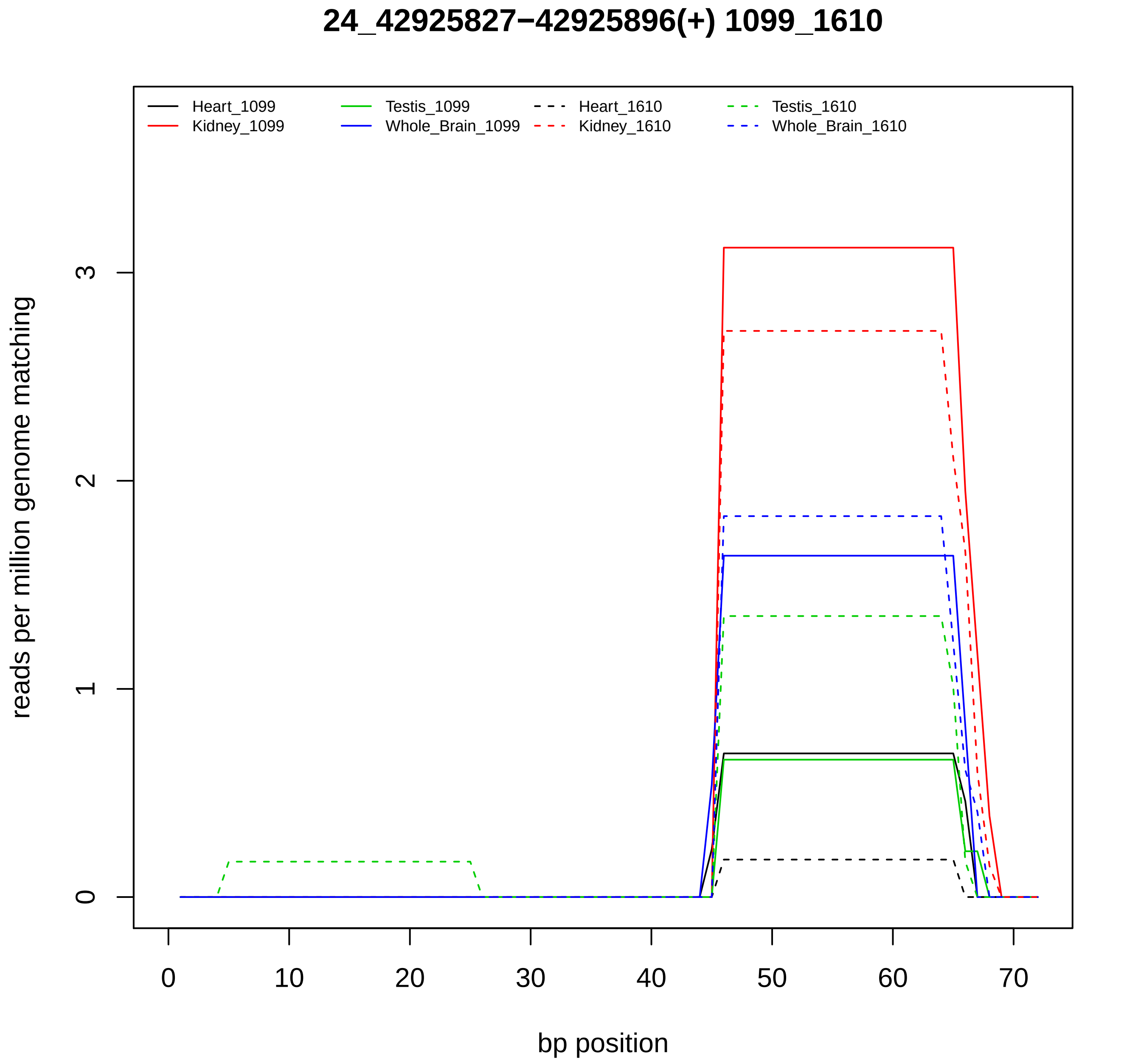

24_42925827−42925896(+) 1099_1610
Heart_1099
Kidney_1099
Testis_1099
Whole_Brain_1099
Heart_1610
Kidney_1610
Testis_1610
Whole_Brain_1610
reads per million genome matching
0
1
2
3
0
10
20
30
40
50
60
70
bp position

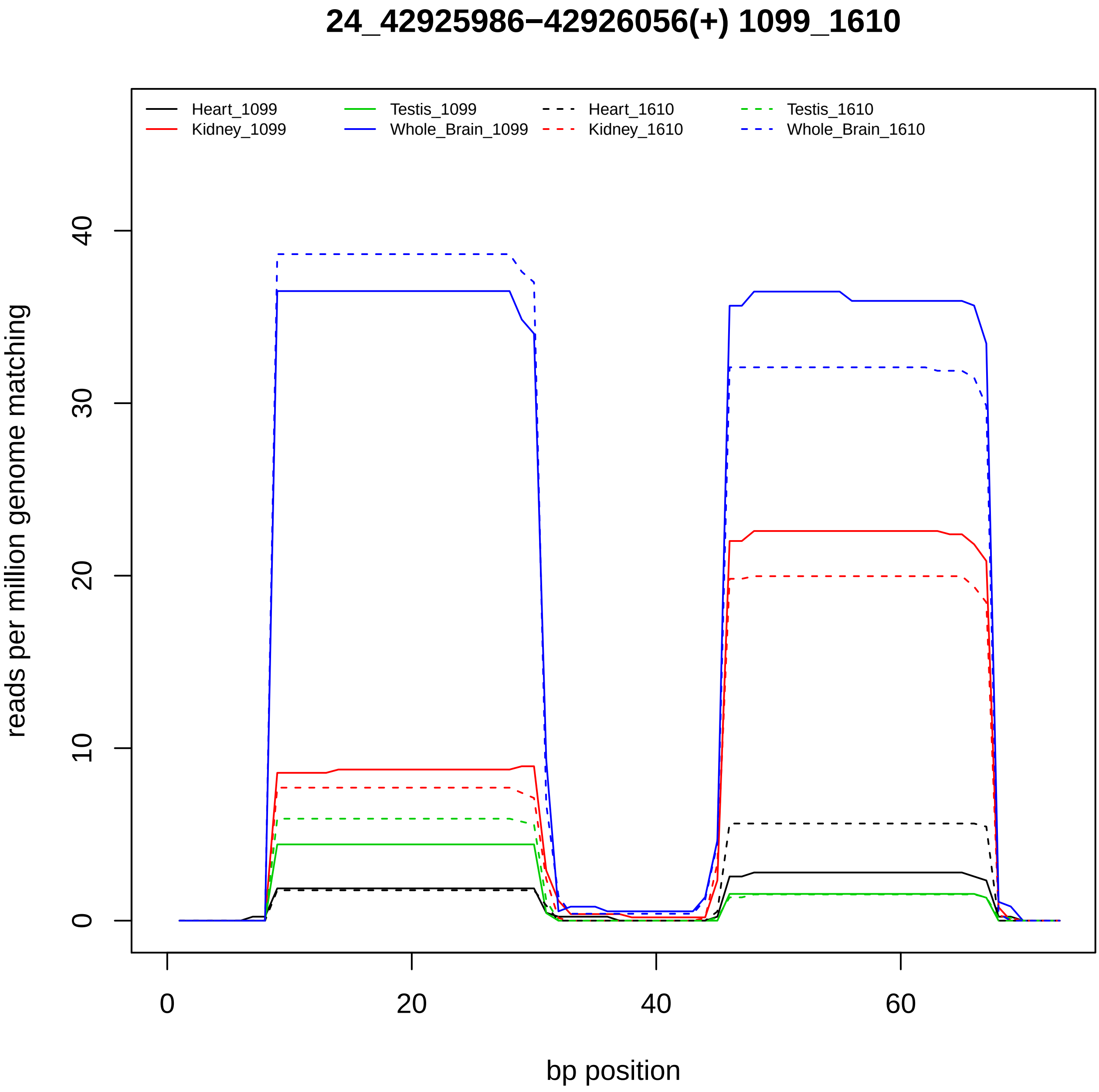

24_42925986−42926056(+) 1099_1610
Heart_1099
Kidney_1099
Testis_1099
Whole_Brain_1099
Heart_1610
Kidney_1610
Testis_1610
Whole_Brain_1610
reads per million genome matching
bp position
0
10
20
30
40
0
20
40
60

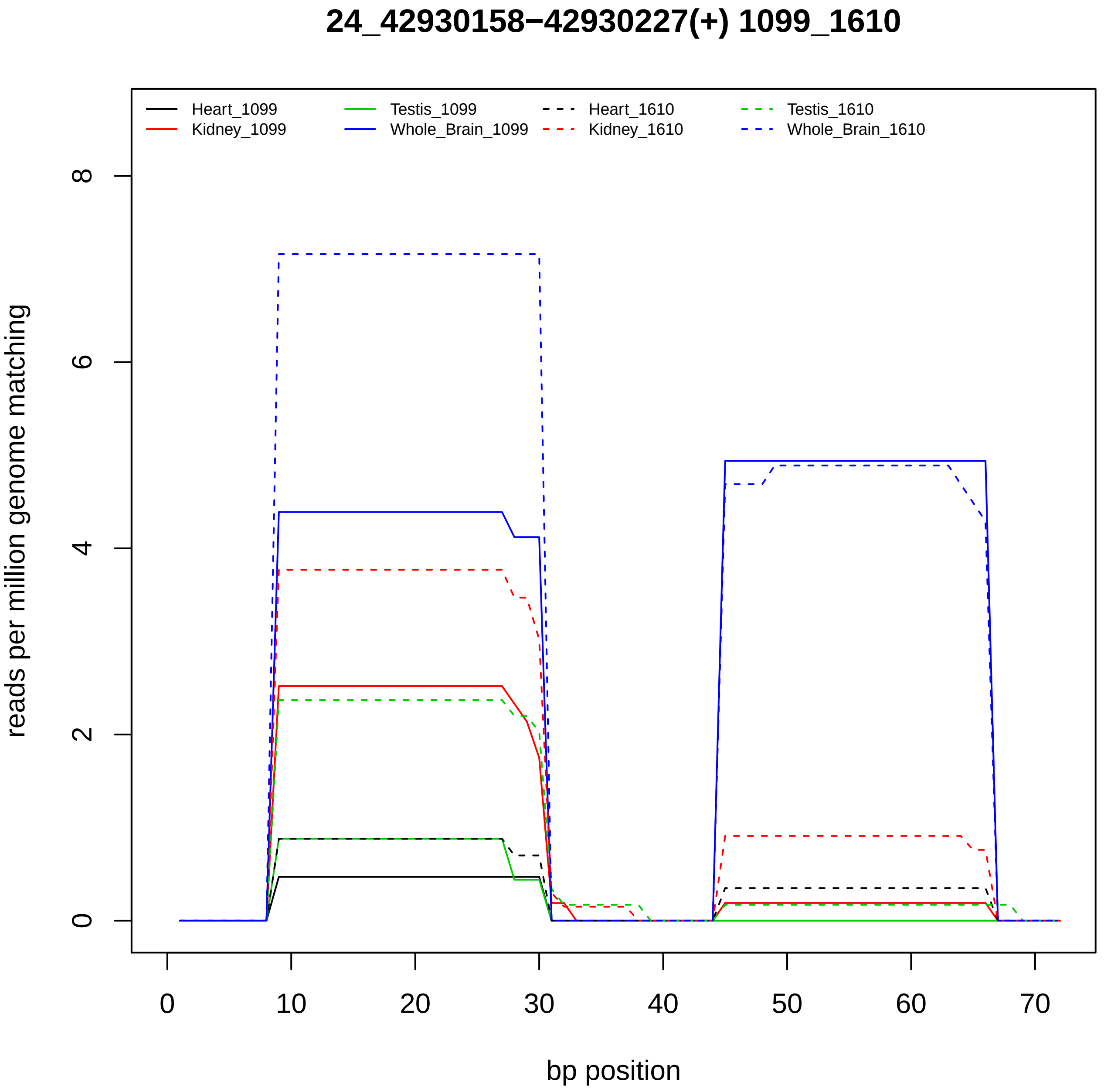

24_42930158−42930227(+) 1099_1610
Heart_1099
Kidney_1099
Testis_1099
Whole_Brain_1099
Heart_1610
Kidney_1610
Testis_1610
Whole_Brain_1610
reads per million genome matching
0
2
4
6
8
0
10
20
30
40
50
60
70
bp position

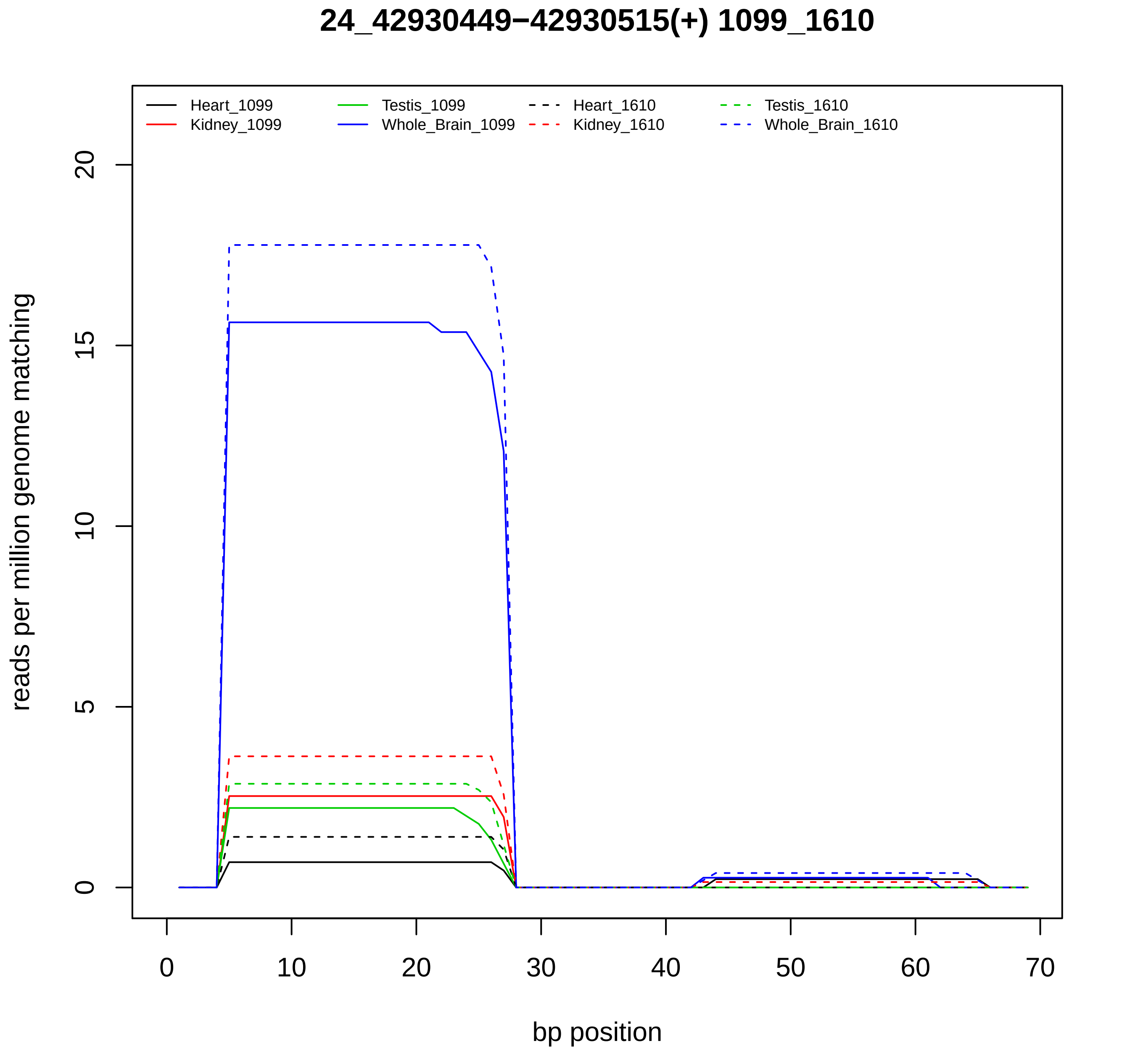
24_42930449−42930515(+) 1099_1610
Heart_1099
Kidney_1099
Testis_1099
Whole_Brain_1099
Heart_1610
Kidney_1610
Testis_1610
Whole_Brain_1610
reads per million genome matching
0
5
10
15
20
0
10
20
30
40
50
60
70
bp position

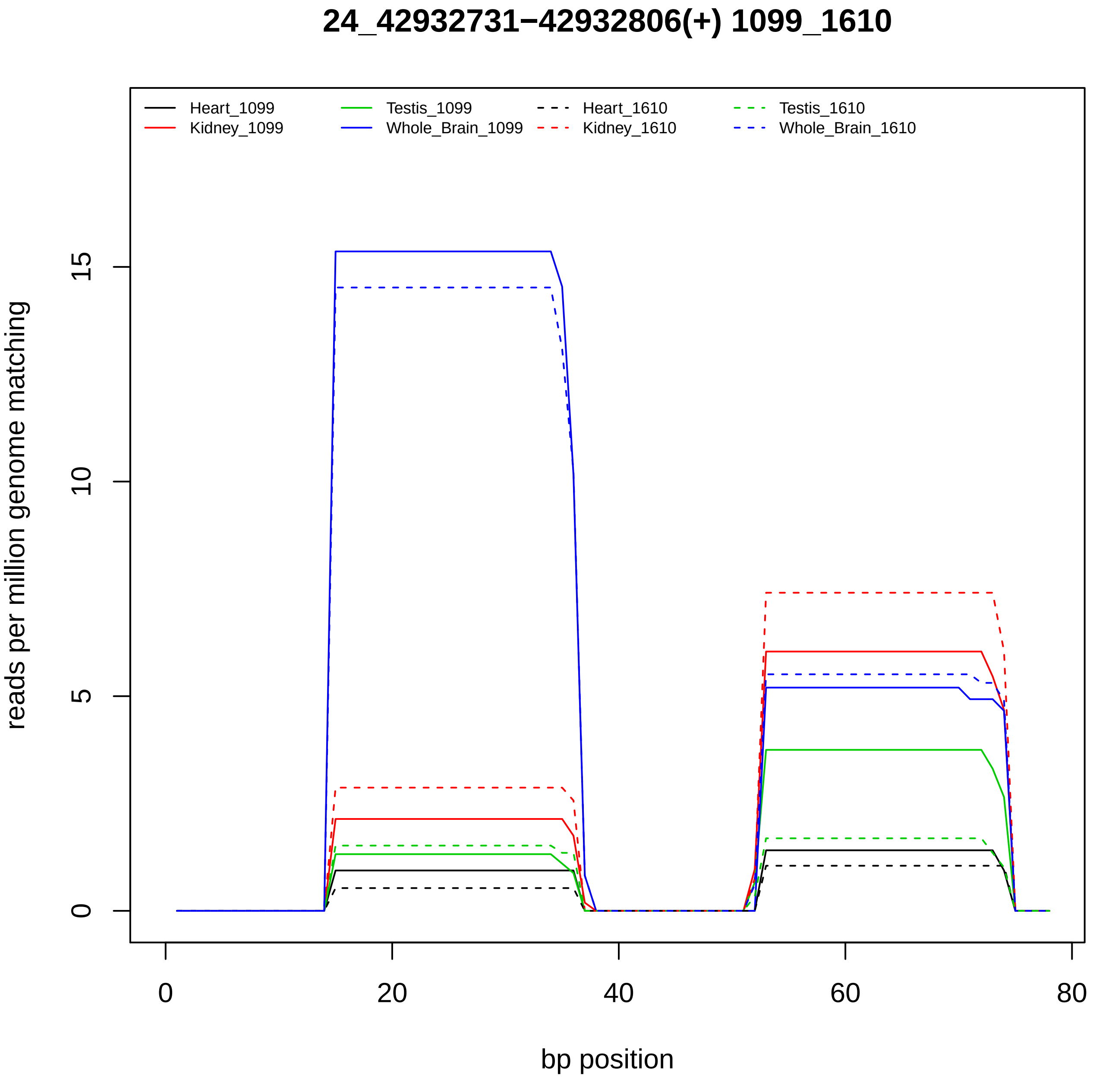

24_42932731−42932806(+) 1099_1610
Heart_1099
Kidney_1099
Testis_1099
Whole_Brain_1099
Heart_1610
Kidney_1610
Testis_1610
Whole_Brain_1610
reads per million genome matching
0
5
10
15
0
20
40
60
80
bp position

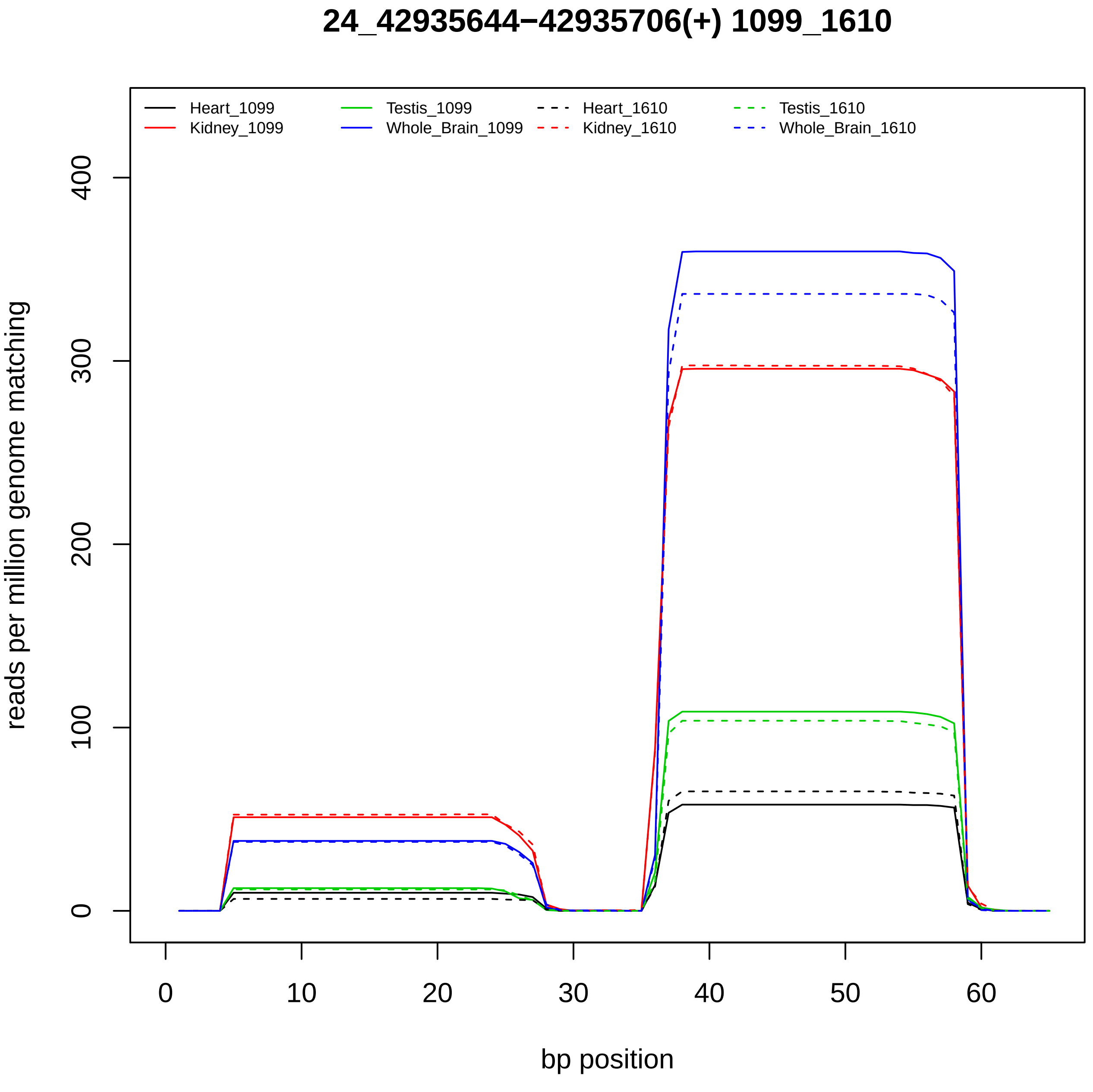

24_42935644−42935706(+) 1099_1610
Heart_1099
Kidney_1099
Testis_1099
Whole_Brain_1099
Heart_1610
Kidney_1610
Testis_1610
Whole_Brain_1610
reads per million genome matching
0
100
200
300
400
bp position
0
10
20
30
40
50
60

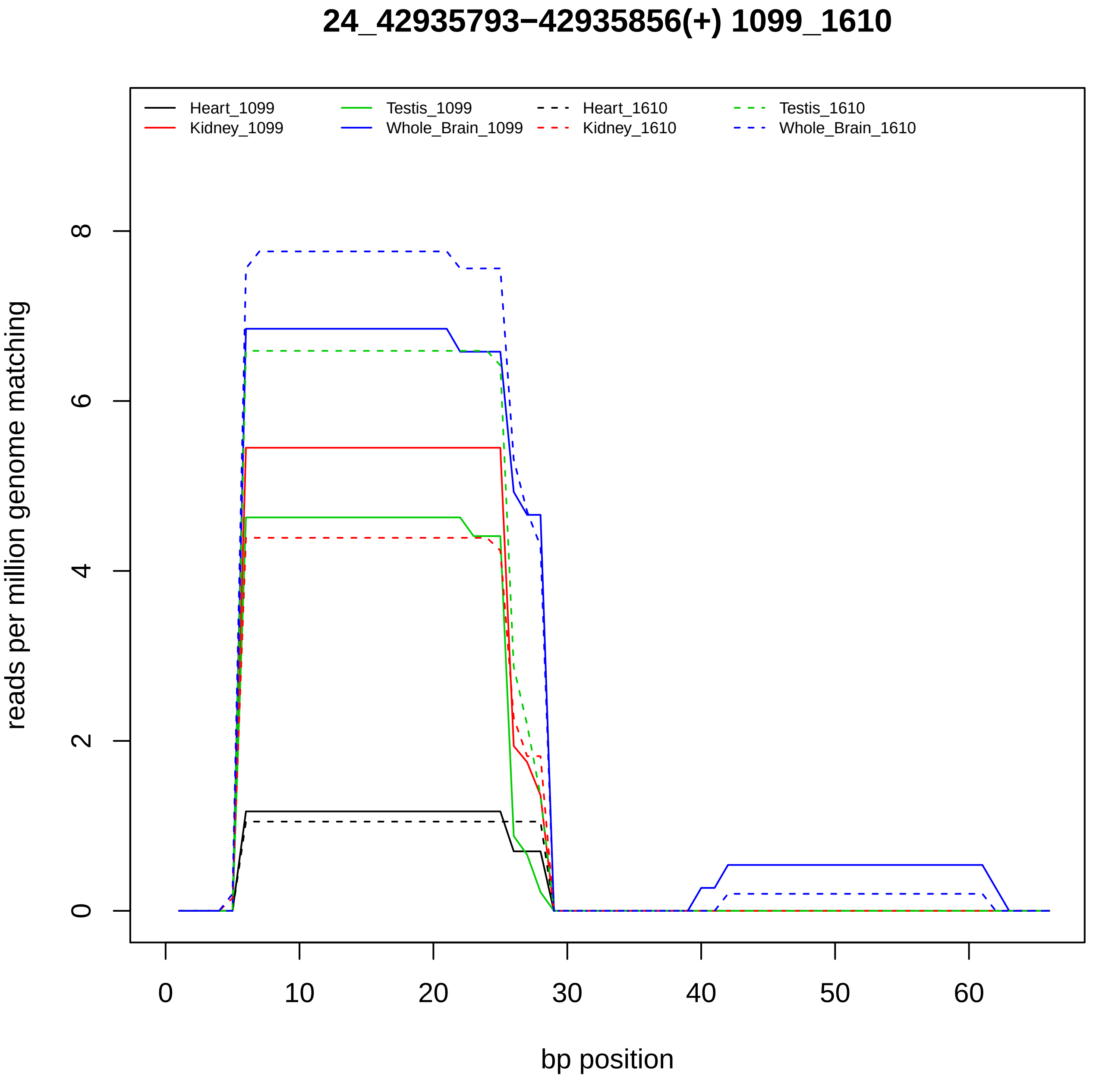

24_42935793−42935856(+) 1099_1610
Heart_1099
Kidney_1099
Testis_1099
Whole_Brain_1099
Heart_1610
Kidney_1610
Testis_1610
Whole_Brain_1610
reads per million genome matching
0
2
4
6
8
0
10
20
30
40
50
60
bp position

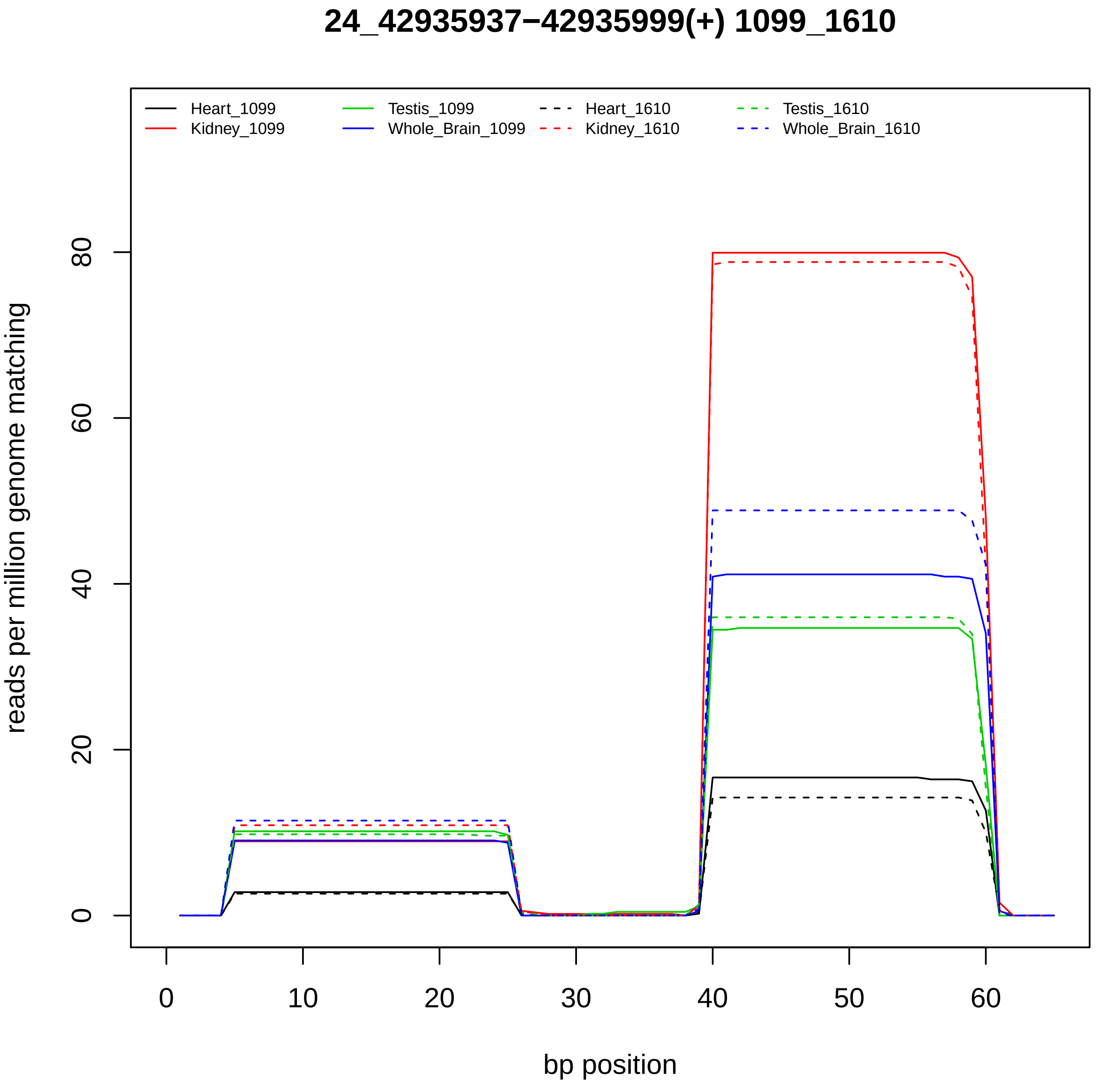

24_42935937−42935999(+) 1099_1610
Heart_1099
Kidney_1099
Testis_1099
Whole_Brain_1099
Heart_1610
Kidney_1610
Testis_1610
Whole_Brain_1610
reads per million genome matching
0
20
40
60
80
0
10
20
30
40
50
60
bp position

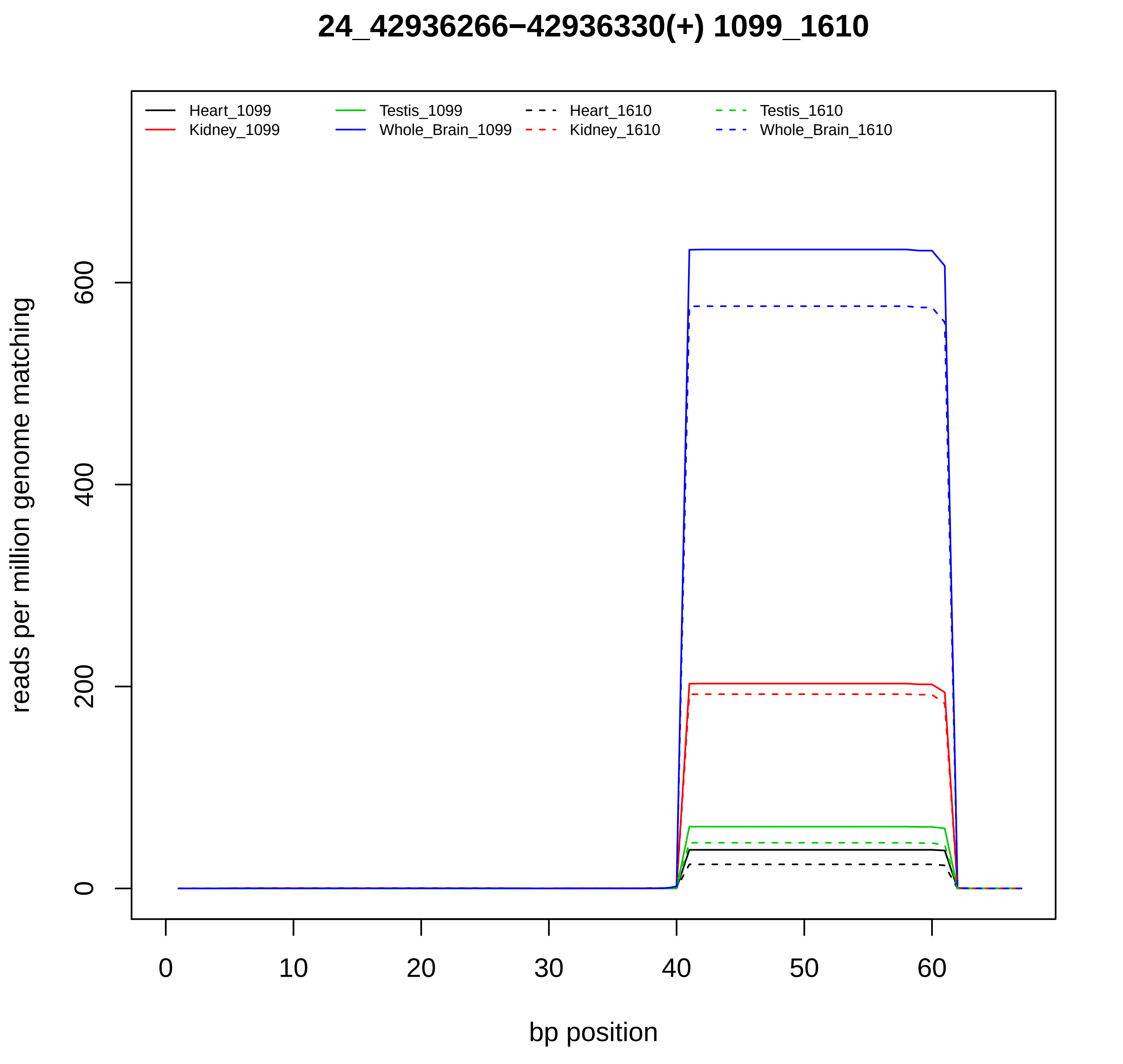
24_42936266−42936330(+) 1099_1610
Heart_1099
Kidney_1099
Testis_1099
Whole_Brain_1099
Heart_1610
Kidney_1610
Testis_1610
Whole_Brain_1610
reads per million genome matching
0
200
400
600
0
10
20
30
40
50
60
bp position

24_42937013−42937076(+) 1099_1610

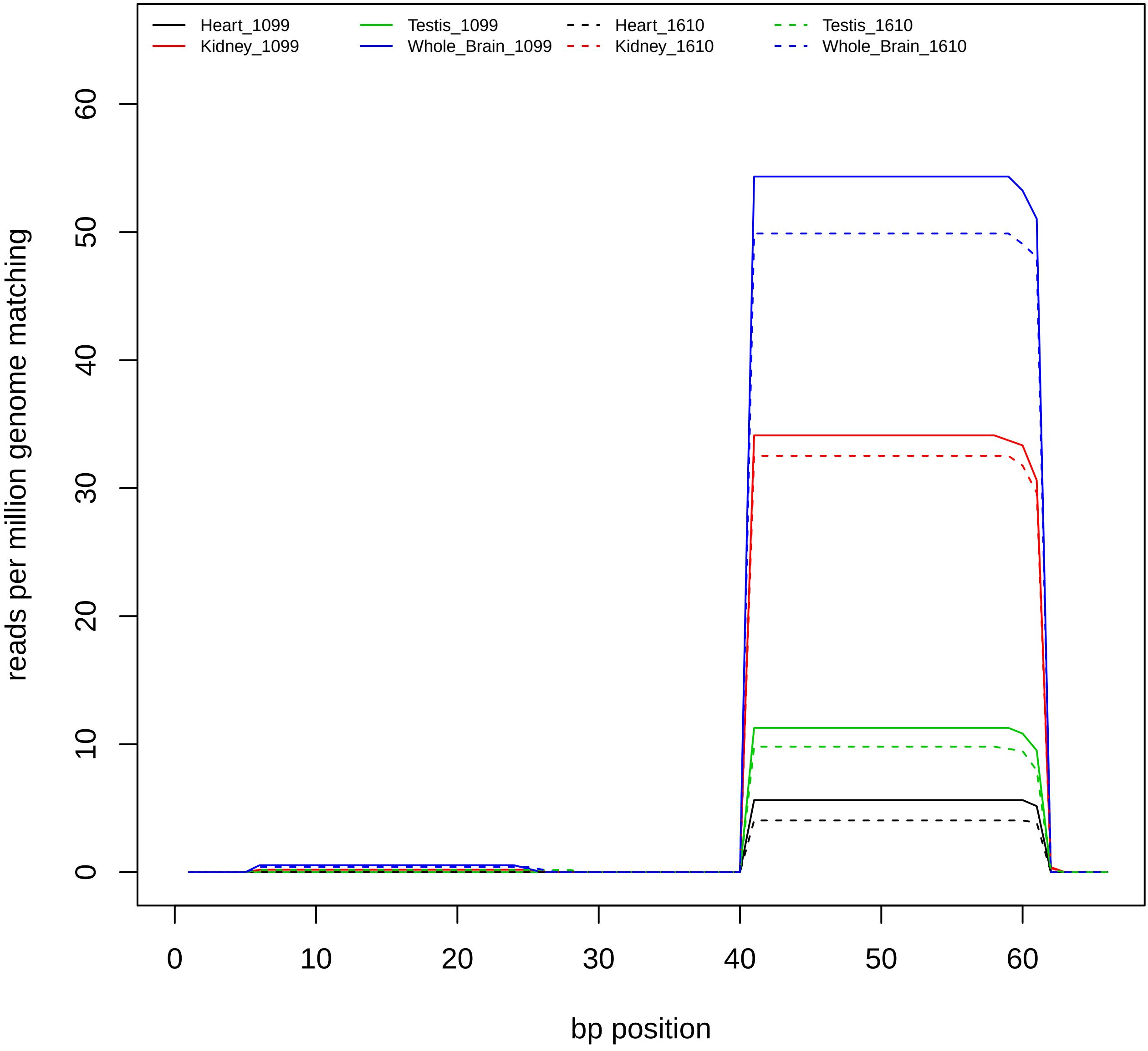

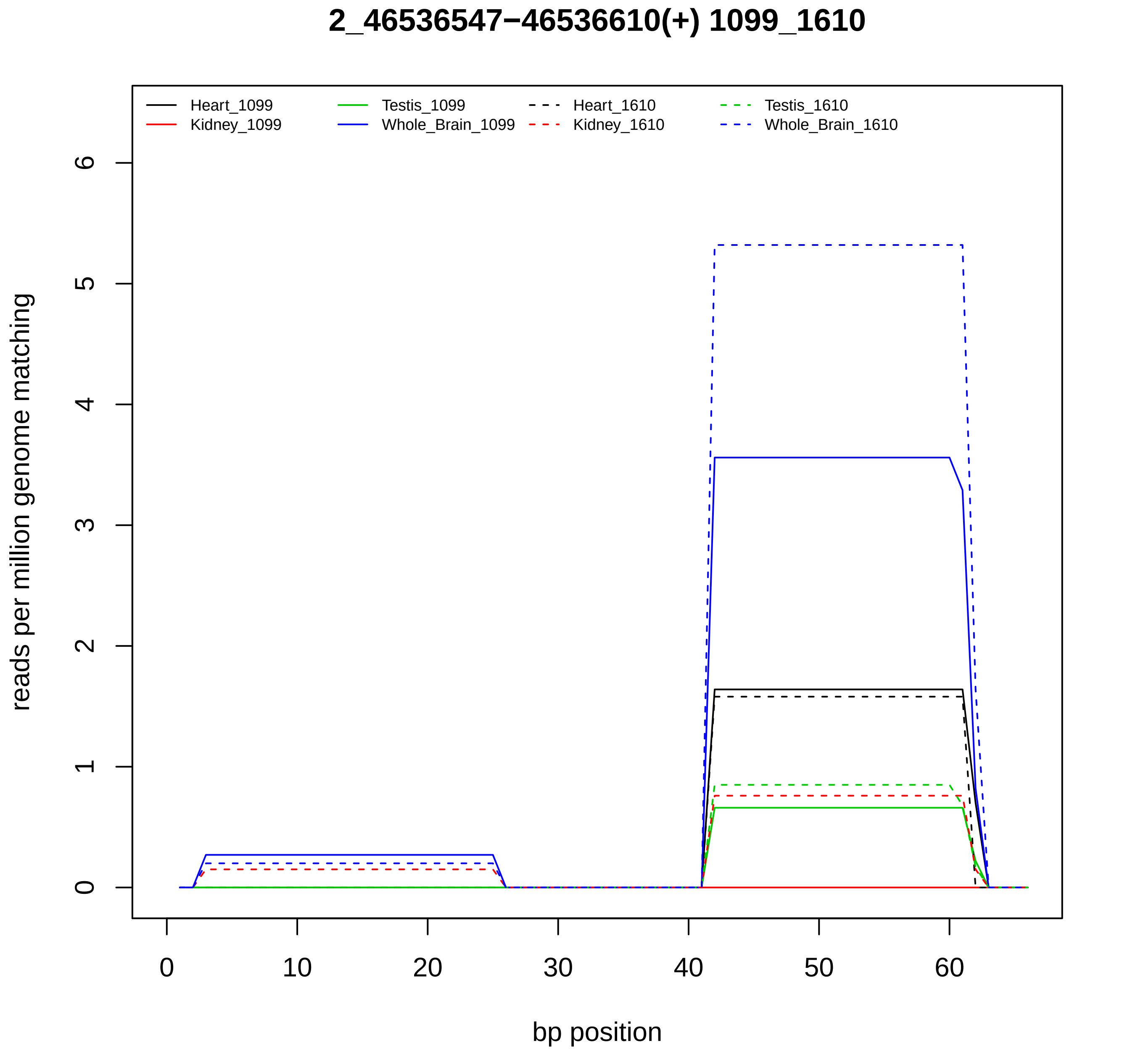

2_46536547−46536610(+) 1099_1610
Heart_1099
Kidney_1099
Testis_1099
Whole_Brain_1099
Heart_1610
Kidney_1610
Testis_1610
Whole_Brain_1610
reads per million genome matching
bp position
0
1
2
3
4
5
6
0
10
20
30
40
50
60

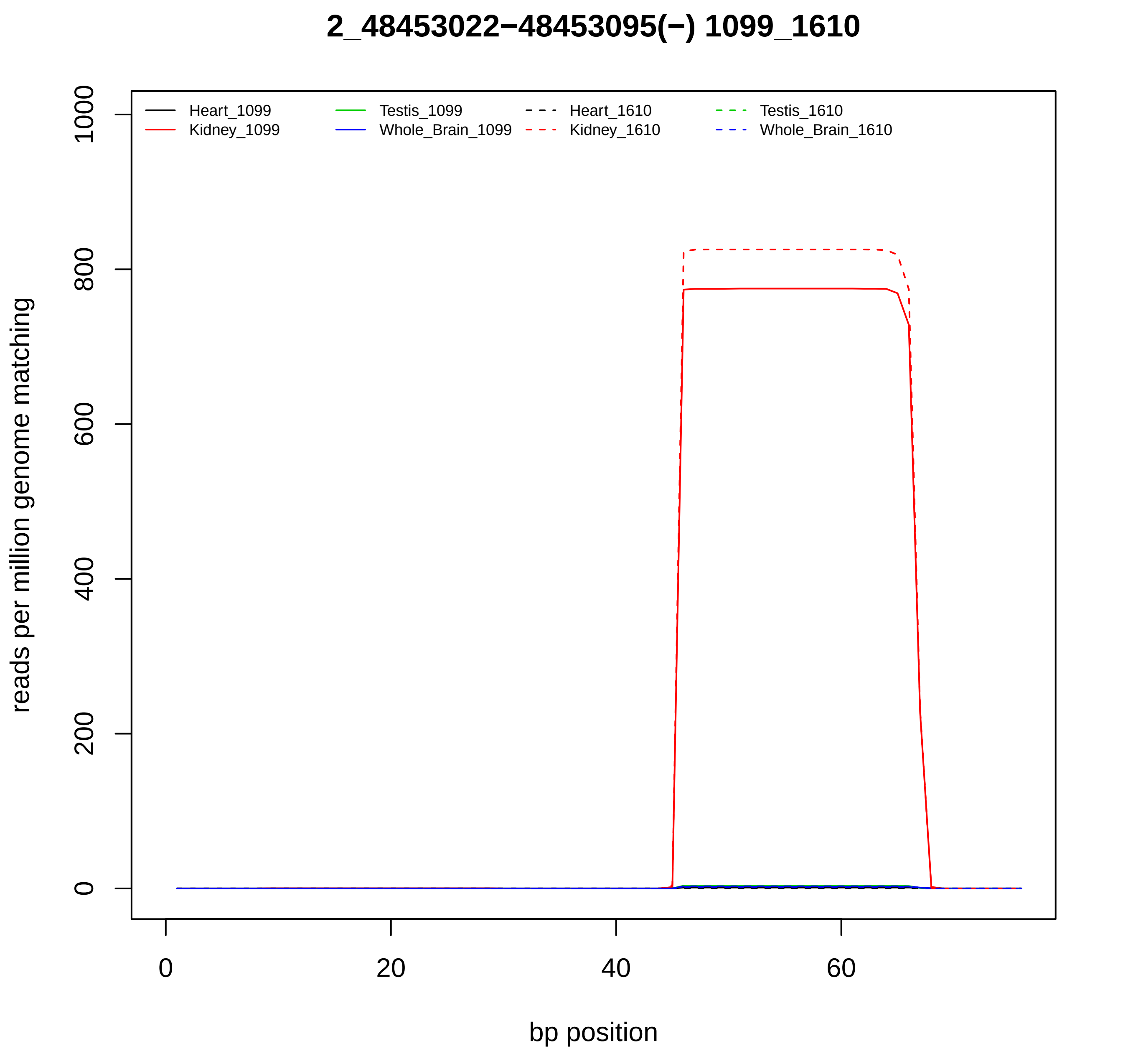

2_48453022−48453095(−) 1099_1610
Heart_1099
Kidney_1099
Testis_1099
Whole_Brain_1099
Heart_1610
Kidney_1610
Testis_1610
Whole_Brain_1610
reads per million genome matching
bp position
0
200
400
600
800
1000
0
20
40
60

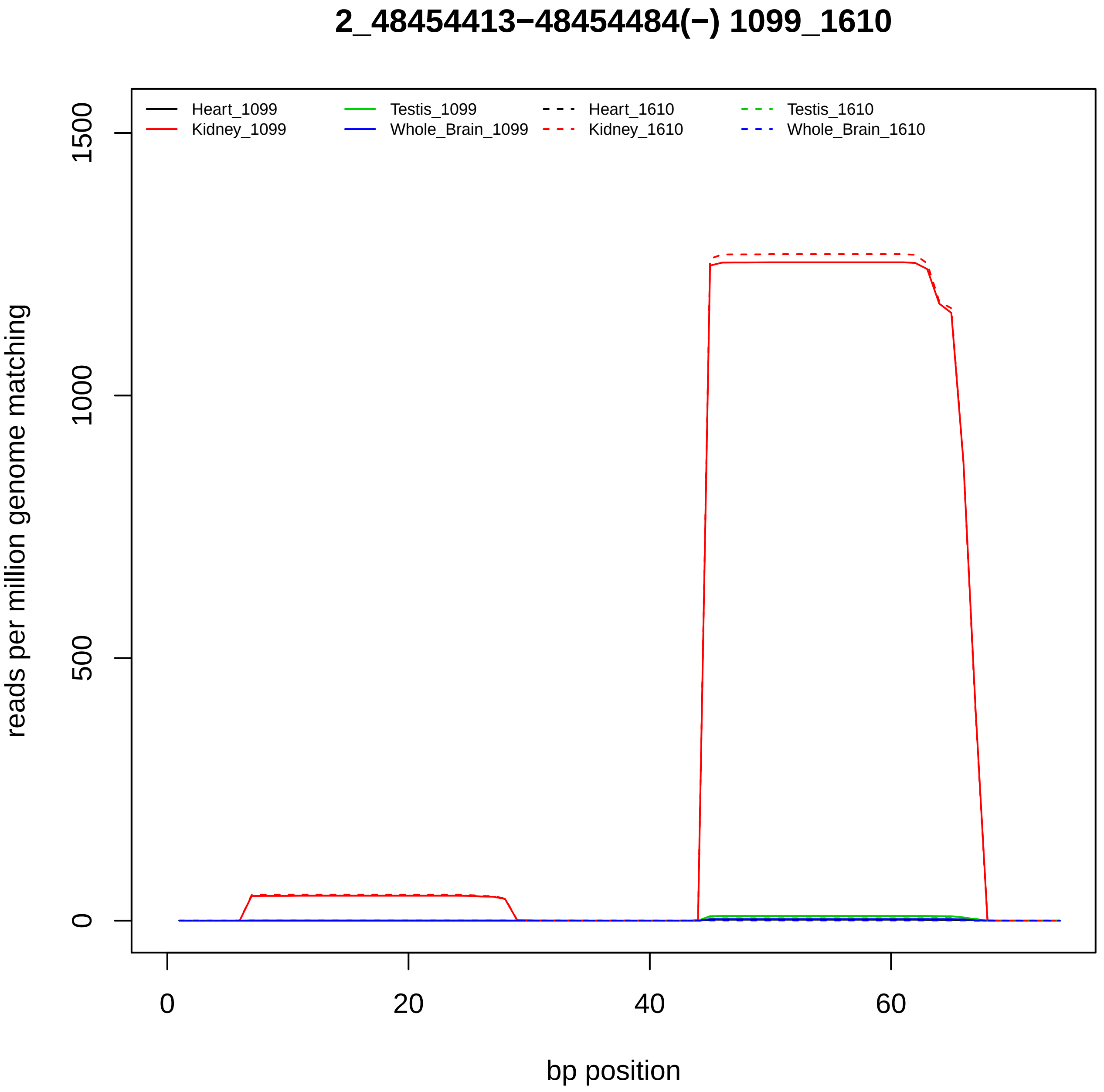
2_48454413−48454484(−) 1099_1610
Heart_1099
Kidney_1099
Testis_1099
Whole_Brain_1099
Heart_1610
Kidney_1610
Testis_1610
Whole_Brain_1610
reads per million genome matching
0
500
1000
1500
0
20
40
60
bp position

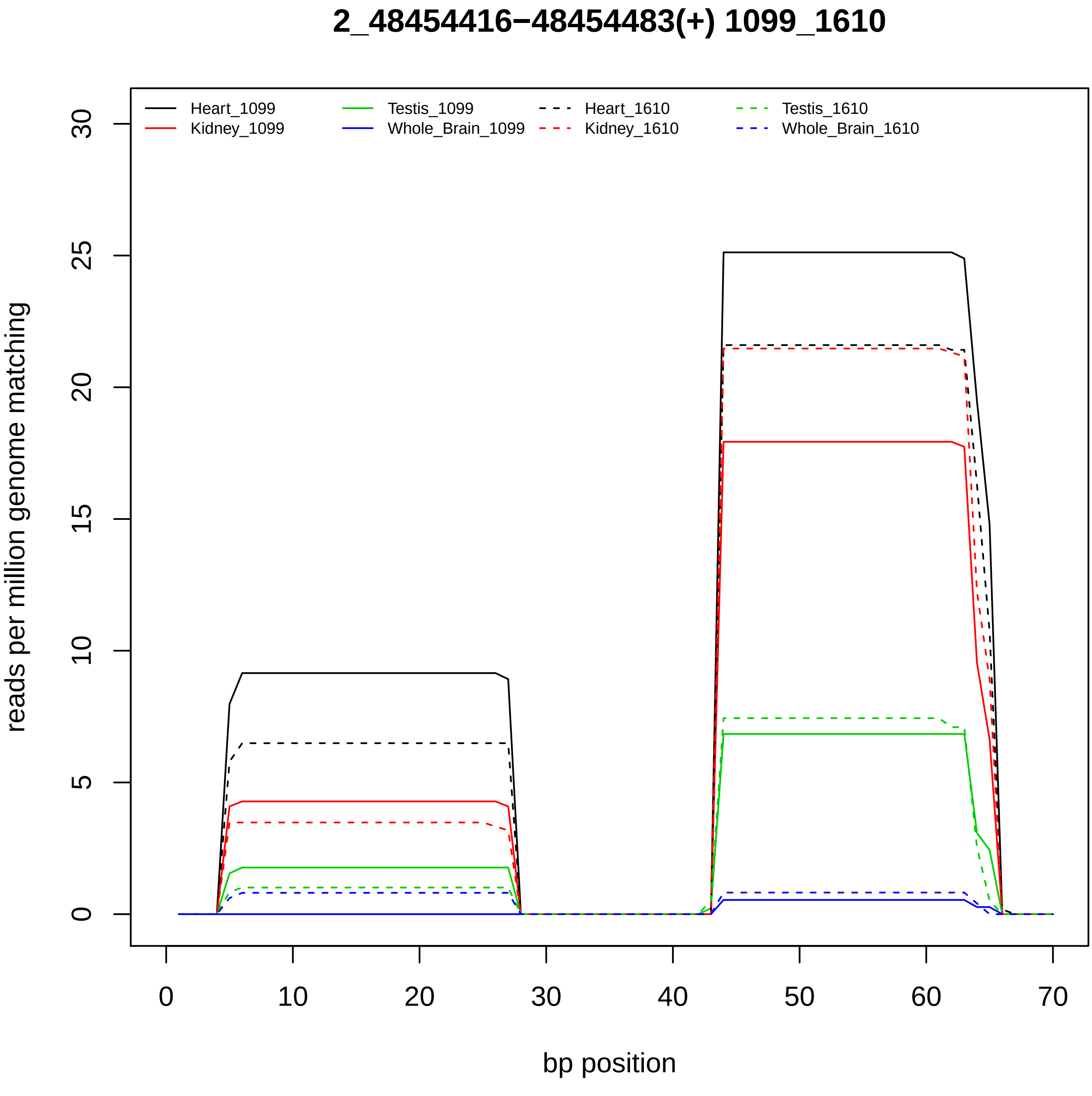

2_48454416−48454483(+) 1099_1610
Heart_1099
Kidney_1099
Testis_1099
Whole_Brain_1099
Heart_1610
Kidney_1610
Testis_1610
Whole_Brain_1610
reads per million genome matching
0
5
10
15
20
25
30
bp position
0
10
20
30
40
50
60
70

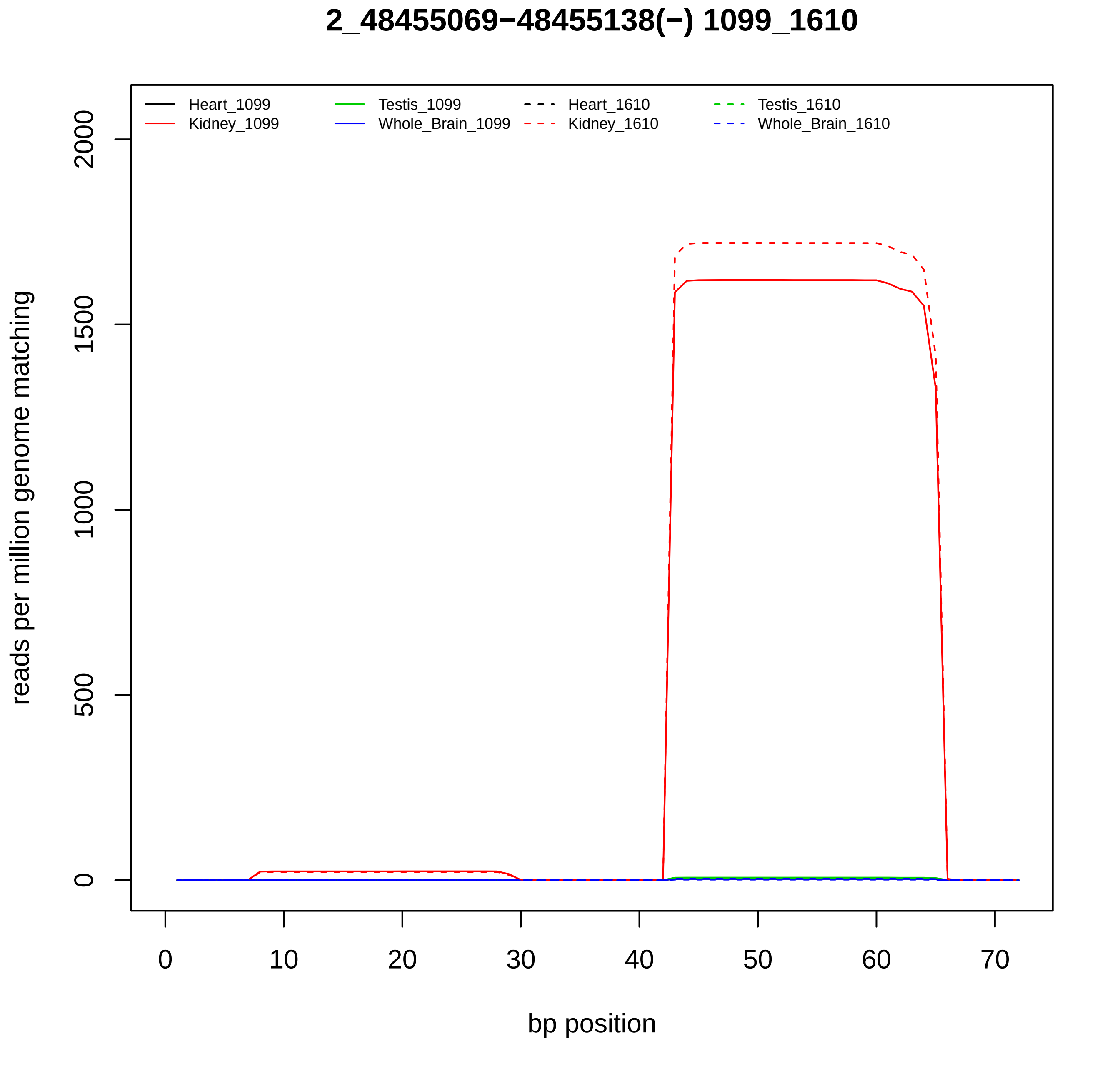
2_48455069−48455138(−) 1099_1610
Heart_1099
Kidney_1099
Testis_1099
Whole_Brain_1099
Heart_1610
Kidney_1610
Testis_1610
Whole_Brain_1610
reads per million genome matching
0
500
1000
1500
2000
bp position
0
10
20
30
40
50
60
70

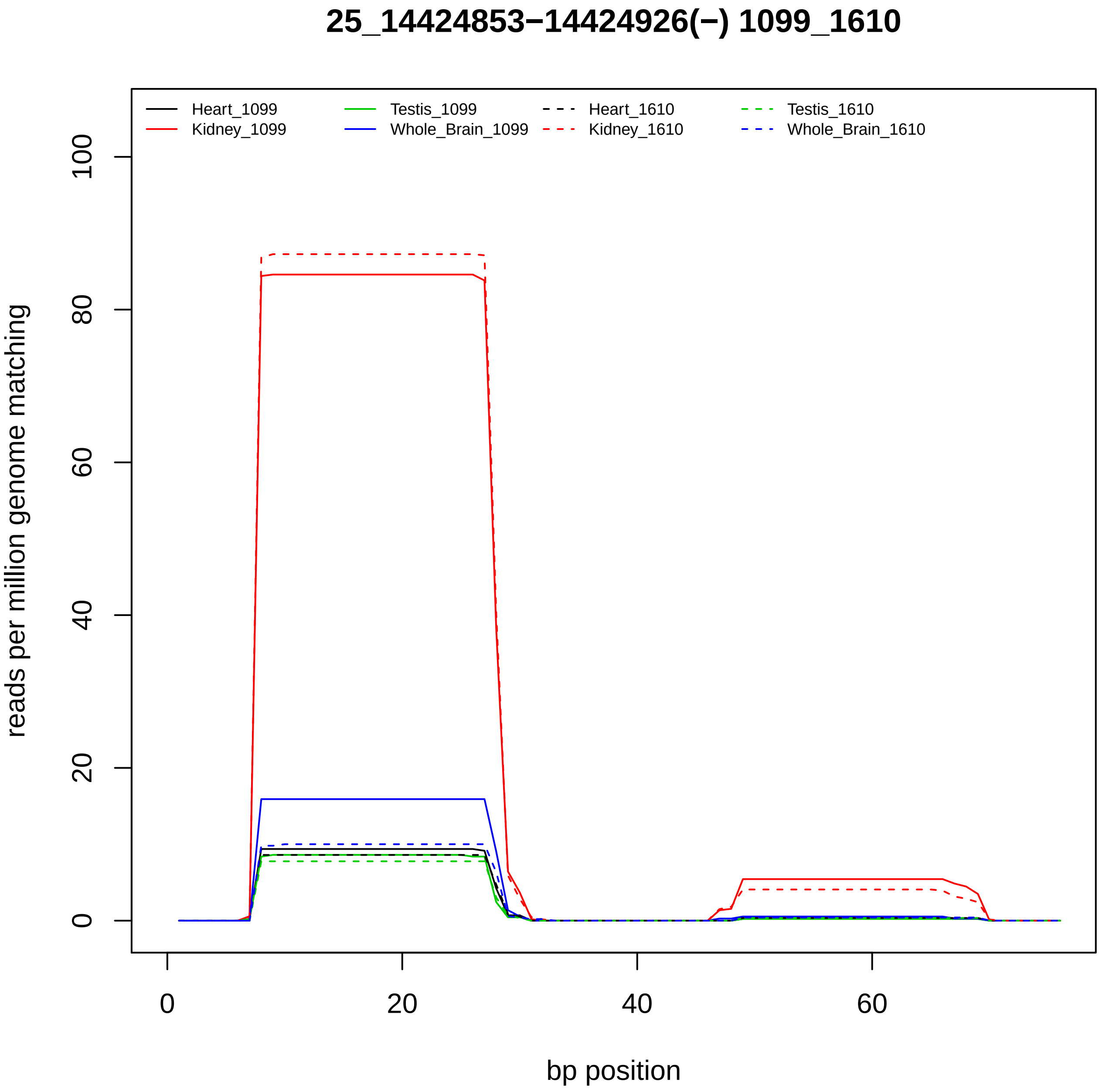

25_14424853−14424926(−) 1099_1610
Heart_1099
Kidney_1099
Testis_1099
Whole_Brain_1099
Heart_1610
Kidney_1610
Testis_1610
Whole_Brain_1610
reads per million genome matching
0
20
40
60
80
100
0
20
40
60
bp position

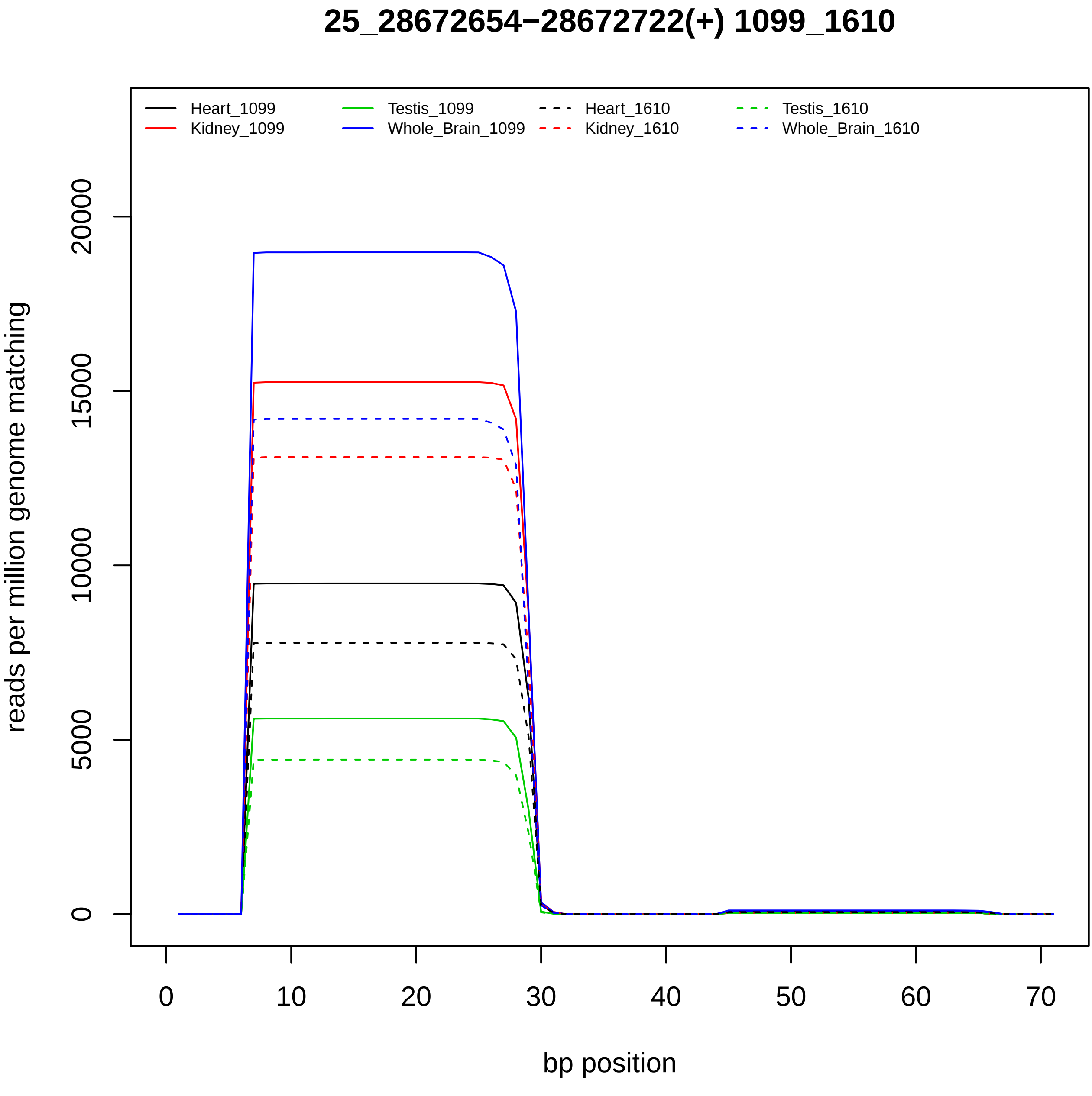
25_28672654−28672722(+) 1099_1610
Heart_1099
Kidney_1099
Testis_1099
Whole_Brain_1099
Heart_1610
Kidney_1610
Testis_1610
Whole_Brain_1610
reads per million genome matching
0
5000
10000
15000
20000
0
10
20
30
40
50
60
70
bp position

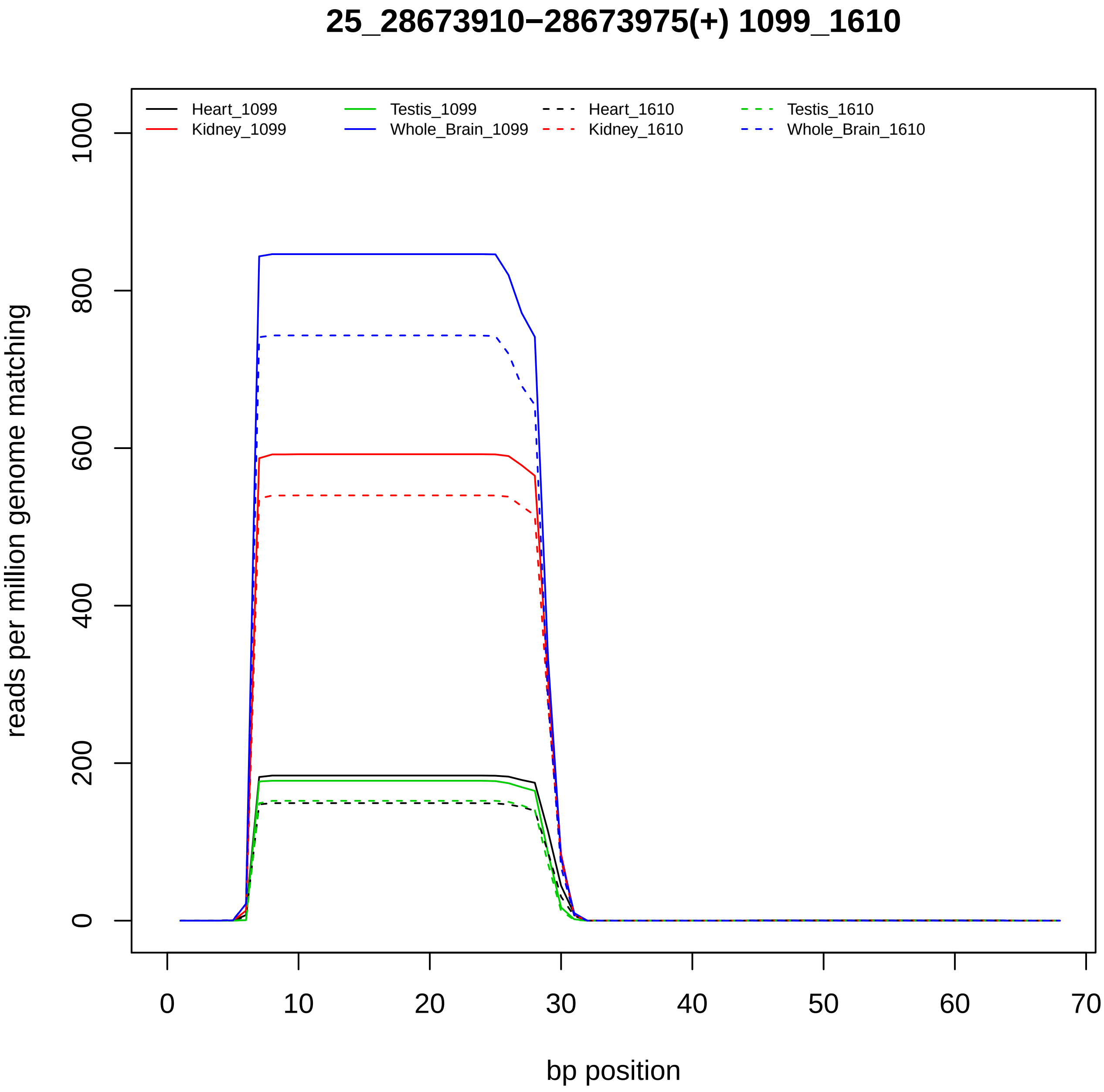
25_28673910−28673975(+) 1099_1610
Heart_1099
Kidney_1099
Testis_1099
Whole_Brain_1099
Heart_1610
Kidney_1610
Testis_1610
Whole_Brain_1610
reads per million genome matching
bp position
0
200
400
600
800
1000
0
10
20
30
40
50
60
70

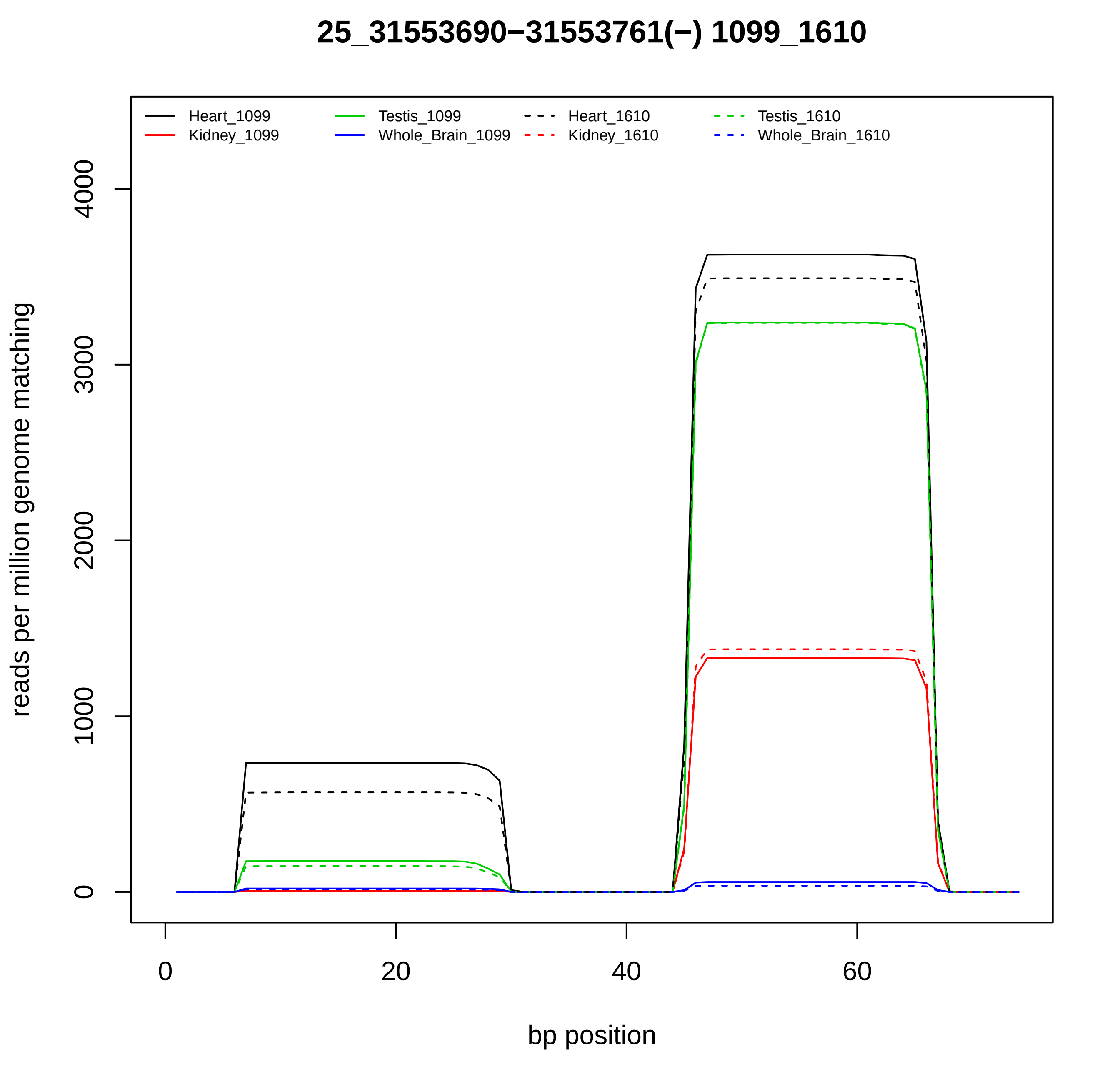

25_31553690−31553761(−) 1099_1610
Heart_1099
Kidney_1099
Testis_1099
Whole_Brain_1099
Heart_1610
Kidney_1610
Testis_1610
Whole_Brain_1610
reads per million genome matching
0
1000
2000
3000
4000
0
20
40
60
bp position

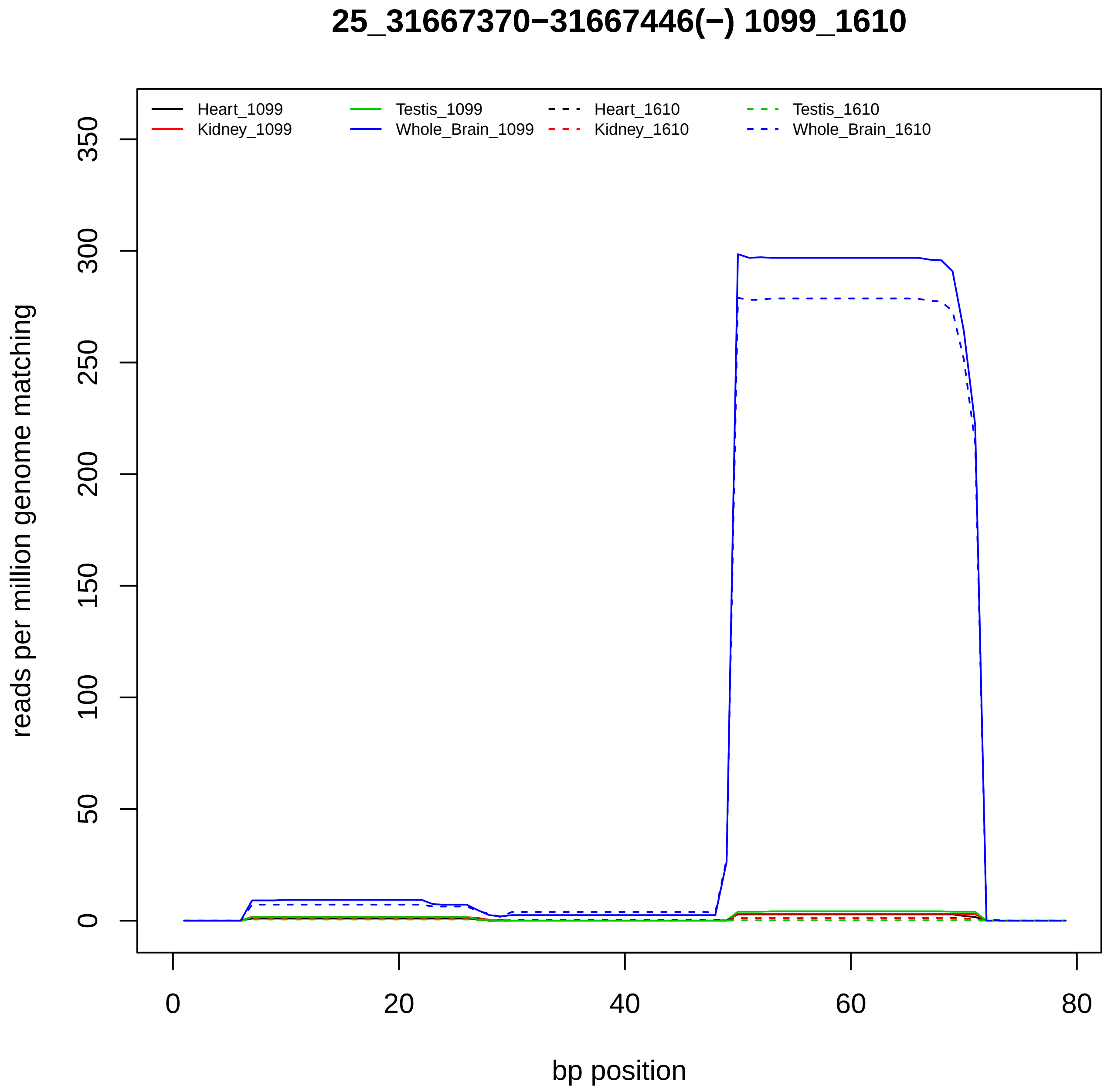

25_31667370−31667446(−) 1099_1610
Heart_1099
Kidney_1099
Testis_1099
Whole_Brain_1099
Heart_1610
Kidney_1610
Testis_1610
Whole_Brain_1610
reads per million genome matching
bp position
0
50
100
150
200
250
300
350
0
20
40
60
80

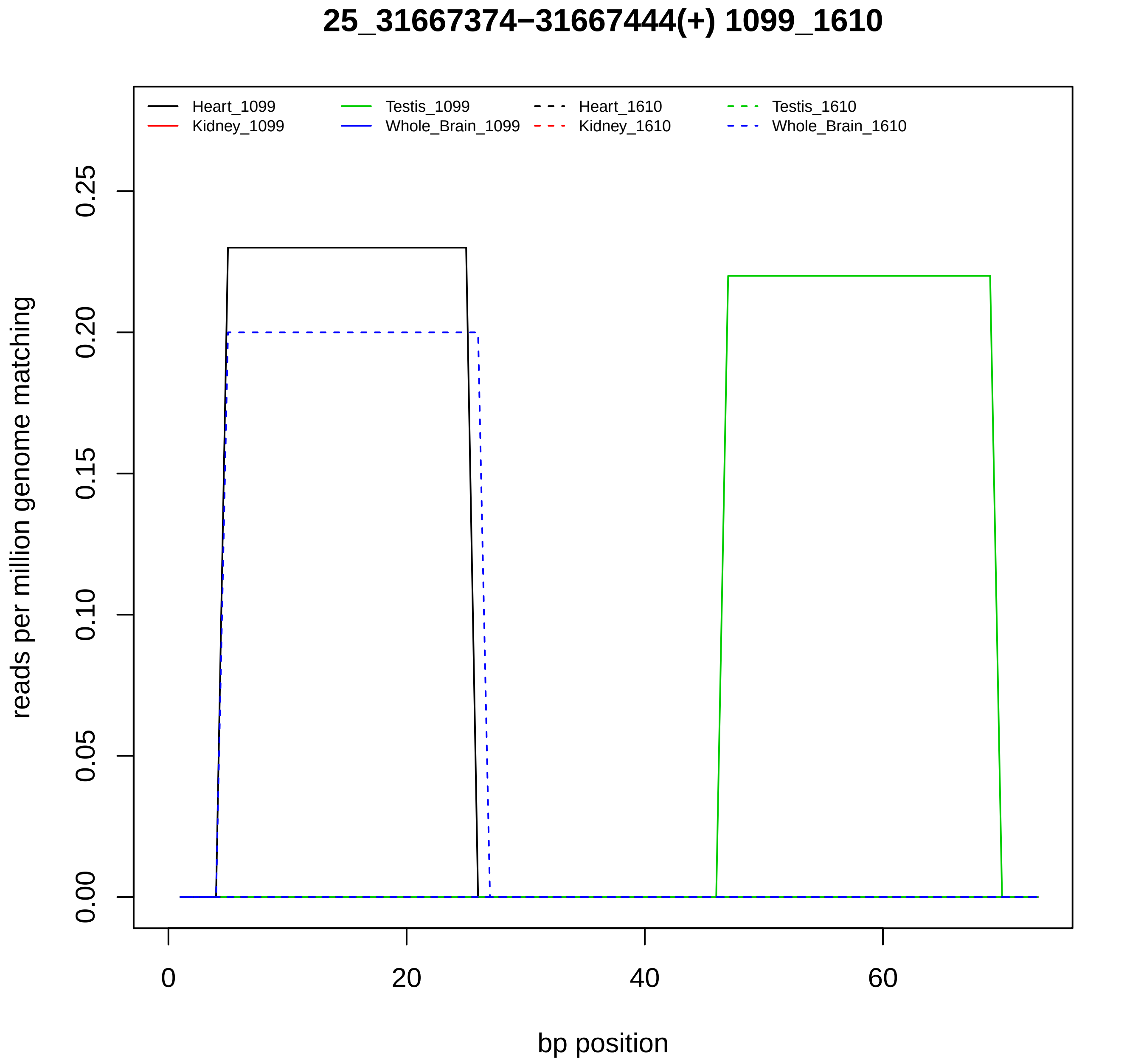
25_31667374−31667444(+) 1099_1610
Heart_1099
Kidney_1099
Testis_1099
Whole_Brain_1099
Heart_1610
Kidney_1610
Testis_1610
Whole_Brain_1610
reads per million genome matching
0.00
0.05
0.10
0.15
0.20
0.25
0
20
40
60
bp position

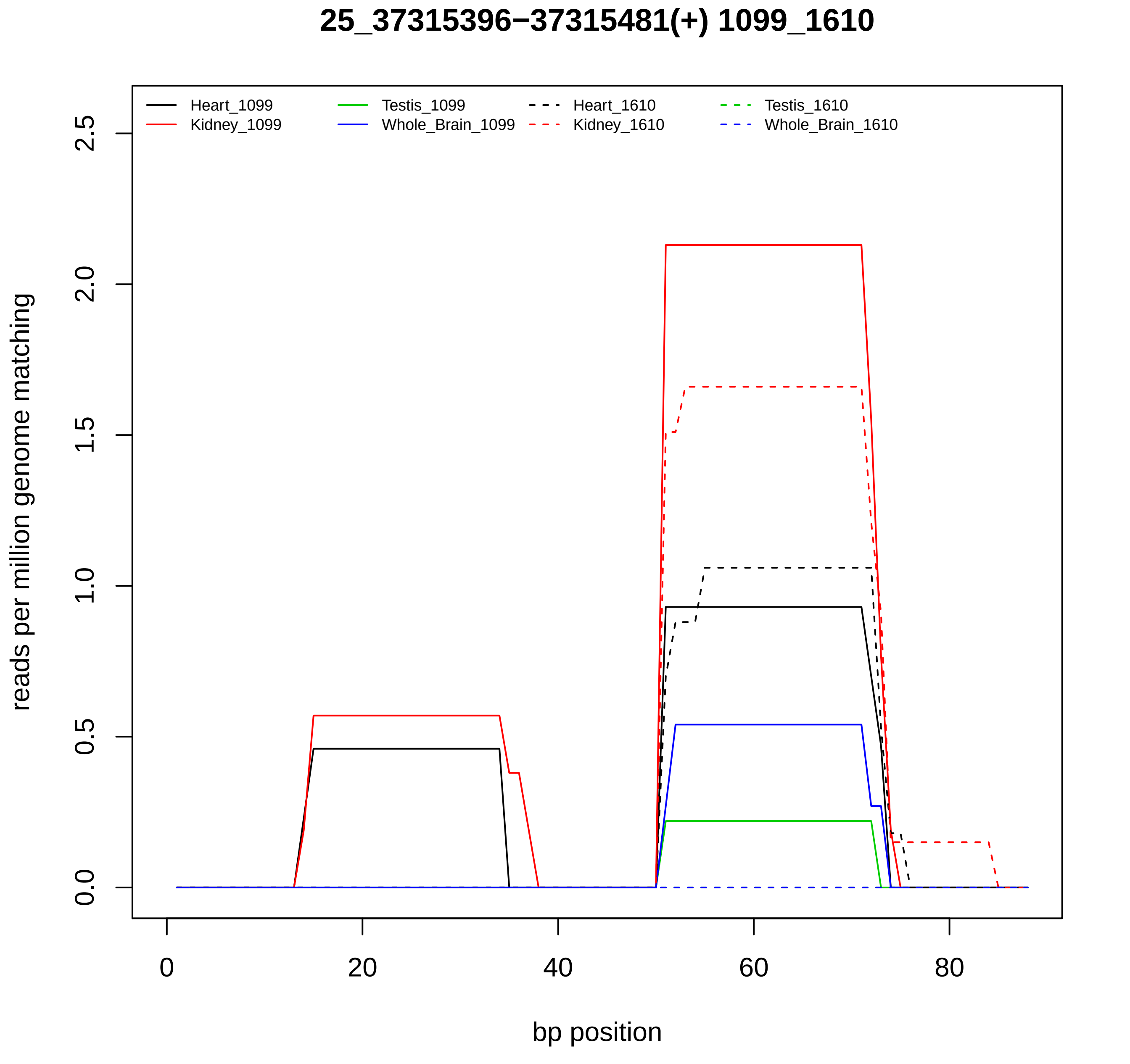
25_37315396−37315481(+) 1099_1610
Heart_1099
Kidney_1099
Testis_1099
Whole_Brain_1099
Heart_1610
Kidney_1610
Testis_1610
Whole_Brain_1610
reads per million genome matching
bp position
0.0
0.5
1.0
1.5
2.0
2.5
0
20
40
60
80

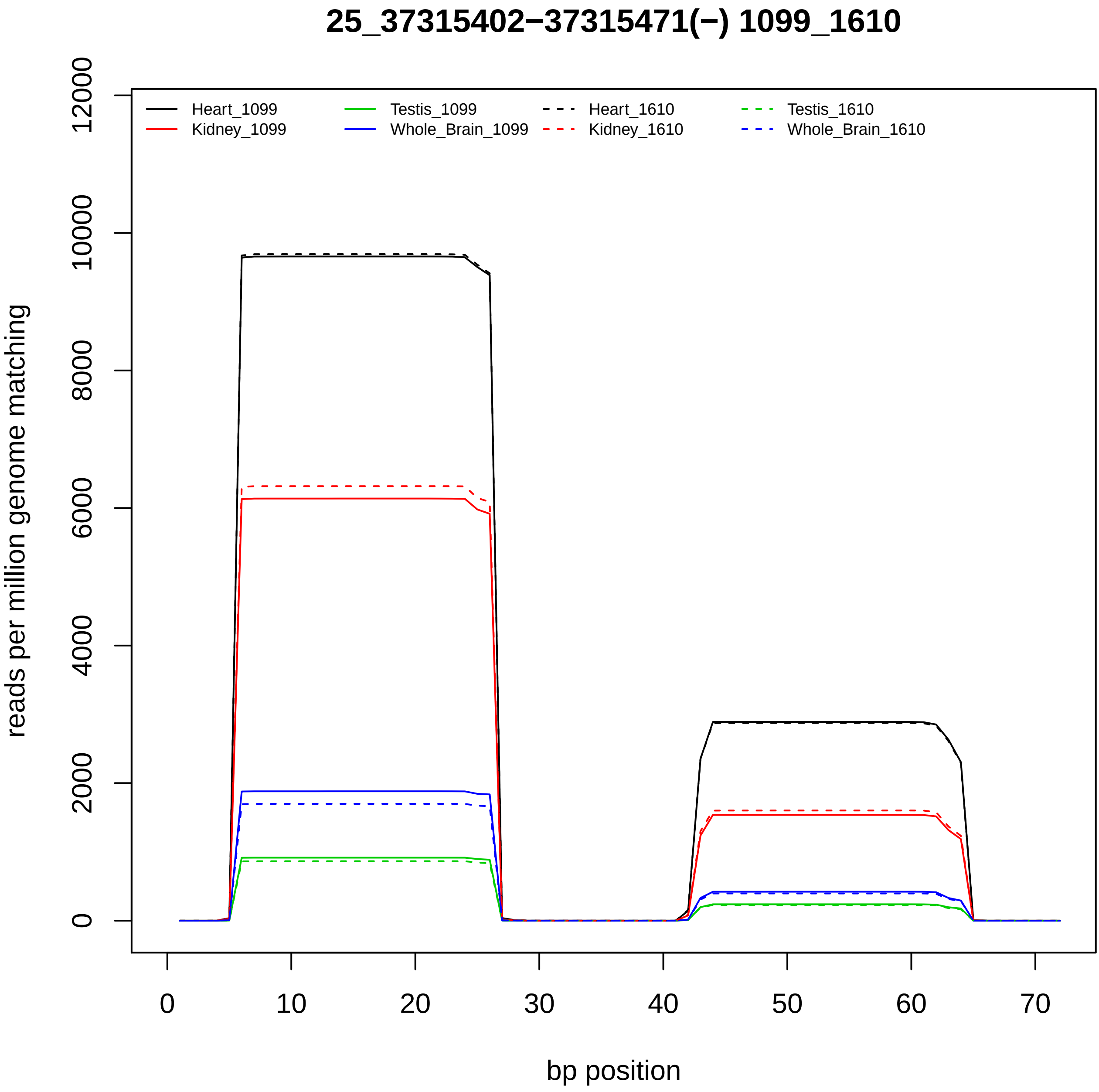

25_37315402−37315471(−) 1099_1610
Heart_1099
Kidney_1099
Testis_1099
Whole_Brain_1099
Heart_1610
Kidney_1610
Testis_1610
Whole_Brain_1610
reads per million genome matching
0
2000
4000
6000
8000
10000
12000
bp position
0
10
20
30
40
50
60
70

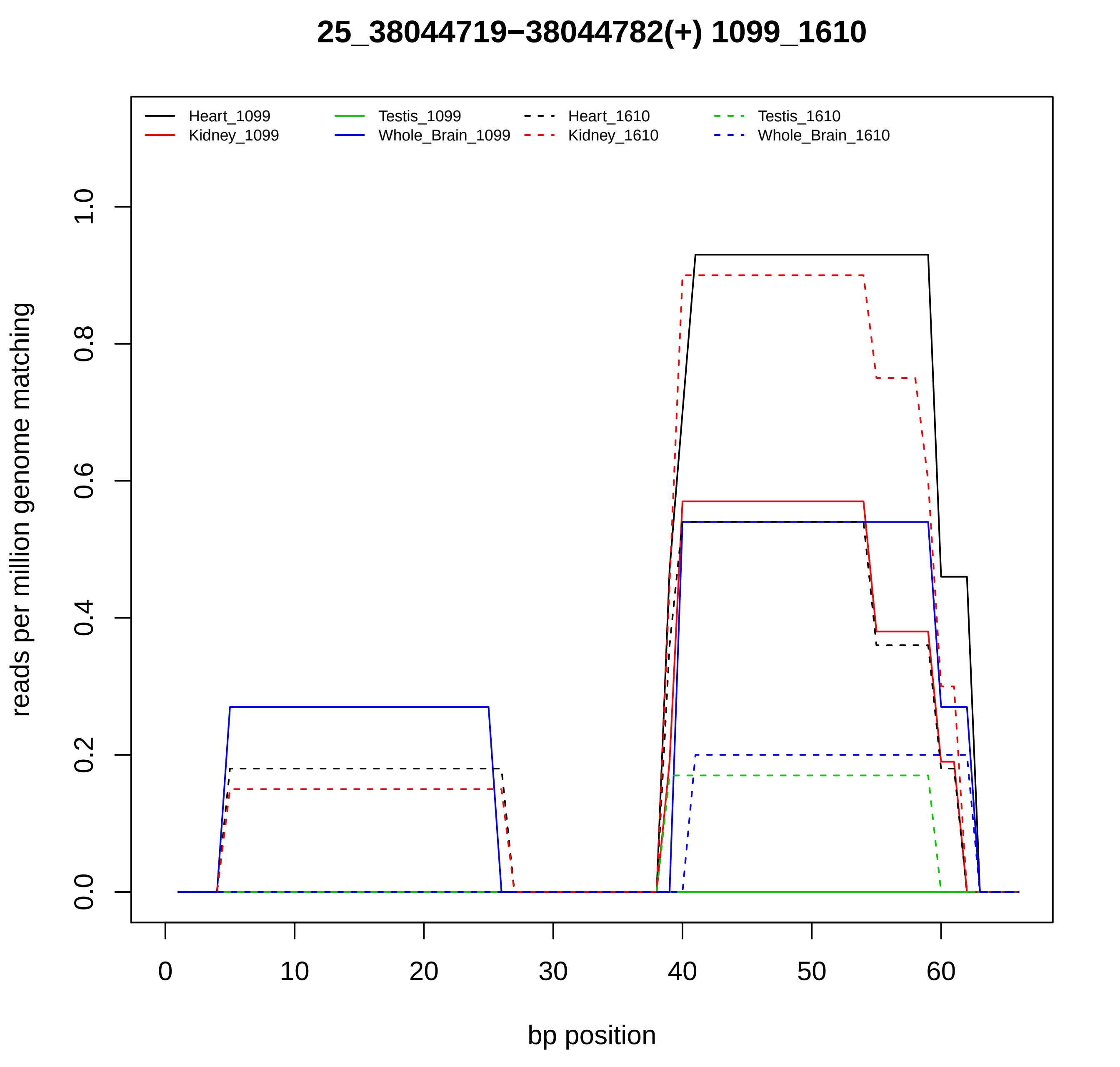

25_38044719−38044782(+) 1099_1610
Heart_1099
Kidney_1099
Testis_1099
Whole_Brain_1099
Heart_1610
Kidney_1610
Testis_1610
Whole_Brain_1610
reads per million genome matching
0.0
0.2
0.4
0.6
0.8
1.0
0
10
20
30
40
50
60
bp position

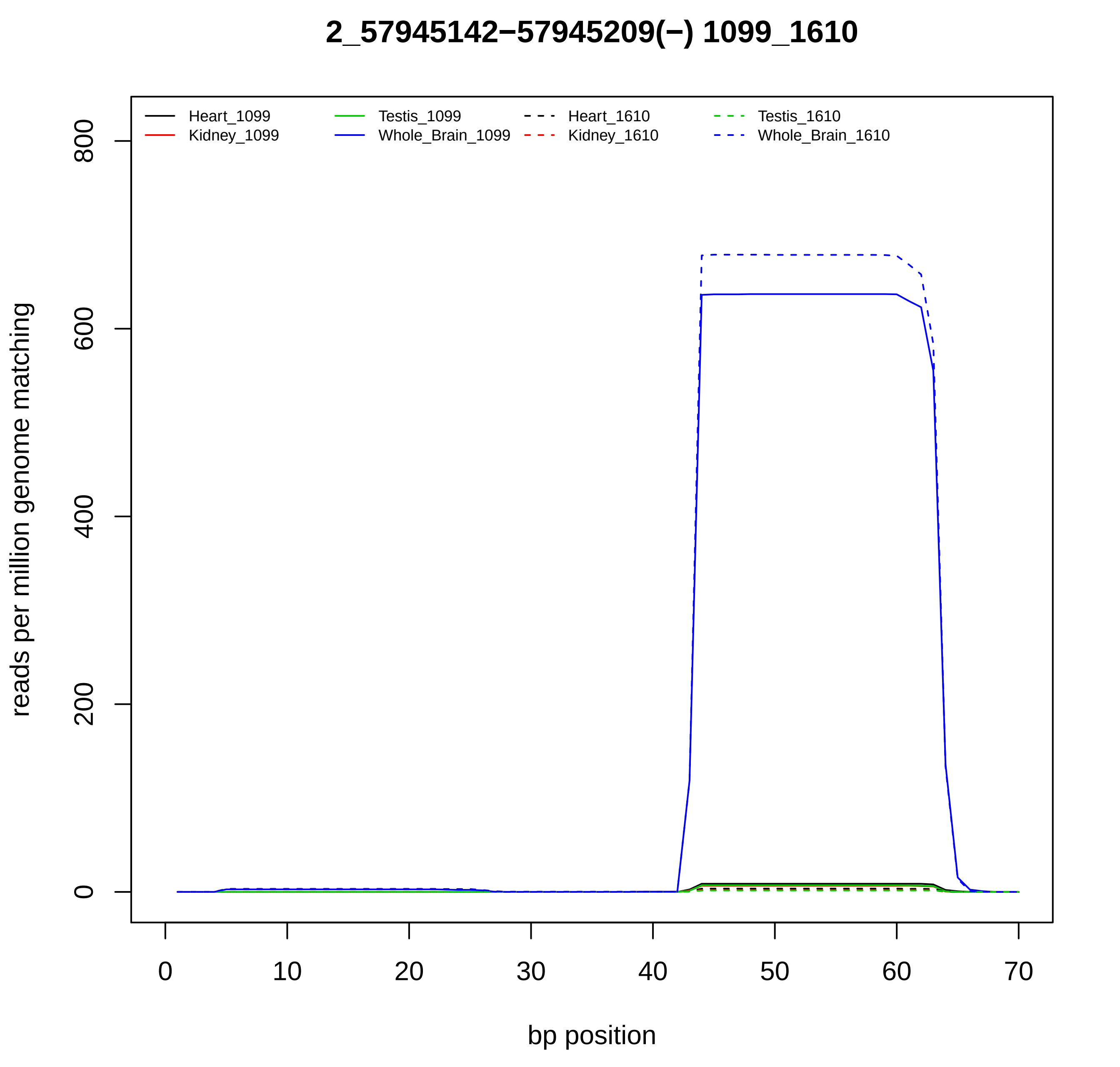

2_57945142−57945209(−) 1099_1610
Heart_1099
Kidney_1099
Testis_1099
Whole_Brain_1099
Heart_1610
Kidney_1610
Testis_1610
Whole_Brain_1610
reads per million genome matching
0
200
400
600
800
0
10
20
30
40
50
60
70
bp position

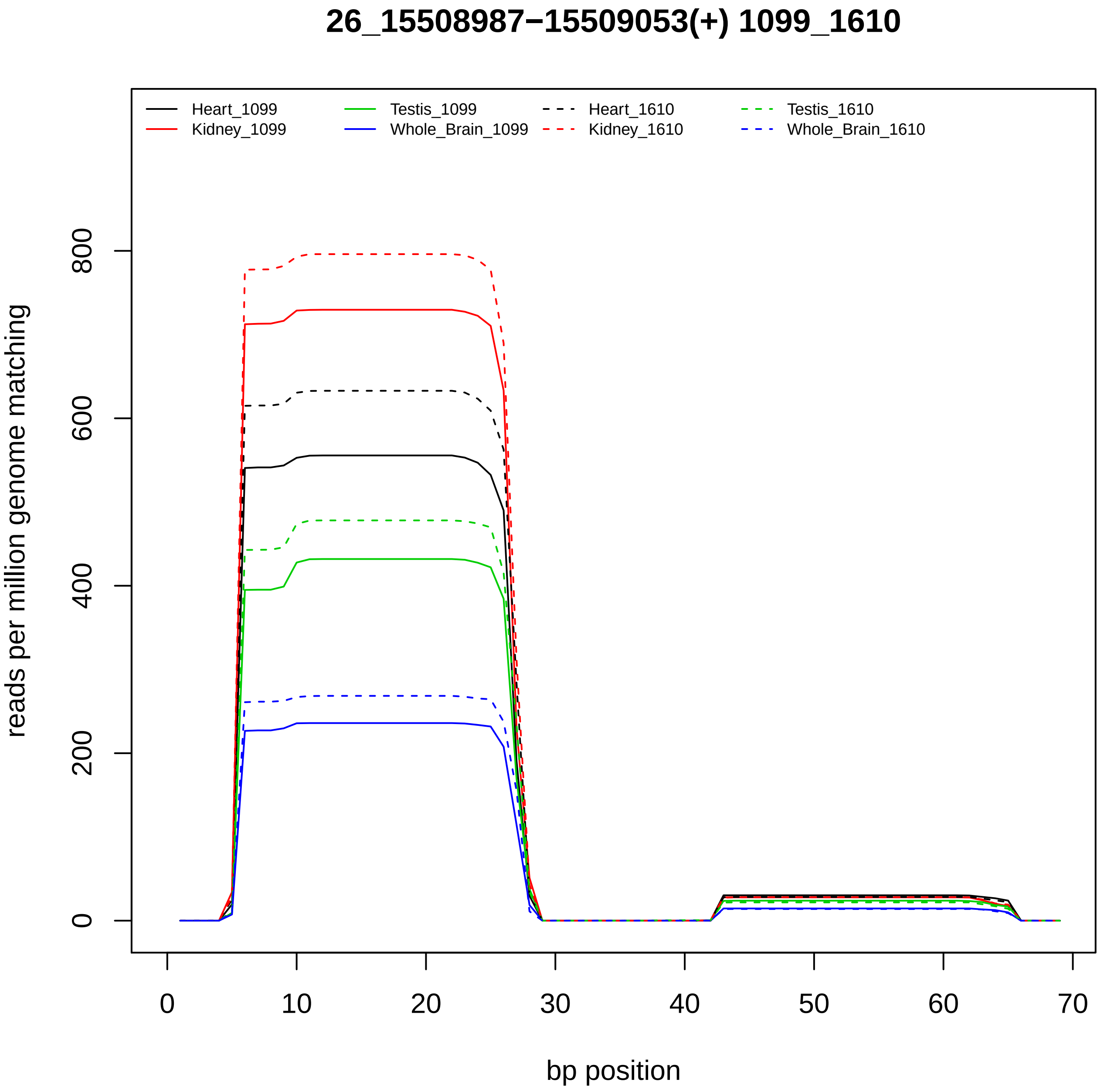
26_15508987−15509053(+) 1099_1610
Heart_1099
Kidney_1099
Testis_1099
Whole_Brain_1099
Heart_1610
Kidney_1610
Testis_1610
Whole_Brain_1610
reads per million genome matching
0
200
400
600
800
0
10
20
30
40
50
60
70
bp position

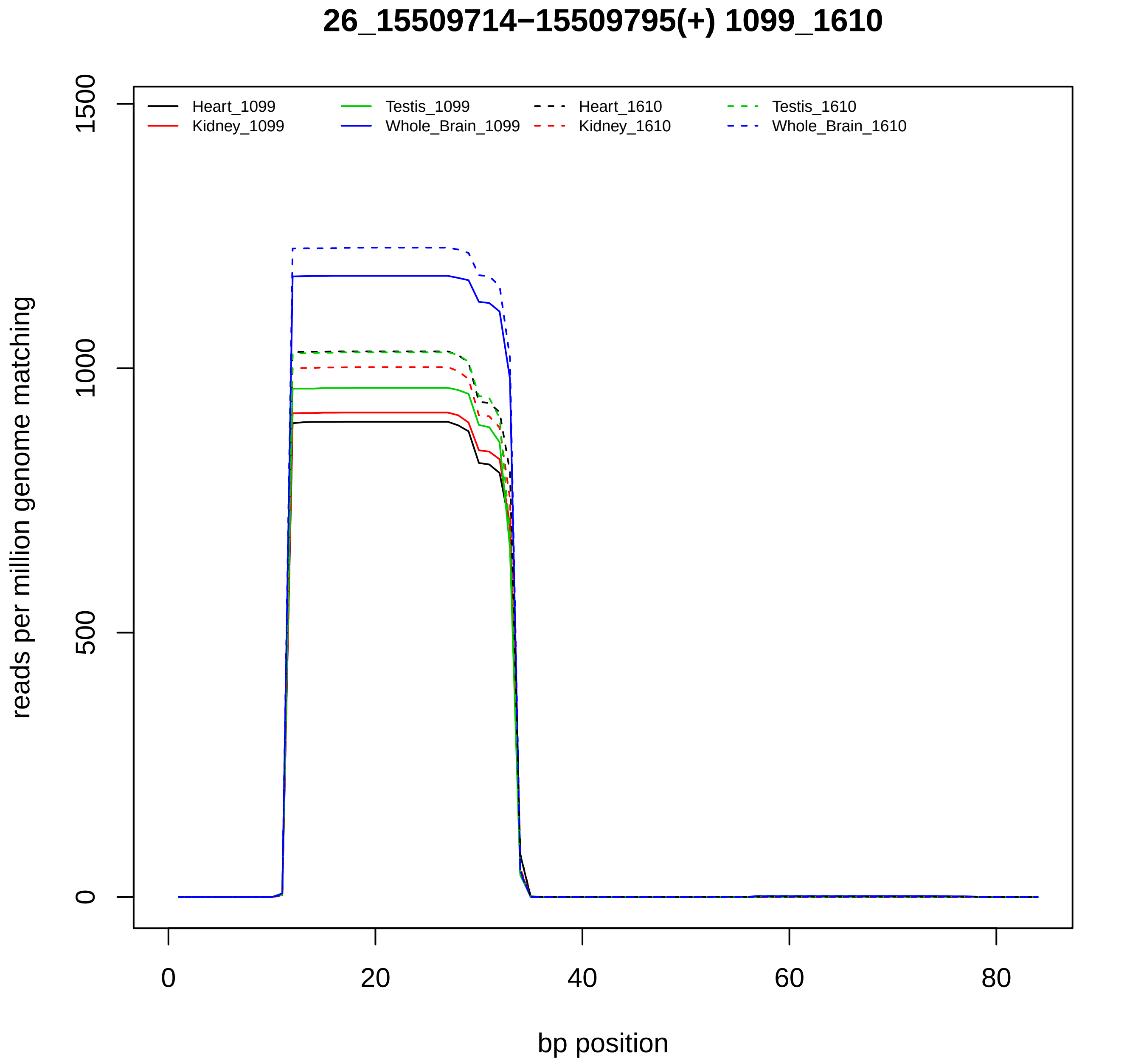

26_15509714−15509795(+) 1099_1610
Heart_1099
Kidney_1099
Testis_1099
Whole_Brain_1099
Heart_1610
Kidney_1610
Testis_1610
Whole_Brain_1610
reads per million genome matching
bp position
0
500
1000
1500
0
20
40
60
80

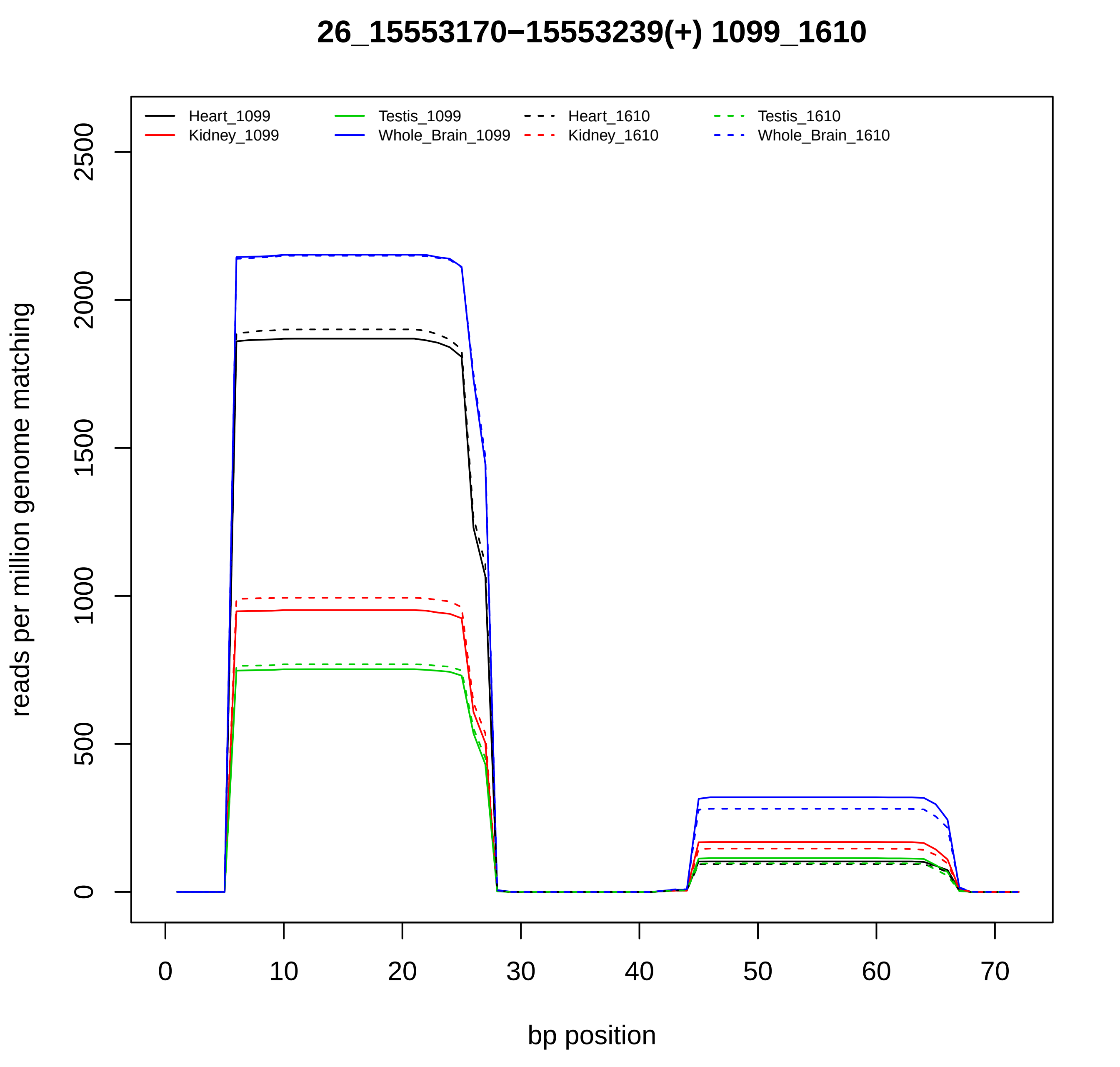
26_15553170−15553239(+) 1099_1610
Heart_1099
Kidney_1099
Testis_1099
Whole_Brain_1099
Heart_1610
Kidney_1610
Testis_1610
Whole_Brain_1610
reads per million genome matching
0
500
1000
1500
2000
2500
bp position
0
10
20
30
40
50
60
70

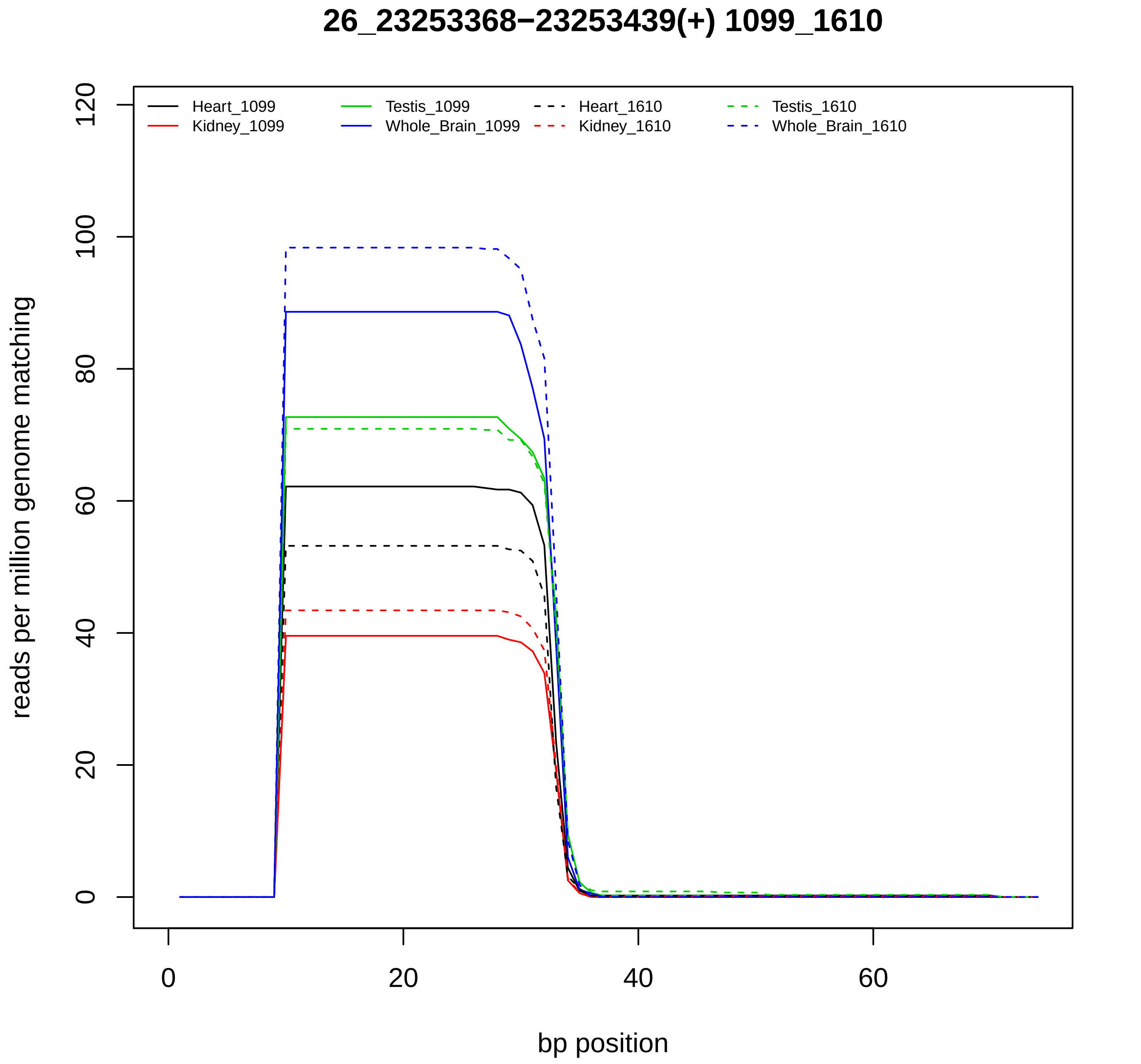

26_23253368−23253439(+) 1099_1610
Heart_1099
Kidney_1099
Testis_1099
Whole_Brain_1099
Heart_1610
Kidney_1610
Testis_1610
Whole_Brain_1610
reads per million genome matching
bp position
0
20
40
60
80
100
120
0
20
40
60

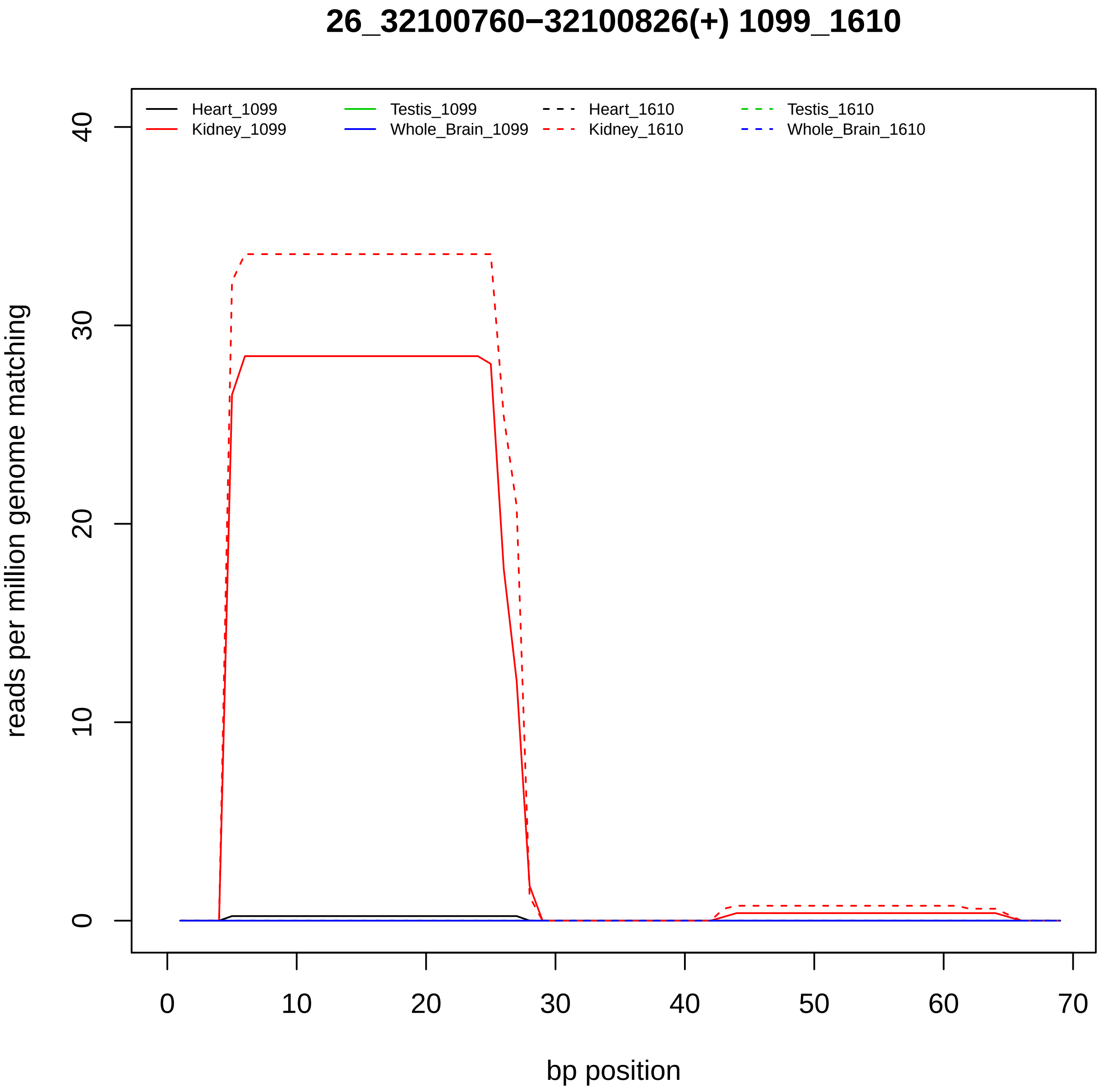

26_32100760−32100826(+) 1099_1610
Heart_1099
Kidney_1099
Testis_1099
Whole_Brain_1099
Heart_1610
Kidney_1610
Testis_1610
Whole_Brain_1610
reads per million genome matching
0
10
20
30
40
bp position
0
10
20
30
40
50
60
70

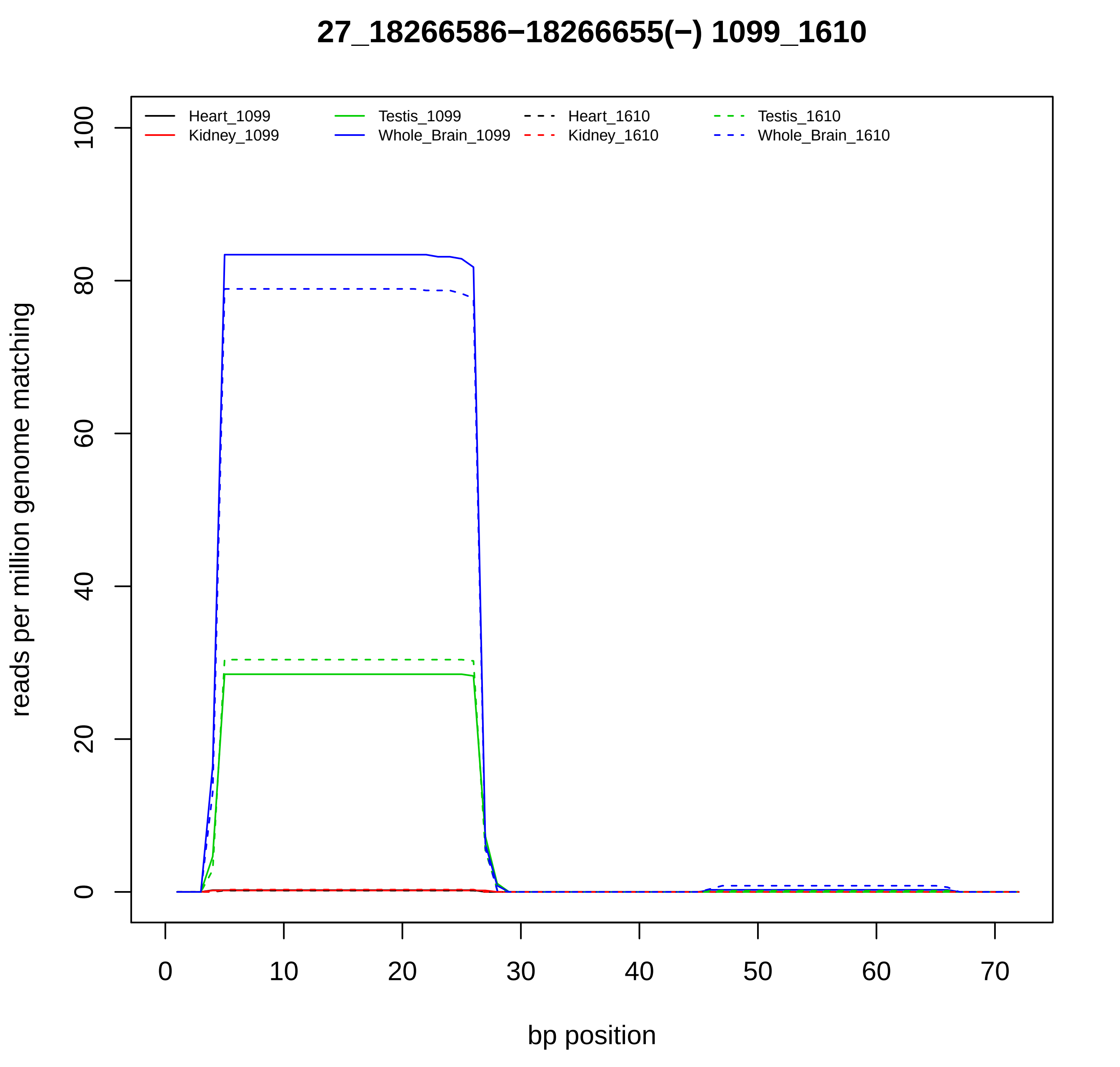

27_18266586−18266655(−) 1099_1610
Heart_1099
Kidney_1099
Testis_1099
Whole_Brain_1099
Heart_1610
Kidney_1610
Testis_1610
Whole_Brain_1610
reads per million genome matching
bp position
0
20
40
60
80
100
0
10
20
30
40
50
60
70

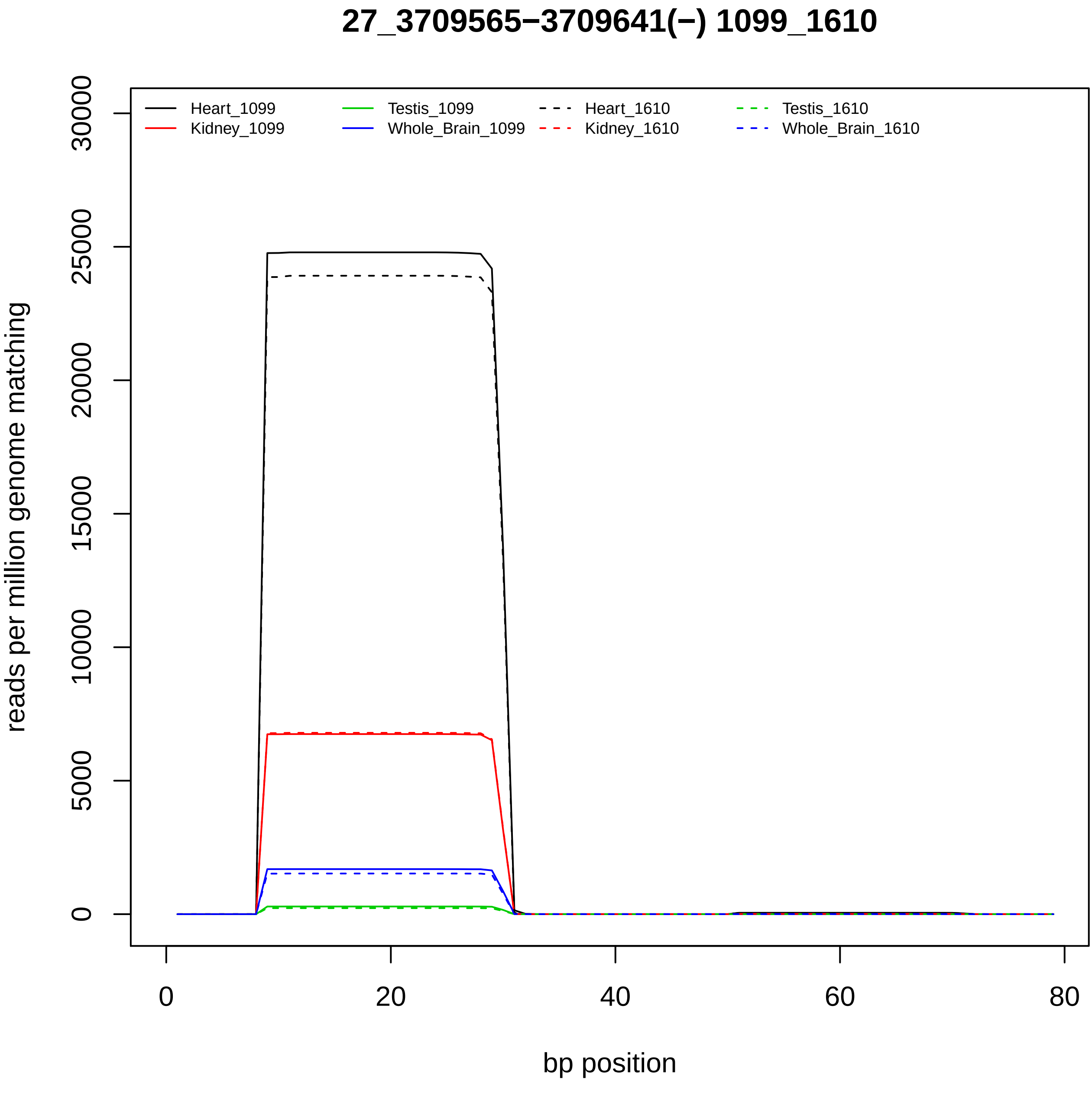
27_3709565−3709641(−) 1099_1610
Heart_1099
Kidney_1099
Testis_1099
Whole_Brain_1099
Heart_1610
Kidney_1610
Testis_1610
Whole_Brain_1610
reads per million genome matching
0
5000
10000
15000
20000
25000
30000
bp position
0
20
40
60
80

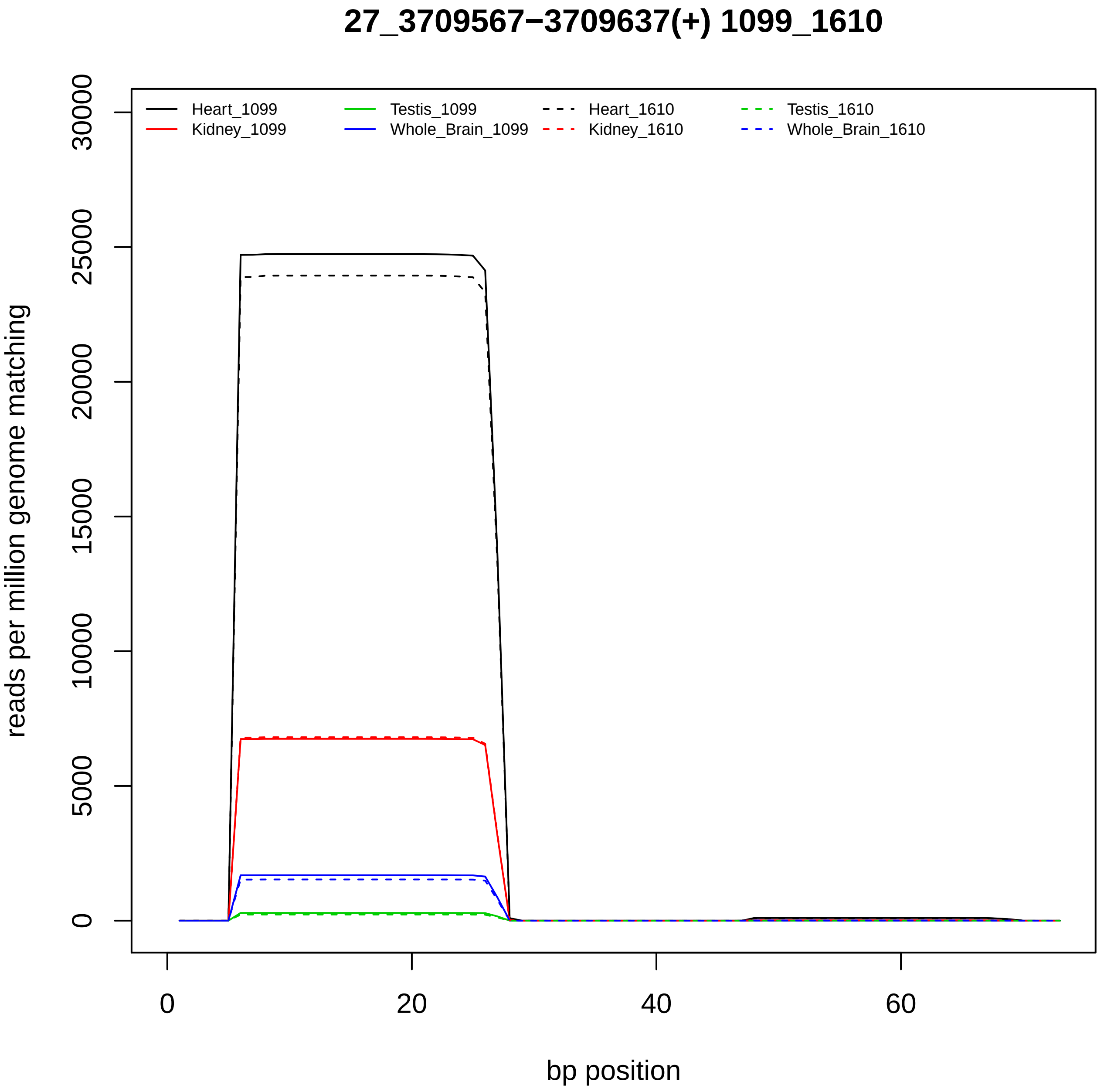

27_3709567−3709637(+) 1099_1610
Heart_1099
Kidney_1099
Testis_1099
Whole_Brain_1099
Heart_1610
Kidney_1610
Testis_1610
Whole_Brain_1610
reads per million genome matching
0
5000
10000
15000
20000
25000
30000
0
20
40
60
bp position

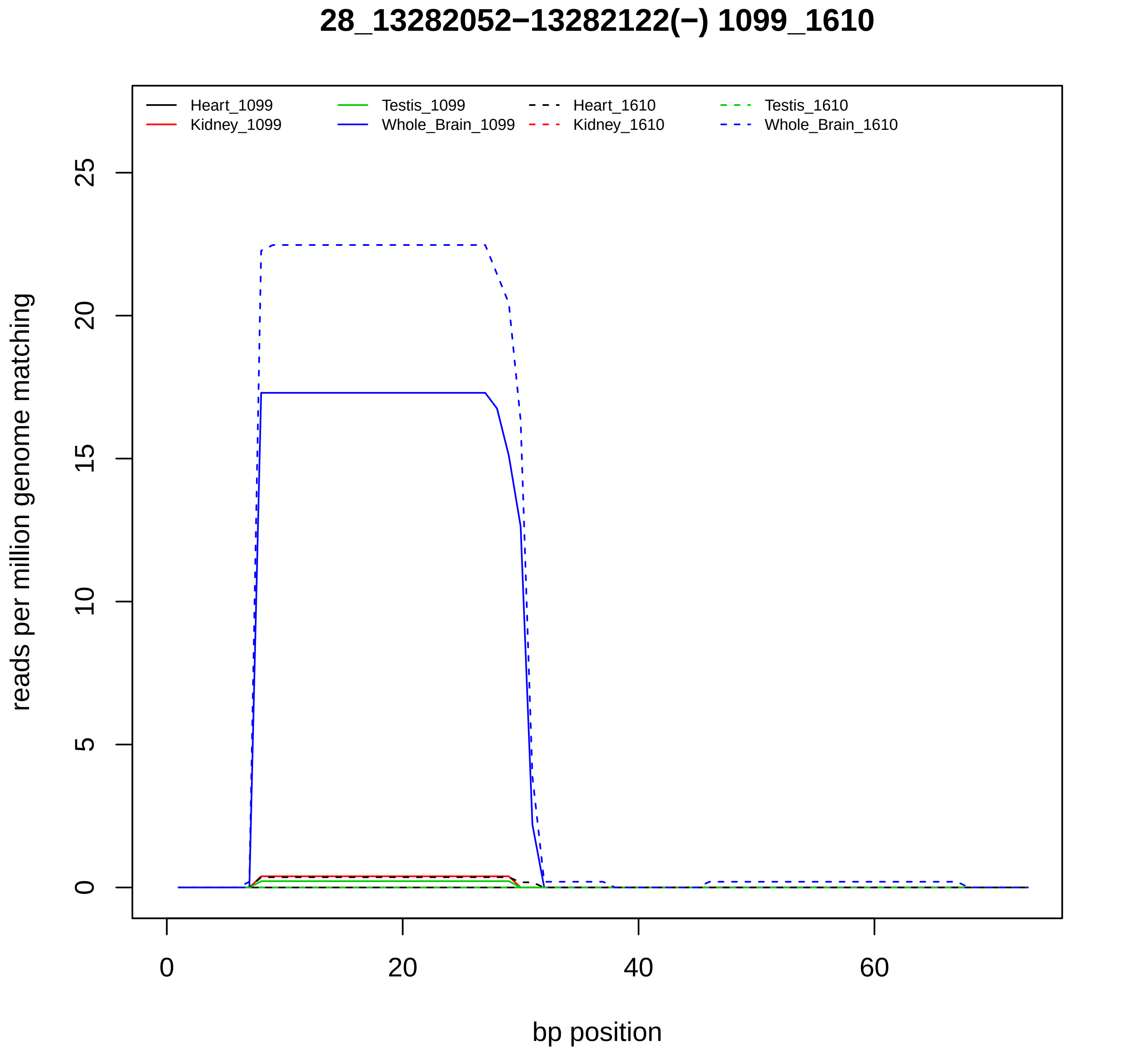

28_13282052−13282122(−) 1099_1610
Heart_1099
Kidney_1099
Testis_1099
Whole_Brain_1099
Heart_1610
Kidney_1610
Testis_1610
Whole_Brain_1610
reads per million genome matching
bp position
0
5
10
15
20
25
0
20
40
60

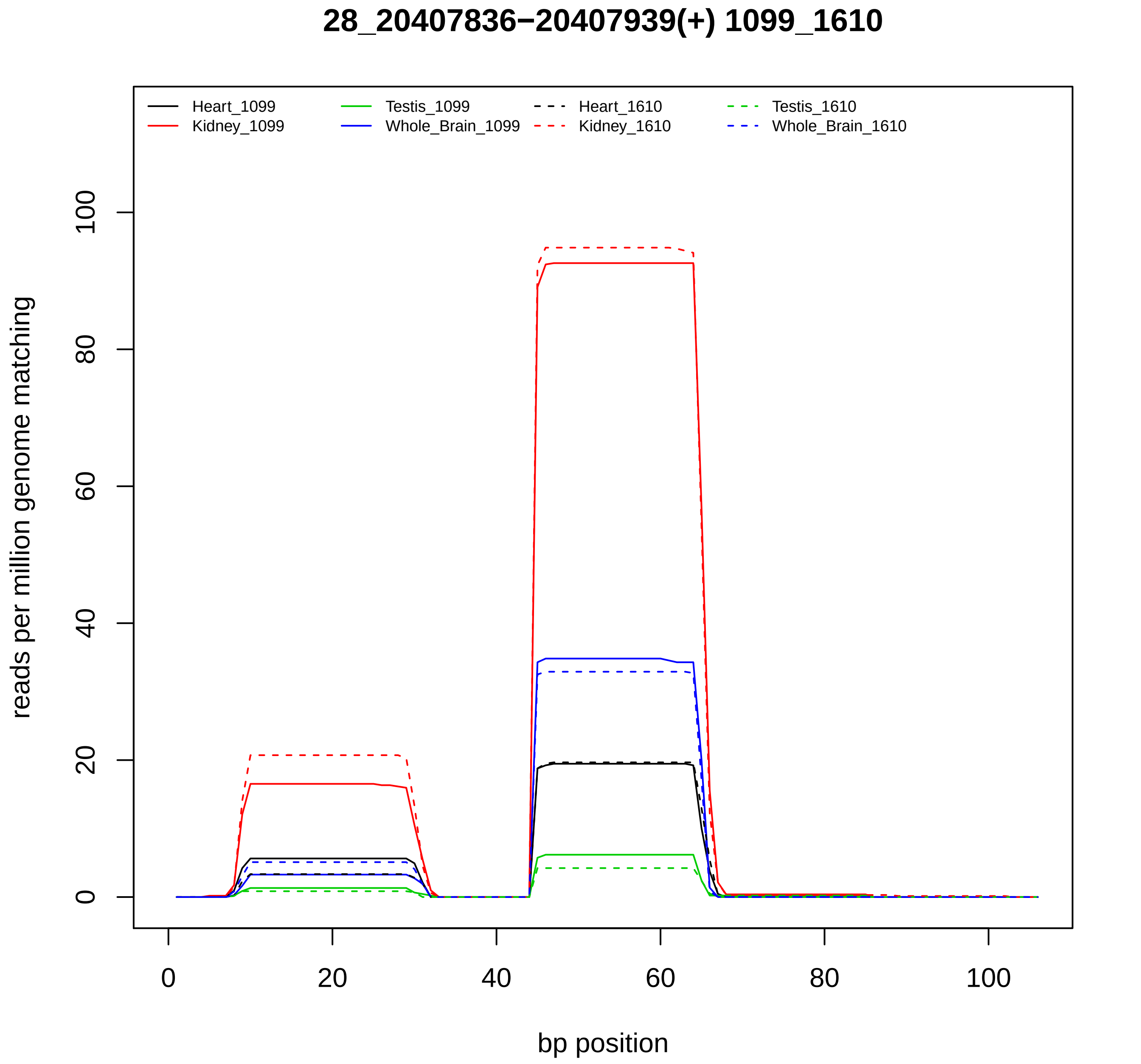

28_20407836−20407939(+) 1099_1610
Heart_1099
Kidney_1099
Testis_1099
Whole_Brain_1099
Heart_1610
Kidney_1610
Testis_1610
Whole_Brain_1610
reads per million genome matching
bp position
0
20
40
60
80
100

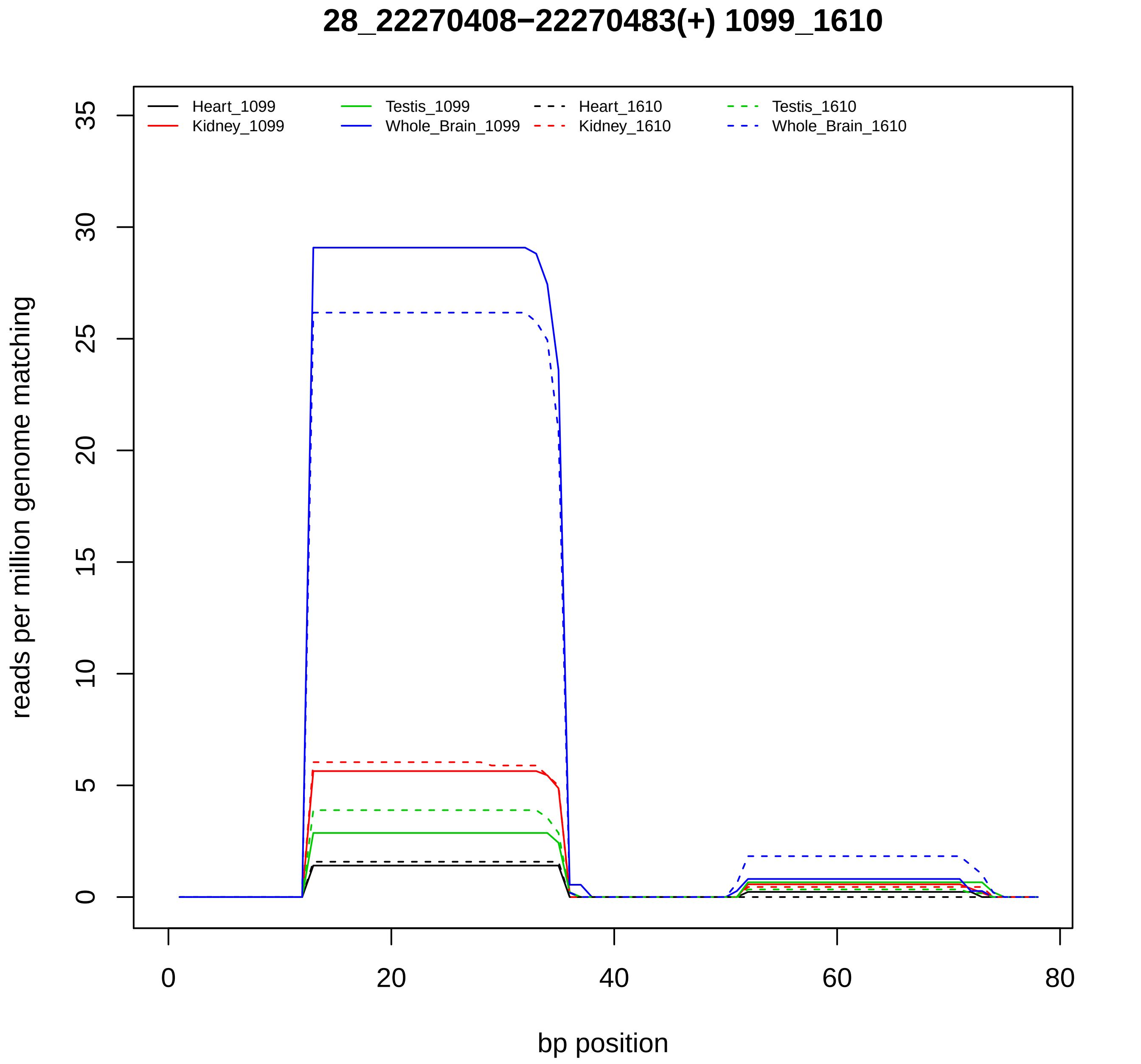

28_22270408−22270483(+) 1099_1610
Heart_1099
Kidney_1099
Testis_1099
Whole_Brain_1099
Heart_1610
Kidney_1610
Testis_1610
Whole_Brain_1610
reads per million genome matching
0
5
10
15
20
25
30
35
0
20
40
60
80
bp position

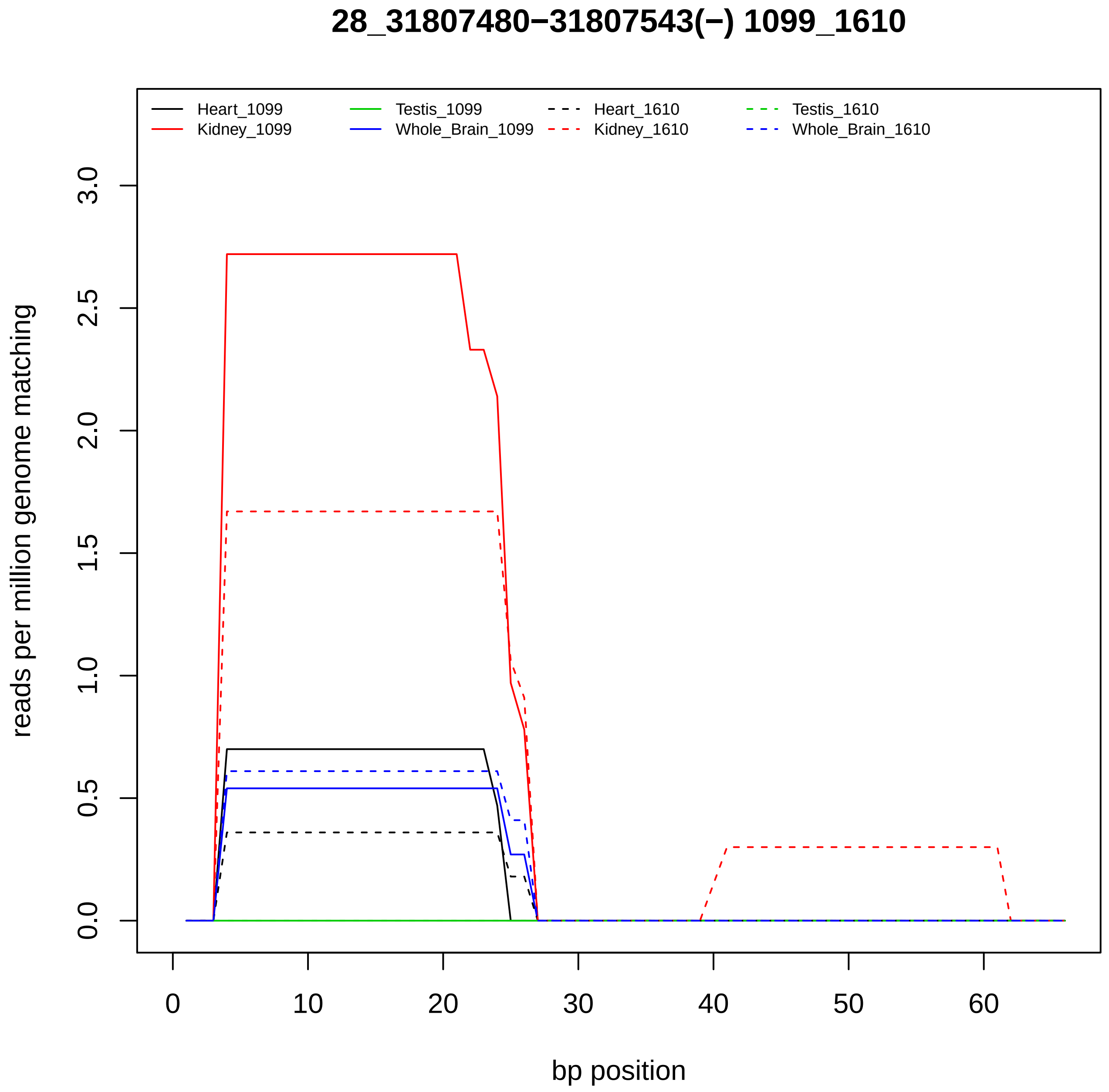

28_31807480−31807543(−) 1099_1610
Heart_1099
Kidney_1099
Testis_1099
Whole_Brain_1099
Heart_1610
Kidney_1610
Testis_1610
Whole_Brain_1610
reads per million genome matching
0.0
0.5
1.0
1.5
2.0
2.5
3.0
0
10
20
30
40
50
60
bp position

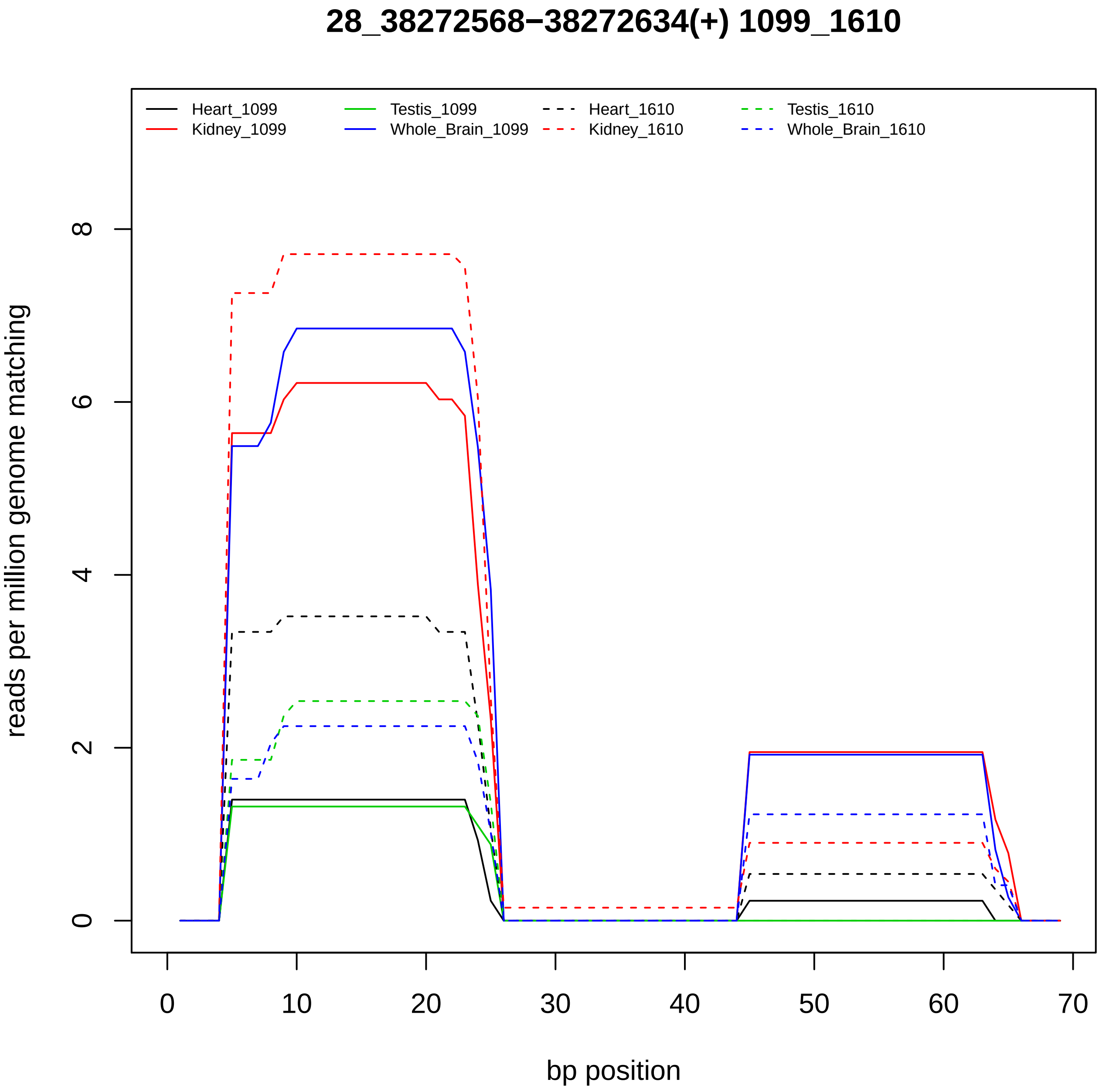

28_38272568−38272634(+) 1099_1610
Heart_1099
Kidney_1099
Testis_1099
Whole_Brain_1099
Heart_1610
Kidney_1610
Testis_1610
Whole_Brain_1610
reads per million genome matching
0
2
4
6
8
bp position
0
10
20
30
40
50
60
70

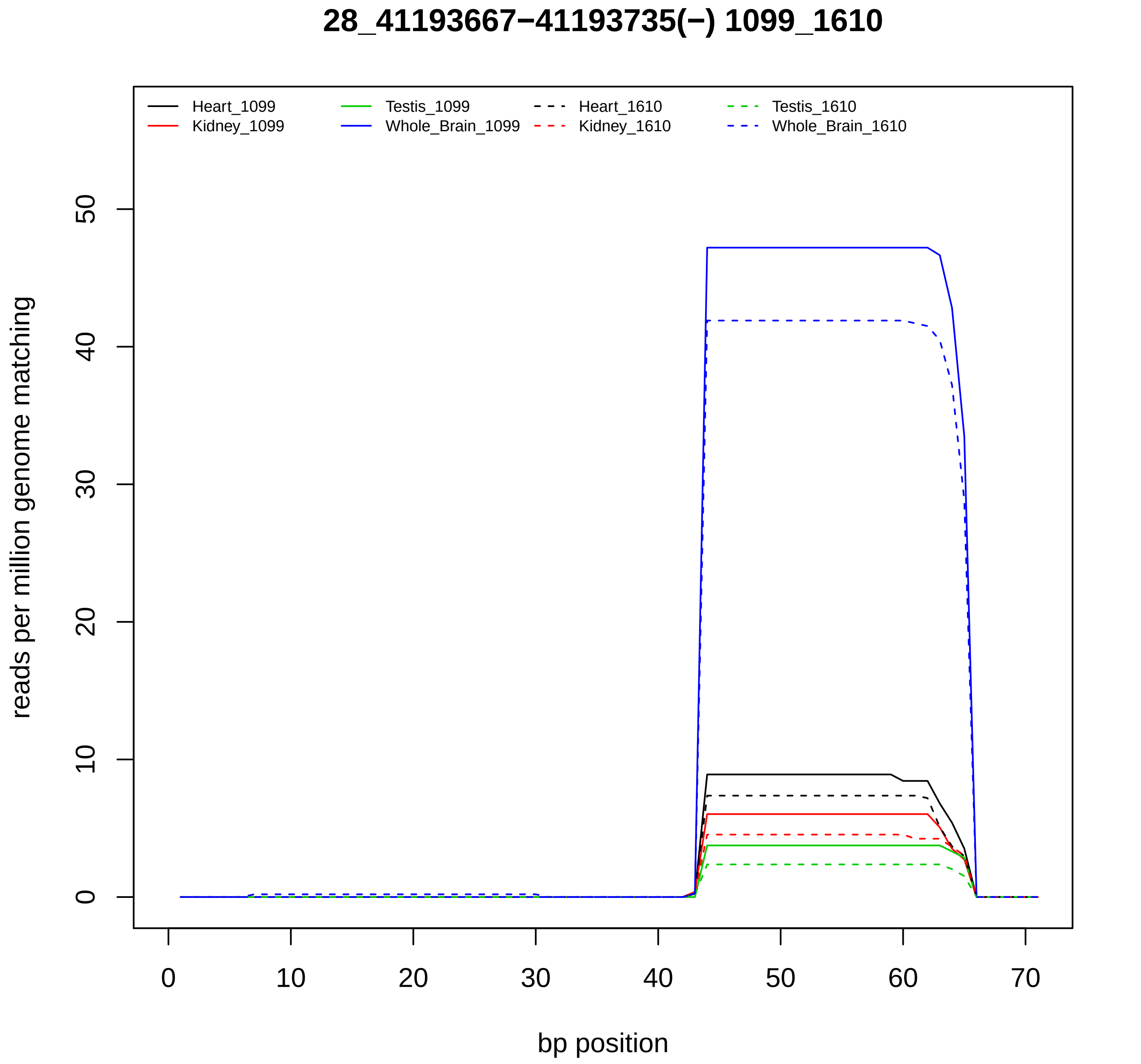

28_41193667−41193735(−) 1099_1610
Heart_1099
Kidney_1099
Testis_1099
Whole_Brain_1099
Heart_1610
Kidney_1610
Testis_1610
Whole_Brain_1610
reads per million genome matching
bp position
0
10
20
30
40
50
60
70

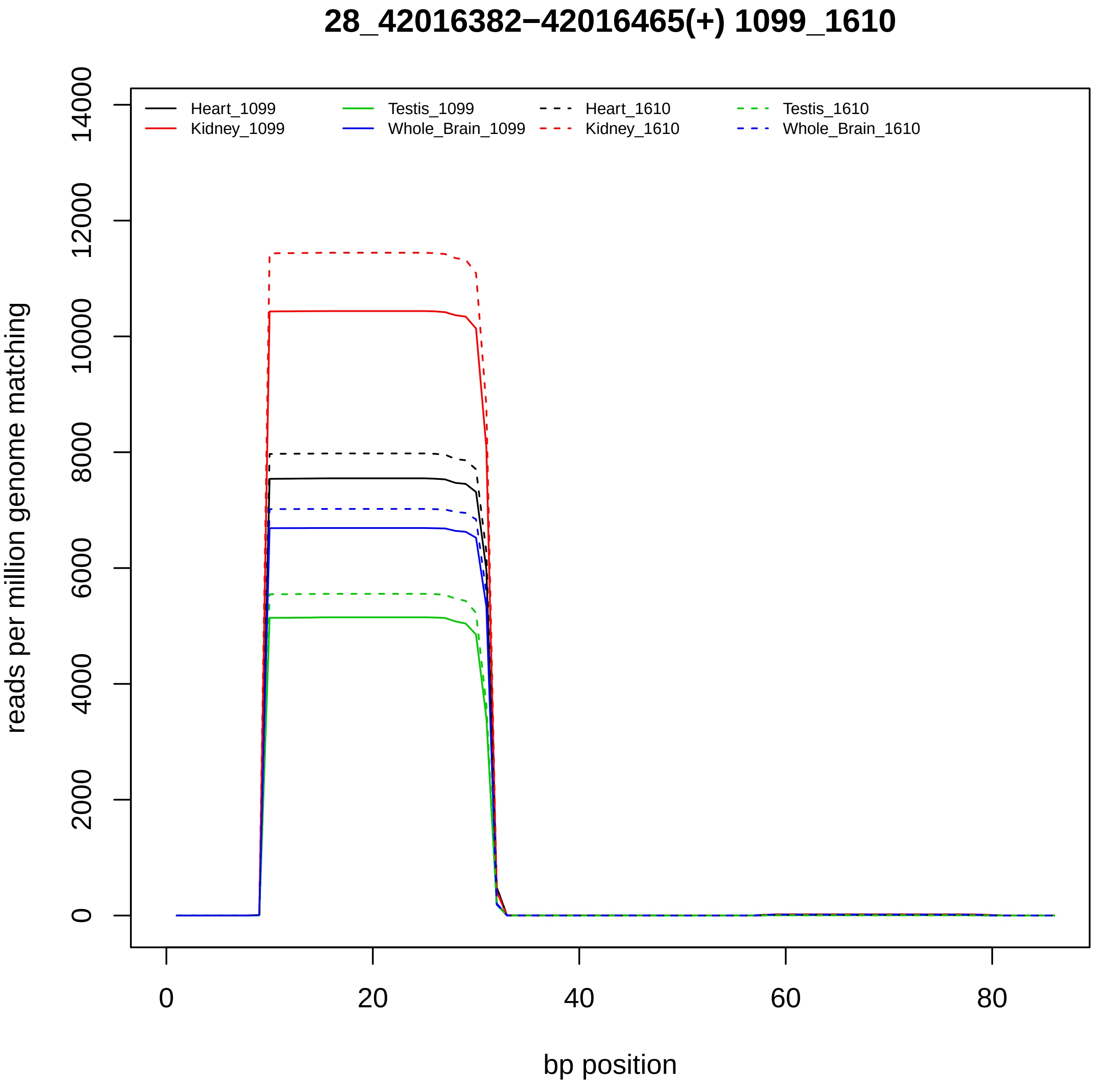

28_42016382−42016465(+) 1099_1610
Heart_1099
Kidney_1099
Testis_1099
Whole_Brain_1099
Heart_1610
Kidney_1610
Testis_1610
Whole_Brain_1610
reads per million genome matching
0
2000
4000
6000
8000
10000
12000
14000
0
20
40
60
80
bp position

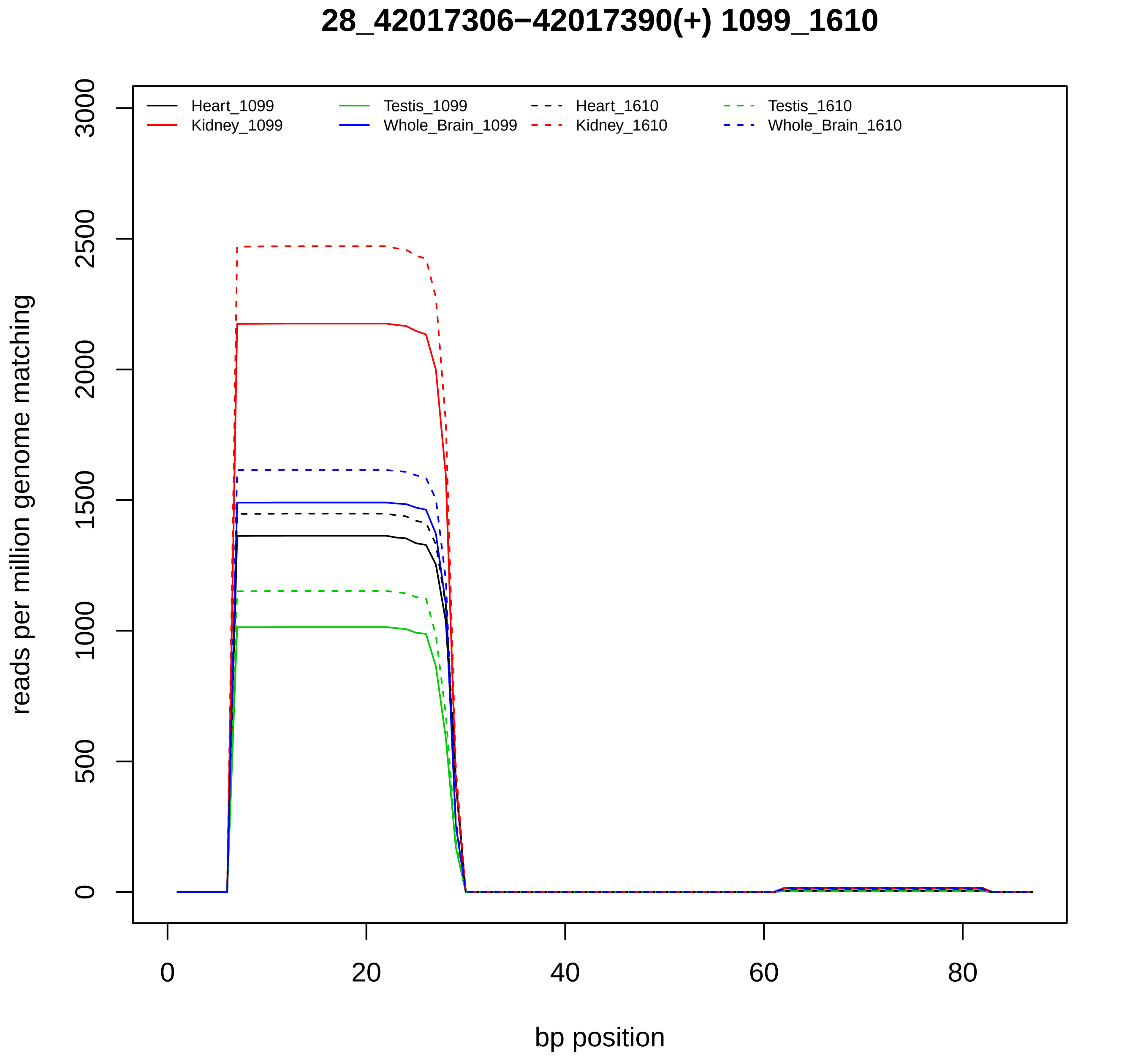

28_42017306−42017390(+) 1099_1610
Heart_1099
Kidney_1099
Testis_1099
Whole_Brain_1099
Heart_1610
Kidney_1610
Testis_1610
Whole_Brain_1610
reads per million genome matching
bp position
0
500
1000
1500
2000
2500
3000
0
20
40
60
80

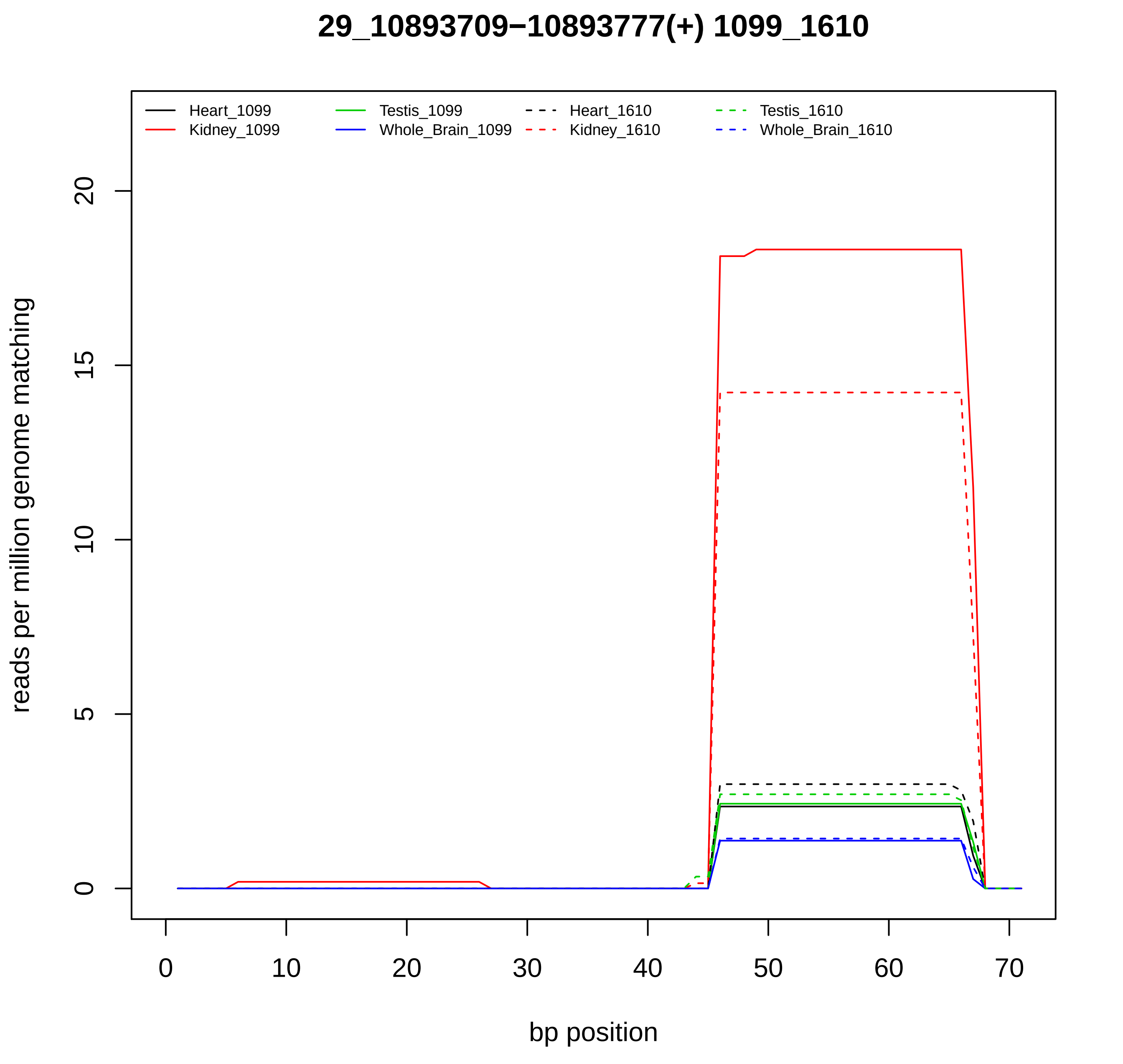

29_10893709−10893777(+) 1099_1610
Heart_1099
Kidney_1099
Testis_1099
Whole_Brain_1099
Heart_1610
Kidney_1610
Testis_1610
Whole_Brain_1610
reads per million genome matching
bp position
0
5
10
15
20
0
10
20
30
40
50
60
70

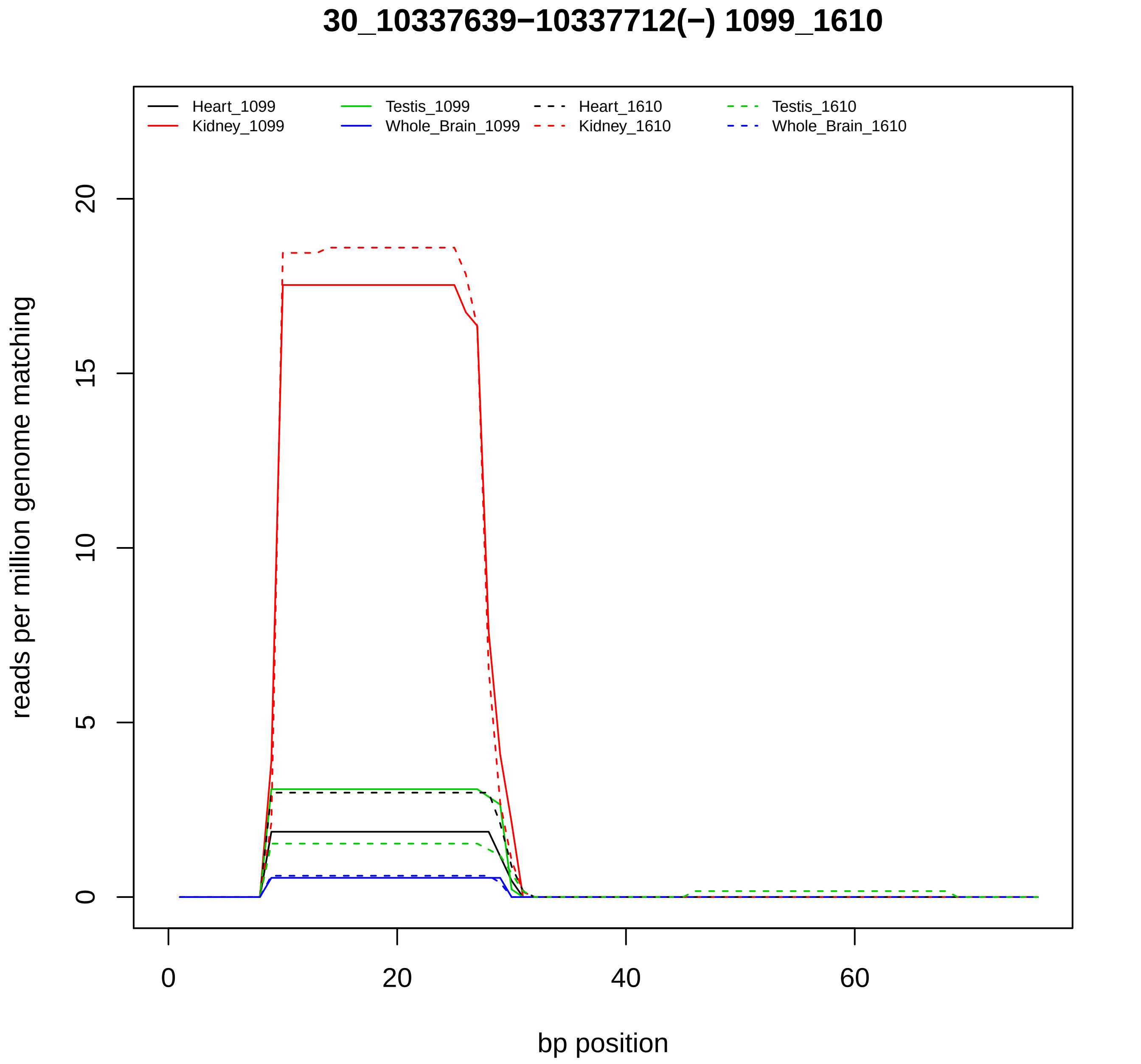

30_10337639−10337712(−) 1099_1610
Heart_1099
Kidney_1099
Testis_1099
Whole_Brain_1099
Heart_1610
Kidney_1610
Testis_1610
Whole_Brain_1610
reads per million genome matching
bp position
0
5
10
15
20
0
20
40
60

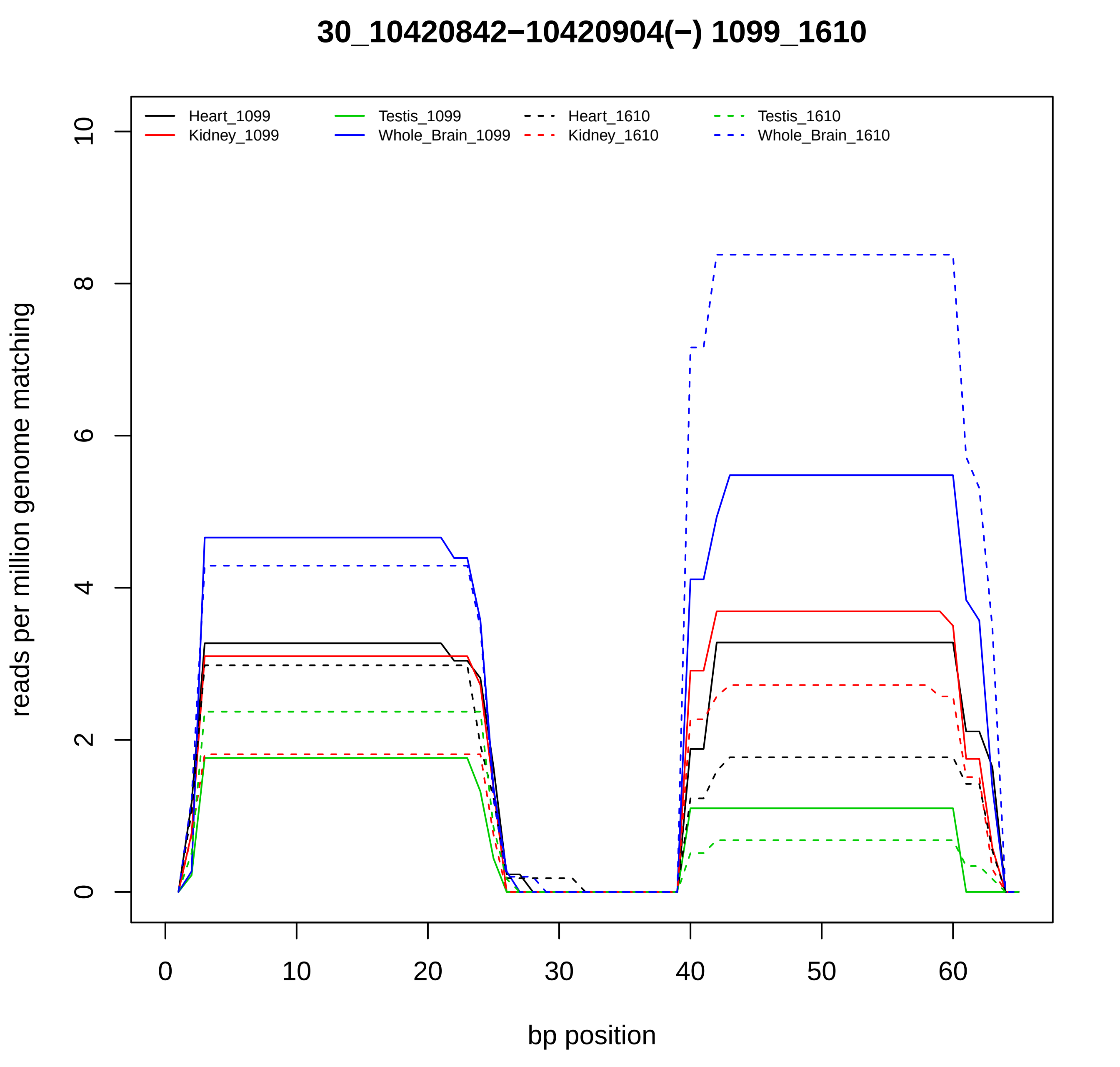
30_10420842−10420904(−) 1099_1610
Heart_1099
Kidney_1099
Testis_1099
Whole_Brain_1099
Heart_1610
Kidney_1610
Testis_1610
Whole_Brain_1610
reads per million genome matching
bp position
0
2
4
6
8
10
0
10
20
30
40
50
60

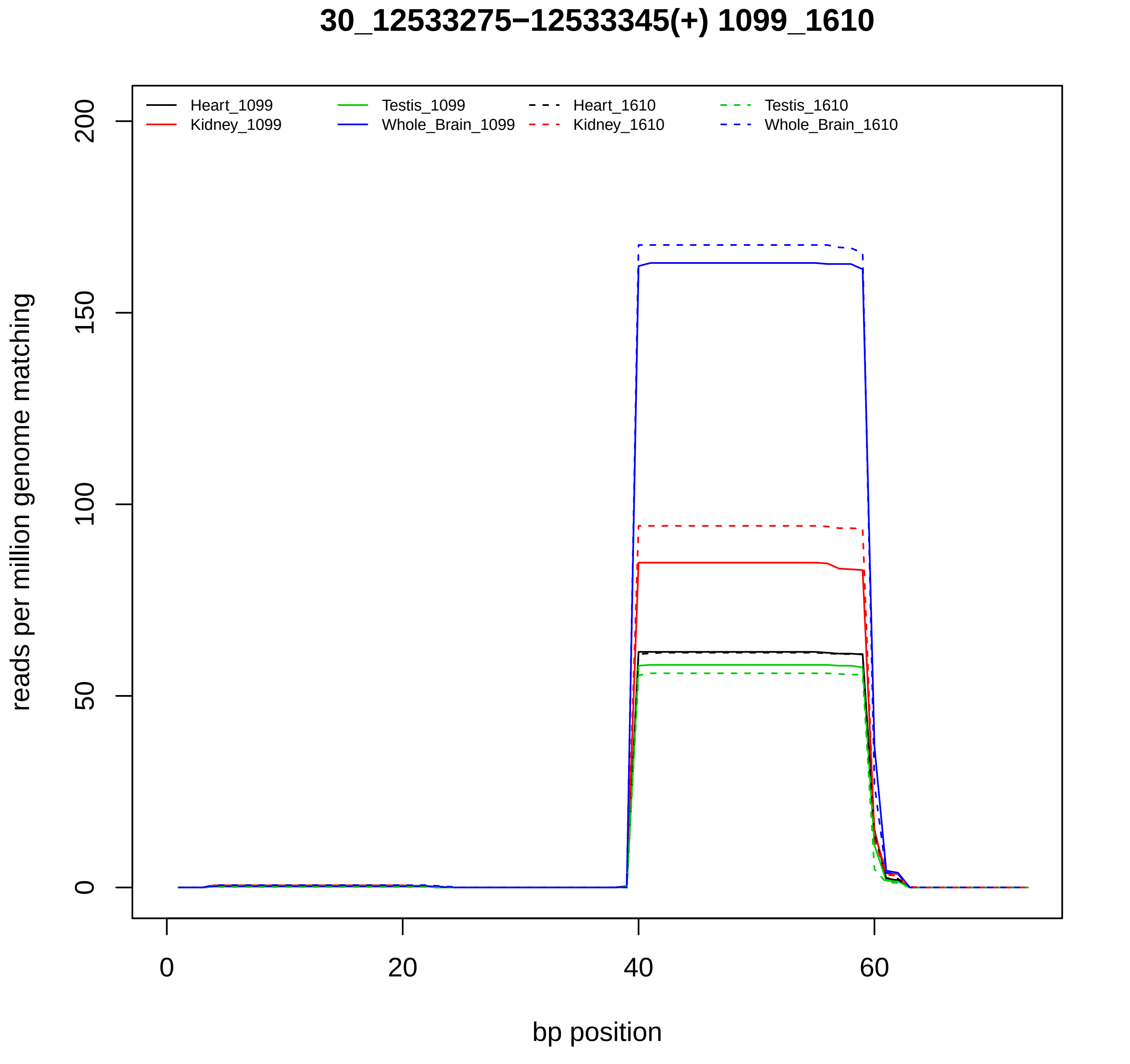

30_12533275−12533345(+) 1099_1610
Heart_1099
Kidney_1099
Testis_1099
Whole_Brain_1099
Heart_1610
Kidney_1610
Testis_1610
Whole_Brain_1610
reads per million genome matching
bp position
0
50
100
150
200
0
20
40
60

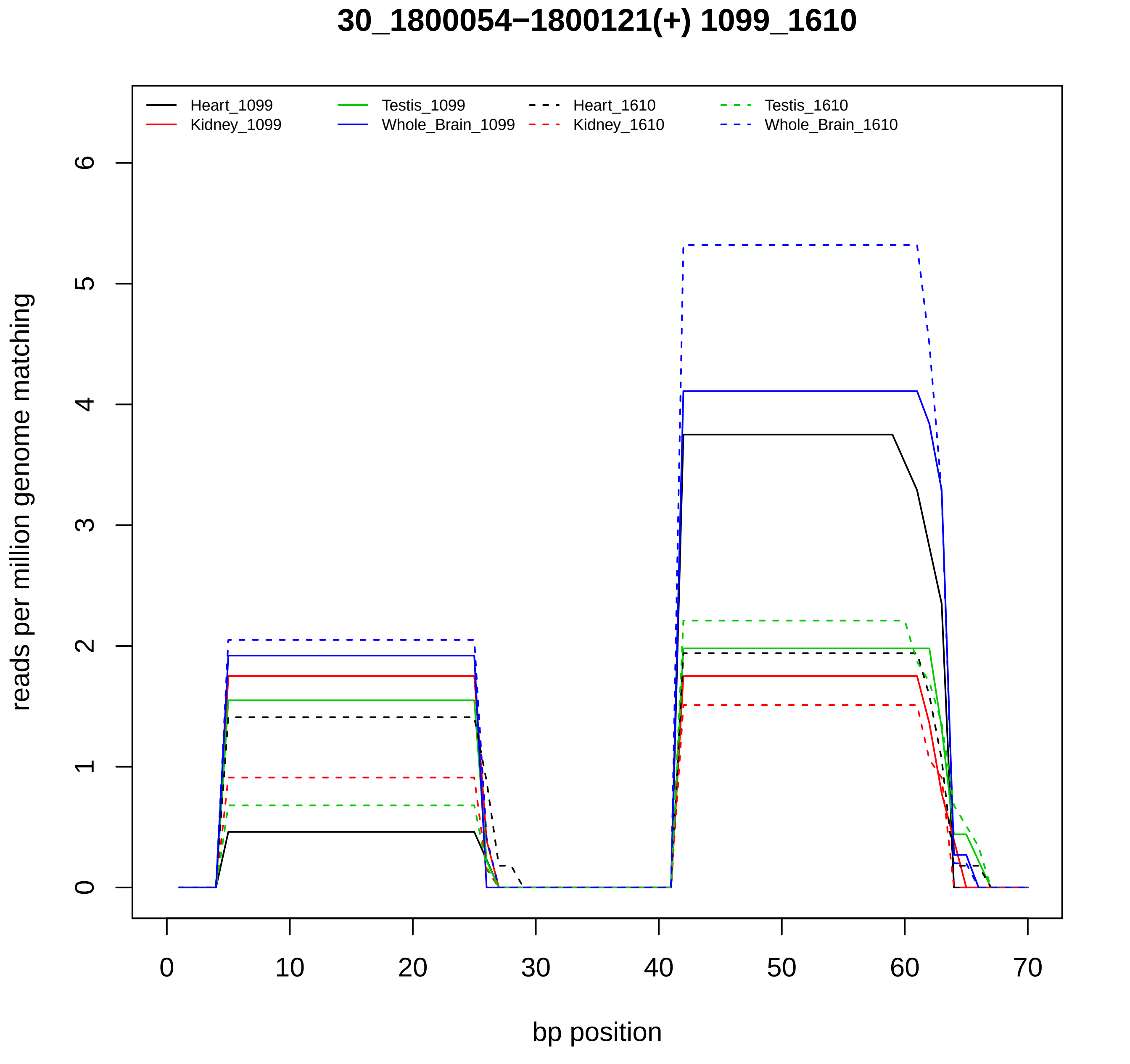
30_1800054−1800121(+) 1099_1610
Heart_1099
Kidney_1099
Testis_1099
Whole_Brain_1099
Heart_1610
Kidney_1610
Testis_1610
Whole_Brain_1610
reads per million genome matching
0
1
2
3
4
5
6
0
10
20
30
40
50
60
70
bp position

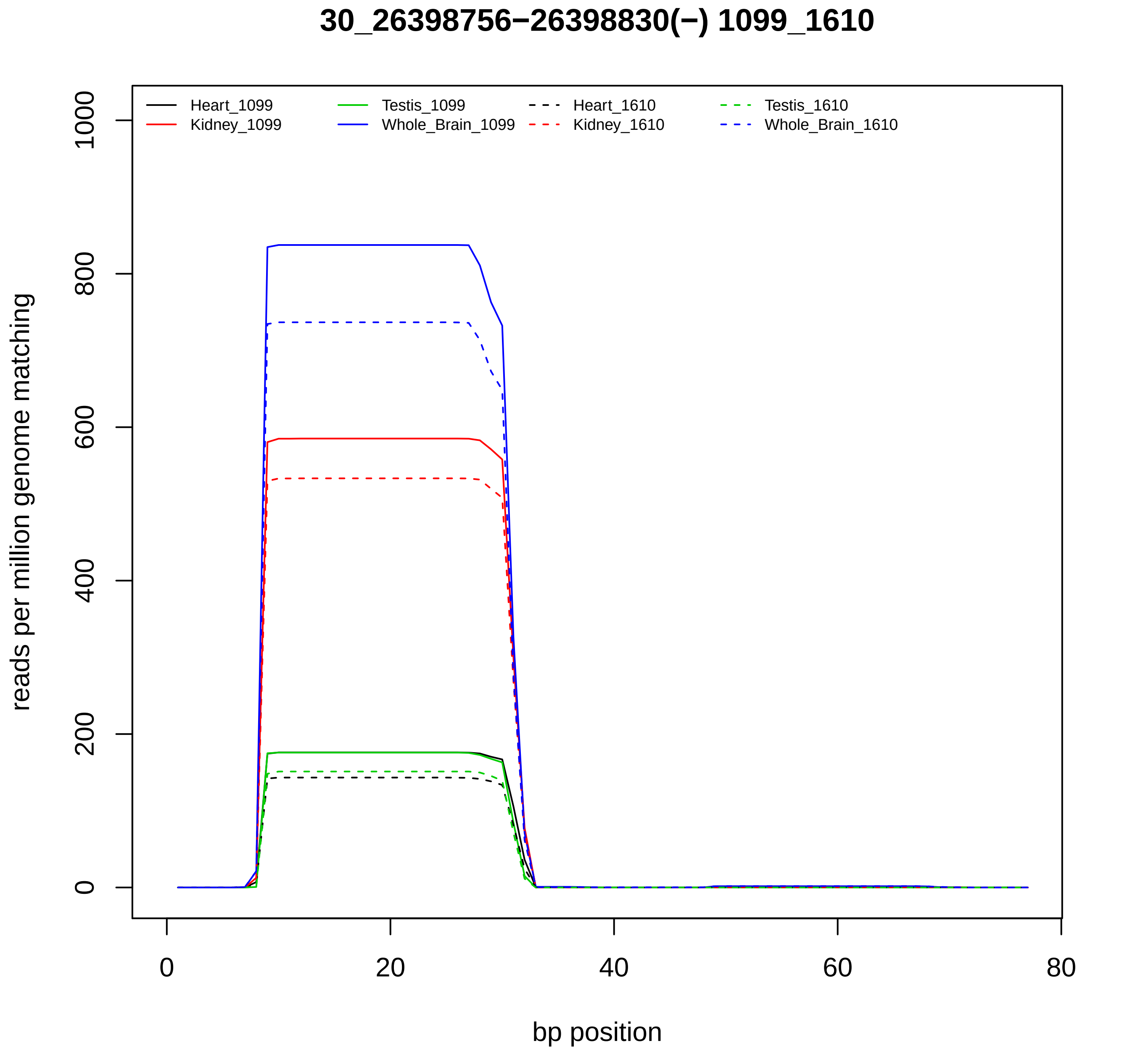

30_26398756−26398830(−) 1099_1610
Heart_1099
Kidney_1099
Testis_1099
Whole_Brain_1099
Heart_1610
Kidney_1610
Testis_1610
Whole_Brain_1610
reads per million genome matching
bp position
0
200
400
600
800
1000
0
20
40
60
80

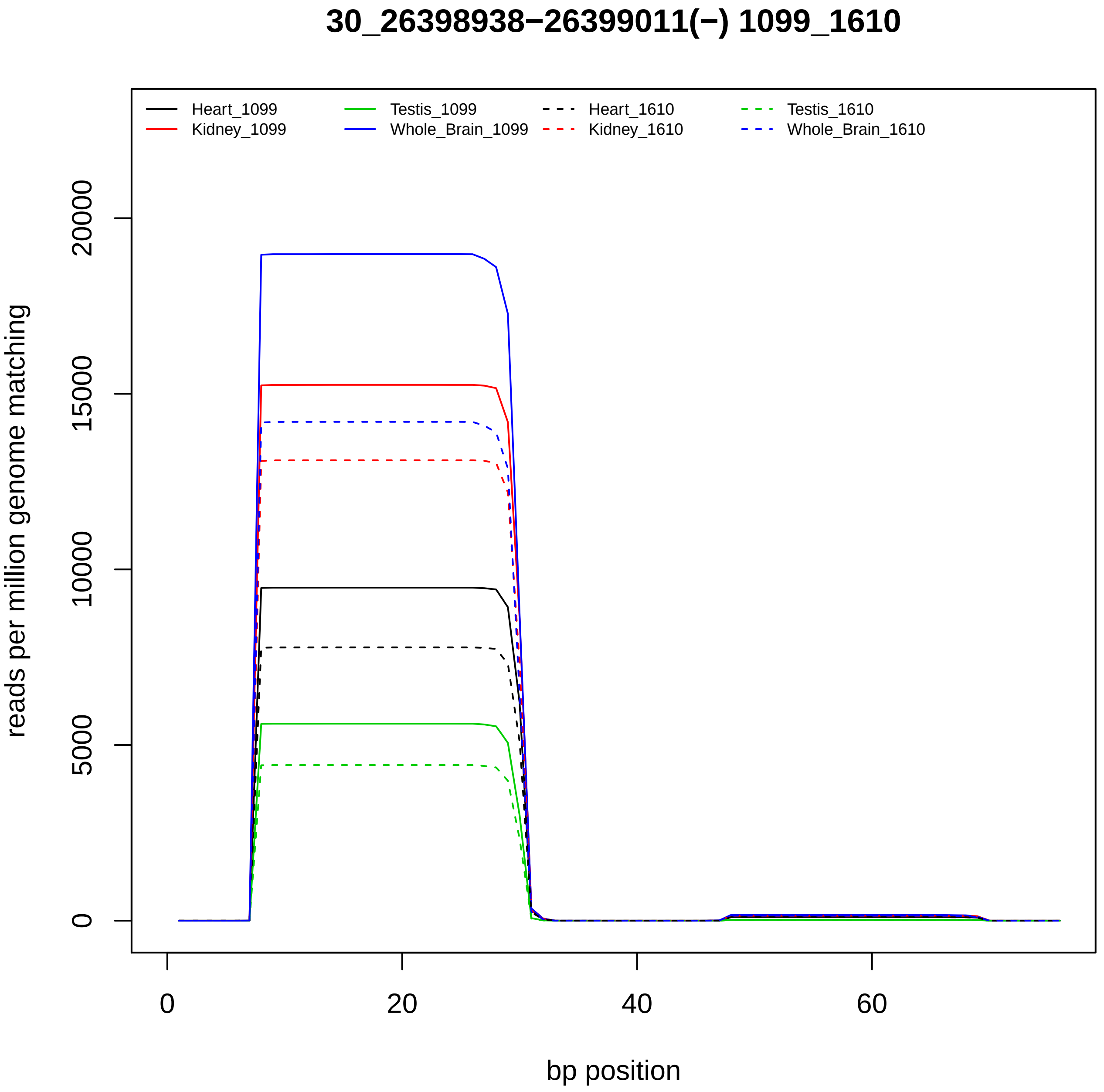

30_26398938−26399011(−) 1099_1610
Heart_1099
Kidney_1099
Testis_1099
Whole_Brain_1099
Heart_1610
Kidney_1610
Testis_1610
Whole_Brain_1610
reads per million genome matching
0
5000
10000
15000
20000
0
20
40
60
bp position

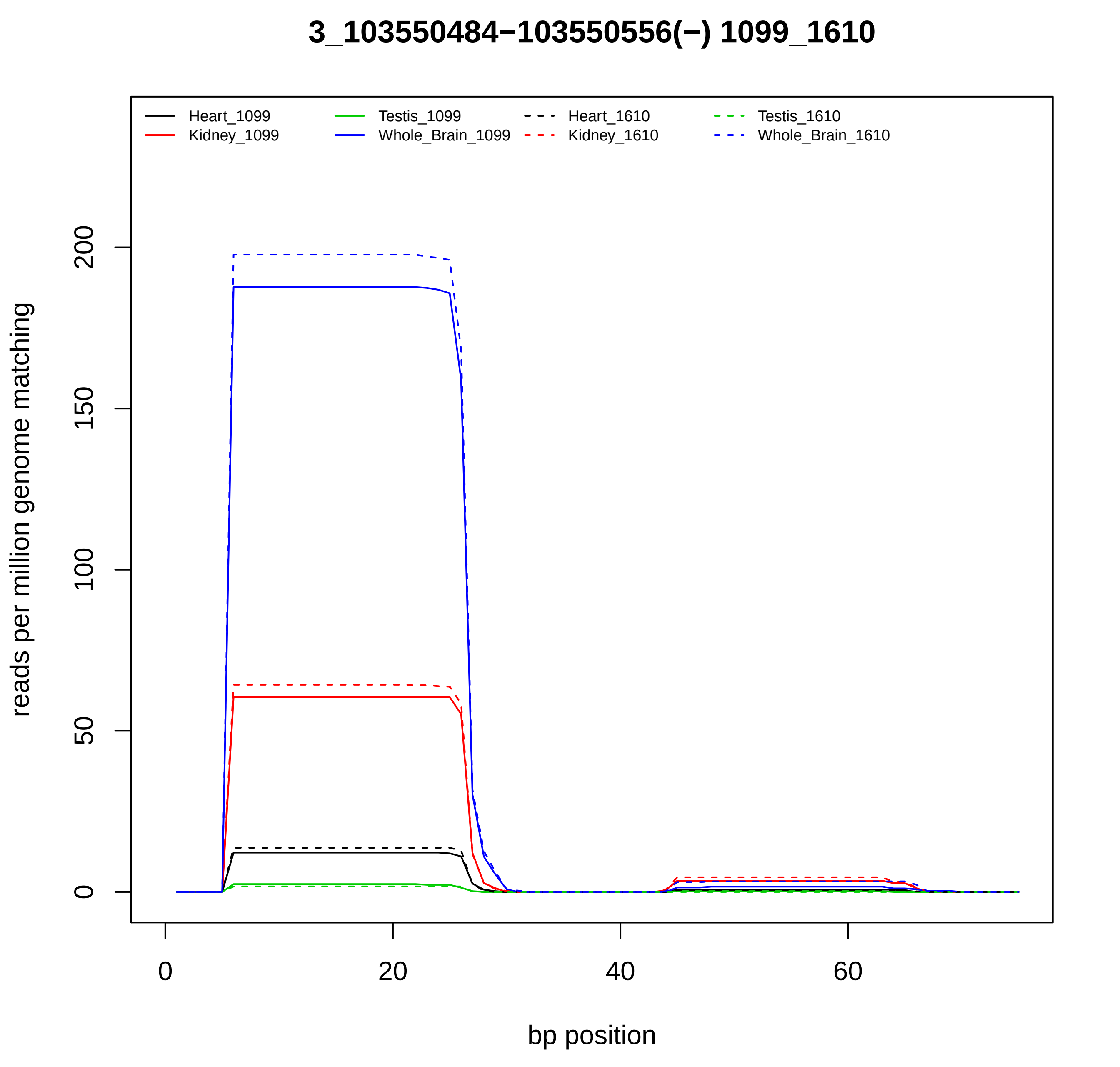

3_103550484−103550556(−) 1099_1610
Heart_1099
Kidney_1099
Testis_1099
Whole_Brain_1099
Heart_1610
Kidney_1610
Testis_1610
Whole_Brain_1610
reads per million genome matching
0
50
100
150
200
0
20
40
60
bp position

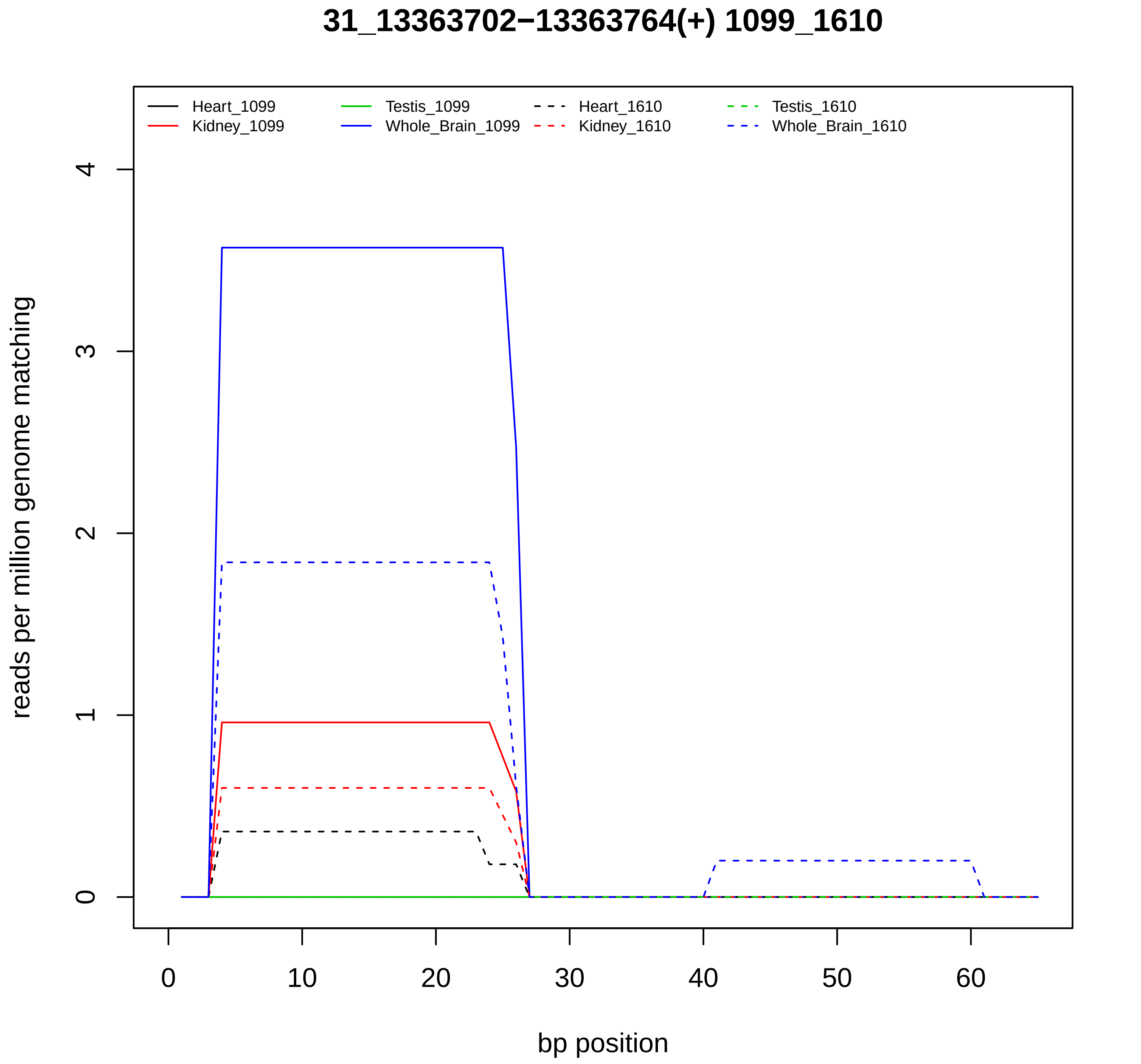

31_13363702−13363764(+) 1099_1610
Heart_1099
Kidney_1099
Testis_1099
Whole_Brain_1099
Heart_1610
Kidney_1610
Testis_1610
Whole_Brain_1610
reads per million genome matching
0
1
2
3
4
0
10
20
30
40
50
60
bp position

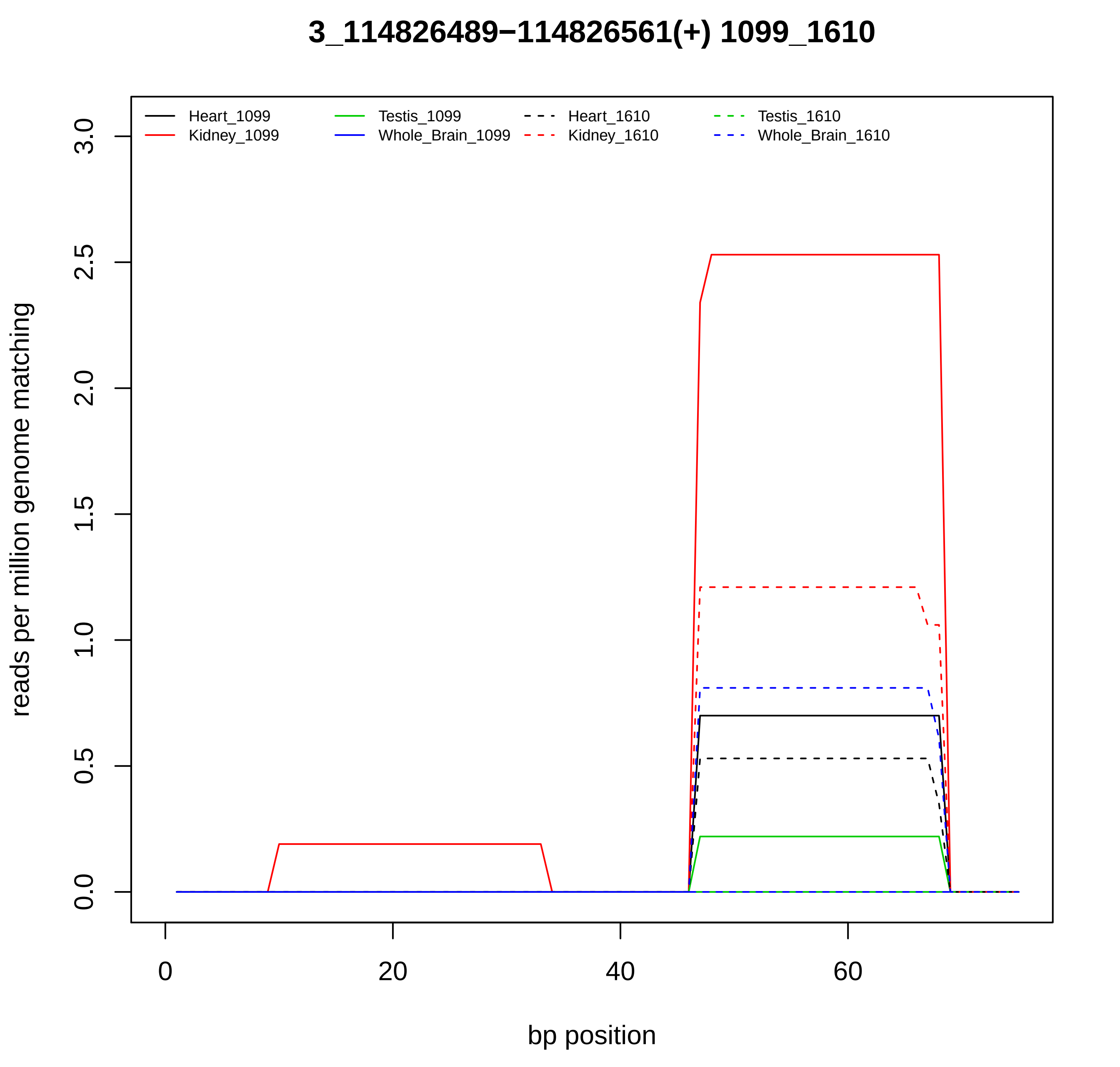

3_114826489−114826561(+) 1099_1610
Heart_1099
Kidney_1099
Testis_1099
Whole_Brain_1099
Heart_1610
Kidney_1610
Testis_1610
Whole_Brain_1610
reads per million genome matching
bp position
0.0
0.5
1.0
1.5
2.0
2.5
3.0
0
20
40
60

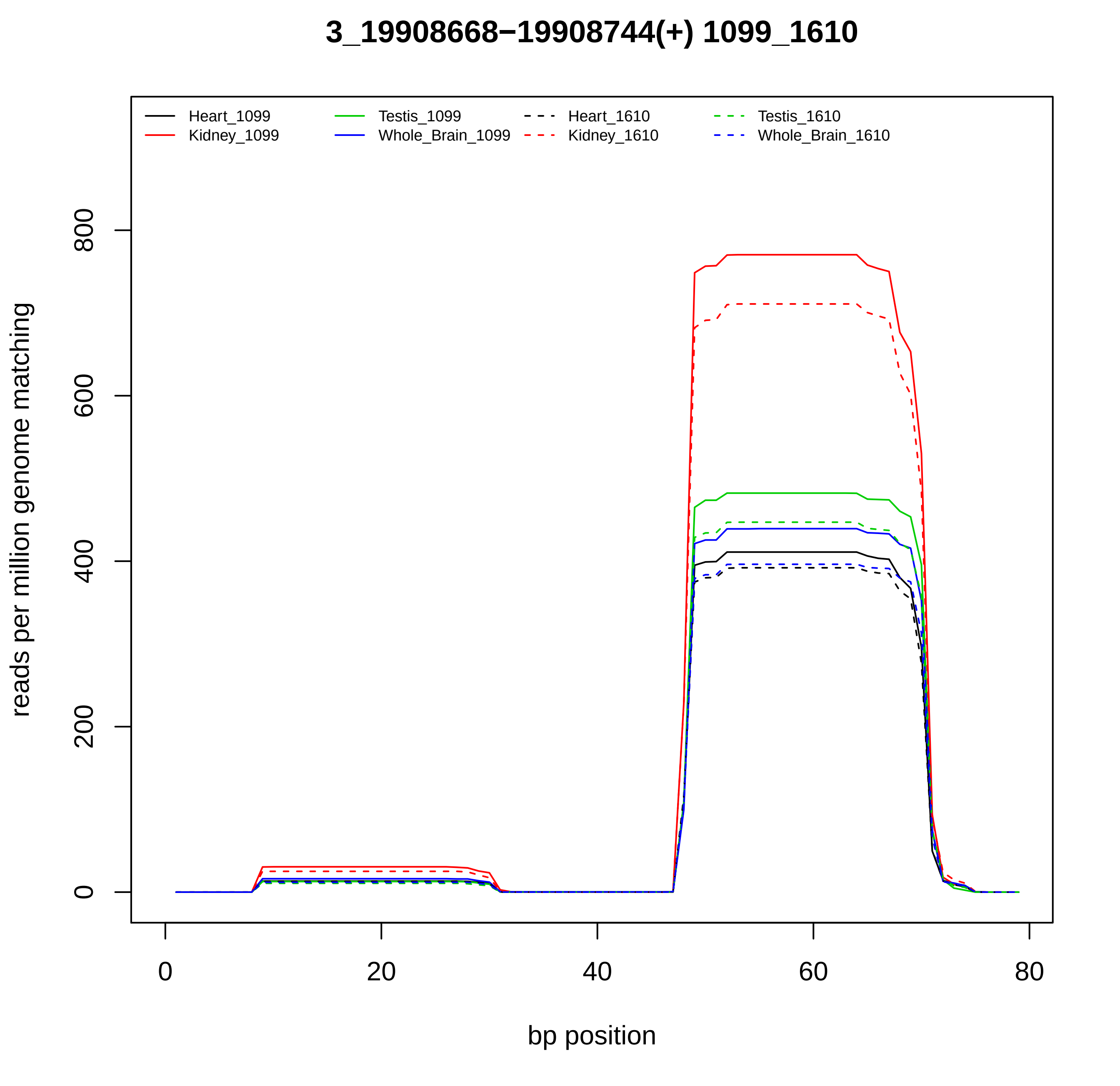

3_19908668−19908744(+) 1099_1610
Heart_1099
Kidney_1099
Testis_1099
Whole_Brain_1099
Heart_1610
Kidney_1610
Testis_1610
Whole_Brain_1610
reads per million genome matching
bp position
0
200
400
600
800
0
20
40
60
80

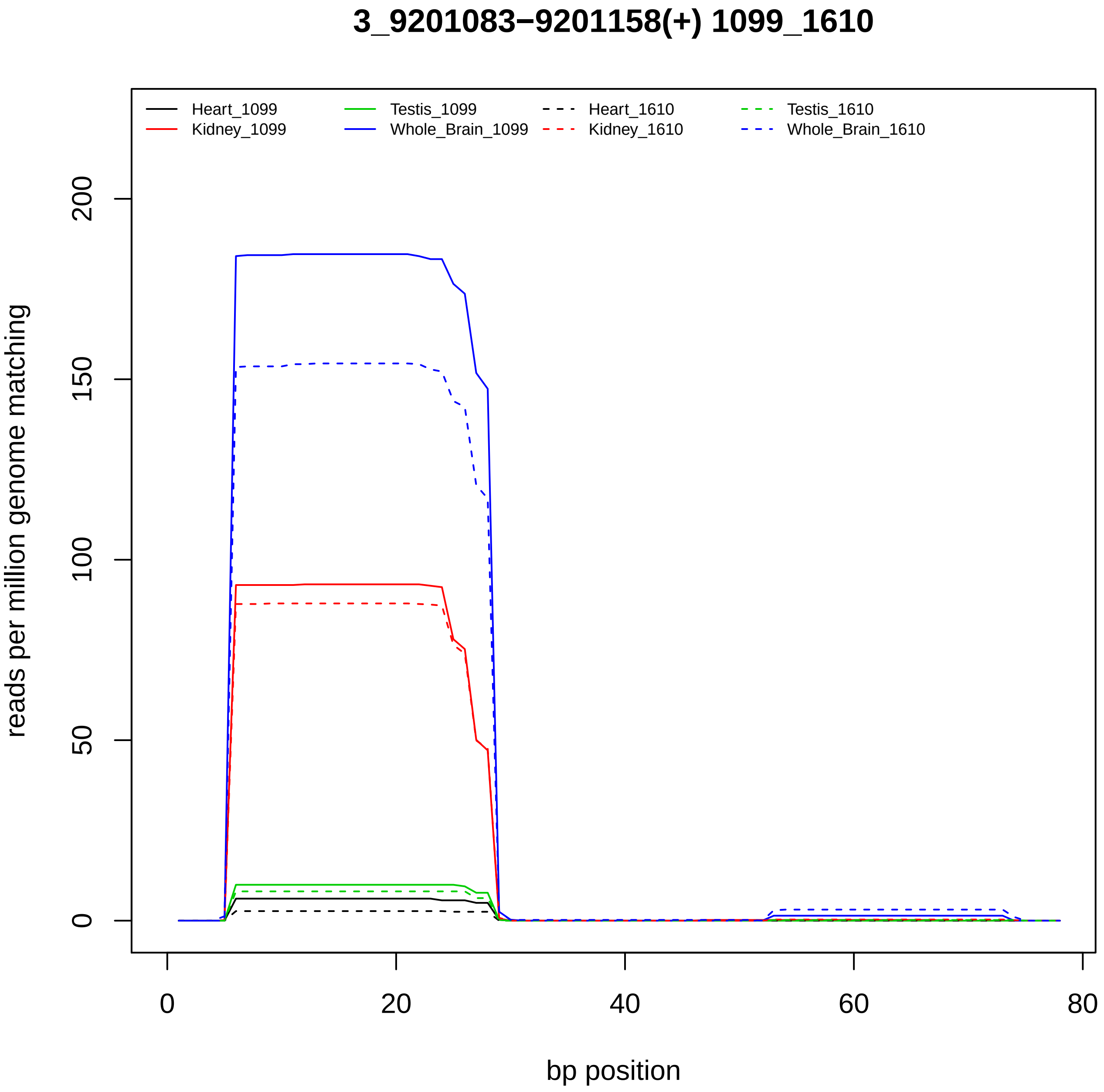

3_9201083−9201158(+) 1099_1610
Heart_1099
Kidney_1099
Testis_1099
Whole_Brain_1099
Heart_1610
Kidney_1610
Testis_1610
Whole_Brain_1610
reads per million genome matching
0
50
100
150
200
bp position
0
20
40
60
80

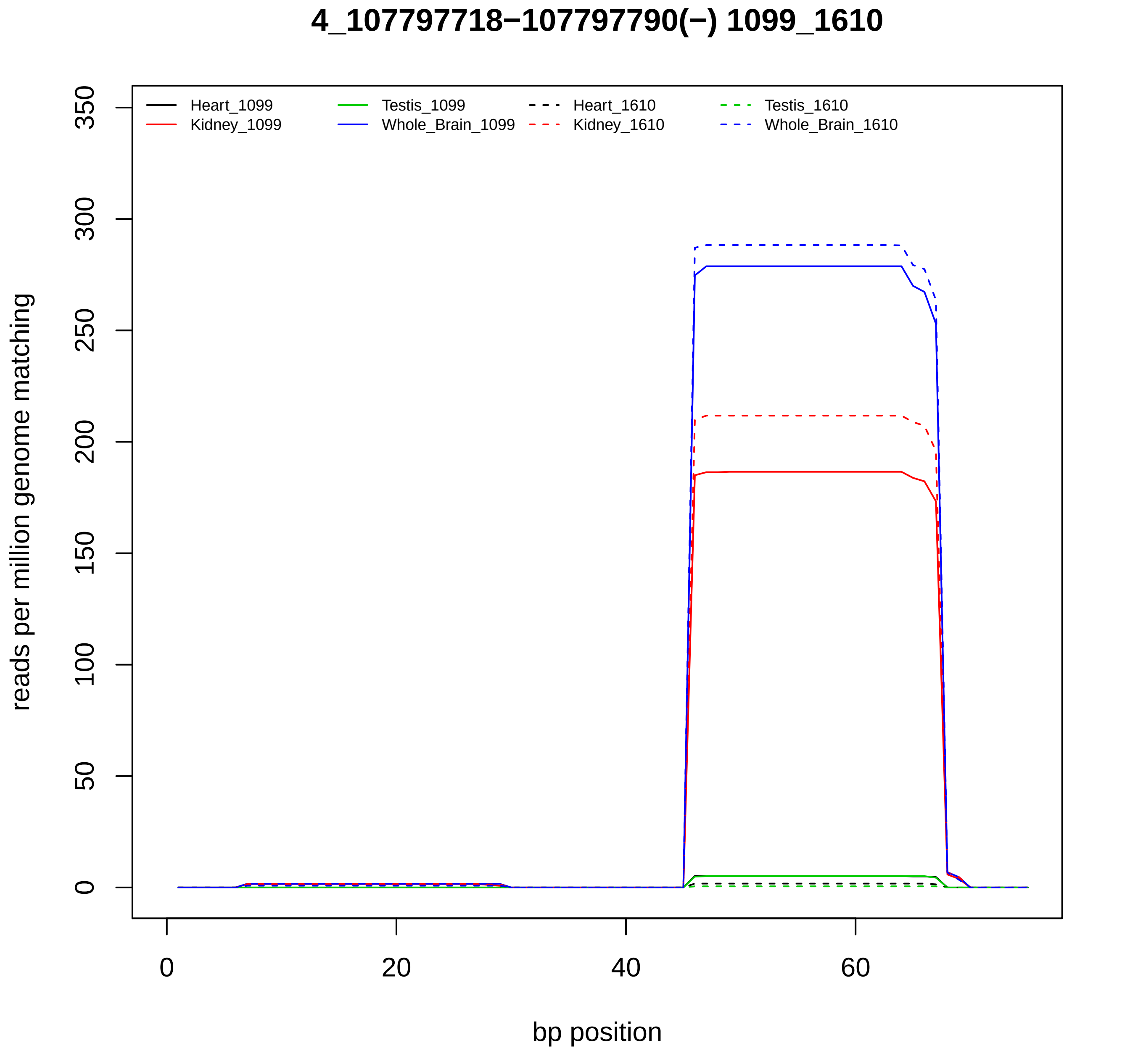

4_107797718−107797790(−) 1099_1610
Heart_1099
Kidney_1099
Testis_1099
Whole_Brain_1099
Heart_1610
Kidney_1610
Testis_1610
Whole_Brain_1610
reads per million genome matching
0
50
100
150
200
250
300
350
0
20
40
60
bp position

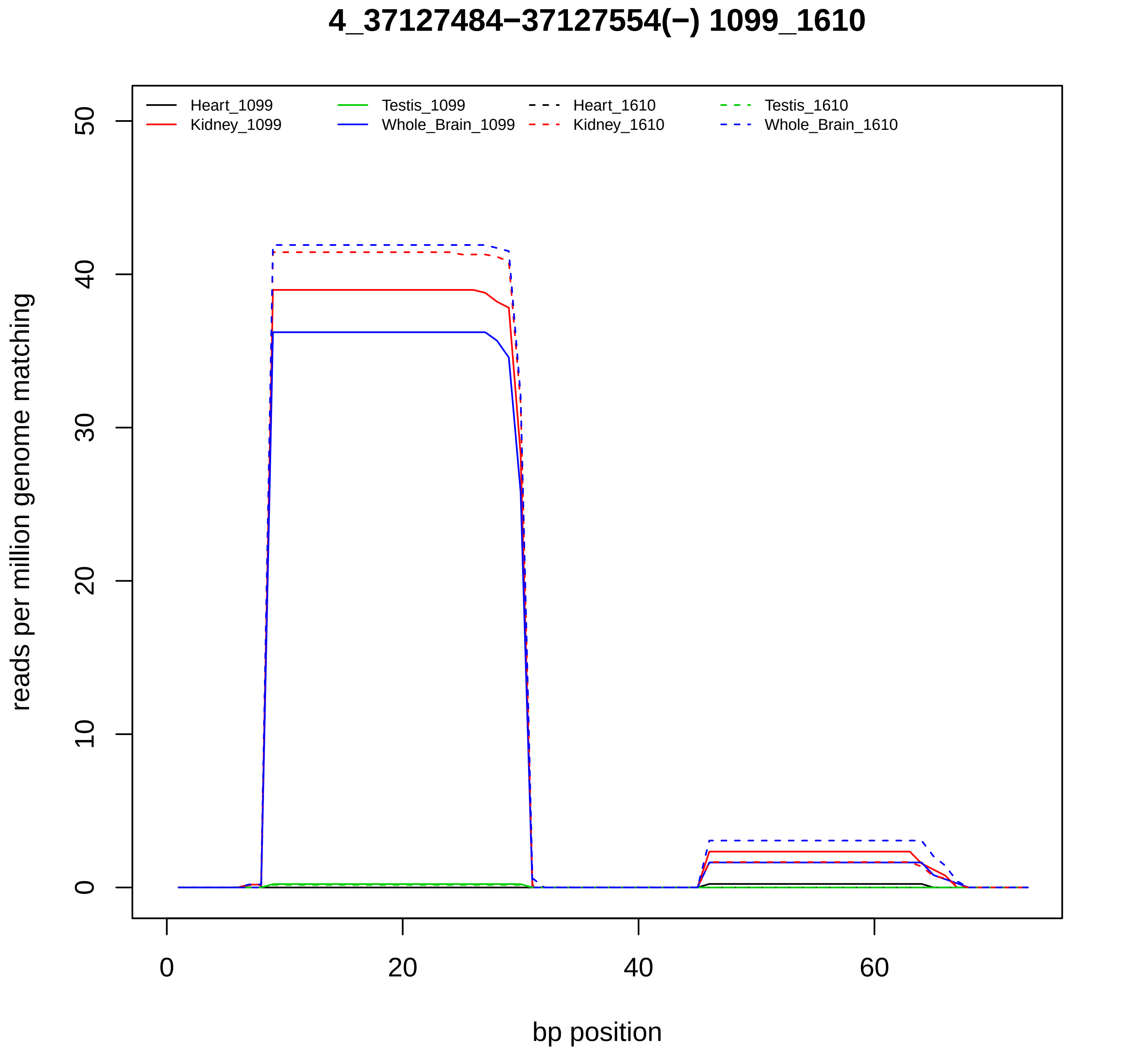

4_37127484−37127554(−) 1099_1610
Heart_1099
Kidney_1099
Testis_1099
Whole_Brain_1099
Heart_1610
Kidney_1610
Testis_1610
Whole_Brain_1610
reads per million genome matching
bp position
0
10
20
30
40
50
0
20
40
60

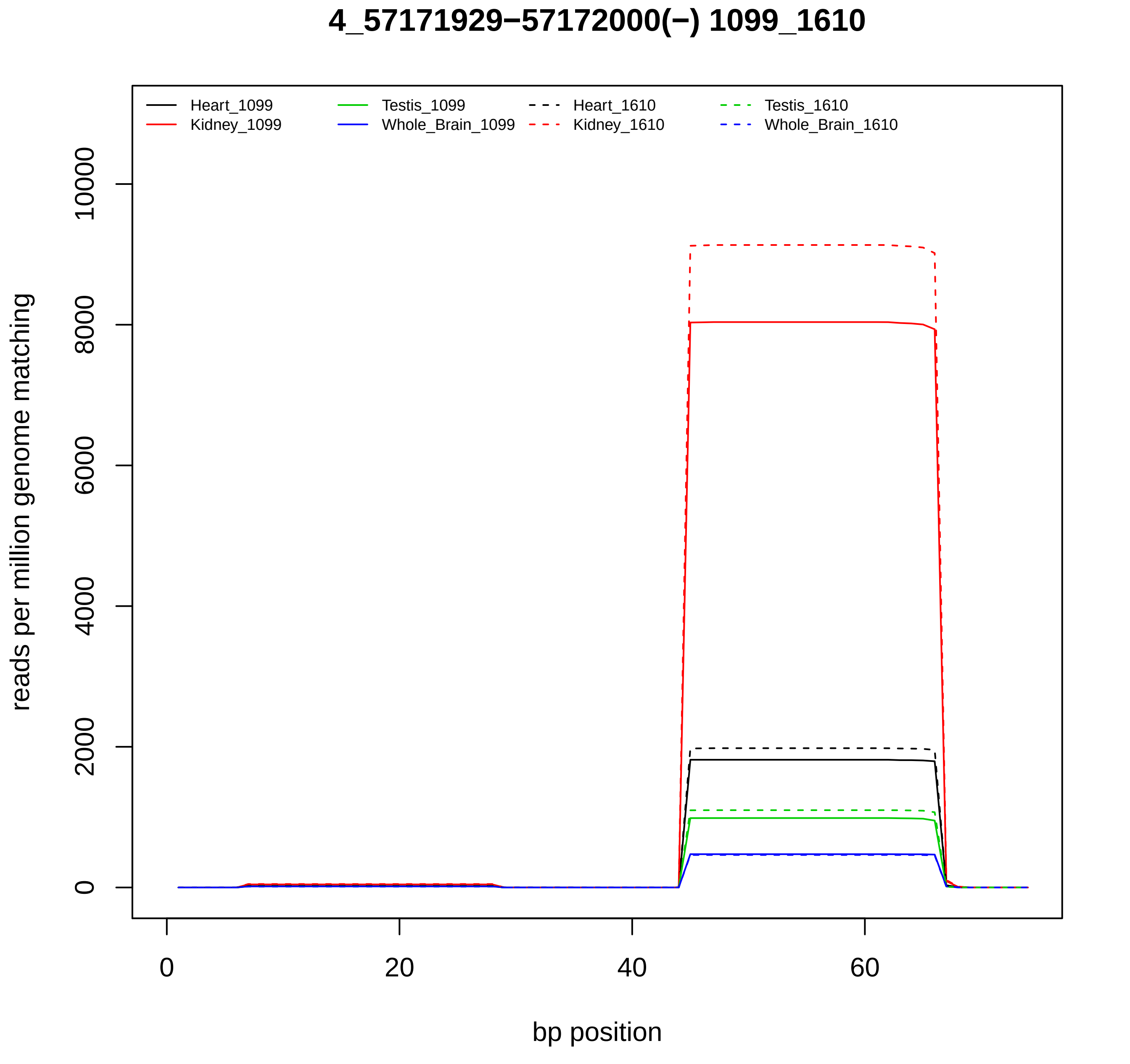

4_57171929−57172000(−) 1099_1610
Heart_1099
Kidney_1099
Testis_1099
Whole_Brain_1099
Heart_1610
Kidney_1610
Testis_1610
Whole_Brain_1610
reads per million genome matching
0
2000
4000
6000
8000
10000
0
20
40
60
bp position

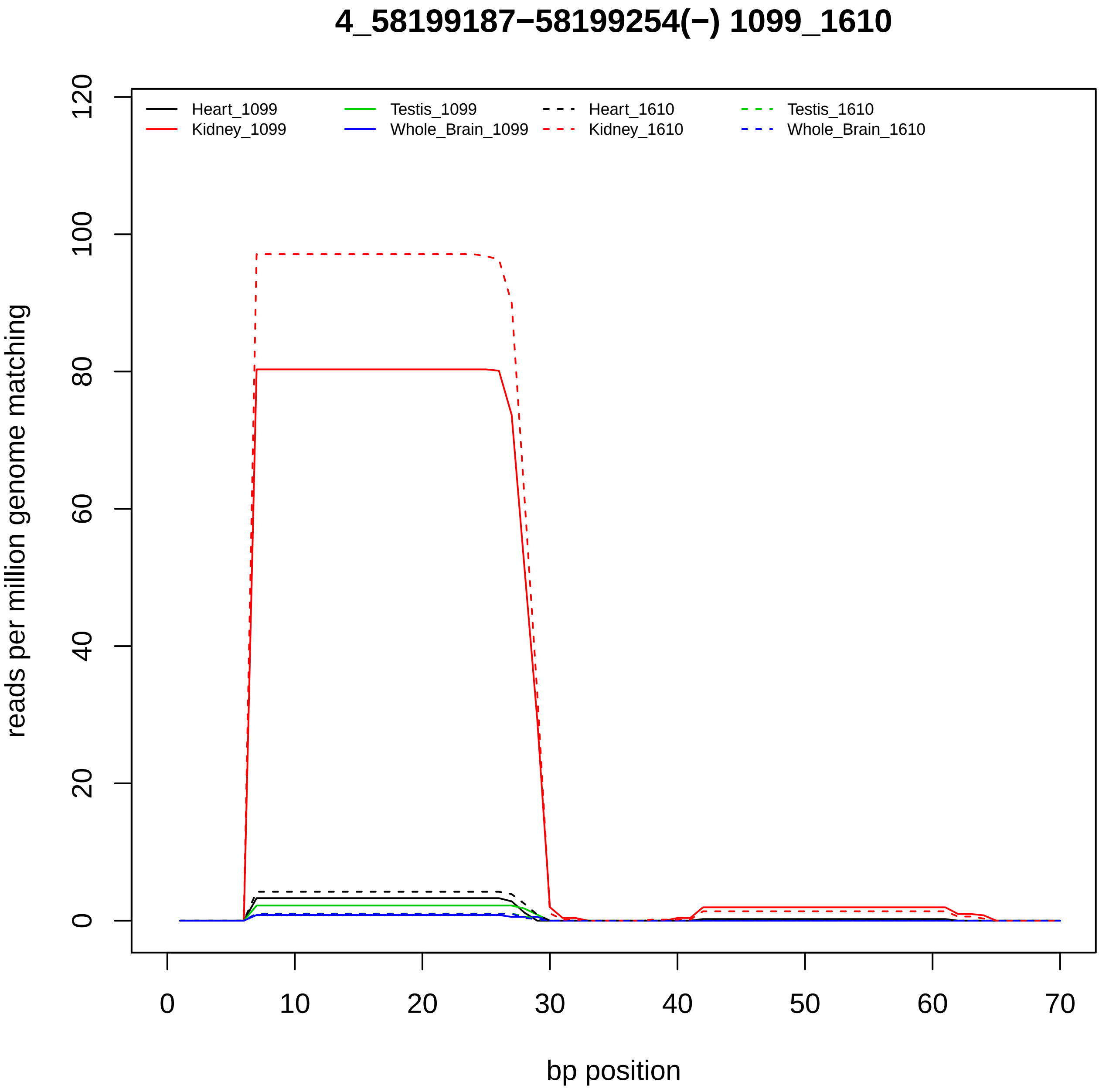

4_58199187−58199254(−) 1099_1610
Heart_1099
Kidney_1099
Testis_1099
Whole_Brain_1099
Heart_1610
Kidney_1610
Testis_1610
Whole_Brain_1610
reads per million genome matching
bp position
0
20
40
60
80
100
120
0
10
20
30
40
50
60
70

4_83387805−83387877(+) 1099_1610

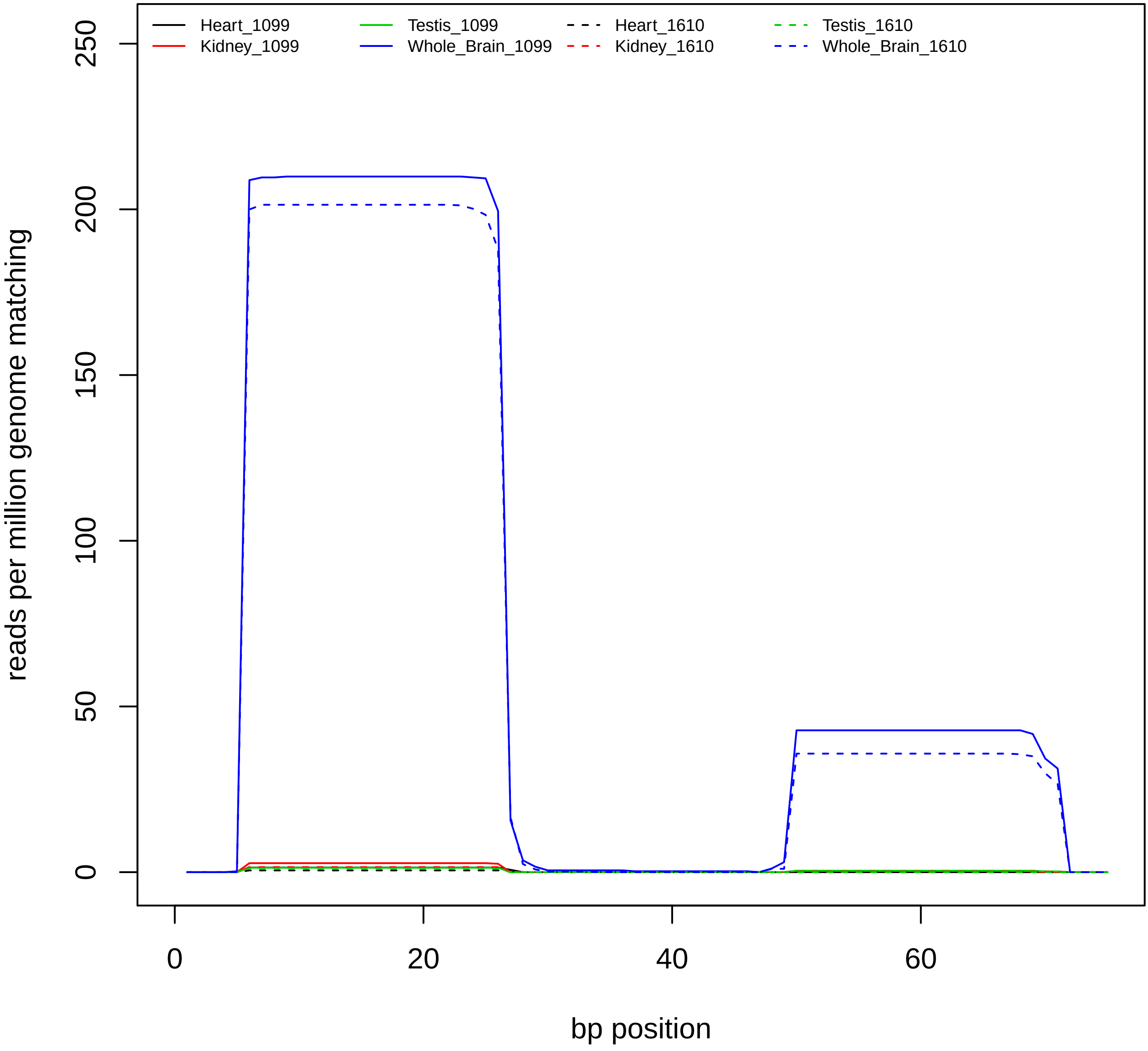

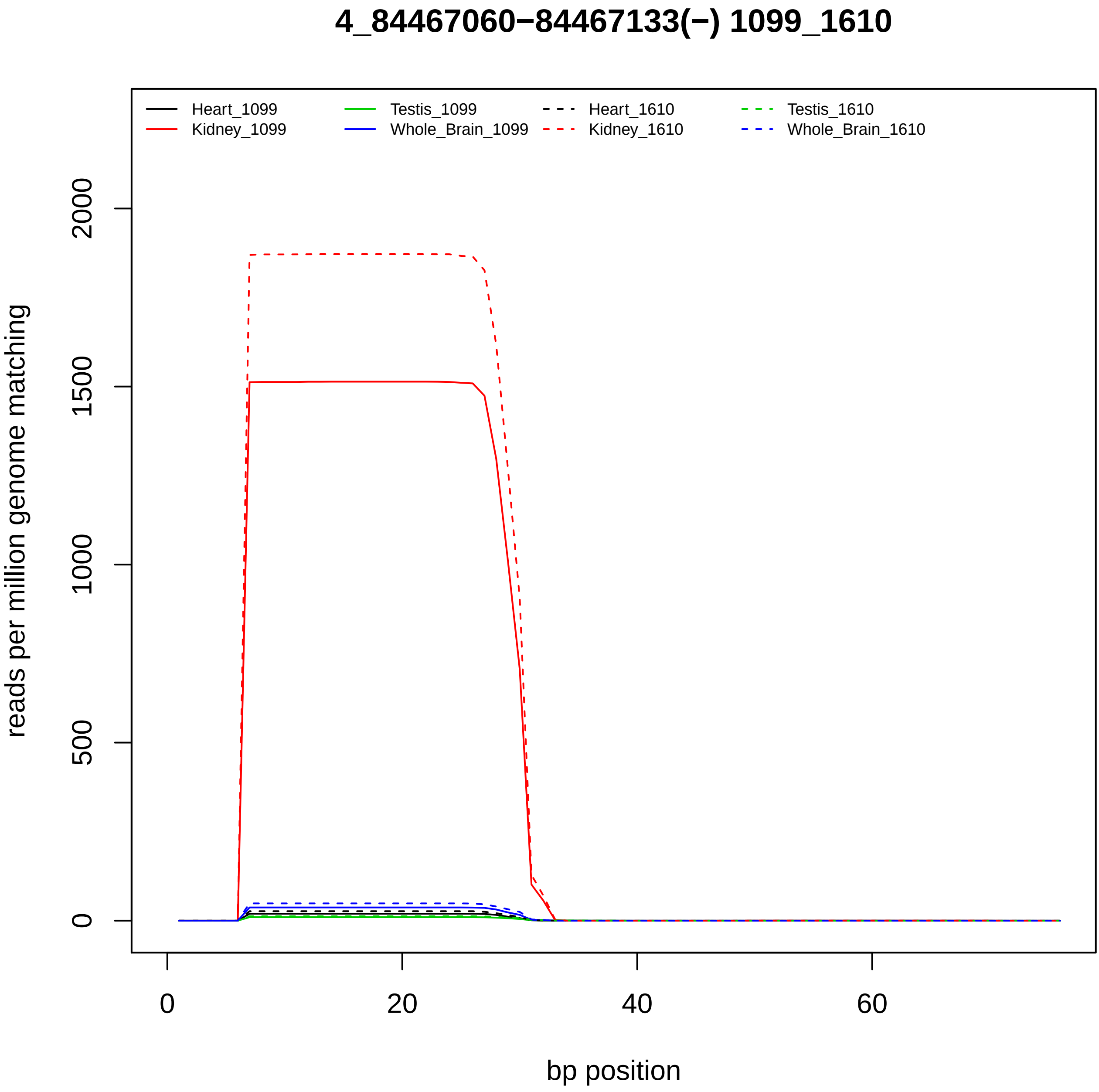

4_84467060−84467133(−) 1099_1610
Heart_1099
Kidney_1099
Testis_1099
Whole_Brain_1099
Heart_1610
Kidney_1610
Testis_1610
Whole_Brain_1610
reads per million genome matching
0
500
1000
1500
2000
0
20
40
60
bp position

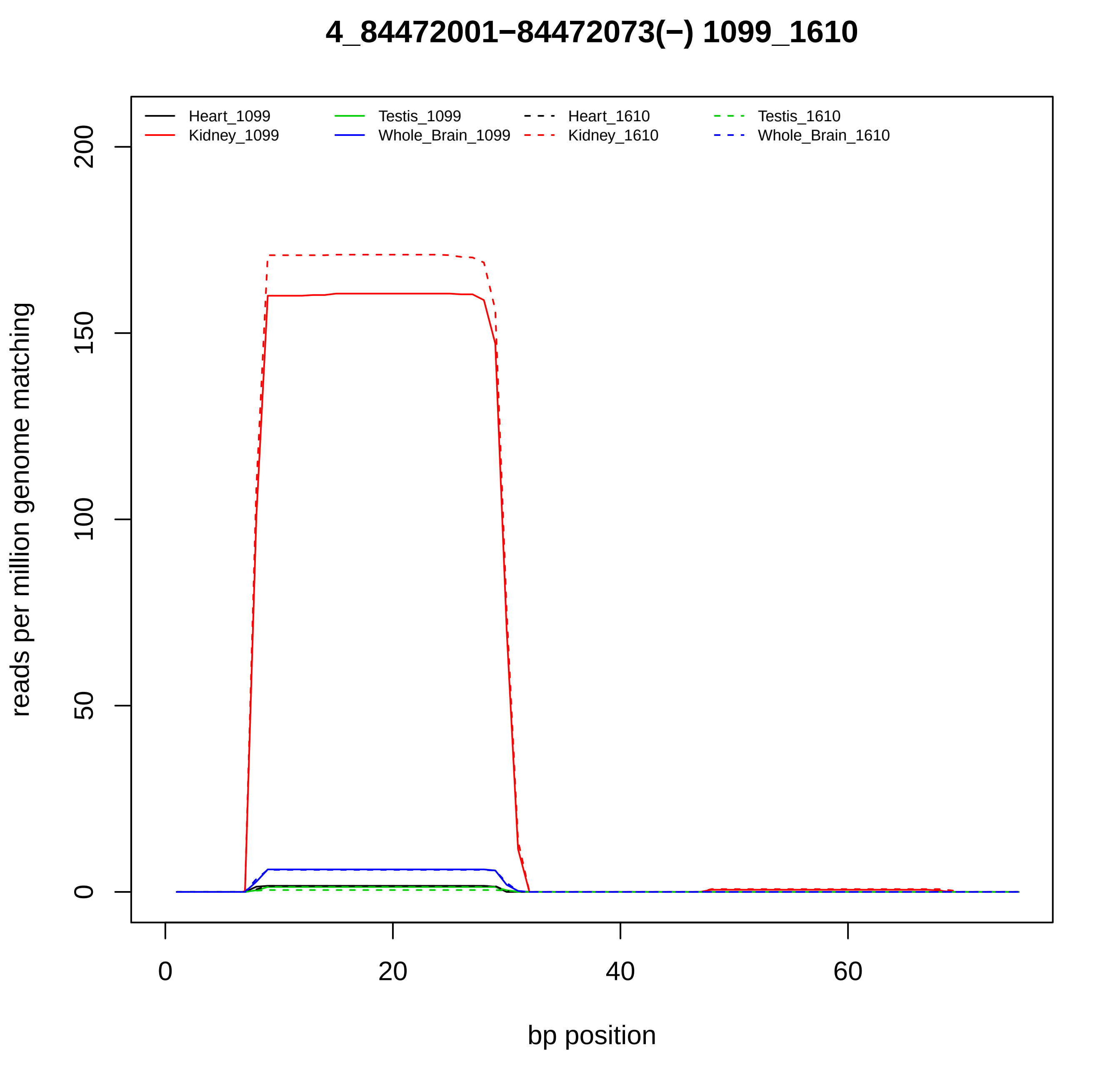
4_84472001−84472073(−) 1099_1610
Heart_1099
Kidney_1099
Testis_1099
Whole_Brain_1099
Heart_1610
Kidney_1610
Testis_1610
Whole_Brain_1610
reads per million genome matching
0
50
100
150
200
0
20
40
60
bp position

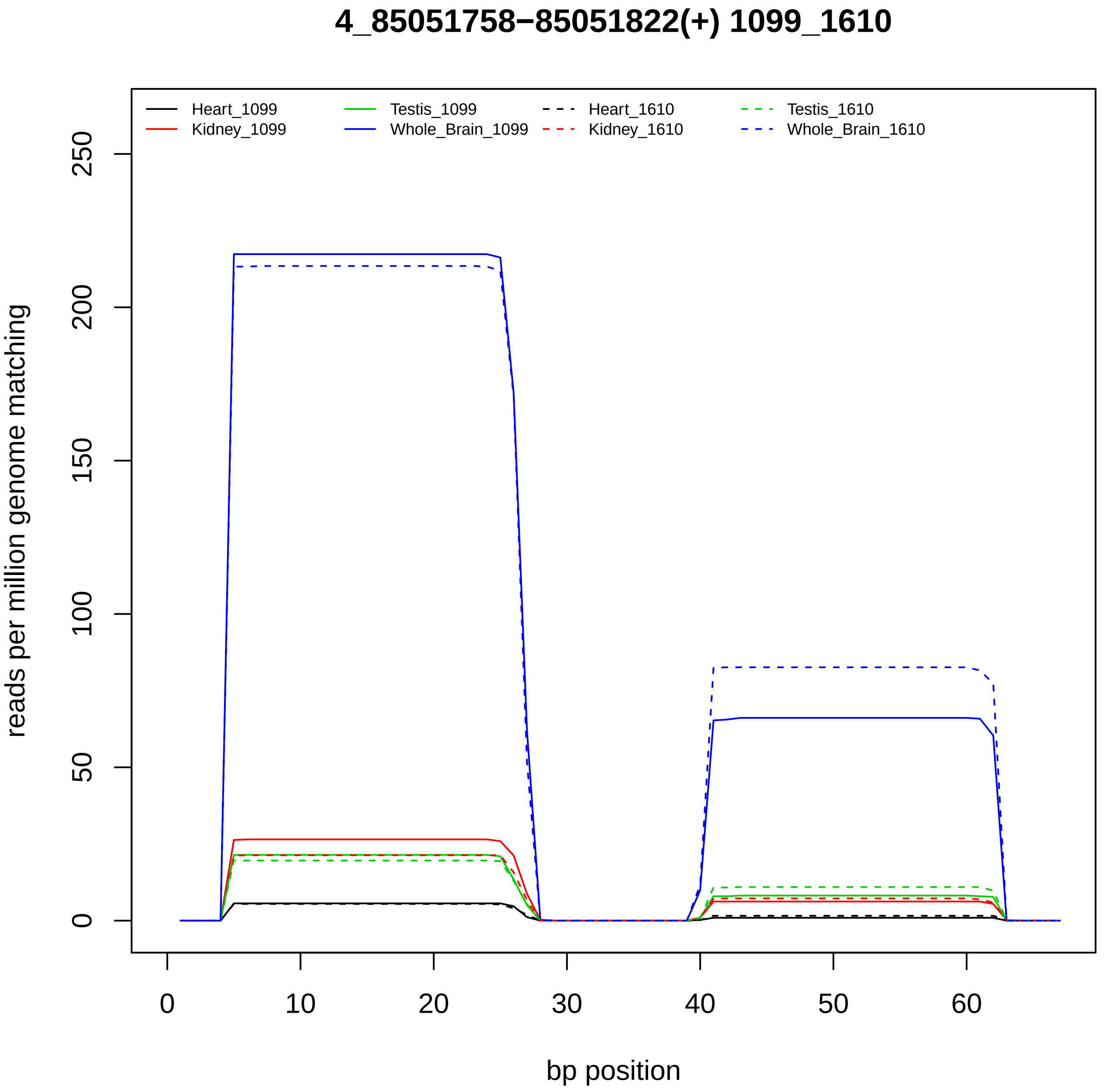

4_85051758−85051822(+) 1099_1610
Heart_1099
Kidney_1099
Testis_1099
Whole_Brain_1099
Heart_1610
Kidney_1610
Testis_1610
Whole_Brain_1610
reads per million genome matching
0
50
100
150
200
250
0
10
20
30
40
50
60
bp position

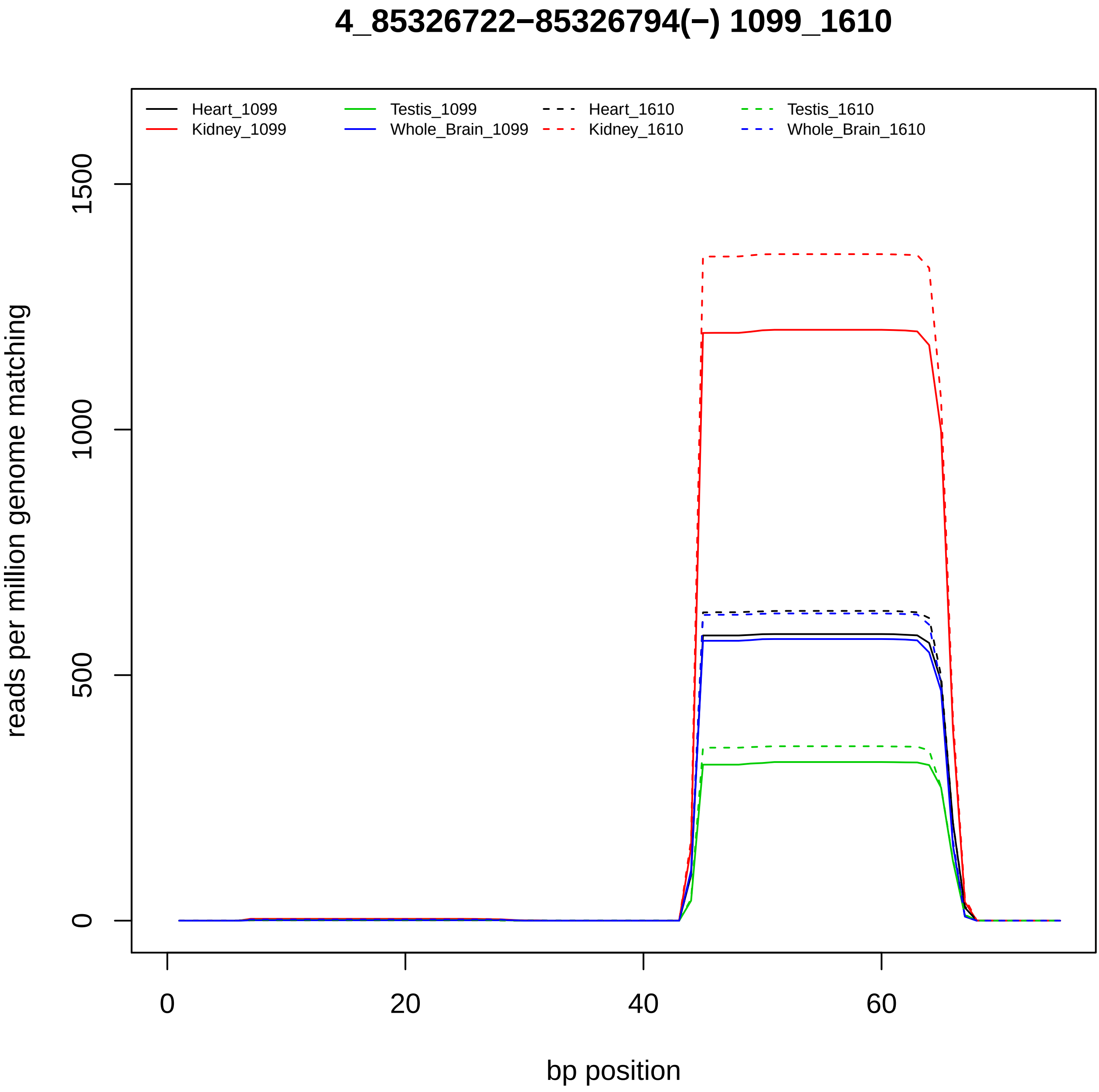
4_85326722−85326794(−) 1099_1610
Heart_1099
Kidney_1099
Testis_1099
Whole_Brain_1099
Heart_1610
Kidney_1610
Testis_1610
Whole_Brain_1610
reads per million genome matching
0
500
1000
1500
0
20
40
60
bp position

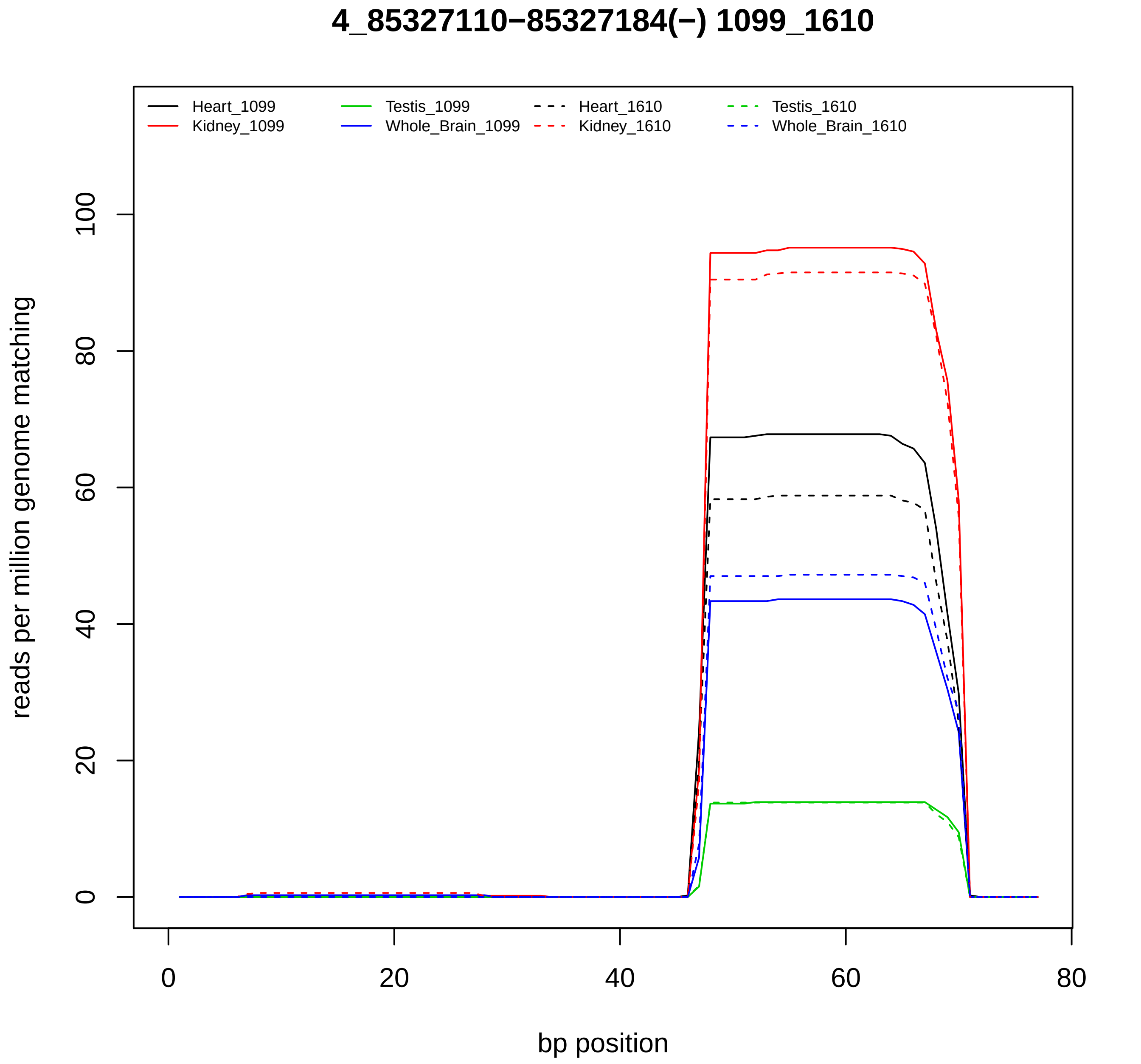

4_85327110−85327184(−) 1099_1610
Heart_1099
Kidney_1099
Testis_1099
Whole_Brain_1099
Heart_1610
Kidney_1610
Testis_1610
Whole_Brain_1610
reads per million genome matching
0
20
40
60
80
100
0
20
40
60
80
bp position

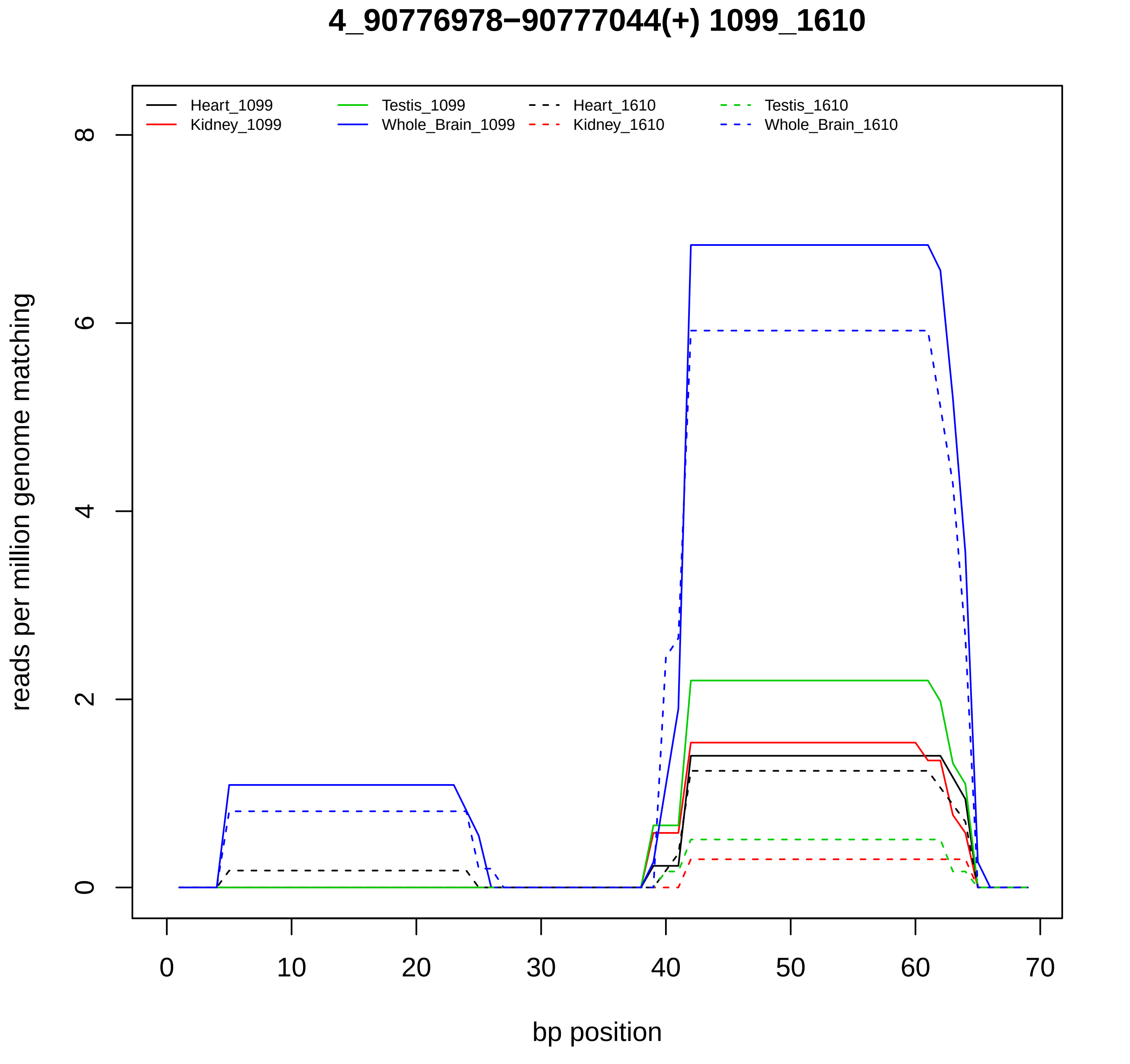
4_90776978−90777044(+) 1099_1610
Heart_1099
Kidney_1099
Testis_1099
Whole_Brain_1099
Heart_1610
Kidney_1610
Testis_1610
Whole_Brain_1610
reads per million genome matching
0
2
4
6
8
0
10
20
30
40
50
60
70
bp position

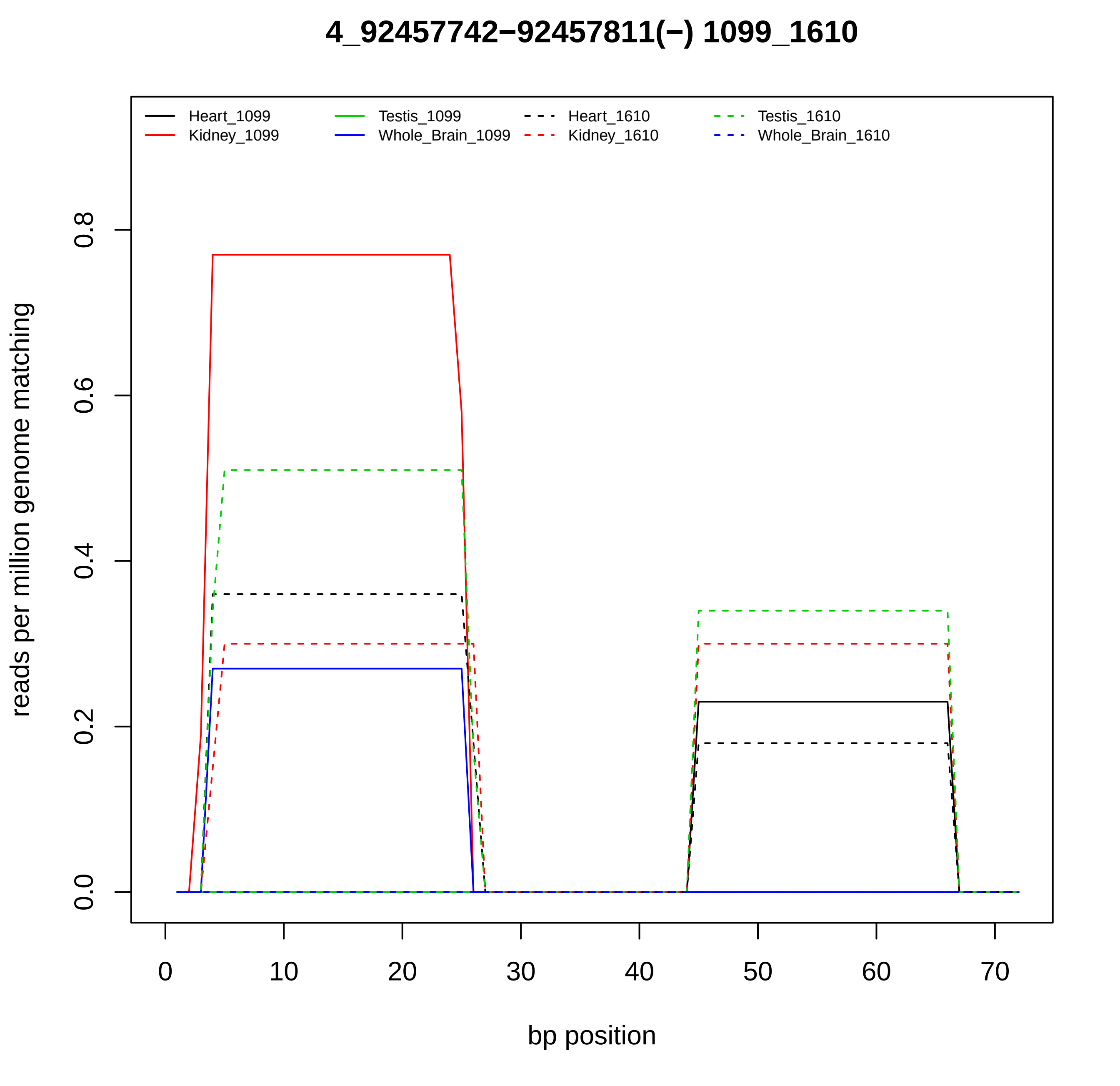

4_92457742−92457811(−) 1099_1610
Heart_1099
Kidney_1099
Testis_1099
Whole_Brain_1099
Heart_1610
Kidney_1610
Testis_1610
Whole_Brain_1610
reads per million genome matching
0.0
0.2
0.4
0.6
0.8
bp position
0
10
20
30
40
50
60
70

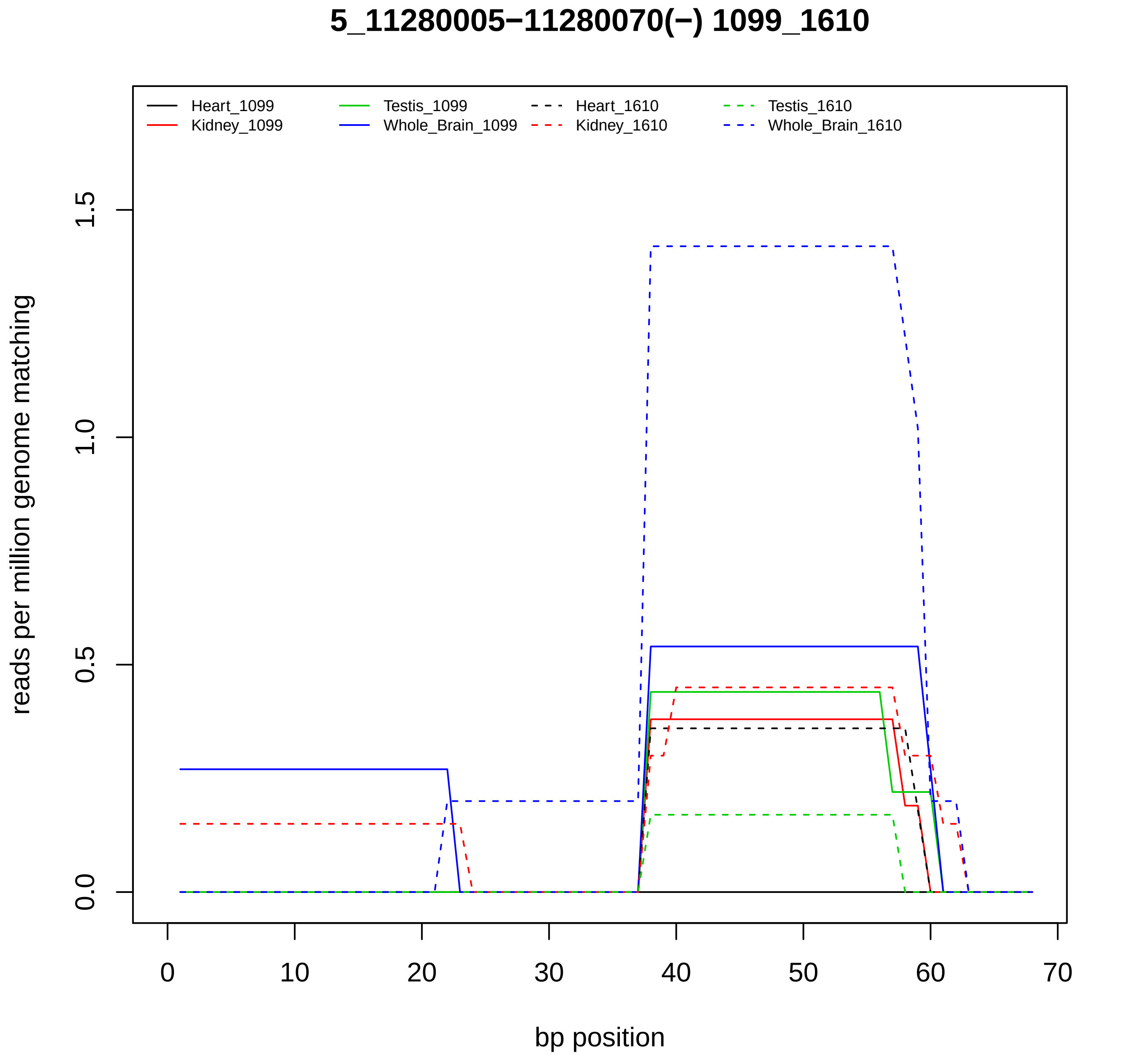

5_11280005−11280070(−) 1099_1610
Heart_1099
Kidney_1099
Testis_1099
Whole_Brain_1099
Heart_1610
Kidney_1610
Testis_1610
Whole_Brain_1610
reads per million genome matching
0.0
0.5
1.0
1.5
0
10
20
30
40
50
60
70
bp position

5_12172414−12172482(−) 1099_1610

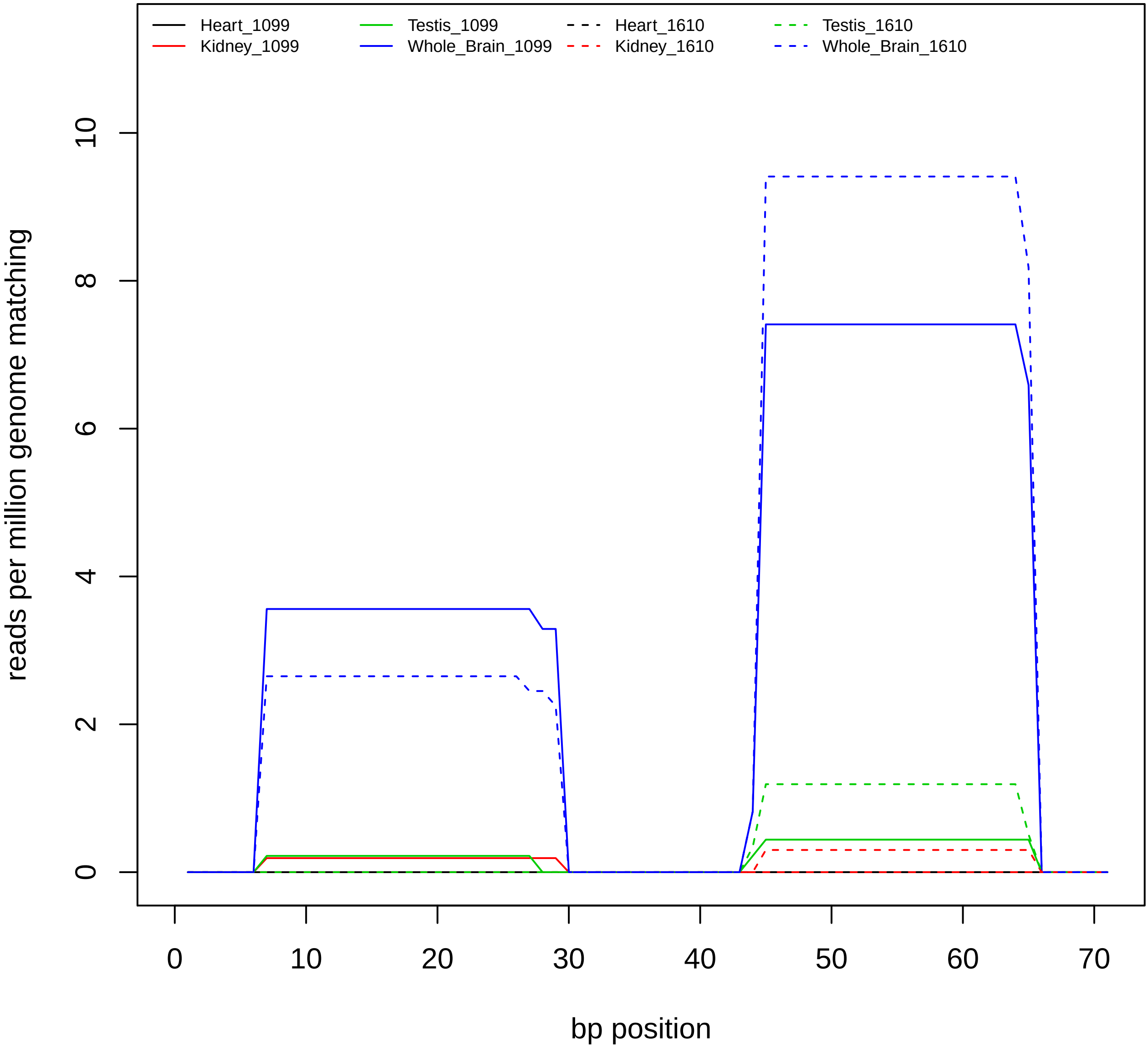

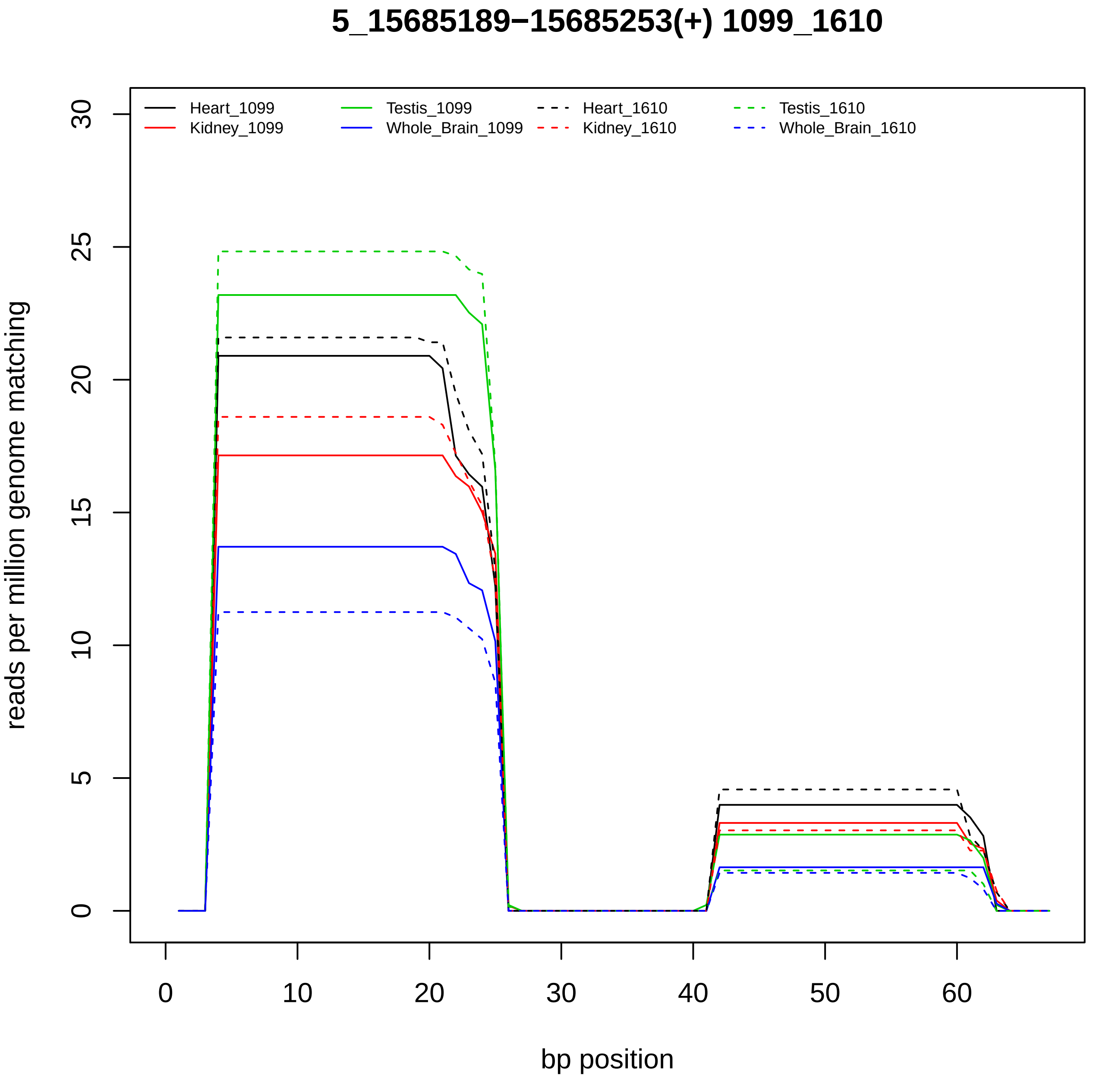

5_15685189−15685253(+) 1099_1610
Heart_1099
Kidney_1099
Testis_1099
Whole_Brain_1099
Heart_1610
Kidney_1610
Testis_1610
Whole_Brain_1610
reads per million genome matching
bp position
0
5
10
15
20
25
30
0
10
20
30
40
50
60

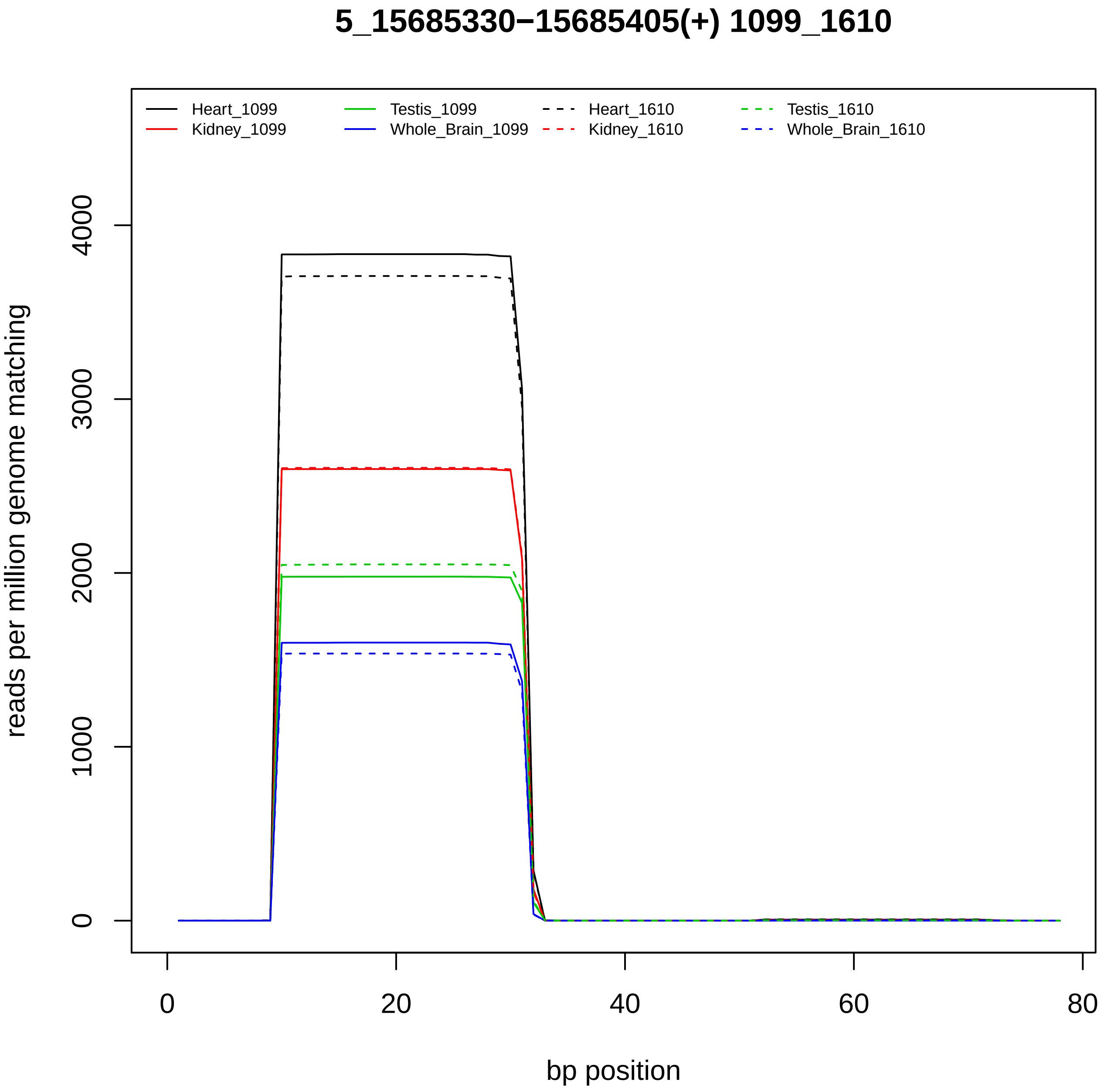
5_15685330−15685405(+) 1099_1610
Heart_1099
Kidney_1099
Testis_1099
Whole_Brain_1099
Heart_1610
Kidney_1610
Testis_1610
Whole_Brain_1610
reads per million genome matching
0
1000
2000
3000
4000
0
20
40
60
80
bp position

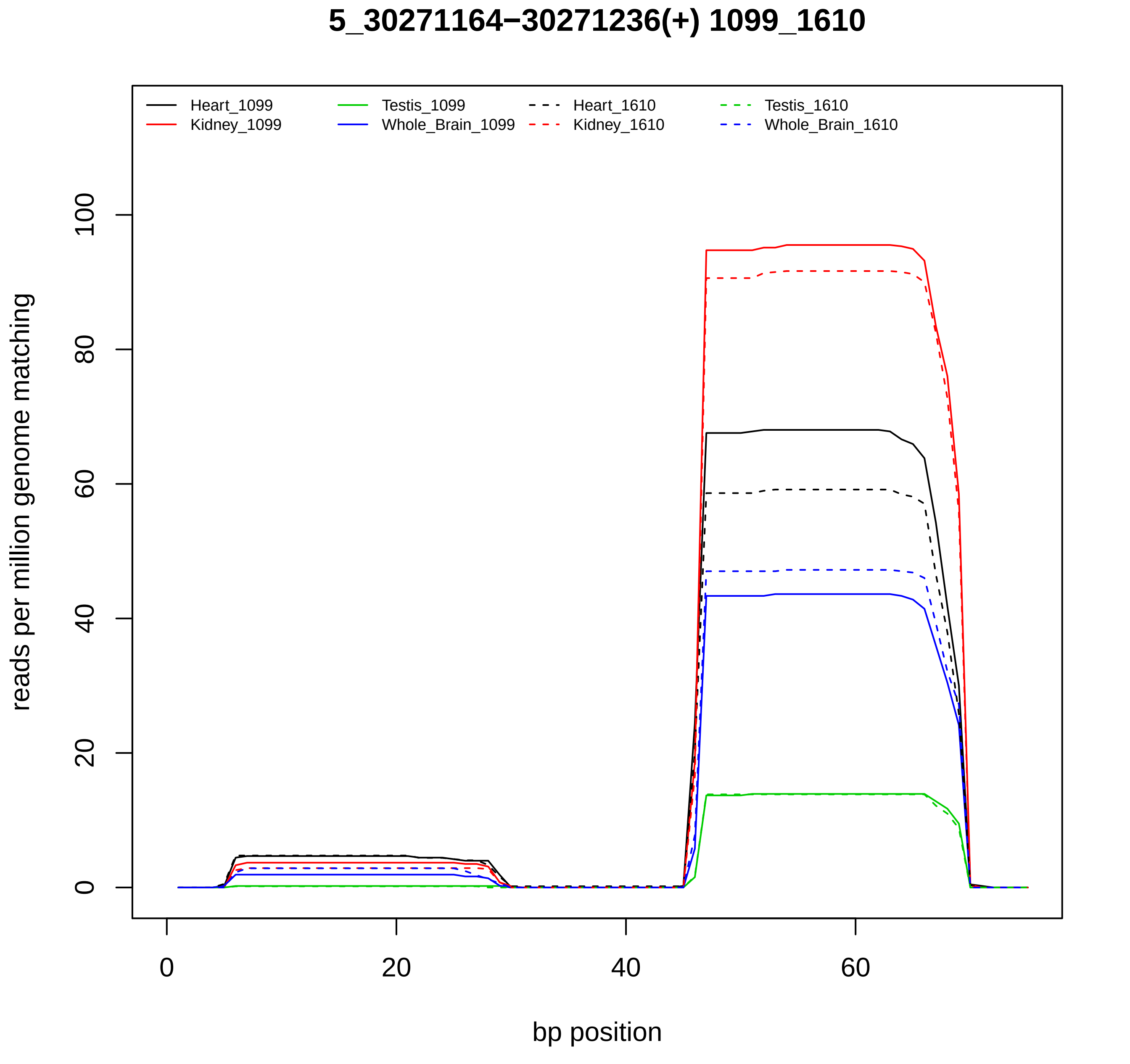

5_30271164−30271236(+) 1099_1610
Heart_1099
Kidney_1099
Testis_1099
Whole_Brain_1099
Heart_1610
Kidney_1610
Testis_1610
Whole_Brain_1610
reads per million genome matching
bp position
0
20
40
60
80
100

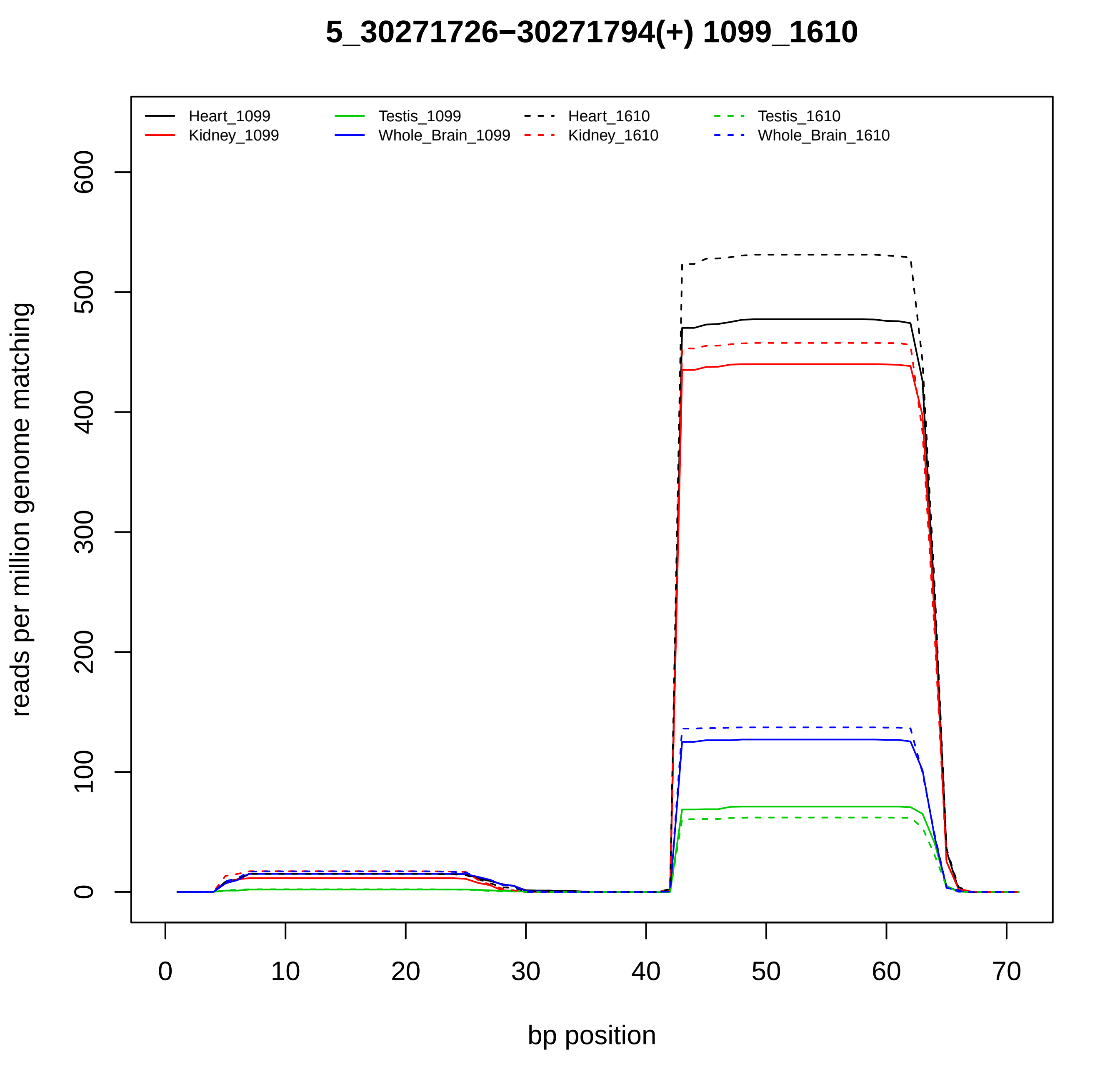

5_30271726−30271794(+) 1099_1610
Heart_1099
Kidney_1099
Testis_1099
Whole_Brain_1099
Heart_1610
Kidney_1610
Testis_1610
Whole_Brain_1610
reads per million genome matching
0
100
200
300
400
500
600
0
10
20
30
40
50
60
70
bp position

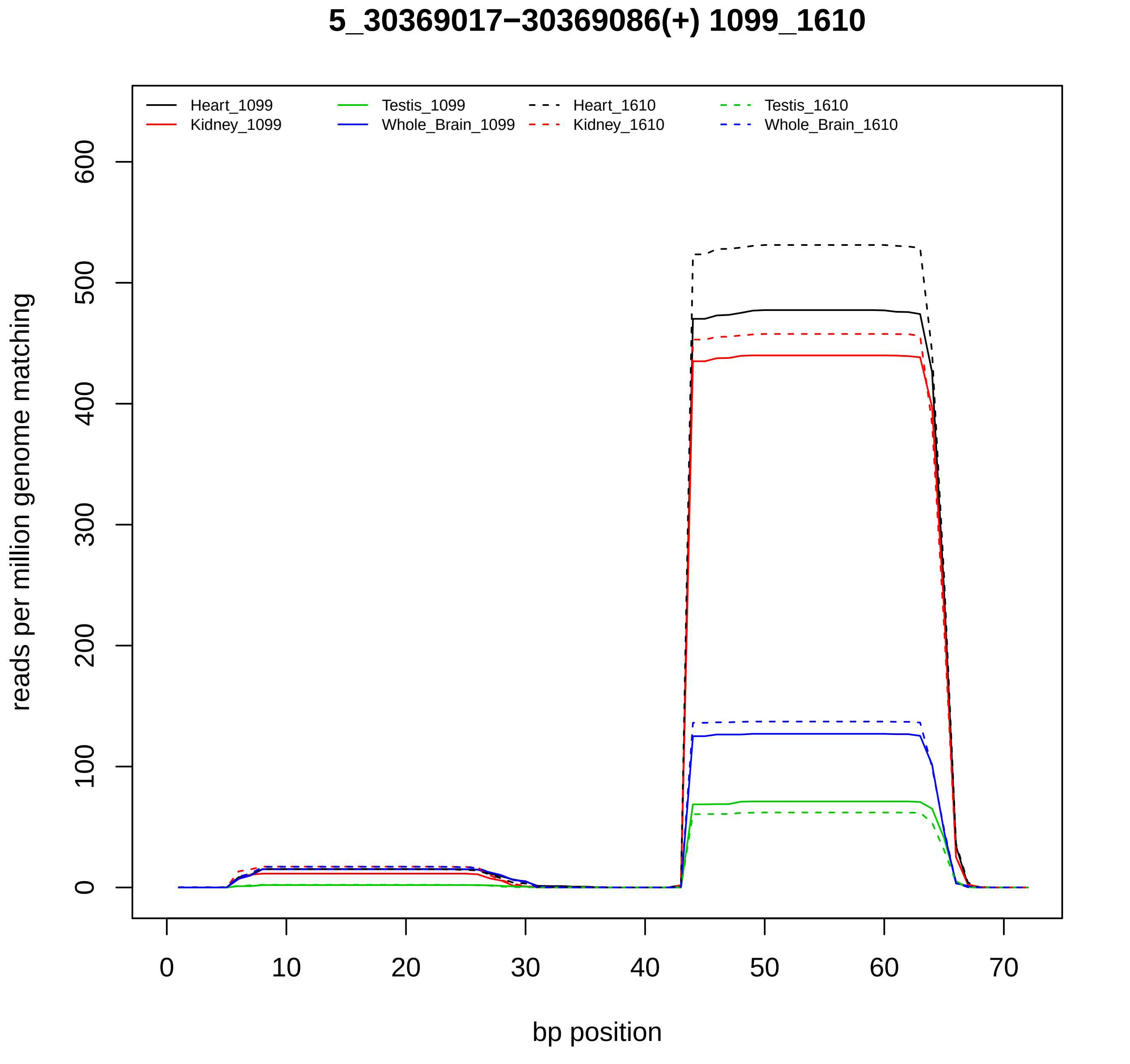

5_30369017−30369086(+) 1099_1610
Heart_1099
Kidney_1099
Testis_1099
Whole_Brain_1099
Heart_1610
Kidney_1610
Testis_1610
Whole_Brain_1610
reads per million genome matching
0
100
200
300
400
500
600
bp position
0
10
20
30
40
50
60
70

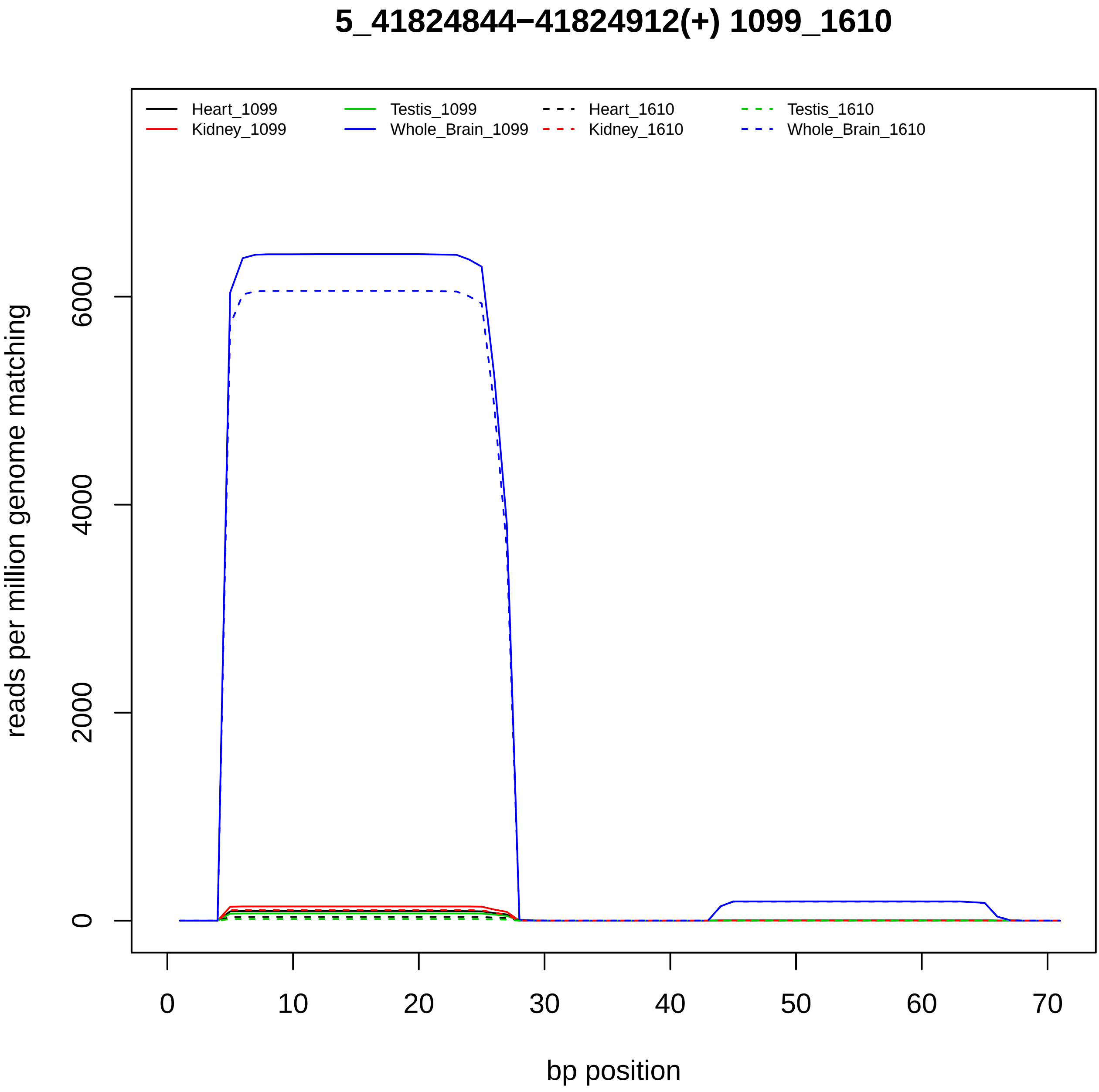

5_41824844−41824912(+) 1099_1610
Heart_1099
Kidney_1099
Testis_1099
Whole_Brain_1099
Heart_1610
Kidney_1610
Testis_1610
Whole_Brain_1610
reads per million genome matching
0
2000
4000
6000
0
10
20
30
40
50
60
70
bp position

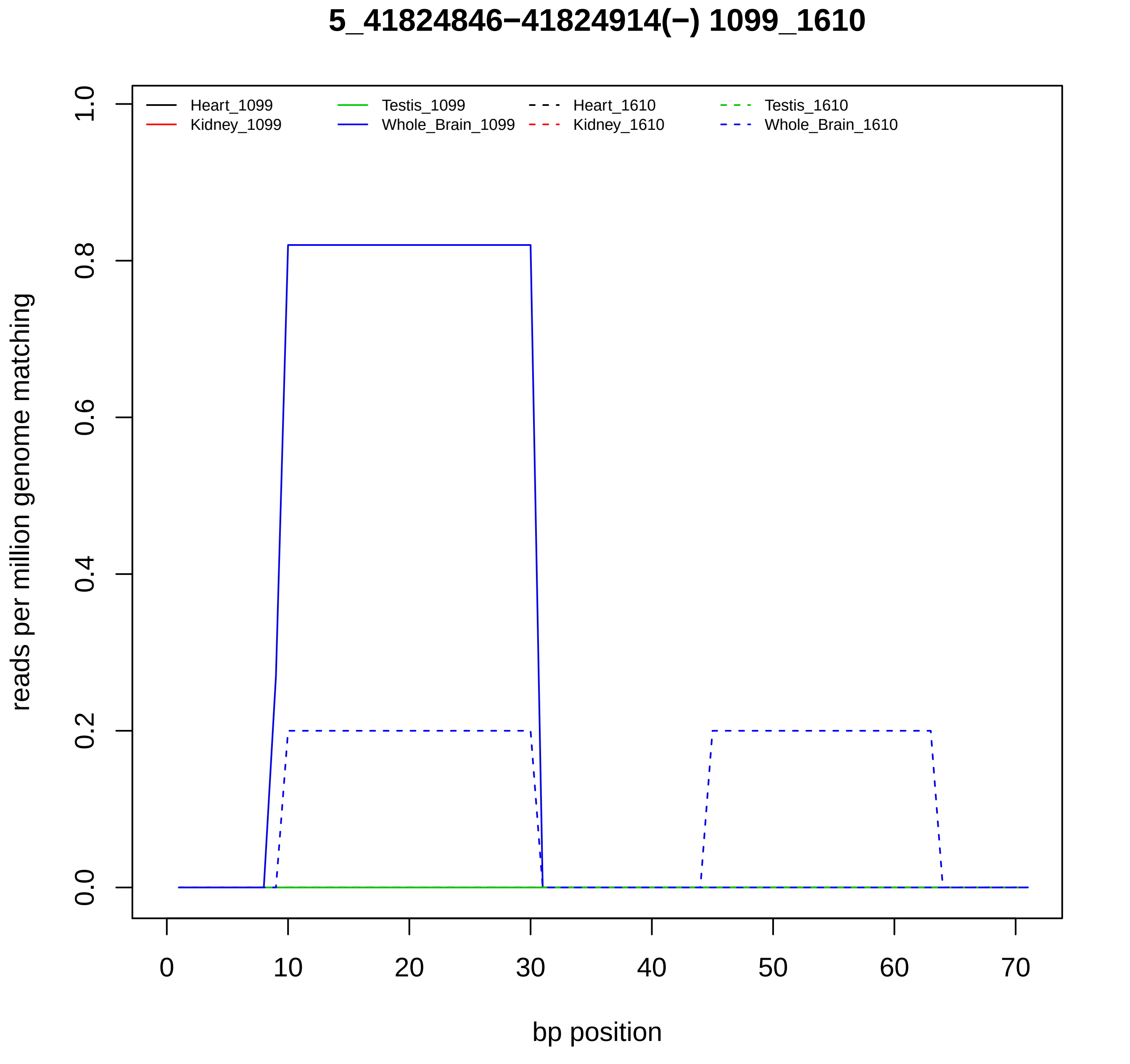

5_41824846−41824914(−) 1099_1610
Heart_1099
Kidney_1099
Testis_1099
Whole_Brain_1099
Heart_1610
Kidney_1610
Testis_1610
Whole_Brain_1610
reads per million genome matching
0.0
0.2
0.4
0.6
0.8
1.0
bp position
0
10
20
30
40
50
60
70

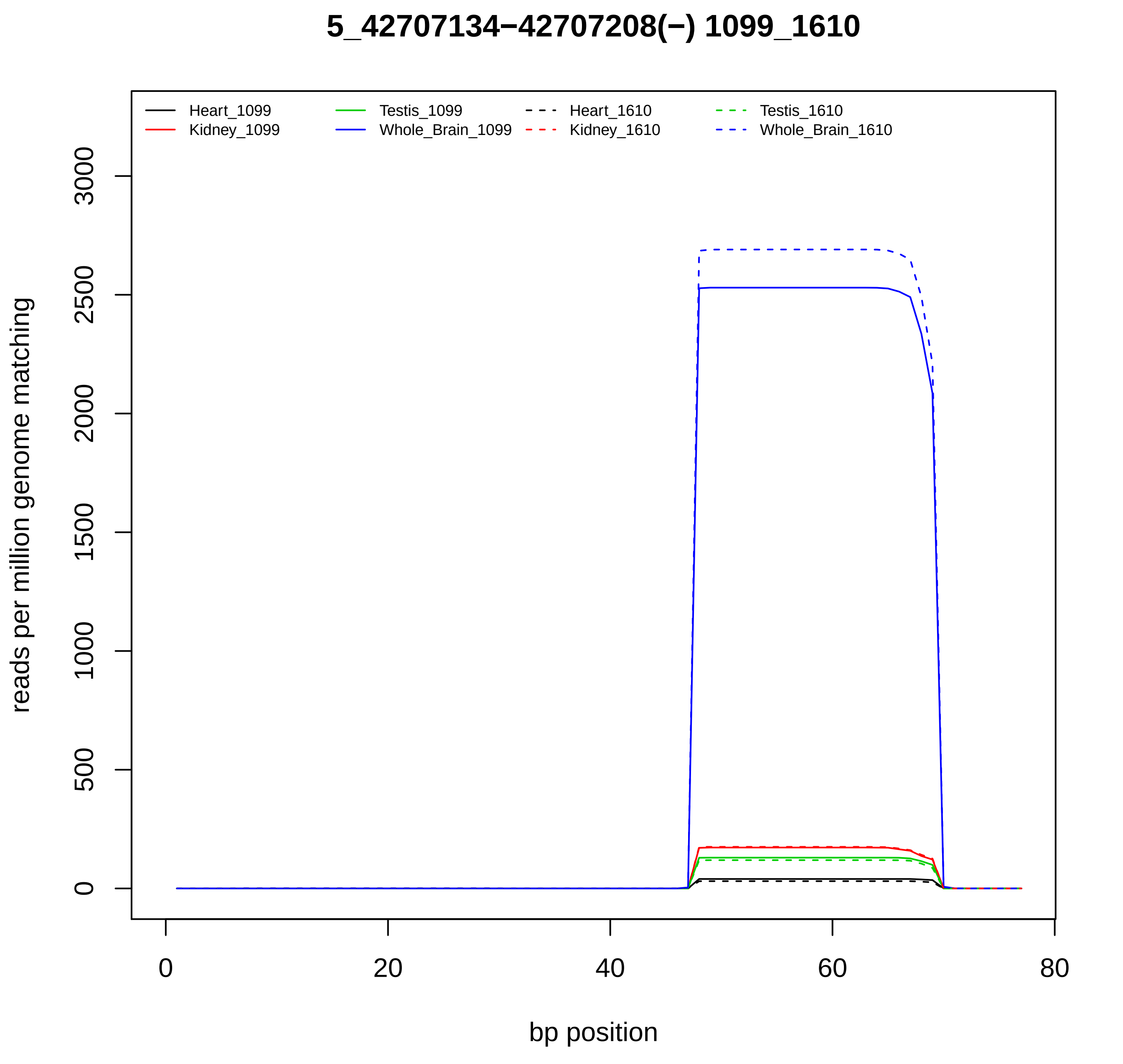

5_42707134−42707208(−) 1099_1610
Heart_1099
Kidney_1099
Testis_1099
Whole_Brain_1099
Heart_1610
Kidney_1610
Testis_1610
Whole_Brain_1610
reads per million genome matching
0
500
1000
1500
2000
2500
3000
bp position
0
20
40
60
80

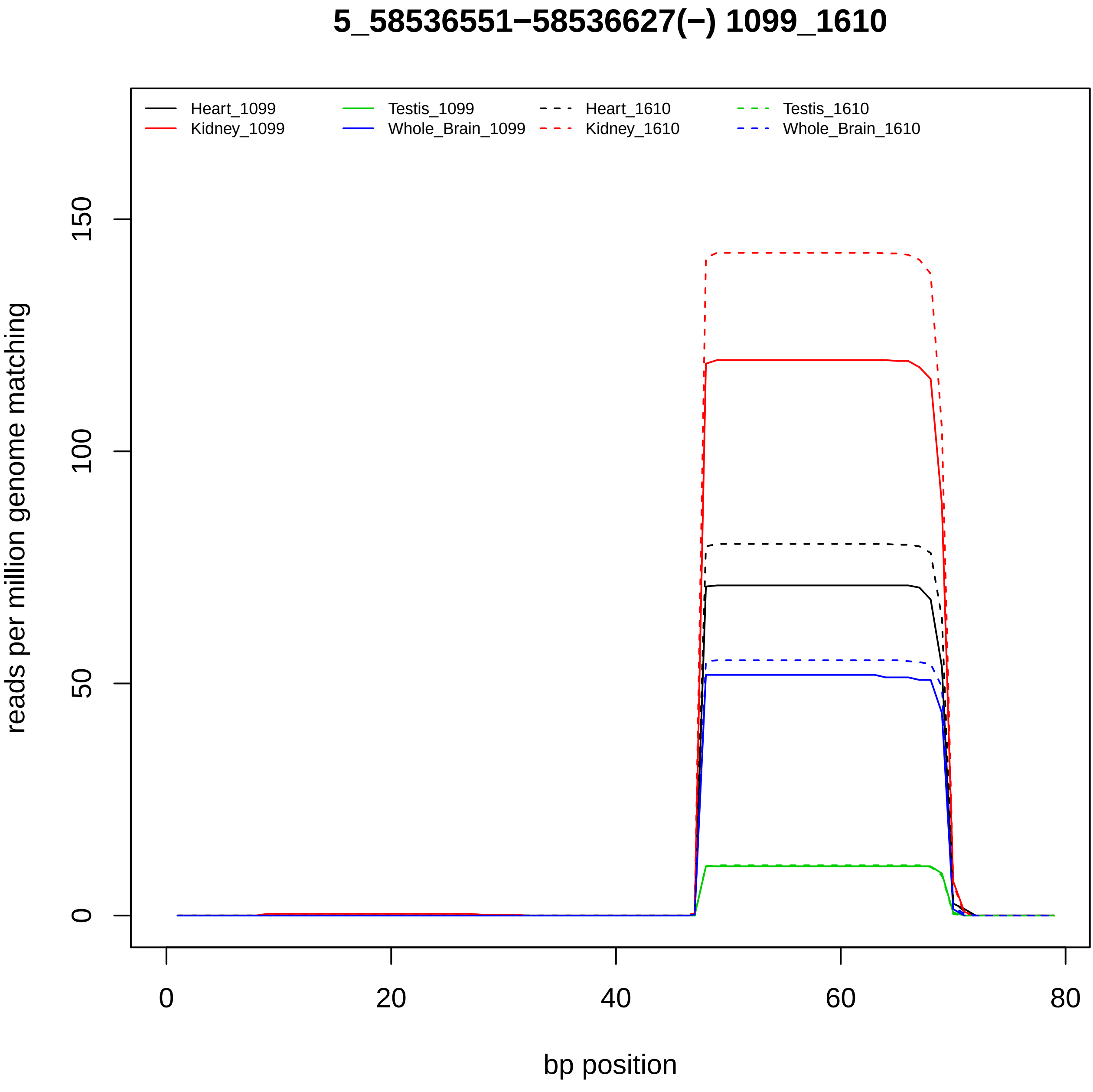

5_58536551−58536627(−) 1099_1610
Heart_1099
Kidney_1099
Testis_1099
Whole_Brain_1099
Heart_1610
Kidney_1610
Testis_1610
Whole_Brain_1610
reads per million genome matching
bp position
0
50
100
150
0
20
40
60
80

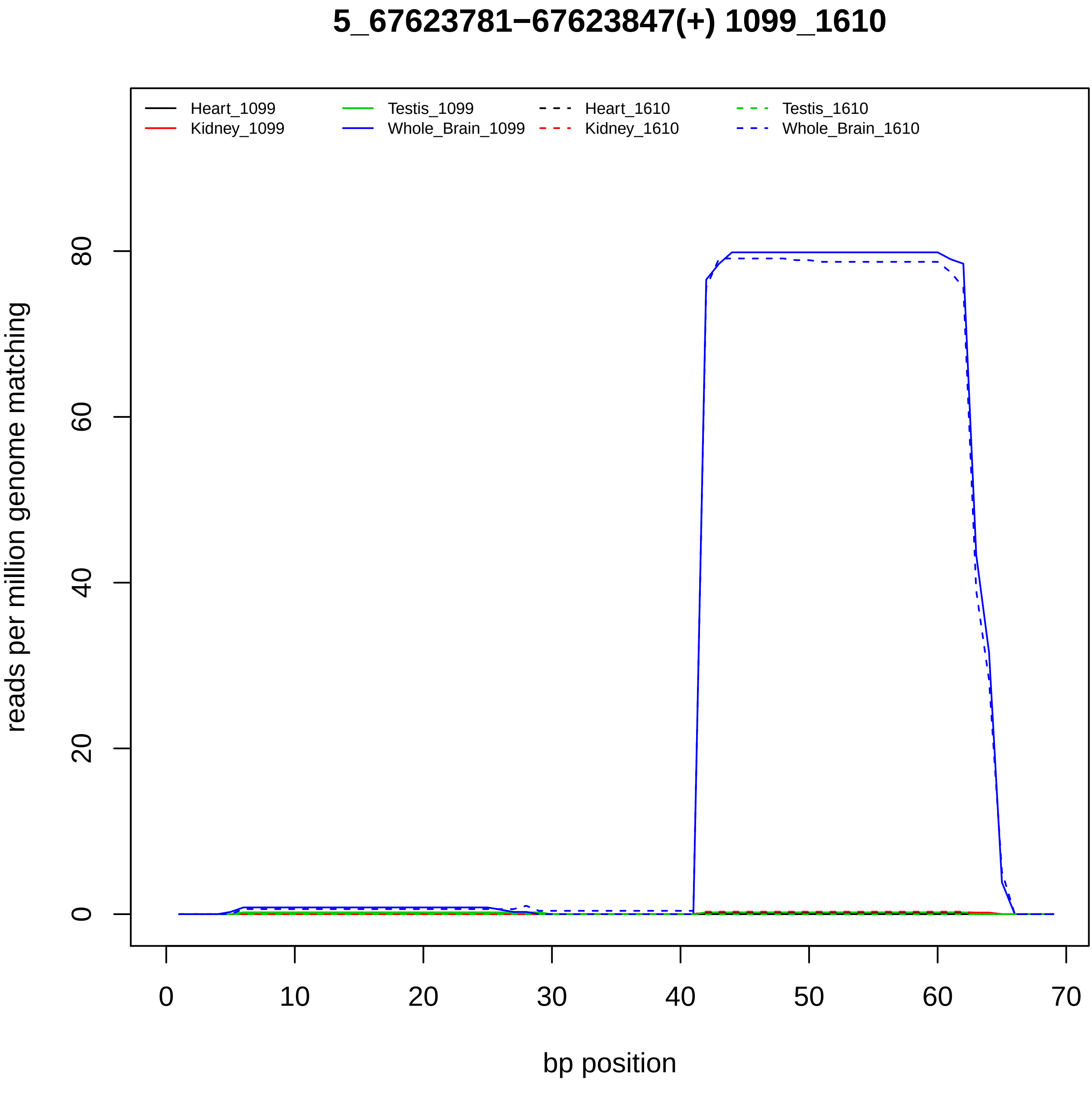

5_67623781−67623847(+) 1099_1610
Heart_1099
Kidney_1099
Testis_1099
Whole_Brain_1099
Heart_1610
Kidney_1610
Testis_1610
Whole_Brain_1610
reads per million genome matching
bp position
0
20
40
60
80
0
10
20
30
40
50
60
70

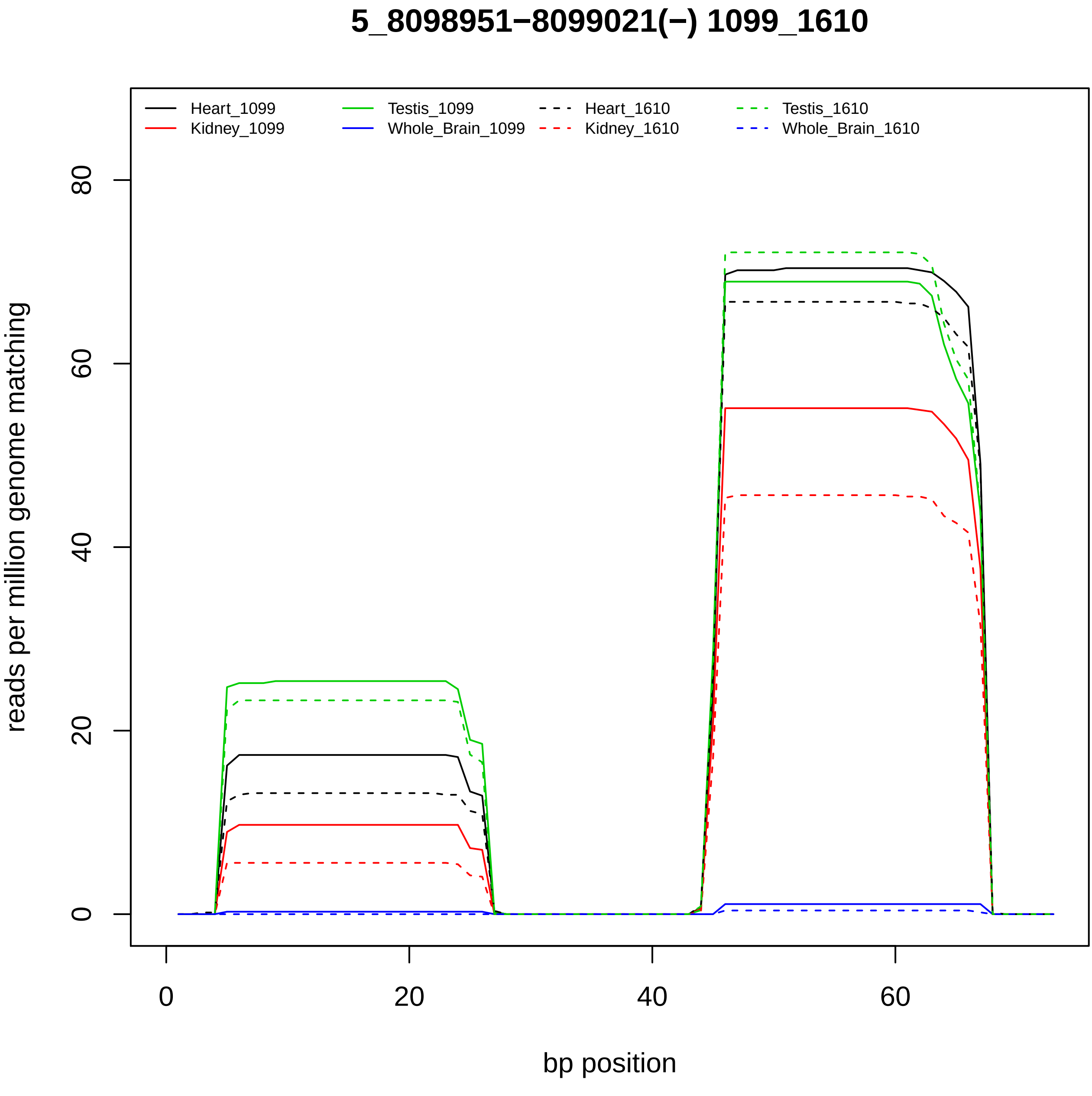
5_8098951−8099021(−) 1099_1610
Heart_1099
Kidney_1099
Testis_1099
Whole_Brain_1099
Heart_1610
Kidney_1610
Testis_1610
Whole_Brain_1610
reads per million genome matching
0
20
40
60
80
bp position
0
20
40
60

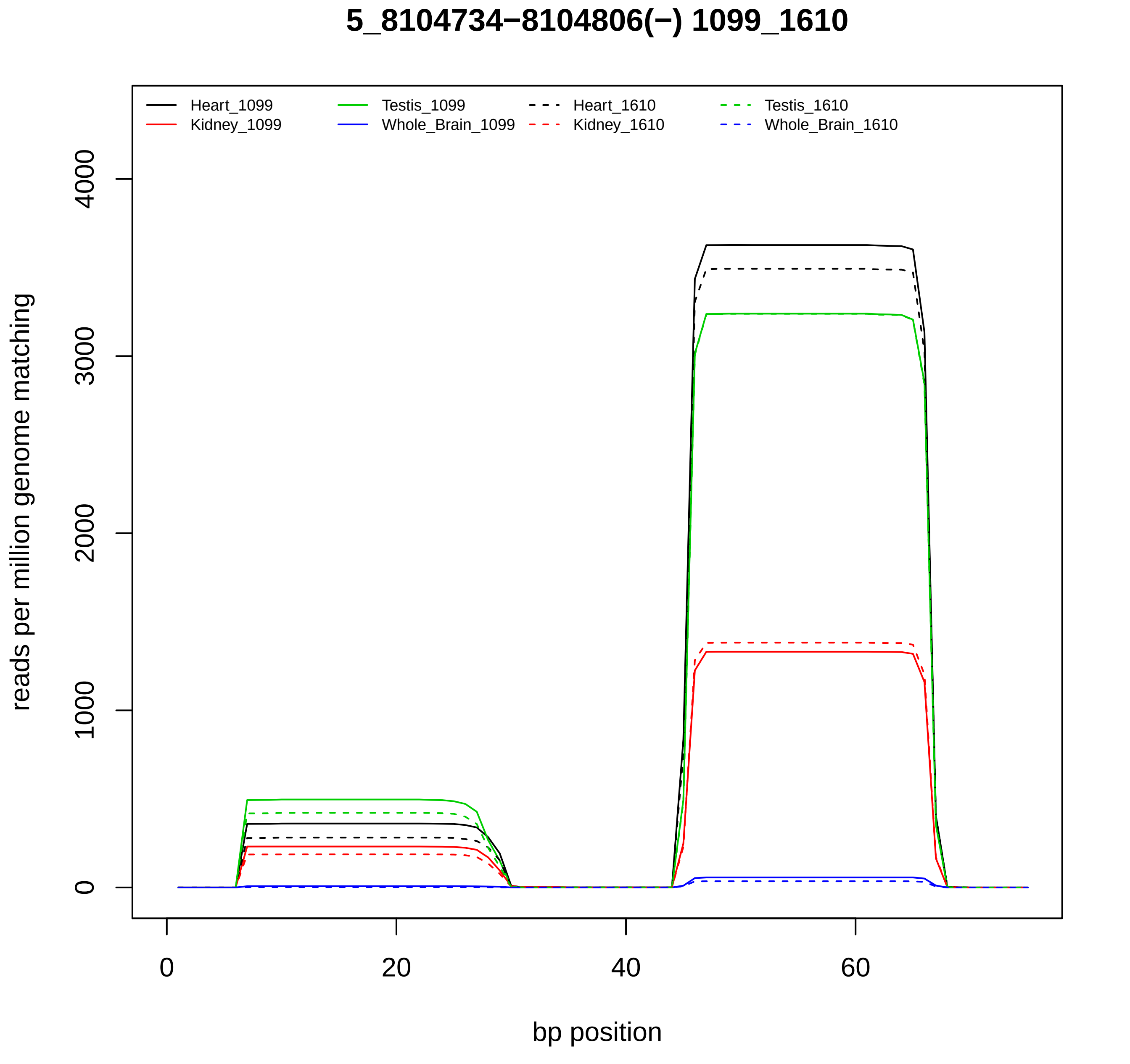

5_8104734−8104806(−) 1099_1610
Heart_1099
Kidney_1099
Testis_1099
Whole_Brain_1099
Heart_1610
Kidney_1610
Testis_1610
Whole_Brain_1610
reads per million genome matching
0
1000
2000
3000
4000
0
20
40
60
bp position

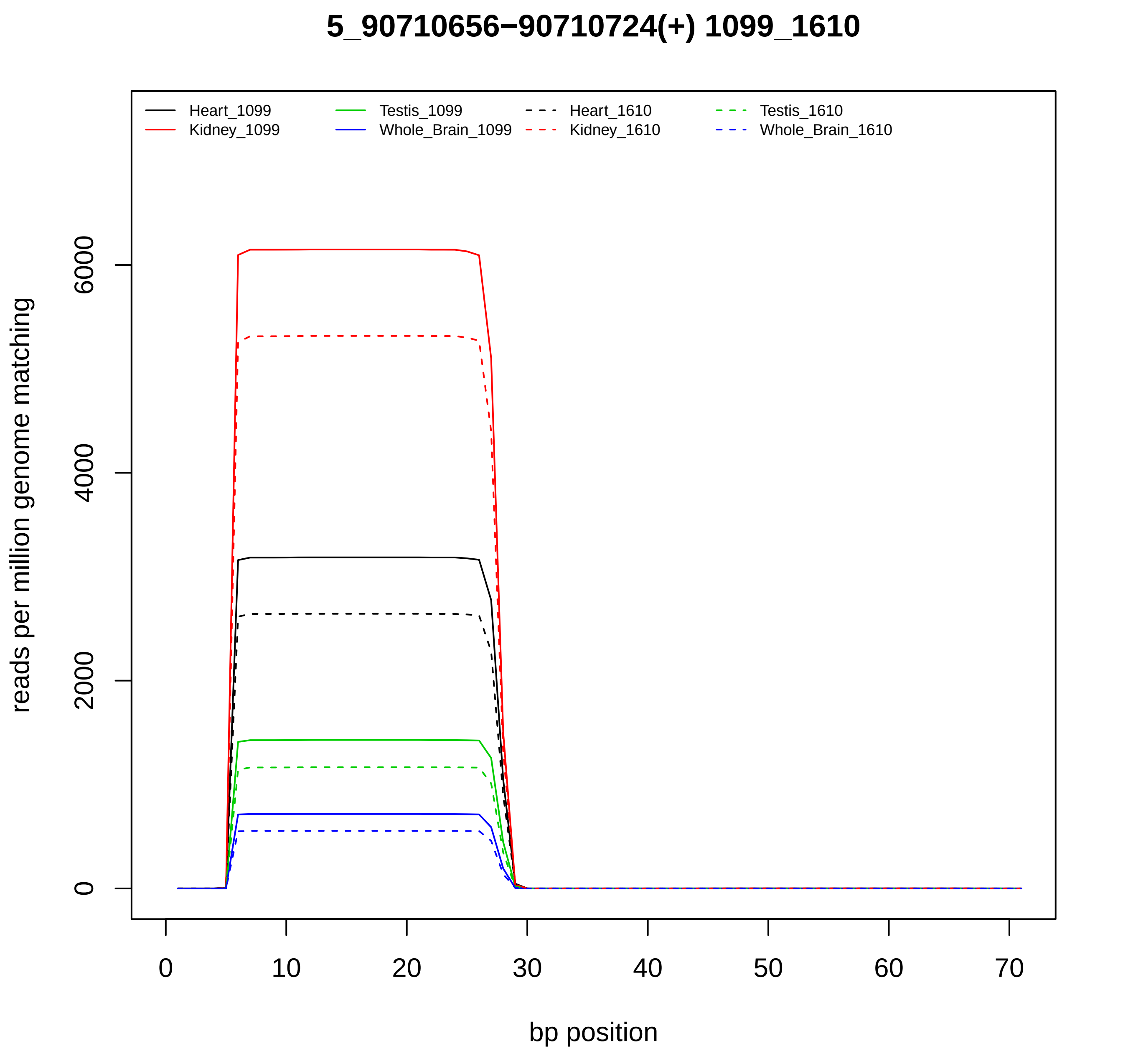

5_90710656−90710724(+) 1099_1610
Heart_1099
Kidney_1099
Testis_1099
Whole_Brain_1099
Heart_1610
Kidney_1610
Testis_1610
Whole_Brain_1610
reads per million genome matching
0
2000
4000
6000
0
10
20
30
40
50
60
70
bp position

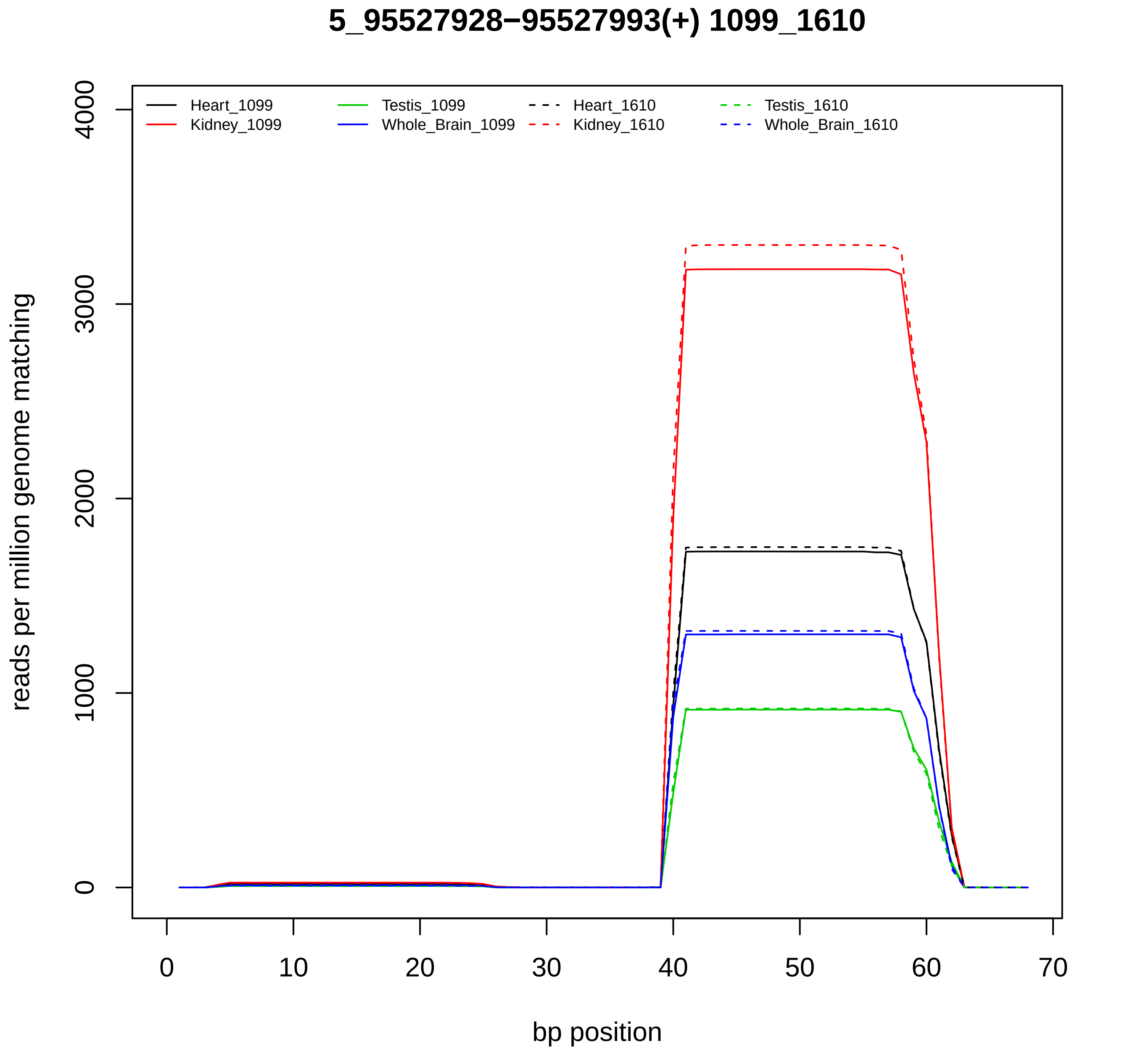

5_95527928−95527993(+) 1099_1610
Heart_1099
Kidney_1099
Testis_1099
Whole_Brain_1099
Heart_1610
Kidney_1610
Testis_1610
Whole_Brain_1610
reads per million genome matching
0
1000
2000
3000
4000
bp position
0
10
20
30
40
50
60
70

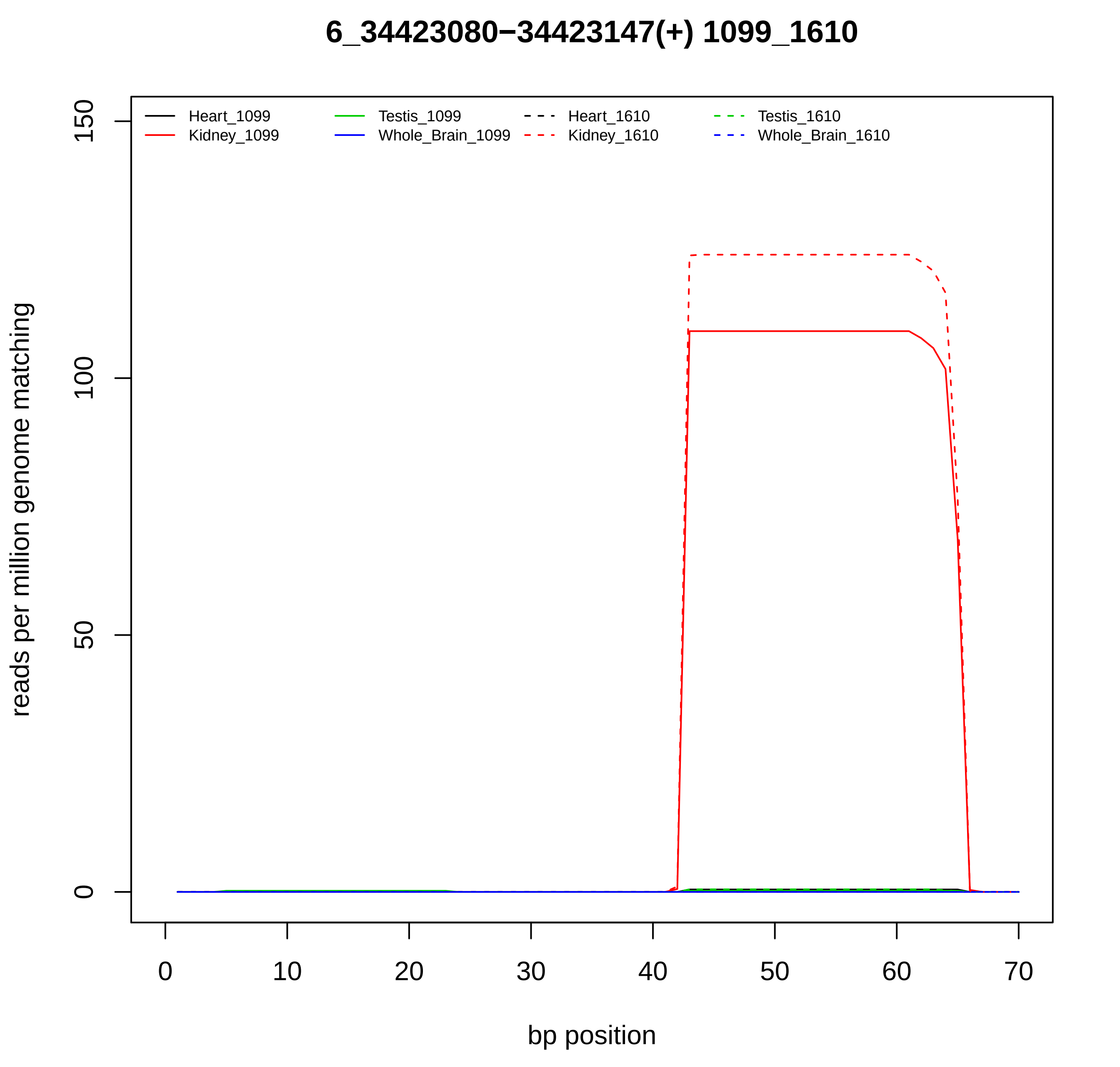
6_34423080−34423147(+) 1099_1610
Heart_1099
Kidney_1099
Testis_1099
Whole_Brain_1099
Heart_1610
Kidney_1610
Testis_1610
Whole_Brain_1610
reads per million genome matching
bp position
0
50
100
150
0
10
20
30
40
50
60
70

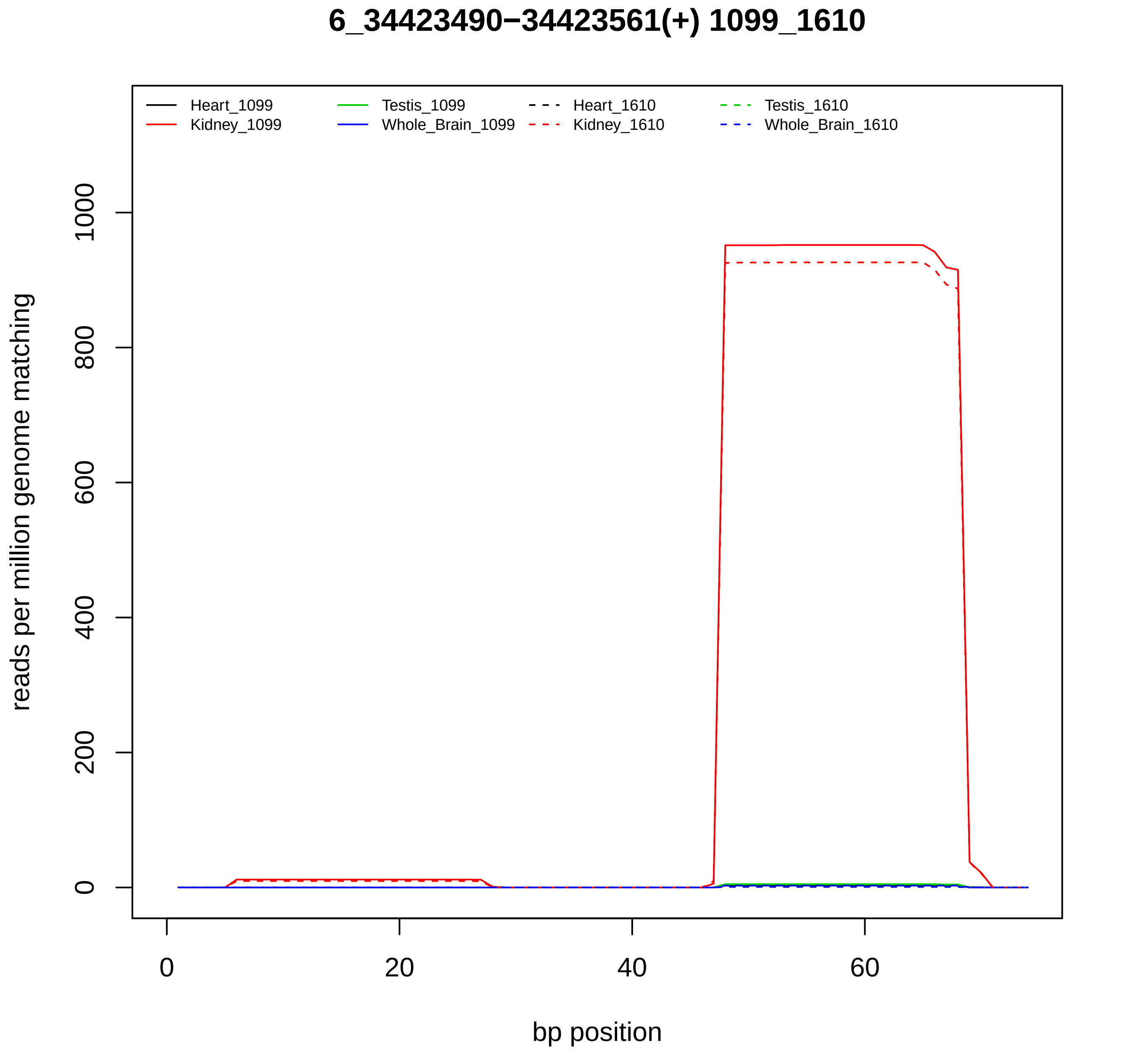

6_34423490−34423561(+) 1099_1610
Heart_1099
Kidney_1099
Testis_1099
Whole_Brain_1099
Heart_1610
Kidney_1610
Testis_1610
Whole_Brain_1610
reads per million genome matching
0
200
400
600
800
1000
0
20
40
60
bp position

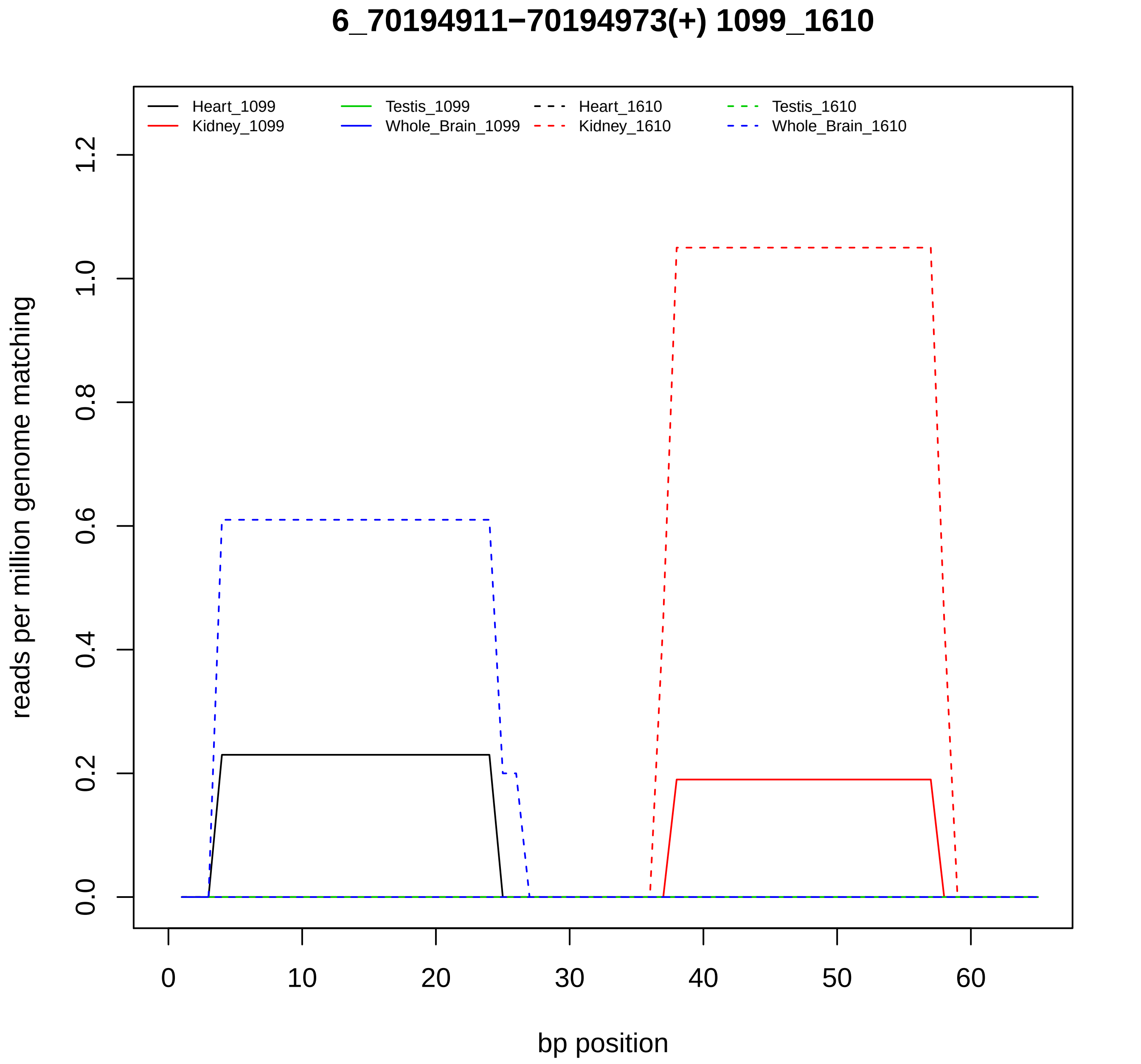

6_70194911−70194973(+) 1099_1610
Heart_1099
Kidney_1099
Testis_1099
Whole_Brain_1099
Heart_1610
Kidney_1610
Testis_1610
Whole_Brain_1610
reads per million genome matching
0.0
0.2
0.4
0.6
0.8
1.0
1.2
0
10
20
30
40
50
60
bp position

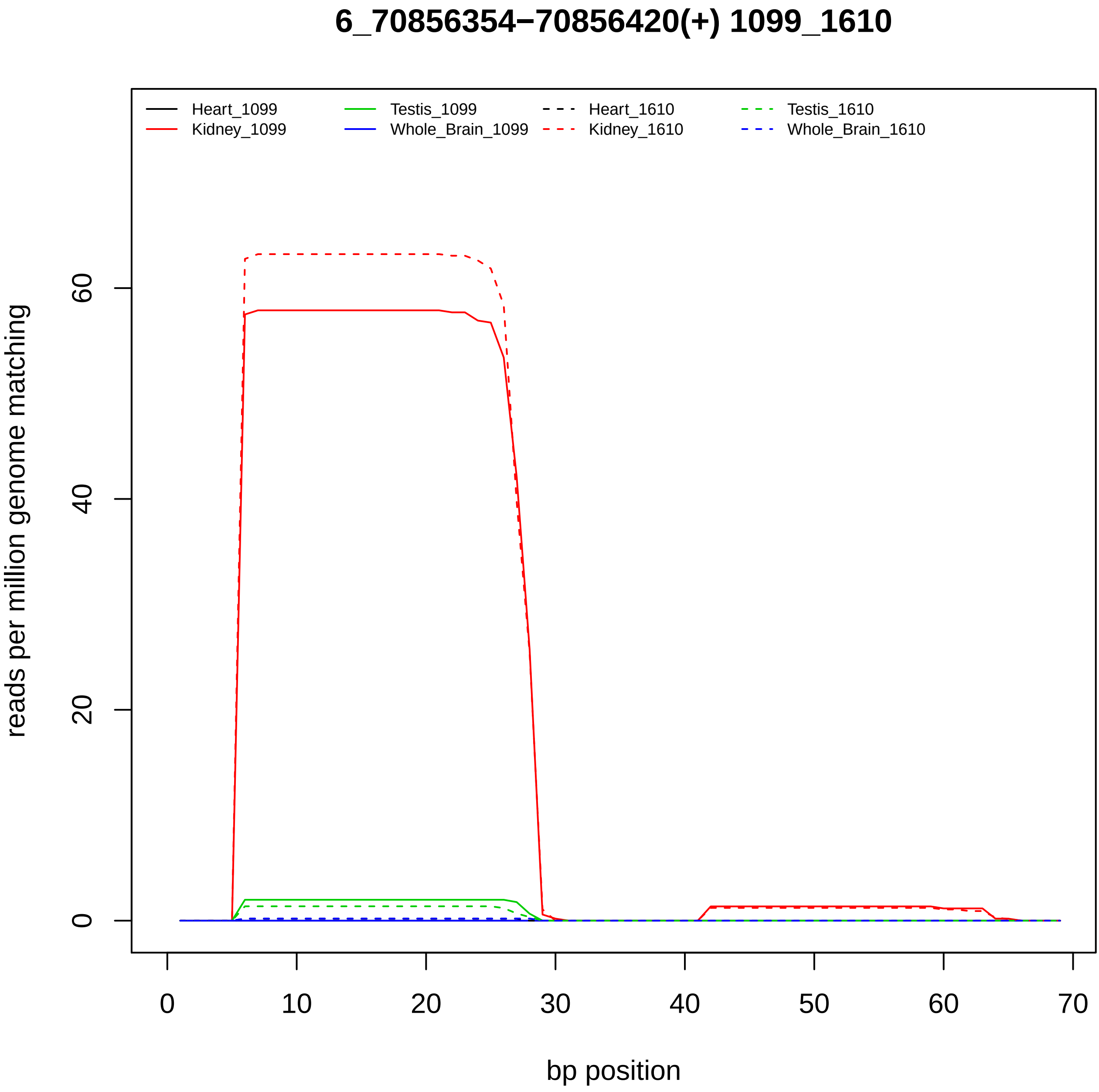

6_70856354−70856420(+) 1099_1610
Heart_1099
Kidney_1099
Testis_1099
Whole_Brain_1099
Heart_1610
Kidney_1610
Testis_1610
Whole_Brain_1610
reads per million genome matching
0
20
40
60
bp position
0
10
20
30
40
50
60
70

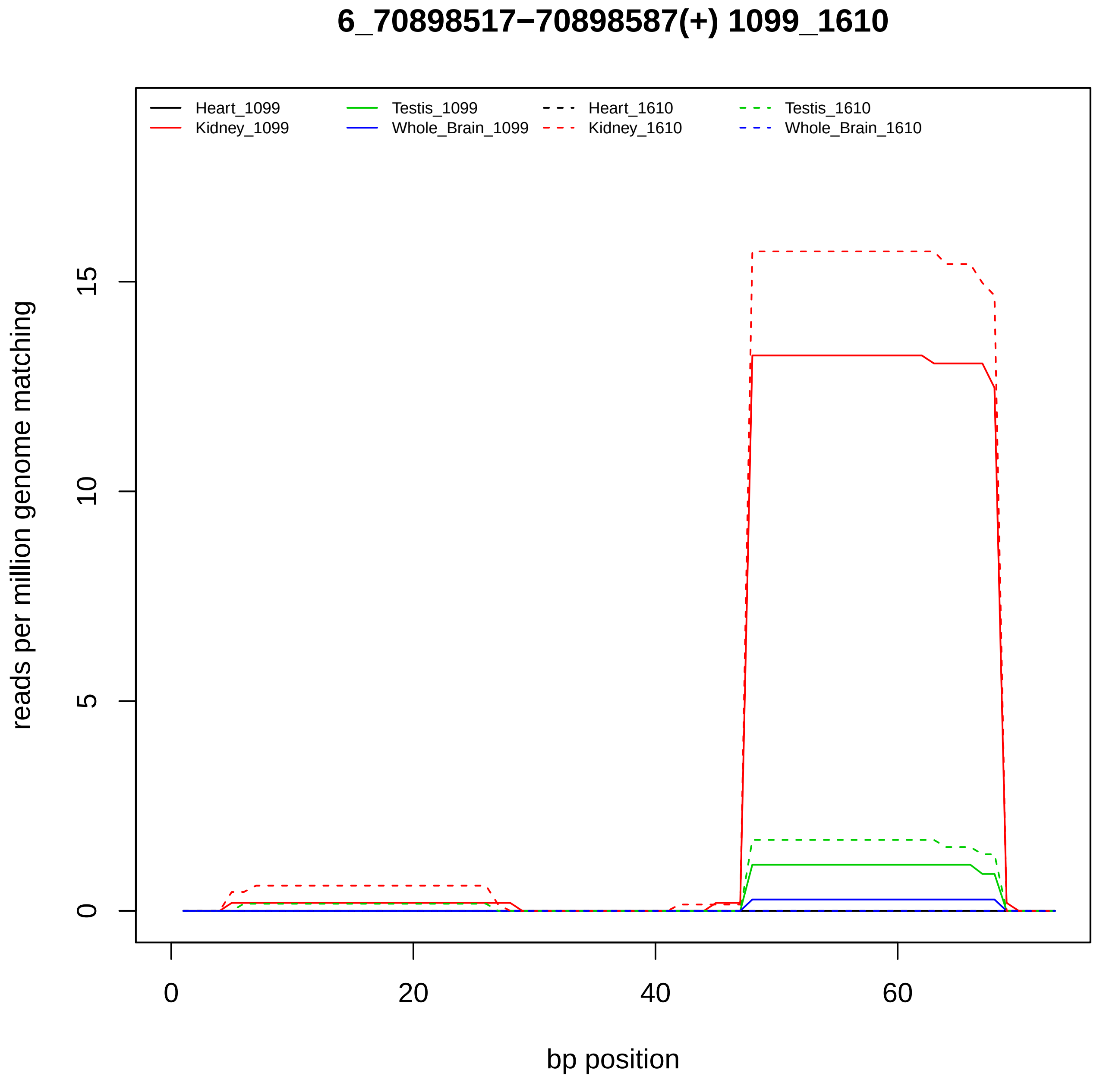

6_70898517−70898587(+) 1099_1610
Heart_1099
Kidney_1099
Testis_1099
Whole_Brain_1099
Heart_1610
Kidney_1610
Testis_1610
Whole_Brain_1610
reads per million genome matching
0
5
10
15
0
20
40
60
bp position

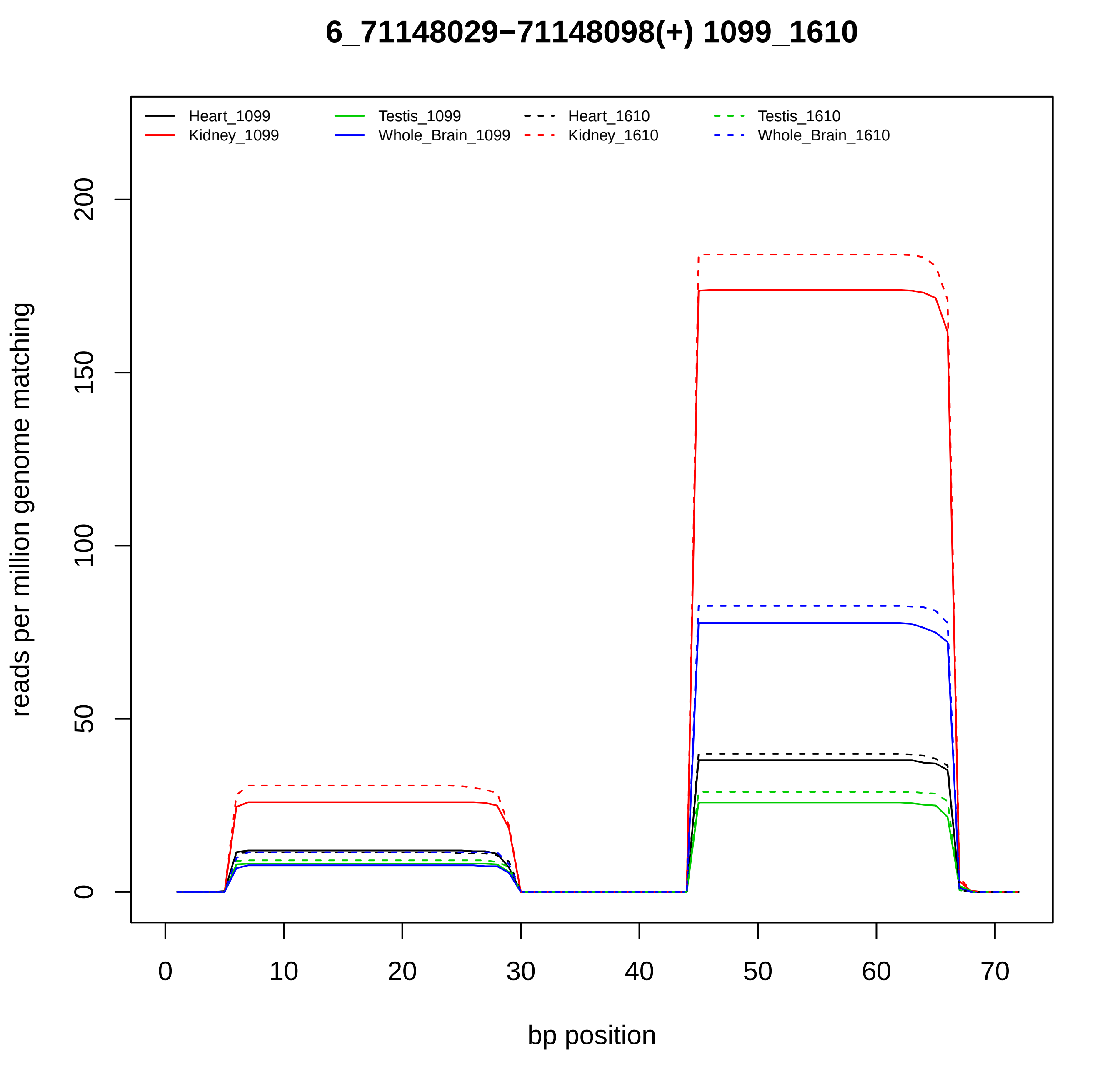
6_71148029−71148098(+) 1099_1610
Heart_1099
Kidney_1099
Testis_1099
Whole_Brain_1099
Heart_1610
Kidney_1610
Testis_1610
Whole_Brain_1610
reads per million genome matching
0
50
100
150
200
bp position
0
10
20
30
40
50
60
70

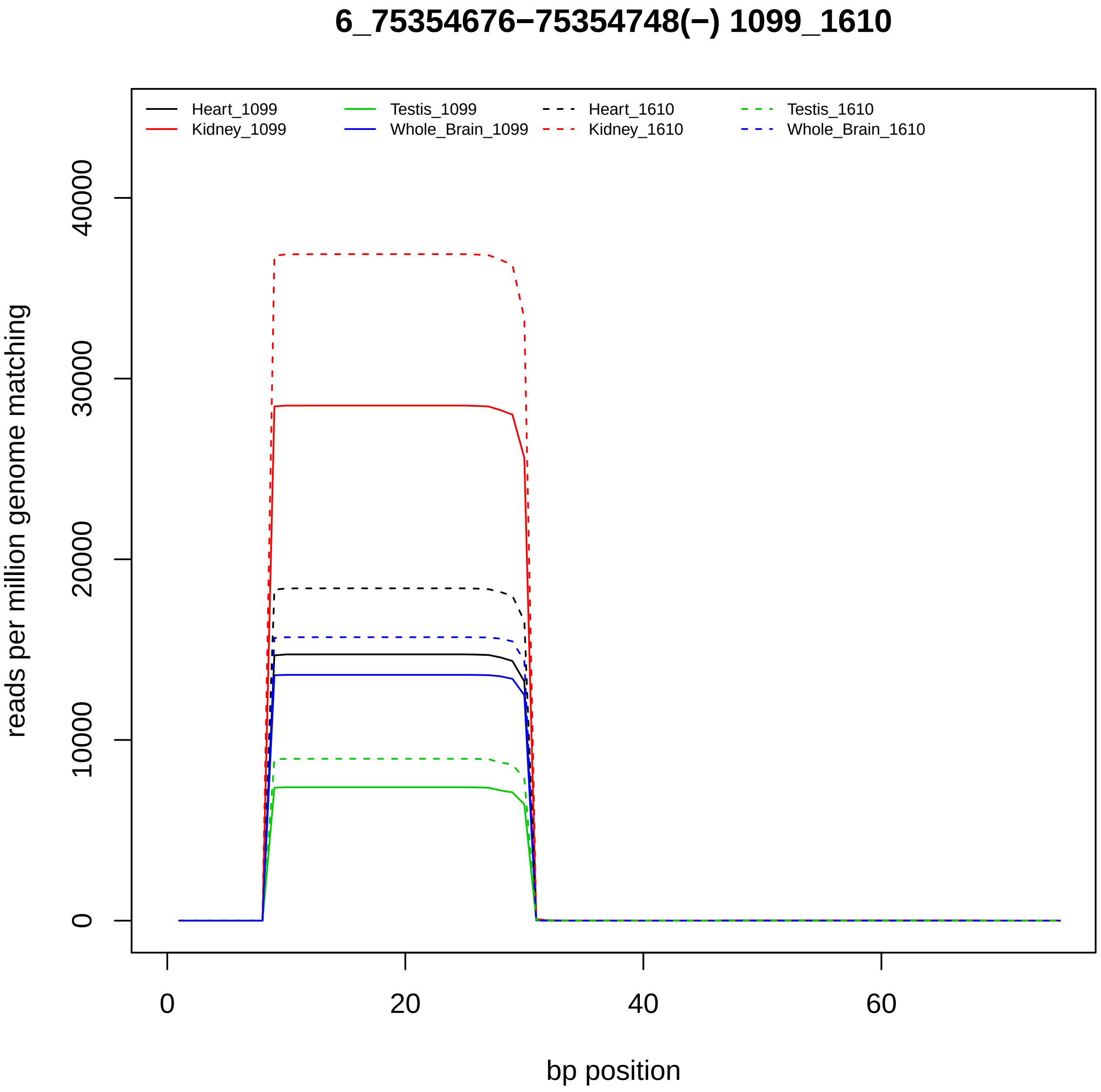

6_75354676−75354748(−) 1099_1610
Heart_1099
Kidney_1099
Testis_1099
Whole_Brain_1099
Heart_1610
Kidney_1610
Testis_1610
Whole_Brain_1610
reads per million genome matching
0
10000
20000
30000
40000
0
20
40
60
bp position

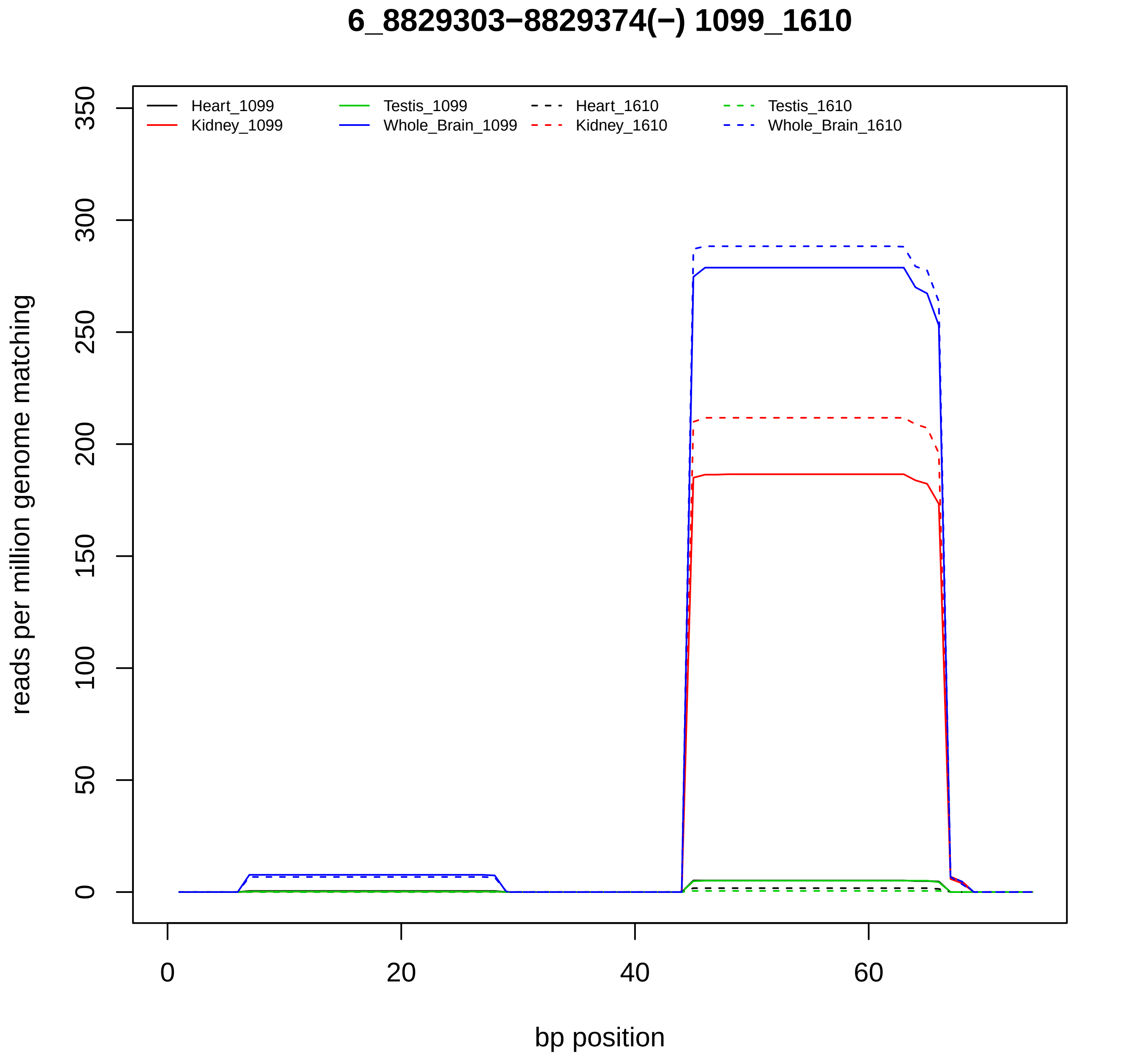

6_8829303−8829374(−) 1099_1610
Heart_1099
Kidney_1099
Testis_1099
Whole_Brain_1099
Heart_1610
Kidney_1610
Testis_1610
Whole_Brain_1610
reads per million genome matching
bp position
0
50
100
150
200
250
300
350
0
20
40
60

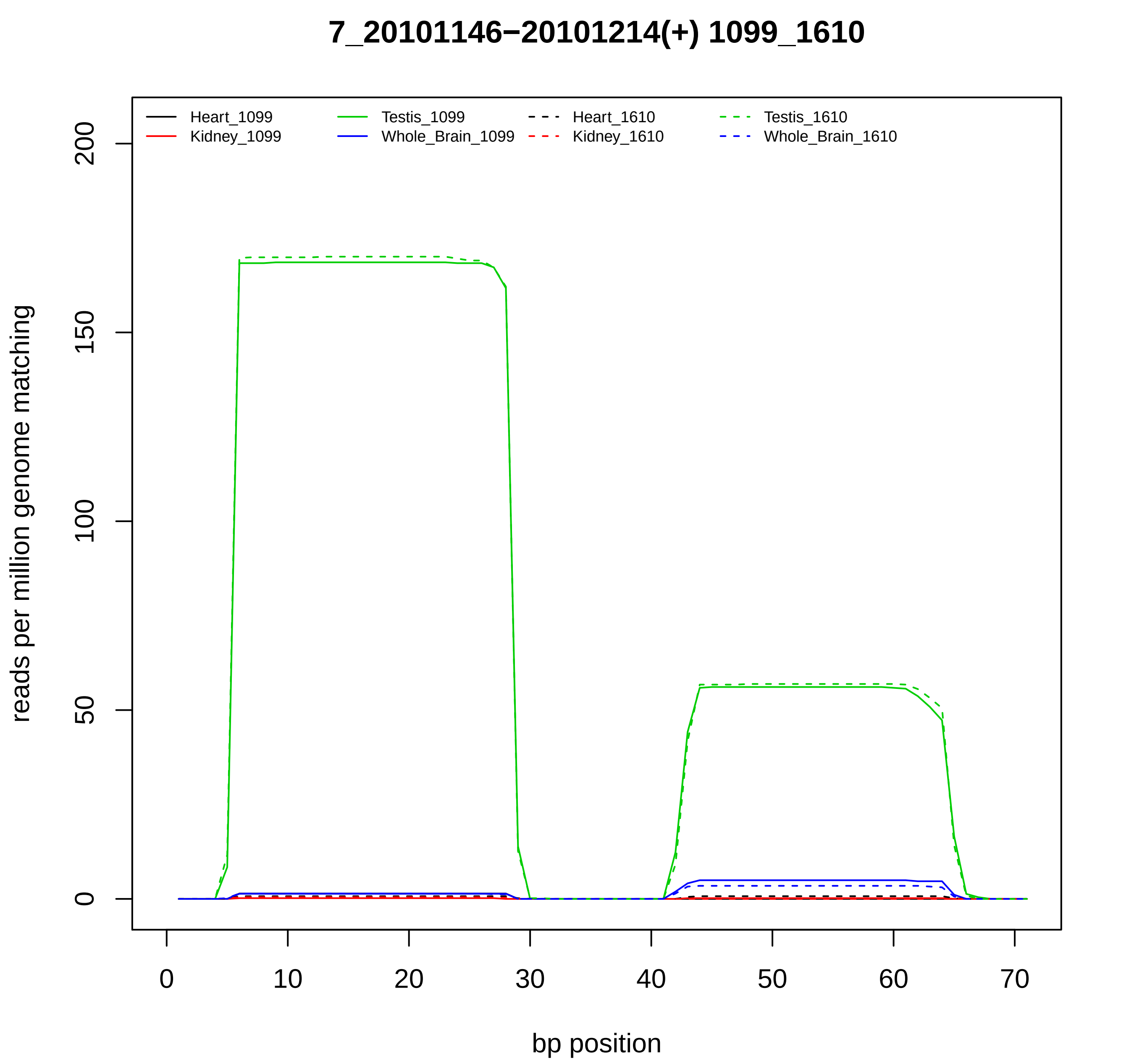
7_20101146−20101214(+) 1099_1610
Heart_1099
Kidney_1099
Testis_1099
Whole_Brain_1099
Heart_1610
Kidney_1610
Testis_1610
Whole_Brain_1610
reads per million genome matching
0
50
100
150
200
bp position
0
10
20
30
40
50
60
70

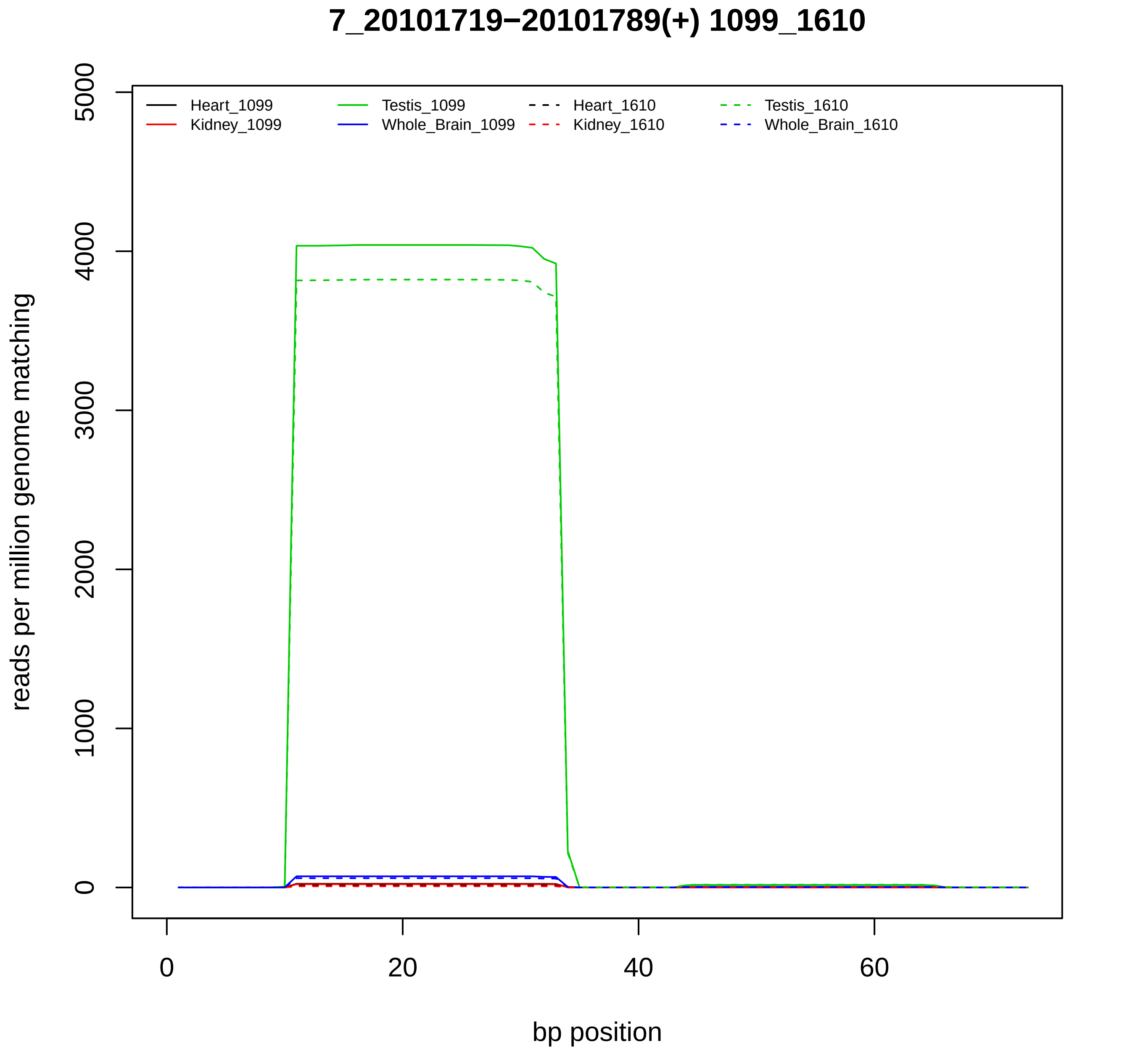

7_20101719−20101789(+) 1099_1610
Heart_1099
Kidney_1099
Testis_1099
Whole_Brain_1099
Heart_1610
Kidney_1610
Testis_1610
Whole_Brain_1610
reads per million genome matching
0
1000
2000
3000
4000
5000
0
20
40
60
bp position

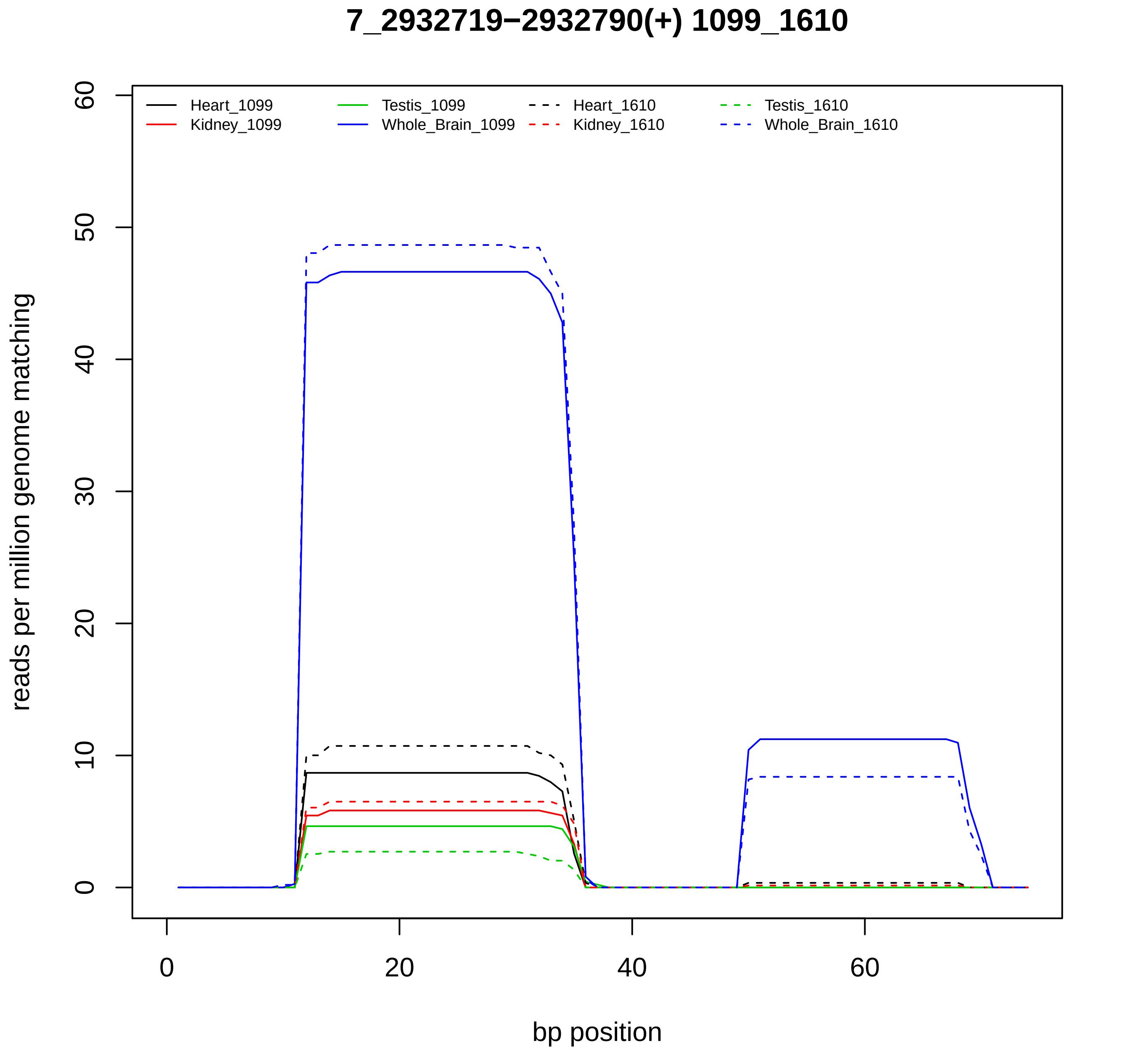
7_2932719−2932790(+) 1099_1610
Heart_1099
Kidney_1099
Testis_1099
Whole_Brain_1099
Heart_1610
Kidney_1610
Testis_1610
Whole_Brain_1610
reads per million genome matching
bp position
0
10
20
30
40
50
60
0
20
40
60

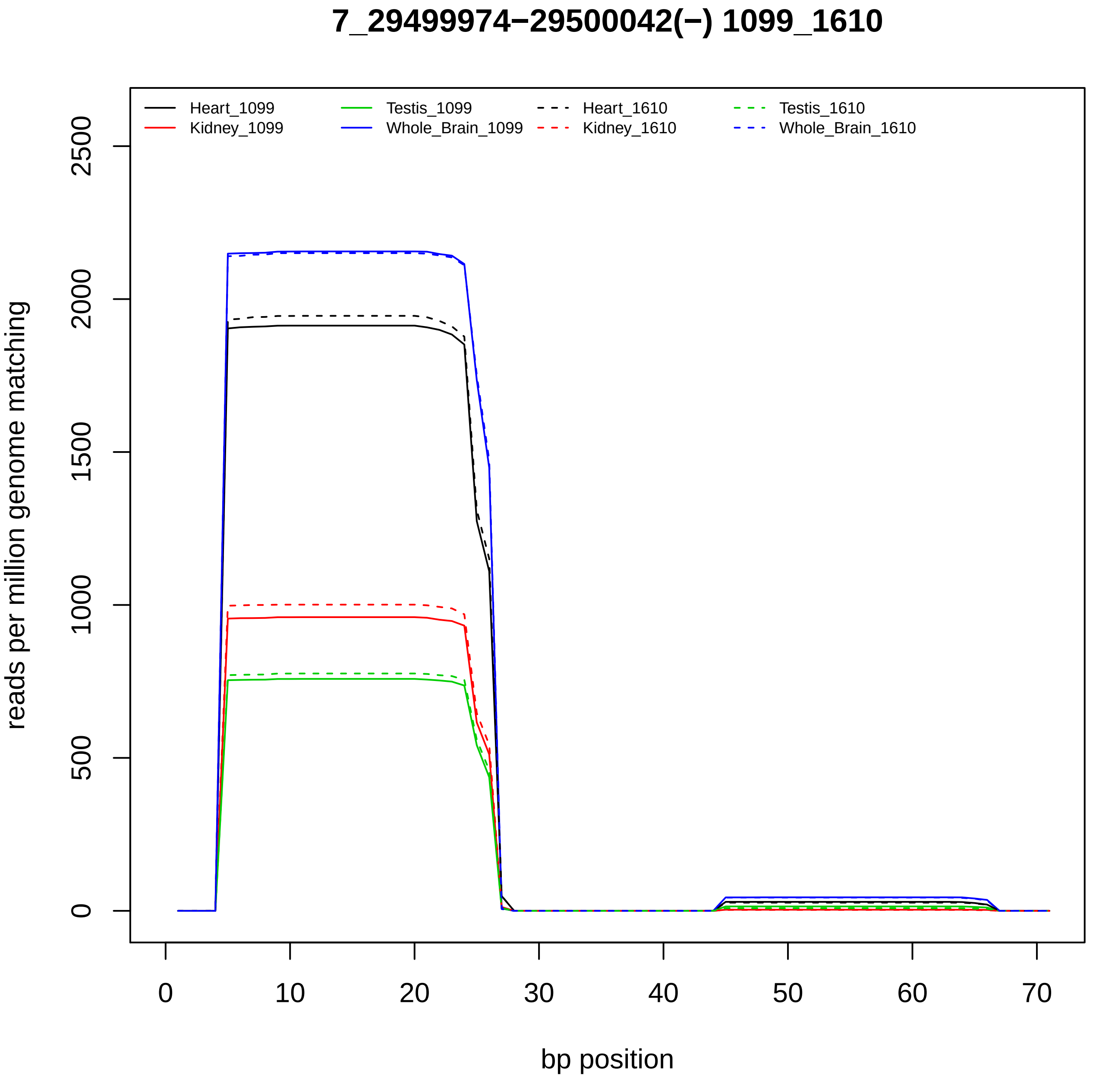

7_29499974−29500042(−) 1099_1610
Heart_1099
Kidney_1099
Testis_1099
Whole_Brain_1099
Heart_1610
Kidney_1610
Testis_1610
Whole_Brain_1610
reads per million genome matching
bp position
0
500
1000
1500
2000
2500
0
10
20
30
40
50
60
70

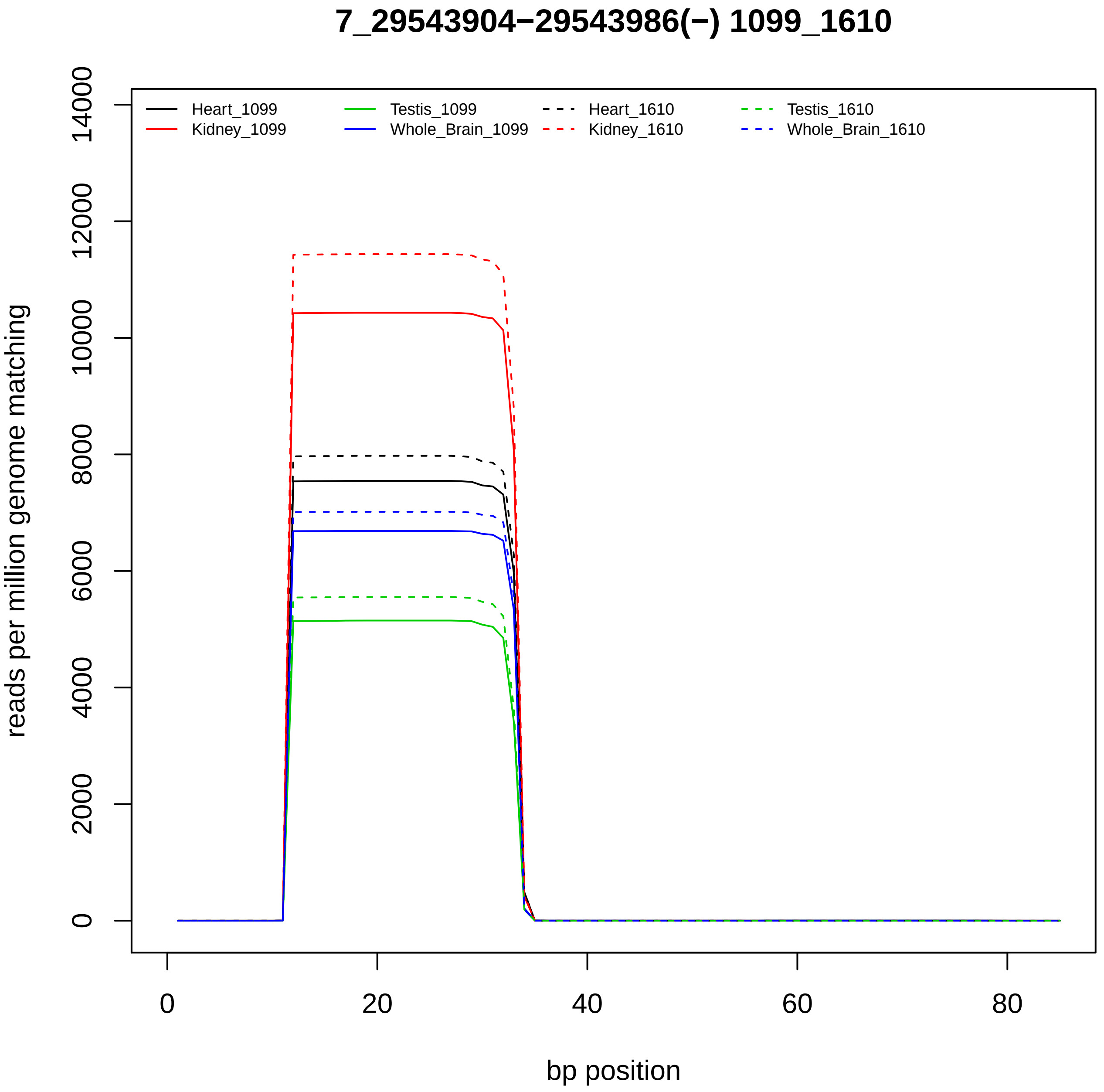
7_29543904−29543986(−) 1099_1610
Heart_1099
Kidney_1099
Testis_1099
Whole_Brain_1099
Heart_1610
Kidney_1610
Testis_1610
Whole_Brain_1610
reads per million genome matching
bp position
0
2000
4000
6000
8000
10000
12000
14000
0
20
40
60
80

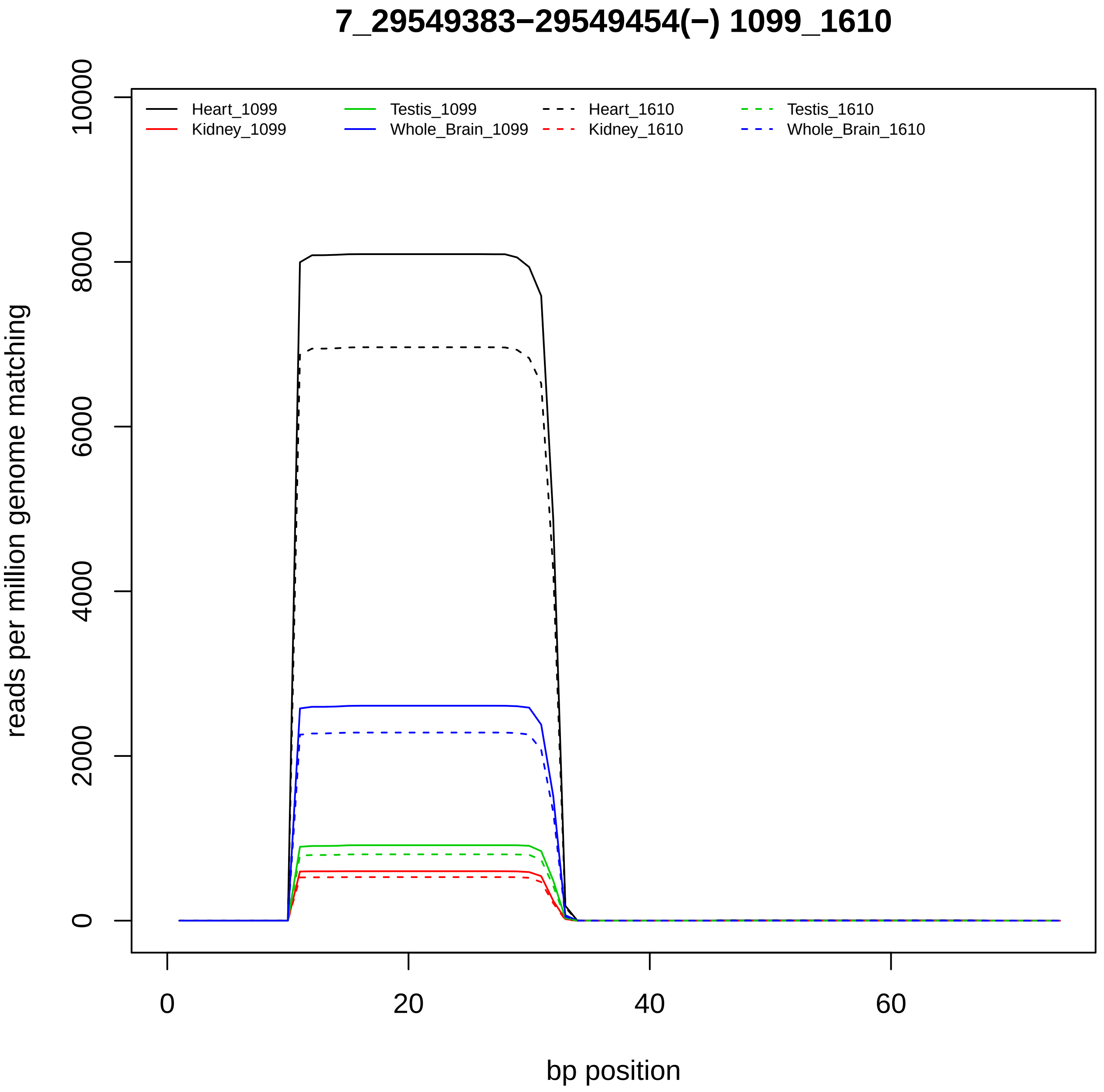
7_29549383−29549454(−) 1099_1610
Heart_1099
Kidney_1099
Testis_1099
Whole_Brain_1099
Heart_1610
Kidney_1610
Testis_1610
Whole_Brain_1610
reads per million genome matching
0
2000
4000
6000
8000
10000
0
20
40
60
bp position

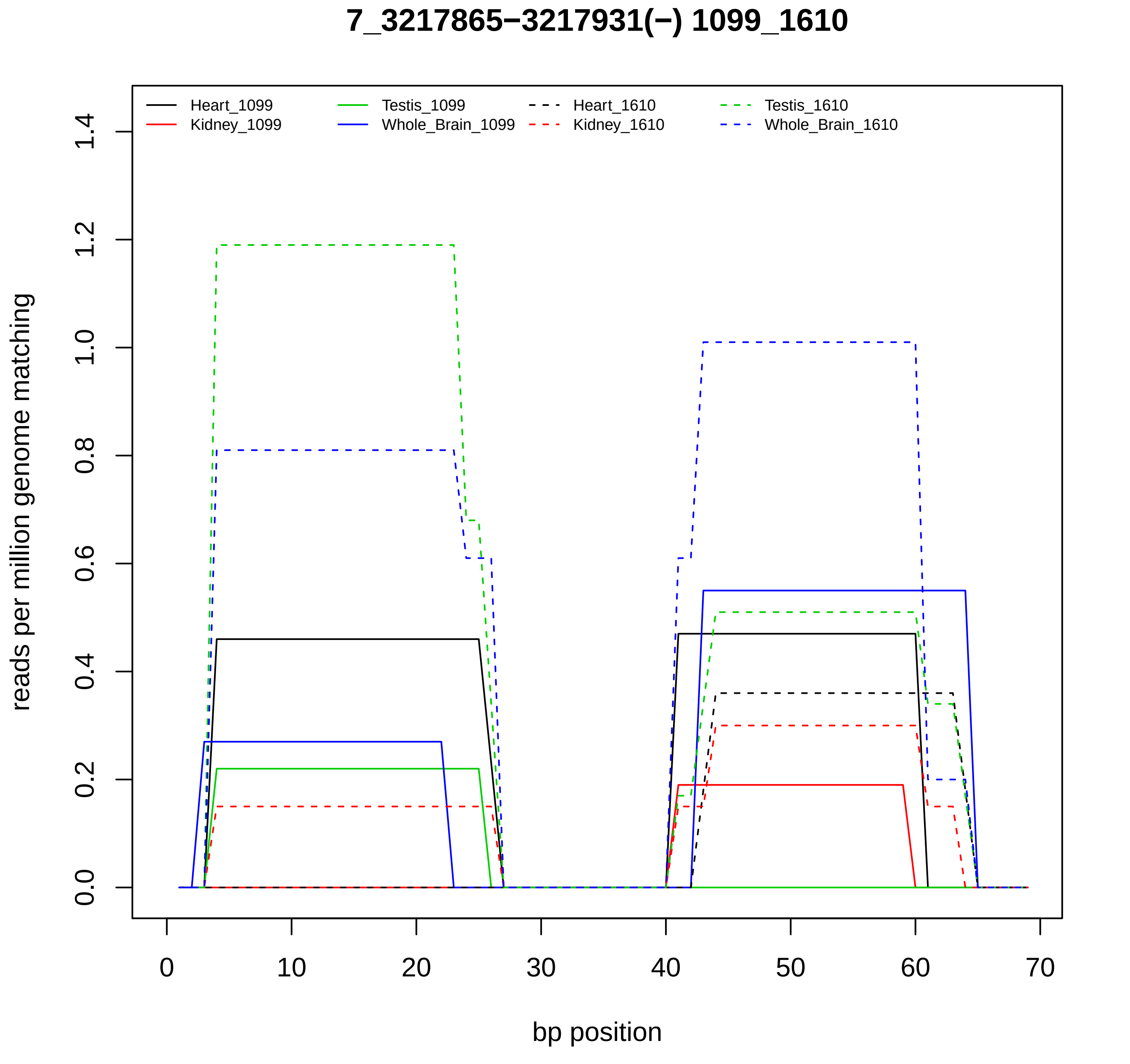

7_3217865−3217931(−) 1099_1610
Heart_1099
Kidney_1099
Testis_1099
Whole_Brain_1099
Heart_1610
Kidney_1610
Testis_1610
Whole_Brain_1610
reads per million genome matching
bp position
0.0
0.2
0.4
0.6
0.8
1.0
1.2
1.4
0
10
20
30
40
50
60
70

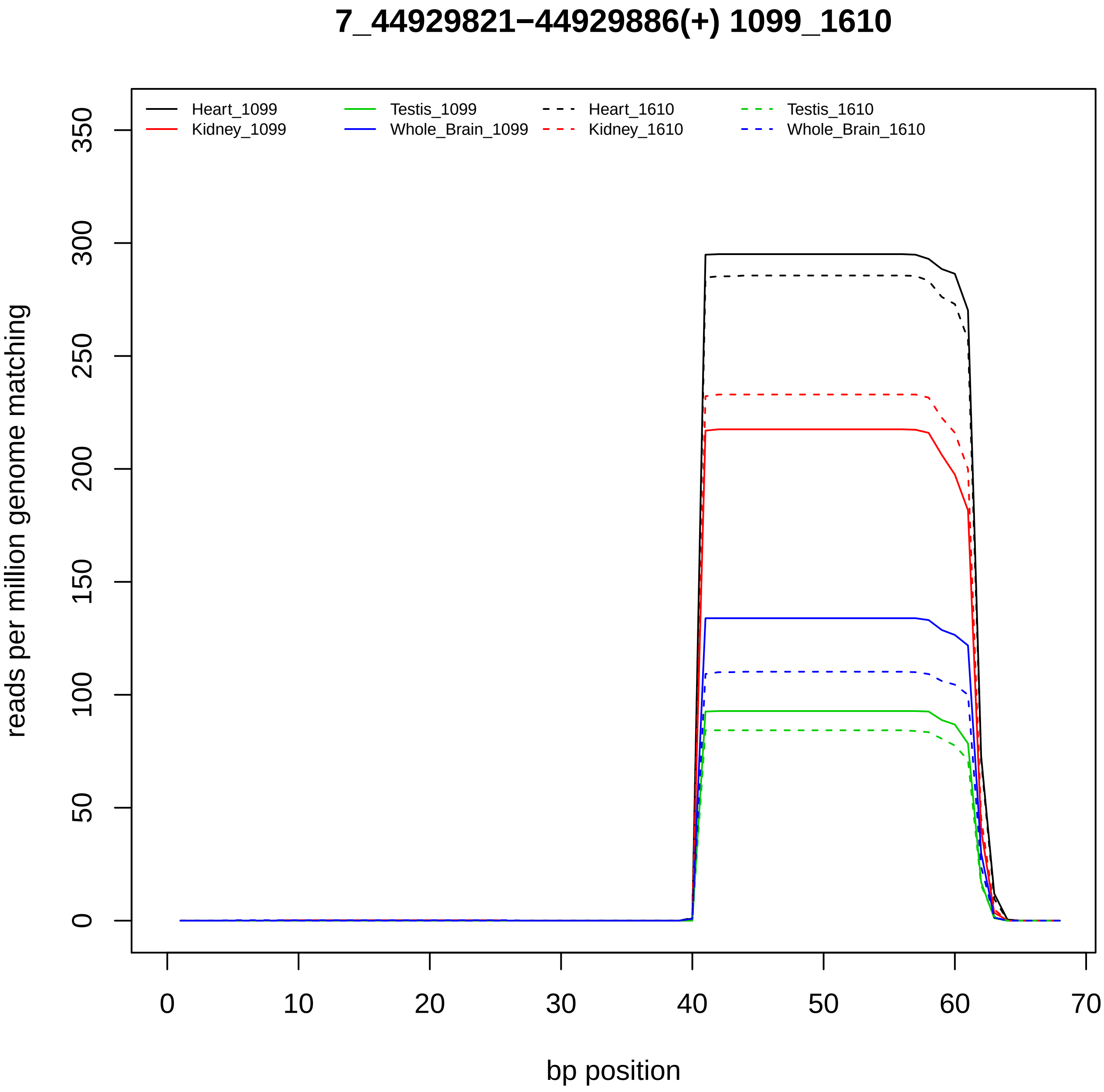

7_44929821−44929886(+) 1099_1610
Heart_1099
Kidney_1099
Testis_1099
Whole_Brain_1099
Heart_1610
Kidney_1610
Testis_1610
Whole_Brain_1610
reads per million genome matching
0
50
100
150
200
250
300
350
bp position
0
10
20
30
40
50
60
70

7_44930017−44930087(+) 1099_1610

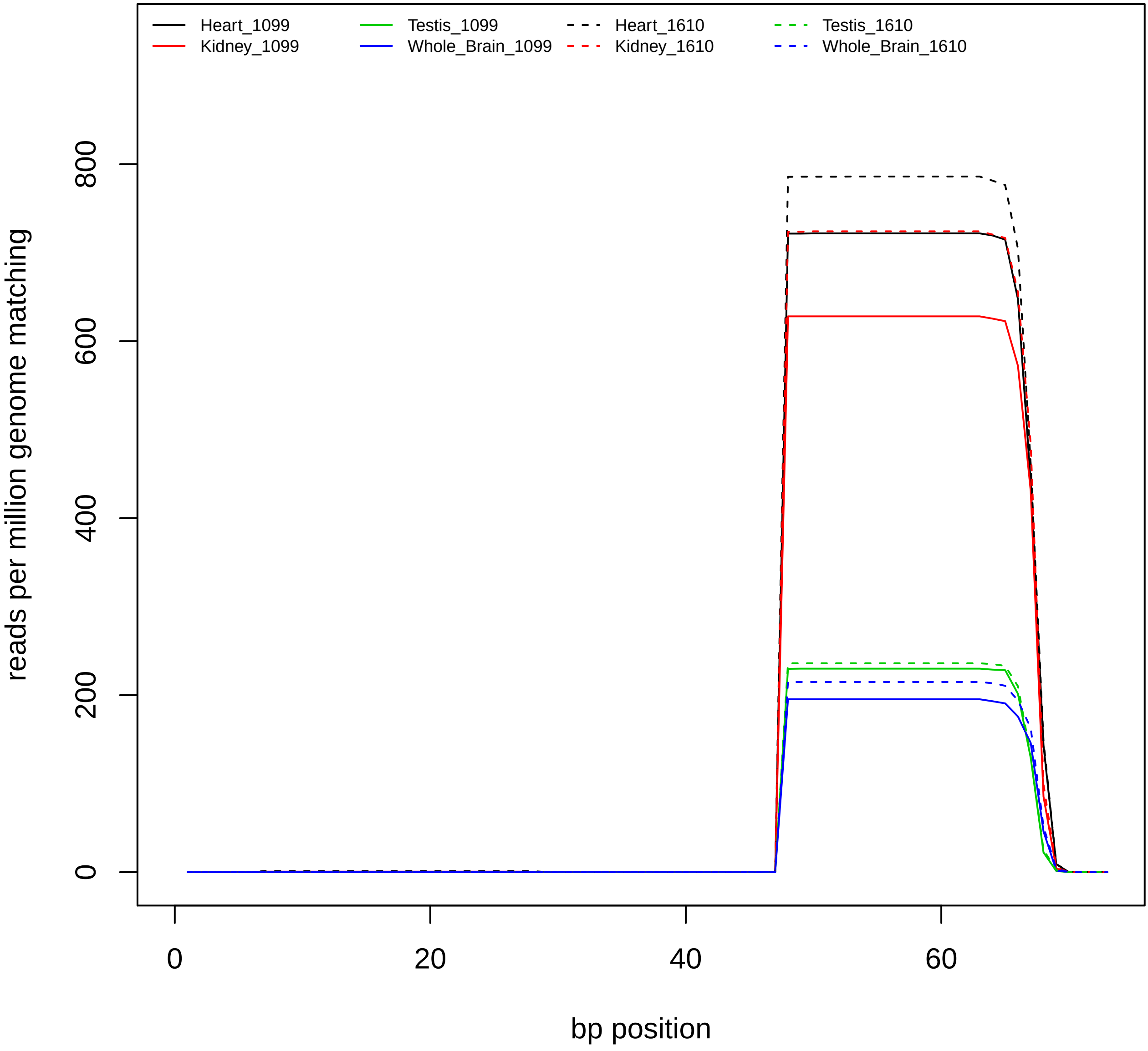

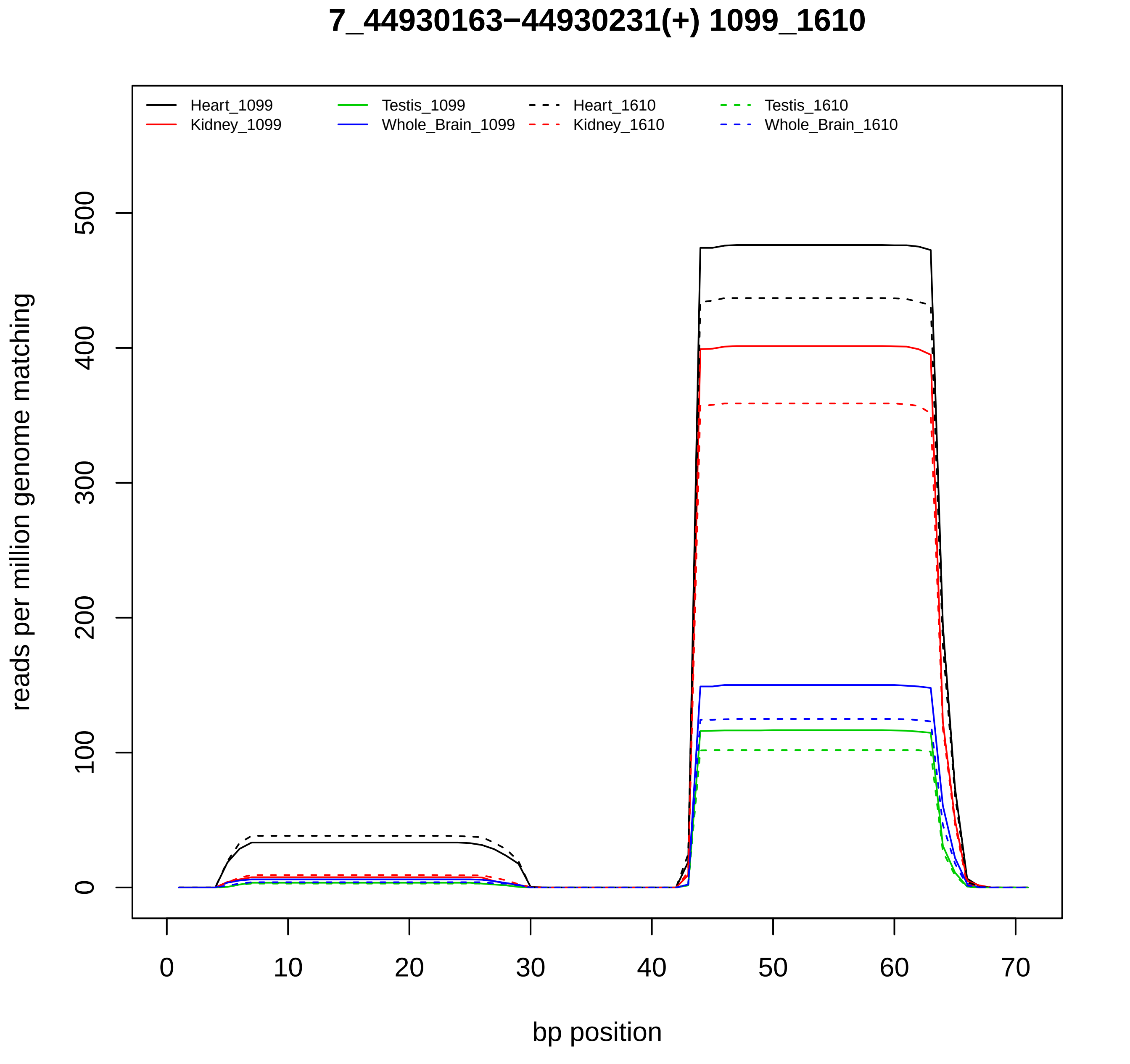

7_44930163−44930231(+) 1099_1610
Heart_1099
Kidney_1099
Testis_1099
Whole_Brain_1099
Heart_1610
Kidney_1610
Testis_1610
Whole_Brain_1610
reads per million genome matching
0
100
200
300
400
500
0
10
20
30
40
50
60
70
bp position

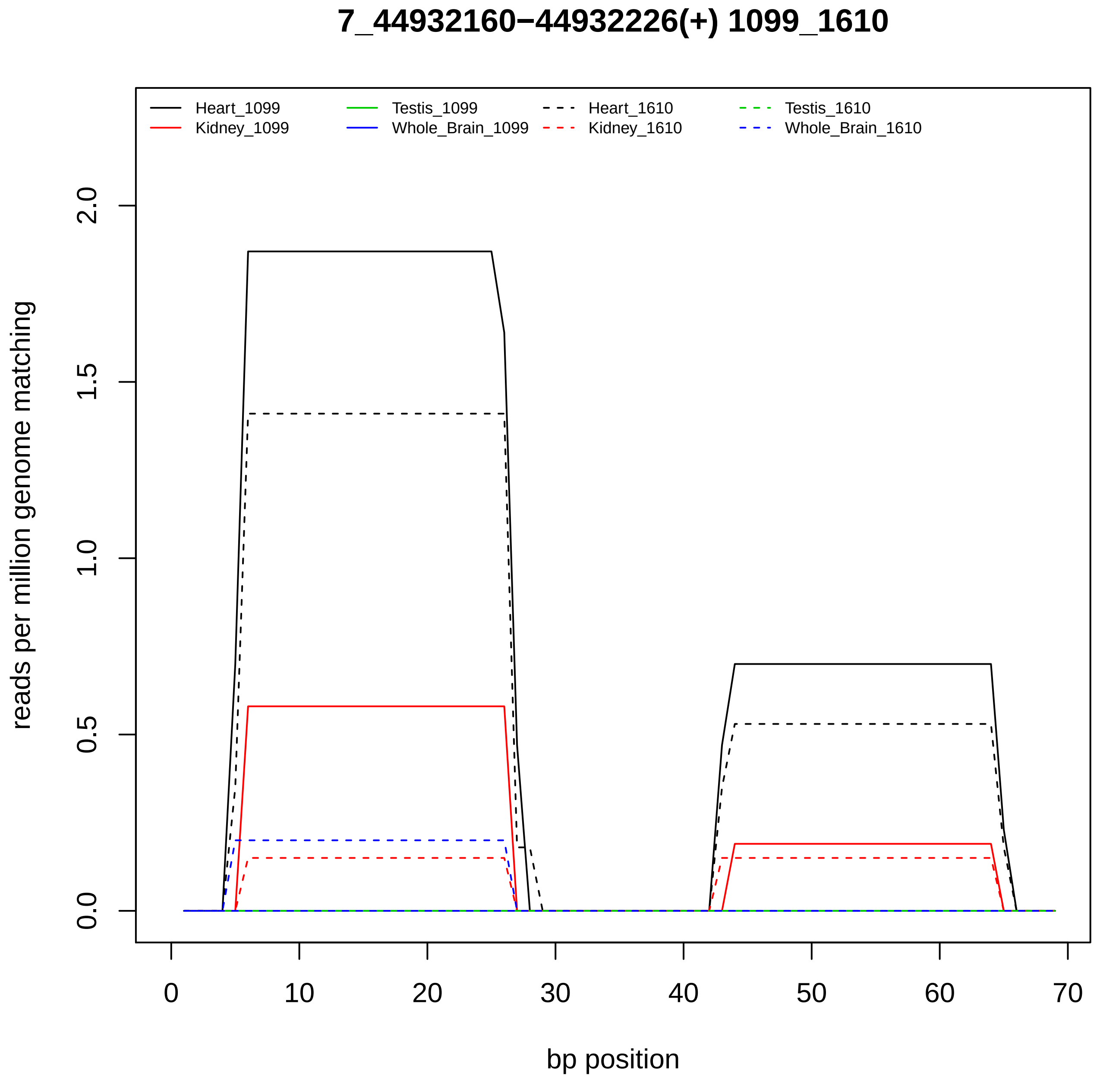

7_44932160−44932226(+) 1099_1610
Heart_1099
Kidney_1099
Testis_1099
Whole_Brain_1099
Heart_1610
Kidney_1610
Testis_1610
Whole_Brain_1610
reads per million genome matching
0.0
0.5
1.0
1.5
2.0
0
10
20
30
40
50
60
70
bp position

7_49327226−49327295(+) 1099_1610

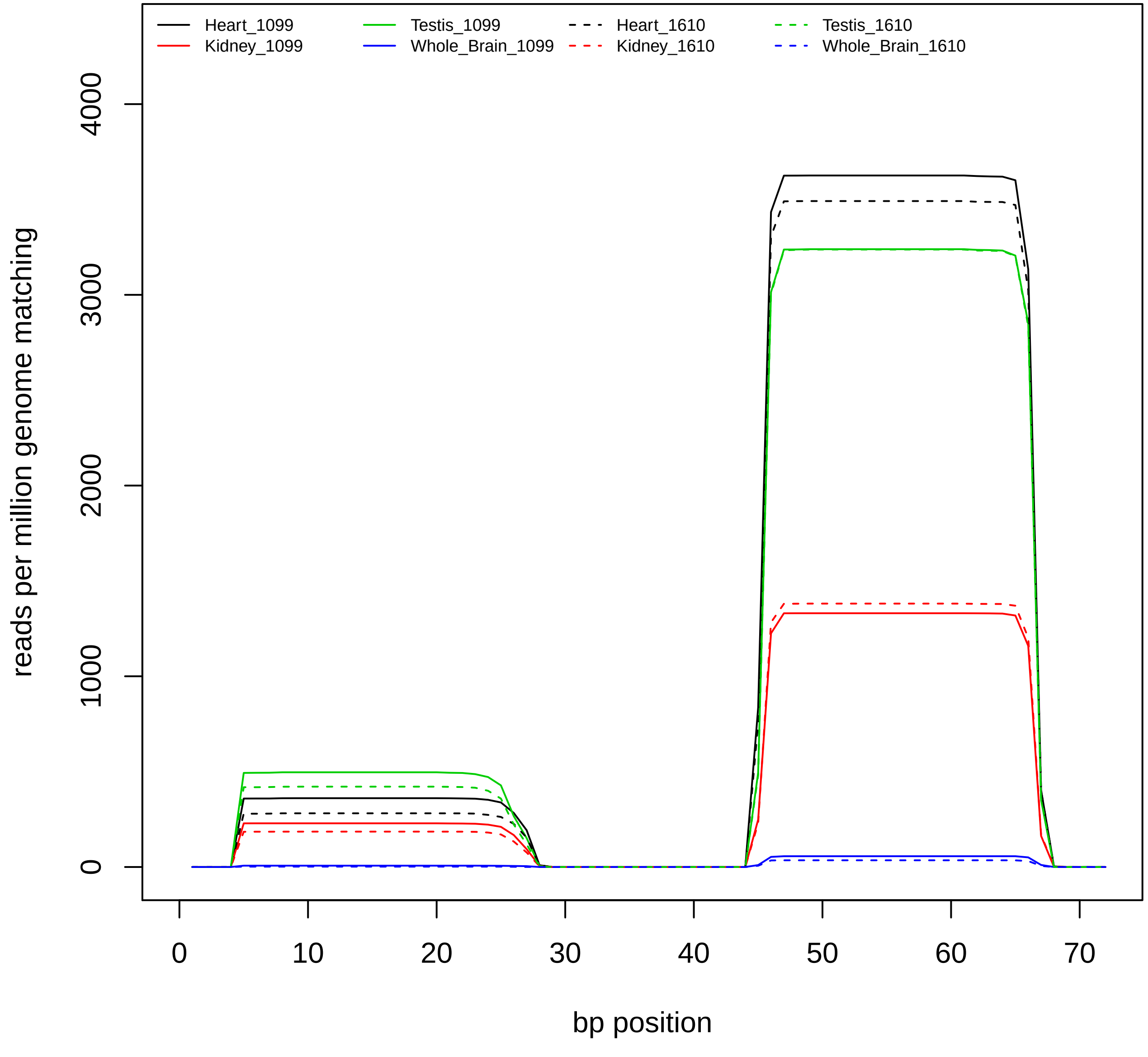

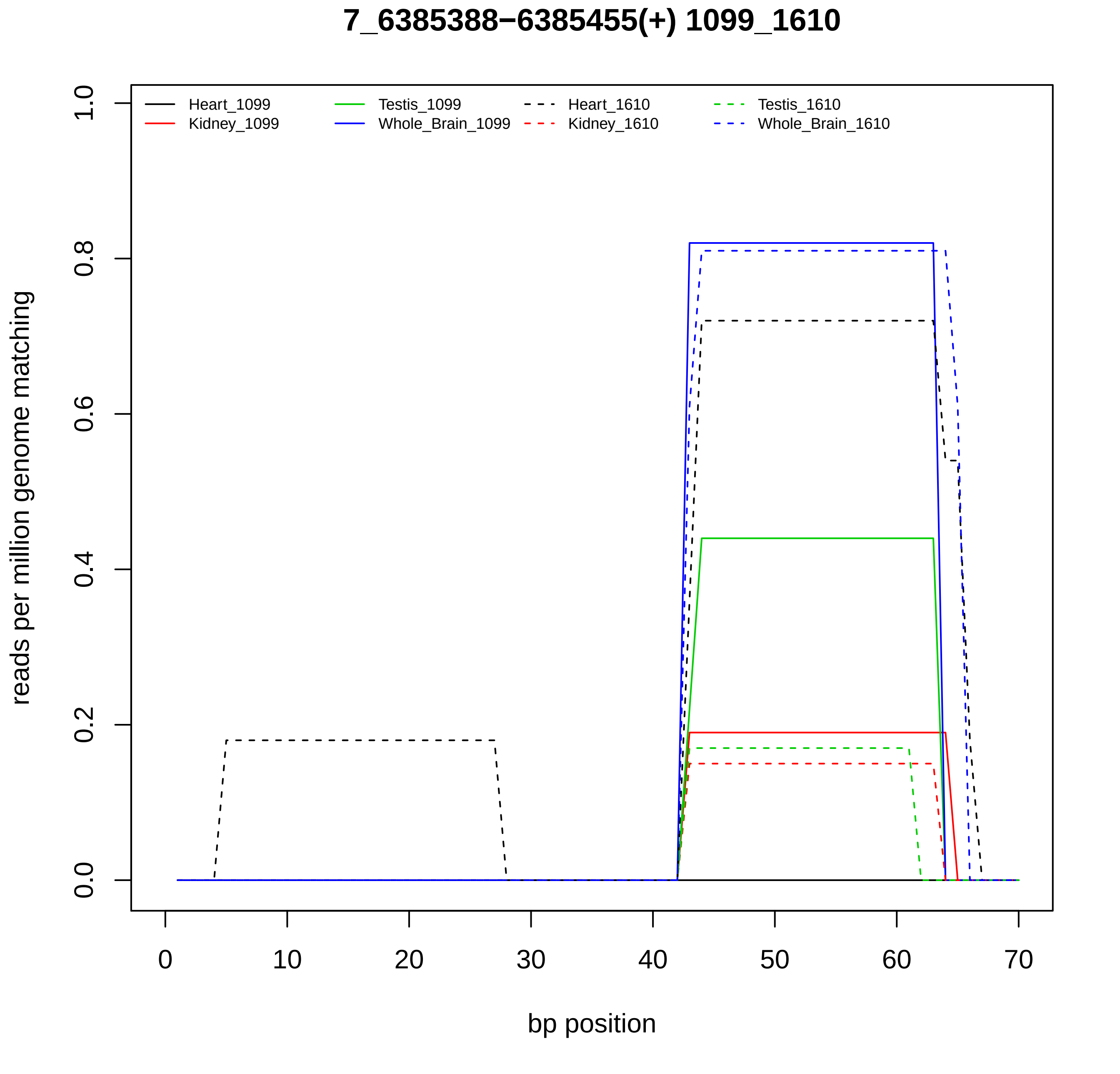

7_6385388−6385455(+) 1099_1610
Heart_1099
Kidney_1099
Testis_1099
Whole_Brain_1099
Heart_1610
Kidney_1610
Testis_1610
Whole_Brain_1610
reads per million genome matching
0.0
0.2
0.4
0.6
0.8
1.0
0
10
20
30
40
50
60
70
bp position

7_65231363−65231439(+) 1099_1610

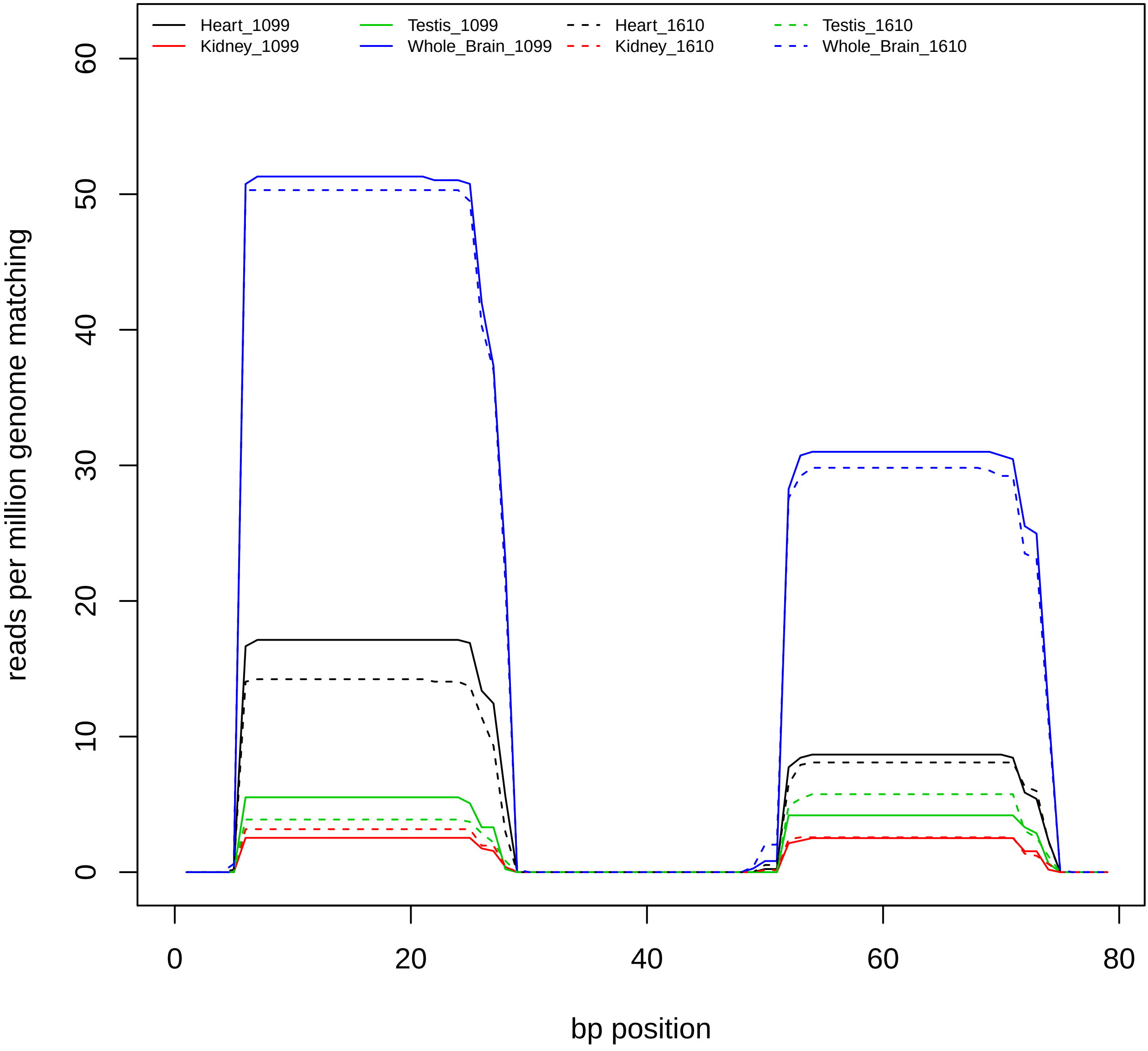

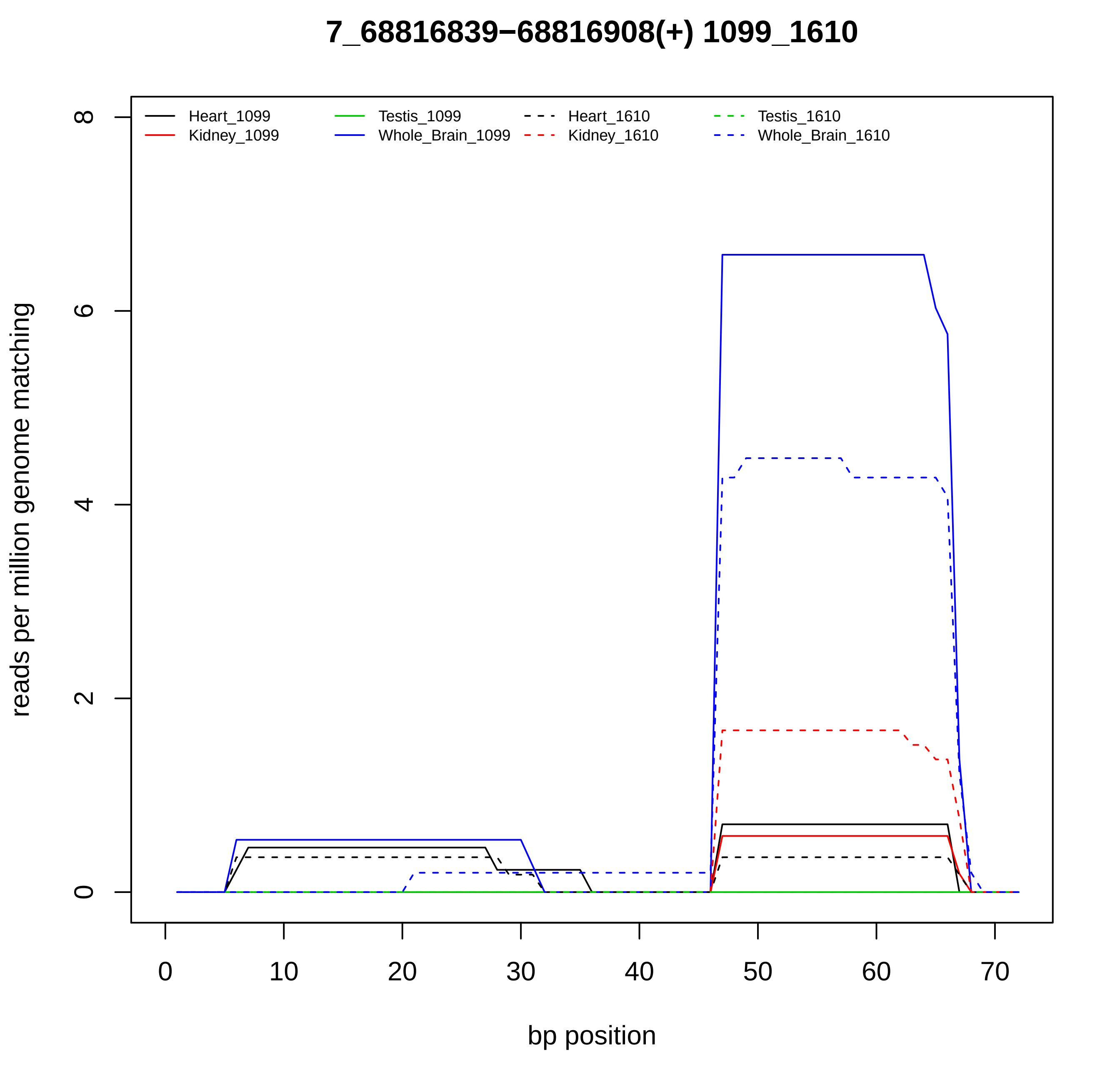
7_68816839−68816908(+) 1099_1610
Heart_1099
Kidney_1099
Testis_1099
Whole_Brain_1099
Heart_1610
Kidney_1610
Testis_1610
Whole_Brain_1610
reads per million genome matching
0
2
4
6
8
0
10
20
30
40
50
60
70
bp position

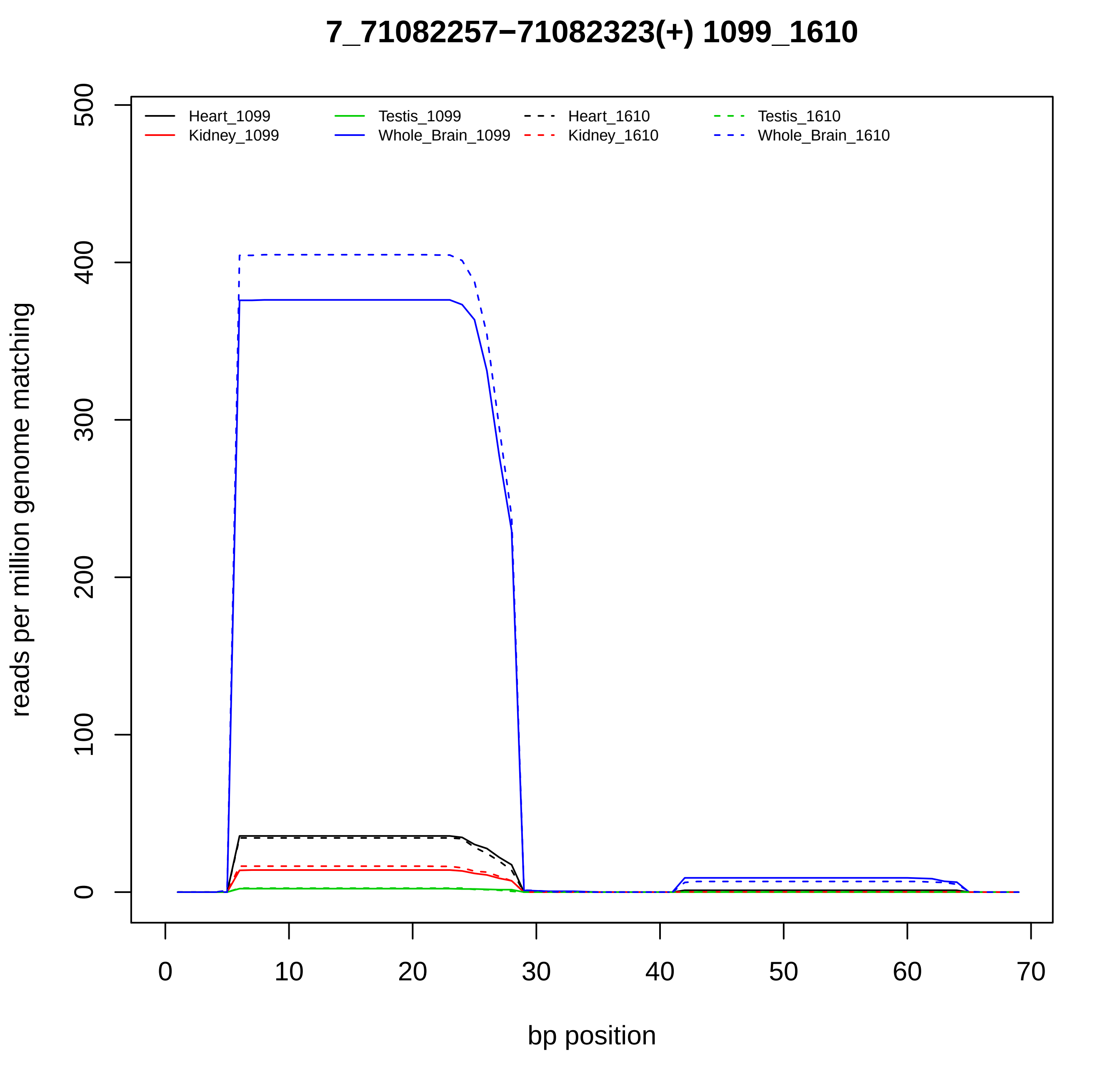

7_71082257−71082323(+) 1099_1610
Heart_1099
Kidney_1099
Testis_1099
Whole_Brain_1099
Heart_1610
Kidney_1610
Testis_1610
Whole_Brain_1610
reads per million genome matching
0
100
200
300
400
500
bp position
0
10
20
30
40
50
60
70

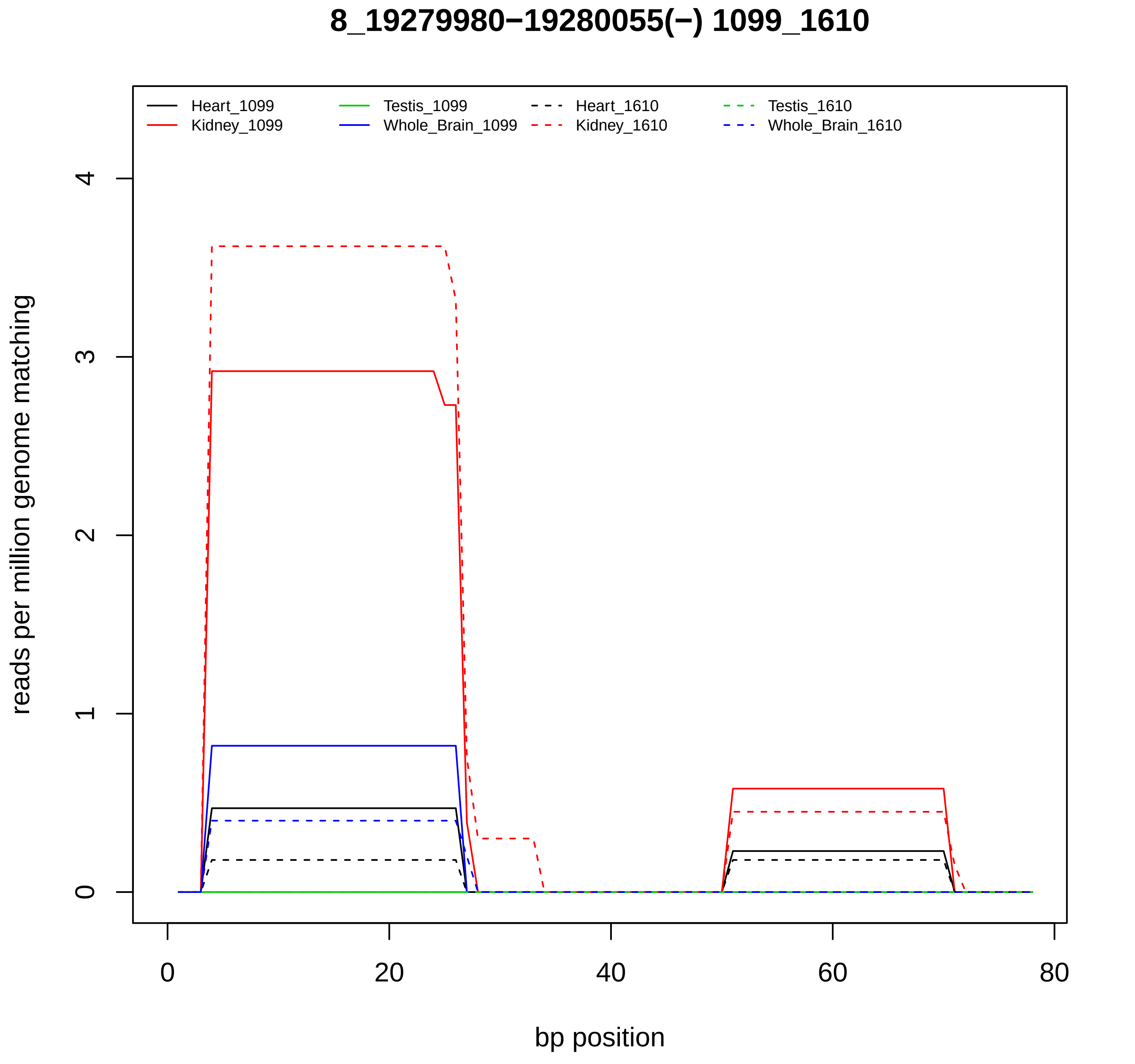

8_19279980−19280055(−) 1099_1610
Heart_1099
Kidney_1099
Testis_1099
Whole_Brain_1099
Heart_1610
Kidney_1610
Testis_1610
Whole_Brain_1610
reads per million genome matching
0
1
2
3
4
0
20
40
60
80
bp position

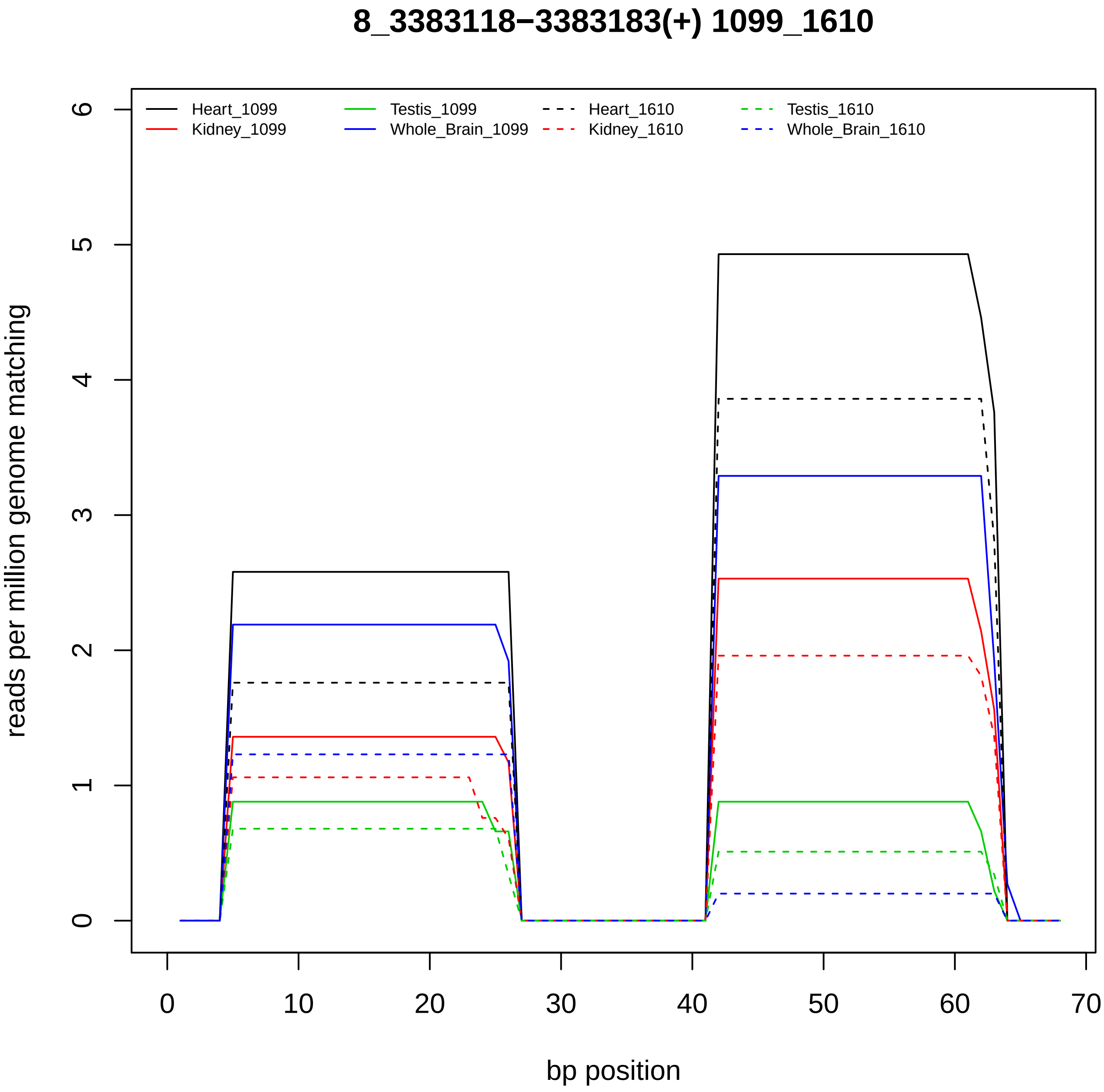

8_3383118−3383183(+) 1099_1610
Heart_1099
Kidney_1099
Testis_1099
Whole_Brain_1099
Heart_1610
Kidney_1610
Testis_1610
Whole_Brain_1610
reads per million genome matching
bp position
0
1
2
3
4
5
6
0
10
20
30
40
50
60
70

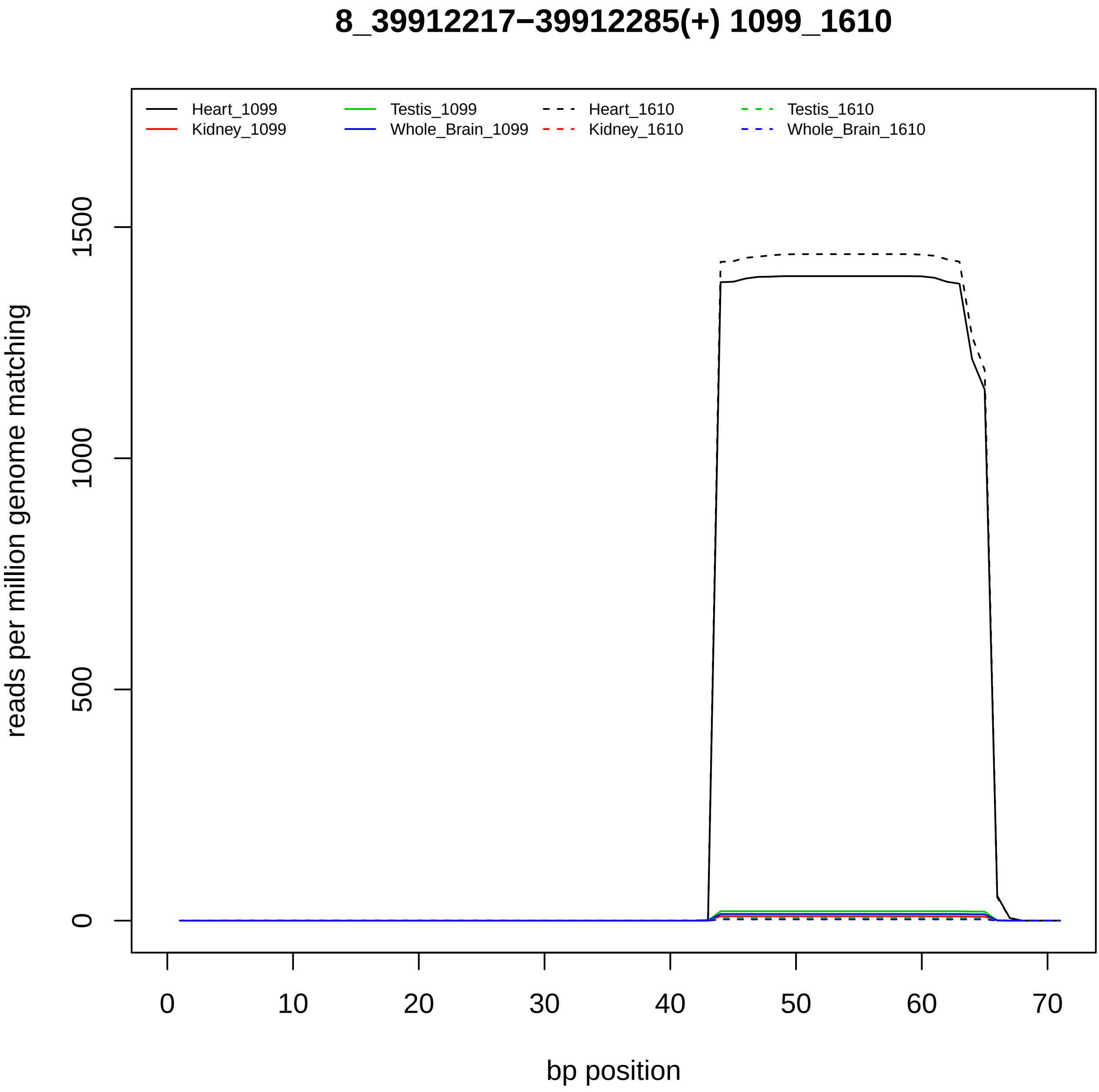

8_39912217−39912285(+) 1099_1610
Heart_1099
Kidney_1099
Testis_1099
Whole_Brain_1099
Heart_1610
Kidney_1610
Testis_1610
Whole_Brain_1610
reads per million genome matching
0
500
1000
1500
0
10
20
30
40
50
60
70
bp position

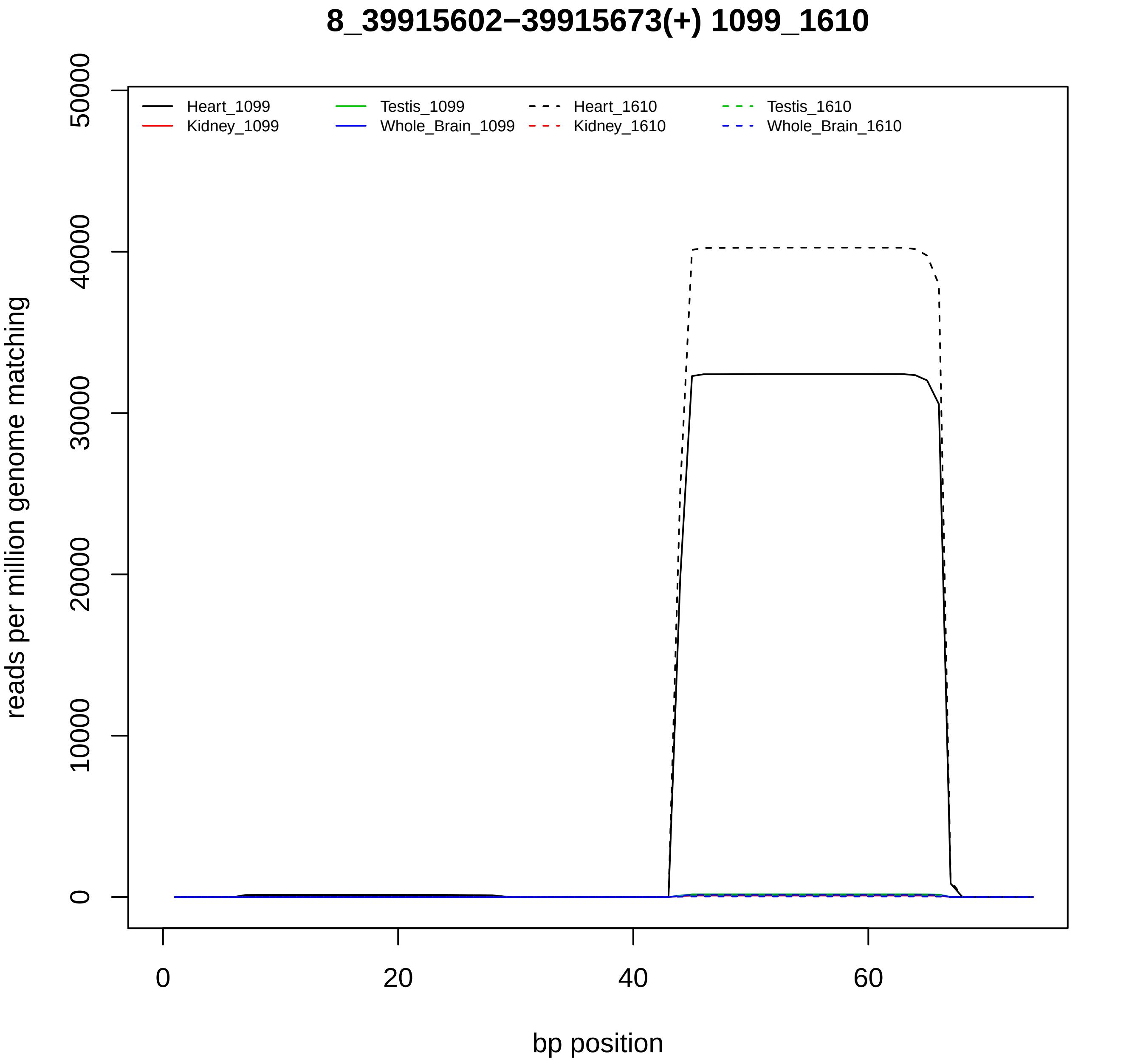

8_39915602−39915673(+) 1099_1610
Heart_1099
Kidney_1099
Testis_1099
Whole_Brain_1099
Heart_1610
Kidney_1610
Testis_1610
Whole_Brain_1610
reads per million genome matching
bp position
0
10000
20000
30000
40000
50000
0
20
40
60

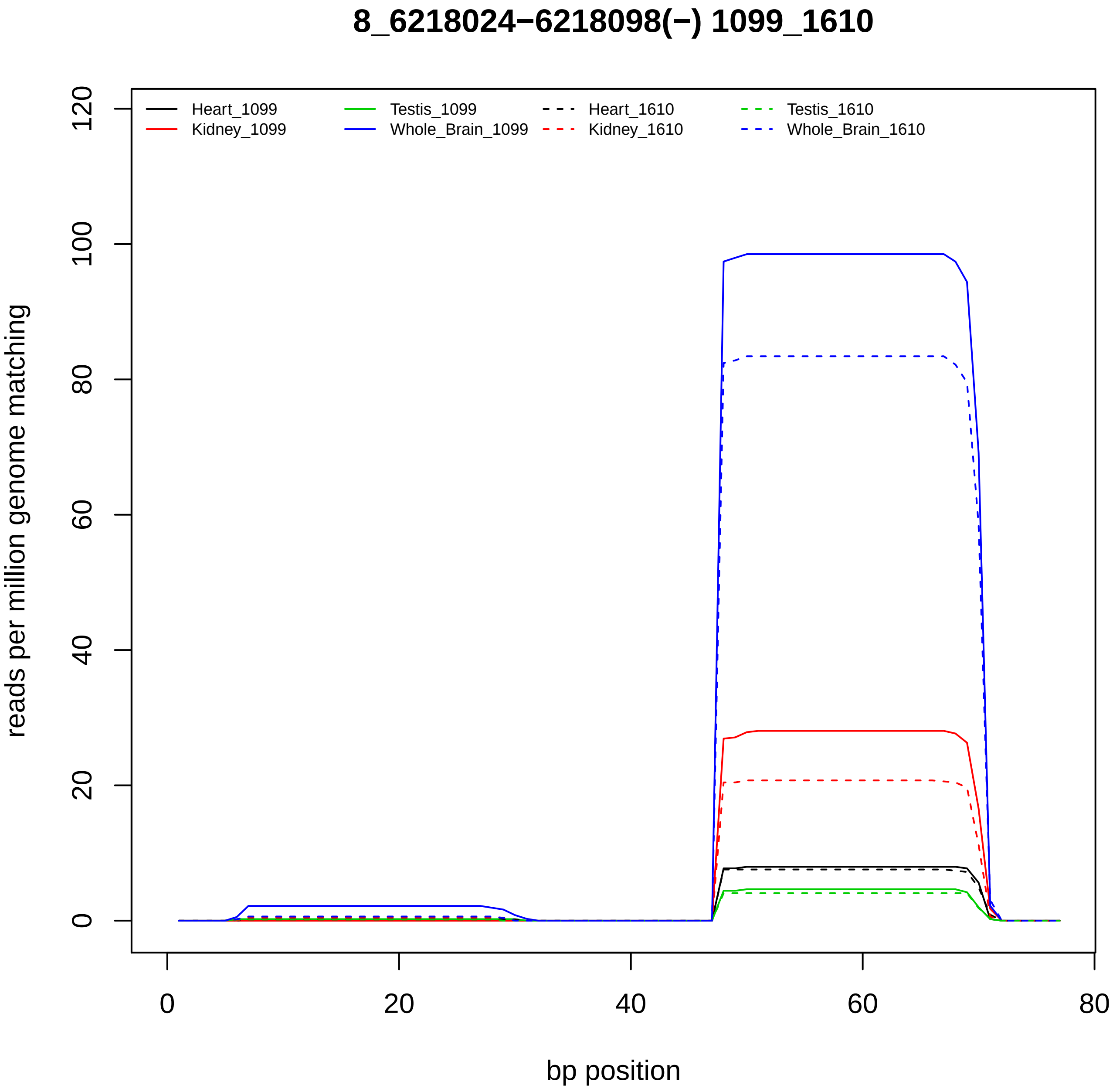

8_6218024−6218098(−) 1099_1610
Heart_1099
Kidney_1099
Testis_1099
Whole_Brain_1099
Heart_1610
Kidney_1610
Testis_1610
Whole_Brain_1610
reads per million genome matching
bp position
0
20
40
60
80
100
120

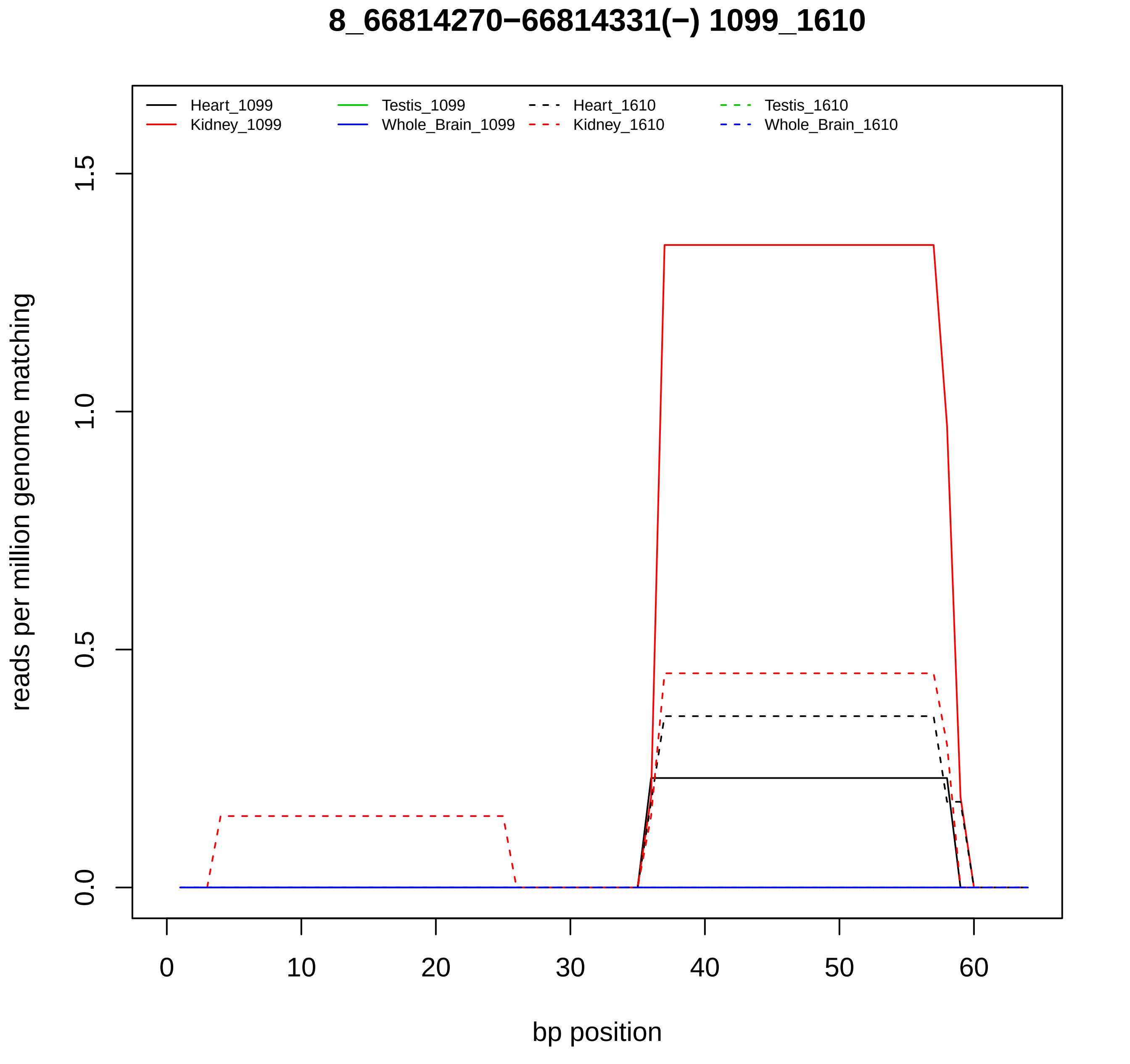

8_66814270−66814331(−) 1099_1610
Heart_1099
Kidney_1099
Testis_1099
Whole_Brain_1099
Heart_1610
Kidney_1610
Testis_1610
Whole_Brain_1610
reads per million genome matching
0.0
0.5
1.0
1.5
0
10
20
30
40
50
60
bp position

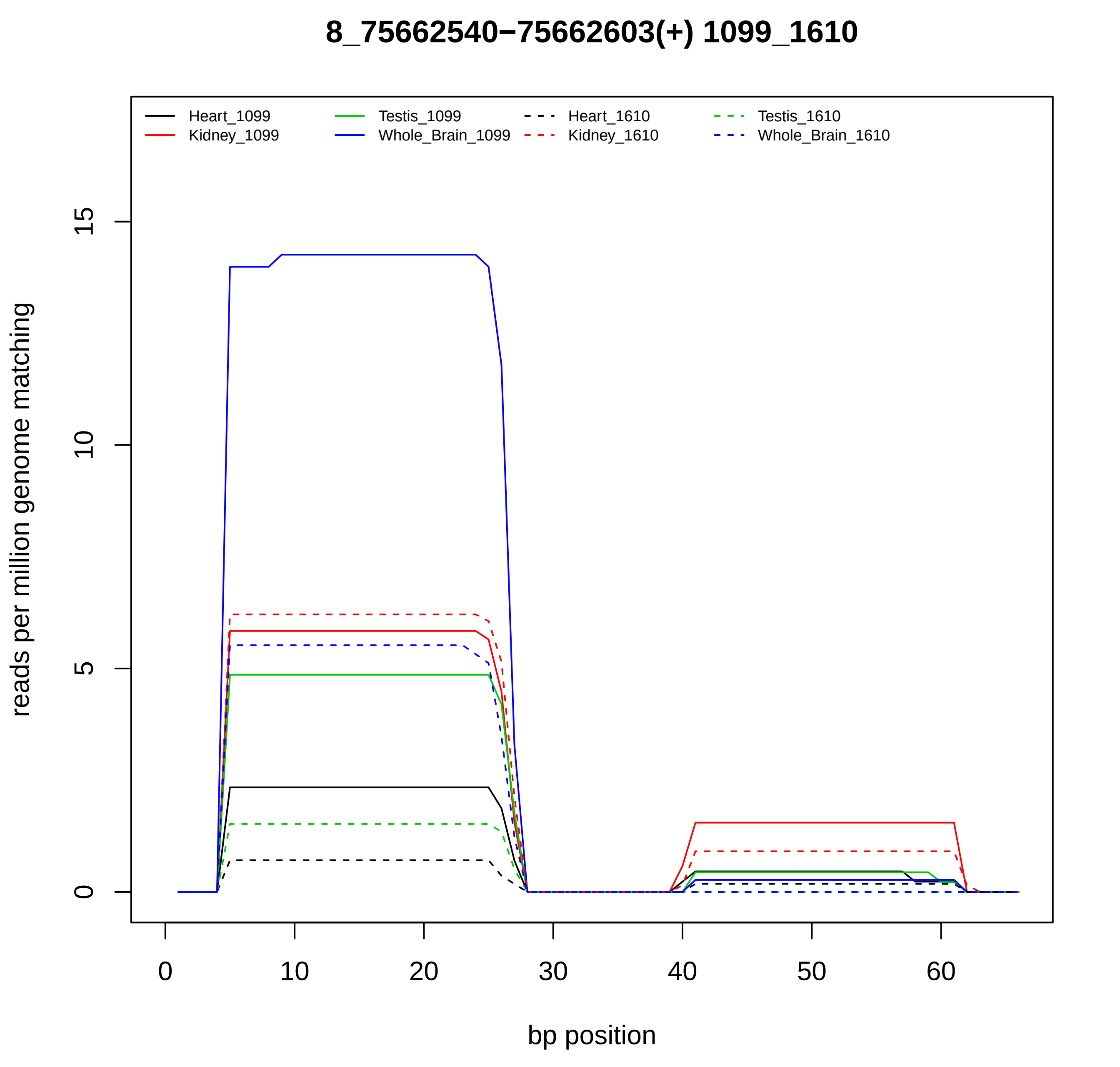

8_75662540−75662603(+) 1099_1610
Heart_1099
Kidney_1099
Testis_1099
Whole_Brain_1099
Heart_1610
Kidney_1610
Testis_1610
Whole_Brain_1610
reads per million genome matching
bp position
0
5
10
15
0
10
20
30
40
50
60

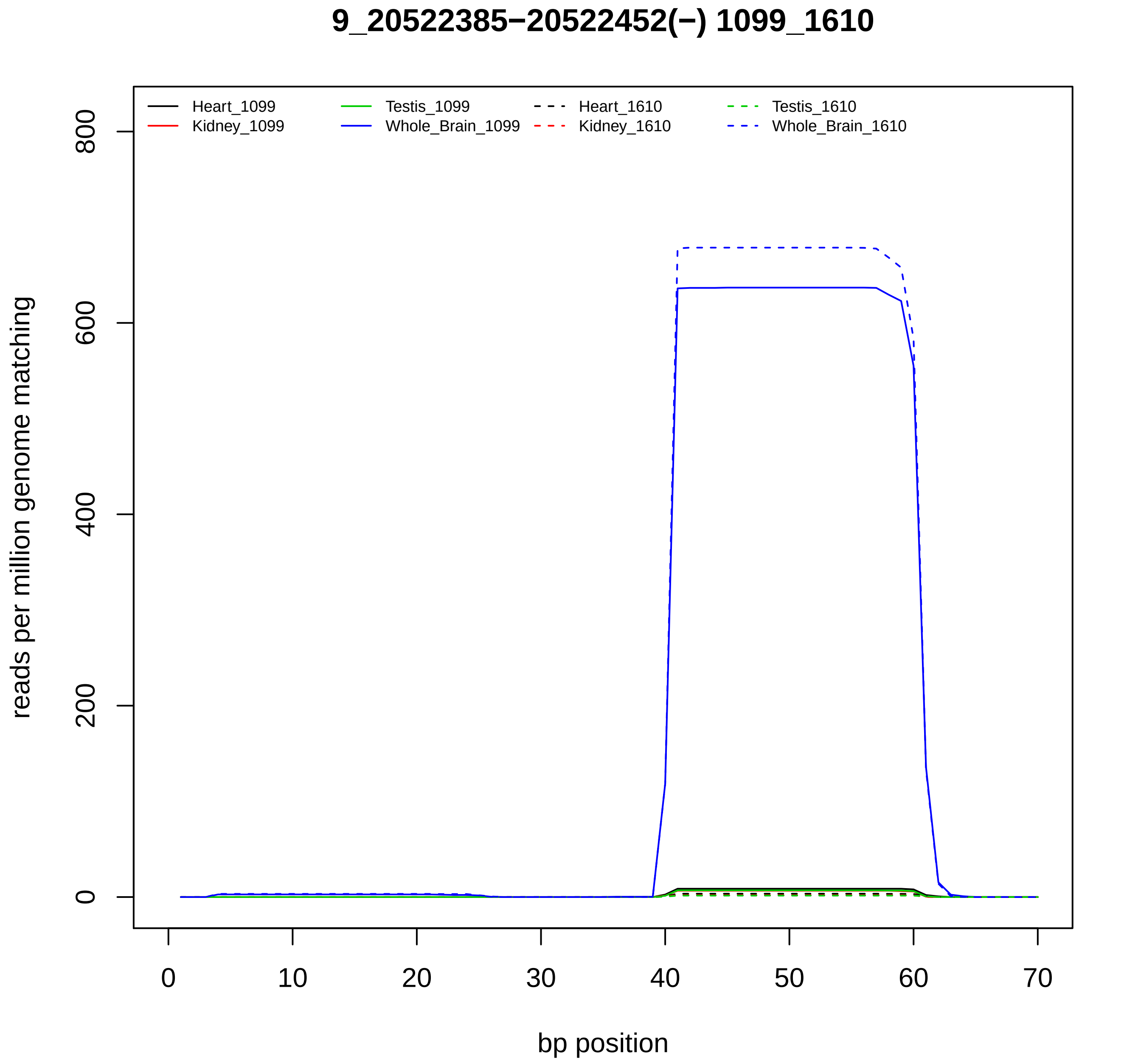

9_20522385−20522452(−) 1099_1610
Heart_1099
Kidney_1099
Testis_1099
Whole_Brain_1099
Heart_1610
Kidney_1610
Testis_1610
Whole_Brain_1610
reads per million genome matching
0
200
400
600
800
0
10
20
30
40
50
60
70
bp position

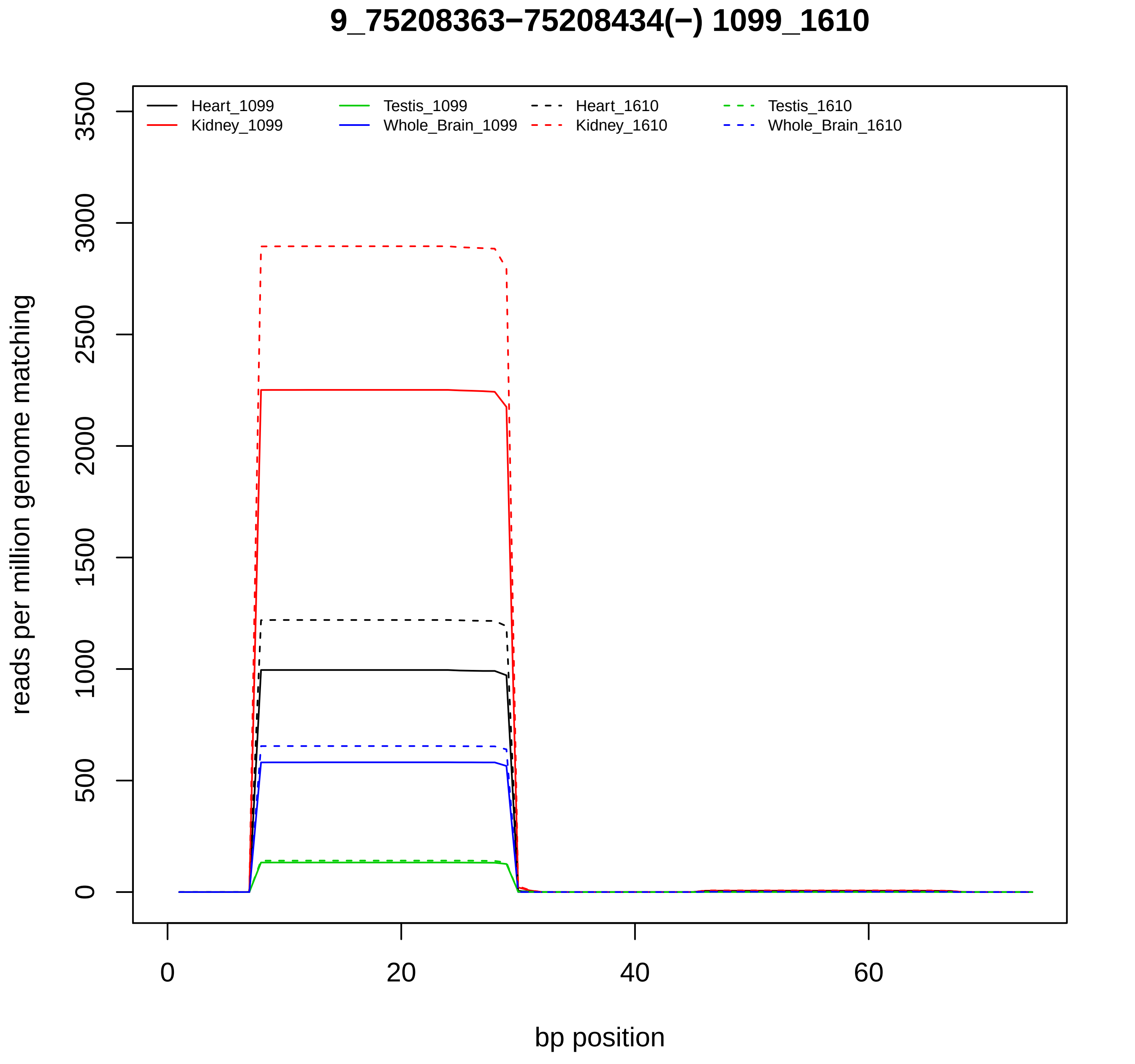
9_75208363−75208434(−) 1099_1610
Heart_1099
Kidney_1099
Testis_1099
Whole_Brain_1099
Heart_1610
Kidney_1610
Testis_1610
Whole_Brain_1610
reads per million genome matching
0
500
1000
1500
2000
2500
3000
3500
bp position
0
20
40
60

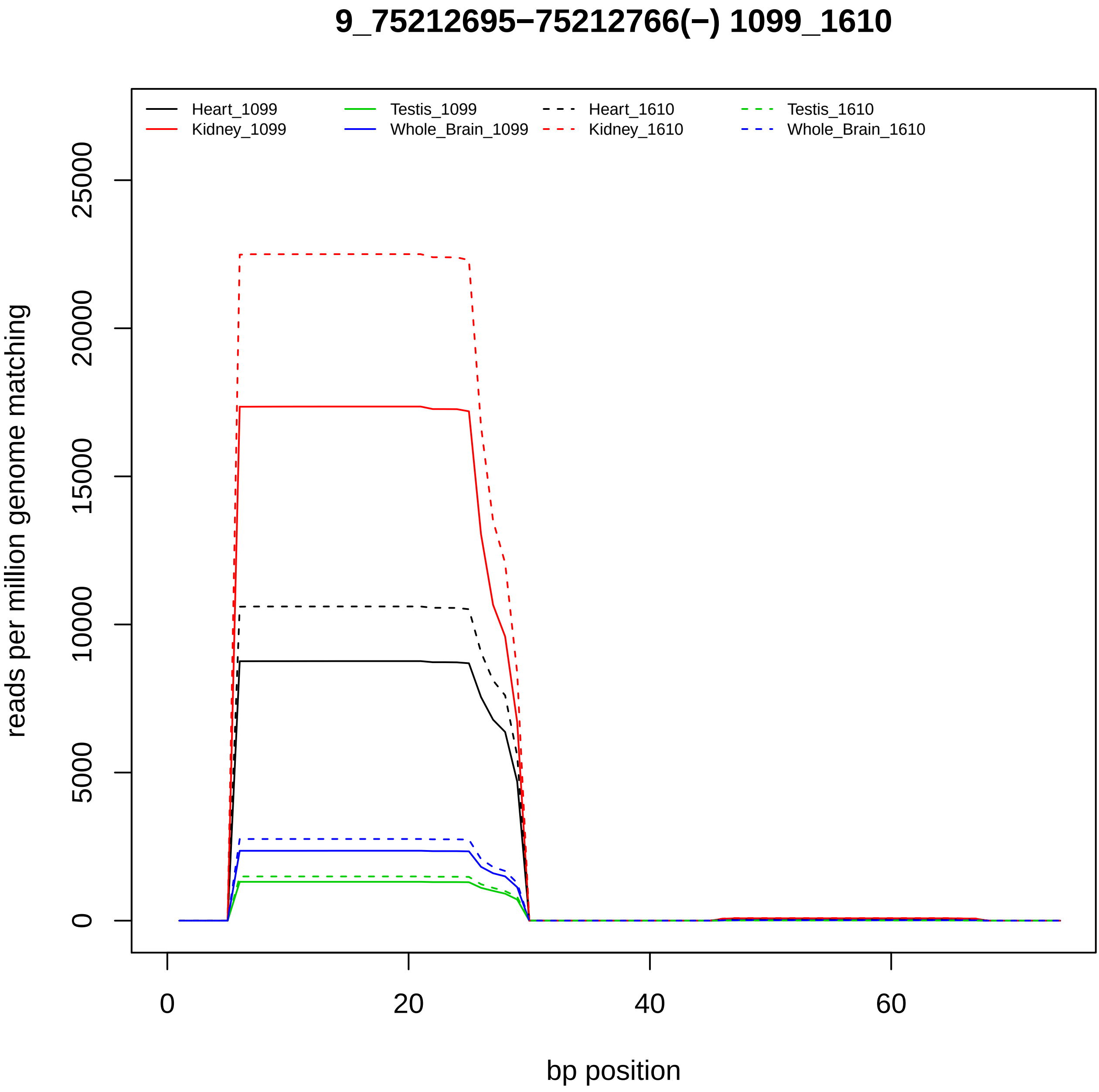
9_75212695−75212766(−) 1099_1610
Heart_1099
Kidney_1099
Testis_1099
Whole_Brain_1099
Heart_1610
Kidney_1610
Testis_1610
Whole_Brain_1610
reads per million genome matching
0
5000
10000
15000
20000
25000
bp position
0
20
40
60

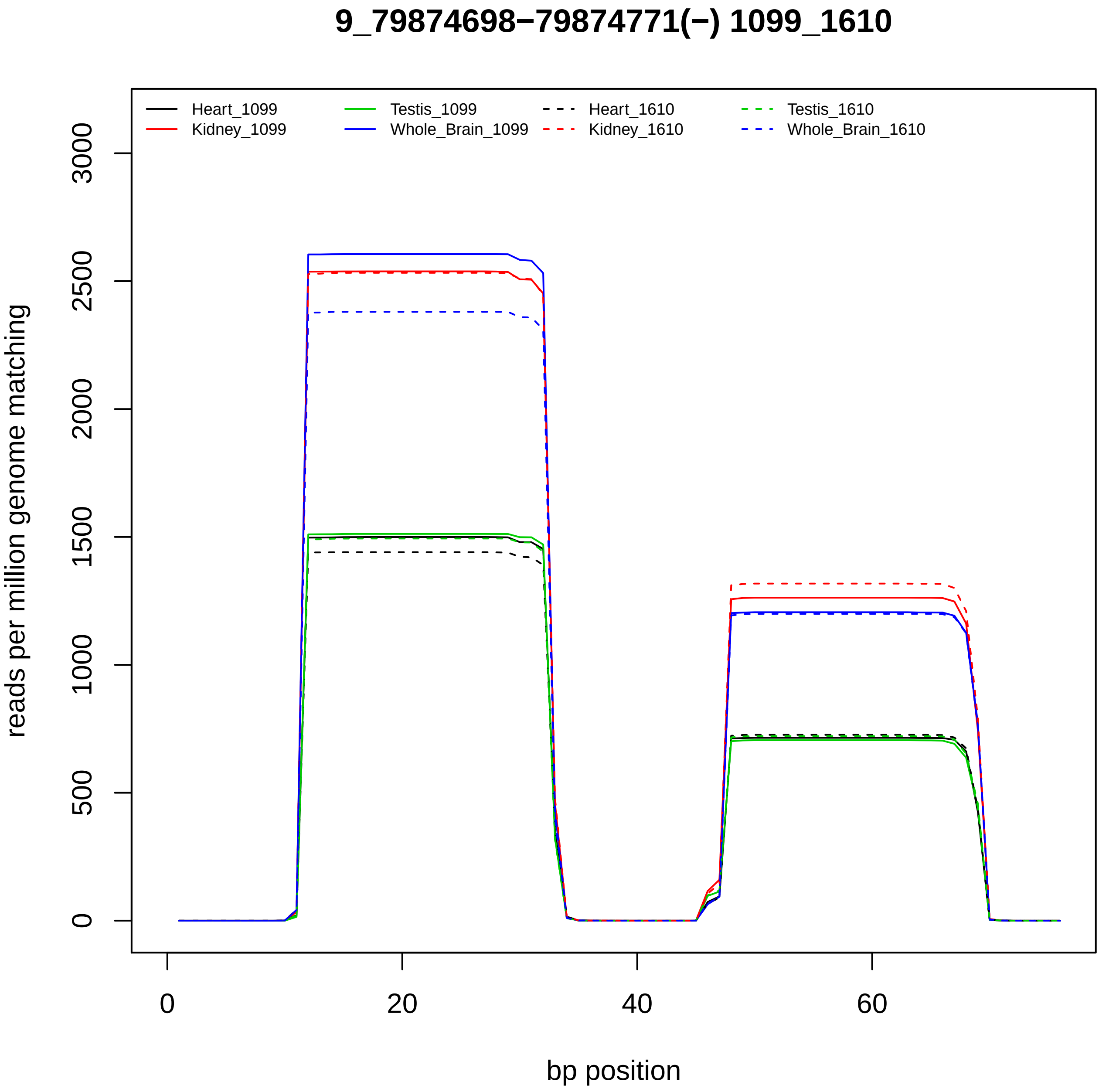

9_79874698−79874771(−) 1099_1610
Heart_1099
Kidney_1099
Testis_1099
Whole_Brain_1099
Heart_1610
Kidney_1610
Testis_1610
Whole_Brain_1610
reads per million genome matching
0
500
1000
1500
2000
2500
3000
bp position
0
20
40
60

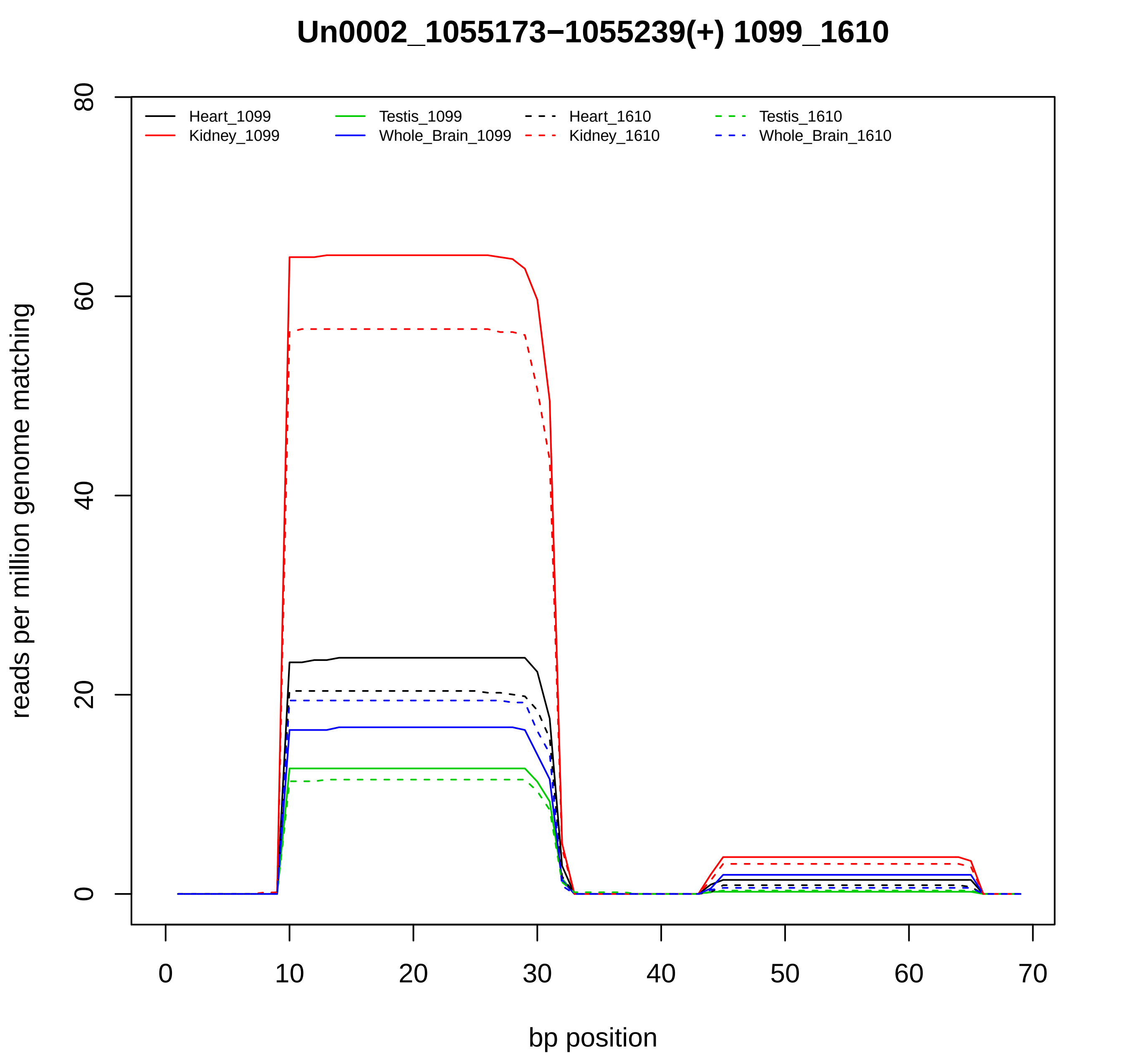

Un0002_1055173−1055239(+) 1099_1610
Heart_1099
Kidney_1099
Testis_1099
Whole_Brain_1099
Heart_1610
Kidney_1610
Testis_1610
Whole_Brain_1610
reads per million genome matching
0
20
40
60
80
bp position
0
10
20
30
40
50
60
70

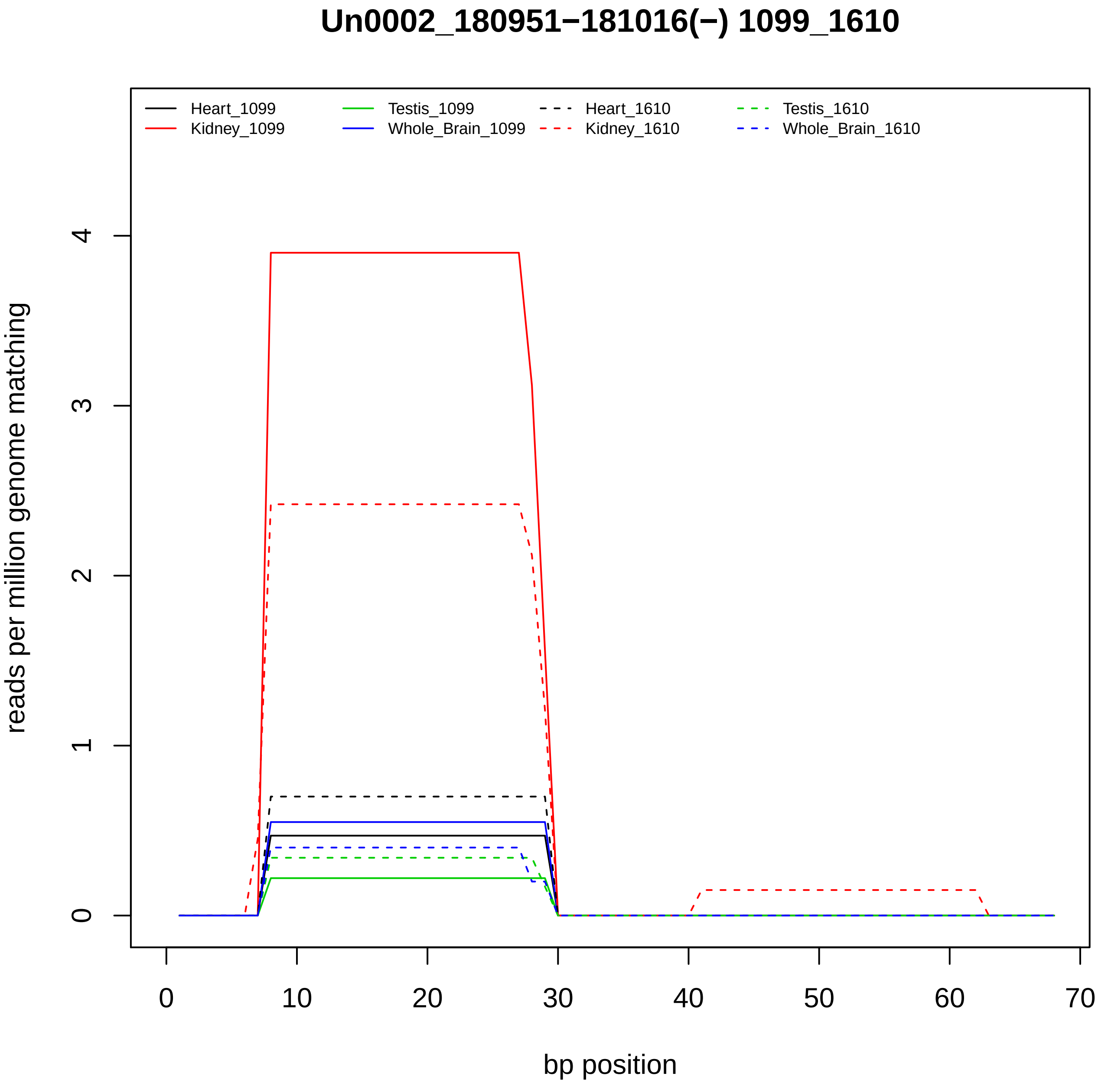

Un0002_180951−181016(−) 1099_1610
Heart_1099
Kidney_1099
Testis_1099
Whole_Brain_1099
Heart_1610
Kidney_1610
Testis_1610
Whole_Brain_1610
reads per million genome matching
0
1
2
3
4
0
10
20
30
40
50
60
70
bp position

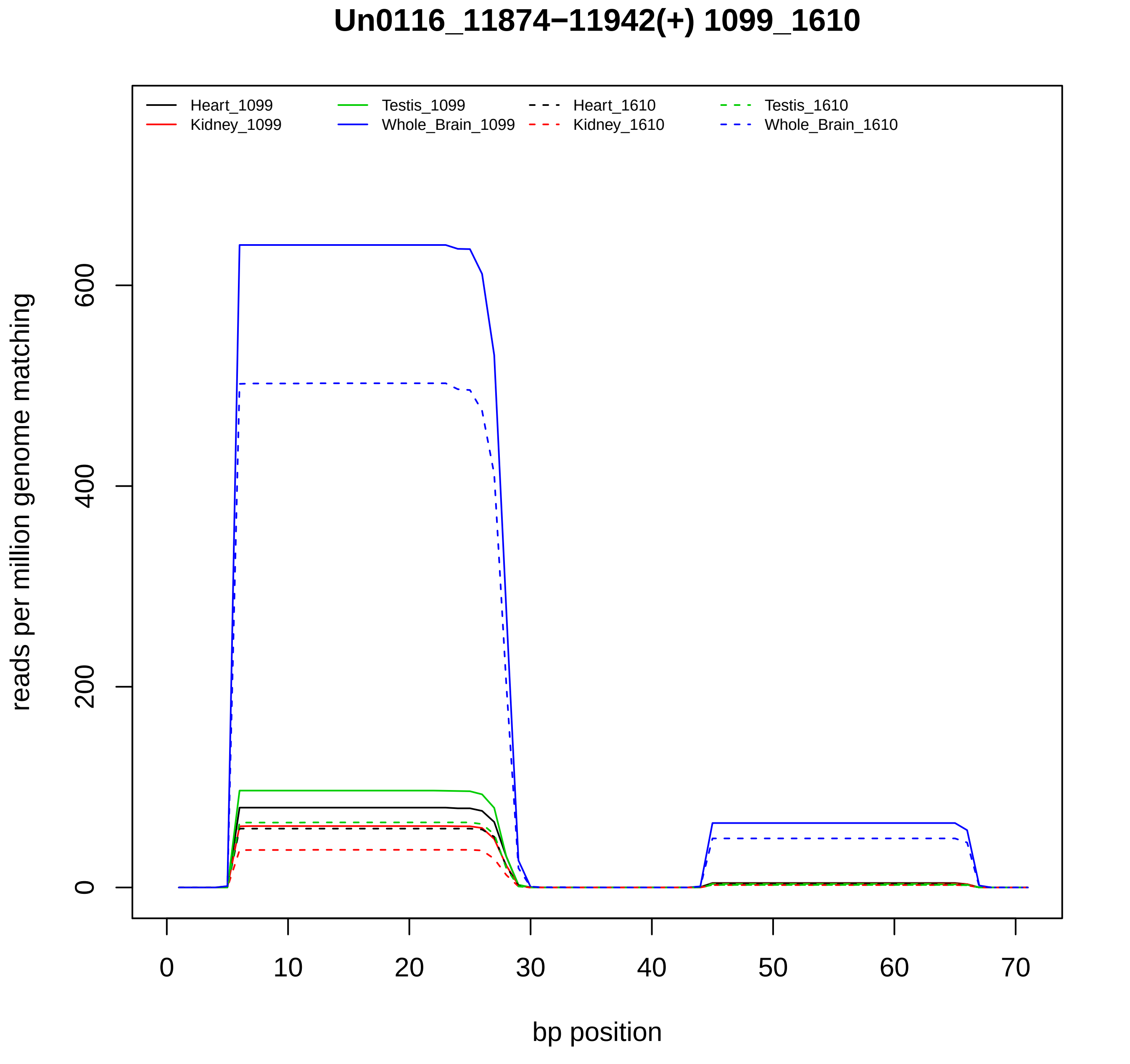

Un0116_11874−11942(+) 1099_1610
Heart_1099
Kidney_1099
Testis_1099
Whole_Brain_1099
Heart_1610
Kidney_1610
Testis_1610
Whole_Brain_1610
reads per million genome matching
0
200
400
600
0
10
20
30
40
50
60
70
bp position

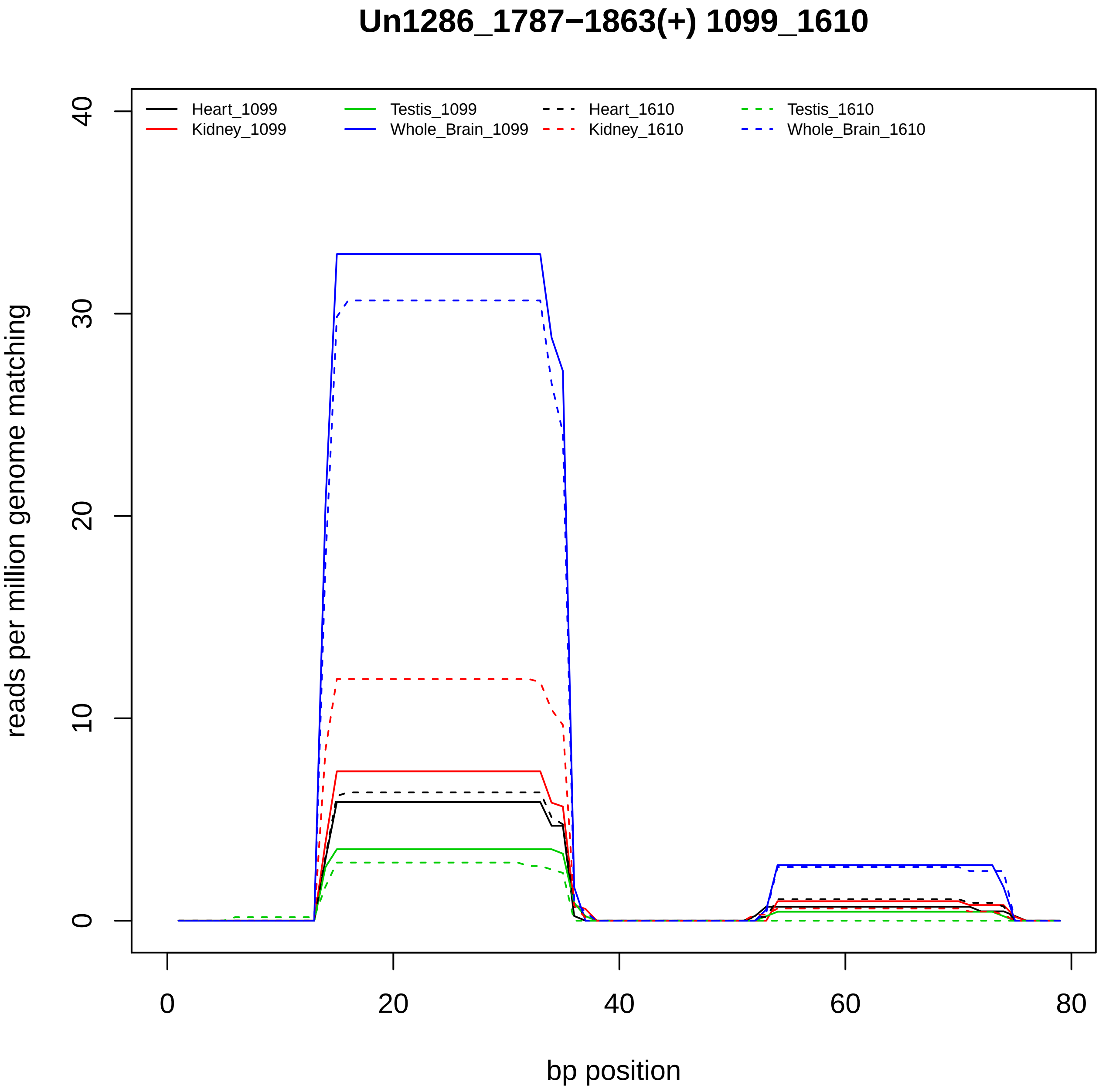

Un1286_1787−1863(+) 1099_1610
Heart_1099
Kidney_1099
Testis_1099
Whole_Brain_1099
Heart_1610
Kidney_1610
Testis_1610
Whole_Brain_1610
reads per million genome matching
bp position
0
10
20
30
40
0
20
40
60
80

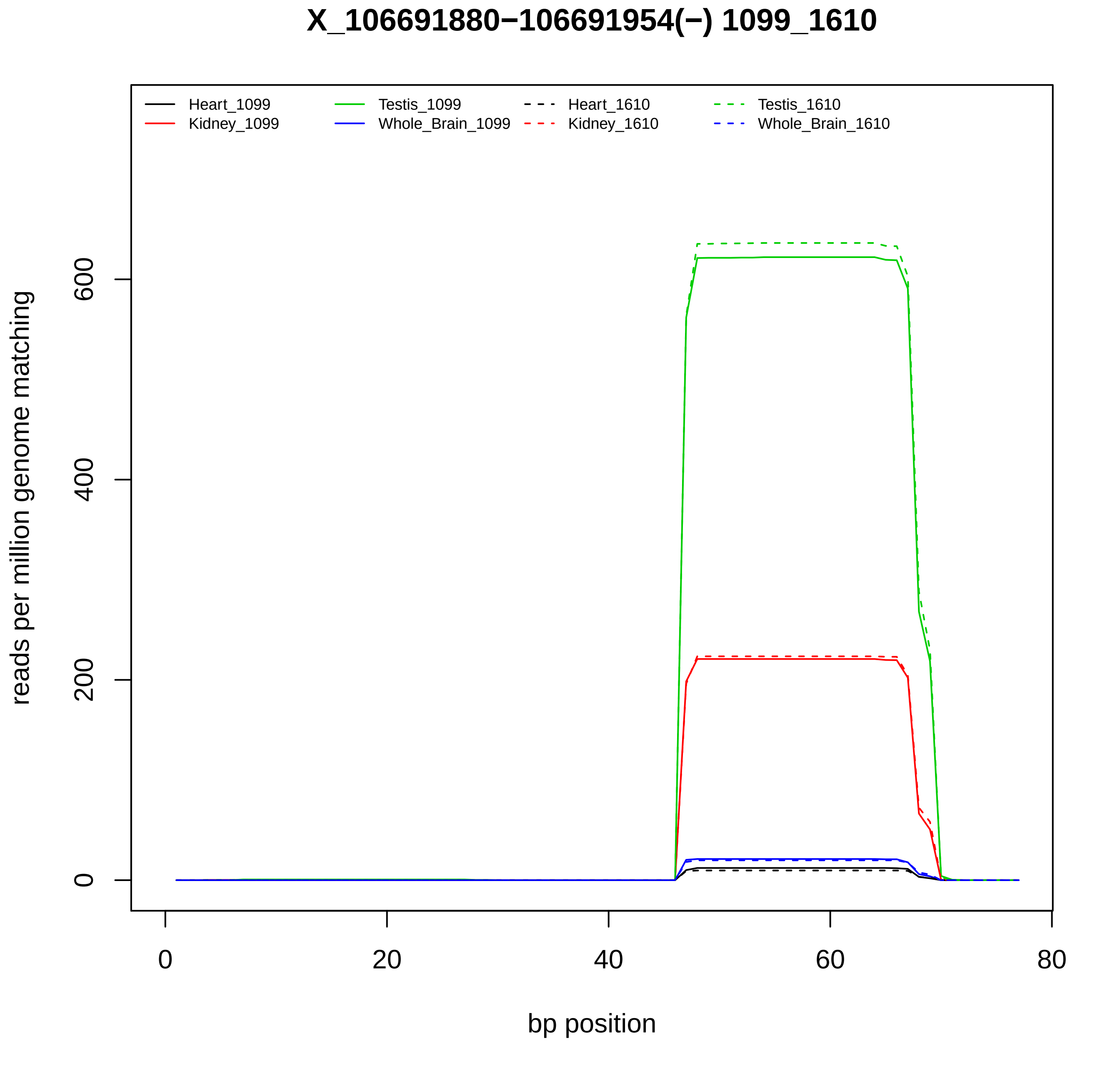

X_106691880−106691954(−) 1099_1610
Heart_1099
Kidney_1099
Testis_1099
Whole_Brain_1099
Heart_1610
Kidney_1610
Testis_1610
Whole_Brain_1610
reads per million genome matching
0
200
400
600
0
20
40
60
80
bp position

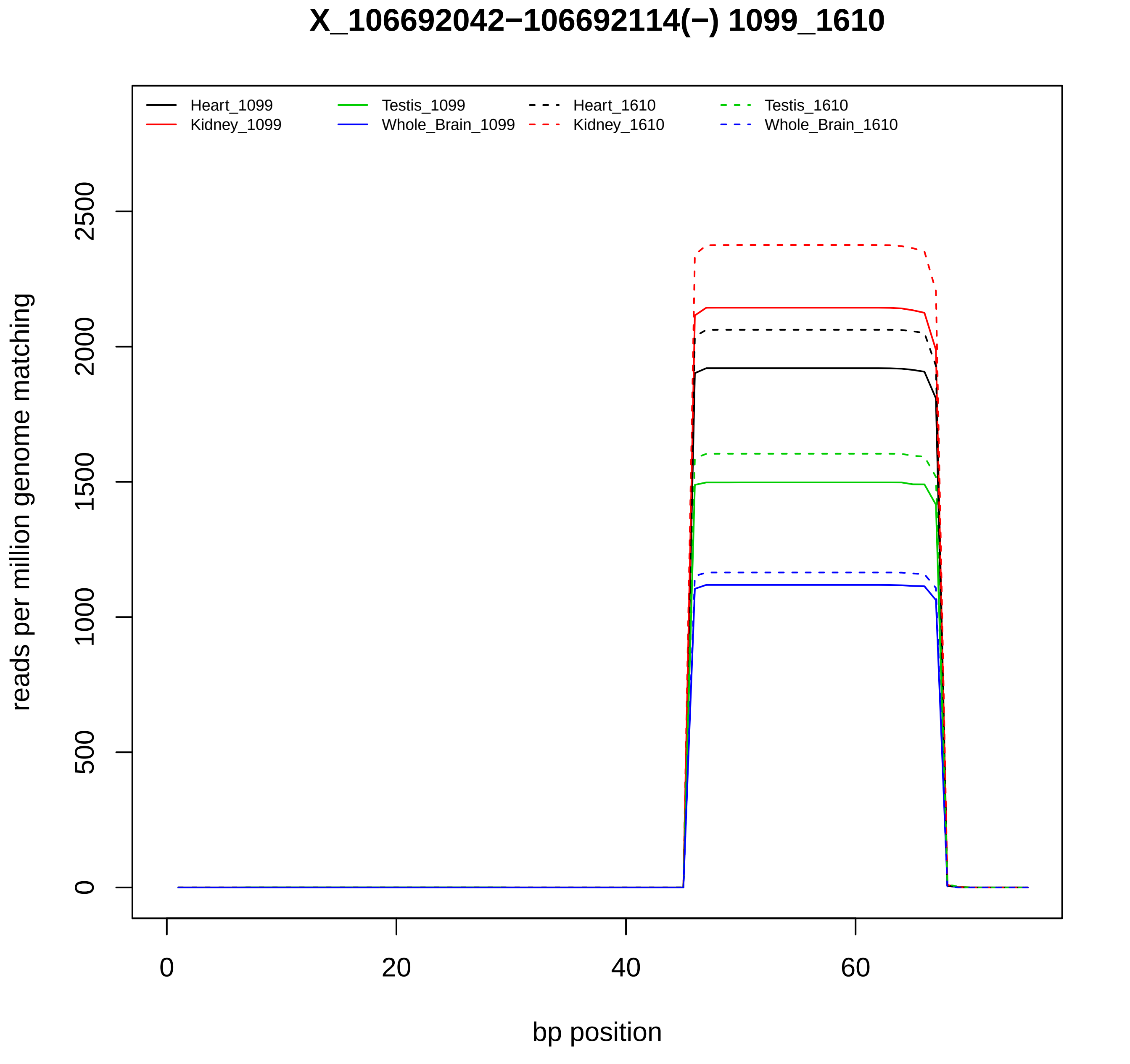
X_106692042−106692114(−) 1099_1610
Heart_1099
Kidney_1099
Testis_1099
Whole_Brain_1099
Heart_1610
Kidney_1610
Testis_1610
Whole_Brain_1610
reads per million genome matching
0
500
1000
1500
2000
2500
0
20
40
60
bp position

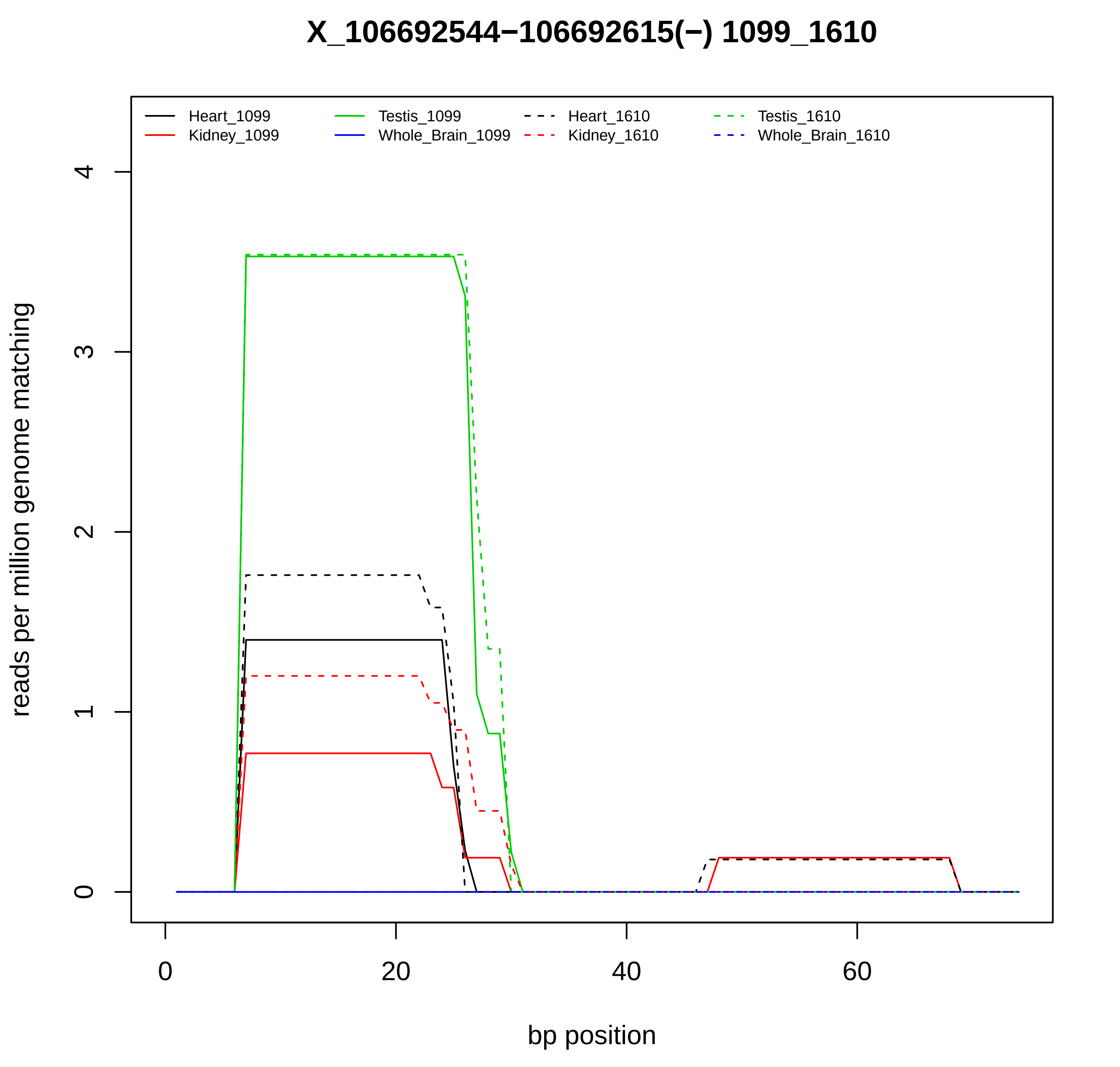

X_106692544−106692615(−) 1099_1610
Heart_1099
Kidney_1099
Testis_1099
Whole_Brain_1099
Heart_1610
Kidney_1610
Testis_1610
Whole_Brain_1610
reads per million genome matching
bp position
0
1
2
3
4
0
20
40
60

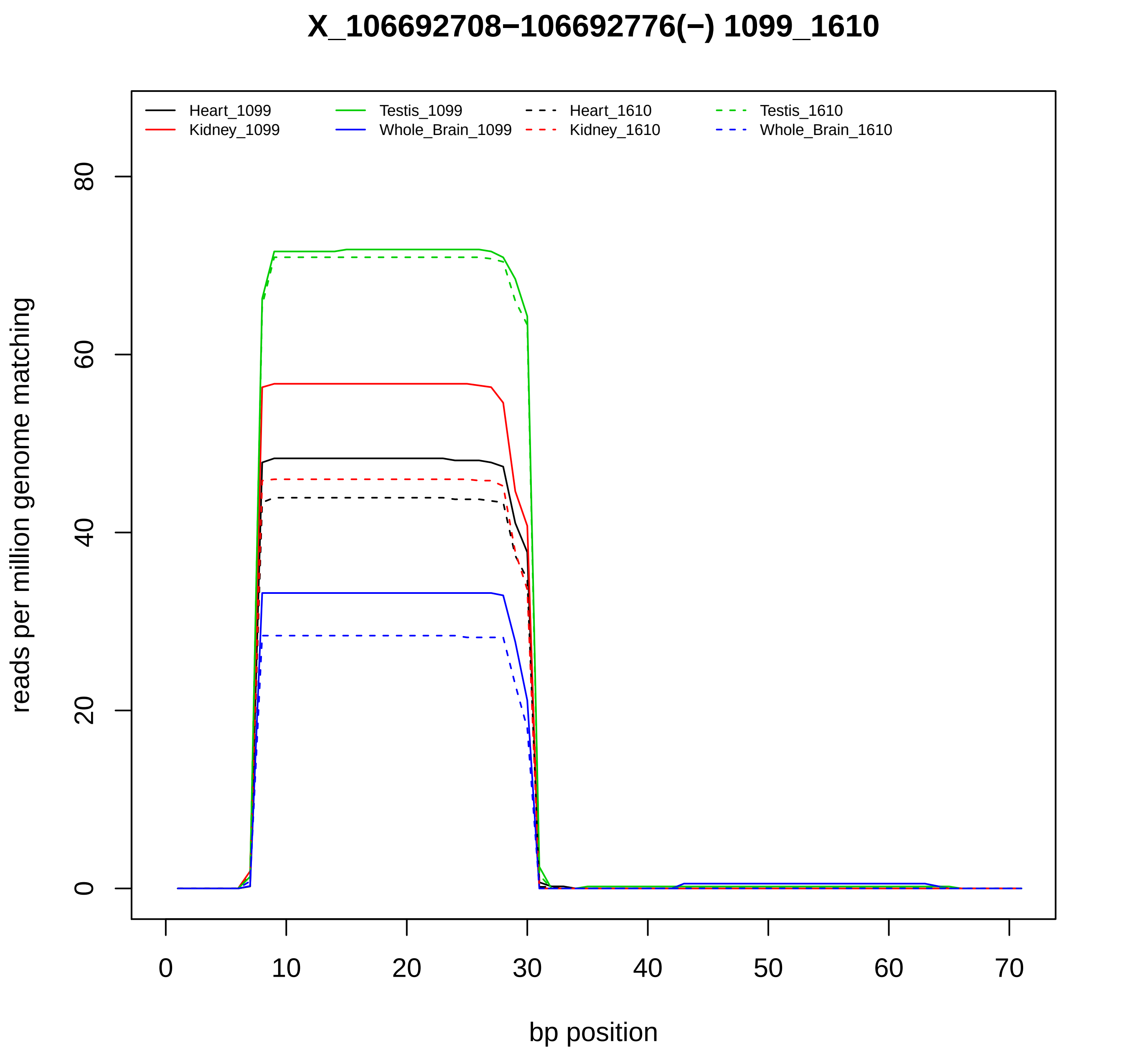

X_106692708−106692776(−) 1099_1610
Heart_1099
Kidney_1099
Testis_1099
Whole_Brain_1099
Heart_1610
Kidney_1610
Testis_1610
Whole_Brain_1610
reads per million genome matching
bp position
0
20
40
60
80
0
10
20
30
40
50
60
70

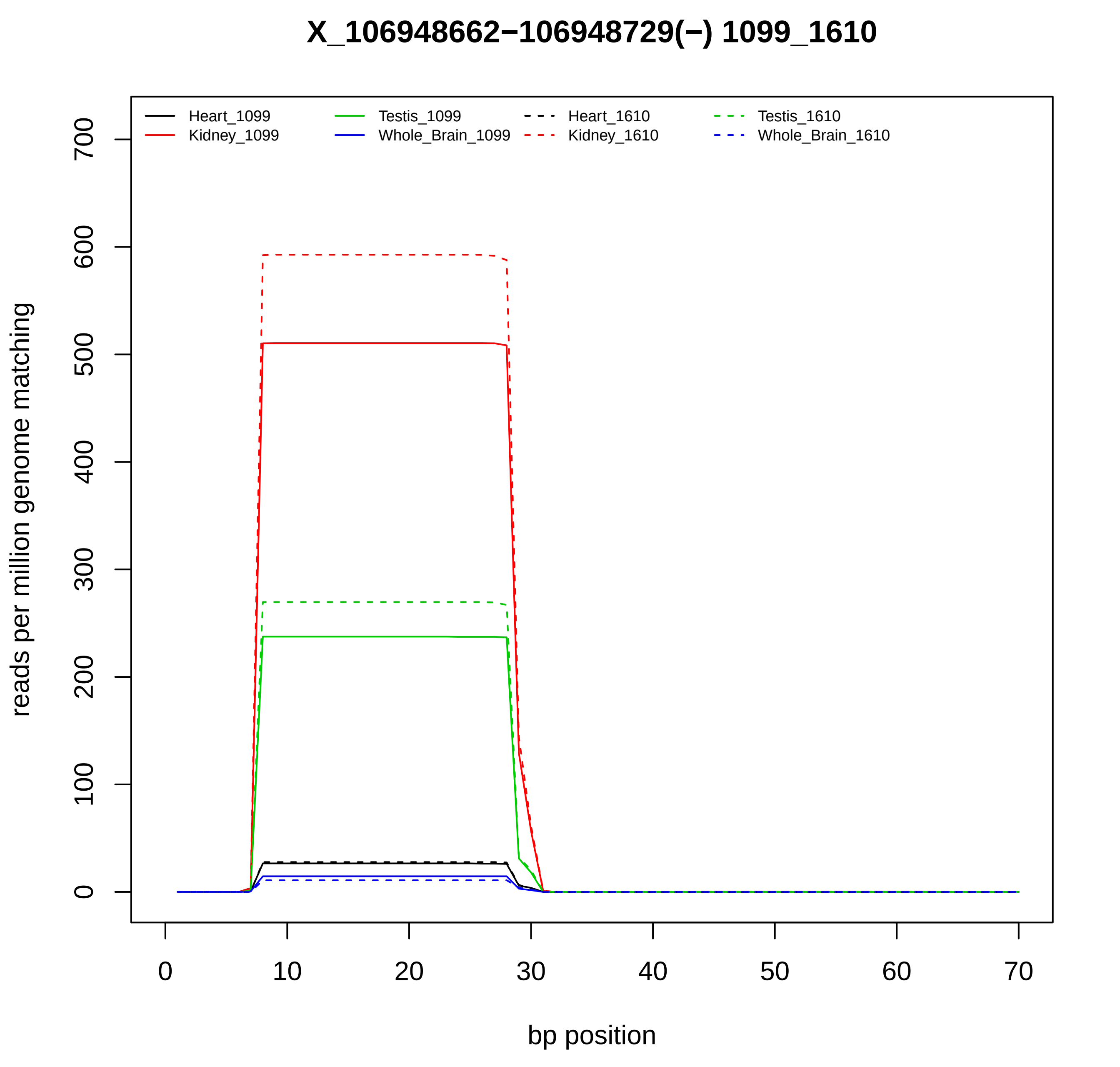

X_106948662−106948729(−) 1099_1610
Heart_1099
Kidney_1099
Testis_1099
Whole_Brain_1099
Heart_1610
Kidney_1610
Testis_1610
Whole_Brain_1610
reads per million genome matching
bp position
0
100
200
300
400
500
600
700
0
10
20
30
40
50
60
70

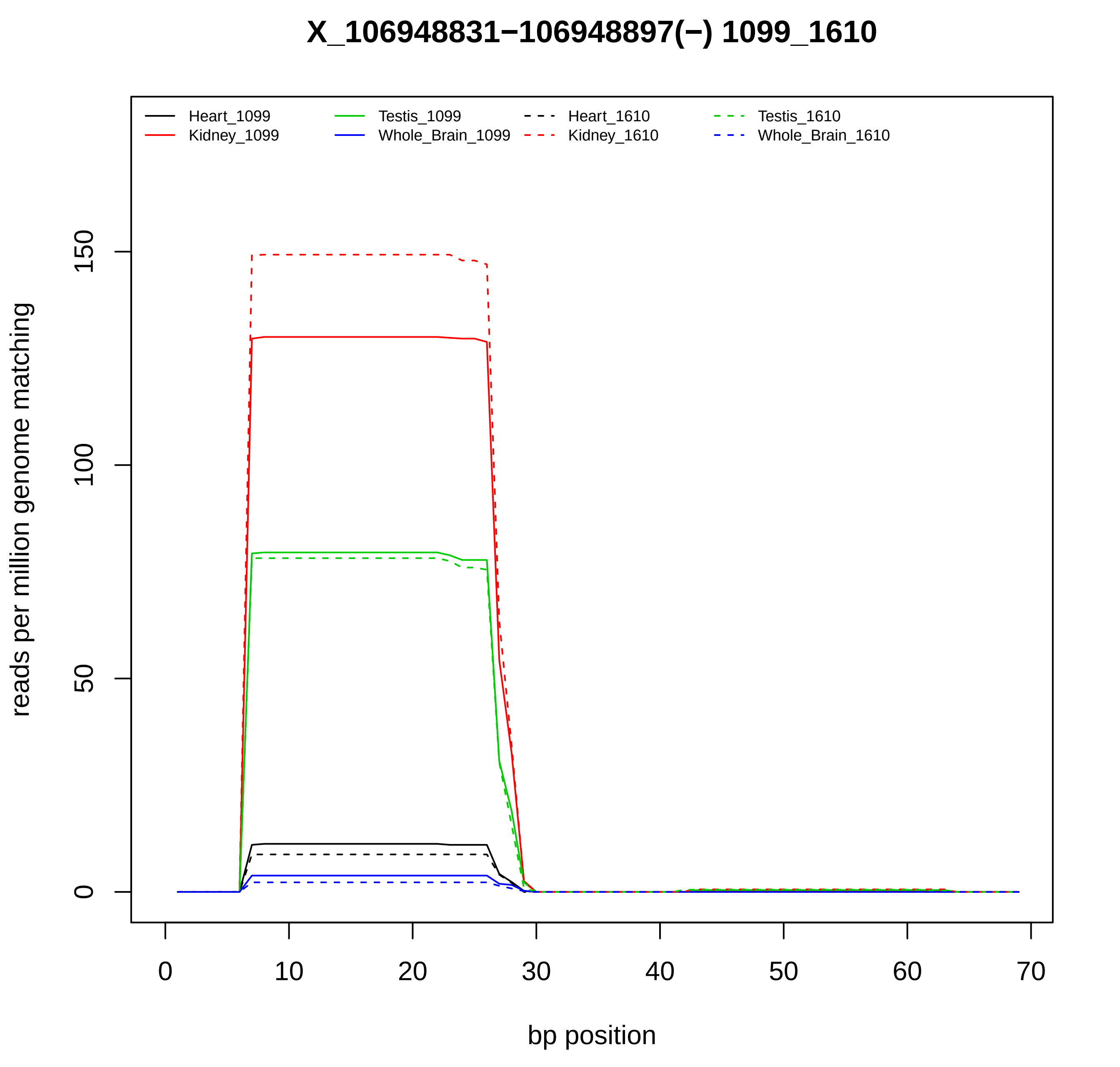
X_106948831−106948897(−) 1099_1610
Heart_1099
Kidney_1099
Testis_1099
Whole_Brain_1099
Heart_1610
Kidney_1610
Testis_1610
Whole_Brain_1610
reads per million genome matching
bp position
0
50
100
150
0
10
20
30
40
50
60
70

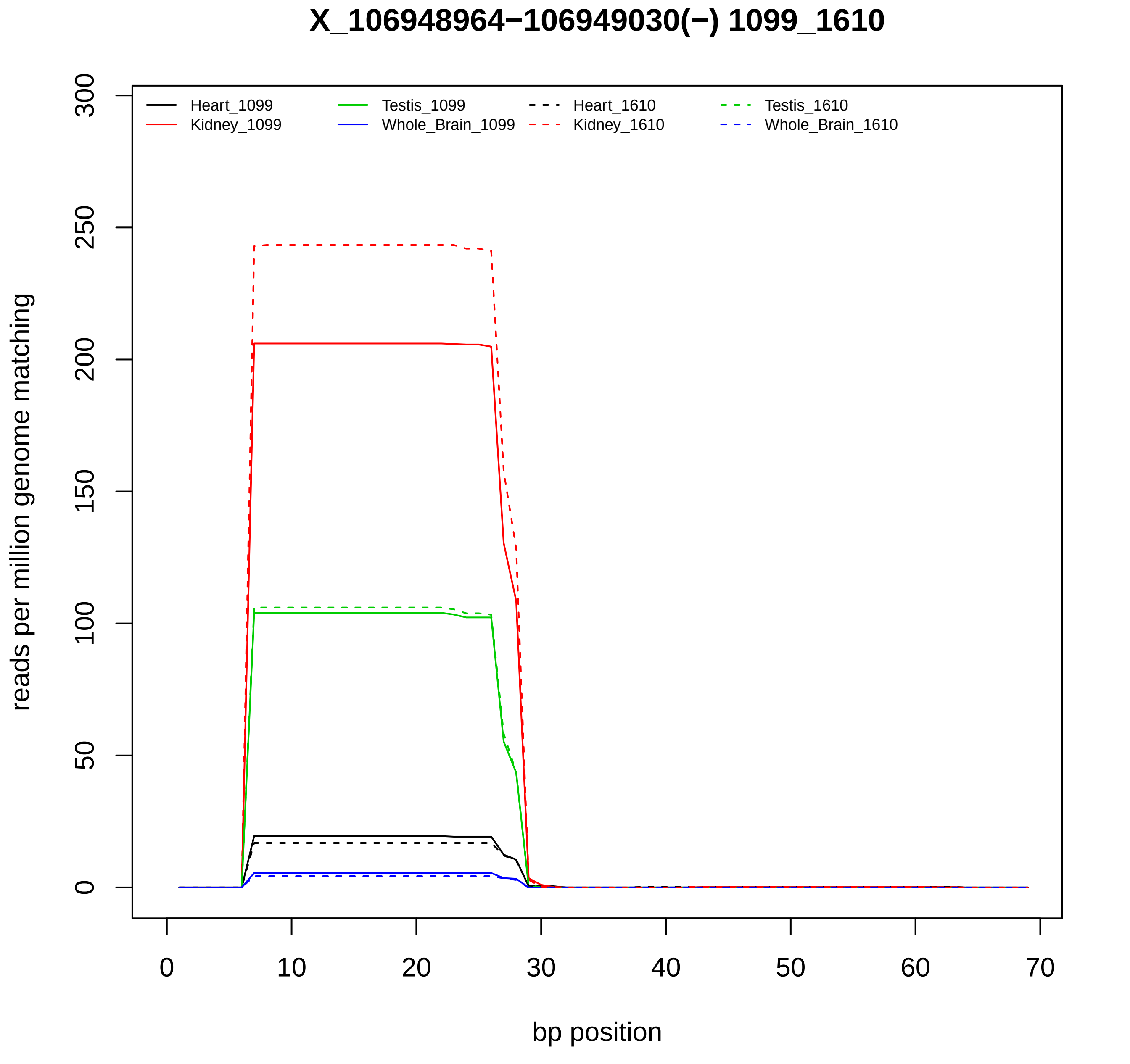

X_106948964−106949030(−) 1099_1610
Heart_1099
Kidney_1099
Testis_1099
Whole_Brain_1099
Heart_1610
Kidney_1610
Testis_1610
Whole_Brain_1610
reads per million genome matching
0
50
100
150
200
250
300
bp position
0
10
20
30
40
50
60
70

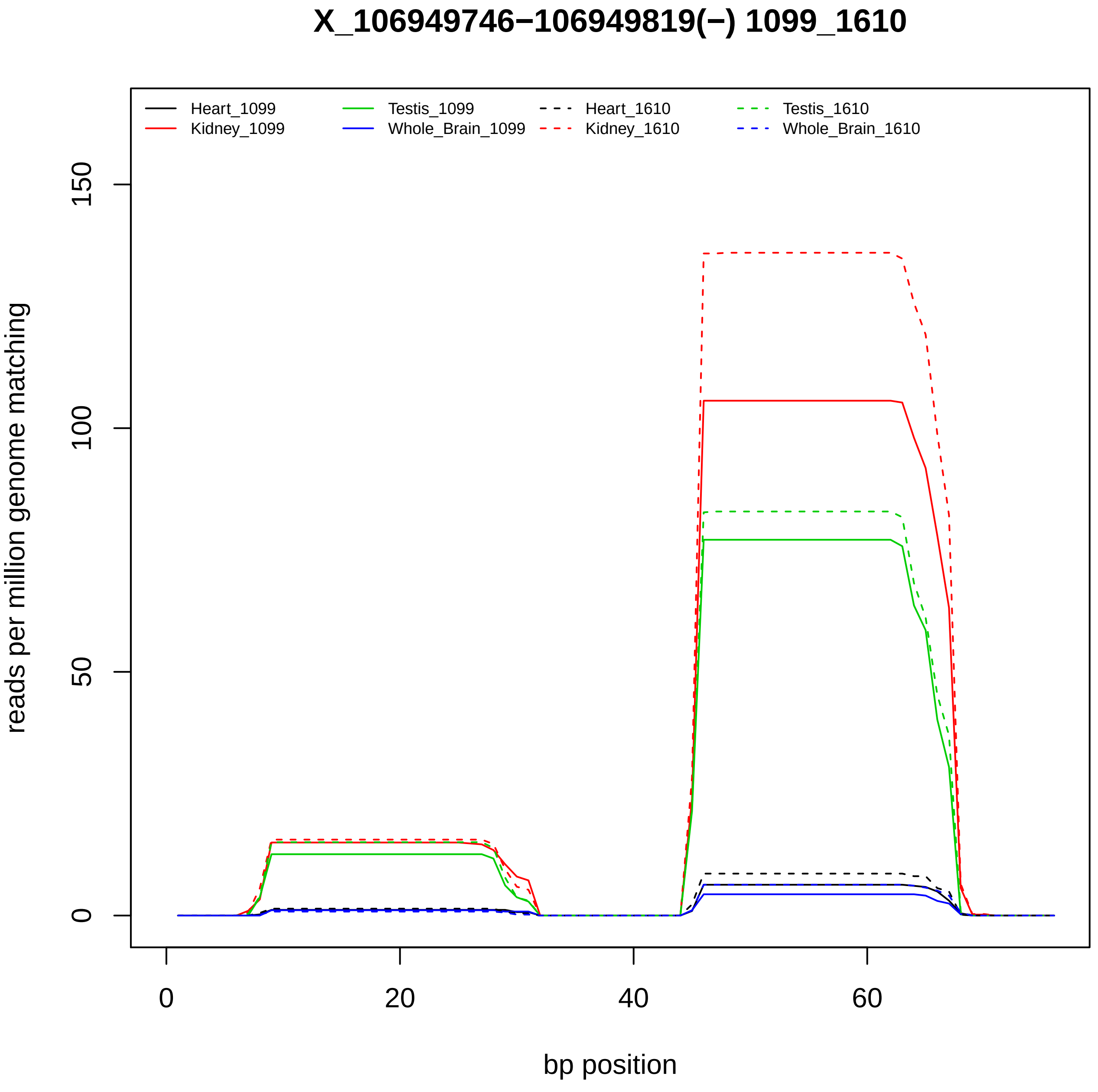

X_106949746−106949819(−) 1099_1610
Heart_1099
Kidney_1099
Testis_1099
Whole_Brain_1099
Heart_1610
Kidney_1610
Testis_1610
Whole_Brain_1610
reads per million genome matching
0
50
100
150
0
20
40
60
bp position

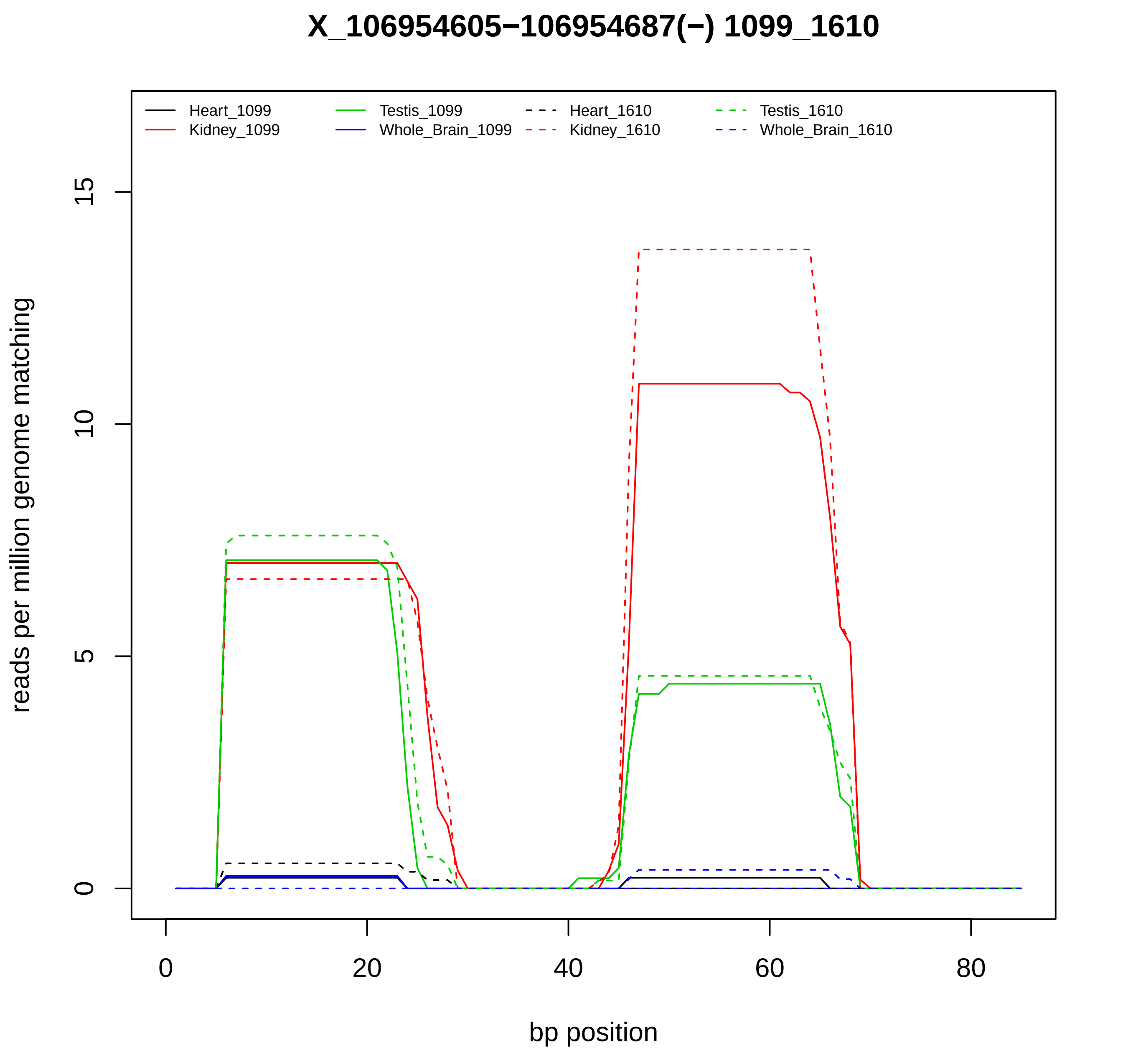

X_106954605−106954687(−) 1099_1610
Heart_1099
Kidney_1099
Testis_1099
Whole_Brain_1099
Heart_1610
Kidney_1610
Testis_1610
Whole_Brain_1610
reads per million genome matching
0
5
10
15
0
20
40
60
80
bp position

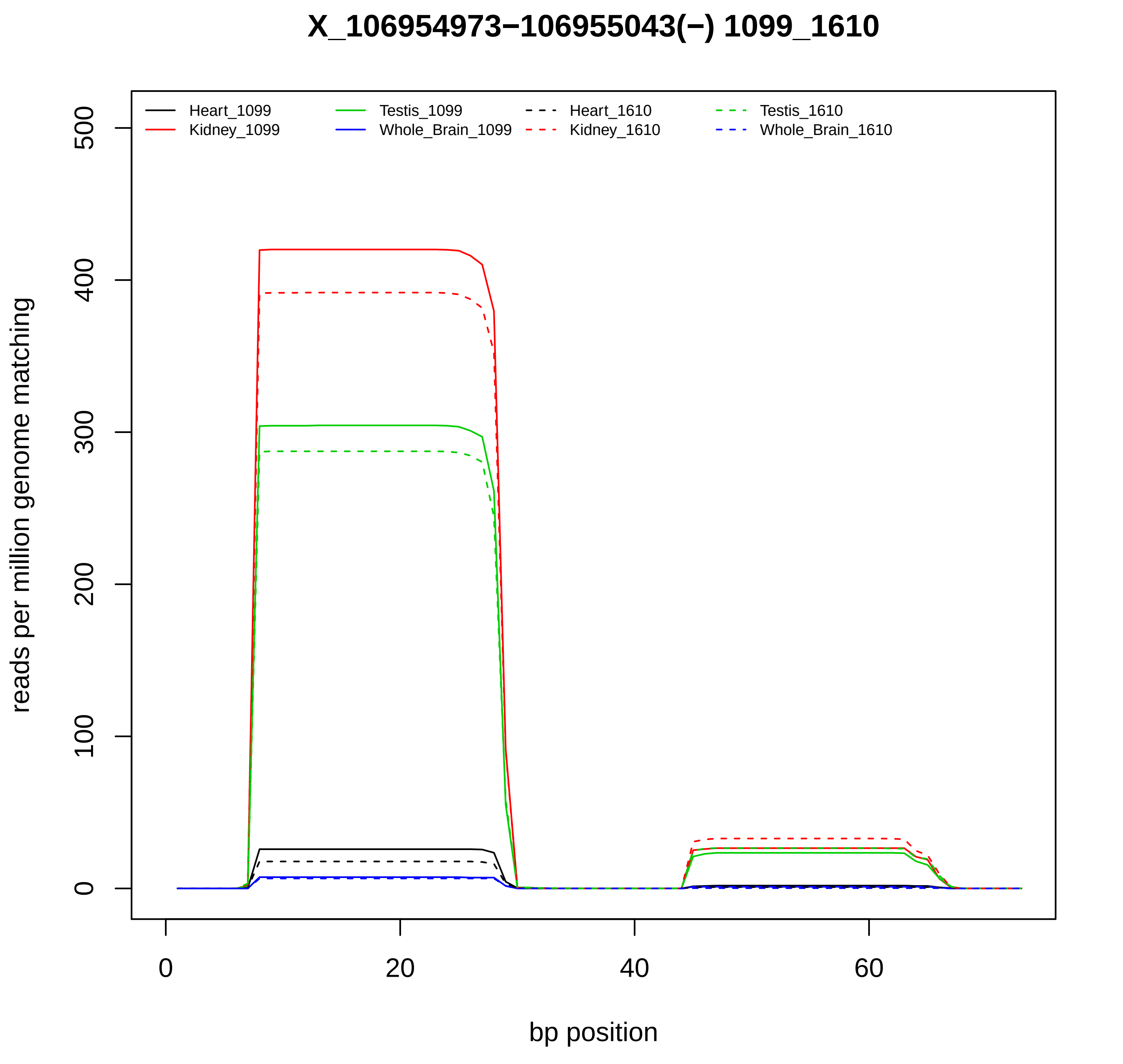

X_106954973−106955043(−) 1099_1610
Heart_1099
Kidney_1099
Testis_1099
Whole_Brain_1099
Heart_1610
Kidney_1610
Testis_1610
Whole_Brain_1610
reads per million genome matching
0
100
200
300
400
500
0
20
40
60
bp position

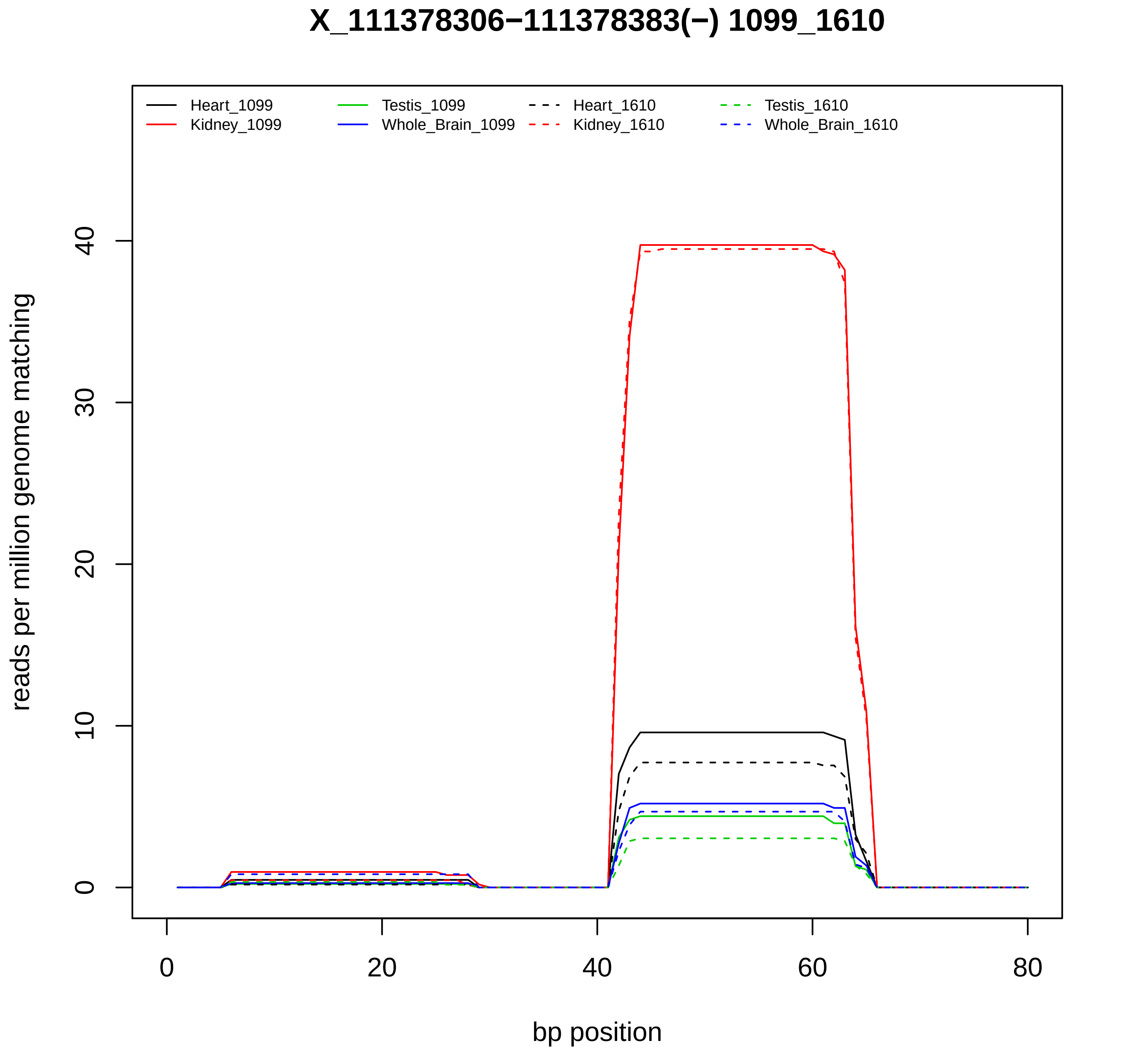

X_111378306−111378383(−) 1099_1610
Heart_1099
Kidney_1099
Testis_1099
Whole_Brain_1099
Heart_1610
Kidney_1610
Testis_1610
Whole_Brain_1610
reads per million genome matching
bp position
0
10
20
30
40
0
20
40
60
80

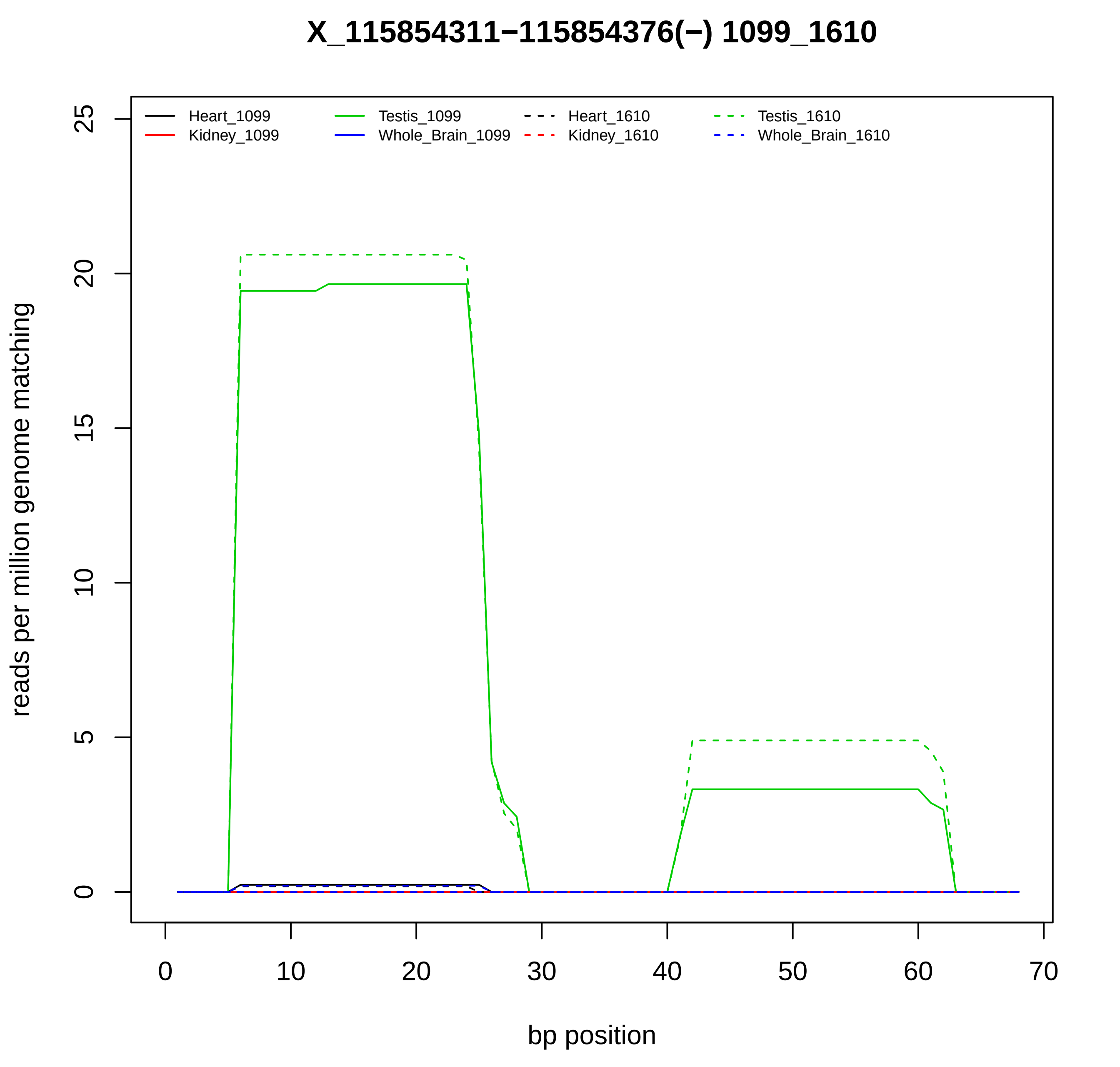
X_115854311−115854376(−) 1099_1610
Heart_1099
Kidney_1099
Testis_1099
Whole_Brain_1099
Heart_1610
Kidney_1610
Testis_1610
Whole_Brain_1610
reads per million genome matching
bp position
0
5
10
15
20
25
0
10
20
30
40
50
60
70

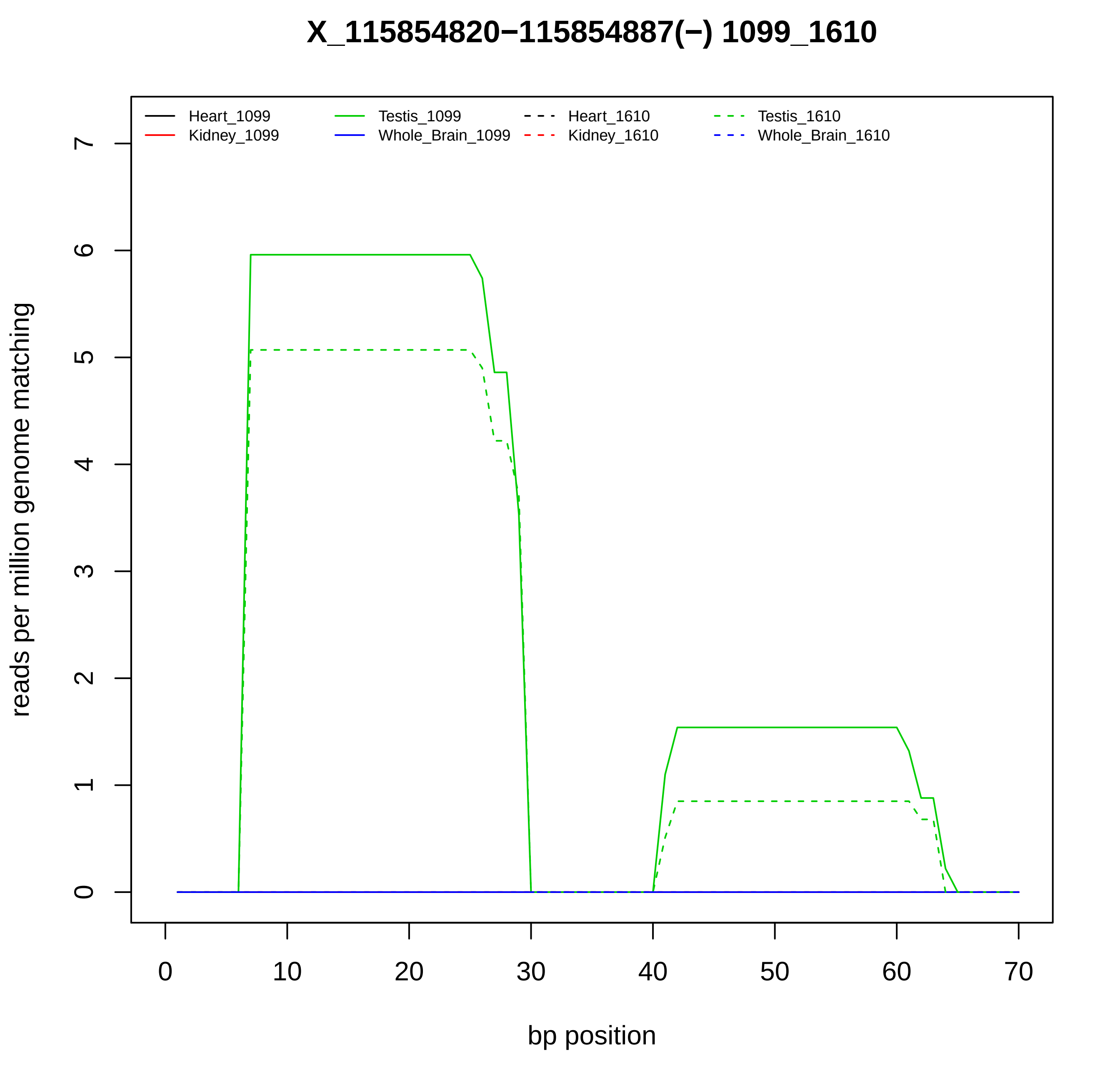
X_115854820−115854887(−) 1099_1610
Heart_1099
Kidney_1099
Testis_1099
Whole_Brain_1099
Heart_1610
Kidney_1610
Testis_1610
Whole_Brain_1610
reads per million genome matching
bp position
0
1
2
3
4
5
6
7
0
10
20
30
40
50
60
70

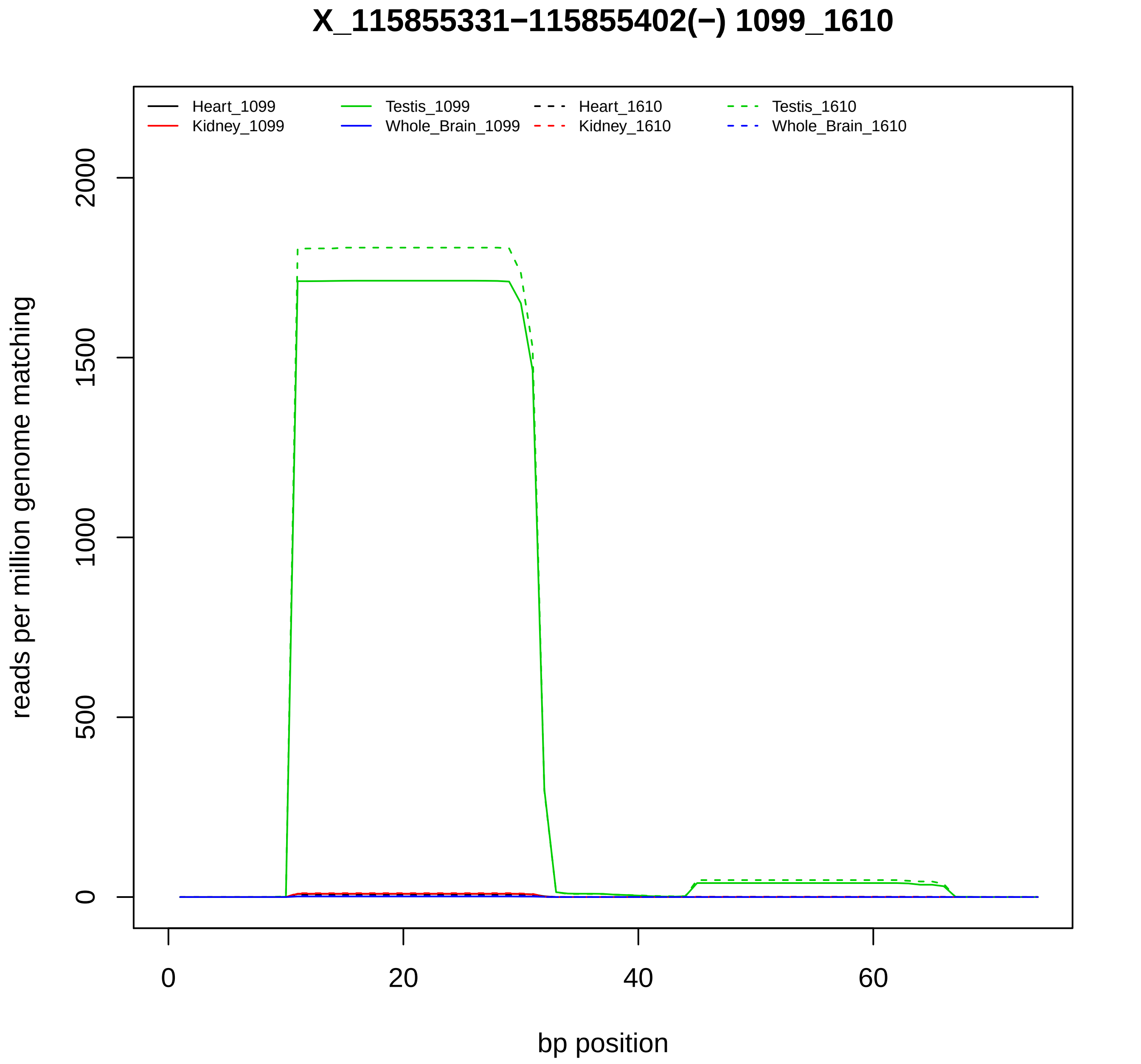

X_115855331−115855402(−) 1099_1610
Heart_1099
Kidney_1099
Testis_1099
Whole_Brain_1099
Heart_1610
Kidney_1610
Testis_1610
Whole_Brain_1610
reads per million genome matching
0
500
1000
1500
2000
bp position
0
20
40
60

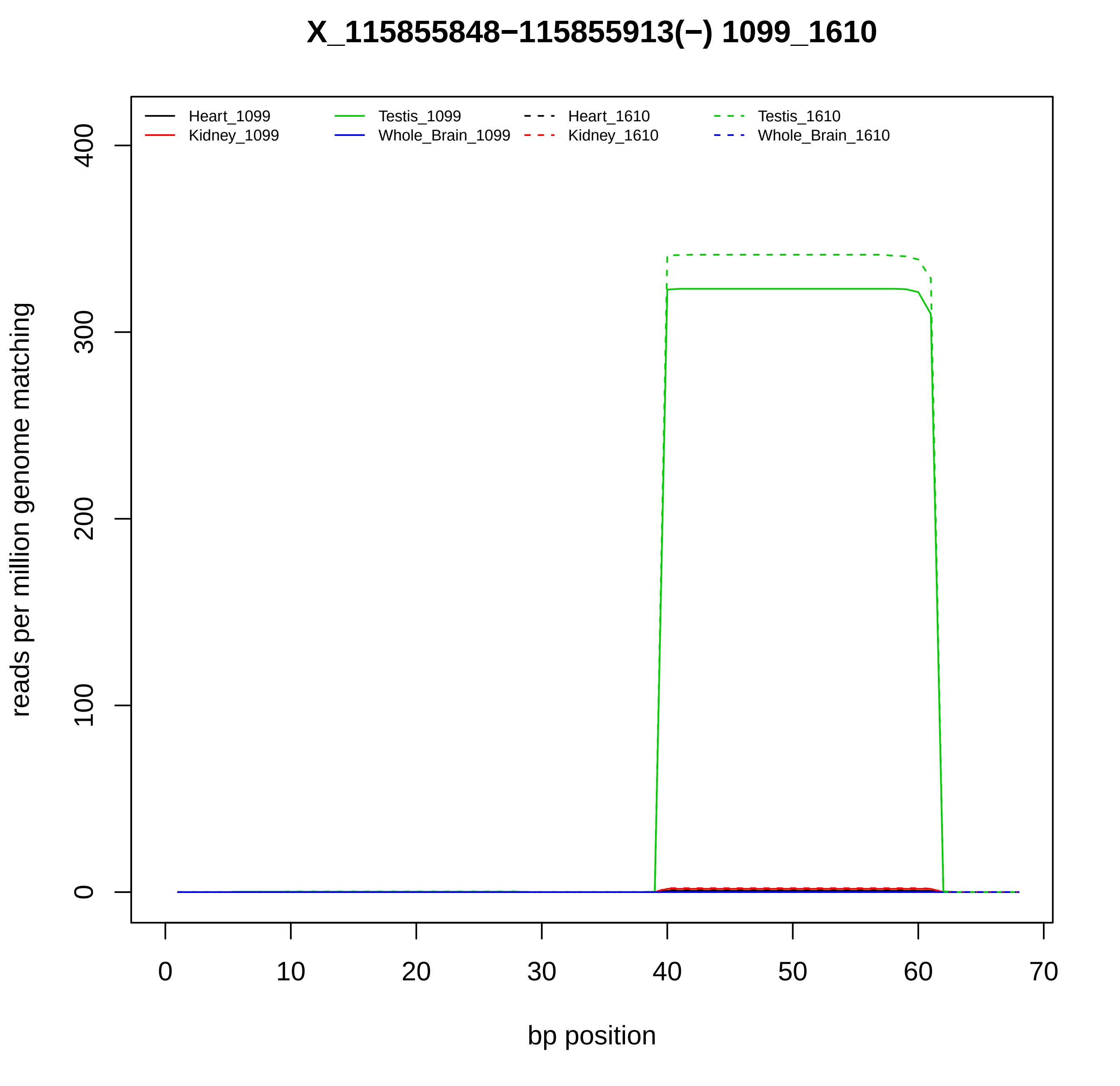

X_115855848−115855913(−) 1099_1610
Heart_1099
Kidney_1099
Testis_1099
Whole_Brain_1099
Heart_1610
Kidney_1610
Testis_1610
Whole_Brain_1610
reads per million genome matching
0
100
200
300
400
0
10
20
30
40
50
60
70
bp position

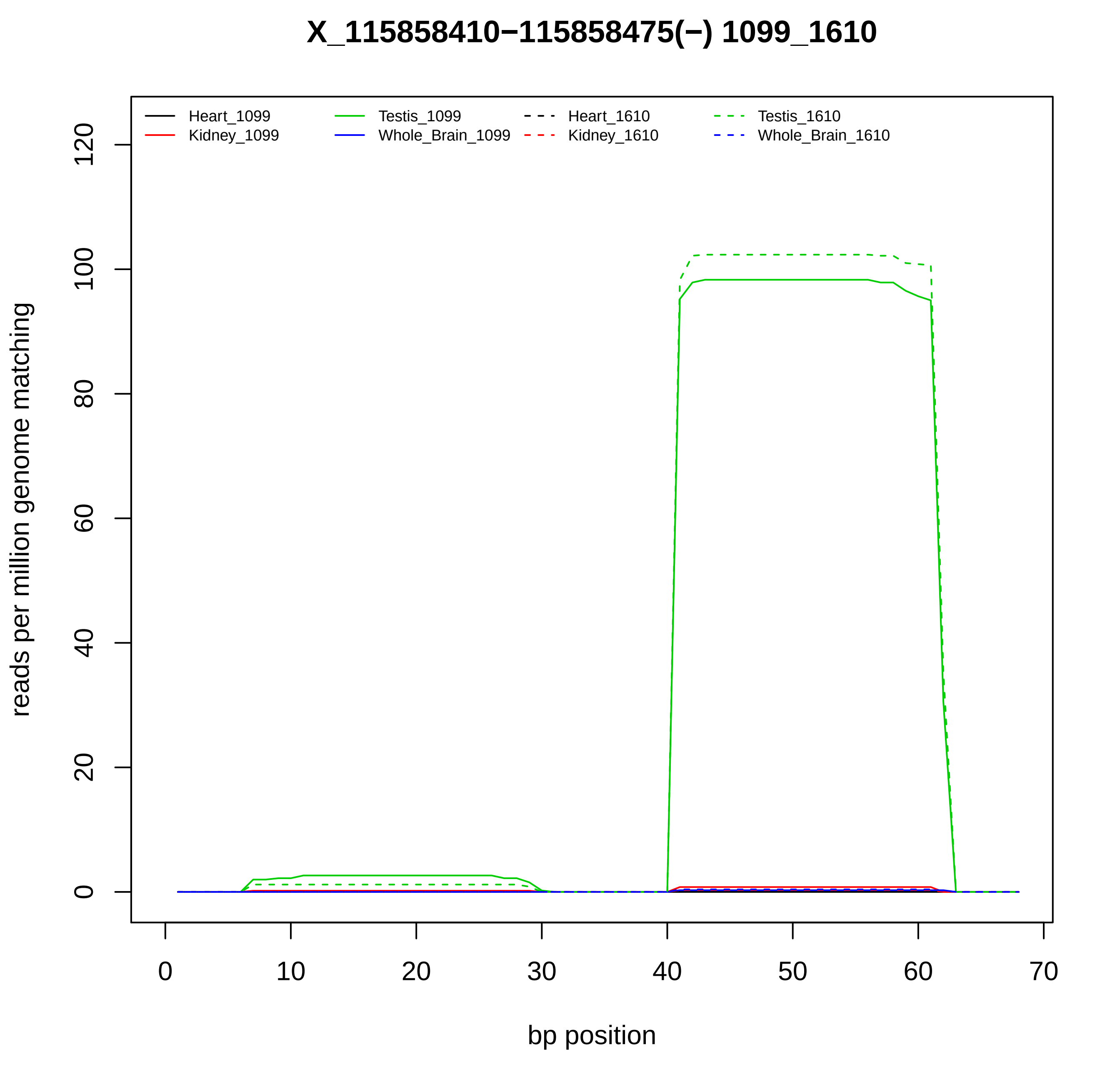

X_115858410−115858475(−) 1099_1610
Heart_1099
Kidney_1099
Testis_1099
Whole_Brain_1099
Heart_1610
Kidney_1610
Testis_1610
Whole_Brain_1610
reads per million genome matching
0
20
40
60
80
100
120
0
10
20
30
40
50
60
70
bp position

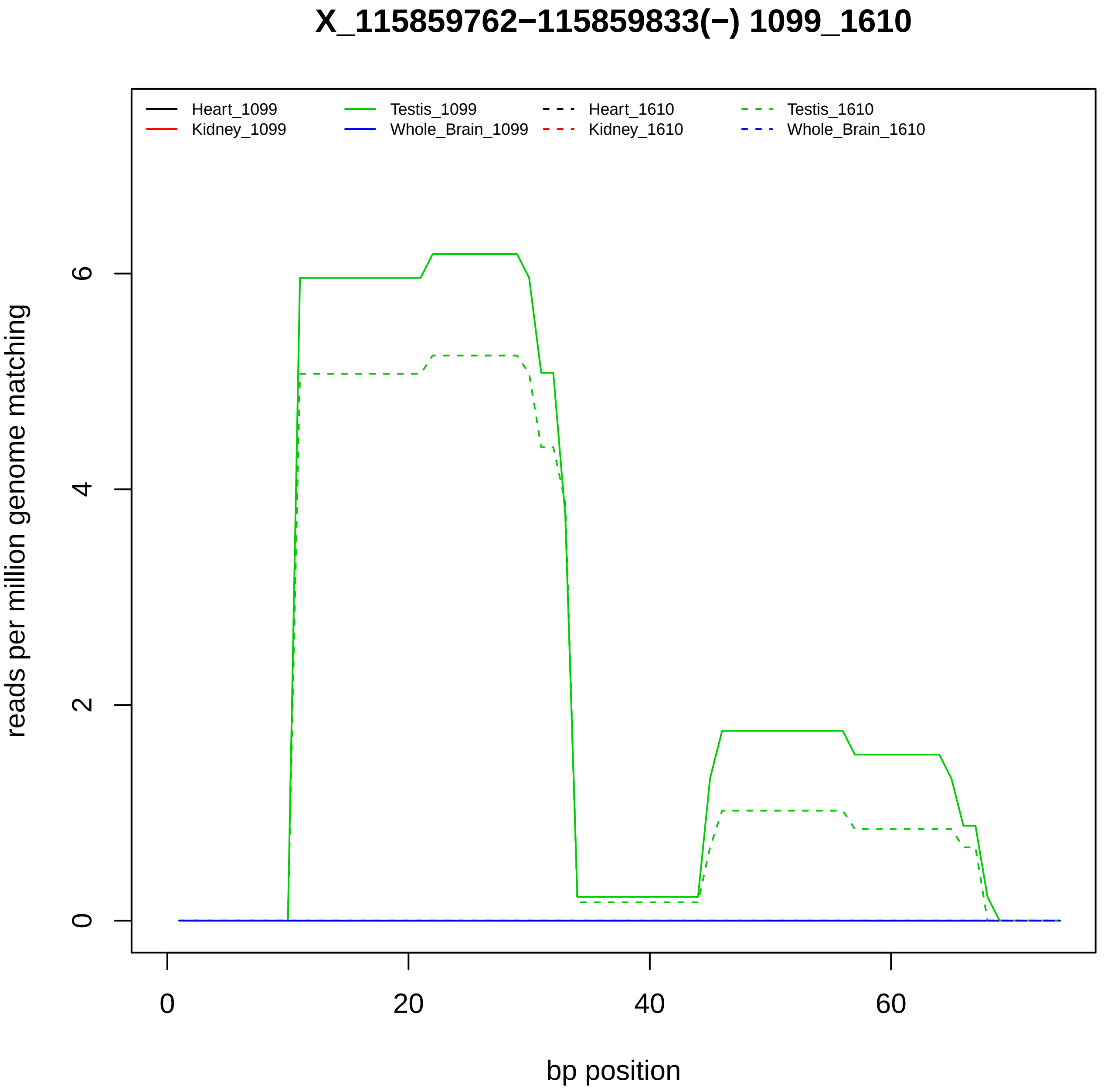
X_115859762−115859833(−) 1099_1610
Heart_1099
Kidney_1099
Testis_1099
Whole_Brain_1099
Heart_1610
Kidney_1610
Testis_1610
Whole_Brain_1610
reads per million genome matching
bp position
0
2
4
6
0
20
40
60

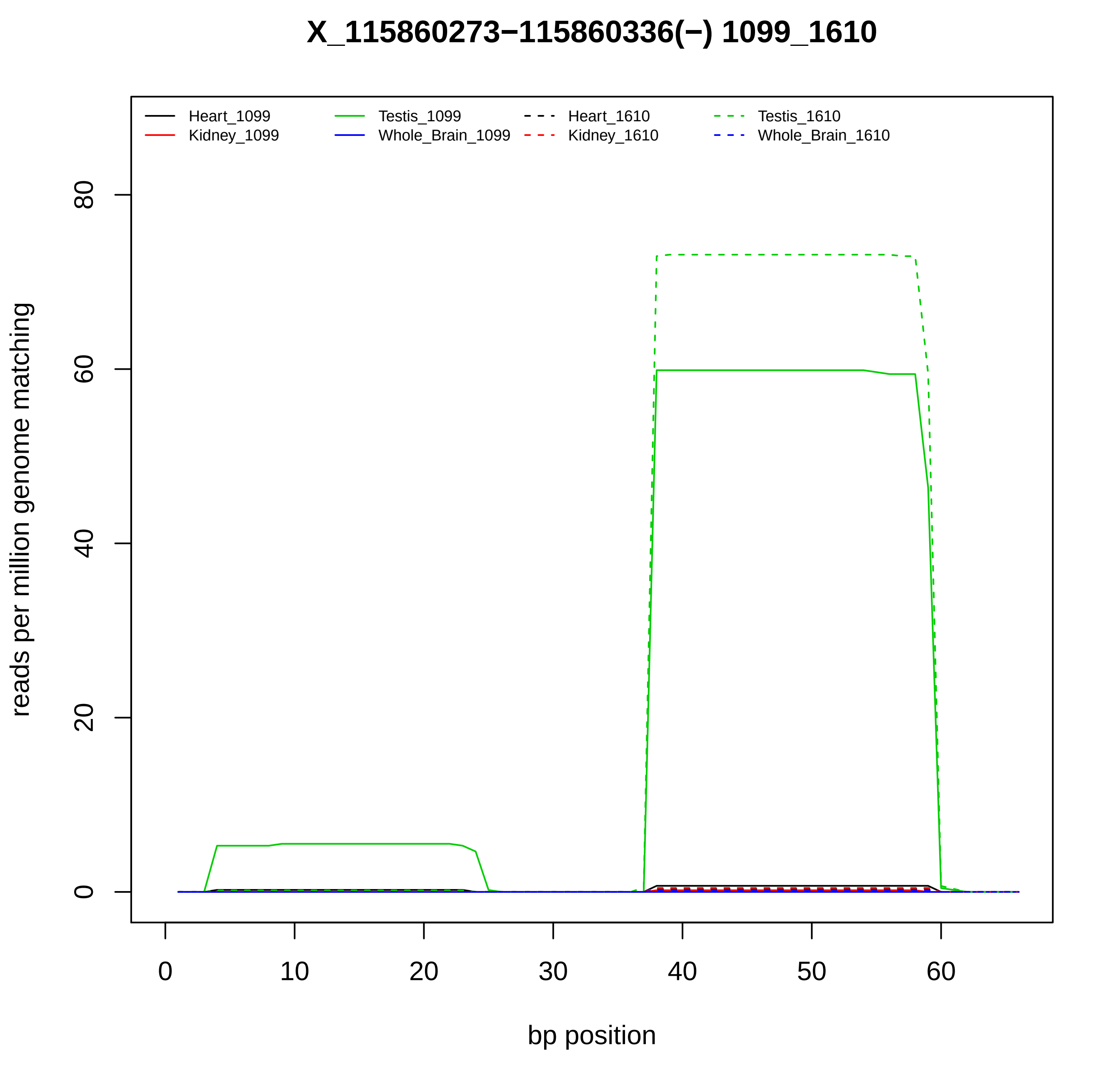

X_115860273−115860336(−) 1099_1610
Heart_1099
Kidney_1099
Testis_1099
Whole_Brain_1099
Heart_1610
Kidney_1610
Testis_1610
Whole_Brain_1610
reads per million genome matching
bp position
0
20
40
60
80
0
10
20
30
40
50
60

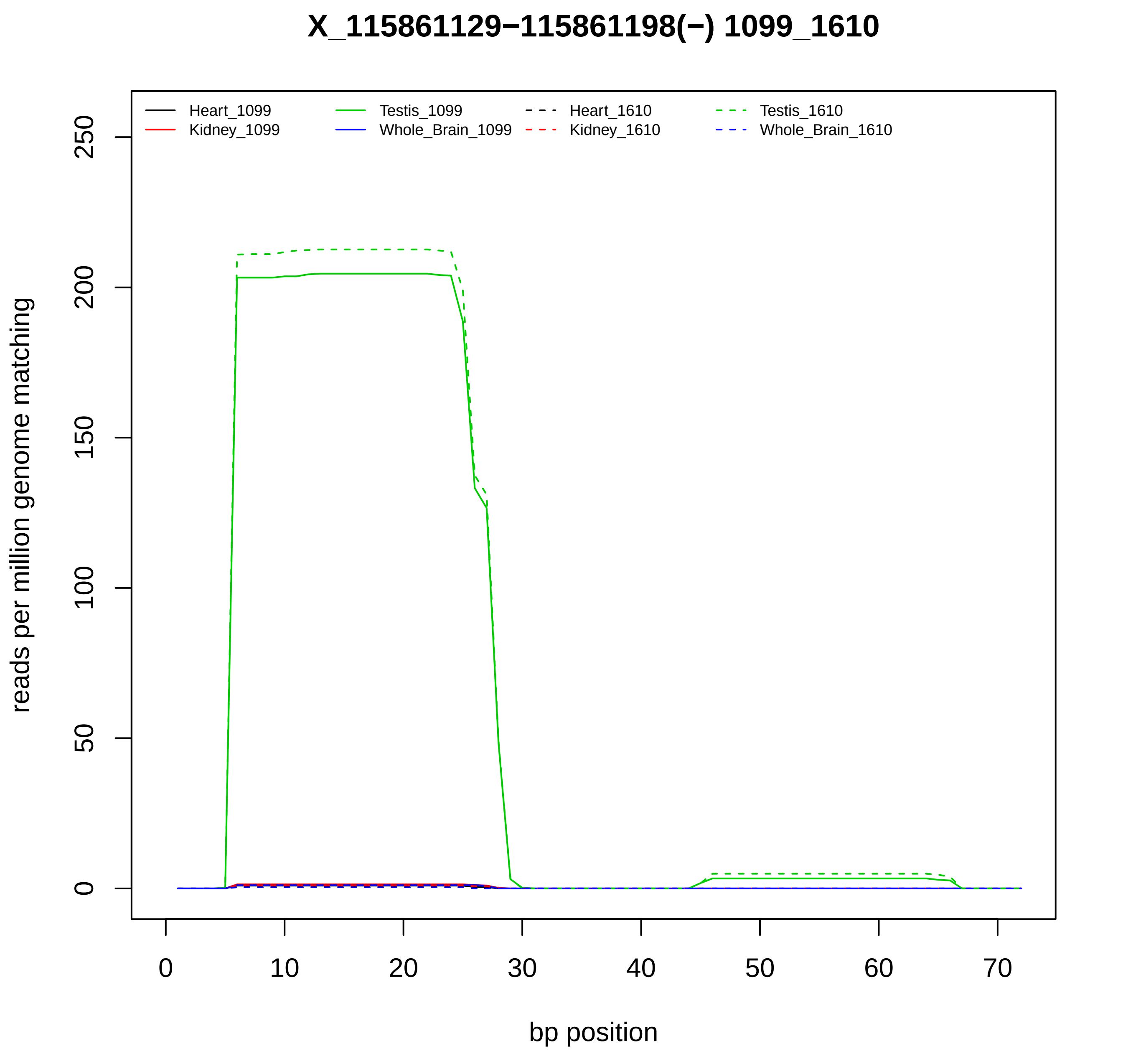

X_115861129−115861198(−) 1099_1610
Heart_1099
Kidney_1099
Testis_1099
Whole_Brain_1099
Heart_1610
Kidney_1610
Testis_1610
Whole_Brain_1610
reads per million genome matching
0
50
100
150
200
250
0
10
20
30
40
50
60
70
bp position

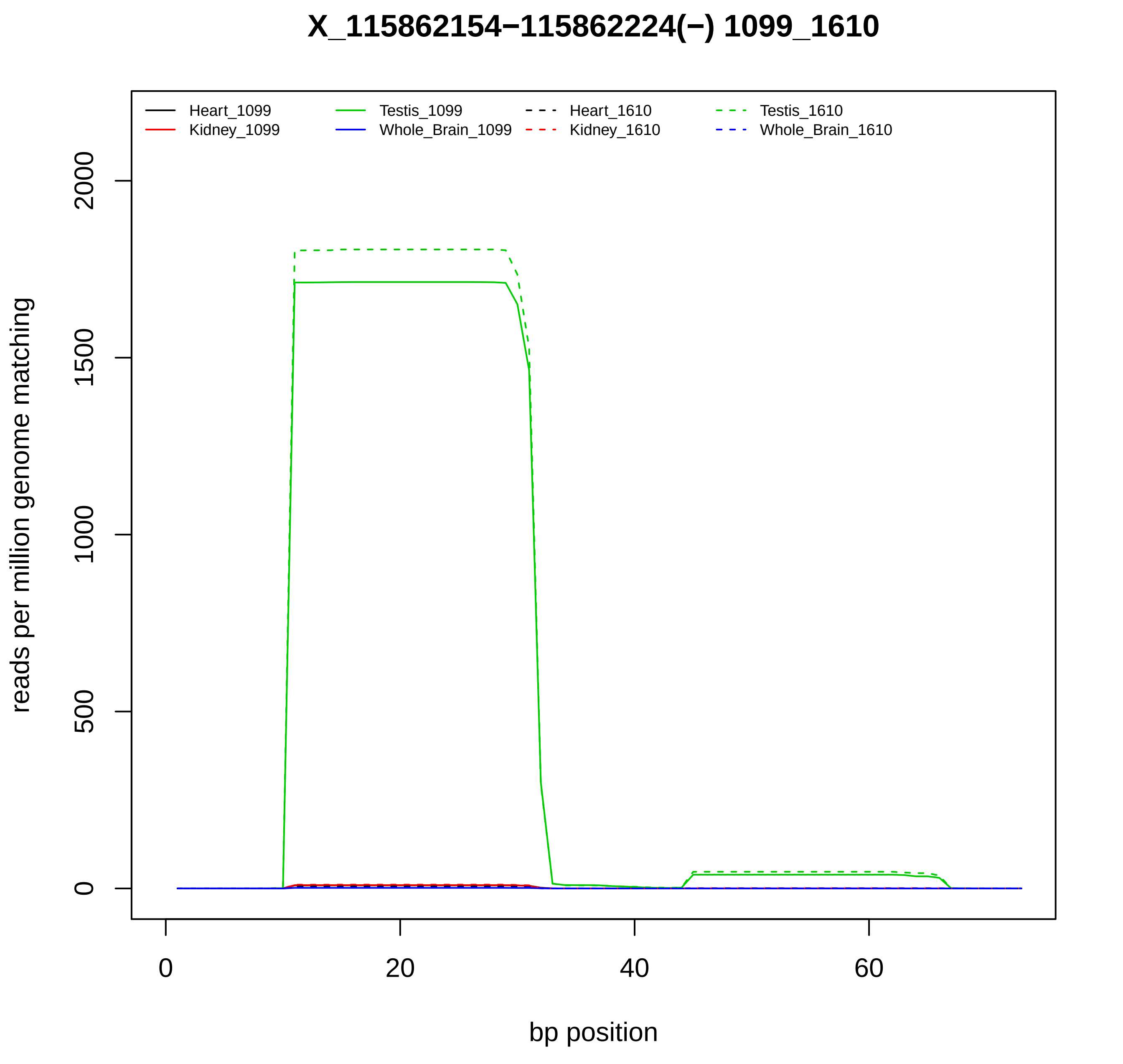

X_115862154−115862224(−) 1099_1610
Heart_1099
Kidney_1099
Testis_1099
Whole_Brain_1099
Heart_1610
Kidney_1610
Testis_1610
Whole_Brain_1610
reads per million genome matching
0
500
1000
1500
2000
bp position
0
20
40
60

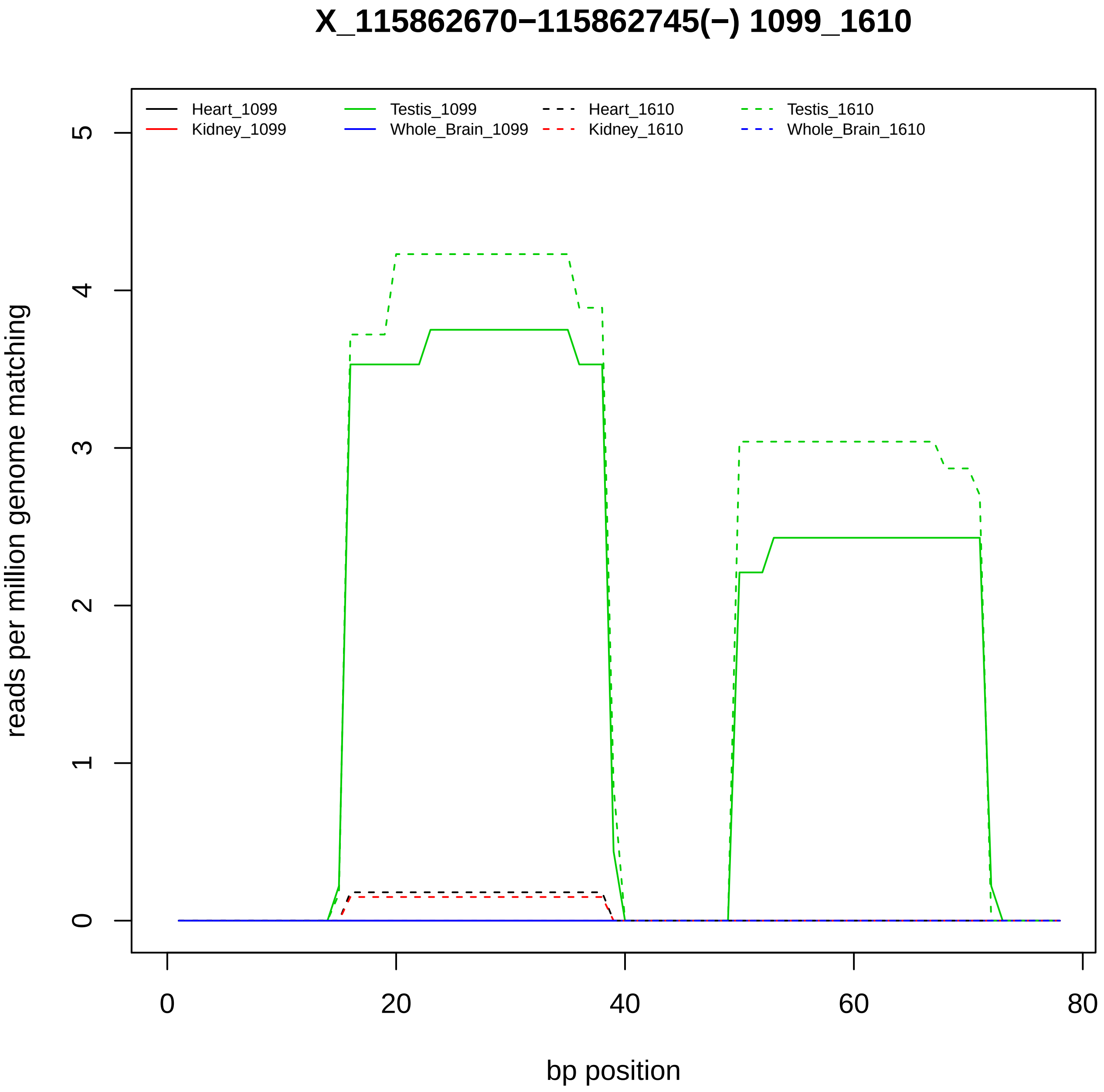

X_115862670−115862745(−) 1099_1610
Heart_1099
Kidney_1099
Testis_1099
Whole_Brain_1099
Heart_1610
Kidney_1610
Testis_1610
Whole_Brain_1610
reads per million genome matching
bp position
0
1
2
3
4
5
0
20
40
60
80

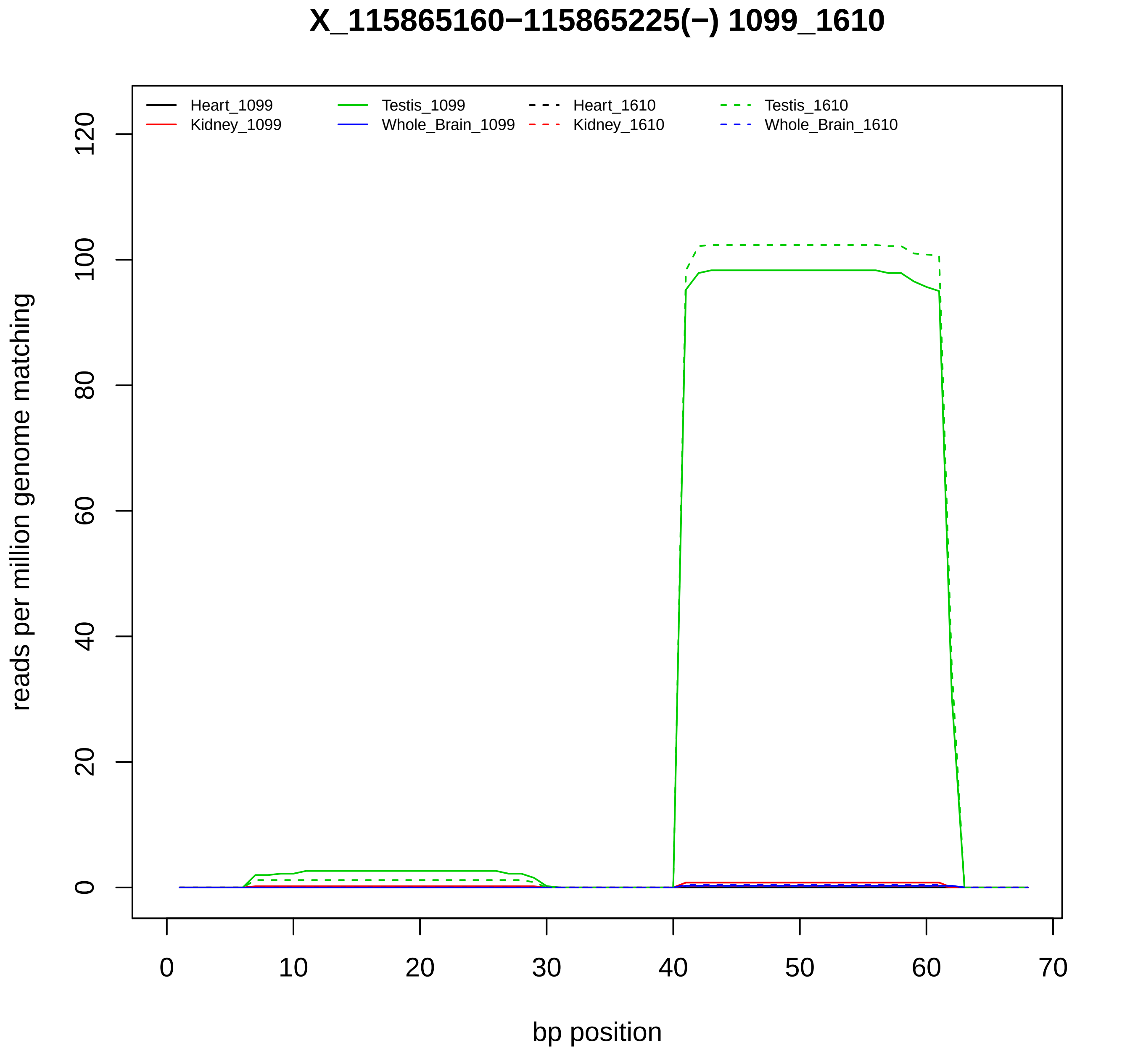

X_115865160−115865225(−) 1099_1610
Heart_1099
Kidney_1099
Testis_1099
Whole_Brain_1099
Heart_1610
Kidney_1610
Testis_1610
Whole_Brain_1610
reads per million genome matching
bp position
0
20
40
60
80
100
120
0
10
20
30
40
50
60
70

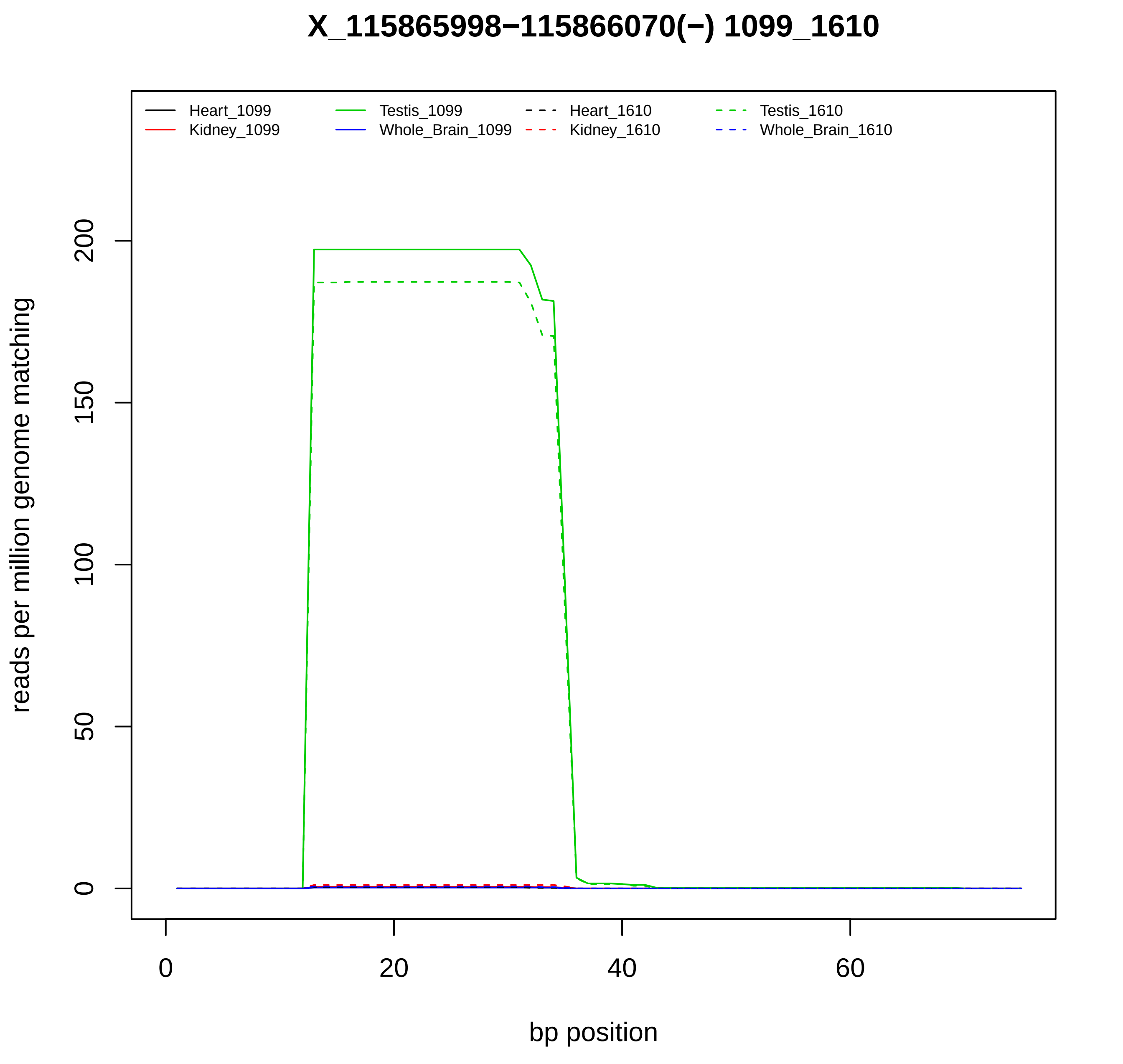

X_115865998−115866070(−) 1099_1610
Heart_1099
Kidney_1099
Testis_1099
Whole_Brain_1099
Heart_1610
Kidney_1610
Testis_1610
Whole_Brain_1610
reads per million genome matching
0
50
100
150
200
0
20
40
60
bp position

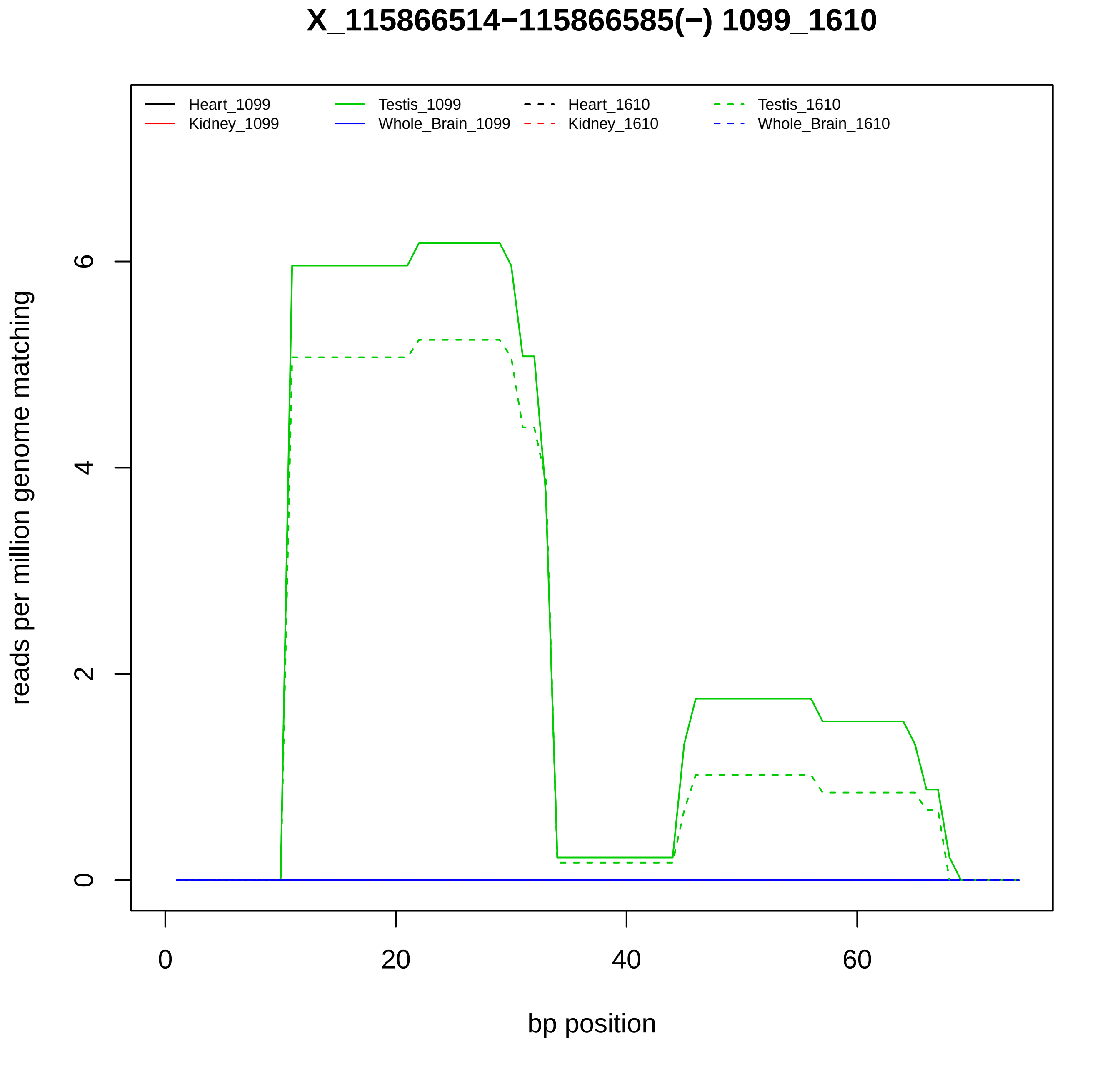
X_115866514−115866585(−) 1099_1610
Heart_1099
Kidney_1099
Testis_1099
Whole_Brain_1099
Heart_1610
Kidney_1610
Testis_1610
Whole_Brain_1610
reads per million genome matching
0
2
4
6
0
20
40
60
bp position

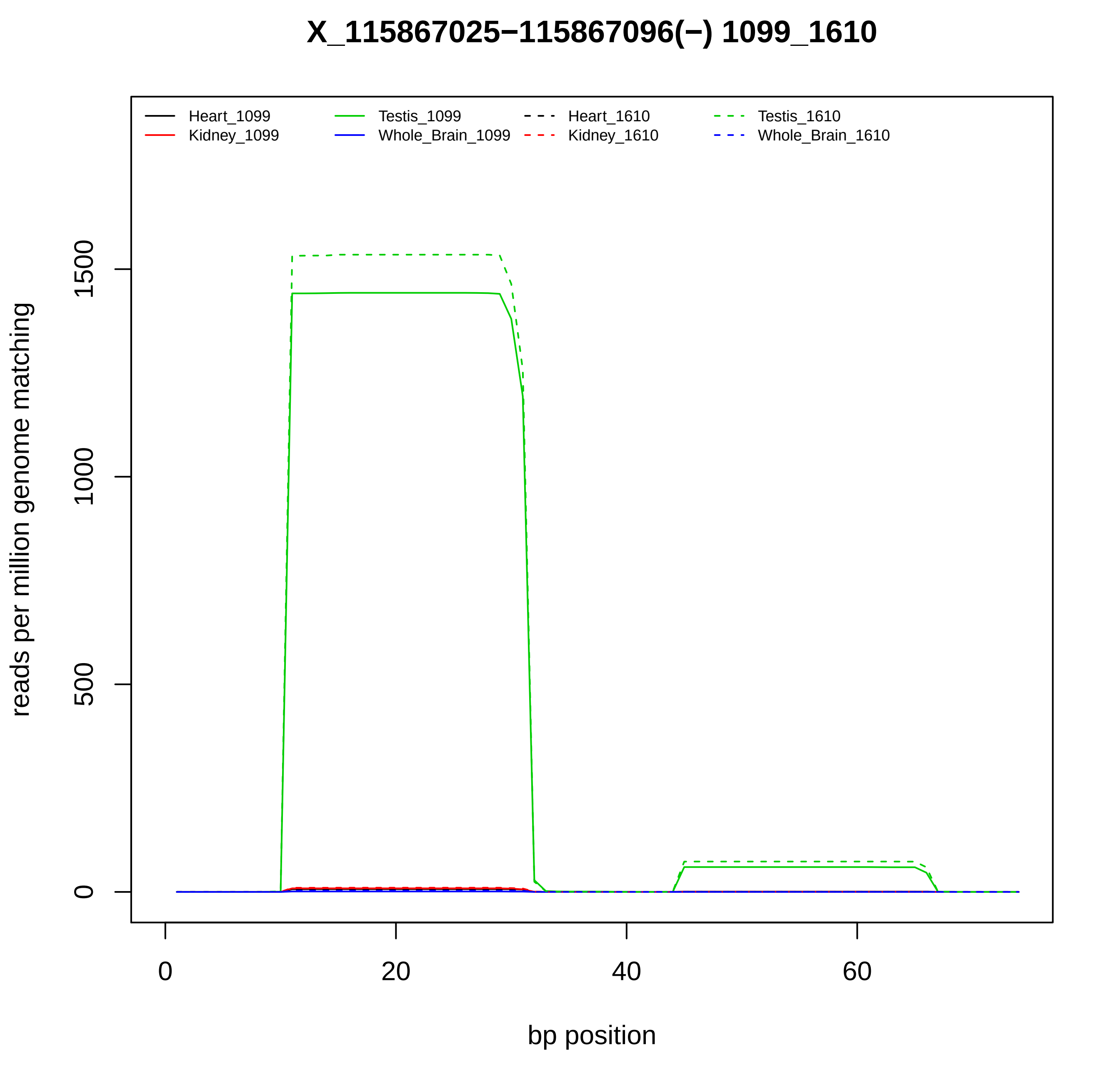

X_115867025−115867096(−) 1099_1610
Heart_1099
Kidney_1099
Testis_1099
Whole_Brain_1099
Heart_1610
Kidney_1610
Testis_1610
Whole_Brain_1610
reads per million genome matching
0
500
1000
1500
0
20
40
60
bp position

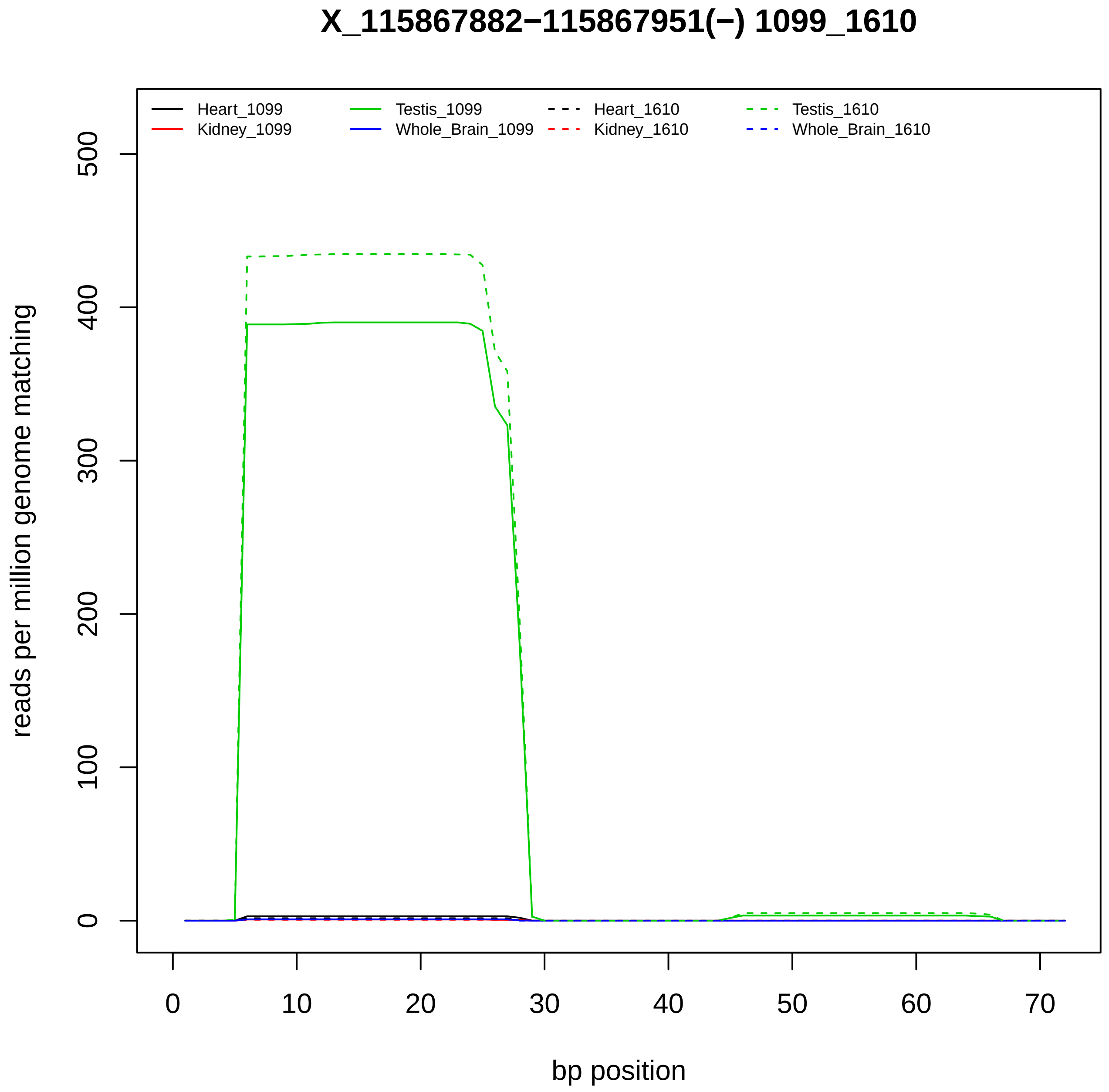

X_115867882−115867951(−) 1099_1610
Heart_1099
Kidney_1099
Testis_1099
Whole_Brain_1099
Heart_1610
Kidney_1610
Testis_1610
Whole_Brain_1610
reads per million genome matching
0
100
200
300
400
500
0
10
20
30
40
50
60
70
bp position

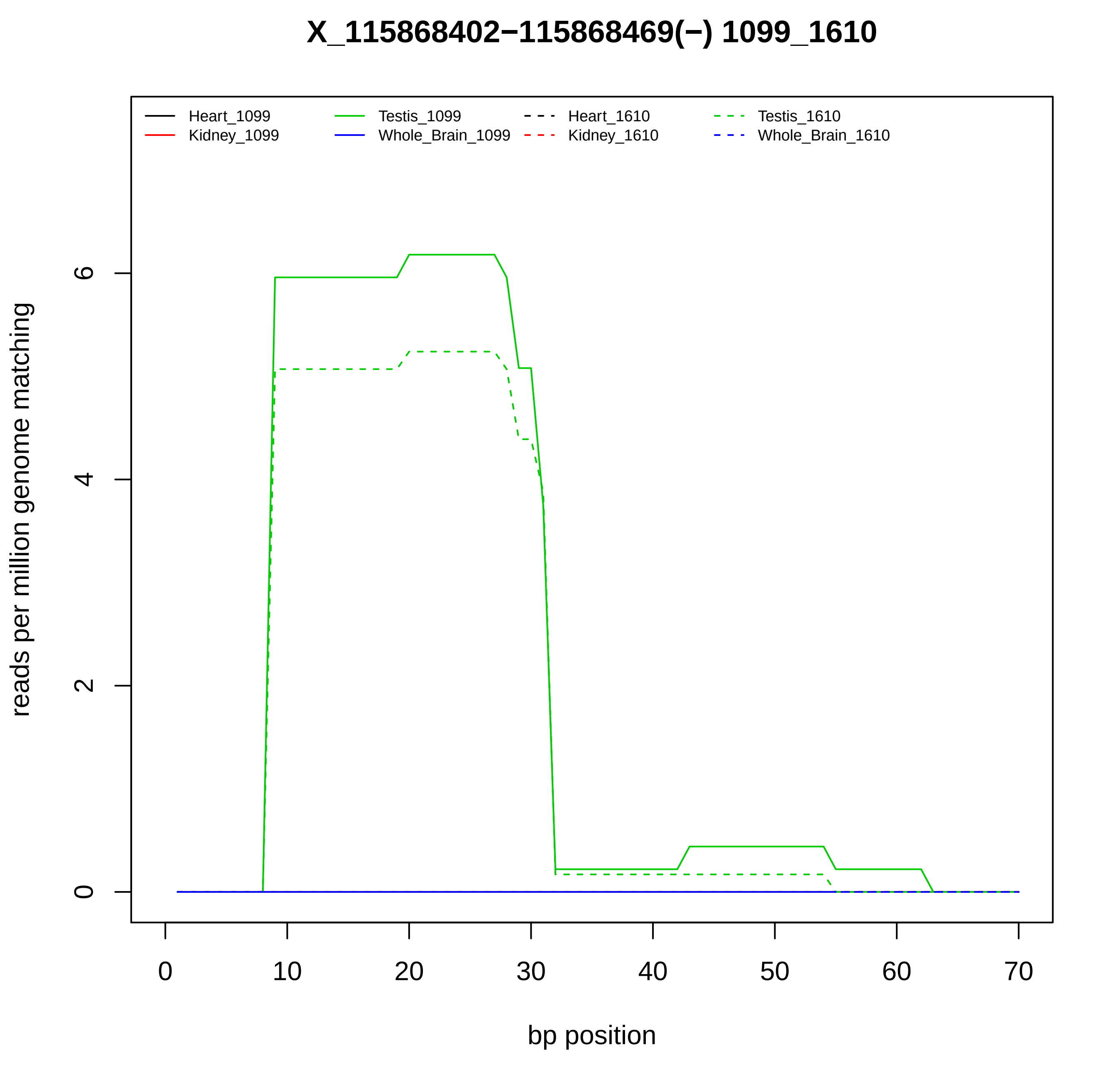

X_115868402−115868469(−) 1099_1610
Heart_1099
Kidney_1099
Testis_1099
Whole_Brain_1099
Heart_1610
Kidney_1610
Testis_1610
Whole_Brain_1610
reads per million genome matching
bp position
0
2
4
6
0
10
20
30
40
50
60
70

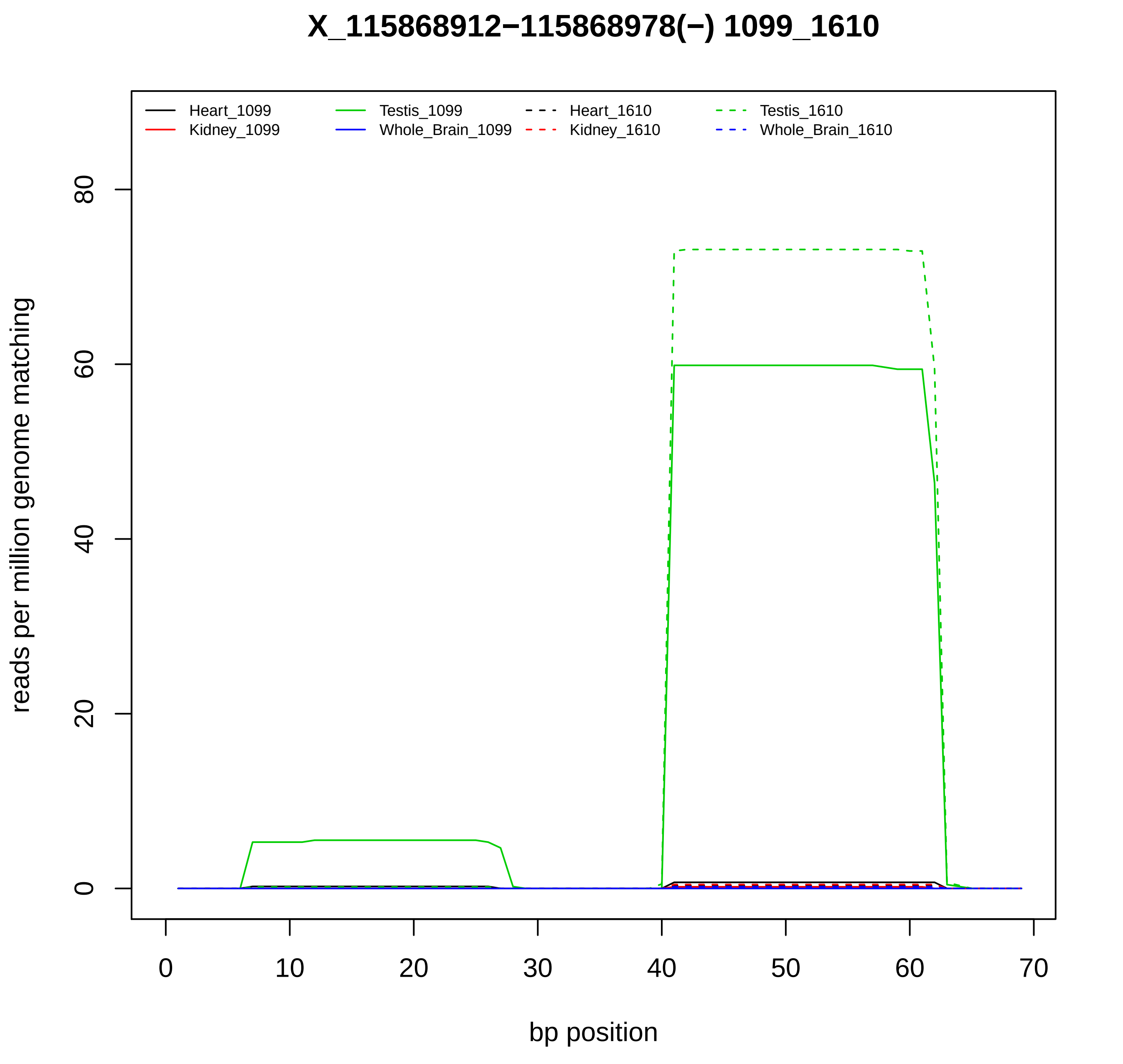

X_115868912−115868978(−) 1099_1610
Heart_1099
Kidney_1099
Testis_1099
Whole_Brain_1099
Heart_1610
Kidney_1610
Testis_1610
Whole_Brain_1610
reads per million genome matching
bp position
0
20
40
60
80
0
10
20
30
40
50
60
70

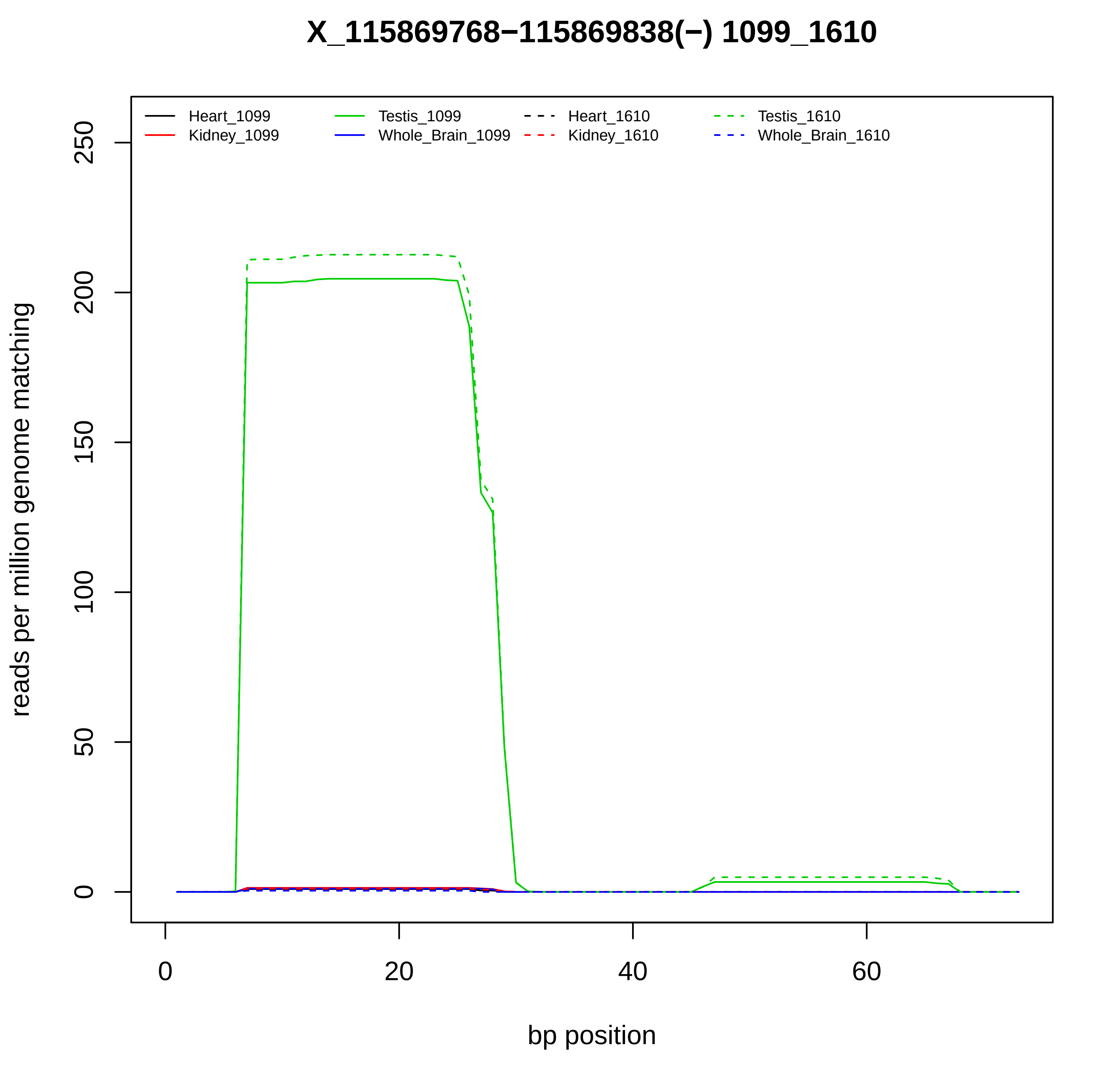

X_115869768−115869838(−) 1099_1610
Heart_1099
Kidney_1099
Testis_1099
Whole_Brain_1099
Heart_1610
Kidney_1610
Testis_1610
Whole_Brain_1610
reads per million genome matching
bp position
0
50
100
150
200
250
0
20
40
60

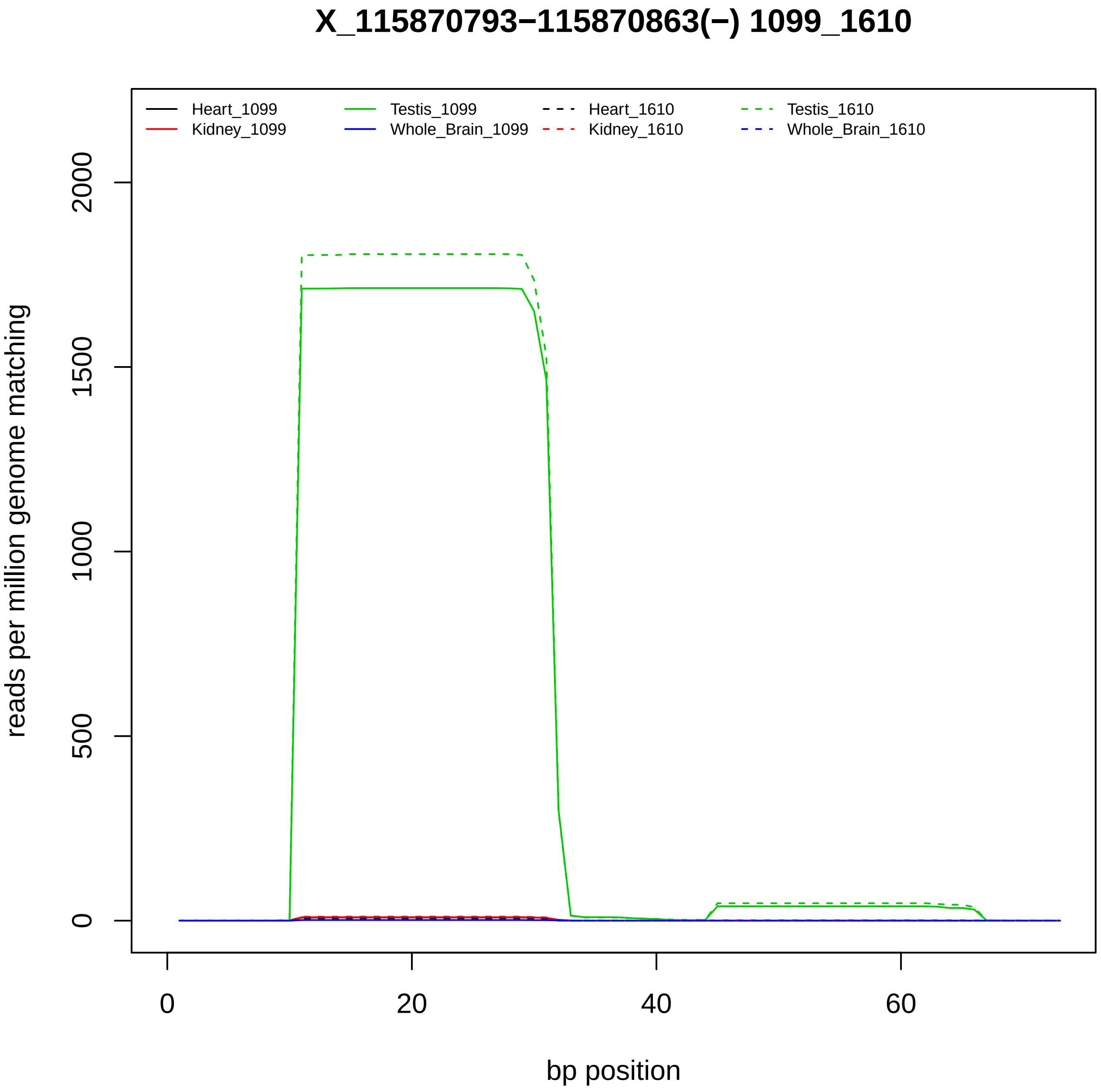

X_115870793−115870863(−) 1099_1610
Heart_1099
Kidney_1099
Testis_1099
Whole_Brain_1099
Heart_1610
Kidney_1610
Testis_1610
Whole_Brain_1610
reads per million genome matching
0
500
1000
1500
2000
bp position
0
20
40
60

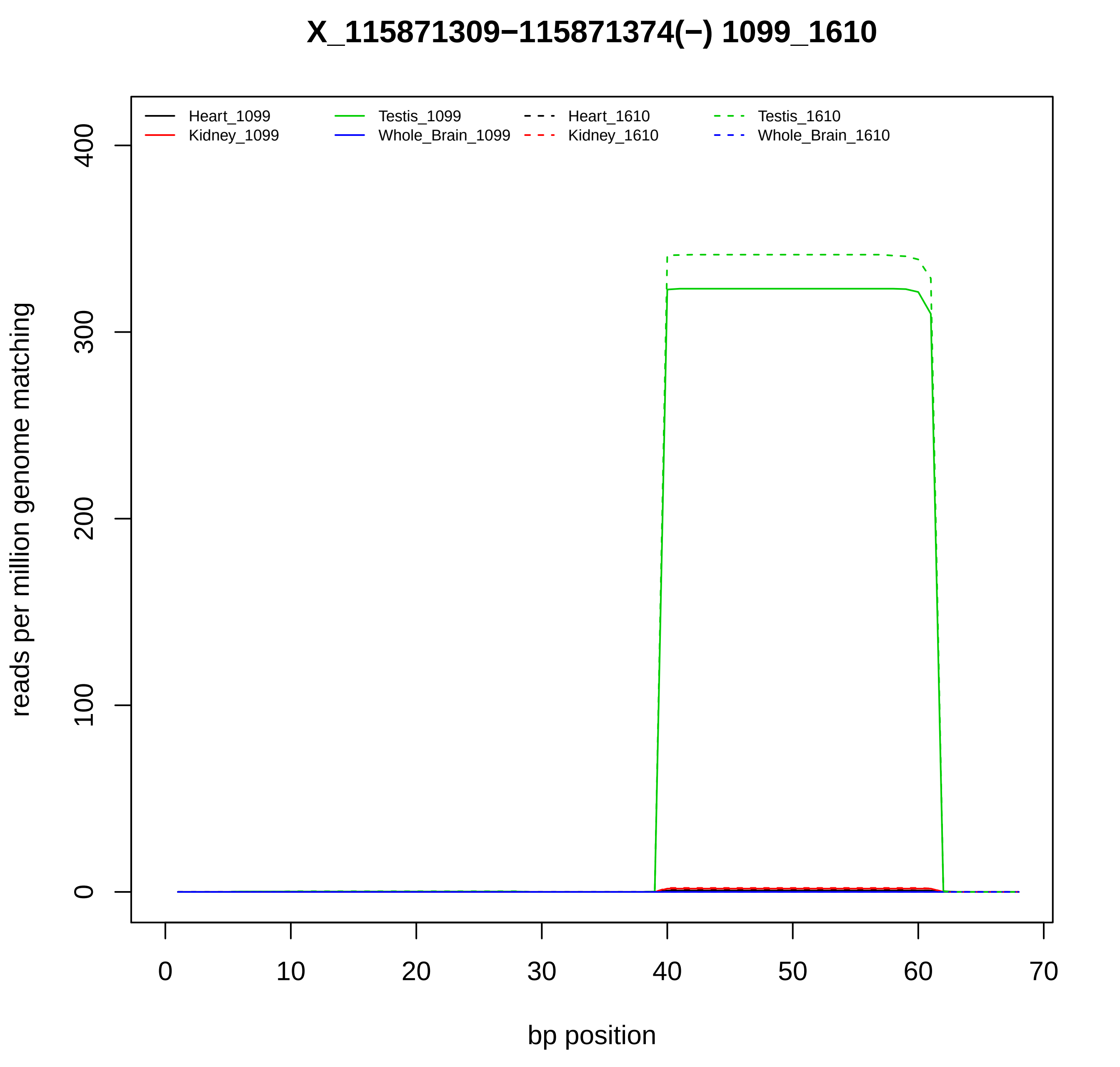

X_115871309−115871374(−) 1099_1610
Heart_1099
Kidney_1099
Testis_1099
Whole_Brain_1099
Heart_1610
Kidney_1610
Testis_1610
Whole_Brain_1610
reads per million genome matching
0
100
200
300
400
0
10
20
30
40
50
60
70
bp position

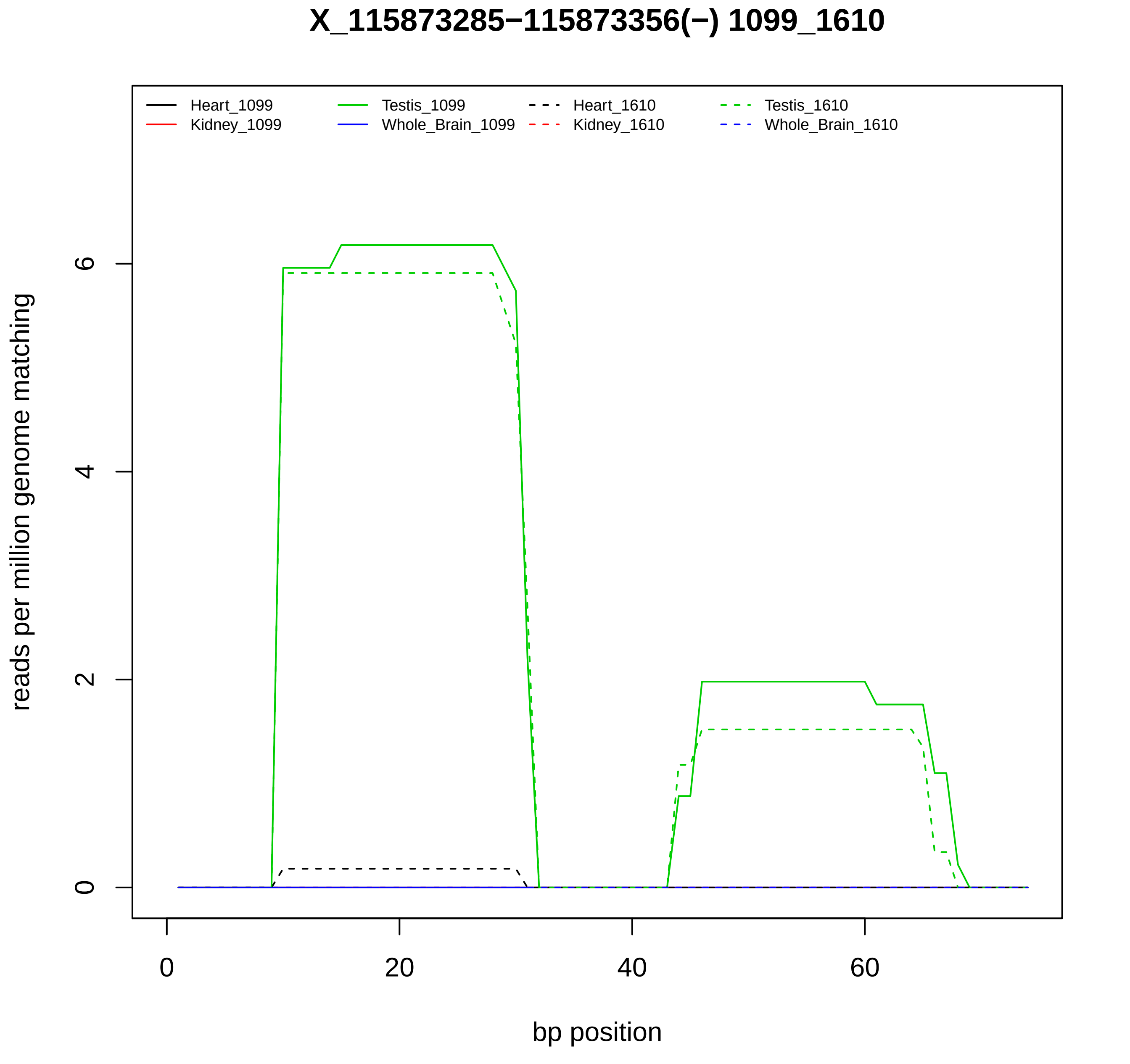
X_115873285−115873356(−) 1099_1610
Heart_1099
Kidney_1099
Testis_1099
Whole_Brain_1099
Heart_1610
Kidney_1610
Testis_1610
Whole_Brain_1610
reads per million genome matching
0
2
4
6
0
20
40
60
bp position

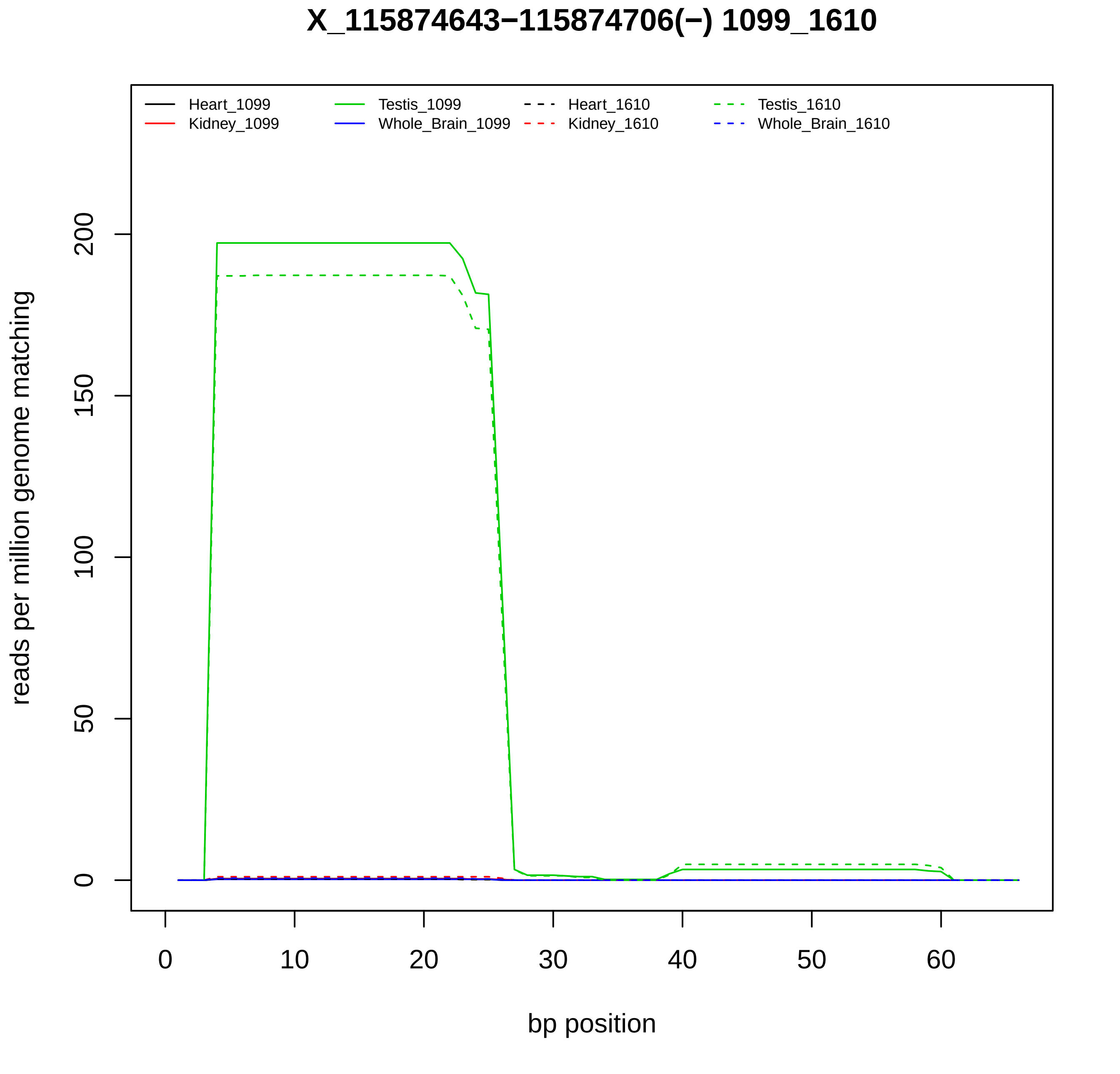

X_115874643−115874706(−) 1099_1610
Heart_1099
Kidney_1099
Testis_1099
Whole_Brain_1099
Heart_1610
Kidney_1610
Testis_1610
Whole_Brain_1610
reads per million genome matching
bp position
0
50
100
150
200
0
10
20
30
40
50
60

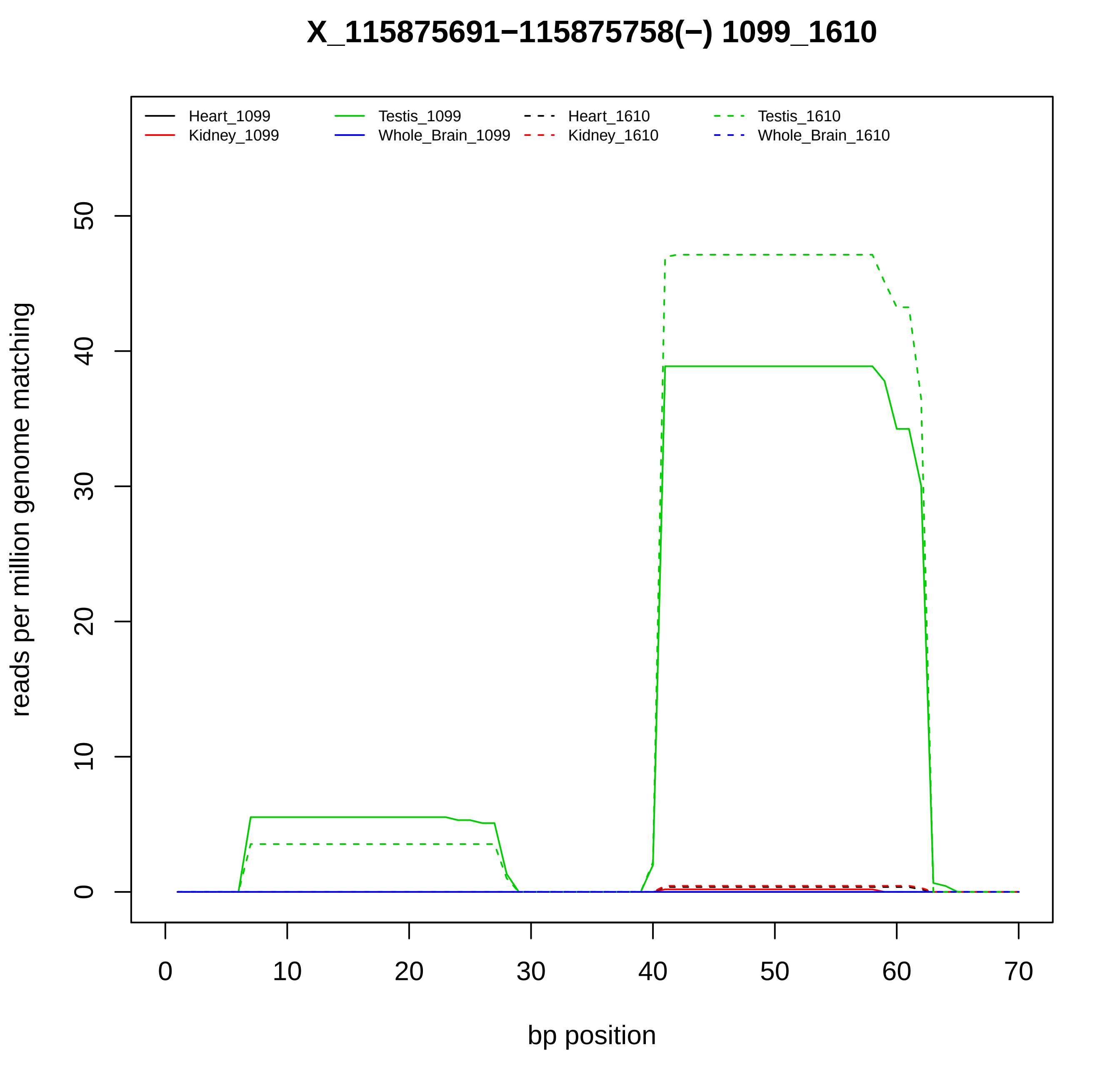

X_115875691−115875758(−) 1099_1610
Heart_1099
Kidney_1099
Testis_1099
Whole_Brain_1099
Heart_1610
Kidney_1610
Testis_1610
Whole_Brain_1610
reads per million genome matching
bp position
0
10
20
30
40
50
60
70

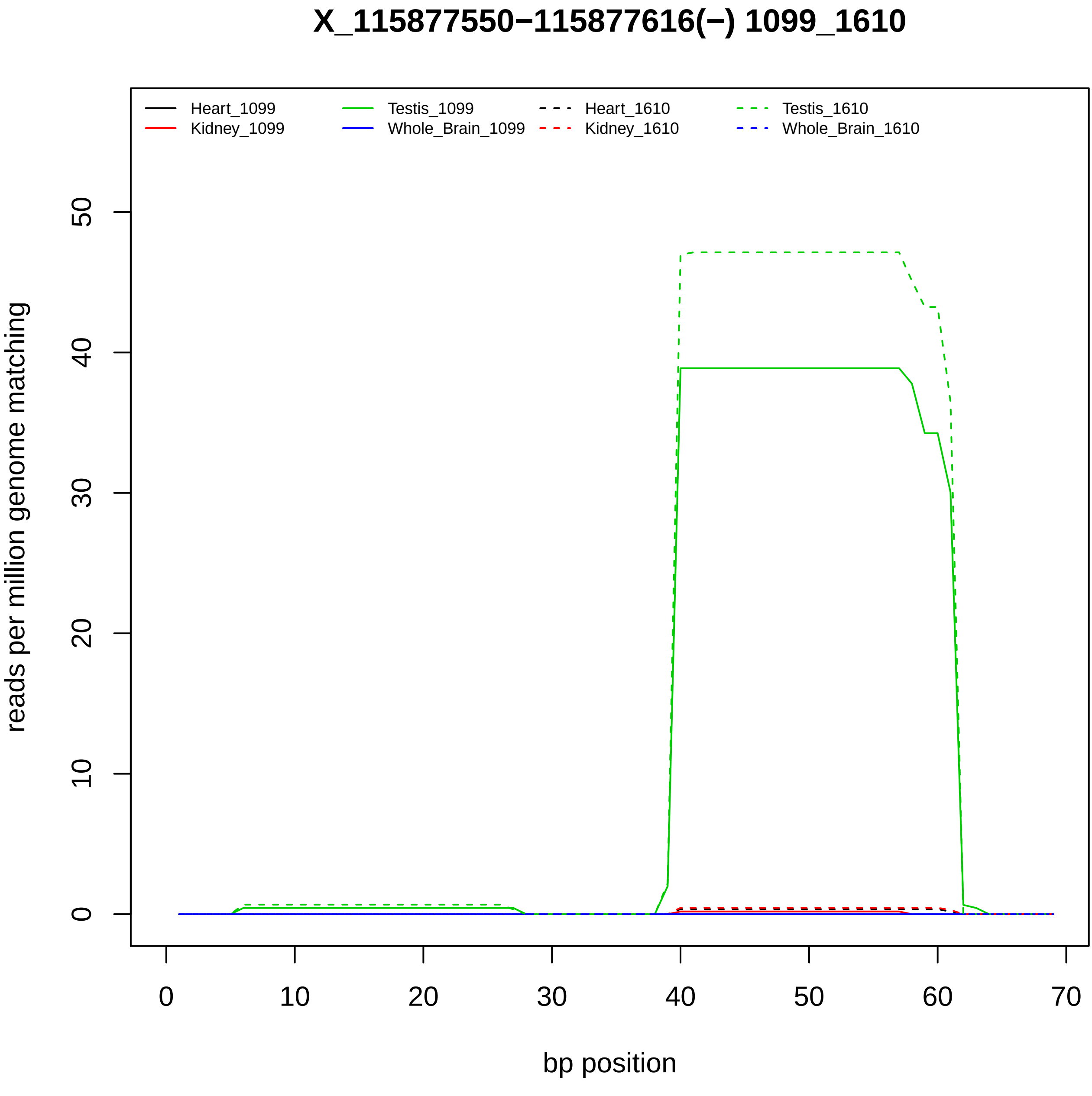

X_115877550−115877616(−) 1099_1610
Heart_1099
Kidney_1099
Testis_1099
Whole_Brain_1099
Heart_1610
Kidney_1610
Testis_1610
Whole_Brain_1610
reads per million genome matching
bp position
0
10
20
30
40
50
60
70

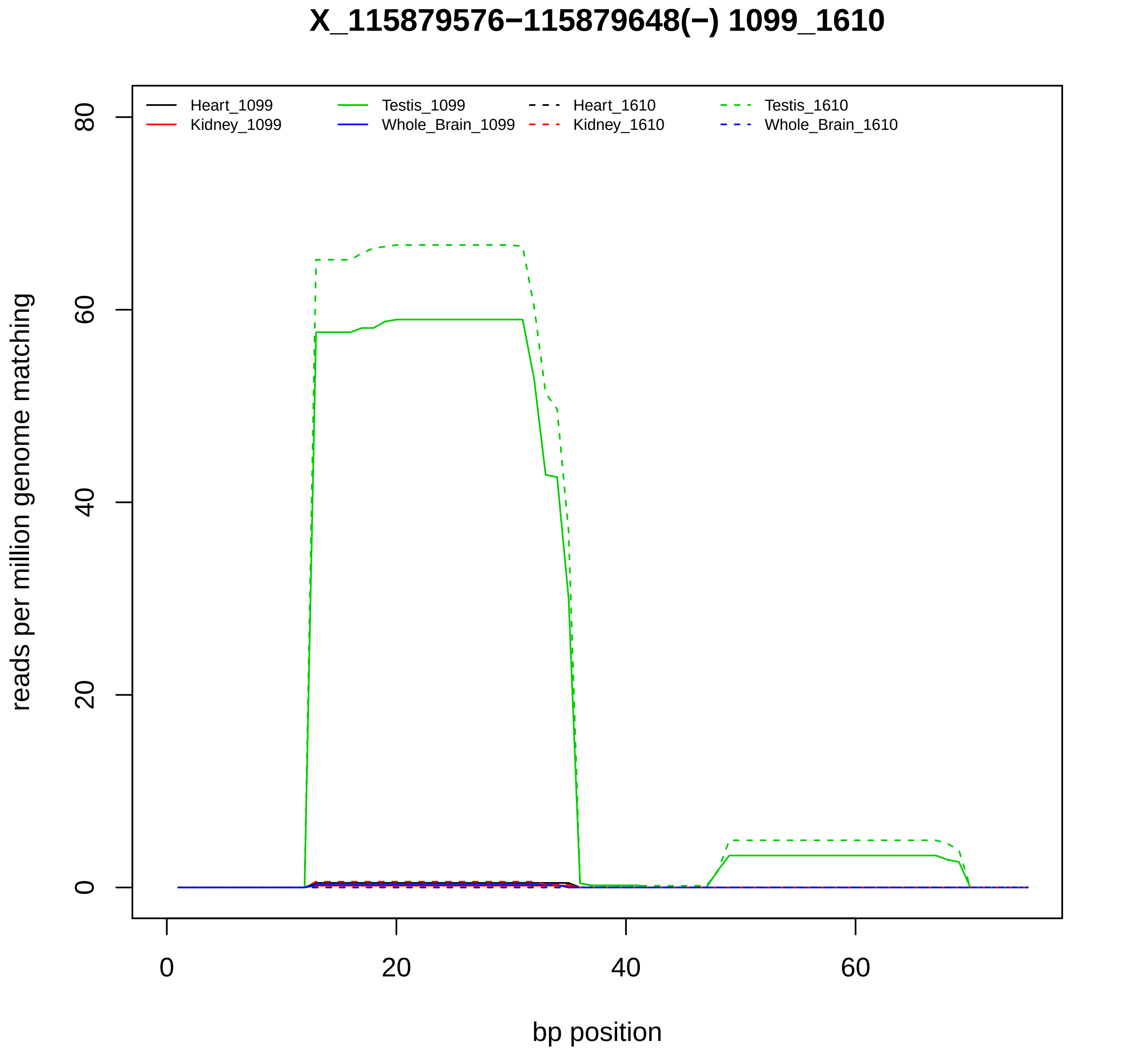

X_115879576−115879648(−) 1099_1610
Heart_1099
Kidney_1099
Testis_1099
Whole_Brain_1099
Heart_1610
Kidney_1610
Testis_1610
Whole_Brain_1610
reads per million genome matching
bp position
0
20
40
60
80

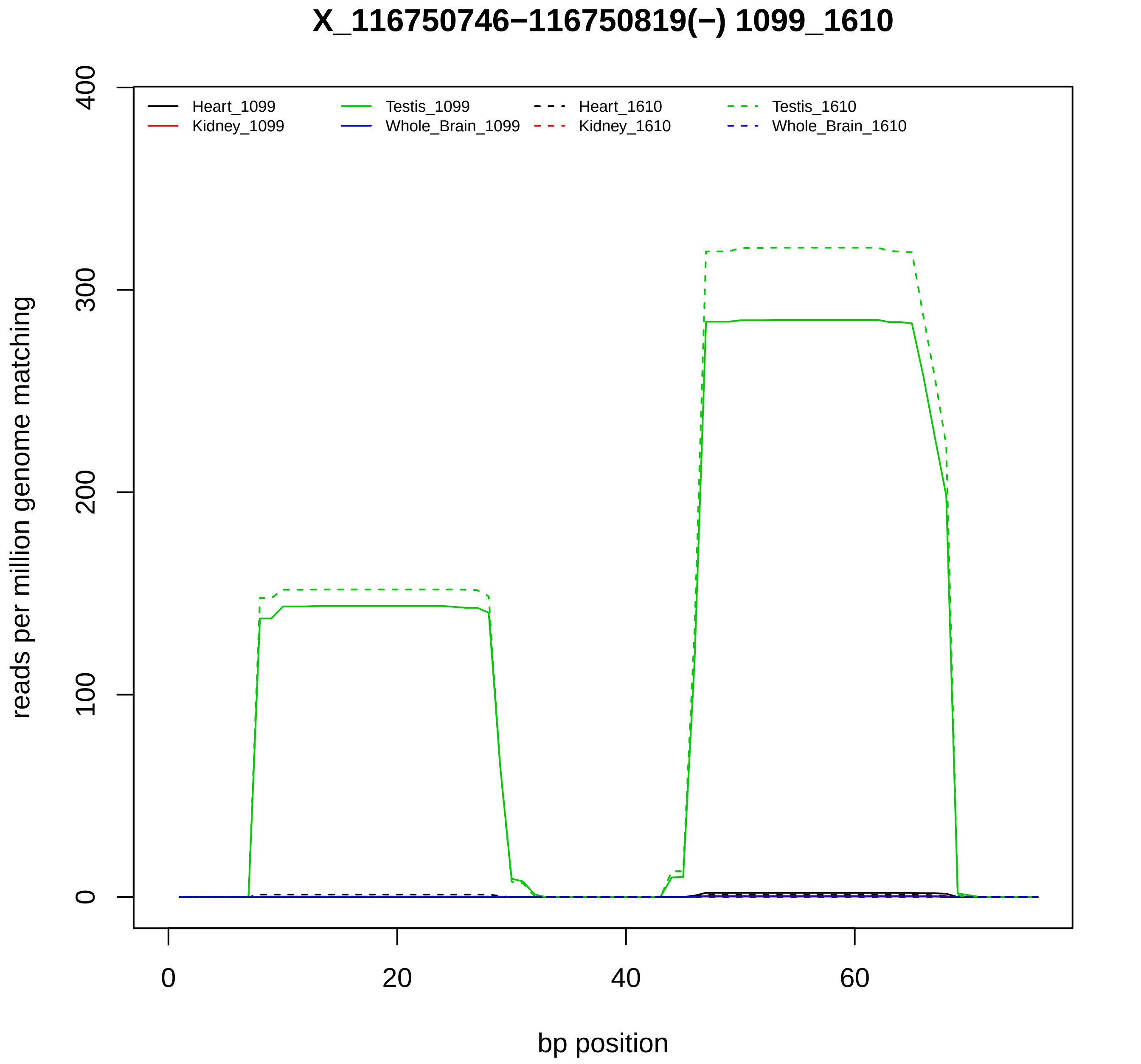
X_116750746−116750819(−) 1099_1610
Heart_1099
Kidney_1099
Testis_1099
Whole_Brain_1099
Heart_1610
Kidney_1610
Testis_1610
Whole_Brain_1610
reads per million genome matching
bp position
0
100
200
300
400
0
20
40
60

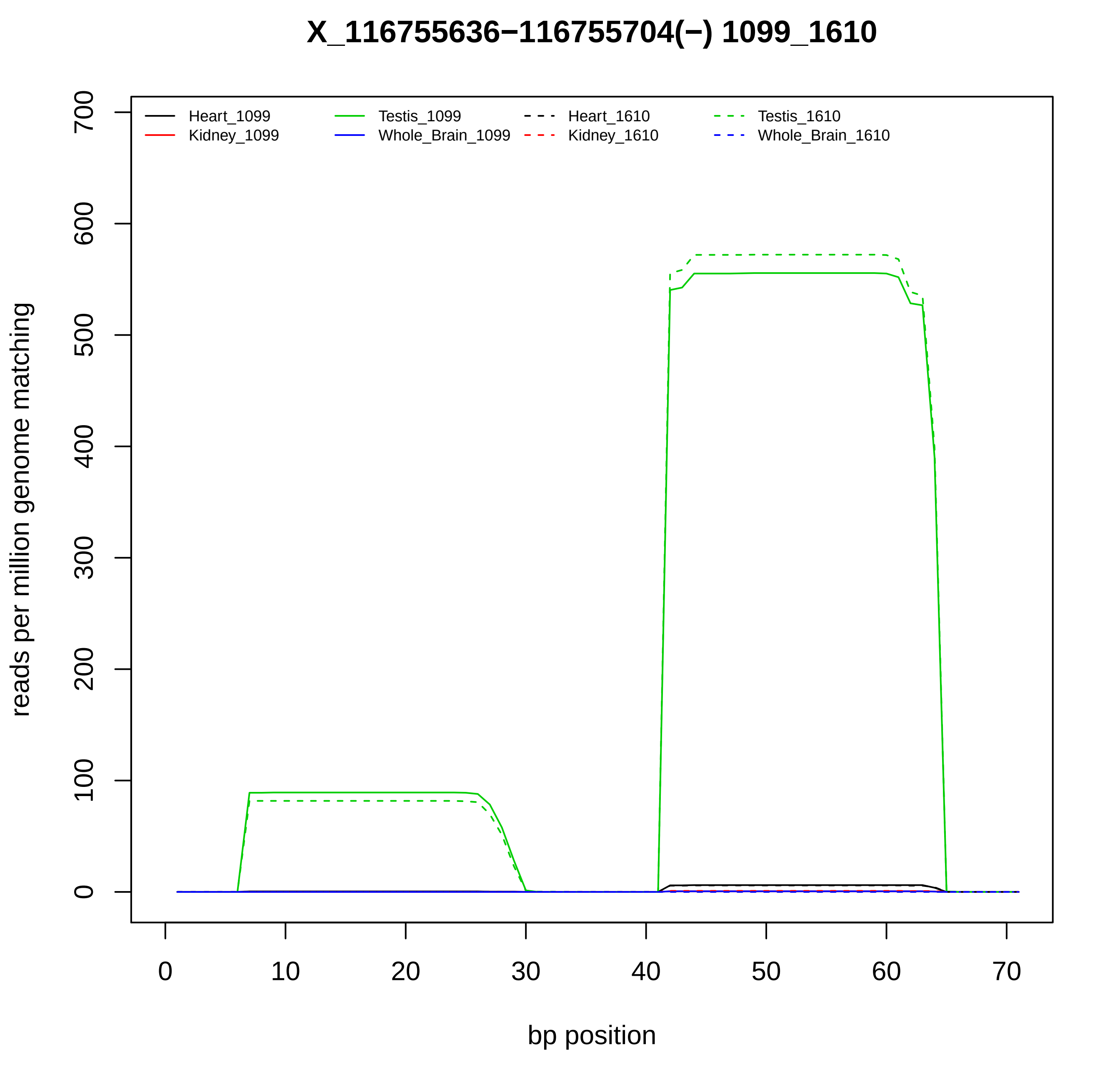
X_116755636−116755704(−) 1099_1610
Heart_1099
Kidney_1099
Testis_1099
Whole_Brain_1099
Heart_1610
Kidney_1610
Testis_1610
Whole_Brain_1610
reads per million genome matching
0
100
200
300
400
500
600
700
bp position
0
10
20
30
40
50
60
70

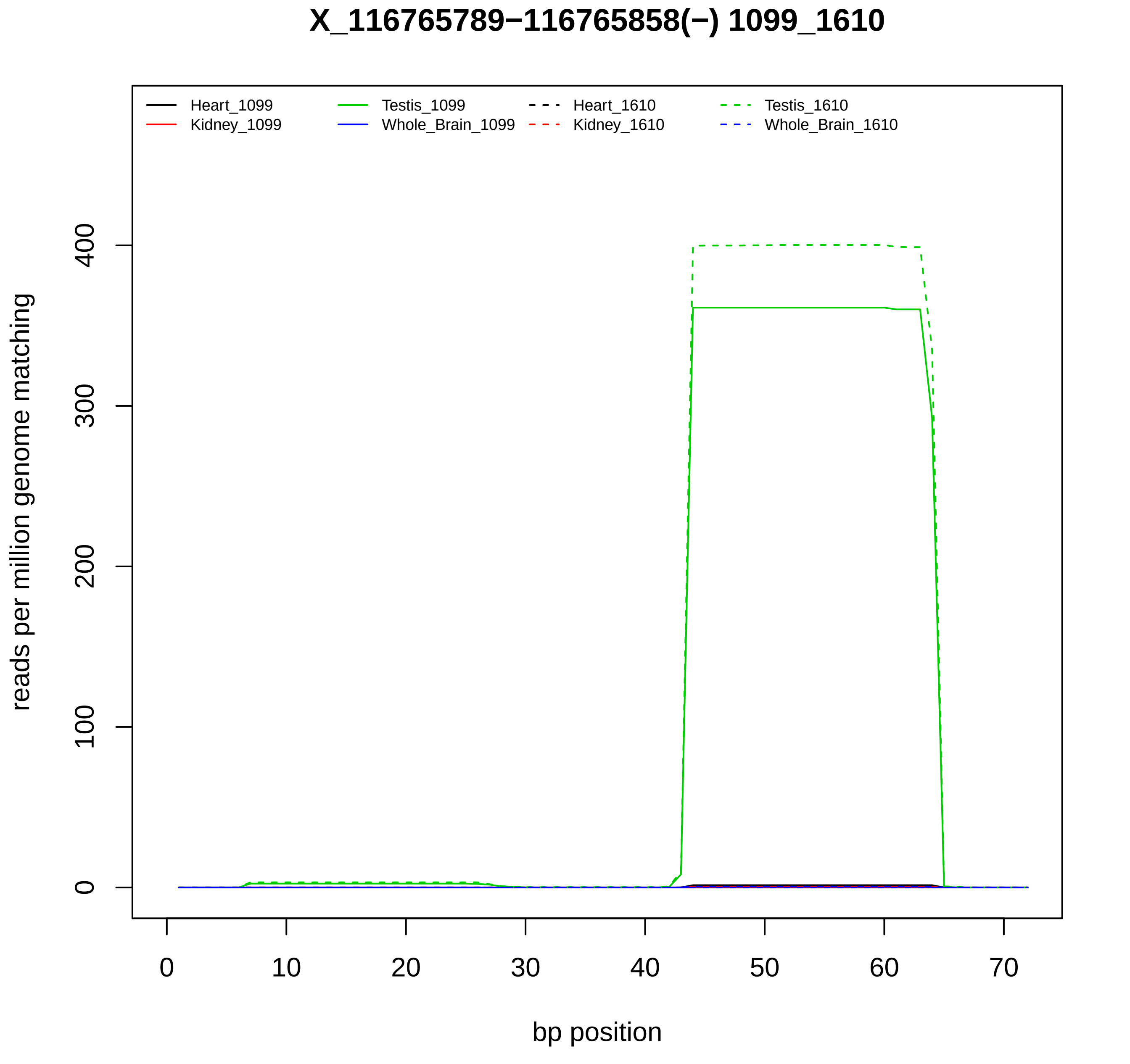

X_116765789−116765858(−) 1099_1610
Heart_1099
Kidney_1099
Testis_1099
Whole_Brain_1099
Heart_1610
Kidney_1610
Testis_1610
Whole_Brain_1610
reads per million genome matching
0
100
200
300
400
0
10
20
30
40
50
60
70
bp position

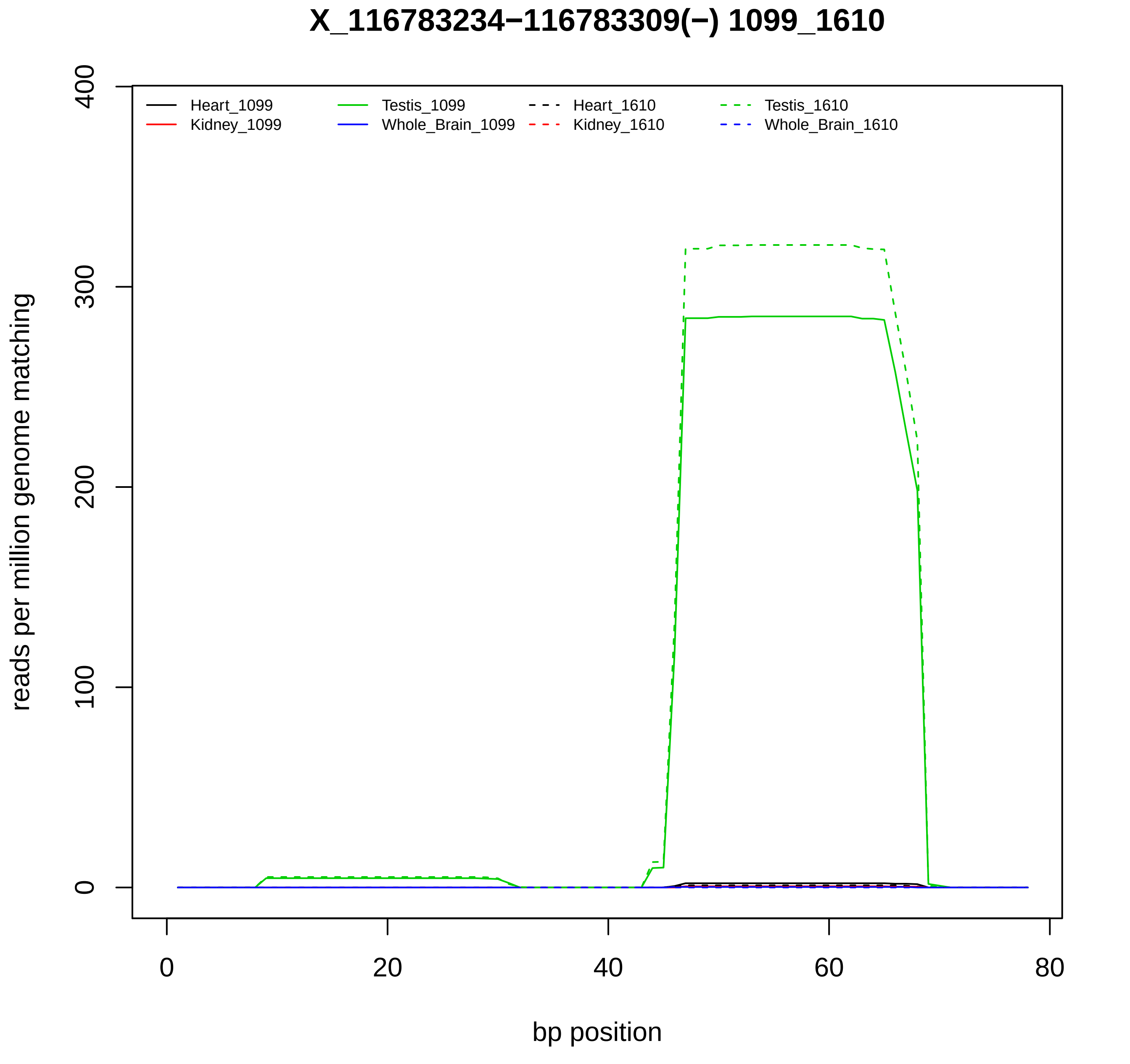

X_116783234−116783309(−) 1099_1610
Heart_1099
Kidney_1099
Testis_1099
Whole_Brain_1099
Heart_1610
Kidney_1610
Testis_1610
Whole_Brain_1610
reads per million genome matching
bp position
0
100
200
300
400
0
20
40
60
80

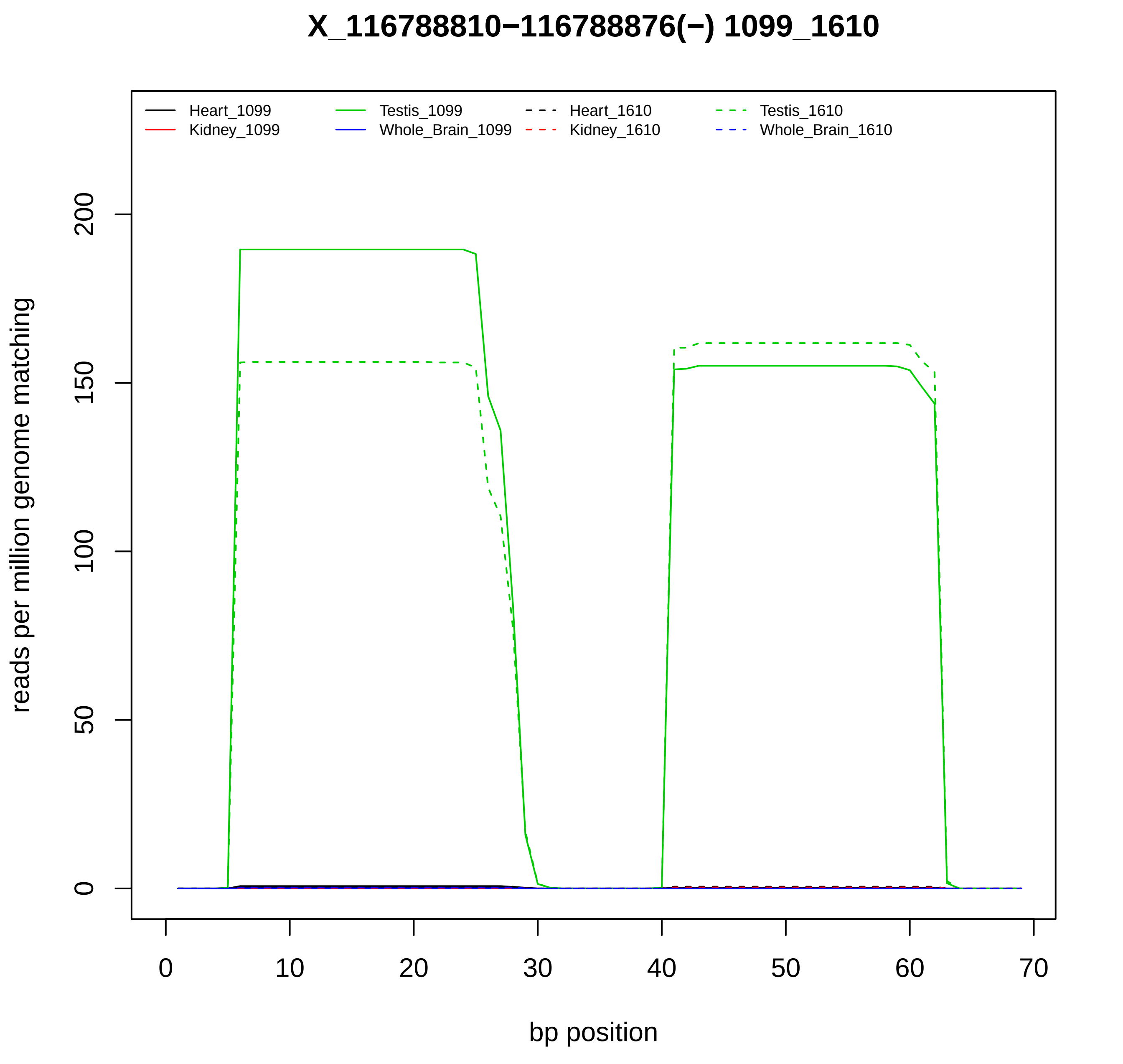
X_116788810−116788876(−) 1099_1610
Heart_1099
Kidney_1099
Testis_1099
Whole_Brain_1099
Heart_1610
Kidney_1610
Testis_1610
Whole_Brain_1610
reads per million genome matching
bp position
0
50
100
150
200
0
10
20
30
40
50
60
70

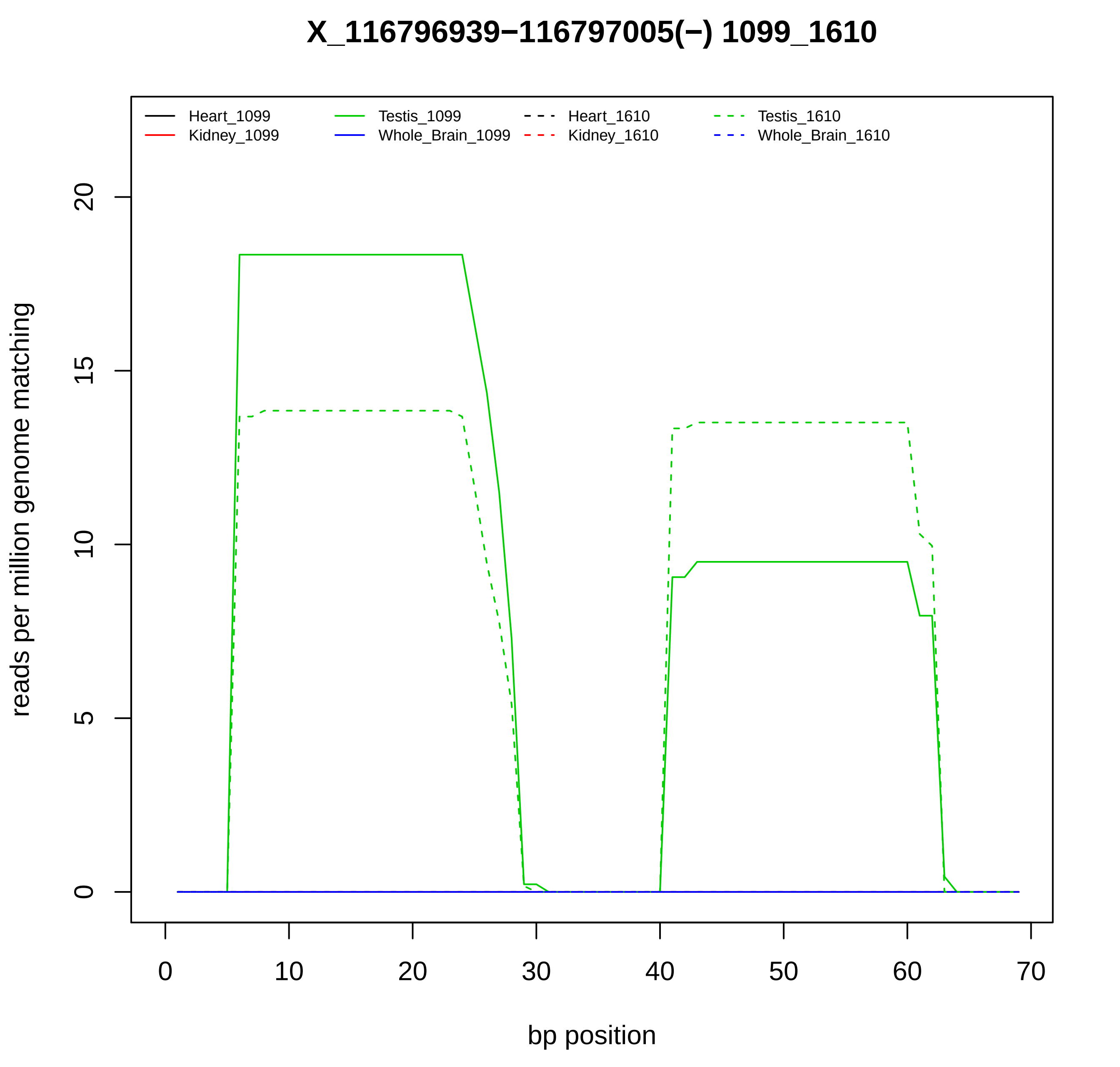
X_116796939−116797005(−) 1099_1610
Heart_1099
Kidney_1099
Testis_1099
Whole_Brain_1099
Heart_1610
Kidney_1610
Testis_1610
Whole_Brain_1610
reads per million genome matching
0
5
10
15
20
0
10
20
30
40
50
60
70
bp position

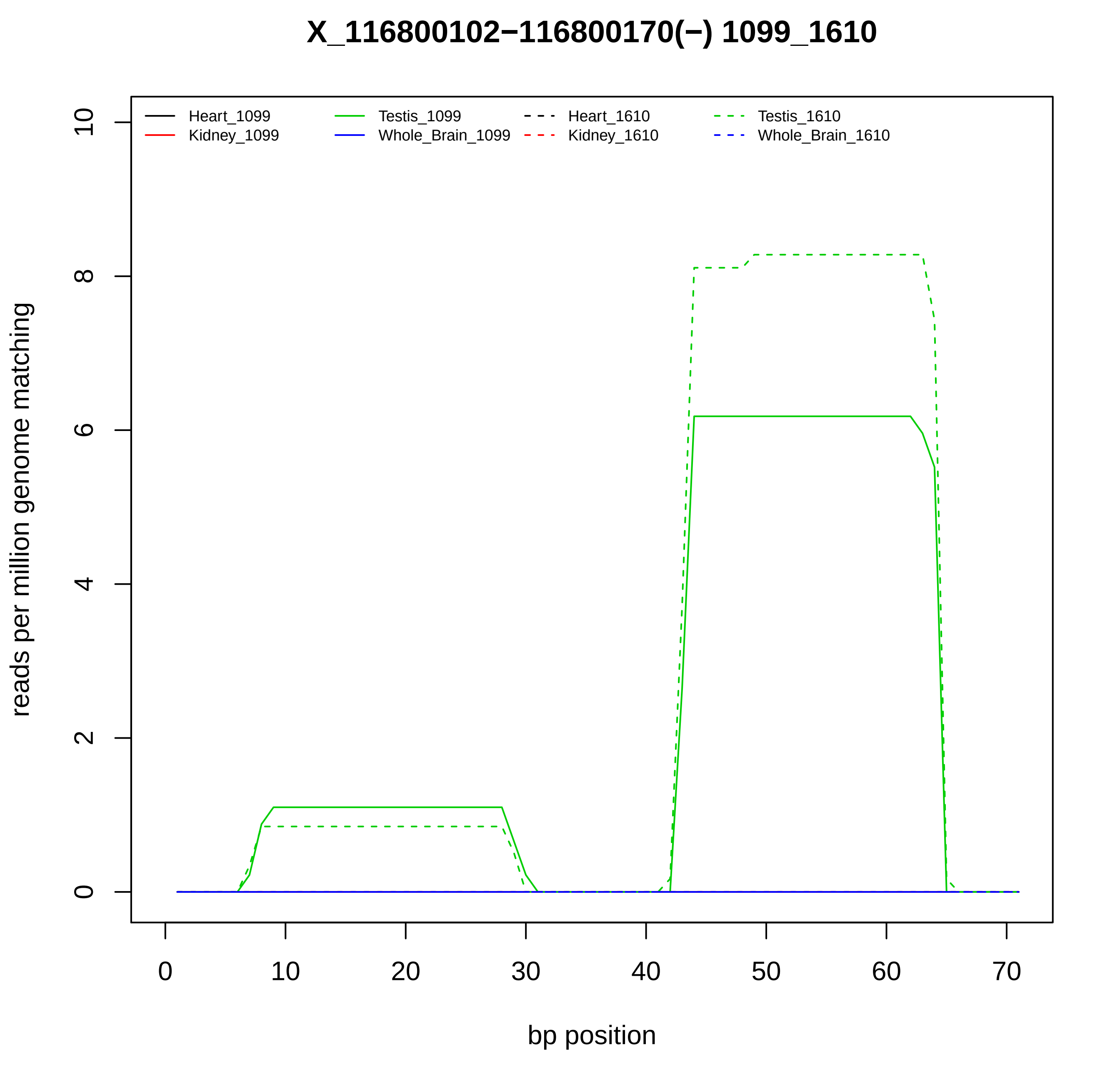

X_116800102−116800170(−) 1099_1610
Heart_1099
Kidney_1099
Testis_1099
Whole_Brain_1099
Heart_1610
Kidney_1610
Testis_1610
Whole_Brain_1610
reads per million genome matching
bp position
0
2
4
6
8
10
0
10
20
30
40
50
60
70

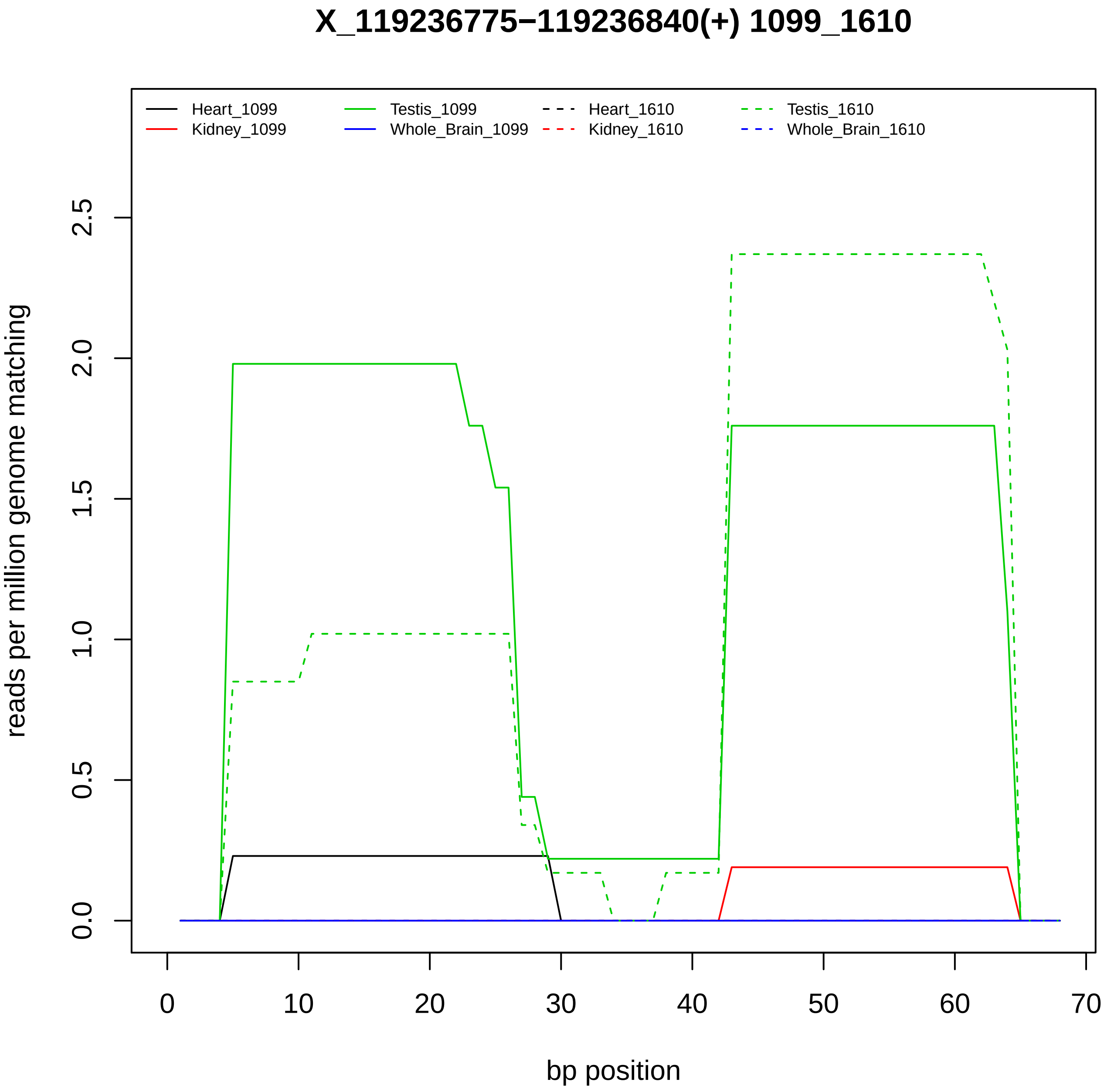

X_119236775−119236840(+) 1099_1610
Heart_1099
Kidney_1099
Testis_1099
Whole_Brain_1099
Heart_1610
Kidney_1610
Testis_1610
Whole_Brain_1610
reads per million genome matching
bp position
0.0
0.5
1.0
1.5
2.0
2.5
0
10
20
30
40
50
60
70

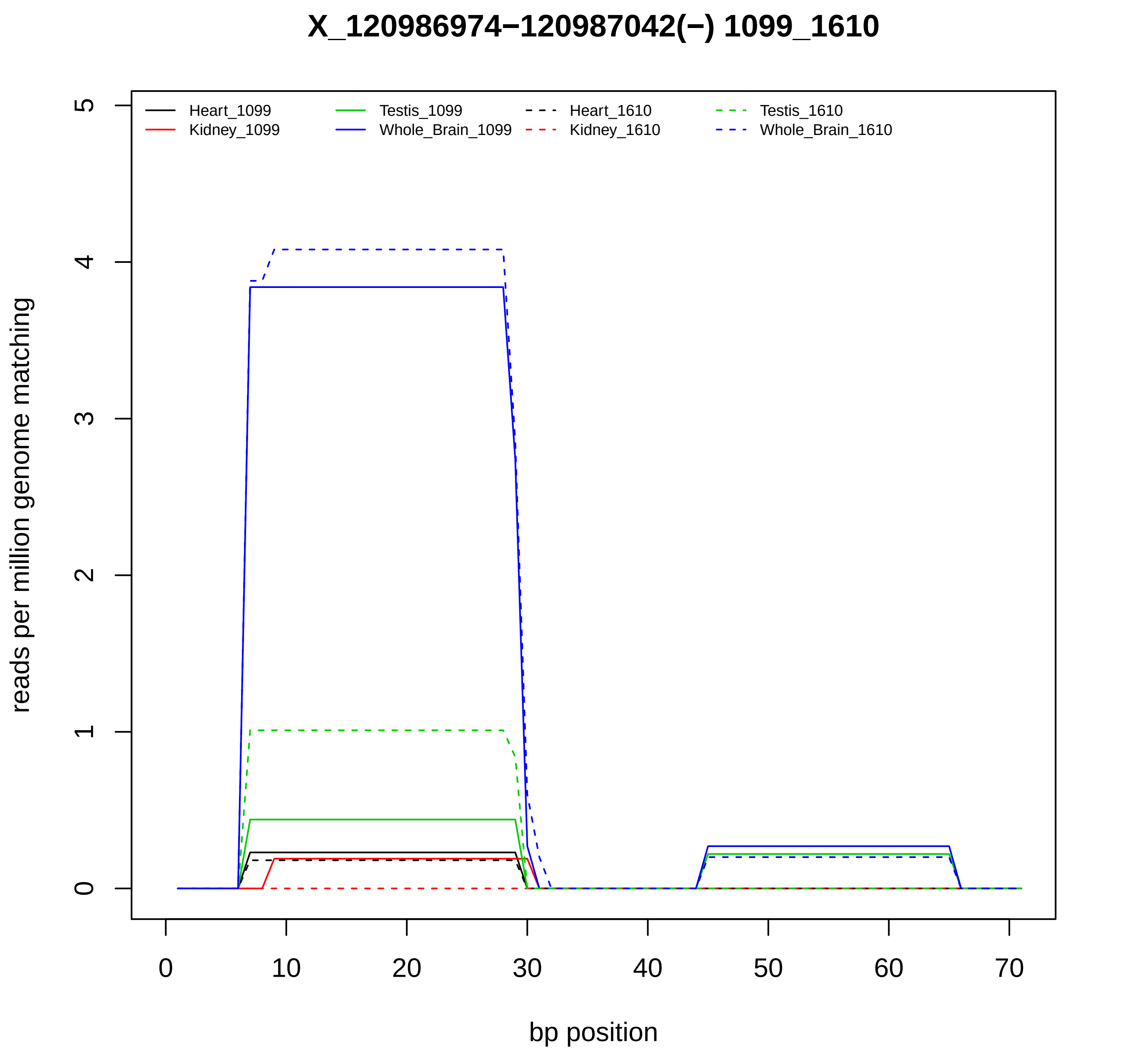

X_120986974−120987042(−) 1099_1610
Heart_1099
Kidney_1099
Testis_1099
Whole_Brain_1099
Heart_1610
Kidney_1610
Testis_1610
Whole_Brain_1610
reads per million genome matching
bp position
0
1
2
3
4
5
0
10
20
30
40
50
60
70

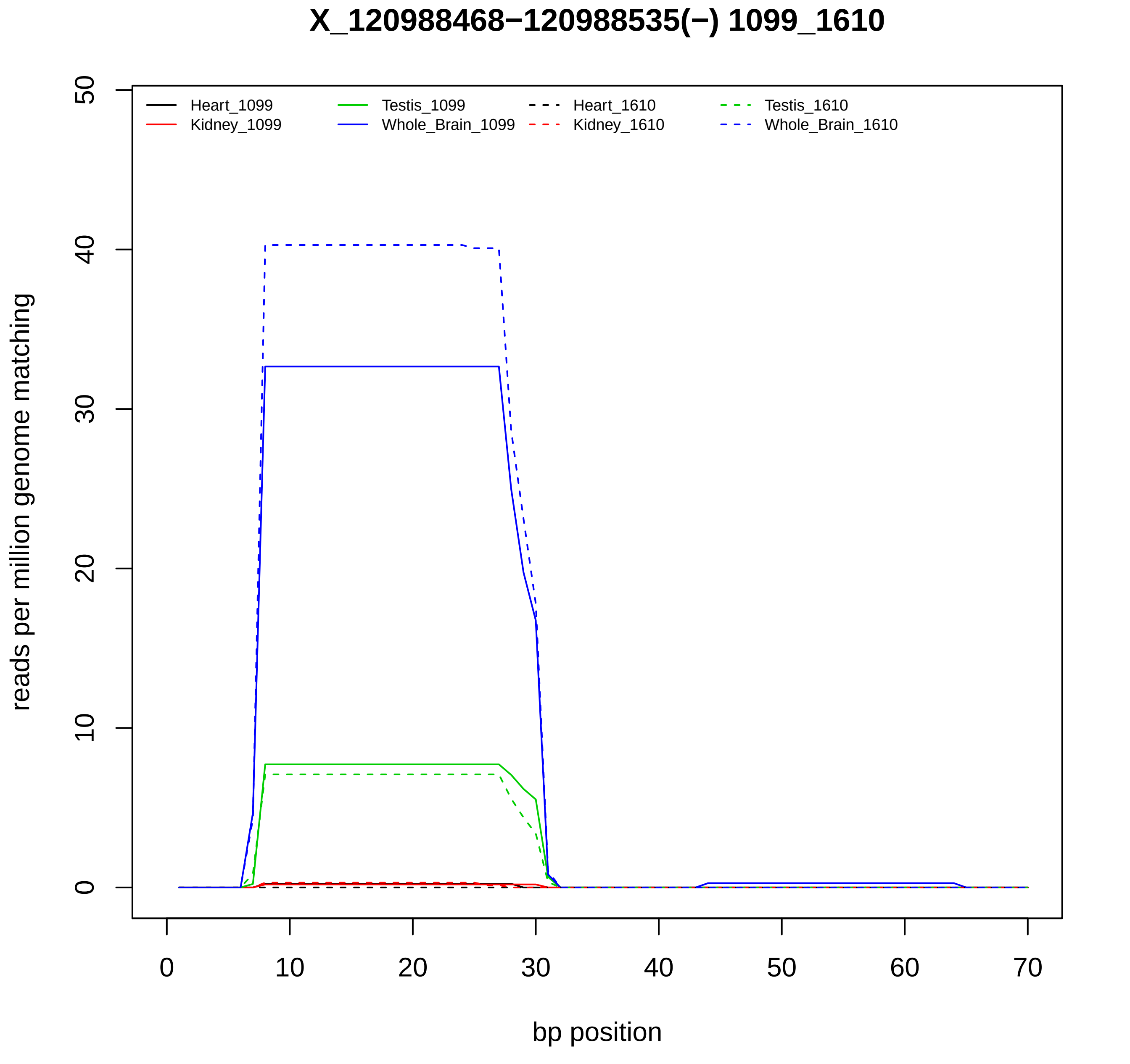
X_120988468−120988535(−) 1099_1610
Heart_1099
Kidney_1099
Testis_1099
Whole_Brain_1099
Heart_1610
Kidney_1610
Testis_1610
Whole_Brain_1610
reads per million genome matching
bp position
0
10
20
30
40
50
60
70

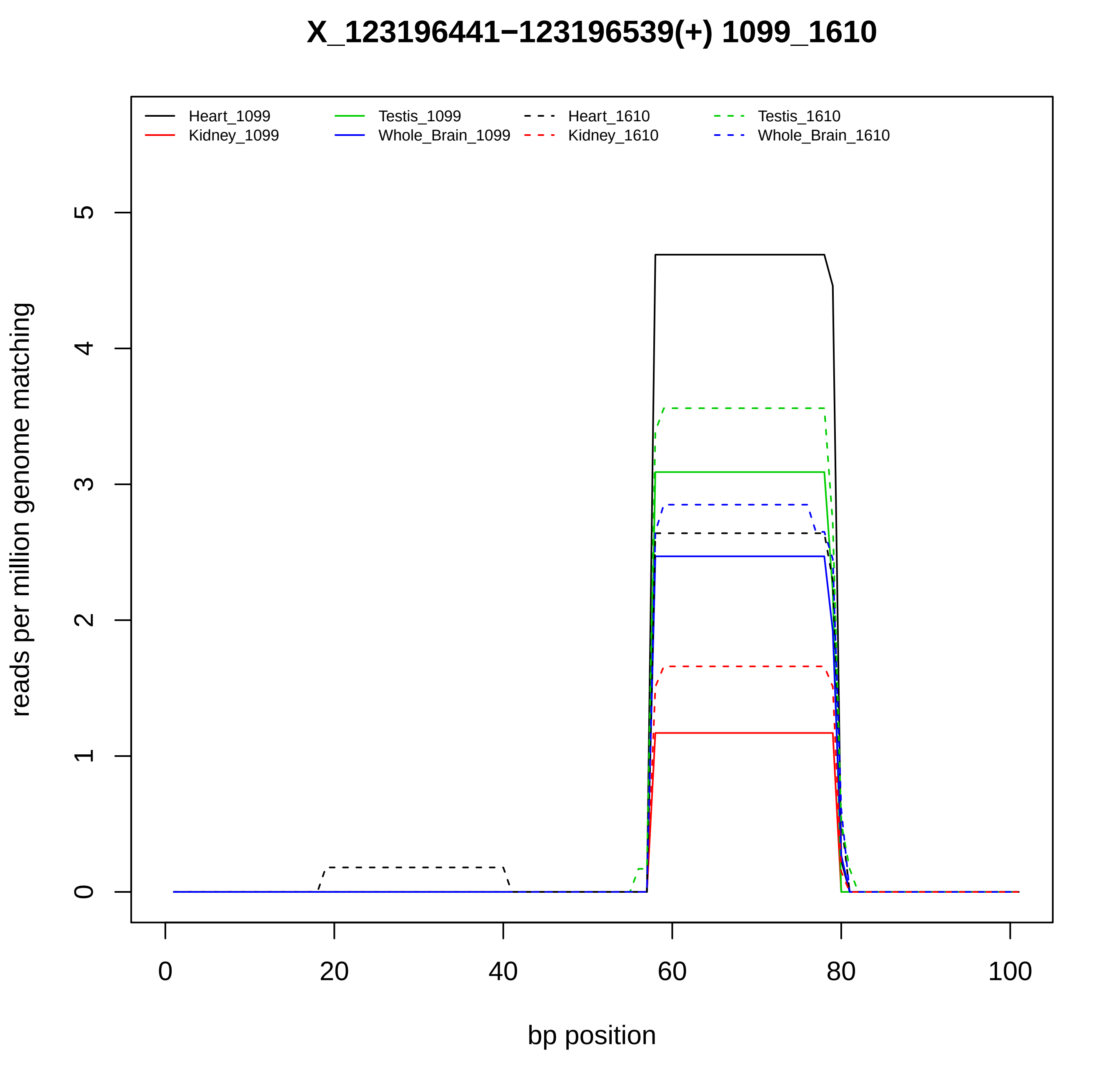
X_123196441−123196539(+) 1099_1610
Heart_1099
Kidney_1099
Testis_1099
Whole_Brain_1099
Heart_1610
Kidney_1610
Testis_1610
Whole_Brain_1610
reads per million genome matching
0
1
2
3
4
5
bp position
0
20
40
60
80
100

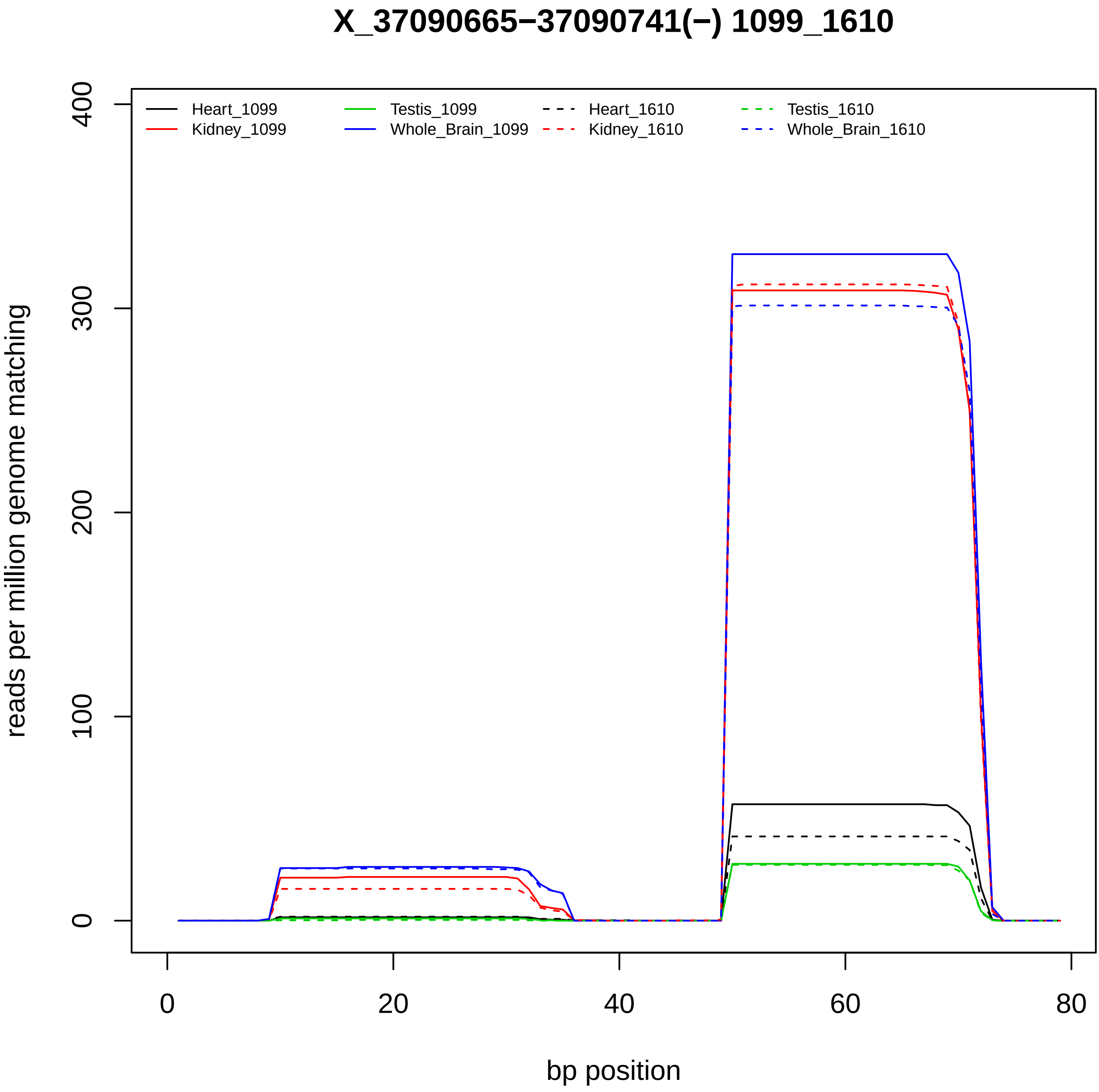
X_37090665−37090741(−) 1099_1610
Heart_1099
Kidney_1099
Testis_1099
Whole_Brain_1099
Heart_1610
Kidney_1610
Testis_1610
Whole_Brain_1610
reads per million genome matching
bp position
0
100
200
300
400
0
20
40
60
80

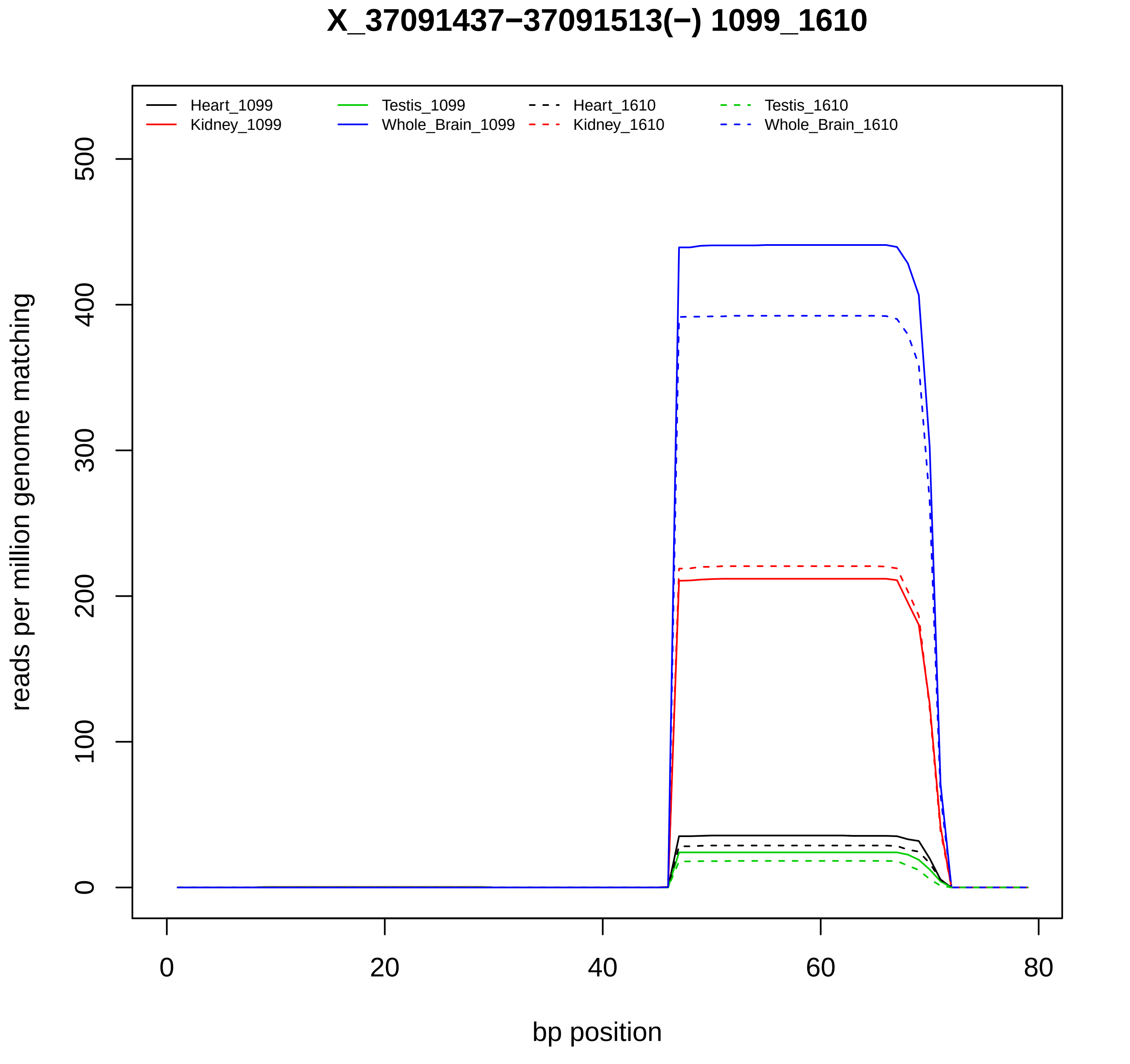

X_37091437−37091513(−) 1099_1610
Heart_1099
Kidney_1099
Testis_1099
Whole_Brain_1099
Heart_1610
Kidney_1610
Testis_1610
Whole_Brain_1610
reads per million genome matching
bp position
0
100
200
300
400
500
0
20
40
60
80

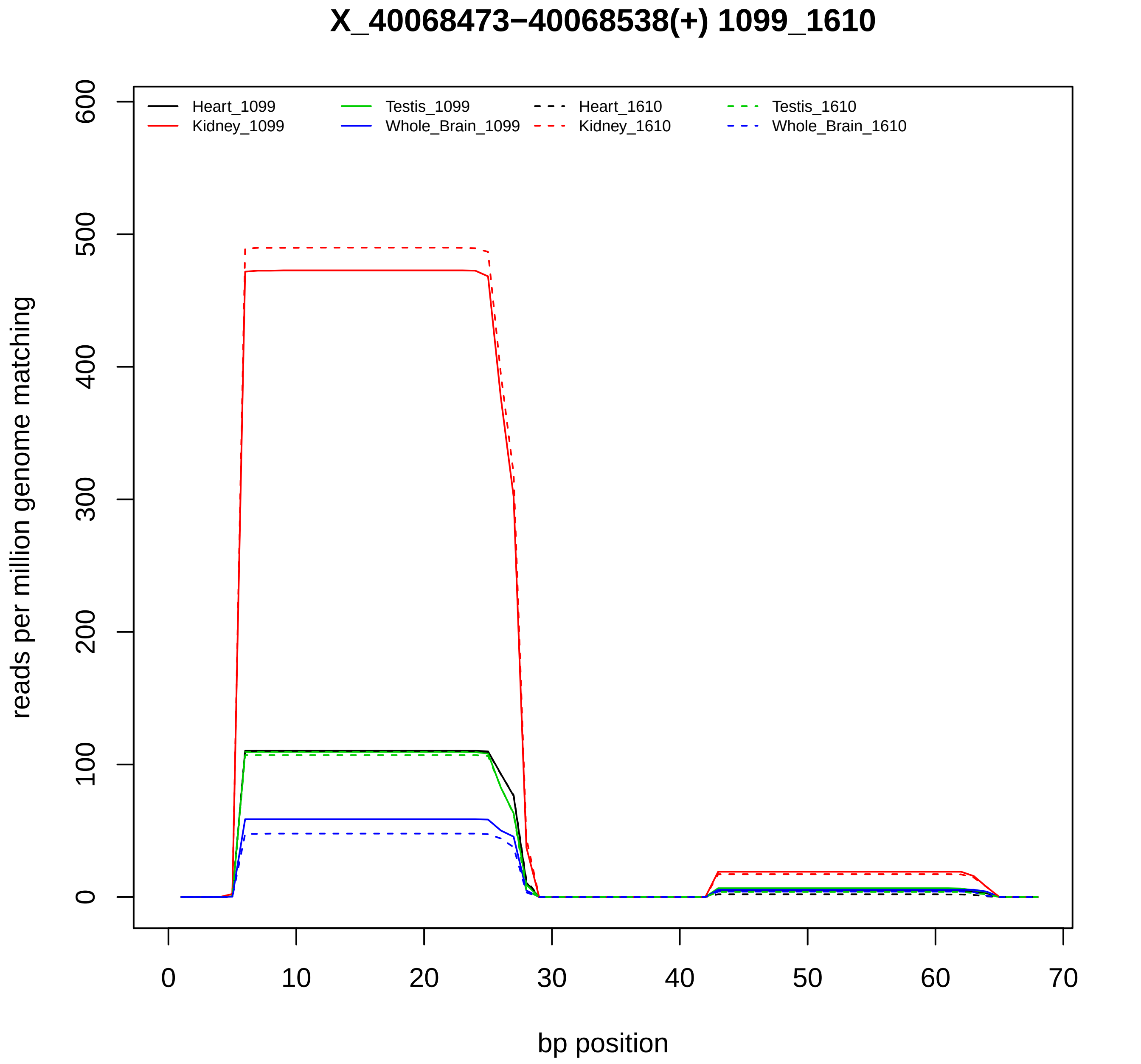

X_40068473−40068538(+) 1099_1610
Heart_1099
Kidney_1099
Testis_1099
Whole_Brain_1099
Heart_1610
Kidney_1610
Testis_1610
Whole_Brain_1610
reads per million genome matching
bp position
0
100
200
300
400
500
600
0
10
20
30
40
50
60
70

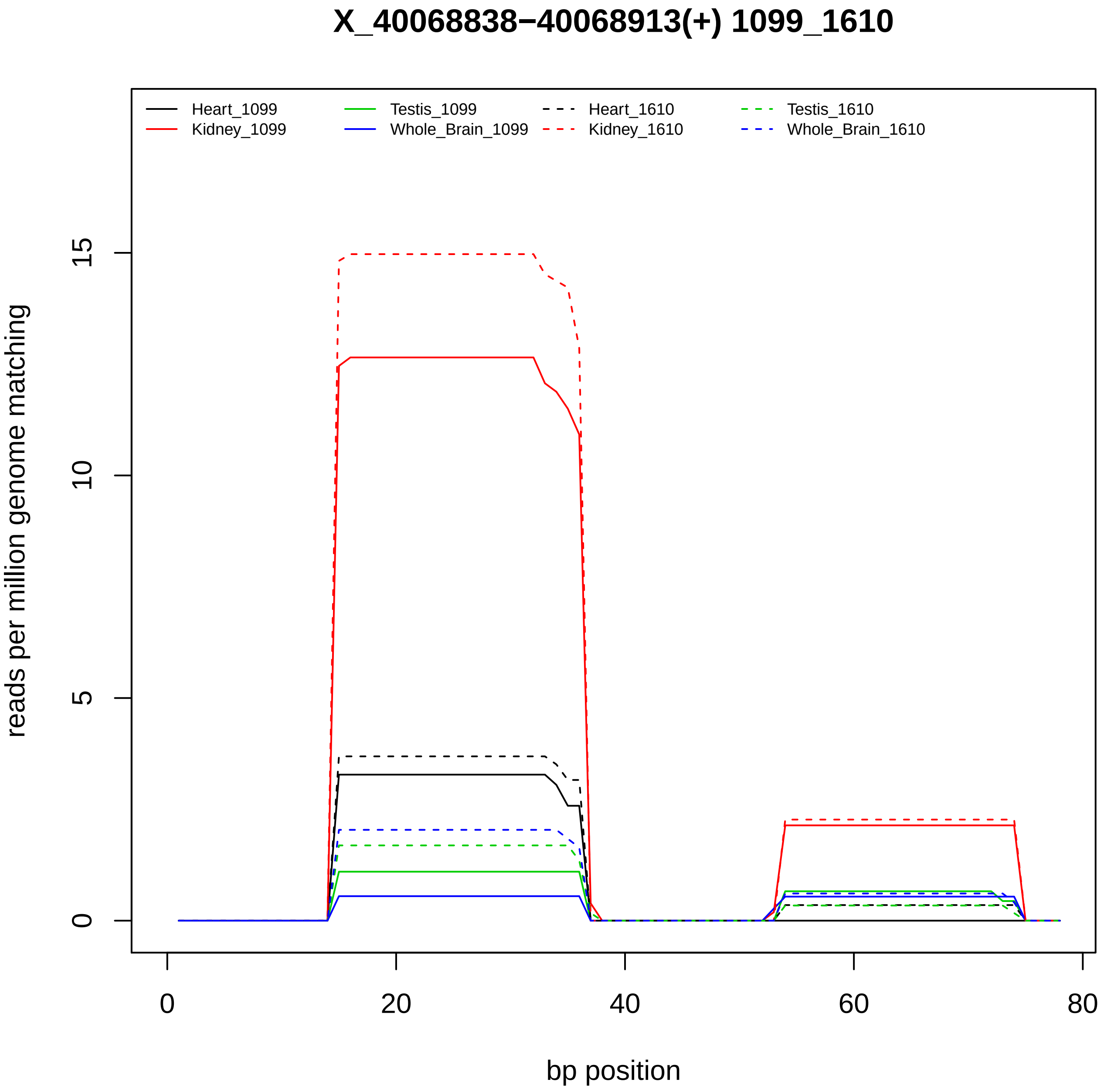

X_40068838−40068913(+) 1099_1610
Heart_1099
Kidney_1099
Testis_1099
Whole_Brain_1099
Heart_1610
Kidney_1610
Testis_1610
Whole_Brain_1610
reads per million genome matching
0
5
10
15
0
20
40
60
80
bp position

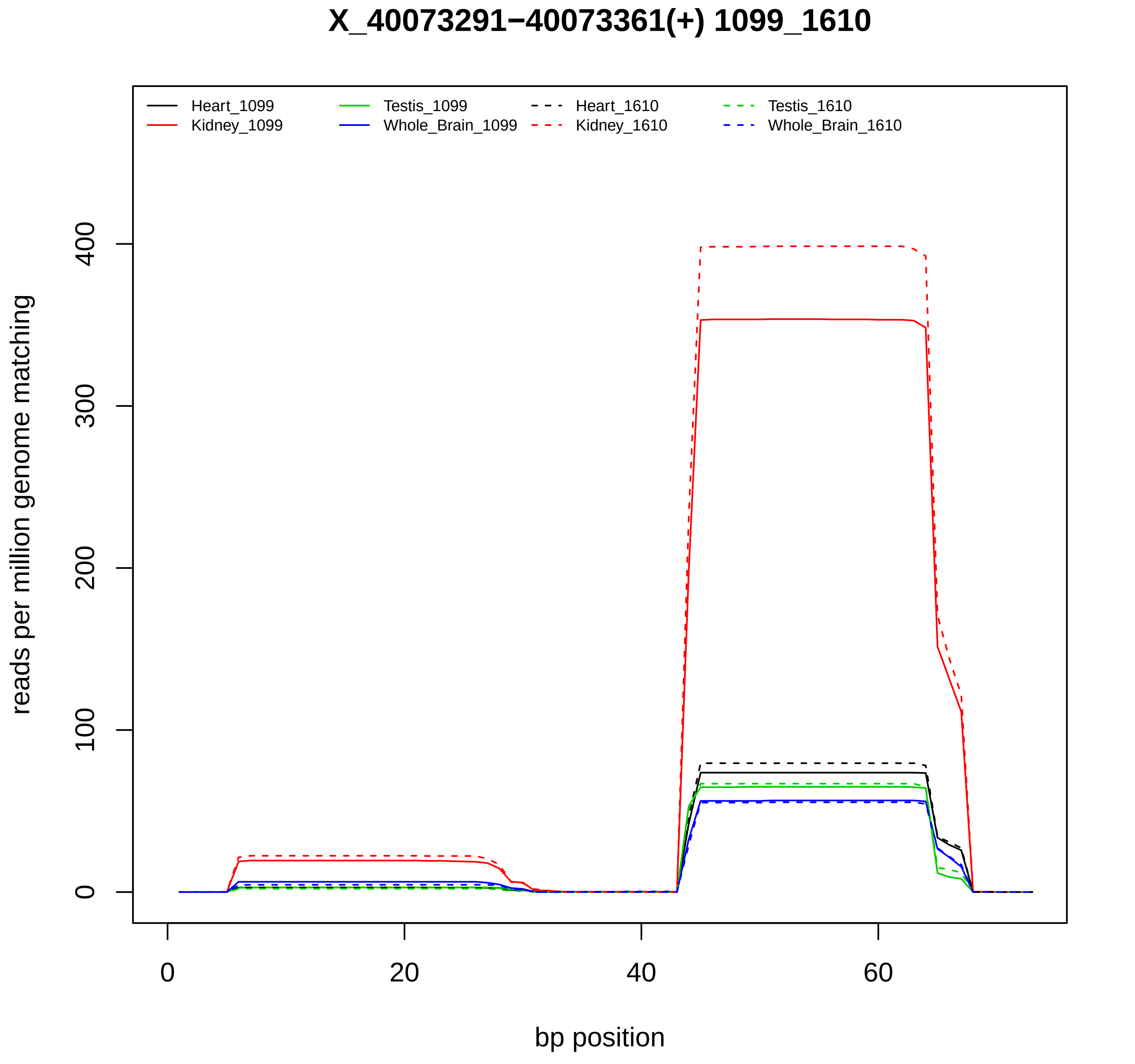
X_40073291−40073361(+) 1099_1610
Heart_1099
Kidney_1099
Testis_1099
Whole_Brain_1099
Heart_1610
Kidney_1610
Testis_1610
Whole_Brain_1610
reads per million genome matching
0
100
200
300
400
0
20
40
60
bp position

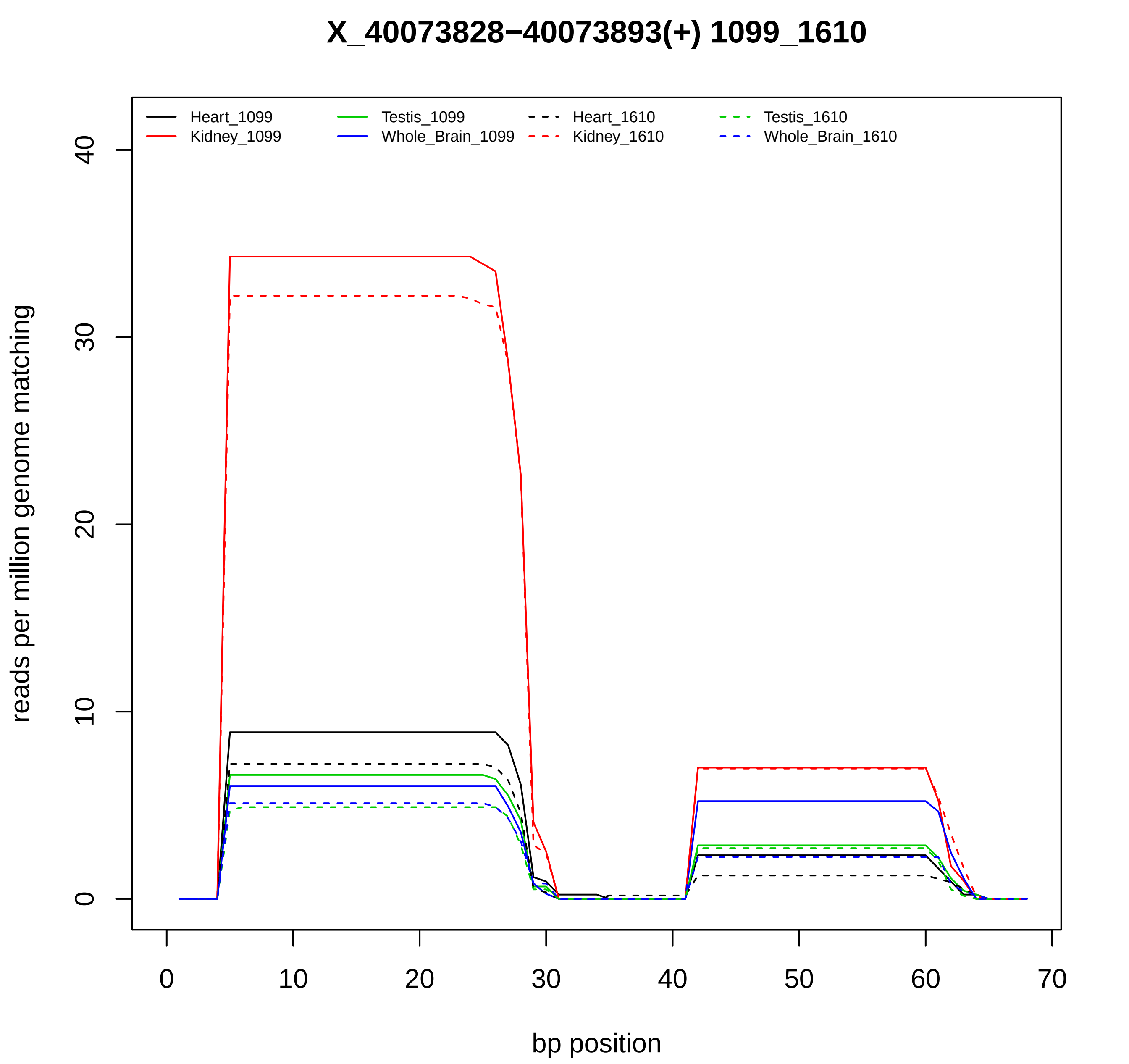
X_40073828−40073893(+) 1099_1610
Heart_1099
Kidney_1099
Testis_1099
Whole_Brain_1099
Heart_1610
Kidney_1610
Testis_1610
Whole_Brain_1610
reads per million genome matching
bp position
0
10
20
30
40
50
60
70

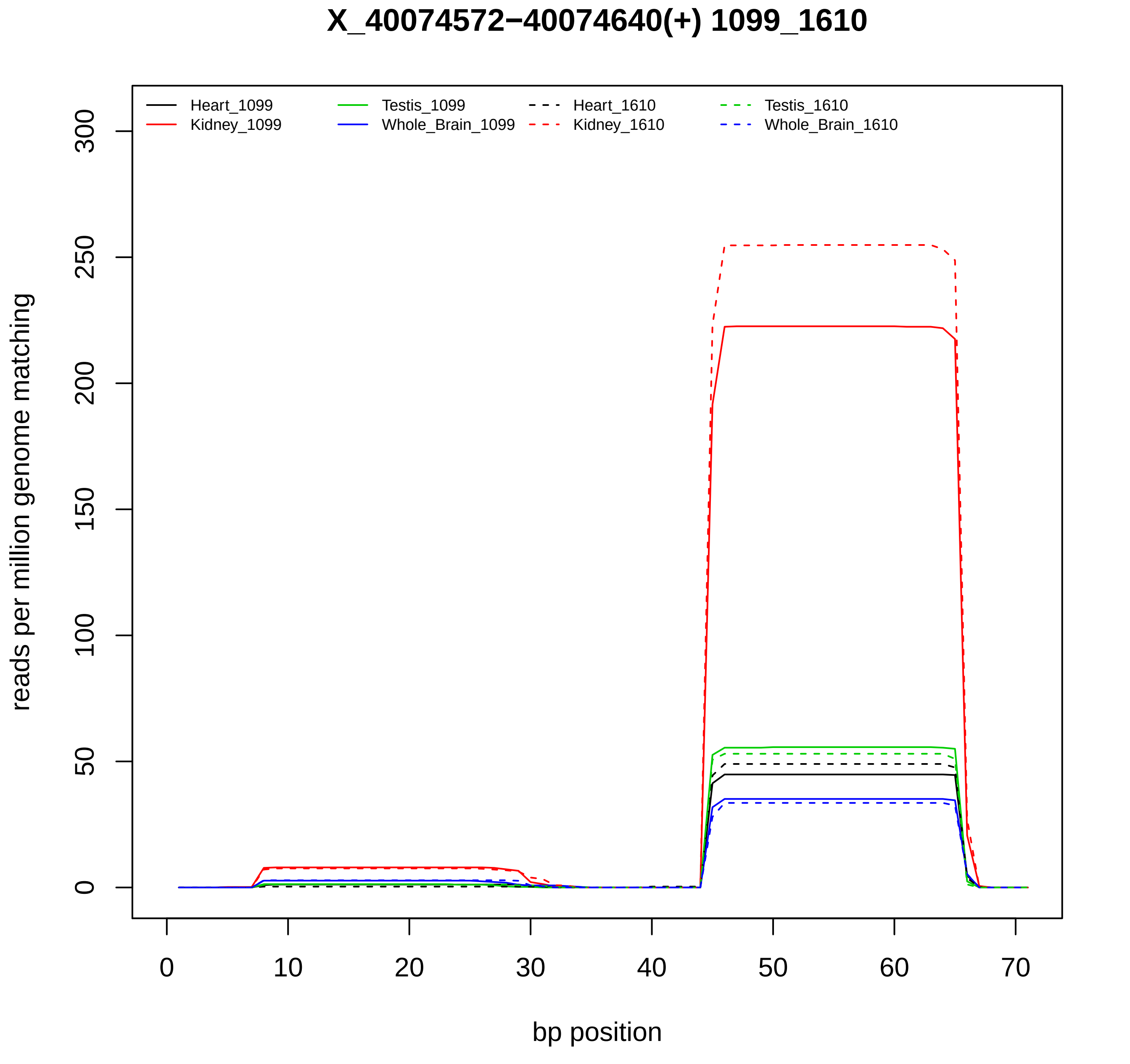
X_40074572−40074640(+) 1099_1610
Heart_1099
Kidney_1099
Testis_1099
Whole_Brain_1099
Heart_1610
Kidney_1610
Testis_1610
Whole_Brain_1610
reads per million genome matching
0
50
100
150
200
250
300
bp position
0
10
20
30
40
50
60
70

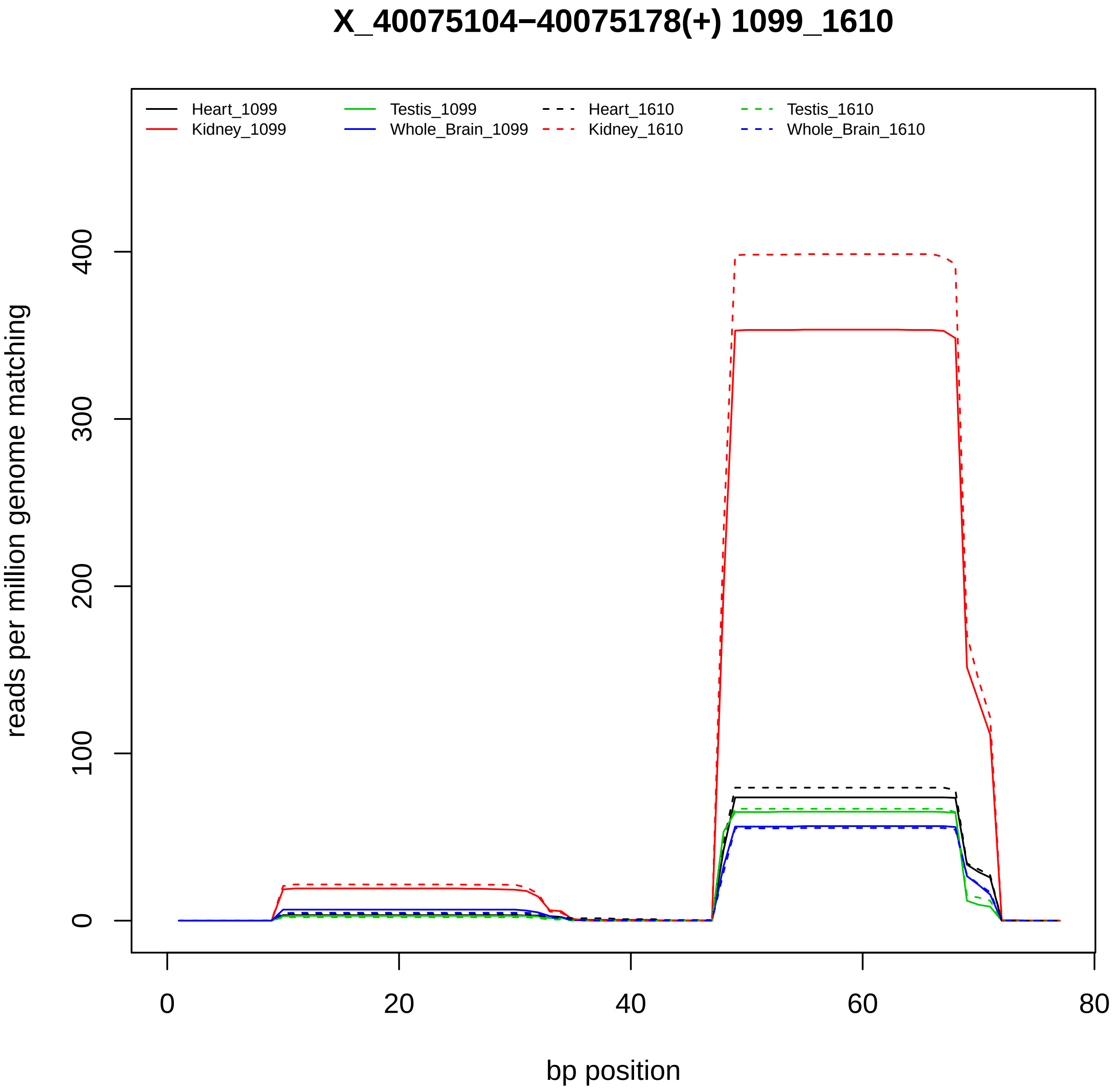

X_40075104−40075178(+) 1099_1610
Heart_1099
Kidney_1099
Testis_1099
Whole_Brain_1099
Heart_1610
Kidney_1610
Testis_1610
Whole_Brain_1610
reads per million genome matching
0
100
200
300
400
0
20
40
60
80
bp position

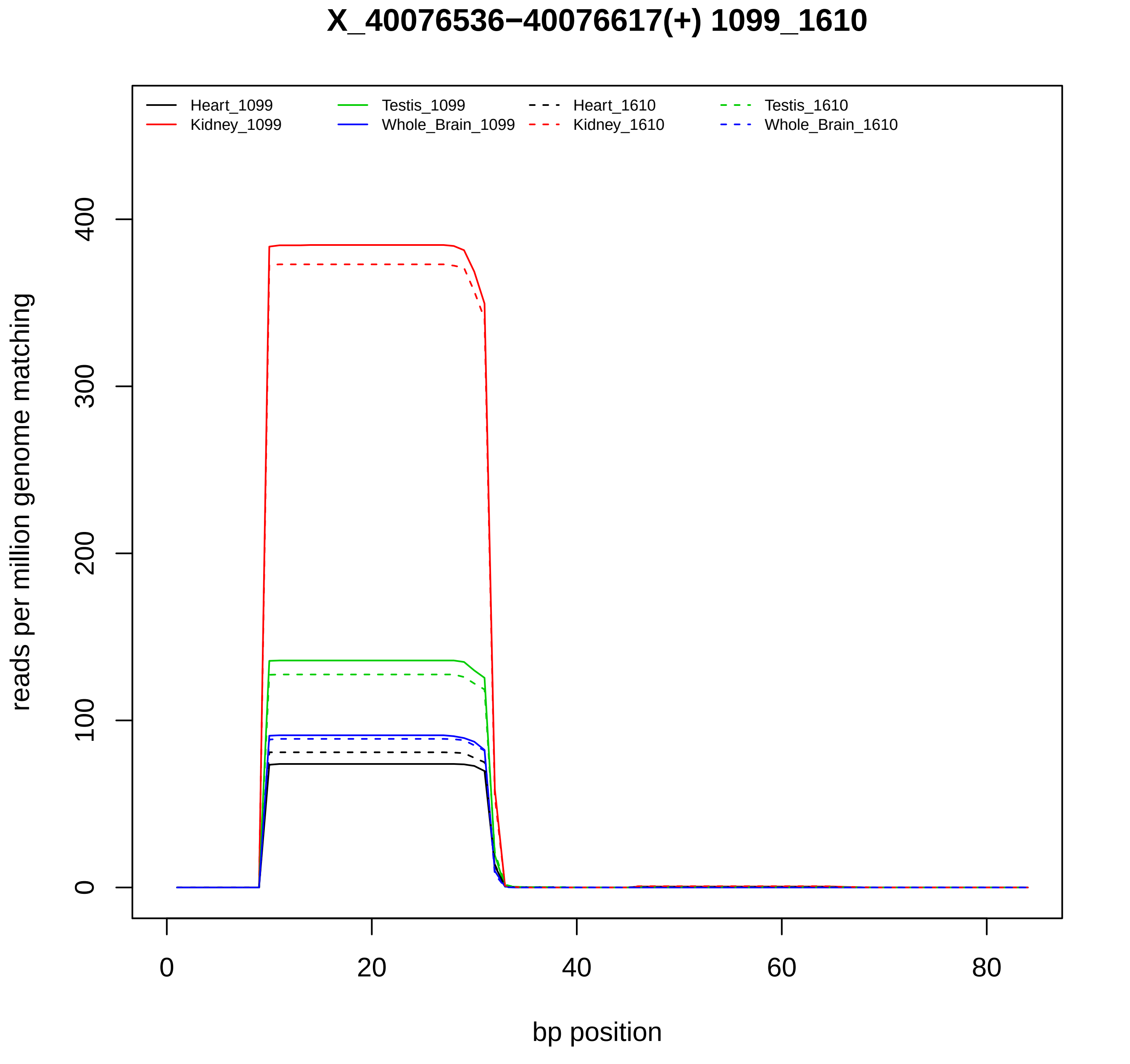

X_40076536−40076617(+) 1099_1610
Heart_1099
Kidney_1099
Testis_1099
Whole_Brain_1099
Heart_1610
Kidney_1610
Testis_1610
Whole_Brain_1610
reads per million genome matching
0
100
200
300
400
0
20
40
60
80
bp position

X_40077414−40077483(+) 1099_1610

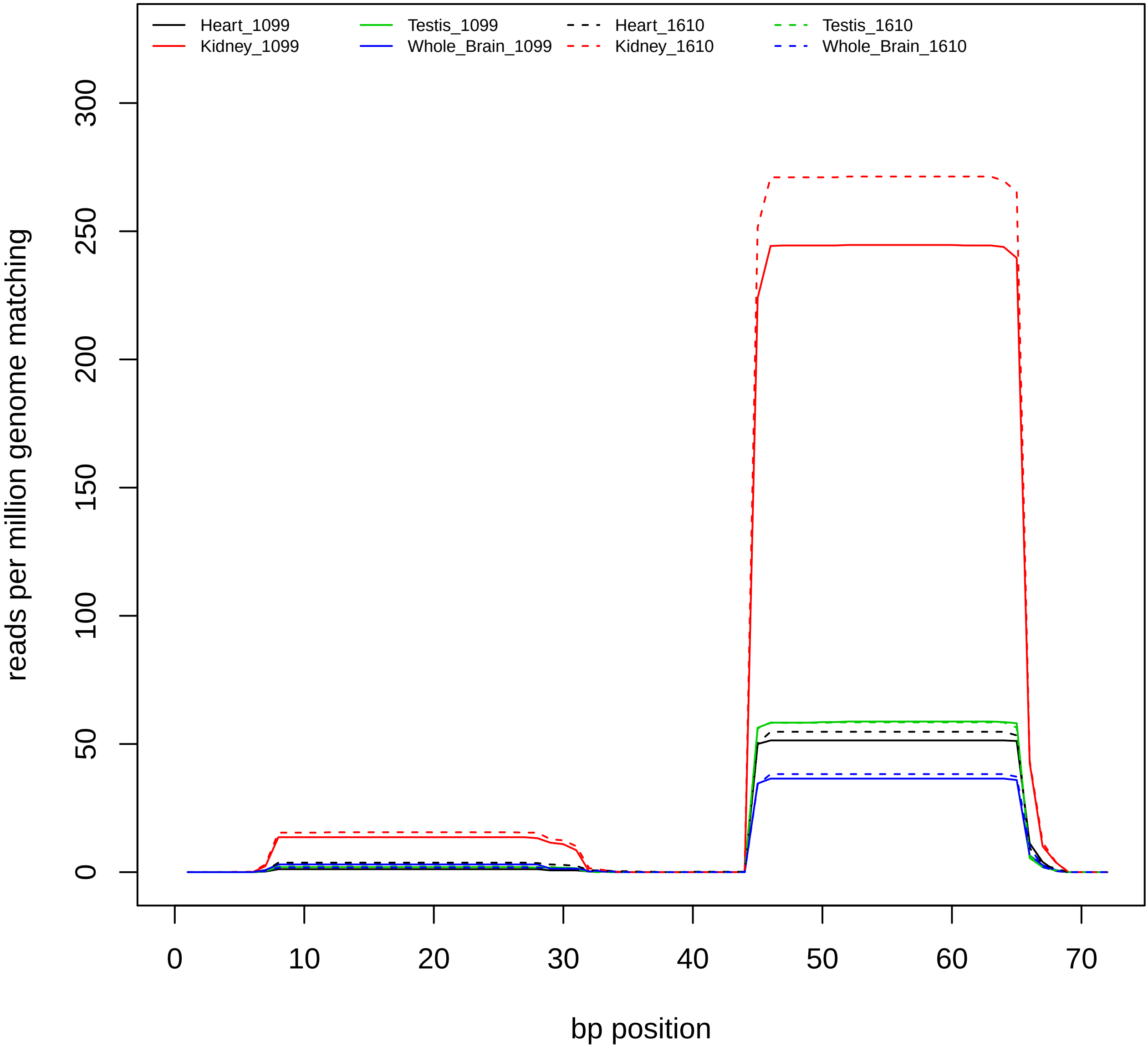

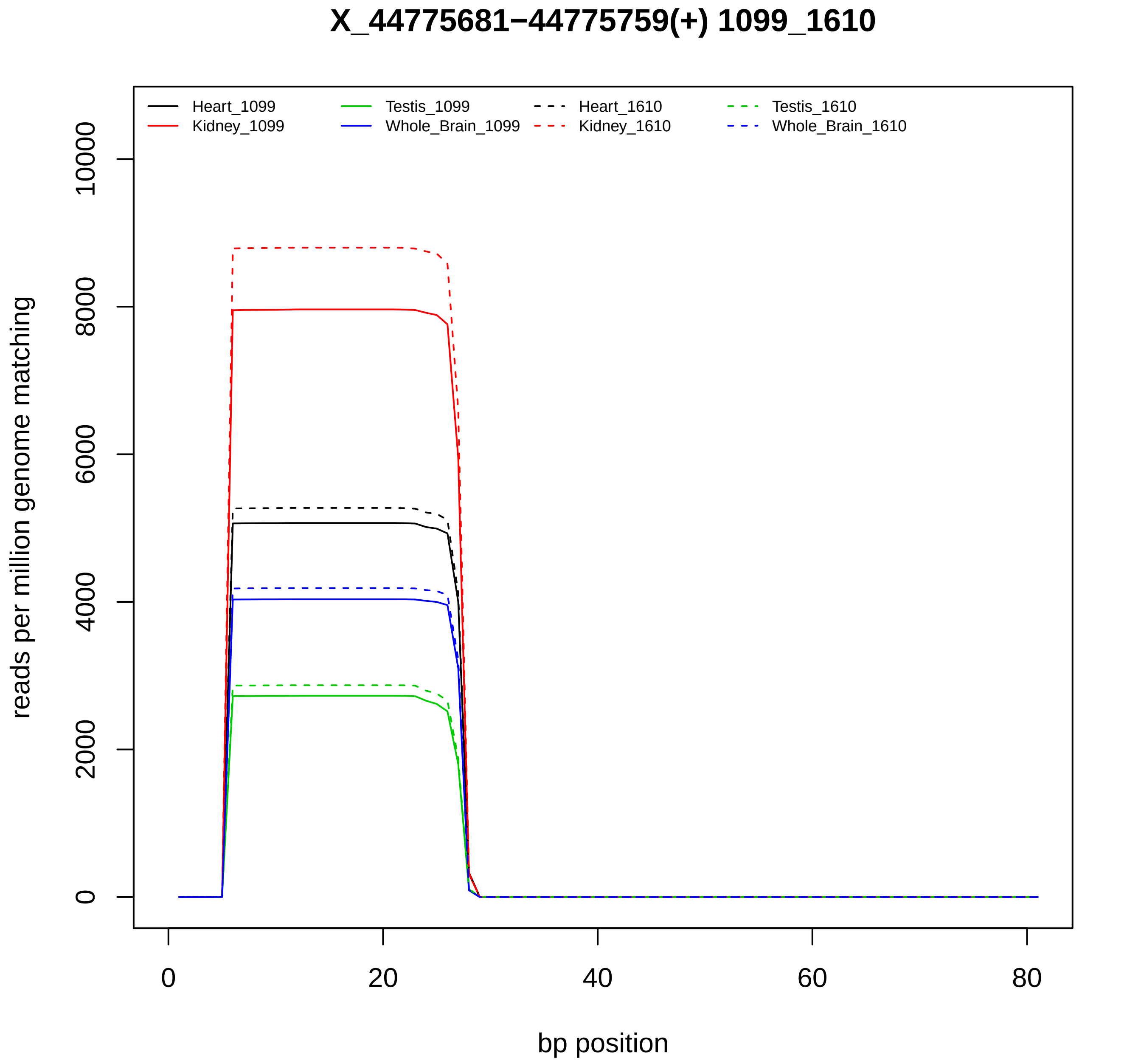

X_44775681−44775759(+) 1099_1610
Heart_1099
Kidney_1099
Testis_1099
Whole_Brain_1099
Heart_1610
Kidney_1610
Testis_1610
Whole_Brain_1610
reads per million genome matching
0
2000
4000
6000
8000
10000
bp position
0
20
40
60
80

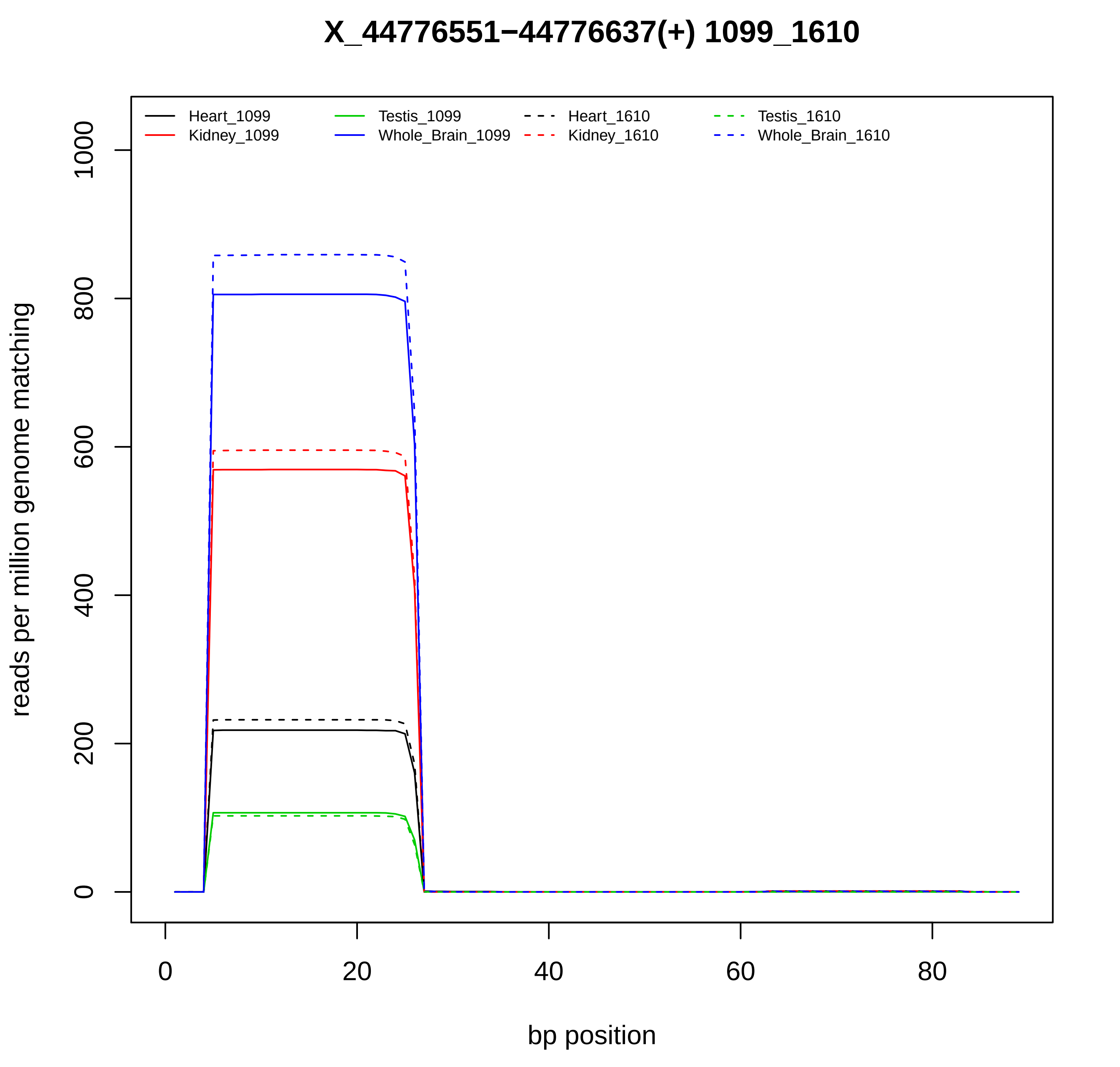

X_44776551−44776637(+) 1099_1610
Heart_1099
Kidney_1099
Testis_1099
Whole_Brain_1099
Heart_1610
Kidney_1610
Testis_1610
Whole_Brain_1610
reads per million genome matching
bp position
0
200
400
600
800
1000
0
20
40
60
80

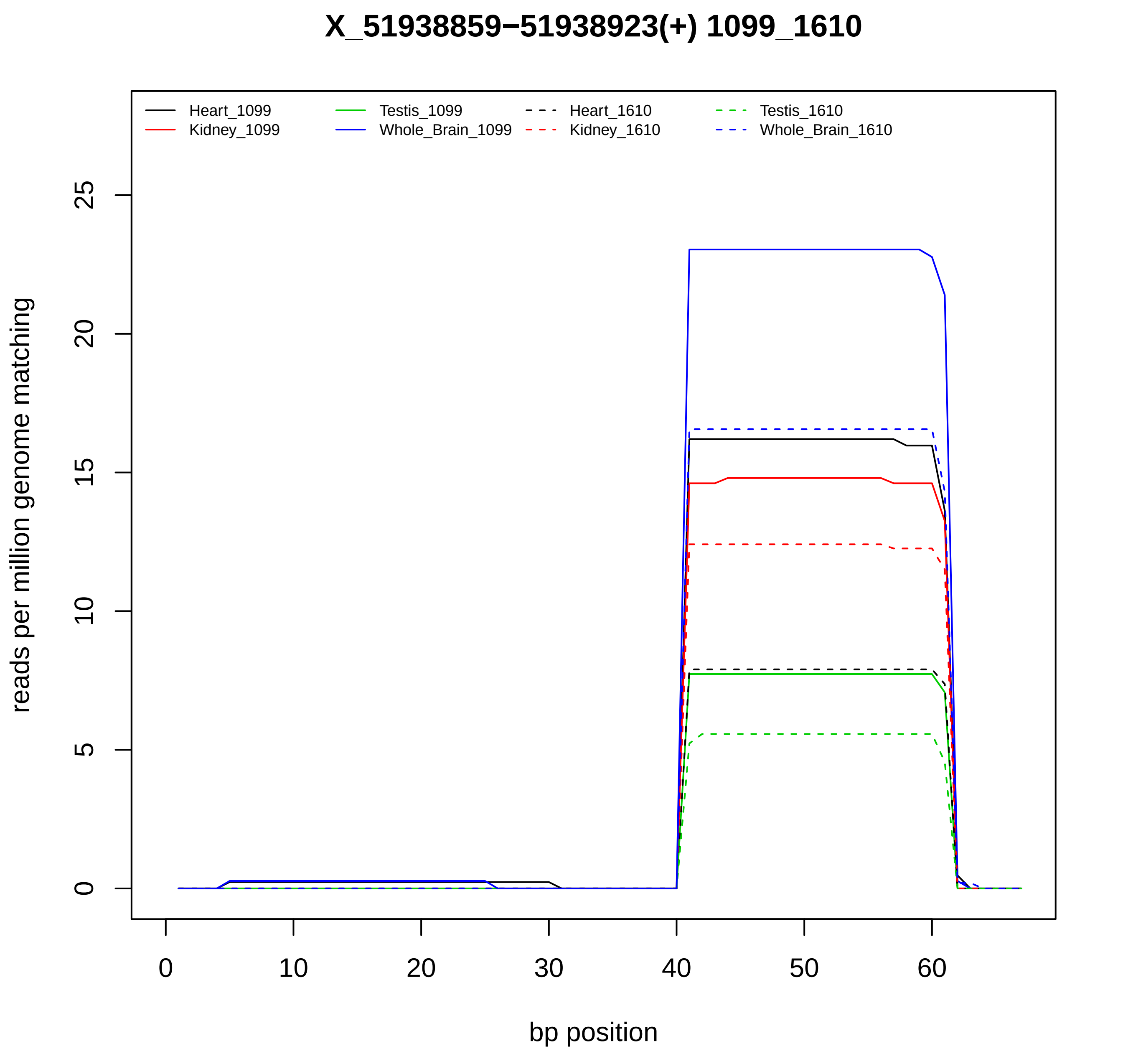

X_51938859−51938923(+) 1099_1610
Heart_1099
Kidney_1099
Testis_1099
Whole_Brain_1099
Heart_1610
Kidney_1610
Testis_1610
Whole_Brain_1610
reads per million genome matching
0
5
10
15
20
25
0
10
20
30
40
50
60
bp position

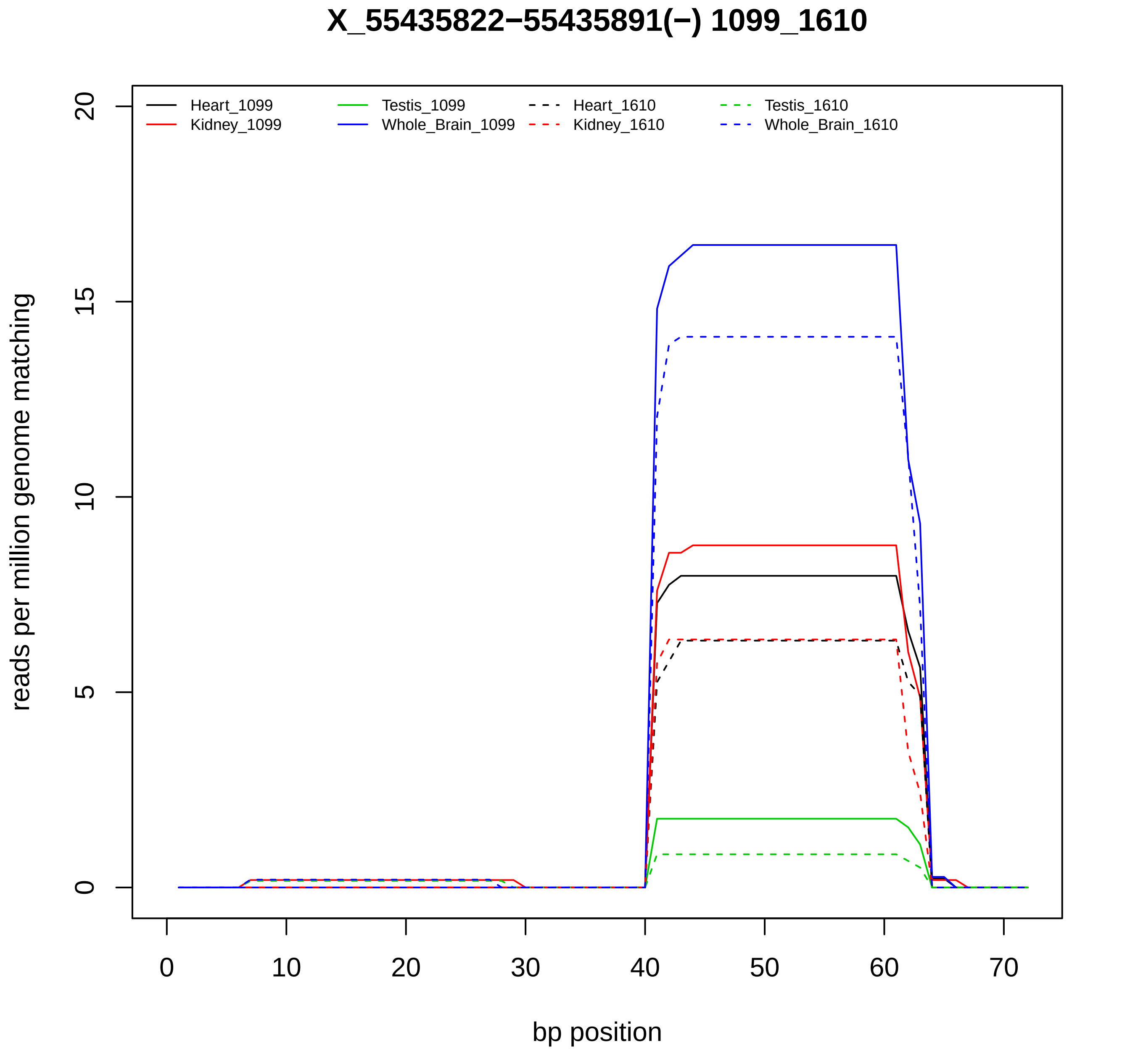

X_55435822−55435891(−) 1099_1610
Heart_1099
Kidney_1099
Testis_1099
Whole_Brain_1099
Heart_1610
Kidney_1610
Testis_1610
Whole_Brain_1610
reads per million genome matching
bp position
0
5
10
15
20
0
10
20
30
40
50
60
70

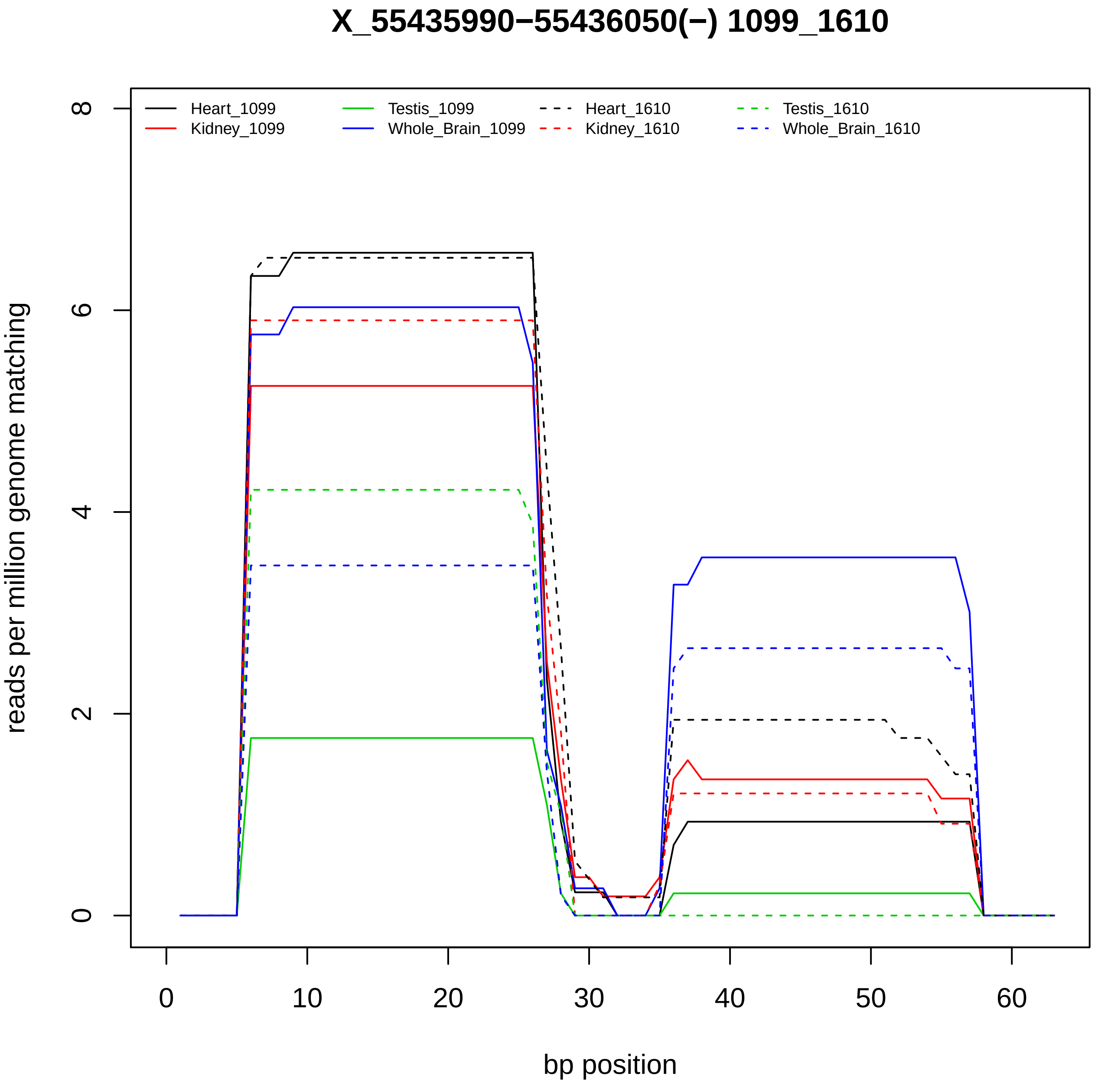
X_55435990−55436050(−) 1099_1610
Heart_1099
Kidney_1099
Testis_1099
Whole_Brain_1099
Heart_1610
Kidney_1610
Testis_1610
Whole_Brain_1610
reads per million genome matching
bp position
0
2
4
6
8
0
10
20
30
40
50
60

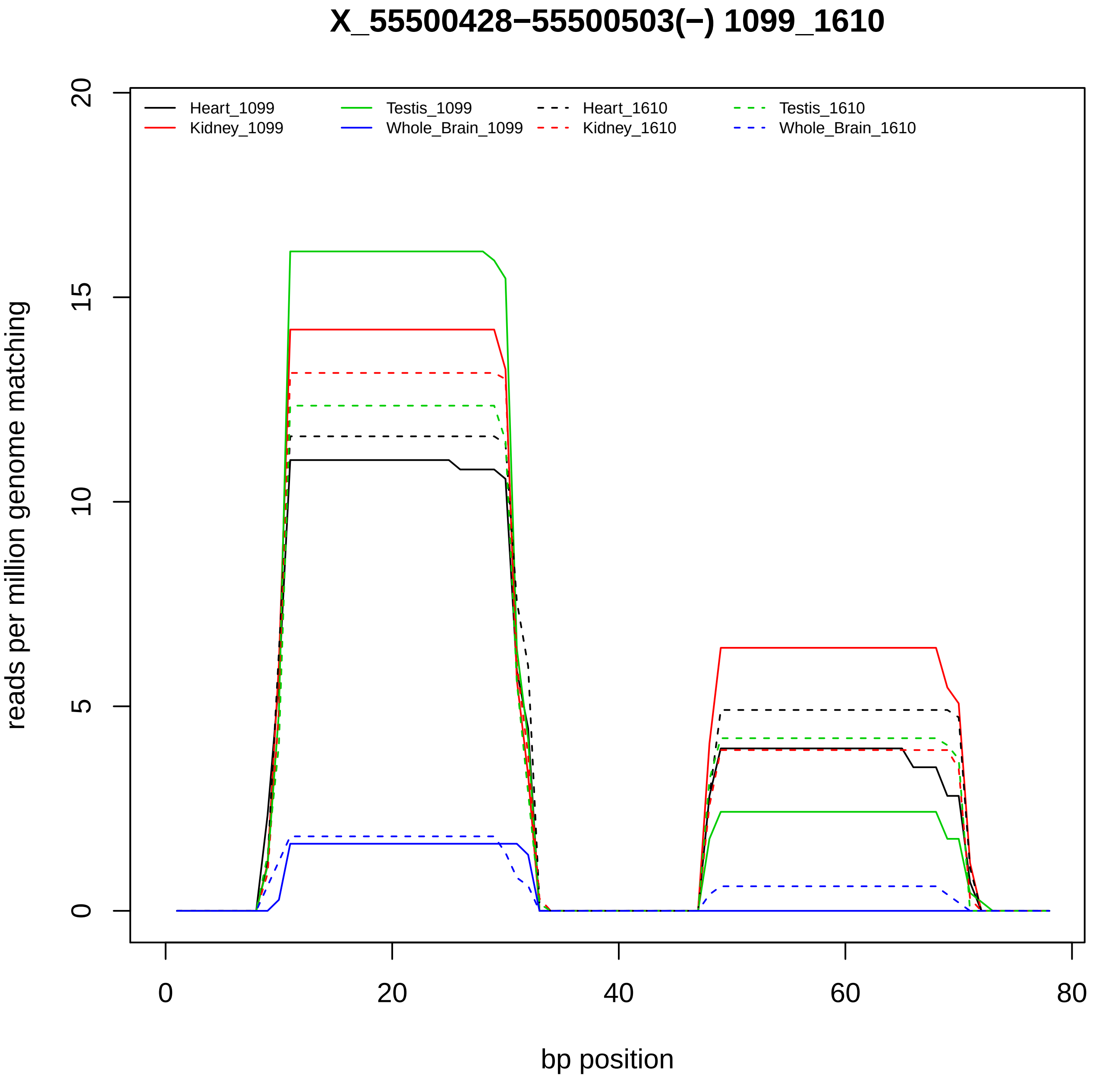

X_55500428−55500503(−) 1099_1610
Heart_1099
Kidney_1099
Testis_1099
Whole_Brain_1099
Heart_1610
Kidney_1610
Testis_1610
Whole_Brain_1610
reads per million genome matching
bp position
0
5
10
15
20
0
20
40
60
80

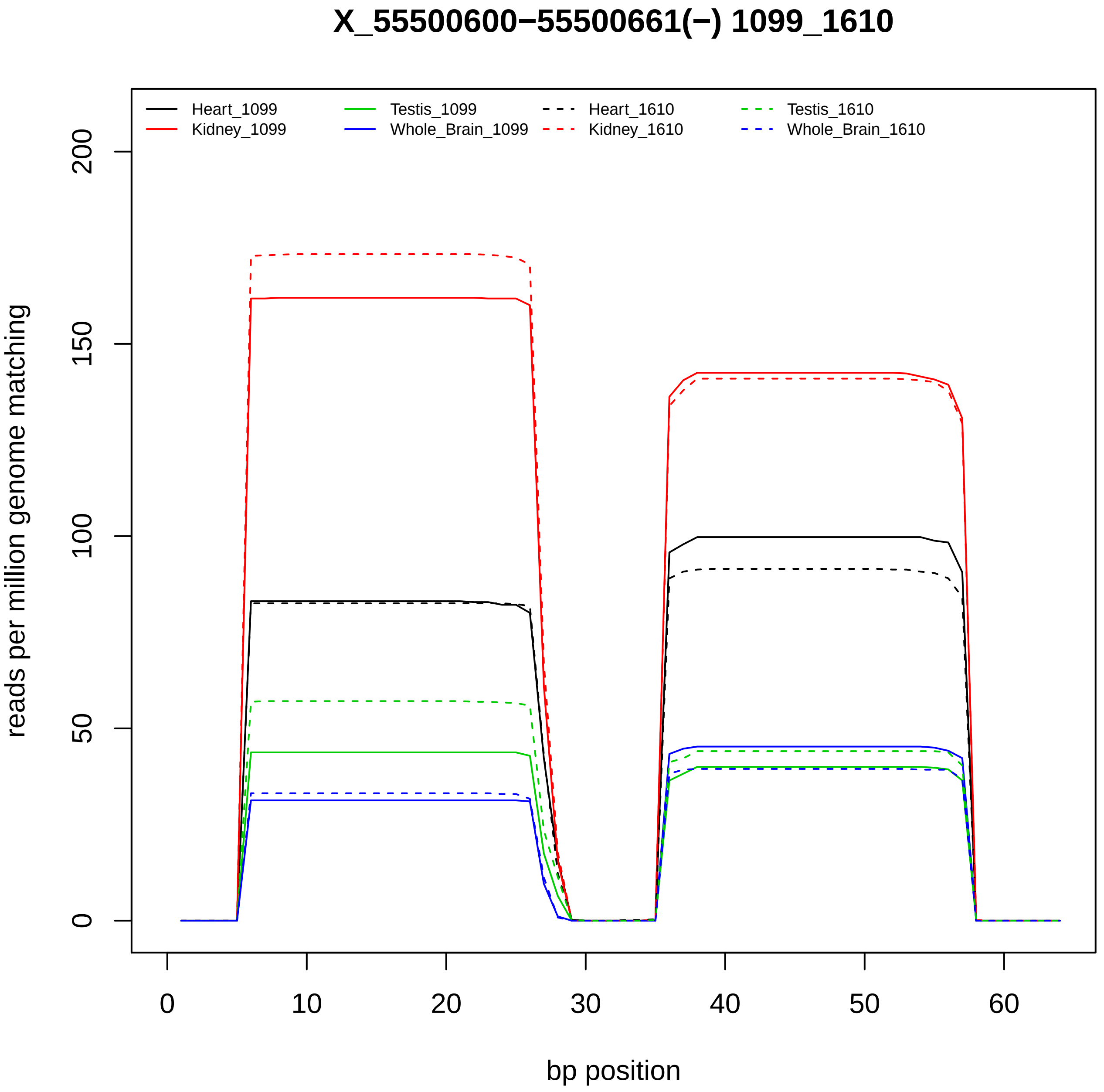

X_55500600−55500661(−) 1099_1610
Heart_1099
Kidney_1099
Testis_1099
Whole_Brain_1099
Heart_1610
Kidney_1610
Testis_1610
Whole_Brain_1610
reads per million genome matching
bp position
0
50
100
150
200
0
10
20
30
40
50
60

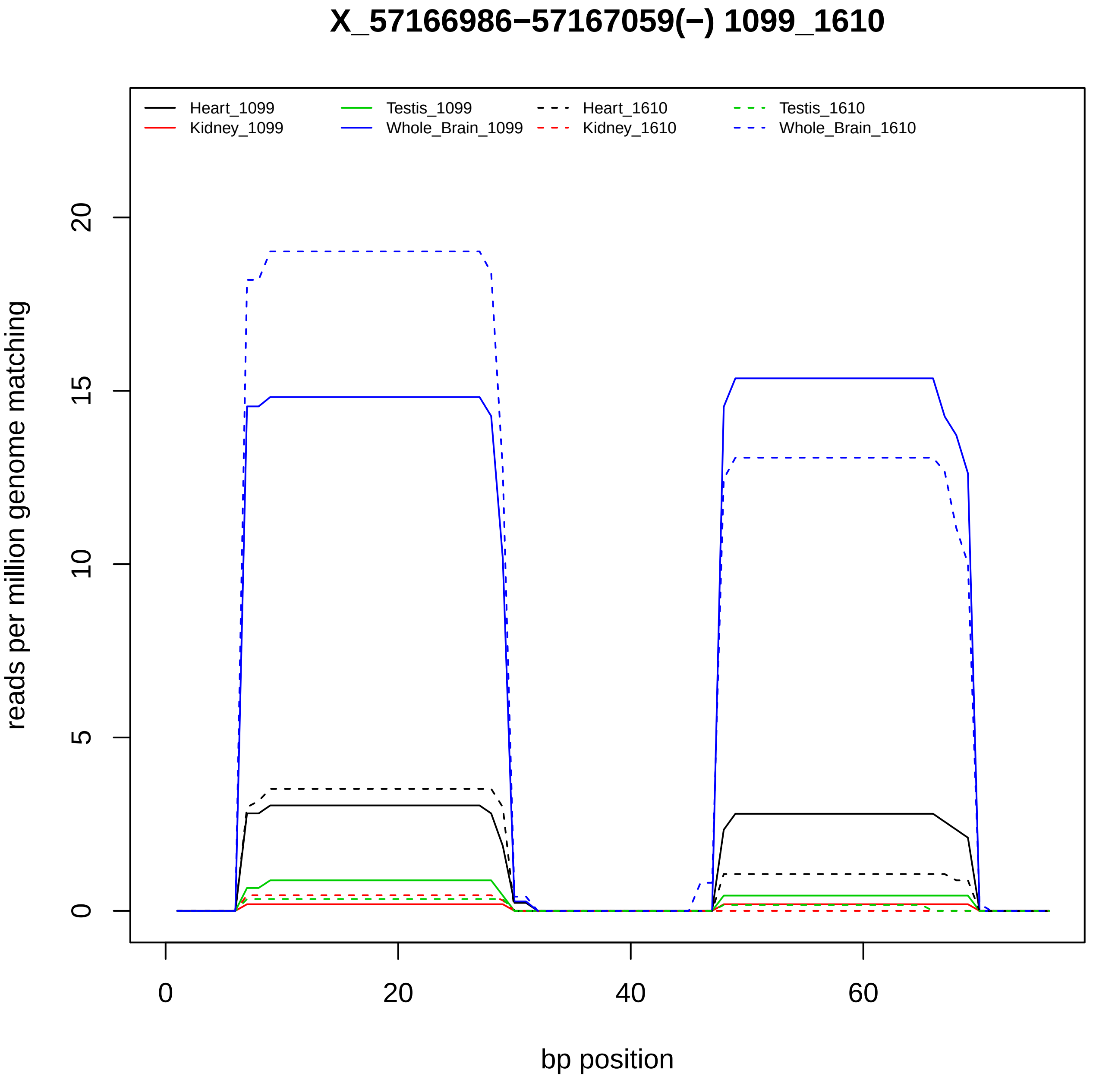

X_57166986−57167059(−) 1099_1610
Heart_1099
Kidney_1099
Testis_1099
Whole_Brain_1099
Heart_1610
Kidney_1610
Testis_1610
Whole_Brain_1610
reads per million genome matching
bp position
0
5
10
15
20
0
20
40
60

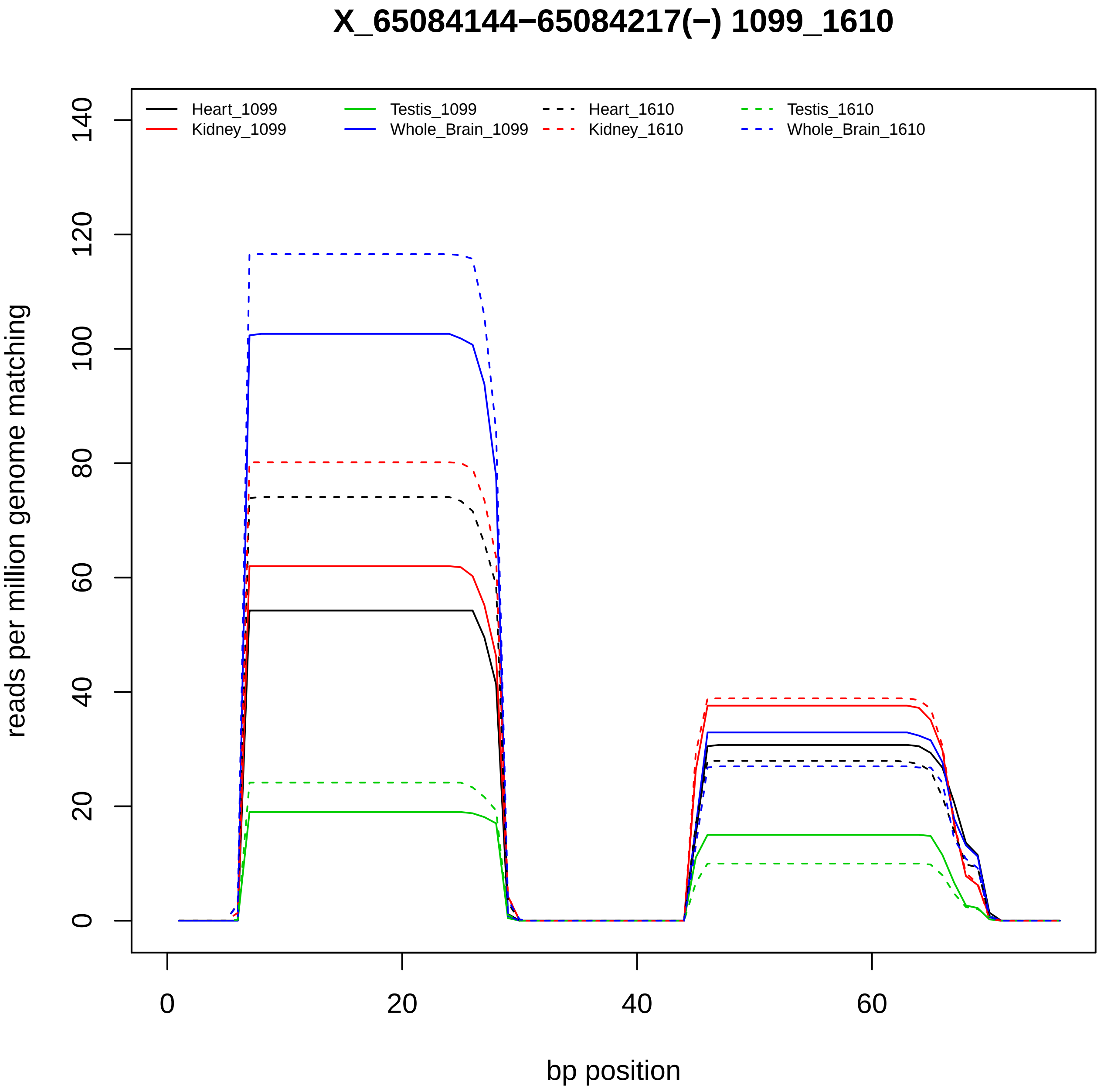

X_65084144−65084217(−) 1099_1610
Heart_1099
Kidney_1099
Testis_1099
Whole_Brain_1099
Heart_1610
Kidney_1610
Testis_1610
Whole_Brain_1610
reads per million genome matching
bp position
0
20
40
60
80
100
120
140

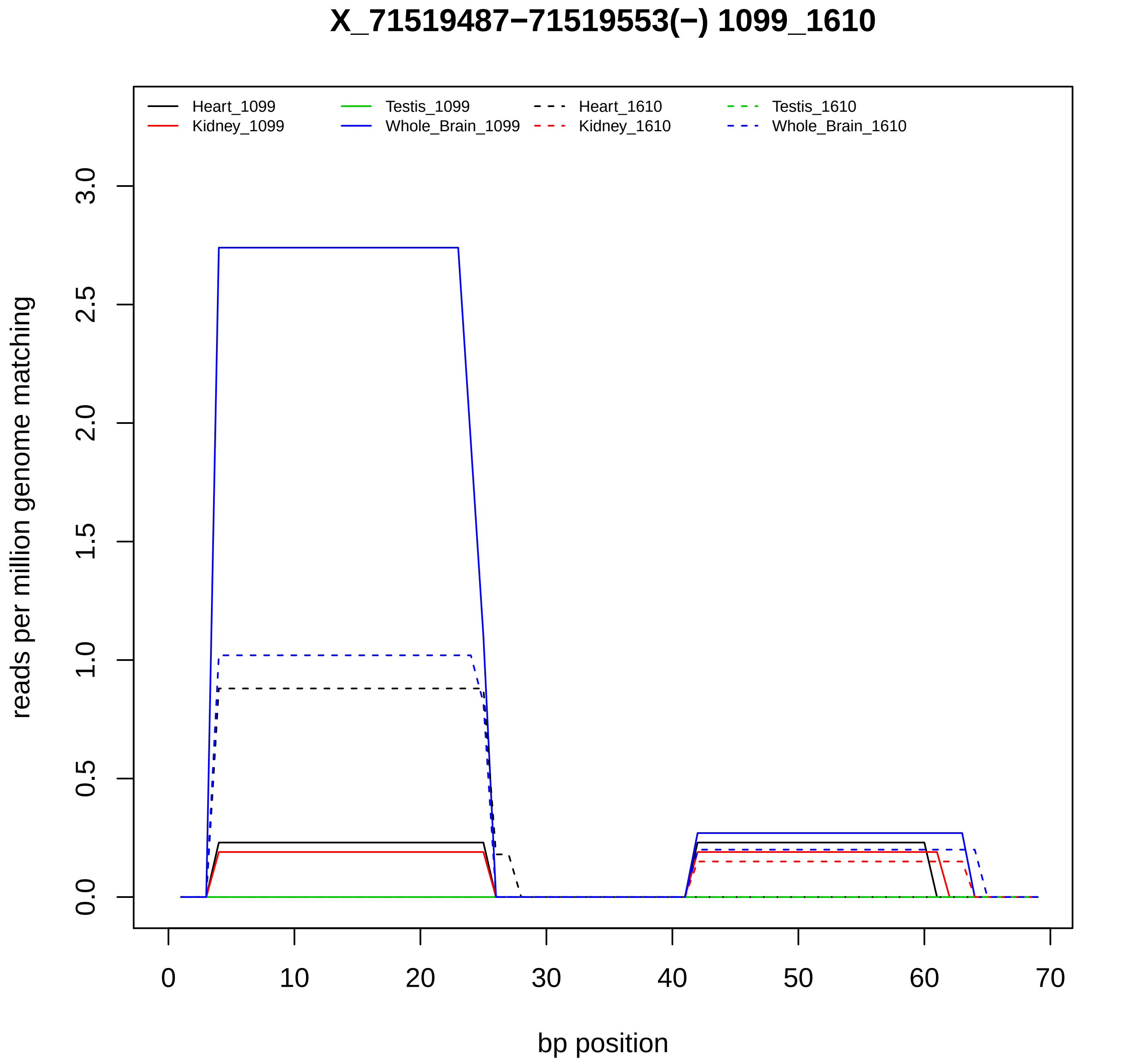

X_71519487−71519553(−) 1099_1610
Heart_1099
Kidney_1099
Testis_1099
Whole_Brain_1099
Heart_1610
Kidney_1610
Testis_1610
Whole_Brain_1610
reads per million genome matching
0.0
0.5
1.0
1.5
2.0
2.5
3.0
0
10
20
30
40
50
60
70
bp position

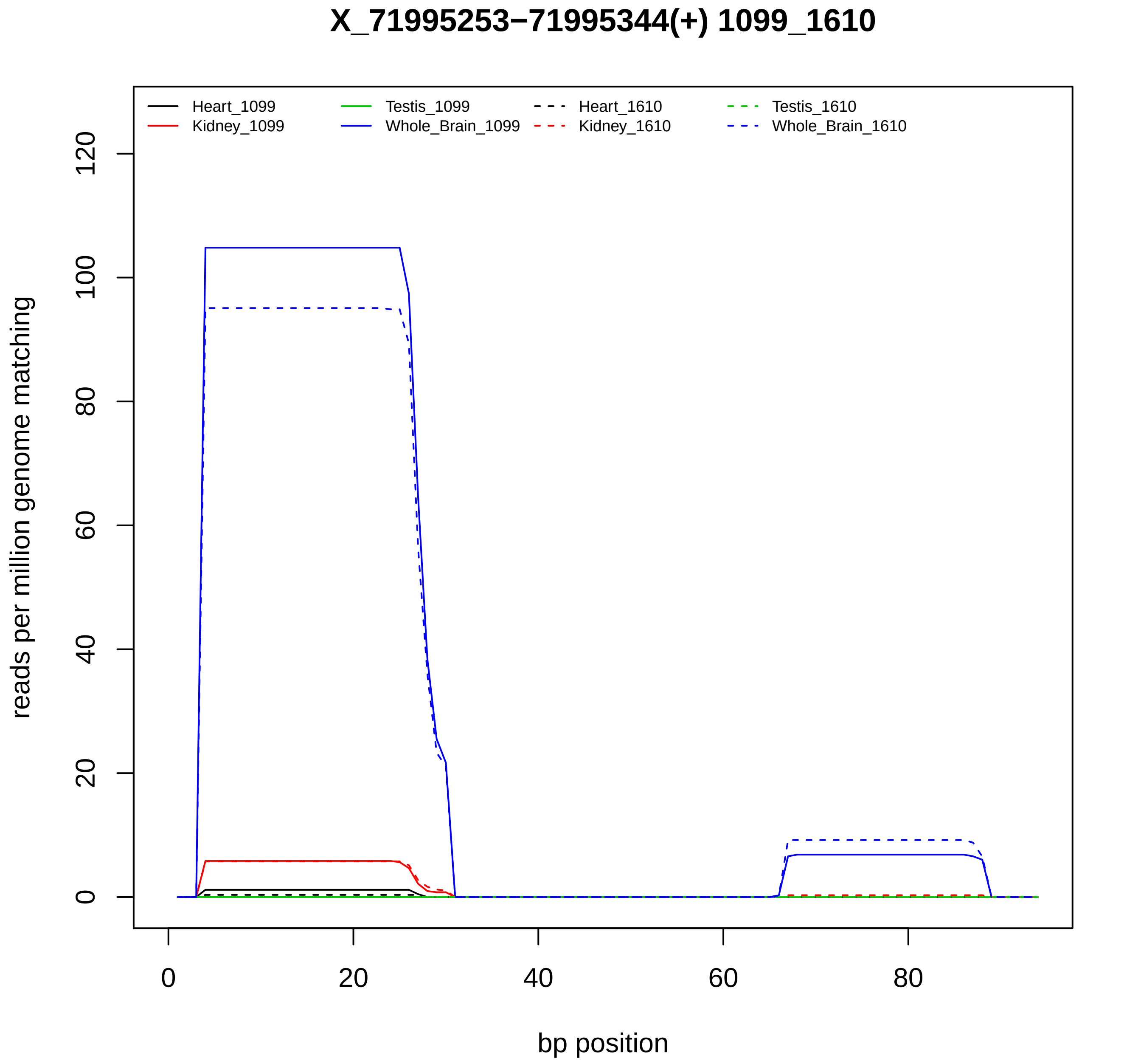

X_71995253−71995344(+) 1099_1610
Heart_1099
Kidney_1099
Testis_1099
Whole_Brain_1099
Heart_1610
Kidney_1610
Testis_1610
Whole_Brain_1610
reads per million genome matching
bp position
0
20
40
60
80
100
120

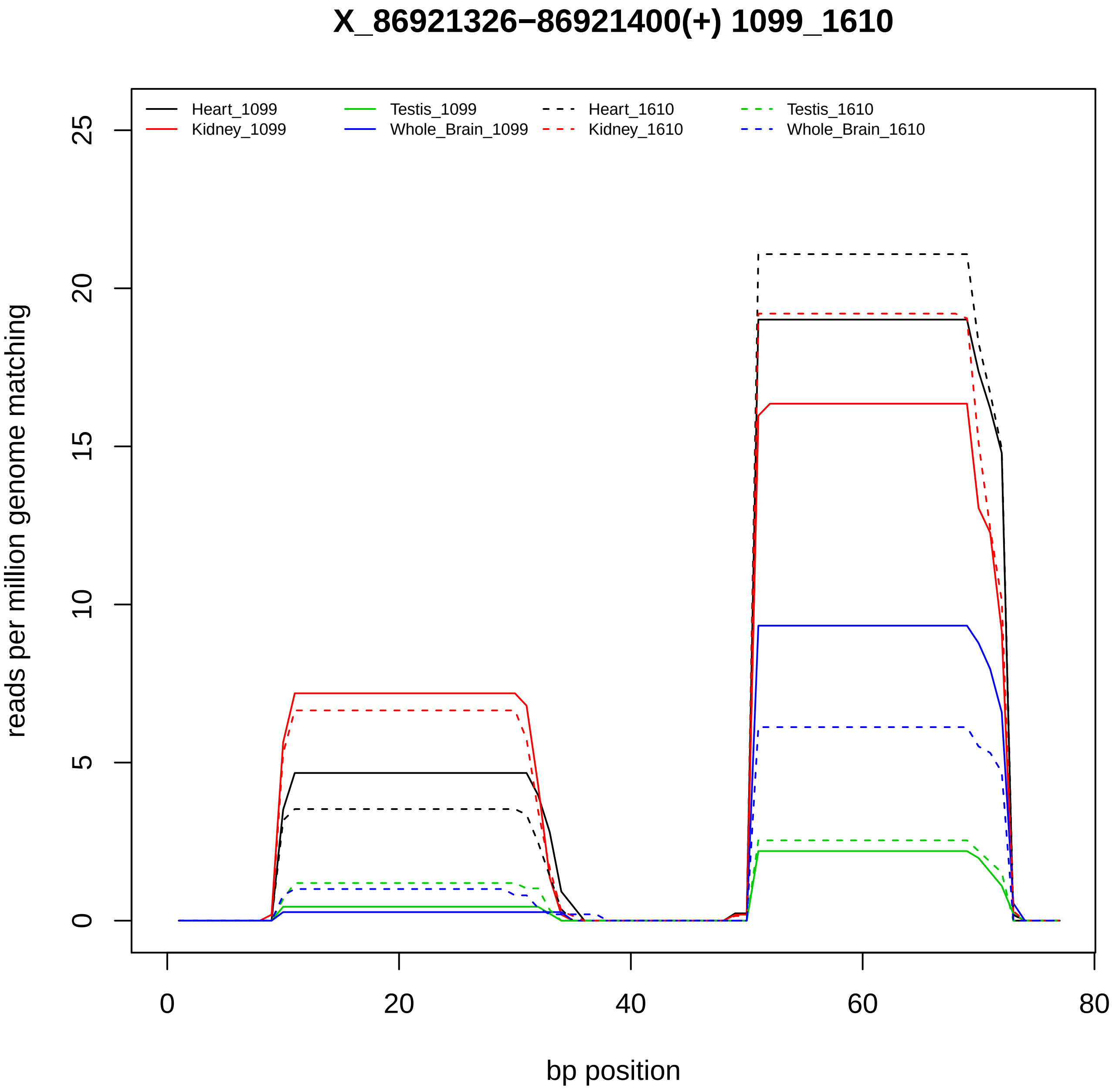
X_86921326−86921400(+) 1099_1610
Heart_1099
Kidney_1099
Testis_1099
Whole_Brain_1099
Heart_1610
Kidney_1610
Testis_1610
Whole_Brain_1610
reads per million genome matching
bp position
0
5
10
15
20
25
0
20
40
60
80

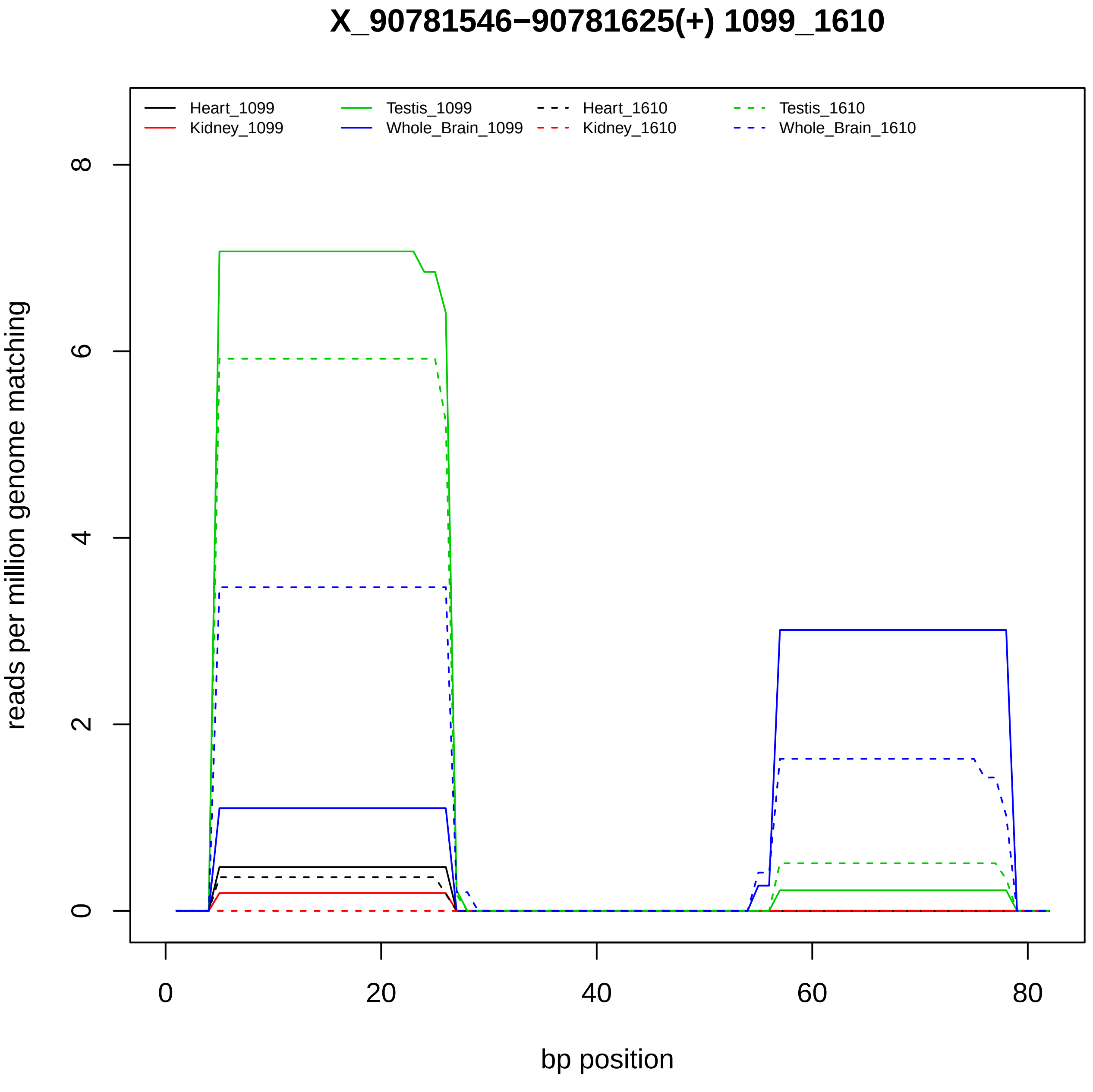
X_90781546−90781625(+) 1099_1610
Heart_1099
Kidney_1099
Testis_1099
Whole_Brain_1099
Heart_1610
Kidney_1610
Testis_1610
Whole_Brain_1610
reads per million genome matching
0
2
4
6
8
0
20
40
60
80
bp position

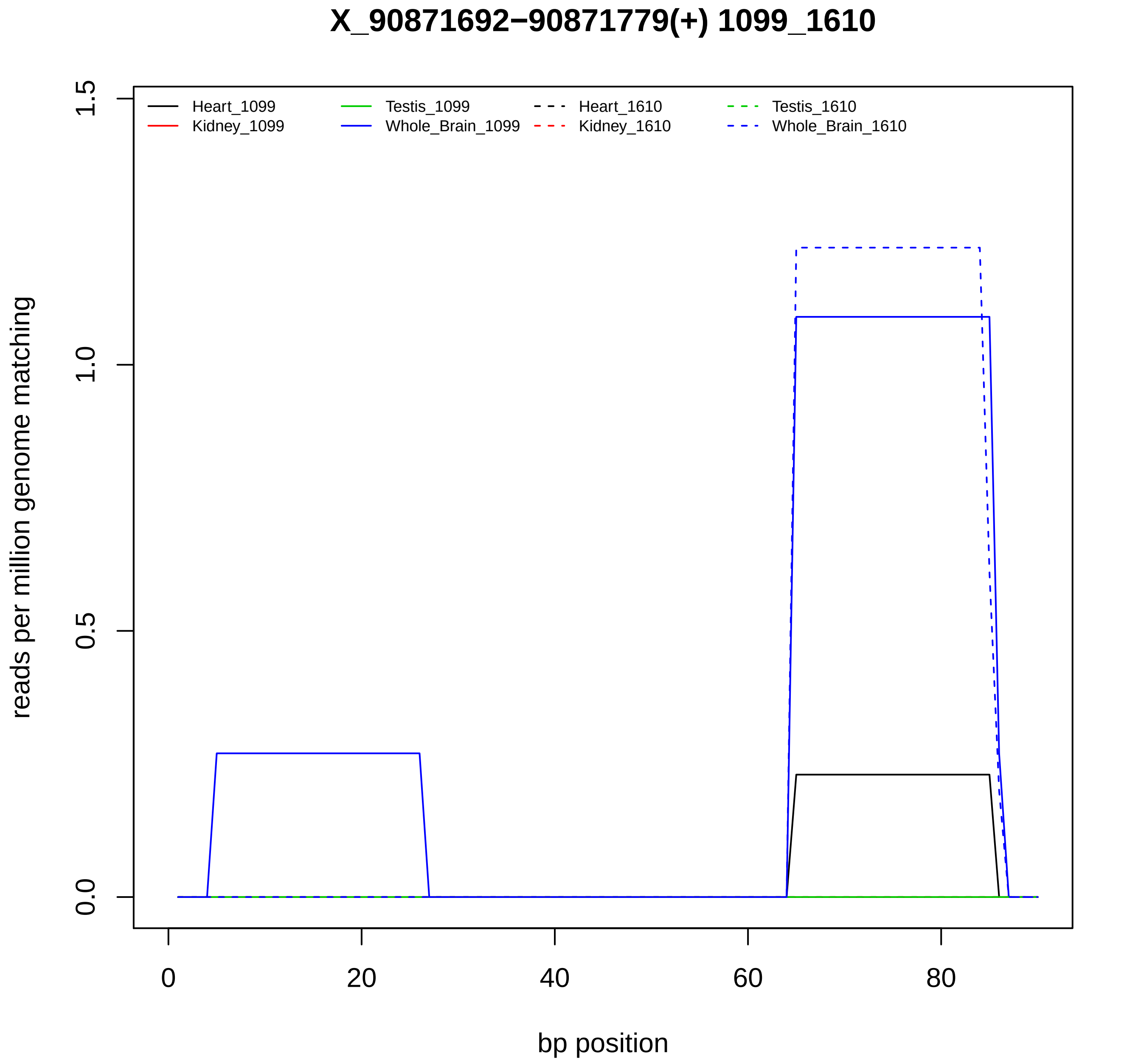

X_90871692−90871779(+) 1099_1610
Heart_1099
Kidney_1099
Testis_1099
Whole_Brain_1099
Heart_1610
Kidney_1610
Testis_1610
Whole_Brain_1610
reads per million genome matching
0.0
0.5
1.0
1.5
0
20
40
60
80
bp position

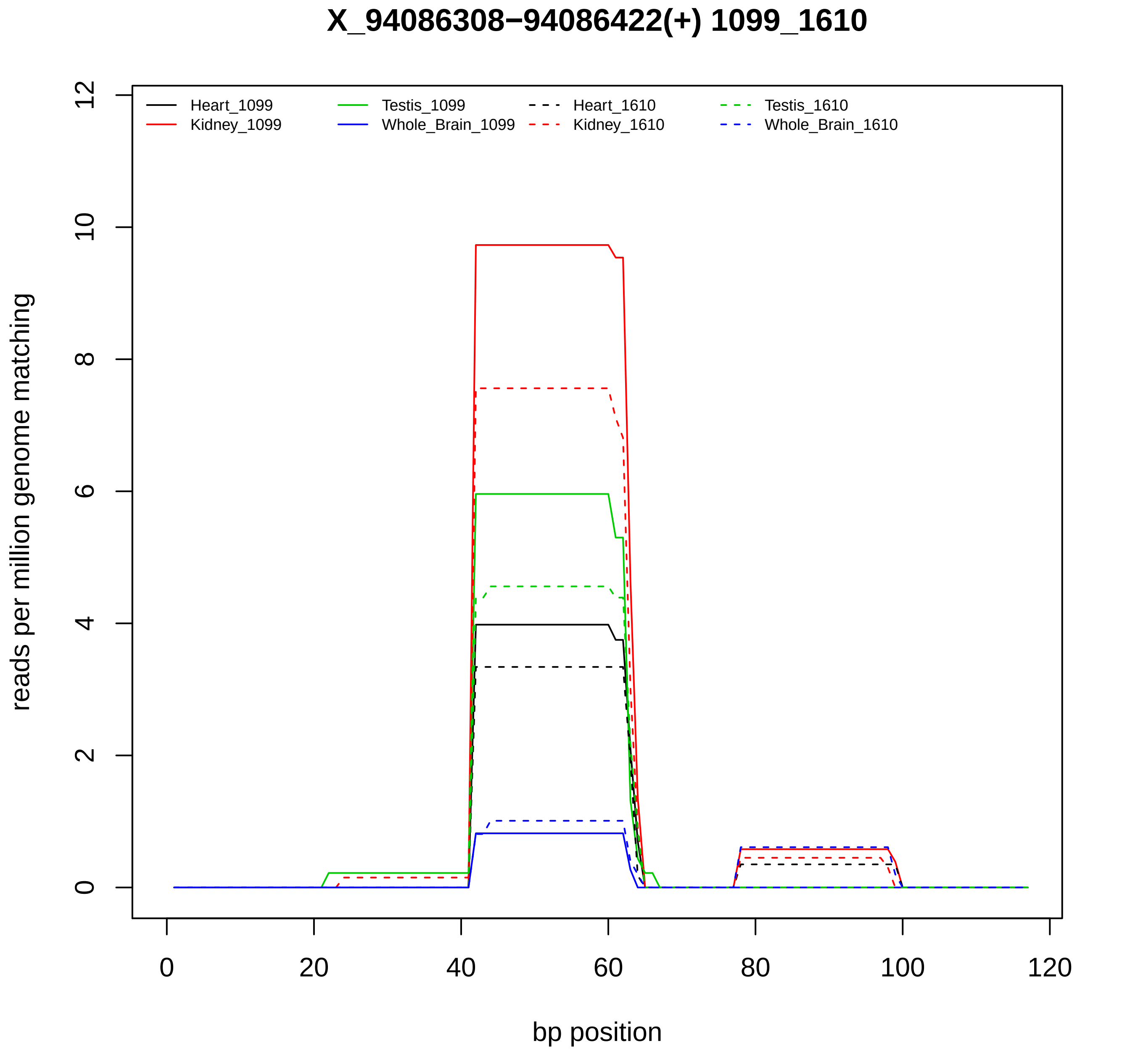

X_94086308−94086422(+) 1099_1610
Heart_1099
Kidney_1099
Testis_1099
Whole_Brain_1099
Heart_1610
Kidney_1610
Testis_1610
Whole_Brain_1610
reads per million genome matching
bp position
0
2
4
6
8
10
12
0
20
40
60
80
100
120

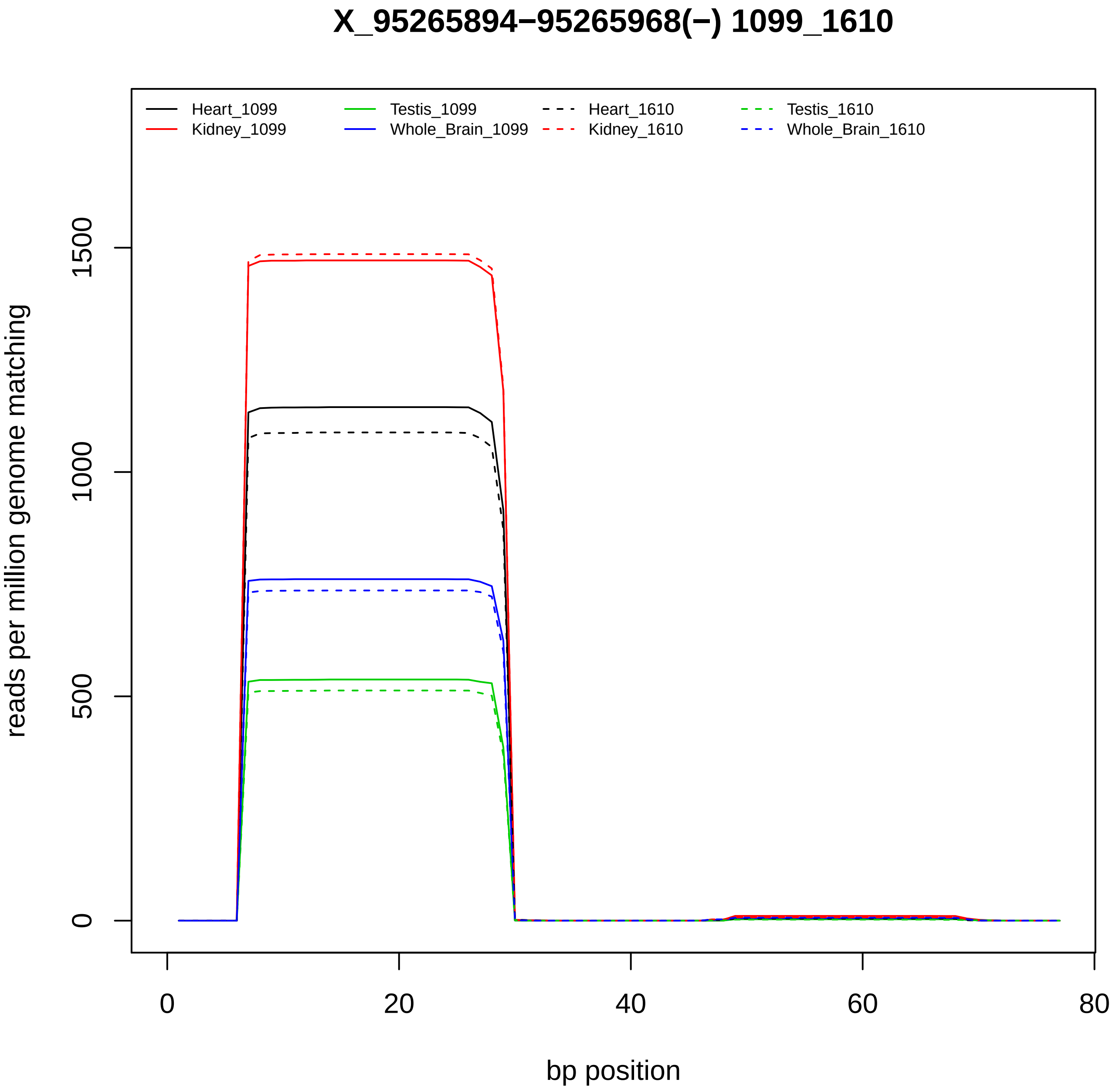

X_95265894−95265968(−) 1099_1610
Heart_1099
Kidney_1099
Testis_1099
Whole_Brain_1099
Heart_1610
Kidney_1610
Testis_1610
Whole_Brain_1610
reads per million genome matching
0
500
1000
1500
bp position
0
20
40
60
80

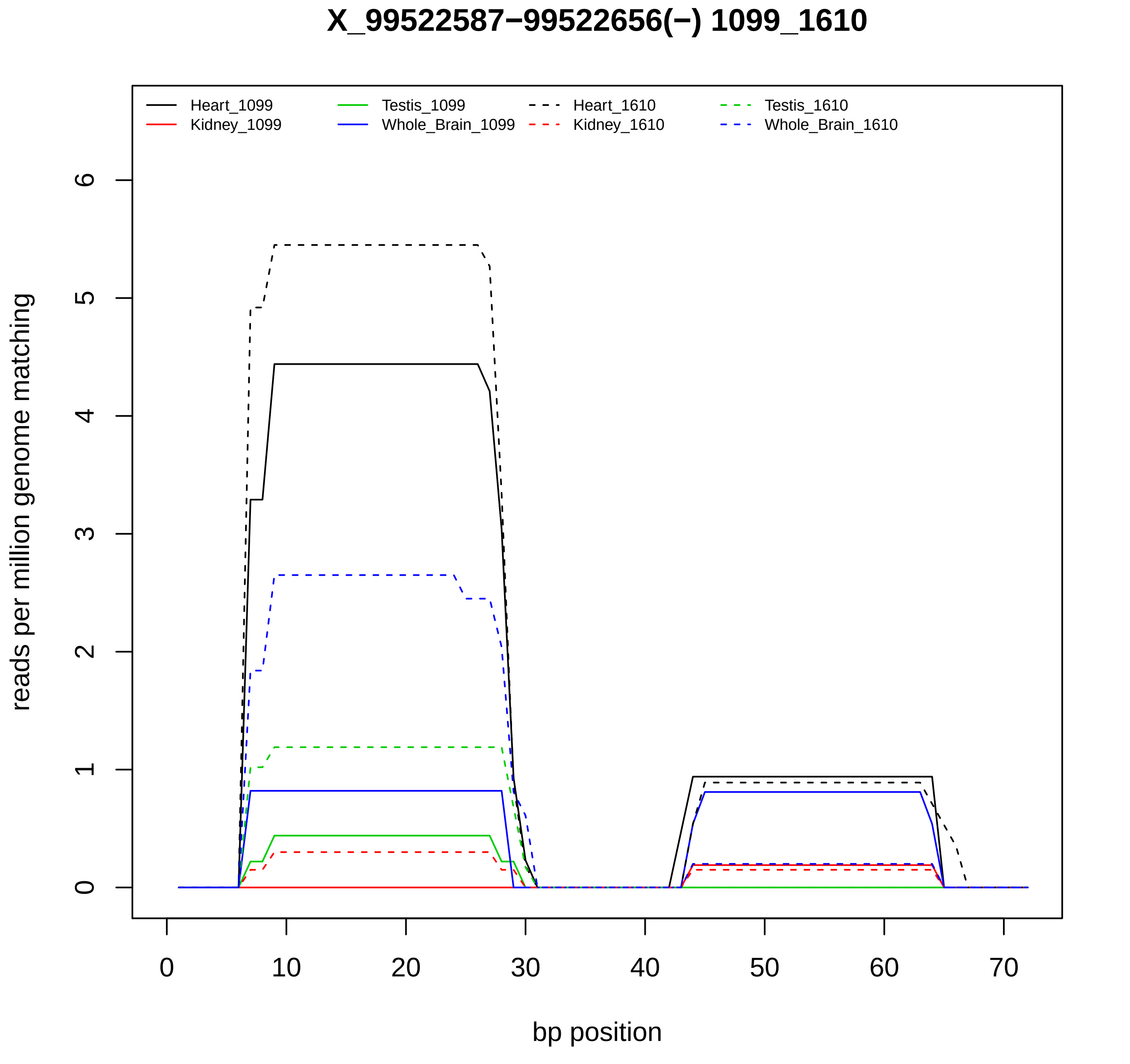

X_99522587−99522656(−) 1099_1610
Heart_1099
Kidney_1099
Testis_1099
Whole_Brain_1099
Heart_1610
Kidney_1610
Testis_1610
Whole_Brain_1610
reads per million genome matching
bp position
0
1
2
3
4
5
6
0
10
20
30
40
50
60
70

Fig. S7

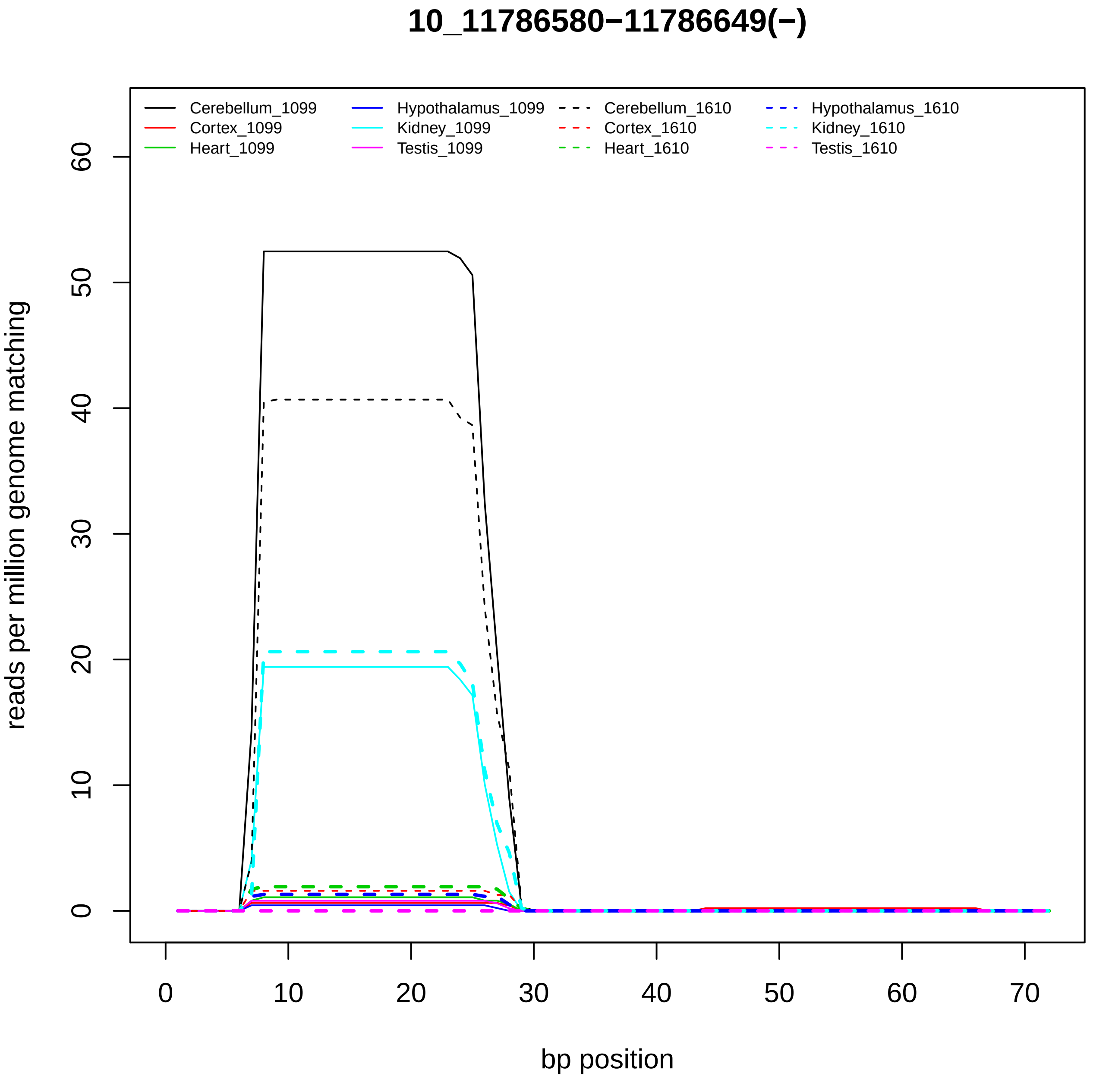

10_11786580−11786649(−)
Cerebellum_1099
Cortex_1099
Heart_1099
Hypothalamus_1099
Kidney_1099
Testis_1099
Cerebellum_1610
Cortex_1610
Heart_1610
Hypothalamus_1610
Kidney_1610
Testis_1610
reads per million genome matching
bp position
0
10
20
30
40
50
60
70

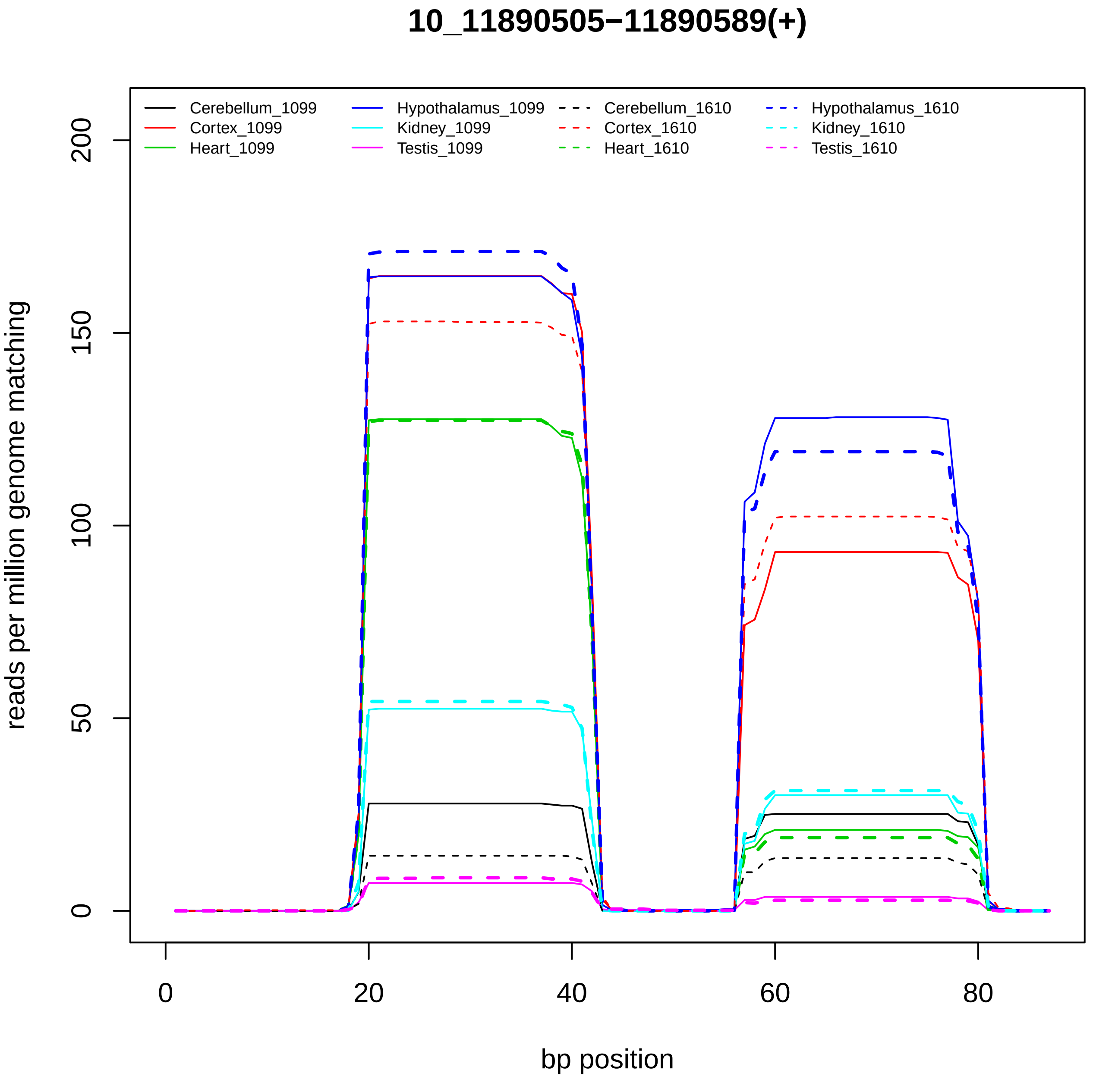
10_11890505−11890589(+)
Cerebellum_1099
Cortex_1099
Heart_1099
Hypothalamus_1099
Kidney_1099
Testis_1099
Cerebellum_1610
Cortex_1610
Heart_1610
Hypothalamus_1610
Kidney_1610
Testis_1610
reads per million genome matching
0
50
100
150
200
bp position
0
20
40
60
80

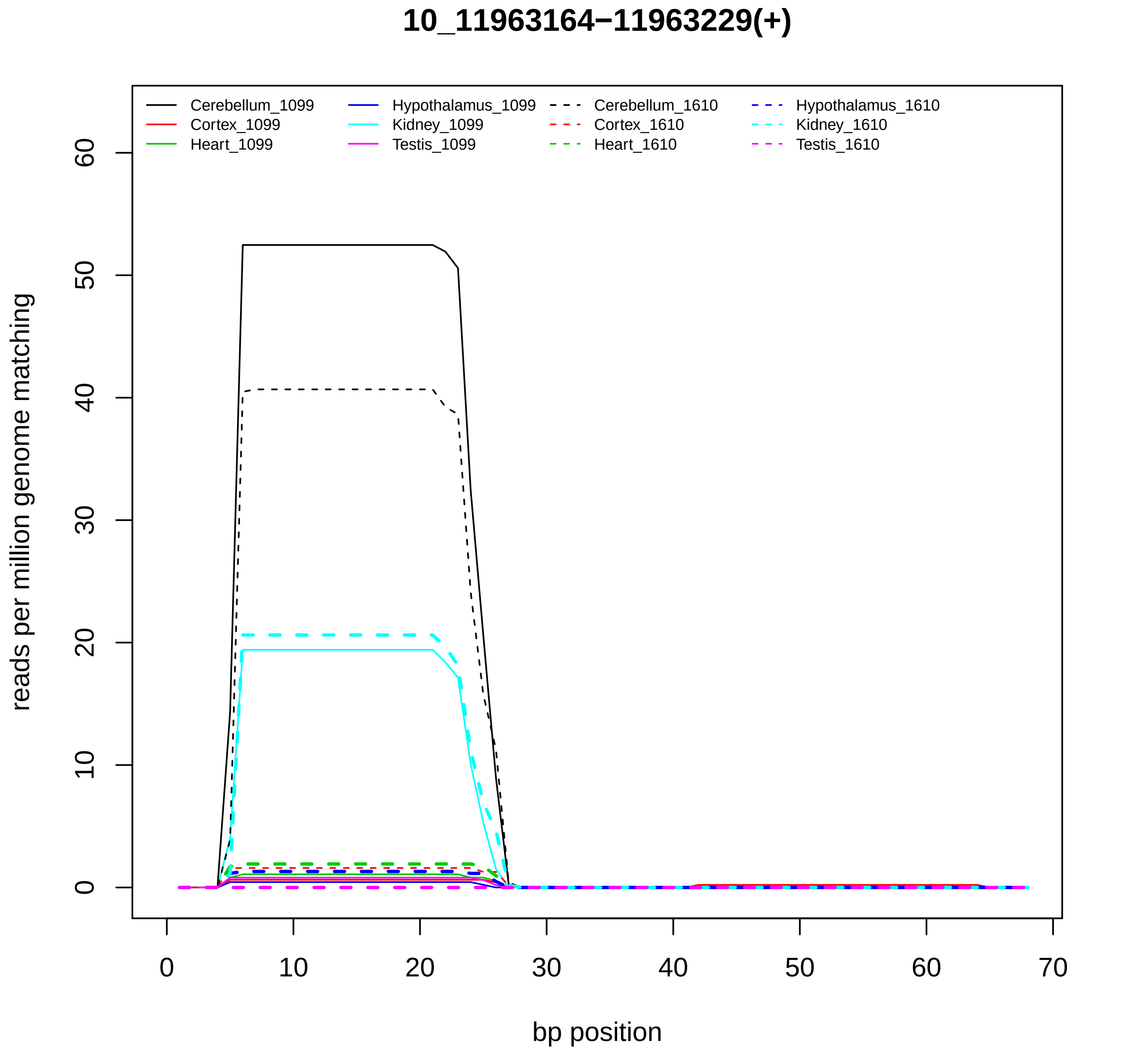
10_11963164−11963229(+)
Cerebellum_1099
Cortex_1099
Heart_1099
Hypothalamus_1099
Kidney_1099
Testis_1099
Cerebellum_1610
Cortex_1610
Heart_1610
Hypothalamus_1610
Kidney_1610
Testis_1610
reads per million genome matching
bp position
0
10
20
30
40
50
60
70

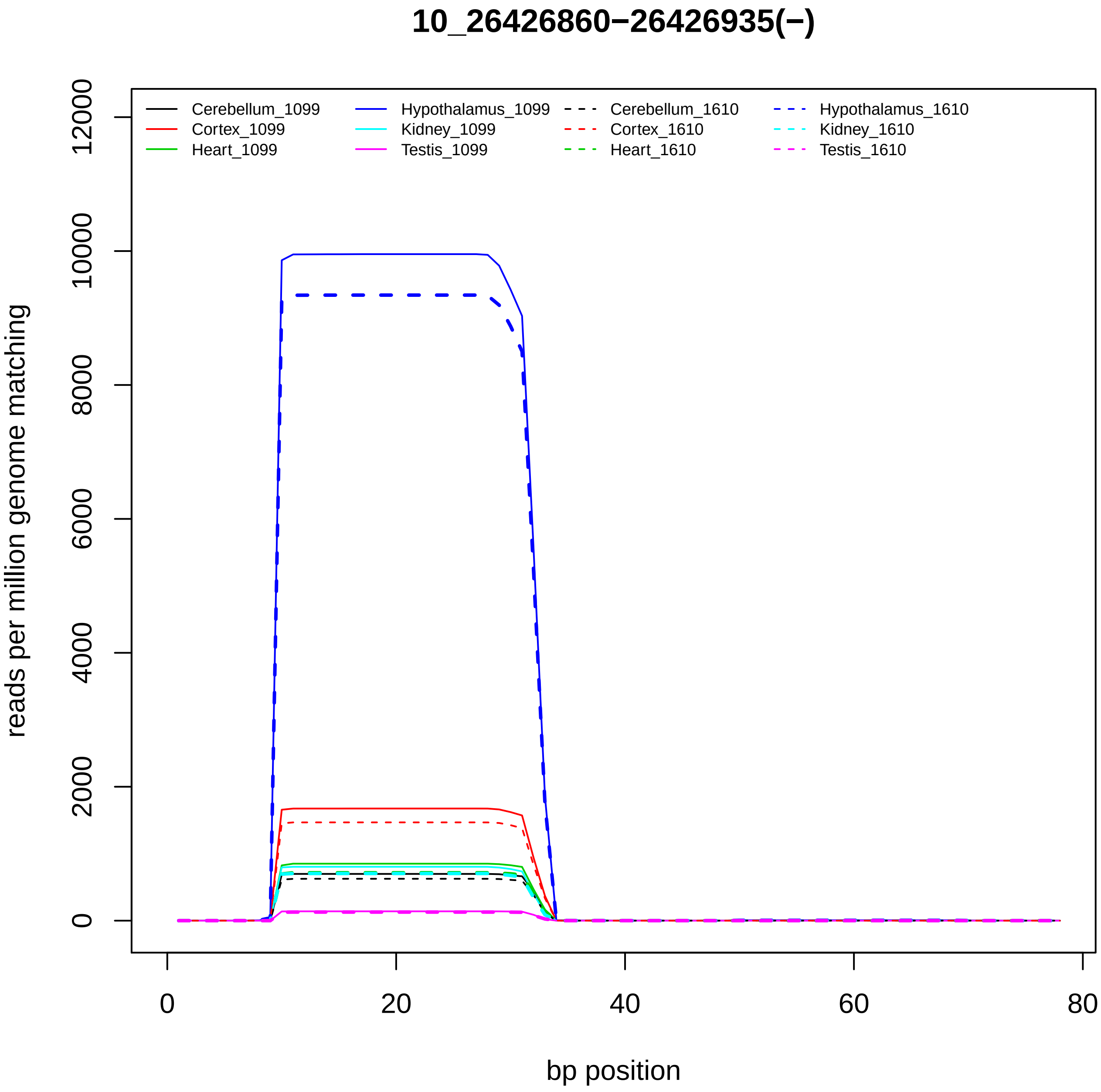
10_26426860−26426935(−)
Cerebellum_1099
Cortex_1099
Heart_1099
Hypothalamus_1099
Kidney_1099
Testis_1099
Cerebellum_1610
Cortex_1610
Heart_1610
Hypothalamus_1610
Kidney_1610
Testis_1610
reads per million genome matching
0
2000
4000
6000
8000
10000
12000
0
20
40
60
80
bp position

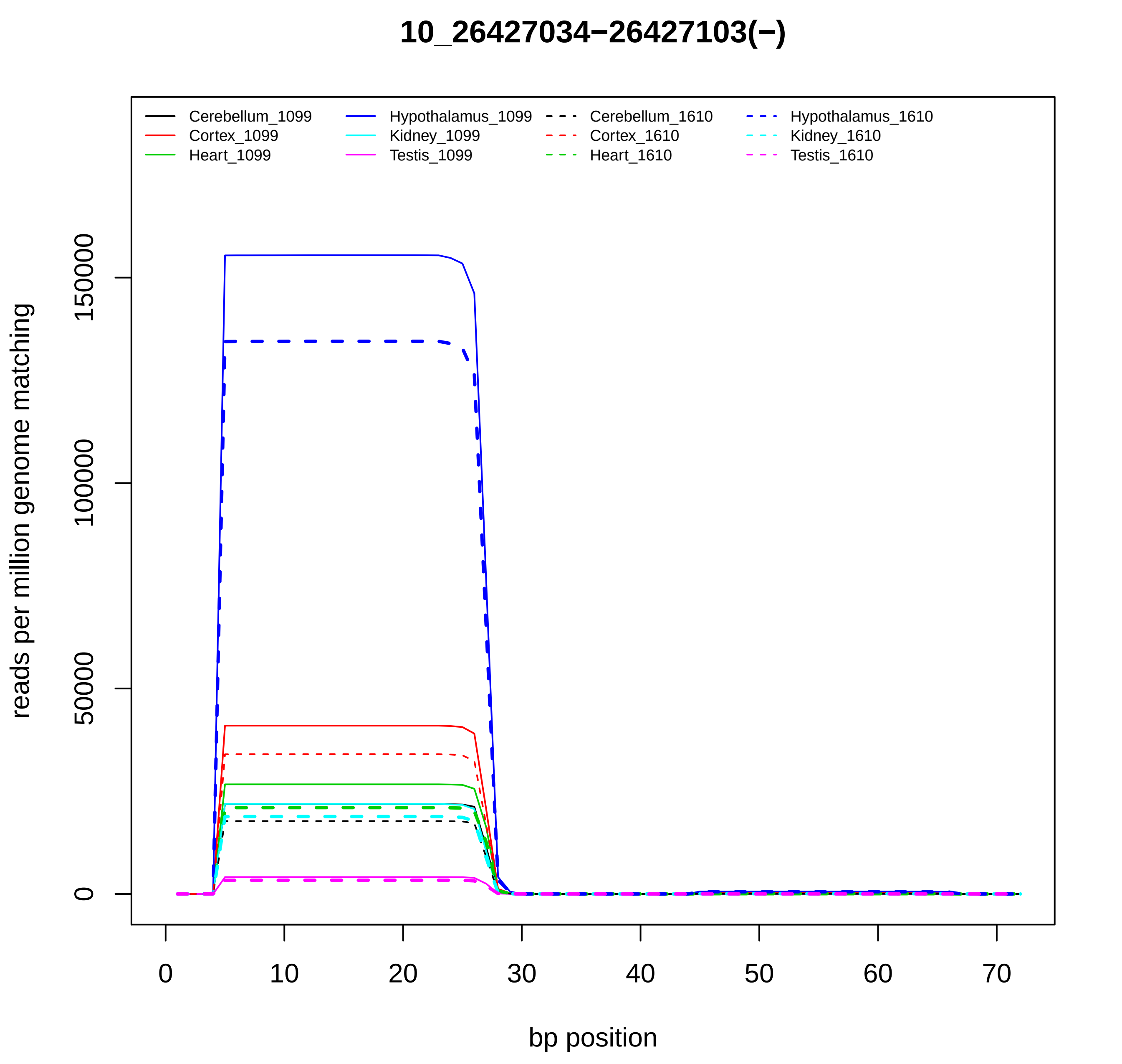

10_26427034−26427103(−)
Cerebellum_1099
Cortex_1099
Heart_1099
Hypothalamus_1099
Kidney_1099
Testis_1099
Cerebellum_1610
Cortex_1610
Heart_1610
Hypothalamus_1610
Kidney_1610
Testis_1610
reads per million genome matching
0
50000
100000
150000
0
10
20
30
40
50
60
70
bp position

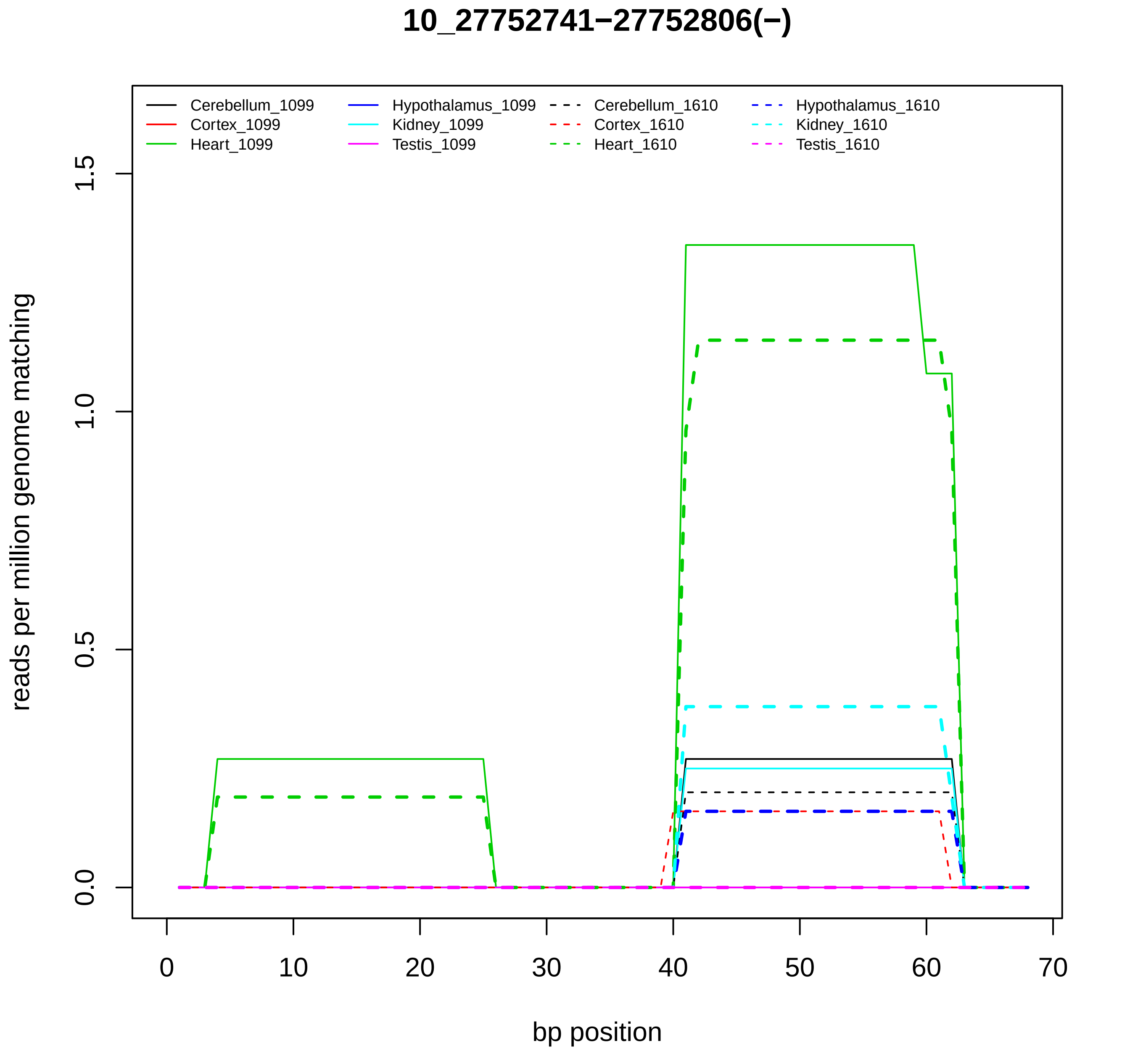

10_27752741−27752806(−)
Cerebellum_1099
Cortex_1099
Heart_1099
Hypothalamus_1099
Kidney_1099
Testis_1099
Cerebellum_1610
Cortex_1610
Heart_1610
Hypothalamus_1610
Kidney_1610
Testis_1610
reads per million genome matching
bp position
0.0
0.5
1.0
1.5
0
10
20
30
40
50
60
70

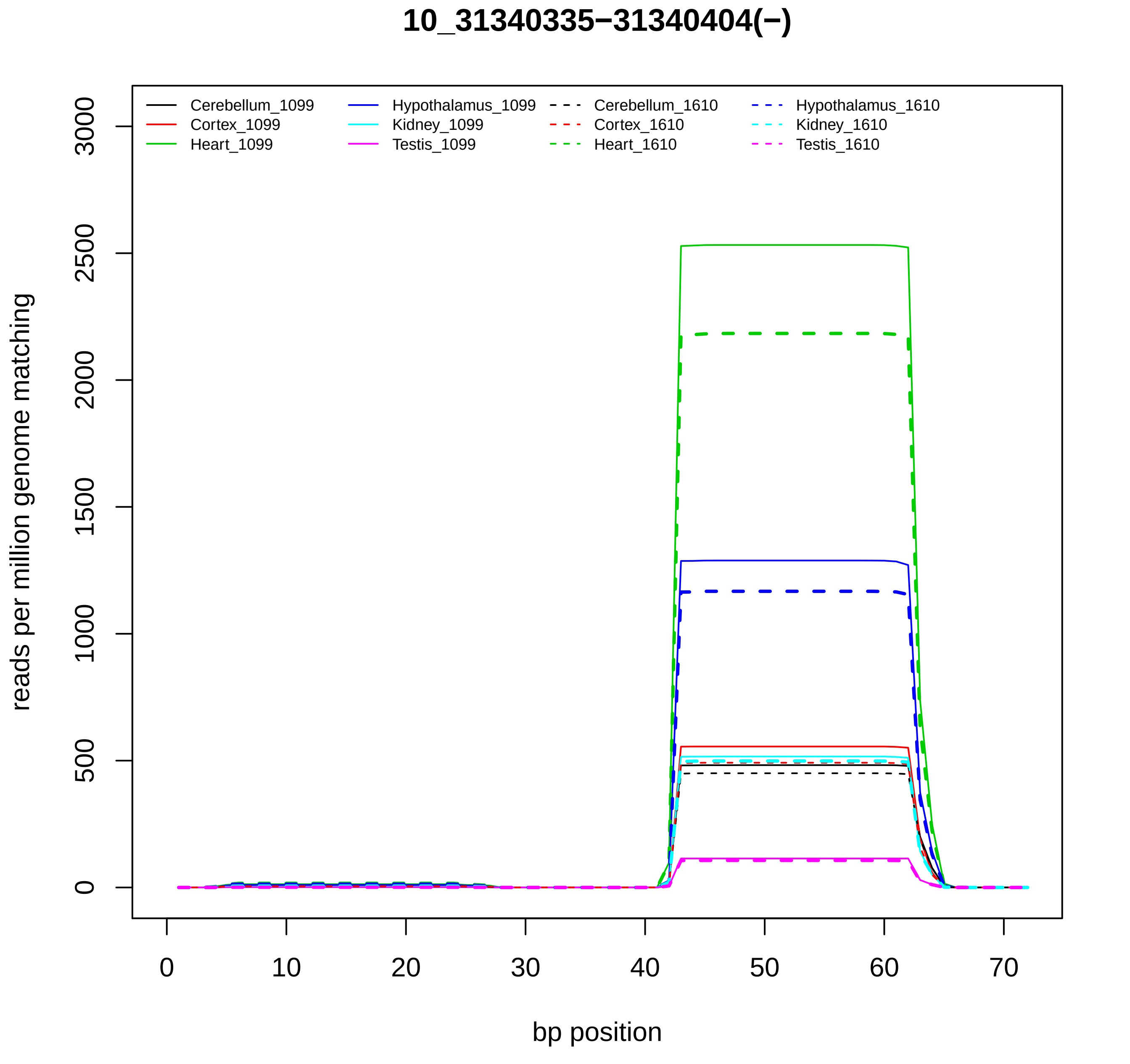

10_31340335−31340404(−)
Cerebellum_1099
Cortex_1099
Heart_1099
Hypothalamus_1099
Kidney_1099
Testis_1099
Cerebellum_1610
Cortex_1610
Heart_1610
Hypothalamus_1610
Kidney_1610
Testis_1610
reads per million genome matching
0
500
1000
1500
2000
2500
3000
bp position
0
10
20
30
40
50
60
70

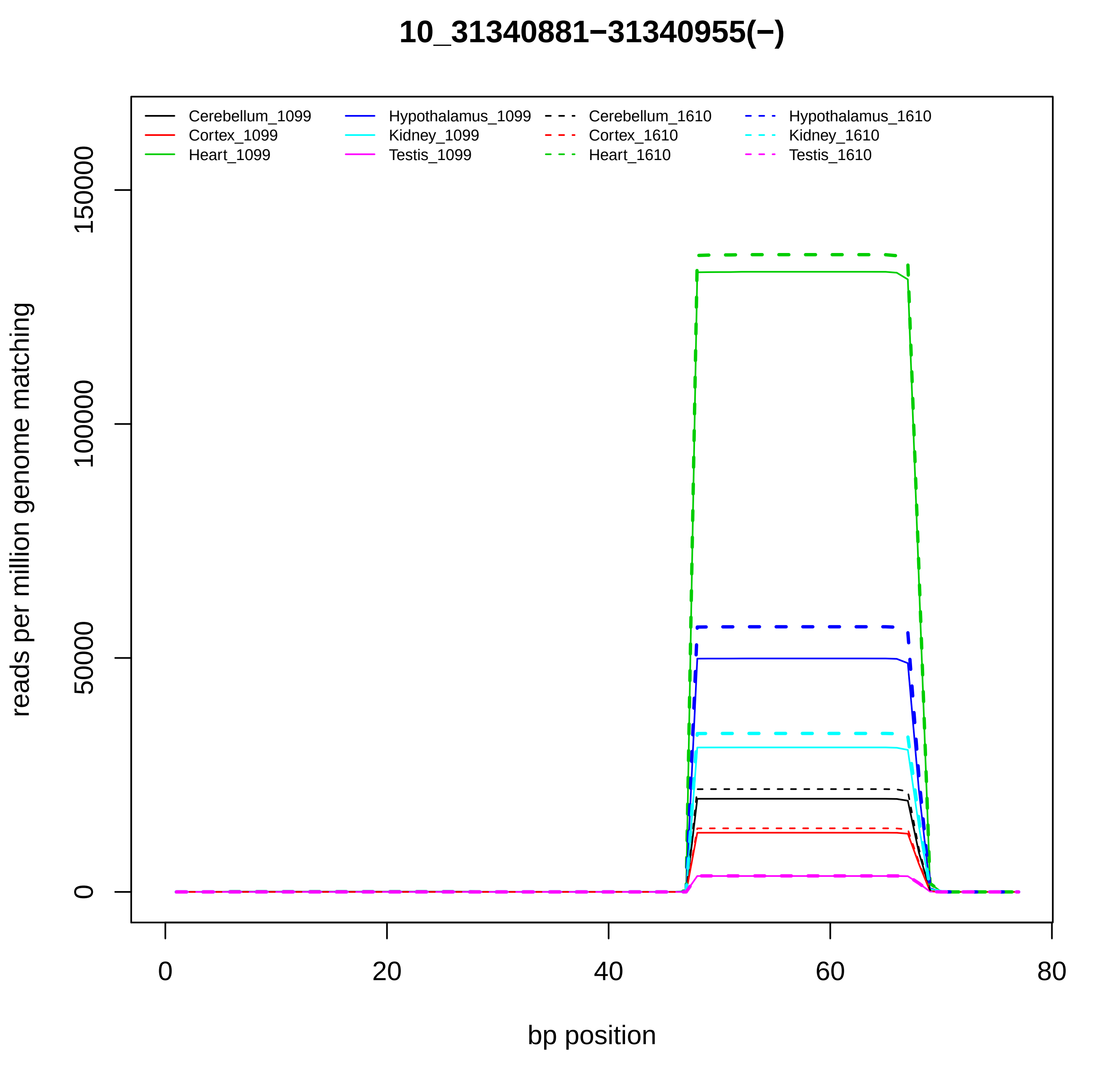
10_31340881−31340955(−)
Cerebellum_1099
Cortex_1099
Heart_1099
Hypothalamus_1099
Kidney_1099
Testis_1099
Cerebellum_1610
Cortex_1610
Heart_1610
Hypothalamus_1610
Kidney_1610
Testis_1610
reads per million genome matching
150000
100000
50000
0
bp position
0
20
40
60
80

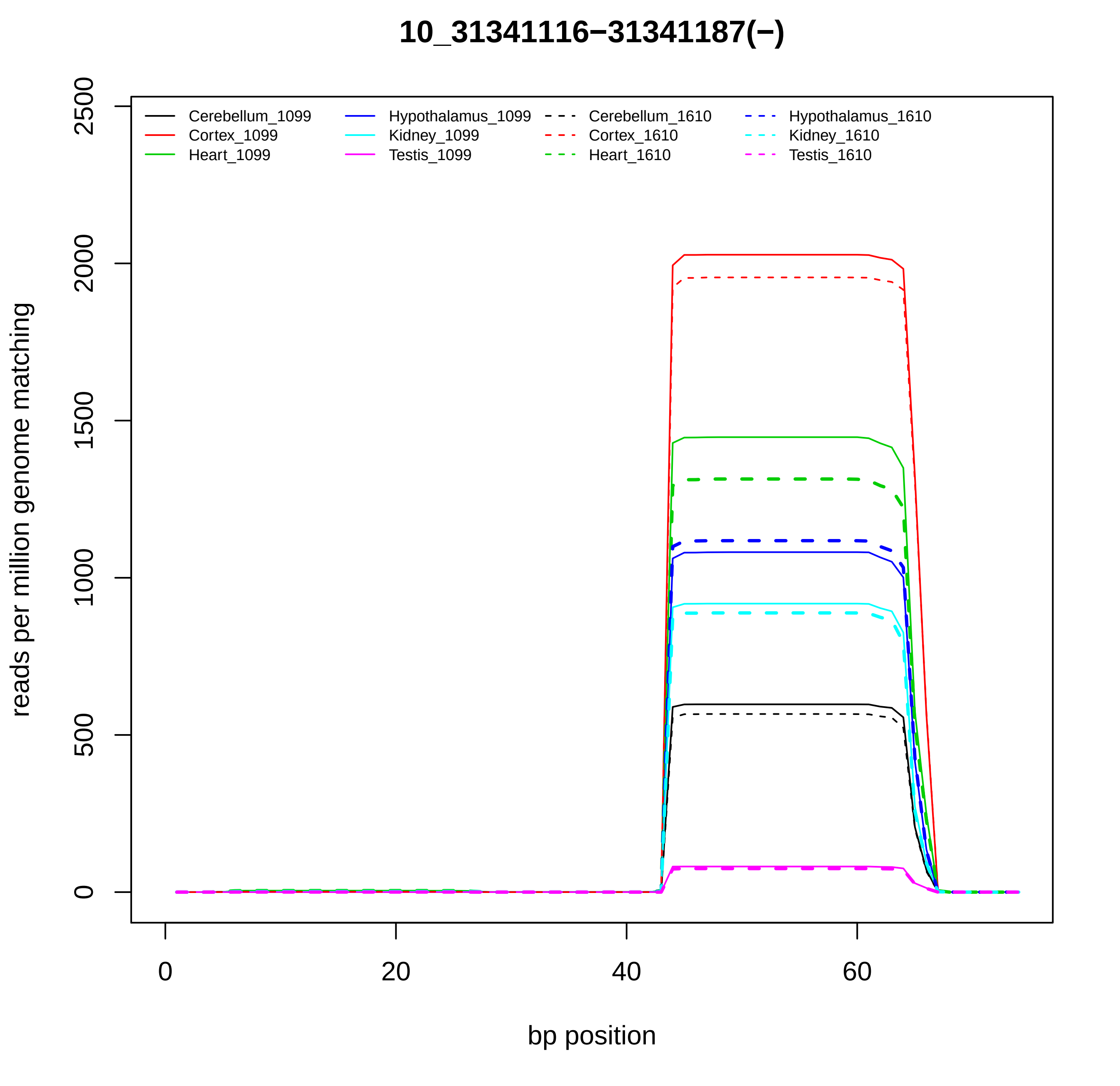

10_31341116−31341187(−)
Cerebellum_1099
Cortex_1099
Heart_1099
Hypothalamus_1099
Kidney_1099
Testis_1099
Cerebellum_1610
Cortex_1610
Heart_1610
Hypothalamus_1610
Kidney_1610
Testis_1610
reads per million genome matching
bp position
0
500
1000
1500
2000
2500
0
20
40
60

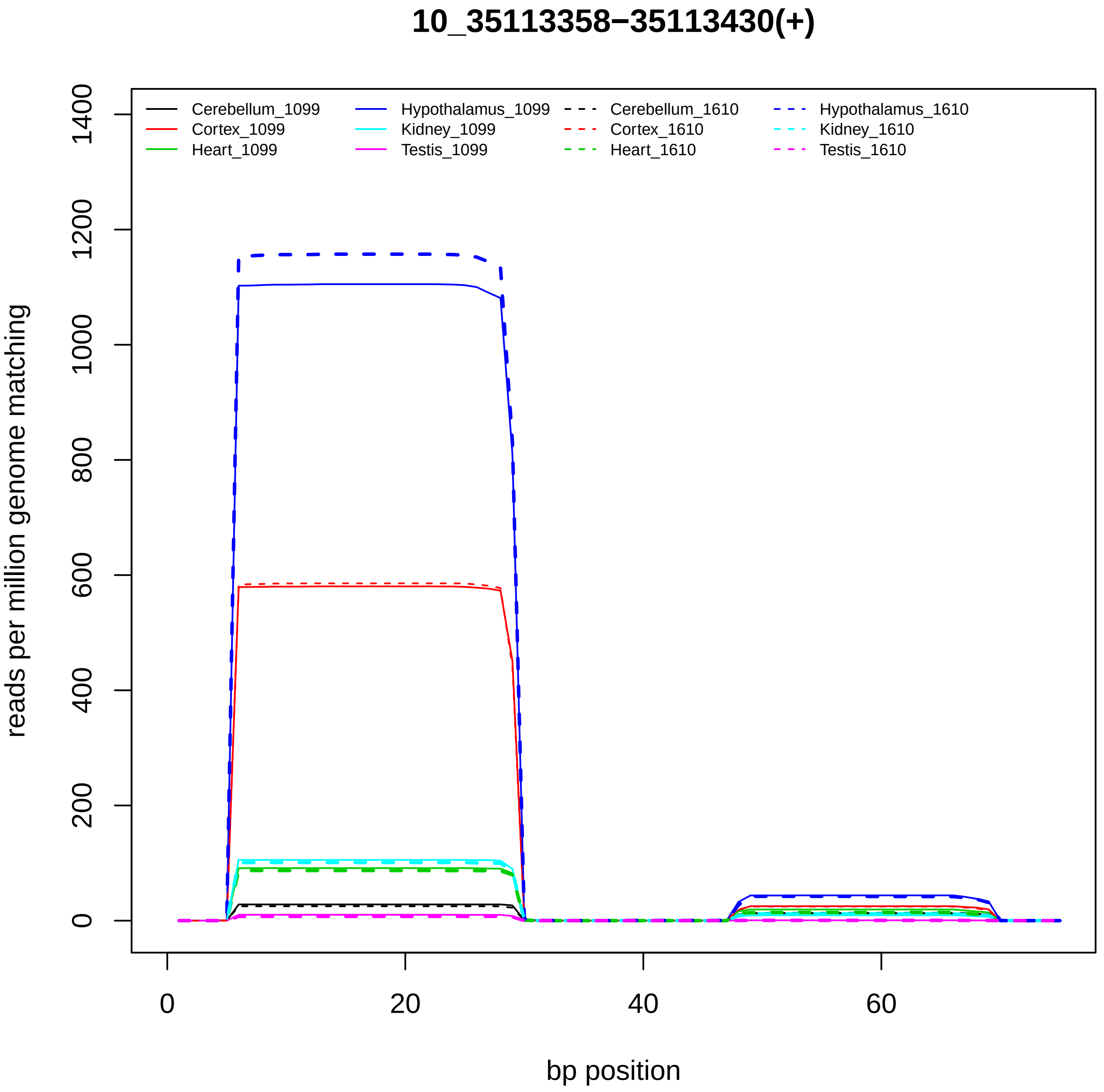
10_35113358−35113430(+)
Cerebellum_1099
Cortex_1099
Heart_1099
Hypothalamus_1099
Kidney_1099
Testis_1099
Cerebellum_1610
Cortex_1610
Heart_1610
Hypothalamus_1610
Kidney_1610
Testis_1610
reads per million genome matching
0
200
400
600
800
1000
1200
1400
0
20
40
60
bp position

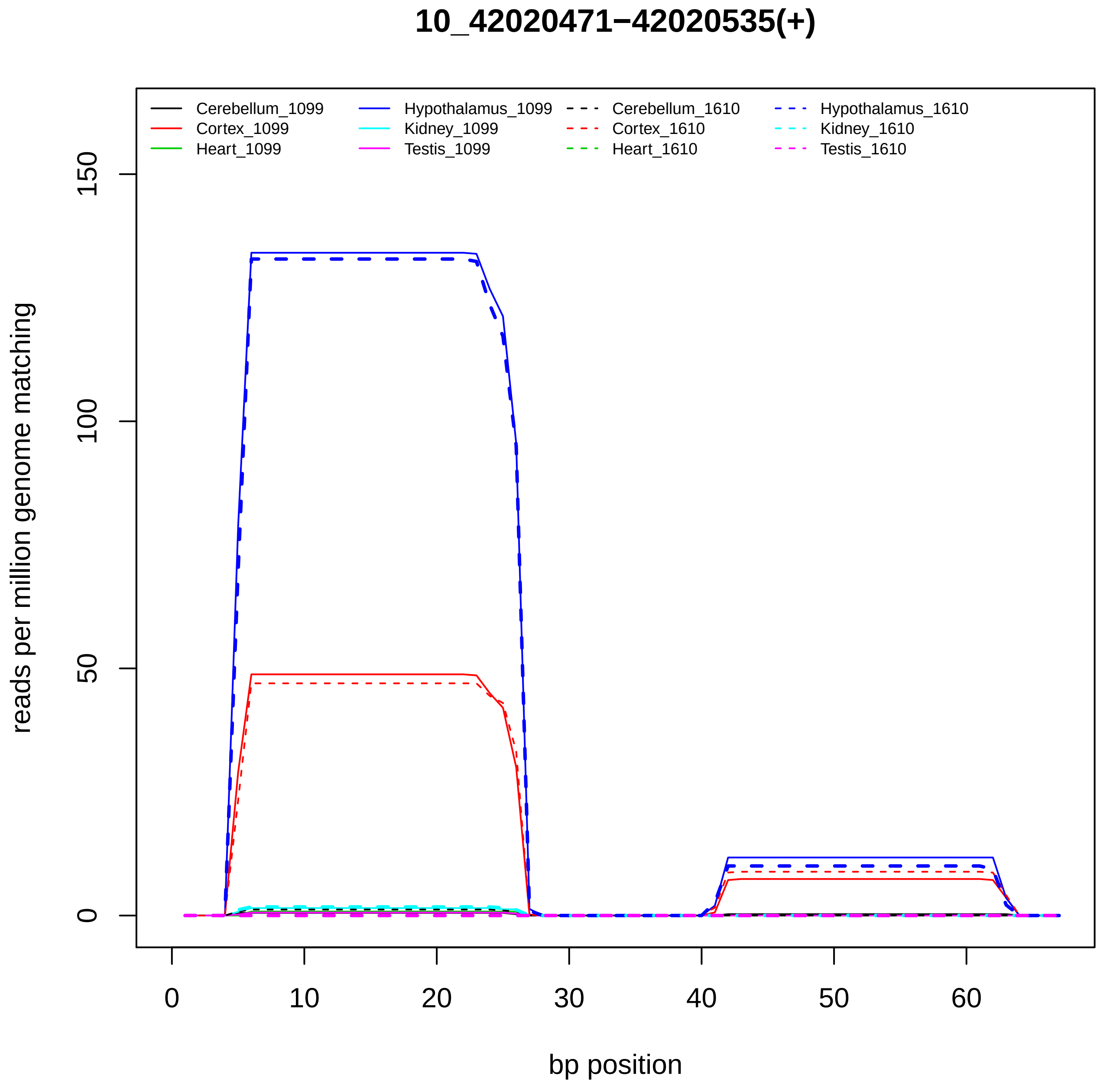

10_42020471−42020535(+)
Cerebellum_1099
Cortex_1099
Heart_1099
Hypothalamus_1099
Kidney_1099
Testis_1099
Cerebellum_1610
Cortex_1610
Heart_1610
Hypothalamus_1610
Kidney_1610
Testis_1610
reads per million genome matching
0
50
100
150
0
10
20
30
40
50
60
bp position

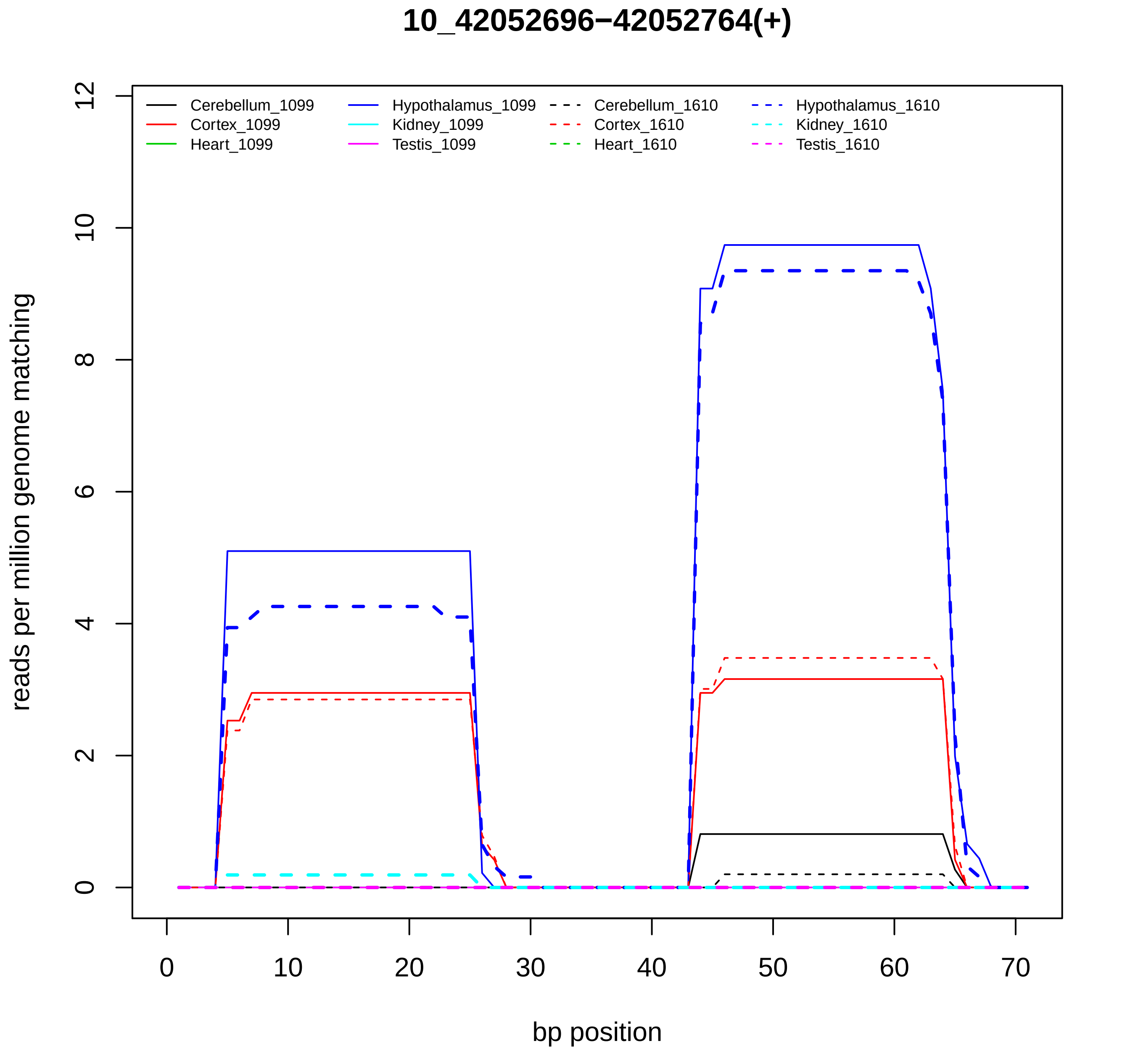

10_42052696−42052764(+)
Cerebellum_1099
Cortex_1099
Heart_1099
Hypothalamus_1099
Kidney_1099
Testis_1099
Cerebellum_1610
Cortex_1610
Heart_1610
Hypothalamus_1610
Kidney_1610
Testis_1610
reads per million genome matching
0
2
4
6
8
10
12
0
10
20
30
40
50
60
70
bp position

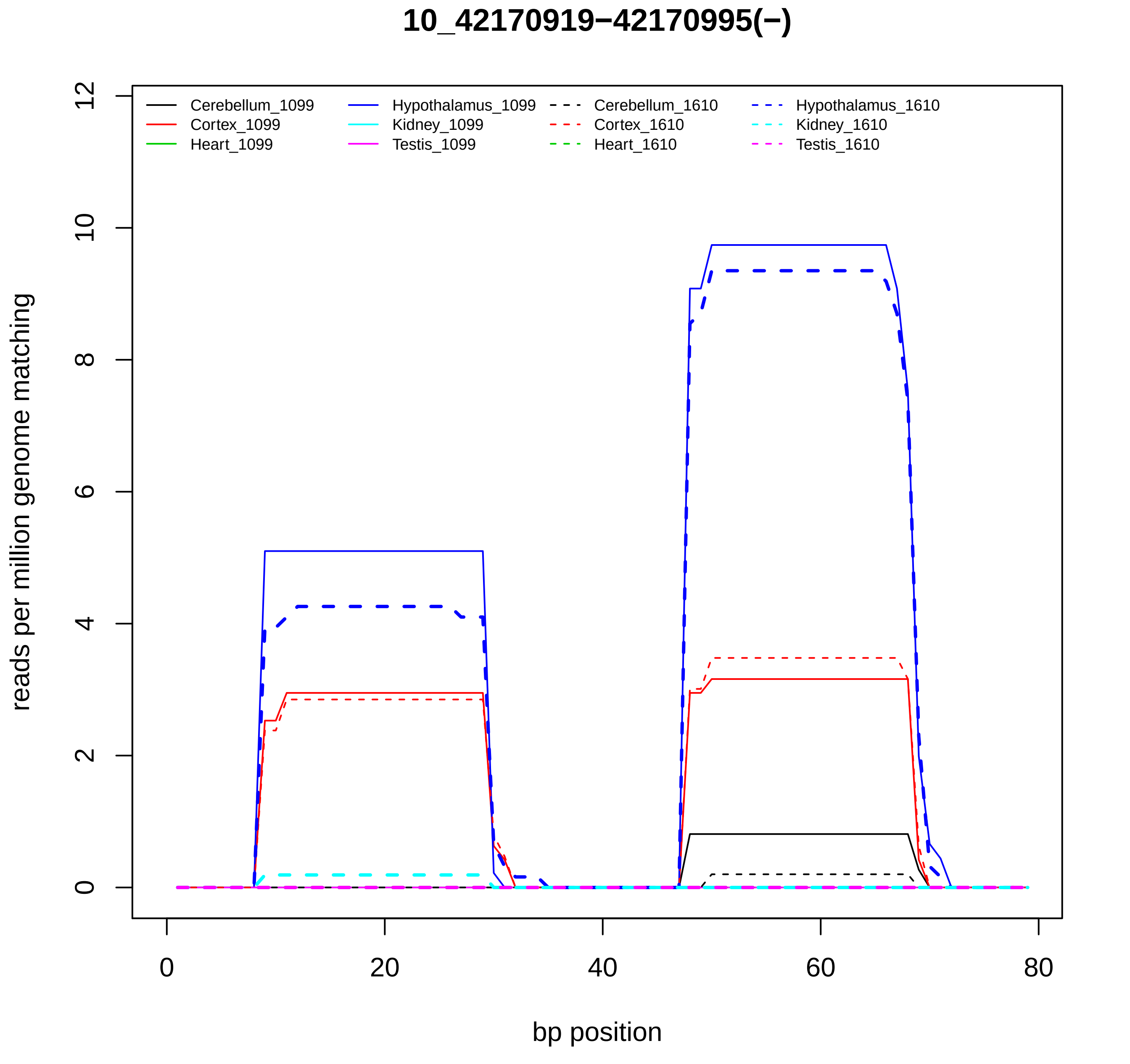

10_42170919−42170995(−)
Cerebellum_1099
Cortex_1099
Heart_1099
Hypothalamus_1099
Kidney_1099
Testis_1099
Cerebellum_1610
Cortex_1610
Heart_1610
Hypothalamus_1610
Kidney_1610
Testis_1610
reads per million genome matching
bp position
0
2
4
6
8
10
12
0
20
40
60
80

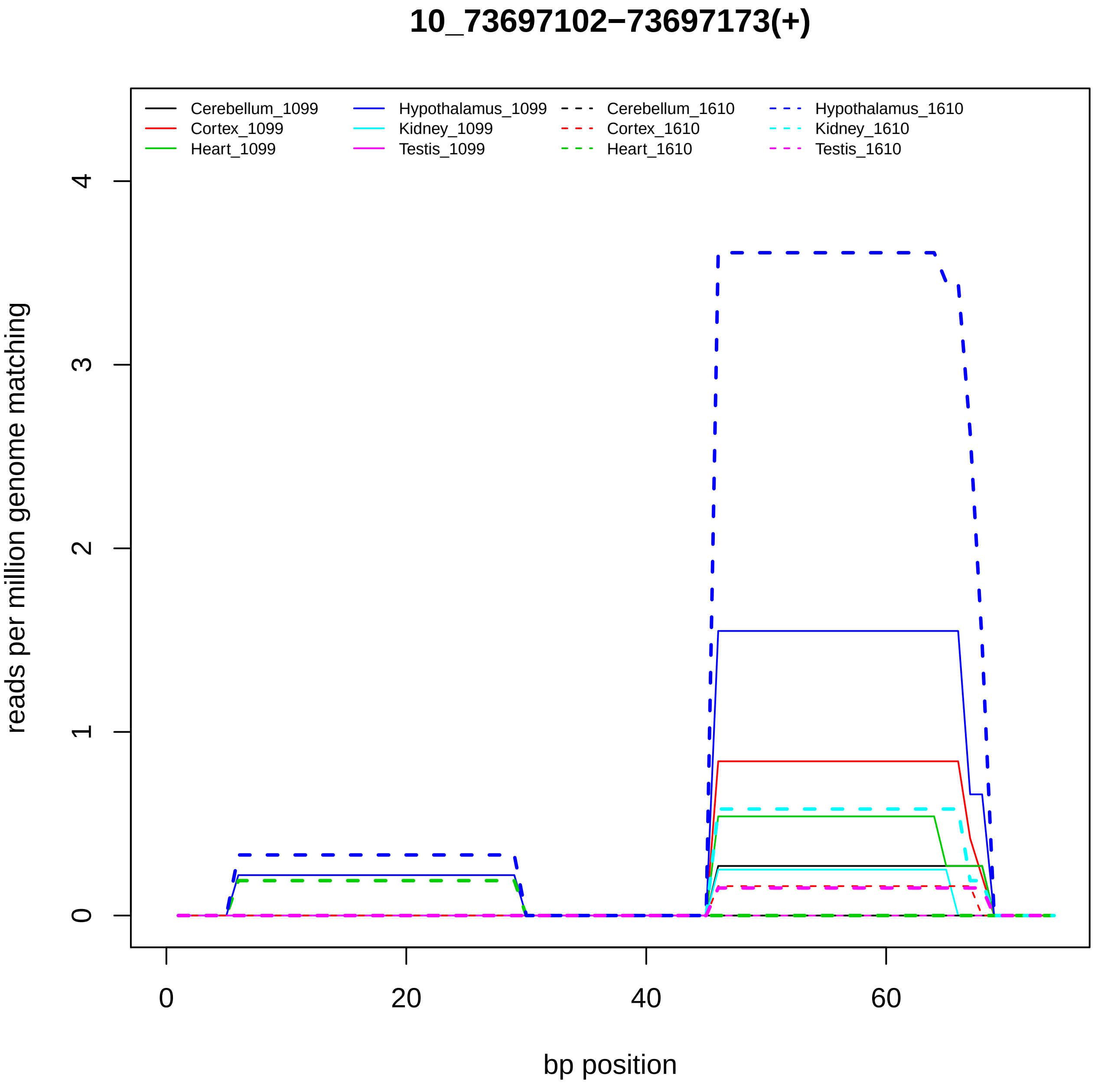

10_73697102−73697173(+)
Cerebellum_1099
Cortex_1099
Heart_1099
Hypothalamus_1099
Kidney_1099
Testis_1099
Cerebellum_1610
Cortex_1610
Heart_1610
Hypothalamus_1610
Kidney_1610
Testis_1610
reads per million genome matching
0
1
2
3
4
0
20
40
60
bp position

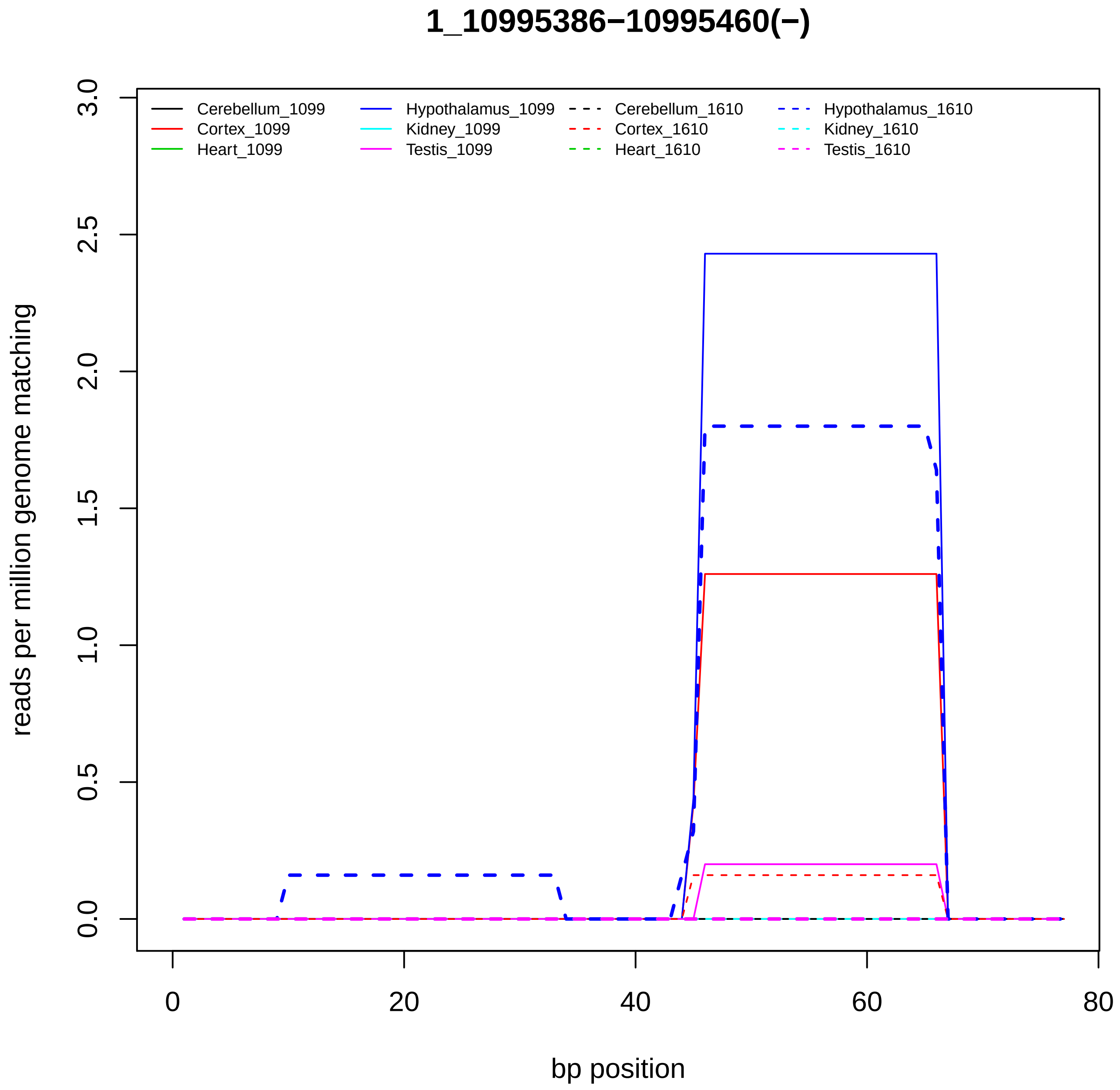

1_10995386−10995460(−)
Cerebellum_1099
Cortex_1099
Heart_1099
Hypothalamus_1099
Kidney_1099
Testis_1099
Cerebellum_1610
Cortex_1610
Heart_1610
Hypothalamus_1610
Kidney_1610
Testis_1610
reads per million genome matching
bp position
0.0
0.5
1.0
1.5
2.0
2.5
3.0
0
20
40
60
80

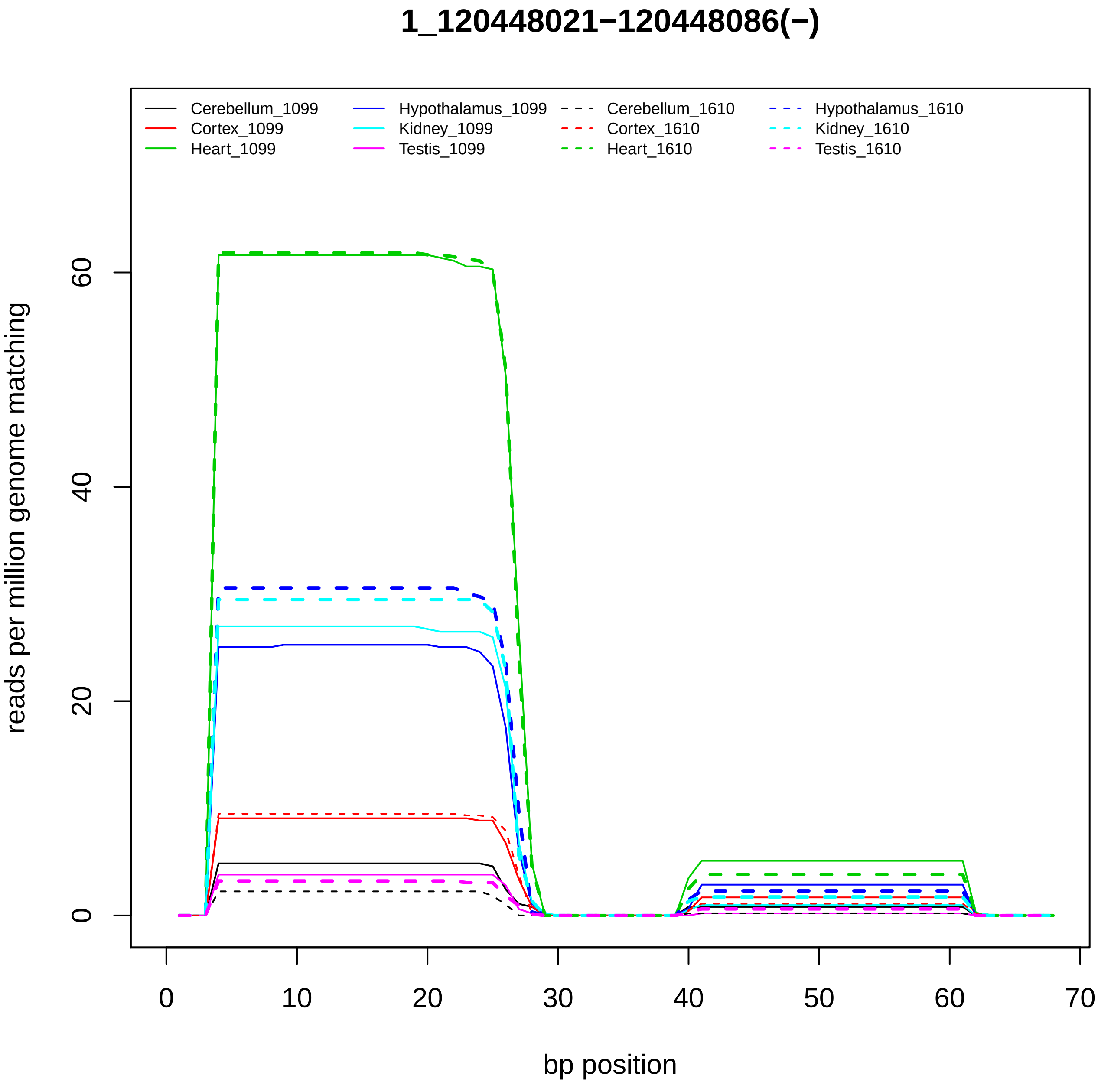

1_120448021−120448086(−)
Cerebellum_1099
Cortex_1099
Heart_1099
Hypothalamus_1099
Kidney_1099
Testis_1099
Cerebellum_1610
Cortex_1610
Heart_1610
Hypothalamus_1610
Kidney_1610
Testis_1610
reads per million genome matching
bp position
0
20
40
60
0
10
20
30
40
50
60
70

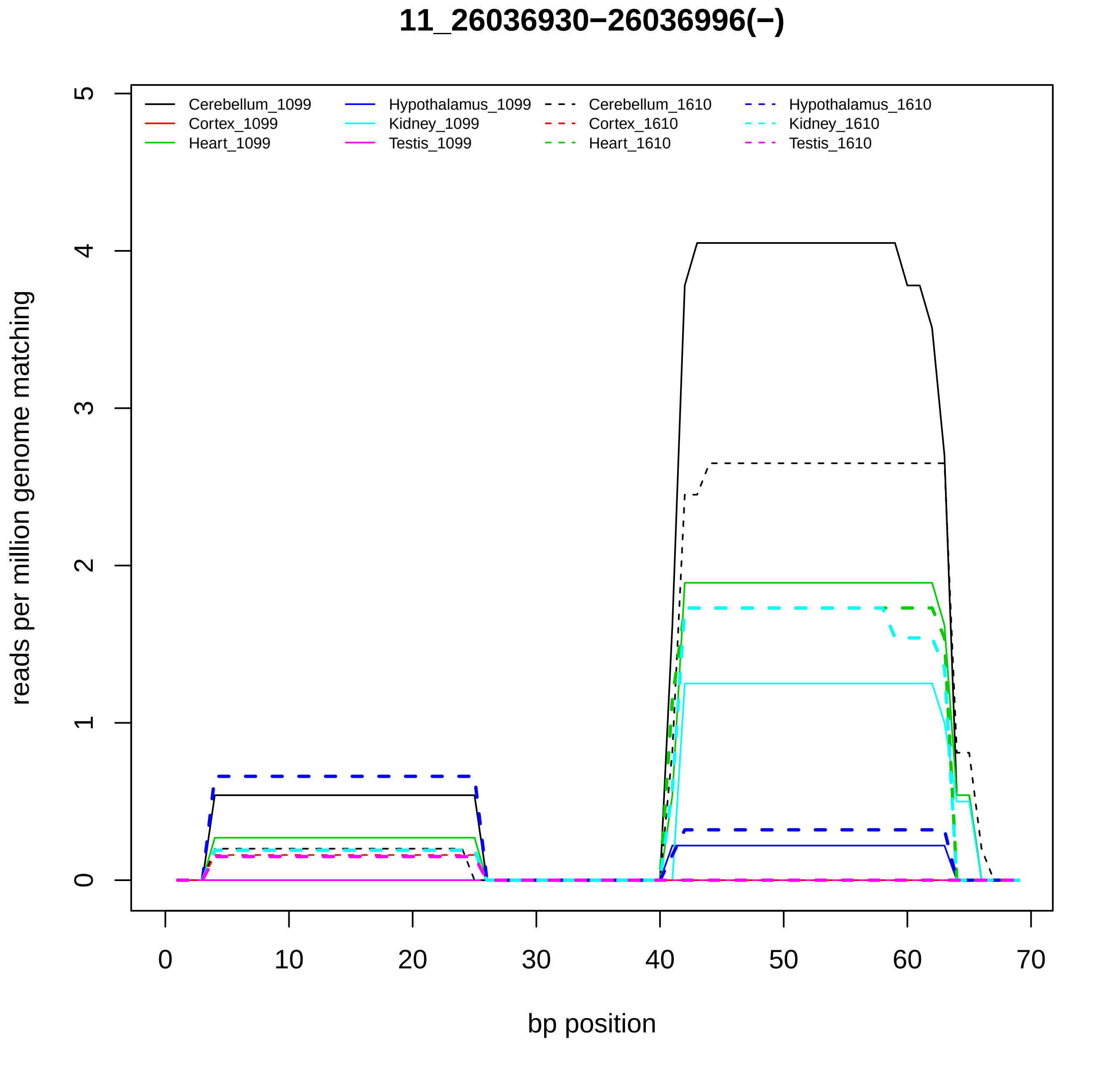

11_26036930−26036996(−)
Cerebellum_1099
Cortex_1099
Heart_1099
Hypothalamus_1099
Kidney_1099
Testis_1099
Cerebellum_1610
Cortex_1610
Heart_1610
Hypothalamus_1610
Kidney_1610
Testis_1610
reads per million genome matching
bp position
0
1
2
3
4
5
0
10
20
30
40
50
60
70

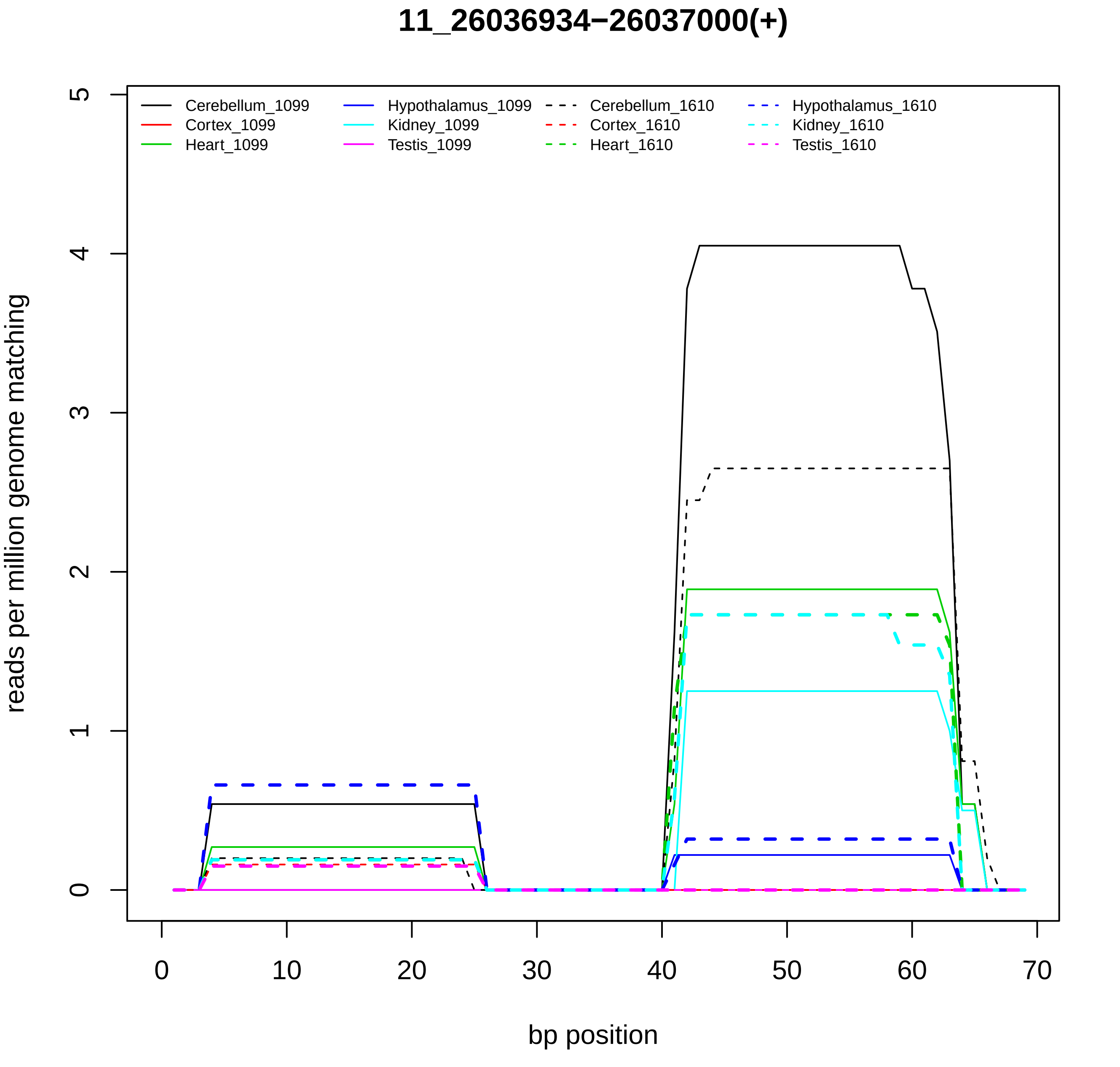

11_26036934−26037000(+)
Cerebellum_1099
Cortex_1099
Heart_1099
Hypothalamus_1099
Kidney_1099
Testis_1099
Cerebellum_1610
Cortex_1610
Heart_1610
Hypothalamus_1610
Kidney_1610
Testis_1610
reads per million genome matching
bp position
0
1
2
3
4
5
0
10
20
30
40
50
60
70

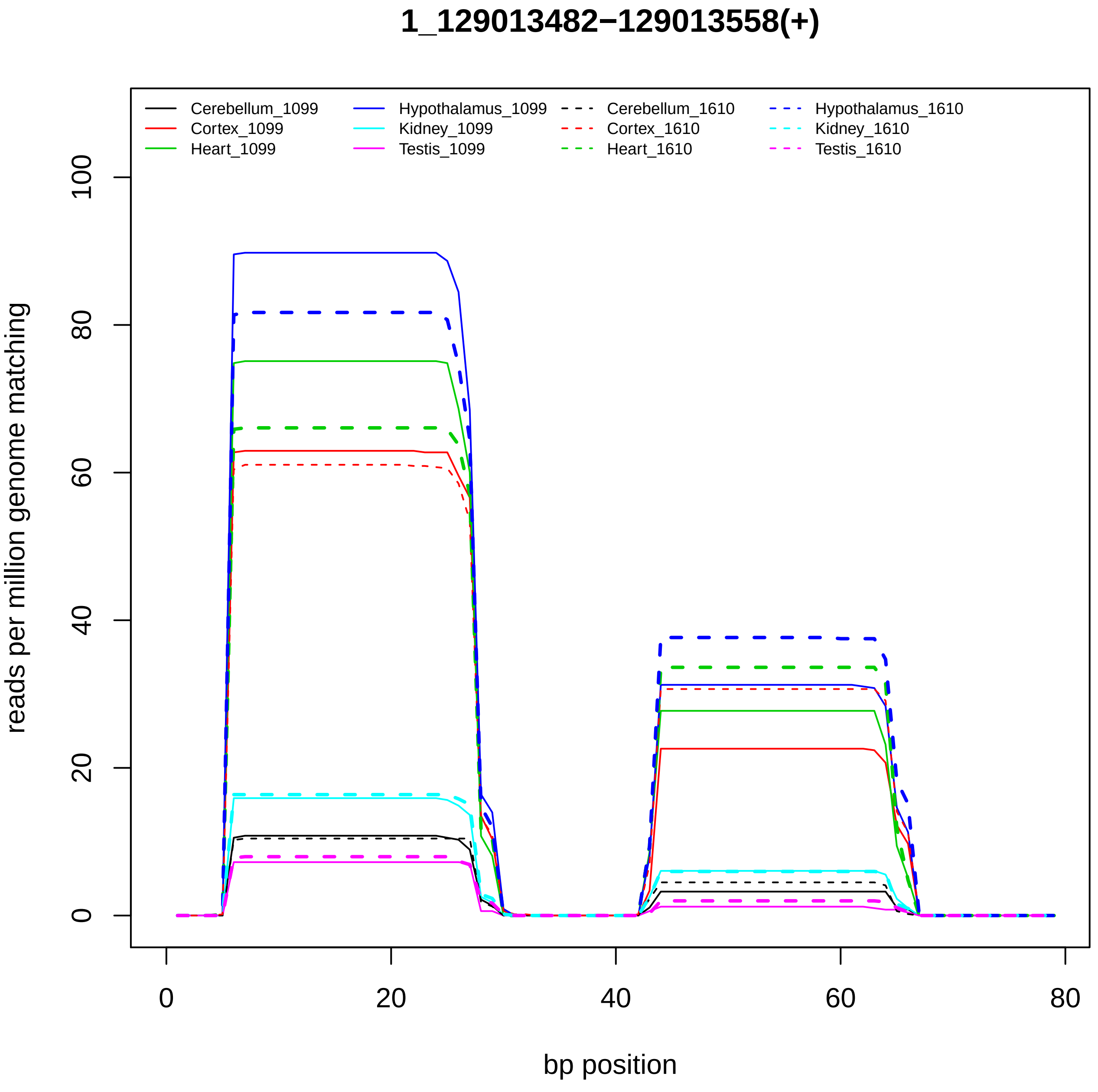

1_129013482−129013558(+)
Cerebellum_1099
Cortex_1099
Heart_1099
Hypothalamus_1099
Kidney_1099
Testis_1099
Cerebellum_1610
Cortex_1610
Heart_1610
Hypothalamus_1610
Kidney_1610
Testis_1610
reads per million genome matching
0
20
40
60
80
100
0
20
40
60
80
bp position

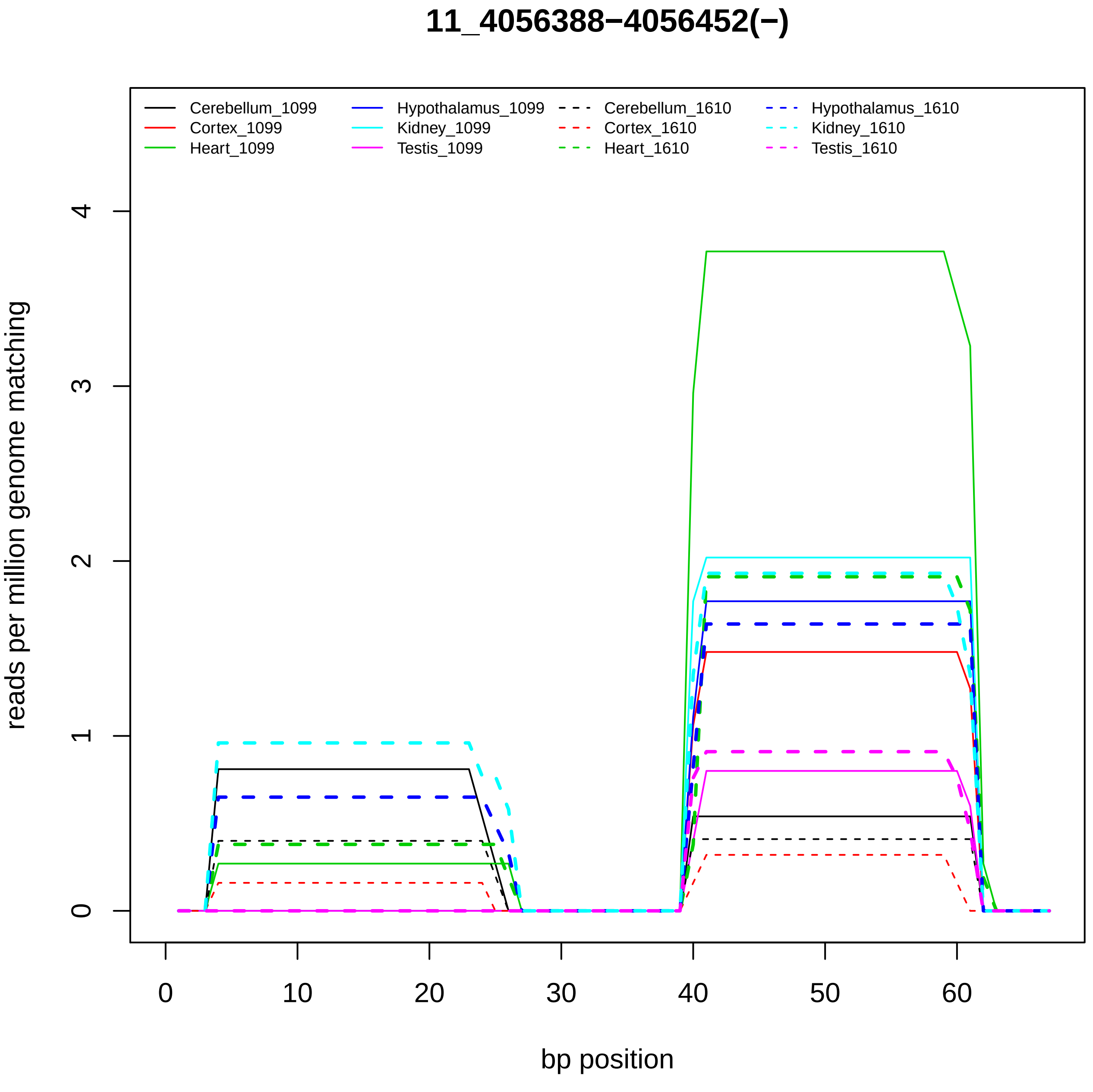
11_4056388−4056452(−)
Cerebellum_1099
Cortex_1099
Heart_1099
Hypothalamus_1099
Kidney_1099
Testis_1099
Cerebellum_1610
Cortex_1610
Heart_1610
Hypothalamus_1610
Kidney_1610
Testis_1610
reads per million genome matching
0
1
2
3
4
0
10
20
30
40
50
60
bp position

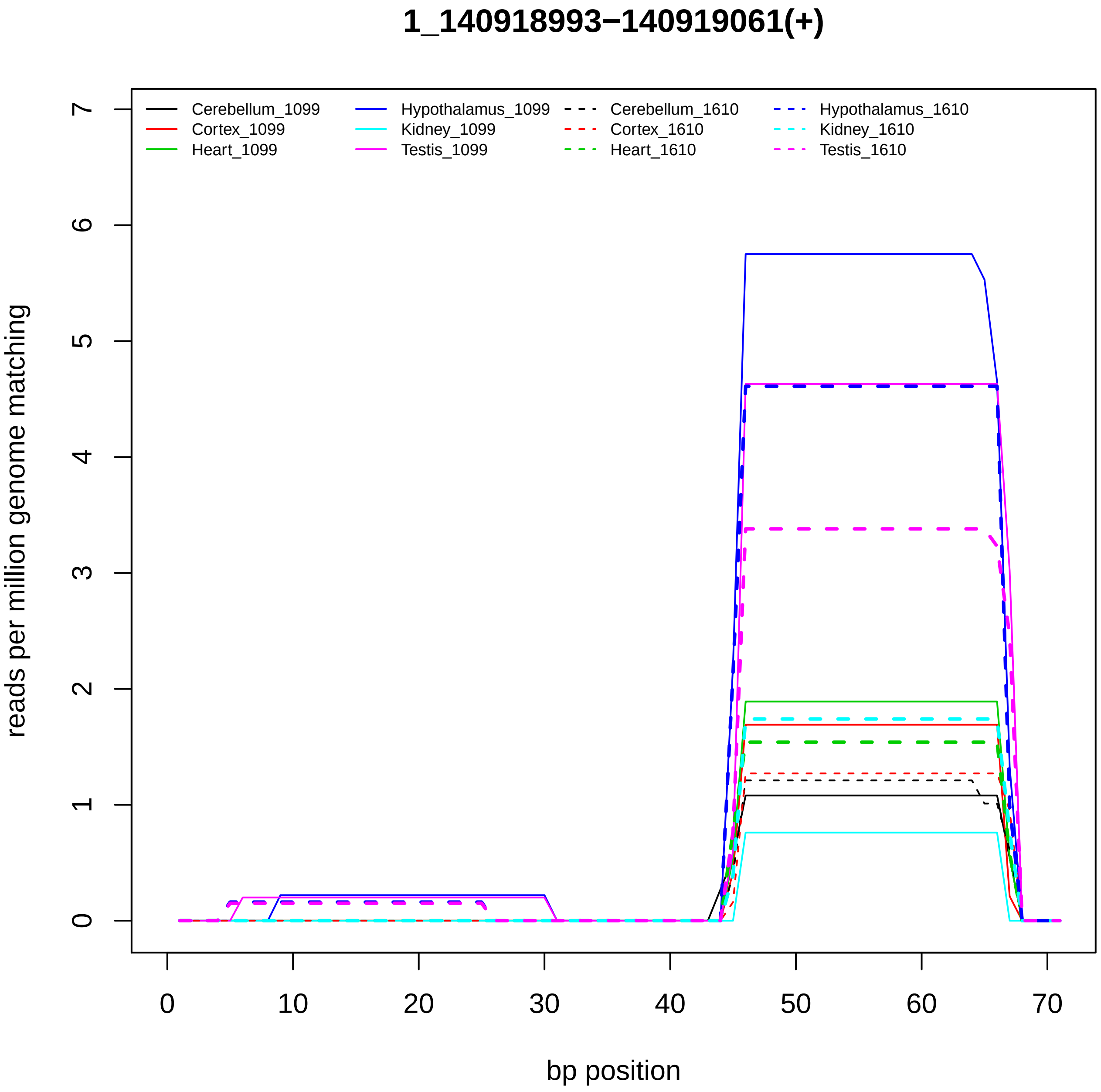

1_140918993−140919061(+)
Cerebellum_1099
Cortex_1099
Heart_1099
Hypothalamus_1099
Kidney_1099
Testis_1099
Cerebellum_1610
Cortex_1610
Heart_1610
Hypothalamus_1610
Kidney_1610
Testis_1610
reads per million genome matching
bp position
0
1
2
3
4
5
6
7
0
10
20
30
40
50
60
70

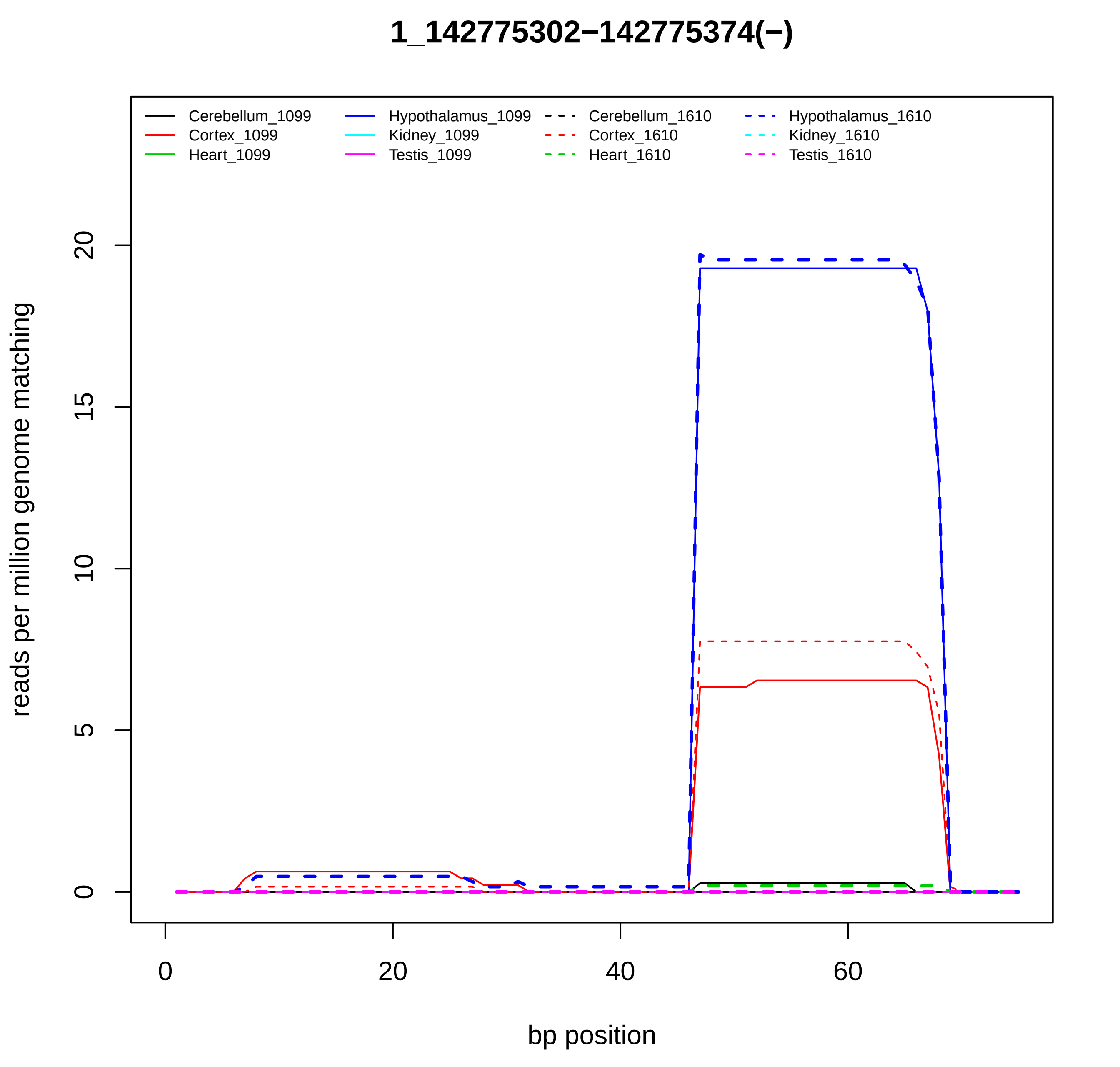

1_142775302−142775374(−)
Cerebellum_1099
Cortex_1099
Heart_1099
Hypothalamus_1099
Kidney_1099
Testis_1099
Cerebellum_1610
Cortex_1610
Heart_1610
Hypothalamus_1610
Kidney_1610
Testis_1610
reads per million genome matching
0
5
10
15
20
0
20
40
60
bp position

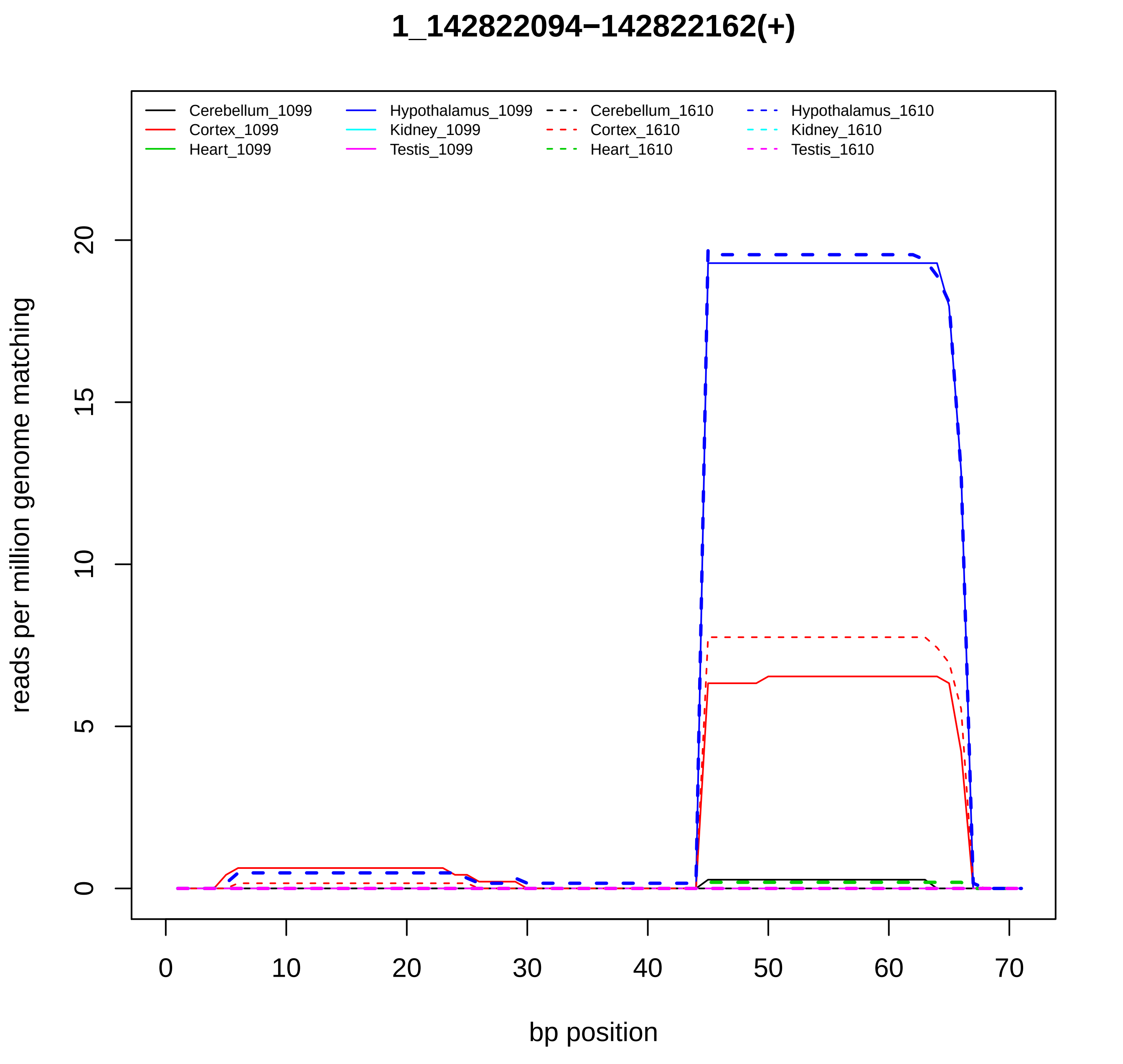

1_142822094−142822162(+)
Cerebellum_1099
Cortex_1099
Heart_1099
Hypothalamus_1099
Kidney_1099
Testis_1099
Cerebellum_1610
Cortex_1610
Heart_1610
Hypothalamus_1610
Kidney_1610
Testis_1610
reads per million genome matching
0
5
10
15
20
0
10
20
30
40
50
60
70
bp position

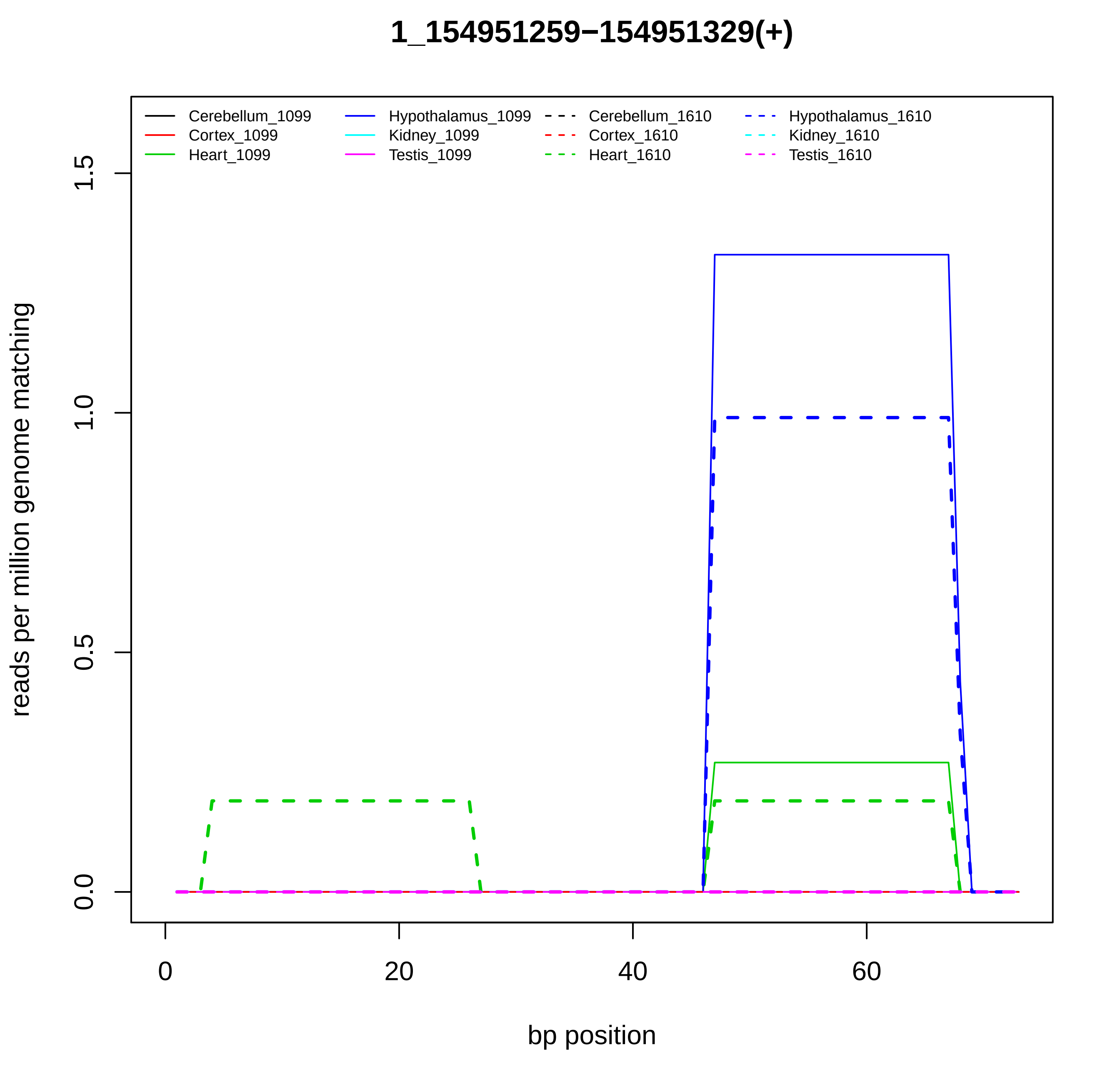
1_154951259−154951329(+)
Cerebellum_1099
Cortex_1099
Heart_1099
Hypothalamus_1099
Kidney_1099
Testis_1099
Cerebellum_1610
Cortex_1610
Heart_1610
Hypothalamus_1610
Kidney_1610
Testis_1610
reads per million genome matching
0.0
0.5
1.0
1.5
0
20
40
60
bp position

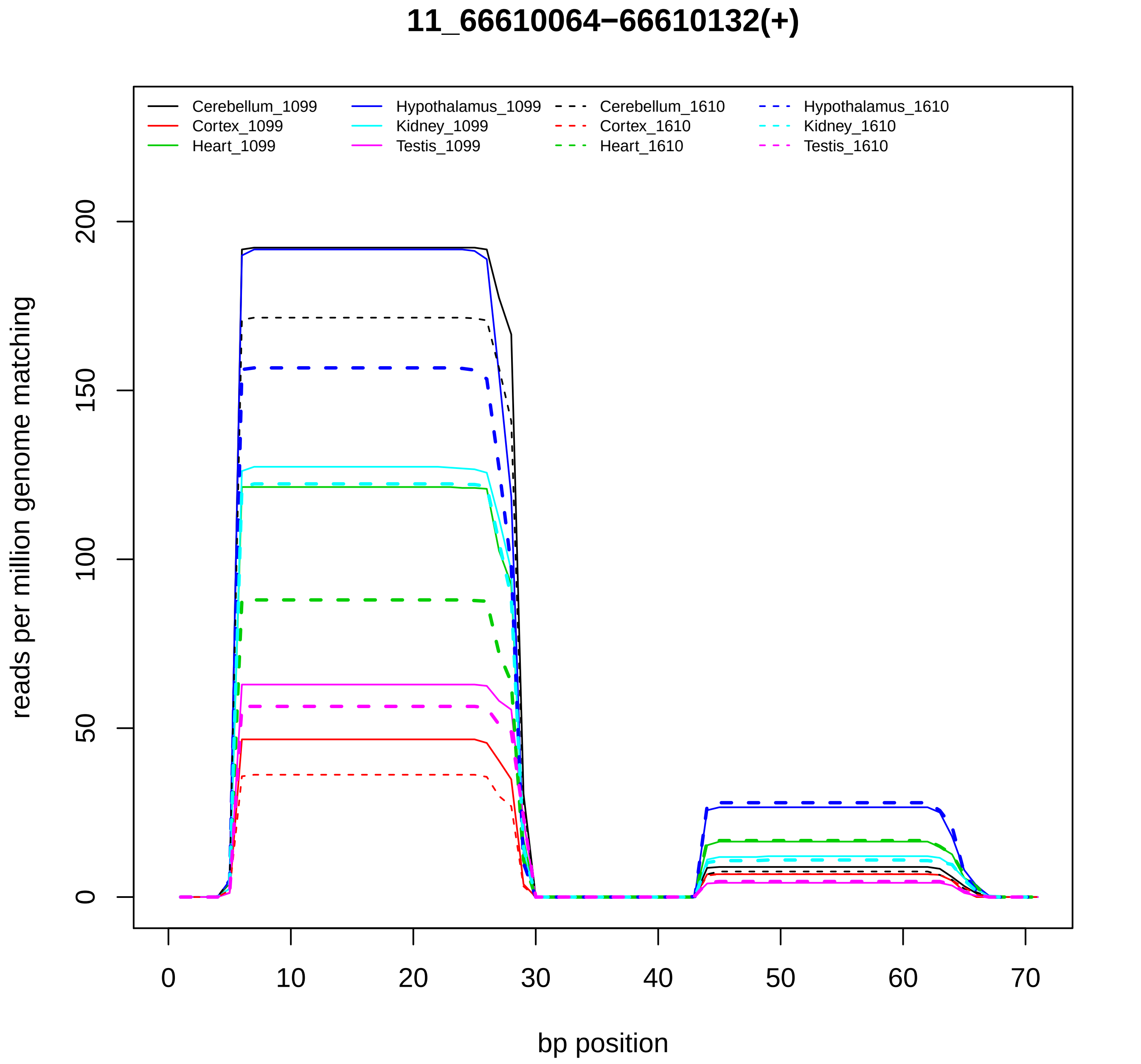

11_66610064−66610132(+)
Cerebellum_1099
Cortex_1099
Heart_1099
Hypothalamus_1099
Kidney_1099
Testis_1099
Cerebellum_1610
Cortex_1610
Heart_1610
Hypothalamus_1610
Kidney_1610
Testis_1610
reads per million genome matching
0
50
100
150
200
0
10
20
30
40
50
60
70
bp position

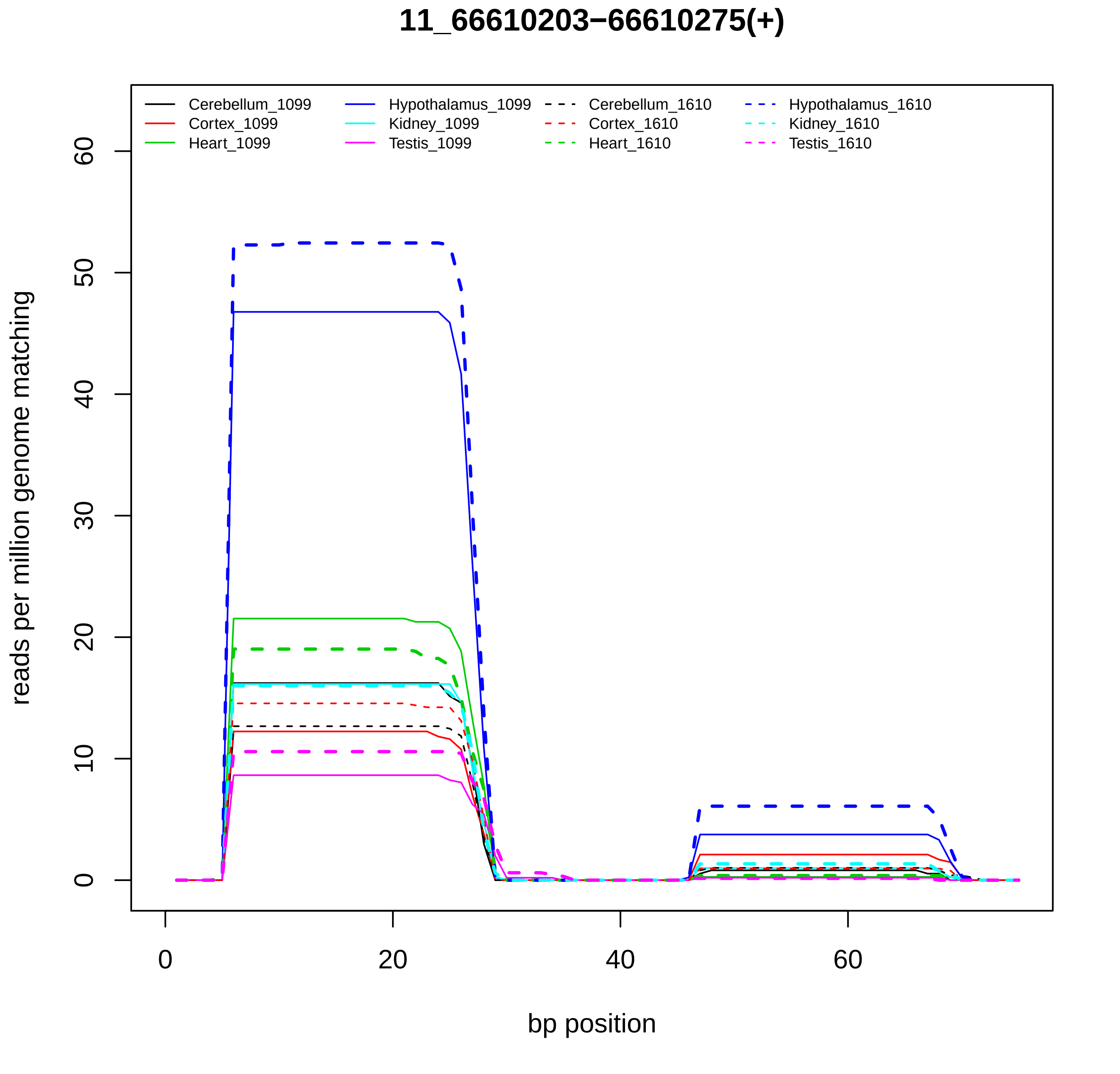

11_66610203−66610275(+)
Cerebellum_1099
Cortex_1099
Heart_1099
Hypothalamus_1099
Kidney_1099
Testis_1099
Cerebellum_1610
Cortex_1610
Heart_1610
Hypothalamus_1610
Kidney_1610
Testis_1610
reads per million genome matching
0
10
20
30
40
50
60
0
20
40
60
bp position

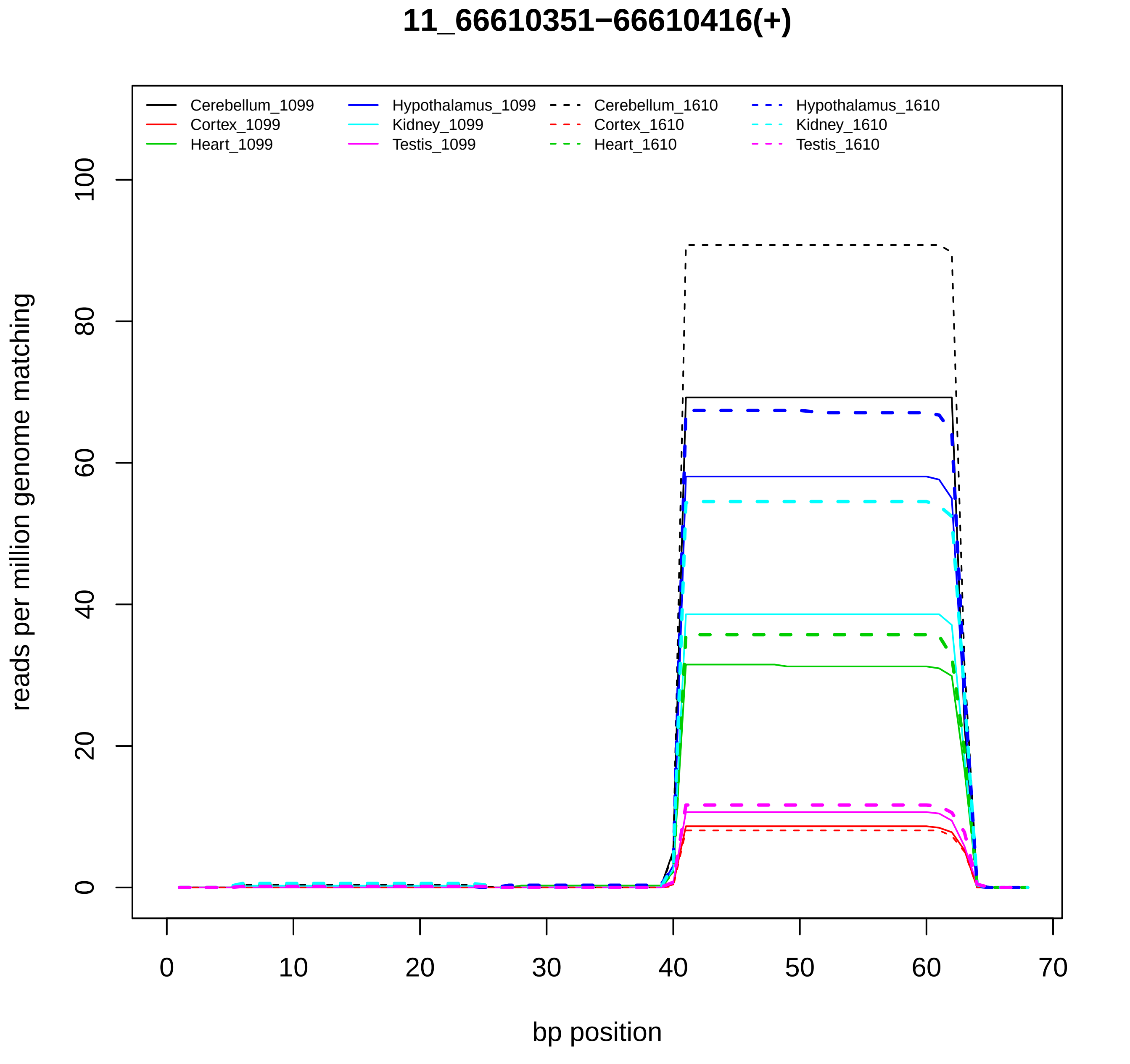

11_66610351−66610416(+)
Cerebellum_1099
Cortex_1099
Heart_1099
Hypothalamus_1099
Kidney_1099
Testis_1099
Cerebellum_1610
Cortex_1610
Heart_1610
Hypothalamus_1610
Kidney_1610
Testis_1610
reads per million genome matching
0
20
40
60
80
100
0
10
20
30
40
50
60
70
bp position

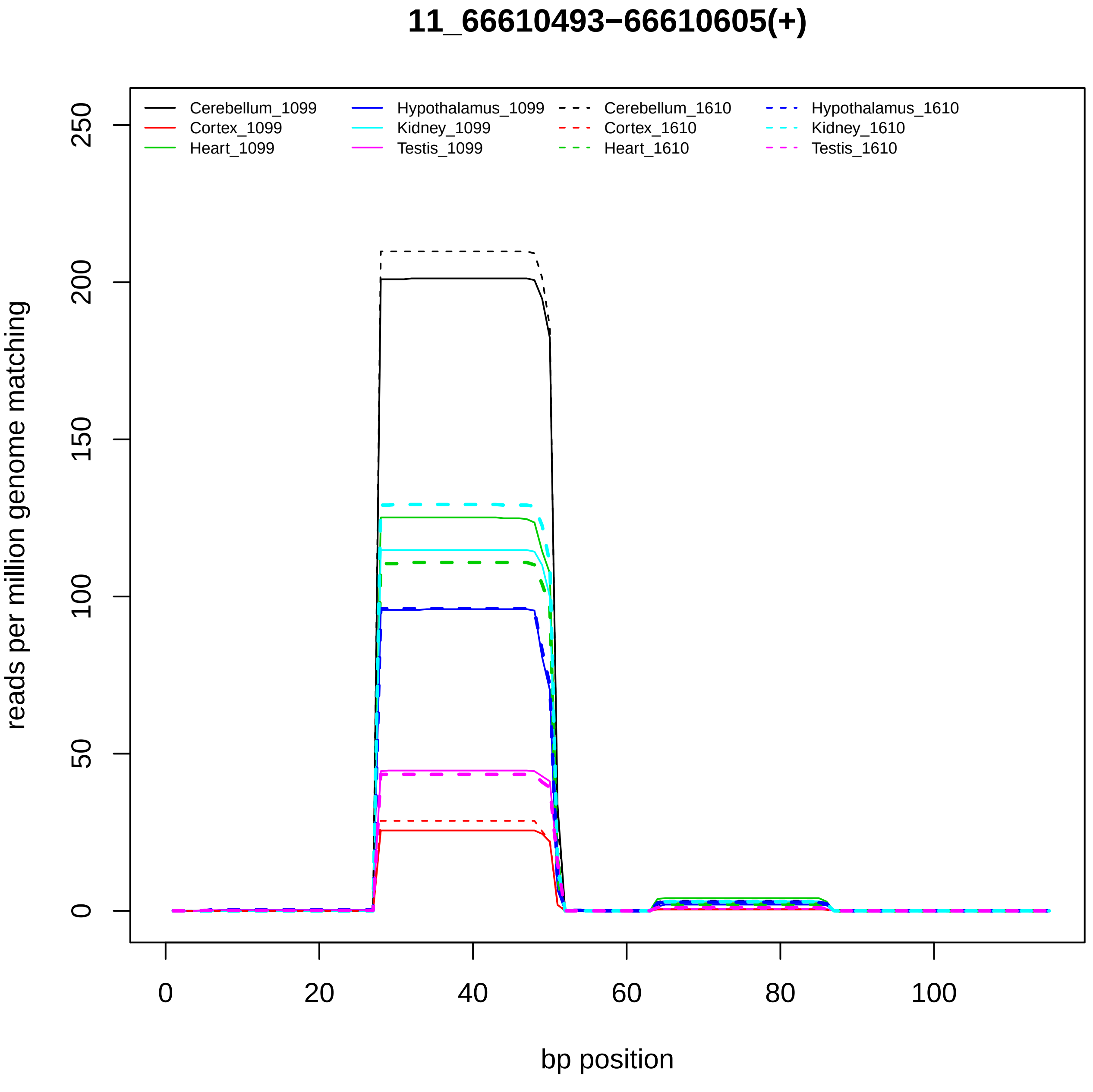

11_66610493−66610605(+)
Cerebellum_1099
Cortex_1099
Heart_1099
Hypothalamus_1099
Kidney_1099
Testis_1099
Cerebellum_1610
Cortex_1610
Heart_1610
Hypothalamus_1610
Kidney_1610
Testis_1610
reads per million genome matching
0
50
100
150
200
250
bp position
0
20
40
60
80
100

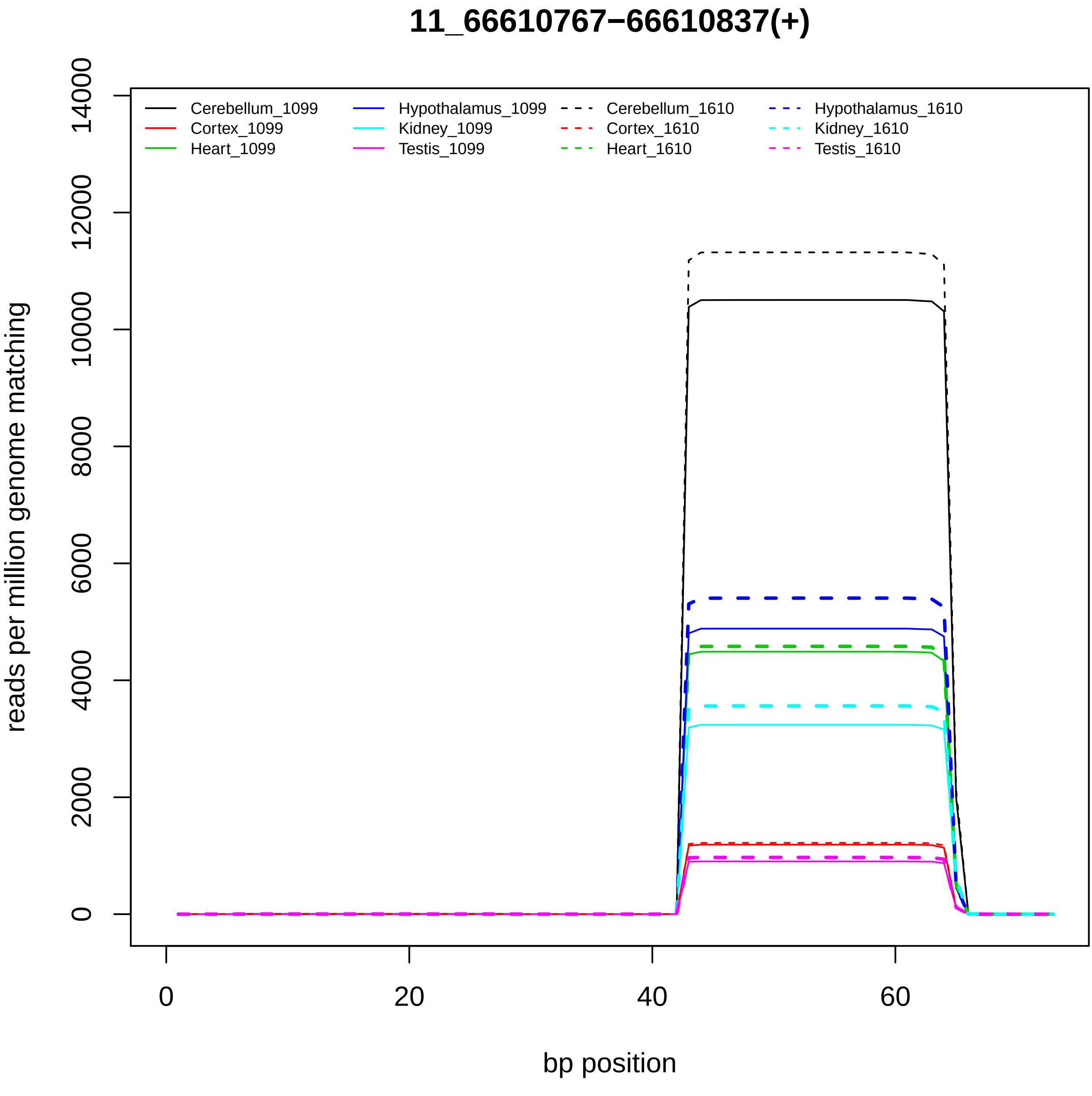

11_66610767−66610837(+)
Cerebellum_1099
Cortex_1099
Heart_1099
Hypothalamus_1099
Kidney_1099
Testis_1099
Cerebellum_1610
Cortex_1610
Heart_1610
Hypothalamus_1610
Kidney_1610
Testis_1610
reads per million genome matching
0
2000
4000
6000
8000
10000
12000
14000
0
20
40
60
bp position

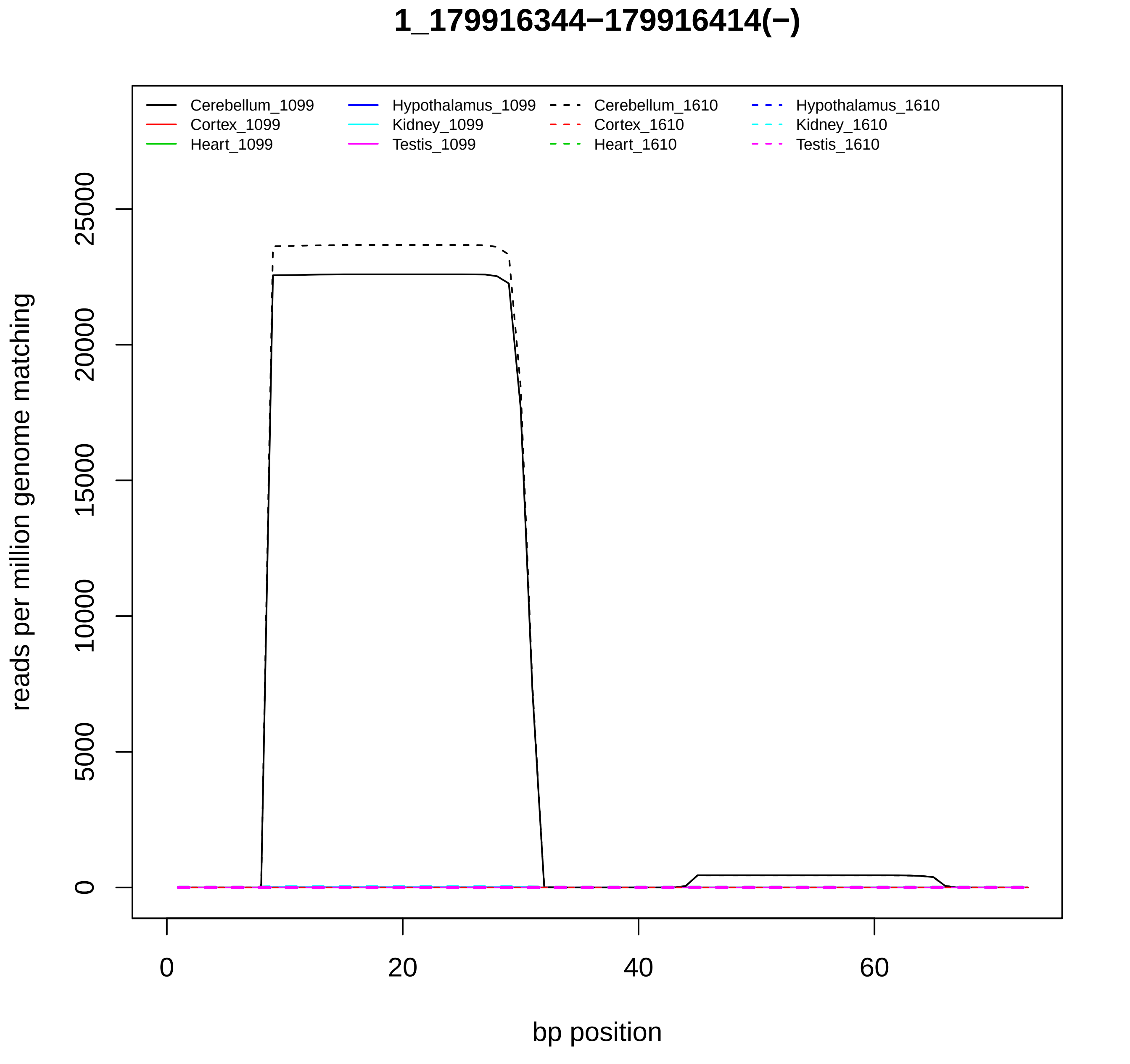
1_179916344−179916414(−)
Cerebellum_1099
Cortex_1099
Heart_1099
Hypothalamus_1099
Kidney_1099
Testis_1099
Cerebellum_1610
Cortex_1610
Heart_1610
Hypothalamus_1610
Kidney_1610
Testis_1610
reads per million genome matching
0
5000
10000
15000
20000
25000
0
20
40
60
bp position

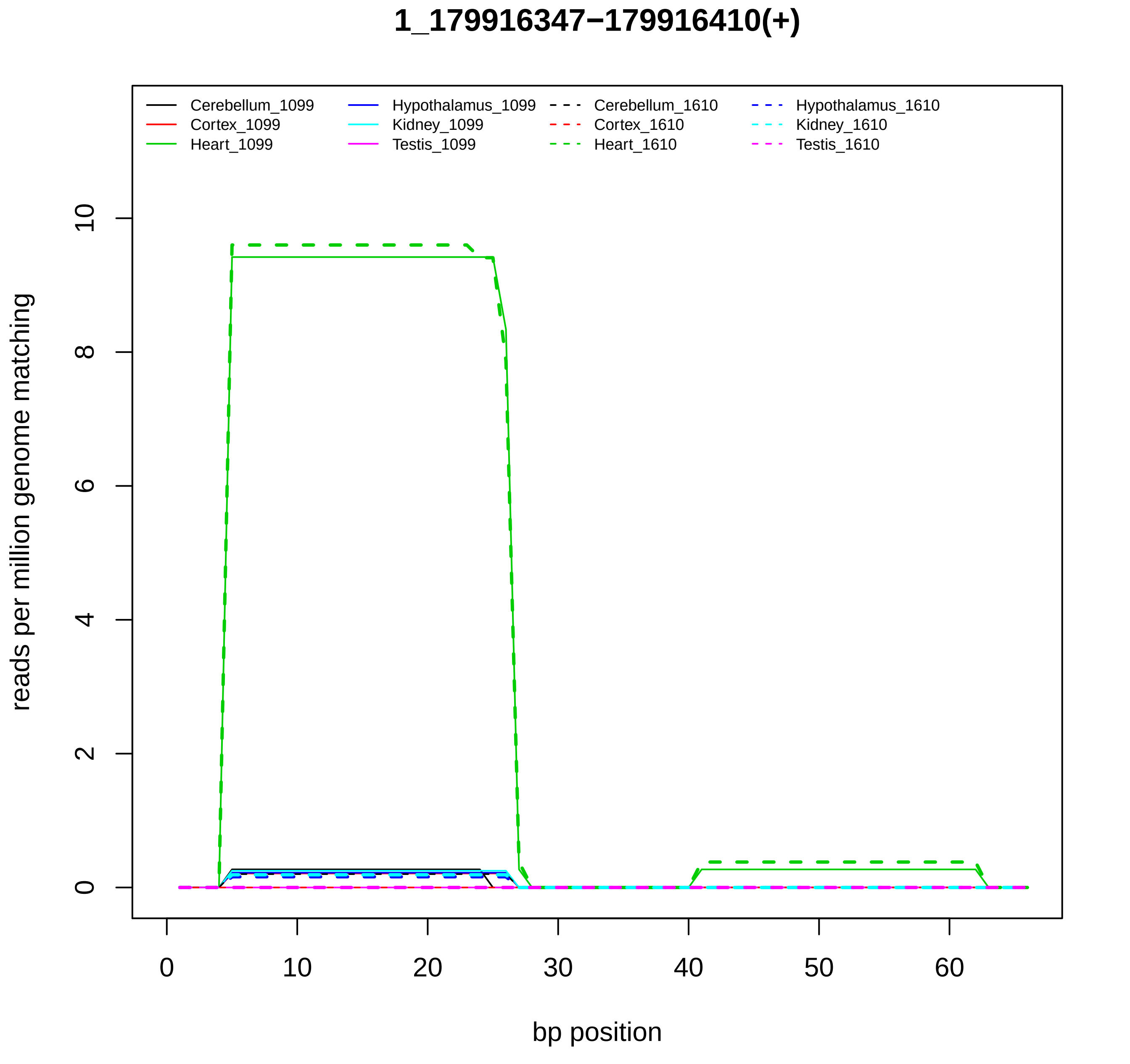

1_179916347−179916410(+)
Cerebellum_1099
Cortex_1099
Heart_1099
Hypothalamus_1099
Kidney_1099
Testis_1099
Cerebellum_1610
Cortex_1610
Heart_1610
Hypothalamus_1610
Kidney_1610
Testis_1610
reads per million genome matching
0
2
4
6
8
10
0
10
20
30
40
50
60
bp position

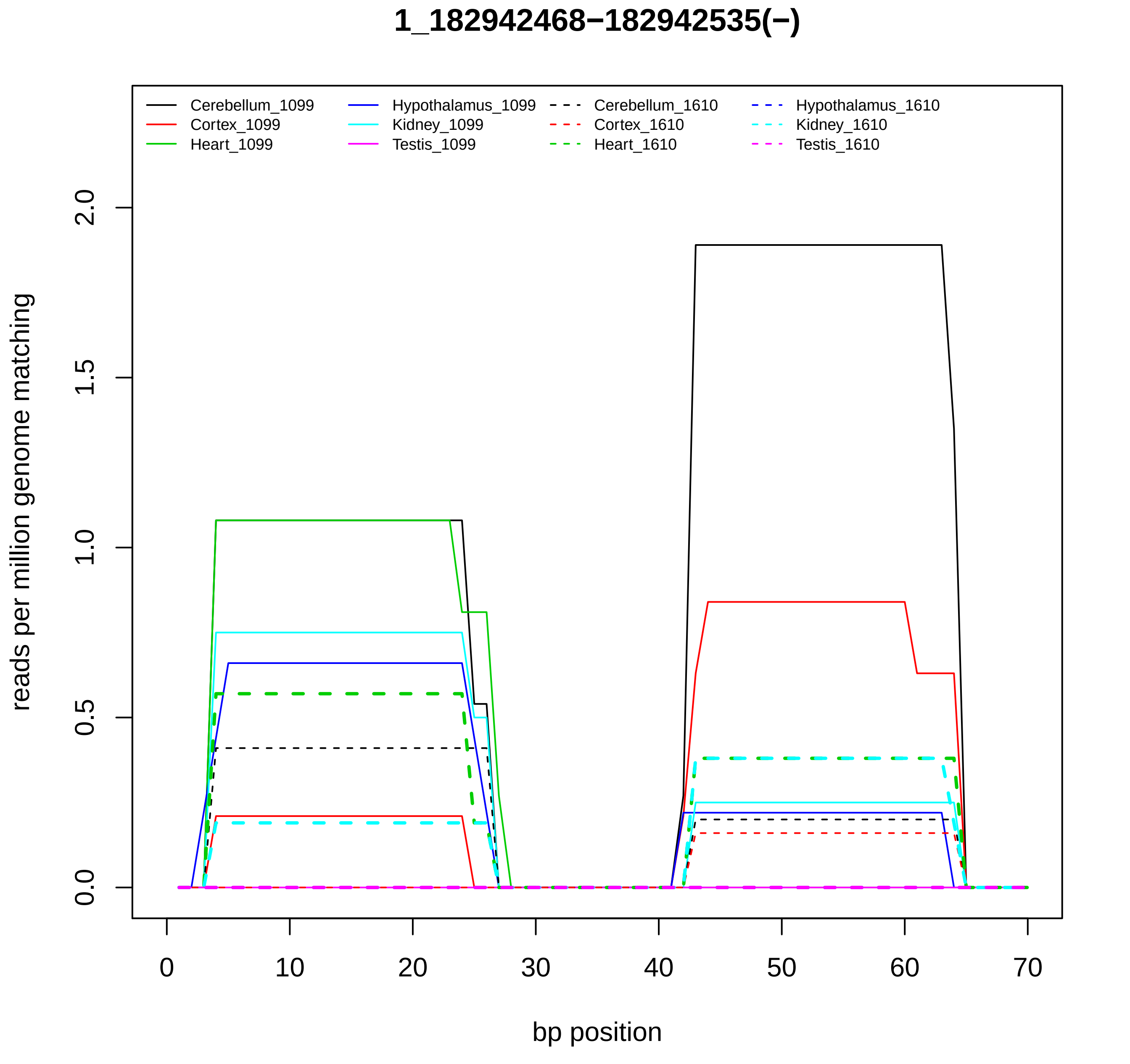

1_182942468−182942535(−)
Cerebellum_1099
Cortex_1099
Heart_1099
Hypothalamus_1099
Kidney_1099
Testis_1099
Cerebellum_1610
Cortex_1610
Heart_1610
Hypothalamus_1610
Kidney_1610
Testis_1610
reads per million genome matching
0.0
0.5
1.0
1.5
2.0
0
10
20
30
40
50
60
70
bp position

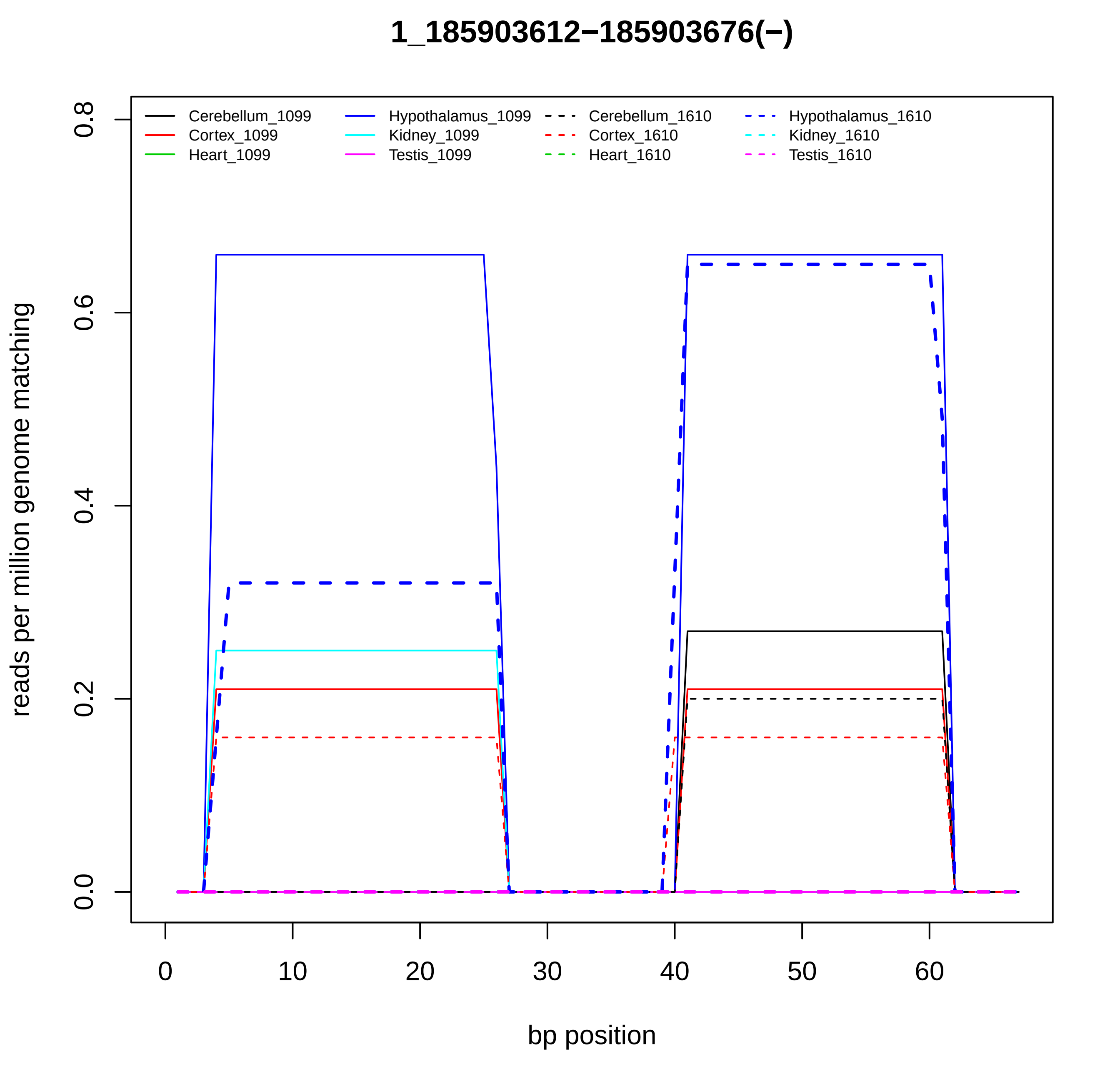

1_185903612−185903676(−)
Cerebellum_1099
Cortex_1099
Heart_1099
Hypothalamus_1099
Kidney_1099
Testis_1099
Cerebellum_1610
Cortex_1610
Heart_1610
Hypothalamus_1610
Kidney_1610
Testis_1610
0.0
0.2
0.4
0.6
0.8
reads per million genome matching
0
10
20
30
40
50
60
bp position

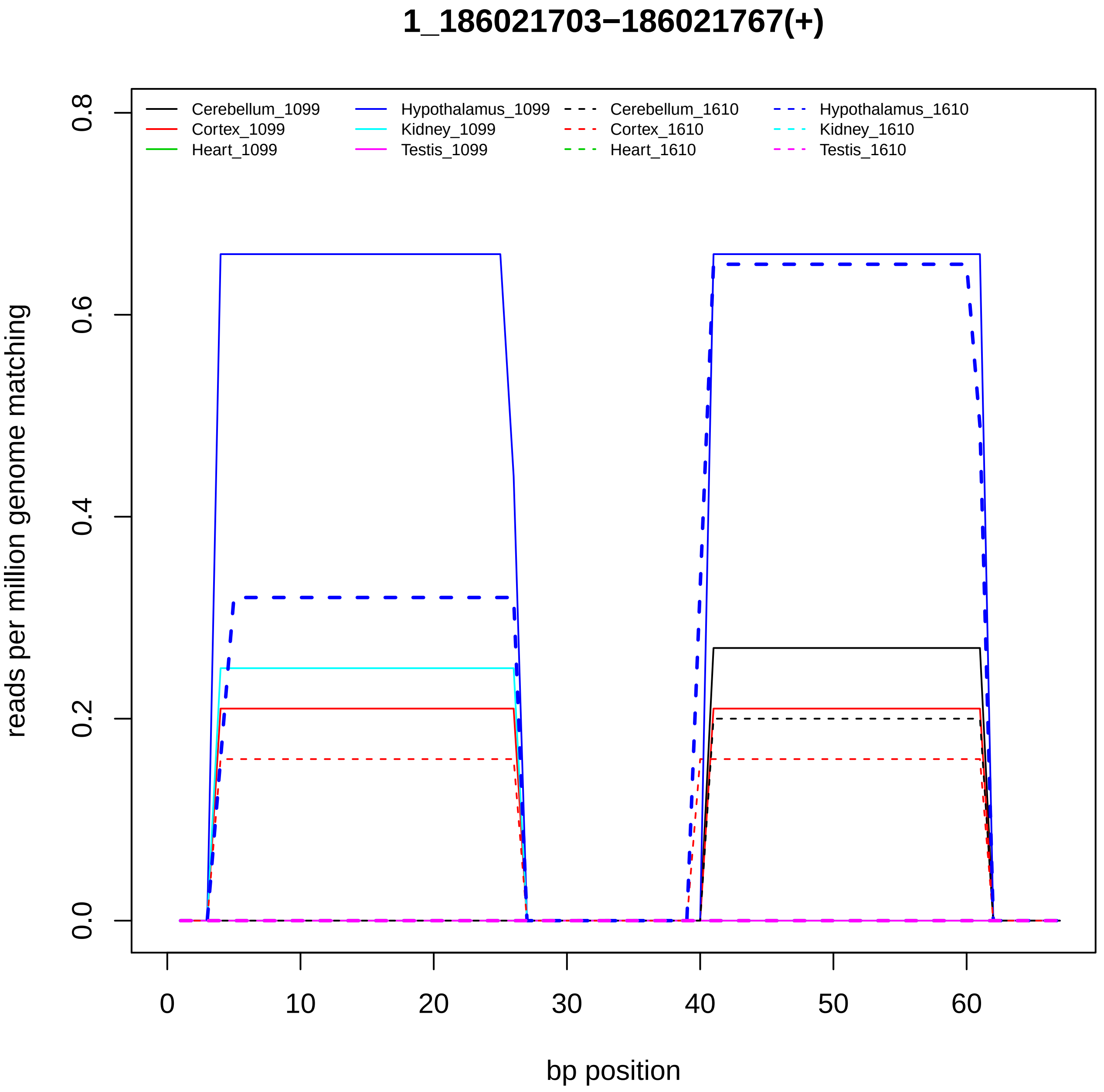

1_186021703−186021767(+)
Cerebellum_1099
Cortex_1099
Heart_1099
Hypothalamus_1099
Kidney_1099
Testis_1099
Cerebellum_1610
Cortex_1610
Heart_1610
Hypothalamus_1610
Kidney_1610
Testis_1610
reads per million genome matching
0.0
0.2
0.4
0.6
0.8
0
10
20
30
40
50
60
bp position

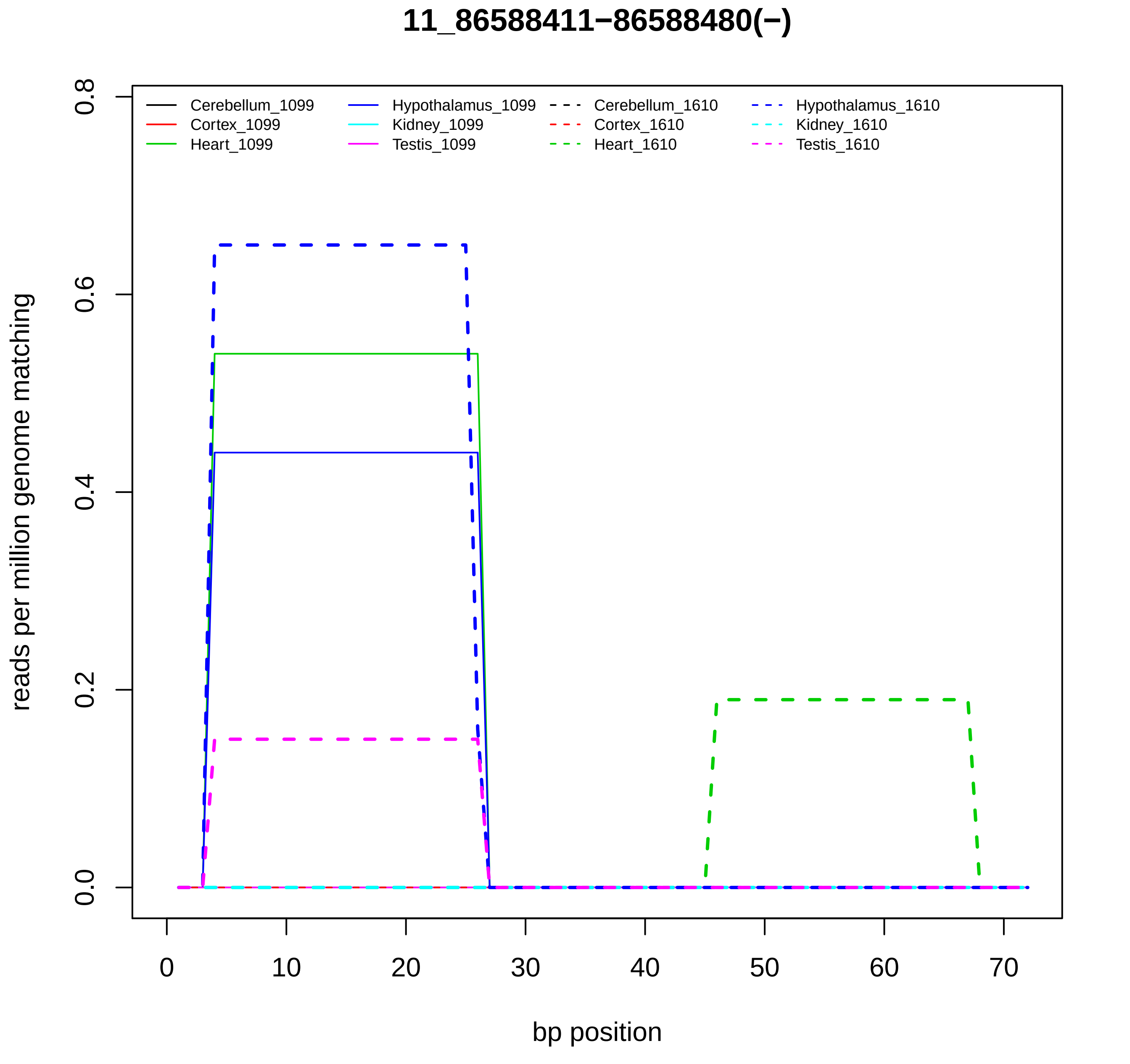

11_86588411−86588480(−)
Cerebellum_1099
Cortex_1099
Heart_1099
Hypothalamus_1099
Kidney_1099
Testis_1099
Cerebellum_1610
Cortex_1610
Heart_1610
Hypothalamus_1610
Kidney_1610
Testis_1610
reads per million genome matching
0.0
0.2
0.4
0.6
0.8
0
10
20
30
40
50
60
70
bp position

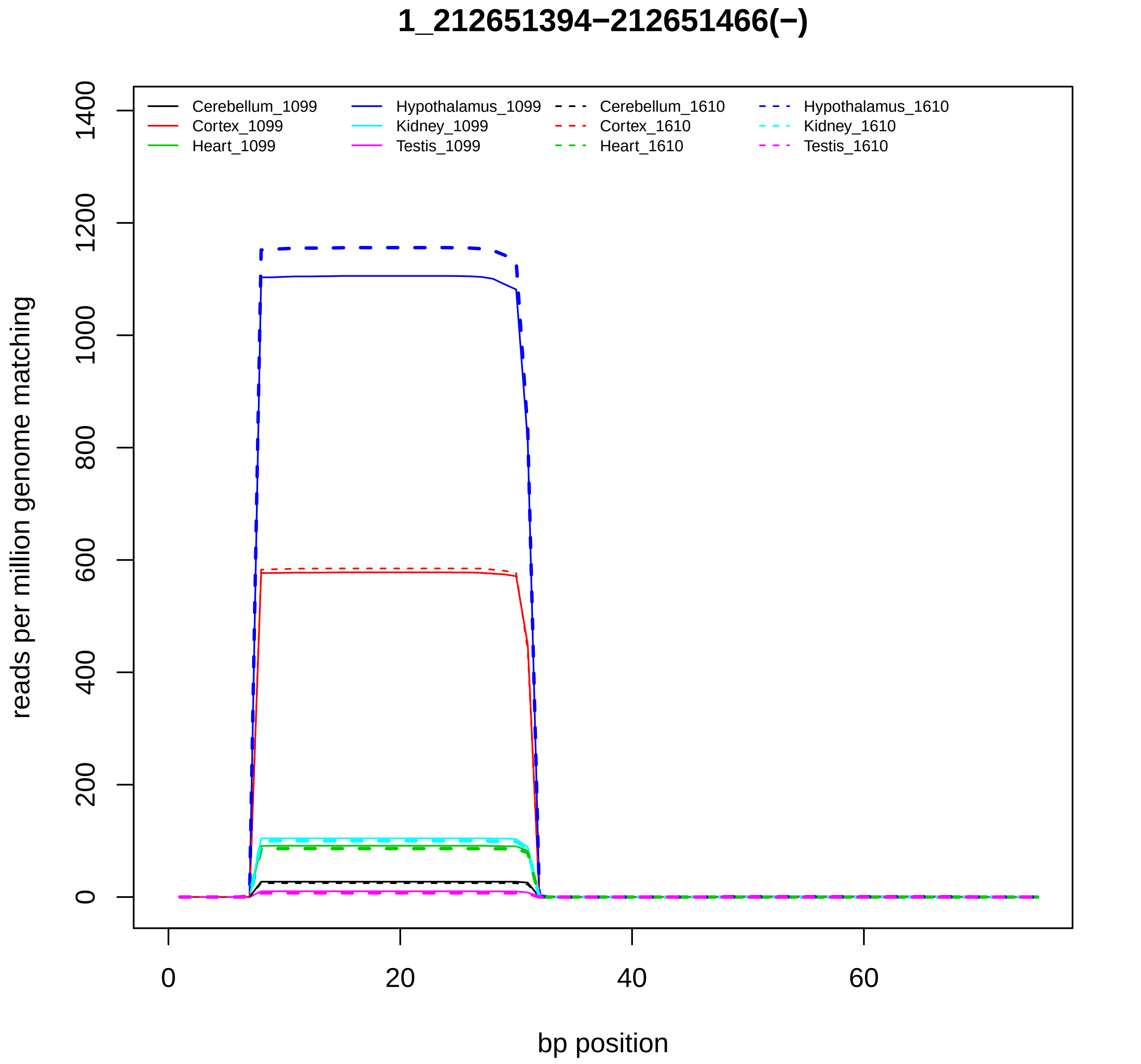

1_212651394−212651466(−)
Cerebellum_1099
Cortex_1099
Heart_1099
Hypothalamus_1099
Kidney_1099
Testis_1099
Cerebellum_1610
Cortex_1610
Heart_1610
Hypothalamus_1610
Kidney_1610
Testis_1610
reads per million genome matching
0
200
400
600
800
1000
1200
1400
0
20
40
60
bp position

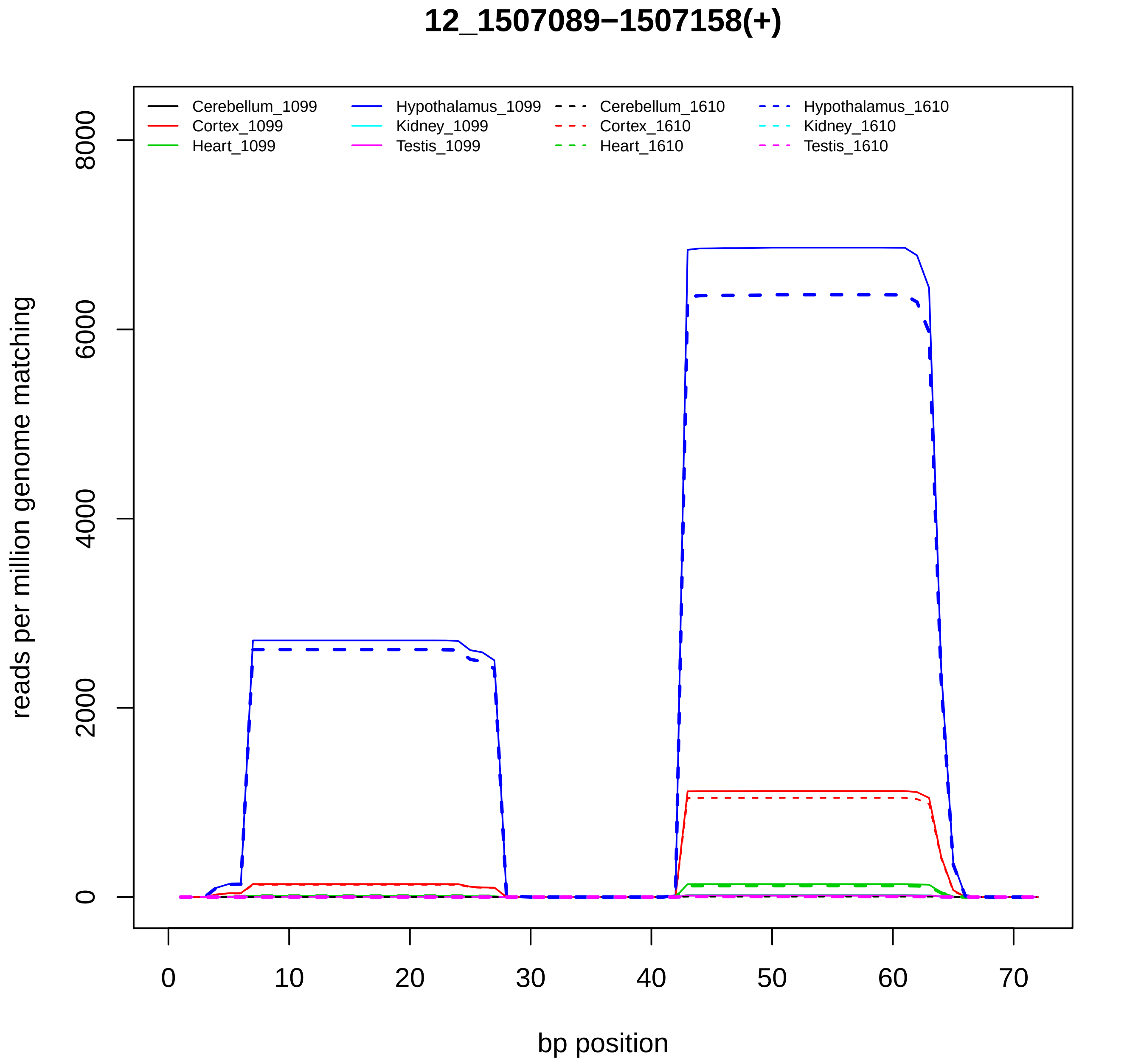

12_1507089−1507158(+)
Cerebellum_1099
Cortex_1099
Heart_1099
Hypothalamus_1099
Kidney_1099
Testis_1099
Cerebellum_1610
Cortex_1610
Heart_1610
Hypothalamus_1610
Kidney_1610
Testis_1610
reads per million genome matching
0
2000
4000
6000
8000
0
10
20
30
40
50
60
70
bp position

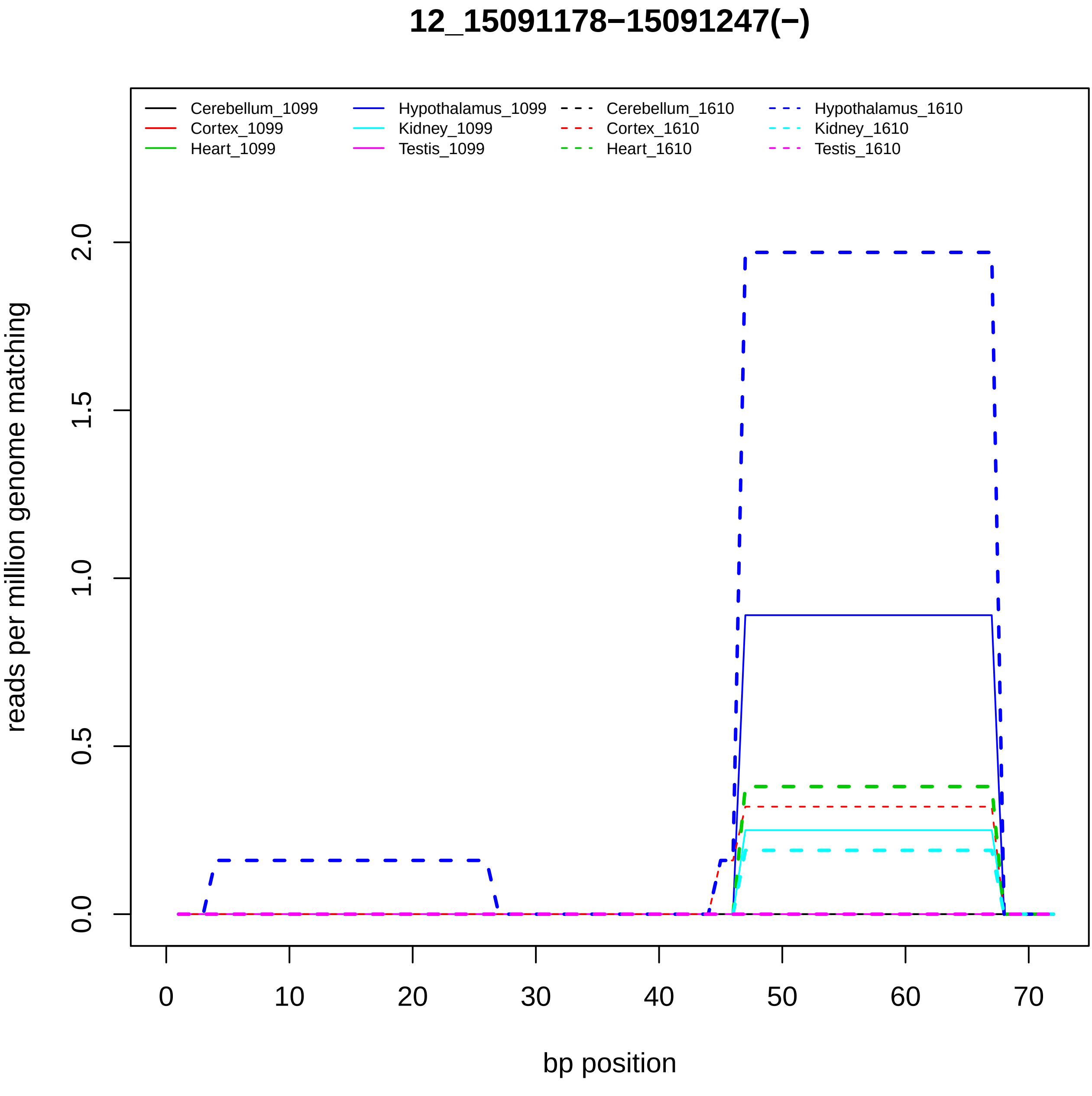

12_15091178−15091247(−)
Cerebellum_1099
Cortex_1099
Heart_1099
Hypothalamus_1099
Kidney_1099
Testis_1099
Cerebellum_1610
Cortex_1610
Heart_1610
Hypothalamus_1610
Kidney_1610
Testis_1610
reads per million genome matching
0.0
0.5
1.0
1.5
2.0
0
10
20
30
40
50
60
70
bp position

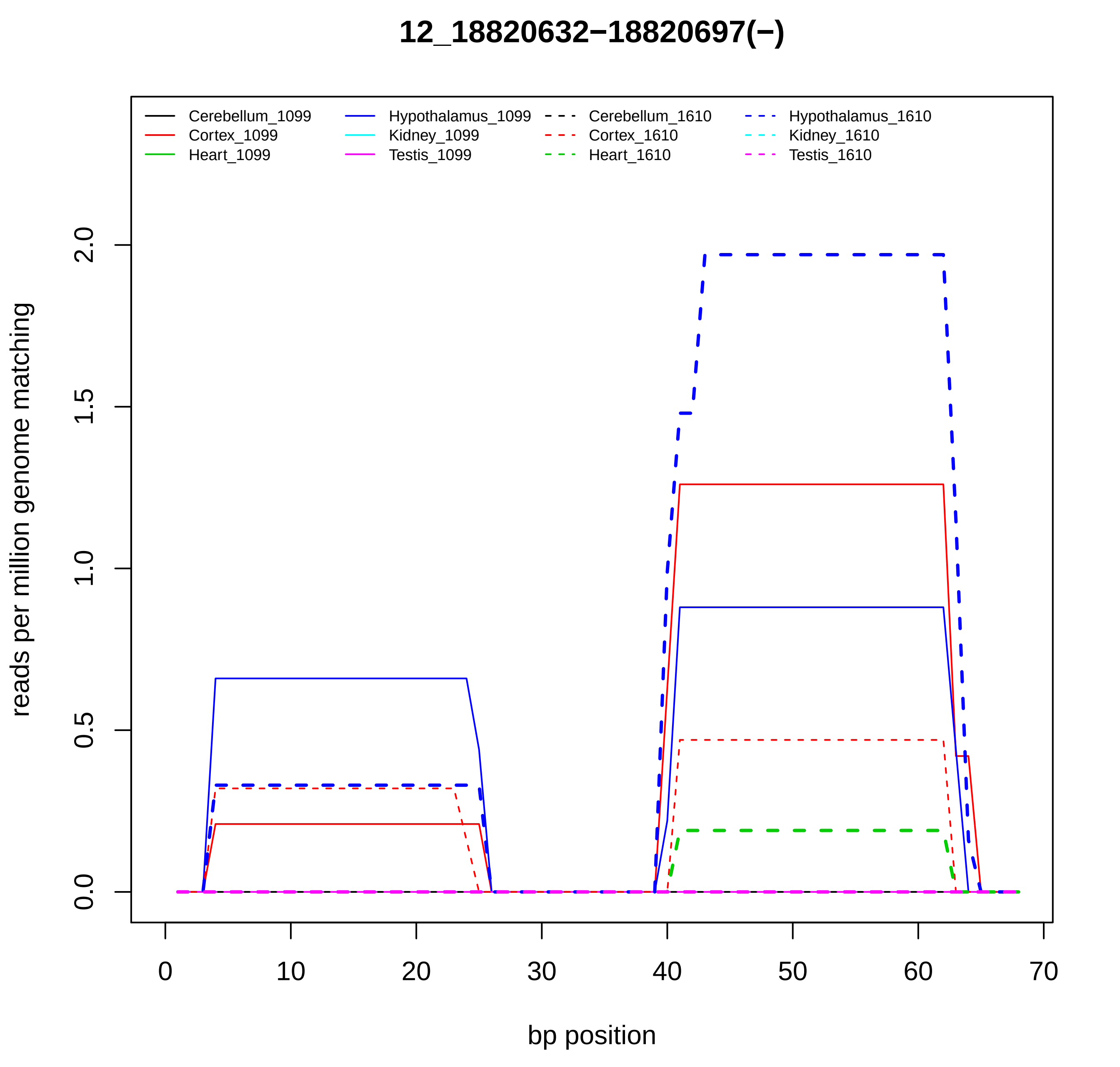

12_18820632−18820697(−)
Cerebellum_1099
Cortex_1099
Heart_1099
Hypothalamus_1099
Kidney_1099
Testis_1099
Cerebellum_1610
Cortex_1610
Heart_1610
Hypothalamus_1610
Kidney_1610
Testis_1610
reads per million genome matching
0.0
0.5
1.0
1.5
2.0
0
10
20
30
40
50
60
70
bp position

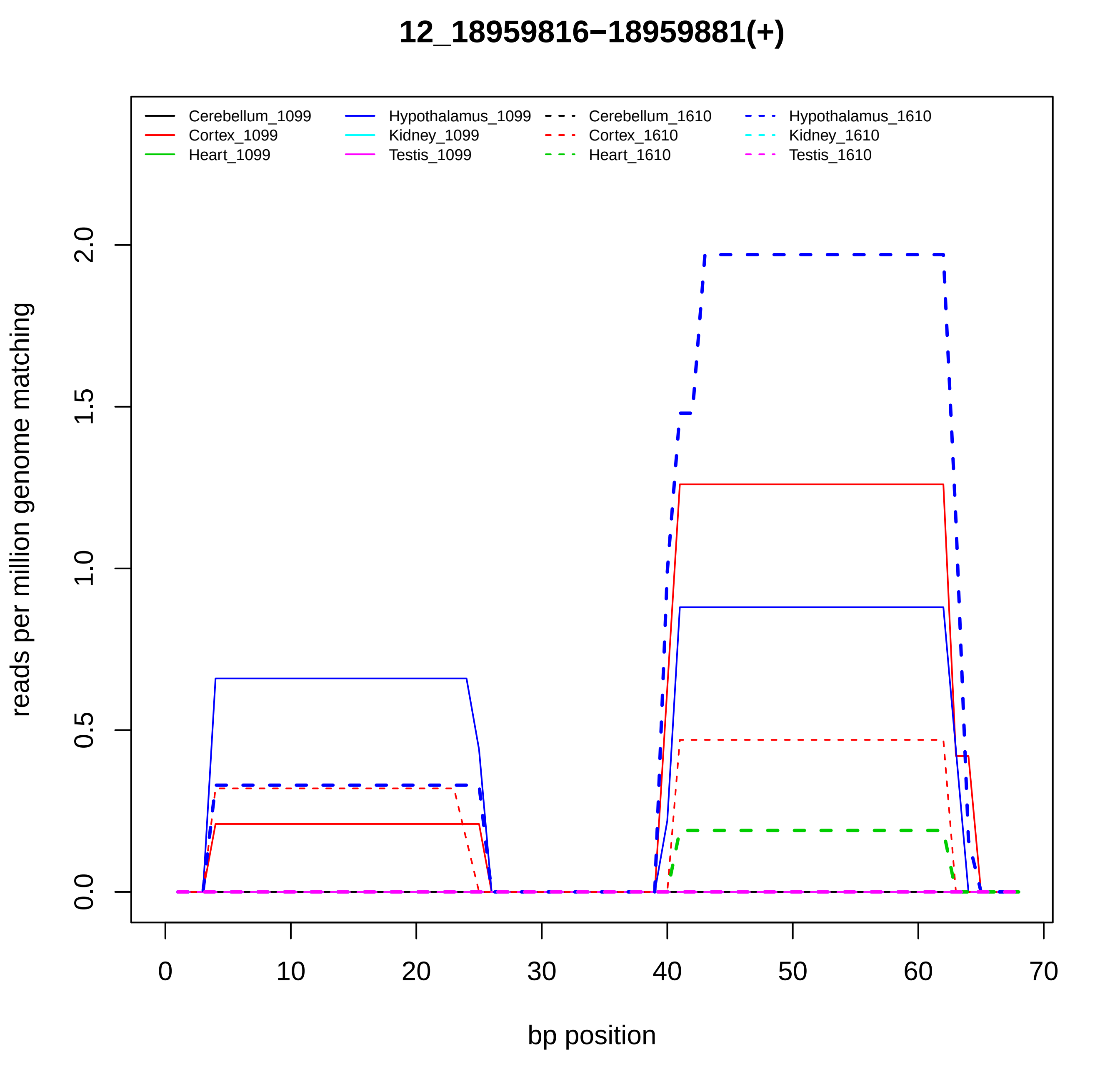

12_18959816−18959881(+)
Cerebellum_1099
Cortex_1099
Heart_1099
Hypothalamus_1099
Kidney_1099
Testis_1099
Cerebellum_1610
Cortex_1610
Heart_1610
Hypothalamus_1610
Kidney_1610
Testis_1610
reads per million genome matching
0.0
0.5
1.0
1.5
2.0
0
10
20
30
40
50
60
70
bp position

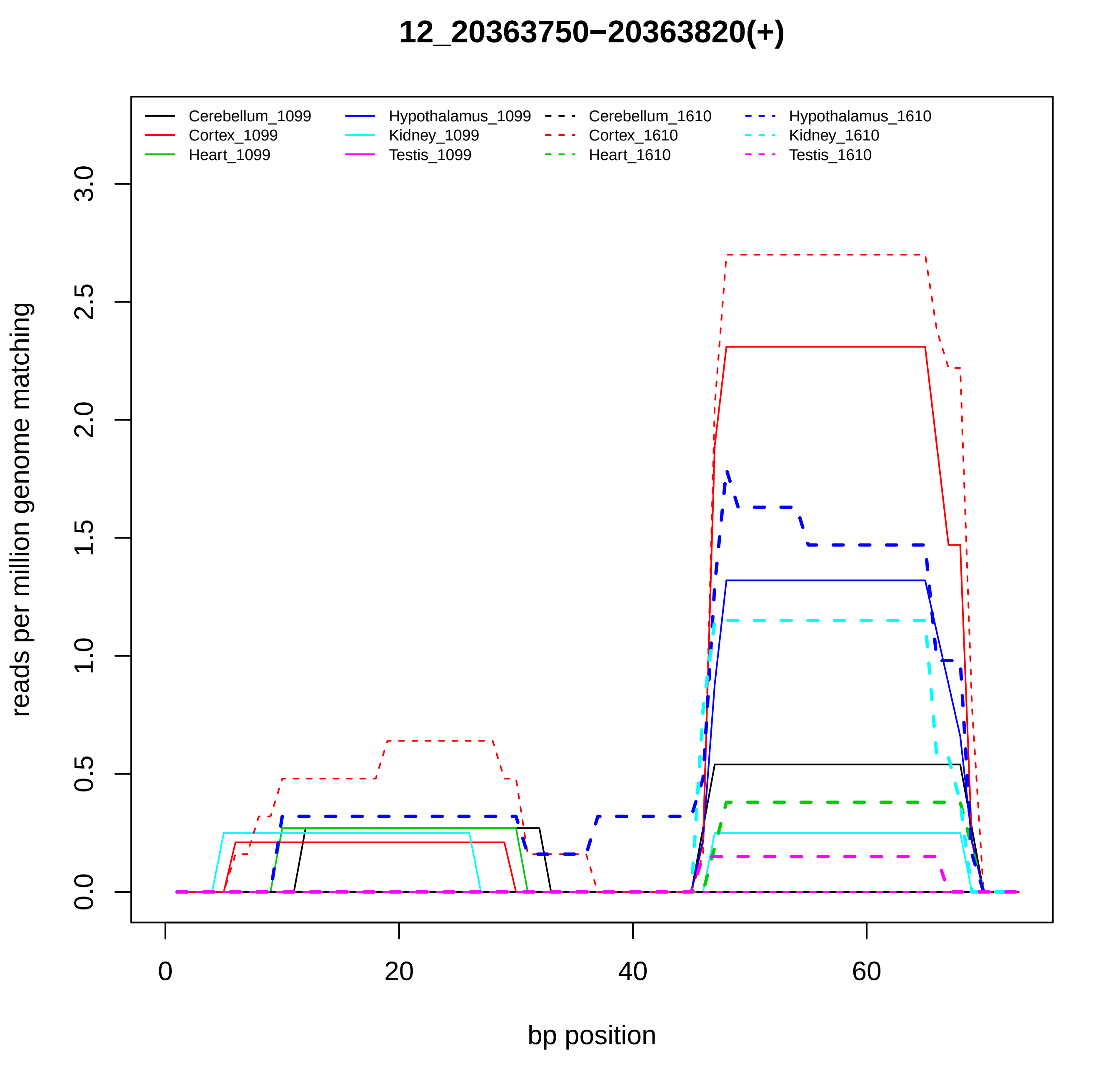
12_20363750−20363820(+)
Cerebellum_1099
Cortex_1099
Heart_1099
Hypothalamus_1099
Kidney_1099
Testis_1099
Cerebellum_1610
Cortex_1610
Heart_1610
Hypothalamus_1610
Kidney_1610
Testis_1610
reads per million genome matching
0.0
0.5
1.0
1.5
2.0
2.5
3.0
bp position
0
20
40
60

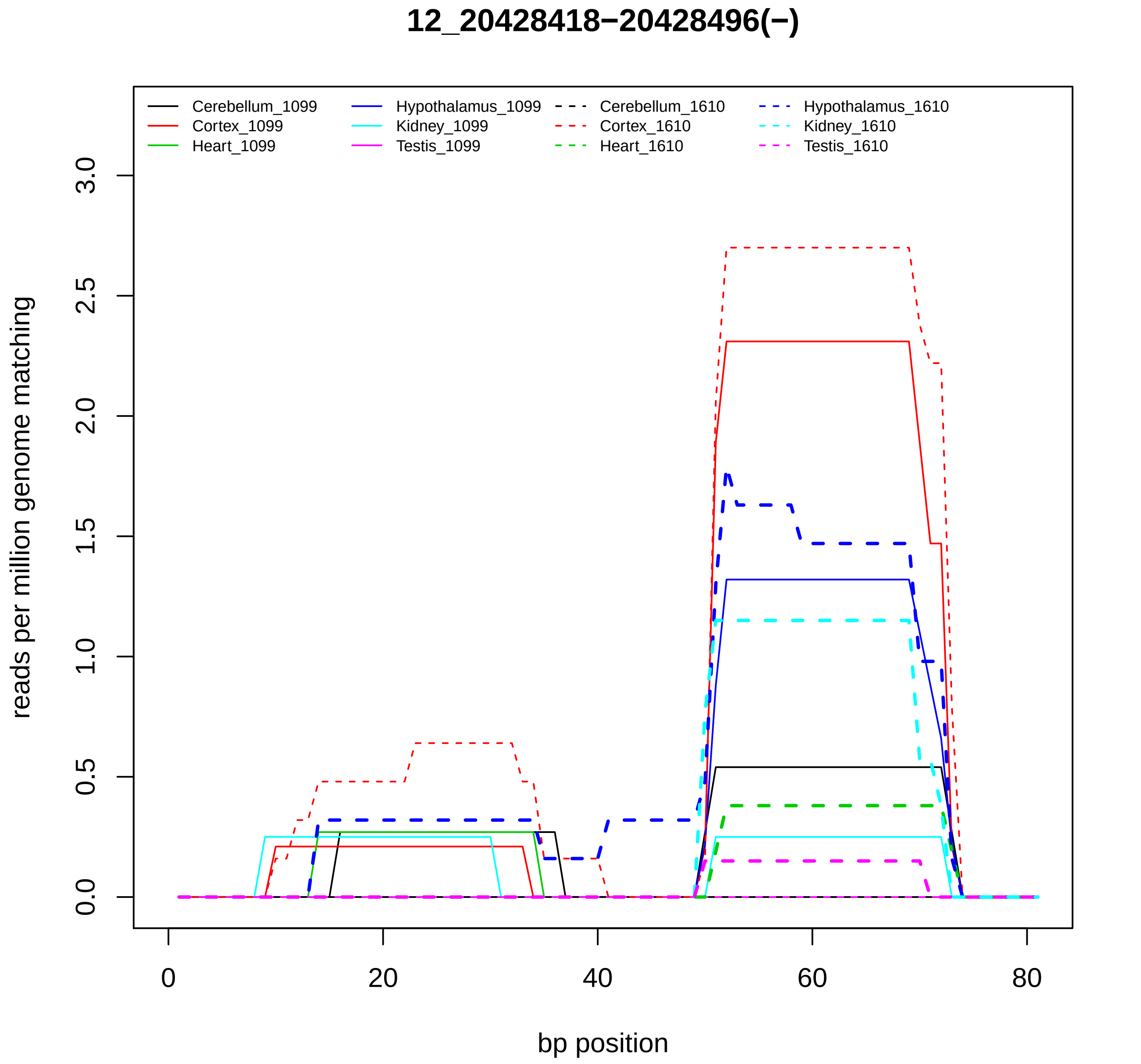

12_20428418−20428496(−)
Cerebellum_1099
Cortex_1099
Heart_1099
Hypothalamus_1099
Kidney_1099
Testis_1099
Cerebellum_1610
Cortex_1610
Heart_1610
Hypothalamus_1610
Kidney_1610
Testis_1610
reads per million genome matching
0.0
0.5
1.0
1.5
2.0
2.5
3.0
0
20
40
60
80
bp position

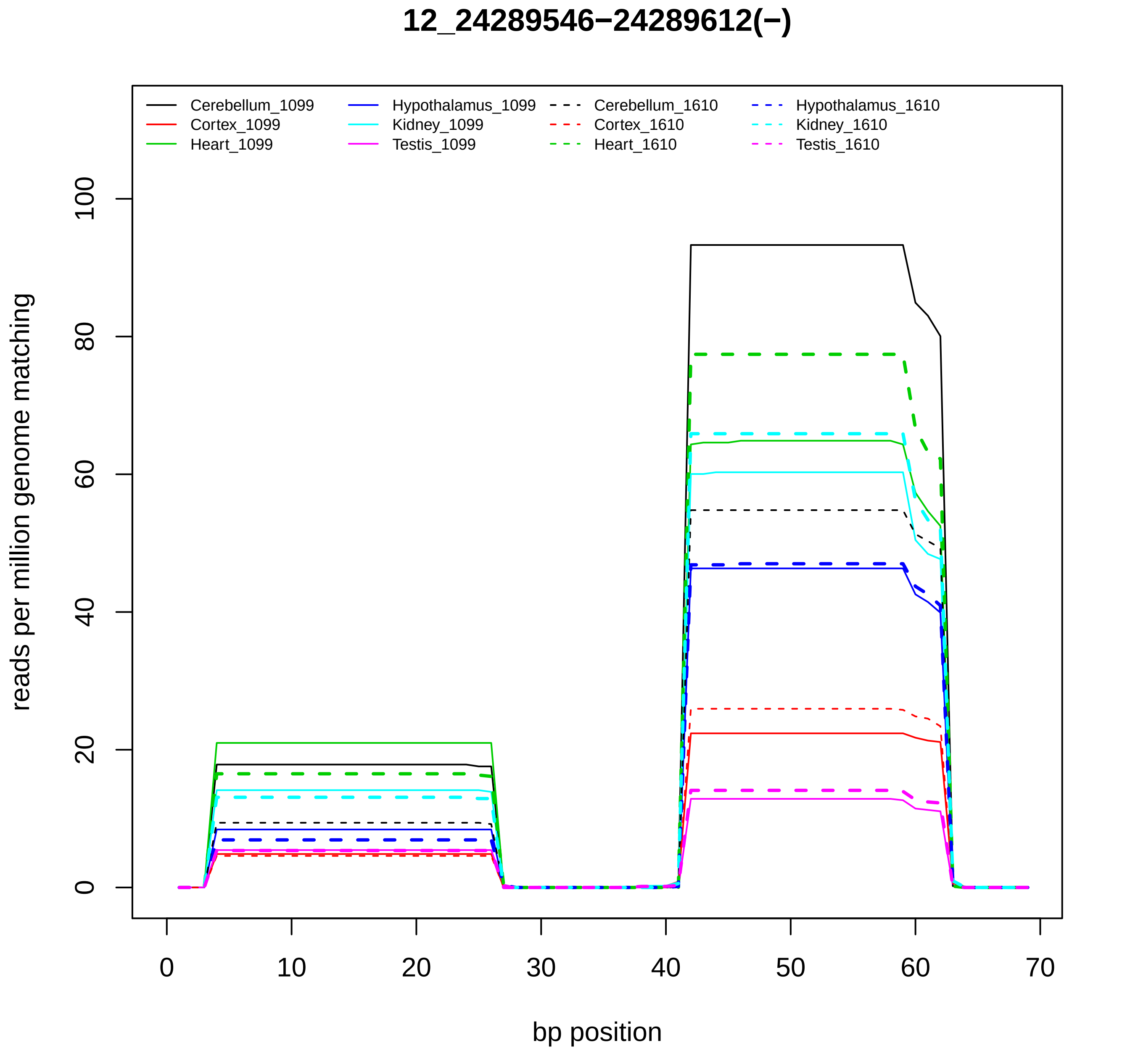

12_24289546−24289612(−)
Cerebellum_1099
Cortex_1099
Heart_1099
Hypothalamus_1099
Kidney_1099
Testis_1099
Cerebellum_1610
Cortex_1610
Heart_1610
Hypothalamus_1610
Kidney_1610
Testis_1610
reads per million genome matching
bp position
0
20
40
60
80
100
0
10
20
30
40
50
60
70

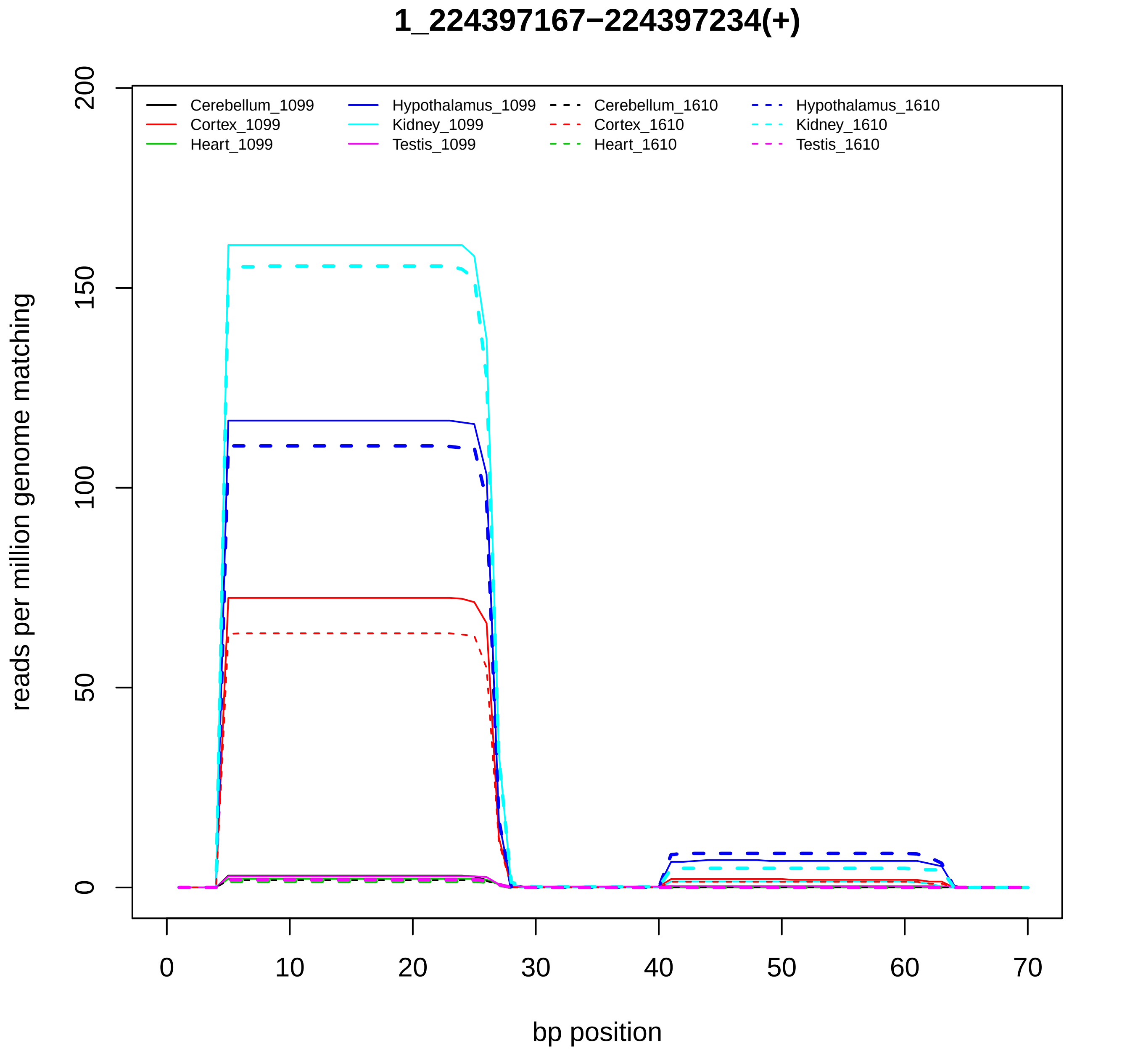

1_224397167−224397234(+)
reads per million genome matching
bp position
0
50
100
150
200
0
10
20
30
40
50
60
70
Cerebellum_1099
Cortex_1099
Heart_1099
Hypothalamus_1099
Kidney_1099
Testis_1099
Cerebellum_1610
Cortex_1610
Heart_1610
Hypothalamus_1610
Kidney_1610
Testis_1610

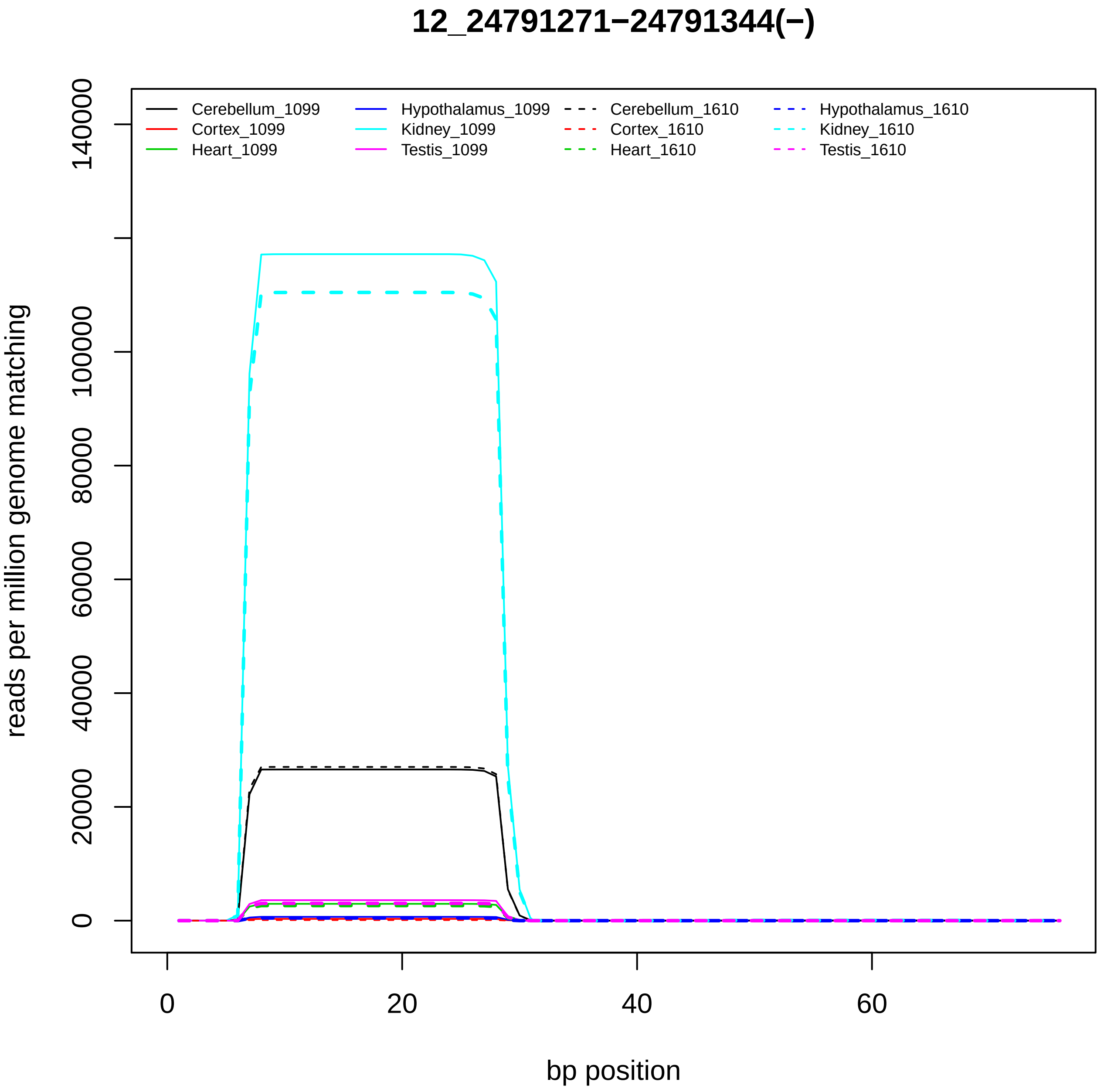

12_24791271−24791344(−)
Cerebellum_1099
Cortex_1099
Heart_1099
Hypothalamus_1099
Kidney_1099
Testis_1099
Cerebellum_1610
Cortex_1610
Heart_1610
Hypothalamus_1610
Kidney_1610
Testis_1610
reads per million genome matching
0
20000
40000
60000
80000
100000
140000
bp position
0
20
40
60

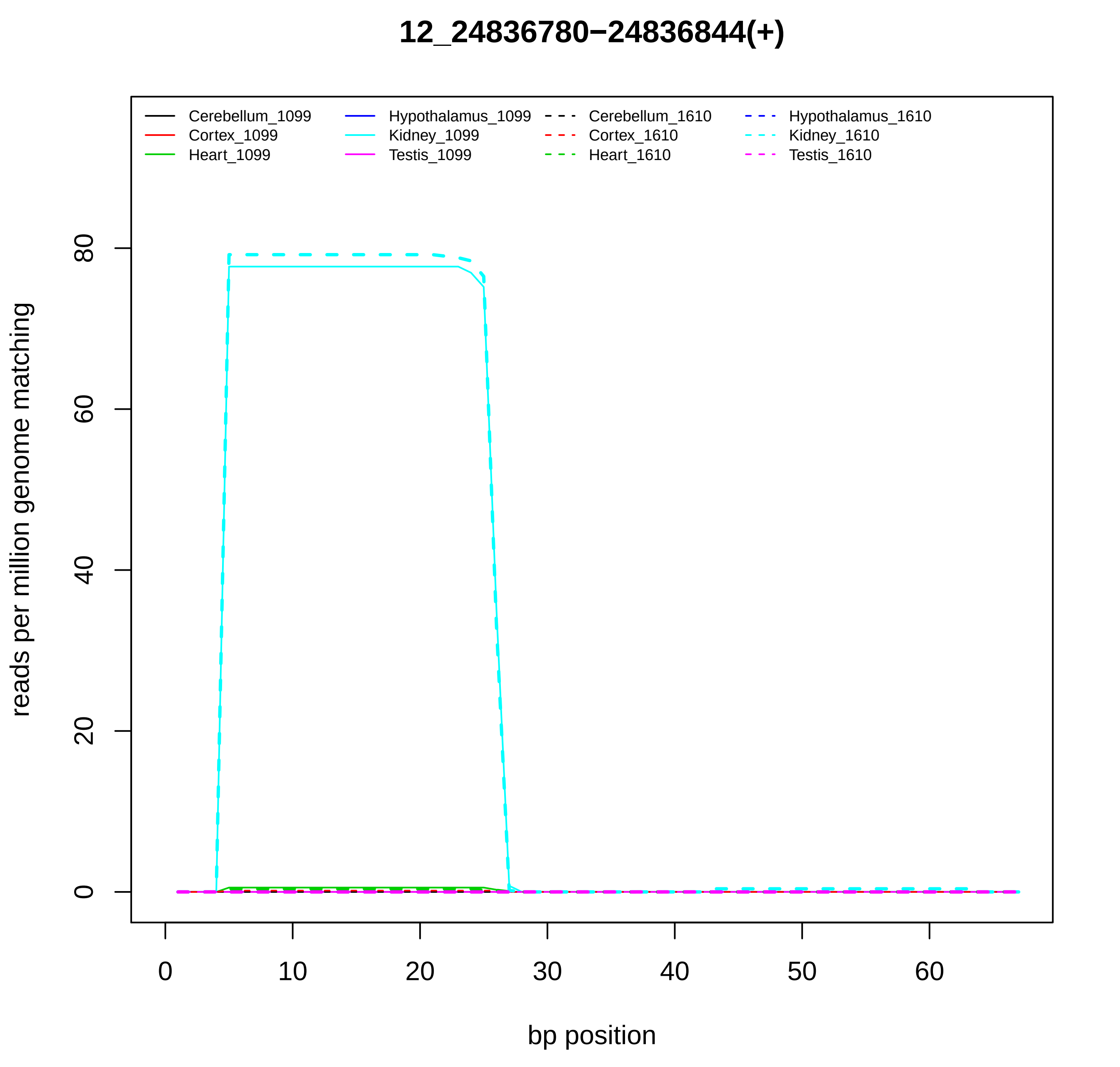

12_24836780−24836844(+)
Cerebellum_1099
Cortex_1099
Heart_1099
Hypothalamus_1099
Kidney_1099
Testis_1099
Cerebellum_1610
Cortex_1610
Heart_1610
Hypothalamus_1610
Kidney_1610
Testis_1610
reads per million genome matching
bp position
0
20
40
60
80
0
10
20
30
40
50
60

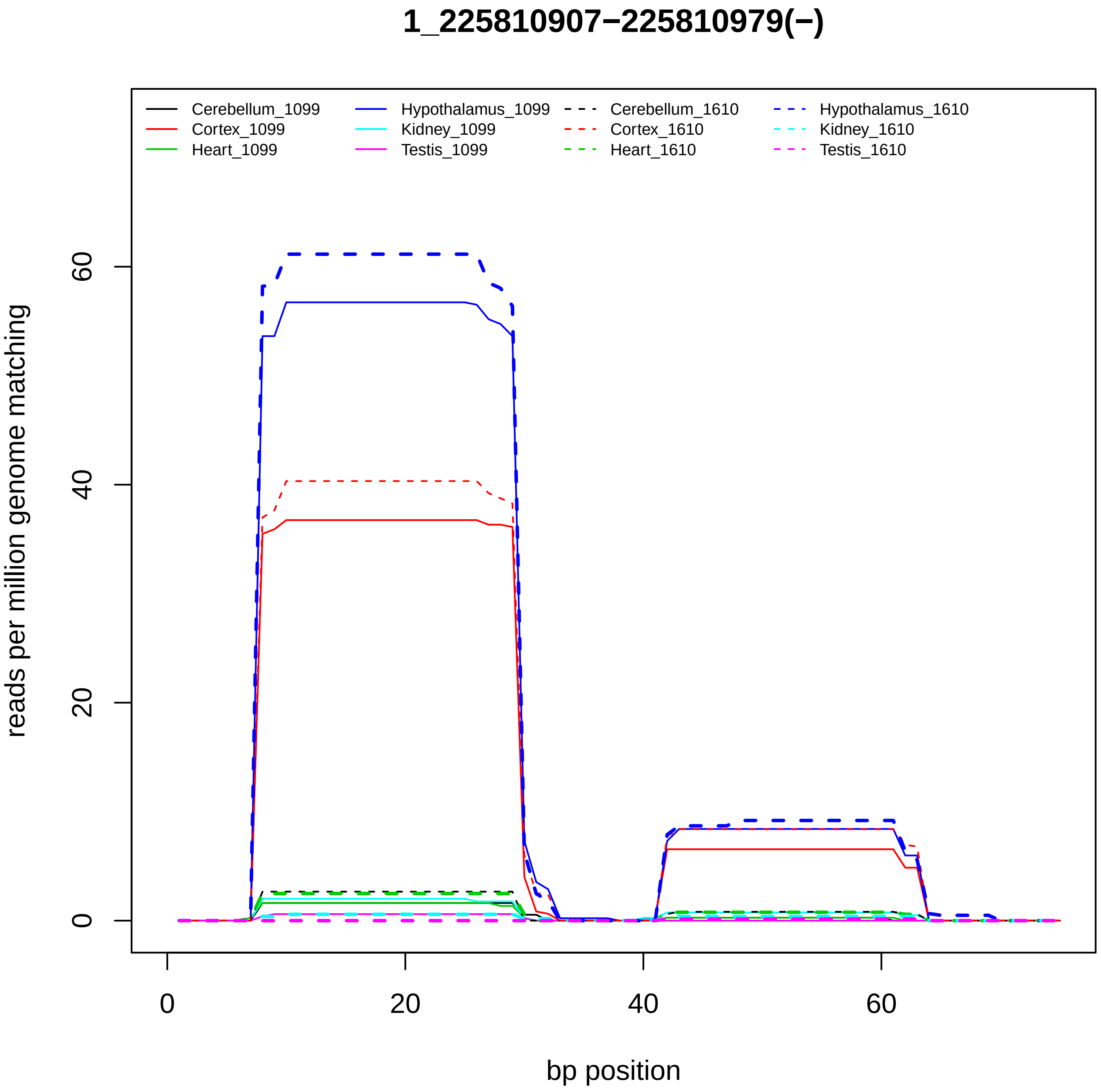

1_225810907−225810979(−)
Cerebellum_1099
Cortex_1099
Heart_1099
Hypothalamus_1099
Kidney_1099
Testis_1099
Cerebellum_1610
Cortex_1610
Heart_1610
Hypothalamus_1610
Kidney_1610
Testis_1610
reads per million genome matching
0
20
40
60
0
20
40
60
bp position

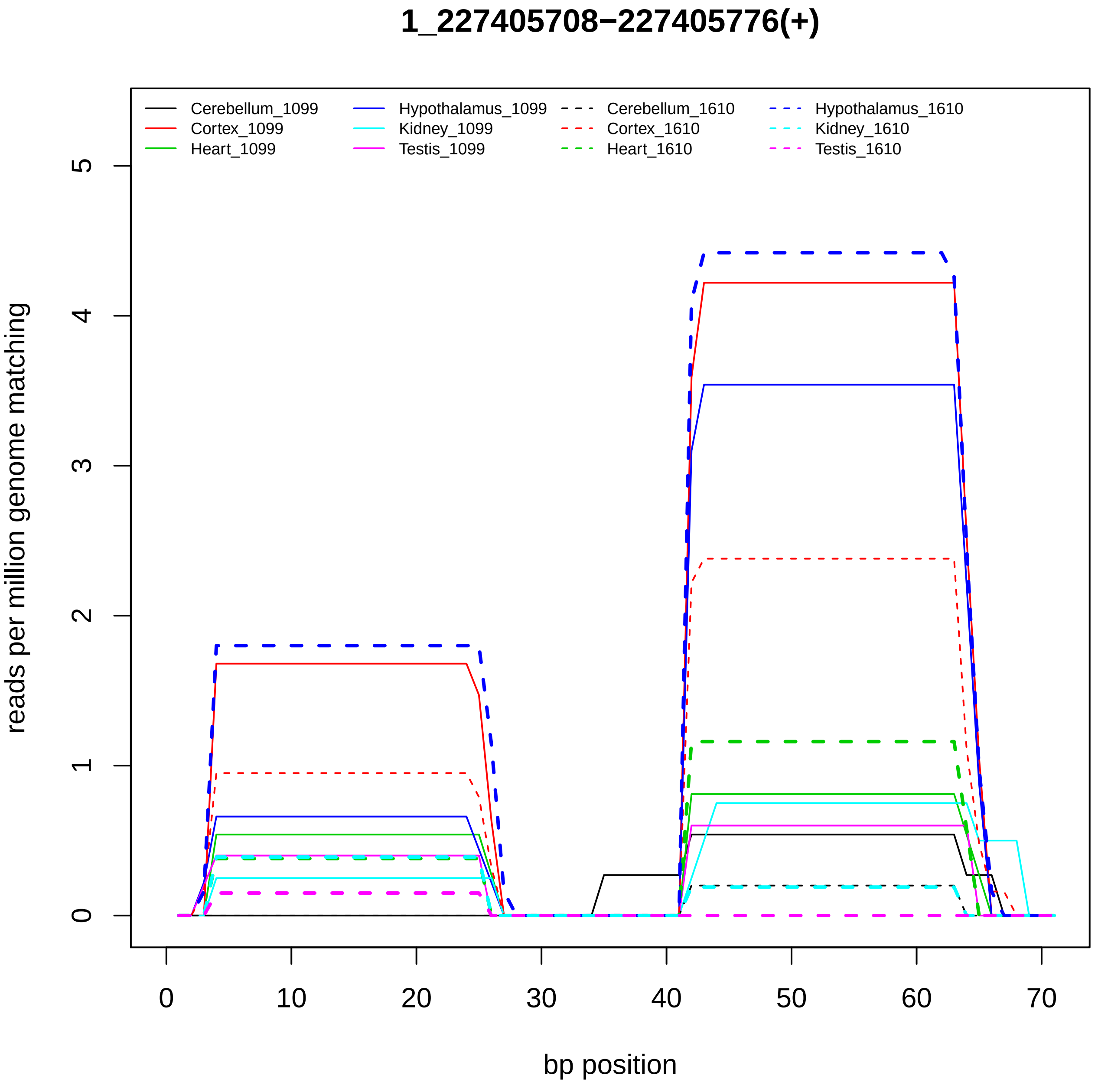

1_227405708−227405776(+)
Cerebellum_1099
Cortex_1099
Heart_1099
Hypothalamus_1099
Kidney_1099
Testis_1099
Cerebellum_1610
Cortex_1610
Heart_1610
Hypothalamus_1610
Kidney_1610
Testis_1610
reads per million genome matching
bp position
0
1
2
3
4
5
0
10
20
30
40
50
60
70

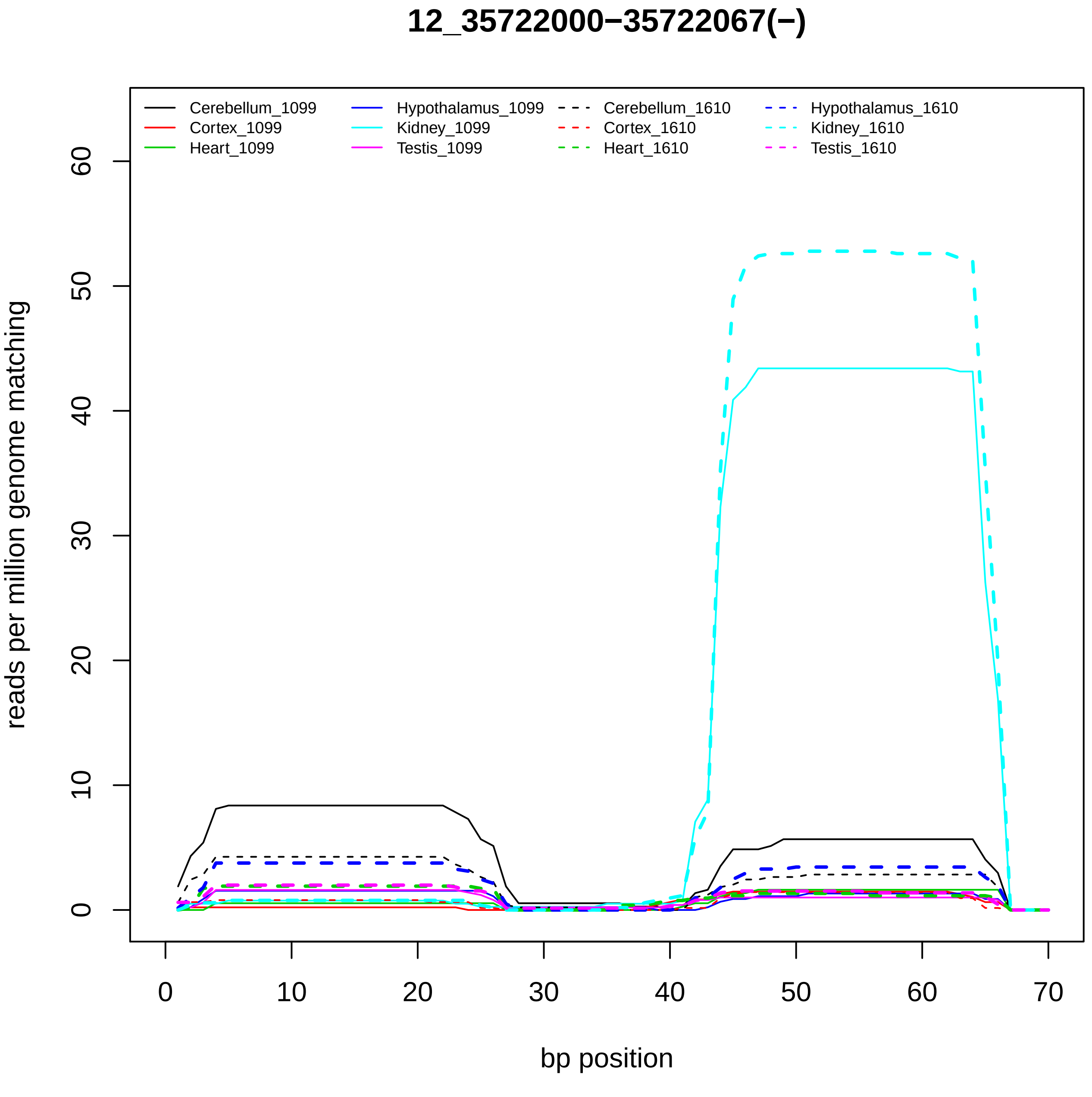

12_35722000−35722067(−)
Cerebellum_1099
Cortex_1099
Heart_1099
Hypothalamus_1099
Kidney_1099
Testis_1099
Cerebellum_1610
Cortex_1610
Heart_1610
Hypothalamus_1610
Kidney_1610
Testis_1610
reads per million genome matching
bp position
0
10
20
30
40
50
60
70

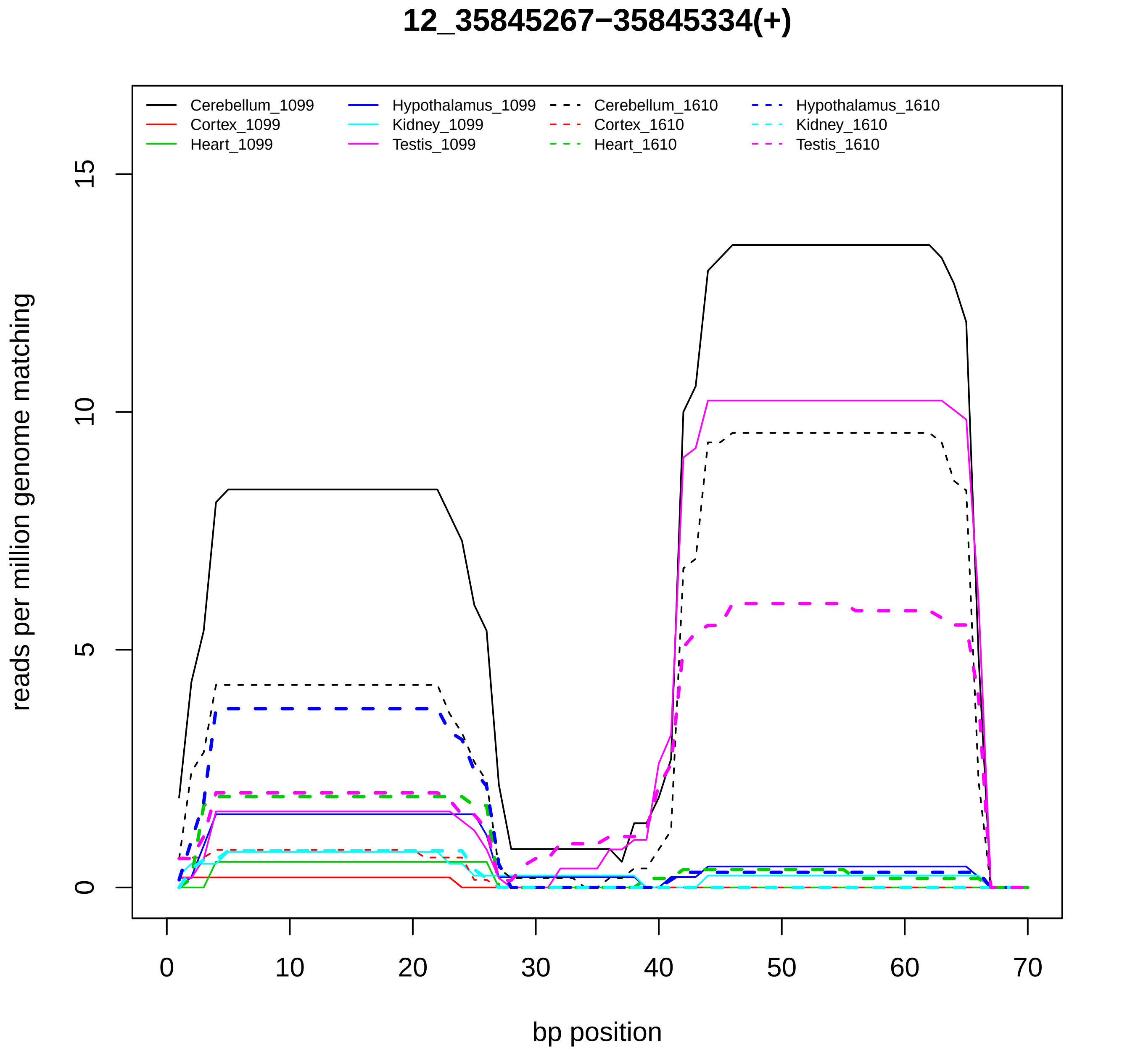

12_35845267−35845334(+)
Cerebellum_1099
Cortex_1099
Heart_1099
Hypothalamus_1099
Kidney_1099
Testis_1099
Cerebellum_1610
Cortex_1610
Heart_1610
Hypothalamus_1610
Kidney_1610
Testis_1610
reads per million genome matching
0
5
10
15
0
10
20
30
40
50
60
70
bp position

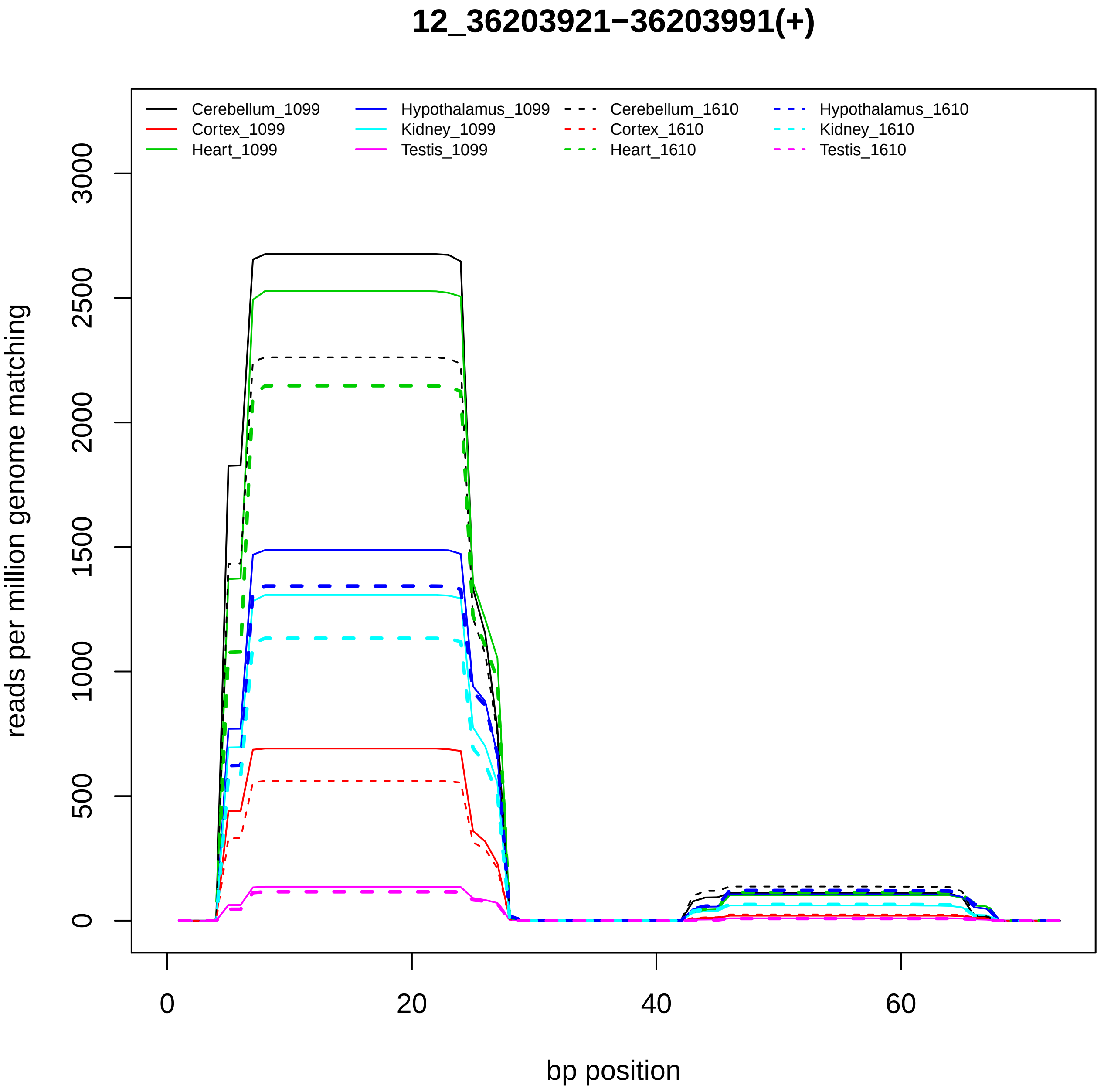
12_36203921−36203991(+)
Cerebellum_1099
Cortex_1099
Heart_1099
Hypothalamus_1099
Kidney_1099
Testis_1099
Cerebellum_1610
Cortex_1610
Heart_1610
Hypothalamus_1610
Kidney_1610
Testis_1610
reads per million genome matching
0
500
1000
1500
2000
2500
3000
bp position
0
20
40
60

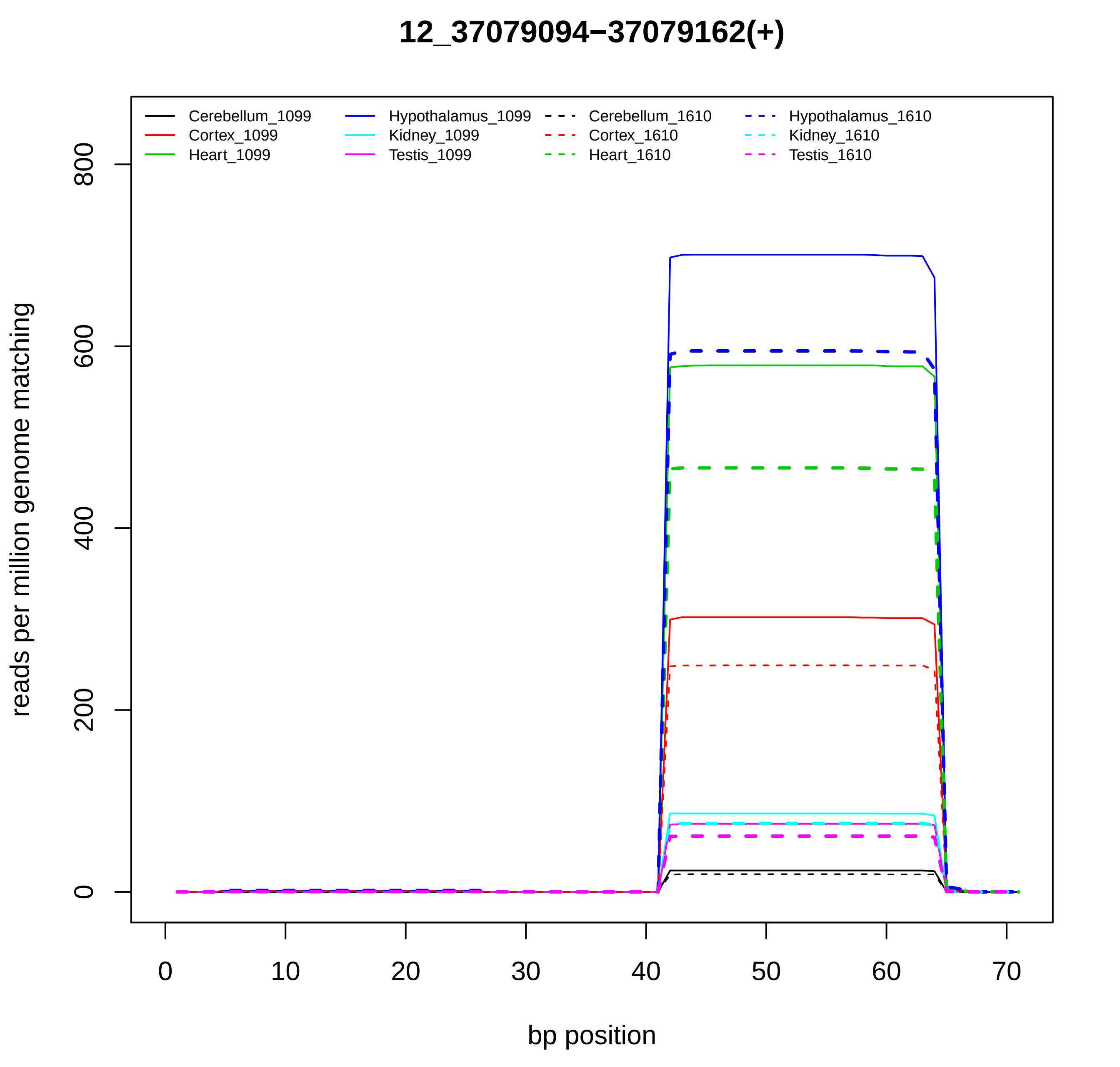
12_37079094−37079162(+)
Cerebellum_1099
Cortex_1099
Heart_1099
Hypothalamus_1099
Kidney_1099
Testis_1099
Cerebellum_1610
Cortex_1610
Heart_1610
Hypothalamus_1610
Kidney_1610
Testis_1610
reads per million genome matching
bp position
0
200
400
600
800
0
10
20
30
40
50
60
70

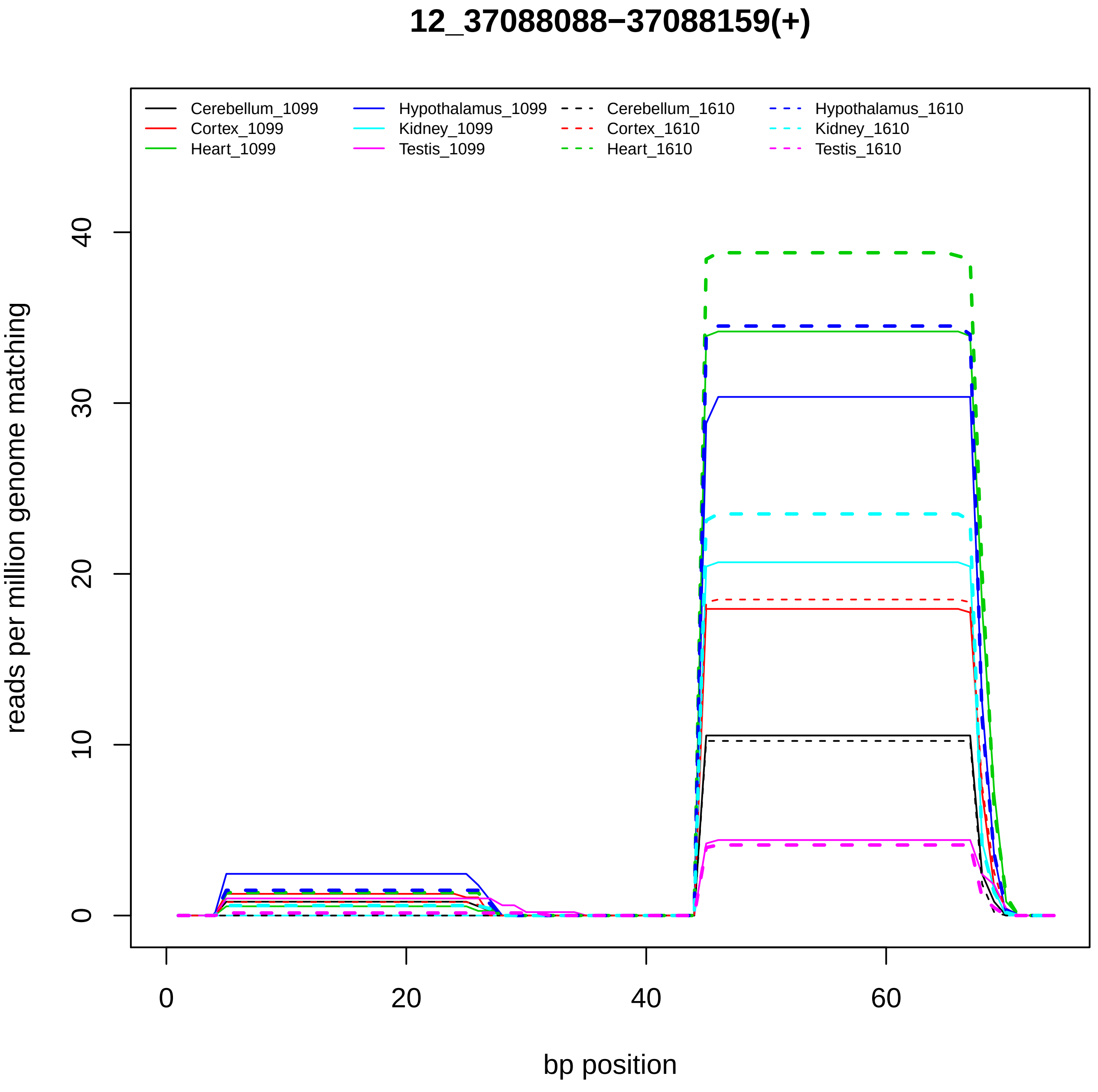

12_37088088−37088159(+)
Cerebellum_1099
Cortex_1099
Heart_1099
Hypothalamus_1099
Kidney_1099
Testis_1099
Cerebellum_1610
Cortex_1610
Heart_1610
Hypothalamus_1610
Kidney_1610
Testis_1610
reads per million genome matching
bp position
0
10
20
30
40
0
20
40
60

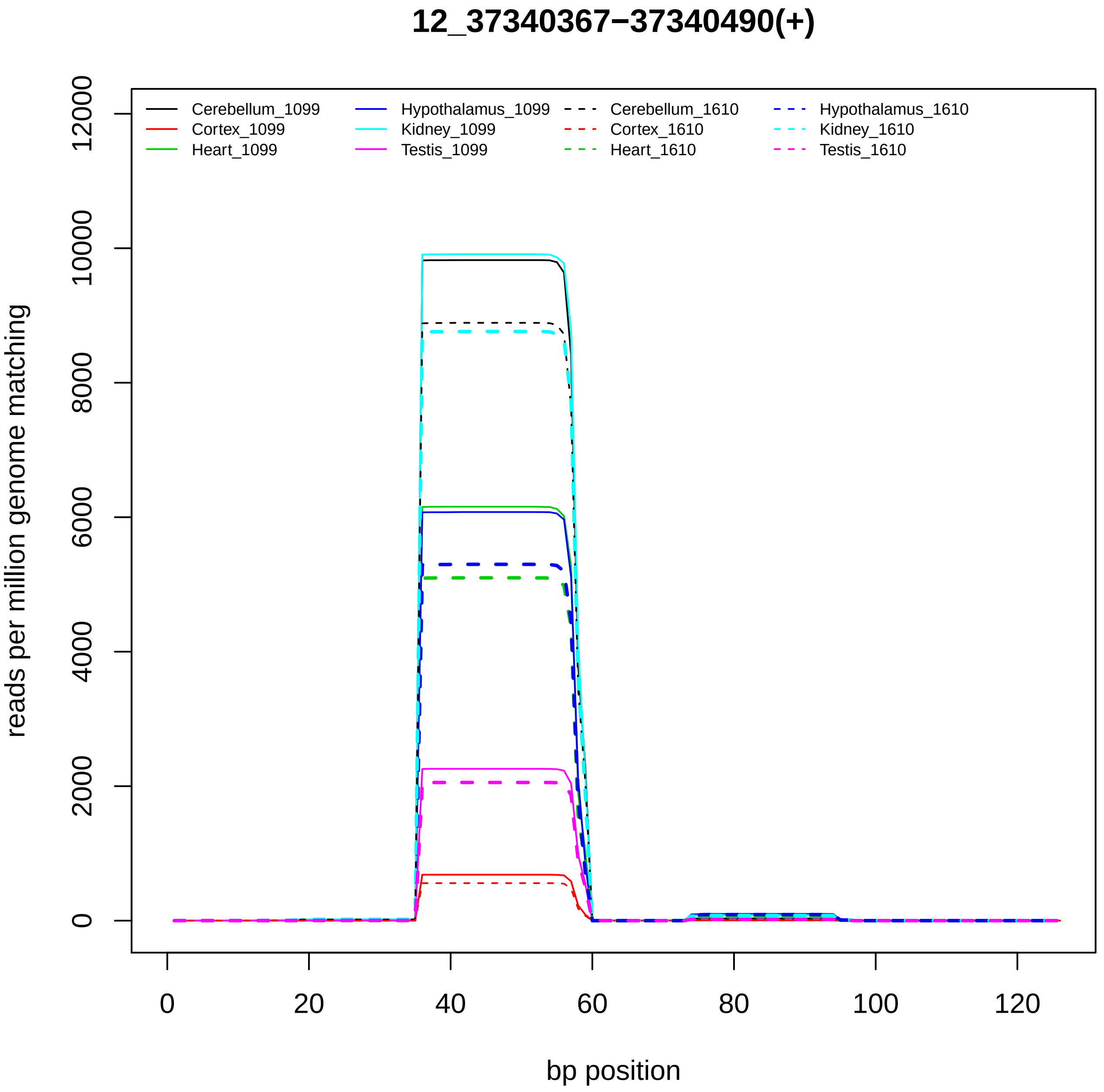

12_37340367−37340490(+)
Cerebellum_1099
Cortex_1099
Heart_1099
Hypothalamus_1099
Kidney_1099
Testis_1099
Cerebellum_1610
Cortex_1610
Heart_1610
Hypothalamus_1610
Kidney_1610
Testis_1610
reads per million genome matching
0
2000
4000
6000
8000
10000
12000
0
20
40
60
80
100
120
bp position

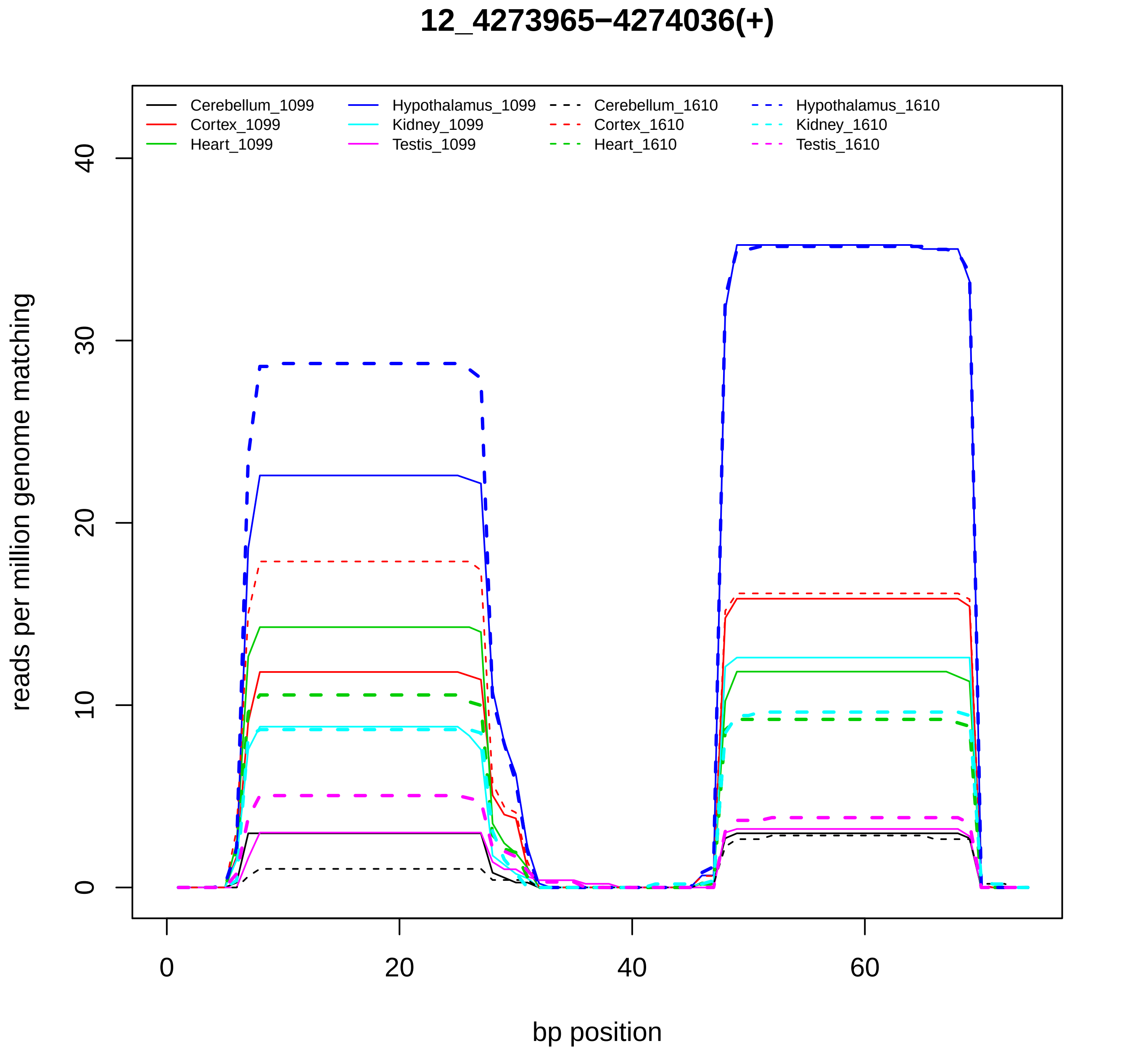

12_4273965−4274036(+)
Cerebellum_1099
Cortex_1099
Heart_1099
Hypothalamus_1099
Kidney_1099
Testis_1099
Cerebellum_1610
Cortex_1610
Heart_1610
Hypothalamus_1610
Kidney_1610
Testis_1610
reads per million genome matching
0
10
20
30
40
0
20
40
60
bp position

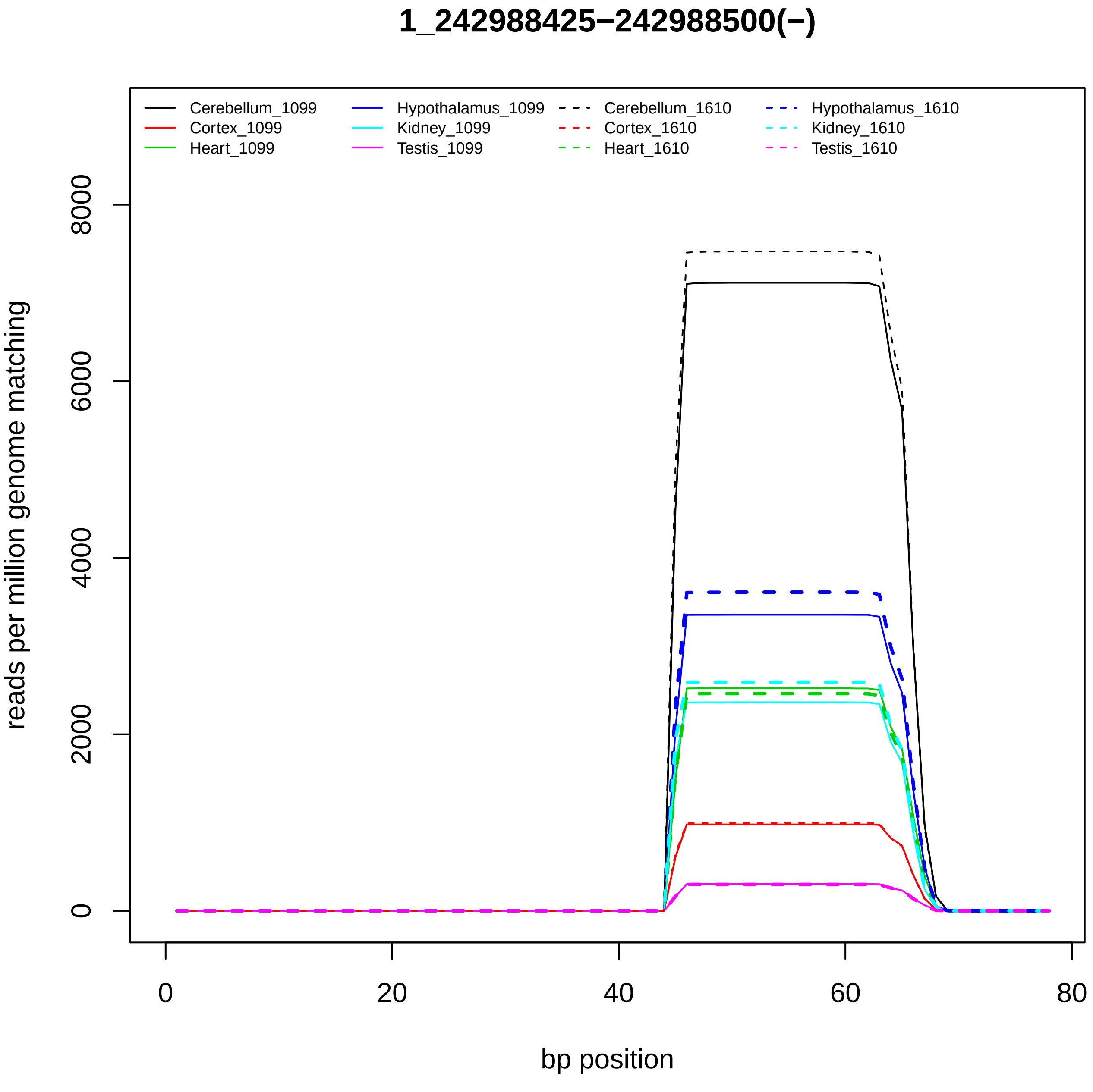

1_242988425−242988500(−)
Cerebellum_1099
Cortex_1099
Heart_1099
Hypothalamus_1099
Kidney_1099
Testis_1099
Cerebellum_1610
Cortex_1610
Heart_1610
Hypothalamus_1610
Kidney_1610
Testis_1610
reads per million genome matching
0
2000
4000
6000
8000
0
20
40
60
80
bp position

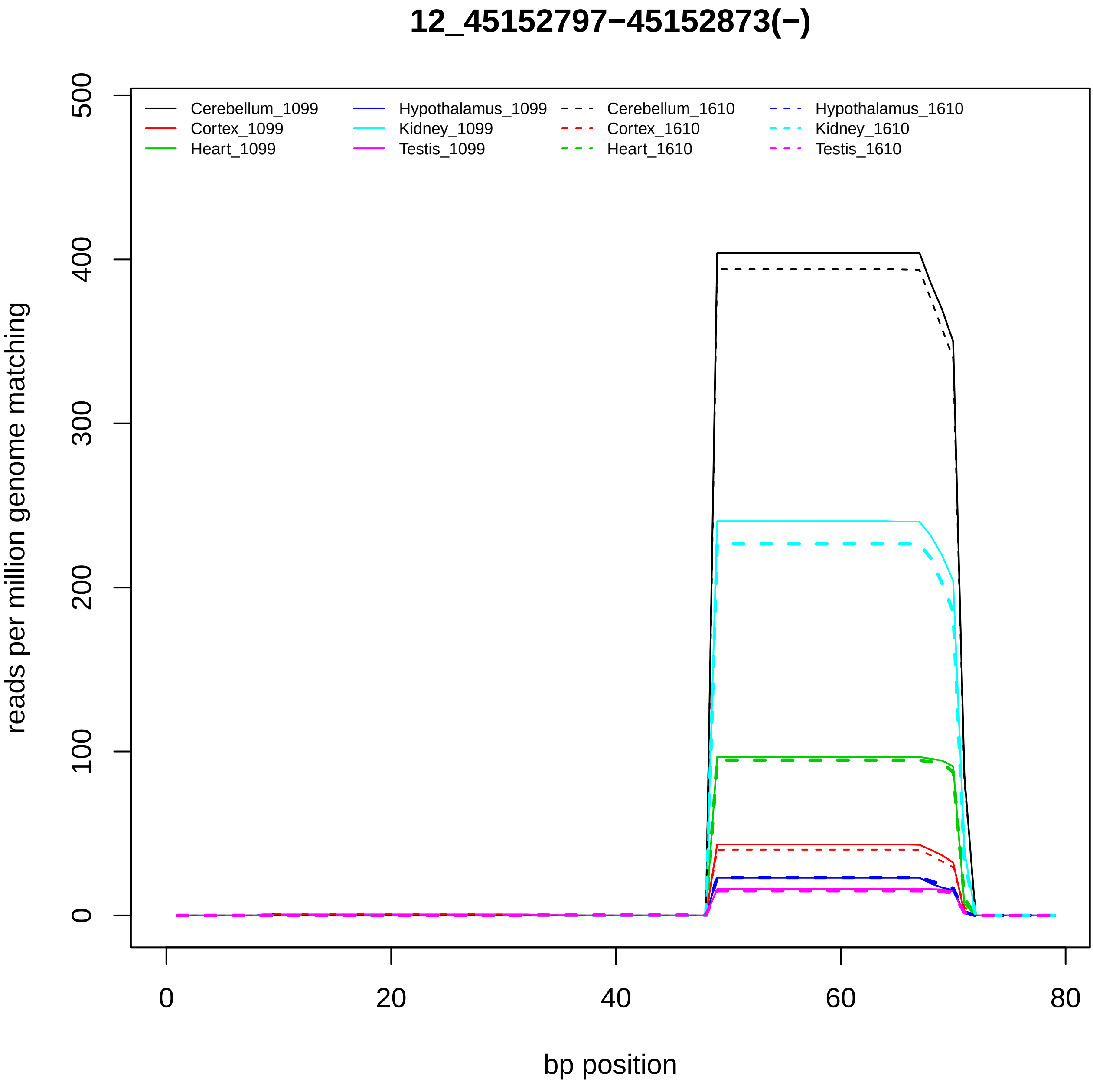
12_45152797−45152873(−)
Cerebellum_1099
Cortex_1099
Heart_1099
Hypothalamus_1099
Kidney_1099
Testis_1099
Cerebellum_1610
Cortex_1610
Heart_1610
Hypothalamus_1610
Kidney_1610
Testis_1610
reads per million genome matching
0
100
200
300
400
500
0
20
40
60
80
bp position

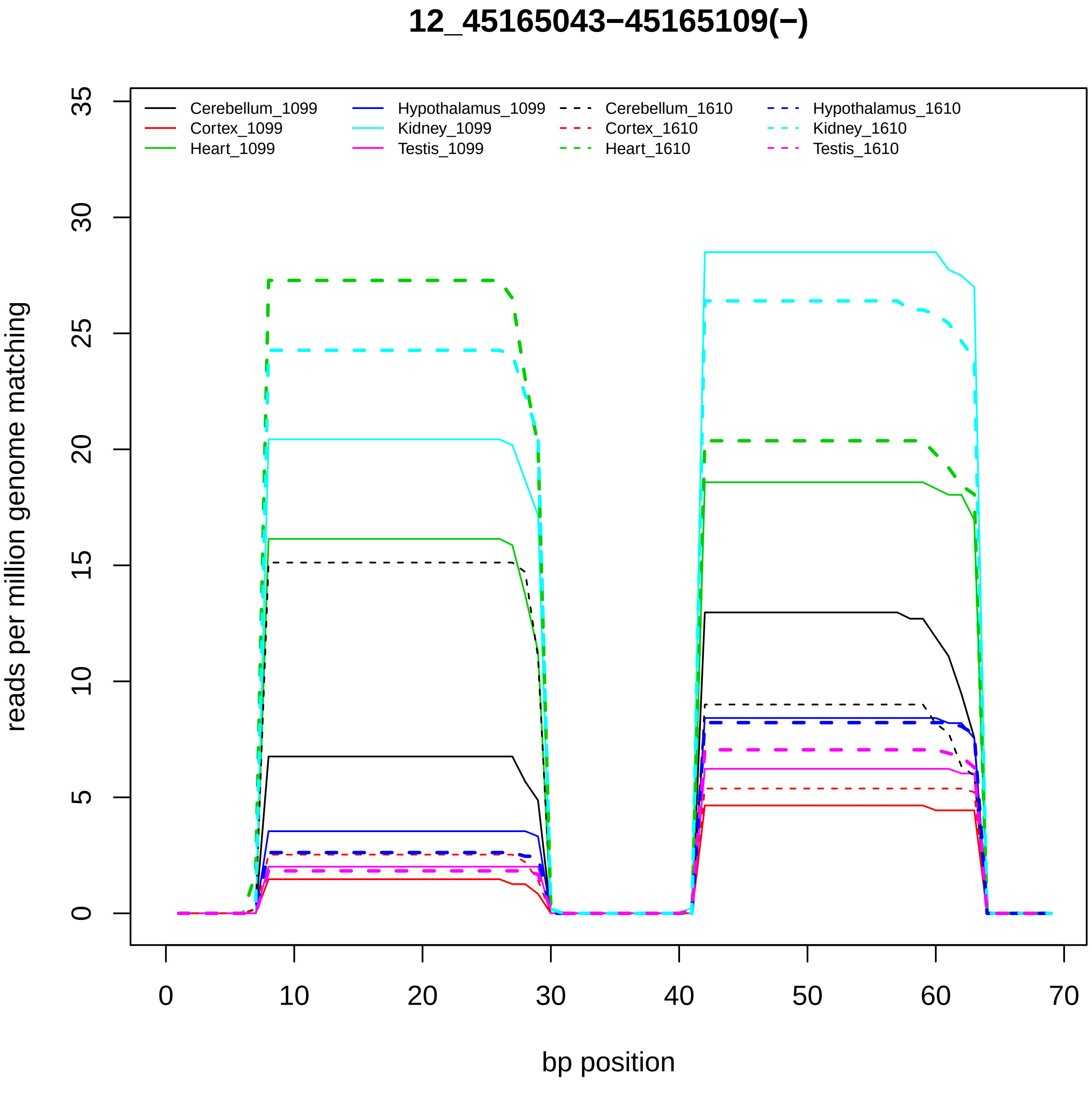
12_45165043−45165109(−)
Cerebellum_1099
Cortex_1099
Heart_1099
Hypothalamus_1099
Kidney_1099
Testis_1099
Cerebellum_1610
Cortex_1610
Heart_1610
Hypothalamus_1610
Kidney_1610
Testis_1610
reads per million genome matching
0
5
10
15
20
25
30
35
0
10
20
30
40
50
60
70
bp position

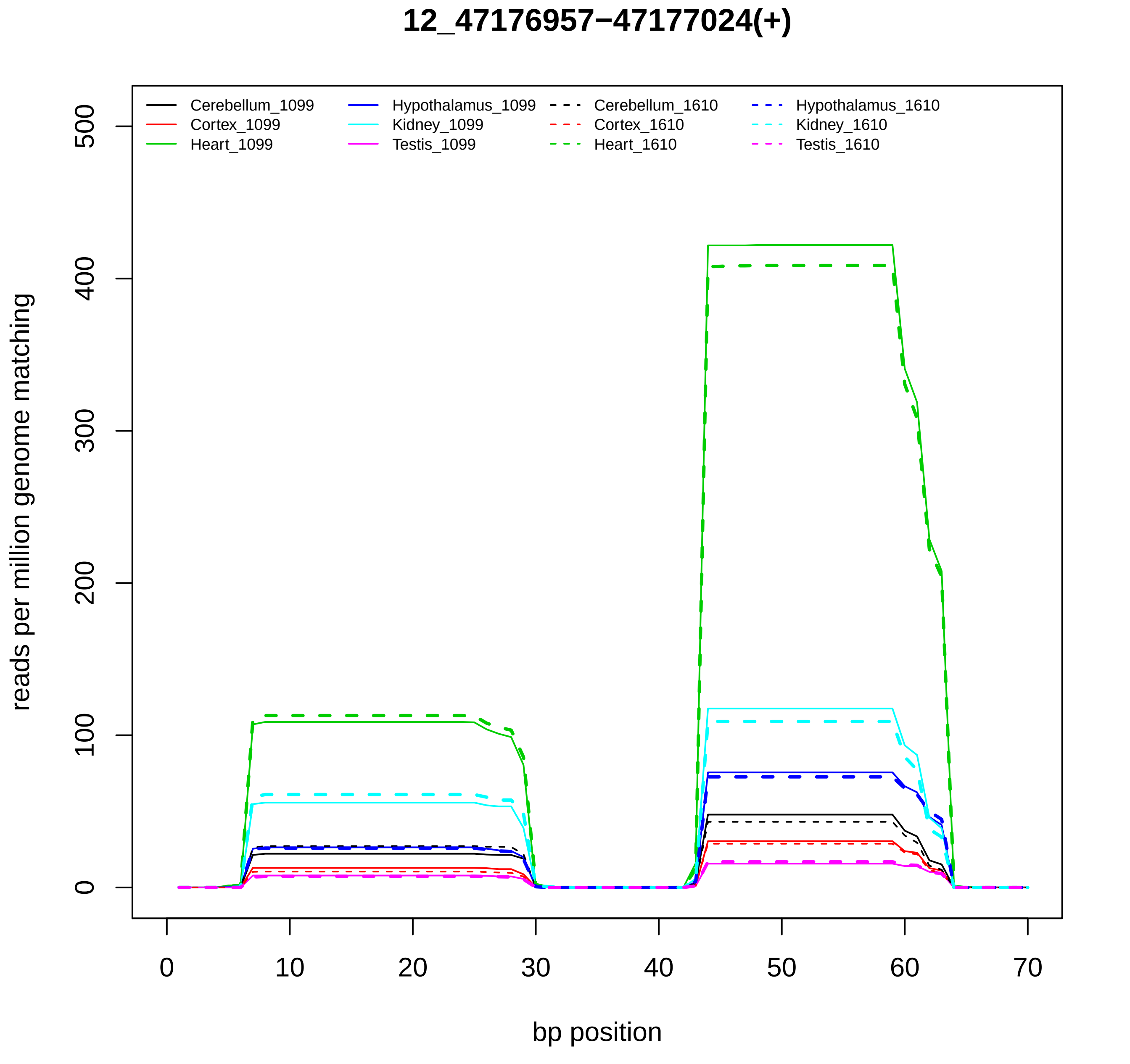
12_47176957−47177024(+)
Cerebellum_1099
Cortex_1099
Heart_1099
Hypothalamus_1099
Kidney_1099
Testis_1099
Cerebellum_1610
Cortex_1610
Heart_1610
Hypothalamus_1610
Kidney_1610
Testis_1610
reads per million genome matching
0
100
200
300
400
500
0
10
20
30
40
50
60
70
bp position

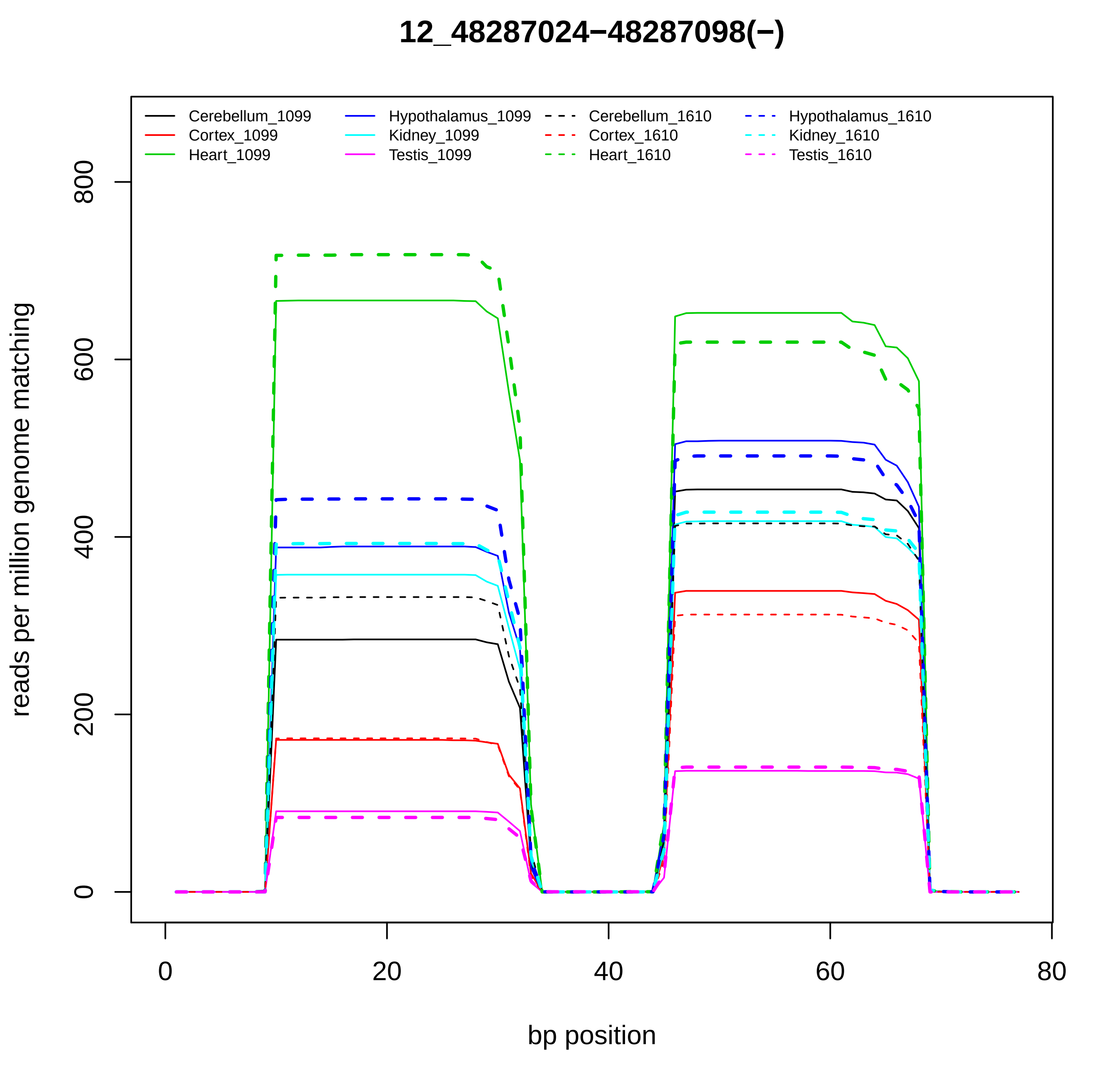
12_48287024−48287098(−)
Cerebellum_1099
Cortex_1099
Heart_1099
Hypothalamus_1099
Kidney_1099
Testis_1099
Cerebellum_1610
Cortex_1610
Heart_1610
Hypothalamus_1610
Kidney_1610
Testis_1610
reads per million genome matching
0
200
400
600
800
0
20
40
60
80
bp position

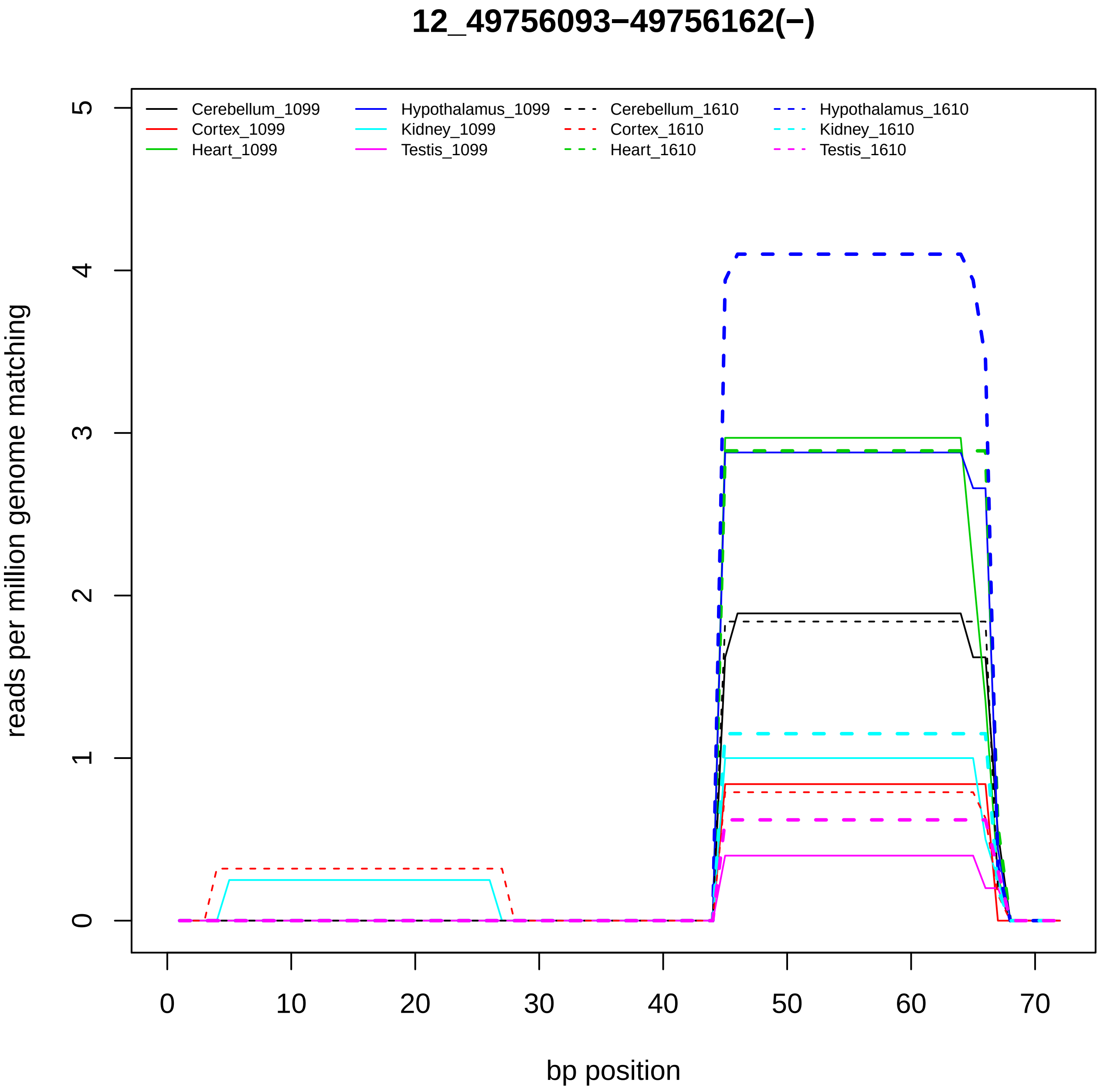

12_49756093−49756162(−)
Cerebellum_1099
Cortex_1099
Heart_1099
Hypothalamus_1099
Kidney_1099
Testis_1099
Cerebellum_1610
Cortex_1610
Heart_1610
Hypothalamus_1610
Kidney_1610
Testis_1610
reads per million genome matching
bp position
0
1
2
3
4
5
0
10
20
30
40
50
60
70

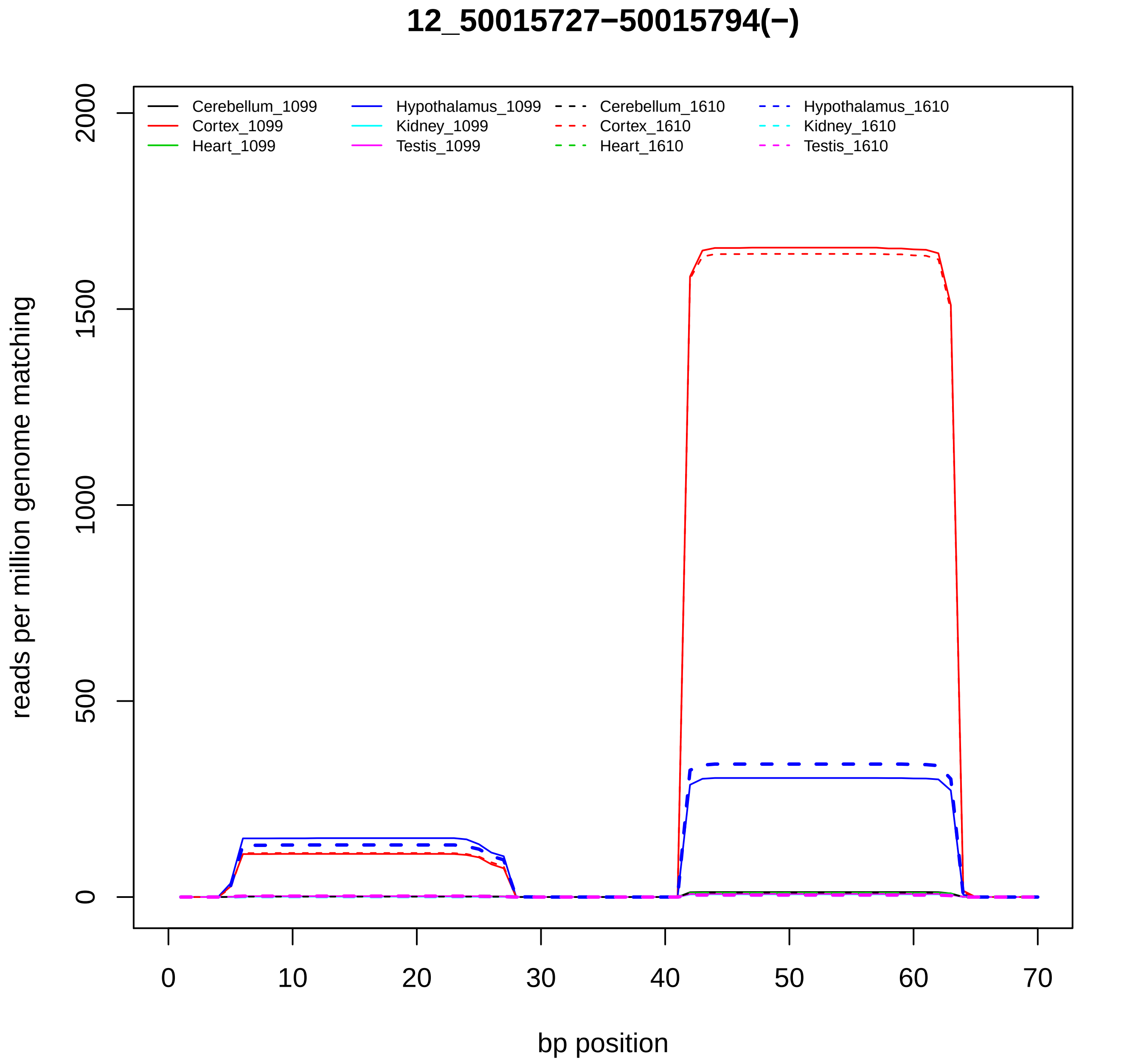

12_50015727−50015794(−)
Cerebellum_1099
Cortex_1099
Heart_1099
Hypothalamus_1099
Kidney_1099
Testis_1099
Cerebellum_1610
Cortex_1610
Heart_1610
Hypothalamus_1610
Kidney_1610
Testis_1610
reads per million genome matching
0
500
1000
1500
2000
bp position
0
10
20
30
40
50
60
70

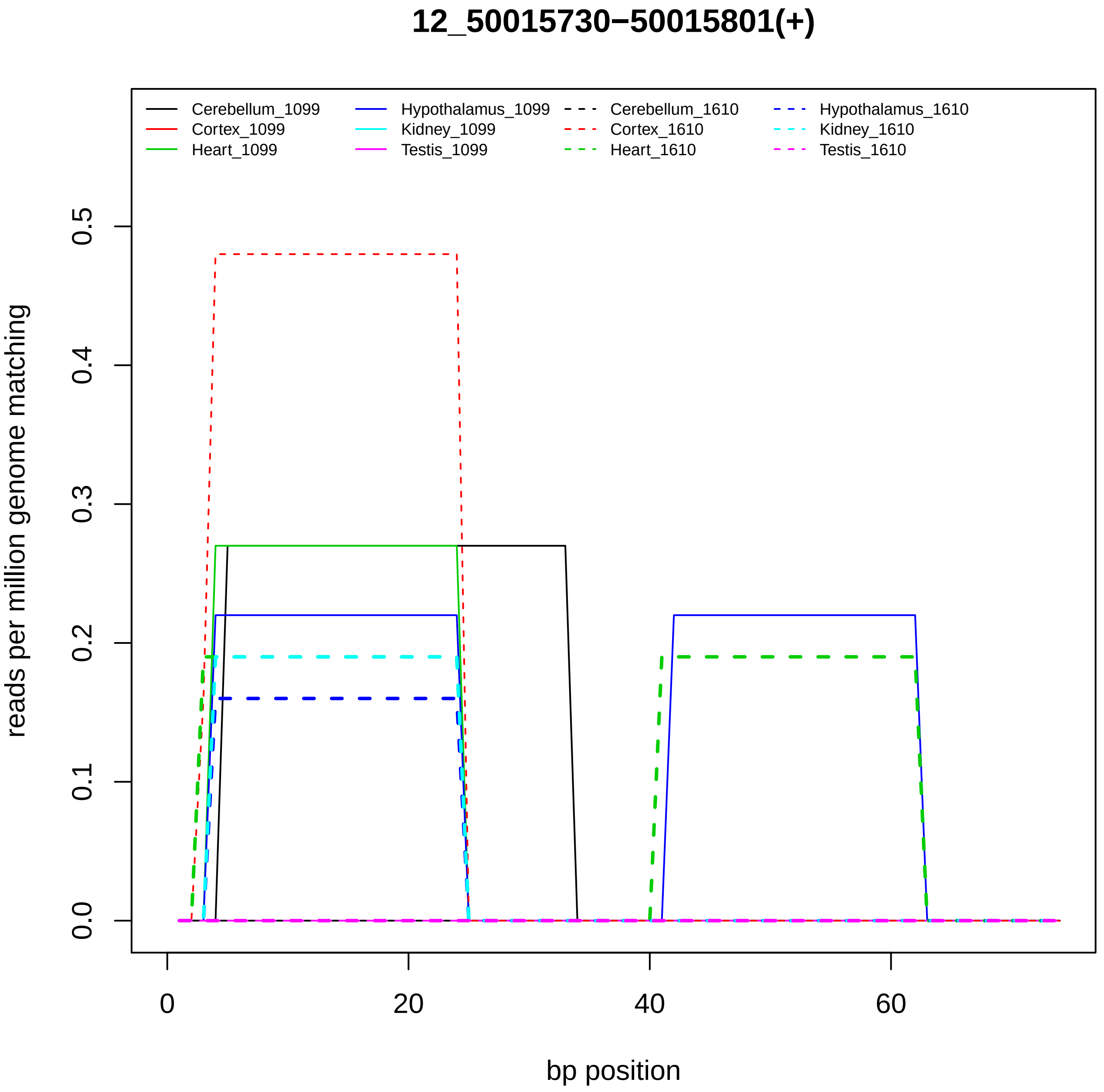

12_50015730−50015801(+)
Cerebellum_1099
Cortex_1099
Heart_1099
Hypothalamus_1099
Kidney_1099
Testis_1099
Cerebellum_1610
Cortex_1610
Heart_1610
Hypothalamus_1610
Kidney_1610
Testis_1610
reads per million genome matching
0.0
0.1
0.2
0.3
0.4
0.5
0
20
40
60
bp position

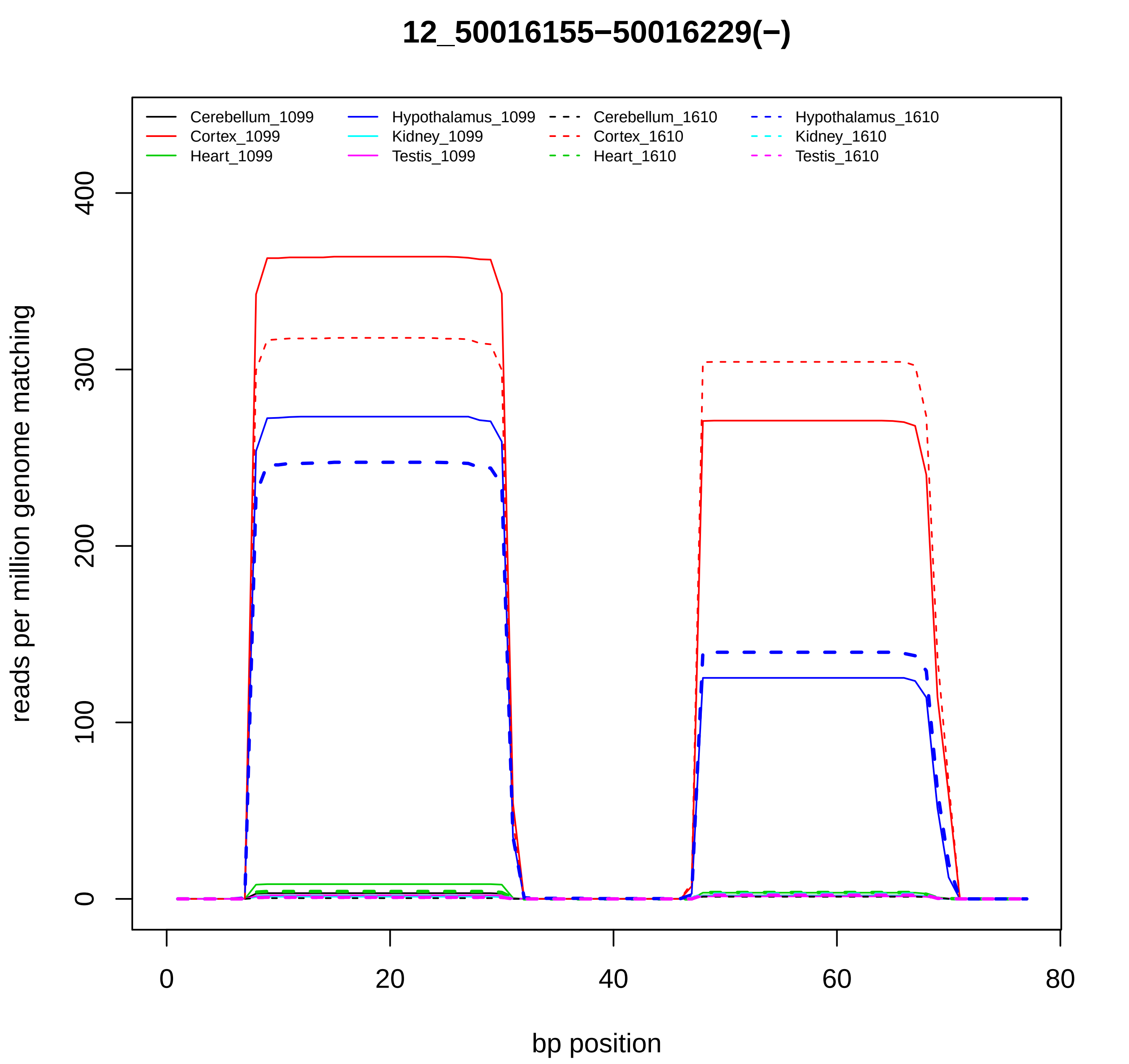

12_50016155−50016229(−)
Cerebellum_1099
Cortex_1099
Heart_1099
Hypothalamus_1099
Kidney_1099
Testis_1099
Cerebellum_1610
Cortex_1610
Heart_1610
Hypothalamus_1610
Kidney_1610
Testis_1610
reads per million genome matching
0
100
200
300
400
0
20
40
60
80
bp position

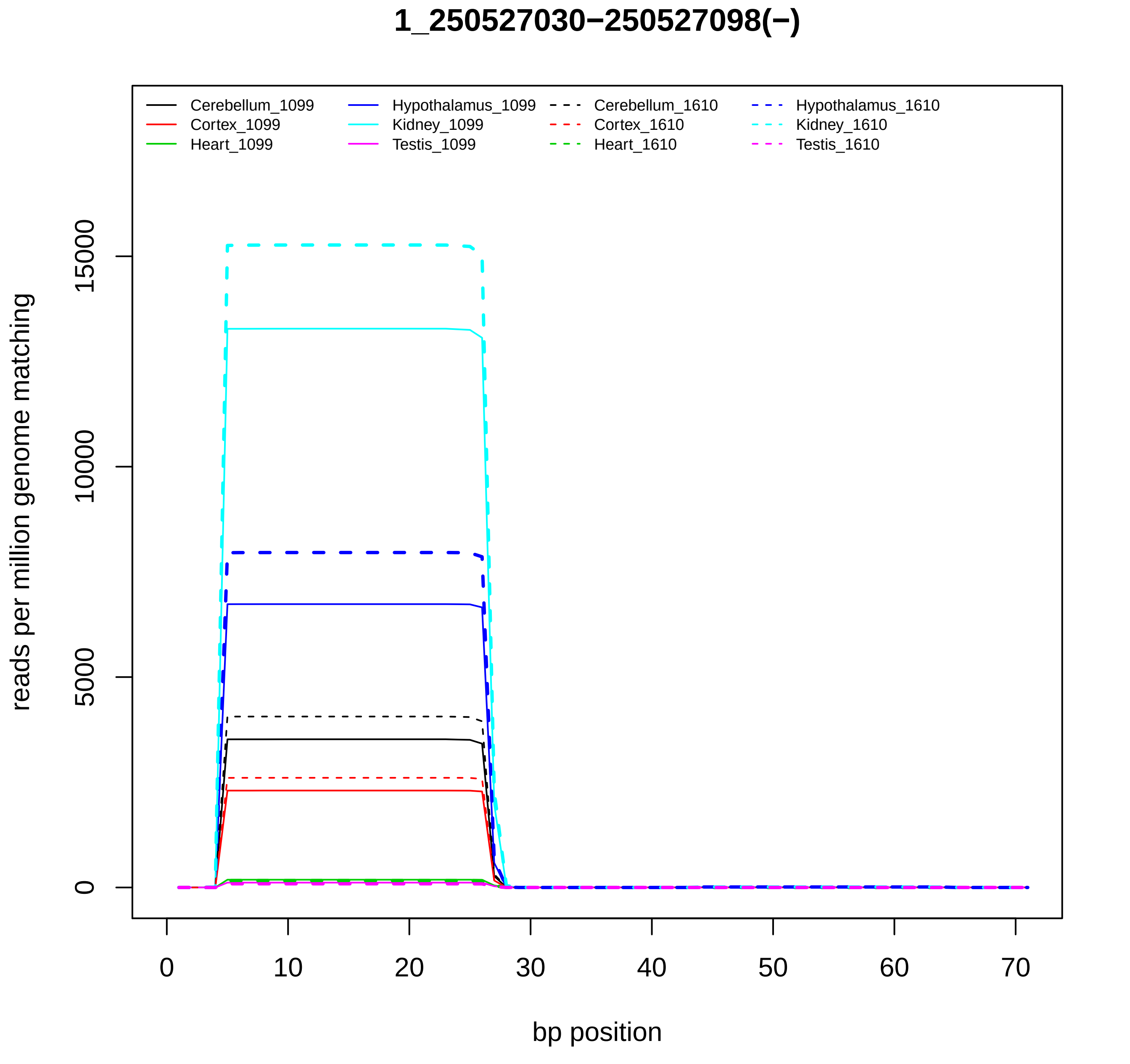

1_250527030−250527098(−)
Cerebellum_1099
Cortex_1099
Heart_1099
Hypothalamus_1099
Kidney_1099
Testis_1099
Cerebellum_1610
Cortex_1610
Heart_1610
Hypothalamus_1610
Kidney_1610
Testis_1610
reads per million genome matching
0
5000
10000
15000
0
10
20
30
40
50
60
70
bp position

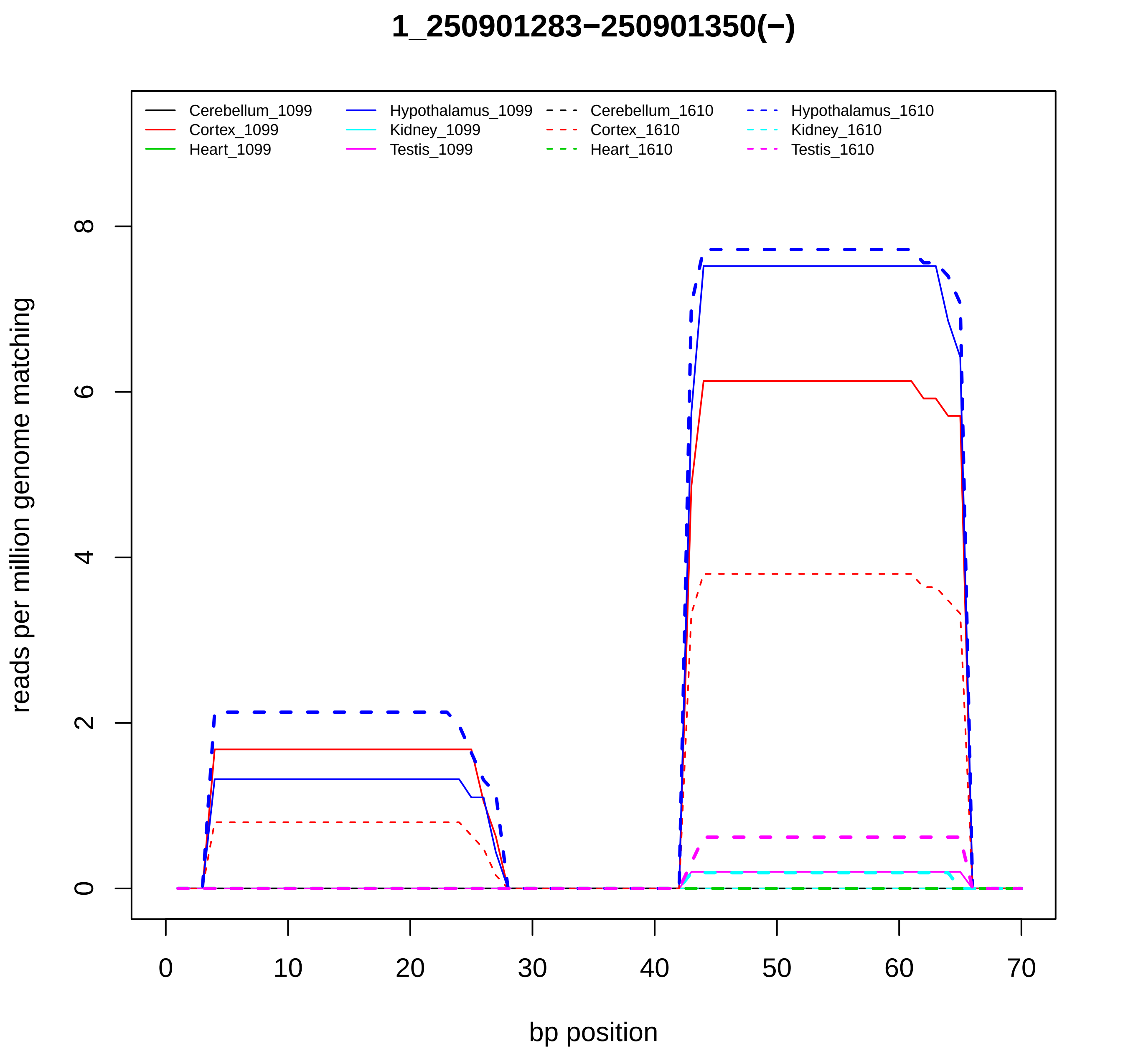
1_250901283−250901350(−)
Cerebellum_1099
Cortex_1099
Heart_1099
Hypothalamus_1099
Kidney_1099
Testis_1099
Cerebellum_1610
Cortex_1610
Heart_1610
Hypothalamus_1610
Kidney_1610
Testis_1610
reads per million genome matching
0
2
4
6
8
0
10
20
30
40
50
60
70
bp position

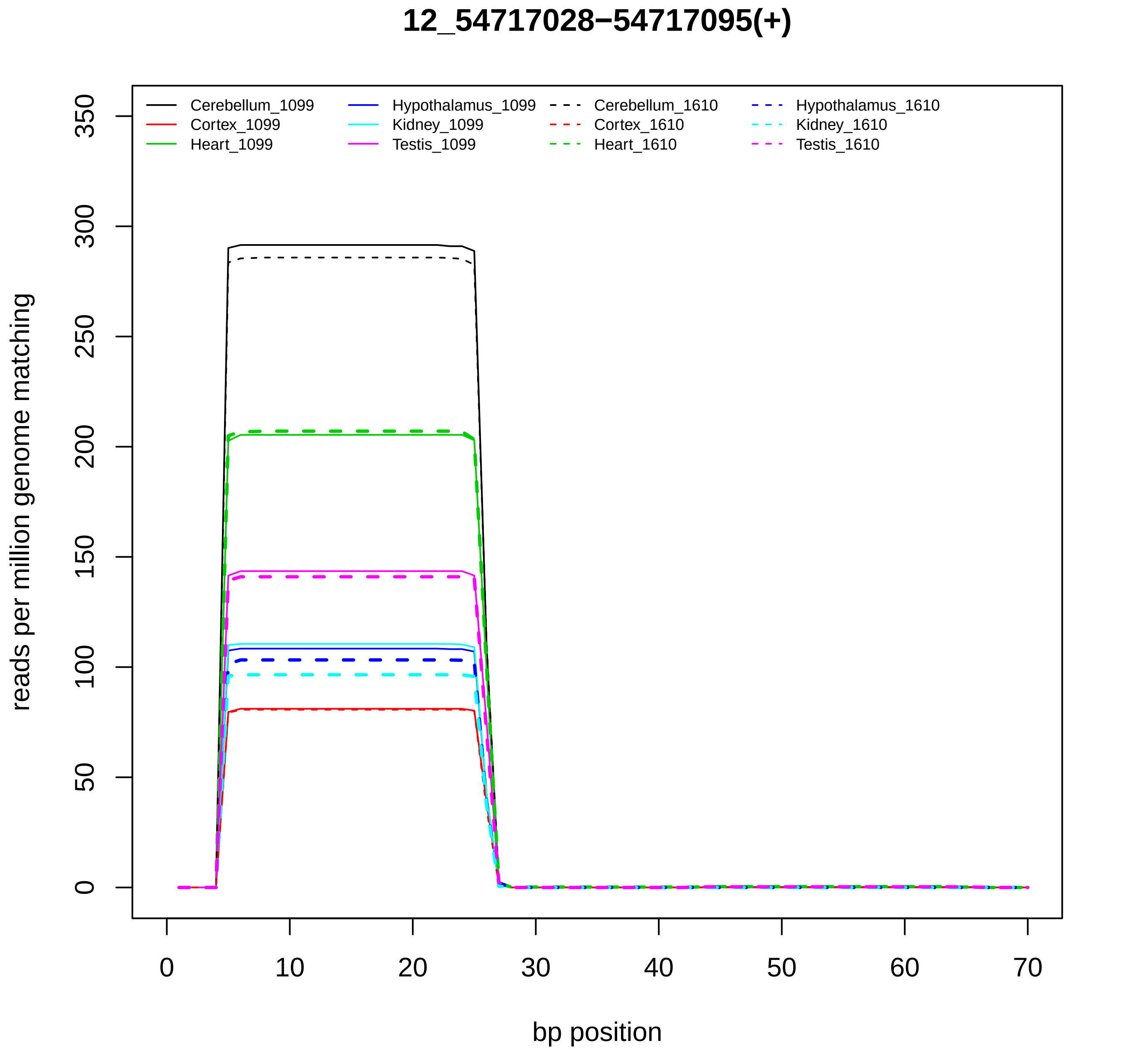
12_54717028−54717095(+)
Cerebellum_1099
Cortex_1099
Heart_1099
Hypothalamus_1099
Kidney_1099
Testis_1099
Cerebellum_1610
Cortex_1610
Heart_1610
Hypothalamus_1610
Kidney_1610
Testis_1610
reads per million genome matching
bp position
0
50
100
150
200
250
300
350
0
10
20
30
40
50
60
70

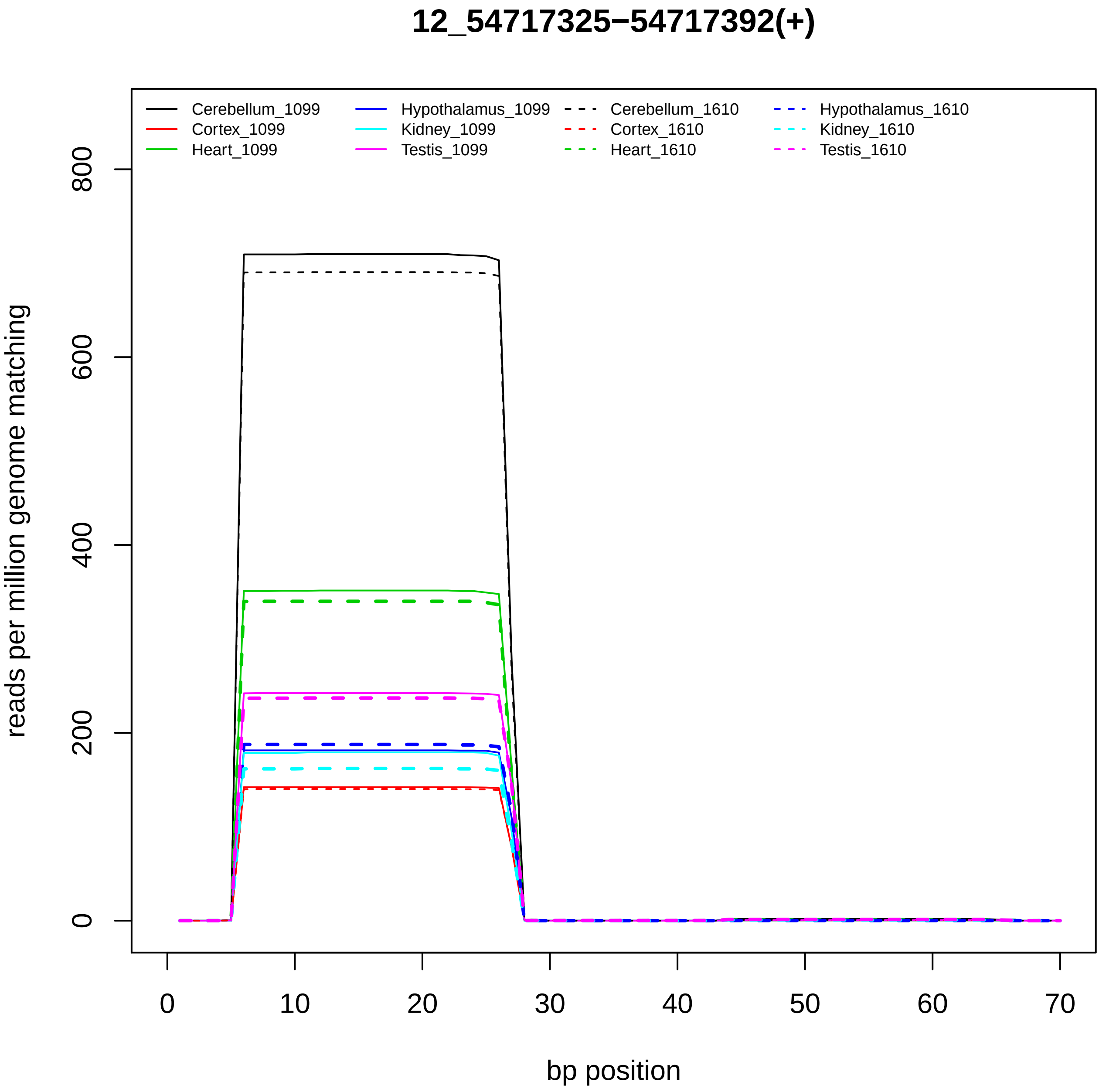
12_54717325−54717392(+)
Cerebellum_1099
Cortex_1099
Heart_1099
Hypothalamus_1099
Kidney_1099
Testis_1099
Cerebellum_1610
Cortex_1610
Heart_1610
Hypothalamus_1610
Kidney_1610
Testis_1610
reads per million genome matching
0
200
400
600
800
0
10
20
30
40
50
60
70
bp position

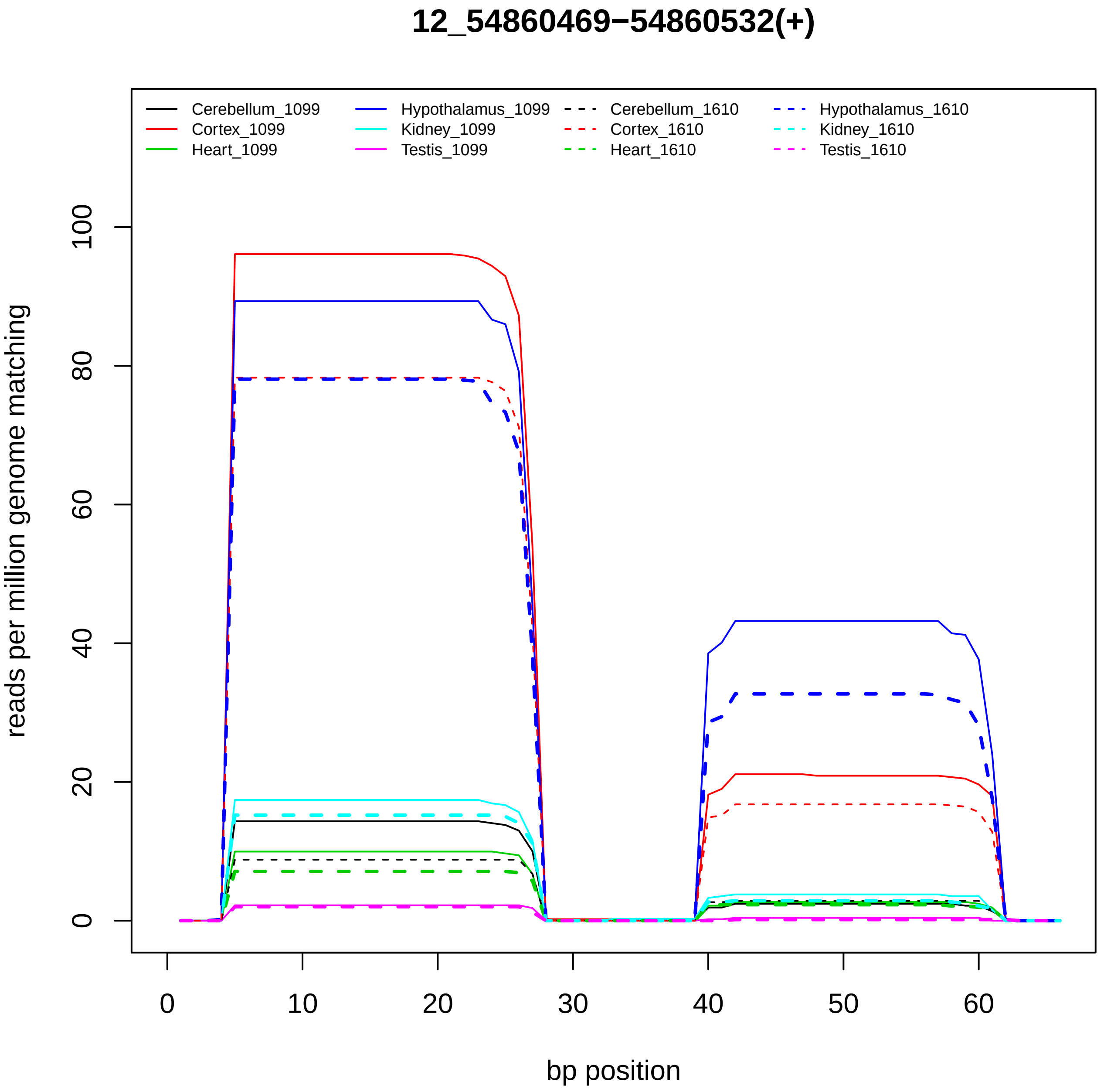
12_54860469−54860532(+)
Cerebellum_1099
Cortex_1099
Heart_1099
Hypothalamus_1099
Kidney_1099
Testis_1099
Cerebellum_1610
Cortex_1610
Heart_1610
Hypothalamus_1610
Kidney_1610
Testis_1610
reads per million genome matching
0
20
40
60
80
100
bp position
0
10
20
30
40
50
60

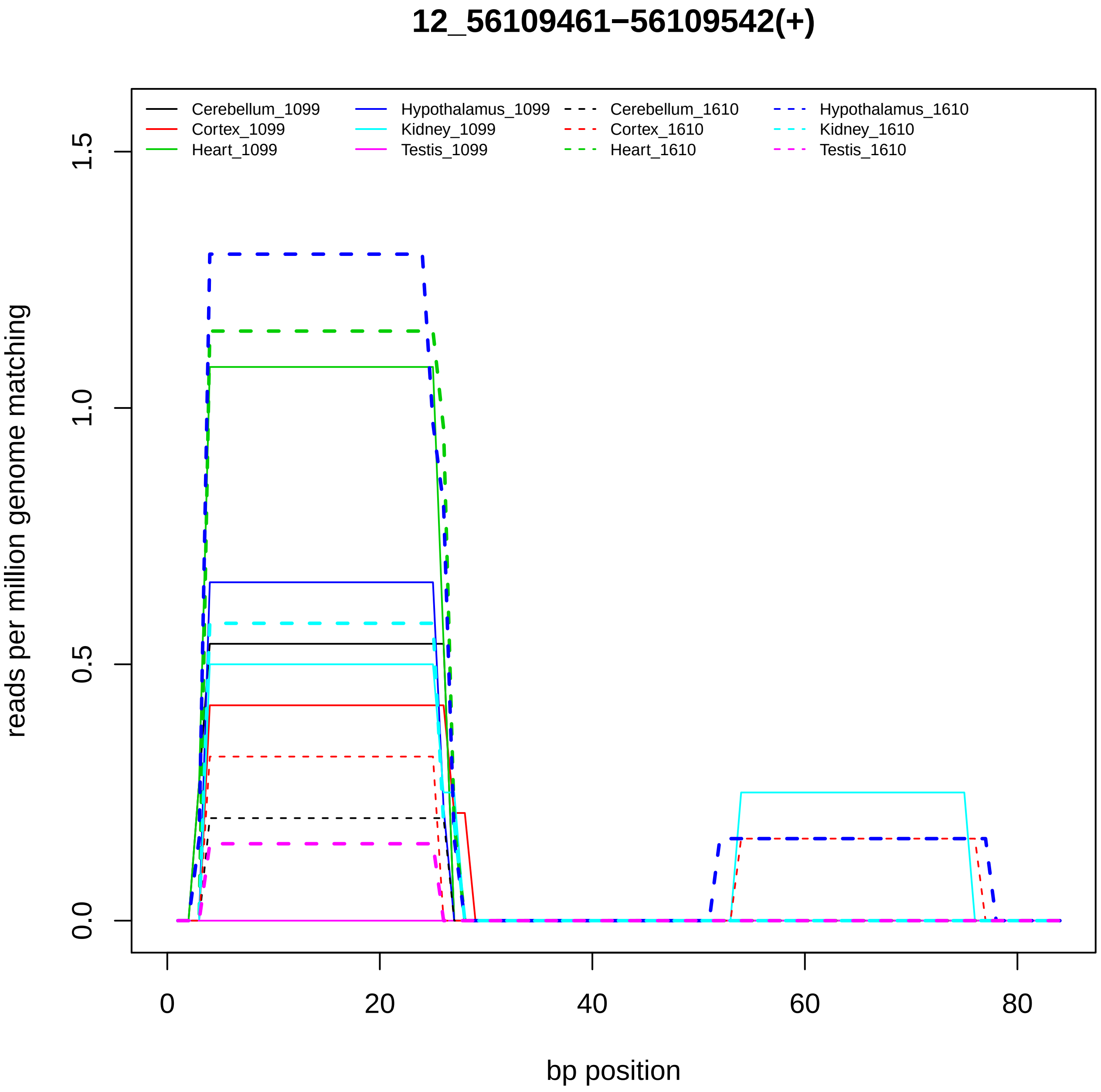

12_56109461−56109542(+)
Cerebellum_1099
Cortex_1099
Heart_1099
Hypothalamus_1099
Kidney_1099
Testis_1099
Cerebellum_1610
Cortex_1610
Heart_1610
Hypothalamus_1610
Kidney_1610
Testis_1610
reads per million genome matching
0.0
0.5
1.0
1.5
bp position
0
20
40
60
80

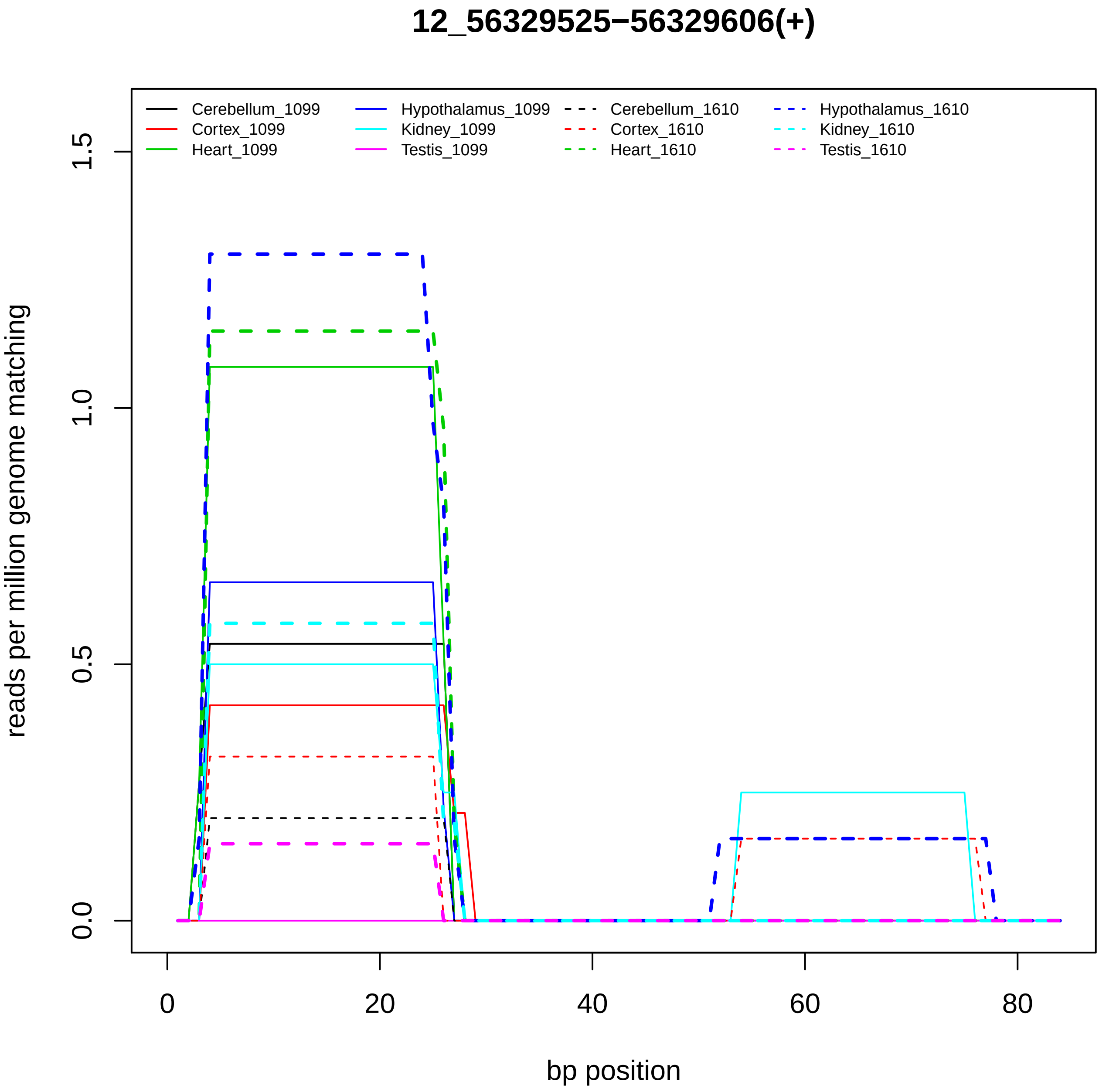

12_56329525−56329606(+)
Cerebellum_1099
Cortex_1099
Heart_1099
Hypothalamus_1099
Kidney_1099
Testis_1099
Cerebellum_1610
Cortex_1610
Heart_1610
Hypothalamus_1610
Kidney_1610
Testis_1610
reads per million genome matching
0.0
0.5
1.0
1.5
bp position
0
20
40
60
80

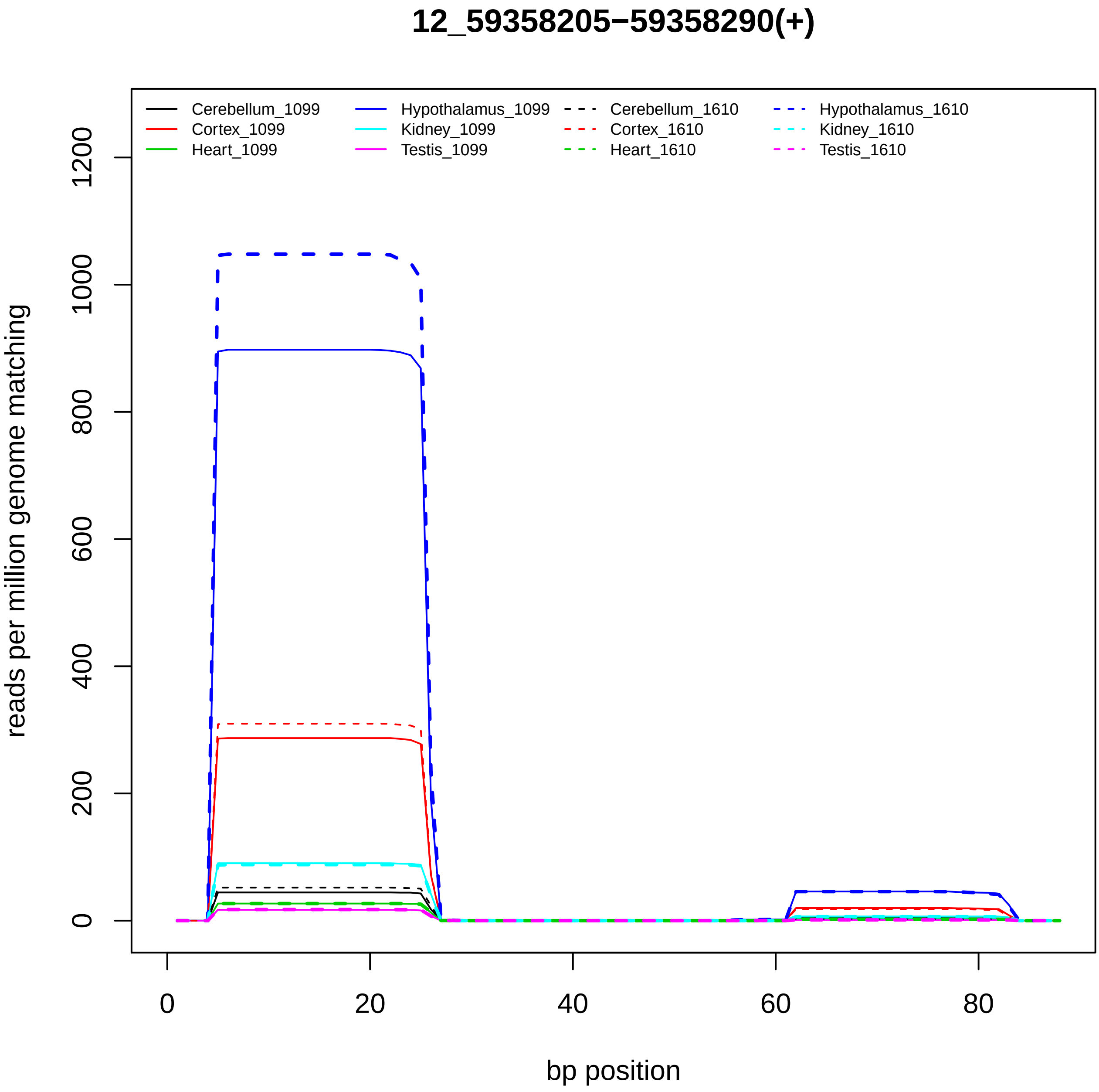

12_59358205−59358290(+)
Cerebellum_1099
Cortex_1099
Heart_1099
Hypothalamus_1099
Kidney_1099
Testis_1099
Cerebellum_1610
Cortex_1610
Heart_1610
Hypothalamus_1610
Kidney_1610
Testis_1610
reads per million genome matching
0
200
400
600
800
1000
1200
0
20
40
60
80
bp position

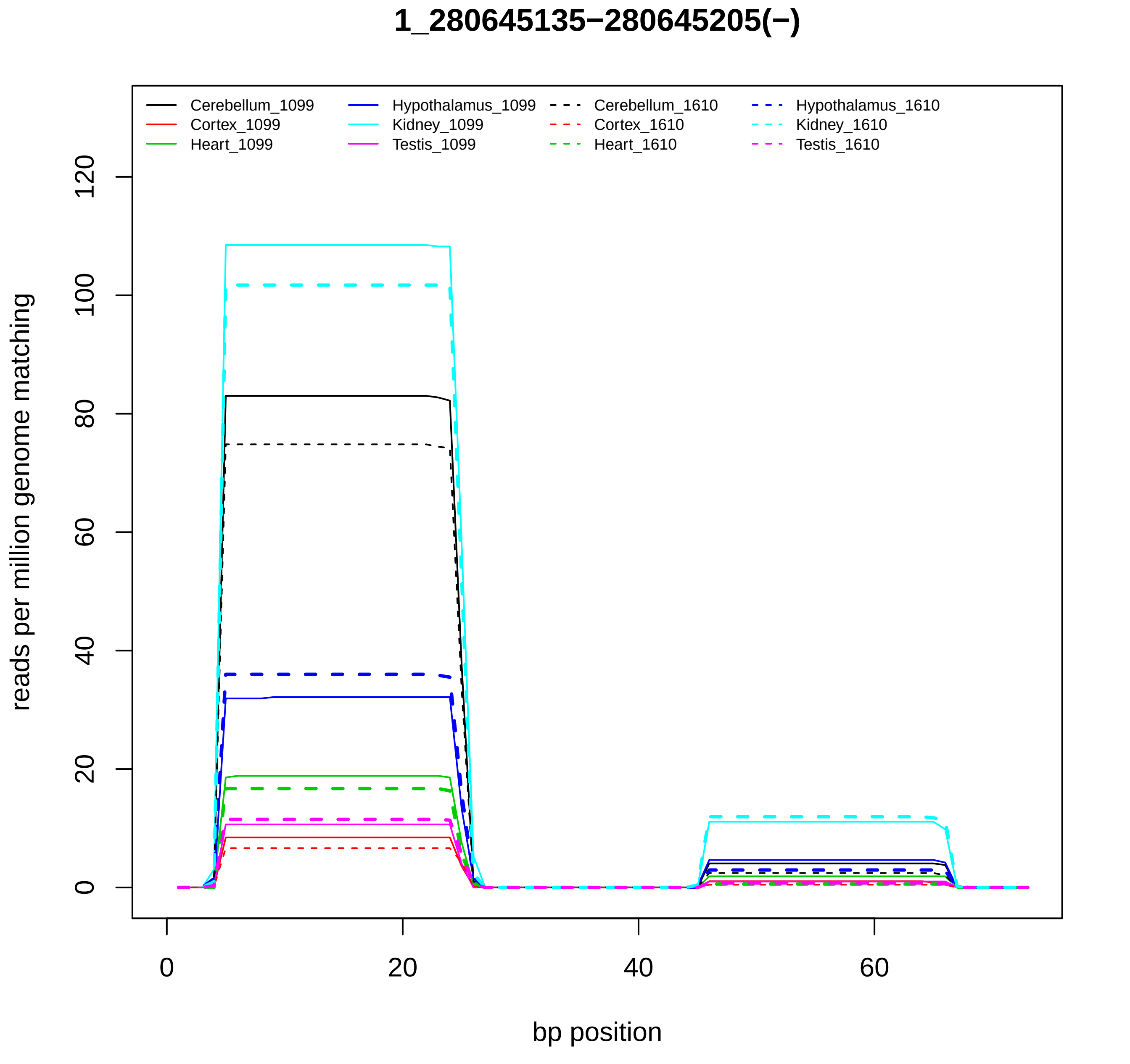

1_280645135−280645205(−)
Cerebellum_1099
Cortex_1099
Heart_1099
Hypothalamus_1099
Kidney_1099
Testis_1099
Cerebellum_1610
Cortex_1610
Heart_1610
Hypothalamus_1610
Kidney_1610
Testis_1610
reads per million genome matching
bp position
0
20
40
60
80
100
120

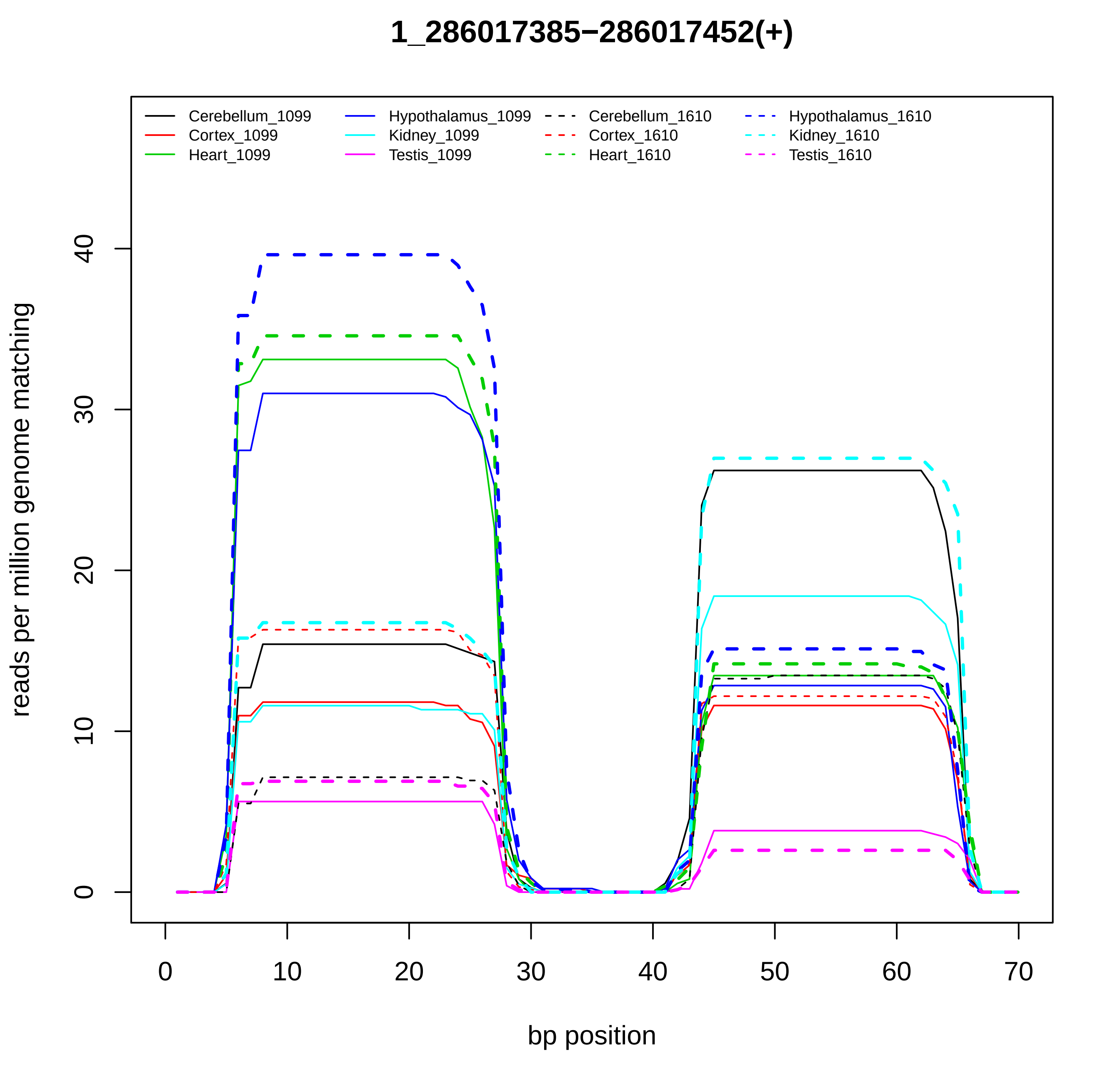
1_286017385−286017452(+)
Cerebellum_1099
Cortex_1099
Heart_1099
Hypothalamus_1099
Kidney_1099
Testis_1099
Cerebellum_1610
Cortex_1610
Heart_1610
Hypothalamus_1610
Kidney_1610
Testis_1610
reads per million genome matching
bp position
0
10
20
30
40
0
10
20
30
40
50
60
70

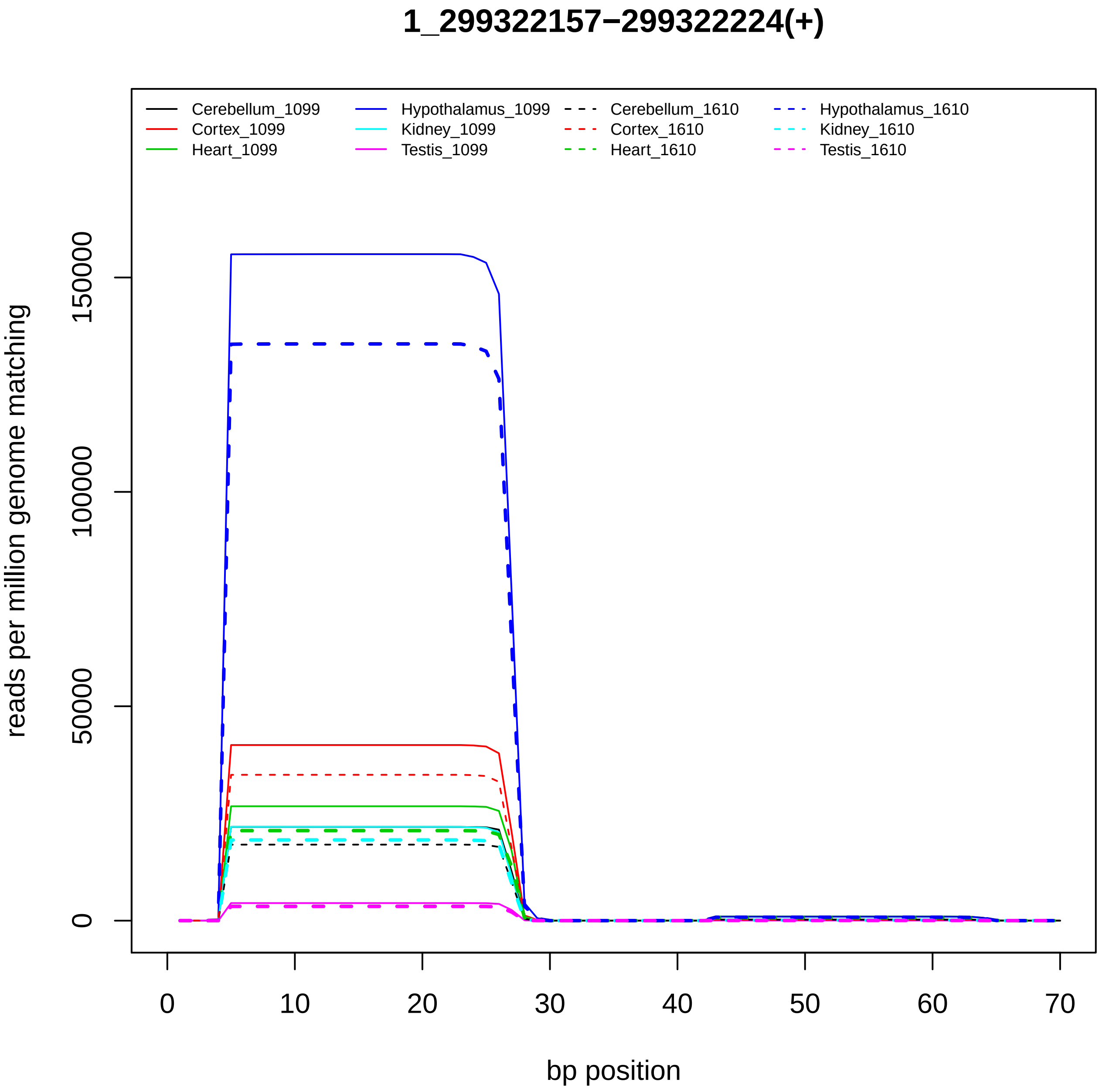

1_299322157−299322224(+)
Cerebellum_1099
Cortex_1099
Heart_1099
Hypothalamus_1099
Kidney_1099
Testis_1099
Cerebellum_1610
Cortex_1610
Heart_1610
Hypothalamus_1610
Kidney_1610
Testis_1610
reads per million genome matching
0
50000
100000
150000
0
10
20
30
40
50
60
70
bp position

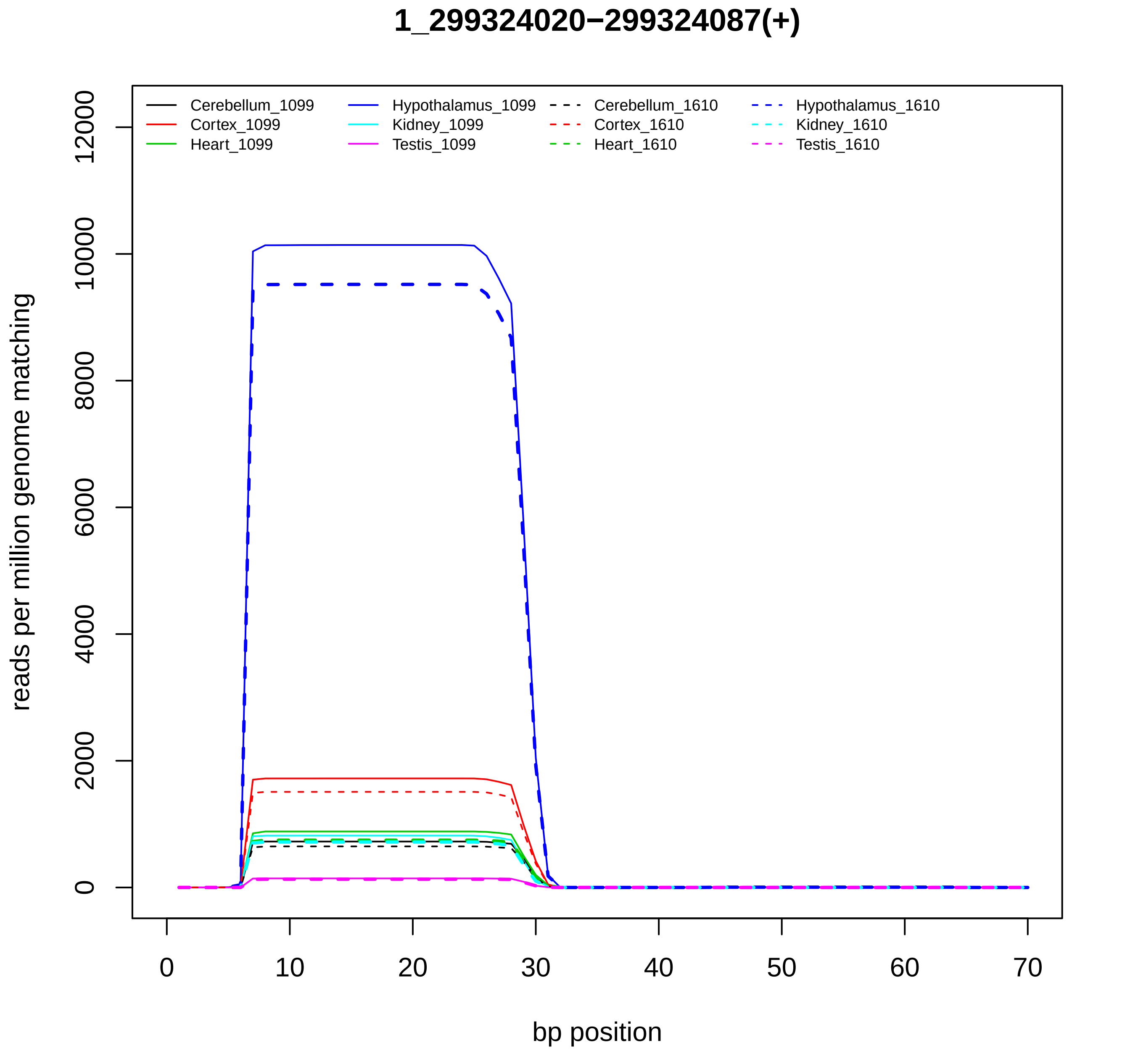

1_299324020−299324087(+)
Cerebellum_1099
Cortex_1099
Heart_1099
Hypothalamus_1099
Kidney_1099
Testis_1099
Cerebellum_1610
Cortex_1610
Heart_1610
Hypothalamus_1610
Kidney_1610
Testis_1610
reads per million genome matching
0
2000
4000
6000
8000
10000
12000
bp position
0
10
20
30
40
50
60
70

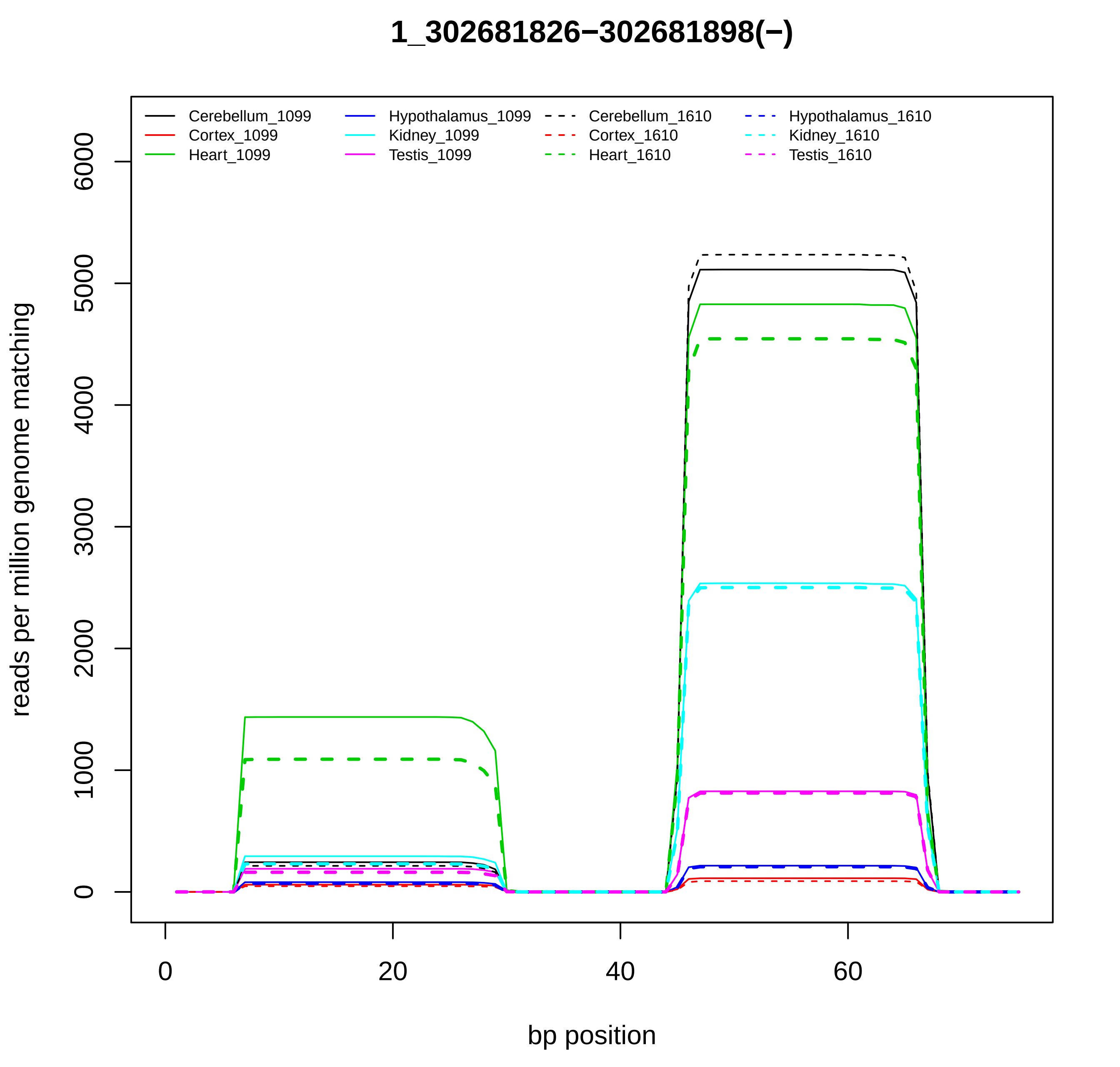
1_302681826−302681898(−)
Cerebellum_1099
Cortex_1099
Heart_1099
Hypothalamus_1099
Kidney_1099
Testis_1099
Cerebellum_1610
Cortex_1610
Heart_1610
Hypothalamus_1610
Kidney_1610
Testis_1610
reads per million genome matching
0
1000
2000
3000
4000
5000
6000
bp position
0
20
40
60

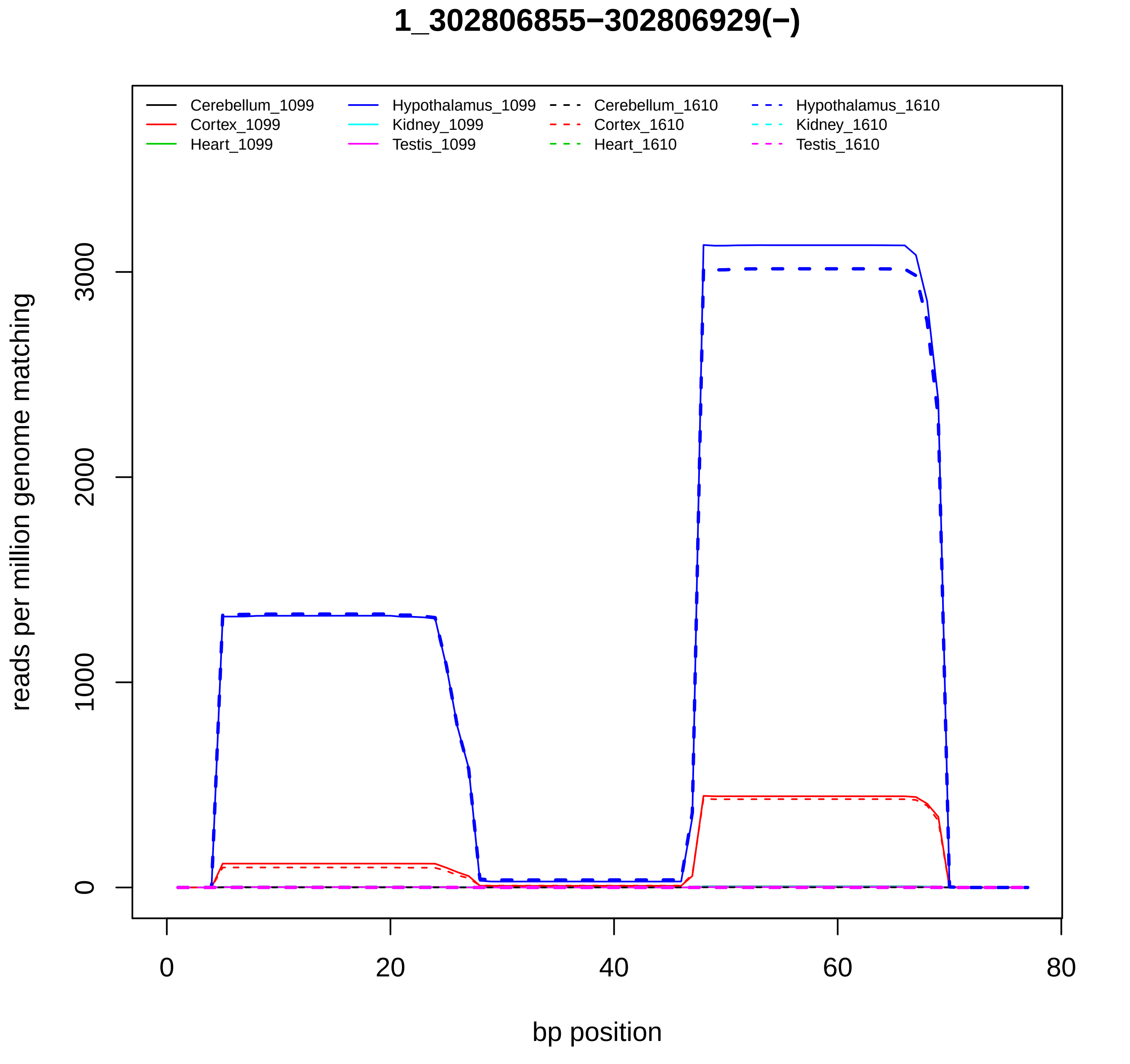

1_302806855−302806929(−)
Cerebellum_1099
Cortex_1099
Heart_1099
Hypothalamus_1099
Kidney_1099
Testis_1099
Cerebellum_1610
Cortex_1610
Heart_1610
Hypothalamus_1610
Kidney_1610
Testis_1610
reads per million genome matching
0
1000
2000
3000
0
20
40
60
80
bp position

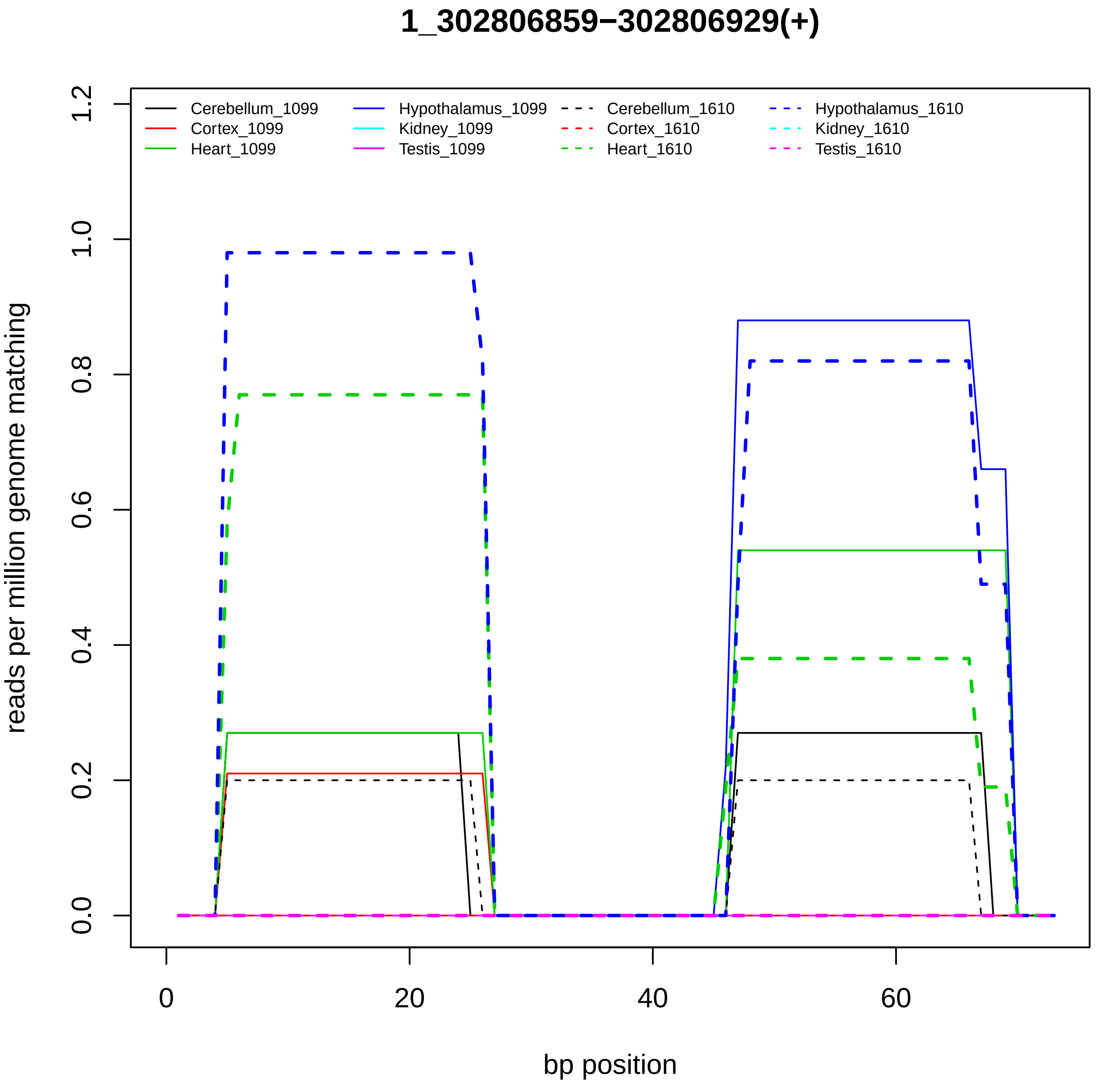
1_302806859−302806929(+)
Cerebellum_1099
Cortex_1099
Heart_1099
Hypothalamus_1099
Kidney_1099
Testis_1099
Cerebellum_1610
Cortex_1610
Heart_1610
Hypothalamus_1610
Kidney_1610
Testis_1610
reads per million genome matching
0.0
0.2
0.4
0.6
0.8
1.0
1.2
0
20
40
60
bp position

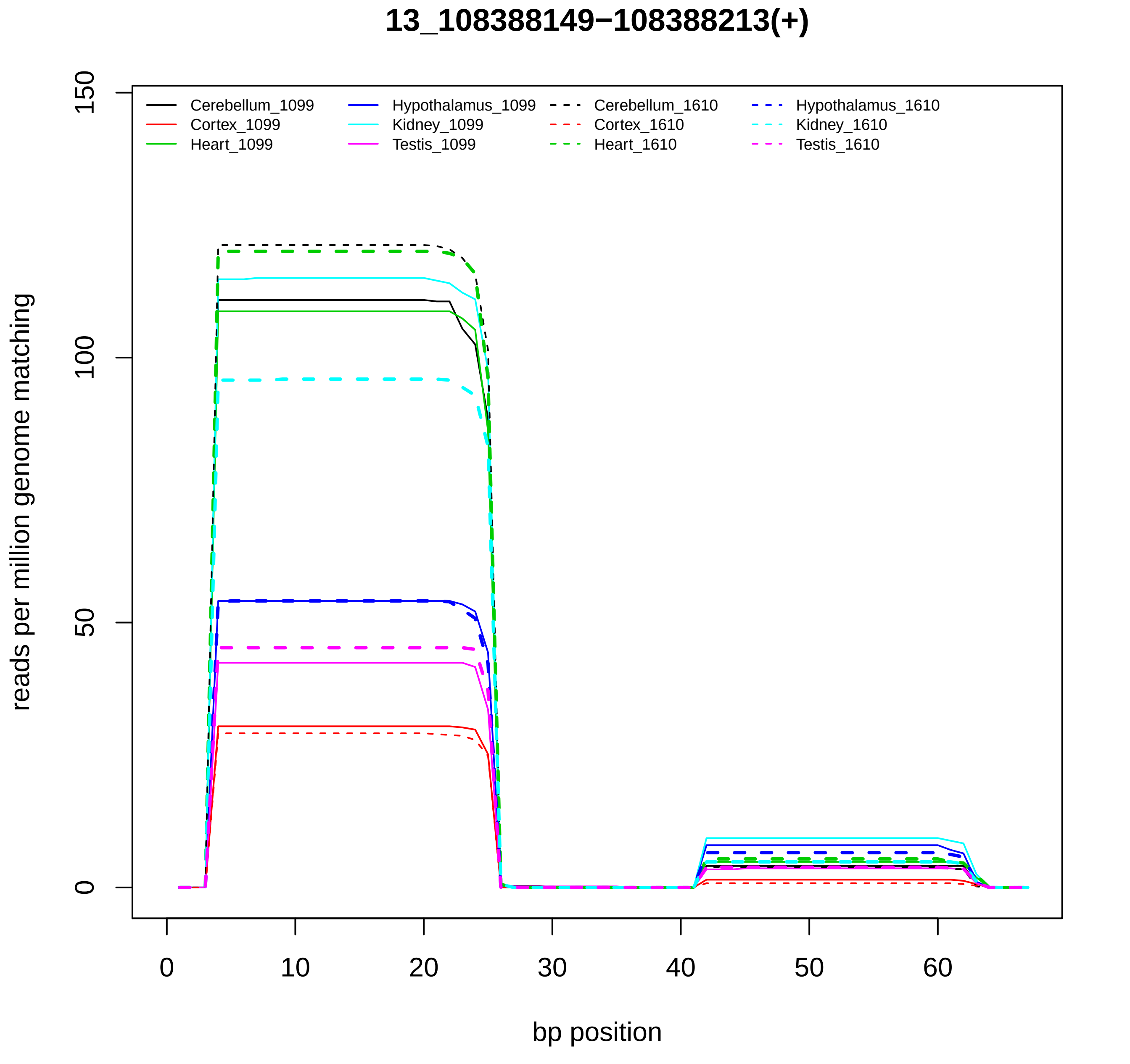

13_108388149−108388213(+)
Cerebellum_1099
Cortex_1099
Heart_1099
Hypothalamus_1099
Kidney_1099
Testis_1099
Cerebellum_1610
Cortex_1610
Heart_1610
Hypothalamus_1610
Kidney_1610
Testis_1610
reads per million genome matching
bp position
0
50
100
150
0
10
20
30
40
50
60

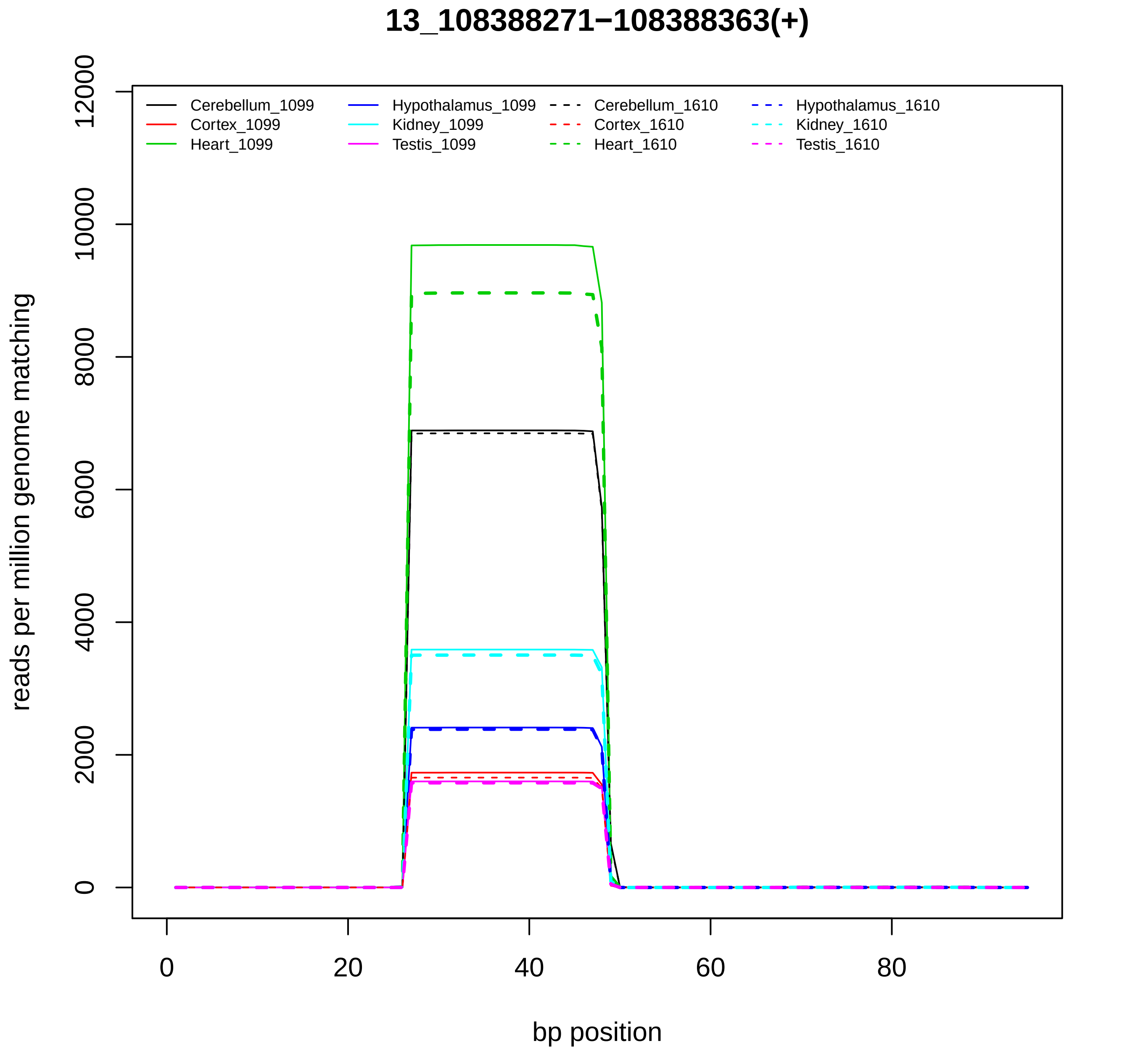
13_108388271−108388363(+)
Cerebellum_1099
Cortex_1099
Heart_1099
Hypothalamus_1099
Kidney_1099
Testis_1099
Cerebellum_1610
Cortex_1610
Heart_1610
Hypothalamus_1610
Kidney_1610
Testis_1610
reads per million genome matching
0
2000
4000
6000
8000
10000
12000
0
20
40
60
80
bp position

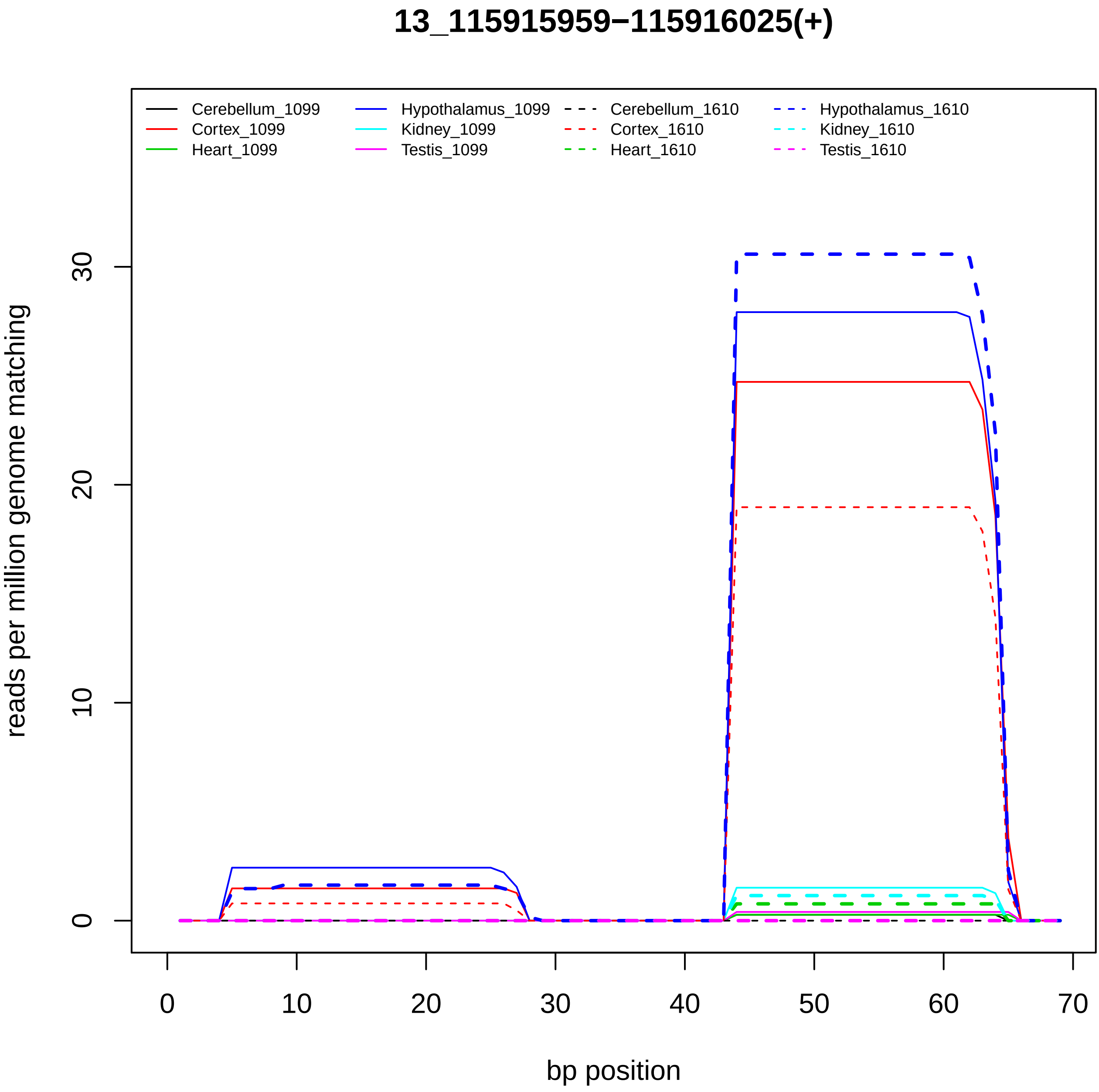

13_115915959−115916025(+)
Cerebellum_1099
Cortex_1099
Heart_1099
Hypothalamus_1099
Kidney_1099
Testis_1099
Cerebellum_1610
Cortex_1610
Heart_1610
Hypothalamus_1610
Kidney_1610
Testis_1610
reads per million genome matching
0
10
20
30
0
10
20
30
40
50
60
70
bp position

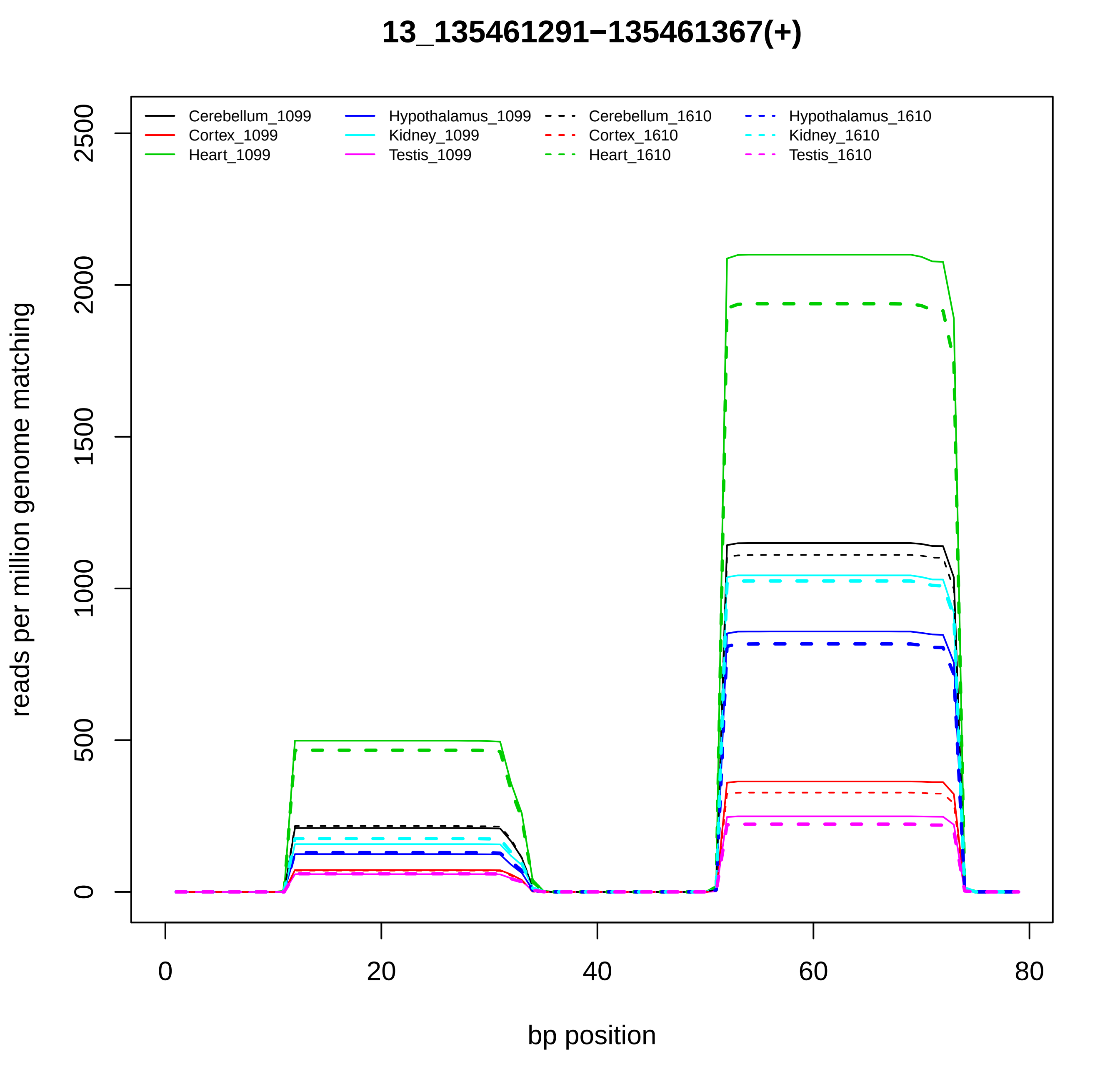
13_135461291−135461367(+)
Cerebellum_1099
Cortex_1099
Heart_1099
Hypothalamus_1099
Kidney_1099
Testis_1099
Cerebellum_1610
Cortex_1610
Heart_1610
Hypothalamus_1610
Kidney_1610
Testis_1610
reads per million genome matching
0
500
1000
1500
2000
2500
bp position
0
20
40
60
80

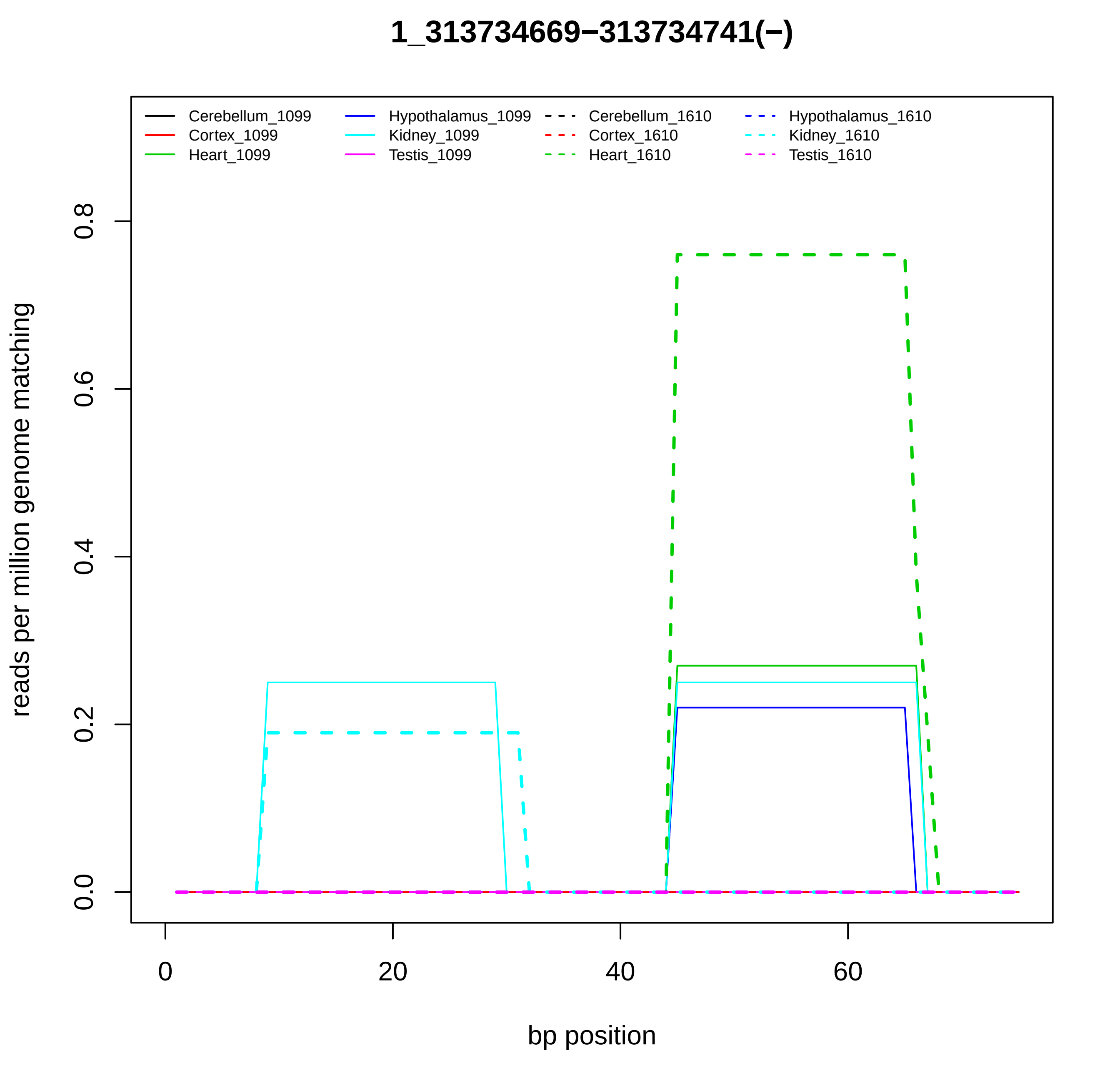

1_313734669−313734741(−)
Cerebellum_1099
Cortex_1099
Heart_1099
Hypothalamus_1099
Kidney_1099
Testis_1099
Cerebellum_1610
Cortex_1610
Heart_1610
Hypothalamus_1610
Kidney_1610
Testis_1610
reads per million genome matching
0.0
0.2
0.4
0.6
0.8
0
20
40
60
bp position

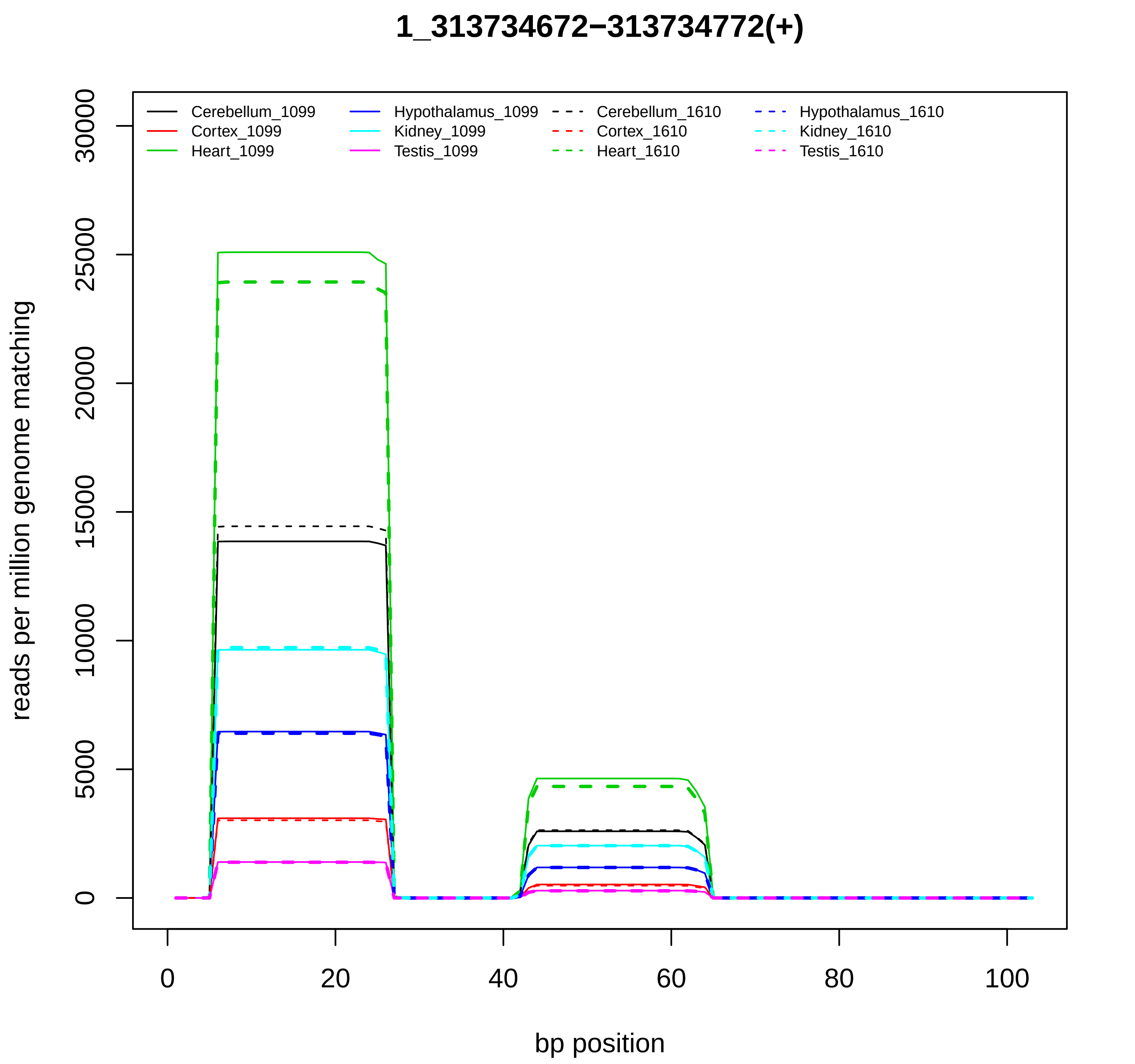

1_313734672−313734772(+)
Cerebellum_1099
Cortex_1099
Heart_1099
Hypothalamus_1099
Kidney_1099
Testis_1099
Cerebellum_1610
Cortex_1610
Heart_1610
Hypothalamus_1610
Kidney_1610
Testis_1610
reads per million genome matching
0
5000
10000
15000
20000
25000
30000
0
20
40
60
80
100
bp position

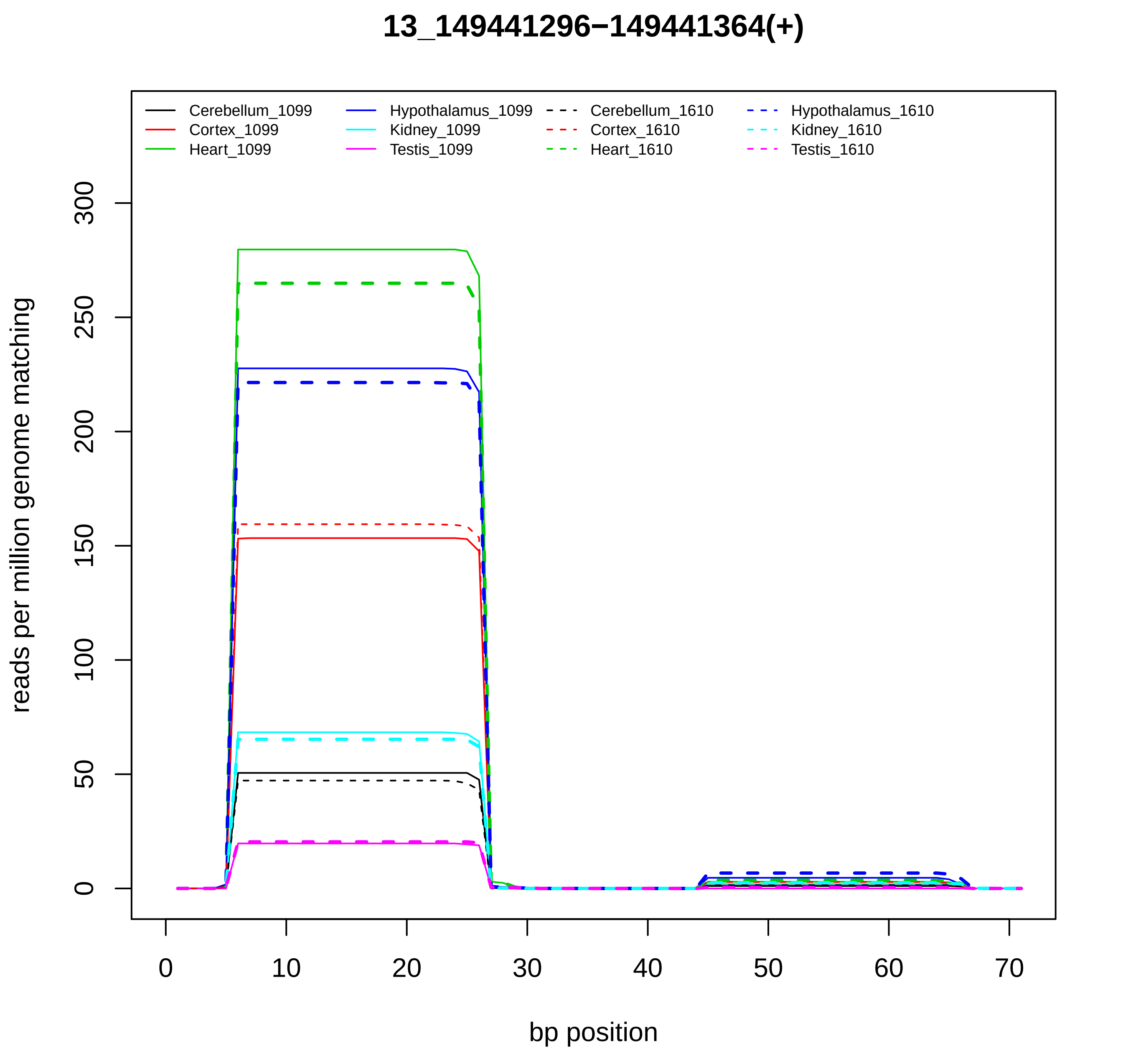
13_149441296−149441364(+)
Cerebellum_1099
Cortex_1099
Heart_1099
Hypothalamus_1099
Kidney_1099
Testis_1099
Cerebellum_1610
Cortex_1610
Heart_1610
Hypothalamus_1610
Kidney_1610
Testis_1610
reads per million genome matching
0
50
100
150
200
250
300
bp position
0
10
20
30
40
50
60
70

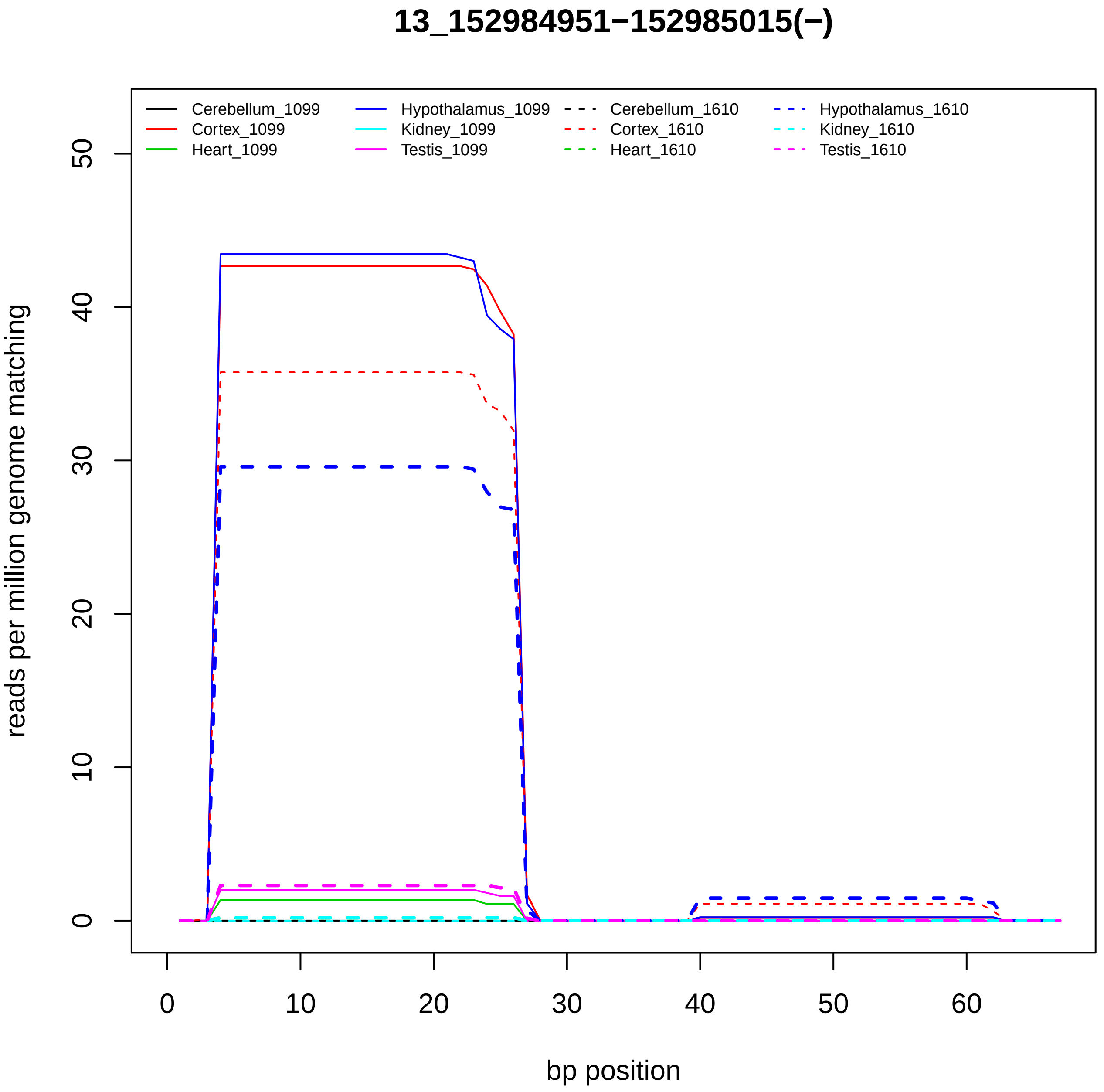

13_152984951−152985015(−)
Cerebellum_1099
Cortex_1099
Heart_1099
Hypothalamus_1099
Kidney_1099
Testis_1099
Cerebellum_1610
Cortex_1610
Heart_1610
Hypothalamus_1610
Kidney_1610
Testis_1610
reads per million genome matching
0
10
20
30
40
50
0
10
20
30
40
50
60
bp position

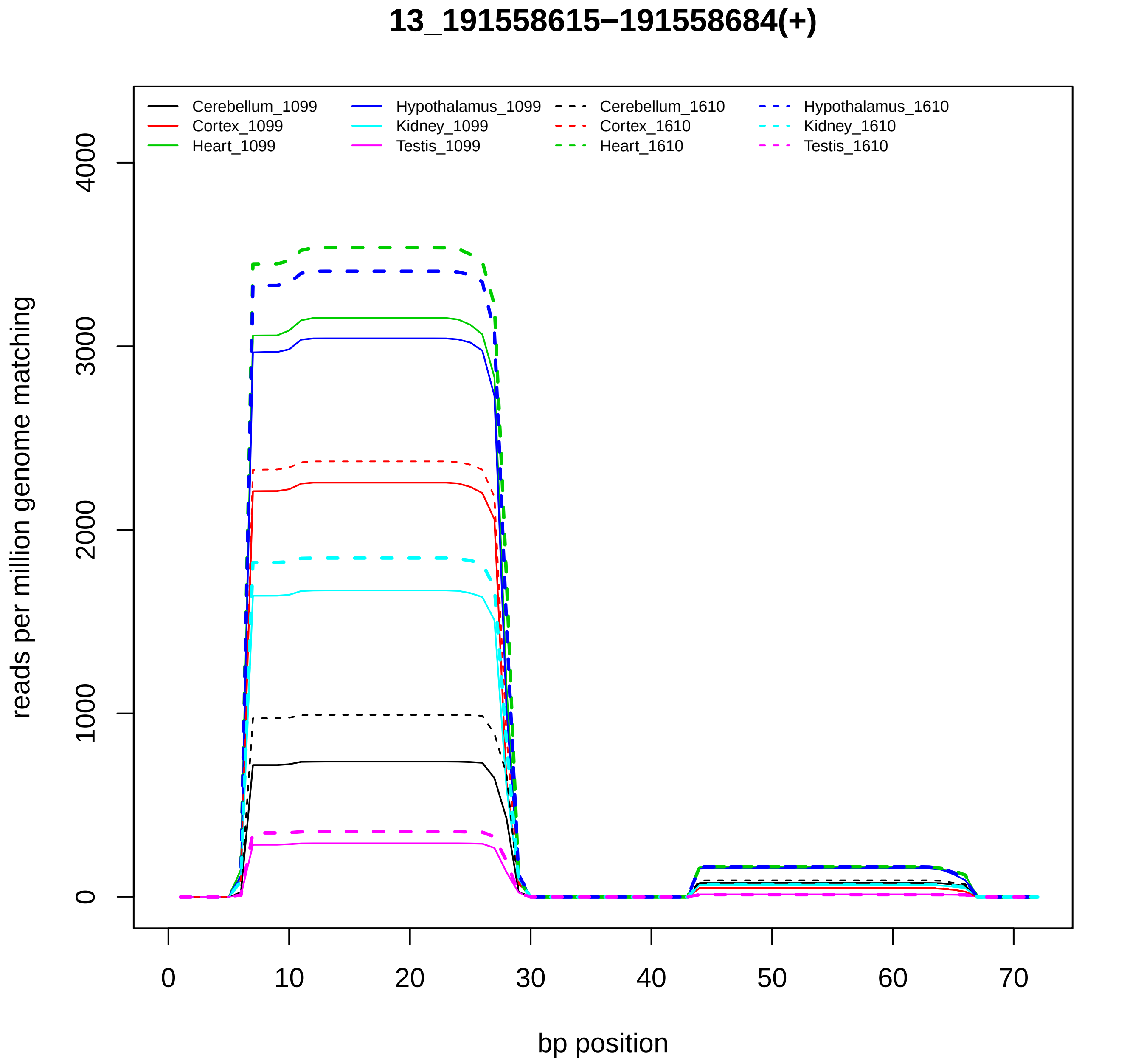

13_191558615−191558684(+)
Cerebellum_1099
Cortex_1099
Heart_1099
Hypothalamus_1099
Kidney_1099
Testis_1099
Cerebellum_1610
Cortex_1610
Heart_1610
Hypothalamus_1610
Kidney_1610
Testis_1610
reads per million genome matching
0
1000
2000
3000
4000
0
10
20
30
40
50
60
70
bp position

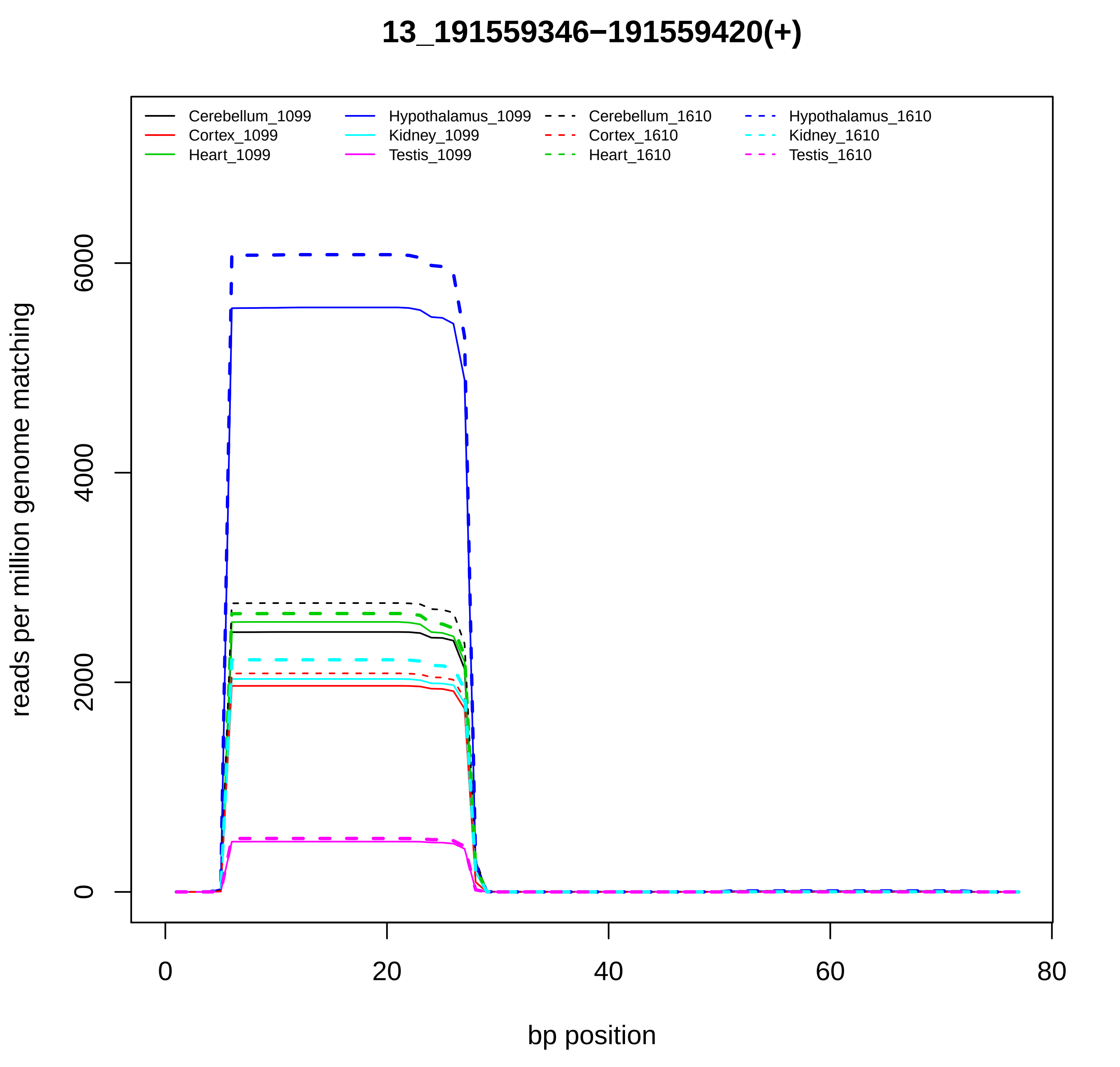
13_191559346−191559420(+)
Cerebellum_1099
Cortex_1099
Heart_1099
Hypothalamus_1099
Kidney_1099
Testis_1099
Cerebellum_1610
Cortex_1610
Heart_1610
Hypothalamus_1610
Kidney_1610
Testis_1610
reads per million genome matching
0
2000
4000
6000
0
20
40
60
80
bp position

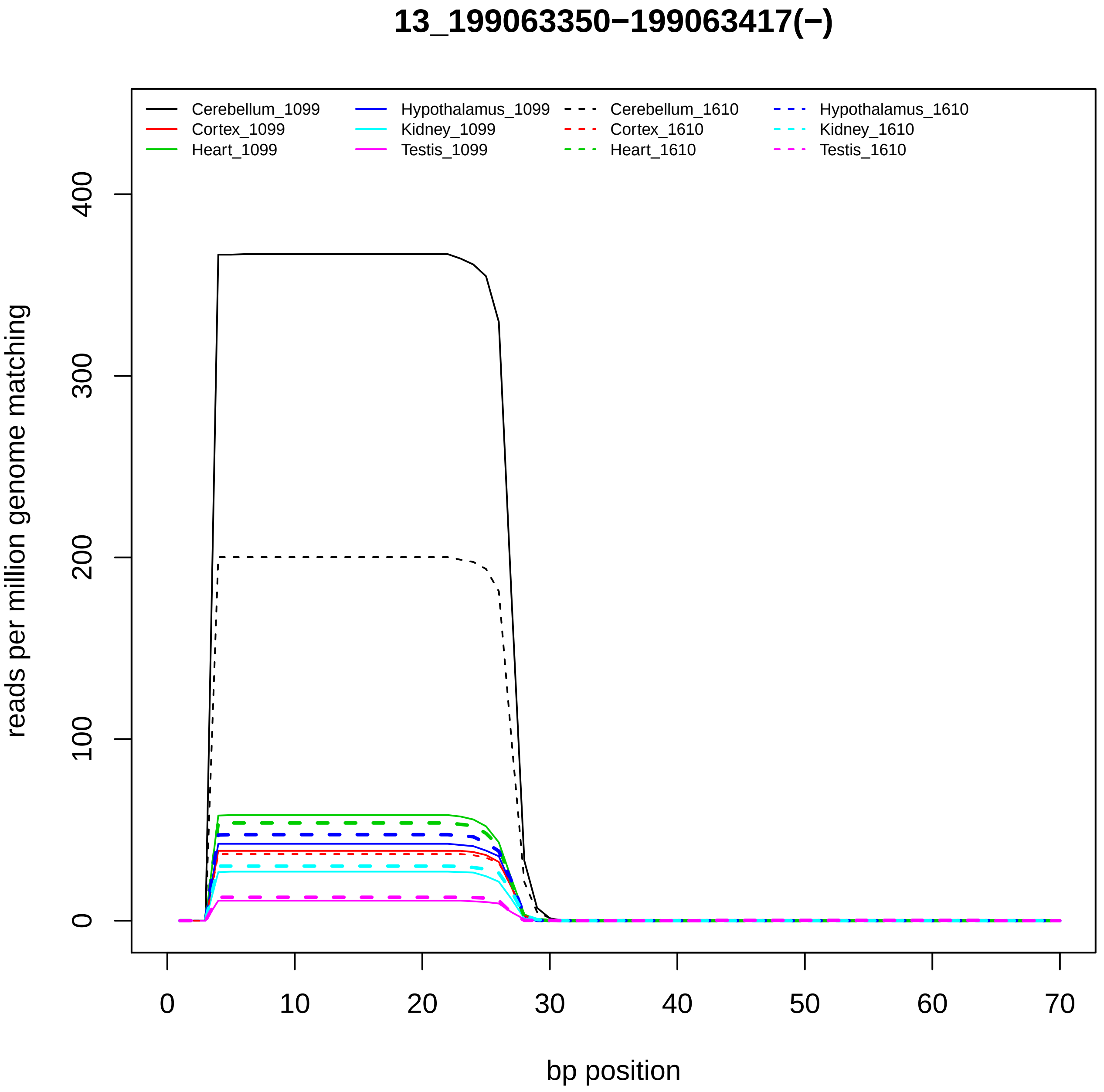
13_199063350−199063417(−)
Cerebellum_1099
Cortex_1099
Heart_1099
Hypothalamus_1099
Kidney_1099
Testis_1099
Cerebellum_1610
Cortex_1610
Heart_1610
Hypothalamus_1610
Kidney_1610
Testis_1610
reads per million genome matching
0
100
200
300
400
bp position
0
10
20
30
40
50
60
70

13_209487901−209487967(+)

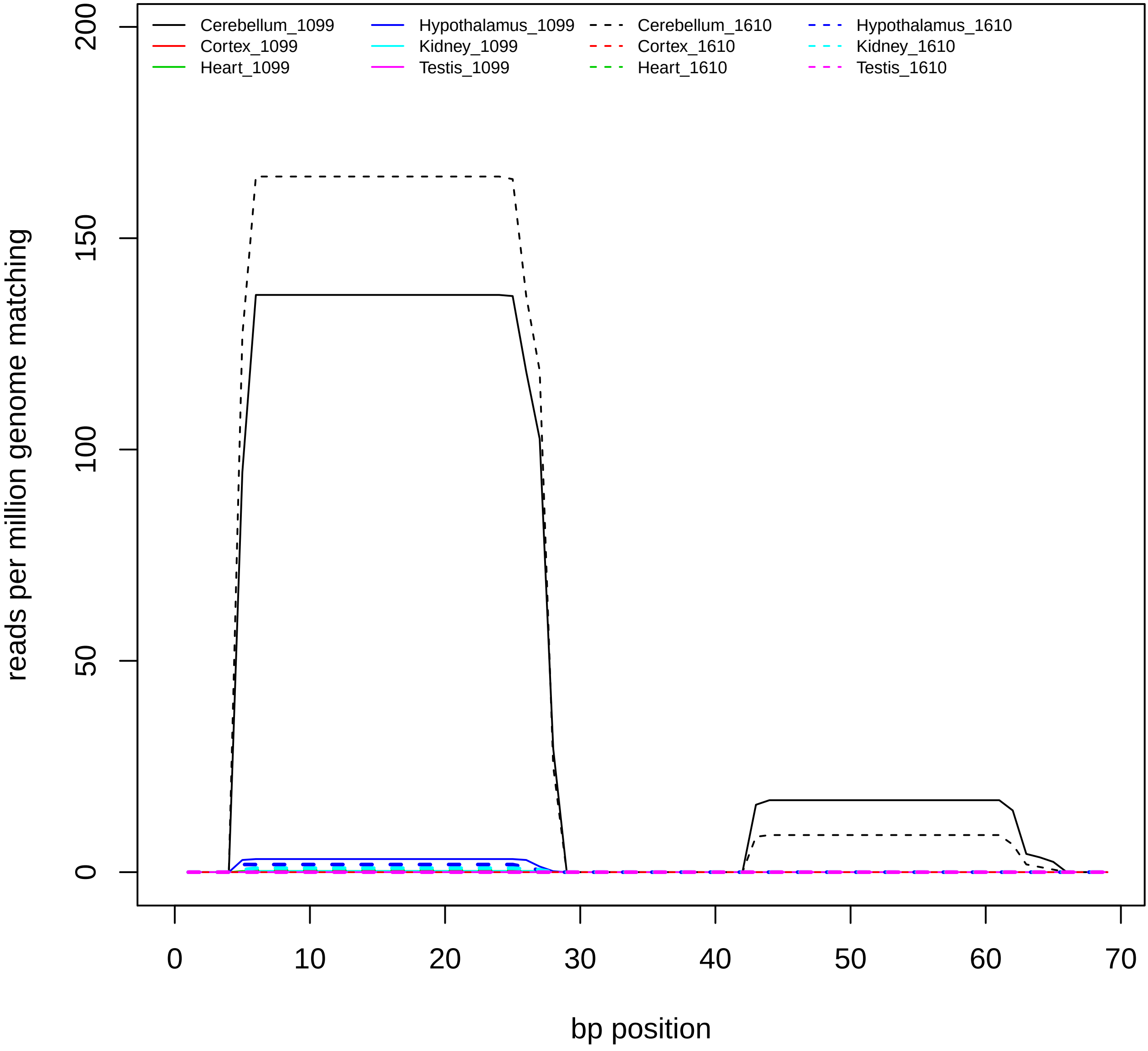

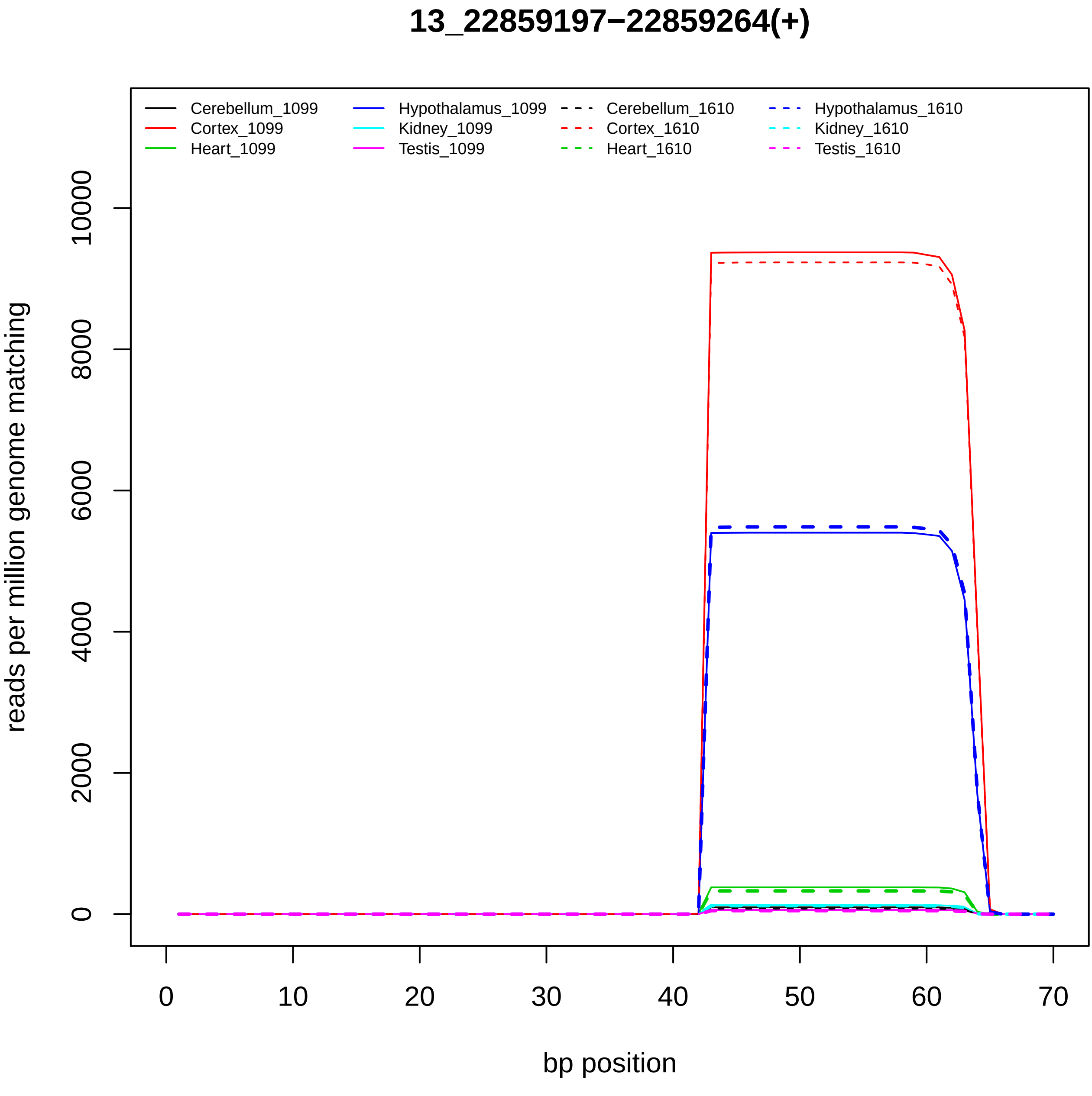
13_22859197−22859264(+)
Cerebellum_1099
Cortex_1099
Heart_1099
Hypothalamus_1099
Kidney_1099
Testis_1099
Cerebellum_1610
Cortex_1610
Heart_1610
Hypothalamus_1610
Kidney_1610
Testis_1610
reads per million genome matching
0
2000
4000
6000
8000
10000
0
10
20
30
40
50
60
70
bp position

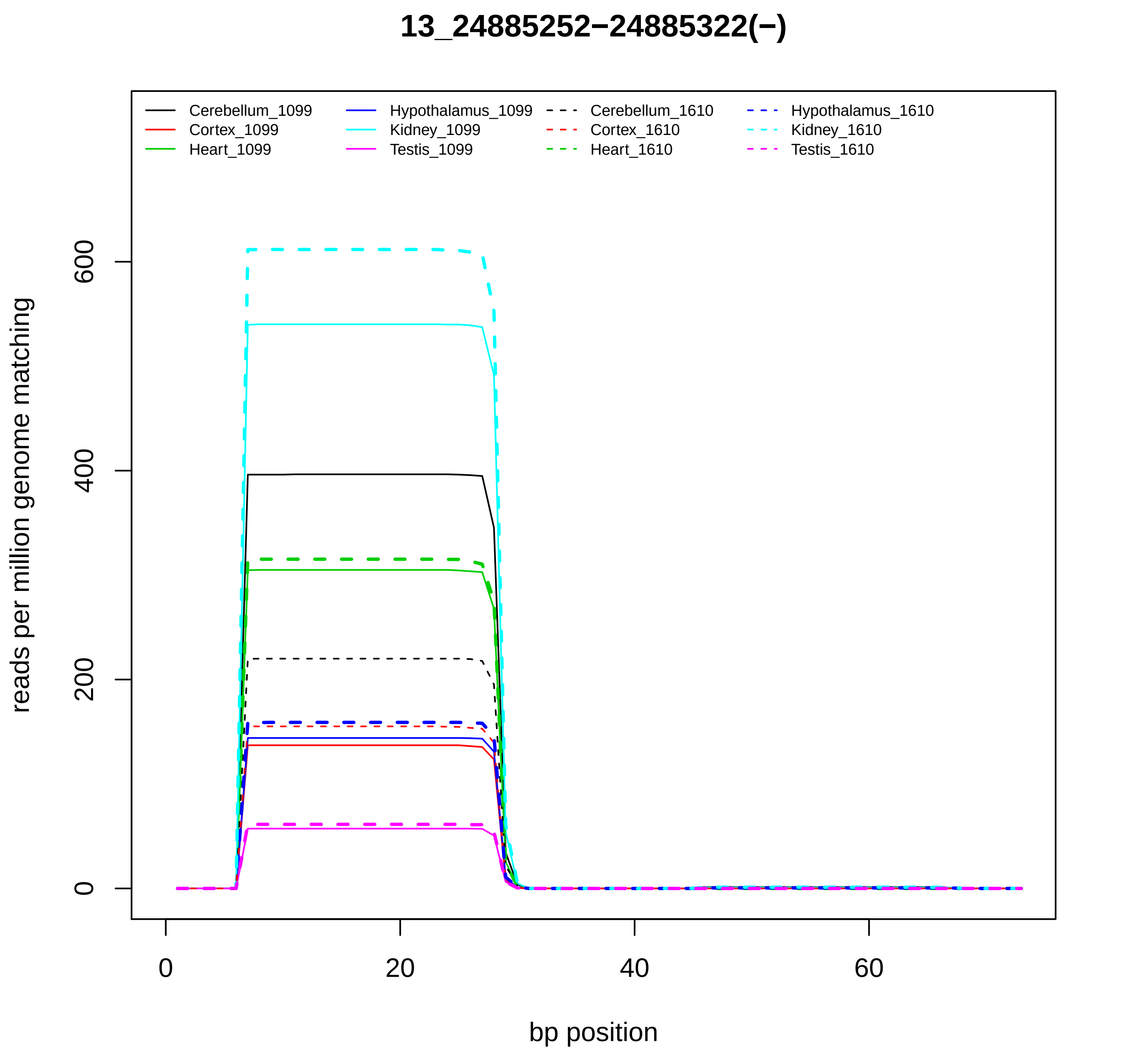

13_24885252−24885322(−)
Cerebellum_1099
Cortex_1099
Heart_1099
Hypothalamus_1099
Kidney_1099
Testis_1099
Cerebellum_1610
Cortex_1610
Heart_1610
Hypothalamus_1610
Kidney_1610
Testis_1610
reads per million genome matching
0
200
400
600
0
20
40
60
bp position

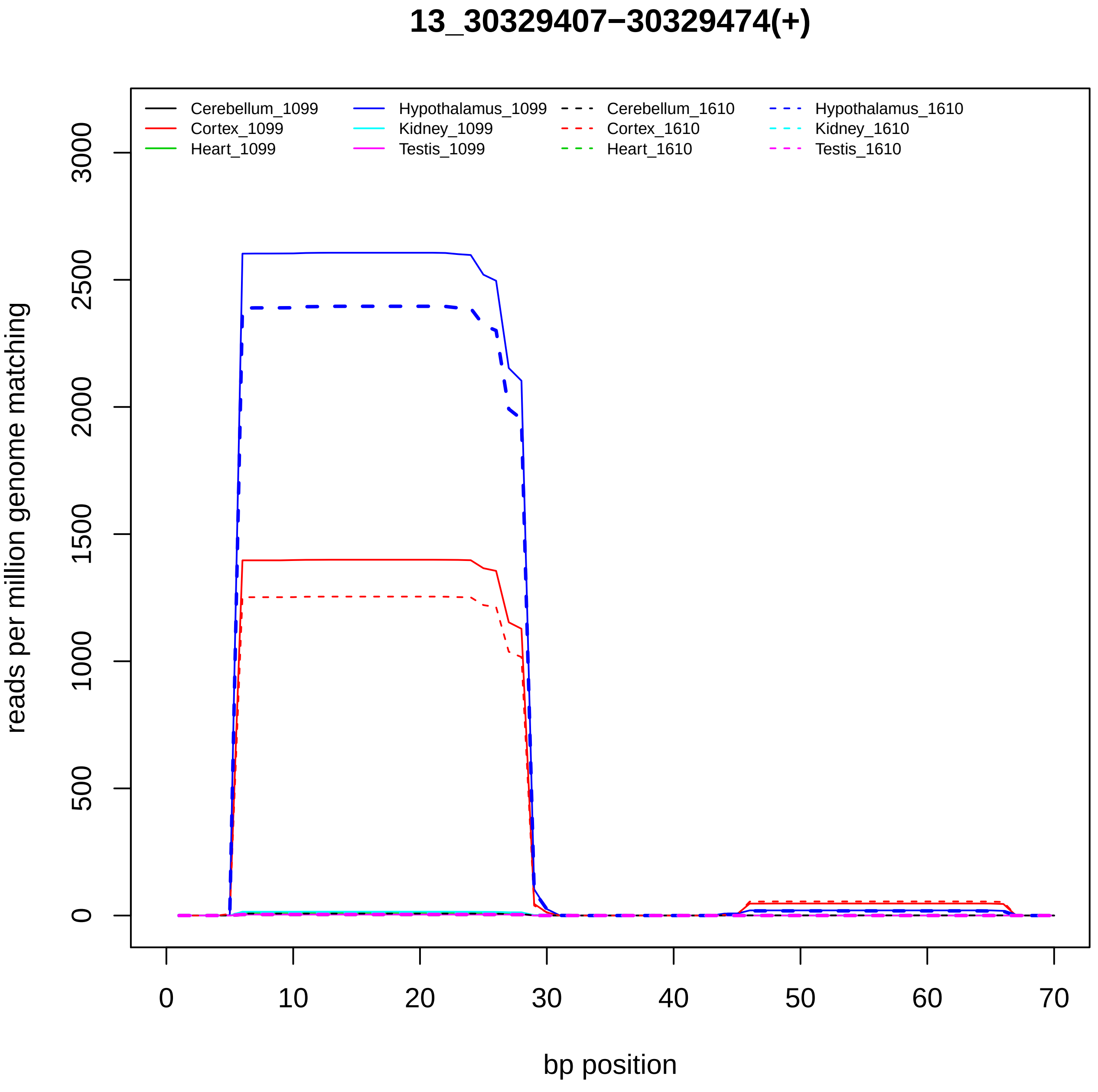

13_30329407−30329474(+)
Cerebellum_1099
Cortex_1099
Heart_1099
Hypothalamus_1099
Kidney_1099
Testis_1099
Cerebellum_1610
Cortex_1610
Heart_1610
Hypothalamus_1610
Kidney_1610
Testis_1610
reads per million genome matching
0
500
1000
1500
2000
2500
3000
0
10
20
30
40
50
60
70
bp position

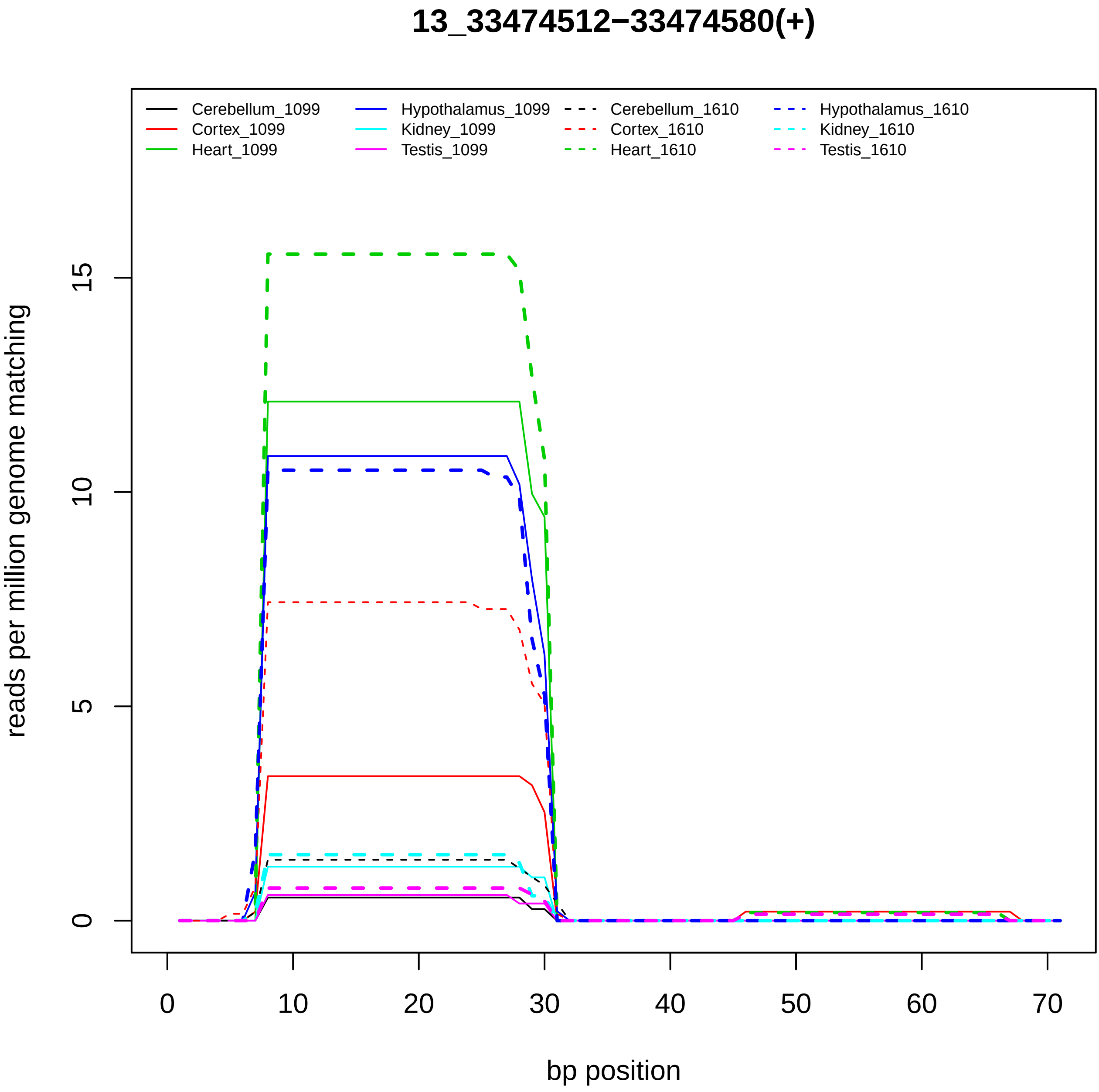
13_33474512−33474580(+)
Cerebellum_1099
Cortex_1099
Heart_1099
Hypothalamus_1099
Kidney_1099
Testis_1099
Cerebellum_1610
Cortex_1610
Heart_1610
Hypothalamus_1610
Kidney_1610
Testis_1610
reads per million genome matching
bp position
0
5
10
15
0
10
20
30
40
50
60
70

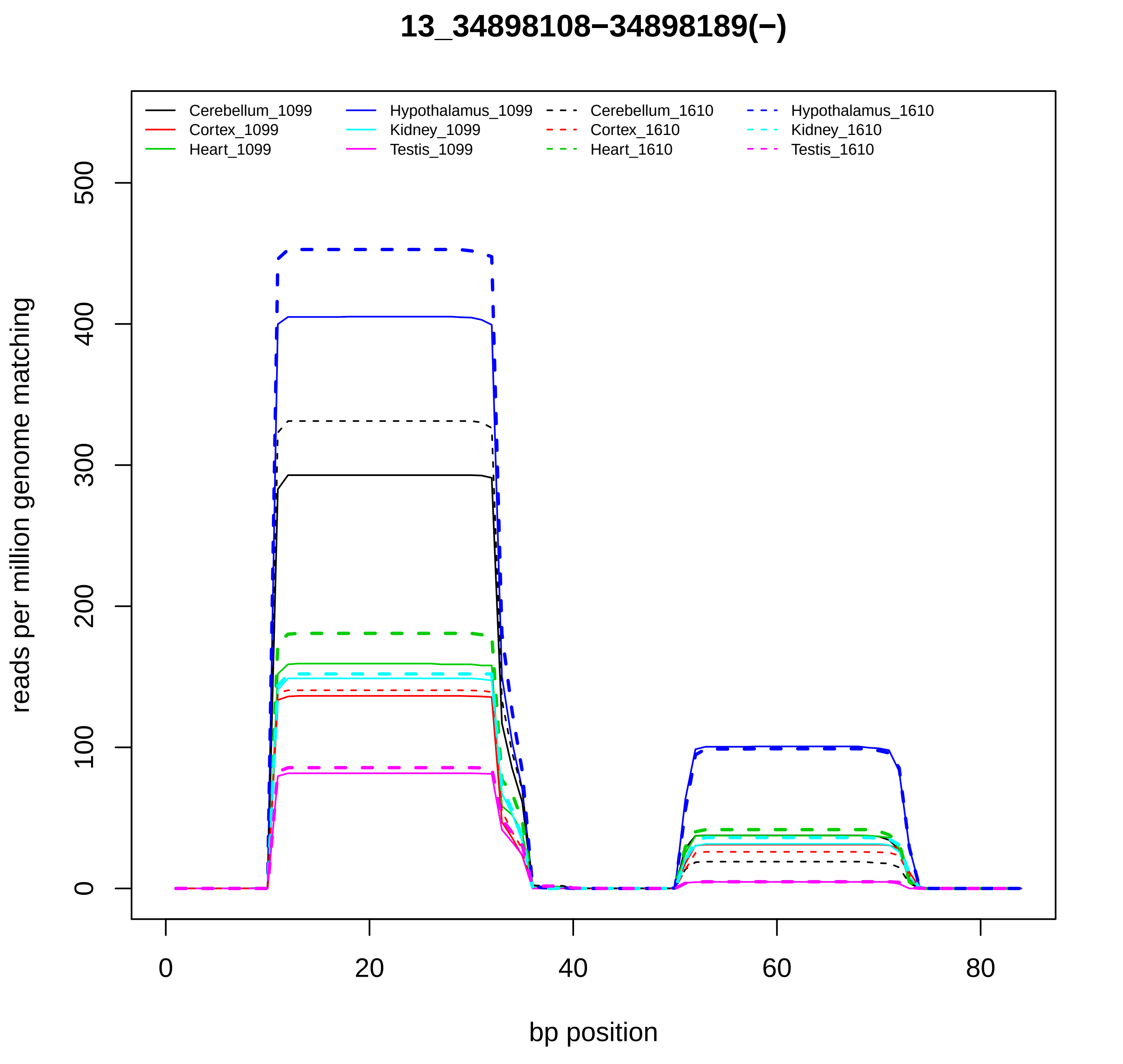
13_34898108−34898189(−)
Cerebellum_1099
Cortex_1099
Heart_1099
Hypothalamus_1099
Kidney_1099
Testis_1099
Cerebellum_1610
Cortex_1610
Heart_1610
Hypothalamus_1610
Kidney_1610
Testis_1610
reads per million genome matching
0
100
200
300
400
500
0
20
40
60
80
bp position

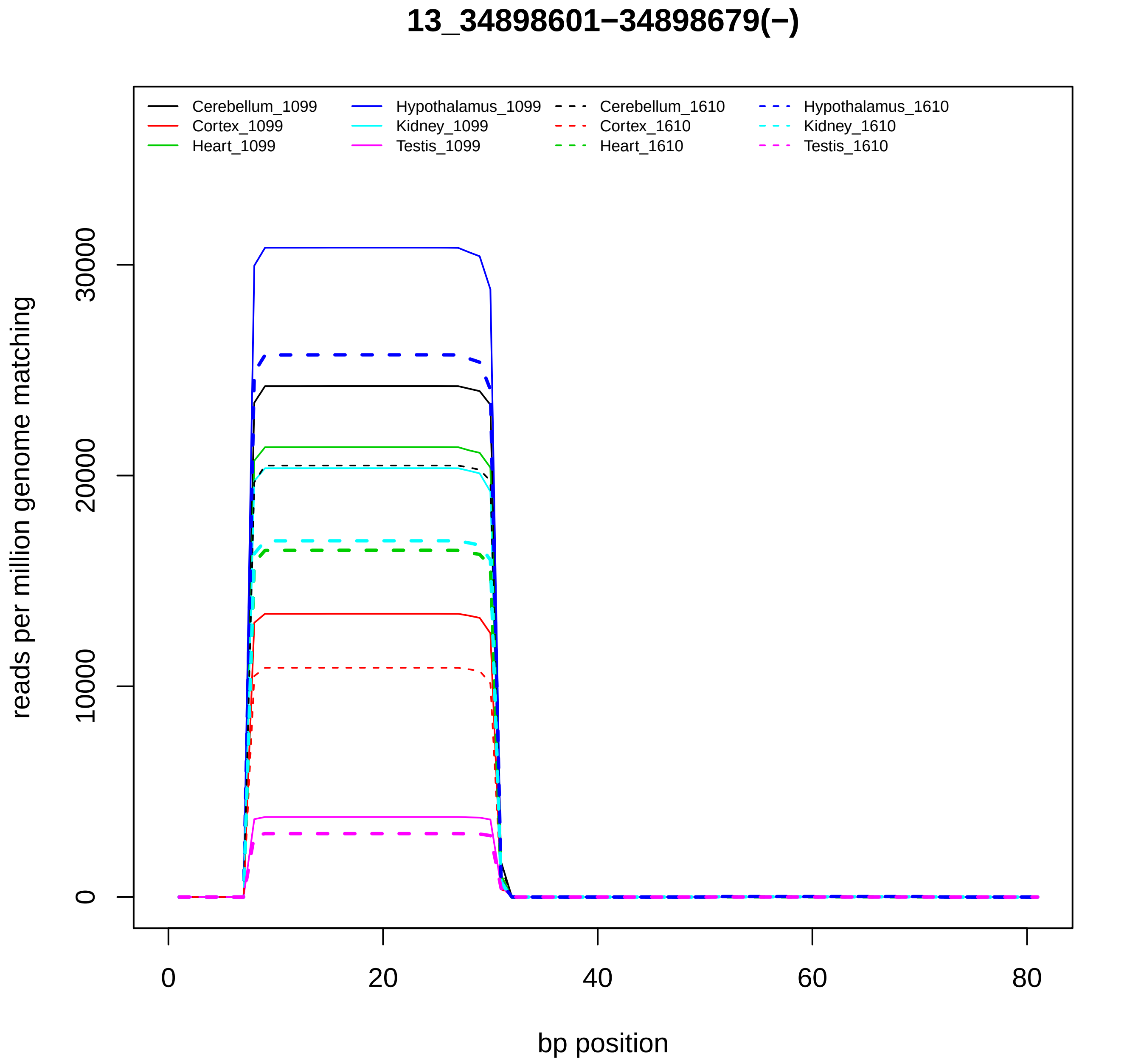
13_34898601−34898679(−)
Cerebellum_1099
Cortex_1099
Heart_1099
Hypothalamus_1099
Kidney_1099
Testis_1099
Cerebellum_1610
Cortex_1610
Heart_1610
Hypothalamus_1610
Kidney_1610
Testis_1610
reads per million genome matching
0
10000
20000
30000
0
20
40
60
80
bp position

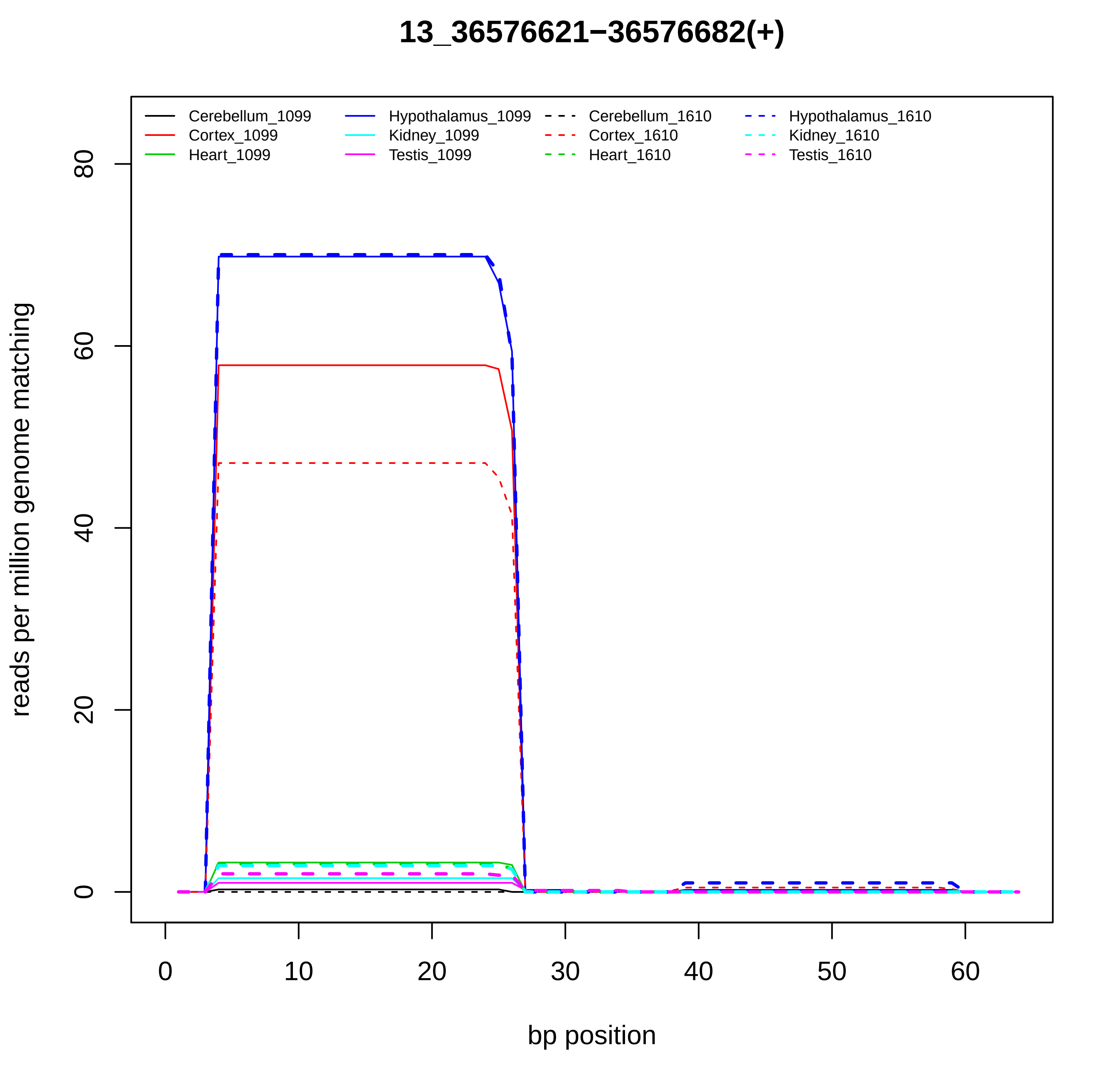

13_36576621−36576682(+)
Cerebellum_1099
Cortex_1099
Heart_1099
Hypothalamus_1099
Kidney_1099
Testis_1099
Cerebellum_1610
Cortex_1610
Heart_1610
Hypothalamus_1610
Kidney_1610
Testis_1610
reads per million genome matching
bp position
0
20
40
60
80
0
10
20
30
40
50
60

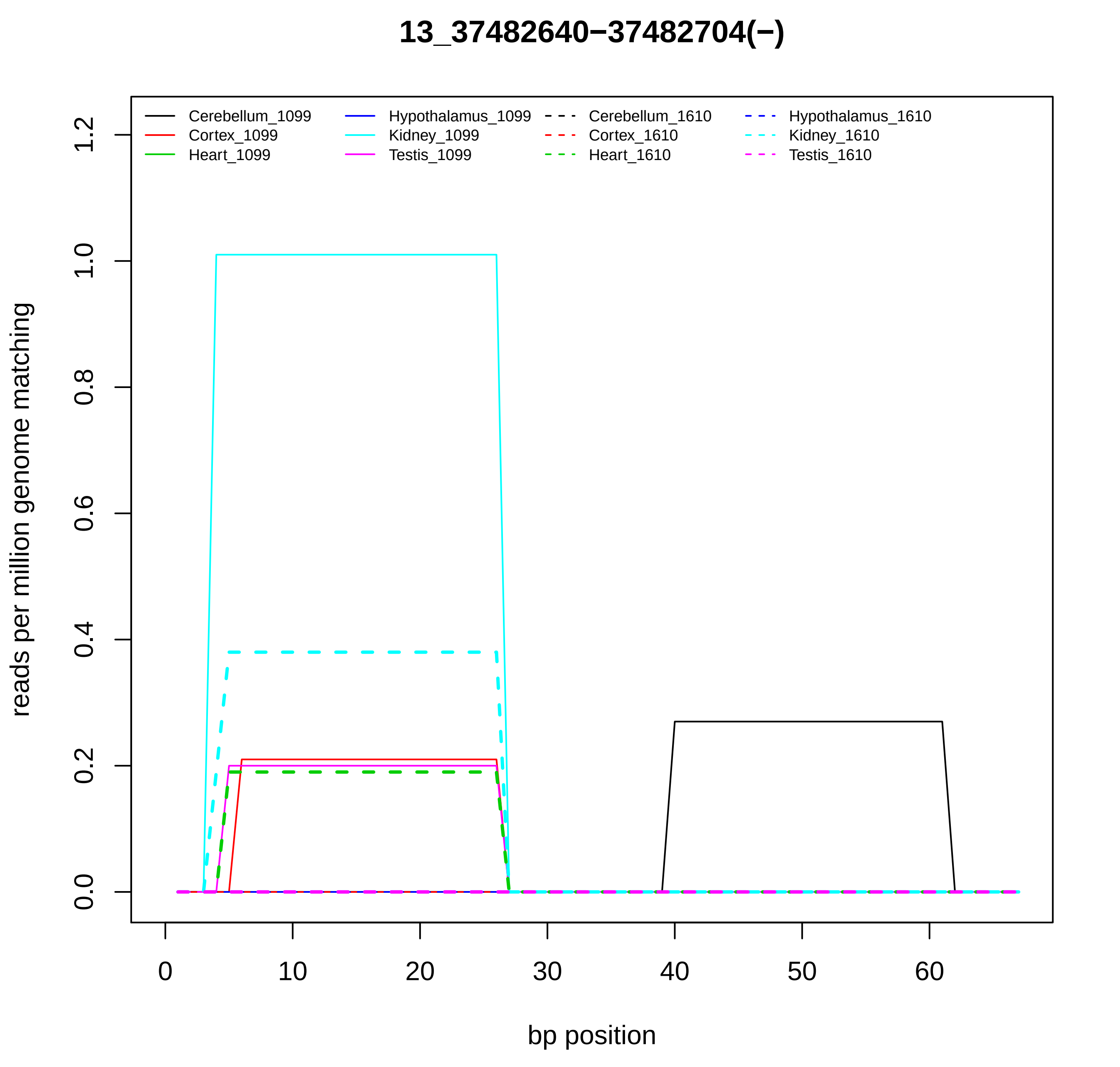

13_37482640−37482704(−)
Cerebellum_1099
Cortex_1099
Heart_1099
Hypothalamus_1099
Kidney_1099
Testis_1099
Cerebellum_1610
Cortex_1610
Heart_1610
Hypothalamus_1610
Kidney_1610
Testis_1610
reads per million genome matching
0.0
0.2
0.4
0.6
0.8
1.0
1.2
0
10
20
30
40
50
60
bp position

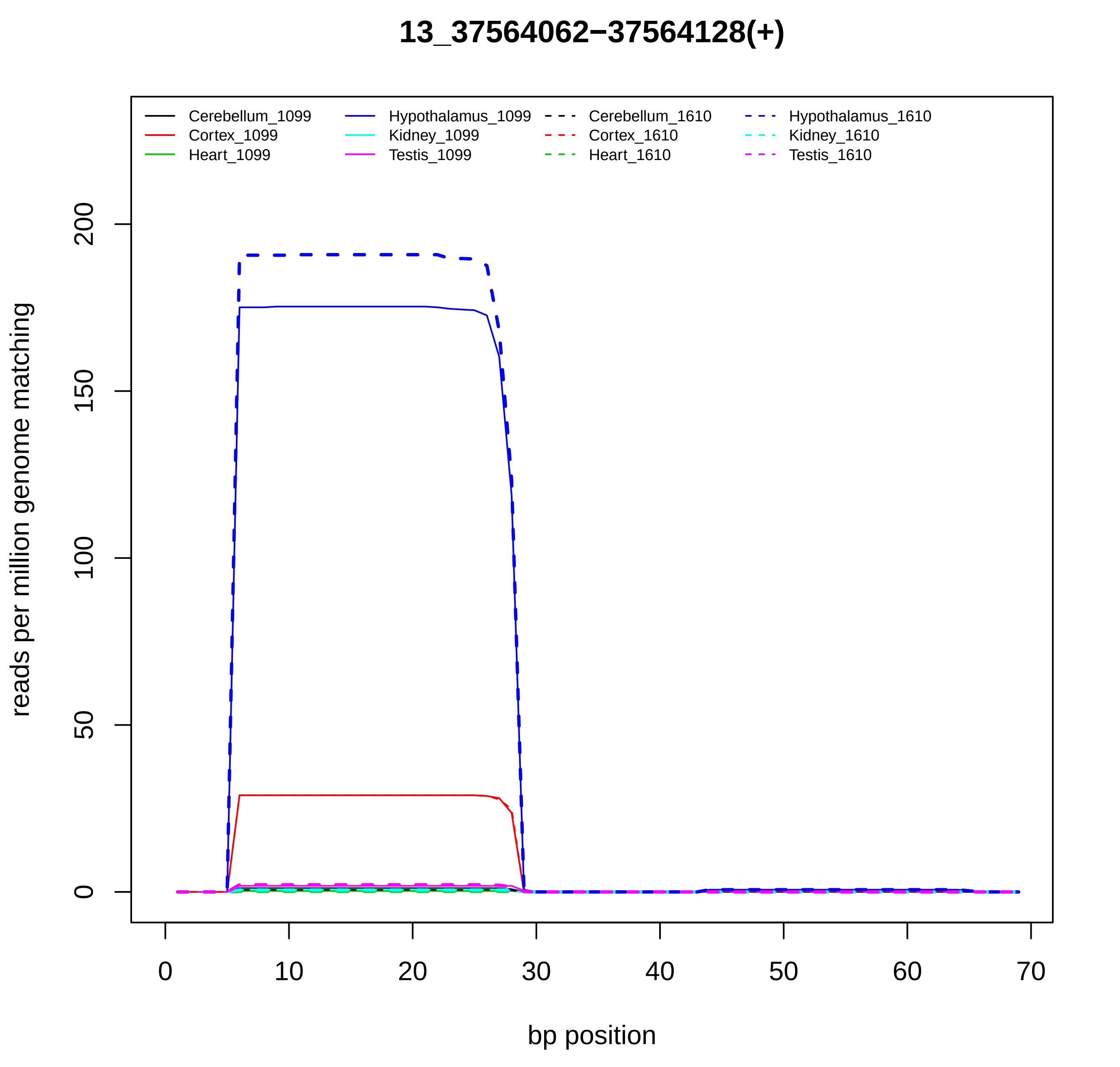

13_37564062−37564128(+)
Cerebellum_1099
Cortex_1099
Heart_1099
Hypothalamus_1099
Kidney_1099
Testis_1099
Cerebellum_1610
Cortex_1610
Heart_1610
Hypothalamus_1610
Kidney_1610
Testis_1610
reads per million genome matching
0
50
100
150
200
0
10
20
30
40
50
60
70
bp position

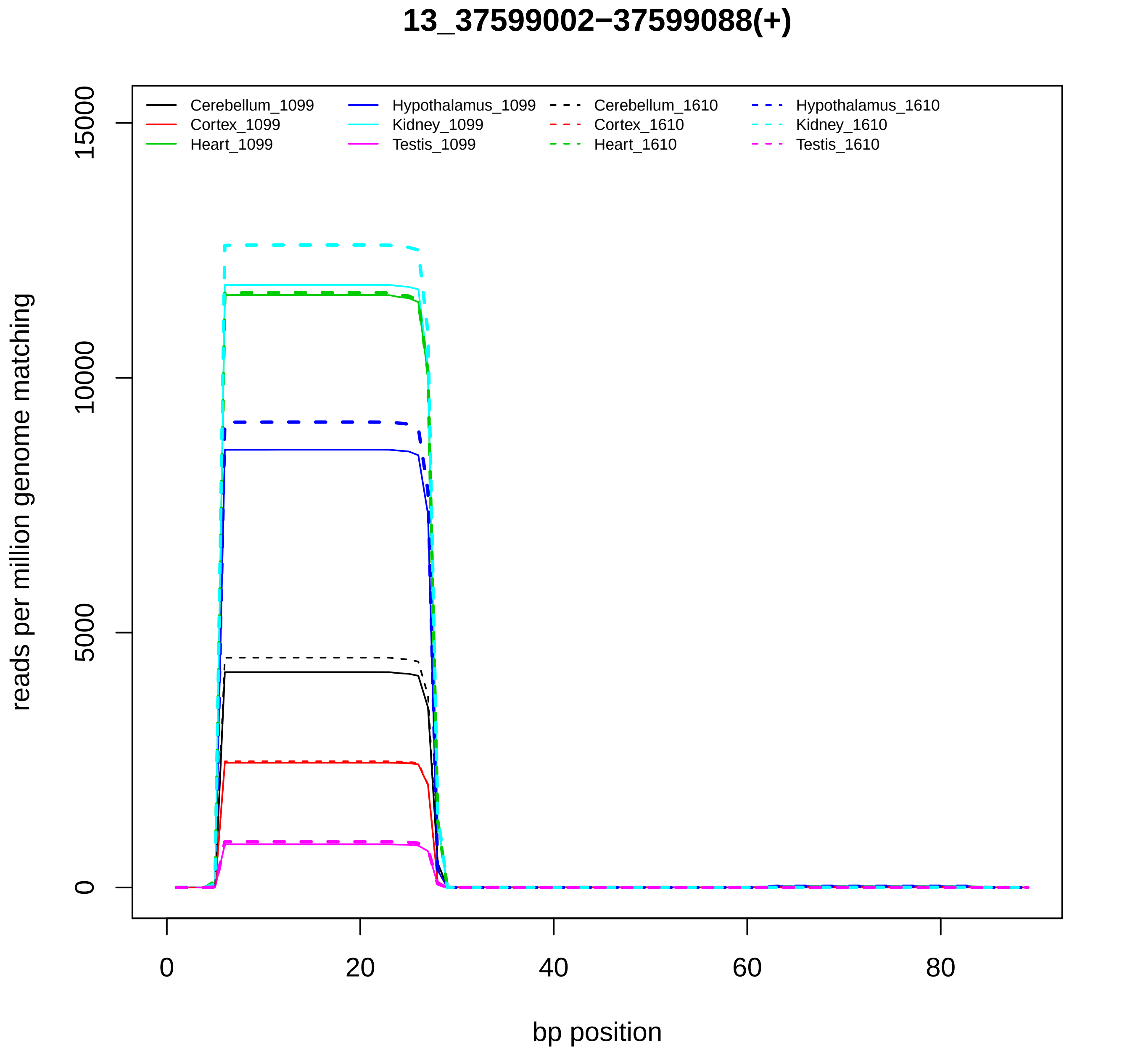

13_37599002−37599088(+)
Cerebellum_1099
Cortex_1099
Heart_1099
Hypothalamus_1099
Kidney_1099
Testis_1099
Cerebellum_1610
Cortex_1610
Heart_1610
Hypothalamus_1610
Kidney_1610
Testis_1610
reads per million genome matching
0
5000
10000
15000
0
20
40
60
80
bp position

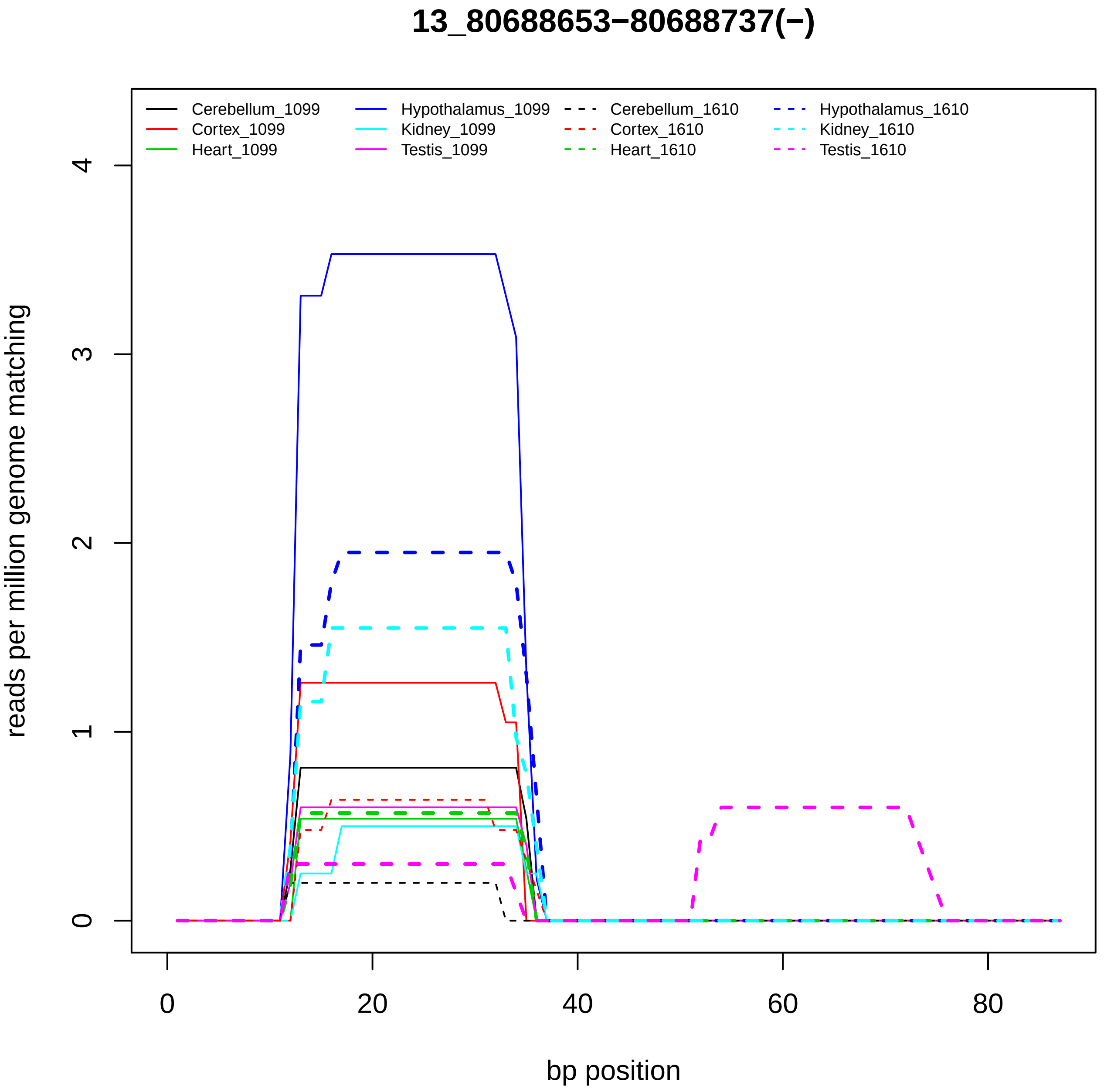

13_80688653−80688737(−)
Cerebellum_1099
Cortex_1099
Heart_1099
Hypothalamus_1099
Kidney_1099
Testis_1099
Cerebellum_1610
Cortex_1610
Heart_1610
Hypothalamus_1610
Kidney_1610
Testis_1610
reads per million genome matching
bp position
0
1
2
3
4
0
20
40
60
80

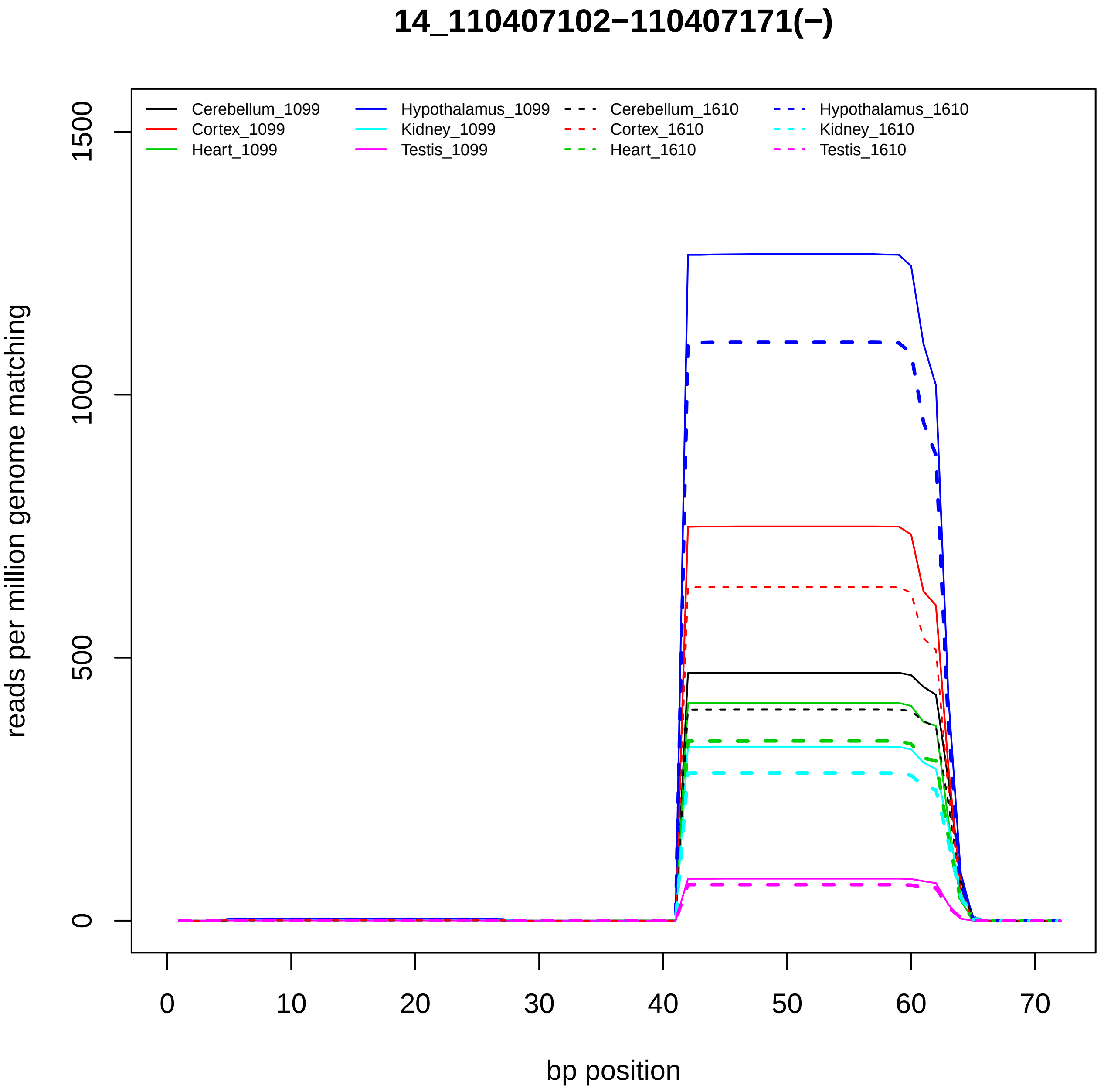

14_110407102−110407171(−)
Cerebellum_1099
Cortex_1099
Heart_1099
Hypothalamus_1099
Kidney_1099
Testis_1099
Cerebellum_1610
Cortex_1610
Heart_1610
Hypothalamus_1610
Kidney_1610
Testis_1610
reads per million genome matching
0
500
1000
1500
bp position
0
10
20
30
40
50
60
70

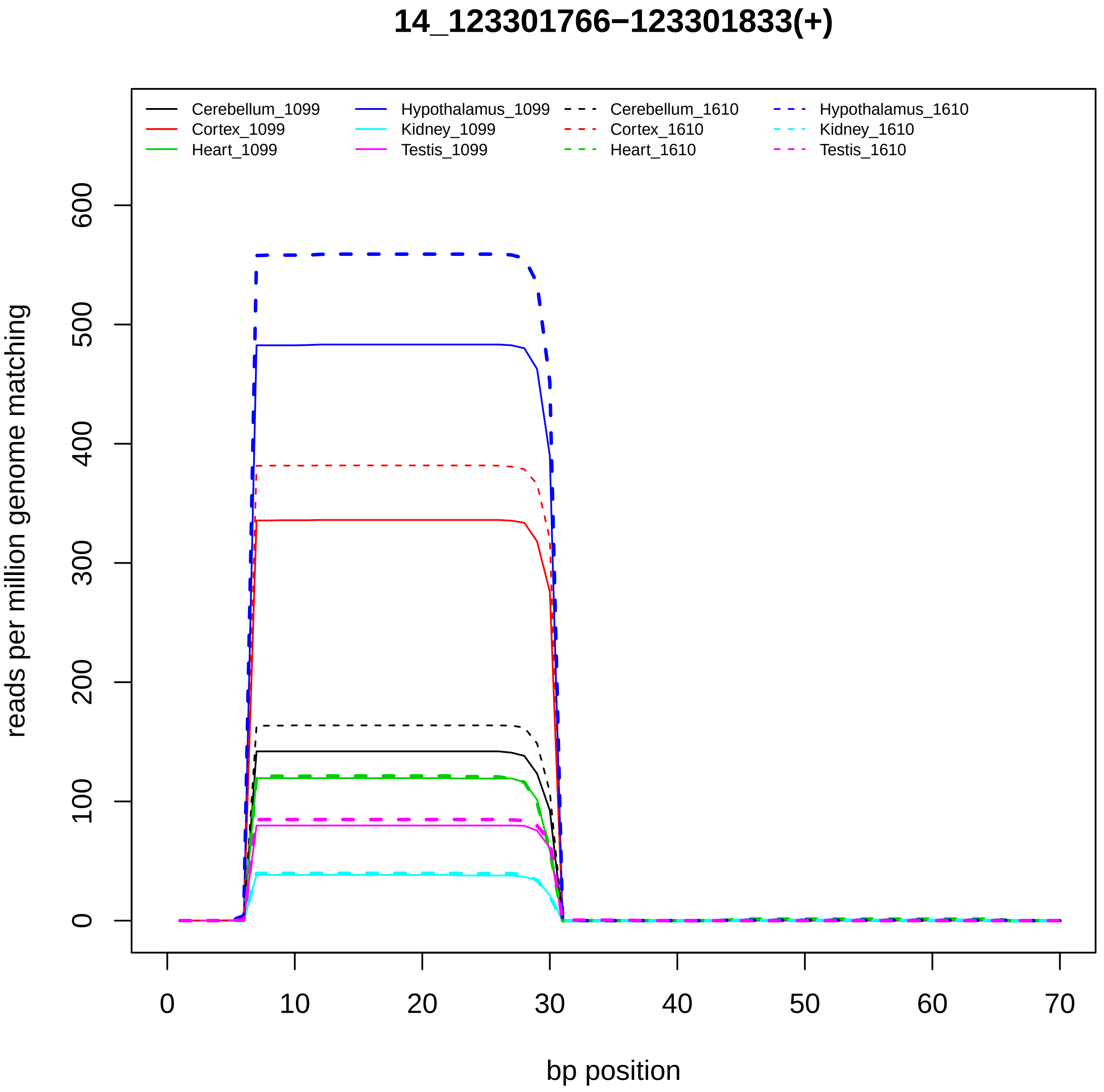

14_123301766−123301833(+)
Cerebellum_1099
Cortex_1099
Heart_1099
Hypothalamus_1099
Kidney_1099
Testis_1099
Cerebellum_1610
Cortex_1610
Heart_1610
Hypothalamus_1610
Kidney_1610
Testis_1610
reads per million genome matching
0
100
200
300
400
500
600
0
10
20
30
40
50
60
70
bp position

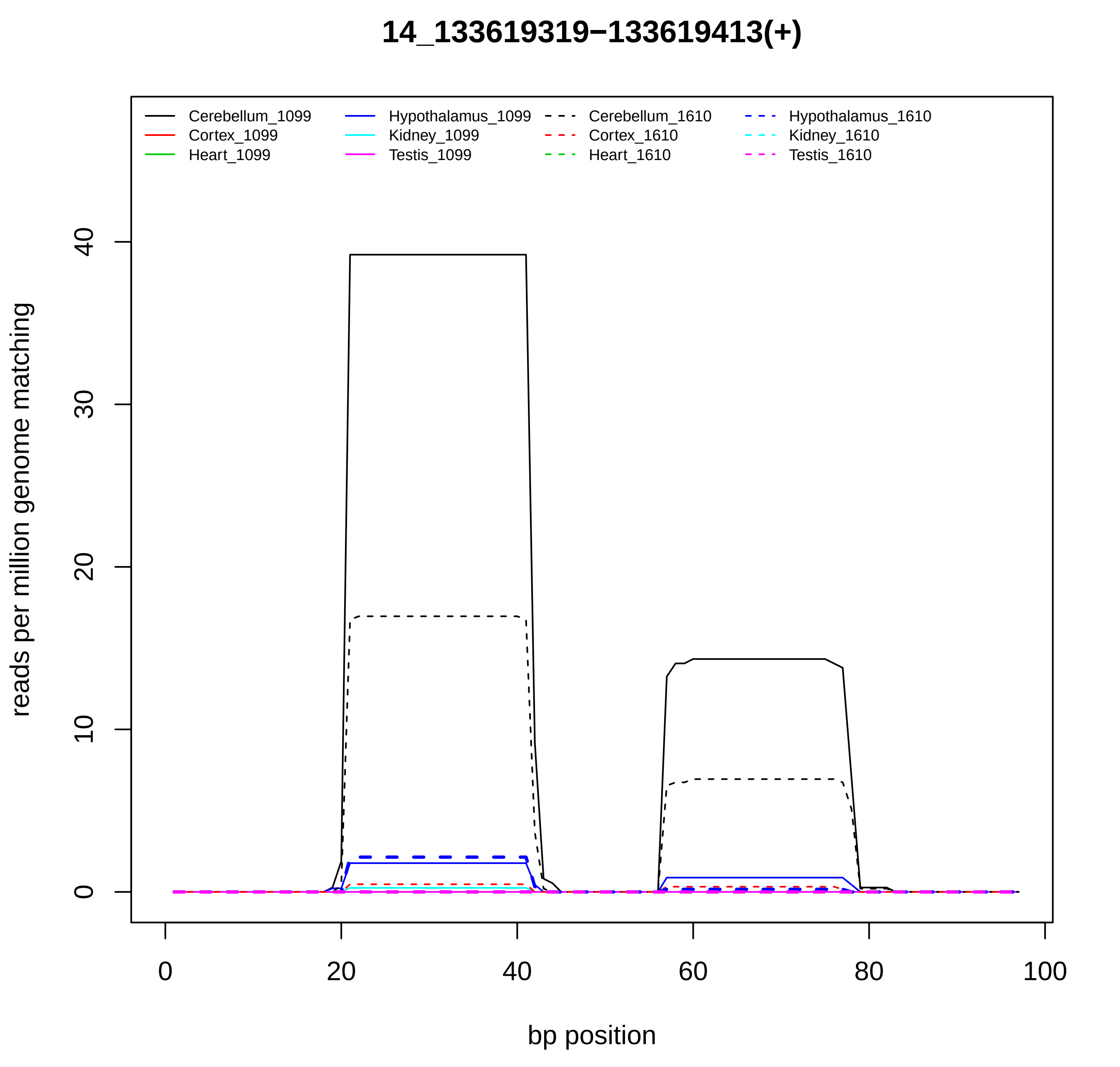

14_133619319−133619413(+)
Cerebellum_1099
Cortex_1099
Heart_1099
Hypothalamus_1099
Kidney_1099
Testis_1099
Cerebellum_1610
Cortex_1610
Heart_1610
Hypothalamus_1610
Kidney_1610
Testis_1610
reads per million genome matching
bp position
0
10
20
30
40
0
20
40
60
80
100

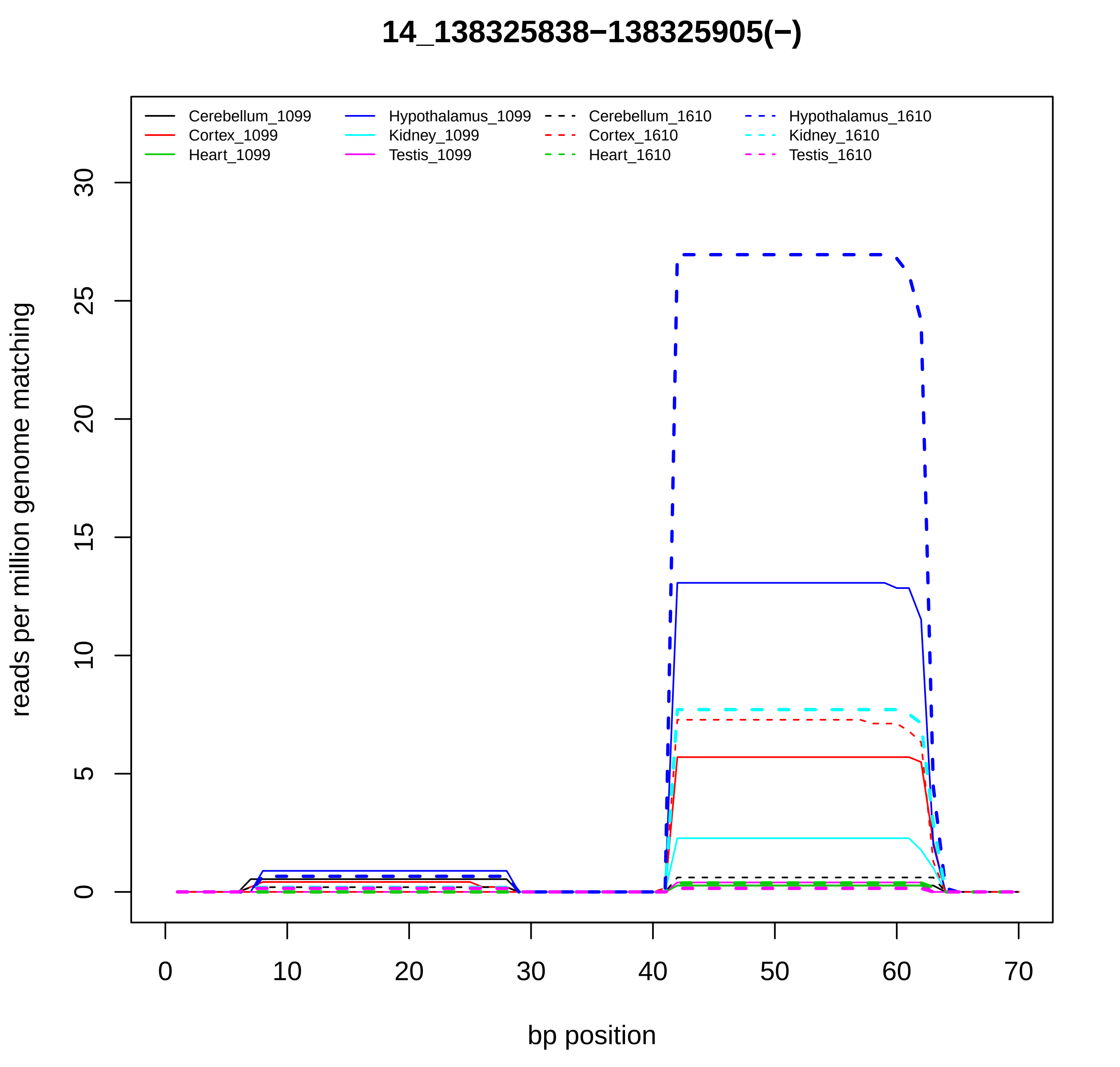

14_138325838−138325905(−)
Cerebellum_1099
Cortex_1099
Heart_1099
Hypothalamus_1099
Kidney_1099
Testis_1099
Cerebellum_1610
Cortex_1610
Heart_1610
Hypothalamus_1610
Kidney_1610
Testis_1610
reads per million genome matching
bp position
0
5
10
15
20
25
30
0
10
20
30
40
50
60
70

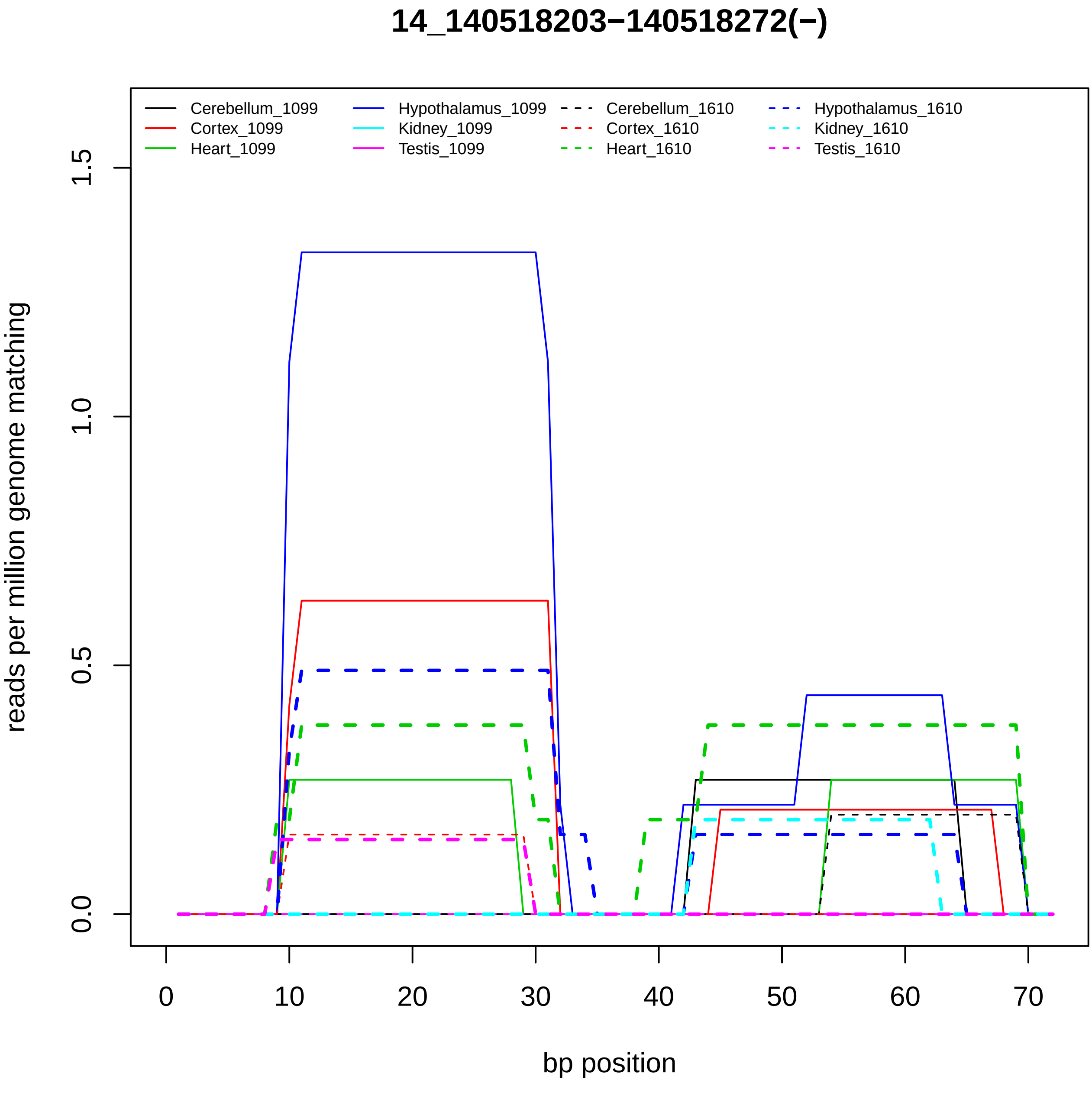

14_140518203−140518272(−)
Cerebellum_1099
Cortex_1099
Heart_1099
Hypothalamus_1099
Kidney_1099
Testis_1099
Cerebellum_1610
Cortex_1610
Heart_1610
Hypothalamus_1610
Kidney_1610
Testis_1610
reads per million genome matching
0.0
0.5
1.0
1.5
0
10
20
30
40
50
60
70
bp position

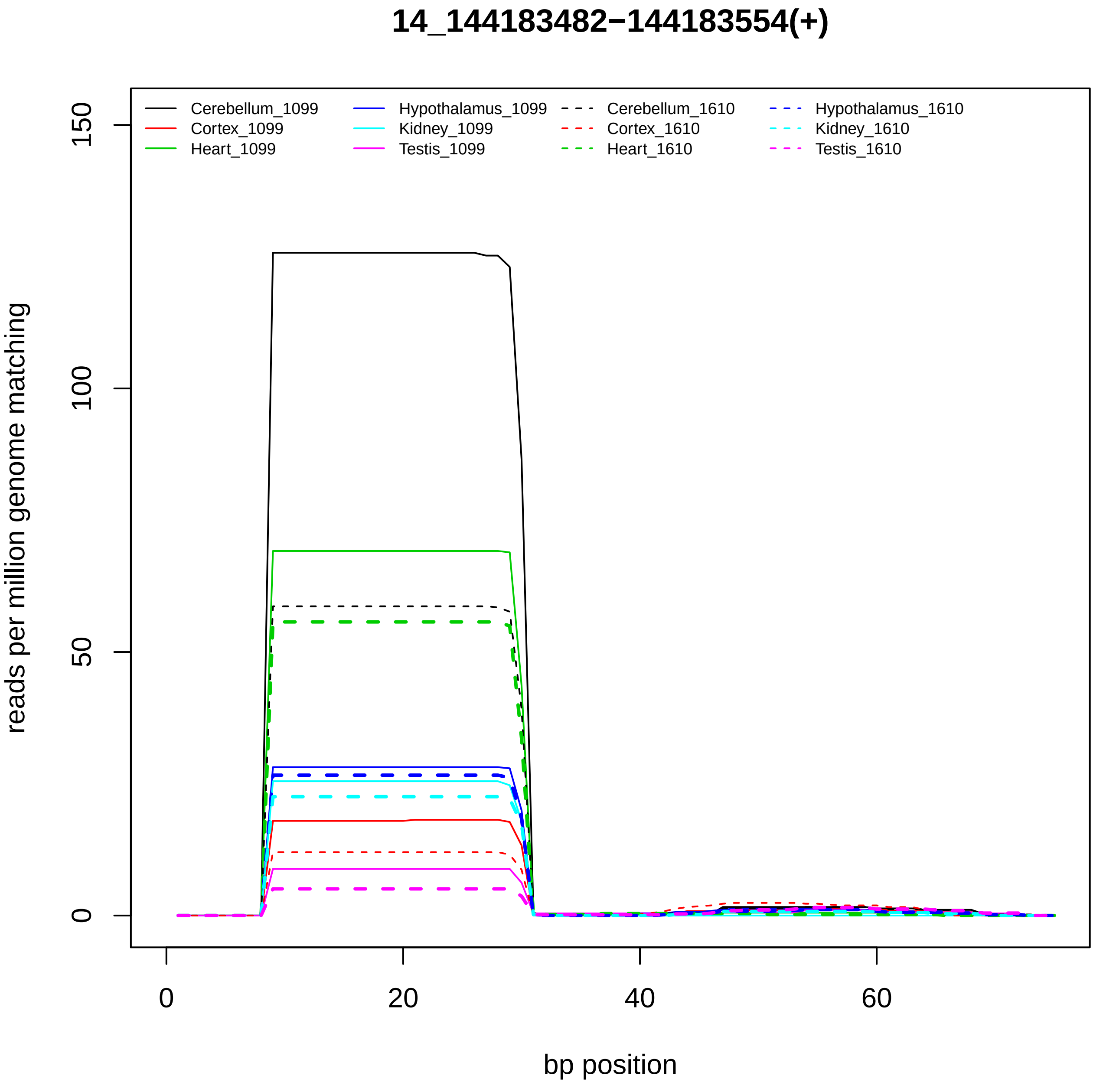

14_144183482−144183554(+)
Cerebellum_1099
Cortex_1099
Heart_1099
Hypothalamus_1099
Kidney_1099
Testis_1099
Cerebellum_1610
Cortex_1610
Heart_1610
Hypothalamus_1610
Kidney_1610
Testis_1610
reads per million genome matching
0
50
100
150
bp position
0
20
40
60

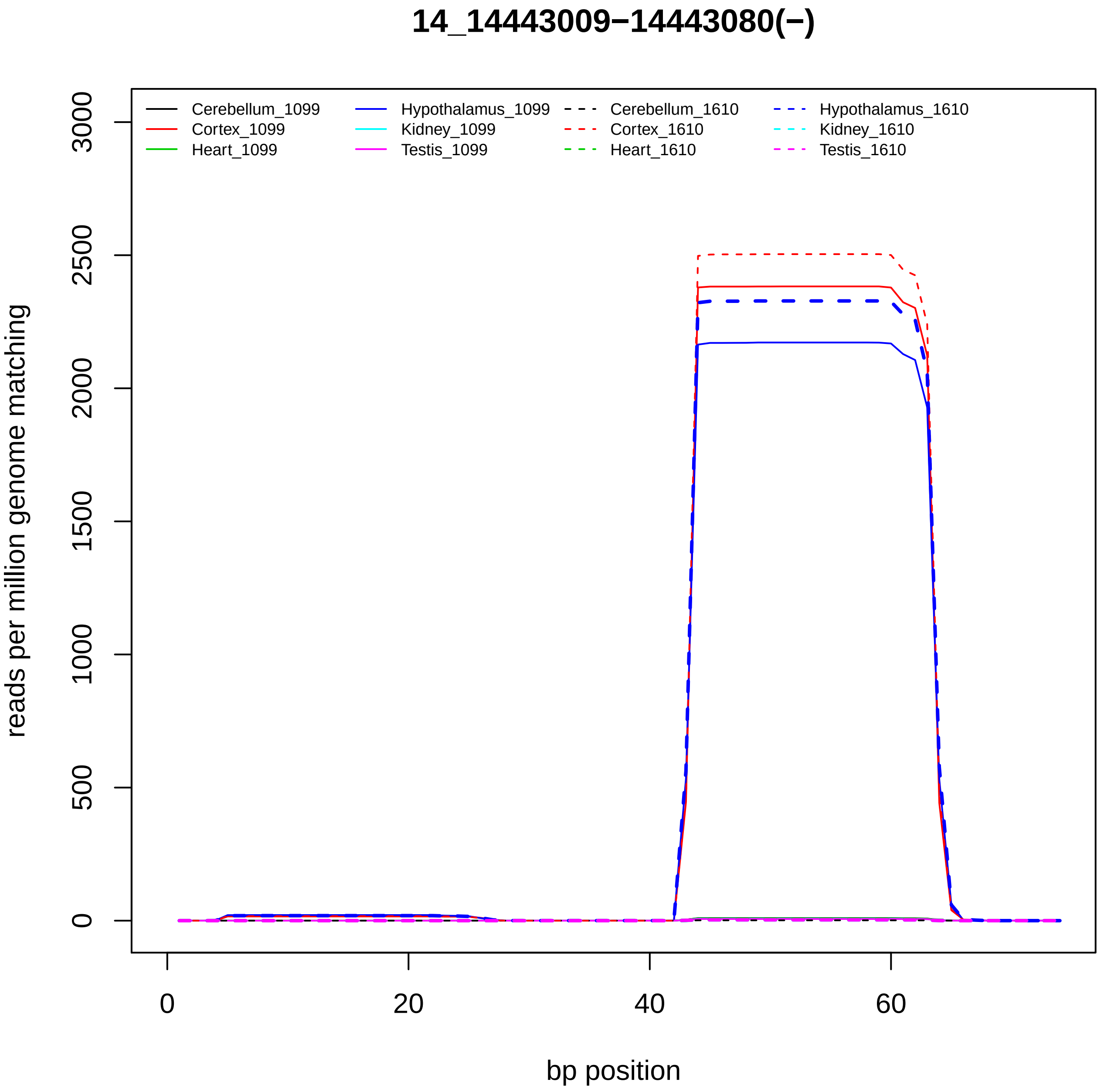

14_14443009−14443080(−)
Cerebellum_1099
Cortex_1099
Heart_1099
Hypothalamus_1099
Kidney_1099
Testis_1099
Cerebellum_1610
Cortex_1610
Heart_1610
Hypothalamus_1610
Kidney_1610
Testis_1610
reads per million genome matching
0
500
1000
1500
2000
2500
3000
0
20
40
60
bp position

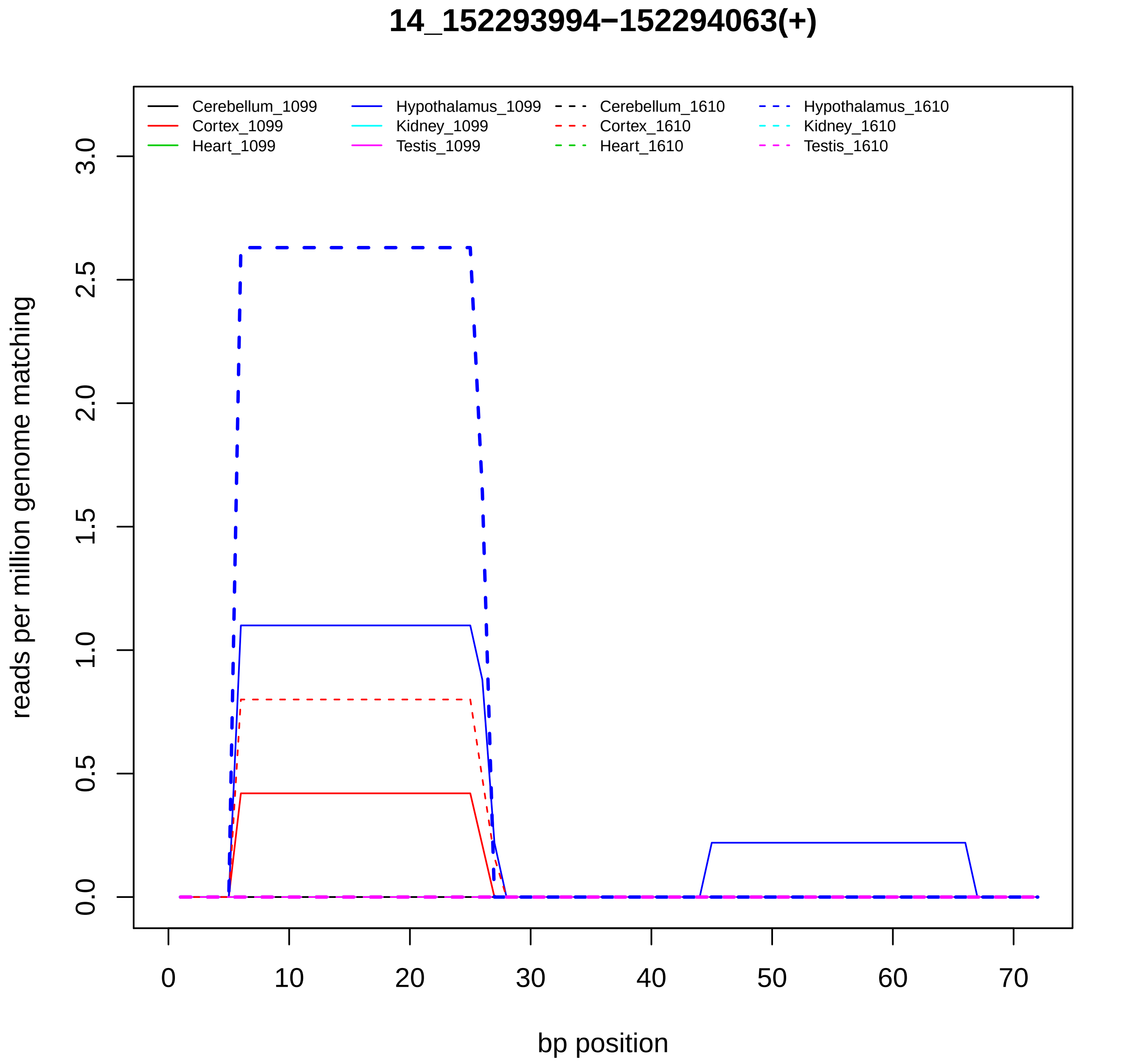

14_152293994−152294063(+)
Cerebellum_1099
Cortex_1099
Heart_1099
Hypothalamus_1099
Kidney_1099
Testis_1099
Cerebellum_1610
Cortex_1610
Heart_1610
Hypothalamus_1610
Kidney_1610
Testis_1610
reads per million genome matching
0.0
0.5
1.0
1.5
2.0
2.5
3.0
0
10
20
30
40
50
60
70
bp position

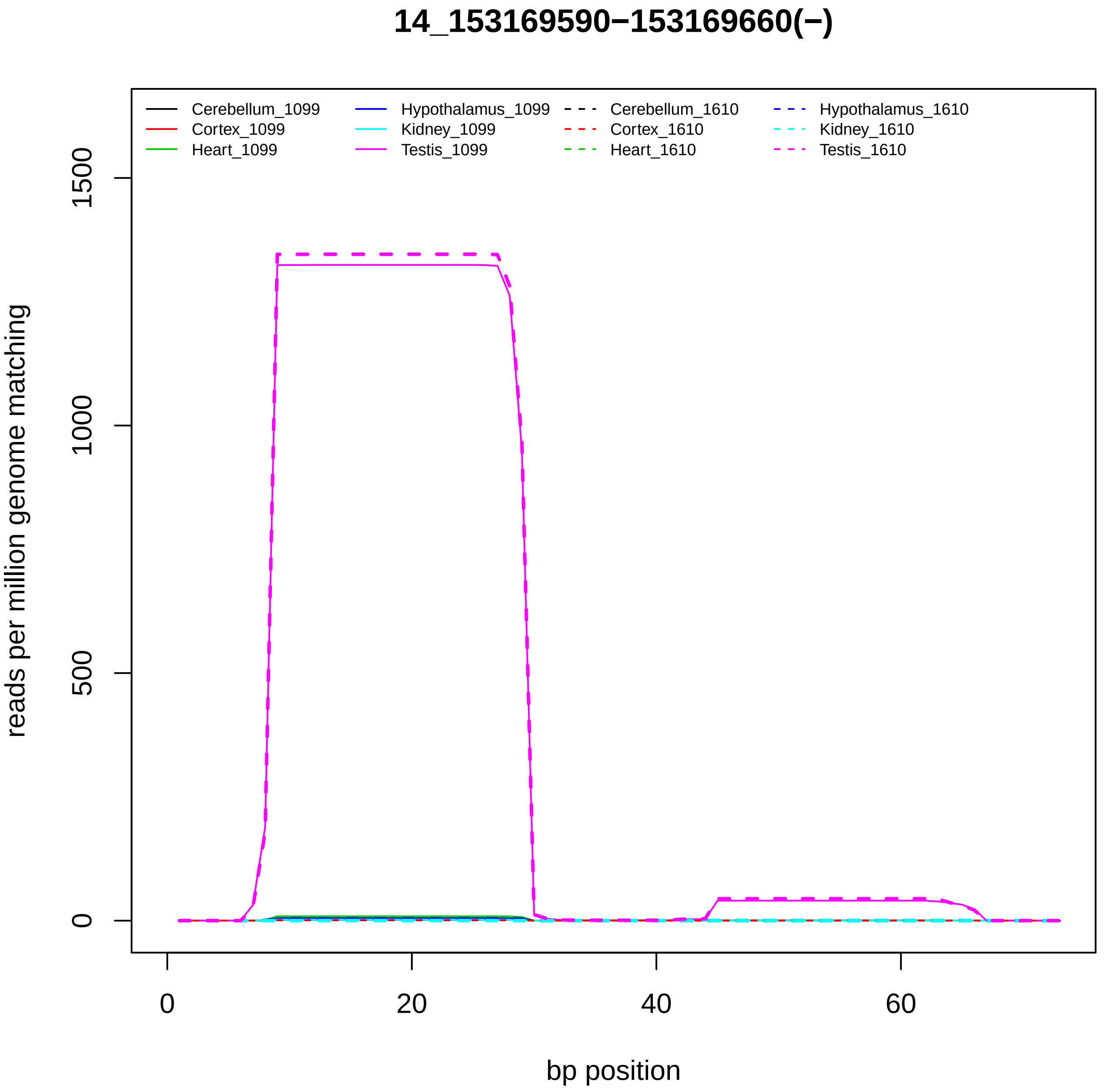

14_153169590−153169660(−)
Cerebellum_1099
Cortex_1099
Heart_1099
Hypothalamus_1099
Kidney_1099
Testis_1099
Cerebellum_1610
Cortex_1610
Heart_1610
Hypothalamus_1610
Kidney_1610
Testis_1610
reads per million genome matching
0
500
1000
1500
0
20
40
60
bp position

14_24697020−24697115(−)

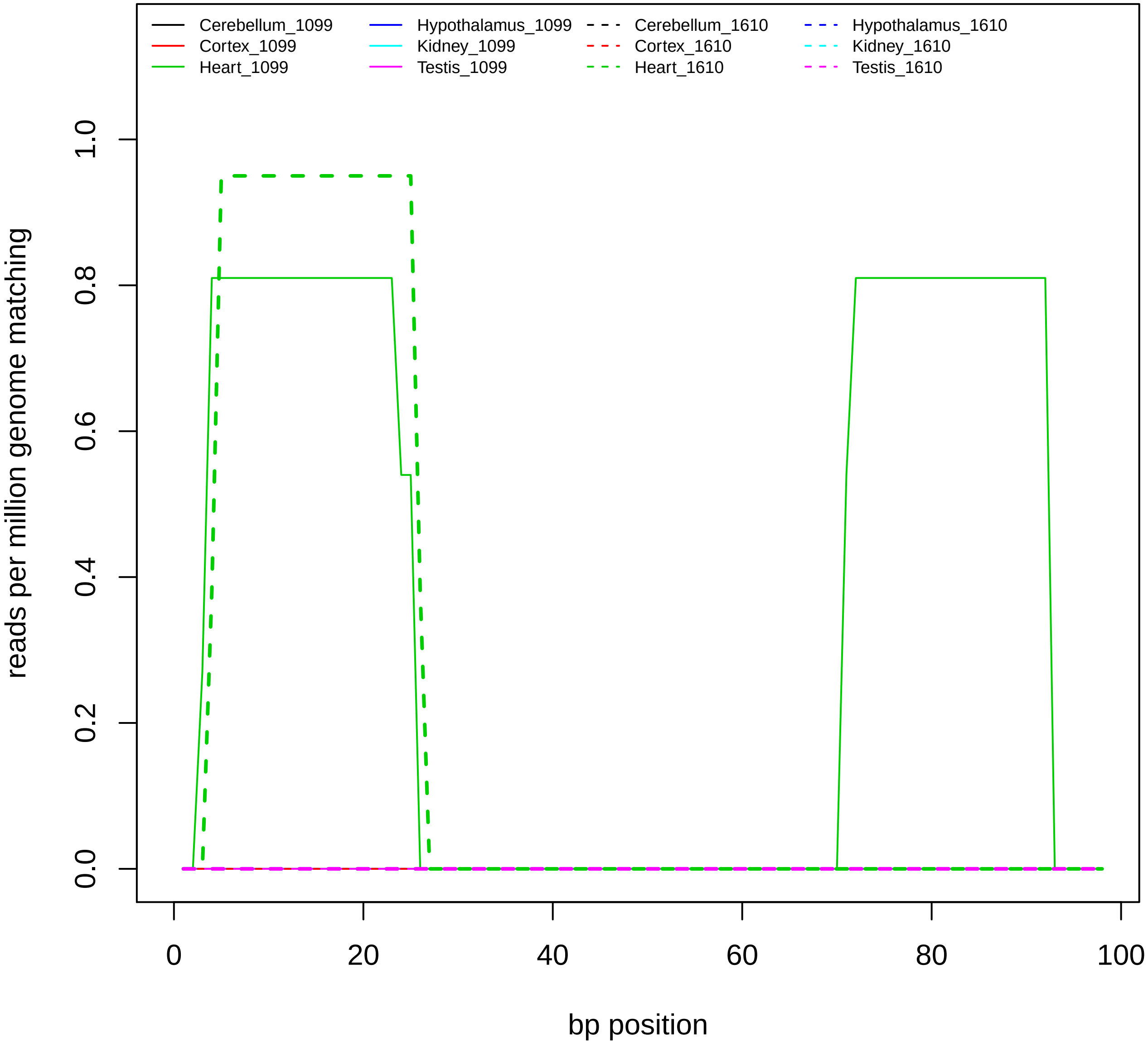

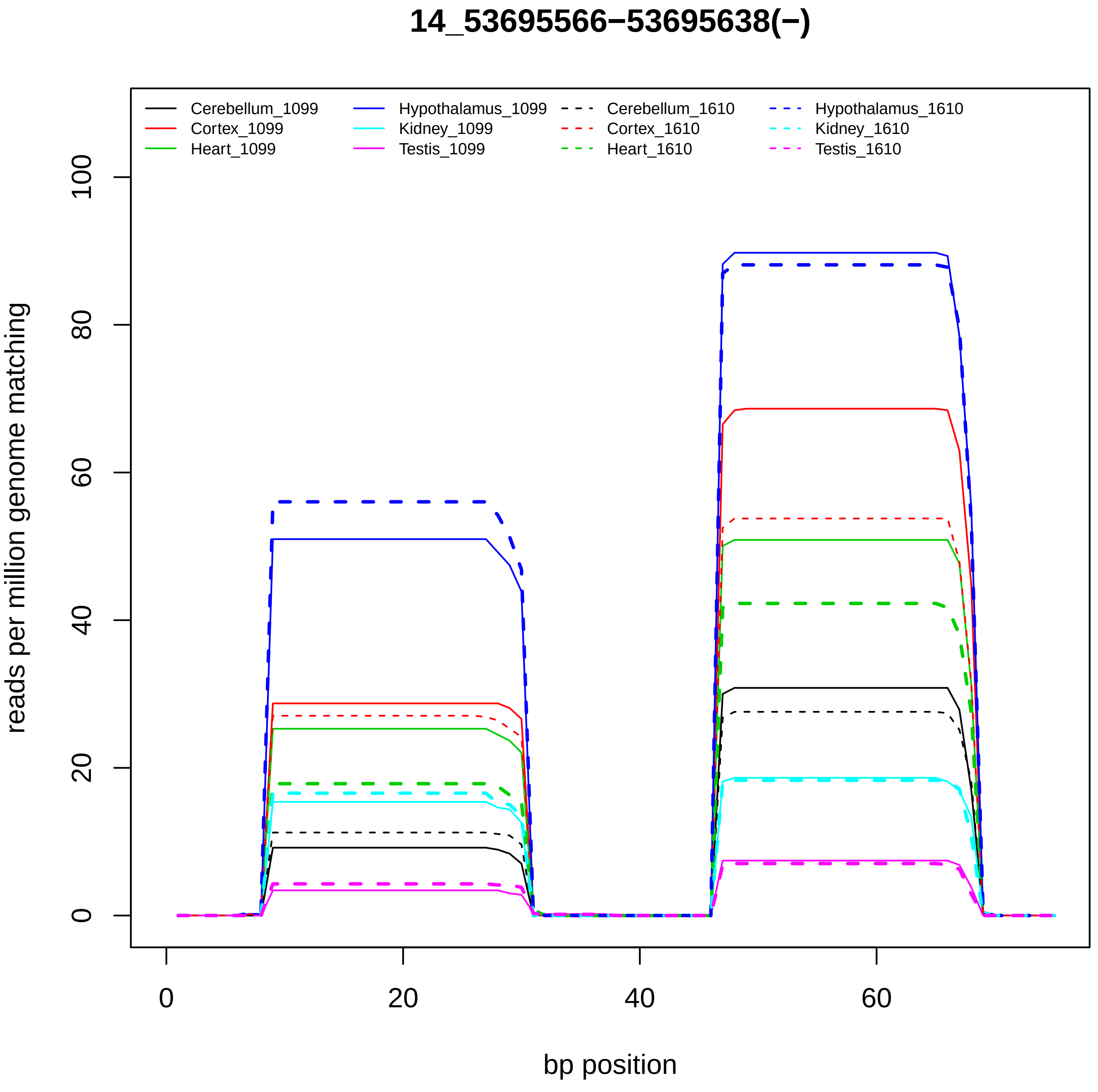
14_53695566−53695638(−)
Cerebellum_1099
Cortex_1099
Heart_1099
Hypothalamus_1099
Kidney_1099
Testis_1099
Cerebellum_1610
Cortex_1610
Heart_1610
Hypothalamus_1610
Kidney_1610
Testis_1610
reads per million genome matching
0
20
40
60
80
100
0
20
40
60
bp position

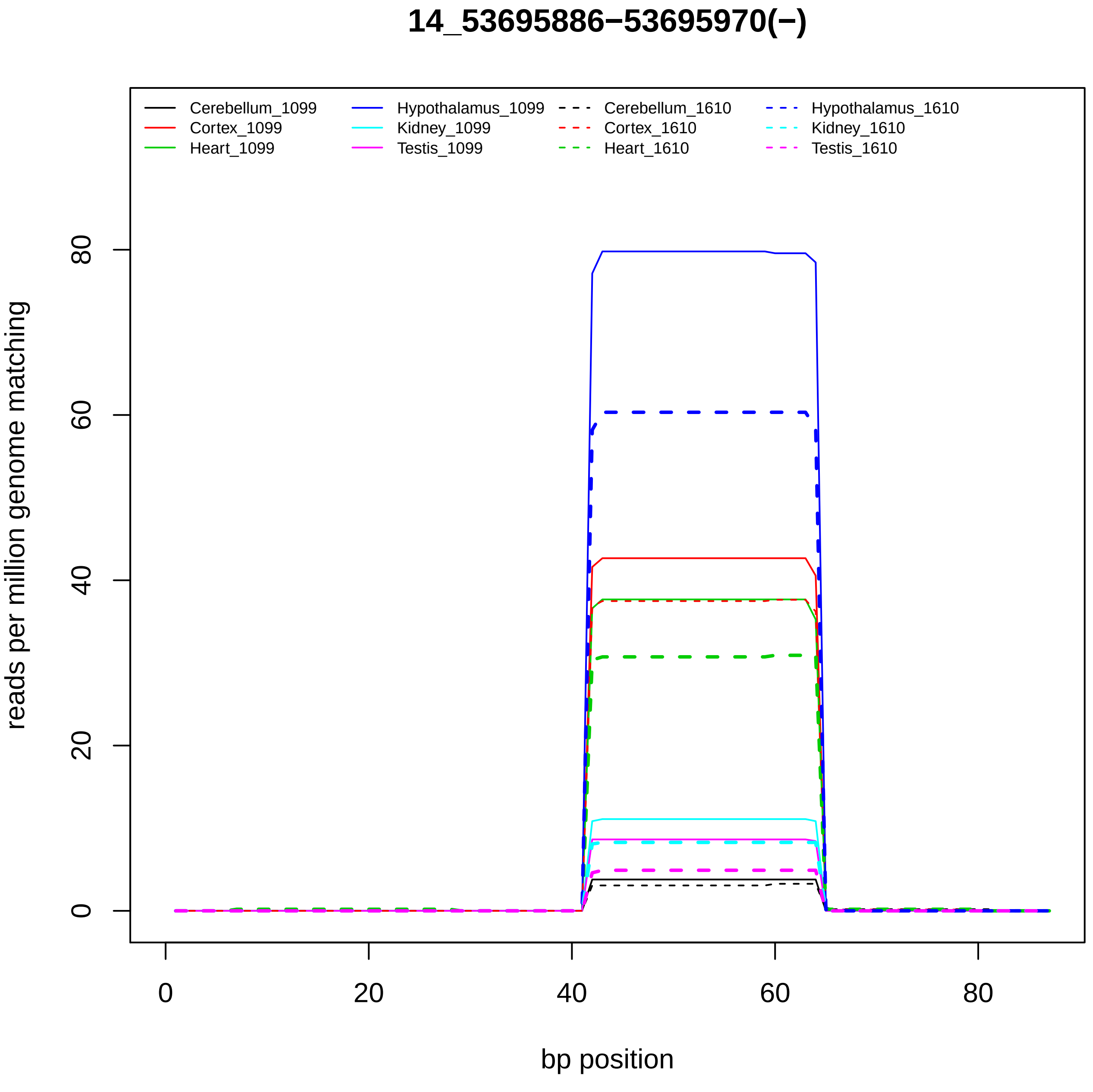

14_53695886−53695970(−)
Cerebellum_1099
Cortex_1099
Heart_1099
Hypothalamus_1099
Kidney_1099
Testis_1099
Cerebellum_1610
Cortex_1610
Heart_1610
Hypothalamus_1610
Kidney_1610
Testis_1610
reads per million genome matching
0
20
40
60
80
bp position
0
20
40
60
80

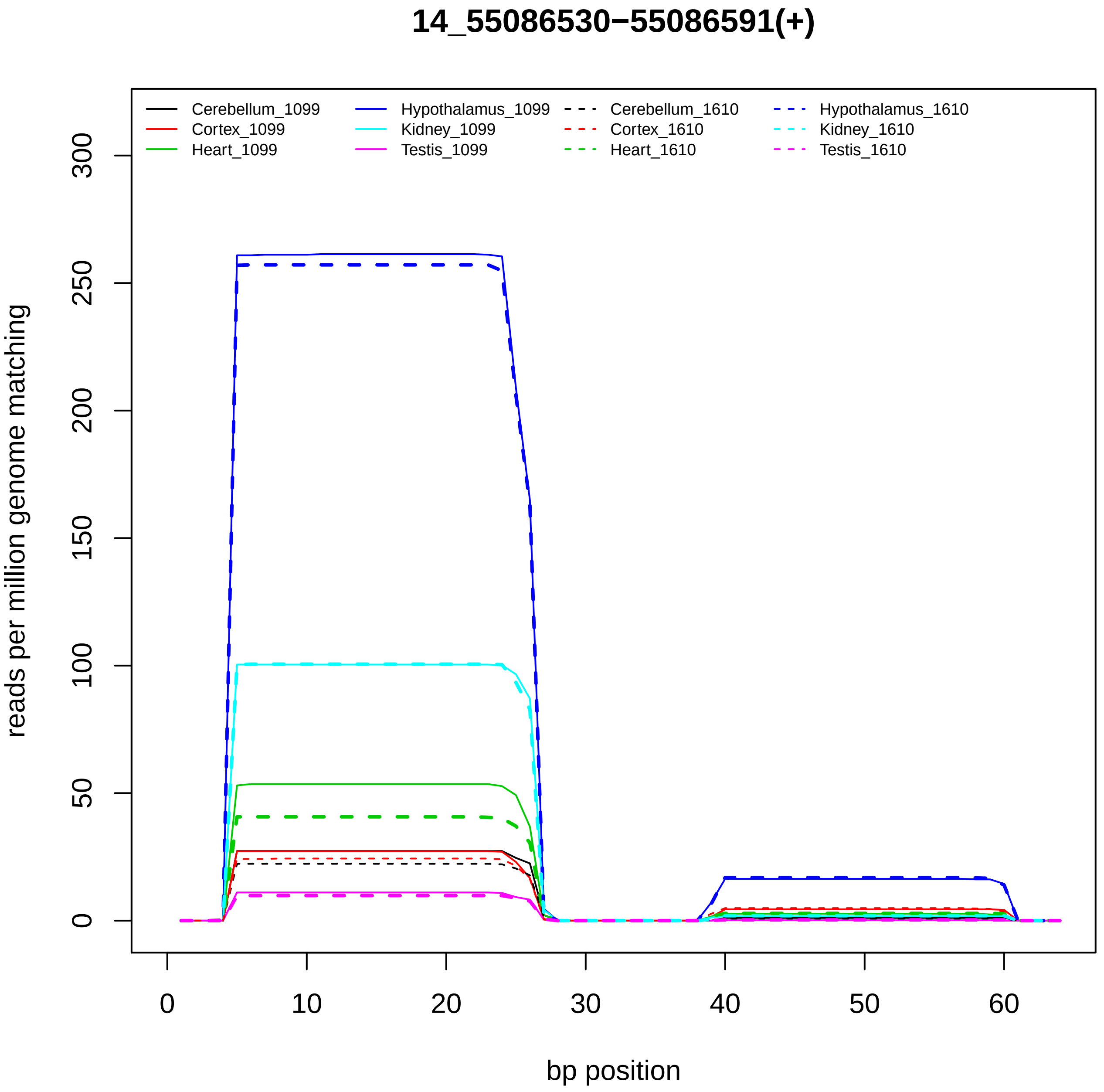

14_55086530−55086591(+)
Cerebellum_1099
Cortex_1099
Heart_1099
Hypothalamus_1099
Kidney_1099
Testis_1099
Cerebellum_1610
Cortex_1610
Heart_1610
Hypothalamus_1610
Kidney_1610
Testis_1610
reads per million genome matching
0
50
100
150
200
250
300
bp position
0
10
20
30
40
50
60

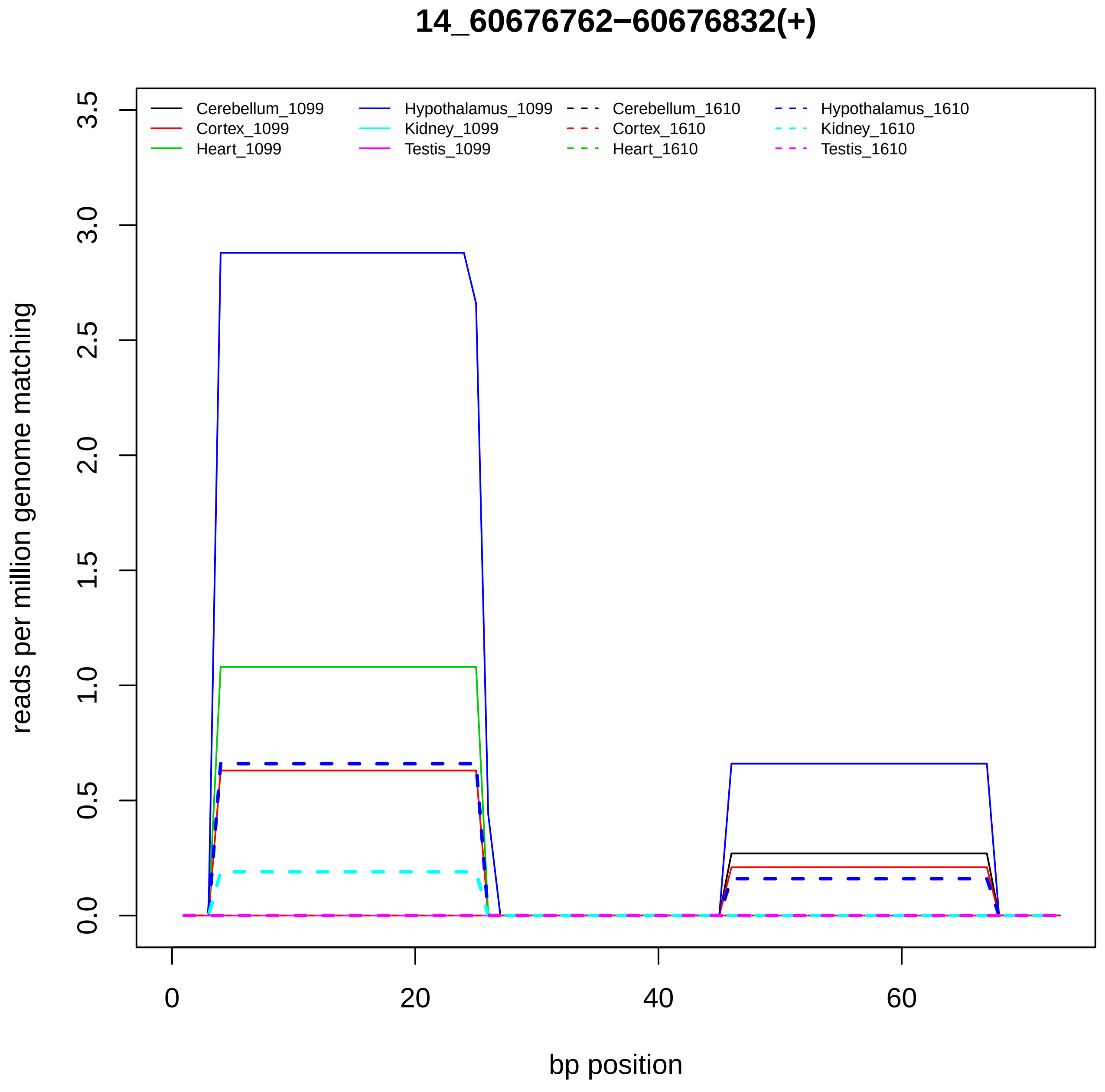

14_60676762−60676832(+)
Cerebellum_1099
Cortex_1099
Heart_1099
Hypothalamus_1099
Kidney_1099
Testis_1099
Cerebellum_1610
Cortex_1610
Heart_1610
Hypothalamus_1610
Kidney_1610
Testis_1610
reads per million genome matching
0.0
0.5
1.0
1.5
2.0
2.5
3.0
3.5
0
20
40
60
bp position

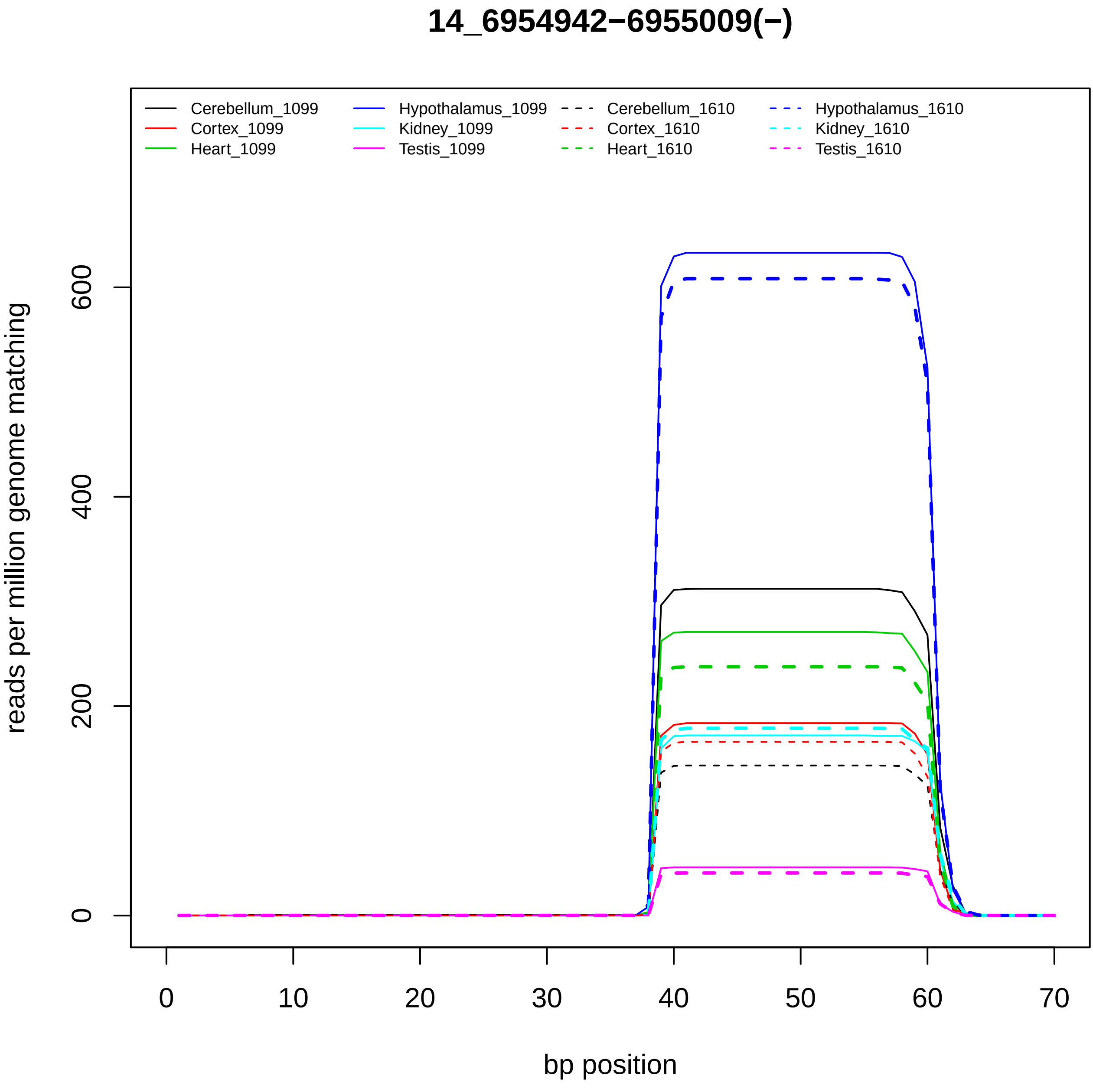

14_6954942−6955009(−)
Cerebellum_1099
Cortex_1099
Heart_1099
Hypothalamus_1099
Kidney_1099
Testis_1099
Cerebellum_1610
Cortex_1610
Heart_1610
Hypothalamus_1610
Kidney_1610
Testis_1610
reads per million genome matching
0
200
400
600
0
10
20
30
40
50
60
70
bp position

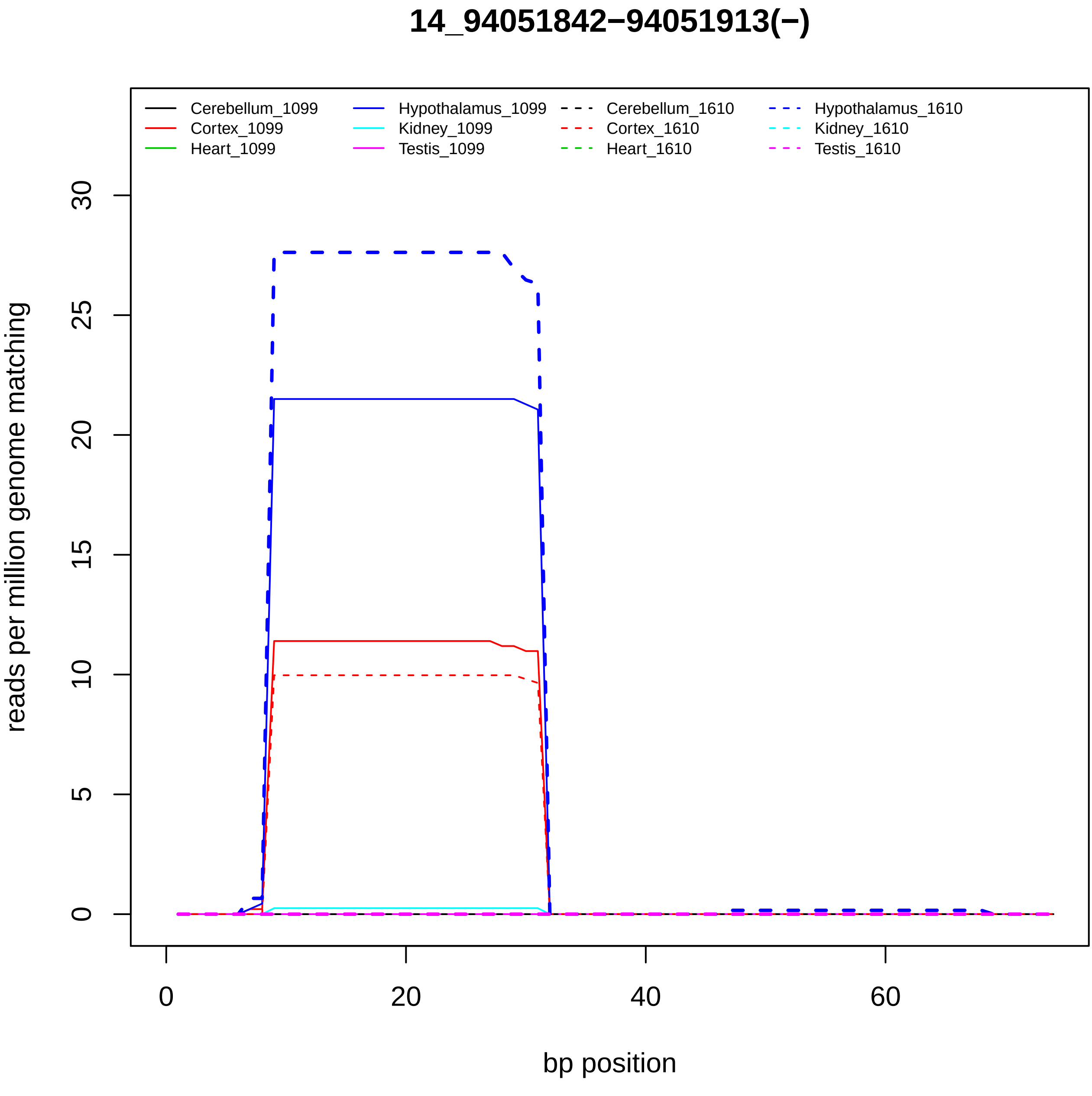

14_94051842−94051913(−)
Cerebellum_1099
Cortex_1099
Heart_1099
Hypothalamus_1099
Kidney_1099
Testis_1099
Cerebellum_1610
Cortex_1610
Heart_1610
Hypothalamus_1610
Kidney_1610
Testis_1610
reads per million genome matching
0
5
10
15
20
25
30
0
20
40
60
bp position

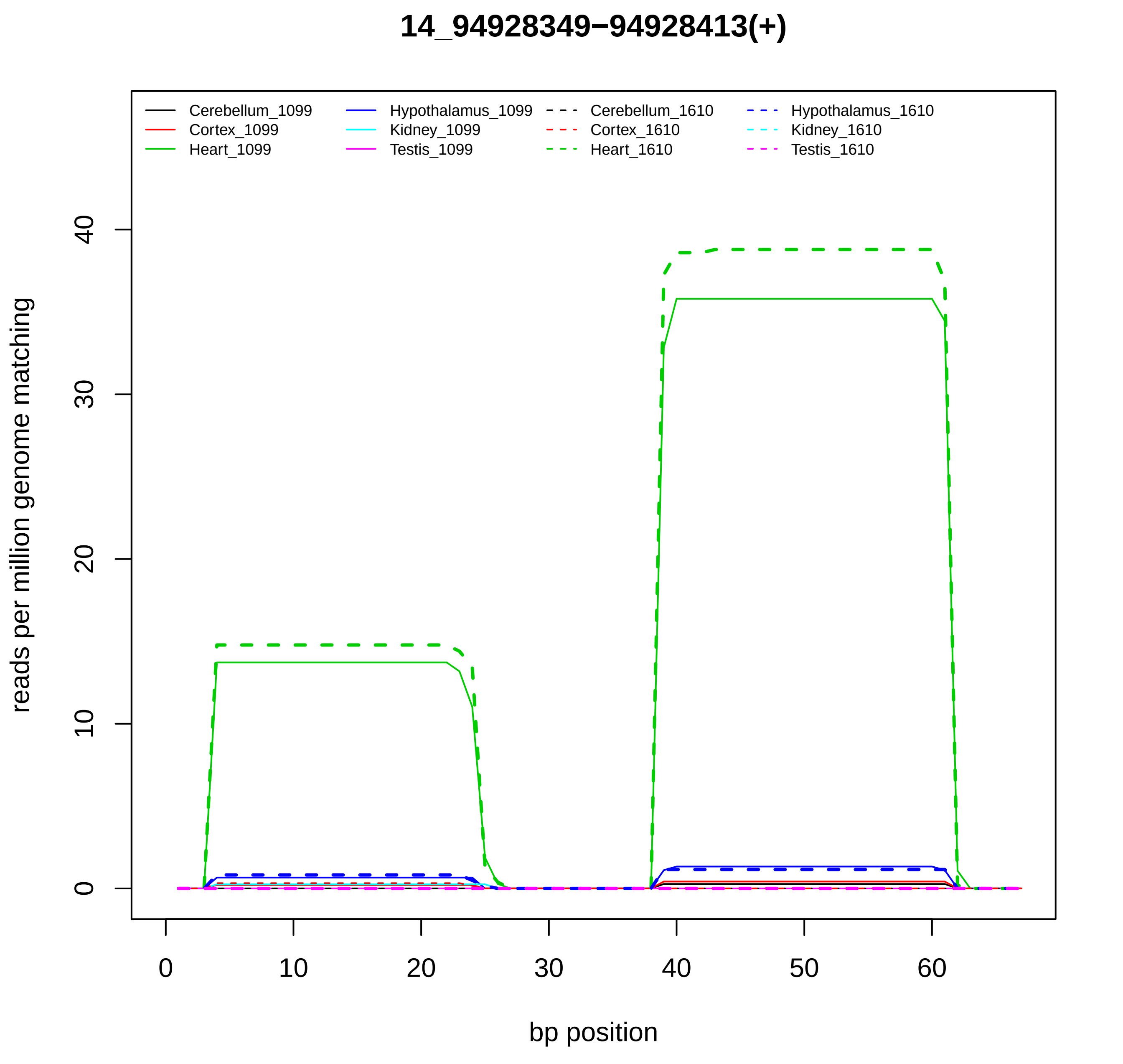

14_94928349−94928413(+)
Cerebellum_1099
Cortex_1099
Heart_1099
Hypothalamus_1099
Kidney_1099
Testis_1099
Cerebellum_1610
Cortex_1610
Heart_1610
Hypothalamus_1610
Kidney_1610
Testis_1610
reads per million genome matching
0
10
20
30
40
0
10
20
30
40
50
60
bp position

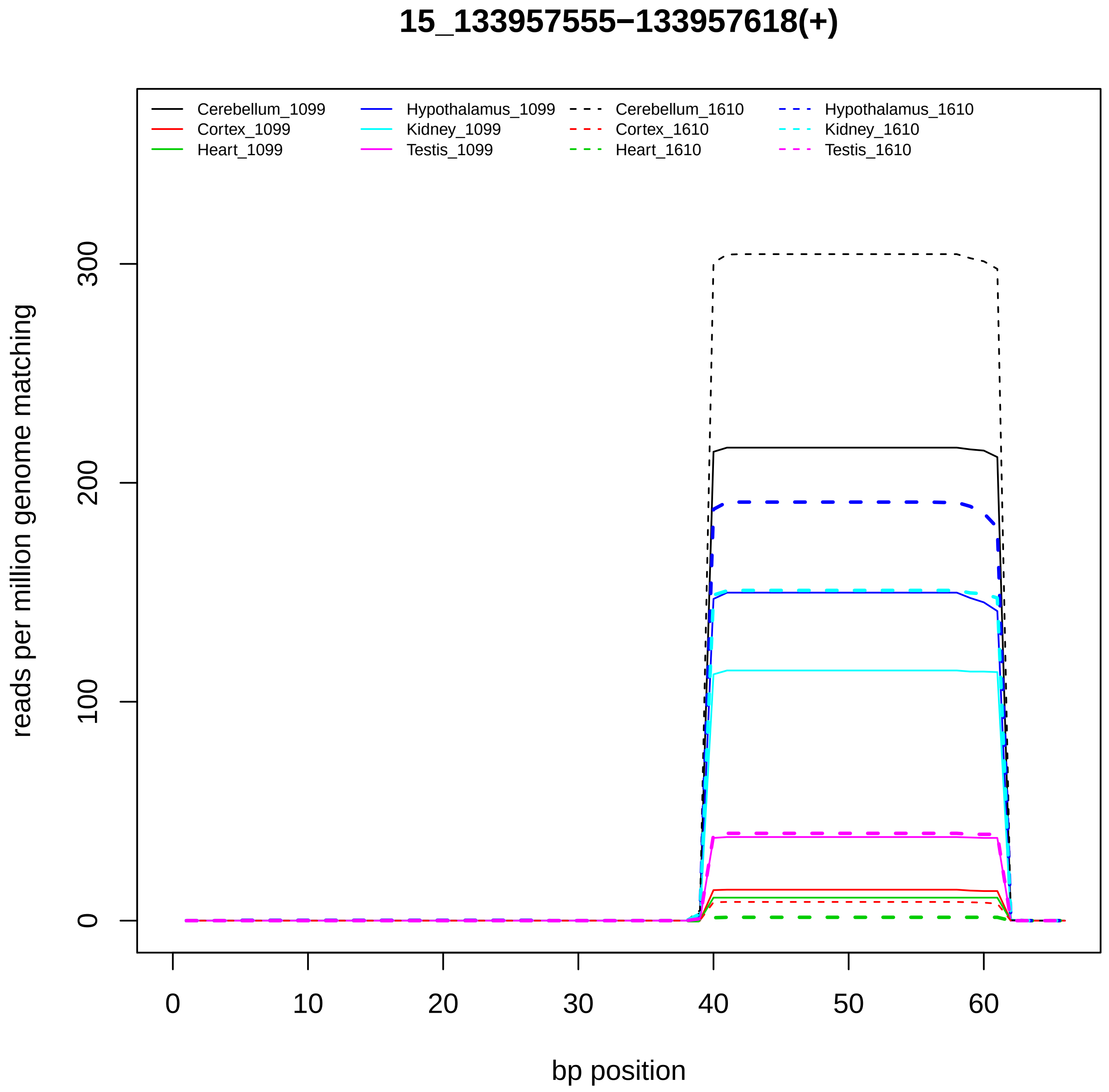
15_133957555−133957618(+)
Cerebellum_1099
Cortex_1099
Heart_1099
Hypothalamus_1099
Kidney_1099
Testis_1099
Cerebellum_1610
Cortex_1610
Heart_1610
Hypothalamus_1610
Kidney_1610
Testis_1610
reads per million genome matching
0
100
200
300
0
10
20
30
40
50
60
bp position

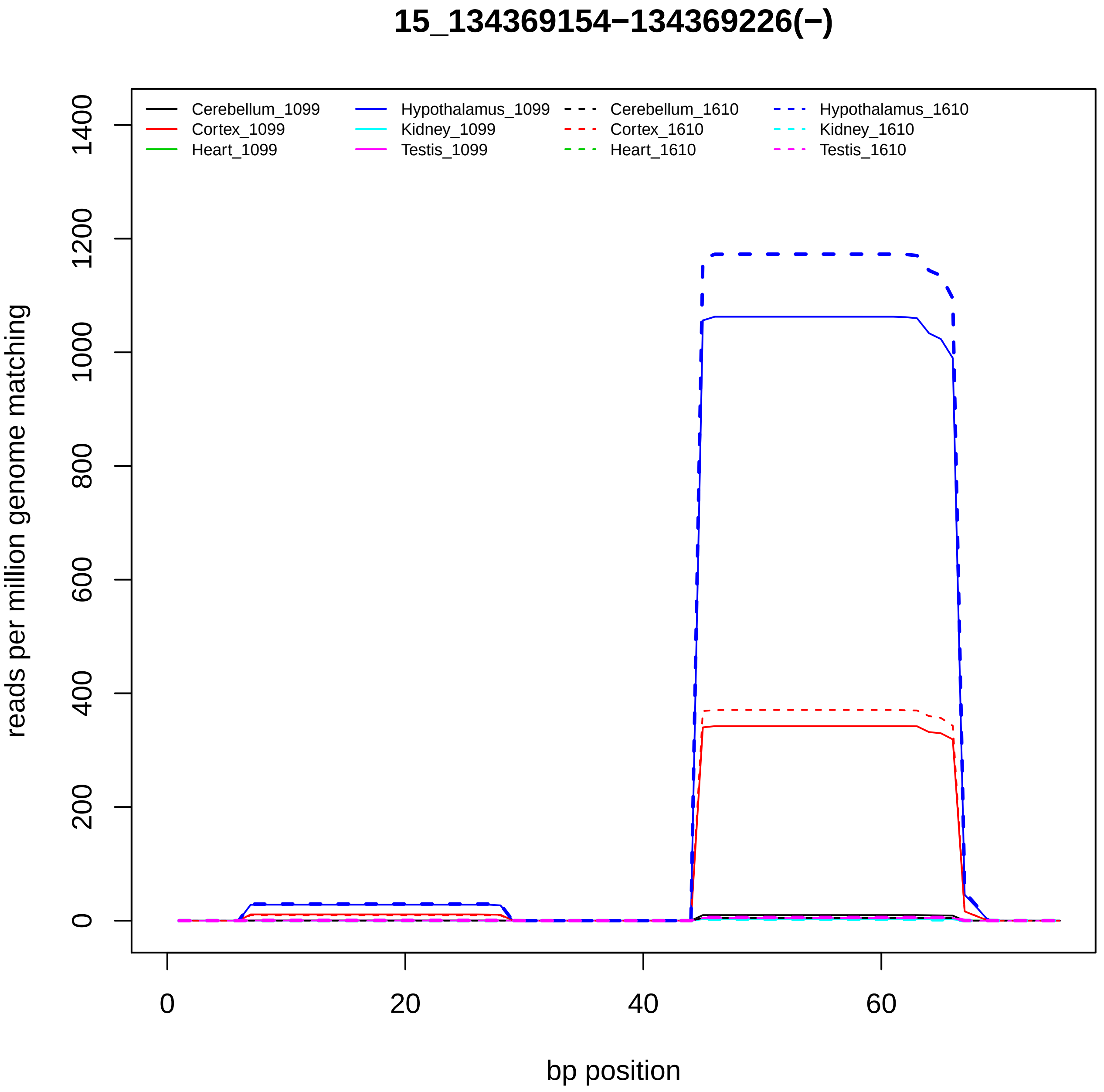

15_134369154−134369226(−)
Cerebellum_1099
Cortex_1099
Heart_1099
Hypothalamus_1099
Kidney_1099
Testis_1099
Cerebellum_1610
Cortex_1610
Heart_1610
Hypothalamus_1610
Kidney_1610
Testis_1610
reads per million genome matching
0
200
400
600
800
1000
1200
1400
0
20
40
60
bp position

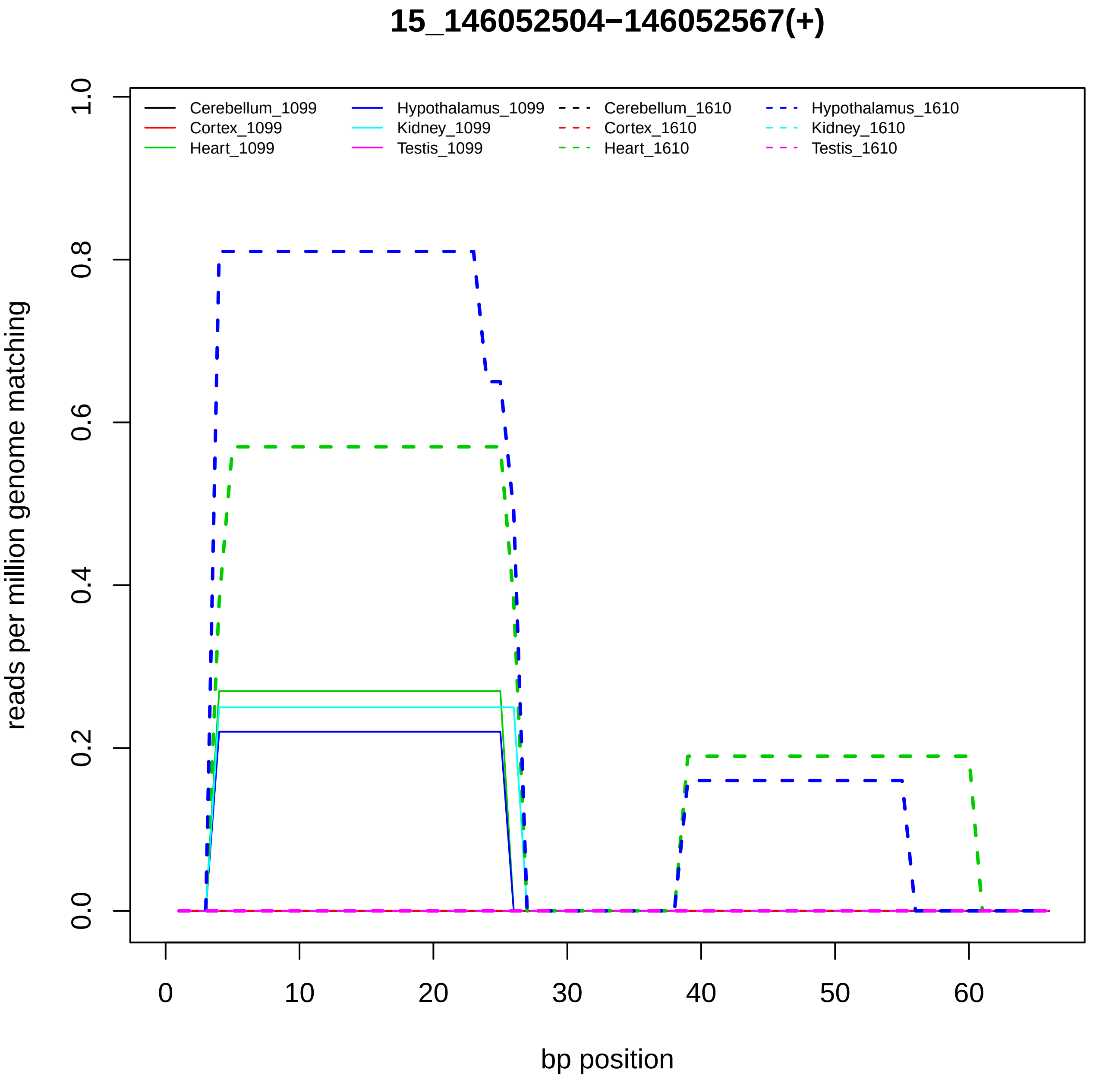
15_146052504−146052567(+)
Cerebellum_1099
Cortex_1099
Heart_1099
Hypothalamus_1099
Kidney_1099
Testis_1099
Cerebellum_1610
Cortex_1610
Heart_1610
Hypothalamus_1610
Kidney_1610
Testis_1610
reads per million genome matching
0.0
0.2
0.4
0.6
0.8
1.0
0
10
20
30
40
50
60
bp position

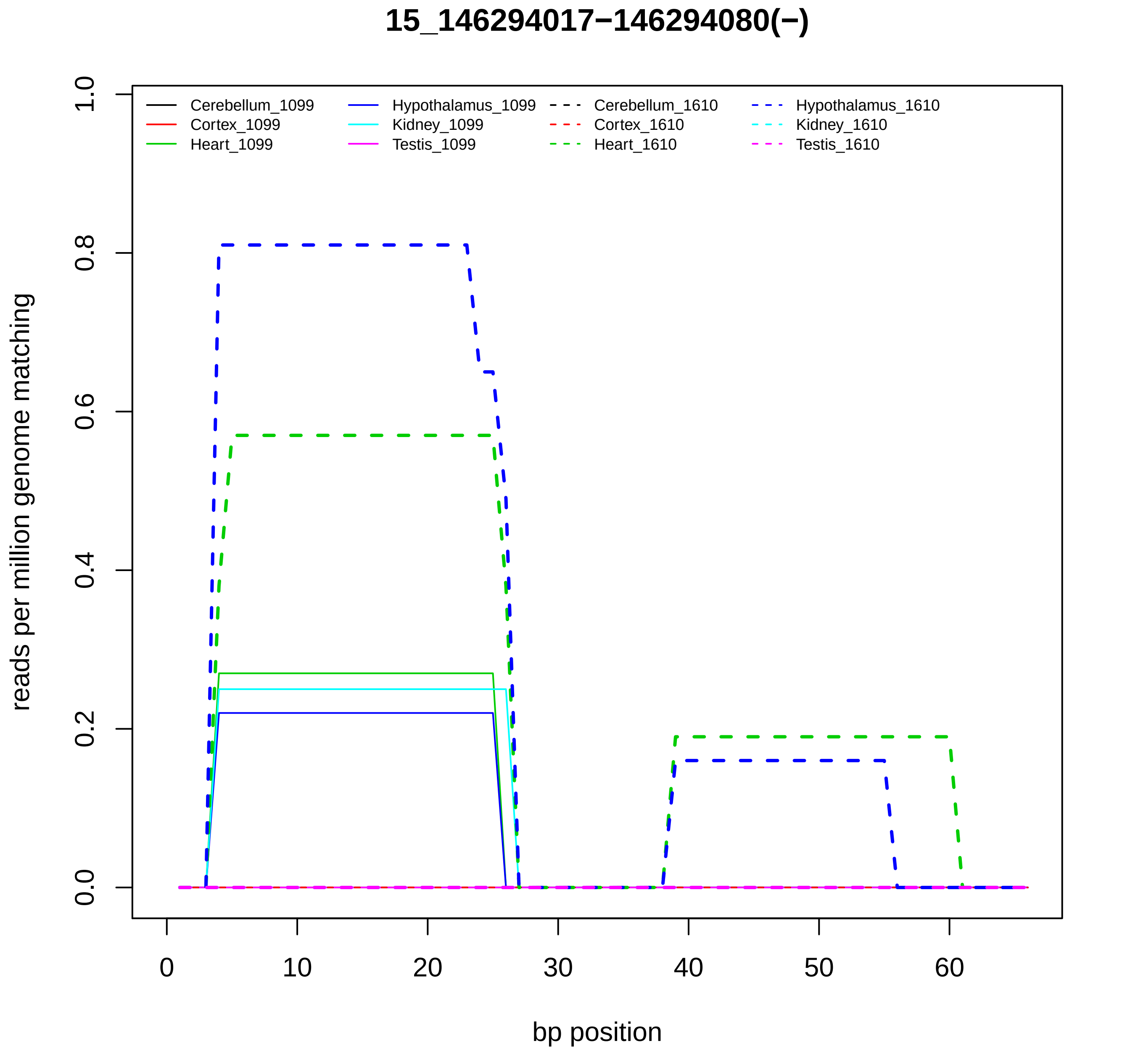

15_146294017−146294080(−)
Cerebellum_1099
Cortex_1099
Heart_1099
Hypothalamus_1099
Kidney_1099
Testis_1099
Cerebellum_1610
Cortex_1610
Heart_1610
Hypothalamus_1610
Kidney_1610
Testis_1610
reads per million genome matching
bp position
0.0
0.2
0.4
0.6
0.8
1.0
0
10
20
30
40
50
60

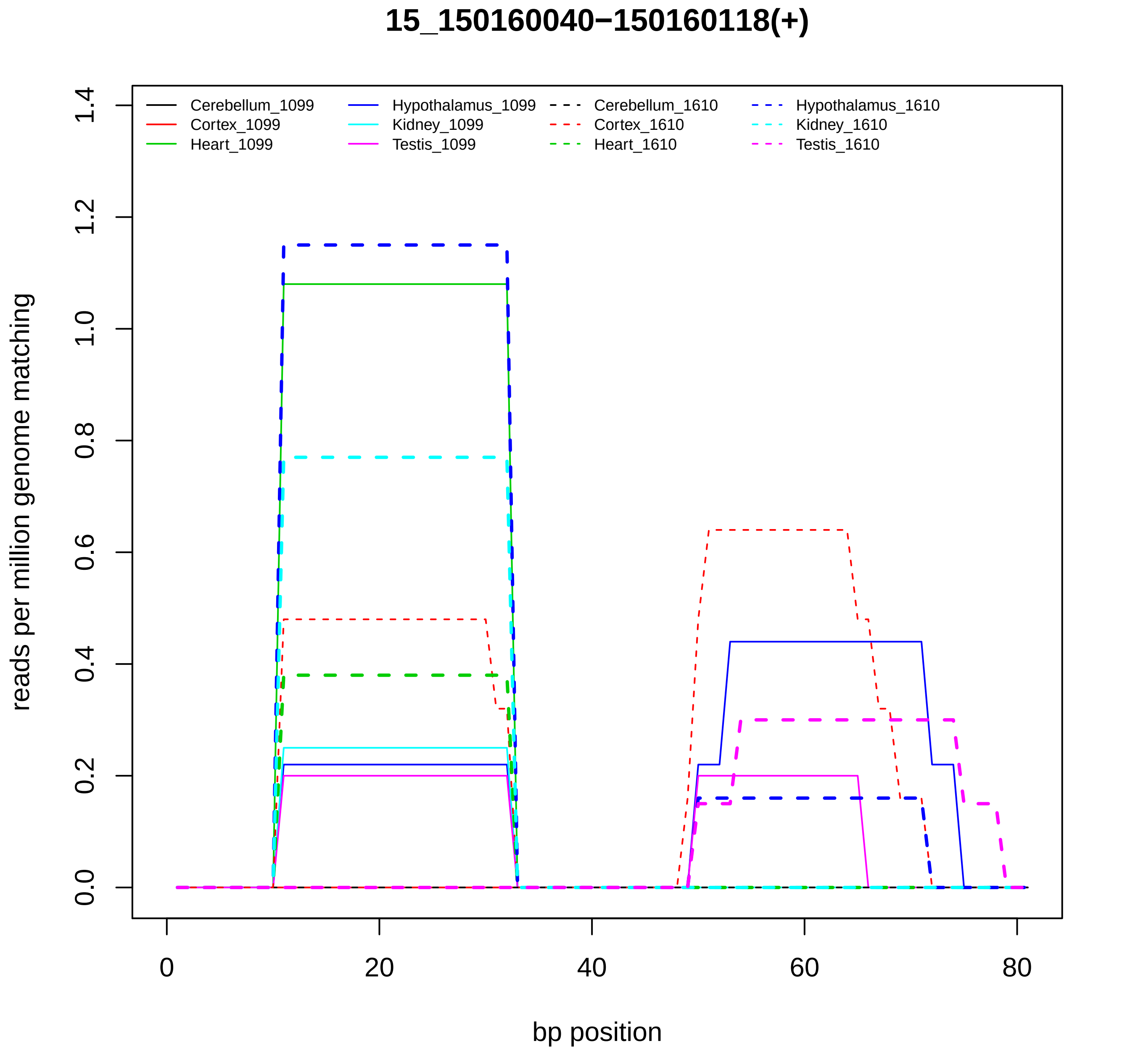
15_150160040−150160118(+)
Cerebellum_1099
Cortex_1099
Heart_1099
Hypothalamus_1099
Kidney_1099
Testis_1099
Cerebellum_1610
Cortex_1610
Heart_1610
Hypothalamus_1610
Kidney_1610
Testis_1610
reads per million genome matching
0.0
0.2
0.4
0.6
0.8
1.0
1.2
1.4
0
20
40
60
80
bp position

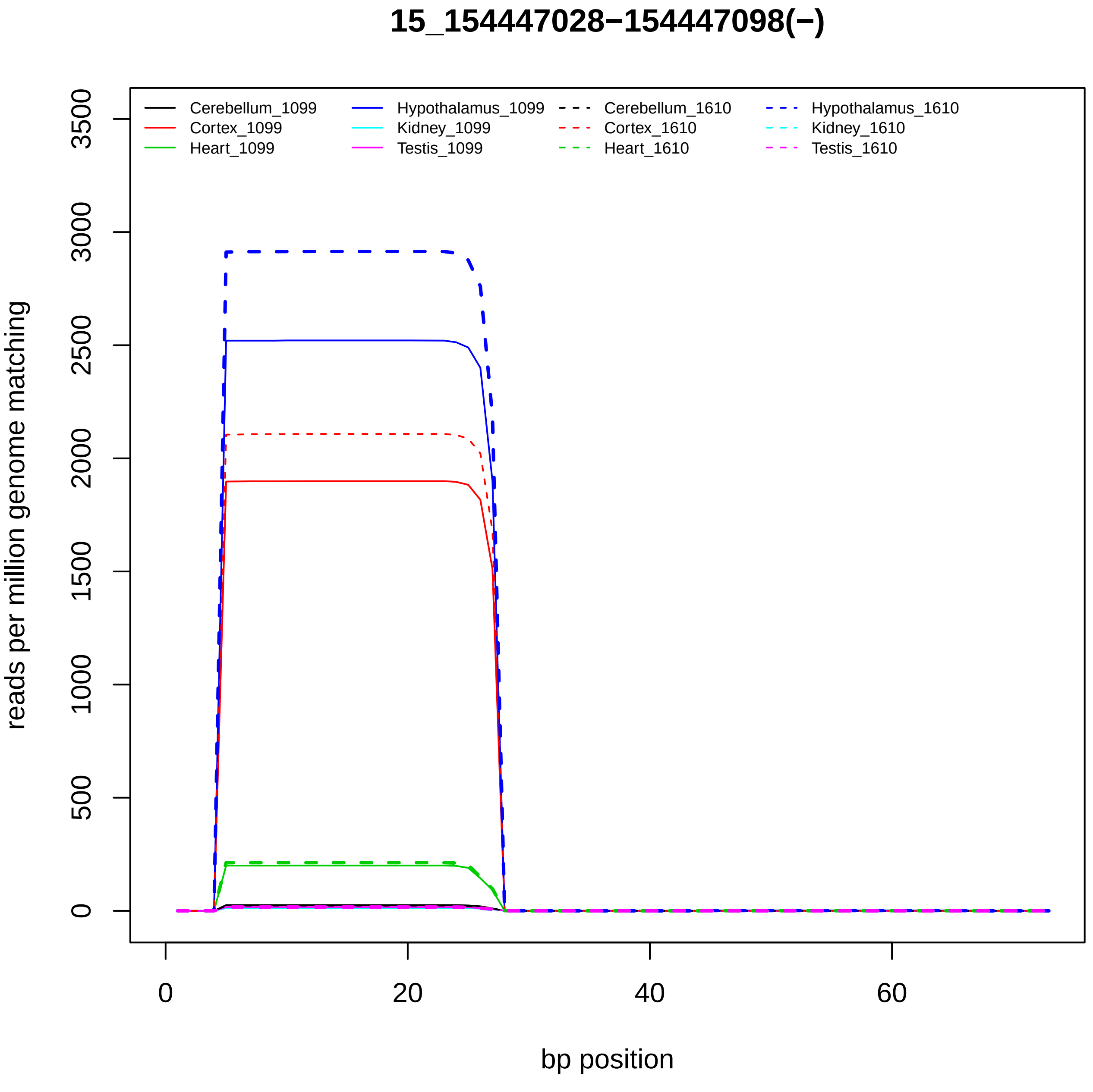

15_154447028−154447098(−)
Cerebellum_1099
Cortex_1099
Heart_1099
Hypothalamus_1099
Kidney_1099
Testis_1099
Cerebellum_1610
Cortex_1610
Heart_1610
Hypothalamus_1610
Kidney_1610
Testis_1610
reads per million genome matching
0
500
1000
1500
2000
2500
3000
3500
0
20
40
60
bp position

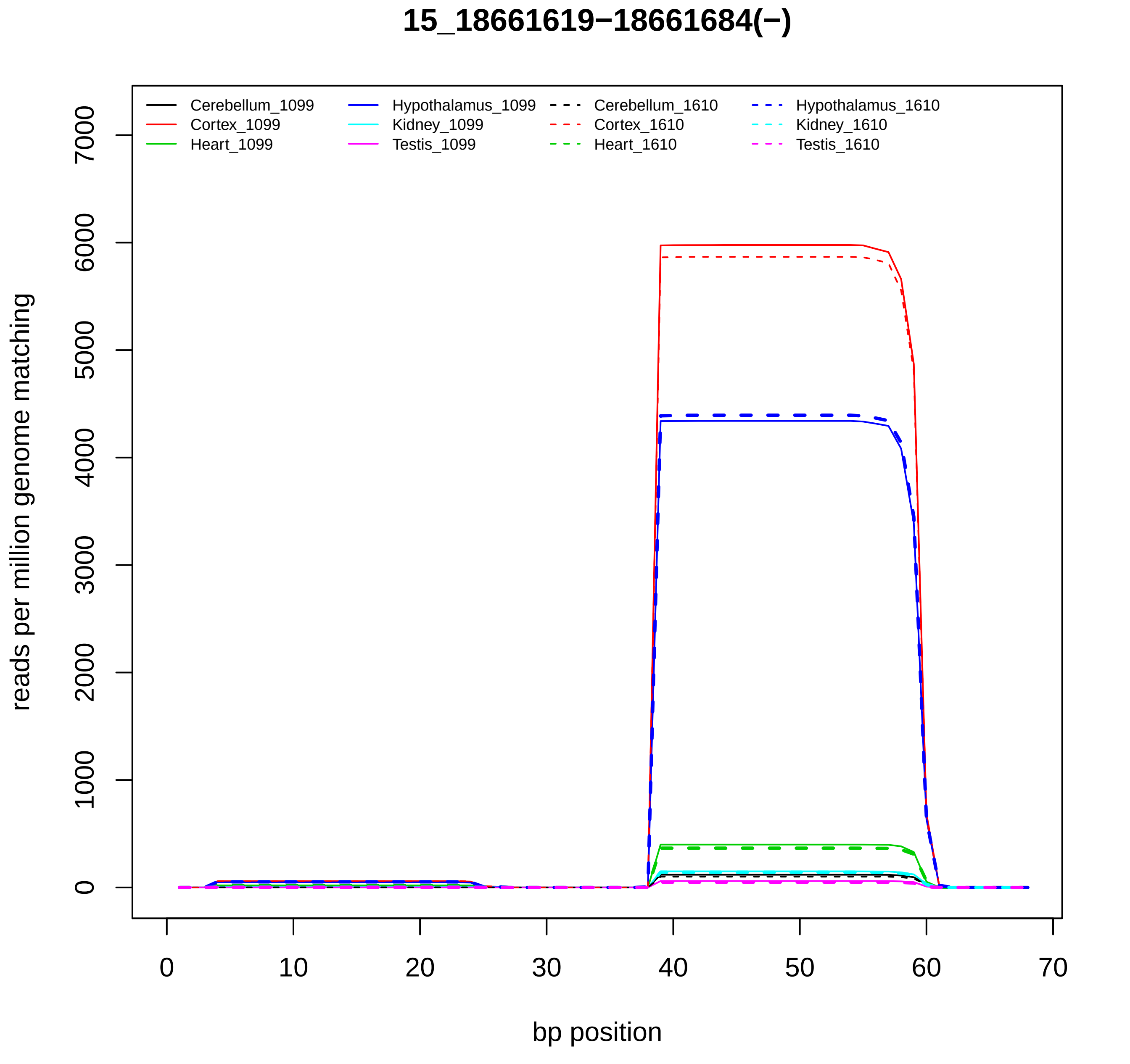

15_18661619−18661684(−)
Cerebellum_1099
Cortex_1099
Heart_1099
Hypothalamus_1099
Kidney_1099
Testis_1099
Cerebellum_1610
Cortex_1610
Heart_1610
Hypothalamus_1610
Kidney_1610
Testis_1610
reads per million genome matching
bp position
0
1000
2000
3000
4000
5000
6000
7000
0
10
20
30
40
50
60
70

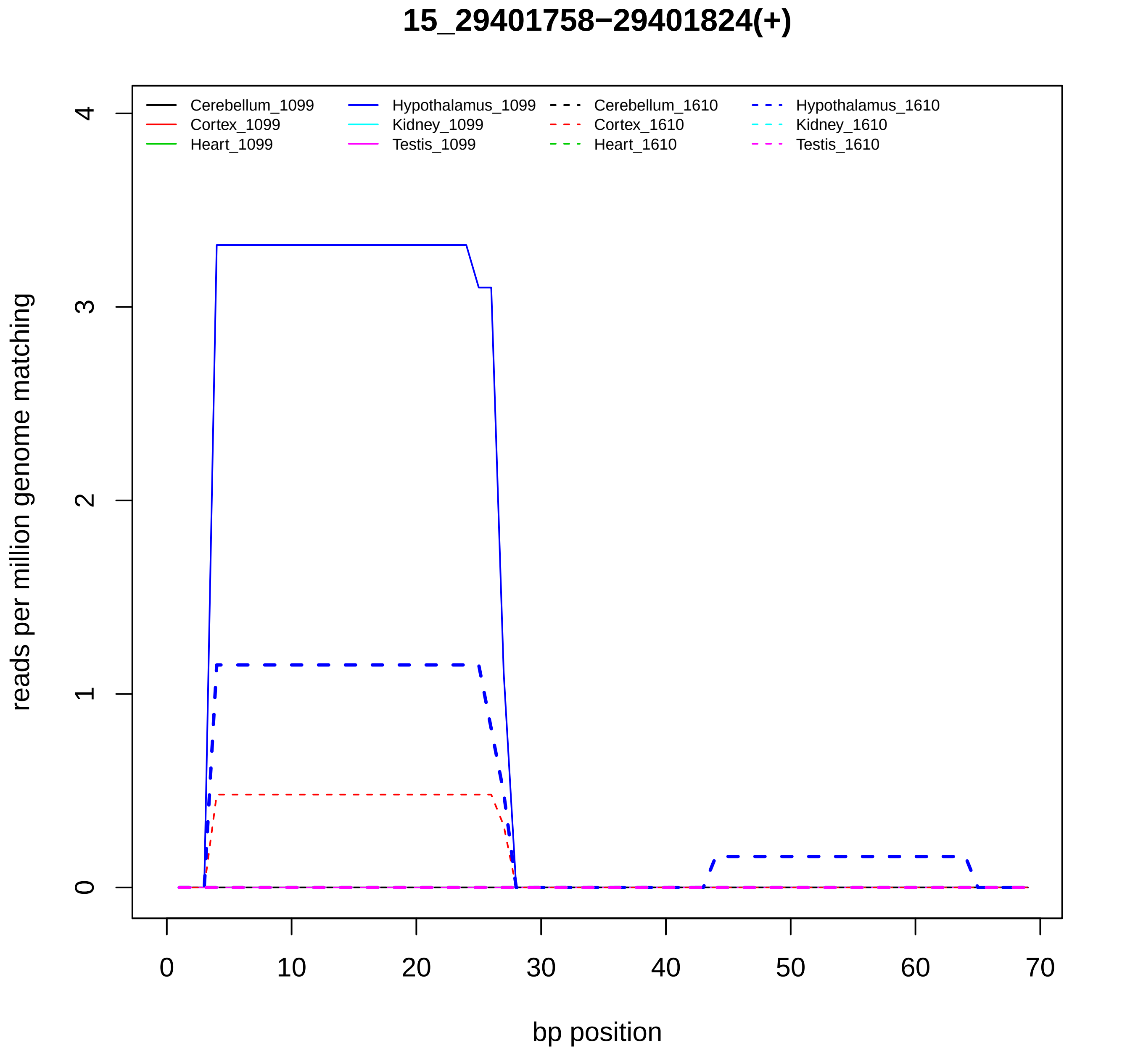

15_29401758−29401824(+)
Cerebellum_1099
Cortex_1099
Heart_1099
Hypothalamus_1099
Kidney_1099
Testis_1099
Cerebellum_1610
Cortex_1610
Heart_1610
Hypothalamus_1610
Kidney_1610
Testis_1610
reads per million genome matching
bp position
0
1
2
3
4
0
10
20
30
40
50
60
70

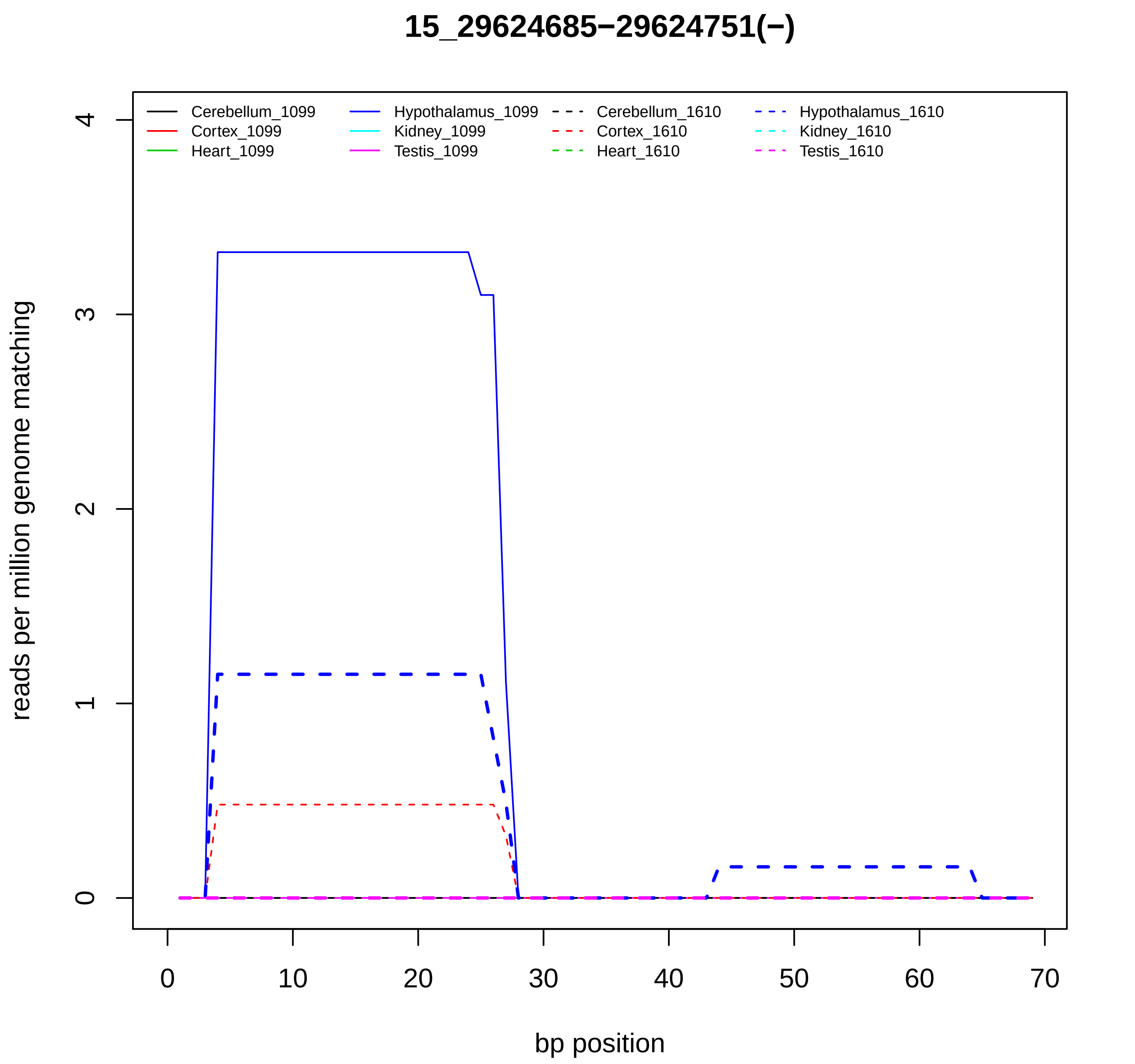

15_29624685−29624751(−)
Cerebellum_1099
Cortex_1099
Heart_1099
Hypothalamus_1099
Kidney_1099
Testis_1099
Cerebellum_1610
Cortex_1610
Heart_1610
Hypothalamus_1610
Kidney_1610
Testis_1610
reads per million genome matching
0
1
2
3
4
0
10
20
30
40
50
60
70
bp position

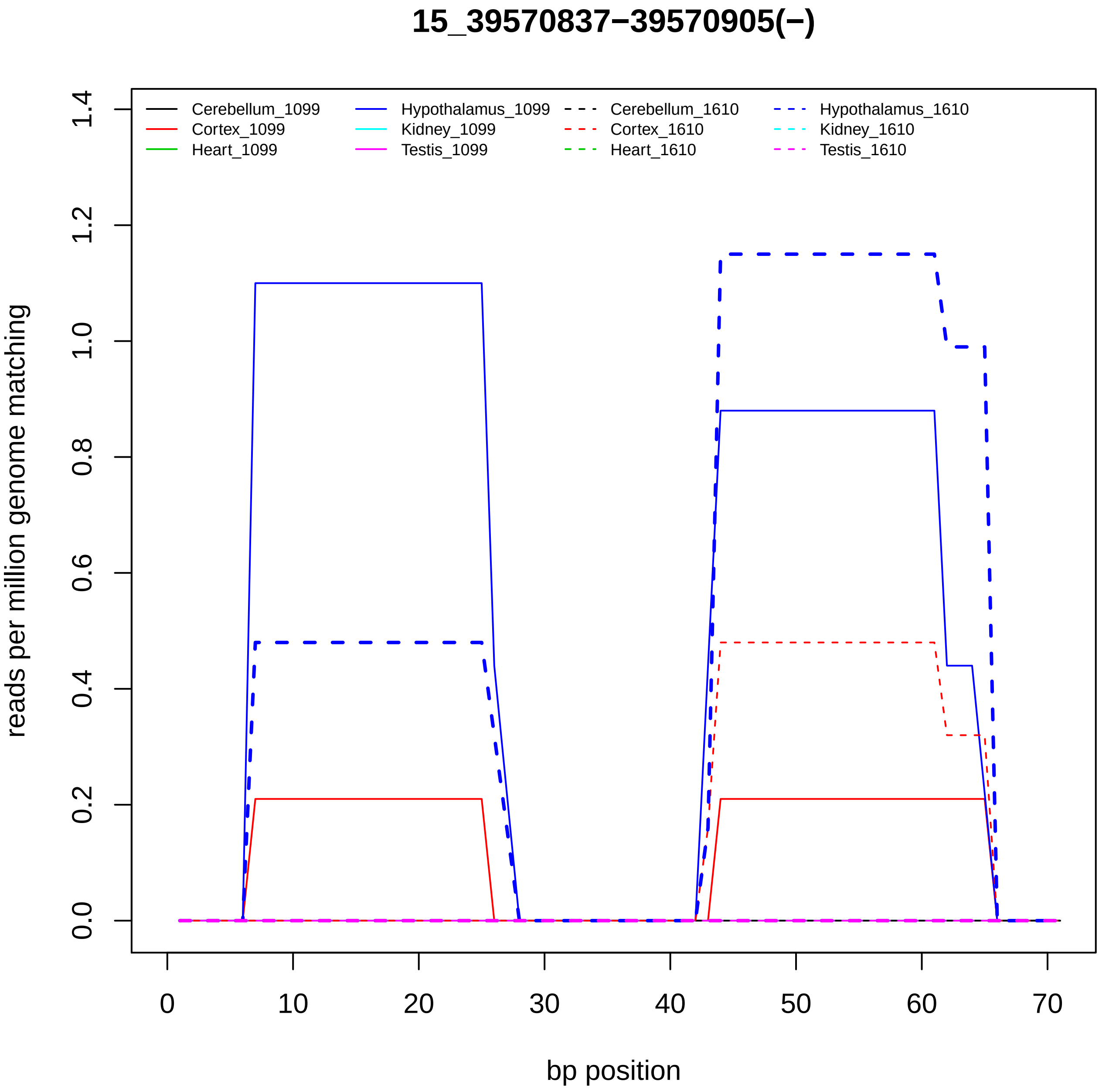
15_39570837−39570905(−)
Cerebellum_1099
Cortex_1099
Heart_1099
Hypothalamus_1099
Kidney_1099
Testis_1099
Cerebellum_1610
Cortex_1610
Heart_1610
Hypothalamus_1610
Kidney_1610
Testis_1610
reads per million genome matching
bp position
0.0
0.2
0.4
0.6
0.8
1.0
1.2
1.4
0
10
20
30
40
50
60
70

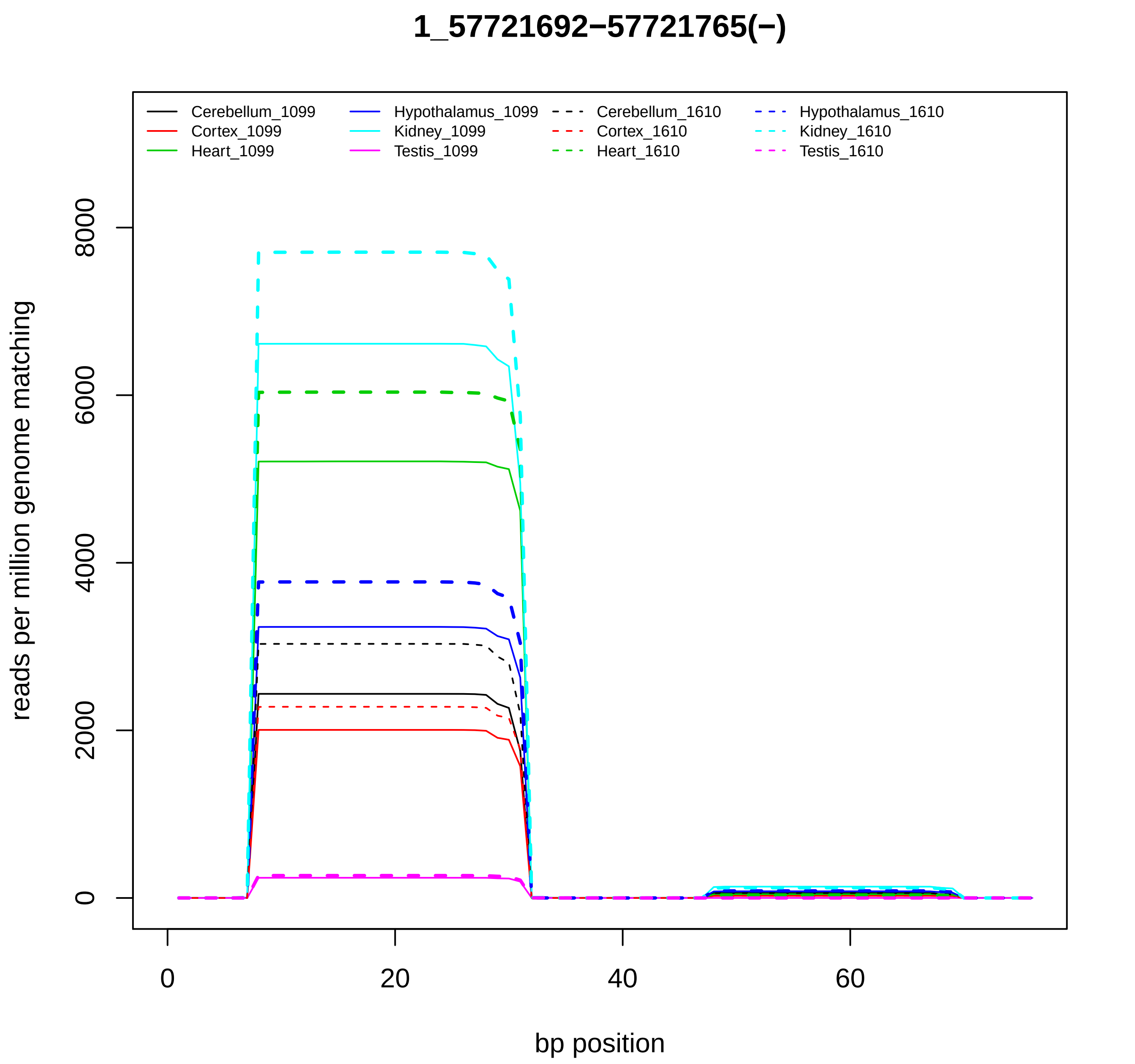

1_57721692−57721765(−)
Cerebellum_1099
Cortex_1099
Heart_1099
Hypothalamus_1099
Kidney_1099
Testis_1099
Cerebellum_1610
Cortex_1610
Heart_1610
Hypothalamus_1610
Kidney_1610
Testis_1610
reads per million genome matching
bp position
0
2000
4000
6000
8000
0
20
40
60

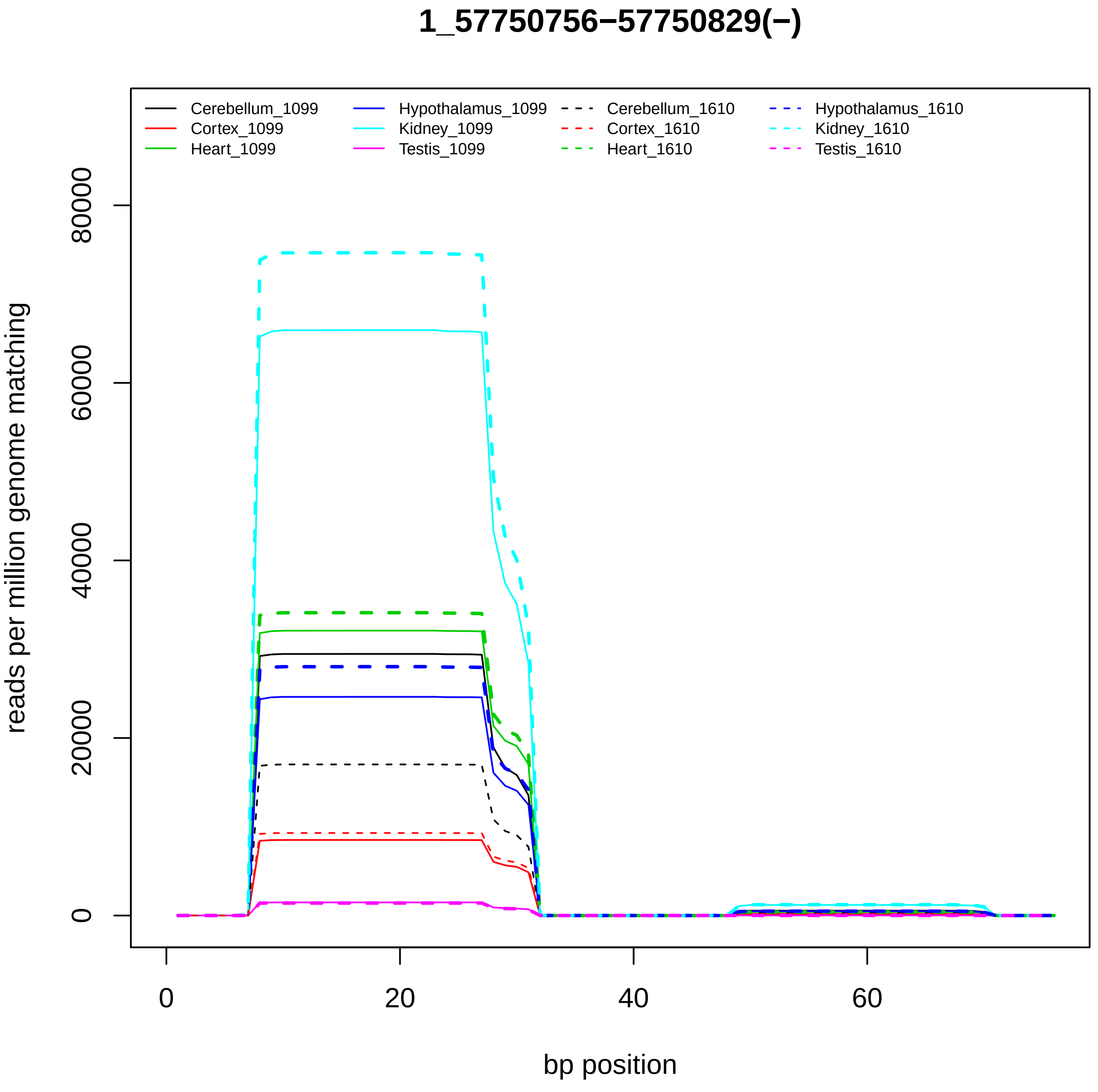

1_57750756−57750829(−)
Cerebellum_1099
Cortex_1099
Heart_1099
Hypothalamus_1099
Kidney_1099
Testis_1099
Cerebellum_1610
Cortex_1610
Heart_1610
Hypothalamus_1610
Kidney_1610
Testis_1610
reads per million genome matching
0
20000
40000
60000
80000
0
20
40
60
bp position

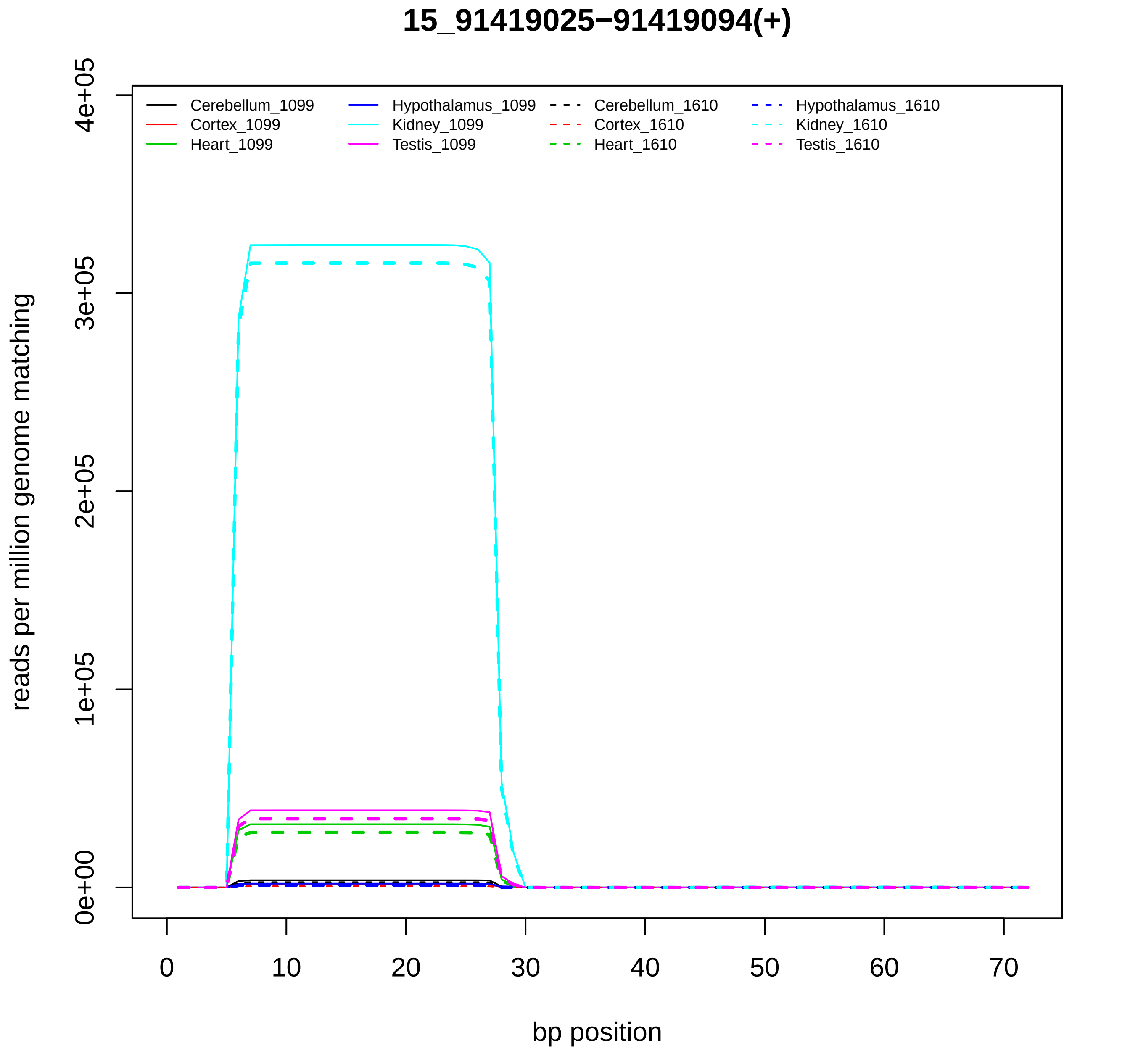

15_91419025−91419094(+)
Cerebellum_1099
Cortex_1099
Heart_1099
Hypothalamus_1099
Kidney_1099
Testis_1099
Cerebellum_1610
Cortex_1610
Heart_1610
Hypothalamus_1610
Kidney_1610
Testis_1610
reads per million genome matching
0e+00
1e+05
2e+05
3e+05
4e+05
bp position
0
10
20
30
40
50
60
70

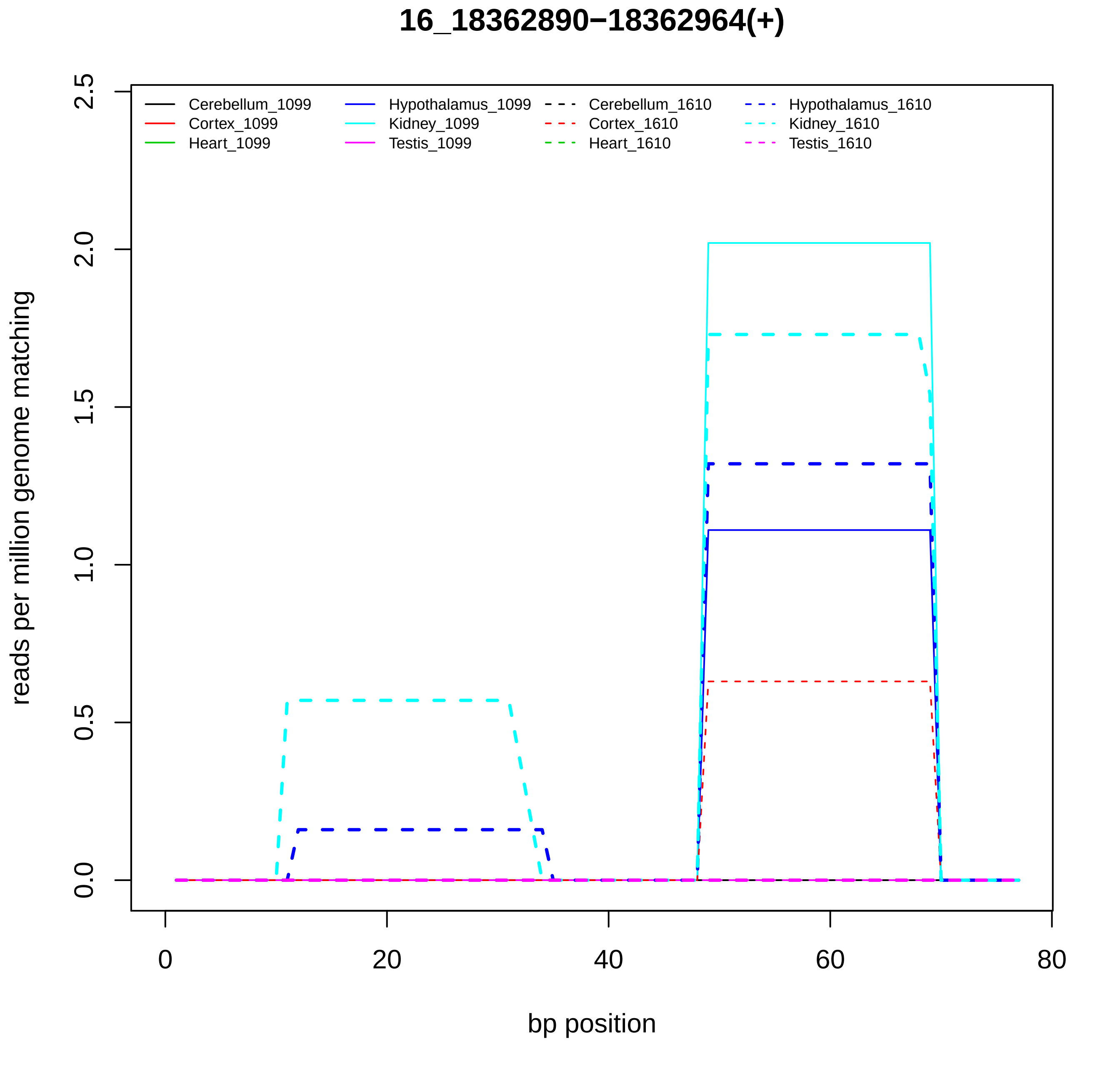

16_18362890−18362964(+)
Cerebellum_1099
Cortex_1099
Heart_1099
Hypothalamus_1099
Kidney_1099
Testis_1099
Cerebellum_1610
Cortex_1610
Heart_1610
Hypothalamus_1610
Kidney_1610
Testis_1610
reads per million genome matching
bp position
0.0
0.5
1.0
1.5
2.0
2.5
0
20
40
60
80

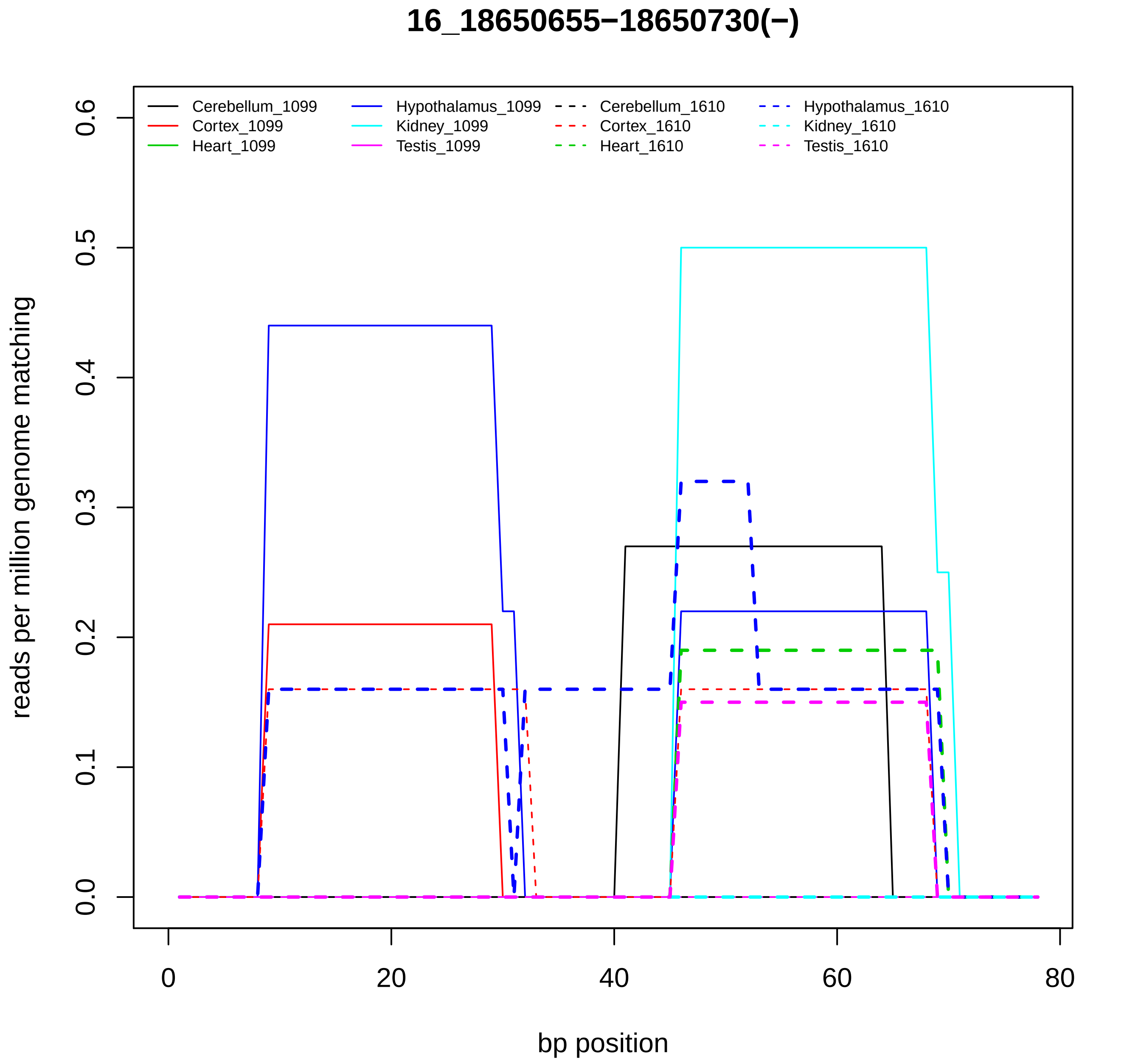

16_18650655−18650730(−)
Cerebellum_1099
Cortex_1099
Heart_1099
Hypothalamus_1099
Kidney_1099
Testis_1099
Cerebellum_1610
Cortex_1610
Heart_1610
Hypothalamus_1610
Kidney_1610
Testis_1610
reads per million genome matching
0.0
0.1
0.2
0.3
0.4
0.5
0.6
0
20
40
60
80
bp position

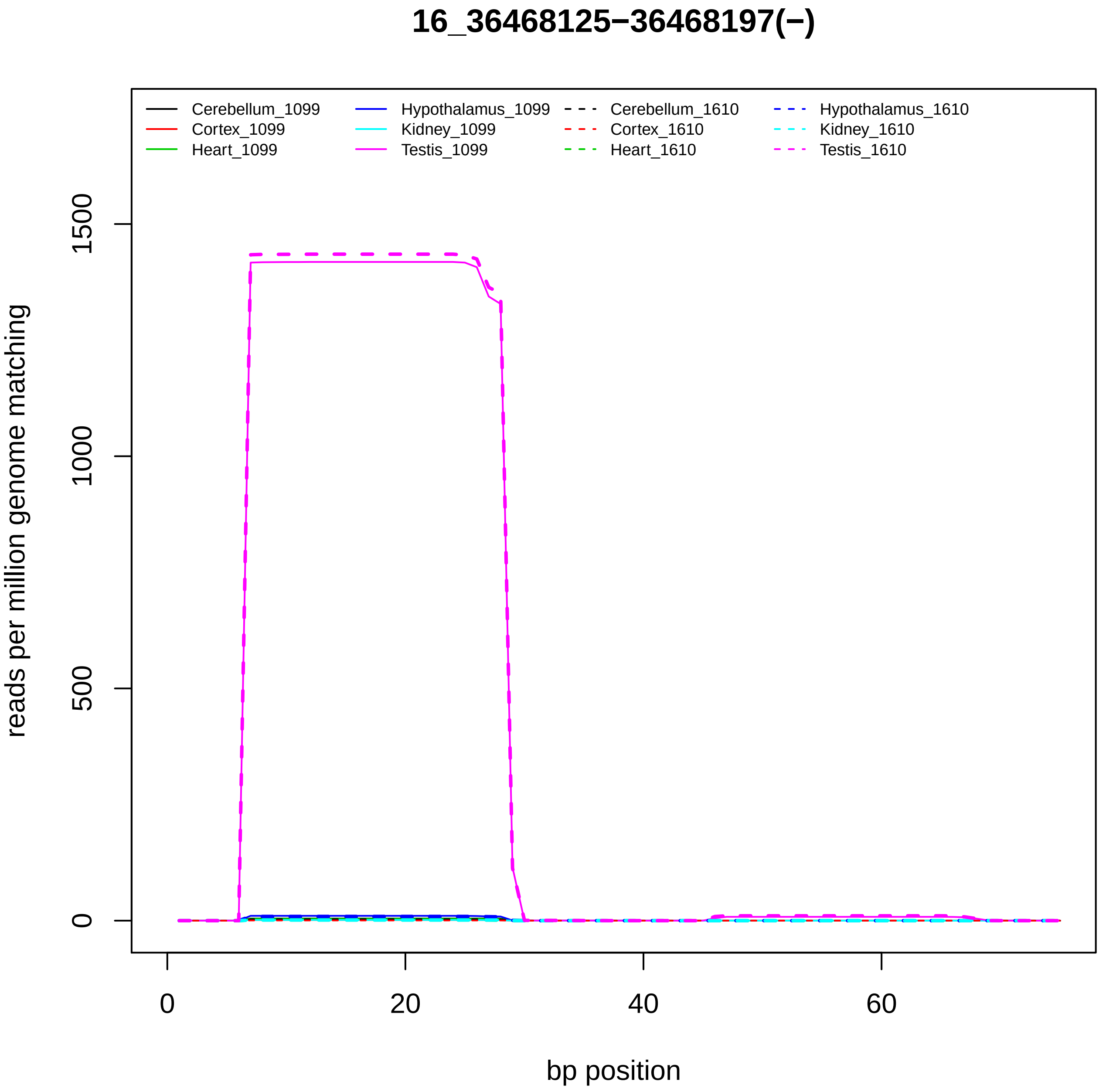

16_36468125−36468197(−)
Cerebellum_1099
Cortex_1099
Heart_1099
Hypothalamus_1099
Kidney_1099
Testis_1099
Cerebellum_1610
Cortex_1610
Heart_1610
Hypothalamus_1610
Kidney_1610
Testis_1610
reads per million genome matching
0
500
1000
1500
0
20
40
60
bp position

16_36468246−36468315(−)

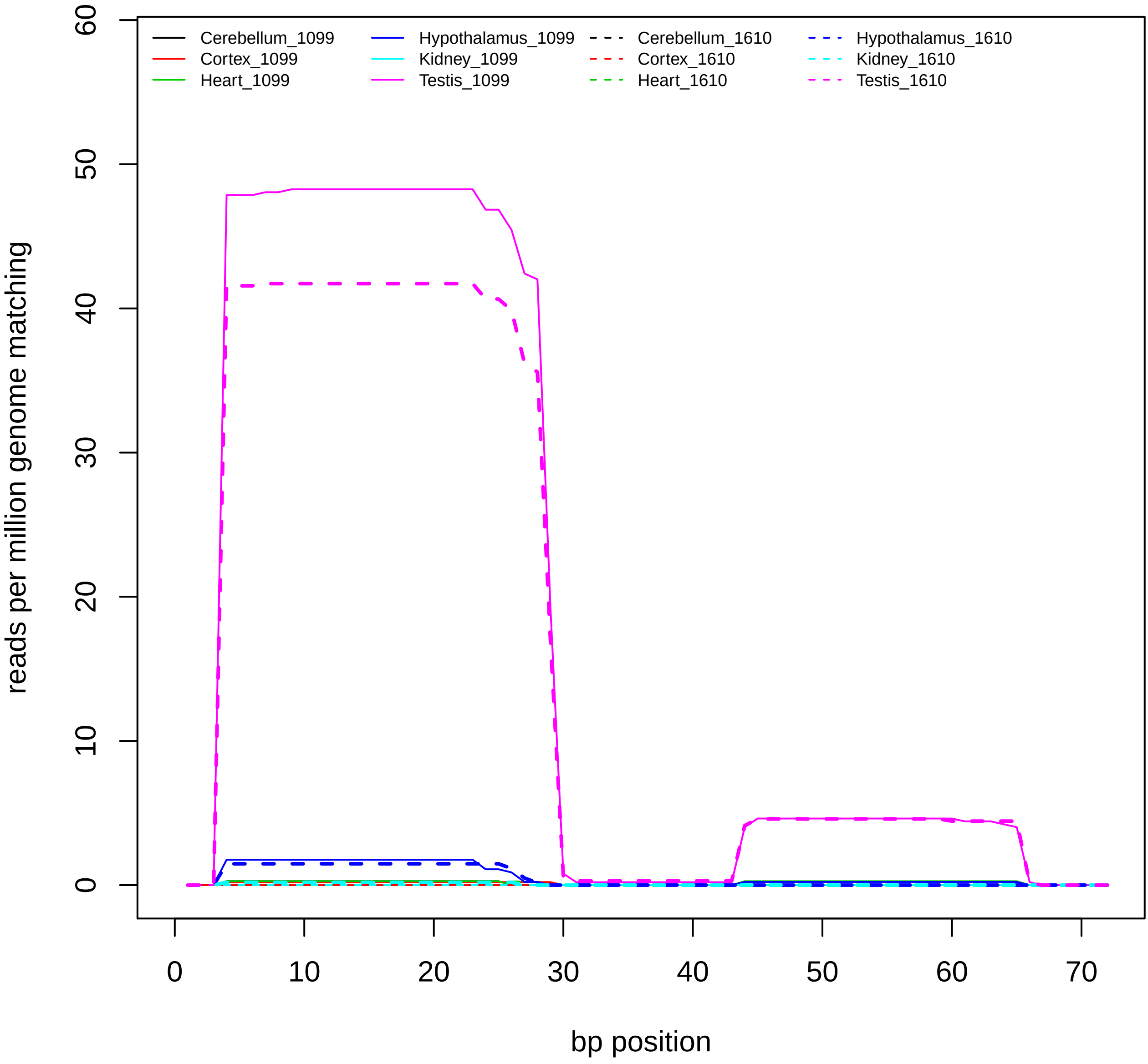

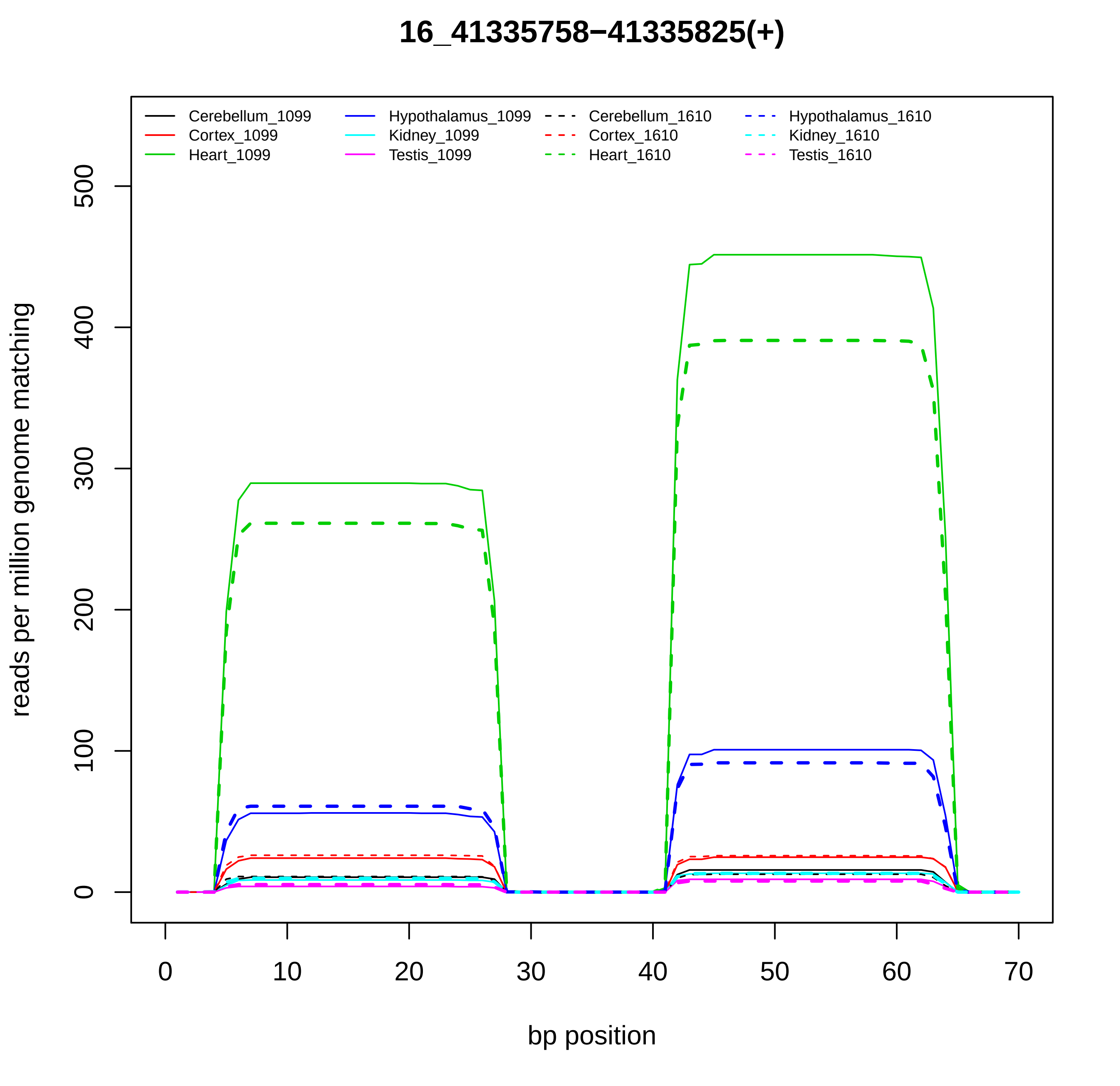

16_41335758−41335825(+)
Cerebellum_1099
Cortex_1099
Heart_1099
Hypothalamus_1099
Kidney_1099
Testis_1099
Cerebellum_1610
Cortex_1610
Heart_1610
Hypothalamus_1610
Kidney_1610
Testis_1610
reads per million genome matching
0
100
200
300
400
500
bp position
0
10
20
30
40
50
60
70

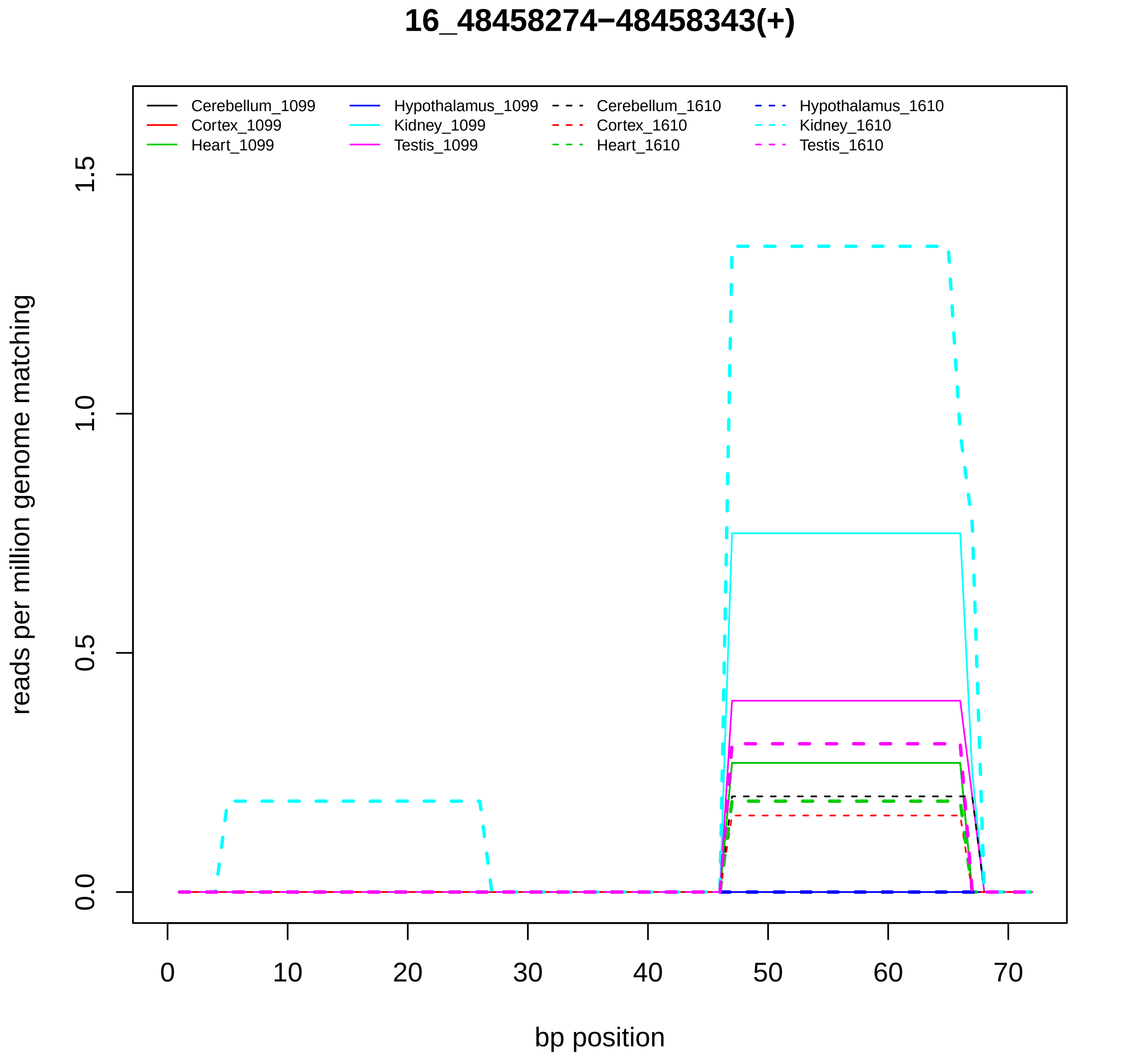

16_48458274−48458343(+)
Cerebellum_1099
Cortex_1099
Heart_1099
Hypothalamus_1099
Kidney_1099
Testis_1099
Cerebellum_1610
Cortex_1610
Heart_1610
Hypothalamus_1610
Kidney_1610
Testis_1610
reads per million genome matching
0.0
0.5
1.0
1.5
0
10
20
30
40
50
60
70
bp position

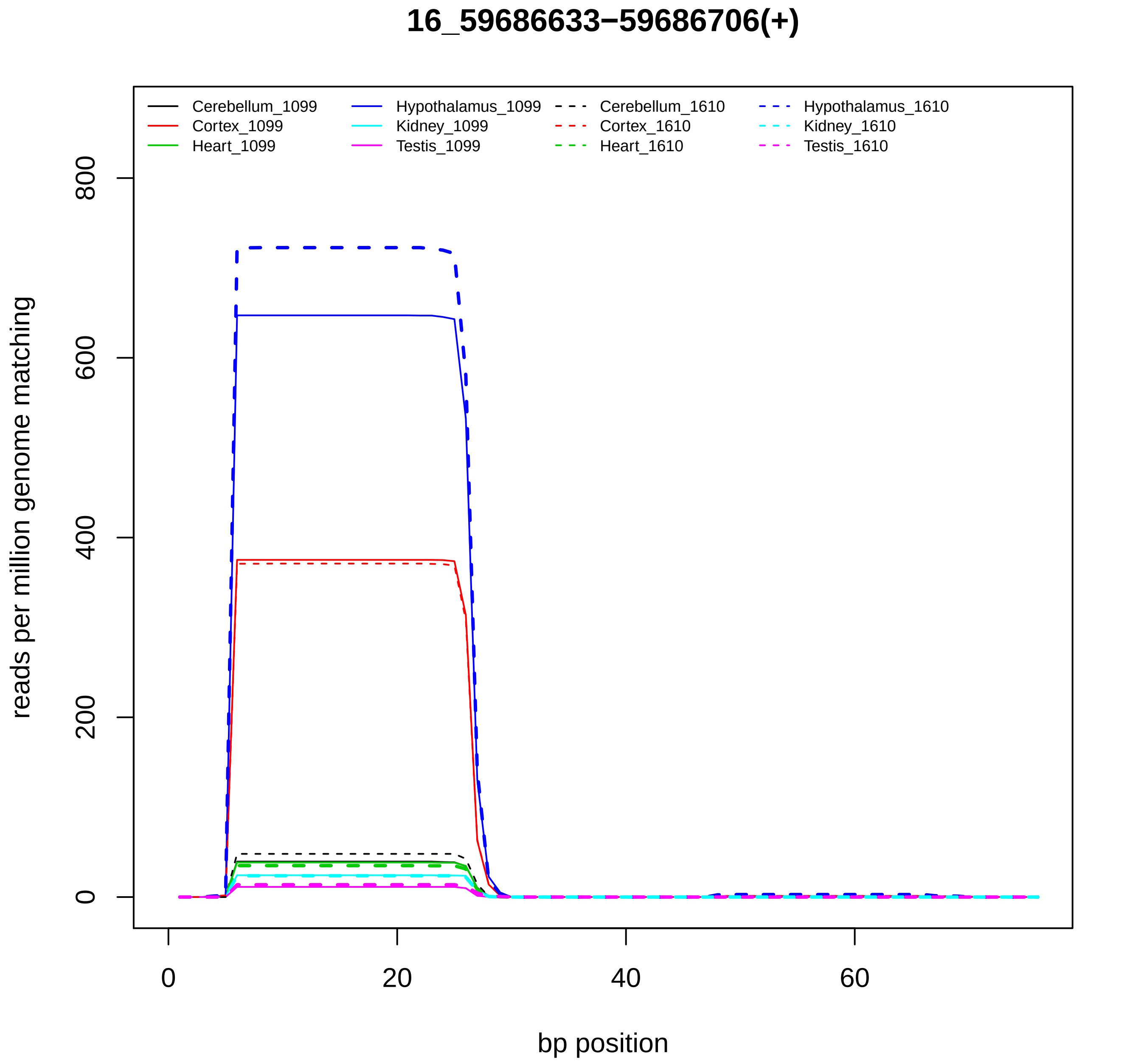

16_59686633−59686706(+)
Cerebellum_1099
Cortex_1099
Heart_1099
Hypothalamus_1099
Kidney_1099
Testis_1099
Cerebellum_1610
Cortex_1610
Heart_1610
Hypothalamus_1610
Kidney_1610
Testis_1610
reads per million genome matching
0
200
400
600
800
0
20
40
60
bp position

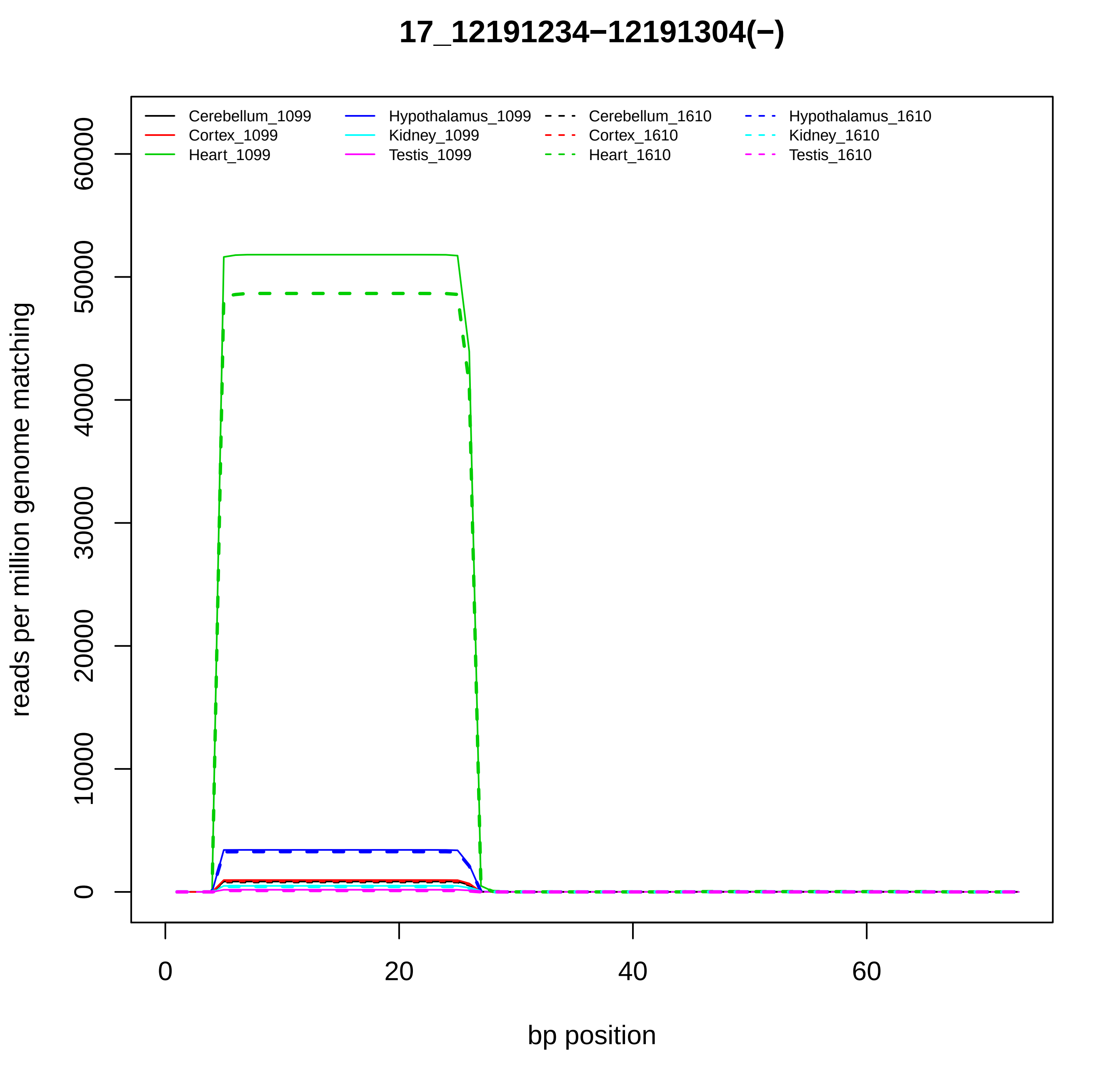
17_12191234−12191304(−)
Cerebellum_1099
Cortex_1099
Heart_1099
Hypothalamus_1099
Kidney_1099
Testis_1099
Cerebellum_1610
Cortex_1610
Heart_1610
Hypothalamus_1610
Kidney_1610
Testis_1610
reads per million genome matching
0
10000
20000
30000
40000
50000
60000
0
20
40
60
bp position

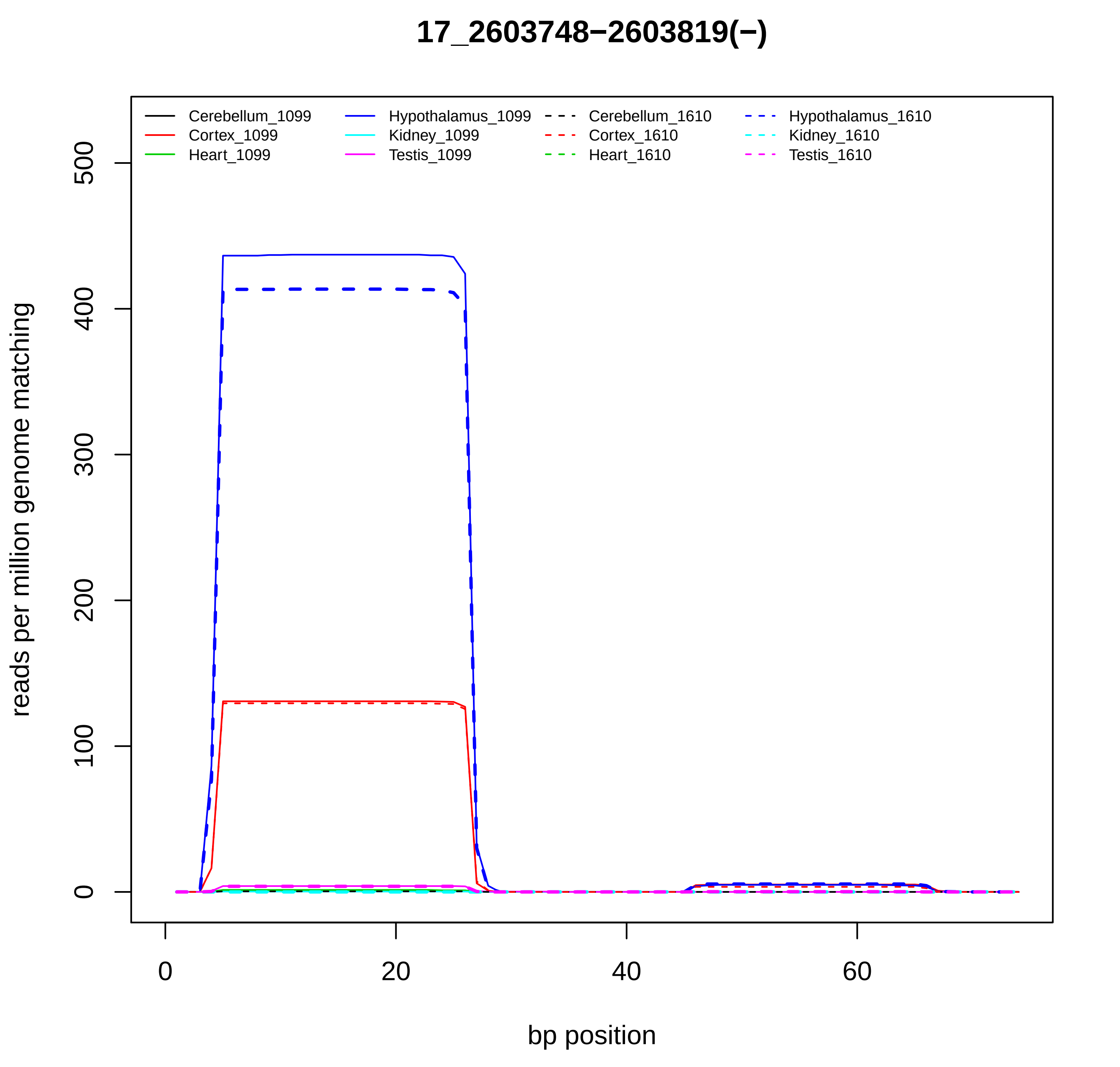

17_2603748−2603819(−)
Cerebellum_1099
Cortex_1099
Heart_1099
Hypothalamus_1099
Kidney_1099
Testis_1099
Cerebellum_1610
Cortex_1610
Heart_1610
Hypothalamus_1610
Kidney_1610
Testis_1610
reads per million genome matching
0
100
200
300
400
500
0
20
40
60
bp position

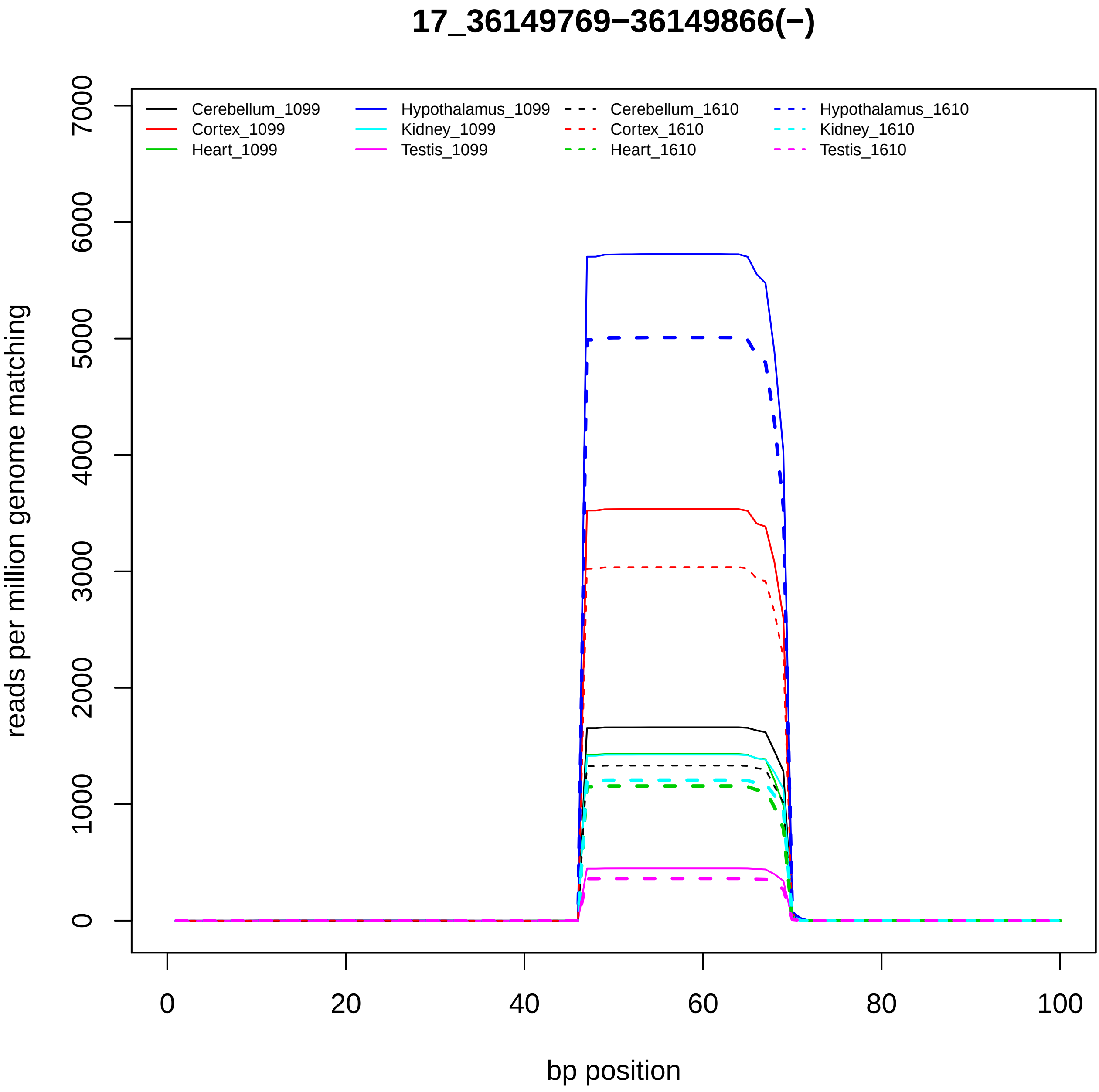
17_36149769−36149866(−)
Cerebellum_1099
Cortex_1099
Heart_1099
Hypothalamus_1099
Kidney_1099
Testis_1099
Cerebellum_1610
Cortex_1610
Heart_1610
Hypothalamus_1610
Kidney_1610
Testis_1610
reads per million genome matching
bp position
0
1000
2000
3000
4000
5000
6000
7000
0
20
40
60
80
100

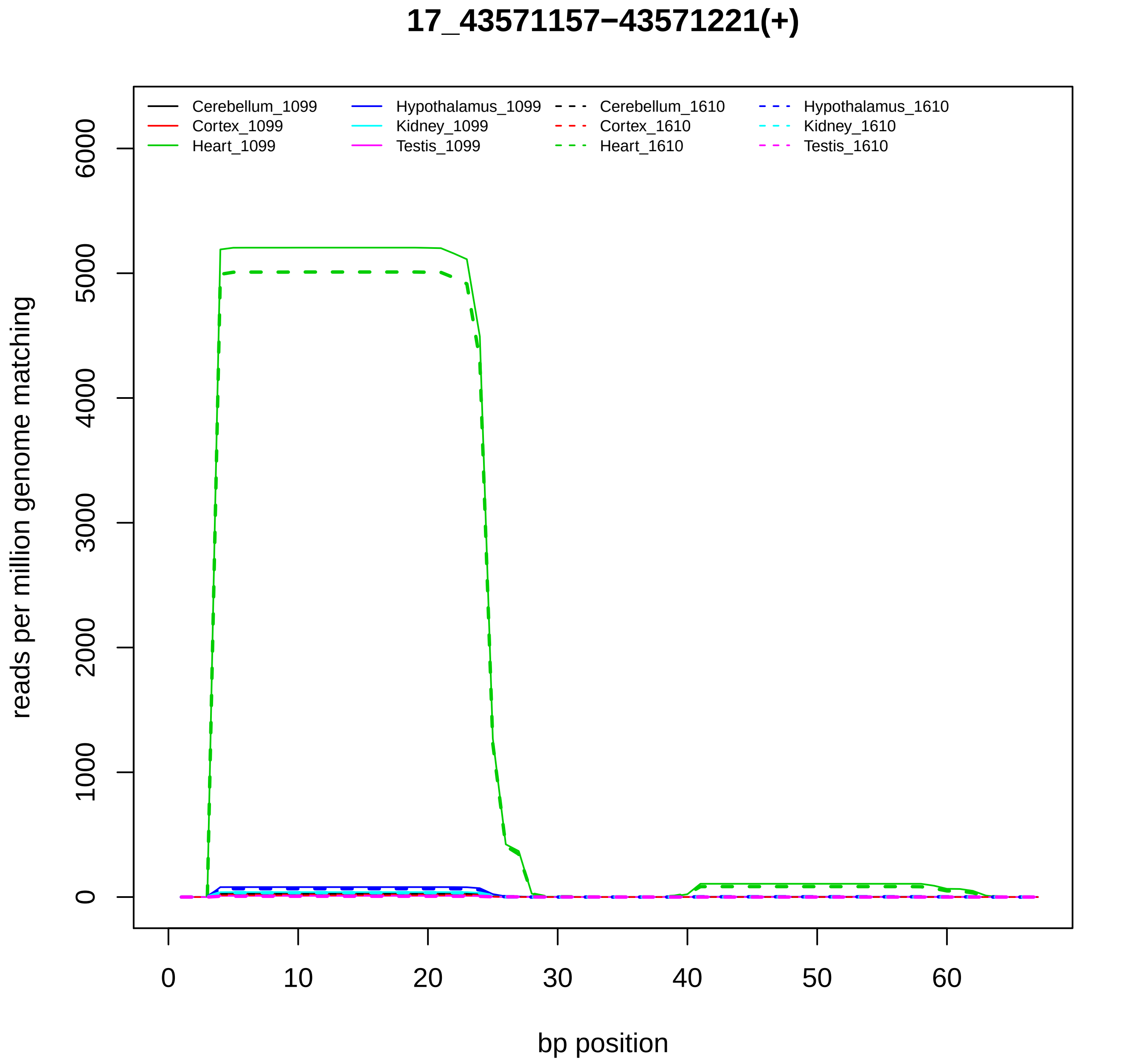

17_43571157−43571221(+)
Cerebellum_1099
Cortex_1099
Heart_1099
Hypothalamus_1099
Kidney_1099
Testis_1099
Cerebellum_1610
Cortex_1610
Heart_1610
Hypothalamus_1610
Kidney_1610
Testis_1610
reads per million genome matching
0
1000
2000
3000
4000
5000
6000
0
10
20
30
40
50
60
bp position

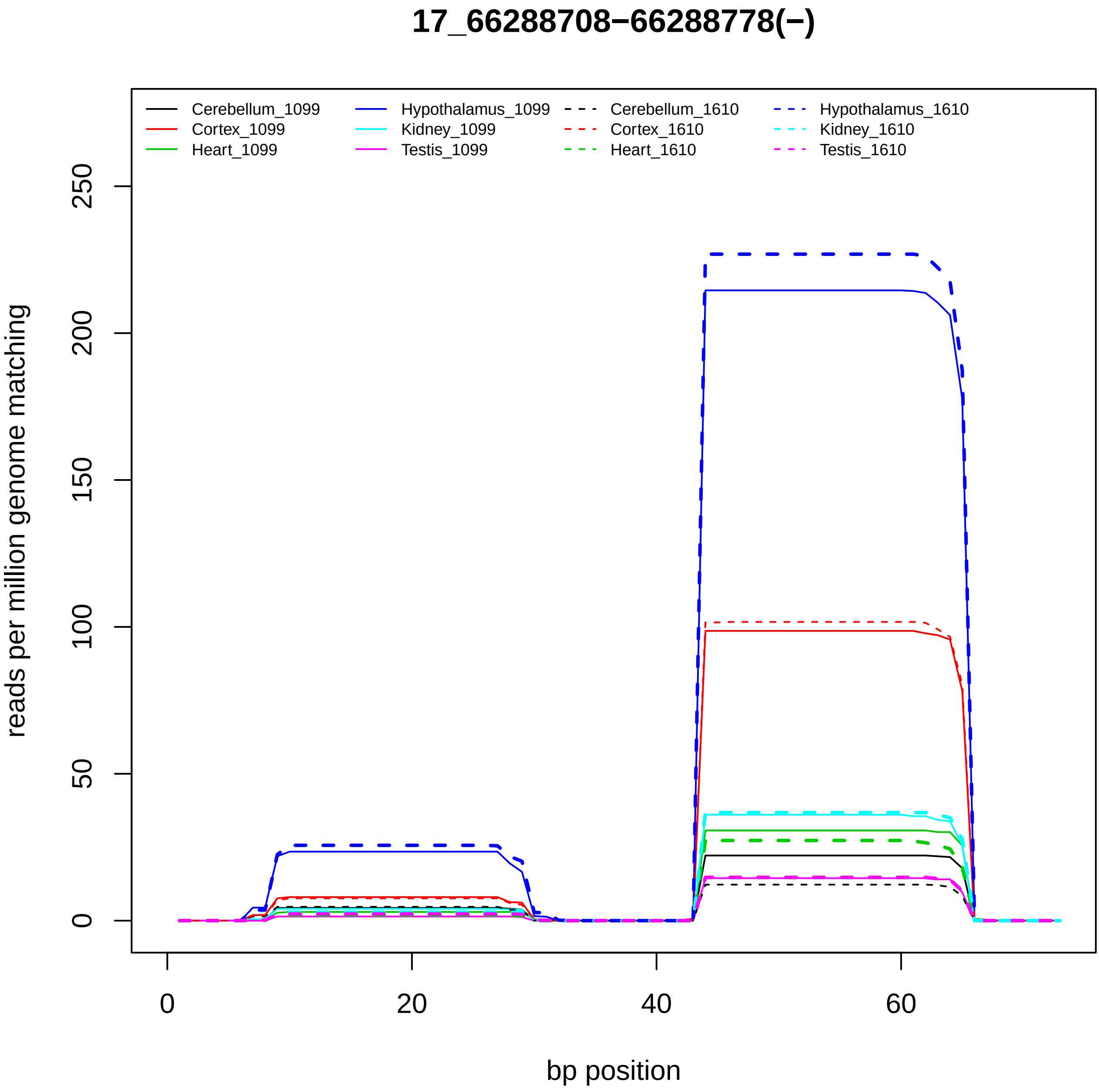

17_66288708−66288778(−)
Cerebellum_1099
Cortex_1099
Heart_1099
Hypothalamus_1099
Kidney_1099
Testis_1099
Cerebellum_1610
Cortex_1610
Heart_1610
Hypothalamus_1610
Kidney_1610
Testis_1610
reads per million genome matching
0
50
100
150
200
250
0
20
40
60
bp position

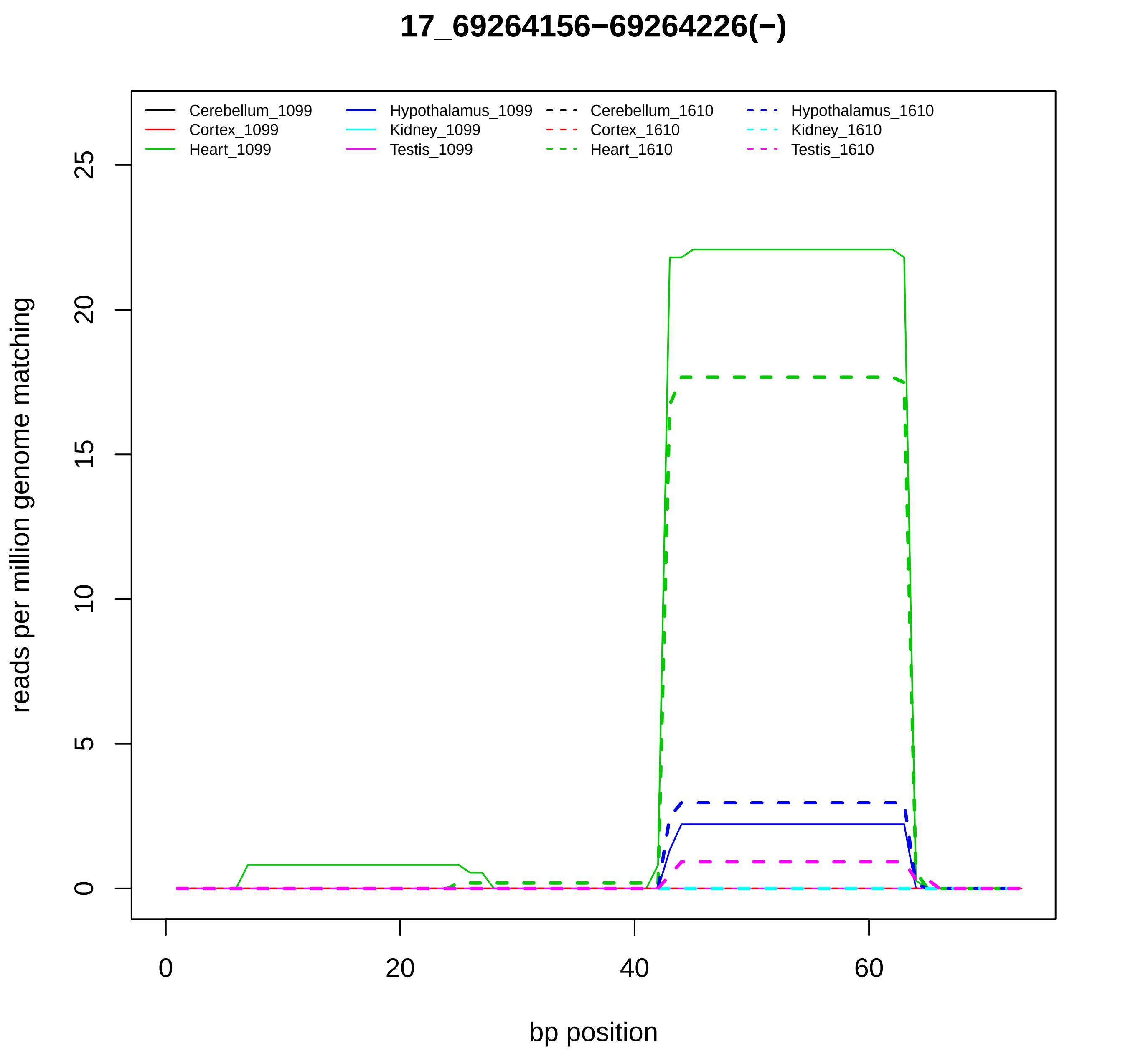

17_69264156−69264226(−)
Cerebellum_1099
Cortex_1099
Heart_1099
Hypothalamus_1099
Kidney_1099
Testis_1099
Cerebellum_1610
Cortex_1610
Heart_1610
Hypothalamus_1610
Kidney_1610
Testis_1610
reads per million genome matching
0
5
10
15
20
25
0
20
40
60
bp position

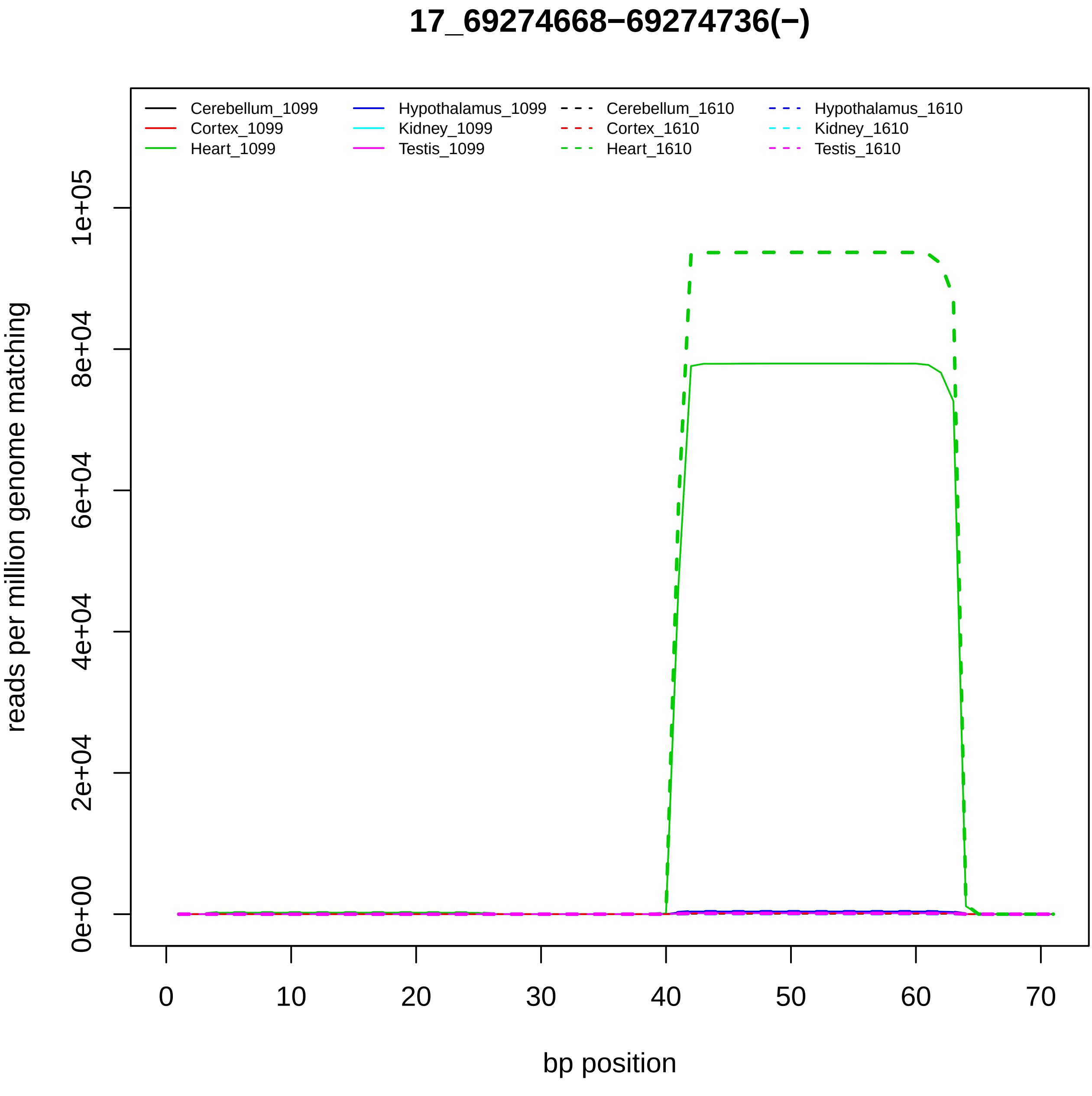

17_69274668−69274736(−)
Cerebellum_1099
Cortex_1099
Heart_1099
Hypothalamus_1099
Kidney_1099
Testis_1099
Cerebellum_1610
Cortex_1610
Heart_1610
Hypothalamus_1610
Kidney_1610
Testis_1610
reads per million genome matching
0e+00
2e+04
4e+04
6e+04
8e+04
1e+05
0
10
20
30
40
50
60
70
bp position

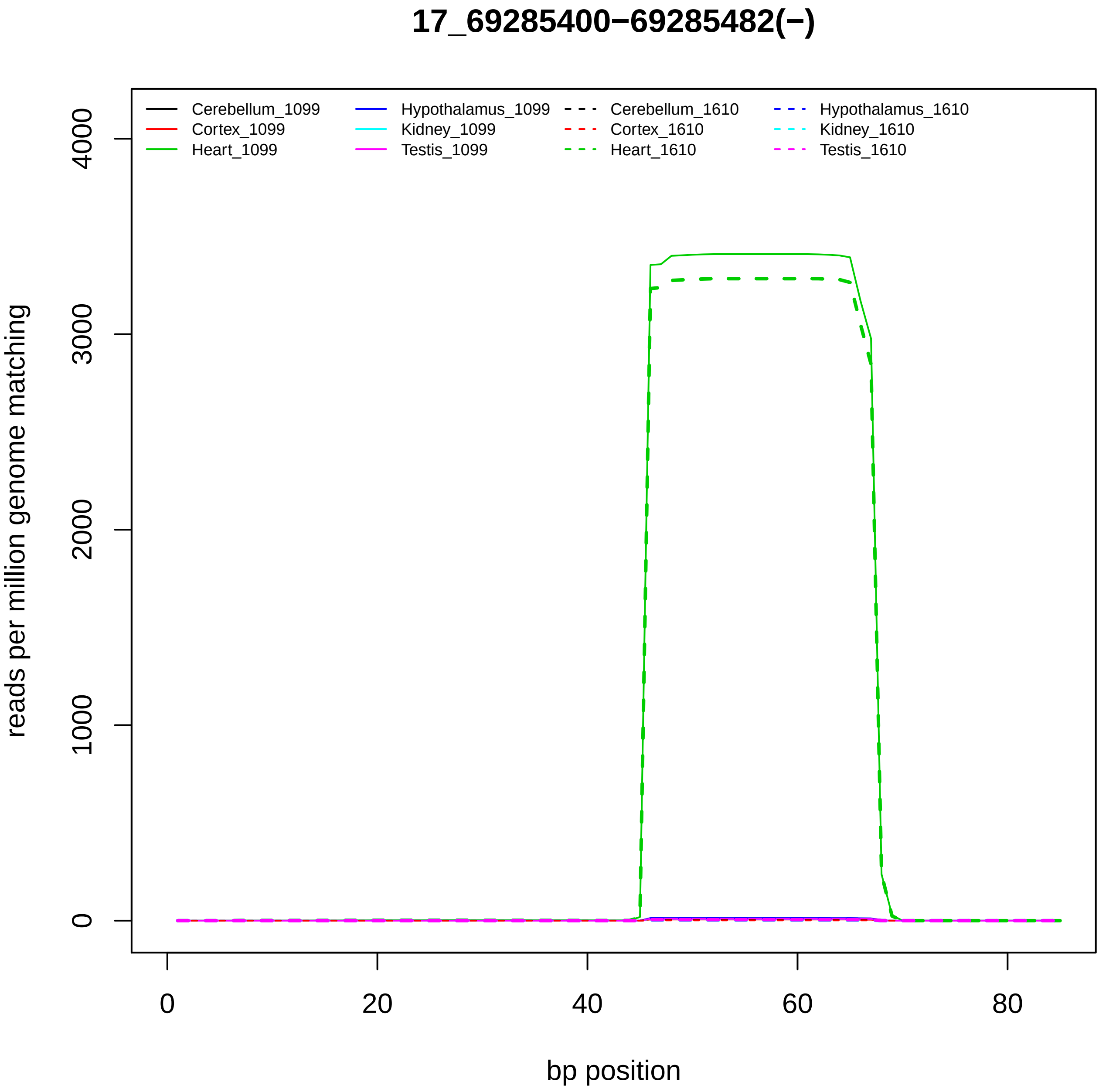
17_69285400−69285482(−)
Cerebellum_1099
Cortex_1099
Heart_1099
Hypothalamus_1099
Kidney_1099
Testis_1099
Cerebellum_1610
Cortex_1610
Heart_1610
Hypothalamus_1610
Kidney_1610
Testis_1610
reads per million genome matching
0
1000
2000
3000
4000
bp position
0
20
40
60
80

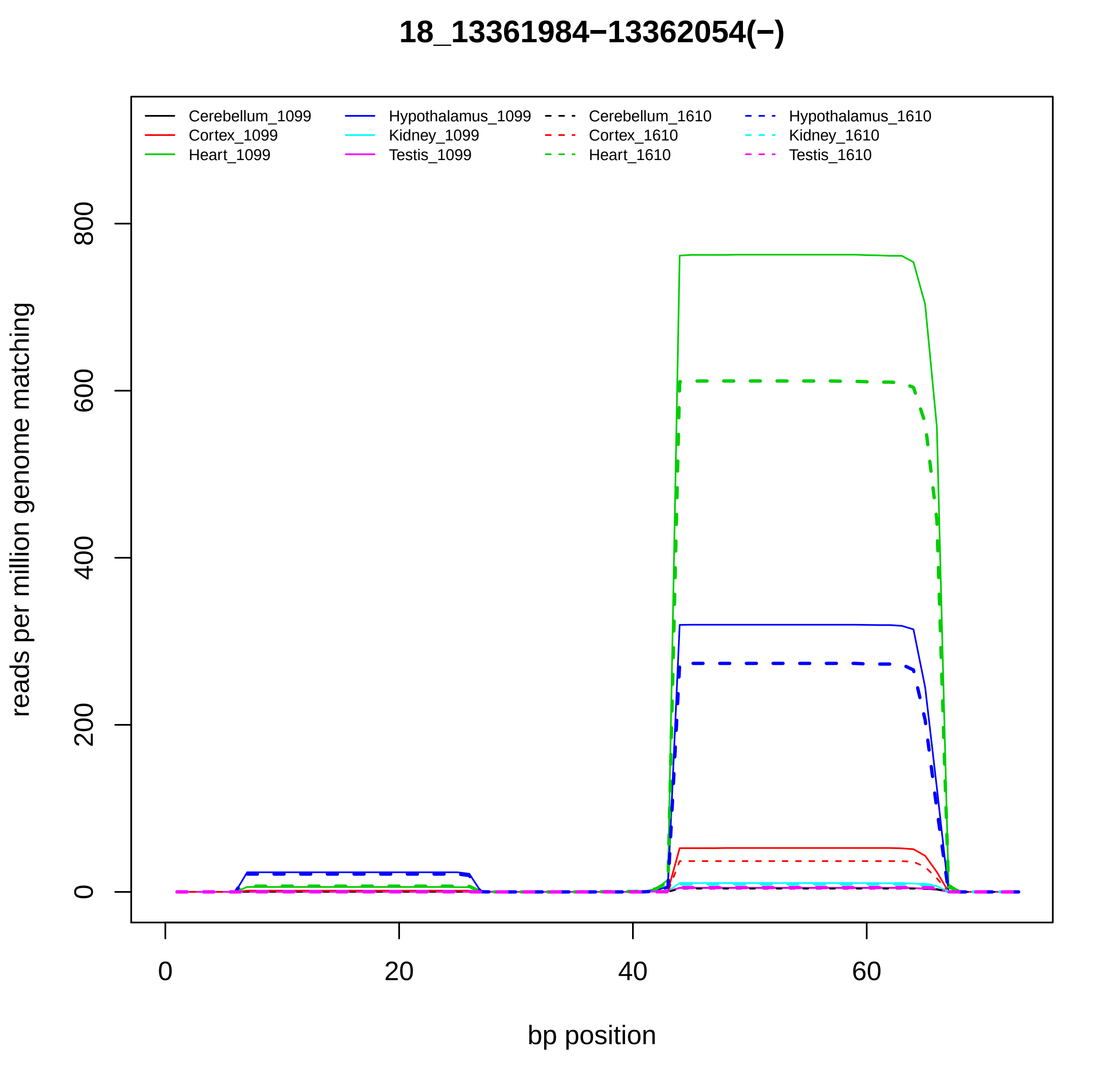
18_13361984−13362054(−)
Cerebellum_1099
Cortex_1099
Heart_1099
Hypothalamus_1099
Kidney_1099
Testis_1099
Cerebellum_1610
Cortex_1610
Heart_1610
Hypothalamus_1610
Kidney_1610
Testis_1610
reads per million genome matching
0
200
400
600
800
0
20
40
60
bp position

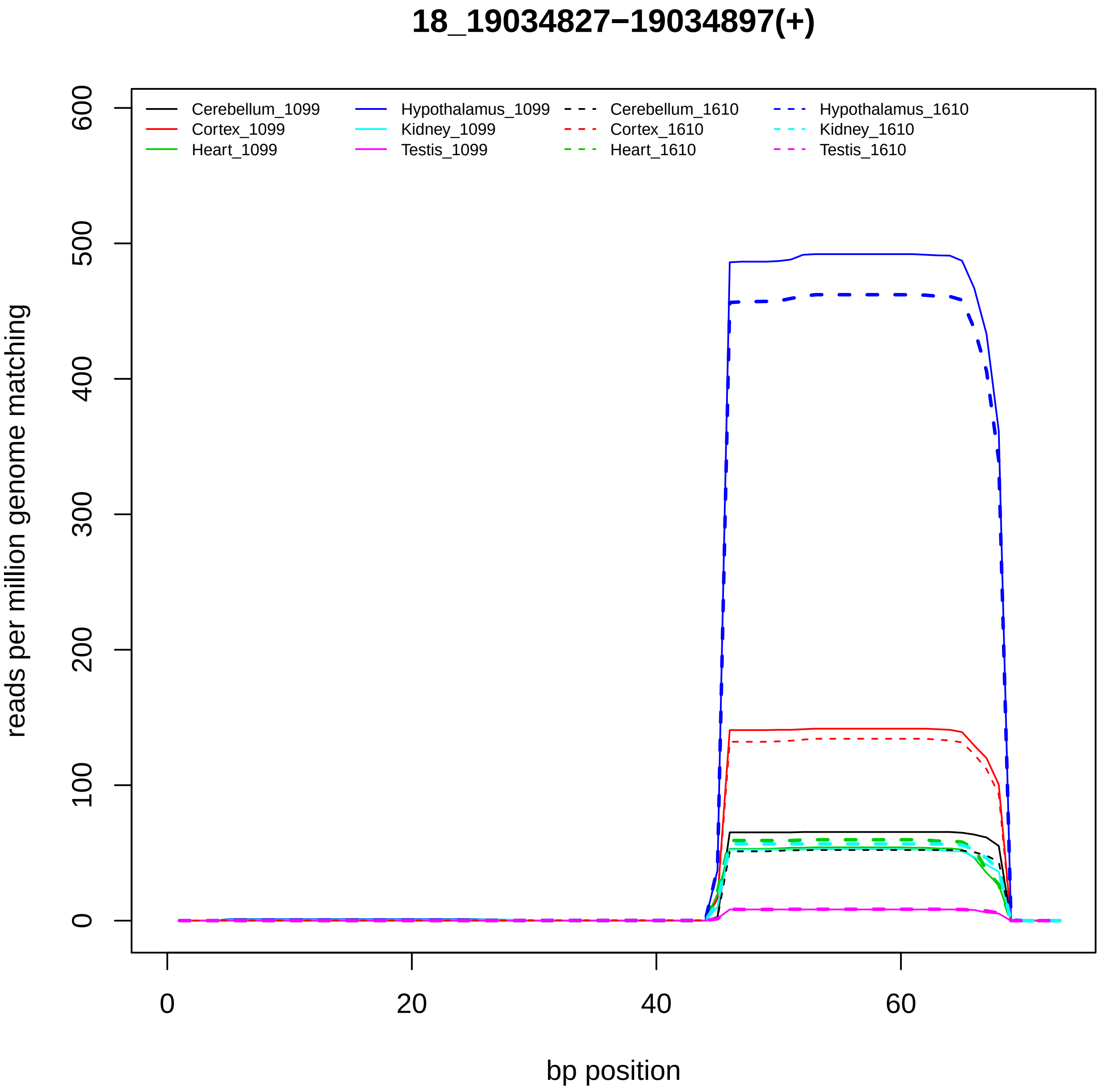

18_19034827−19034897(+)
Cerebellum_1099
Cortex_1099
Heart_1099
Hypothalamus_1099
Kidney_1099
Testis_1099
Cerebellum_1610
Cortex_1610
Heart_1610
Hypothalamus_1610
Kidney_1610
Testis_1610
reads per million genome matching
0
100
200
300
400
500
600
0
20
40
60
bp position

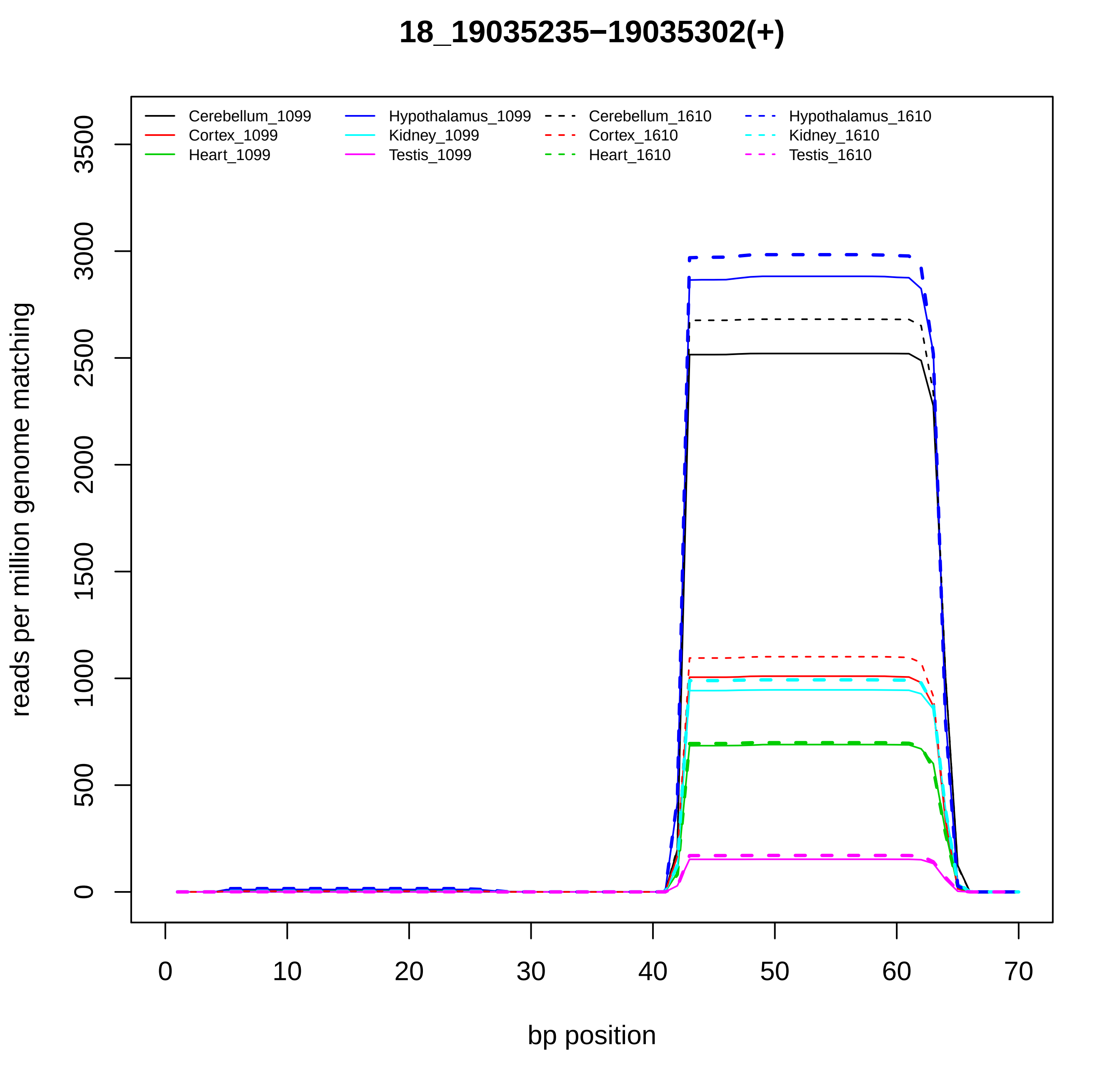
18_19035235−19035302(+)
Cerebellum_1099
Cortex_1099
Heart_1099
Hypothalamus_1099
Kidney_1099
Testis_1099
Cerebellum_1610
Cortex_1610
Heart_1610
Hypothalamus_1610
Kidney_1610
Testis_1610
reads per million genome matching
0
500
1000
1500
2000
2500
3000
3500
bp position
0
10
20
30
40
50
60
70

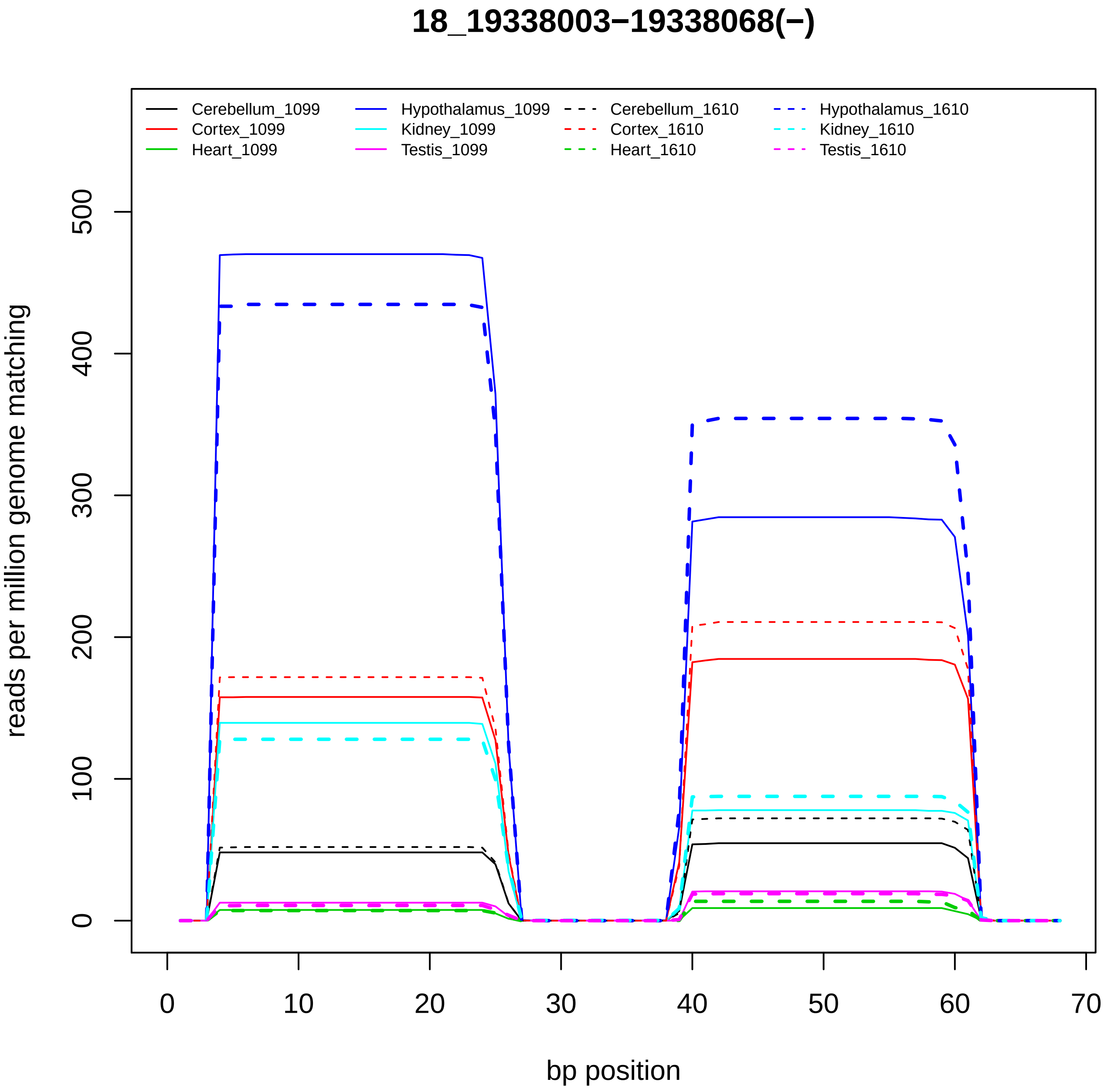
18_19338003−19338068(−)
Cerebellum_1099
Cortex_1099
Heart_1099
Hypothalamus_1099
Kidney_1099
Testis_1099
Cerebellum_1610
Cortex_1610
Heart_1610
Hypothalamus_1610
Kidney_1610
Testis_1610
reads per million genome matching
0
100
200
300
400
500
0
10
20
30
40
50
60
70
bp position

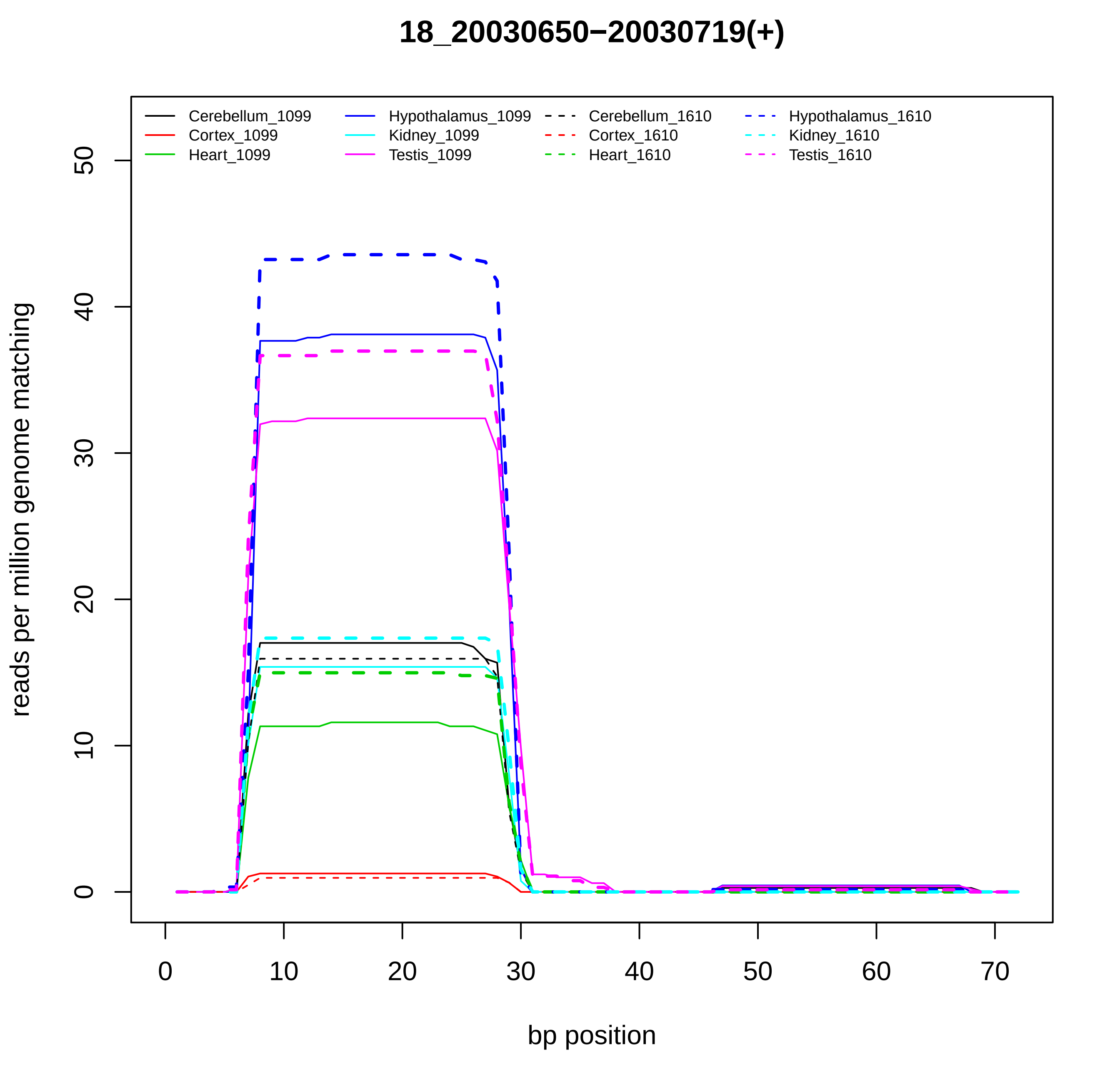

18_20030650−20030719(+)
Cerebellum_1099
Cortex_1099
Heart_1099
Hypothalamus_1099
Kidney_1099
Testis_1099
Cerebellum_1610
Cortex_1610
Heart_1610
Hypothalamus_1610
Kidney_1610
Testis_1610
reads per million genome matching
0
10
20
30
40
50
0
10
20
30
40
50
60
70
bp position

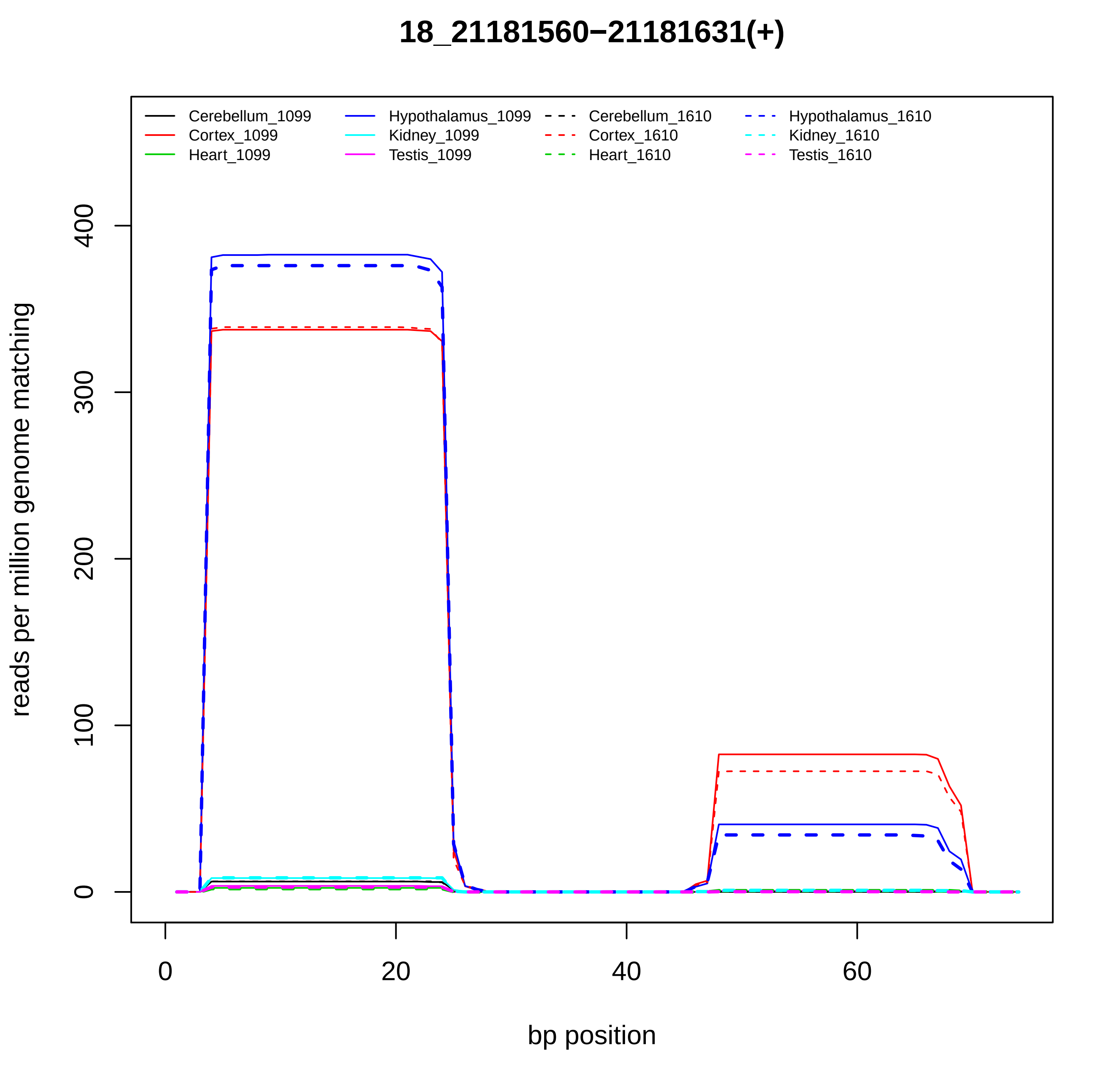

18_21181560−21181631(+)
Cerebellum_1099
Cortex_1099
Heart_1099
Hypothalamus_1099
Kidney_1099
Testis_1099
Cerebellum_1610
Cortex_1610
Heart_1610
Hypothalamus_1610
Kidney_1610
Testis_1610
reads per million genome matching
0
100
200
300
400
0
20
40
60
bp position

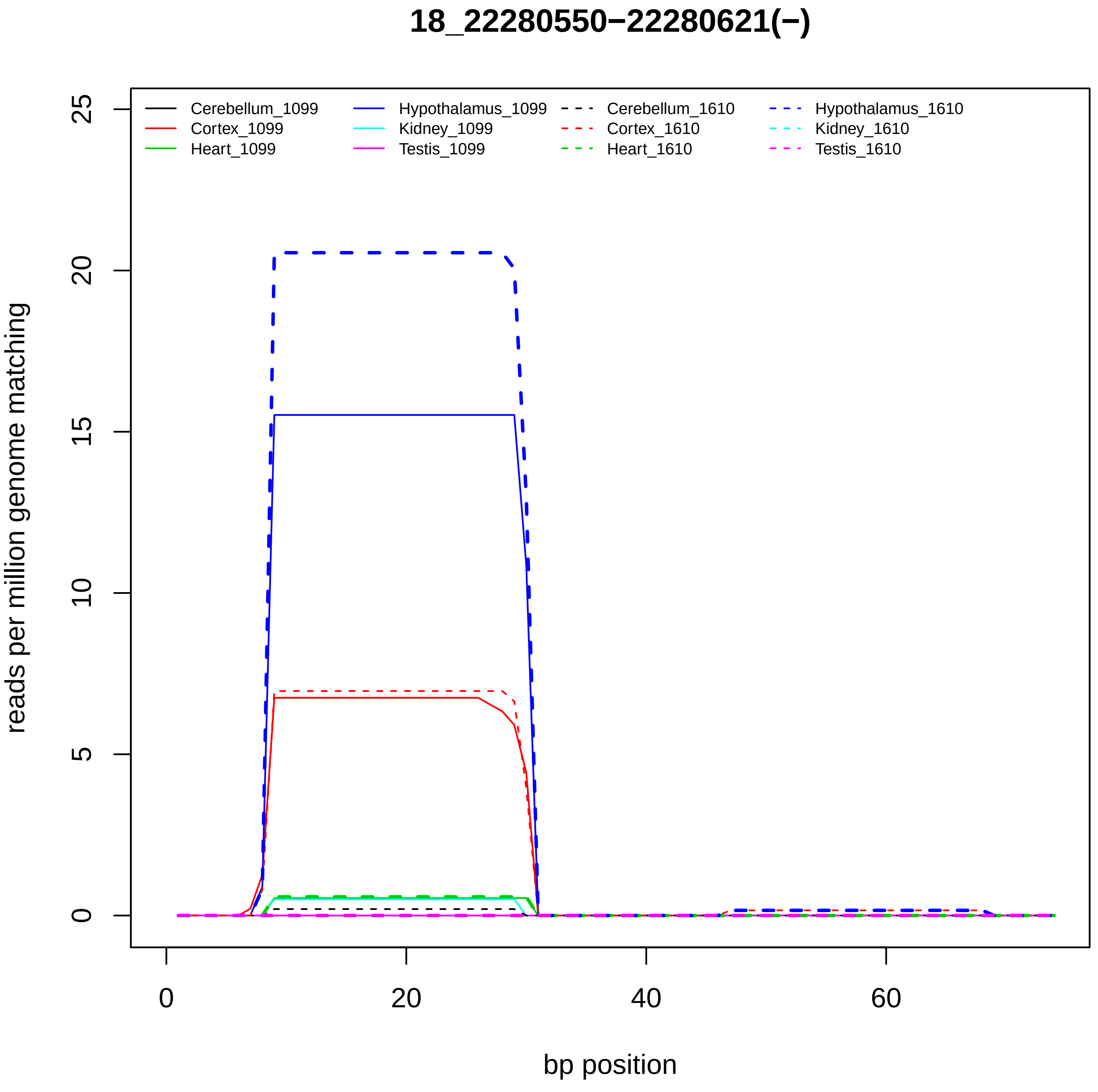

18_22280550−22280621(−)
Cerebellum_1099
Cortex_1099
Heart_1099
Hypothalamus_1099
Kidney_1099
Testis_1099
Cerebellum_1610
Cortex_1610
Heart_1610
Hypothalamus_1610
Kidney_1610
Testis_1610
reads per million genome matching
bp position
0
5
10
15
20
25
0
20
40
60

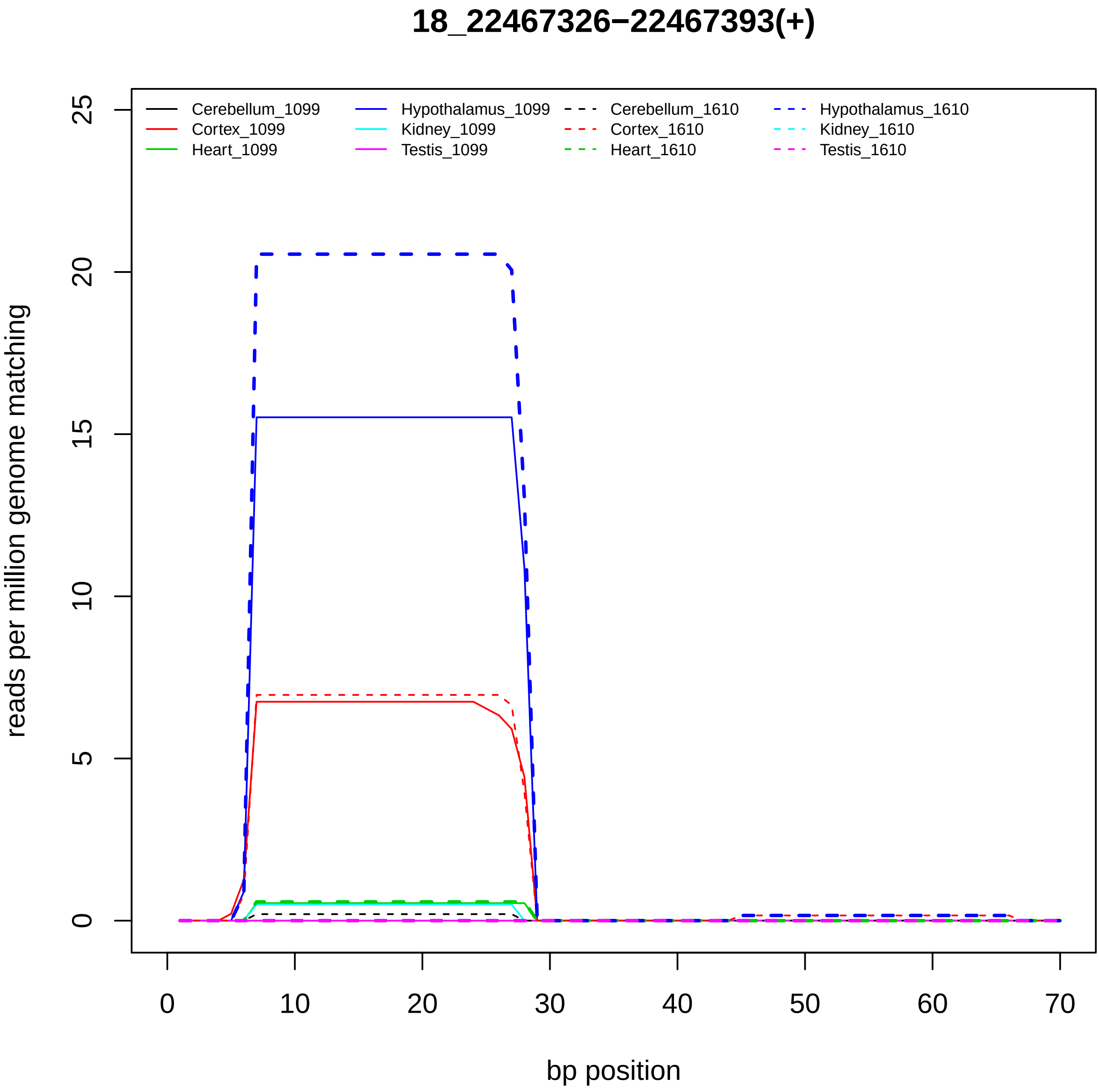

18_22467326−22467393(+)
Cerebellum_1099
Cortex_1099
Heart_1099
Hypothalamus_1099
Kidney_1099
Testis_1099
Cerebellum_1610
Cortex_1610
Heart_1610
Hypothalamus_1610
Kidney_1610
Testis_1610
reads per million genome matching
0
5
10
15
20
25
0
10
20
30
40
50
60
70
bp position

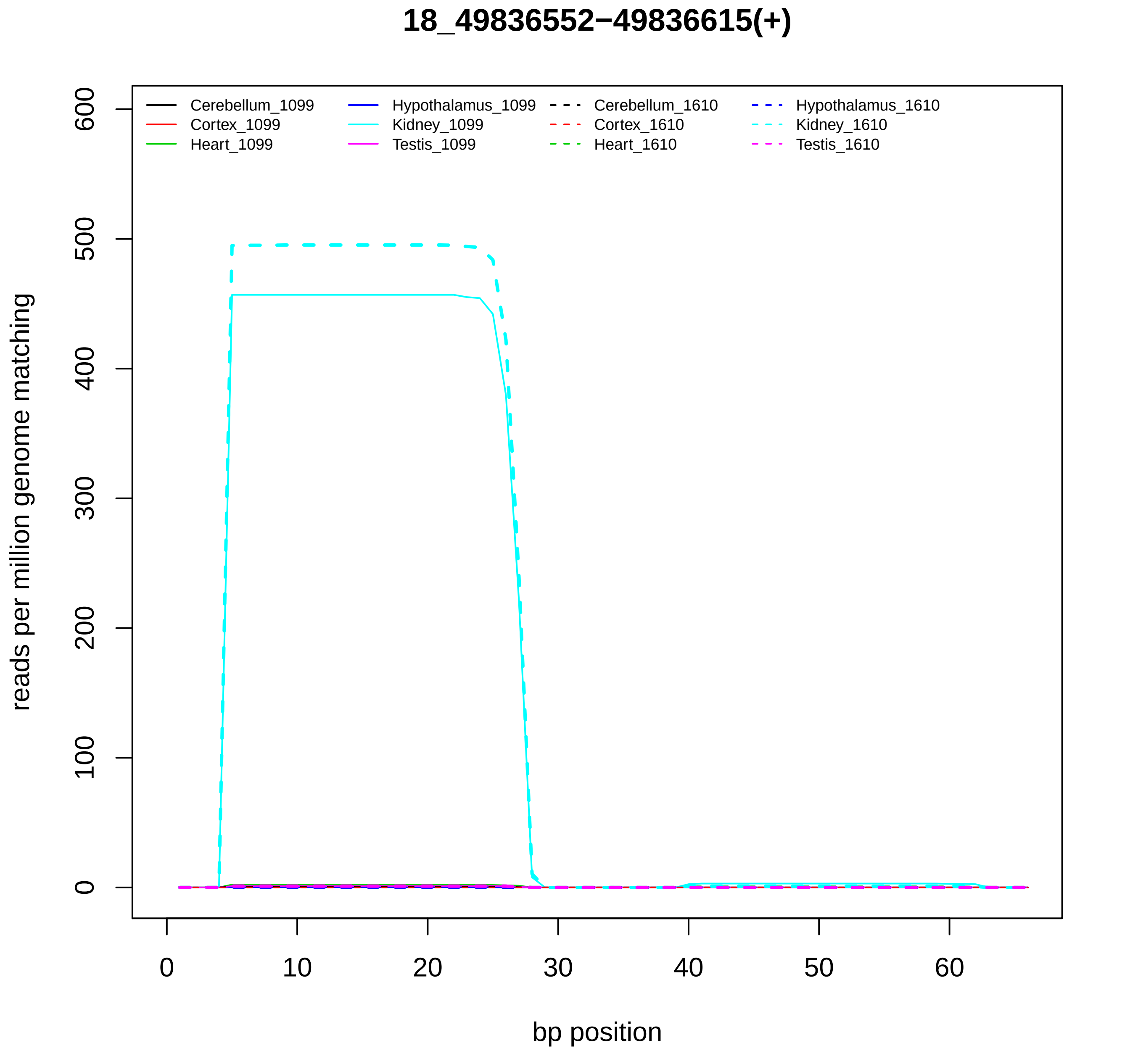

18_49836552−49836615(+)
Cerebellum_1099
Cortex_1099
Heart_1099
Hypothalamus_1099
Kidney_1099
Testis_1099
Cerebellum_1610
Cortex_1610
Heart_1610
Hypothalamus_1610
Kidney_1610
Testis_1610
reads per million genome matching
0
100
200
300
400
500
600
bp position
0
10
20
30
40
50
60

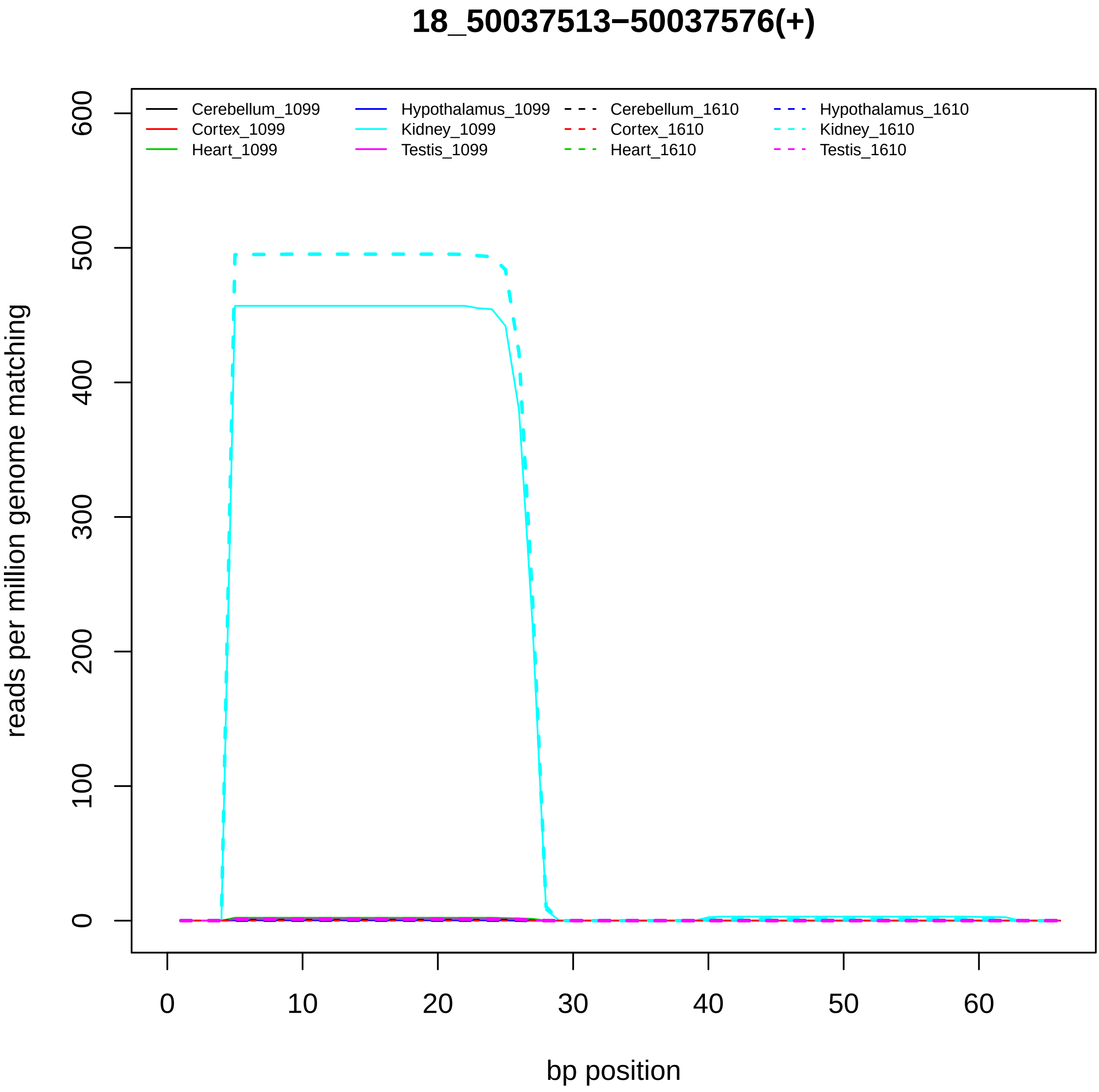
18_50037513−50037576(+)
Cerebellum_1099
Cortex_1099
Heart_1099
Hypothalamus_1099
Kidney_1099
Testis_1099
Cerebellum_1610
Cortex_1610
Heart_1610
Hypothalamus_1610
Kidney_1610
Testis_1610
reads per million genome matching
0
100
200
300
400
500
600
0
10
20
30
40
50
60
bp position

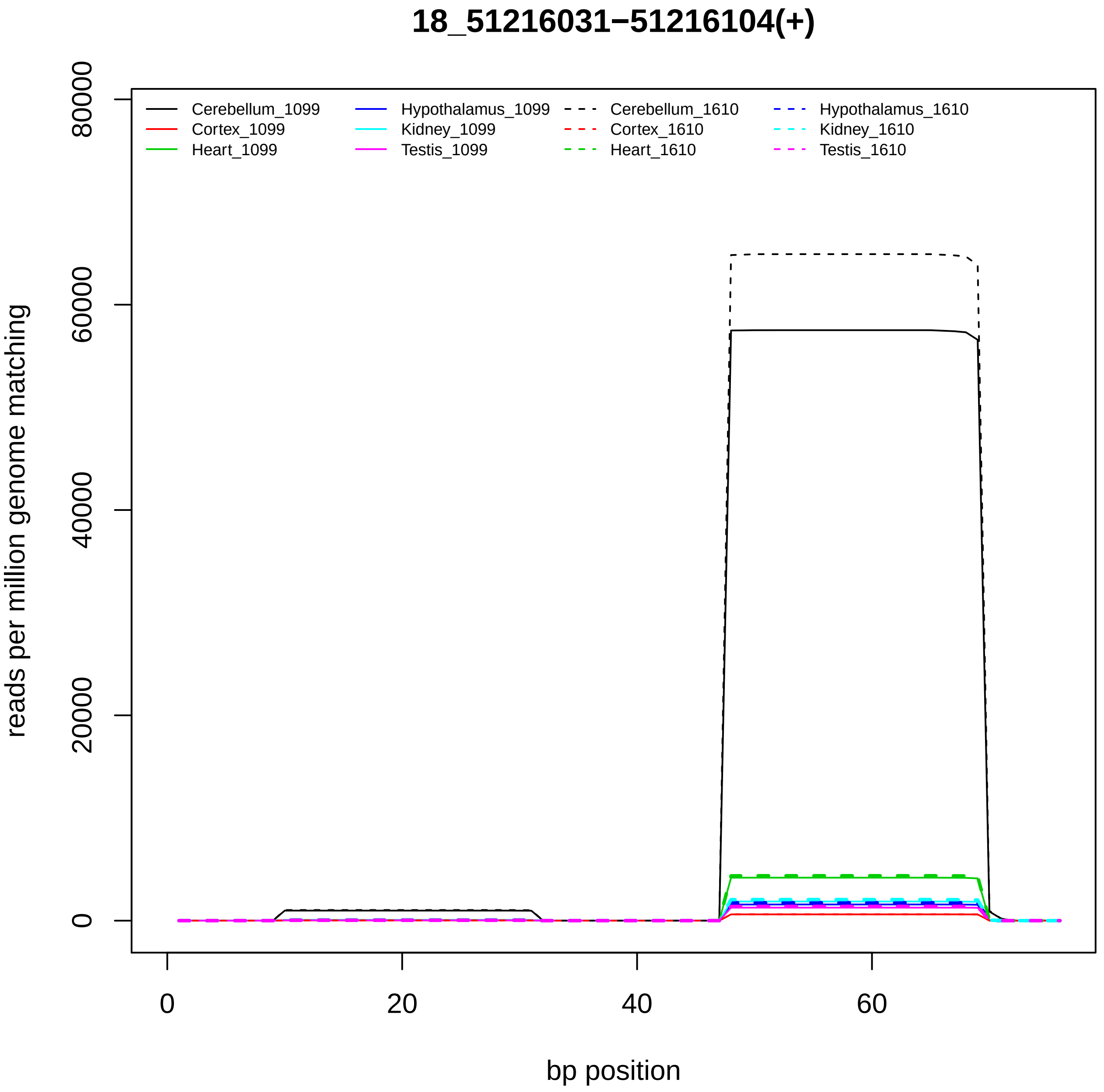

18_51216031−51216104(+)
Cerebellum_1099
Cortex_1099
Heart_1099
Hypothalamus_1099
Kidney_1099
Testis_1099
Cerebellum_1610
Cortex_1610
Heart_1610
Hypothalamus_1610
Kidney_1610
Testis_1610
reads per million genome matching
0
20000
40000
60000
80000
0
20
40
60
bp position

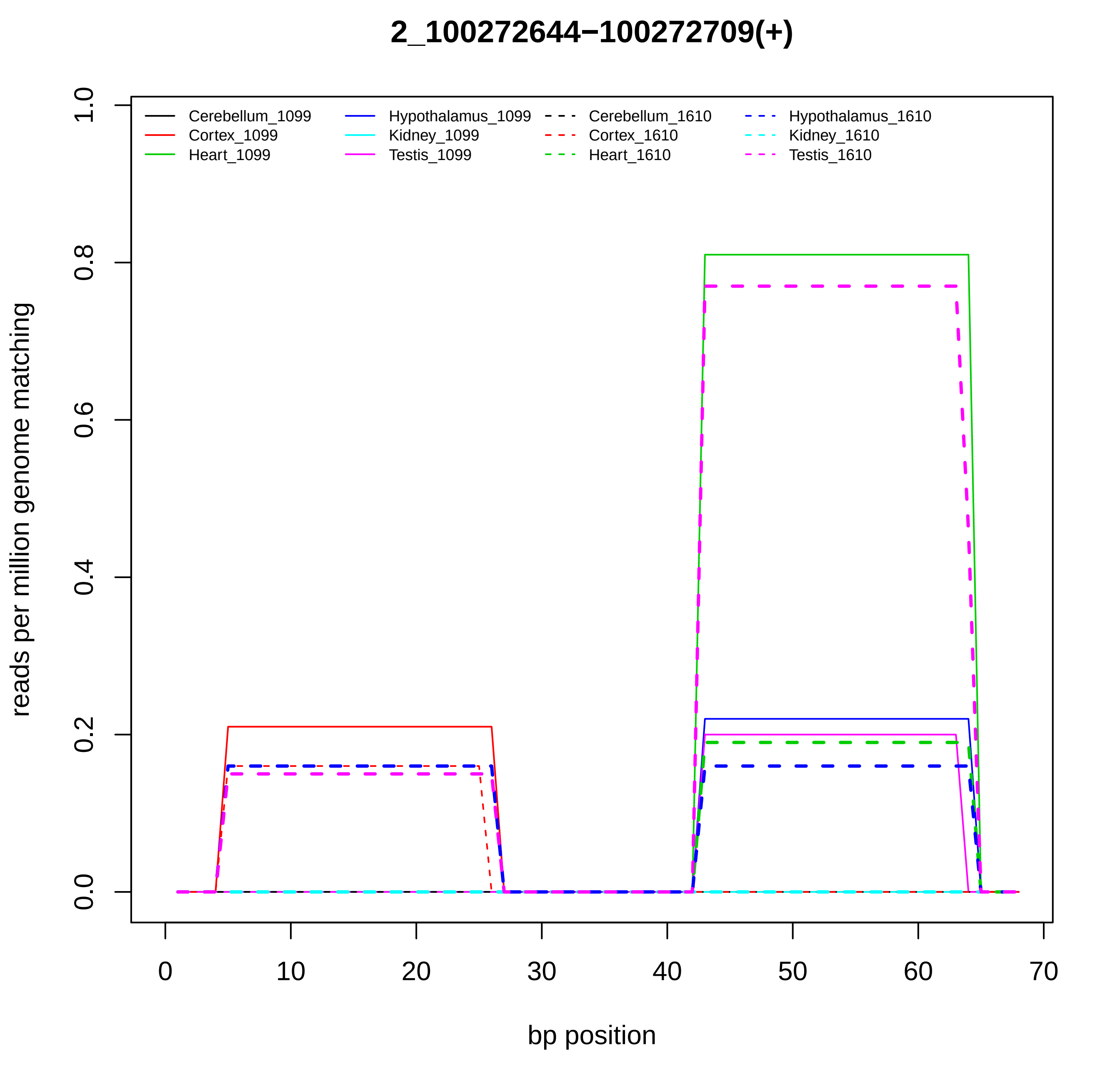
2_100272644−100272709(+)
Cerebellum_1099
Cortex_1099
Heart_1099
Hypothalamus_1099
Kidney_1099
Testis_1099
Cerebellum_1610
Cortex_1610
Heart_1610
Hypothalamus_1610
Kidney_1610
Testis_1610
reads per million genome matching
0.0
0.2
0.4
0.6
0.8
1.0
0
10
20
30
40
50
60
70
bp position

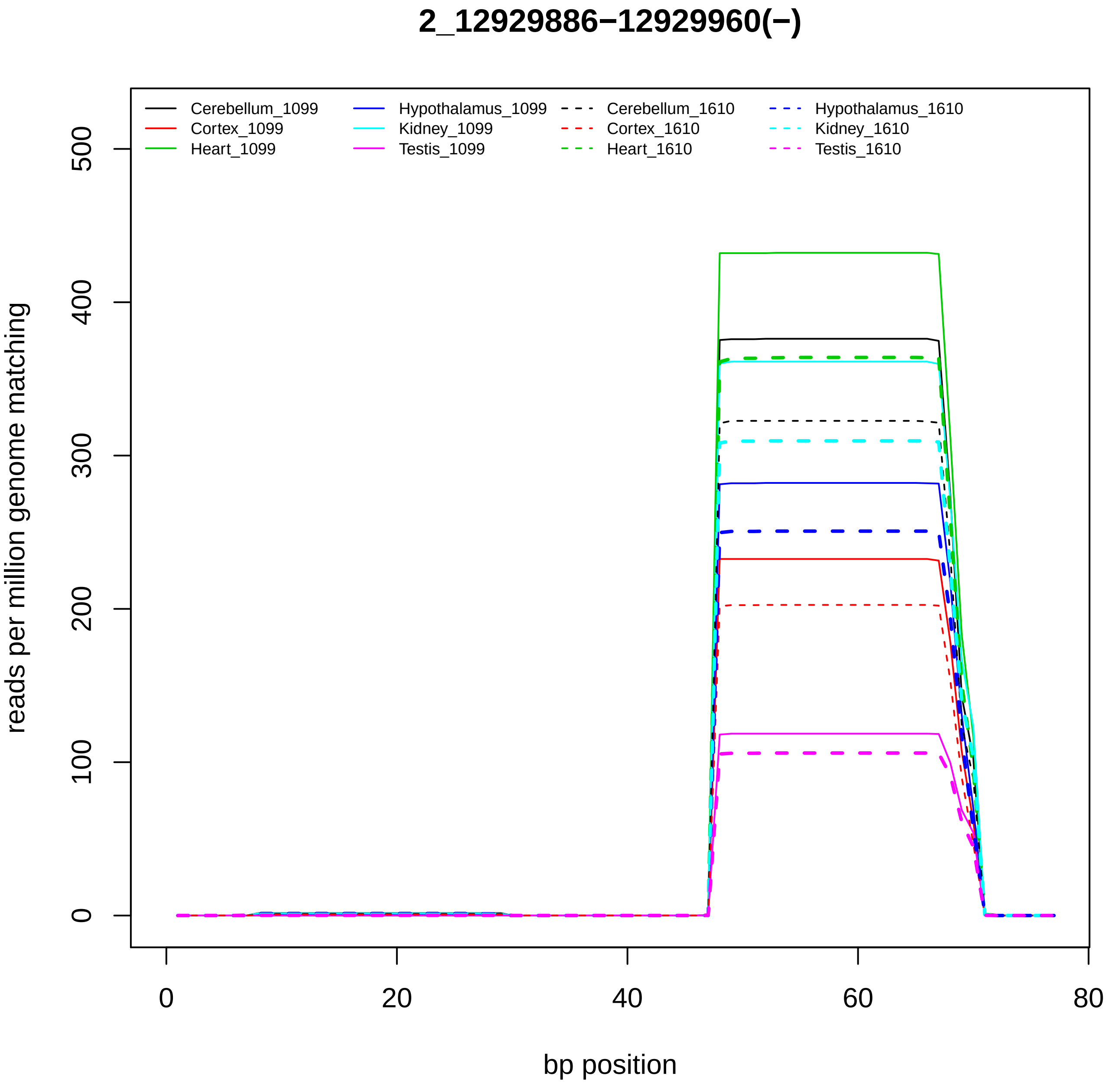

2_12929886−12929960(−)
Cerebellum_1099
Cortex_1099
Heart_1099
Hypothalamus_1099
Kidney_1099
Testis_1099
Cerebellum_1610
Cortex_1610
Heart_1610
Hypothalamus_1610
Kidney_1610
Testis_1610
reads per million genome matching
bp position
0
100
200
300
400
500
0
20
40
60
80

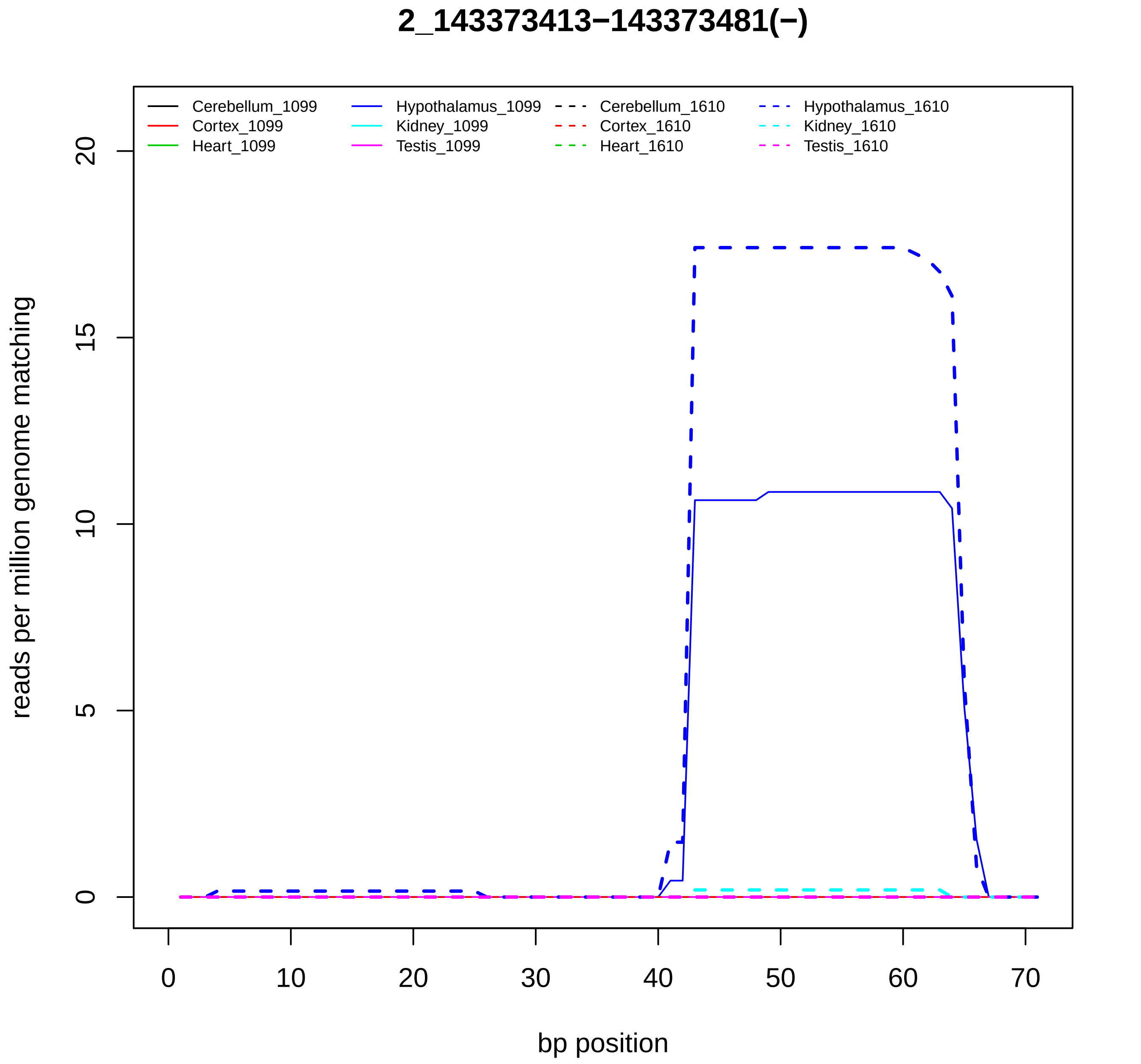

2_143373413−143373481(−)
Cerebellum_1099
Cortex_1099
Heart_1099
Hypothalamus_1099
Kidney_1099
Testis_1099
Cerebellum_1610
Cortex_1610
Heart_1610
Hypothalamus_1610
Kidney_1610
Testis_1610
reads per million genome matching
0
5
10
15
20
0
10
20
30
40
50
60
70
bp position

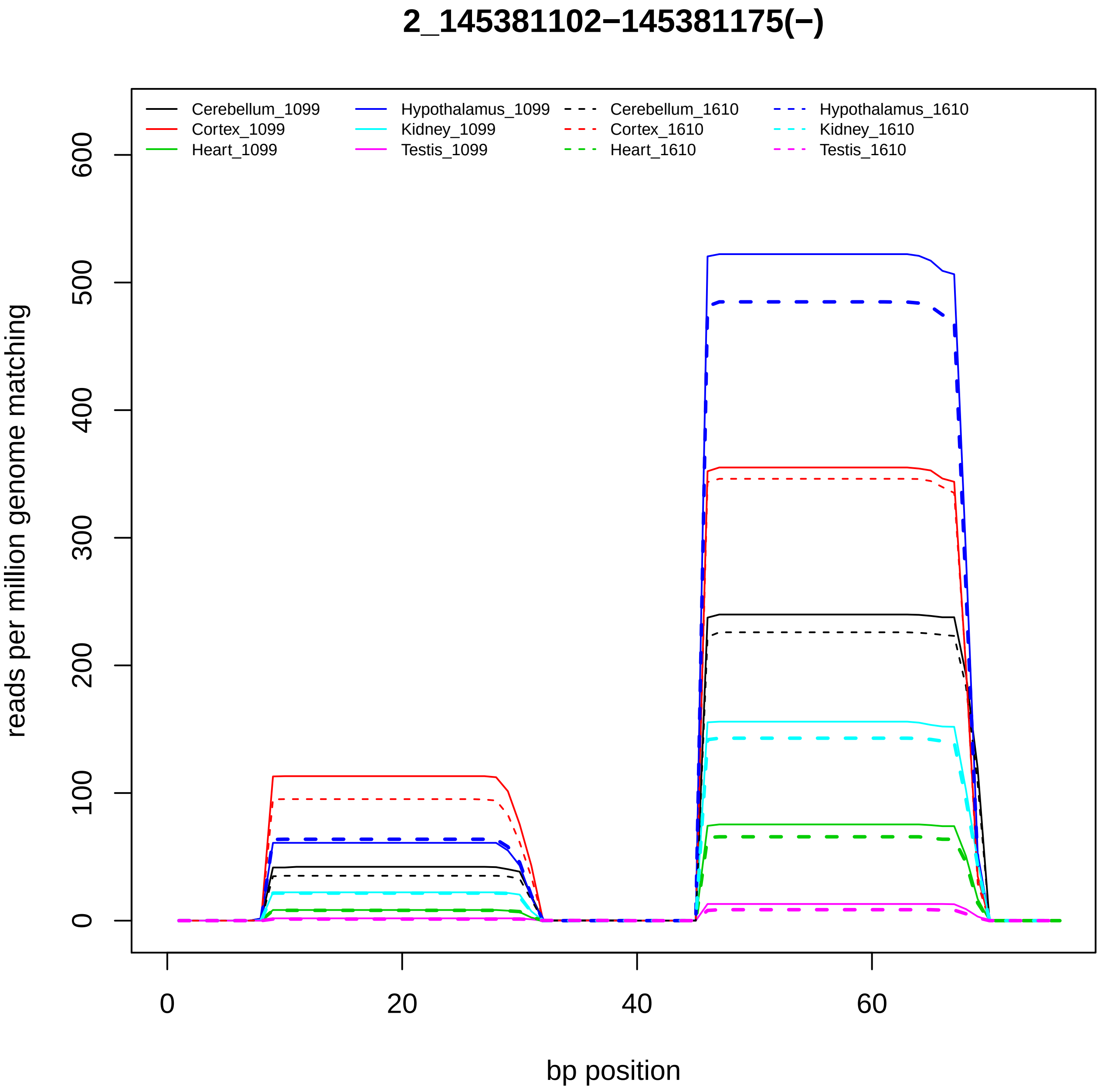
2_145381102−145381175(−)
Cerebellum_1099
Cortex_1099
Heart_1099
Hypothalamus_1099
Kidney_1099
Testis_1099
Cerebellum_1610
Cortex_1610
Heart_1610
Hypothalamus_1610
Kidney_1610
Testis_1610
reads per million genome matching
0
100
200
300
400
500
600
0
20
40
60
bp position

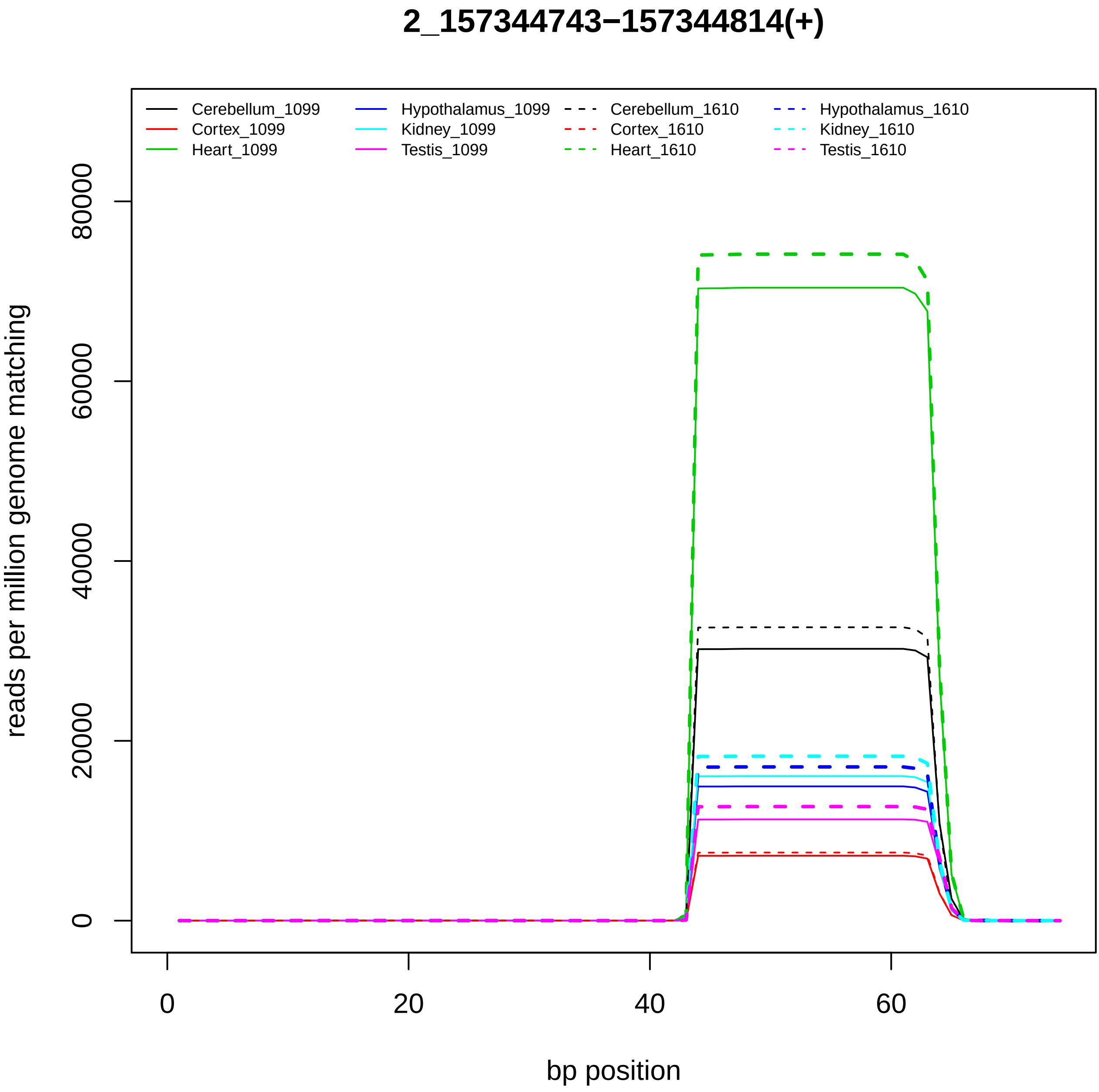
2_157344743−157344814(+)
Cerebellum_1099
Cortex_1099
Heart_1099
Hypothalamus_1099
Kidney_1099
Testis_1099
Cerebellum_1610
Cortex_1610
Heart_1610
Hypothalamus_1610
Kidney_1610
Testis_1610
reads per million genome matching
0
20000
40000
60000
80000
0
20
40
60
bp position

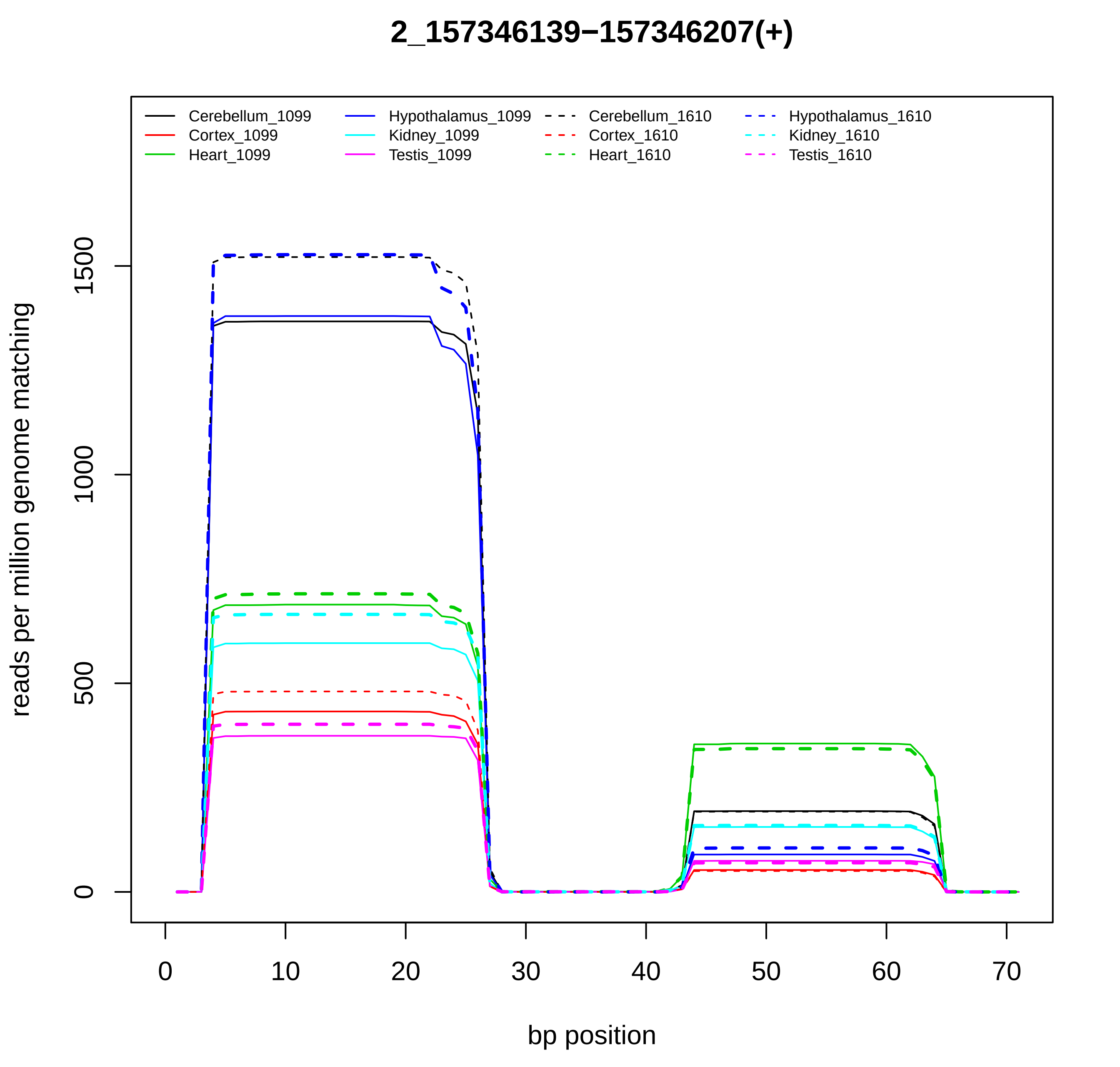

2_157346139−157346207(+)
Cerebellum_1099
Cortex_1099
Heart_1099
Hypothalamus_1099
Kidney_1099
Testis_1099
Cerebellum_1610
Cortex_1610
Heart_1610
Hypothalamus_1610
Kidney_1610
Testis_1610
reads per million genome matching
0
500
1000
1500
bp position
0
10
20
30
40
50
60
70

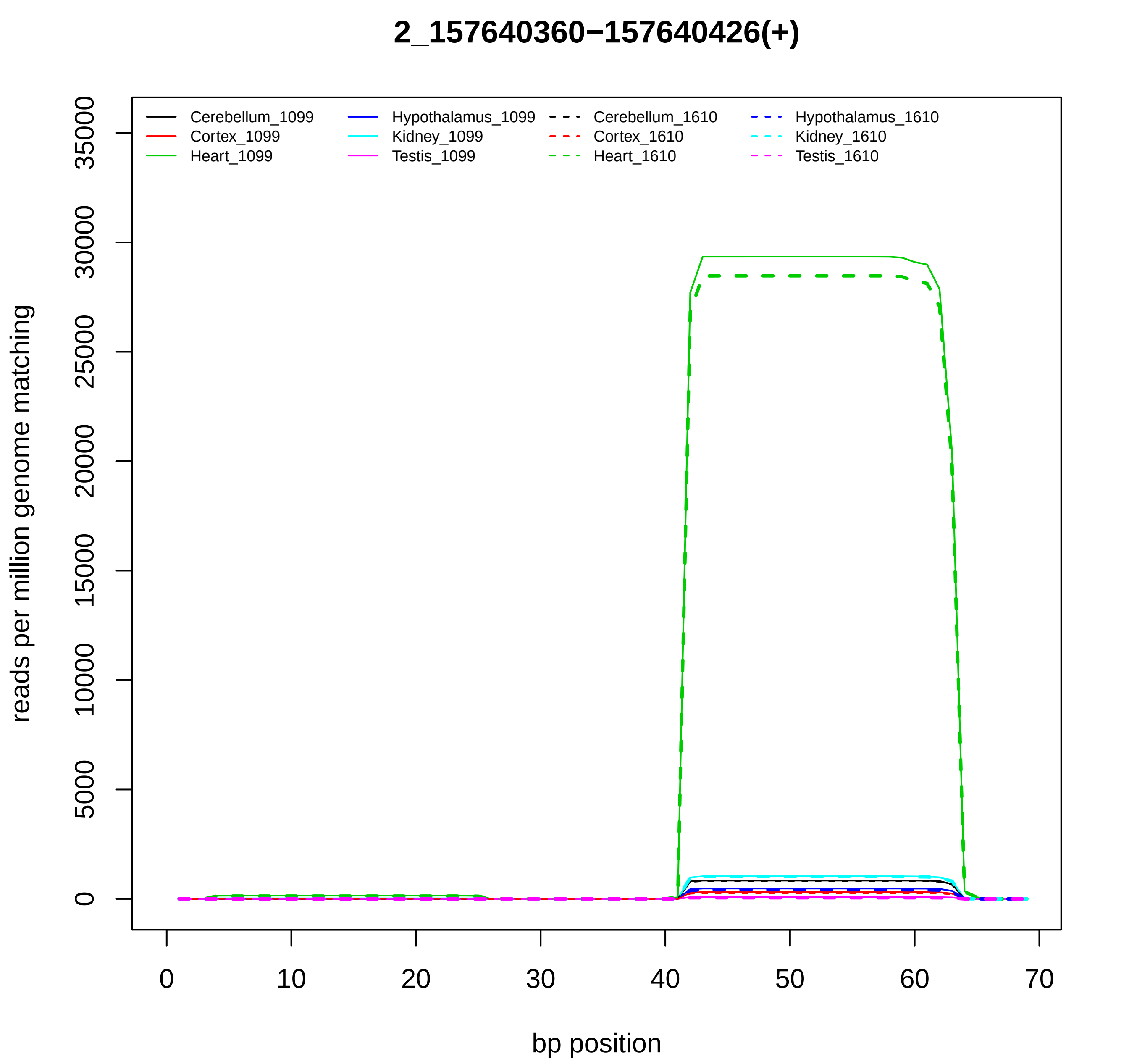

2_157640360−157640426(+)
Cerebellum_1099
Cortex_1099
Heart_1099
Hypothalamus_1099
Kidney_1099
Testis_1099
Cerebellum_1610
Cortex_1610
Heart_1610
Hypothalamus_1610
Kidney_1610
Testis_1610
reads per million genome matching
0
5000
10000
15000
20000
25000
30000
35000
0
10
20
30
40
50
60
70
bp position

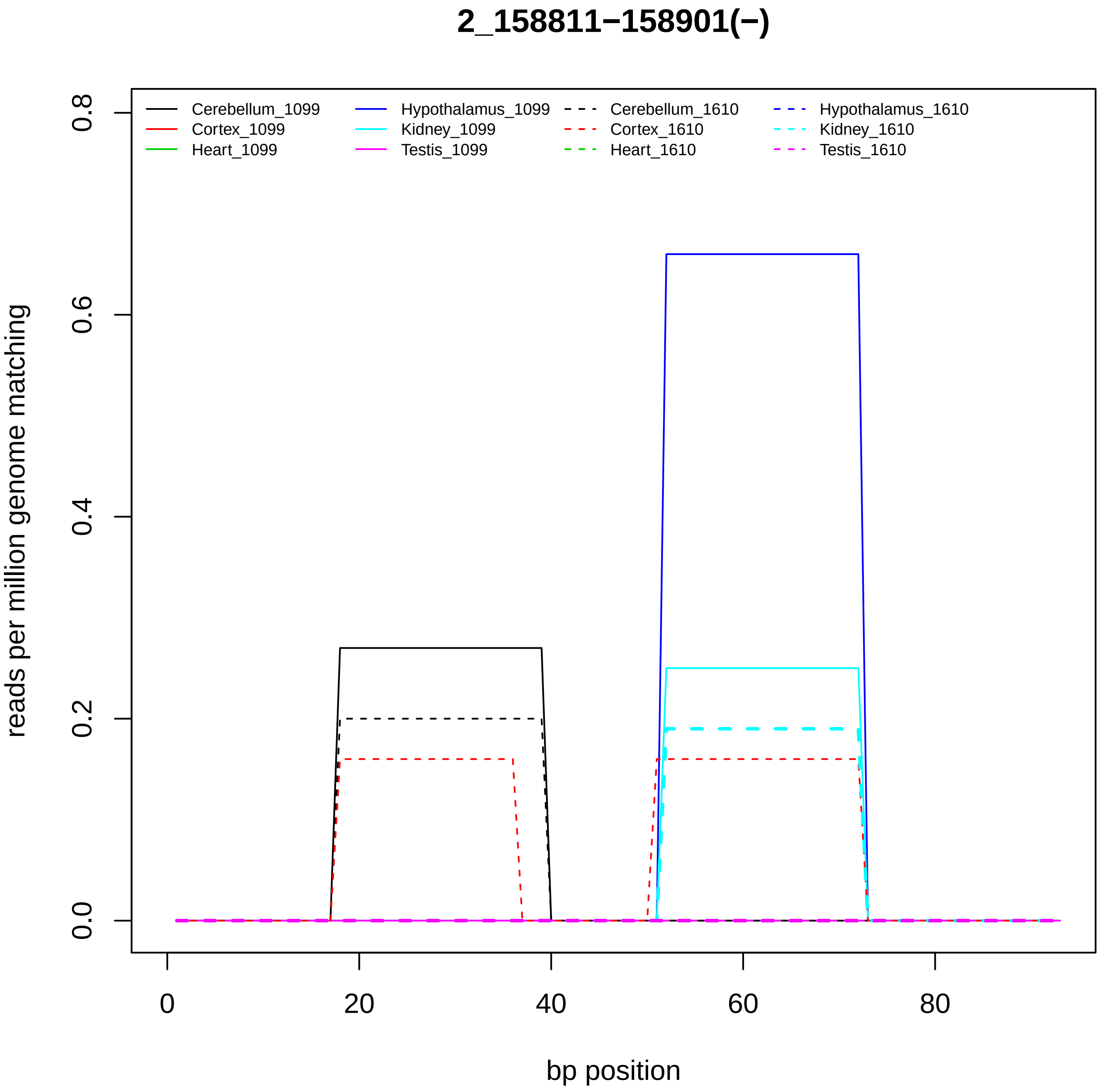

2_158811−158901(−)
Cerebellum_1099
Cortex_1099
Heart_1099
Hypothalamus_1099
Kidney_1099
Testis_1099
Cerebellum_1610
Cortex_1610
Heart_1610
Hypothalamus_1610
Kidney_1610
Testis_1610
reads per million genome matching
0.0
0.2
0.4
0.6
0.8
0
20
40
60
80
bp position

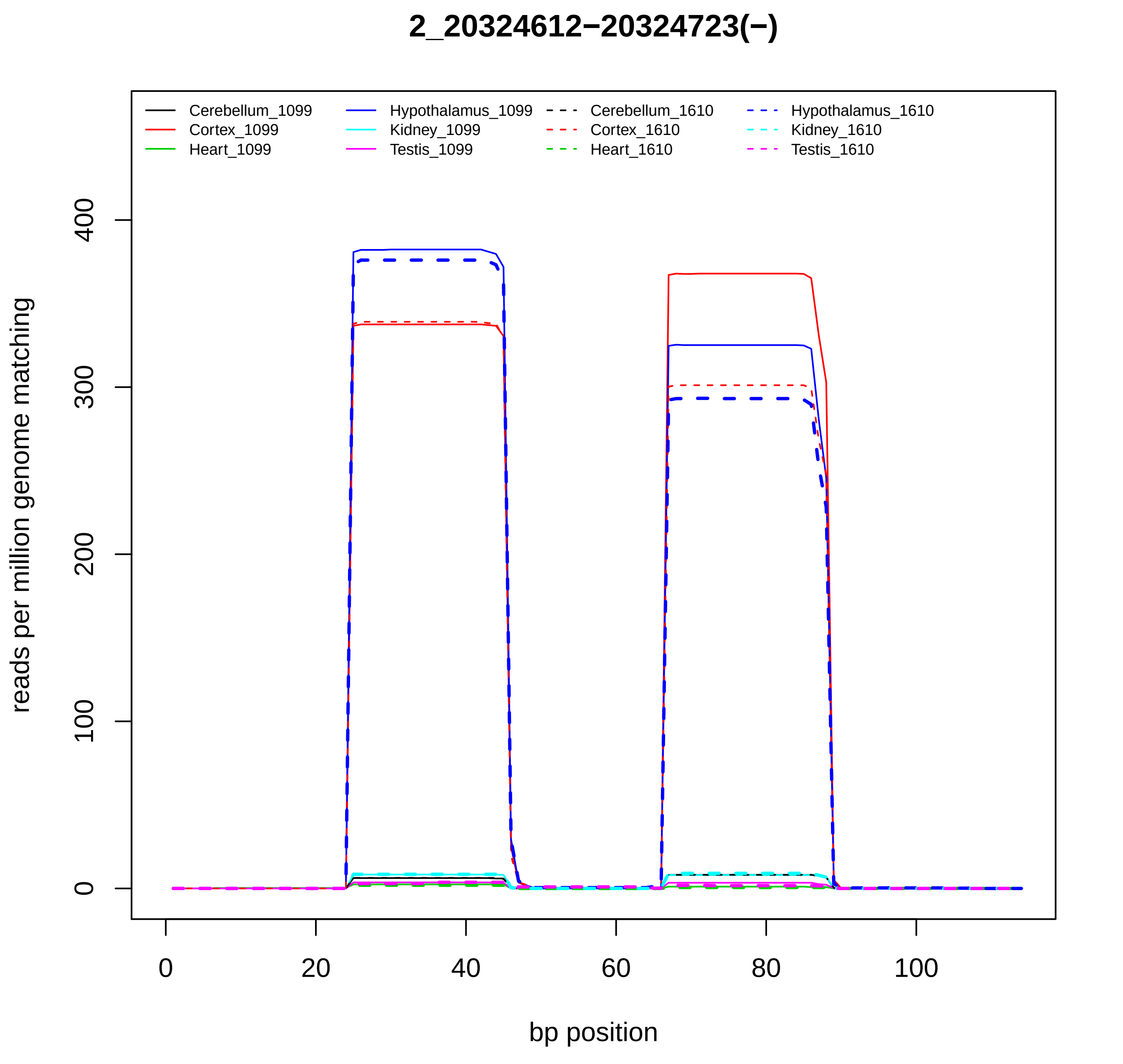

2_20324612−20324723(−)
Cerebellum_1099
Cortex_1099
Heart_1099
Hypothalamus_1099
Kidney_1099
Testis_1099
Cerebellum_1610
Cortex_1610
Heart_1610
Hypothalamus_1610
Kidney_1610
Testis_1610
reads per million genome matching
0
100
200
300
400
0
20
40
60
80
100
bp position

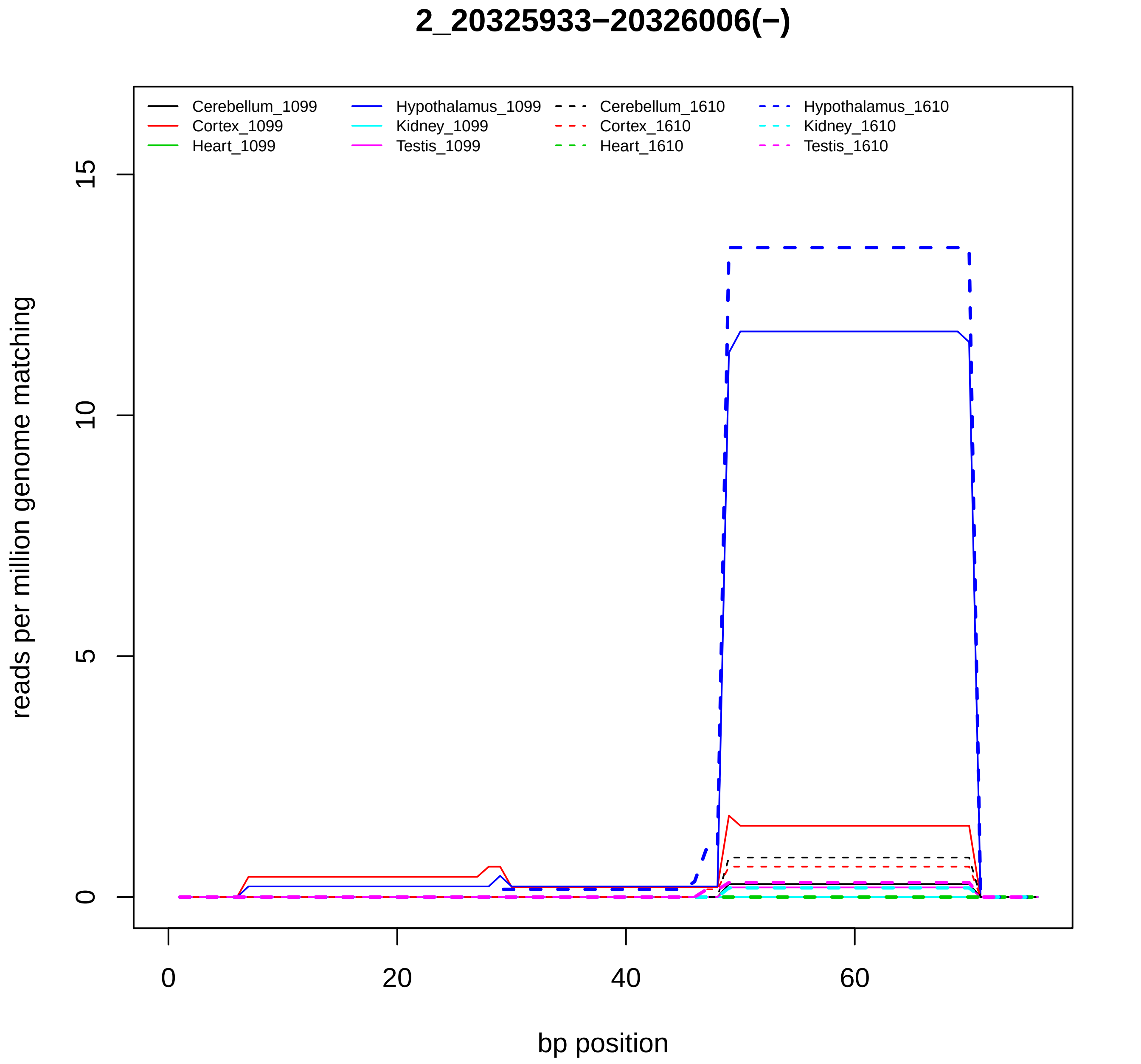

2_20325933−20326006(−)
Cerebellum_1099
Cortex_1099
Heart_1099
Hypothalamus_1099
Kidney_1099
Testis_1099
Cerebellum_1610
Cortex_1610
Heart_1610
Hypothalamus_1610
Kidney_1610
Testis_1610
reads per million genome matching
0
5
10
15
0
20
40
60
bp position

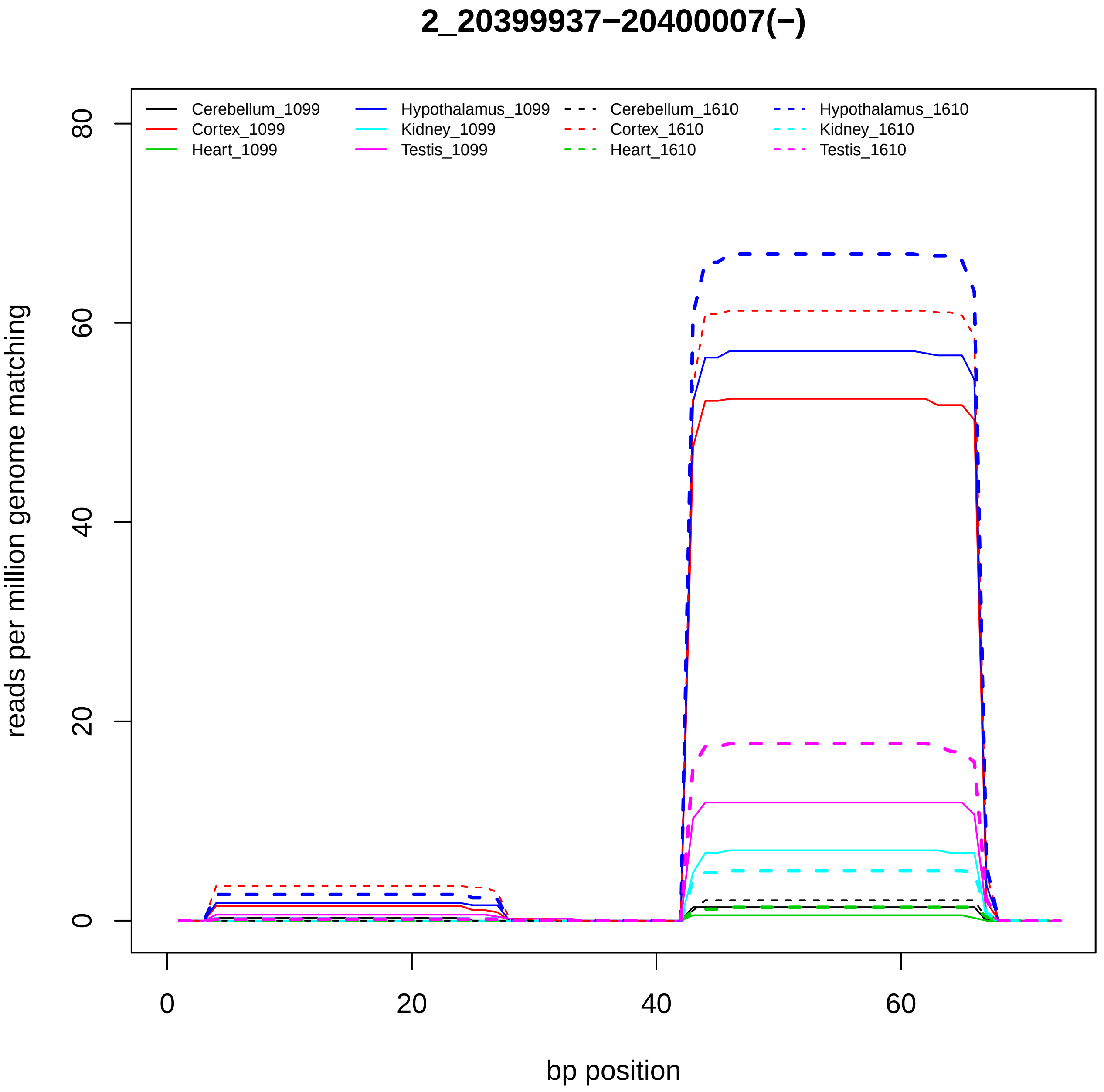

2_20399937−20400007(−)
Cerebellum_1099
Cortex_1099
Heart_1099
Hypothalamus_1099
Kidney_1099
Testis_1099
Cerebellum_1610
Cortex_1610
Heart_1610
Hypothalamus_1610
Kidney_1610
Testis_1610
reads per million genome matching
0
20
40
60
80
bp position
0
20
40
60

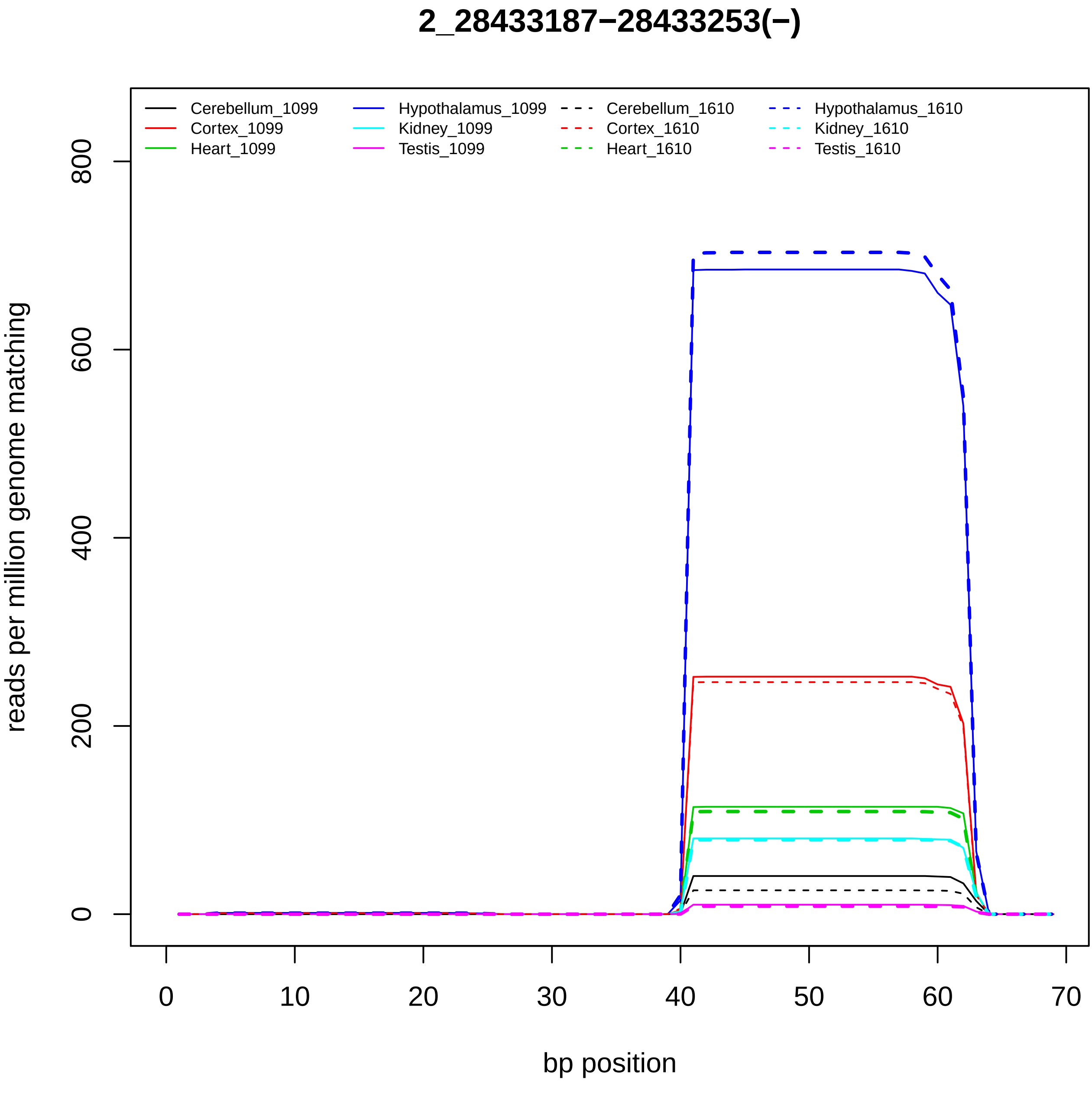

2_28433187−28433253(−)
Cerebellum_1099
Cortex_1099
Heart_1099
Hypothalamus_1099
Kidney_1099
Testis_1099
Cerebellum_1610
Cortex_1610
Heart_1610
Hypothalamus_1610
Kidney_1610
Testis_1610
reads per million genome matching
0
200
400
600
800
0
10
20
30
40
50
60
70
bp position

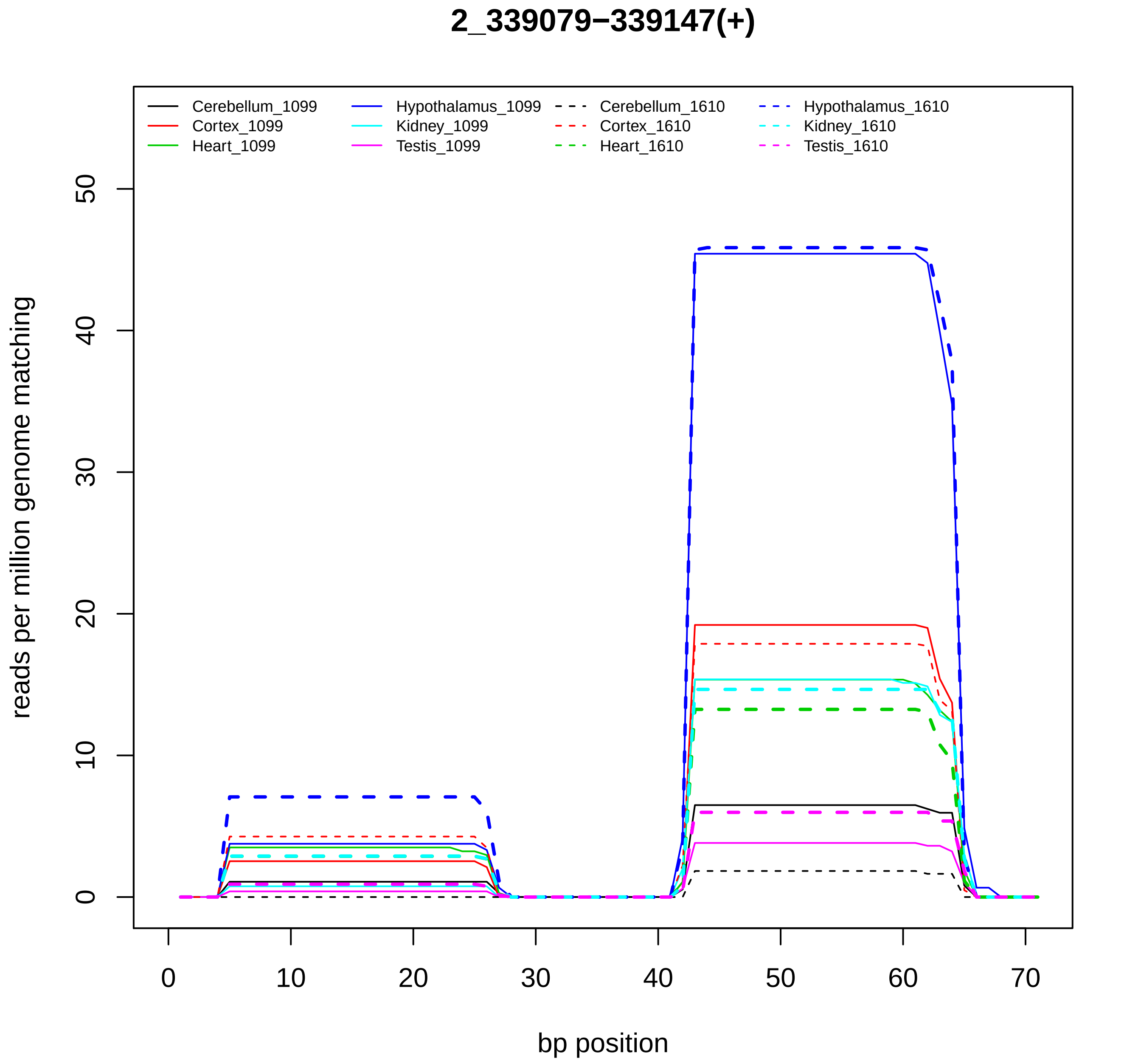

2_339079−339147(+)
Cerebellum_1099
Cortex_1099
Heart_1099
Hypothalamus_1099
Kidney_1099
Testis_1099
Cerebellum_1610
Cortex_1610
Heart_1610
Hypothalamus_1610
Kidney_1610
Testis_1610
reads per million genome matching
bp position
0
10
20
30
40
50
60
70

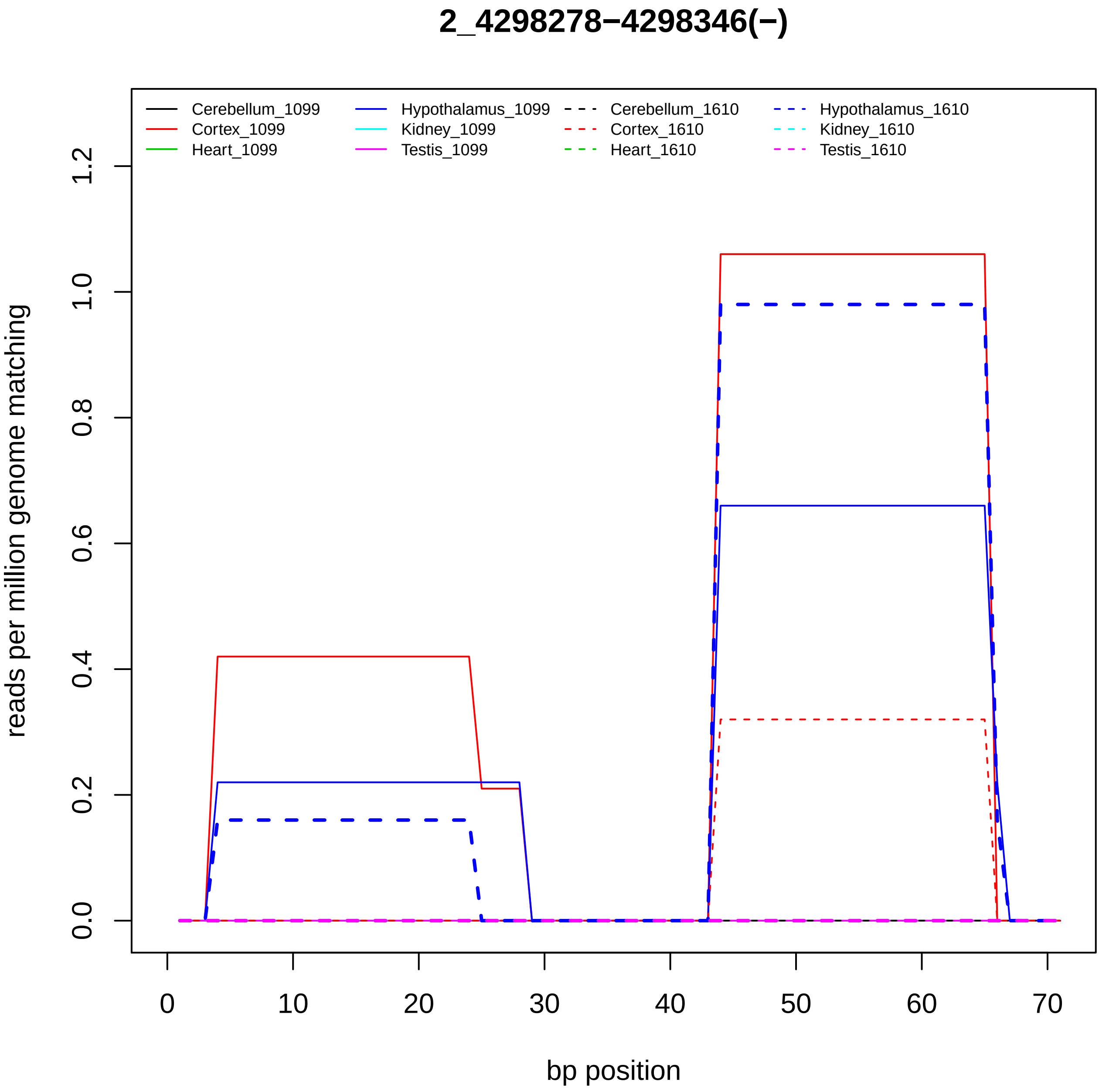

2_4298278−4298346(−)
Cerebellum_1099
Cortex_1099
Heart_1099
Hypothalamus_1099
Kidney_1099
Testis_1099
Cerebellum_1610
Cortex_1610
Heart_1610
Hypothalamus_1610
Kidney_1610
Testis_1610
reads per million genome matching
0.0
0.2
0.4
0.6
0.8
1.0
1.2
0
10
20
30
40
50
60
70
bp position

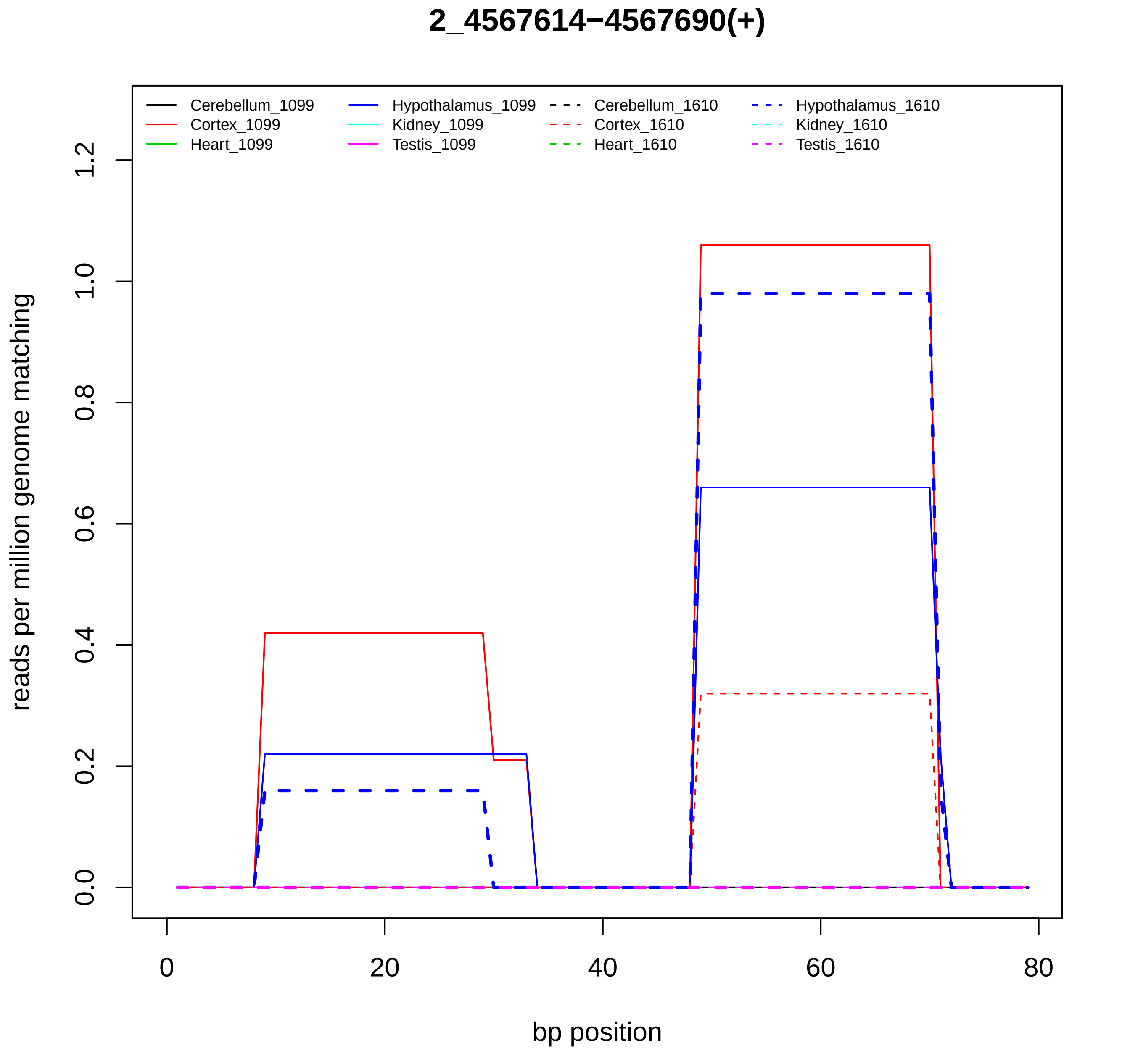

2_4567614−4567690(+)
Cerebellum_1099
Cortex_1099
Heart_1099
Hypothalamus_1099
Kidney_1099
Testis_1099
Cerebellum_1610
Cortex_1610
Heart_1610
Hypothalamus_1610
Kidney_1610
Testis_1610
reads per million genome matching
bp position
0.0
0.2
0.4
0.6
0.8
1.0
1.2
0
20
40
60
80

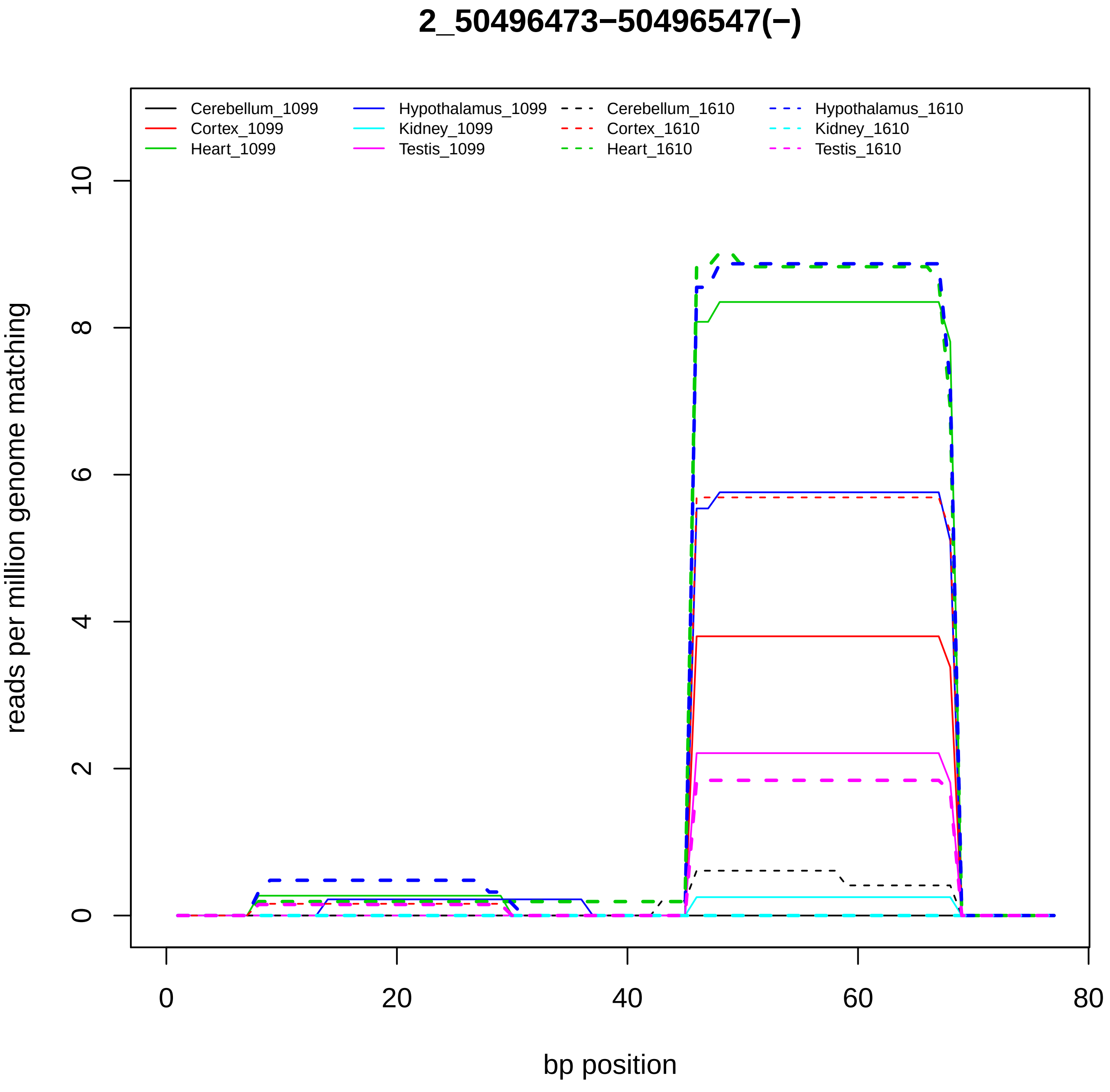

2_50496473−50496547(−)
Cerebellum_1099
Cortex_1099
Heart_1099
Hypothalamus_1099
Kidney_1099
Testis_1099
Cerebellum_1610
Cortex_1610
Heart_1610
Hypothalamus_1610
Kidney_1610
Testis_1610
reads per million genome matching
bp position
0
2
4
6
8
10
0
20
40
60
80

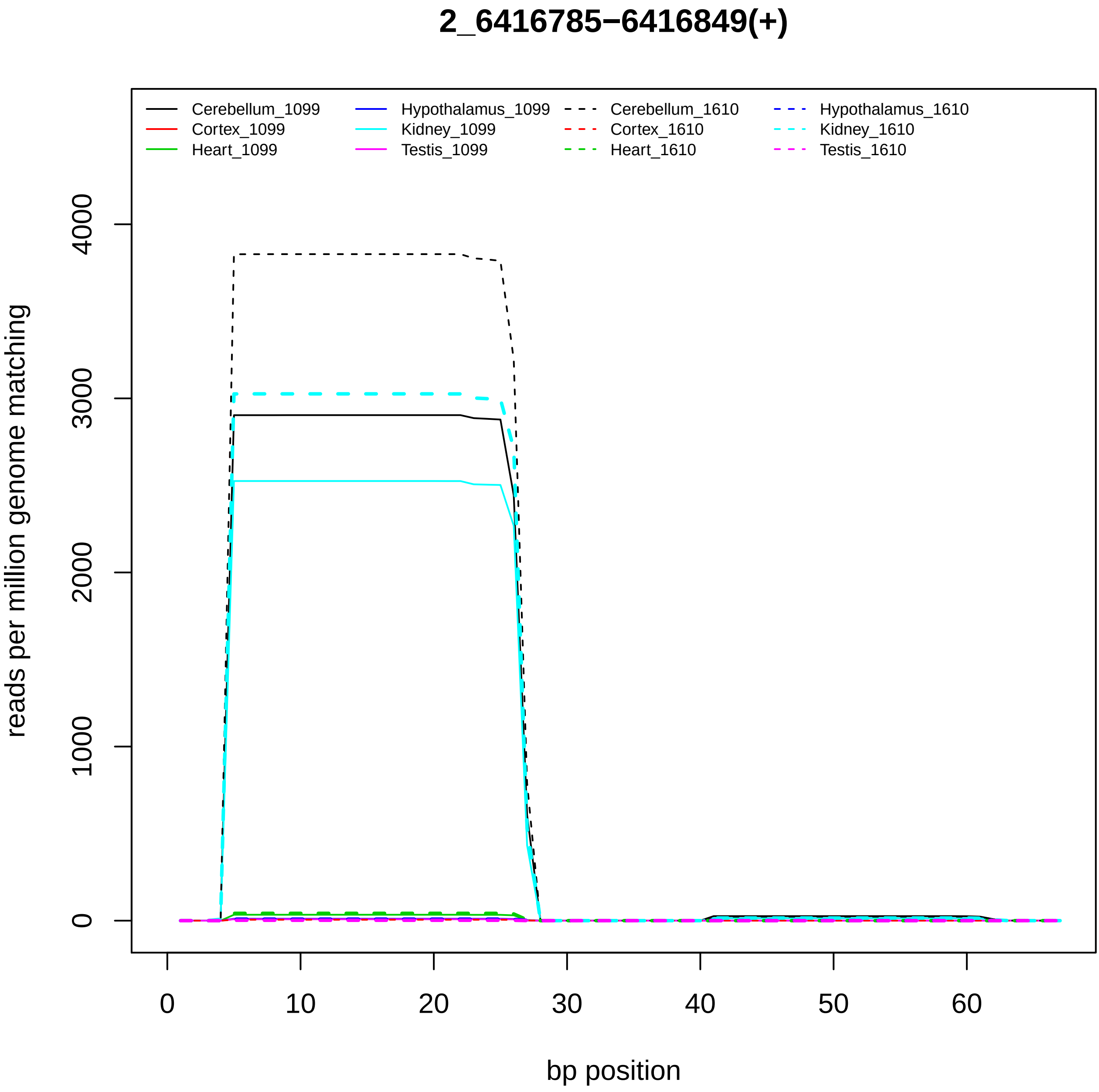
2_6416785−6416849(+)
Cerebellum_1099
Cortex_1099
Heart_1099
Hypothalamus_1099
Kidney_1099
Testis_1099
Cerebellum_1610
Cortex_1610
Heart_1610
Hypothalamus_1610
Kidney_1610
Testis_1610
reads per million genome matching
0
1000
2000
3000
4000
0
10
20
30
40
50
60
bp position

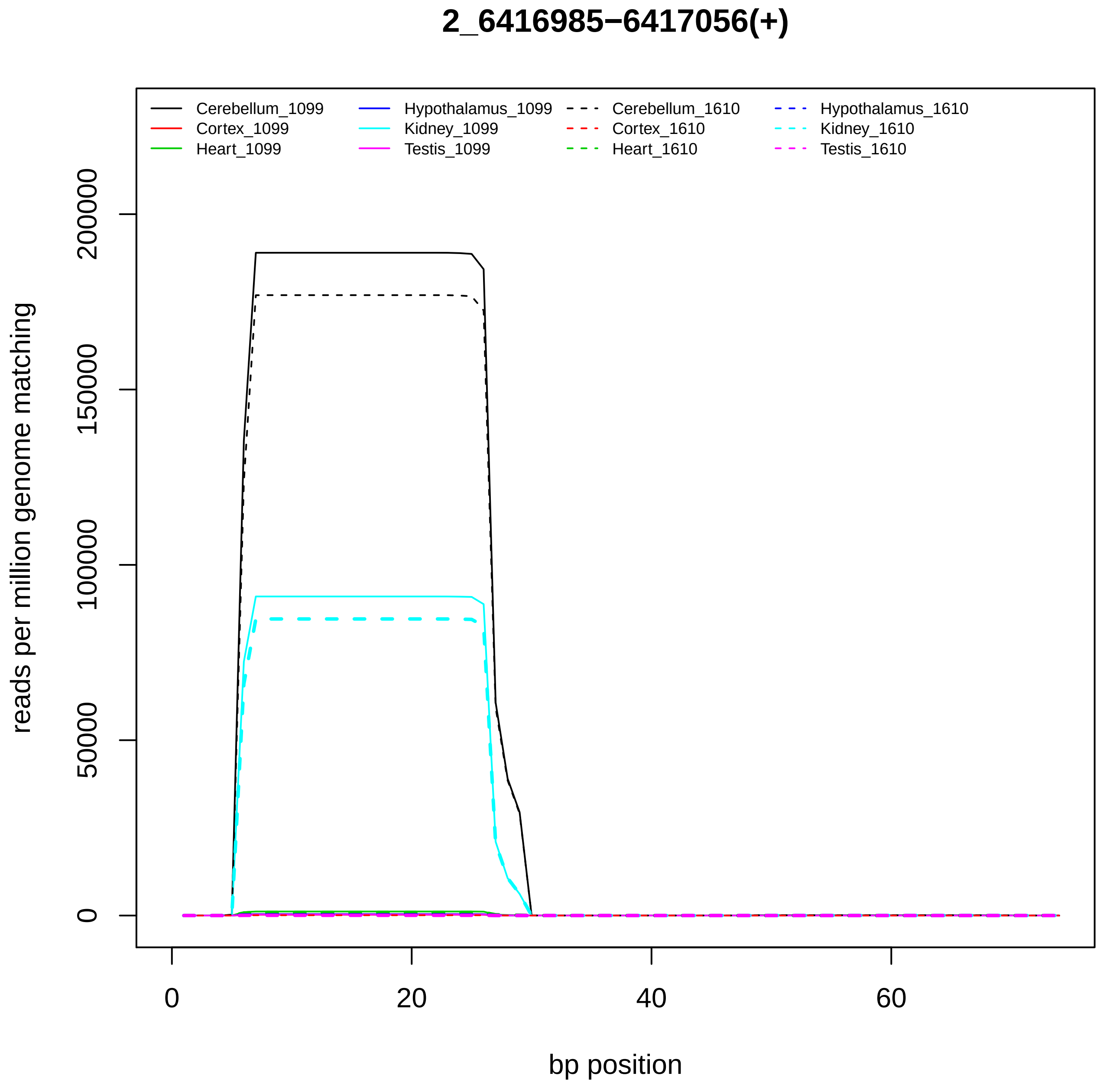

2_6416985−6417056(+)
Cerebellum_1099
Cortex_1099
Heart_1099
Hypothalamus_1099
Kidney_1099
Testis_1099
Cerebellum_1610
Cortex_1610
Heart_1610
Hypothalamus_1610
Kidney_1610
Testis_1610
reads per million genome matching
0
50000
100000
150000
200000
0
20
40
60
bp position

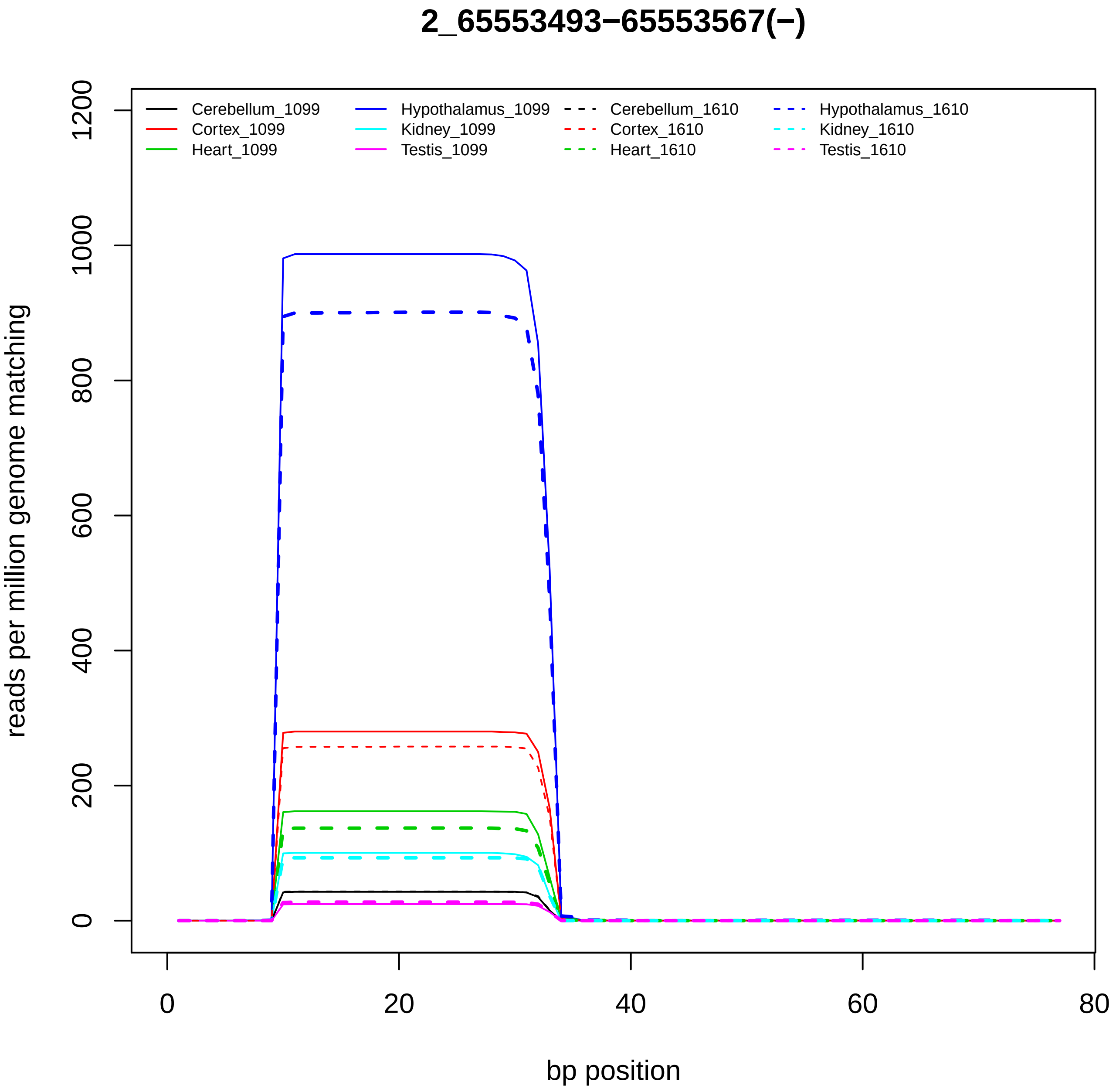
2_65553493−65553567(−)
Cerebellum_1099
Cortex_1099
Heart_1099
Hypothalamus_1099
Kidney_1099
Testis_1099
Cerebellum_1610
Cortex_1610
Heart_1610
Hypothalamus_1610
Kidney_1610
Testis_1610
reads per million genome matching
0
200
400
600
800
1000
1200
bp position
0
20
40
60
80

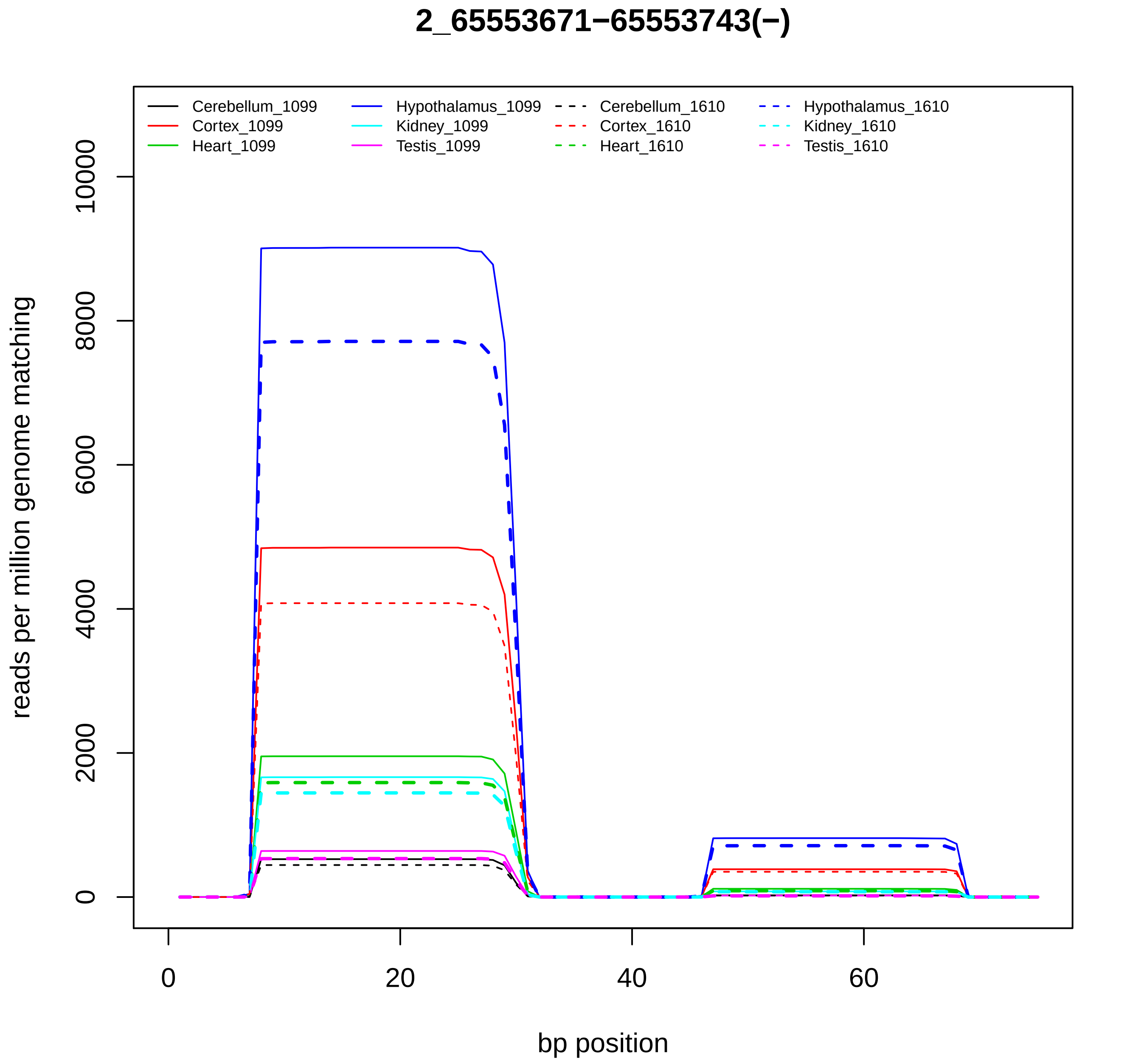

2_65553671−65553743(−)
Cerebellum_1099
Cortex_1099
Heart_1099
Hypothalamus_1099
Kidney_1099
Testis_1099
Cerebellum_1610
Cortex_1610
Heart_1610
Hypothalamus_1610
Kidney_1610
Testis_1610
reads per million genome matching
0
2000
4000
6000
8000
10000
0
20
40
60
bp position

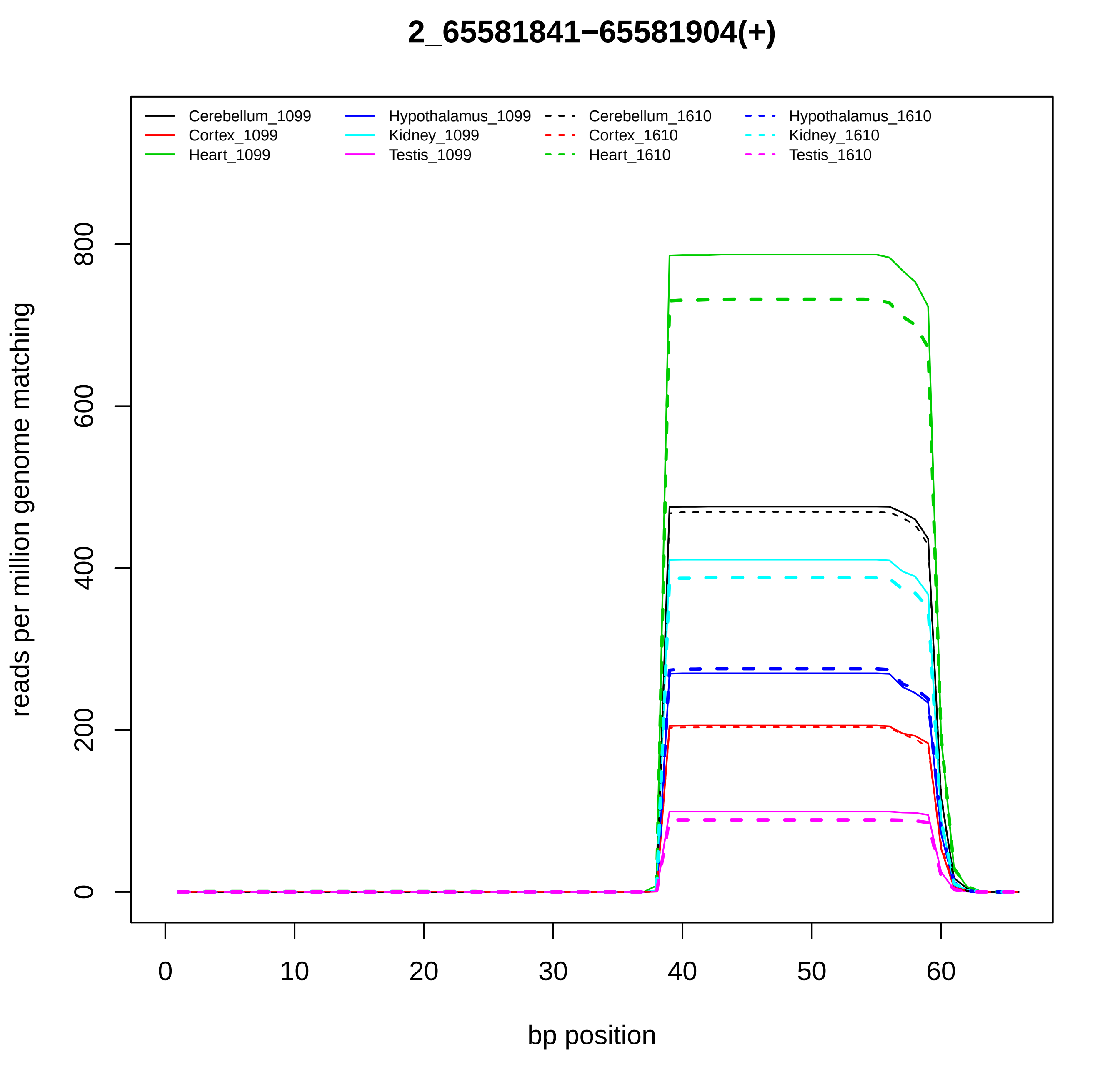
2_65581841−65581904(+)
Cerebellum_1099
Cortex_1099
Heart_1099
Hypothalamus_1099
Kidney_1099
Testis_1099
Cerebellum_1610
Cortex_1610
Heart_1610
Hypothalamus_1610
Kidney_1610
Testis_1610
reads per million genome matching
0
200
400
600
800
0
10
20
30
40
50
60
bp position

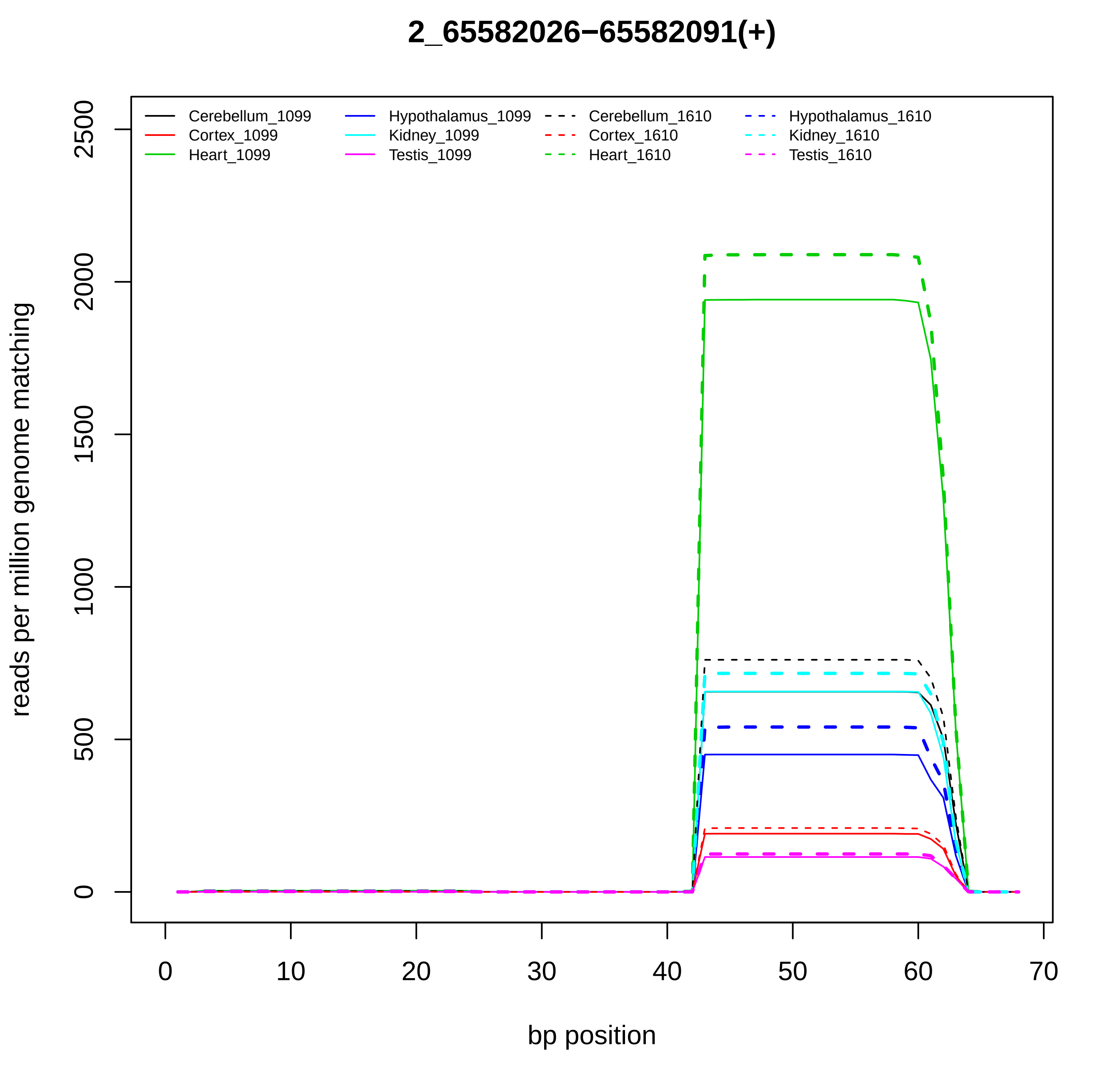
2_65582026−65582091(+)
Cerebellum_1099
Cortex_1099
Heart_1099
Hypothalamus_1099
Kidney_1099
Testis_1099
Cerebellum_1610
Cortex_1610
Heart_1610
Hypothalamus_1610
Kidney_1610
Testis_1610
reads per million genome matching
0
500
1000
1500
2000
2500
0
10
20
30
40
50
60
70
bp position

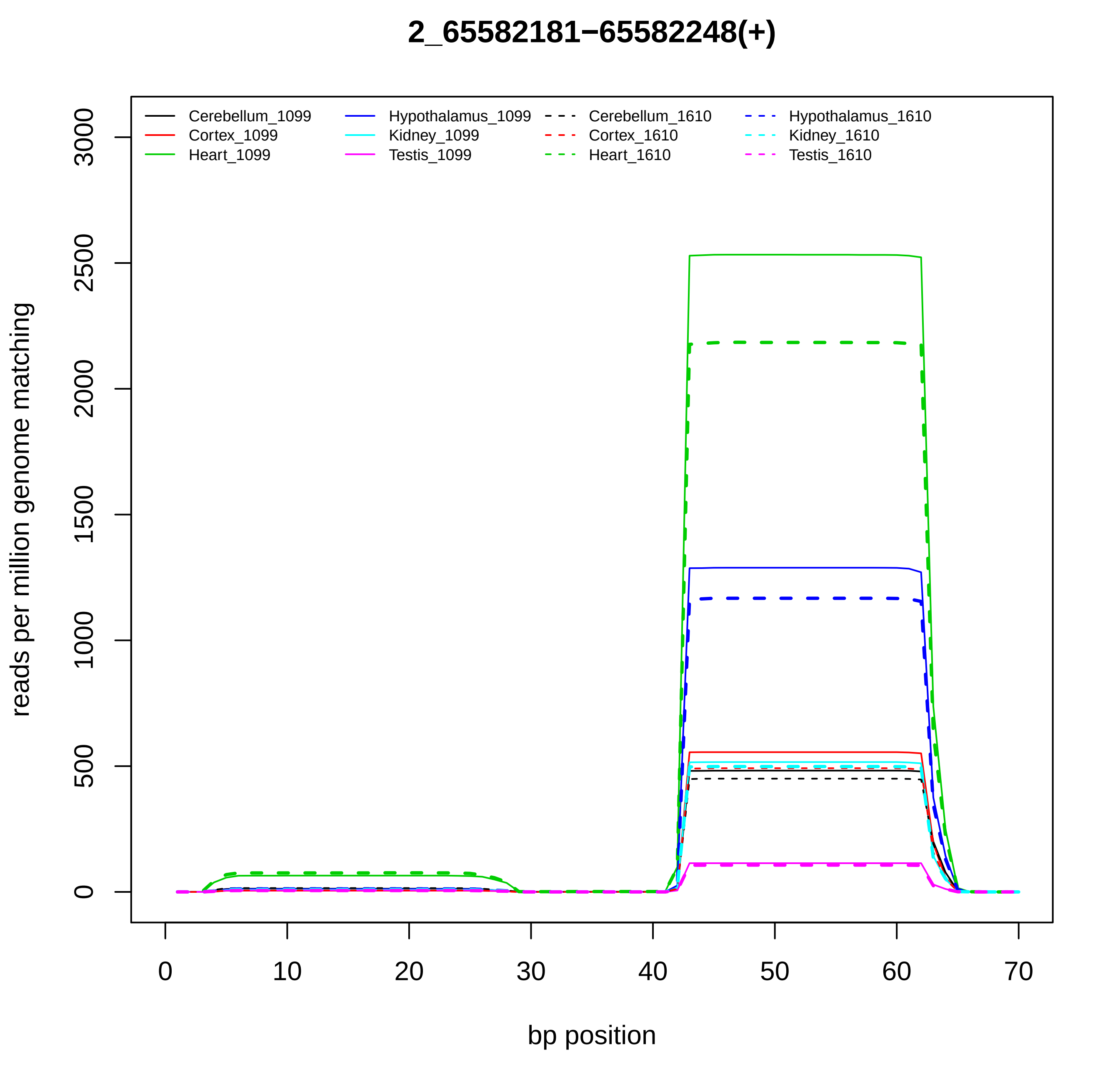

2_65582181−65582248(+)
Cerebellum_1099
Cortex_1099
Heart_1099
Hypothalamus_1099
Kidney_1099
Testis_1099
Cerebellum_1610
Cortex_1610
Heart_1610
Hypothalamus_1610
Kidney_1610
Testis_1610
reads per million genome matching
bp position
0
500
1000
1500
2000
2500
3000
0
10
20
30
40
50
60
70

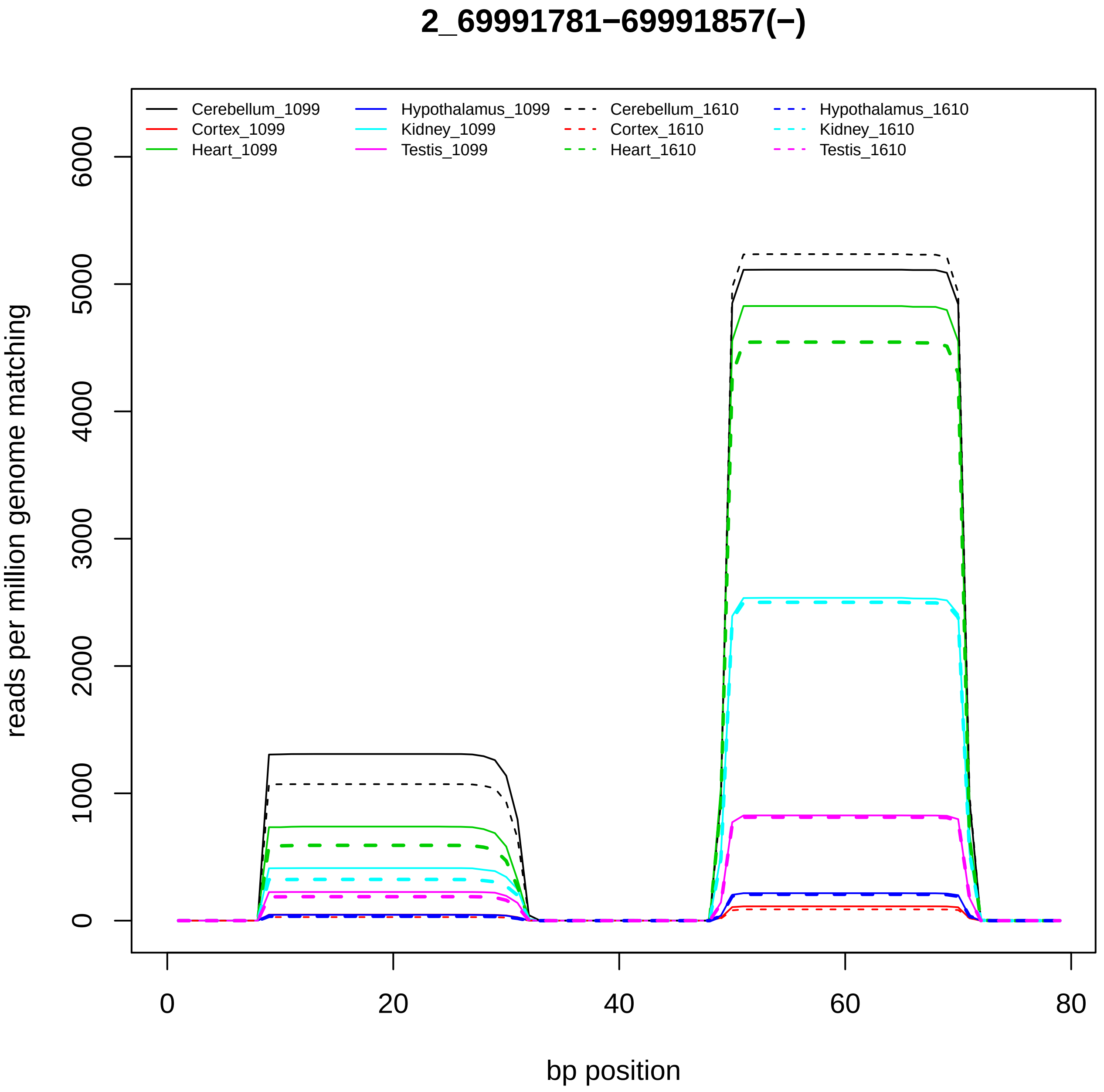

2_69991781−69991857(−)
Cerebellum_1099
Cortex_1099
Heart_1099
Hypothalamus_1099
Kidney_1099
Testis_1099
Cerebellum_1610
Cortex_1610
Heart_1610
Hypothalamus_1610
Kidney_1610
Testis_1610
reads per million genome matching
0
1000
2000
3000
4000
5000
6000
bp position
0
20
40
60
80

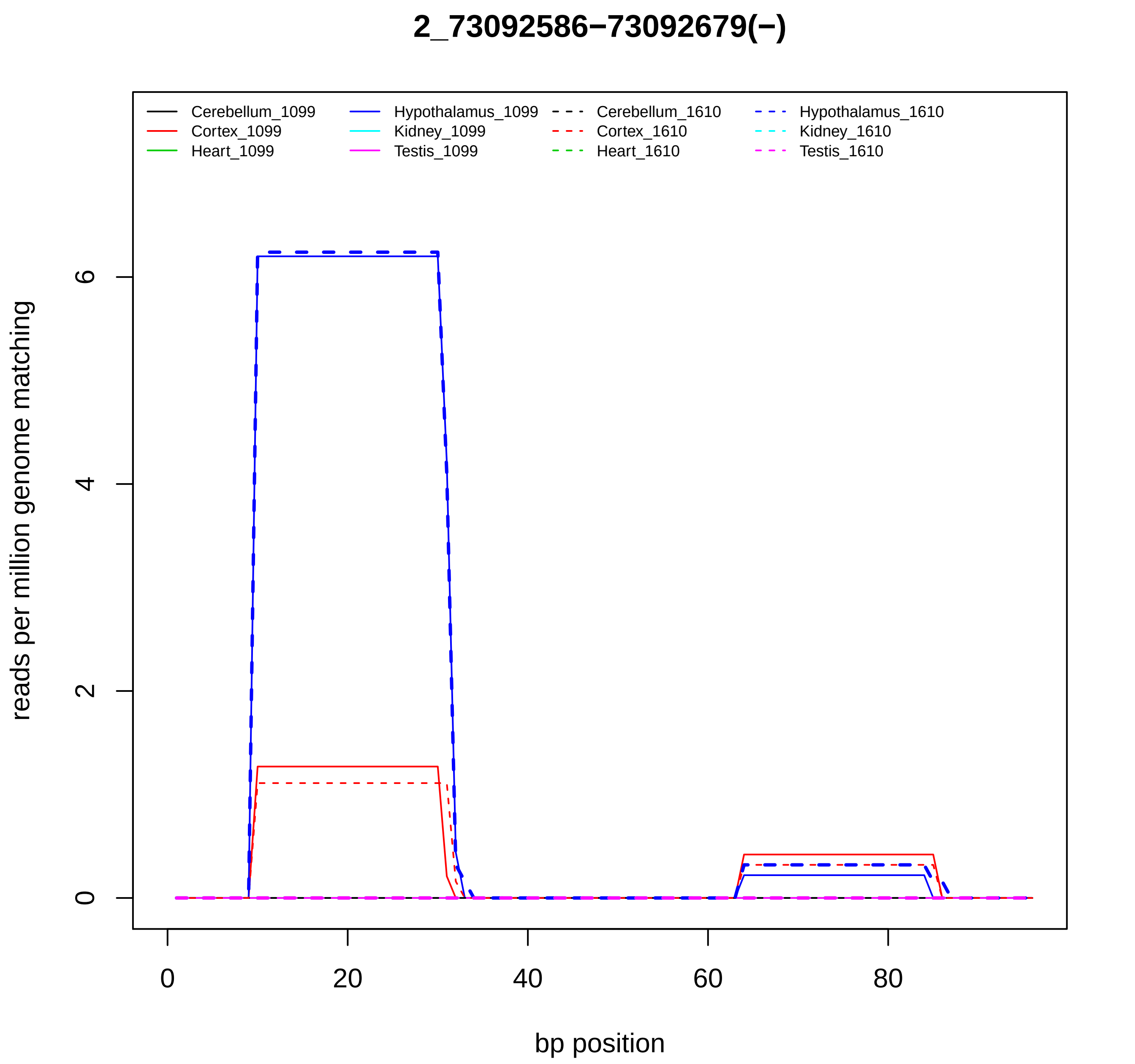
2_73092586−73092679(−)
Cerebellum_1099
Cortex_1099
Heart_1099
Hypothalamus_1099
Kidney_1099
Testis_1099
Cerebellum_1610
Cortex_1610
Heart_1610
Hypothalamus_1610
Kidney_1610
Testis_1610
reads per million genome matching
0
2
4
6
0
20
40
60
80
bp position

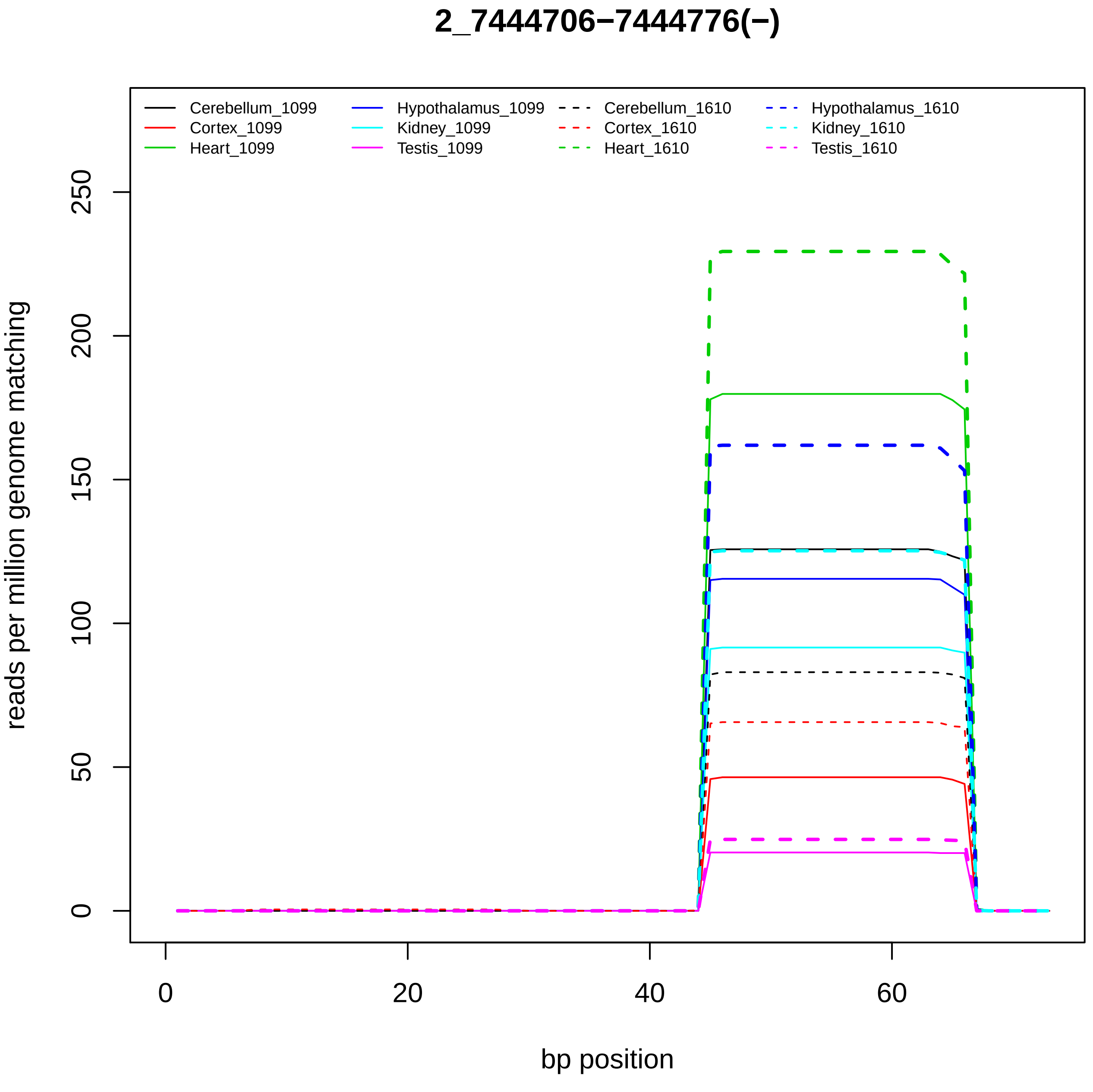

2_7444706−7444776(−)
Cerebellum_1099
Cortex_1099
Heart_1099
Hypothalamus_1099
Kidney_1099
Testis_1099
Cerebellum_1610
Cortex_1610
Heart_1610
Hypothalamus_1610
Kidney_1610
Testis_1610
reads per million genome matching
0
50
100
150
200
250
0
20
40
60
bp position

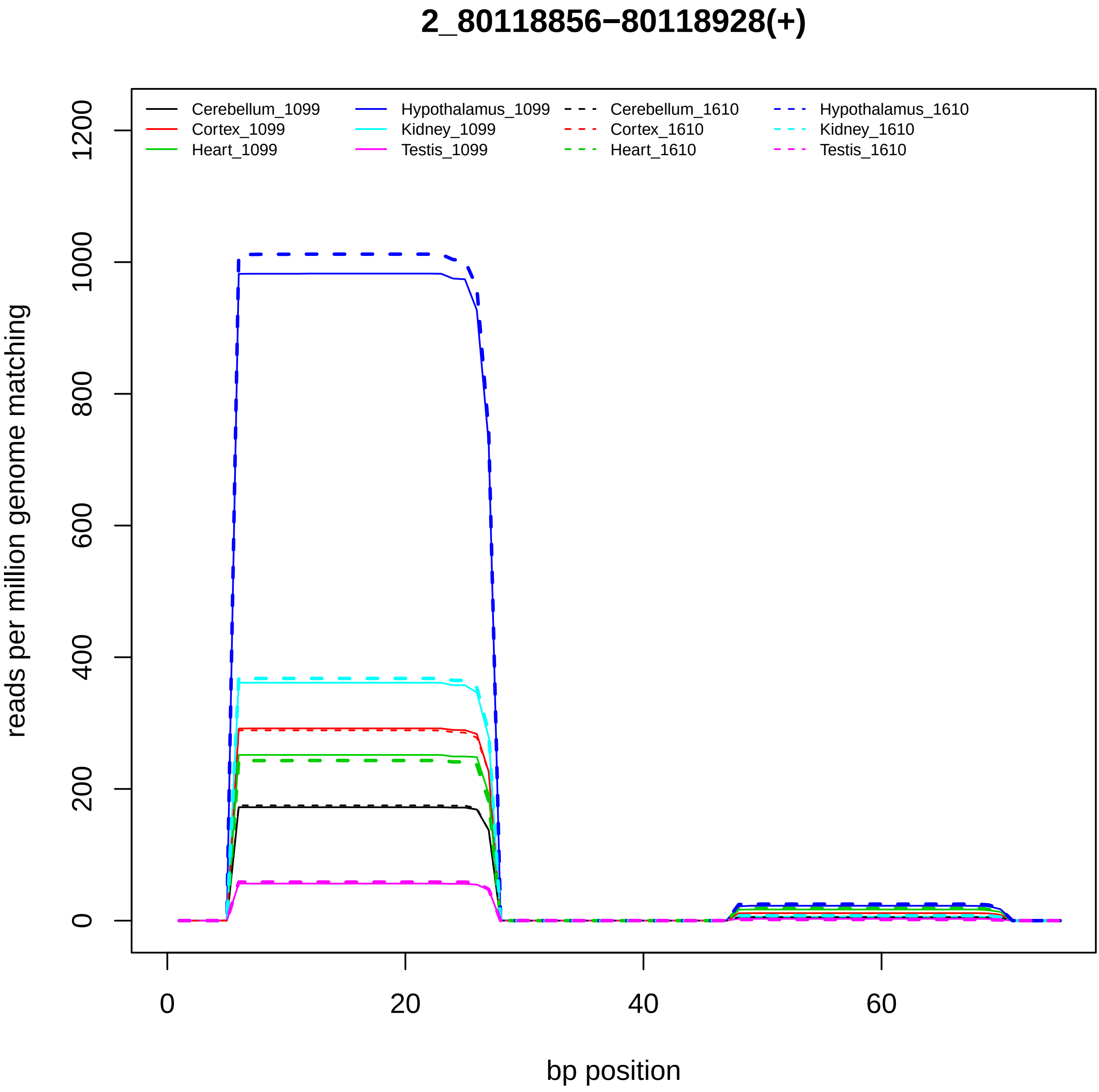

2_80118856−80118928(+)
Cerebellum_1099
Cortex_1099
Heart_1099
Hypothalamus_1099
Kidney_1099
Testis_1099
Cerebellum_1610
Cortex_1610
Heart_1610
Hypothalamus_1610
Kidney_1610
Testis_1610
reads per million genome matching
0
200
400
600
800
1000
1200
0
20
40
60
bp position

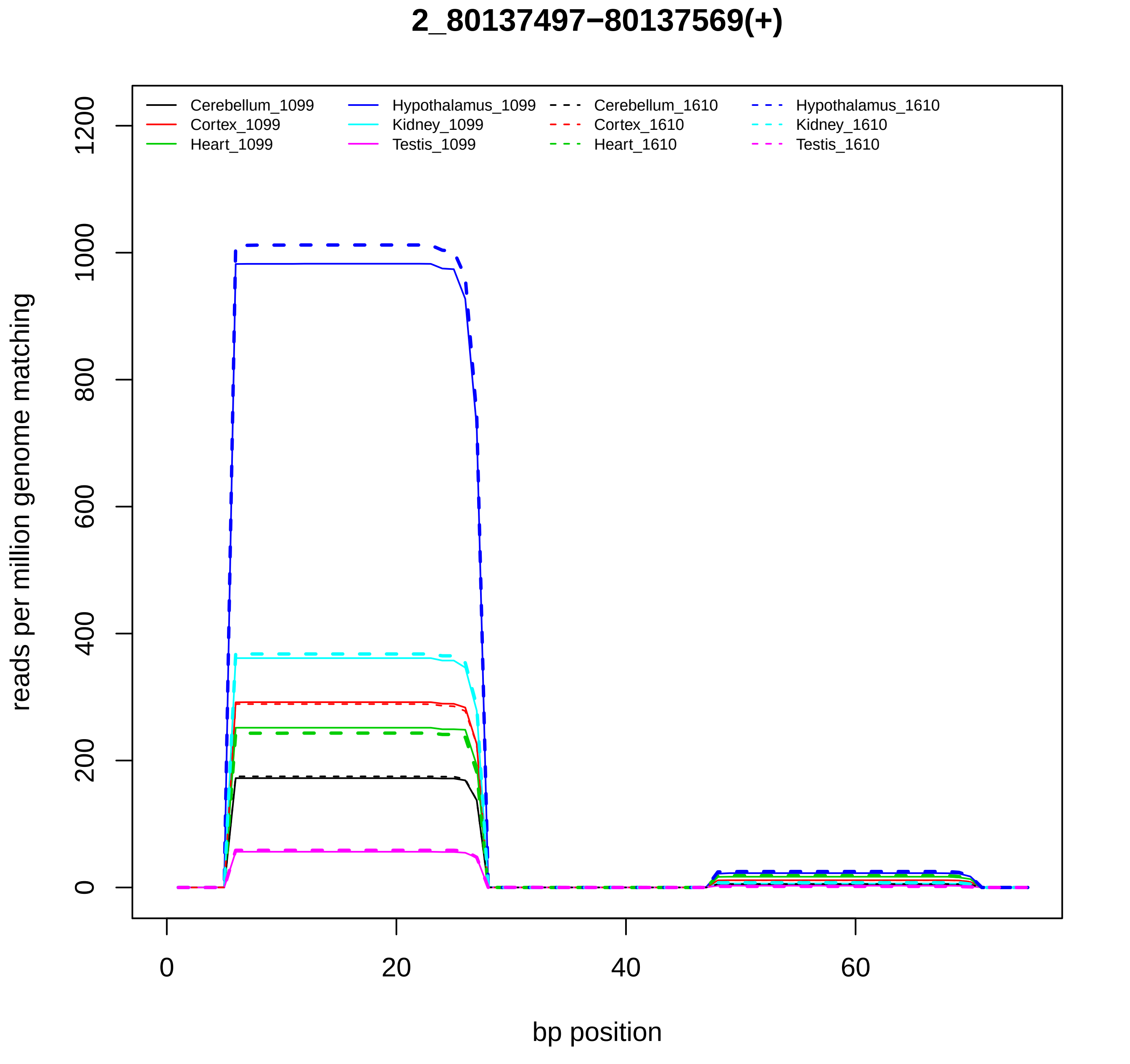

2_80137497−80137569(+)
Cerebellum_1099
Cortex_1099
Heart_1099
Hypothalamus_1099
Kidney_1099
Testis_1099
Cerebellum_1610
Cortex_1610
Heart_1610
Hypothalamus_1610
Kidney_1610
Testis_1610
reads per million genome matching
0
200
400
600
800
1000
1200
0
20
40
60
bp position

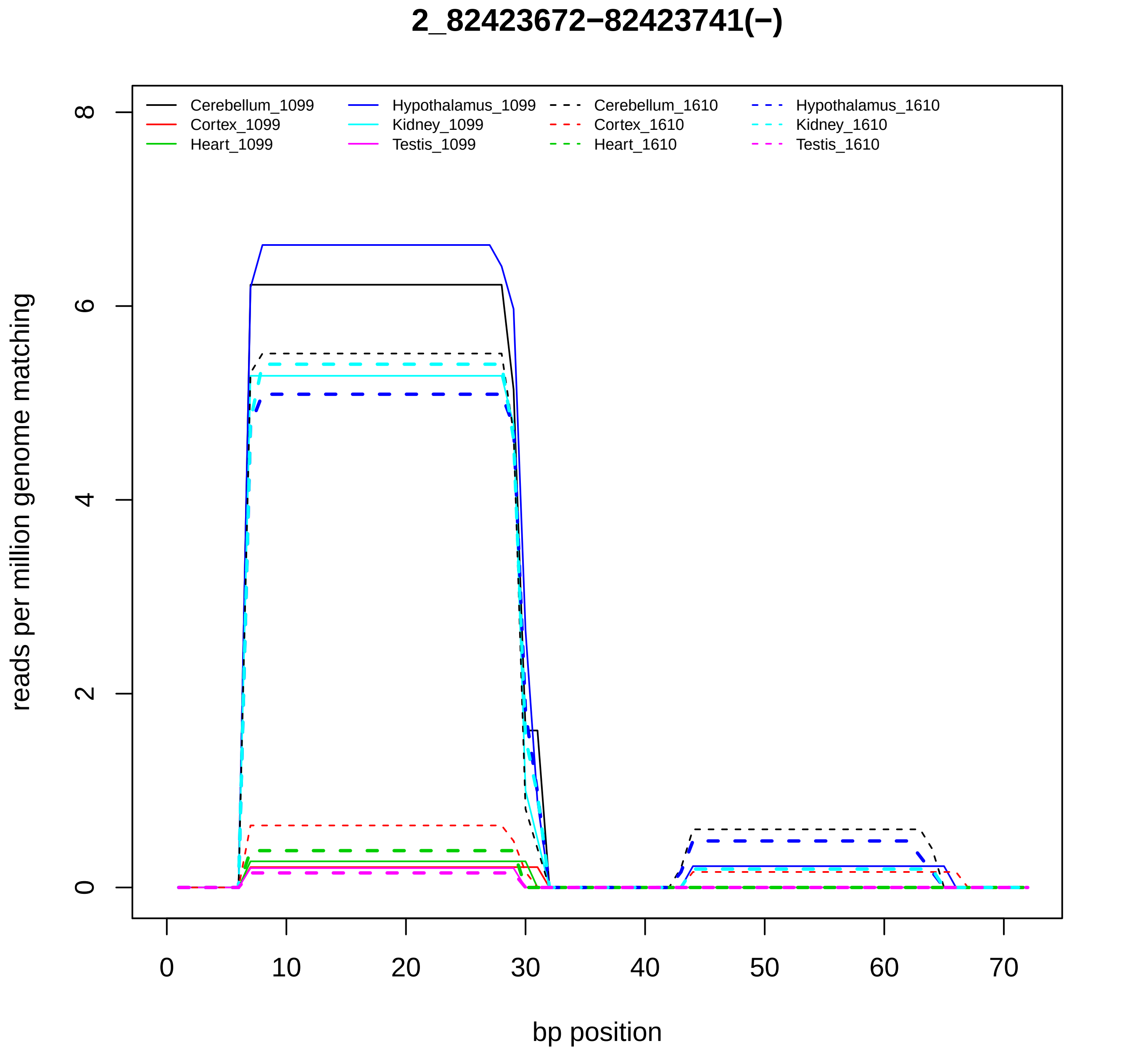

2_82423672−82423741(−)
Cerebellum_1099
Cortex_1099
Heart_1099
Hypothalamus_1099
Kidney_1099
Testis_1099
Cerebellum_1610
Cortex_1610
Heart_1610
Hypothalamus_1610
Kidney_1610
Testis_1610
reads per million genome matching
bp position
0
2
4
6
8
0
10
20
30
40
50
60
70

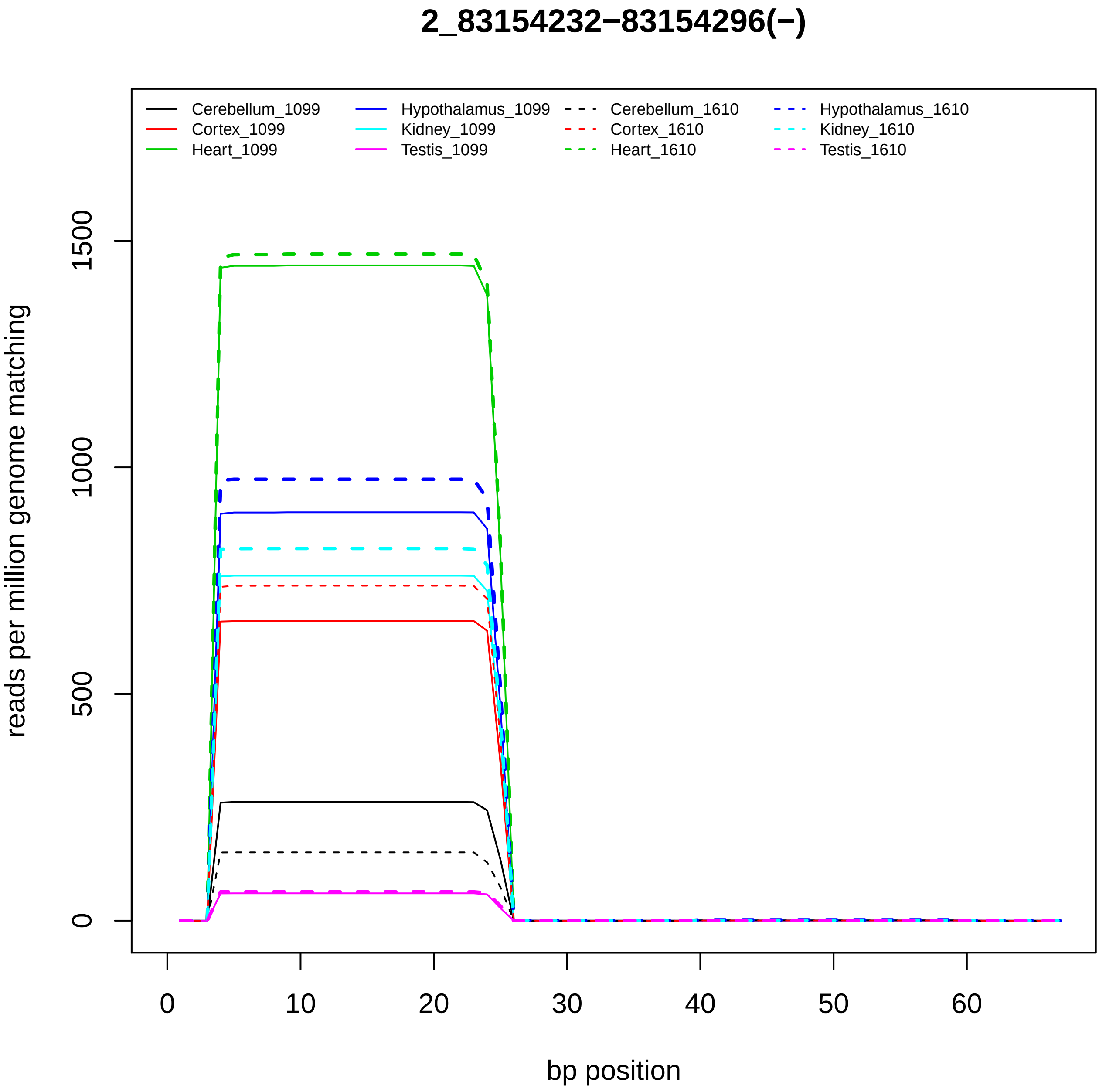

2_83154232−83154296(−)
Cerebellum_1099
Cortex_1099
Heart_1099
Hypothalamus_1099
Kidney_1099
Testis_1099
Cerebellum_1610
Cortex_1610
Heart_1610
Hypothalamus_1610
Kidney_1610
Testis_1610
reads per million genome matching
0
500
1000
1500
0
10
20
30
40
50
60
bp position

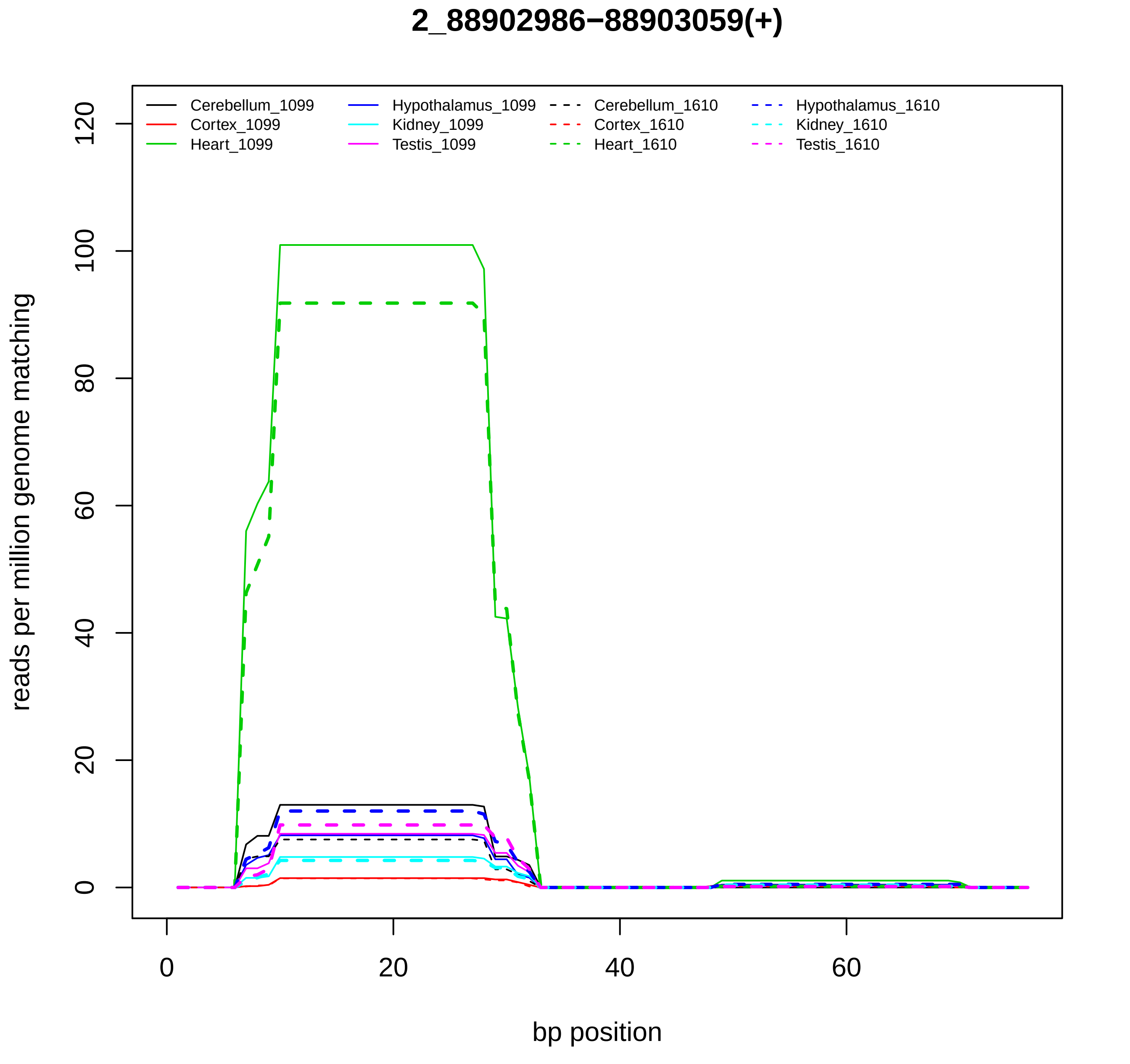

2_88902986−88903059(+)
Cerebellum_1099
Cortex_1099
Heart_1099
Hypothalamus_1099
Kidney_1099
Testis_1099
Cerebellum_1610
Cortex_1610
Heart_1610
Hypothalamus_1610
Kidney_1610
Testis_1610
reads per million genome matching
0
20
40
60
80
100
120
0
20
40
60
bp position

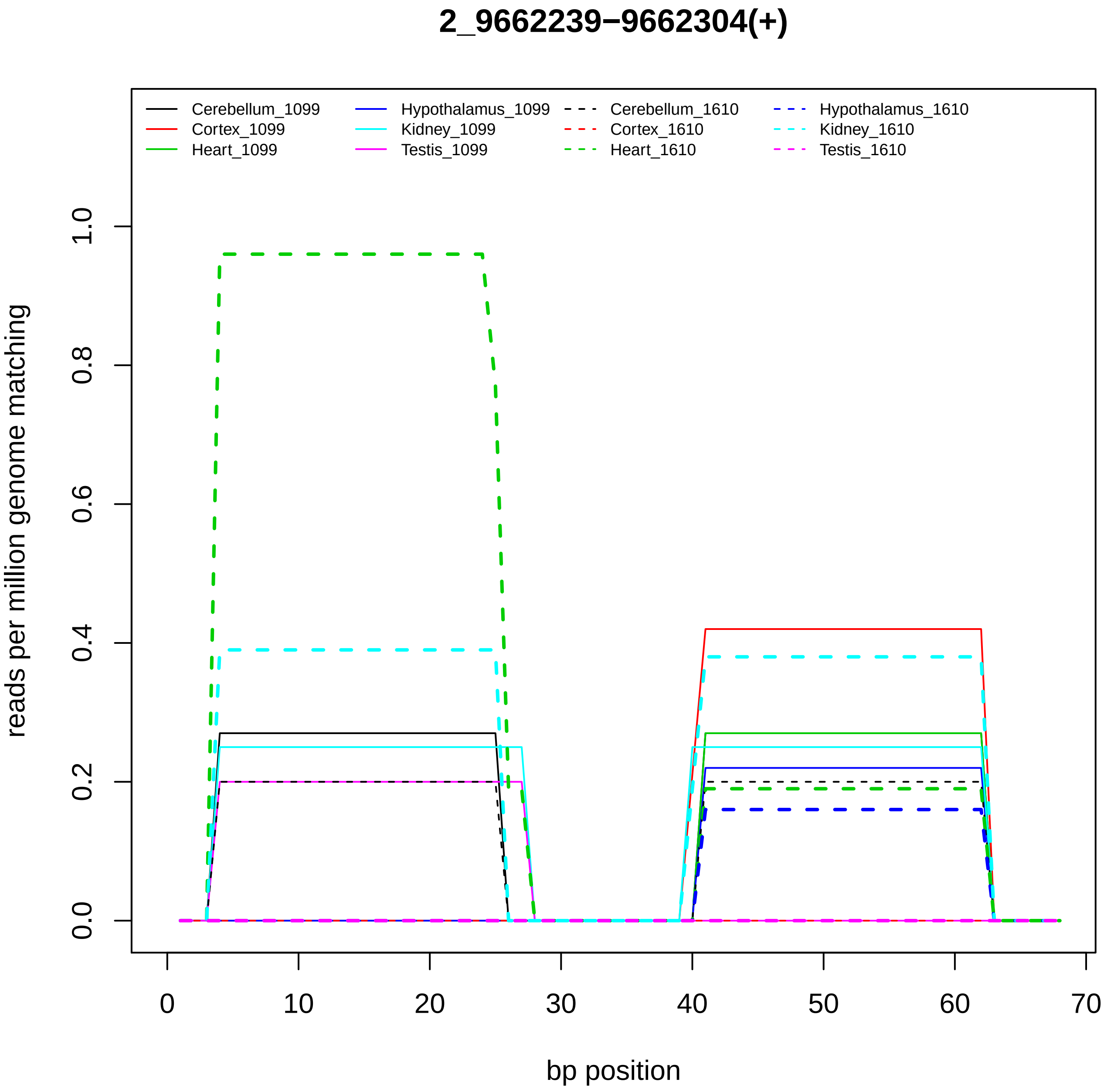
2_9662239−9662304(+)
Cerebellum_1099
Cortex_1099
Heart_1099
Hypothalamus_1099
Kidney_1099
Testis_1099
Cerebellum_1610
Cortex_1610
Heart_1610
Hypothalamus_1610
Kidney_1610
Testis_1610
reads per million genome matching
0.0
0.2
0.4
0.6
0.8
1.0
0
10
20
30
40
50
60
70
bp position

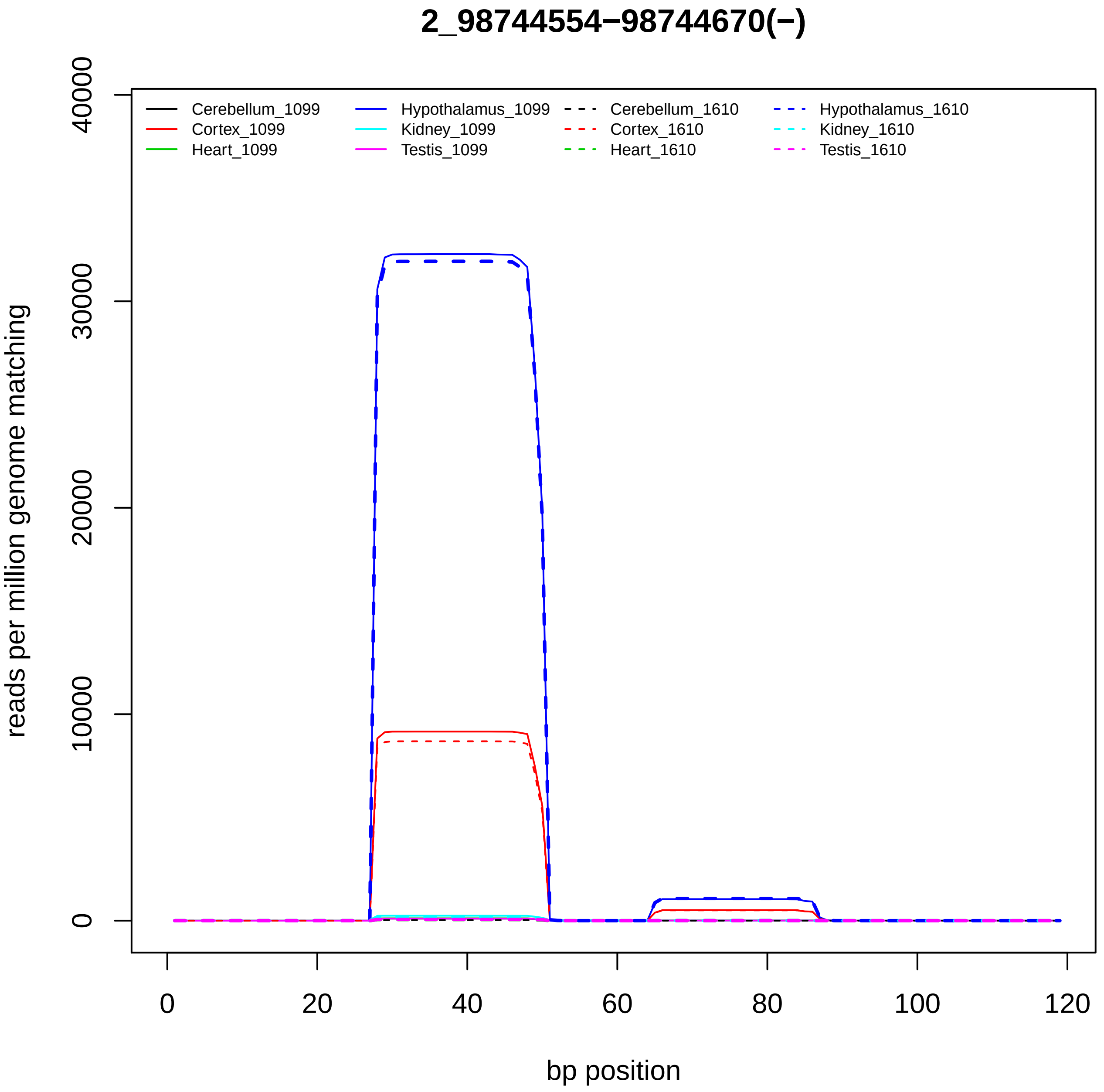

2_98744554−98744670(−)
Cerebellum_1099
Cortex_1099
Heart_1099
Hypothalamus_1099
Kidney_1099
Testis_1099
Cerebellum_1610
Cortex_1610
Heart_1610
Hypothalamus_1610
Kidney_1610
Testis_1610
reads per million genome matching
0
10000
20000
30000
40000
bp position
0
20
40
60
80
100
120

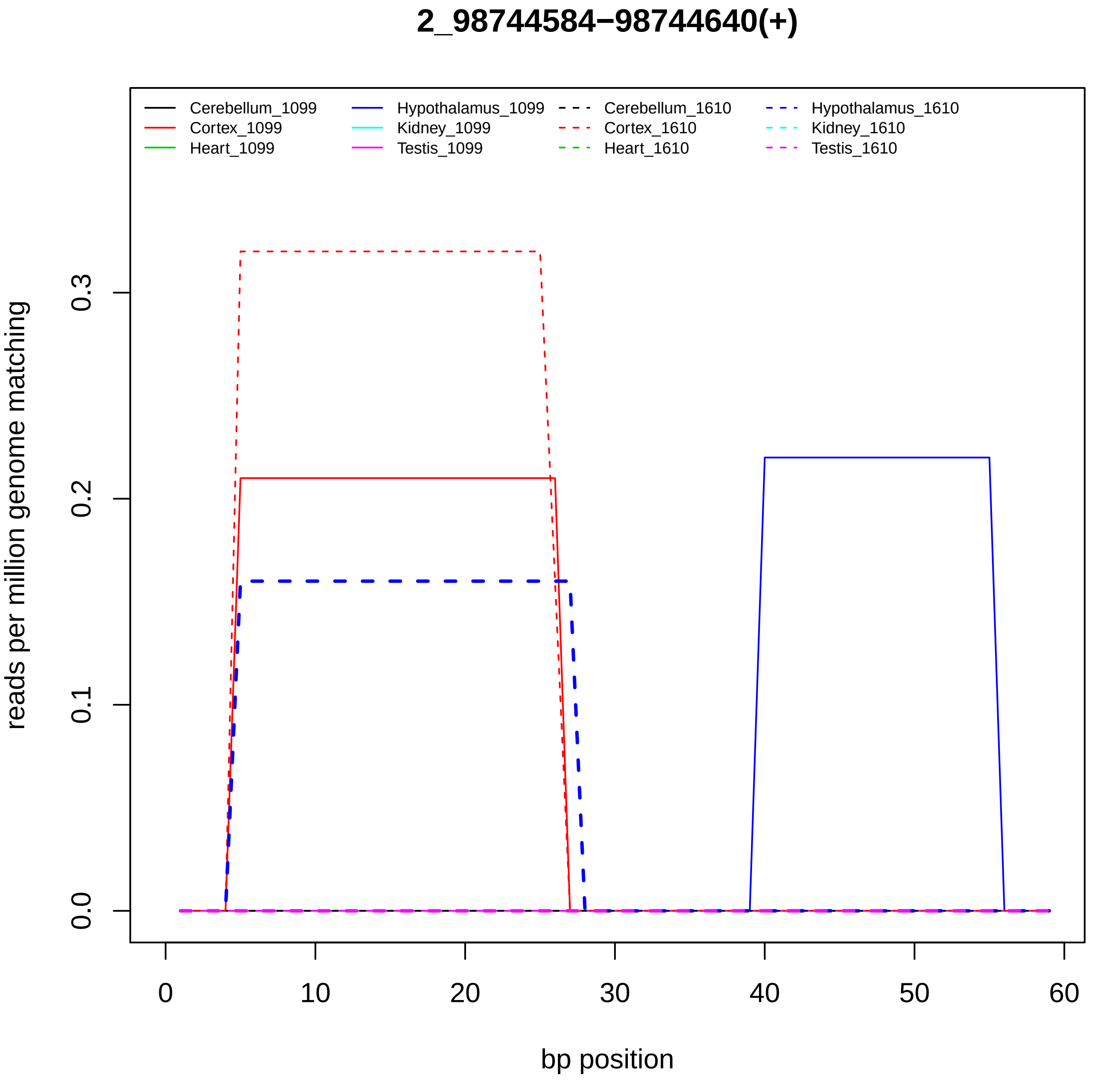
2_98744584−98744640(+)
Cerebellum_1099
Cortex_1099
Heart_1099
Hypothalamus_1099
Kidney_1099
Testis_1099
Cerebellum_1610
Cortex_1610
Heart_1610
Hypothalamus_1610
Kidney_1610
Testis_1610
reads per million genome matching
0.0
0.1
0.2
0.3
0
10
20
30
40
50
60
bp position

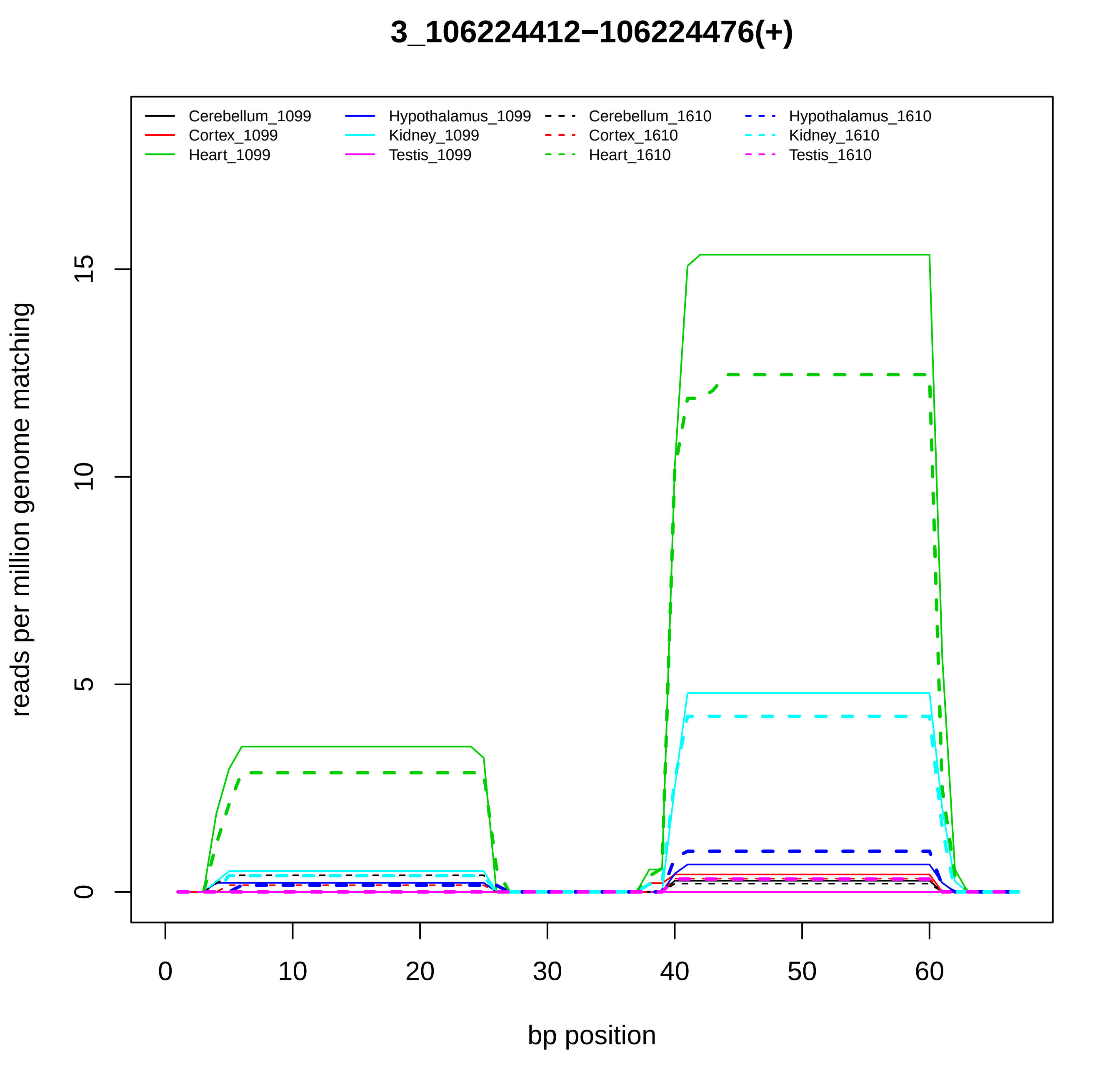

3_106224412−106224476(+)
Cerebellum_1099
Cortex_1099
Heart_1099
Hypothalamus_1099
Kidney_1099
Testis_1099
Cerebellum_1610
Cortex_1610
Heart_1610
Hypothalamus_1610
Kidney_1610
Testis_1610
reads per million genome matching
0
5
10
15
0
10
20
30
40
50
60
bp position

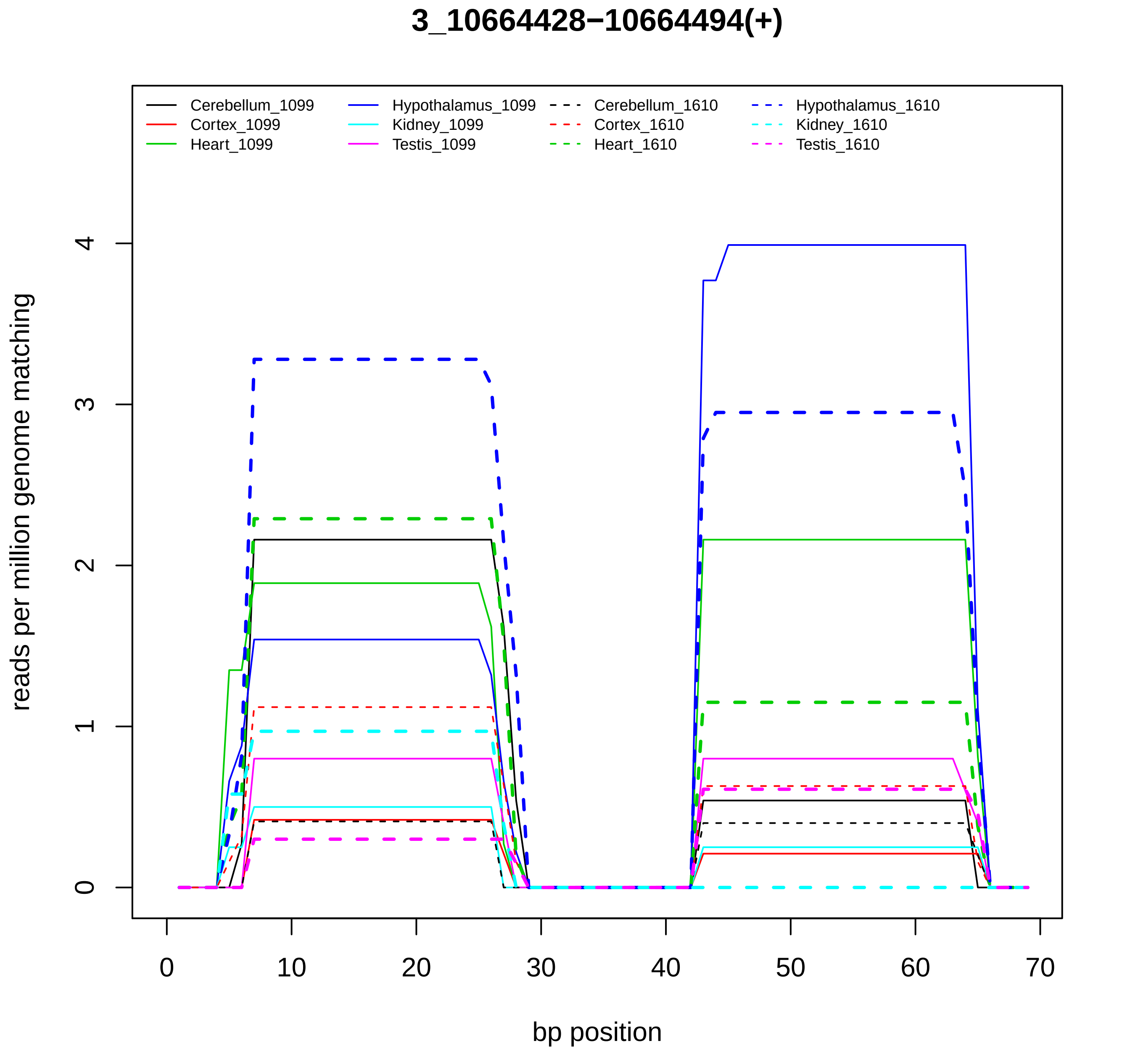

3_10664428−10664494(+)
Cerebellum_1099
Cortex_1099
Heart_1099
Hypothalamus_1099
Kidney_1099
Testis_1099
Cerebellum_1610
Cortex_1610
Heart_1610
Hypothalamus_1610
Kidney_1610
Testis_1610
reads per million genome matching
bp position
0
1
2
3
4
0
10
20
30
40
50
60
70

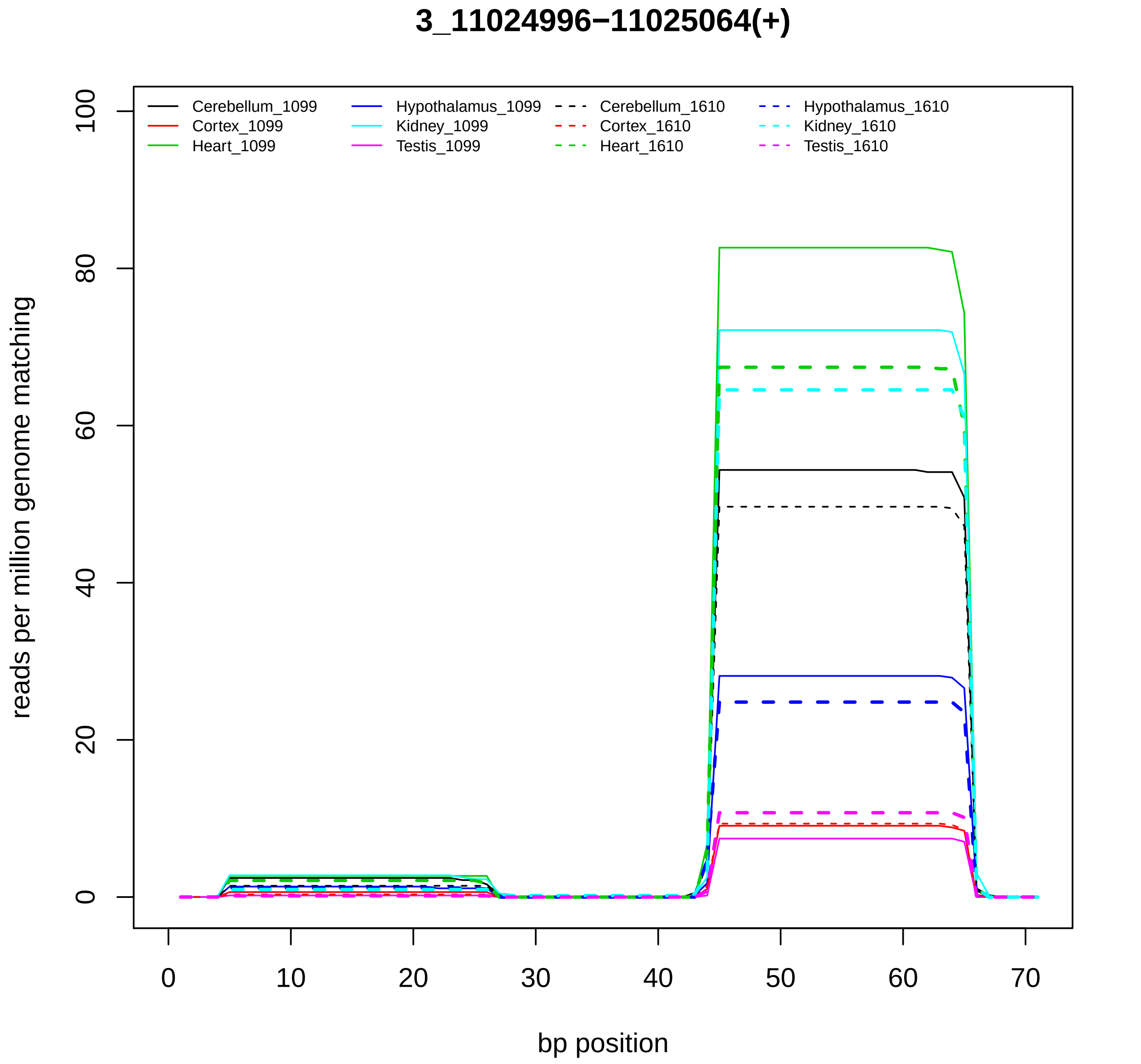

3_11024996−11025064(+)
Cerebellum_1099
Cortex_1099
Heart_1099
Hypothalamus_1099
Kidney_1099
Testis_1099
Cerebellum_1610
Cortex_1610
Heart_1610
Hypothalamus_1610
Kidney_1610
Testis_1610
reads per million genome matching
bp position
0
20
40
60
80
100
0
10
20
30
40
50
60
70

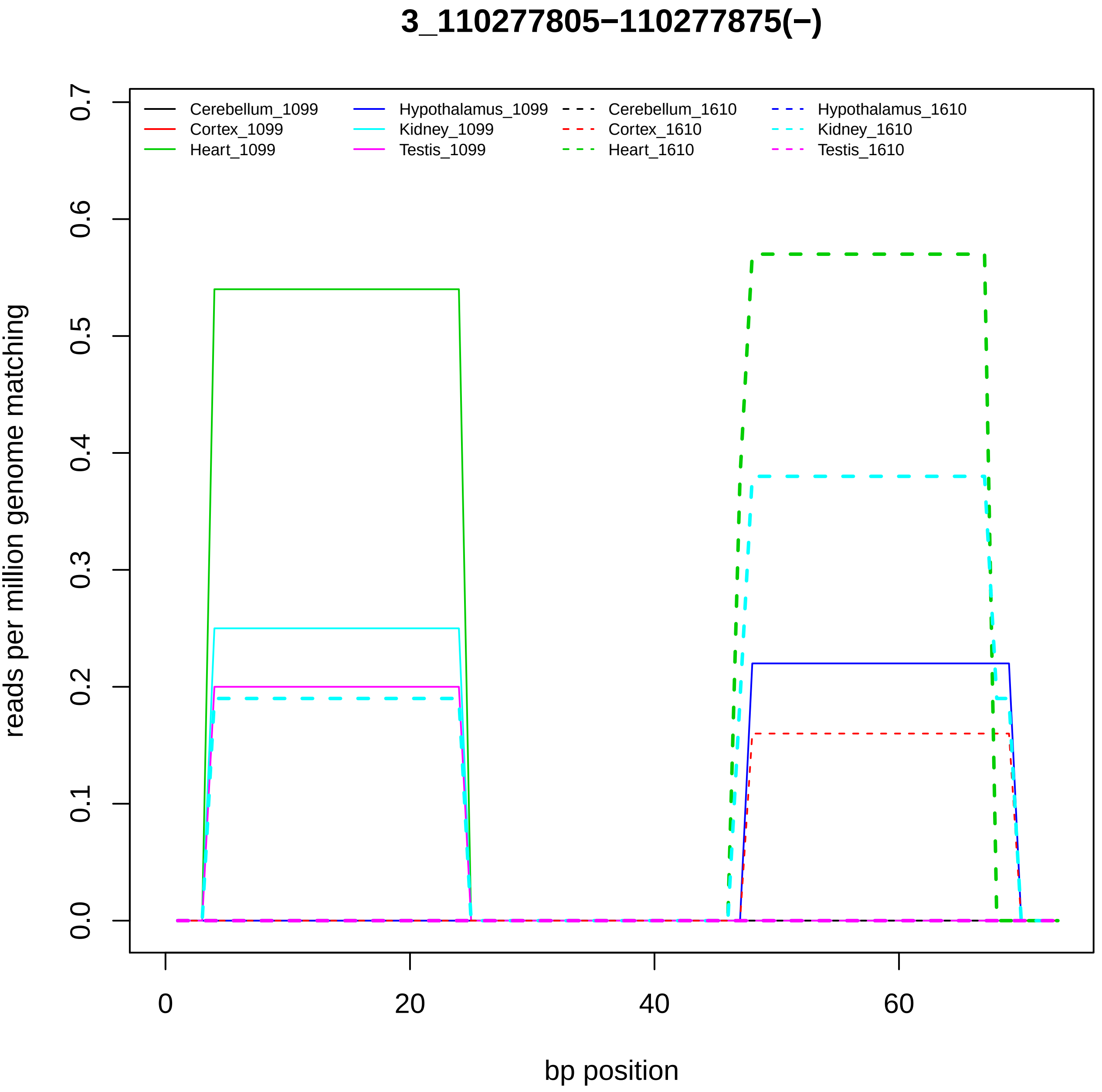
3_110277805−110277875(−)
Cerebellum_1099
Cortex_1099
Heart_1099
Hypothalamus_1099
Kidney_1099
Testis_1099
Cerebellum_1610
Cortex_1610
Heart_1610
Hypothalamus_1610
Kidney_1610
Testis_1610
reads per million genome matching
0.0
0.1
0.2
0.3
0.4
0.5
0.6
0.7
0
20
40
60
bp position

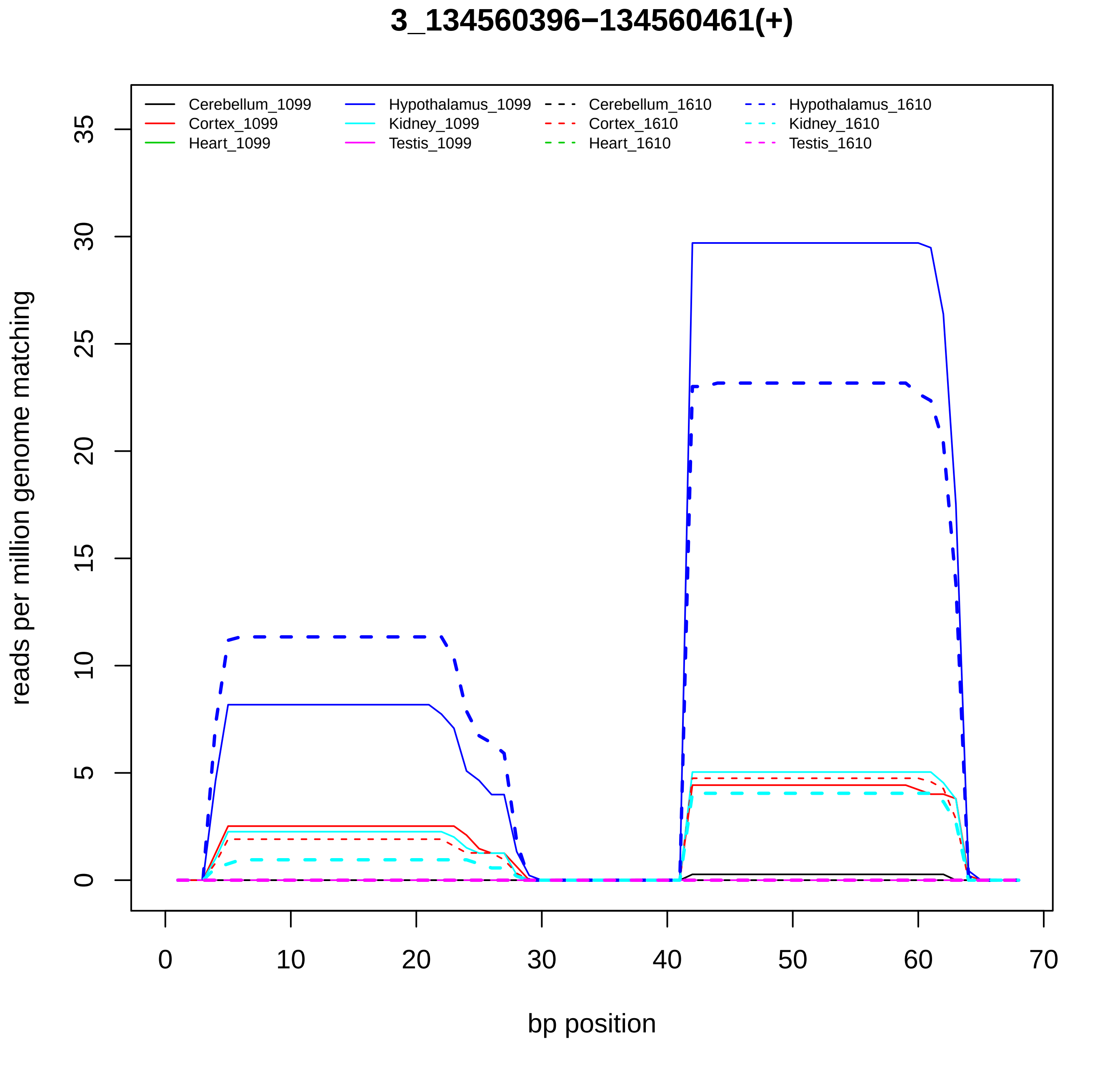
3_134560396−134560461(+)
Cerebellum_1099
Cortex_1099
Heart_1099
Hypothalamus_1099
Kidney_1099
Testis_1099
Cerebellum_1610
Cortex_1610
Heart_1610
Hypothalamus_1610
Kidney_1610
Testis_1610
reads per million genome matching
bp position
0
5
10
15
20
25
30
35
0
10
20
30
40
50
60
70

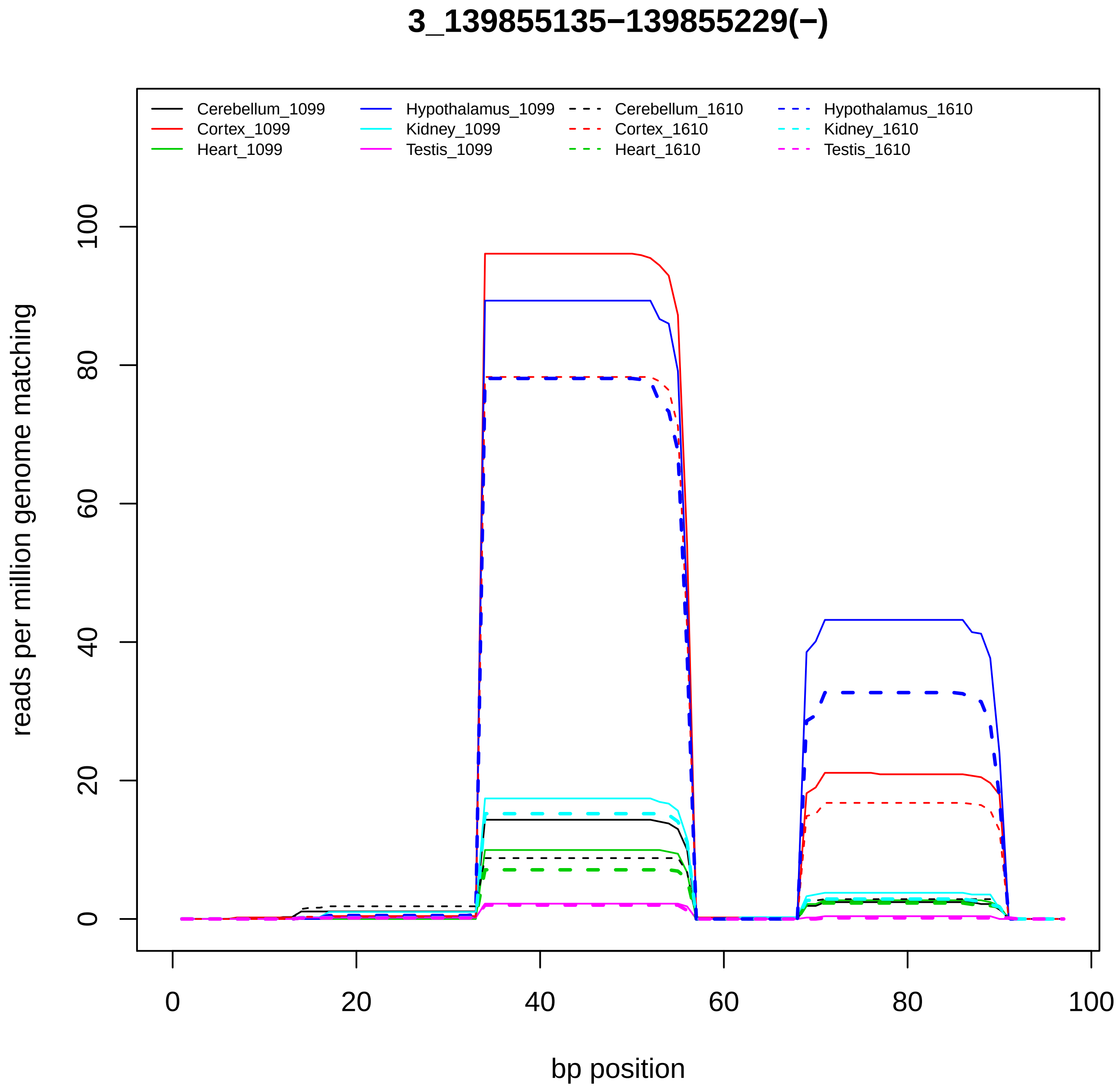
3_139855135−139855229(−)
Cerebellum_1099
Cortex_1099
Heart_1099
Hypothalamus_1099
Kidney_1099
Testis_1099
Cerebellum_1610
Cortex_1610
Heart_1610
Hypothalamus_1610
Kidney_1610
Testis_1610
reads per million genome matching
bp position
0
20
40
60
80
100

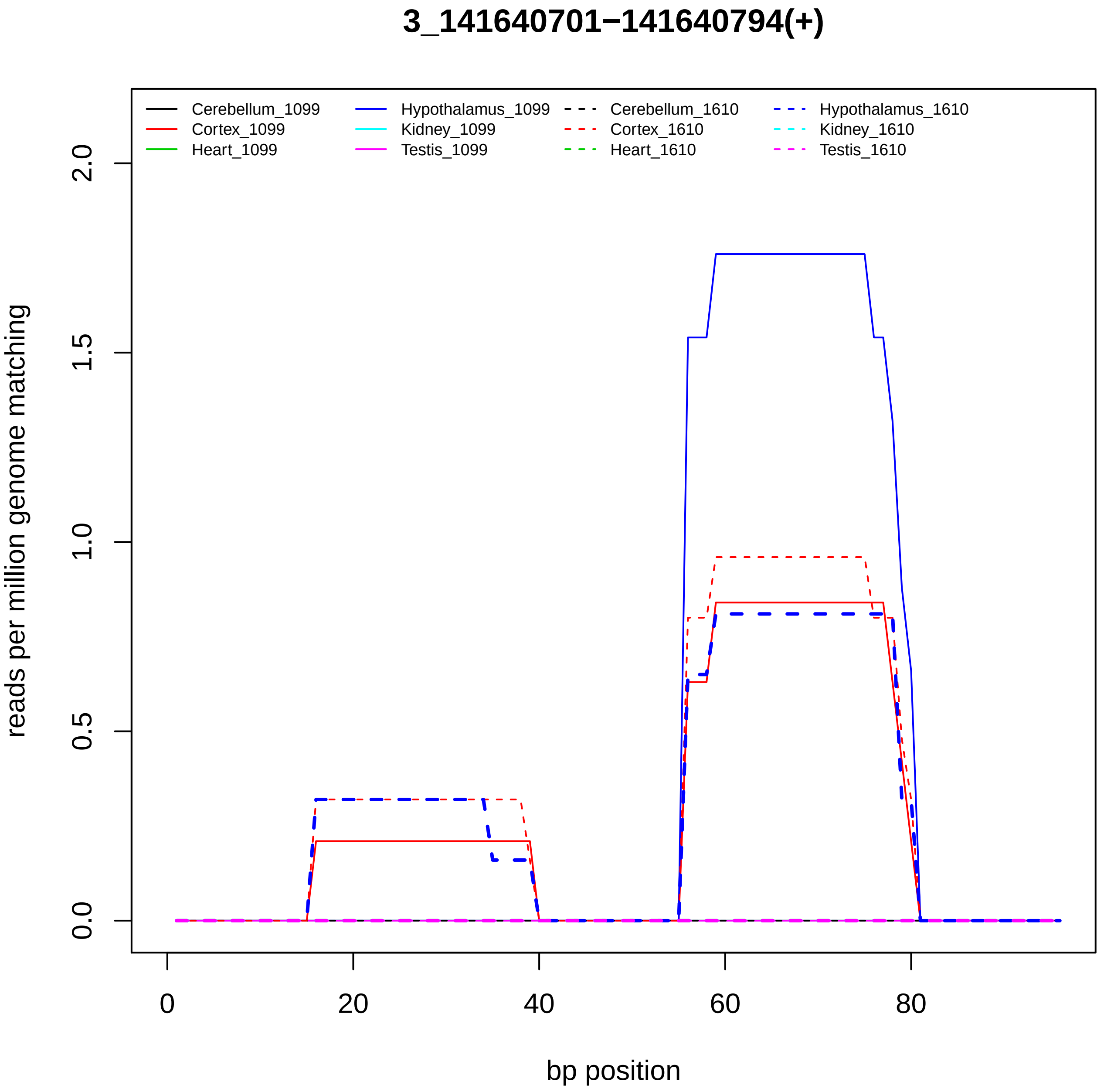

3_141640701−141640794(+)
Cerebellum_1099
Cortex_1099
Heart_1099
Hypothalamus_1099
Kidney_1099
Testis_1099
Cerebellum_1610
Cortex_1610
Heart_1610
Hypothalamus_1610
Kidney_1610
Testis_1610
reads per million genome matching
0.0
0.5
1.0
1.5
2.0
0
20
40
60
80
bp position

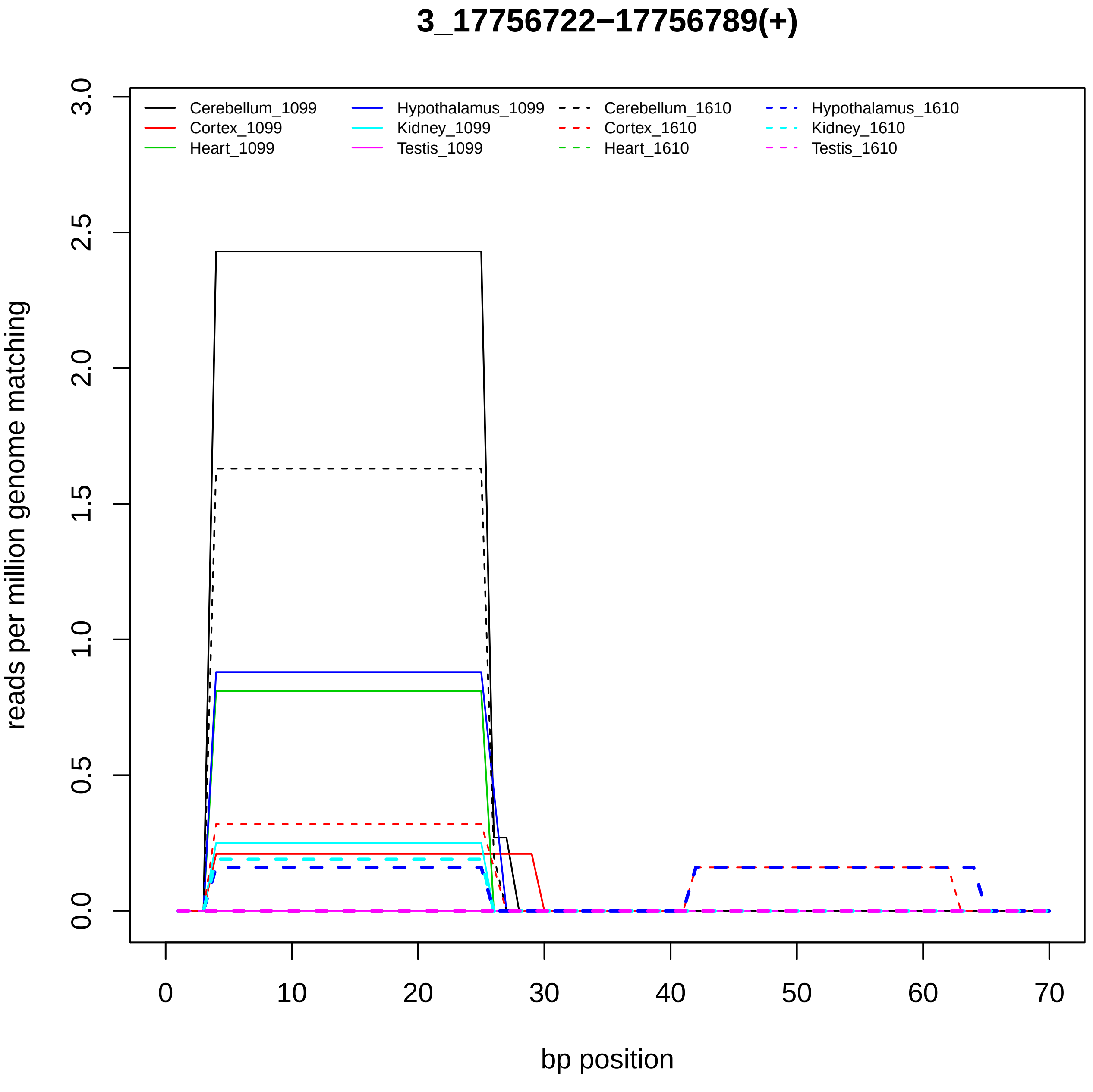

3_17756722−17756789(+)
Cerebellum_1099
Cortex_1099
Heart_1099
Hypothalamus_1099
Kidney_1099
Testis_1099
Cerebellum_1610
Cortex_1610
Heart_1610
Hypothalamus_1610
Kidney_1610
Testis_1610
reads per million genome matching
bp position
0.0
0.5
1.0
1.5
2.0
2.5
3.0
0
10
20
30
40
50
60
70

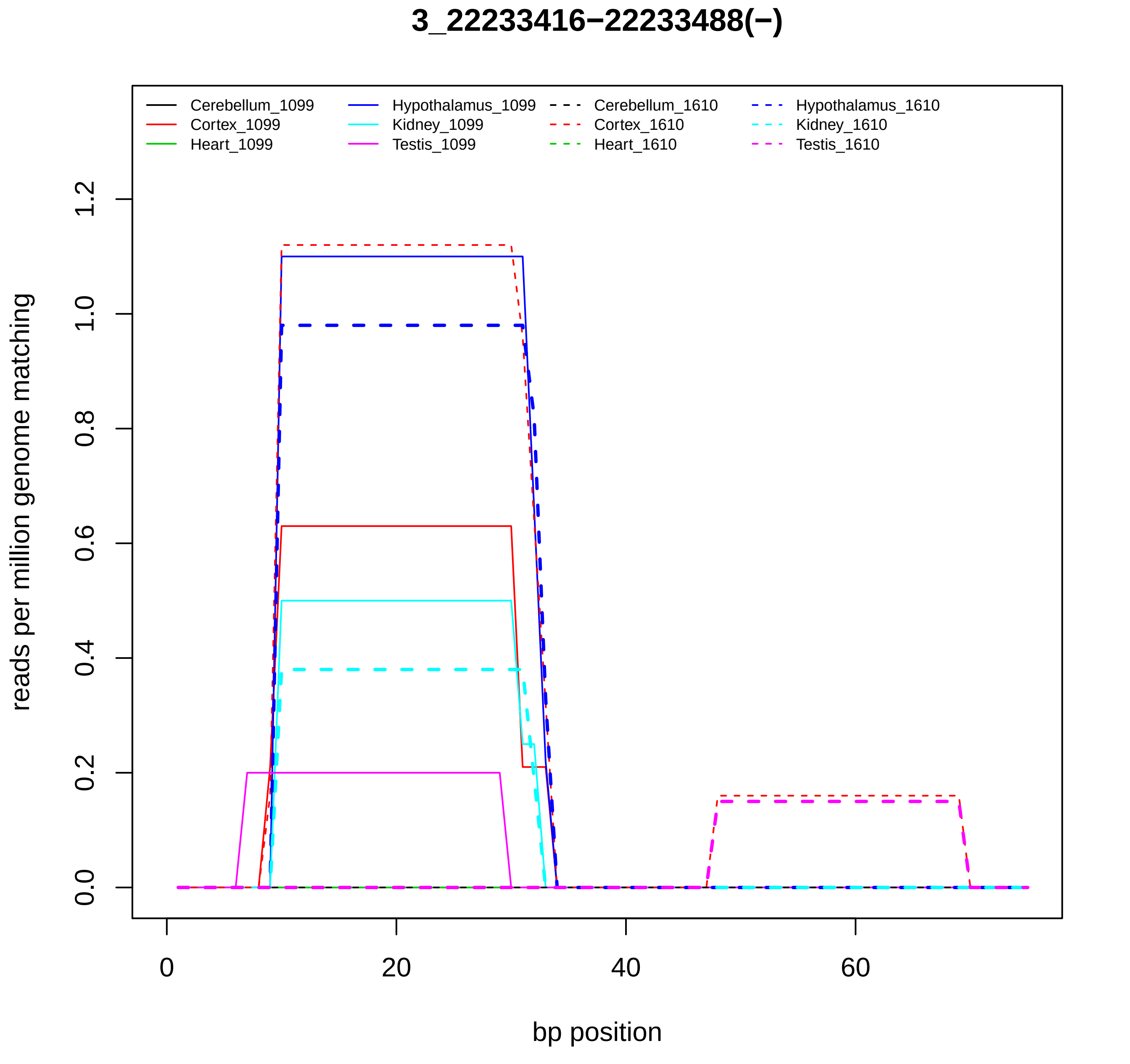

3_22233416−22233488(−)
Cerebellum_1099
Cortex_1099
Heart_1099
Hypothalamus_1099
Kidney_1099
Testis_1099
Cerebellum_1610
Cortex_1610
Heart_1610
Hypothalamus_1610
Kidney_1610
Testis_1610
reads per million genome matching
0.0
0.2
0.4
0.6
0.8
1.0
1.2
0
20
40
60
bp position

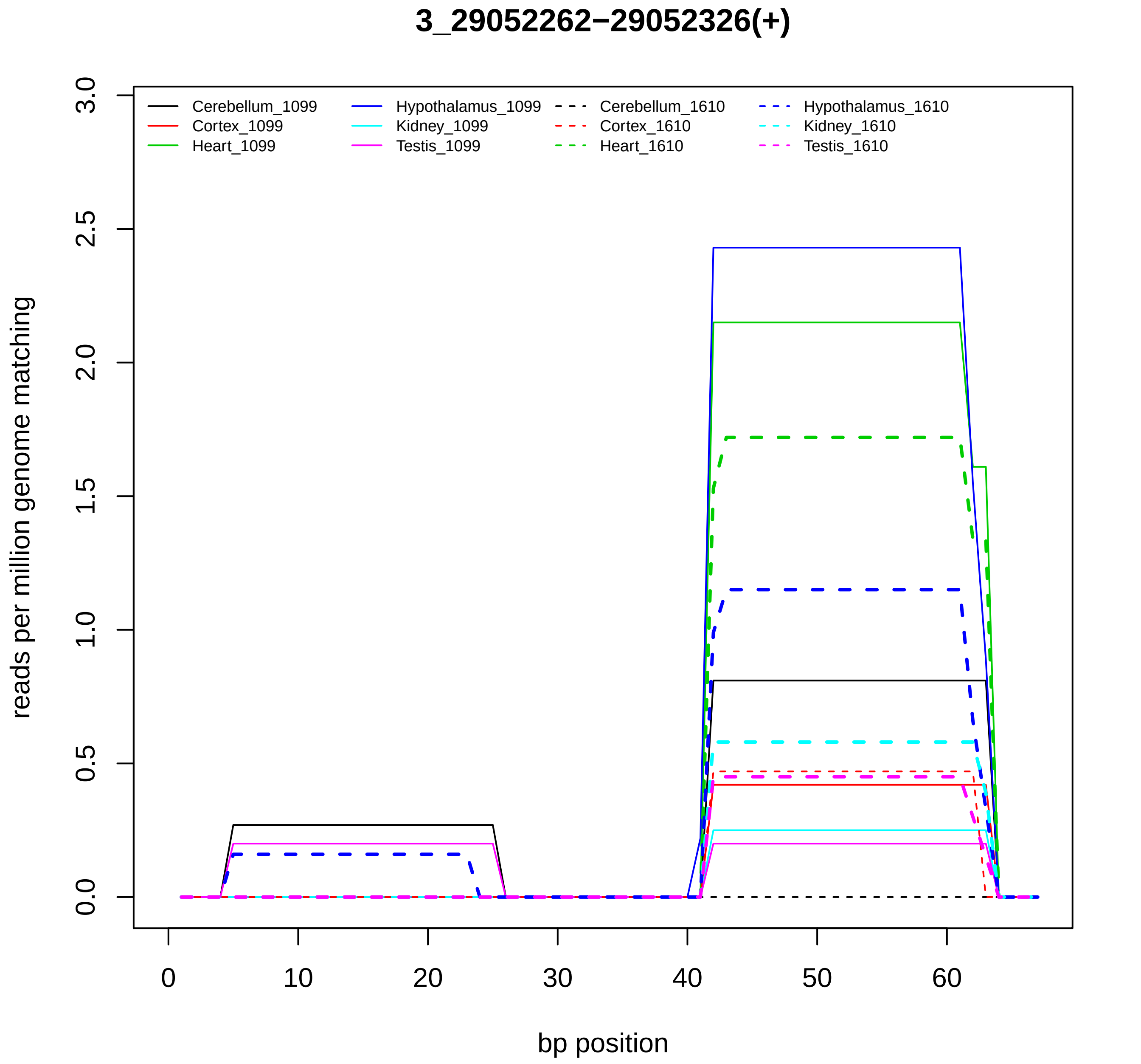
3_29052262−29052326(+)
Cerebellum_1099
Cortex_1099
Heart_1099
Hypothalamus_1099
Kidney_1099
Testis_1099
Cerebellum_1610
Cortex_1610
Heart_1610
Hypothalamus_1610
Kidney_1610
Testis_1610
reads per million genome matching
0.0
0.5
1.0
1.5
2.0
2.5
3.0
0
10
20
30
40
50
60
bp position

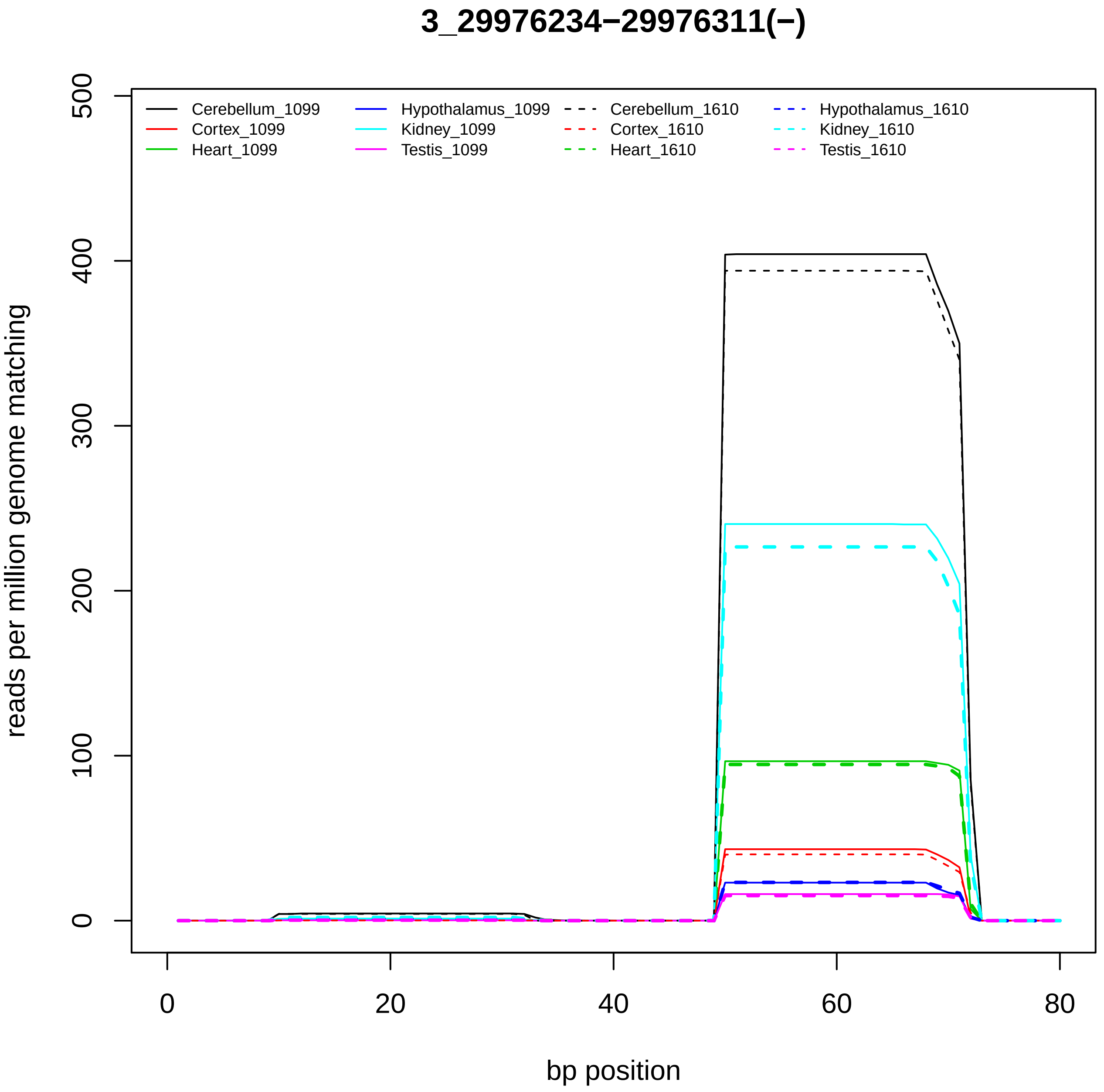

3_29976234−29976311(−)
Cerebellum_1099
Cortex_1099
Heart_1099
Hypothalamus_1099
Kidney_1099
Testis_1099
Cerebellum_1610
Cortex_1610
Heart_1610
Hypothalamus_1610
Kidney_1610
Testis_1610
reads per million genome matching
0
100
200
300
400
500
0
20
40
60
80
bp position

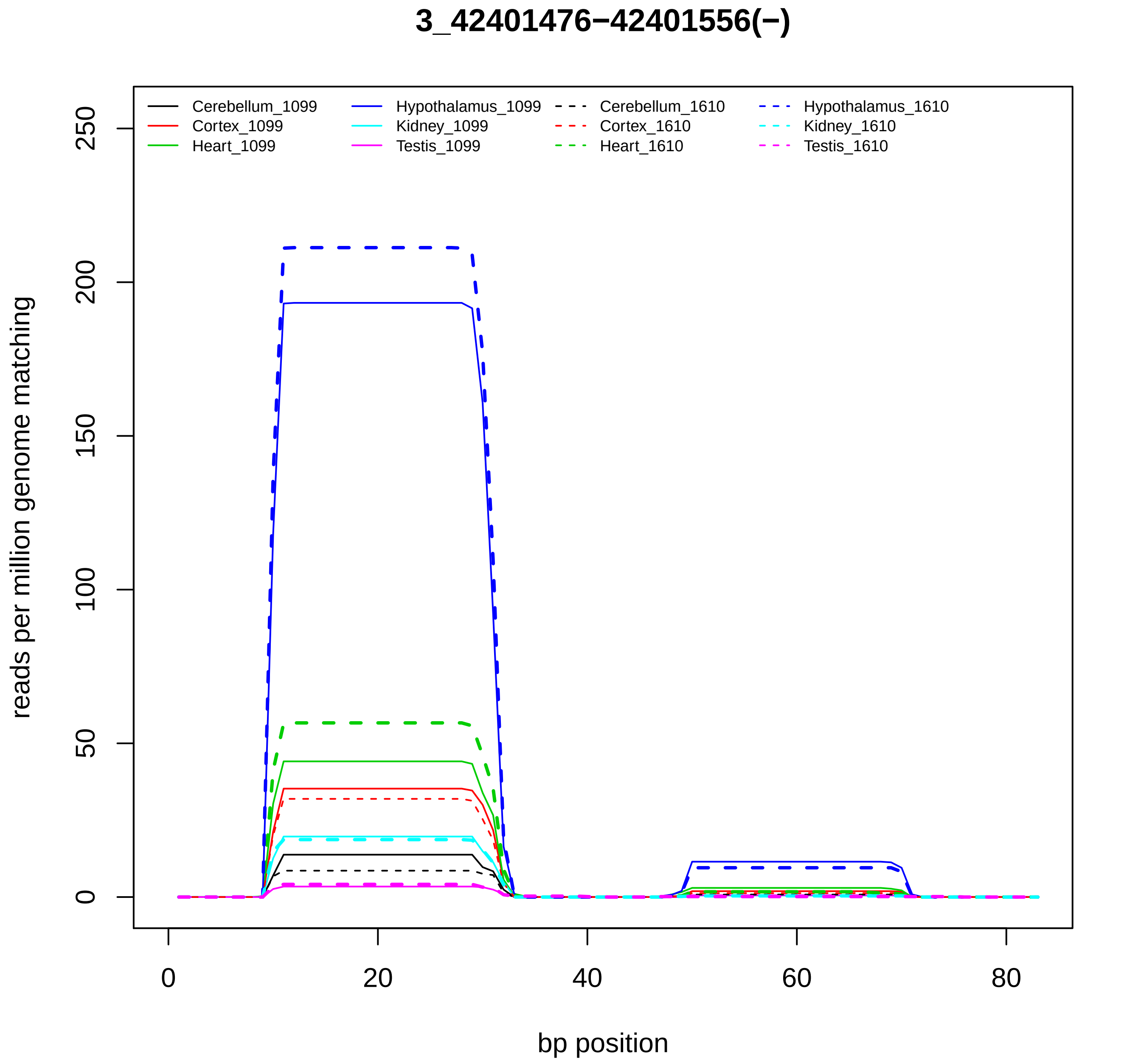
3_42401476−42401556(−)
Cerebellum_1099
Cortex_1099
Heart_1099
Hypothalamus_1099
Kidney_1099
Testis_1099
Cerebellum_1610
Cortex_1610
Heart_1610
Hypothalamus_1610
Kidney_1610
Testis_1610
reads per million genome matching
0
50
100
150
200
250
bp position
0
20
40
60
80

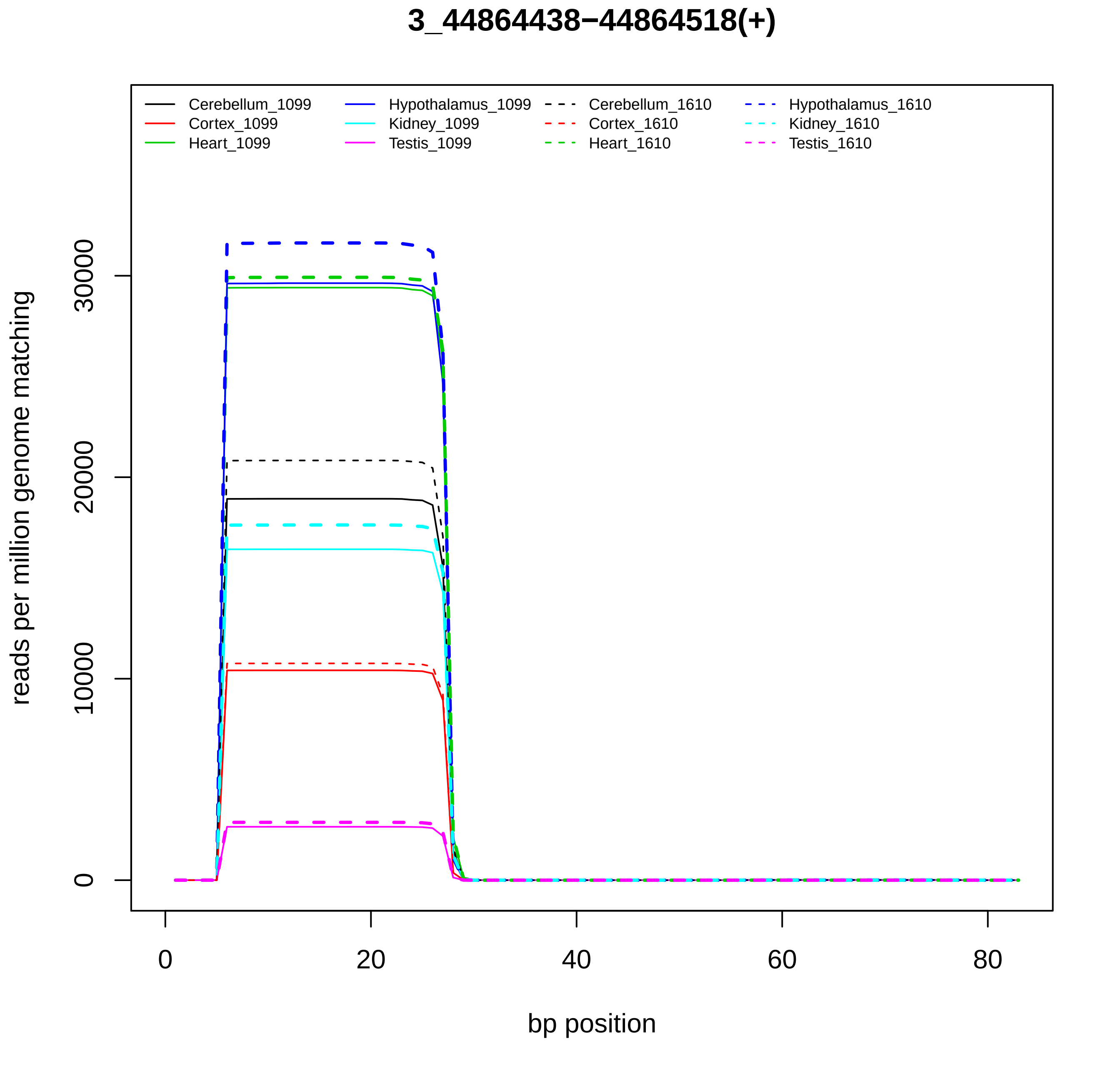
3_44864438−44864518(+)
Cerebellum_1099
Cortex_1099
Heart_1099
Hypothalamus_1099
Kidney_1099
Testis_1099
Cerebellum_1610
Cortex_1610
Heart_1610
Hypothalamus_1610
Kidney_1610
Testis_1610
reads per million genome matching
0
10000
20000
30000
0
20
40
60
80
bp position

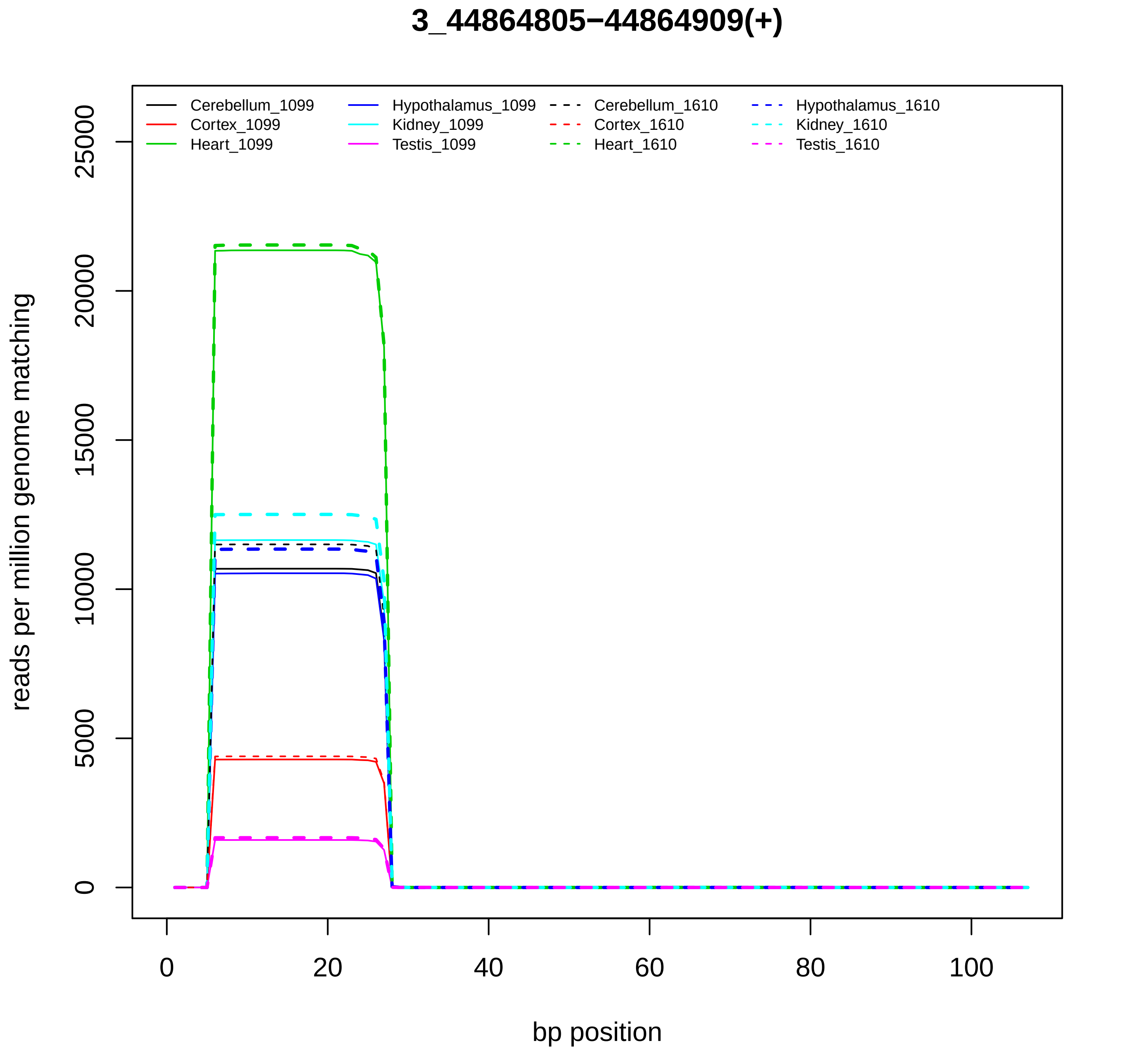

3_44864805−44864909(+)
Cerebellum_1099
Cortex_1099
Heart_1099
Hypothalamus_1099
Kidney_1099
Testis_1099
Cerebellum_1610
Cortex_1610
Heart_1610
Hypothalamus_1610
Kidney_1610
Testis_1610
reads per million genome matching
0
5000
10000
15000
20000
25000
0
20
40
60
80
100
bp position

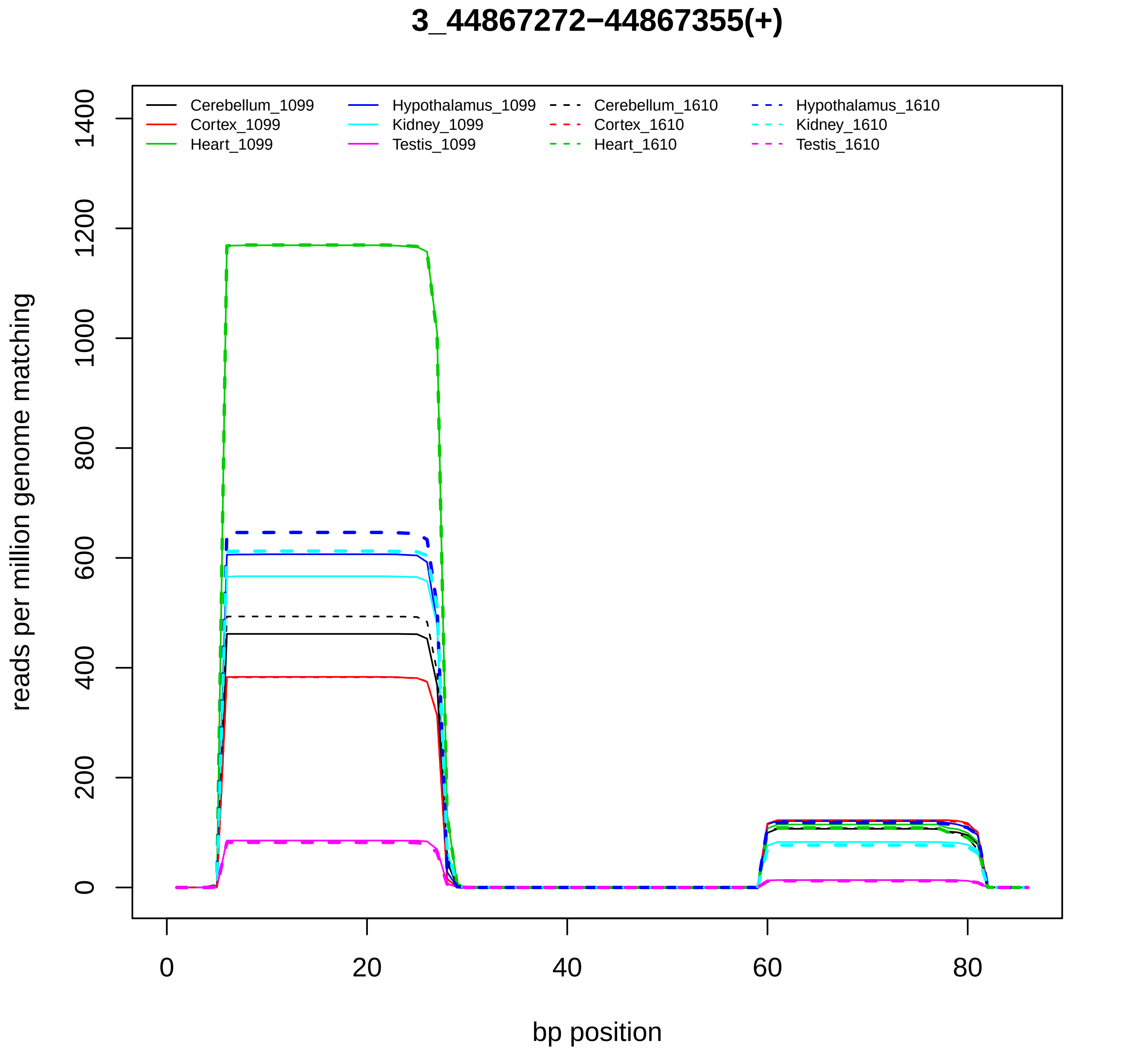

3_44867272−44867355(+)
Cerebellum_1099
Cortex_1099
Heart_1099
Hypothalamus_1099
Kidney_1099
Testis_1099
Cerebellum_1610
Cortex_1610
Heart_1610
Hypothalamus_1610
Kidney_1610
Testis_1610
reads per million genome matching
0
200
400
600
800
1000
1200
1400
0
20
40
60
80
bp position

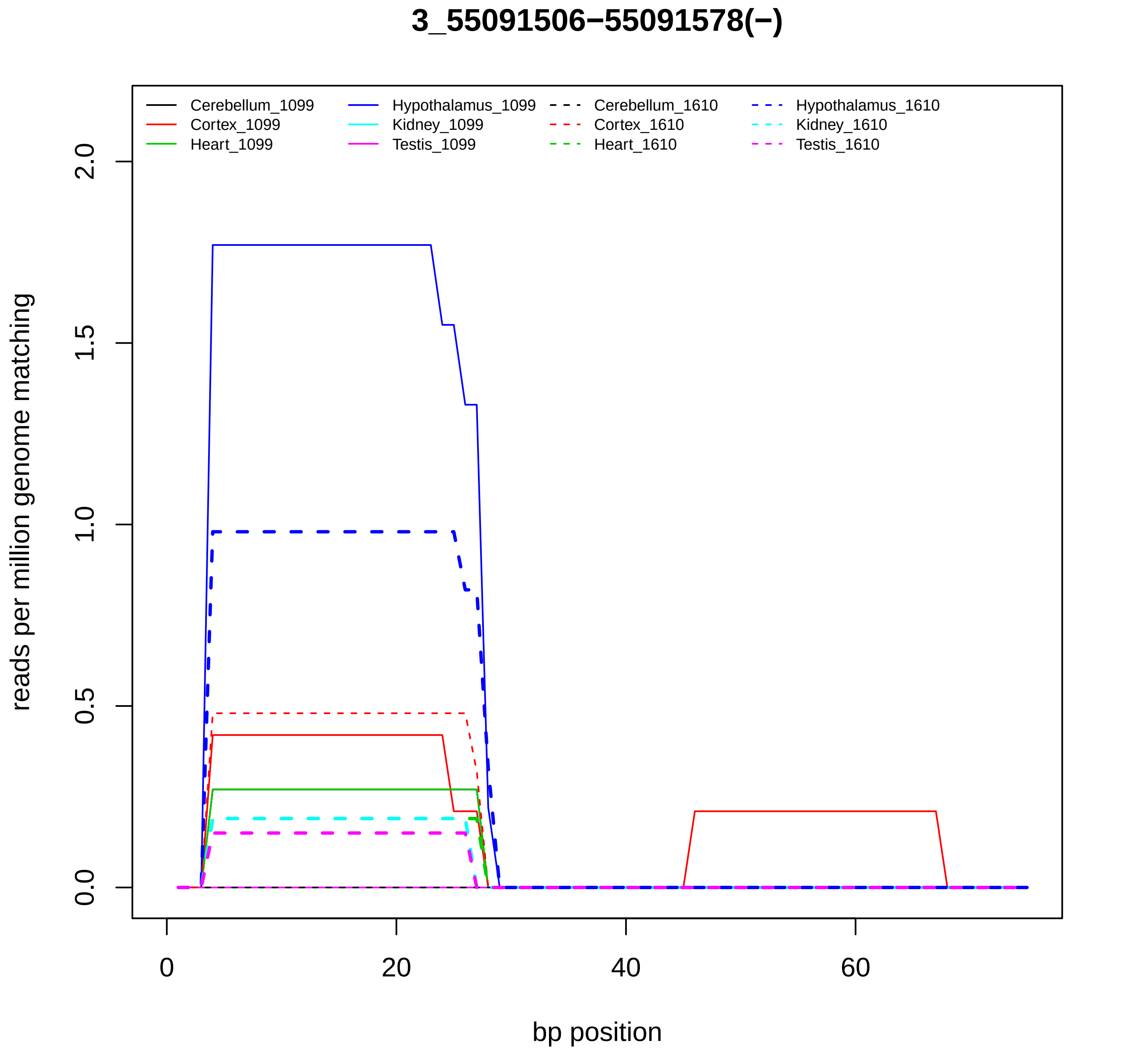
3_55091506−55091578(−)
Cerebellum_1099
Cortex_1099
Heart_1099
Hypothalamus_1099
Kidney_1099
Testis_1099
Cerebellum_1610
Cortex_1610
Heart_1610
Hypothalamus_1610
Kidney_1610
Testis_1610
reads per million genome matching
0.0
0.5
1.0
1.5
2.0
0
20
40
60
bp position

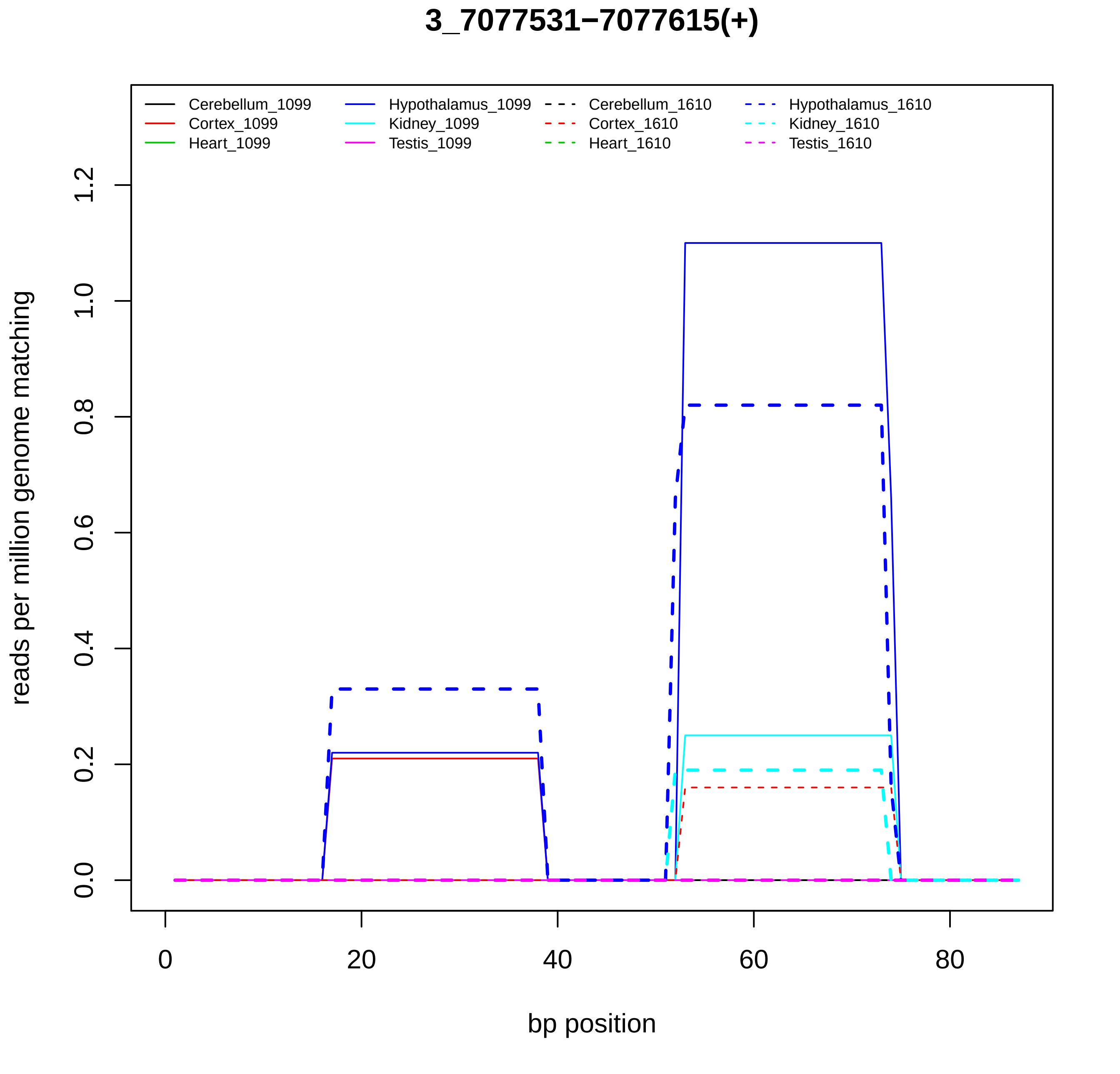
3_7077531−7077615(+)
Cerebellum_1099
Cortex_1099
Heart_1099
Hypothalamus_1099
Kidney_1099
Testis_1099
Cerebellum_1610
Cortex_1610
Heart_1610
Hypothalamus_1610
Kidney_1610
Testis_1610
reads per million genome matching
0.0
0.2
0.4
0.6
0.8
1.0
1.2
0
20
40
60
80
bp position

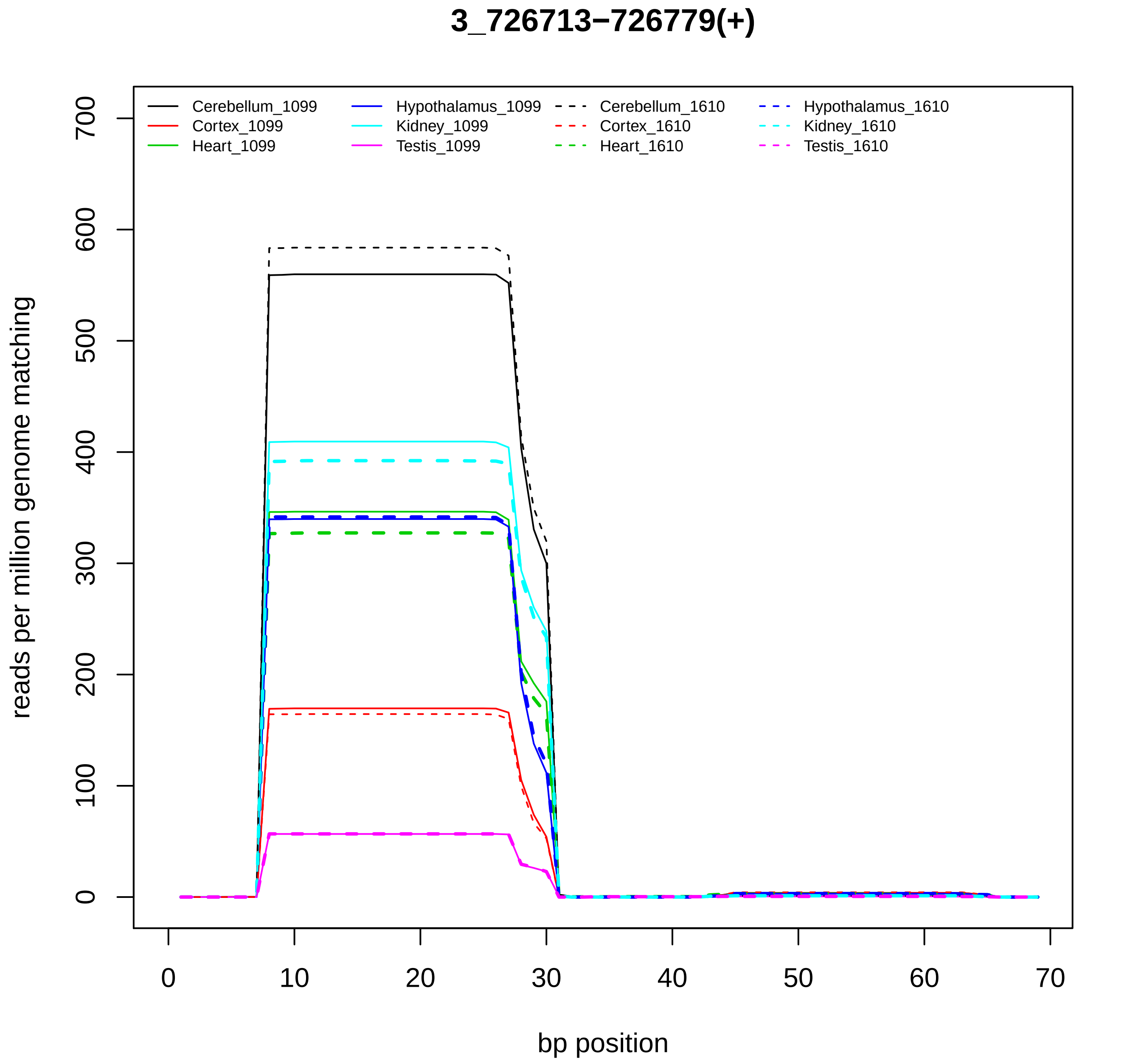

3_726713−726779(+)
Cerebellum_1099
Cortex_1099
Heart_1099
Hypothalamus_1099
Kidney_1099
Testis_1099
Cerebellum_1610
Cortex_1610
Heart_1610
Hypothalamus_1610
Kidney_1610
Testis_1610
reads per million genome matching
bp position
0
100
200
300
400
500
600
700
0
10
20
30
40
50
60
70

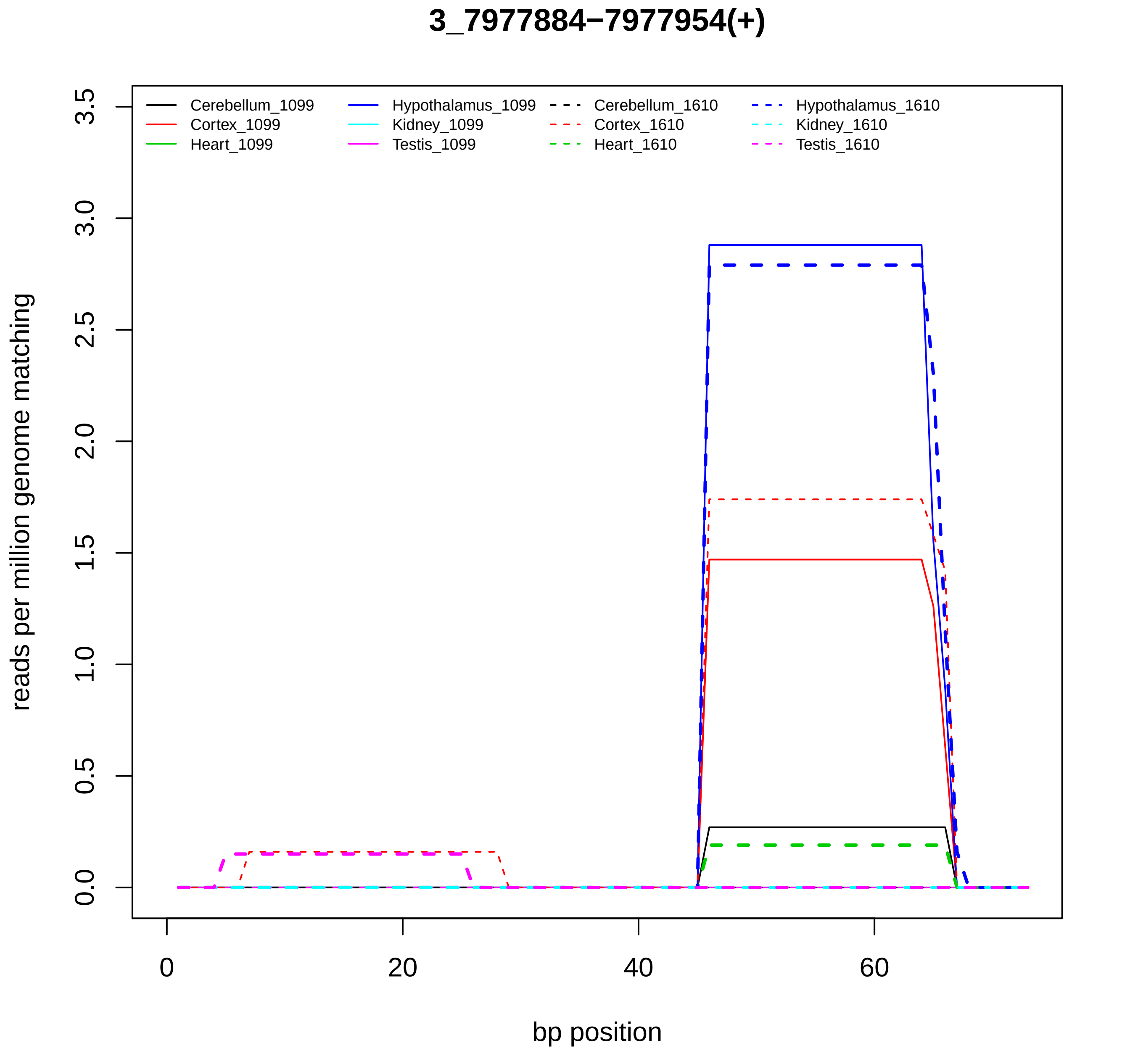

3_7977884−7977954(+)
Cerebellum_1099
Cortex_1099
Heart_1099
Hypothalamus_1099
Kidney_1099
Testis_1099
Cerebellum_1610
Cortex_1610
Heart_1610
Hypothalamus_1610
Kidney_1610
Testis_1610
reads per million genome matching
0.0
0.5
1.0
1.5
2.0
2.5
3.0
3.5
0
20
40
60
bp position

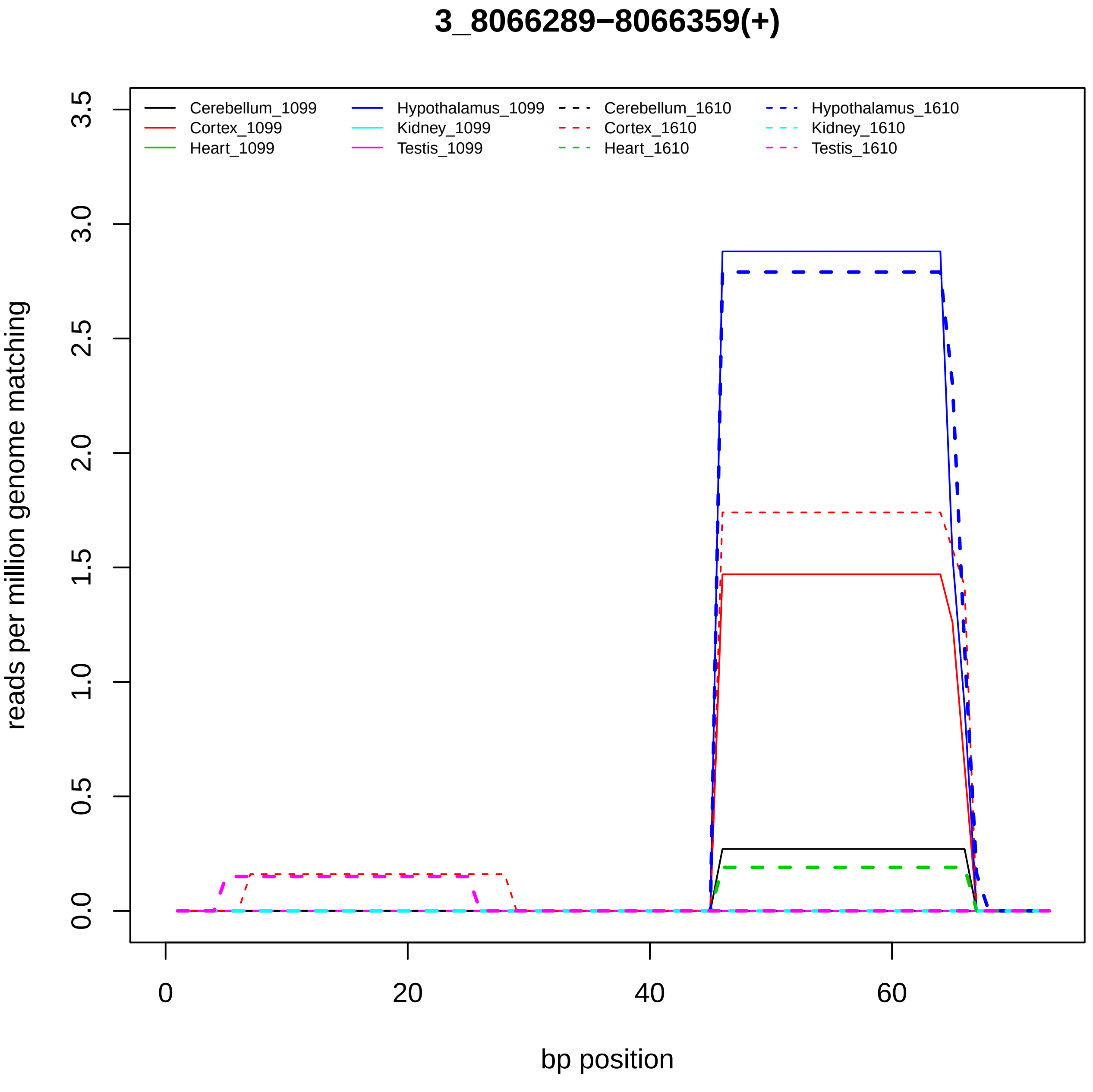

3_8066289−8066359(+)
Cerebellum_1099
Cortex_1099
Heart_1099
Hypothalamus_1099
Kidney_1099
Testis_1099
Cerebellum_1610
Cortex_1610
Heart_1610
Hypothalamus_1610
Kidney_1610
Testis_1610
reads per million genome matching
0.0
0.5
1.0
1.5
2.0
2.5
3.0
3.5
0
20
40
60
bp position

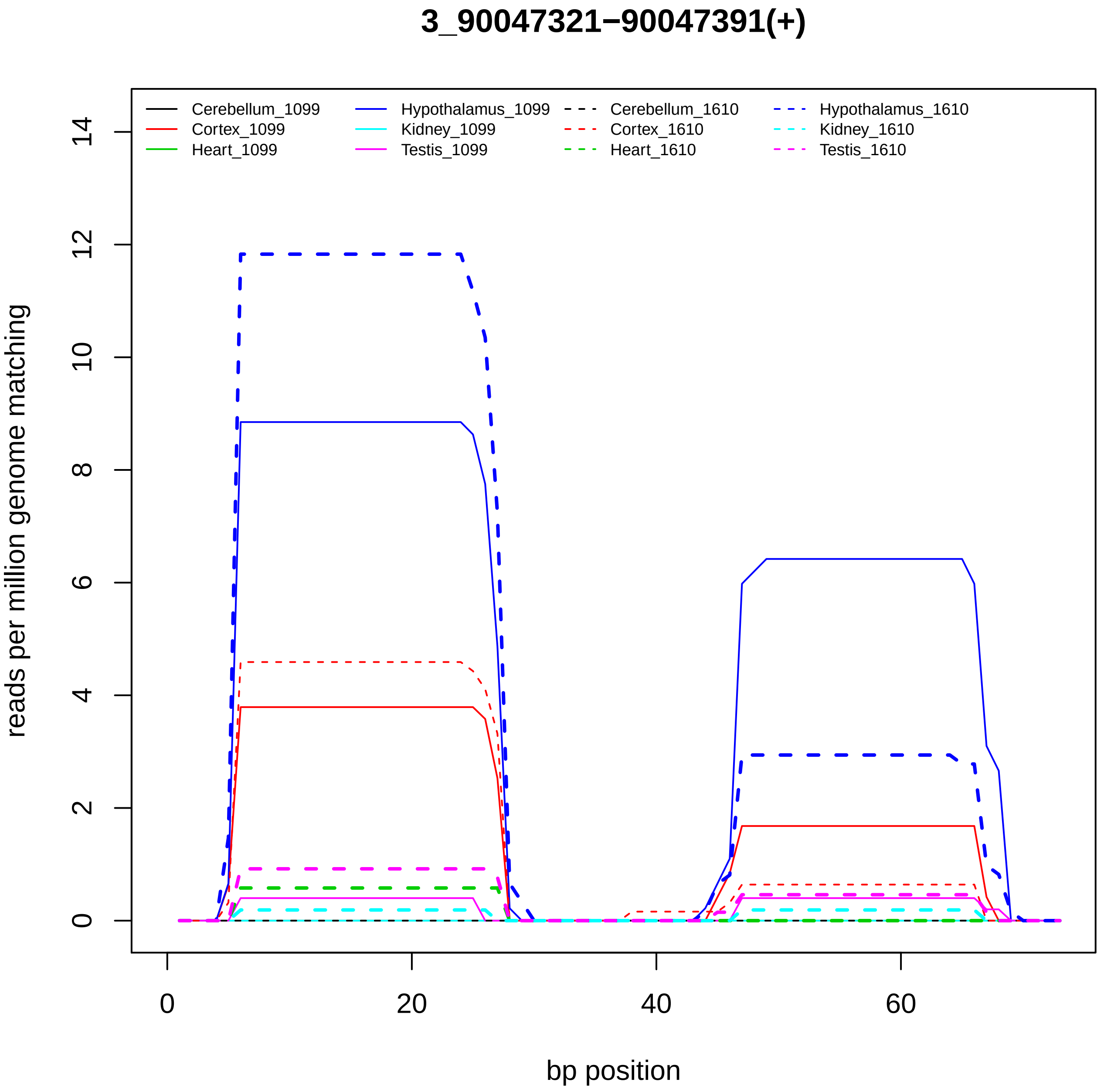

3_90047321−90047391(+)
Cerebellum_1099
Cortex_1099
Heart_1099
Hypothalamus_1099
Kidney_1099
Testis_1099
Cerebellum_1610
Cortex_1610
Heart_1610
Hypothalamus_1610
Kidney_1610
Testis_1610
reads per million genome matching
bp position
0
2
4
6
8
10
12
14
0
20
40
60

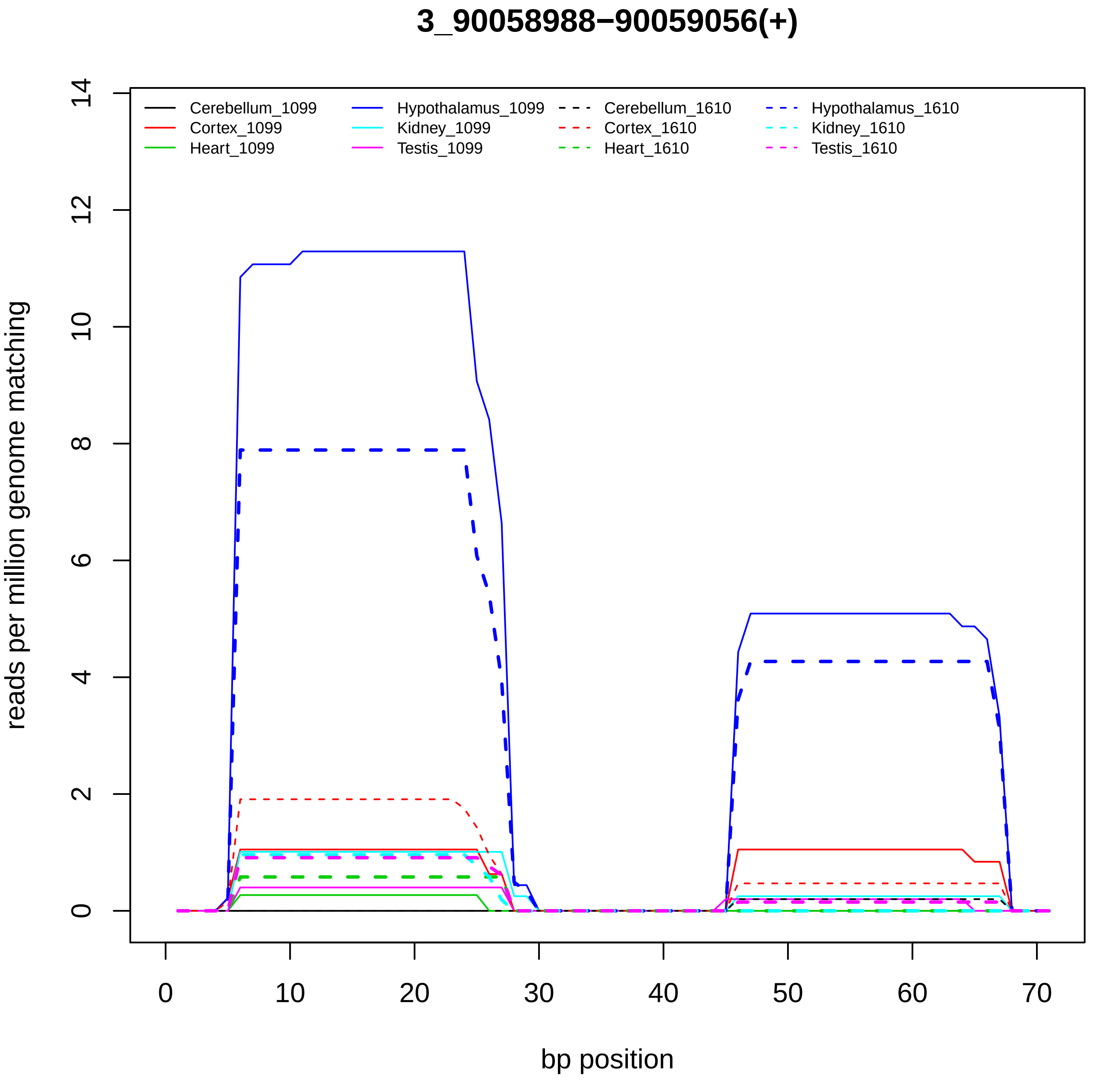

3_90058988−90059056(+)
Cerebellum_1099
Cortex_1099
Heart_1099
Hypothalamus_1099
Kidney_1099
Testis_1099
Cerebellum_1610
Cortex_1610
Heart_1610
Hypothalamus_1610
Kidney_1610
Testis_1610
reads per million genome matching
bp position
0
2
4
6
8
10
12
14
0
10
20
30
40
50
60
70

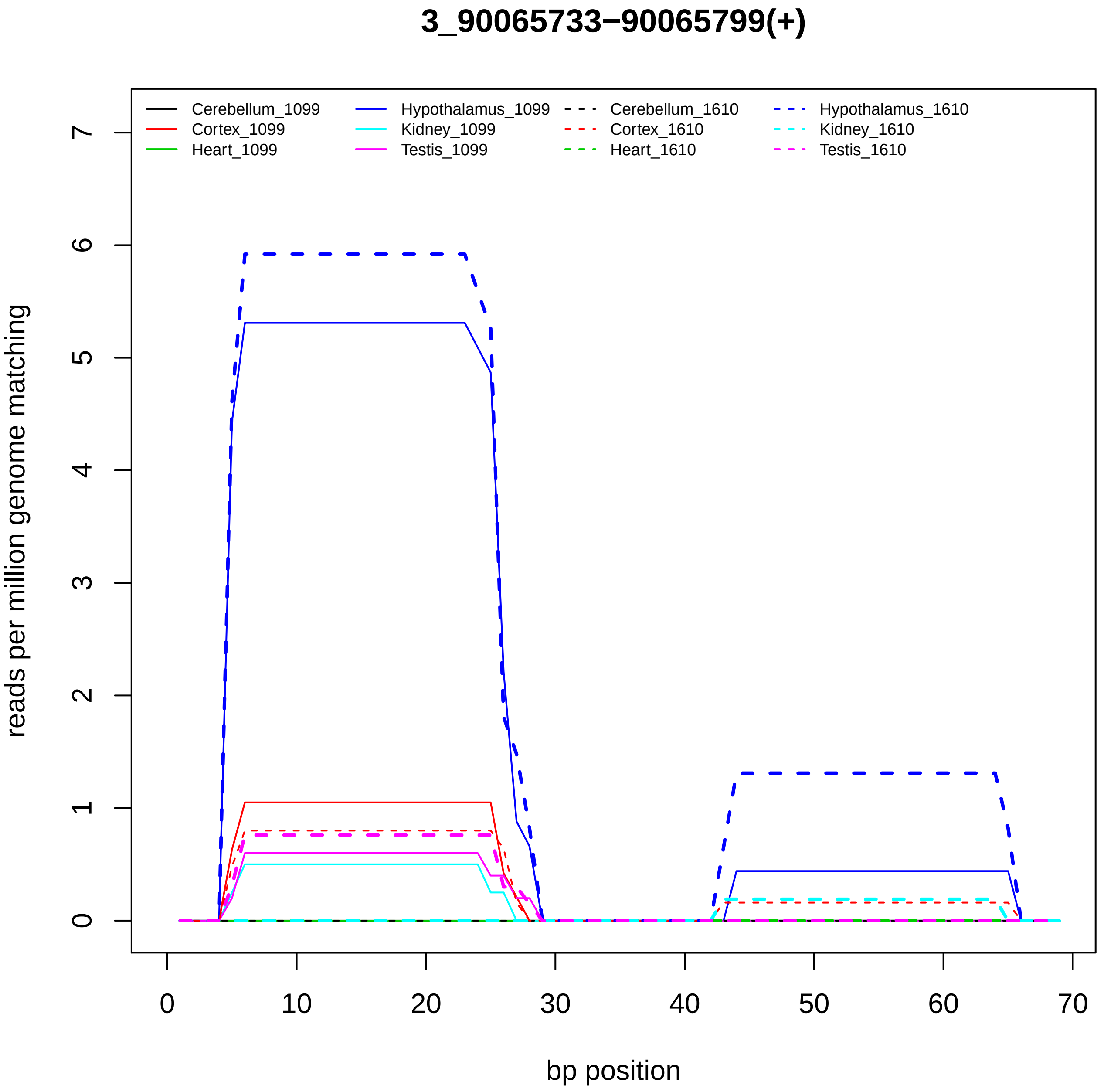

3_90065733−90065799(+)
Cerebellum_1099
Cortex_1099
Heart_1099
Hypothalamus_1099
Kidney_1099
Testis_1099
Cerebellum_1610
Cortex_1610
Heart_1610
Hypothalamus_1610
Kidney_1610
Testis_1610
reads per million genome matching
bp position
0
1
2
3
4
5
6
7
0
10
20
30
40
50
60
70

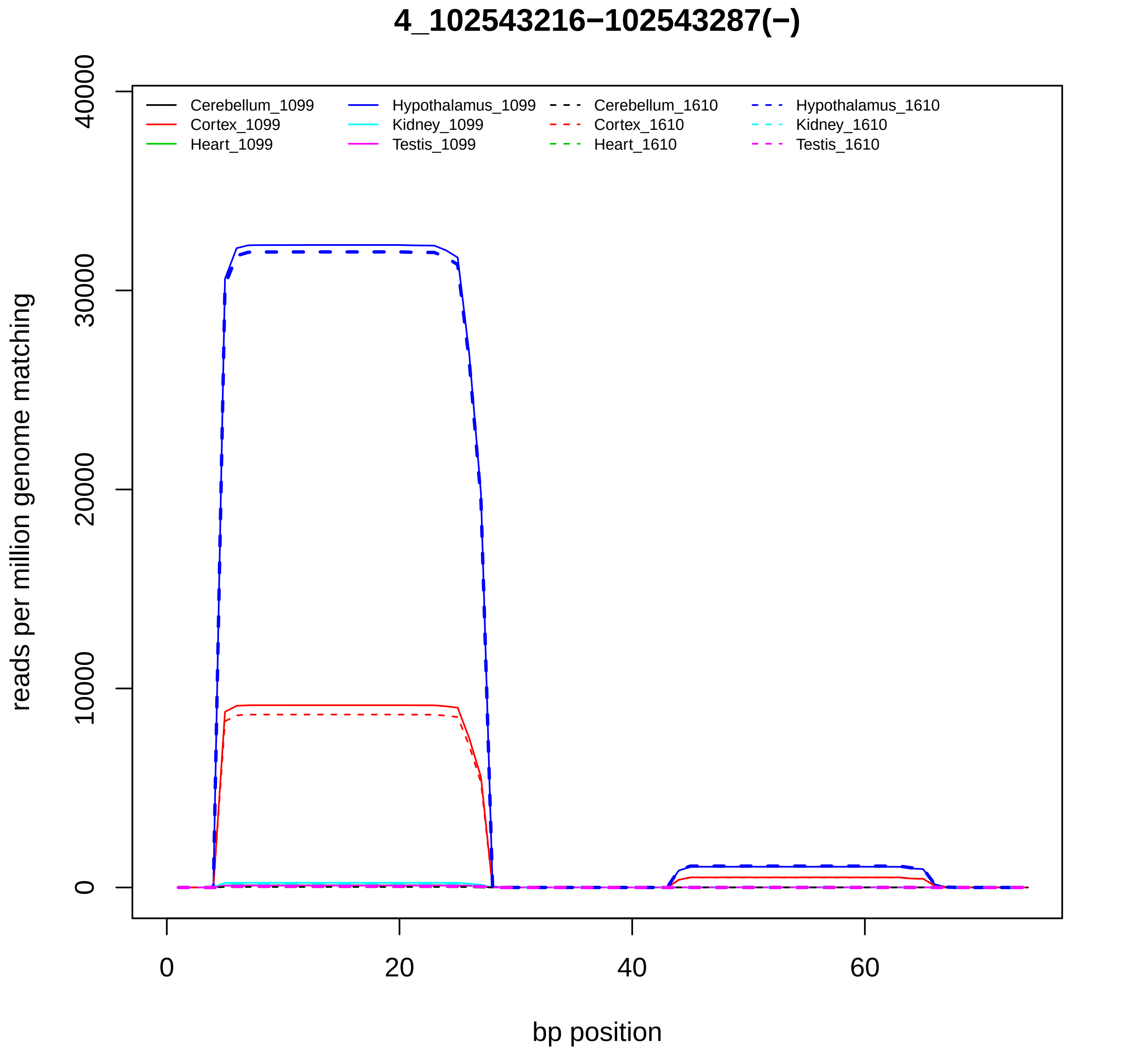

4_102543216−102543287(−)
Cerebellum_1099
Cortex_1099
Heart_1099
Hypothalamus_1099
Kidney_1099
Testis_1099
Cerebellum_1610
Cortex_1610
Heart_1610
Hypothalamus_1610
Kidney_1610
Testis_1610
0
10000
20000
30000
40000
reads per million genome matching
0
20
40
60
bp position

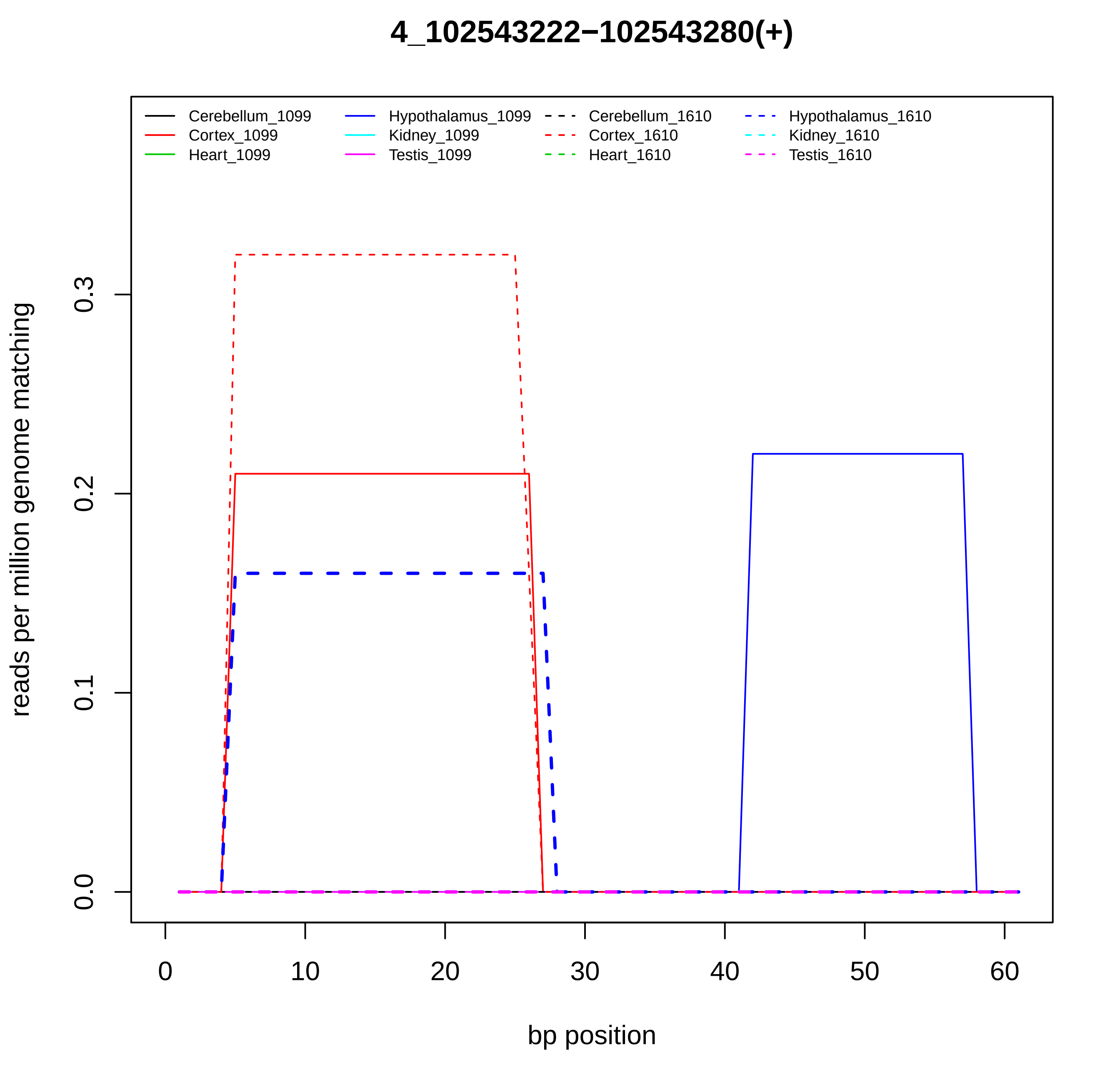

4_102543222−102543280(+)
Cerebellum_1099
Cortex_1099
Heart_1099
Hypothalamus_1099
Kidney_1099
Testis_1099
Cerebellum_1610
Cortex_1610
Heart_1610
Hypothalamus_1610
Kidney_1610
Testis_1610
reads per million genome matching
0.0
0.1
0.2
0.3
0
10
20
30
40
50
60
bp position

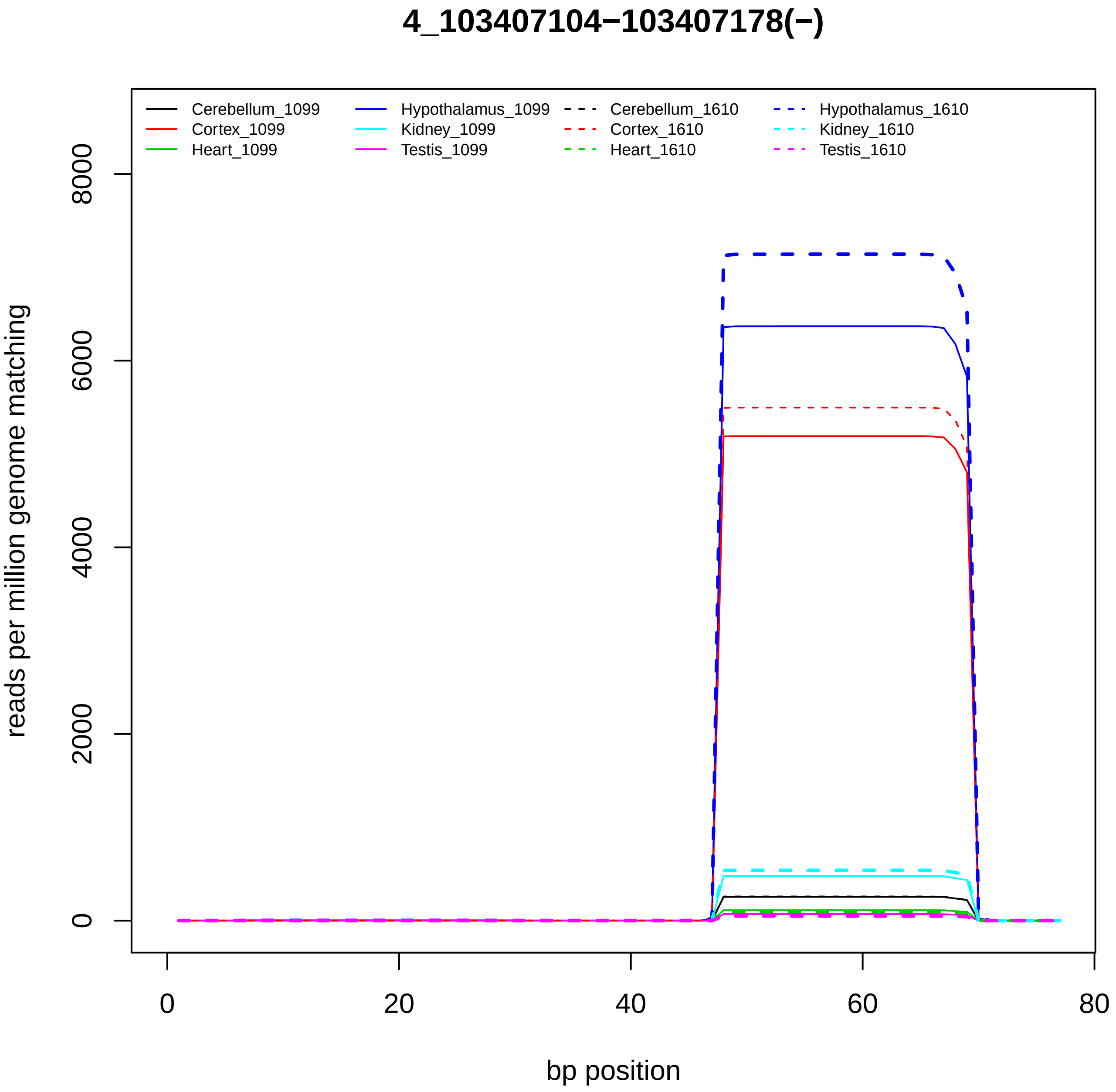

4_103407104−103407178(−)
Cerebellum_1099
Cortex_1099
Heart_1099
Hypothalamus_1099
Kidney_1099
Testis_1099
Cerebellum_1610
Cortex_1610
Heart_1610
Hypothalamus_1610
Kidney_1610
Testis_1610
reads per million genome matching
0
2000
4000
6000
8000
0
20
40
60
80
bp position

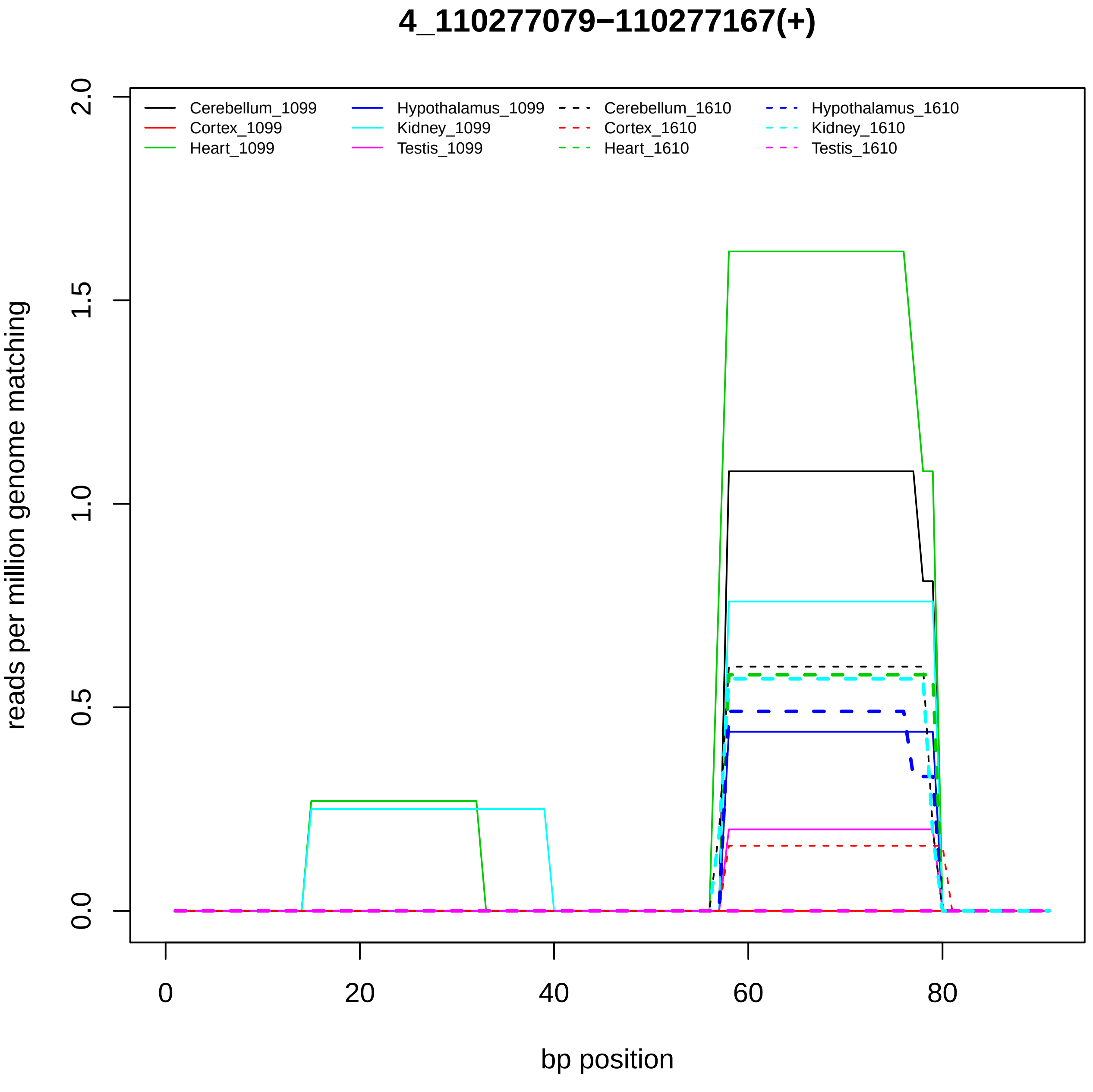
4_110277079−110277167(+)
Cerebellum_1099
Cortex_1099
Heart_1099
Hypothalamus_1099
Kidney_1099
Testis_1099
Cerebellum_1610
Cortex_1610
Heart_1610
Hypothalamus_1610
Kidney_1610
Testis_1610
reads per million genome matching
bp position
0.0
0.5
1.0
1.5
2.0
0
20
40
60
80

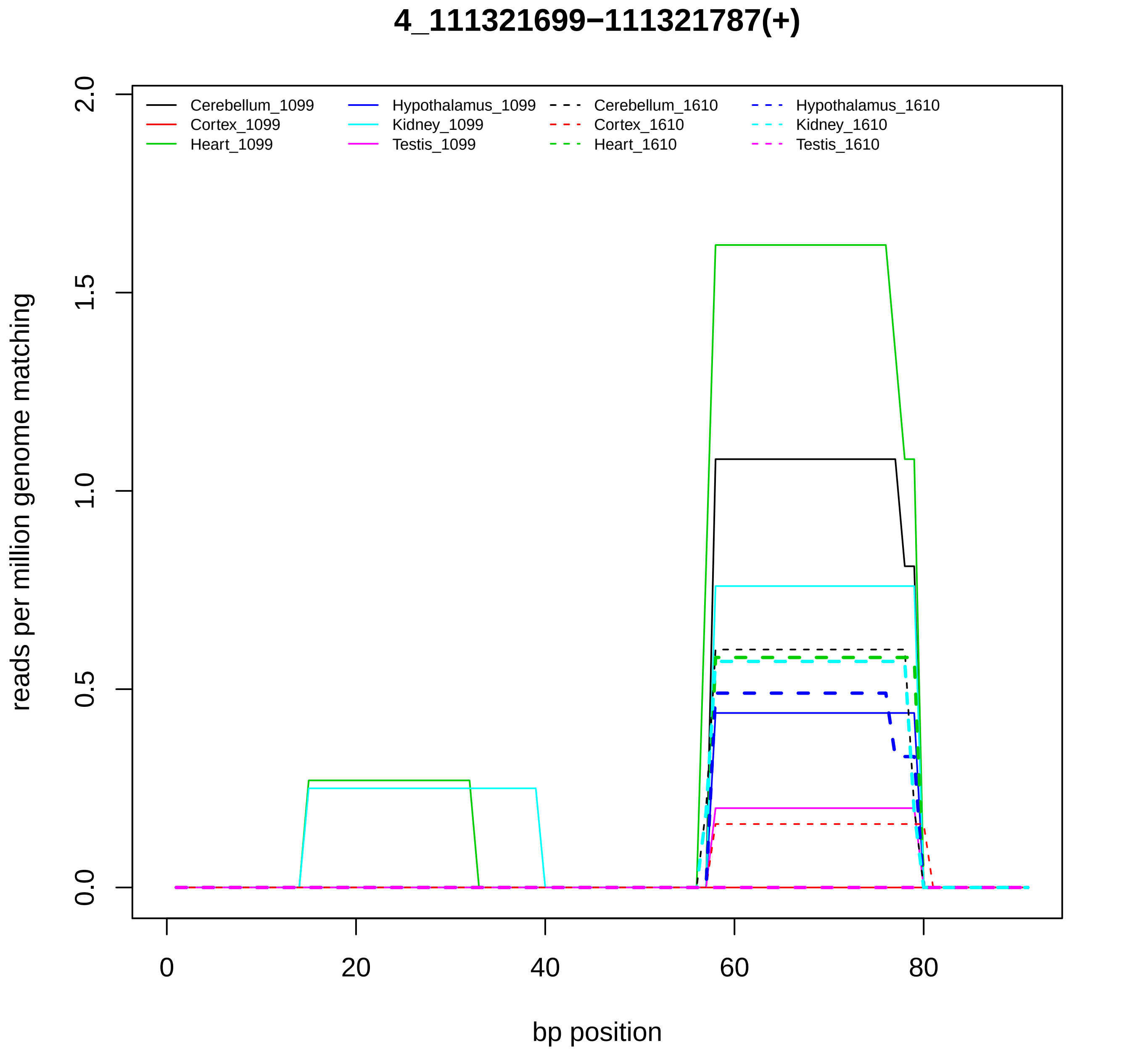

4_111321699−111321787(+)
Cerebellum_1099
Cortex_1099
Heart_1099
Hypothalamus_1099
Kidney_1099
Testis_1099
Cerebellum_1610
Cortex_1610
Heart_1610
Hypothalamus_1610
Kidney_1610
Testis_1610
reads per million genome matching
bp position
0.0
0.5
1.0
1.5
2.0
0
20
40
60
80

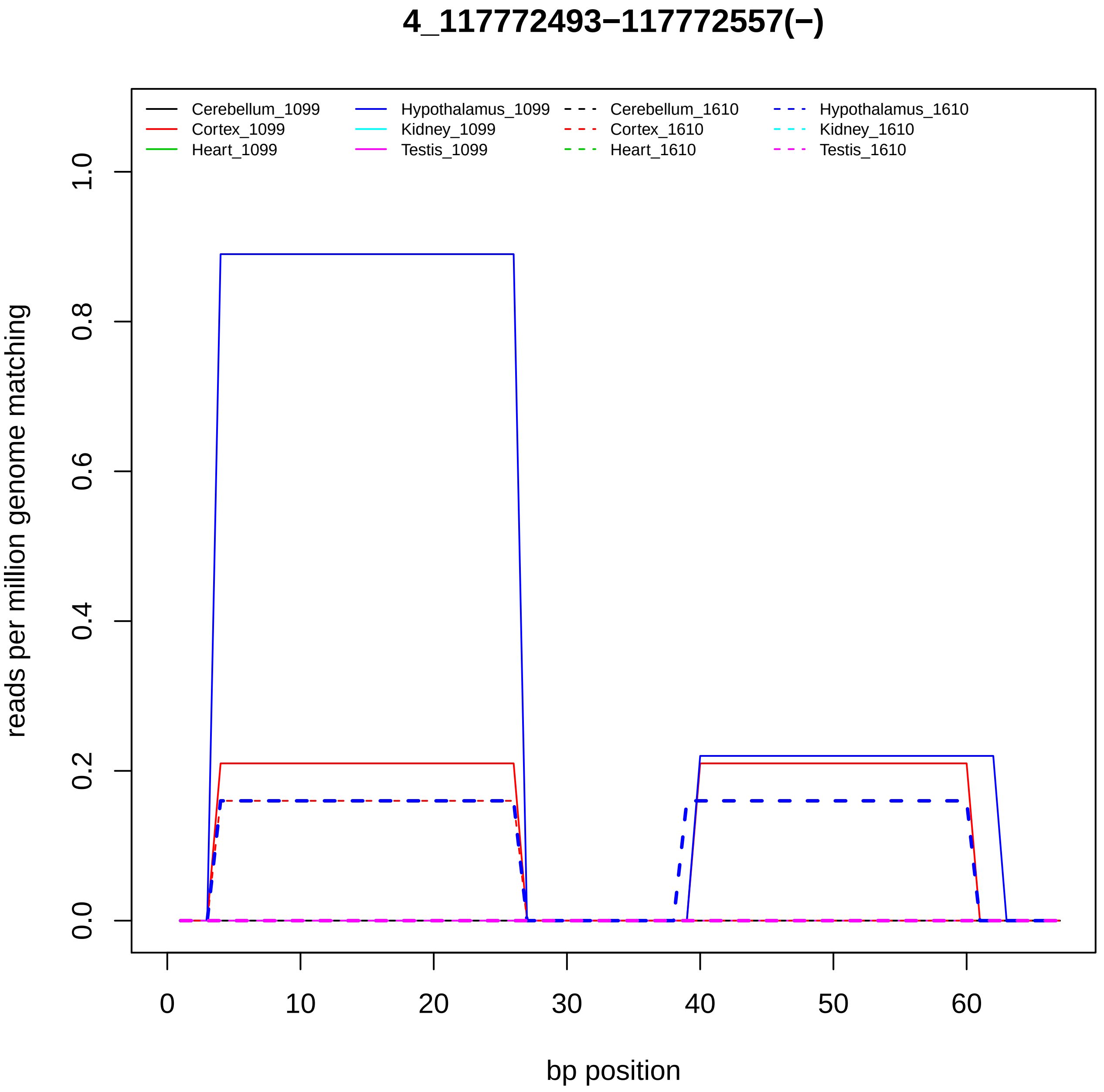

4_117772493−117772557(−)
Cerebellum_1099
Cortex_1099
Heart_1099
Hypothalamus_1099
Kidney_1099
Testis_1099
Cerebellum_1610
Cortex_1610
Heart_1610
Hypothalamus_1610
Kidney_1610
Testis_1610
reads per million genome matching
0.0
0.2
0.4
0.6
0.8
1.0
0
10
20
30
40
50
60
bp position

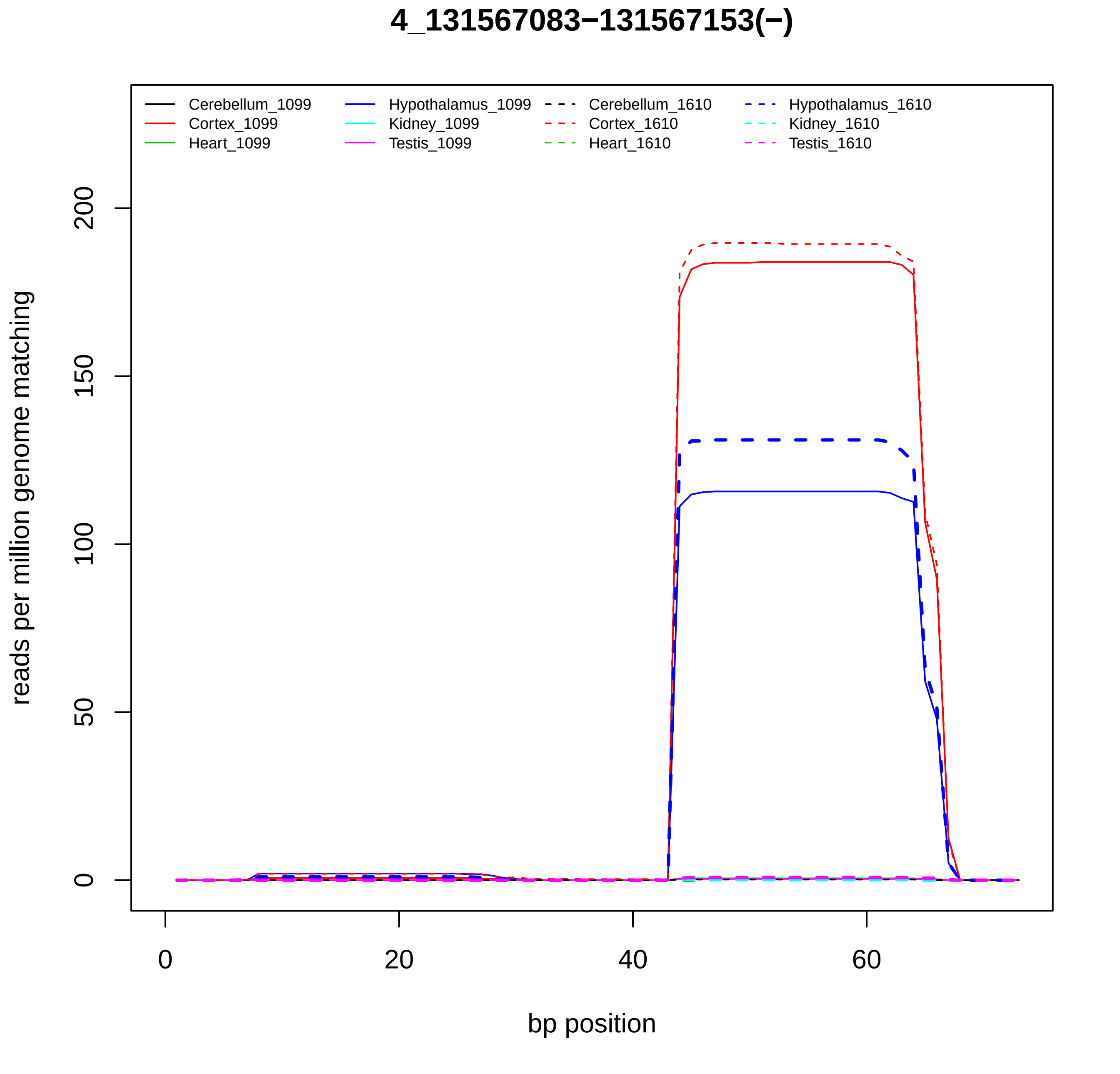

4_131567083−131567153(−)
Cerebellum_1099
Cortex_1099
Heart_1099
Hypothalamus_1099
Kidney_1099
Testis_1099
Cerebellum_1610
Cortex_1610
Heart_1610
Hypothalamus_1610
Kidney_1610
Testis_1610
reads per million genome matching
0
50
100
150
200
0
20
40
60
bp position

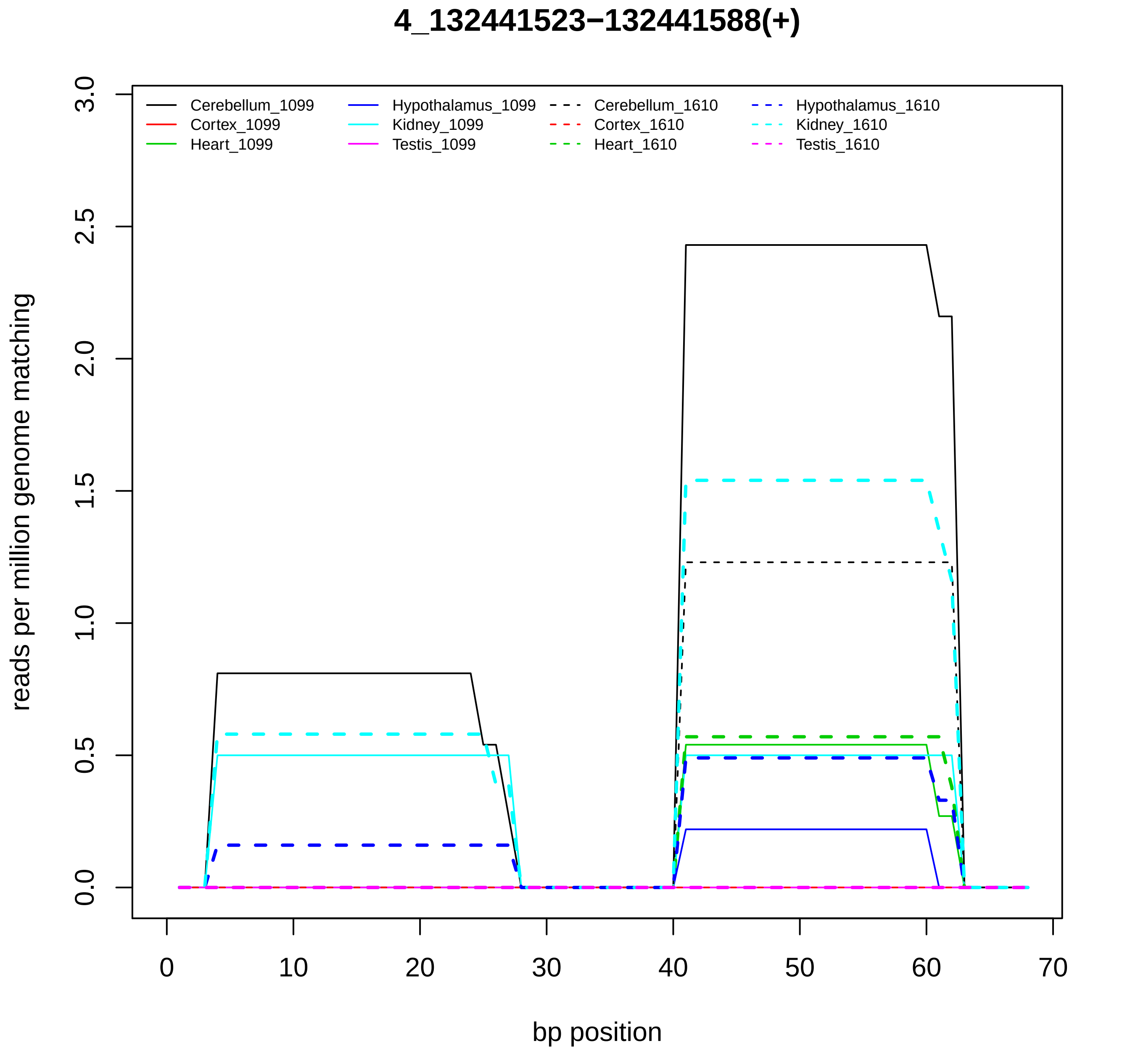

4_132441523−132441588(+)
Cerebellum_1099
Cortex_1099
Heart_1099
Hypothalamus_1099
Kidney_1099
Testis_1099
Cerebellum_1610
Cortex_1610
Heart_1610
Hypothalamus_1610
Kidney_1610
Testis_1610
reads per million genome matching
bp position
0.0
0.5
1.0
1.5
2.0
2.5
3.0
0
10
20
30
40
50
60
70

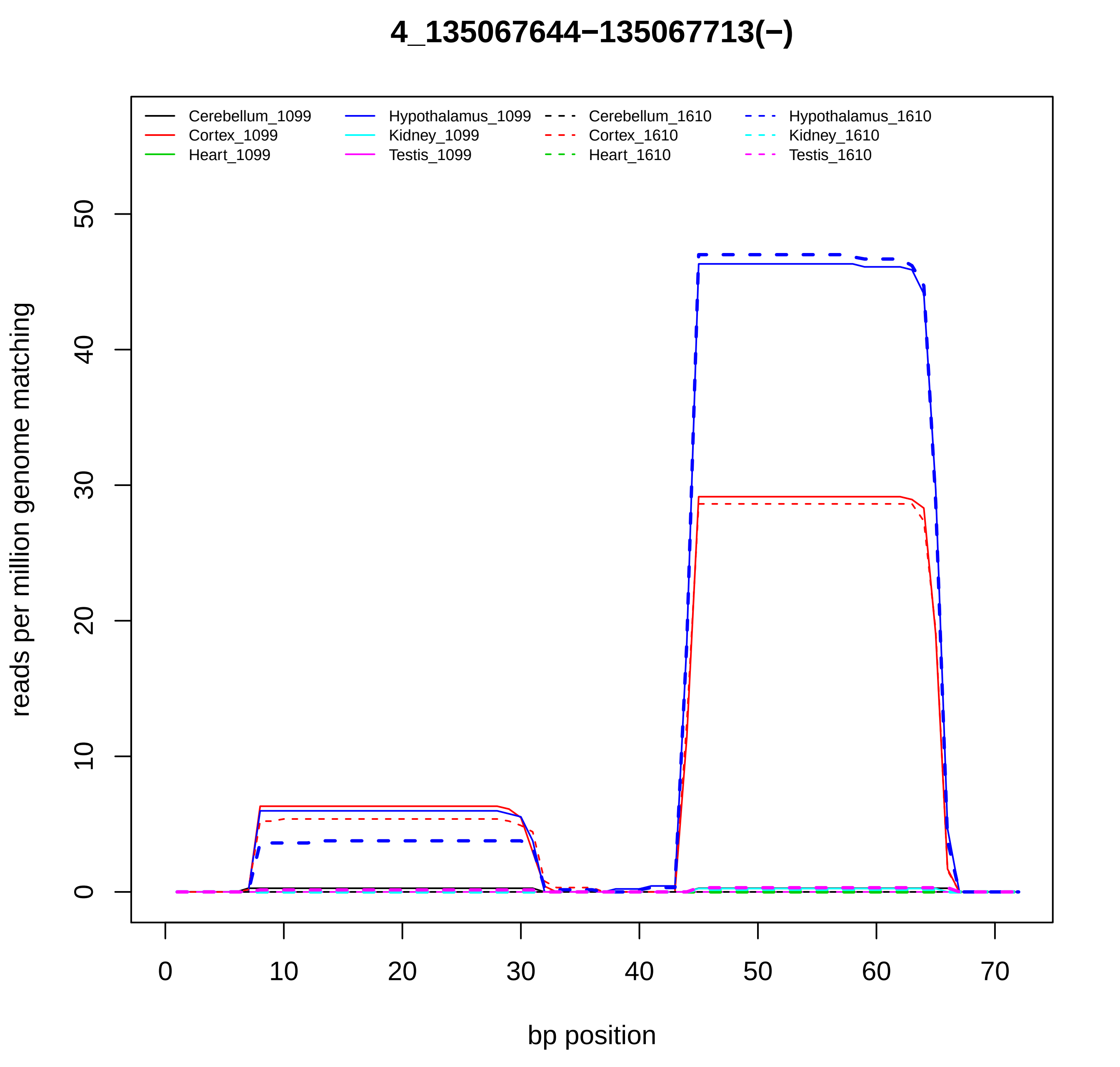

4_135067644−135067713(−)
Cerebellum_1099
Cortex_1099
Heart_1099
Hypothalamus_1099
Kidney_1099
Testis_1099
Cerebellum_1610
Cortex_1610
Heart_1610
Hypothalamus_1610
Kidney_1610
Testis_1610
reads per million genome matching
0
10
20
30
40
50
0
10
20
30
40
50
60
70
bp position

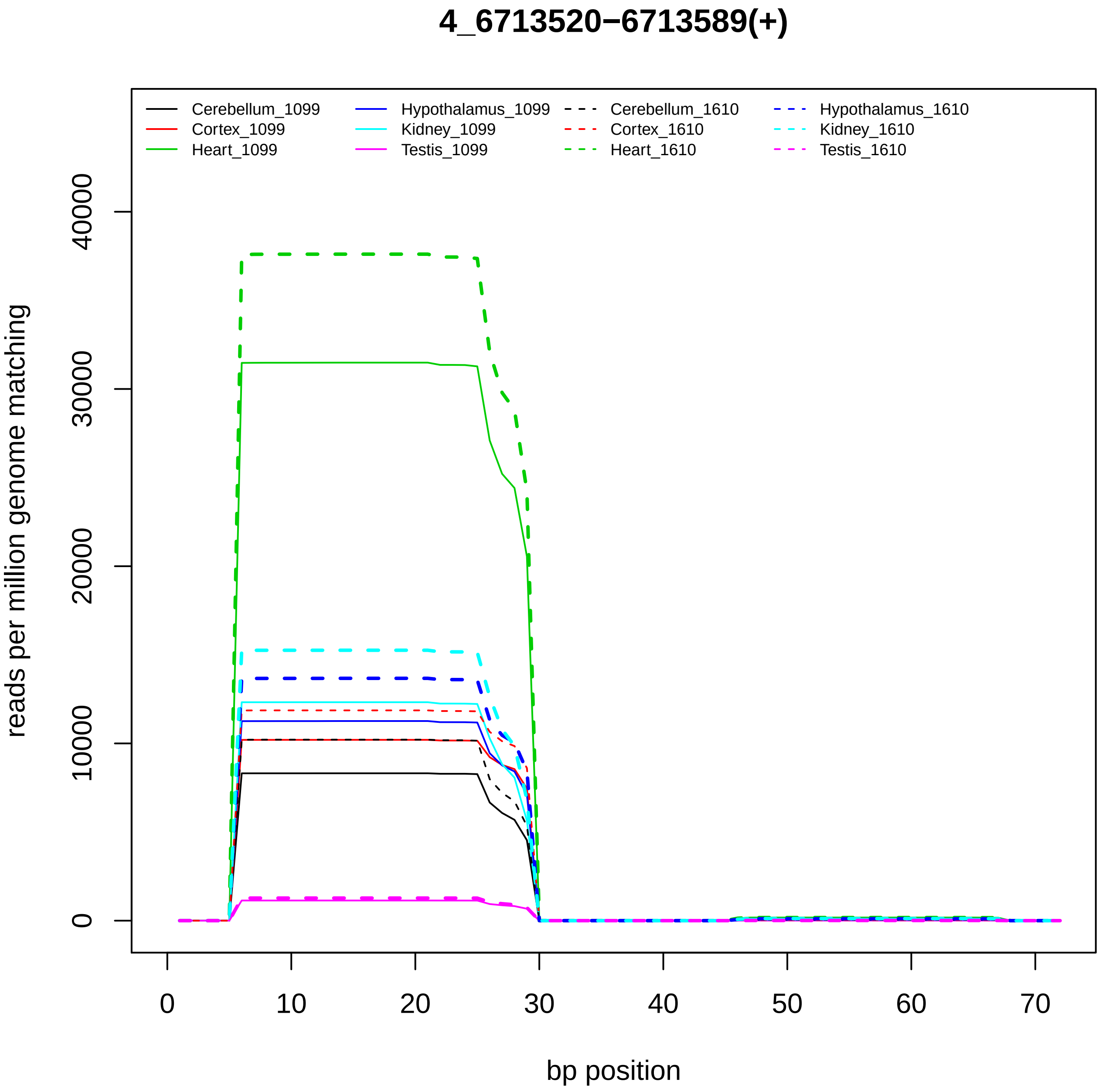

4_6713520−6713589(+)
Cerebellum_1099
Cortex_1099
Heart_1099
Hypothalamus_1099
Kidney_1099
Testis_1099
Cerebellum_1610
Cortex_1610
Heart_1610
Hypothalamus_1610
Kidney_1610
Testis_1610
reads per million genome matching
0
10000
20000
30000
40000
0
10
20
30
40
50
60
70
bp position

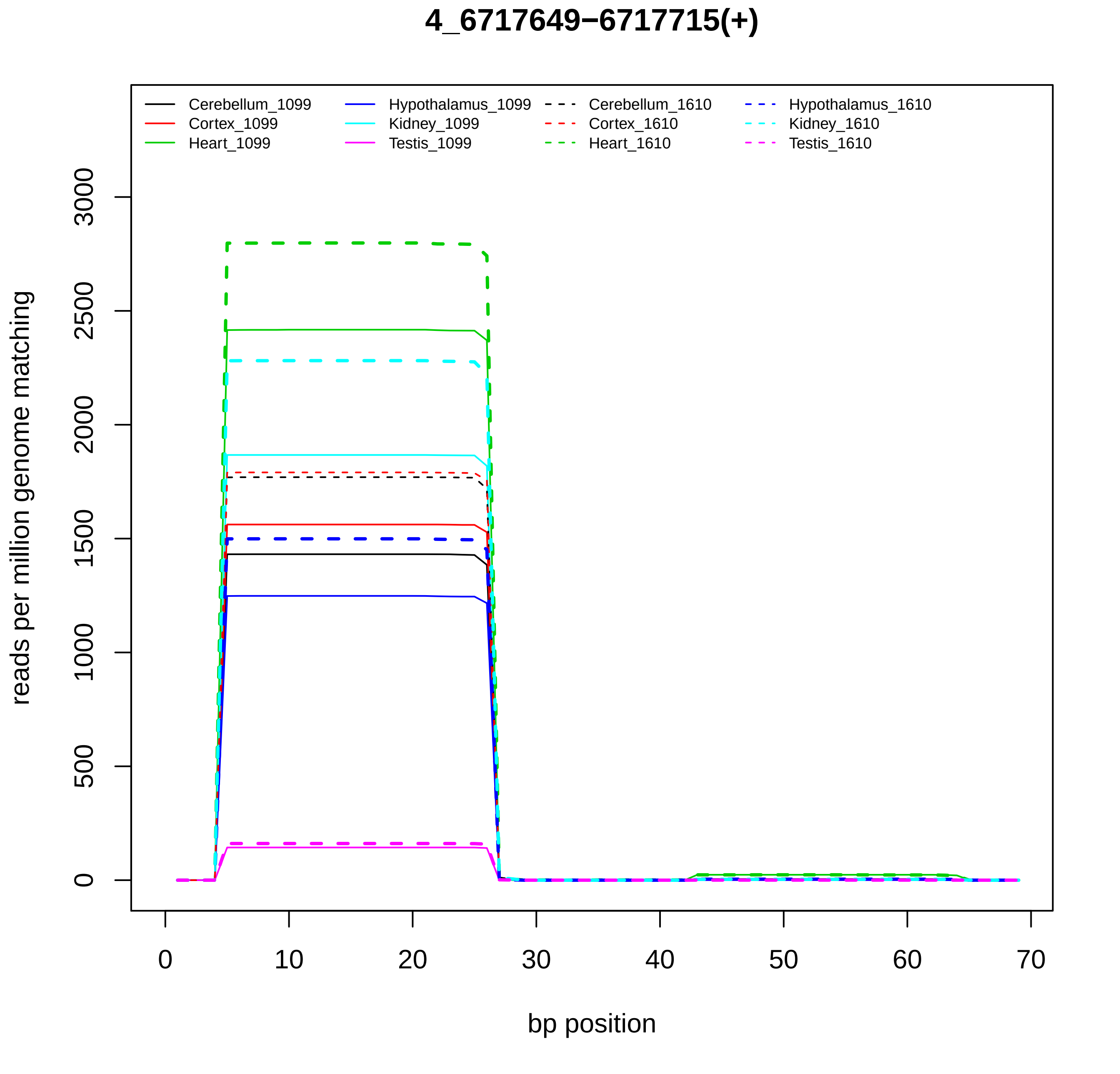

4_6717649−6717715(+)
Cerebellum_1099
Cortex_1099
Heart_1099
Hypothalamus_1099
Kidney_1099
Testis_1099
Cerebellum_1610
Cortex_1610
Heart_1610
Hypothalamus_1610
Kidney_1610
Testis_1610
reads per million genome matching
0
500
1000
1500
2000
2500
3000
0
10
20
30
40
50
60
70
bp position

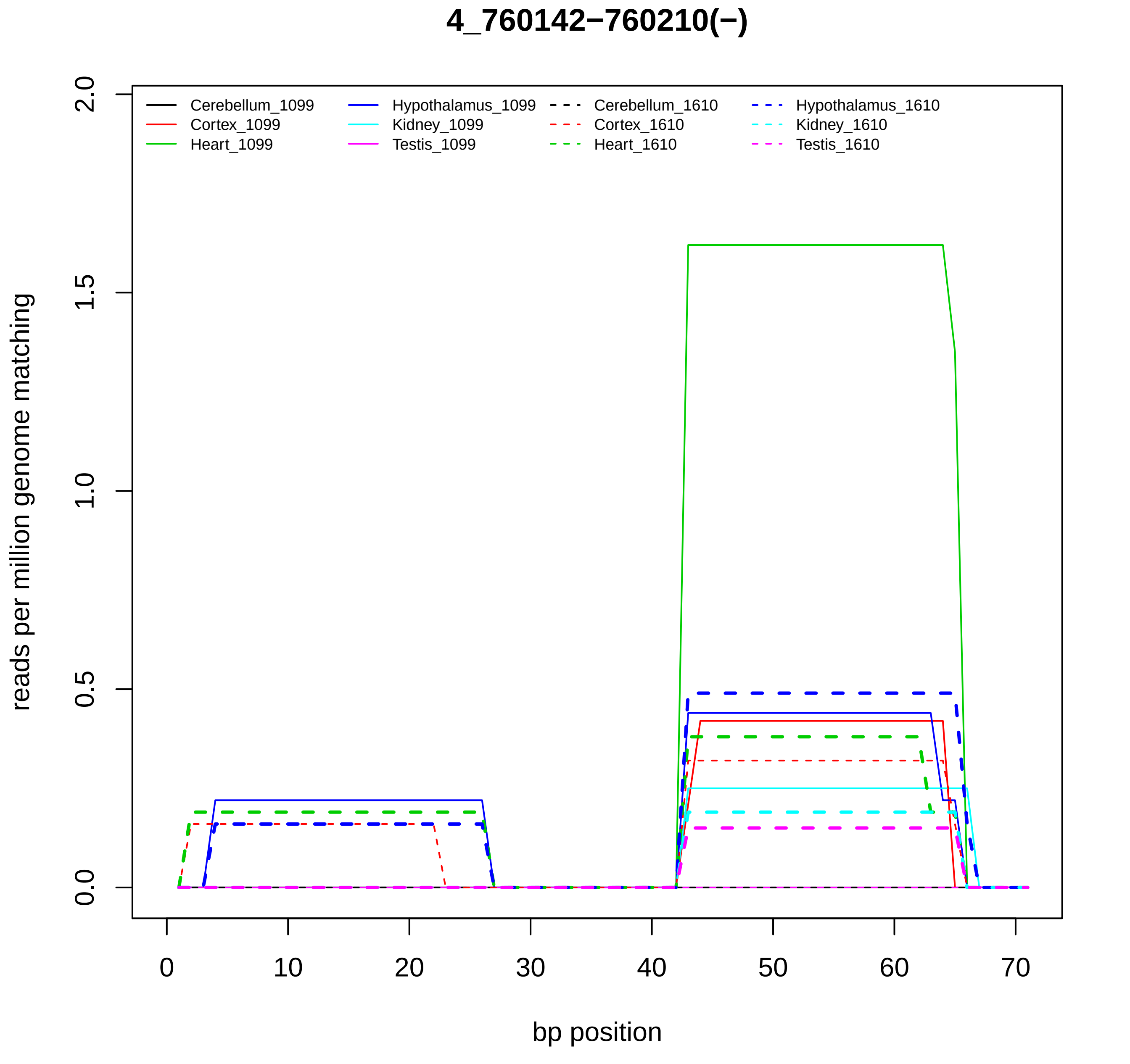

4_760142−760210(−)
Cerebellum_1099
Cortex_1099
Heart_1099
Hypothalamus_1099
Kidney_1099
Testis_1099
Cerebellum_1610
Cortex_1610
Heart_1610
Hypothalamus_1610
Kidney_1610
Testis_1610
reads per million genome matching
bp position
0.0
0.5
1.0
1.5
2.0
0
10
20
30
40
50
60
70

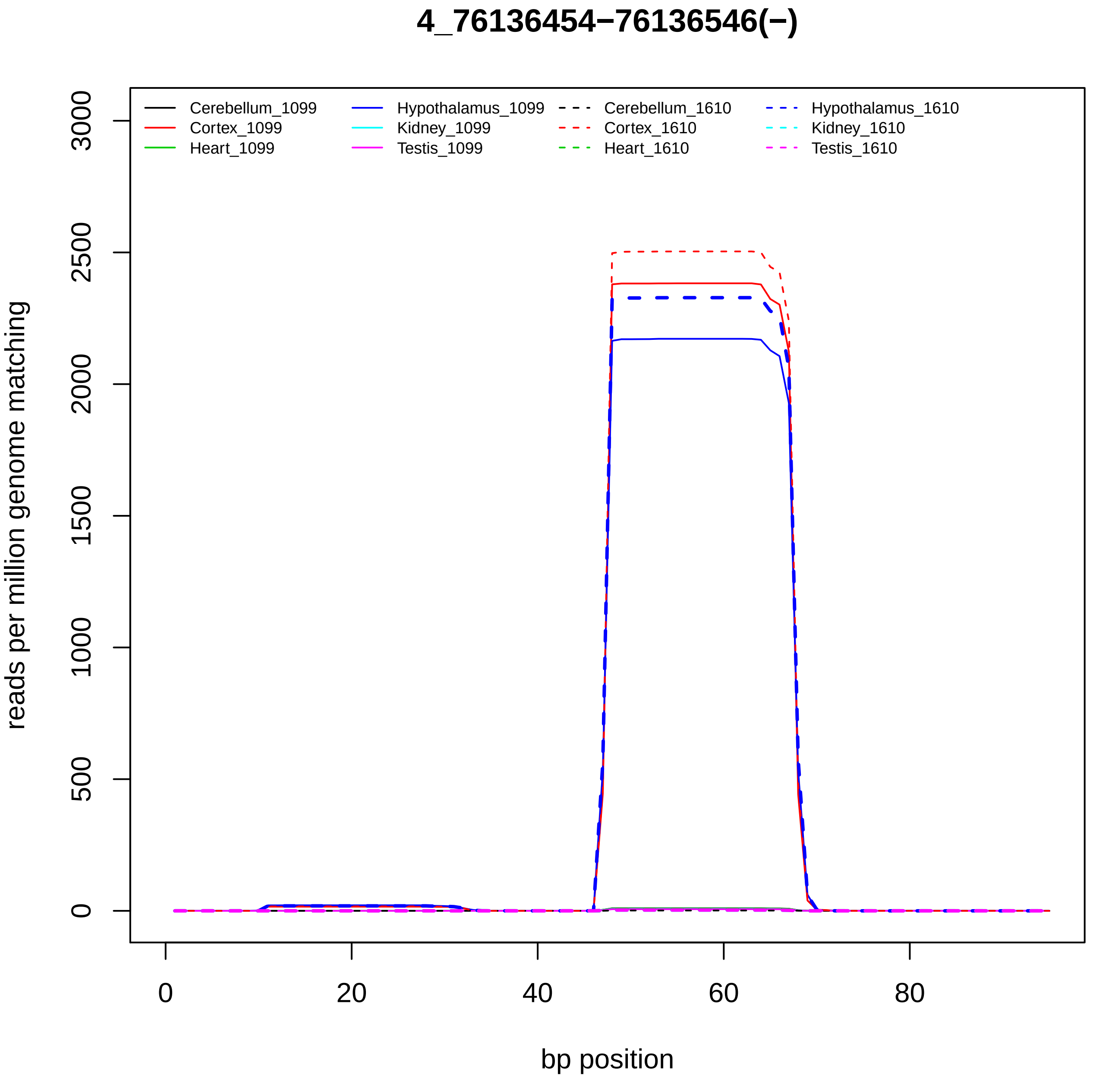

4_76136454−76136546(−)
Cerebellum_1099
Cortex_1099
Heart_1099
Hypothalamus_1099
Kidney_1099
Testis_1099
Cerebellum_1610
Cortex_1610
Heart_1610
Hypothalamus_1610
Kidney_1610
Testis_1610
reads per million genome matching
0
500
1000
1500
2000
2500
3000
bp position
0
20
40
60
80

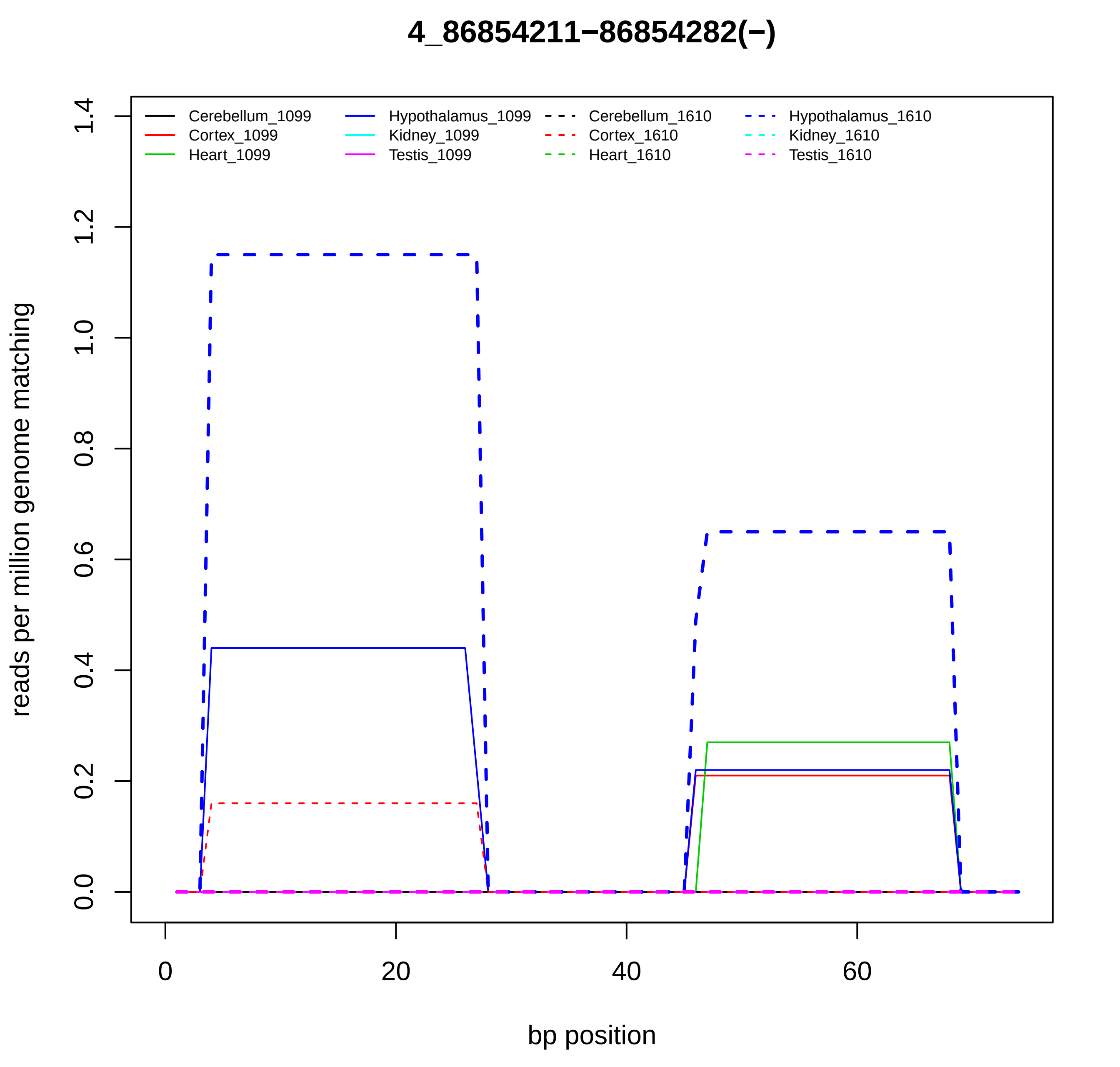

4_86854211−86854282(−)
Cerebellum_1099
Cortex_1099
Heart_1099
Hypothalamus_1099
Kidney_1099
Testis_1099
Cerebellum_1610
Cortex_1610
Heart_1610
Hypothalamus_1610
Kidney_1610
Testis_1610
reads per million genome matching
0.0
0.2
0.4
0.6
0.8
1.0
1.2
1.4
0
20
40
60
bp position

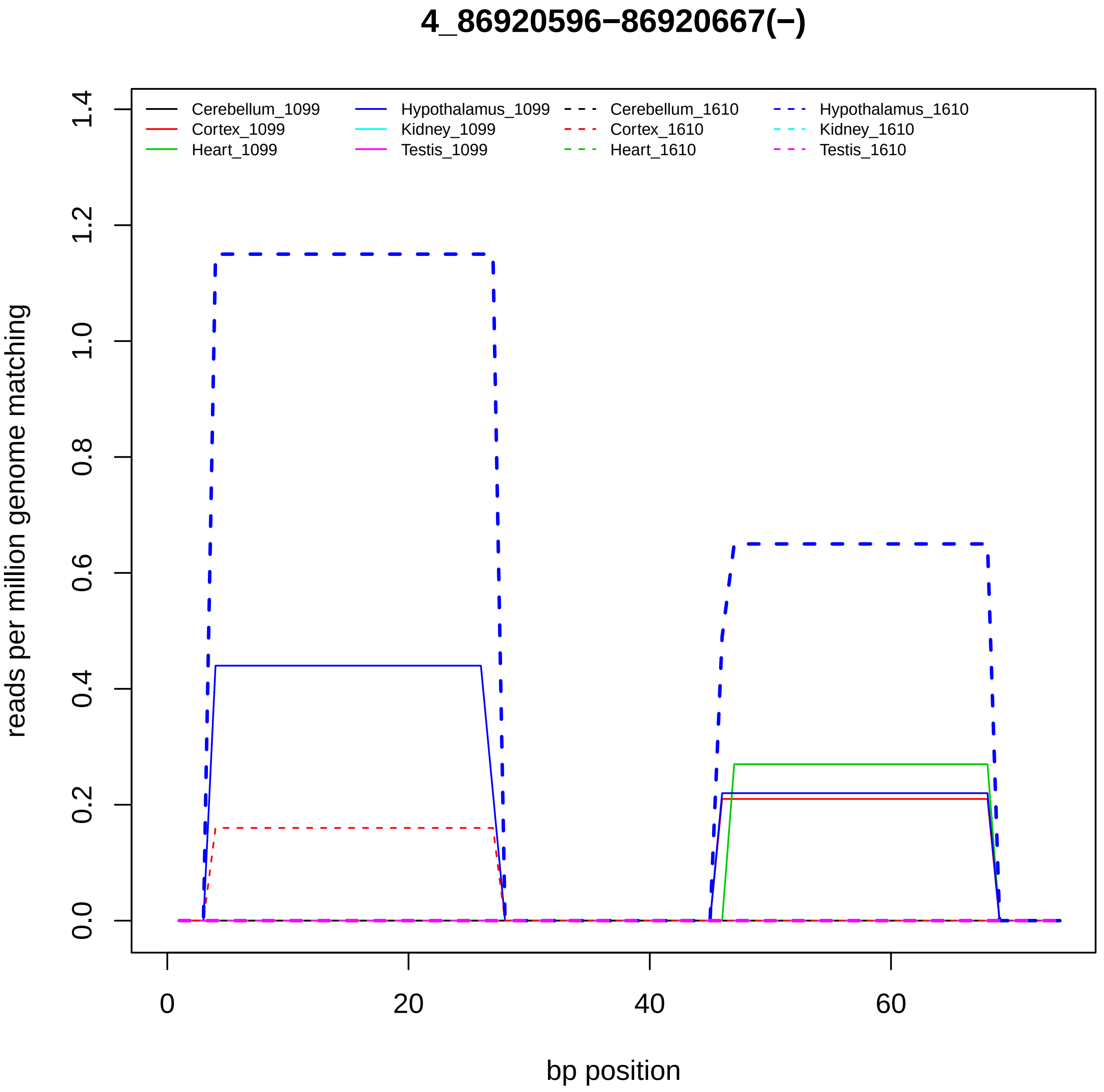

4_86920596−86920667(−)
Cerebellum_1099
Cortex_1099
Heart_1099
Hypothalamus_1099
Kidney_1099
Testis_1099
Cerebellum_1610
Cortex_1610
Heart_1610
Hypothalamus_1610
Kidney_1610
Testis_1610
reads per million genome matching
bp position
0.0
0.2
0.4
0.6
0.8
1.0
1.2
1.4
0
20
40
60

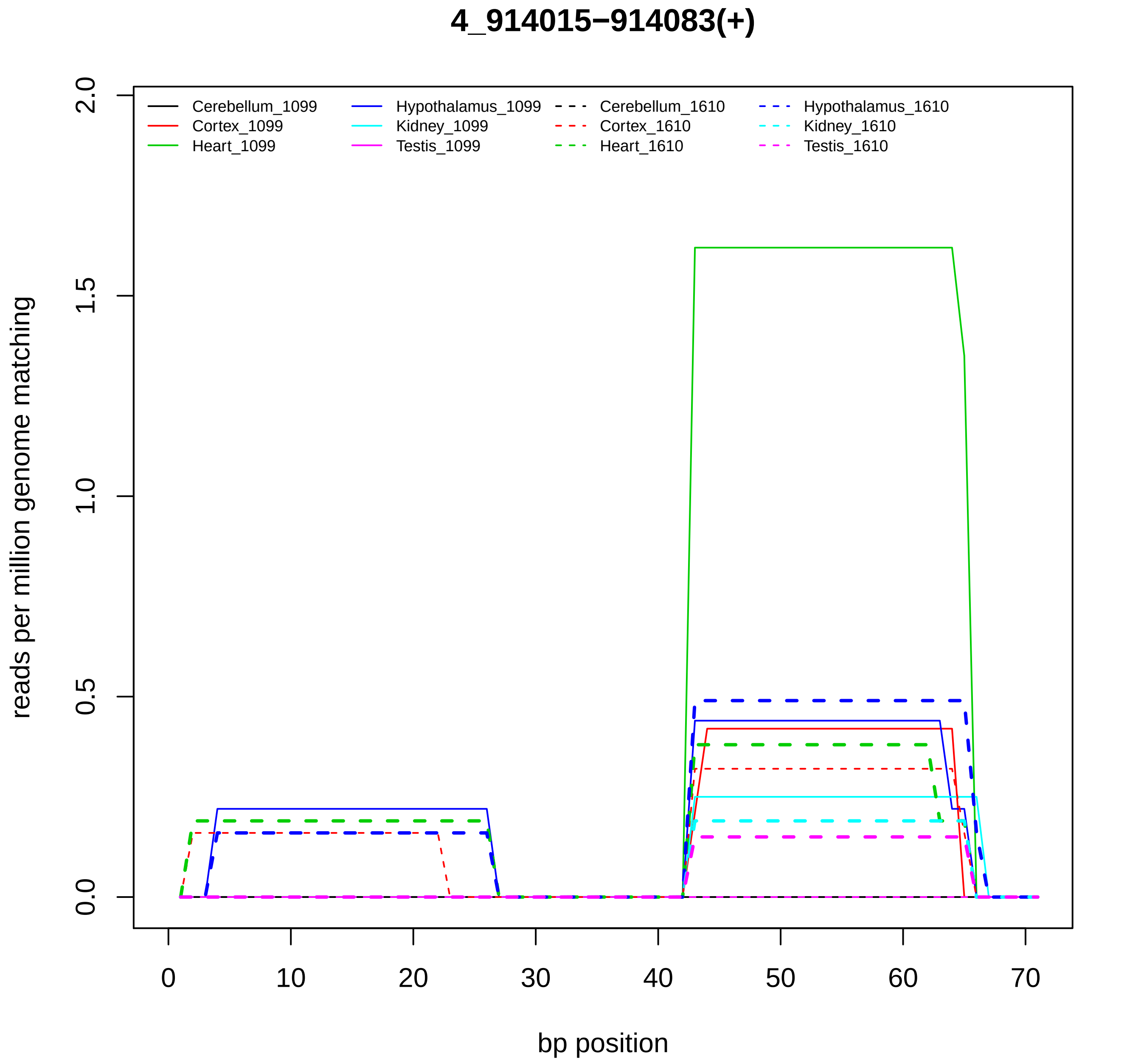

4_914015−914083(+)
Cerebellum_1099
Cortex_1099
Heart_1099
Hypothalamus_1099
Kidney_1099
Testis_1099
Cerebellum_1610
Cortex_1610
Heart_1610
Hypothalamus_1610
Kidney_1610
Testis_1610
reads per million genome matching
bp position
0.0
0.5
1.0
1.5
2.0
0
10
20
30
40
50
60
70

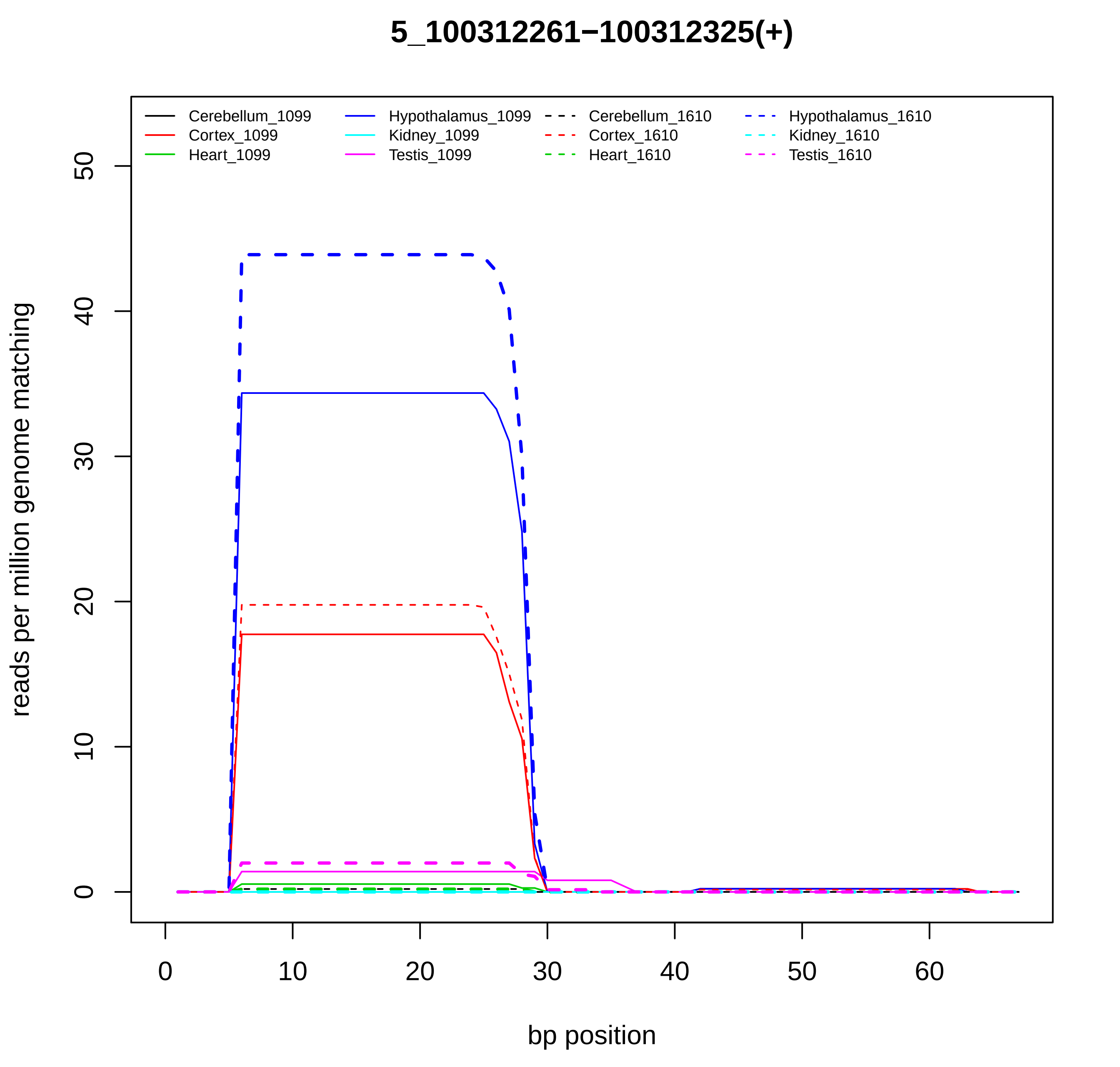

5_100312261−100312325(+)
Cerebellum_1099
Cortex_1099
Heart_1099
Hypothalamus_1099
Kidney_1099
Testis_1099
Cerebellum_1610
Cortex_1610
Heart_1610
Hypothalamus_1610
Kidney_1610
Testis_1610
reads per million genome matching
0
10
20
30
40
50
0
10
20
30
40
50
60
bp position

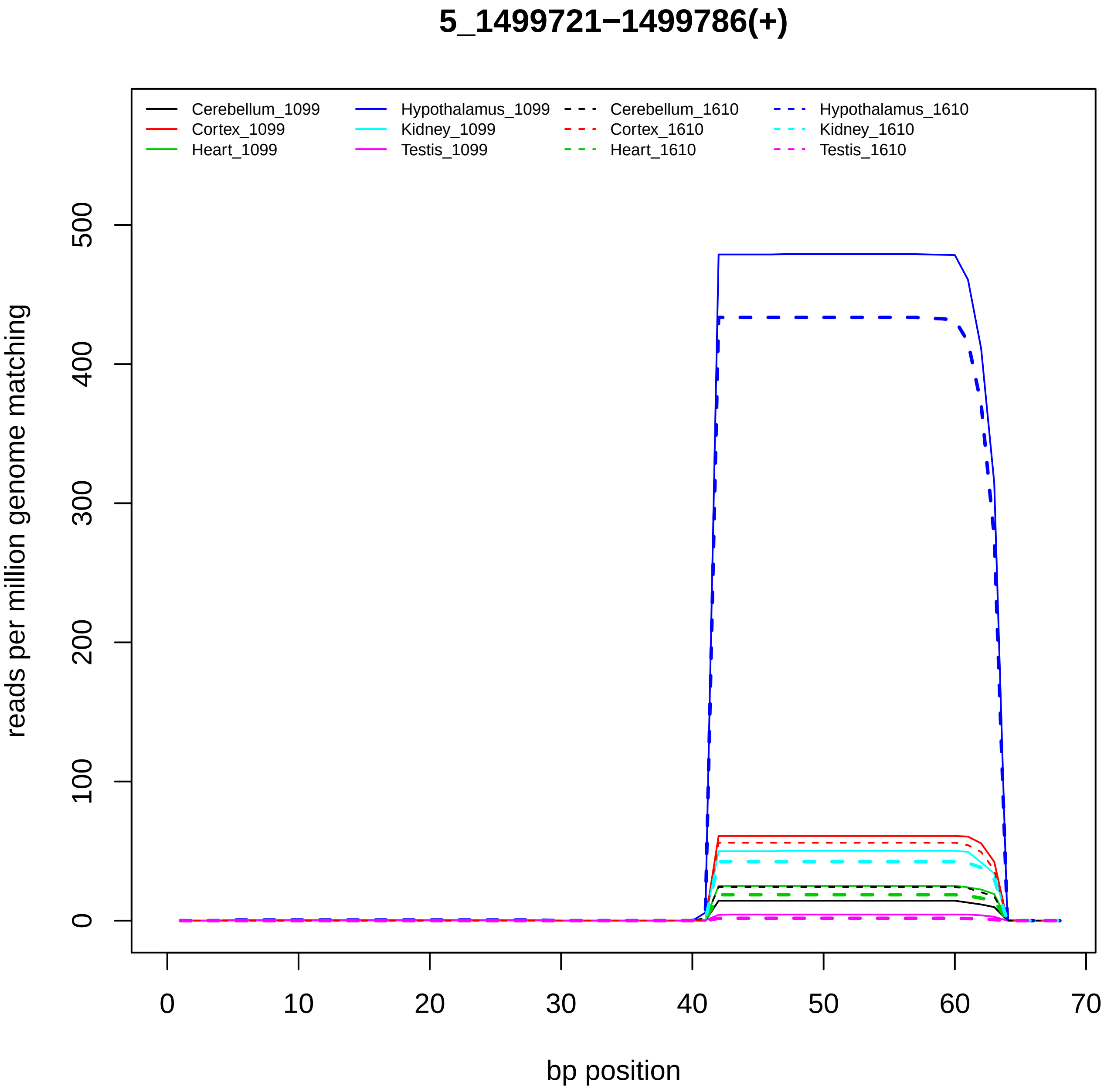

5_1499721−1499786(+)
Cerebellum_1099
Cortex_1099
Heart_1099
Hypothalamus_1099
Kidney_1099
Testis_1099
Cerebellum_1610
Cortex_1610
Heart_1610
Hypothalamus_1610
Kidney_1610
Testis_1610
reads per million genome matching
0
100
200
300
400
500
0
10
20
30
40
50
60
70
bp position

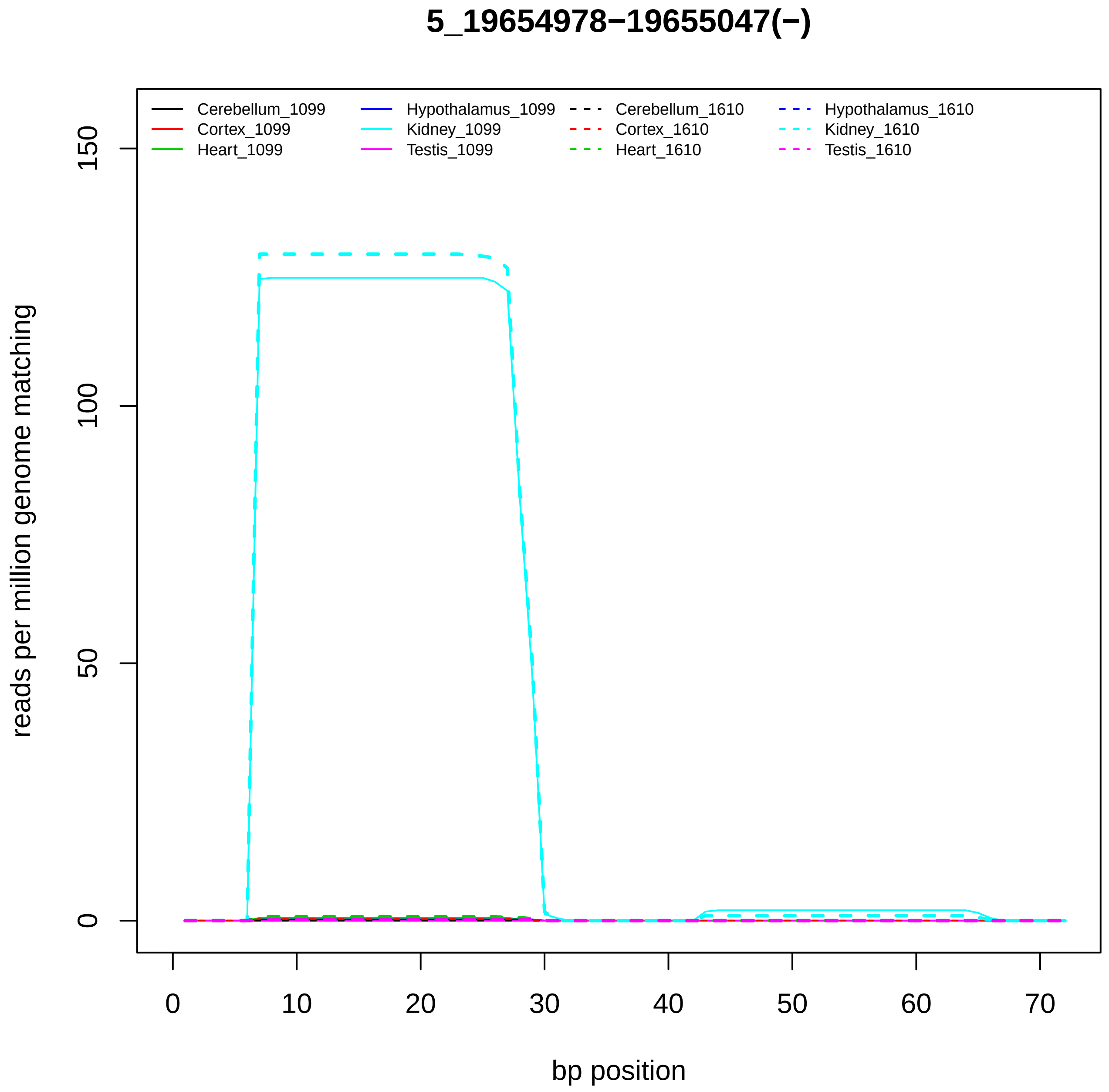

5_19654978−19655047(−)
Cerebellum_1099
Cortex_1099
Heart_1099
Hypothalamus_1099
Kidney_1099
Testis_1099
Cerebellum_1610
Cortex_1610
Heart_1610
Hypothalamus_1610
Kidney_1610
Testis_1610
reads per million genome matching
bp position
0
50
100
150
0
10
20
30
40
50
60
70

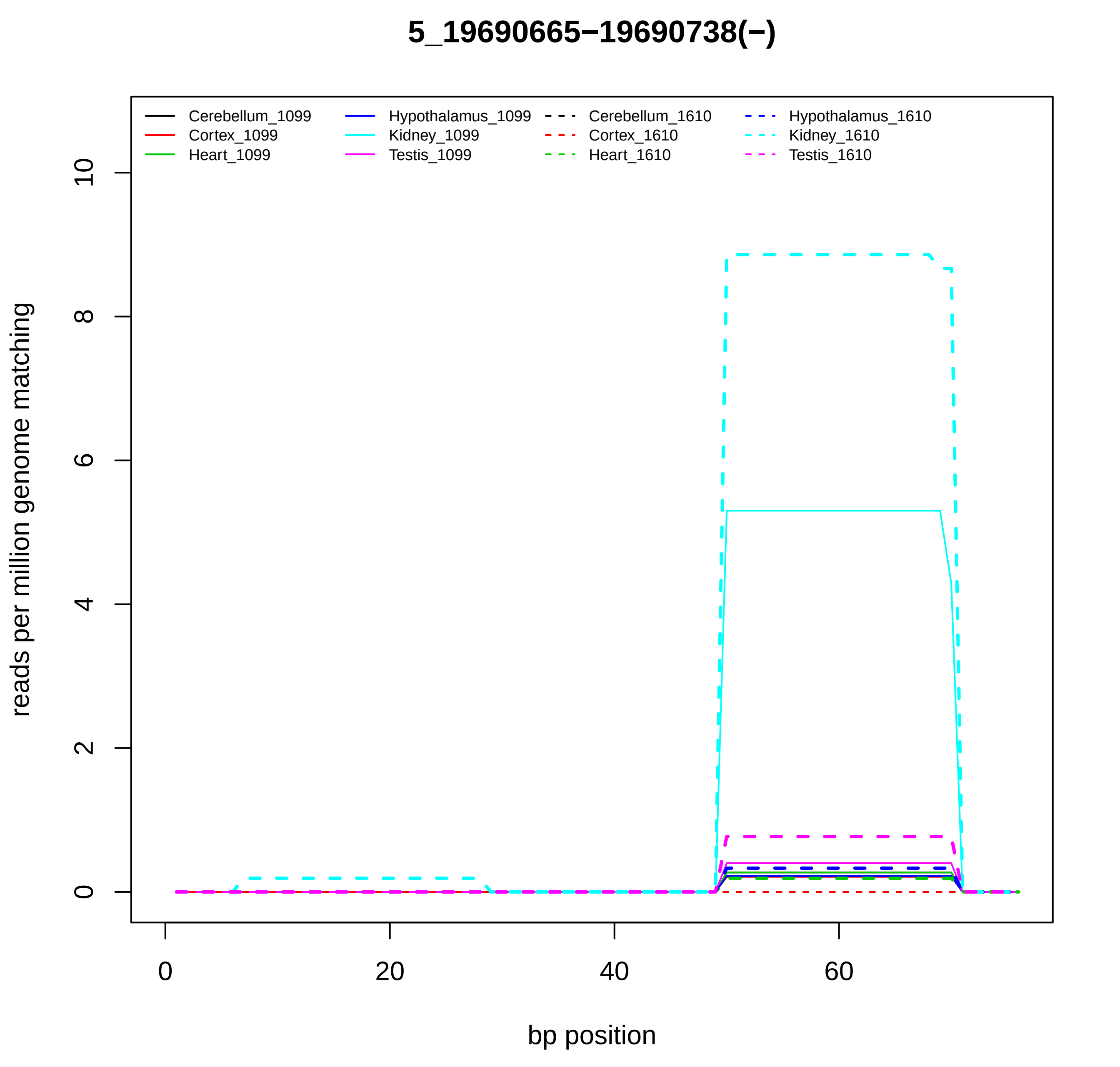
5_19690665−19690738(−)
Cerebellum_1099
Cortex_1099
Heart_1099
Hypothalamus_1099
Kidney_1099
Testis_1099
Cerebellum_1610
Cortex_1610
Heart_1610
Hypothalamus_1610
Kidney_1610
Testis_1610
reads per million genome matching
0
2
4
6
8
10
0
20
40
60
bp position

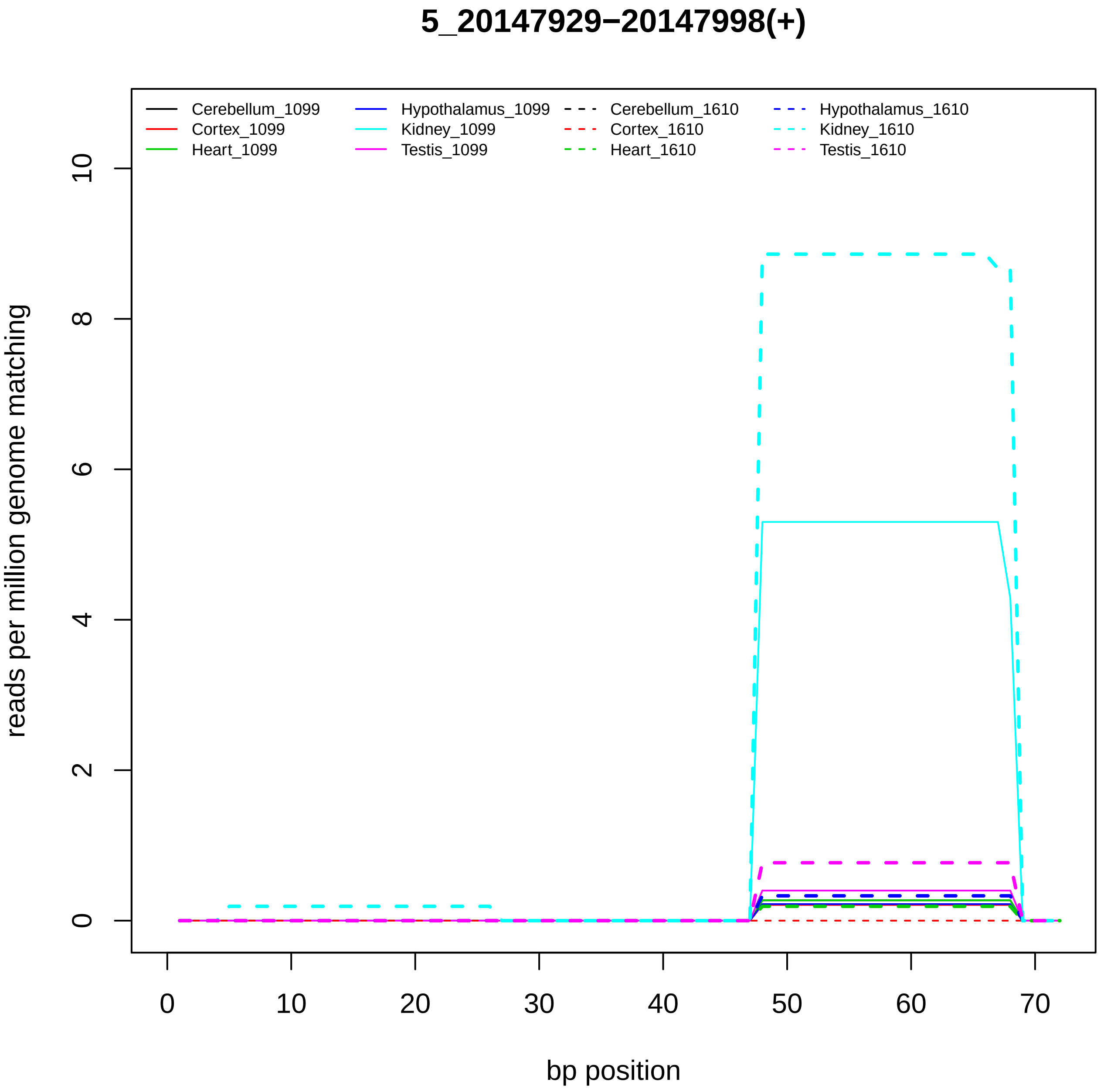

5_20147929−20147998(+)
Cerebellum_1099
Cortex_1099
Heart_1099
Hypothalamus_1099
Kidney_1099
Testis_1099
Cerebellum_1610
Cortex_1610
Heart_1610
Hypothalamus_1610
Kidney_1610
Testis_1610
reads per million genome matching
0
2
4
6
8
10
0
10
20
30
40
50
60
70
bp position

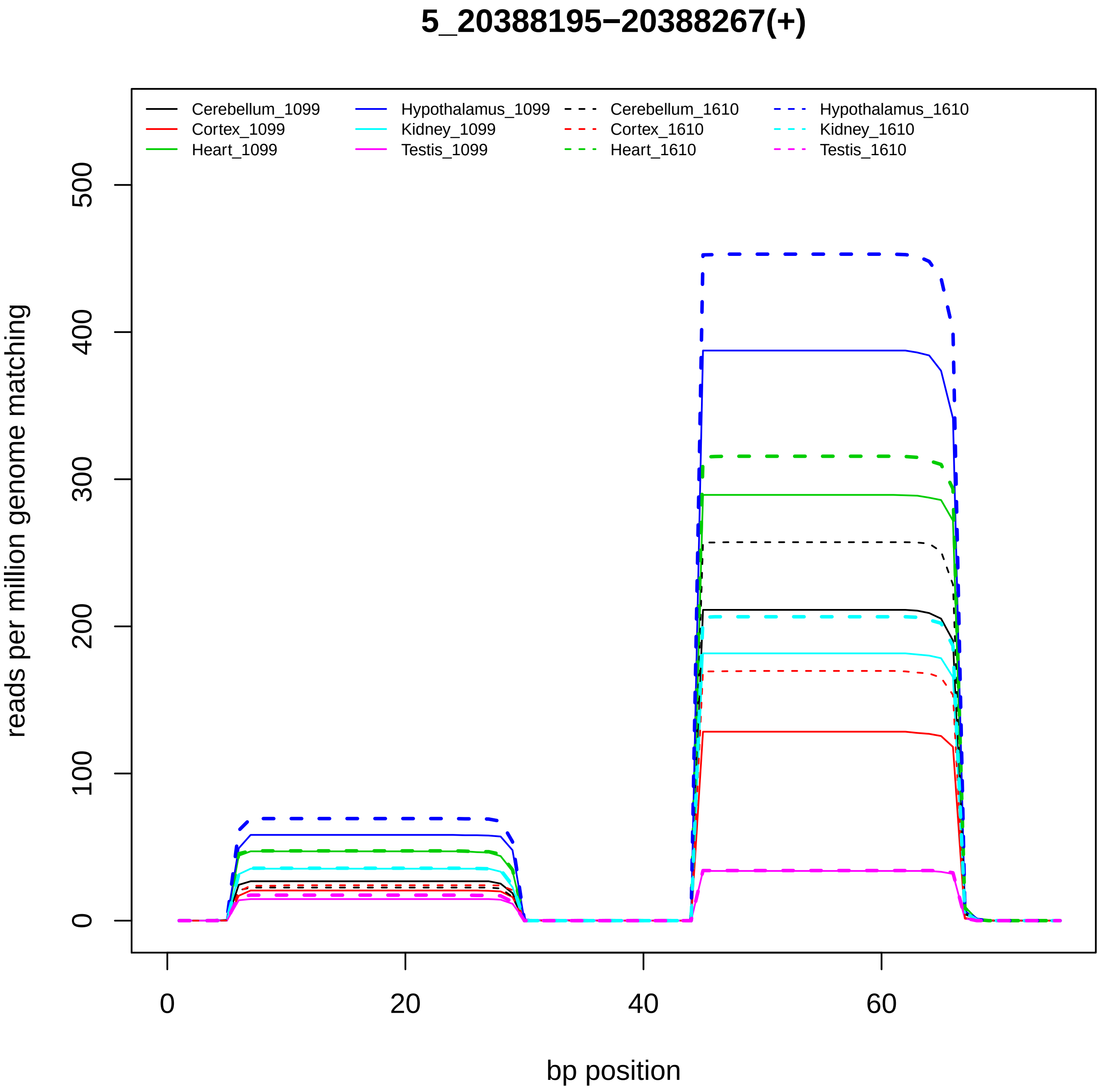

5_20388195−20388267(+)
Cerebellum_1099
Cortex_1099
Heart_1099
Hypothalamus_1099
Kidney_1099
Testis_1099
Cerebellum_1610
Cortex_1610
Heart_1610
Hypothalamus_1610
Kidney_1610
Testis_1610
reads per million genome matching
0
100
200
300
400
500
0
20
40
60
bp position

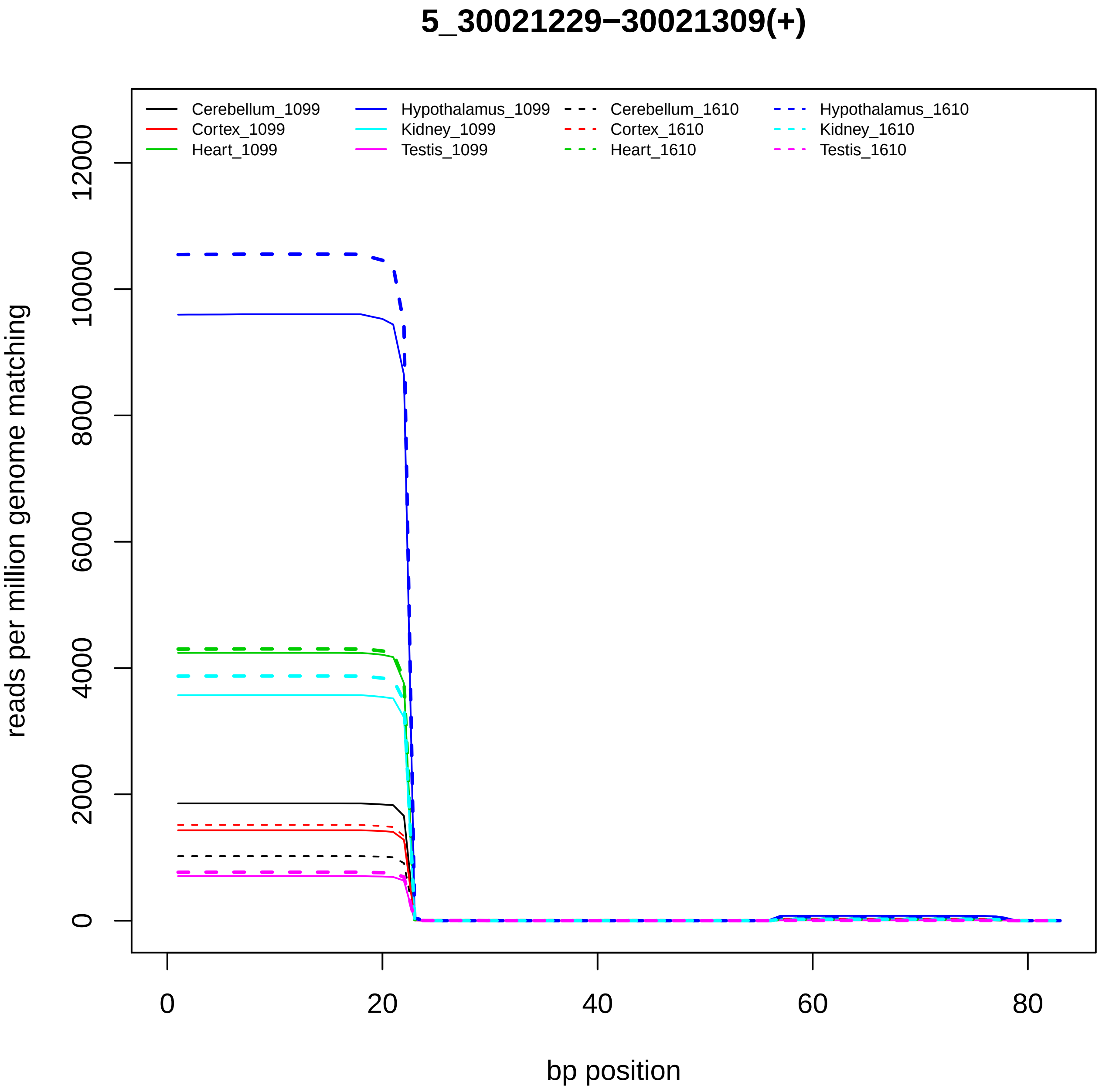

5_30021229−30021309(+)
Cerebellum_1099
Cortex_1099
Heart_1099
Hypothalamus_1099
Kidney_1099
Testis_1099
Cerebellum_1610
Cortex_1610
Heart_1610
Hypothalamus_1610
Kidney_1610
Testis_1610
reads per million genome matching
0
2000
4000
6000
8000
10000
12000
bp position
0
20
40
60
80

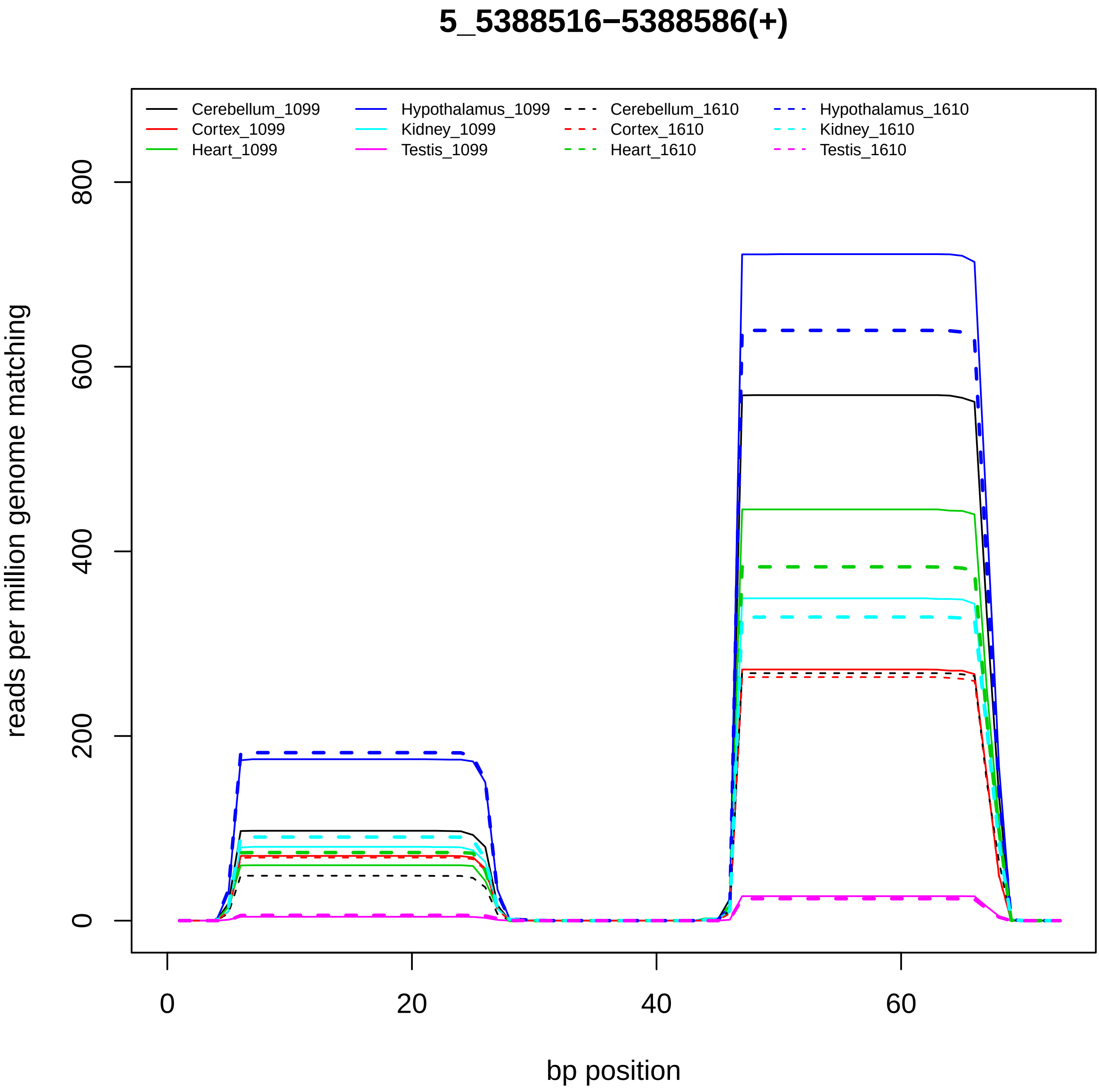
5_5388516−5388586(+)
Cerebellum_1099
Cortex_1099
Heart_1099
Hypothalamus_1099
Kidney_1099
Testis_1099
Cerebellum_1610
Cortex_1610
Heart_1610
Hypothalamus_1610
Kidney_1610
Testis_1610
reads per million genome matching
0
200
400
600
800
0
20
40
60
bp position

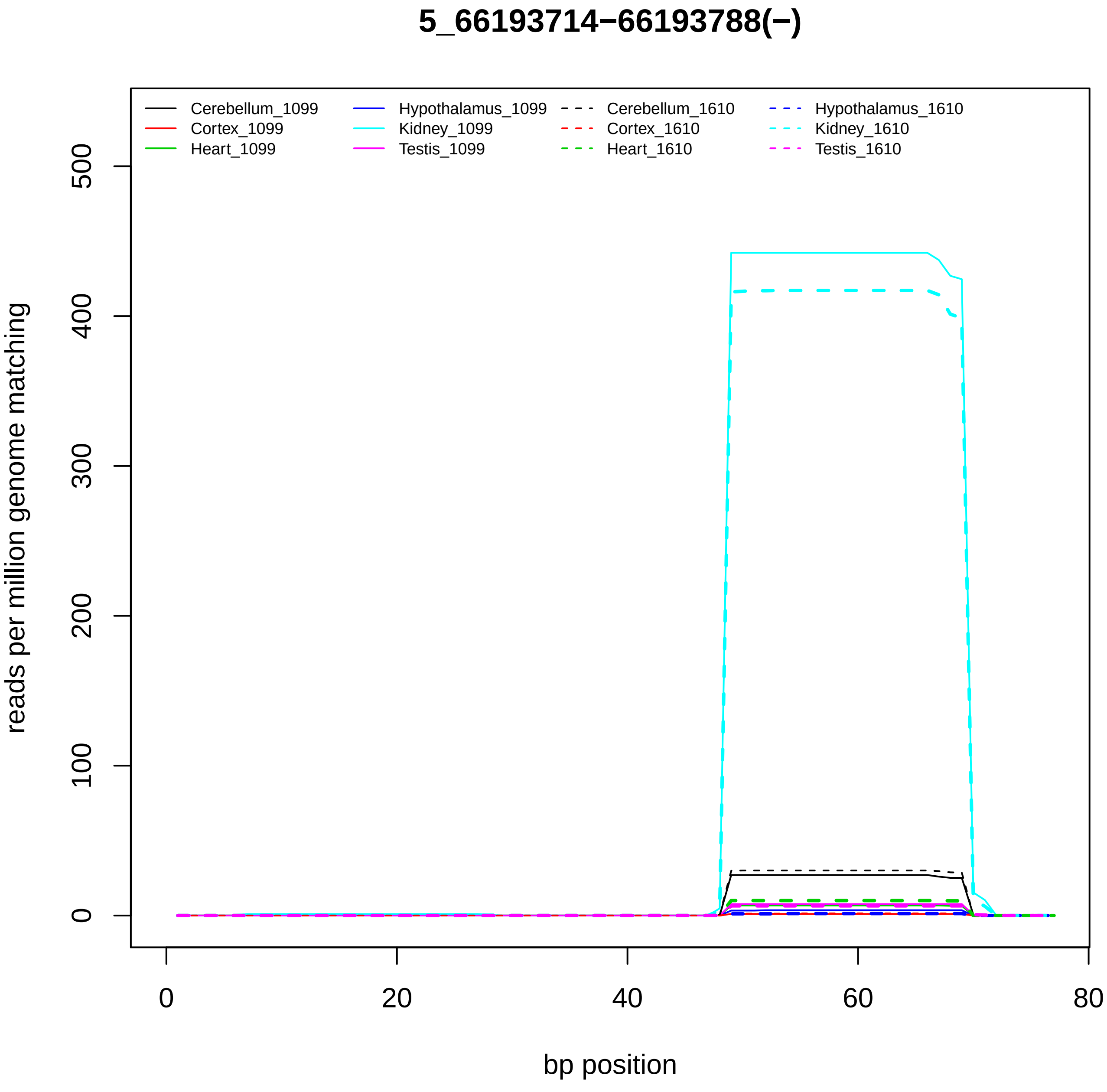

5_66193714−66193788(−)
Cerebellum_1099
Cortex_1099
Heart_1099
Hypothalamus_1099
Kidney_1099
Testis_1099
Cerebellum_1610
Cortex_1610
Heart_1610
Hypothalamus_1610
Kidney_1610
Testis_1610
reads per million genome matching
0
100
200
300
400
500
0
20
40
60
80
bp position

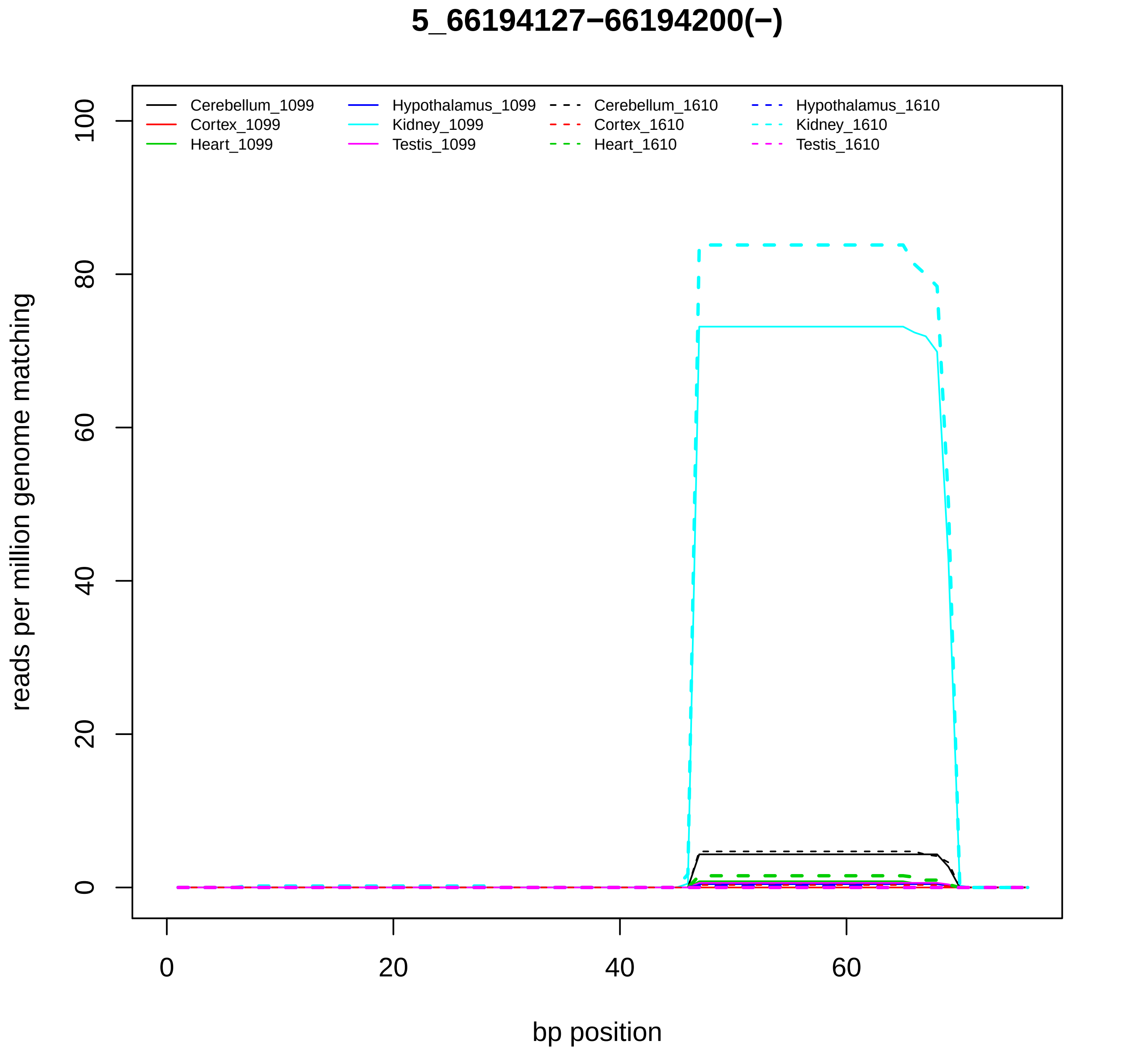

5_66194127−66194200(−)
Cerebellum_1099
Cortex_1099
Heart_1099
Hypothalamus_1099
Kidney_1099
Testis_1099
Cerebellum_1610
Cortex_1610
Heart_1610
Hypothalamus_1610
Kidney_1610
Testis_1610
reads per million genome matching
0
20
40
60
80
100
0
20
40
60
bp position

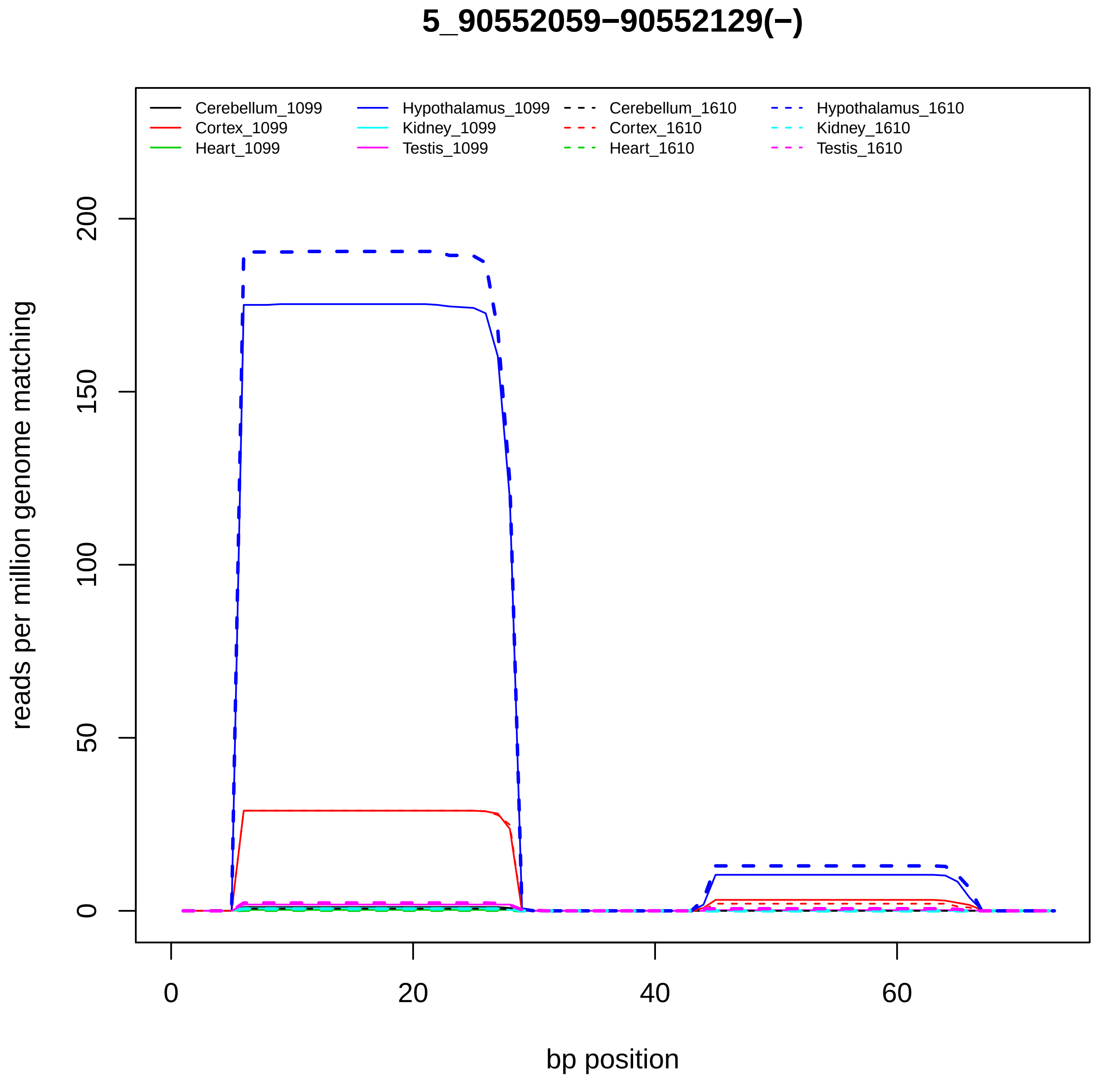

5_90552059−90552129(−)
Cerebellum_1099
Cortex_1099
Heart_1099
Hypothalamus_1099
Kidney_1099
Testis_1099
Cerebellum_1610
Cortex_1610
Heart_1610
Hypothalamus_1610
Kidney_1610
Testis_1610
reads per million genome matching
0
50
100
150
200
0
20
40
60
bp position

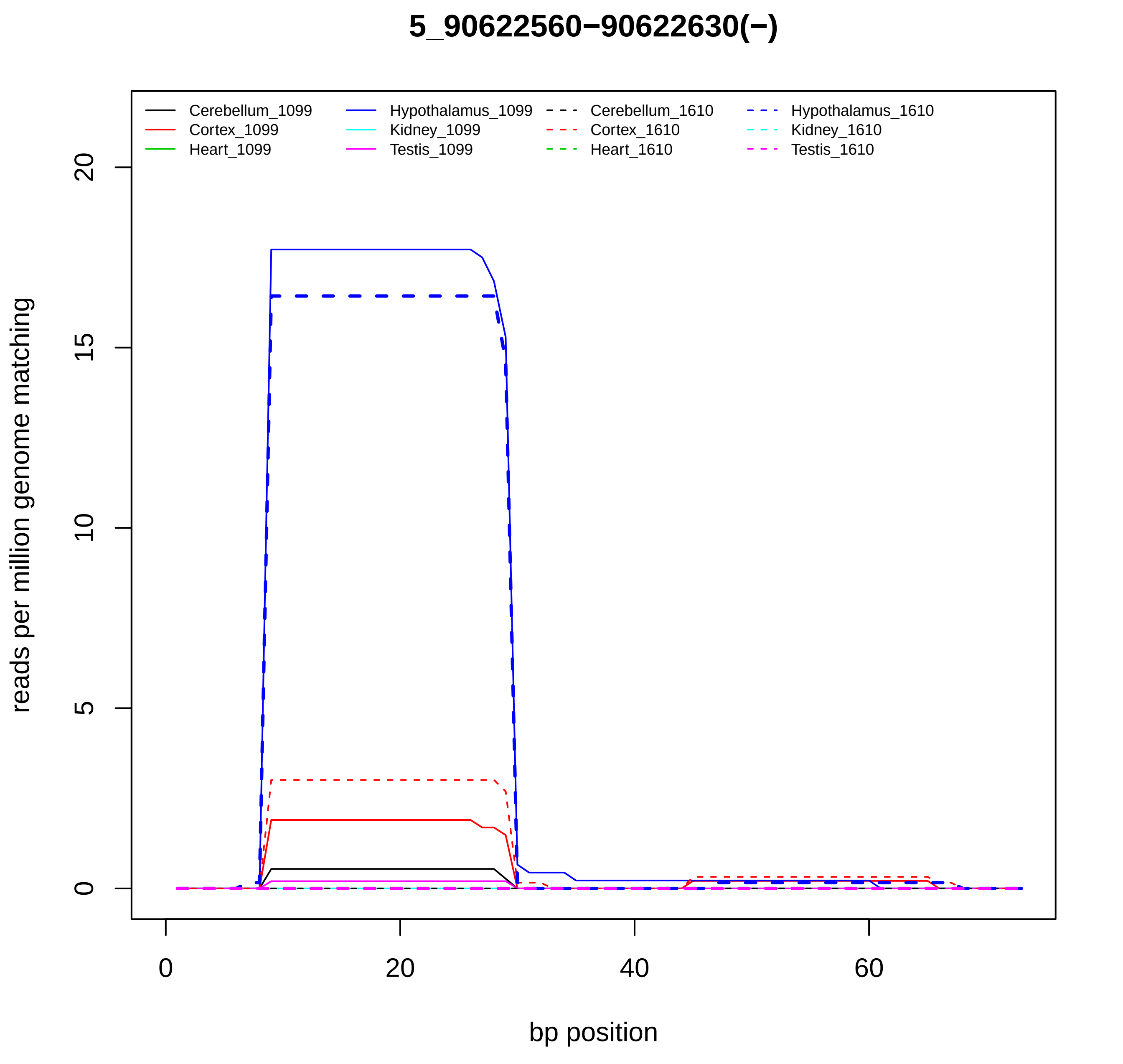

5_90622560−90622630(−)
Cerebellum_1099
Cortex_1099
Heart_1099
Hypothalamus_1099
Kidney_1099
Testis_1099
Cerebellum_1610
Cortex_1610
Heart_1610
Hypothalamus_1610
Kidney_1610
Testis_1610
reads per million genome matching
0
5
10
15
20
0
20
40
60
bp position

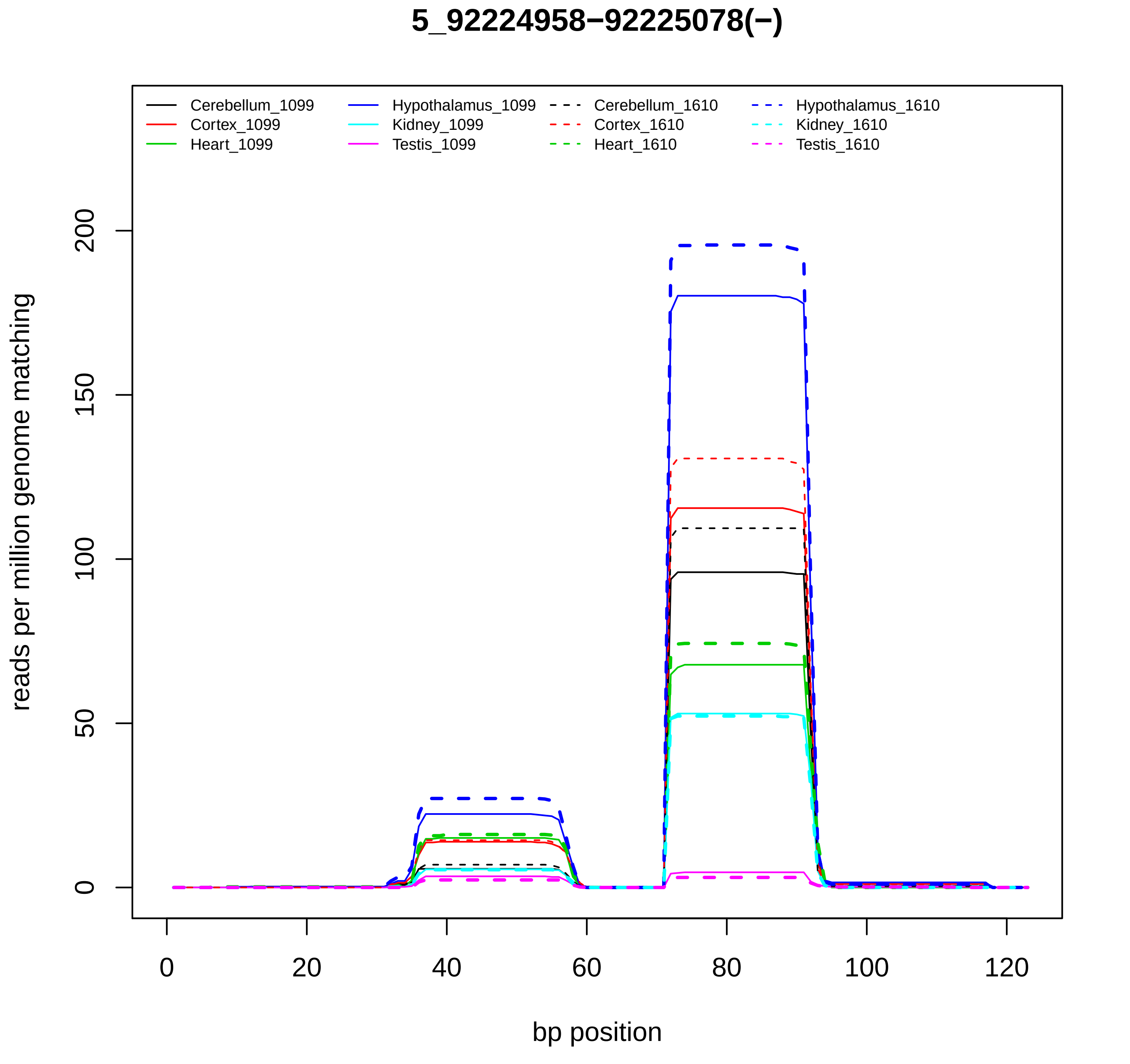

5_92224958−92225078(−)
Cerebellum_1099
Cortex_1099
Heart_1099
Hypothalamus_1099
Kidney_1099
Testis_1099
Cerebellum_1610
Cortex_1610
Heart_1610
Hypothalamus_1610
Kidney_1610
Testis_1610
reads per million genome matching
0
50
100
150
200
0
20
40
60
80
100
120
bp position

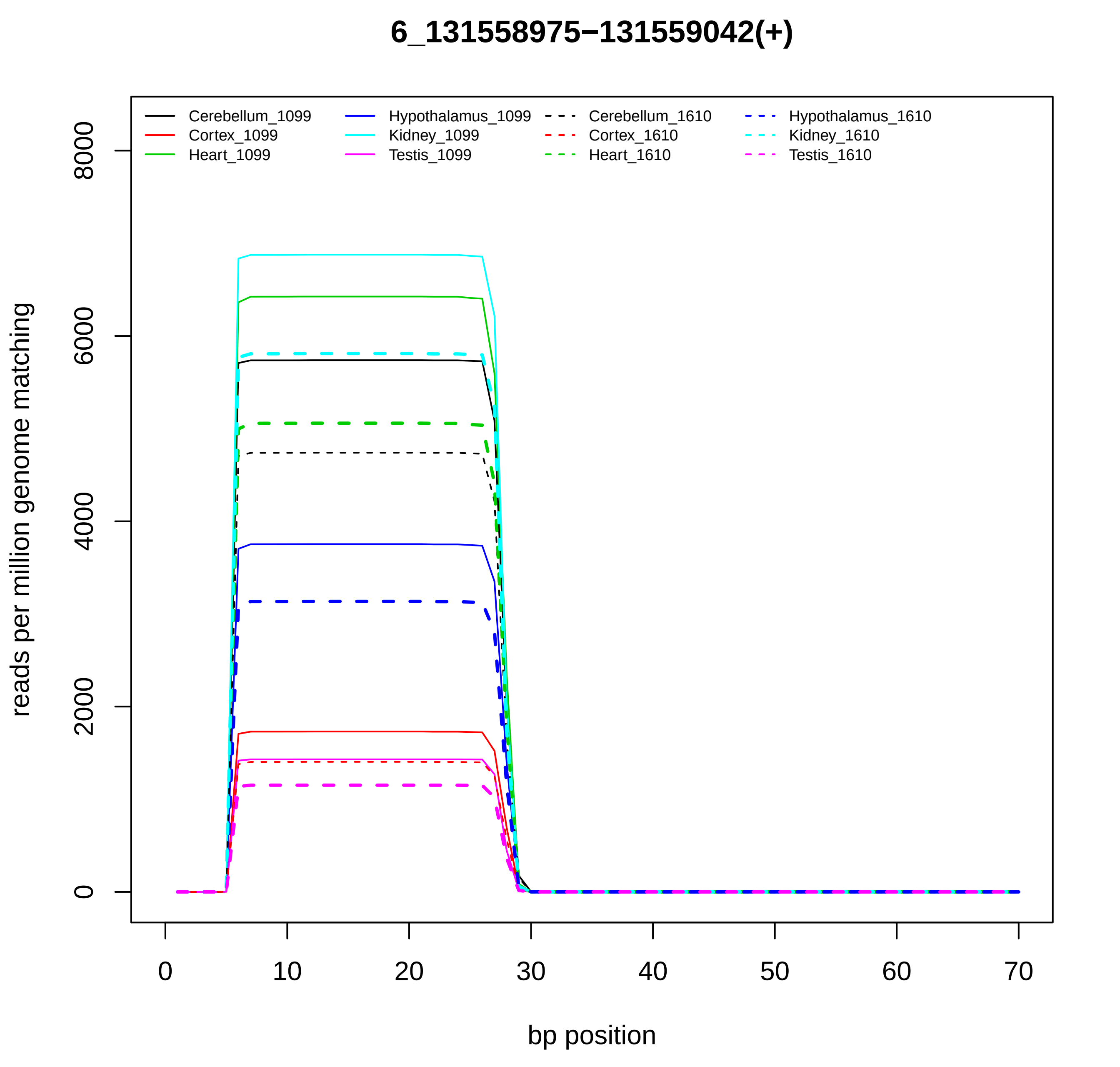
6_131558975−131559042(+)
Cerebellum_1099
Cortex_1099
Heart_1099
Hypothalamus_1099
Kidney_1099
Testis_1099
Cerebellum_1610
Cortex_1610
Heart_1610
Hypothalamus_1610
Kidney_1610
Testis_1610
reads per million genome matching
0
2000
4000
6000
8000
0
10
20
30
40
50
60
70
bp position

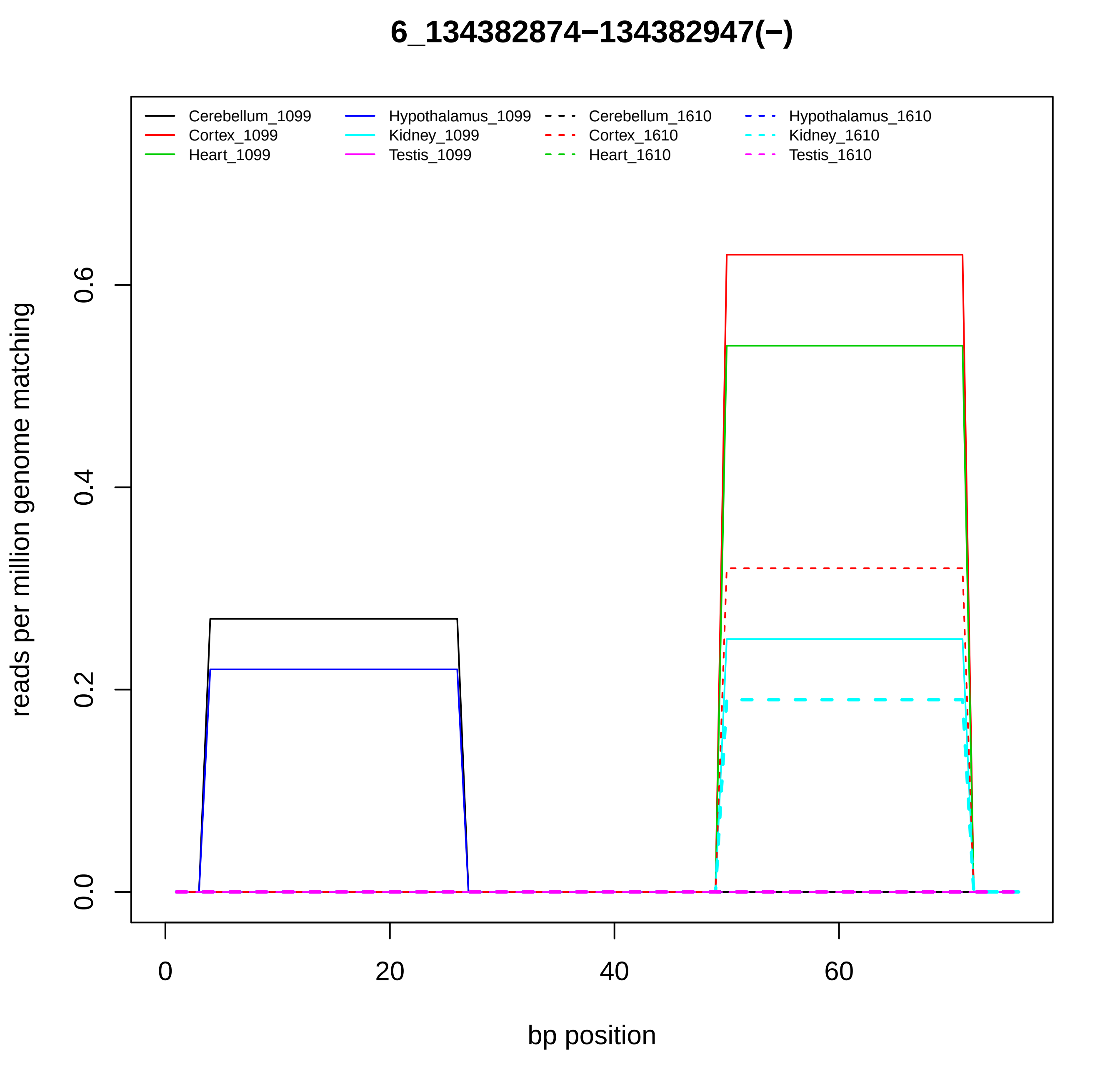

6_134382874−134382947(−)
Cerebellum_1099
Cortex_1099
Heart_1099
Hypothalamus_1099
Kidney_1099
Testis_1099
Cerebellum_1610
Cortex_1610
Heart_1610
Hypothalamus_1610
Kidney_1610
Testis_1610
reads per million genome matching
0.0
0.2
0.4
0.6
0
20
40
60
bp position

6_135736120−135736189(+)

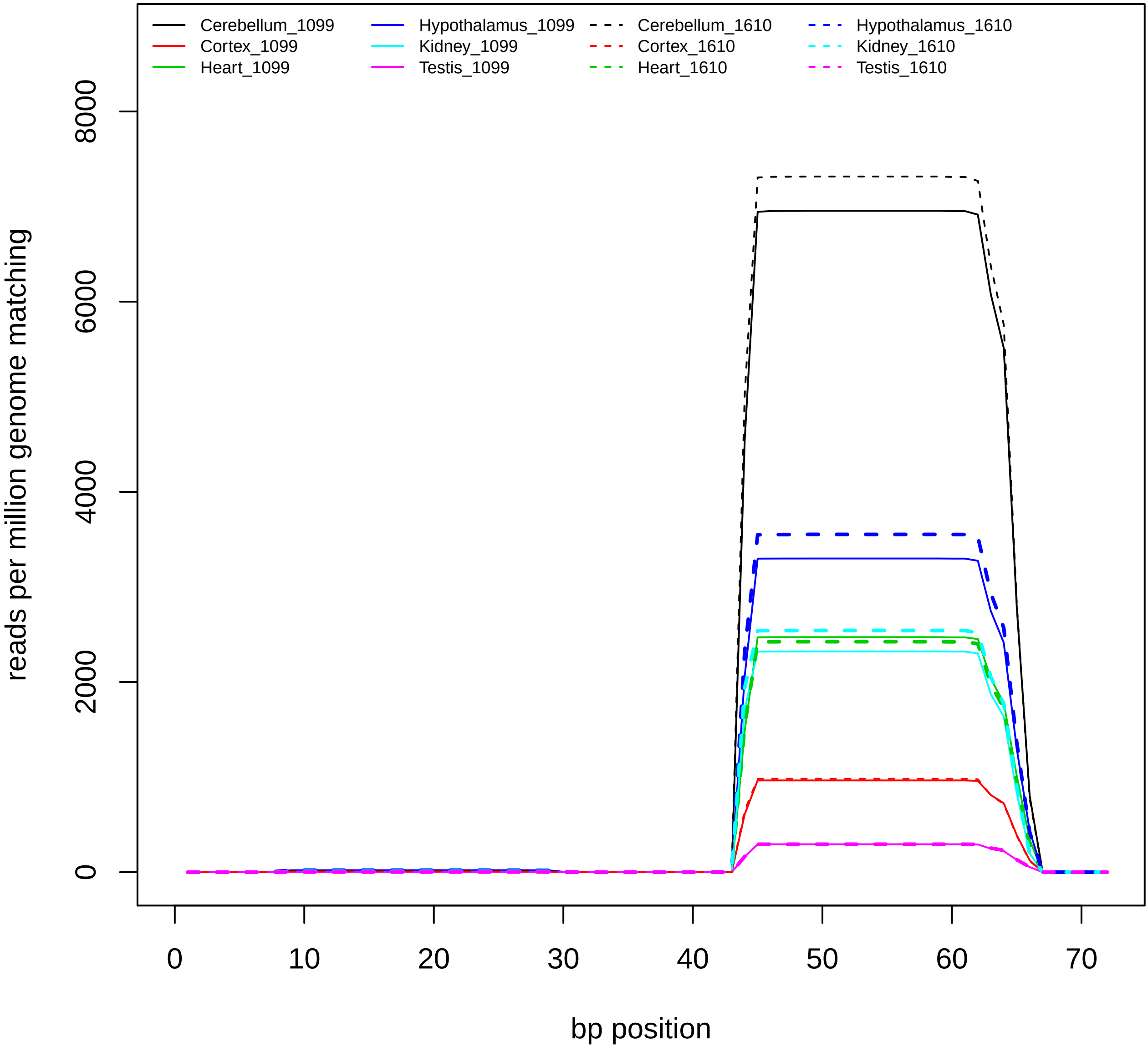

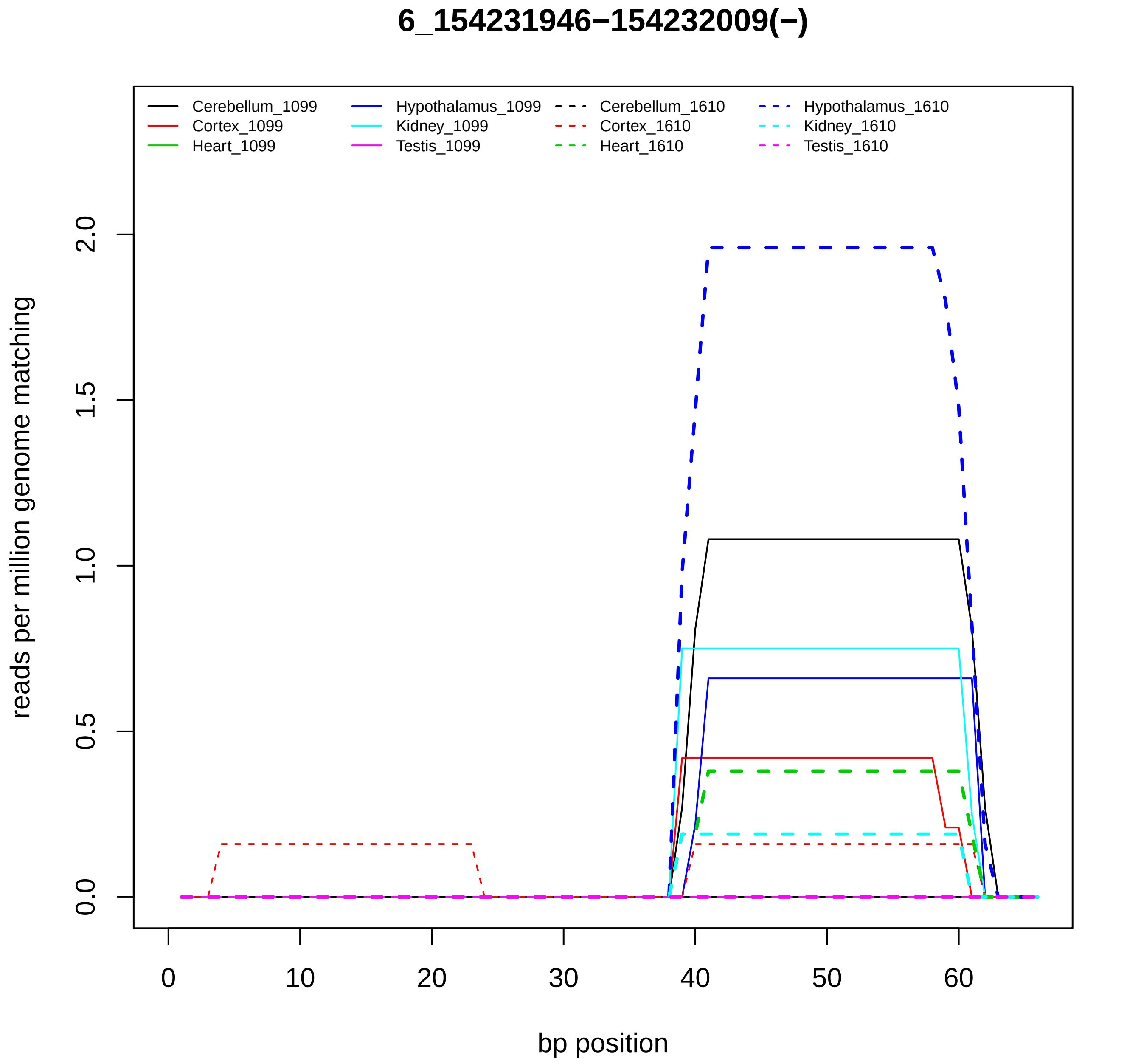

6_154231946−154232009(−)
Cerebellum_1099
Cortex_1099
Heart_1099
Hypothalamus_1099
Kidney_1099
Testis_1099
Cerebellum_1610
Cortex_1610
Heart_1610
Hypothalamus_1610
Kidney_1610
Testis_1610
reads per million genome matching
0.0
0.5
1.0
1.5
2.0
0
10
20
30
40
50
60
bp position

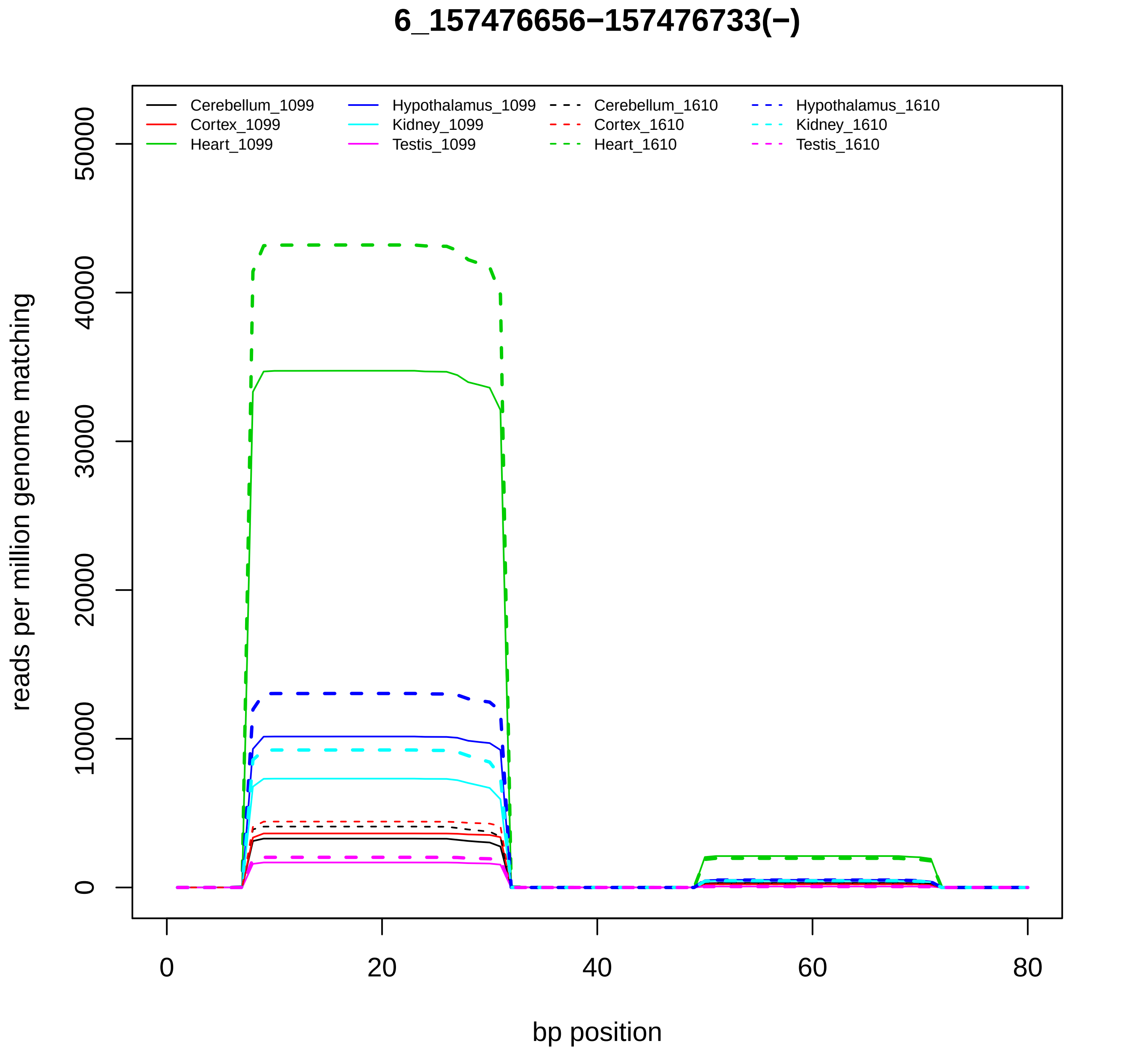

6_157476656−157476733(−)
Cerebellum_1099
Cortex_1099
Heart_1099
Hypothalamus_1099
Kidney_1099
Testis_1099
Cerebellum_1610
Cortex_1610
Heart_1610
Hypothalamus_1610
Kidney_1610
Testis_1610
reads per million genome matching
0
10000
20000
30000
40000
50000
0
20
40
60
80
bp position

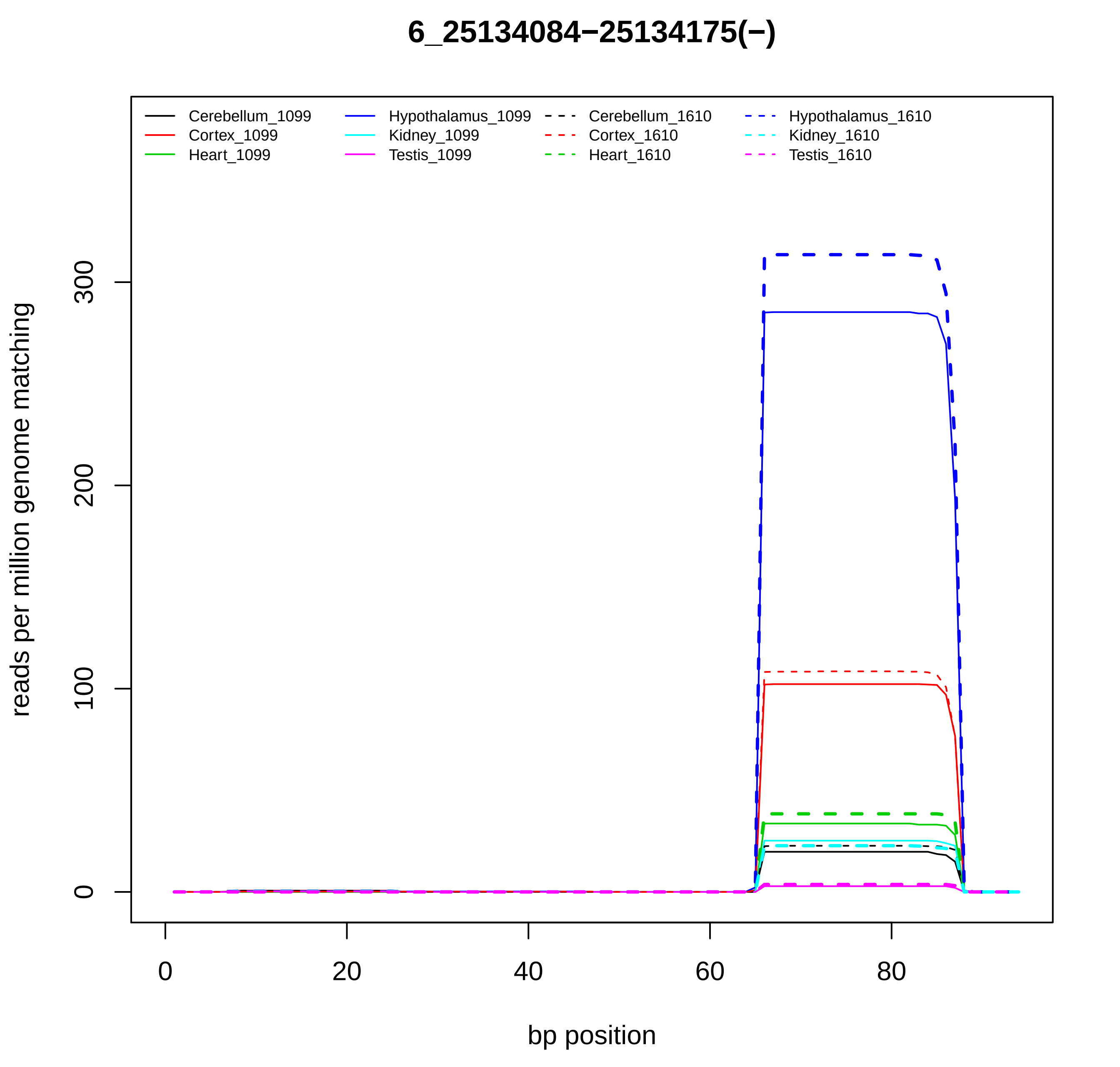

6_25134084−25134175(−)
Cerebellum_1099
Cortex_1099
Heart_1099
Hypothalamus_1099
Kidney_1099
Testis_1099
Cerebellum_1610
Cortex_1610
Heart_1610
Hypothalamus_1610
Kidney_1610
Testis_1610
reads per million genome matching
0
100
200
300
0
20
40
60
80
bp position

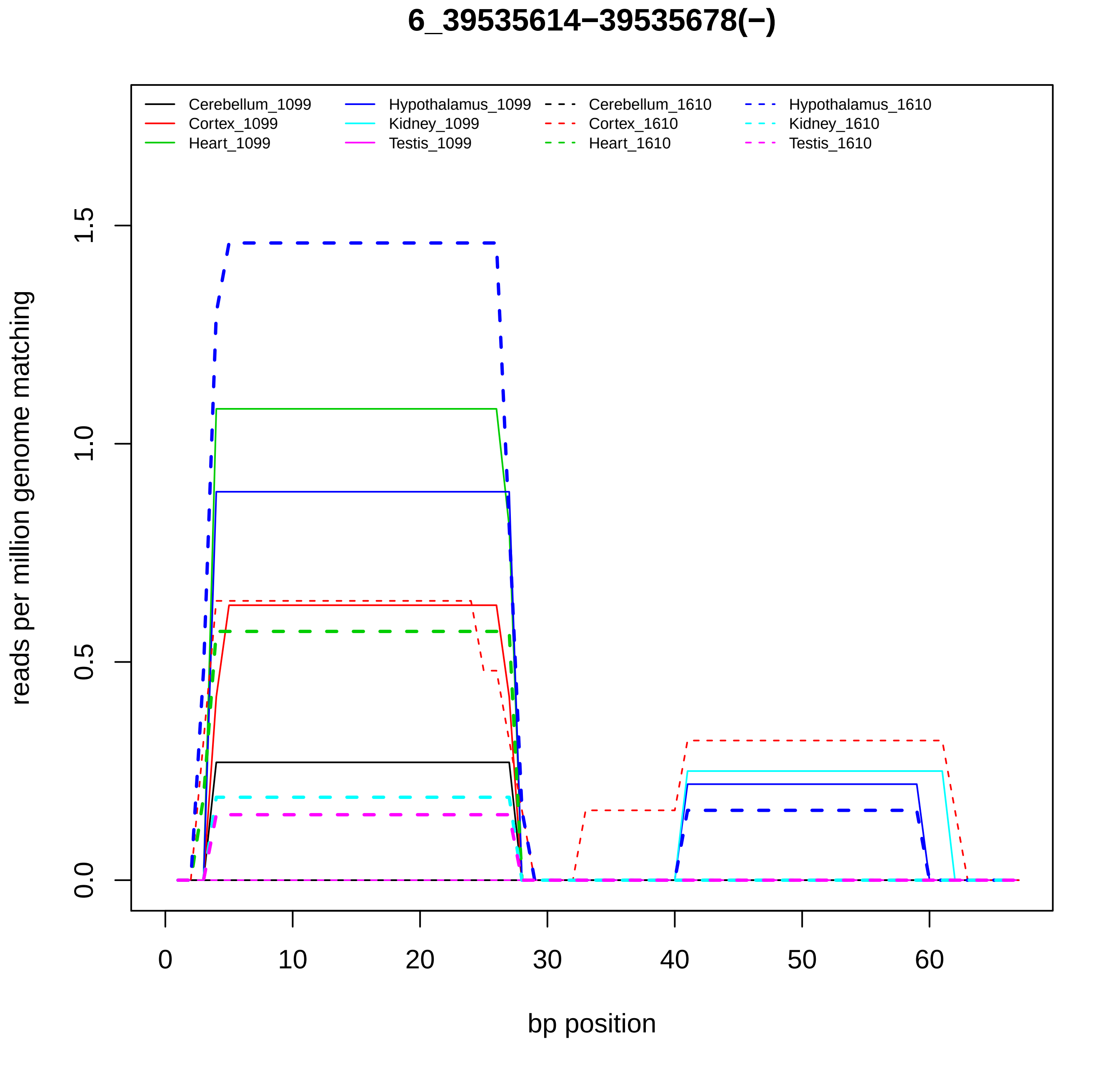
6_39535614−39535678(−)
Cerebellum_1099
Cortex_1099
Heart_1099
Hypothalamus_1099
Kidney_1099
Testis_1099
Cerebellum_1610
Cortex_1610
Heart_1610
Hypothalamus_1610
Kidney_1610
Testis_1610
reads per million genome matching
0.0
0.5
1.0
1.5
0
10
20
30
40
50
60
bp position

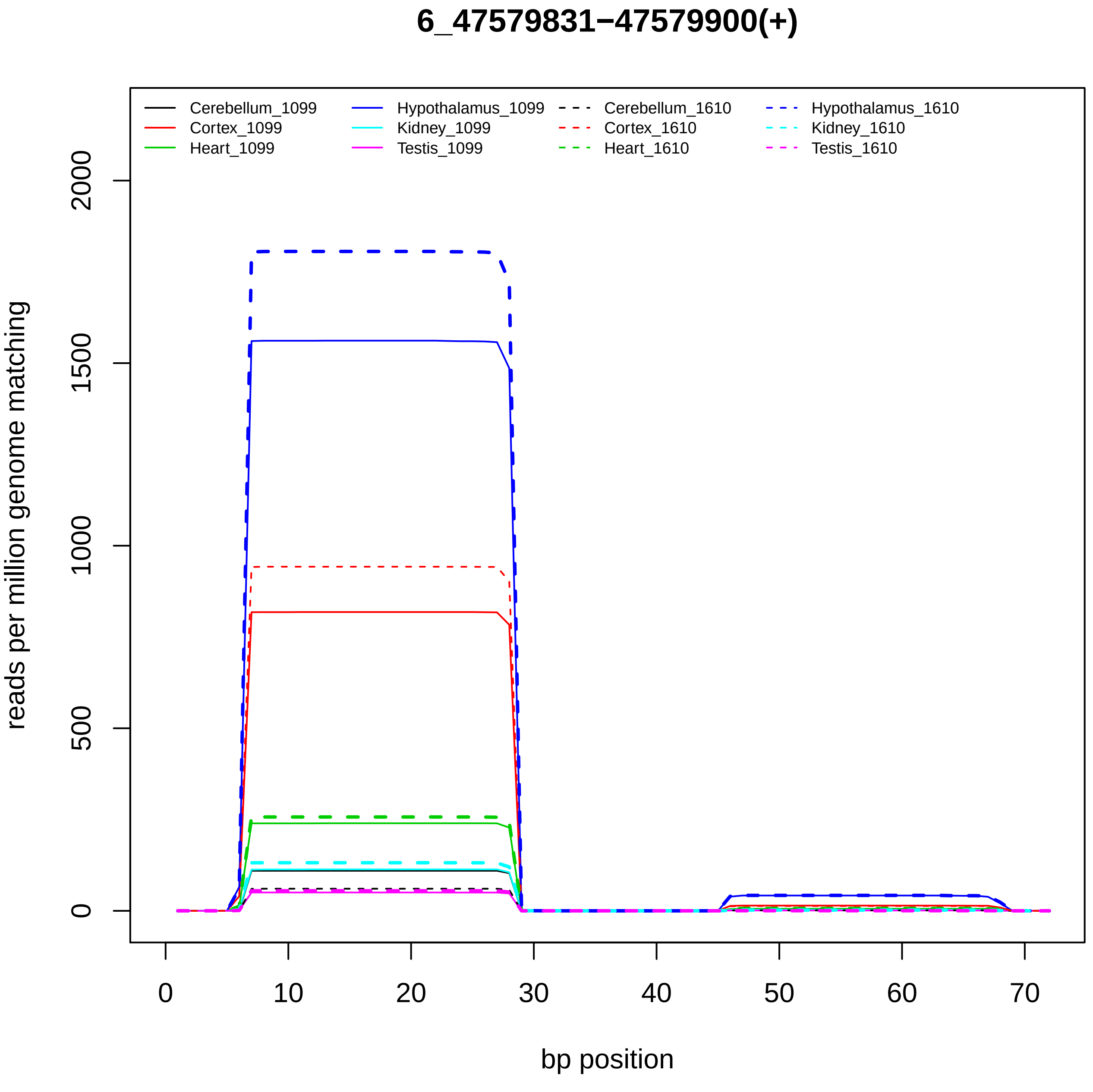

6_47579831−47579900(+)
Cerebellum_1099
Cortex_1099
Heart_1099
Hypothalamus_1099
Kidney_1099
Testis_1099
Cerebellum_1610
Cortex_1610
Heart_1610
Hypothalamus_1610
Kidney_1610
Testis_1610
reads per million genome matching
0
500
1000
1500
2000
0
10
20
30
40
50
60
70
bp position

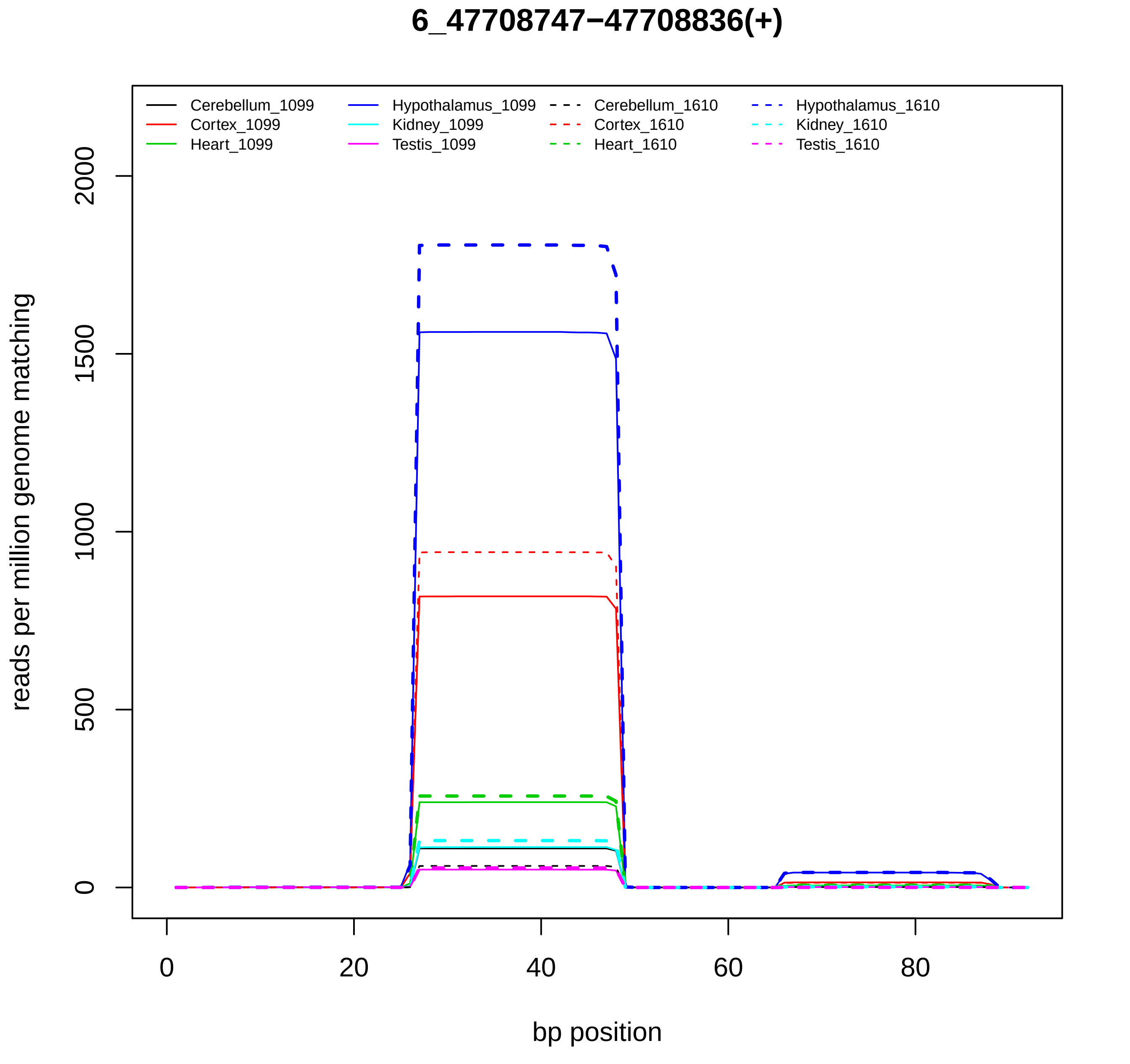
6_47708747−47708836(+)
Cerebellum_1099
Cortex_1099
Heart_1099
Hypothalamus_1099
Kidney_1099
Testis_1099
Cerebellum_1610
Cortex_1610
Heart_1610
Hypothalamus_1610
Kidney_1610
Testis_1610
reads per million genome matching
0
500
1000
1500
2000
0
20
40
60
80
bp position

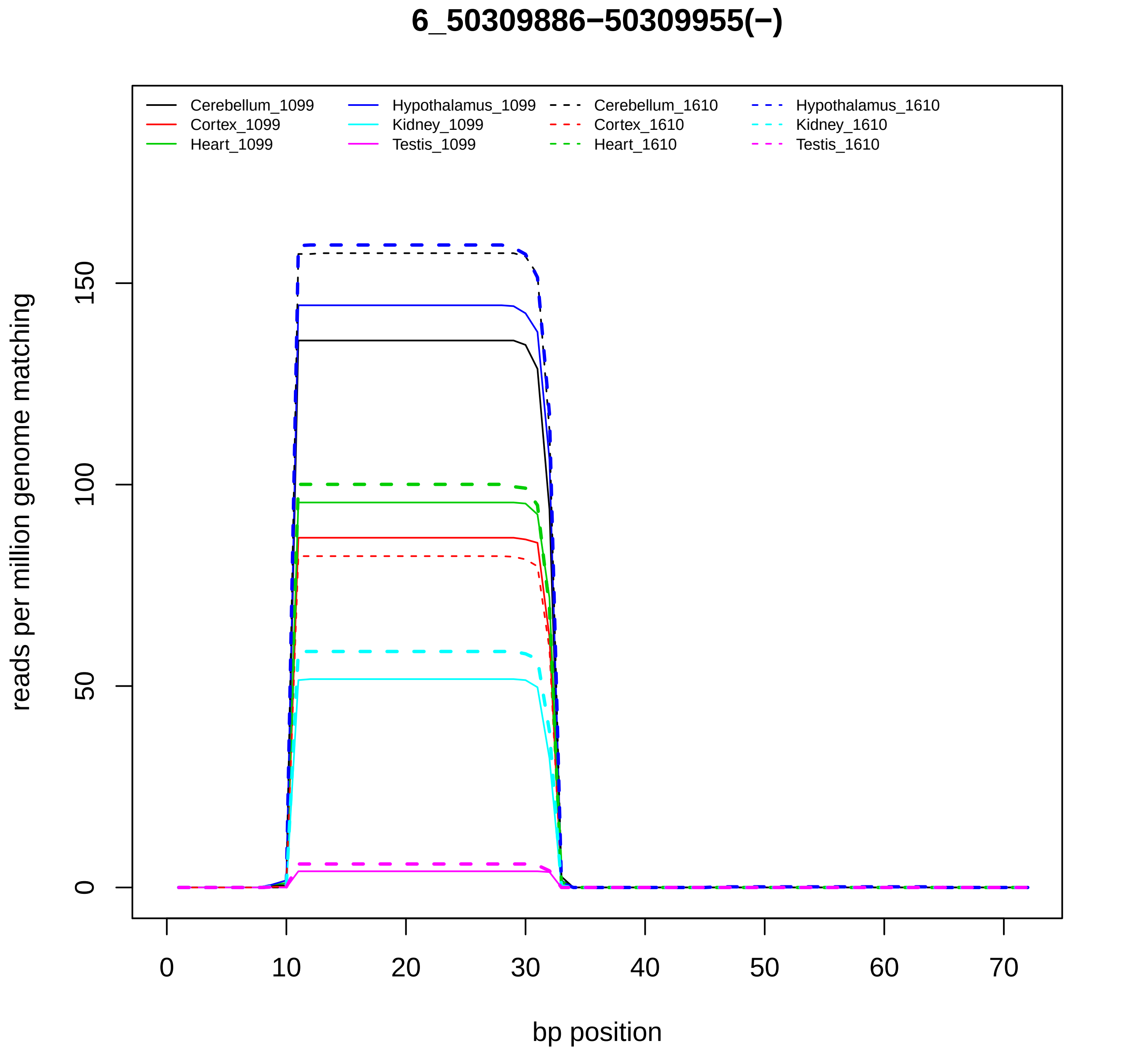

6_50309886−50309955(−)
Cerebellum_1099
Cortex_1099
Heart_1099
Hypothalamus_1099
Kidney_1099
Testis_1099
Cerebellum_1610
Cortex_1610
Heart_1610
Hypothalamus_1610
Kidney_1610
Testis_1610
reads per million genome matching
0
50
100
150
0
10
20
30
40
50
60
70
bp position

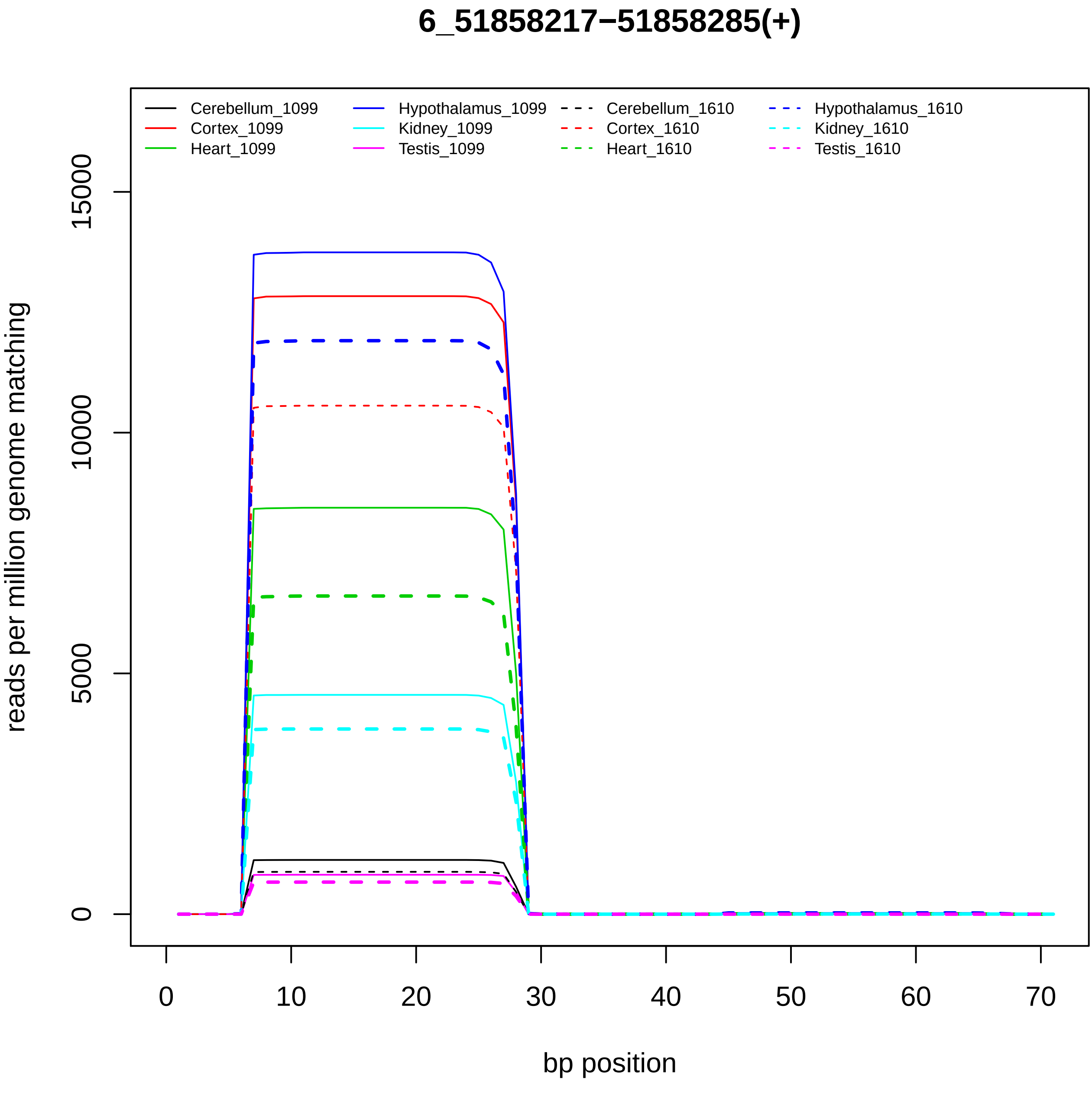

6_51858217−51858285(+)
Cerebellum_1099
Cortex_1099
Heart_1099
Hypothalamus_1099
Kidney_1099
Testis_1099
Cerebellum_1610
Cortex_1610
Heart_1610
Hypothalamus_1610
Kidney_1610
Testis_1610
reads per million genome matching
0
5000
10000
15000
0
10
20
30
40
50
60
70
bp position

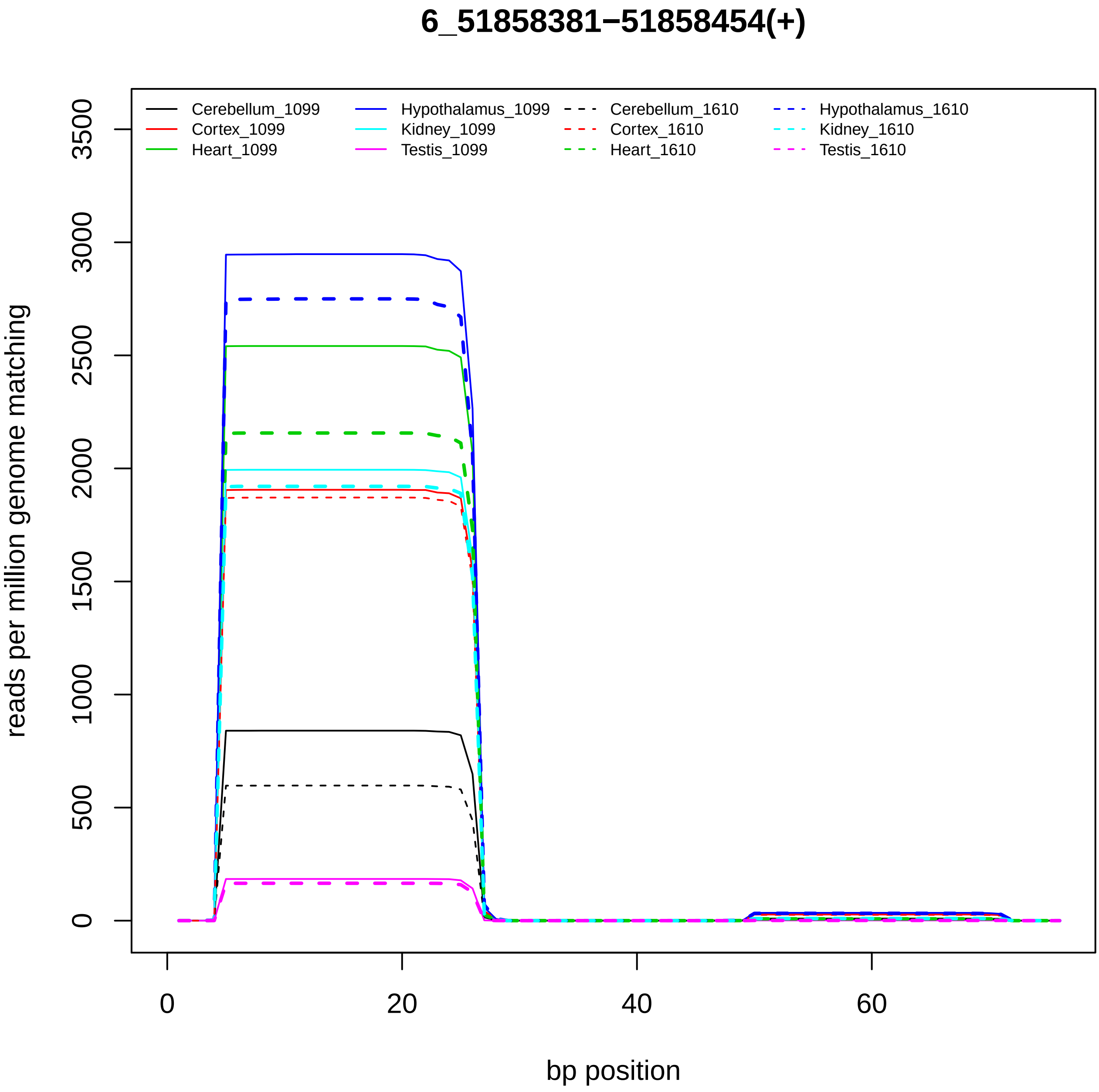
6_51858381−51858454(+)
Cerebellum_1099
Cortex_1099
Heart_1099
Hypothalamus_1099
Kidney_1099
Testis_1099
Cerebellum_1610
Cortex_1610
Heart_1610
Hypothalamus_1610
Kidney_1610
Testis_1610
reads per million genome matching
0
500
1000
1500
2000
2500
3000
3500
bp position
0
20
40
60

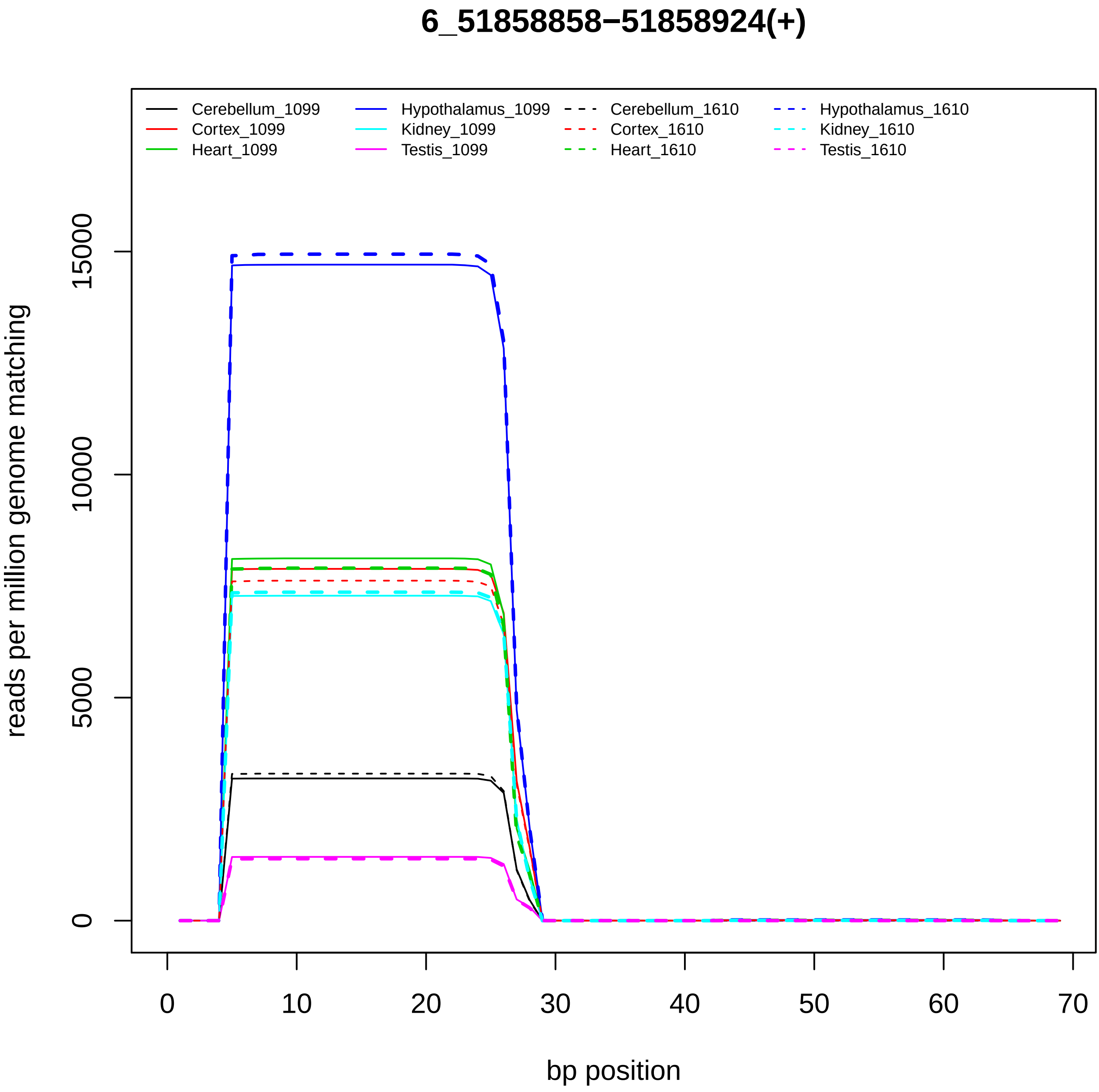

6_51858858−51858924(+)
Cerebellum_1099
Cortex_1099
Heart_1099
Hypothalamus_1099
Kidney_1099
Testis_1099
Cerebellum_1610
Cortex_1610
Heart_1610
Hypothalamus_1610
Kidney_1610
Testis_1610
reads per million genome matching
0
5000
10000
15000
bp position
0
10
20
30
40
50
60
70

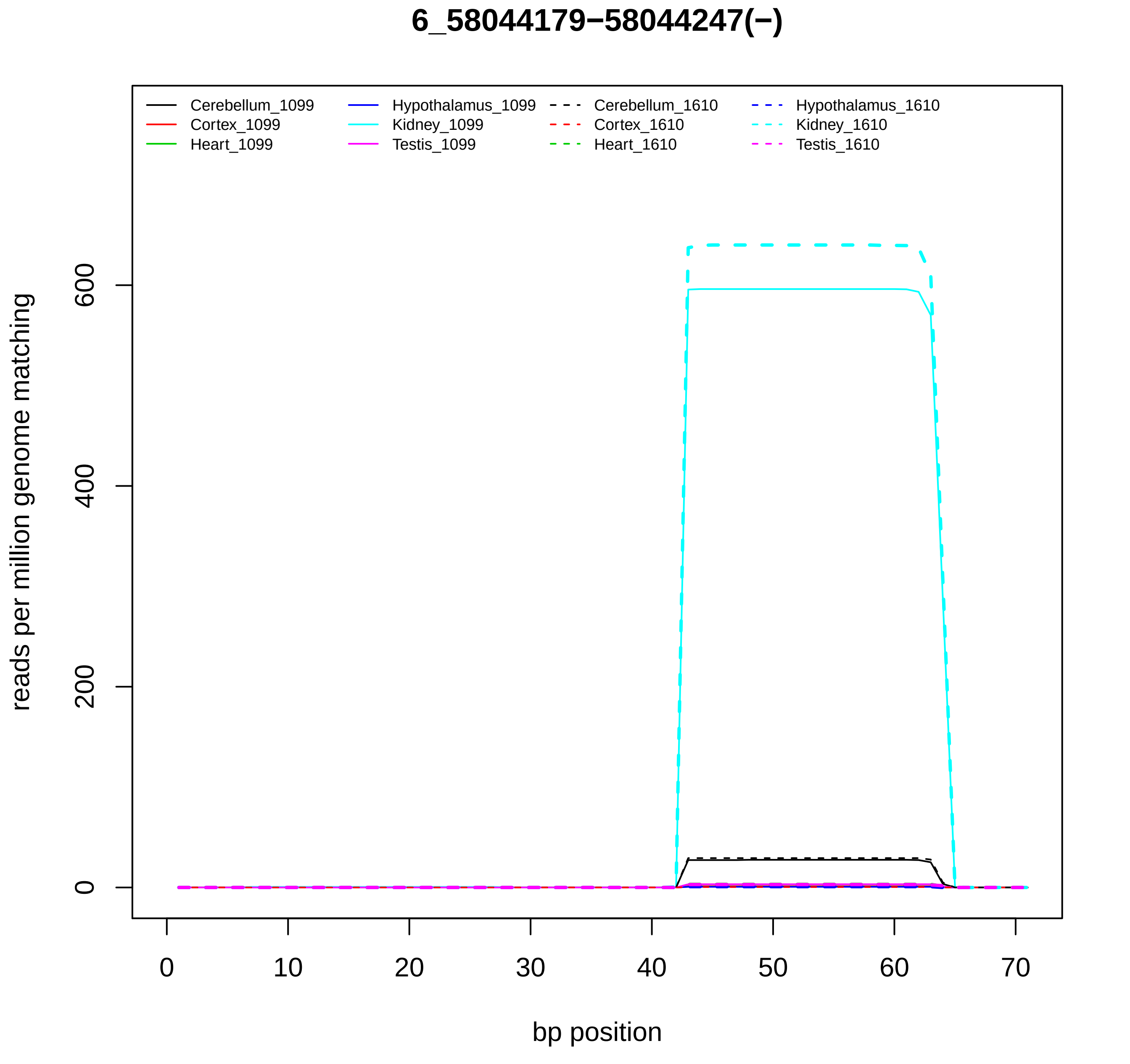
6_58044179−58044247(−)
Cerebellum_1099
Cortex_1099
Heart_1099
Hypothalamus_1099
Kidney_1099
Testis_1099
Cerebellum_1610
Cortex_1610
Heart_1610
Hypothalamus_1610
Kidney_1610
Testis_1610
reads per million genome matching
0
200
400
600
0
10
20
30
40
50
60
70
bp position

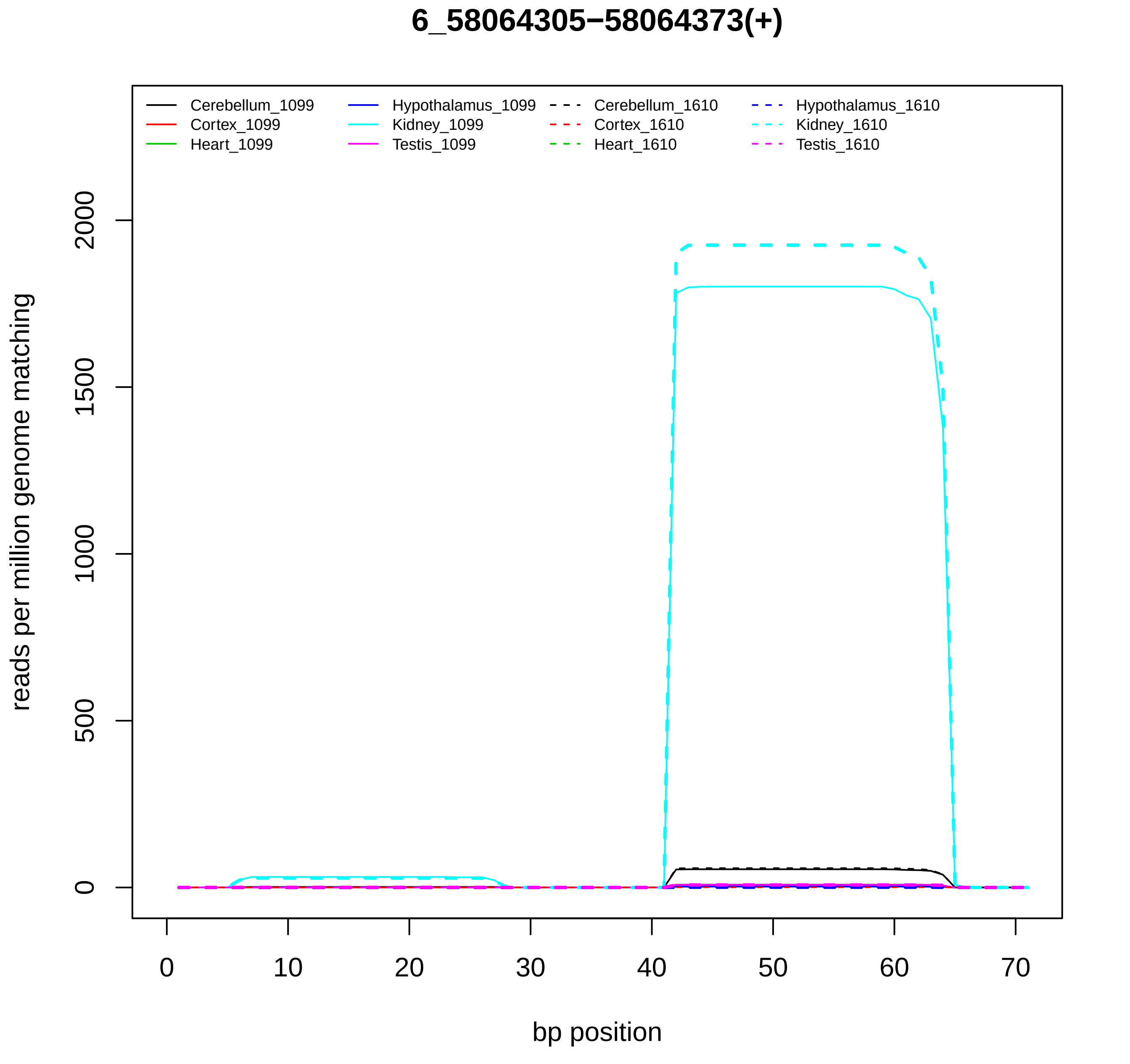

6_58064305−58064373(+)
Cerebellum_1099
Cortex_1099
Heart_1099
Hypothalamus_1099
Kidney_1099
Testis_1099
Cerebellum_1610
Cortex_1610
Heart_1610
Hypothalamus_1610
Kidney_1610
Testis_1610
reads per million genome matching
0
500
1000
1500
2000
0
10
20
30
40
50
60
70
bp position

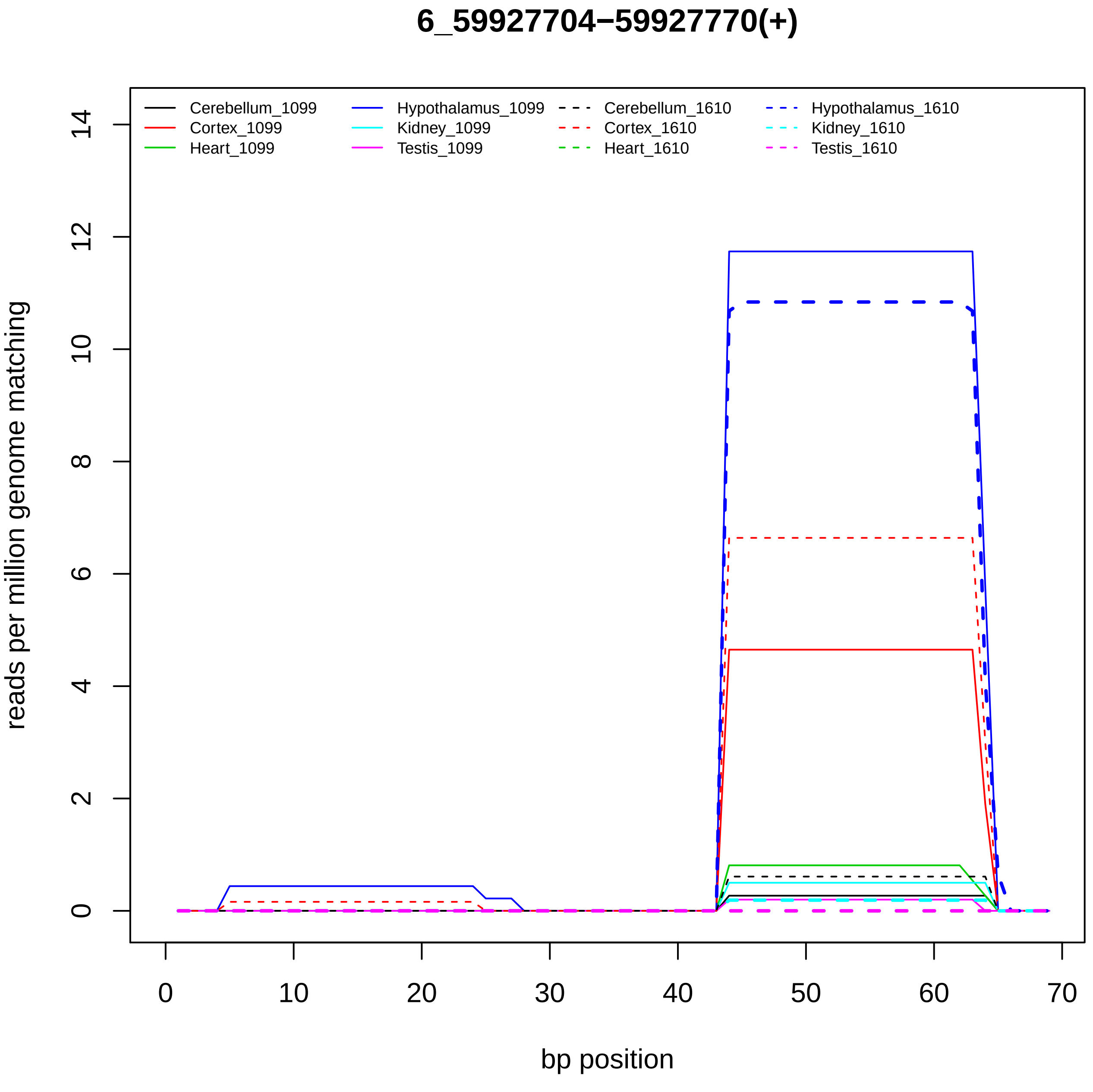
6_59927704−59927770(+)
Cerebellum_1099
Cortex_1099
Heart_1099
Hypothalamus_1099
Kidney_1099
Testis_1099
Cerebellum_1610
Cortex_1610
Heart_1610
Hypothalamus_1610
Kidney_1610
Testis_1610
reads per million genome matching
bp position
0
2
4
6
8
10
12
14
0
10
20
30
40
50
60
70

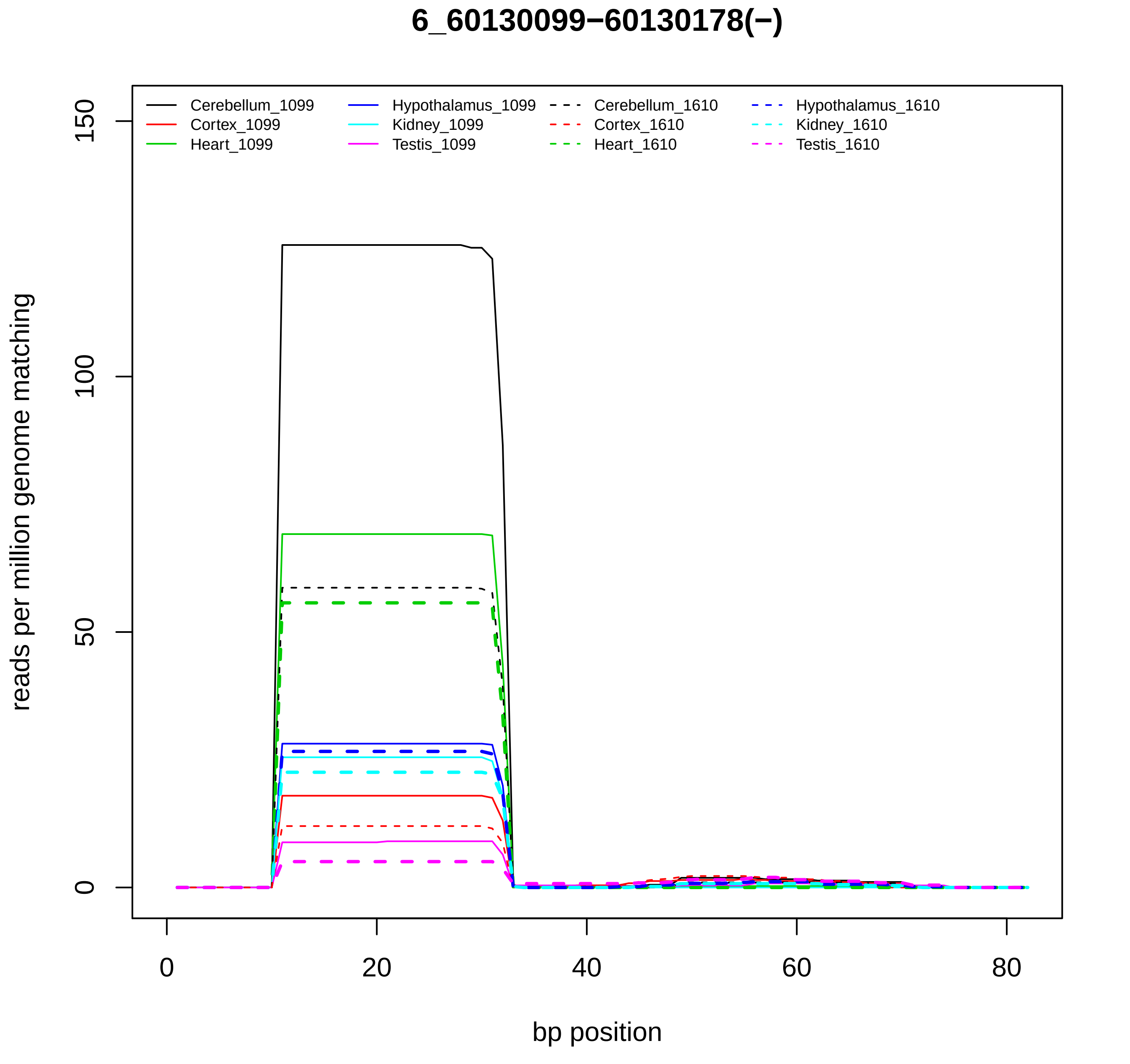

6_60130099−60130178(−)
reads per million genome matching
bp position
0
50
100
150
0
20
40
60
80
Cerebellum_1099
Cortex_1099
Heart_1099
Hypothalamus_1099
Kidney_1099
Testis_1099
Cerebellum_1610
Cortex_1610
Heart_1610
Hypothalamus_1610
Kidney_1610
Testis_1610

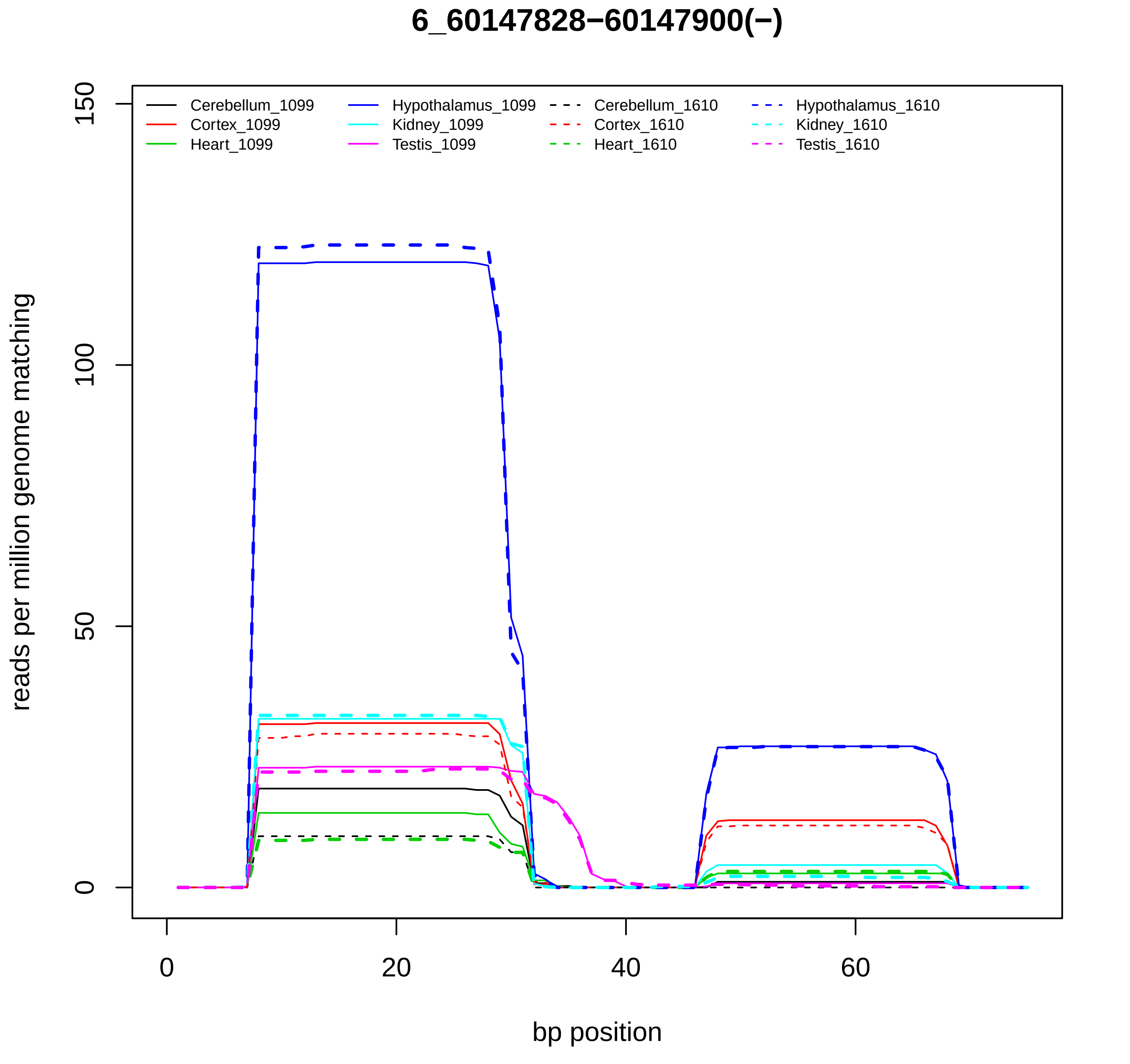
6_60147828−60147900(−)
Cerebellum_1099
Cortex_1099
Heart_1099
Hypothalamus_1099
Kidney_1099
Testis_1099
Cerebellum_1610
Cortex_1610
Heart_1610
Hypothalamus_1610
Kidney_1610
Testis_1610
reads per million genome matching
0
50
100
150
bp position
0
20
40
60

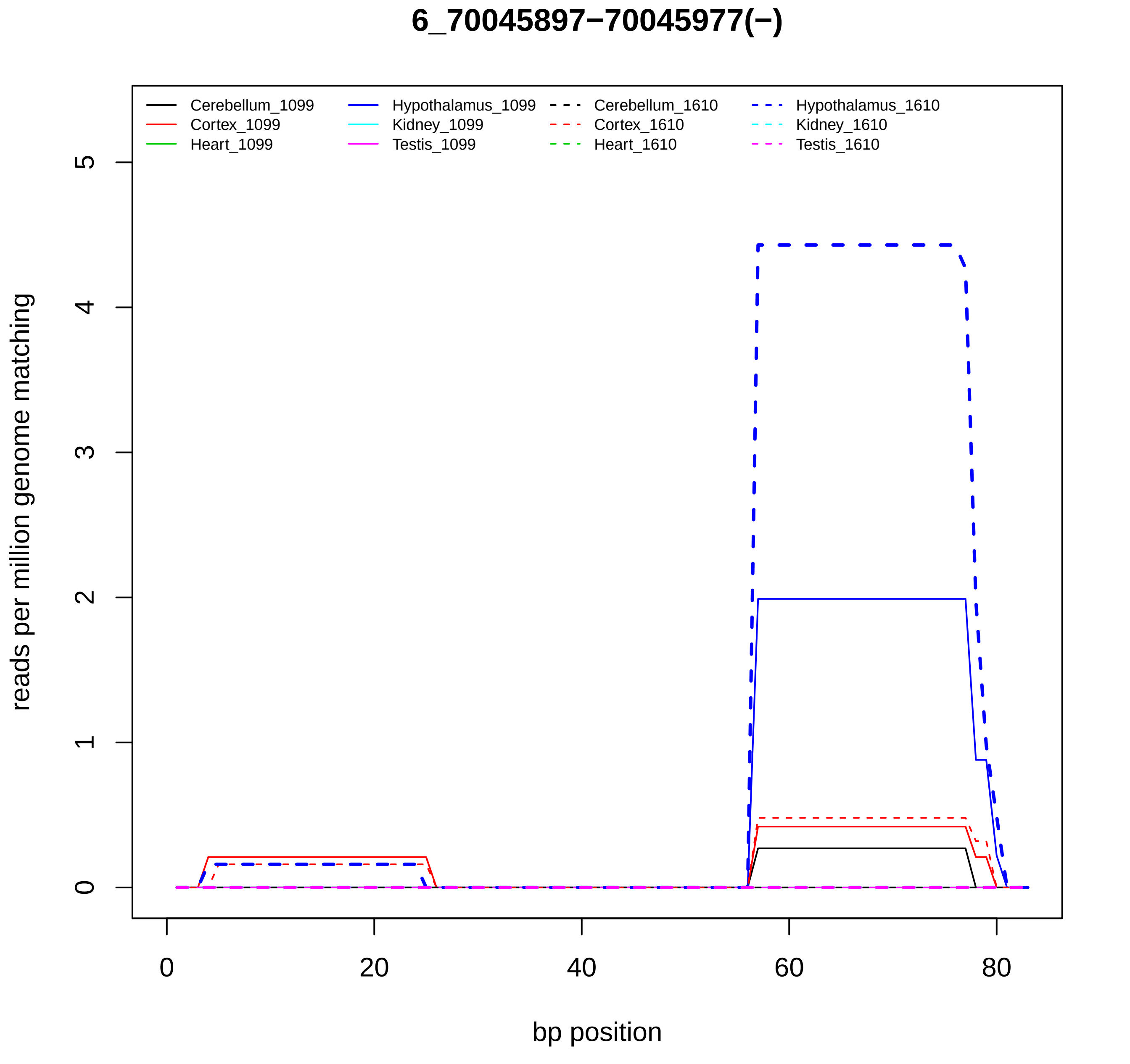

6_70045897−70045977(−)
Cerebellum_1099
Cortex_1099
Heart_1099
Hypothalamus_1099
Kidney_1099
Testis_1099
Cerebellum_1610
Cortex_1610
Heart_1610
Hypothalamus_1610
Kidney_1610
Testis_1610
reads per million genome matching
bp position
0
1
2
3
4
5
0
20
40
60
80

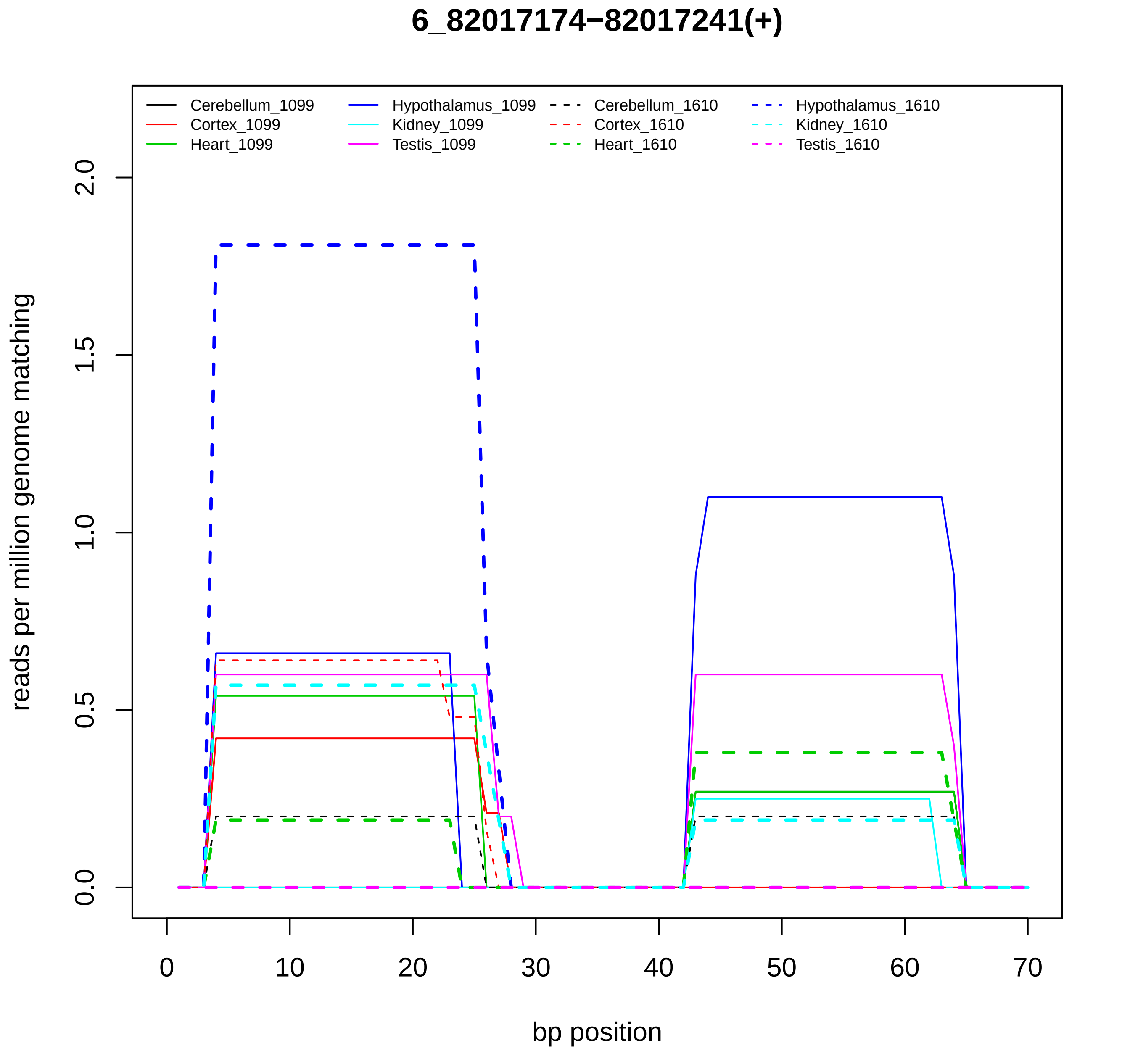

6_82017174−82017241(+)
Cerebellum_1099
Cortex_1099
Heart_1099
Hypothalamus_1099
Kidney_1099
Testis_1099
Cerebellum_1610
Cortex_1610
Heart_1610
Hypothalamus_1610
Kidney_1610
Testis_1610
reads per million genome matching
0.0
0.5
1.0
1.5
2.0
0
10
20
30
40
50
60
70
bp position

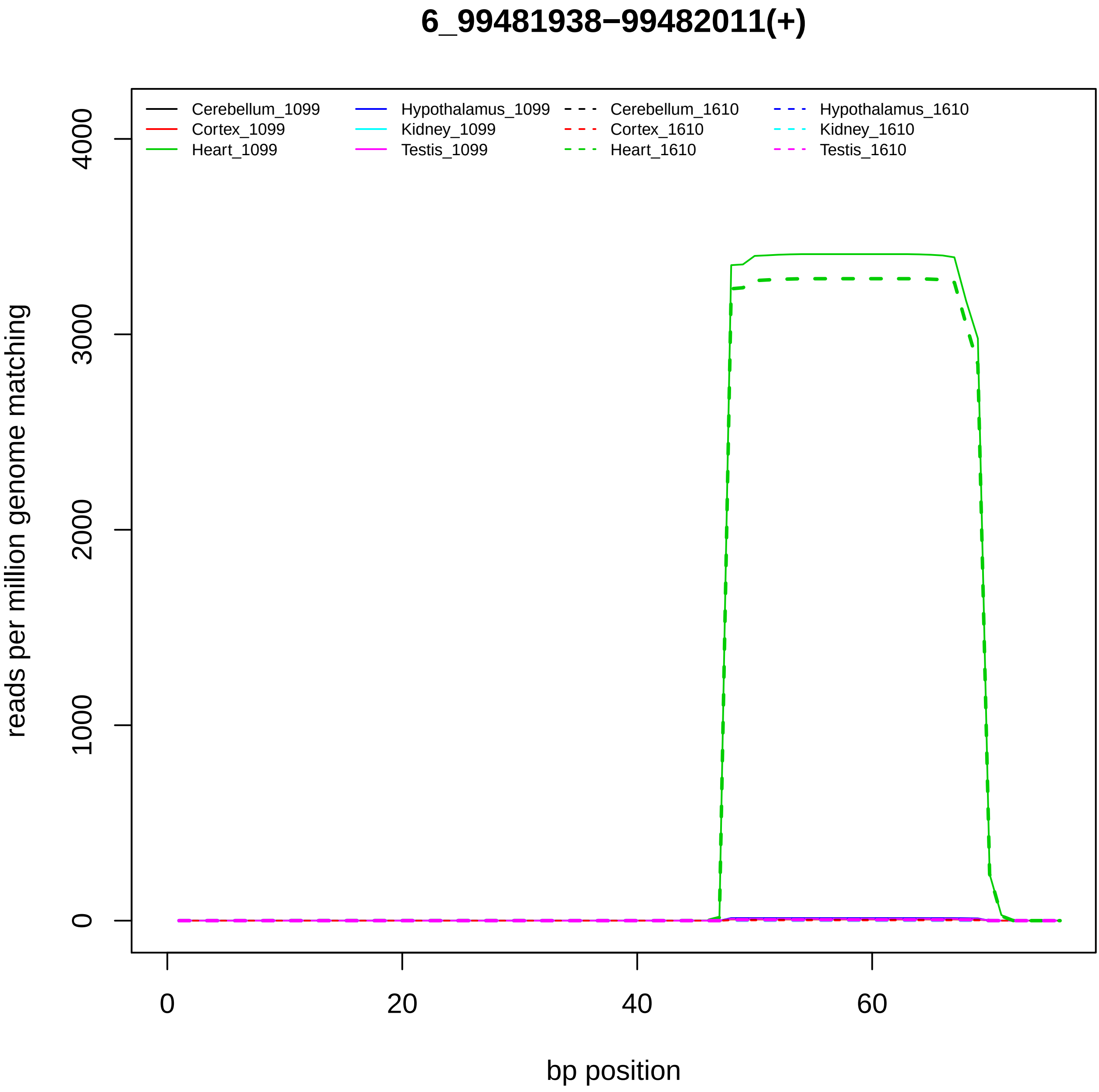
6_99481938−99482011(+)
Cerebellum_1099
Cortex_1099
Heart_1099
Hypothalamus_1099
Kidney_1099
Testis_1099
Cerebellum_1610
Cortex_1610
Heart_1610
Hypothalamus_1610
Kidney_1610
Testis_1610
reads per million genome matching
0
1000
2000
3000
4000
0
20
40
60
bp position

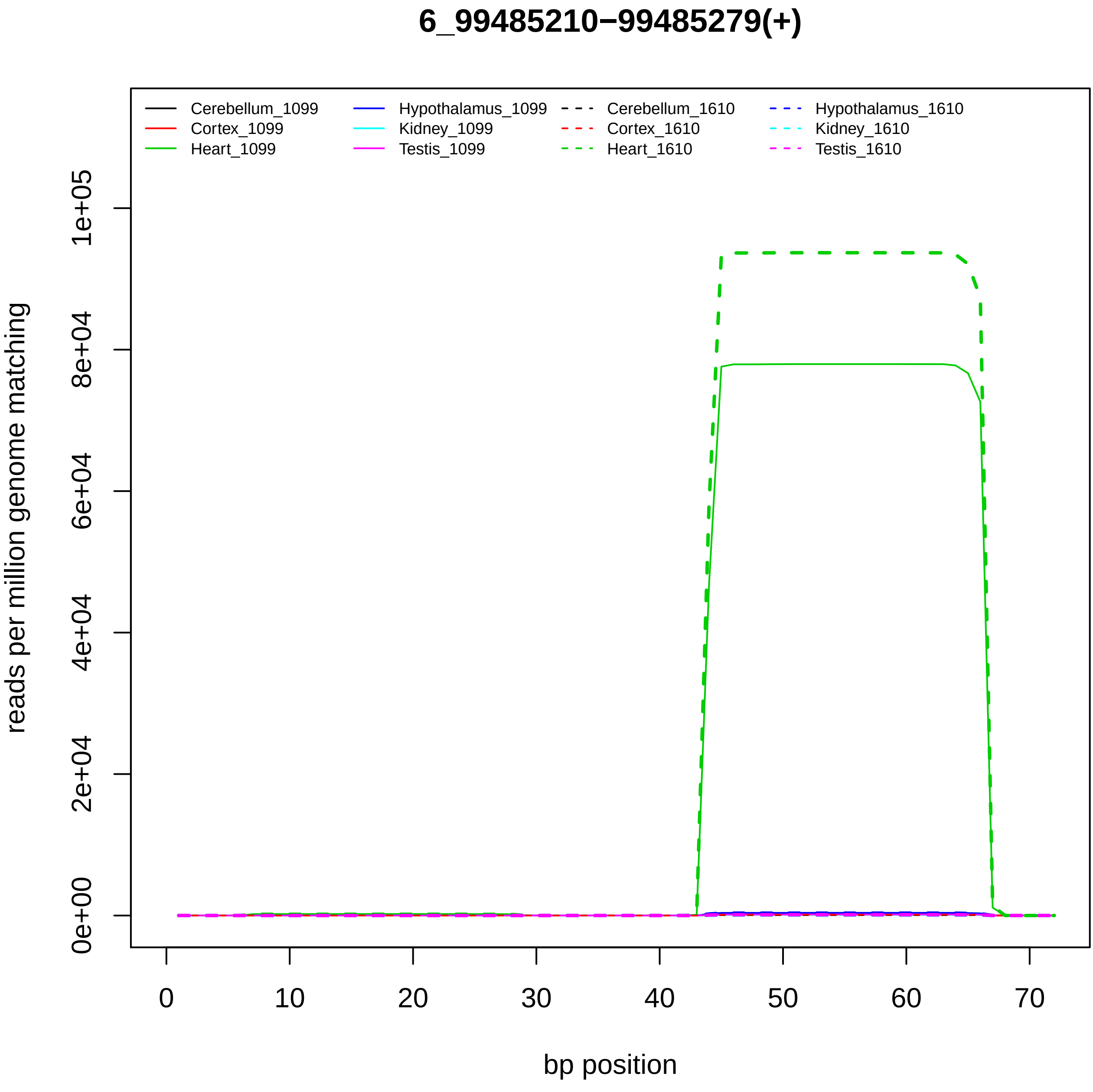

6_99485210−99485279(+)
Cerebellum_1099
Cortex_1099
Heart_1099
Hypothalamus_1099
Kidney_1099
Testis_1099
Cerebellum_1610
Cortex_1610
Heart_1610
Hypothalamus_1610
Kidney_1610
Testis_1610
reads per million genome matching
0e+00
2e+04
4e+04
6e+04
8e+04
1e+05
0
10
20
30
40
50
60
70
bp position

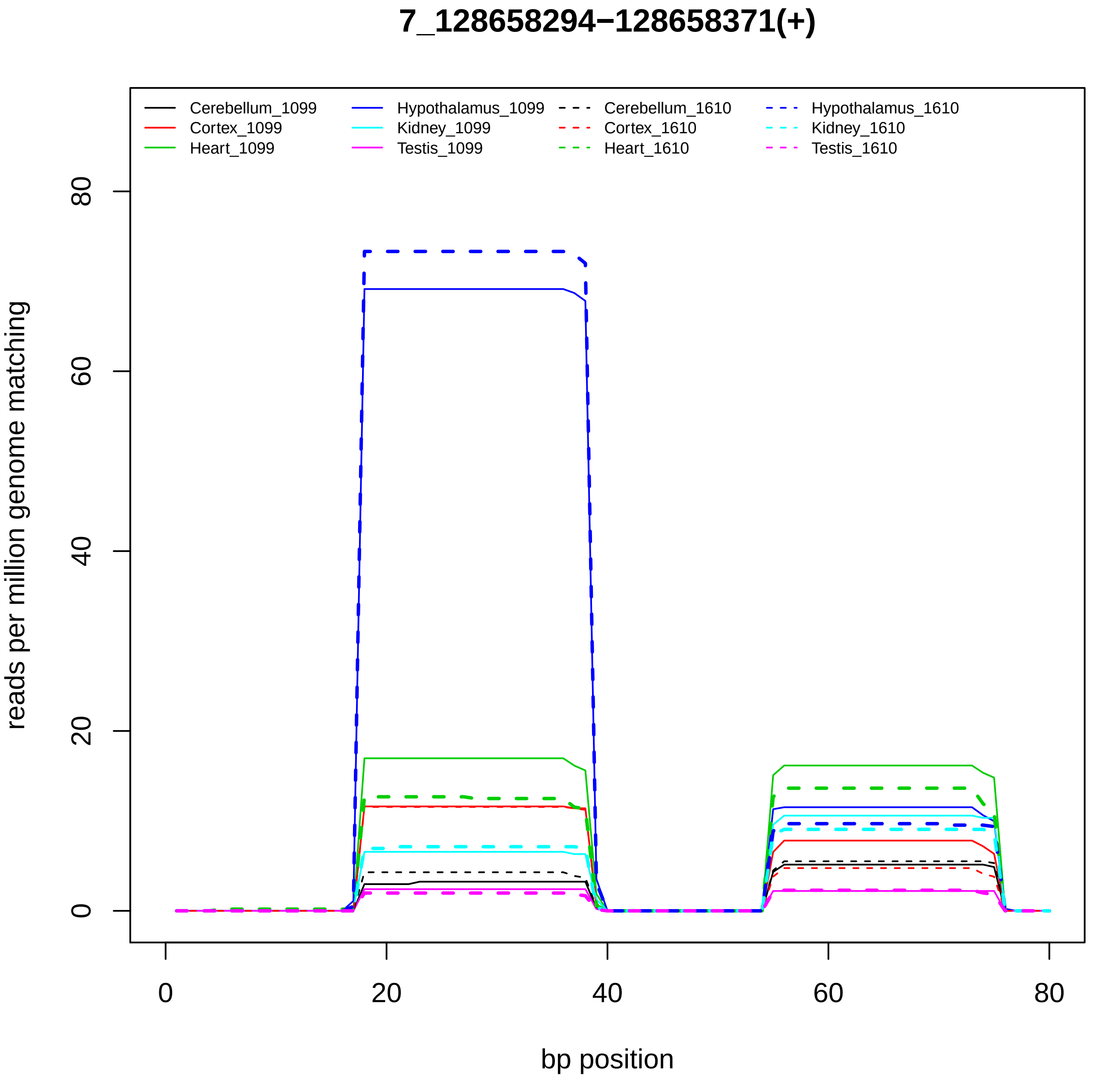

7_128658294−128658371(+)
Cerebellum_1099
Cortex_1099
Heart_1099
Hypothalamus_1099
Kidney_1099
Testis_1099
Cerebellum_1610
Cortex_1610
Heart_1610
Hypothalamus_1610
Kidney_1610
Testis_1610
reads per million genome matching
0
20
40
60
80
bp position
0
20
40
60
80

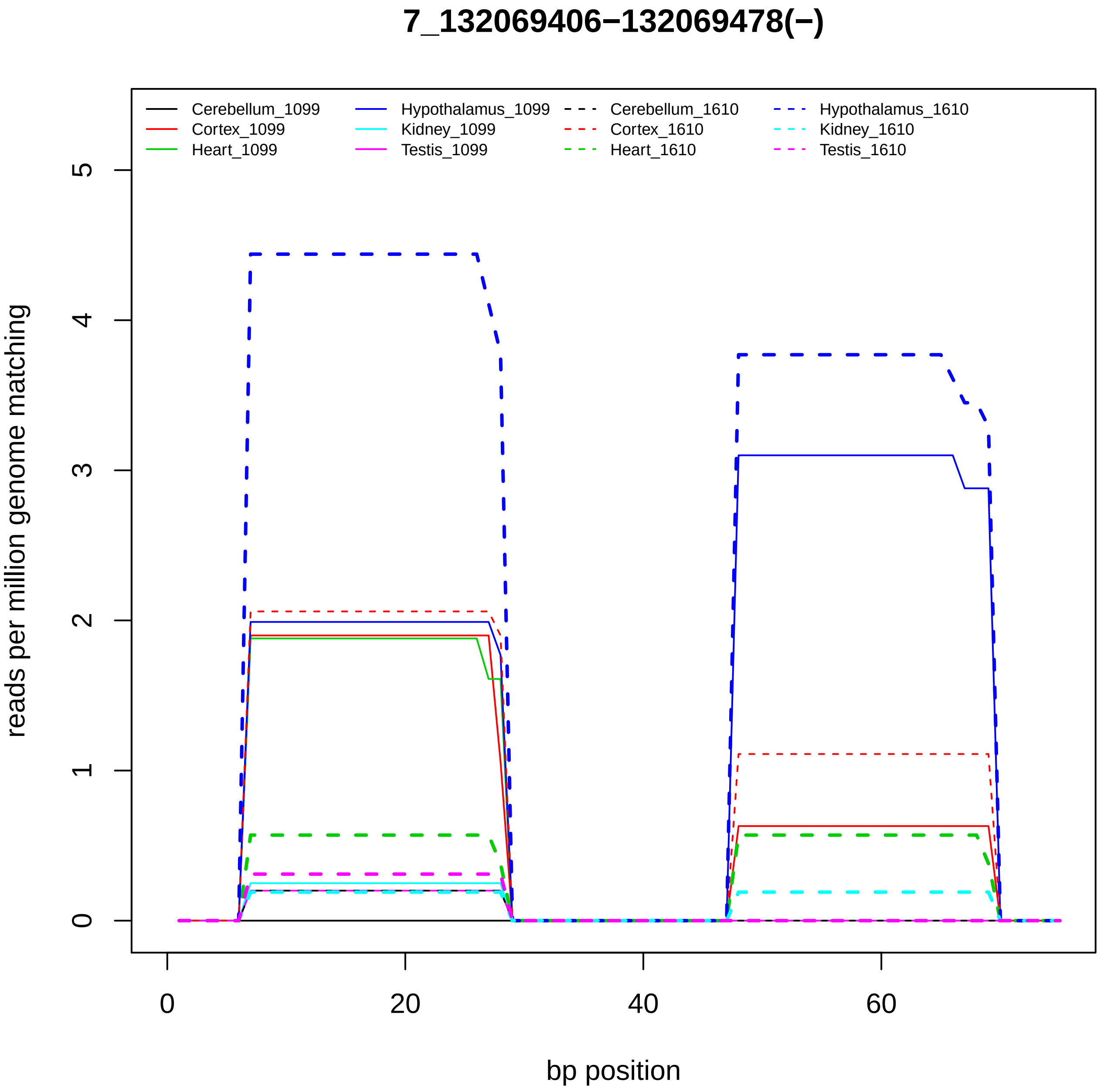
7_132069406−132069478(−)
Cerebellum_1099
Cortex_1099
Heart_1099
Hypothalamus_1099
Kidney_1099
Testis_1099
Cerebellum_1610
Cortex_1610
Heart_1610
Hypothalamus_1610
Kidney_1610
Testis_1610
reads per million genome matching
0
1
2
3
4
5
0
20
40
60
bp position

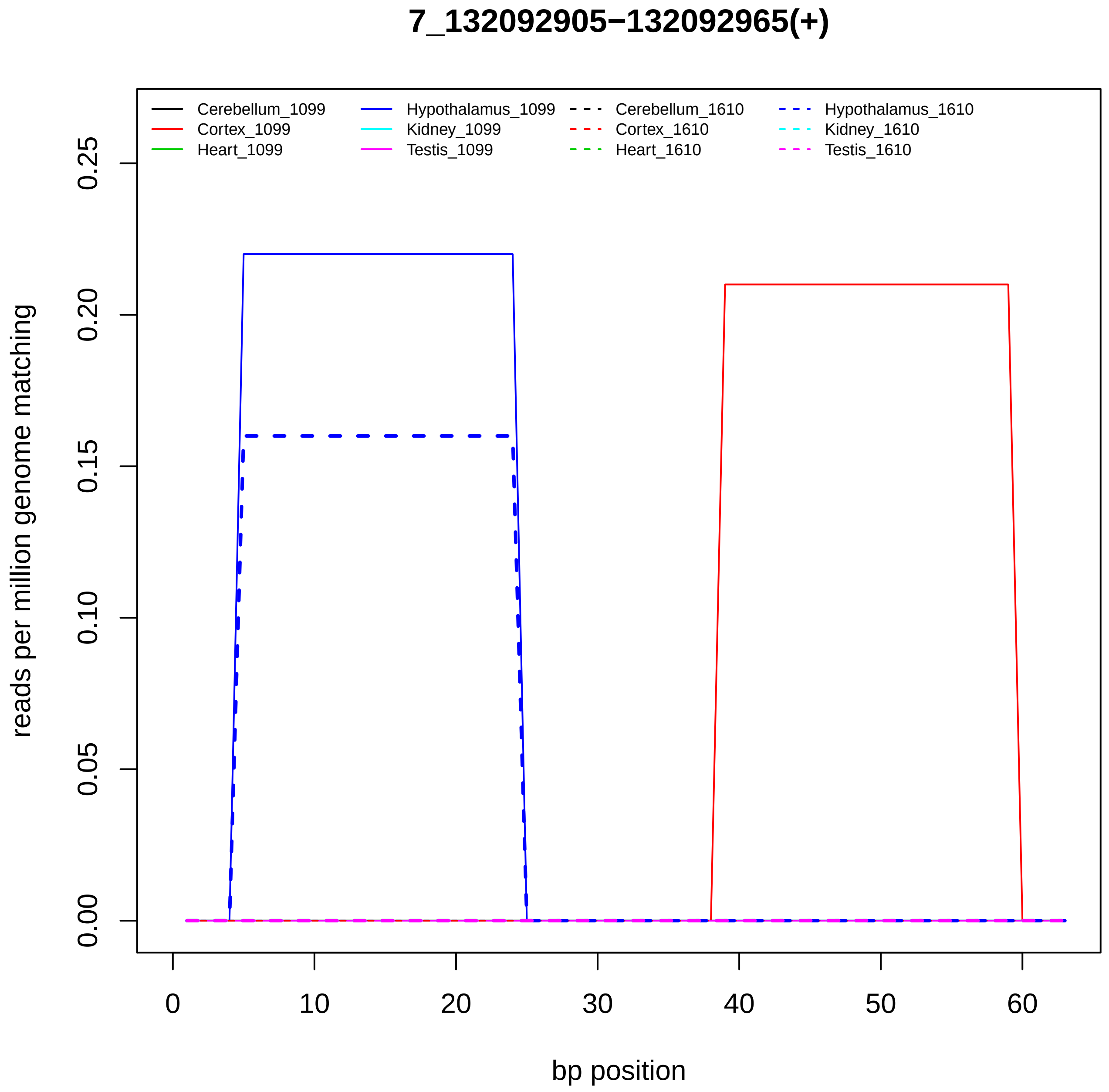

7_132092905−132092965(+)
Cerebellum_1099
Cortex_1099
Heart_1099
Hypothalamus_1099
Kidney_1099
Testis_1099
Cerebellum_1610
Cortex_1610
Heart_1610
Hypothalamus_1610
Kidney_1610
Testis_1610
reads per million genome matching
0.00
0.05
0.10
0.15
0.20
0.25
0
10
20
30
40
50
60
bp position

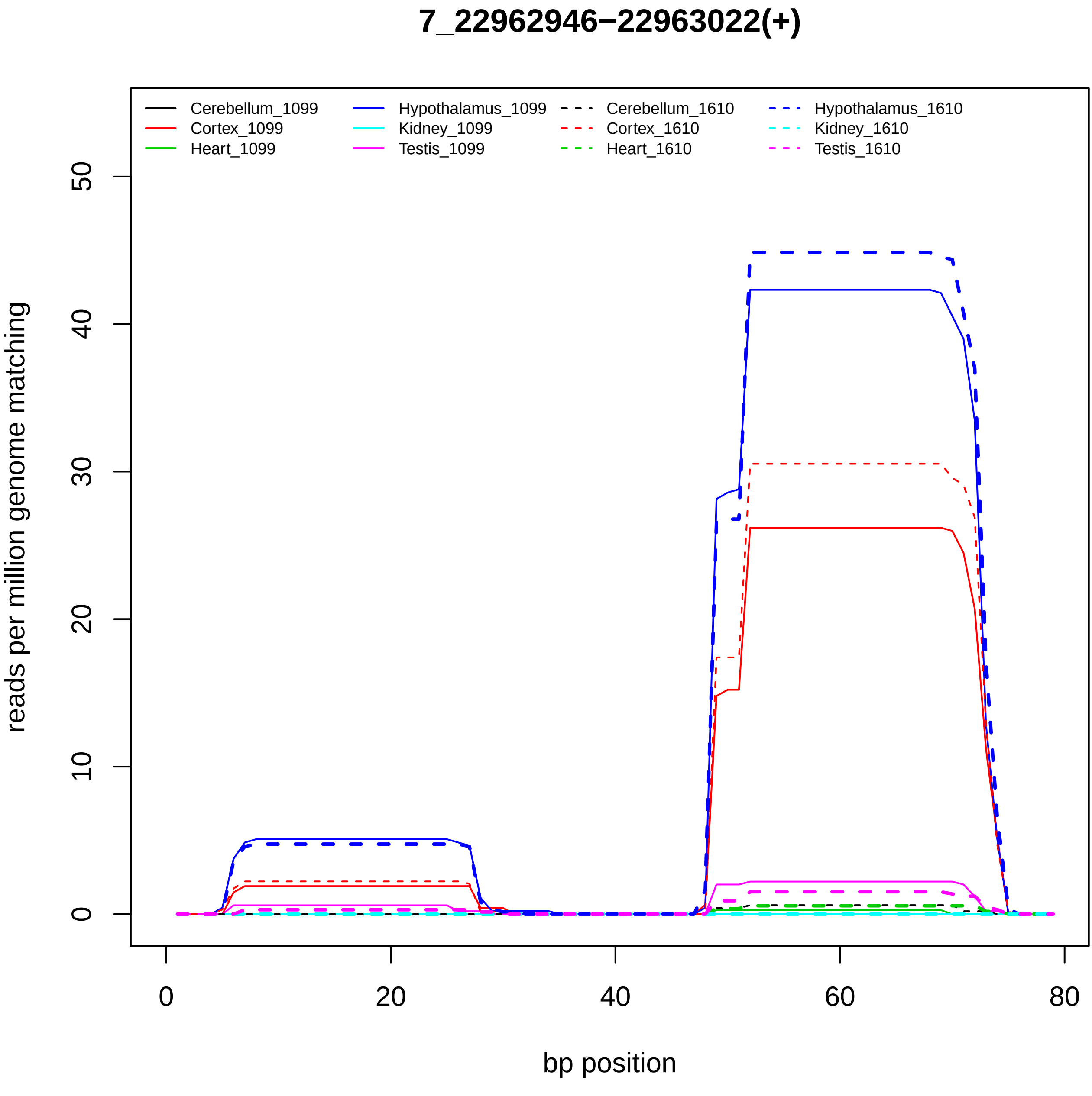
7_22962946−22963022(+)
Cerebellum_1099
Cortex_1099
Heart_1099
Hypothalamus_1099
Kidney_1099
Testis_1099
Cerebellum_1610
Cortex_1610
Heart_1610
Hypothalamus_1610
Kidney_1610
Testis_1610
reads per million genome matching
0
10
20
30
40
50
bp position
0
20
40
60
80

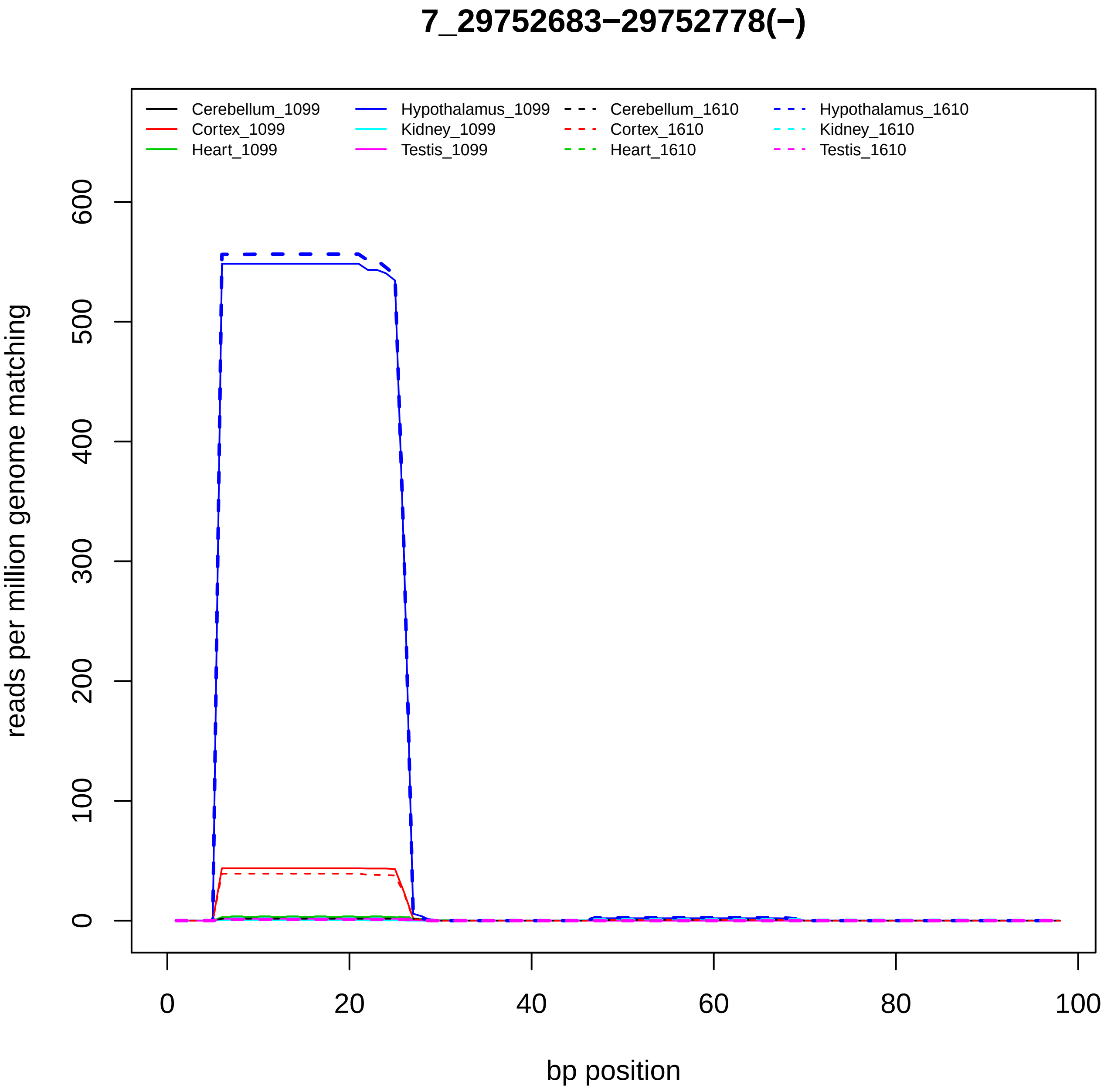

7_29752683−29752778(−)
Cerebellum_1099
Cortex_1099
Heart_1099
Hypothalamus_1099
Kidney_1099
Testis_1099
Cerebellum_1610
Cortex_1610
Heart_1610
Hypothalamus_1610
Kidney_1610
Testis_1610
reads per million genome matching
0
100
200
300
400
500
600
0
20
40
60
80
100
bp position

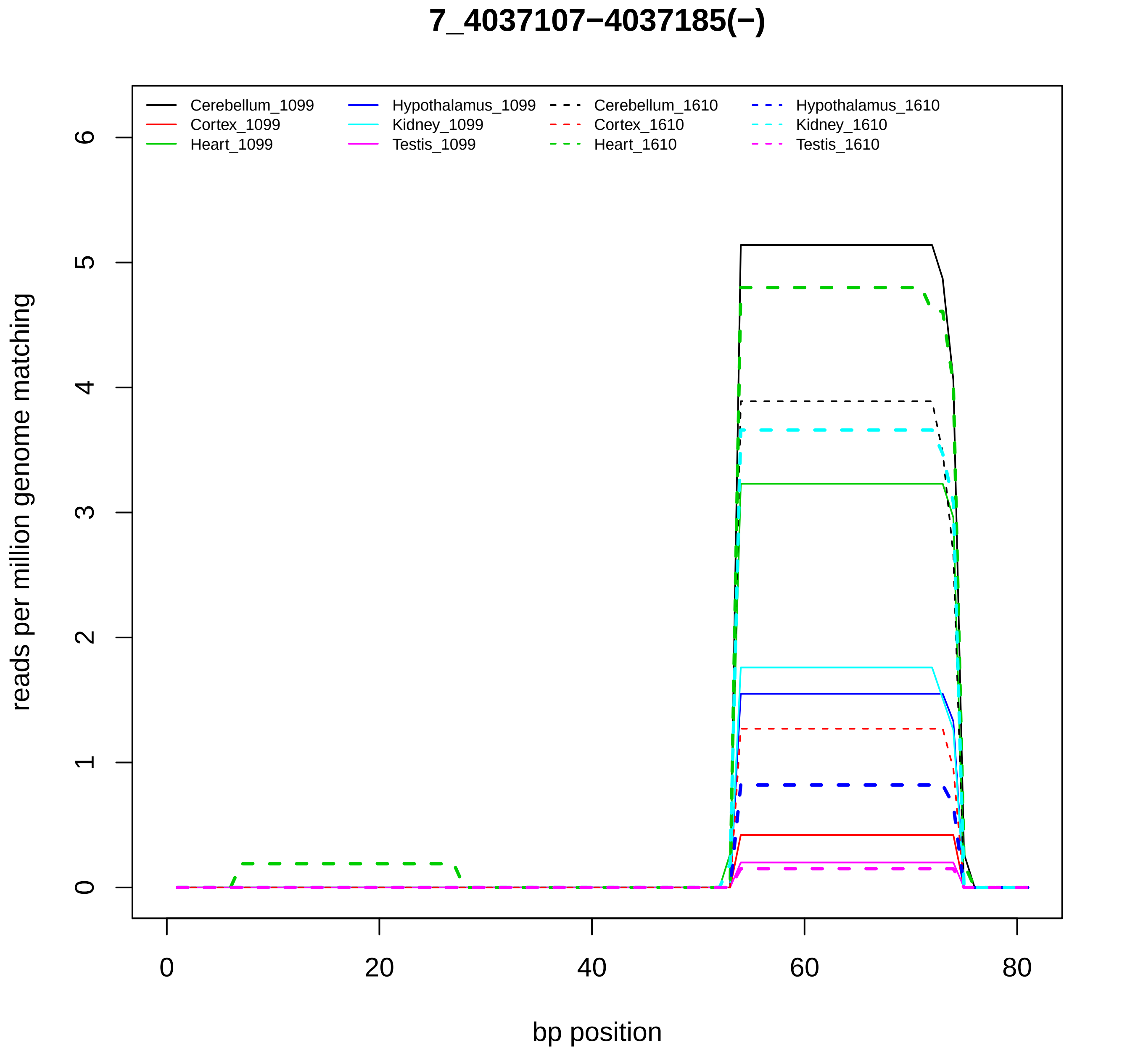

7_4037107−4037185(−)
Cerebellum_1099
Cortex_1099
Heart_1099
Hypothalamus_1099
Kidney_1099
Testis_1099
Cerebellum_1610
Cortex_1610
Heart_1610
Hypothalamus_1610
Kidney_1610
Testis_1610
reads per million genome matching
bp position
0
1
2
3
4
5
6
0
20
40
60
80

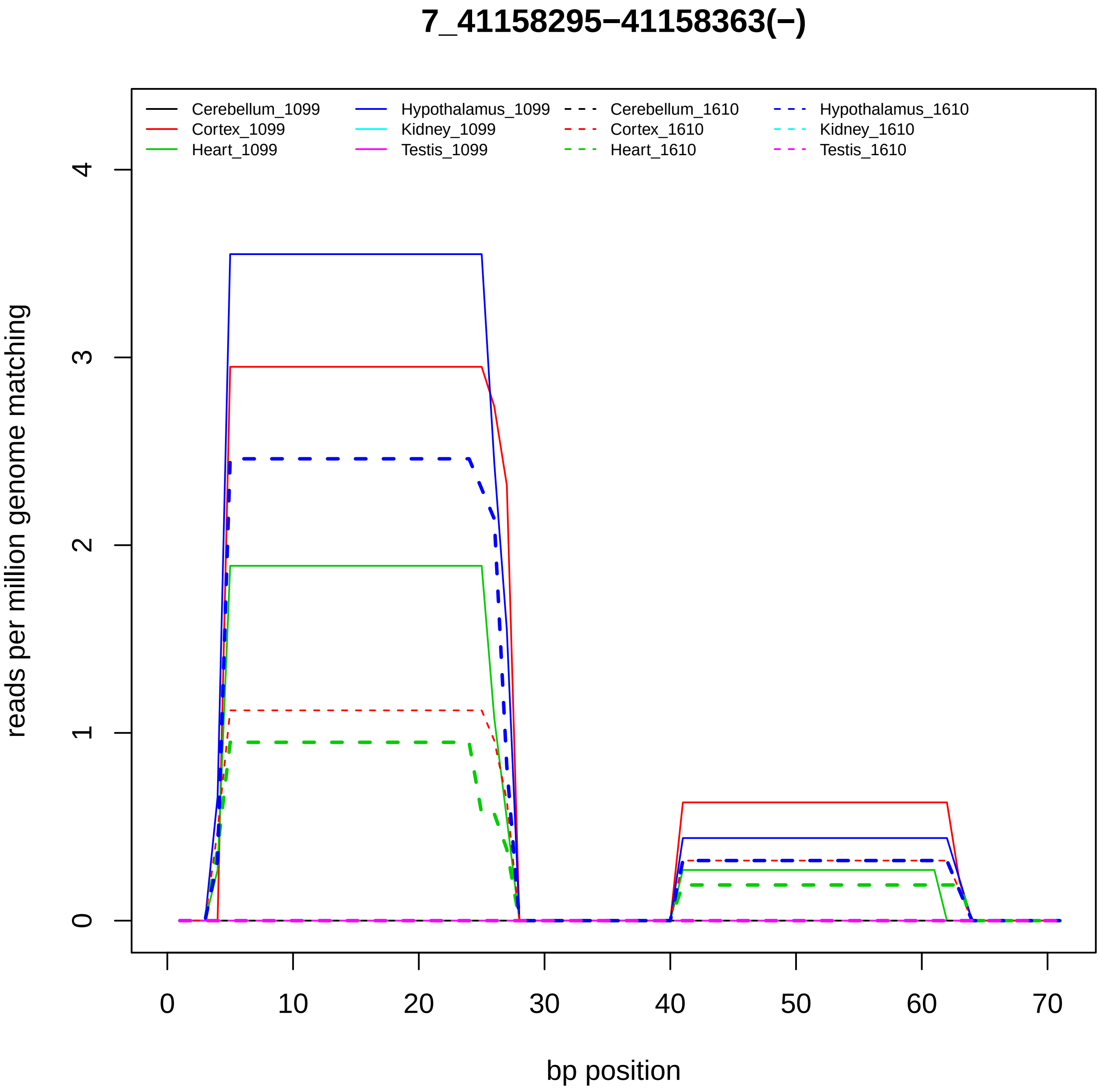

7_41158295−41158363(−)
Cerebellum_1099
Cortex_1099
Heart_1099
Hypothalamus_1099
Kidney_1099
Testis_1099
Cerebellum_1610
Cortex_1610
Heart_1610
Hypothalamus_1610
Kidney_1610
Testis_1610
reads per million genome matching
0
1
2
3
4
0
10
20
30
40
50
60
70
bp position

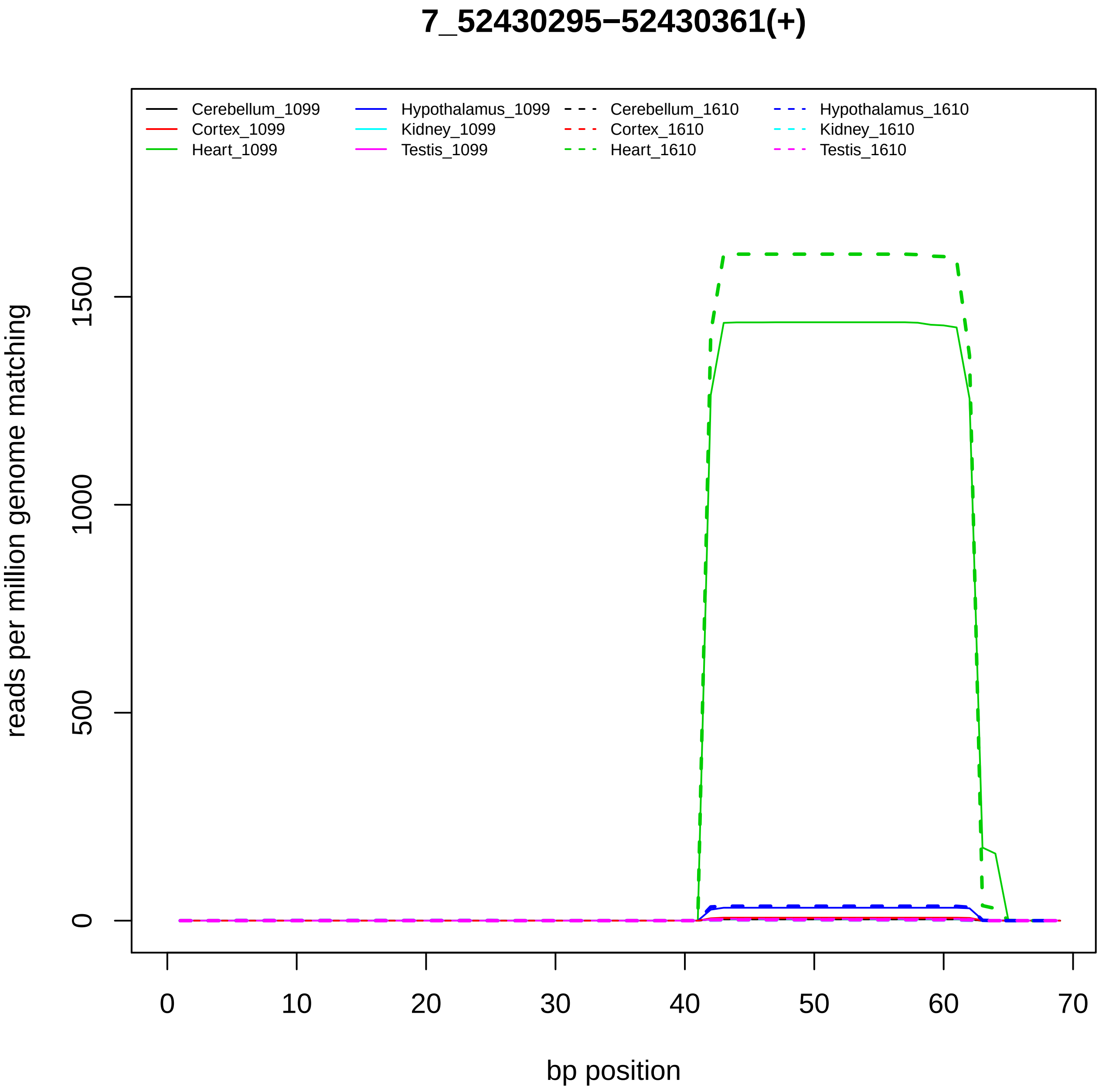

7_52430295−52430361(+)
Cerebellum_1099
Cortex_1099
Heart_1099
Hypothalamus_1099
Kidney_1099
Testis_1099
Cerebellum_1610
Cortex_1610
Heart_1610
Hypothalamus_1610
Kidney_1610
Testis_1610
reads per million genome matching
0
500
1000
1500
0
10
20
30
40
50
60
70
bp position

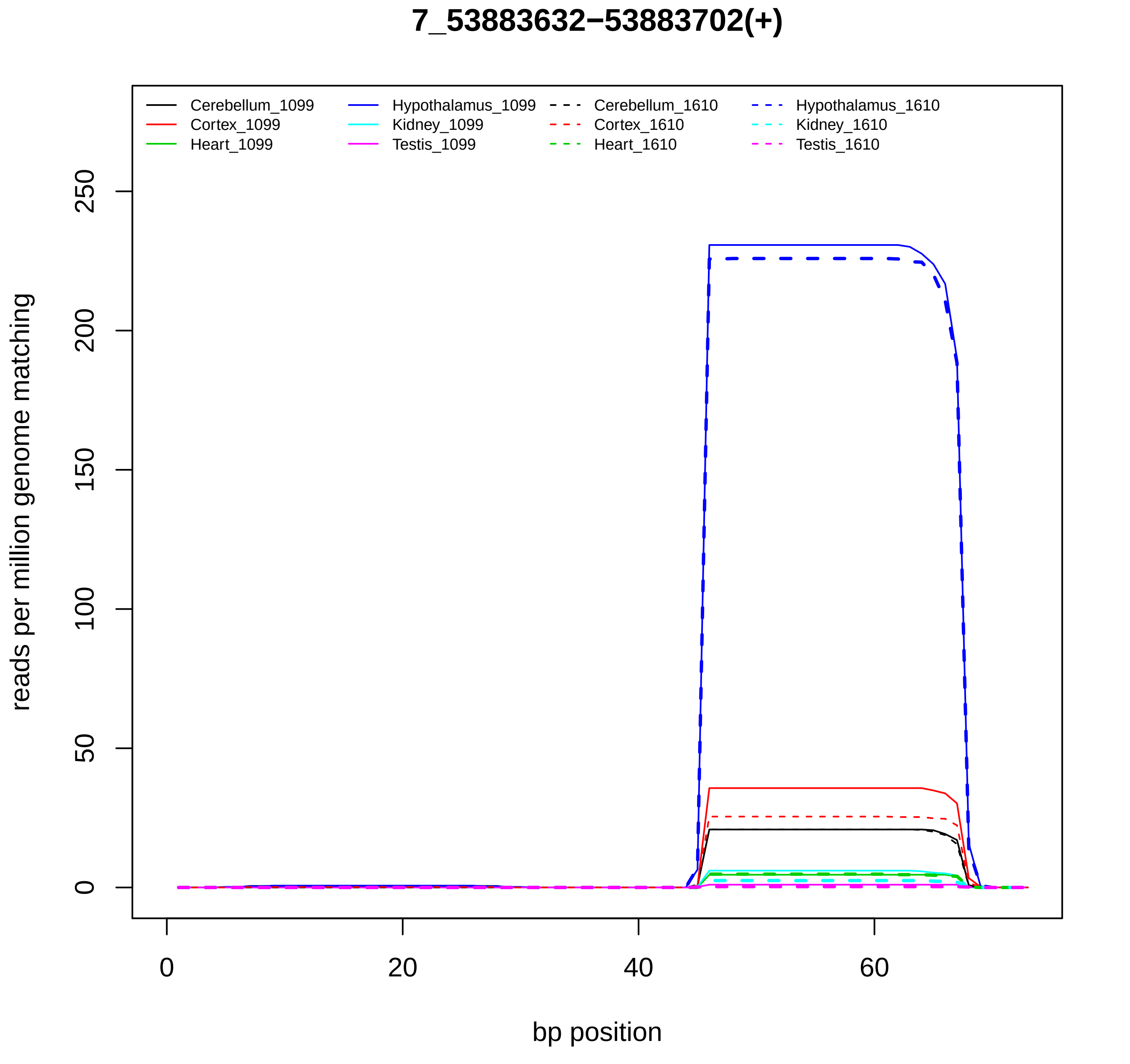

7_53883632−53883702(+)
Cerebellum_1099
Cortex_1099
Heart_1099
Hypothalamus_1099
Kidney_1099
Testis_1099
Cerebellum_1610
Cortex_1610
Heart_1610
Hypothalamus_1610
Kidney_1610
Testis_1610
reads per million genome matching
0
50
100
150
200
250
0
20
40
60
bp position

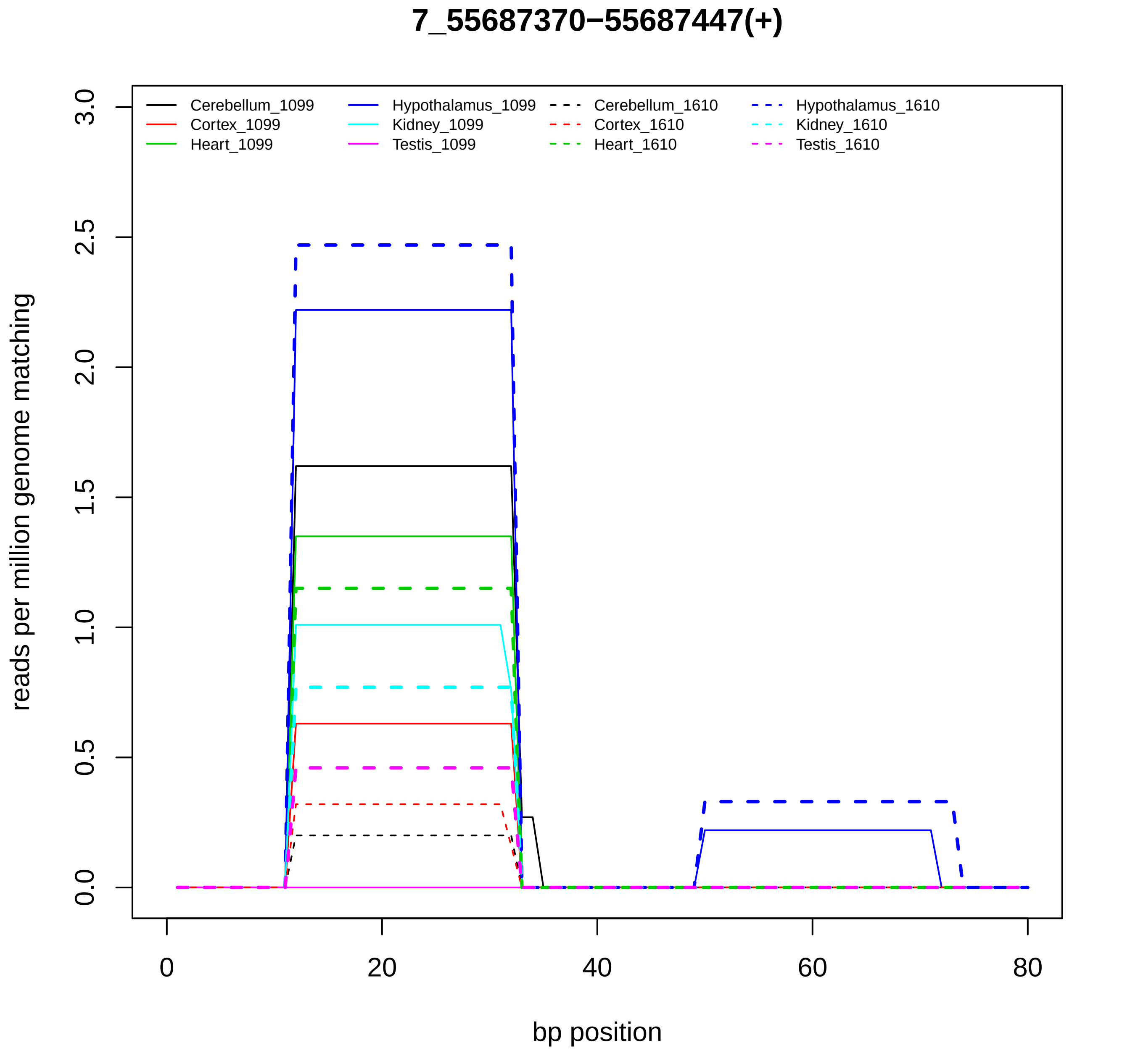

7_55687370−55687447(+)
Cerebellum_1099
Cortex_1099
Heart_1099
Hypothalamus_1099
Kidney_1099
Testis_1099
Cerebellum_1610
Cortex_1610
Heart_1610
Hypothalamus_1610
Kidney_1610
Testis_1610
reads per million genome matching
0.0
0.5
1.0
1.5
2.0
2.5
3.0
0
20
40
60
80
bp position

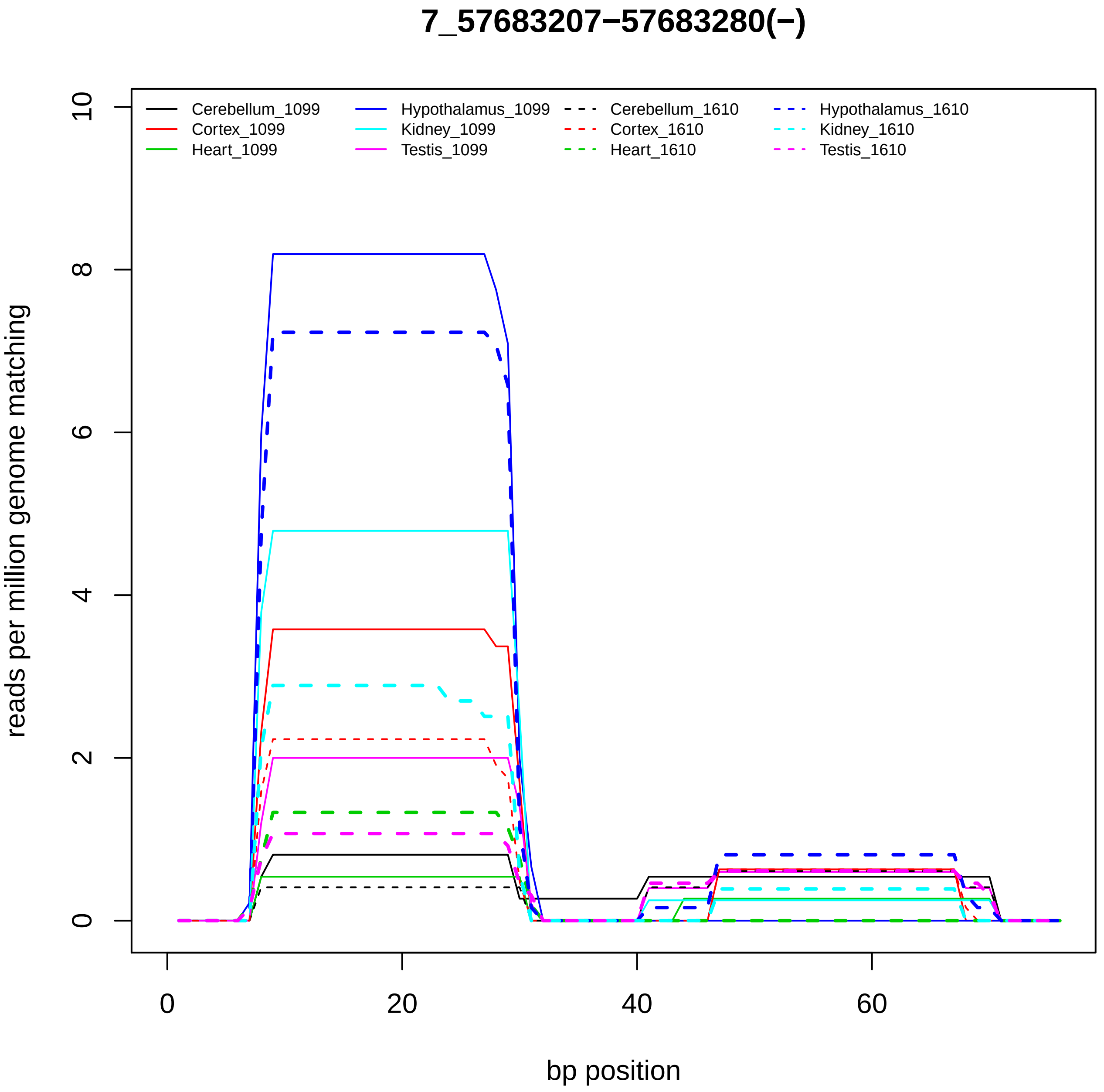

7_57683207−57683280(−)
Cerebellum_1099
Cortex_1099
Heart_1099
Hypothalamus_1099
Kidney_1099
Testis_1099
Cerebellum_1610
Cortex_1610
Heart_1610
Hypothalamus_1610
Kidney_1610
Testis_1610
reads per million genome matching
0
2
4
6
8
10
0
20
40
60
bp position

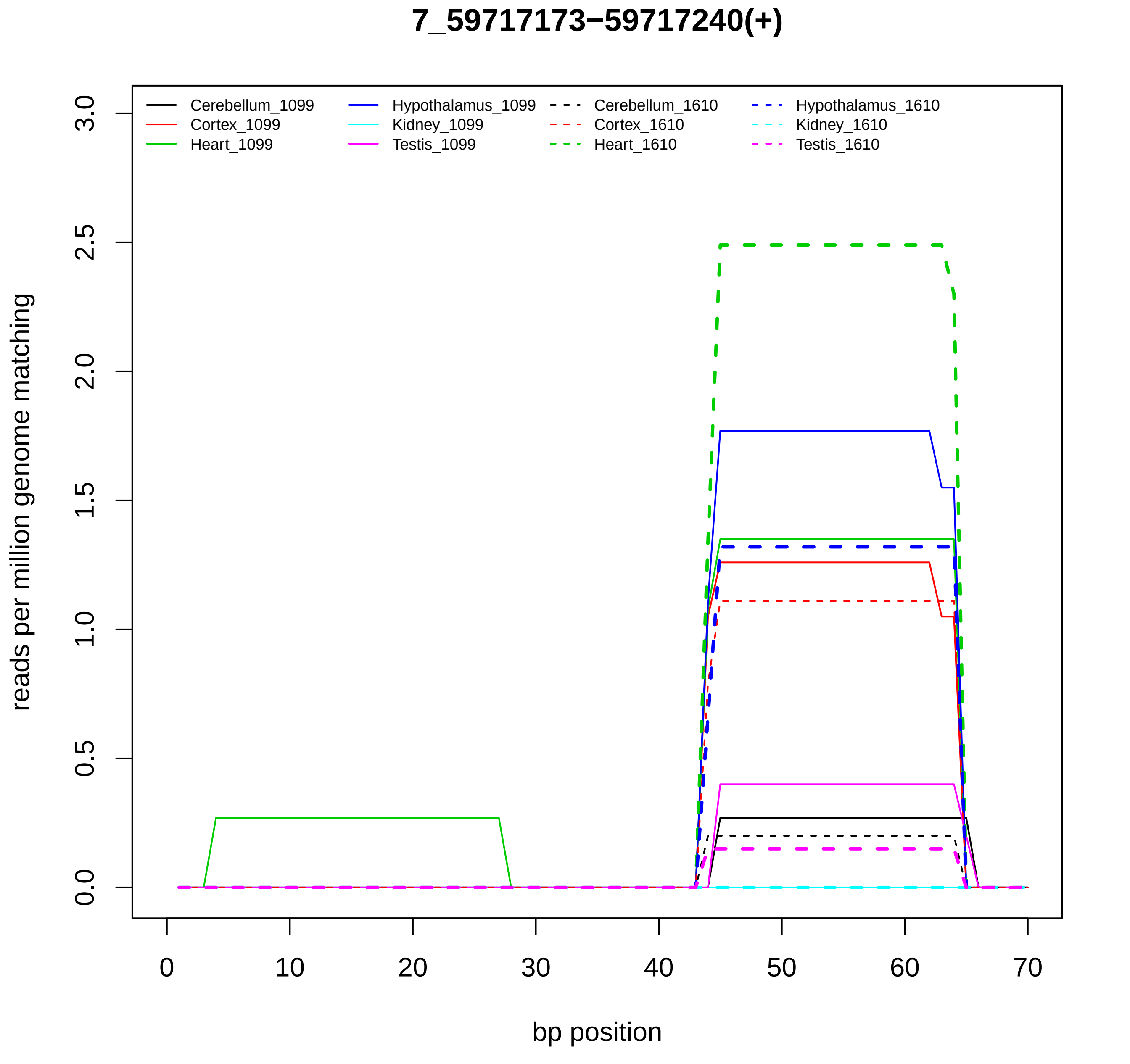

7_59717173−59717240(+)
Cerebellum_1099
Cortex_1099
Heart_1099
Hypothalamus_1099
Kidney_1099
Testis_1099
Cerebellum_1610
Cortex_1610
Heart_1610
Hypothalamus_1610
Kidney_1610
Testis_1610
reads per million genome matching
bp position
0.0
0.5
1.0
1.5
2.0
2.5
3.0
0
10
20
30
40
50
60
70

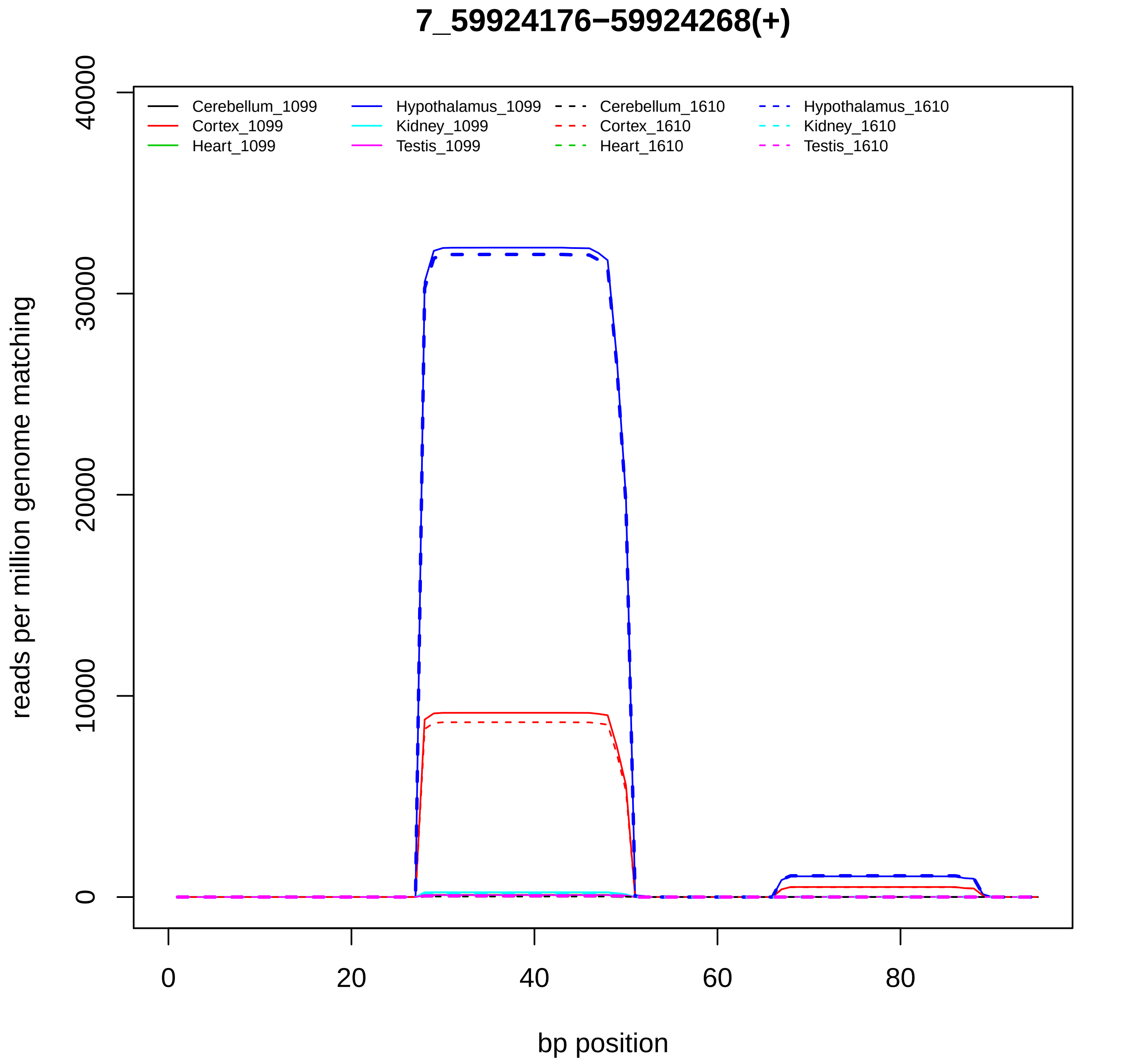

7_59924176−59924268(+)
Cerebellum_1099
Cortex_1099
Heart_1099
Hypothalamus_1099
Kidney_1099
Testis_1099
Cerebellum_1610
Cortex_1610
Heart_1610
Hypothalamus_1610
Kidney_1610
Testis_1610
reads per million genome matching
0
10000
20000
30000
40000
0
20
40
60
80
bp position

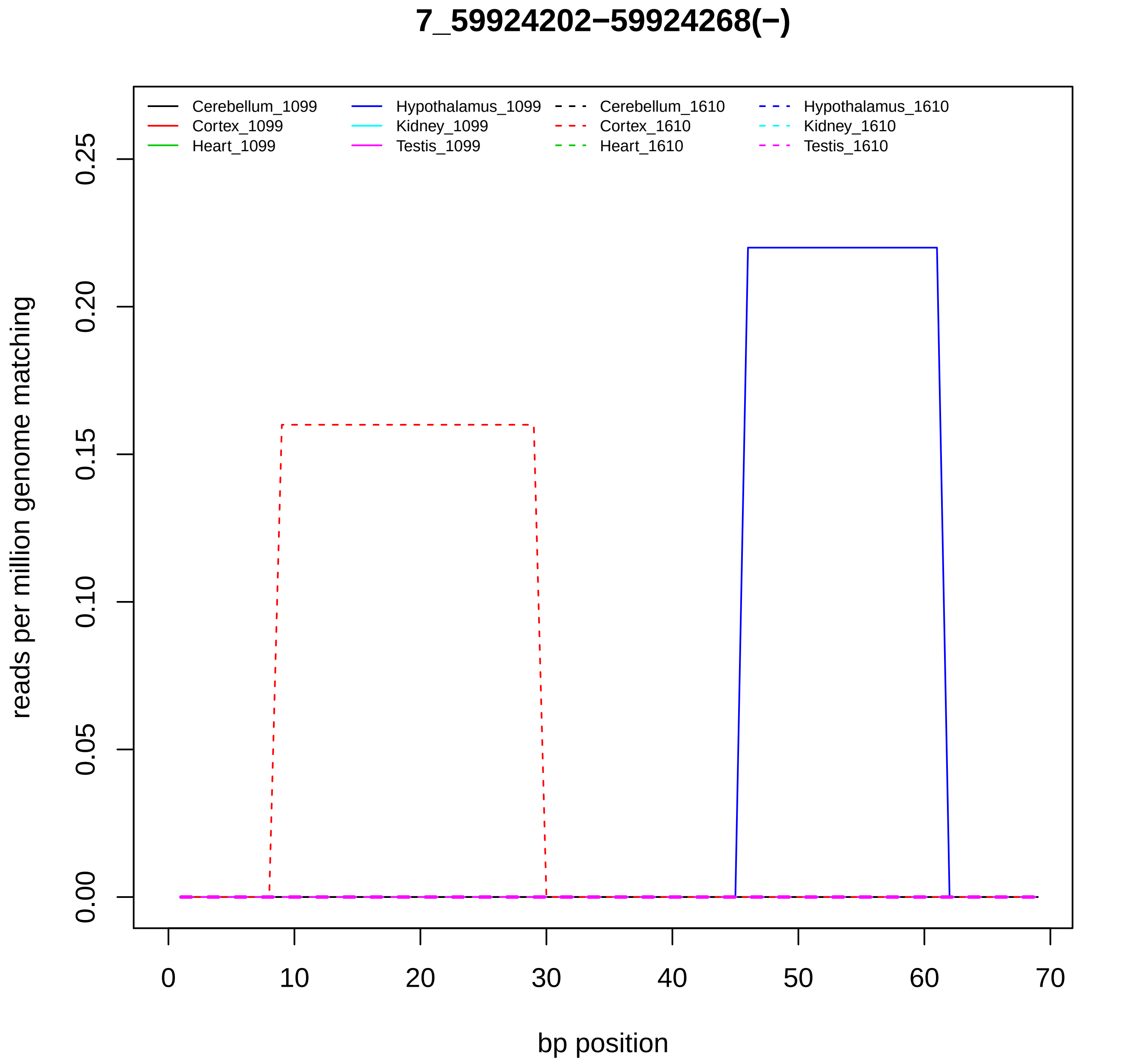

7_59924202−59924268(−)
Cerebellum_1099
Cortex_1099
Heart_1099
Hypothalamus_1099
Kidney_1099
Testis_1099
Cerebellum_1610
Cortex_1610
Heart_1610
Hypothalamus_1610
Kidney_1610
Testis_1610
reads per million genome matching
0.00
0.05
0.10
0.15
0.20
0.25
0
10
20
30
40
50
60
70
bp position

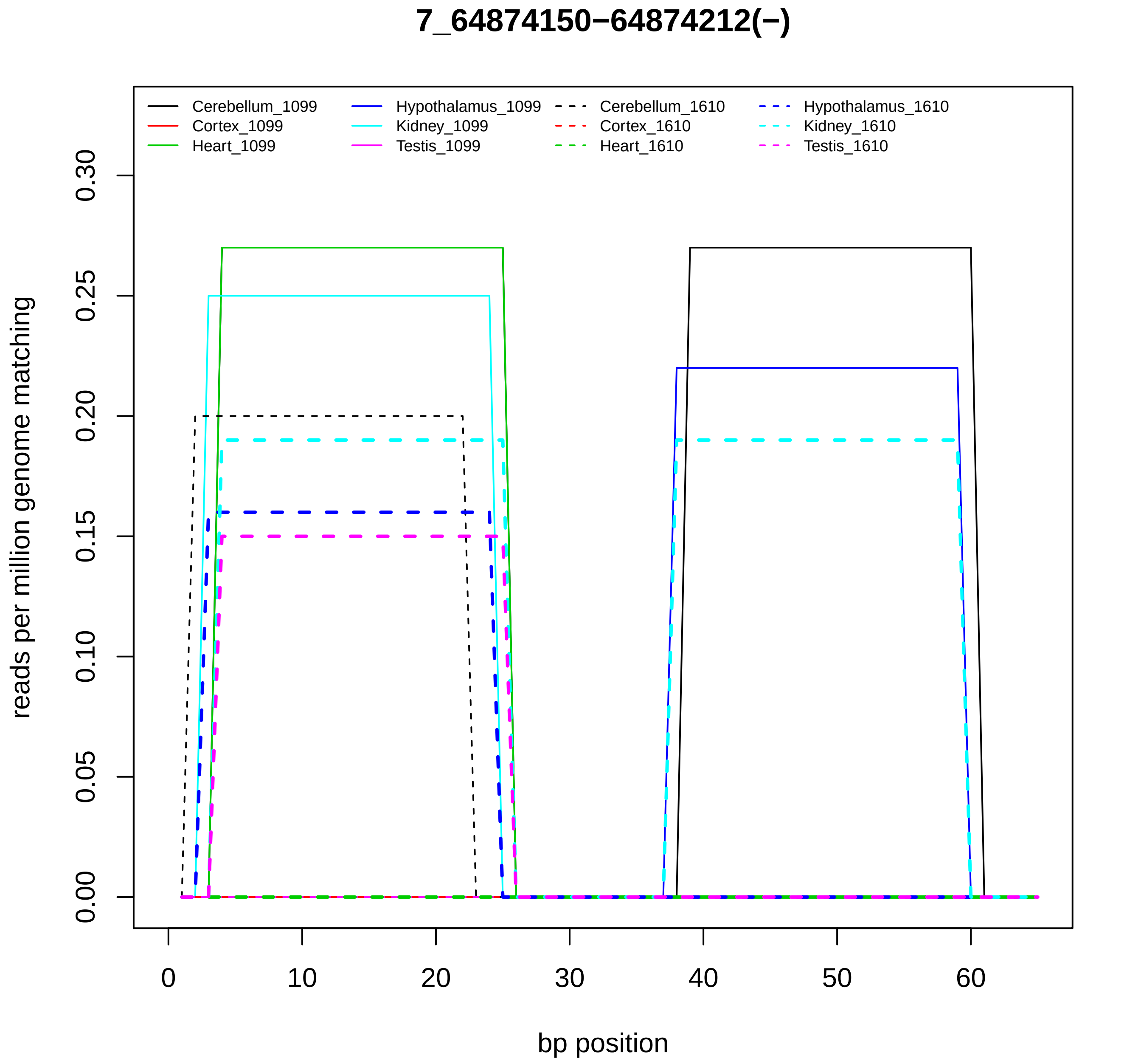

7_64874150−64874212(−)
Cerebellum_1099
Cortex_1099
Heart_1099
Hypothalamus_1099
Kidney_1099
Testis_1099
Cerebellum_1610
Cortex_1610
Heart_1610
Hypothalamus_1610
Kidney_1610
Testis_1610
reads per million genome matching
0.00
0.05
0.10
0.15
0.20
0.25
0.30
0
10
20
30
40
50
60
bp position

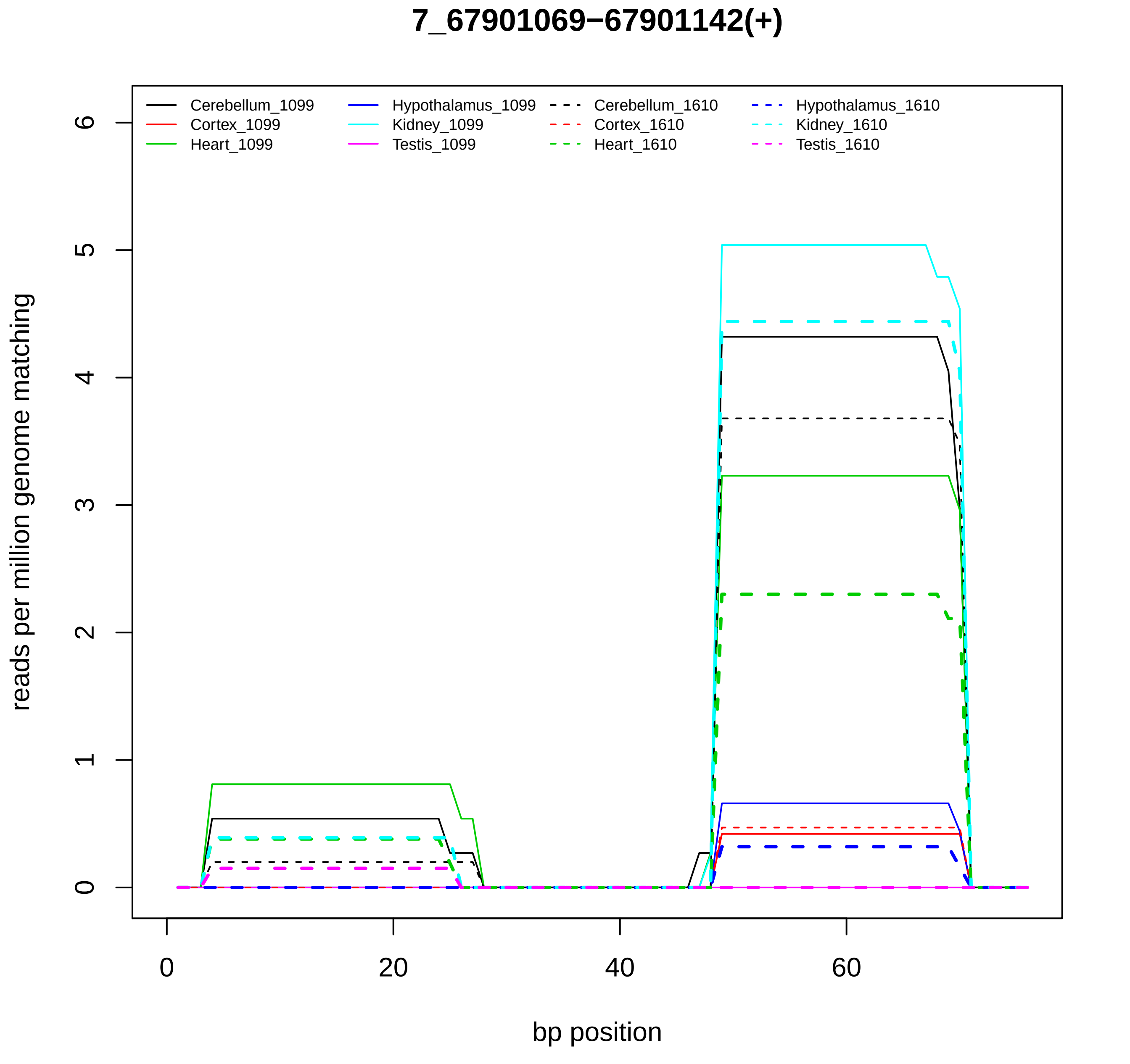
7_67901069−67901142(+)
Cerebellum_1099
Cortex_1099
Heart_1099
Hypothalamus_1099
Kidney_1099
Testis_1099
Cerebellum_1610
Cortex_1610
Heart_1610
Hypothalamus_1610
Kidney_1610
Testis_1610
reads per million genome matching
0
1
2
3
4
5
6
0
20
40
60
bp position

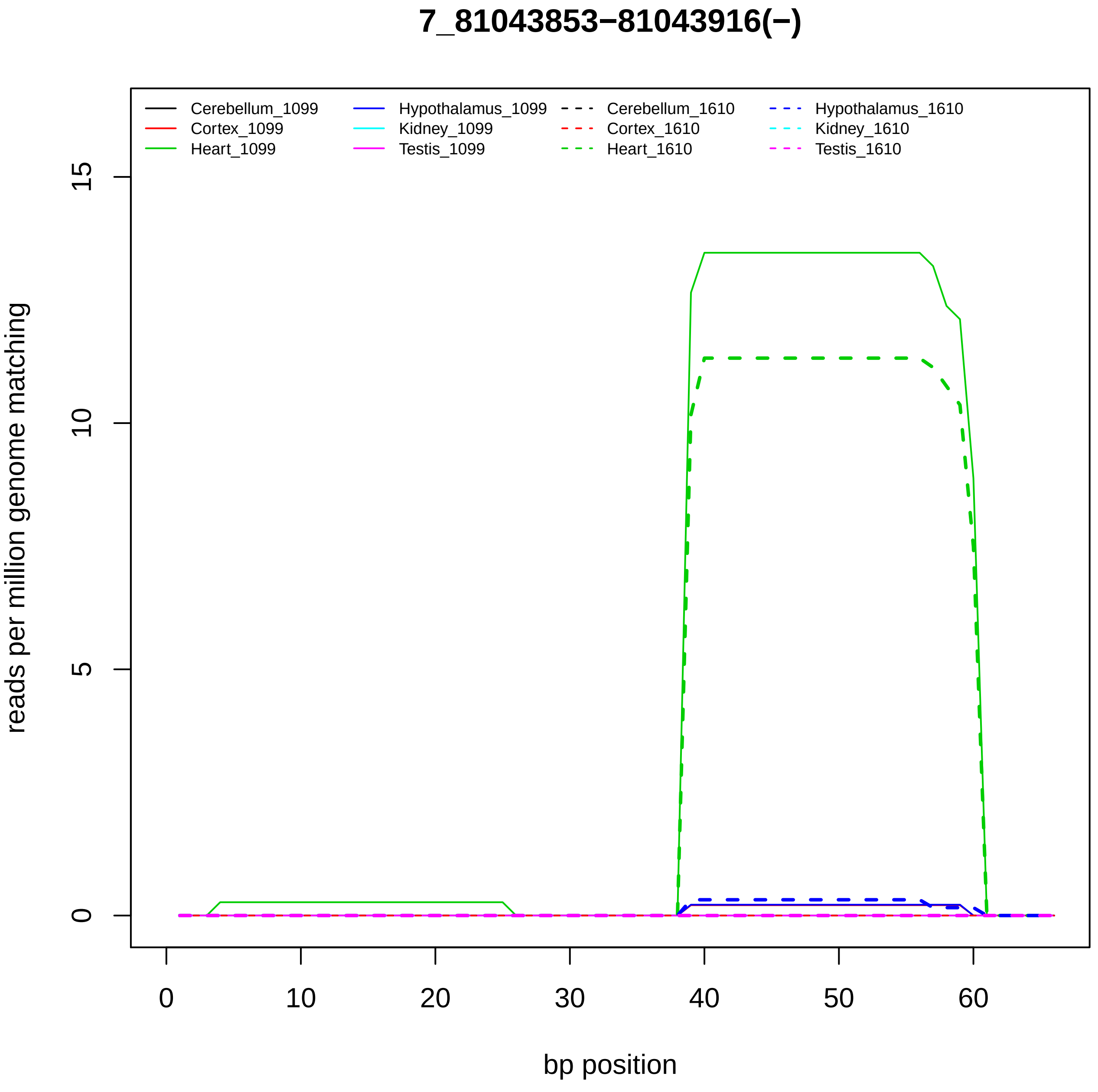
7_81043853−81043916(−)
Cerebellum_1099
Cortex_1099
Heart_1099
Hypothalamus_1099
Kidney_1099
Testis_1099
Cerebellum_1610
Cortex_1610
Heart_1610
Hypothalamus_1610
Kidney_1610
Testis_1610
reads per million genome matching
0
5
10
15
0
10
20
30
40
50
60
bp position

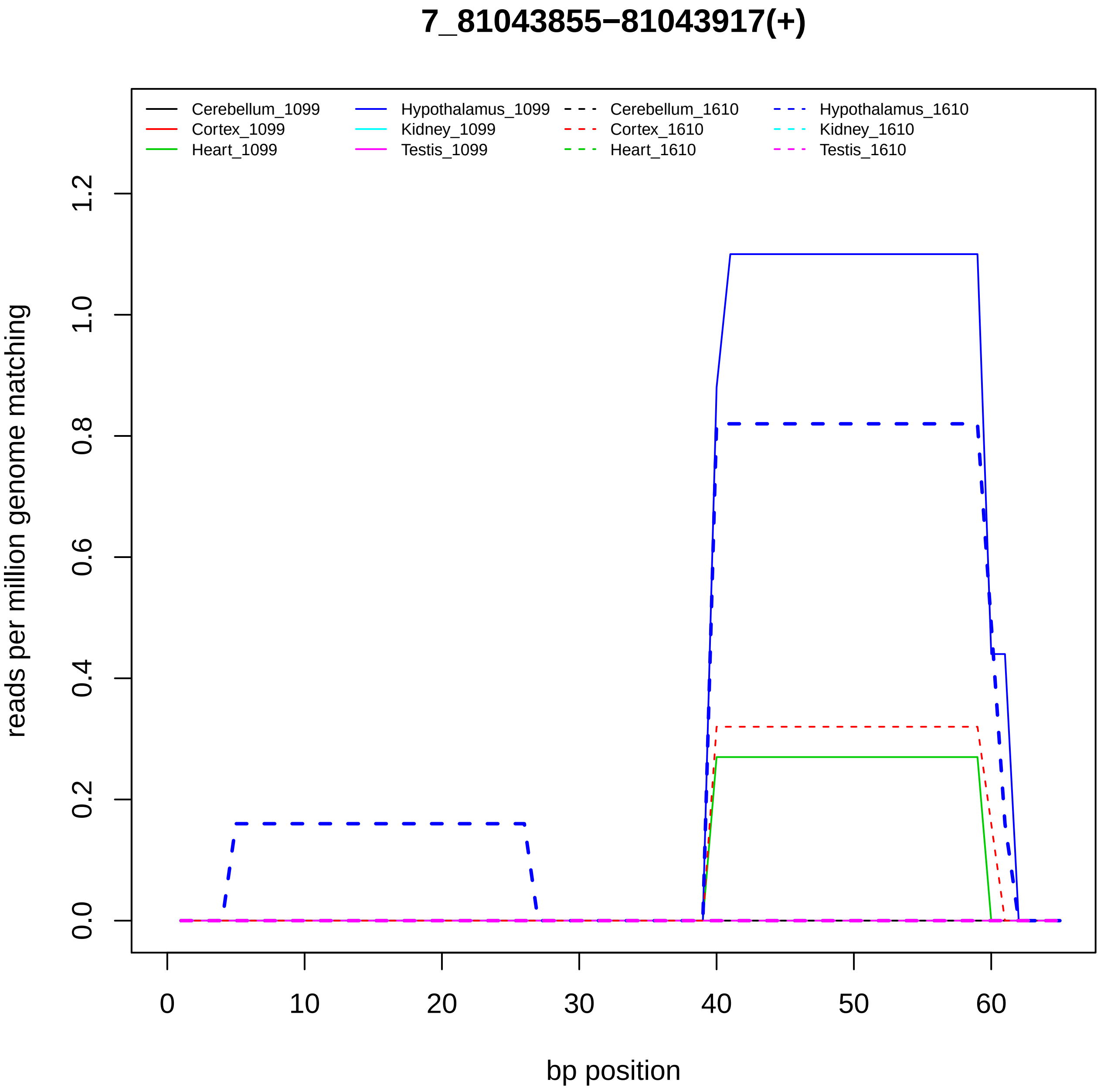

7_81043855−81043917(+)
Cerebellum_1099
Cortex_1099
Heart_1099
Hypothalamus_1099
Kidney_1099
Testis_1099
Cerebellum_1610
Cortex_1610
Heart_1610
Hypothalamus_1610
Kidney_1610
Testis_1610
reads per million genome matching
0.0
0.2
0.4
0.6
0.8
1.0
1.2
0
10
20
30
40
50
60
bp position

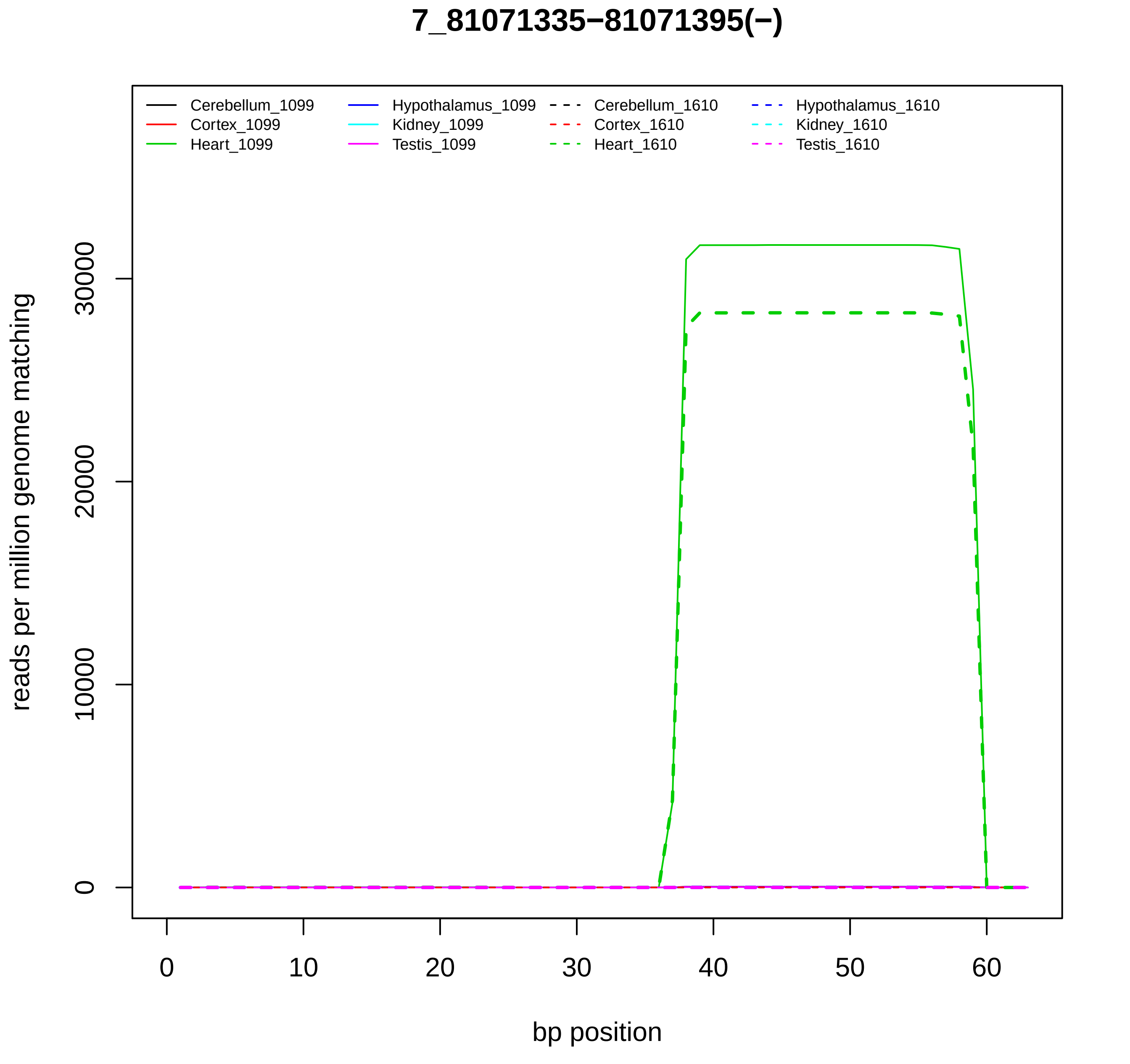

7_81071335−81071395(−)
Cerebellum_1099
Cortex_1099
Heart_1099
Hypothalamus_1099
Kidney_1099
Testis_1099
Cerebellum_1610
Cortex_1610
Heart_1610
Hypothalamus_1610
Kidney_1610
Testis_1610
reads per million genome matching
0
10000
20000
30000
0
10
20
30
40
50
60
bp position

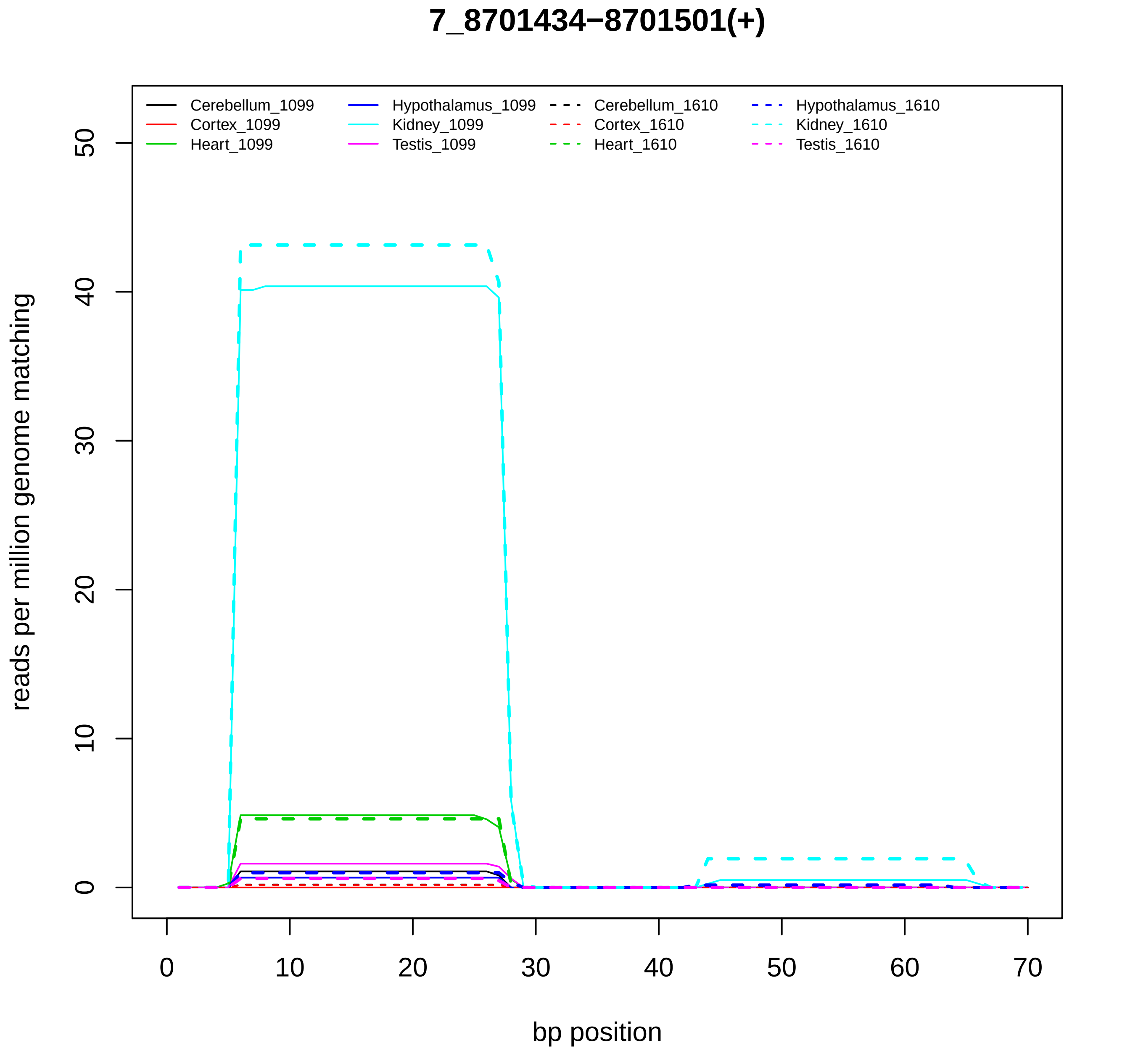

7_8701434−8701501(+)
Cerebellum_1099
Cortex_1099
Heart_1099
Hypothalamus_1099
Kidney_1099
Testis_1099
Cerebellum_1610
Cortex_1610
Heart_1610
Hypothalamus_1610
Kidney_1610
Testis_1610
reads per million genome matching
bp position
0
10
20
30
40
50
60
70

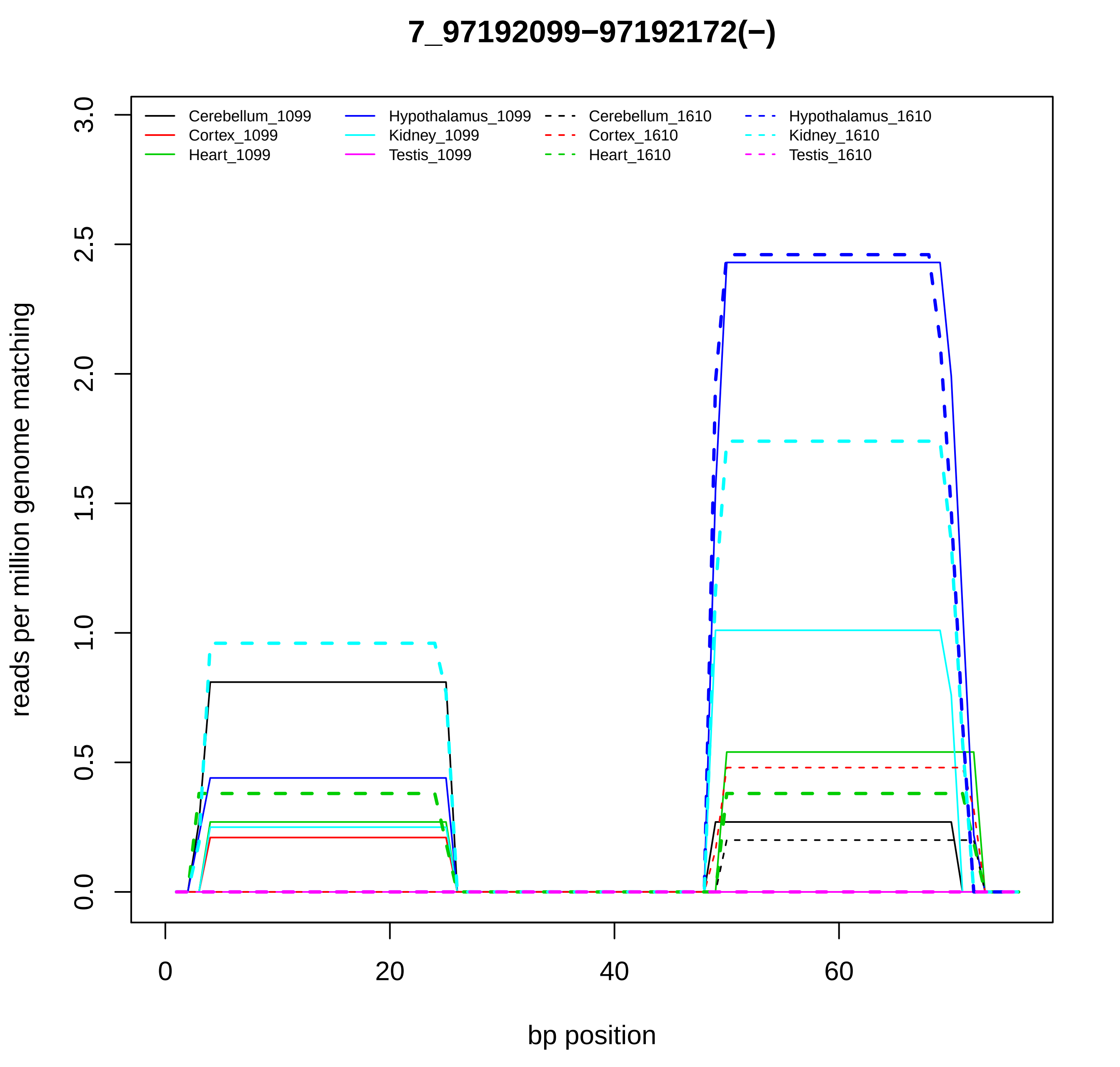

7_97192099−97192172(−)
Cerebellum_1099
Cortex_1099
Heart_1099
Hypothalamus_1099
Kidney_1099
Testis_1099
Cerebellum_1610
Cortex_1610
Heart_1610
Hypothalamus_1610
Kidney_1610
Testis_1610
reads per million genome matching
0.0
0.5
1.0
1.5
2.0
2.5
3.0
0
20
40
60
bp position

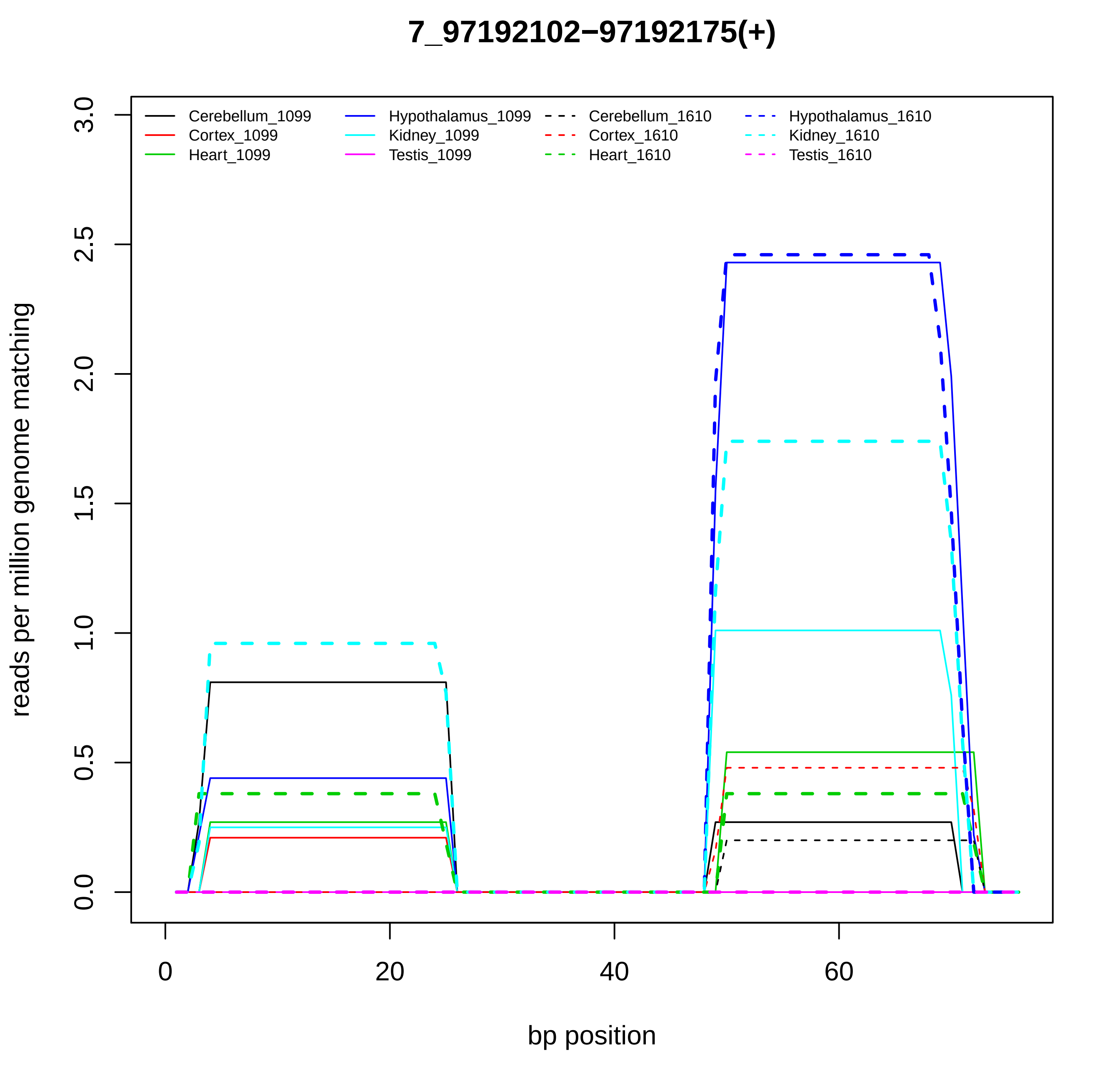

7_97192102−97192175(+)
Cerebellum_1099
Cortex_1099
Heart_1099
Hypothalamus_1099
Kidney_1099
Testis_1099
Cerebellum_1610
Cortex_1610
Heart_1610
Hypothalamus_1610
Kidney_1610
Testis_1610
reads per million genome matching
0.0
0.5
1.0
1.5
2.0
2.5
3.0
0
20
40
60
bp position

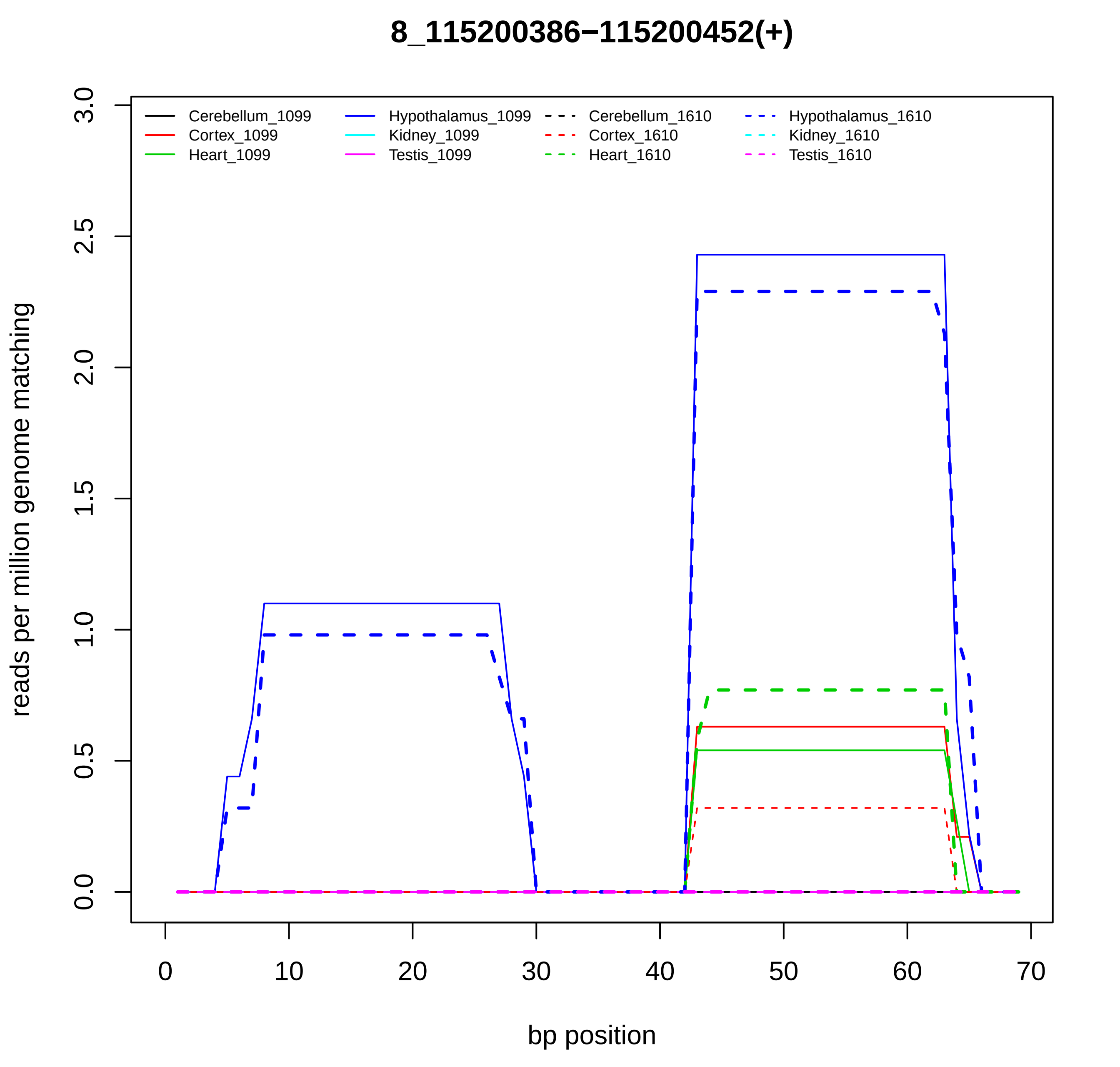

8_115200386−115200452(+)
Cerebellum_1099
Cortex_1099
Heart_1099
Hypothalamus_1099
Kidney_1099
Testis_1099
Cerebellum_1610
Cortex_1610
Heart_1610
Hypothalamus_1610
Kidney_1610
Testis_1610
reads per million genome matching
bp position
0.0
0.5
1.0
1.5
2.0
2.5
3.0
0
10
20
30
40
50
60
70

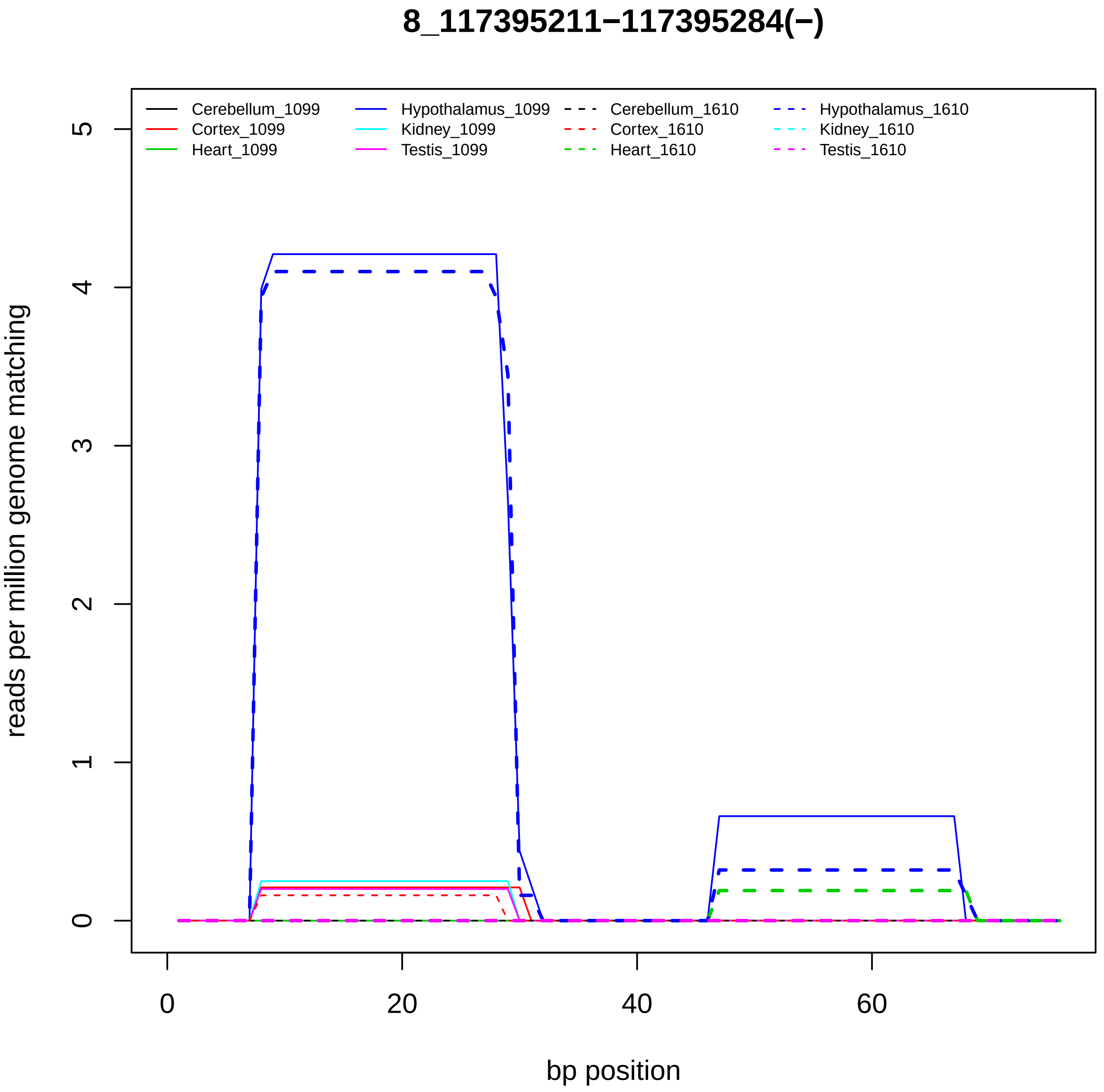

8_117395211−117395284(−)
Cerebellum_1099
Cortex_1099
Heart_1099
Hypothalamus_1099
Kidney_1099
Testis_1099
Cerebellum_1610
Cortex_1610
Heart_1610
Hypothalamus_1610
Kidney_1610
Testis_1610
reads per million genome matching
bp position
0
1
2
3
4
5
0
20
40
60

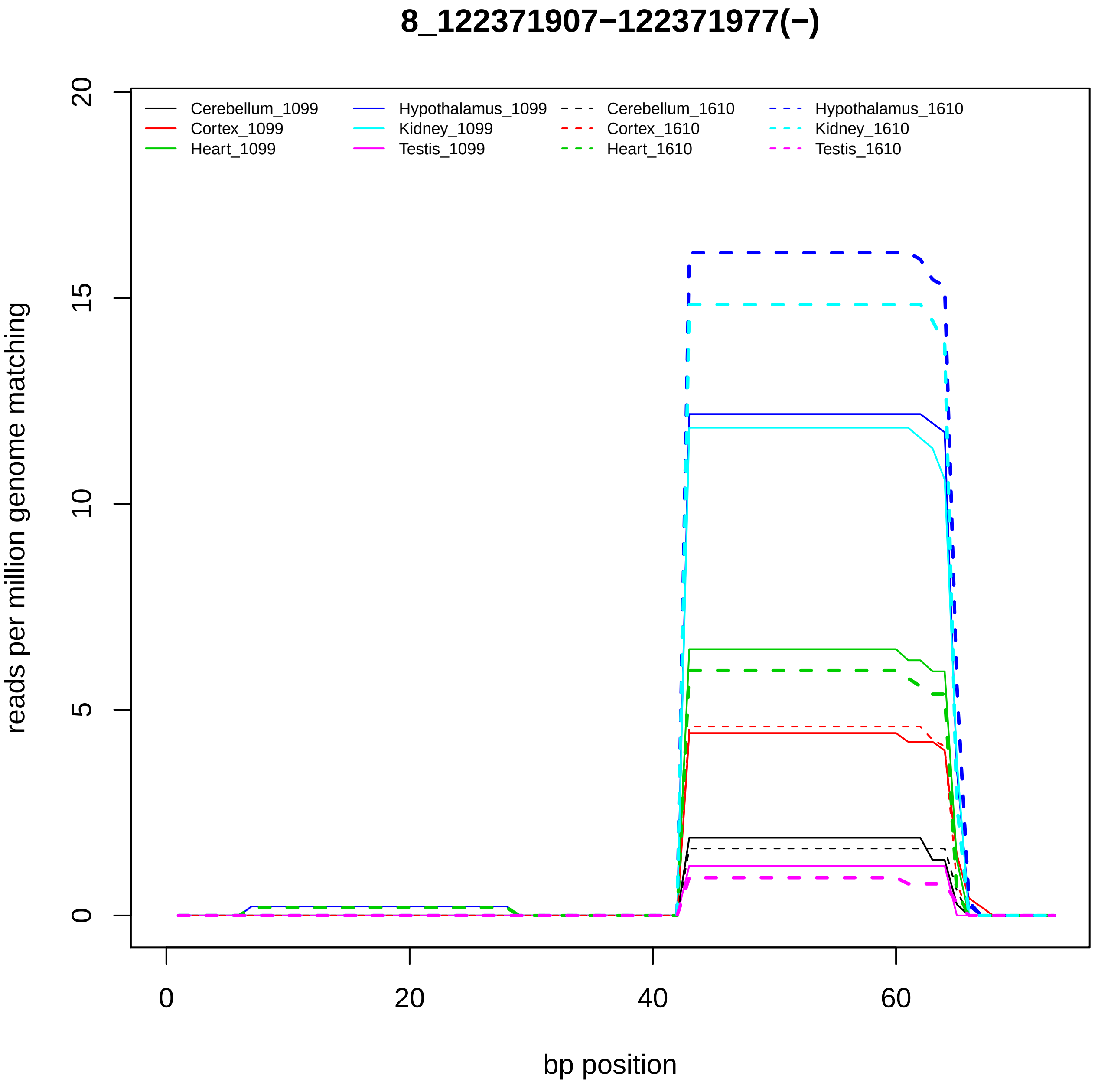

8_122371907−122371977(−)
Cerebellum_1099
Cortex_1099
Heart_1099
Hypothalamus_1099
Kidney_1099
Testis_1099
Cerebellum_1610
Cortex_1610
Heart_1610
Hypothalamus_1610
Kidney_1610
Testis_1610
reads per million genome matching
bp position
0
5
10
15
20
0
20
40
60

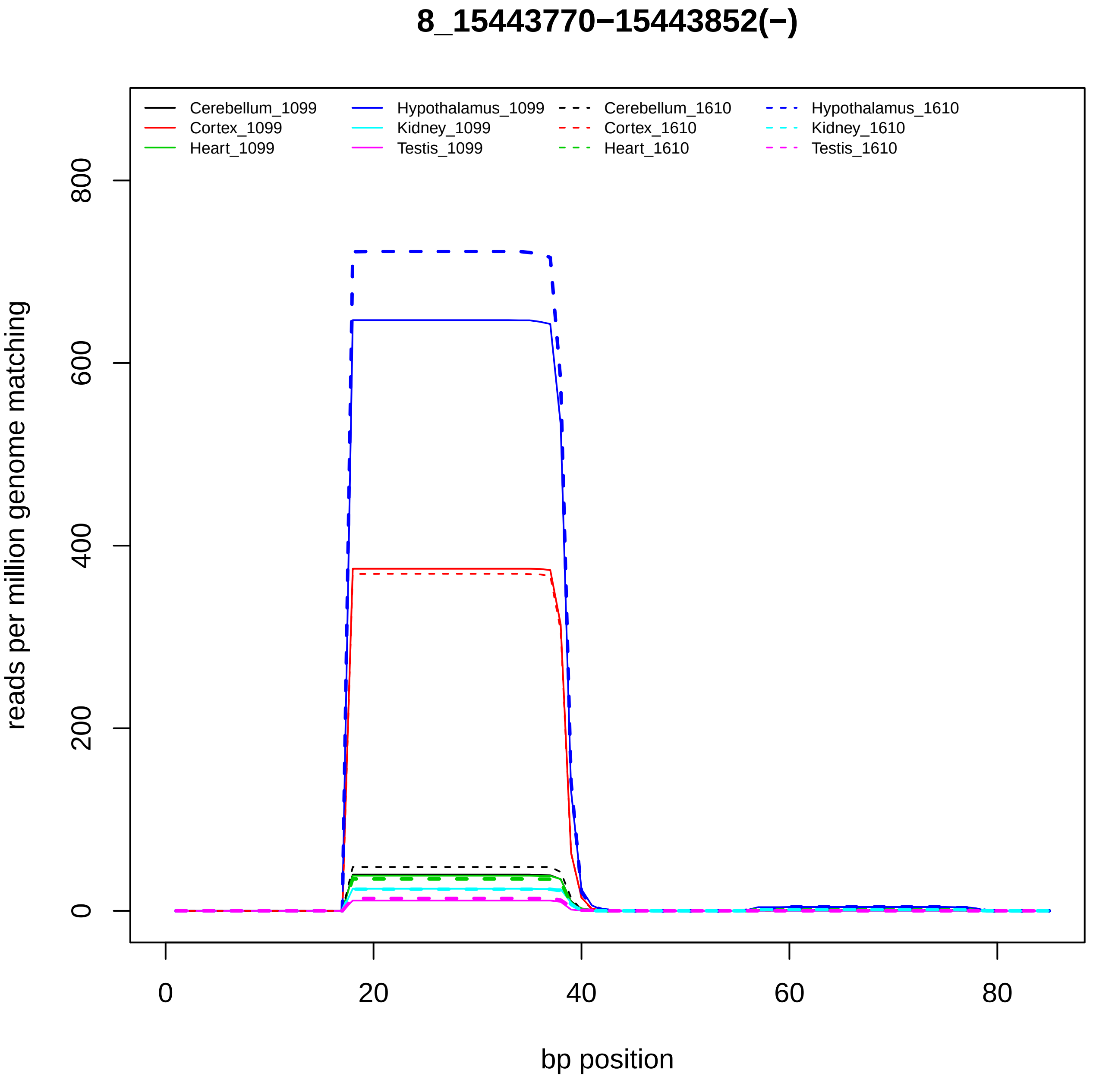

8_15443770−15443852(−)
Cerebellum_1099
Cortex_1099
Heart_1099
Hypothalamus_1099
Kidney_1099
Testis_1099
Cerebellum_1610
Cortex_1610
Heart_1610
Hypothalamus_1610
Kidney_1610
Testis_1610
reads per million genome matching
0
200
400
600
800
bp position
0
20
40
60
80

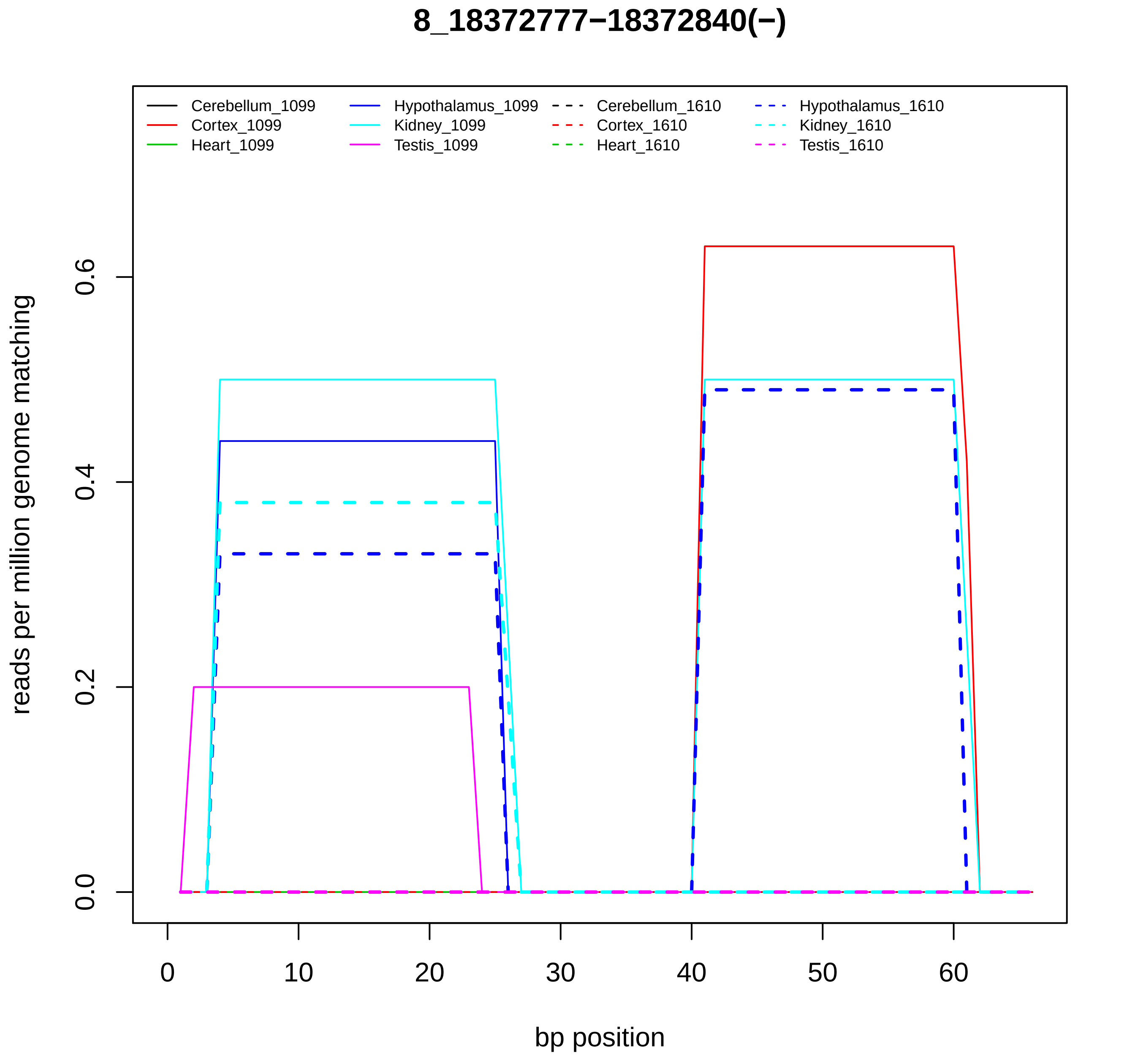
8_18372777−18372840(−)
Cerebellum_1099
Cortex_1099
Heart_1099
Hypothalamus_1099
Kidney_1099
Testis_1099
Cerebellum_1610
Cortex_1610
Heart_1610
Hypothalamus_1610
Kidney_1610
Testis_1610
reads per million genome matching
0.0
0.2
0.4
0.6
0
10
20
30
40
50
60
bp position

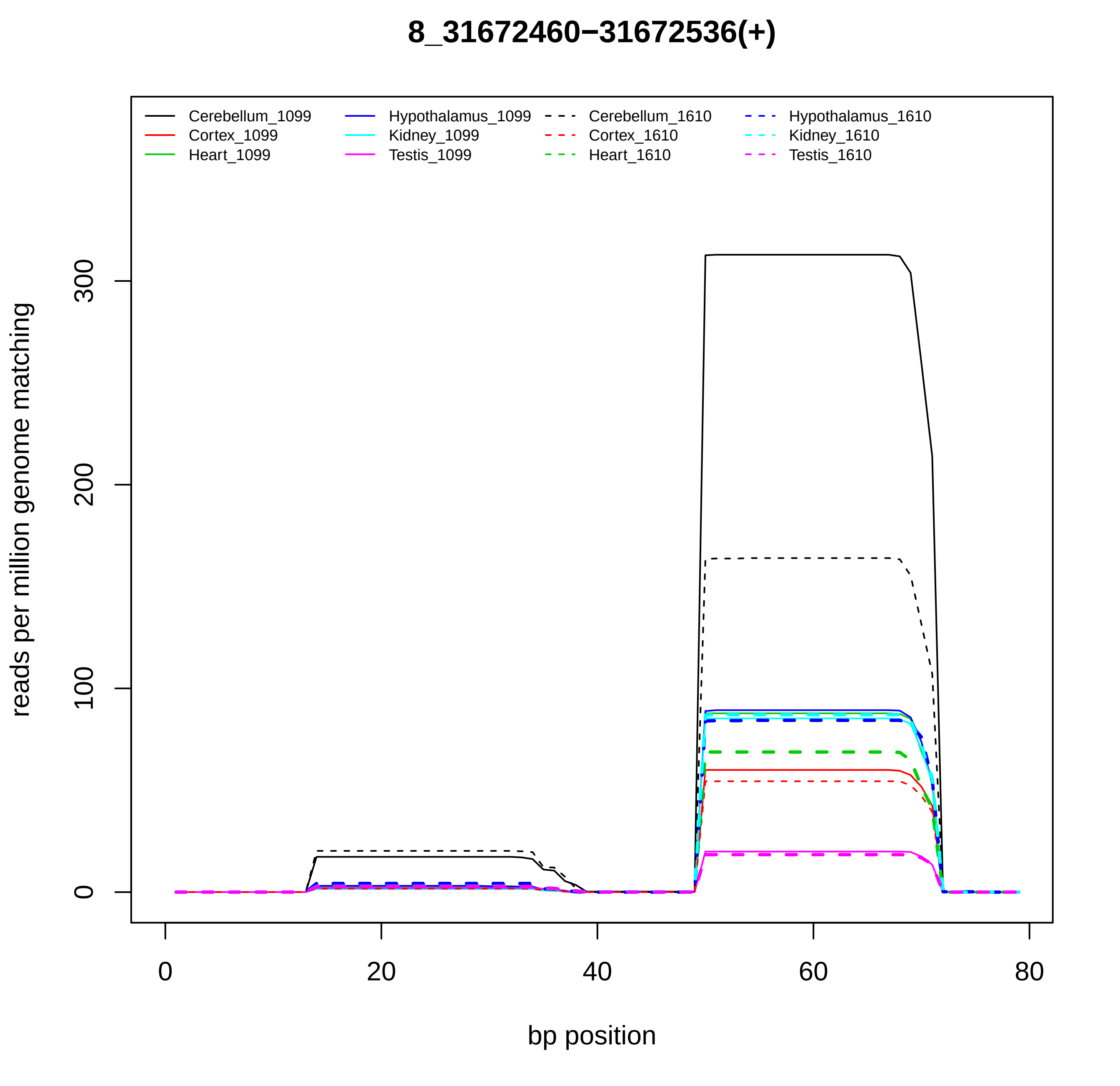
8_31672460−31672536(+)
Cerebellum_1099
Cortex_1099
Heart_1099
Hypothalamus_1099
Kidney_1099
Testis_1099
Cerebellum_1610
Cortex_1610
Heart_1610
Hypothalamus_1610
Kidney_1610
Testis_1610
reads per million genome matching
0
100
200
300
bp position
0
20
40
60
80

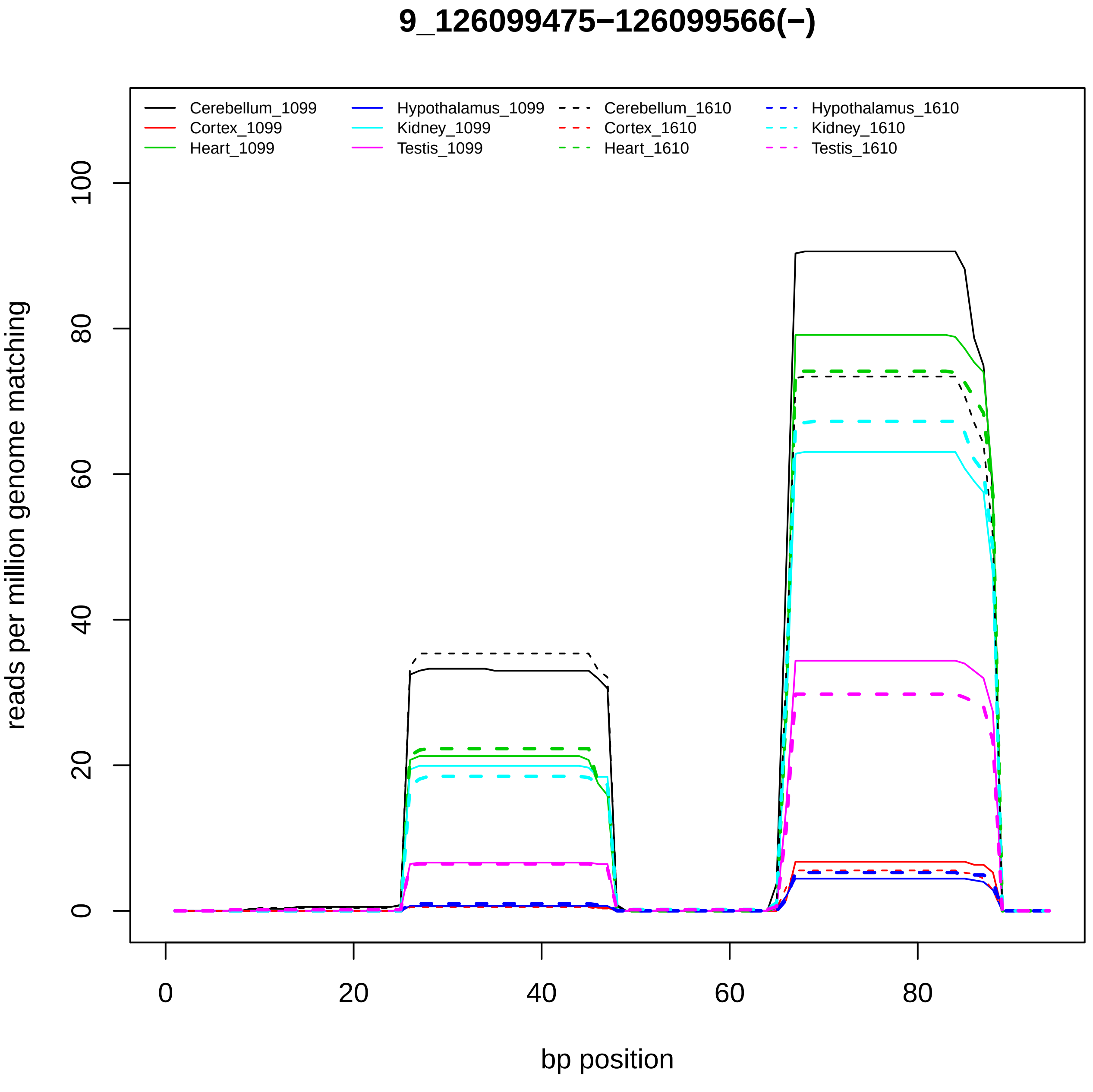
9_126099475−126099566(−)
Cerebellum_1099
Cortex_1099
Heart_1099
Hypothalamus_1099
Kidney_1099
Testis_1099
Cerebellum_1610
Cortex_1610
Heart_1610
Hypothalamus_1610
Kidney_1610
Testis_1610
reads per million genome matching
0
20
40
60
80
100
bp position

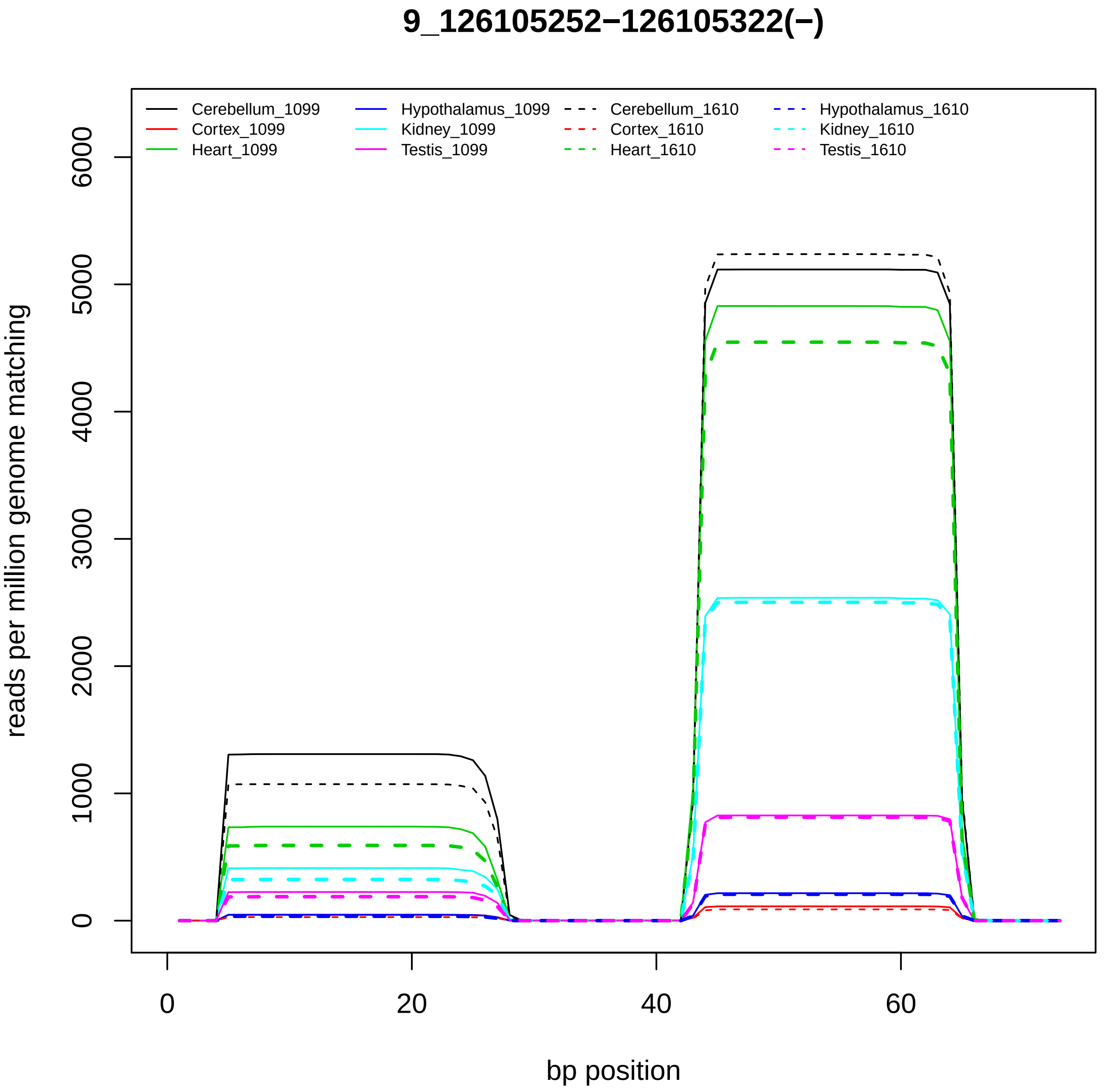
9_126105252−126105322(−)
Cerebellum_1099
Cortex_1099
Heart_1099
Hypothalamus_1099
Kidney_1099
Testis_1099
Cerebellum_1610
Cortex_1610
Heart_1610
Hypothalamus_1610
Kidney_1610
Testis_1610
reads per million genome matching
0
1000
2000
3000
4000
5000
6000
0
20
40
60
bp position

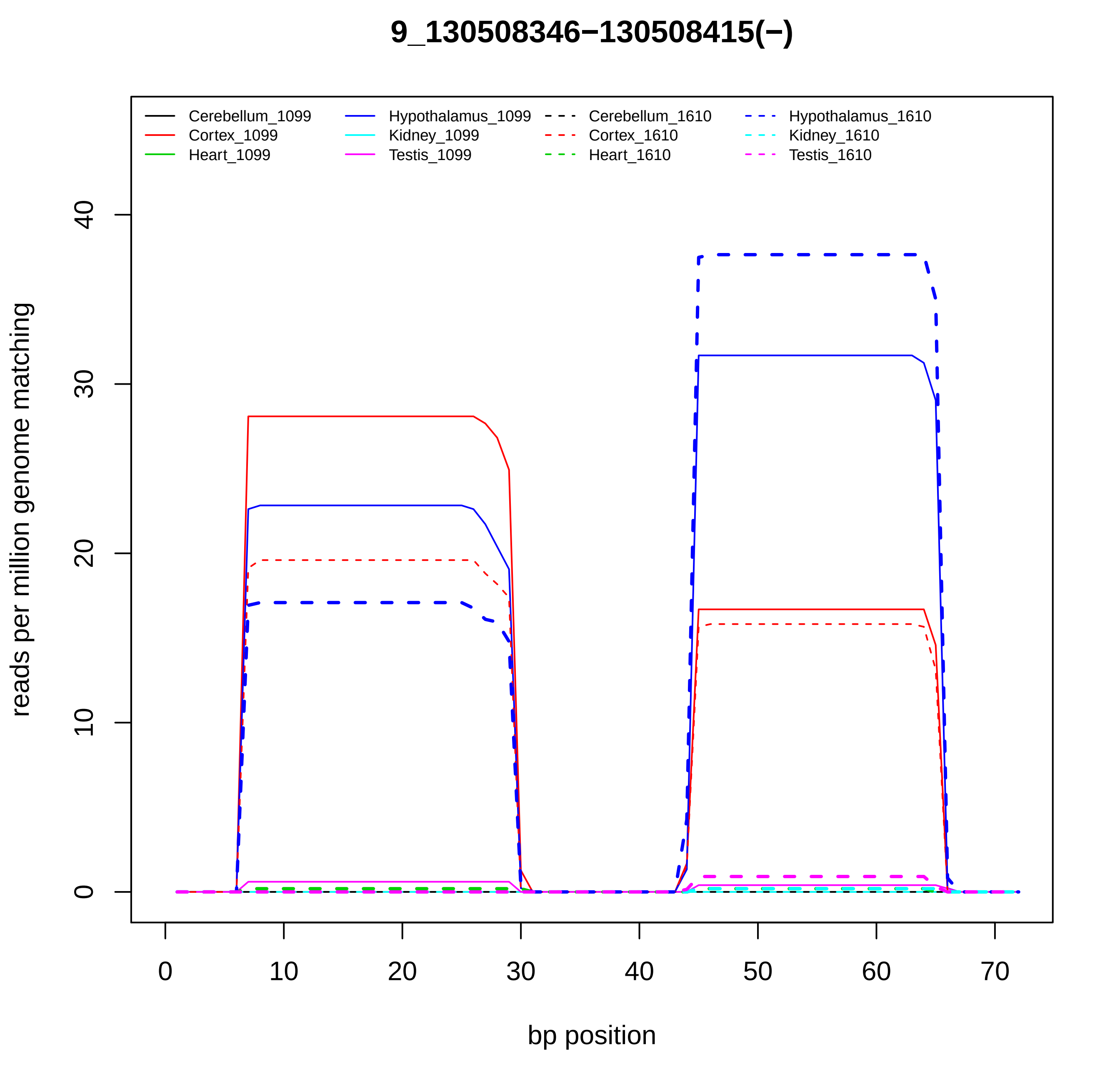

9_130508346−130508415(−)
Cerebellum_1099
Cortex_1099
Heart_1099
Hypothalamus_1099
Kidney_1099
Testis_1099
Cerebellum_1610
Cortex_1610
Heart_1610
Hypothalamus_1610
Kidney_1610
Testis_1610
reads per million genome matching
bp position
0
10
20
30
40
50
60
70

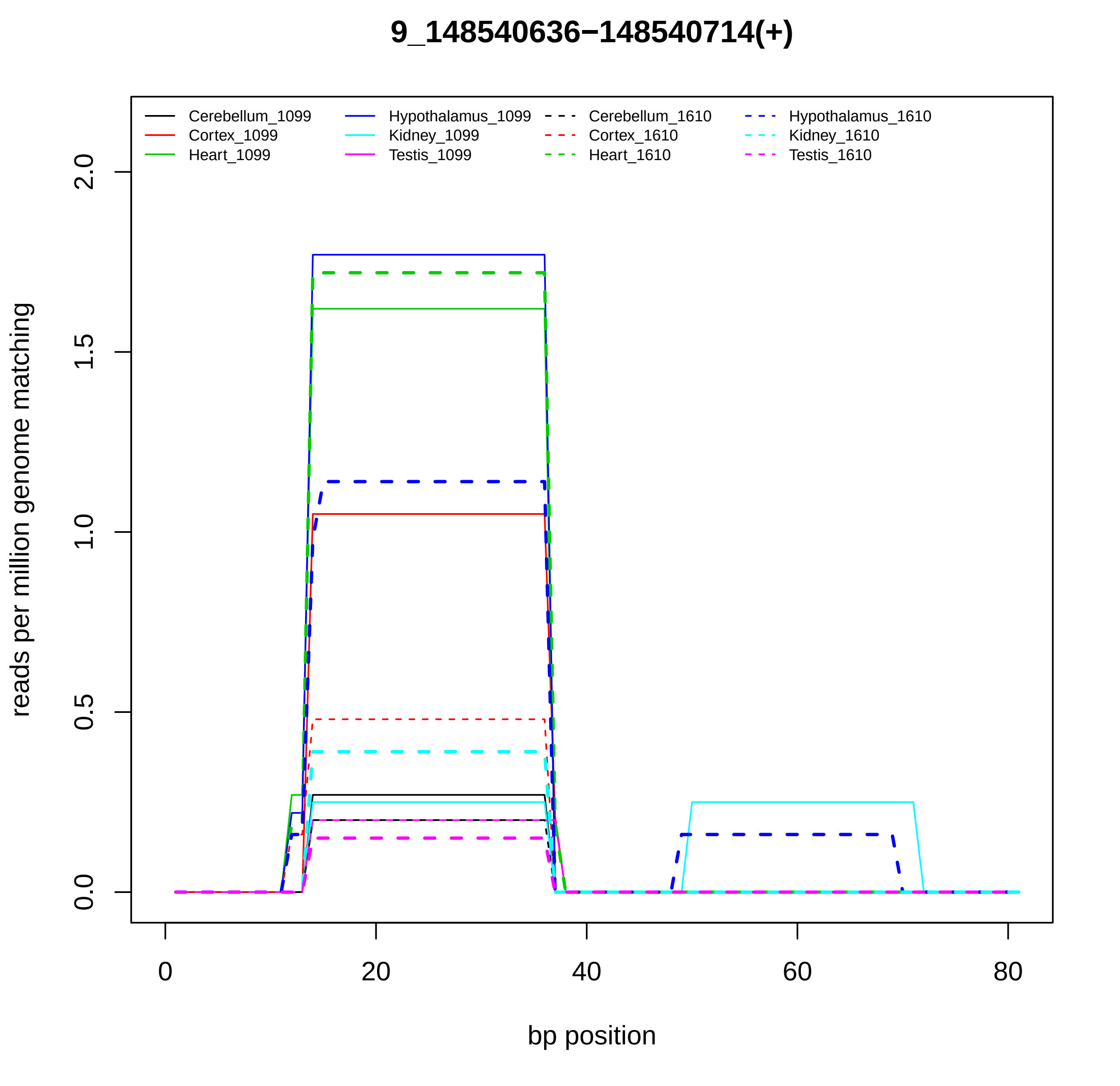
9_148540636−148540714(+)
Cerebellum_1099
Cortex_1099
Heart_1099
Hypothalamus_1099
Kidney_1099
Testis_1099
Cerebellum_1610
Cortex_1610
Heart_1610
Hypothalamus_1610
Kidney_1610
Testis_1610
reads per million genome matching
0.0
0.5
1.0
1.5
2.0
0
20
40
60
80
bp position

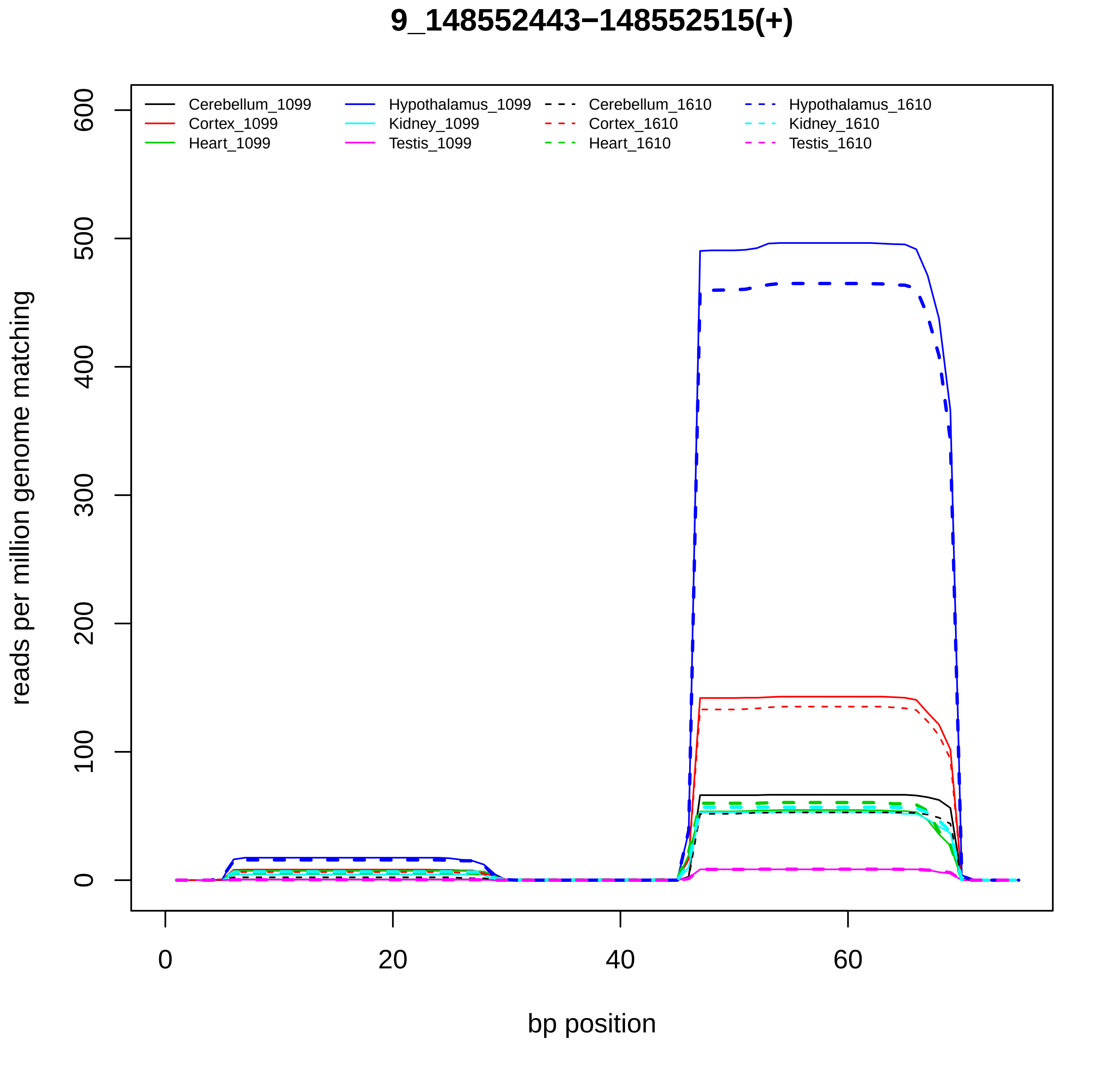

9_148552443−148552515(+)
Cerebellum_1099
Cortex_1099
Heart_1099
Hypothalamus_1099
Kidney_1099
Testis_1099
Cerebellum_1610
Cortex_1610
Heart_1610
Hypothalamus_1610
Kidney_1610
Testis_1610
reads per million genome matching
0
100
200
300
400
500
600
bp position
0
20
40
60

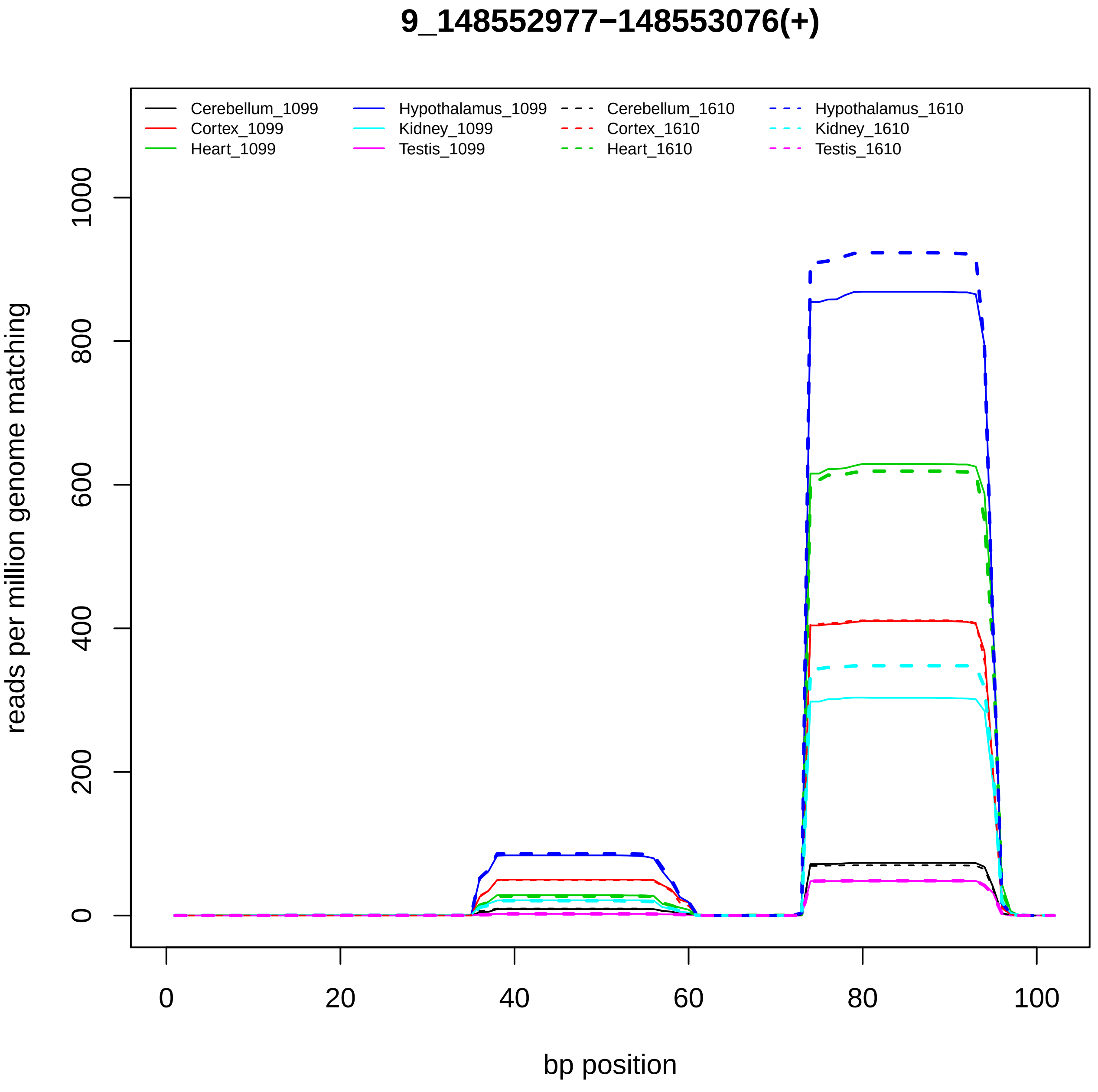
9_148552977−148553076(+)
Cerebellum_1099
Cortex_1099
Heart_1099
Hypothalamus_1099
Kidney_1099
Testis_1099
Cerebellum_1610
Cortex_1610
Heart_1610
Hypothalamus_1610
Kidney_1610
Testis_1610
reads per million genome matching
0
200
400
600
800
1000
0
20
40
60
80
100
bp position

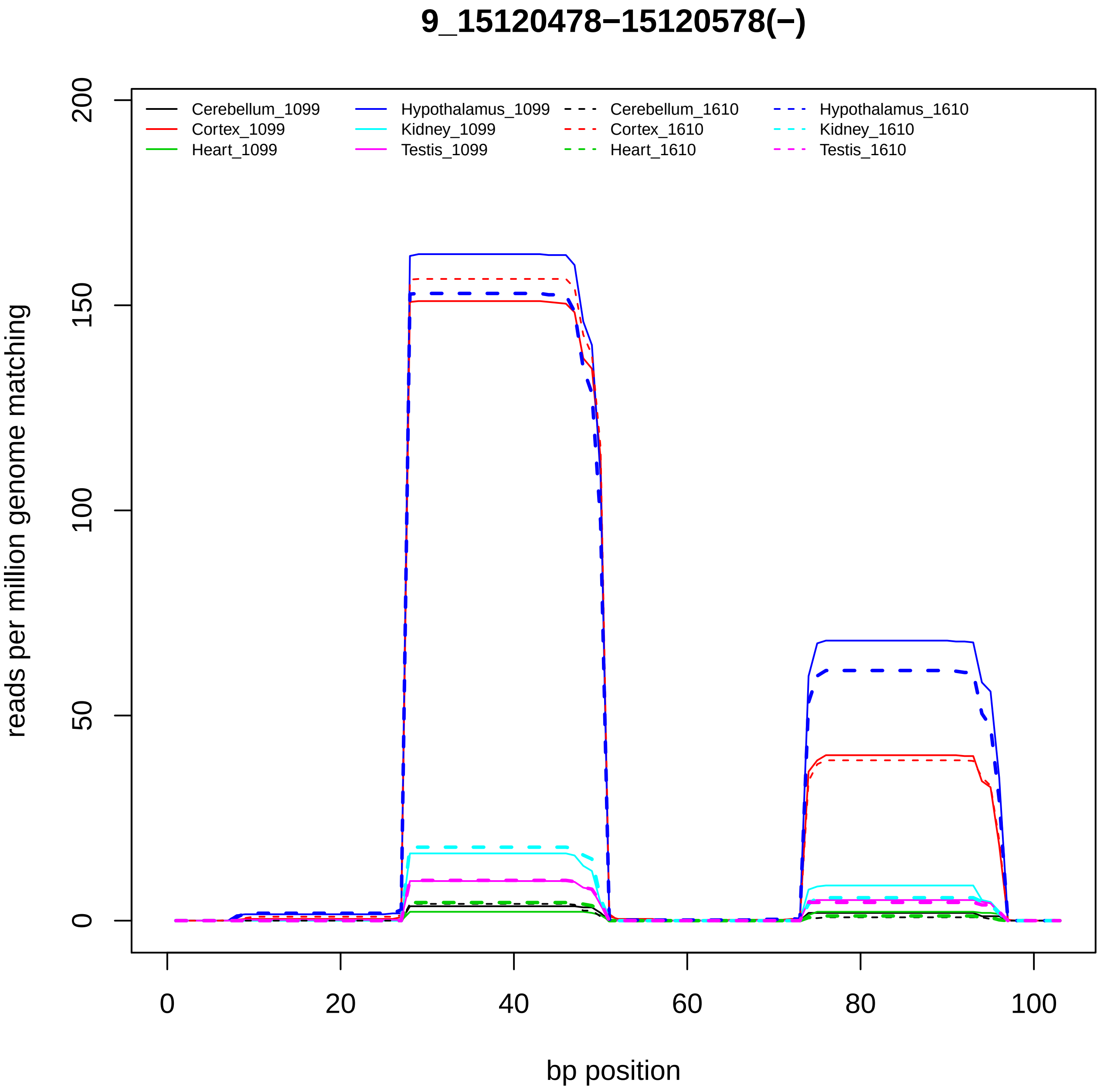

9_15120478−15120578(−)
Cerebellum_1099
Cortex_1099
Heart_1099
Hypothalamus_1099
Kidney_1099
Testis_1099
Cerebellum_1610
Cortex_1610
Heart_1610
Hypothalamus_1610
Kidney_1610
Testis_1610
reads per million genome matching
bp position
0
50
100
150
200
0
20
40
60
80
100

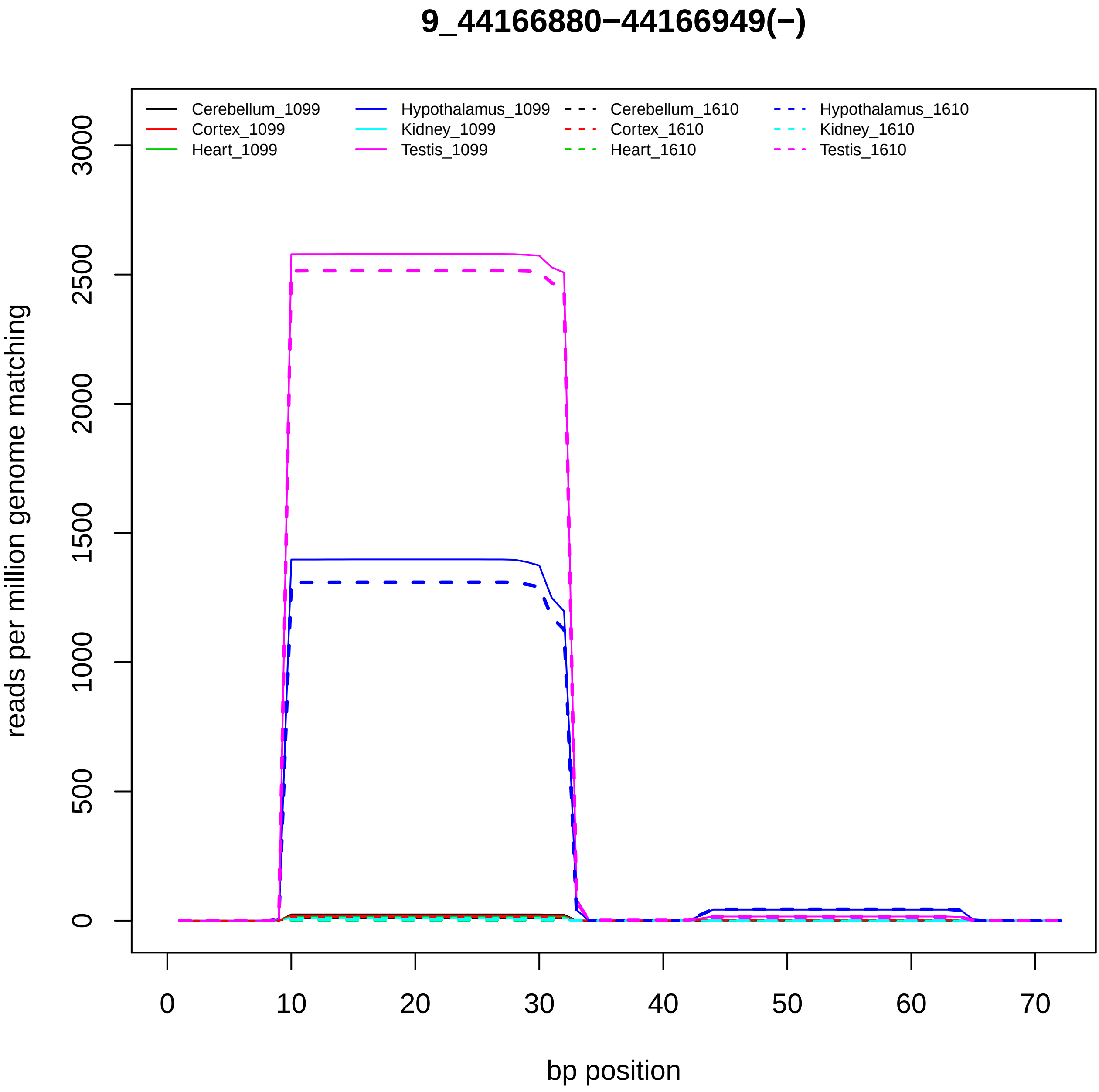

9_44166880−44166949(−)
Cerebellum_1099
Cortex_1099
Heart_1099
Hypothalamus_1099
Kidney_1099
Testis_1099
Cerebellum_1610
Cortex_1610
Heart_1610
Hypothalamus_1610
Kidney_1610
Testis_1610
reads per million genome matching
0
500
1000
1500
2000
2500
3000
0
10
20
30
40
50
60
70
bp position

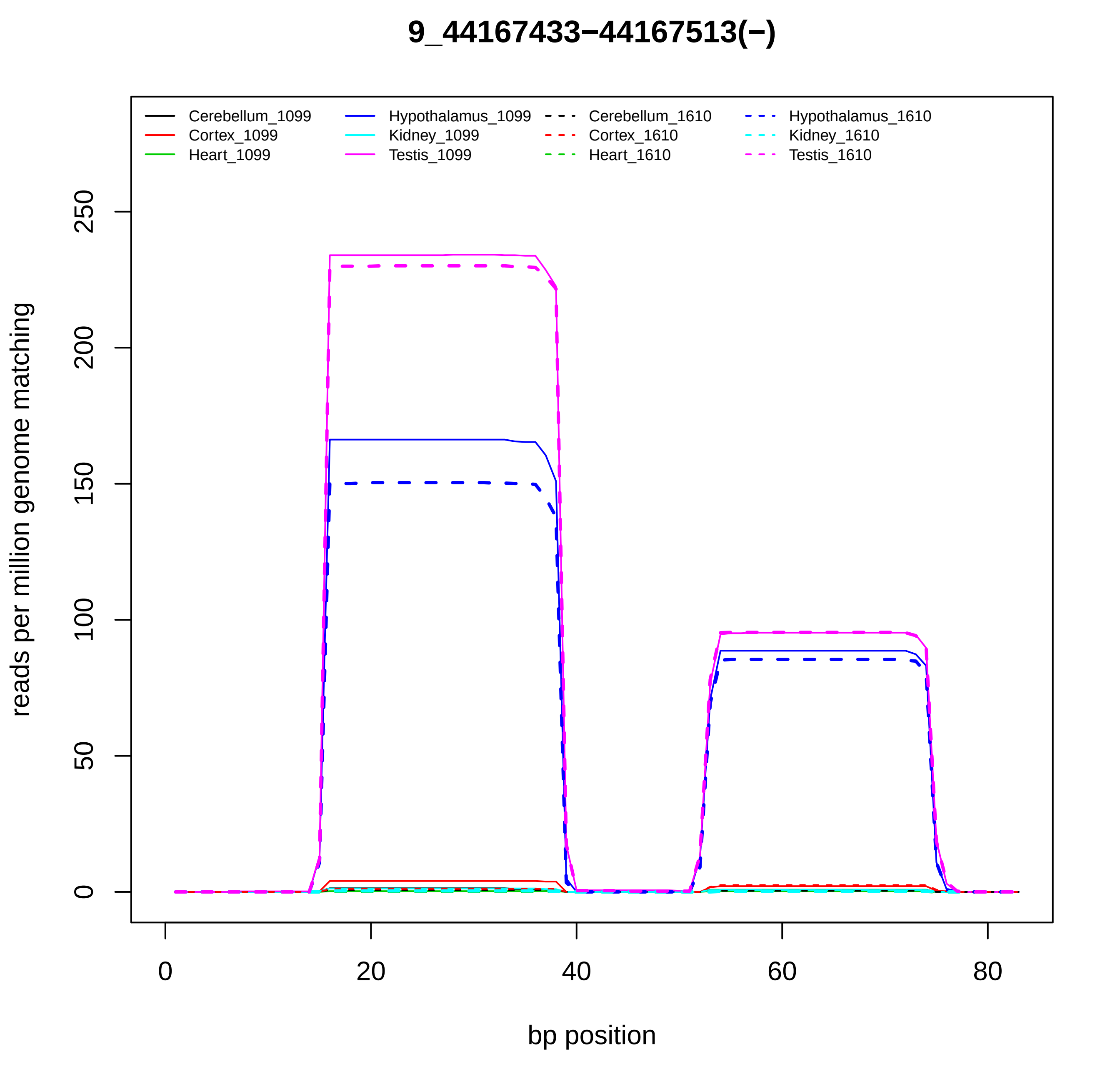
9_44167433−44167513(−)
Cerebellum_1099
Cortex_1099
Heart_1099
Hypothalamus_1099
Kidney_1099
Testis_1099
Cerebellum_1610
Cortex_1610
Heart_1610
Hypothalamus_1610
Kidney_1610
Testis_1610
reads per million genome matching
0
50
100
150
200
250
0
20
40
60
80
bp position

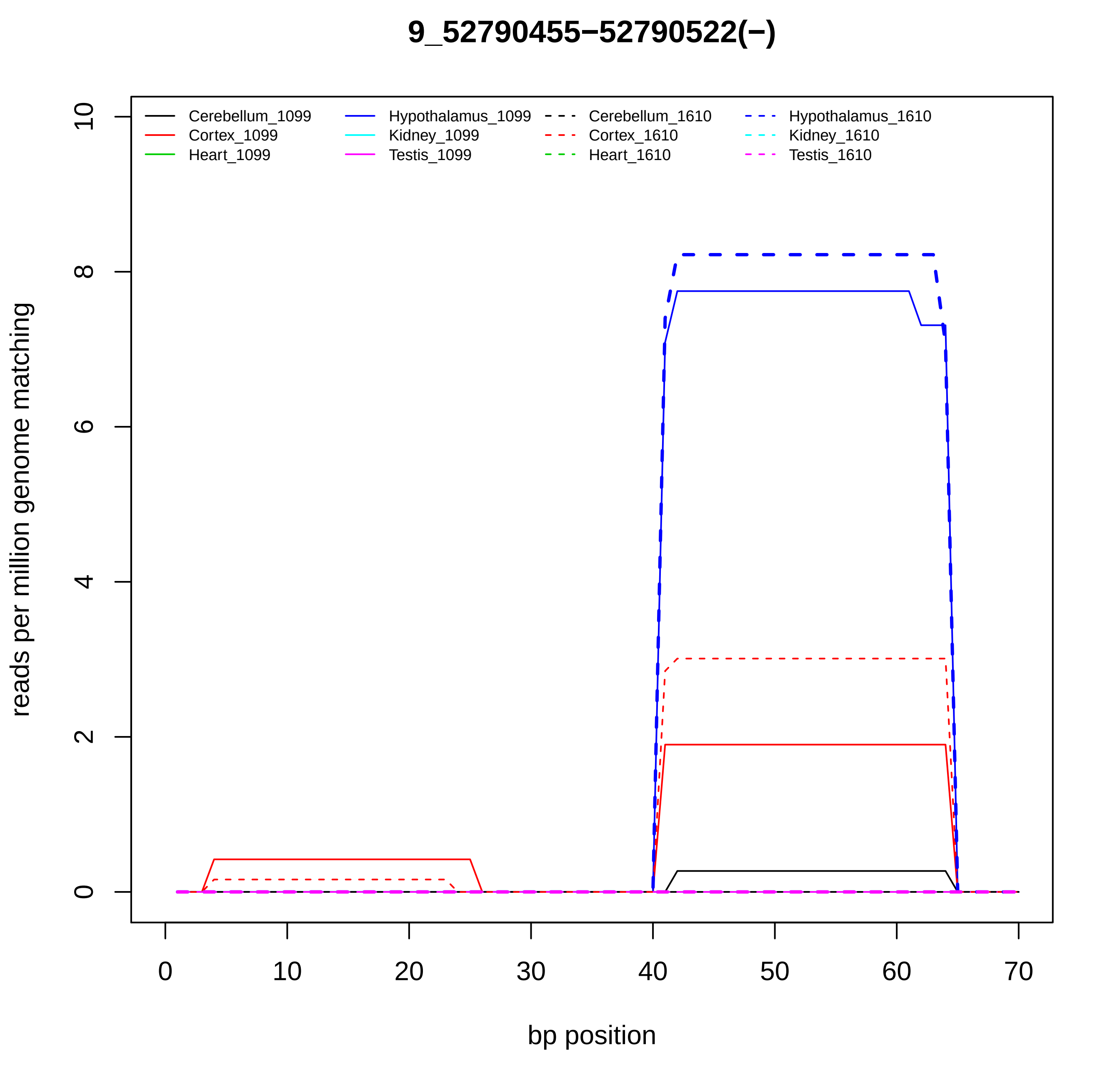
9_52790455−52790522(−)
Cerebellum_1099
Cortex_1099
Heart_1099
Hypothalamus_1099
Kidney_1099
Testis_1099
Cerebellum_1610
Cortex_1610
Heart_1610
Hypothalamus_1610
Kidney_1610
Testis_1610
reads per million genome matching
bp position
0
2
4
6
8
10
0
10
20
30
40
50
60
70

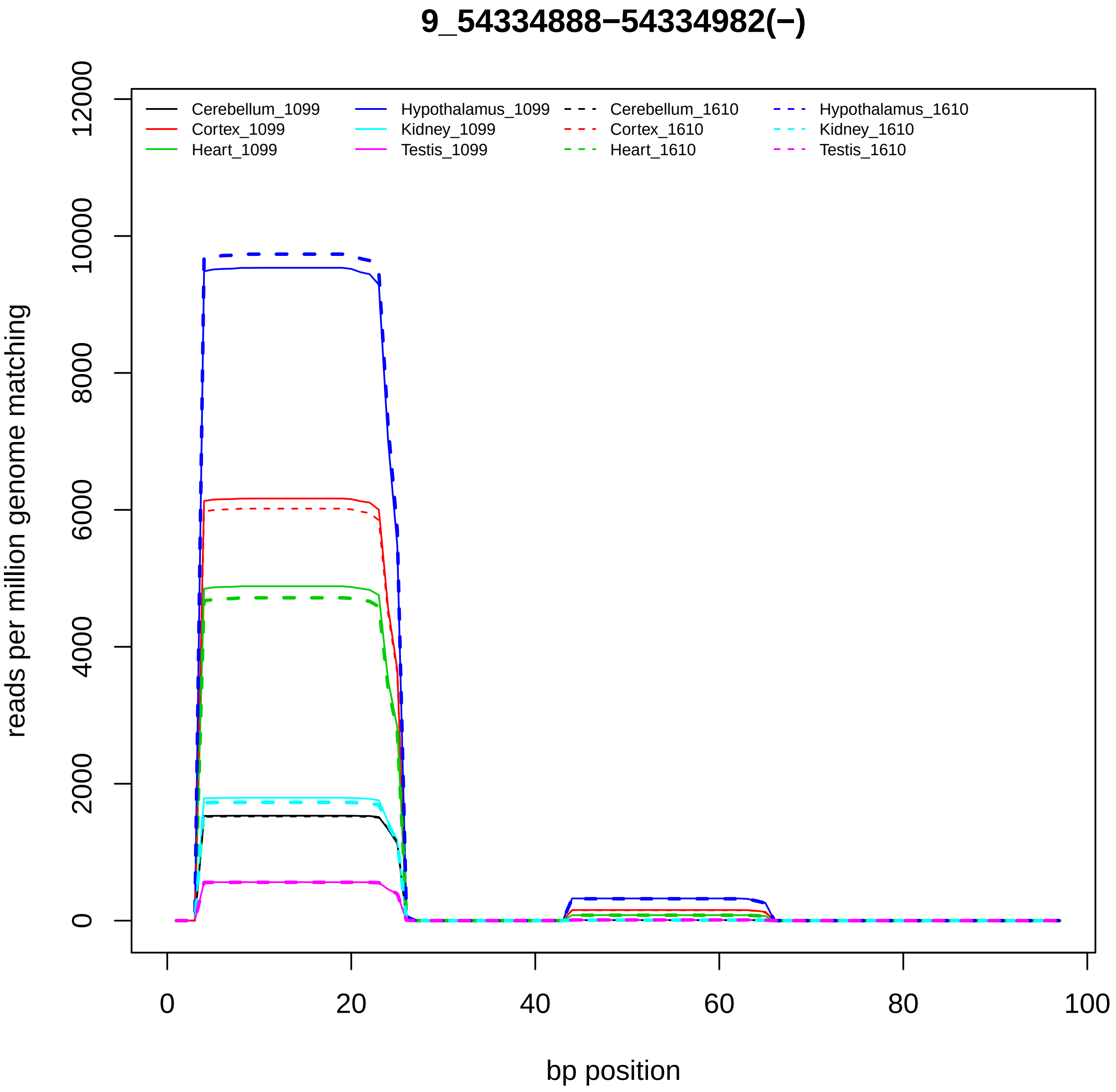
9_54334888−54334982(−)
reads per million genome matching
bp position
0
2000
4000
6000
8000
10000
12000
0
20
40
60
80
100
Cerebellum_1099
Cortex_1099
Heart_1099
Hypothalamus_1099
Kidney_1099
Testis_1099
Cerebellum_1610
Cortex_1610
Heart_1610
Hypothalamus_1610
Kidney_1610
Testis_1610

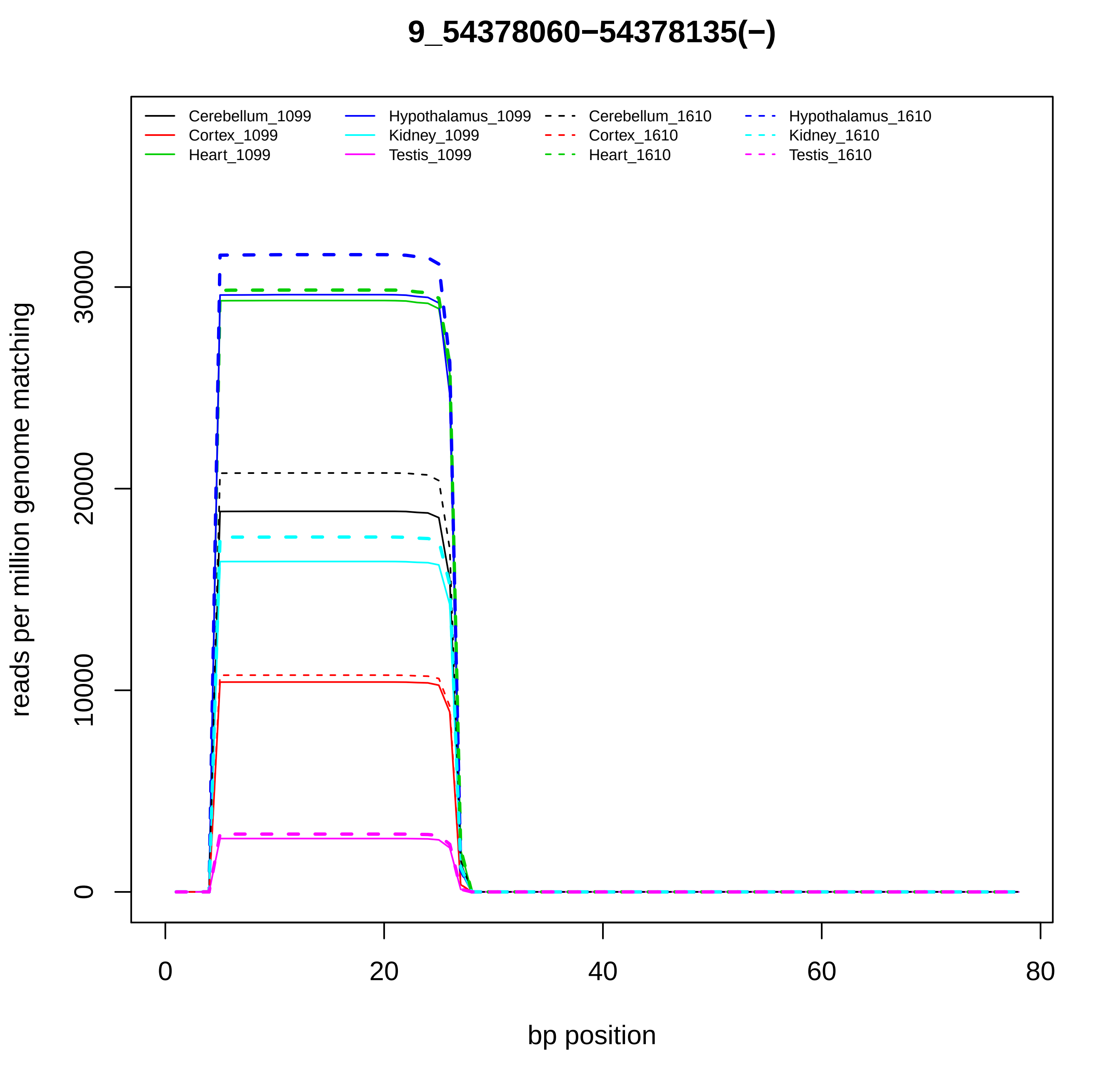
9_54378060−54378135(−)
Cerebellum_1099
Cortex_1099
Heart_1099
Hypothalamus_1099
Kidney_1099
Testis_1099
Cerebellum_1610
Cortex_1610
Heart_1610
Hypothalamus_1610
Kidney_1610
Testis_1610
reads per million genome matching
0
10000
20000
30000
bp position
0
20
40
60
80

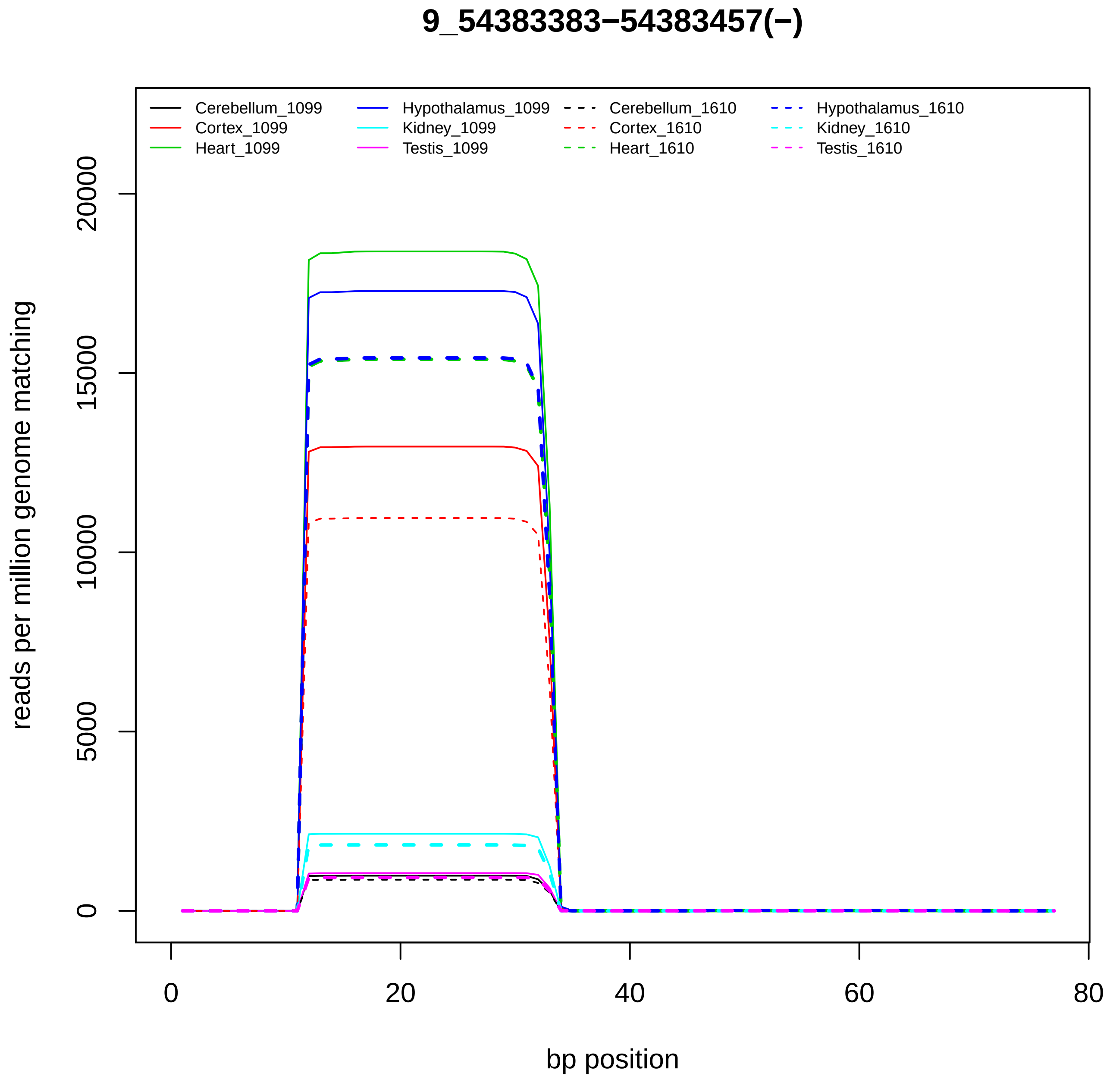
9_54383383−54383457(−)
Cerebellum_1099
Cortex_1099
Heart_1099
Hypothalamus_1099
Kidney_1099
Testis_1099
Cerebellum_1610
Cortex_1610
Heart_1610
Hypothalamus_1610
Kidney_1610
Testis_1610
reads per million genome matching
0
5000
10000
15000
20000
bp position
0
20
40
60
80

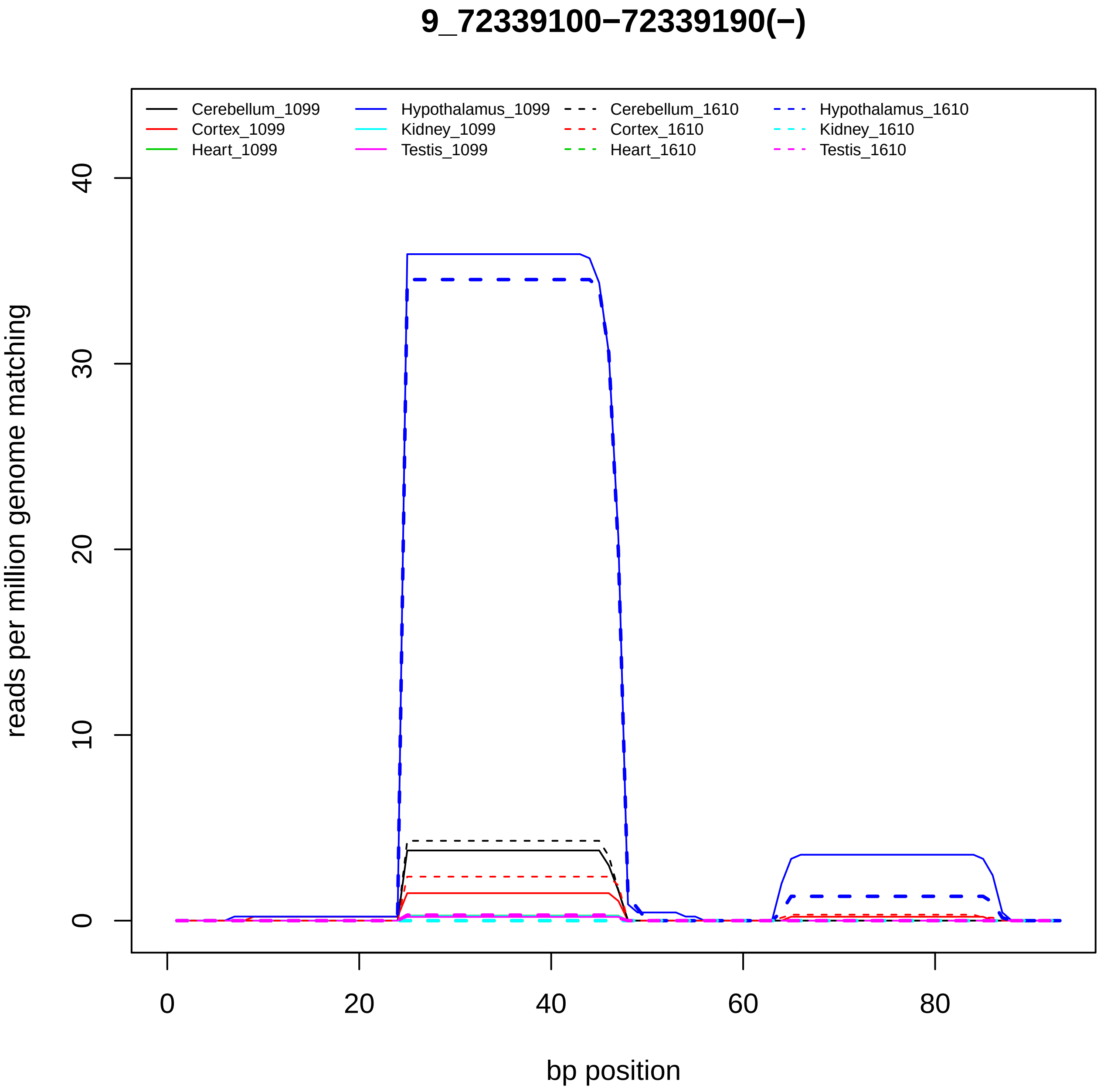

9_72339100−72339190(−)
Cerebellum_1099
Cortex_1099
Heart_1099
Hypothalamus_1099
Kidney_1099
Testis_1099
Cerebellum_1610
Cortex_1610
Heart_1610
Hypothalamus_1610
Kidney_1610
Testis_1610
reads per million genome matching
0
10
20
30
40
0
20
40
60
80
bp position

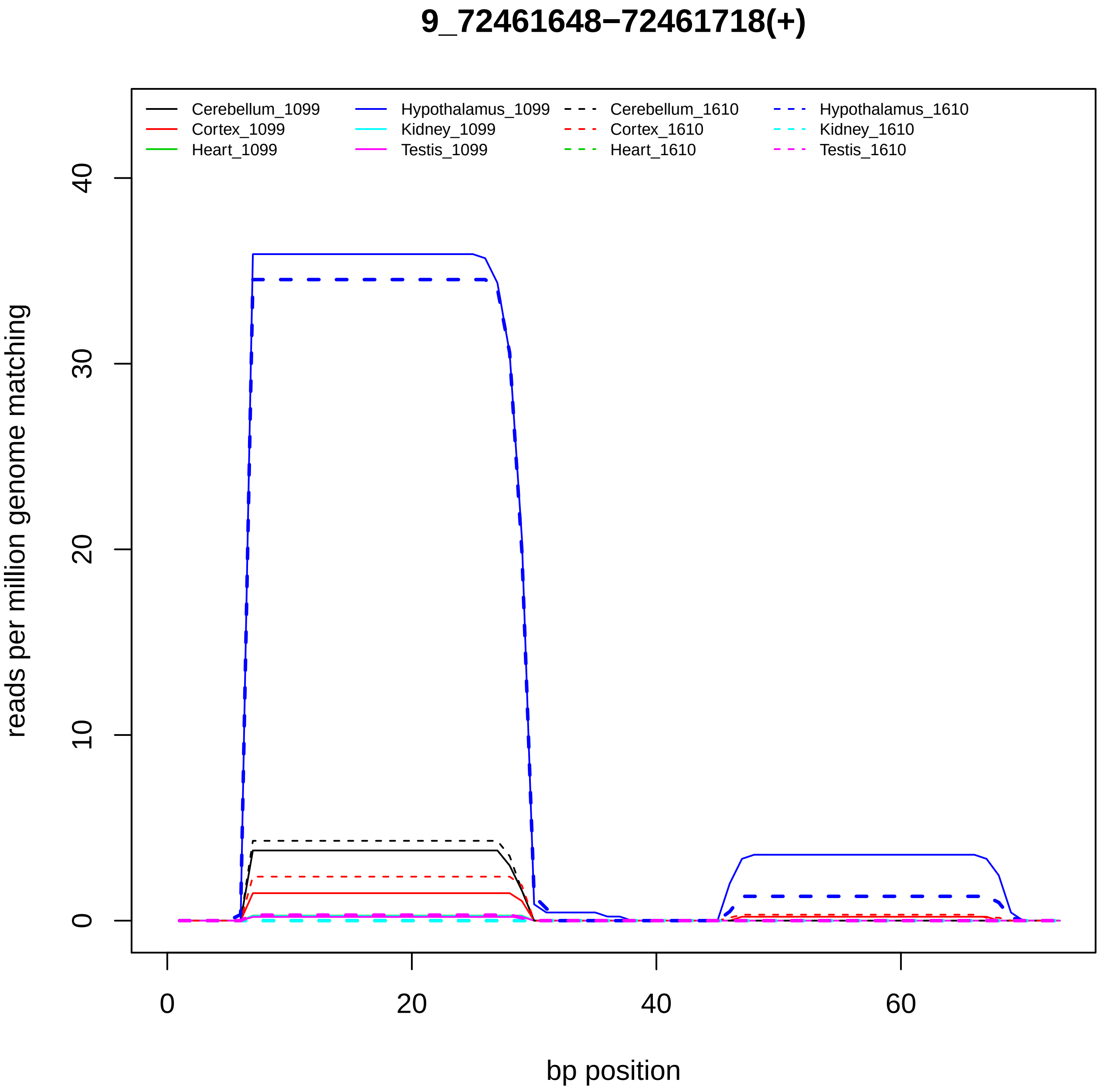
9_72461648−72461718(+)
Cerebellum_1099
Cortex_1099
Heart_1099
Hypothalamus_1099
Kidney_1099
Testis_1099
Cerebellum_1610
Cortex_1610
Heart_1610
Hypothalamus_1610
Kidney_1610
Testis_1610
reads per million genome matching
0
10
20
30
40
0
20
40
60
bp position

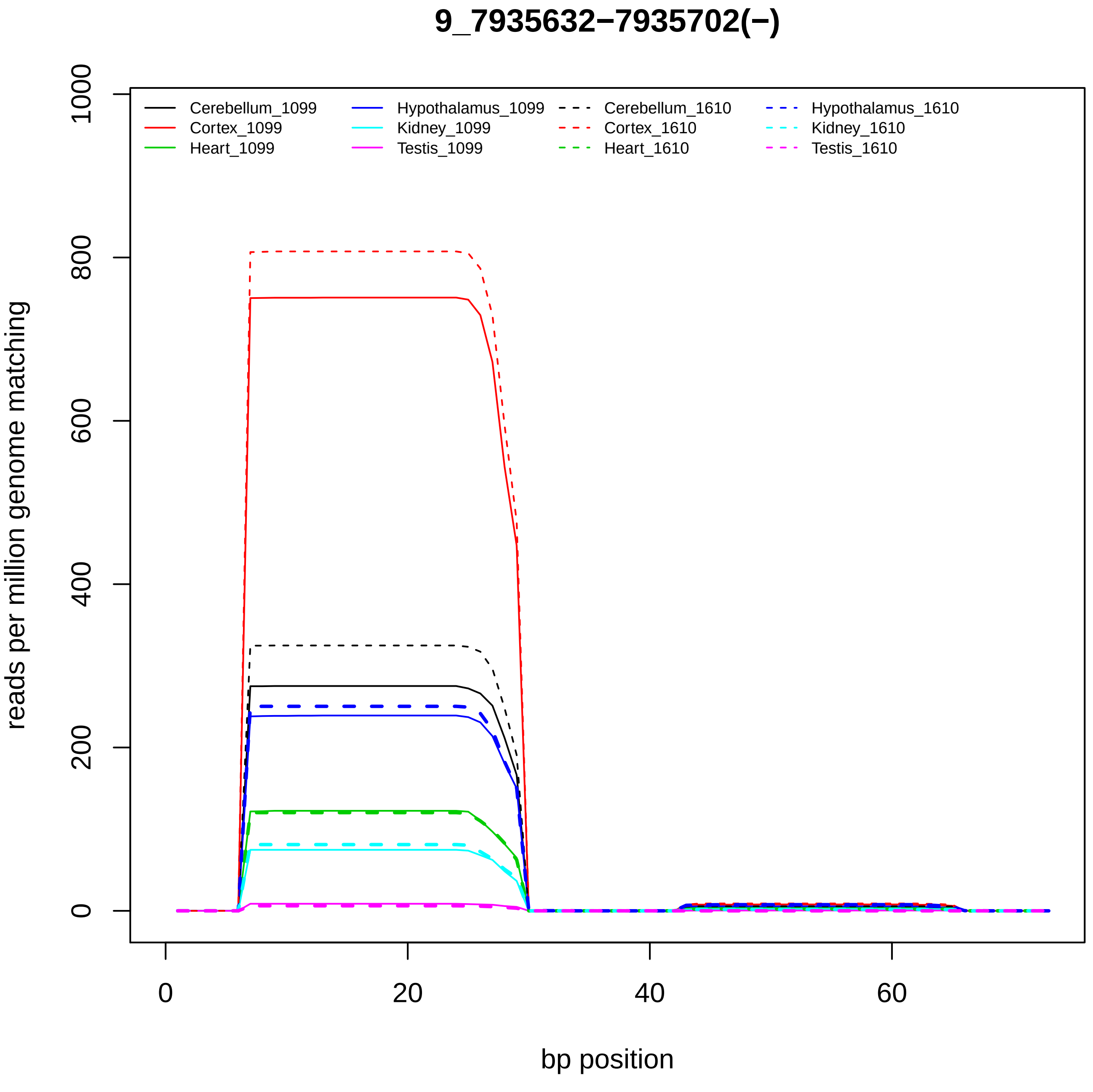

9_7935632−7935702(−)
Cerebellum_1099
Cortex_1099
Heart_1099
Hypothalamus_1099
Kidney_1099
Testis_1099
Cerebellum_1610
Cortex_1610
Heart_1610
Hypothalamus_1610
Kidney_1610
Testis_1610
reads per million genome matching
bp position
0
200
400
600
800
1000
0
20
40
60

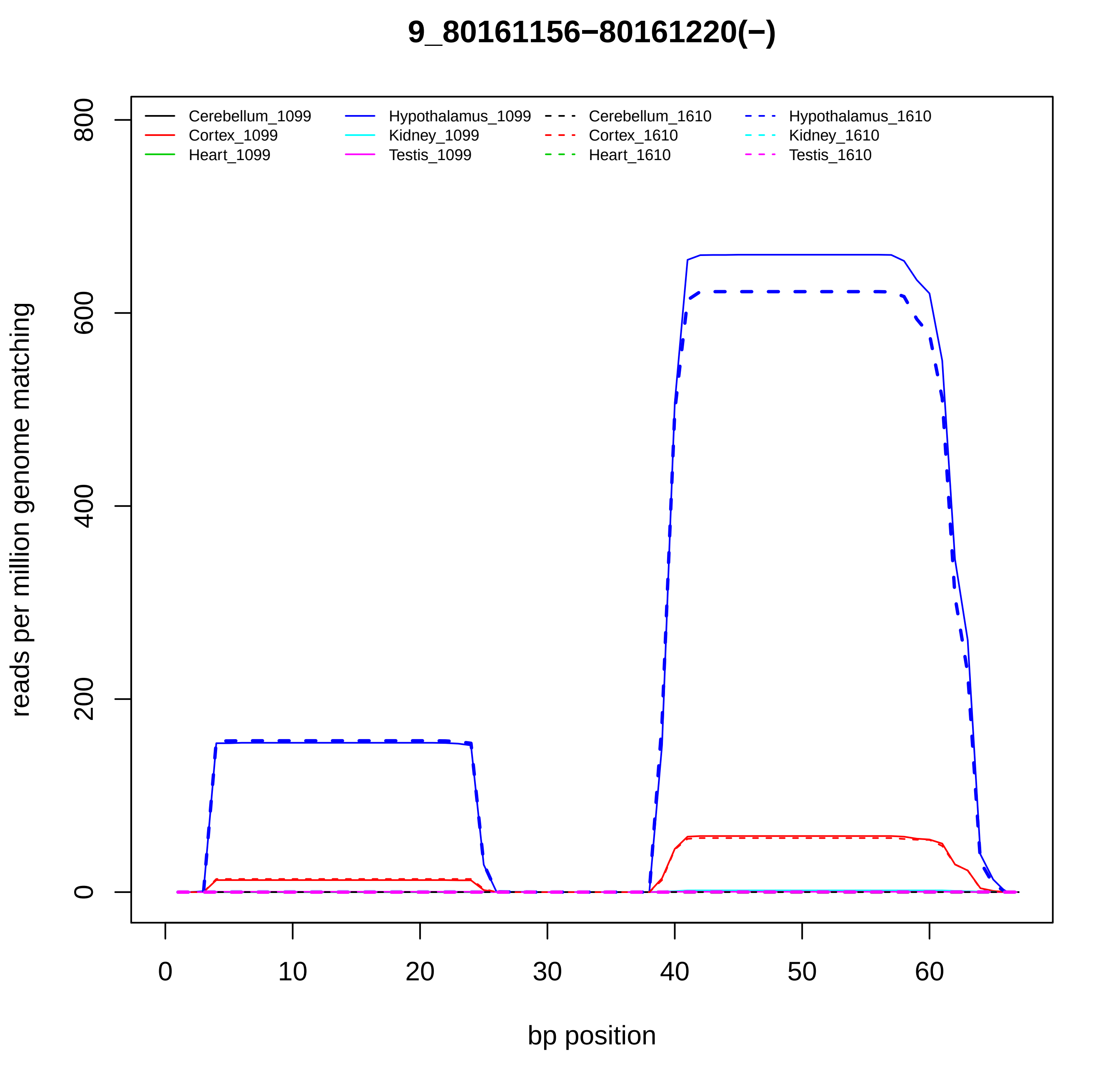

9_80161156−80161220(−)
Cerebellum_1099
Cortex_1099
Heart_1099
Hypothalamus_1099
Kidney_1099
Testis_1099
Cerebellum_1610
Cortex_1610
Heart_1610
Hypothalamus_1610
Kidney_1610
Testis_1610
reads per million genome matching
0
200
400
600
800
bp position
0
10
20
30
40
50
60

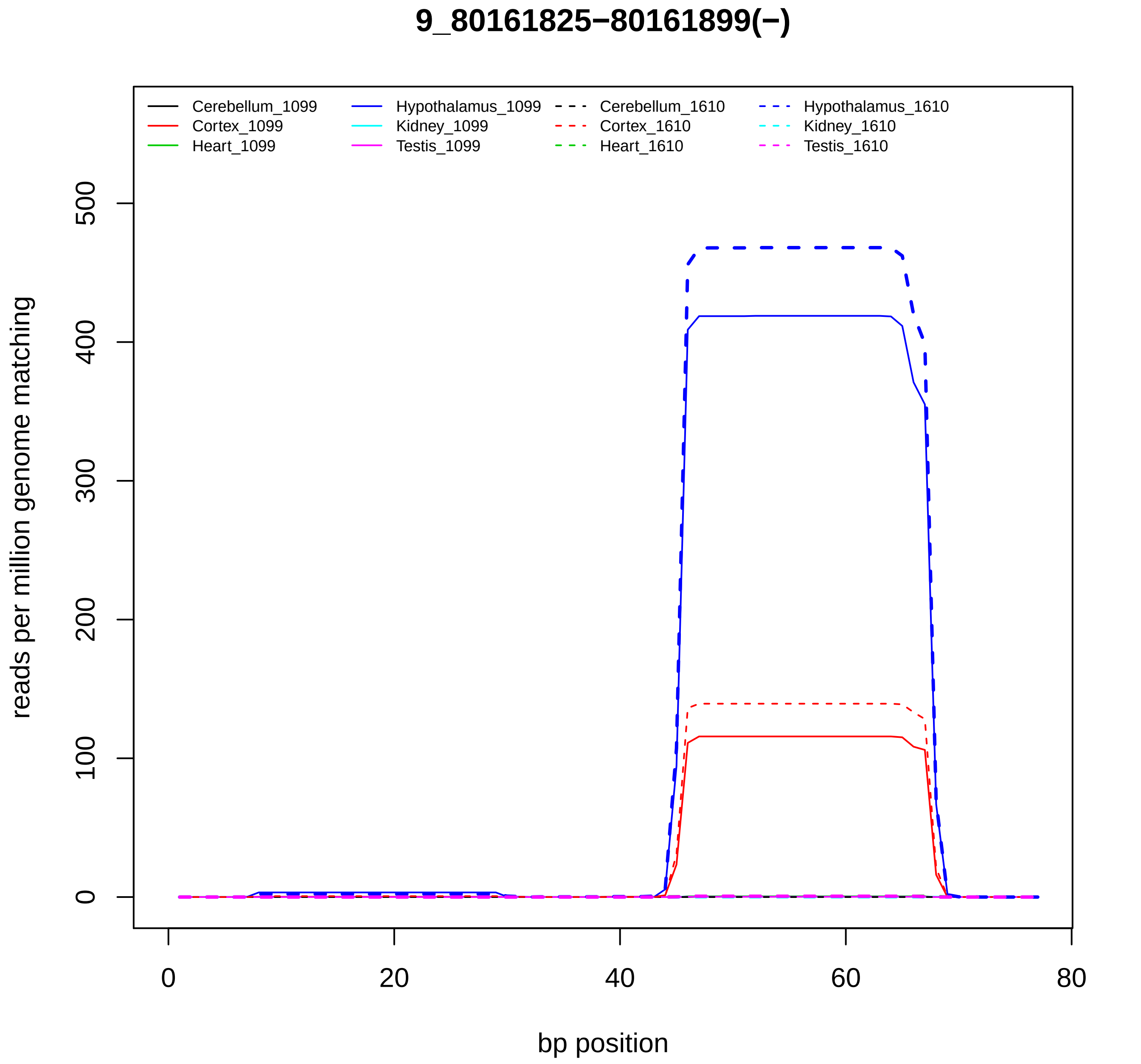

9_80161825−80161899(−)
Cerebellum_1099
Cortex_1099
Heart_1099
Hypothalamus_1099
Kidney_1099
Testis_1099
Cerebellum_1610
Cortex_1610
Heart_1610
Hypothalamus_1610
Kidney_1610
Testis_1610
reads per million genome matching
0
100
200
300
400
500
0
20
40
60
80
bp position

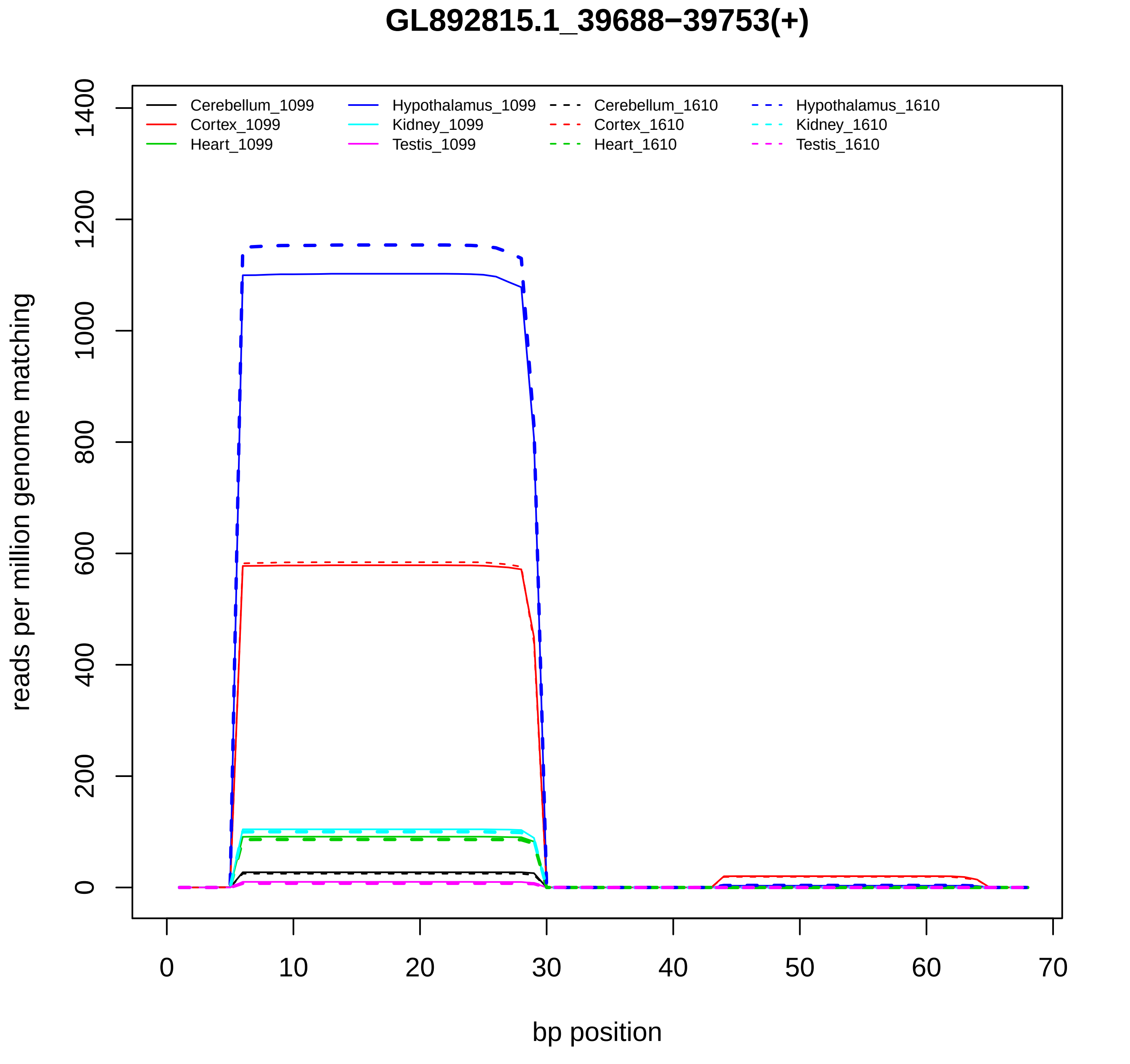
GL892815.1_39688−39753(+)
Cerebellum_1099
Cortex_1099
Heart_1099
Hypothalamus_1099
Kidney_1099
Testis_1099
Cerebellum_1610
Cortex_1610
Heart_1610
Hypothalamus_1610
Kidney_1610
Testis_1610
reads per million genome matching
0
200
400
600
800
1000
1200
1400
0
10
20
30
40
50
60
70
bp position

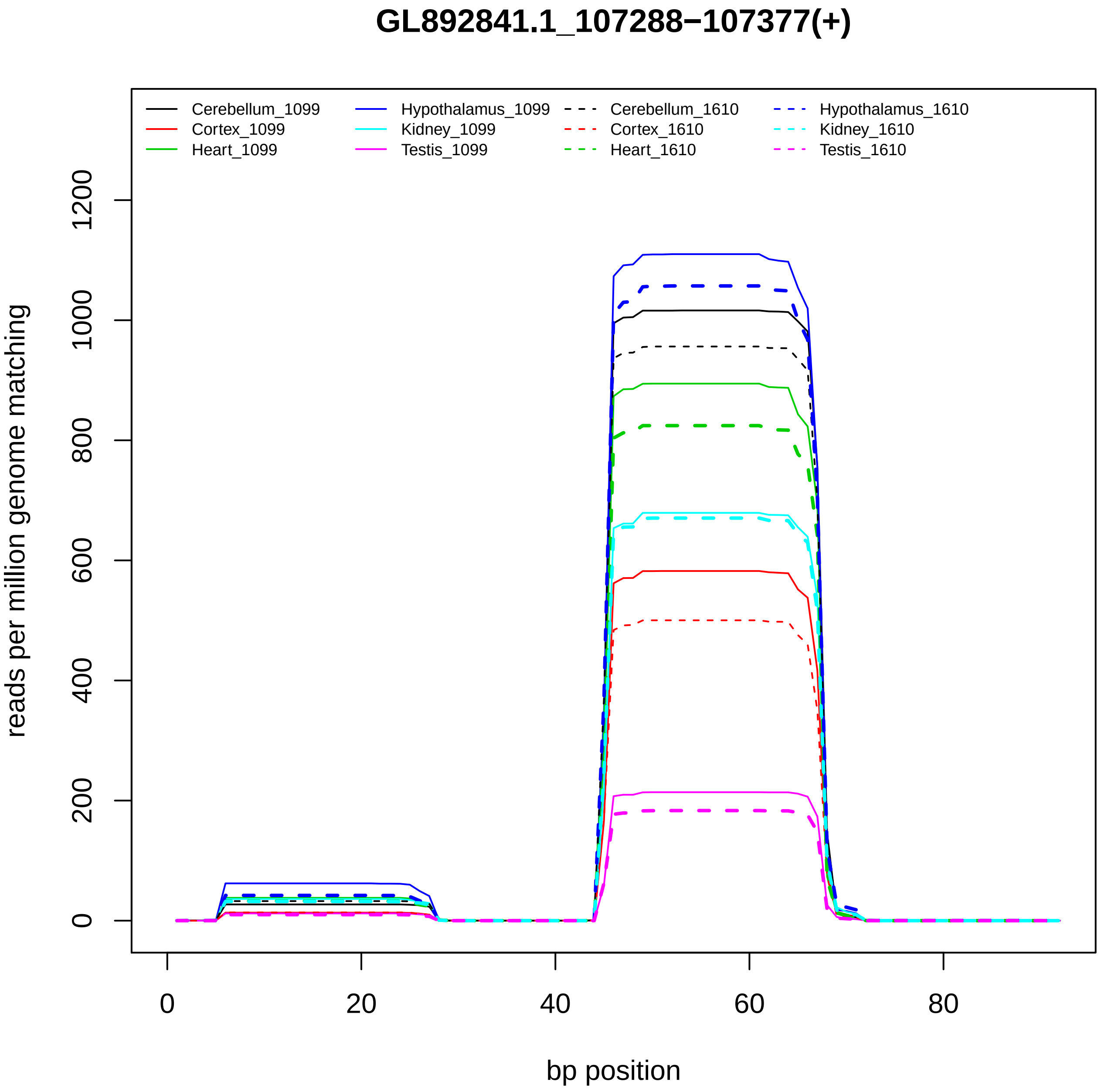
GL892841.1_107288−107377(+)
Cerebellum_1099
Cortex_1099
Heart_1099
Hypothalamus_1099
Kidney_1099
Testis_1099
Cerebellum_1610
Cortex_1610
Heart_1610
Hypothalamus_1610
Kidney_1610
Testis_1610
reads per million genome matching
bp position
0
200
400
600
800
1000
1200
0
20
40
60
80

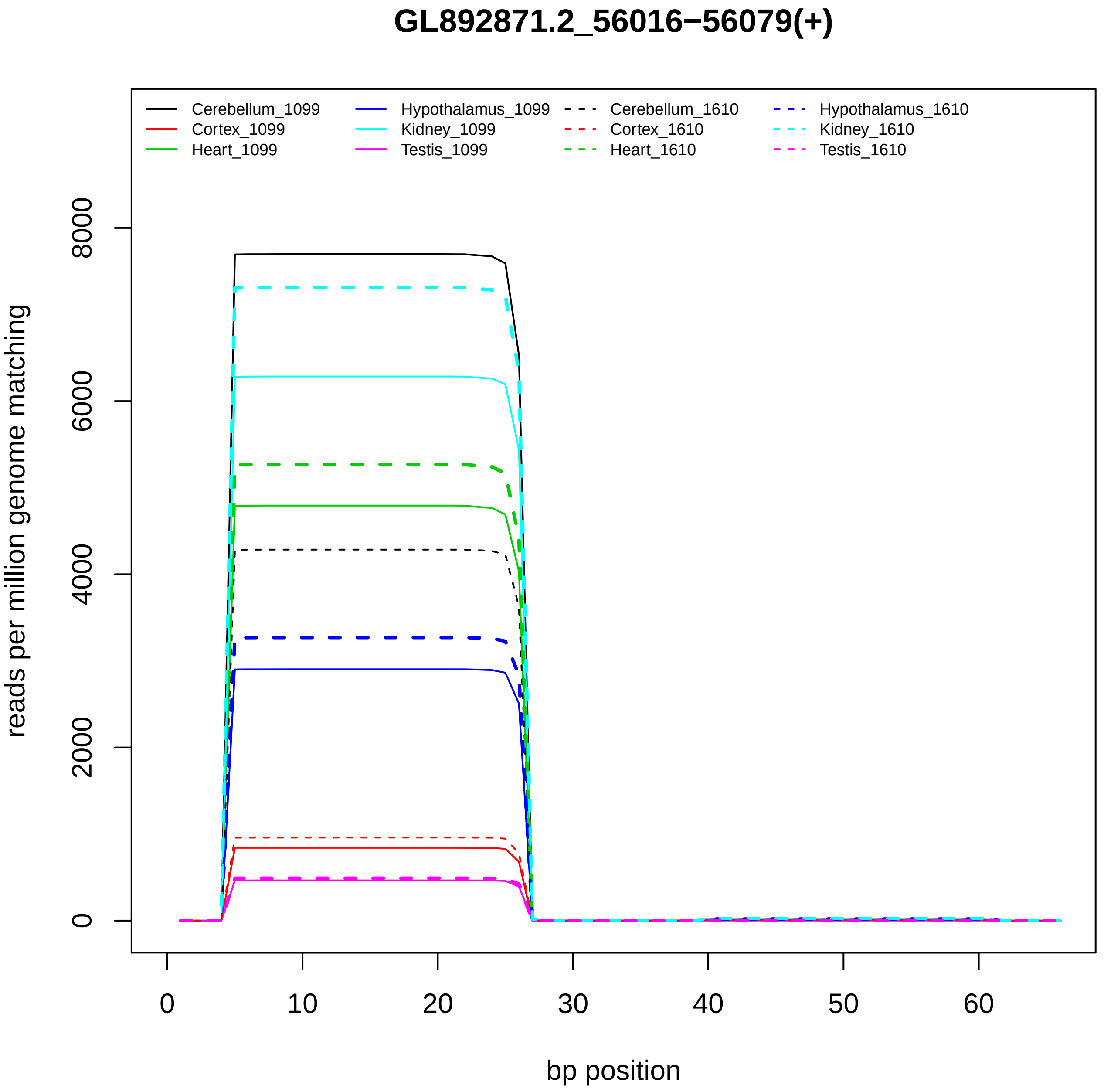

GL892871.2_56016−56079(+)
Cerebellum_1099
Cortex_1099
Heart_1099
Hypothalamus_1099
Kidney_1099
Testis_1099
Cerebellum_1610
Cortex_1610
Heart_1610
Hypothalamus_1610
Kidney_1610
Testis_1610
reads per million genome matching
0
2000
4000
6000
8000
0
10
20
30
40
50
60
bp position

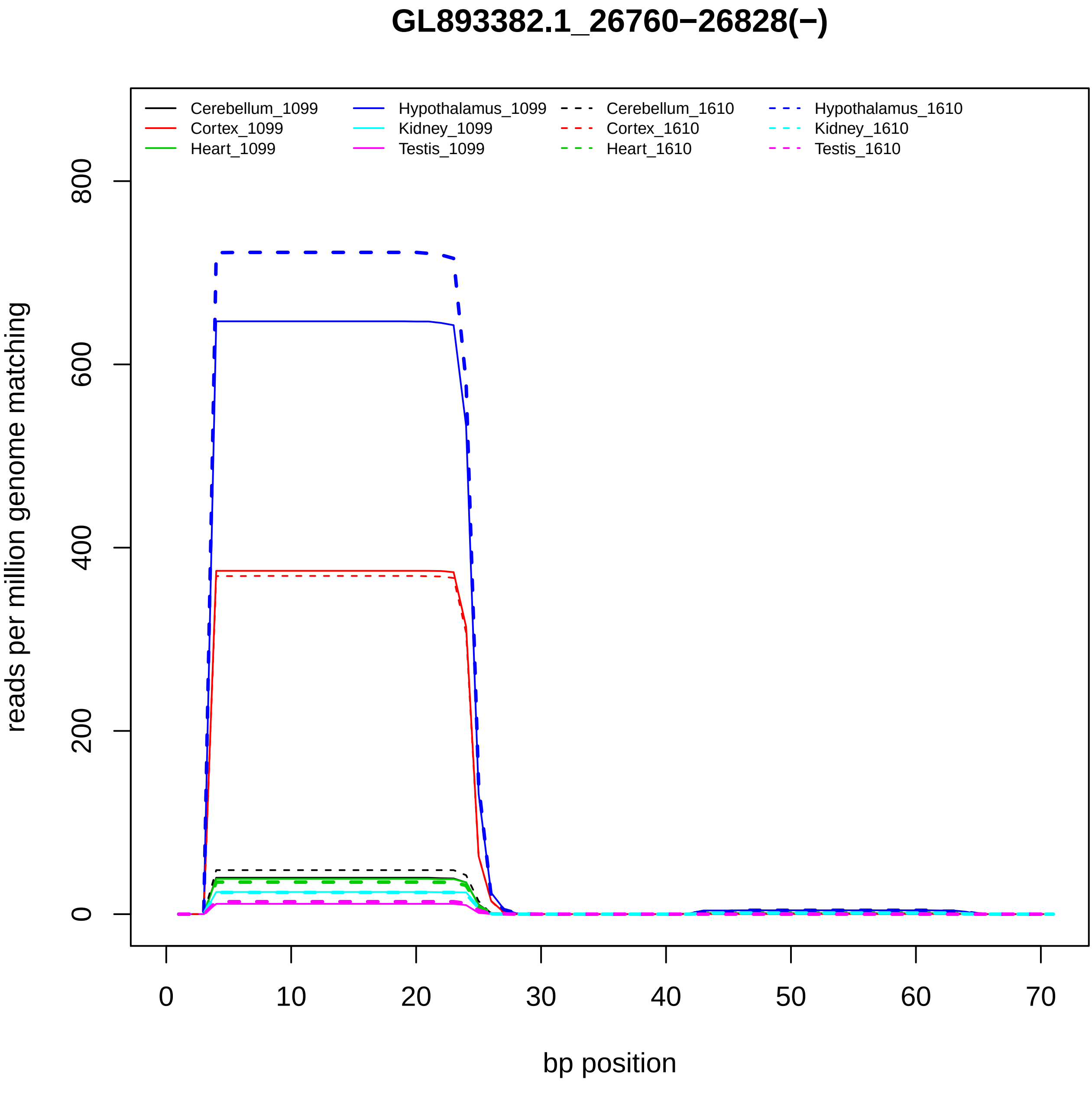
GL893382.1_26760−26828(−)
Cerebellum_1099
Cortex_1099
Heart_1099
Hypothalamus_1099
Kidney_1099
Testis_1099
Cerebellum_1610
Cortex_1610
Heart_1610
Hypothalamus_1610
Kidney_1610
Testis_1610
reads per million genome matching
0
200
400
600
800
0
10
20
30
40
50
60
70
bp position

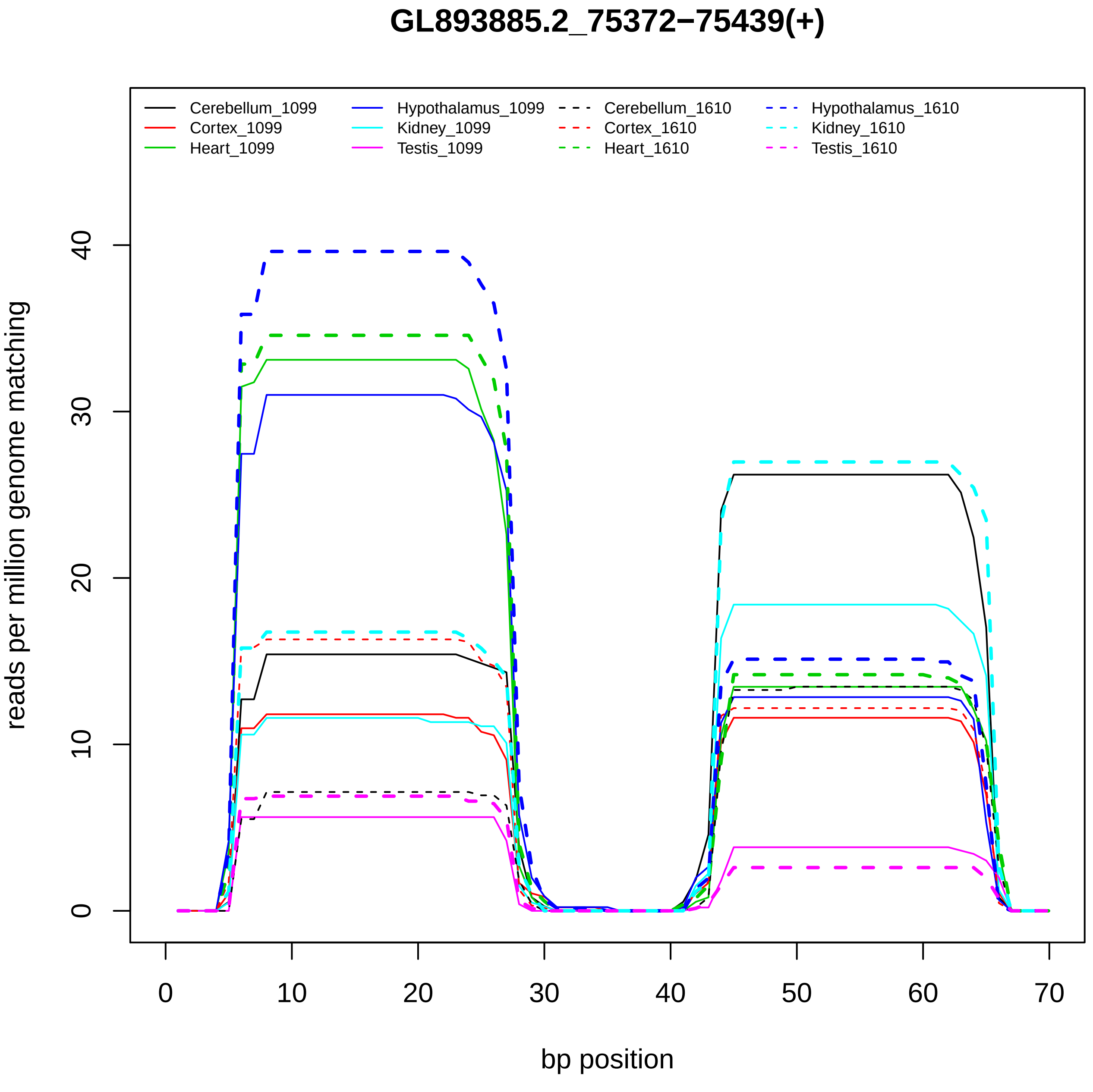
GL893885.2_75372−75439(+)
Cerebellum_1099
Cortex_1099
Heart_1099
Hypothalamus_1099
Kidney_1099
Testis_1099
Cerebellum_1610
Cortex_1610
Heart_1610
Hypothalamus_1610
Kidney_1610
Testis_1610
reads per million genome matching
0
10
20
30
40
0
10
20
30
40
50
60
70
bp position

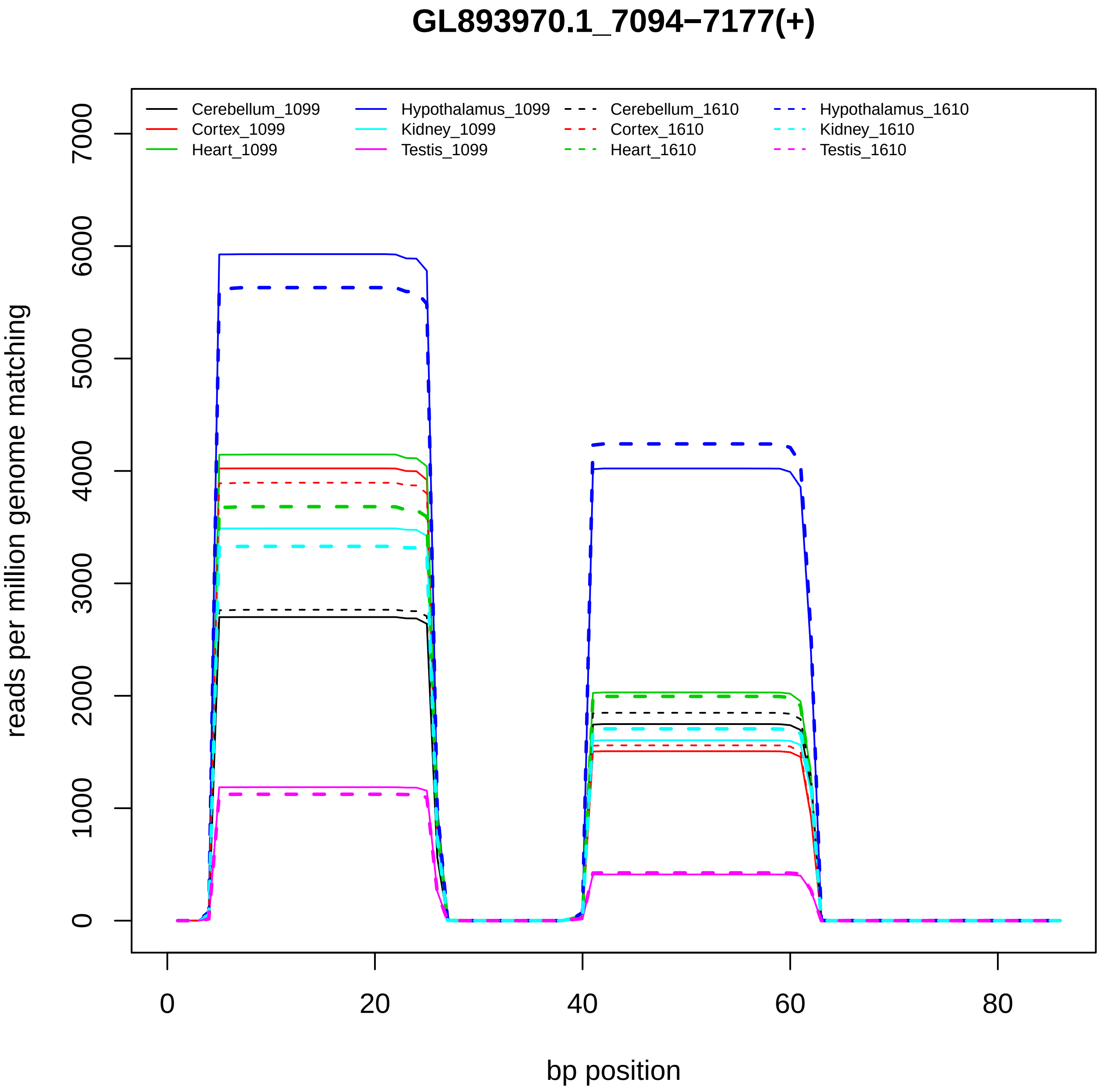
GL893970.1_7094−7177(+)
Cerebellum_1099
Cortex_1099
Heart_1099
Hypothalamus_1099
Kidney_1099
Testis_1099
Cerebellum_1610
Cortex_1610
Heart_1610
Hypothalamus_1610
Kidney_1610
Testis_1610
reads per million genome matching
0
1000
2000
3000
4000
5000
6000
7000
0
20
40
60
80
bp position

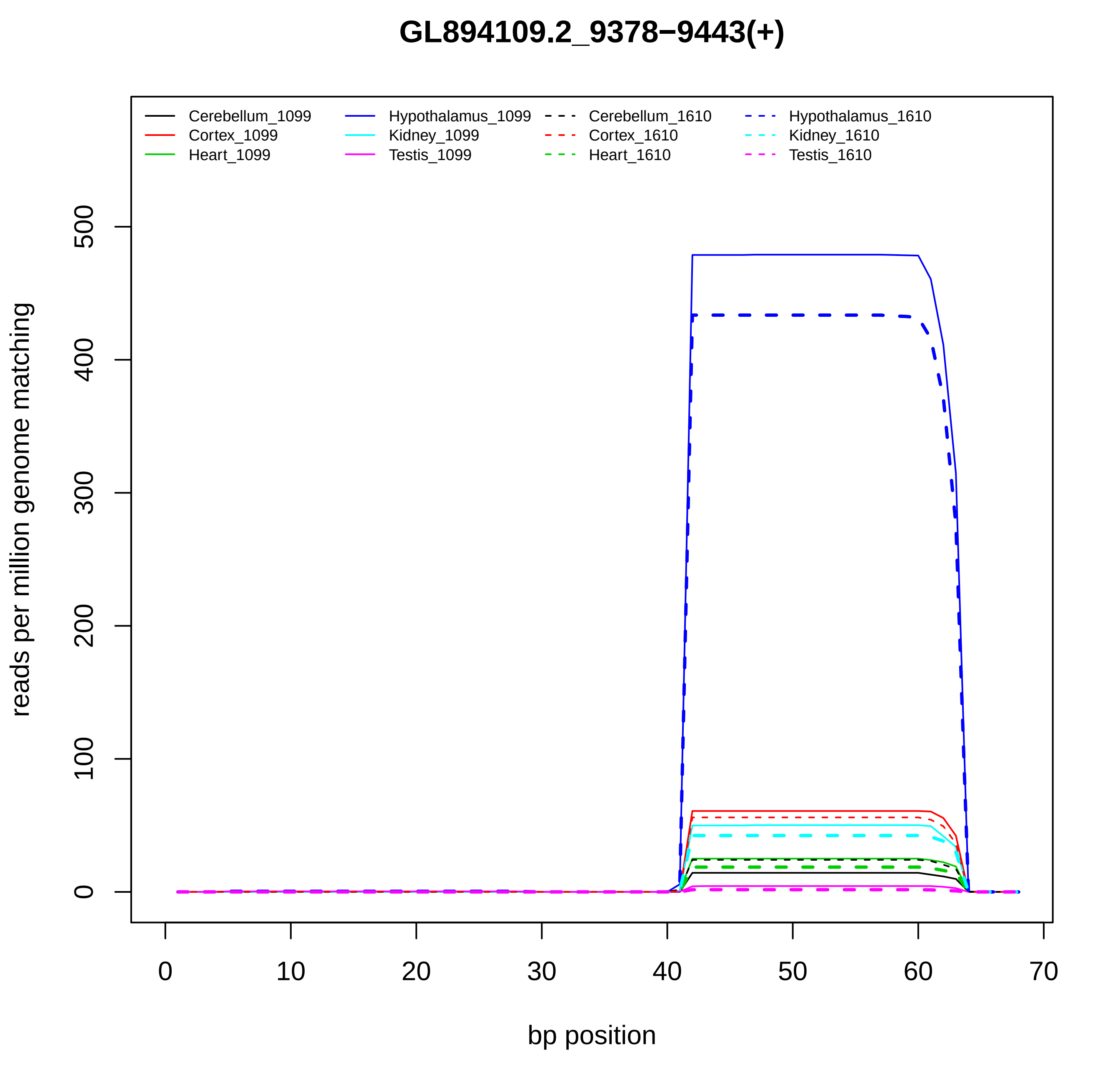

GL894109.2_9378−9443(+)
Cerebellum_1099
Cortex_1099
Heart_1099
Hypothalamus_1099
Kidney_1099
Testis_1099
Cerebellum_1610
Cortex_1610
Heart_1610
Hypothalamus_1610
Kidney_1610
Testis_1610
reads per million genome matching
0
100
200
300
400
500
0
10
20
30
40
50
60
70
bp position

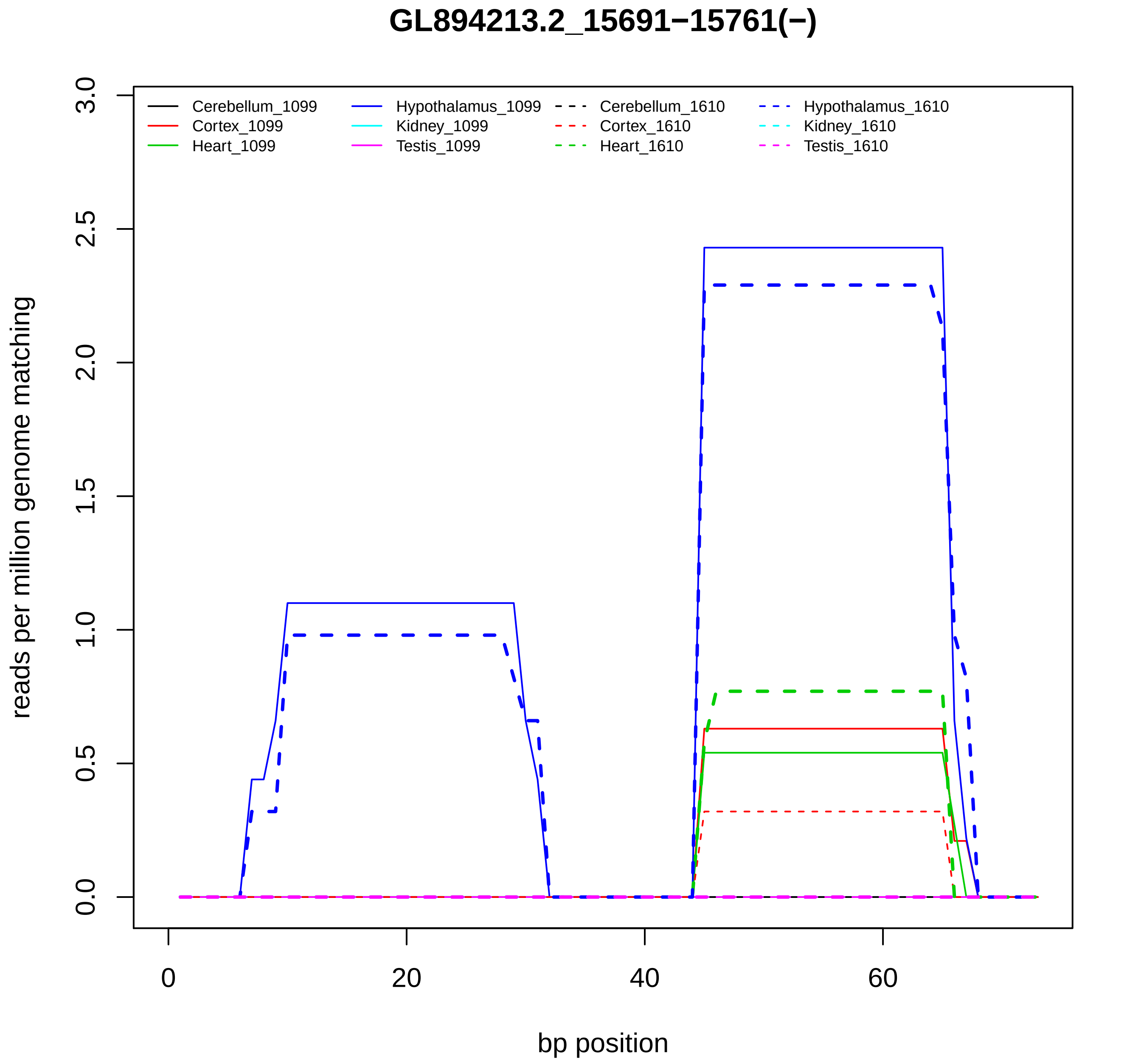
GL894213.2_15691−15761(−)
Cerebellum_1099
Cortex_1099
Heart_1099
Hypothalamus_1099
Kidney_1099
Testis_1099
Cerebellum_1610
Cortex_1610
Heart_1610
Hypothalamus_1610
Kidney_1610
Testis_1610
reads per million genome matching
0.0
0.5
1.0
1.5
2.0
2.5
3.0
0
20
40
60
bp position

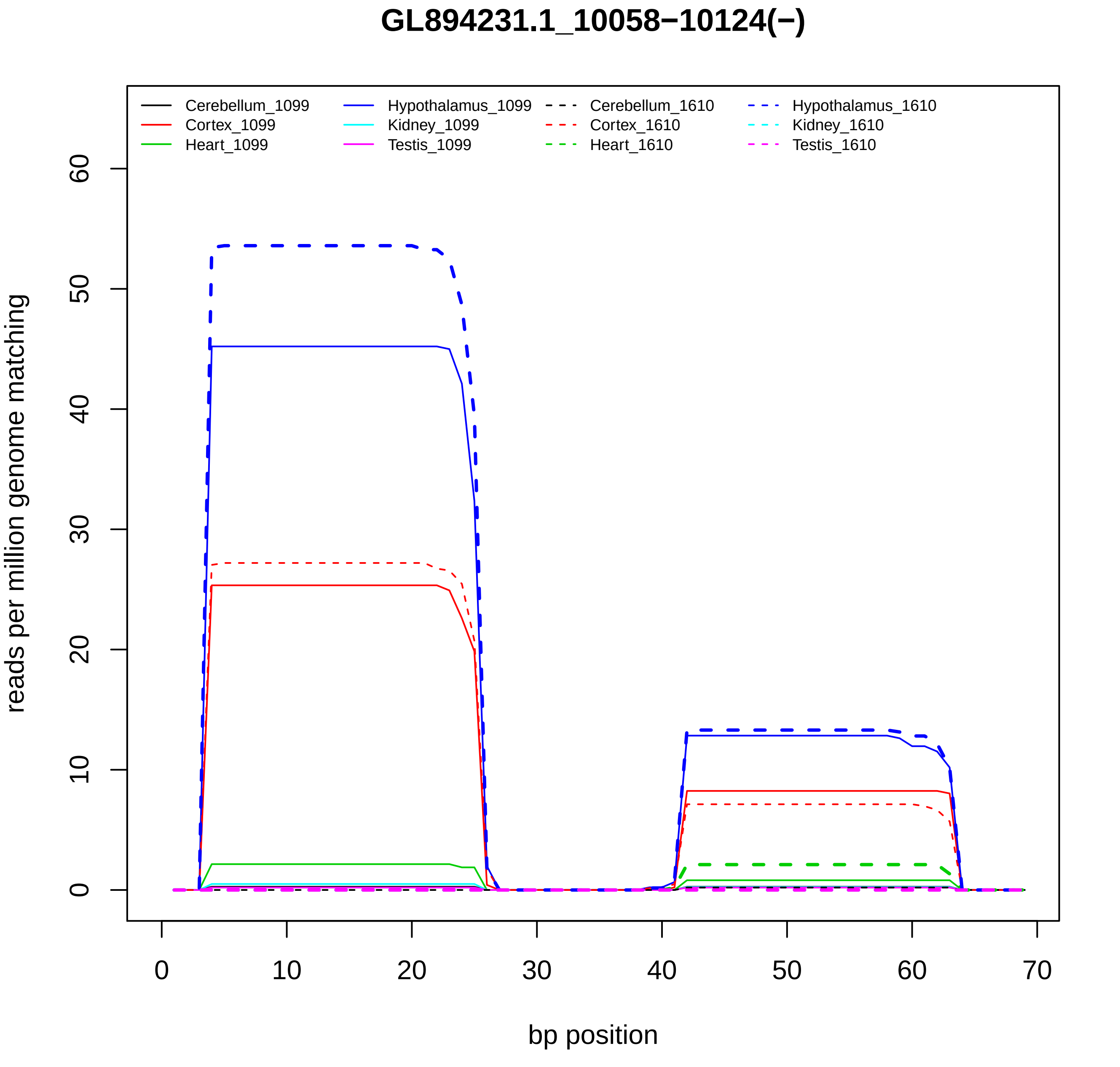
GL894231.1_10058−10124(−)
Cerebellum_1099
Cortex_1099
Heart_1099
Hypothalamus_1099
Kidney_1099
Testis_1099
Cerebellum_1610
Cortex_1610
Heart_1610
Hypothalamus_1610
Kidney_1610
Testis_1610
reads per million genome matching
bp position
0
10
20
30
40
50
60
70

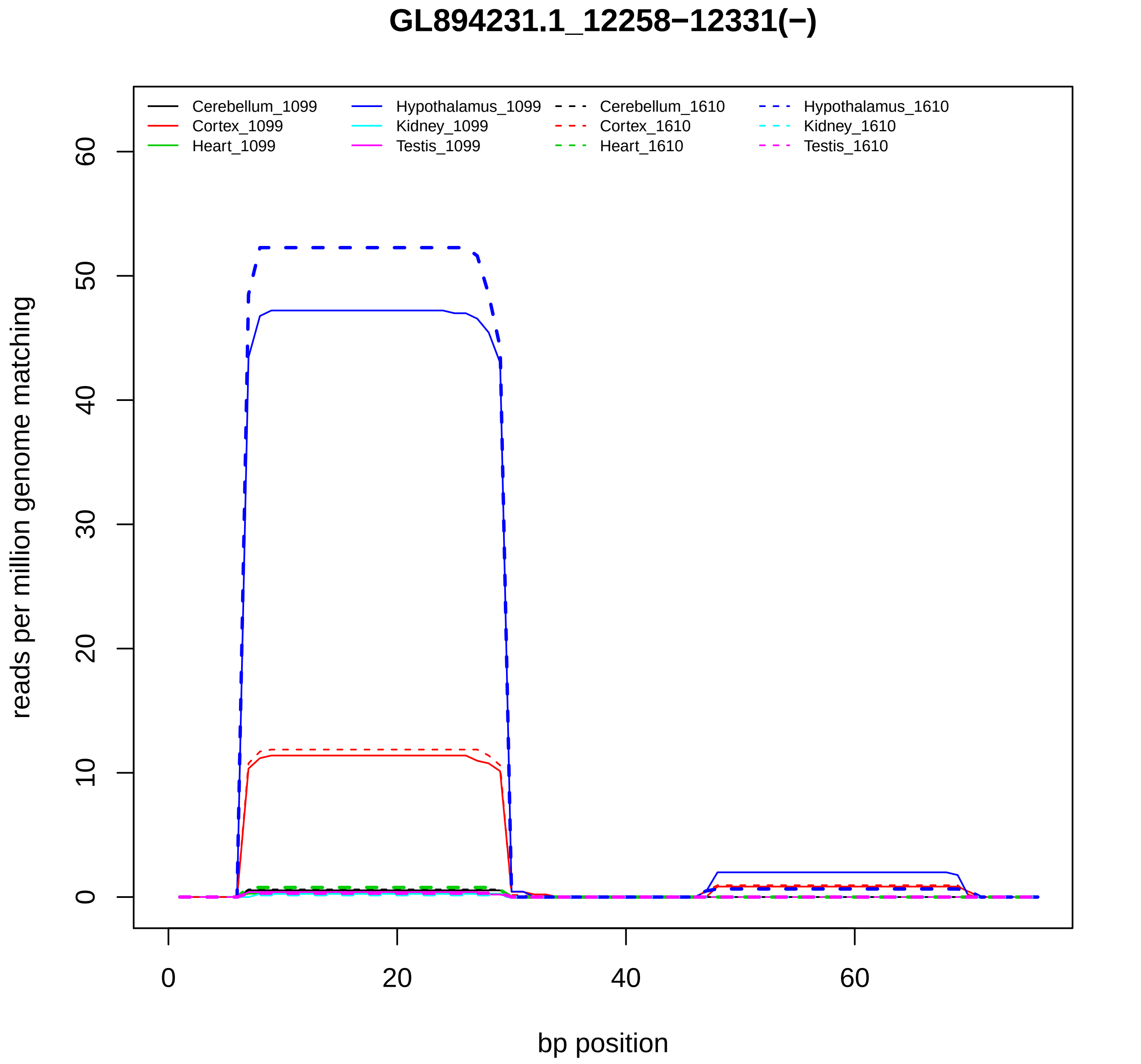

GL894231.1_12258−12331(−)
Cerebellum_1099
Cortex_1099
Heart_1099
Hypothalamus_1099
Kidney_1099
Testis_1099
Cerebellum_1610
Cortex_1610
Heart_1610
Hypothalamus_1610
Kidney_1610
Testis_1610
reads per million genome matching
0
10
20
30
40
50
60
bp position
0
20
40
60

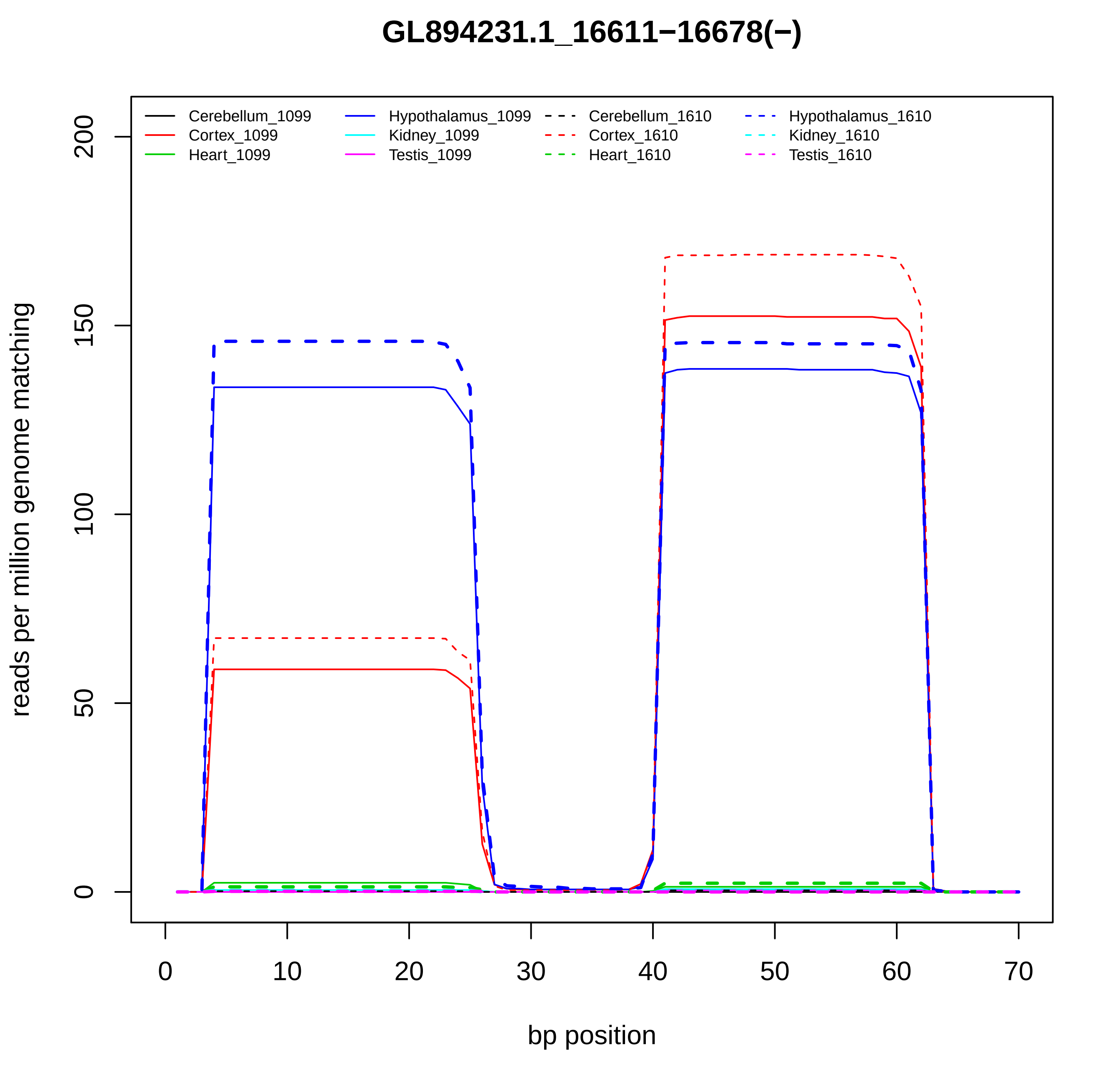

GL894231.1_16611−16678(−)
Cerebellum_1099
Cortex_1099
Heart_1099
Hypothalamus_1099
Kidney_1099
Testis_1099
Cerebellum_1610
Cortex_1610
Heart_1610
Hypothalamus_1610
Kidney_1610
Testis_1610
reads per million genome matching
0
50
100
150
200
0
10
20
30
40
50
60
70
bp position

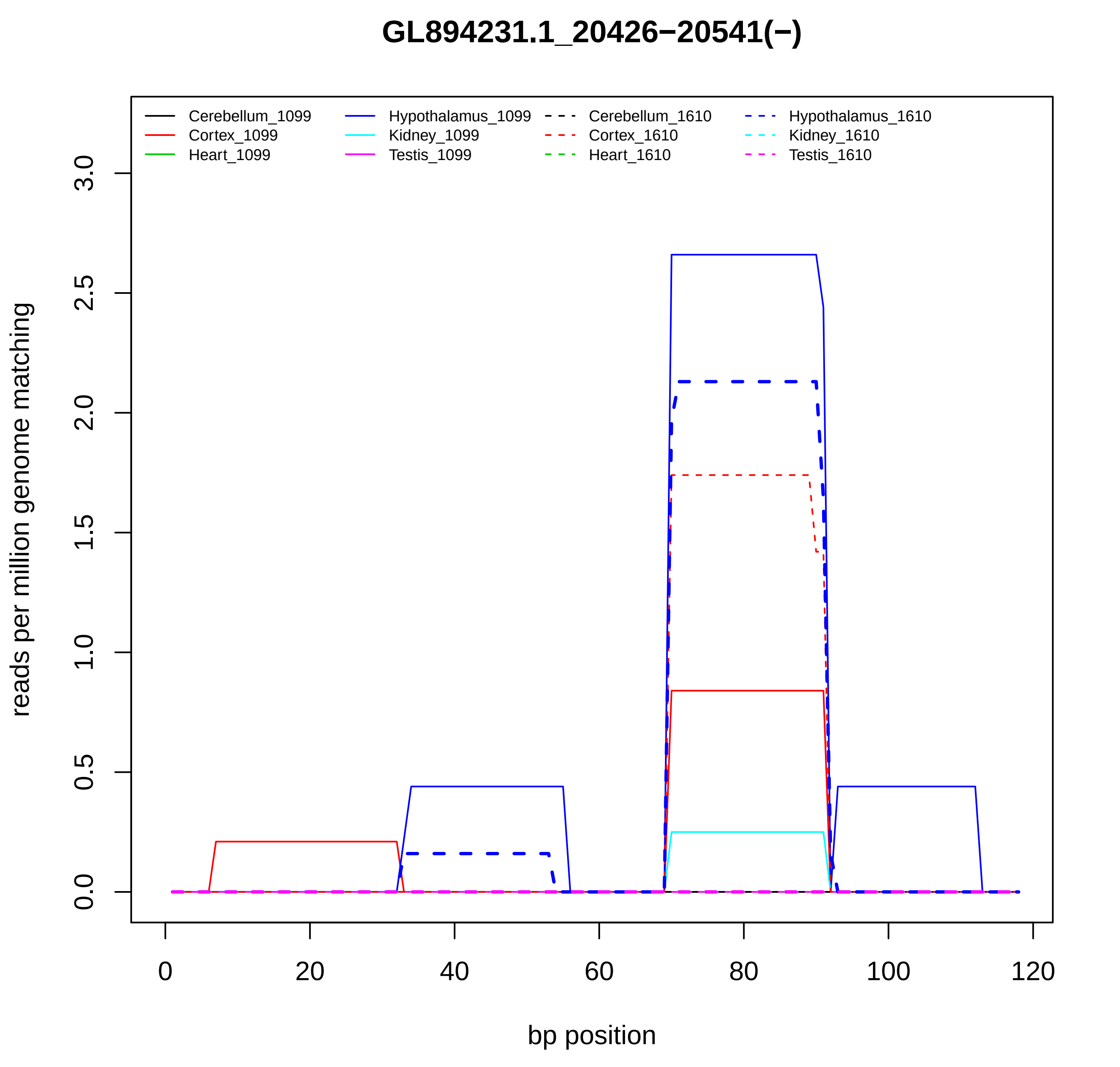

GL894231.1_20426−20541(−)
Cerebellum_1099
Cortex_1099
Heart_1099
Hypothalamus_1099
Kidney_1099
Testis_1099
Cerebellum_1610
Cortex_1610
Heart_1610
Hypothalamus_1610
Kidney_1610
Testis_1610
reads per million genome matching
0.0
0.5
1.0
1.5
2.0
2.5
3.0
0
20
40
60
80
100
120
bp position

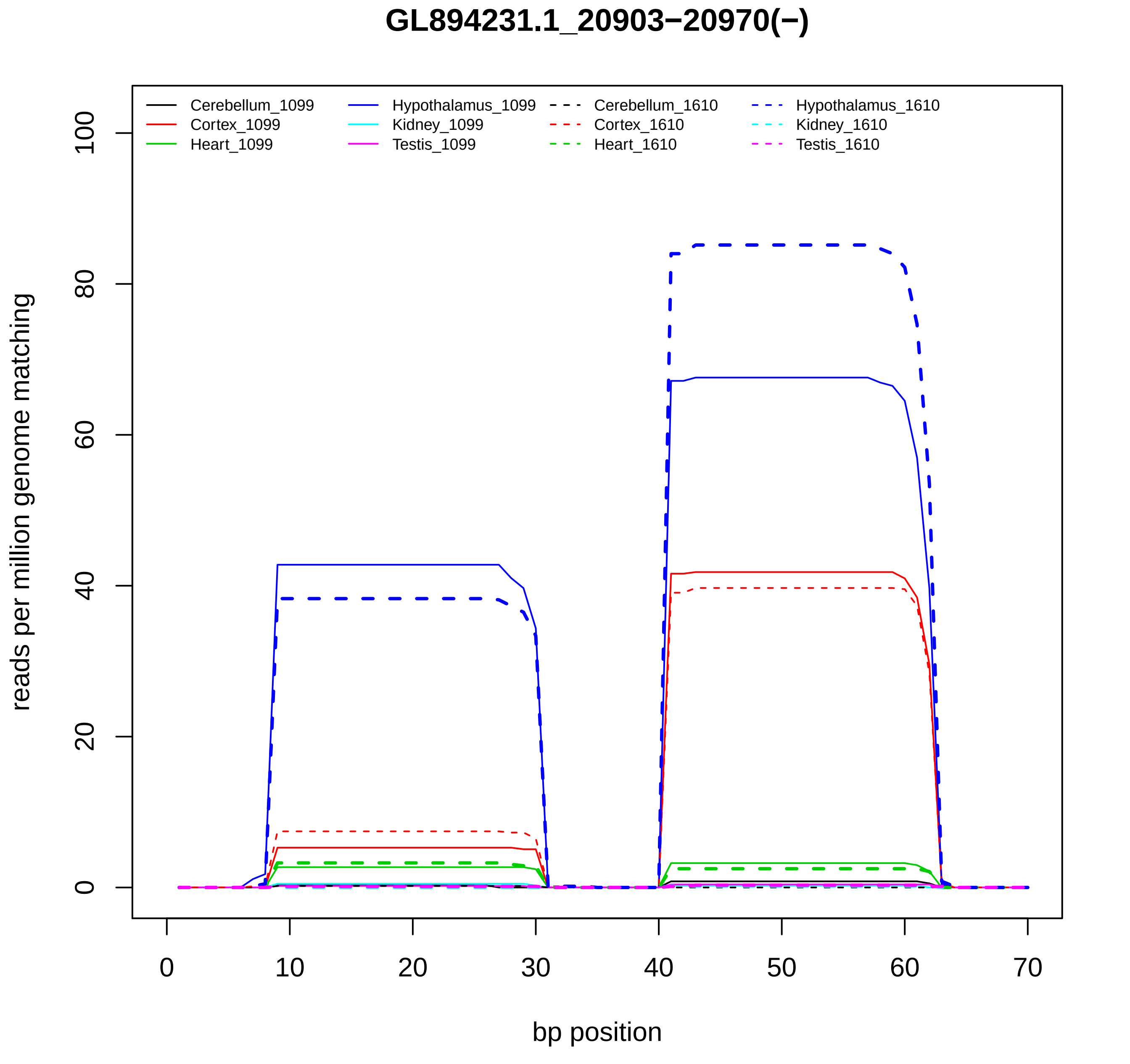

GL894231.1_20903−20970(−)
Cerebellum_1099
Cortex_1099
Heart_1099
Hypothalamus_1099
Kidney_1099
Testis_1099
Cerebellum_1610
Cortex_1610
Heart_1610
Hypothalamus_1610
Kidney_1610
Testis_1610
reads per million genome matching
bp position
0
20
40
60
80
100
0
10
20
30
40
50
60
70

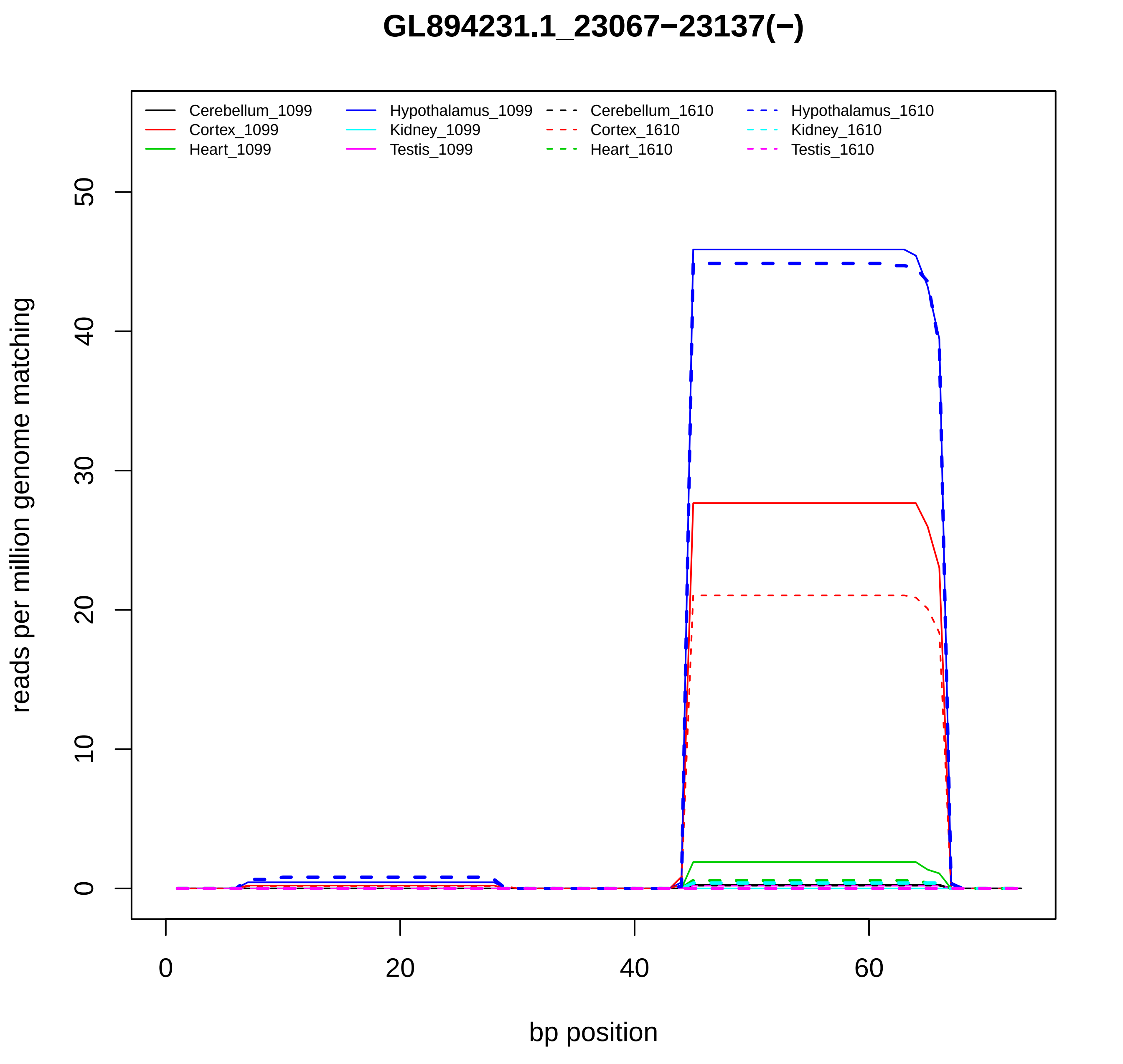

GL894231.1_23067−23137(−)
Cerebellum_1099
Cortex_1099
Heart_1099
Hypothalamus_1099
Kidney_1099
Testis_1099
Cerebellum_1610
Cortex_1610
Heart_1610
Hypothalamus_1610
Kidney_1610
Testis_1610
reads per million genome matching
0
10
20
30
40
50
0
20
40
60
bp position

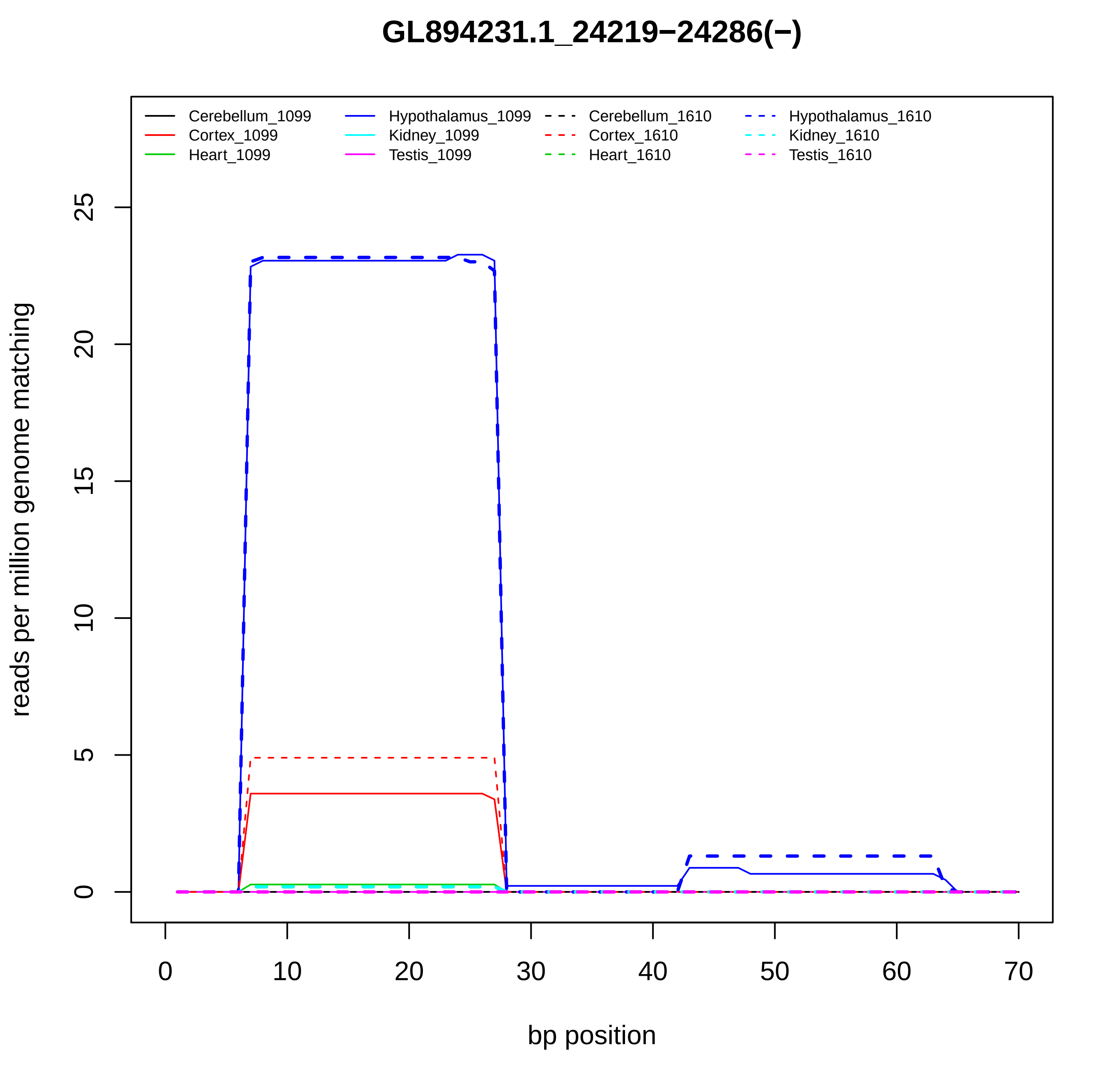

GL894231.1_24219−24286(−)
Cerebellum_1099
Cortex_1099
Heart_1099
Hypothalamus_1099
Kidney_1099
Testis_1099
Cerebellum_1610
Cortex_1610
Heart_1610
Hypothalamus_1610
Kidney_1610
Testis_1610
reads per million genome matching
0
5
10
15
20
25
0
10
20
30
40
50
60
70
bp position

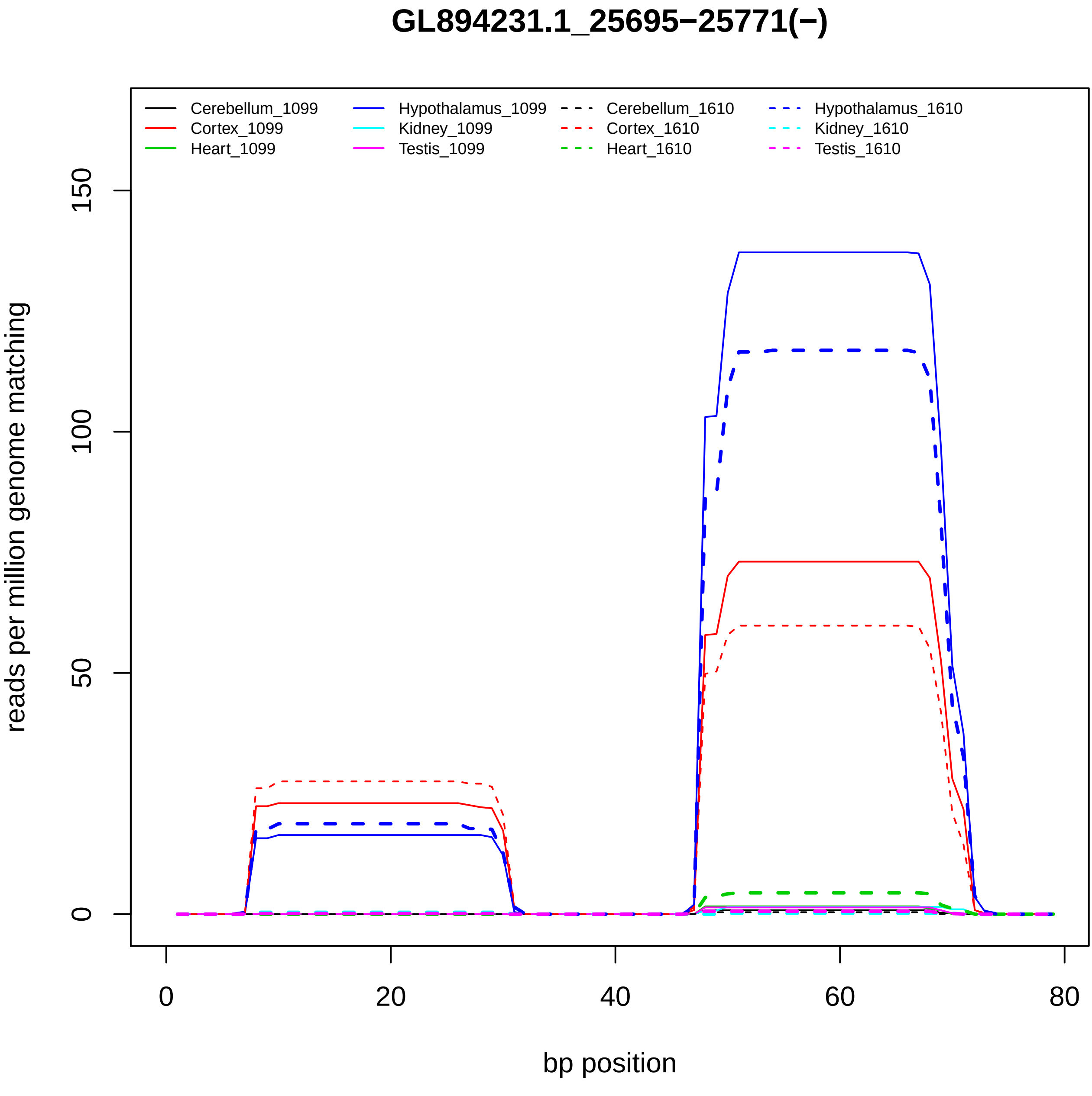
GL894231.1_25695−25771(−)
Cerebellum_1099
Cortex_1099
Heart_1099
Hypothalamus_1099
Kidney_1099
Testis_1099
Cerebellum_1610
Cortex_1610
Heart_1610
Hypothalamus_1610
Kidney_1610
Testis_1610
reads per million genome matching
0
50
100
150
bp position
0
20
40
60
80

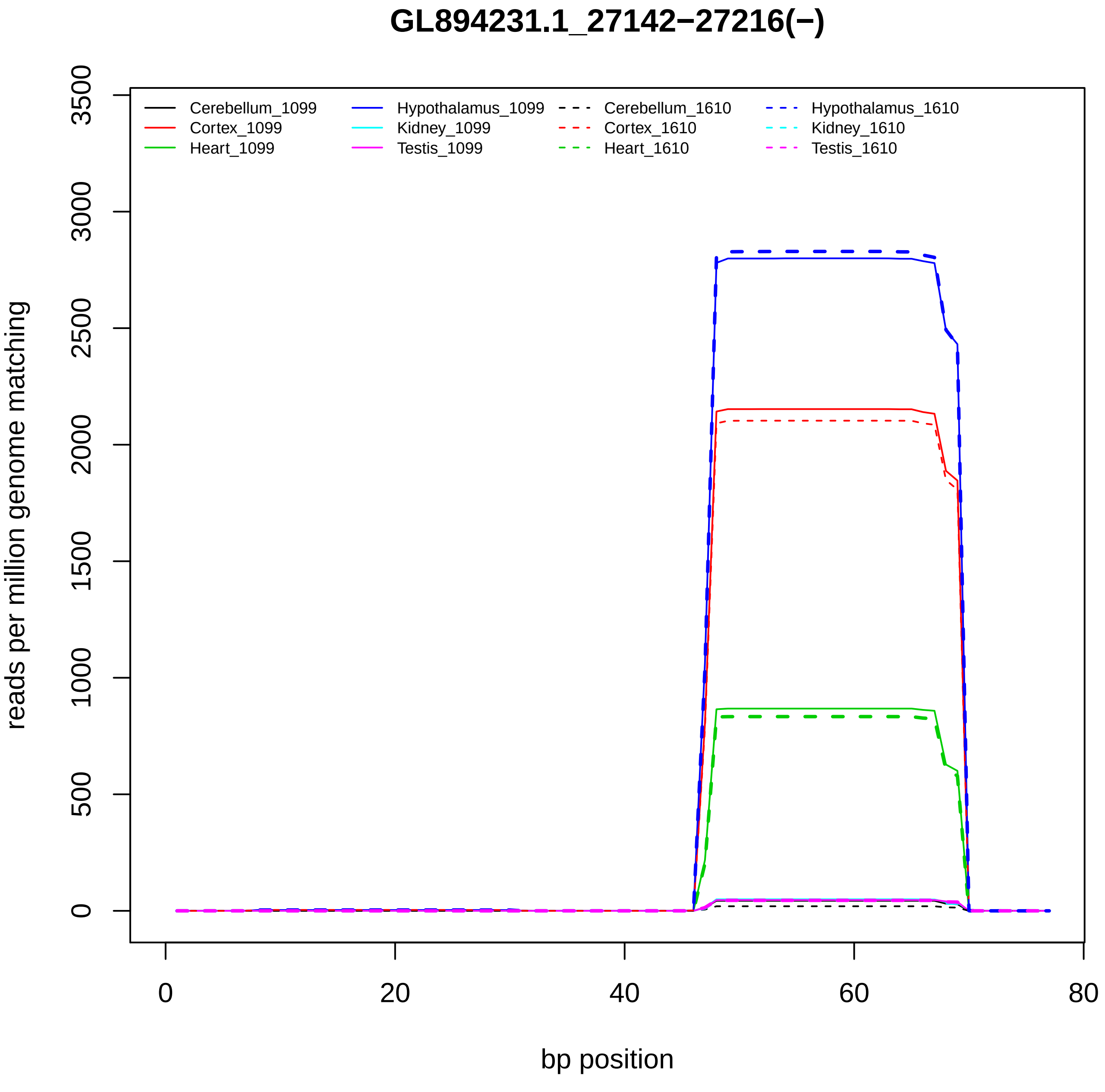

GL894231.1_27142−27216(−)
Cerebellum_1099
Cortex_1099
Heart_1099
Hypothalamus_1099
Kidney_1099
Testis_1099
Cerebellum_1610
Cortex_1610
Heart_1610
Hypothalamus_1610
Kidney_1610
Testis_1610
reads per million genome matching
bp position
0
500
1000
1500
2000
2500
3000
3500
0
20
40
60
80

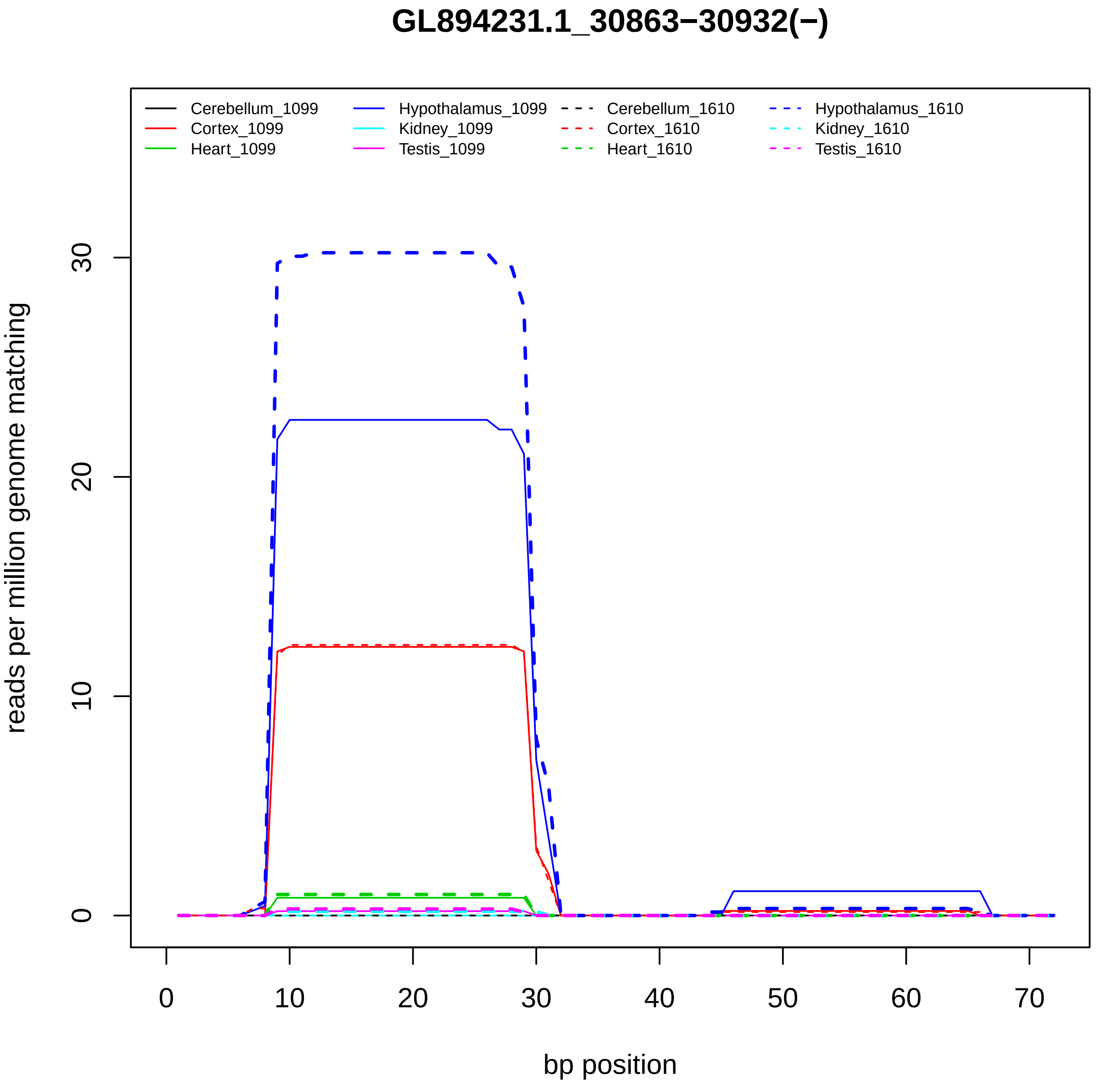
GL894231.1_30863−30932(−)
Cerebellum_1099
Cortex_1099
Heart_1099
Hypothalamus_1099
Kidney_1099
Testis_1099
Cerebellum_1610
Cortex_1610
Heart_1610
Hypothalamus_1610
Kidney_1610
Testis_1610
reads per million genome matching
0
10
20
30
0
10
20
30
40
50
60
70
bp position

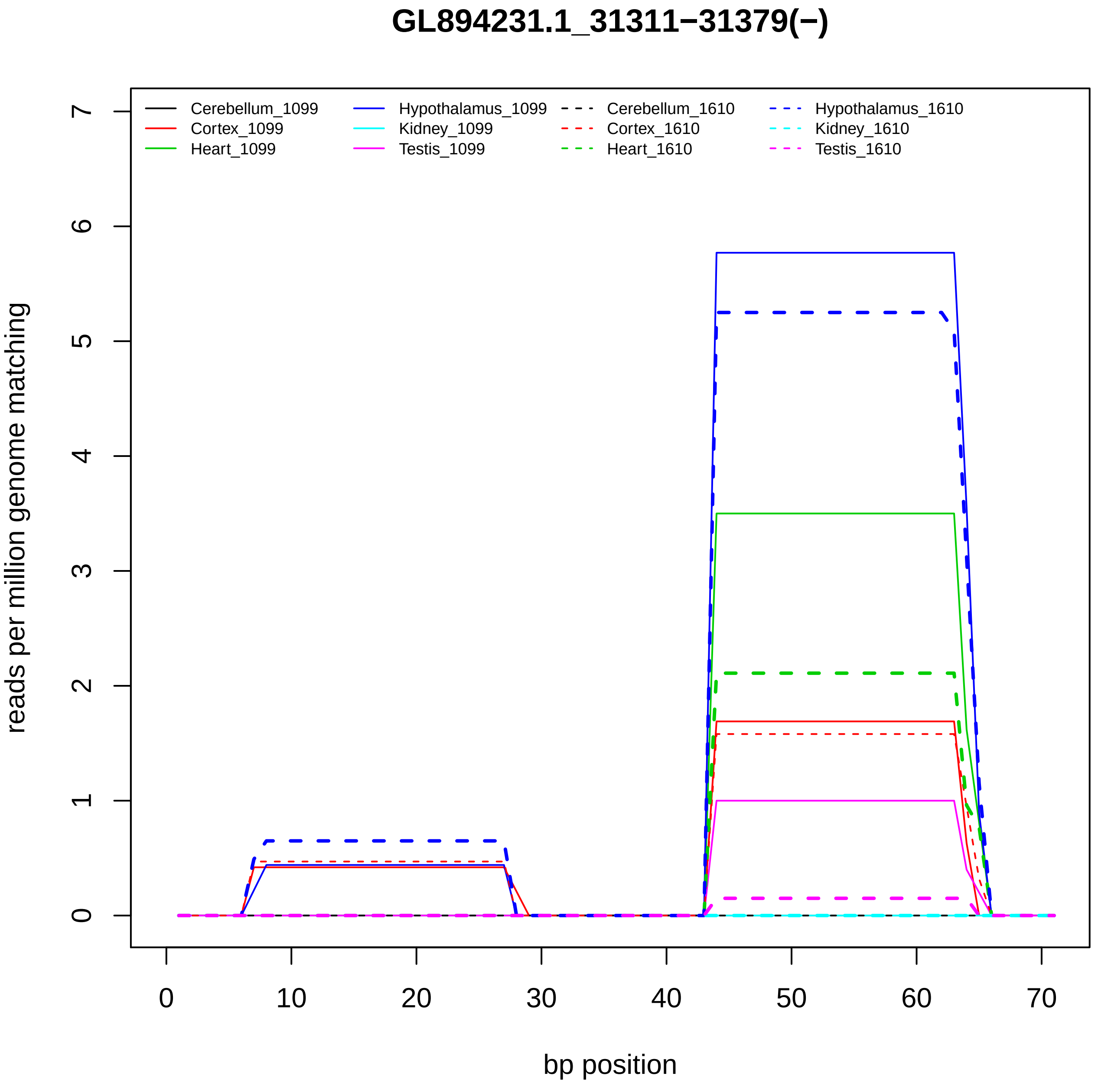

GL894231.1_31311−31379(−)
Cerebellum_1099
Cortex_1099
Heart_1099
Hypothalamus_1099
Kidney_1099
Testis_1099
Cerebellum_1610
Cortex_1610
Heart_1610
Hypothalamus_1610
Kidney_1610
Testis_1610
reads per million genome matching
bp position
0
1
2
3
4
5
6
7
0
10
20
30
40
50
60
70

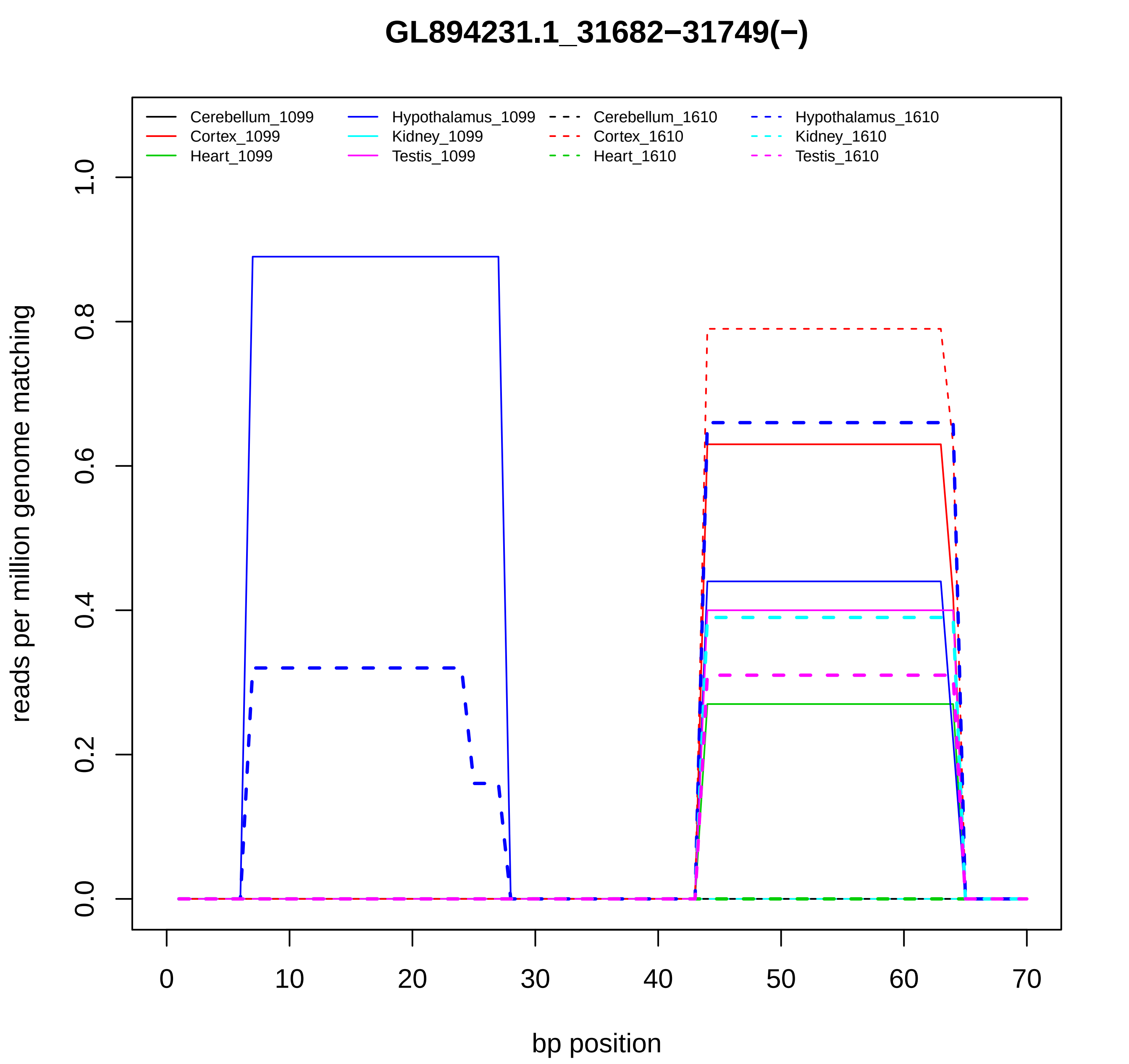

GL894231.1_31682−31749(−)
Cerebellum_1099
Cortex_1099
Heart_1099
Hypothalamus_1099
Kidney_1099
Testis_1099
Cerebellum_1610
Cortex_1610
Heart_1610
Hypothalamus_1610
Kidney_1610
Testis_1610
reads per million genome matching
0.0
0.2
0.4
0.6
0.8
1.0
0
10
20
30
40
50
60
70
bp position

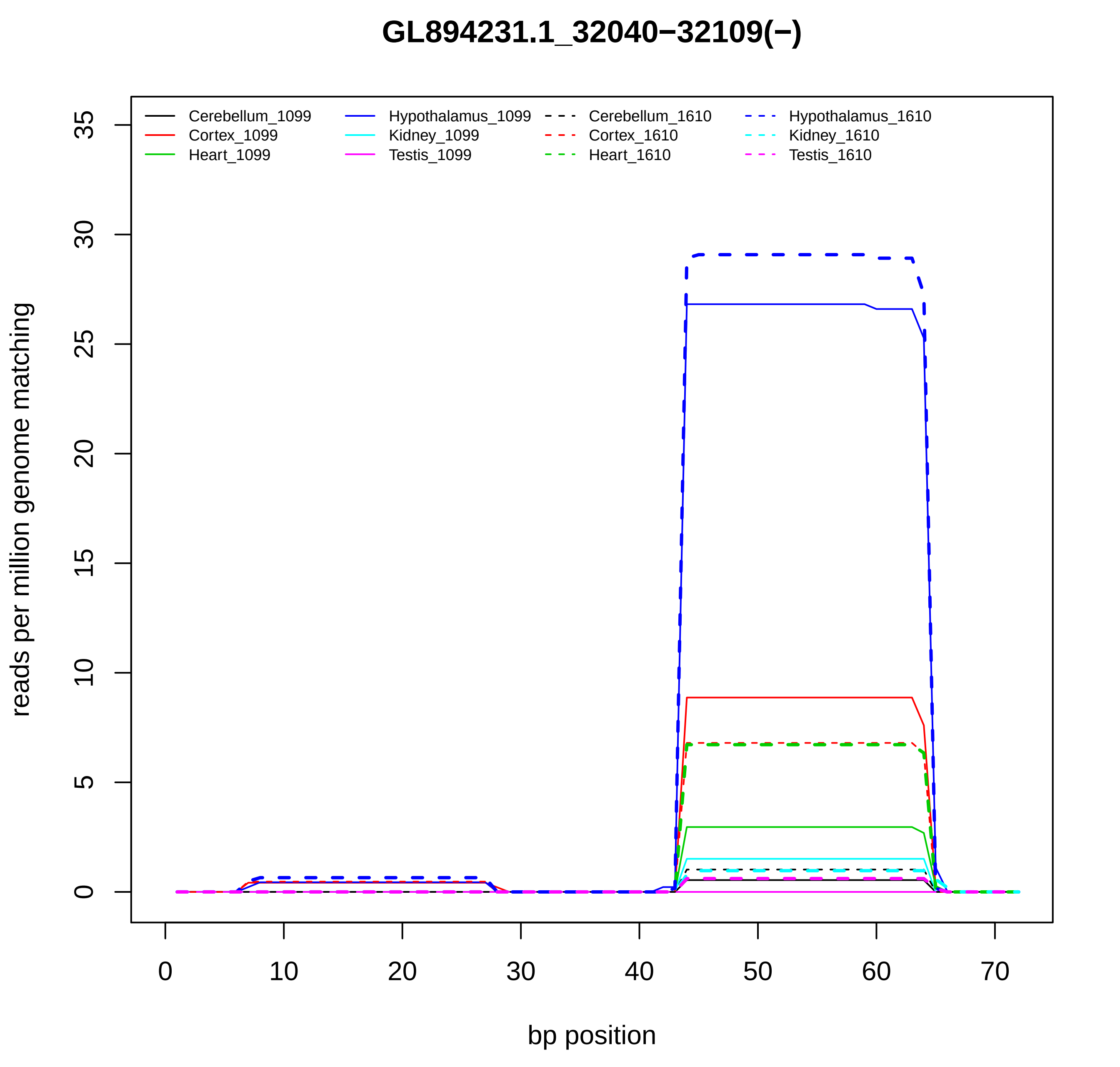
GL894231.1_32040−32109(−)
Cerebellum_1099
Cortex_1099
Heart_1099
Hypothalamus_1099
Kidney_1099
Testis_1099
Cerebellum_1610
Cortex_1610
Heart_1610
Hypothalamus_1610
Kidney_1610
Testis_1610
reads per million genome matching
bp position
0
5
10
15
20
25
30
35
0
10
20
30
40
50
60
70

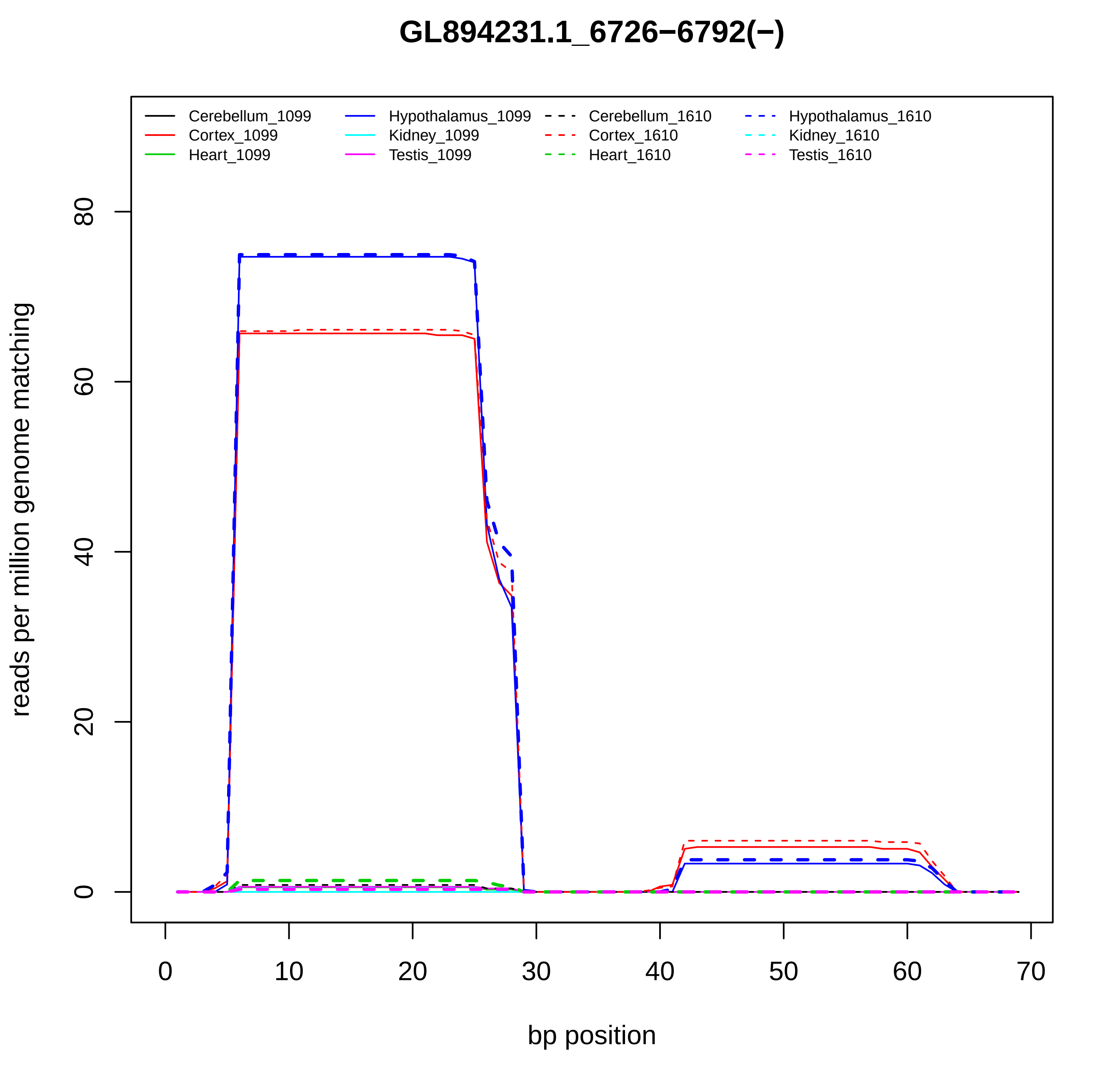

GL894231.1_6726−6792(−)
Cerebellum_1099
Cortex_1099
Heart_1099
Hypothalamus_1099
Kidney_1099
Testis_1099
Cerebellum_1610
Cortex_1610
Heart_1610
Hypothalamus_1610
Kidney_1610
Testis_1610
reads per million genome matching
0
20
40
60
80
bp position
0
10
20
30
40
50
60
70

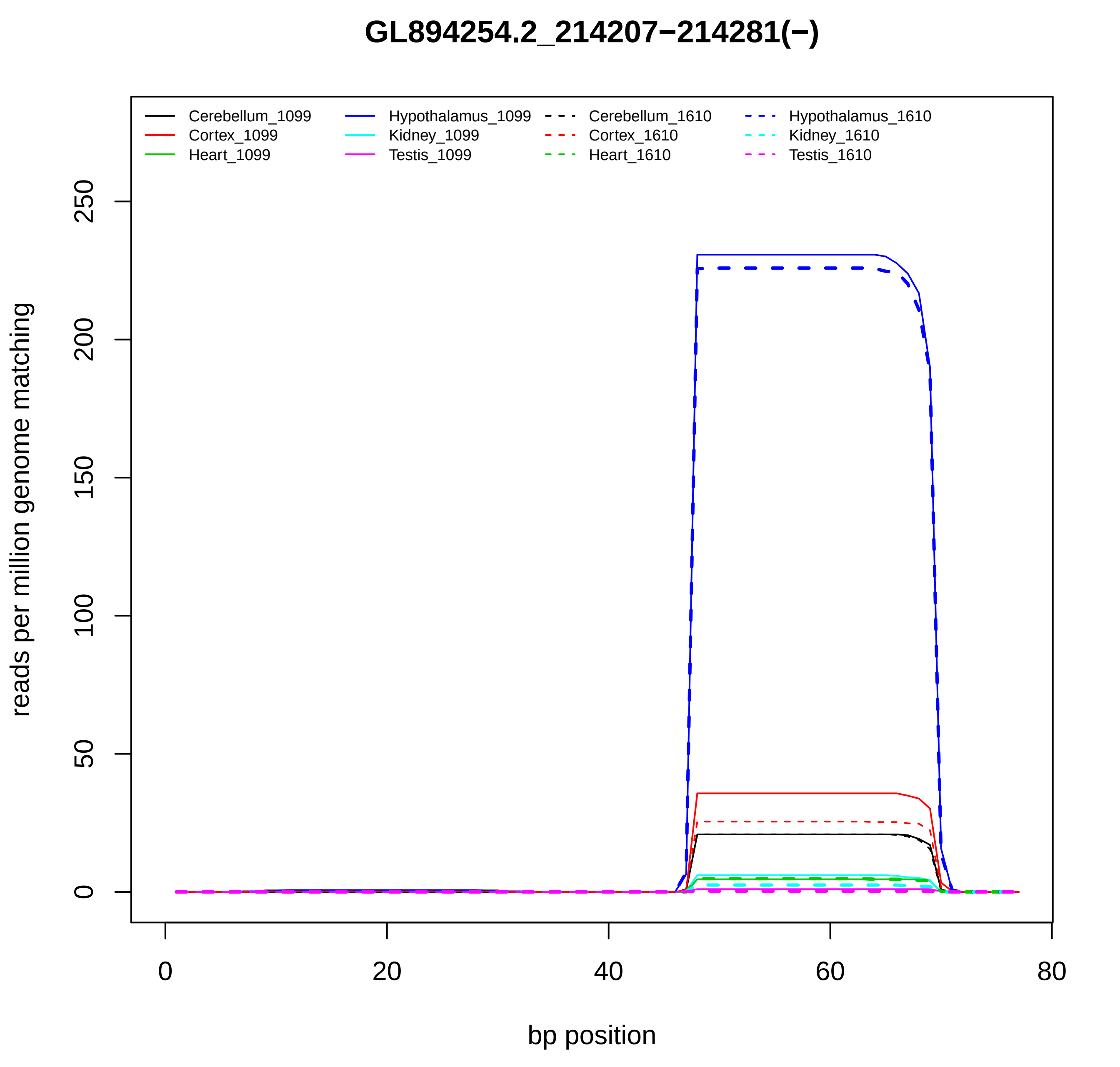
GL894254.2_214207−214281(−)
Cerebellum_1099
Cortex_1099
Heart_1099
Hypothalamus_1099
Kidney_1099
Testis_1099
Cerebellum_1610
Cortex_1610
Heart_1610
Hypothalamus_1610
Kidney_1610
Testis_1610
reads per million genome matching
0
50
100
150
200
250
0
20
40
60
80
bp position

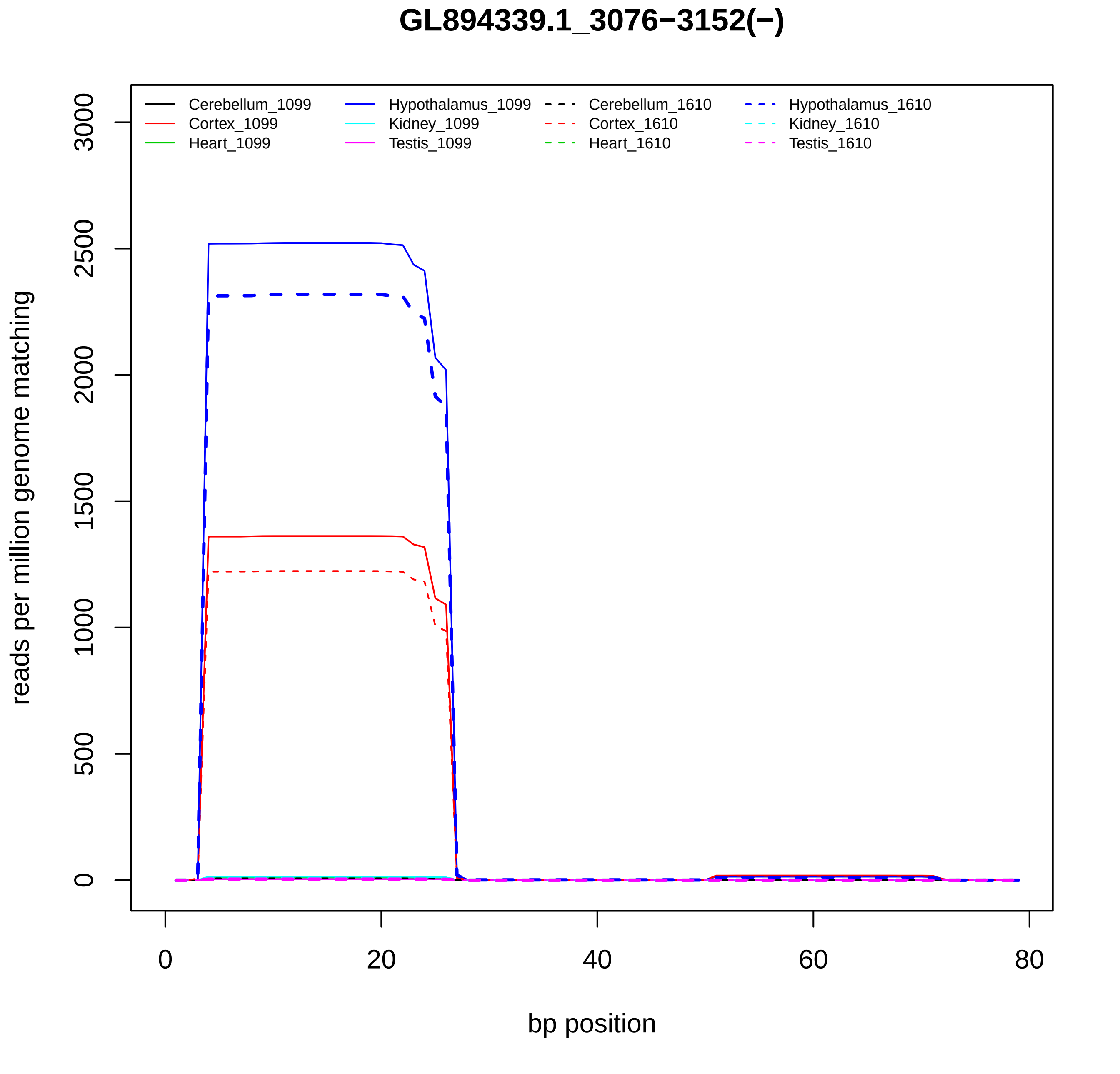

GL894339.1_3076−3152(−)
Cerebellum_1099
Cortex_1099
Heart_1099
Hypothalamus_1099
Kidney_1099
Testis_1099
Cerebellum_1610
Cortex_1610
Heart_1610
Hypothalamus_1610
Kidney_1610
Testis_1610
reads per million genome matching
0
500
1000
1500
2000
2500
3000
0
20
40
60
80
bp position

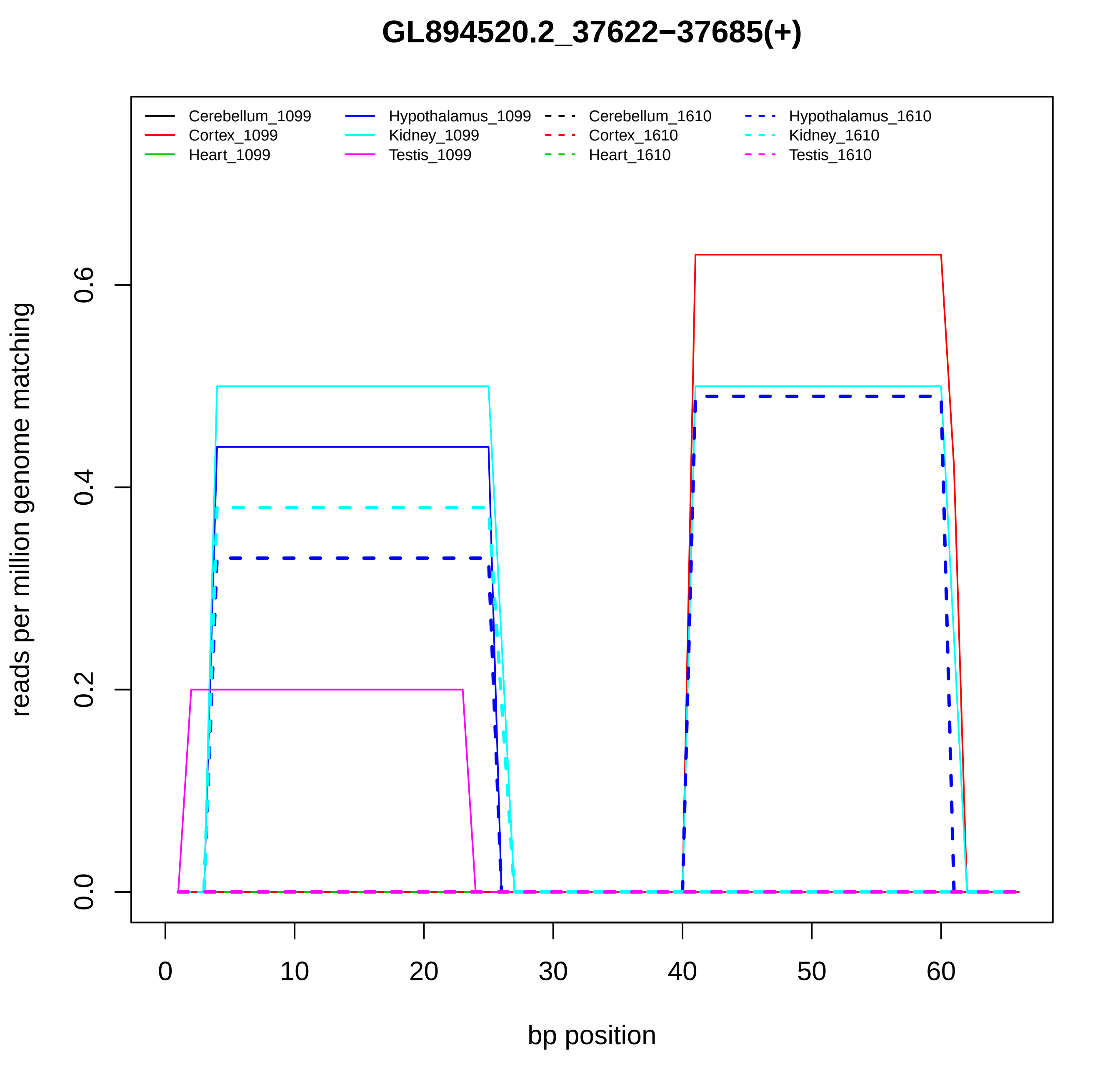
GL894520.2_37622−37685(+)
Cerebellum_1099
Cortex_1099
Heart_1099
Hypothalamus_1099
Kidney_1099
Testis_1099
Cerebellum_1610
Cortex_1610
Heart_1610
Hypothalamus_1610
Kidney_1610
Testis_1610
reads per million genome matching
0.0
0.2
0.4
0.6
0
10
20
30
40
50
60
bp position

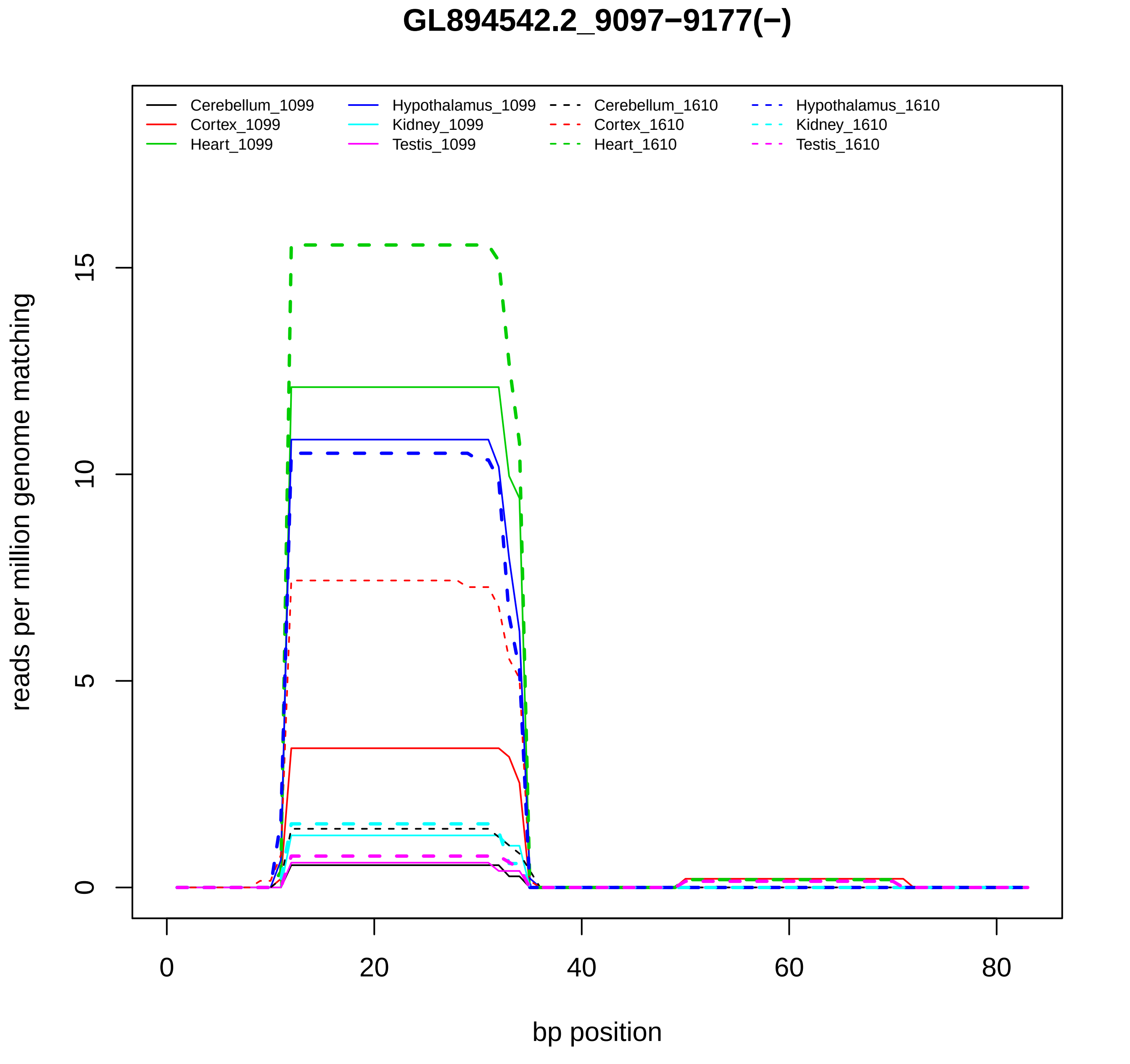

GL894542.2_9097−9177(−)
Cerebellum_1099
Cortex_1099
Heart_1099
Hypothalamus_1099
Kidney_1099
Testis_1099
Cerebellum_1610
Cortex_1610
Heart_1610
Hypothalamus_1610
Kidney_1610
Testis_1610
reads per million genome matching
bp position
0
5
10
15
0
20
40
60
80

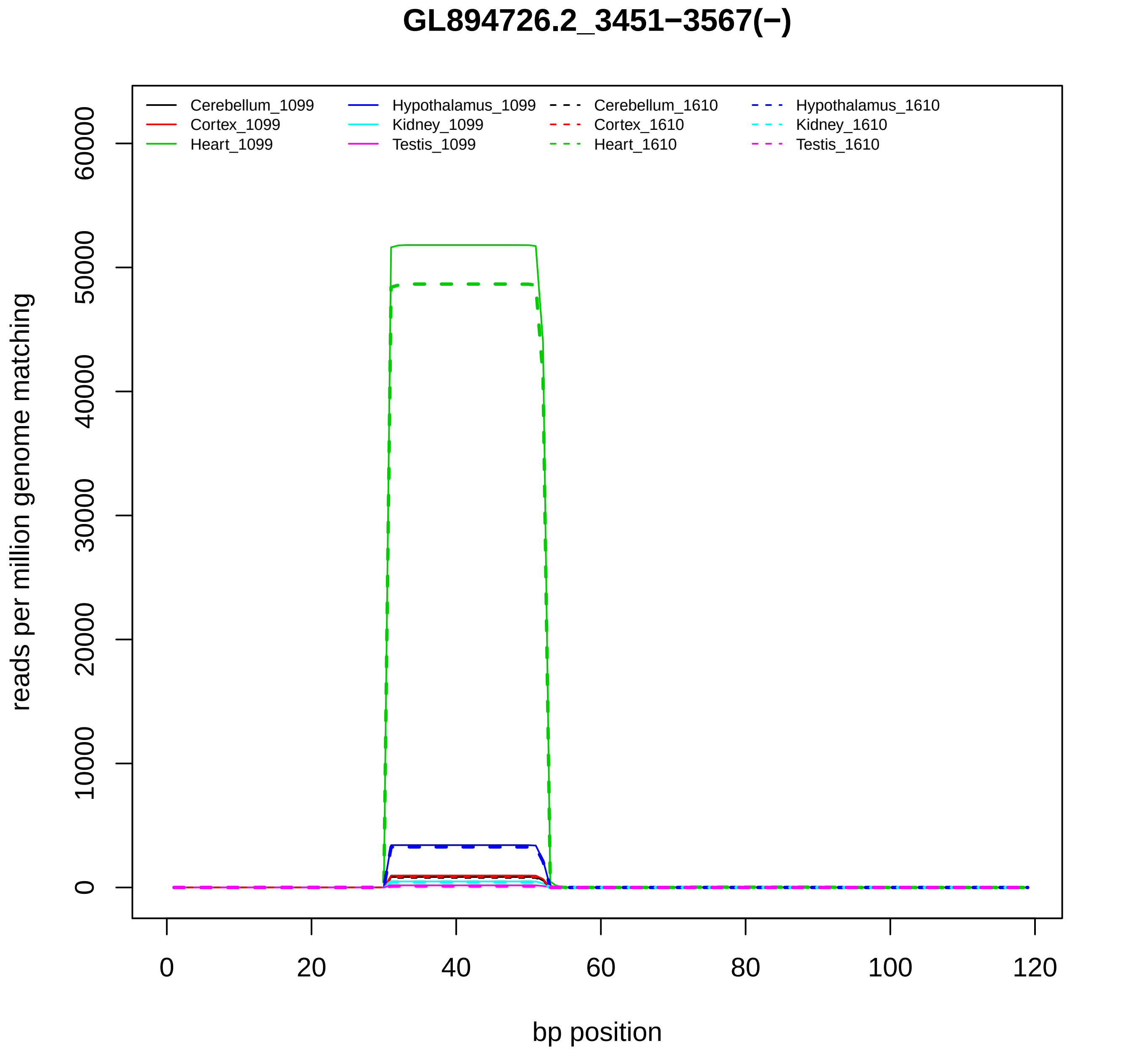

GL894726.2_3451−3567(−)
Cerebellum_1099
Cortex_1099
Heart_1099
Hypothalamus_1099
Kidney_1099
Testis_1099
Cerebellum_1610
Cortex_1610
Heart_1610
Hypothalamus_1610
Kidney_1610
Testis_1610
reads per million genome matching
0
10000
20000
30000
40000
50000
60000
0
20
40
60
80
100
120
bp position

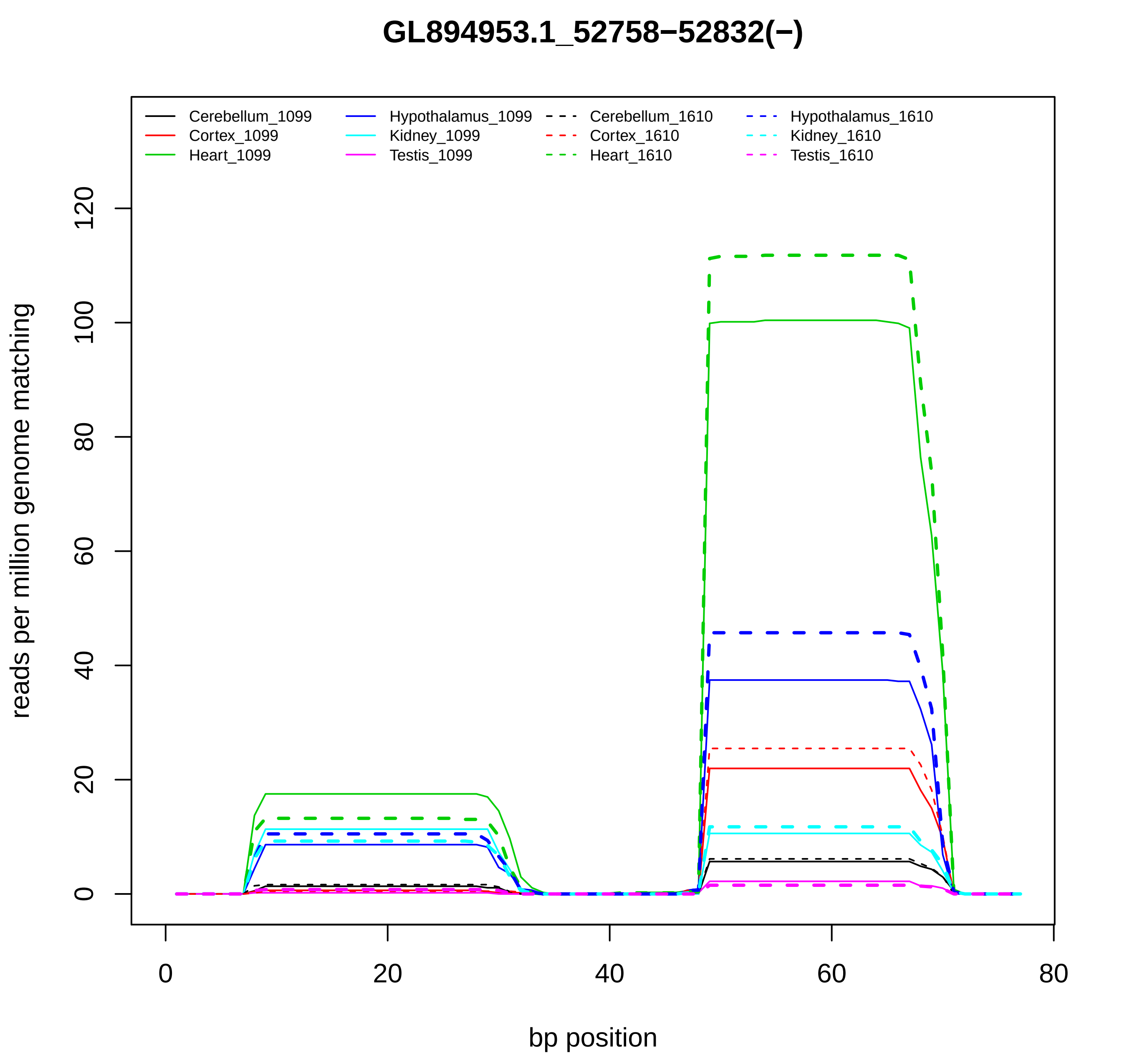

GL894953.1_52758−52832(−)
Cerebellum_1099
Cortex_1099
Heart_1099
Hypothalamus_1099
Kidney_1099
Testis_1099
Cerebellum_1610
Cortex_1610
Heart_1610
Hypothalamus_1610
Kidney_1610
Testis_1610
reads per million genome matching
0
20
40
60
80
100
120
0
20
40
60
80
bp position

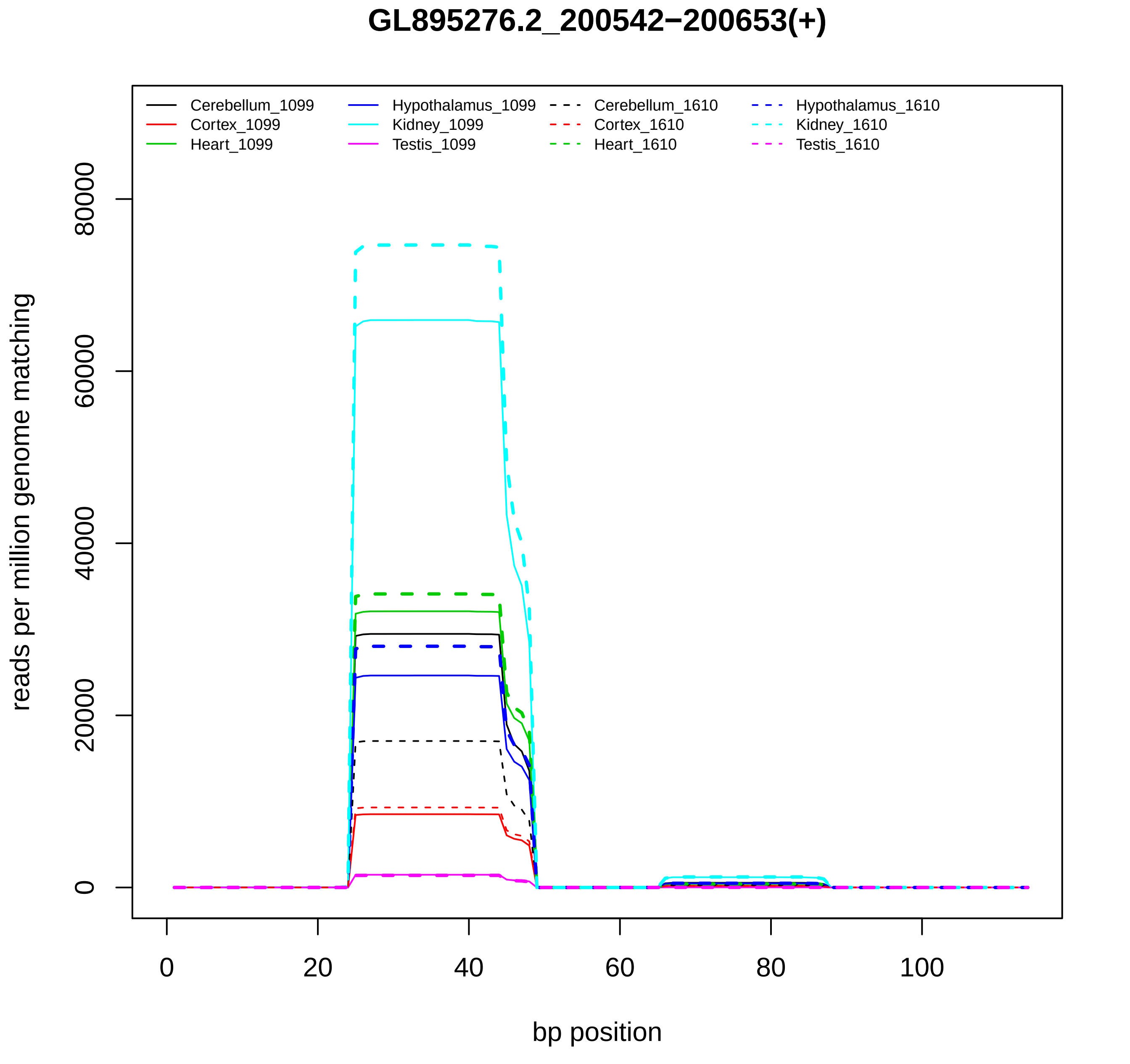
GL895276.2_200542−200653(+)
Cerebellum_1099
Cortex_1099
Heart_1099
Hypothalamus_1099
Kidney_1099
Testis_1099
Cerebellum_1610
Cortex_1610
Heart_1610
Hypothalamus_1610
Kidney_1610
Testis_1610
reads per million genome matching
0
20000
40000
60000
80000
0
20
40
60
80
100
bp position

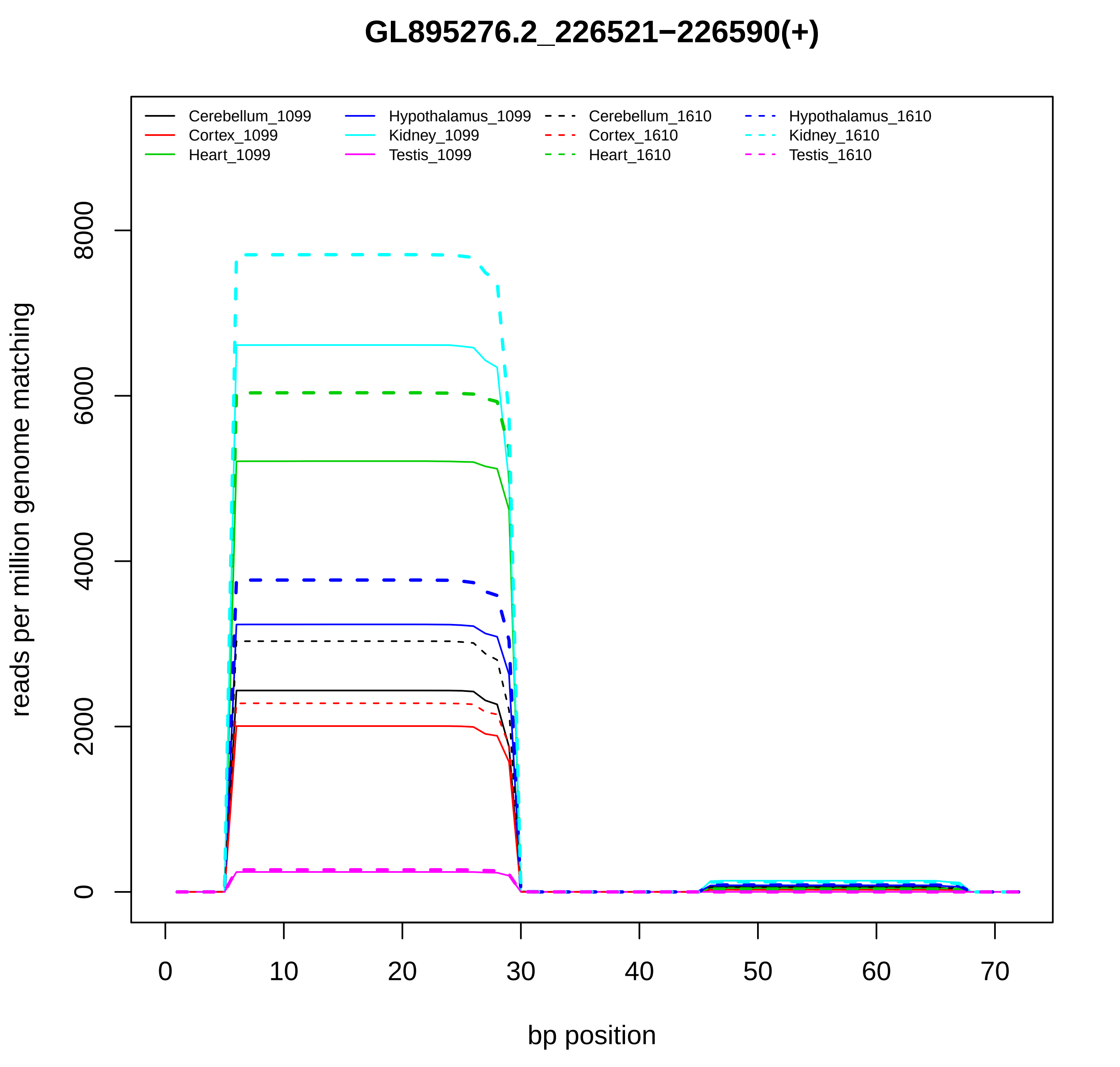

GL895276.2_226521−226590(+)
Cerebellum_1099
Cortex_1099
Heart_1099
Hypothalamus_1099
Kidney_1099
Testis_1099
Cerebellum_1610
Cortex_1610
Heart_1610
Hypothalamus_1610
Kidney_1610
Testis_1610
reads per million genome matching
0
2000
4000
6000
8000
0
10
20
30
40
50
60
70
bp position

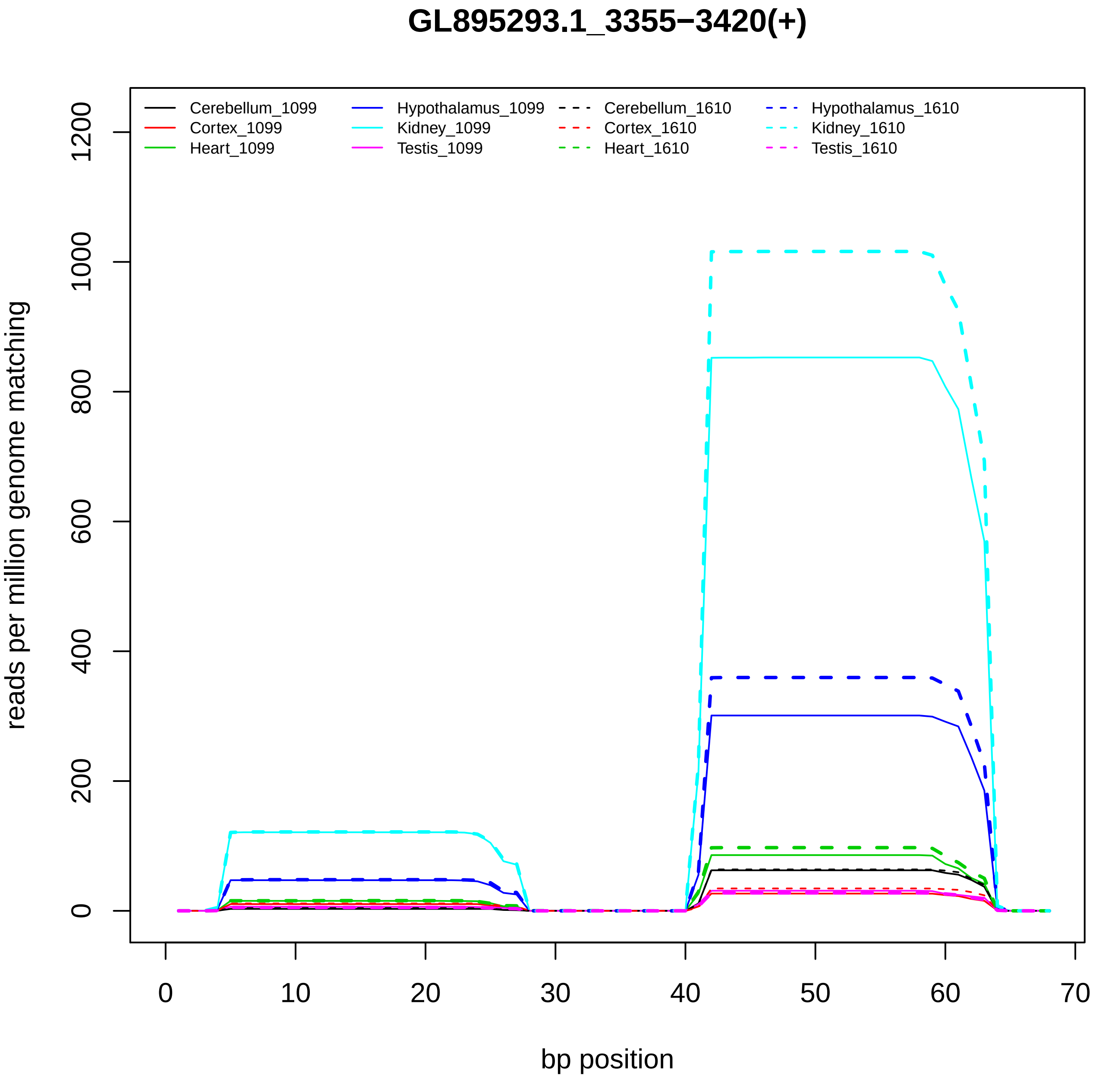

GL895293.1_3355−3420(+)
Cerebellum_1099
Cortex_1099
Heart_1099
Hypothalamus_1099
Kidney_1099
Testis_1099
Cerebellum_1610
Cortex_1610
Heart_1610
Hypothalamus_1610
Kidney_1610
Testis_1610
reads per million genome matching
0
200
400
600
800
1000
1200
bp position
0
10
20
30
40
50
60
70

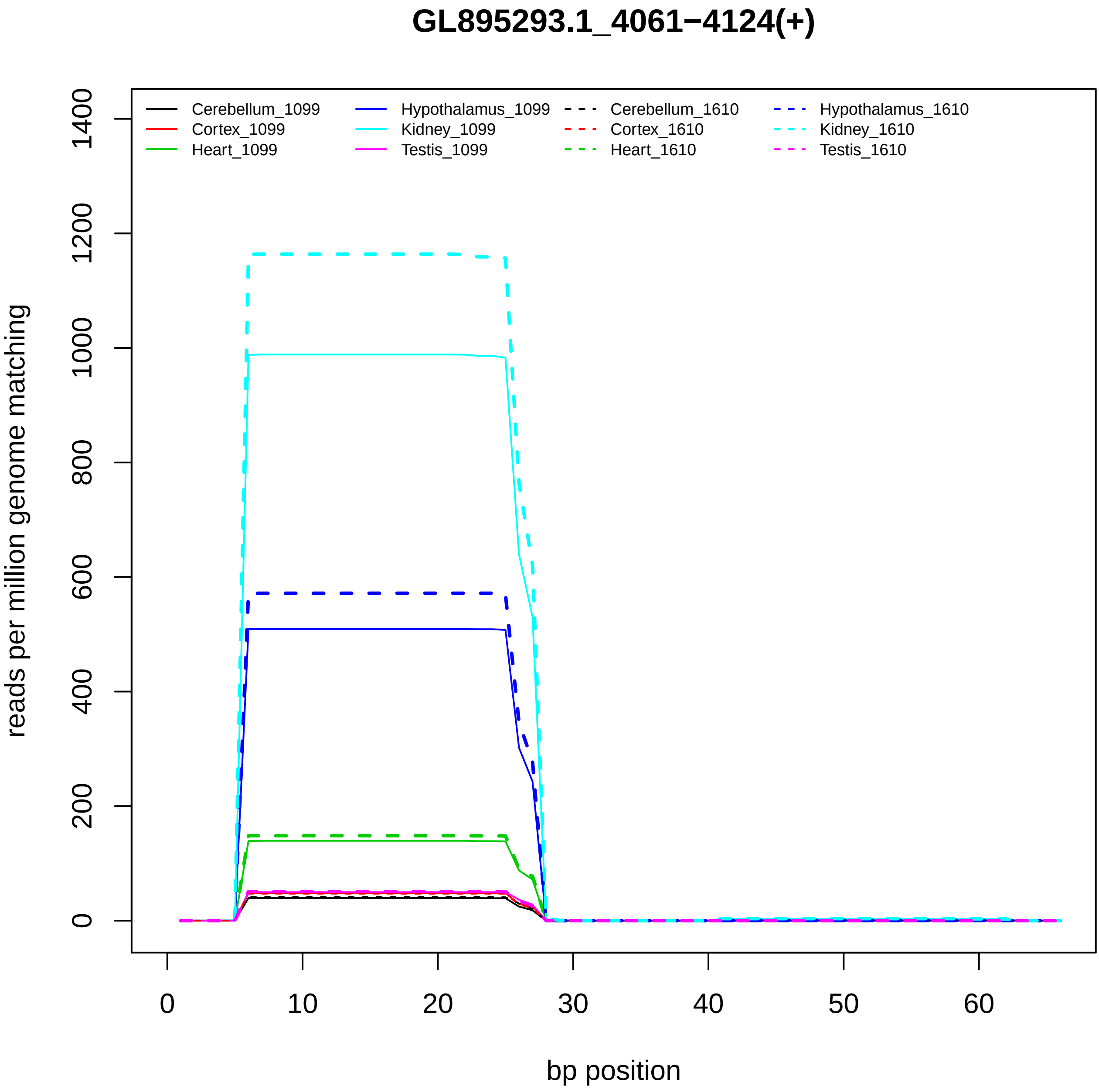

GL895293.1_4061−4124(+)
Cerebellum_1099
Cortex_1099
Heart_1099
Hypothalamus_1099
Kidney_1099
Testis_1099
Cerebellum_1610
Cortex_1610
Heart_1610
Hypothalamus_1610
Kidney_1610
Testis_1610
reads per million genome matching
0
200
400
600
800
1000
1200
1400
0
10
20
30
40
50
60
bp position

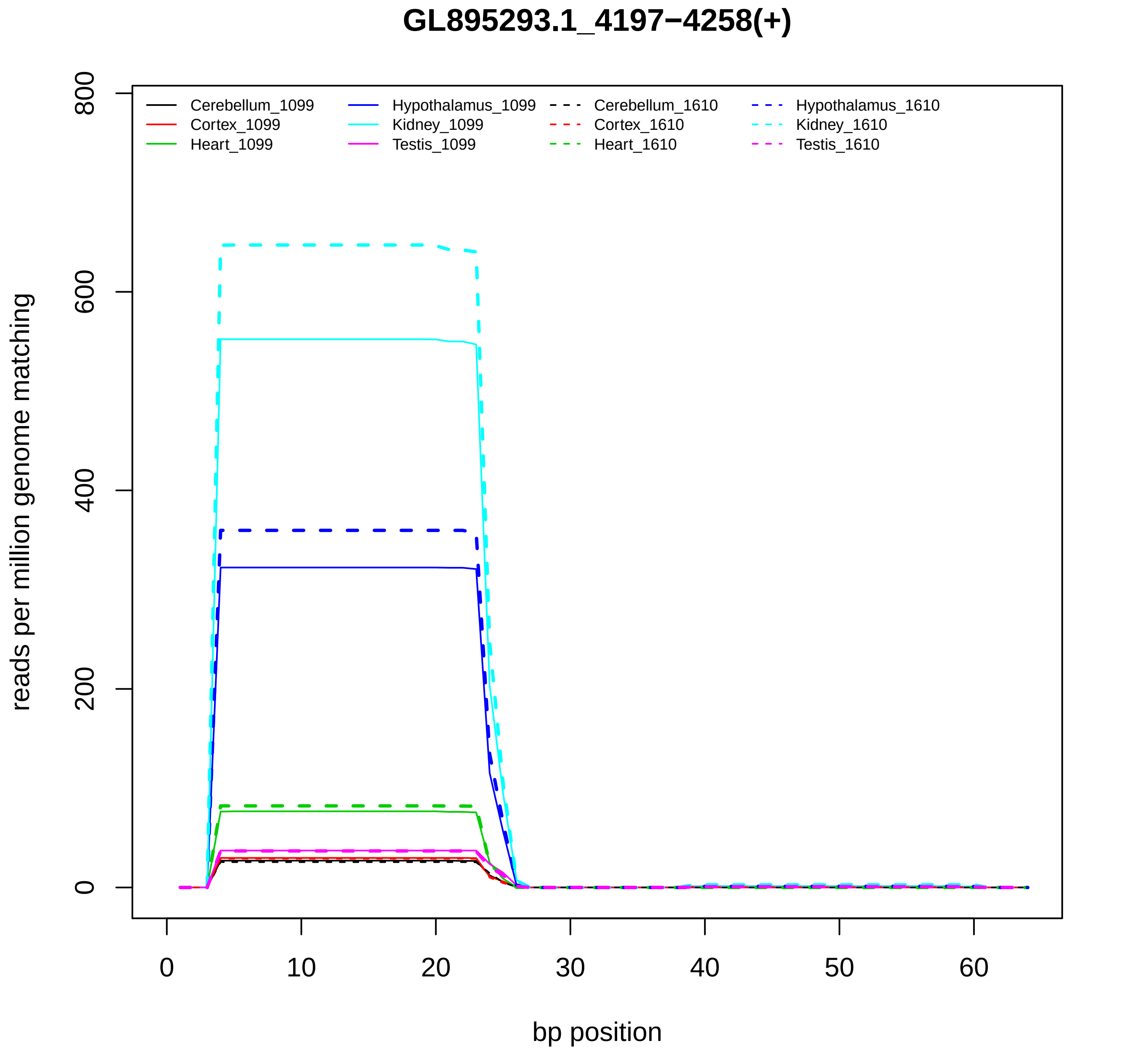

GL895293.1_4197−4258(+)
Cerebellum_1099
Cortex_1099
Heart_1099
Hypothalamus_1099
Kidney_1099
Testis_1099
Cerebellum_1610
Cortex_1610
Heart_1610
Hypothalamus_1610
Kidney_1610
Testis_1610
reads per million genome matching
bp position
0
200
400
600
800
0
10
20
30
40
50
60

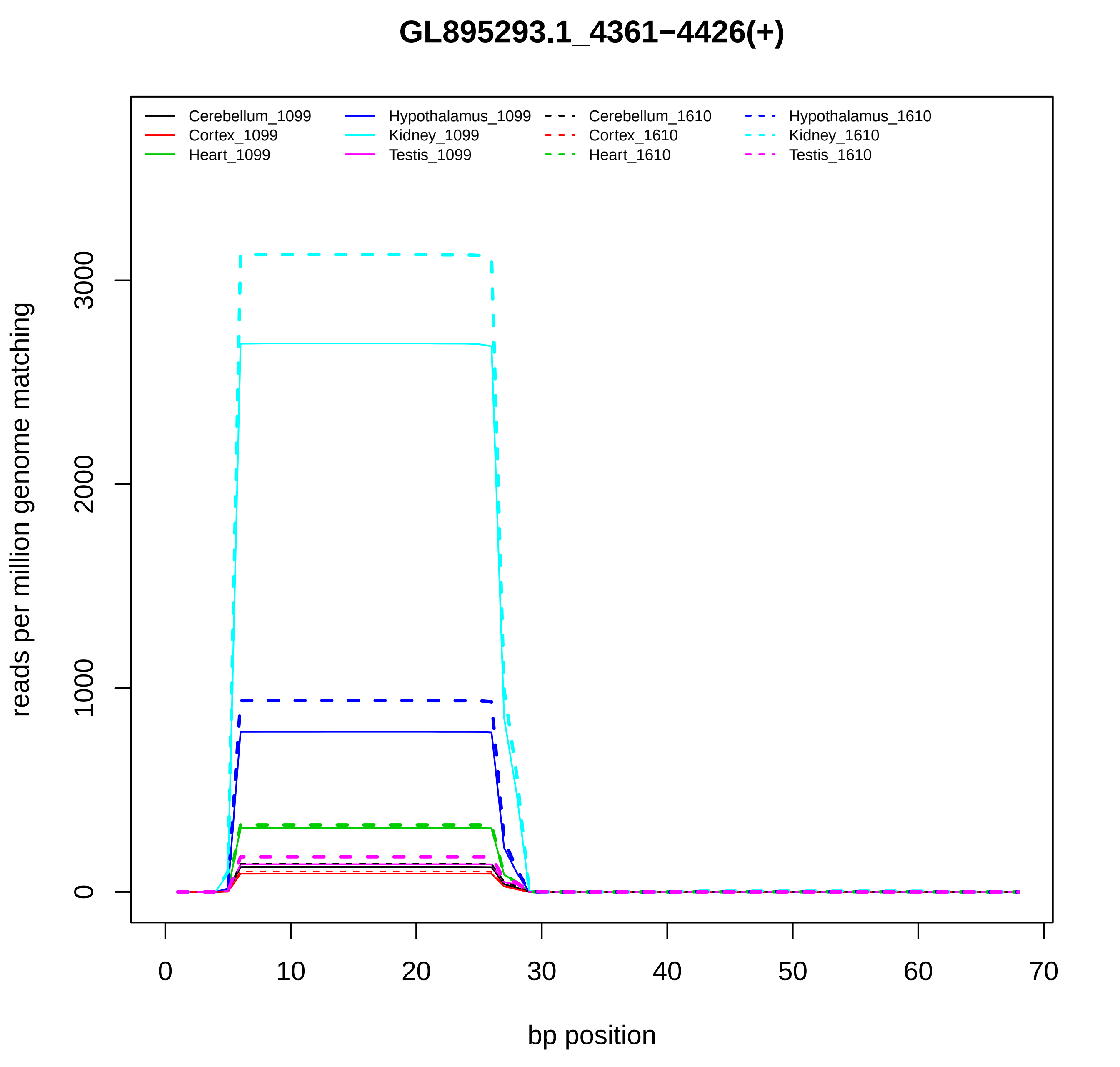

GL895293.1_4361−4426(+)
Cerebellum_1099
Cortex_1099
Heart_1099
Hypothalamus_1099
Kidney_1099
Testis_1099
Cerebellum_1610
Cortex_1610
Heart_1610
Hypothalamus_1610
Kidney_1610
Testis_1610
reads per million genome matching
0
1000
2000
3000
0
10
20
30
40
50
60
70
bp position

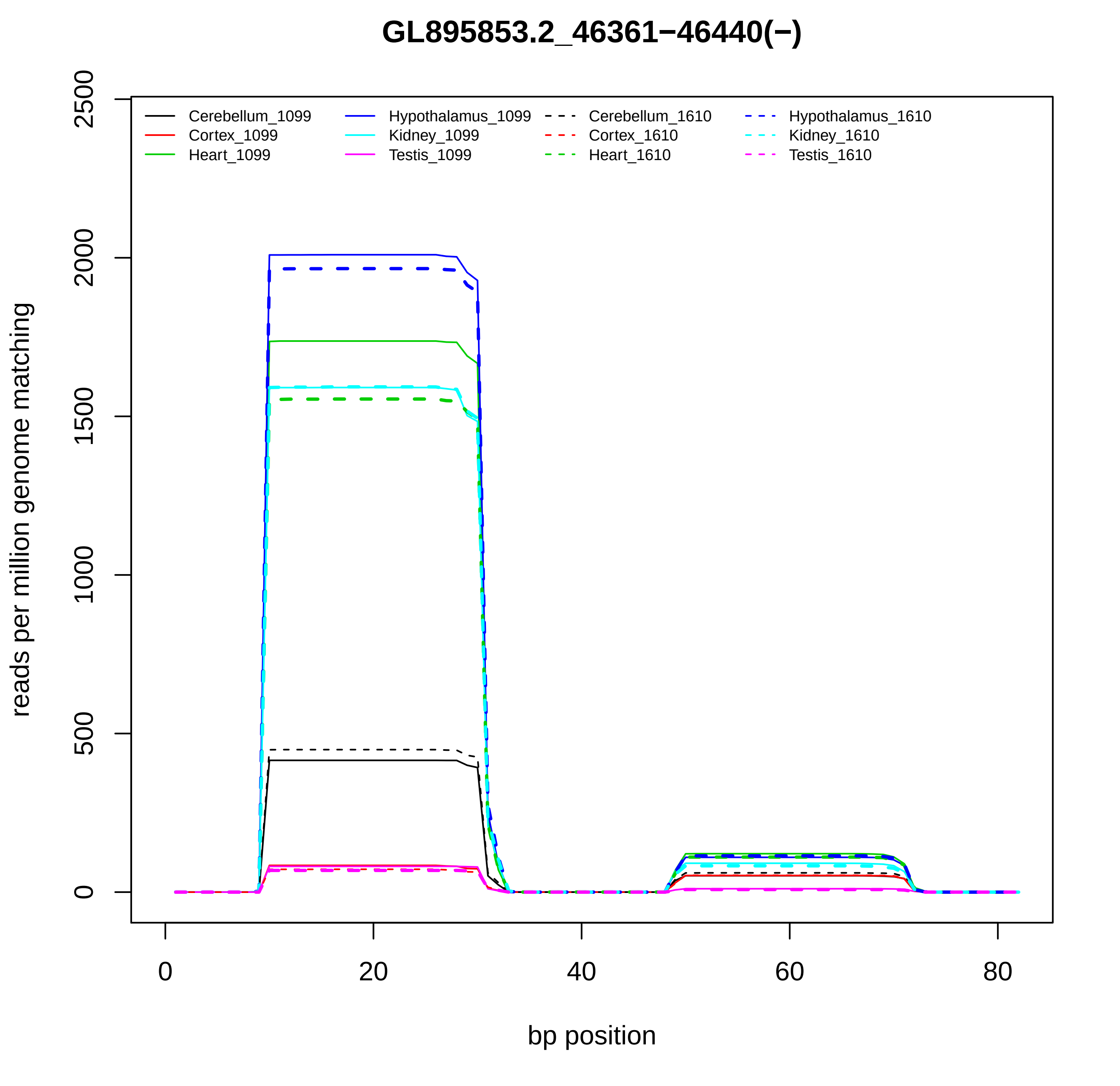
GL895853.2_46361−46440(−)
reads per million genome matching
bp position
0
500
1000
1500
2000
2500
0
20
40
60
80
Cerebellum_1099
Cortex_1099
Heart_1099
Hypothalamus_1099
Kidney_1099
Testis_1099
Cerebellum_1610
Cortex_1610
Heart_1610
Hypothalamus_1610
Kidney_1610
Testis_1610

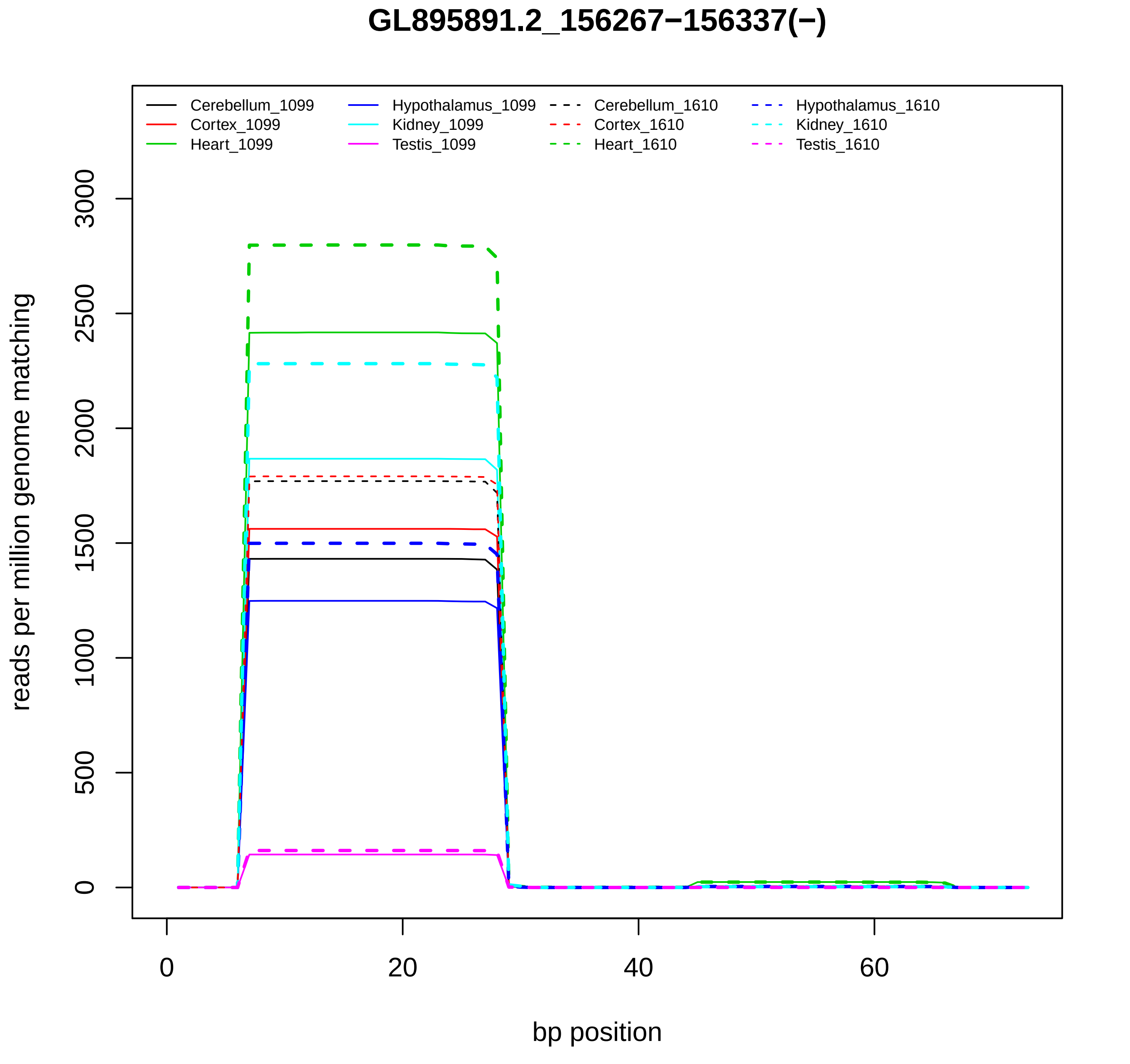
GL895891.2_156267−156337(−)
Cerebellum_1099
Cortex_1099
Heart_1099
Hypothalamus_1099
Kidney_1099
Testis_1099
Cerebellum_1610
Cortex_1610
Heart_1610
Hypothalamus_1610
Kidney_1610
Testis_1610
reads per million genome matching
0
500
1000
1500
2000
2500
3000
0
20
40
60
bp position

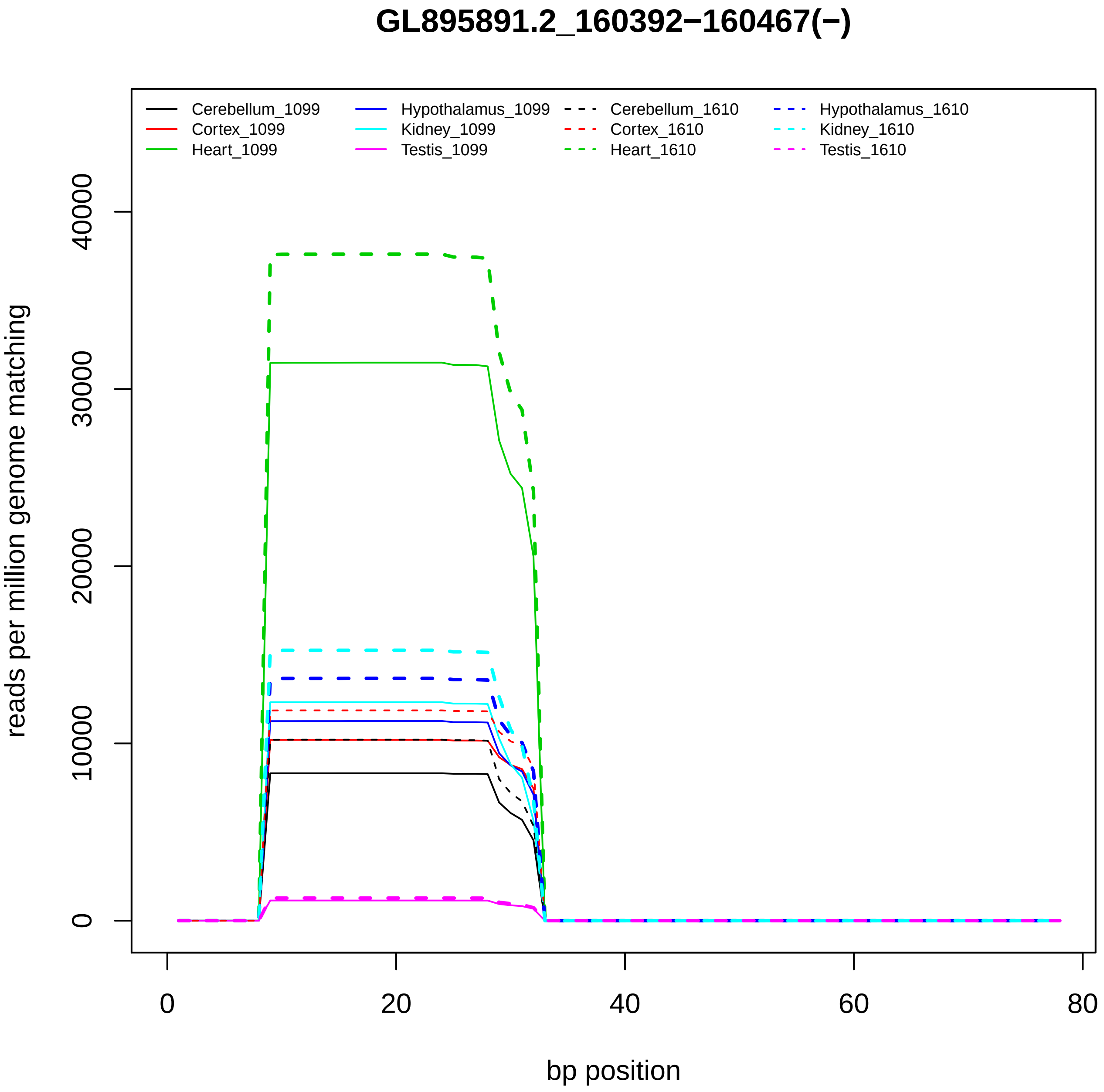

GL895891.2_160392−160467(−)
Cerebellum_1099
Cortex_1099
Heart_1099
Hypothalamus_1099
Kidney_1099
Testis_1099
Cerebellum_1610
Cortex_1610
Heart_1610
Hypothalamus_1610
Kidney_1610
Testis_1610
reads per million genome matching
0
10000
20000
30000
40000
0
20
40
60
80
bp position

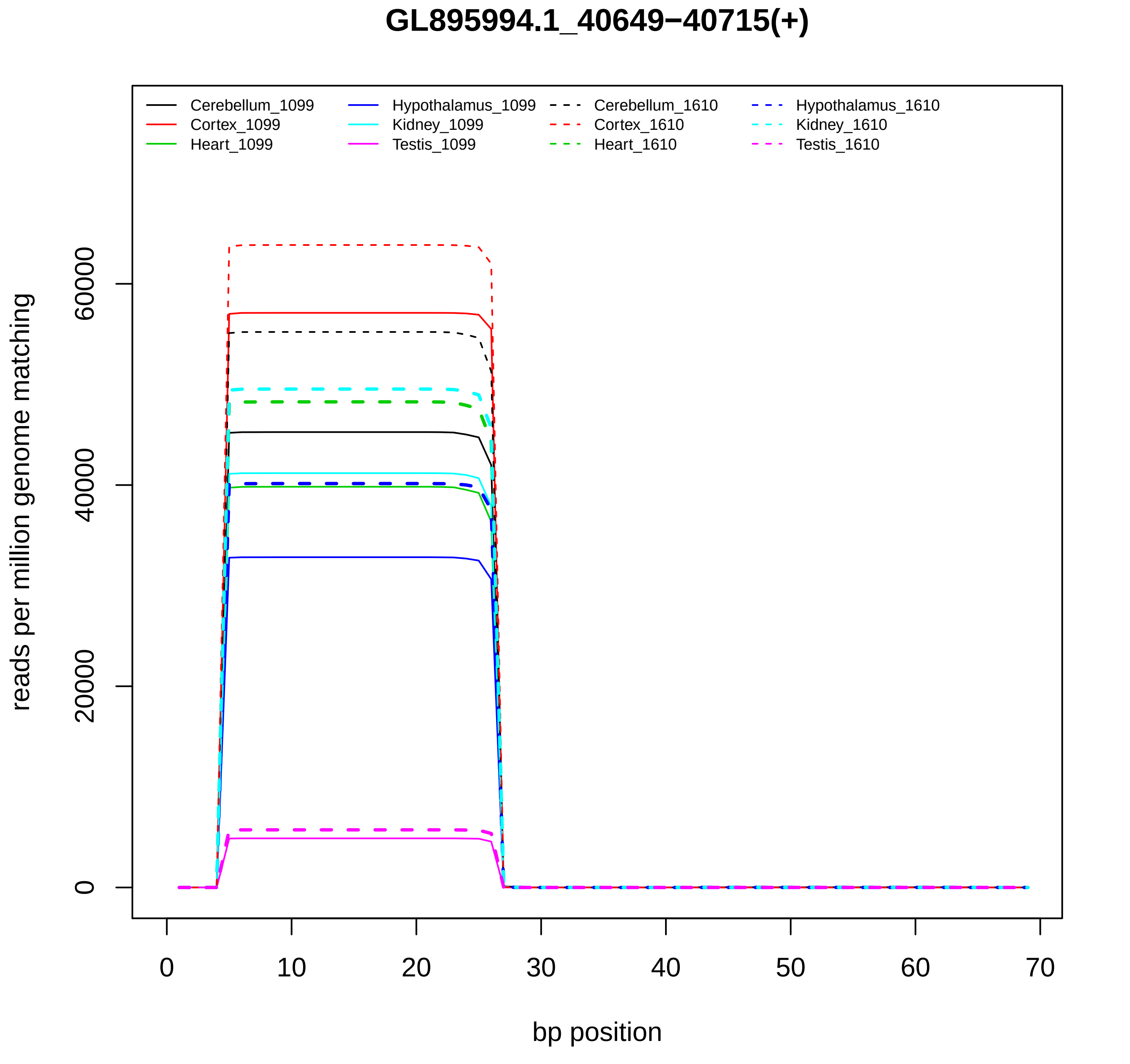

GL895994.1_40649−40715(+)
Cerebellum_1099
Cortex_1099
Heart_1099
Hypothalamus_1099
Kidney_1099
Testis_1099
Cerebellum_1610
Cortex_1610
Heart_1610
Hypothalamus_1610
Kidney_1610
Testis_1610
reads per million genome matching
0
20000
40000
60000
0
10
20
30
40
50
60
70
bp position

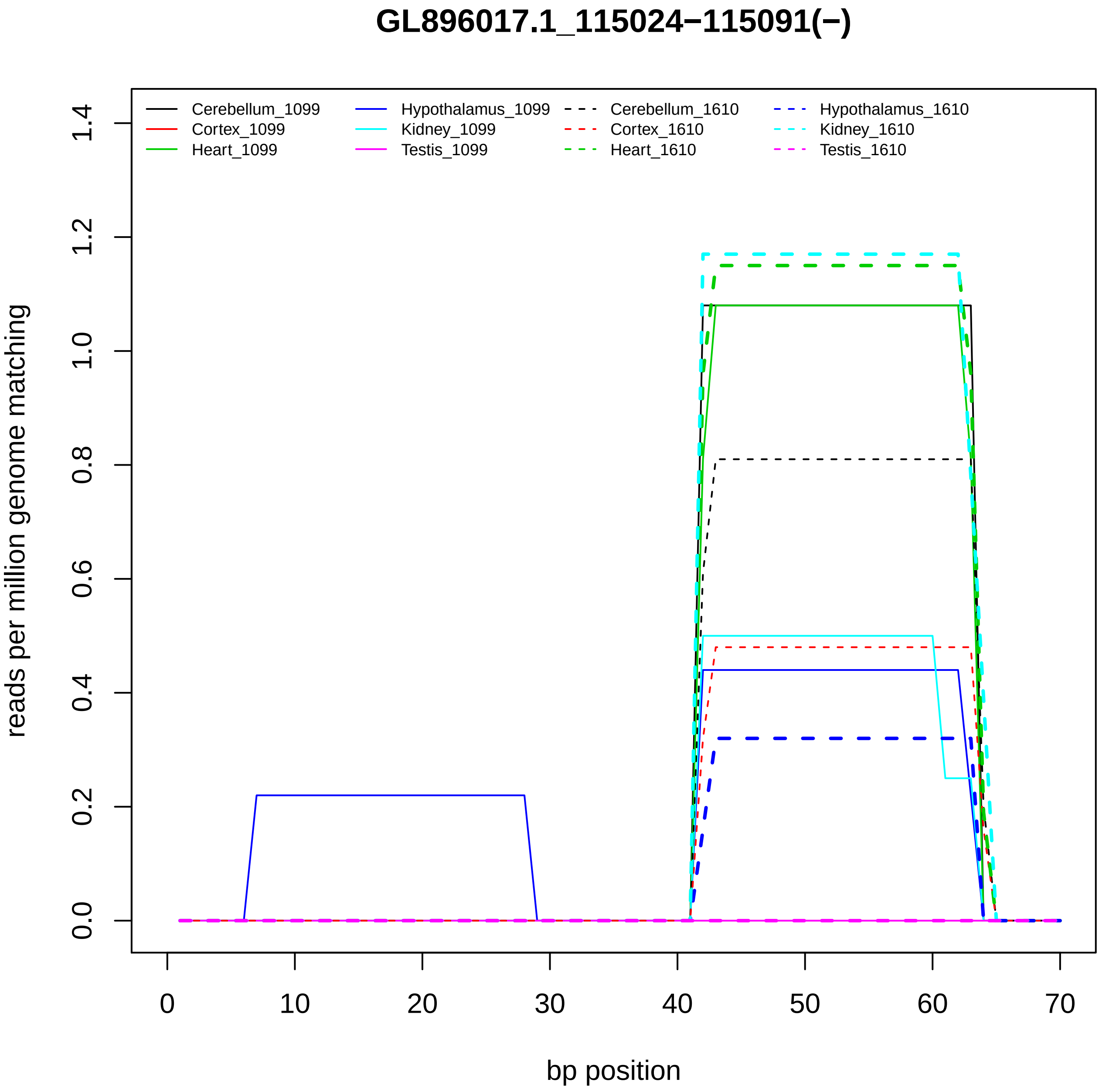

GL896017.1_115024−115091(−)
Cerebellum_1099
Cortex_1099
Heart_1099
Hypothalamus_1099
Kidney_1099
Testis_1099
Cerebellum_1610
Cortex_1610
Heart_1610
Hypothalamus_1610
Kidney_1610
Testis_1610
reads per million genome matching
bp position
0.0
0.2
0.4
0.6
0.8
1.0
1.2
1.4
0
10
20
30
40
50
60
70

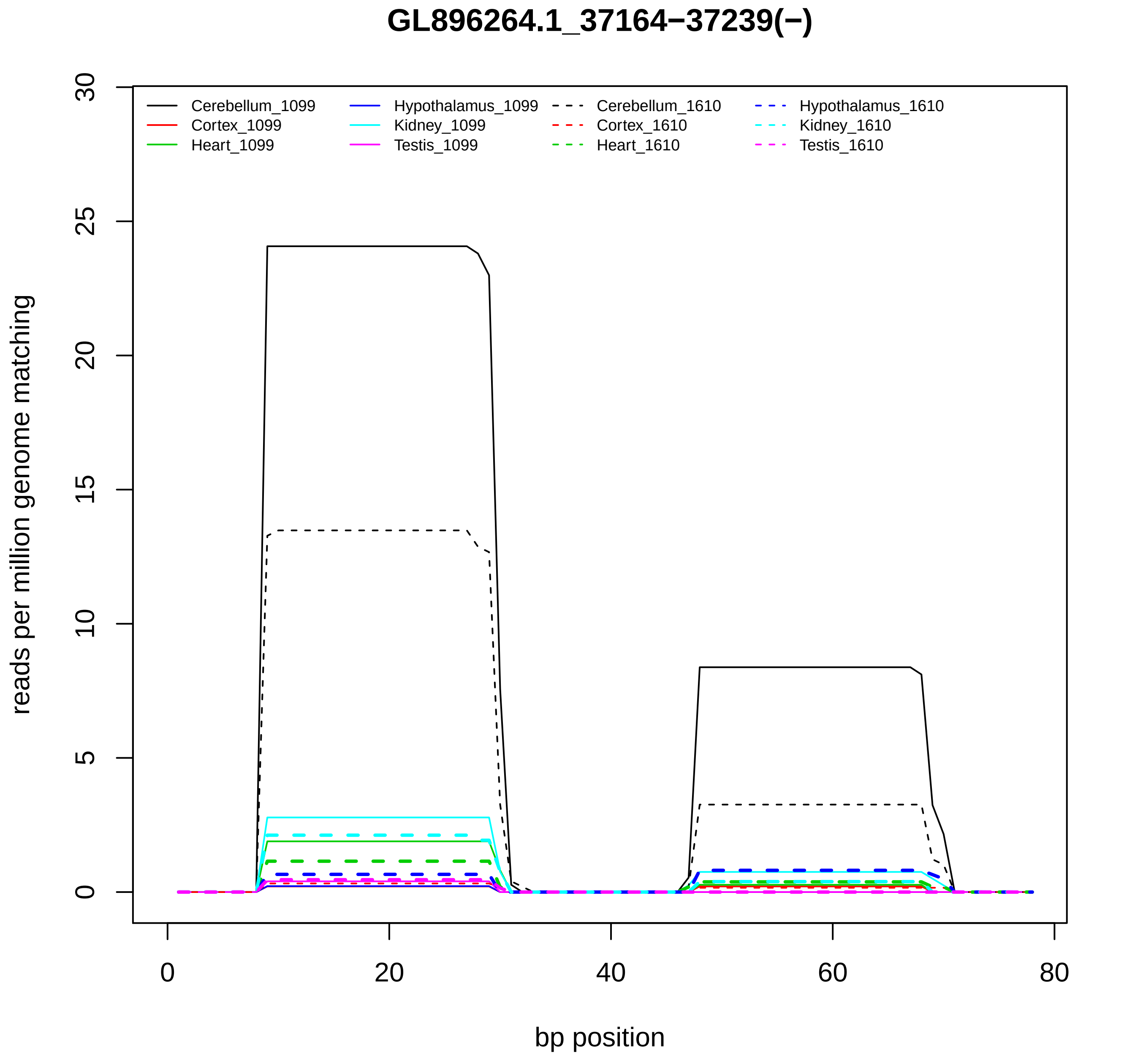
GL896264.1_37164−37239(−)
Cerebellum_1099
Cortex_1099
Heart_1099
Hypothalamus_1099
Kidney_1099
Testis_1099
Cerebellum_1610
Cortex_1610
Heart_1610
Hypothalamus_1610
Kidney_1610
Testis_1610
reads per million genome matching
bp position
0
5
10
15
20
25
30
0
20
40
60
80

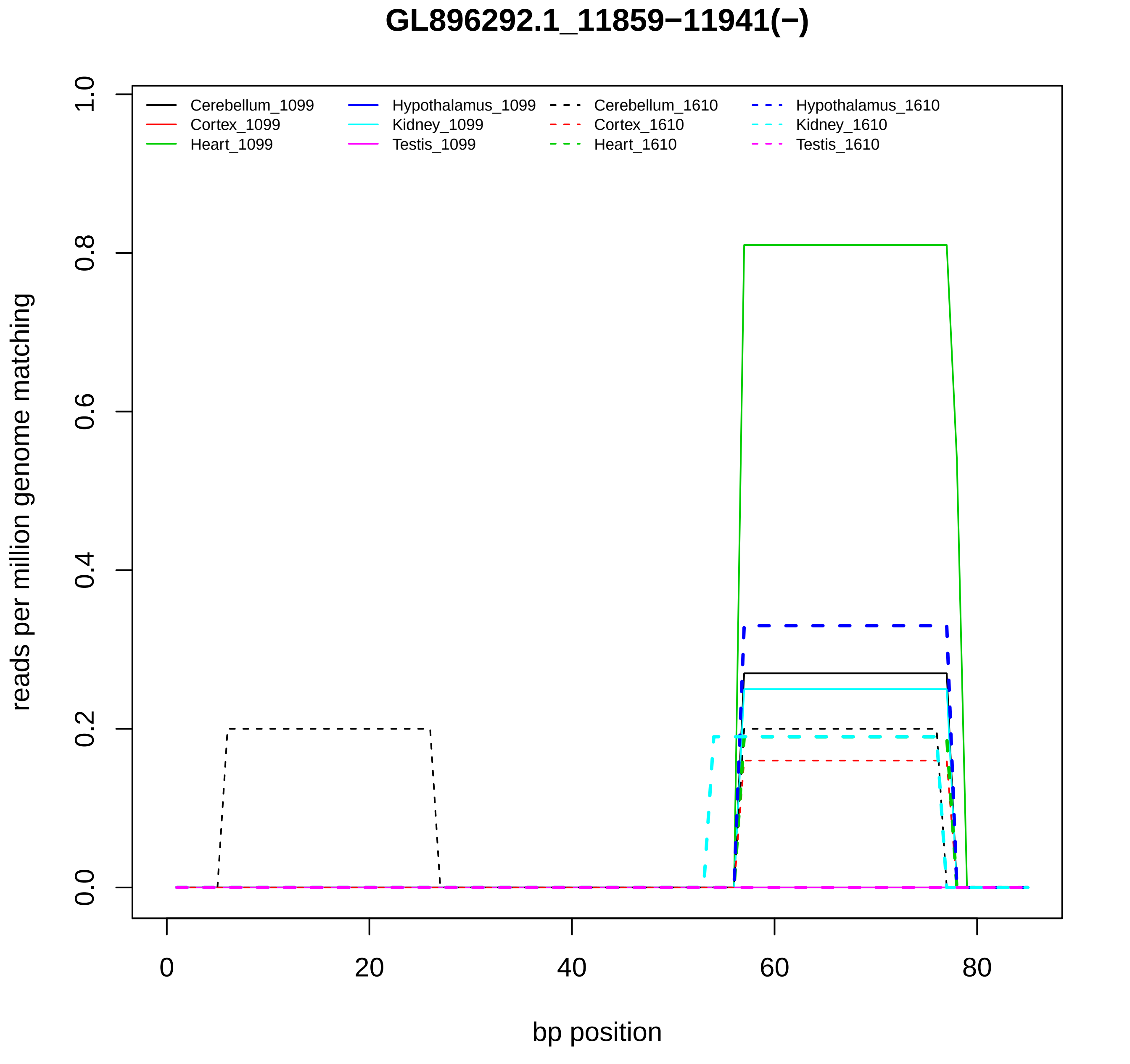

GL896292.1_11859−11941(−)
Cerebellum_1099
Cortex_1099
Heart_1099
Hypothalamus_1099
Kidney_1099
Testis_1099
Cerebellum_1610
Cortex_1610
Heart_1610
Hypothalamus_1610
Kidney_1610
Testis_1610
reads per million genome matching
bp position
0.0
0.2
0.4
0.6
0.8
1.0
0
20
40
60
80

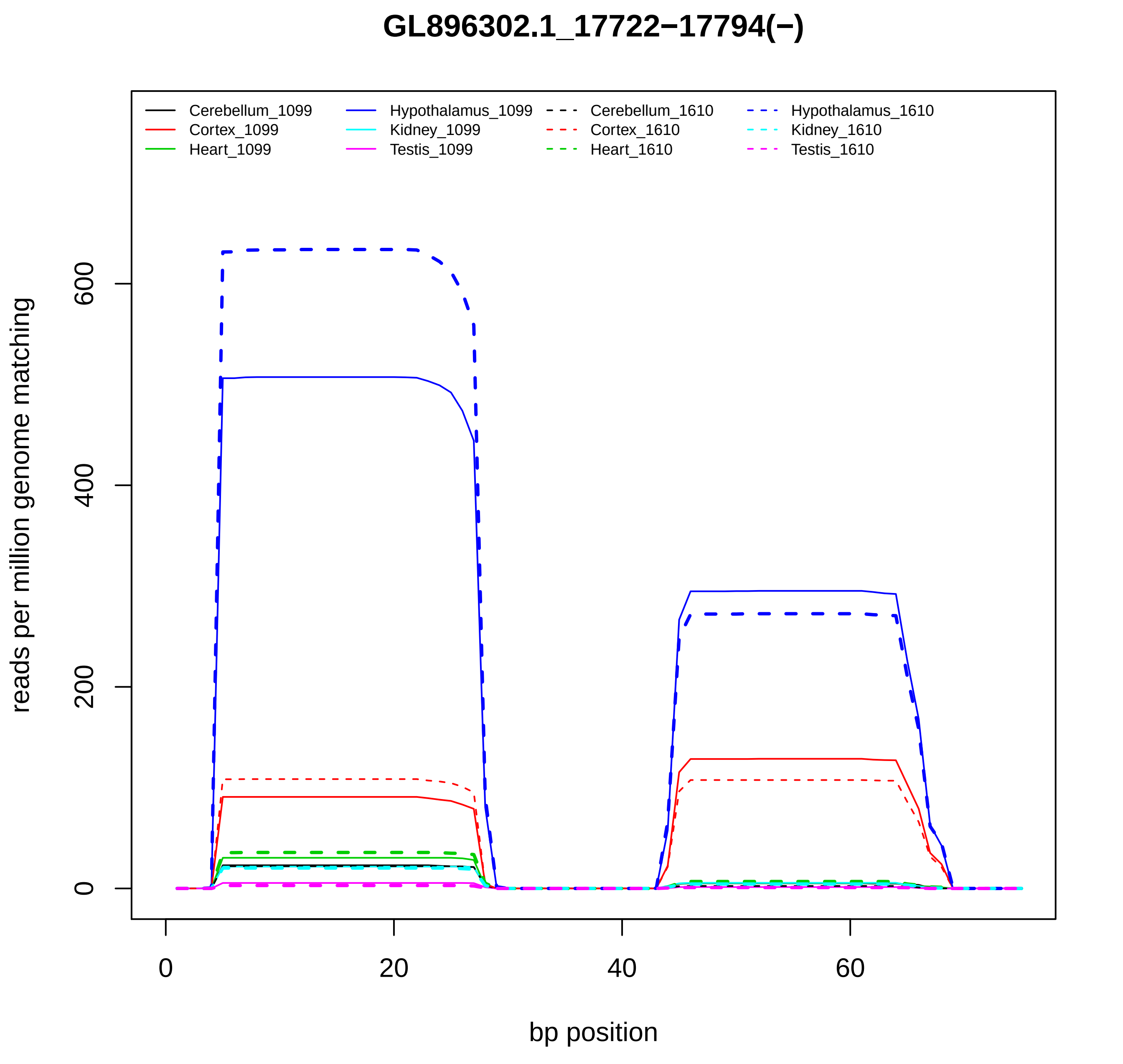
GL896302.1_17722−17794(−)
Cerebellum_1099
Cortex_1099
Heart_1099
Hypothalamus_1099
Kidney_1099
Testis_1099
Cerebellum_1610
Cortex_1610
Heart_1610
Hypothalamus_1610
Kidney_1610
Testis_1610
reads per million genome matching
0
200
400
600
0
20
40
60
bp position

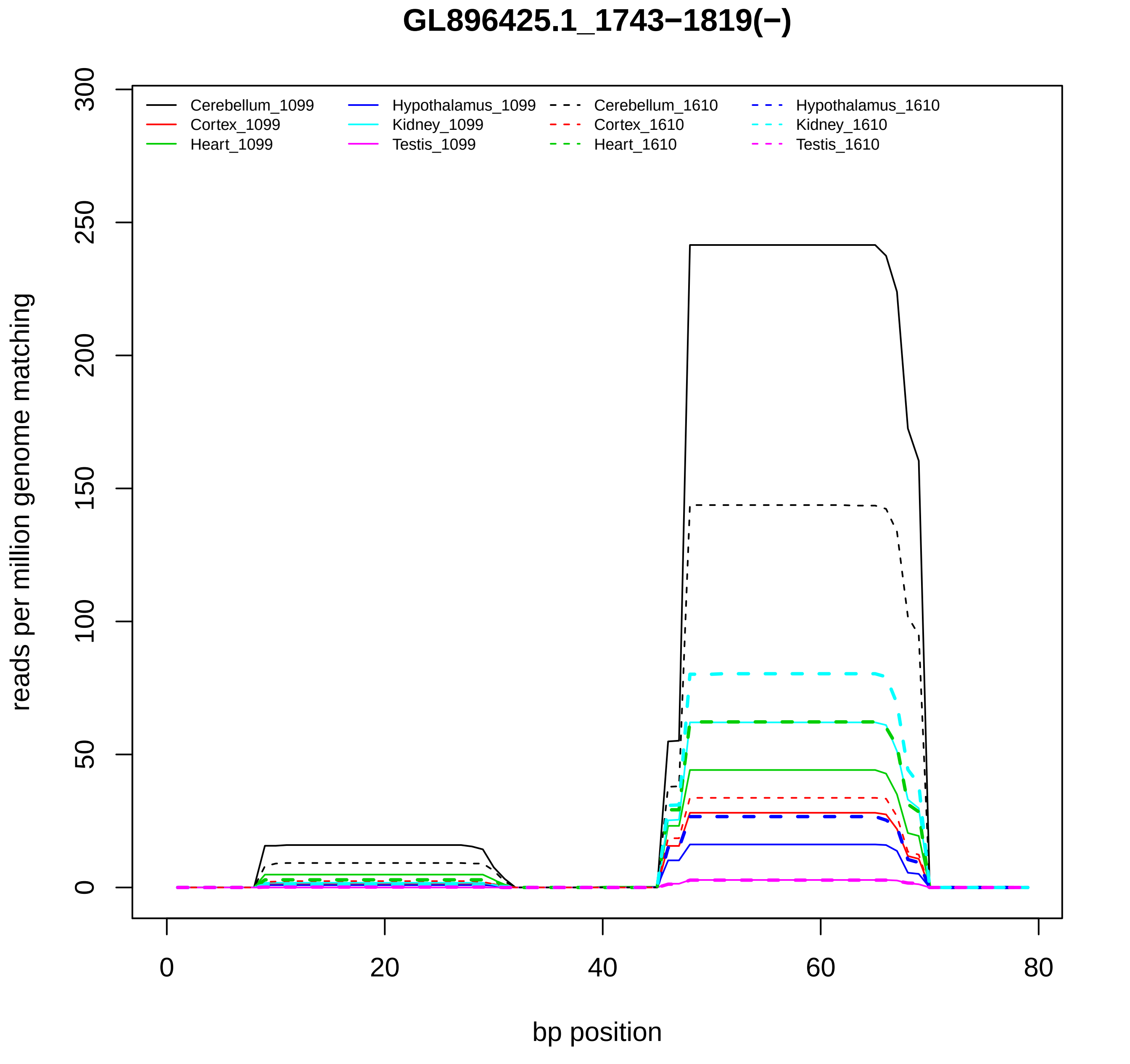
GL896425.1_1743−1819(−)
Cerebellum_1099
Cortex_1099
Heart_1099
Hypothalamus_1099
Kidney_1099
Testis_1099
Cerebellum_1610
Cortex_1610
Heart_1610
Hypothalamus_1610
Kidney_1610
Testis_1610
reads per million genome matching
bp position
0
50
100
150
200
250
300
0
20
40
60
80

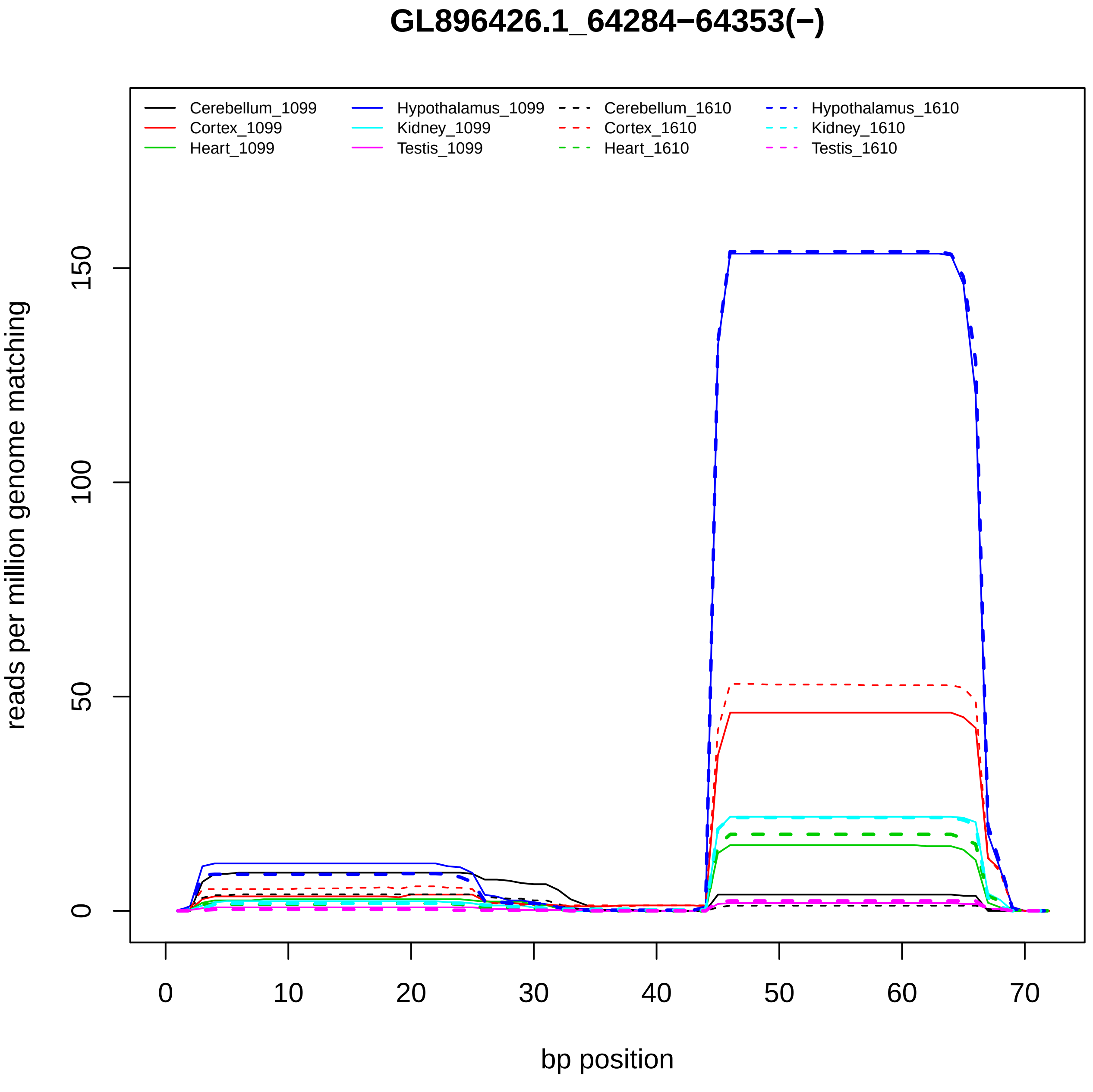

GL896426.1_64284−64353(−)
Cerebellum_1099
Cortex_1099
Heart_1099
Hypothalamus_1099
Kidney_1099
Testis_1099
Cerebellum_1610
Cortex_1610
Heart_1610
Hypothalamus_1610
Kidney_1610
Testis_1610
reads per million genome matching
0
50
100
150
0
10
20
30
40
50
60
70
bp position

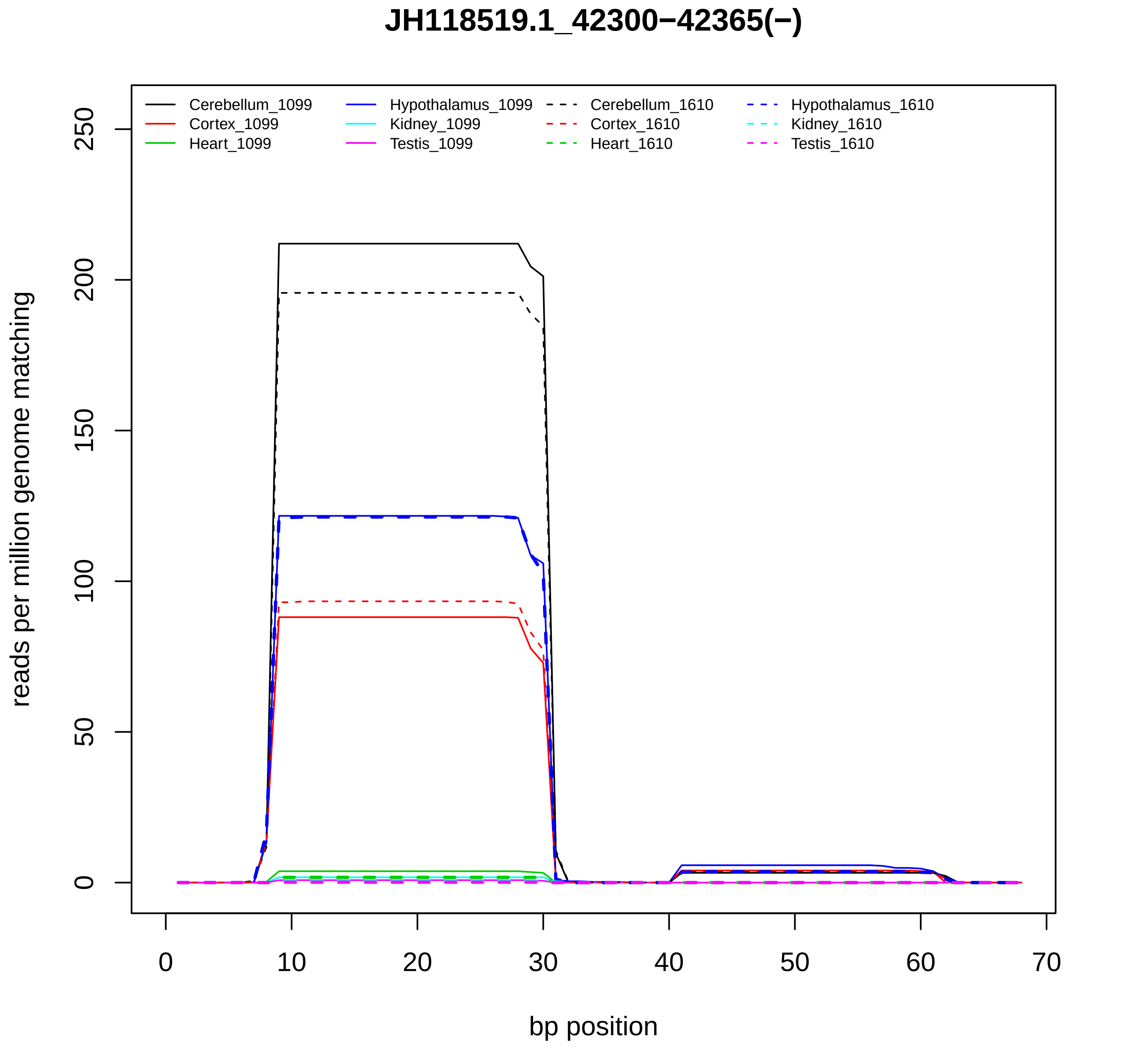

JH118519.1_42300−42365(−)
Cerebellum_1099
Cortex_1099
Heart_1099
Hypothalamus_1099
Kidney_1099
Testis_1099
Cerebellum_1610
Cortex_1610
Heart_1610
Hypothalamus_1610
Kidney_1610
Testis_1610
reads per million genome matching
0
50
100
150
200
250
0
10
20
30
40
50
60
70
bp position

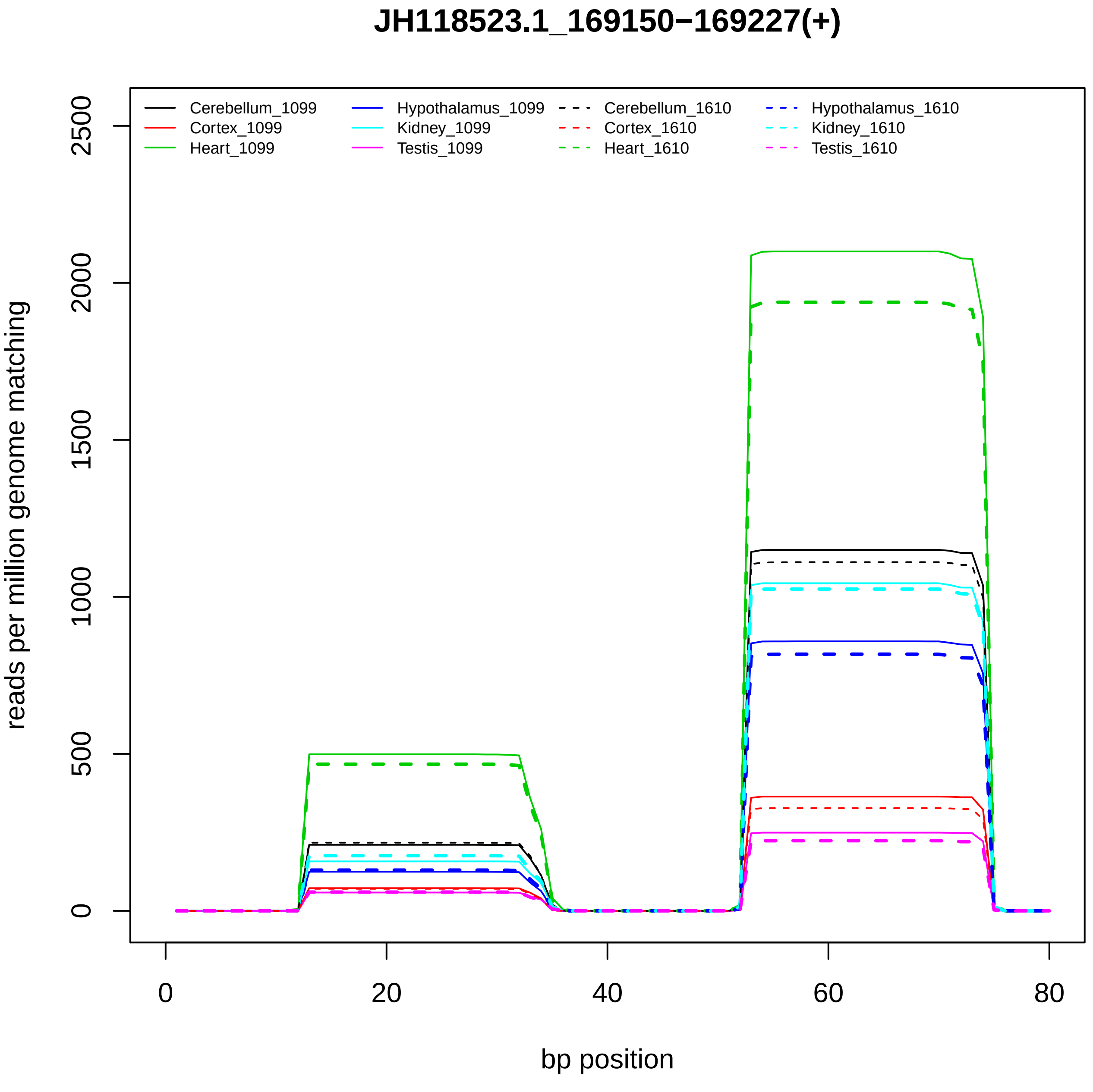

JH118523.1_169150−169227(+)
Cerebellum_1099
Cortex_1099
Heart_1099
Hypothalamus_1099
Kidney_1099
Testis_1099
Cerebellum_1610
Cortex_1610
Heart_1610
Hypothalamus_1610
Kidney_1610
Testis_1610
reads per million genome matching
bp position
0
500
1000
1500
2000
2500
0
20
40
60
80

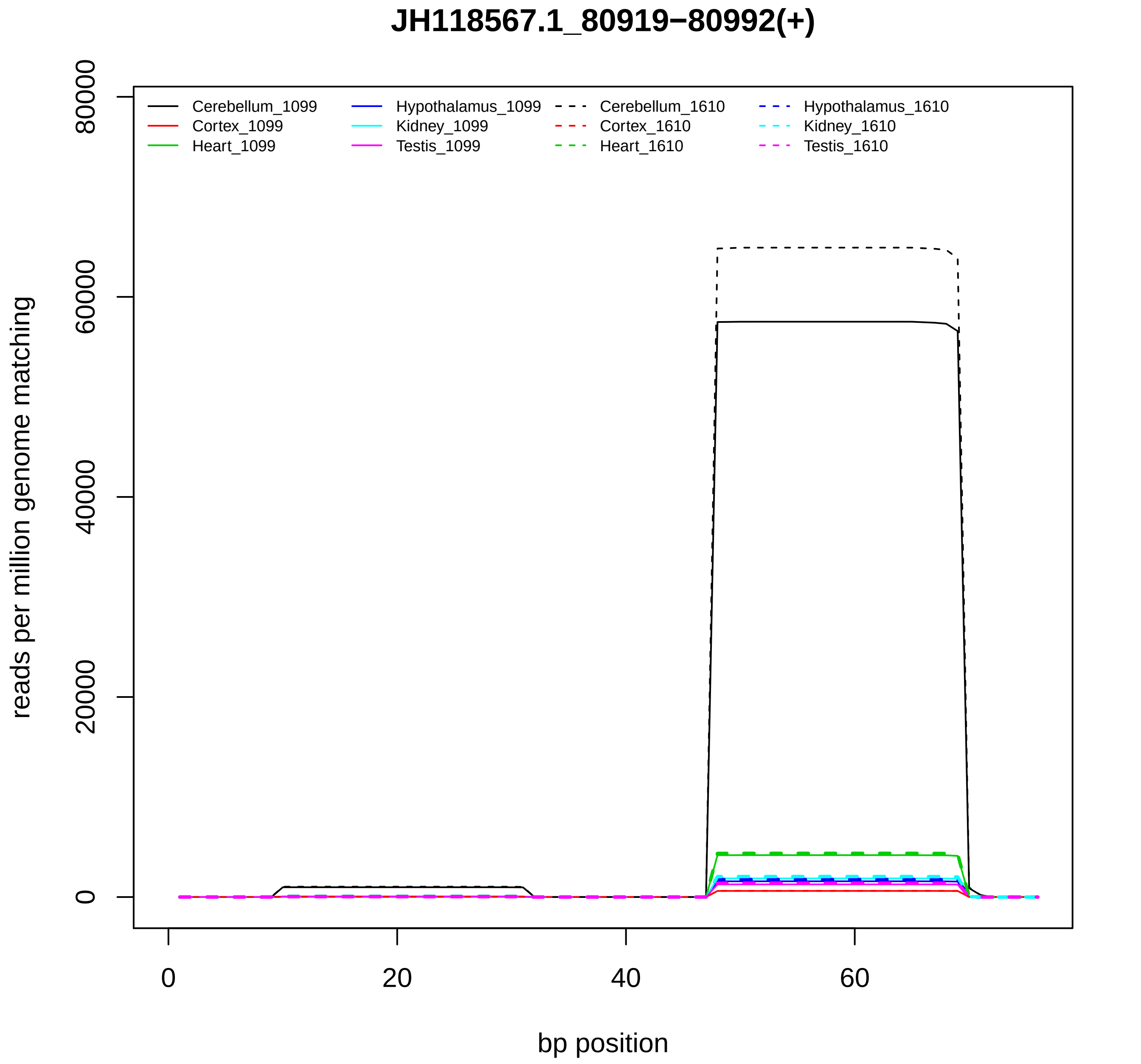

JH118567.1_80919−80992(+)
Cerebellum_1099
Cortex_1099
Heart_1099
Hypothalamus_1099
Kidney_1099
Testis_1099
Cerebellum_1610
Cortex_1610
Heart_1610
Hypothalamus_1610
Kidney_1610
Testis_1610
reads per million genome matching
0
20000
40000
60000
80000
0
20
40
60
bp position

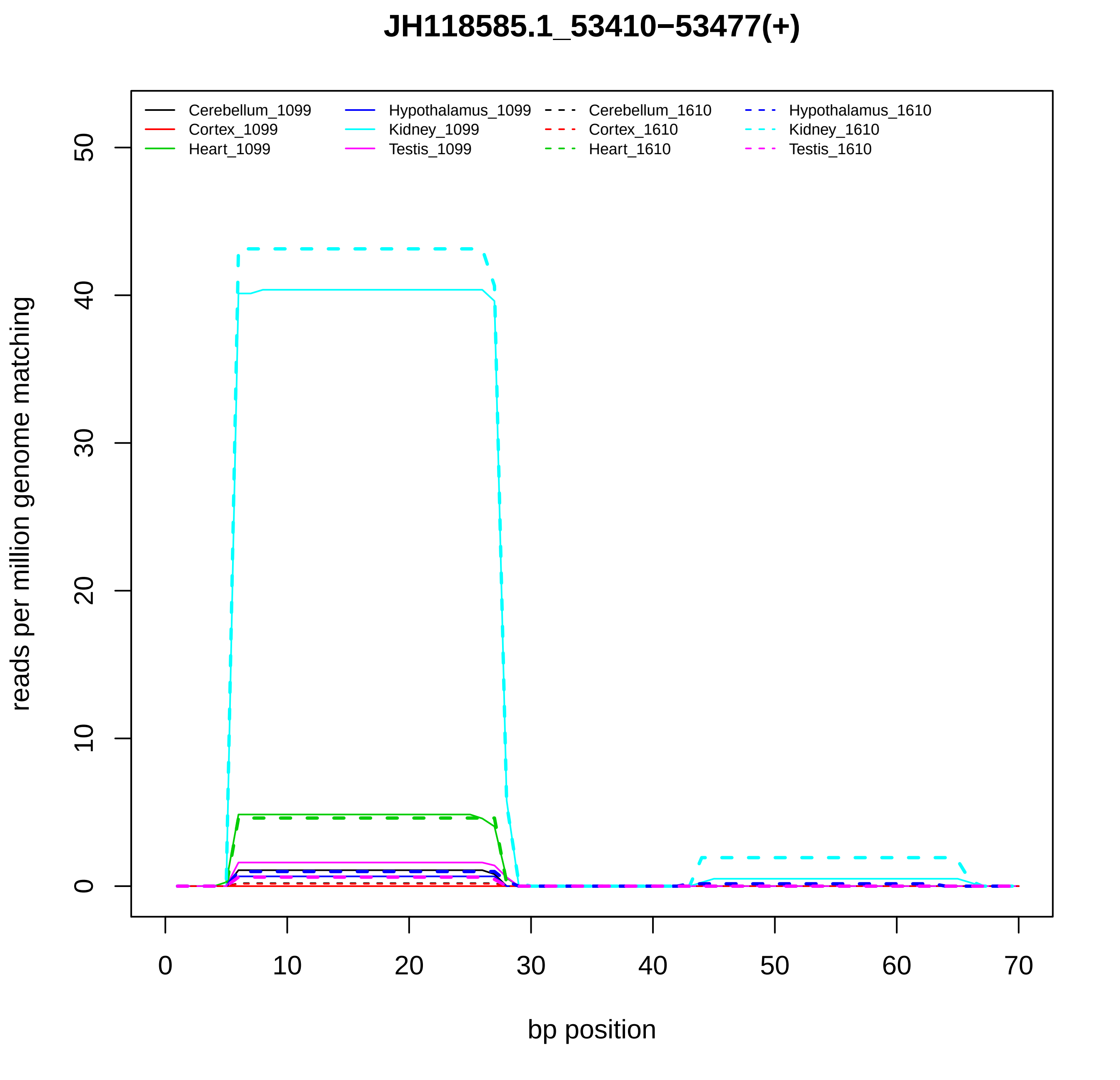

JH118585.1_53410−53477(+)
Cerebellum_1099
Cortex_1099
Heart_1099
Hypothalamus_1099
Kidney_1099
Testis_1099
Cerebellum_1610
Cortex_1610
Heart_1610
Hypothalamus_1610
Kidney_1610
Testis_1610
reads per million genome matching
bp position
0
10
20
30
40
50
60
70

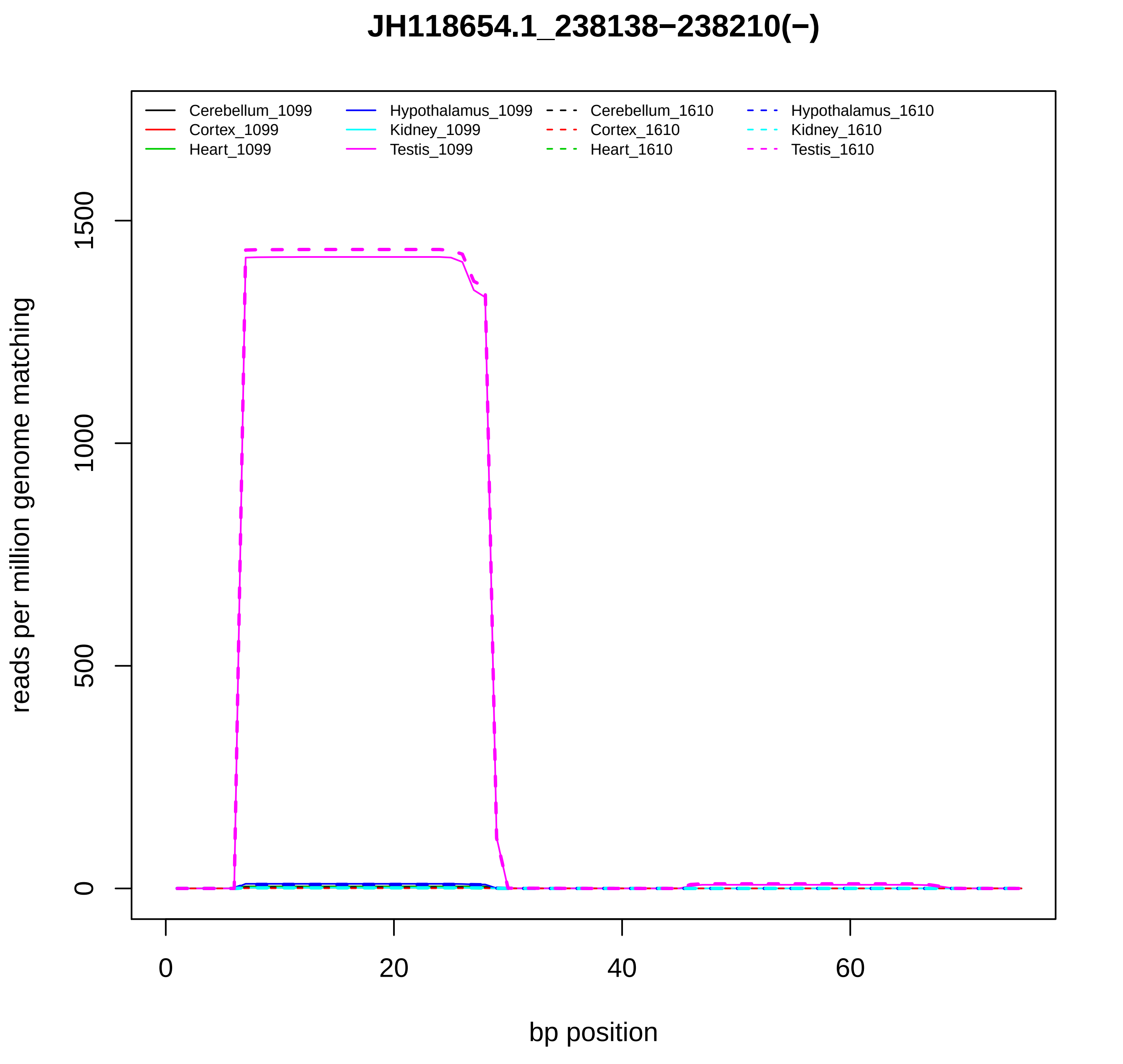

JH118654.1_238138−238210(−)
Cerebellum_1099
Cortex_1099
Heart_1099
Hypothalamus_1099
Kidney_1099
Testis_1099
Cerebellum_1610
Cortex_1610
Heart_1610
Hypothalamus_1610
Kidney_1610
Testis_1610
reads per million genome matching
0
500
1000
1500
bp position
0
20
40
60

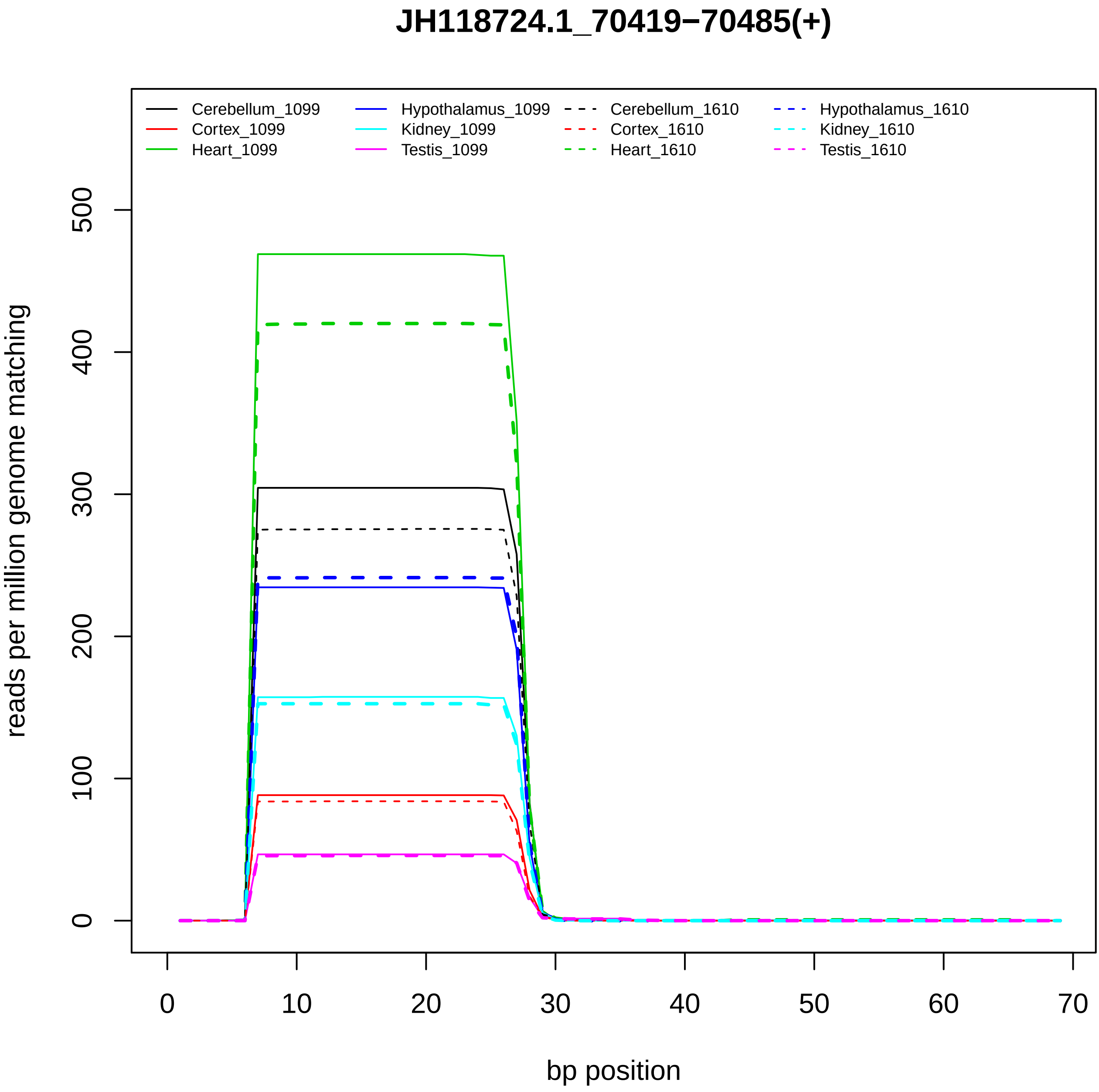

JH118724.1_70419−70485(+)
Cerebellum_1099
Cortex_1099
Heart_1099
Hypothalamus_1099
Kidney_1099
Testis_1099
Cerebellum_1610
Cortex_1610
Heart_1610
Hypothalamus_1610
Kidney_1610
Testis_1610
reads per million genome matching
0
100
200
300
400
500
0
10
20
30
40
50
60
70
bp position

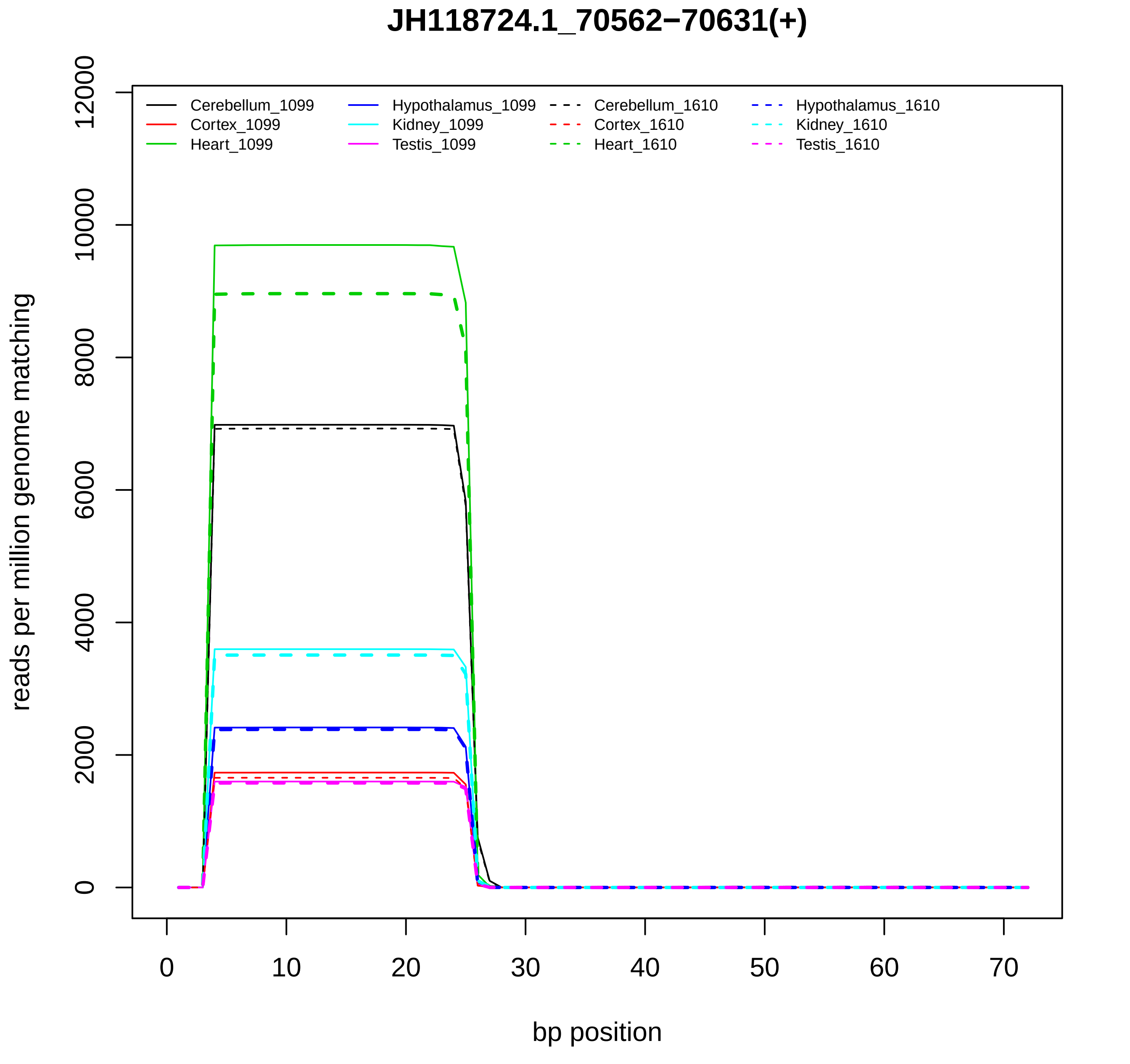

JH118724.1_70562−70631(+)
Cerebellum_1099
Cortex_1099
Heart_1099
Hypothalamus_1099
Kidney_1099
Testis_1099
Cerebellum_1610
Cortex_1610
Heart_1610
Hypothalamus_1610
Kidney_1610
Testis_1610
reads per million genome matching
0
2000
4000
6000
8000
10000
12000
bp position
0
10
20
30
40
50
60
70

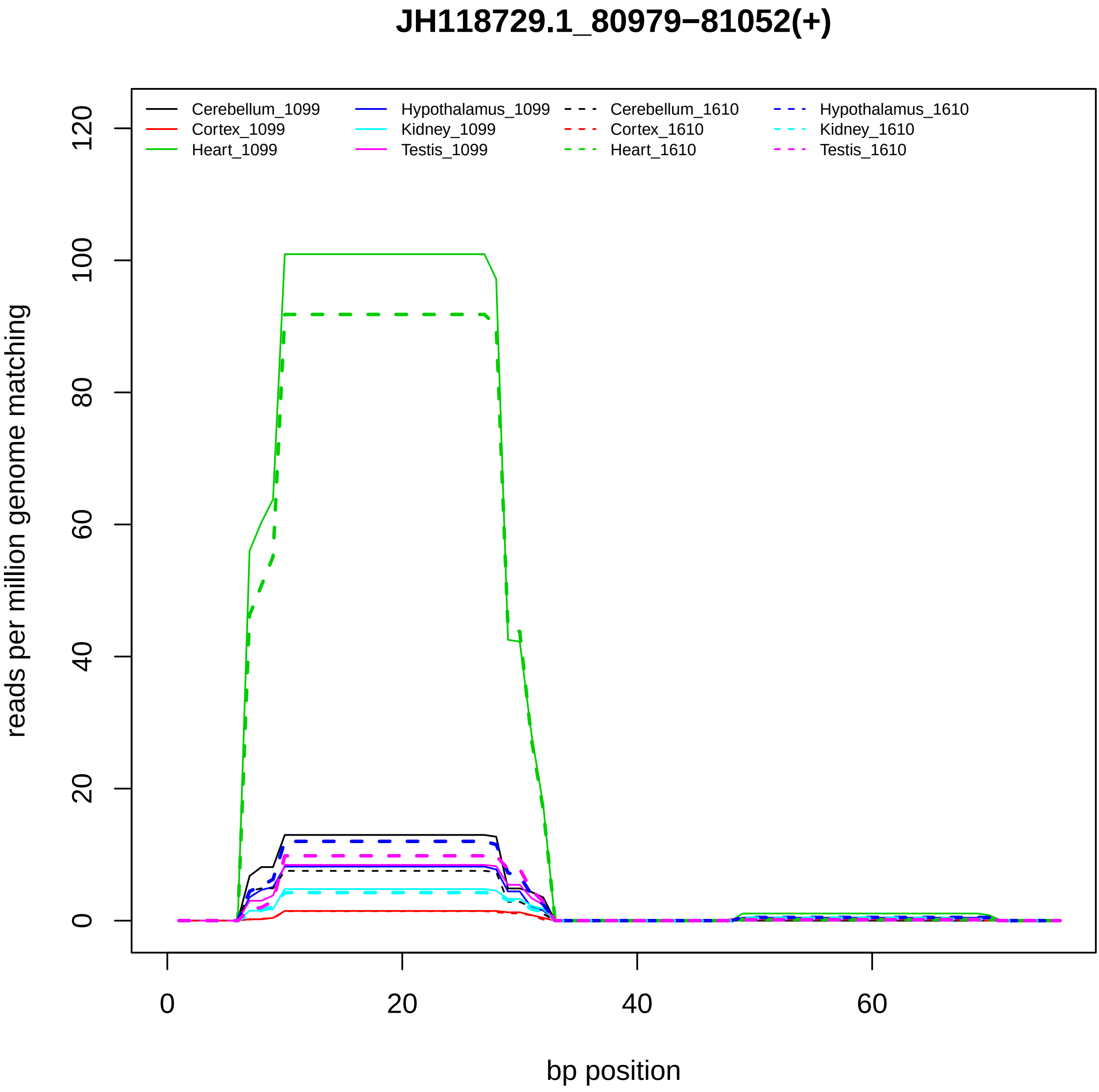
JH118729.1_80979−81052(+)
Cerebellum_1099
Cortex_1099
Heart_1099
Hypothalamus_1099
Kidney_1099
Testis_1099
Cerebellum_1610
Cortex_1610
Heart_1610
Hypothalamus_1610
Kidney_1610
Testis_1610
reads per million genome matching
bp position
0
20
40
60
80
100
120

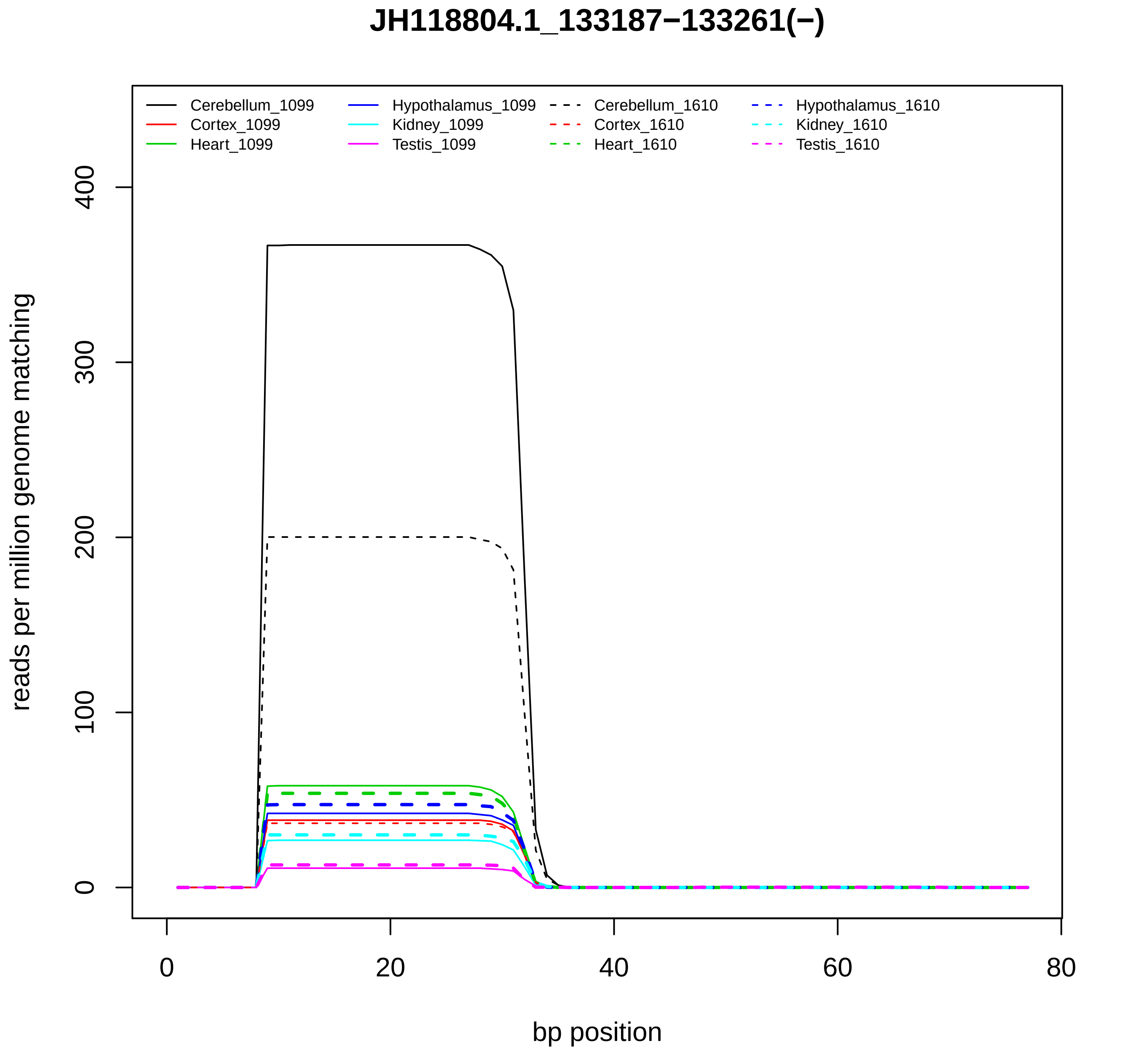
JH118804.1_133187−133261(−)
Cerebellum_1099
Cortex_1099
Heart_1099
Hypothalamus_1099
Kidney_1099
Testis_1099
Cerebellum_1610
Cortex_1610
Heart_1610
Hypothalamus_1610
Kidney_1610
Testis_1610
reads per million genome matching
0
100
200
300
400
bp position
0
20
40
60
80

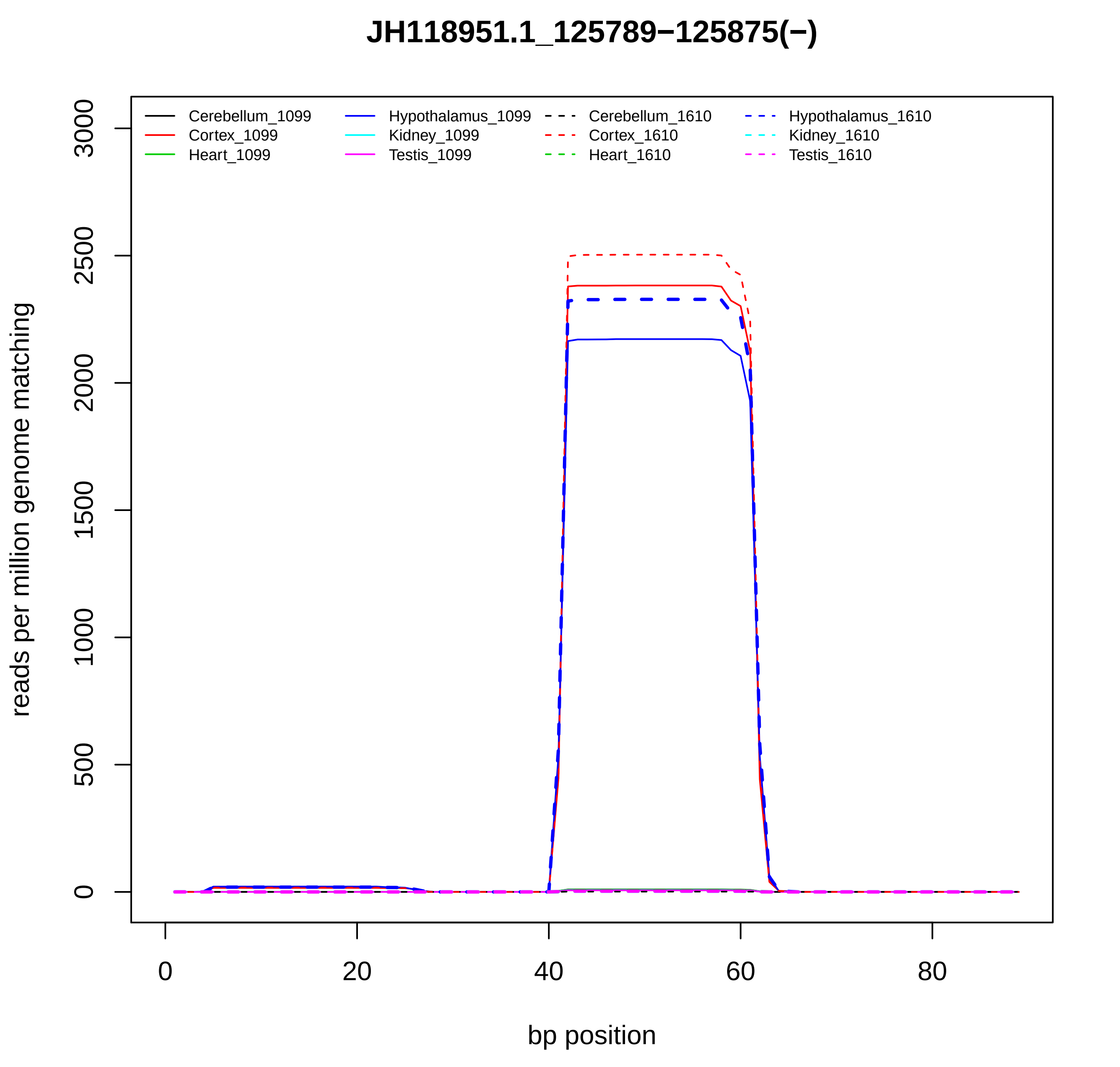

JH118951.1_125789−125875(−)
Cerebellum_1099
Cortex_1099
Heart_1099
Hypothalamus_1099
Kidney_1099
Testis_1099
Cerebellum_1610
Cortex_1610
Heart_1610
Hypothalamus_1610
Kidney_1610
Testis_1610
reads per million genome matching
0
500
1000
1500
2000
2500
3000
bp position
0
20
40
60
80

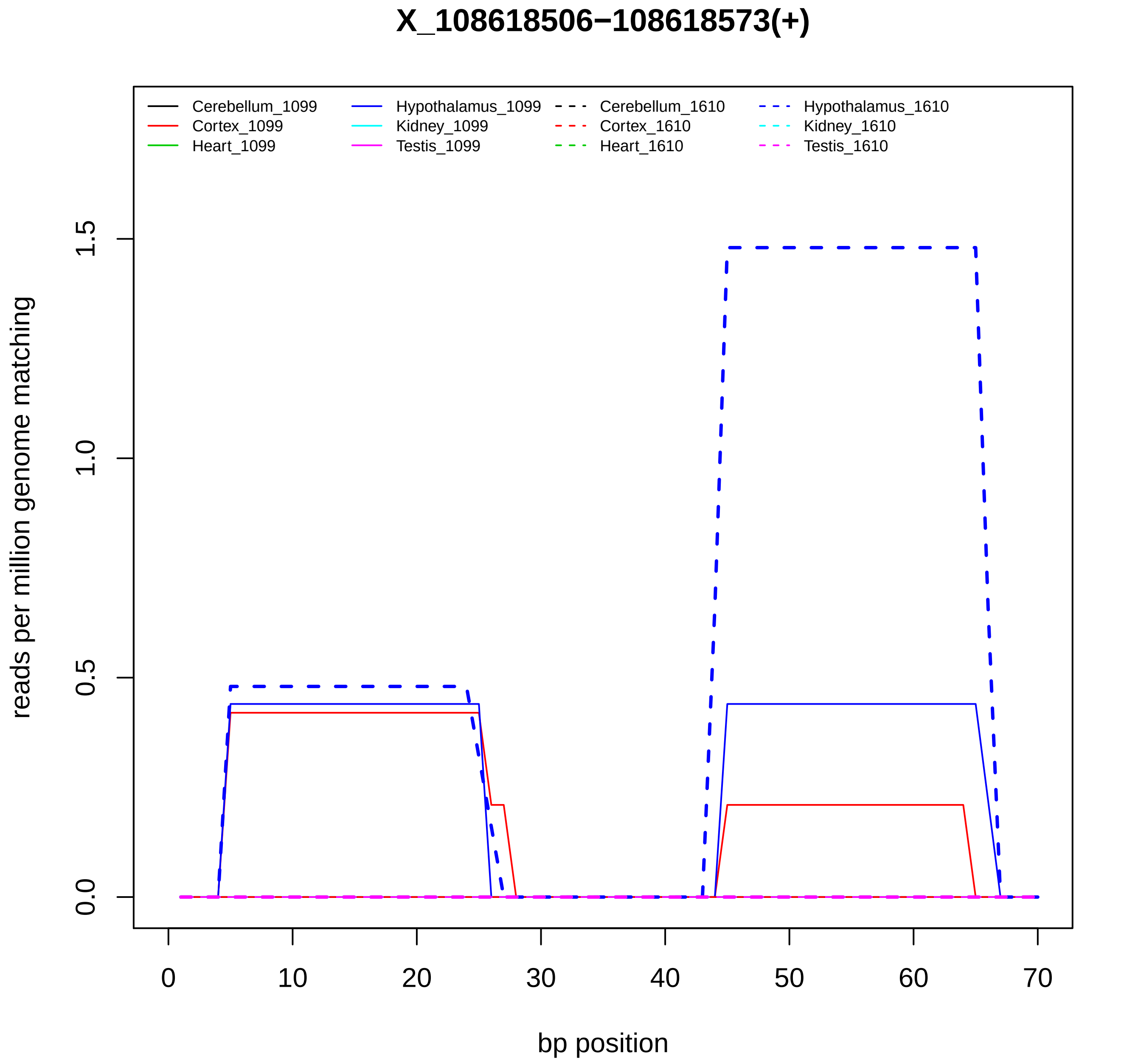

X_108618506−108618573(+)
Cerebellum_1099
Cortex_1099
Heart_1099
Hypothalamus_1099
Kidney_1099
Testis_1099
Cerebellum_1610
Cortex_1610
Heart_1610
Hypothalamus_1610
Kidney_1610
Testis_1610
reads per million genome matching
0.0
0.5
1.0
1.5
0
10
20
30
40
50
60
70
bp position

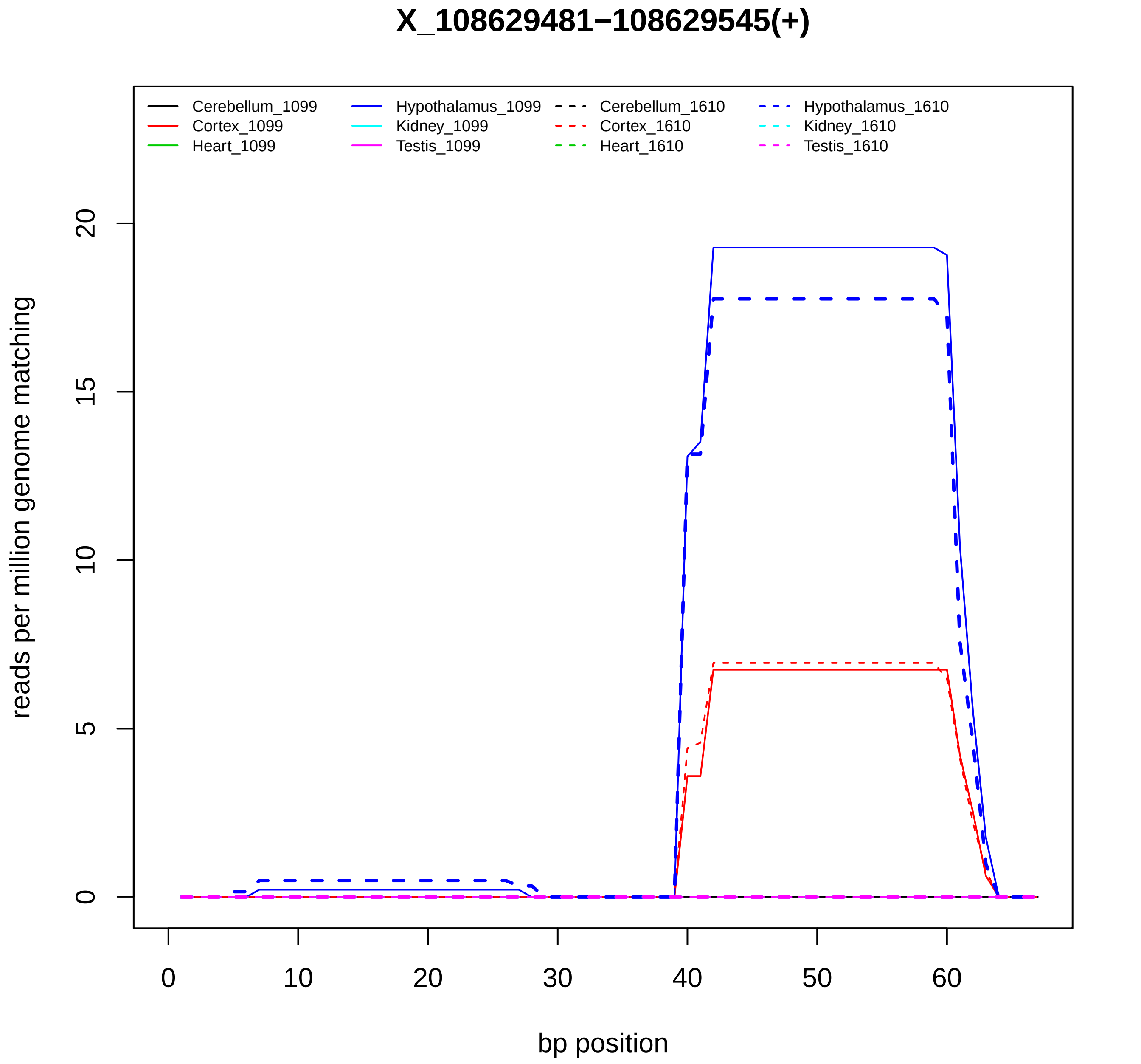

X_108629481−108629545(+)
Cerebellum_1099
Cortex_1099
Heart_1099
Hypothalamus_1099
Kidney_1099
Testis_1099
Cerebellum_1610
Cortex_1610
Heart_1610
Hypothalamus_1610
Kidney_1610
Testis_1610
reads per million genome matching
0
5
10
15
20
0
10
20
30
40
50
60
bp position

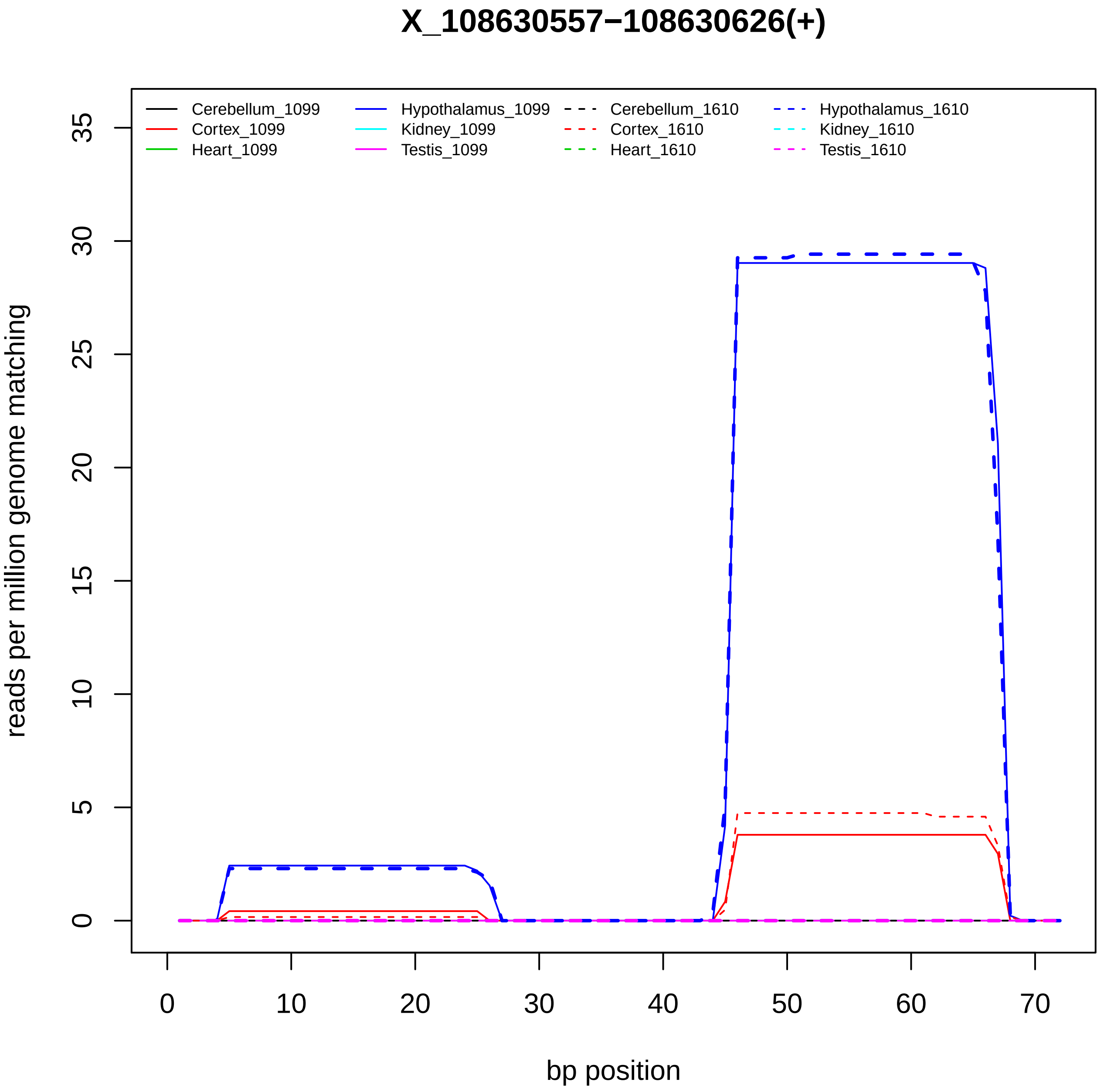
X_108630557−108630626(+)
Cerebellum_1099
Cortex_1099
Heart_1099
Hypothalamus_1099
Kidney_1099
Testis_1099
Cerebellum_1610
Cortex_1610
Heart_1610
Hypothalamus_1610
Kidney_1610
Testis_1610
reads per million genome matching
0
5
10
15
20
25
30
35
0
10
20
30
40
50
60
70
bp position

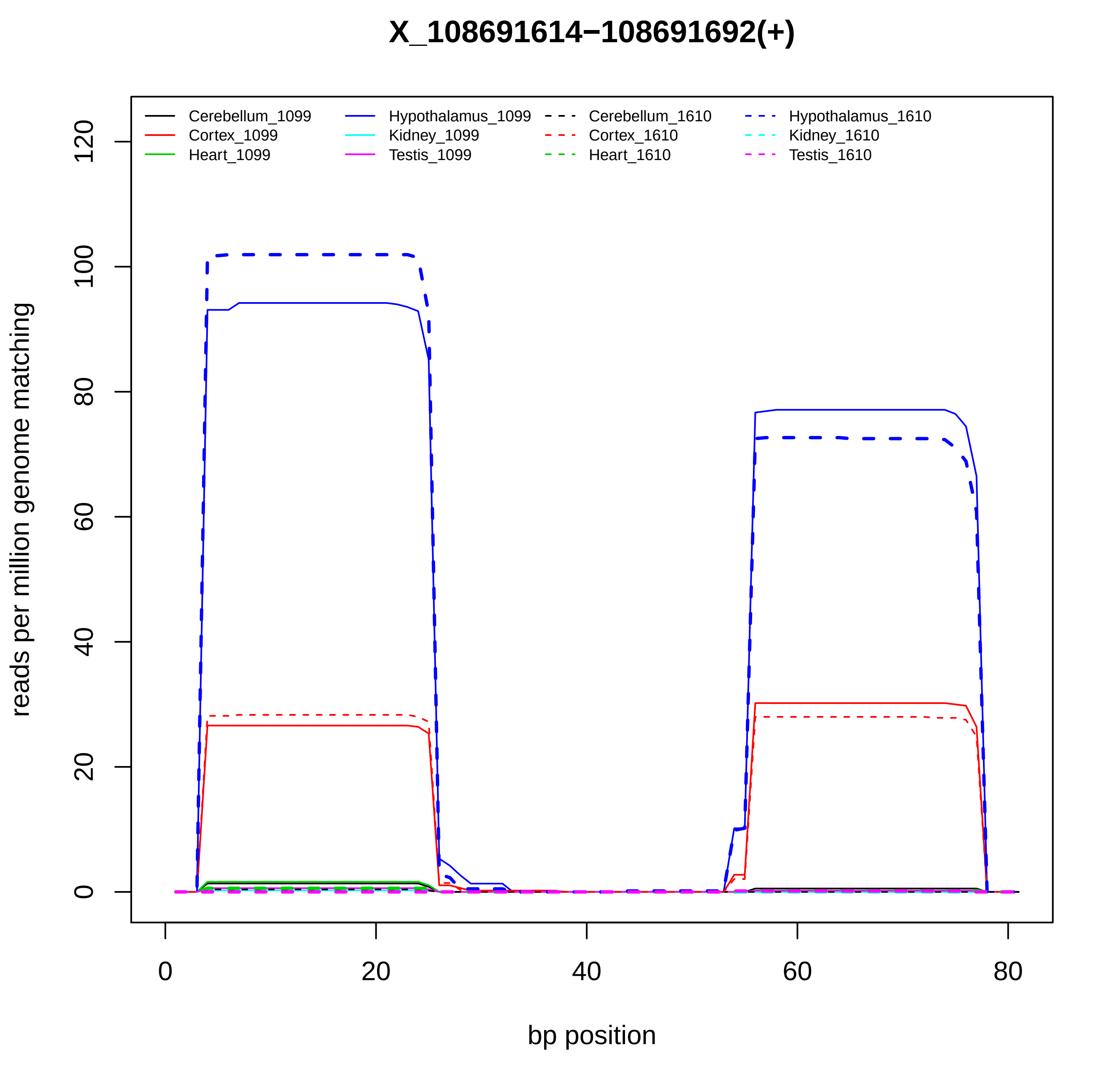

X_108691614−108691692(+)
Cerebellum_1099
Cortex_1099
Heart_1099
Hypothalamus_1099
Kidney_1099
Testis_1099
Cerebellum_1610
Cortex_1610
Heart_1610
Hypothalamus_1610
Kidney_1610
Testis_1610
reads per million genome matching
0
20
40
60
80
100
120
bp position
0
20
40
60
80

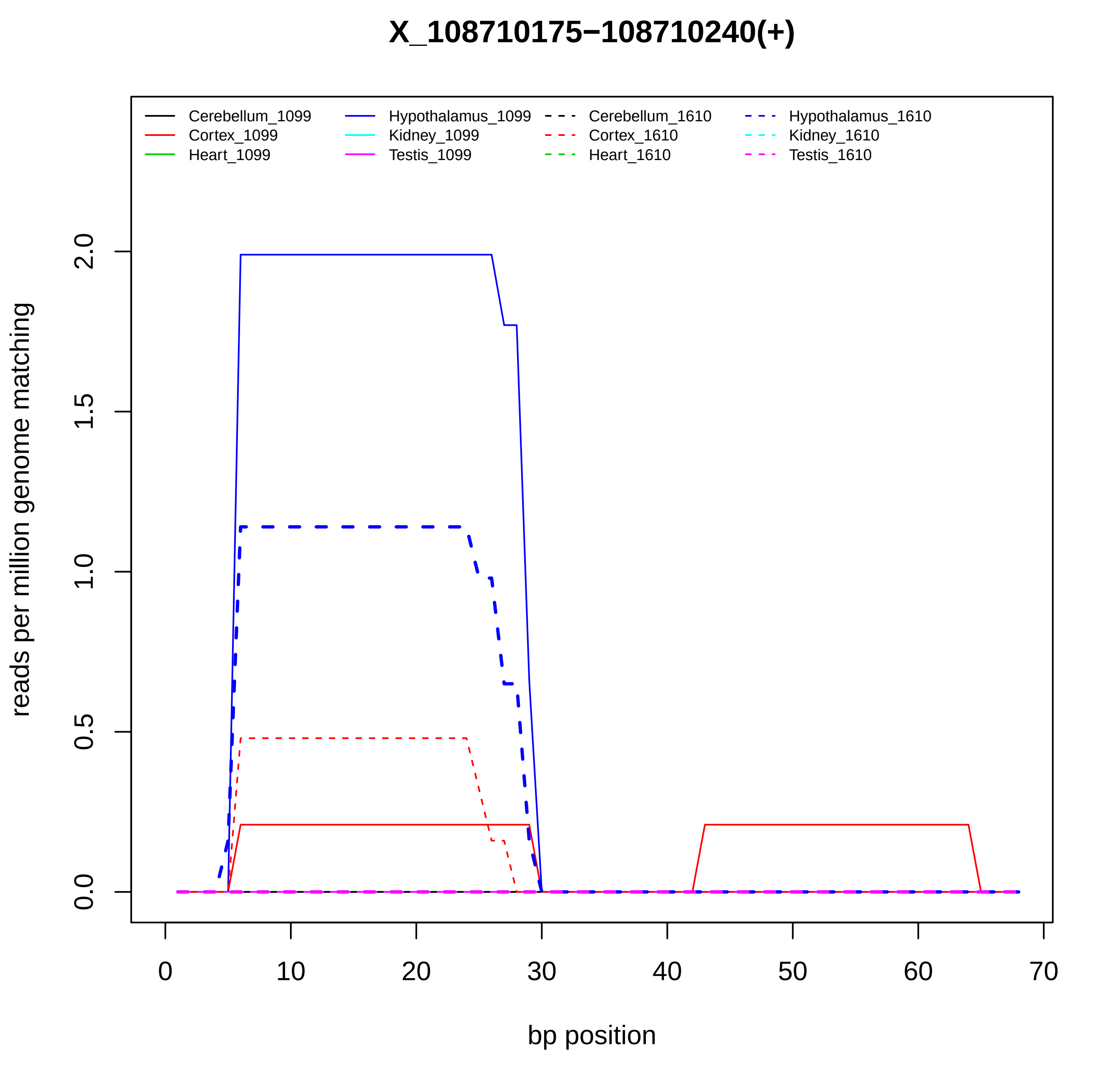
X_108710175−108710240(+)
Cerebellum_1099
Cortex_1099
Heart_1099
Hypothalamus_1099
Kidney_1099
Testis_1099
Cerebellum_1610
Cortex_1610
Heart_1610
Hypothalamus_1610
Kidney_1610
Testis_1610
reads per million genome matching
0.0
0.5
1.0
1.5
2.0
0
10
20
30
40
50
60
70
bp position

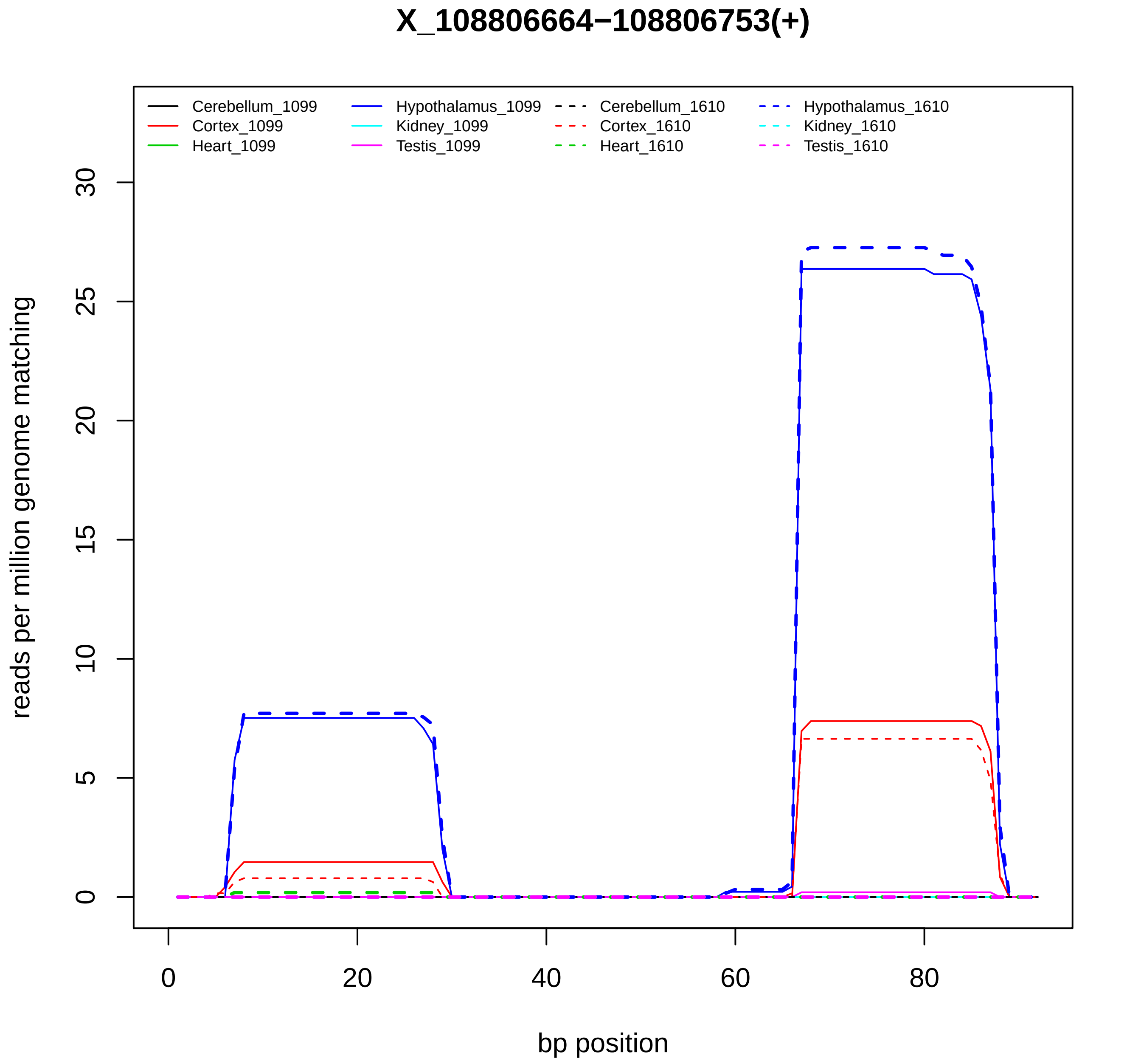

X_108806664−108806753(+)
Cerebellum_1099
Cortex_1099
Heart_1099
Hypothalamus_1099
Kidney_1099
Testis_1099
Cerebellum_1610
Cortex_1610
Heart_1610
Hypothalamus_1610
Kidney_1610
Testis_1610
reads per million genome matching
bp position
0
5
10
15
20
25
30
0
20
40
60
80

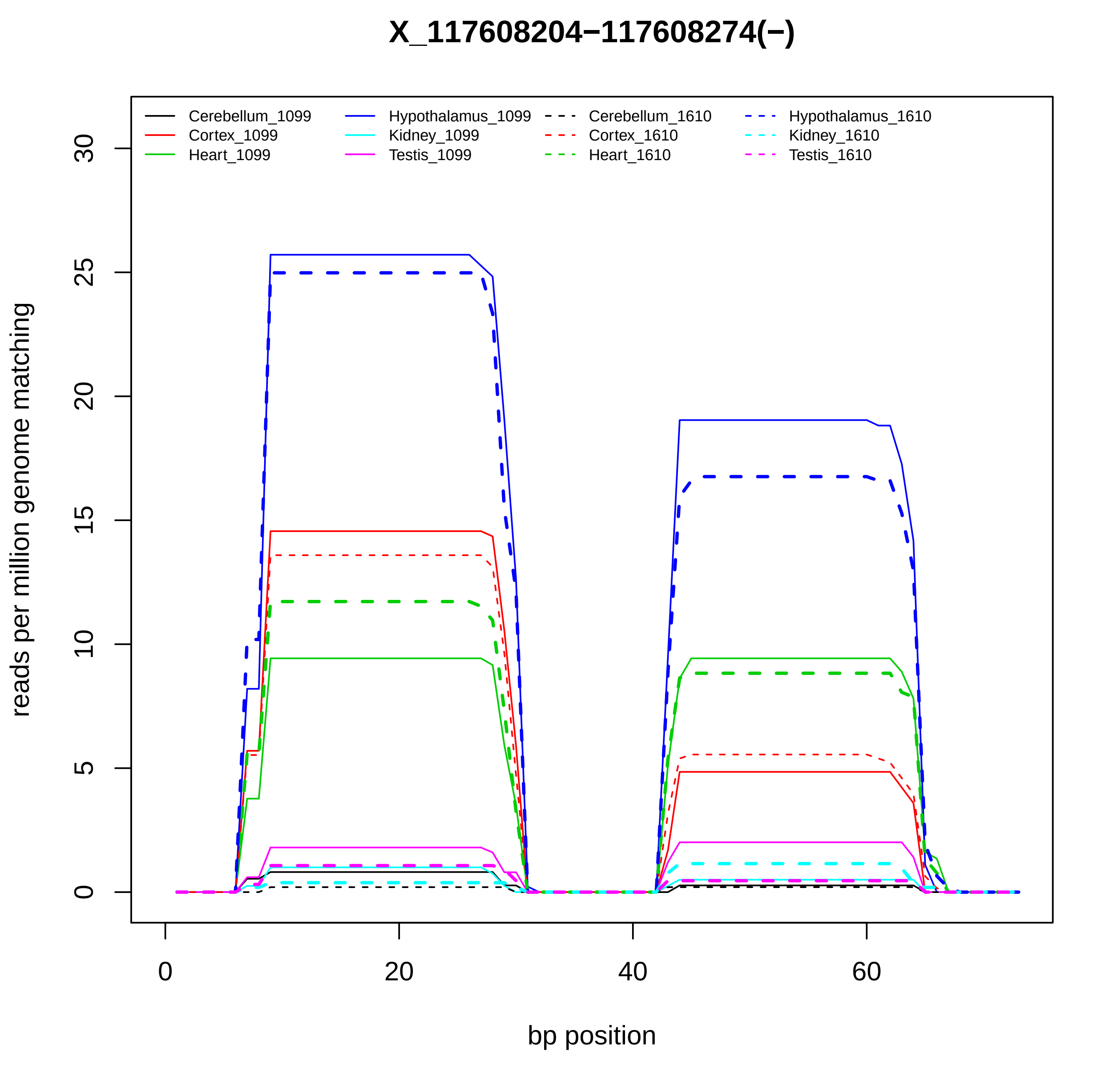
X_117608204−117608274(−)
Cerebellum_1099
Cortex_1099
Heart_1099
Hypothalamus_1099
Kidney_1099
Testis_1099
Cerebellum_1610
Cortex_1610
Heart_1610
Hypothalamus_1610
Kidney_1610
Testis_1610
reads per million genome matching
bp position
0
5
10
15
20
25
30
0
20
40
60

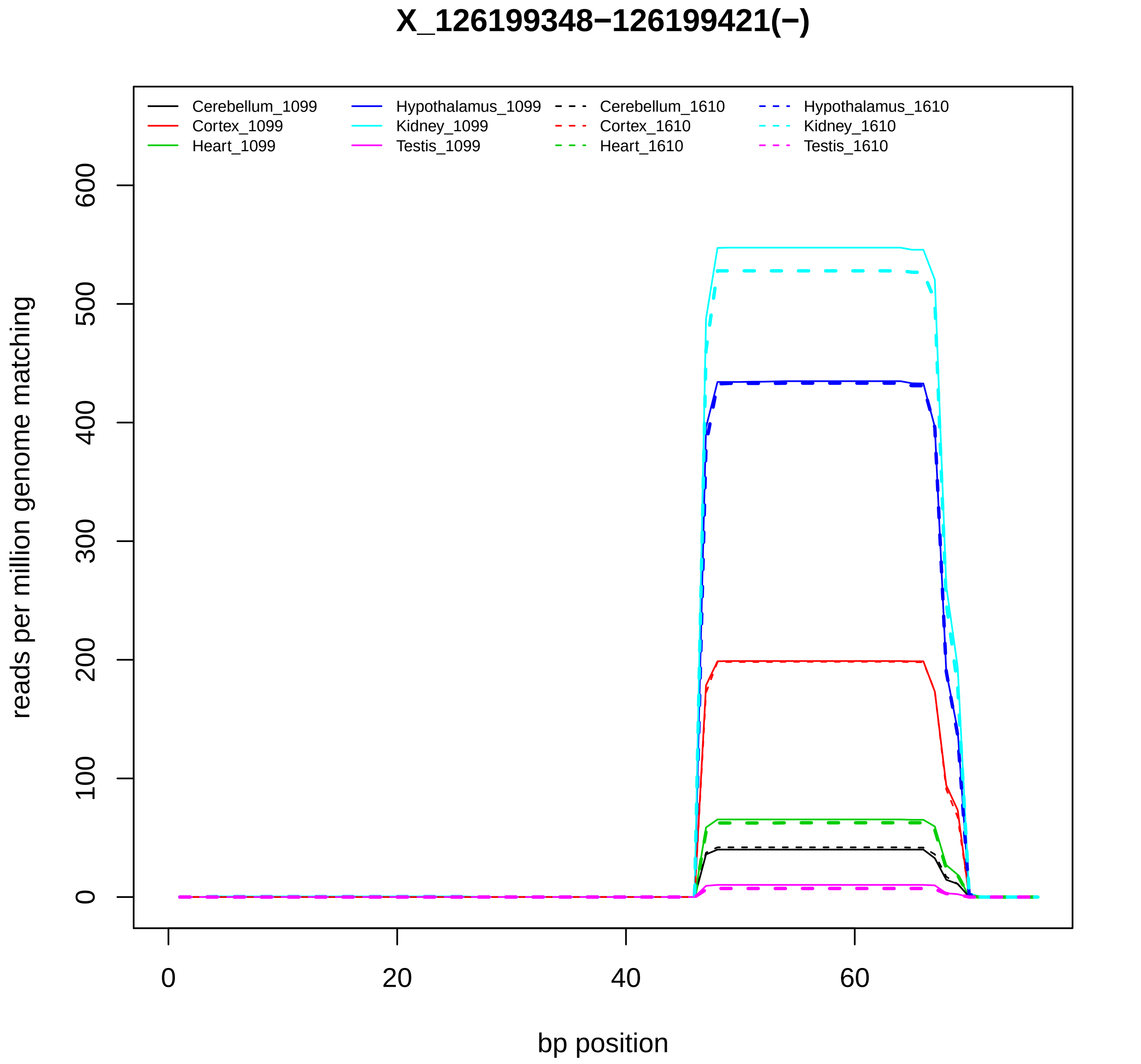

X_126199348−126199421(−)
Cerebellum_1099
Cortex_1099
Heart_1099
Hypothalamus_1099
Kidney_1099
Testis_1099
Cerebellum_1610
Cortex_1610
Heart_1610
Hypothalamus_1610
Kidney_1610
Testis_1610
reads per million genome matching
0
100
200
300
400
500
600
0
20
40
60
bp position

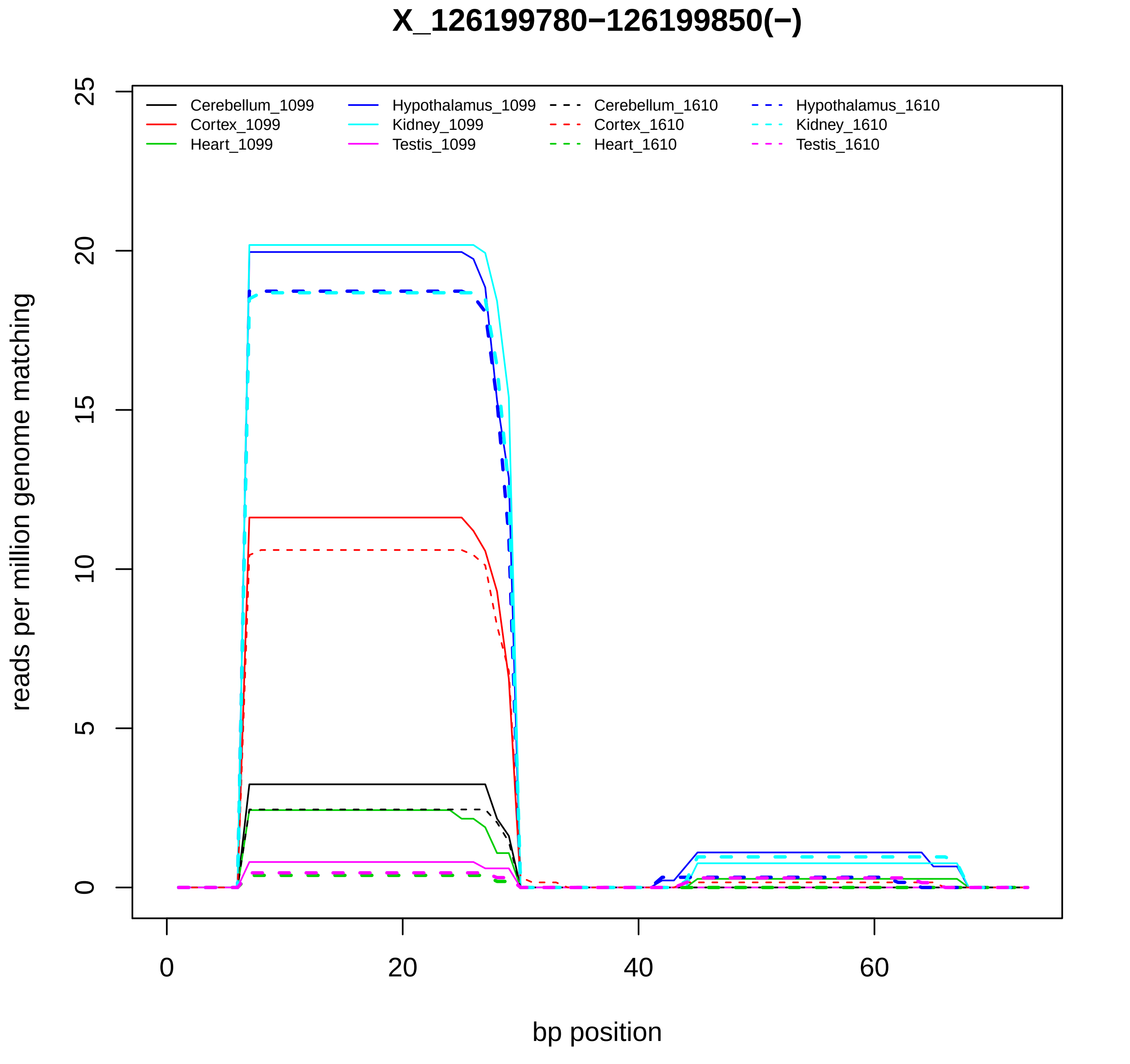

X_126199780−126199850(−)
Cerebellum_1099
Cortex_1099
Heart_1099
Hypothalamus_1099
Kidney_1099
Testis_1099
Cerebellum_1610
Cortex_1610
Heart_1610
Hypothalamus_1610
Kidney_1610
Testis_1610
reads per million genome matching
bp position
0
5
10
15
20
25
0
20
40
60

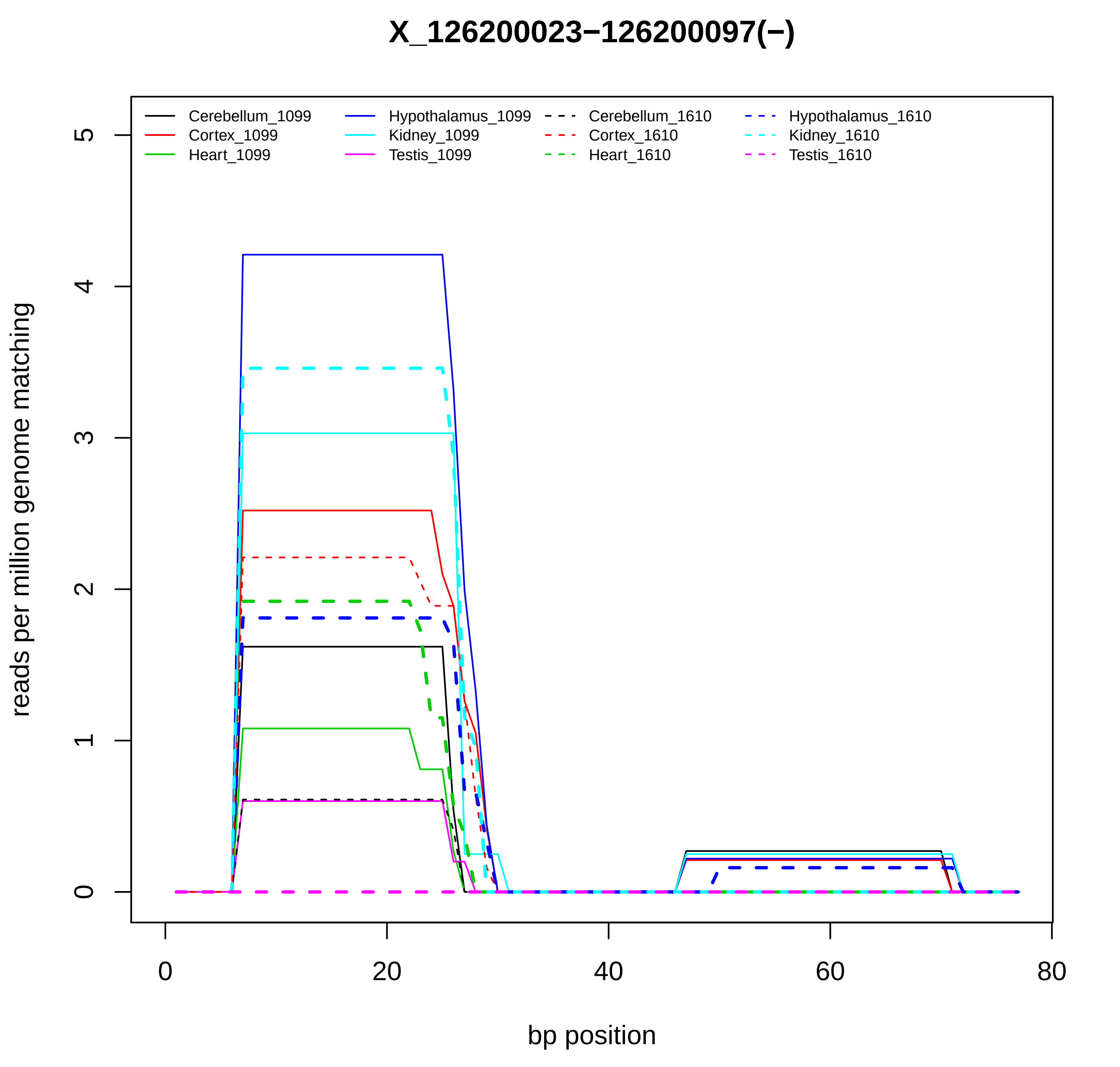

X_126200023−126200097(−)
Cerebellum_1099
Cortex_1099
Heart_1099
Hypothalamus_1099
Kidney_1099
Testis_1099
Cerebellum_1610
Cortex_1610
Heart_1610
Hypothalamus_1610
Kidney_1610
Testis_1610
reads per million genome matching
0
1
2
3
4
5
0
20
40
60
80
bp position

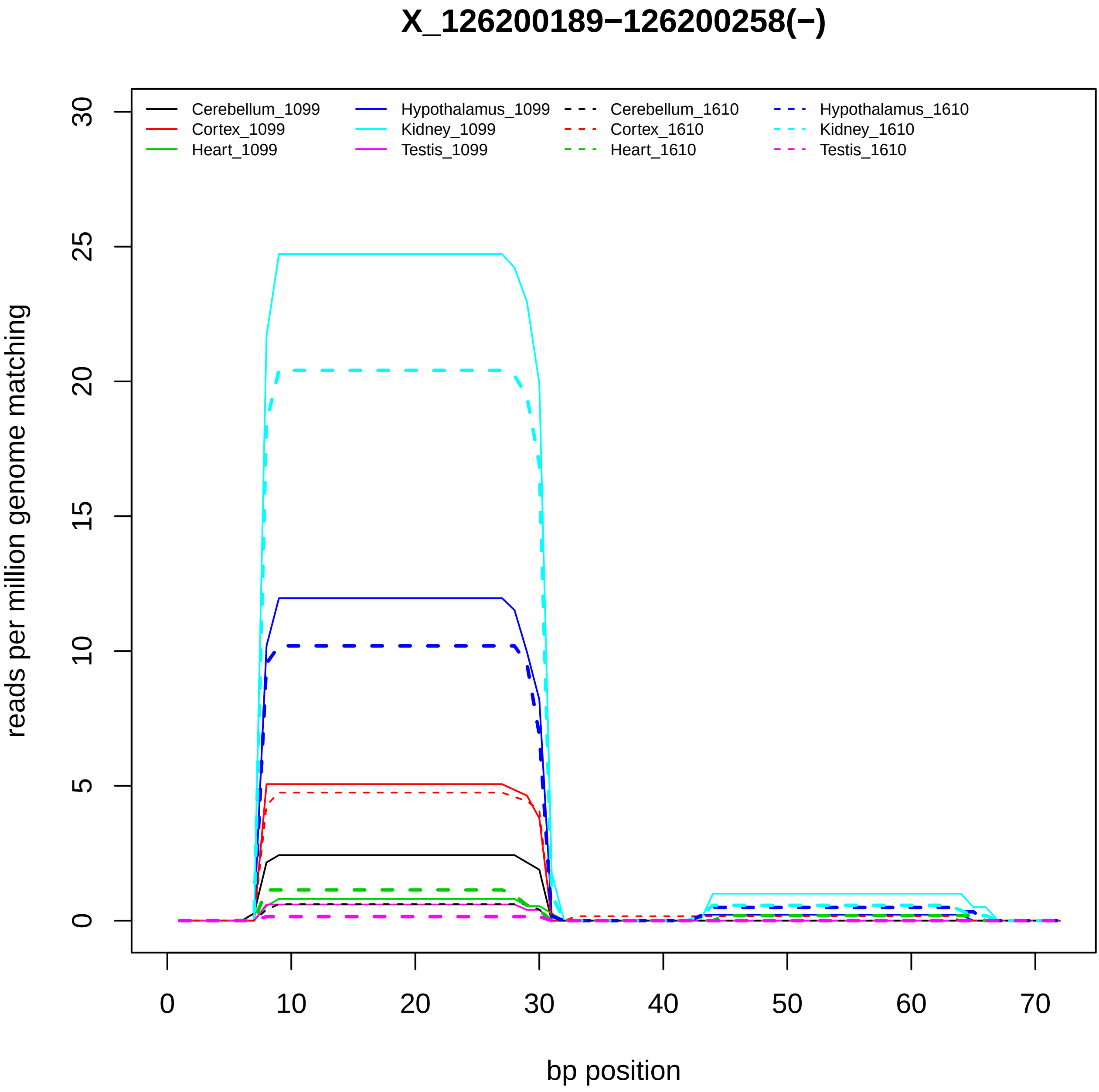
X_126200189−126200258(−)
Cerebellum_1099
Cortex_1099
Heart_1099
Hypothalamus_1099
Kidney_1099
Testis_1099
Cerebellum_1610
Cortex_1610
Heart_1610
Hypothalamus_1610
Kidney_1610
Testis_1610
reads per million genome matching
bp position
0
5
10
15
20
25
30
0
10
20
30
40
50
60
70

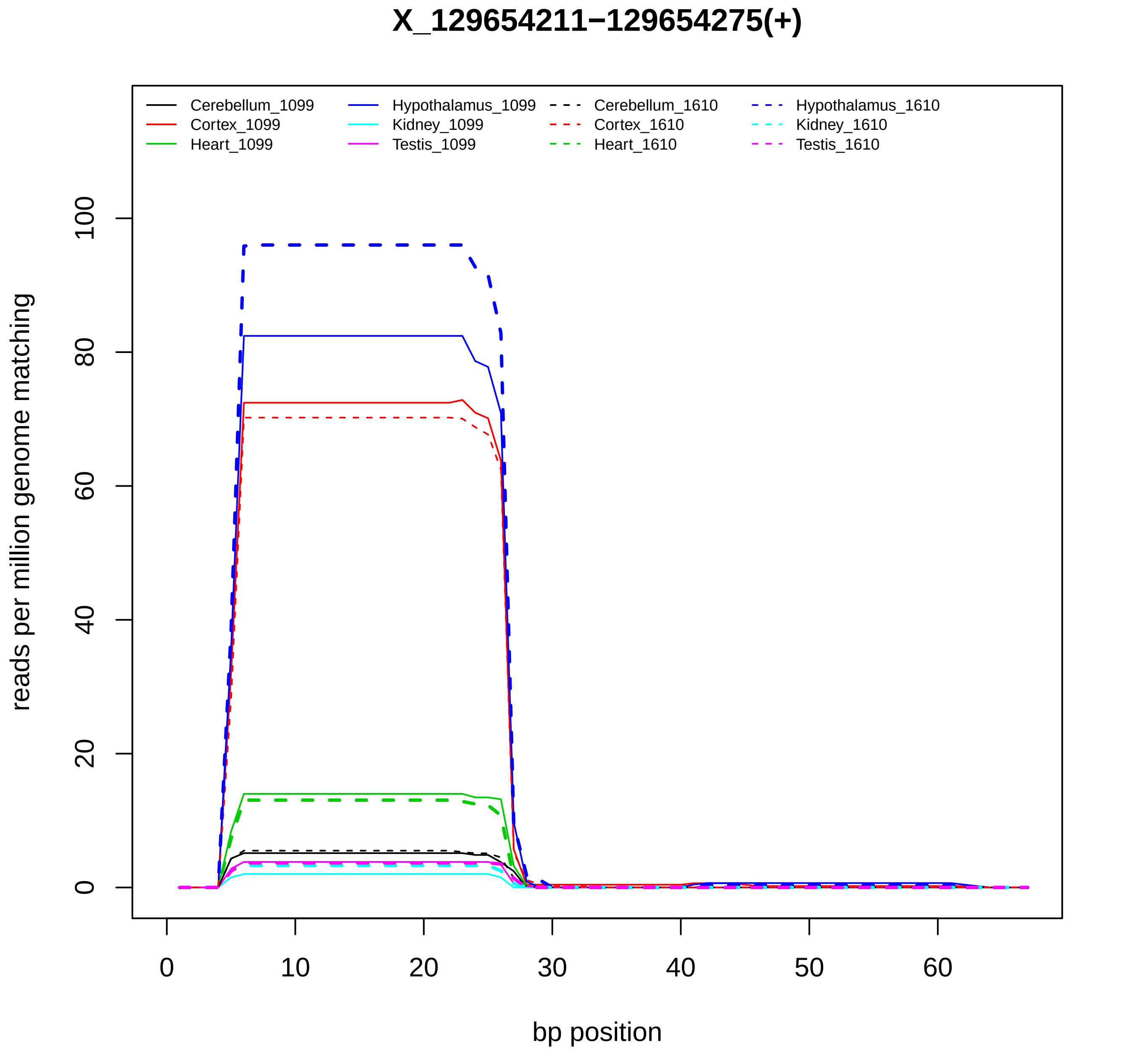

X_129654211−129654275(+)
Cerebellum_1099
Cortex_1099
Heart_1099
Hypothalamus_1099
Kidney_1099
Testis_1099
Cerebellum_1610
Cortex_1610
Heart_1610
Hypothalamus_1610
Kidney_1610
Testis_1610
reads per million genome matching
0
20
40
60
80
100
0
10
20
30
40
50
60
bp position

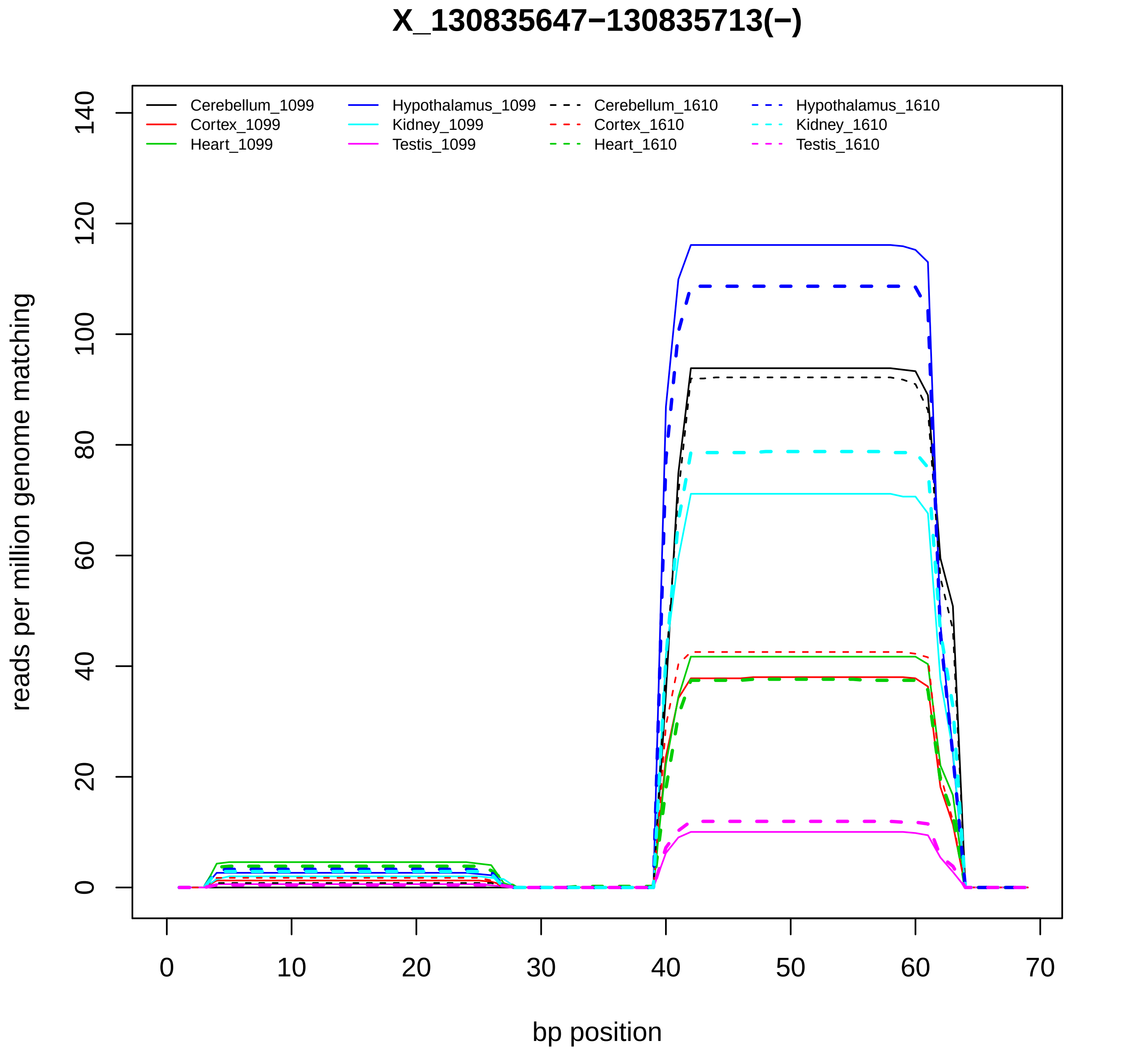
X_130835647−130835713(−)
Cerebellum_1099
Cortex_1099
Heart_1099
Hypothalamus_1099
Kidney_1099
Testis_1099
Cerebellum_1610
Cortex_1610
Heart_1610
Hypothalamus_1610
Kidney_1610
Testis_1610
reads per million genome matching
bp position
0
20
40
60
80
100
120
140
0
10
20
30
40
50
60
70

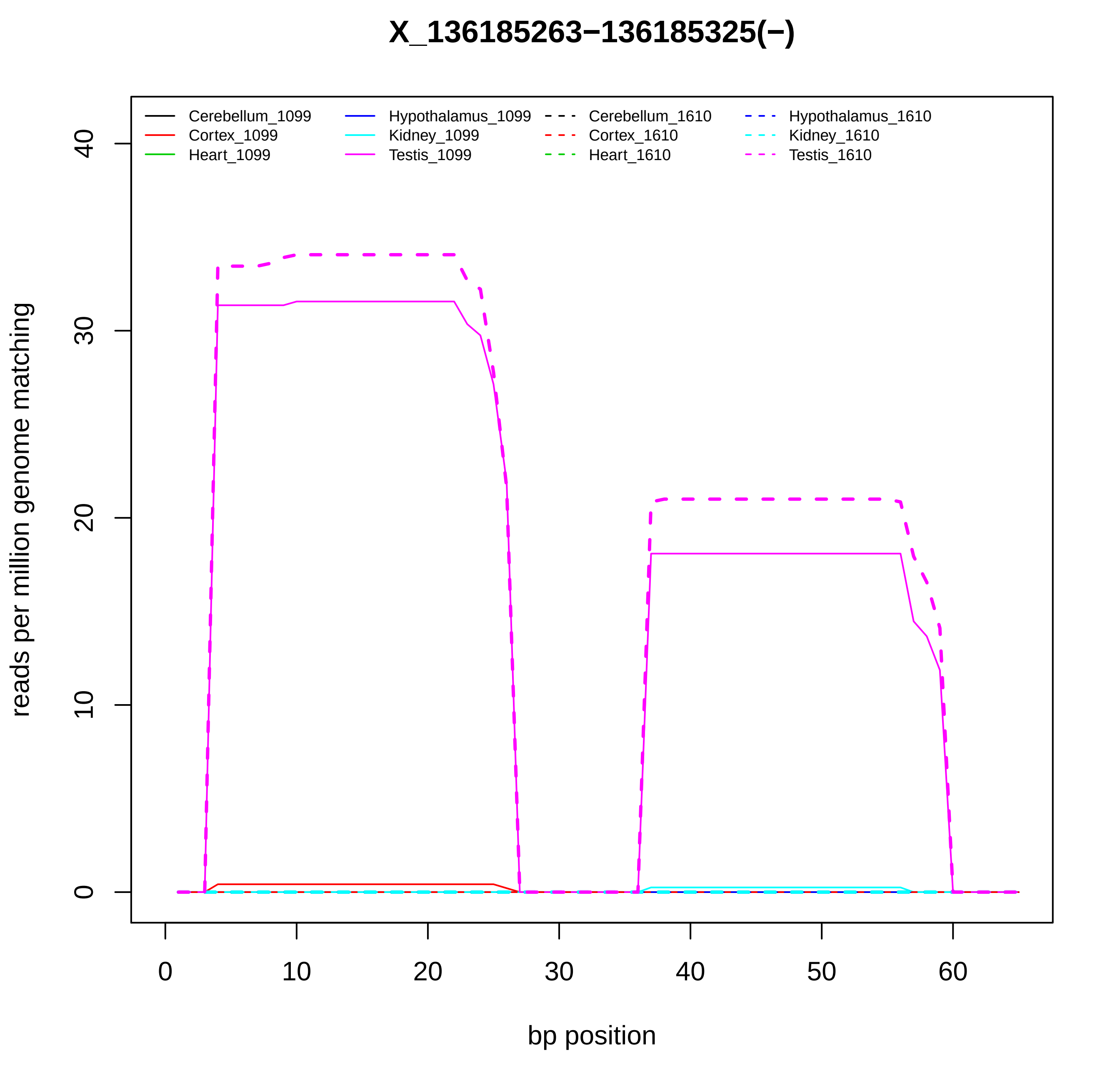
X_136185263−136185325(−)
Cerebellum_1099
Cortex_1099
Heart_1099
Hypothalamus_1099
Kidney_1099
Testis_1099
Cerebellum_1610
Cortex_1610
Heart_1610
Hypothalamus_1610
Kidney_1610
Testis_1610
reads per million genome matching
bp position
0
10
20
30
40
0
10
20
30
40
50
60

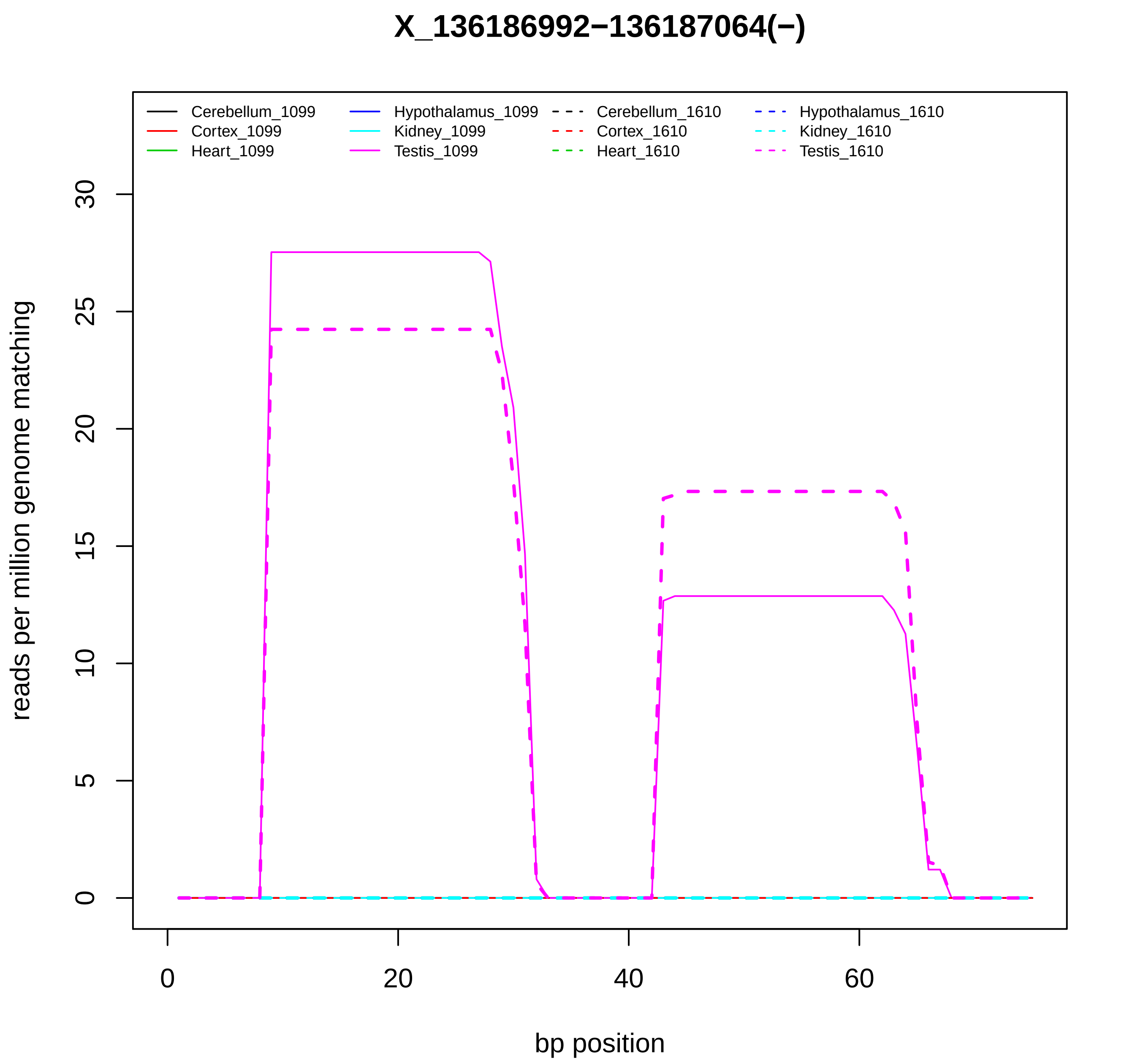

X_136186992−136187064(−)
Cerebellum_1099
Cortex_1099
Heart_1099
Hypothalamus_1099
Kidney_1099
Testis_1099
Cerebellum_1610
Cortex_1610
Heart_1610
Hypothalamus_1610
Kidney_1610
Testis_1610
reads per million genome matching
bp position
0
5
10
15
20
25
30
0
20
40
60

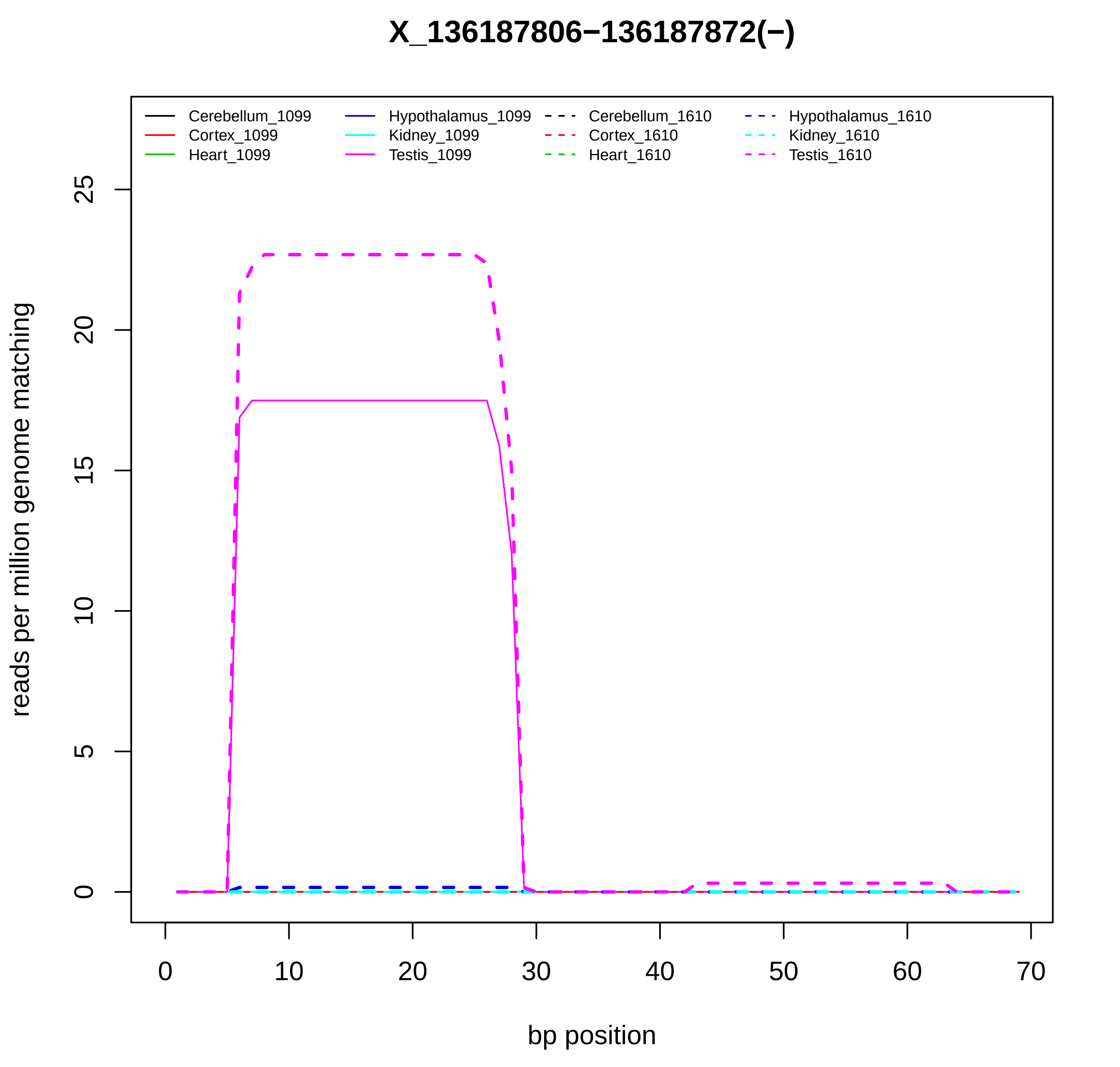

X_136187806−136187872(−)
Cerebellum_1099
Cortex_1099
Heart_1099
Hypothalamus_1099
Kidney_1099
Testis_1099
Cerebellum_1610
Cortex_1610
Heart_1610
Hypothalamus_1610
Kidney_1610
Testis_1610
reads per million genome matching
0
5
10
15
20
25
0
10
20
30
40
50
60
70
bp position

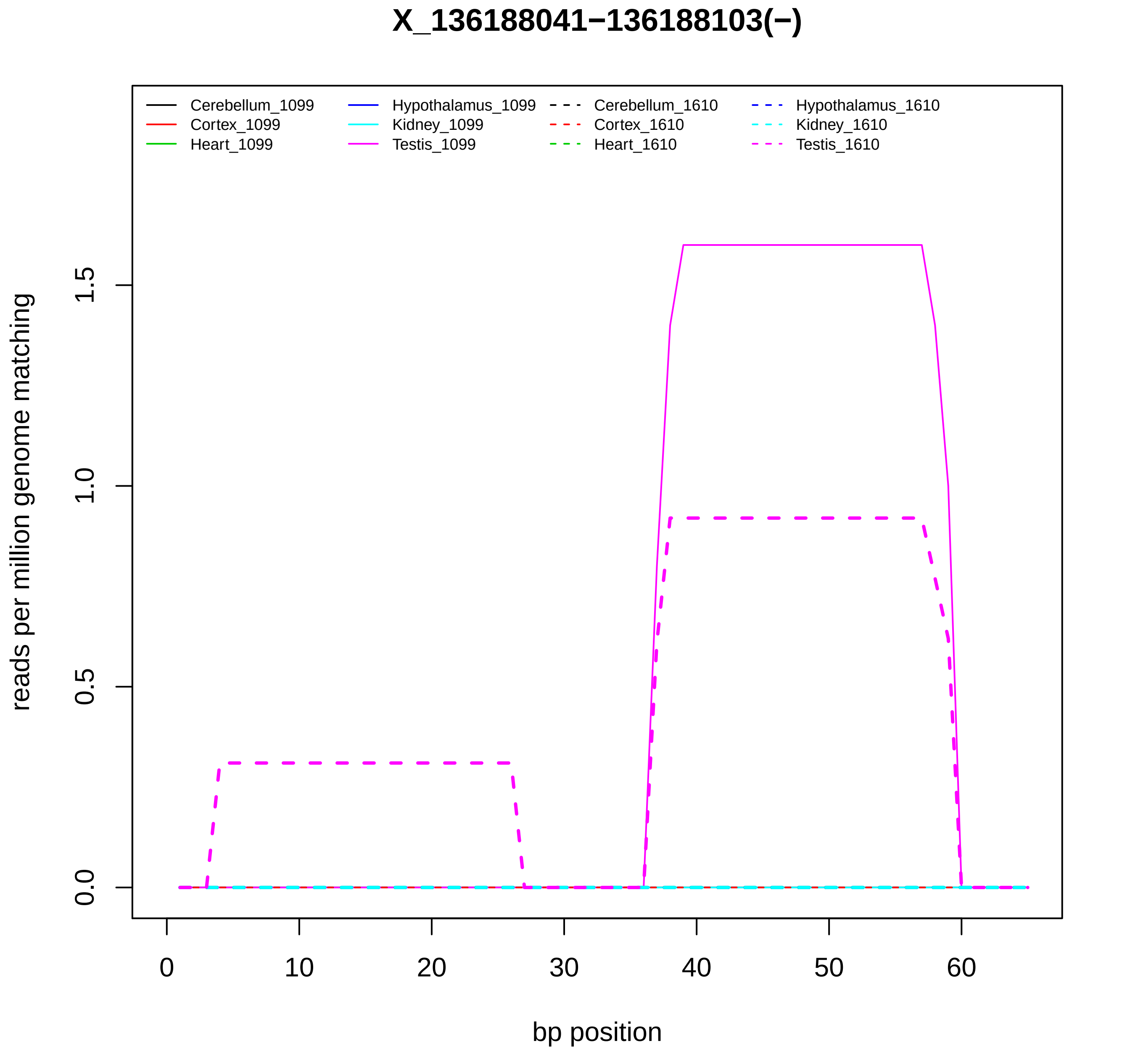

X_136188041−136188103(−)
Cerebellum_1099
Cortex_1099
Heart_1099
Hypothalamus_1099
Kidney_1099
Testis_1099
Cerebellum_1610
Cortex_1610
Heart_1610
Hypothalamus_1610
Kidney_1610
Testis_1610
reads per million genome matching
0.0
0.5
1.0
1.5
0
10
20
30
40
50
60
bp position

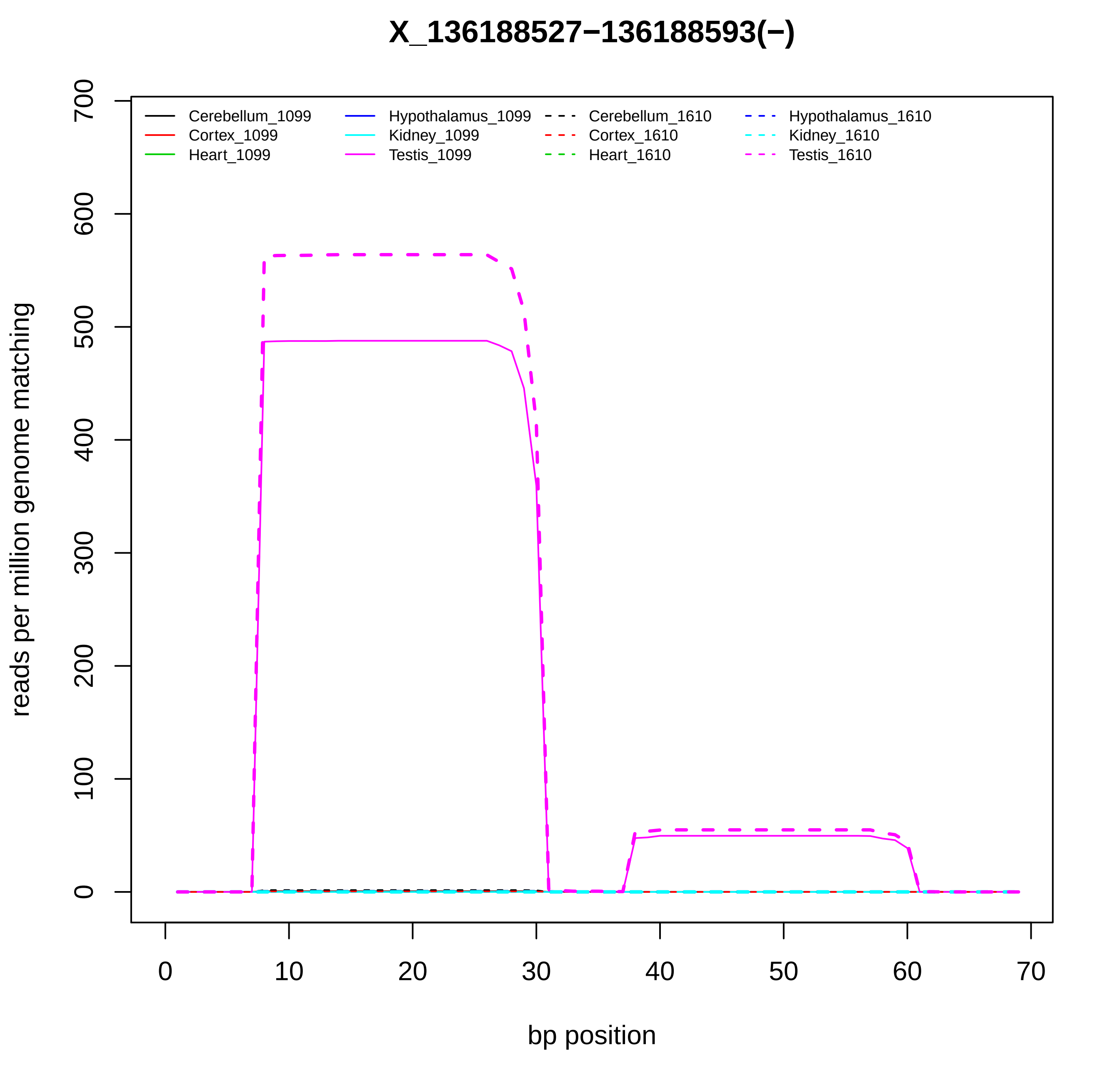

X_136188527−136188593(−)
Cerebellum_1099
Cortex_1099
Heart_1099
Hypothalamus_1099
Kidney_1099
Testis_1099
Cerebellum_1610
Cortex_1610
Heart_1610
Hypothalamus_1610
Kidney_1610
Testis_1610
reads per million genome matching
bp position
0
100
200
300
400
500
600
700
0
10
20
30
40
50
60
70

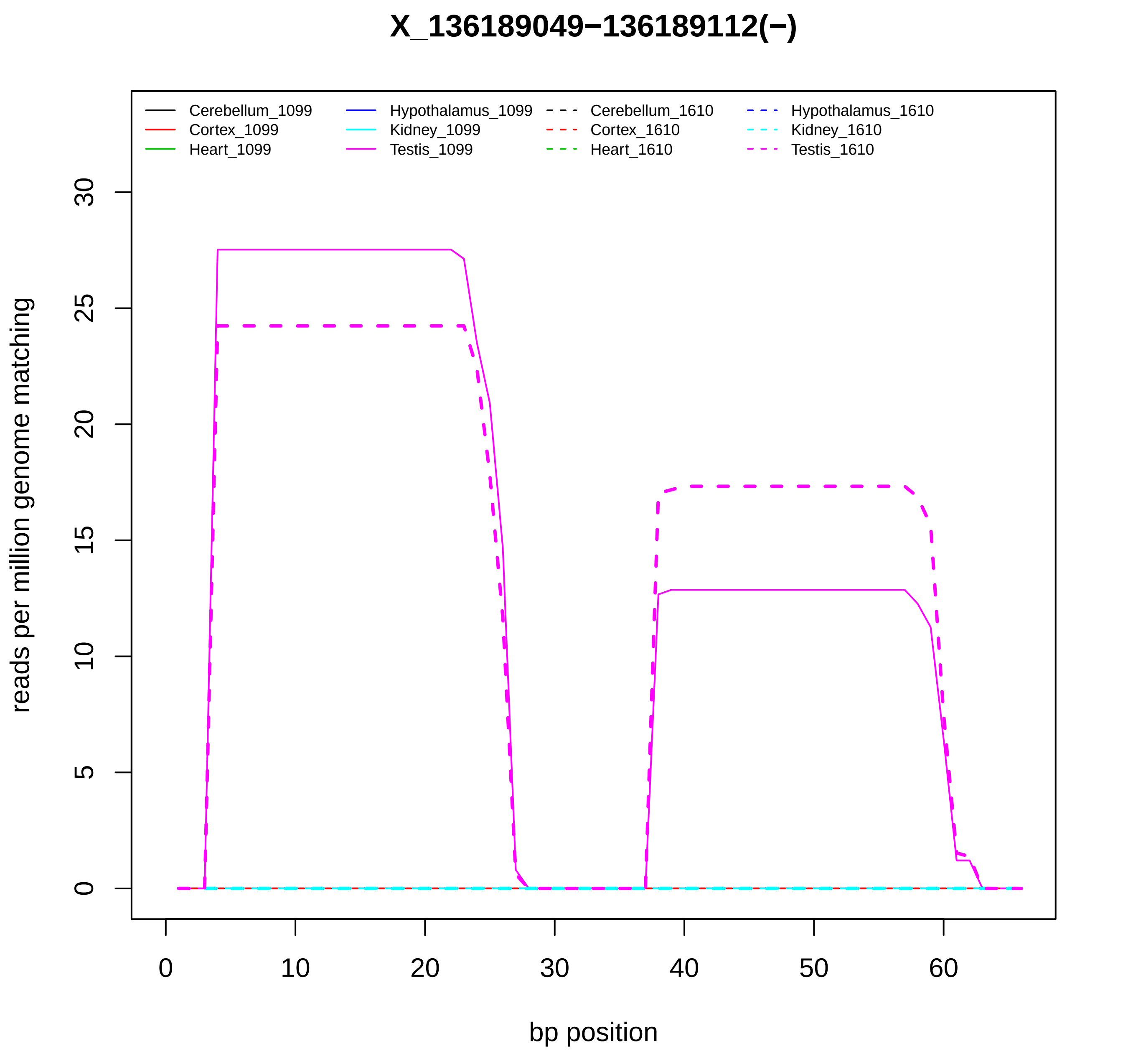

X_136189049−136189112(−)
Cerebellum_1099
Cortex_1099
Heart_1099
Hypothalamus_1099
Kidney_1099
Testis_1099
Cerebellum_1610
Cortex_1610
Heart_1610
Hypothalamus_1610
Kidney_1610
Testis_1610
reads per million genome matching
0
5
10
15
20
25
30
0
10
20
30
40
50
60
bp position

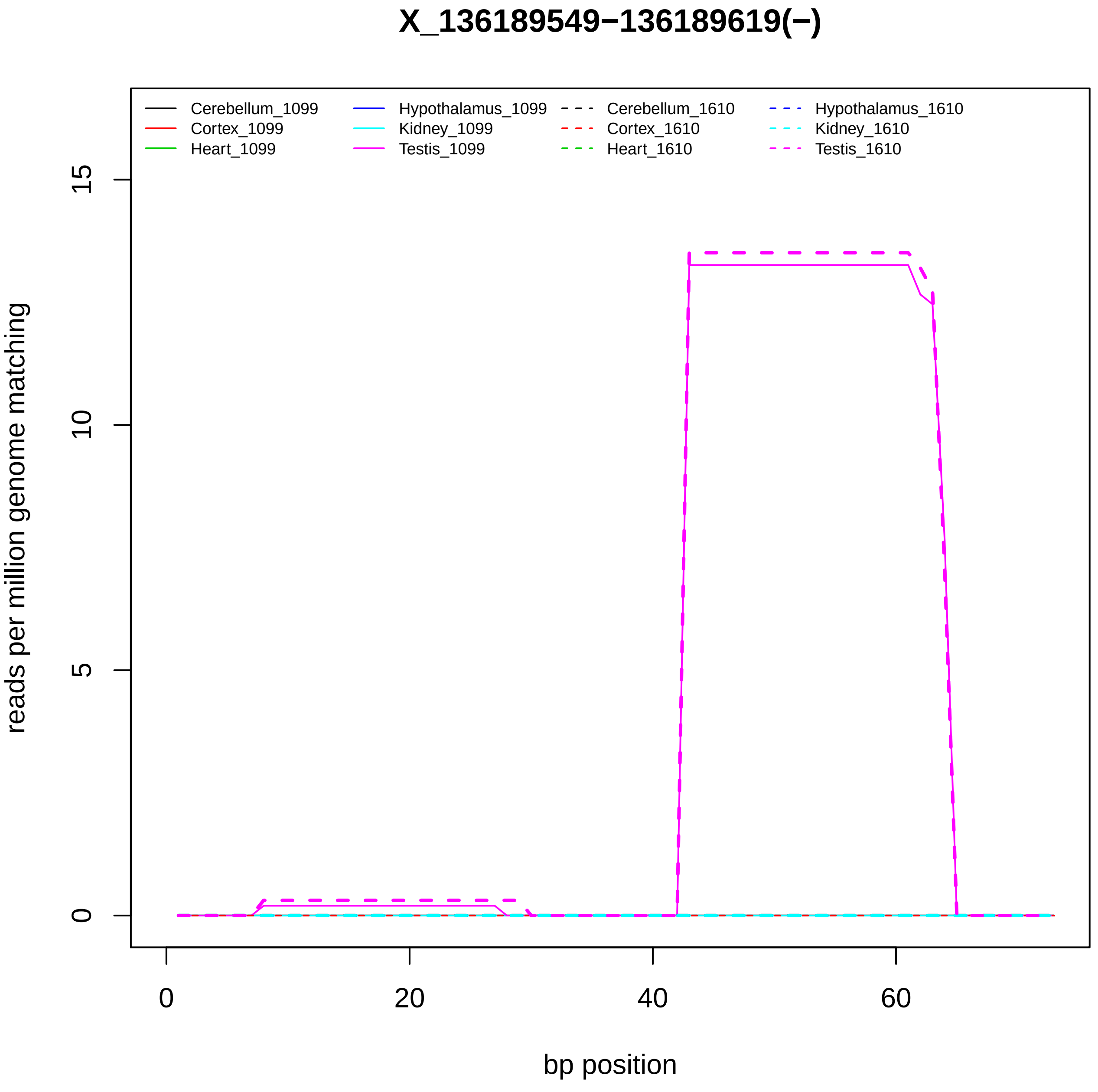

X_136189549−136189619(−)
Cerebellum_1099
Cortex_1099
Heart_1099
Hypothalamus_1099
Kidney_1099
Testis_1099
Cerebellum_1610
Cortex_1610
Heart_1610
Hypothalamus_1610
Kidney_1610
Testis_1610
reads per million genome matching
bp position
0
5
10
15
0
20
40
60

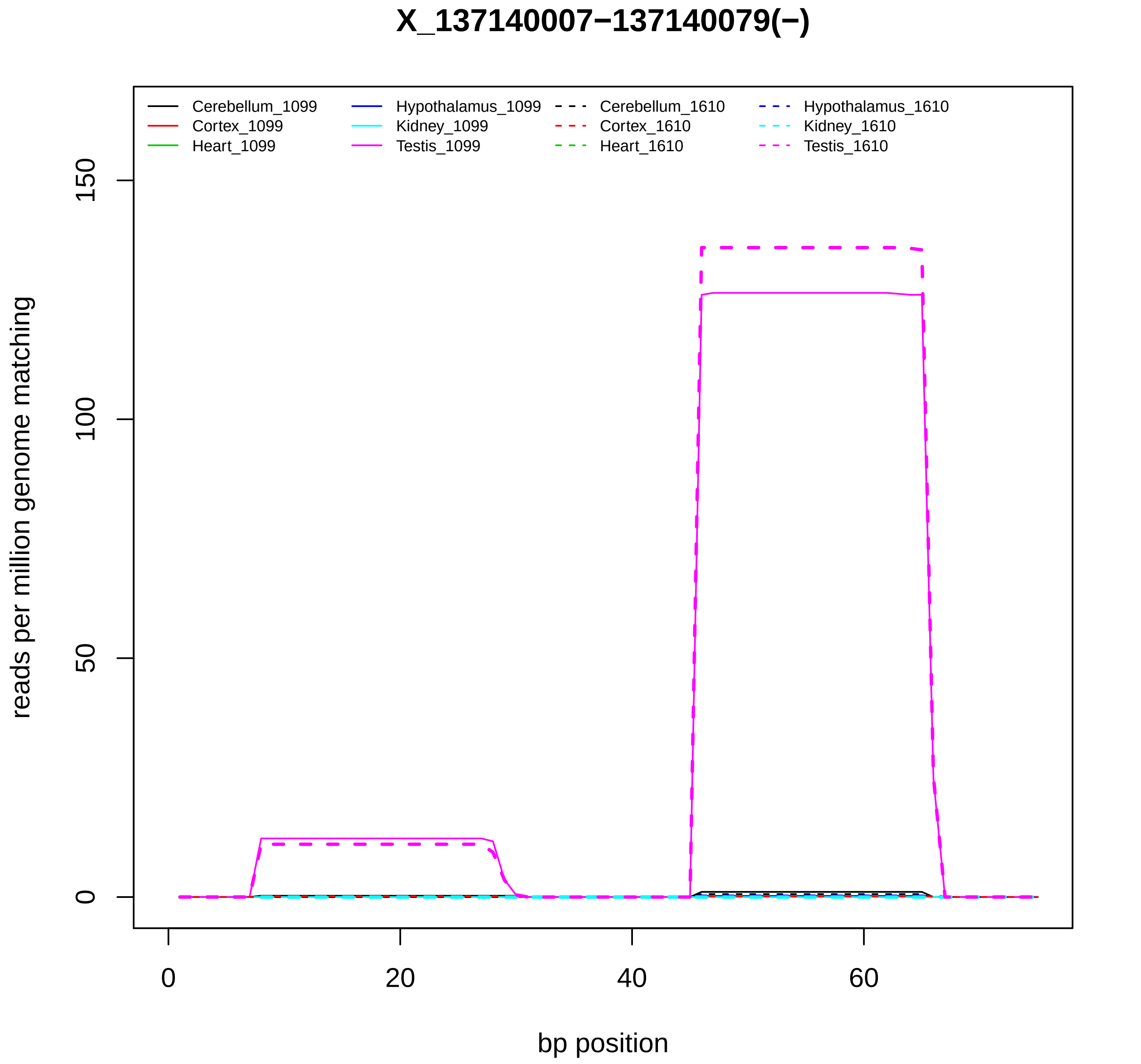
X_137140007−137140079(−)
Cerebellum_1099
Cortex_1099
Heart_1099
Hypothalamus_1099
Kidney_1099
Testis_1099
Cerebellum_1610
Cortex_1610
Heart_1610
Hypothalamus_1610
Kidney_1610
Testis_1610
reads per million genome matching
0
50
100
150
0
20
40
60
bp position

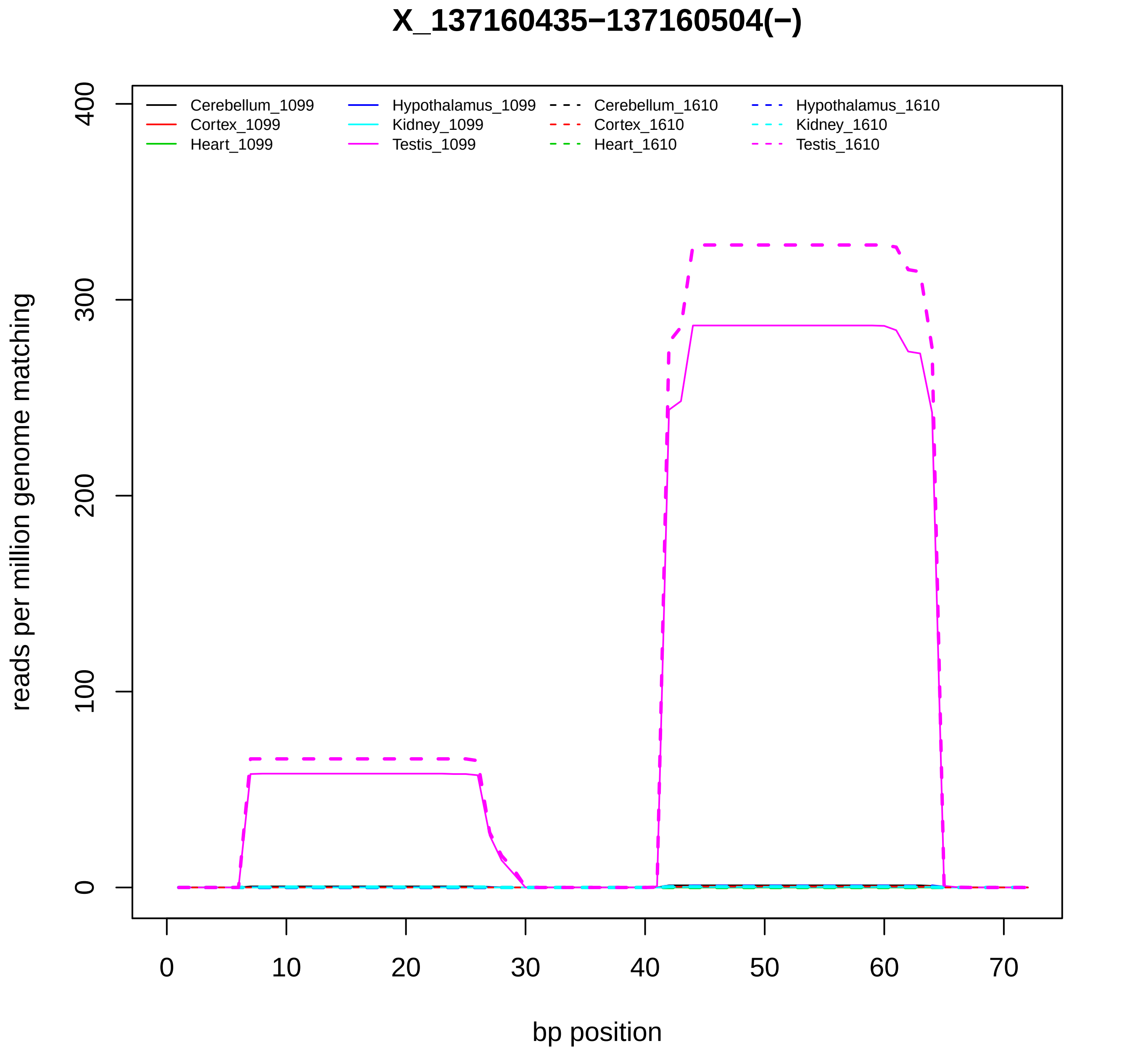

X_137160435−137160504(−)
Cerebellum_1099
Cortex_1099
Heart_1099
Hypothalamus_1099
Kidney_1099
Testis_1099
Cerebellum_1610
Cortex_1610
Heart_1610
Hypothalamus_1610
Kidney_1610
Testis_1610
reads per million genome matching
0
100
200
300
400
0
10
20
30
40
50
60
70
bp position

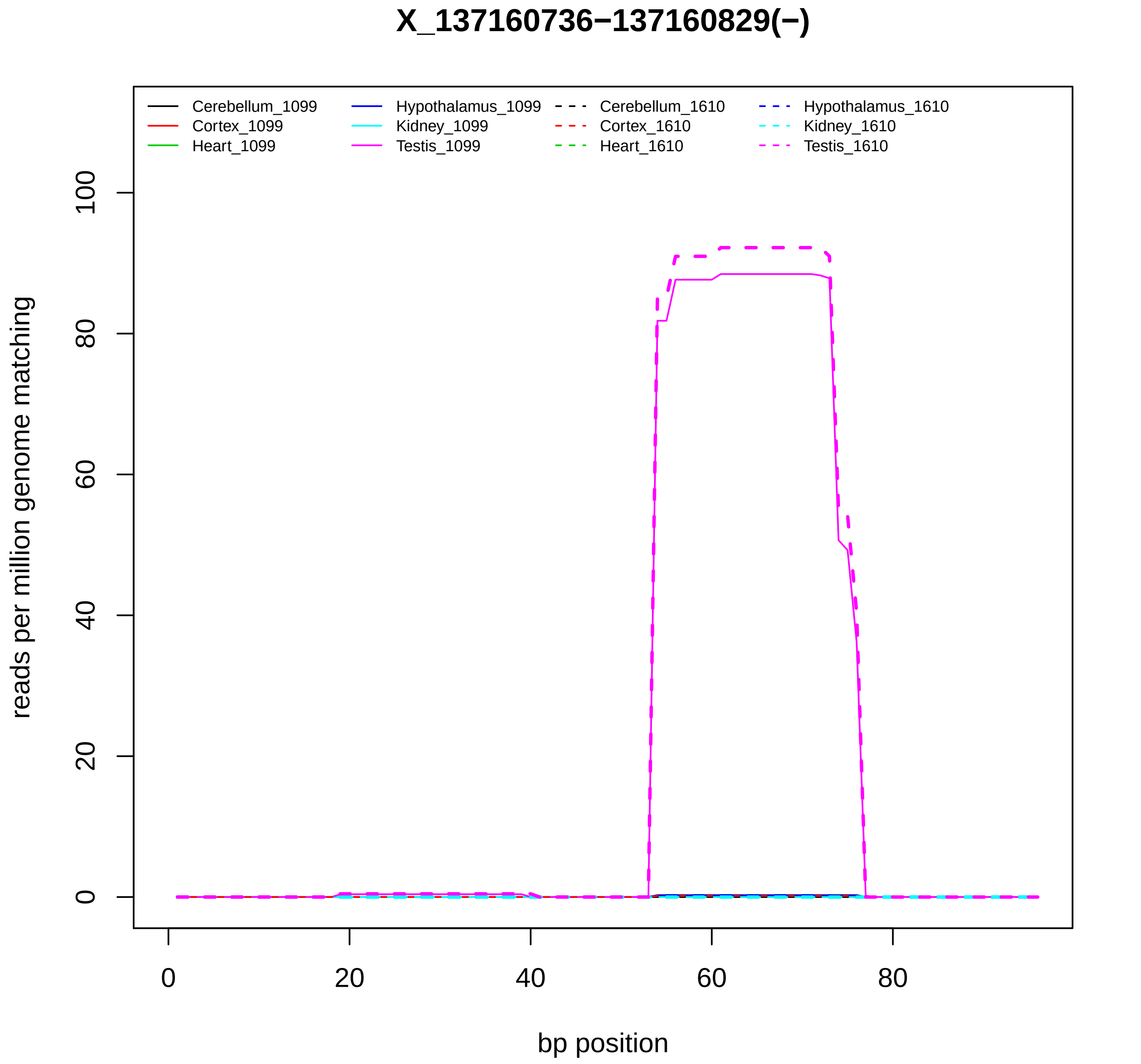

X_137160736−137160829(−)
Cerebellum_1099
Cortex_1099
Heart_1099
Hypothalamus_1099
Kidney_1099
Testis_1099
Cerebellum_1610
Cortex_1610
Heart_1610
Hypothalamus_1610
Kidney_1610
Testis_1610
reads per million genome matching
0
20
40
60
80
100
bp position

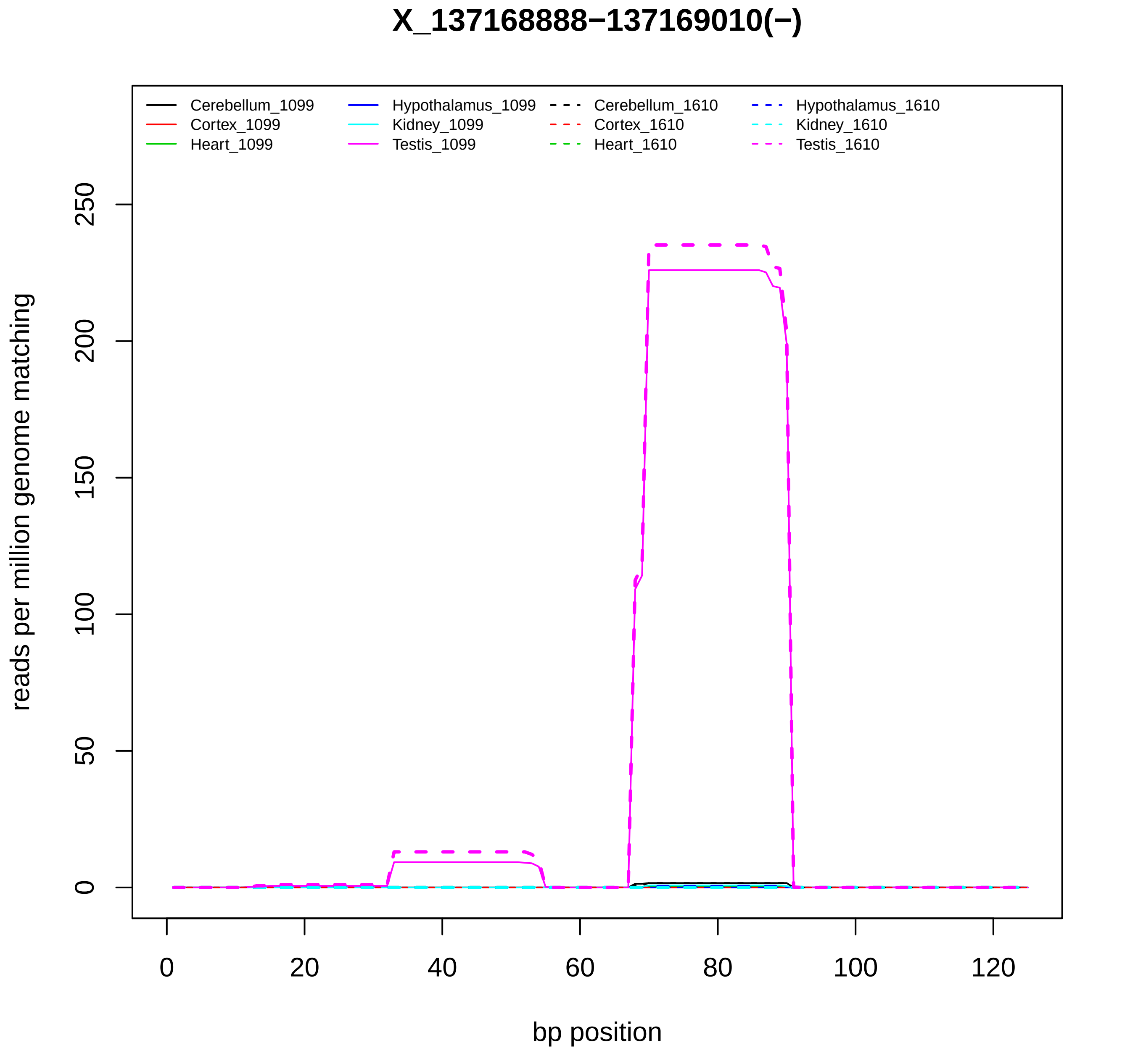

X_137168888−137169010(−)
Cerebellum_1099
Cortex_1099
Heart_1099
Hypothalamus_1099
Kidney_1099
Testis_1099
Cerebellum_1610
Cortex_1610
Heart_1610
Hypothalamus_1610
Kidney_1610
Testis_1610
reads per million genome matching
0
50
100
150
200
250
0
20
40
60
80
100
120
bp position

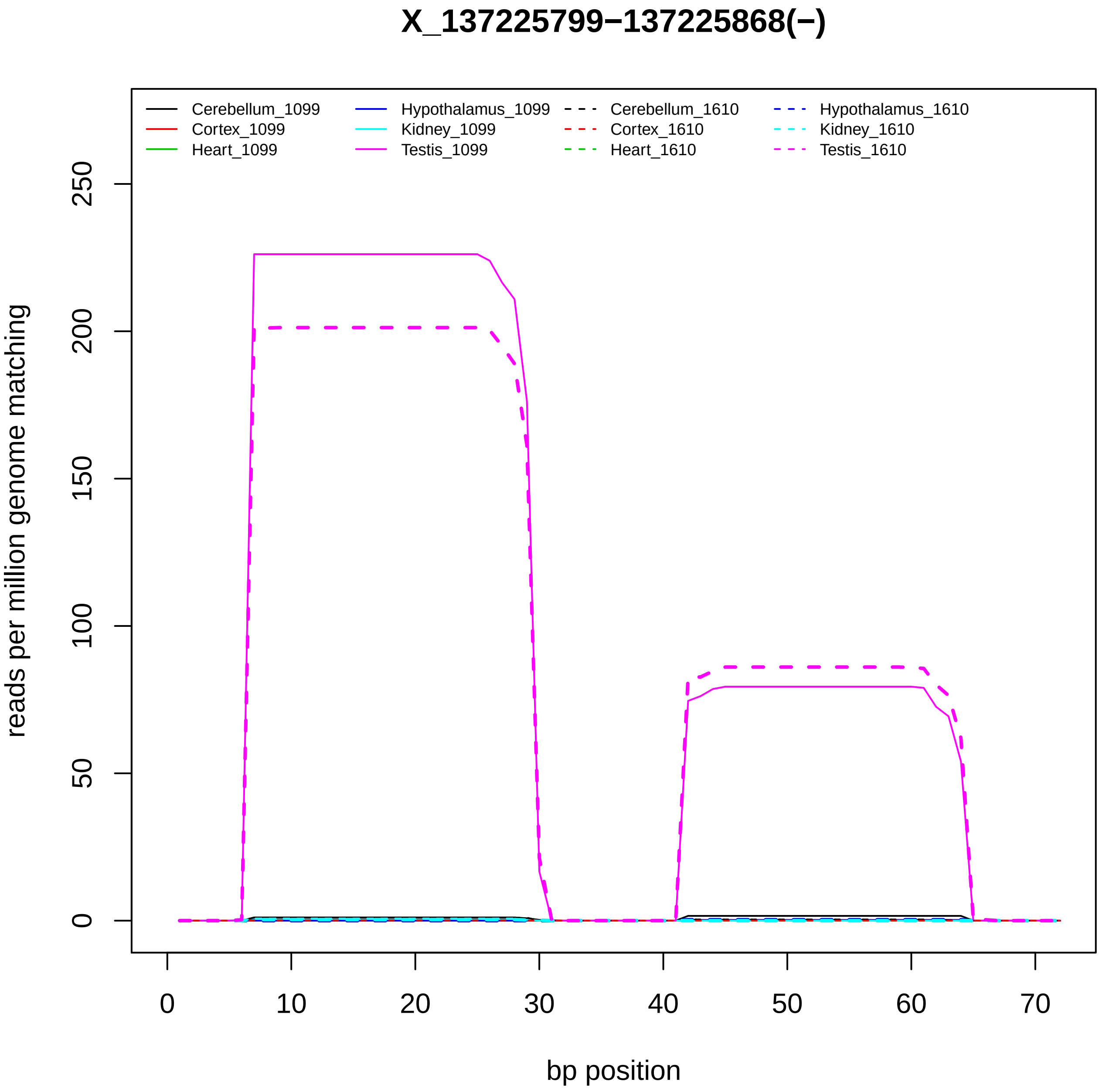

X_137225799−137225868(−)
Cerebellum_1099
Cortex_1099
Heart_1099
Hypothalamus_1099
Kidney_1099
Testis_1099
Cerebellum_1610
Cortex_1610
Heart_1610
Hypothalamus_1610
Kidney_1610
Testis_1610
reads per million genome matching
0
50
100
150
200
250
0
10
20
30
40
50
60
70
bp position

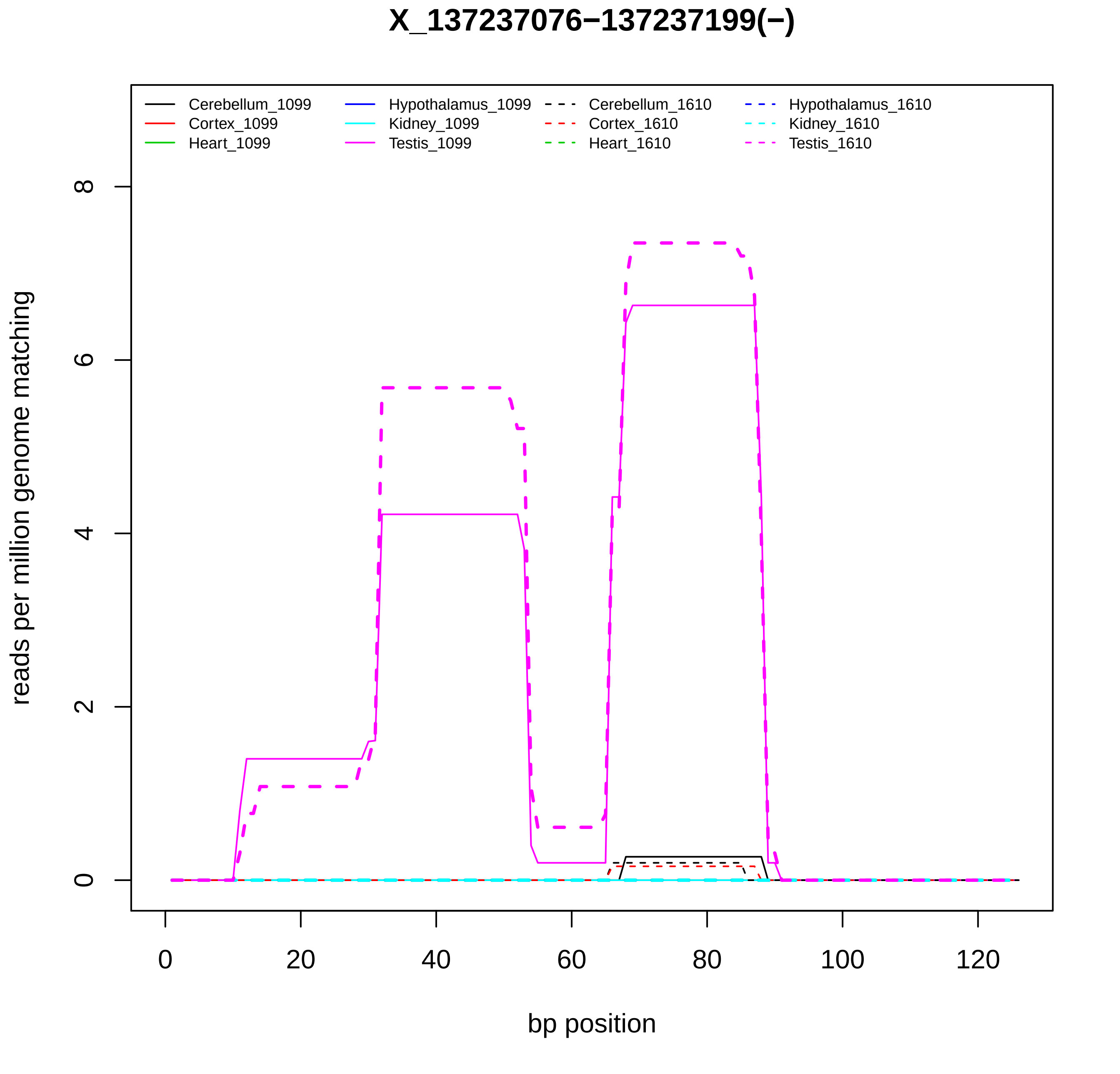

X_137237076−137237199(−)
Cerebellum_1099
Cortex_1099
Heart_1099
Hypothalamus_1099
Kidney_1099
Testis_1099
Cerebellum_1610
Cortex_1610
Heart_1610
Hypothalamus_1610
Kidney_1610
Testis_1610
reads per million genome matching
bp position
0
2
4
6
8
0
20
40
60
80
100
120

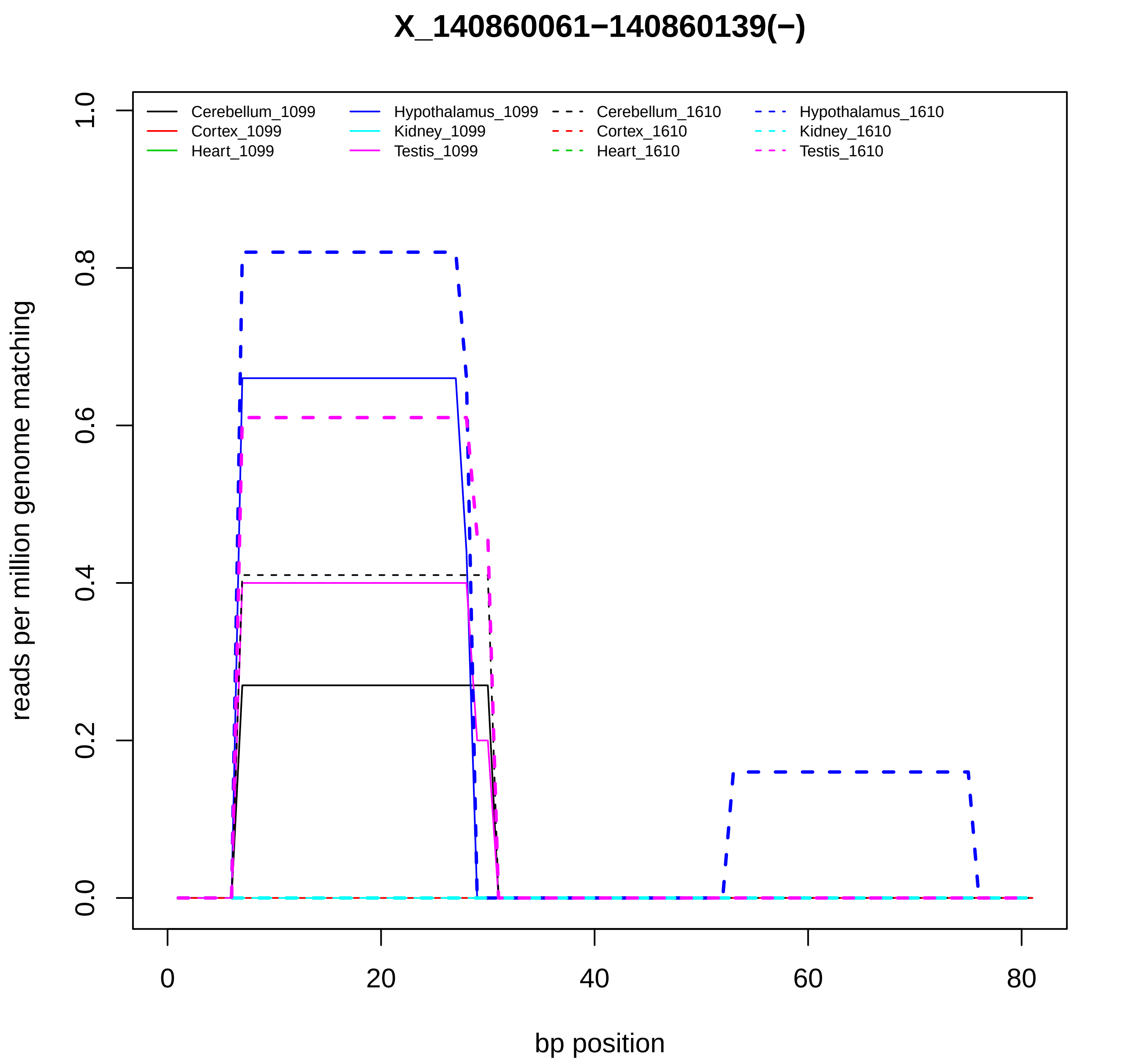
X_140860061−140860139(−)
Cerebellum_1099
Cortex_1099
Heart_1099
Hypothalamus_1099
Kidney_1099
Testis_1099
Cerebellum_1610
Cortex_1610
Heart_1610
Hypothalamus_1610
Kidney_1610
Testis_1610
reads per million genome matching
bp position
0.0
0.2
0.4
0.6
0.8
1.0
0
20
40
60
80

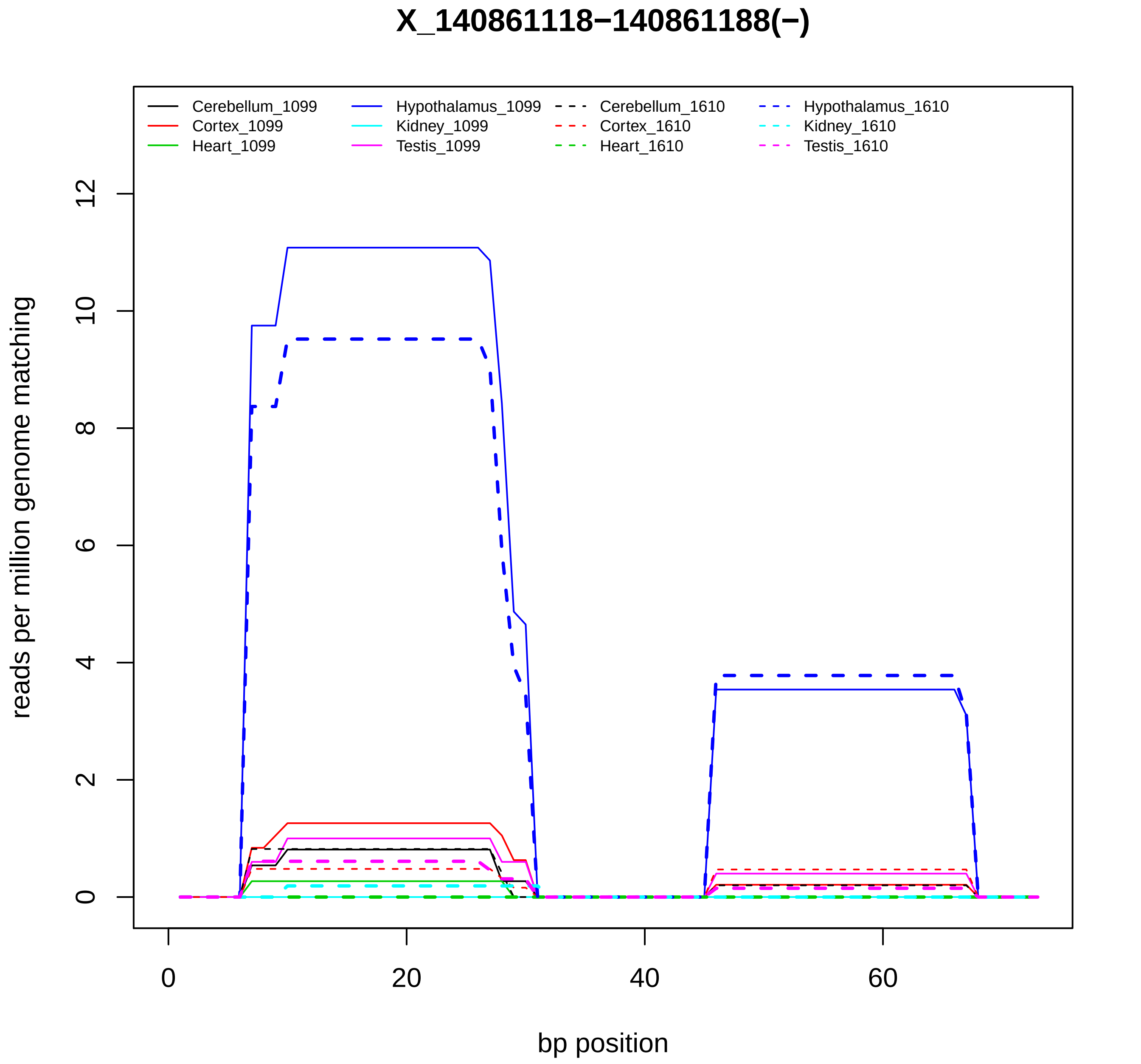

X_140861118−140861188(−)
Cerebellum_1099
Cortex_1099
Heart_1099
Hypothalamus_1099
Kidney_1099
Testis_1099
Cerebellum_1610
Cortex_1610
Heart_1610
Hypothalamus_1610
Kidney_1610
Testis_1610
reads per million genome matching
0
2
4
6
8
10
12
0
20
40
60
bp position

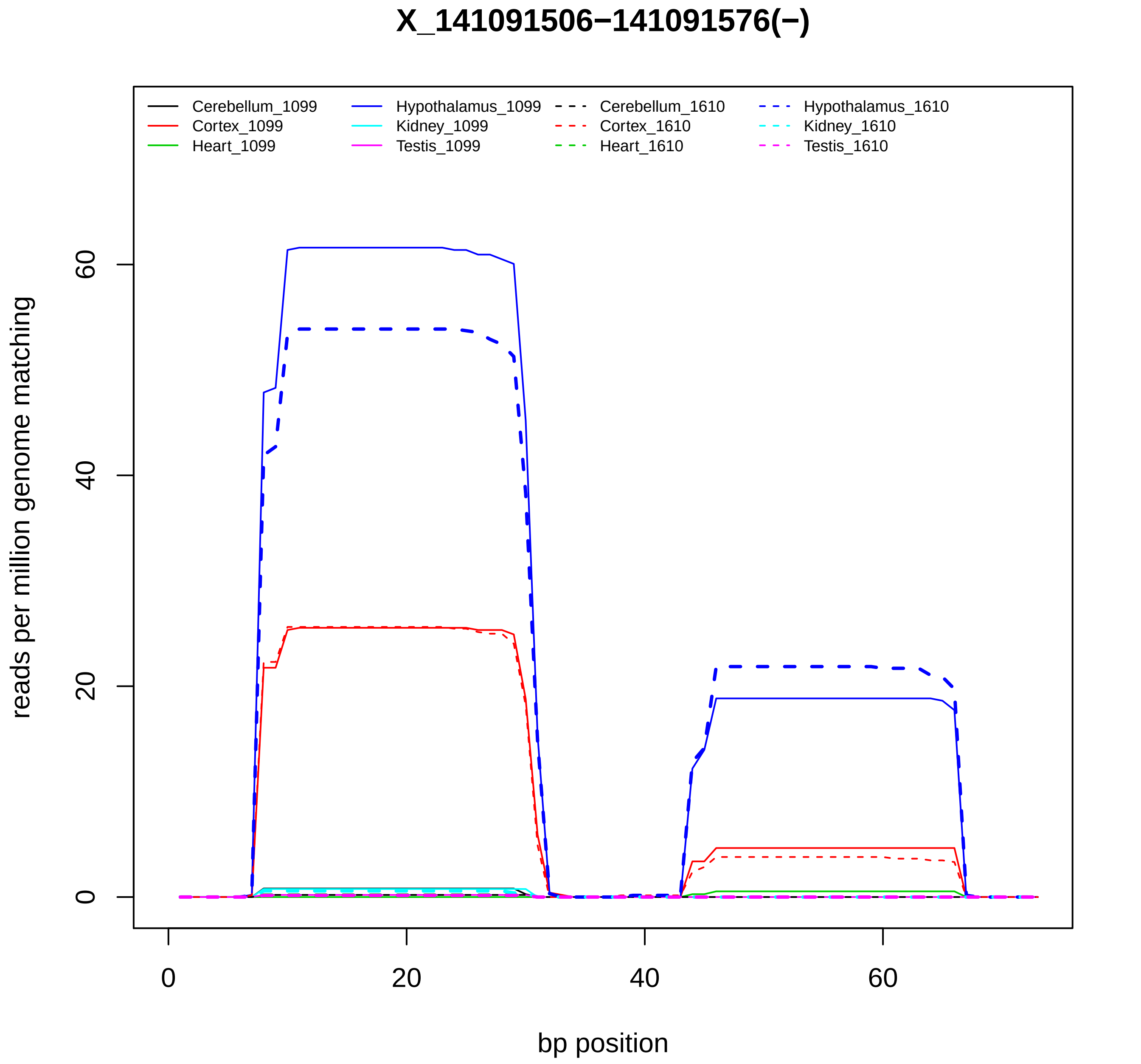
X_141091506−141091576(−)
Cerebellum_1099
Cortex_1099
Heart_1099
Hypothalamus_1099
Kidney_1099
Testis_1099
Cerebellum_1610
Cortex_1610
Heart_1610
Hypothalamus_1610
Kidney_1610
Testis_1610
reads per million genome matching
bp position
0
20
40
60
0
20
40
60

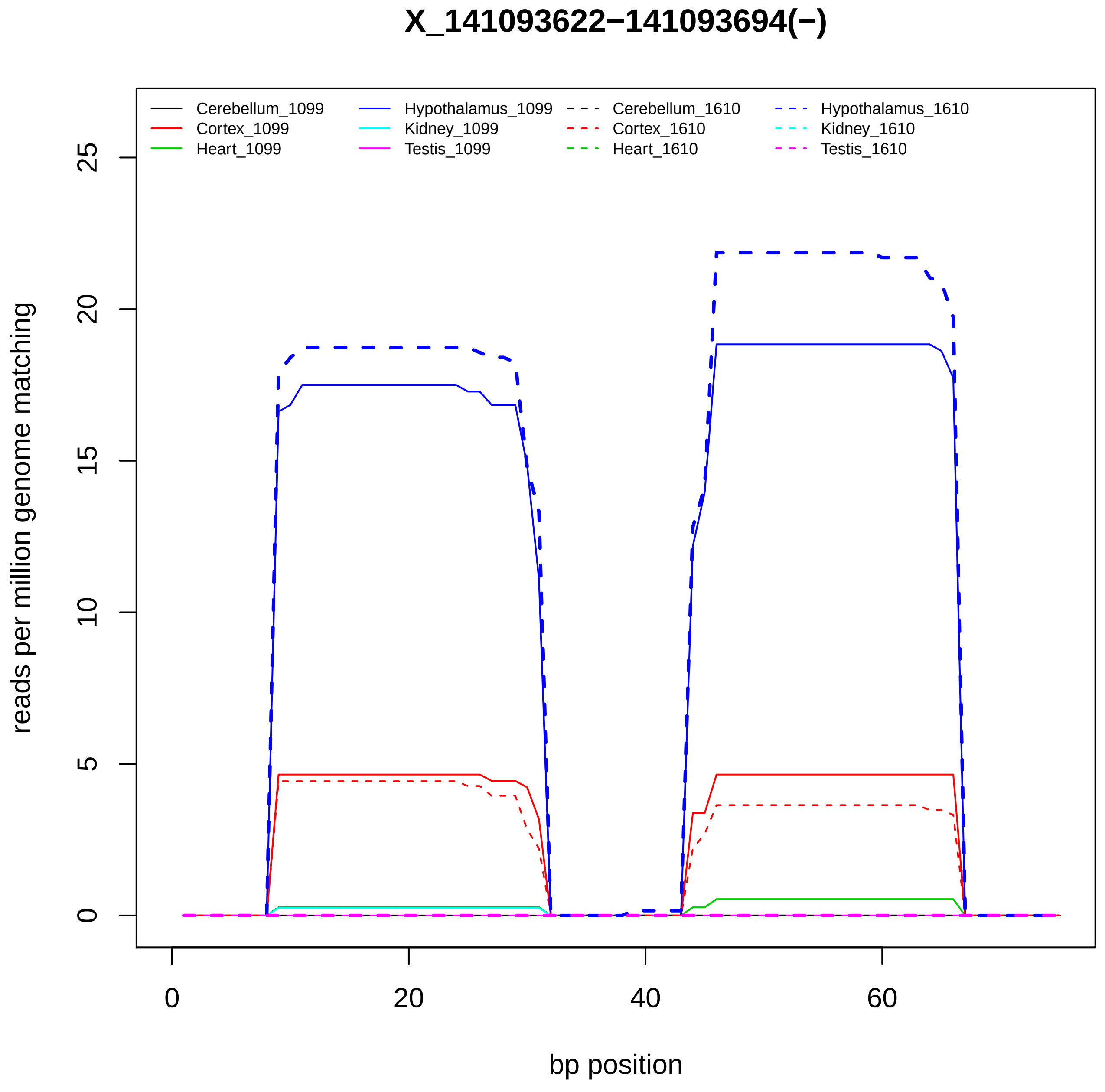
X_141093622−141093694(−)
Cerebellum_1099
Cortex_1099
Heart_1099
Hypothalamus_1099
Kidney_1099
Testis_1099
Cerebellum_1610
Cortex_1610
Heart_1610
Hypothalamus_1610
Kidney_1610
Testis_1610
reads per million genome matching
0
5
10
15
20
25
0
20
40
60
bp position

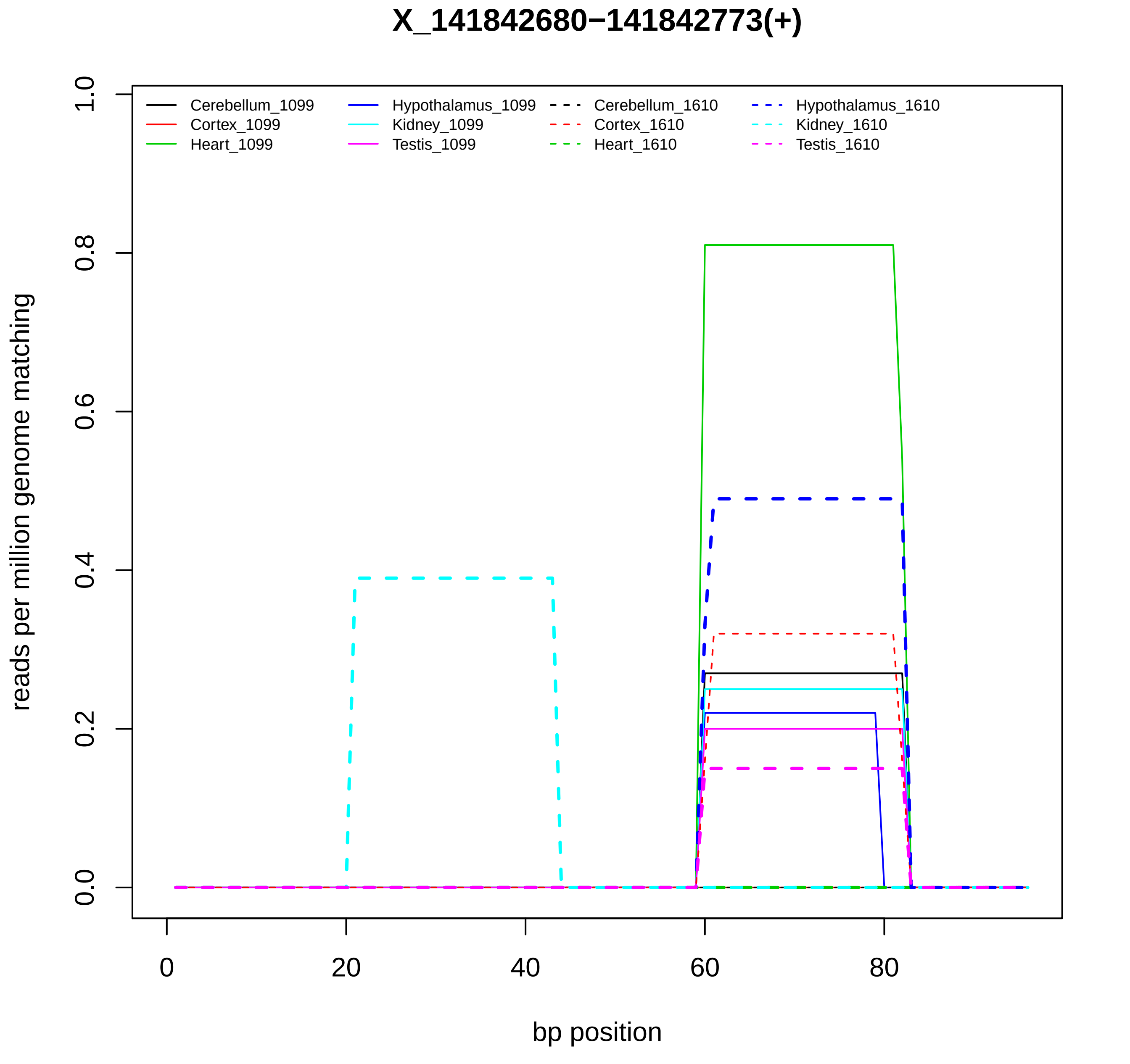

X_141842680−141842773(+)
Cerebellum_1099
Cortex_1099
Heart_1099
Hypothalamus_1099
Kidney_1099
Testis_1099
Cerebellum_1610
Cortex_1610
Heart_1610
Hypothalamus_1610
Kidney_1610
Testis_1610
reads per million genome matching
0.0
0.2
0.4
0.6
0.8
1.0
0
20
40
60
80
bp position

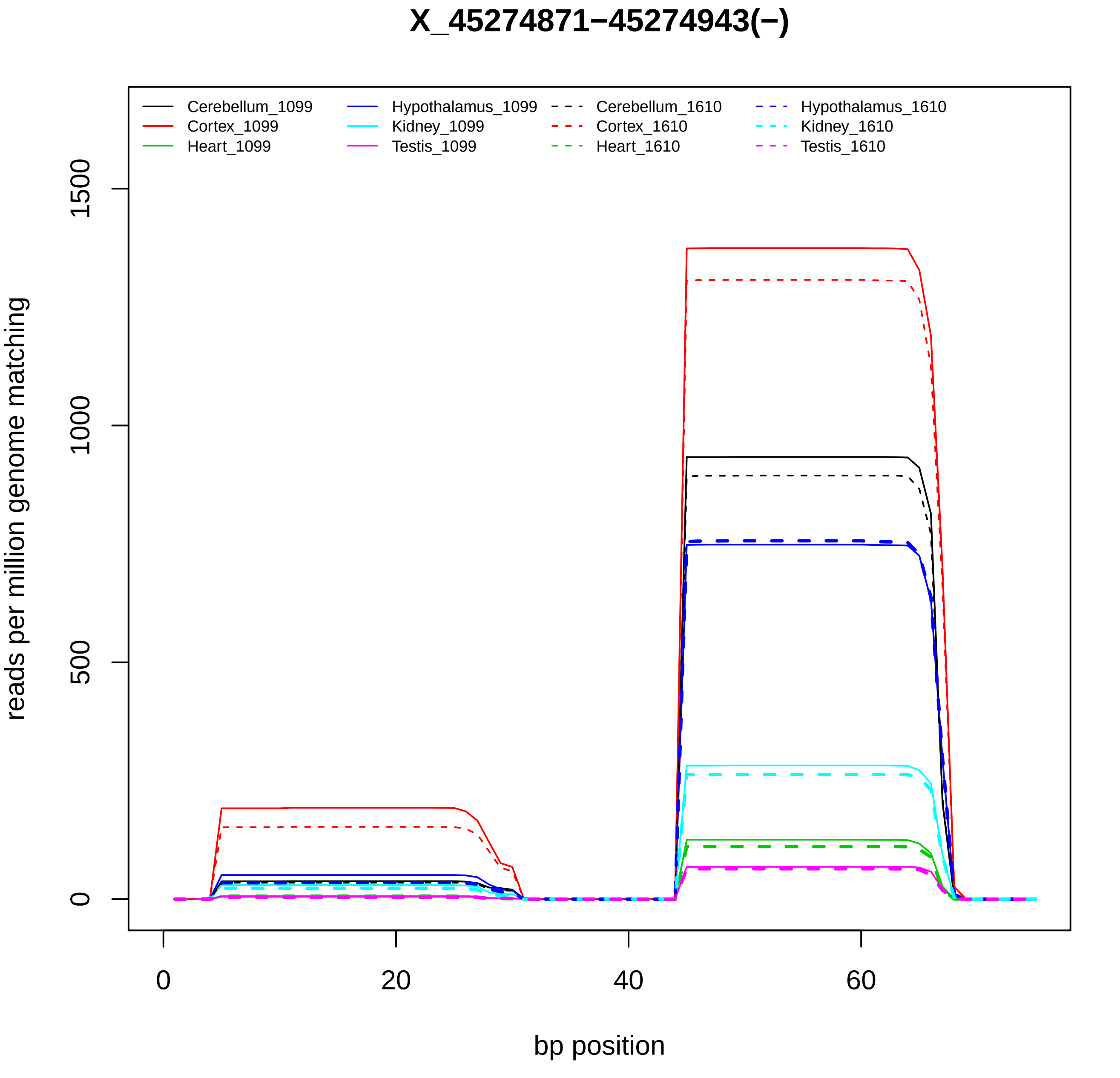

X_45274871−45274943(−)
Cerebellum_1099
Cortex_1099
Heart_1099
Hypothalamus_1099
Kidney_1099
Testis_1099
Cerebellum_1610
Cortex_1610
Heart_1610
Hypothalamus_1610
Kidney_1610
Testis_1610
reads per million genome matching
0
500
1000
1500
0
20
40
60
bp position

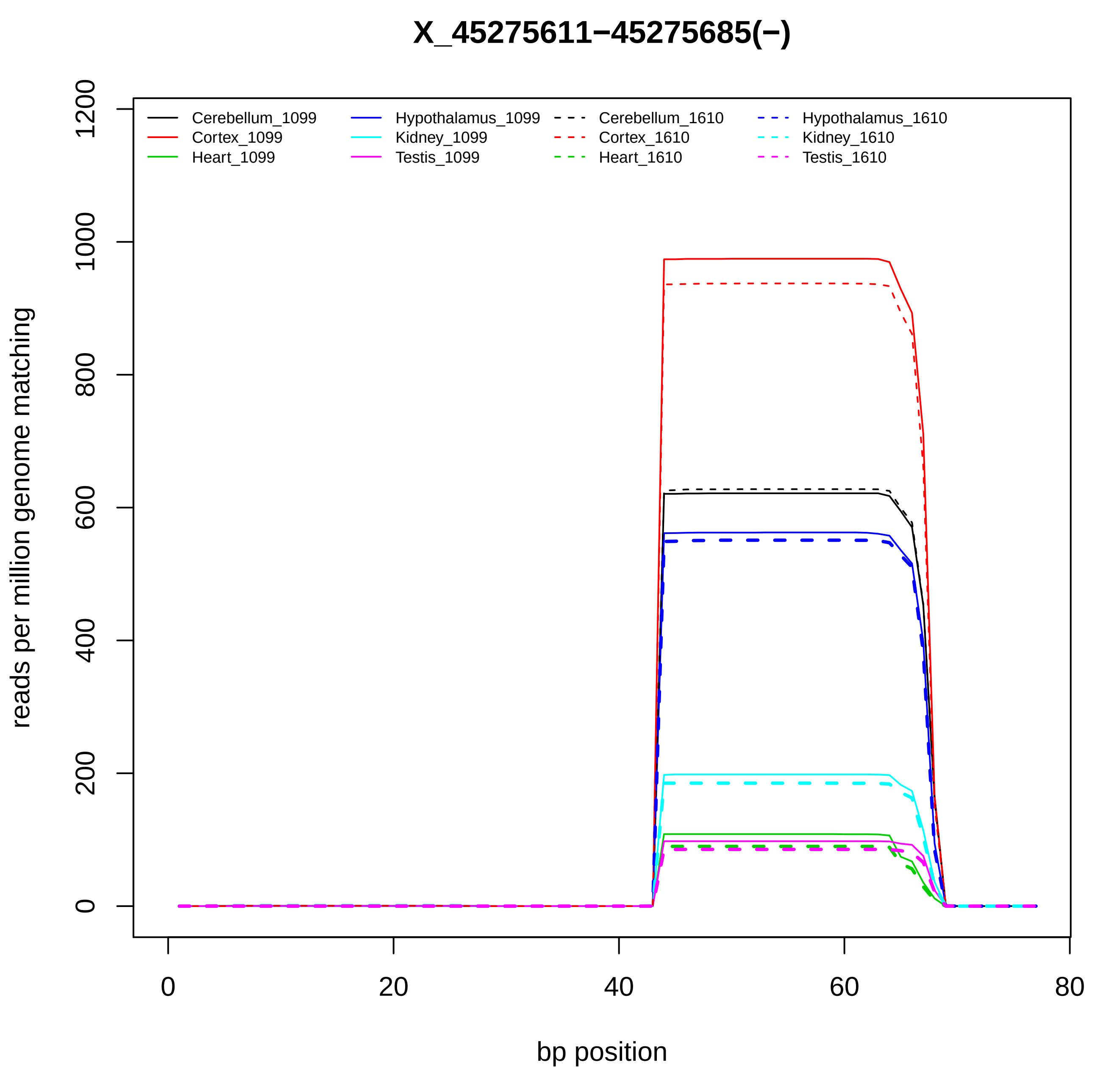
X_45275611−45275685(−)
Cerebellum_1099
Cortex_1099
Heart_1099
Hypothalamus_1099
Kidney_1099
Testis_1099
Cerebellum_1610
Cortex_1610
Heart_1610
Hypothalamus_1610
Kidney_1610
Testis_1610
reads per million genome matching
bp position
0
200
400
600
800
1000
1200
0
20
40
60
80

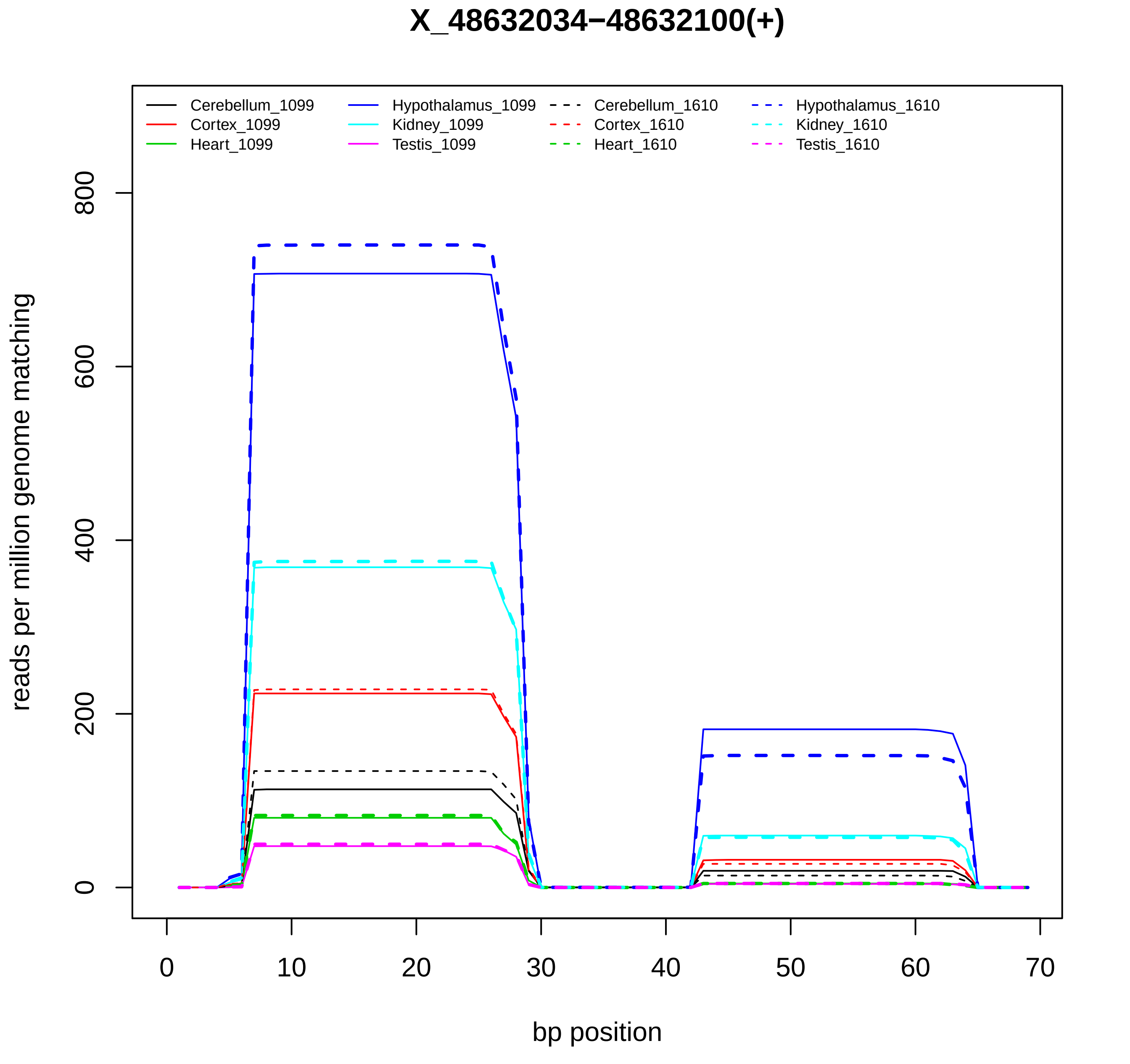

X_48632034−48632100(+)
Cerebellum_1099
Cortex_1099
Heart_1099
Hypothalamus_1099
Kidney_1099
Testis_1099
Cerebellum_1610
Cortex_1610
Heart_1610
Hypothalamus_1610
Kidney_1610
Testis_1610
reads per million genome matching
0
200
400
600
800
0
10
20
30
40
50
60
70
bp position

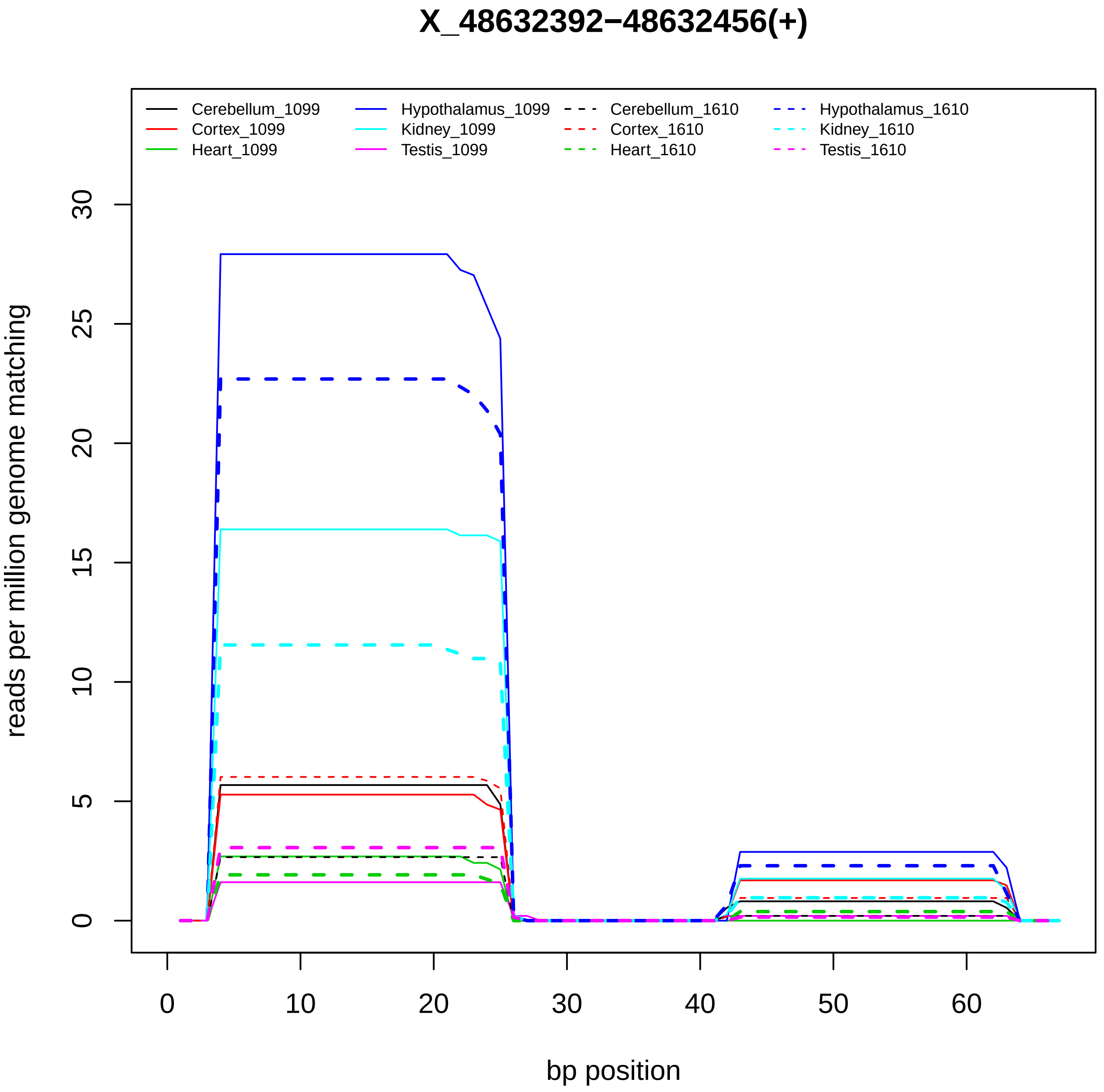

X_48632392−48632456(+)
Cerebellum_1099
Cortex_1099
Heart_1099
Hypothalamus_1099
Kidney_1099
Testis_1099
Cerebellum_1610
Cortex_1610
Heart_1610
Hypothalamus_1610
Kidney_1610
Testis_1610
reads per million genome matching
0
5
10
15
20
25
30
0
10
20
30
40
50
60
bp position

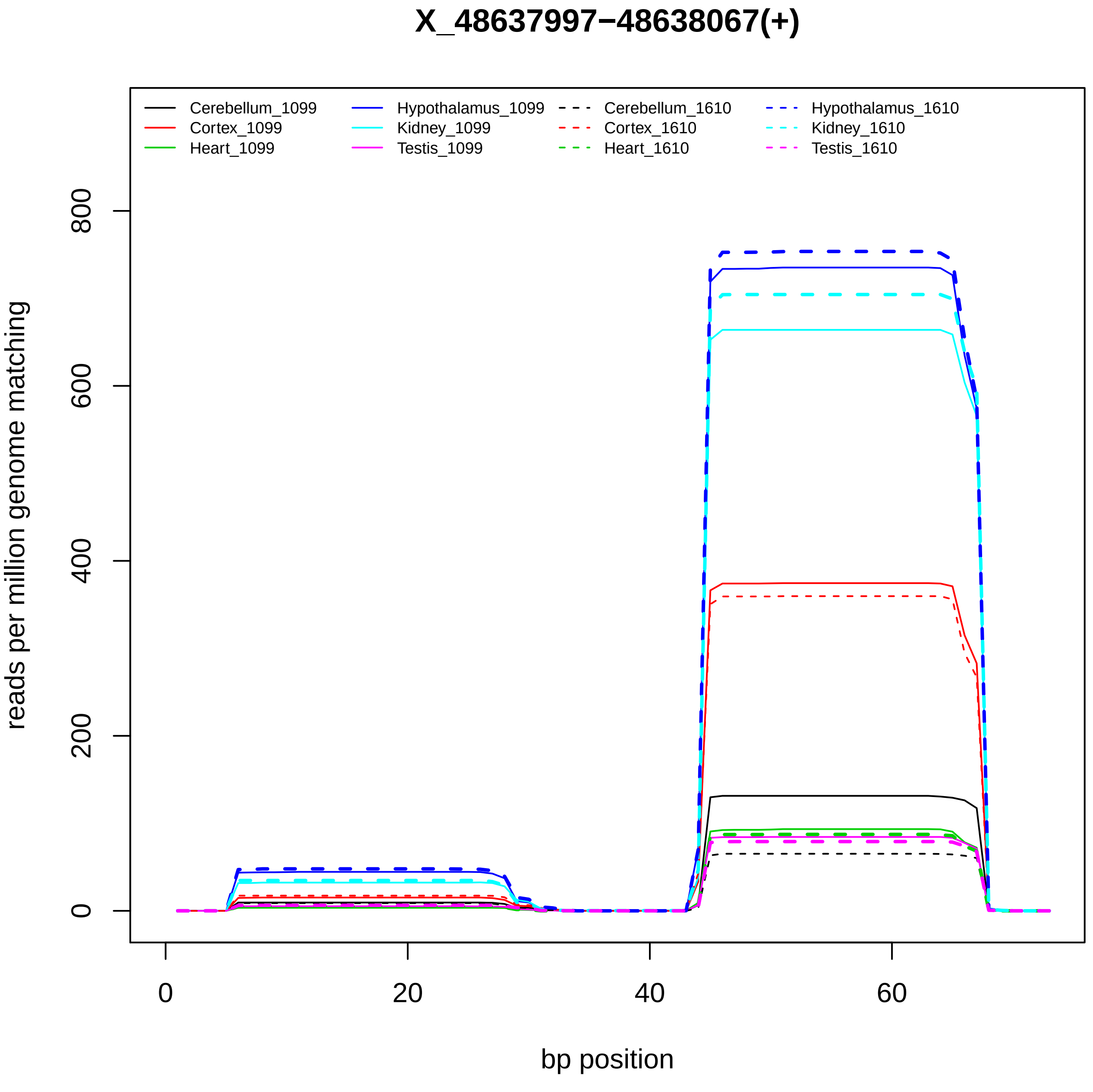

X_48637997−48638067(+)
Cerebellum_1099
Cortex_1099
Heart_1099
Hypothalamus_1099
Kidney_1099
Testis_1099
Cerebellum_1610
Cortex_1610
Heart_1610
Hypothalamus_1610
Kidney_1610
Testis_1610
reads per million genome matching
0
200
400
600
800
0
20
40
60
bp position

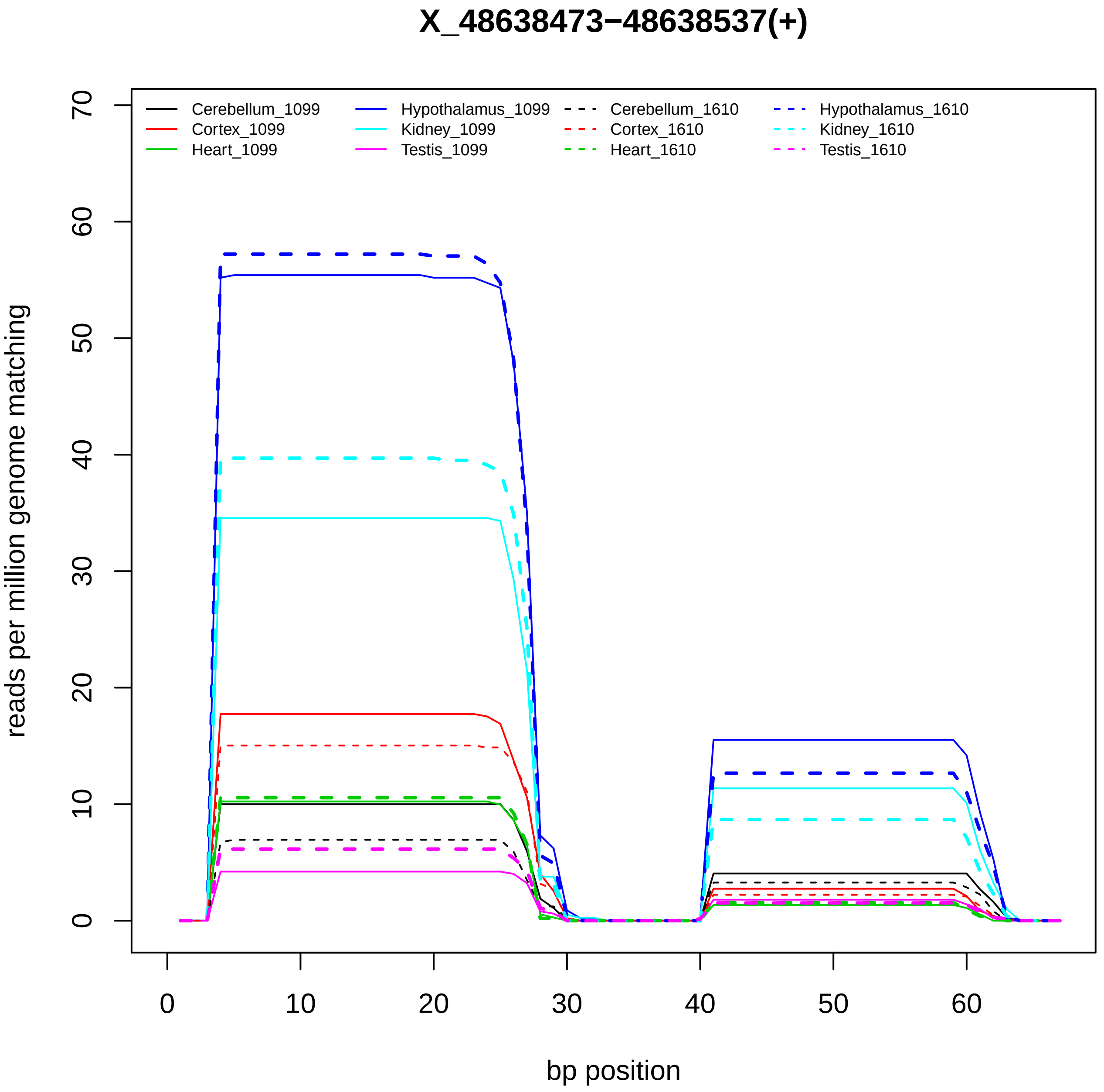

X_48638473−48638537(+)
Cerebellum_1099
Cortex_1099
Heart_1099
Hypothalamus_1099
Kidney_1099
Testis_1099
Cerebellum_1610
Cortex_1610
Heart_1610
Hypothalamus_1610
Kidney_1610
Testis_1610
reads per million genome matching
bp position
0
10
20
30
40
50
60
70

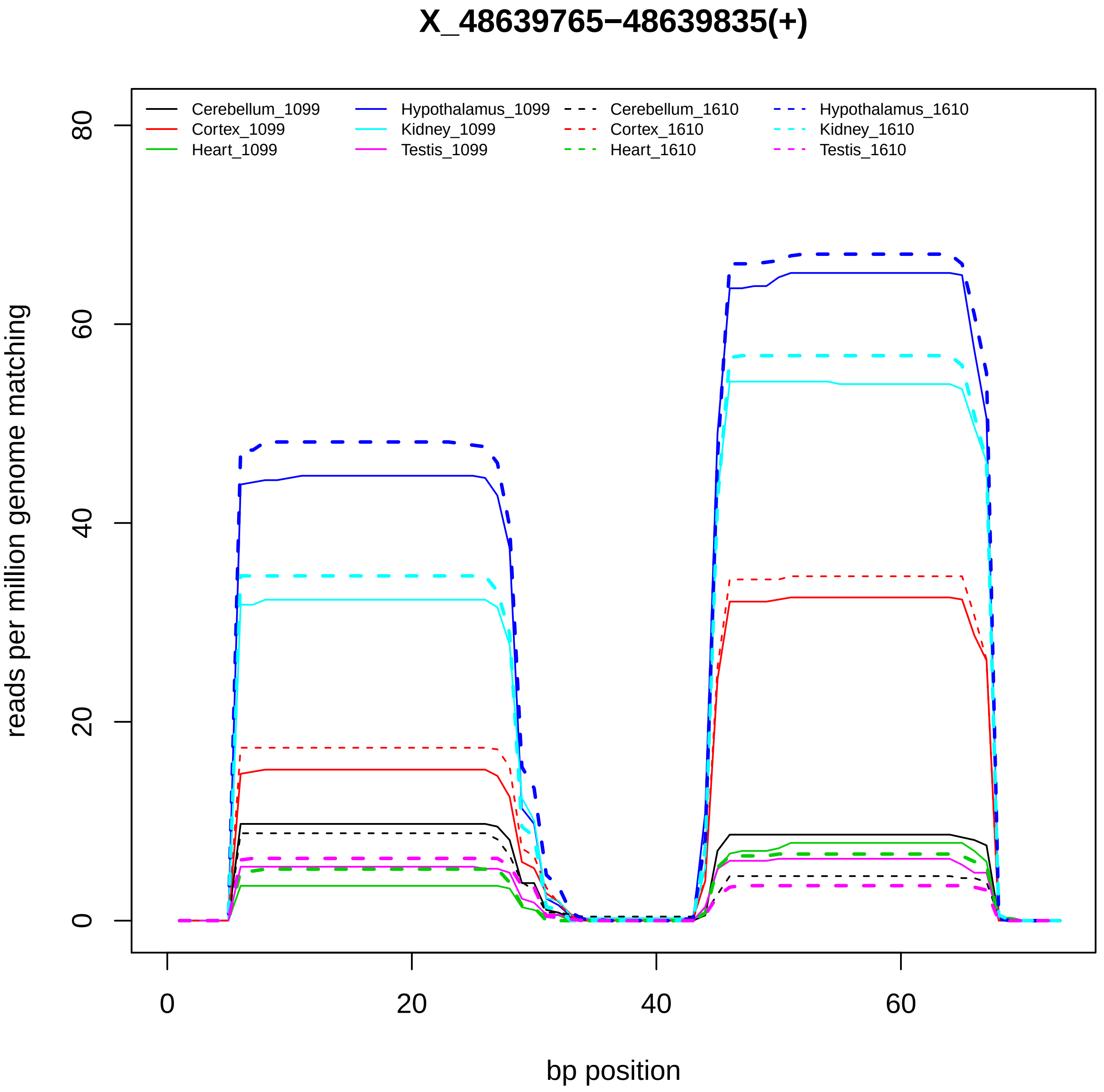
X_48639765−48639835(+)
Cerebellum_1099
Cortex_1099
Heart_1099
Hypothalamus_1099
Kidney_1099
Testis_1099
Cerebellum_1610
Cortex_1610
Heart_1610
Hypothalamus_1610
Kidney_1610
Testis_1610
reads per million genome matching
0
20
40
60
80
bp position
0
20
40
60

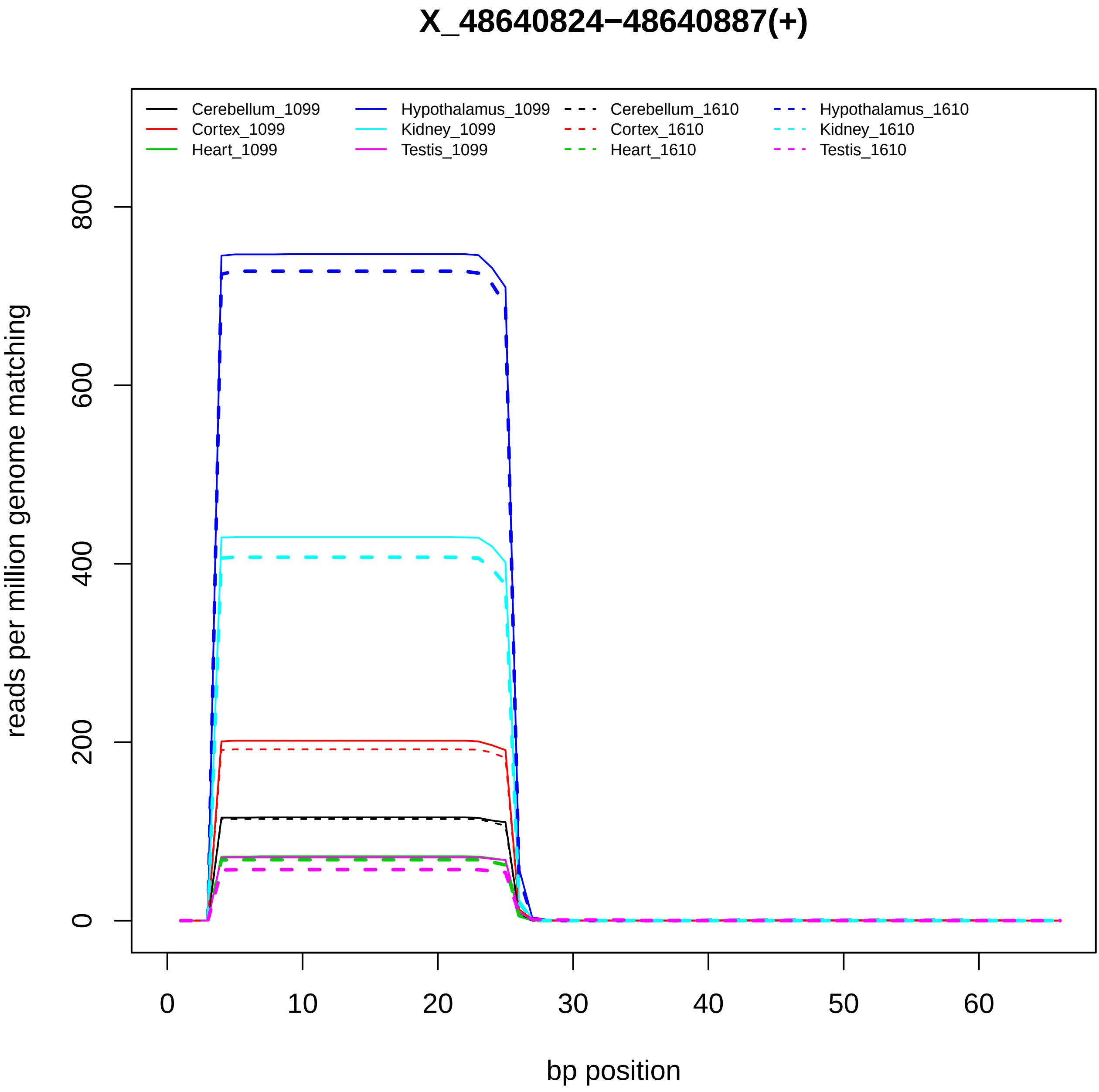
X_48640824−48640887(+)
Cerebellum_1099
Cortex_1099
Heart_1099
Hypothalamus_1099
Kidney_1099
Testis_1099
Cerebellum_1610
Cortex_1610
Heart_1610
Hypothalamus_1610
Kidney_1610
Testis_1610
reads per million genome matching
0
200
400
600
800
bp position
0
10
20
30
40
50
60

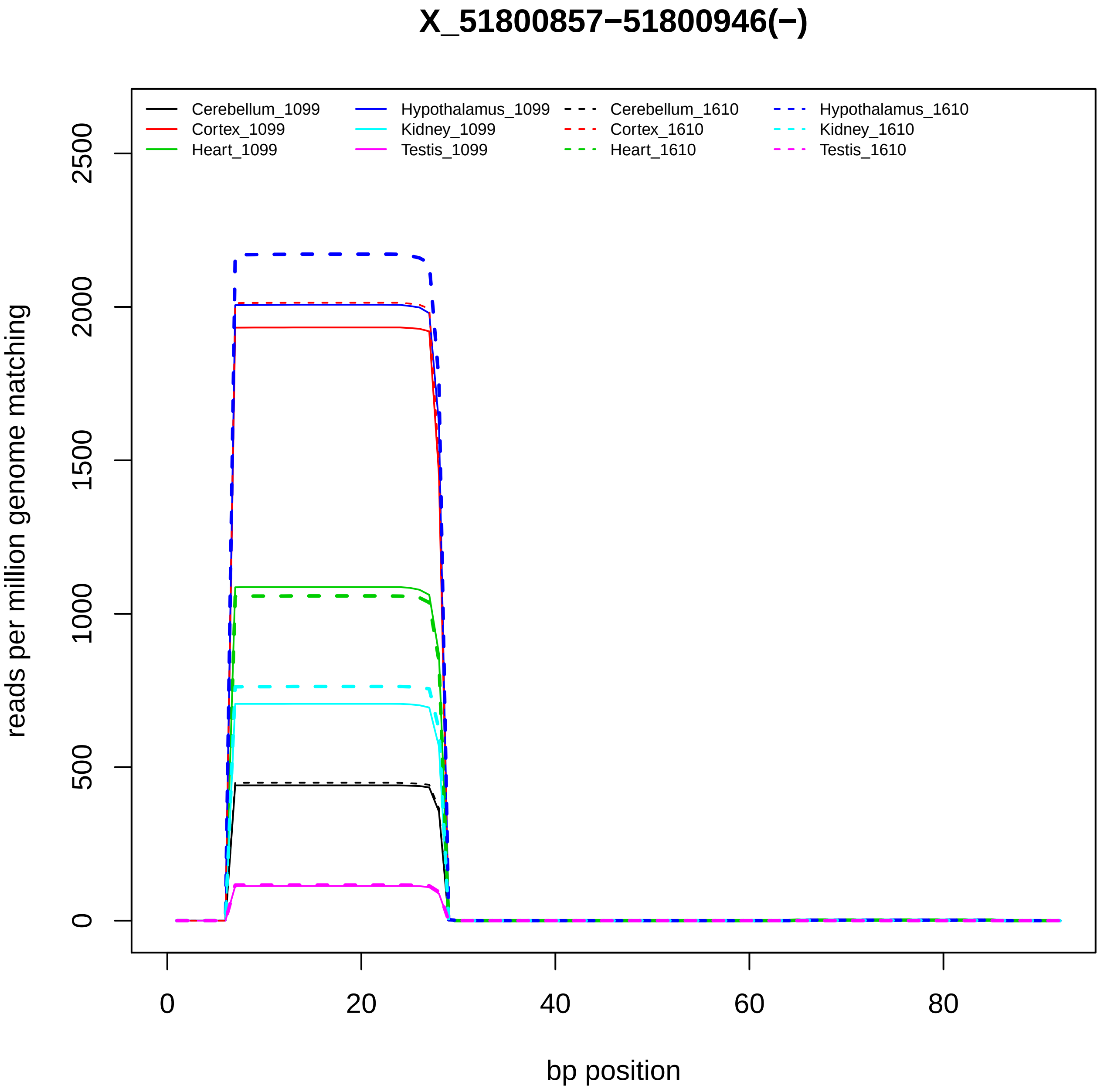

X_51800857−51800946(−)
Cerebellum_1099
Cortex_1099
Heart_1099
Hypothalamus_1099
Kidney_1099
Testis_1099
Cerebellum_1610
Cortex_1610
Heart_1610
Hypothalamus_1610
Kidney_1610
Testis_1610
reads per million genome matching
0
500
1000
1500
2000
2500
0
20
40
60
80
bp position

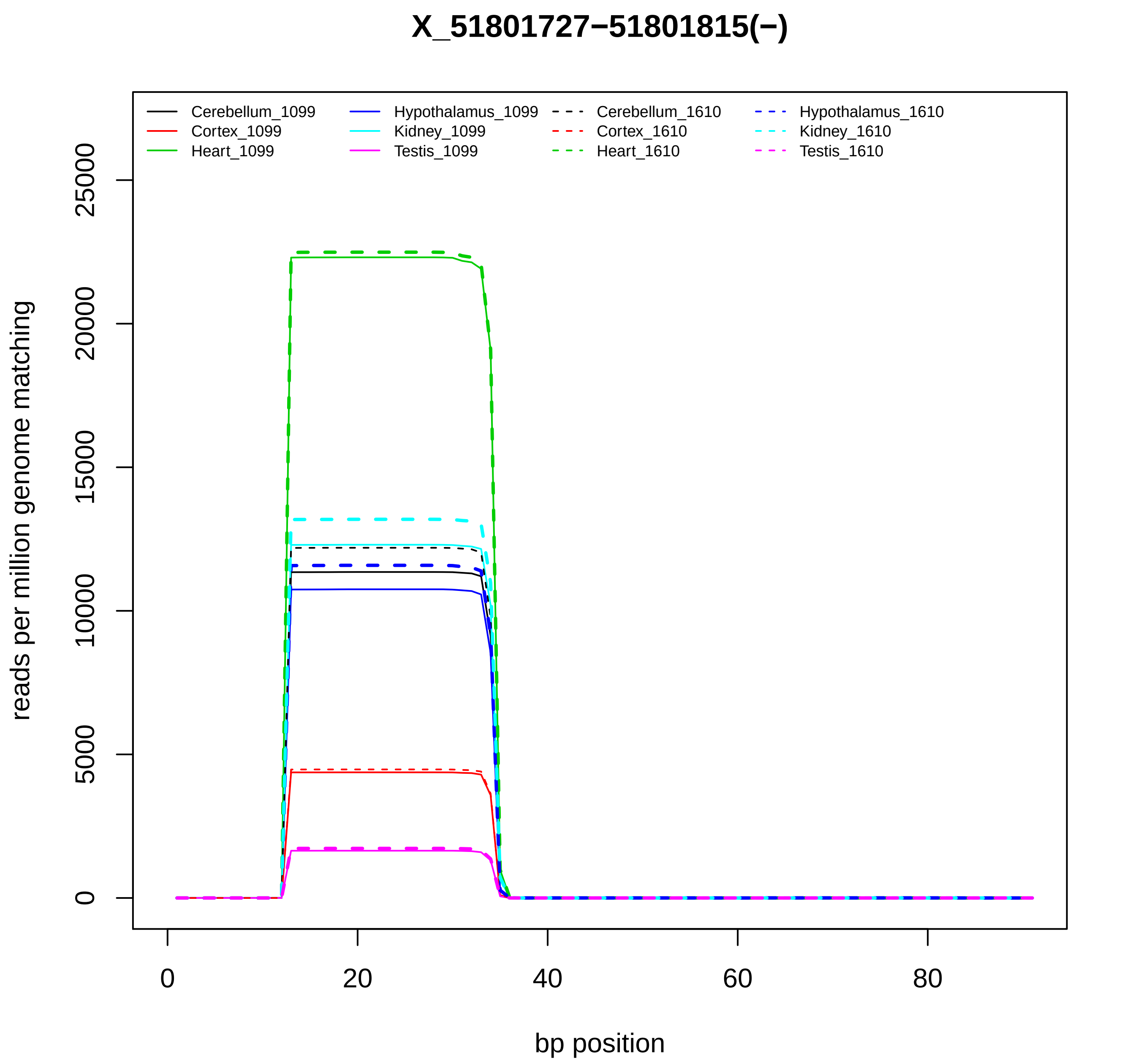
X_51801727−51801815(−)
Cerebellum_1099
Cortex_1099
Heart_1099
Hypothalamus_1099
Kidney_1099
Testis_1099
Cerebellum_1610
Cortex_1610
Heart_1610
Hypothalamus_1610
Kidney_1610
Testis_1610
reads per million genome matching
0
5000
10000
15000
20000
25000
0
20
40
60
80
bp position

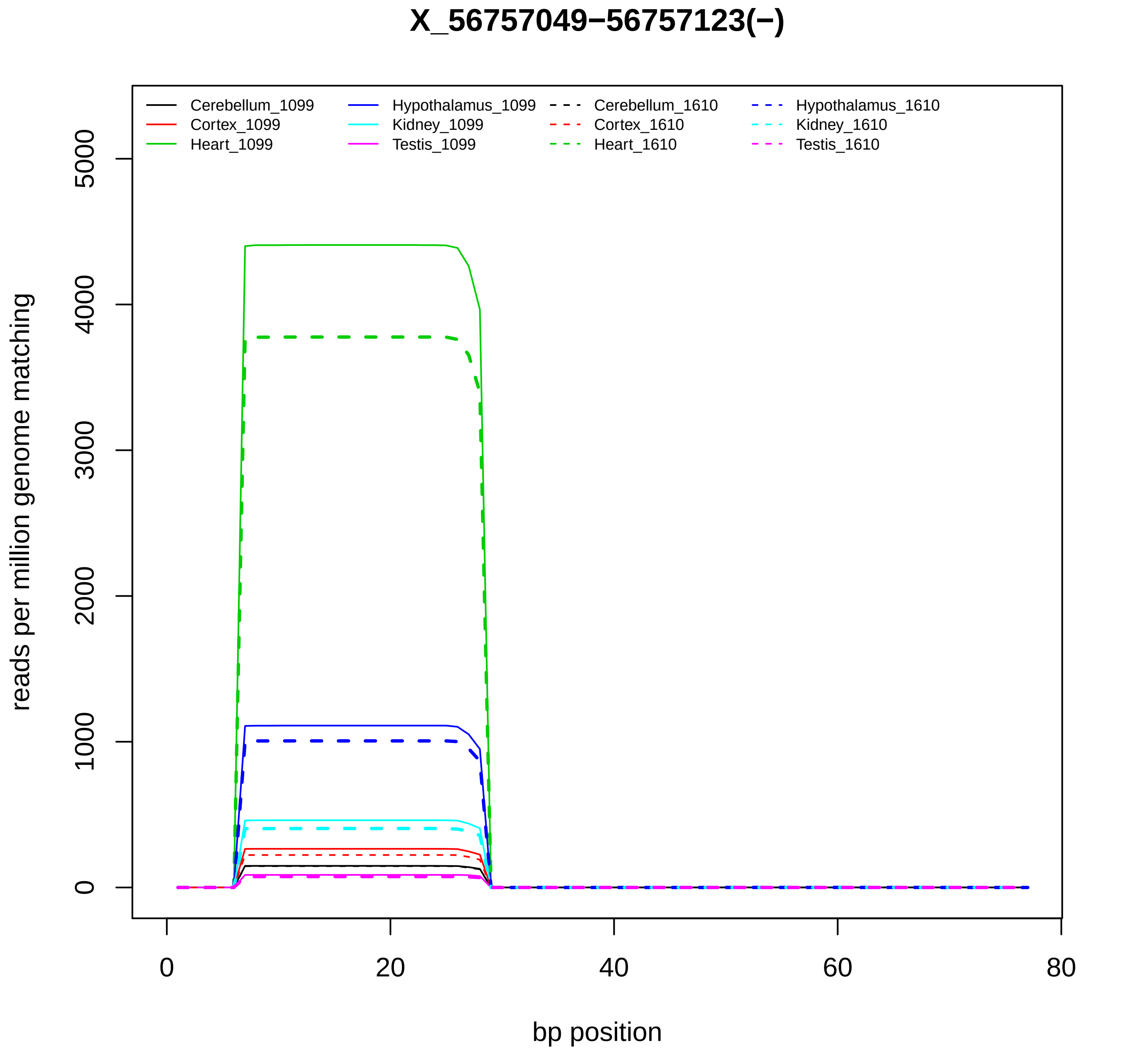

X_56757049−56757123(−)
Cerebellum_1099
Cortex_1099
Heart_1099
Hypothalamus_1099
Kidney_1099
Testis_1099
Cerebellum_1610
Cortex_1610
Heart_1610
Hypothalamus_1610
Kidney_1610
Testis_1610
reads per million genome matching
0
1000
2000
3000
4000
5000
0
20
40
60
80
bp position

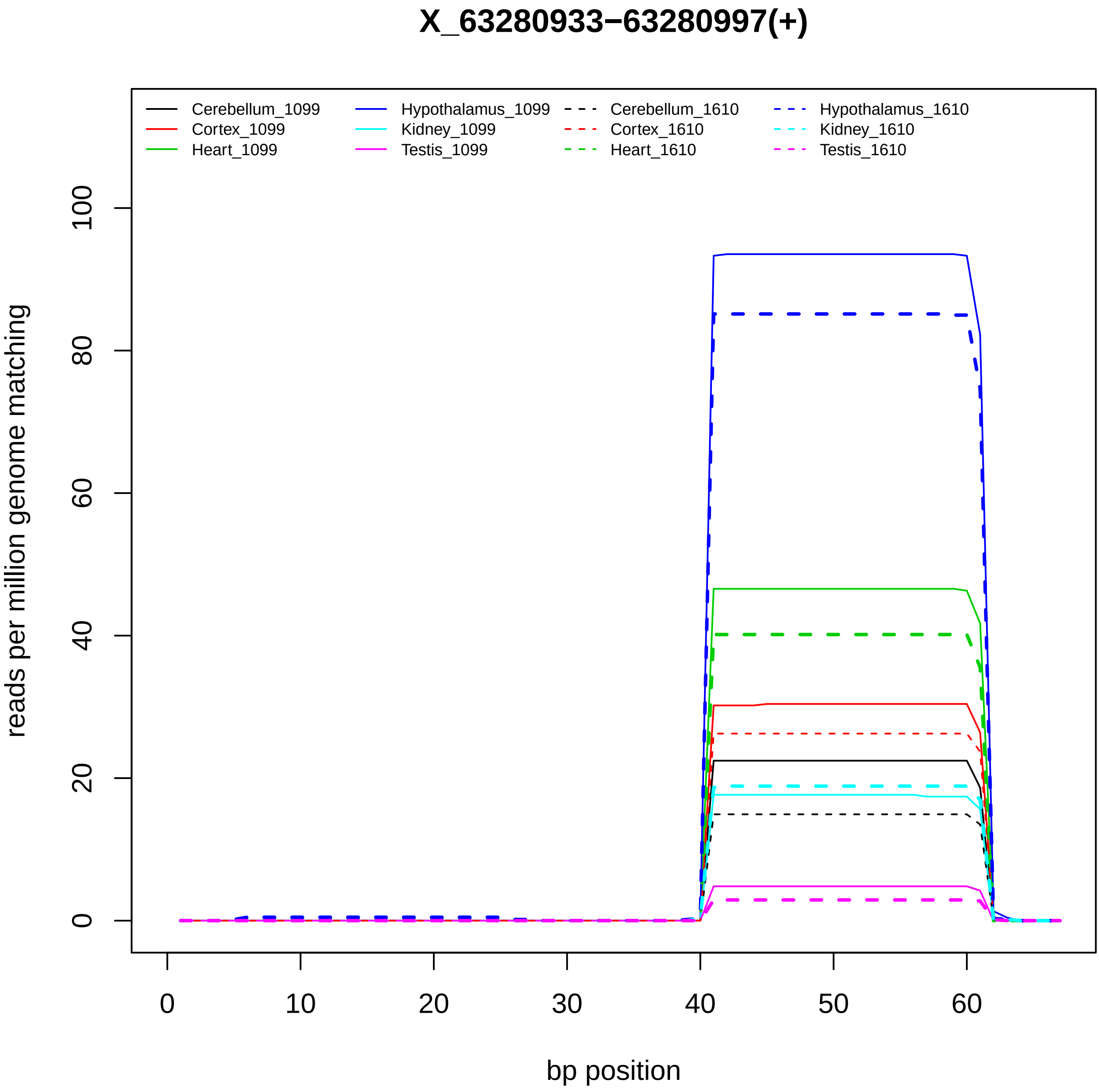

X_63280933−63280997(+)
Cerebellum_1099
Cortex_1099
Heart_1099
Hypothalamus_1099
Kidney_1099
Testis_1099
Cerebellum_1610
Cortex_1610
Heart_1610
Hypothalamus_1610
Kidney_1610
Testis_1610
reads per million genome matching
0
20
40
60
80
100
0
10
20
30
40
50
60
bp position

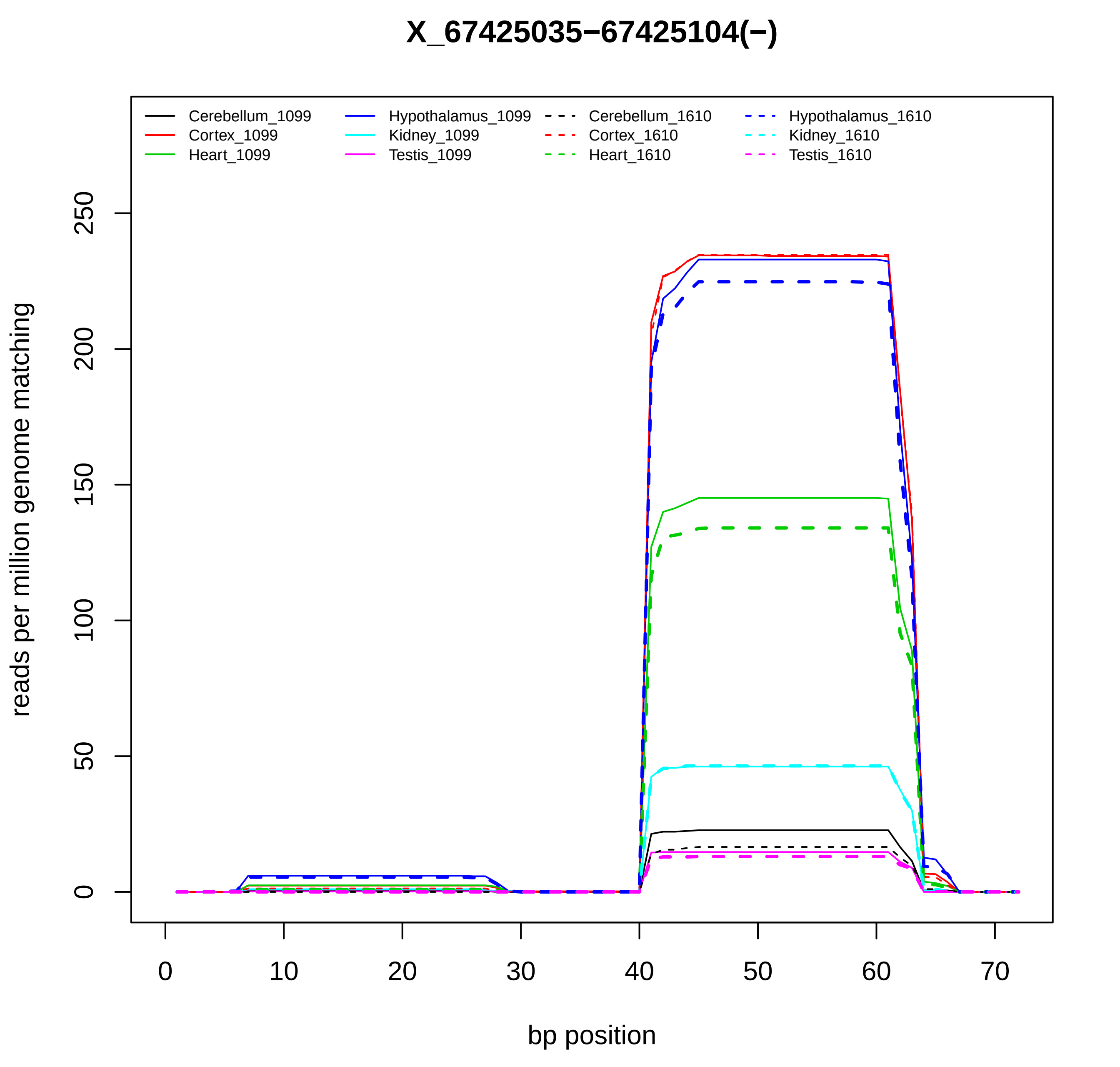
X_67425035−67425104(−)
Cerebellum_1099
Cortex_1099
Heart_1099
Hypothalamus_1099
Kidney_1099
Testis_1099
Cerebellum_1610
Cortex_1610
Heart_1610
Hypothalamus_1610
Kidney_1610
Testis_1610
reads per million genome matching
0
50
100
150
200
250
0
10
20
30
40
50
60
70
bp position

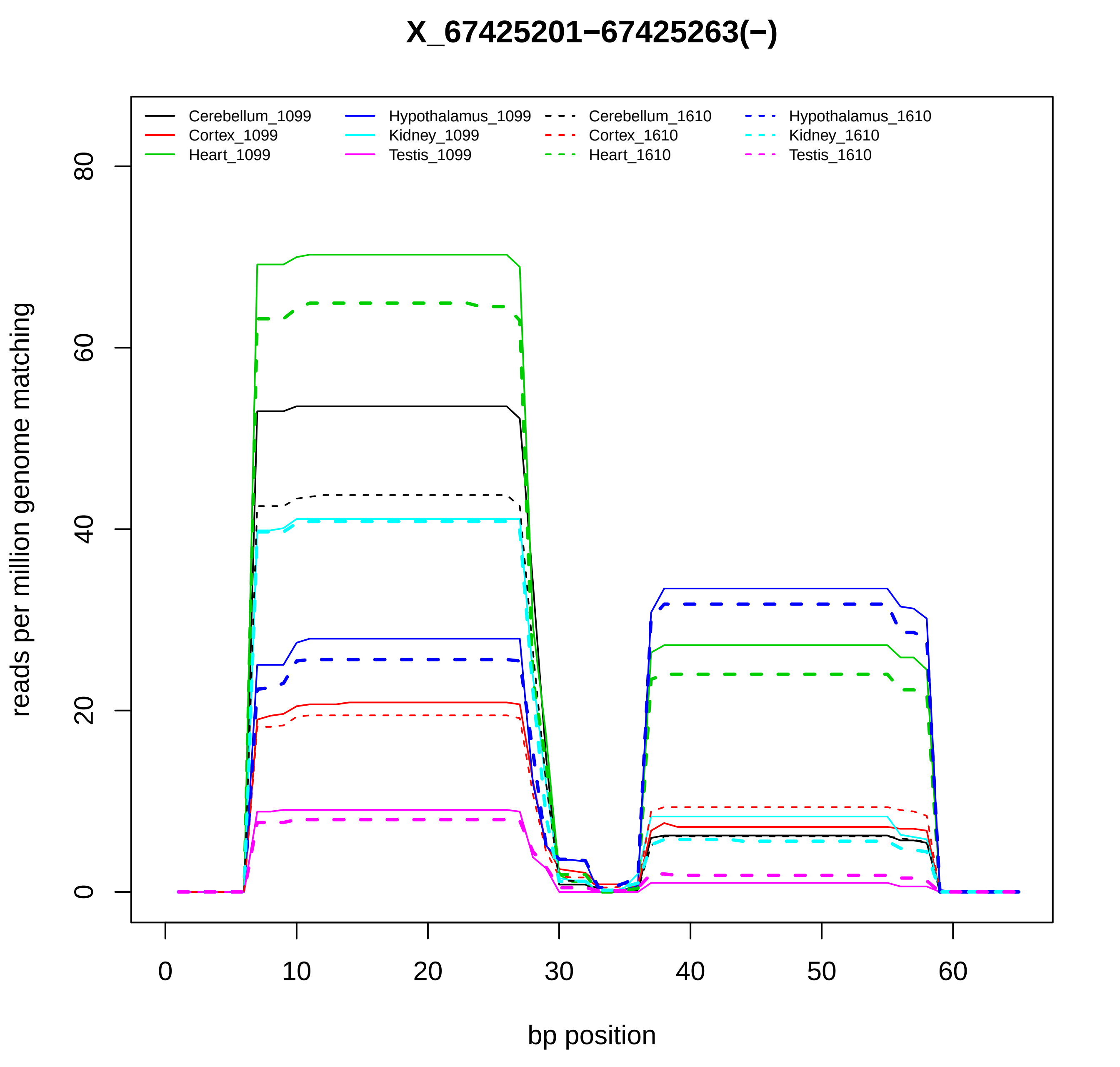

X_67425201−67425263(−)
Cerebellum_1099
Cortex_1099
Heart_1099
Hypothalamus_1099
Kidney_1099
Testis_1099
Cerebellum_1610
Cortex_1610
Heart_1610
Hypothalamus_1610
Kidney_1610
Testis_1610
reads per million genome matching
0
20
40
60
80
0
10
20
30
40
50
60
bp position

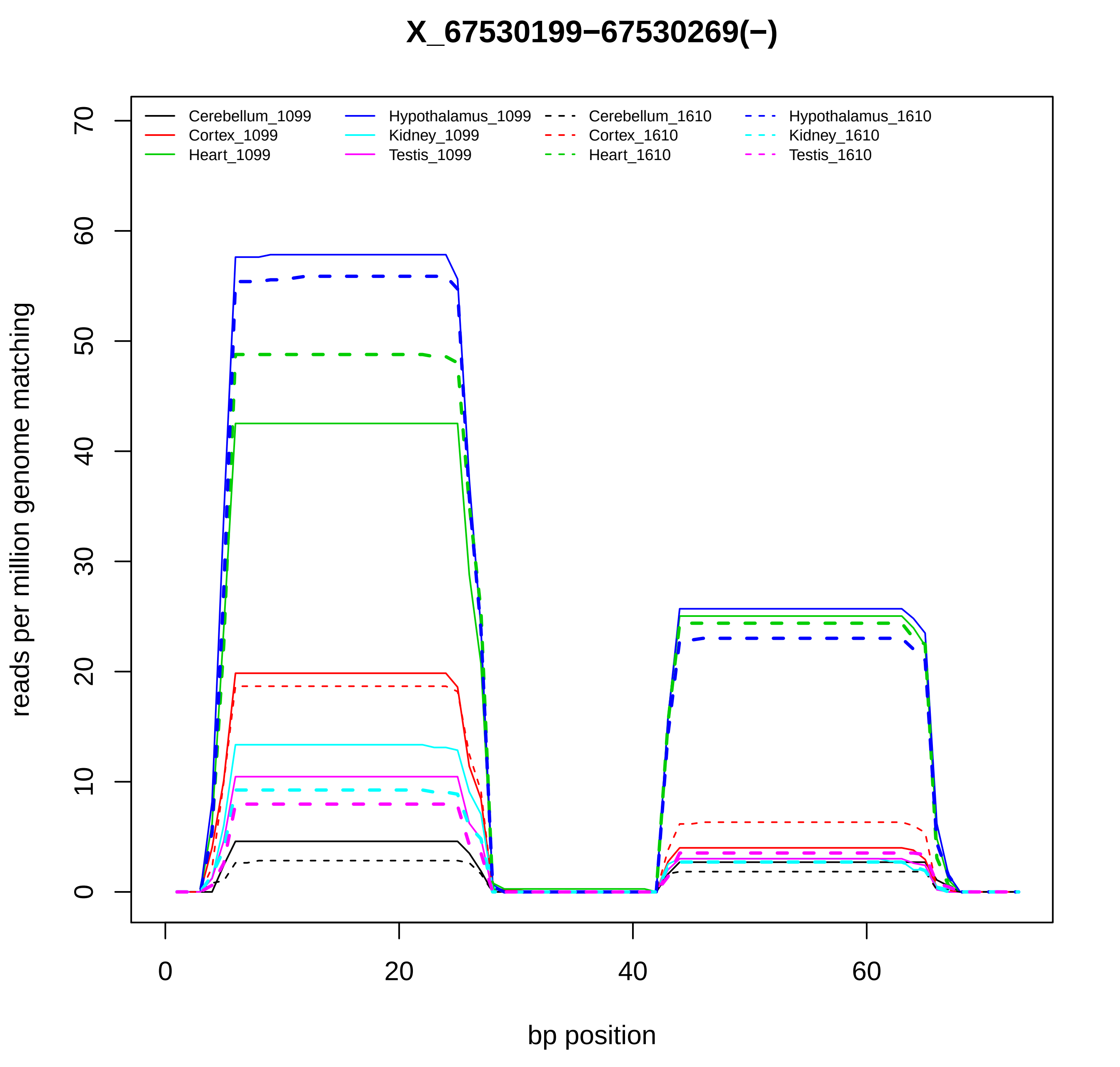

X_67530199−67530269(−)
reads per million genome matching
bp position
Cerebellum_1099
Cortex_1099
Heart_1099
Hypothalamus_1099
Kidney_1099
Testis_1099
Cerebellum_1610
Cortex_1610
Heart_1610
Hypothalamus_1610
Kidney_1610
Testis_1610
0
10
20
30
40
50
60
70
0
20
40
60

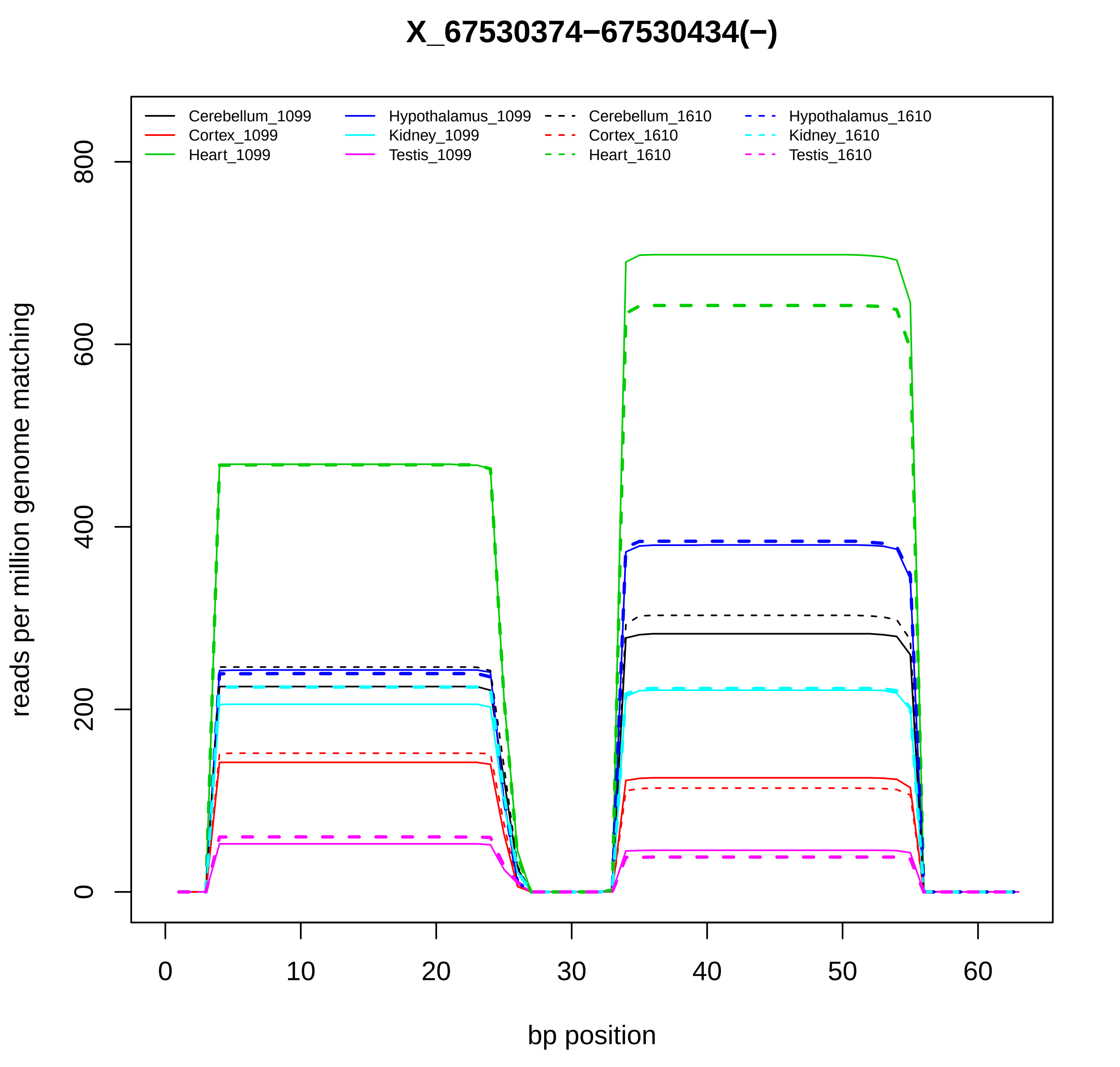

X_67530374−67530434(−)
Cerebellum_1099
Cortex_1099
Heart_1099
Hypothalamus_1099
Kidney_1099
Testis_1099
Cerebellum_1610
Cortex_1610
Heart_1610
Hypothalamus_1610
Kidney_1610
Testis_1610
reads per million genome matching
0
200
400
600
800
0
10
20
30
40
50
60
bp position

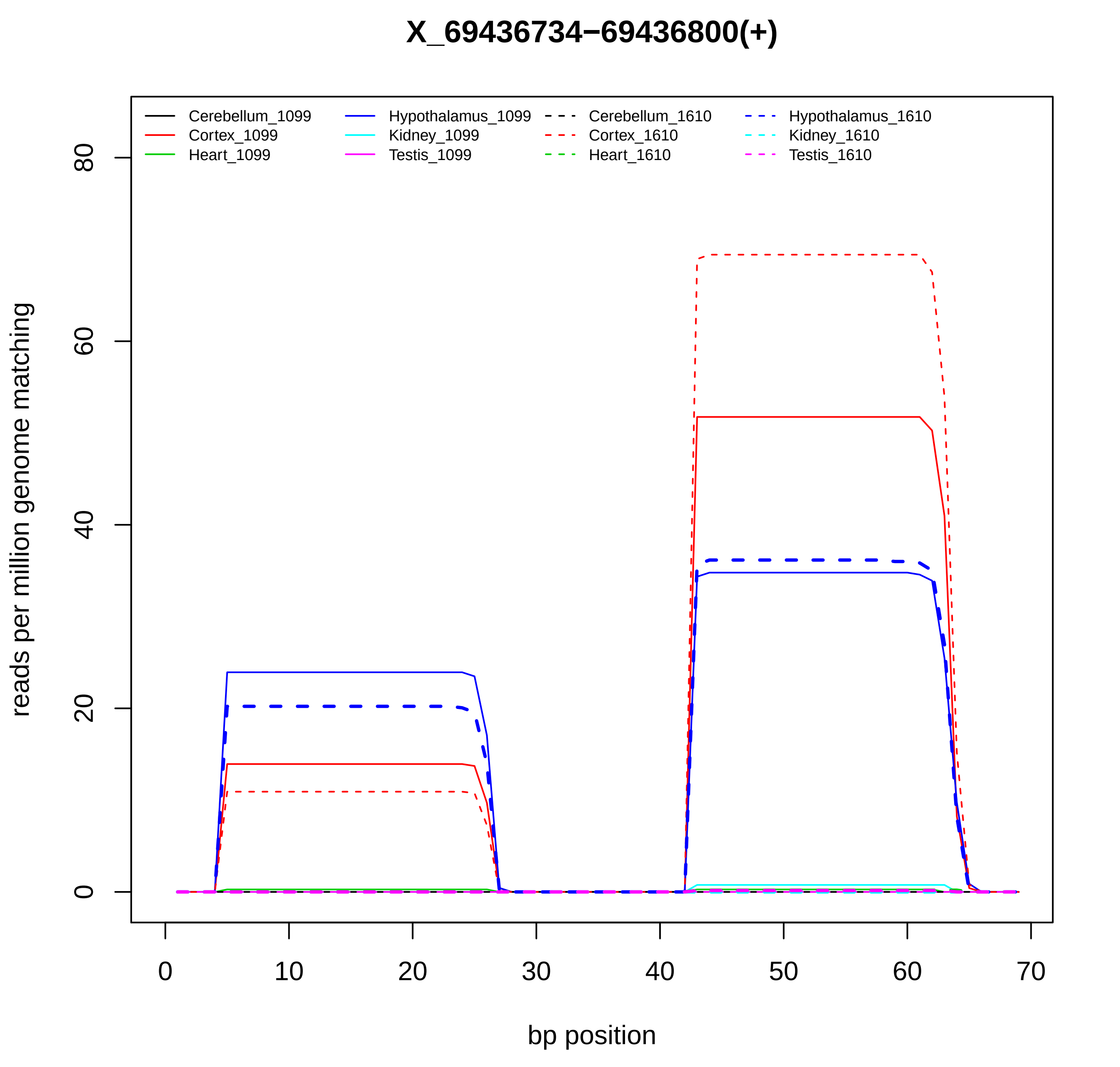
X_69436734−69436800(+)
Cerebellum_1099
Cortex_1099
Heart_1099
Hypothalamus_1099
Kidney_1099
Testis_1099
Cerebellum_1610
Cortex_1610
Heart_1610
Hypothalamus_1610
Kidney_1610
Testis_1610
reads per million genome matching
0
20
40
60
80
0
10
20
30
40
50
60
70
bp position

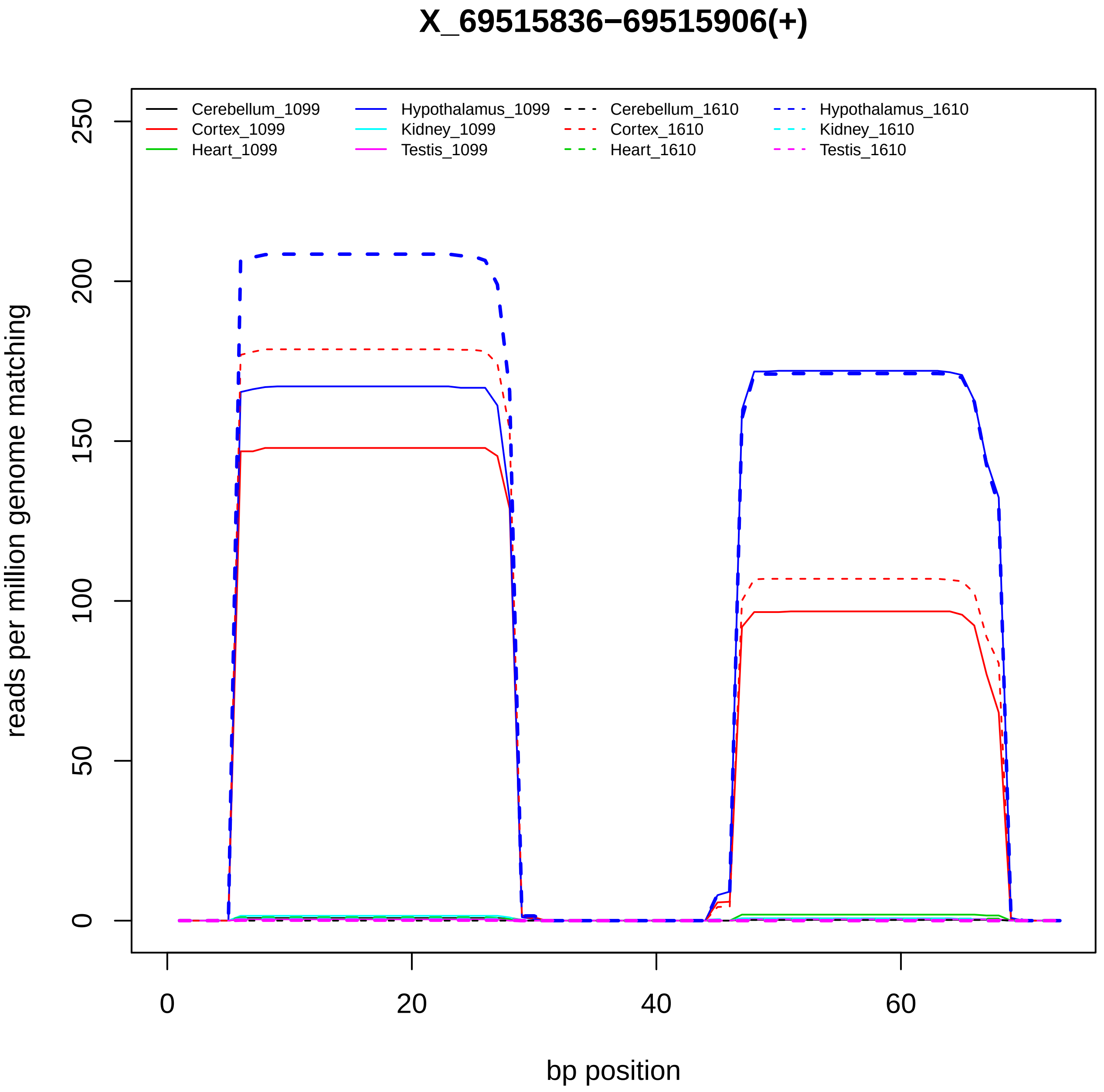

X_69515836−69515906(+)
Cerebellum_1099
Cortex_1099
Heart_1099
Hypothalamus_1099
Kidney_1099
Testis_1099
Cerebellum_1610
Cortex_1610
Heart_1610
Hypothalamus_1610
Kidney_1610
Testis_1610
reads per million genome matching
0
50
100
150
200
250
bp position
0
20
40
60

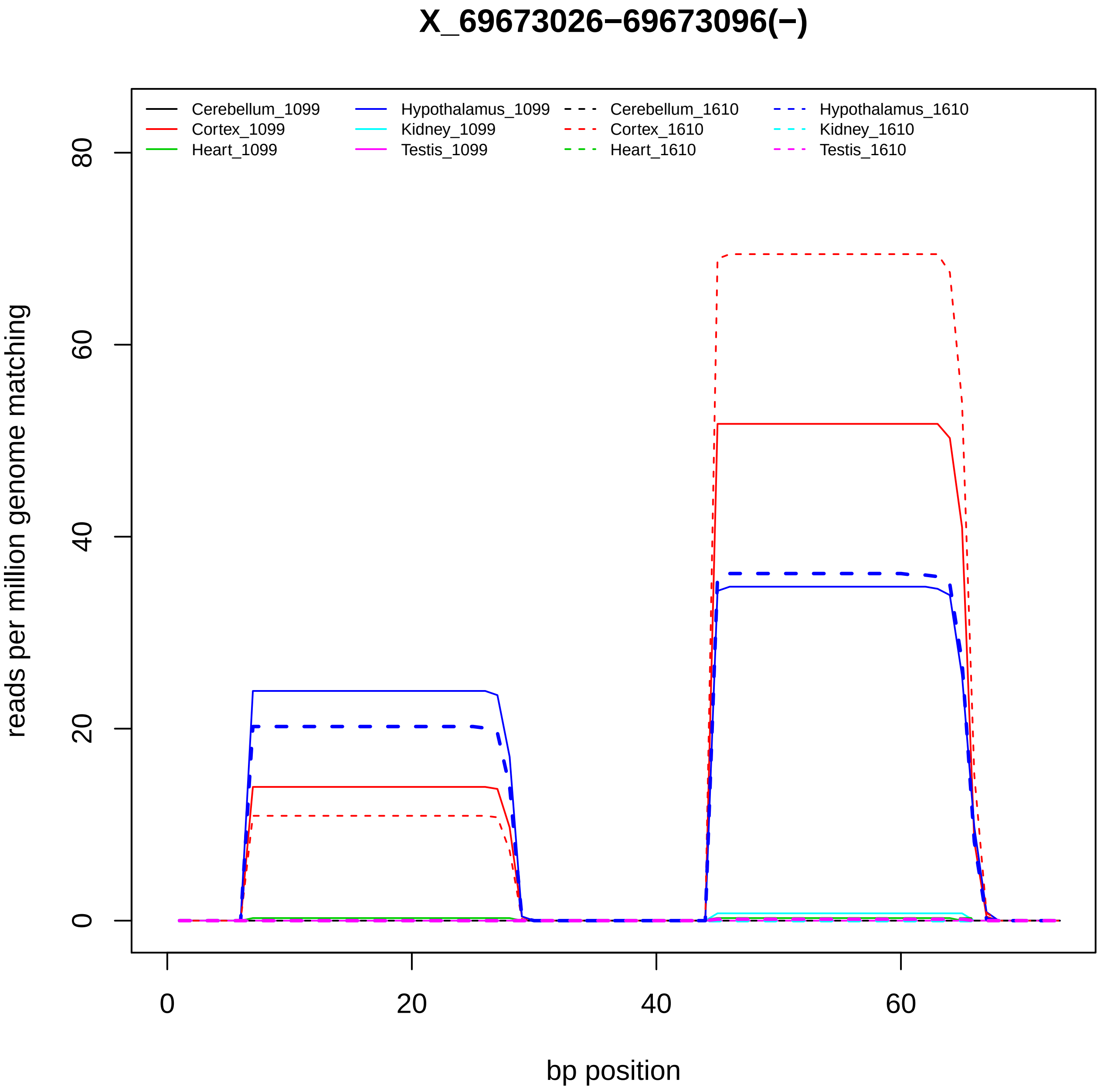

X_69673026−69673096(−)
Cerebellum_1099
Cortex_1099
Heart_1099
Hypothalamus_1099
Kidney_1099
Testis_1099
Cerebellum_1610
Cortex_1610
Heart_1610
Hypothalamus_1610
Kidney_1610
Testis_1610
reads per million genome matching
0
20
40
60
80
bp position
0
20
40
60

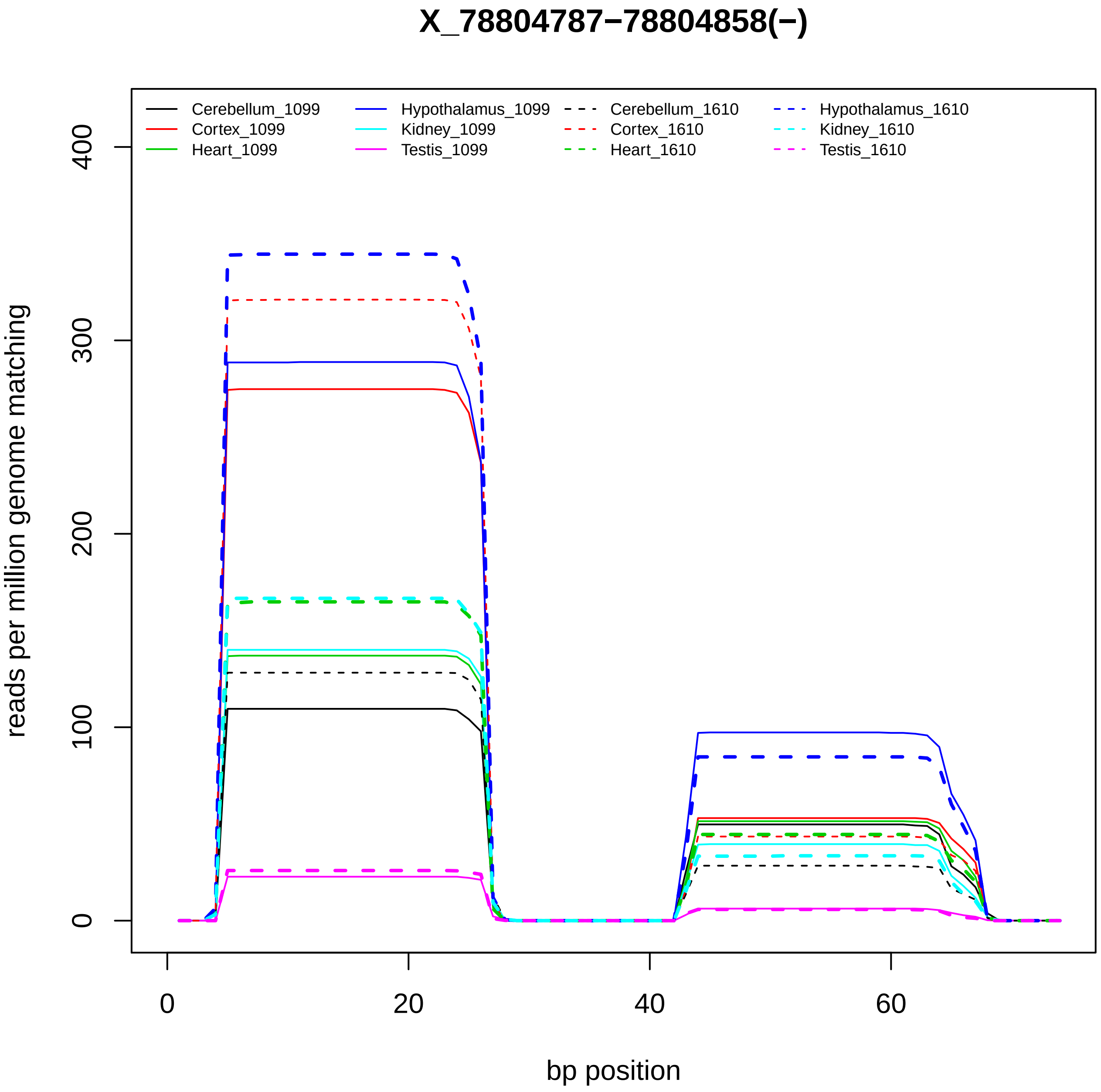
X_78804787−78804858(−)
Cerebellum_1099
Cortex_1099
Heart_1099
Hypothalamus_1099
Kidney_1099
Testis_1099
Cerebellum_1610
Cortex_1610
Heart_1610
Hypothalamus_1610
Kidney_1610
Testis_1610
reads per million genome matching
0
100
200
300
400
0
20
40
60
bp position

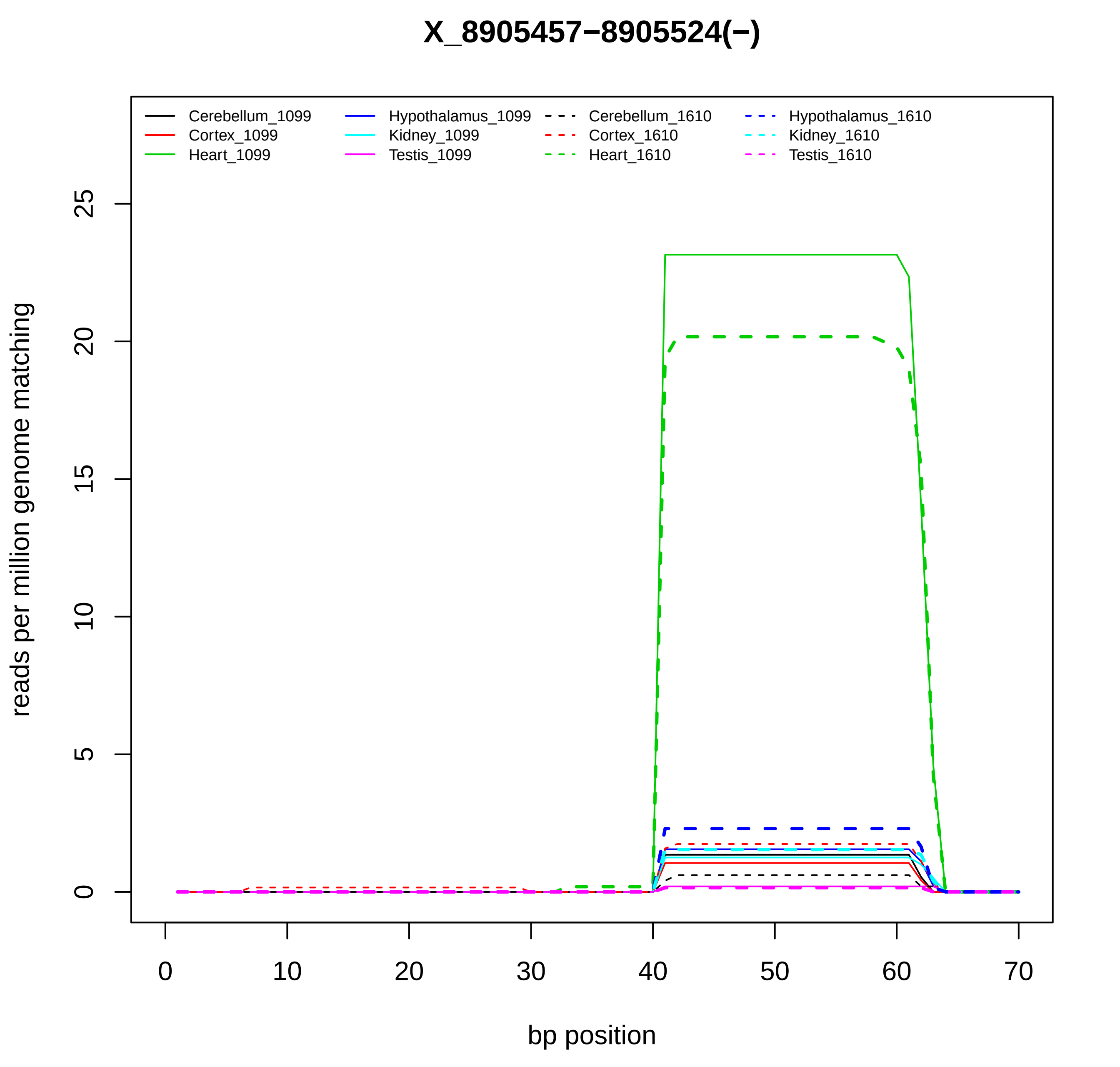
X_8905457−8905524(−)
Cerebellum_1099
Cortex_1099
Heart_1099
Hypothalamus_1099
Kidney_1099
Testis_1099
Cerebellum_1610
Cortex_1610
Heart_1610
Hypothalamus_1610
Kidney_1610
Testis_1610
reads per million genome matching
0
5
10
15
20
25
0
10
20
30
40
50
60
70
bp position

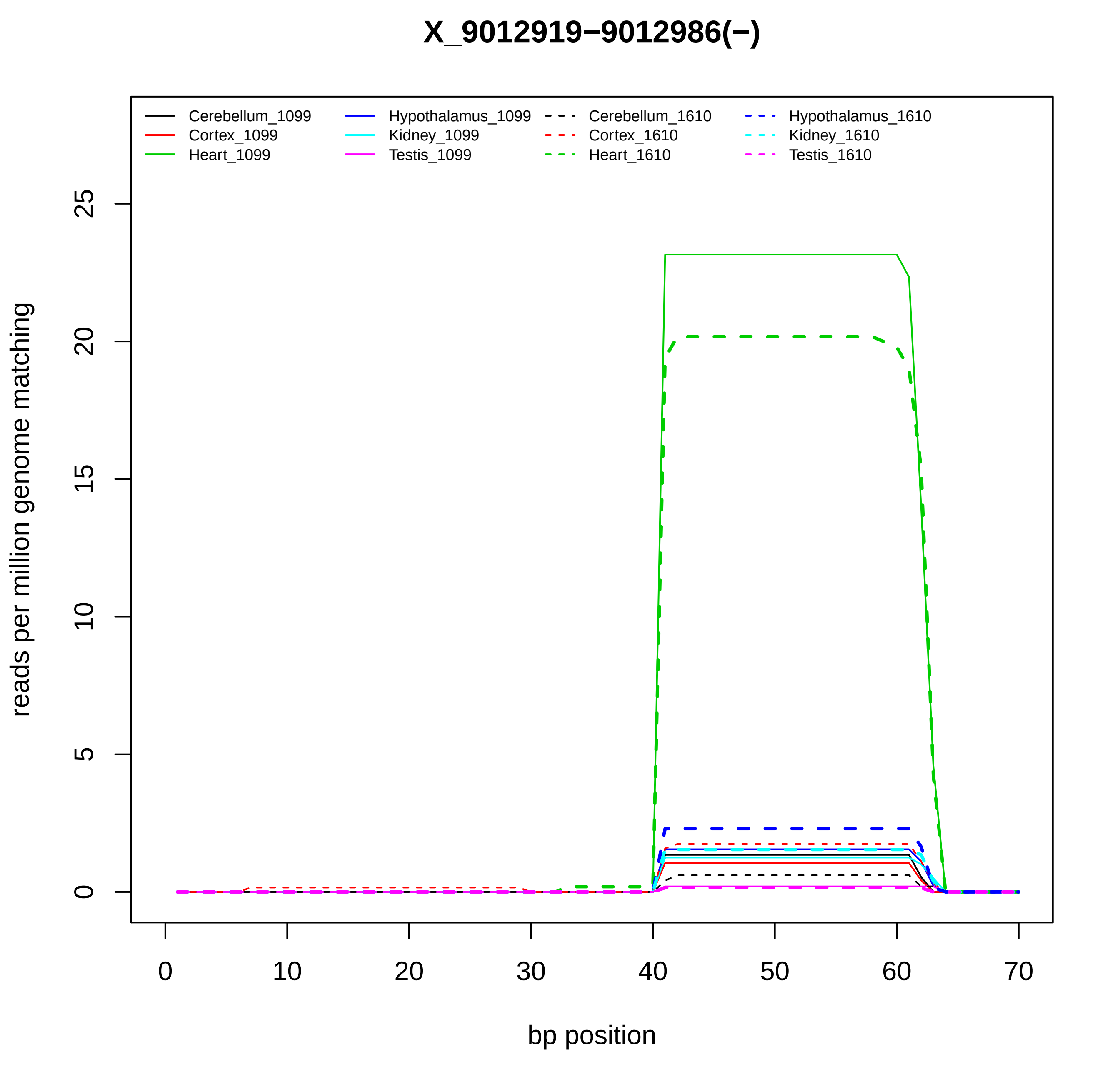

X_9012919−9012986(−)
Cerebellum_1099
Cortex_1099
Heart_1099
Hypothalamus_1099
Kidney_1099
Testis_1099
Cerebellum_1610
Cortex_1610
Heart_1610
Hypothalamus_1610
Kidney_1610
Testis_1610
reads per million genome matching
bp position
0
5
10
15
20
25
0
10
20
30
40
50
60
70

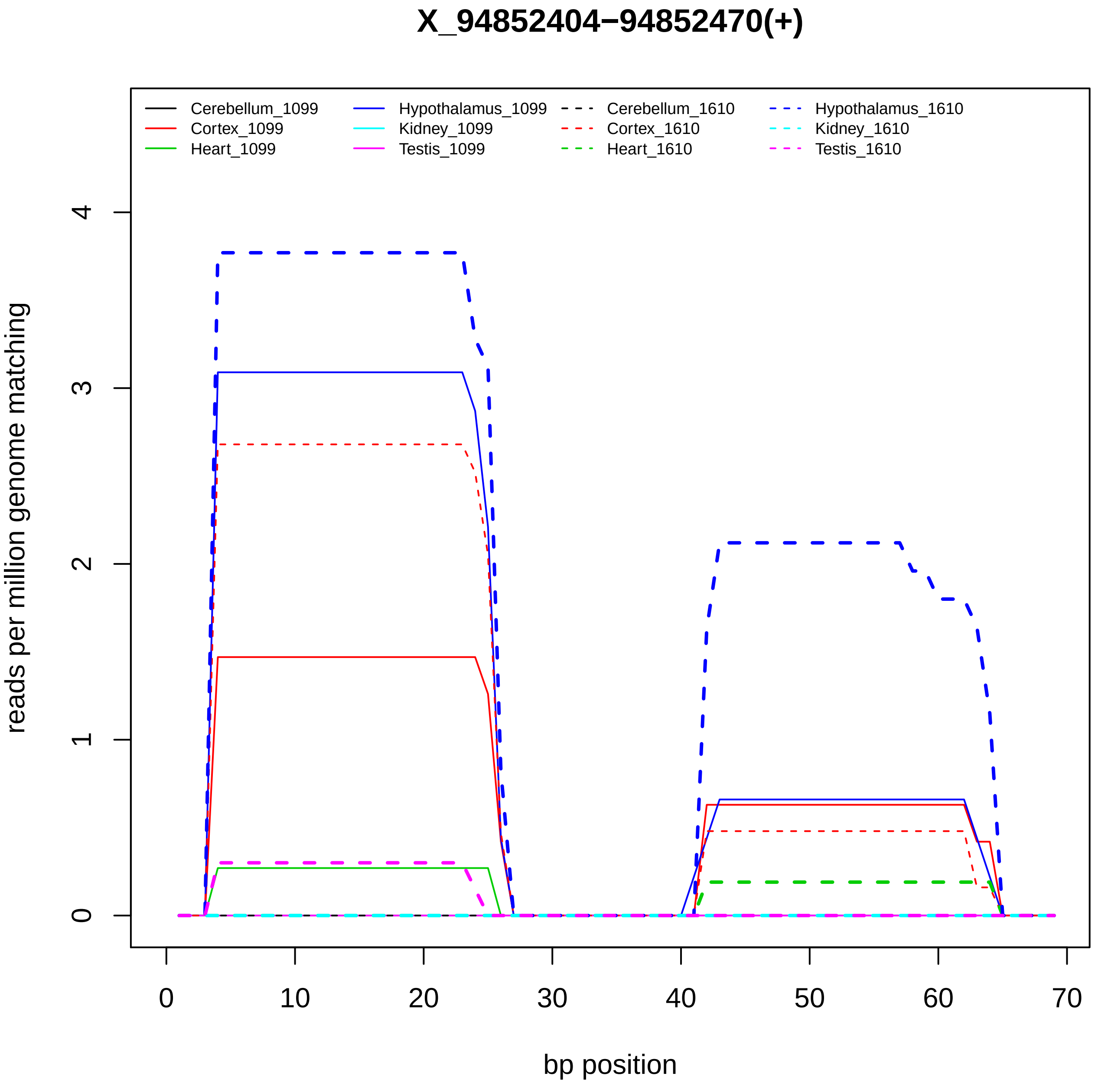

X_94852404−94852470(+)
Cerebellum_1099
Cortex_1099
Heart_1099
Hypothalamus_1099
Kidney_1099
Testis_1099
Cerebellum_1610
Cortex_1610
Heart_1610
Hypothalamus_1610
Kidney_1610
Testis_1610
reads per million genome matching
0
1
2
3
4
0
10
20
30
40
50
60
70
bp position

Fig. S8

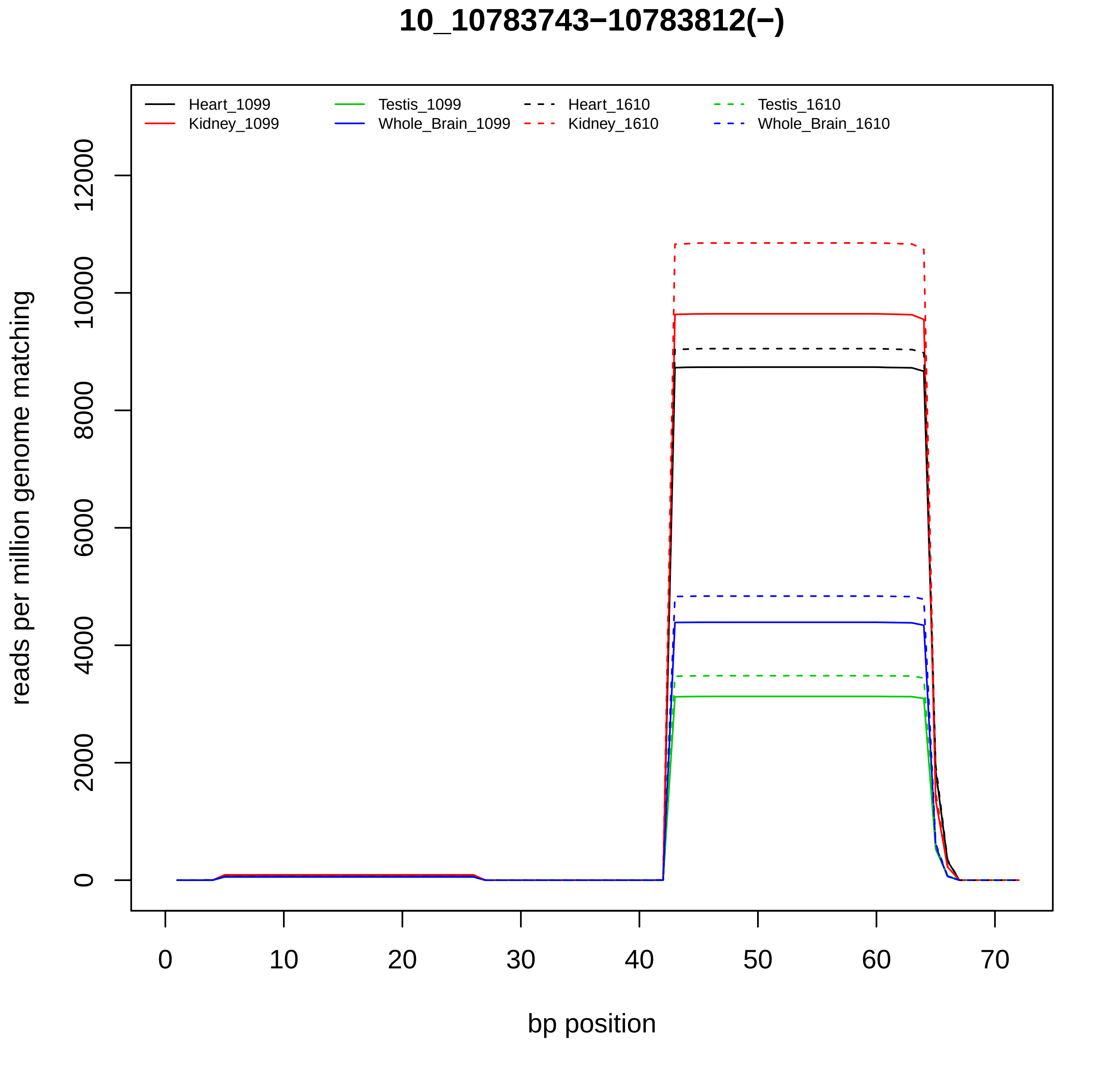

10_10783743−10783812(−)
Heart_1099
Kidney_1099
Testis_1099
Whole_Brain_1099
Heart_1610
Kidney_1610
Testis_1610
Whole_Brain_1610
reads per million genome matching
0
2000
4000
6000
8000
10000
12000
0
10
20
30
40
50
60
70
bp position

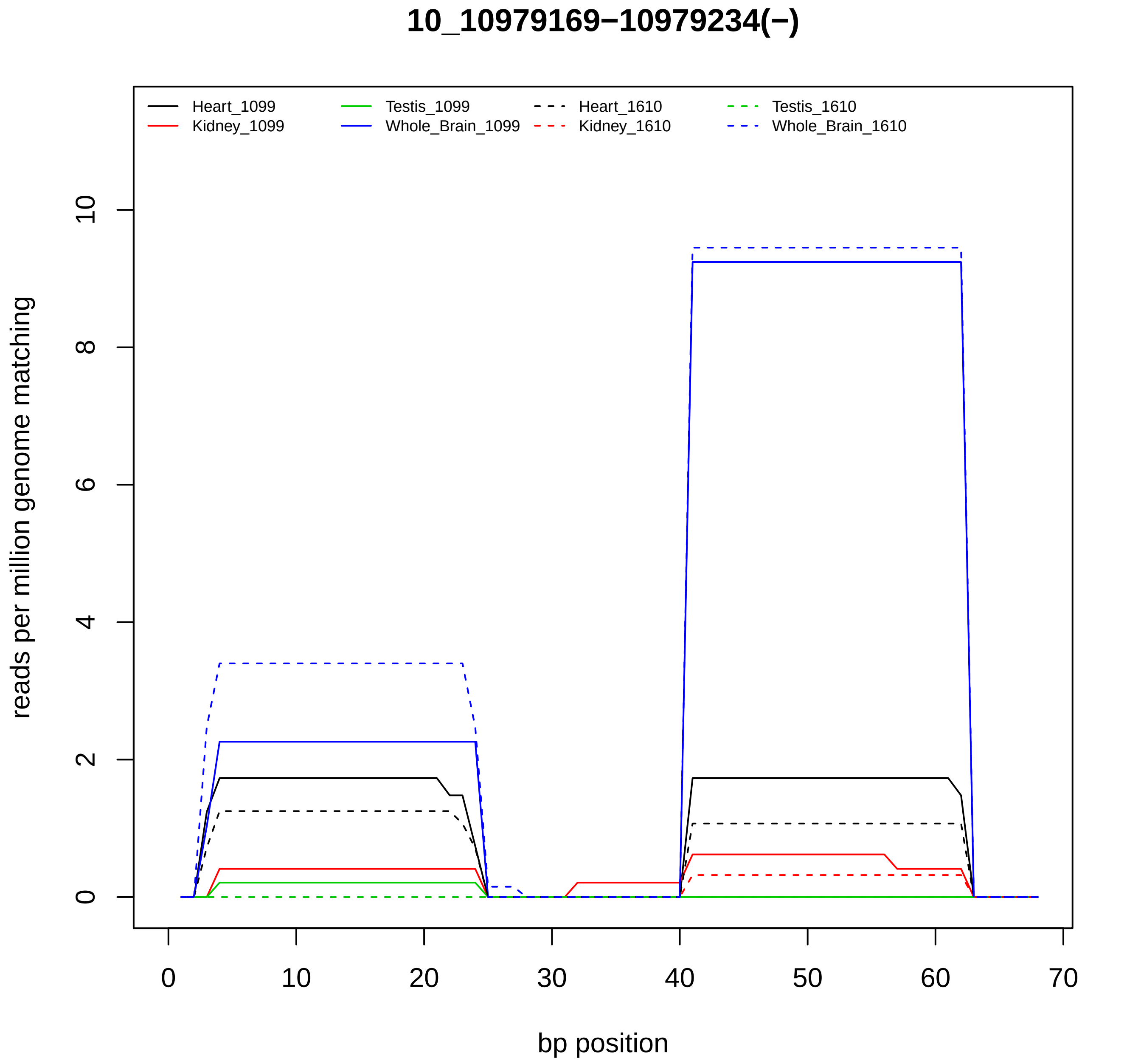

10_10979169−10979234(−)
Heart_1099
Kidney_1099
Testis_1099
Whole_Brain_1099
Heart_1610
Kidney_1610
Testis_1610
Whole_Brain_1610
reads per million genome matching
0
2
4
6
8
10
bp position
0
10
20
30
40
50
60
70

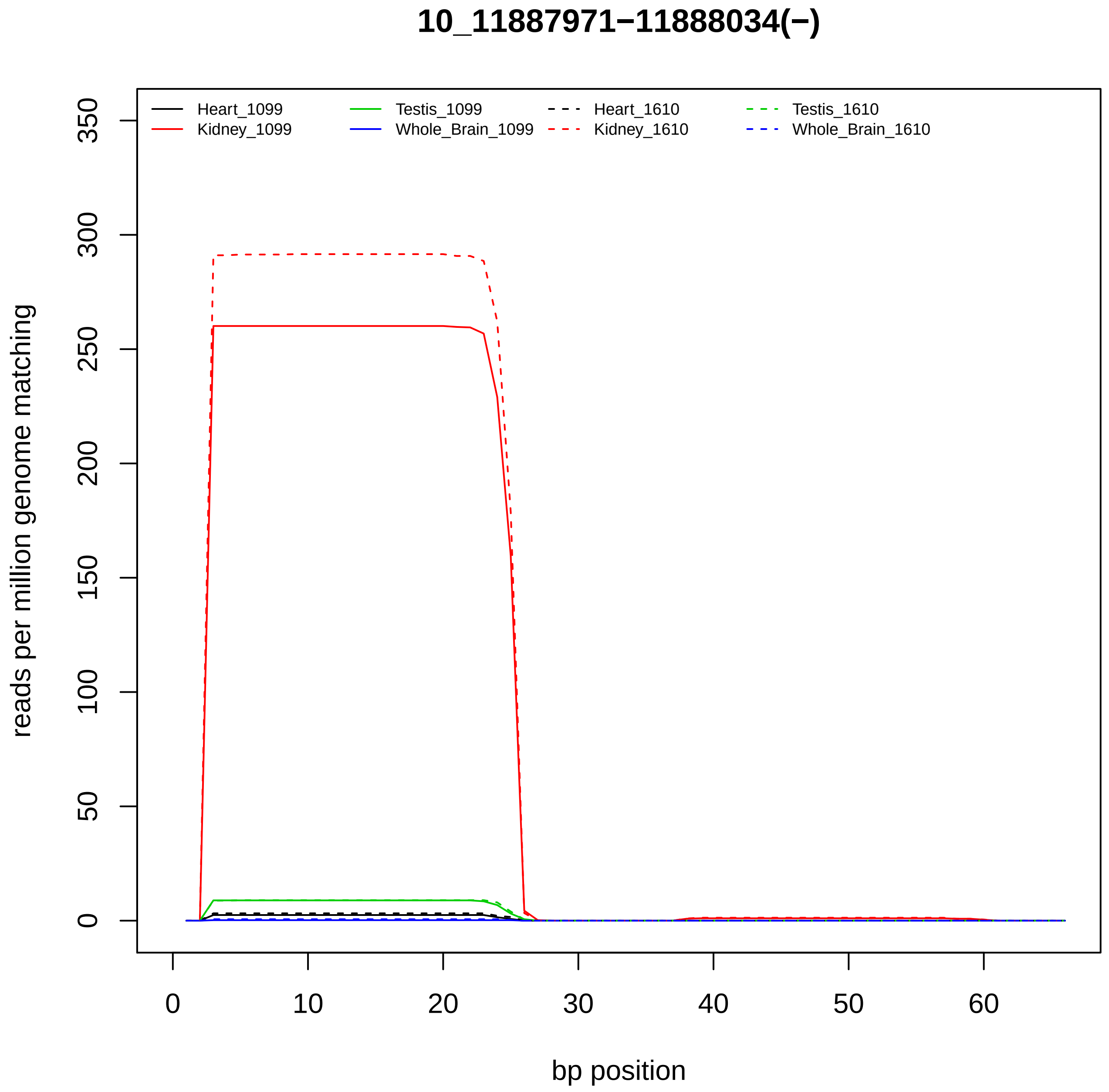

10_11887971−11888034(−)
Heart_1099
Kidney_1099
Testis_1099
Whole_Brain_1099
Heart_1610
Kidney_1610
Testis_1610
Whole_Brain_1610
reads per million genome matching
bp position
0
50
100
150
200
250
300
350
0
10
20
30
40
50
60

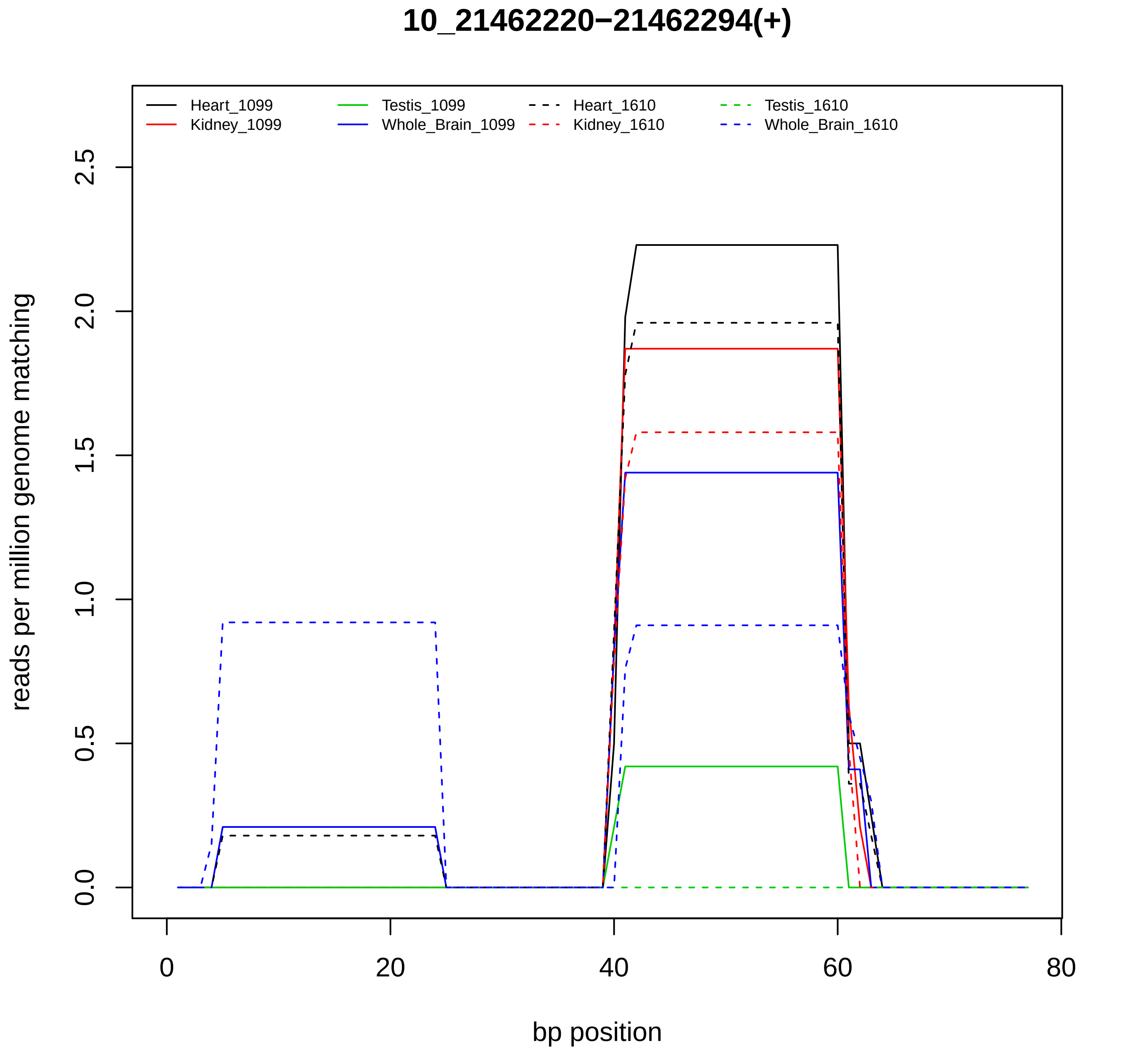

10_21462220−21462294(+)
Heart_1099
Kidney_1099
Testis_1099
Whole_Brain_1099
Heart_1610
Kidney_1610
Testis_1610
Whole_Brain_1610
reads per million genome matching
0.0
0.5
1.0
1.5
2.0
2.5
0
20
40
60
80
bp position

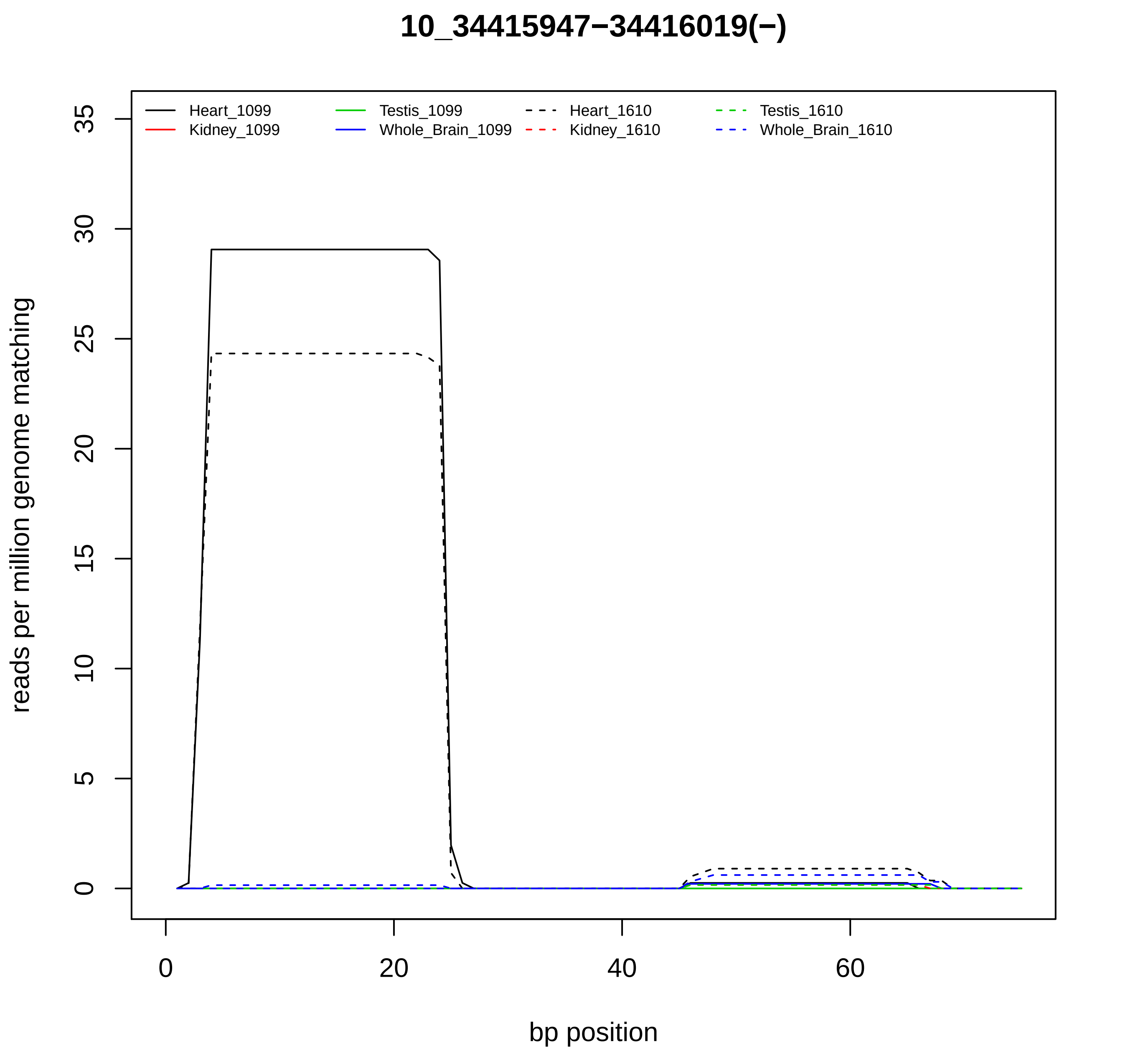

10_34415947−34416019(−)
Heart_1099
Kidney_1099
Testis_1099
Whole_Brain_1099
Heart_1610
Kidney_1610
Testis_1610
Whole_Brain_1610
reads per million genome matching
bp position
0
5
10
15
20
25
30
35
0
20
40
60

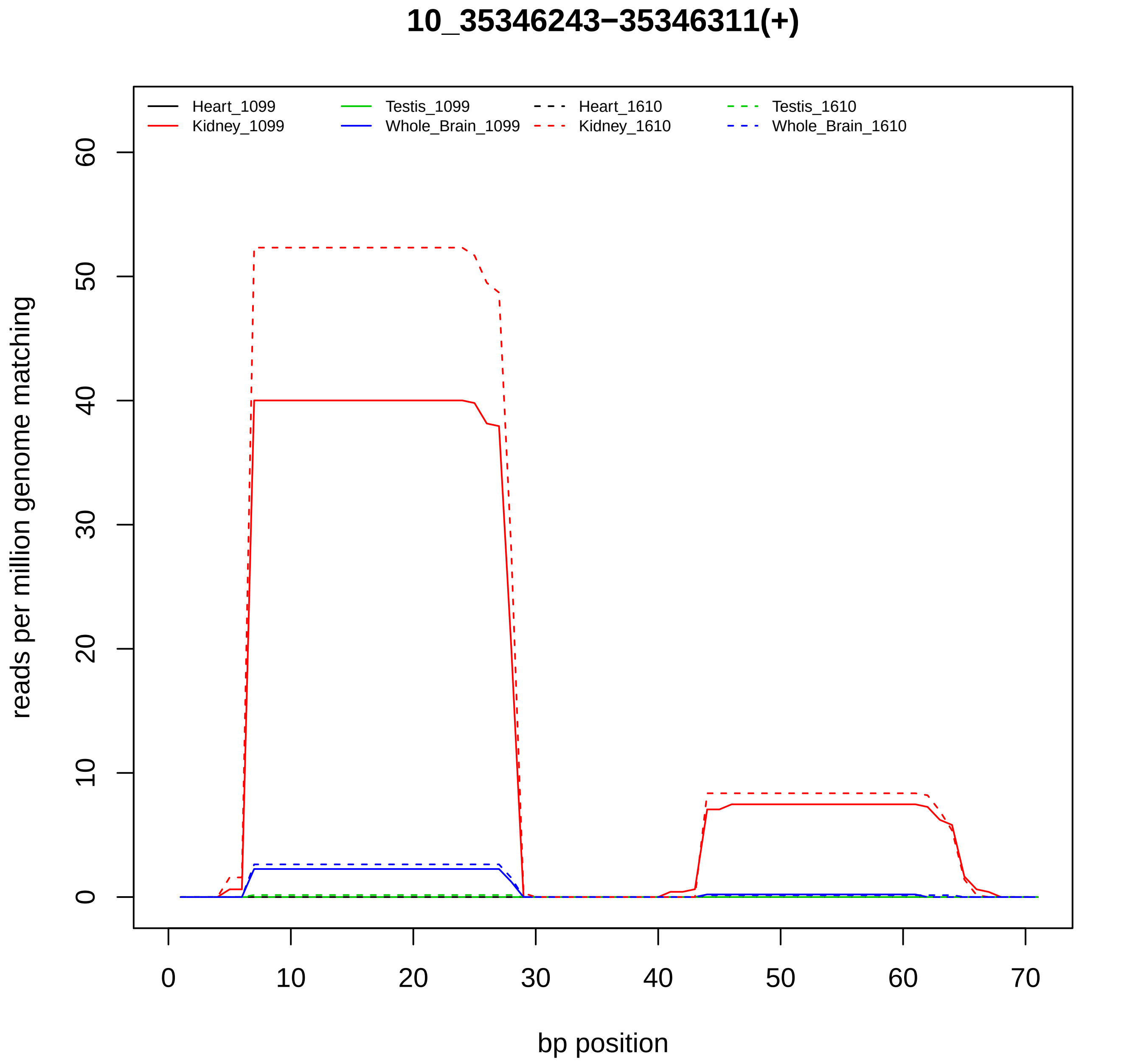

10_35346243−35346311(+)
Heart_1099
Kidney_1099
Testis_1099
Whole_Brain_1099
Heart_1610
Kidney_1610
Testis_1610
Whole_Brain_1610
reads per million genome matching
bp position
0
10
20
30
40
50
60
70

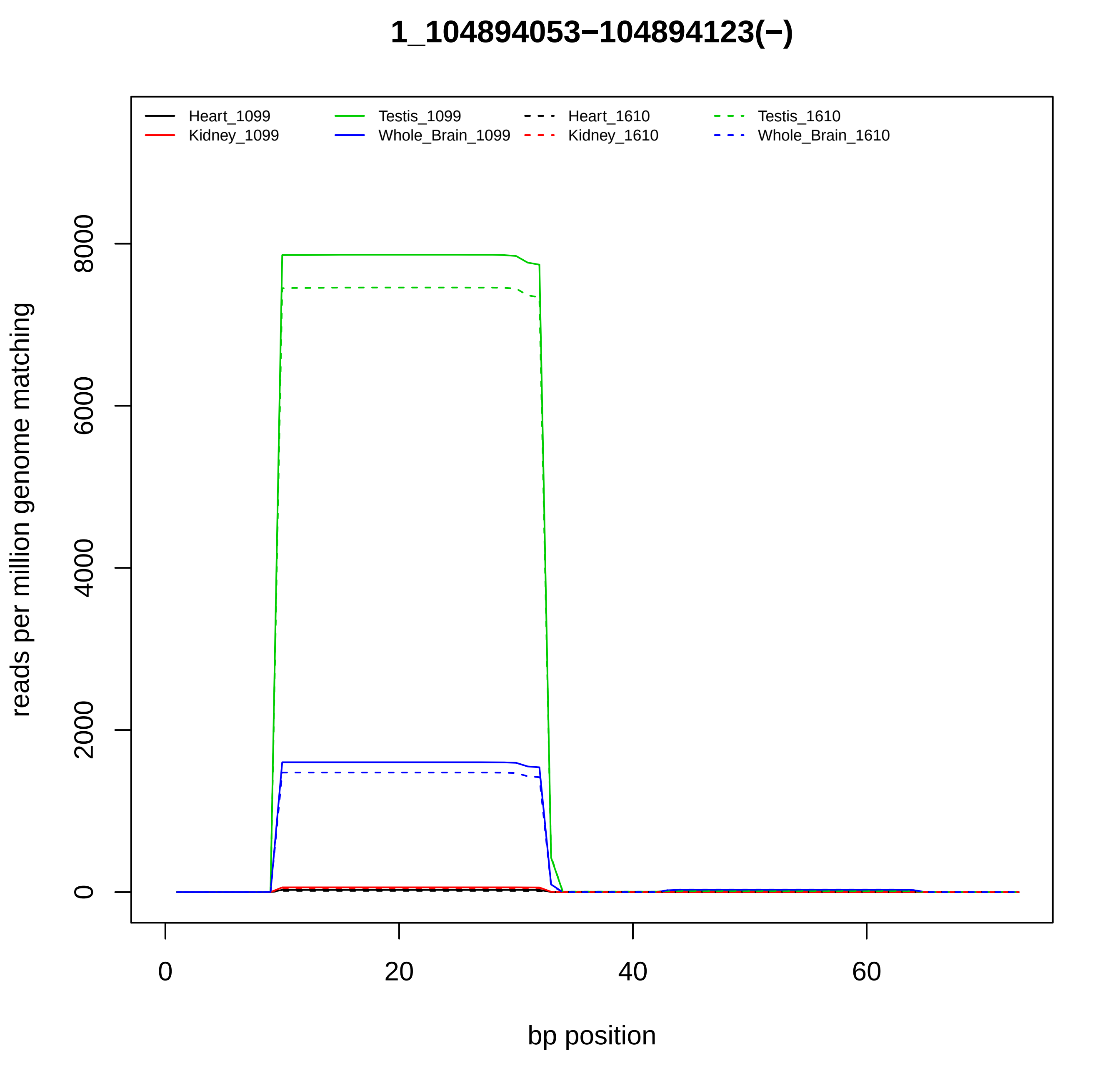

1_104894053−104894123(−)
Heart_1099
Kidney_1099
Testis_1099
Whole_Brain_1099
Heart_1610
Kidney_1610
Testis_1610
Whole_Brain_1610
reads per million genome matching
0
2000
4000
6000
8000
0
20
40
60
bp position

1_104894564−104894637(−)

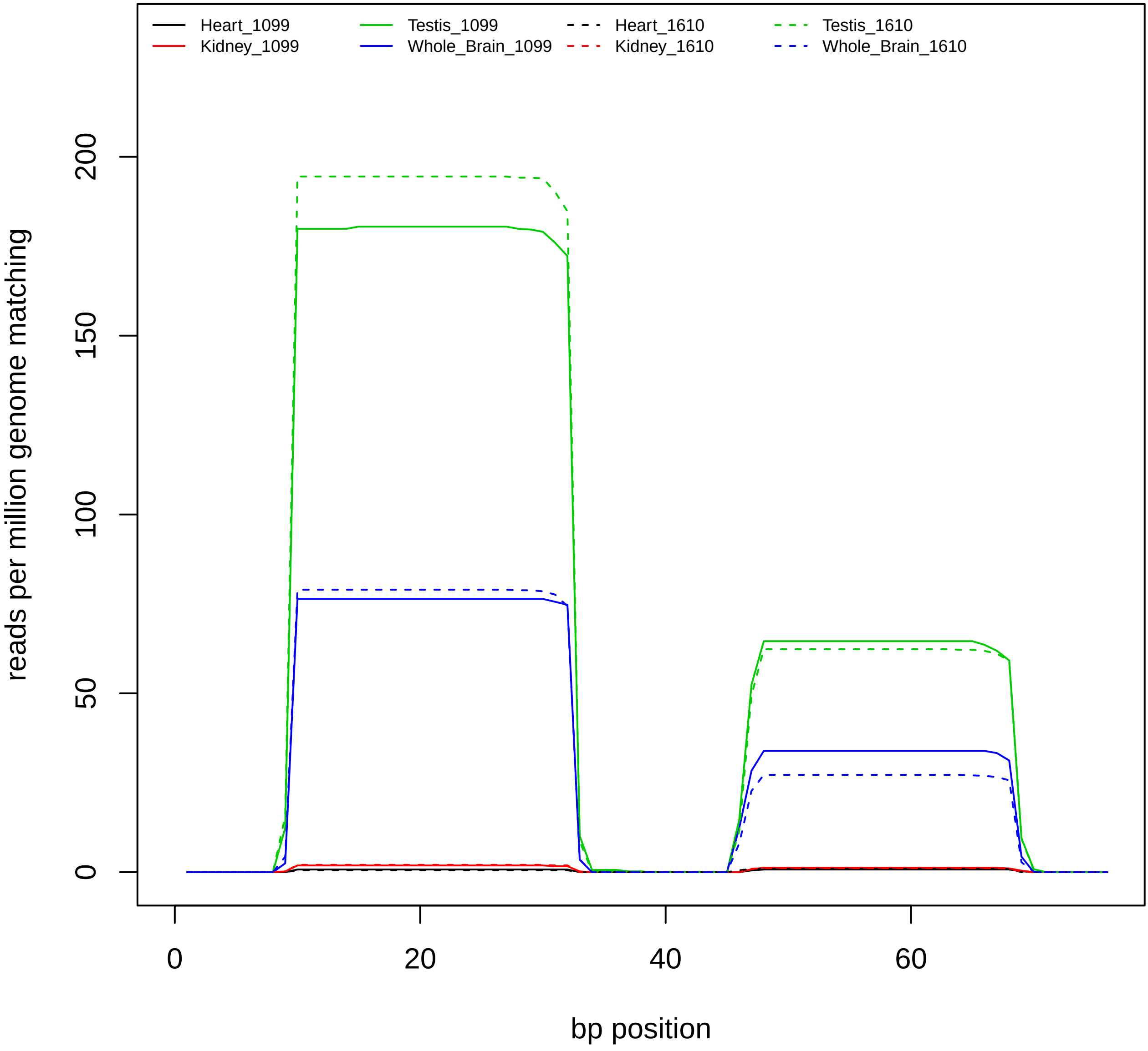

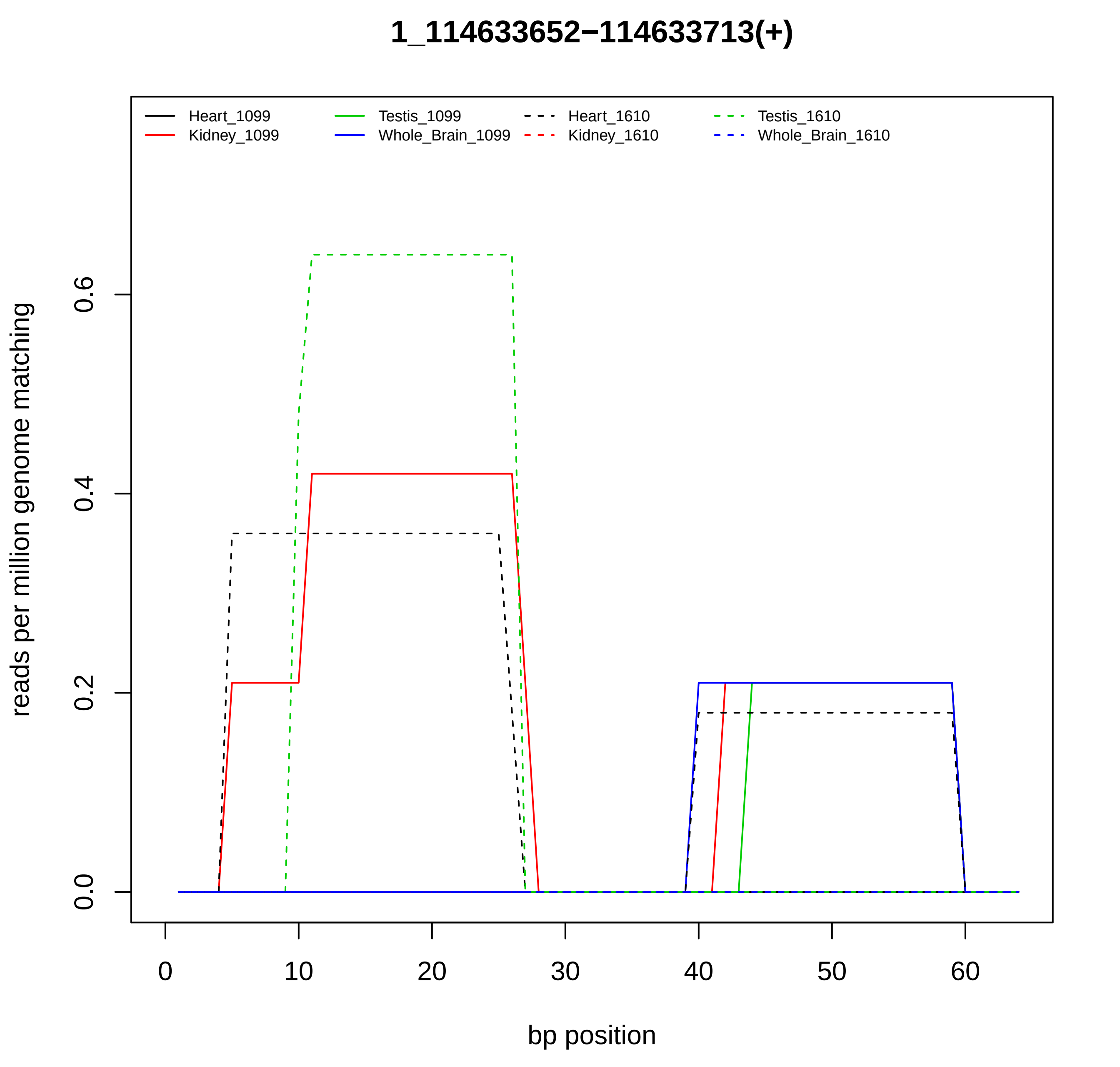

1_114633652−114633713(+)
Heart_1099
Kidney_1099
Testis_1099
Whole_Brain_1099
Heart_1610
Kidney_1610
Testis_1610
Whole_Brain_1610
reads per million genome matching
0.0
0.2
0.4
0.6
0
10
20
30
40
50
60
bp position

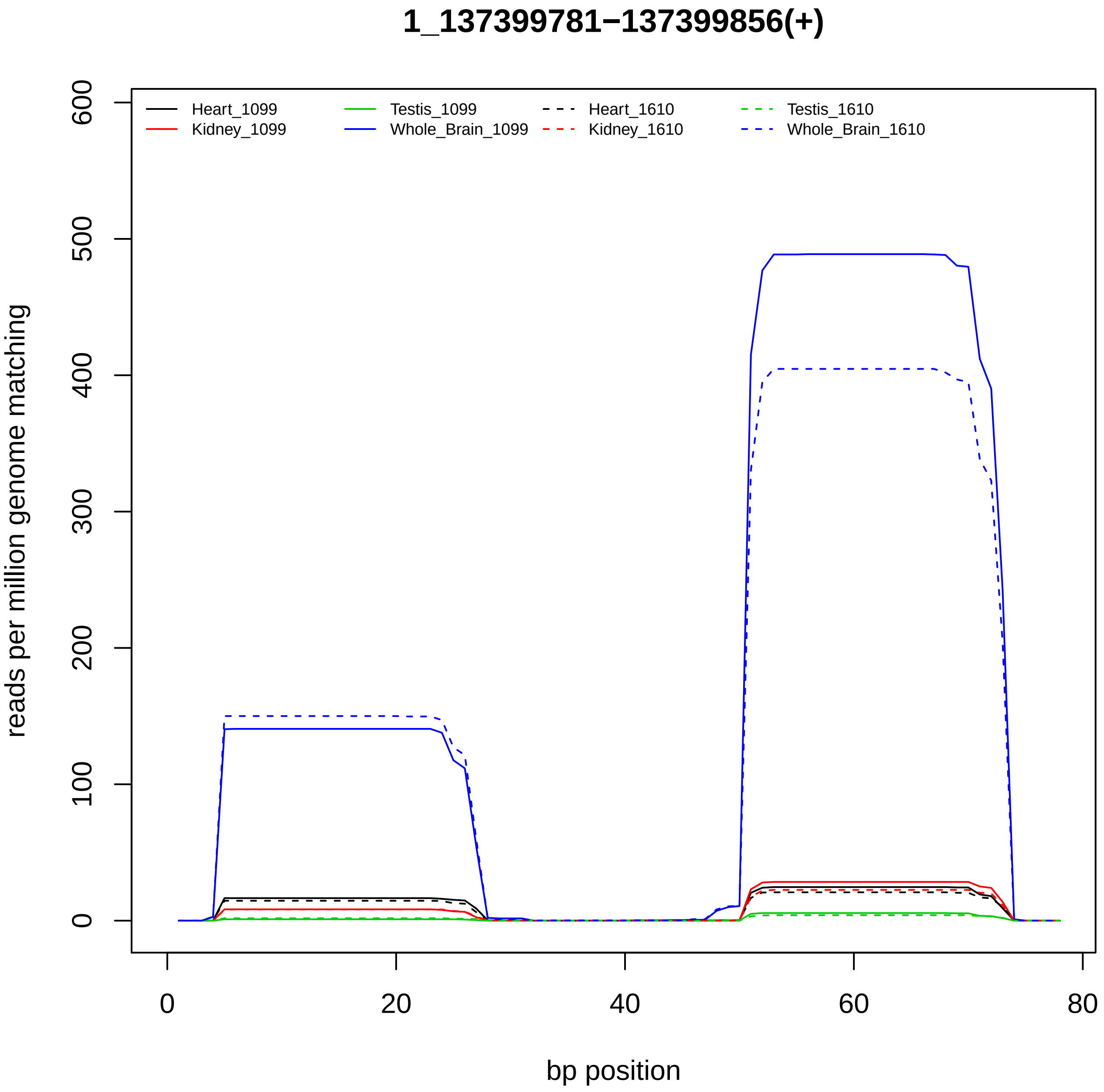

1_137399781−137399856(+)
Heart_1099
Kidney_1099
Testis_1099
Whole_Brain_1099
Heart_1610
Kidney_1610
Testis_1610
Whole_Brain_1610
reads per million genome matching
0
100
200
300
400
500
600
0
20
40
60
80
bp position

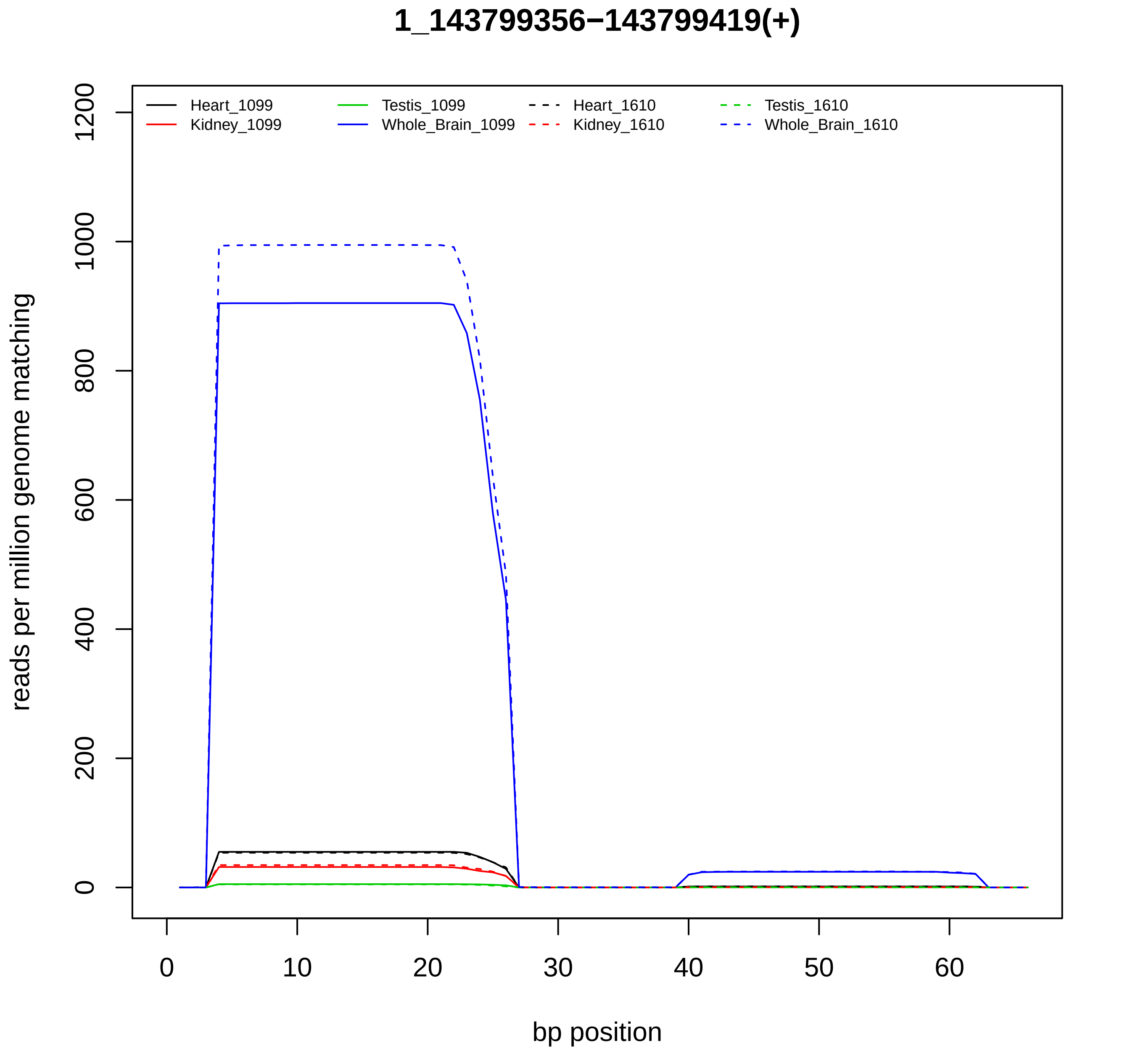

1_143799356−143799419(+)
Heart_1099
Kidney_1099
Testis_1099
Whole_Brain_1099
Heart_1610
Kidney_1610
Testis_1610
Whole_Brain_1610
reads per million genome matching
bp position
0
200
400
600
800
1000
1200
0
10
20
30
40
50
60

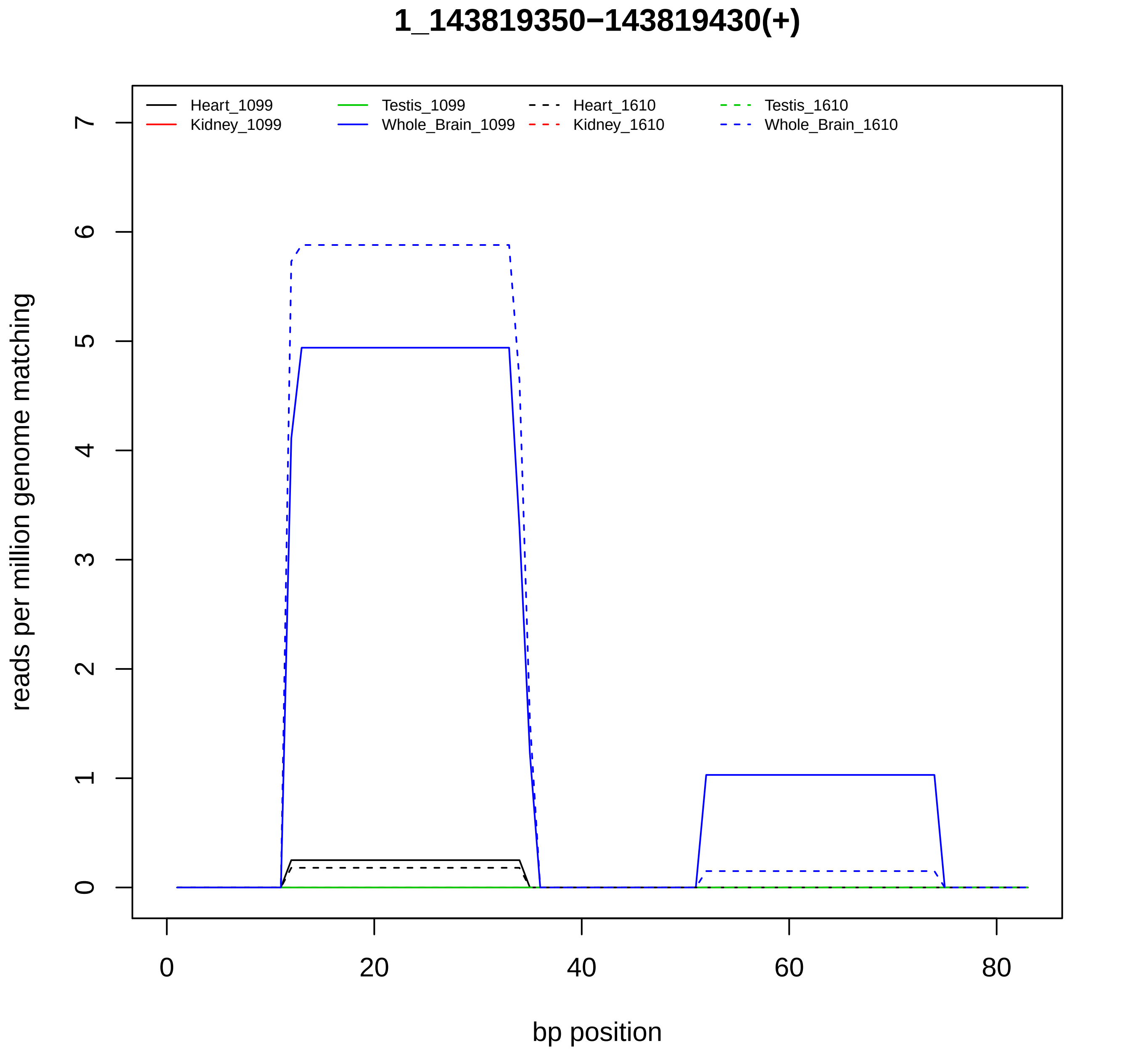

1_143819350−143819430(+)
Heart_1099
Kidney_1099
Testis_1099
Whole_Brain_1099
Heart_1610
Kidney_1610
Testis_1610
Whole_Brain_1610
reads per million genome matching
bp position
0
1
2
3
4
5
6
7
0
20
40
60
80

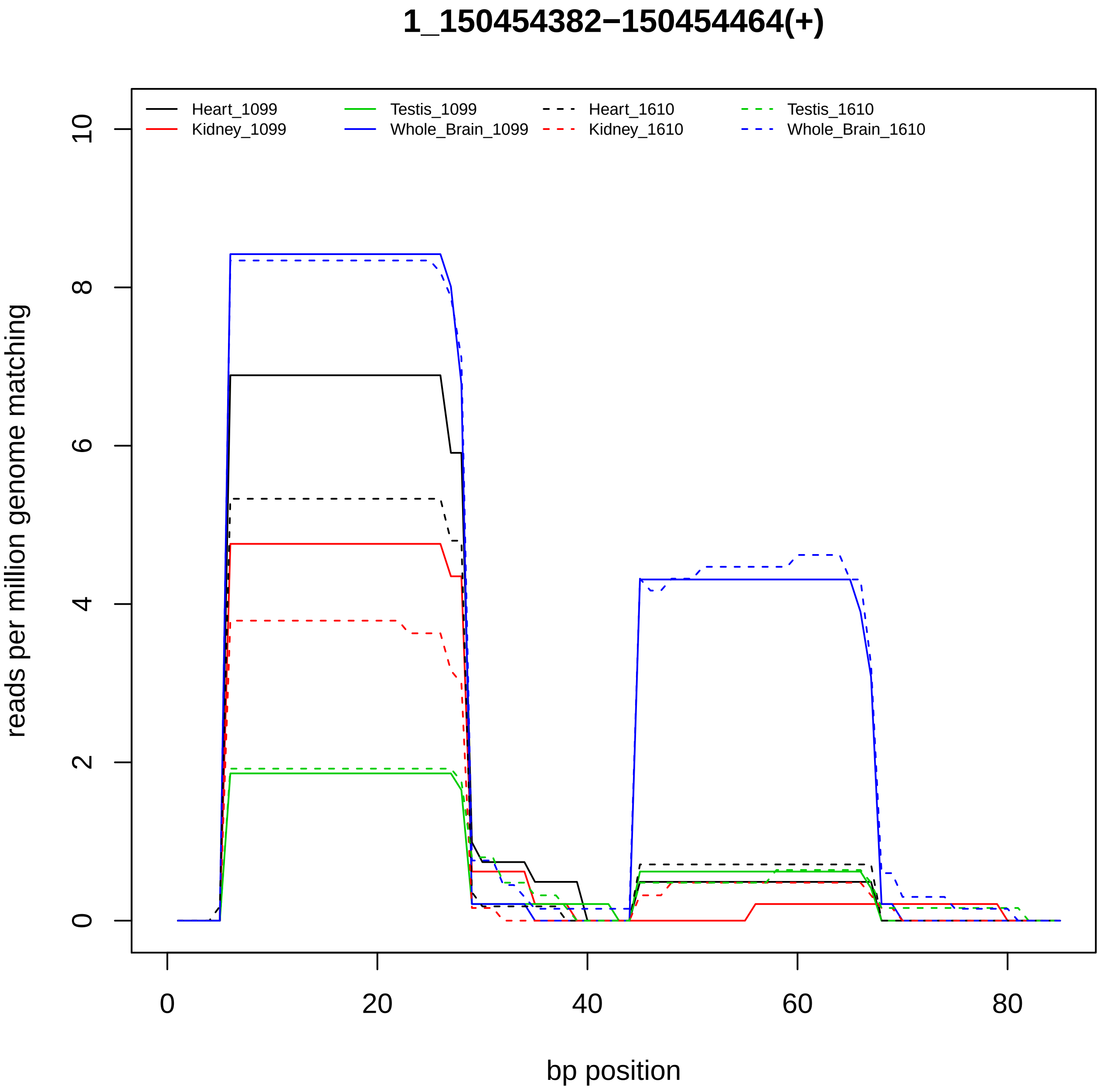
1_150454382−150454464(+)
Heart_1099
Kidney_1099
Testis_1099
Whole_Brain_1099
Heart_1610
Kidney_1610
Testis_1610
Whole_Brain_1610
reads per million genome matching
bp position
0
2
4
6
8
10
0
20
40
60
80

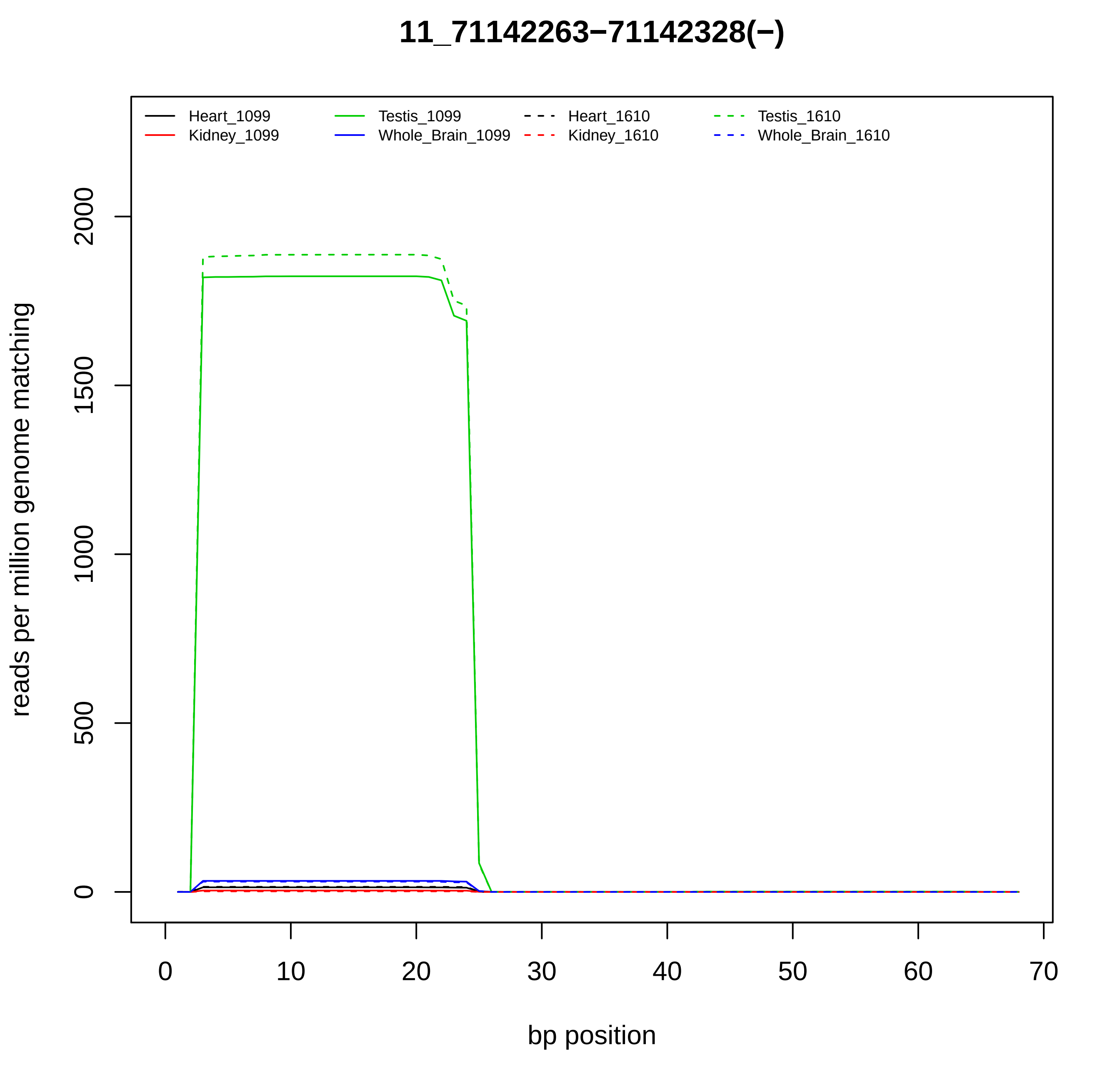

11_71142263−71142328(−)
Heart_1099
Kidney_1099
Testis_1099
Whole_Brain_1099
Heart_1610
Kidney_1610
Testis_1610
Whole_Brain_1610
reads per million genome matching
0
500
1000
1500
2000
0
10
20
30
40
50
60
70
bp position

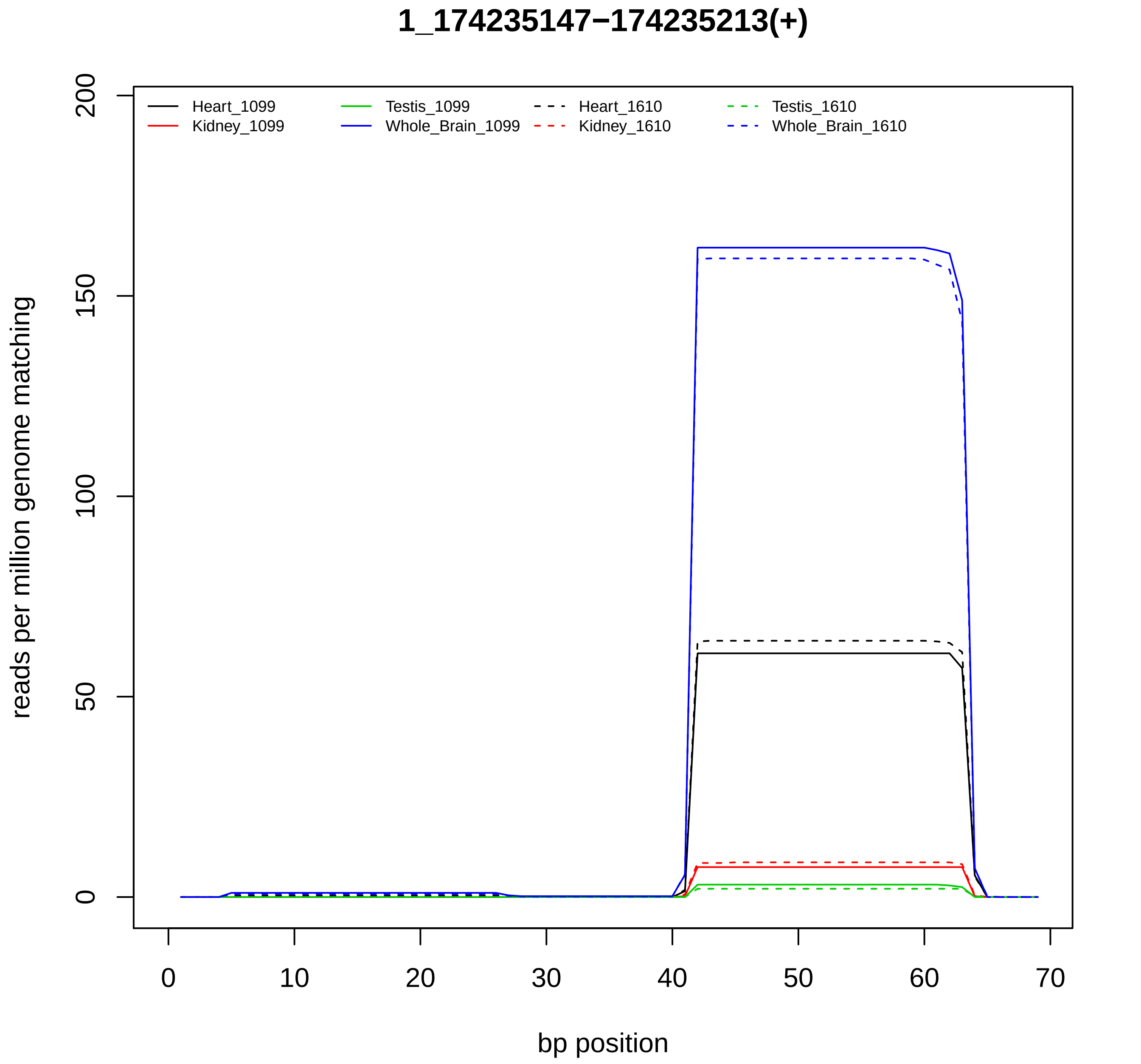
1_174235147−174235213(+)
Heart_1099
Kidney_1099
Testis_1099
Whole_Brain_1099
Heart_1610
Kidney_1610
Testis_1610
Whole_Brain_1610
reads per million genome matching
0
50
100
150
200
bp position
0
10
20
30
40
50
60
70

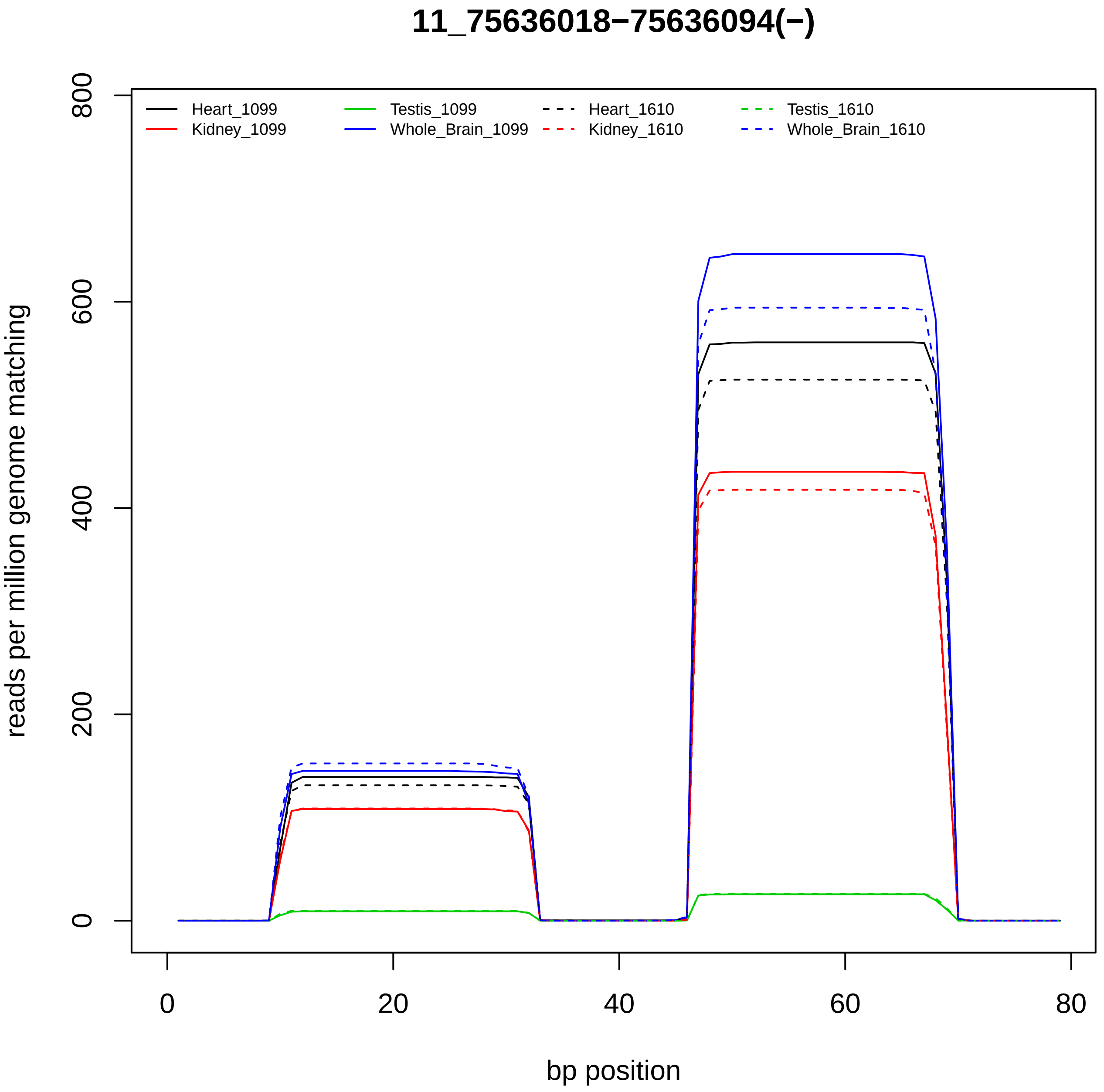

11_75636018−75636094(−)
Heart_1099
Kidney_1099
Testis_1099
Whole_Brain_1099
Heart_1610
Kidney_1610
Testis_1610
Whole_Brain_1610
reads per million genome matching
0
200
400
600
800
0
20
40
60
80
bp position

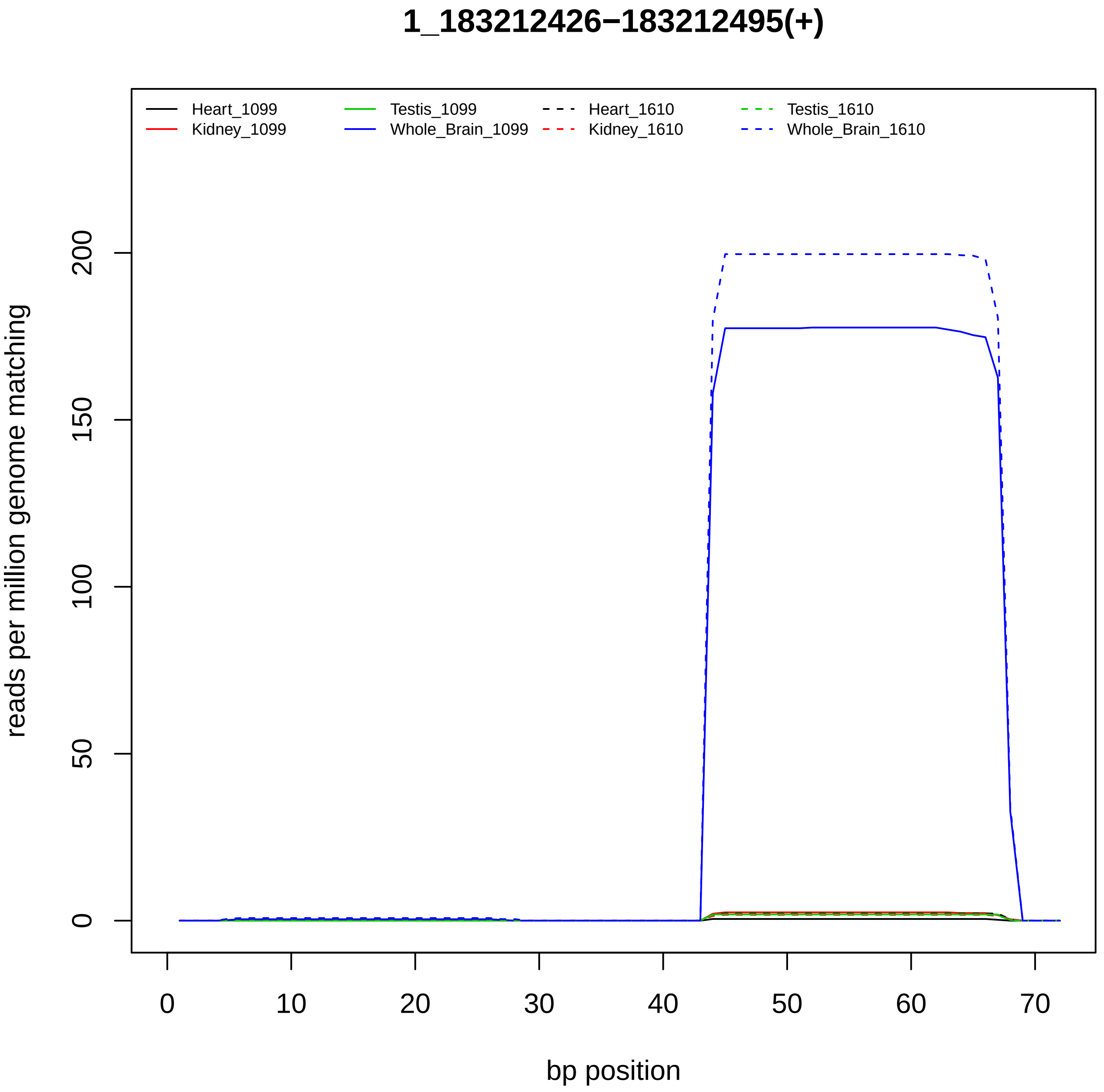

1_183212426−183212495(+)
Heart_1099
Kidney_1099
Testis_1099
Whole_Brain_1099
Heart_1610
Kidney_1610
Testis_1610
Whole_Brain_1610
reads per million genome matching
0
50
100
150
200
0
10
20
30
40
50
60
70
bp position

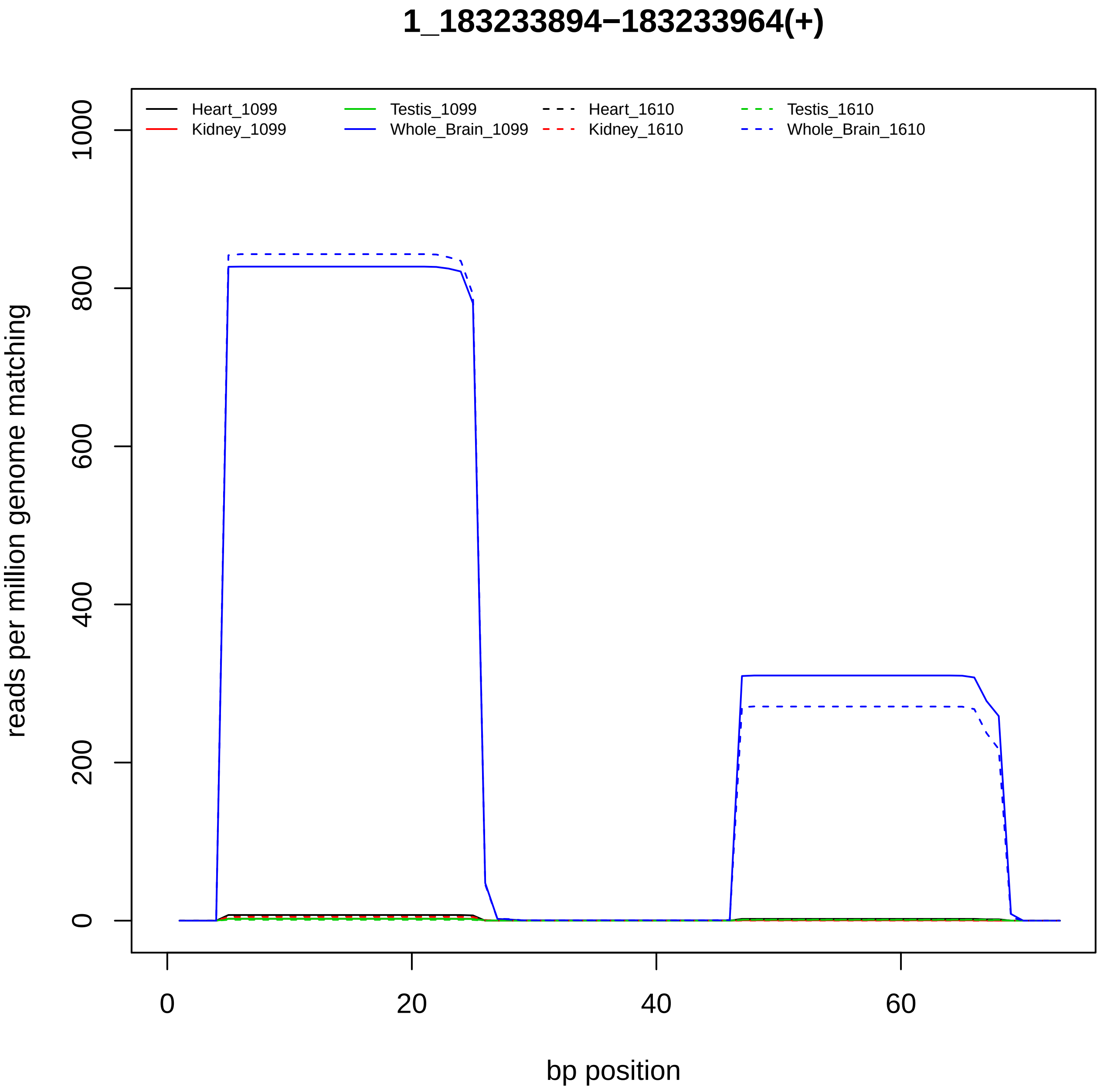
1_183233894−183233964(+)
Heart_1099
Kidney_1099
Testis_1099
Whole_Brain_1099
Heart_1610
Kidney_1610
Testis_1610
Whole_Brain_1610
reads per million genome matching
0
200
400
600
800
1000
0
20
40
60
bp position

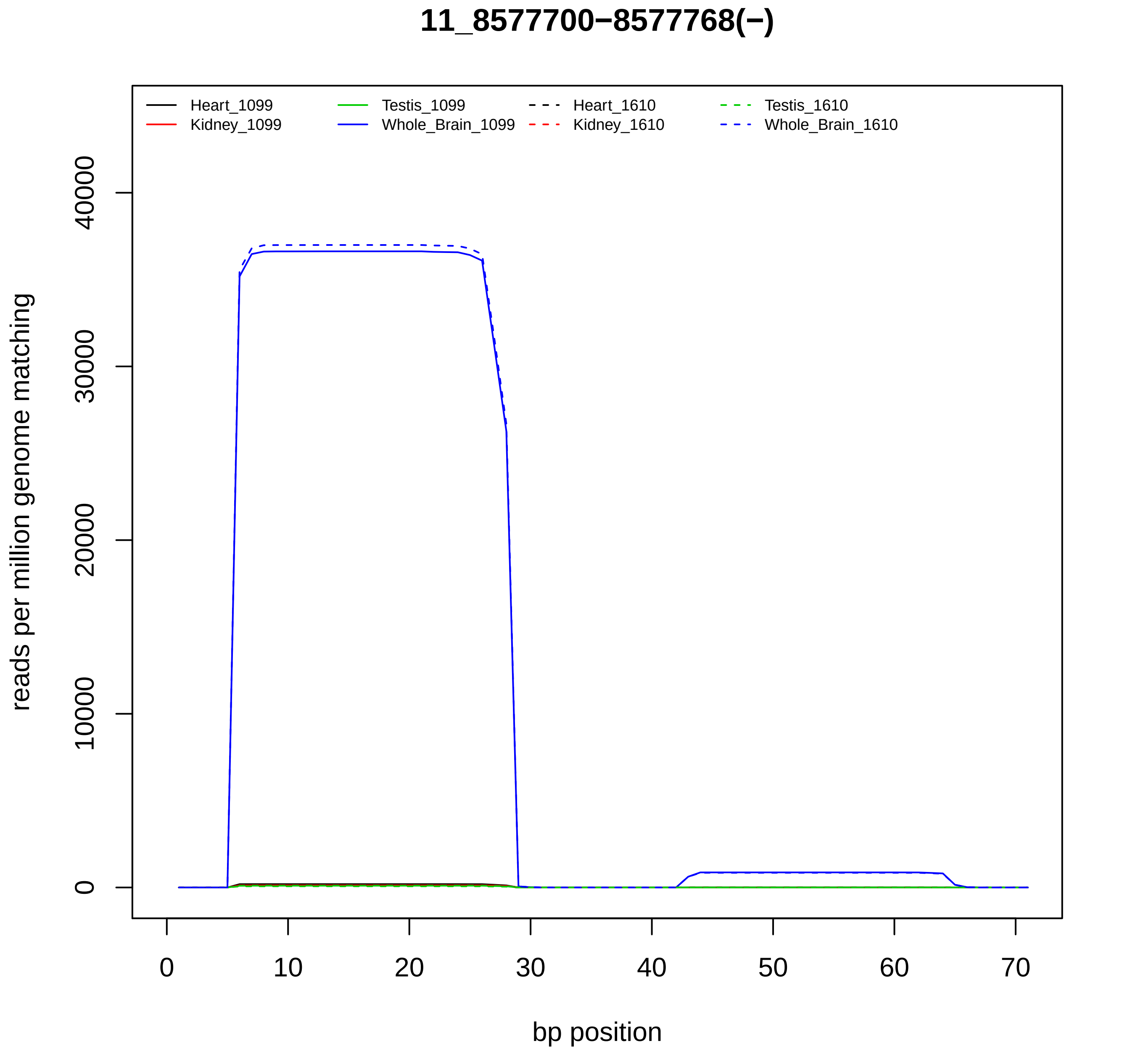
11_8577700−8577768(−)
Heart_1099
Kidney_1099
Testis_1099
Whole_Brain_1099
Heart_1610
Kidney_1610
Testis_1610
Whole_Brain_1610
reads per million genome matching
0
10000
20000
30000
40000
0
10
20
30
40
50
60
70
bp position

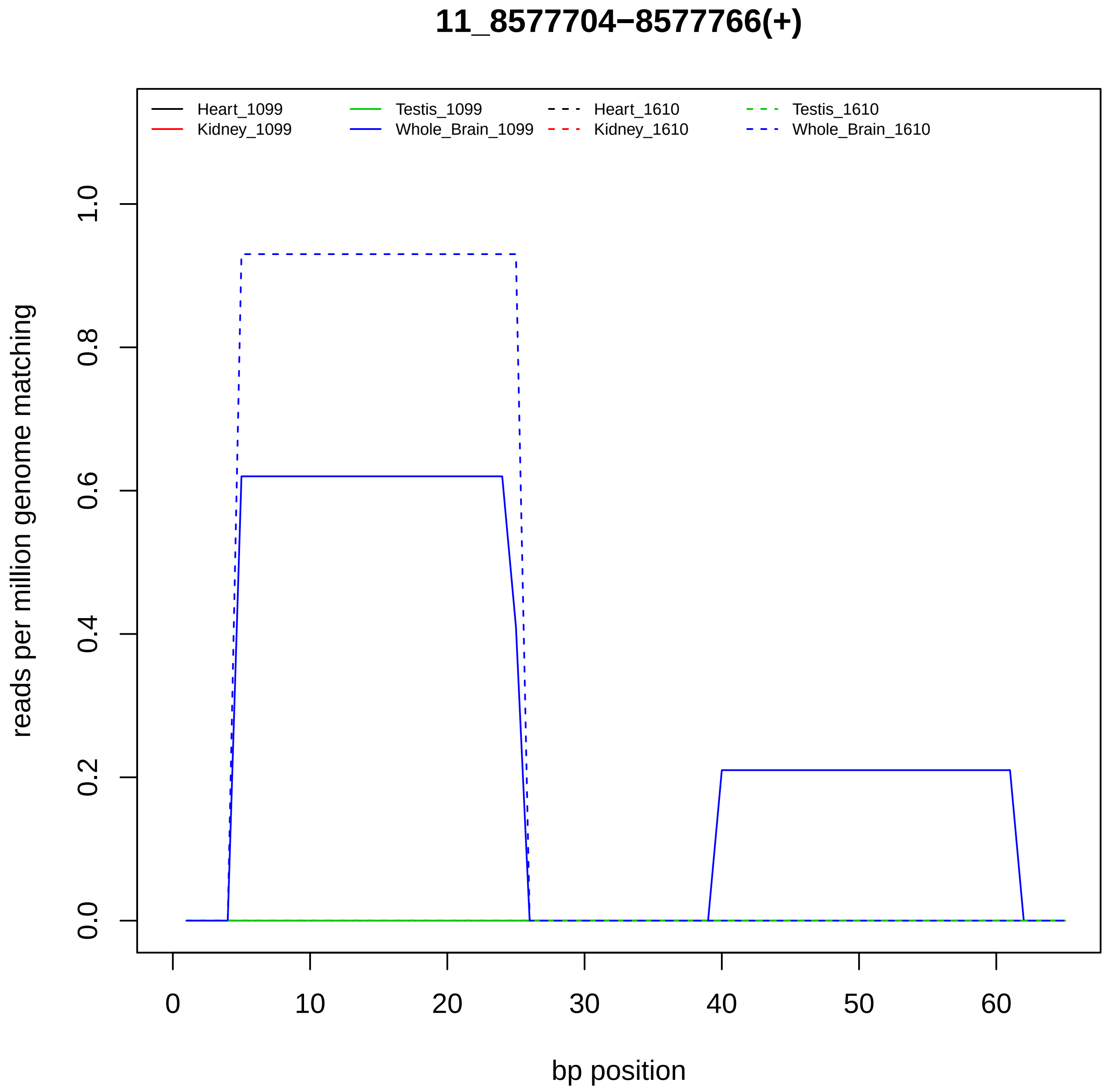

11_8577704−8577766(+)
Heart_1099
Kidney_1099
Testis_1099
Whole_Brain_1099
Heart_1610
Kidney_1610
Testis_1610
Whole_Brain_1610
reads per million genome matching
0.0
0.2
0.4
0.6
0.8
1.0
0
10
20
30
40
50
60
bp position

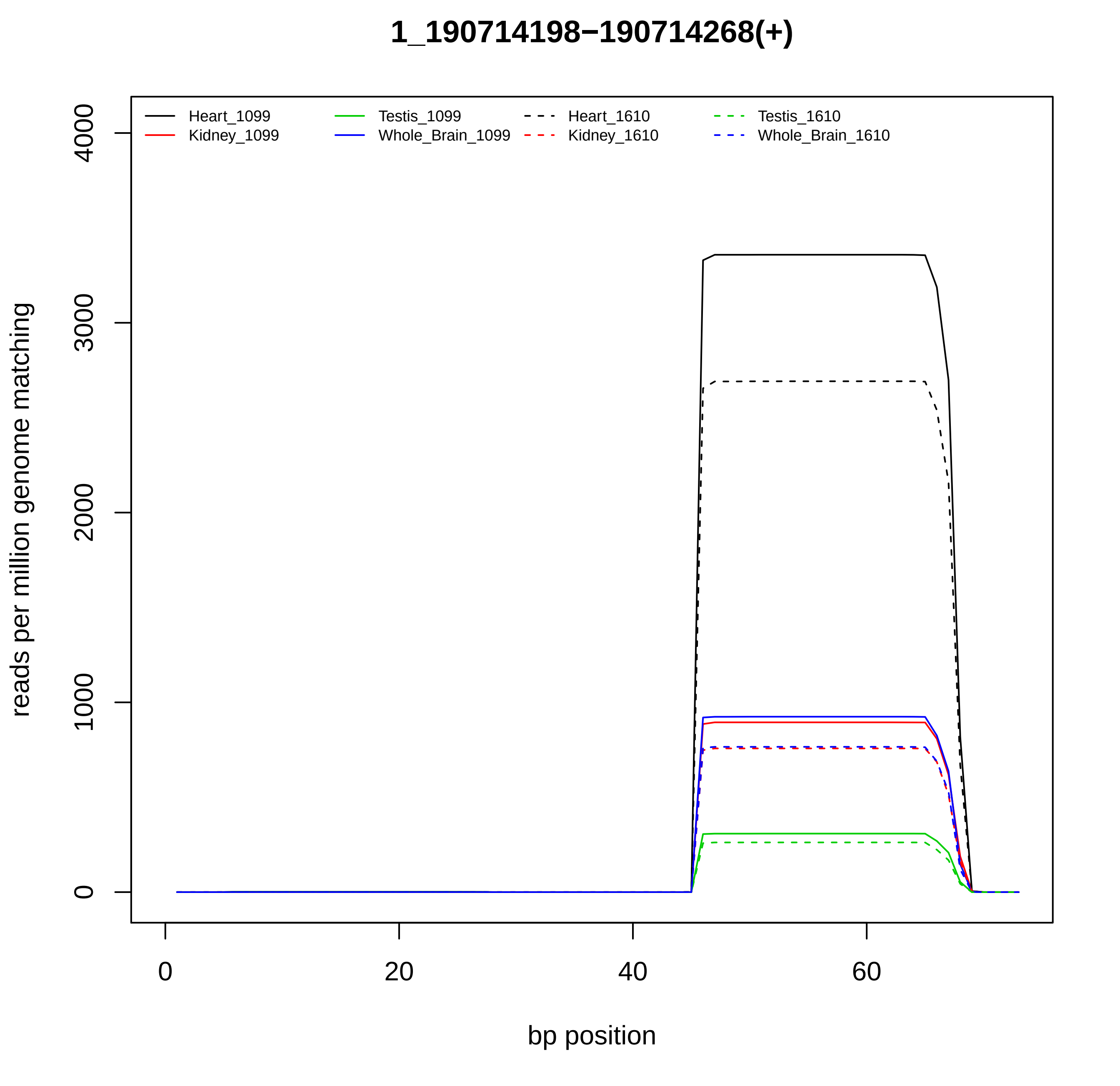

1_190714198−190714268(+)
Heart_1099
Kidney_1099
Testis_1099
Whole_Brain_1099
Heart_1610
Kidney_1610
Testis_1610
Whole_Brain_1610
reads per million genome matching
bp position
0
1000
2000
3000
4000
0
20
40
60

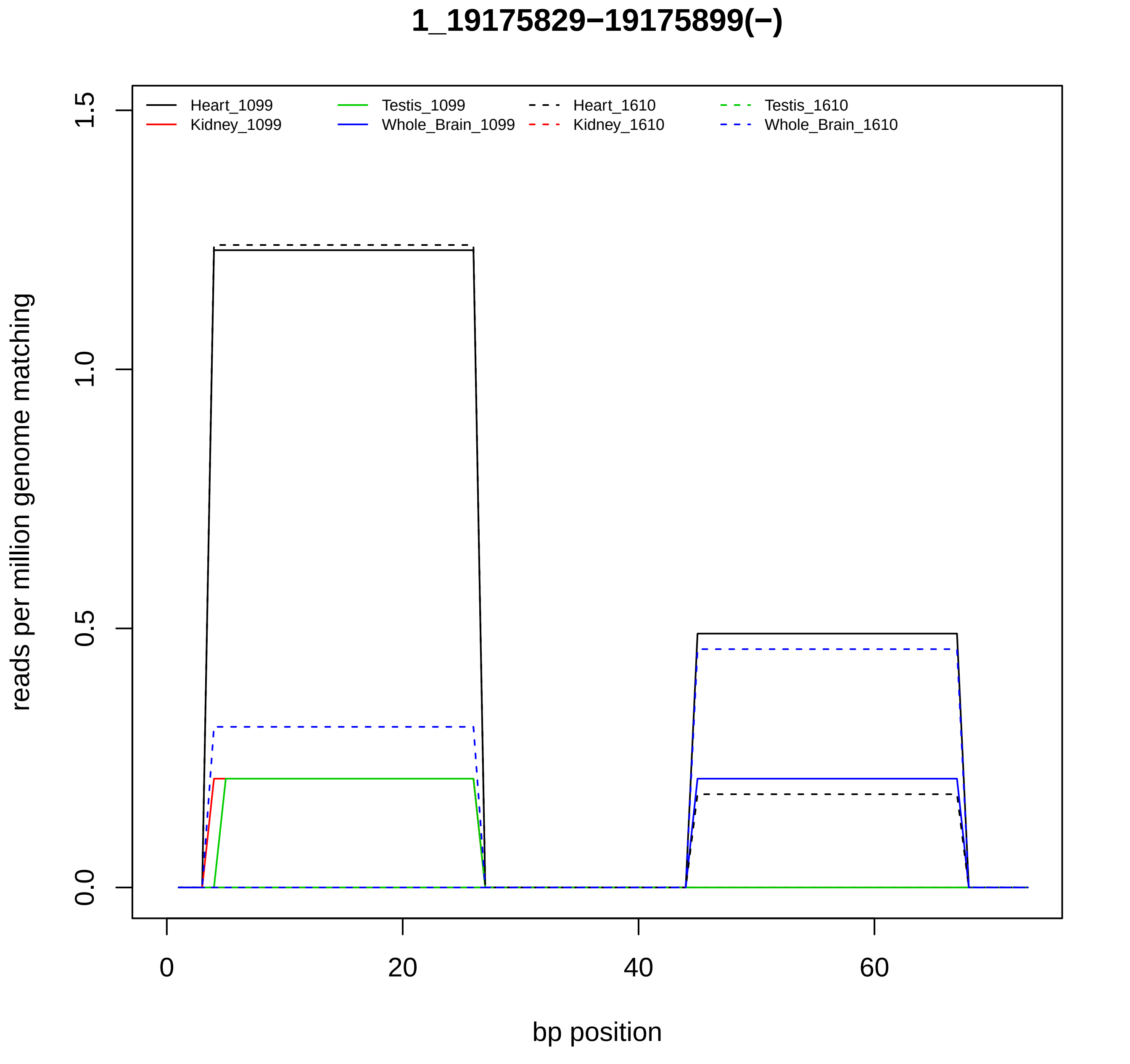

1_19175829−19175899(−)
Heart_1099
Kidney_1099
Testis_1099
Whole_Brain_1099
Heart_1610
Kidney_1610
Testis_1610
Whole_Brain_1610
reads per million genome matching
bp position
0.0
0.5
1.0
1.5
0
20
40
60

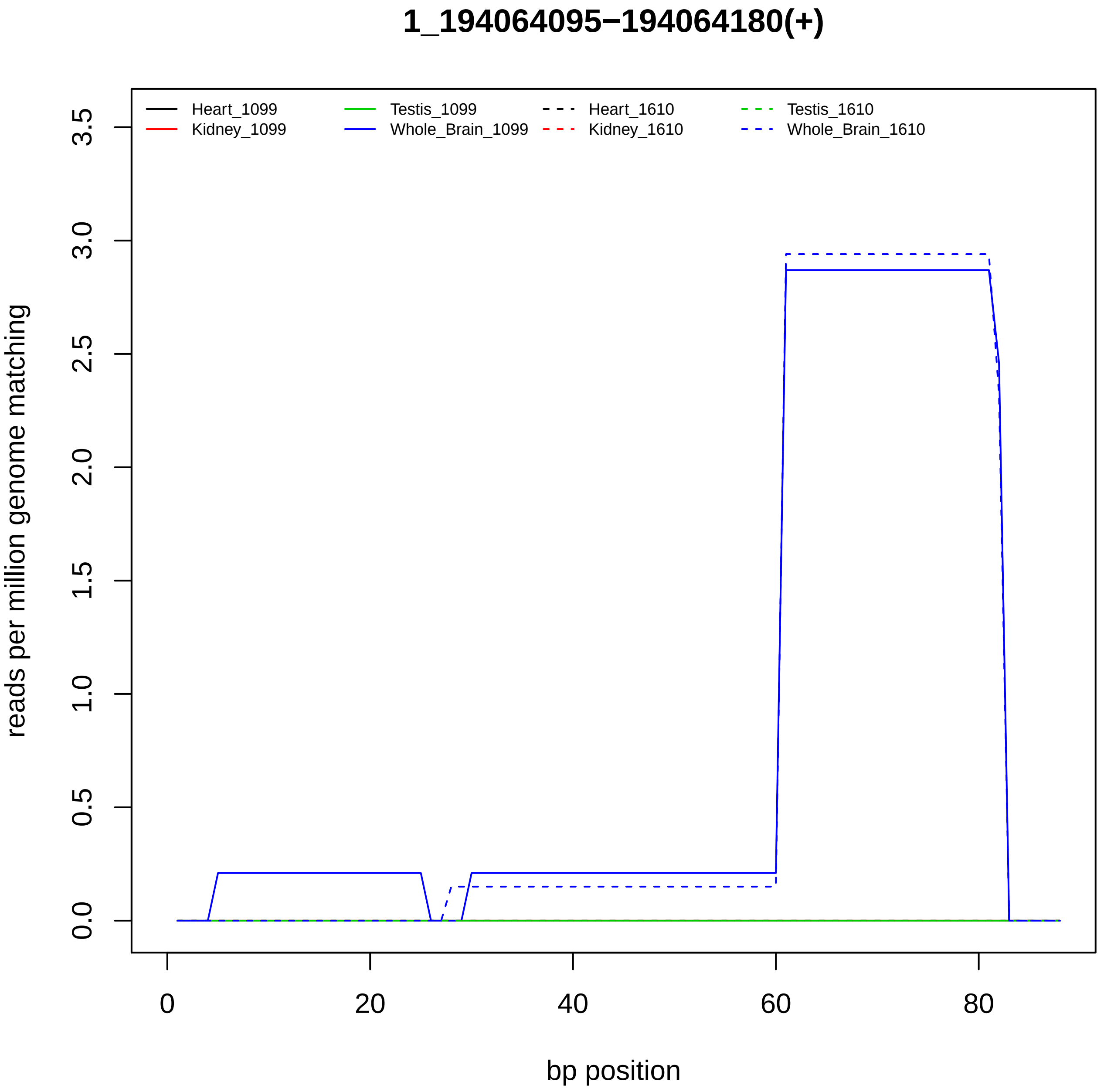

1_194064095−194064180(+)
Heart_1099
Kidney_1099
Testis_1099
Whole_Brain_1099
Heart_1610
Kidney_1610
Testis_1610
Whole_Brain_1610
reads per million genome matching
bp position
0.0
0.5
1.0
1.5
2.0
2.5
3.0
3.5
0
20
40
60
80

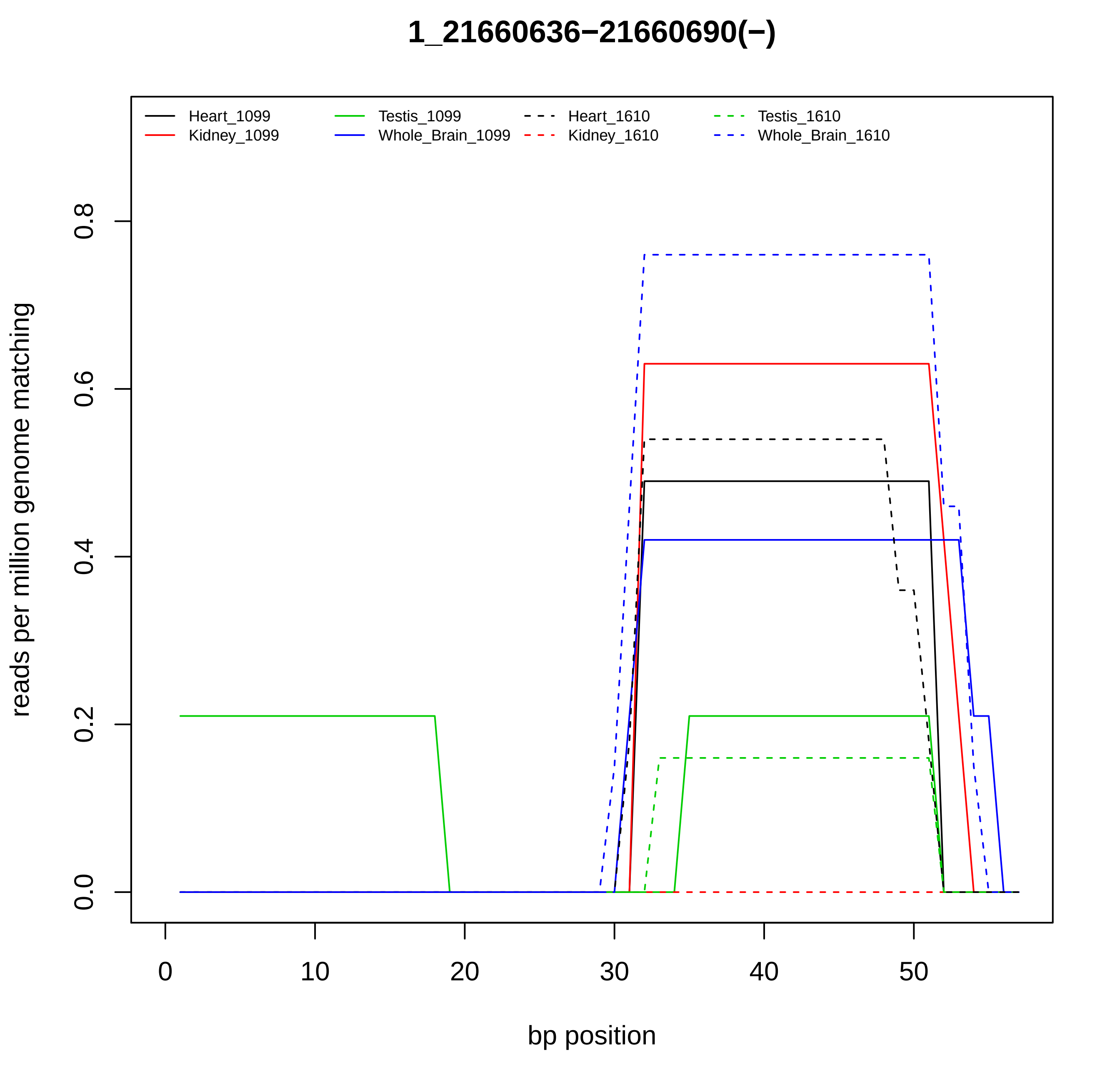
1_21660636−21660690(−)
Heart_1099
Kidney_1099
Testis_1099
Whole_Brain_1099
Heart_1610
Kidney_1610
Testis_1610
Whole_Brain_1610
reads per million genome matching
0.0
0.2
0.4
0.6
0.8
0
10
20
30
40
50
bp position

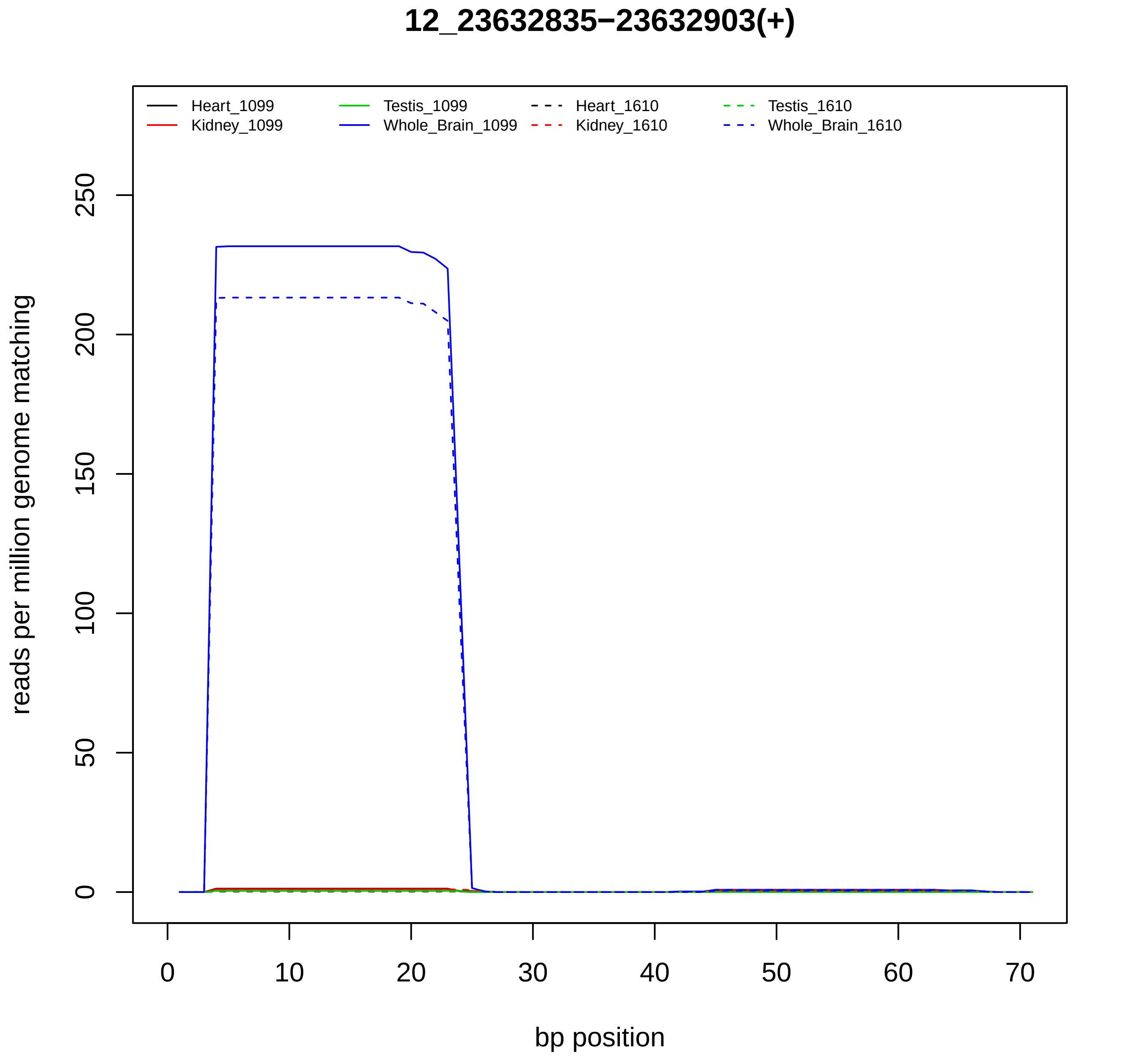

12_23632835−23632903(+)
Heart_1099
Kidney_1099
Testis_1099
Whole_Brain_1099
Heart_1610
Kidney_1610
Testis_1610
Whole_Brain_1610
reads per million genome matching
bp position
0
50
100
150
200
250
0
10
20
30
40
50
60
70

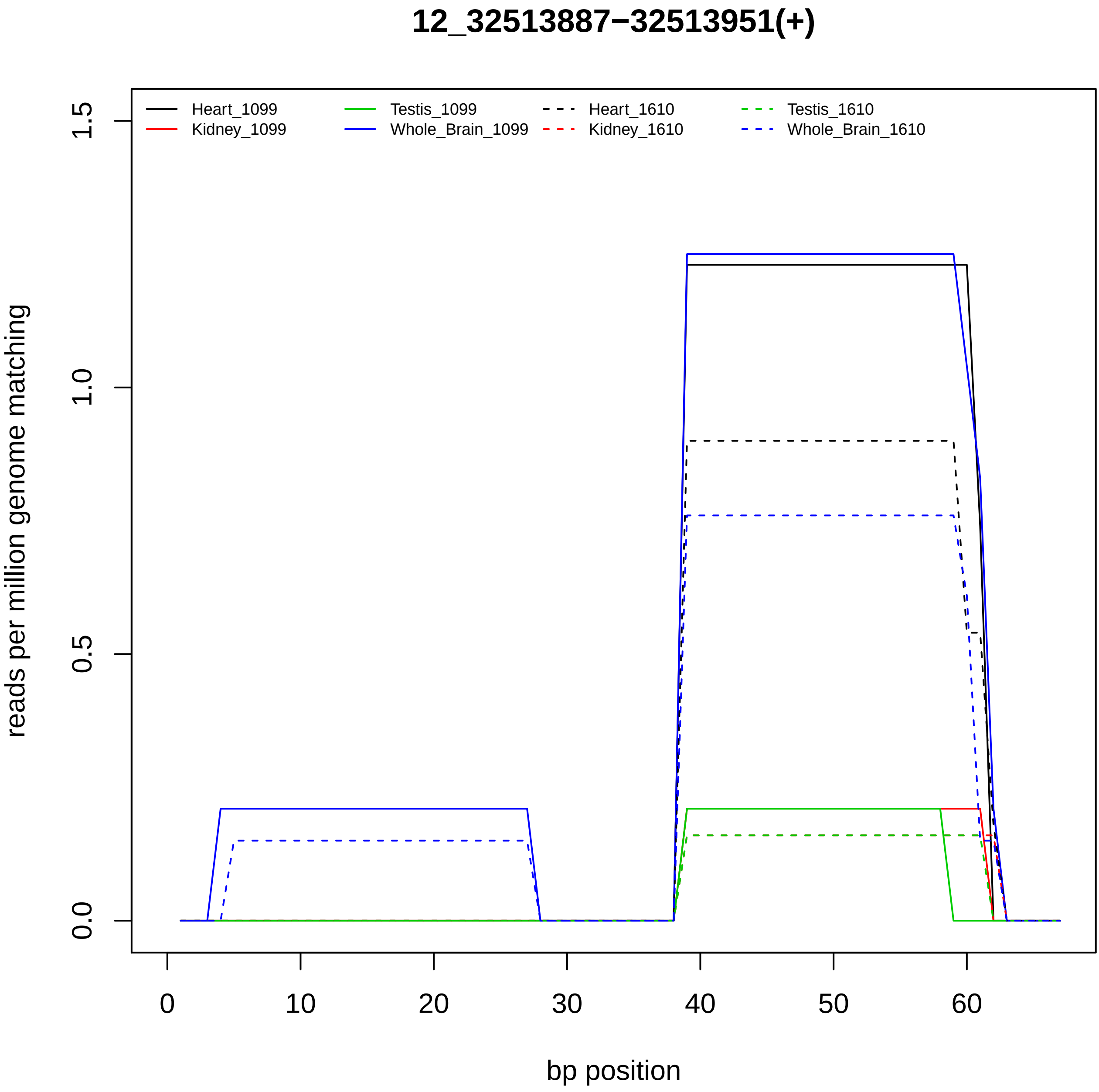

12_32513887−32513951(+)
Heart_1099
Kidney_1099
Testis_1099
Whole_Brain_1099
Heart_1610
Kidney_1610
Testis_1610
Whole_Brain_1610
reads per million genome matching
0.0
0.5
1.0
1.5
bp position
0
10
20
30
40
50
60

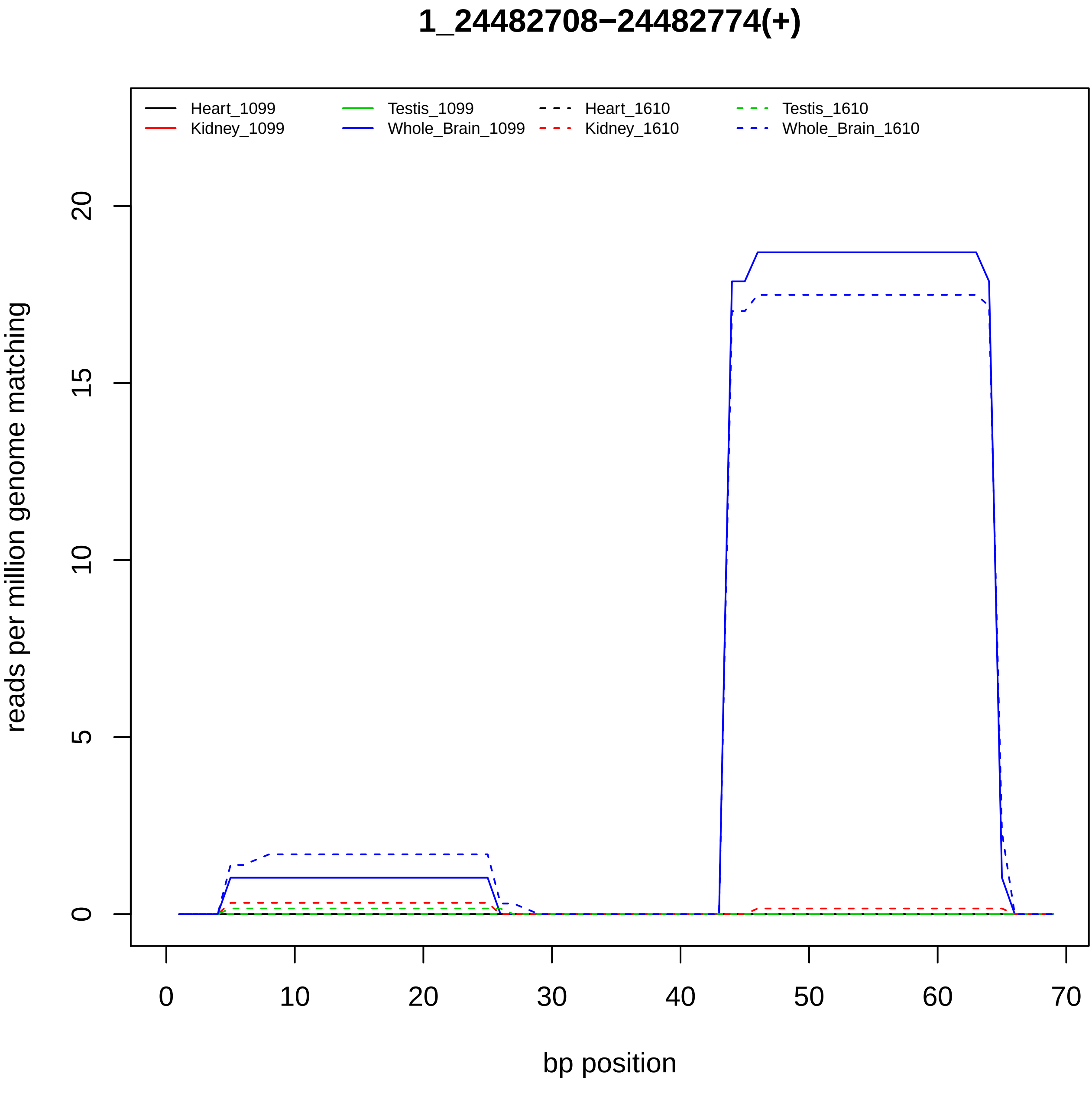
1_24482708−24482774(+)
Heart_1099
Kidney_1099
Testis_1099
Whole_Brain_1099
Heart_1610
Kidney_1610
Testis_1610
Whole_Brain_1610
reads per million genome matching
bp position
0
5
10
15
20
0
10
20
30
40
50
60
70

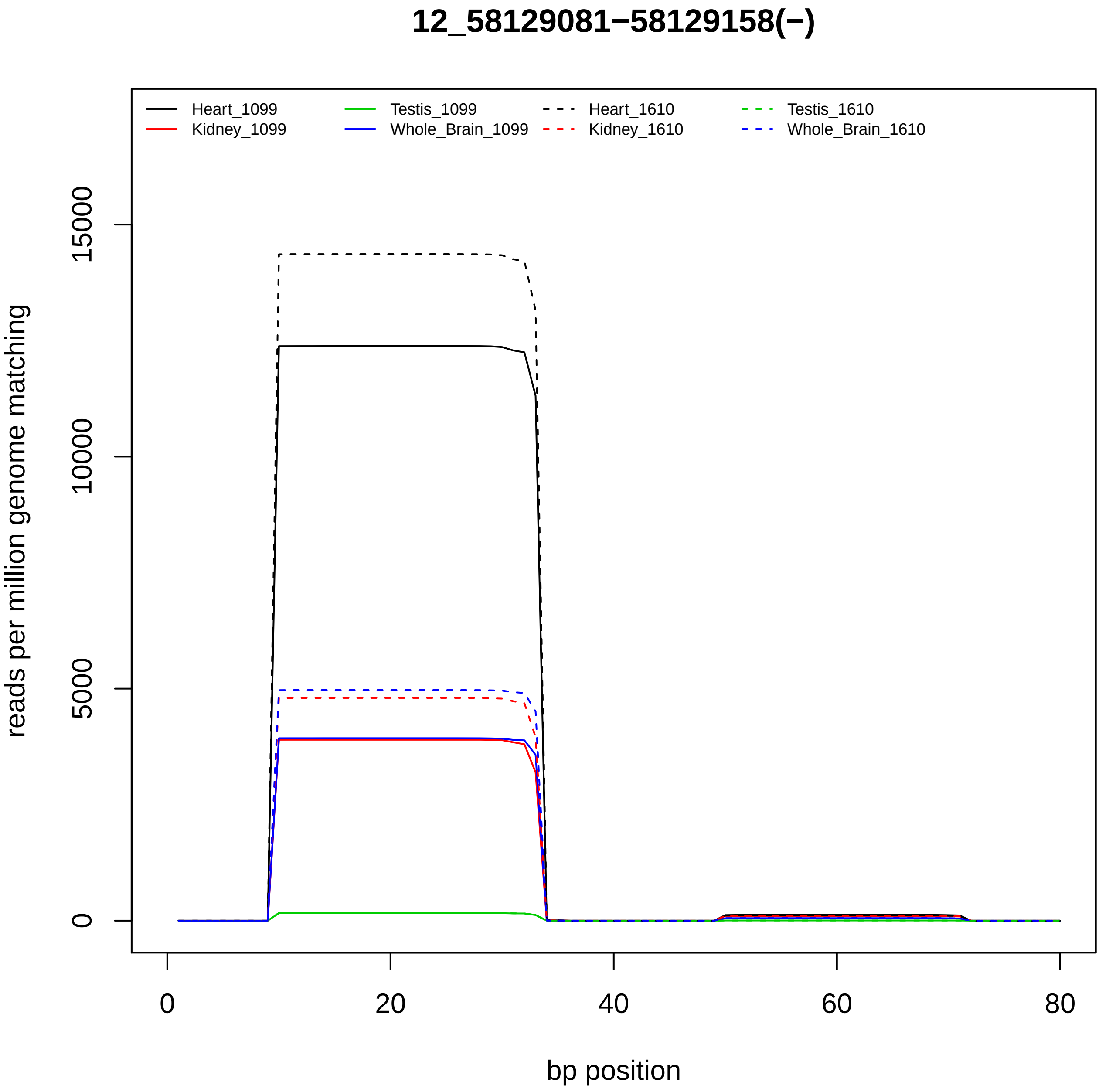

12_58129081−58129158(−)
Heart_1099
Kidney_1099
Testis_1099
Whole_Brain_1099
Heart_1610
Kidney_1610
Testis_1610
Whole_Brain_1610
reads per million genome matching
0
5000
10000
15000
bp position
0
20
40
60
80

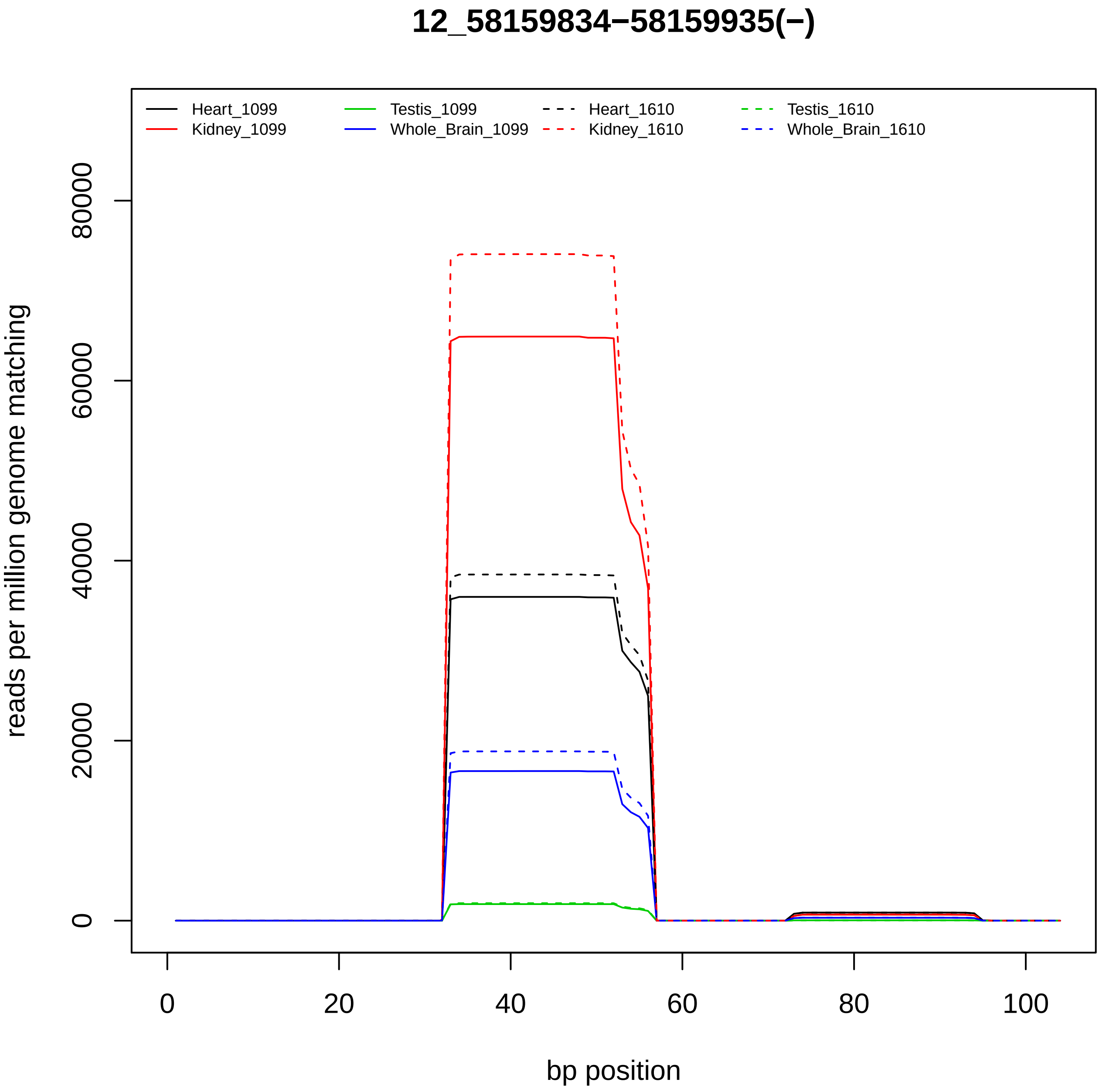

12_58159834−58159935(−)
Heart_1099
Kidney_1099
Testis_1099
Whole_Brain_1099
Heart_1610
Kidney_1610
Testis_1610
Whole_Brain_1610
reads per million genome matching
0
20000
40000
60000
80000
0
20
40
60
80
100
bp position

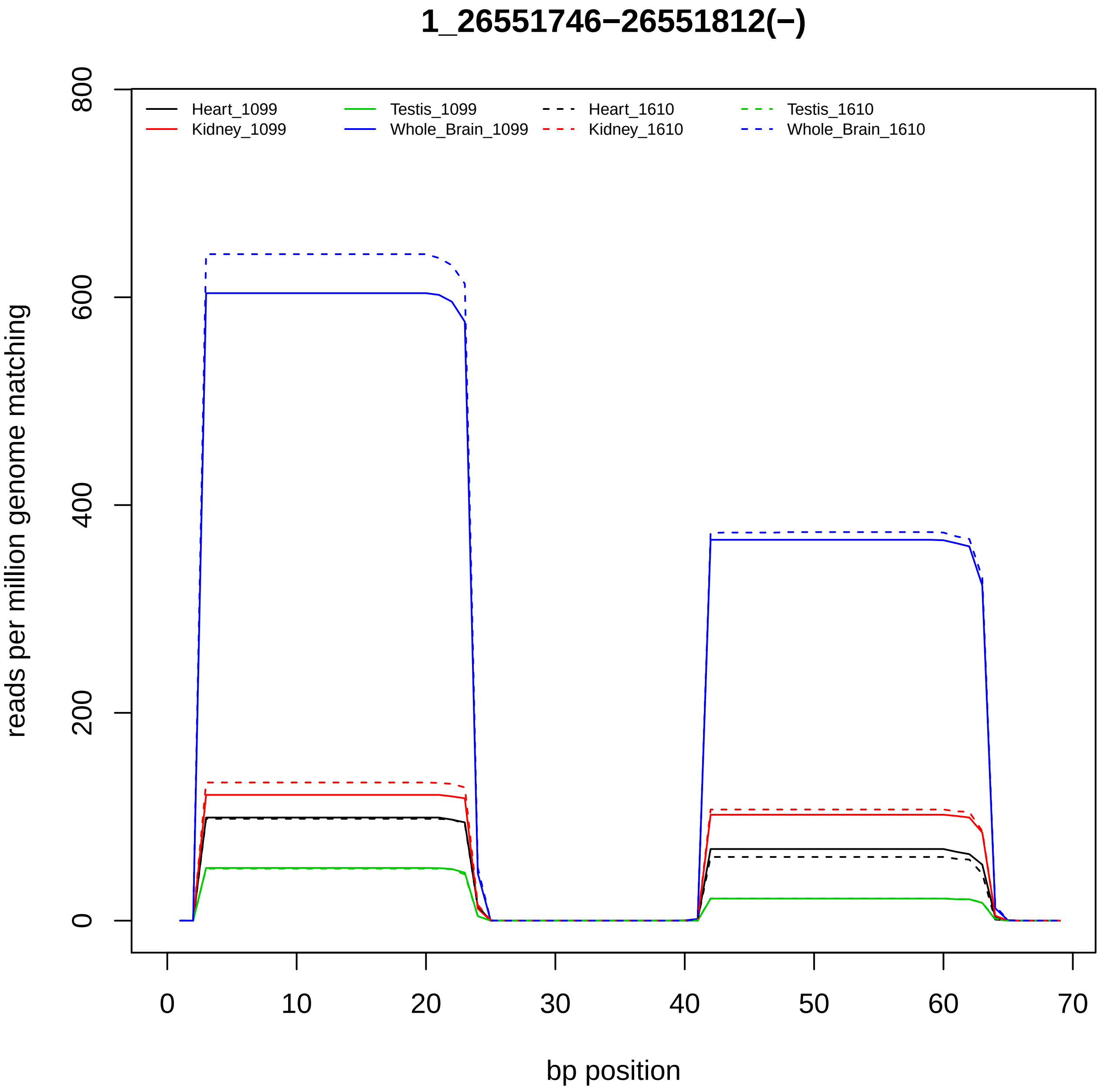
1_26551746−26551812(−)
Heart_1099
Kidney_1099
Testis_1099
Whole_Brain_1099
Heart_1610
Kidney_1610
Testis_1610
Whole_Brain_1610
reads per million genome matching
0
200
400
600
800
0
10
20
30
40
50
60
70
bp position

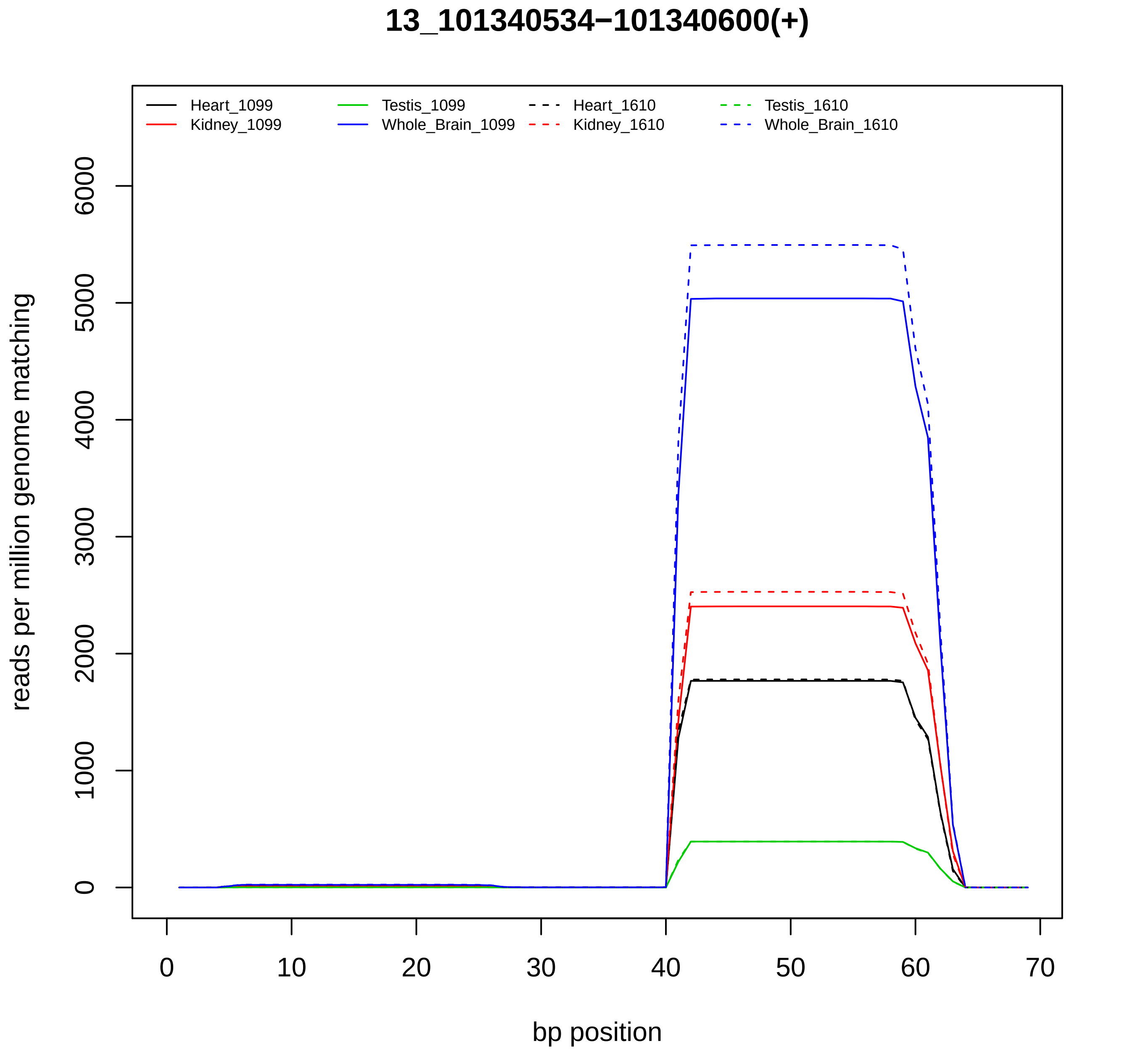

13_101340534−101340600(+)
Heart_1099
Kidney_1099
Testis_1099
Whole_Brain_1099
Heart_1610
Kidney_1610
Testis_1610
Whole_Brain_1610
reads per million genome matching
0
1000
2000
3000
4000
5000
6000
0
10
20
30
40
50
60
70
bp position

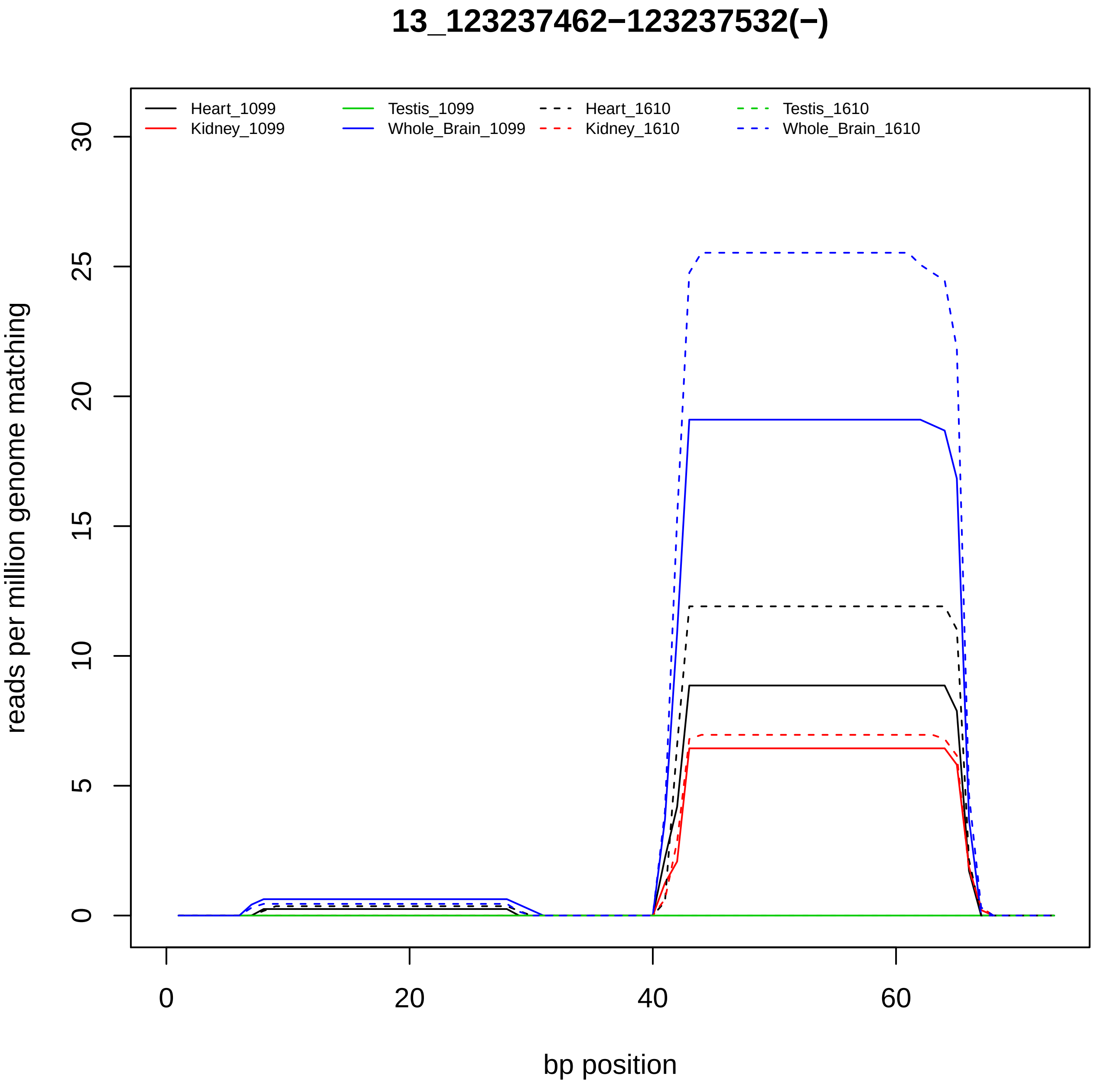

13_123237462−123237532(−)
Heart_1099
Kidney_1099
Testis_1099
Whole_Brain_1099
Heart_1610
Kidney_1610
Testis_1610
Whole_Brain_1610
reads per million genome matching
bp position
0
5
10
15
20
25
30
0
20
40
60

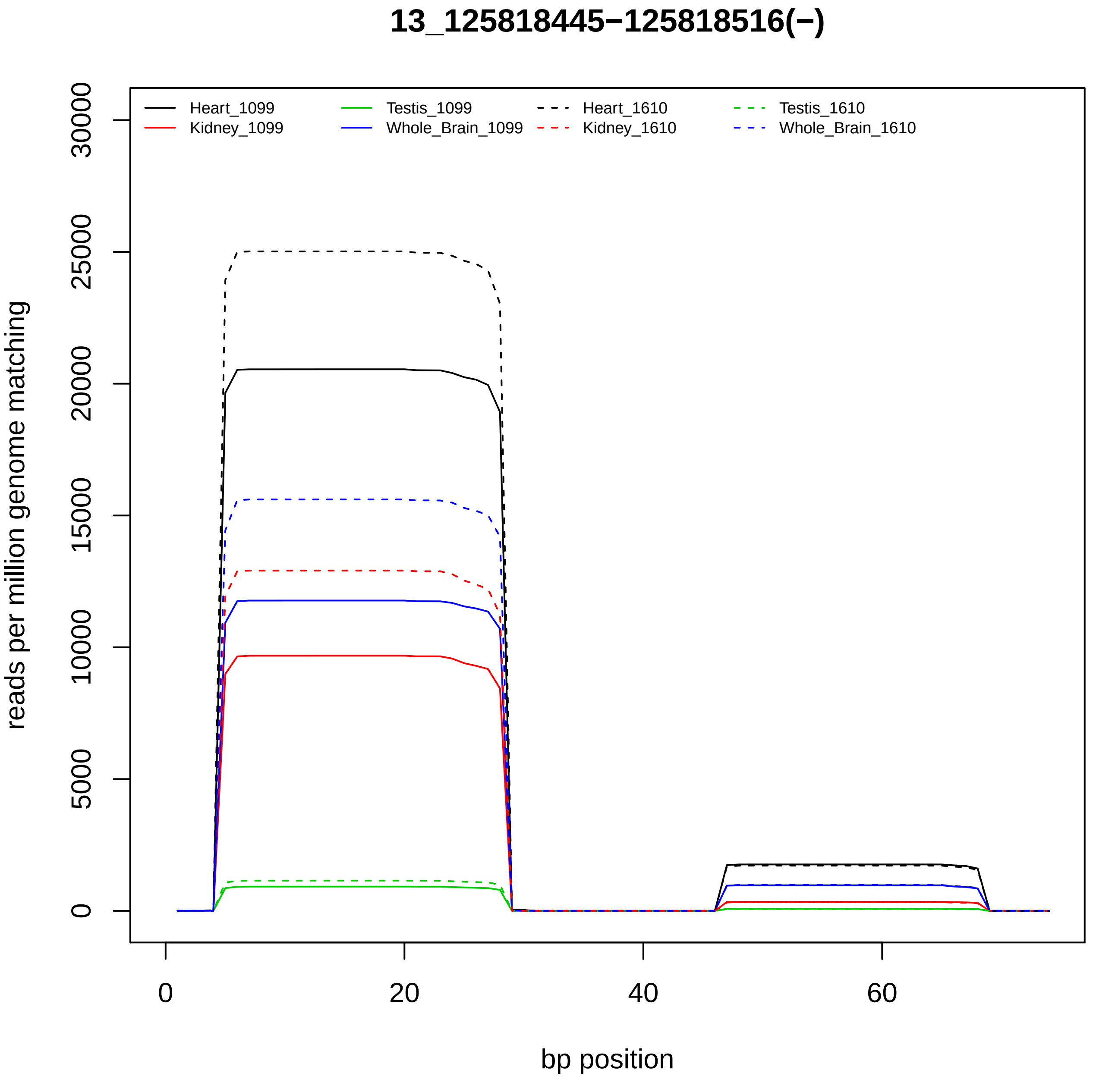

13_125818445−125818516(−)
Heart_1099
Kidney_1099
Testis_1099
Whole_Brain_1099
Heart_1610
Kidney_1610
Testis_1610
Whole_Brain_1610
reads per million genome matching
0
5000
10000
15000
20000
25000
30000
0
20
40
60
bp position

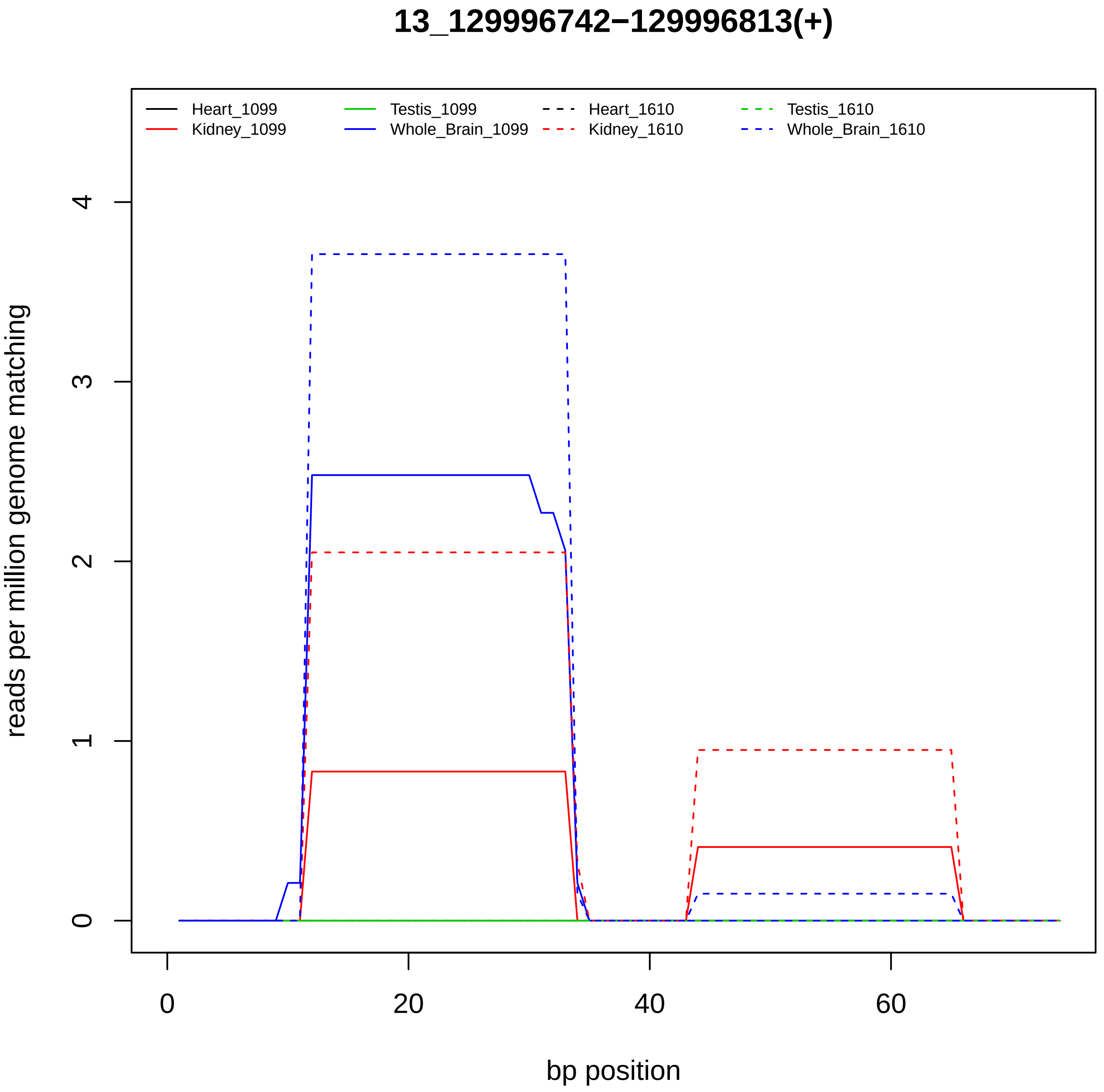
13_129996742−129996813(+)
Heart_1099
Kidney_1099
Testis_1099
Whole_Brain_1099
Heart_1610
Kidney_1610
Testis_1610
Whole_Brain_1610
reads per million genome matching
bp position
0
1
2
3
4
0
20
40
60

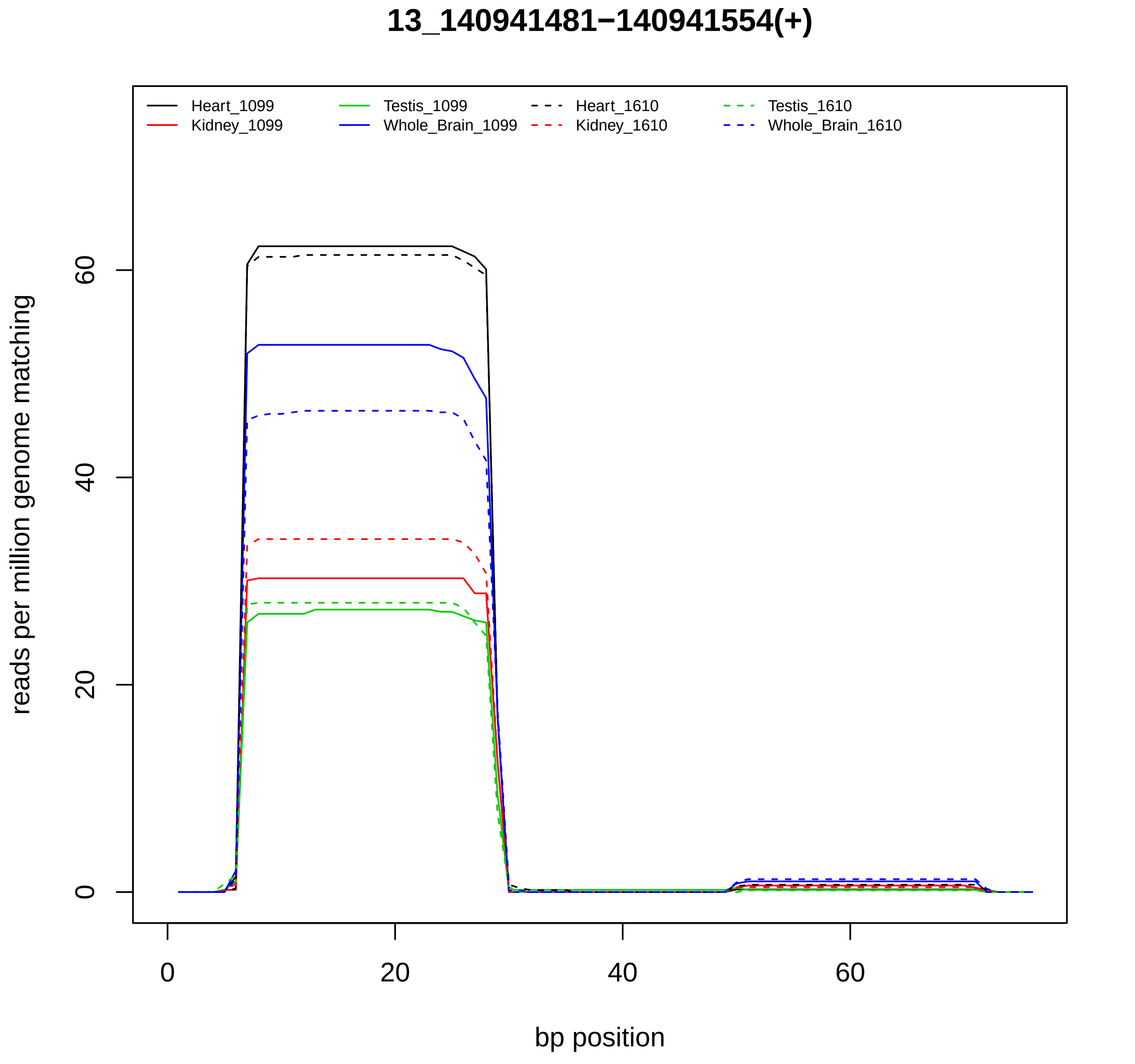

13_140941481−140941554(+)
Heart_1099
Kidney_1099
Testis_1099
Whole_Brain_1099
Heart_1610
Kidney_1610
Testis_1610
Whole_Brain_1610
reads per million genome matching
0
20
40
60
bp position
0
20
40
60

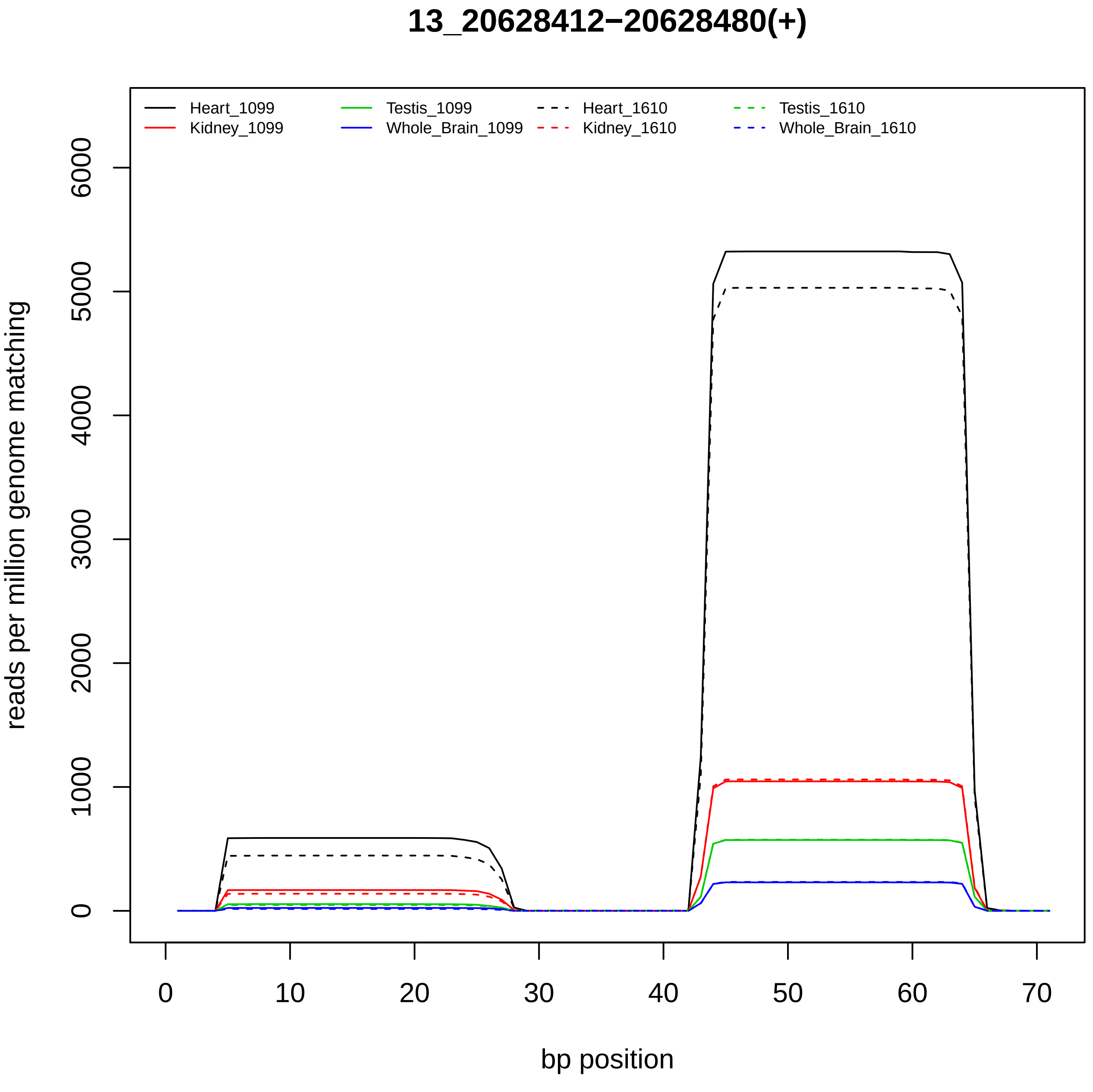
13_20628412−20628480(+)
Heart_1099
Kidney_1099
Testis_1099
Whole_Brain_1099
Heart_1610
Kidney_1610
Testis_1610
Whole_Brain_1610
reads per million genome matching
0
1000
2000
3000
4000
5000
6000
bp position
0
10
20
30
40
50
60
70

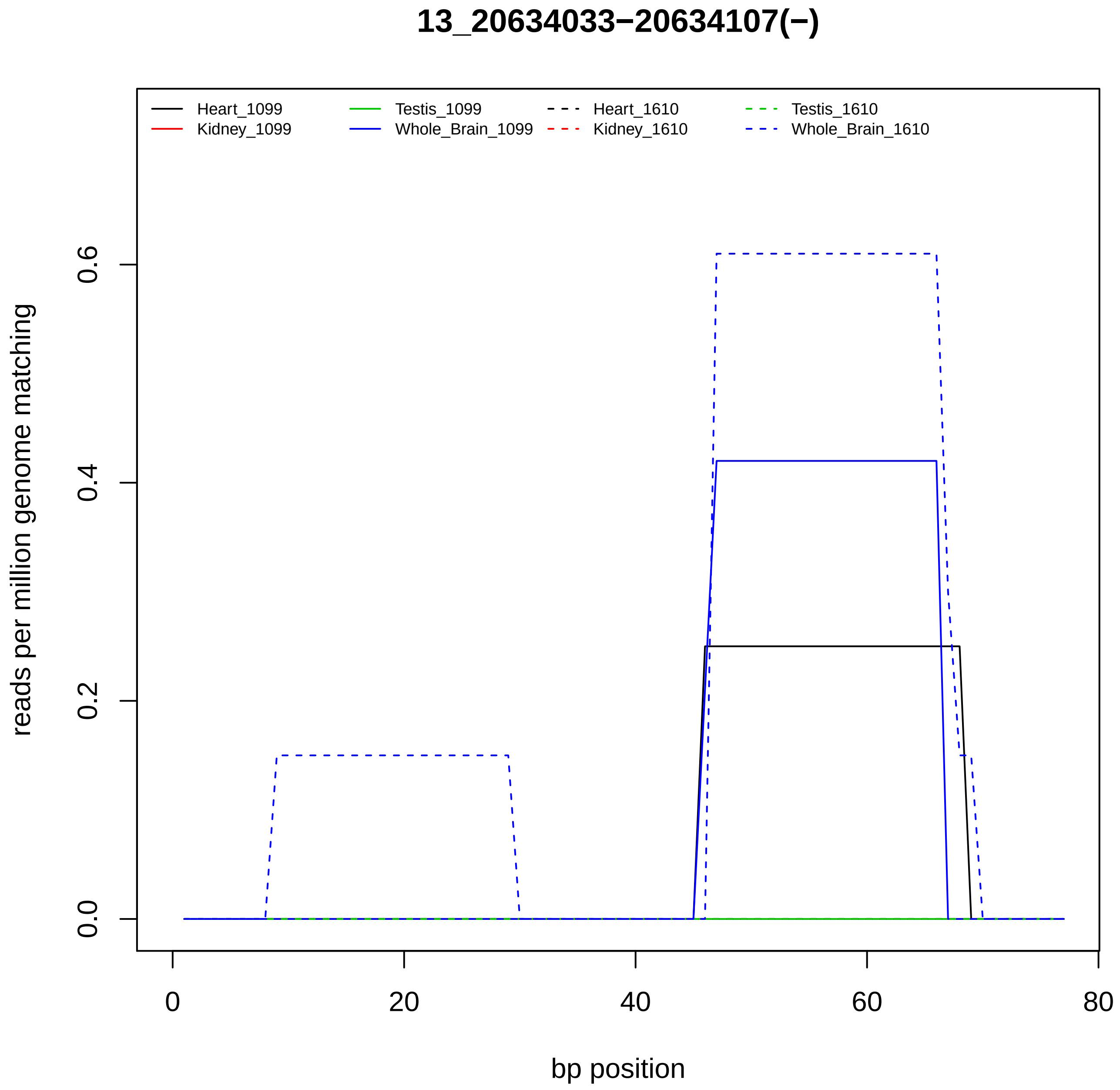
13_20634033−20634107(−)
Heart_1099
Kidney_1099
Testis_1099
Whole_Brain_1099
Heart_1610
Kidney_1610
Testis_1610
Whole_Brain_1610
reads per million genome matching
0.0
0.2
0.4
0.6
0
20
40
60
80
bp position

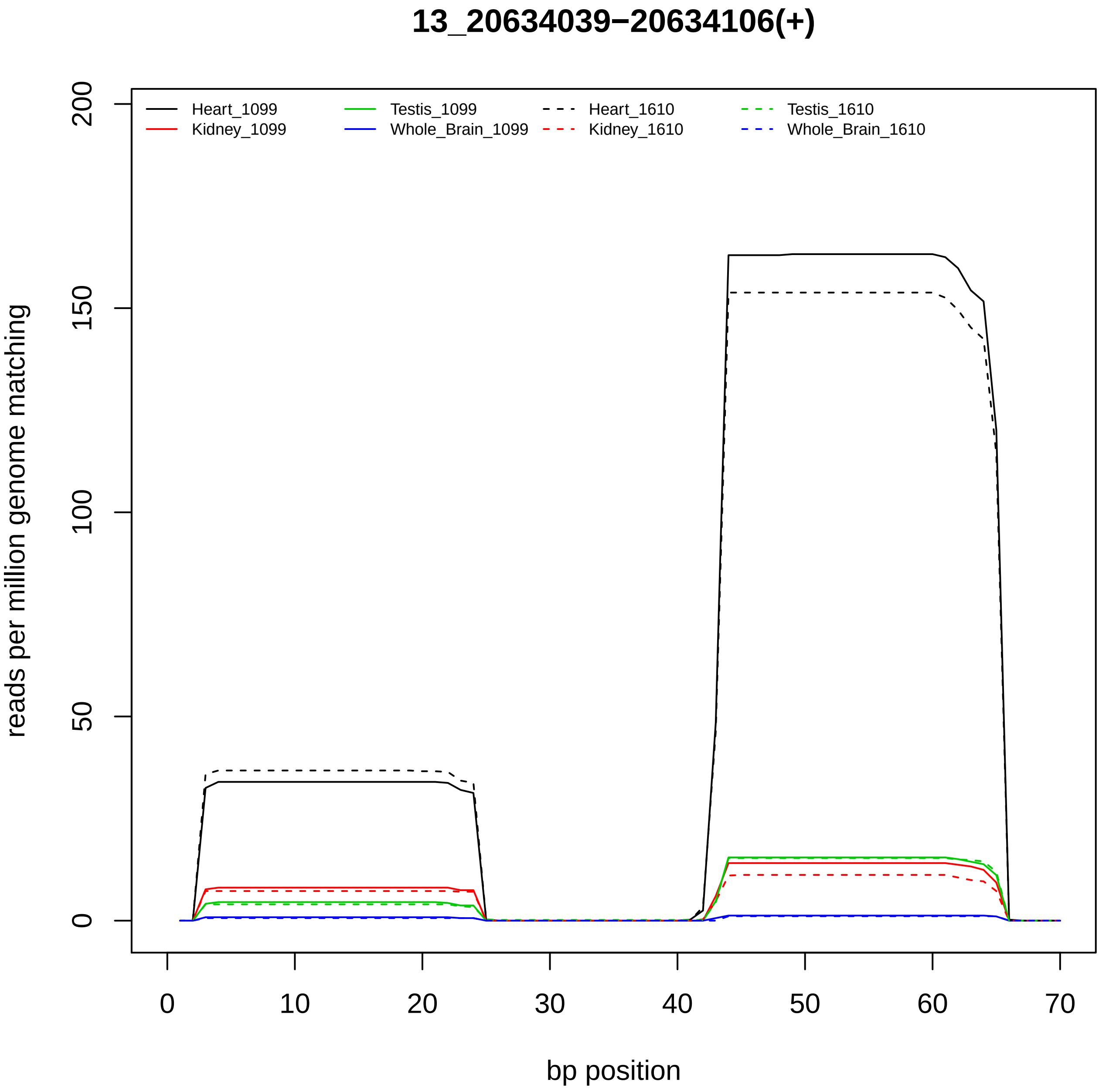

13_20634039−20634106(+)
Heart_1099
Kidney_1099
Testis_1099
Whole_Brain_1099
Heart_1610
Kidney_1610
Testis_1610
Whole_Brain_1610
reads per million genome matching
bp position
0
50
100
150
200
0
10
20
30
40
50
60
70

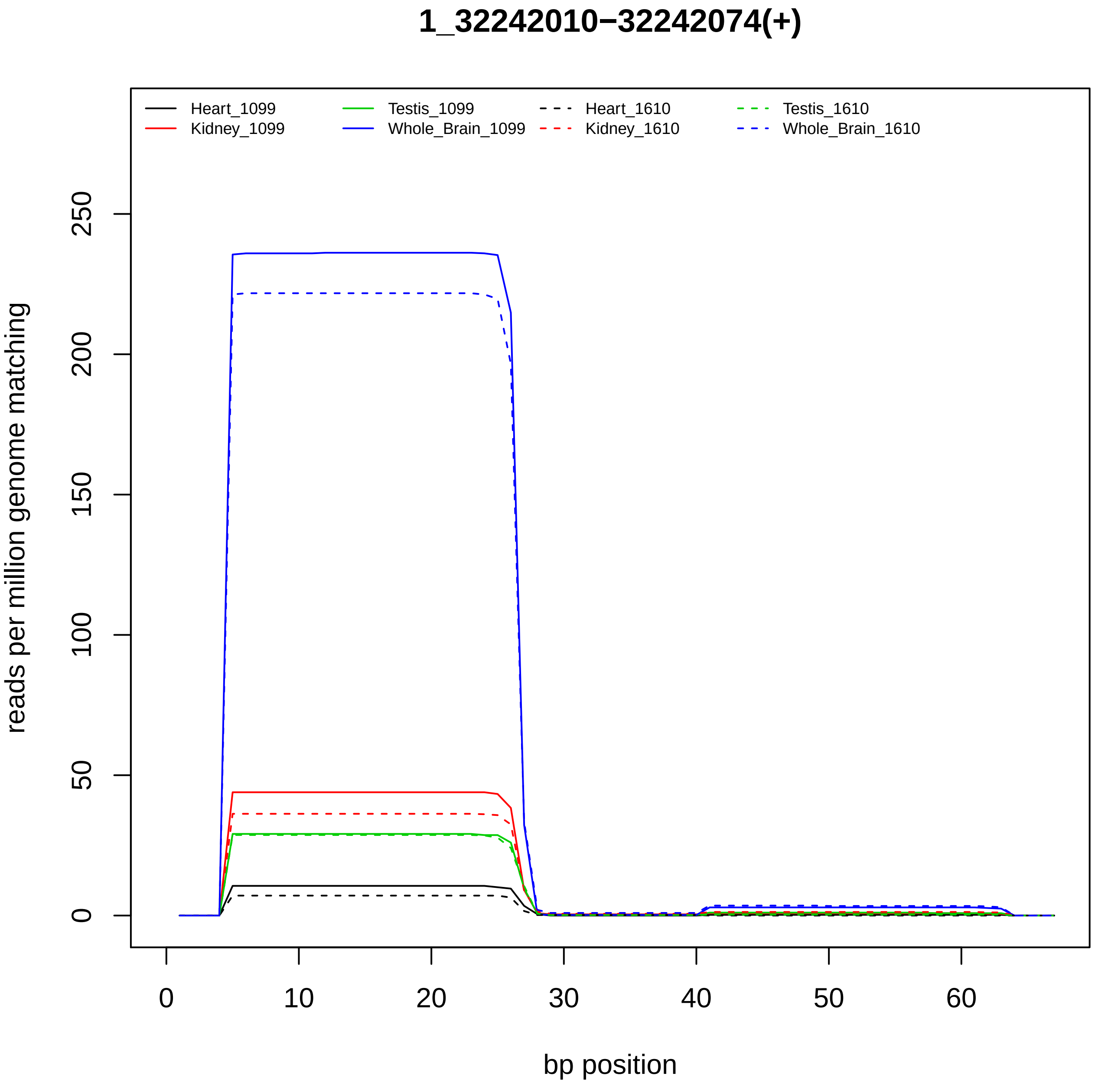

1_32242010−32242074(+)
Heart_1099
Kidney_1099
Testis_1099
Whole_Brain_1099
Heart_1610
Kidney_1610
Testis_1610
Whole_Brain_1610
reads per million genome matching
0
50
100
150
200
250
bp position
0
10
20
30
40
50
60

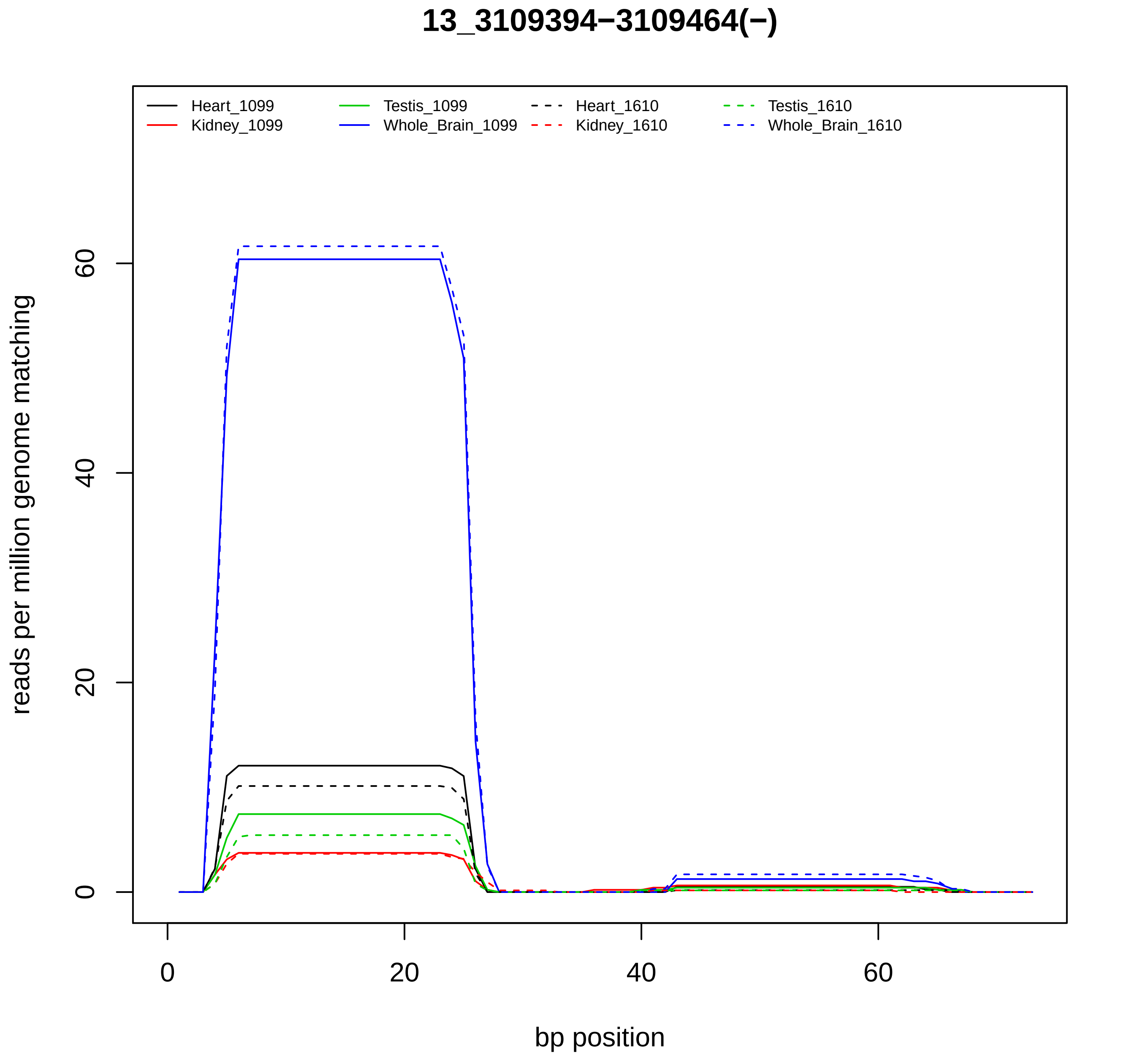
13_3109394−3109464(−)
Heart_1099
Kidney_1099
Testis_1099
Whole_Brain_1099
Heart_1610
Kidney_1610
Testis_1610
Whole_Brain_1610
reads per million genome matching
bp position
0
20
40
60
0
20
40
60

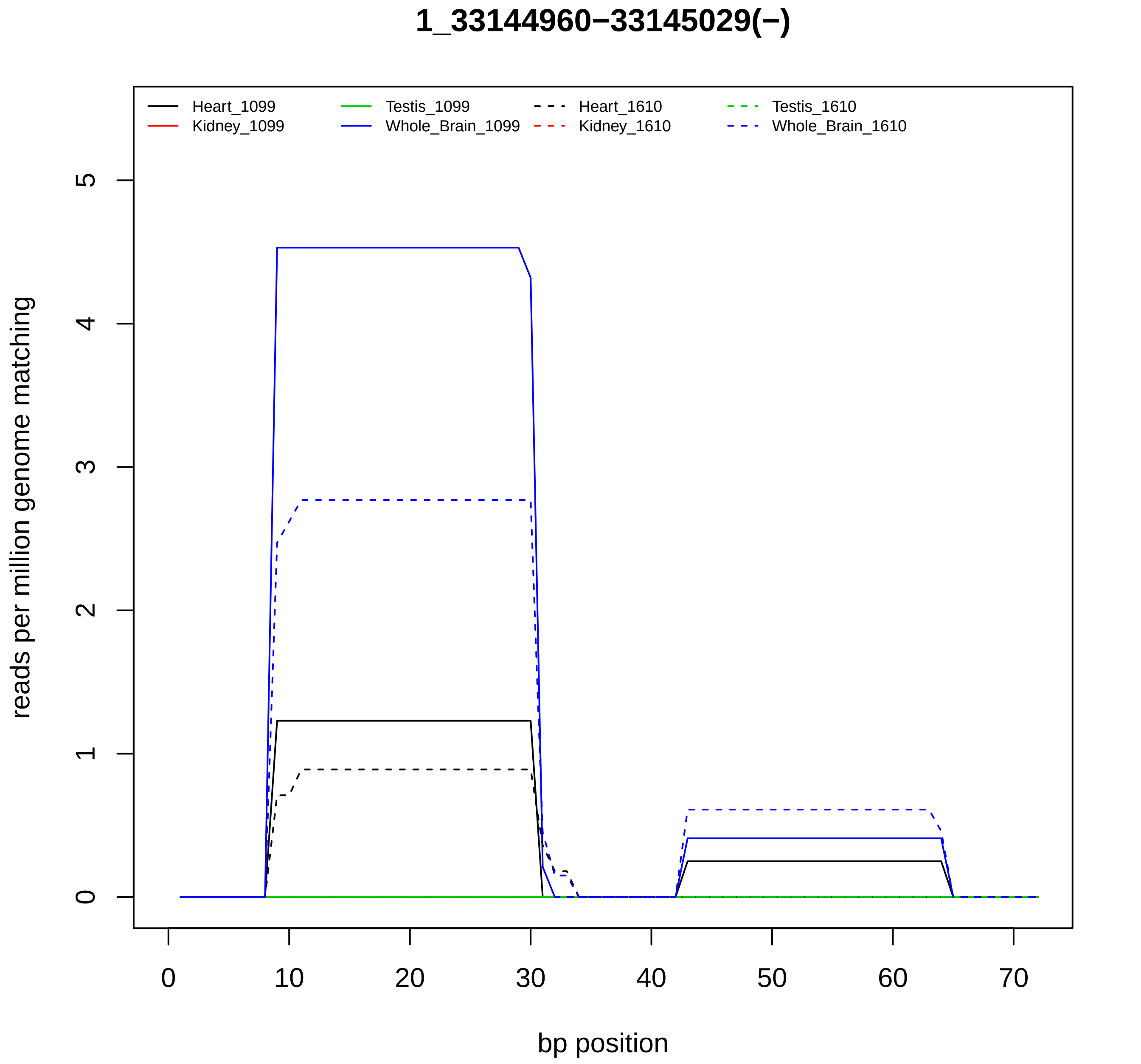

1_33144960−33145029(−)
Heart_1099
Kidney_1099
Testis_1099
Whole_Brain_1099
Heart_1610
Kidney_1610
Testis_1610
Whole_Brain_1610
reads per million genome matching
bp position
0
1
2
3
4
5
0
10
20
30
40
50
60
70

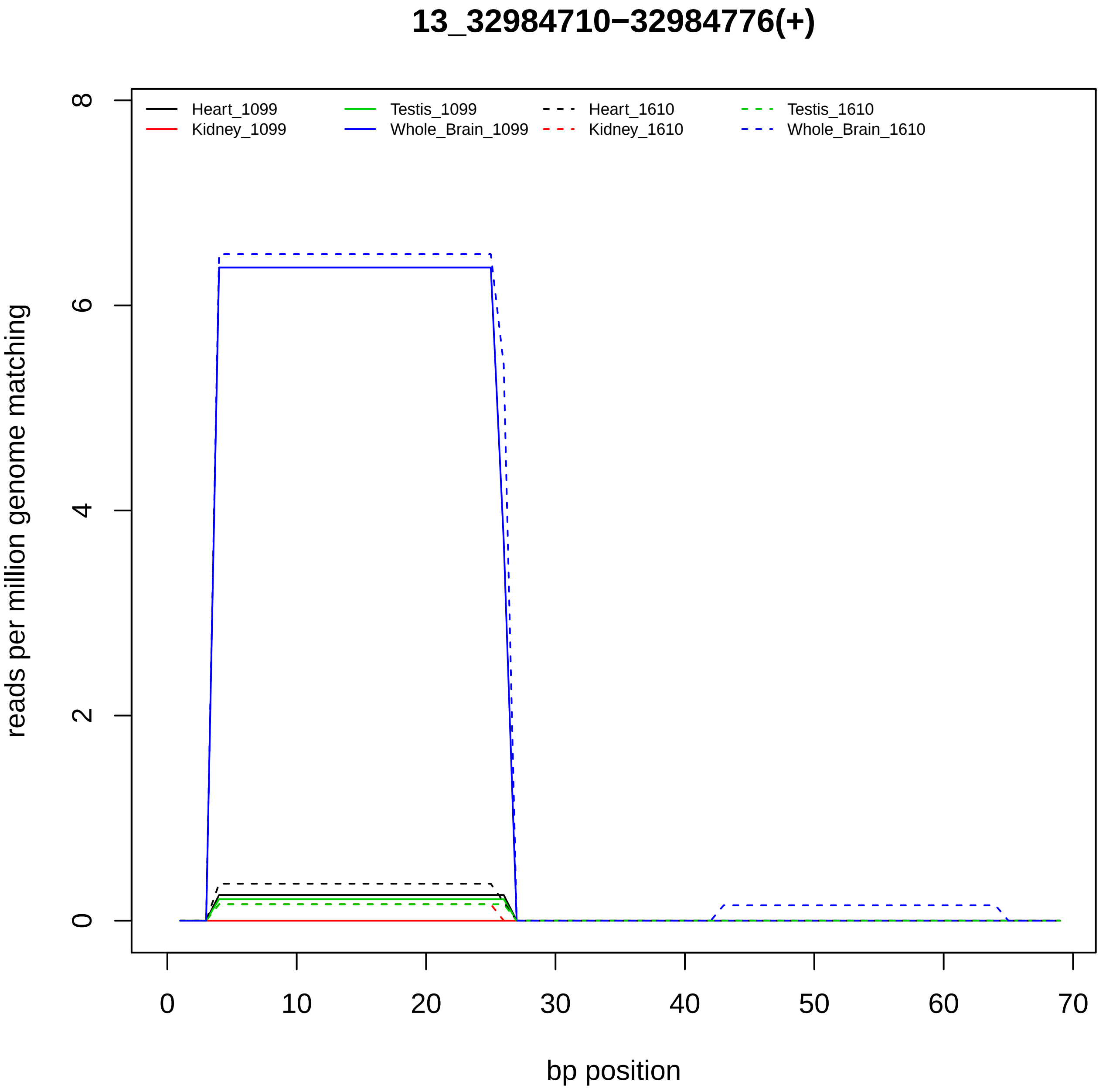

13_32984710−32984776(+)
Heart_1099
Kidney_1099
Testis_1099
Whole_Brain_1099
Heart_1610
Kidney_1610
Testis_1610
Whole_Brain_1610
reads per million genome matching
0
2
4
6
8
0
10
20
30
40
50
60
70
bp position

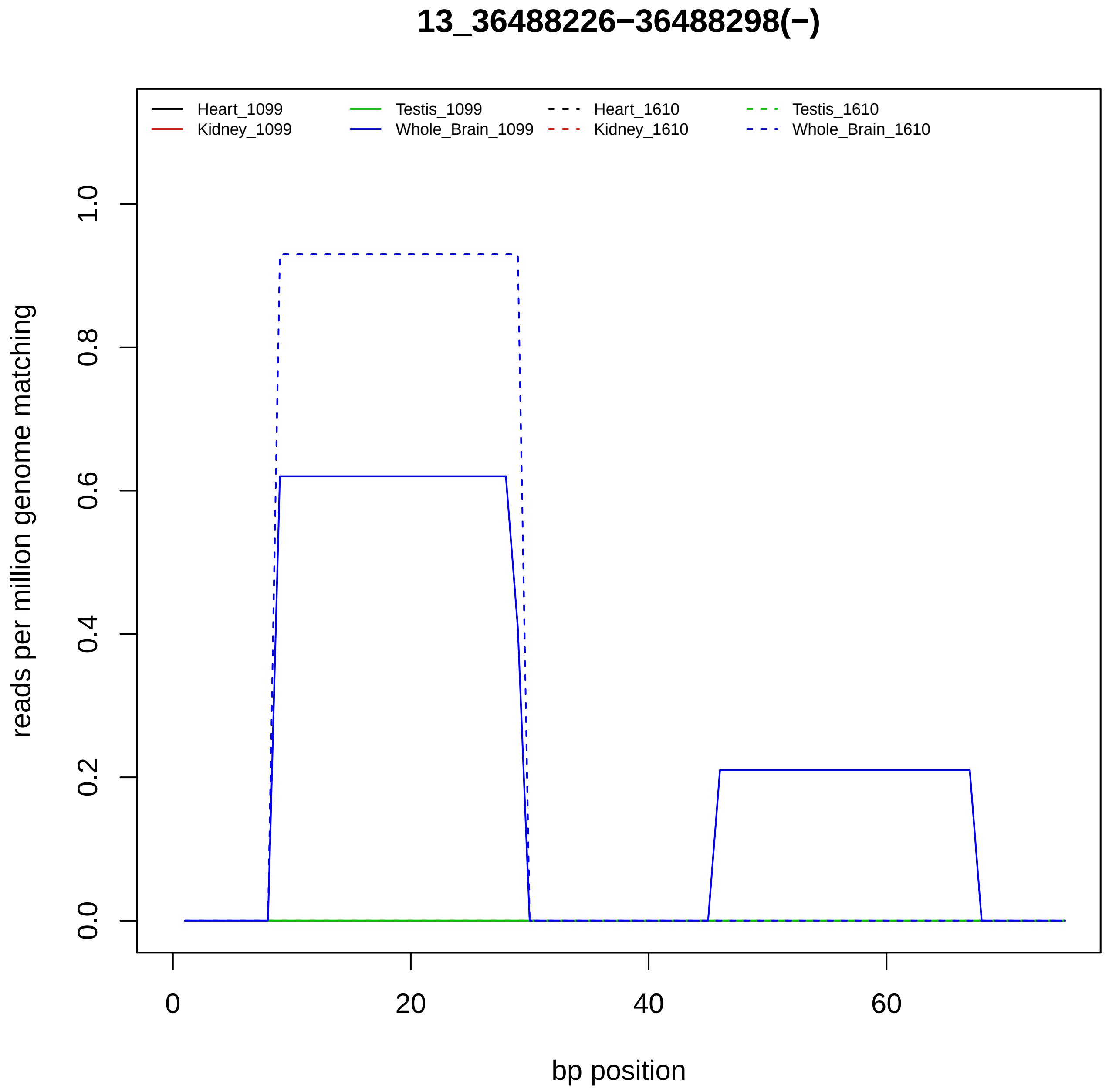

13_36488226−36488298(−)
Heart_1099
Kidney_1099
Testis_1099
Whole_Brain_1099
Heart_1610
Kidney_1610
Testis_1610
Whole_Brain_1610
reads per million genome matching
0.0
0.2
0.4
0.6
0.8
1.0
0
20
40
60
bp position

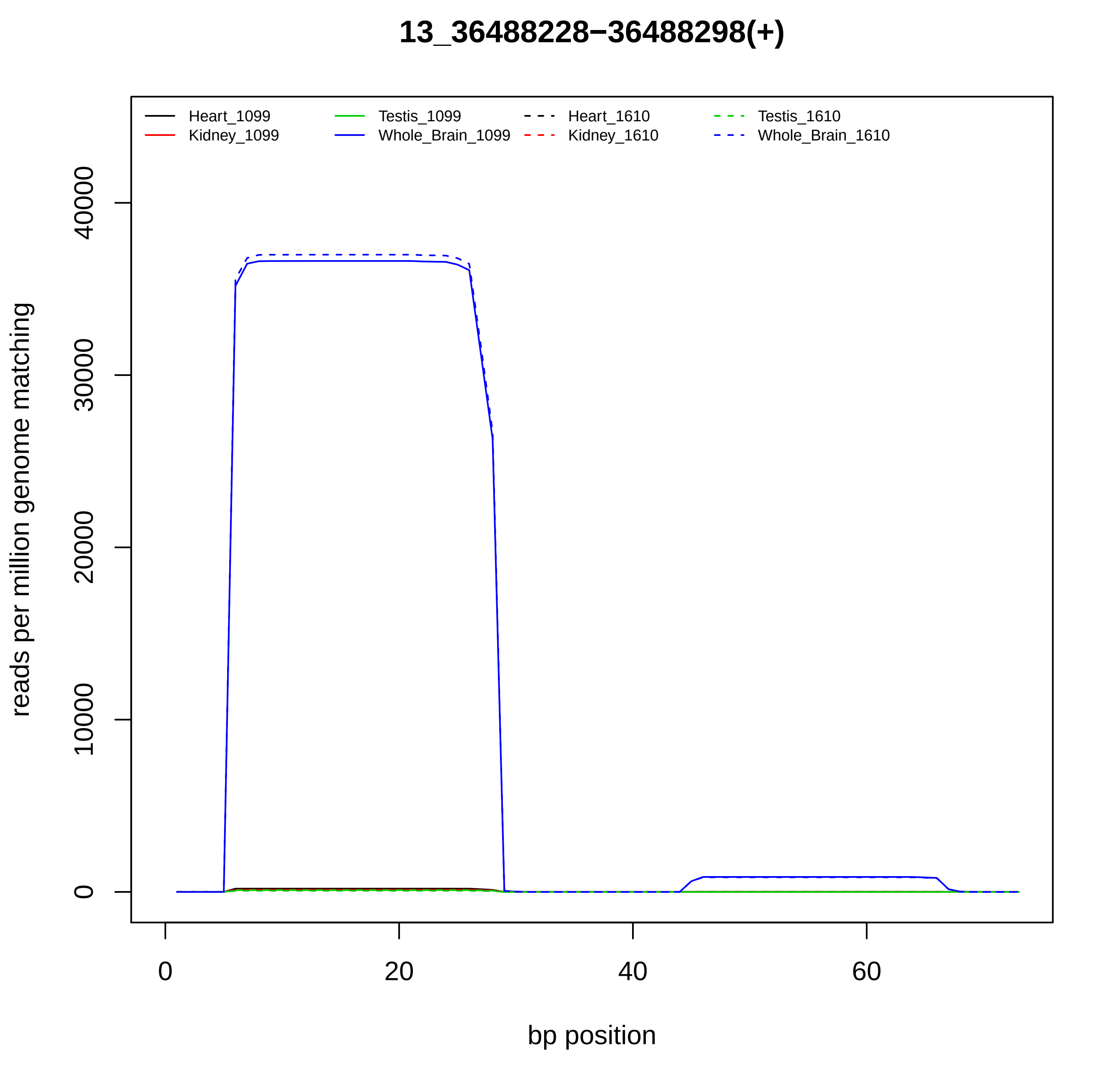

13_36488228−36488298(+)
Heart_1099
Kidney_1099
Testis_1099
Whole_Brain_1099
Heart_1610
Kidney_1610
Testis_1610
Whole_Brain_1610
reads per million genome matching
0
10000
20000
30000
40000
bp position
0
20
40
60

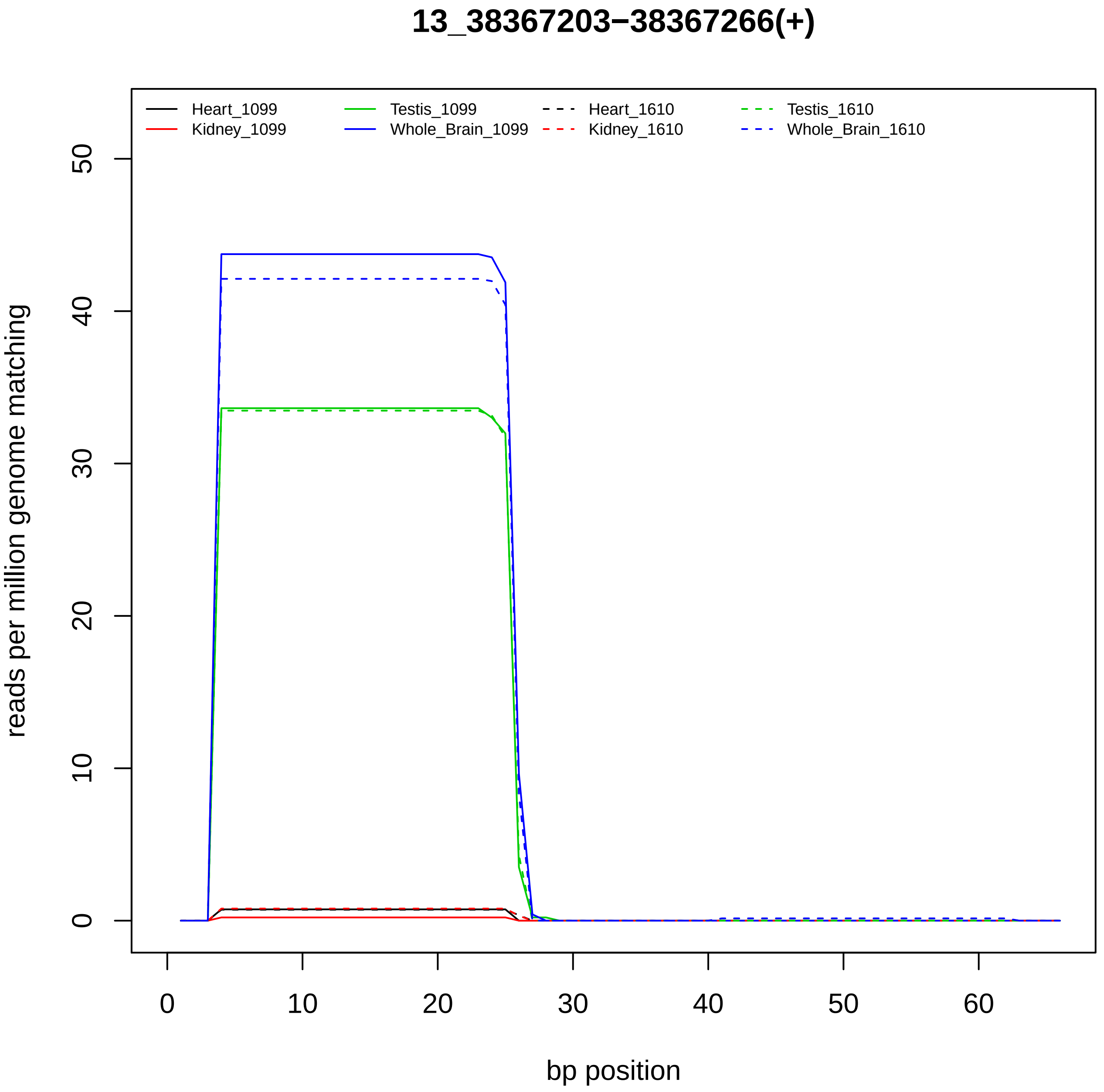

13_38367203−38367266(+)
Heart_1099
Kidney_1099
Testis_1099
Whole_Brain_1099
Heart_1610
Kidney_1610
Testis_1610
Whole_Brain_1610
reads per million genome matching
bp position
0
10
20
30
40
50
60

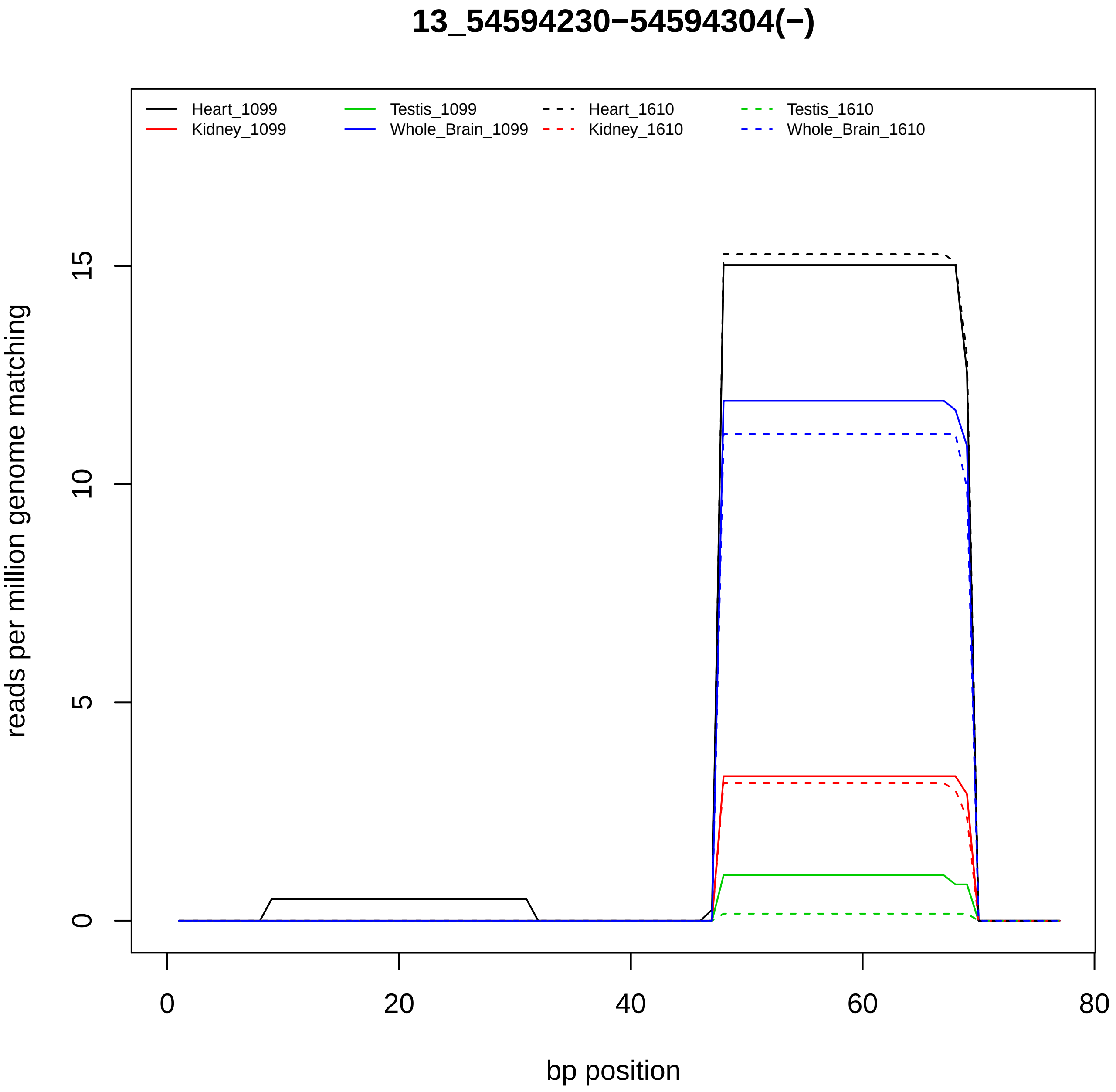
13_54594230−54594304(−)
Heart_1099
Kidney_1099
Testis_1099
Whole_Brain_1099
Heart_1610
Kidney_1610
Testis_1610
Whole_Brain_1610
reads per million genome matching
bp position
0
5
10
15
0
20
40
60
80

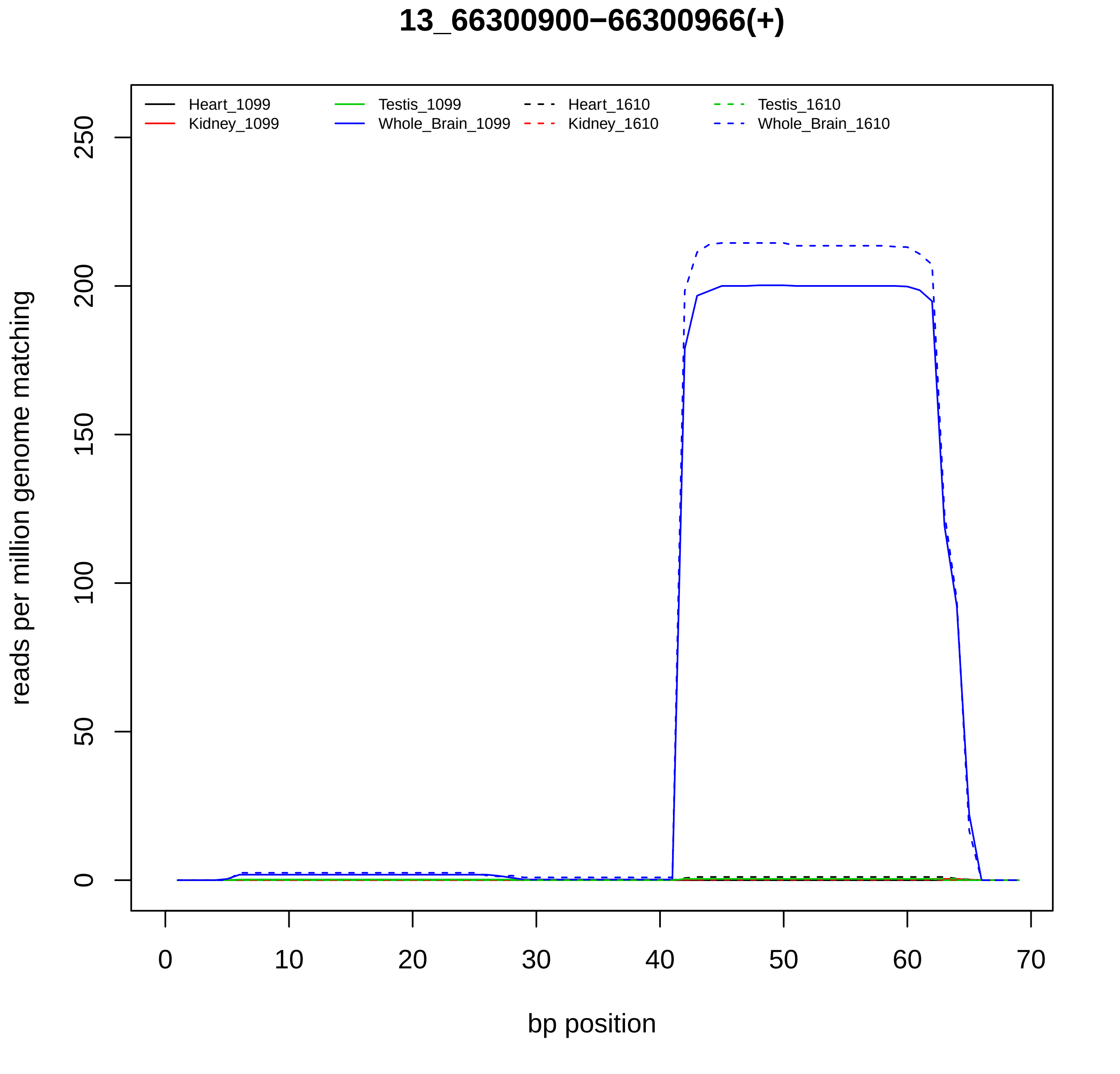

13_66300900−66300966(+)
Heart_1099
Kidney_1099
Testis_1099
Whole_Brain_1099
Heart_1610
Kidney_1610
Testis_1610
Whole_Brain_1610
reads per million genome matching
bp position
0
50
100
150
200
250
0
10
20
30
40
50
60
70

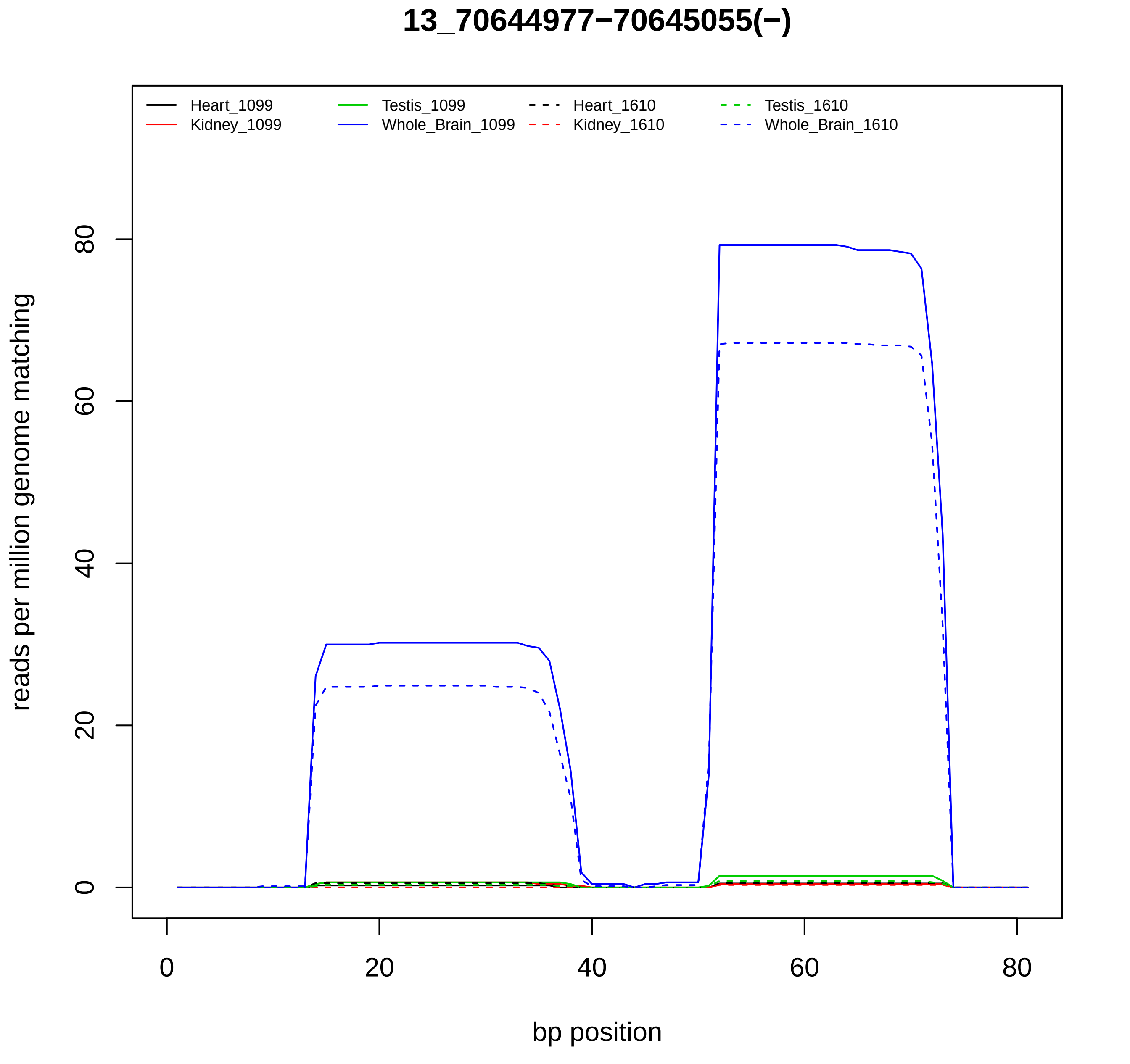

13_70644977−70645055(−)
Heart_1099
Kidney_1099
Testis_1099
Whole_Brain_1099
Heart_1610
Kidney_1610
Testis_1610
Whole_Brain_1610
reads per million genome matching
0
20
40
60
80
bp position
0
20
40
60
80

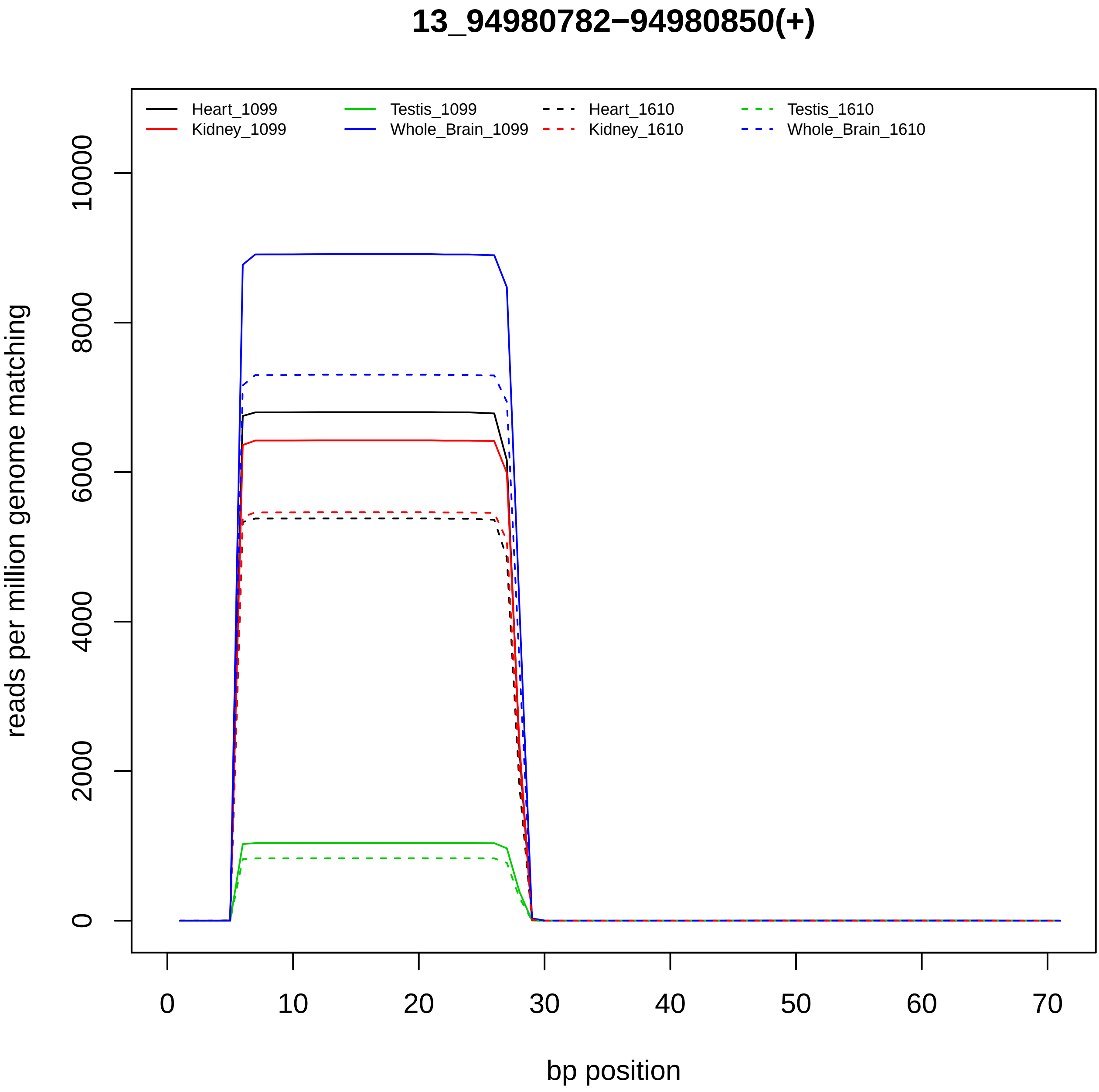

13_94980782−94980850(+)
Heart_1099
Kidney_1099
Testis_1099
Whole_Brain_1099
Heart_1610
Kidney_1610
Testis_1610
Whole_Brain_1610
reads per million genome matching
0
2000
4000
6000
8000
10000
bp position
0
10
20
30
40
50
60
70

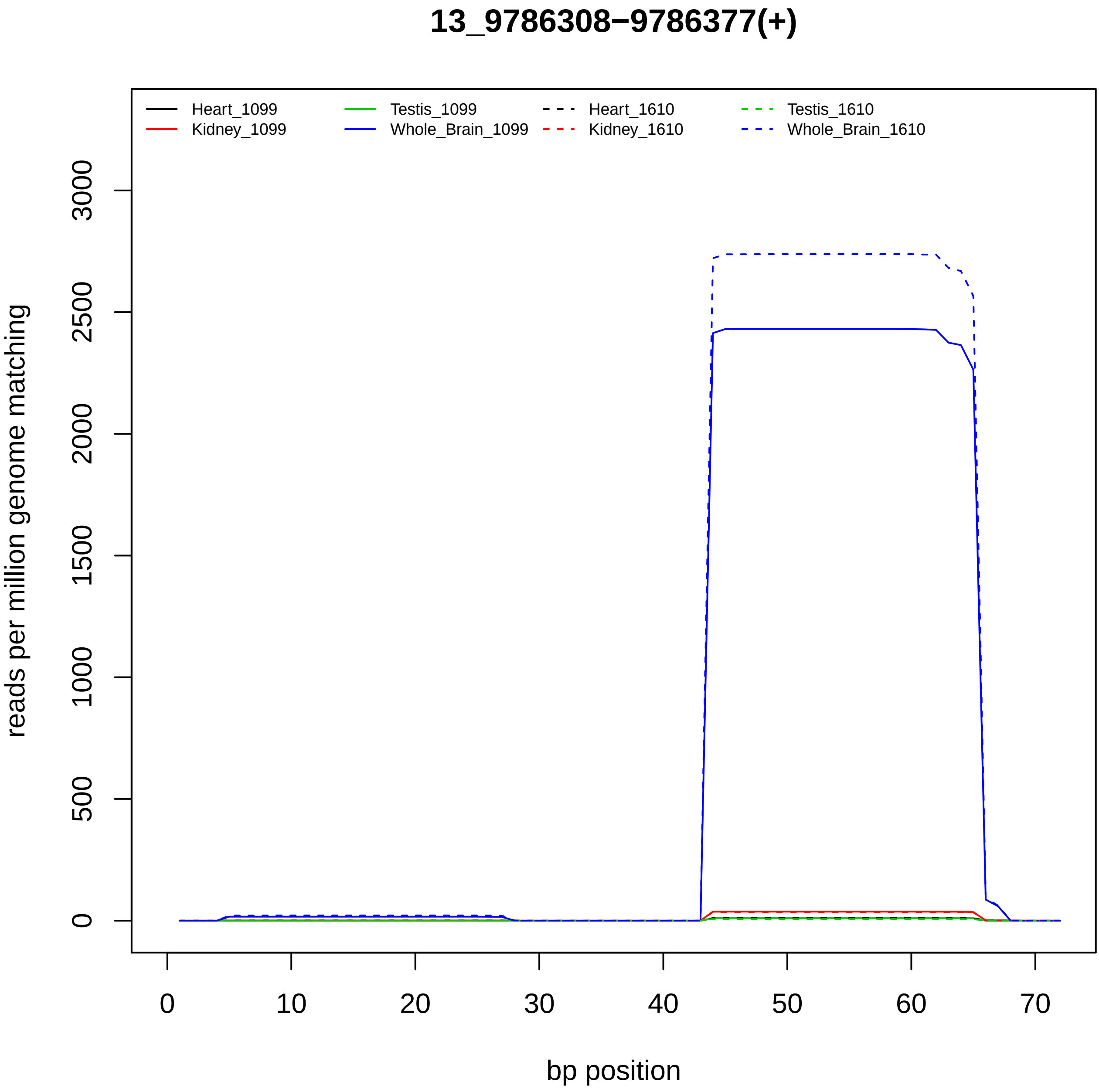
13_9786308−9786377(+)
Heart_1099
Kidney_1099
Testis_1099
Whole_Brain_1099
Heart_1610
Kidney_1610
Testis_1610
Whole_Brain_1610
reads per million genome matching
0
500
1000
1500
2000
2500
3000
0
10
20
30
40
50
60
70
bp position

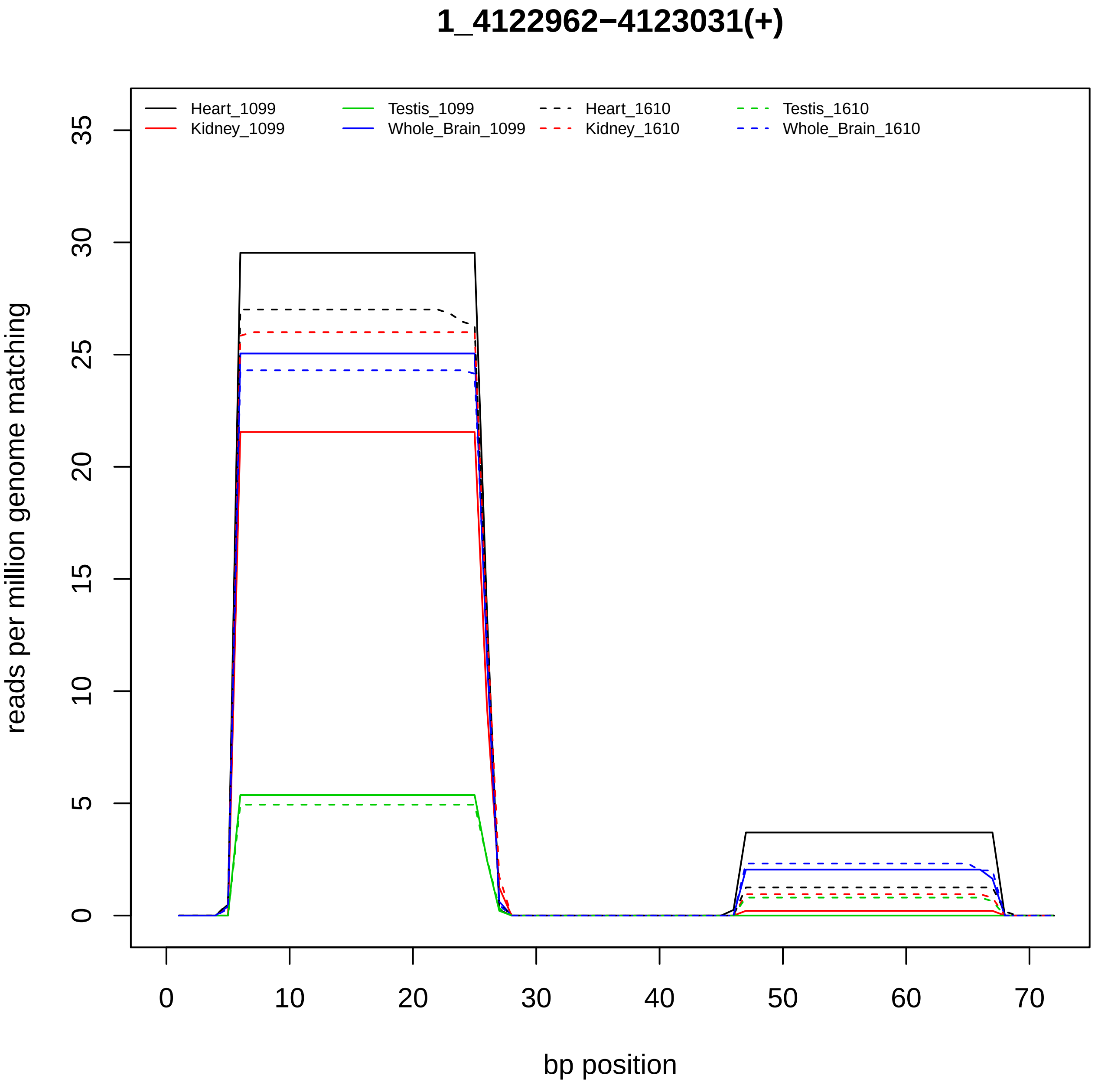

1_4122962−4123031(+)
Heart_1099
Kidney_1099
Testis_1099
Whole_Brain_1099
Heart_1610
Kidney_1610
Testis_1610
Whole_Brain_1610
reads per million genome matching
bp position
0
5
10
15
20
25
30
35
0
10
20
30
40
50
60
70

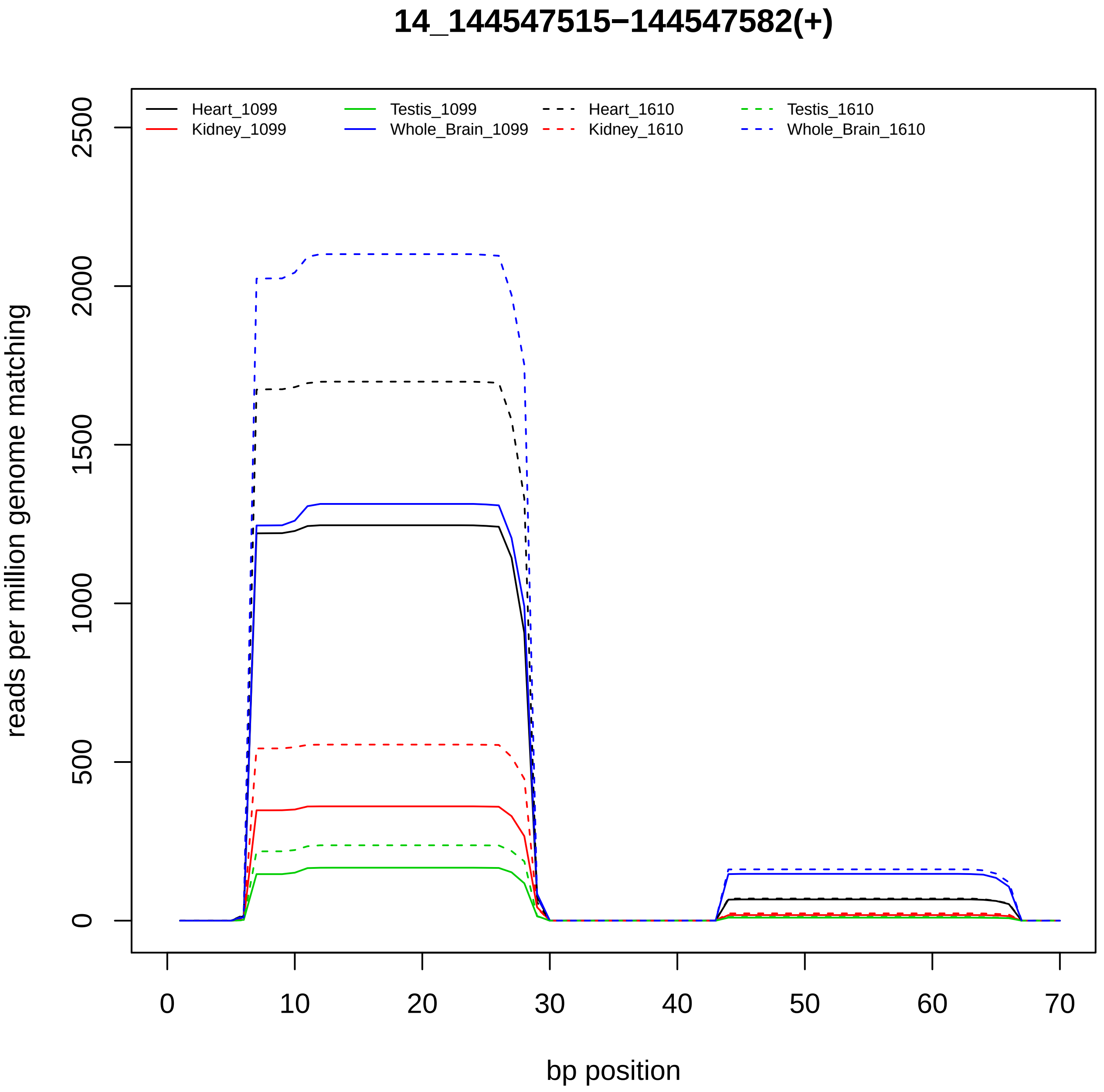
14_144547515−144547582(+)
Heart_1099
Kidney_1099
Testis_1099
Whole_Brain_1099
Heart_1610
Kidney_1610
Testis_1610
Whole_Brain_1610
reads per million genome matching
0
500
1000
1500
2000
2500
bp position
0
10
20
30
40
50
60
70

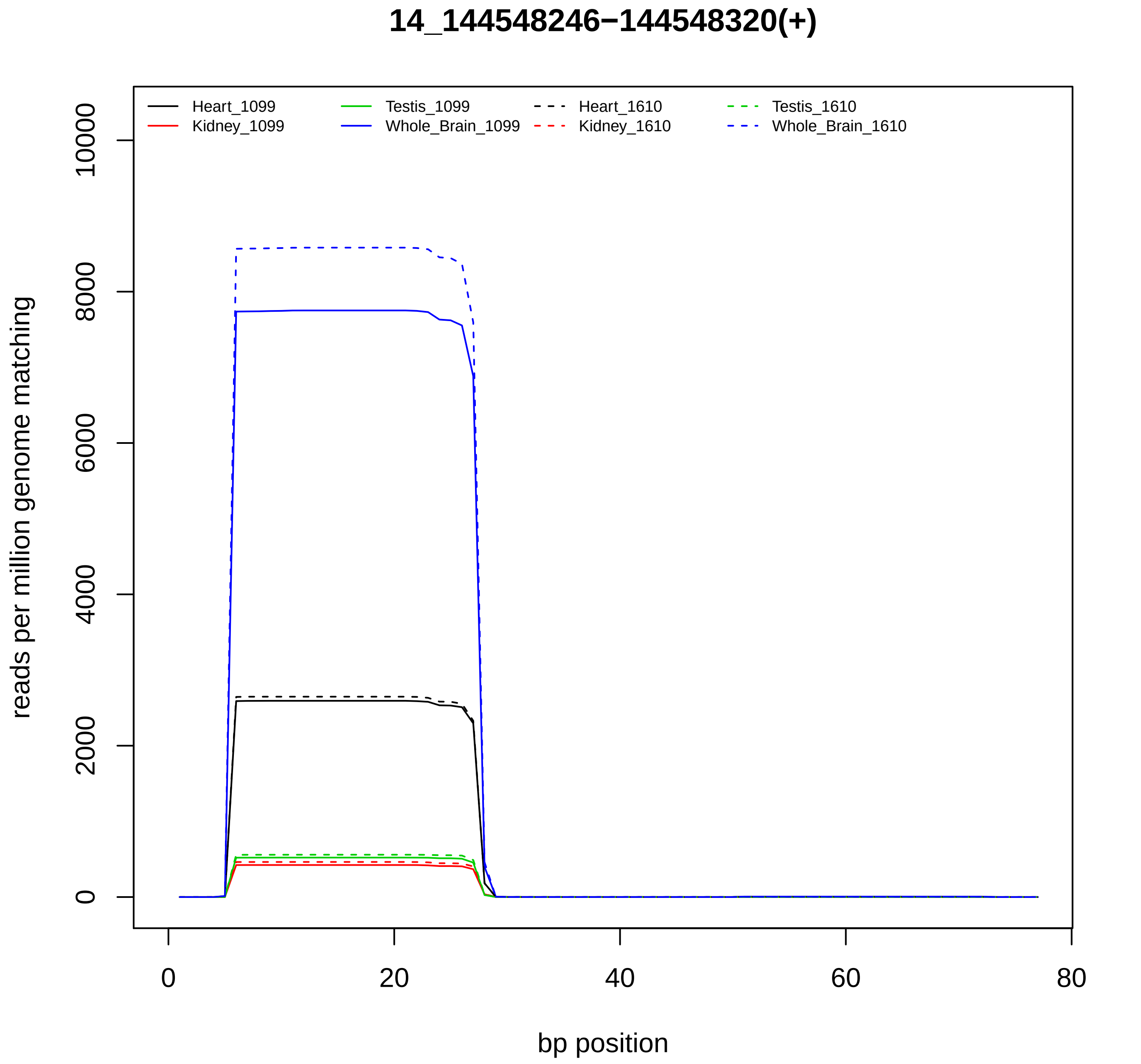

14_144548246−144548320(+)
Heart_1099
Kidney_1099
Testis_1099
Whole_Brain_1099
Heart_1610
Kidney_1610
Testis_1610
Whole_Brain_1610
reads per million genome matching
0
2000
4000
6000
8000
10000
0
20
40
60
80
bp position

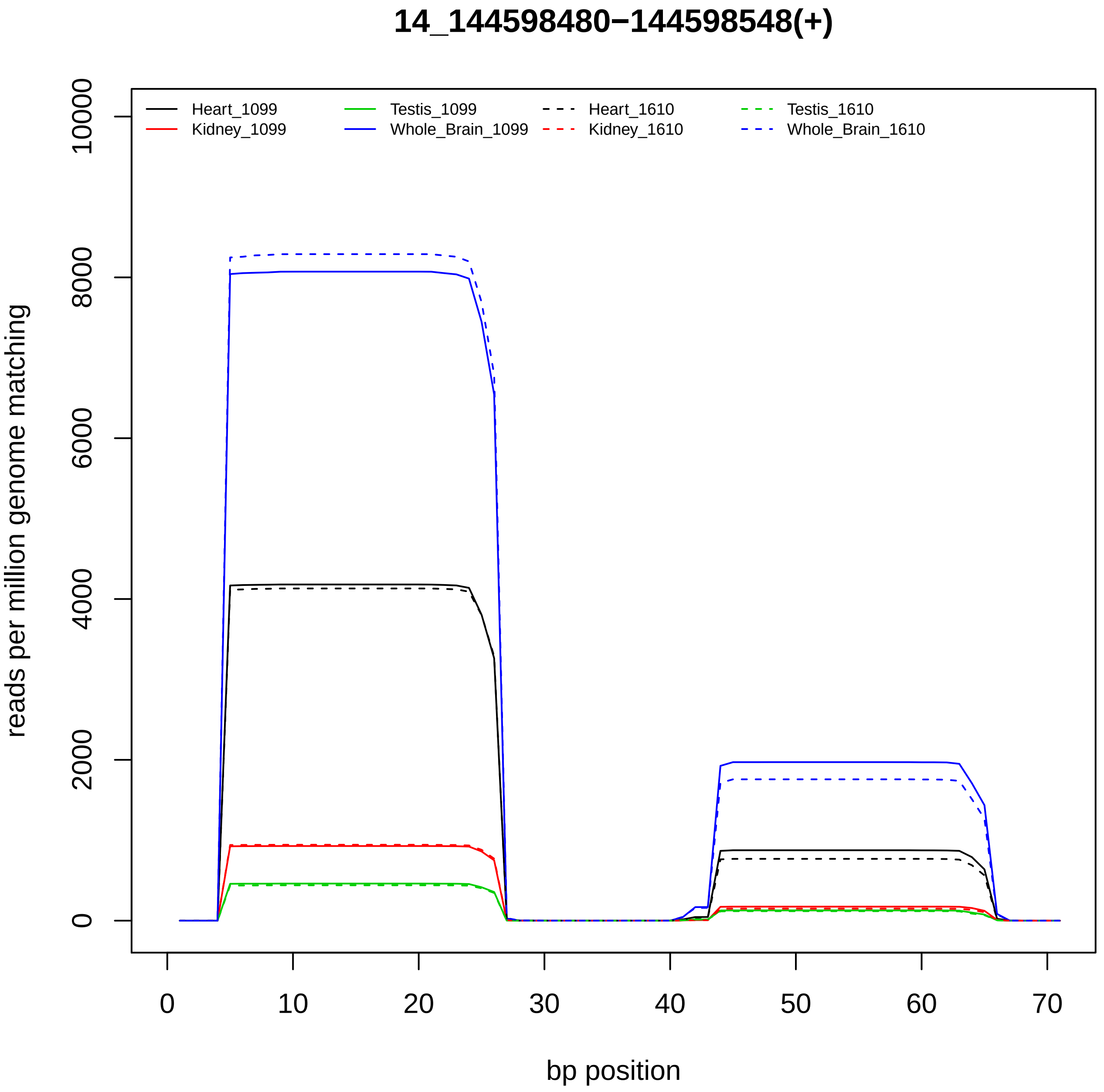
14_144598480−144598548(+)
Heart_1099
Kidney_1099
Testis_1099
Whole_Brain_1099
Heart_1610
Kidney_1610
Testis_1610
Whole_Brain_1610
reads per million genome matching
0
2000
4000
6000
8000
10000
0
10
20
30
40
50
60
70
bp position

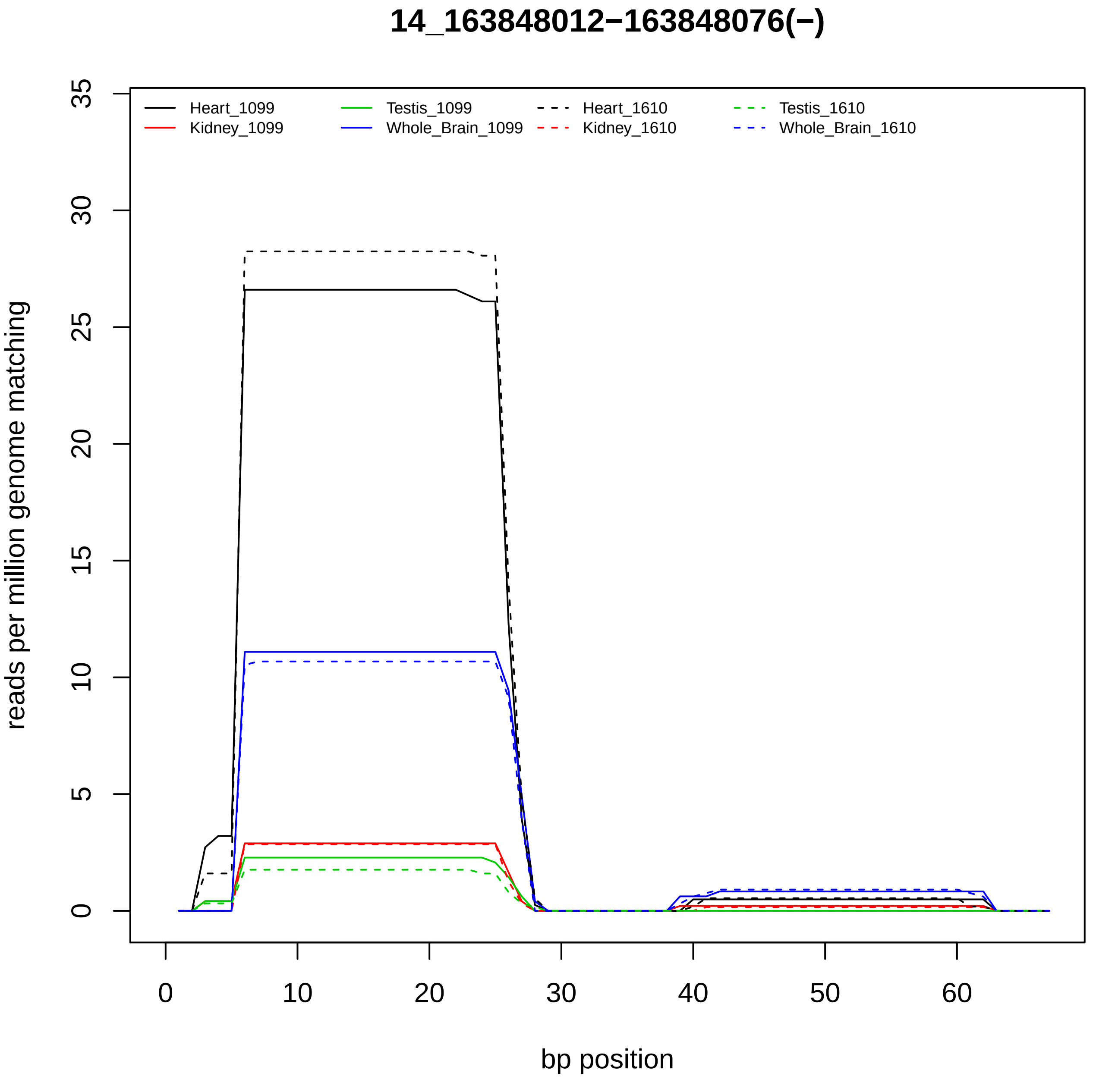
14_163848012−163848076(−)
Heart_1099
Kidney_1099
Testis_1099
Whole_Brain_1099
Heart_1610
Kidney_1610
Testis_1610
Whole_Brain_1610
reads per million genome matching
bp position
0
5
10
15
20
25
30
35
0
10
20
30
40
50
60

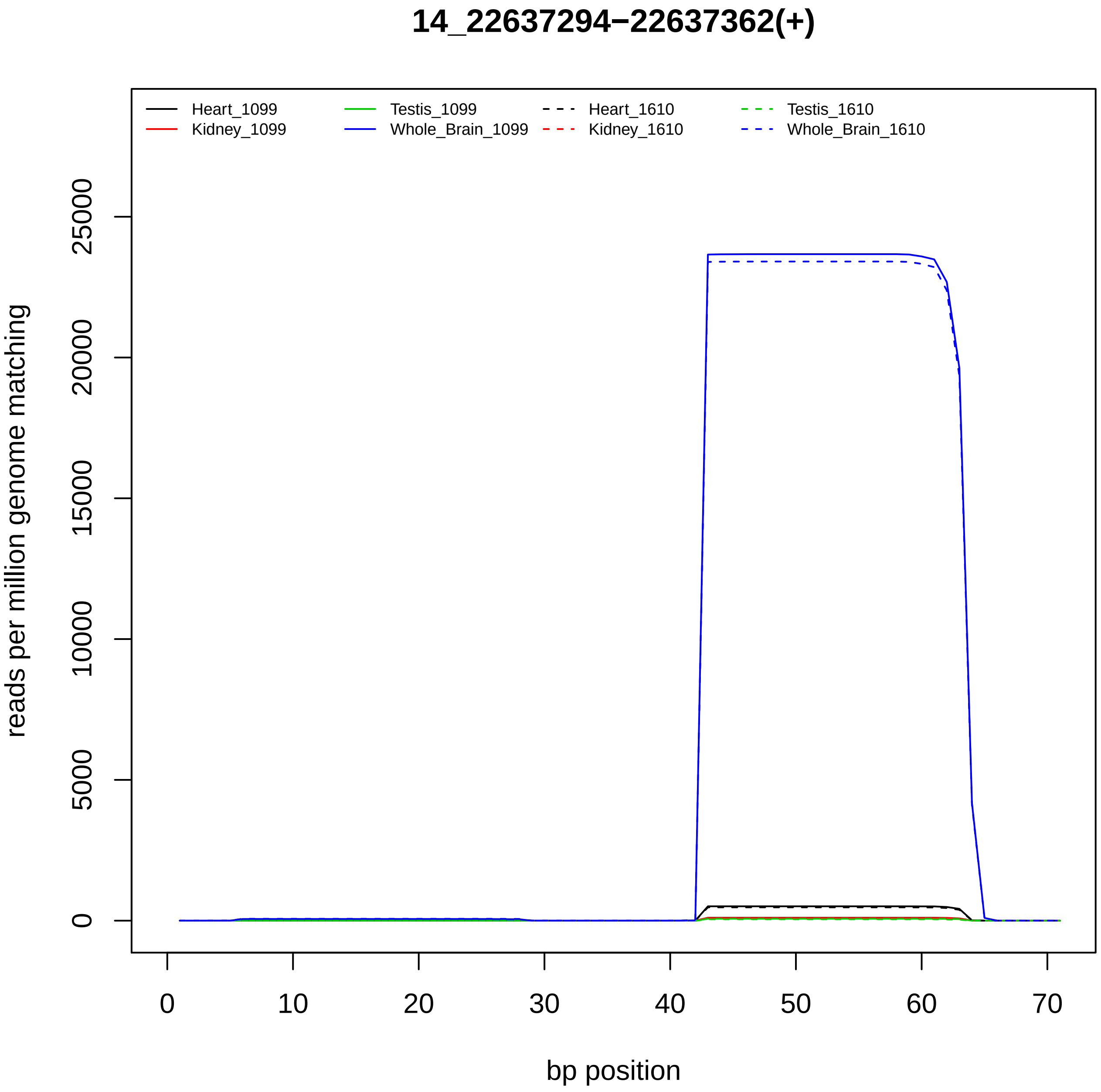

14_22637294−22637362(+)
Heart_1099
Kidney_1099
Testis_1099
Whole_Brain_1099
Heart_1610
Kidney_1610
Testis_1610
Whole_Brain_1610
reads per million genome matching
0
5000
10000
15000
20000
25000
0
10
20
30
40
50
60
70
bp position

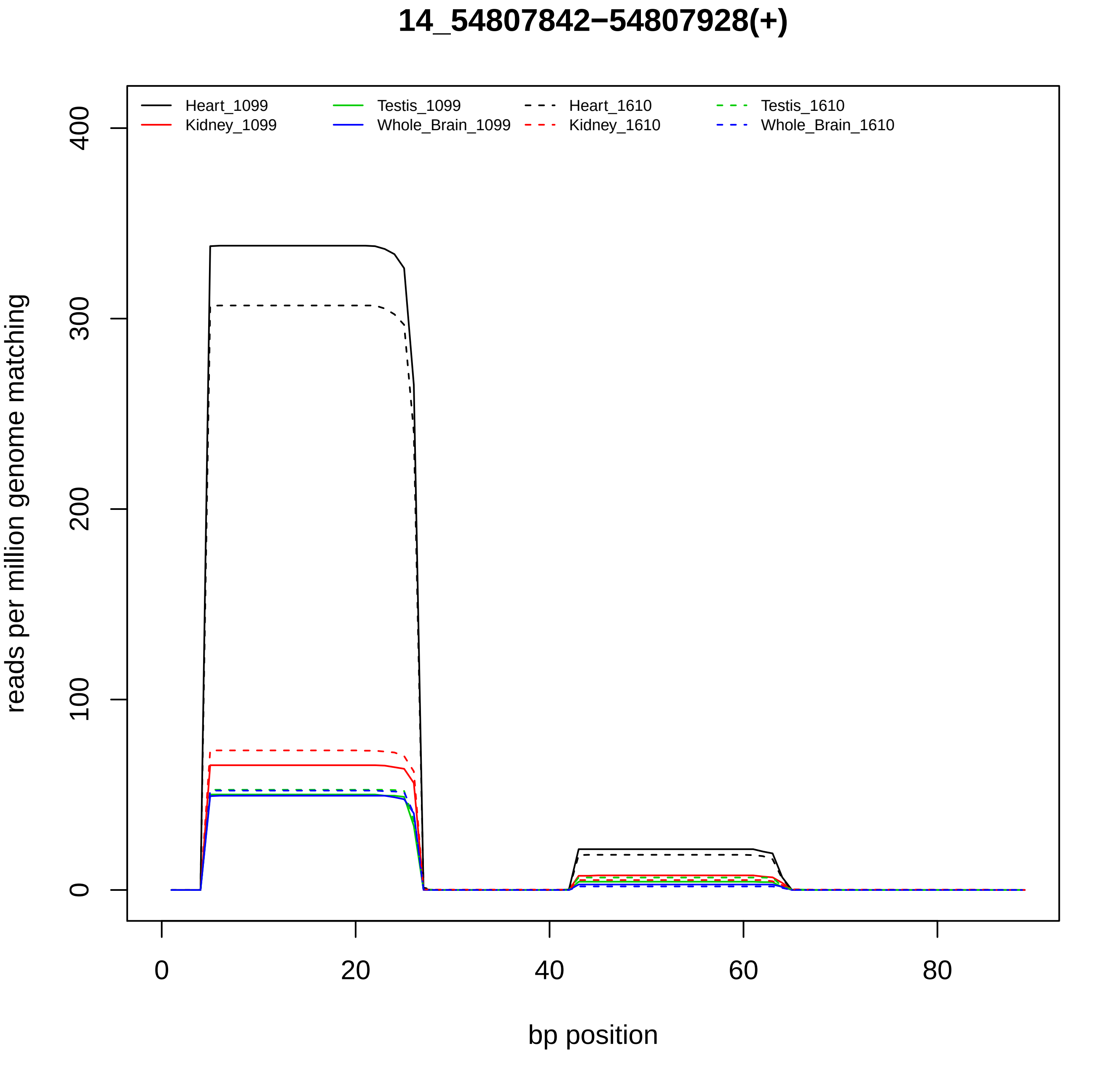

14_54807842−54807928(+)
Heart_1099
Kidney_1099
Testis_1099
Whole_Brain_1099
Heart_1610
Kidney_1610
Testis_1610
Whole_Brain_1610
reads per million genome matching
0
100
200
300
400
bp position
0
20
40
60
80

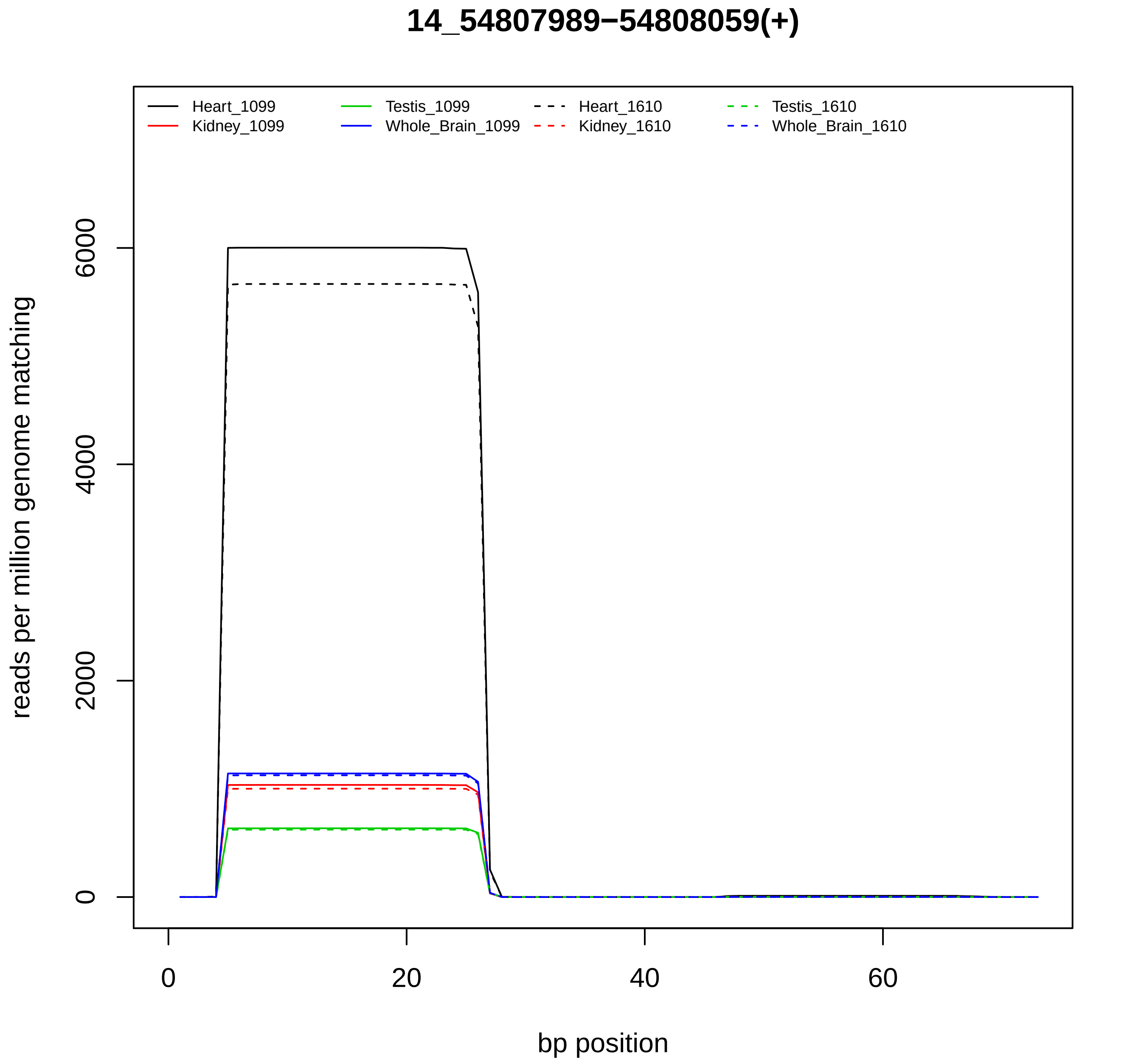
14_54807989−54808059(+)
Heart_1099
Kidney_1099
Testis_1099
Whole_Brain_1099
Heart_1610
Kidney_1610
Testis_1610
Whole_Brain_1610
reads per million genome matching
0
2000
4000
6000
0
20
40
60
bp position

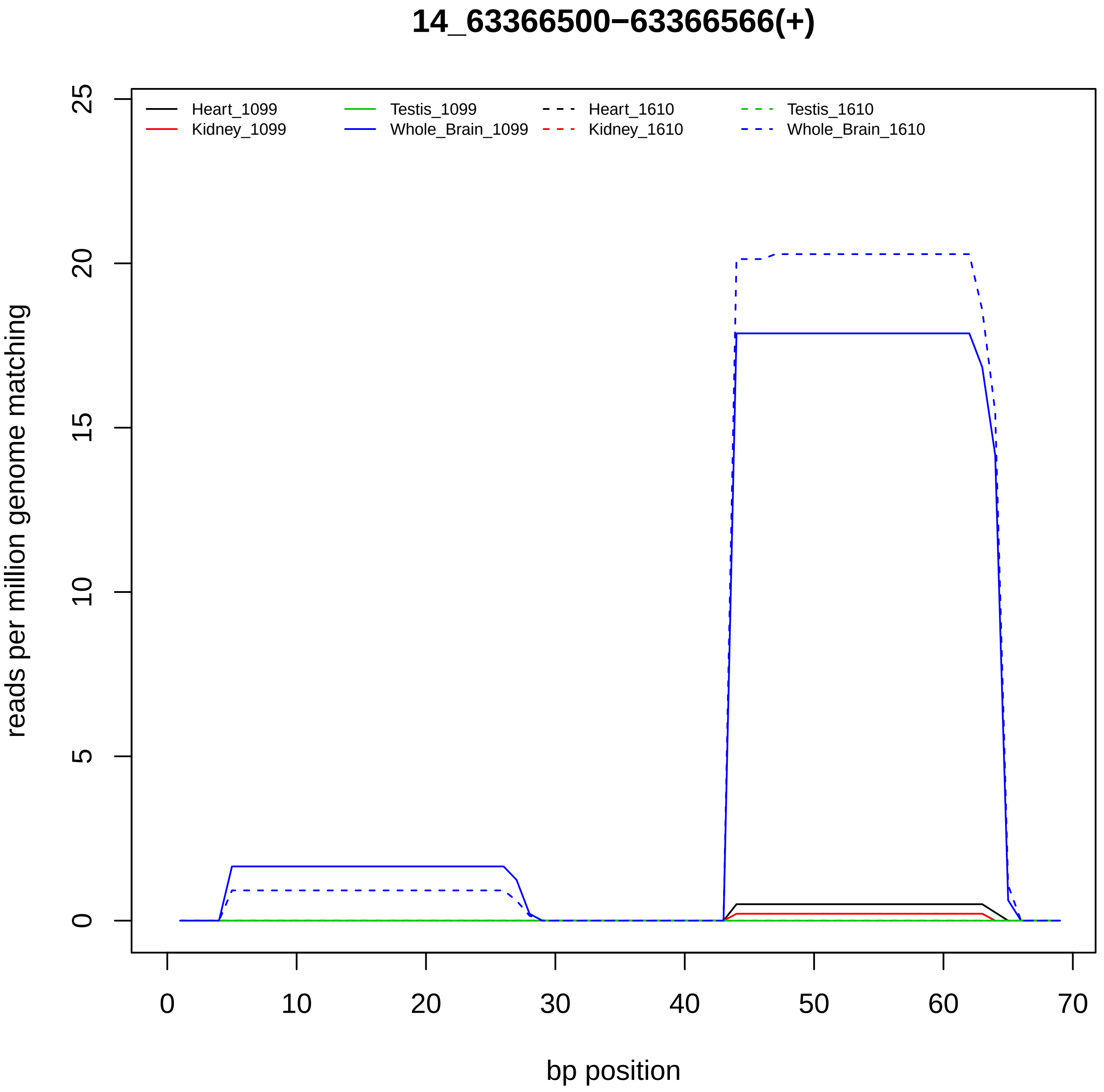

14_63366500−63366566(+)
Heart_1099
Kidney_1099
Testis_1099
Whole_Brain_1099
Heart_1610
Kidney_1610
Testis_1610
Whole_Brain_1610
reads per million genome matching
bp position
0
5
10
15
20
25
0
10
20
30
40
50
60
70

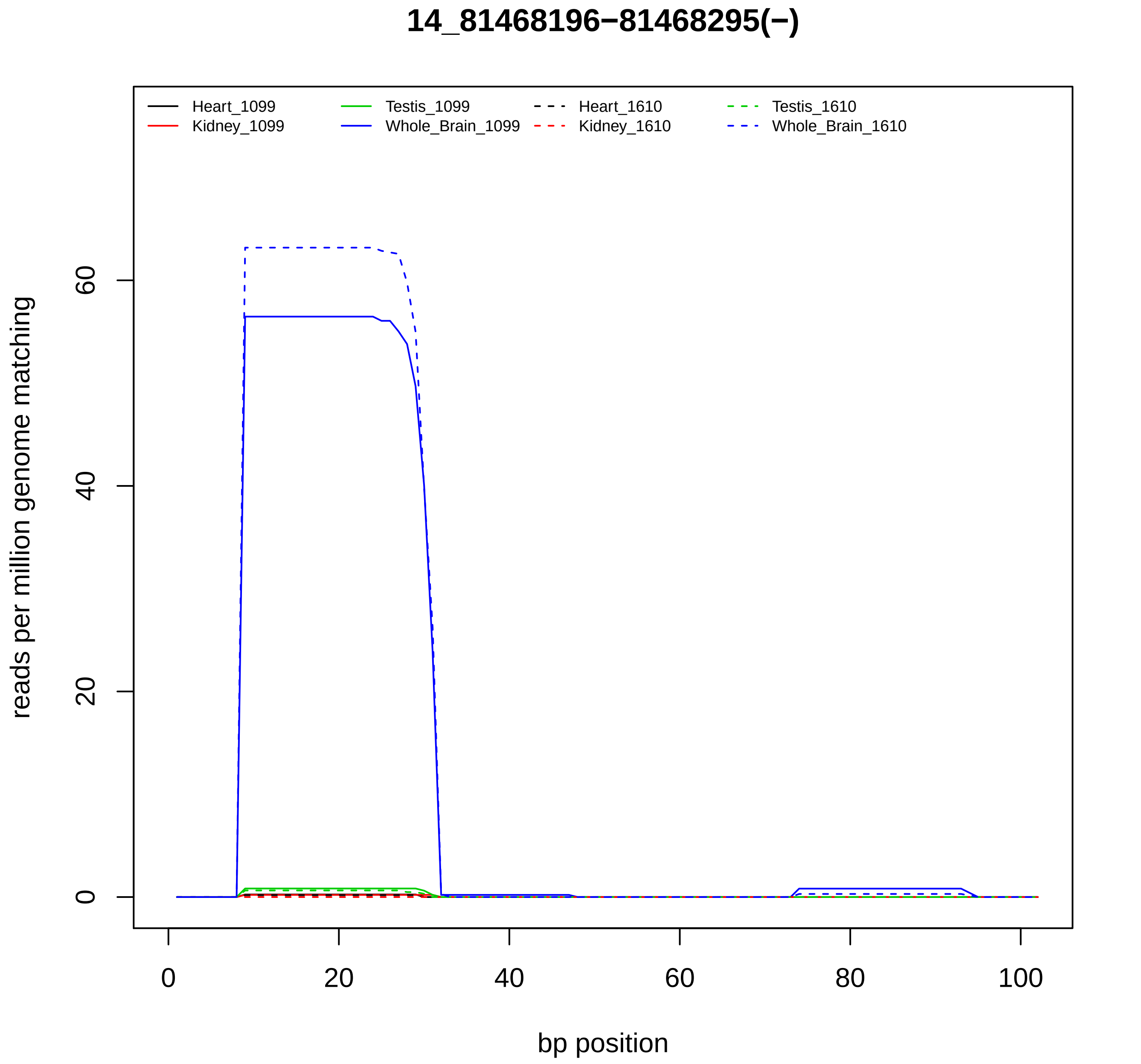

14_81468196−81468295(−)
Heart_1099
Kidney_1099
Testis_1099
Whole_Brain_1099
Heart_1610
Kidney_1610
Testis_1610
Whole_Brain_1610
reads per million genome matching
0
20
40
60
bp position
0
20
40
60
80
100

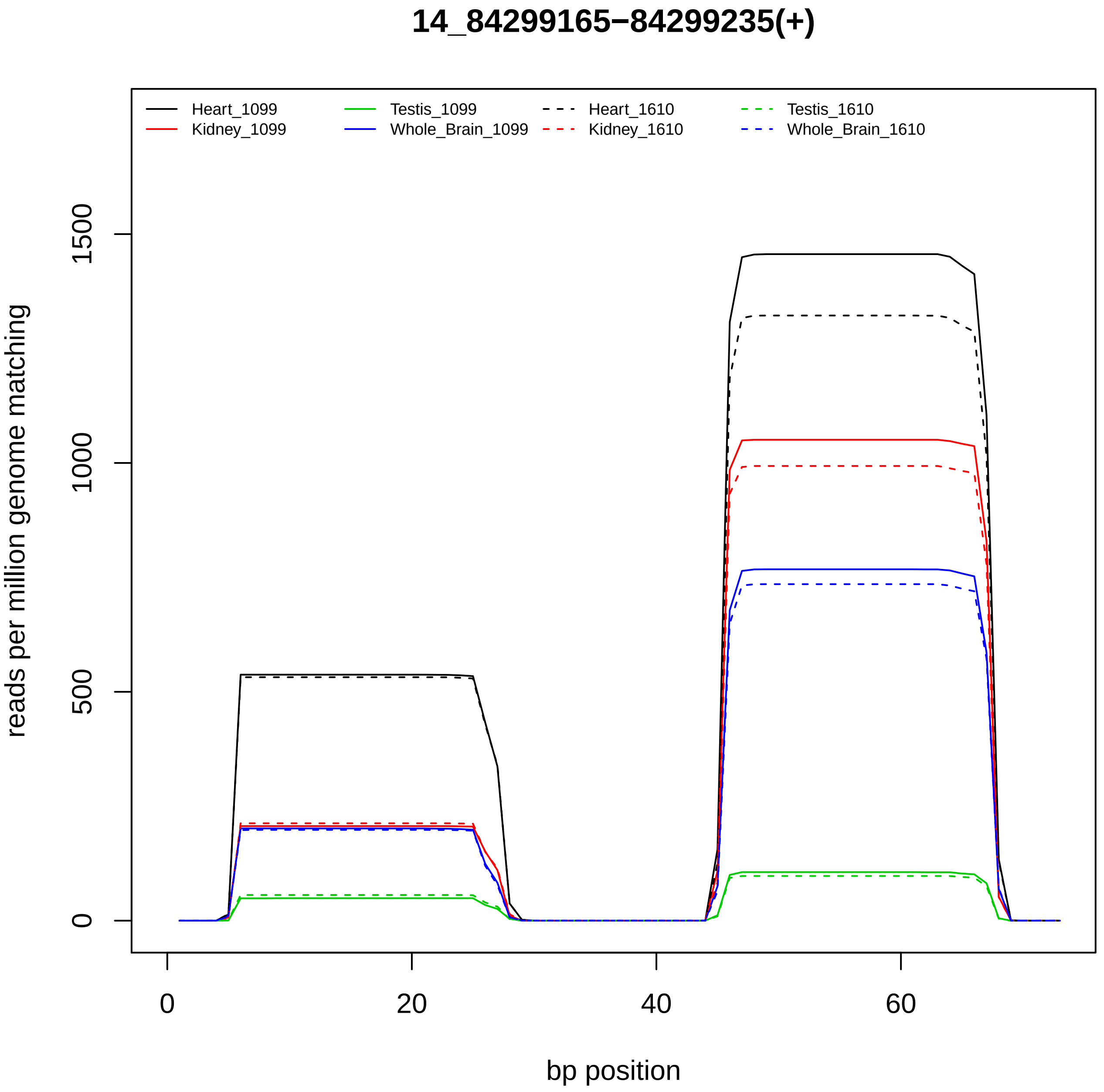

14_84299165−84299235(+)
Heart_1099
Kidney_1099
Testis_1099
Whole_Brain_1099
Heart_1610
Kidney_1610
Testis_1610
Whole_Brain_1610
reads per million genome matching
0
500
1000
1500
0
20
40
60
bp position

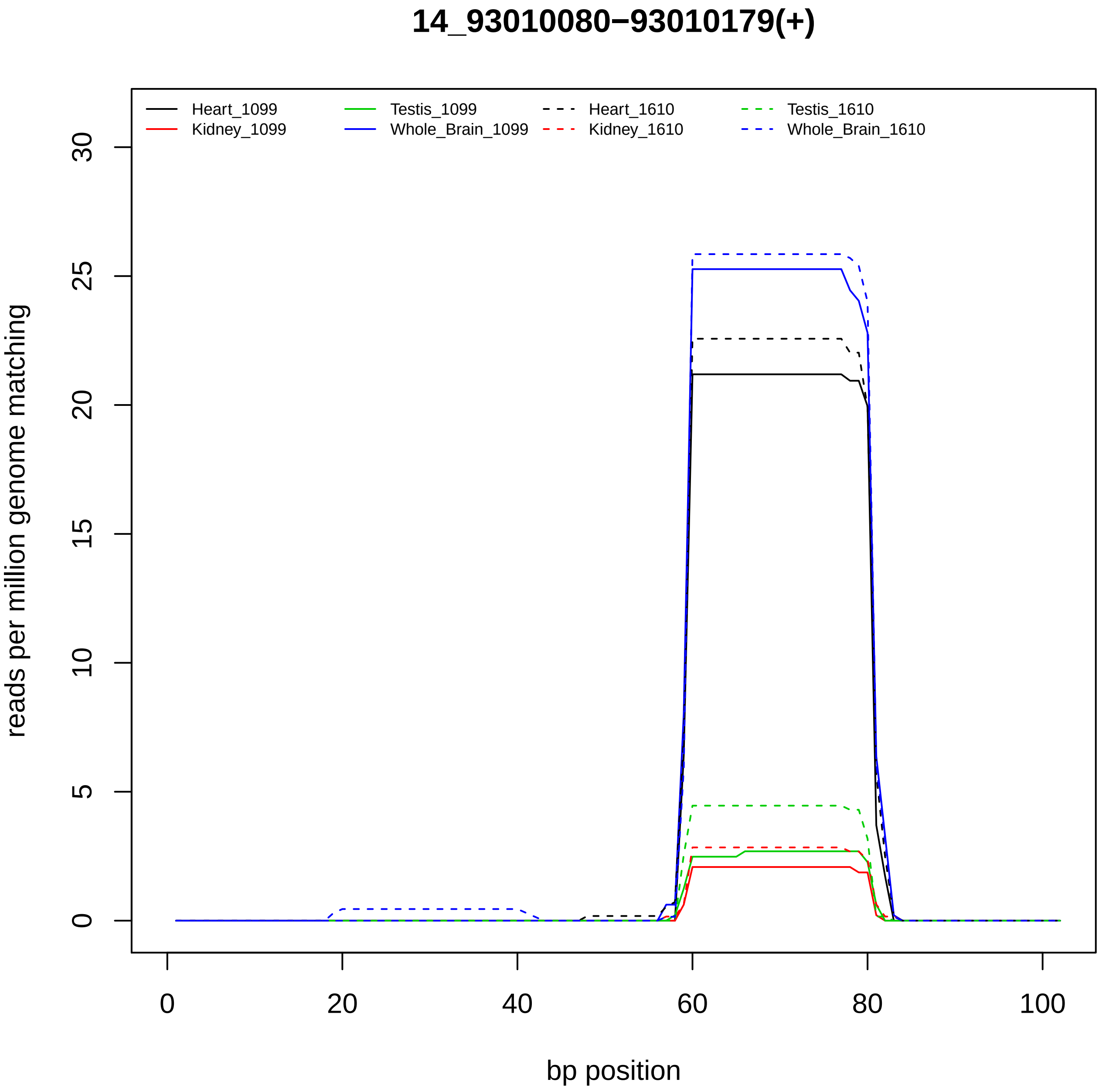
14_93010080−93010179(+)
Heart_1099
Kidney_1099
Testis_1099
Whole_Brain_1099
Heart_1610
Kidney_1610
Testis_1610
Whole_Brain_1610
reads per million genome matching
bp position
0
5
10
15
20
25
30
0
20
40
60
80
100

1_49384664−49384729(−)

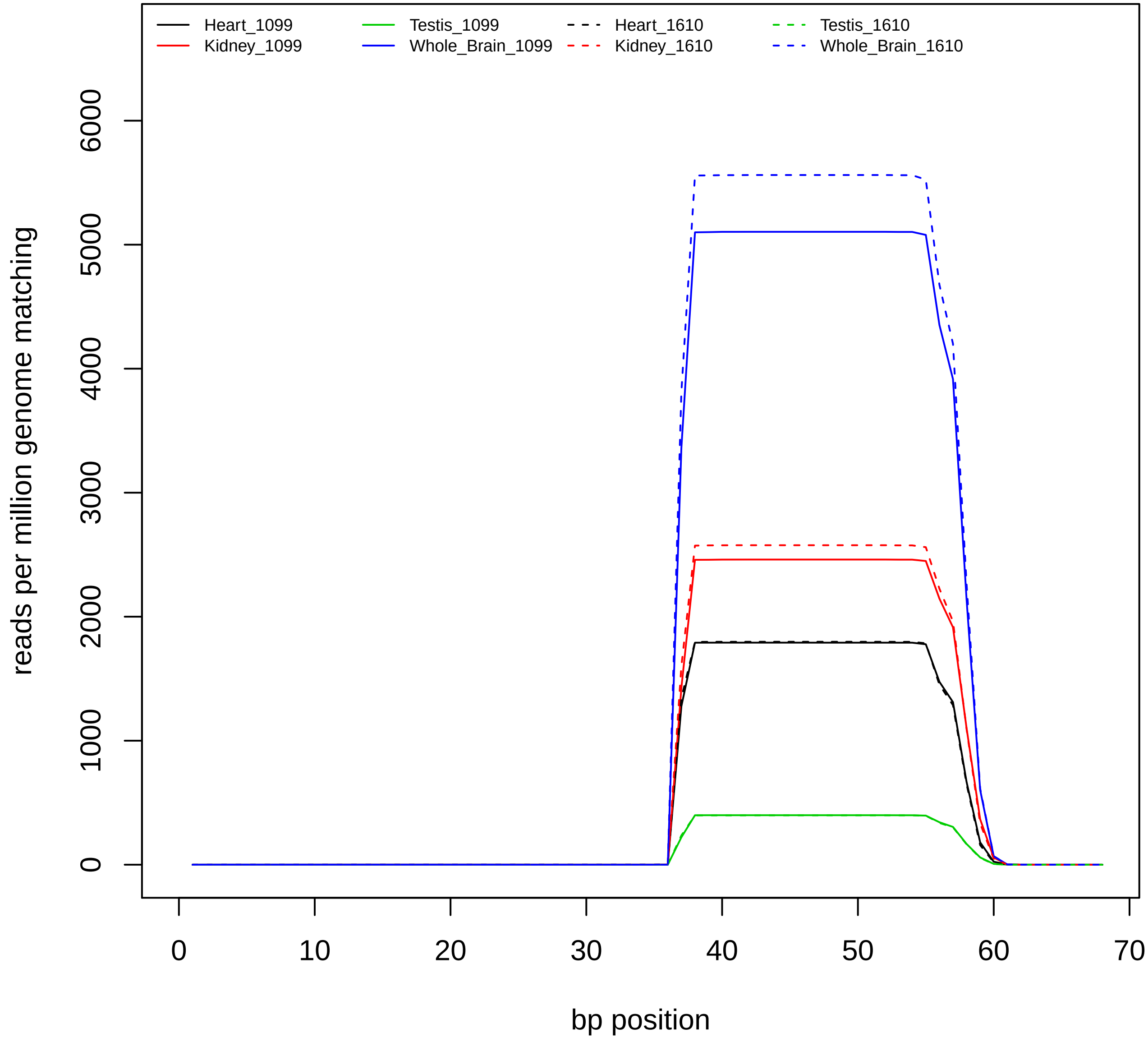

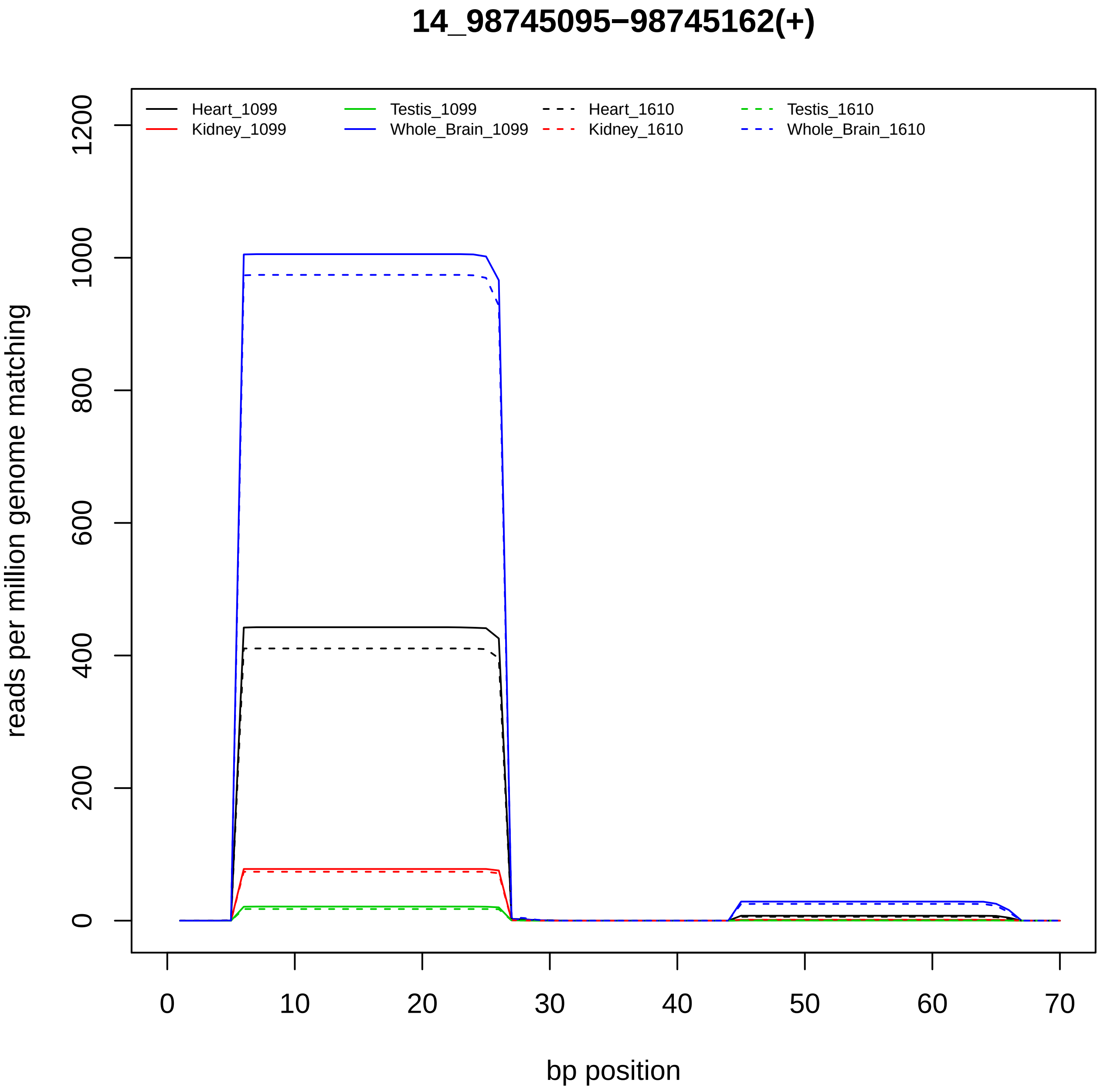
14_98745095−98745162(+)
Heart_1099
Kidney_1099
Testis_1099
Whole_Brain_1099
Heart_1610
Kidney_1610
Testis_1610
Whole_Brain_1610
reads per million genome matching
0
200
400
600
800
1000
1200
bp position
0
10
20
30
40
50
60
70

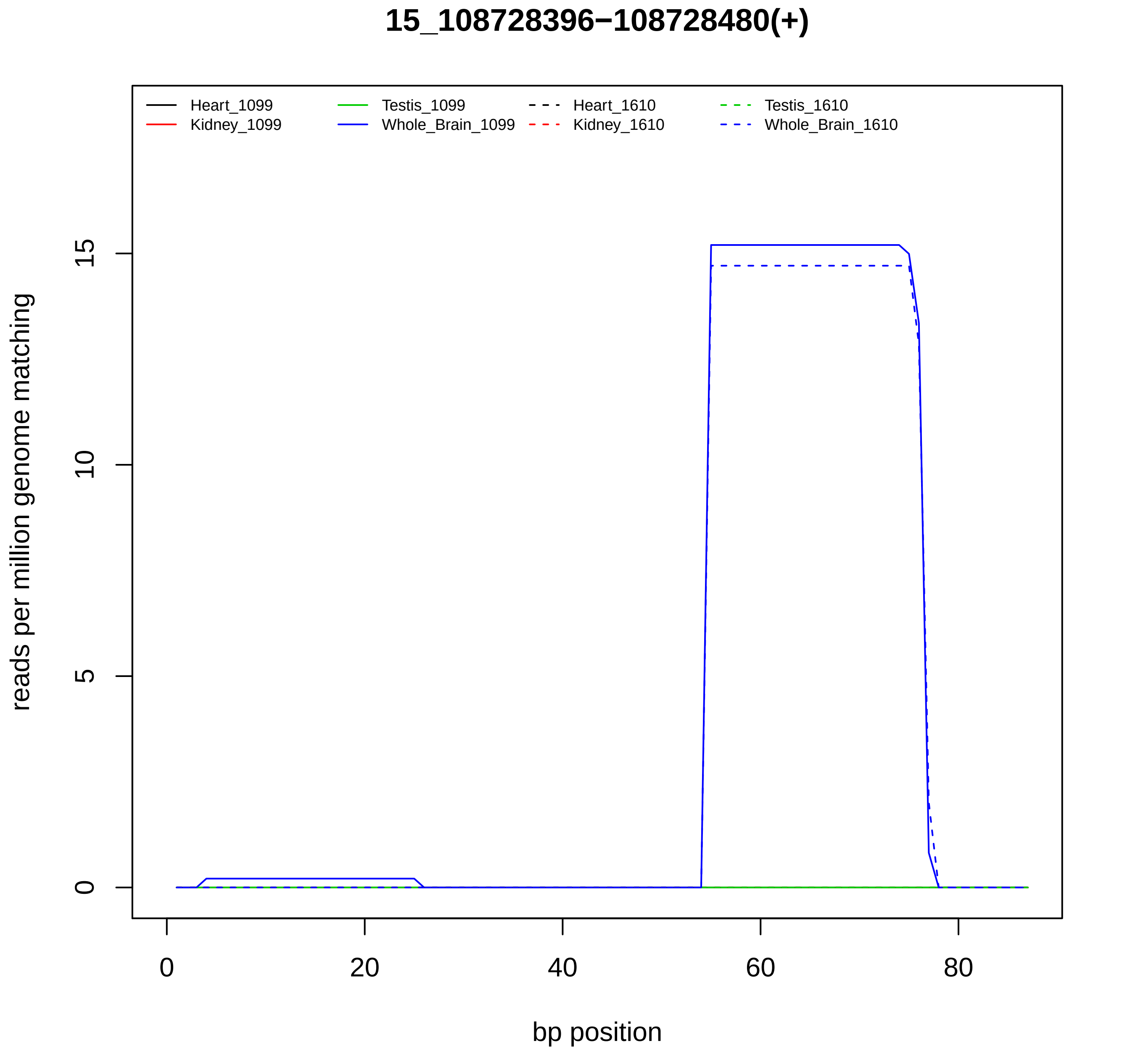

15_108728396−108728480(+)
Heart_1099
Kidney_1099
Testis_1099
Whole_Brain_1099
Heart_1610
Kidney_1610
Testis_1610
Whole_Brain_1610
reads per million genome matching
0
5
10
15
0
20
40
60
80
bp position

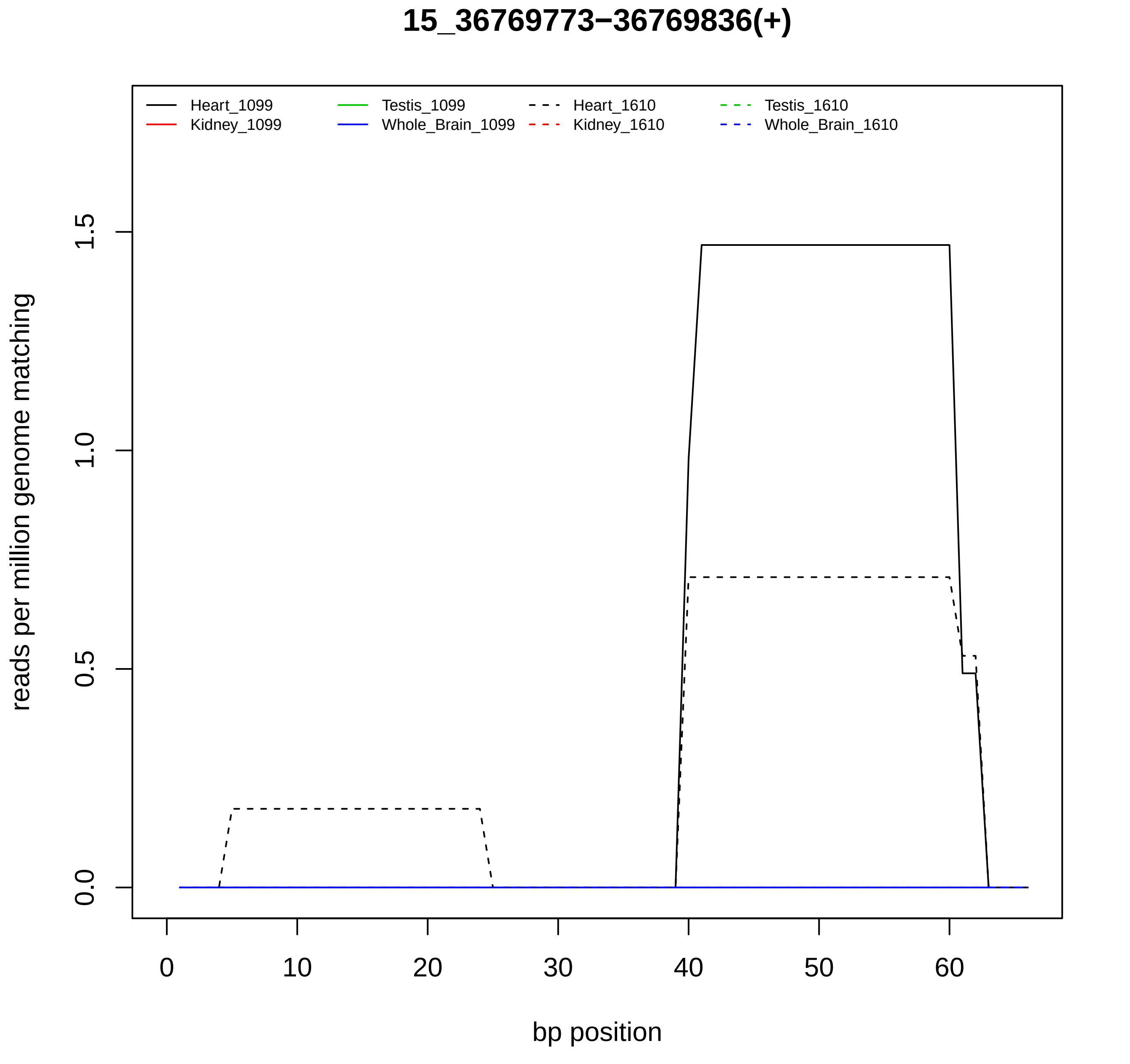
15_36769773−36769836(+)
Heart_1099
Kidney_1099
Testis_1099
Whole_Brain_1099
Heart_1610
Kidney_1610
Testis_1610
Whole_Brain_1610
reads per million genome matching
bp position
0.0
0.5
1.0
1.5
0
10
20
30
40
50
60

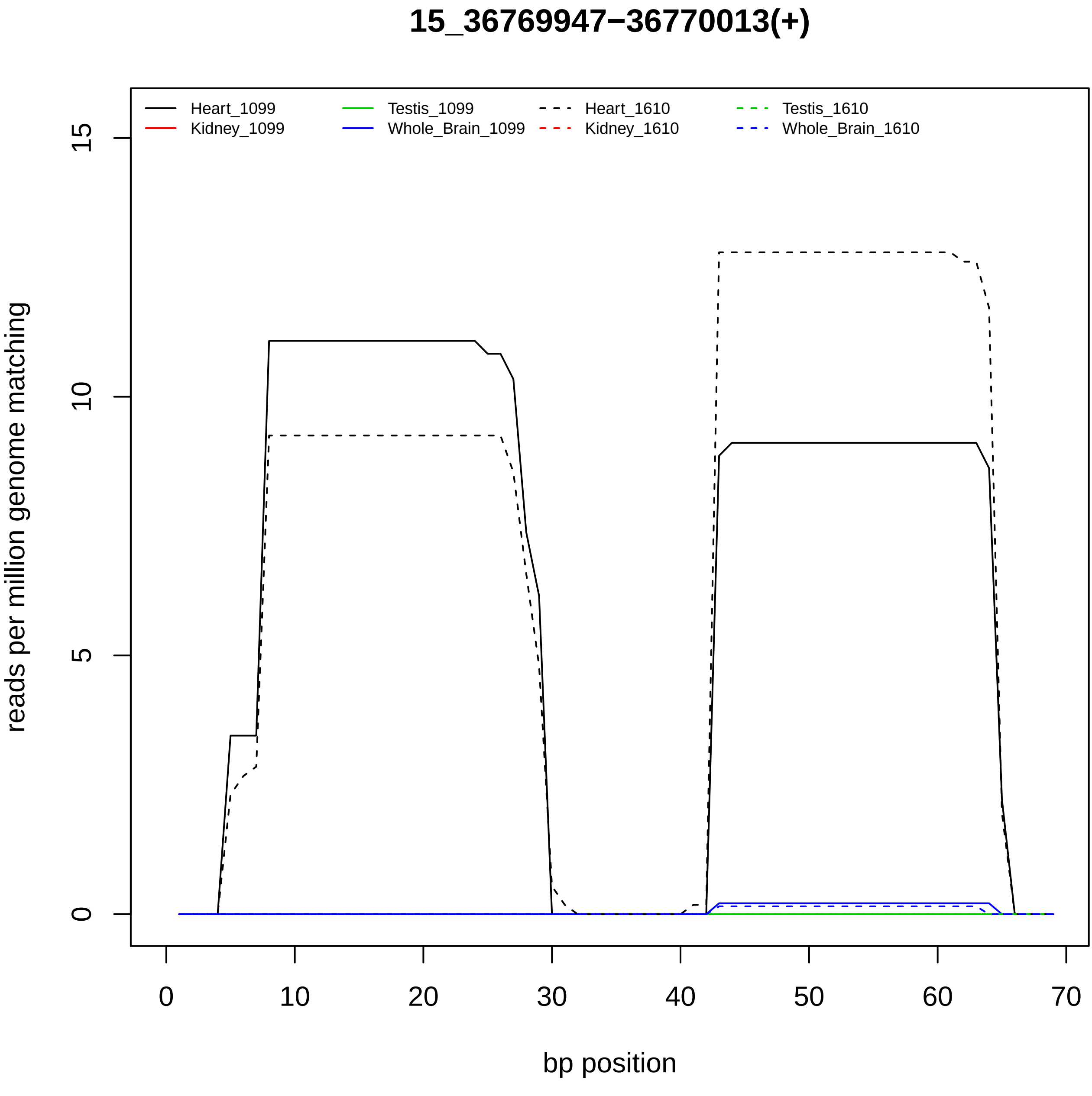

15_36769947−36770013(+)
Heart_1099
Kidney_1099
Testis_1099
Whole_Brain_1099
Heart_1610
Kidney_1610
Testis_1610
Whole_Brain_1610
reads per million genome matching
bp position
0
5
10
15
0
10
20
30
40
50
60
70

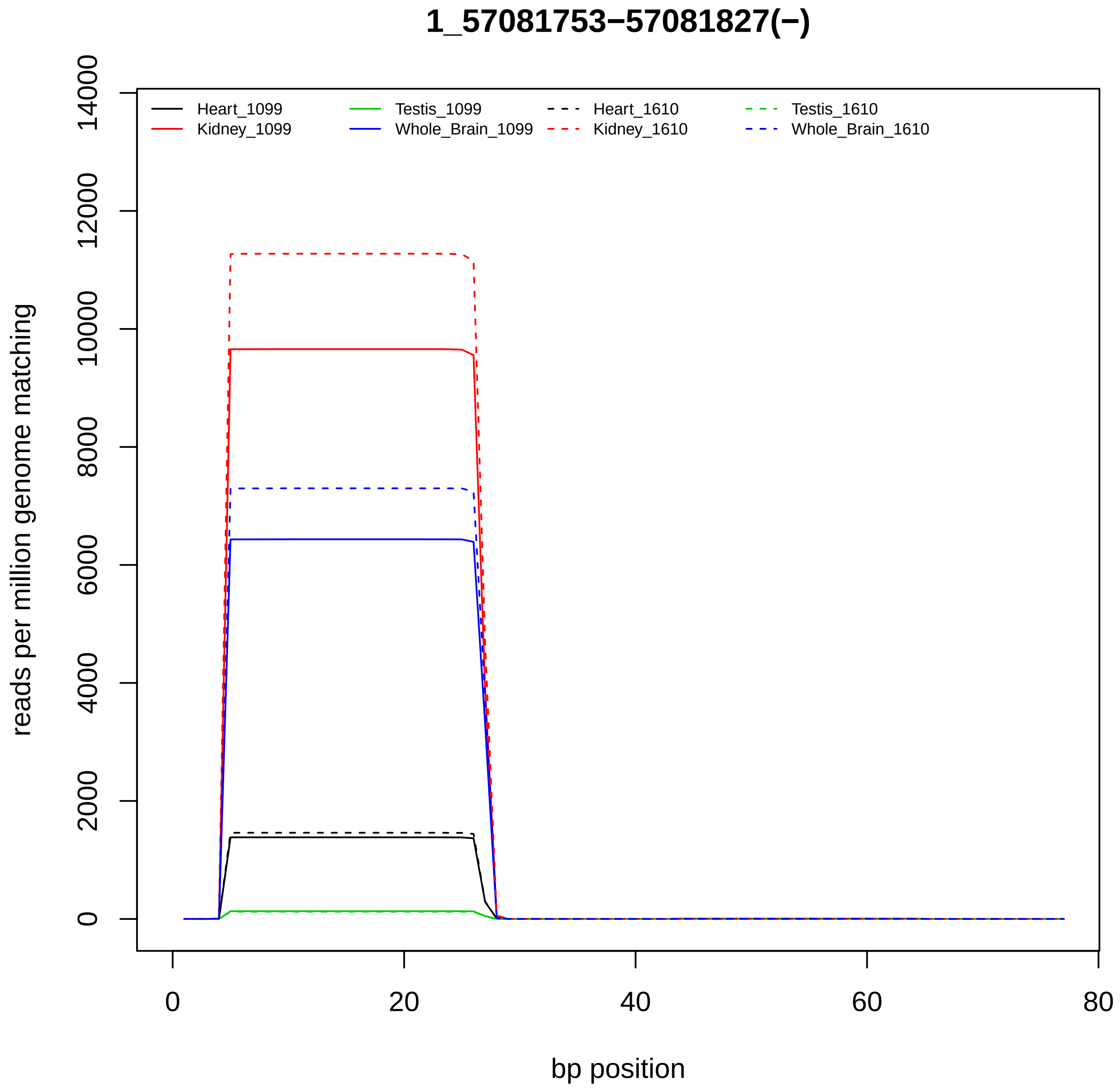

1_57081753−57081827(−)
Heart_1099
Kidney_1099
Testis_1099
Whole_Brain_1099
Heart_1610
Kidney_1610
Testis_1610
Whole_Brain_1610
reads per million genome matching
bp position
0
2000
4000
6000
8000
10000
12000
14000
0
20
40
60
80

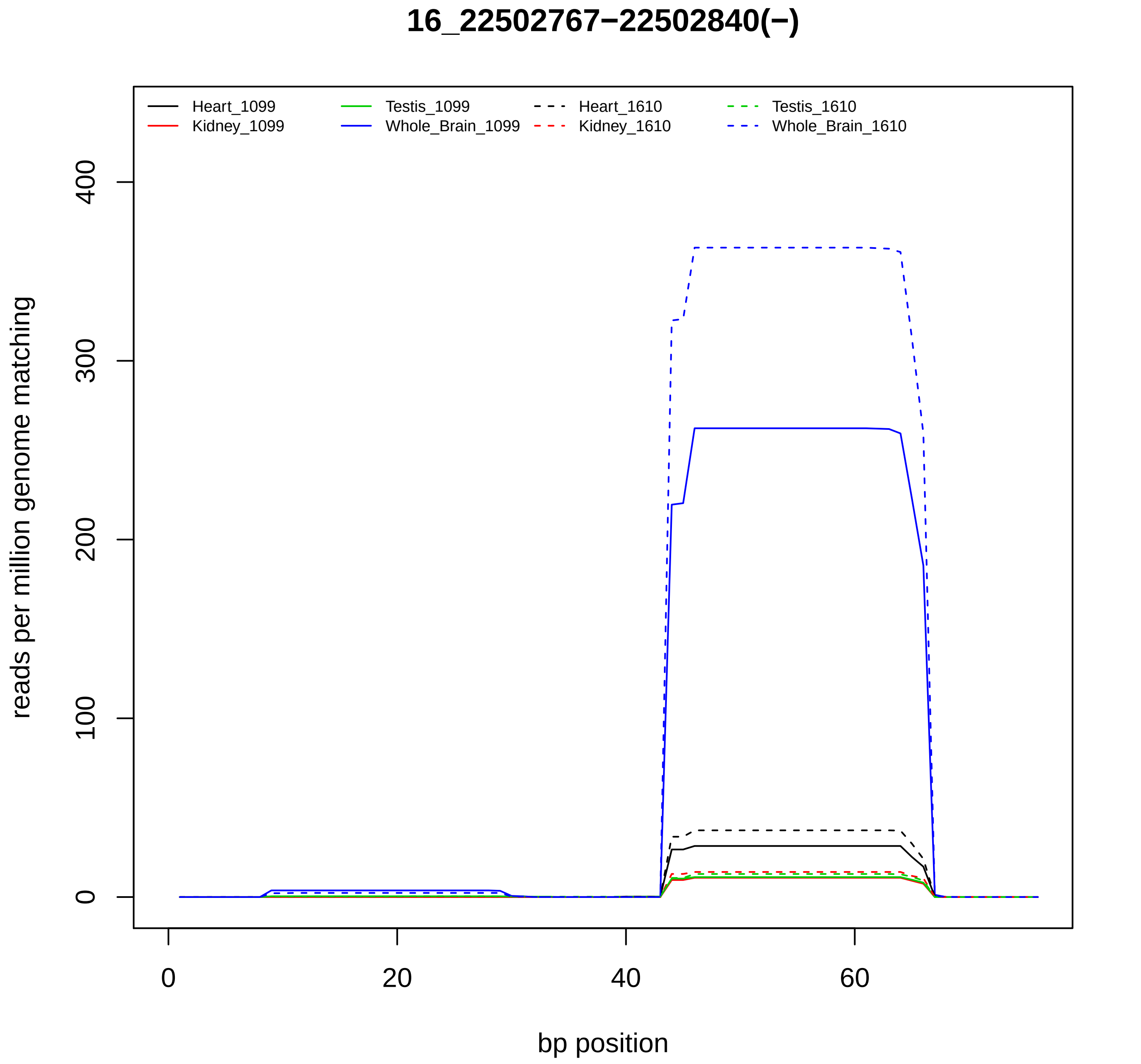
16_22502767−22502840(−)
Heart_1099
Kidney_1099
Testis_1099
Whole_Brain_1099
Heart_1610
Kidney_1610
Testis_1610
Whole_Brain_1610
reads per million genome matching
0
100
200
300
400
0
20
40
60
bp position

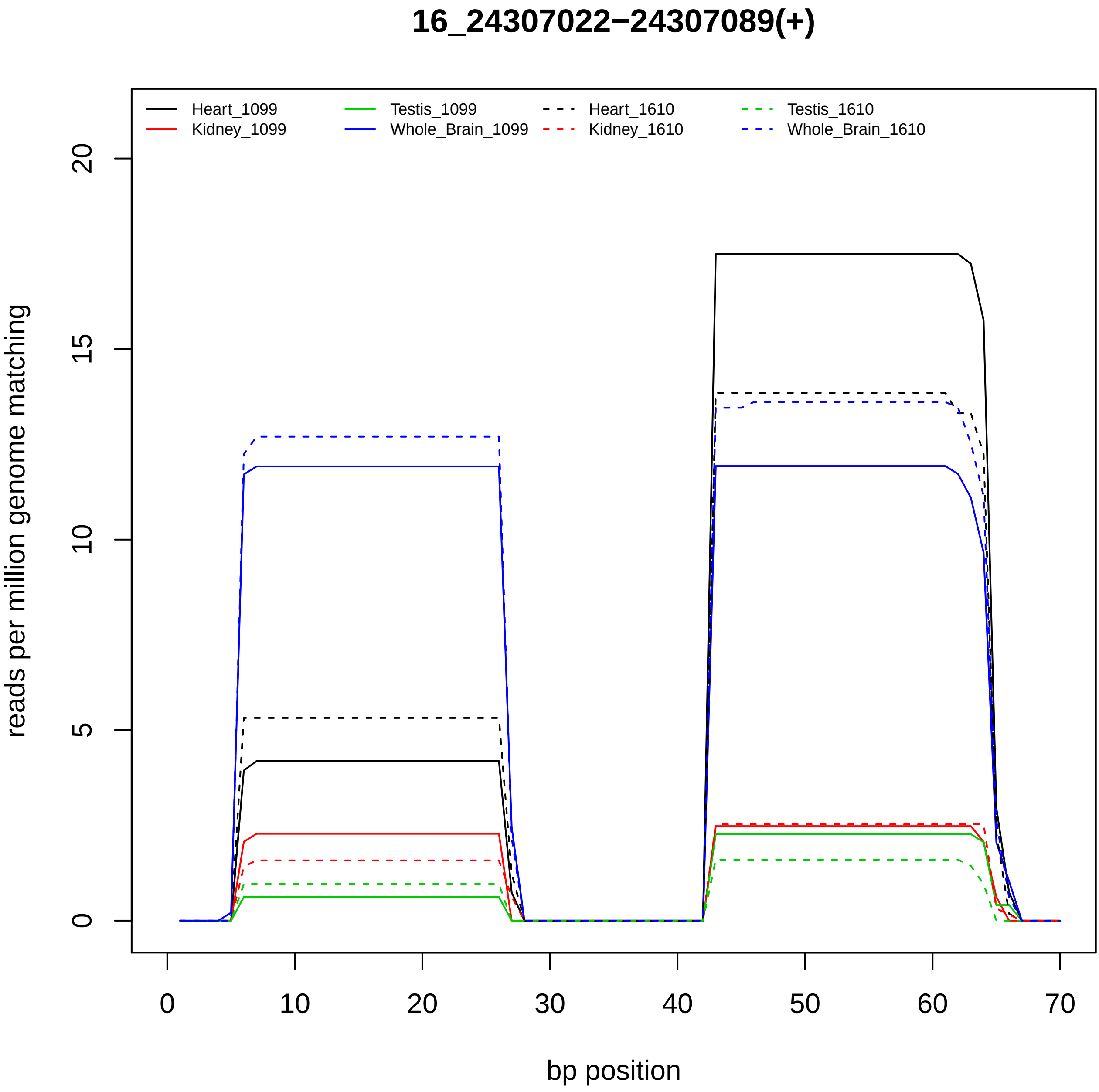

16_24307022−24307089(+)
Heart_1099
Kidney_1099
Testis_1099
Whole_Brain_1099
Heart_1610
Kidney_1610
Testis_1610
Whole_Brain_1610
reads per million genome matching
0
5
10
15
20
0
10
20
30
40
50
60
70
bp position

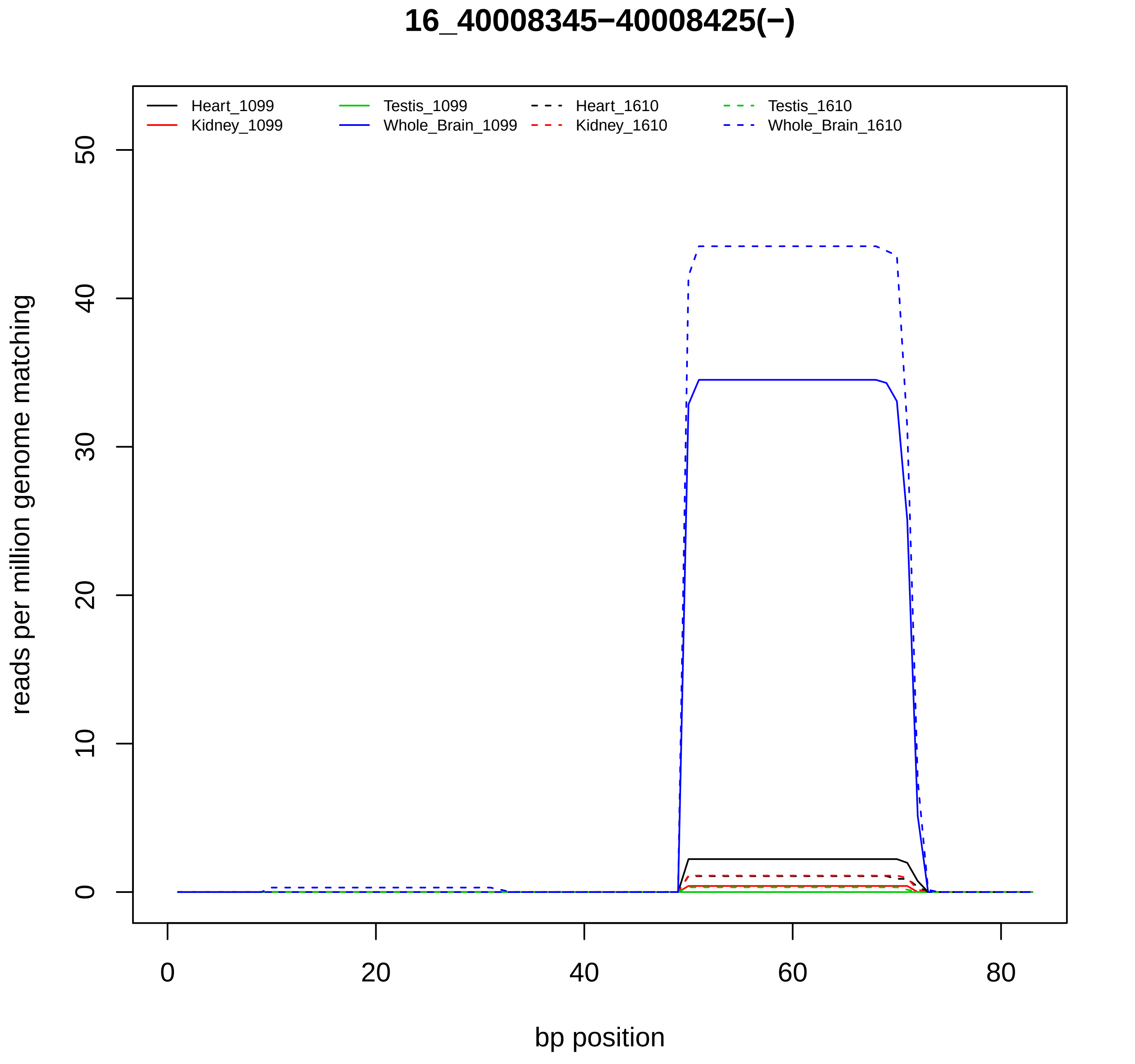
16_40008345−40008425(−)
Heart_1099
Kidney_1099
Testis_1099
Whole_Brain_1099
Heart_1610
Kidney_1610
Testis_1610
Whole_Brain_1610
reads per million genome matching
0
10
20
30
40
50
0
20
40
60
80
bp position

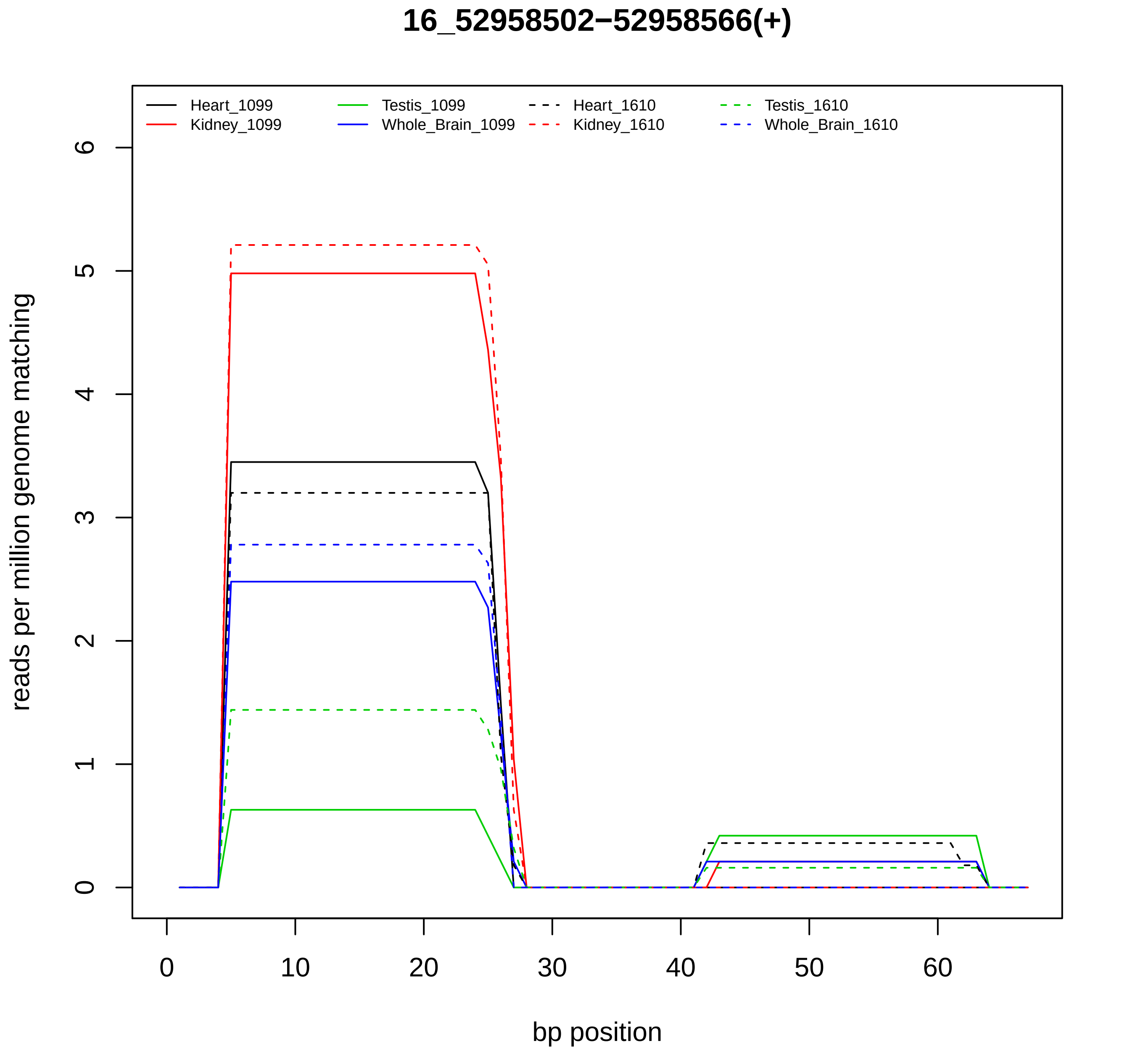
16_52958502−52958566(+)
Heart_1099
Kidney_1099
Testis_1099
Whole_Brain_1099
Heart_1610
Kidney_1610
Testis_1610
Whole_Brain_1610
reads per million genome matching
0
1
2
3
4
5
6
0
10
20
30
40
50
60
bp position

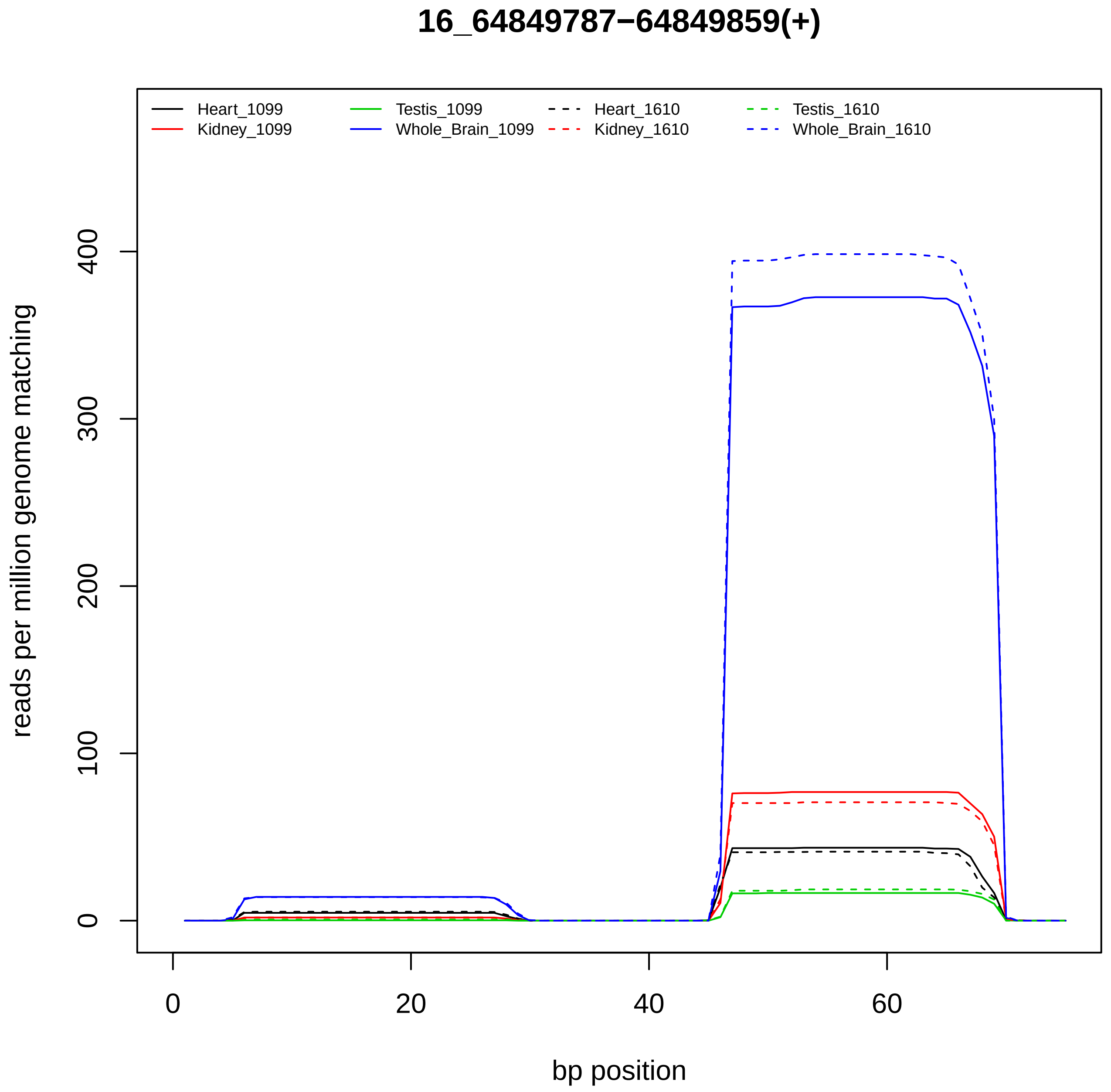

16_64849787−64849859(+)
Heart_1099
Kidney_1099
Testis_1099
Whole_Brain_1099
Heart_1610
Kidney_1610
Testis_1610
Whole_Brain_1610
reads per million genome matching
0
100
200
300
400
0
20
40
60
bp position

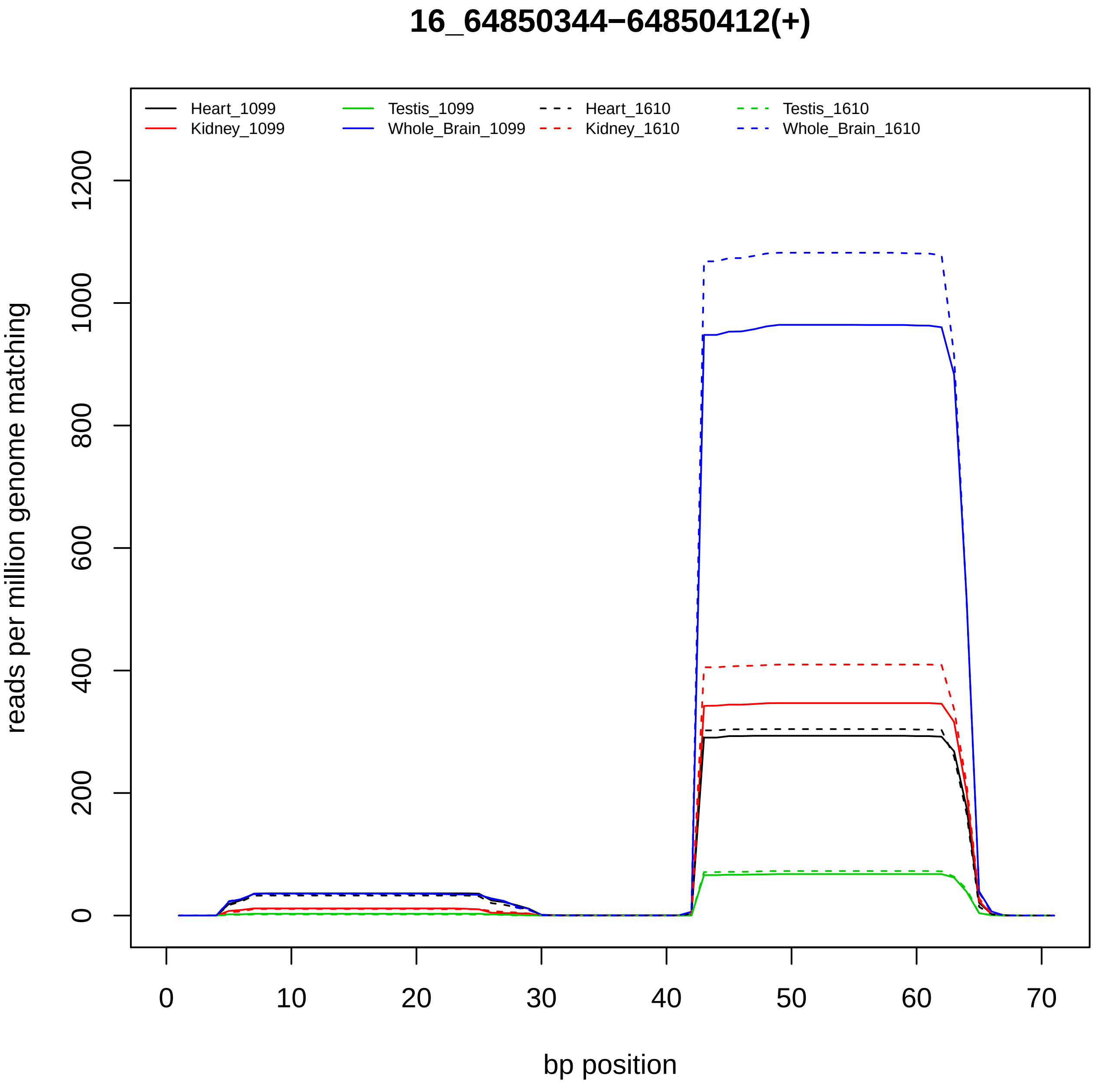
16_64850344−64850412(+)
Heart_1099
Kidney_1099
Testis_1099
Whole_Brain_1099
Heart_1610
Kidney_1610
Testis_1610
Whole_Brain_1610
reads per million genome matching
0
200
400
600
800
1000
1200
0
10
20
30
40
50
60
70
bp position

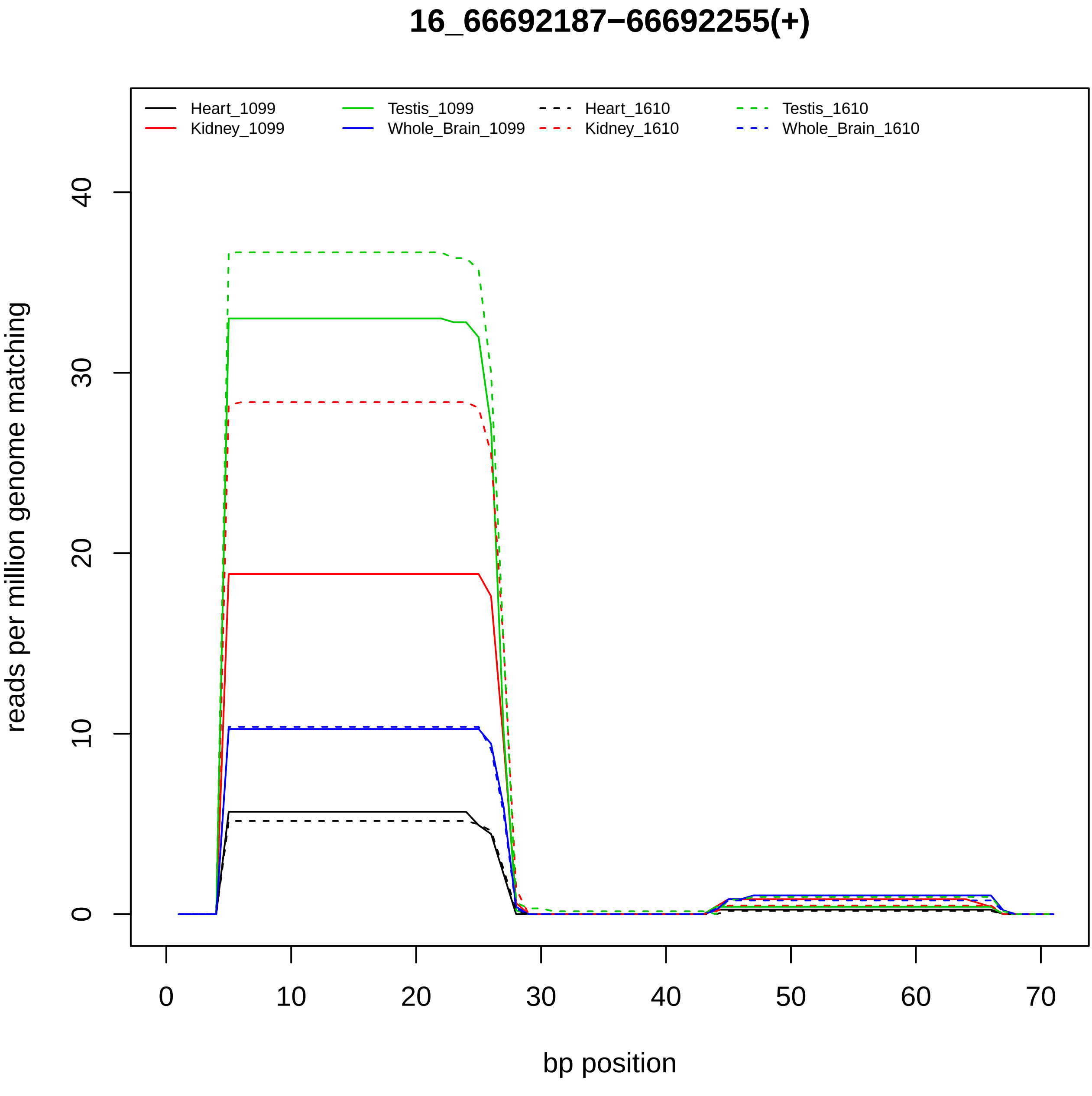
16_66692187−66692255(+)
Heart_1099
Kidney_1099
Testis_1099
Whole_Brain_1099
Heart_1610
Kidney_1610
Testis_1610
Whole_Brain_1610
reads per million genome matching
0
10
20
30
40
bp position
0
10
20
30
40
50
60
70

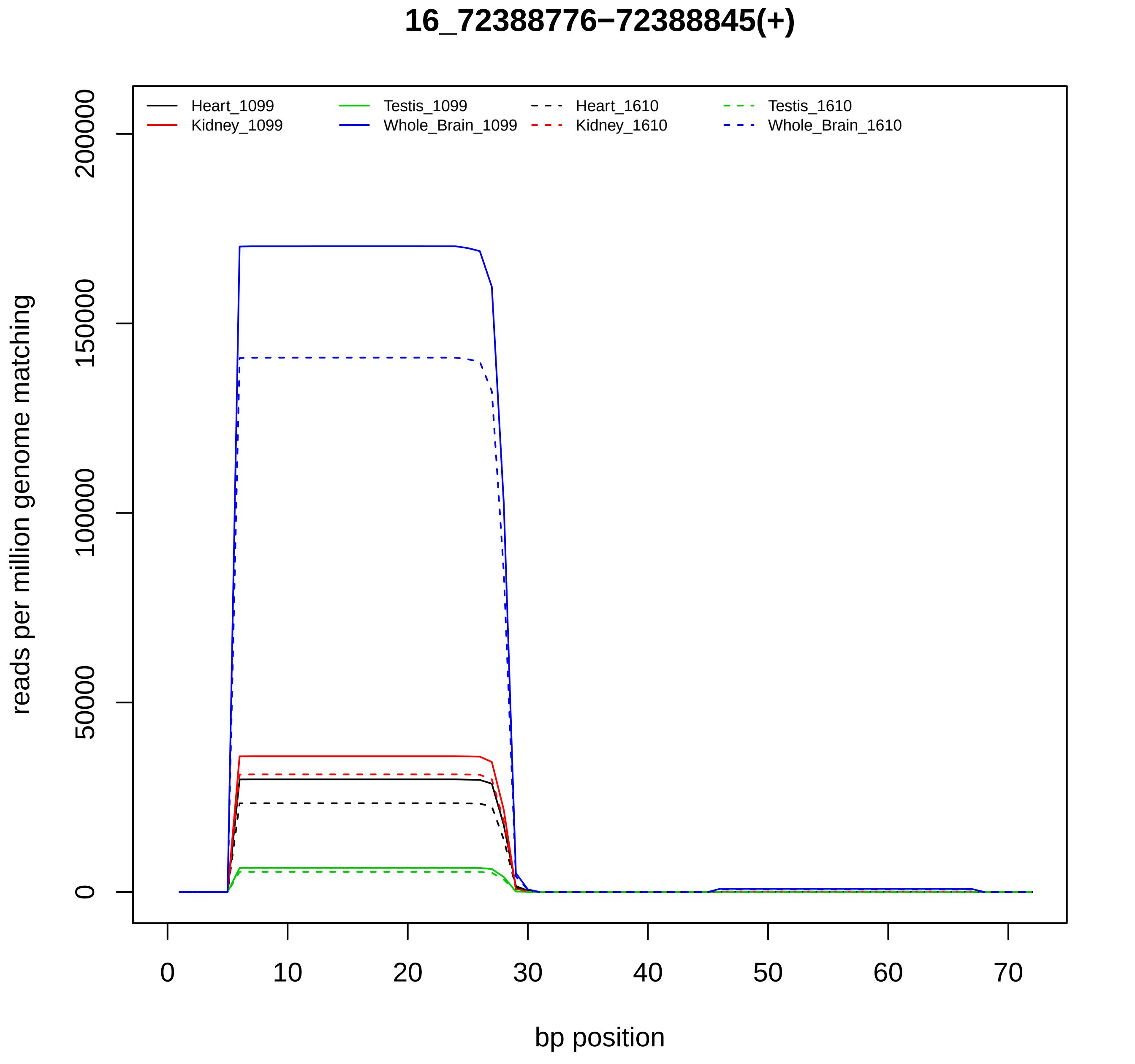

16_72388776−72388845(+)
Heart_1099
Kidney_1099
Testis_1099
Whole_Brain_1099
Heart_1610
Kidney_1610
Testis_1610
Whole_Brain_1610
reads per million genome matching
0
50000
100000
150000
200000
0
10
20
30
40
50
60
70
bp position

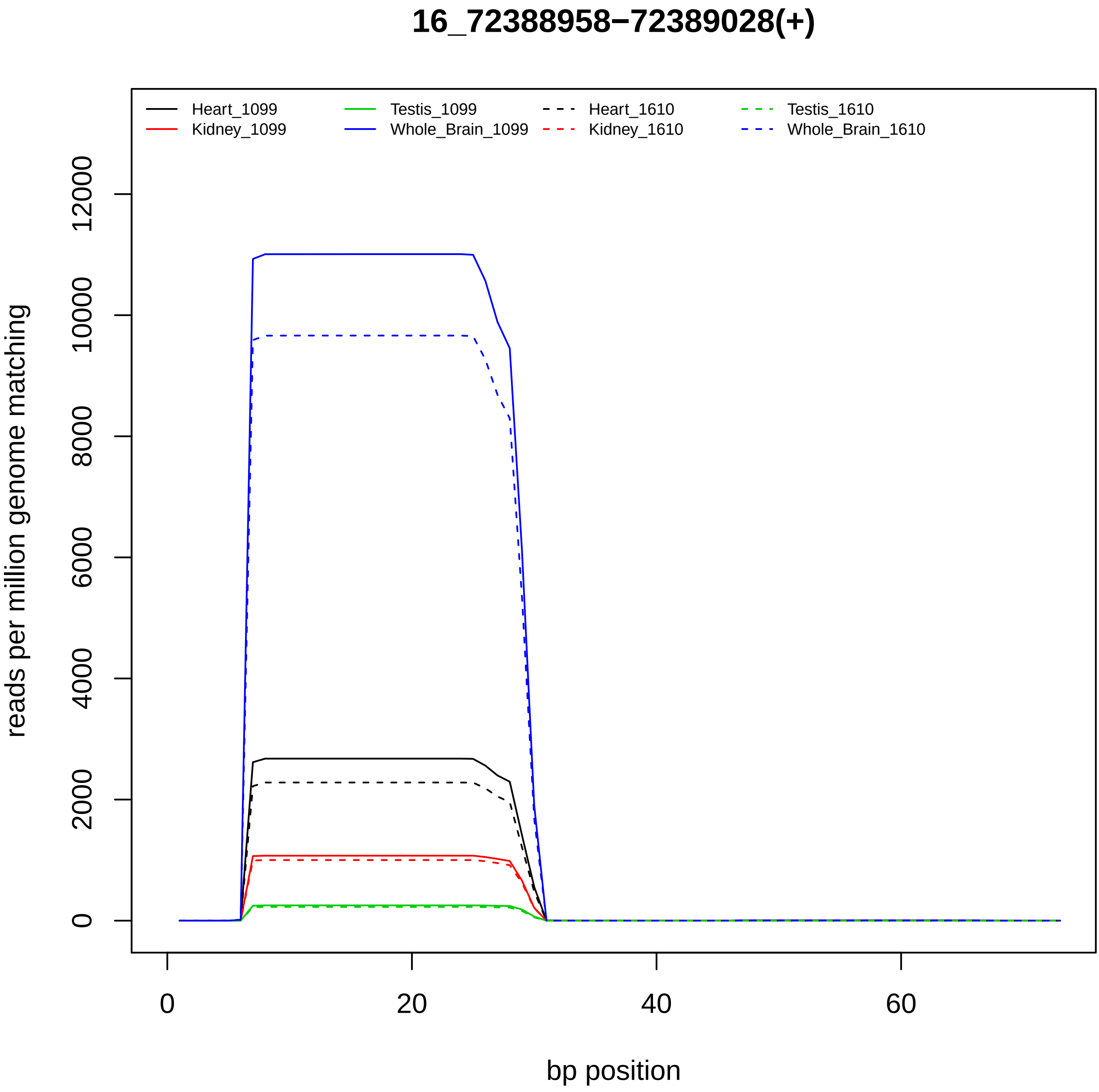

16_72388958−72389028(+)
Heart_1099
Kidney_1099
Testis_1099
Whole_Brain_1099
Heart_1610
Kidney_1610
Testis_1610
Whole_Brain_1610
reads per million genome matching
0
2000
4000
6000
8000
10000
12000
0
20
40
60
bp position

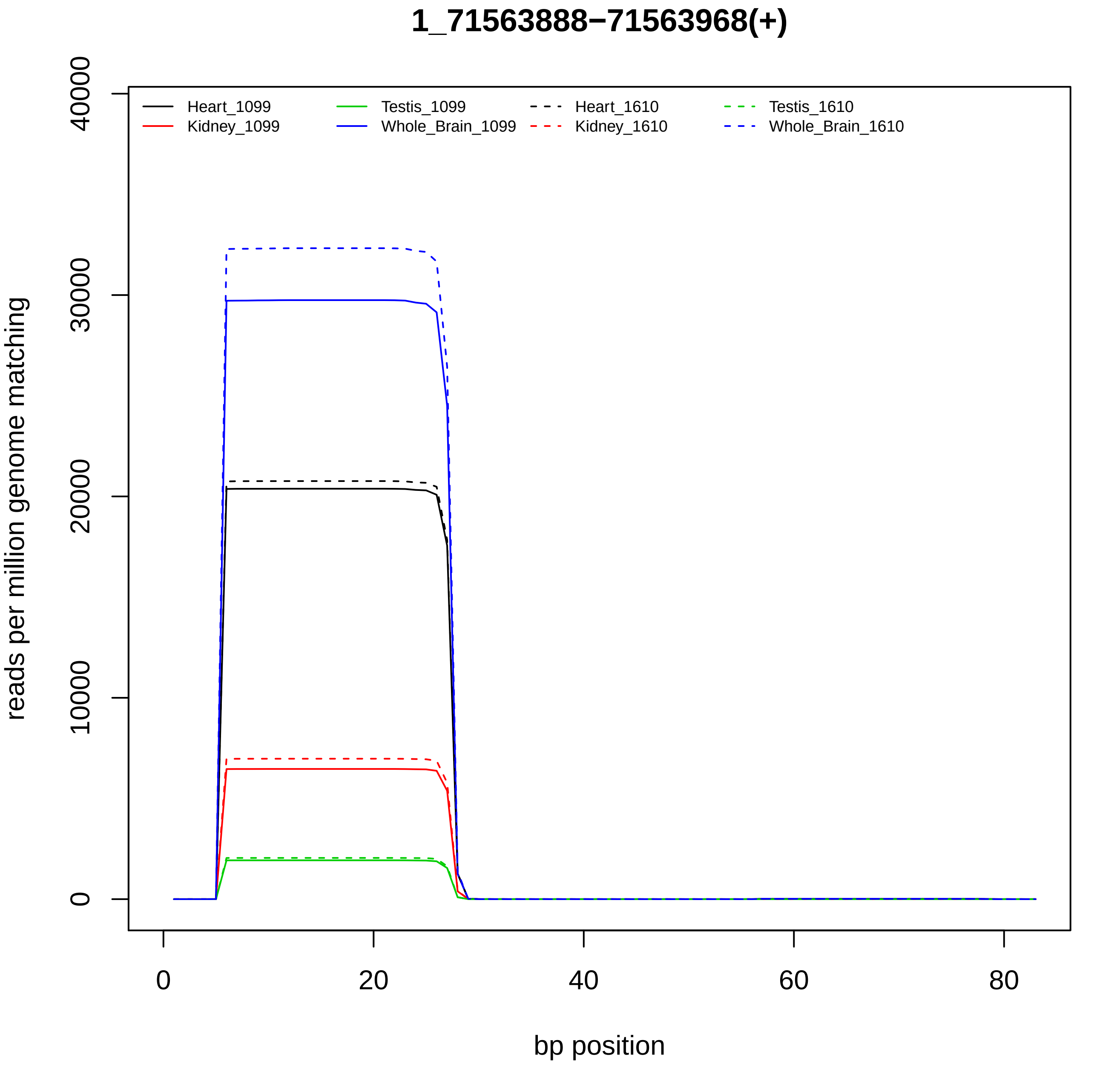
1_71563888−71563968(+)
Heart_1099
Kidney_1099
Testis_1099
Whole_Brain_1099
Heart_1610
Kidney_1610
Testis_1610
Whole_Brain_1610
reads per million genome matching
0
10000
20000
30000
40000
bp position
0
20
40
60
80

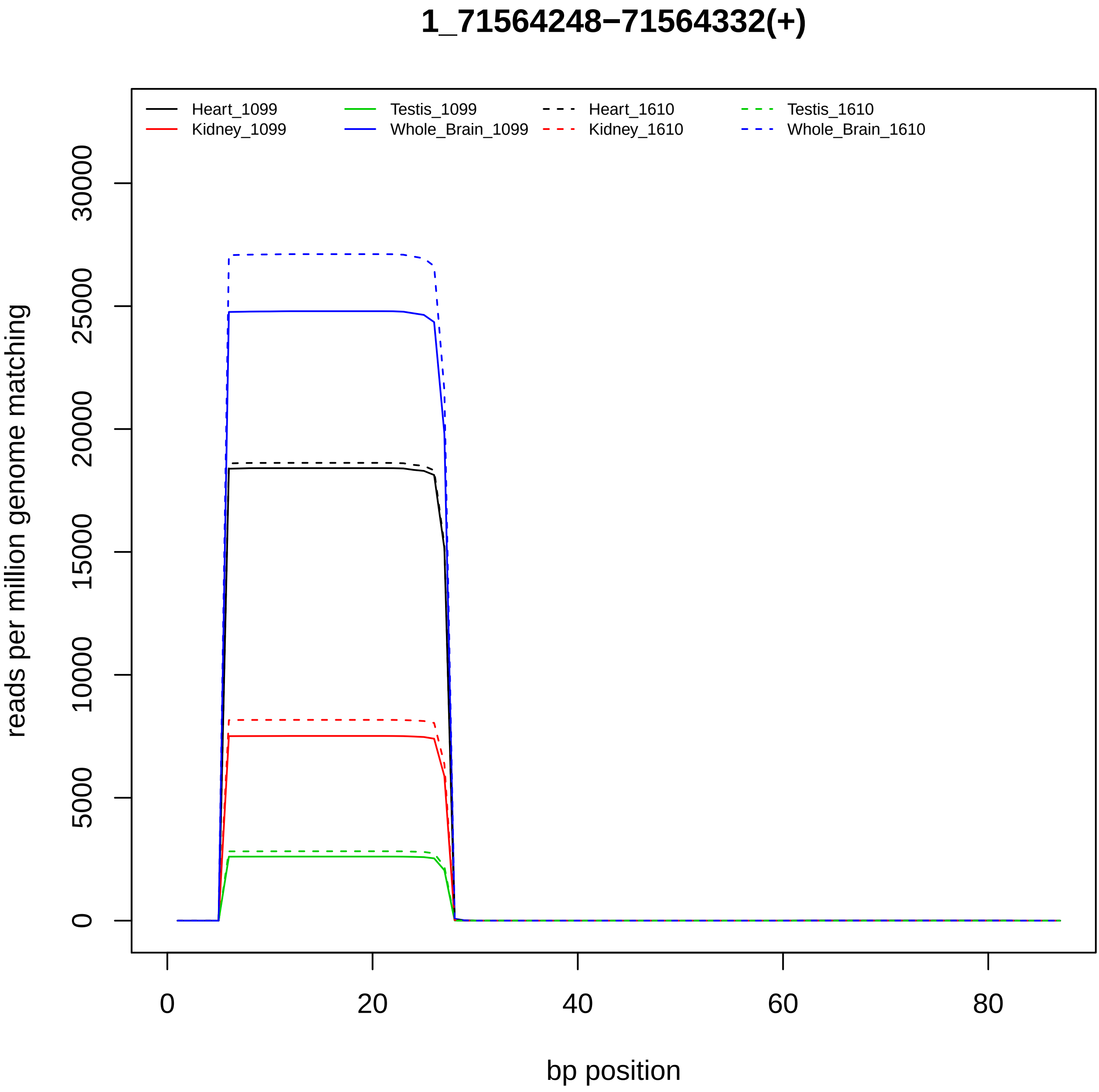

1_71564248−71564332(+)
Heart_1099
Kidney_1099
Testis_1099
Whole_Brain_1099
Heart_1610
Kidney_1610
Testis_1610
Whole_Brain_1610
reads per million genome matching
0
5000
10000
15000
20000
25000
30000
0
20
40
60
80
bp position

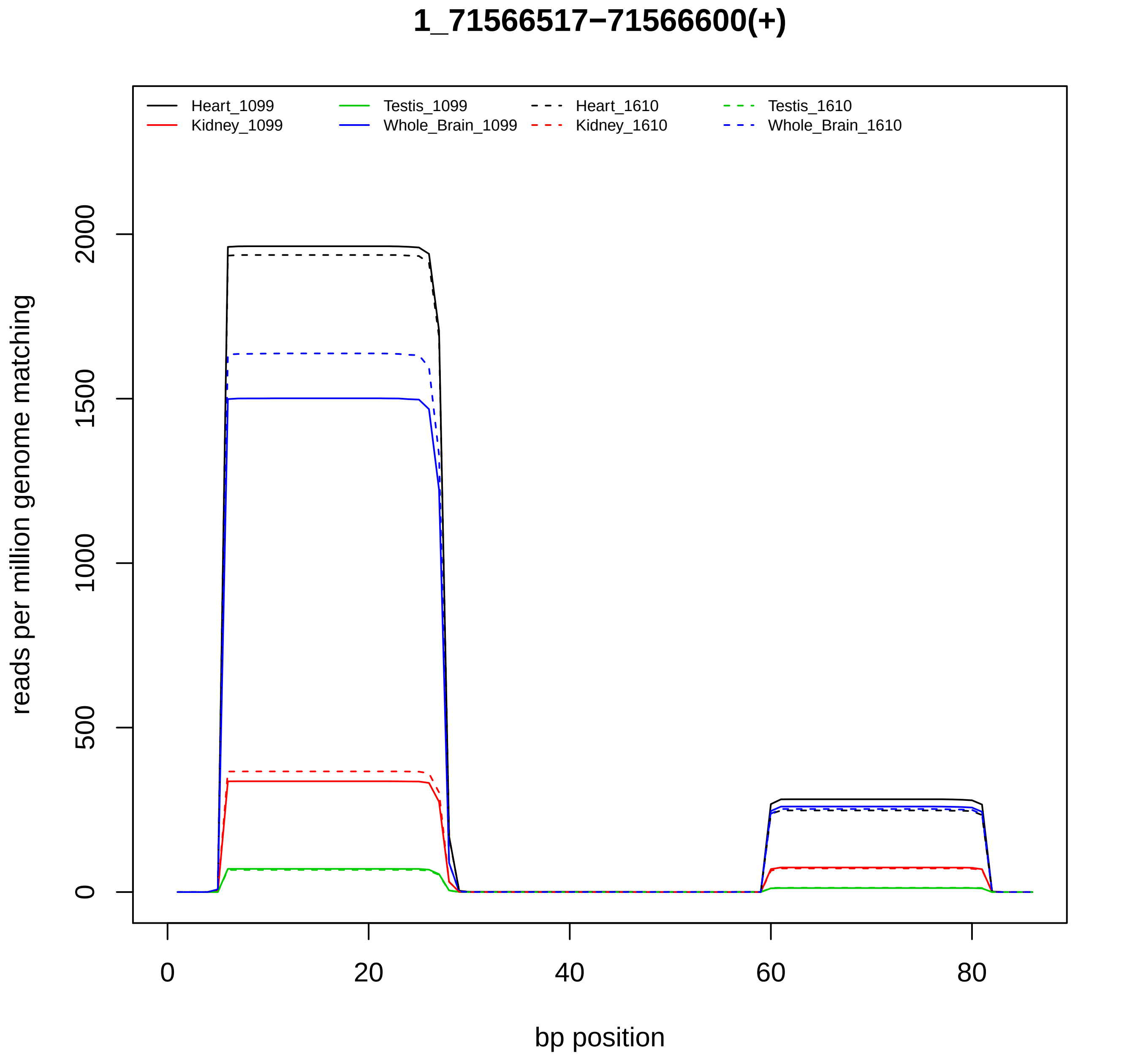
1_71566517−71566600(+)
Heart_1099
Kidney_1099
Testis_1099
Whole_Brain_1099
Heart_1610
Kidney_1610
Testis_1610
Whole_Brain_1610
reads per million genome matching
0
500
1000
1500
2000
0
20
40
60
80
bp position

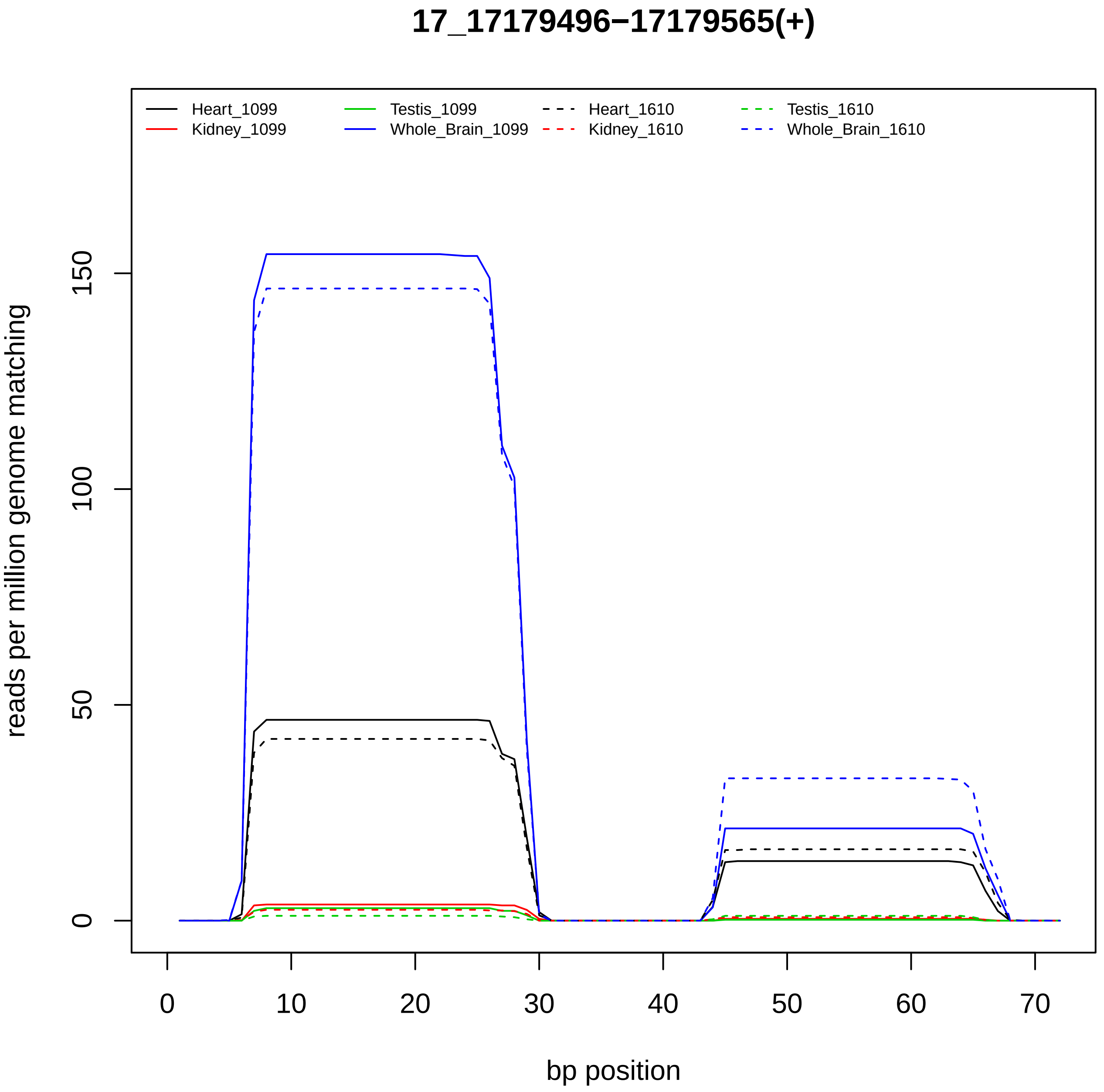

17_17179496−17179565(+)
Heart_1099
Kidney_1099
Testis_1099
Whole_Brain_1099
Heart_1610
Kidney_1610
Testis_1610
Whole_Brain_1610
reads per million genome matching
0
50
100
150
0
10
20
30
40
50
60
70
bp position

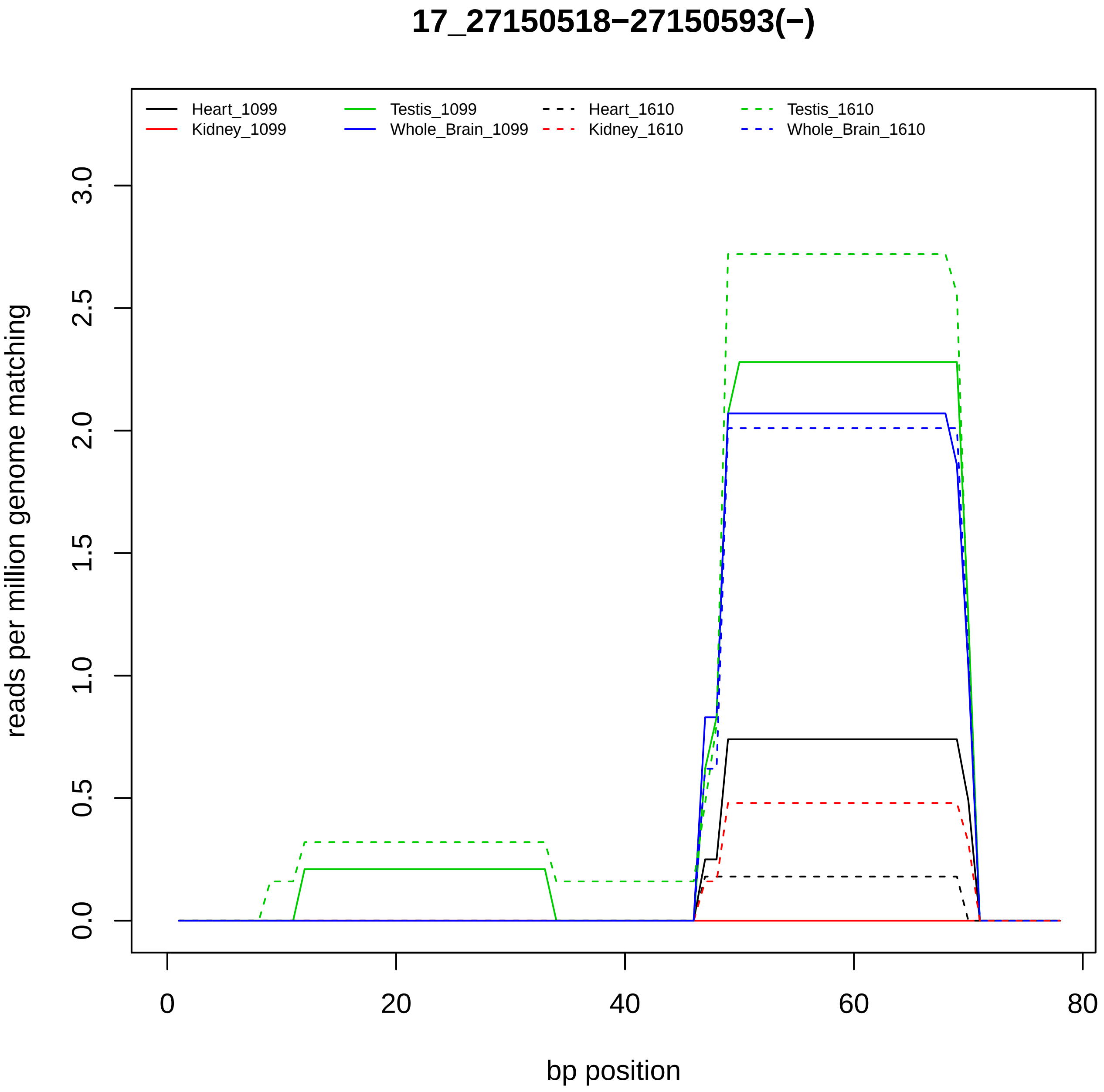

17_27150518−27150593(−)
Heart_1099
Kidney_1099
Testis_1099
Whole_Brain_1099
Heart_1610
Kidney_1610
Testis_1610
Whole_Brain_1610
reads per million genome matching
0.0
0.5
1.0
1.5
2.0
2.5
3.0
0
20
40
60
80
bp position

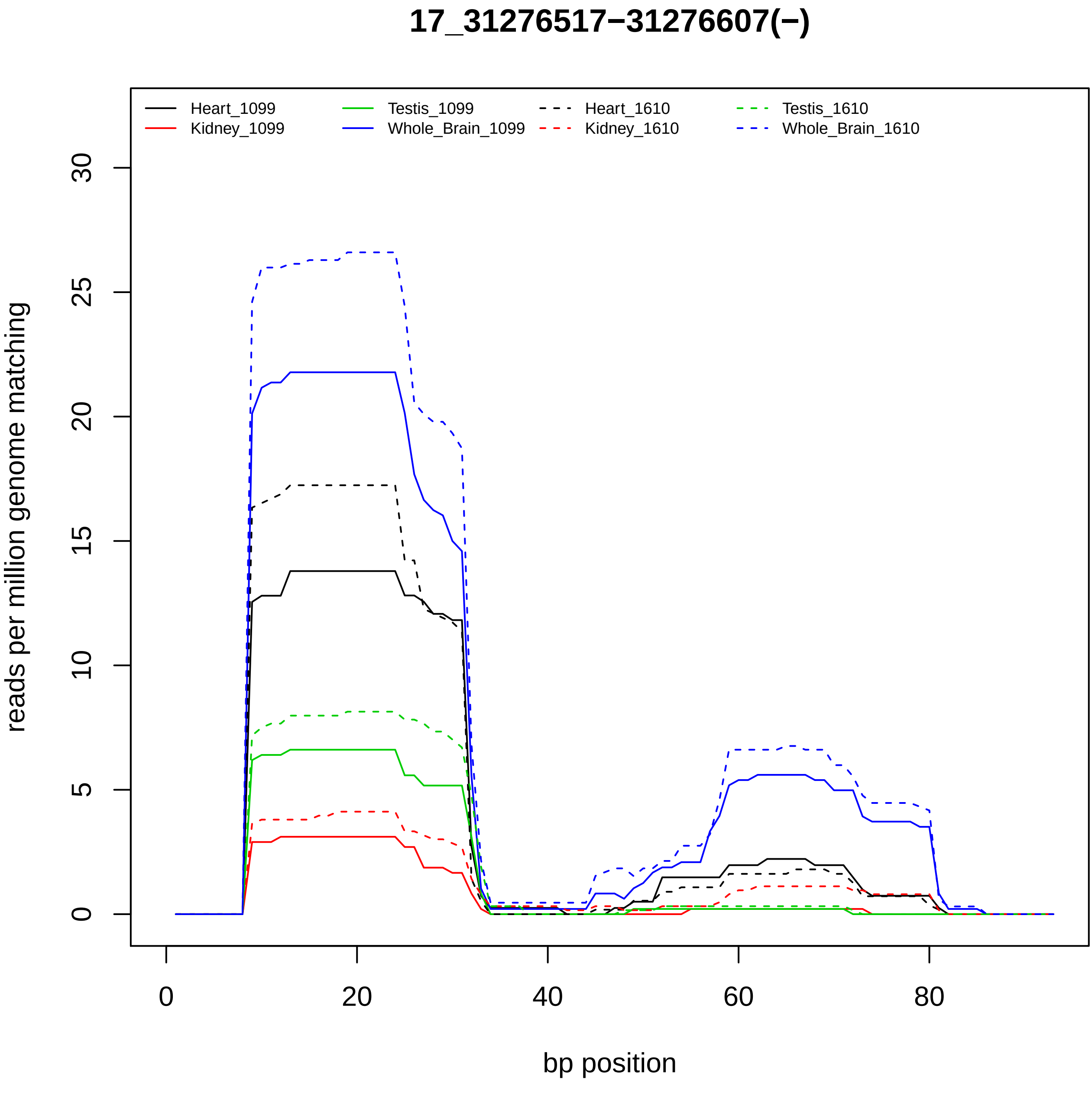

17_31276517−31276607(−)
Heart_1099
Kidney_1099
Testis_1099
Whole_Brain_1099
Heart_1610
Kidney_1610
Testis_1610
Whole_Brain_1610
reads per million genome matching
bp position
0
5
10
15
20
25
30
0
20
40
60
80

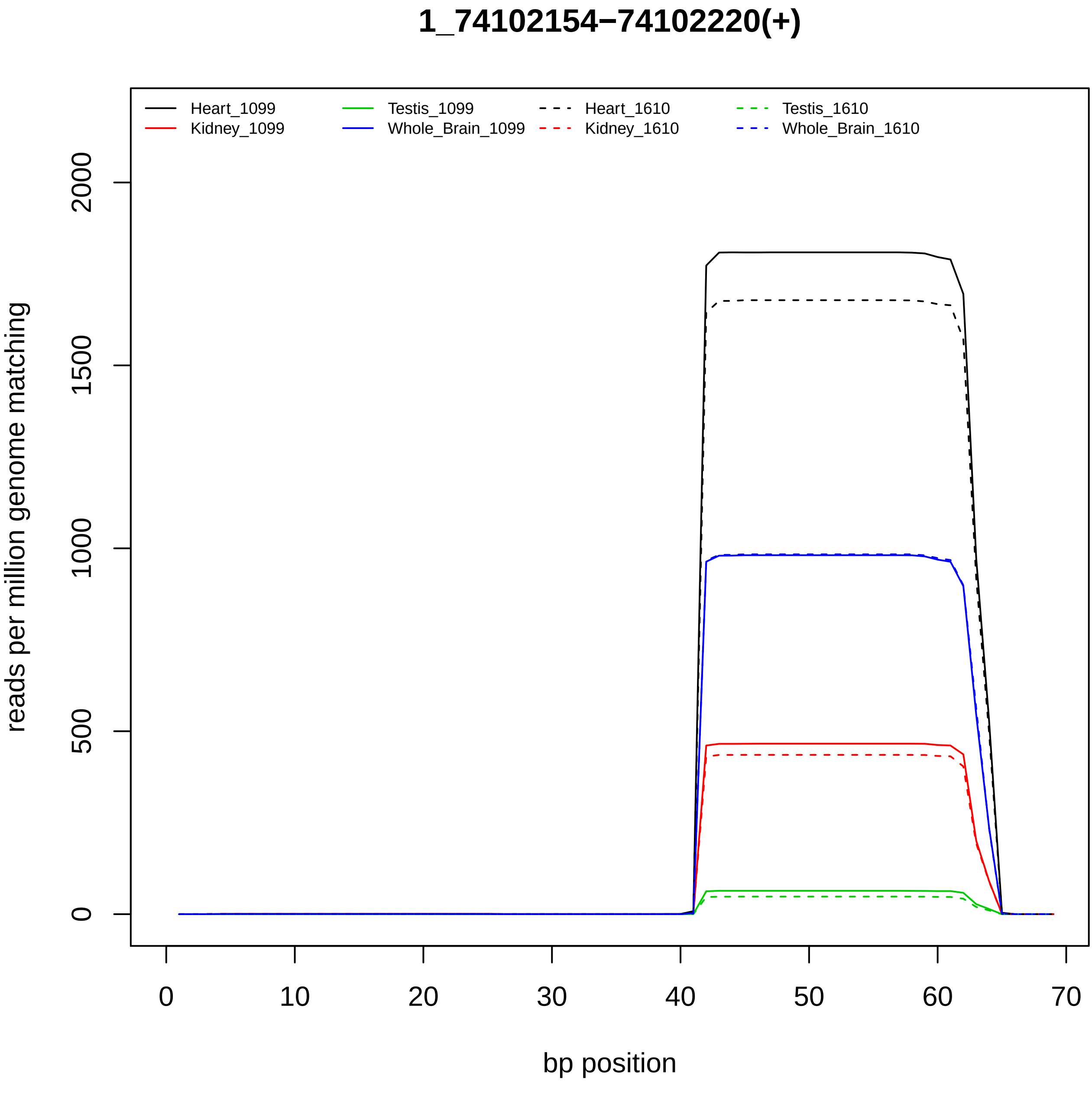

1_74102154−74102220(+)
Heart_1099
Kidney_1099
Testis_1099
Whole_Brain_1099
Heart_1610
Kidney_1610
Testis_1610
Whole_Brain_1610
reads per million genome matching
0
500
1000
1500
2000
0
10
20
30
40
50
60
70
bp position

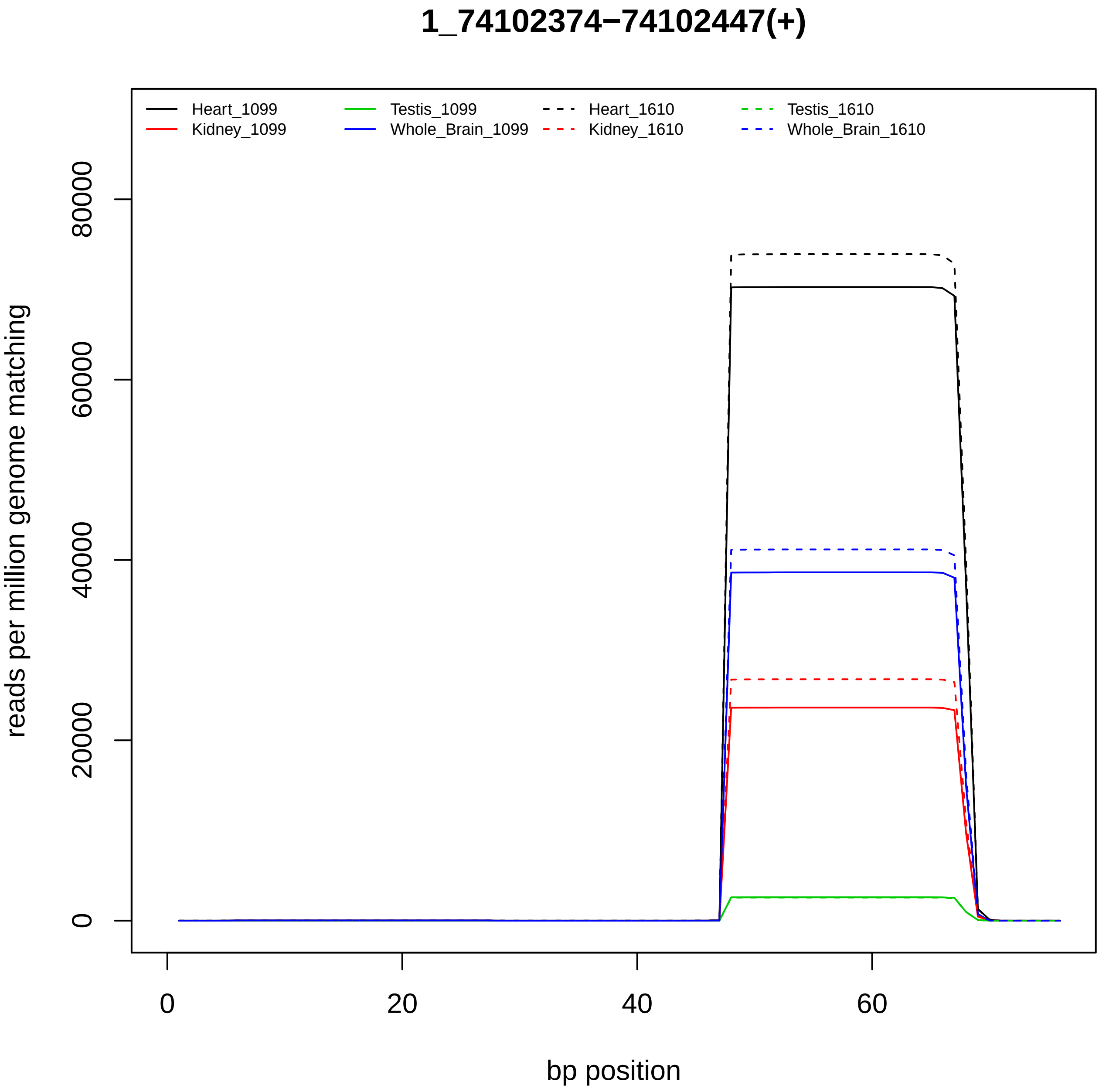
1_74102374−74102447(+)
Heart_1099
Kidney_1099
Testis_1099
Whole_Brain_1099
Heart_1610
Kidney_1610
Testis_1610
Whole_Brain_1610
reads per million genome matching
0
20000
40000
60000
80000
0
20
40
60
bp position

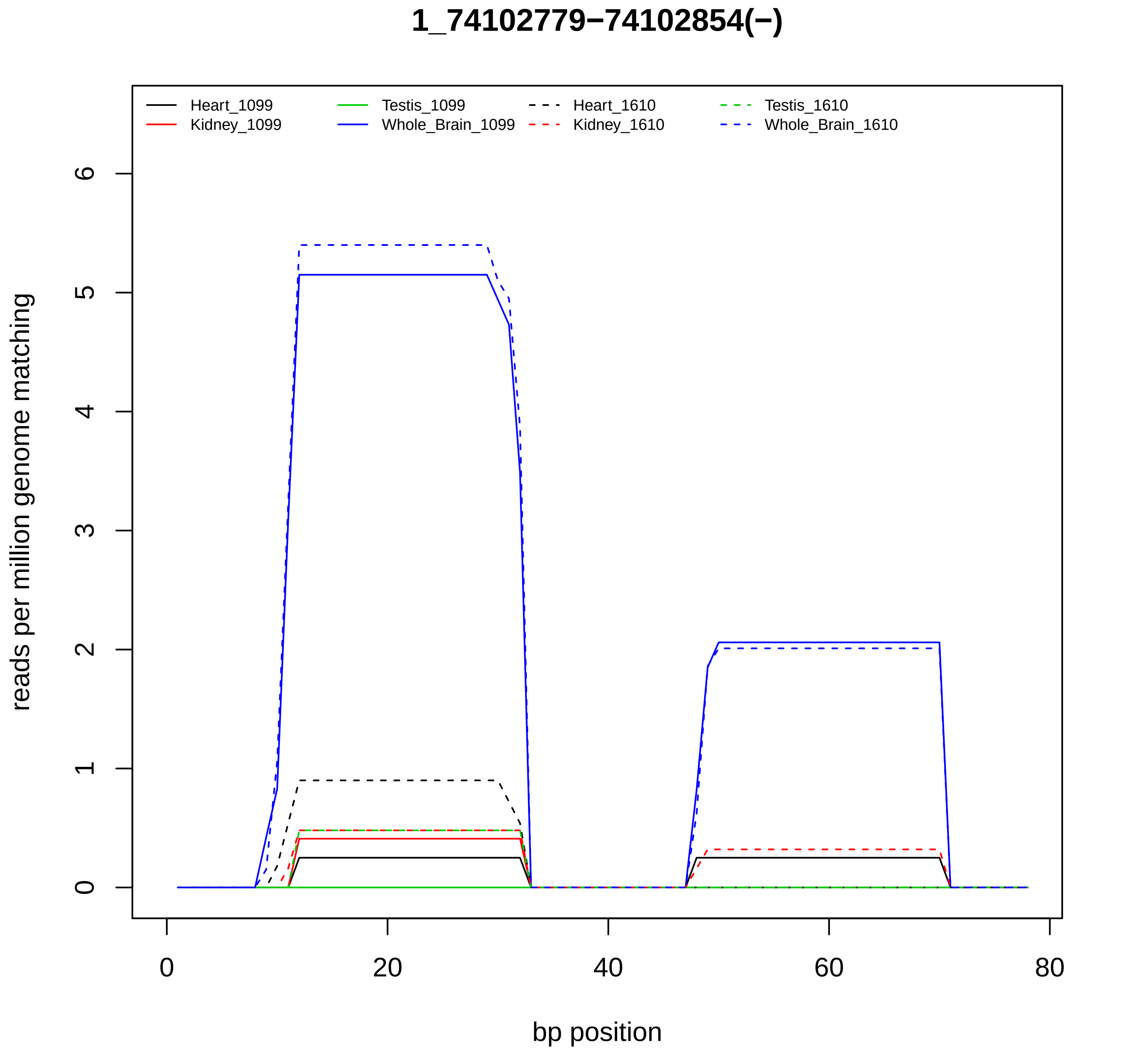

1_74102779−74102854(−)
Heart_1099
Kidney_1099
Testis_1099
Whole_Brain_1099
Heart_1610
Kidney_1610
Testis_1610
Whole_Brain_1610
reads per million genome matching
bp position
0
1
2
3
4
5
6
0
20
40
60
80

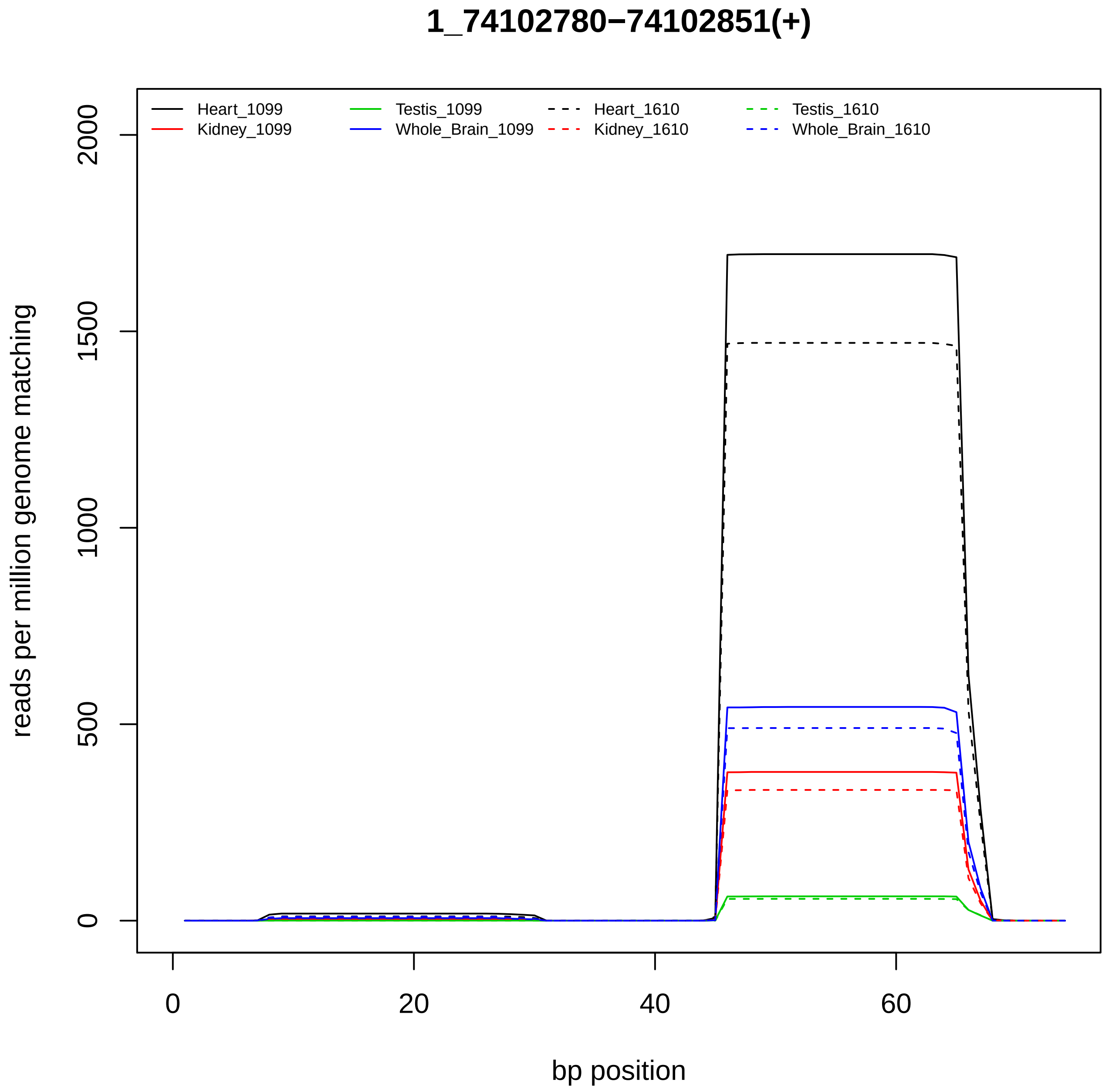

1_74102780−74102851(+)
Heart_1099
Kidney_1099
Testis_1099
Whole_Brain_1099
Heart_1610
Kidney_1610
Testis_1610
Whole_Brain_1610
reads per million genome matching
bp position
0
500
1000
1500
2000
0
20
40
60

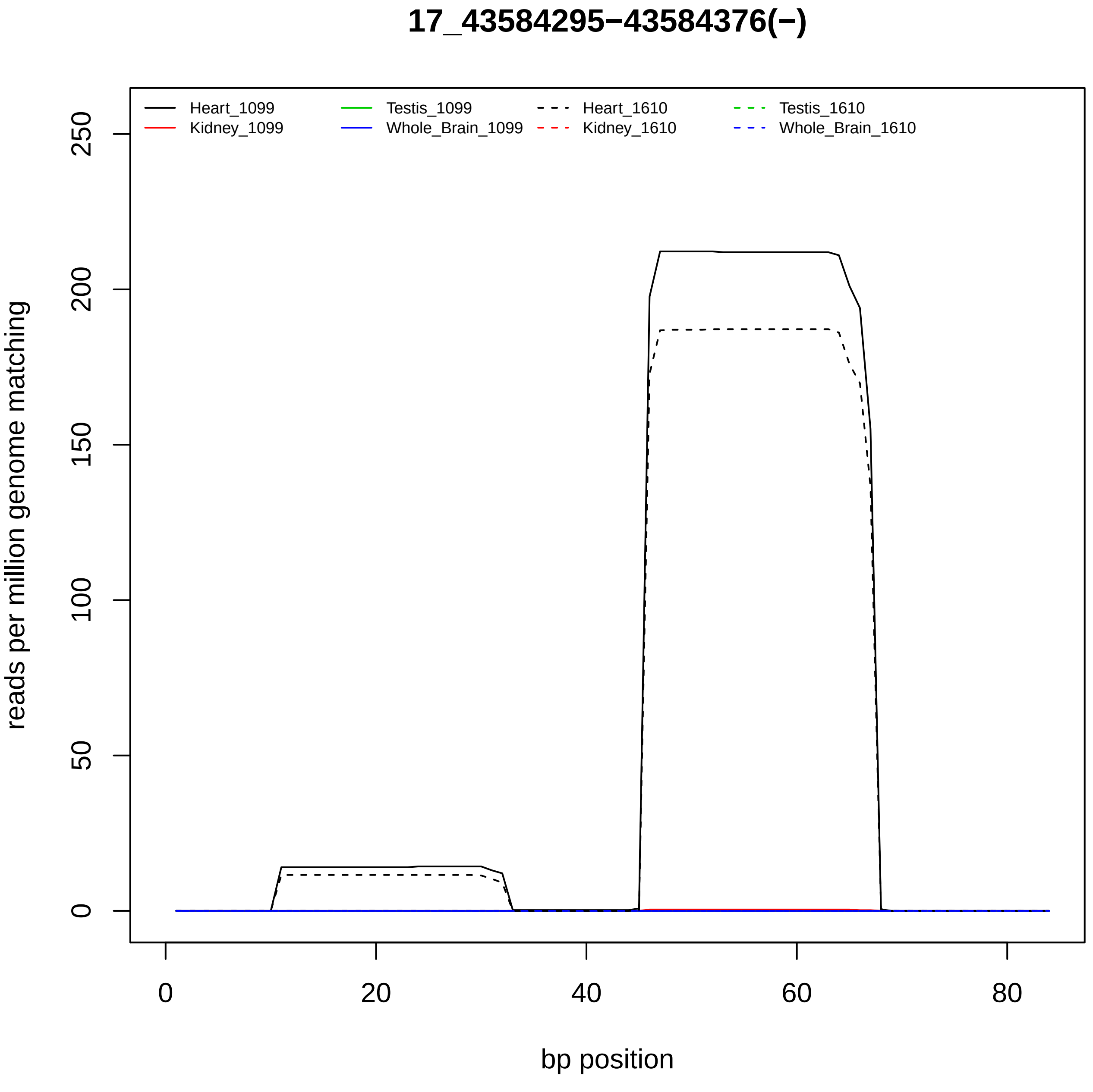

17_43584295−43584376(−)
Heart_1099
Kidney_1099
Testis_1099
Whole_Brain_1099
Heart_1610
Kidney_1610
Testis_1610
Whole_Brain_1610
reads per million genome matching
bp position
0
50
100
150
200
250
0
20
40
60
80

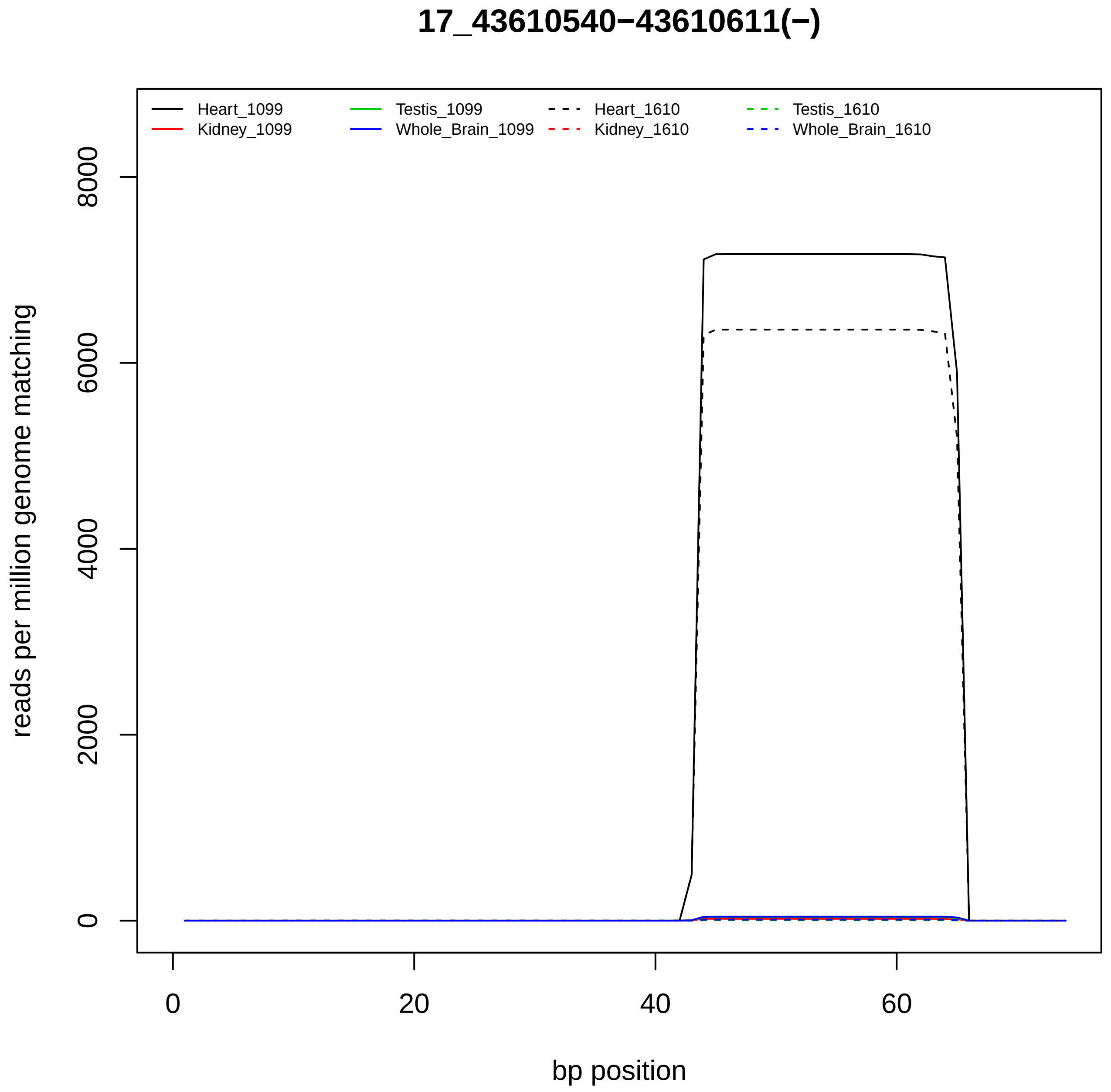
17_43610540−43610611(−)
Heart_1099
Kidney_1099
Testis_1099
Whole_Brain_1099
Heart_1610
Kidney_1610
Testis_1610
Whole_Brain_1610
reads per million genome matching
0
2000
4000
6000
8000
0
20
40
60
bp position

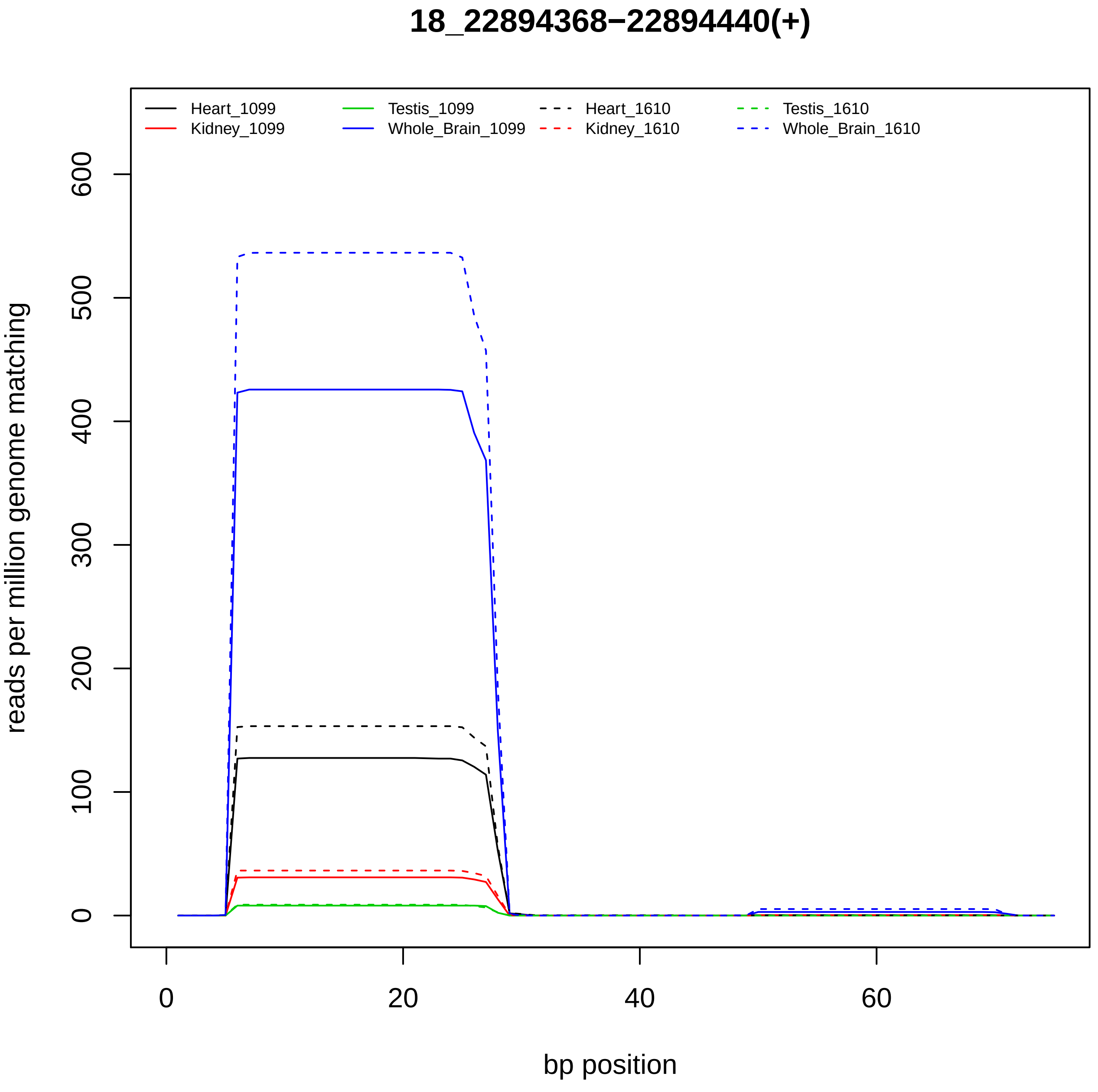

18_22894368−22894440(+)
Heart_1099
Kidney_1099
Testis_1099
Whole_Brain_1099
Heart_1610
Kidney_1610
Testis_1610
Whole_Brain_1610
reads per million genome matching
0
100
200
300
400
500
600
0
20
40
60
bp position

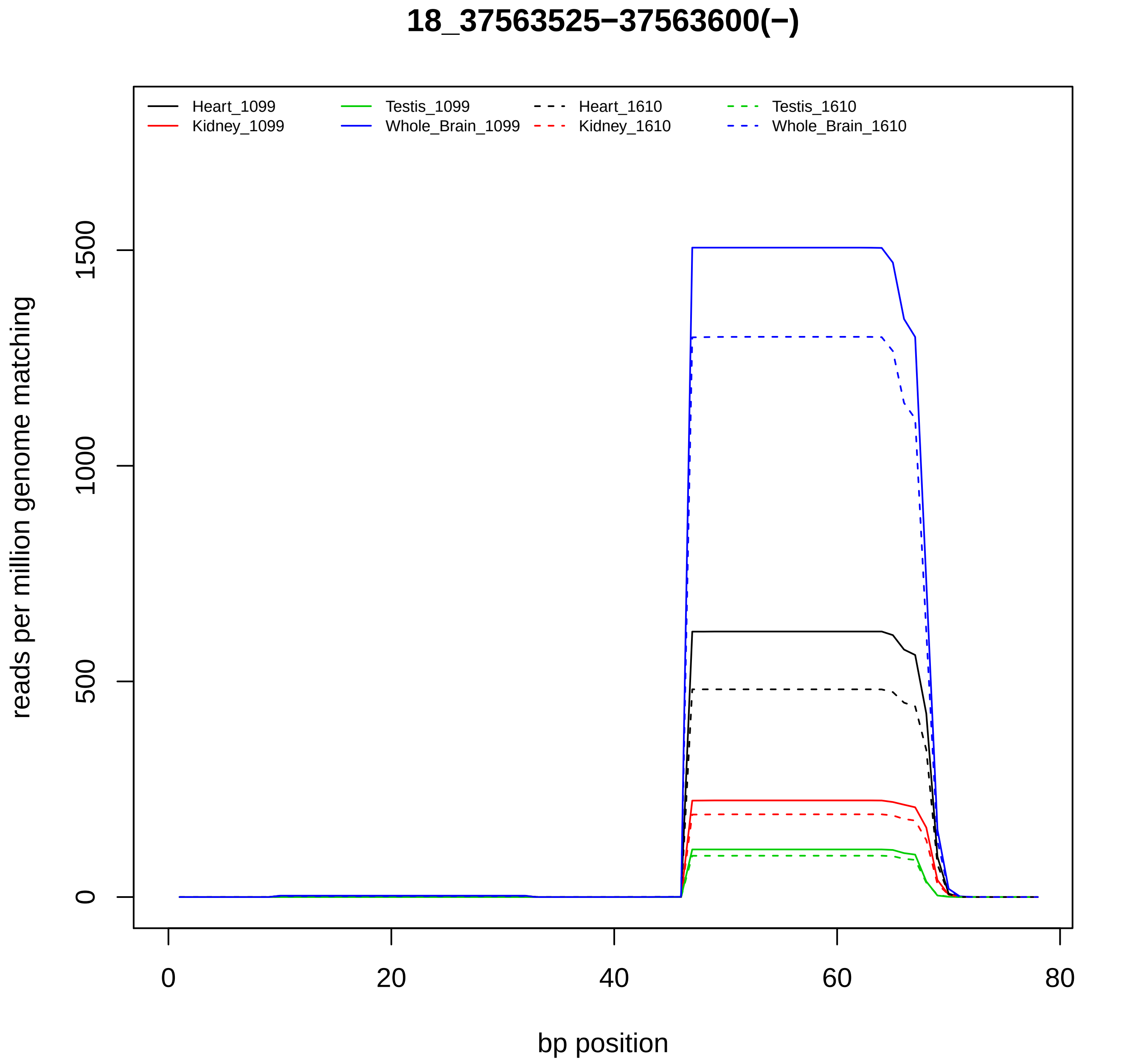

18_37563525−37563600(−)
Heart_1099
Kidney_1099
Testis_1099
Whole_Brain_1099
Heart_1610
Kidney_1610
Testis_1610
Whole_Brain_1610
reads per million genome matching
0
500
1000
1500
0
20
40
60
80
bp position

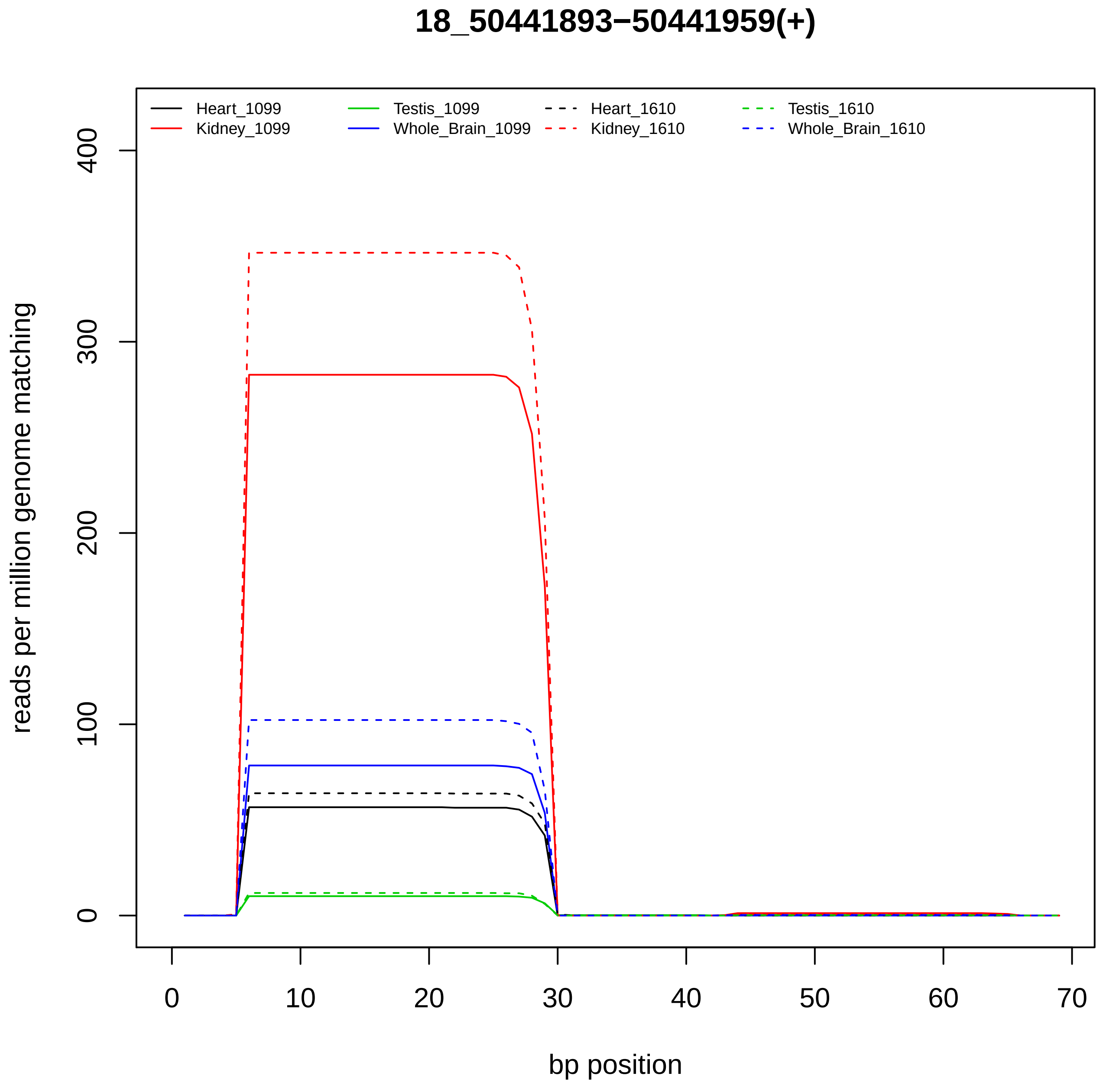

18_50441893−50441959(+)
Heart_1099
Kidney_1099
Testis_1099
Whole_Brain_1099
Heart_1610
Kidney_1610
Testis_1610
Whole_Brain_1610
reads per million genome matching
0
100
200
300
400
0
10
20
30
40
50
60
70
bp position

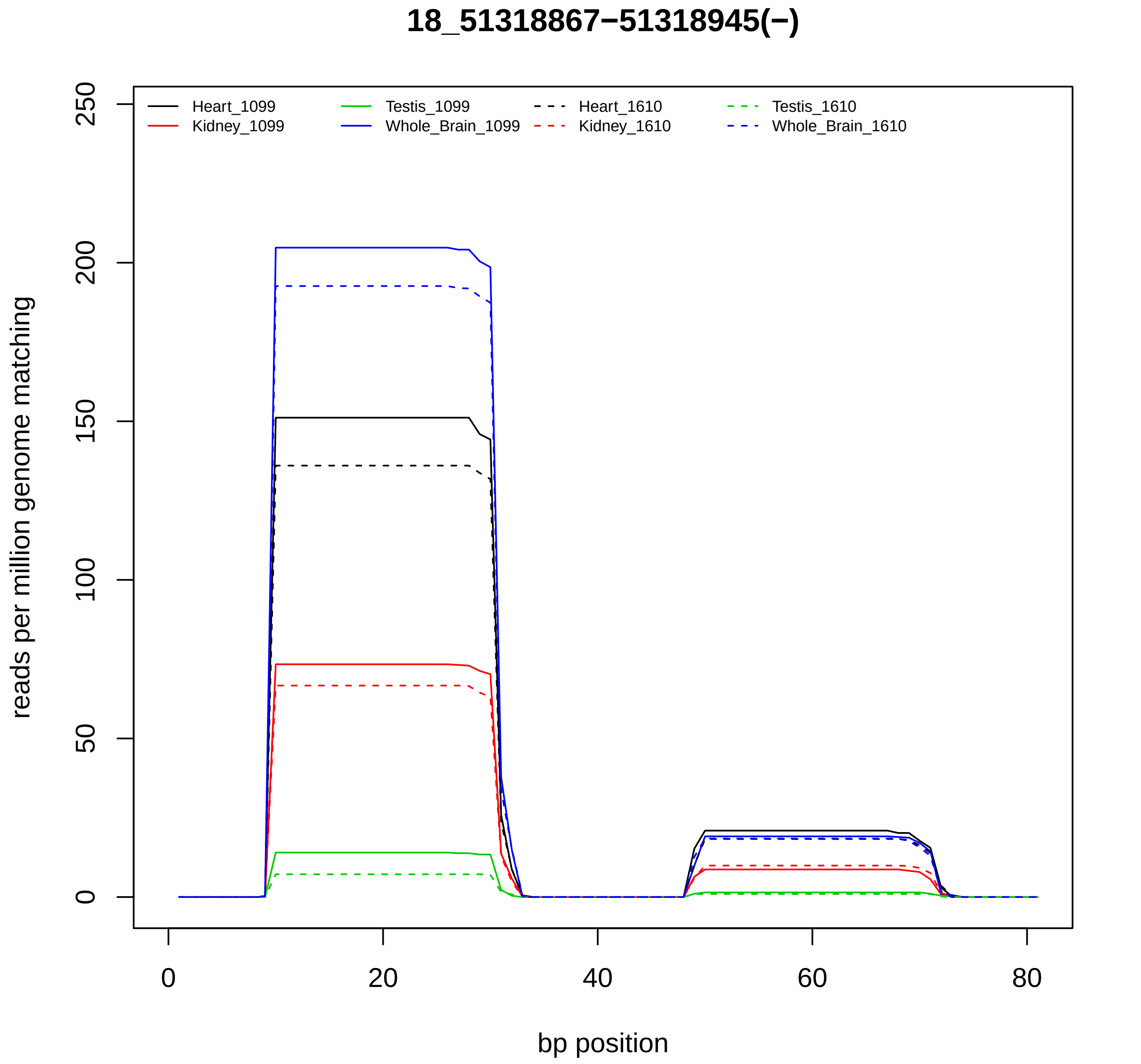

18_51318867−51318945(−)
Heart_1099
Kidney_1099
Testis_1099
Whole_Brain_1099
Heart_1610
Kidney_1610
Testis_1610
Whole_Brain_1610
reads per million genome matching
0
50
100
150
200
250
0
20
40
60
80
bp position

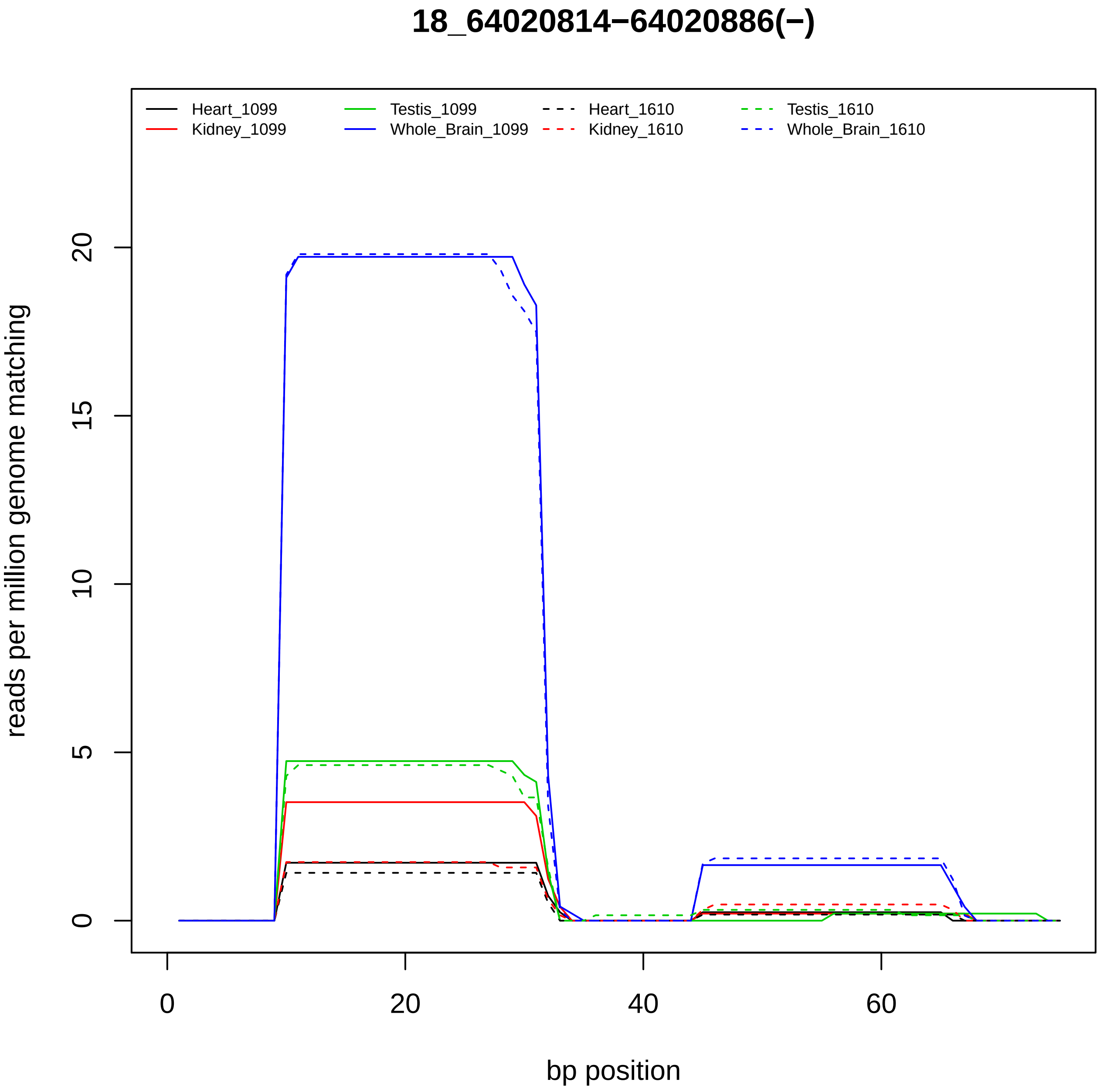

18_64020814−64020886(−)
Heart_1099
Kidney_1099
Testis_1099
Whole_Brain_1099
Heart_1610
Kidney_1610
Testis_1610
Whole_Brain_1610
reads per million genome matching
0
5
10
15
20
0
20
40
60
bp position

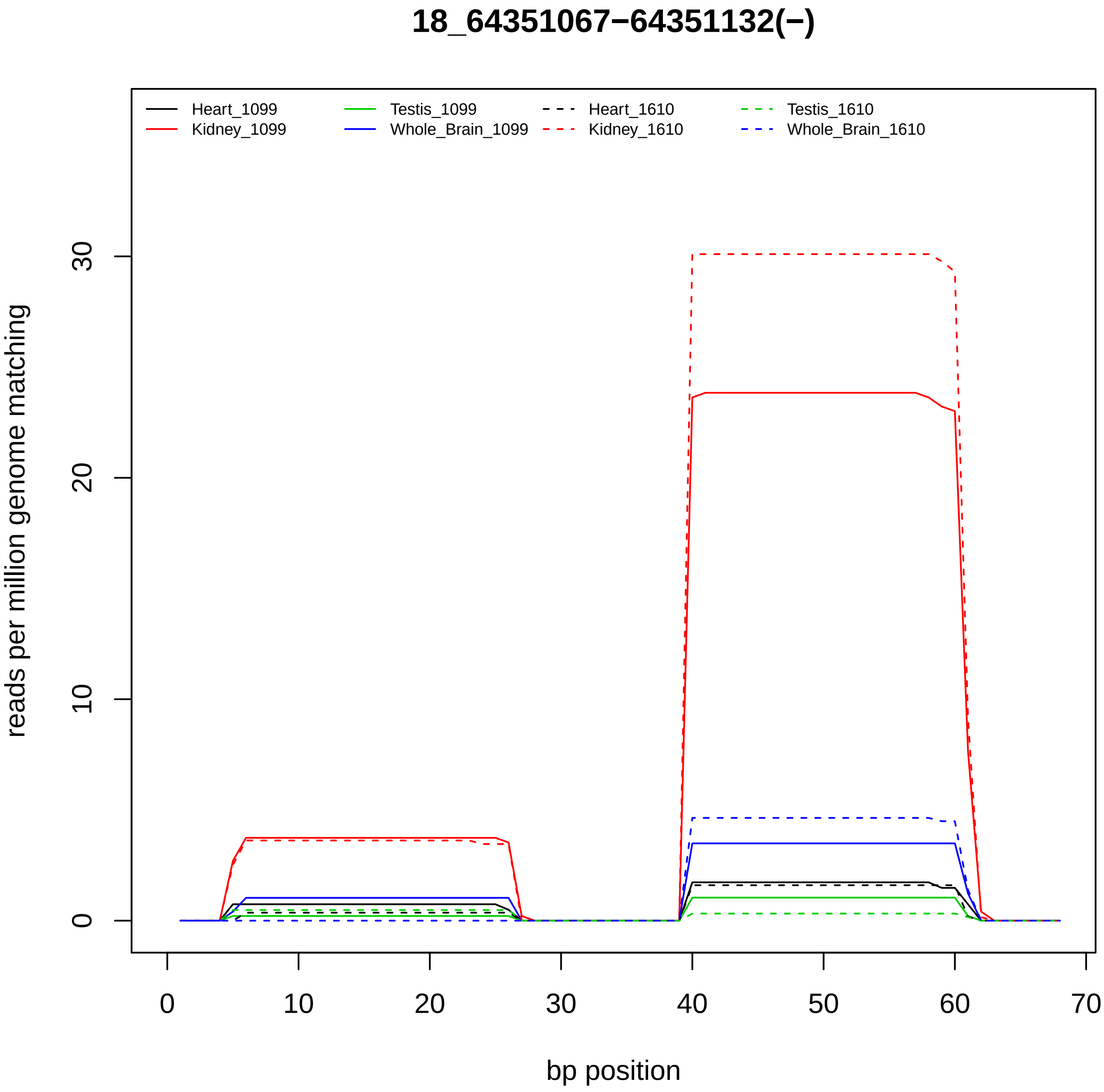
18_64351067−64351132(−)
Heart_1099
Kidney_1099
Testis_1099
Whole_Brain_1099
Heart_1610
Kidney_1610
Testis_1610
Whole_Brain_1610
reads per million genome matching
bp position
0
10
20
30
0
10
20
30
40
50
60
70

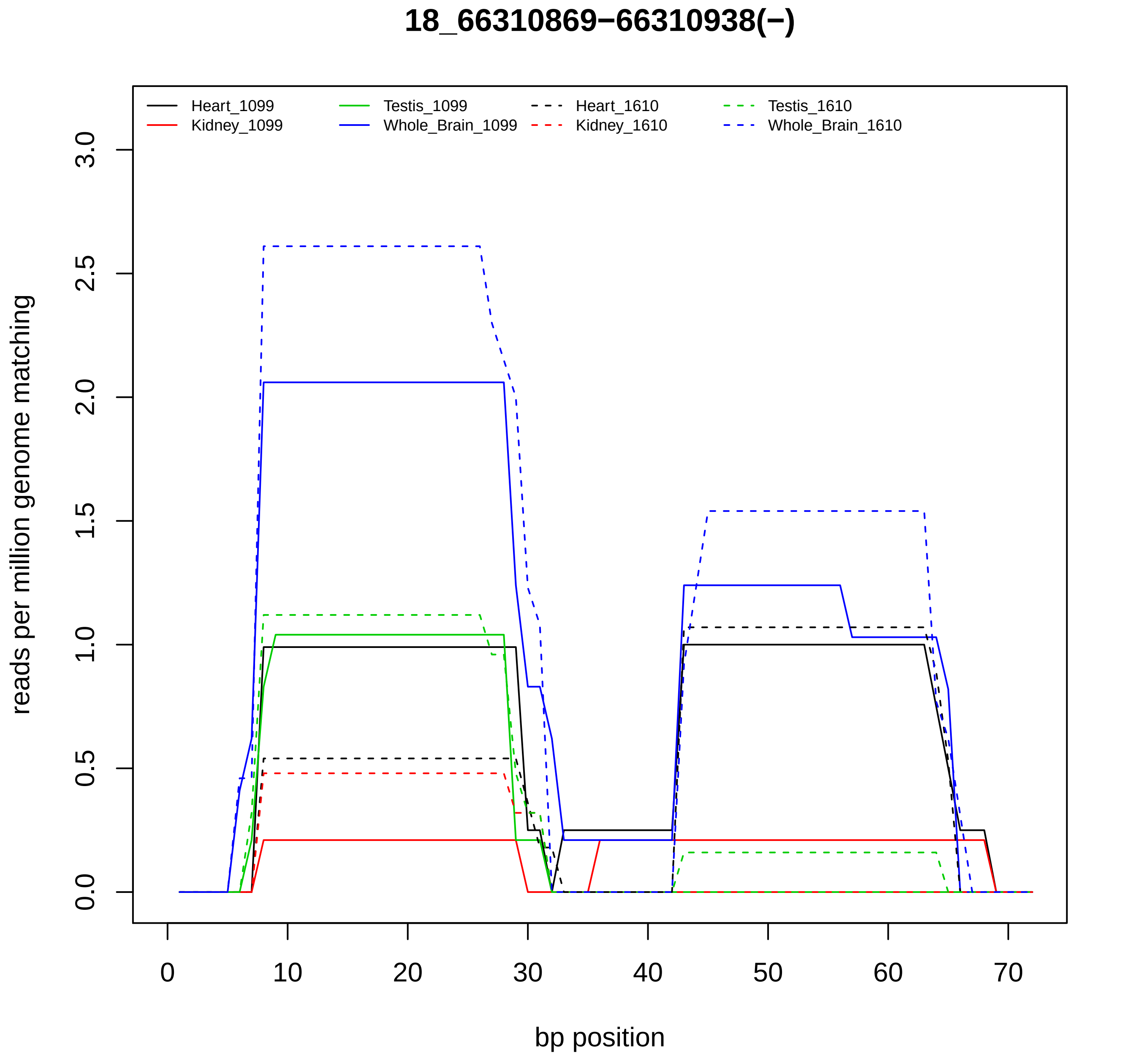
18_66310869−66310938(−)
Heart_1099
Kidney_1099
Testis_1099
Whole_Brain_1099
Heart_1610
Kidney_1610
Testis_1610
Whole_Brain_1610
reads per million genome matching
0.0
0.5
1.0
1.5
2.0
2.5
3.0
0
10
20
30
40
50
60
70
bp position

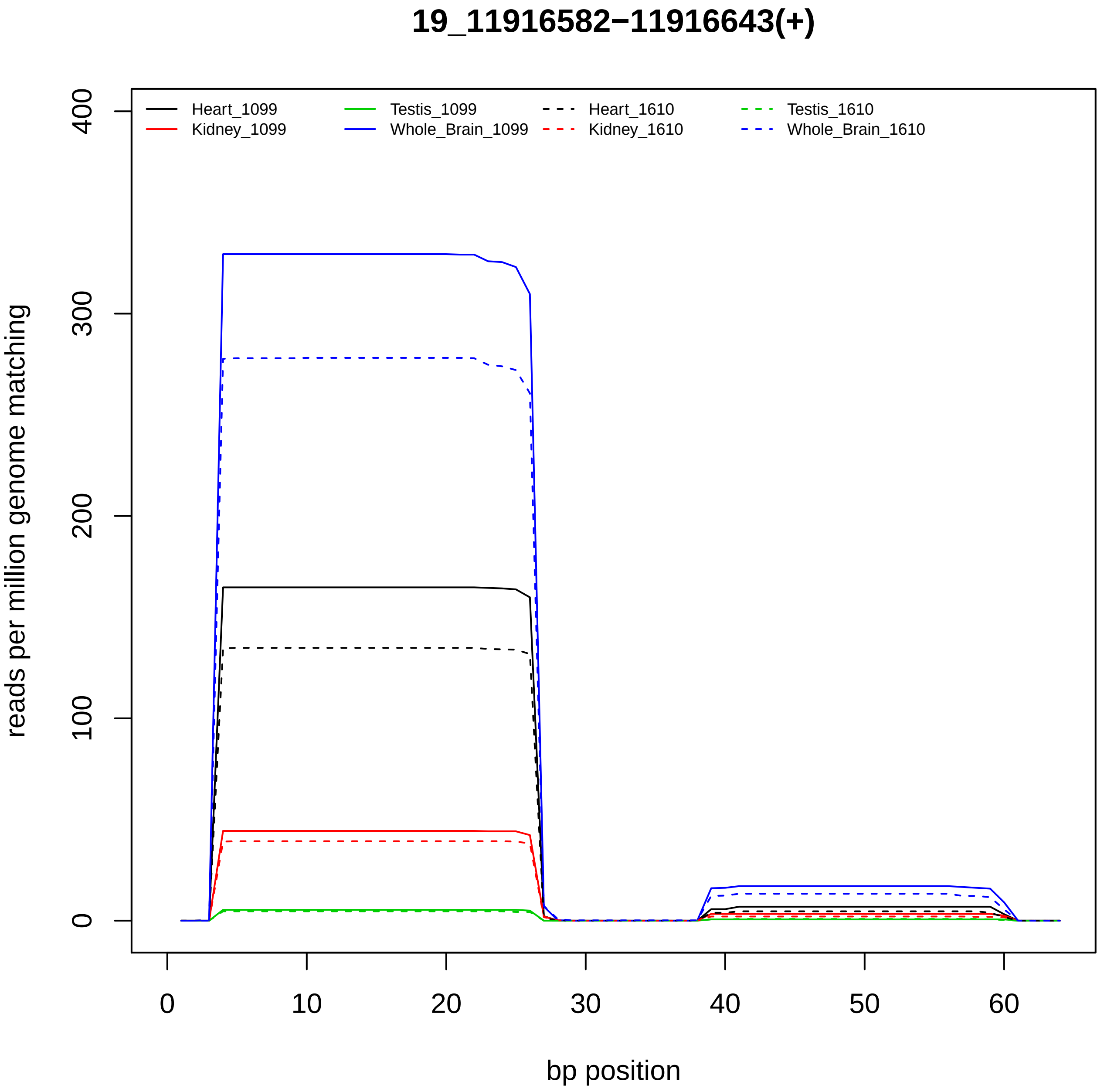

19_11916582−11916643(+)
Heart_1099
Kidney_1099
Testis_1099
Whole_Brain_1099
Heart_1610
Kidney_1610
Testis_1610
Whole_Brain_1610
reads per million genome matching
0
100
200
300
400
0
10
20
30
40
50
60
bp position

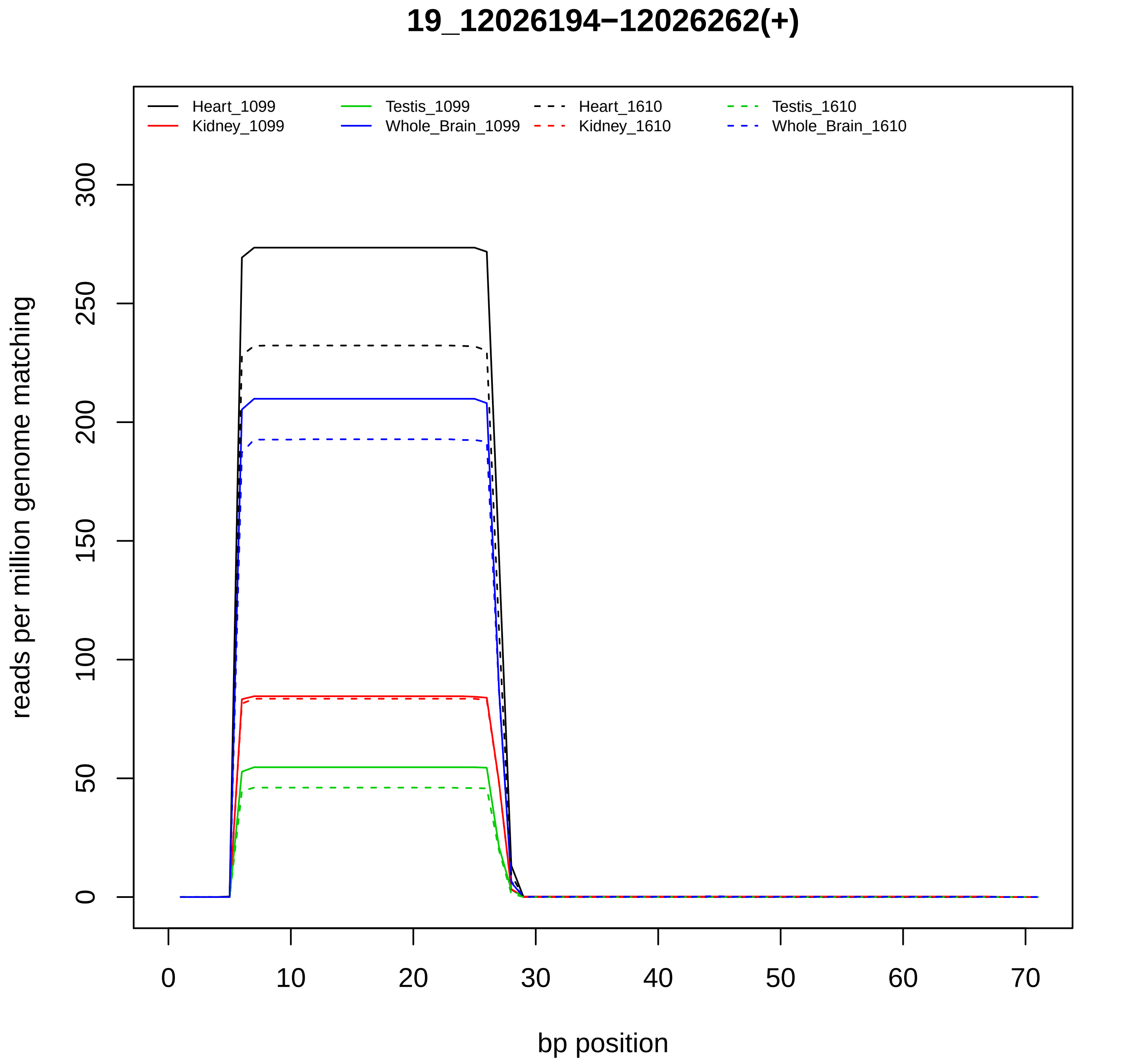

19_12026194−12026262(+)
Heart_1099
Kidney_1099
Testis_1099
Whole_Brain_1099
Heart_1610
Kidney_1610
Testis_1610
Whole_Brain_1610
reads per million genome matching
bp position
0
50
100
150
200
250
300
0
10
20
30
40
50
60
70

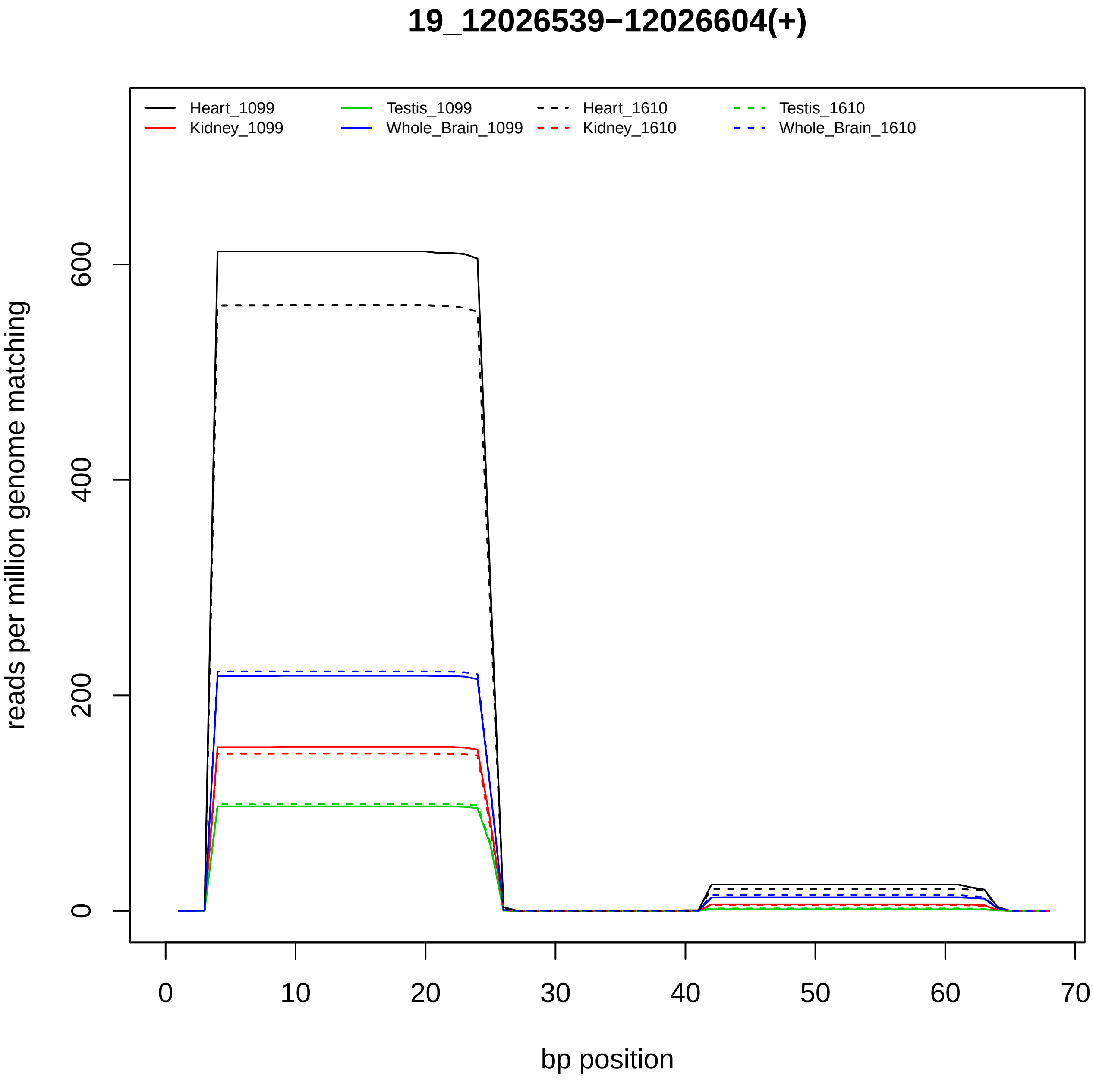

19_12026539−12026604(+)
Heart_1099
Kidney_1099
Testis_1099
Whole_Brain_1099
Heart_1610
Kidney_1610
Testis_1610
Whole_Brain_1610
reads per million genome matching
0
200
400
600
bp position
0
10
20
30
40
50
60
70

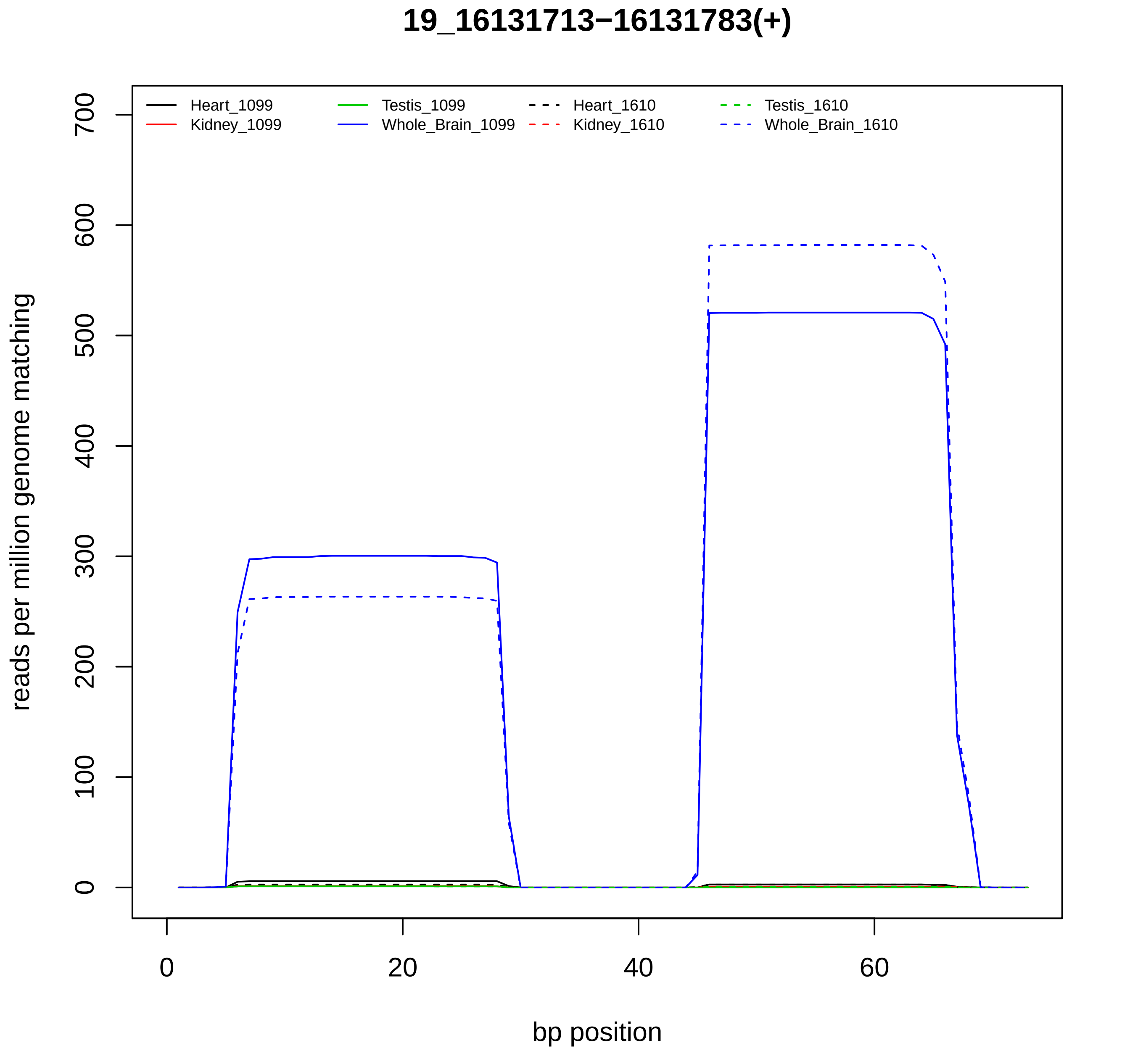
19_16131713−16131783(+)
Heart_1099
Kidney_1099
Testis_1099
Whole_Brain_1099
Heart_1610
Kidney_1610
Testis_1610
Whole_Brain_1610
reads per million genome matching
0
100
200
300
400
500
600
700
0
20
40
60
bp position

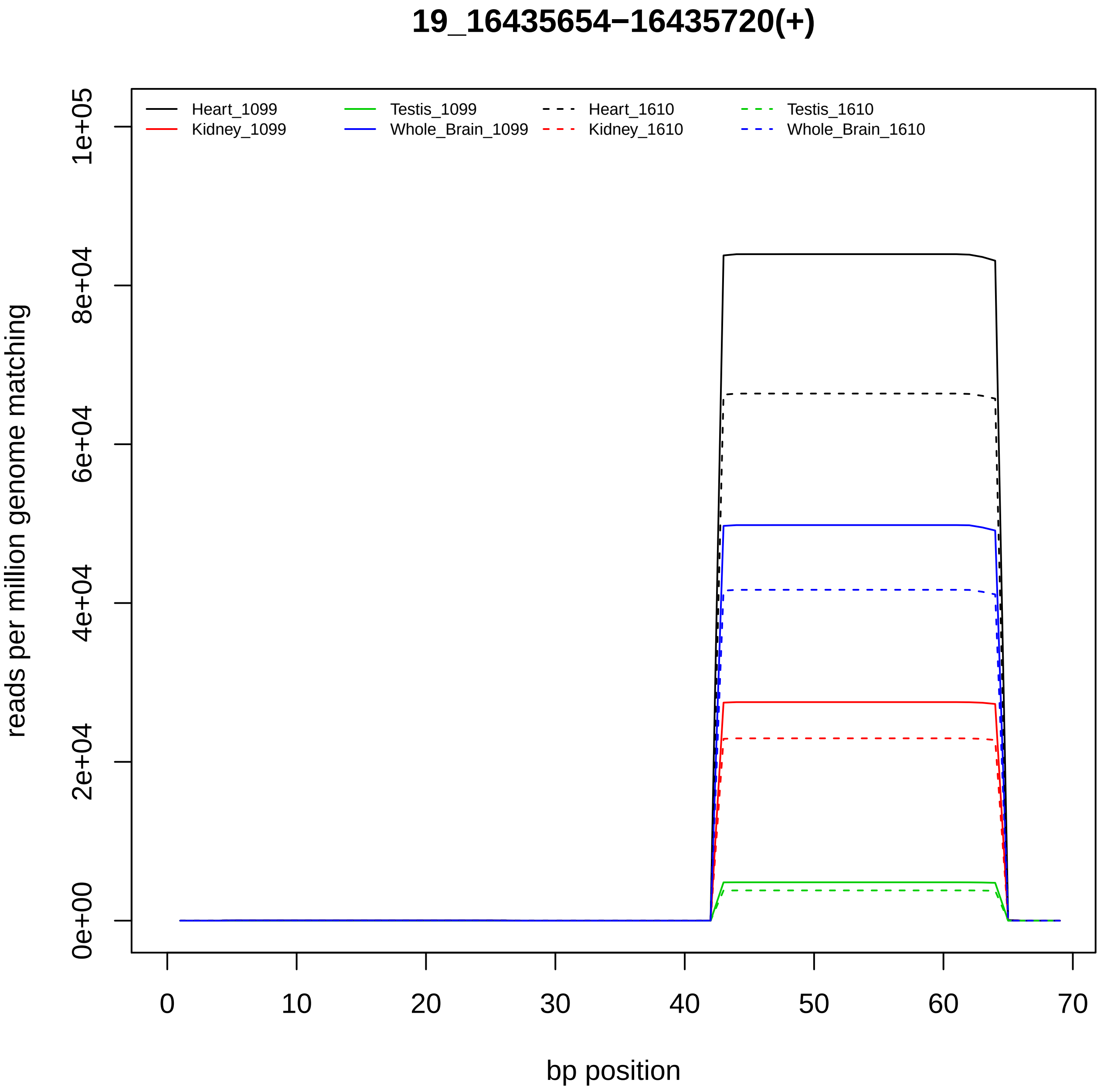

19_16435654−16435720(+)
Heart_1099
Kidney_1099
Testis_1099
Whole_Brain_1099
Heart_1610
Kidney_1610
Testis_1610
Whole_Brain_1610
reads per million genome matching
0e+00
2e+04
4e+04
6e+04
8e+04
1e+05
0
10
20
30
40
50
60
70
bp position

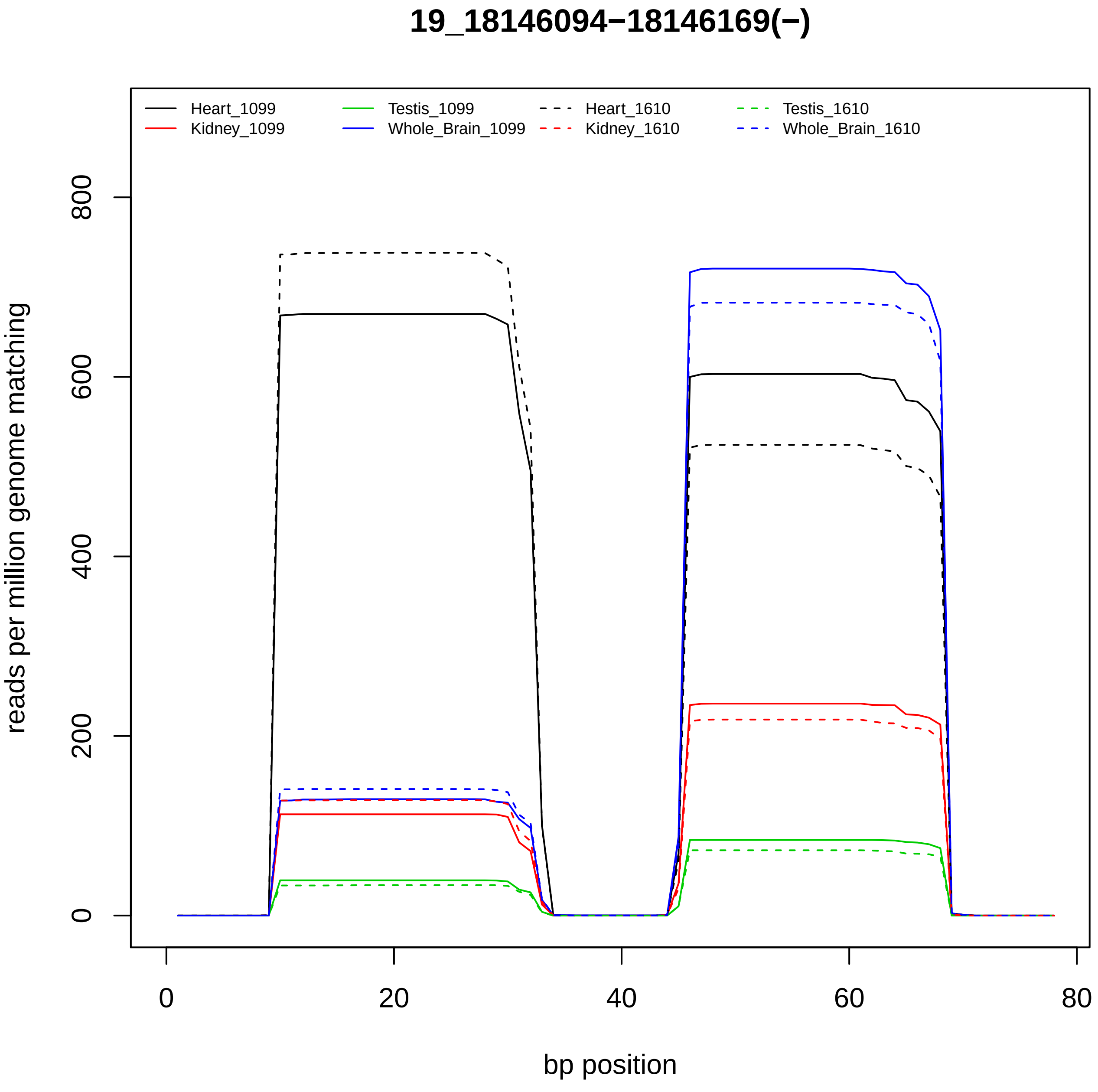
19_18146094−18146169(−)
Heart_1099
Kidney_1099
Testis_1099
Whole_Brain_1099
Heart_1610
Kidney_1610
Testis_1610
Whole_Brain_1610
reads per million genome matching
0
200
400
600
800
bp position
0
20
40
60
80

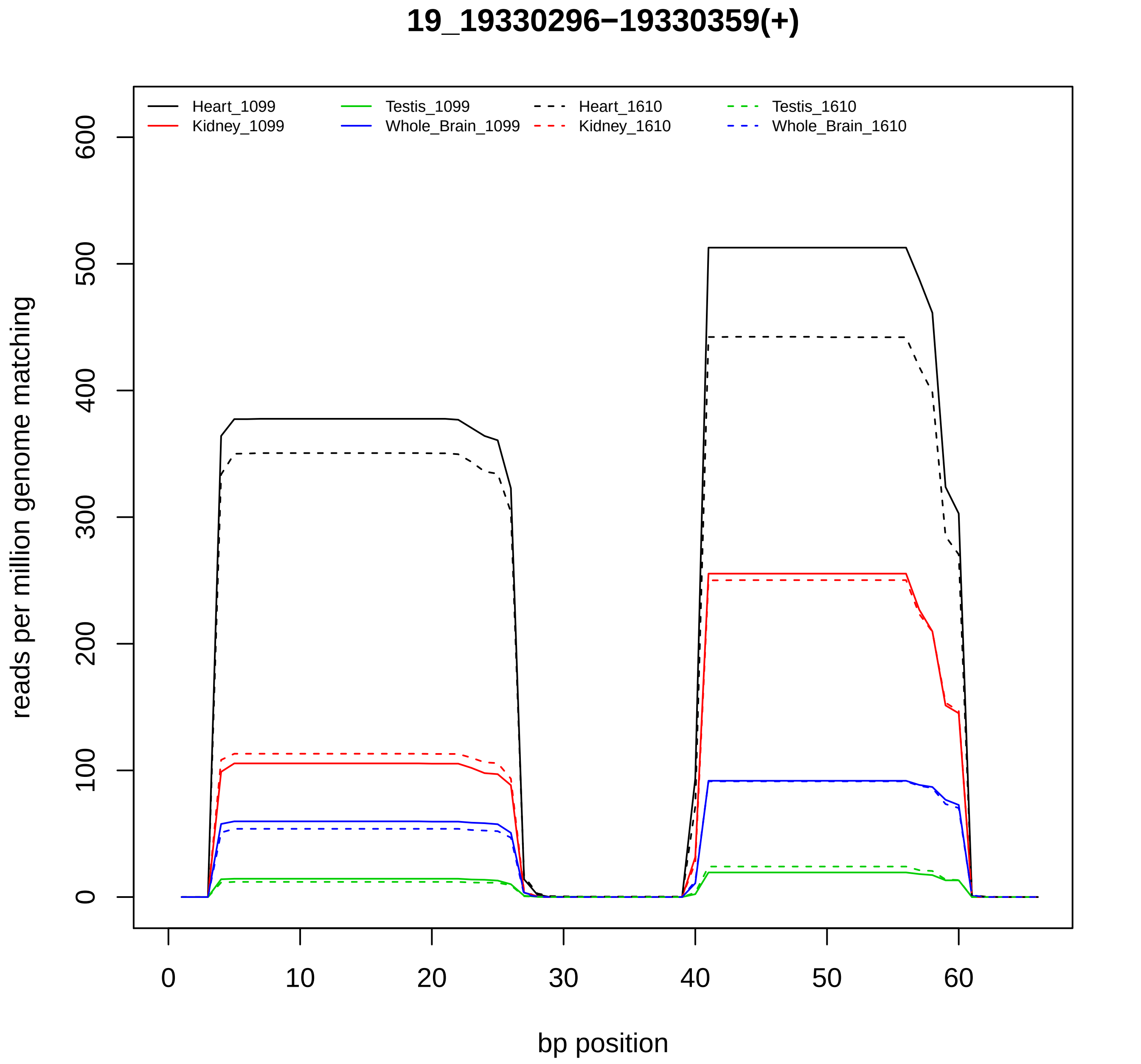

19_19330296−19330359(+)
Heart_1099
Kidney_1099
Testis_1099
Whole_Brain_1099
Heart_1610
Kidney_1610
Testis_1610
Whole_Brain_1610
reads per million genome matching
0
100
200
300
400
500
600
0
10
20
30
40
50
60
bp position

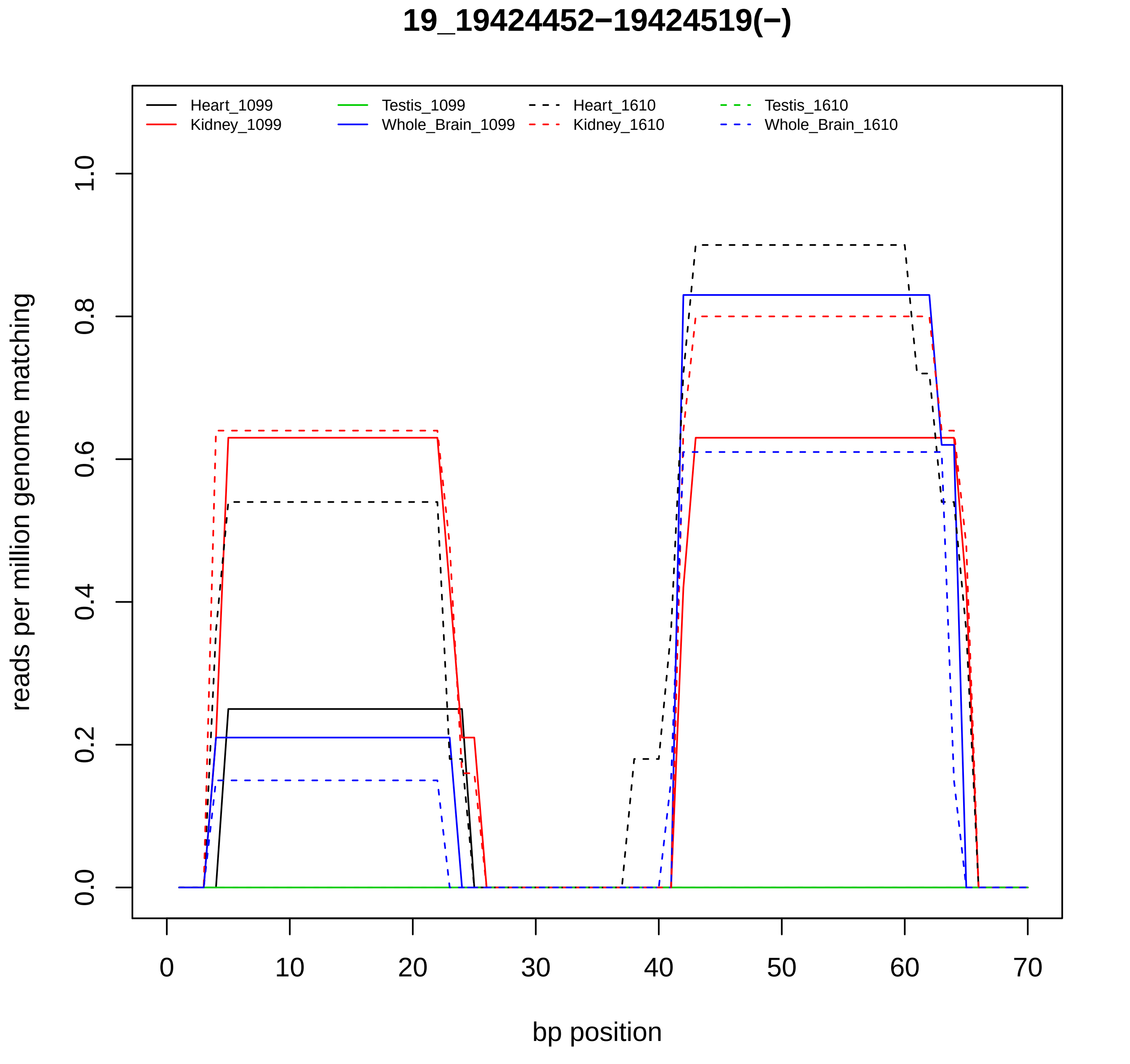

19_19424452−19424519(−)
Heart_1099
Kidney_1099
Testis_1099
Whole_Brain_1099
Heart_1610
Kidney_1610
Testis_1610
Whole_Brain_1610
reads per million genome matching
0.0
0.2
0.4
0.6
0.8
1.0
0
10
20
30
40
50
60
70
bp position

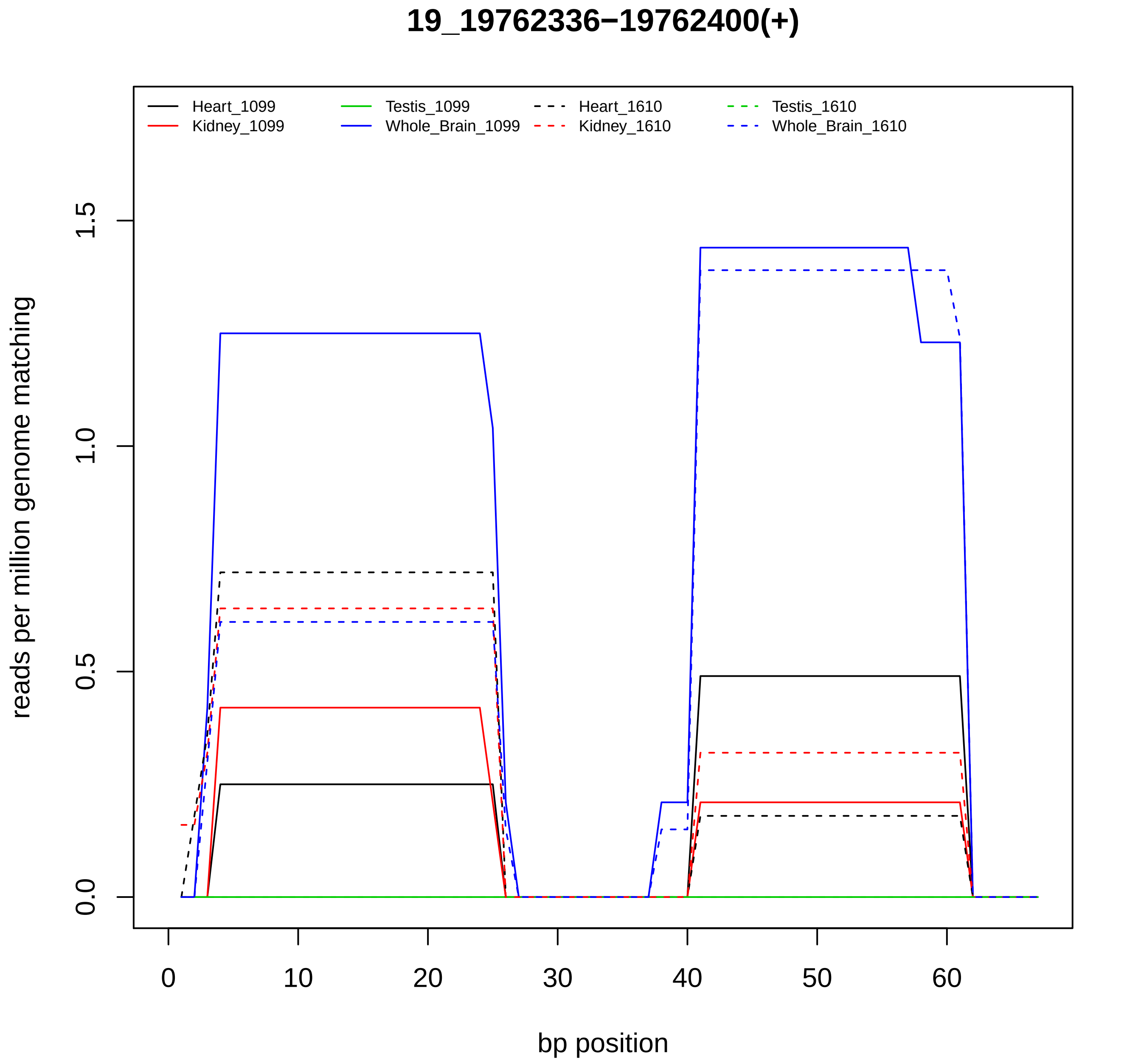
19_19762336−19762400(+)
Heart_1099
Kidney_1099
Testis_1099
Whole_Brain_1099
Heart_1610
Kidney_1610
Testis_1610
Whole_Brain_1610
reads per million genome matching
0.0
0.5
1.0
1.5
0
10
20
30
40
50
60
bp position

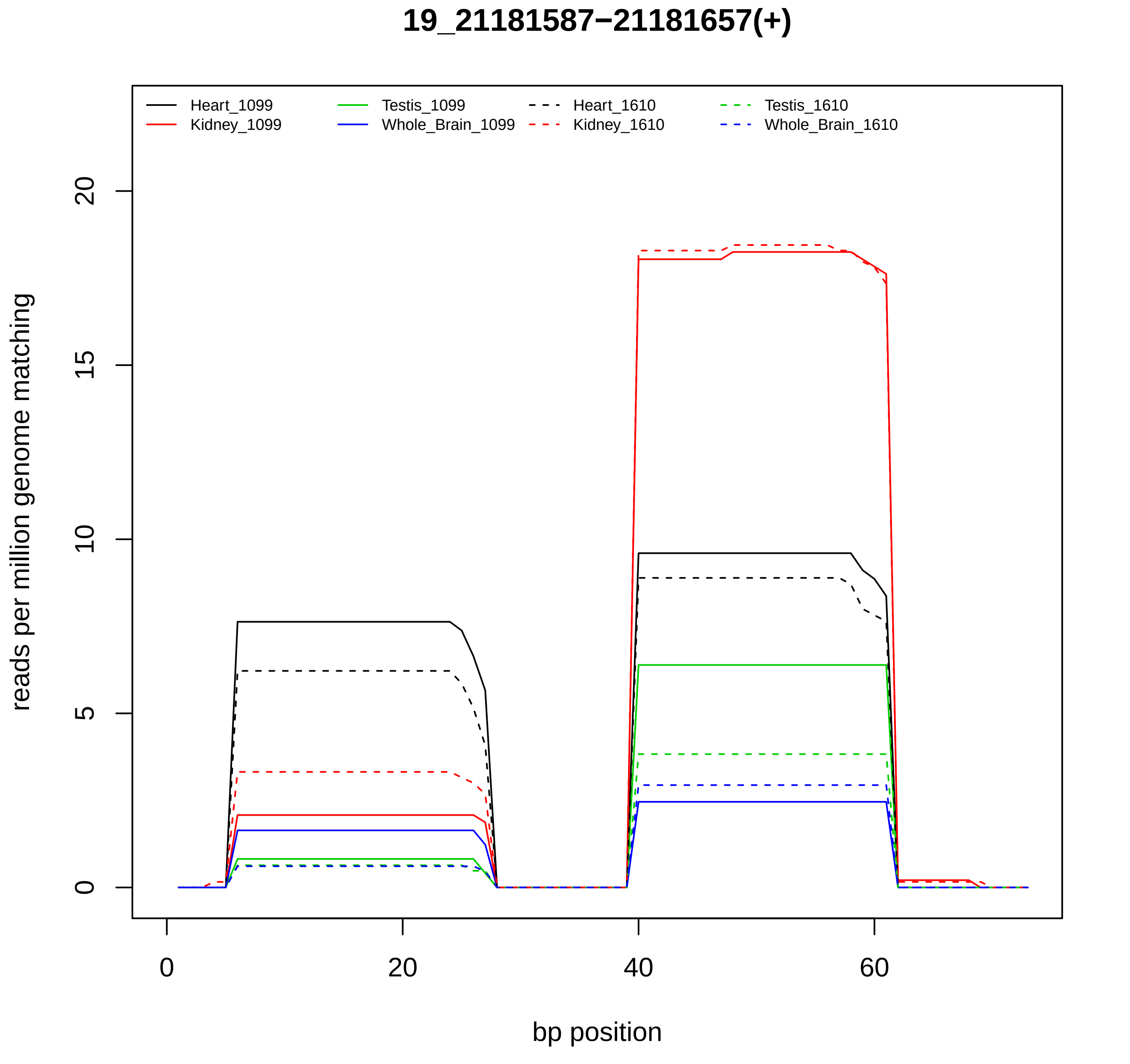

19_21181587−21181657(+)
Heart_1099
Kidney_1099
Testis_1099
Whole_Brain_1099
Heart_1610
Kidney_1610
Testis_1610
Whole_Brain_1610
reads per million genome matching
bp position
0
5
10
15
20
0
20
40
60

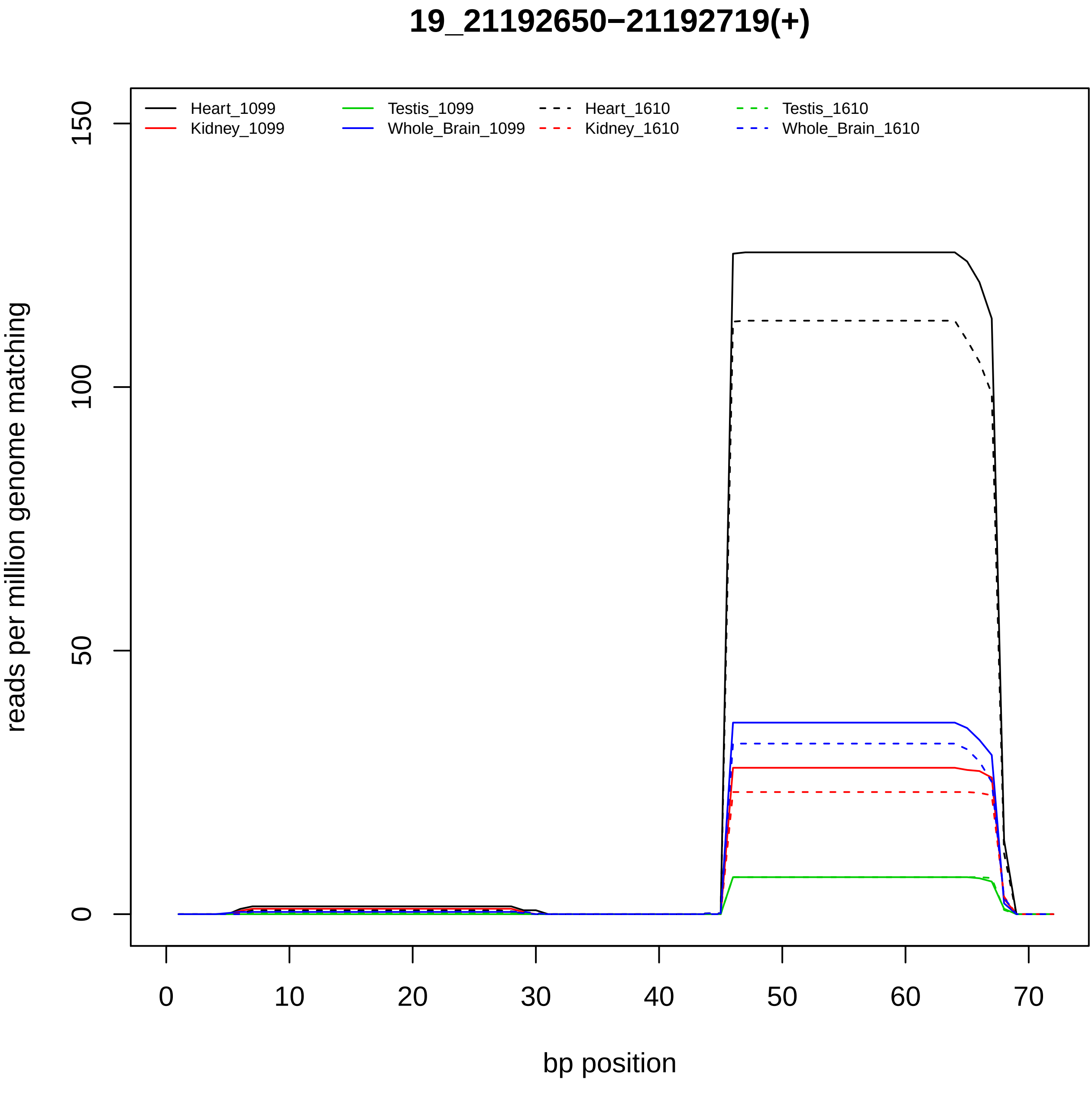

19_21192650−21192719(+)
Heart_1099
Kidney_1099
Testis_1099
Whole_Brain_1099
Heart_1610
Kidney_1610
Testis_1610
Whole_Brain_1610
reads per million genome matching
0
50
100
150
bp position
0
10
20
30
40
50
60
70

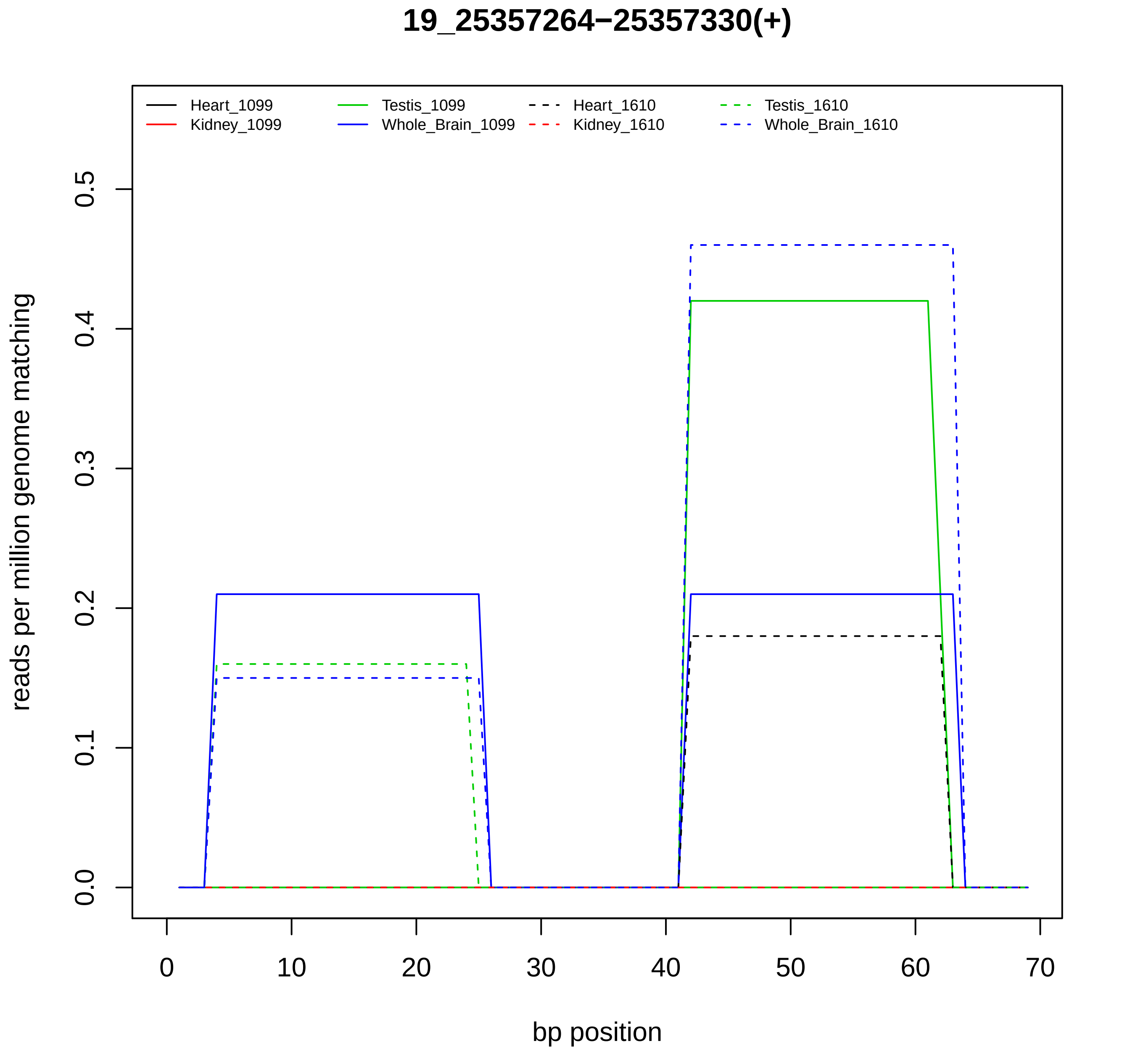
19_25357264−25357330(+)
Heart_1099
Kidney_1099
Testis_1099
Whole_Brain_1099
Heart_1610
Kidney_1610
Testis_1610
Whole_Brain_1610
reads per million genome matching
0.0
0.1
0.2
0.3
0.4
0.5
0
10
20
30
40
50
60
70
bp position

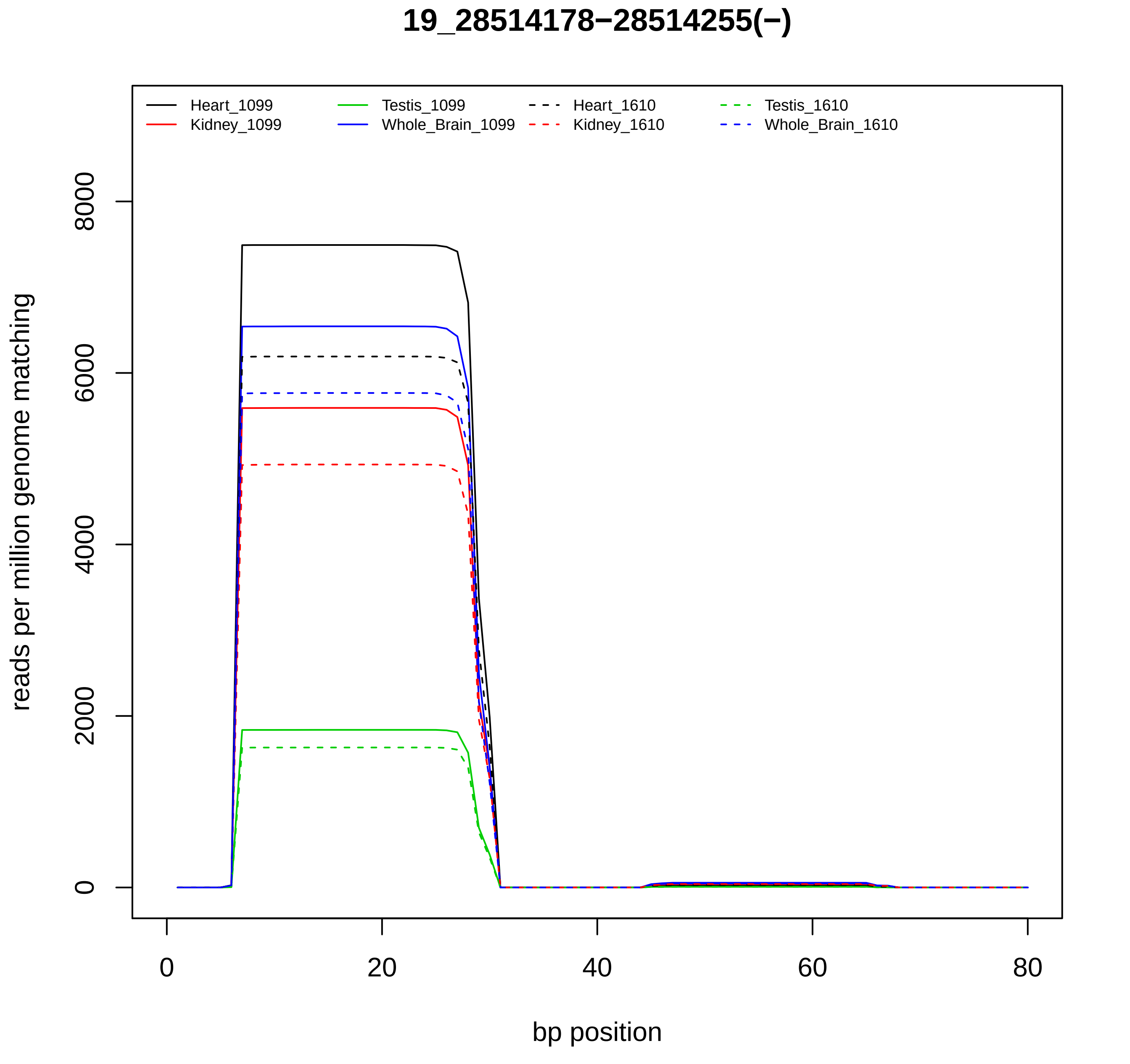

19_28514178−28514255(−)
Heart_1099
Kidney_1099
Testis_1099
Whole_Brain_1099
Heart_1610
Kidney_1610
Testis_1610
Whole_Brain_1610
reads per million genome matching
0
2000
4000
6000
8000
0
20
40
60
80
bp position

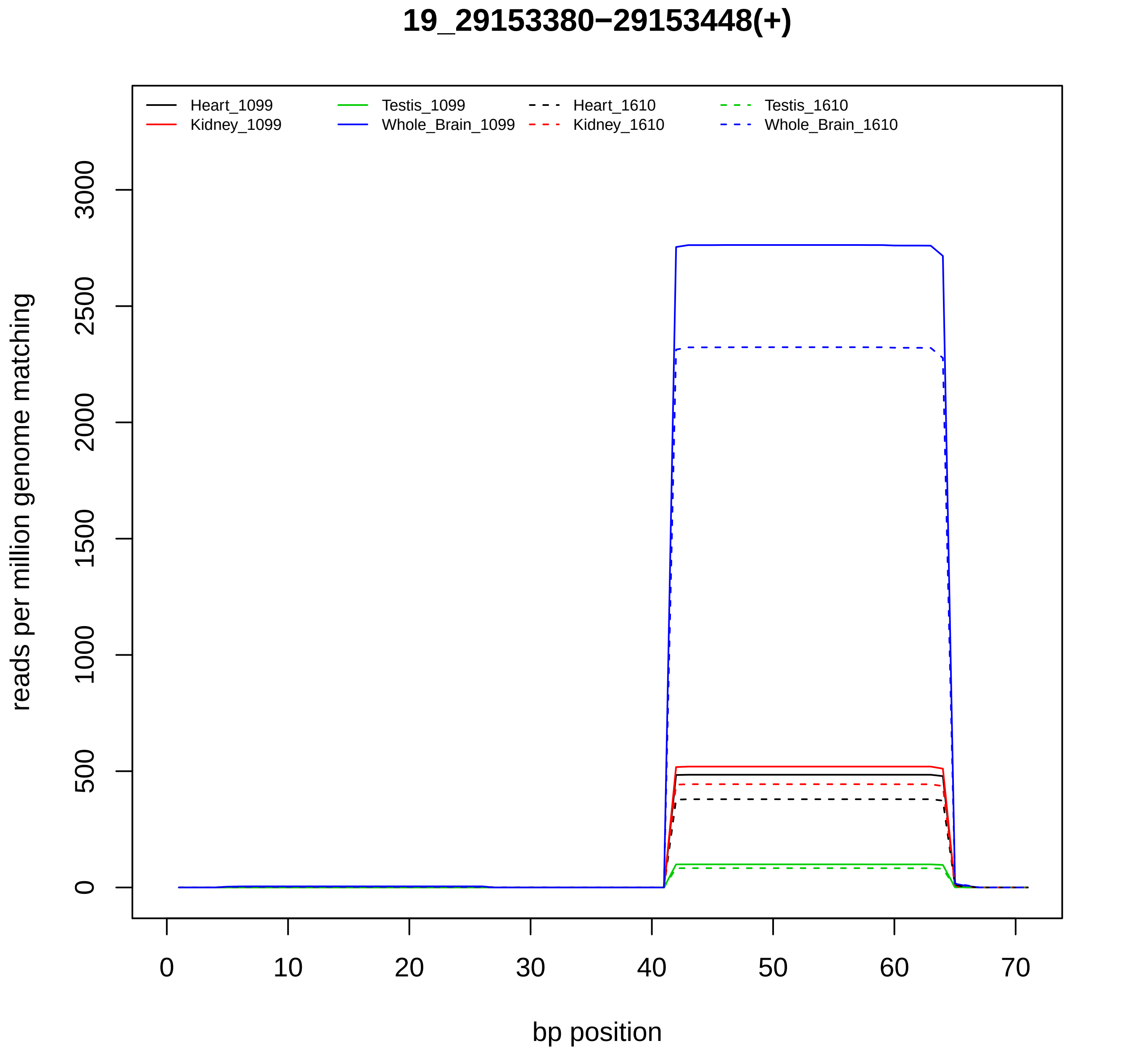

19_29153380−29153448(+)
Heart_1099
Kidney_1099
Testis_1099
Whole_Brain_1099
Heart_1610
Kidney_1610
Testis_1610
Whole_Brain_1610
reads per million genome matching
0
500
1000
1500
2000
2500
3000
0
10
20
30
40
50
60
70
bp position

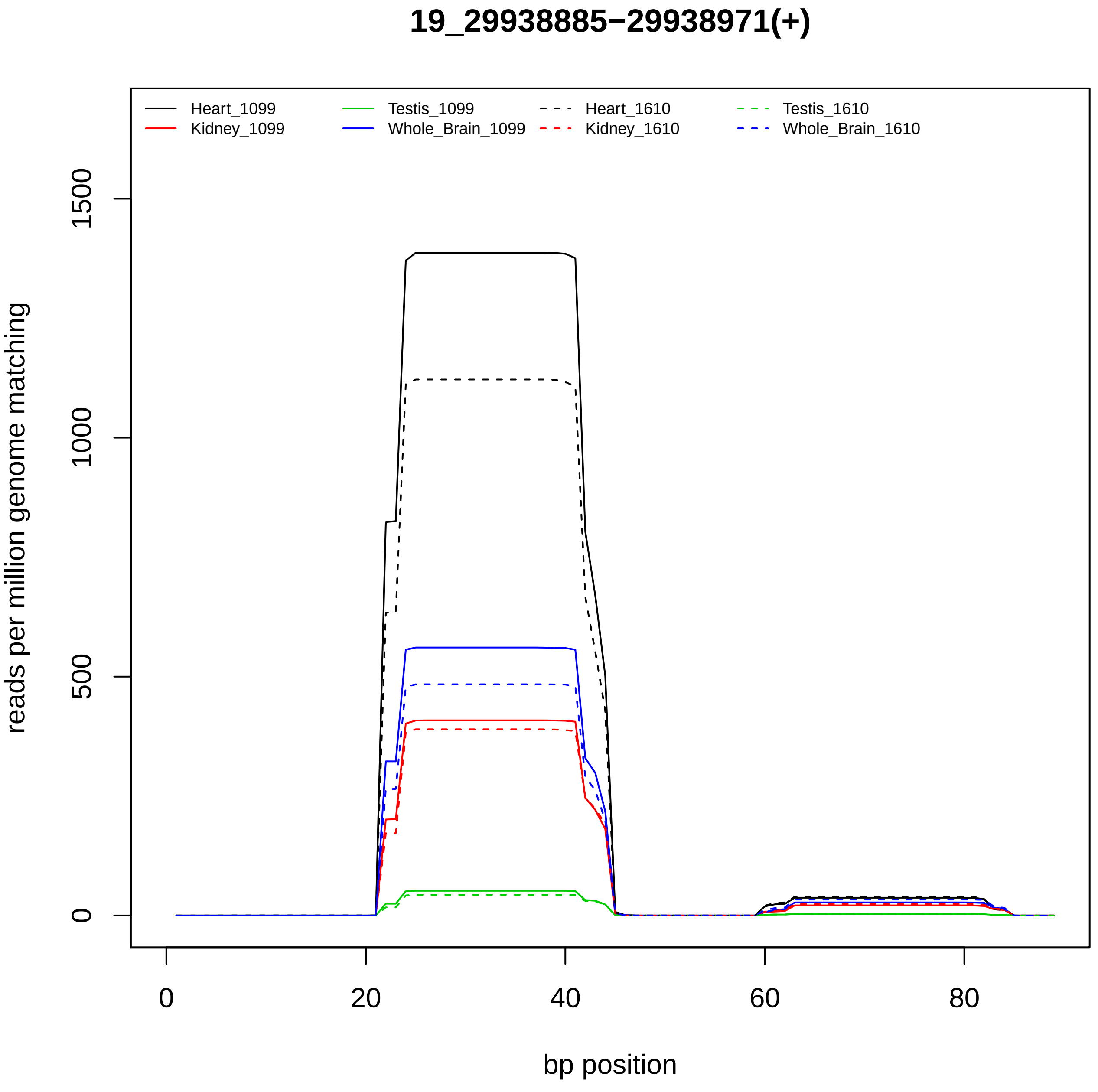

19_29938885−29938971(+)
Heart_1099
Kidney_1099
Testis_1099
Whole_Brain_1099
Heart_1610
Kidney_1610
Testis_1610
Whole_Brain_1610
reads per million genome matching
0
500
1000
1500
0
20
40
60
80
bp position

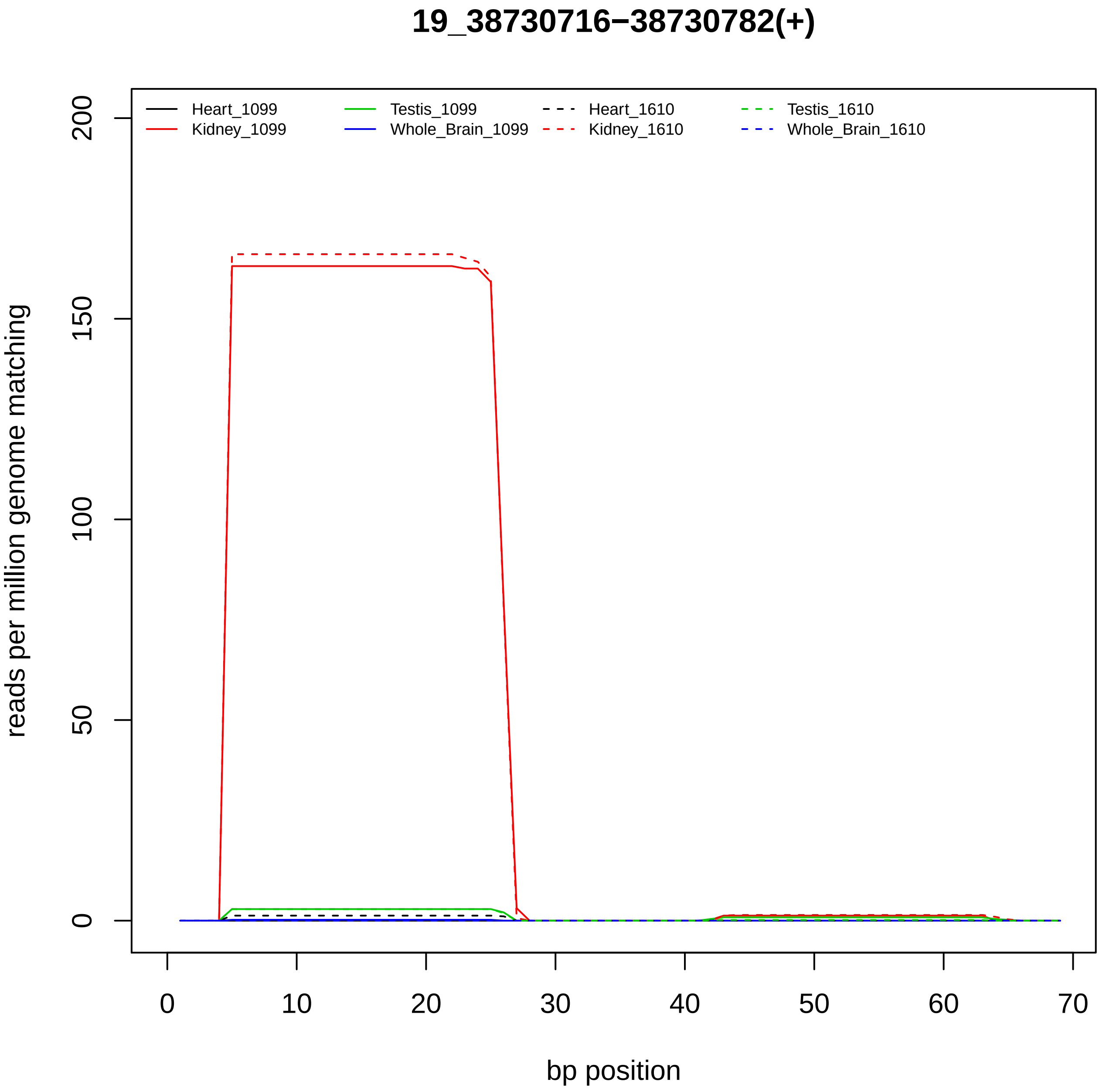

19_38730716−38730782(+)
Heart_1099
Kidney_1099
Testis_1099
Whole_Brain_1099
Heart_1610
Kidney_1610
Testis_1610
Whole_Brain_1610
reads per million genome matching
0
50
100
150
200
0
10
20
30
40
50
60
70
bp position

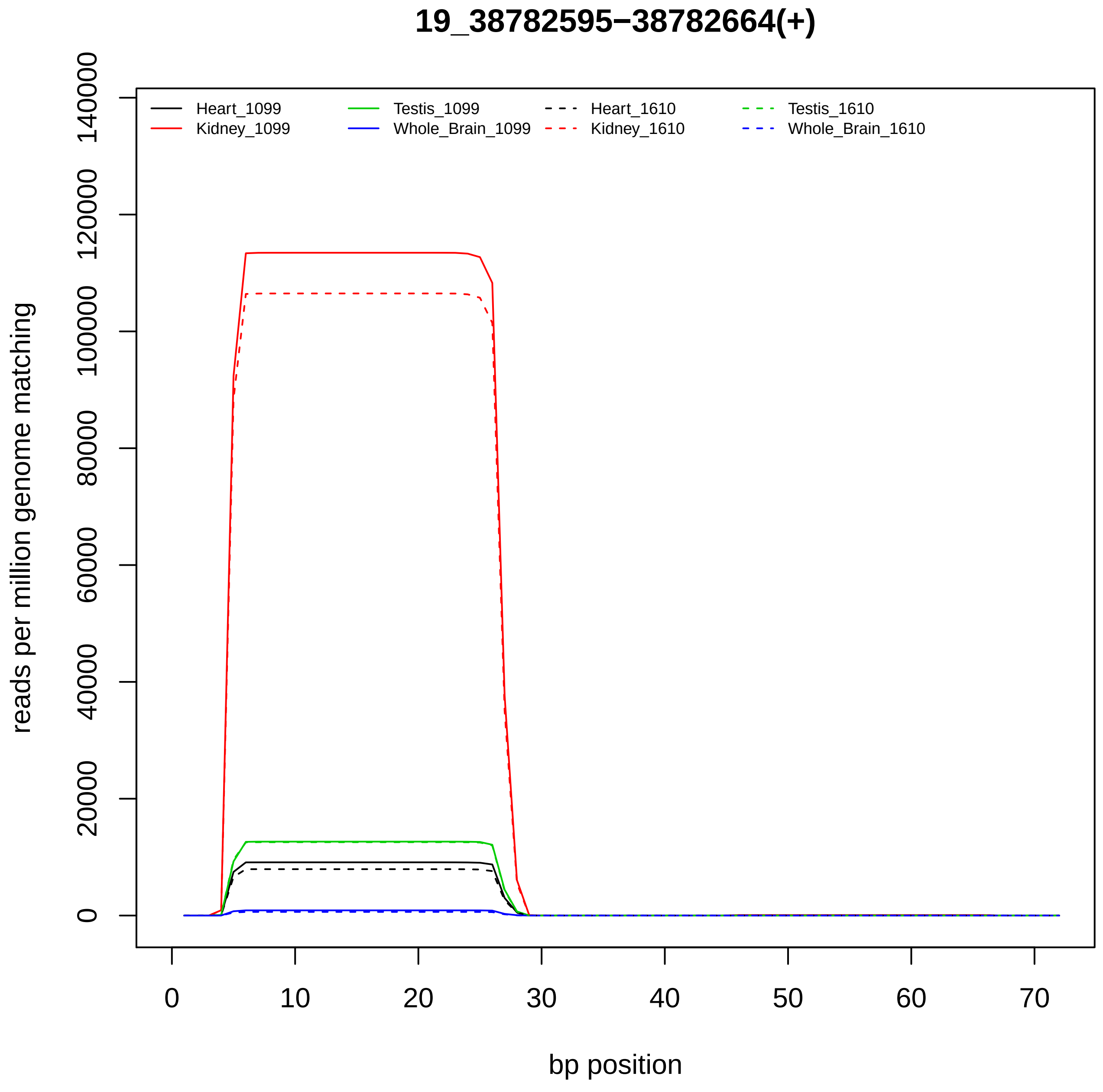

19_38782595−38782664(+)
Heart_1099
Kidney_1099
Testis_1099
Whole_Brain_1099
Heart_1610
Kidney_1610
Testis_1610
Whole_Brain_1610
reads per million genome matching
0
20000
40000
60000
80000
100000
120000
140000
0
10
20
30
40
50
60
70
bp position

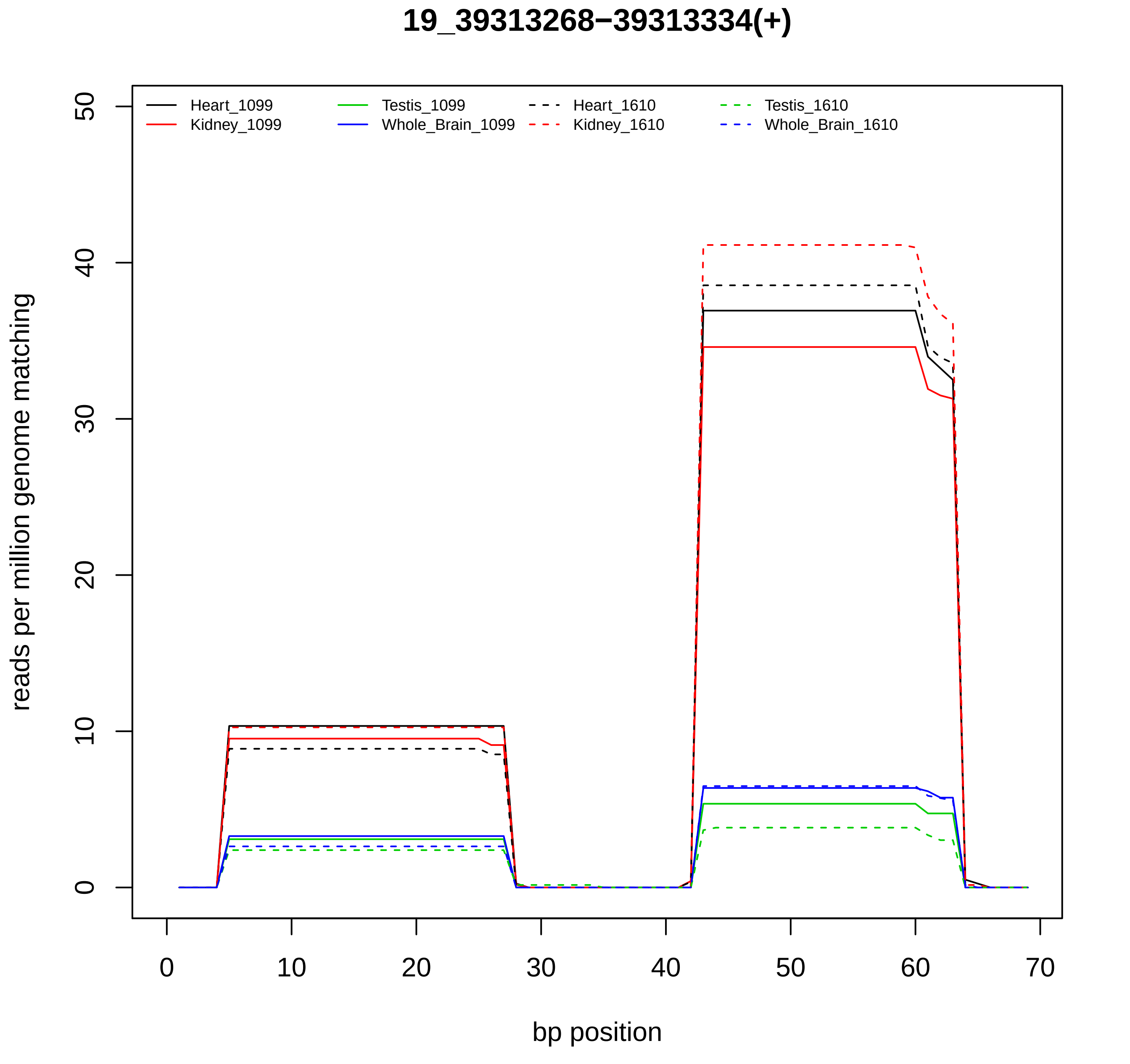

19_39313268−39313334(+)
Heart_1099
Kidney_1099
Testis_1099
Whole_Brain_1099
Heart_1610
Kidney_1610
Testis_1610
Whole_Brain_1610
reads per million genome matching
bp position
0
10
20
30
40
50
60
70

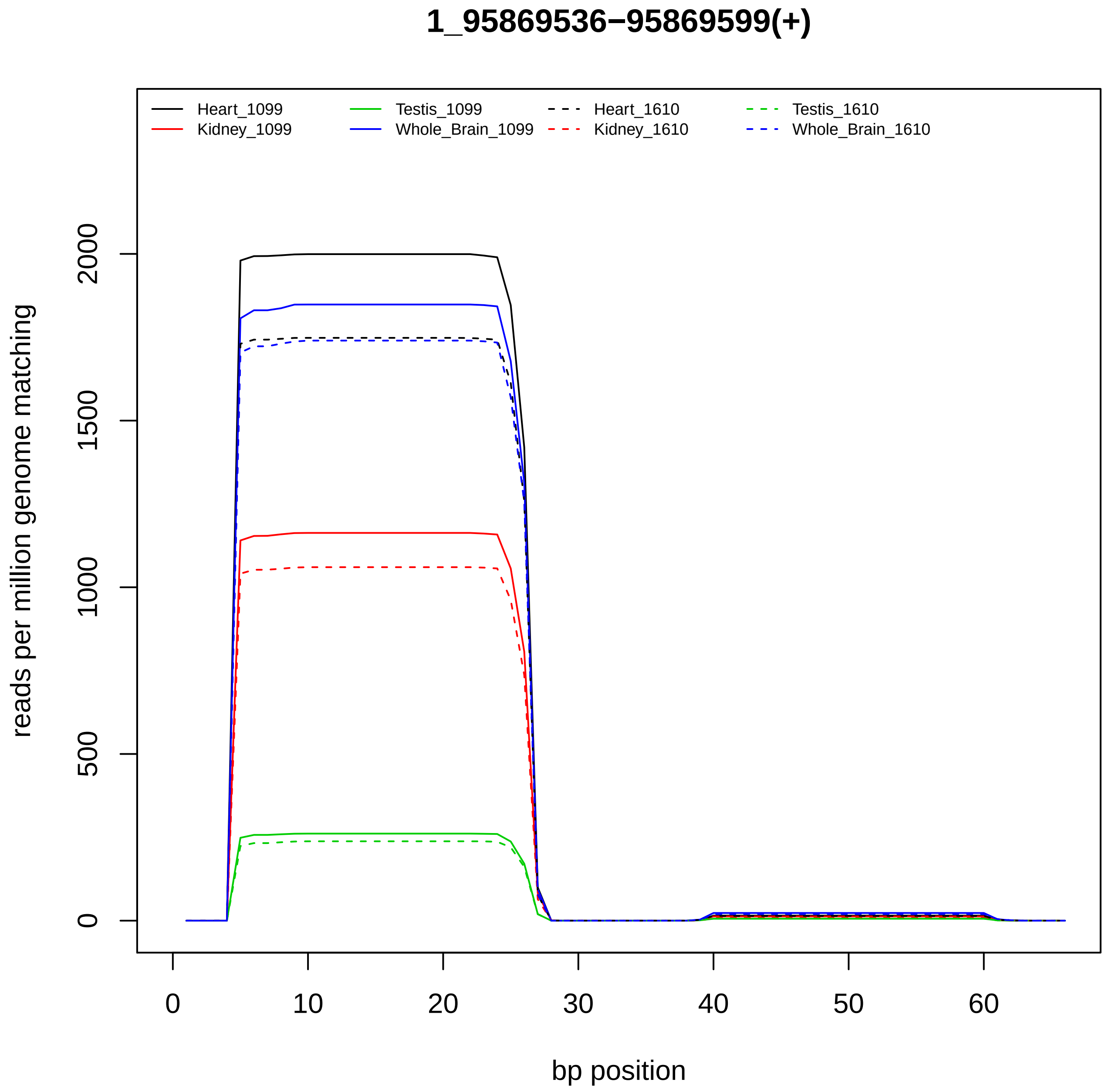

1_95869536−95869599(+)
Heart_1099
Kidney_1099
Testis_1099
Whole_Brain_1099
Heart_1610
Kidney_1610
Testis_1610
Whole_Brain_1610
reads per million genome matching
0
500
1000
1500
2000
0
10
20
30
40
50
60
bp position

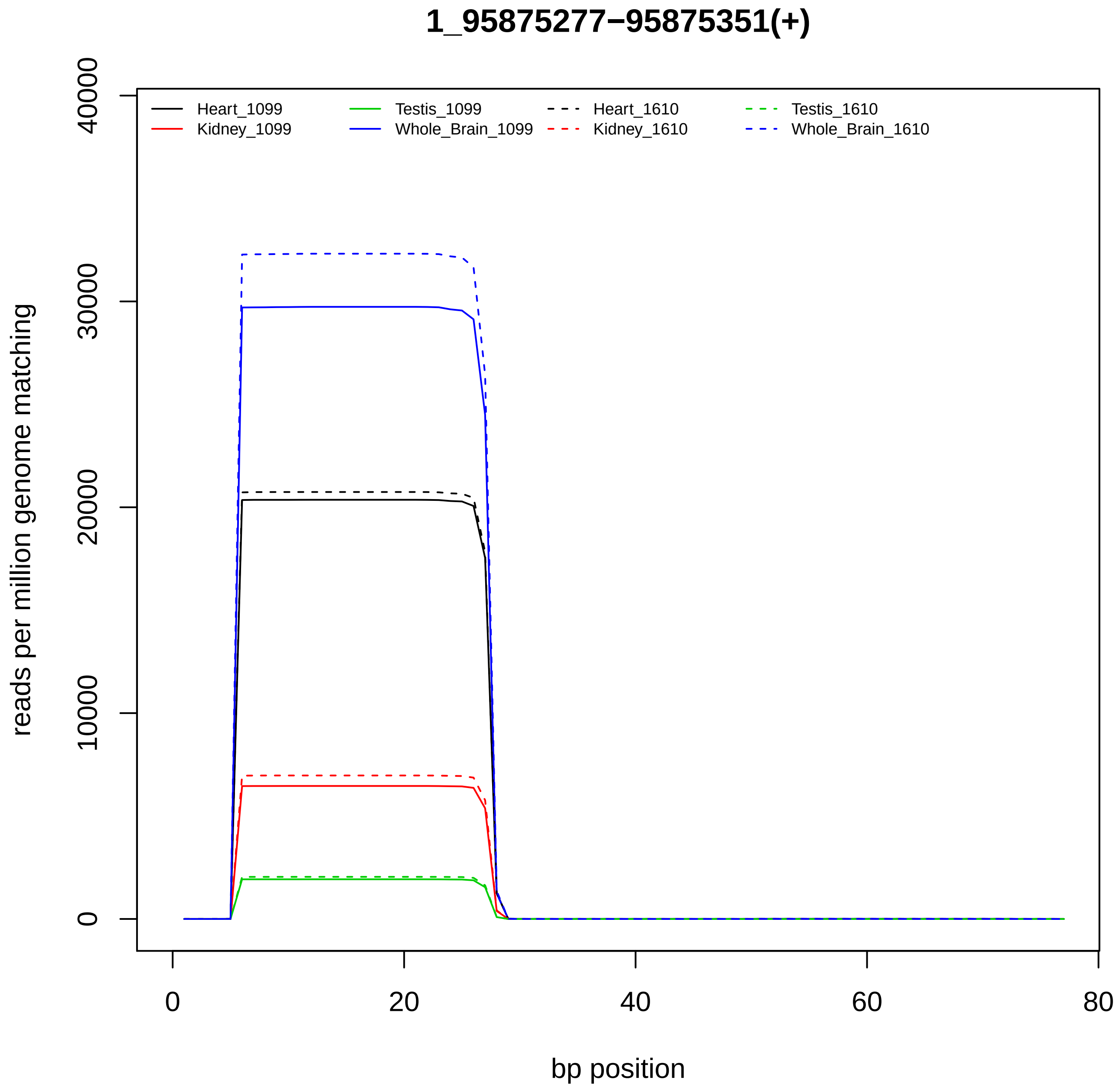

1_95875277−95875351(+)
Heart_1099
Kidney_1099
Testis_1099
Whole_Brain_1099
Heart_1610
Kidney_1610
Testis_1610
Whole_Brain_1610
reads per million genome matching
bp position
0
10000
20000
30000
40000
0
20
40
60
80

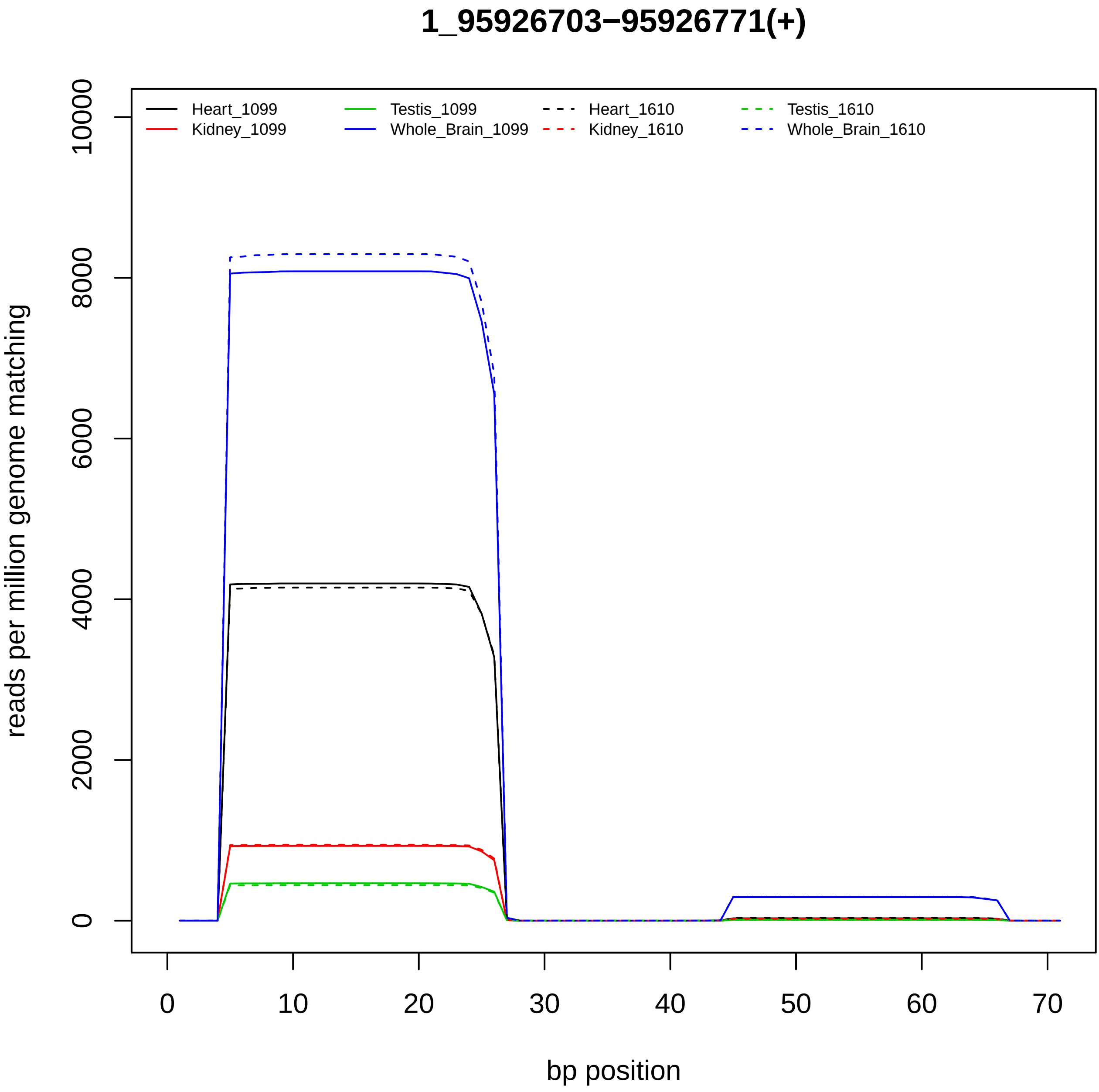

1_95926703−95926771(+)
Heart_1099
Kidney_1099
Testis_1099
Whole_Brain_1099
Heart_1610
Kidney_1610
Testis_1610
Whole_Brain_1610
reads per million genome matching
0
2000
4000
6000
8000
10000
0
10
20
30
40
50
60
70
bp position

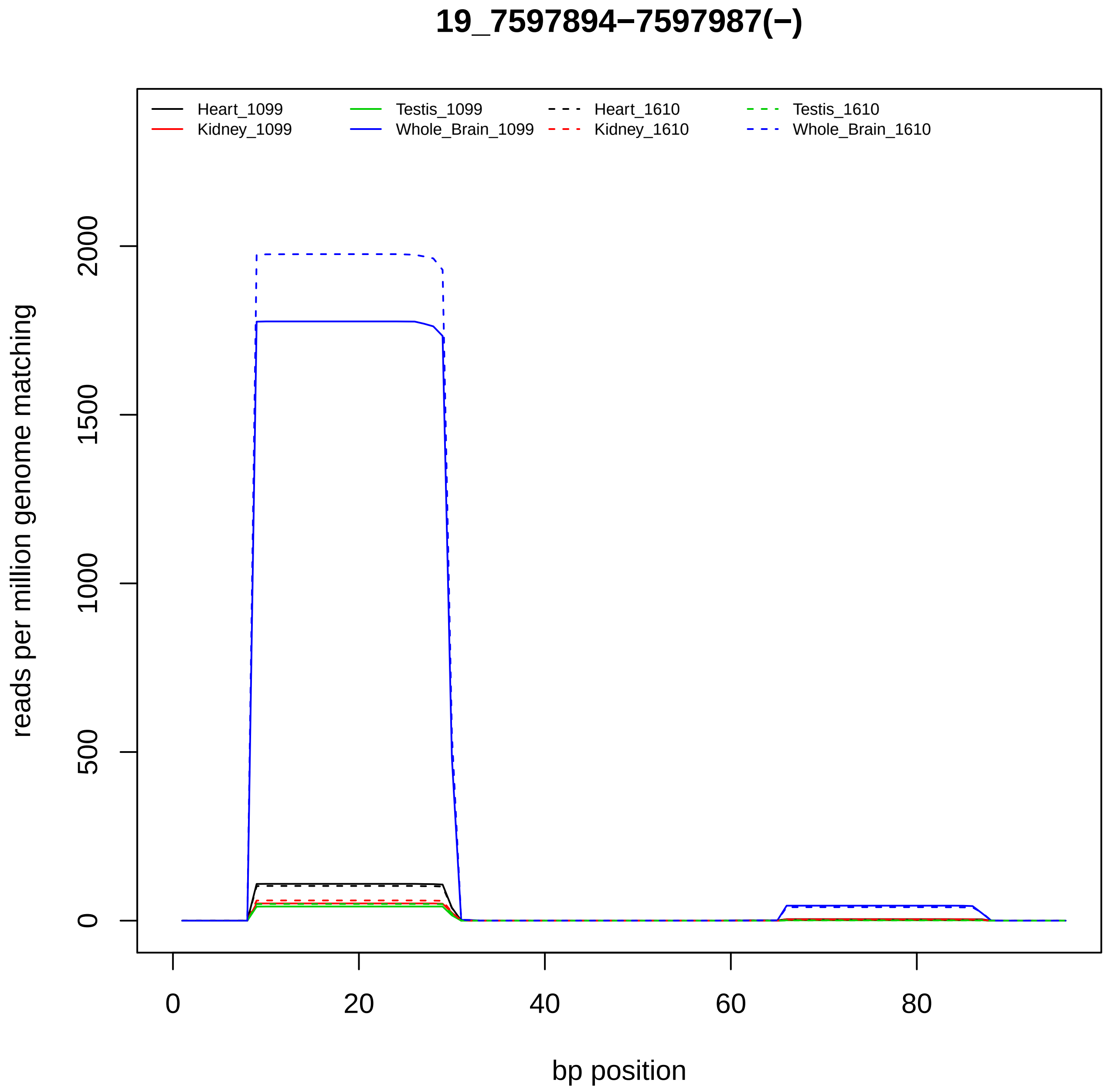

19_7597894−7597987(−)
Heart_1099
Kidney_1099
Testis_1099
Whole_Brain_1099
Heart_1610
Kidney_1610
Testis_1610
Whole_Brain_1610
reads per million genome matching
0
500
1000
1500
2000
0
20
40
60
80
bp position

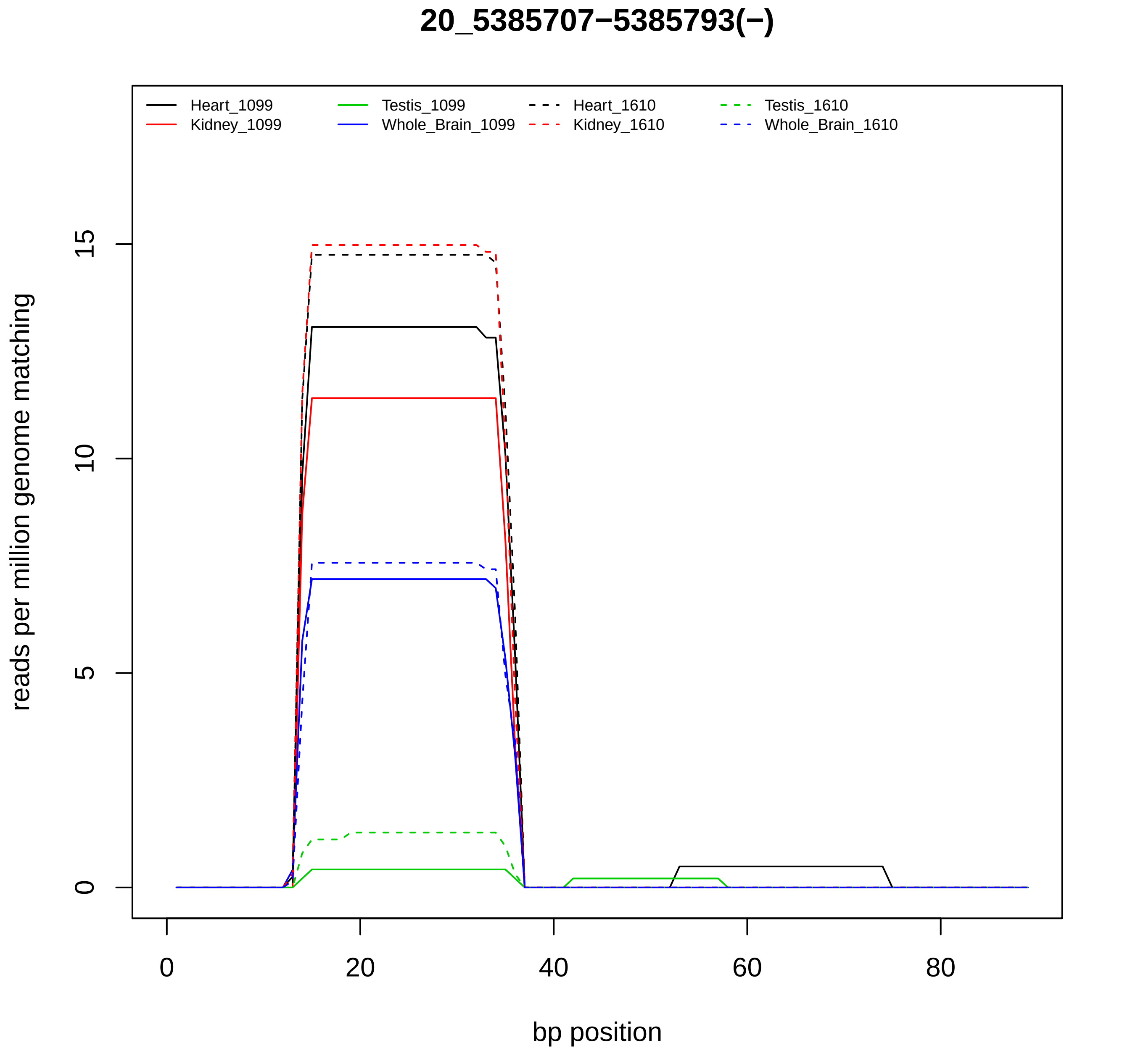

20_5385707−5385793(−)
Heart_1099
Kidney_1099
Testis_1099
Whole_Brain_1099
Heart_1610
Kidney_1610
Testis_1610
Whole_Brain_1610
reads per million genome matching
bp position
0
5
10
15
0
20
40
60
80

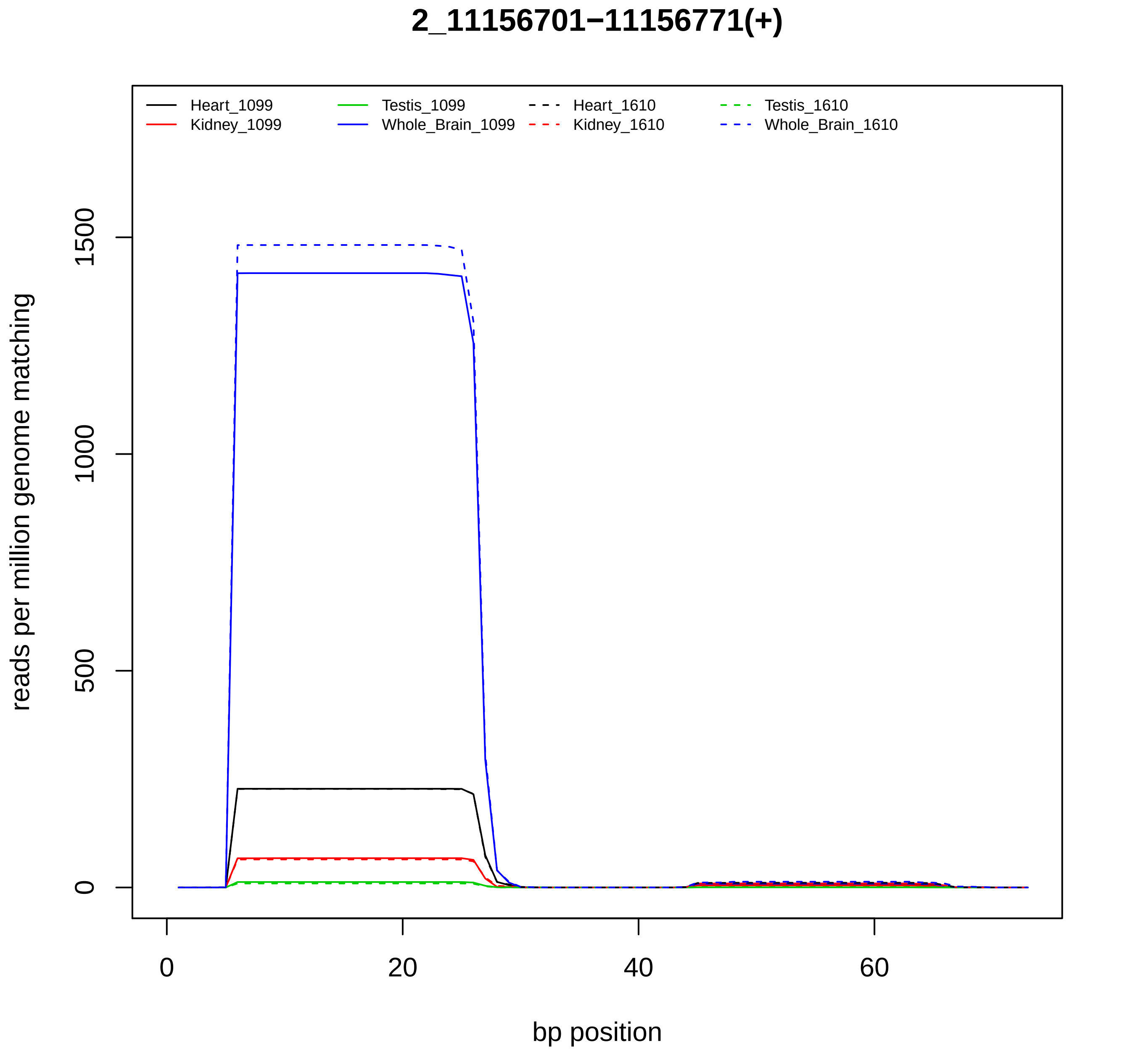

2_11156701−11156771(+)
Heart_1099
Kidney_1099
Testis_1099
Whole_Brain_1099
Heart_1610
Kidney_1610
Testis_1610
Whole_Brain_1610
reads per million genome matching
0
500
1000
1500
0
20
40
60
bp position

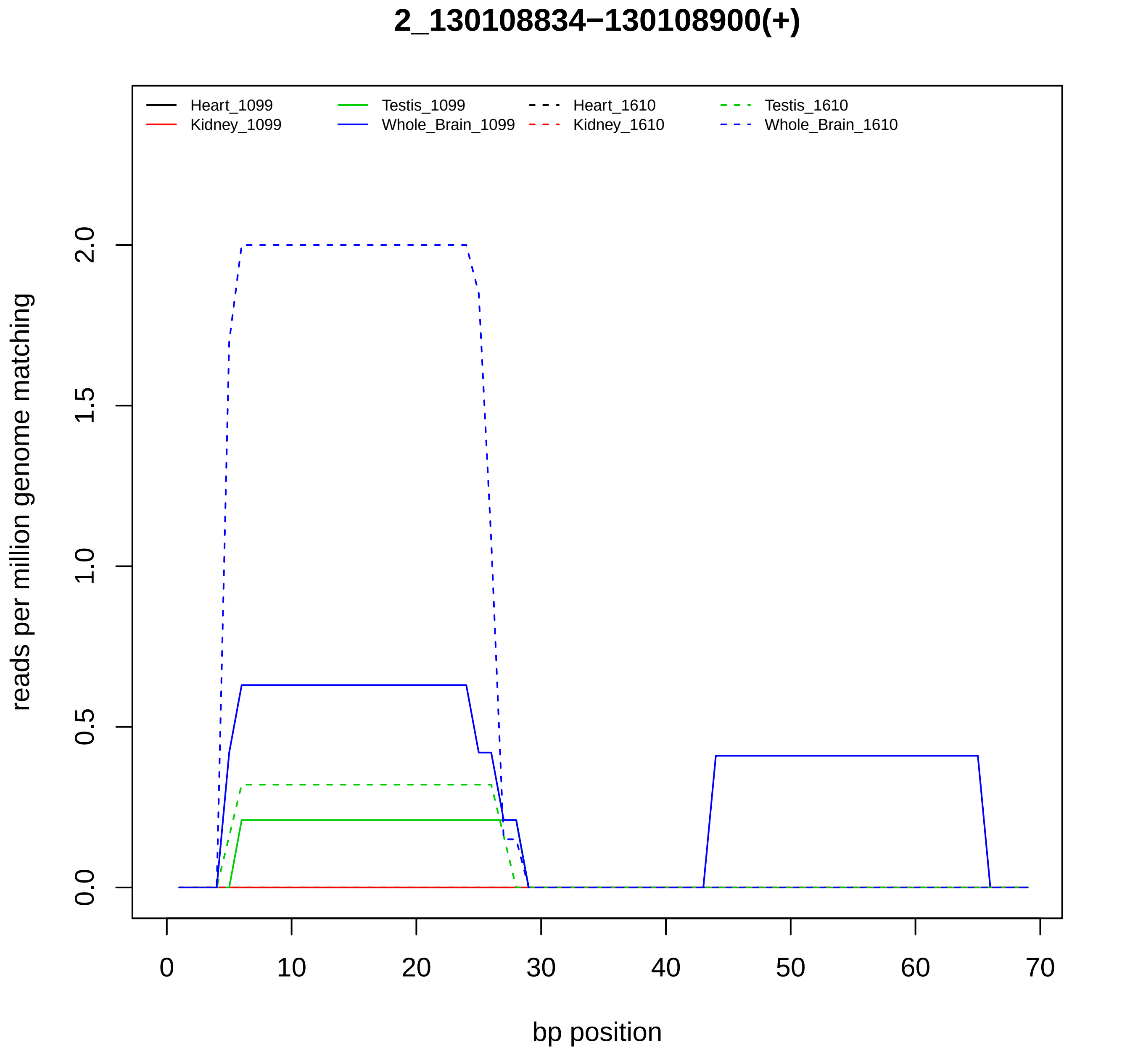

2_130108834−130108900(+)
Heart_1099
Kidney_1099
Testis_1099
Whole_Brain_1099
Heart_1610
Kidney_1610
Testis_1610
Whole_Brain_1610
reads per million genome matching
bp position
0.0
0.5
1.0
1.5
2.0
0
10
20
30
40
50
60
70

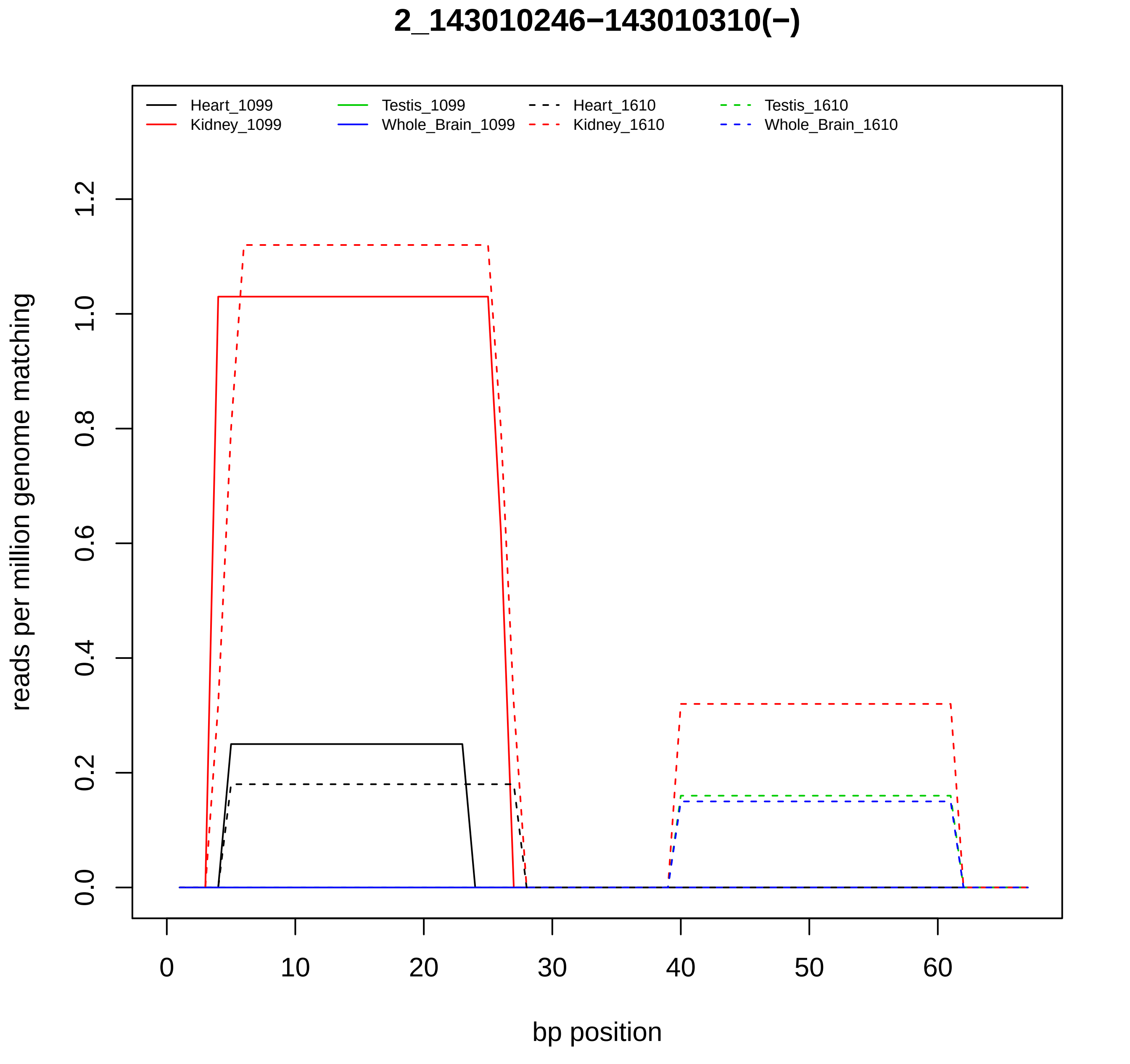
2_143010246−143010310(−)
Heart_1099
Kidney_1099
Testis_1099
Whole_Brain_1099
Heart_1610
Kidney_1610
Testis_1610
Whole_Brain_1610
reads per million genome matching
0.0
0.2
0.4
0.6
0.8
1.0
1.2
0
10
20
30
40
50
60
bp position

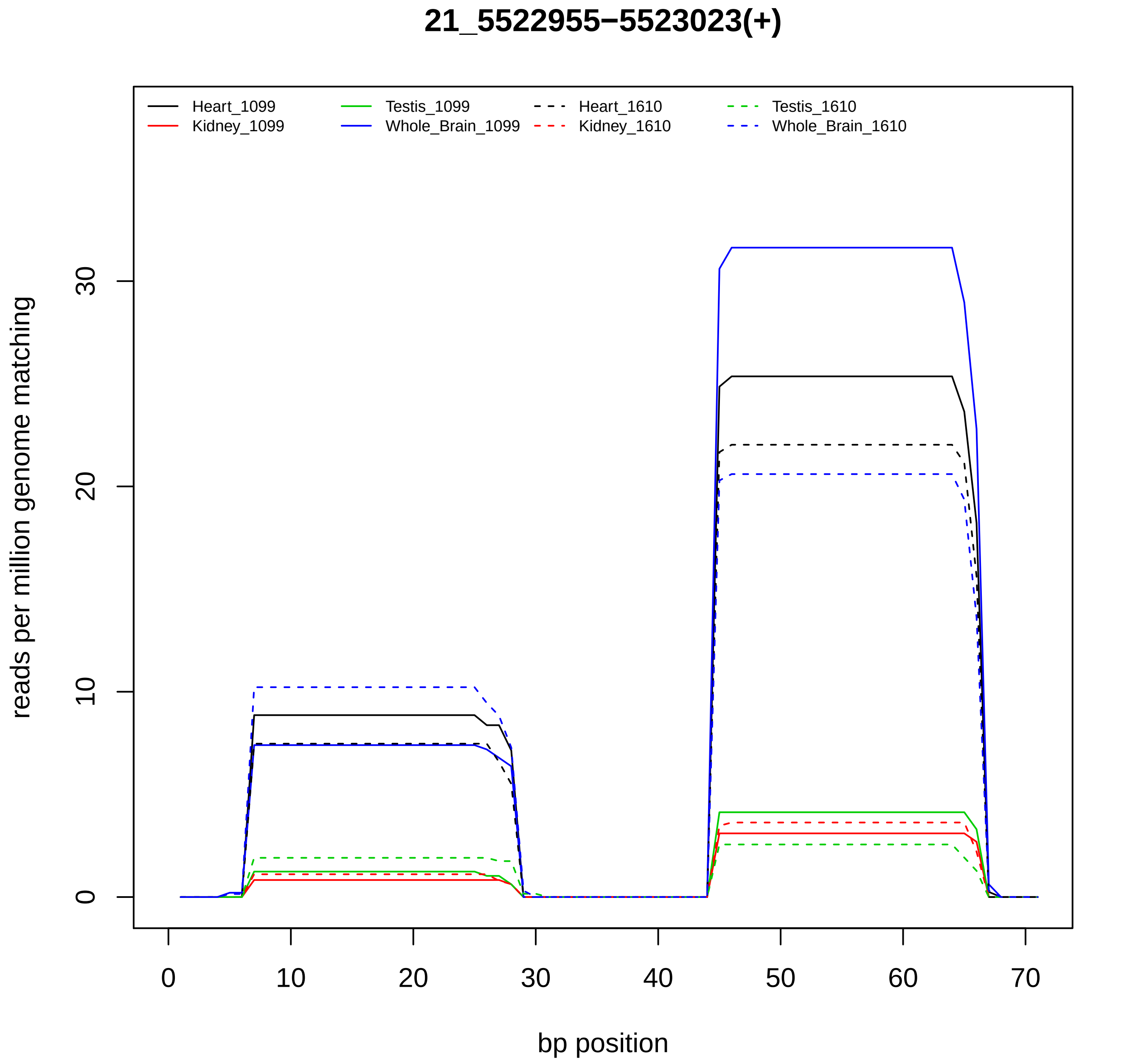
21_5522955−5523023(+)
Heart_1099
Kidney_1099
Testis_1099
Whole_Brain_1099
Heart_1610
Kidney_1610
Testis_1610
Whole_Brain_1610
reads per million genome matching
bp position
0
10
20
30
40
50
60
70

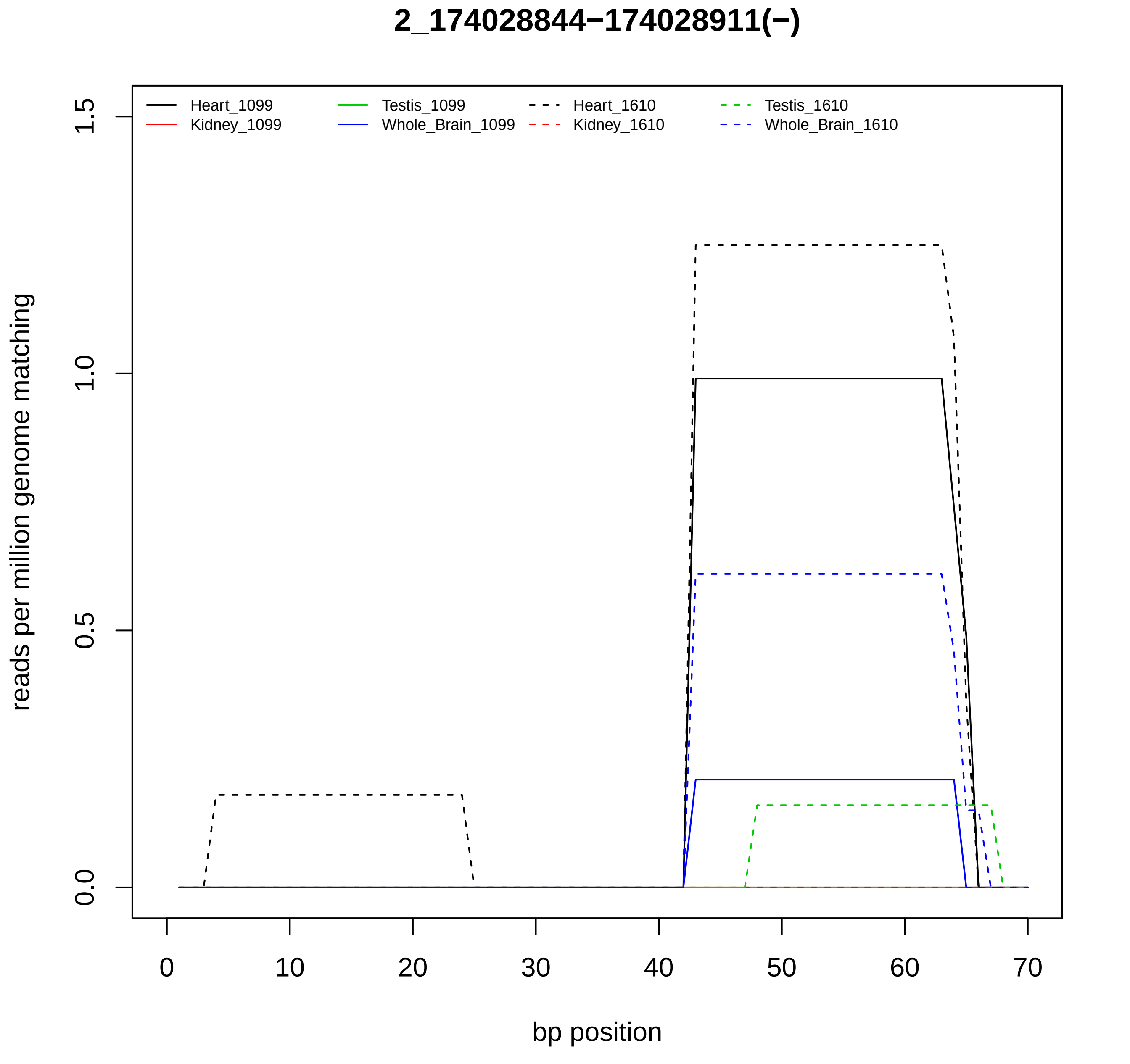
2_174028844−174028911(−)
Heart_1099
Kidney_1099
Testis_1099
Whole_Brain_1099
Heart_1610
Kidney_1610
Testis_1610
Whole_Brain_1610
reads per million genome matching
0.0
0.5
1.0
1.5
0
10
20
30
40
50
60
70
bp position

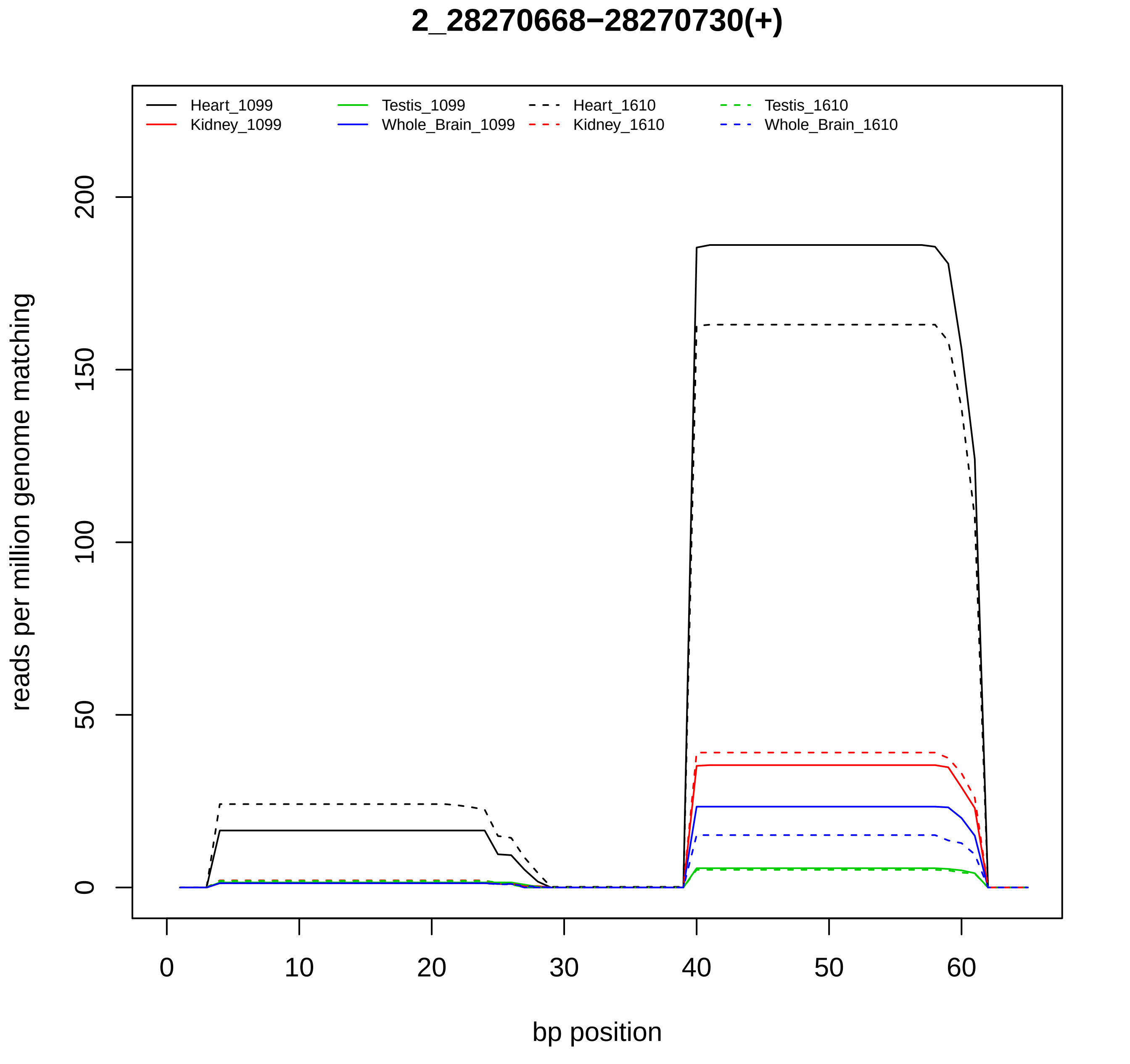

2_28270668−28270730(+)
Heart_1099
Kidney_1099
Testis_1099
Whole_Brain_1099
Heart_1610
Kidney_1610
Testis_1610
Whole_Brain_1610
reads per million genome matching
0
50
100
150
200
bp position
0
10
20
30
40
50
60

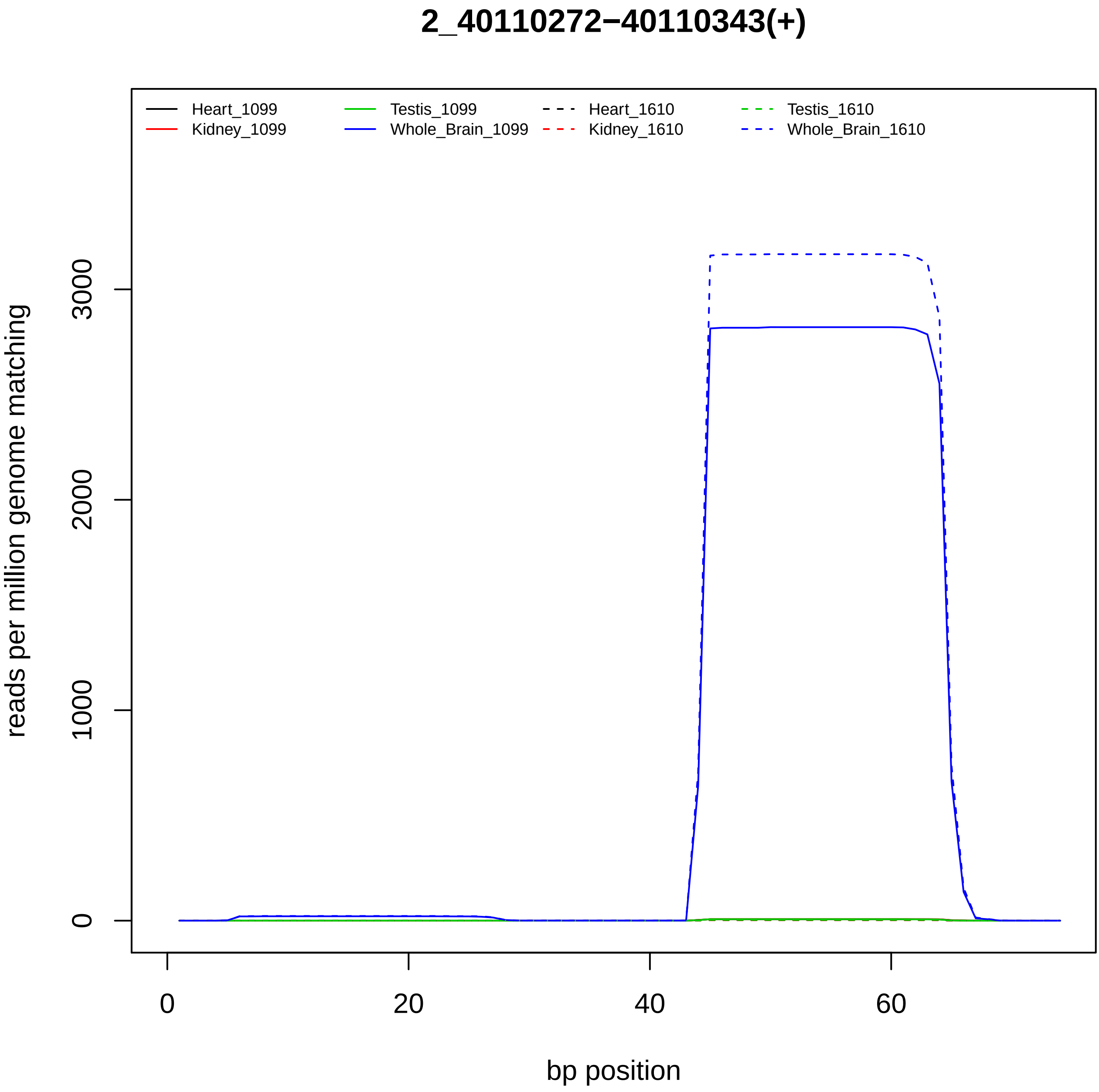
2_40110272−40110343(+)
Heart_1099
Kidney_1099
Testis_1099
Whole_Brain_1099
Heart_1610
Kidney_1610
Testis_1610
Whole_Brain_1610
reads per million genome matching
0
1000
2000
3000
0
20
40
60
bp position

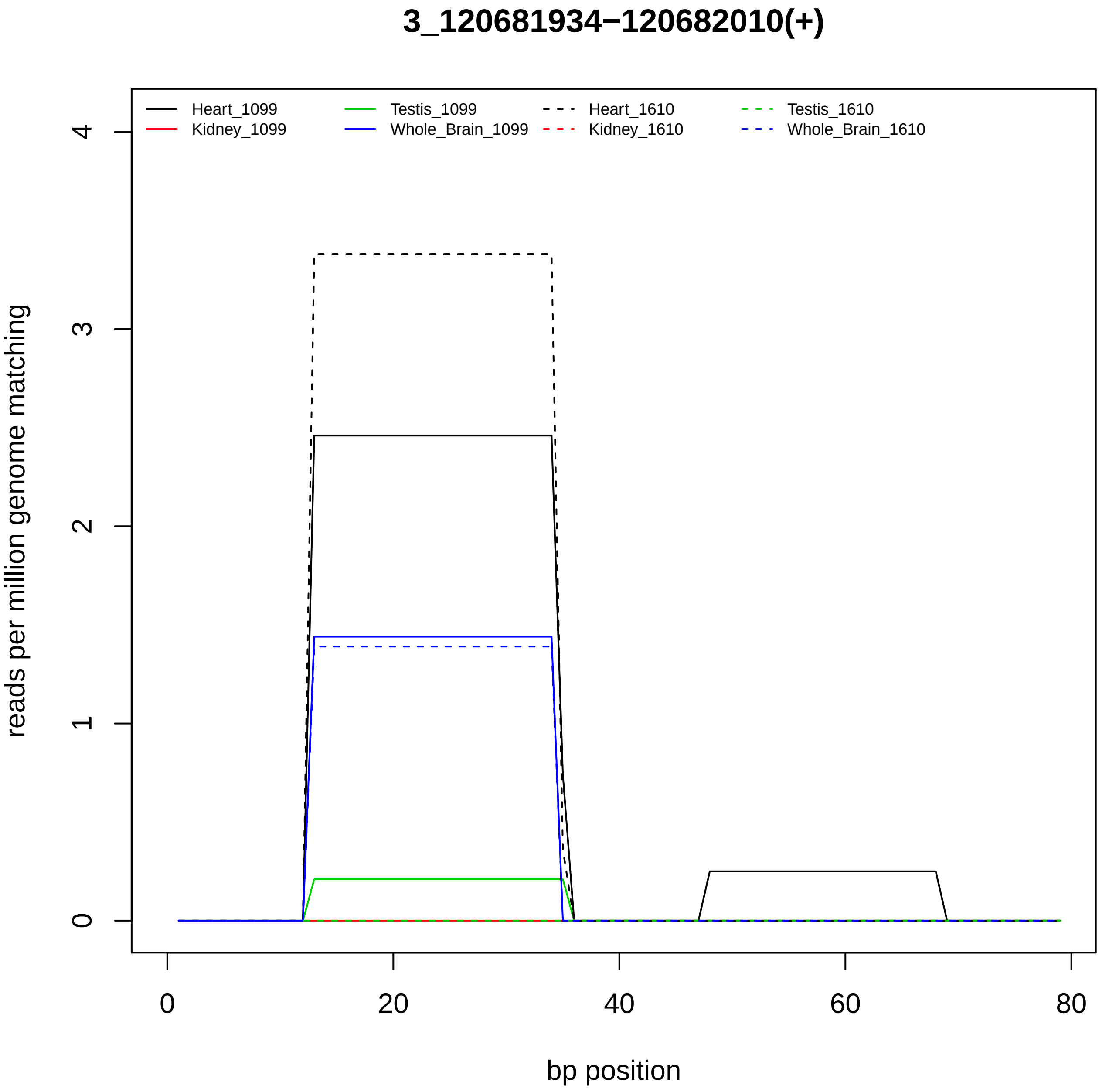

3_120681934−120682010(+)
Heart_1099
Kidney_1099
Testis_1099
Whole_Brain_1099
Heart_1610
Kidney_1610
Testis_1610
Whole_Brain_1610
reads per million genome matching
0
1
2
3
4
0
20
40
60
80
bp position

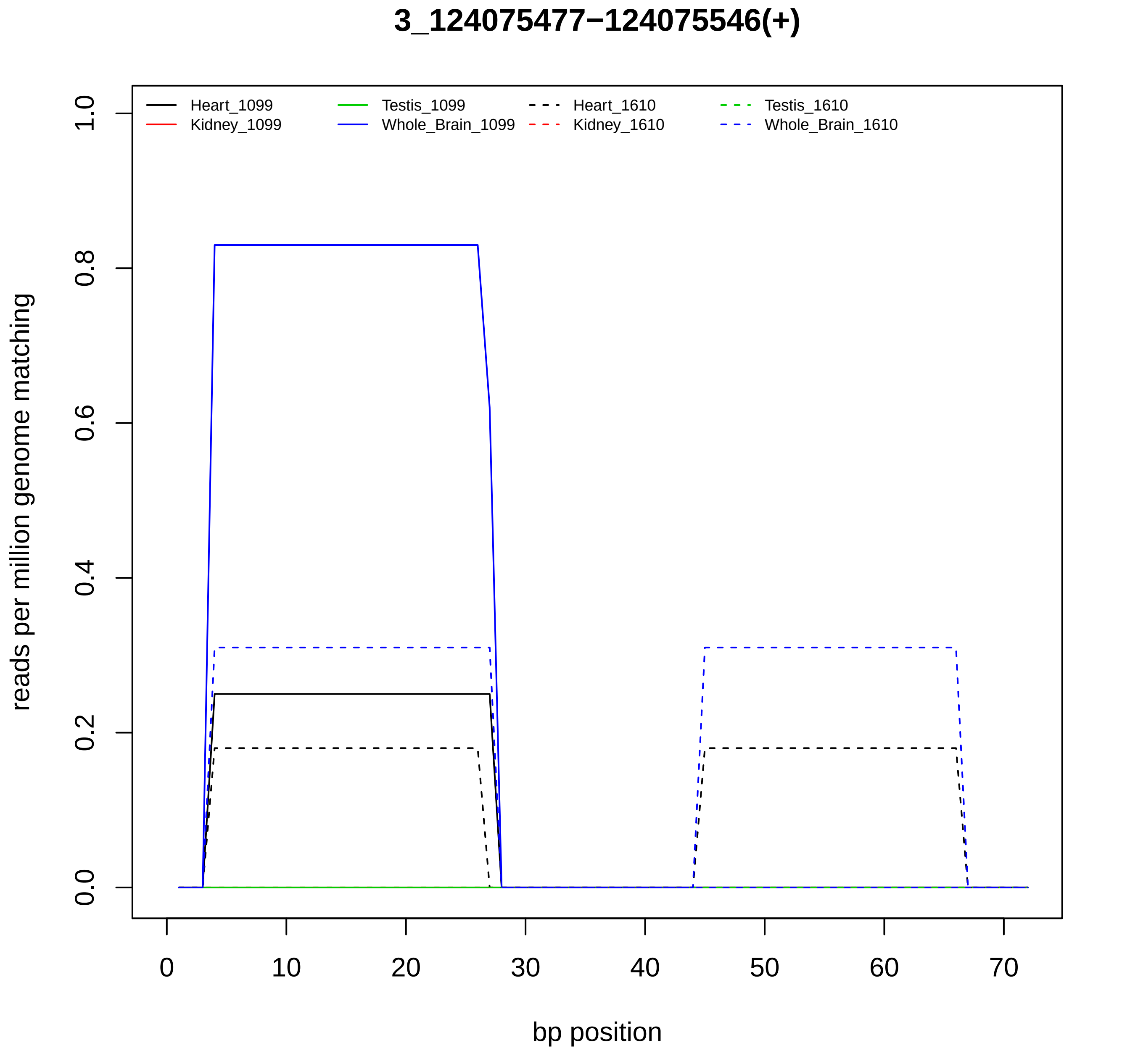

3_124075477−124075546(+)
Heart_1099
Kidney_1099
Testis_1099
Whole_Brain_1099
Heart_1610
Kidney_1610
Testis_1610
Whole_Brain_1610
reads per million genome matching
0.0
0.2
0.4
0.6
0.8
1.0
bp position
0
10
20
30
40
50
60
70

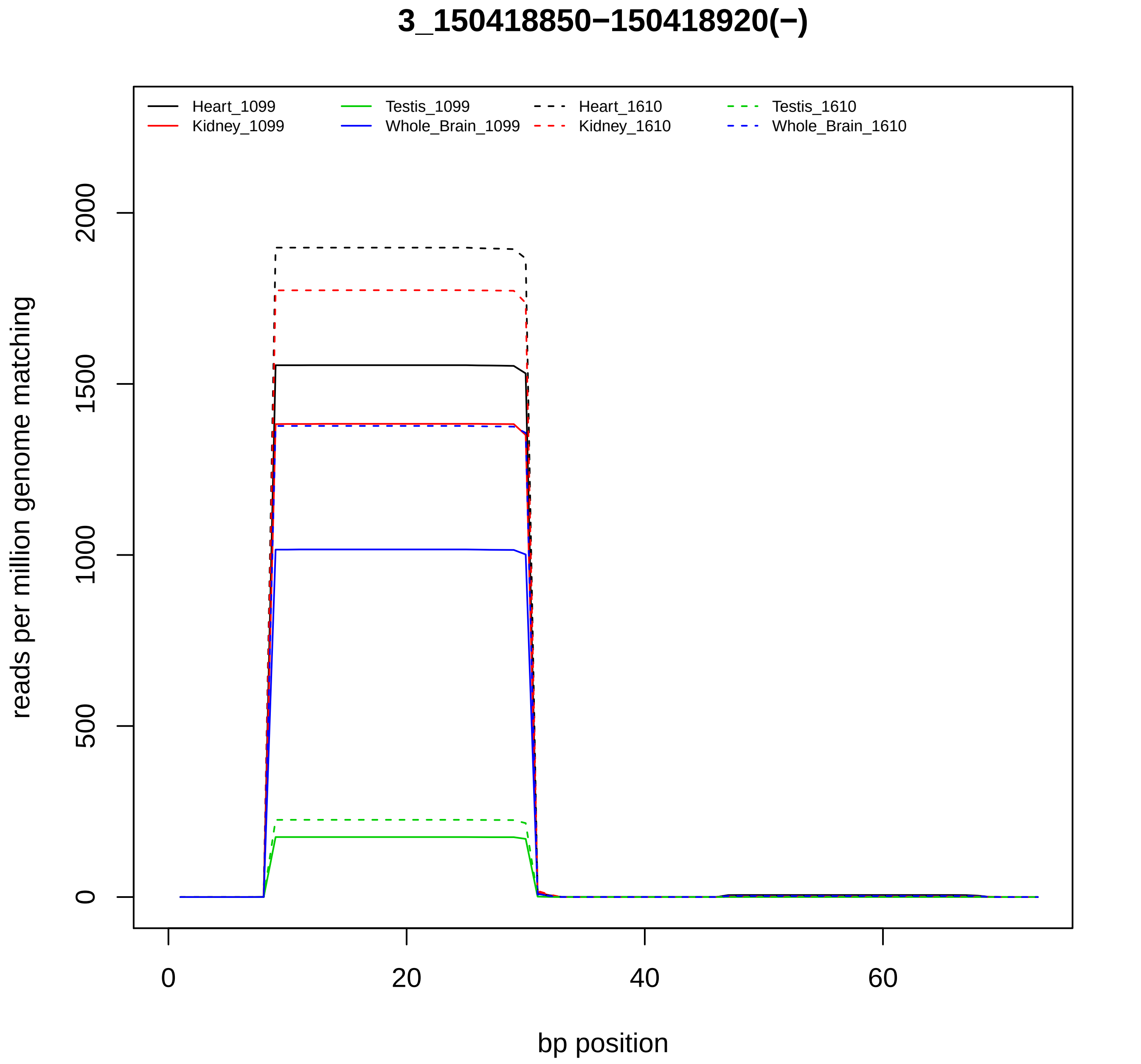
3_150418850−150418920(−)
Heart_1099
Kidney_1099
Testis_1099
Whole_Brain_1099
Heart_1610
Kidney_1610
Testis_1610
Whole_Brain_1610
reads per million genome matching
0
500
1000
1500
2000
0
20
40
60
bp position

3_150424938−150425013(−)

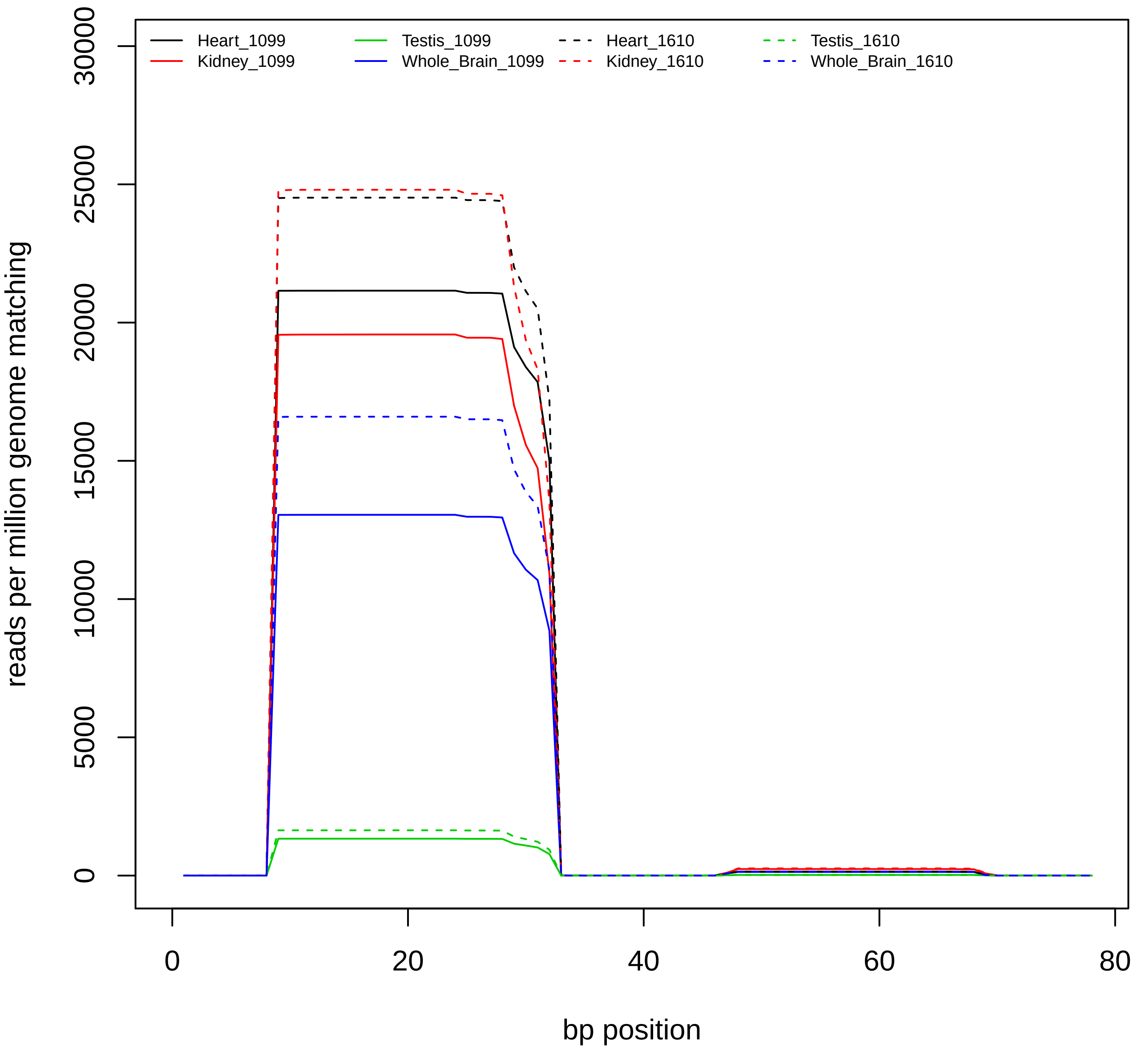

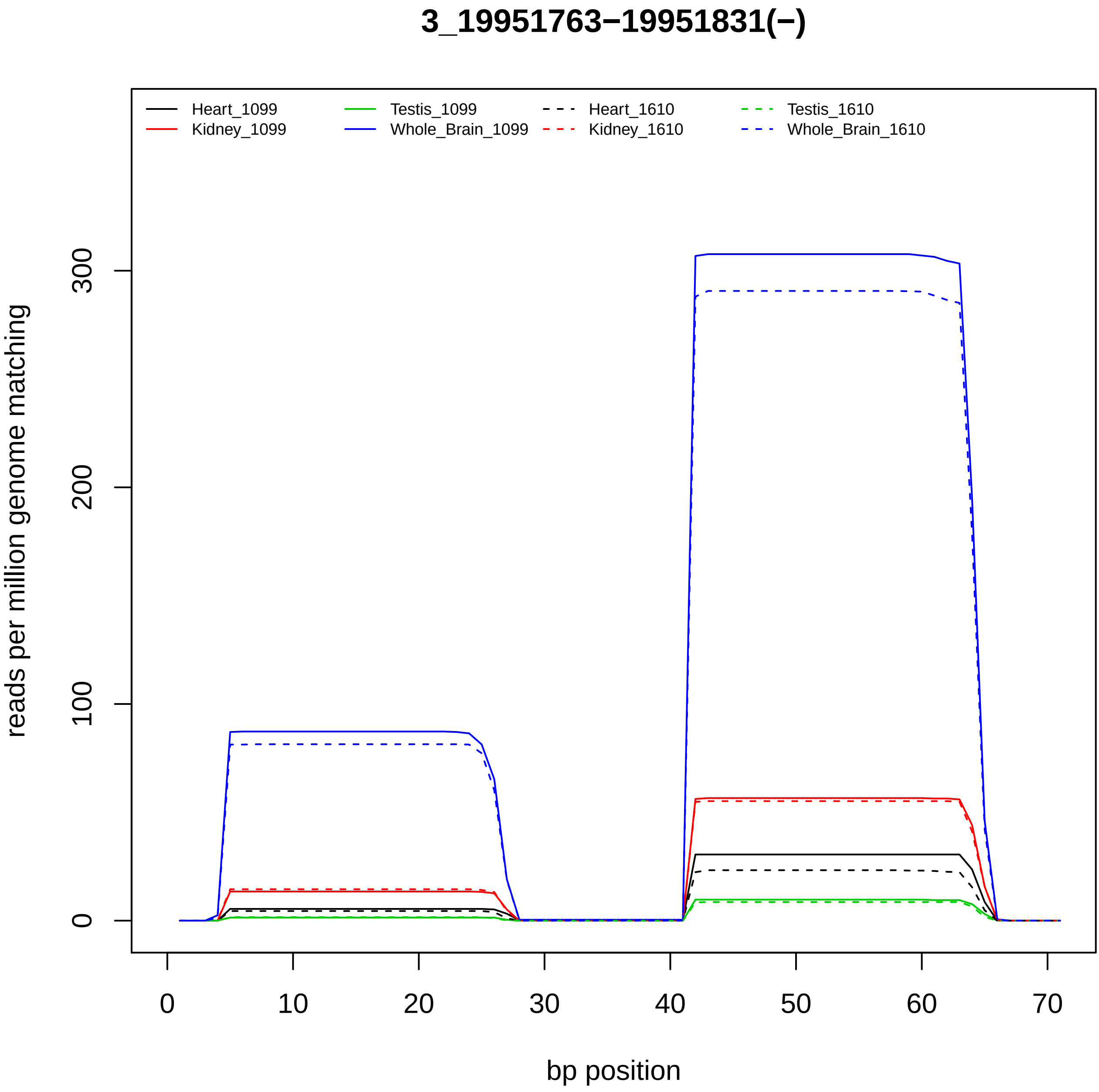

3_19951763−19951831(−)
Heart_1099
Kidney_1099
Testis_1099
Whole_Brain_1099
Heart_1610
Kidney_1610
Testis_1610
Whole_Brain_1610
reads per million genome matching
0
100
200
300
0
10
20
30
40
50
60
70
bp position

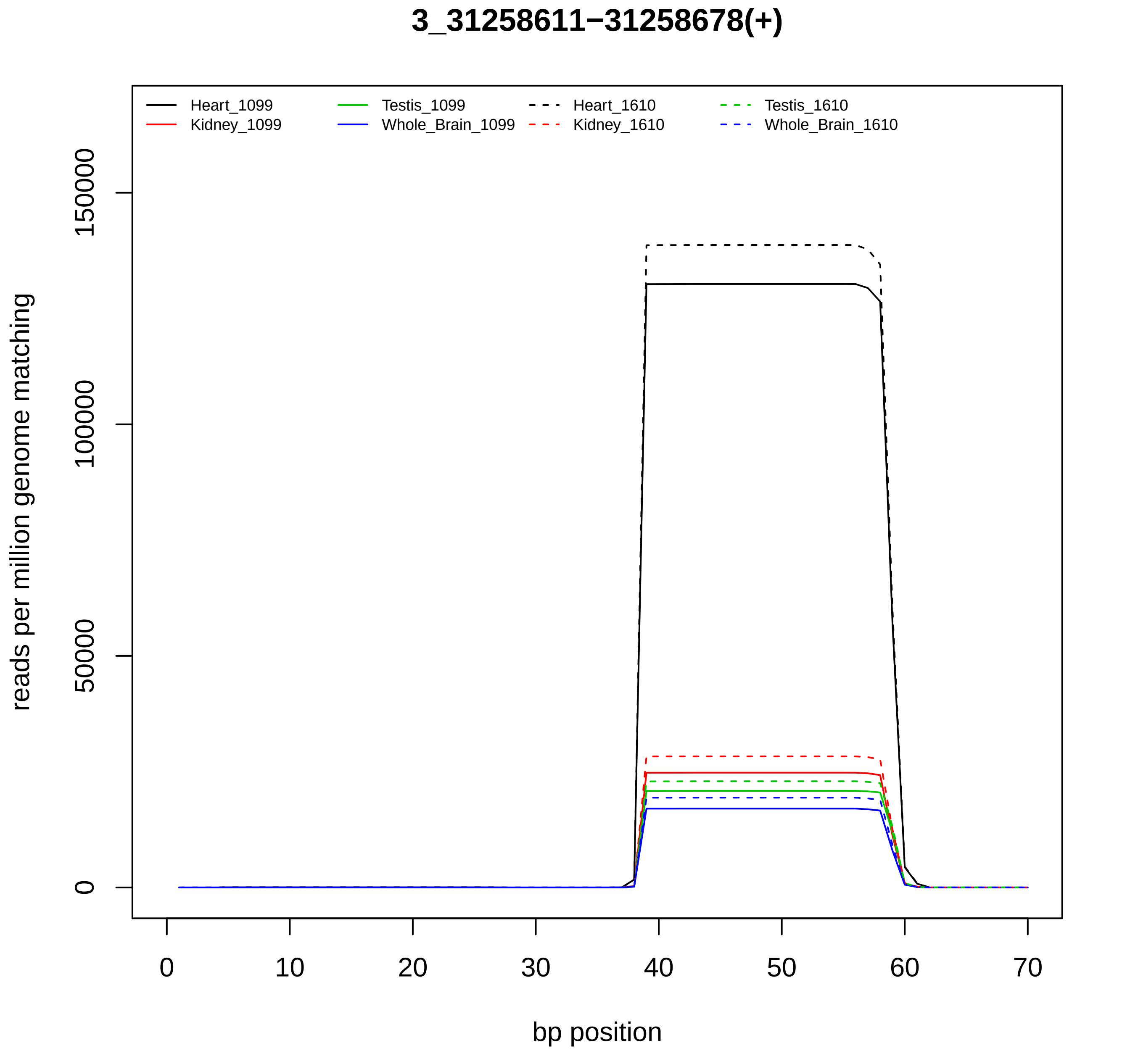

3_31258611−31258678(+)
Heart_1099
Kidney_1099
Testis_1099
Whole_Brain_1099
Heart_1610
Kidney_1610
Testis_1610
Whole_Brain_1610
reads per million genome matching
0
50000
100000
150000
0
10
20
30
40
50
60
70
bp position

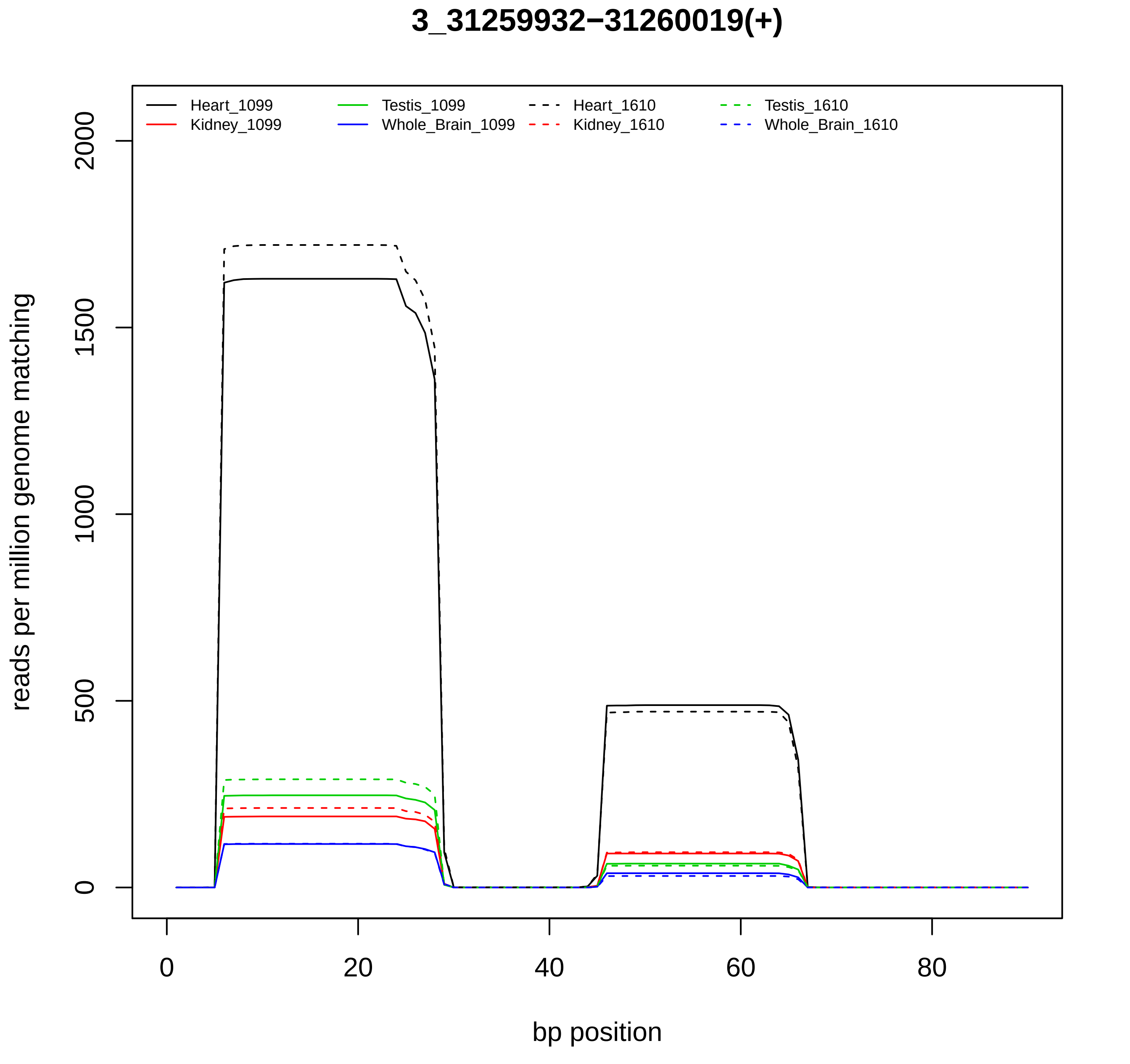

3_31259932−31260019(+)
Heart_1099
Kidney_1099
Testis_1099
Whole_Brain_1099
Heart_1610
Kidney_1610
Testis_1610
Whole_Brain_1610
reads per million genome matching
bp position
0
500
1000
1500
2000
0
20
40
60
80

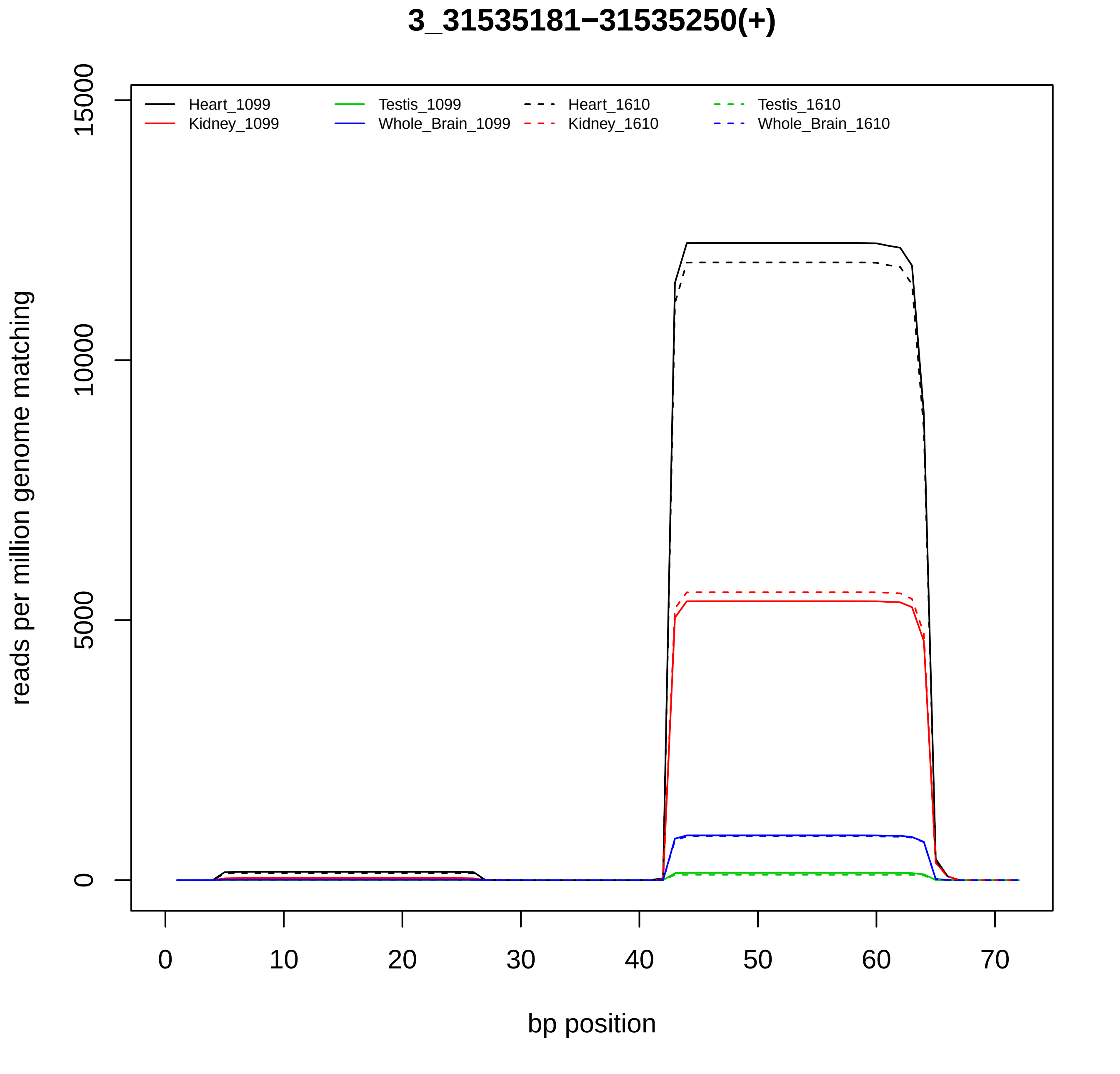

3_31535181−31535250(+)
Heart_1099
Kidney_1099
Testis_1099
Whole_Brain_1099
Heart_1610
Kidney_1610
Testis_1610
Whole_Brain_1610
reads per million genome matching
bp position
0
5000
10000
15000
0
10
20
30
40
50
60
70

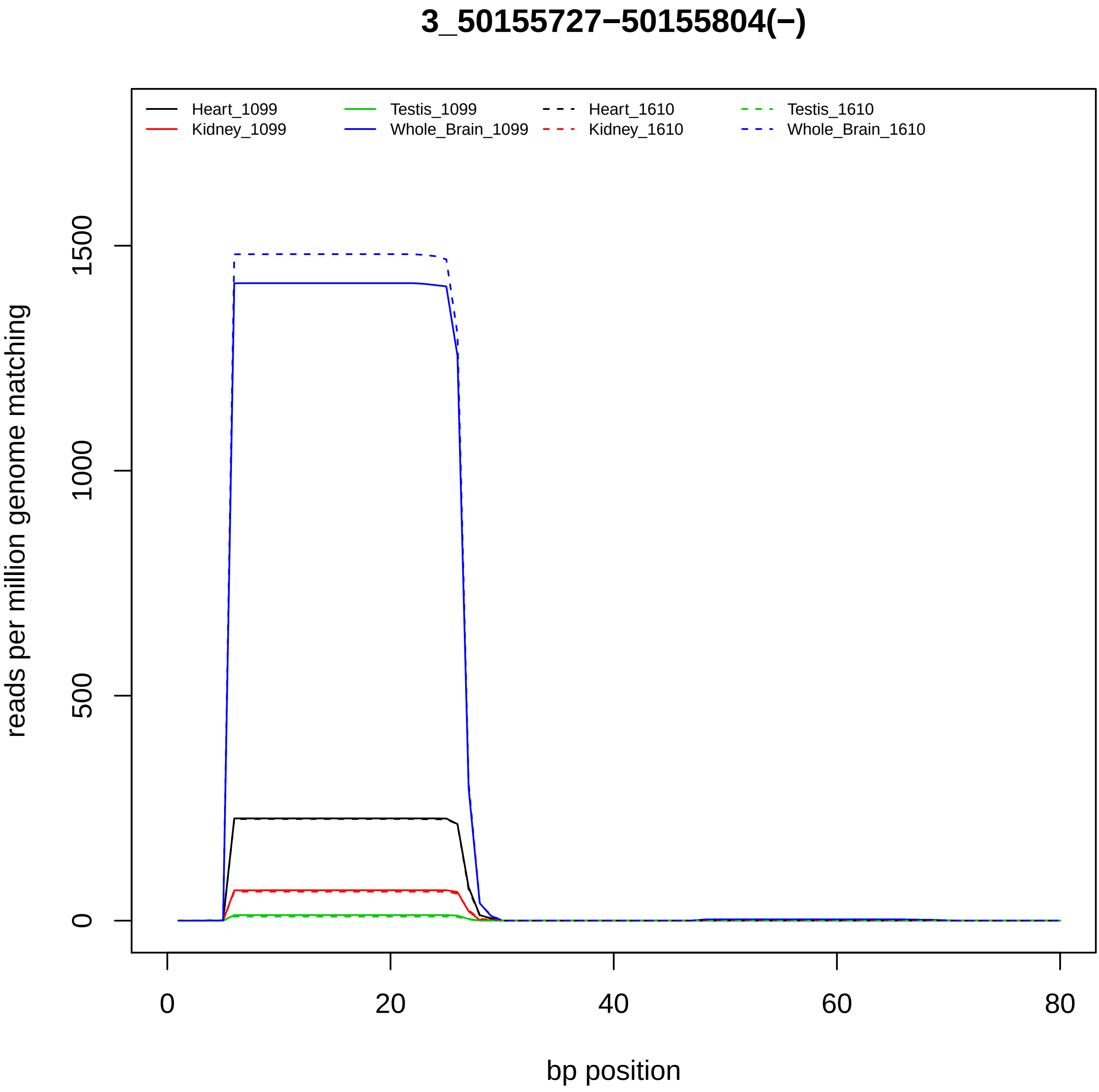

3_50155727−50155804(−)
Heart_1099
Kidney_1099
Testis_1099
Whole_Brain_1099
Heart_1610
Kidney_1610
Testis_1610
Whole_Brain_1610
reads per million genome matching
0
500
1000
1500
0
20
40
60
80
bp position

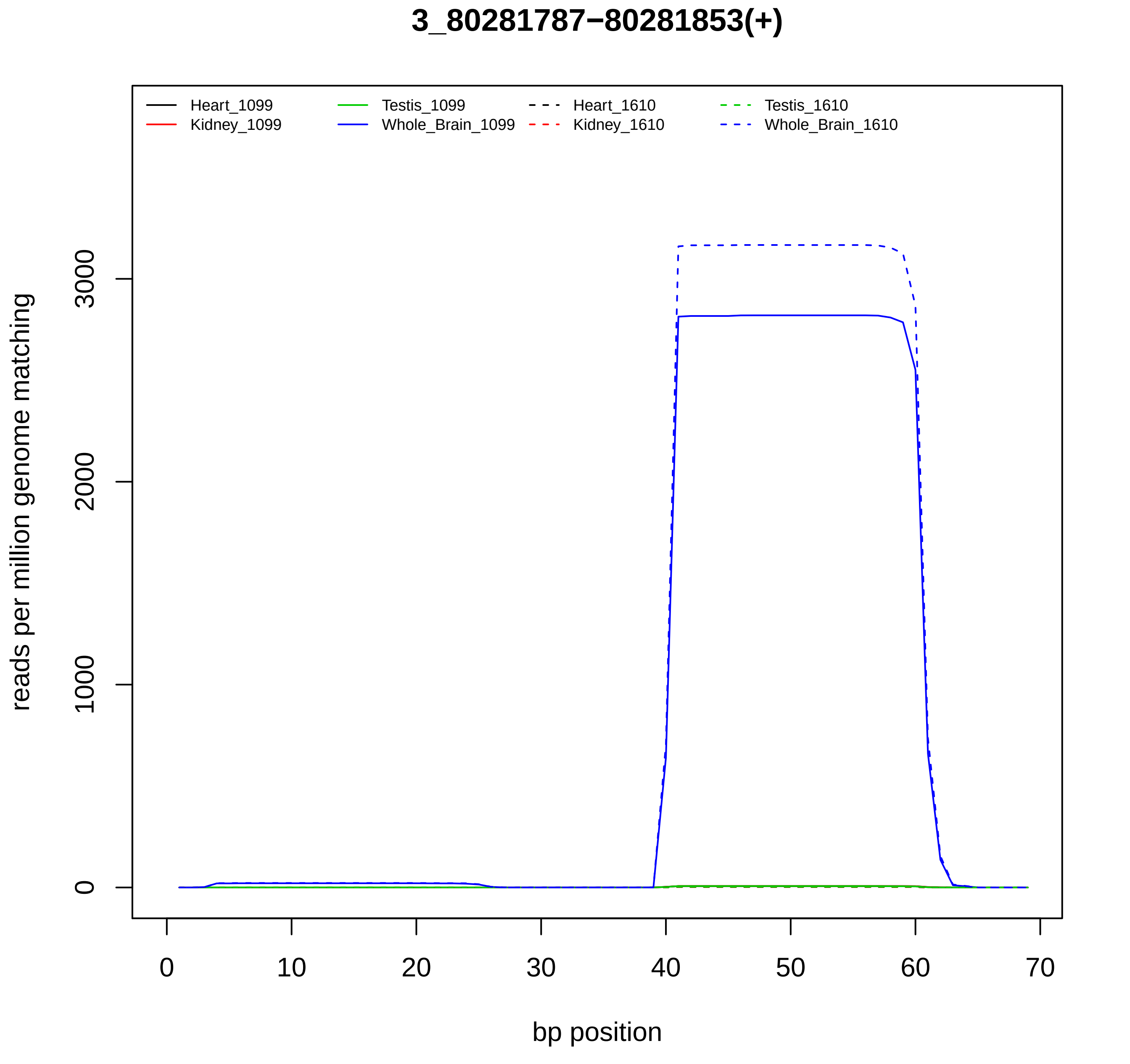

3_80281787−80281853(+)
Heart_1099
Kidney_1099
Testis_1099
Whole_Brain_1099
Heart_1610
Kidney_1610
Testis_1610
Whole_Brain_1610
reads per million genome matching
0
1000
2000
3000
bp position
0
10
20
30
40
50
60
70

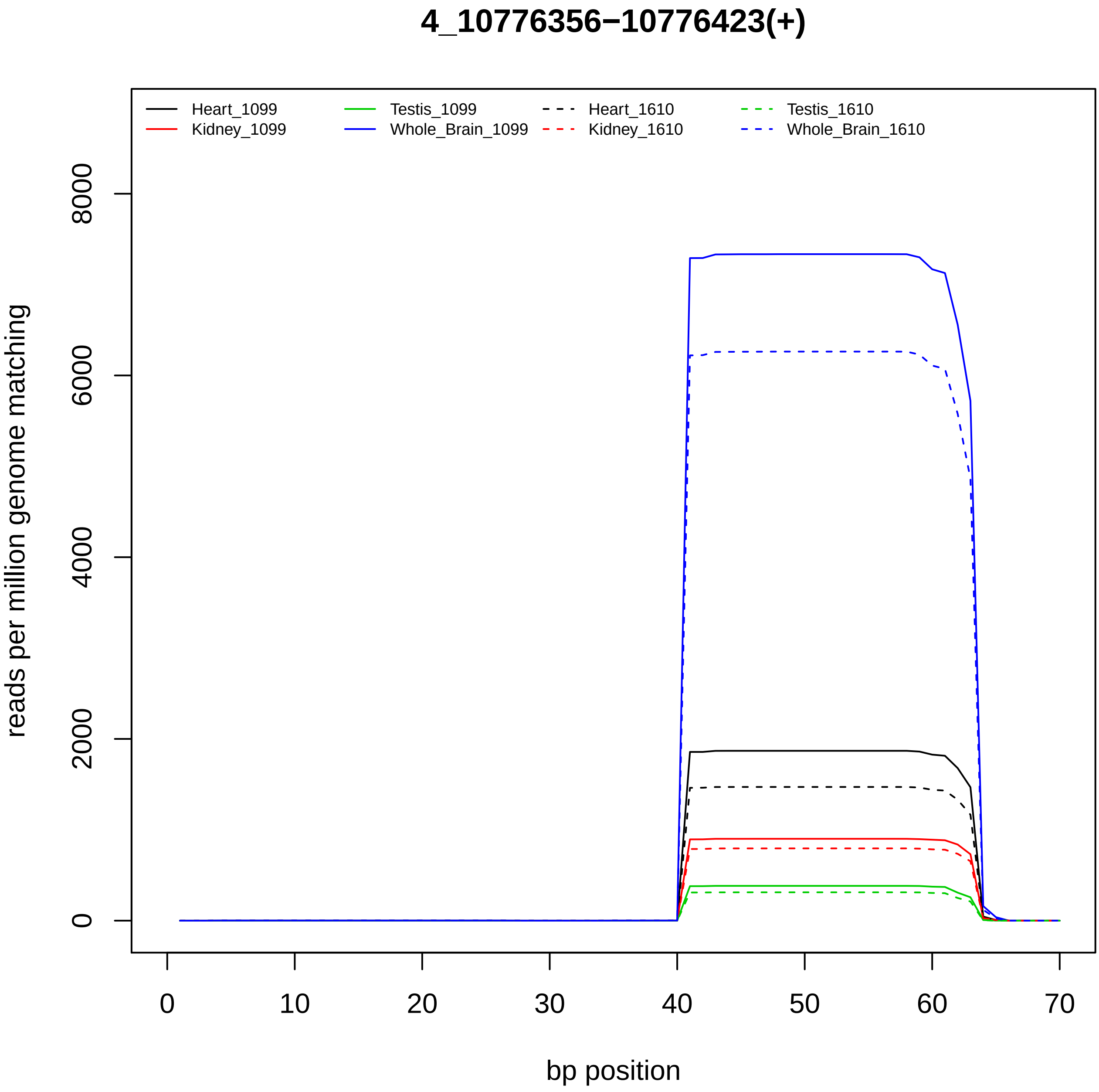
4_10776356−10776423(+)
Heart_1099
Kidney_1099
Testis_1099
Whole_Brain_1099
Heart_1610
Kidney_1610
Testis_1610
Whole_Brain_1610
reads per million genome matching
0
2000
4000
6000
8000
0
10
20
30
40
50
60
70
bp position

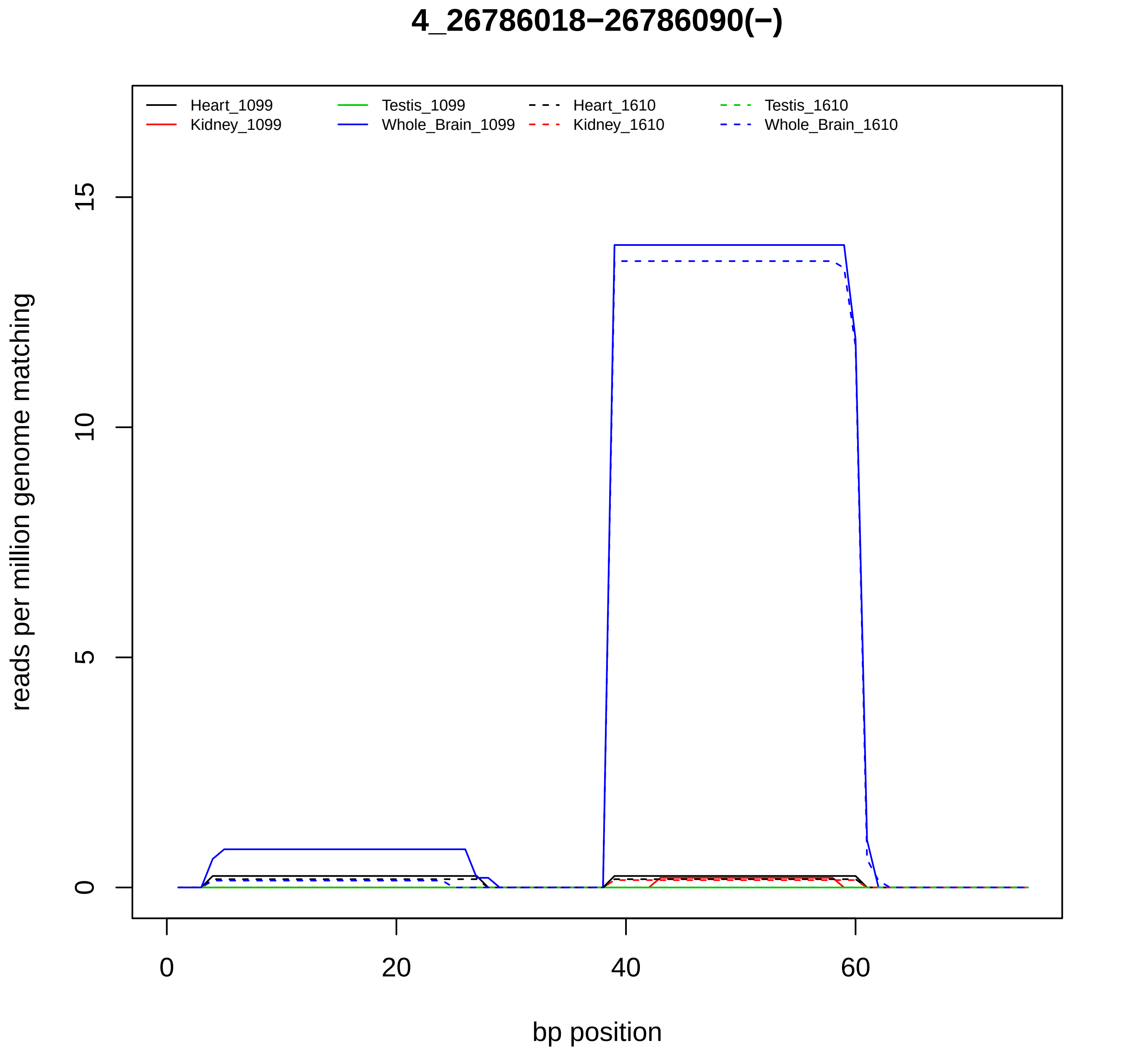
4_26786018−26786090(−)
Heart_1099
Kidney_1099
Testis_1099
Whole_Brain_1099
Heart_1610
Kidney_1610
Testis_1610
Whole_Brain_1610
reads per million genome matching
0
5
10
15
0
20
40
60
bp position

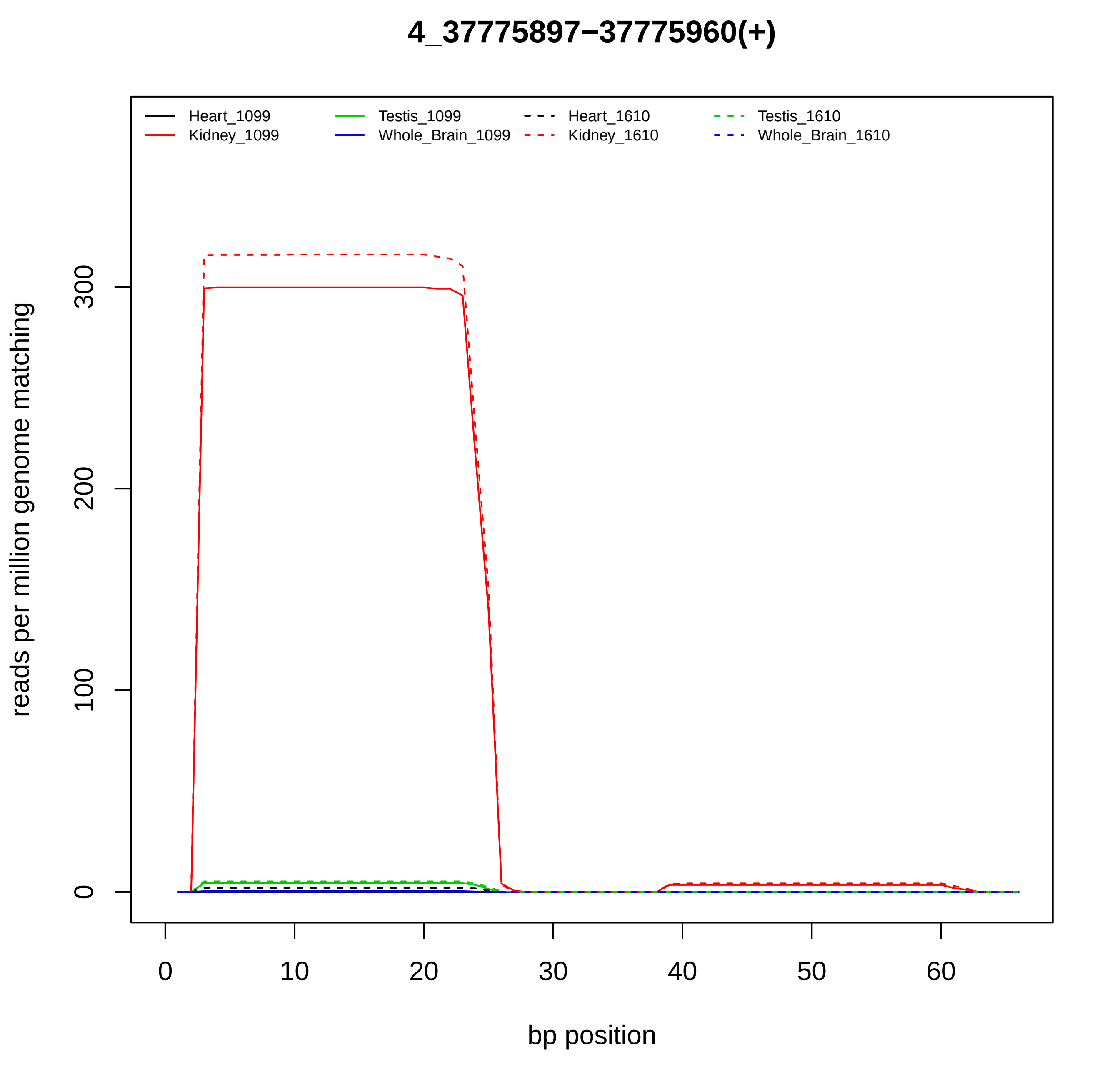
4_37775897−37775960(+)
Heart_1099
Kidney_1099
Testis_1099
Whole_Brain_1099
Heart_1610
Kidney_1610
Testis_1610
Whole_Brain_1610
reads per million genome matching
0
100
200
300
0
10
20
30
40
50
60
bp position

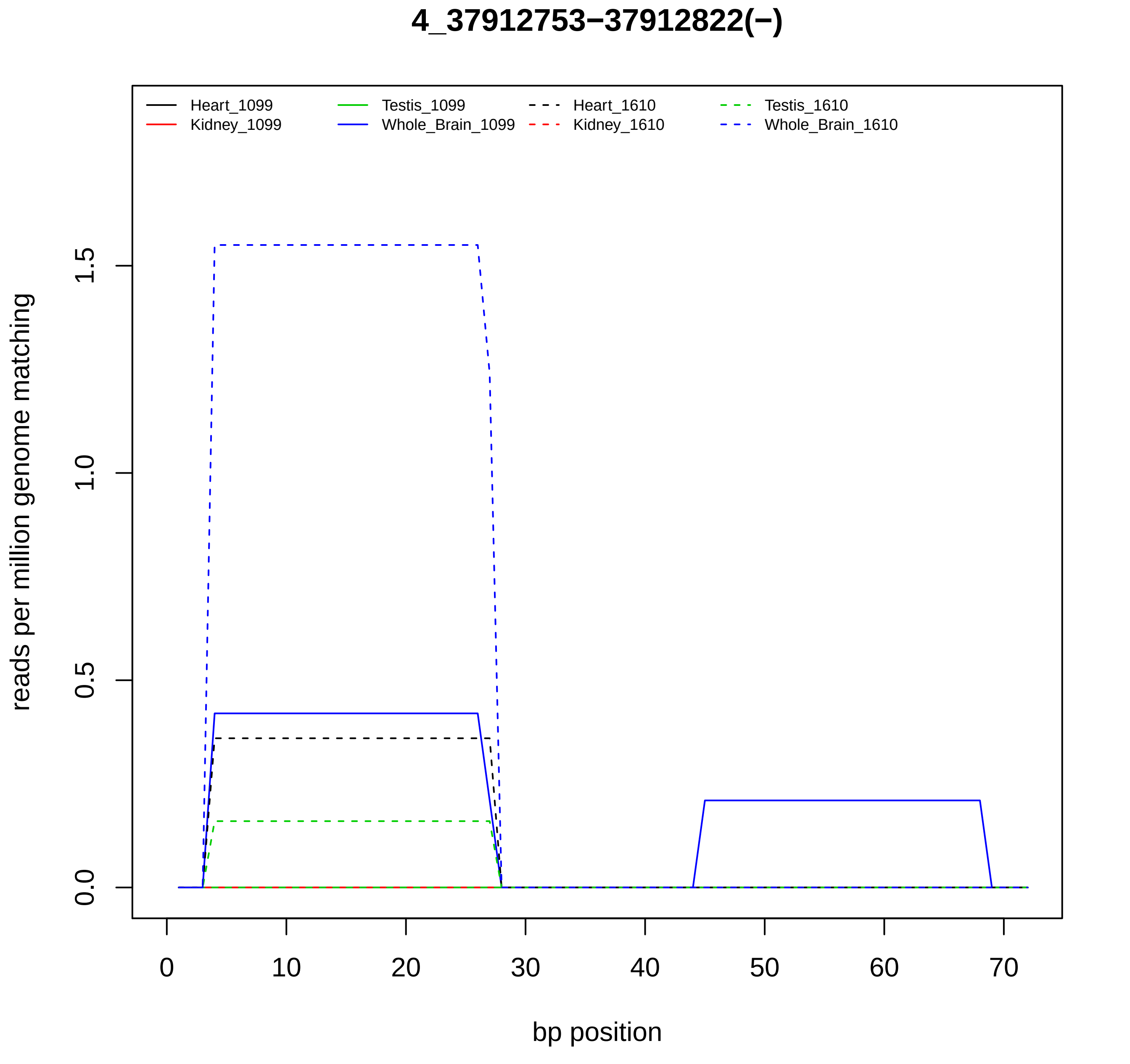

4_37912753−37912822(−)
Heart_1099
Kidney_1099
Testis_1099
Whole_Brain_1099
Heart_1610
Kidney_1610
Testis_1610
Whole_Brain_1610
reads per million genome matching
0.0
0.5
1.0
1.5
0
10
20
30
40
50
60
70
bp position

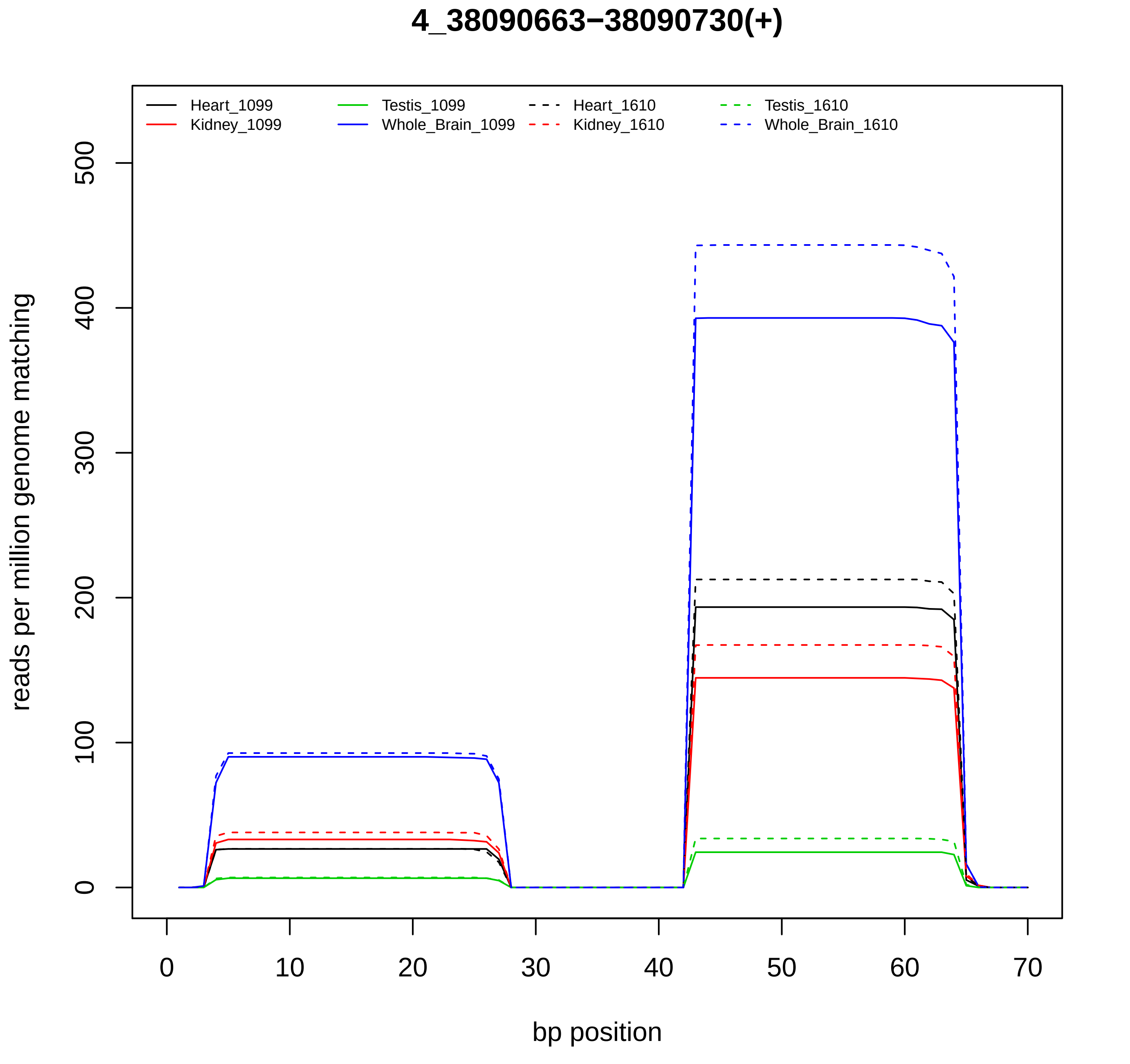

4_38090663−38090730(+)
Heart_1099
Kidney_1099
Testis_1099
Whole_Brain_1099
Heart_1610
Kidney_1610
Testis_1610
Whole_Brain_1610
reads per million genome matching
bp position
0
100
200
300
400
500
0
10
20
30
40
50
60
70

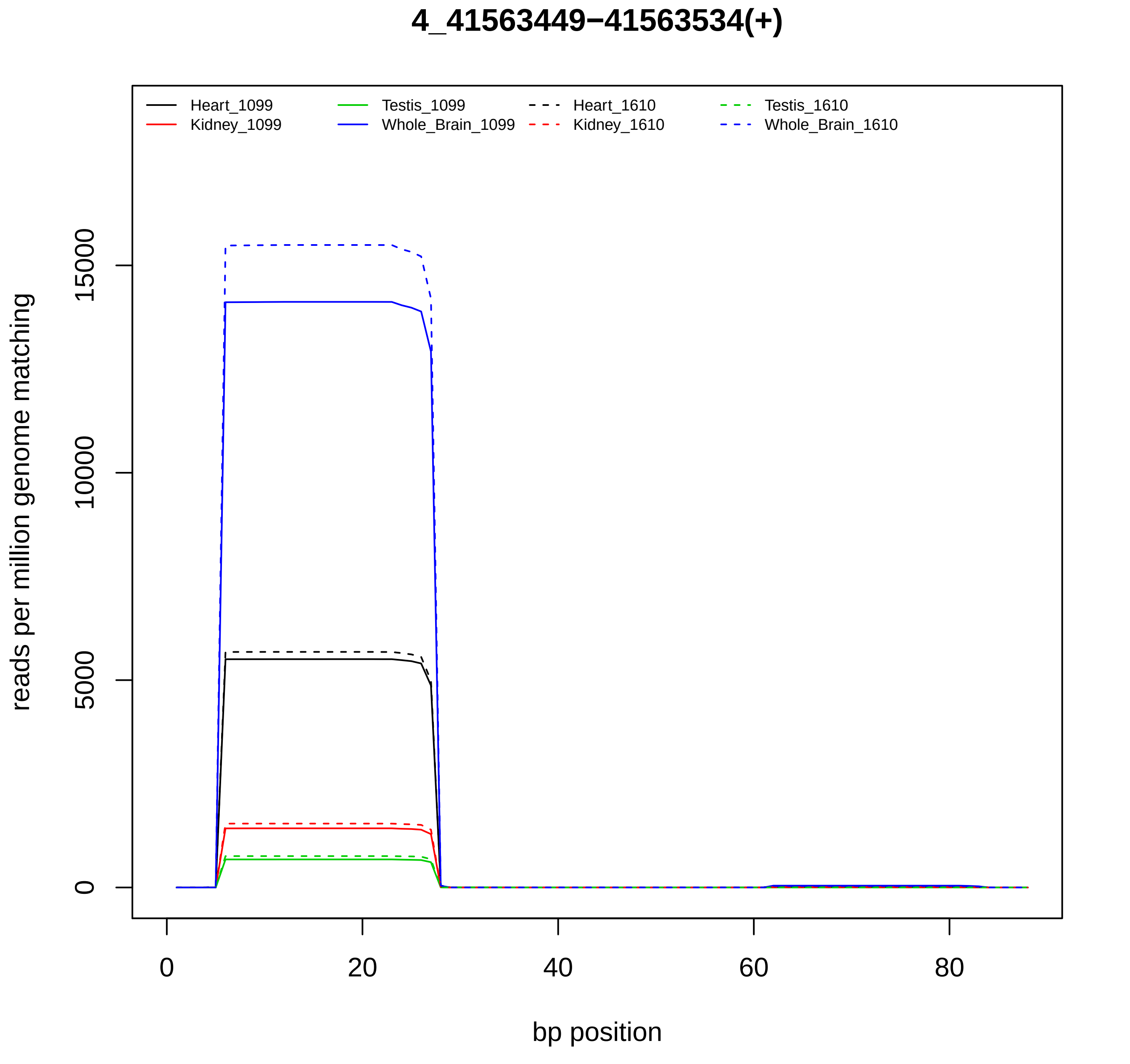

4_41563449−41563534(+)
Heart_1099
Kidney_1099
Testis_1099
Whole_Brain_1099
Heart_1610
Kidney_1610
Testis_1610
Whole_Brain_1610
reads per million genome matching
0
5000
10000
15000
0
20
40
60
80
bp position

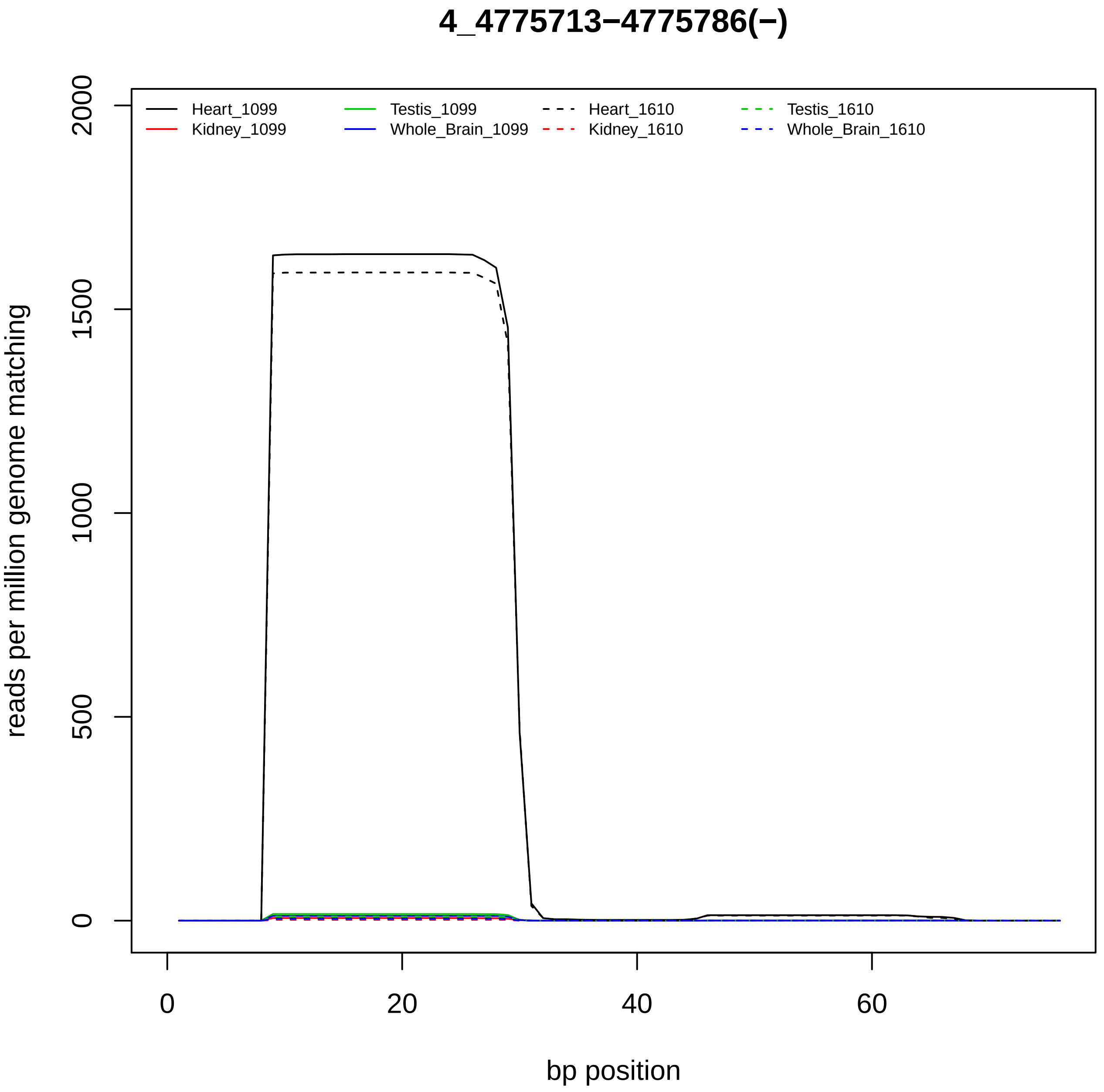

4_4775713−4775786(−)
Heart_1099
Kidney_1099
Testis_1099
Whole_Brain_1099
Heart_1610
Kidney_1610
Testis_1610
Whole_Brain_1610
reads per million genome matching
0
500
1000
1500
2000
0
20
40
60
bp position

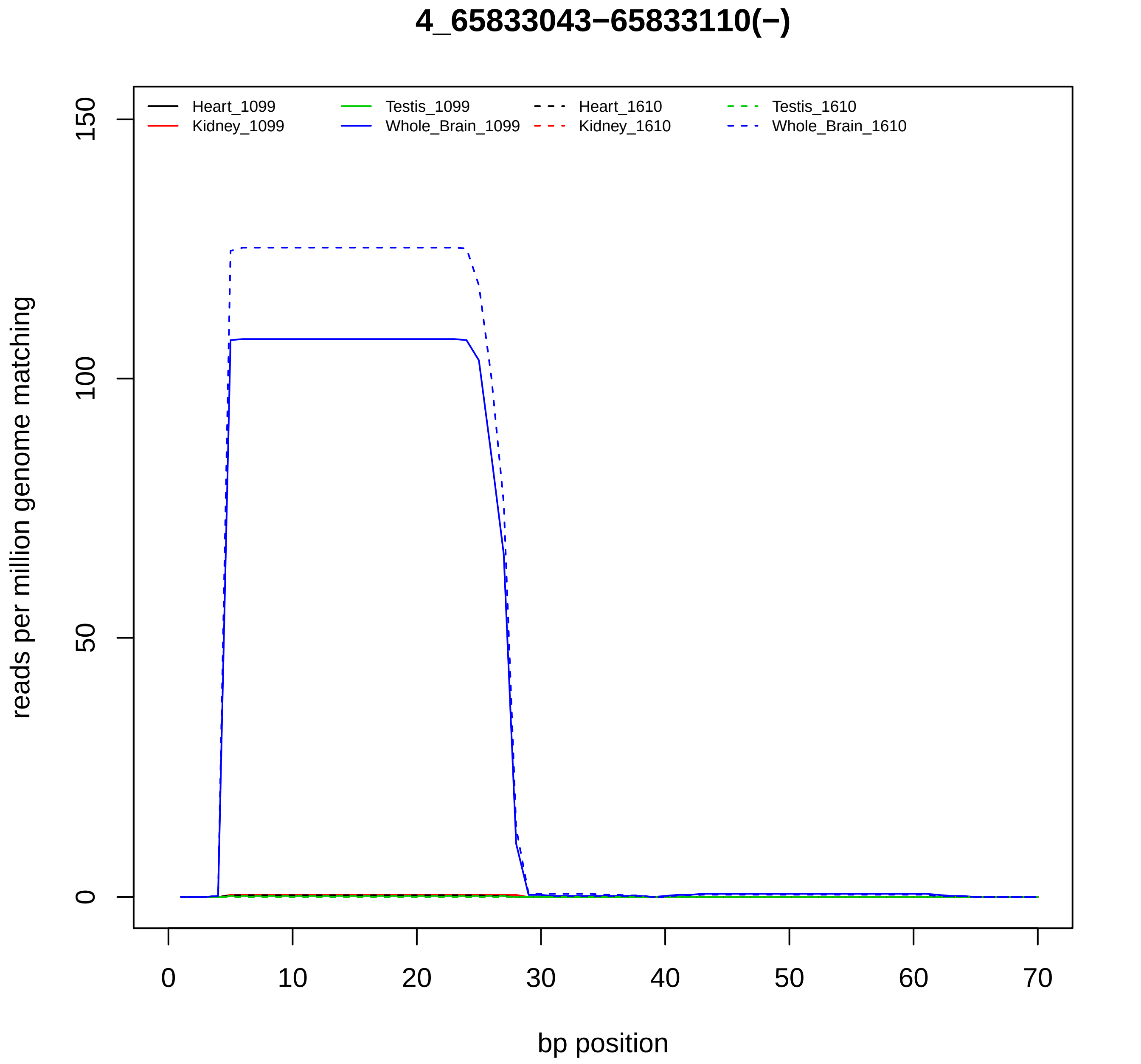

4_65833043−65833110(−)
Heart_1099
Kidney_1099
Testis_1099
Whole_Brain_1099
Heart_1610
Kidney_1610
Testis_1610
Whole_Brain_1610
reads per million genome matching
0
50
100
150
0
10
20
30
40
50
60
70
bp position

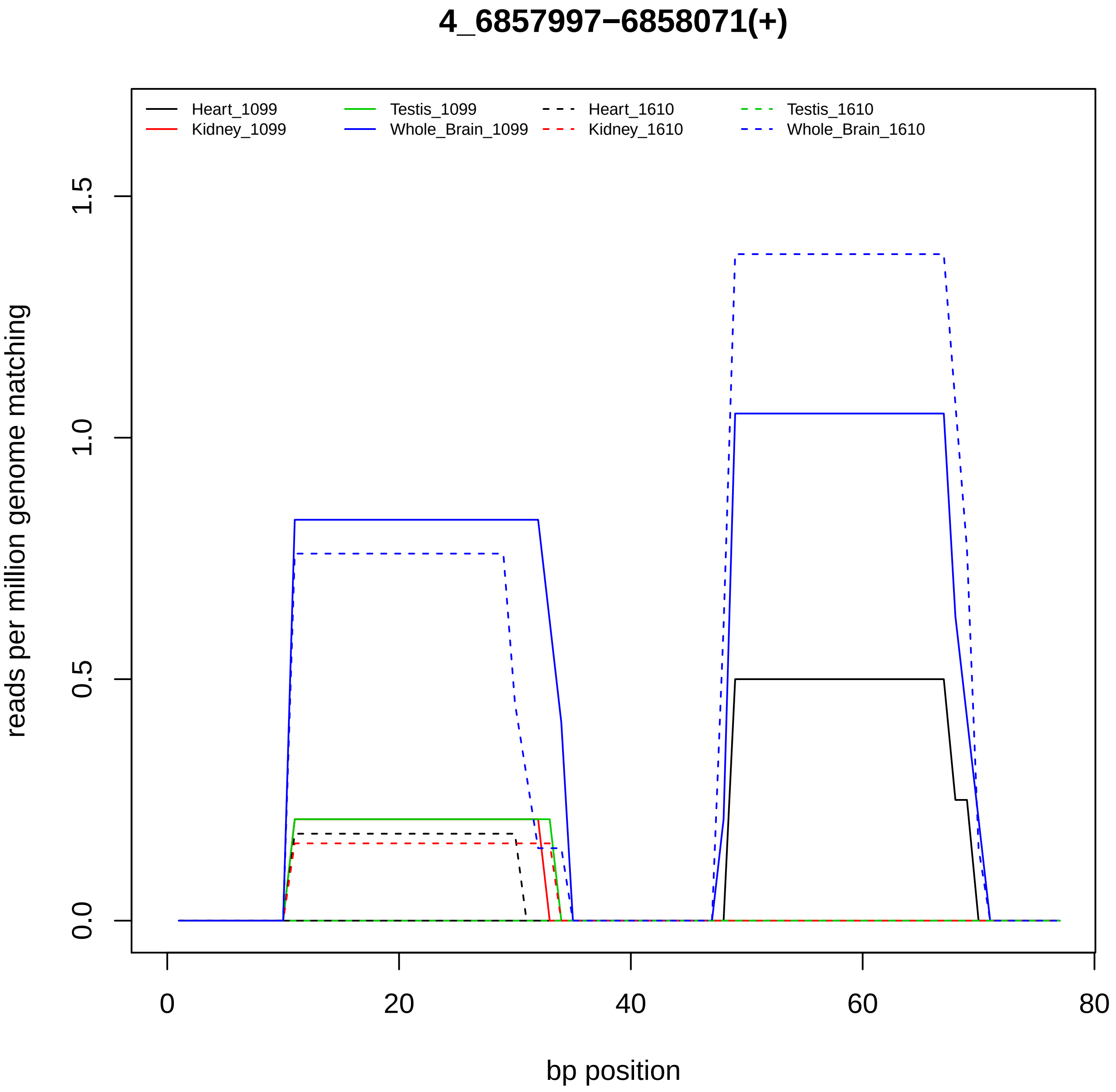

4_6857997−6858071(+)
Heart_1099
Kidney_1099
Testis_1099
Whole_Brain_1099
Heart_1610
Kidney_1610
Testis_1610
Whole_Brain_1610
reads per million genome matching
0.0
0.5
1.0
1.5
0
20
40
60
80
bp position

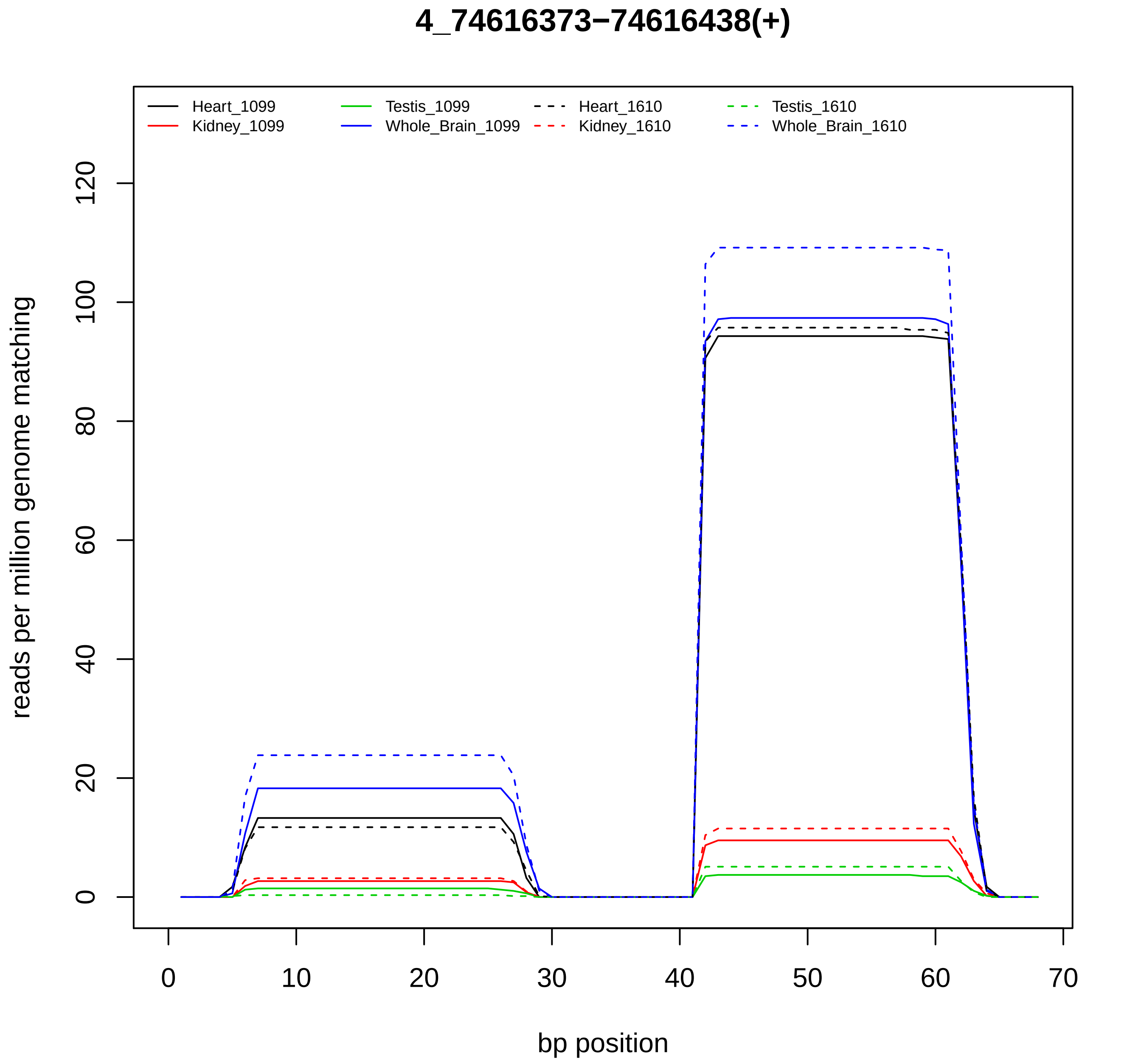

4_74616373−74616438(+)
Heart_1099
Kidney_1099
Testis_1099
Whole_Brain_1099
Heart_1610
Kidney_1610
Testis_1610
Whole_Brain_1610
reads per million genome matching
bp position
0
20
40
60
80
100
120
0
10
20
30
40
50
60
70

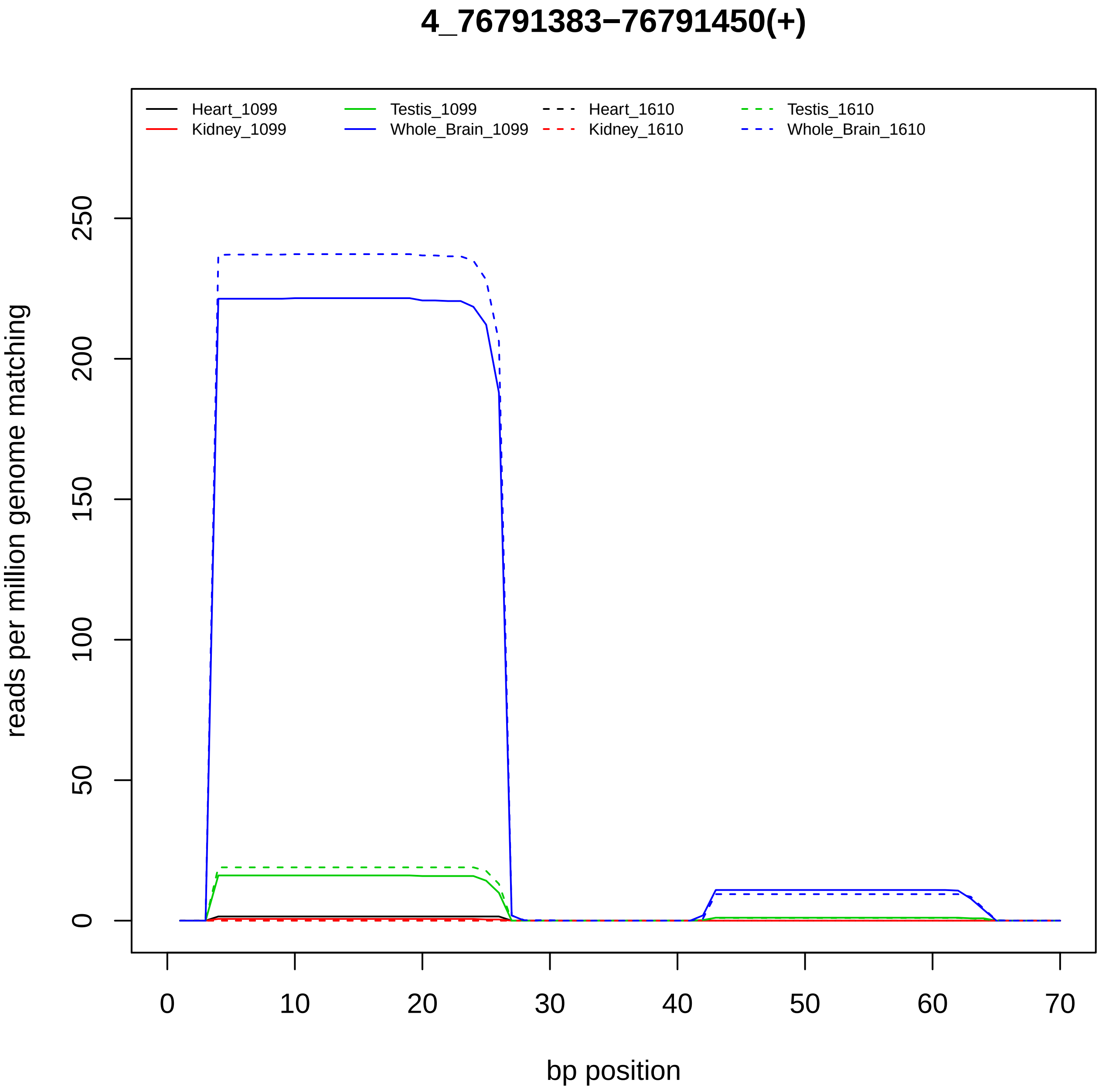
4_76791383−76791450(+)
Heart_1099
Kidney_1099
Testis_1099
Whole_Brain_1099
Heart_1610
Kidney_1610
Testis_1610
Whole_Brain_1610
reads per million genome matching
0
50
100
150
200
250
0
10
20
30
40
50
60
70
bp position

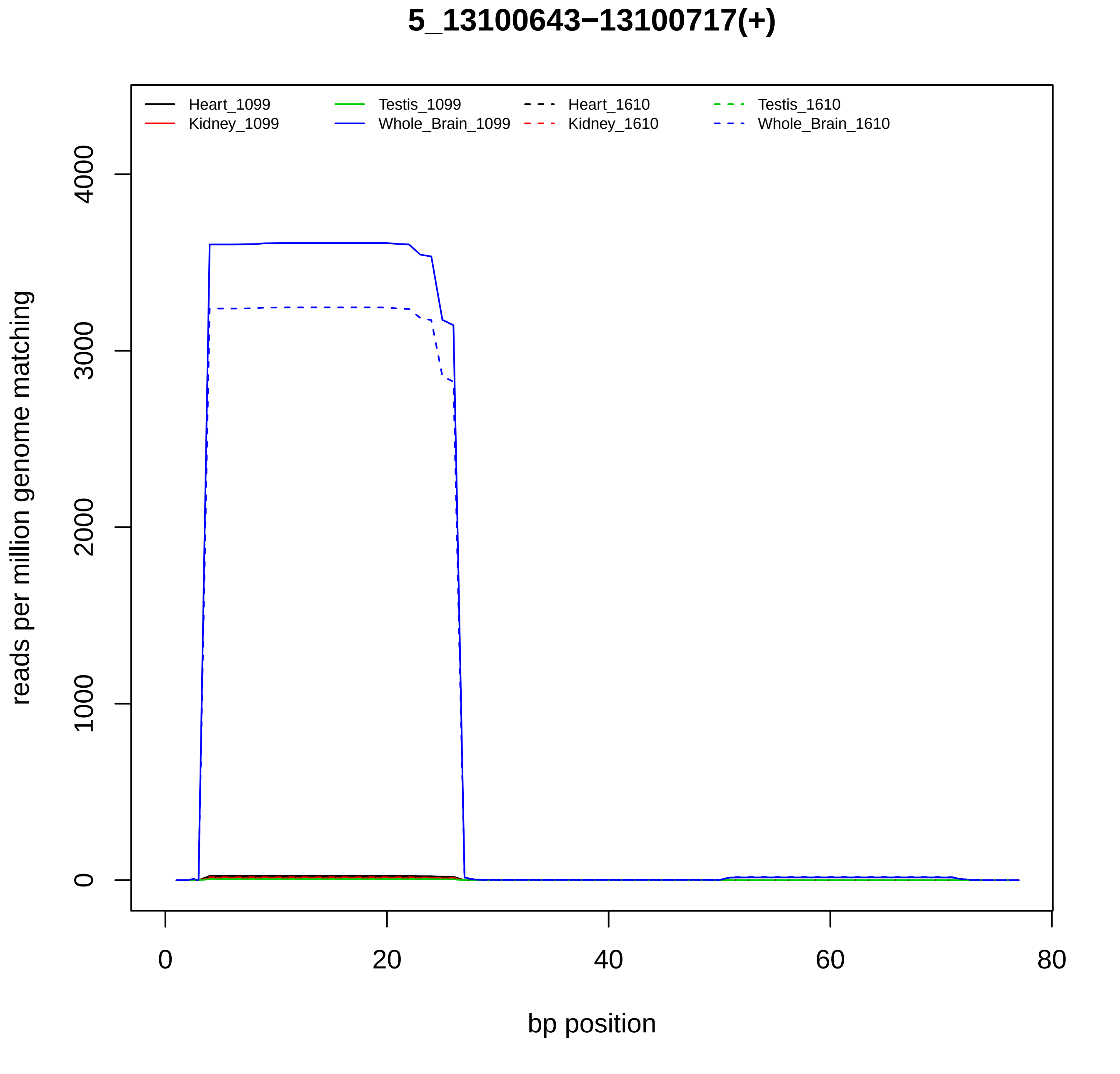

5_13100643−13100717(+)
Heart_1099
Kidney_1099
Testis_1099
Whole_Brain_1099
Heart_1610
Kidney_1610
Testis_1610
Whole_Brain_1610
reads per million genome matching
0
1000
2000
3000
4000
0
20
40
60
80
bp position

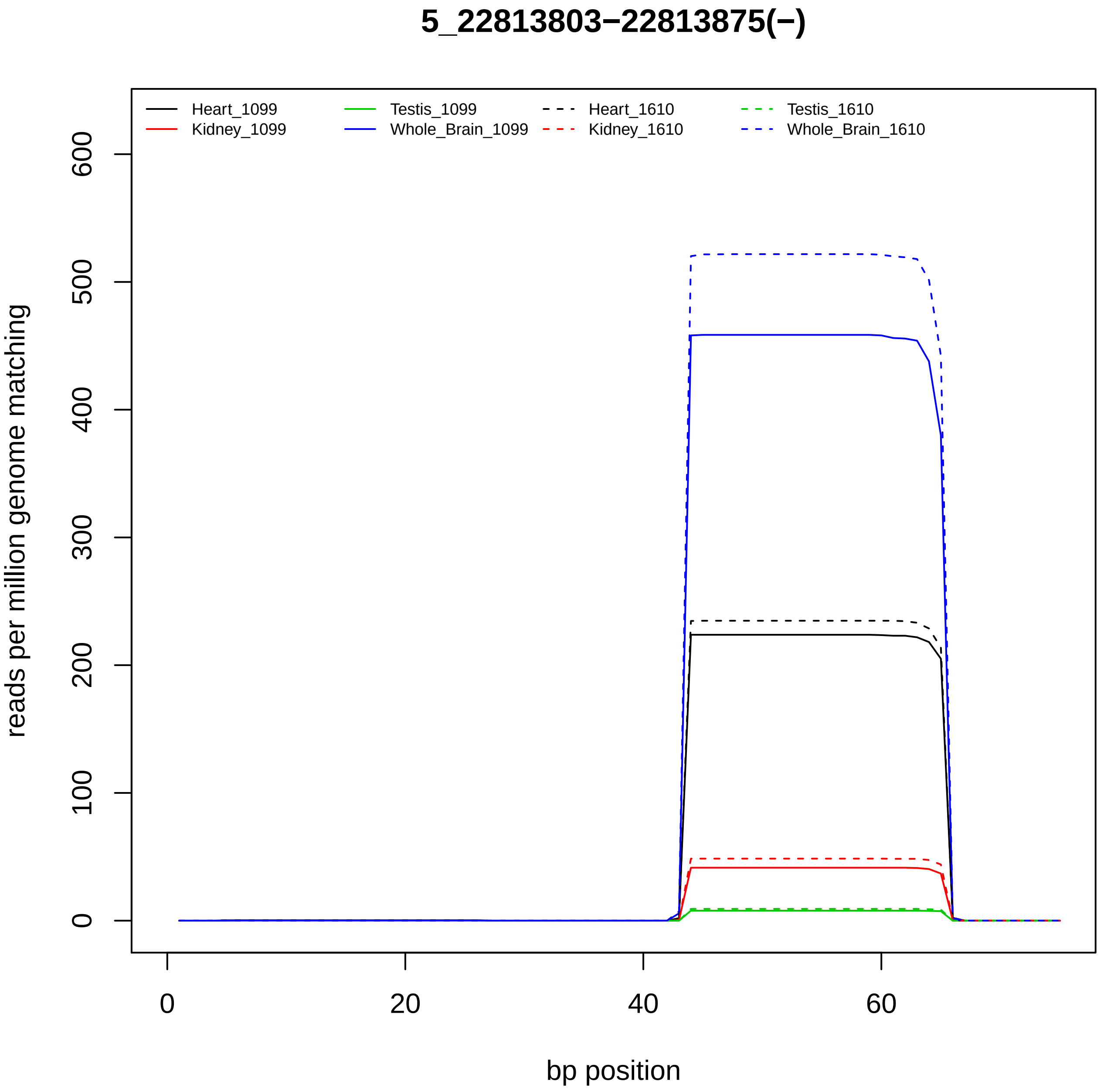

5_22813803−22813875(−)
Heart_1099
Kidney_1099
Testis_1099
Whole_Brain_1099
Heart_1610
Kidney_1610
Testis_1610
Whole_Brain_1610
reads per million genome matching
0
100
200
300
400
500
600
0
20
40
60
bp position

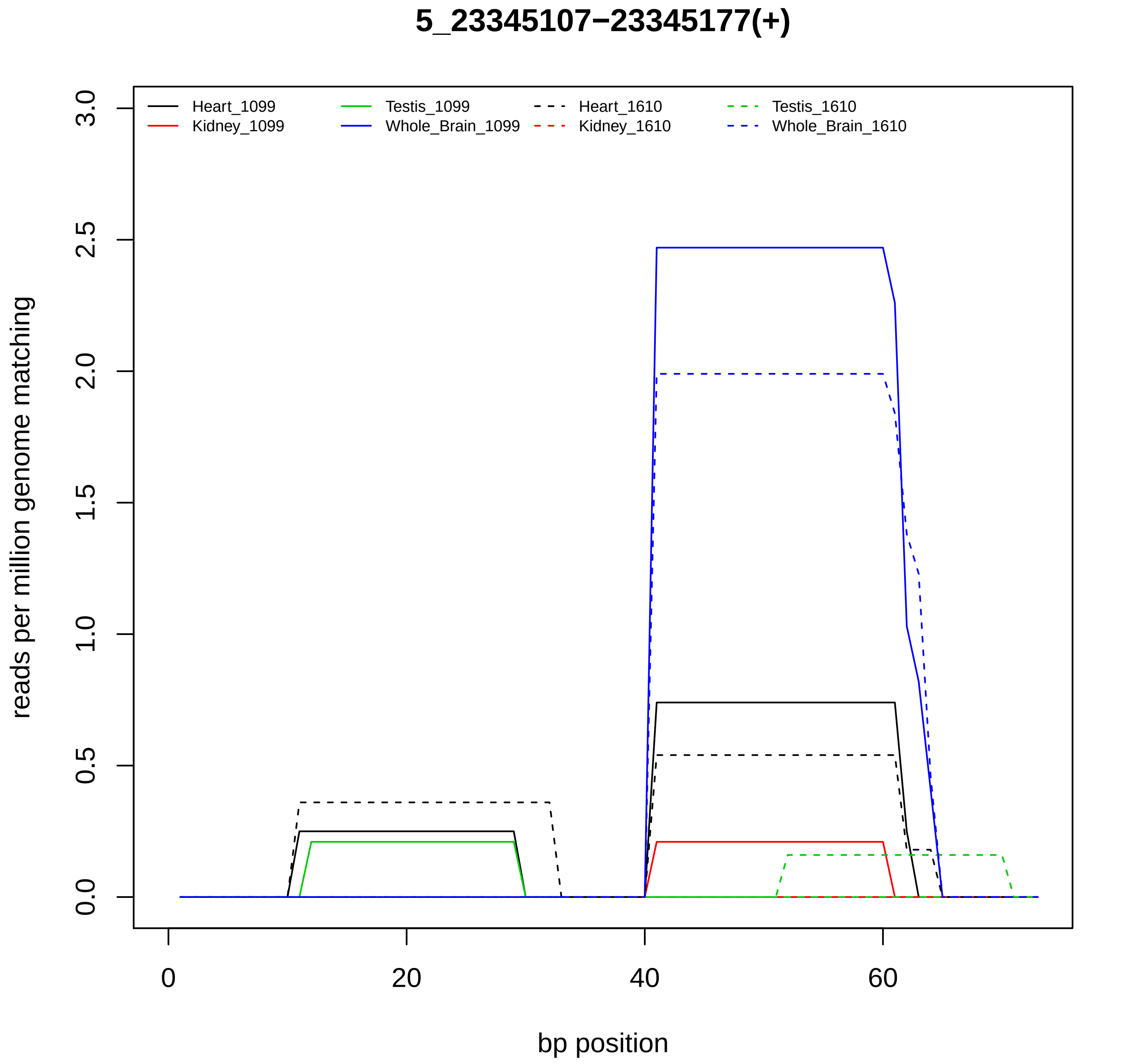

5_23345107−23345177(+)
Heart_1099
Kidney_1099
Testis_1099
Whole_Brain_1099
Heart_1610
Kidney_1610
Testis_1610
Whole_Brain_1610
reads per million genome matching
0.0
0.5
1.0
1.5
2.0
2.5
3.0
bp position
0
20
40
60

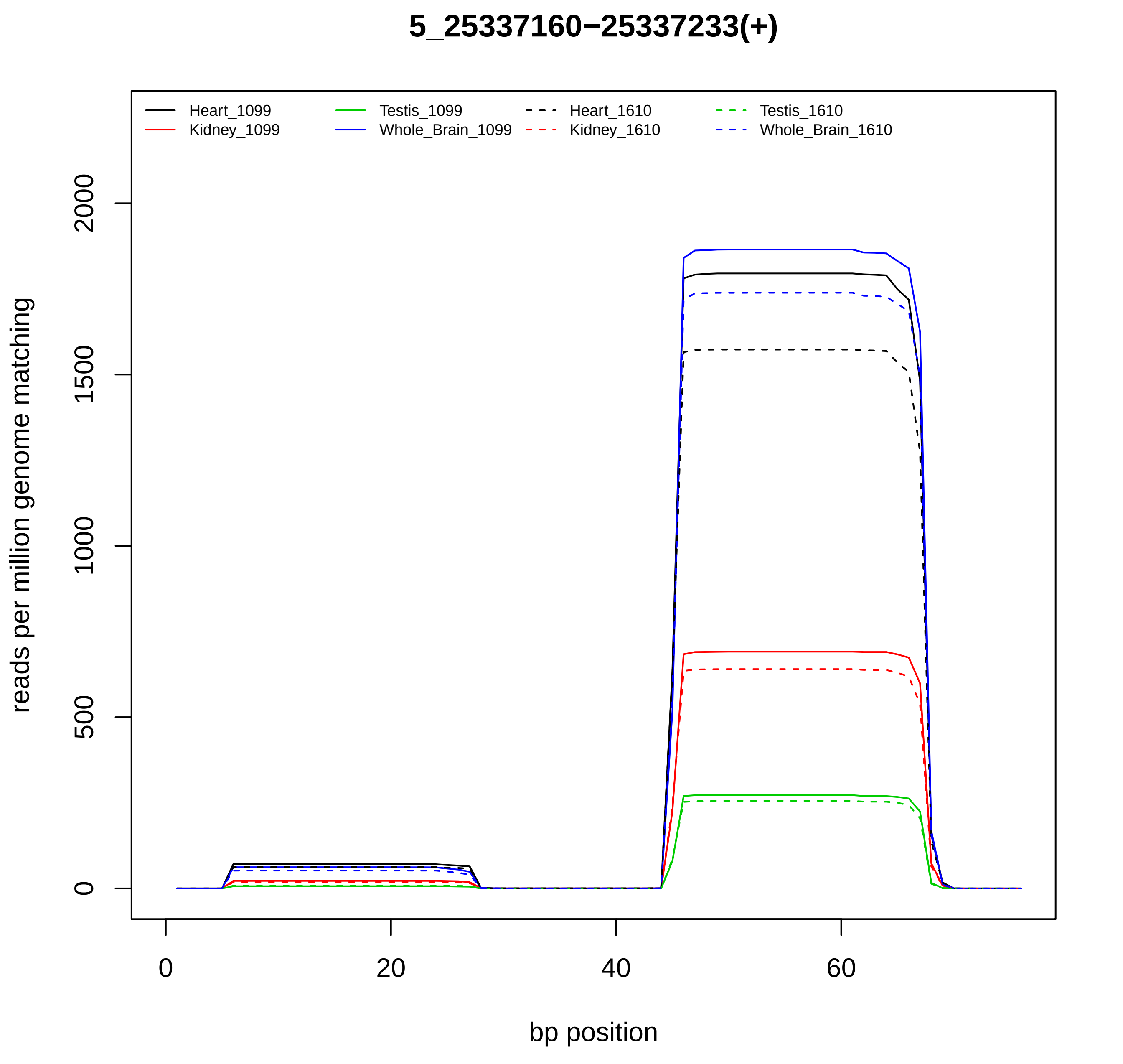

5_25337160−25337233(+)
Heart_1099
Kidney_1099
Testis_1099
Whole_Brain_1099
Heart_1610
Kidney_1610
Testis_1610
Whole_Brain_1610
reads per million genome matching
bp position
0
500
1000
1500
2000
0
20
40
60

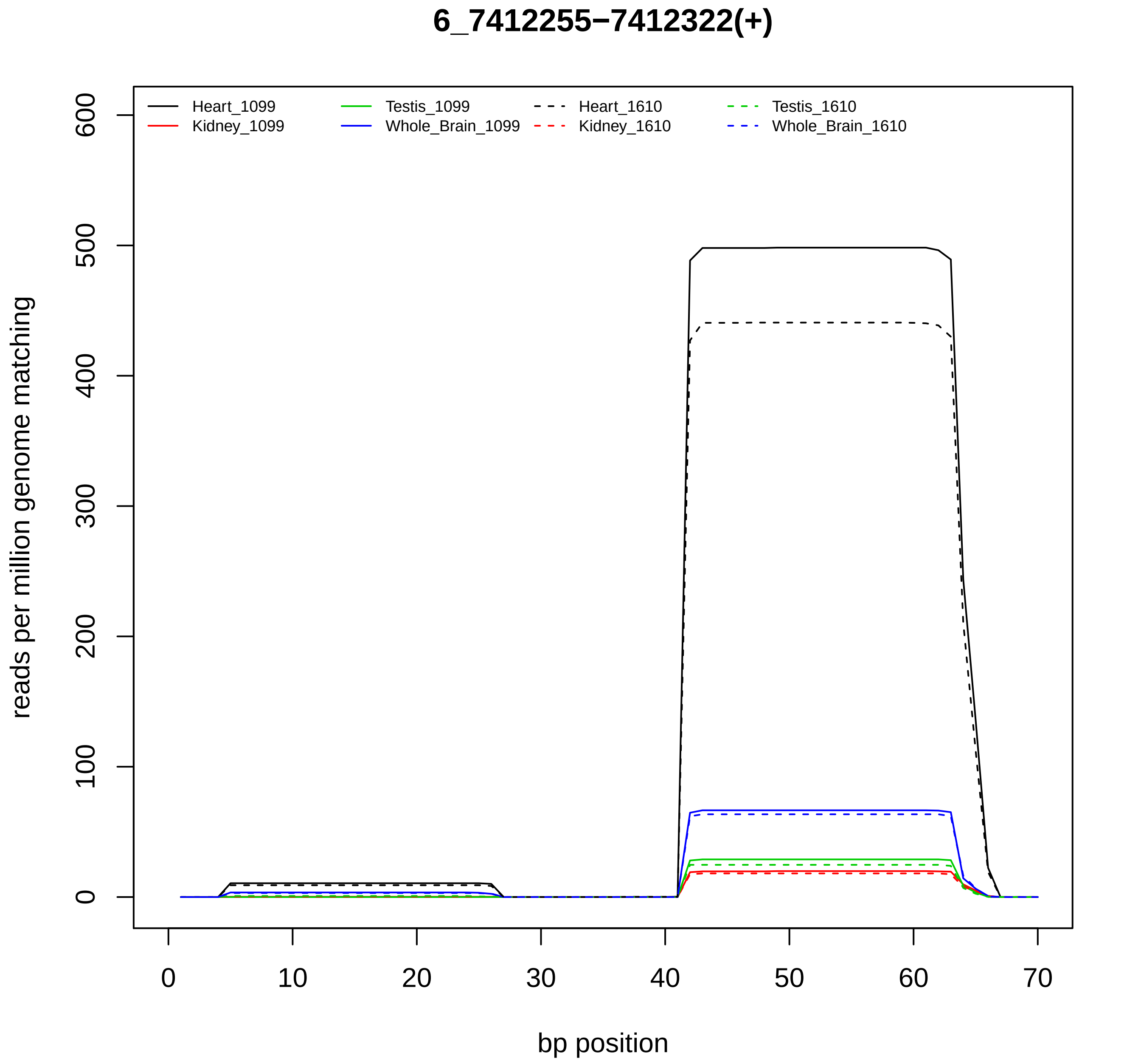

6_7412255−7412322(+)
Heart_1099
Kidney_1099
Testis_1099
Whole_Brain_1099
Heart_1610
Kidney_1610
Testis_1610
Whole_Brain_1610
reads per million genome matching
0
100
200
300
400
500
600
0
10
20
30
40
50
60
70
bp position

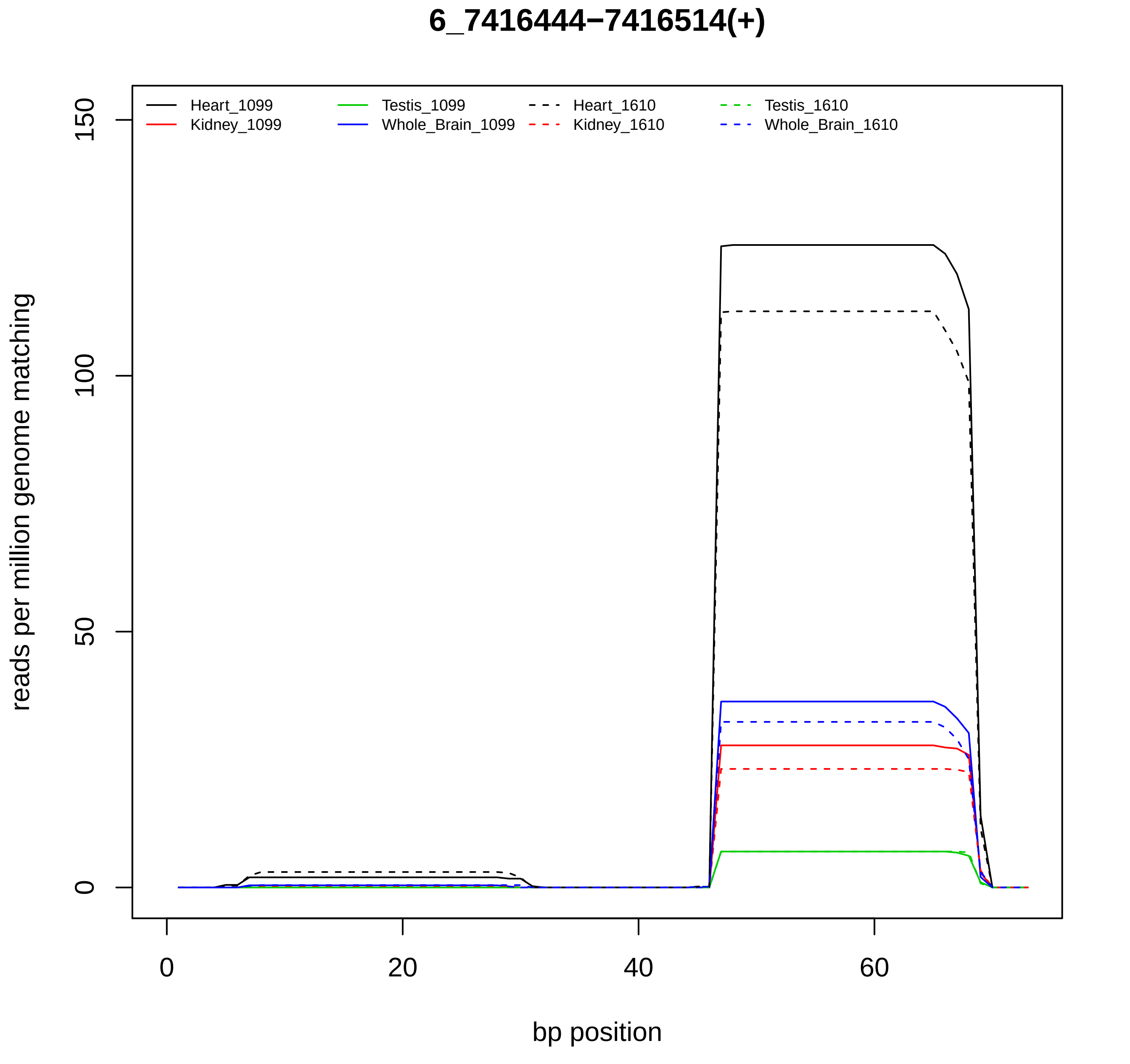

6_7416444−7416514(+)
Heart_1099
Kidney_1099
Testis_1099
Whole_Brain_1099
Heart_1610
Kidney_1610
Testis_1610
Whole_Brain_1610
150
100
50
0
reads per million genome matching
0
20
40
60
bp position

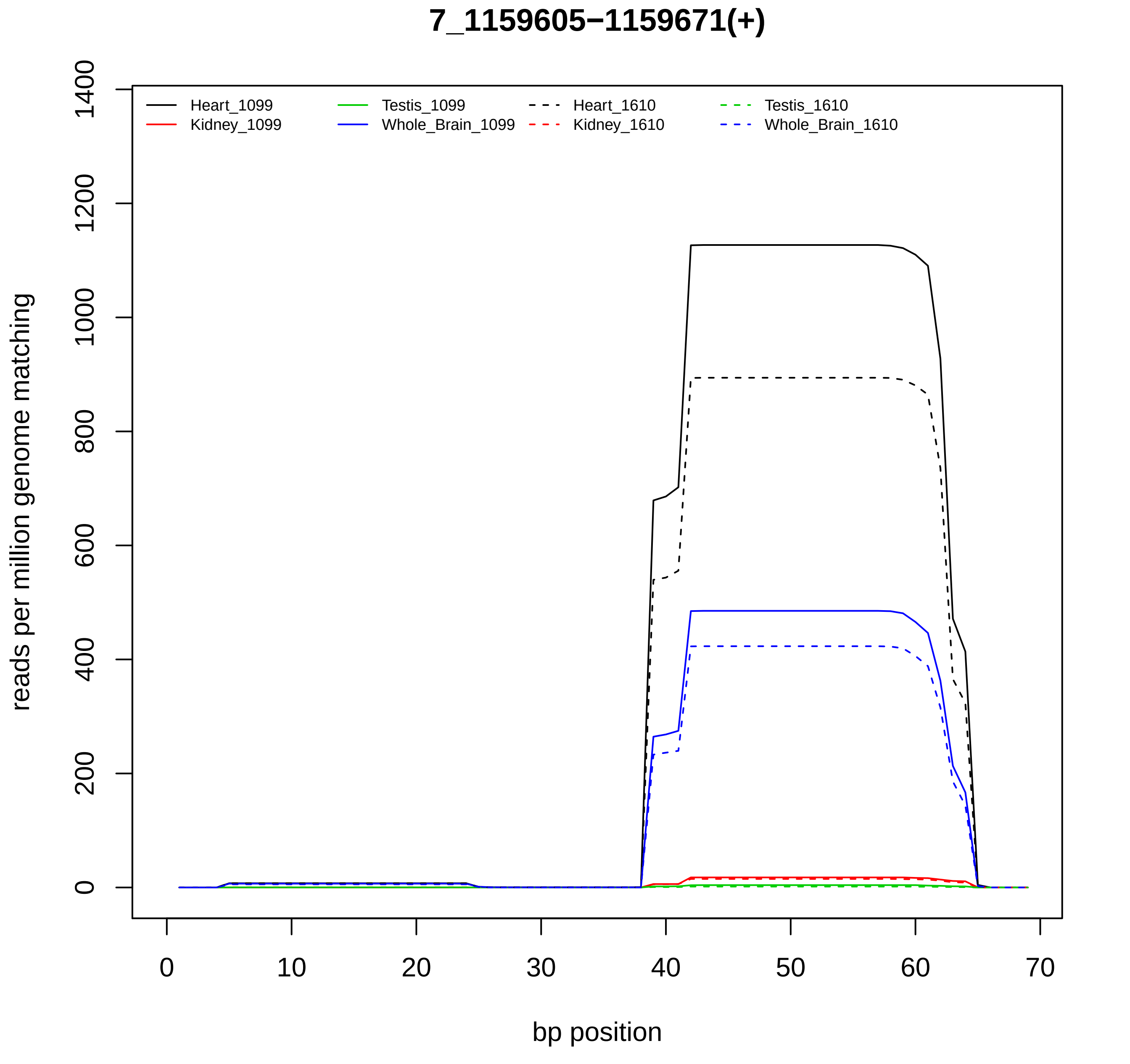
7_1159605−1159671(+)
Heart_1099
Kidney_1099
Testis_1099
Whole_Brain_1099
Heart_1610
Kidney_1610
Testis_1610
Whole_Brain_1610
reads per million genome matching
bp position
0
200
400
600
800
1000
1200
1400
0
10
20
30
40
50
60
70

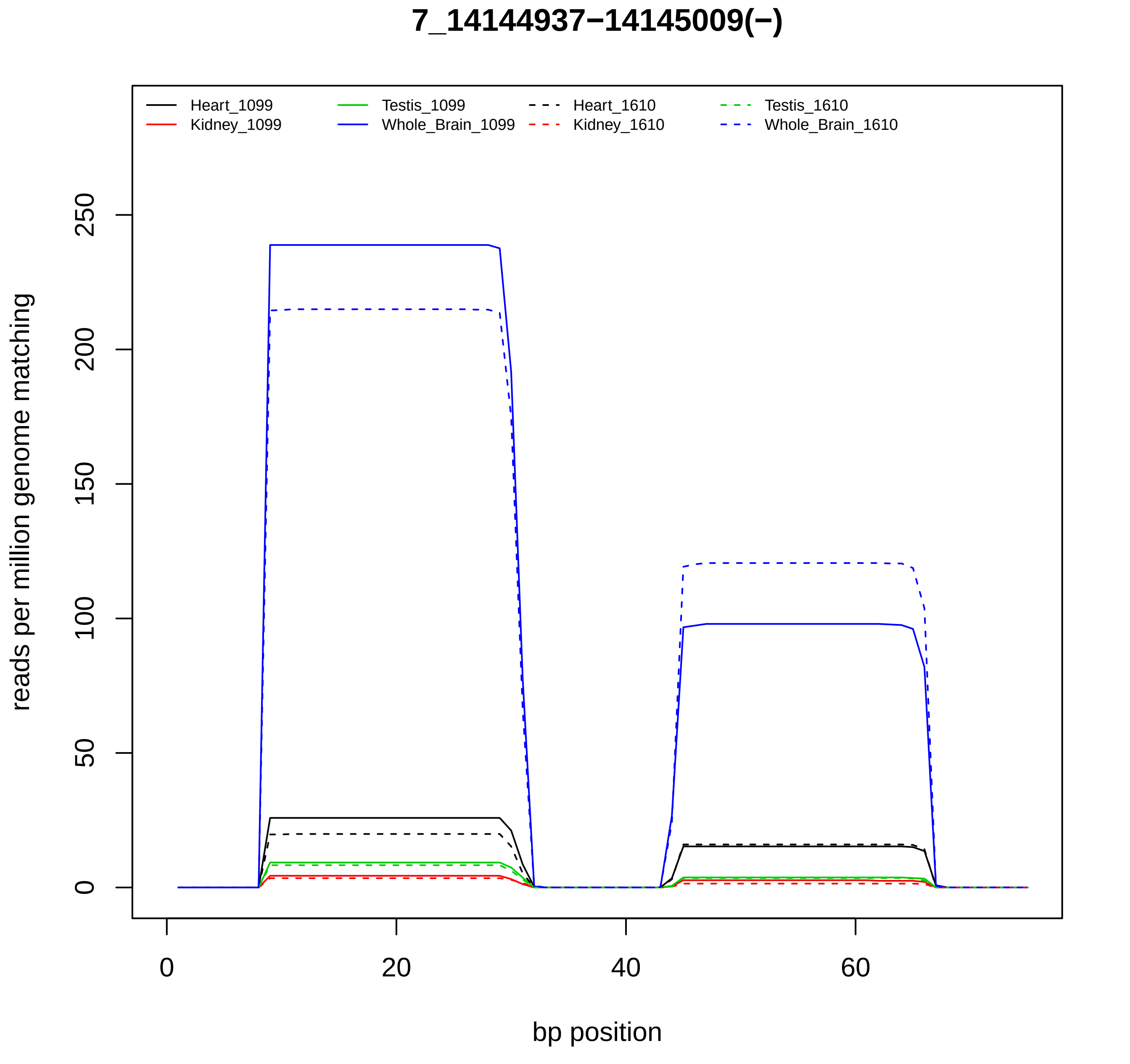
7_14144937−14145009(−)
Heart_1099
Kidney_1099
Testis_1099
Whole_Brain_1099
Heart_1610
Kidney_1610
Testis_1610
Whole_Brain_1610
reads per million genome matching
bp position
0
50
100
150
200
250
0
20
40
60

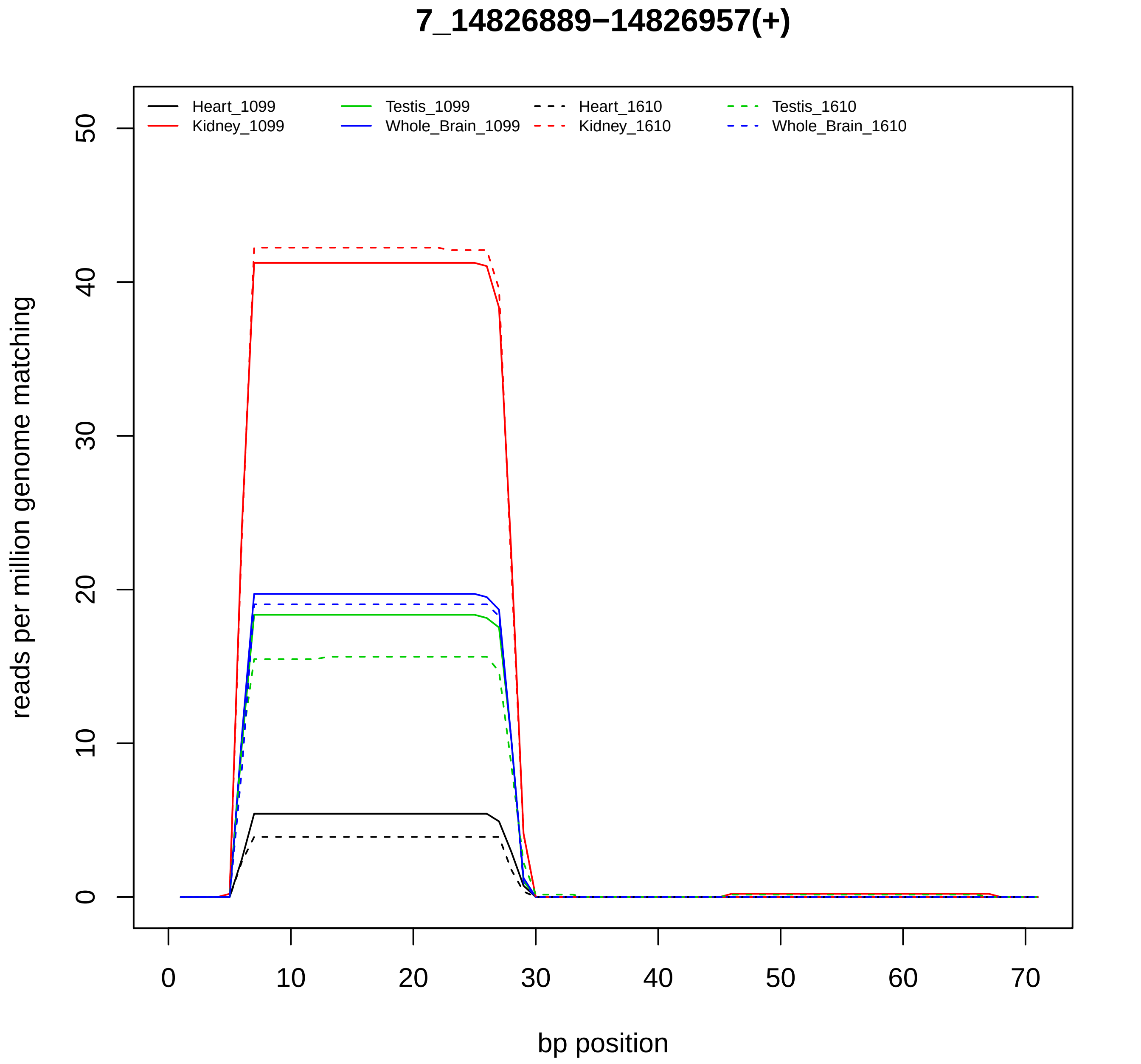
7_14826889−14826957(+)
Heart_1099
Kidney_1099
Testis_1099
Whole_Brain_1099
Heart_1610
Kidney_1610
Testis_1610
Whole_Brain_1610
reads per million genome matching
0
10
20
30
40
50
0
10
20
30
40
50
60
70
bp position

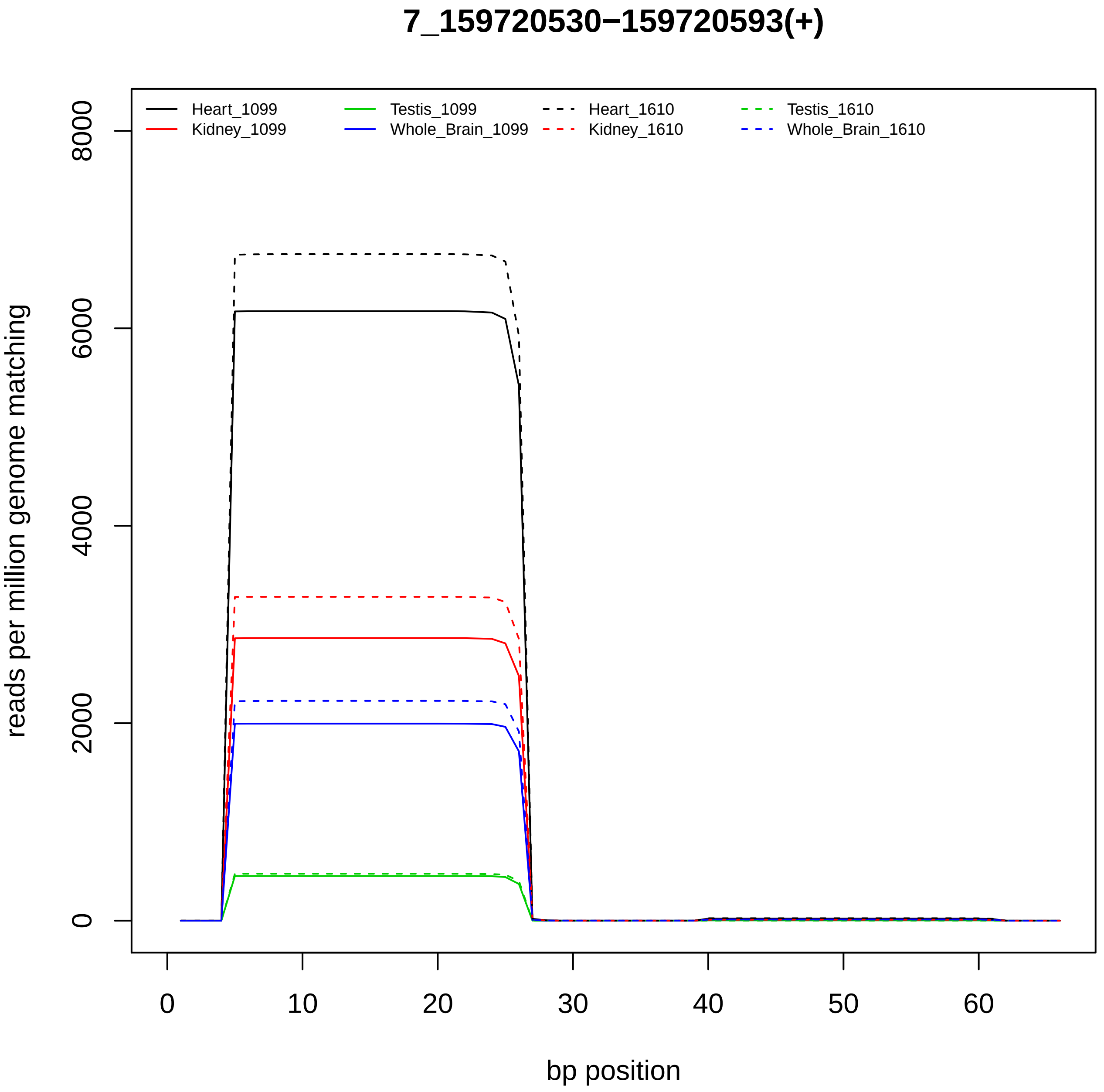
7_159720530−159720593(+)
Heart_1099
Kidney_1099
Testis_1099
Whole_Brain_1099
Heart_1610
Kidney_1610
Testis_1610
Whole_Brain_1610
reads per million genome matching
0
2000
4000
6000
8000
bp position
0
10
20
30
40
50
60

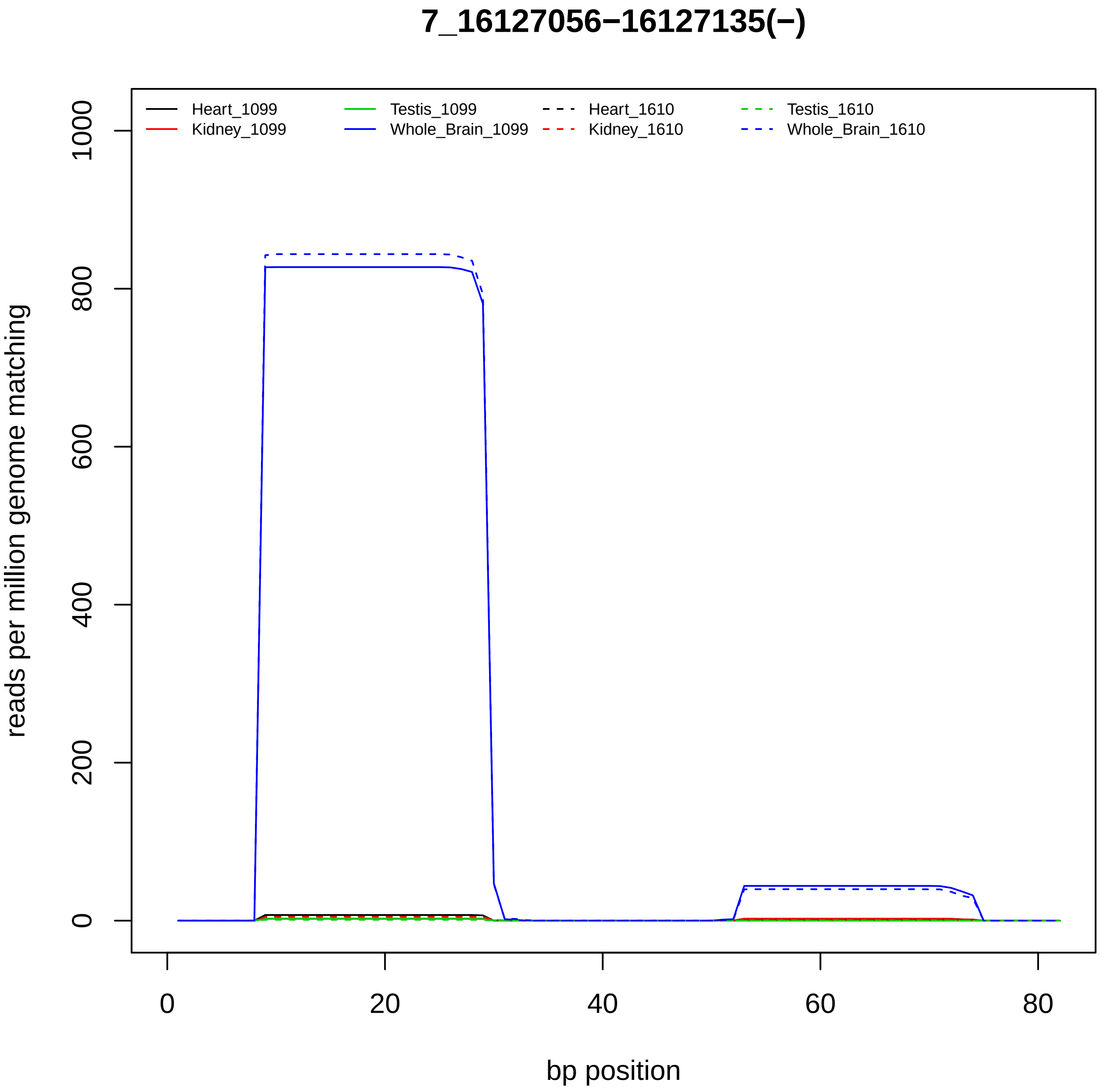
7_16127056−16127135(−)
Heart_1099
Kidney_1099
Testis_1099
Whole_Brain_1099
Heart_1610
Kidney_1610
Testis_1610
Whole_Brain_1610
reads per million genome matching
0
200
400
600
800
1000
bp position
0
20
40
60
80

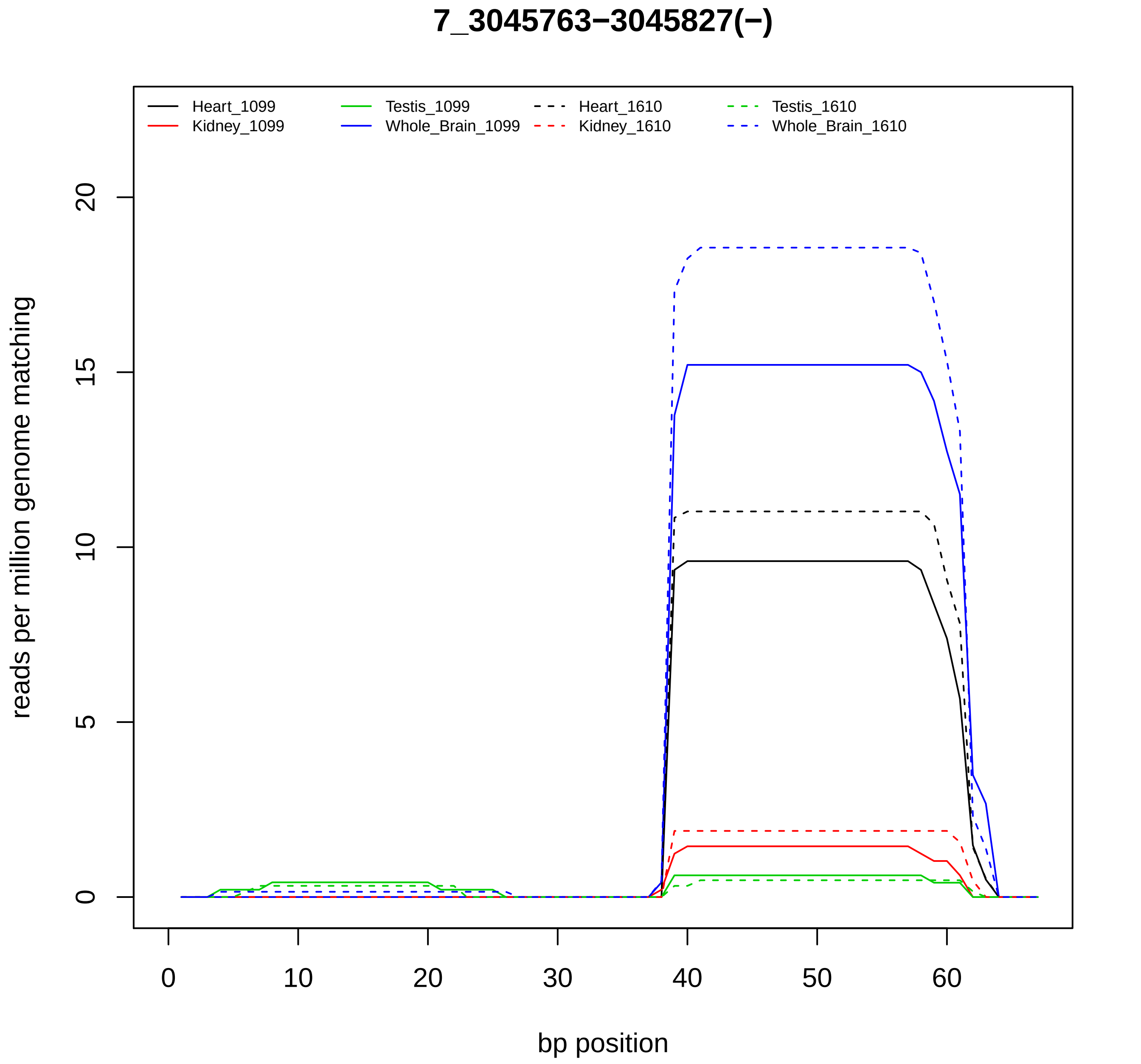

7_3045763−3045827(−)
Heart_1099
Kidney_1099
Testis_1099
Whole_Brain_1099
Heart_1610
Kidney_1610
Testis_1610
Whole_Brain_1610
reads per million genome matching
bp position
0
5
10
15
20
0
10
20
30
40
50
60

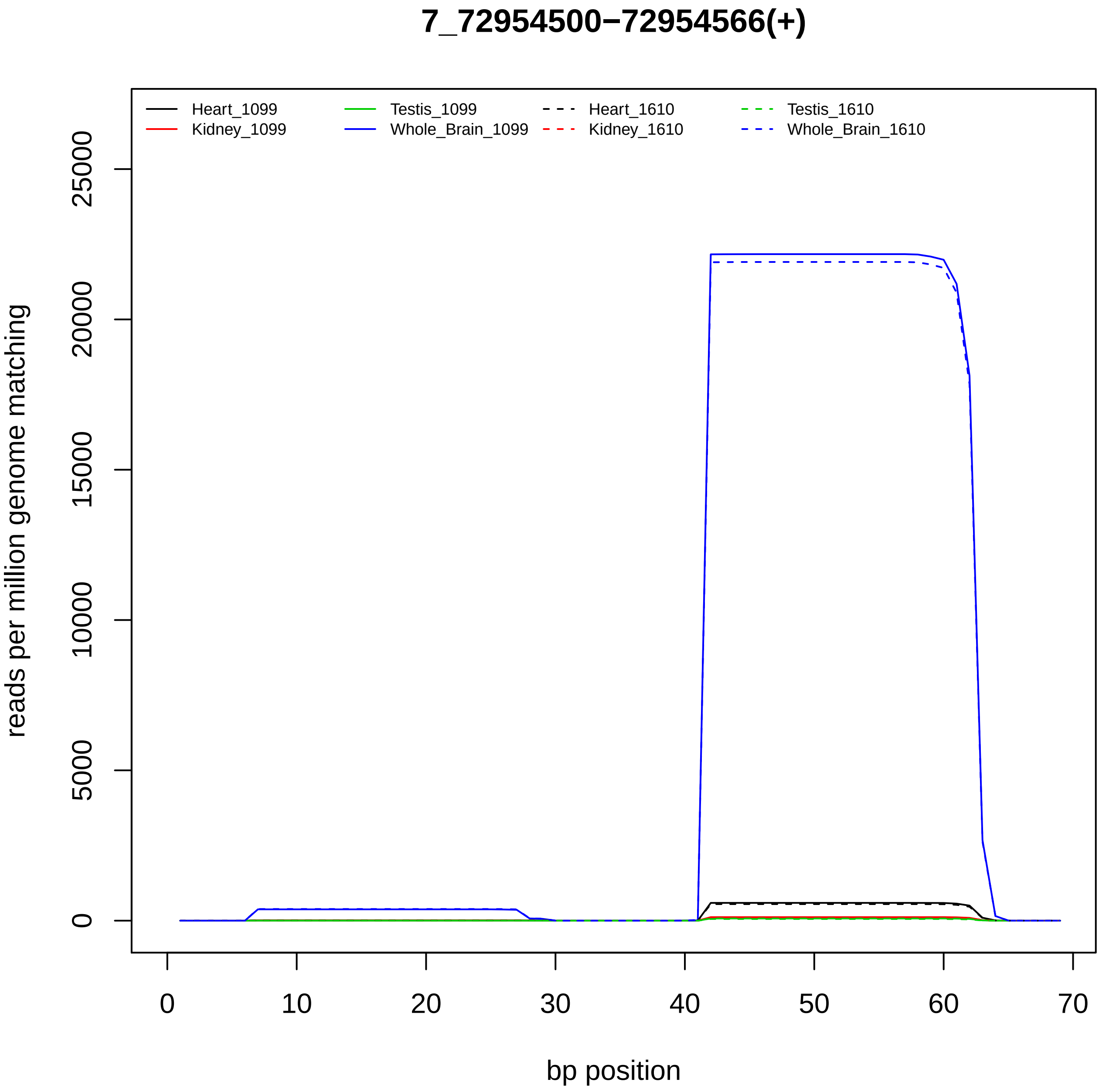

7_72954500−72954566(+)
Heart_1099
Kidney_1099
Testis_1099
Whole_Brain_1099
Heart_1610
Kidney_1610
Testis_1610
Whole_Brain_1610
reads per million genome matching
0
5000
10000
15000
20000
25000
0
10
20
30
40
50
60
70
bp position

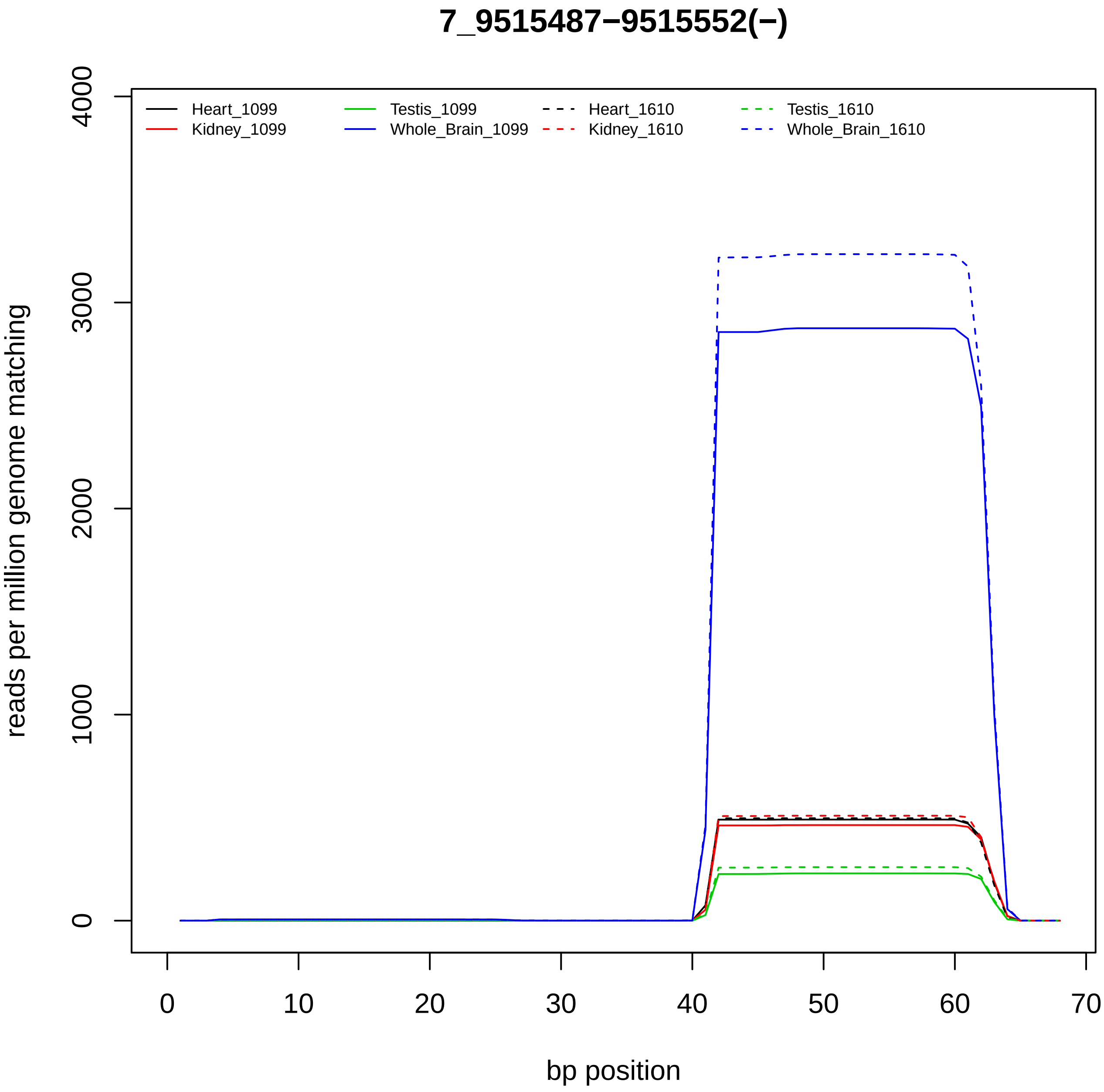

7_9515487−9515552(−)
Heart_1099
Kidney_1099
Testis_1099
Whole_Brain_1099
Heart_1610
Kidney_1610
Testis_1610
Whole_Brain_1610
reads per million genome matching
0
1000
2000
3000
4000
0
10
20
30
40
50
60
70
bp position

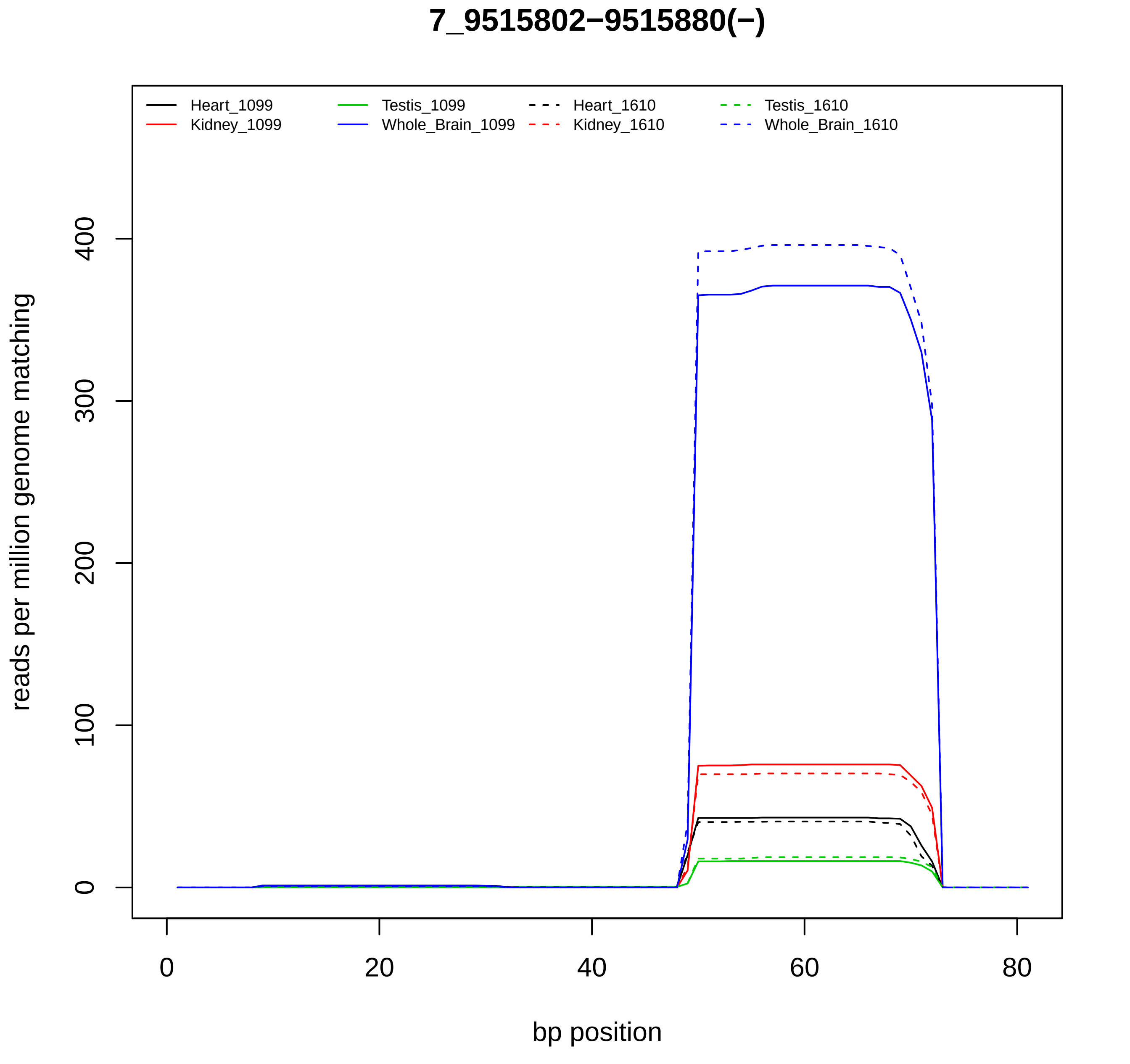

7_9515802−9515880(−)
Heart_1099
Kidney_1099
Testis_1099
Whole_Brain_1099
Heart_1610
Kidney_1610
Testis_1610
Whole_Brain_1610
reads per million genome matching
0
100
200
300
400
0
20
40
60
80
bp position

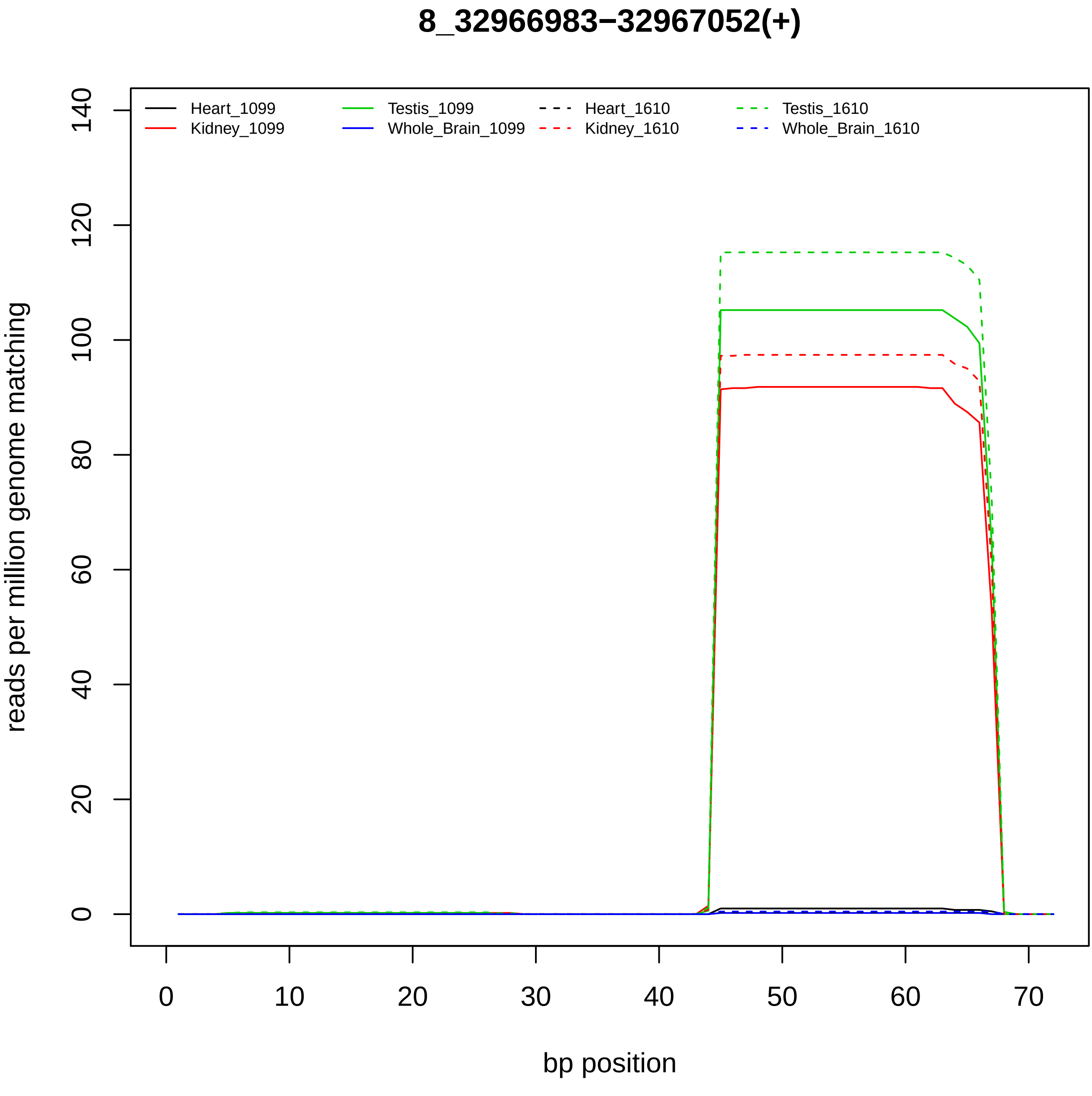

8_32966983−32967052(+)
Heart_1099
Kidney_1099
Testis_1099
Whole_Brain_1099
Heart_1610
Kidney_1610
Testis_1610
Whole_Brain_1610
reads per million genome matching
bp position
0
20
40
60
80
100
120
140
0
10
20
30
40
50
60
70

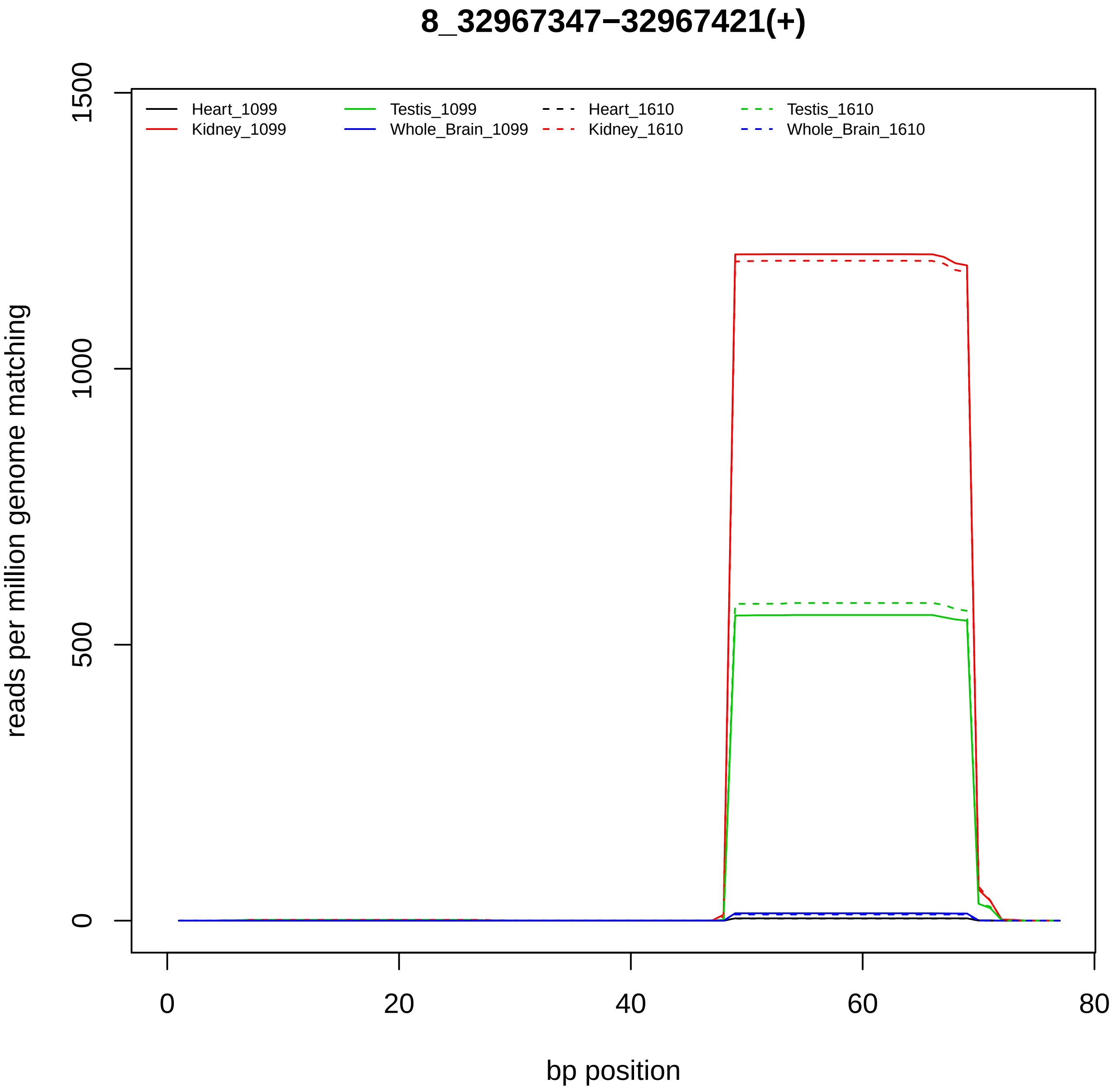

8_32967347−32967421(+)
Heart_1099
Kidney_1099
Testis_1099
Whole_Brain_1099
Heart_1610
Kidney_1610
Testis_1610
Whole_Brain_1610
reads per million genome matching
0
500
1000
1500
0
20
40
60
80
bp position

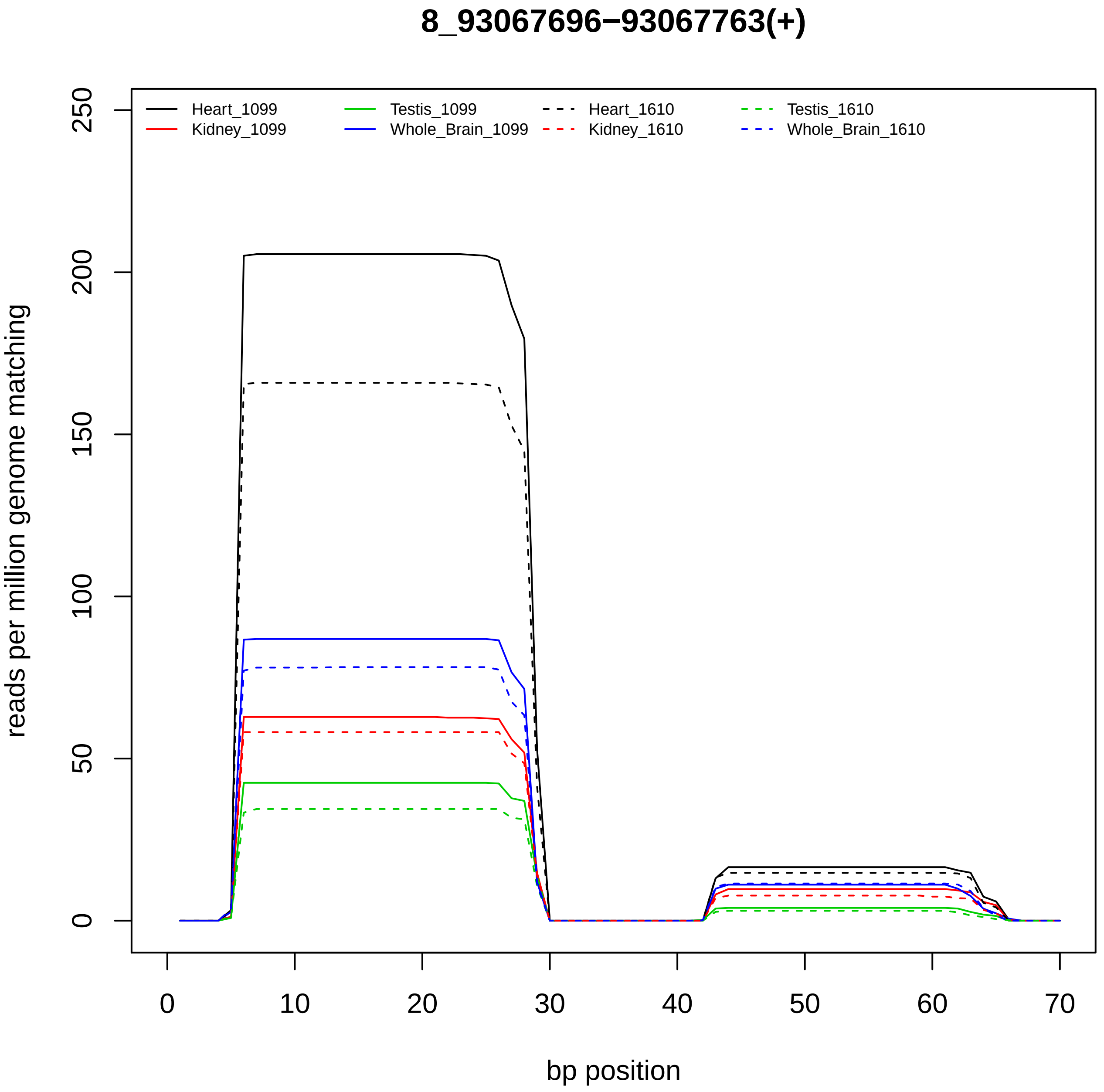
8_93067696−93067763(+)
Heart_1099
Kidney_1099
Testis_1099
Whole_Brain_1099
Heart_1610
Kidney_1610
Testis_1610
Whole_Brain_1610
reads per million genome matching
bp position
0
50
100
150
200
250
0
10
20
30
40
50
60
70

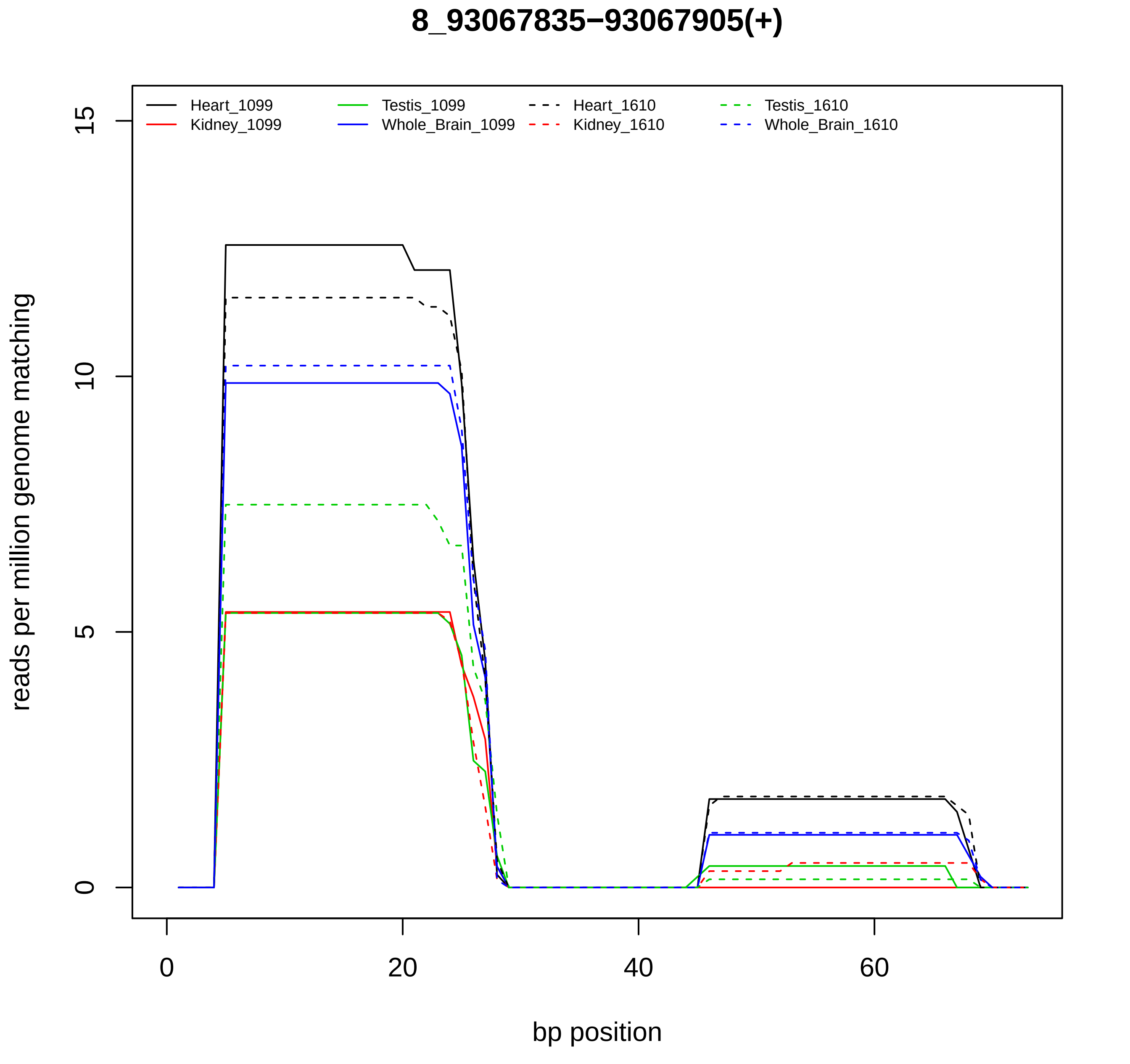
8_93067835−93067905(+)
Heart_1099
Kidney_1099
Testis_1099
Whole_Brain_1099
Heart_1610
Kidney_1610
Testis_1610
Whole_Brain_1610
reads per million genome matching
bp position
0
5
10
15
0
20
40
60

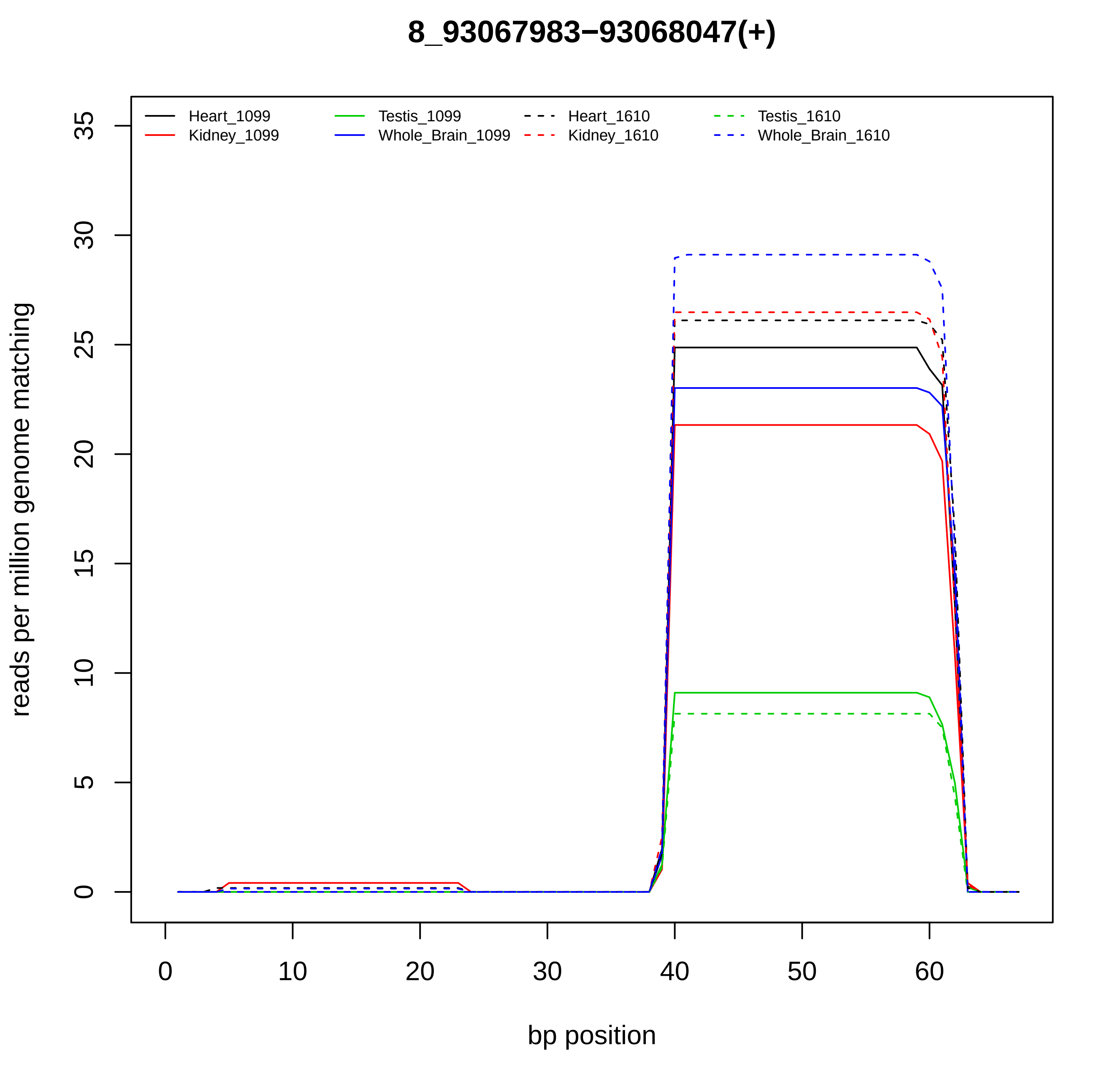

8_93067983−93068047(+)
Heart_1099
Kidney_1099
Testis_1099
Whole_Brain_1099
Heart_1610
Kidney_1610
Testis_1610
Whole_Brain_1610
reads per million genome matching
bp position
0
5
10
15
20
25
30
35
0
10
20
30
40
50
60

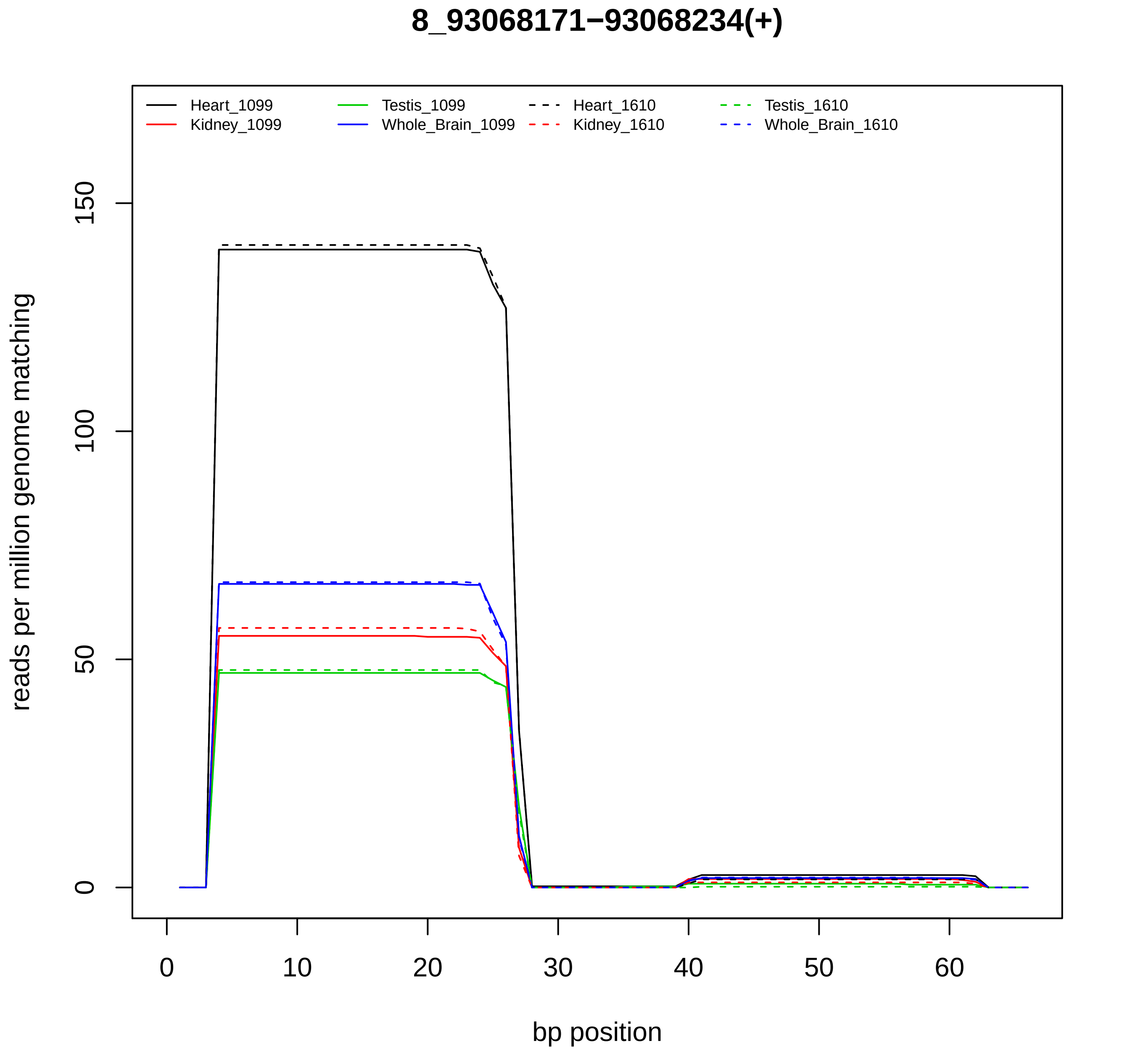
8_93068171−93068234(+)
Heart_1099
Kidney_1099
Testis_1099
Whole_Brain_1099
Heart_1610
Kidney_1610
Testis_1610
Whole_Brain_1610
reads per million genome matching
0
50
100
150
bp position
0
10
20
30
40
50
60

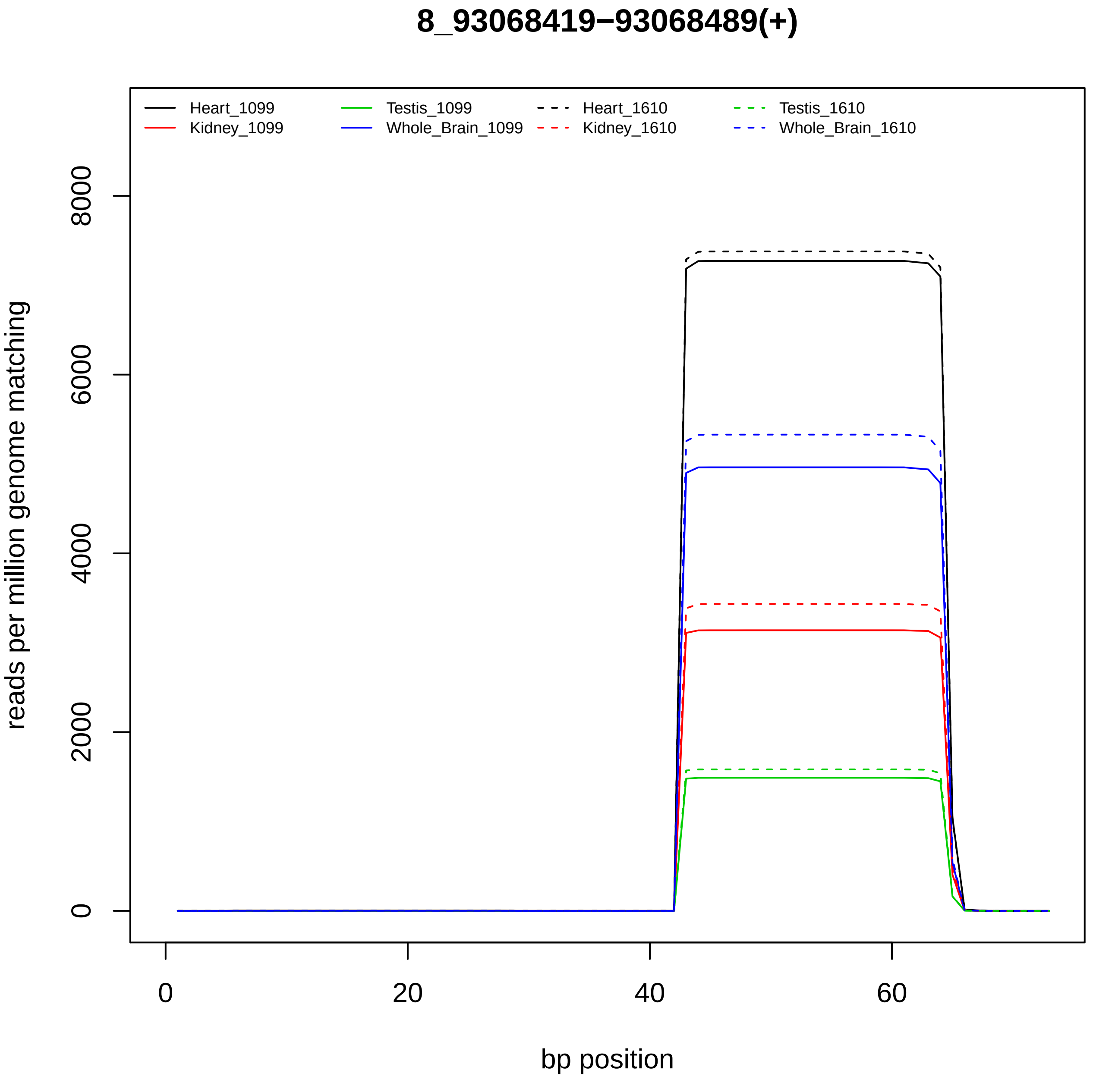
8_93068419−93068489(+)
Heart_1099
Kidney_1099
Testis_1099
Whole_Brain_1099
Heart_1610
Kidney_1610
Testis_1610
Whole_Brain_1610
reads per million genome matching
0
2000
4000
6000
8000
0
20
40
60
bp position

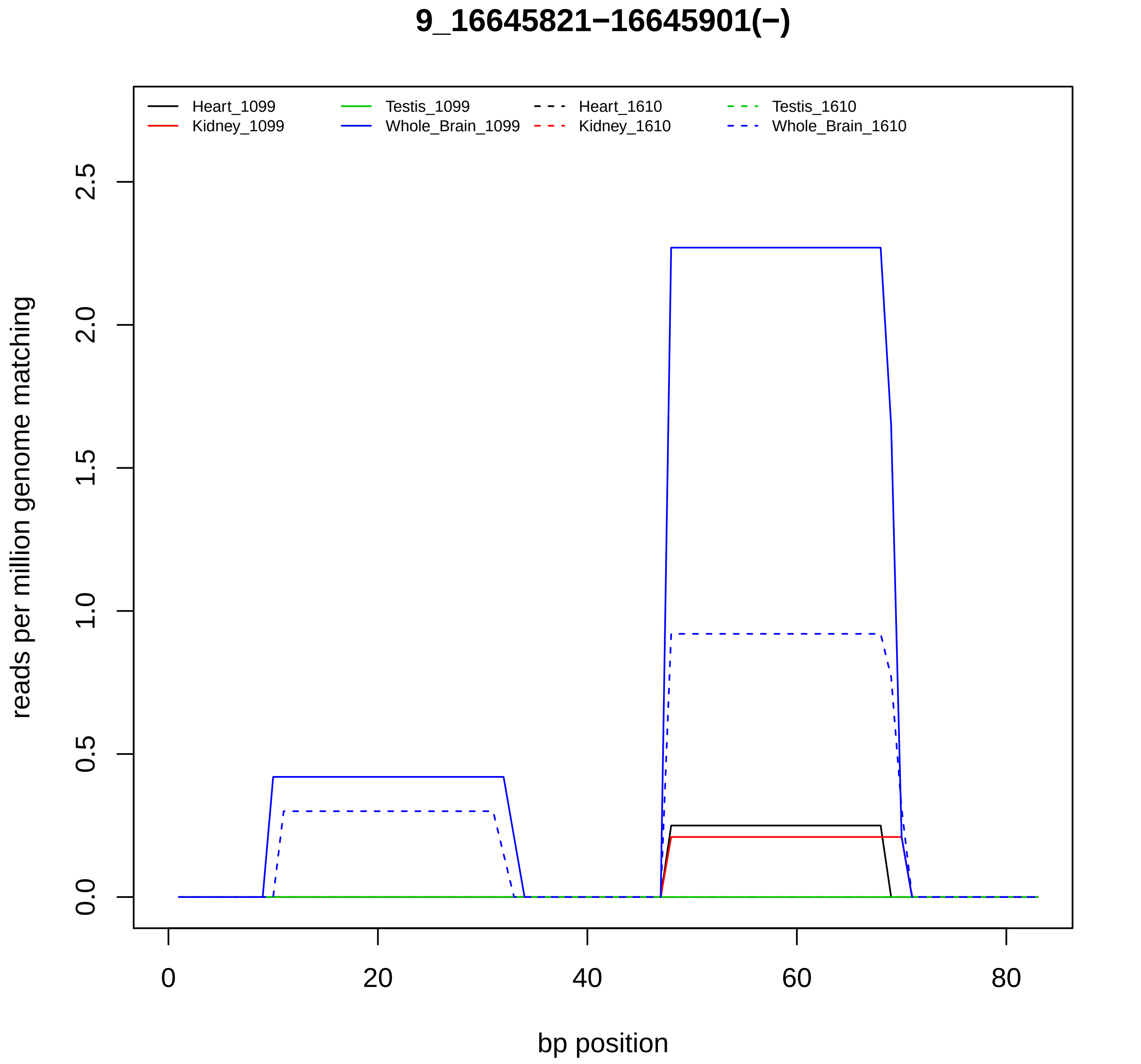

9_16645821−16645901(−)
Heart_1099
Kidney_1099
Testis_1099
Whole_Brain_1099
Heart_1610
Kidney_1610
Testis_1610
Whole_Brain_1610
reads per million genome matching
0.0
0.5
1.0
1.5
2.0
2.5
0
20
40
60
80
bp position

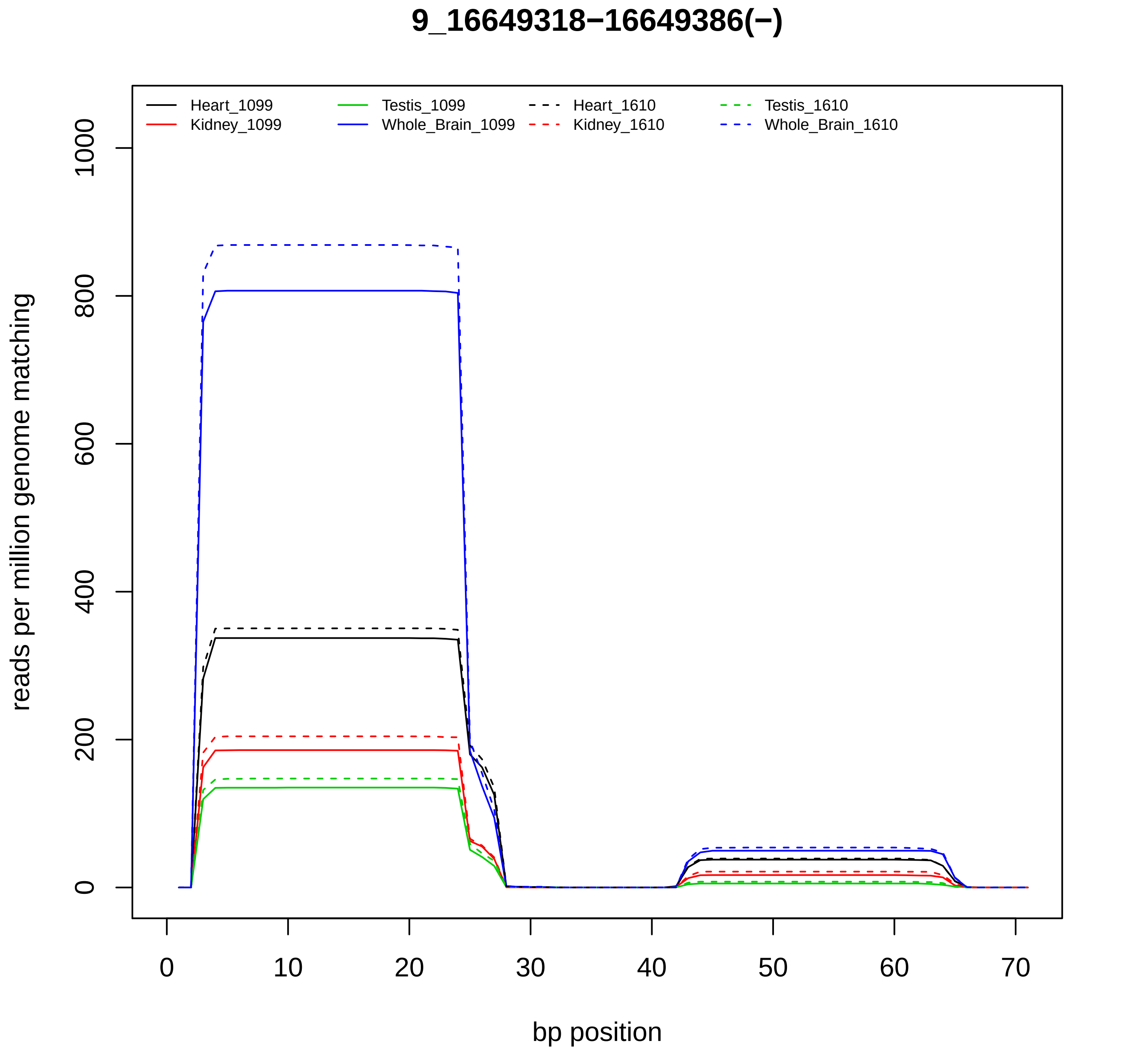
9_16649318−16649386(−)
Heart_1099
Kidney_1099
Testis_1099
Whole_Brain_1099
Heart_1610
Kidney_1610
Testis_1610
Whole_Brain_1610
reads per million genome matching
bp position
0
200
400
600
800
1000
0
10
20
30
40
50
60
70

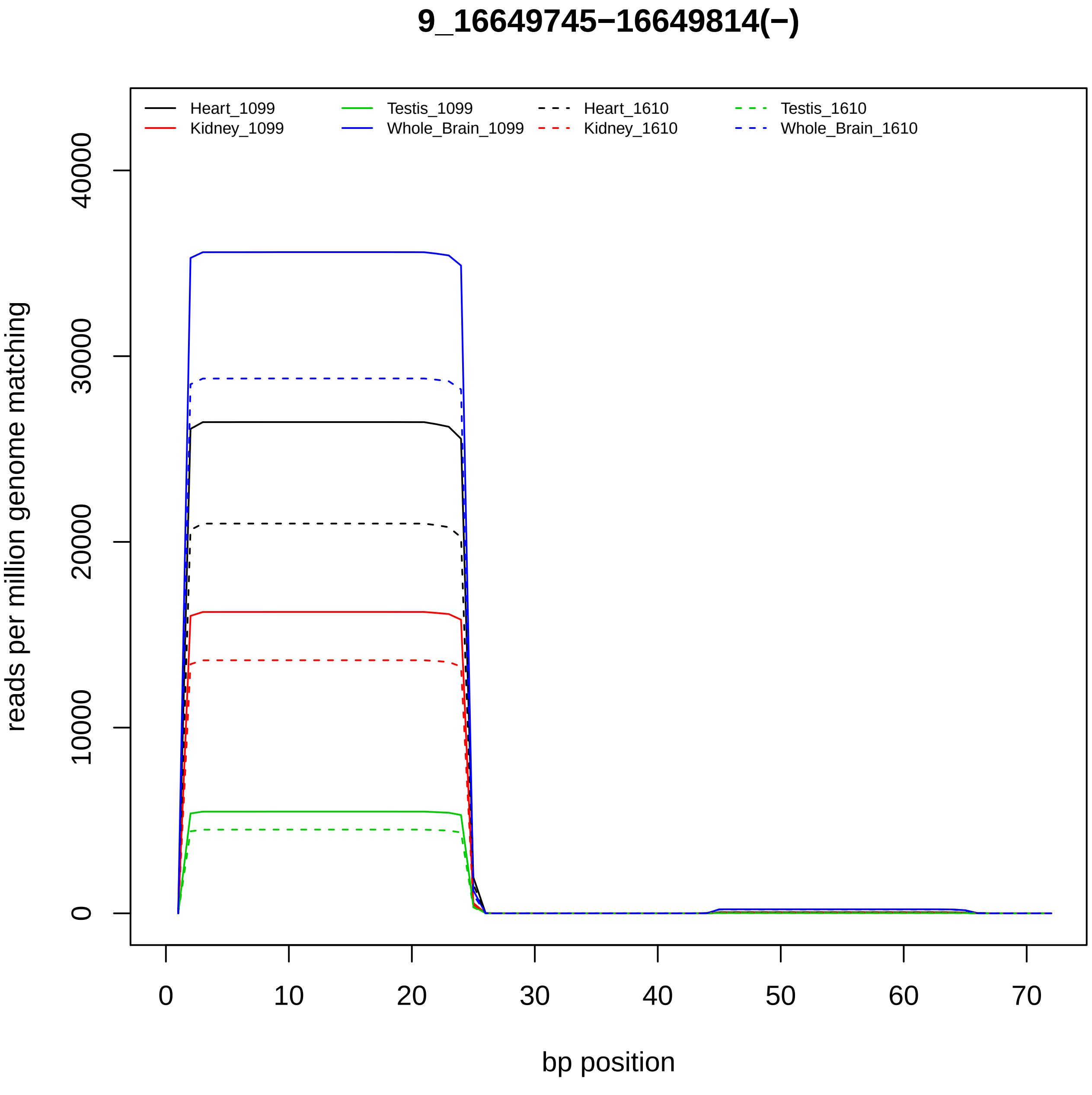

9_16649745−16649814(−)
Heart_1099
Kidney_1099
Testis_1099
Whole_Brain_1099
Heart_1610
Kidney_1610
Testis_1610
Whole_Brain_1610
reads per million genome matching
0
10000
20000
30000
40000
0
10
20
30
40
50
60
70
bp position

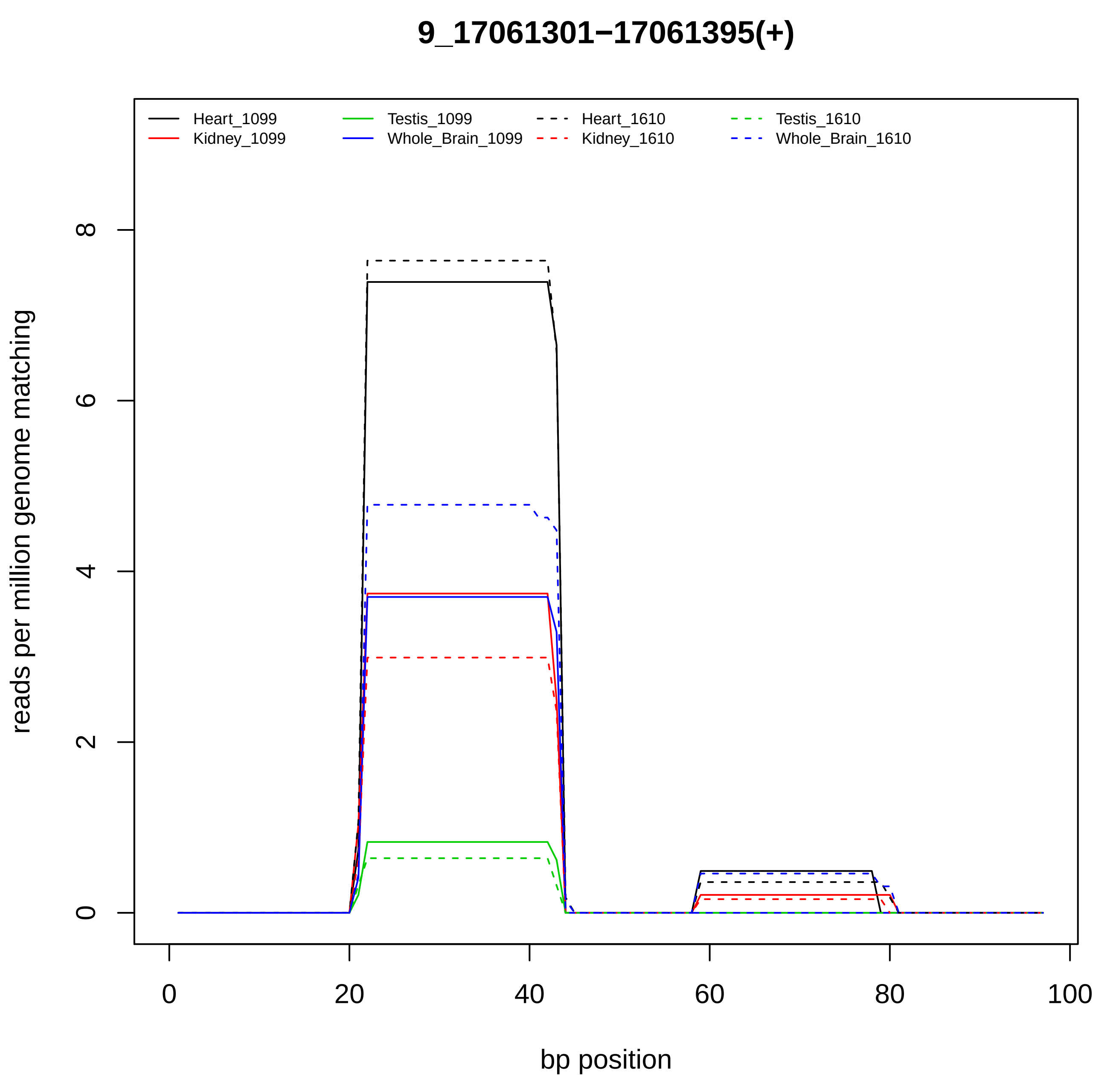
9_17061301−17061395(+)
Heart_1099
Kidney_1099
Testis_1099
Whole_Brain_1099
Heart_1610
Kidney_1610
Testis_1610
Whole_Brain_1610
reads per million genome matching
bp position
0
2
4
6
8
0
20
40
60
80
100

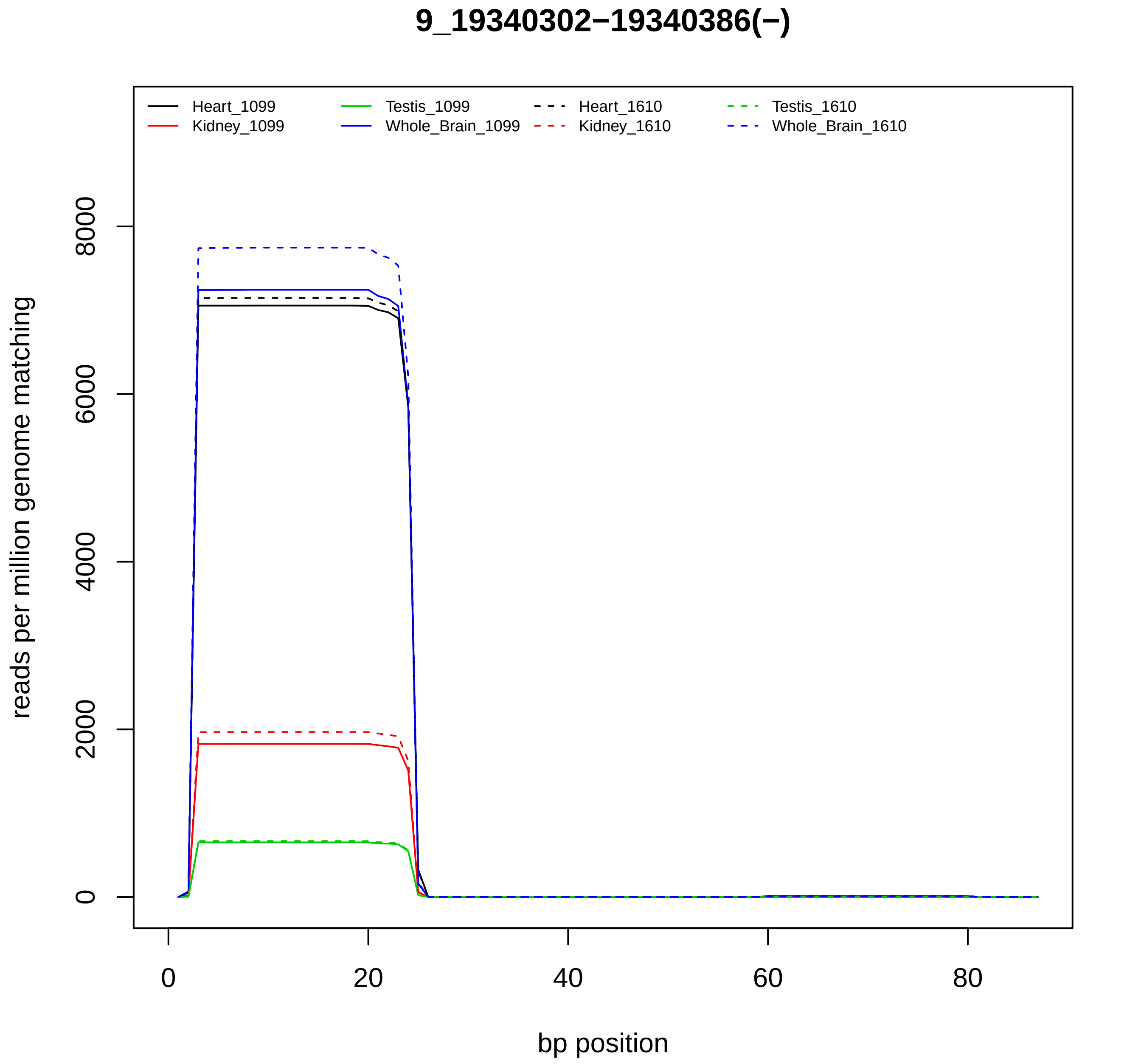

9_19340302−19340386(−)
Heart_1099
Kidney_1099
Testis_1099
Whole_Brain_1099
Heart_1610
Kidney_1610
Testis_1610
Whole_Brain_1610
reads per million genome matching
0
2000
4000
6000
8000
0
20
40
60
80
bp position

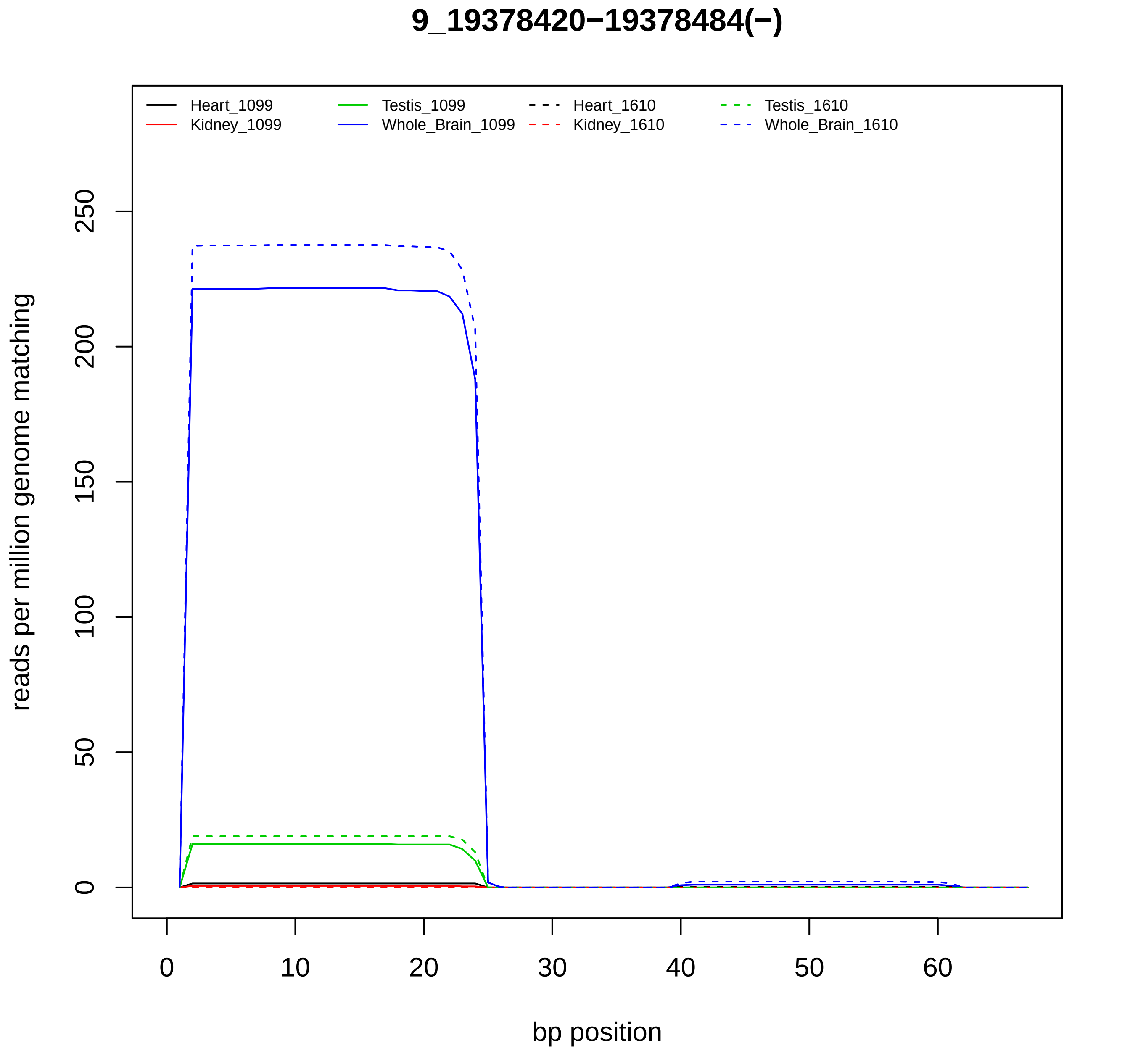

9_19378420−19378484(−)
Heart_1099
Kidney_1099
Testis_1099
Whole_Brain_1099
Heart_1610
Kidney_1610
Testis_1610
Whole_Brain_1610
reads per million genome matching
0
50
100
150
200
250
0
10
20
30
40
50
60
bp position

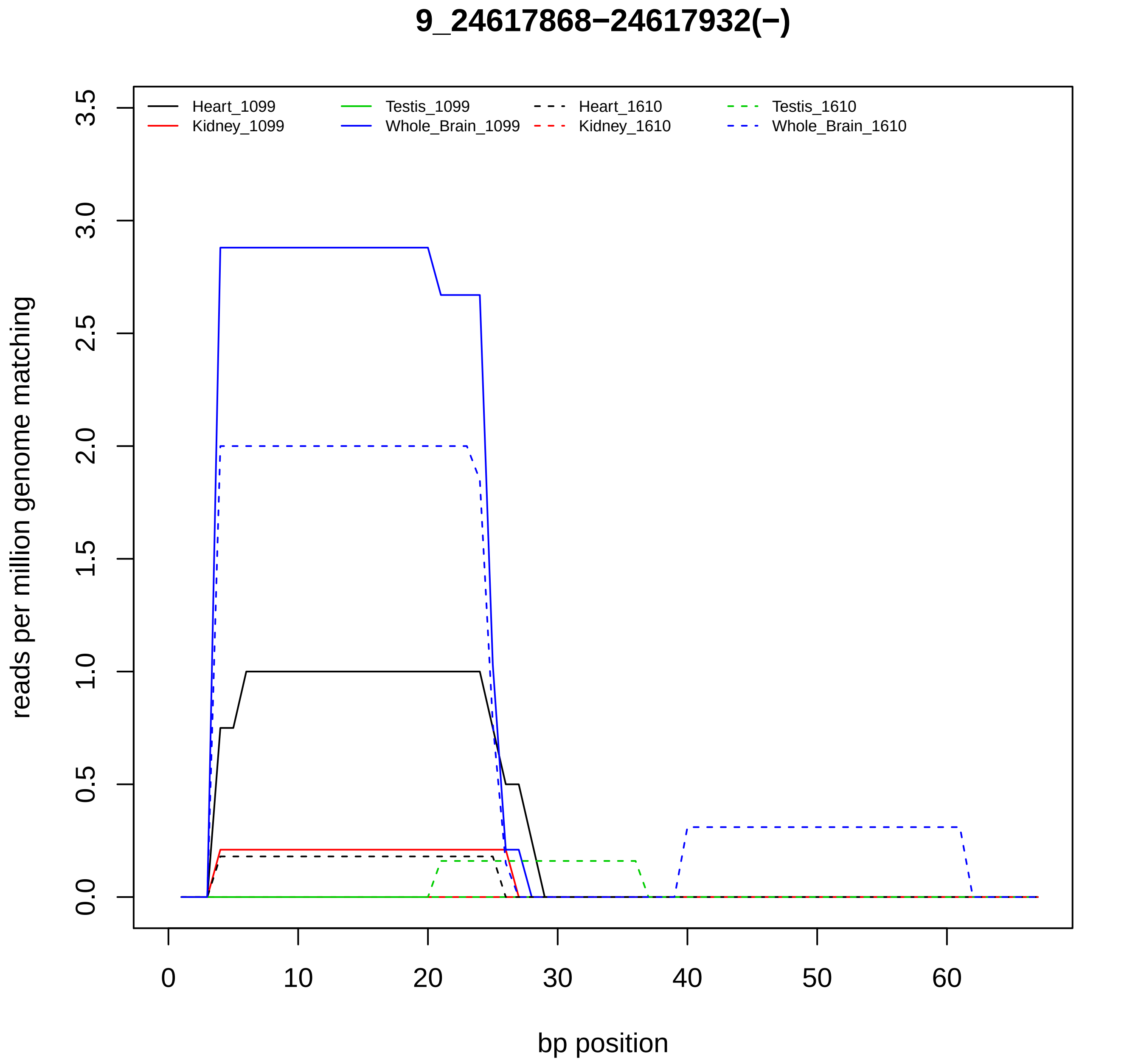

9_24617868−24617932(−)
Heart_1099
Kidney_1099
Testis_1099
Whole_Brain_1099
Heart_1610
Kidney_1610
Testis_1610
Whole_Brain_1610
reads per million genome matching
bp position
0.0
0.5
1.0
1.5
2.0
2.5
3.0
3.5
0
10
20
30
40
50
60

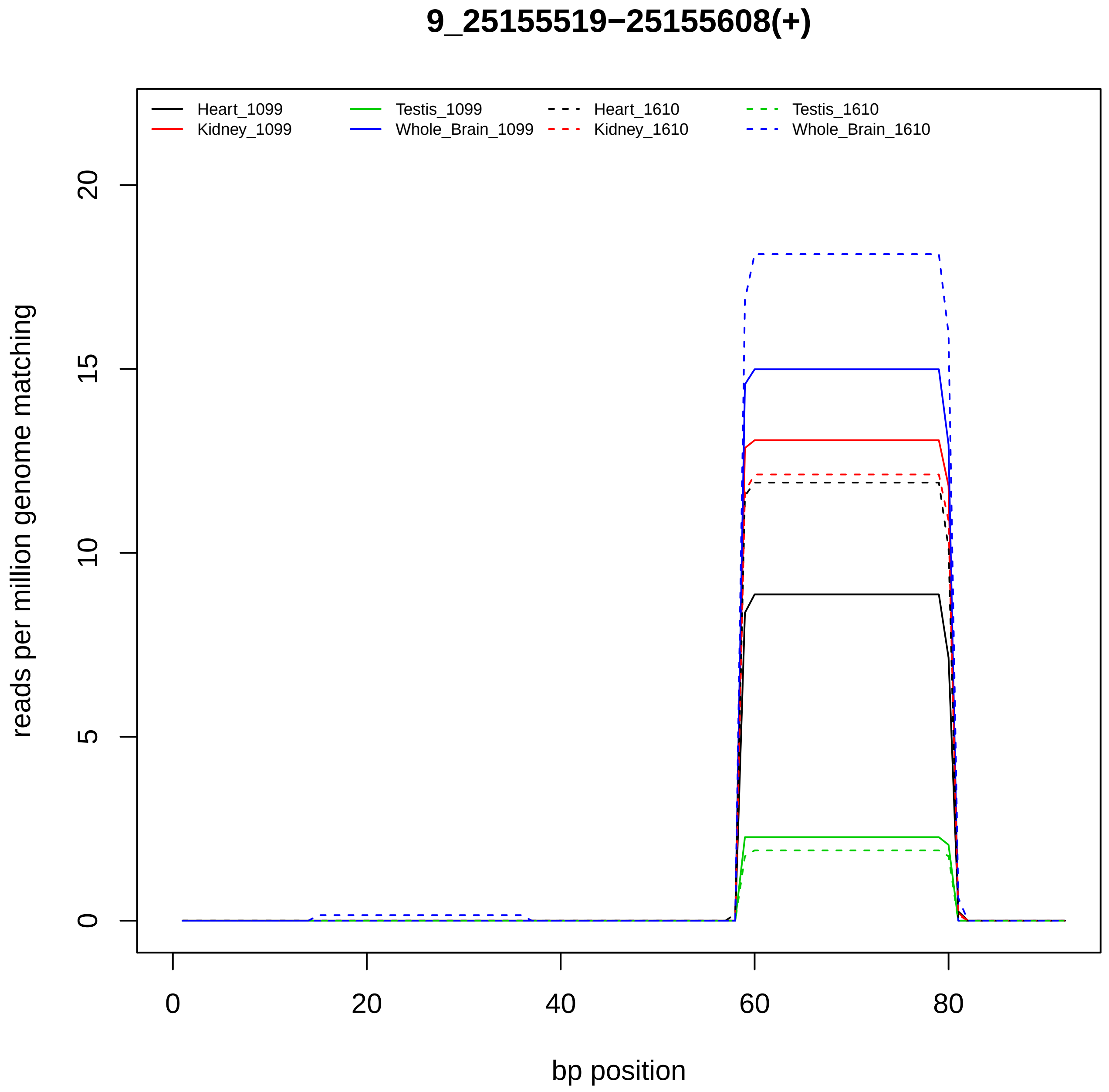

9_25155519−25155608(+)
Heart_1099
Kidney_1099
Testis_1099
Whole_Brain_1099
Heart_1610
Kidney_1610
Testis_1610
Whole_Brain_1610
reads per million genome matching
0
5
10
15
20
0
20
40
60
80
bp position

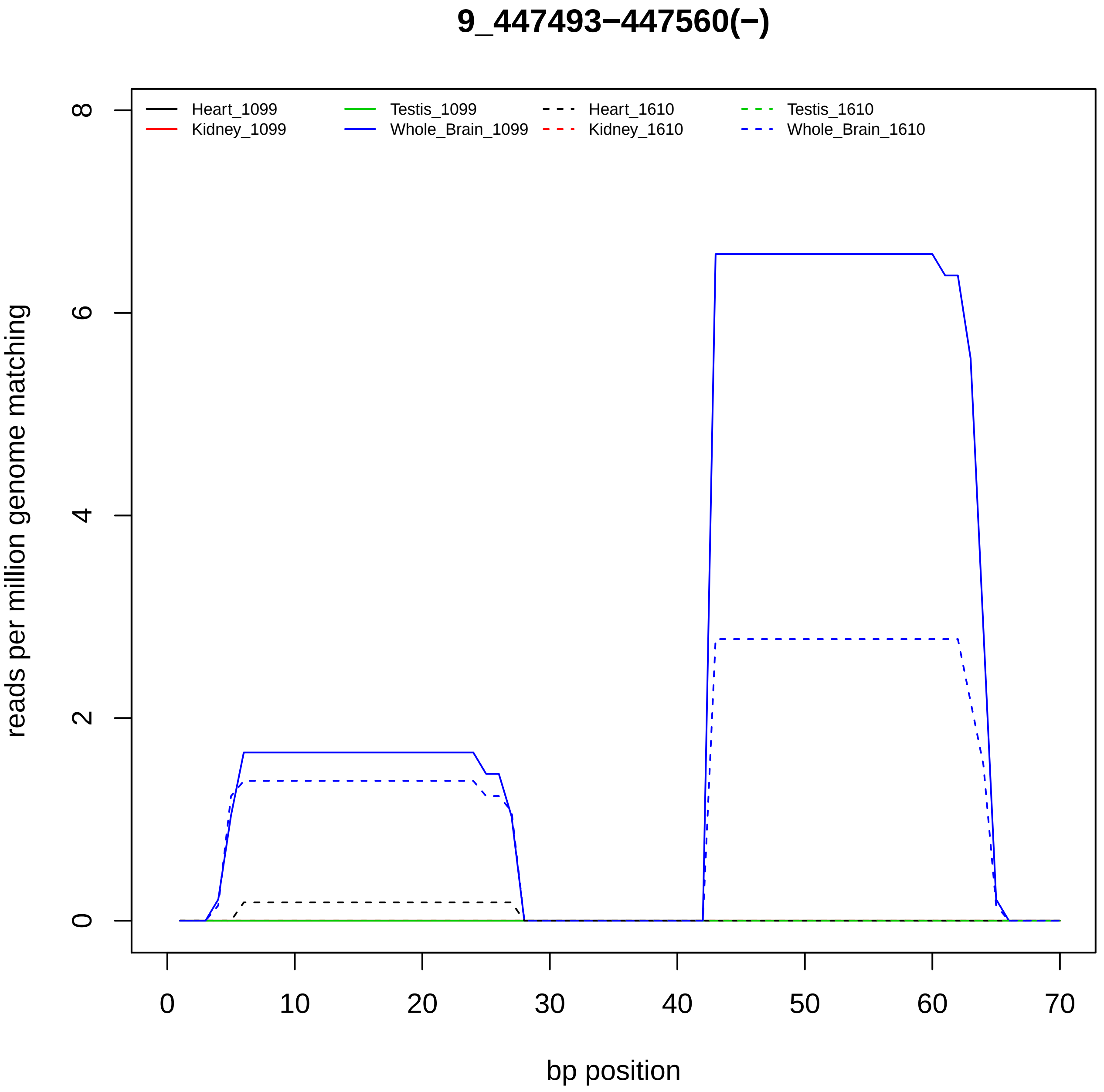

9_447493−447560(−)
Heart_1099
Kidney_1099
Testis_1099
Whole_Brain_1099
Heart_1610
Kidney_1610
Testis_1610
Whole_Brain_1610
reads per million genome matching
bp position
0
2
4
6
8
0
10
20
30
40
50
60
70

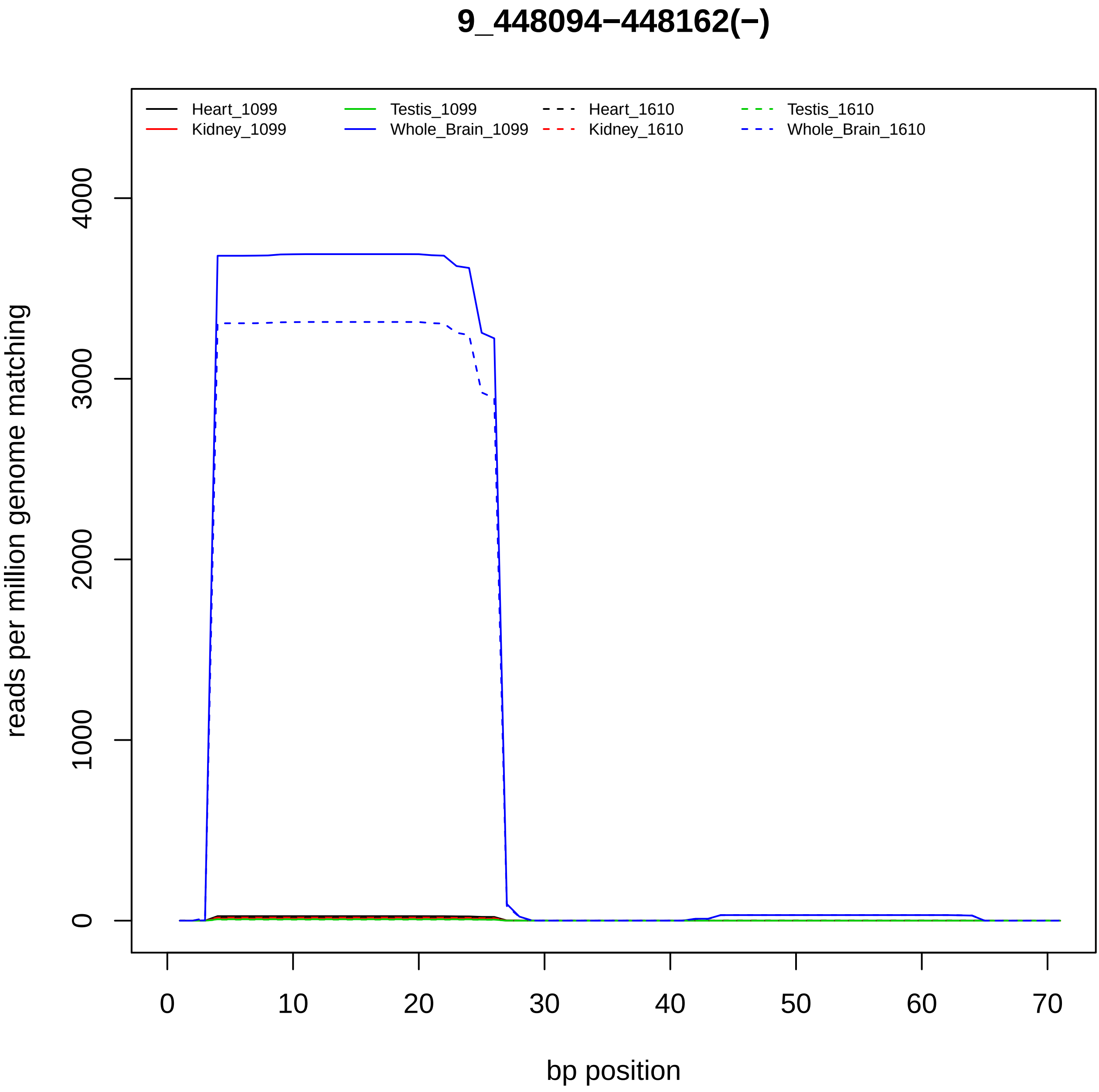

9_448094−448162(−)
Heart_1099
Kidney_1099
Testis_1099
Whole_Brain_1099
Heart_1610
Kidney_1610
Testis_1610
Whole_Brain_1610
reads per million genome matching
0
1000
2000
3000
4000
0
10
20
30
40
50
60
70
bp position

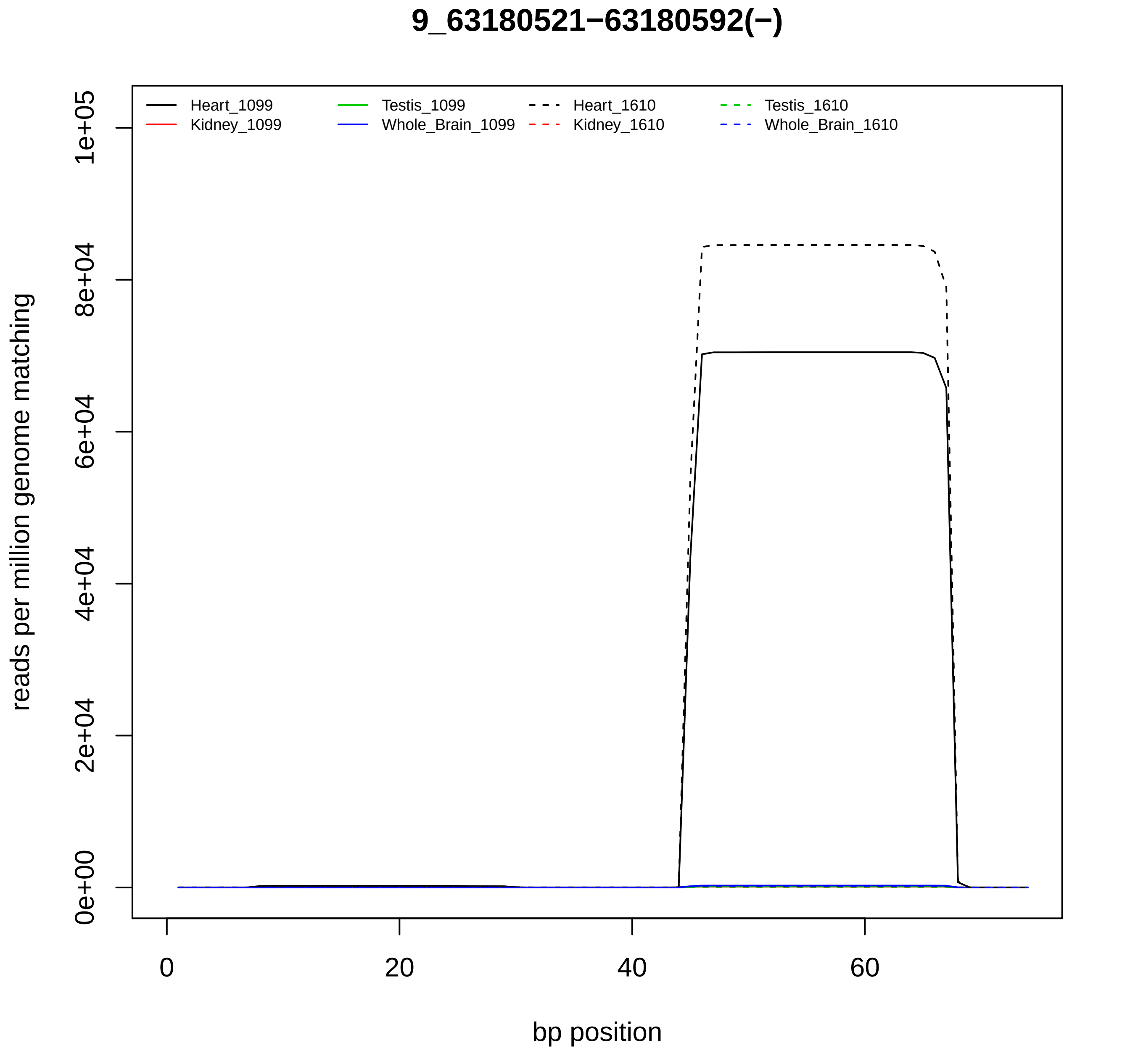

9_63180521−63180592(−)
Heart_1099
Kidney_1099
Testis_1099
Whole_Brain_1099
Heart_1610
Kidney_1610
Testis_1610
Whole_Brain_1610
reads per million genome matching
0e+00
2e+04
4e+04
6e+04
8e+04
1e+05
0
20
40
60
bp position

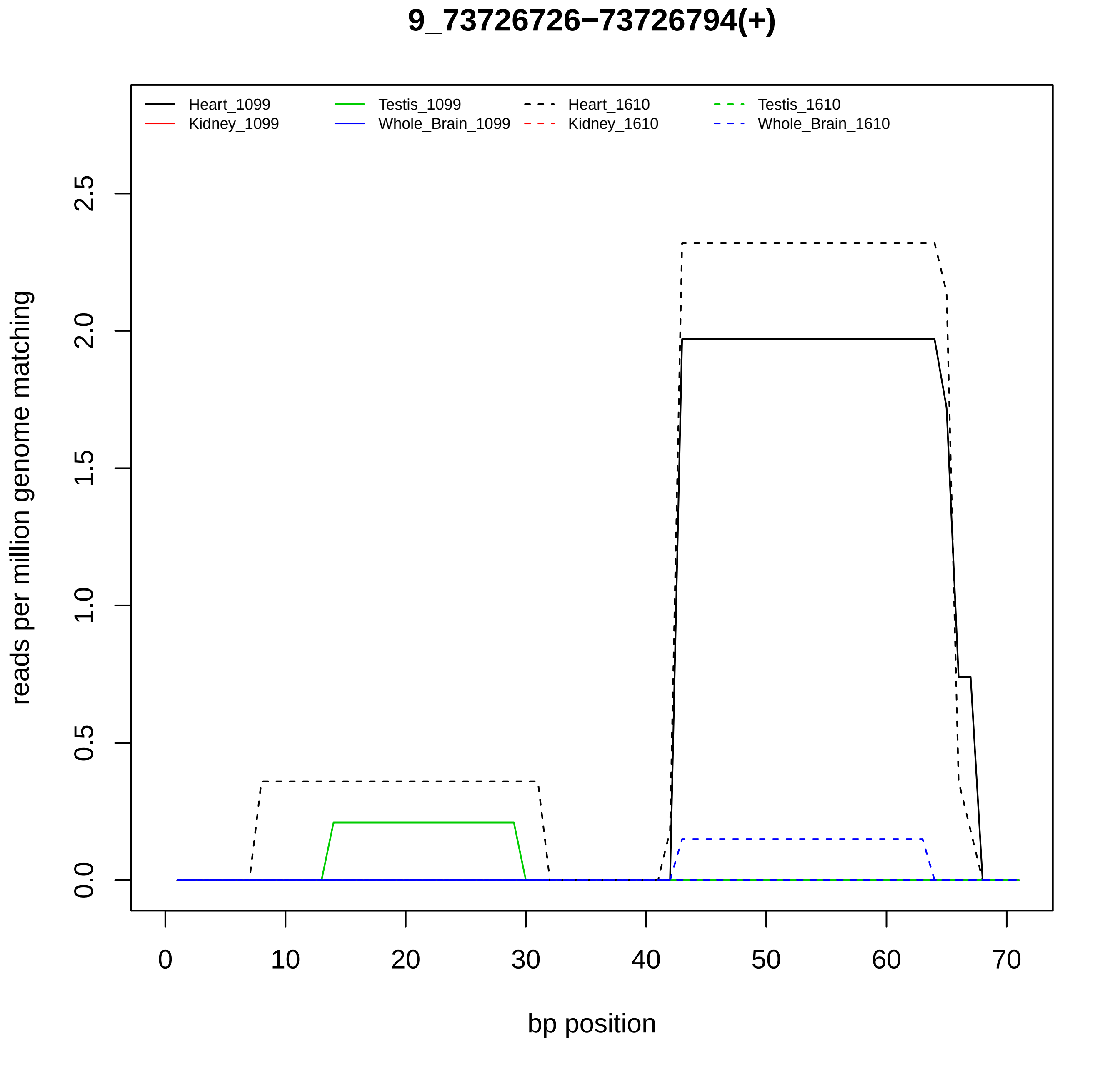
9_73726726−73726794(+)
Heart_1099
Kidney_1099
Testis_1099
Whole_Brain_1099
Heart_1610
Kidney_1610
Testis_1610
Whole_Brain_1610
reads per million genome matching
0.0
0.5
1.0
1.5
2.0
2.5
0
10
20
30
40
50
60
70
bp position

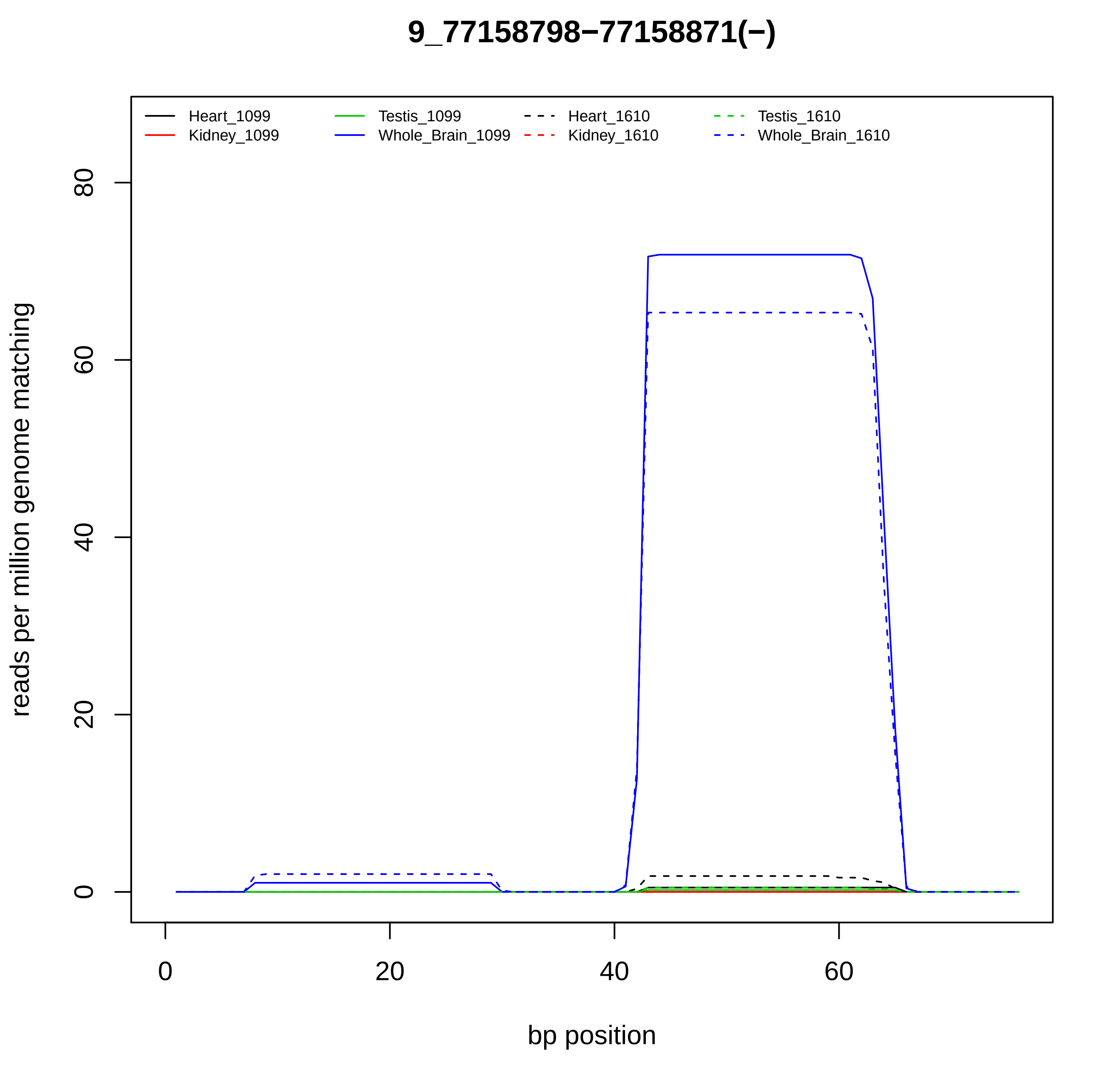

9_77158798−77158871(−)
Heart_1099
Kidney_1099
Testis_1099
Whole_Brain_1099
Heart_1610
Kidney_1610
Testis_1610
Whole_Brain_1610
reads per million genome matching
0
20
40
60
80
0
20
40
60
bp position

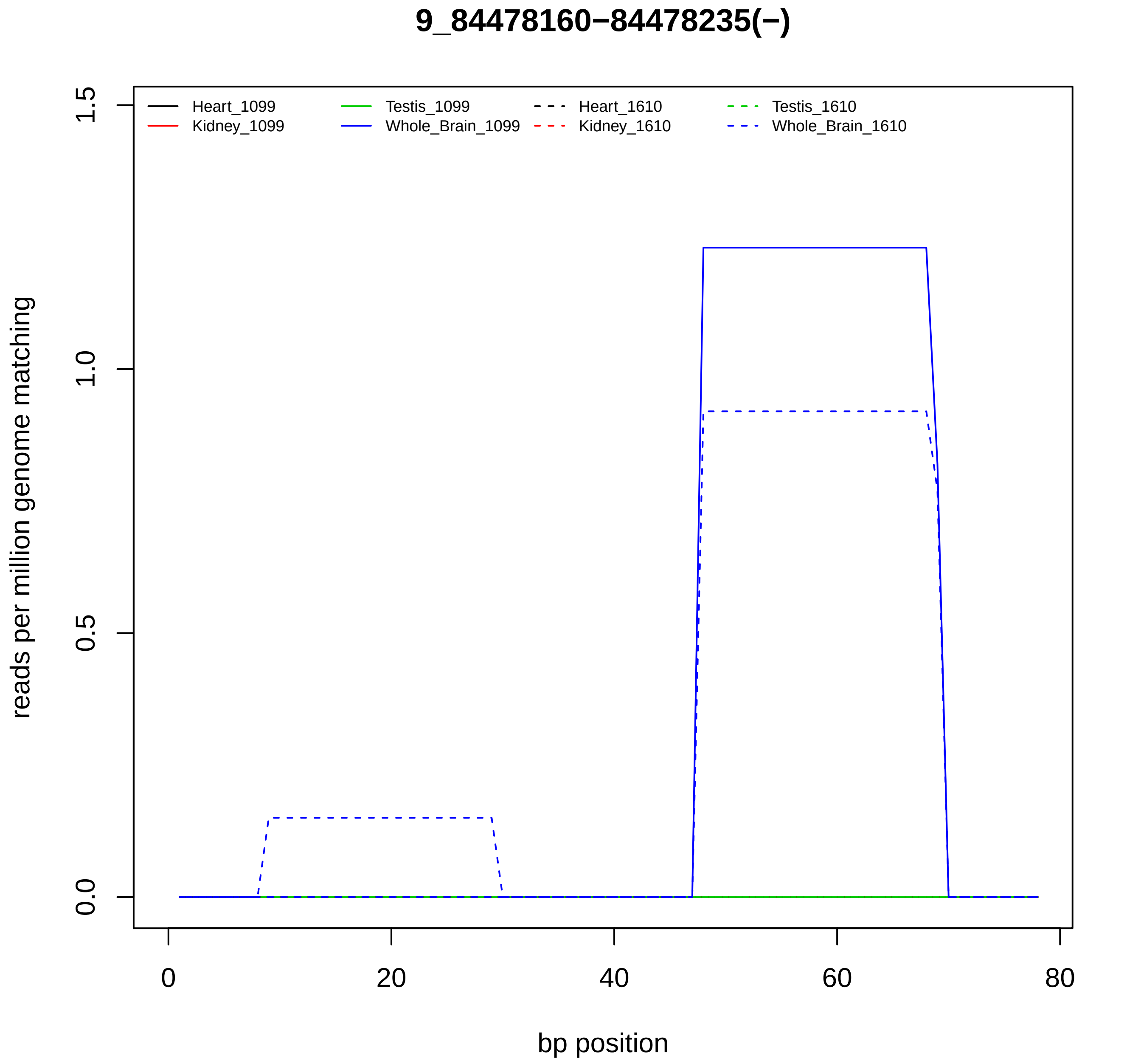
9_84478160−84478235(−)
Heart_1099
Kidney_1099
Testis_1099
Whole_Brain_1099
Heart_1610
Kidney_1610
Testis_1610
Whole_Brain_1610
reads per million genome matching
bp position
0.0
0.5
1.0
1.5
0
20
40
60
80

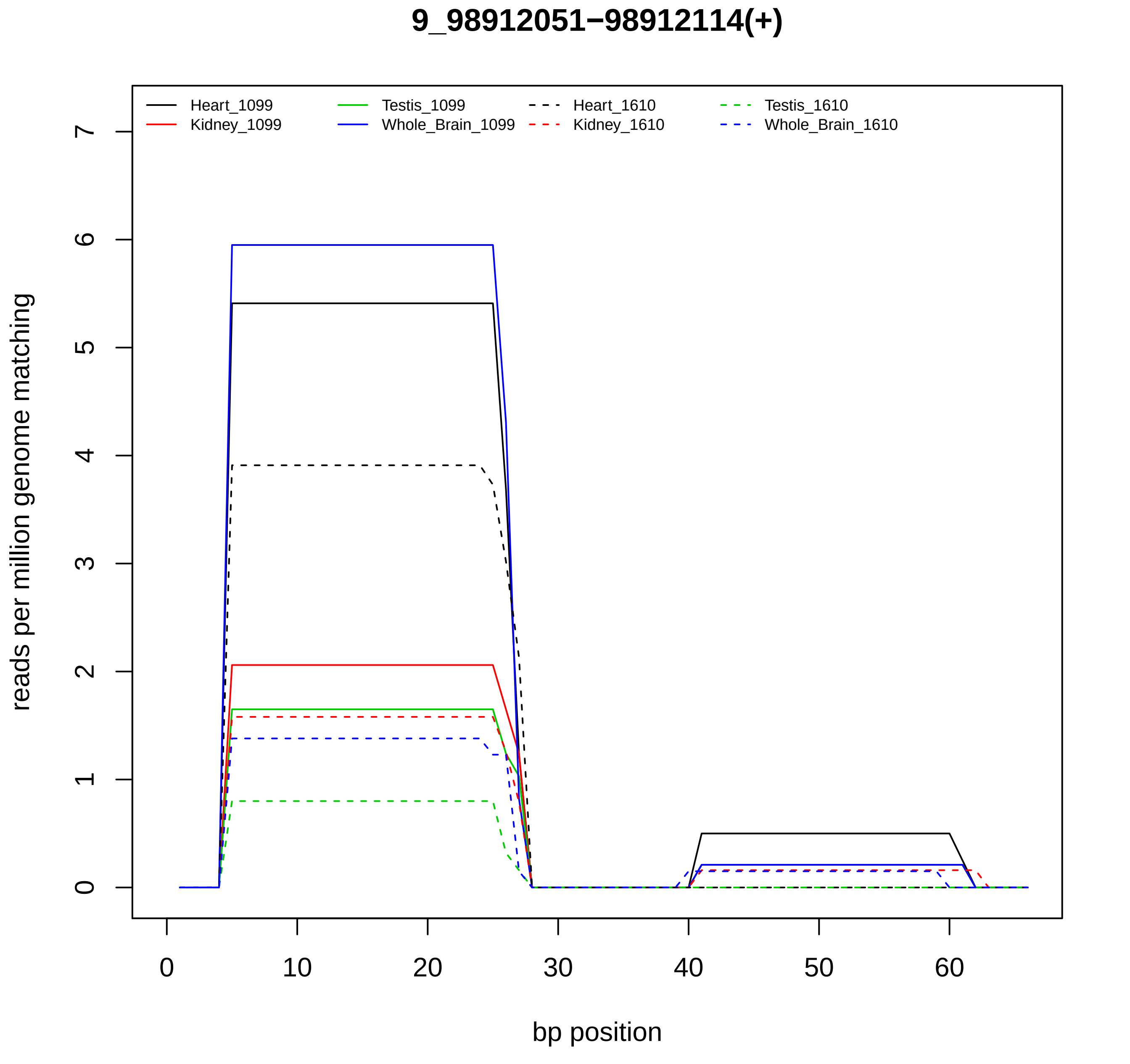

9_98912051−98912114(+)
Heart_1099
Kidney_1099
Testis_1099
Whole_Brain_1099
Heart_1610
Kidney_1610
Testis_1610
Whole_Brain_1610
reads per million genome matching
bp position
0
1
2
3
4
5
6
7
0
10
20
30
40
50
60

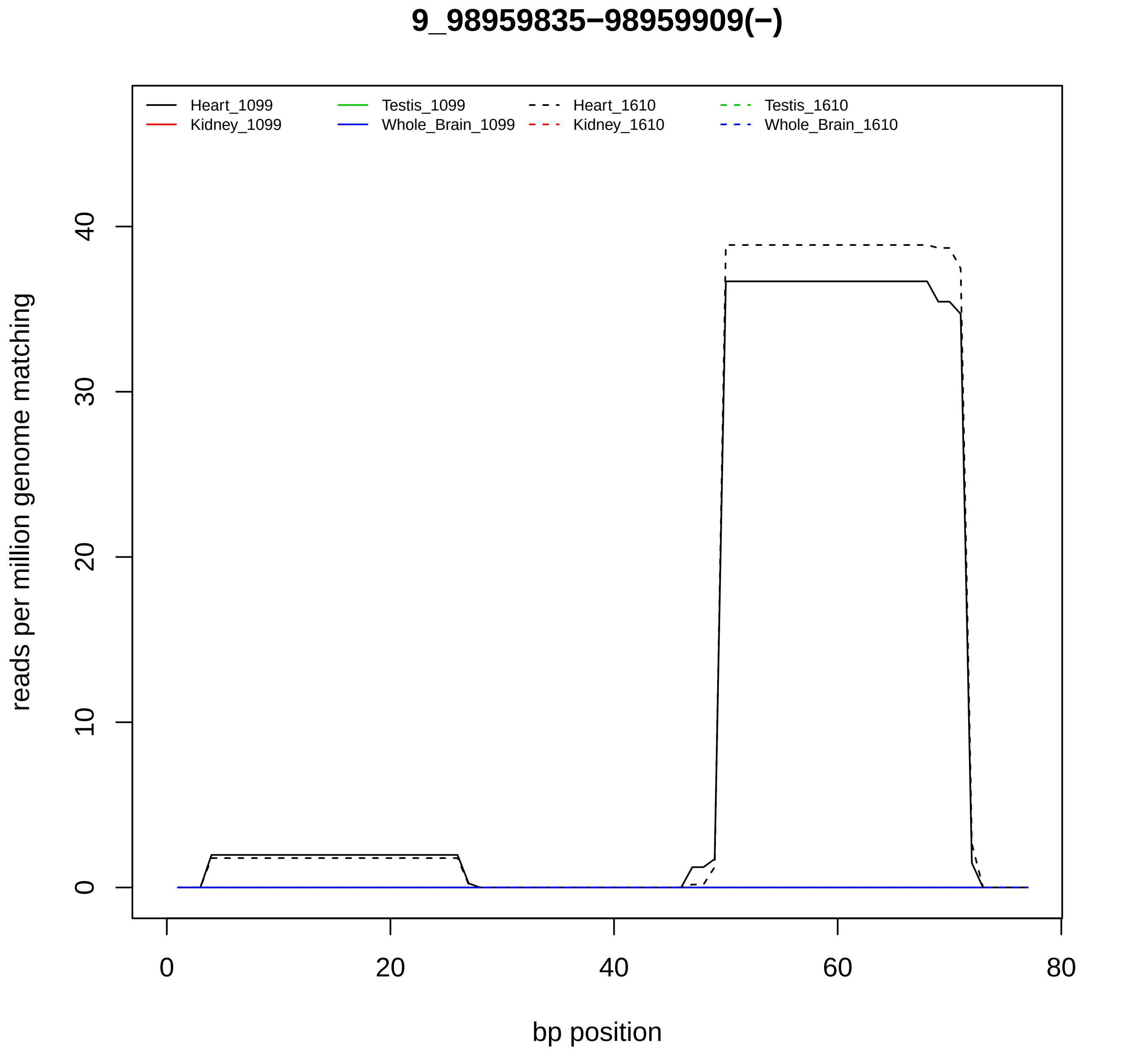

9_98959835−98959909(−)
Heart_1099
Kidney_1099
Testis_1099
Whole_Brain_1099
Heart_1610
Kidney_1610
Testis_1610
Whole_Brain_1610
reads per million genome matching
bp position
0
10
20
30
40
0
20
40
60
80

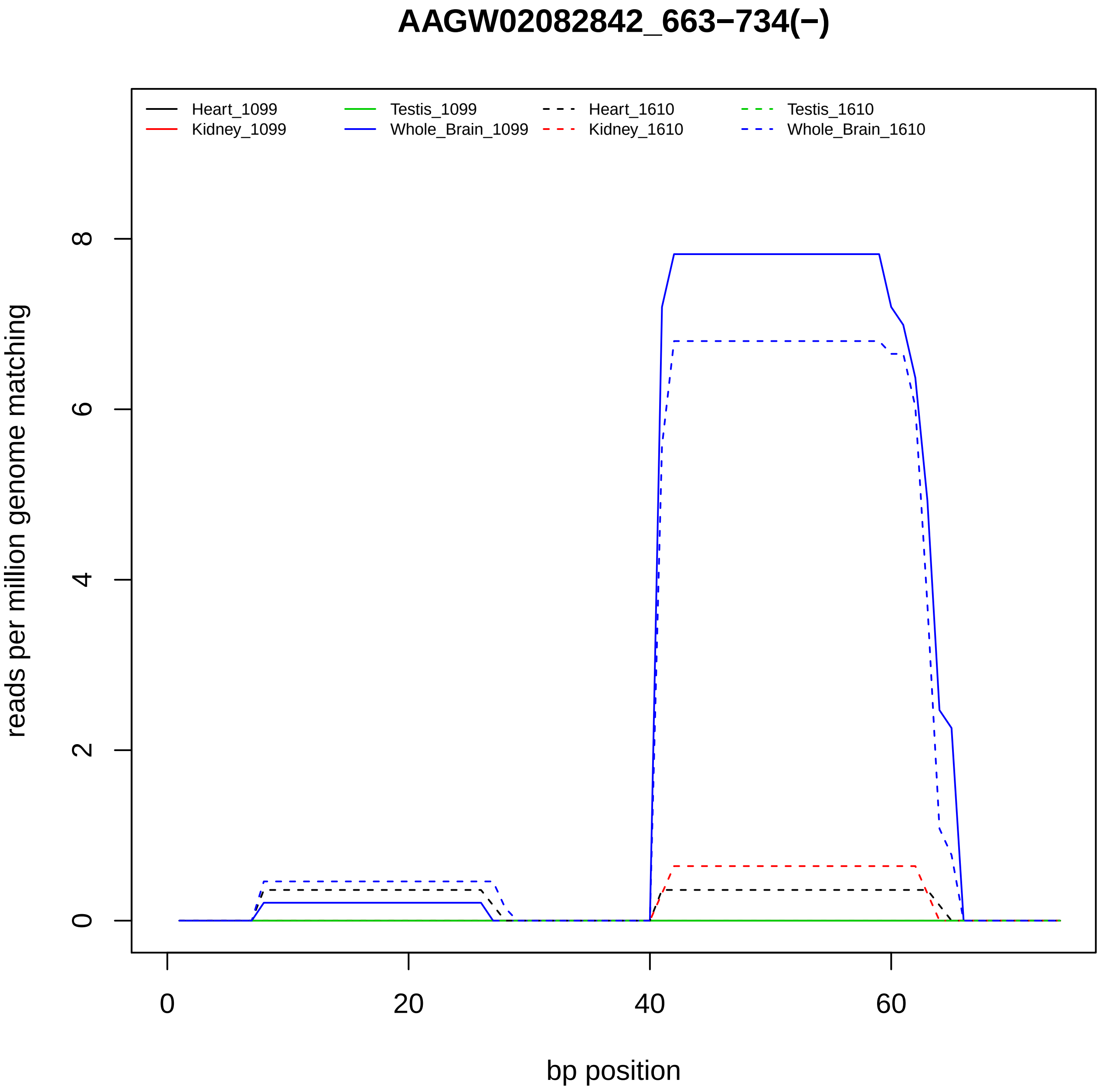
AAGW02082842_663−734(−)
Heart_1099
Kidney_1099
Testis_1099
Whole_Brain_1099
Heart_1610
Kidney_1610
Testis_1610
Whole_Brain_1610
reads per million genome matching
0
2
4
6
8
0
20
40
60
bp position

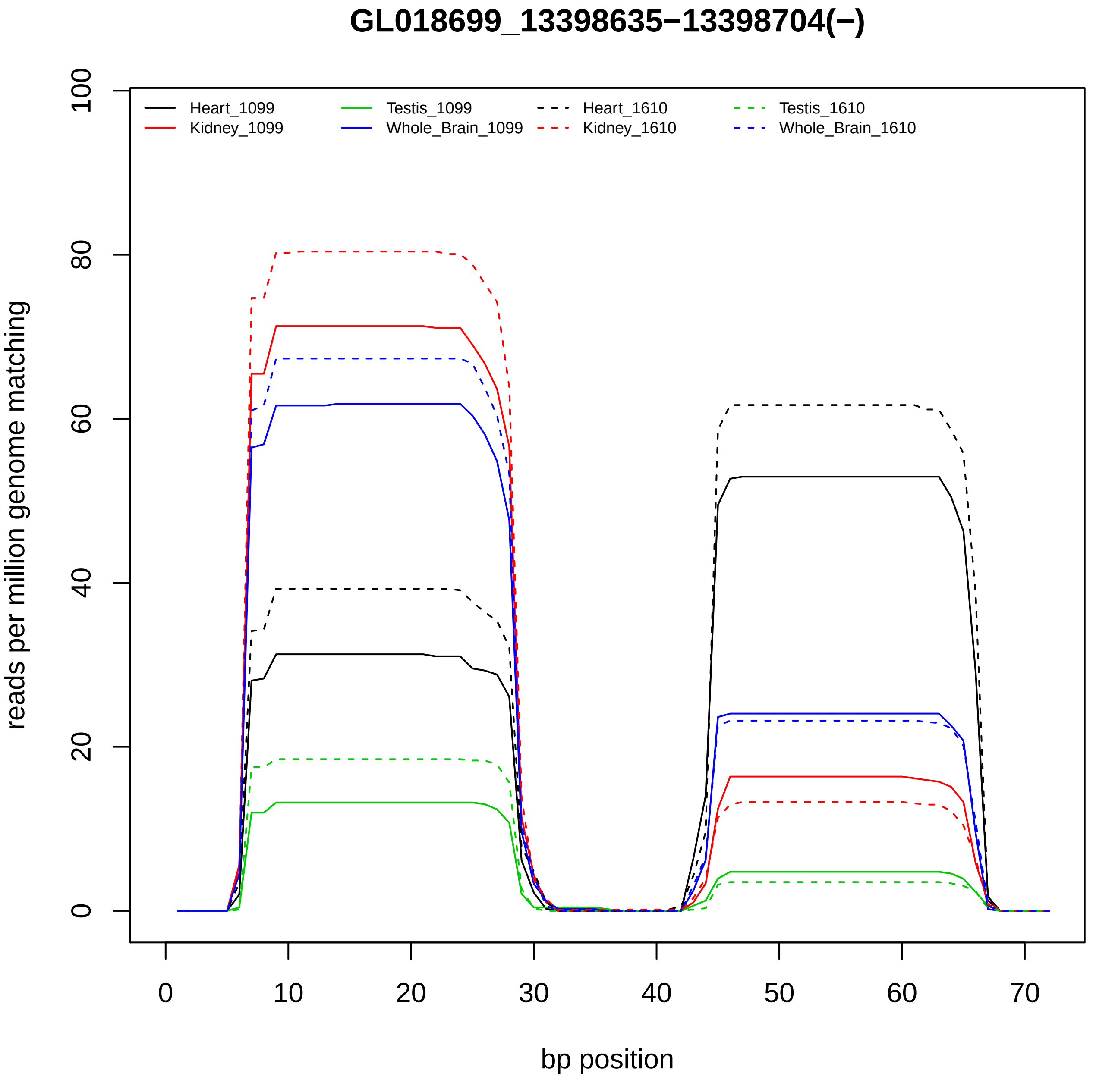
GL018699_13398635−13398704(−)
Heart_1099
Kidney_1099
Testis_1099
Whole_Brain_1099
Heart_1610
Kidney_1610
Testis_1610
Whole_Brain_1610
reads per million genome matching
bp position
0
20
40
60
80
100
0
10
20
30
40
50
60
70

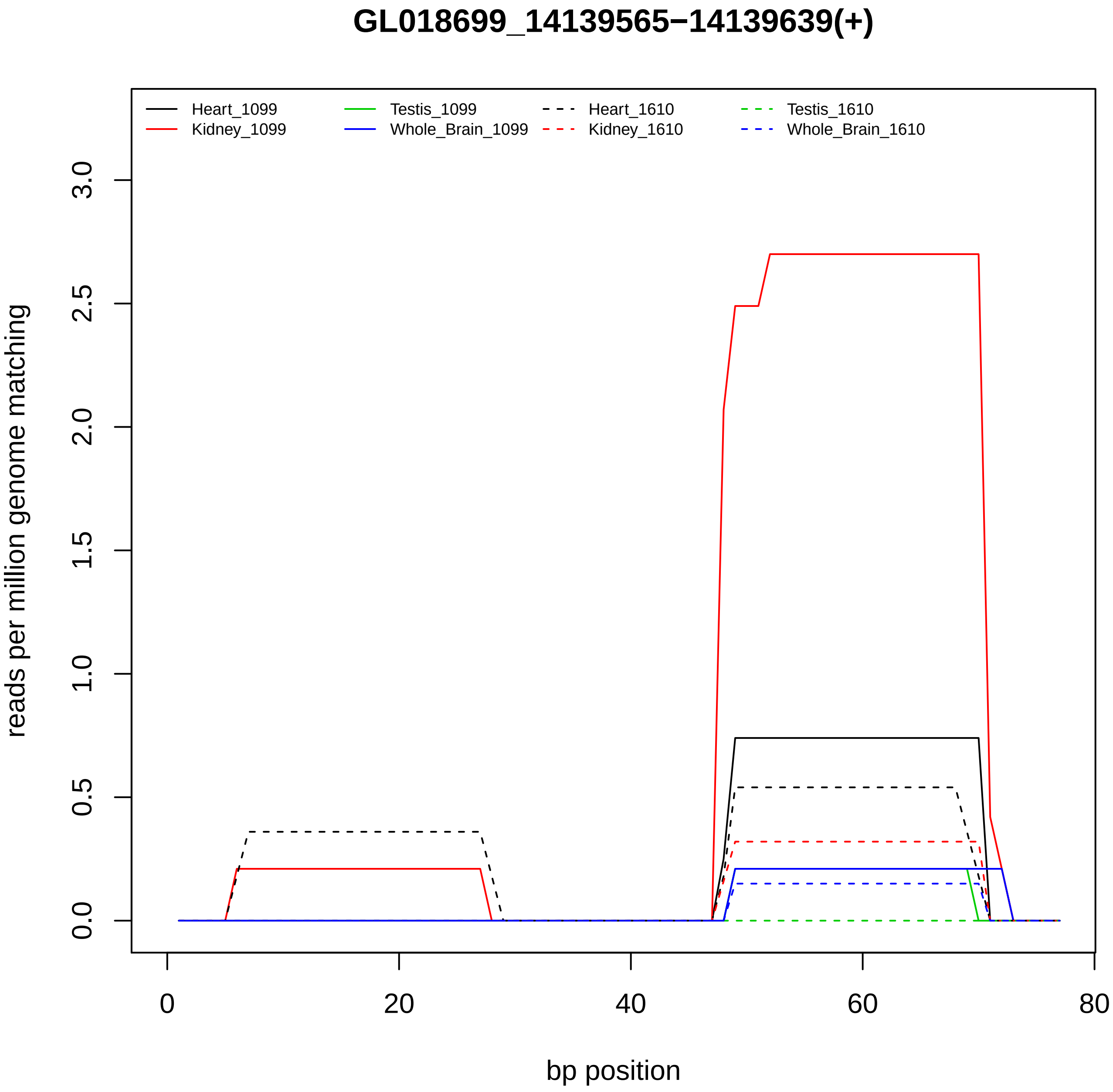
GL018699_14139565−14139639(+)
Heart_1099
Kidney_1099
Testis_1099
Whole_Brain_1099
Heart_1610
Kidney_1610
Testis_1610
Whole_Brain_1610
reads per million genome matching
0.0
0.5
1.0
1.5
2.0
2.5
3.0
0
20
40
60
80
bp position

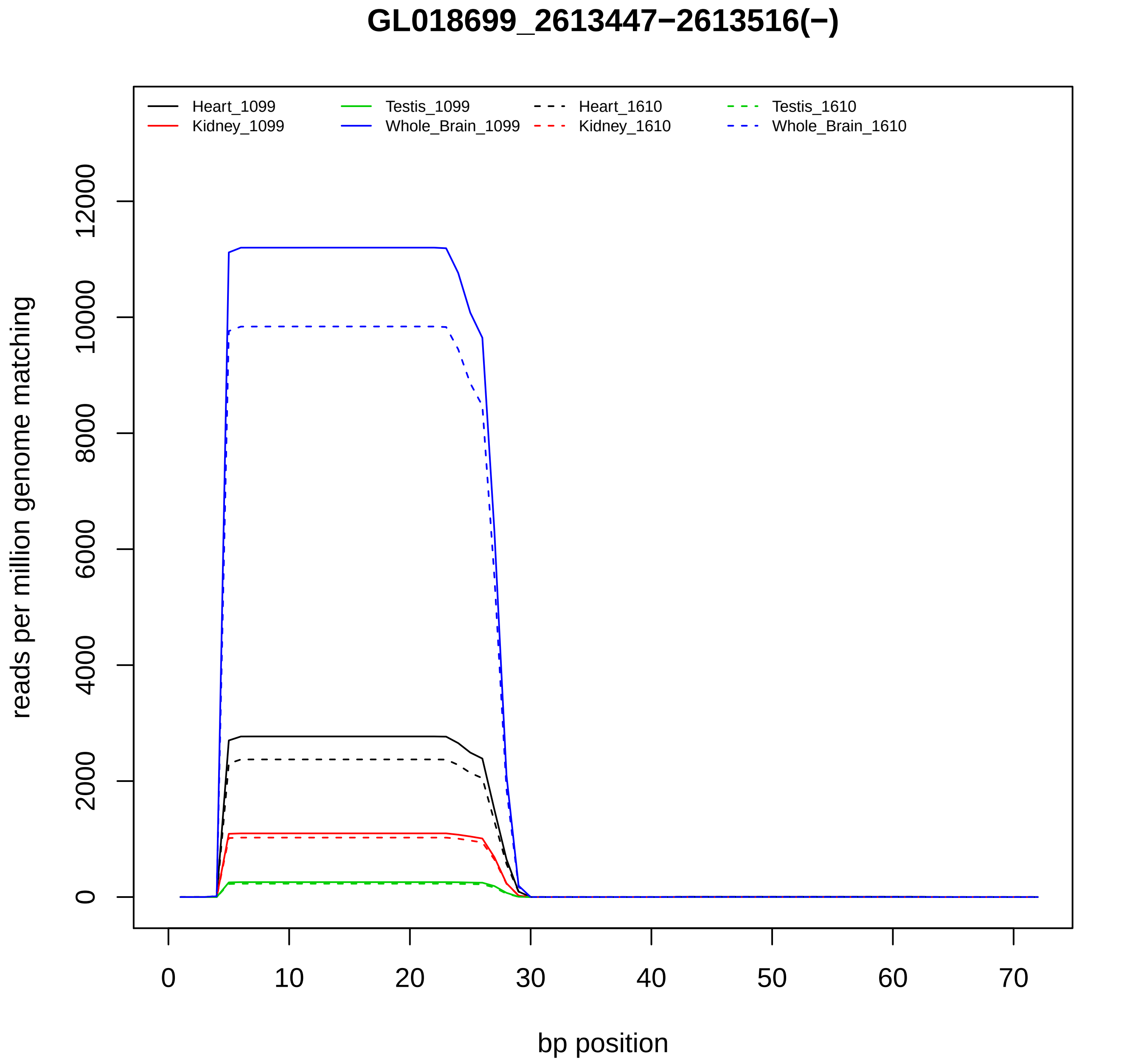
GL018699_2613447−2613516(−)
Heart_1099
Kidney_1099
Testis_1099
Whole_Brain_1099
Heart_1610
Kidney_1610
Testis_1610
Whole_Brain_1610
reads per million genome matching
0
2000
4000
6000
8000
10000
12000
bp position
0
10
20
30
40
50
60
70

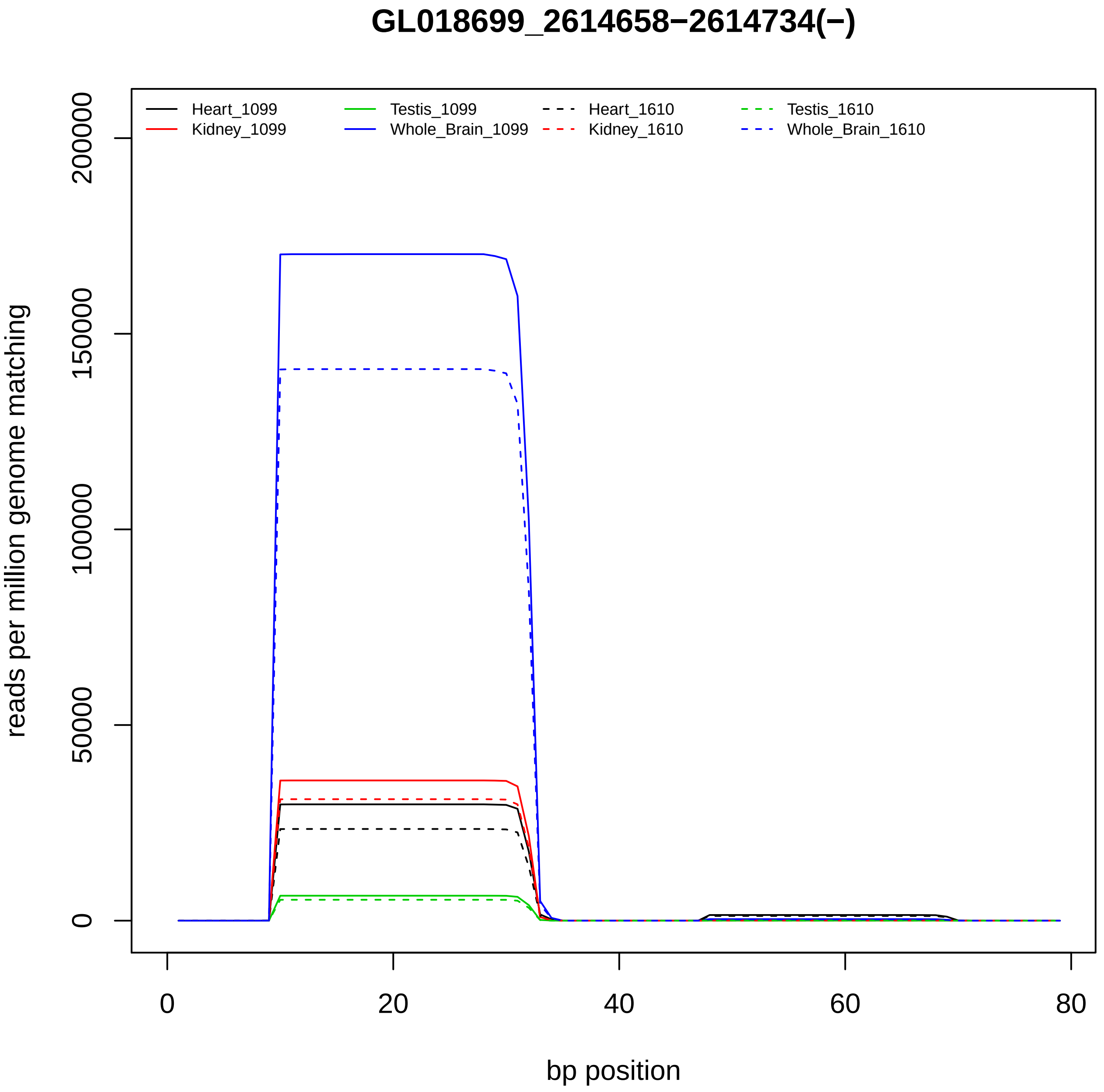

GL018699_2614658−2614734(−)
Heart_1099
Kidney_1099
Testis_1099
Whole_Brain_1099
Heart_1610
Kidney_1610
Testis_1610
Whole_Brain_1610
reads per million genome matching
0
50000
100000
150000
200000
bp position
0
20
40
60
80

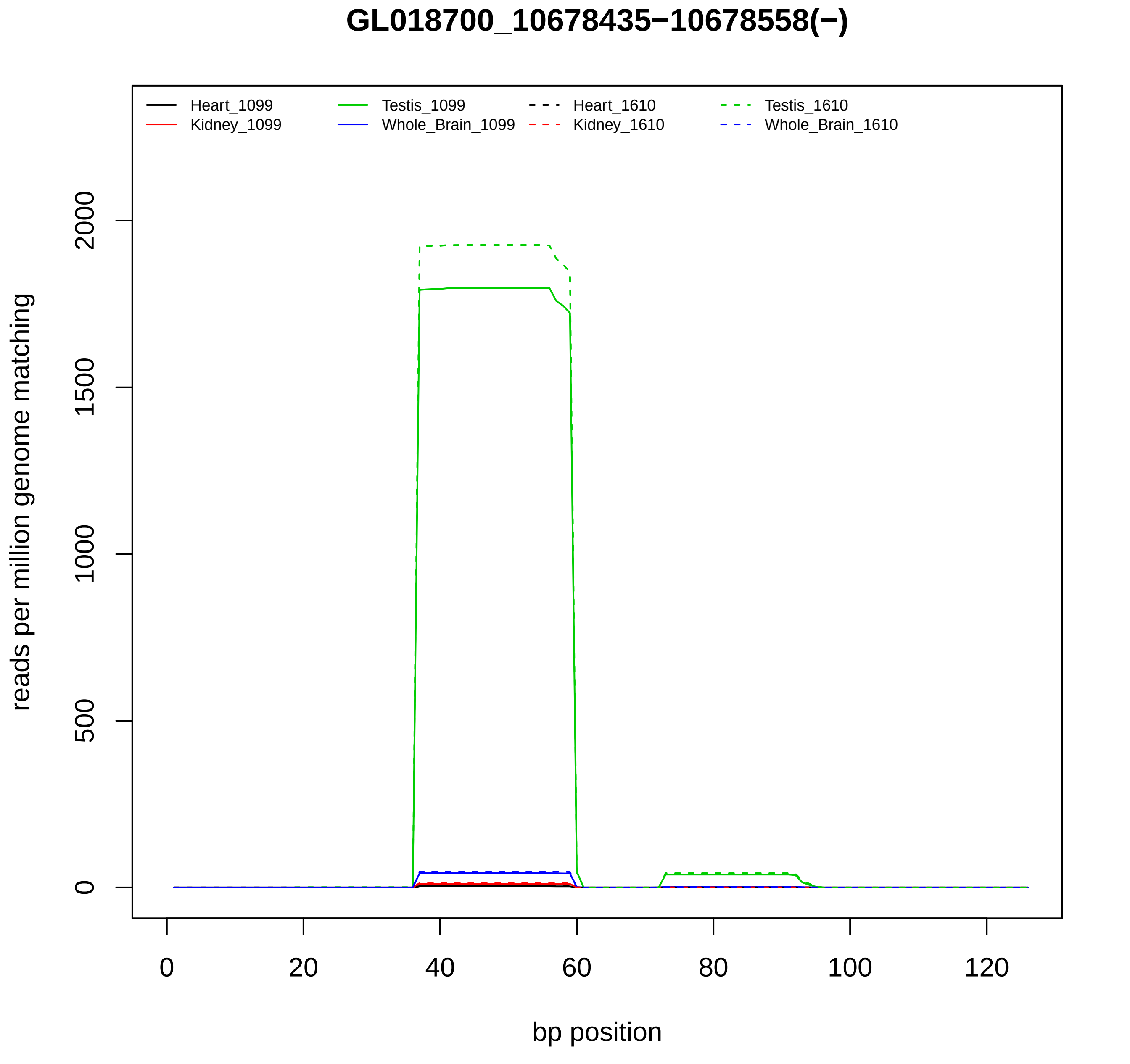
GL018700_10678435−10678558(−)
Heart_1099
Kidney_1099
Testis_1099
Whole_Brain_1099
Heart_1610
Kidney_1610
Testis_1610
Whole_Brain_1610
reads per million genome matching
0
500
1000
1500
2000
0
20
40
60
80
100
120
bp position

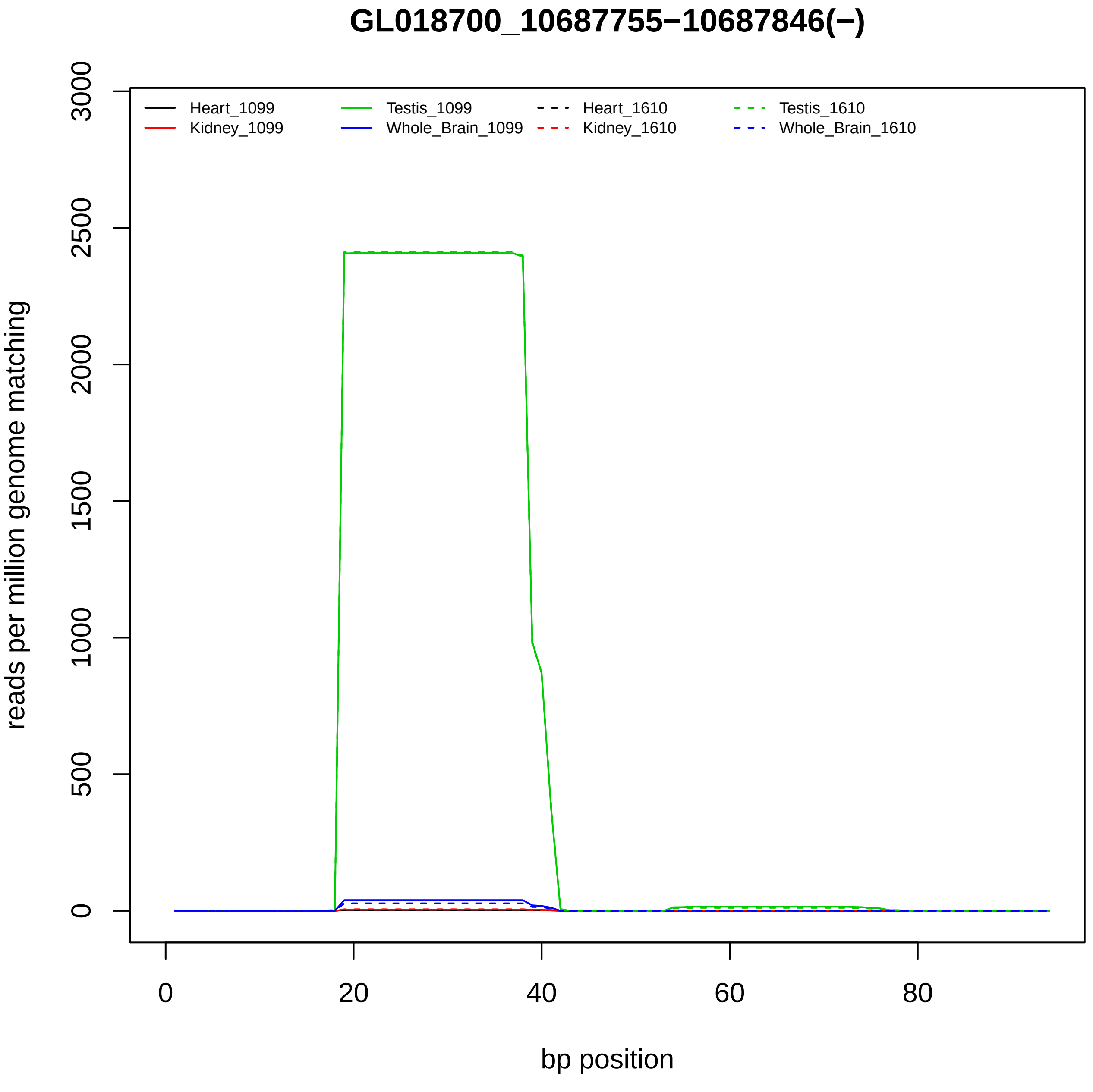
GL018700_10687755−10687846(−)
Heart_1099
Kidney_1099
Testis_1099
Whole_Brain_1099
Heart_1610
Kidney_1610
Testis_1610
Whole_Brain_1610
reads per million genome matching
0
500
1000
1500
2000
2500
3000
bp position
0
20
40
60
80

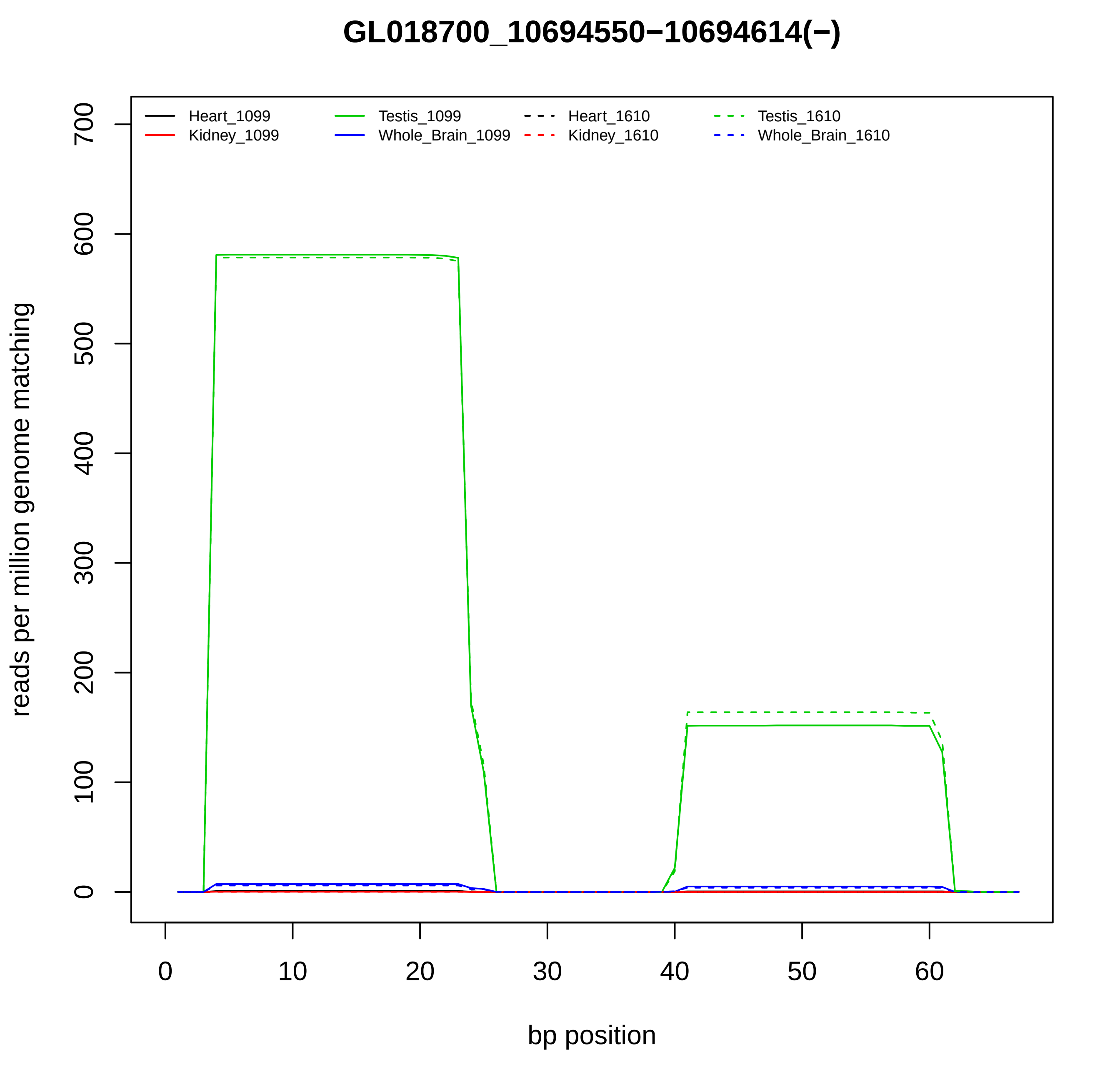

GL018700_10694550−10694614(−)
Heart_1099
Kidney_1099
Testis_1099
Whole_Brain_1099
Heart_1610
Kidney_1610
Testis_1610
Whole_Brain_1610
reads per million genome matching
bp position
0
100
200
300
400
500
600
700
0
10
20
30
40
50
60

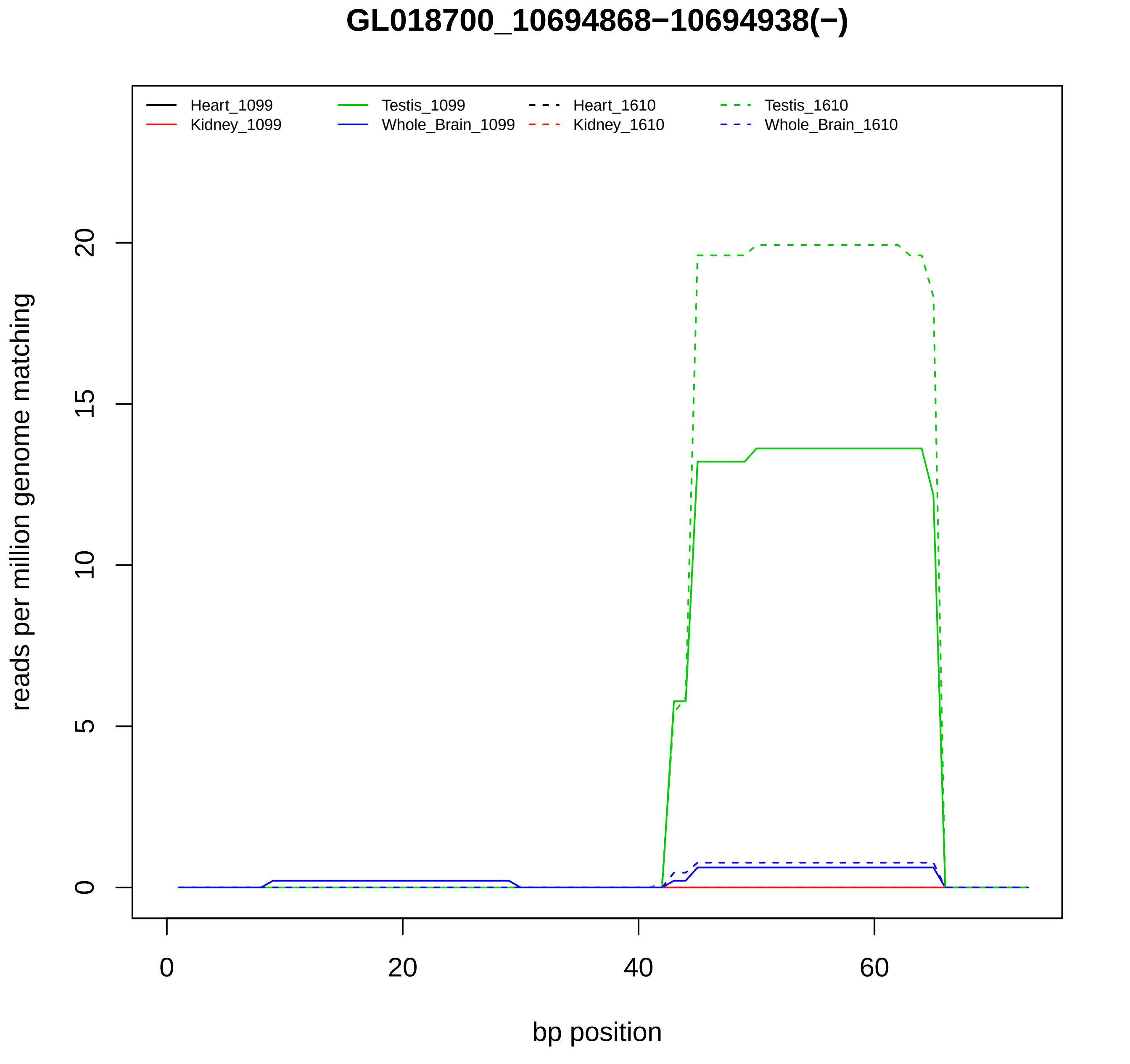
GL018700_10694868−10694938(−)
Heart_1099
Kidney_1099
Testis_1099
Whole_Brain_1099
Heart_1610
Kidney_1610
Testis_1610
Whole_Brain_1610
reads per million genome matching
0
5
10
15
20
0
20
40
60
bp position

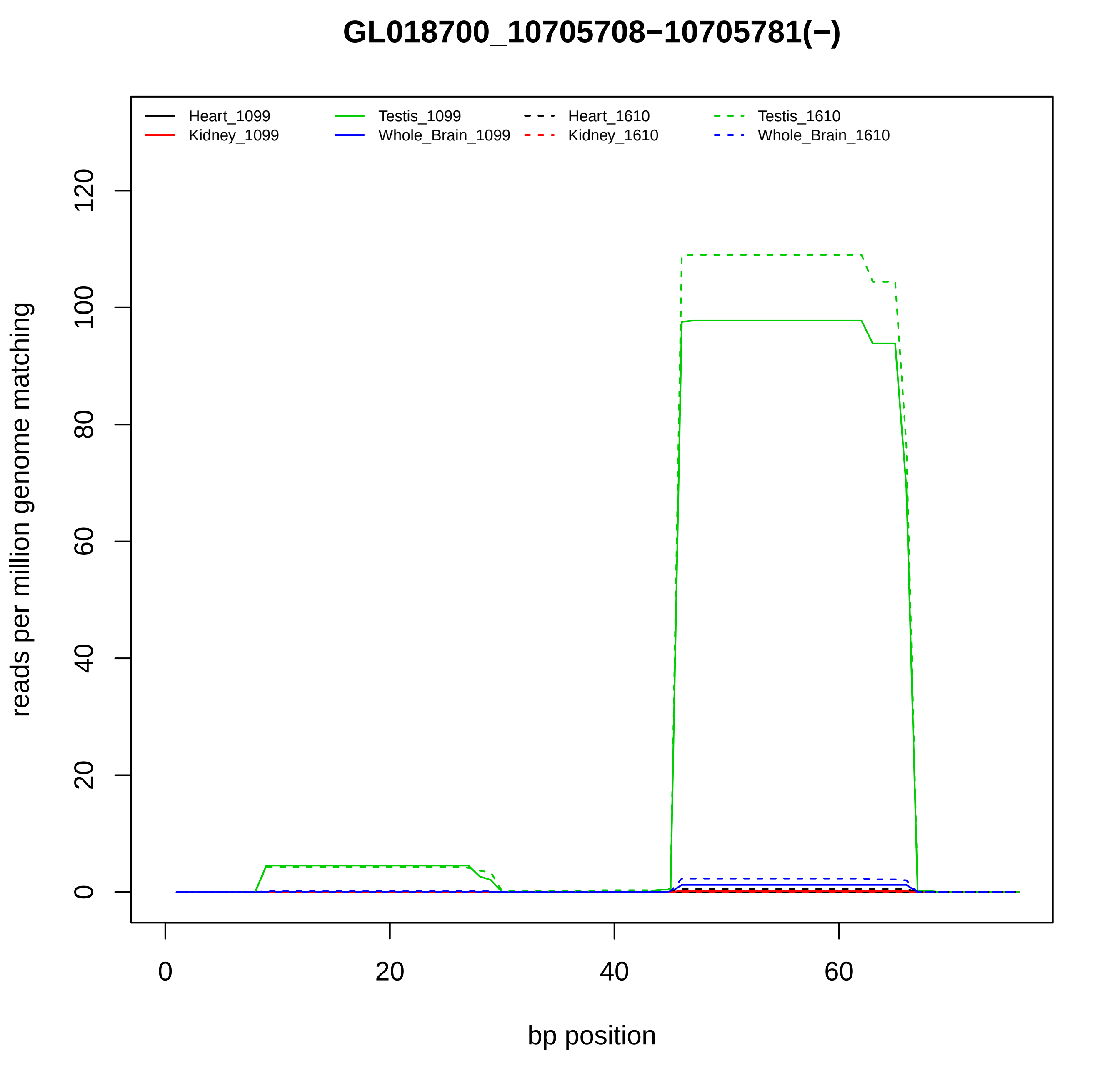

GL018700_10705708−10705781(−)
Heart_1099
Kidney_1099
Testis_1099
Whole_Brain_1099
Heart_1610
Kidney_1610
Testis_1610
Whole_Brain_1610
reads per million genome matching
bp position
0
20
40
60
80
100
120

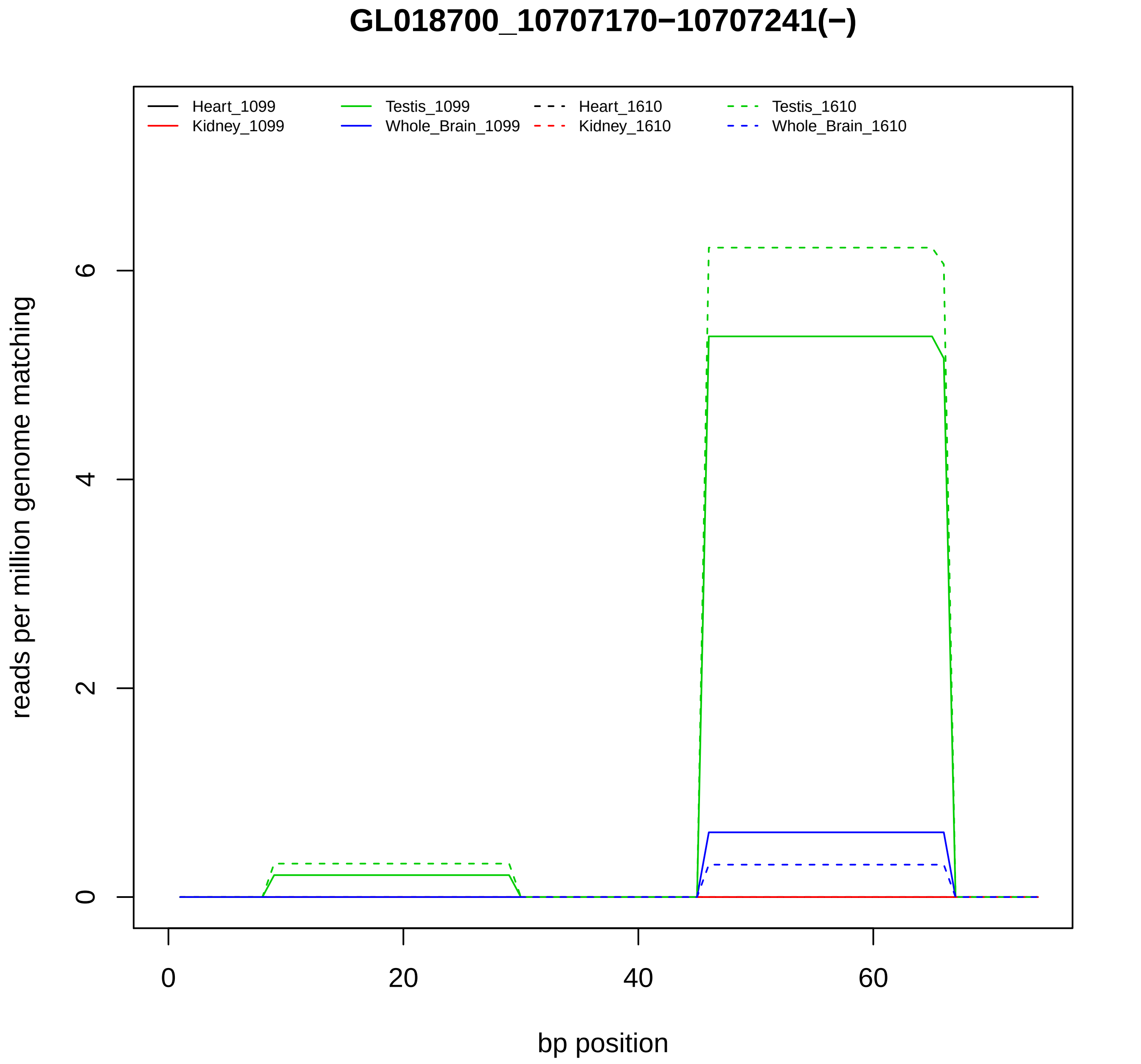

GL018700_10707170−10707241(−)
Heart_1099
Kidney_1099
Testis_1099
Whole_Brain_1099
Heart_1610
Kidney_1610
Testis_1610
Whole_Brain_1610
reads per million genome matching
bp position
0
2
4
6
0
20
40
60

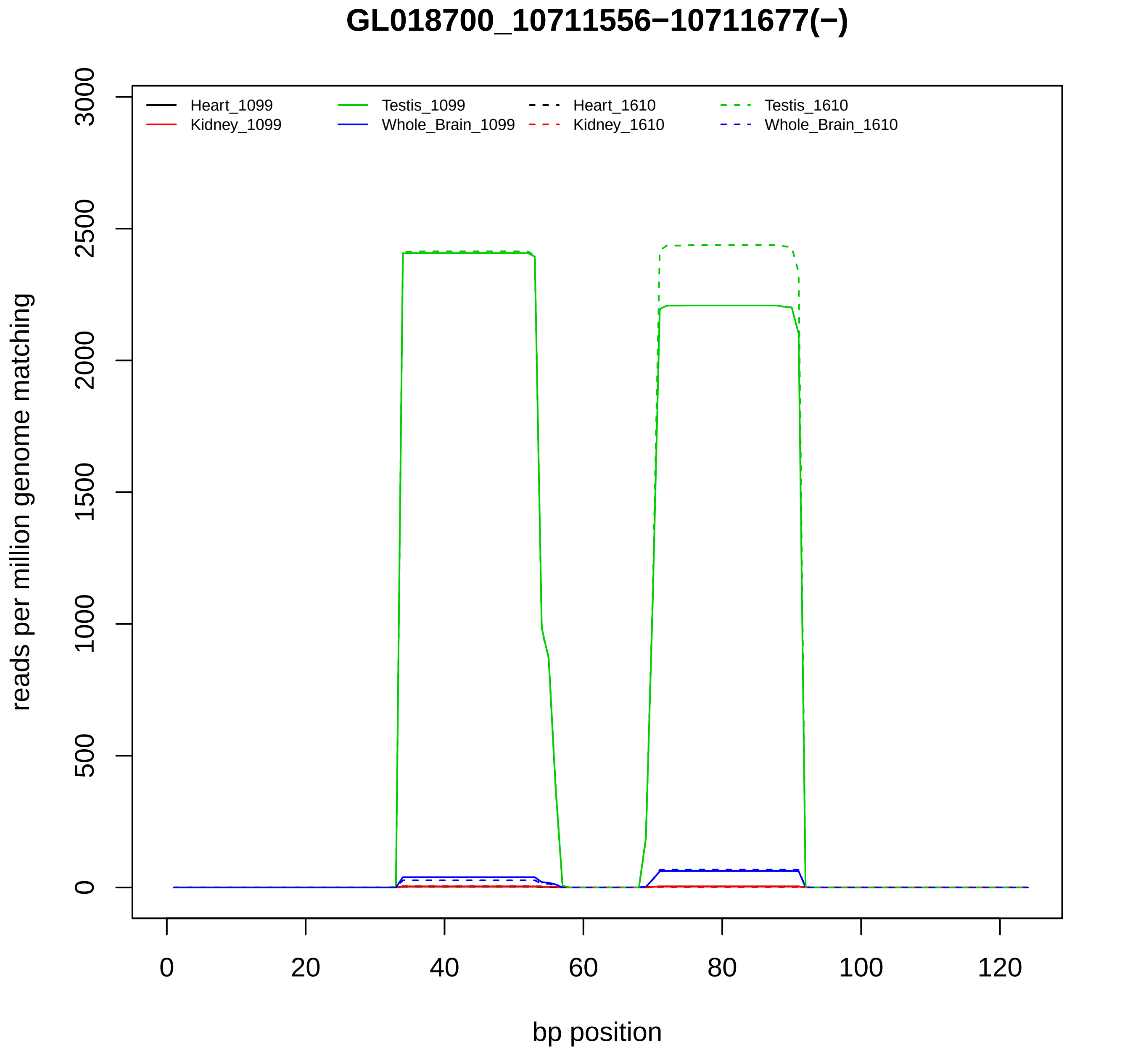

GL018700_10711556−10711677(−)
Heart_1099
Kidney_1099
Testis_1099
Whole_Brain_1099
Heart_1610
Kidney_1610
Testis_1610
Whole_Brain_1610
reads per million genome matching
bp position
0
500
1000
1500
2000
2500
3000
0
20
40
60
80
100
120

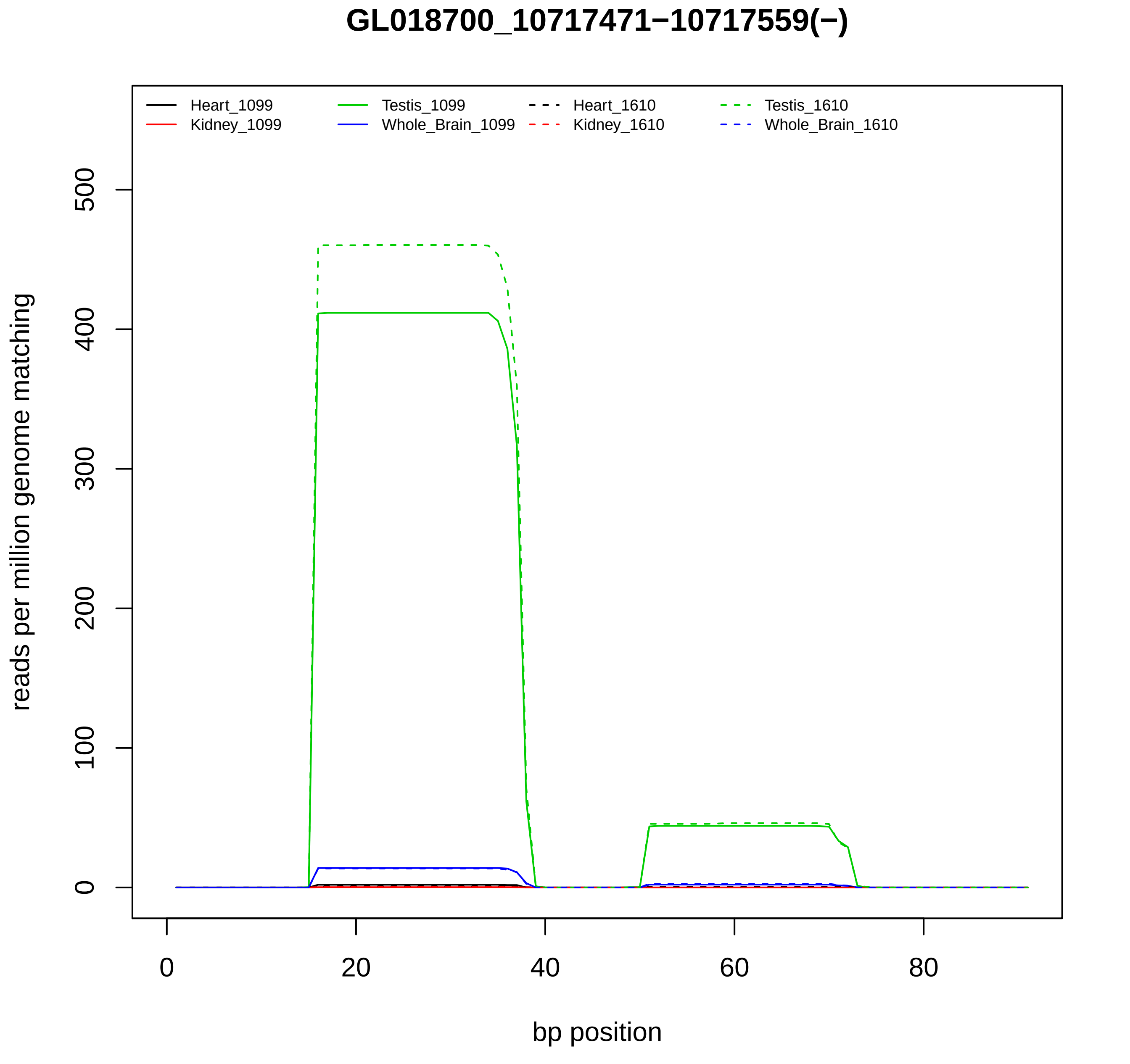
GL018700_10717471−10717559(−)
Heart_1099
Kidney_1099
Testis_1099
Whole_Brain_1099
Heart_1610
Kidney_1610
Testis_1610
Whole_Brain_1610
reads per million genome matching
0
100
200
300
400
500
0
20
40
60
80
bp position

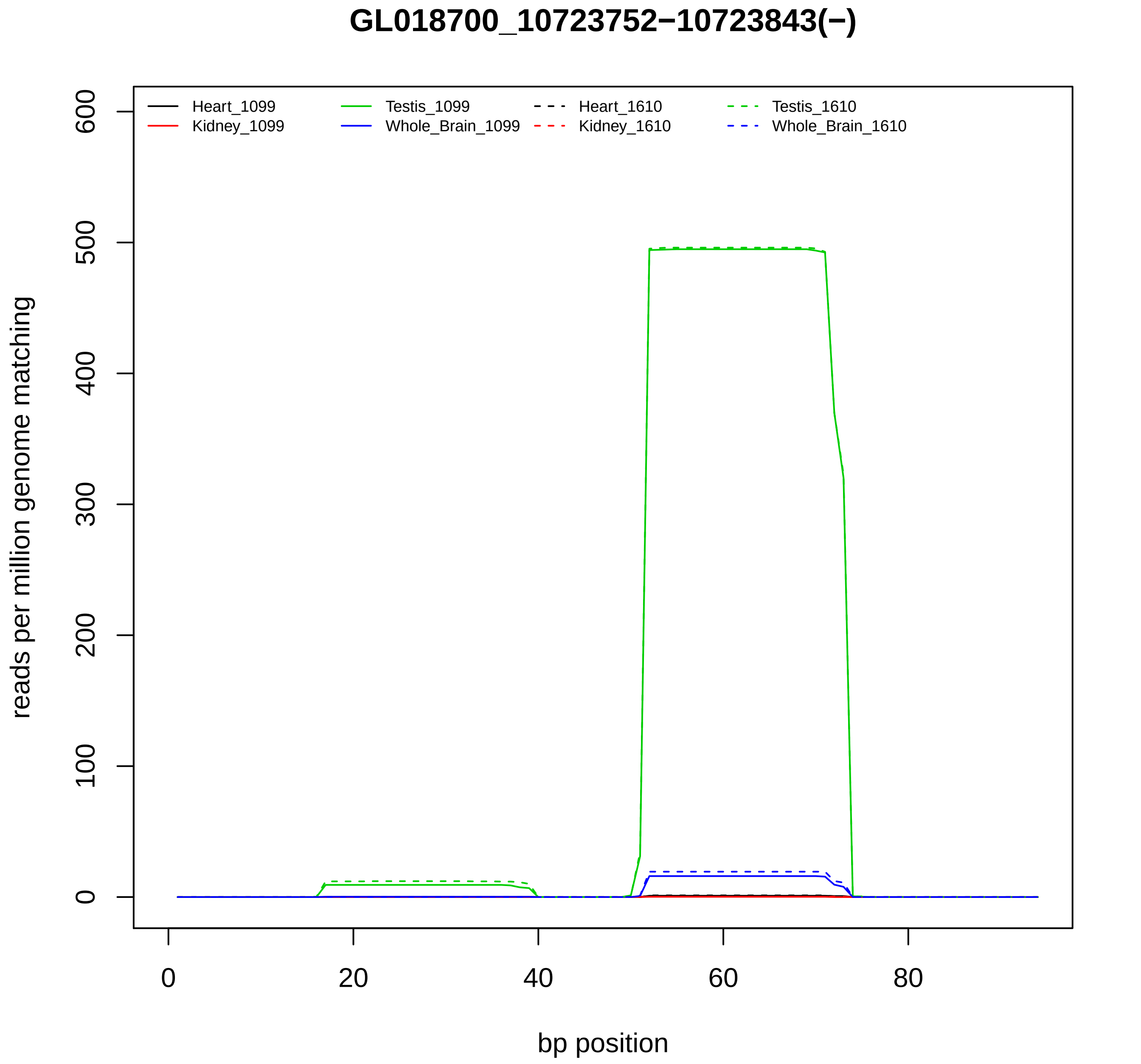

GL018700_10723752−10723843(−)
Heart_1099
Kidney_1099
Testis_1099
Whole_Brain_1099
Heart_1610
Kidney_1610
Testis_1610
Whole_Brain_1610
reads per million genome matching
0
100
200
300
400
500
600
0
20
40
60
80
bp position

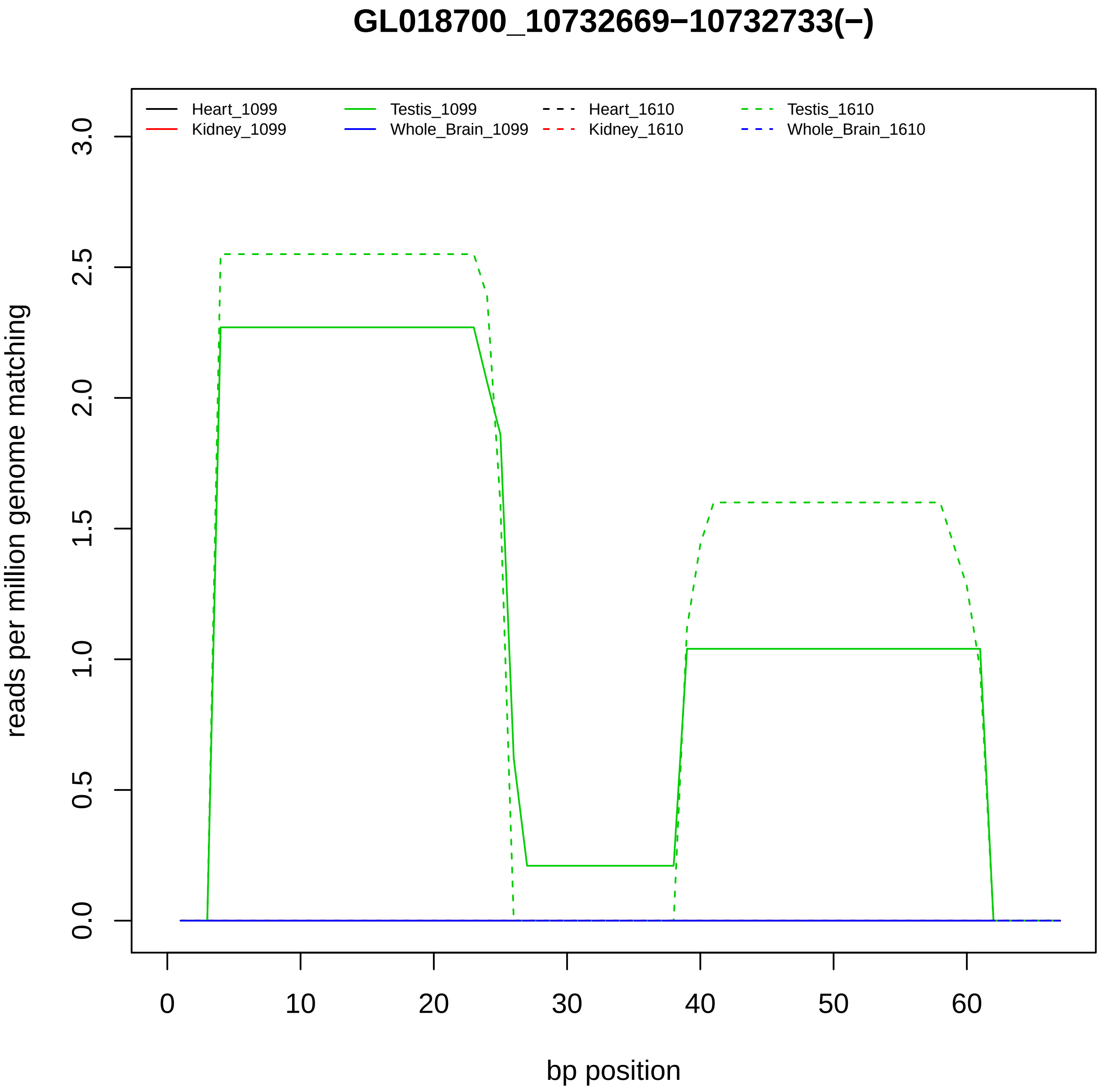

GL018700_10732669−10732733(−)
Heart_1099
Kidney_1099
Testis_1099
Whole_Brain_1099
Heart_1610
Kidney_1610
Testis_1610
Whole_Brain_1610
reads per million genome matching
bp position
0.0
0.5
1.0
1.5
2.0
2.5
3.0
0
10
20
30
40
50
60

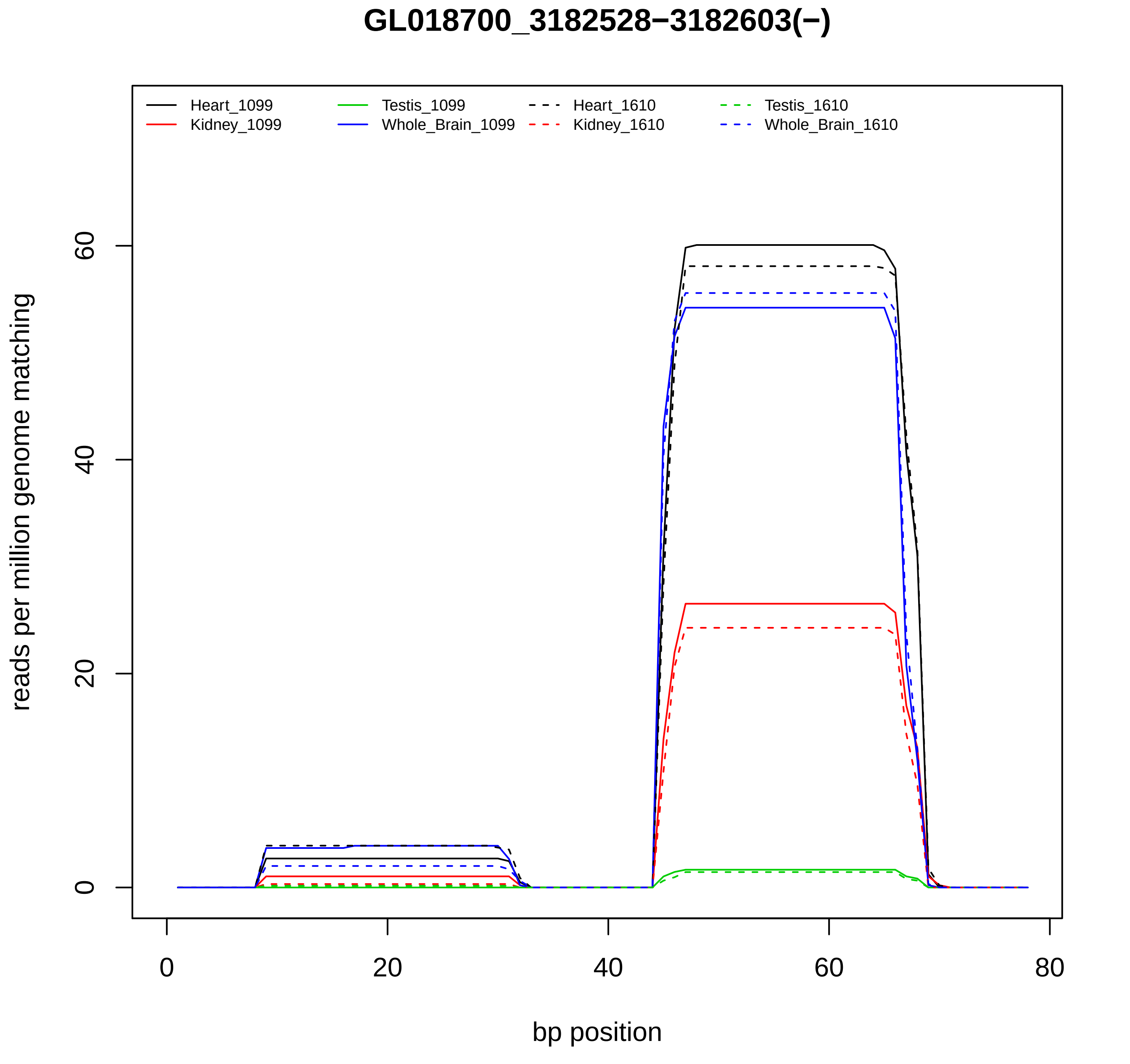

GL018700_3182528−3182603(−)
Heart_1099
Kidney_1099
Testis_1099
Whole_Brain_1099
Heart_1610
Kidney_1610
Testis_1610
Whole_Brain_1610
reads per million genome matching
bp position
0
20
40
60
80

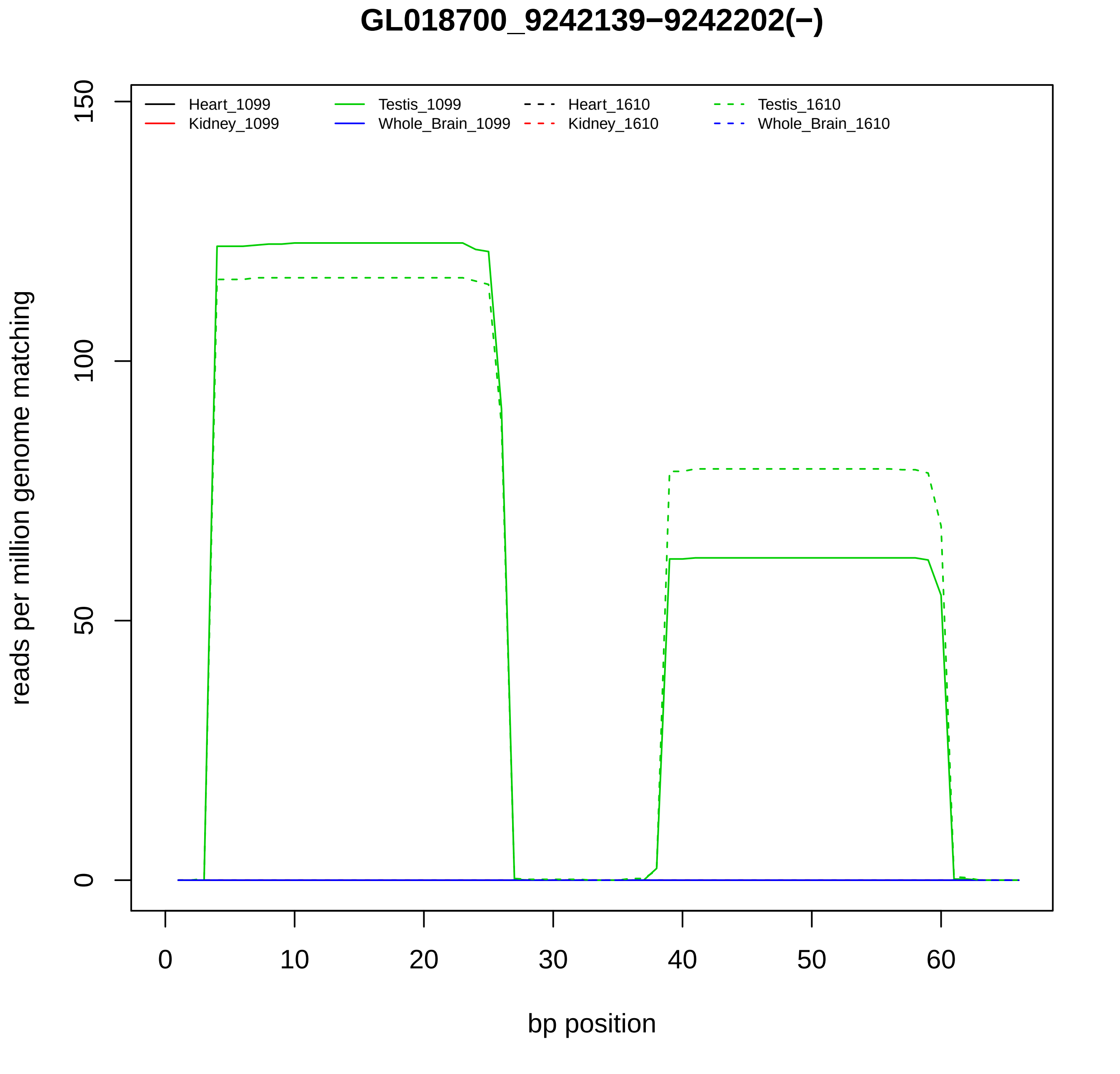

GL018700_9242139−9242202(−)
Heart_1099
Kidney_1099
Testis_1099
Whole_Brain_1099
Heart_1610
Kidney_1610
Testis_1610
Whole_Brain_1610
reads per million genome matching
bp position
0
50
100
150
0
10
20
30
40
50
60

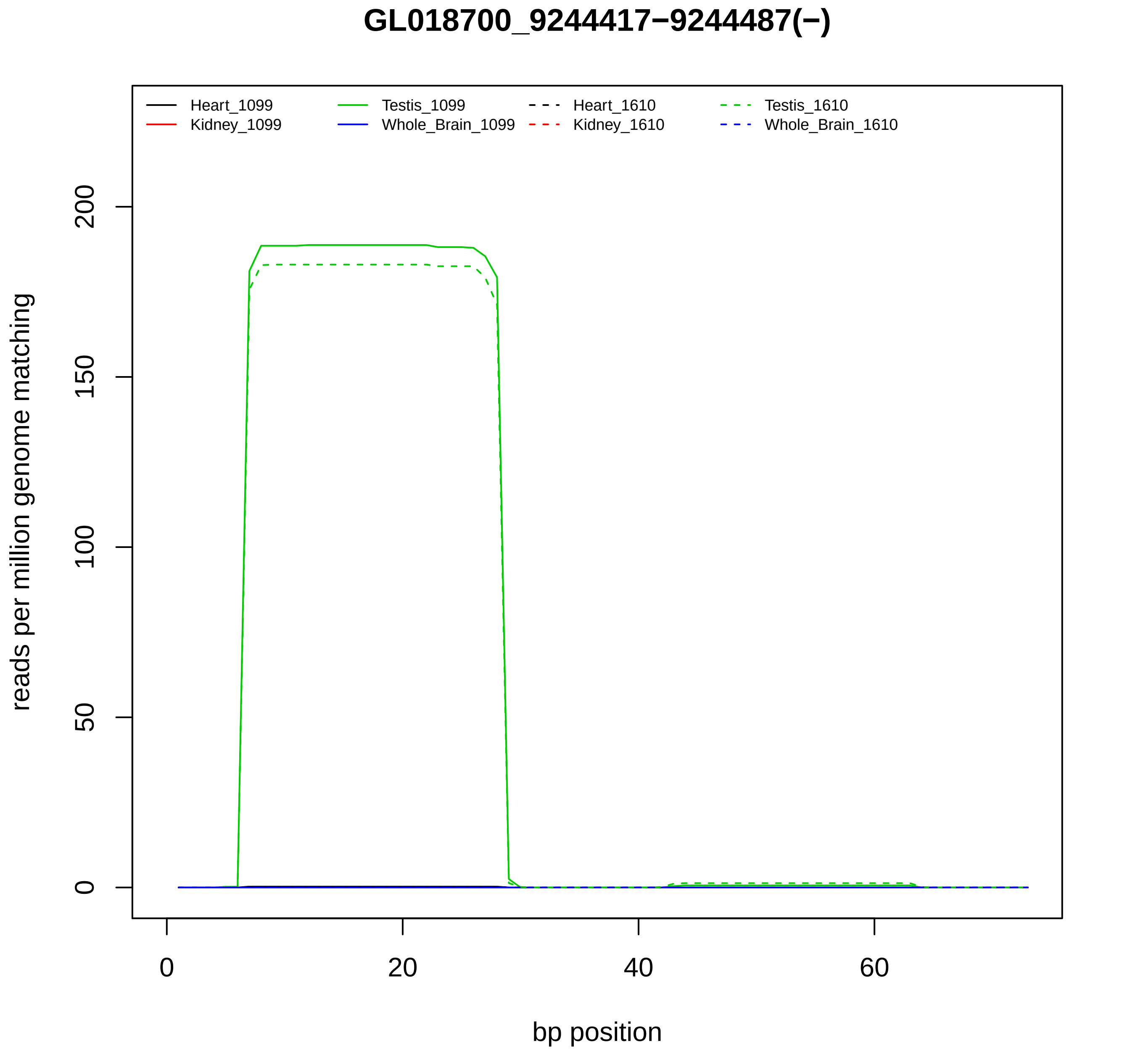

GL018700_9244417−9244487(−)
Heart_1099
Kidney_1099
Testis_1099
Whole_Brain_1099
Heart_1610
Kidney_1610
Testis_1610
Whole_Brain_1610
reads per million genome matching
0
50
100
150
200
0
20
40
60
bp position

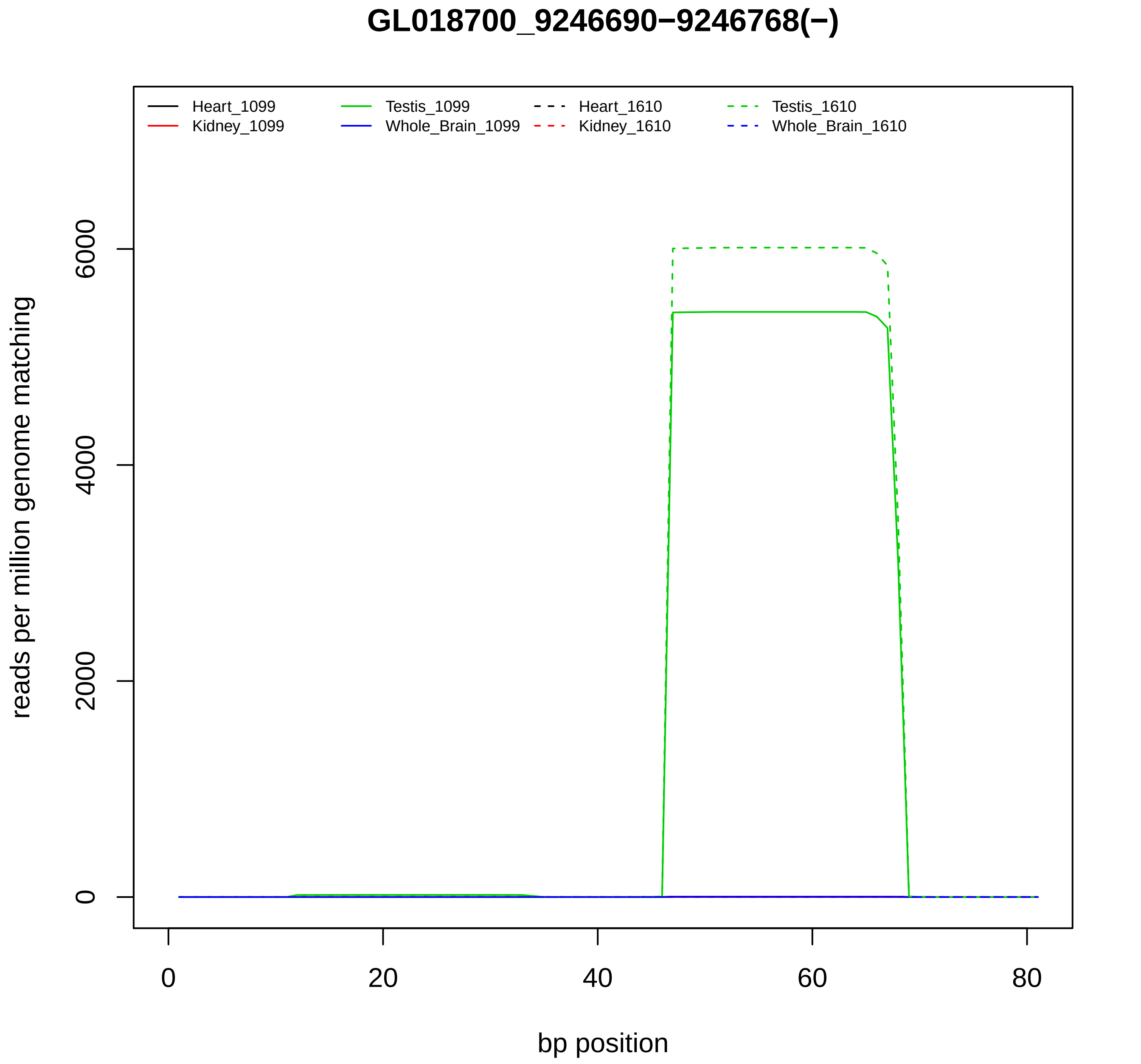
GL018700_9246690−9246768(−)
Heart_1099
Kidney_1099
Testis_1099
Whole_Brain_1099
Heart_1610
Kidney_1610
Testis_1610
Whole_Brain_1610
reads per million genome matching
0
2000
4000
6000
0
20
40
60
80
bp position

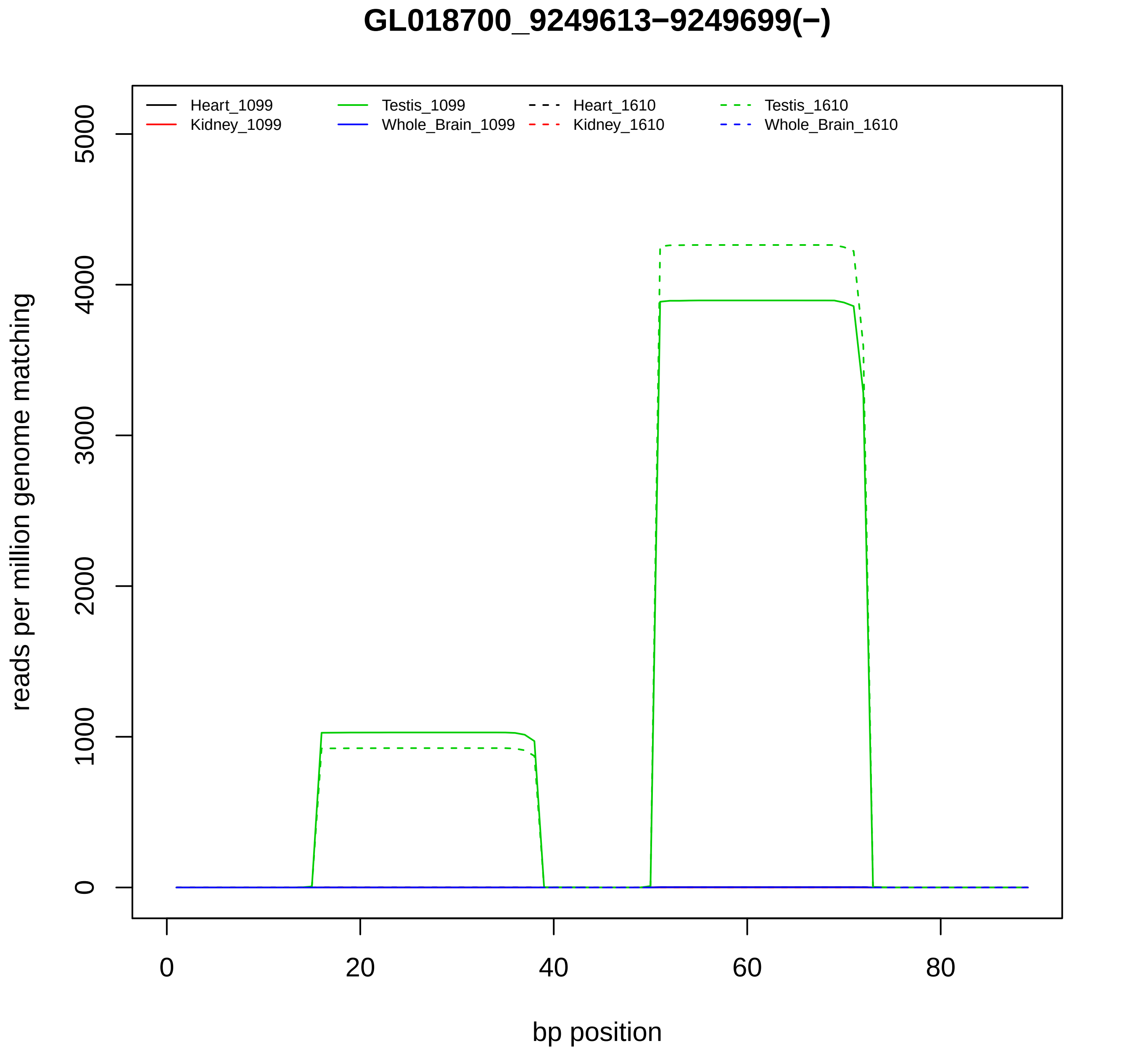

GL018700_9249613−9249699(−)
Heart_1099
Kidney_1099
Testis_1099
Whole_Brain_1099
Heart_1610
Kidney_1610
Testis_1610
Whole_Brain_1610
reads per million genome matching
0
1000
2000
3000
4000
5000
bp position
0
20
40
60
80

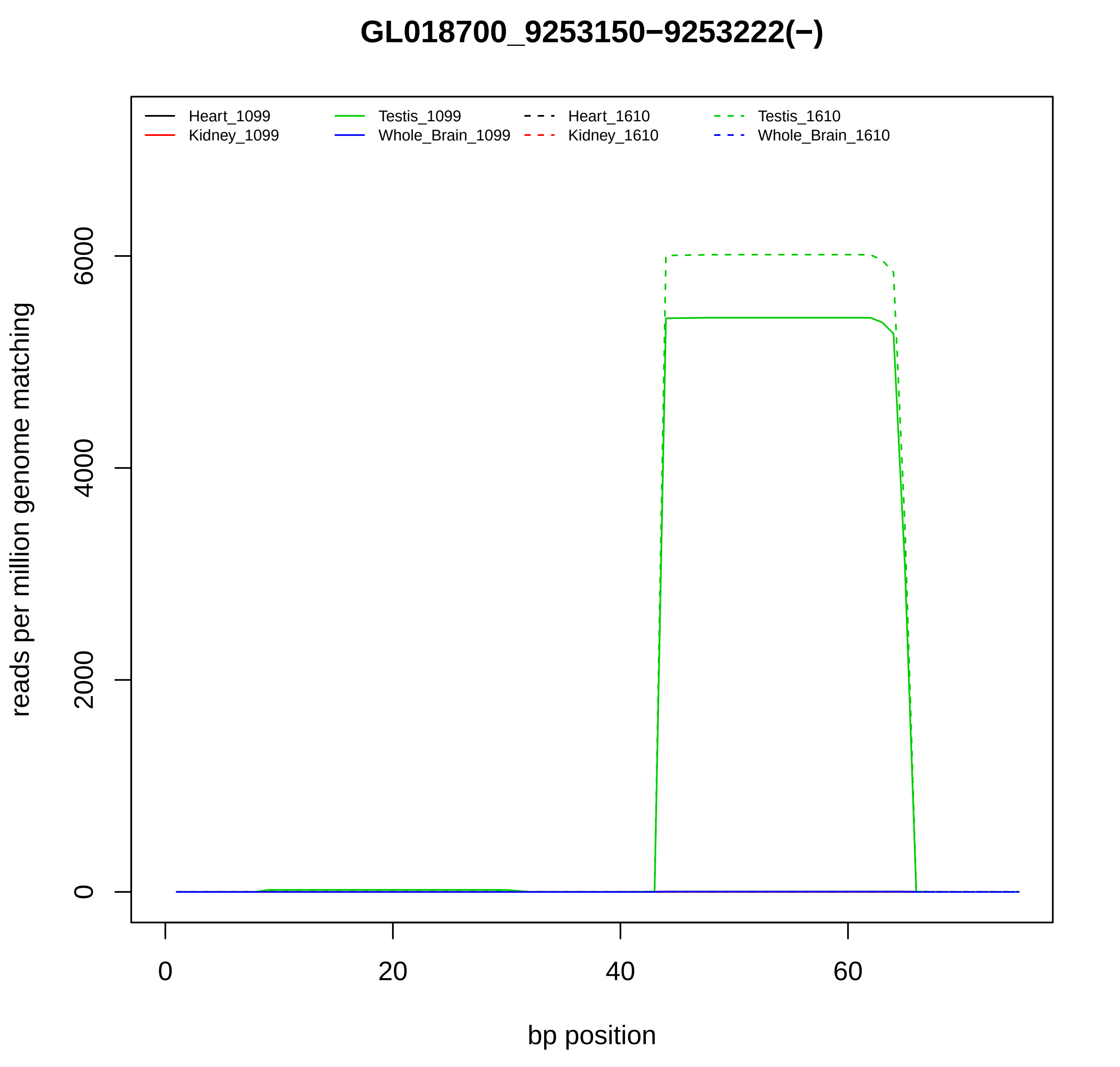
GL018700_9253150−9253222(−)
Heart_1099
Kidney_1099
Testis_1099
Whole_Brain_1099
Heart_1610
Kidney_1610
Testis_1610
Whole_Brain_1610
reads per million genome matching
0
2000
4000
6000
0
20
40
60
bp position

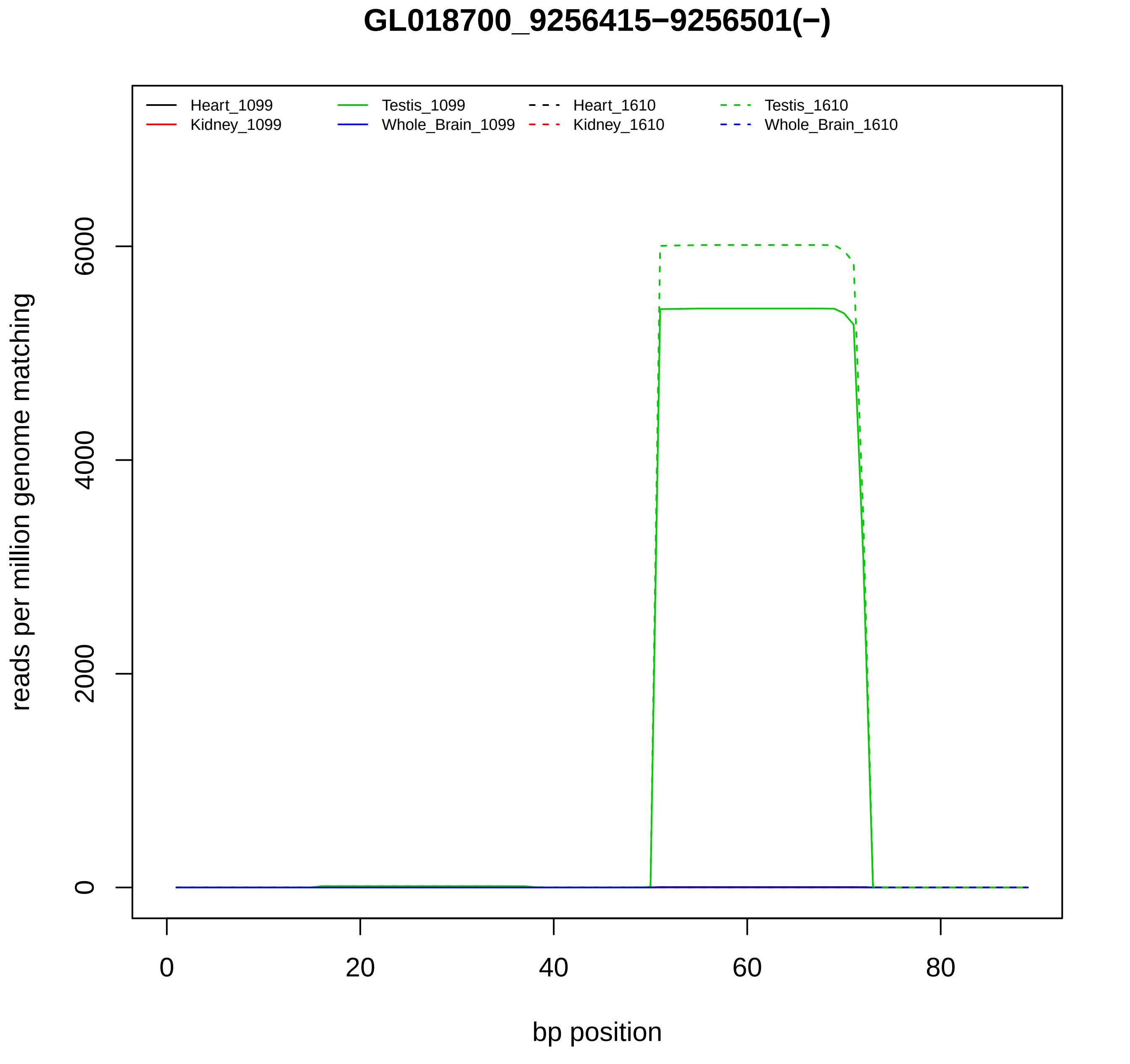

GL018700_9256415−9256501(−)
Heart_1099
Kidney_1099
Testis_1099
Whole_Brain_1099
Heart_1610
Kidney_1610
Testis_1610
Whole_Brain_1610
reads per million genome matching
0
2000
4000
6000
0
20
40
60
80
bp position

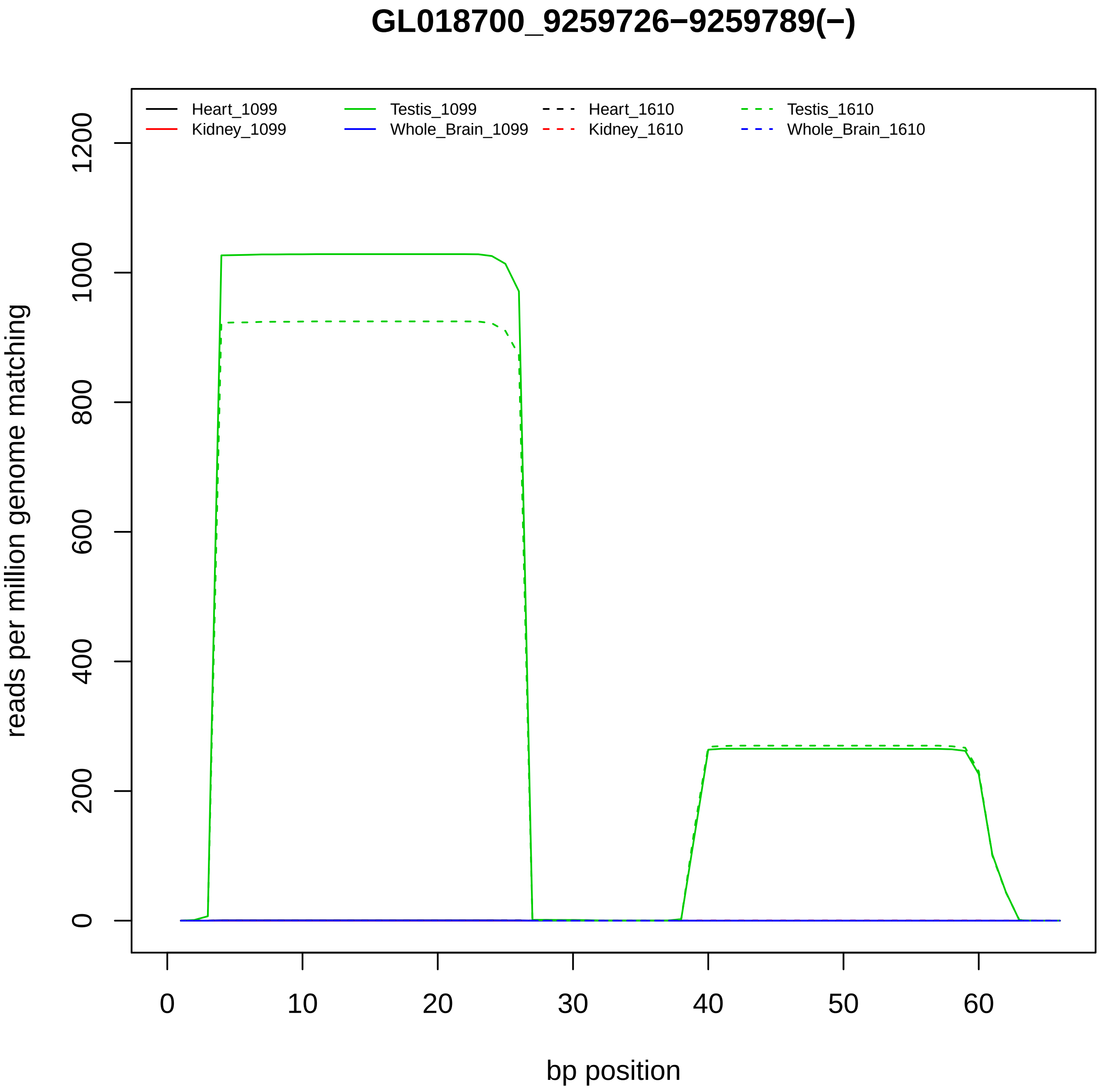
GL018700_9259726−9259789(−)
Heart_1099
Kidney_1099
Testis_1099
Whole_Brain_1099
Heart_1610
Kidney_1610
Testis_1610
Whole_Brain_1610
reads per million genome matching
0
200
400
600
800
1000
1200
0
10
20
30
40
50
60
bp position

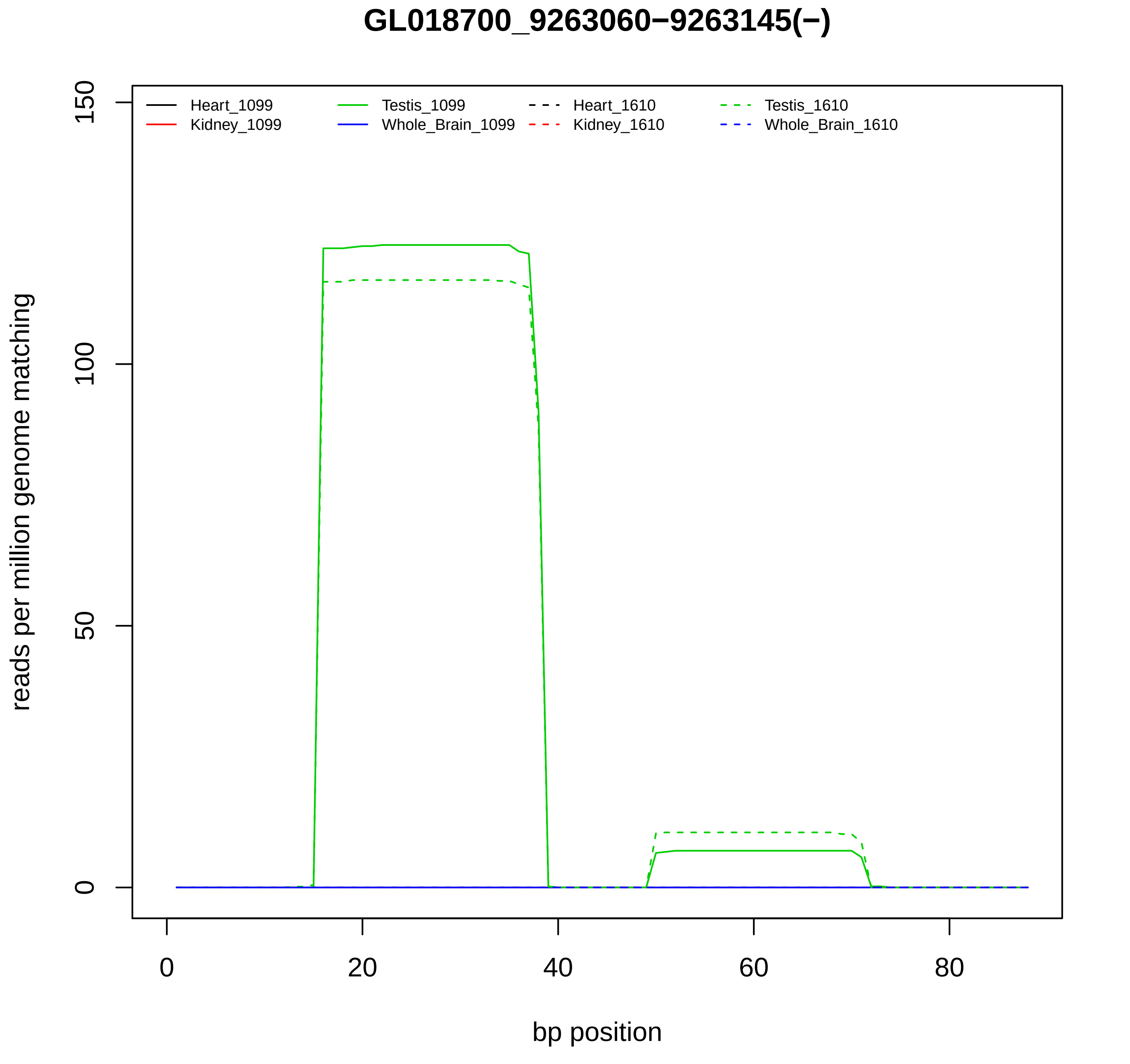

GL018700_9263060−9263145(−)
Heart_1099
Kidney_1099
Testis_1099
Whole_Brain_1099
Heart_1610
Kidney_1610
Testis_1610
Whole_Brain_1610
reads per million genome matching
0
50
100
150
0
20
40
60
80
bp position

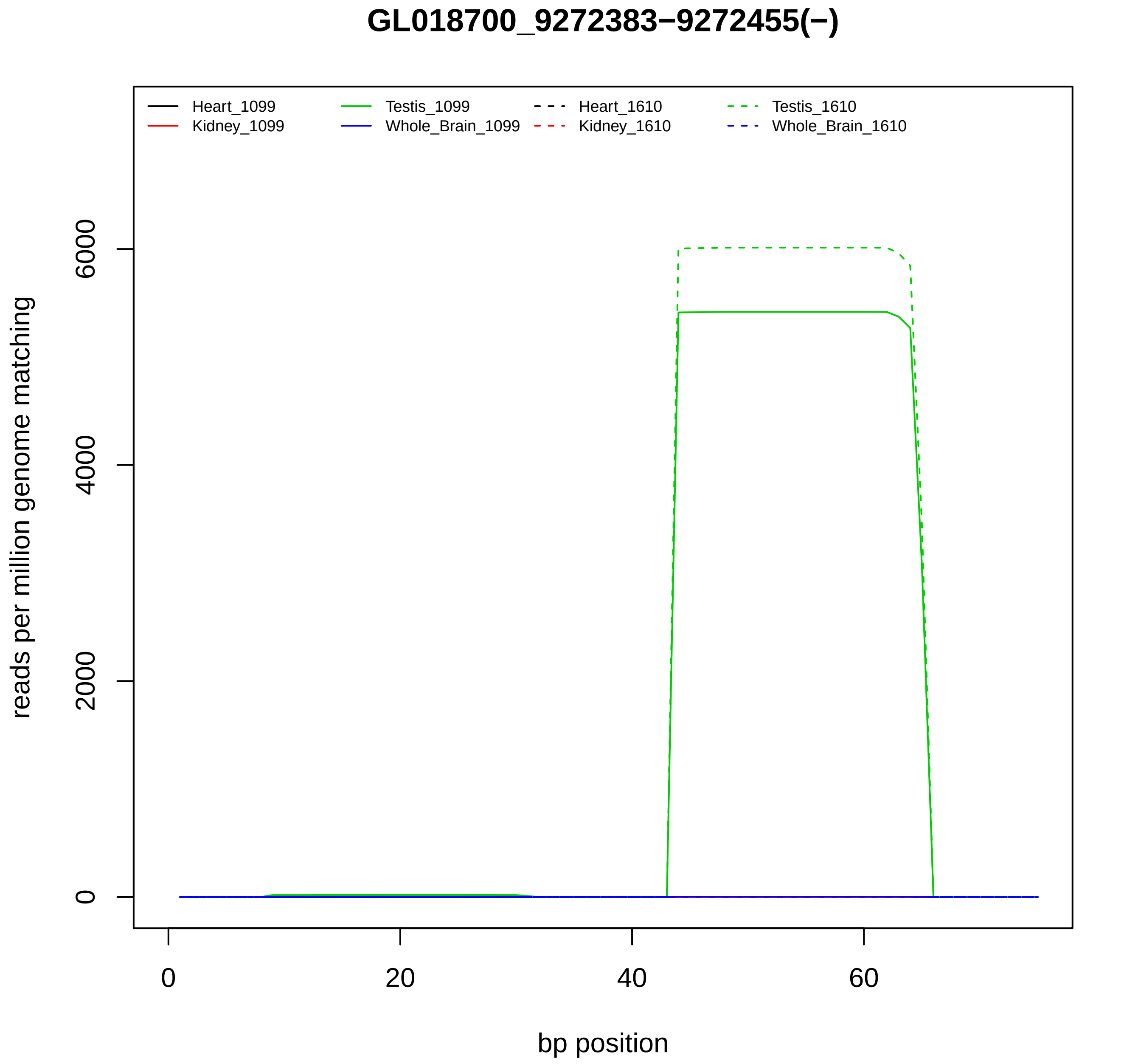
GL018700_9272383−9272455(−)
Heart_1099
Kidney_1099
Testis_1099
Whole_Brain_1099
Heart_1610
Kidney_1610
Testis_1610
Whole_Brain_1610
reads per million genome matching
0
2000
4000
6000
0
20
40
60
bp position

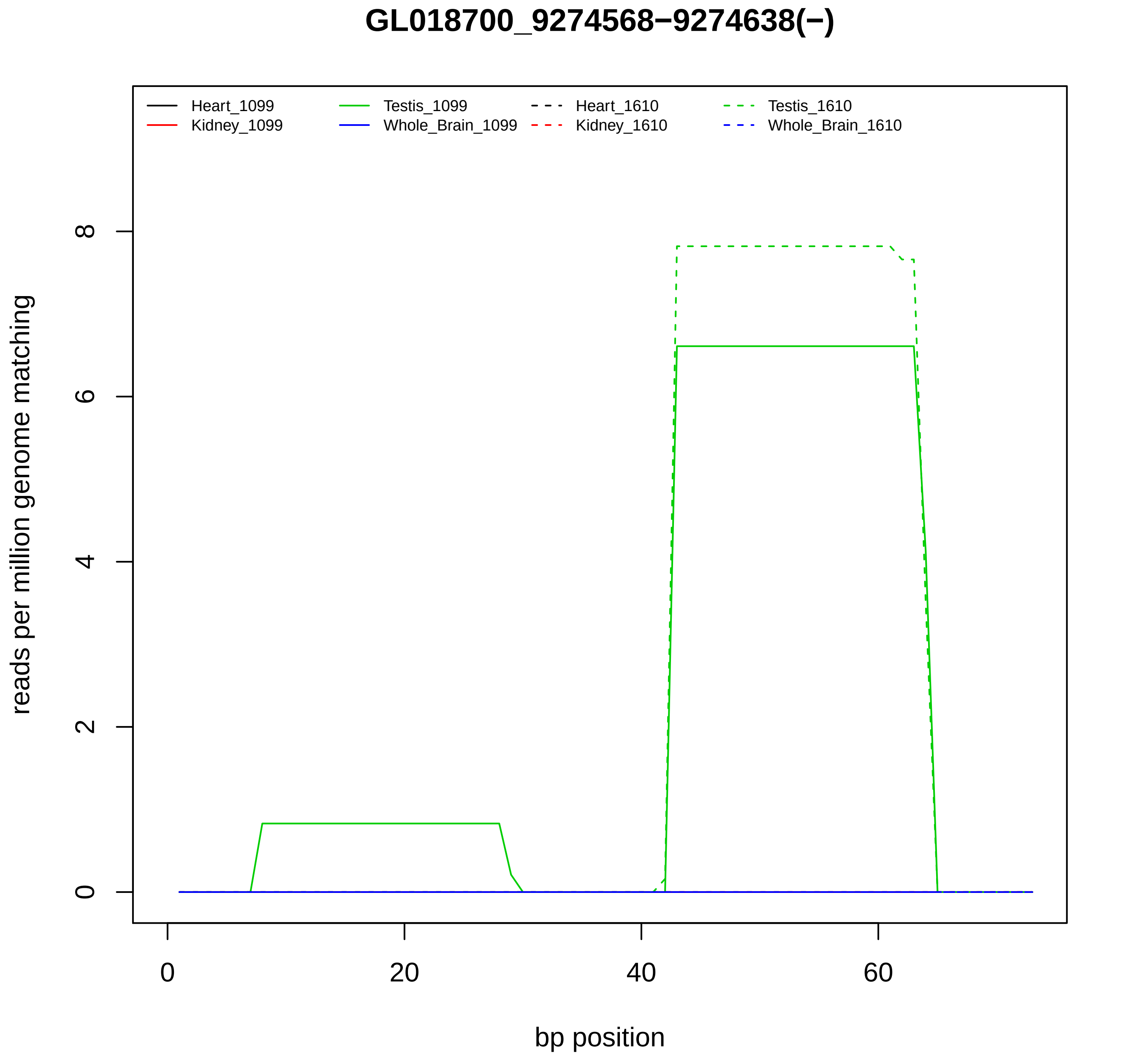

GL018700_9274568−9274638(−)
Heart_1099
Kidney_1099
Testis_1099
Whole_Brain_1099
Heart_1610
Kidney_1610
Testis_1610
Whole_Brain_1610
reads per million genome matching
bp position
0
2
4
6
8
0
20
40
60

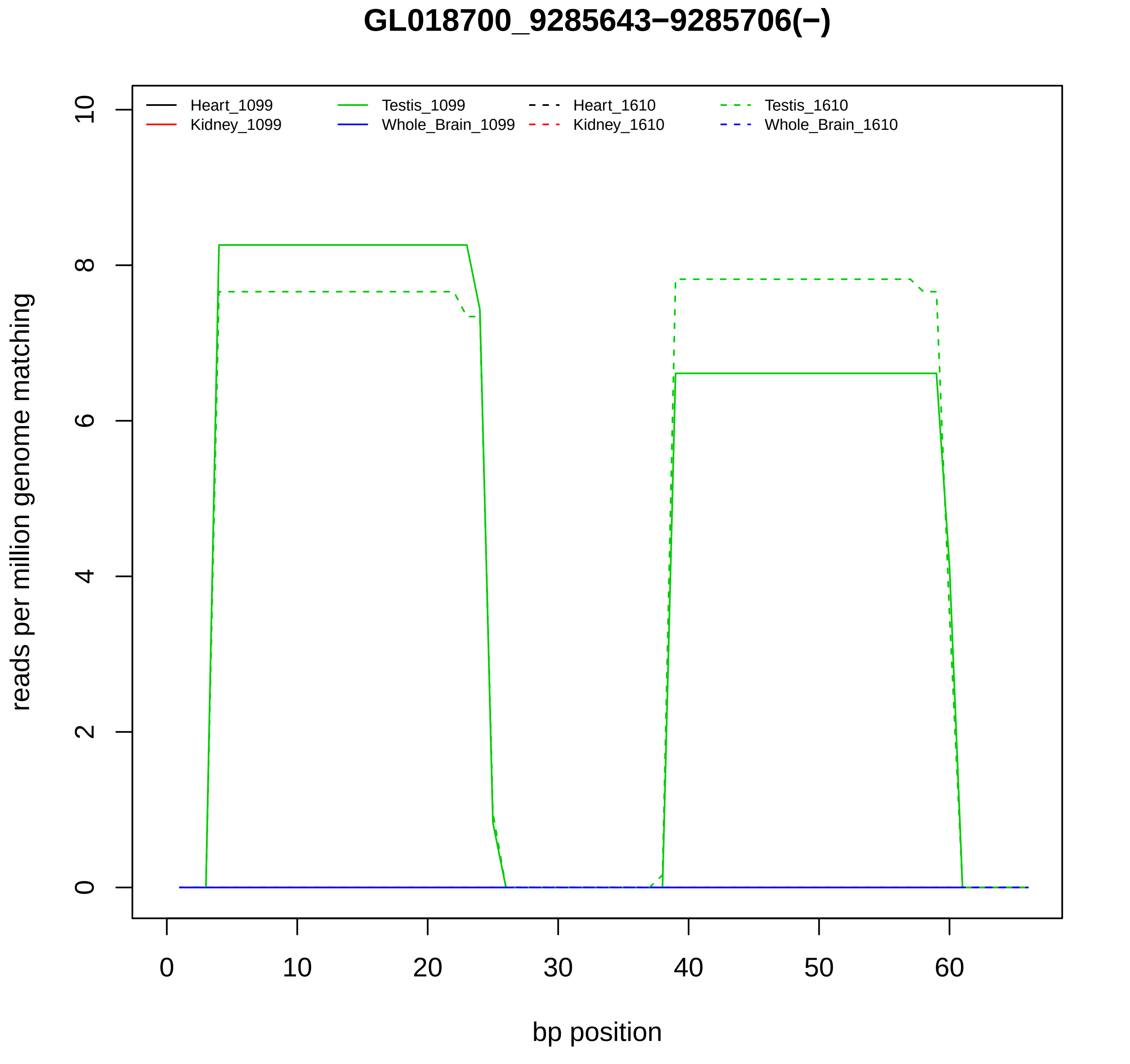

GL018700_9285643−9285706(−)
Heart_1099
Kidney_1099
Testis_1099
Whole_Brain_1099
Heart_1610
Kidney_1610
Testis_1610
Whole_Brain_1610
reads per million genome matching
bp position
0
2
4
6
8
10
0
10
20
30
40
50
60

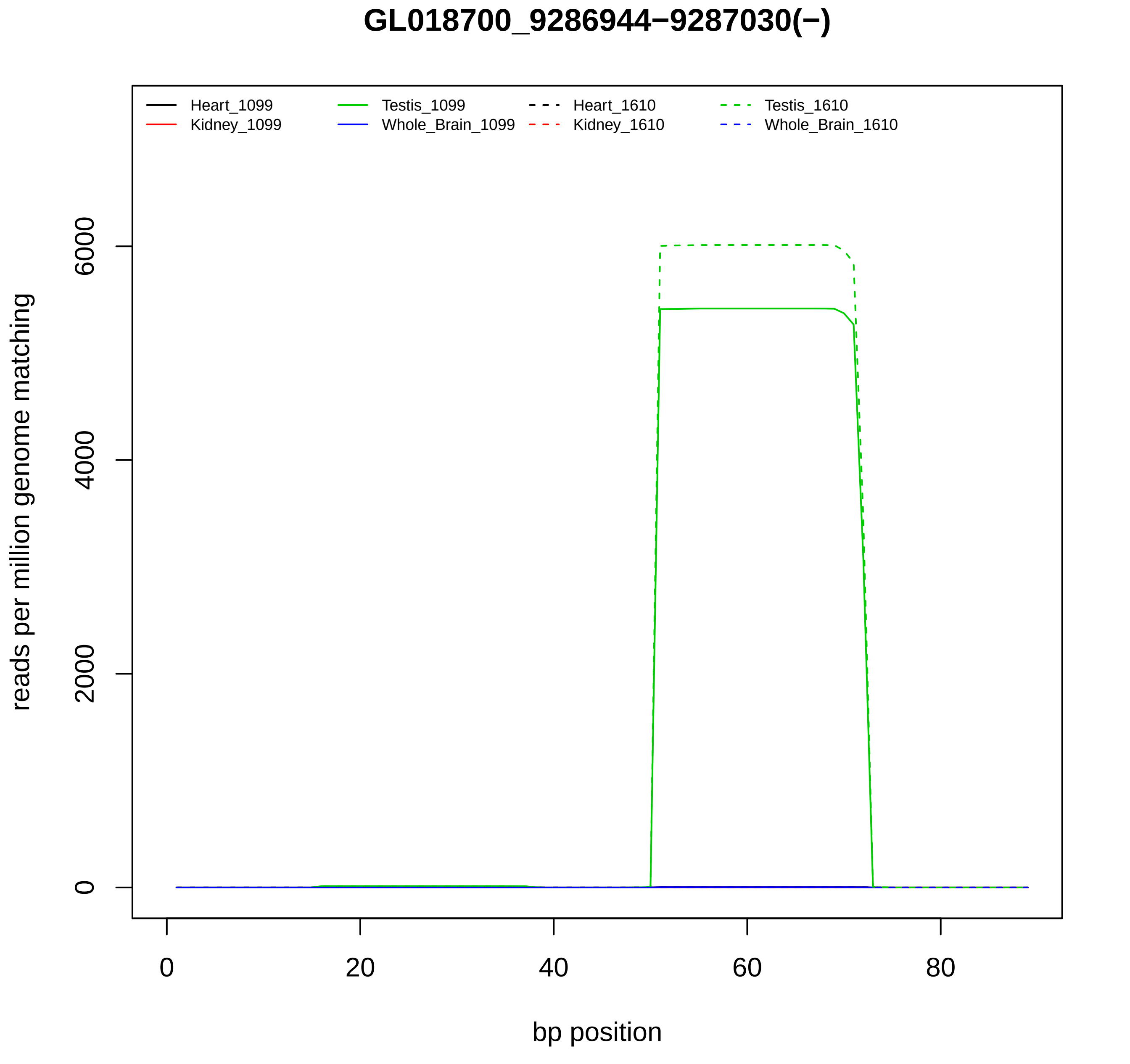

GL018700_9286944−9287030(−)
Heart_1099
Kidney_1099
Testis_1099
Whole_Brain_1099
Heart_1610
Kidney_1610
Testis_1610
Whole_Brain_1610
reads per million genome matching
0
2000
4000
6000
0
20
40
60
80
bp position

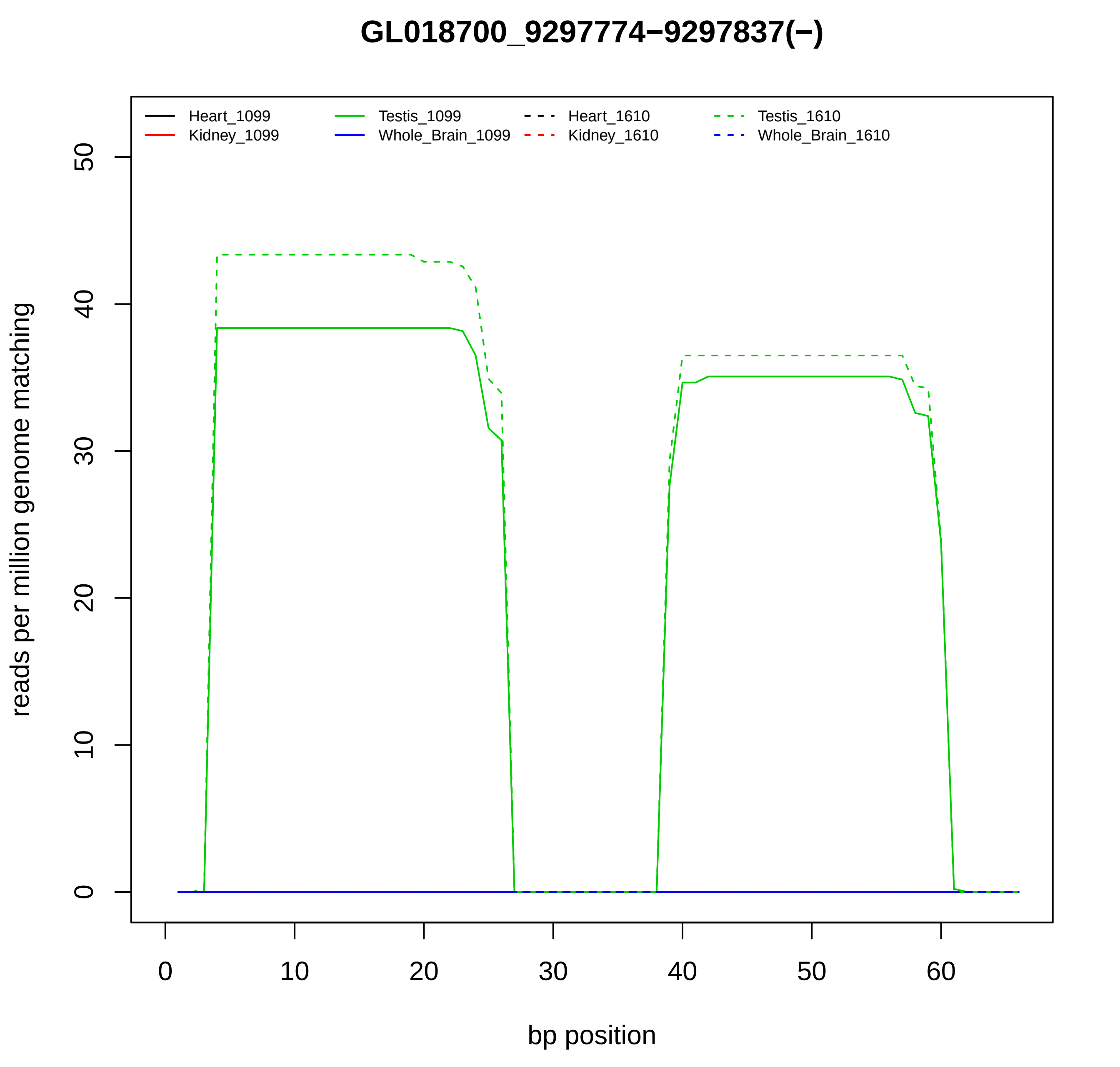
GL018700_9297774−9297837(−)
Heart_1099
Kidney_1099
Testis_1099
Whole_Brain_1099
Heart_1610
Kidney_1610
Testis_1610
Whole_Brain_1610
reads per million genome matching
bp position
0
10
20
30
40
50
60

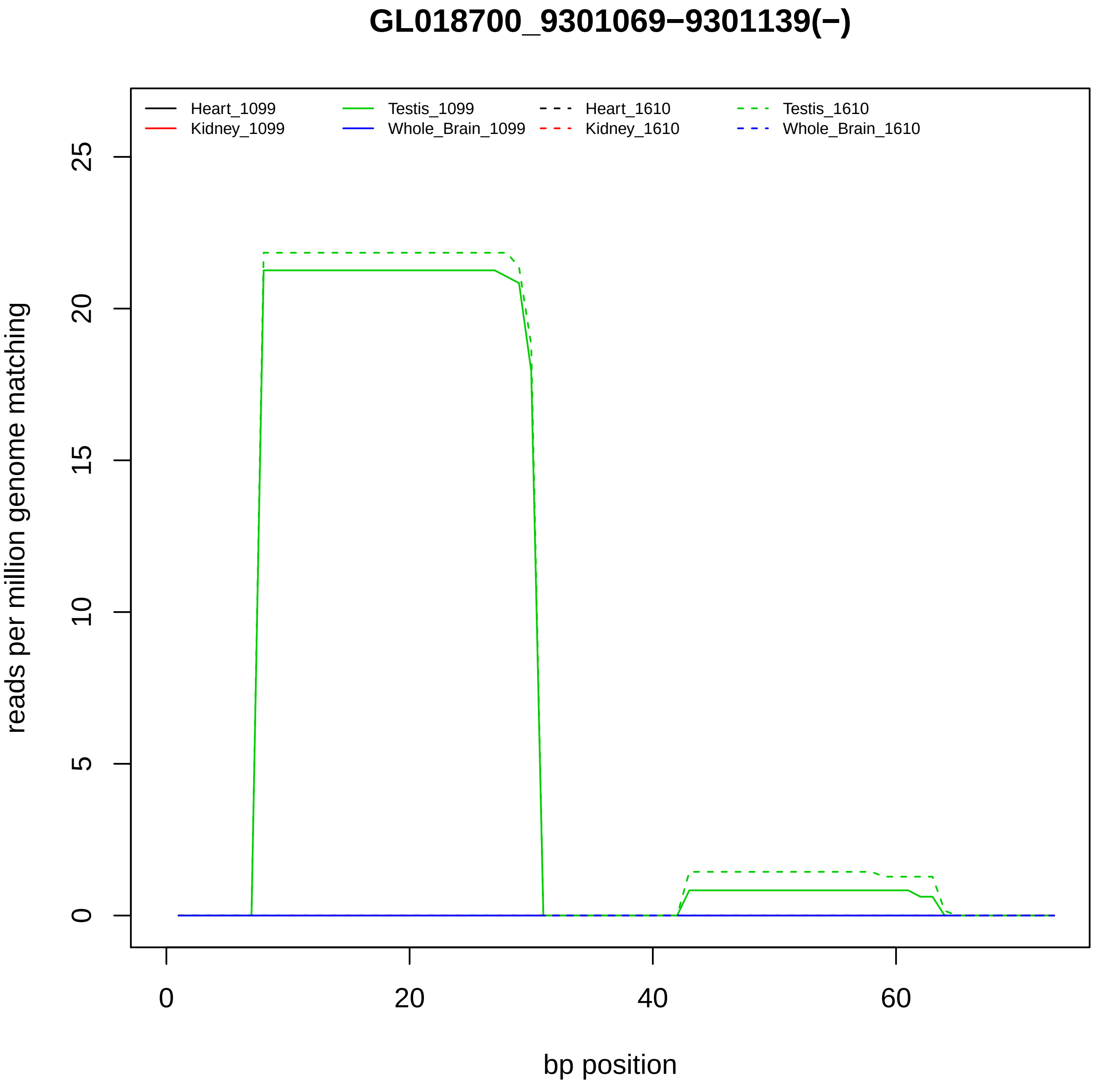
GL018700_9301069−9301139(−)
Heart_1099
Kidney_1099
Testis_1099
Whole_Brain_1099
Heart_1610
Kidney_1610
Testis_1610
Whole_Brain_1610
reads per million genome matching
0
5
10
15
20
25
0
20
40
60
bp position

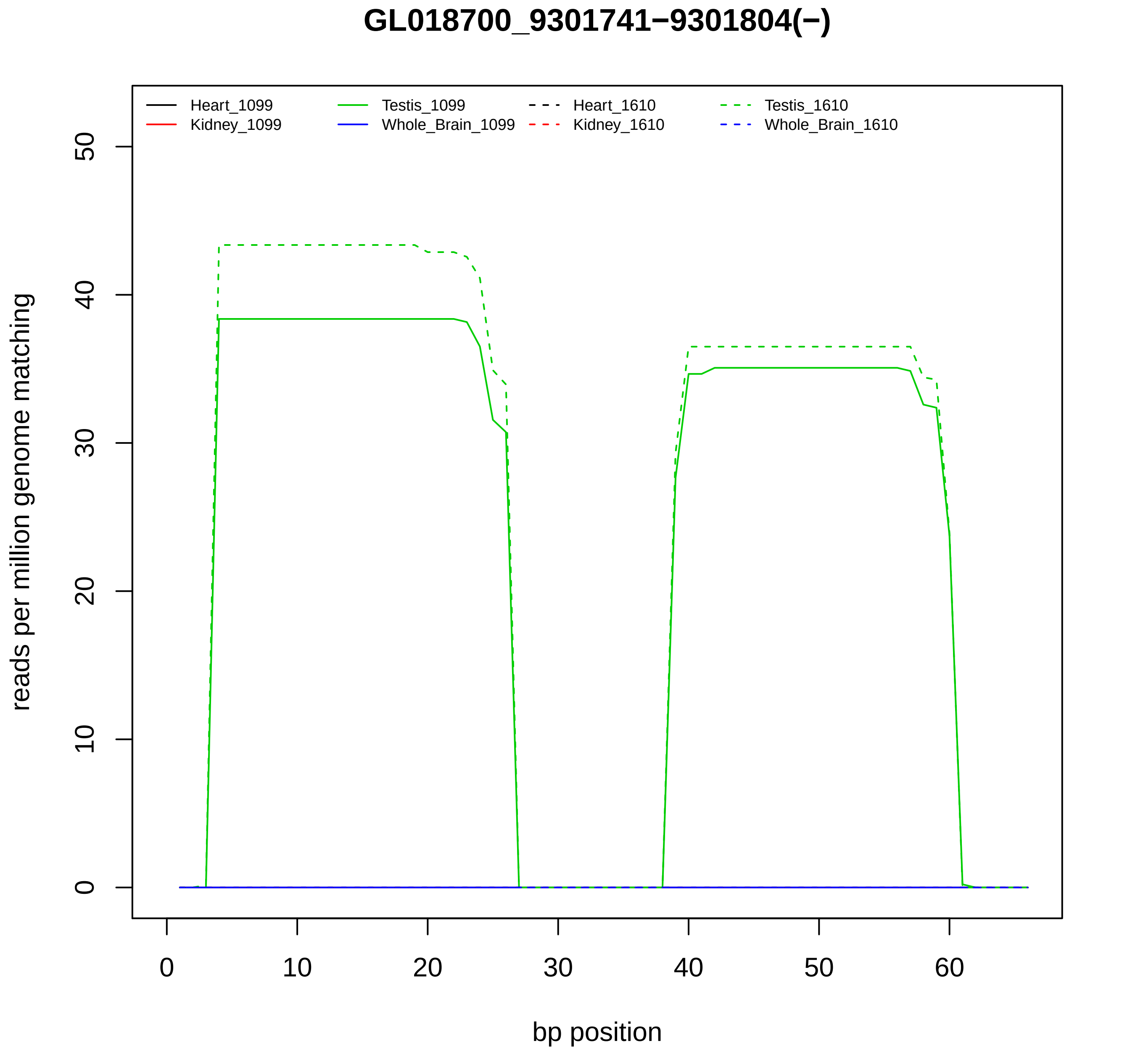
GL018700_9301741−9301804(−)
Heart_1099
Kidney_1099
Testis_1099
Whole_Brain_1099
Heart_1610
Kidney_1610
Testis_1610
Whole_Brain_1610
reads per million genome matching
bp position
0
10
20
30
40
50
60

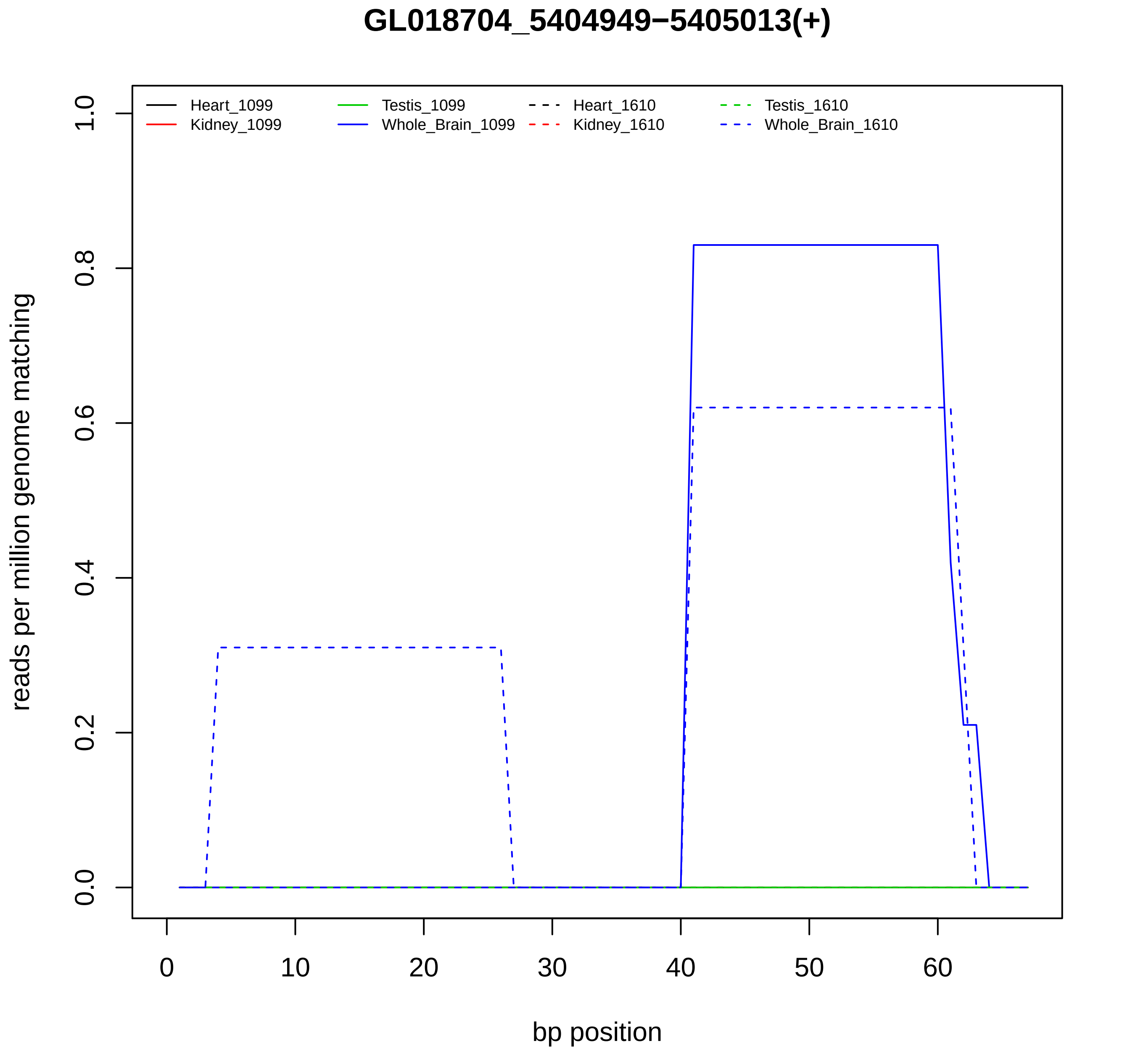
GL018704_5404949−5405013(+)
Heart_1099
Kidney_1099
Testis_1099
Whole_Brain_1099
Heart_1610
Kidney_1610
Testis_1610
Whole_Brain_1610
reads per million genome matching
0.0
0.2
0.4
0.6
0.8
1.0
0
10
20
30
40
50
60
bp position

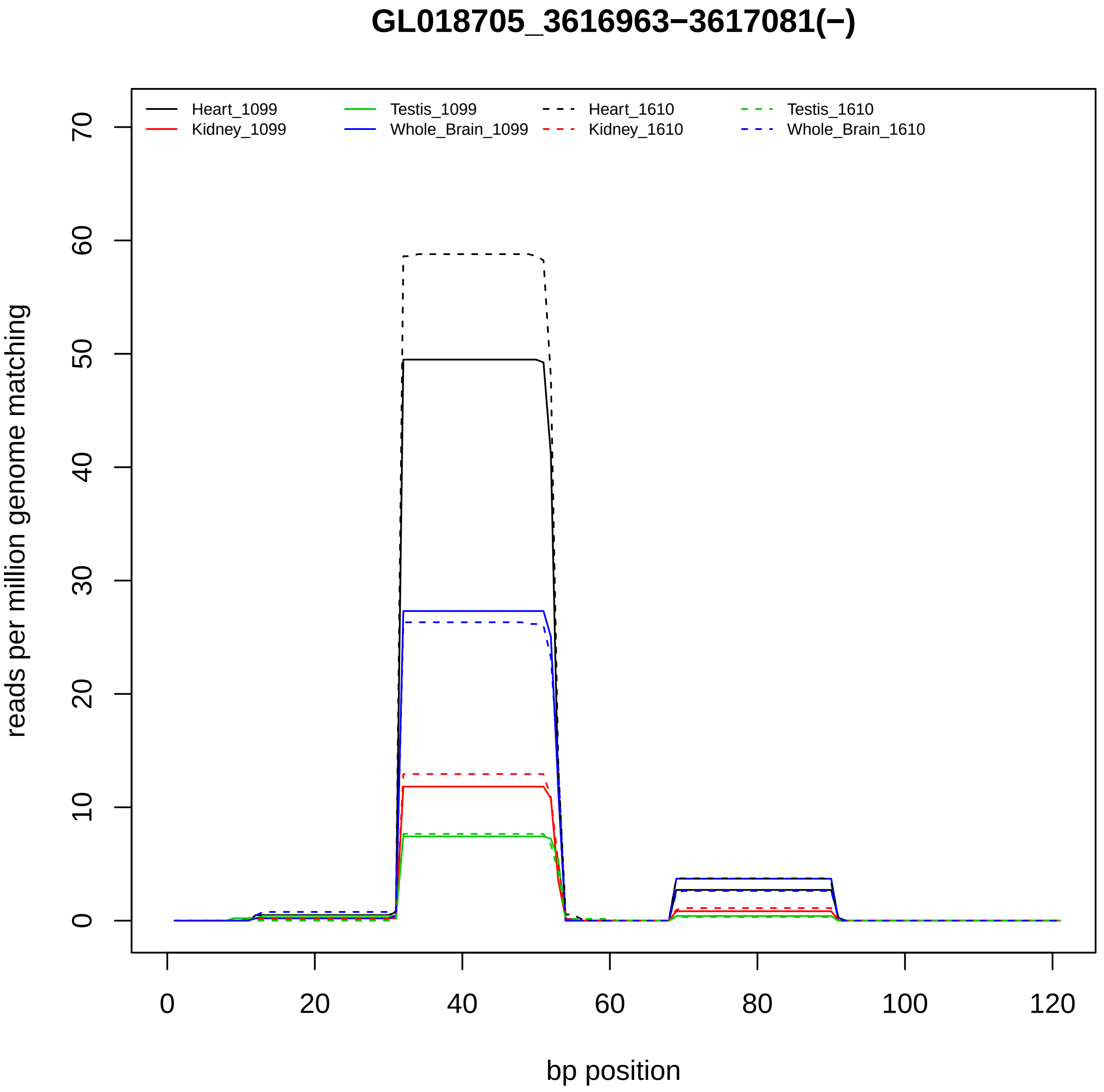

GL018705_3616963−3617081(−)
Heart_1099
Kidney_1099
Testis_1099
Whole_Brain_1099
Heart_1610
Kidney_1610
Testis_1610
Whole_Brain_1610
reads per million genome matching
bp position
0
10
20
30
40
50
60
70
0
20
40
60
80
100
120

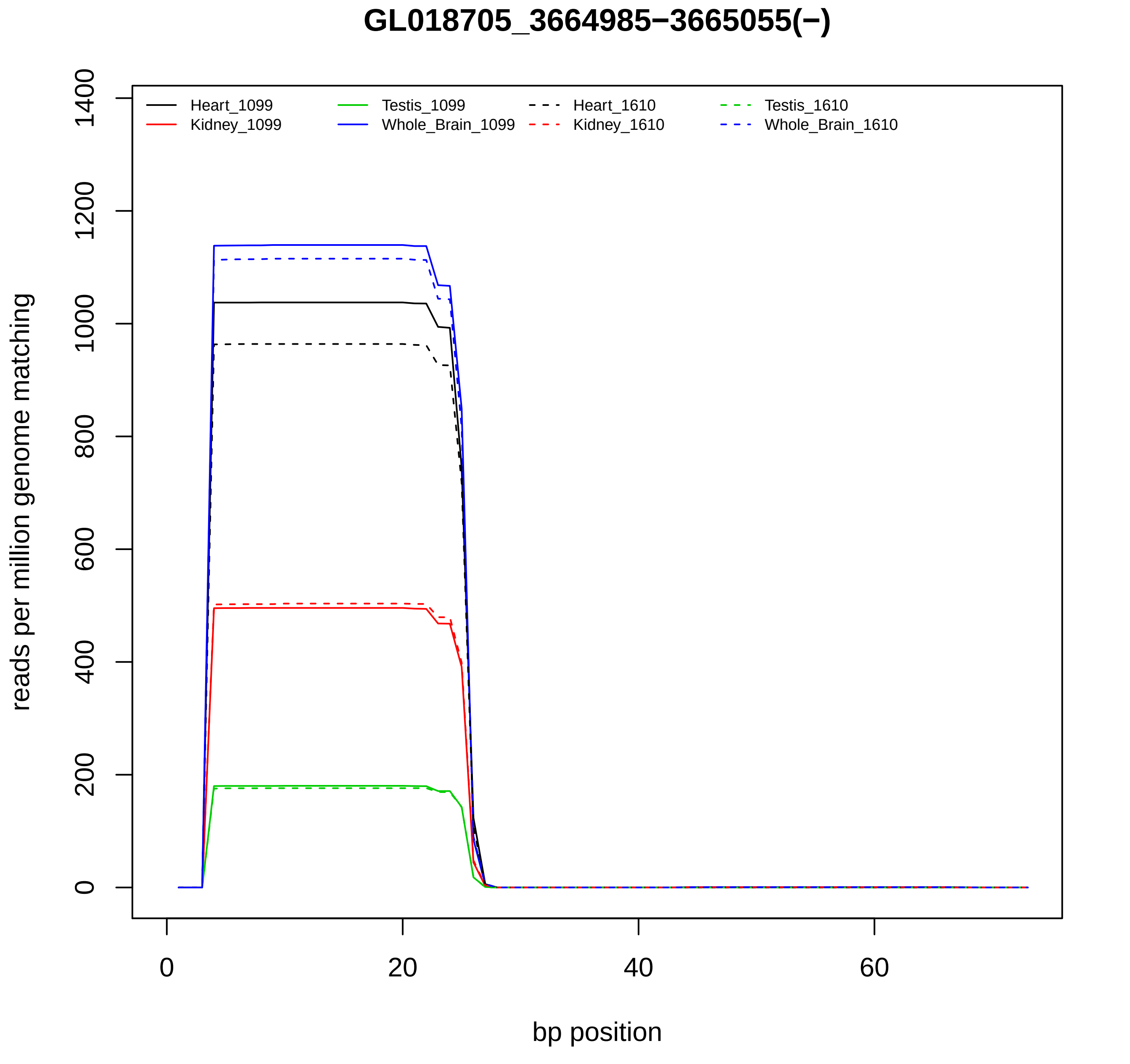

GL018705_3664985−3665055(−)
Heart_1099
Kidney_1099
Testis_1099
Whole_Brain_1099
Heart_1610
Kidney_1610
Testis_1610
Whole_Brain_1610
reads per million genome matching
0
200
400
600
800
1000
1200
1400
bp position
0
20
40
60

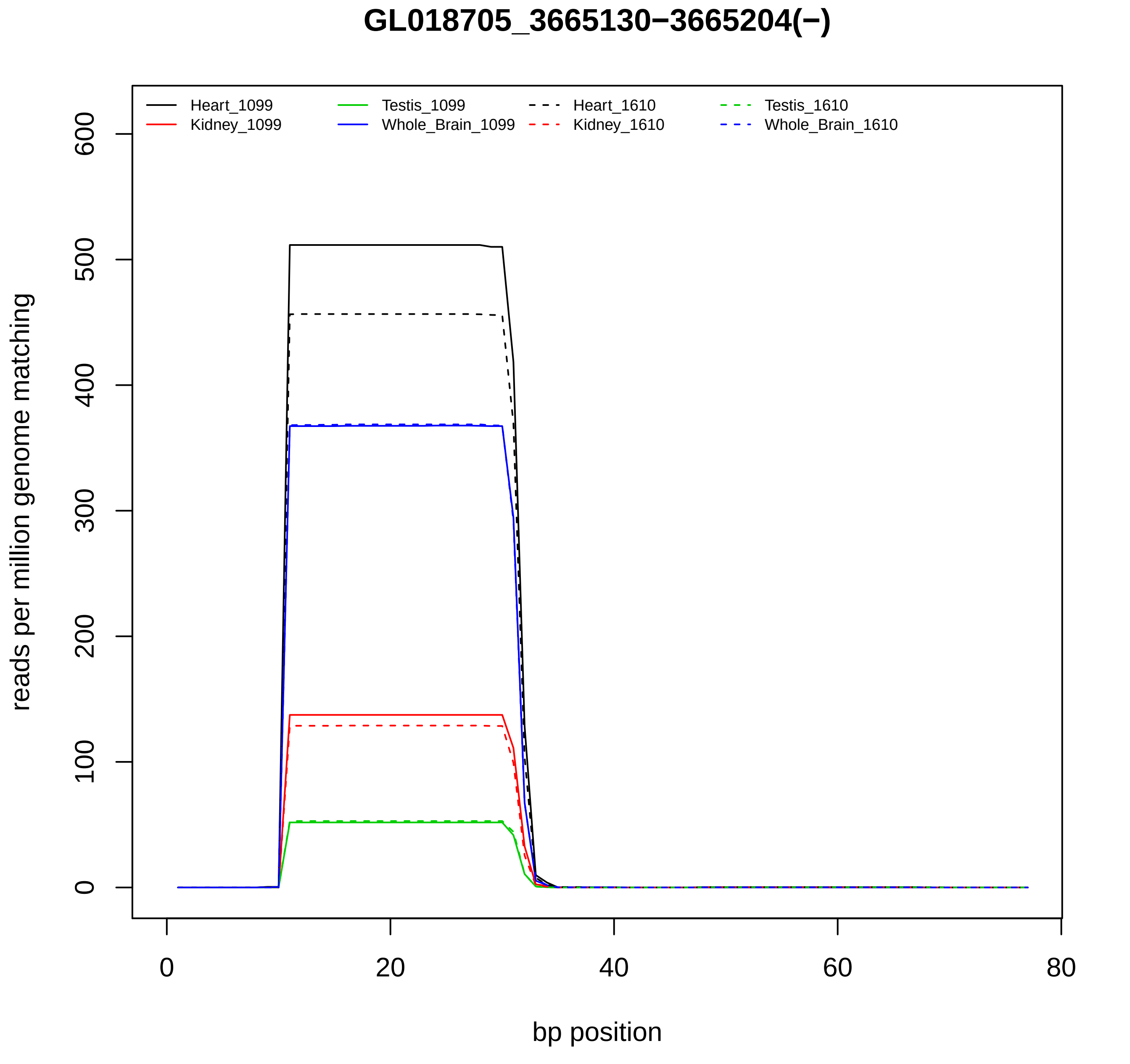

GL018705_3665130−3665204(−)
Heart_1099
Kidney_1099
Testis_1099
Whole_Brain_1099
Heart_1610
Kidney_1610
Testis_1610
Whole_Brain_1610
reads per million genome matching
0
100
200
300
400
500
600
bp position
0
20
40
60
80

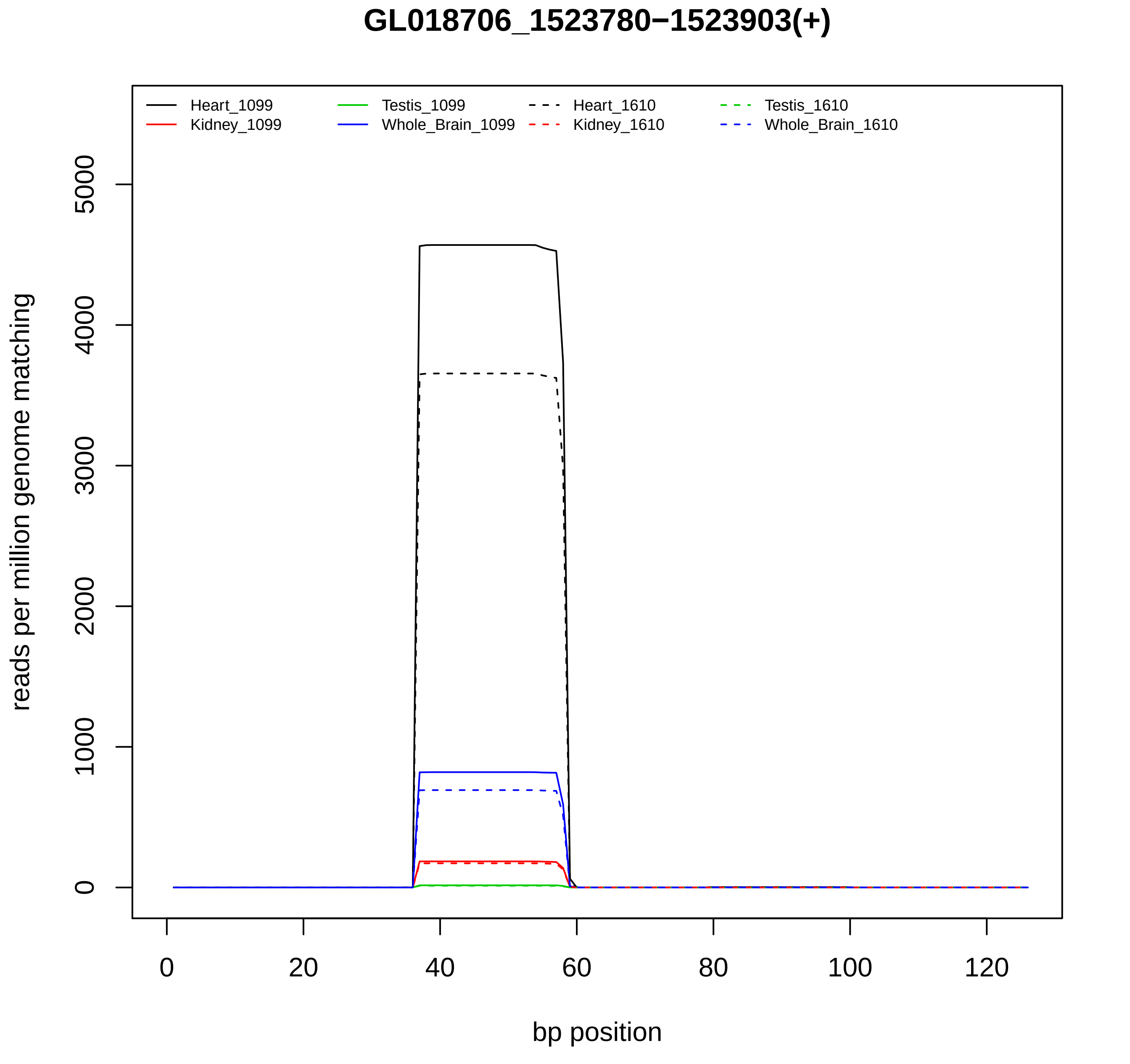

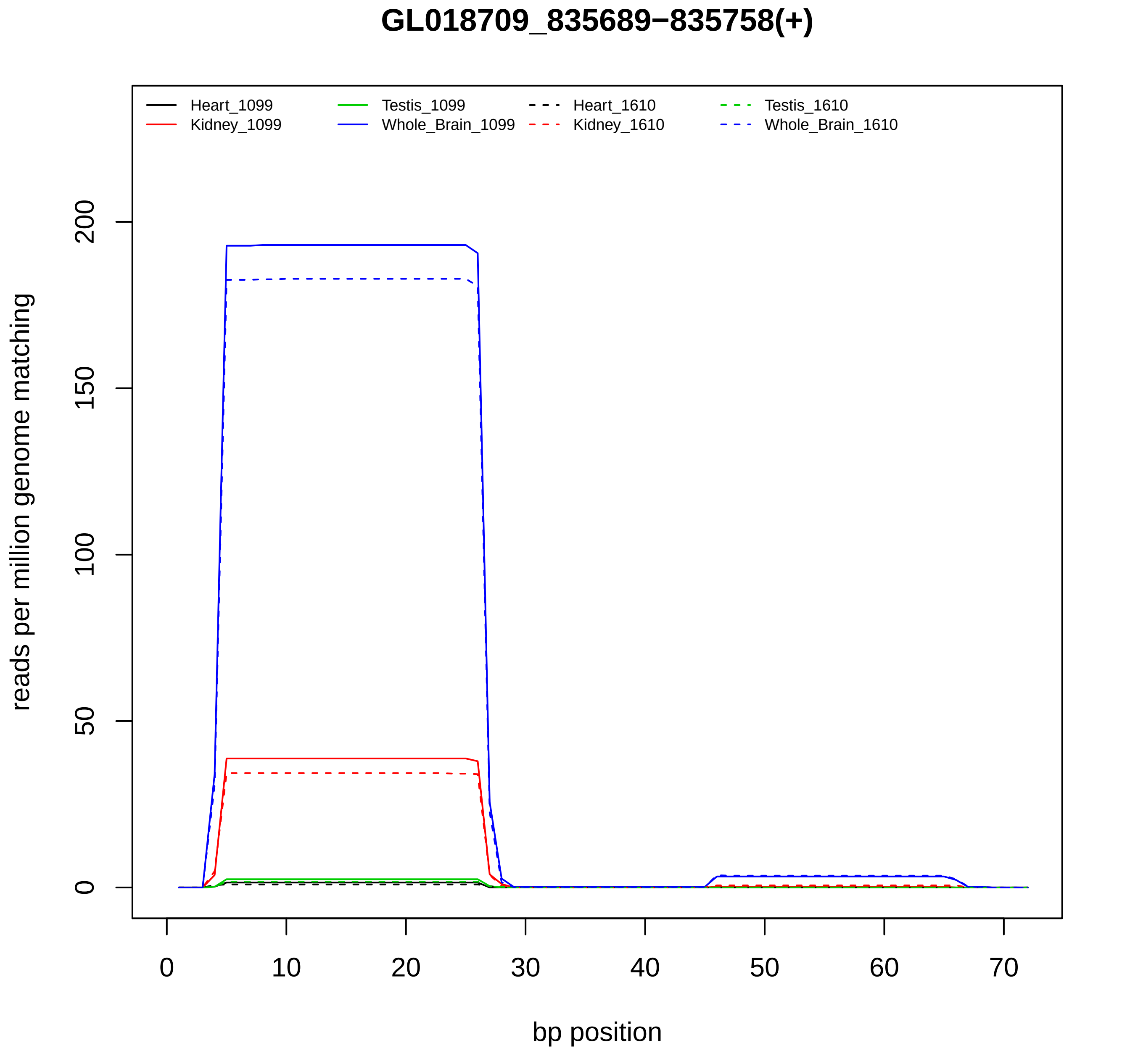

GL018709_835689−835758(+)
Heart_1099
Kidney_1099
Testis_1099
Whole_Brain_1099
Heart_1610
Kidney_1610
Testis_1610
Whole_Brain_1610
reads per million genome matching
bp position
0
50
100
150
200
0
10
20
30
40
50
60
70

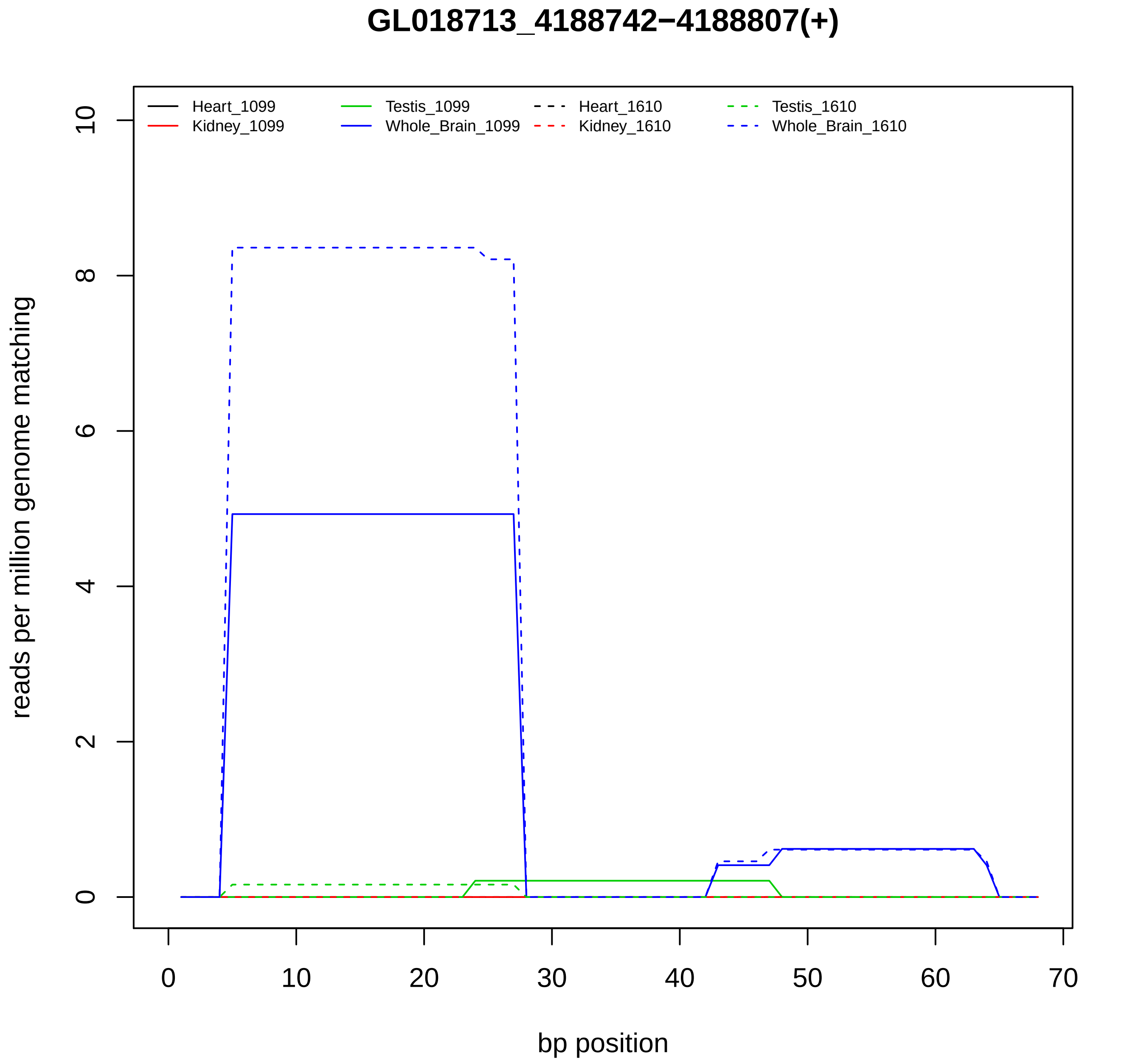
GL018713_4188742−4188807(+)
Heart_1099
Kidney_1099
Testis_1099
Whole_Brain_1099
Heart_1610
Kidney_1610
Testis_1610
Whole_Brain_1610
reads per million genome matching
bp position
0
2
4
6
8
10
0
10
20
30
40
50
60
70

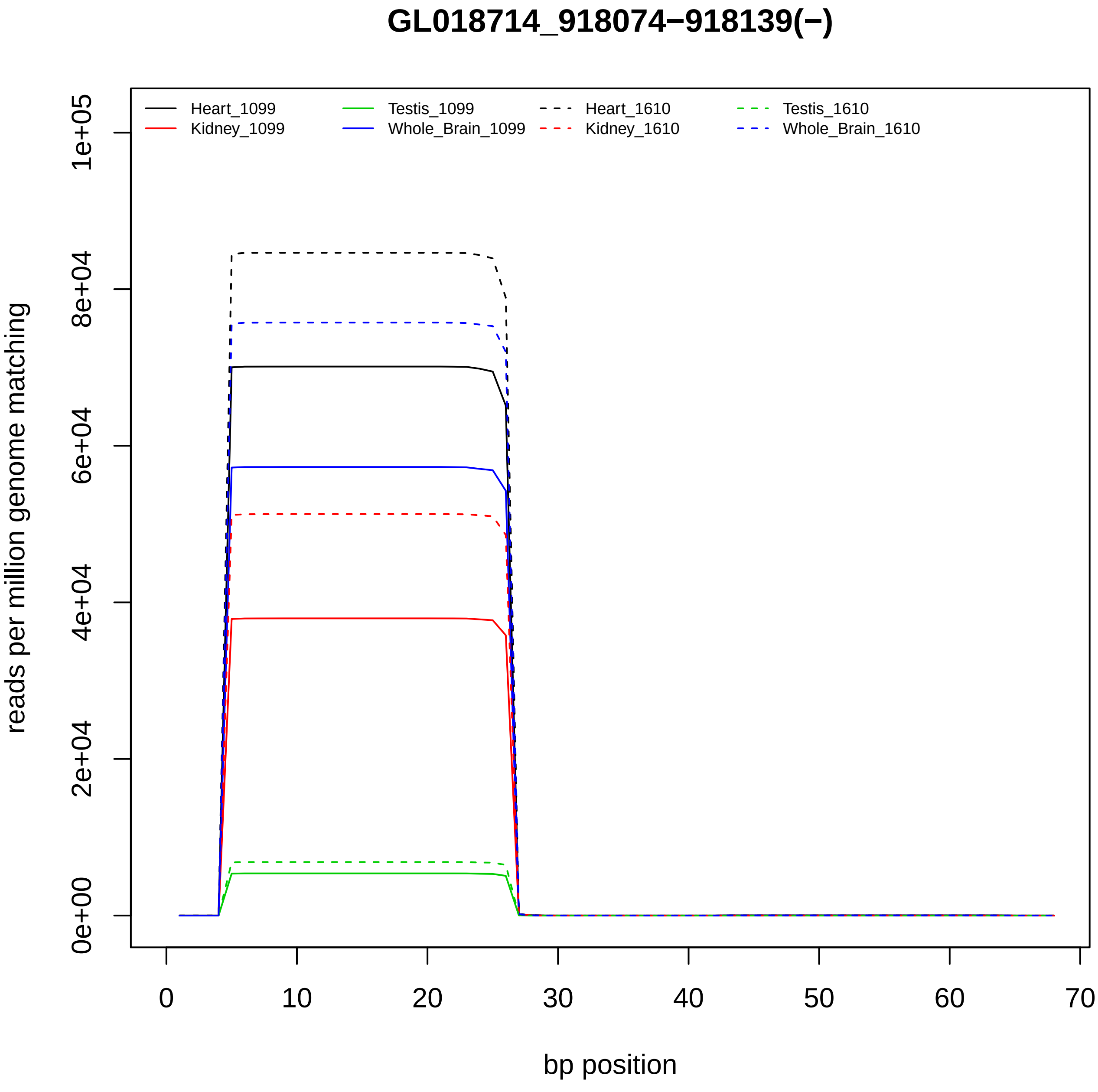

GL018714_918074−918139(−)
Heart_1099
Kidney_1099
Testis_1099
Whole_Brain_1099
Heart_1610
Kidney_1610
Testis_1610
Whole_Brain_1610
reads per million genome matching
0e+00
2e+04
4e+04
6e+04
8e+04
1e+05
bp position
0
10
20
30
40
50
60
70

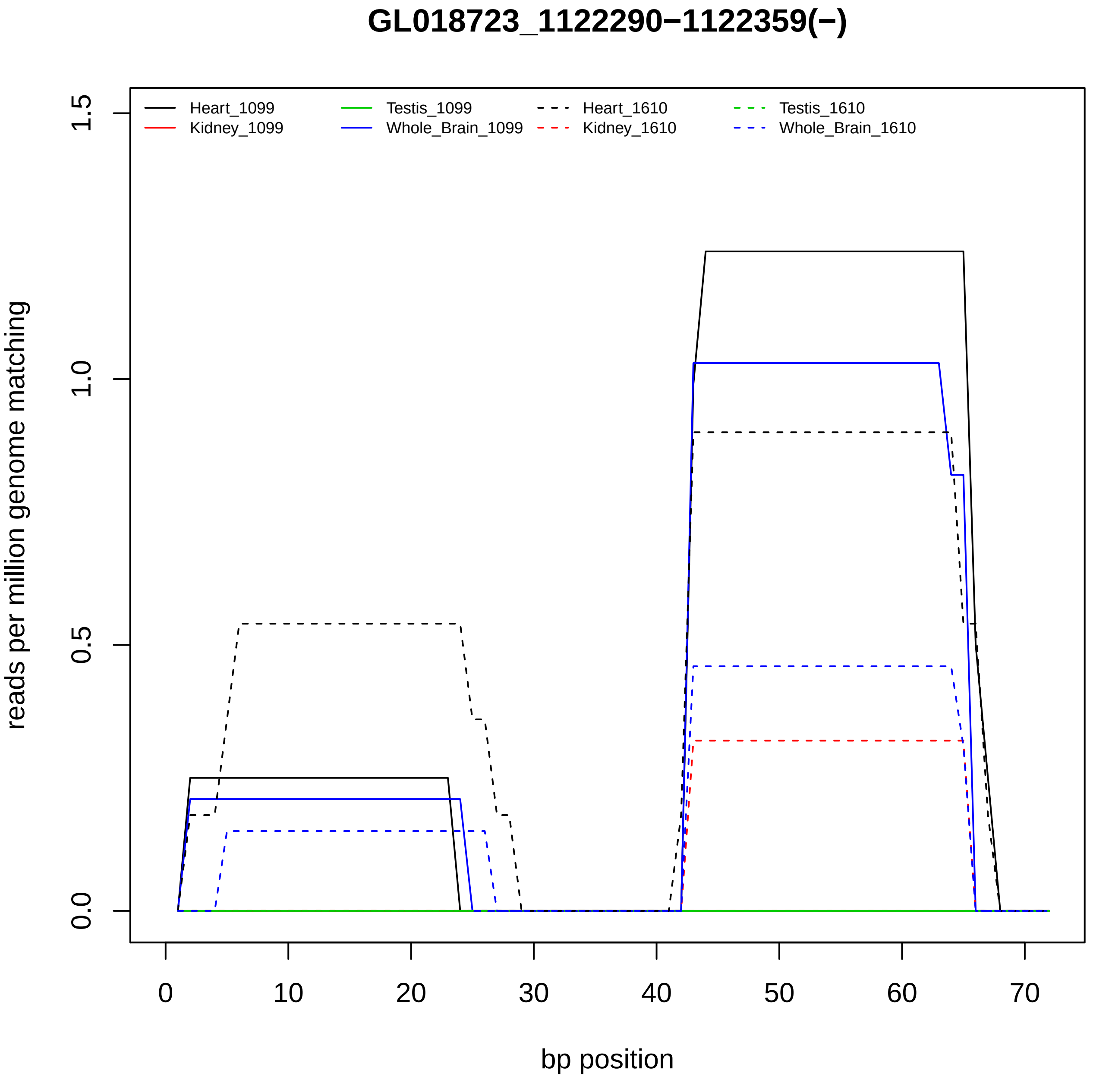

GL018723_1122290−1122359(−)
Heart_1099
Kidney_1099
Testis_1099
Whole_Brain_1099
Heart_1610
Kidney_1610
Testis_1610
Whole_Brain_1610
reads per million genome matching
0.0
0.5
1.0
1.5
bp position
0
10
20
30
40
50
60
70

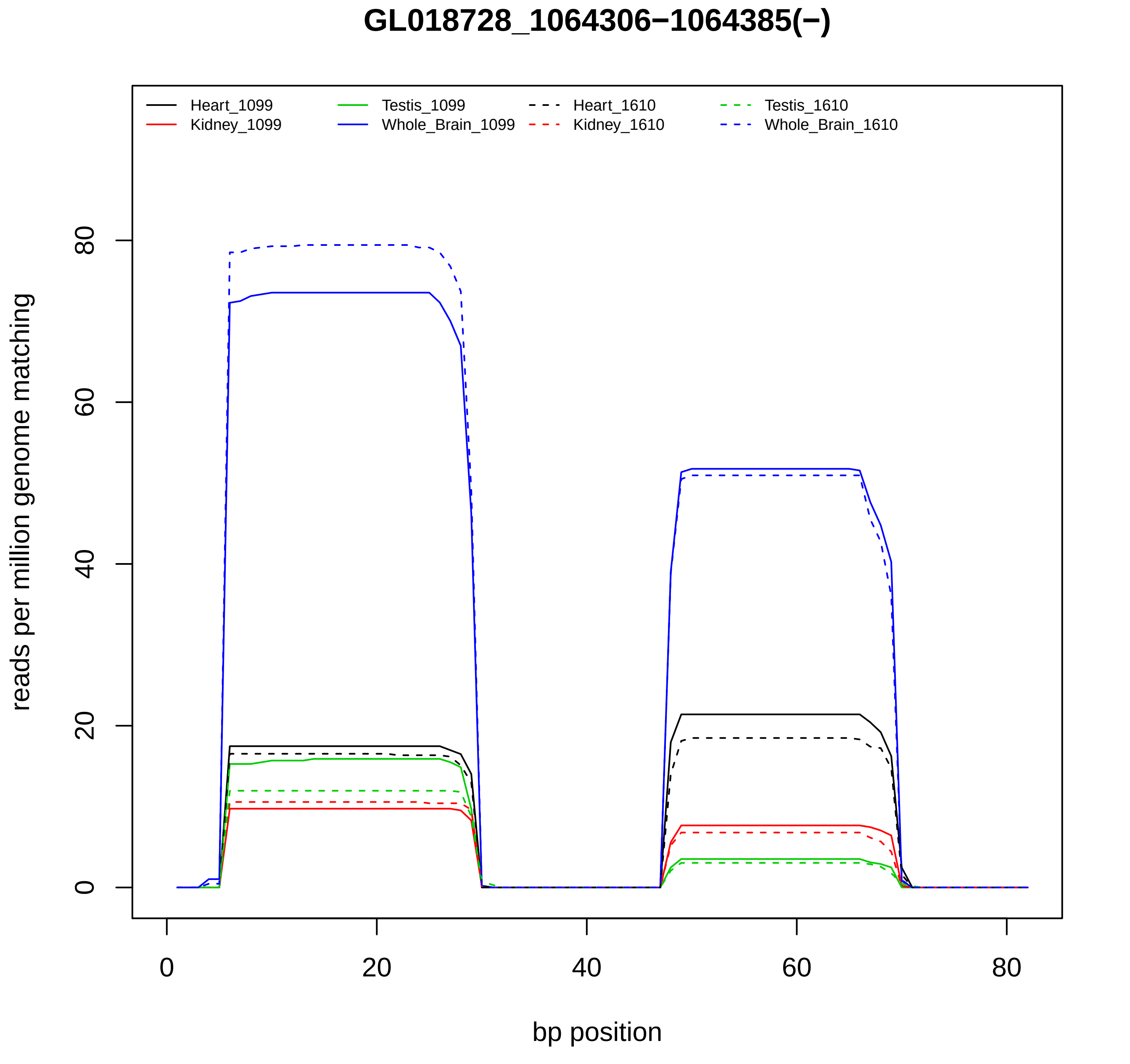

GL018728_1064306−1064385(−)
Heart_1099
Kidney_1099
Testis_1099
Whole_Brain_1099
Heart_1610
Kidney_1610
Testis_1610
Whole_Brain_1610
reads per million genome matching
bp position
0
20
40
60
80
0
20
40
60
80

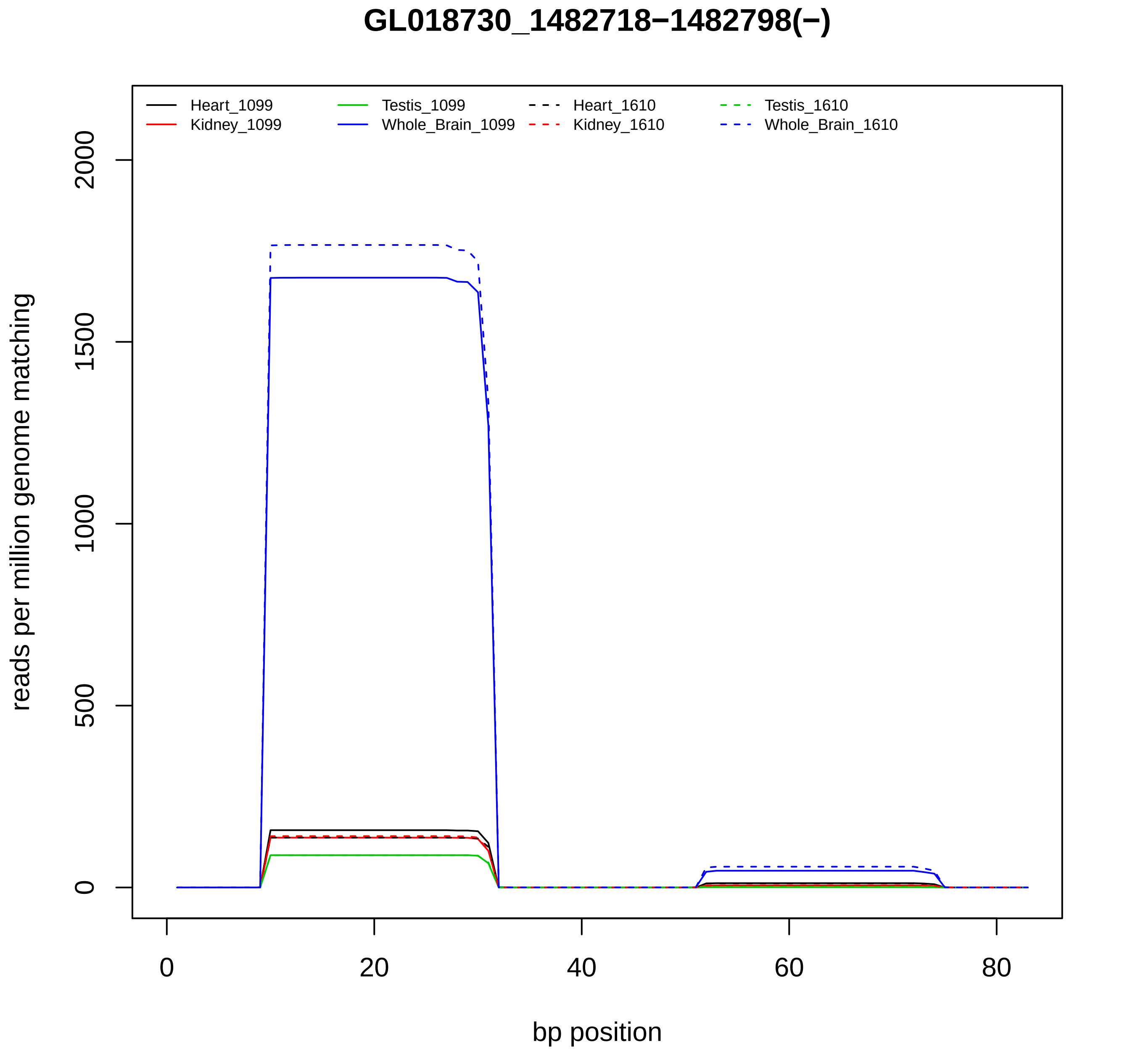
GL018730_1482718−1482798(−)
Heart_1099
Kidney_1099
Testis_1099
Whole_Brain_1099
Heart_1610
Kidney_1610
Testis_1610
Whole_Brain_1610
reads per million genome matching
0
500
1000
1500
2000
bp position
0
20
40
60
80

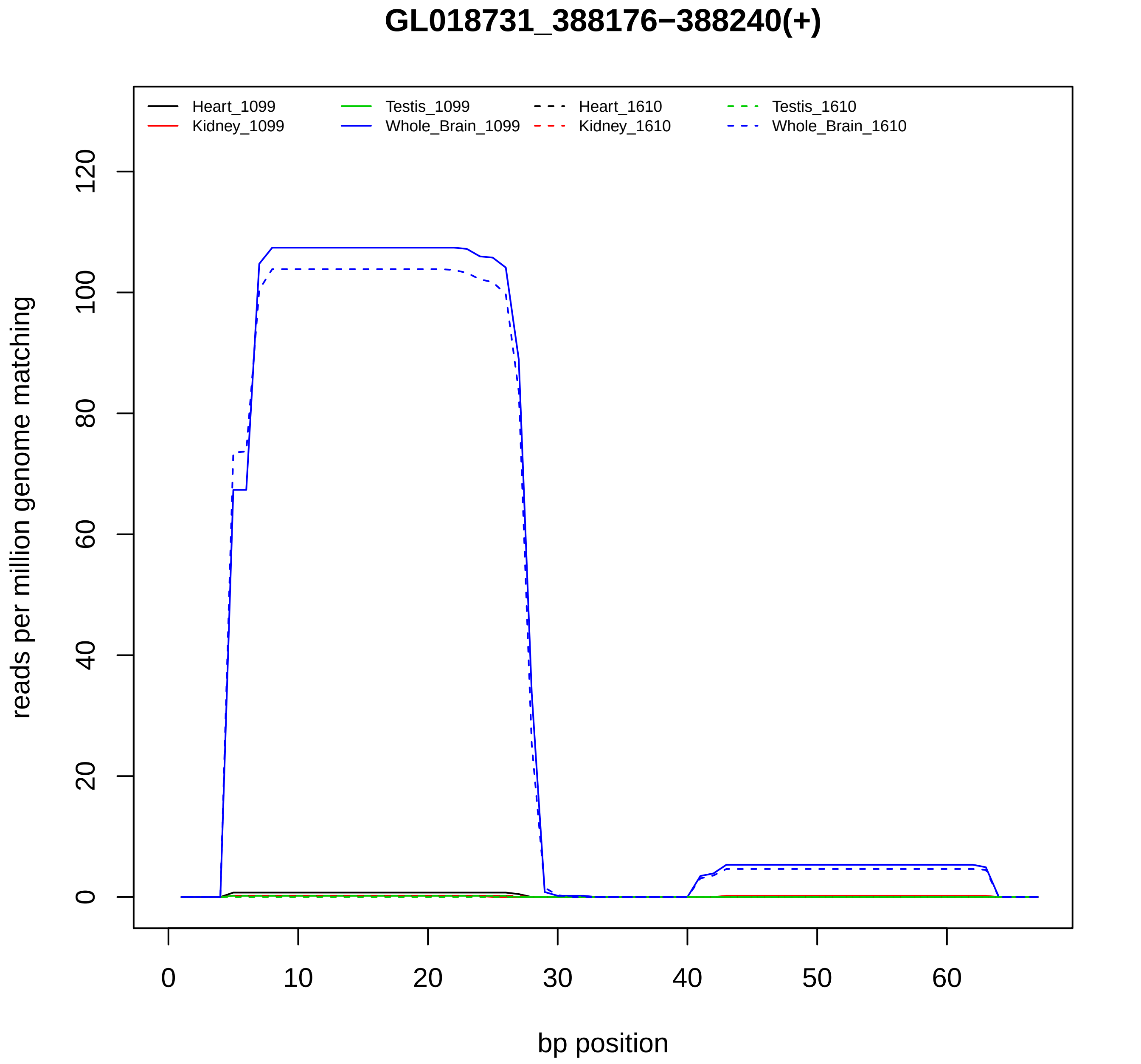

GL018731_388176−388240(+)
Heart_1099
Kidney_1099
Testis_1099
Whole_Brain_1099
Heart_1610
Kidney_1610
Testis_1610
Whole_Brain_1610
reads per million genome matching
bp position
0
20
40
60
80
100
120
0
10
20
30
40
50
60

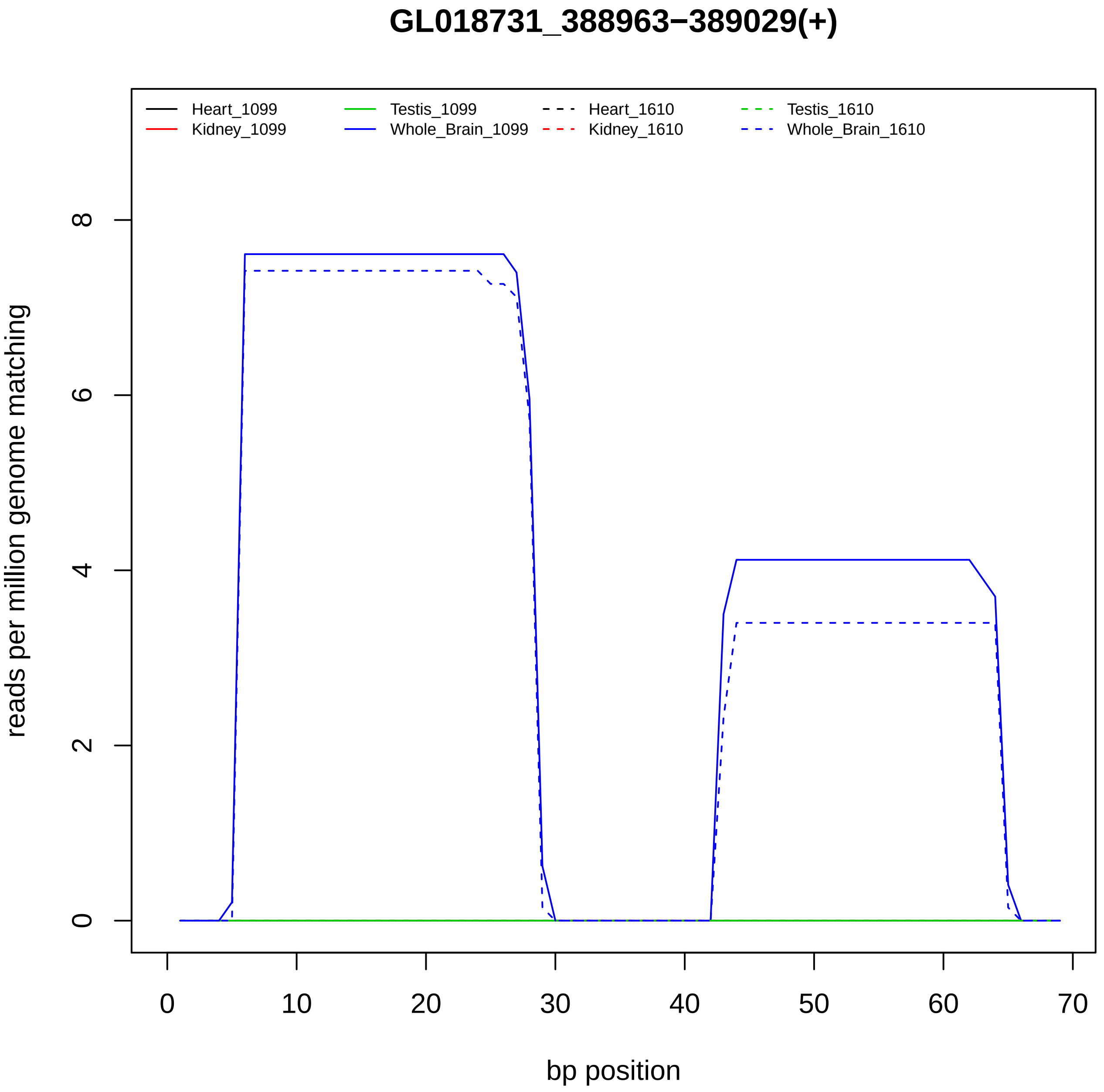
GL018731_388963−389029(+)
Heart_1099
Kidney_1099
Testis_1099
Whole_Brain_1099
Heart_1610
Kidney_1610
Testis_1610
Whole_Brain_1610
reads per million genome matching
bp position
0
2
4
6
8
0
10
20
30
40
50
60
70

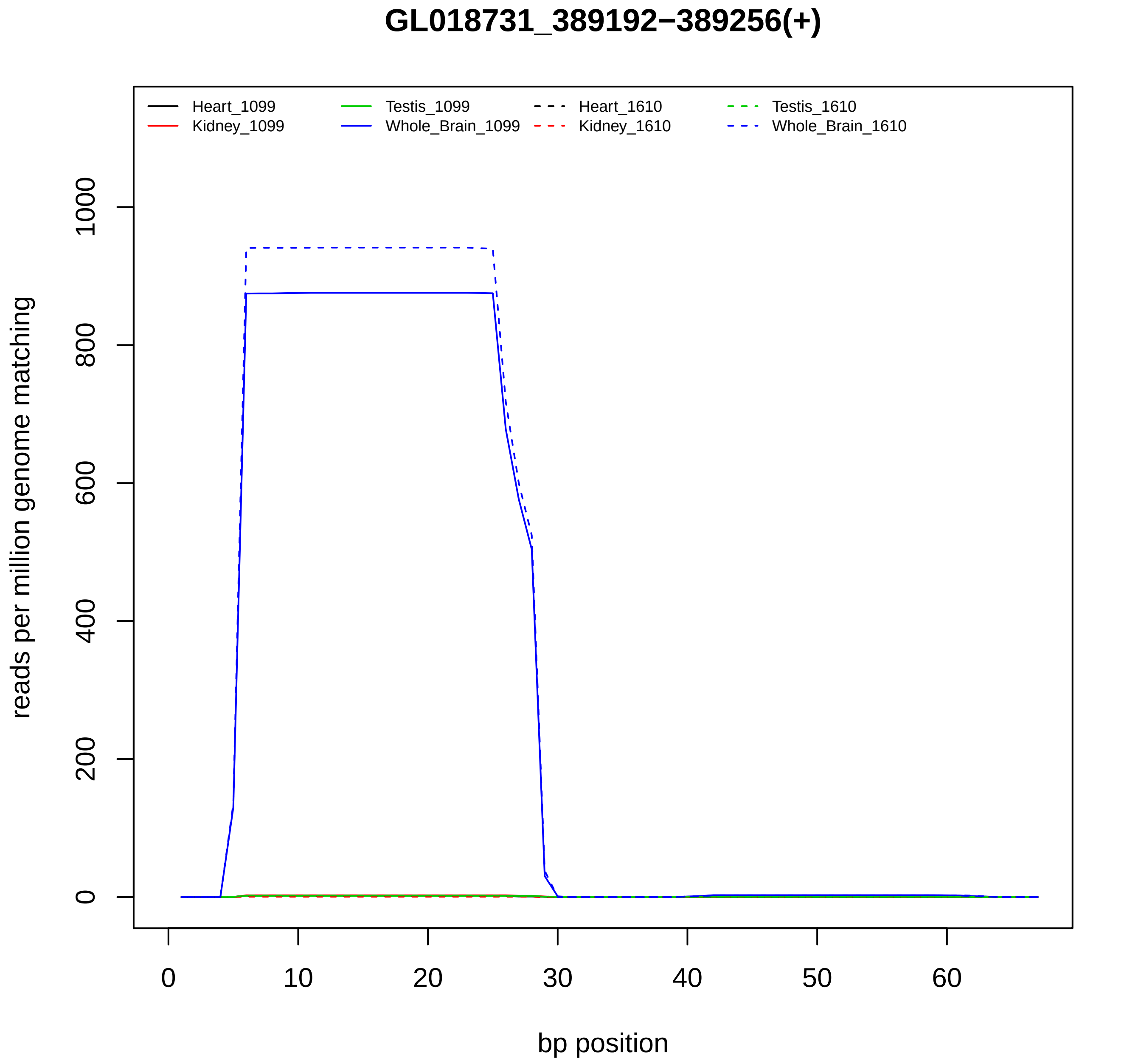

GL018731_389192−389256(+)
Heart_1099
Kidney_1099
Testis_1099
Whole_Brain_1099
Heart_1610
Kidney_1610
Testis_1610
Whole_Brain_1610
reads per million genome matching
0
200
400
600
800
1000
0
10
20
30
40
50
60
bp position

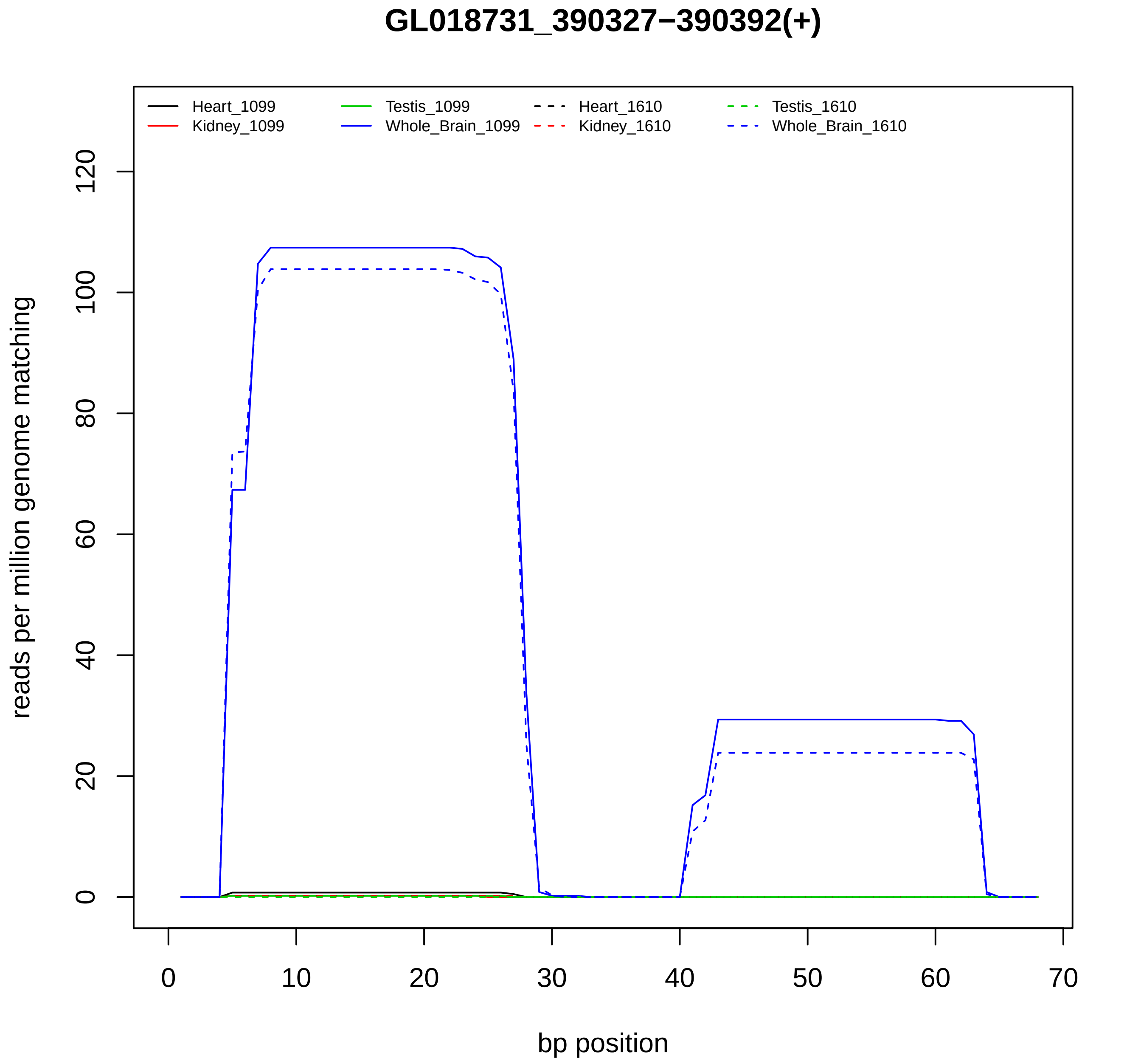

GL018731_390327−390392(+)
Heart_1099
Kidney_1099
Testis_1099
Whole_Brain_1099
Heart_1610
Kidney_1610
Testis_1610
Whole_Brain_1610
reads per million genome matching
0
20
40
60
80
100
120
0
10
20
30
40
50
60
70
bp position

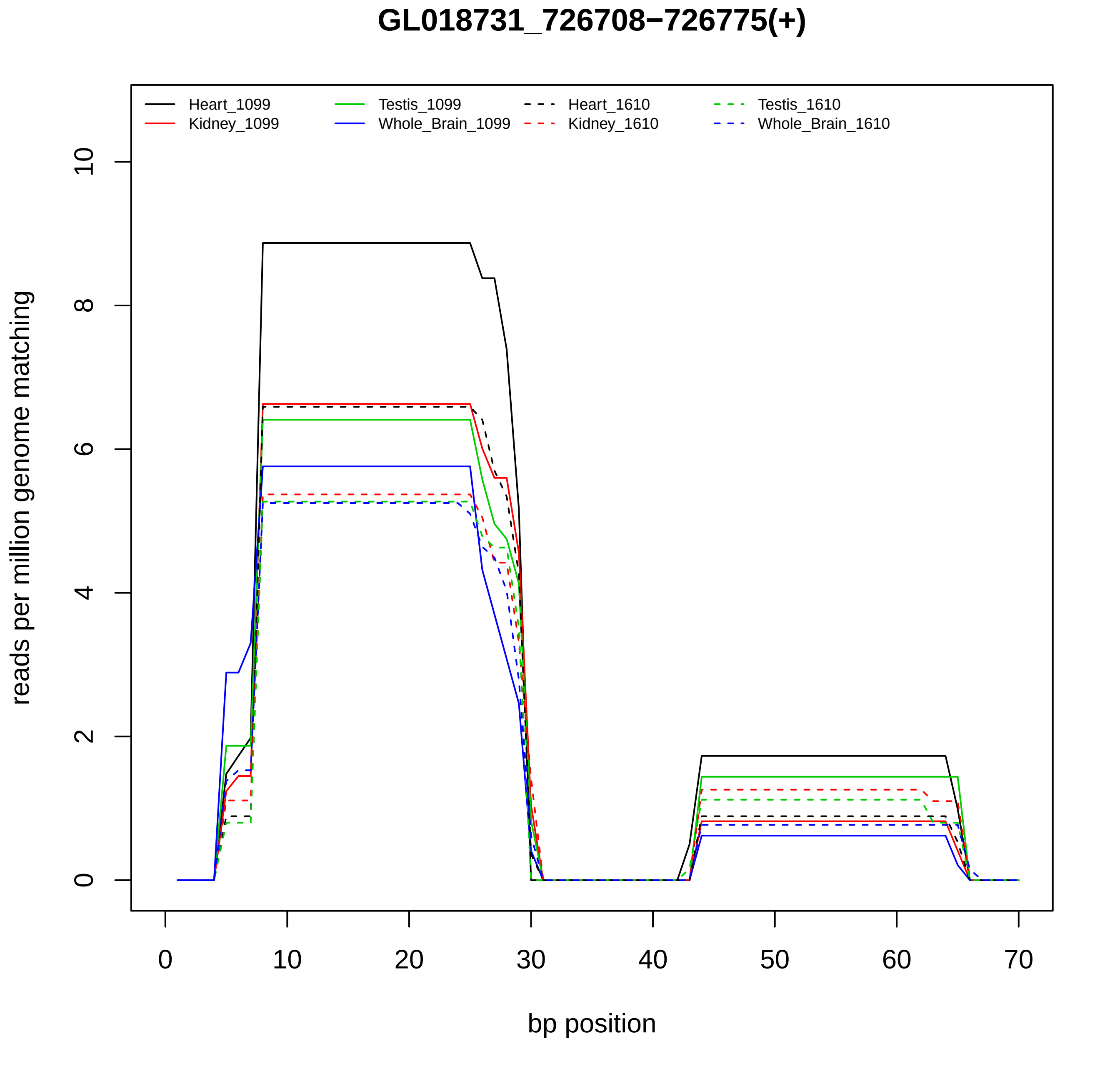
GL018731_726708−726775(+)
Heart_1099
Kidney_1099
Testis_1099
Whole_Brain_1099
Heart_1610
Kidney_1610
Testis_1610
Whole_Brain_1610
reads per million genome matching
0
2
4
6
8
10
0
10
20
30
40
50
60
70
bp position

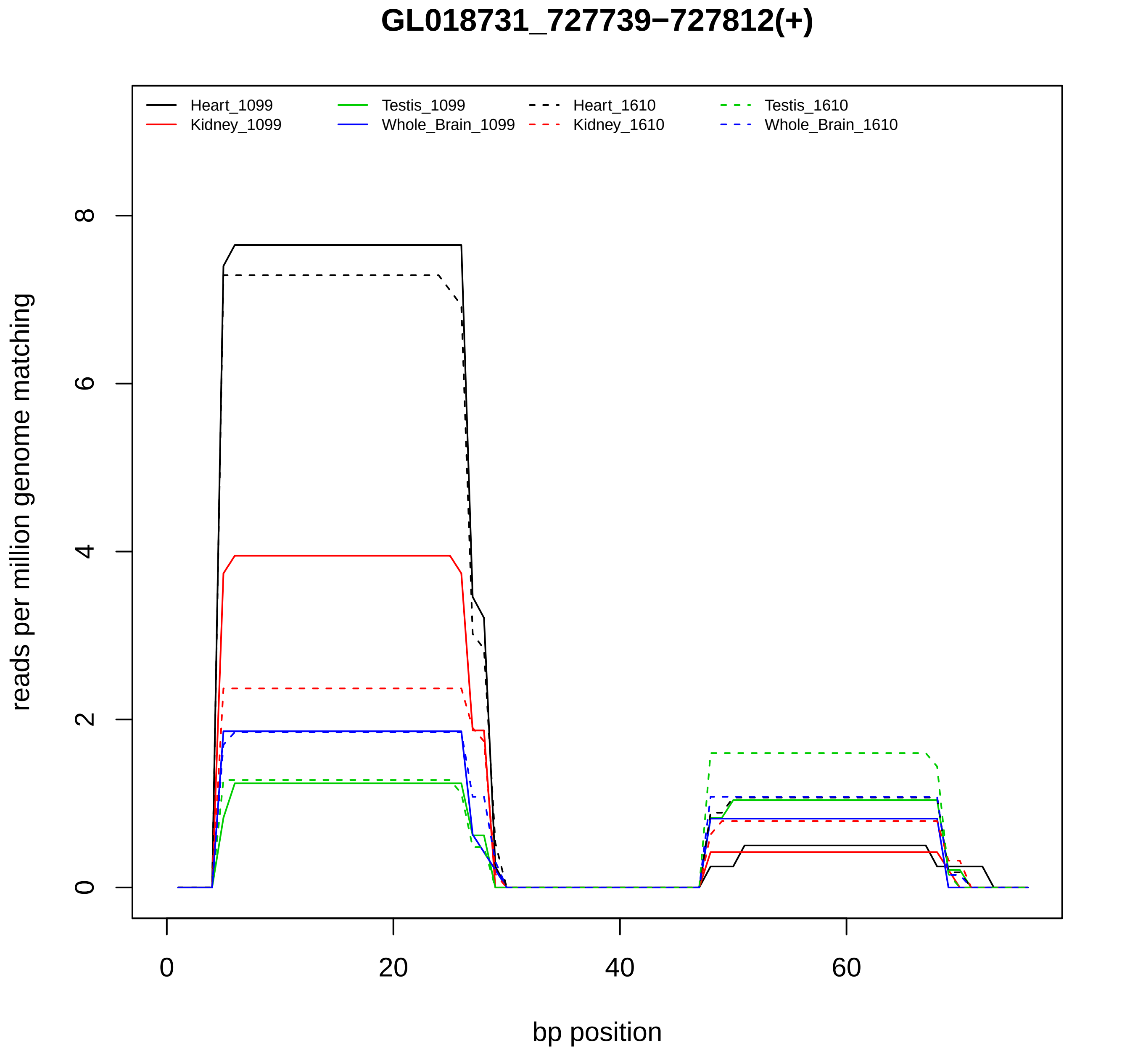

GL018731_727739−727812(+)
Heart_1099
Kidney_1099
Testis_1099
Whole_Brain_1099
Heart_1610
Kidney_1610
Testis_1610
Whole_Brain_1610
reads per million genome matching
bp position
0
2
4
6
8
0
20
40
60

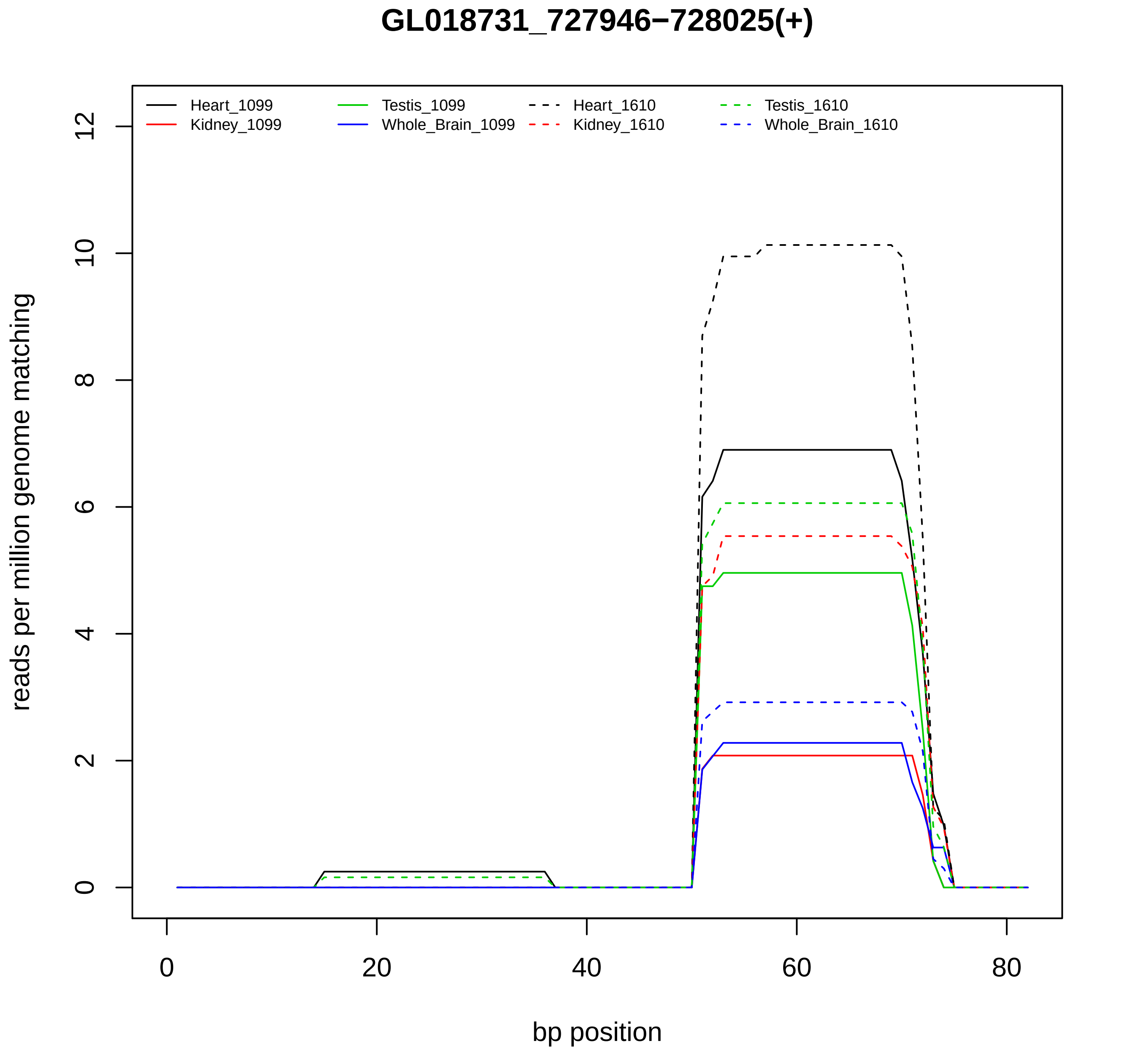

GL018731_727946−728025(+)
Heart_1099
Kidney_1099
Testis_1099
Whole_Brain_1099
Heart_1610
Kidney_1610
Testis_1610
Whole_Brain_1610
reads per million genome matching
bp position
0
2
4
6
8
10
12
0
20
40
60
80

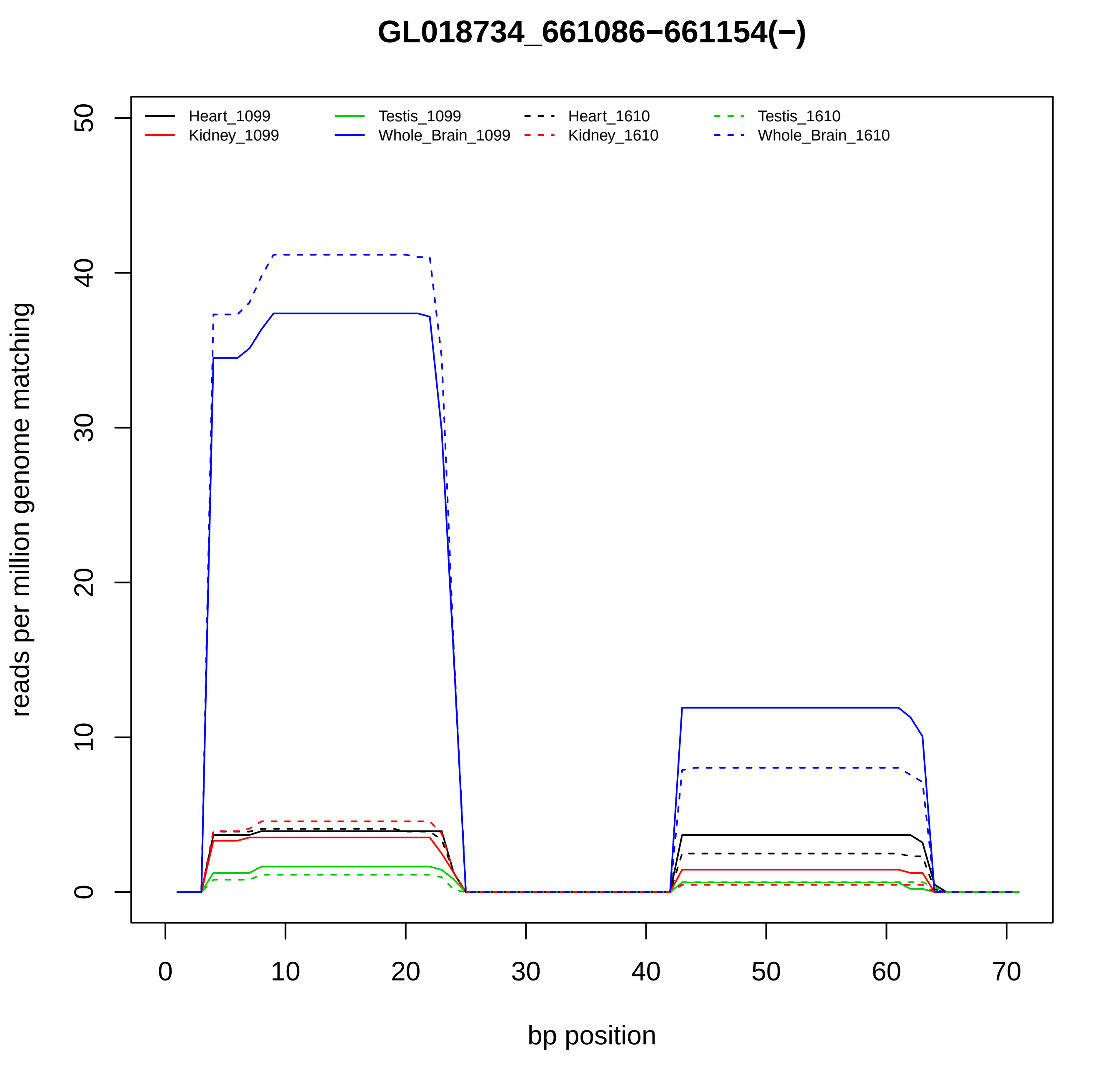
GL018734_661086−661154(−)
Heart_1099
Kidney_1099
Testis_1099
Whole_Brain_1099
Heart_1610
Kidney_1610
Testis_1610
Whole_Brain_1610
reads per million genome matching
bp position
0
10
20
30
40
50
60
70

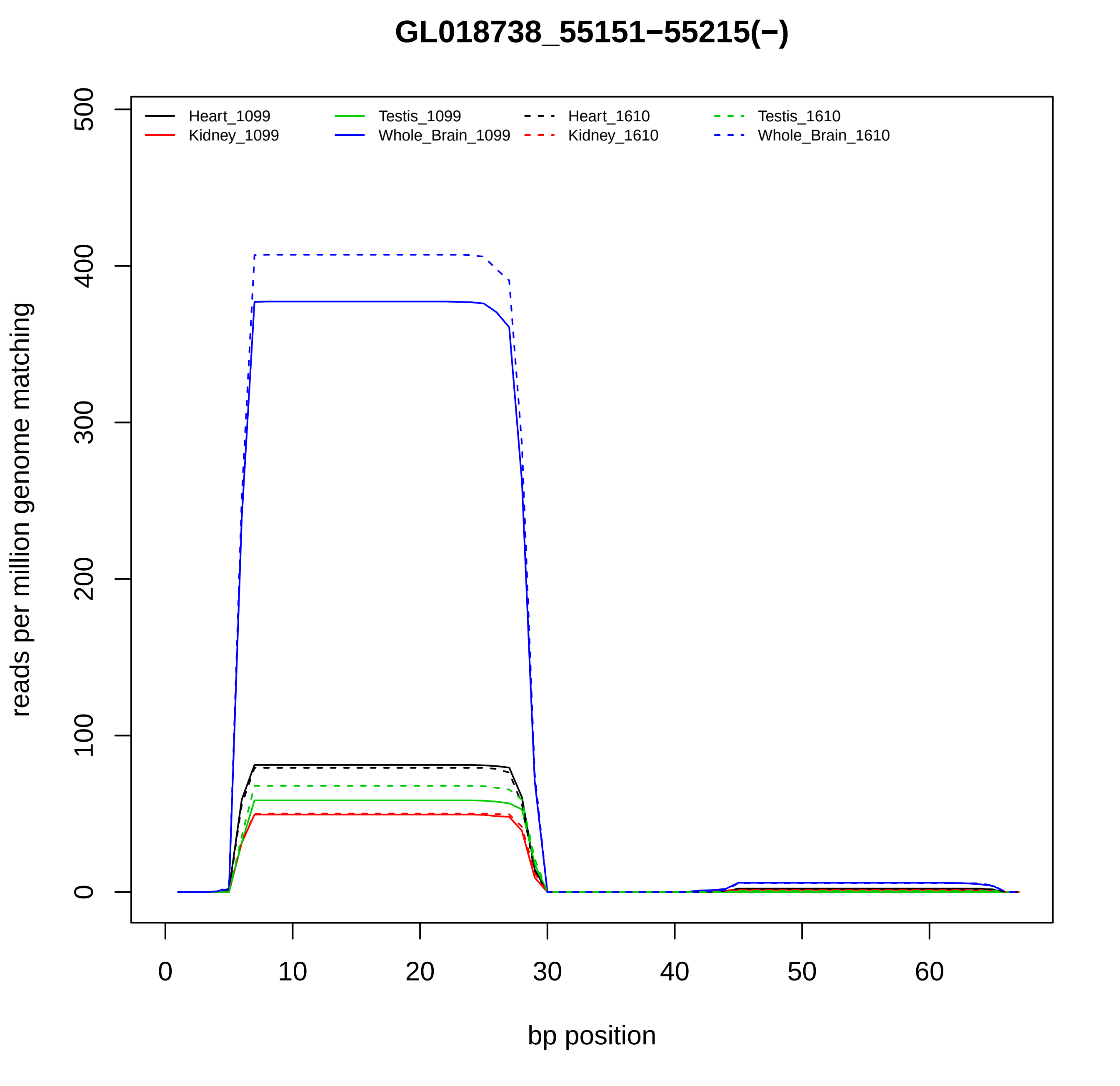

GL018738_55151−55215(−)
Heart_1099
Kidney_1099
Testis_1099
Whole_Brain_1099
Heart_1610
Kidney_1610
Testis_1610
Whole_Brain_1610
reads per million genome matching
bp position
0
100
200
300
400
500
0
10
20
30
40
50
60

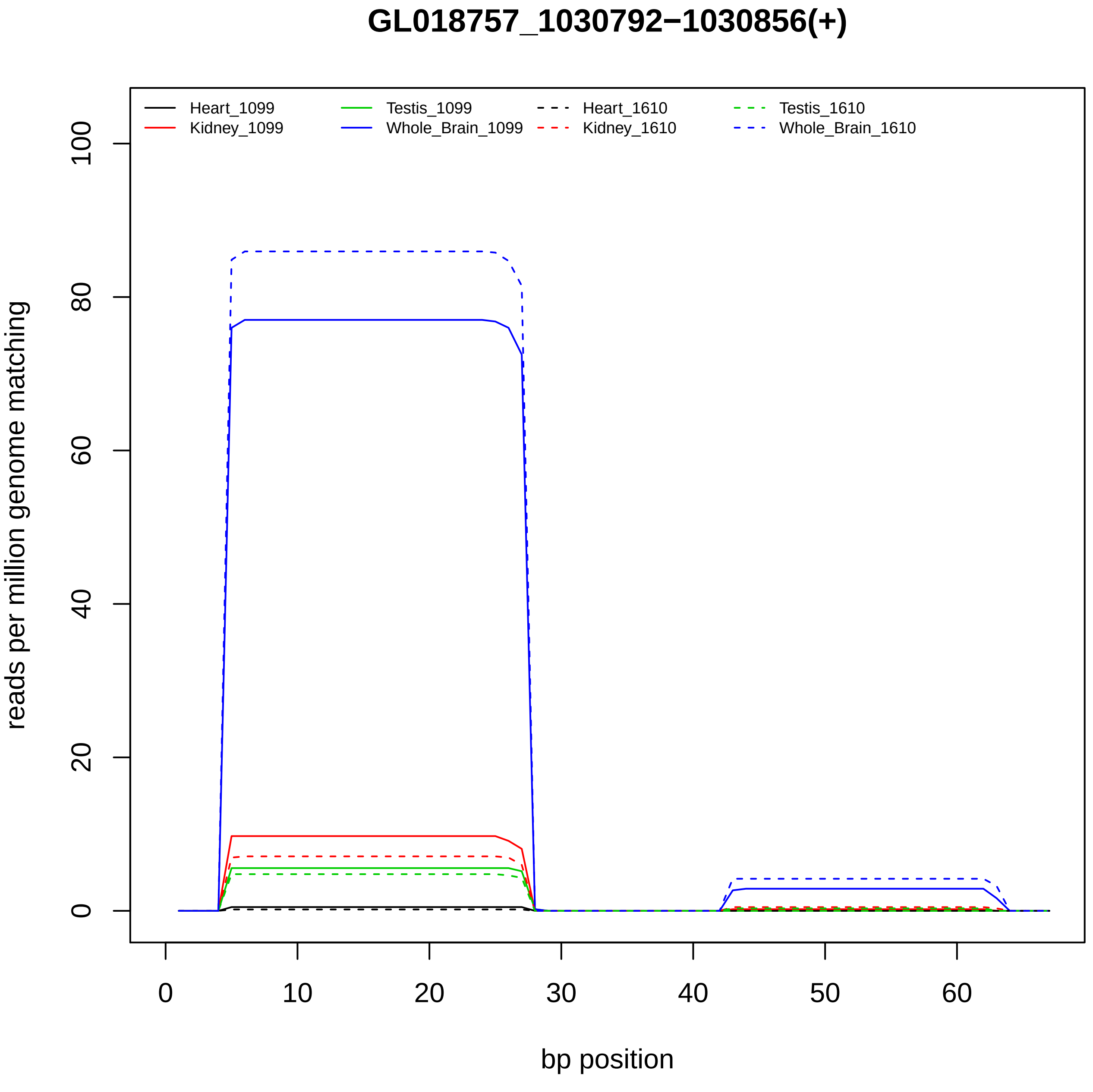
GL018757_1030792−1030856(+)
Heart_1099
Kidney_1099
Testis_1099
Whole_Brain_1099
Heart_1610
Kidney_1610
Testis_1610
Whole_Brain_1610
reads per million genome matching
0
20
40
60
80
100
bp position
0
10
20
30
40
50
60

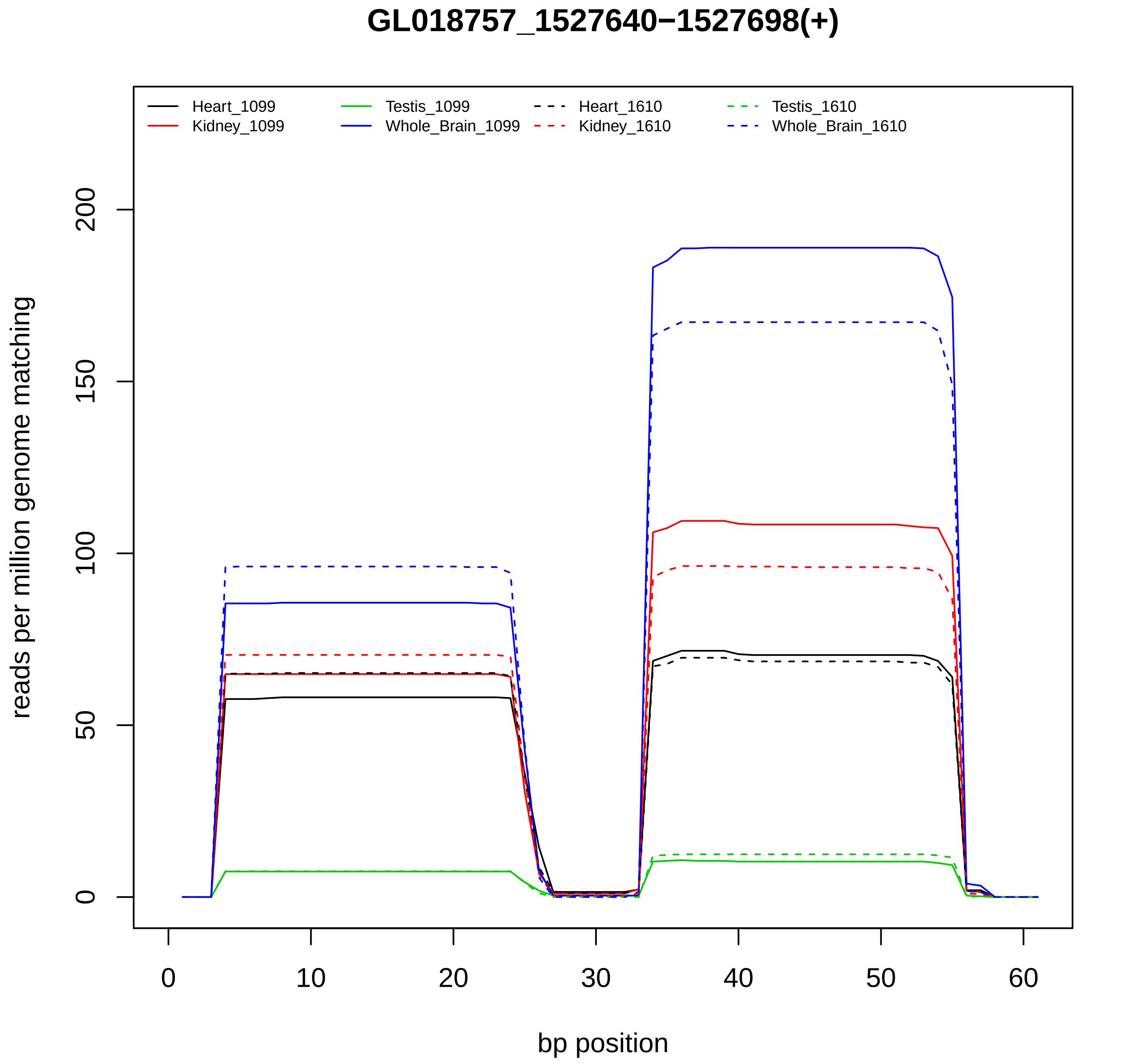

GL018757_1527640−1527698(+)
Heart_1099
Kidney_1099
Testis_1099
Whole_Brain_1099
Heart_1610
Kidney_1610
Testis_1610
Whole_Brain_1610
reads per million genome matching
0
50
100
150
200
0
10
20
30
40
50
60
bp position

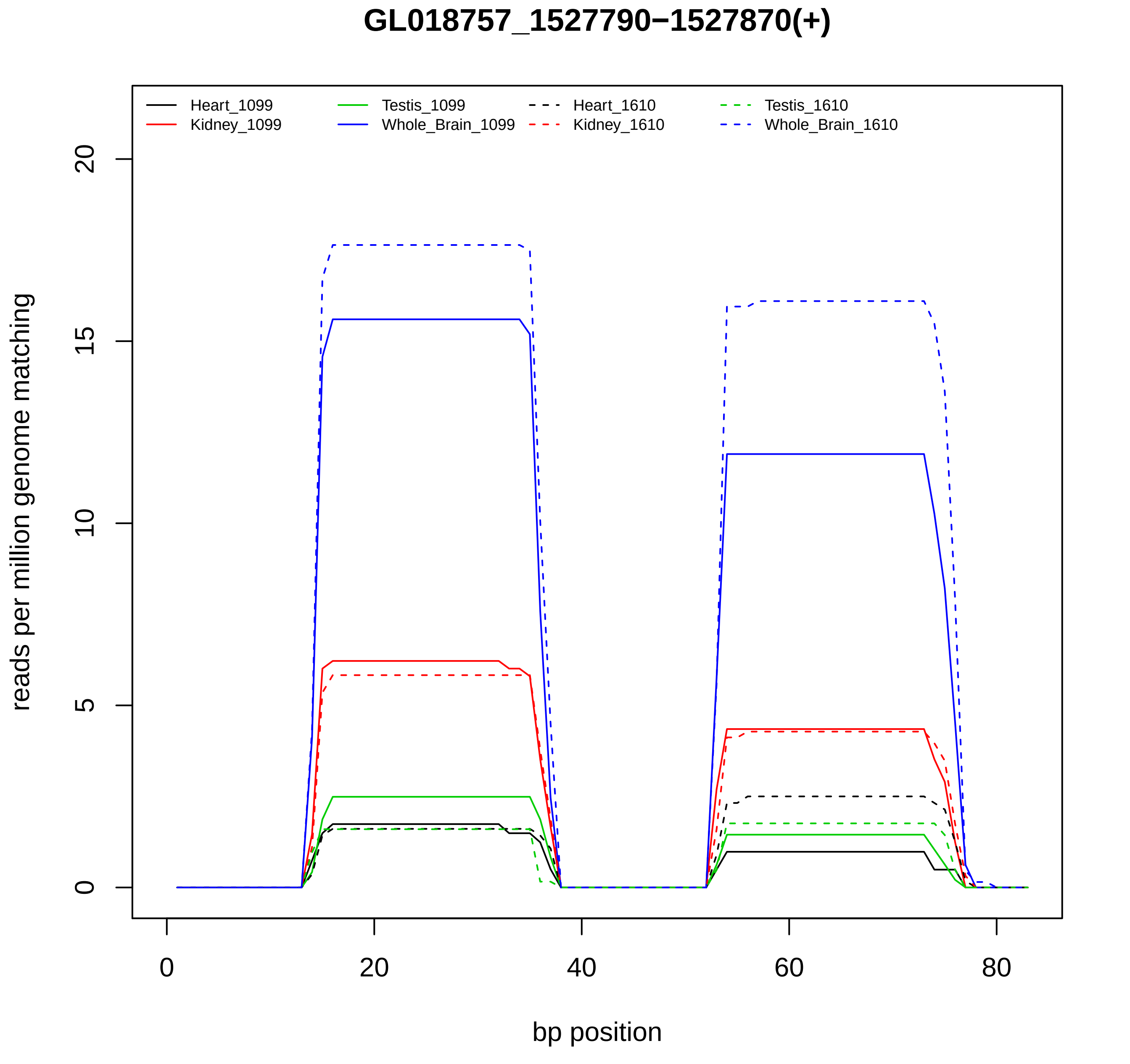

GL018757_1527790−1527870(+)
Heart_1099
Kidney_1099
Testis_1099
Whole_Brain_1099
Heart_1610
Kidney_1610
Testis_1610
Whole_Brain_1610
reads per million genome matching
0
5
10
15
20
0
20
40
60
80
bp position

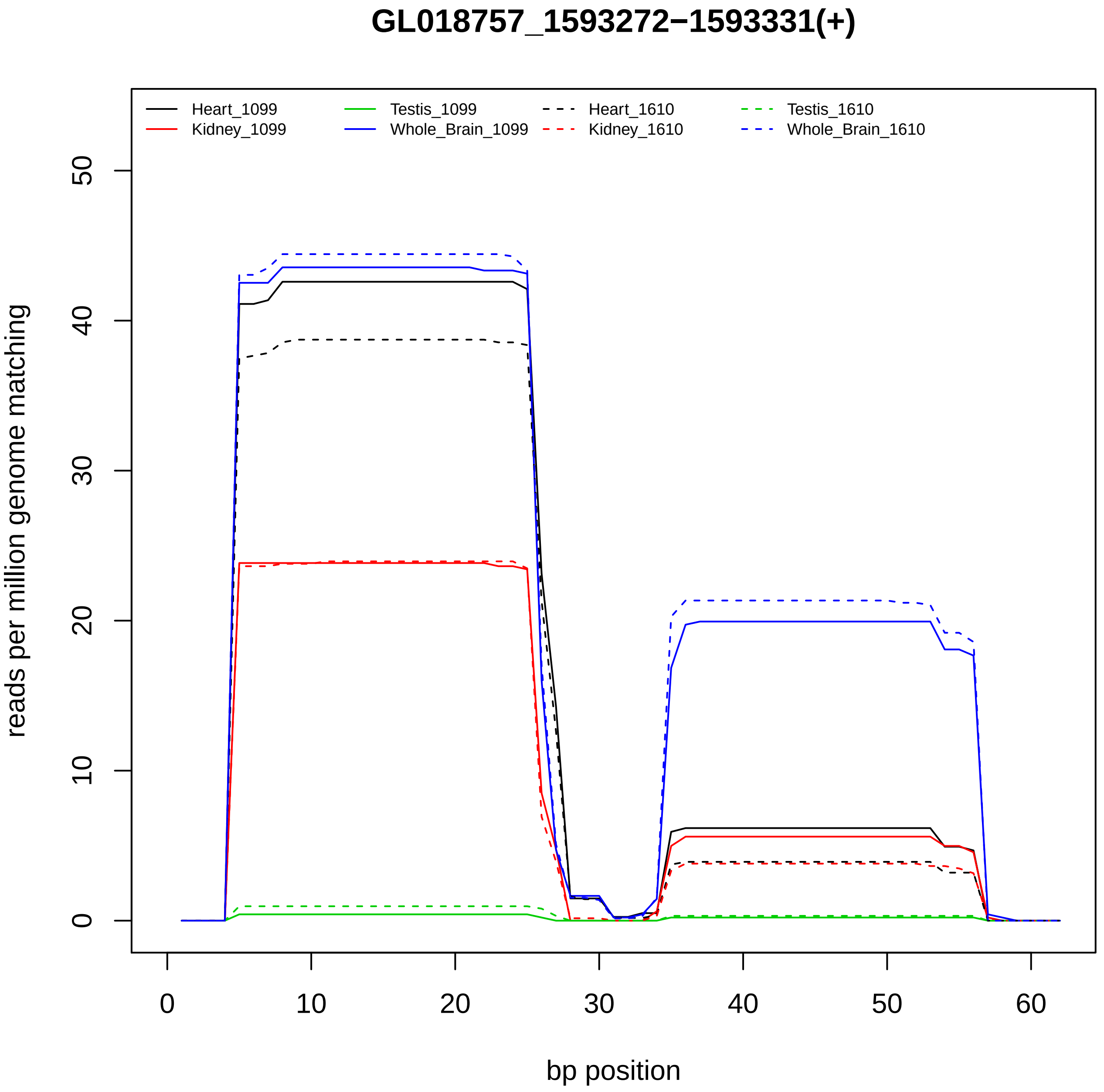
GL018757_1593272−1593331(+)
Heart_1099
Kidney_1099
Testis_1099
Whole_Brain_1099
Heart_1610
Kidney_1610
Testis_1610
Whole_Brain_1610
reads per million genome matching
bp position
0
10
20
30
40
50
60

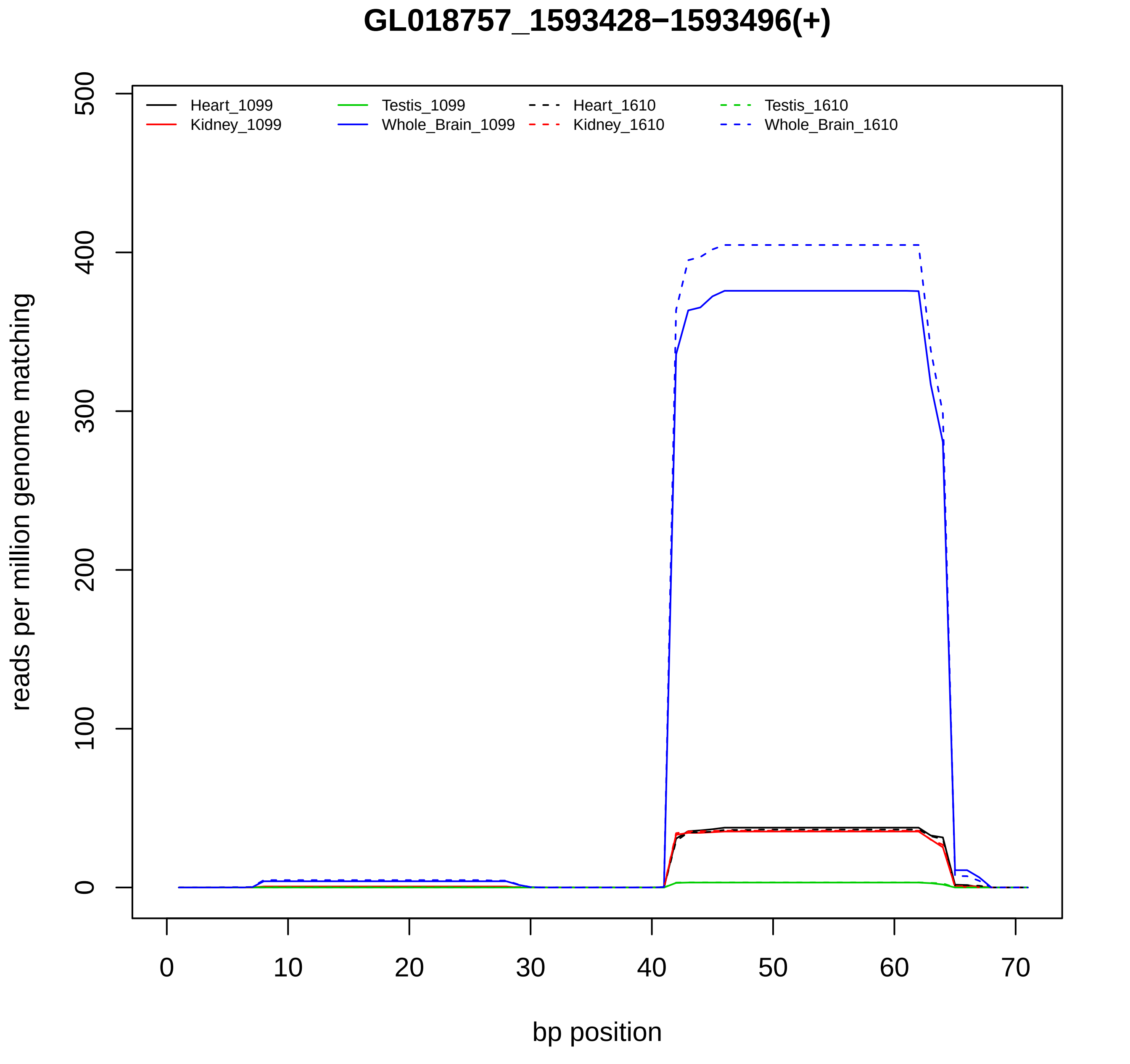

GL018757_1593428−1593496(+)
Heart_1099
Kidney_1099
Testis_1099
Whole_Brain_1099
Heart_1610
Kidney_1610
Testis_1610
Whole_Brain_1610
reads per million genome matching
bp position
0
100
200
300
400
500
0
10
20
30
40
50
60
70

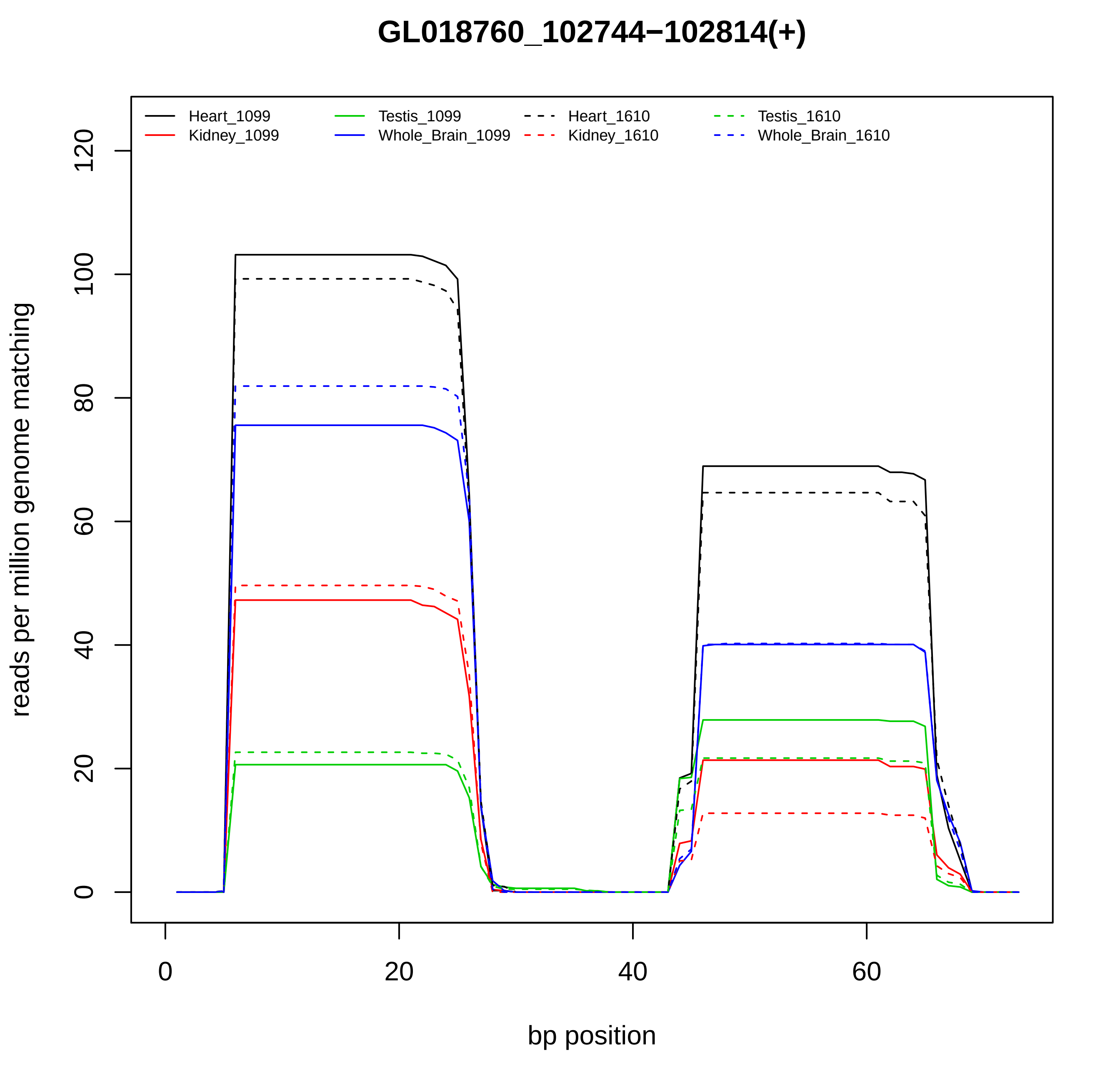

GL018760_102744−102814(+)
Heart_1099
Kidney_1099
Testis_1099
Whole_Brain_1099
Heart_1610
Kidney_1610
Testis_1610
Whole_Brain_1610
reads per million genome matching
bp position
0
20
40
60
80
100
120

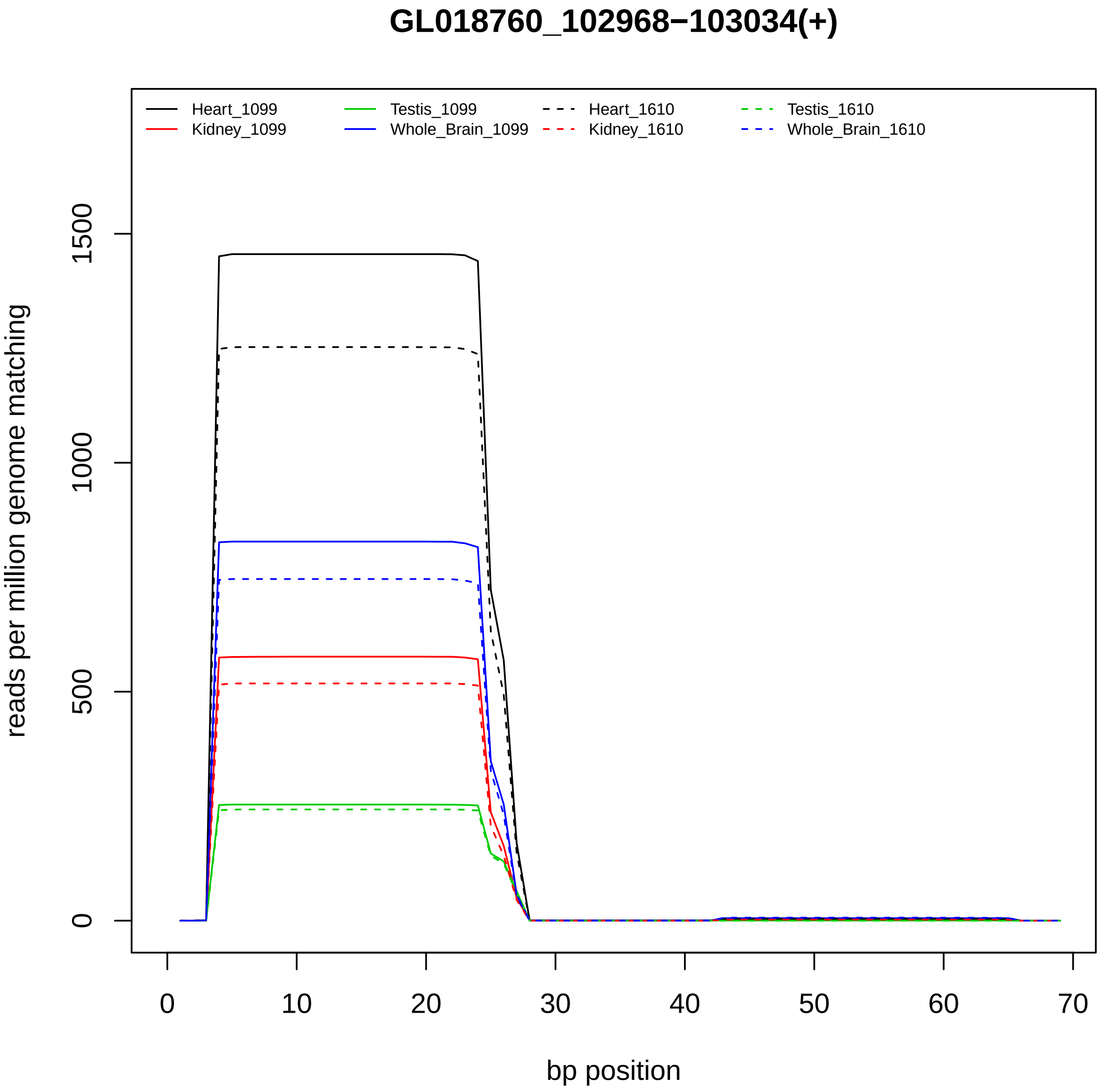

GL018760_102968−103034(+)
Heart_1099
Kidney_1099
Testis_1099
Whole_Brain_1099
Heart_1610
Kidney_1610
Testis_1610
Whole_Brain_1610
reads per million genome matching
0
500
1000
1500
0
10
20
30
40
50
60
70
bp position

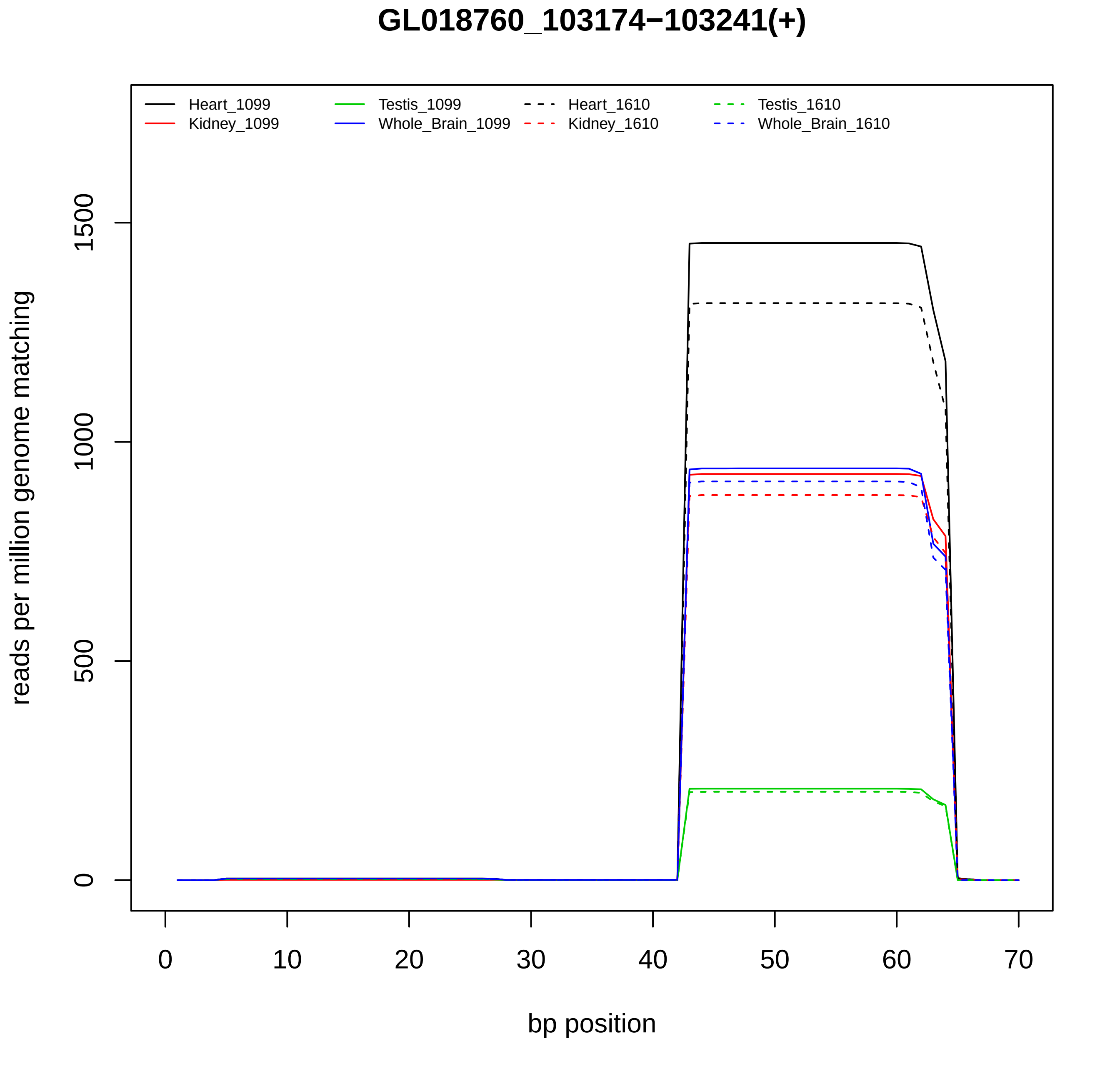
GL018760_103174−103241(+)
Heart_1099
Kidney_1099
Testis_1099
Whole_Brain_1099
Heart_1610
Kidney_1610
Testis_1610
Whole_Brain_1610
reads per million genome matching
bp position
0
500
1000
1500
0
10
20
30
40
50
60
70

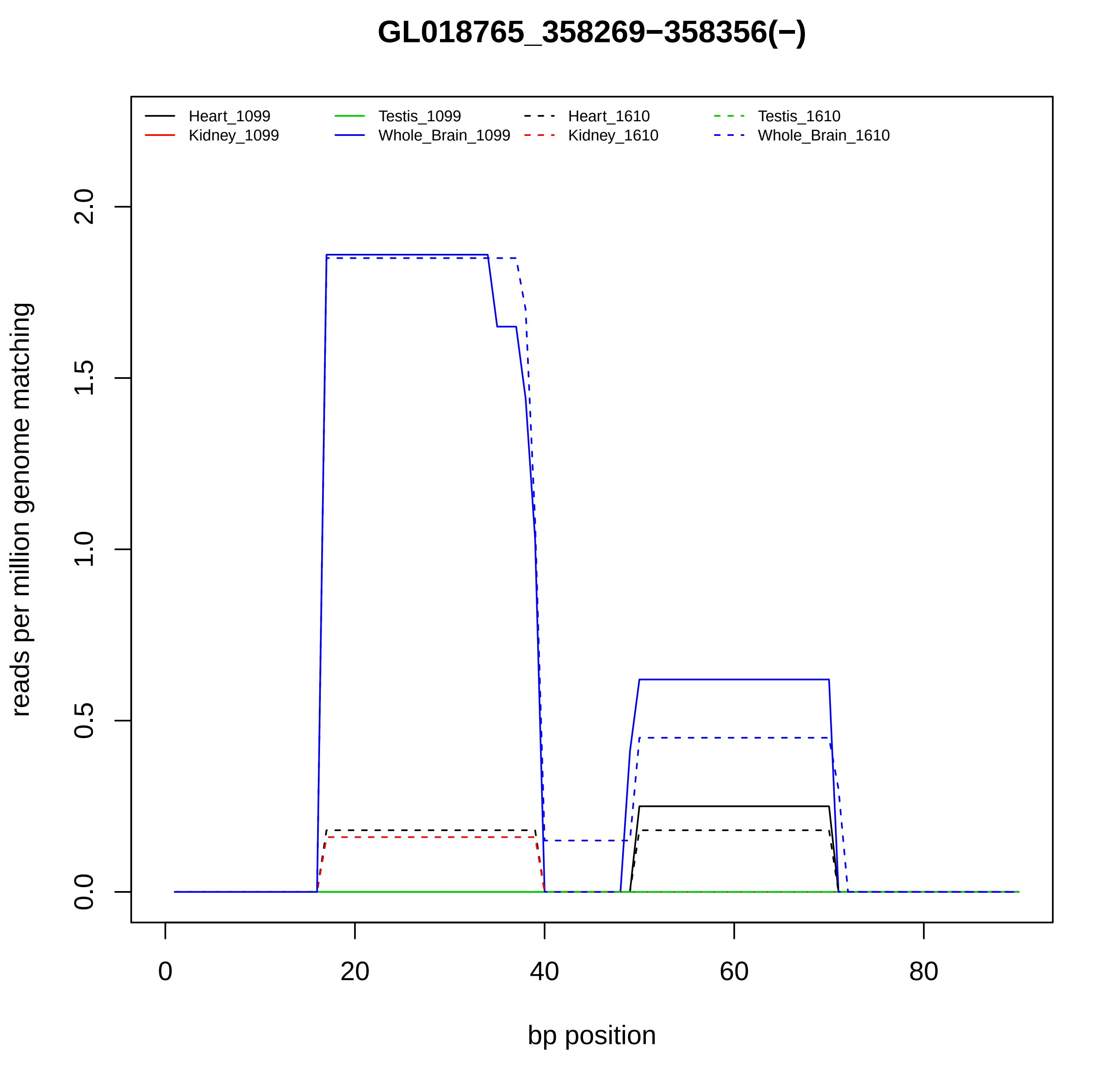

GL018765_358269−358356(−)
Heart_1099
Kidney_1099
Testis_1099
Whole_Brain_1099
Heart_1610
Kidney_1610
Testis_1610
Whole_Brain_1610
reads per million genome matching
0.0
0.5
1.0
1.5
2.0
0
20
40
60
80
bp position

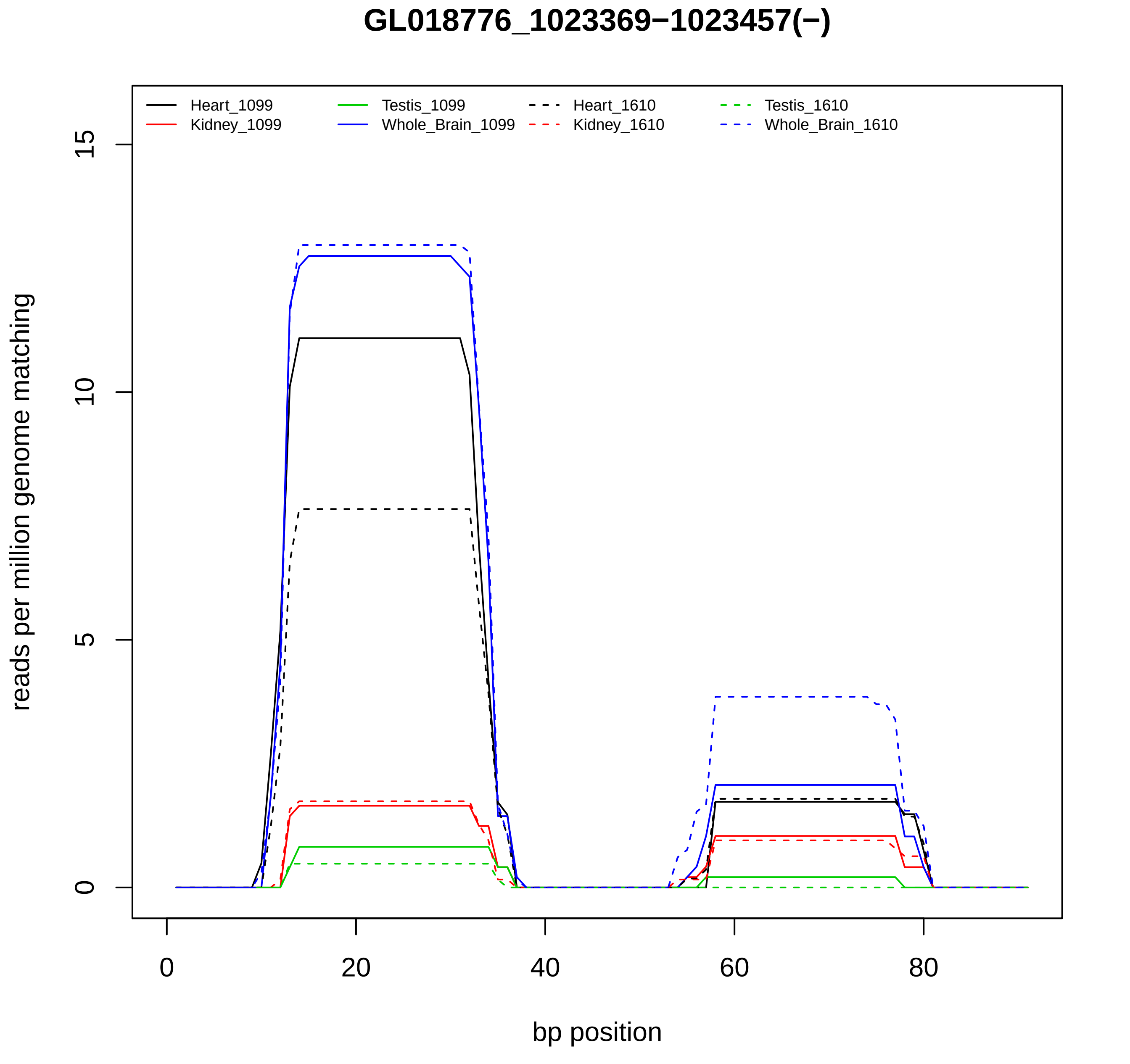
GL018776_1023369−1023457(−)
Heart_1099
Kidney_1099
Testis_1099
Whole_Brain_1099
Heart_1610
Kidney_1610
Testis_1610
Whole_Brain_1610
reads per million genome matching
0
5
10
15
0
20
40
60
80
bp position

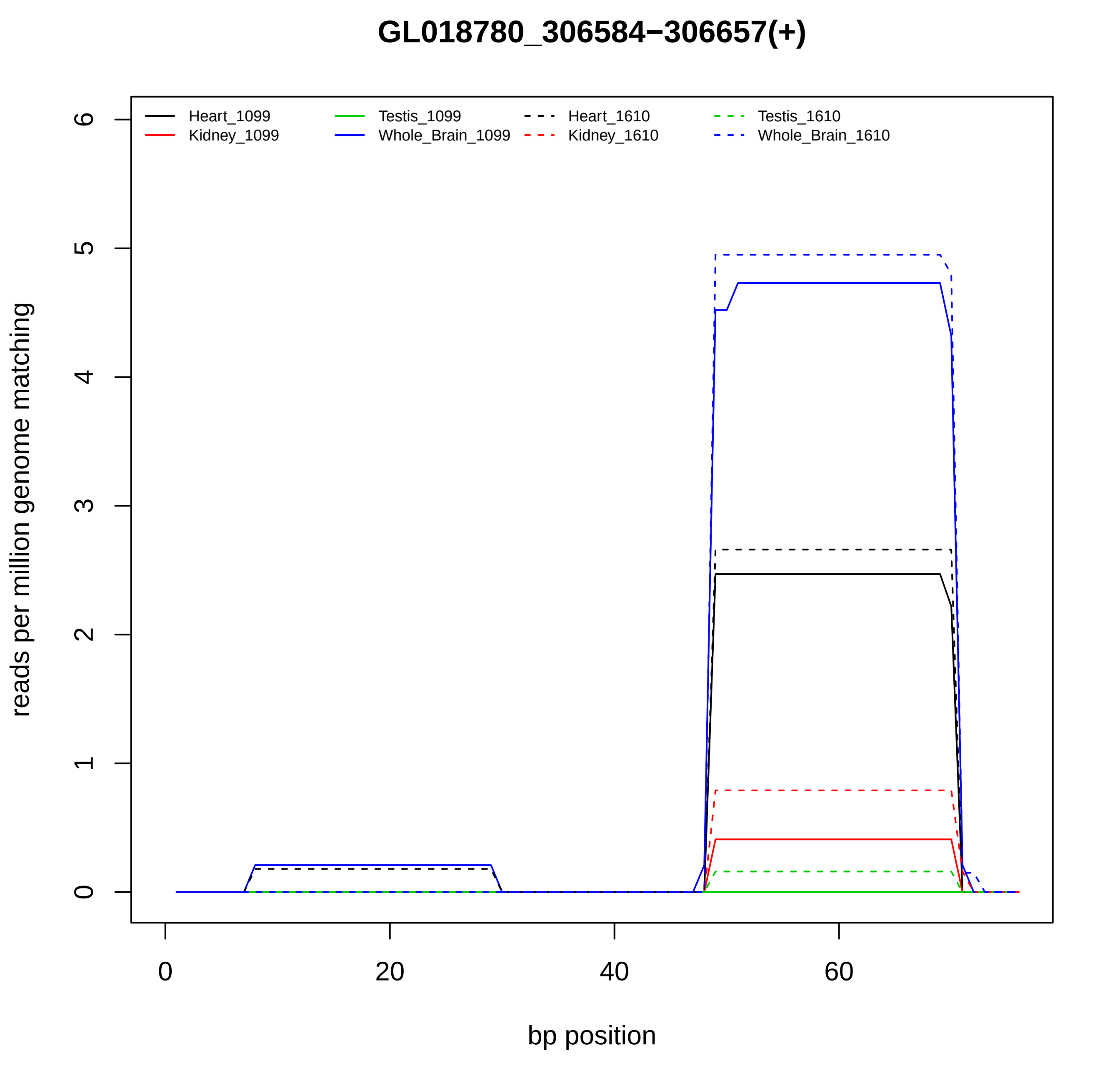
GL018780_306584−306657(+)
Heart_1099
Kidney_1099
Testis_1099
Whole_Brain_1099
Heart_1610
Kidney_1610
Testis_1610
Whole_Brain_1610
reads per million genome matching
bp position
0
1
2
3
4
5
6
0
20
40
60

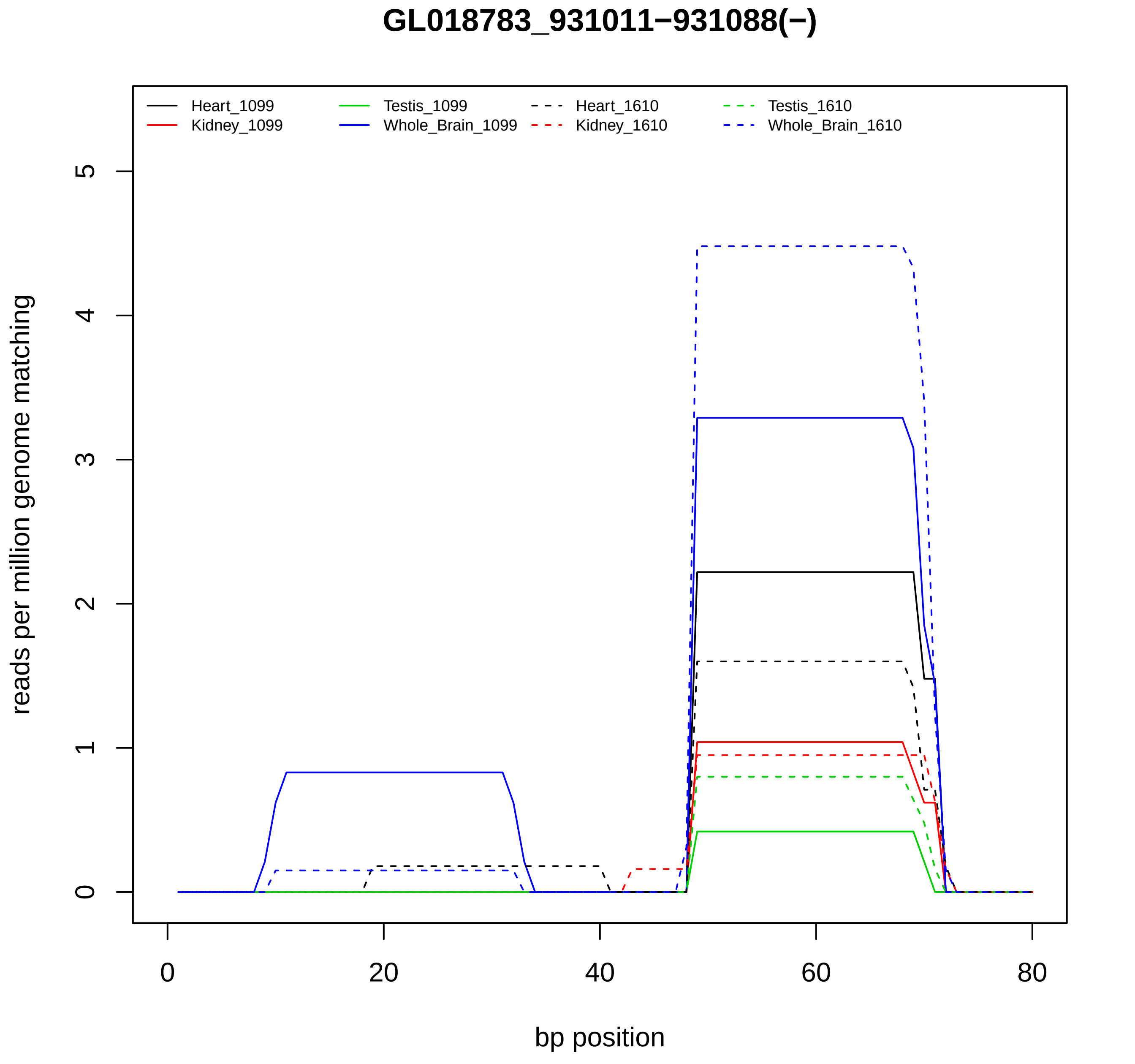

GL018783_931011−931088(−)
Heart_1099
Kidney_1099
Testis_1099
Whole_Brain_1099
Heart_1610
Kidney_1610
Testis_1610
Whole_Brain_1610
reads per million genome matching
bp position
0
1
2
3
4
5
0
20
40
60
80

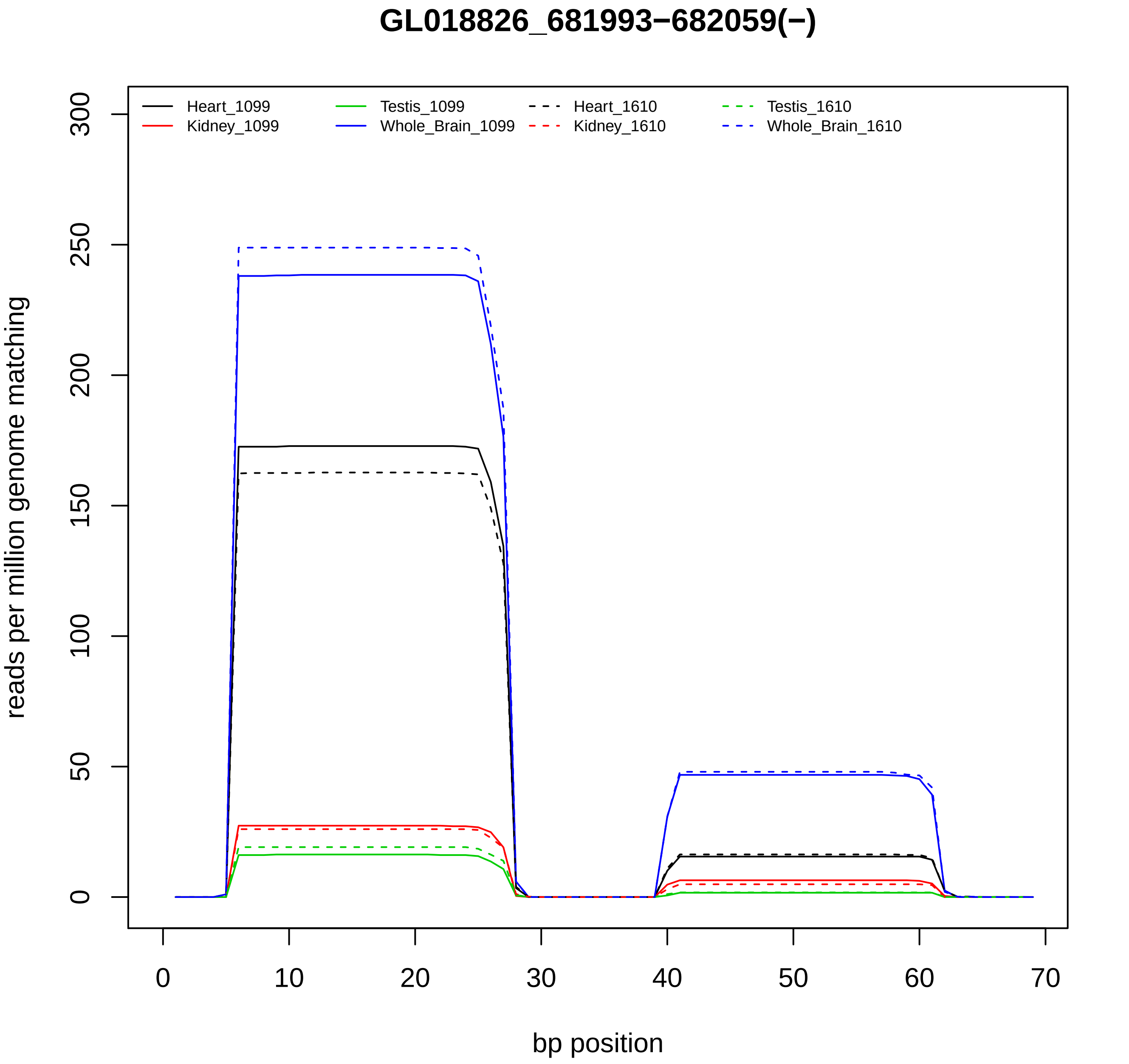

GL018826_681993−682059(−)
Heart_1099
Kidney_1099
Testis_1099
Whole_Brain_1099
Heart_1610
Kidney_1610
Testis_1610
Whole_Brain_1610
reads per million genome matching
0
50
100
150
200
250
300
bp position
0
10
20
30
40
50
60
70

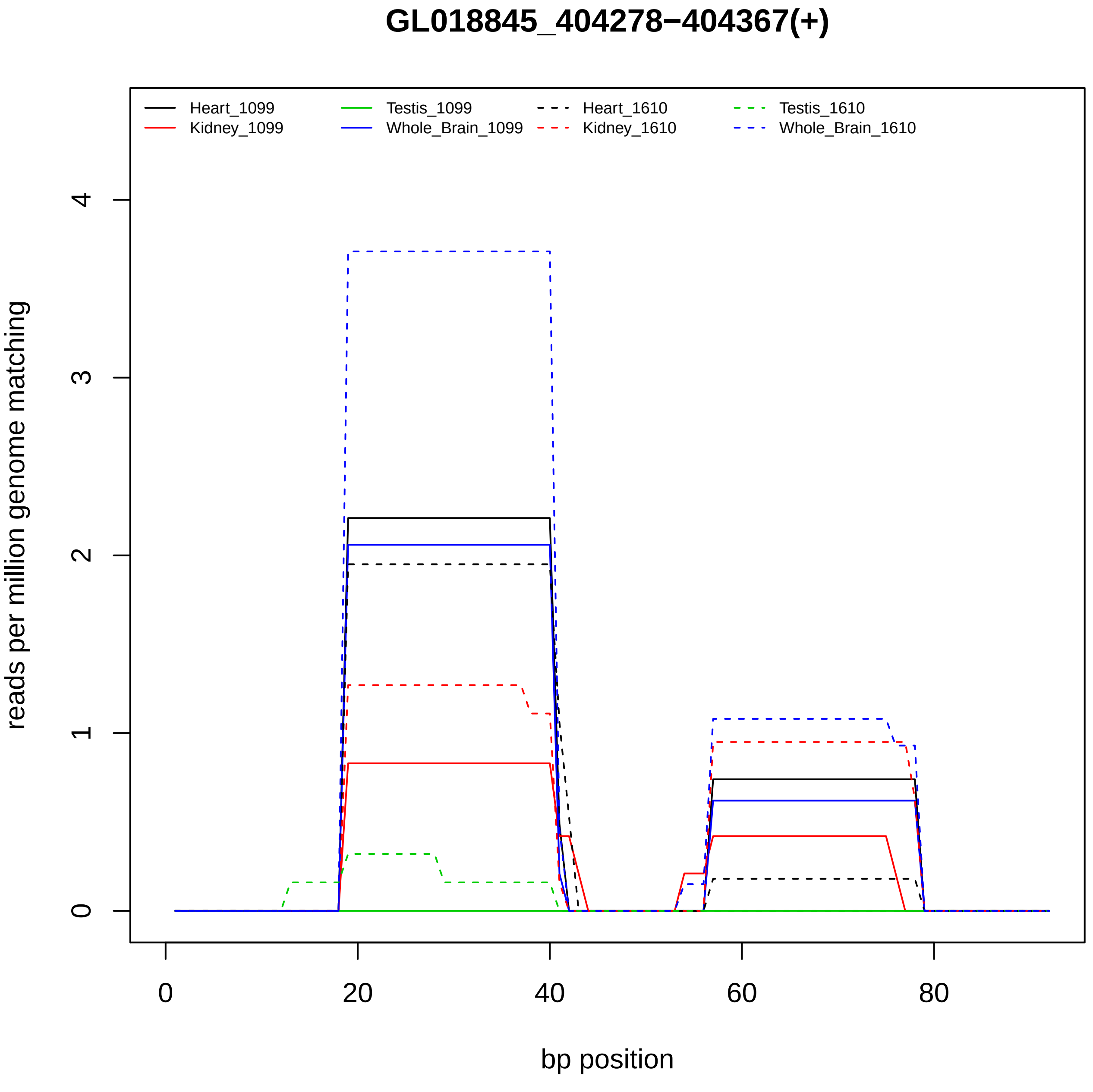

GL018845_404278−404367(+)
Heart_1099
Kidney_1099
Testis_1099
Whole_Brain_1099
Heart_1610
Kidney_1610
Testis_1610
Whole_Brain_1610
reads per million genome matching
bp position
0
1
2
3
4
0
20
40
60
80

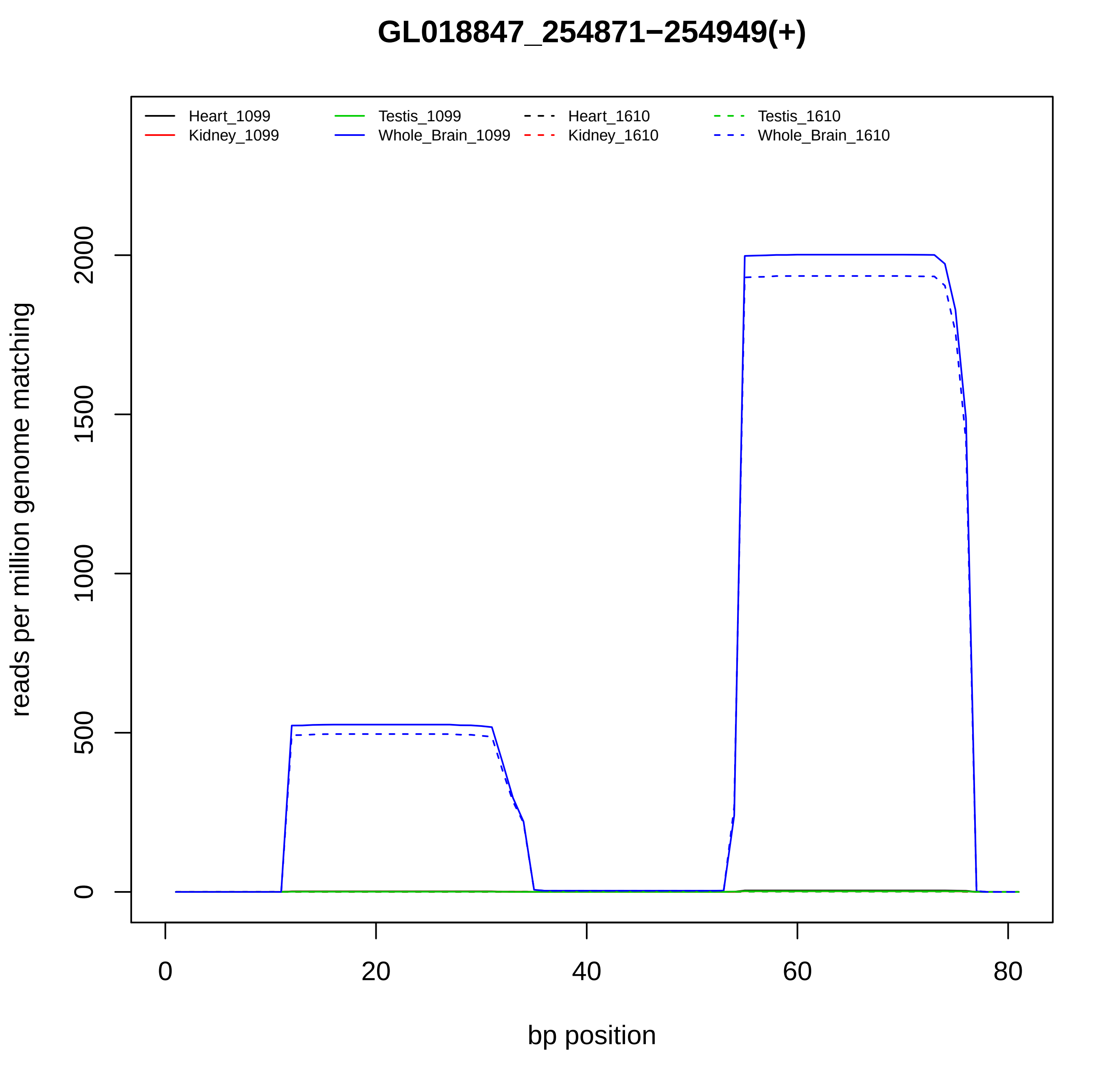

GL018847_254871−254949(+)
Heart_1099
Kidney_1099
Testis_1099
Whole_Brain_1099
Heart_1610
Kidney_1610
Testis_1610
Whole_Brain_1610
reads per million genome matching
0
500
1000
1500
2000
0
20
40
60
80
bp position

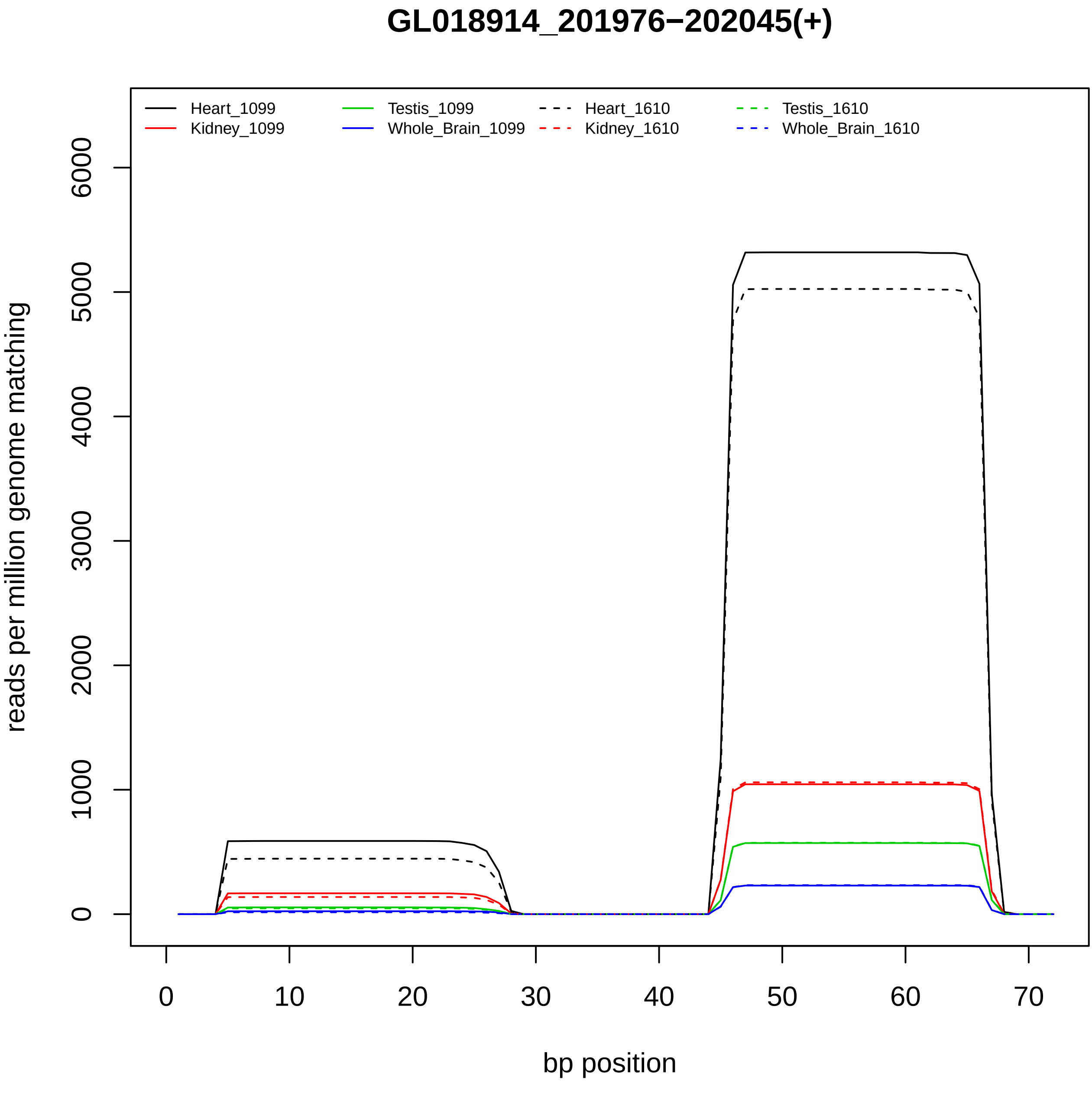
GL018914_201976−202045(+)
Heart_1099
Kidney_1099
Testis_1099
Whole_Brain_1099
Heart_1610
Kidney_1610
Testis_1610
Whole_Brain_1610
reads per million genome matching
0
1000
2000
3000
4000
5000
6000
0
10
20
30
40
50
60
70
bp position

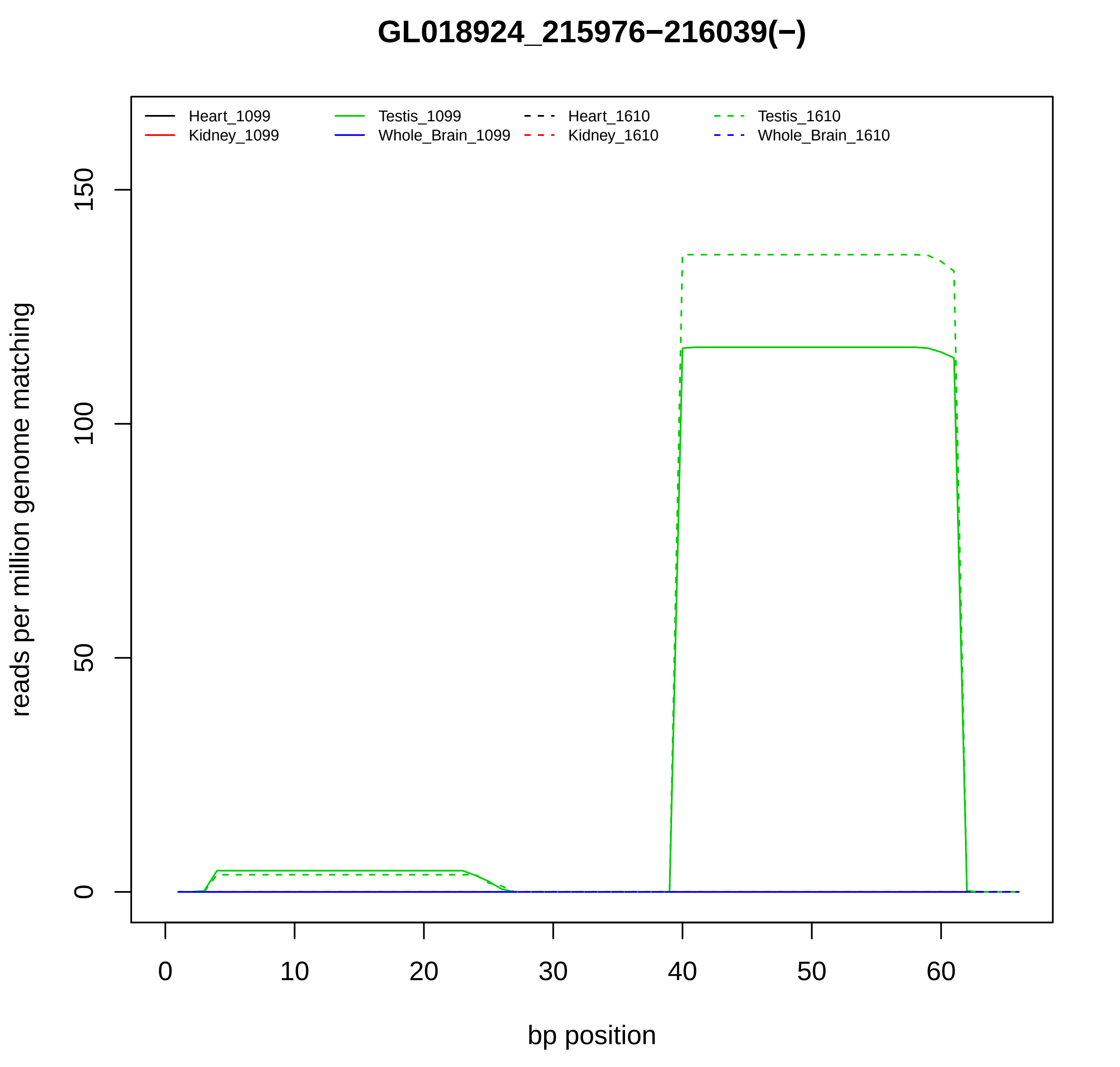
GL018924_215976−216039(−)
Heart_1099
Kidney_1099
Testis_1099
Whole_Brain_1099
Heart_1610
Kidney_1610
Testis_1610
Whole_Brain_1610
reads per million genome matching
0
50
100
150
0
10
20
30
40
50
60
bp position

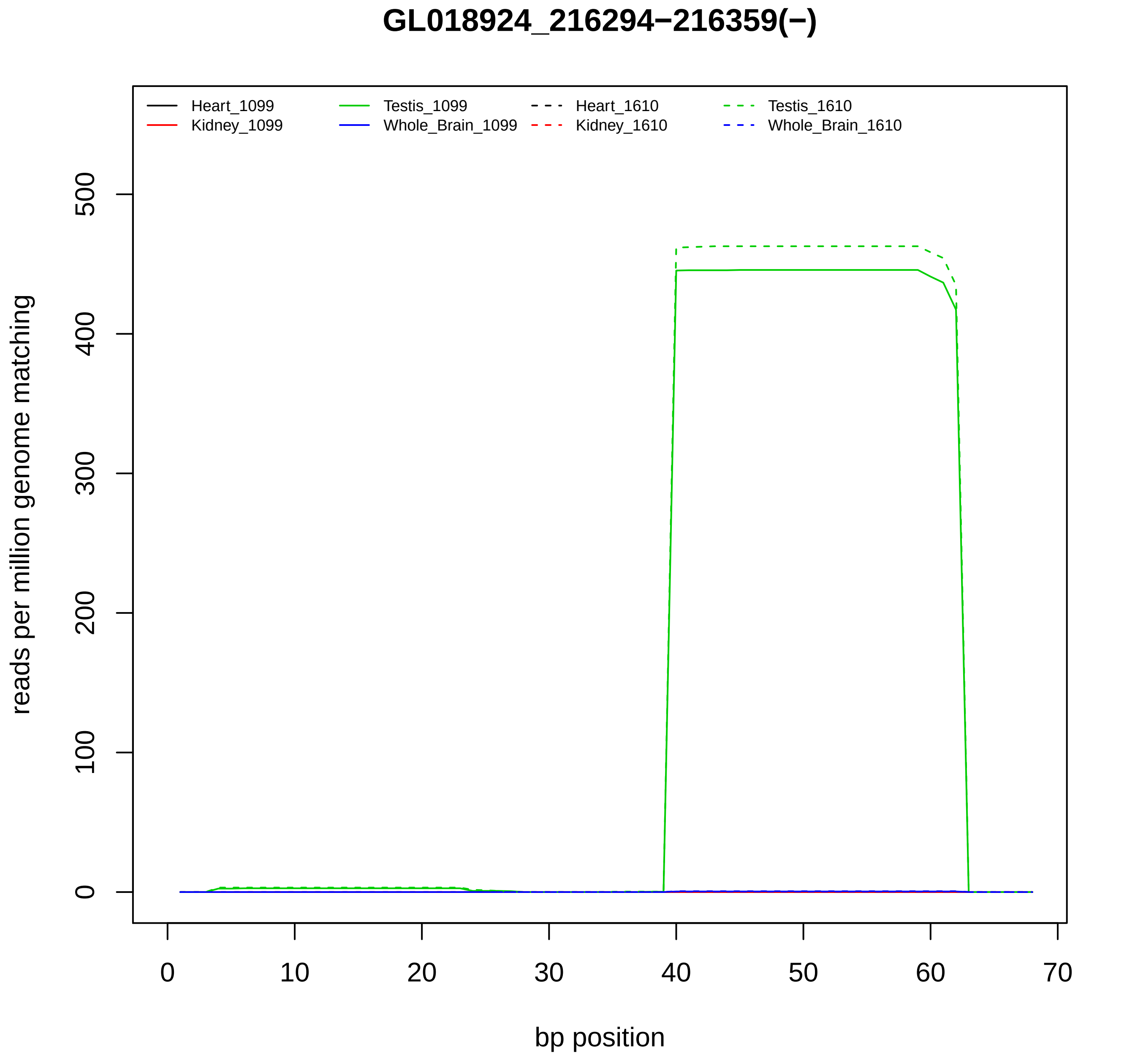

GL018924_216294−216359(−)
Heart_1099
Kidney_1099
Testis_1099
Whole_Brain_1099
Heart_1610
Kidney_1610
Testis_1610
Whole_Brain_1610
reads per million genome matching
0
100
200
300
400
500
bp position
0
10
20
30
40
50
60
70

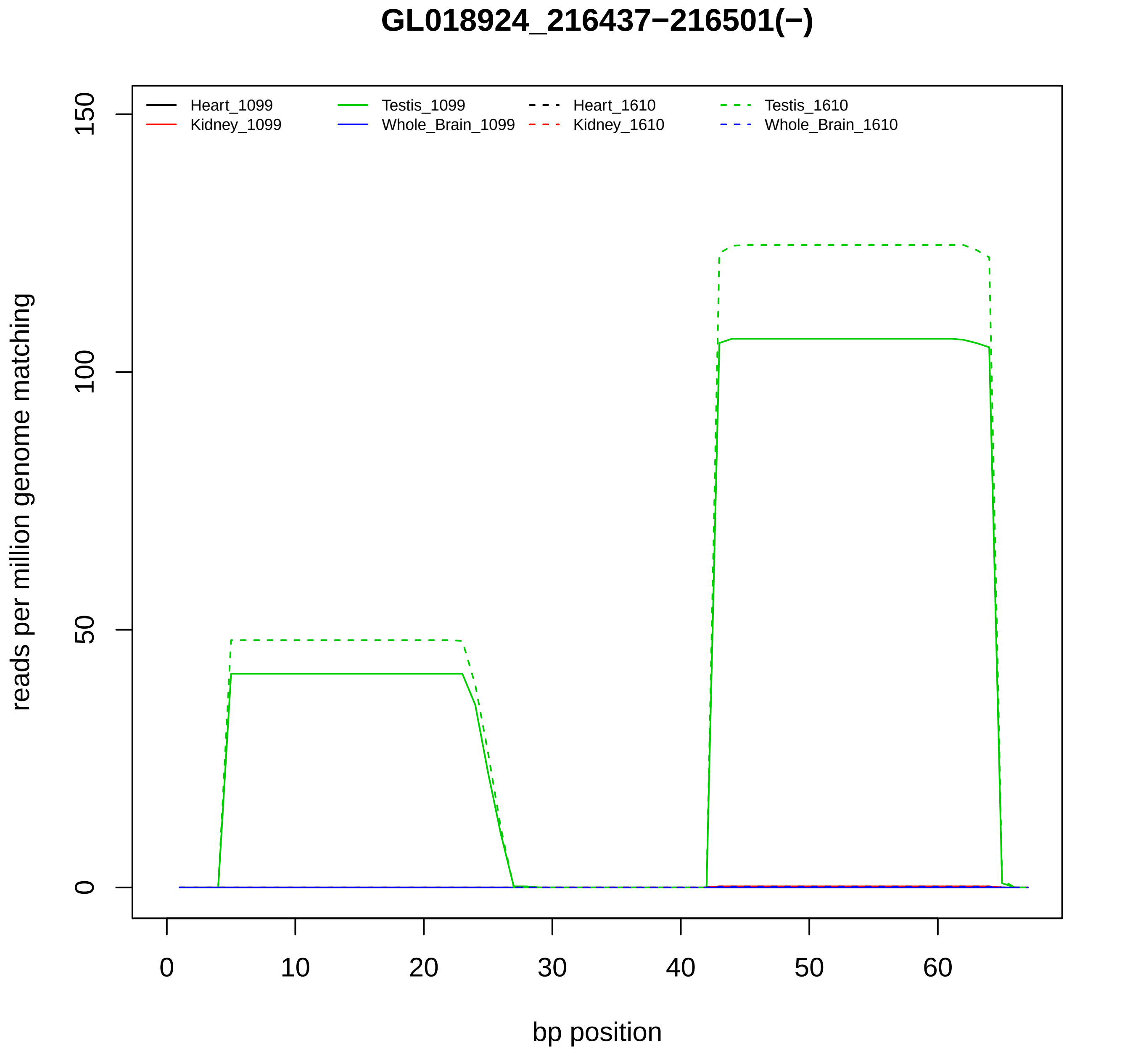

GL018924_216437−216501(−)
Heart_1099
Kidney_1099
Testis_1099
Whole_Brain_1099
Heart_1610
Kidney_1610
Testis_1610
Whole_Brain_1610
reads per million genome matching
bp position
0
50
100
150
0
10
20
30
40
50
60

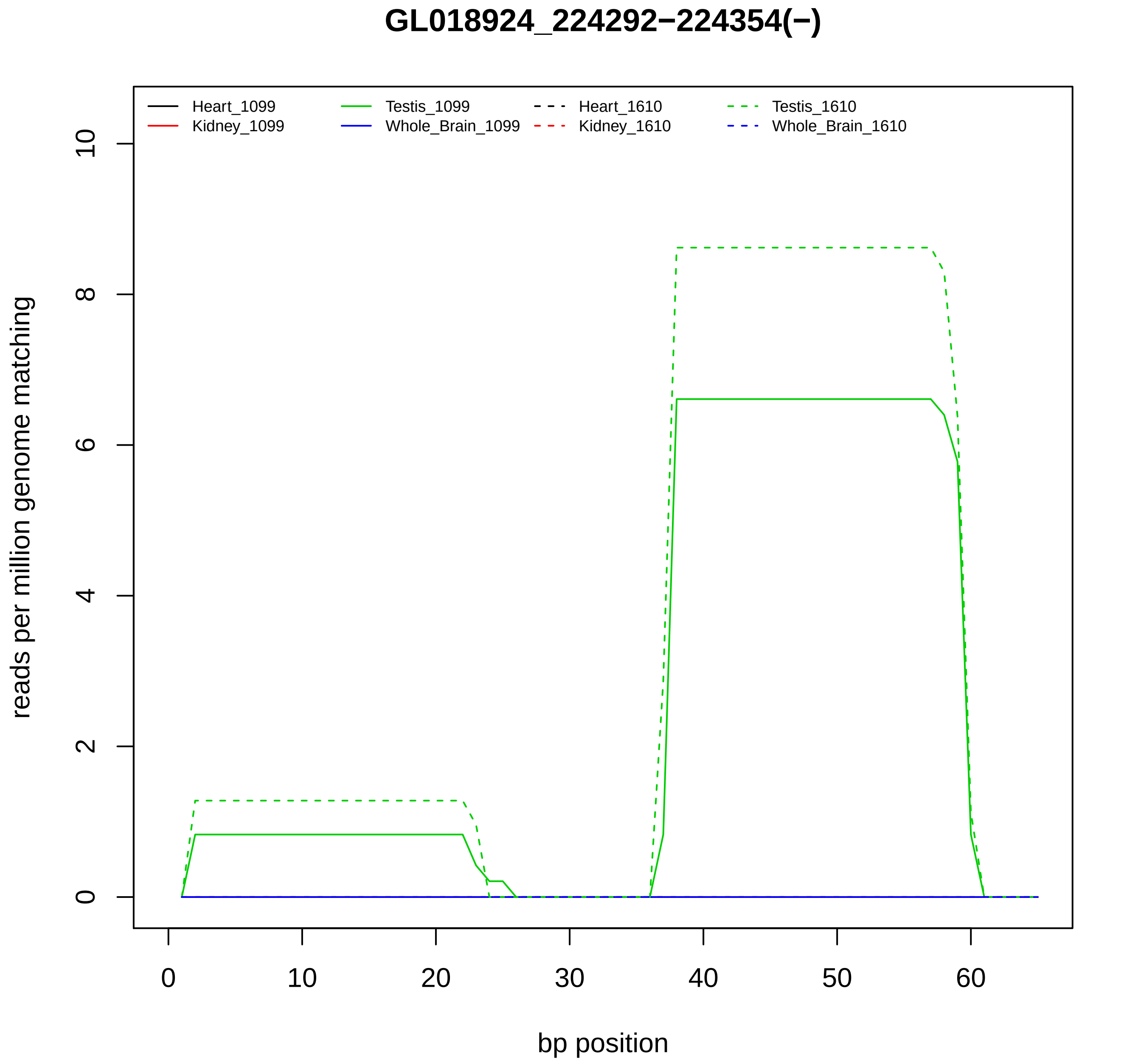

GL018924_224292−224354(−)
Heart_1099
Kidney_1099
Testis_1099
Whole_Brain_1099
Heart_1610
Kidney_1610
Testis_1610
Whole_Brain_1610
reads per million genome matching
0
2
4
6
8
10
0
10
20
30
40
50
60
bp position

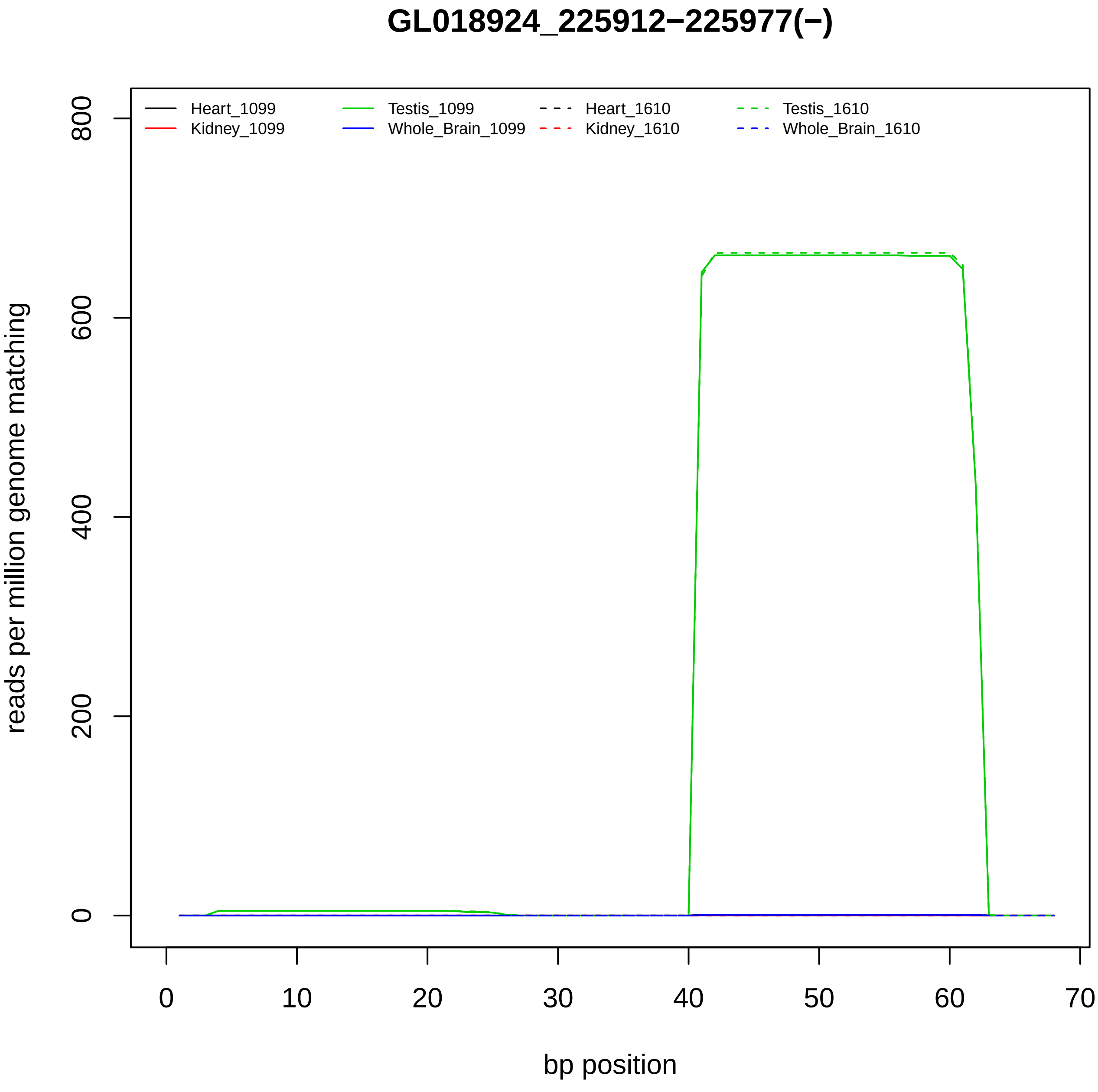

GL018924_225912−225977(−)
Heart_1099
Kidney_1099
Testis_1099
Whole_Brain_1099
Heart_1610
Kidney_1610
Testis_1610
Whole_Brain_1610
reads per million genome matching
bp position
0
200
400
600
800
0
10
20
30
40
50
60
70

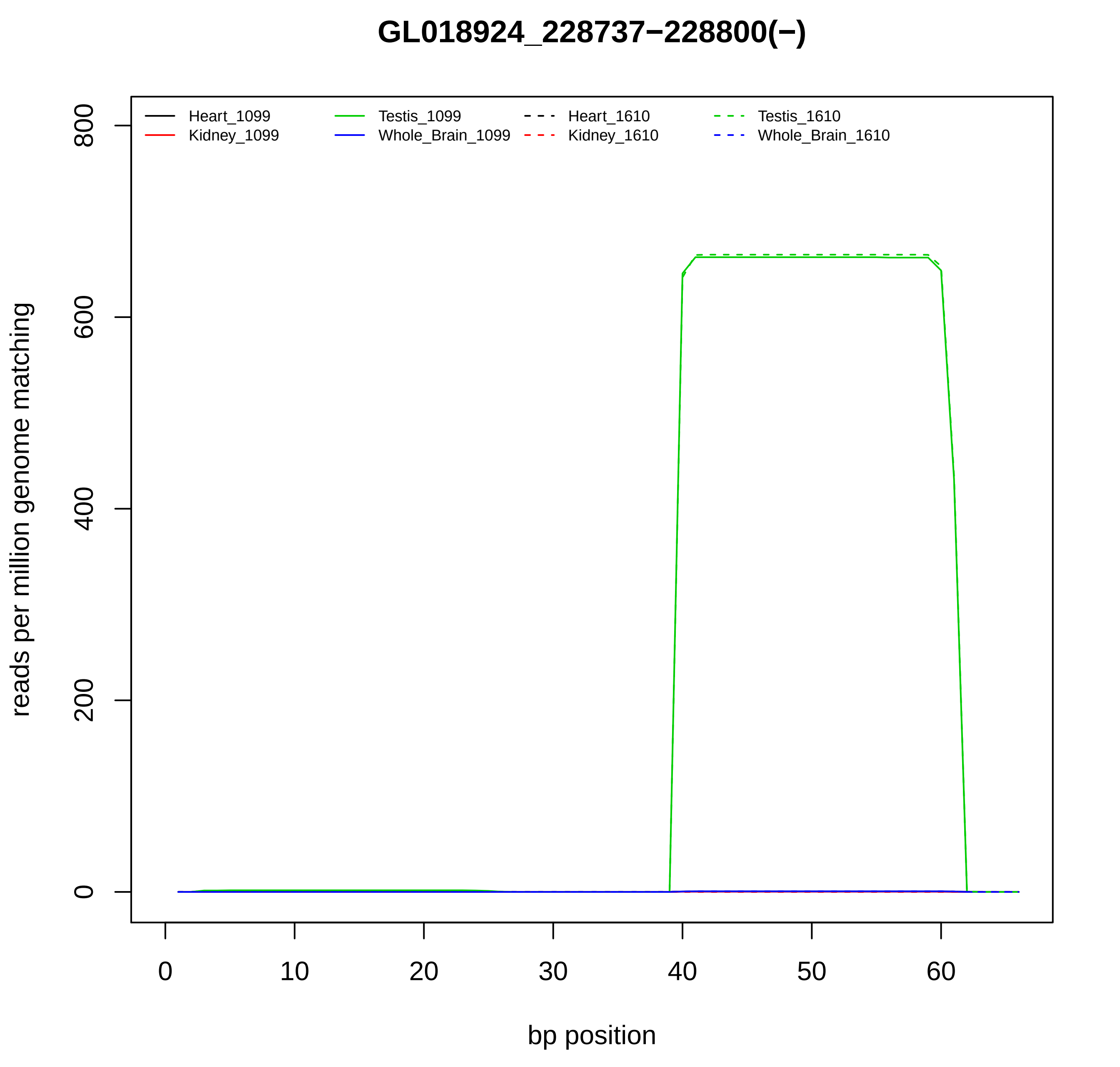

GL018924_228737−228800(−)
Heart_1099
Kidney_1099
Testis_1099
Whole_Brain_1099
Heart_1610
Kidney_1610
Testis_1610
Whole_Brain_1610
reads per million genome matching
0
200
400
600
800
0
10
20
30
40
50
60
bp position

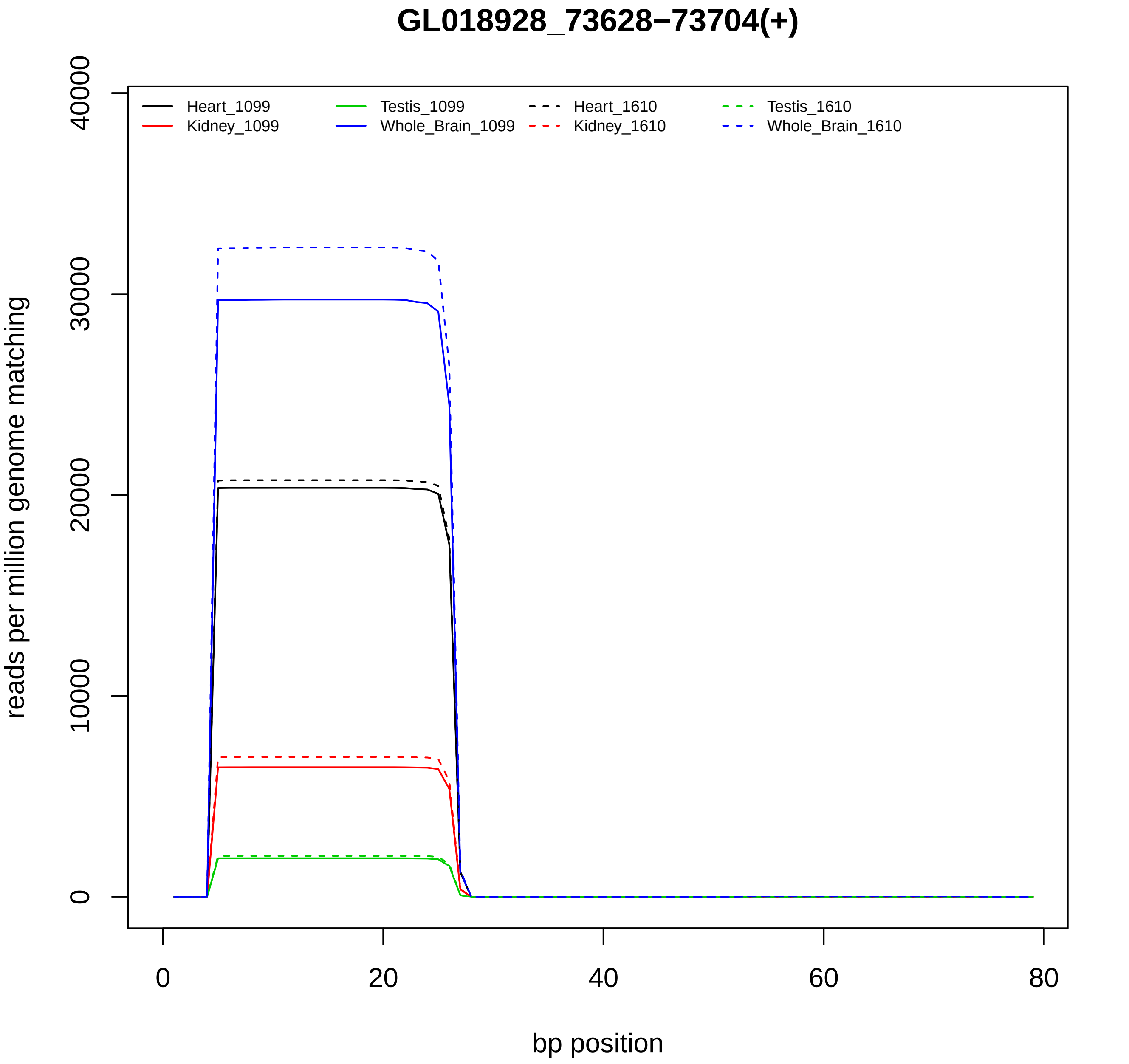

GL018928_73628−73704(+)
Heart_1099
Kidney_1099
Testis_1099
Whole_Brain_1099
Heart_1610
Kidney_1610
Testis_1610
Whole_Brain_1610
reads per million genome matching
bp position
0
10000
20000
30000
40000
0
20
40
60
80

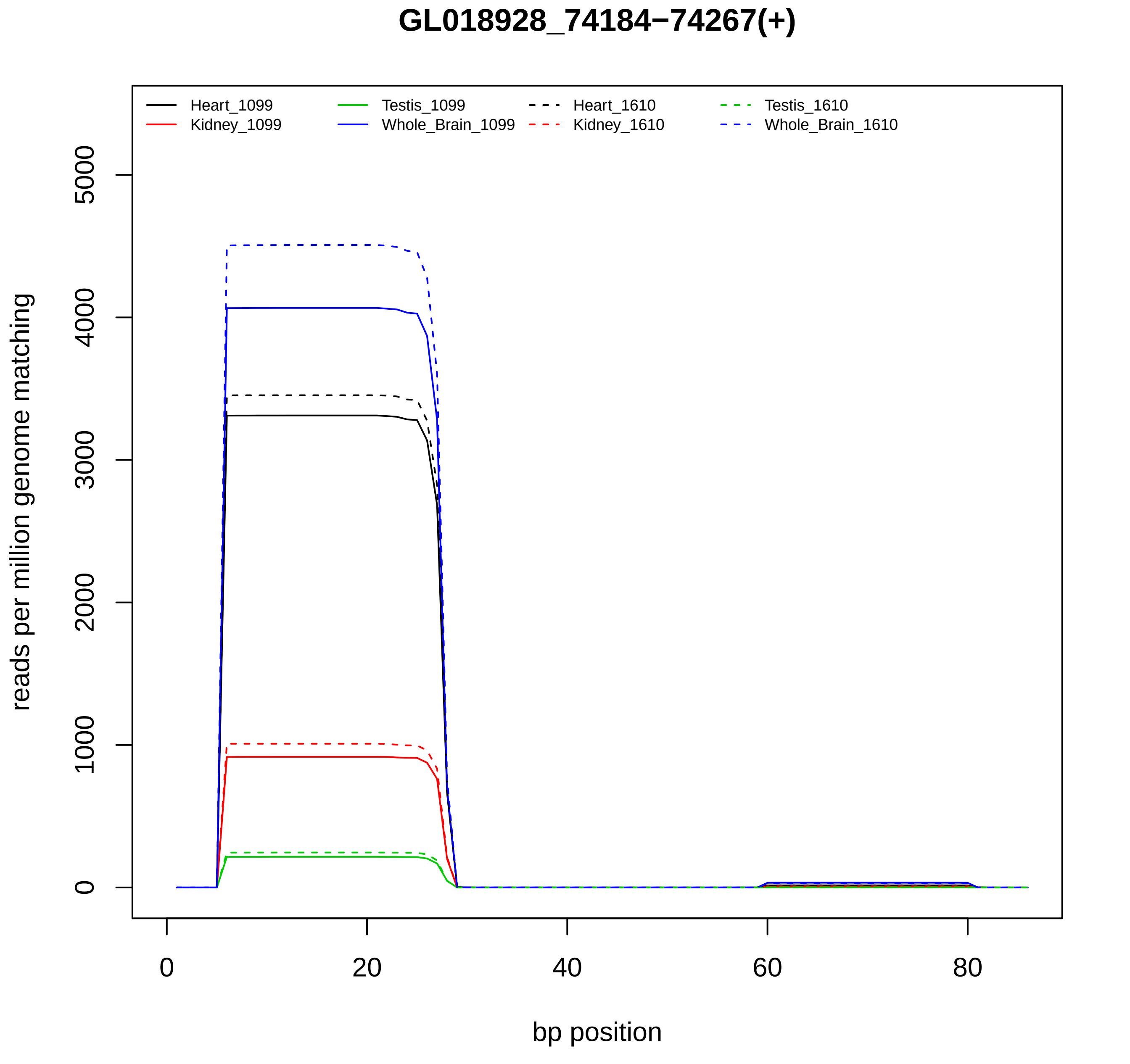
GL018928_74184−74267(+)
Heart_1099
Kidney_1099
Testis_1099
Whole_Brain_1099
Heart_1610
Kidney_1610
Testis_1610
Whole_Brain_1610
reads per million genome matching
0
1000
2000
3000
4000
5000
0
20
40
60
80
bp position

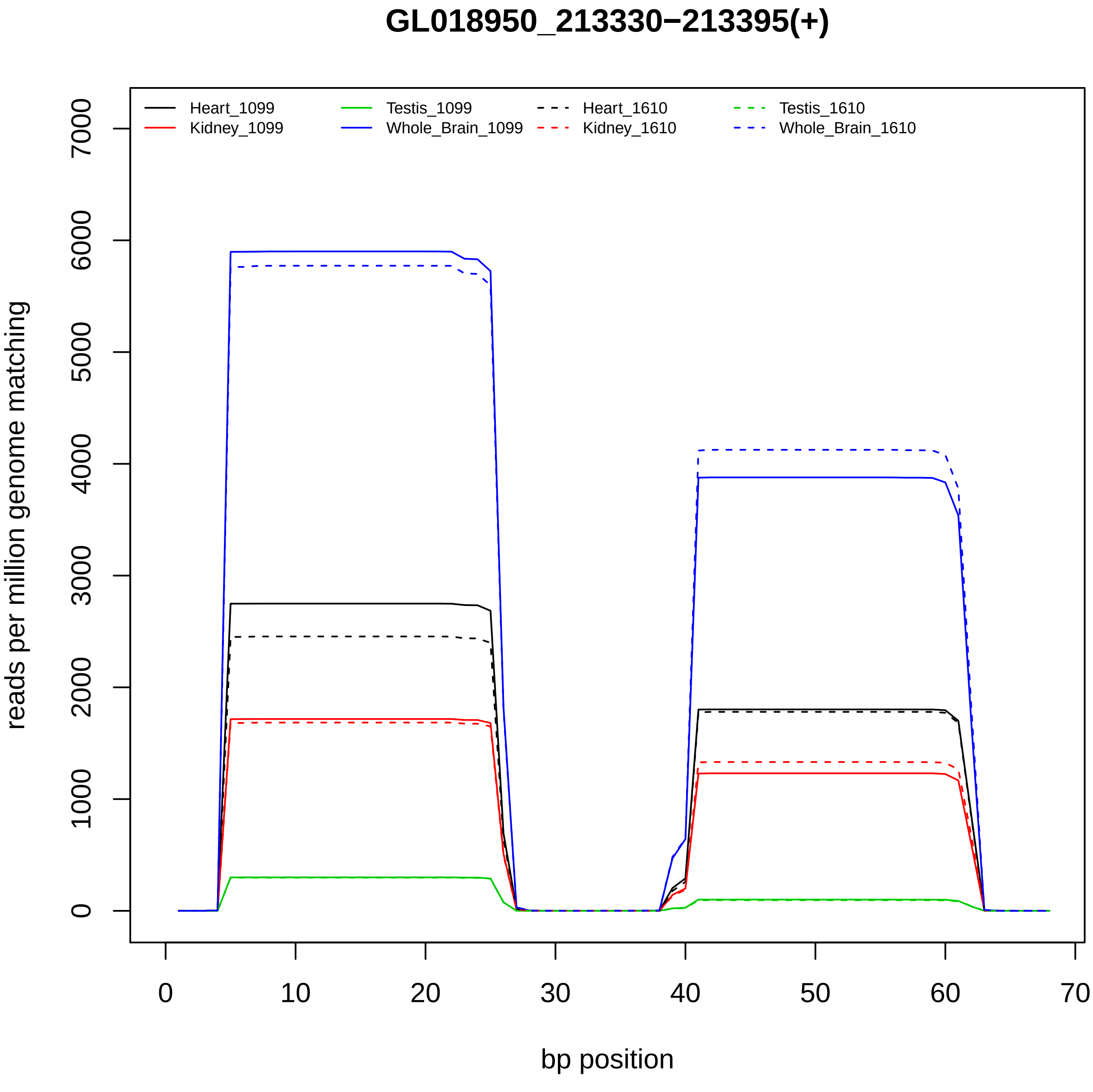

GL018950_213330−213395(+)
Heart_1099
Kidney_1099
Testis_1099
Whole_Brain_1099
Heart_1610
Kidney_1610
Testis_1610
Whole_Brain_1610
reads per million genome matching
0
1000
2000
3000
4000
5000
6000
7000
bp position
0
10
20
30
40
50
60
70

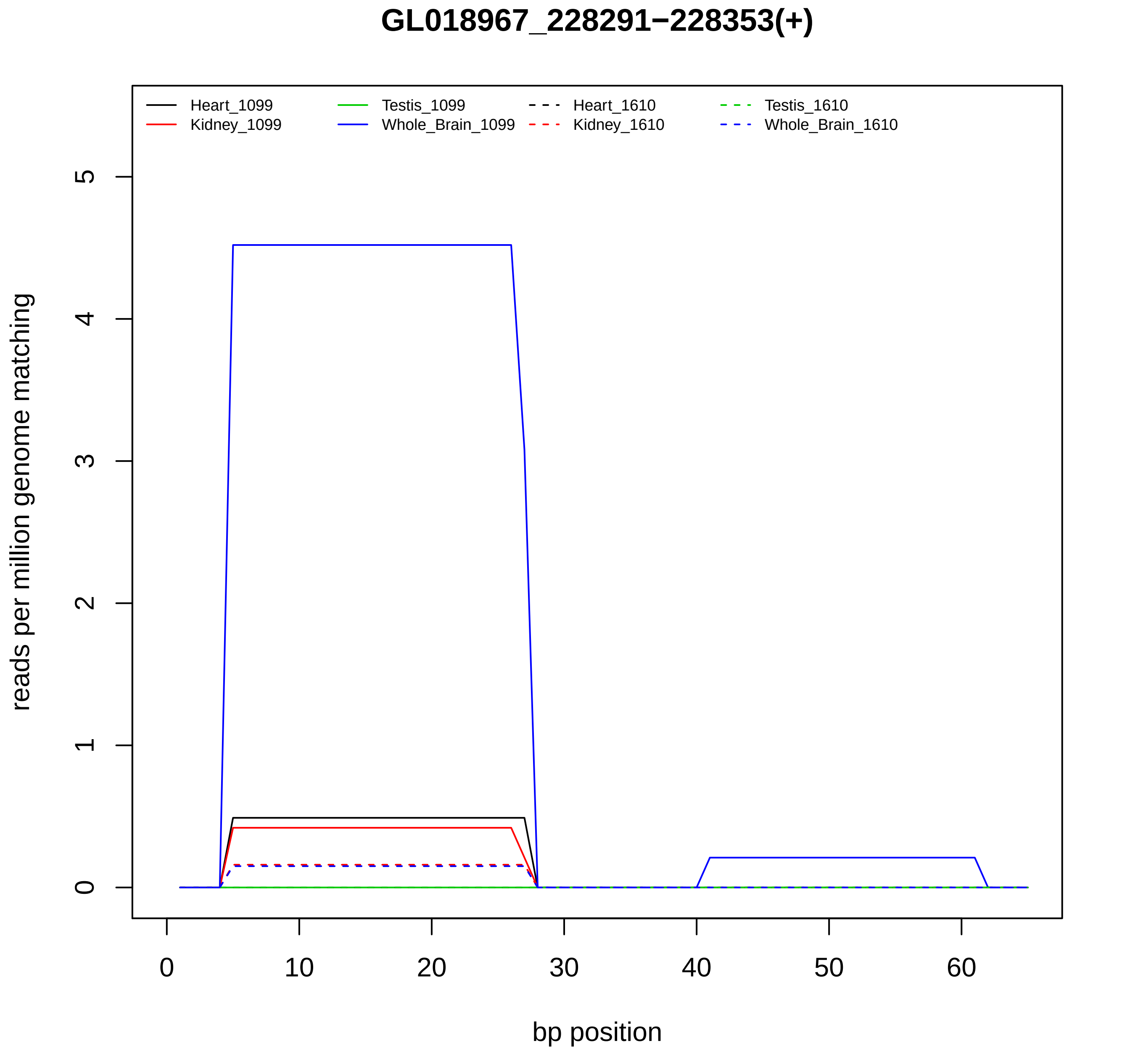

GL018967_228291−228353(+)
Heart_1099
Kidney_1099
Testis_1099
Whole_Brain_1099
Heart_1610
Kidney_1610
Testis_1610
Whole_Brain_1610
reads per million genome matching
0
1
2
3
4
5
0
10
20
30
40
50
60
bp position

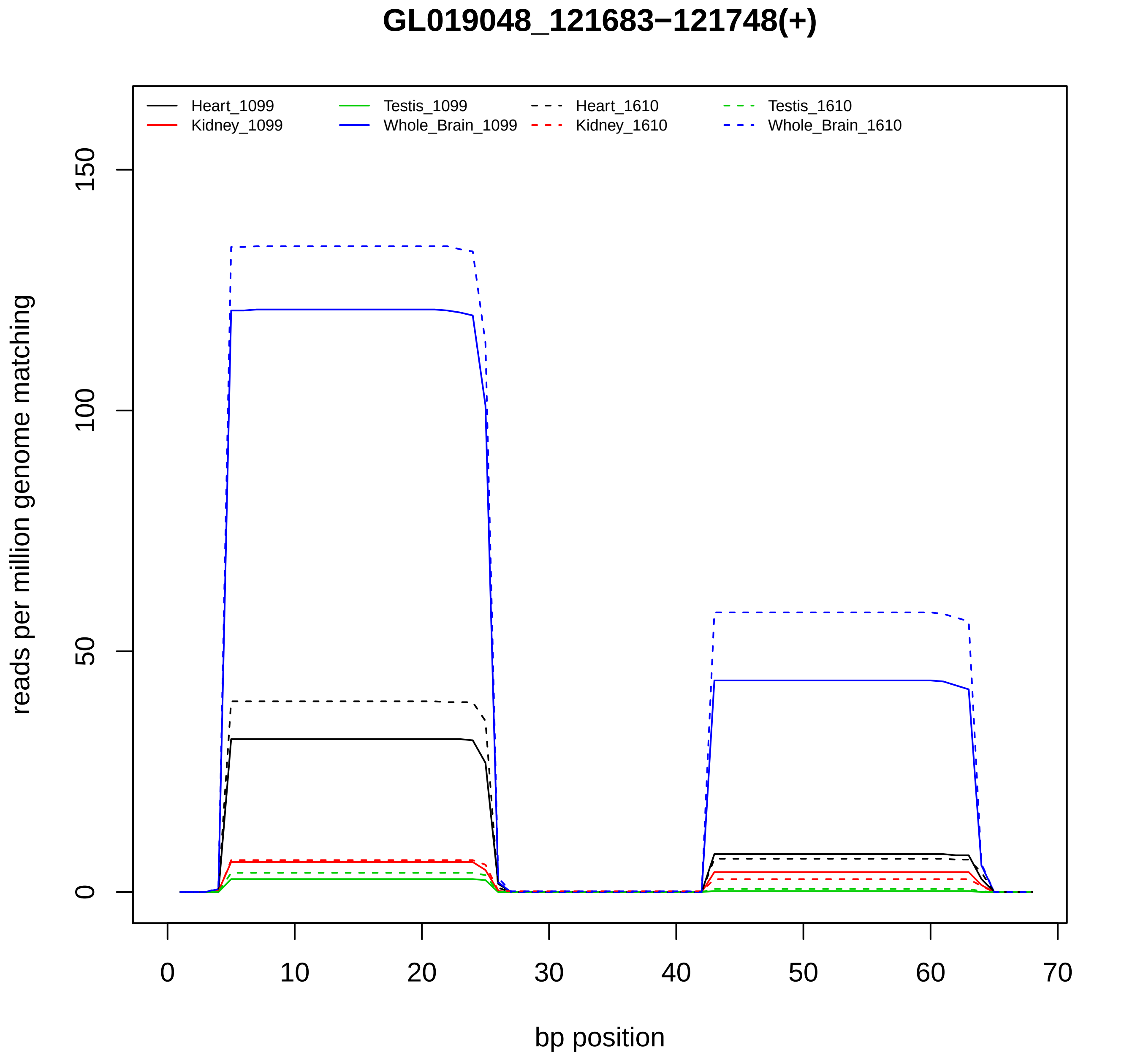
GL019048_121683−121748(+)
Heart_1099
Kidney_1099
Testis_1099
Whole_Brain_1099
Heart_1610
Kidney_1610
Testis_1610
Whole_Brain_1610
reads per million genome matching
0
50
100
150
0
10
20
30
40
50
60
70
bp position

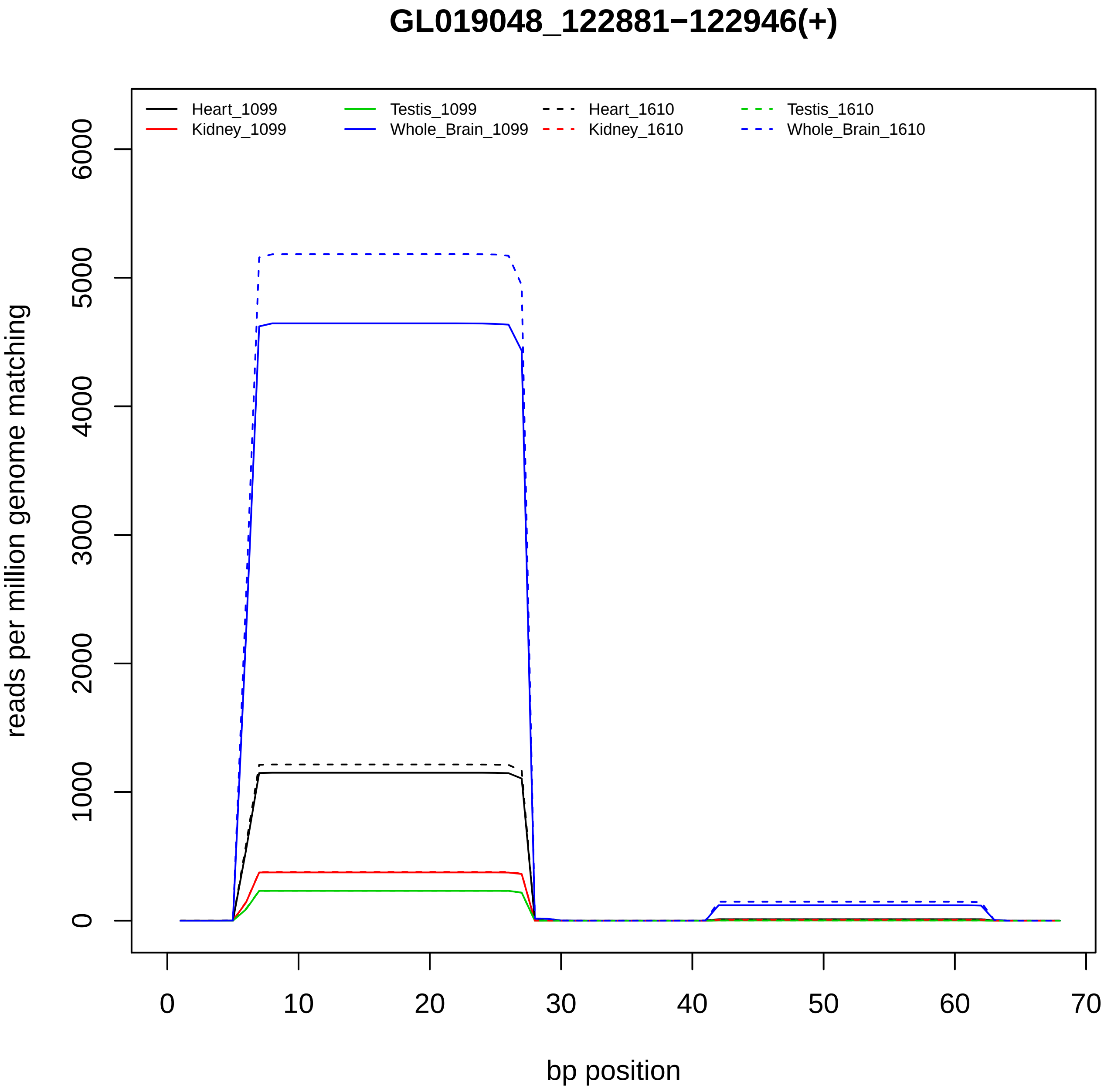
GL019048_122881−122946(+)
Heart_1099
Kidney_1099
Testis_1099
Whole_Brain_1099
Heart_1610
Kidney_1610
Testis_1610
Whole_Brain_1610
reads per million genome matching
0
1000
2000
3000
4000
5000
6000
bp position
0
10
20
30
40
50
60
70

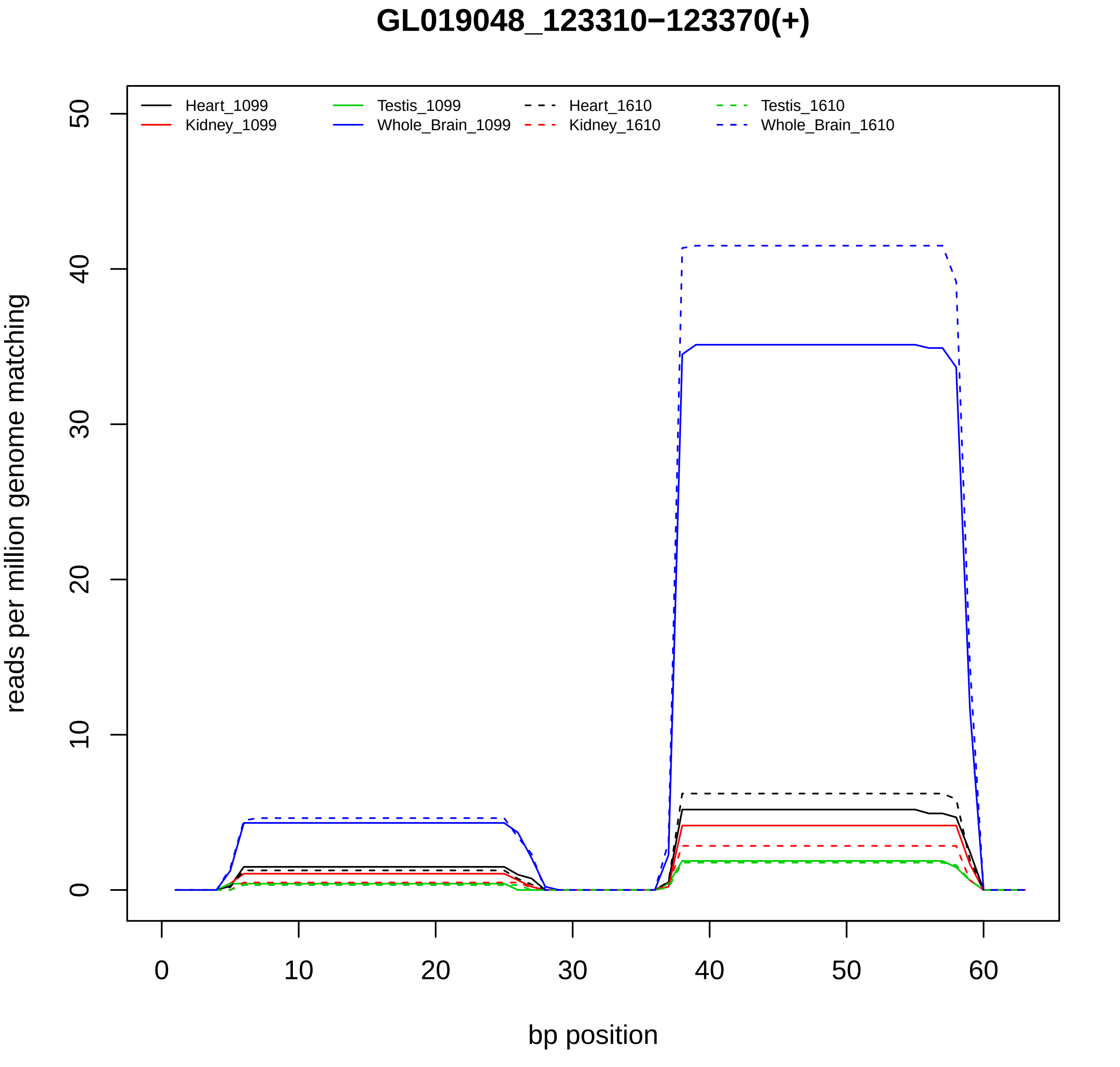
GL019048_123310−123370(+)
Heart_1099
Kidney_1099
Testis_1099
Whole_Brain_1099
Heart_1610
Kidney_1610
Testis_1610
Whole_Brain_1610
reads per million genome matching
0
10
20
30
40
50
0
10
20
30
40
50
60
bp position

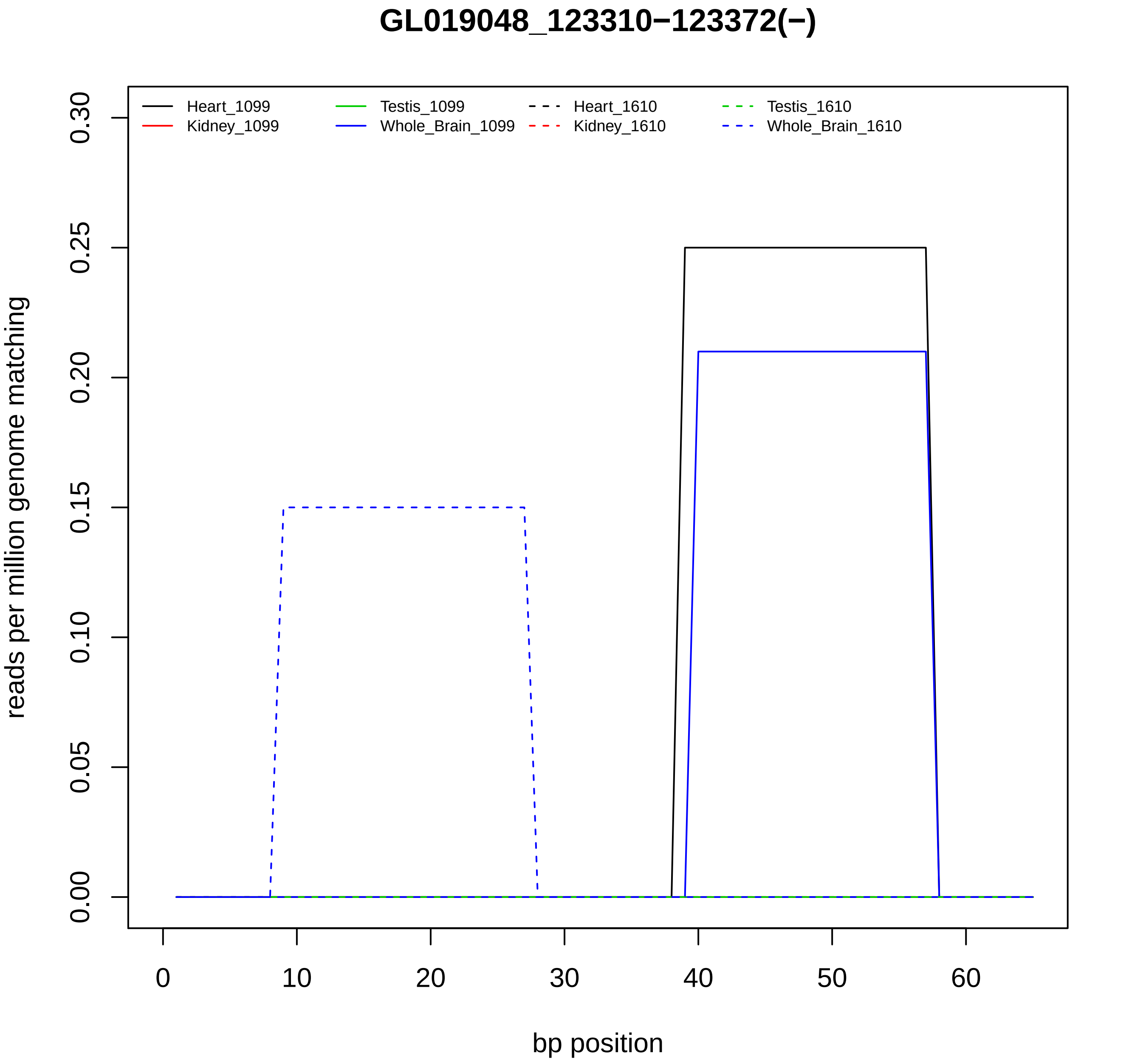

GL019048_123310−123372(−)
Heart_1099
Kidney_1099
Testis_1099
Whole_Brain_1099
Heart_1610
Kidney_1610
Testis_1610
Whole_Brain_1610
reads per million genome matching
0.00
0.05
0.10
0.15
0.20
0.25
0.30
0
10
20
30
40
50
60
bp position

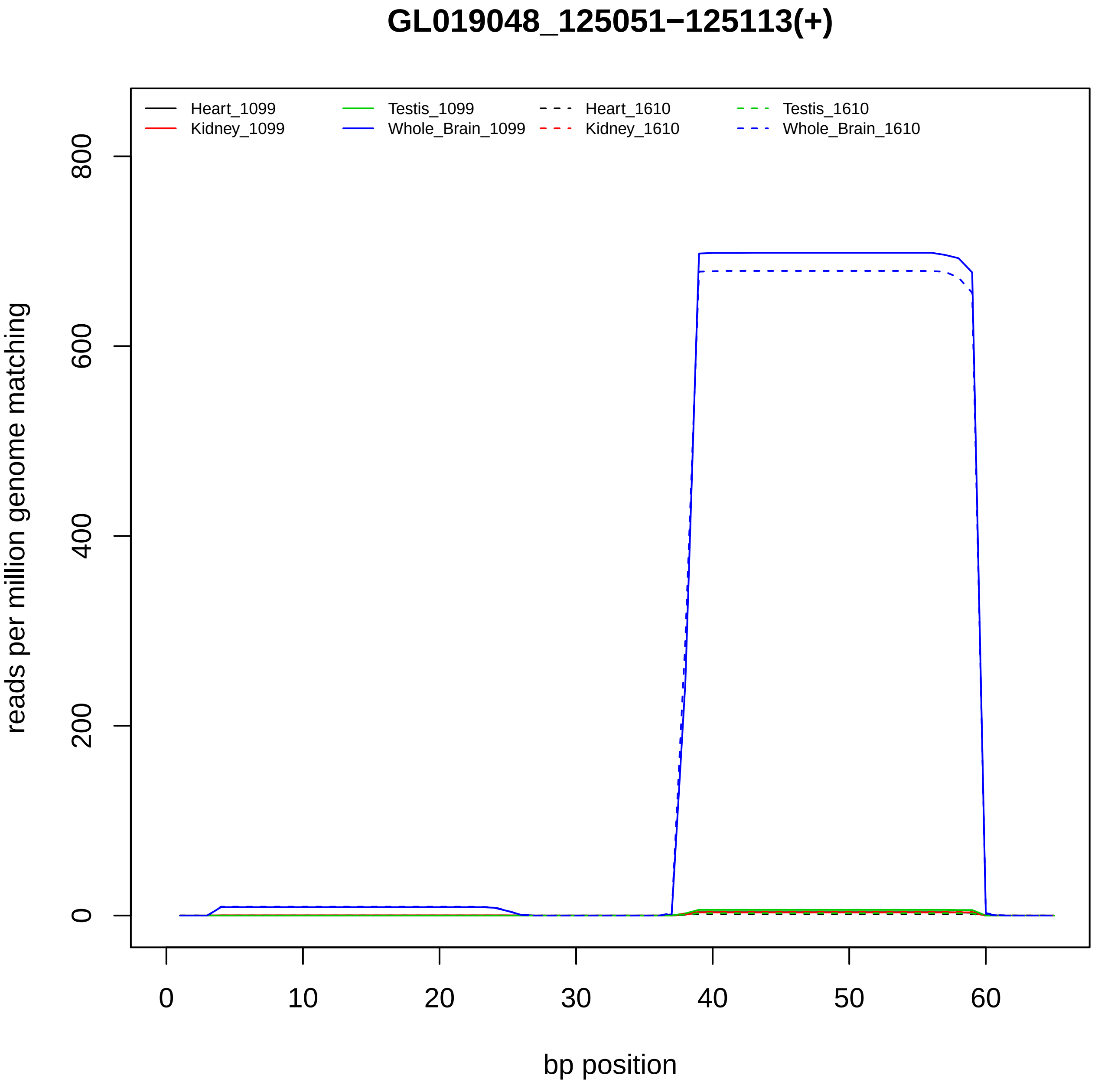
GL019048_125051−125113(+)
Heart_1099
Kidney_1099
Testis_1099
Whole_Brain_1099
Heart_1610
Kidney_1610
Testis_1610
Whole_Brain_1610
reads per million genome matching
bp position
0
200
400
600
800
0
10
20
30
40
50
60

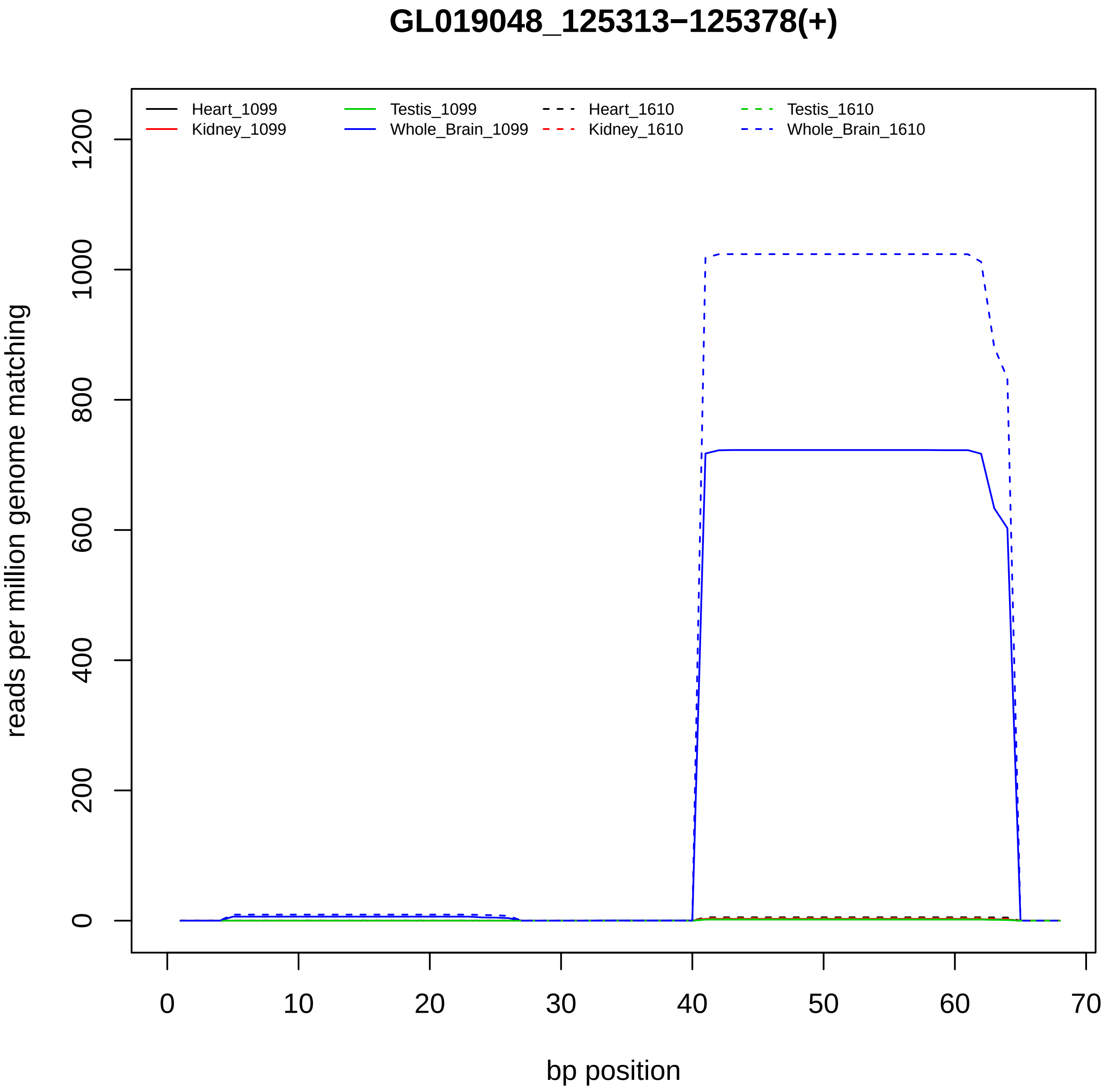

GL019048_125313−125378(+)
Heart_1099
Kidney_1099
Testis_1099
Whole_Brain_1099
Heart_1610
Kidney_1610
Testis_1610
Whole_Brain_1610
reads per million genome matching
0
200
400
600
800
1000
1200
0
10
20
30
40
50
60
70
bp position

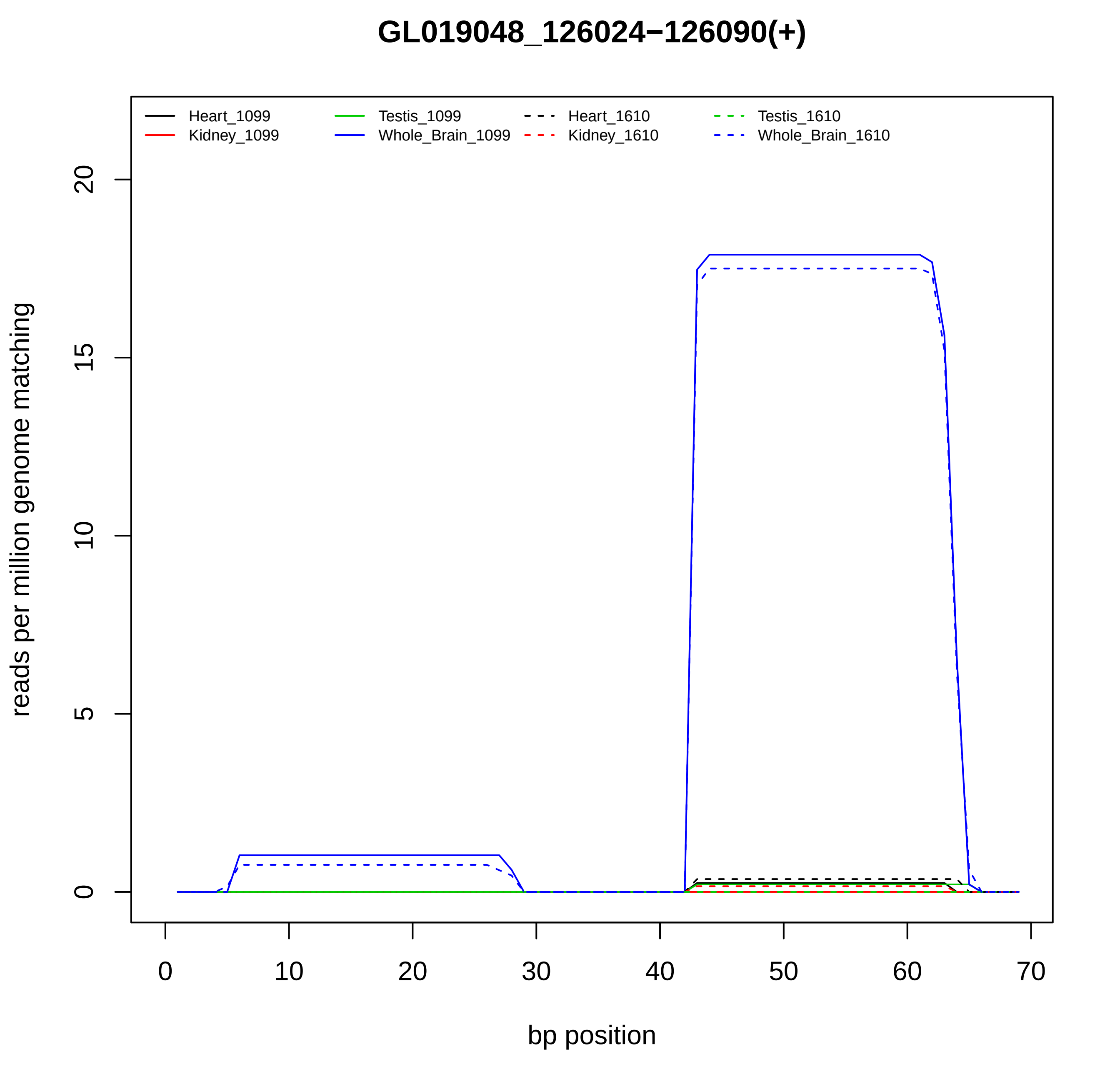

GL019048_126024−126090(+)
Heart_1099
Kidney_1099
Testis_1099
Whole_Brain_1099
Heart_1610
Kidney_1610
Testis_1610
Whole_Brain_1610
reads per million genome matching
0
5
10
15
20
bp position
0
10
20
30
40
50
60
70

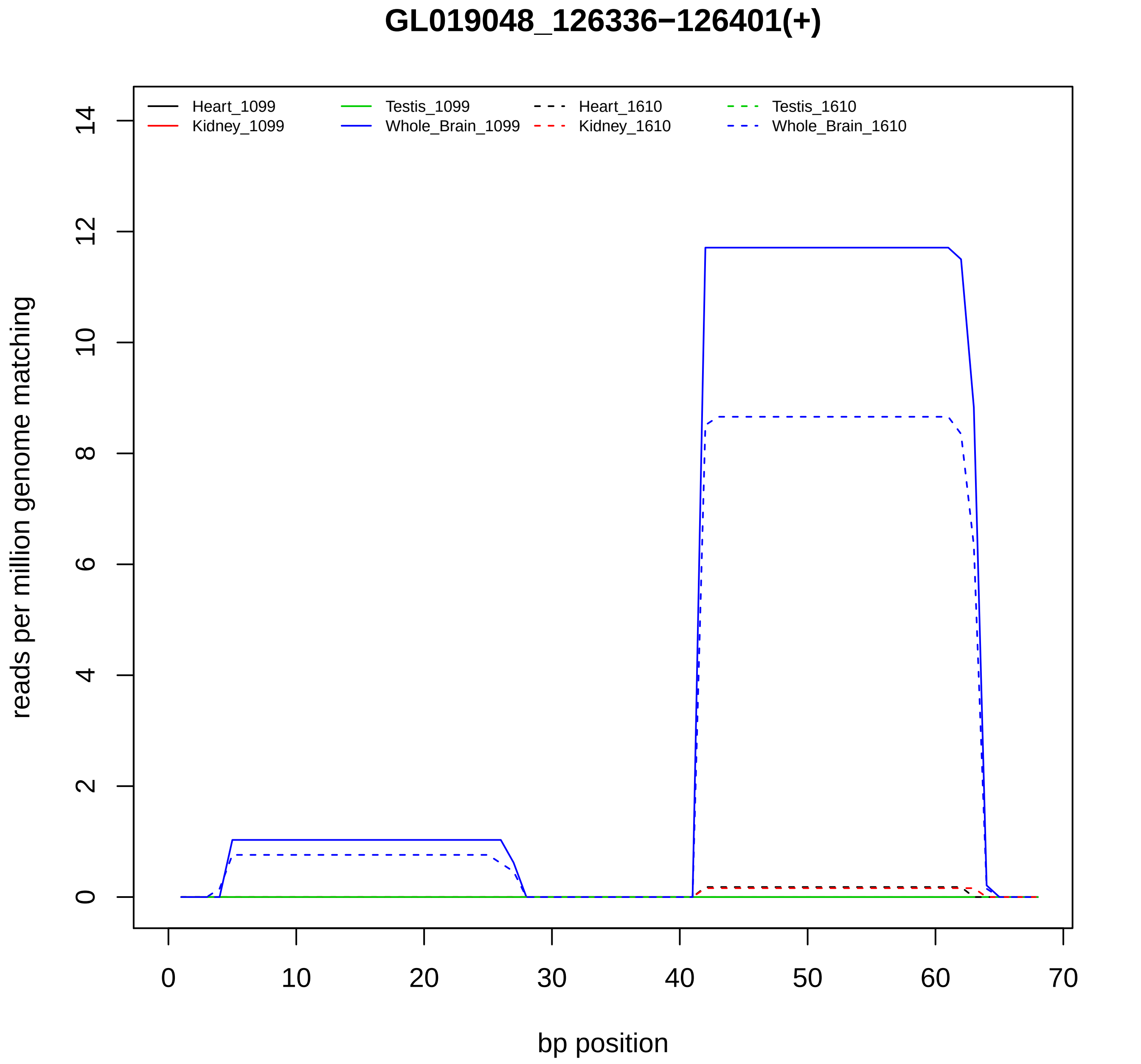
GL019048_126336−126401(+)
Heart_1099
Kidney_1099
Testis_1099
Whole_Brain_1099
Heart_1610
Kidney_1610
Testis_1610
Whole_Brain_1610
reads per million genome matching
bp position
0
2
4
6
8
10
12
14
0
10
20
30
40
50
60
70

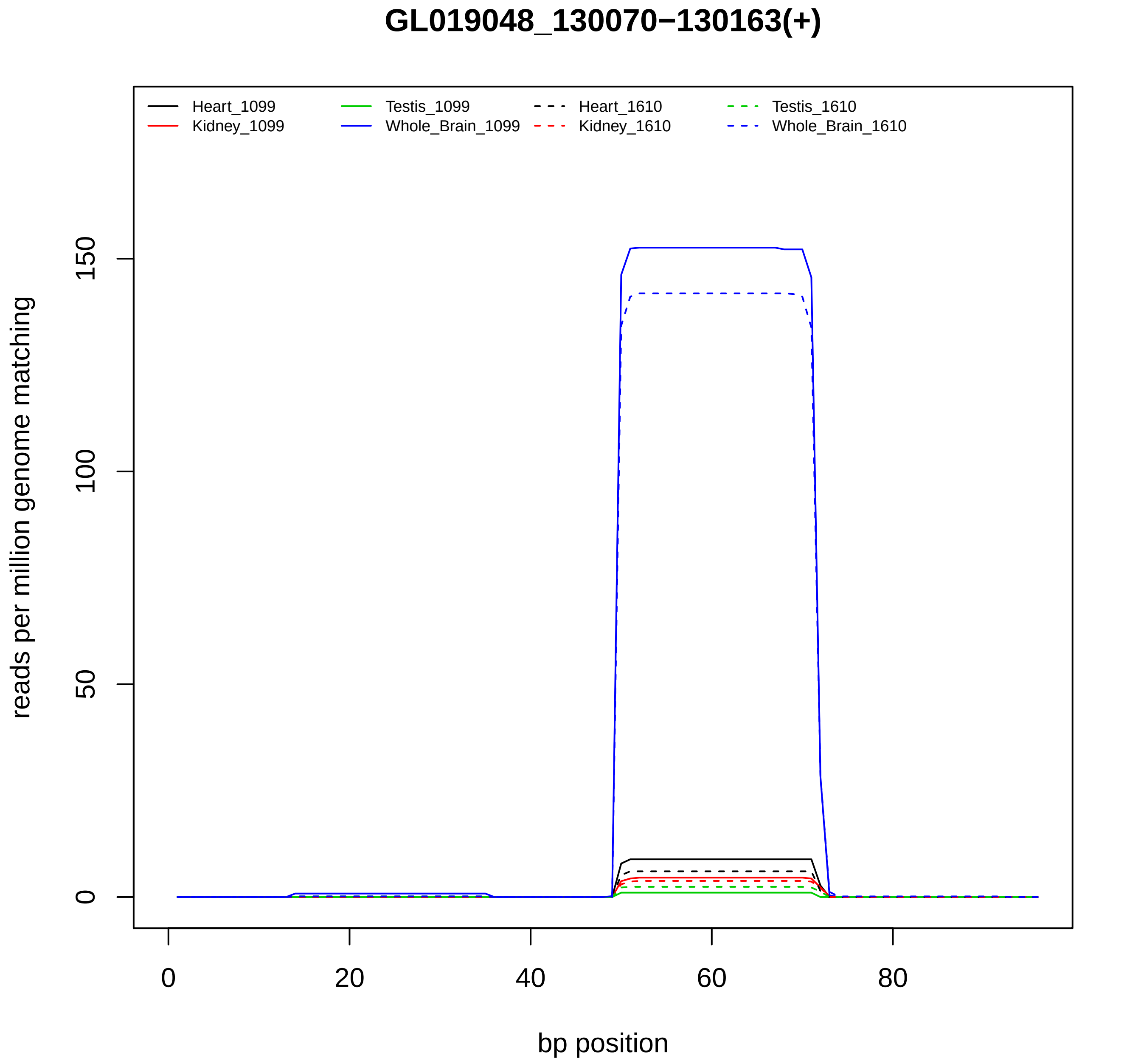

GL019048_130070−130163(+)
Heart_1099
Kidney_1099
Testis_1099
Whole_Brain_1099
Heart_1610
Kidney_1610
Testis_1610
Whole_Brain_1610
reads per million genome matching
0
50
100
150
0
20
40
60
80
bp position

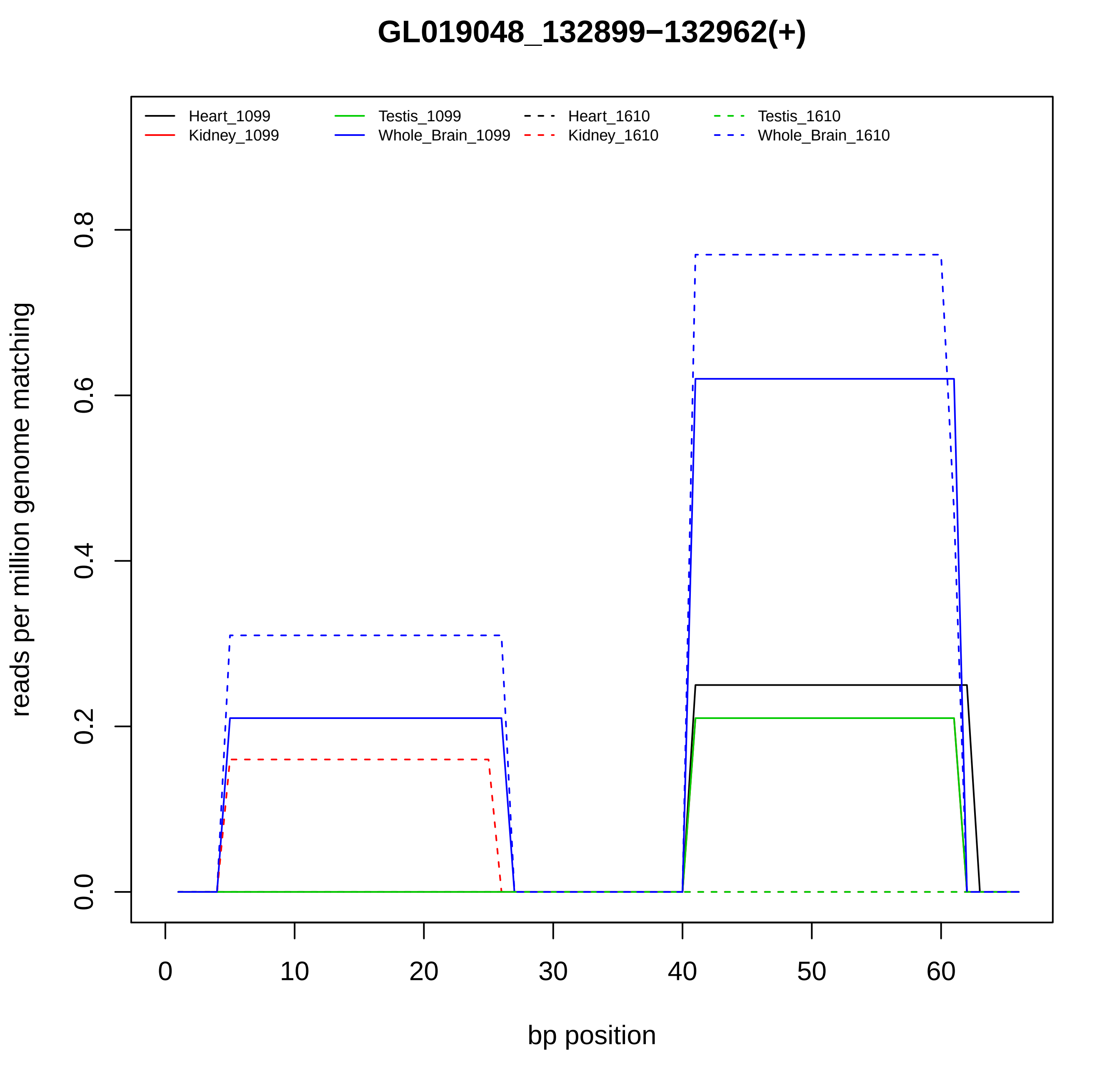

GL019048_132899−132962(+)
Heart_1099
Kidney_1099
Testis_1099
Whole_Brain_1099
Heart_1610
Kidney_1610
Testis_1610
Whole_Brain_1610
reads per million genome matching
0.0
0.2
0.4
0.6
0.8
0
10
20
30
40
50
60
bp position

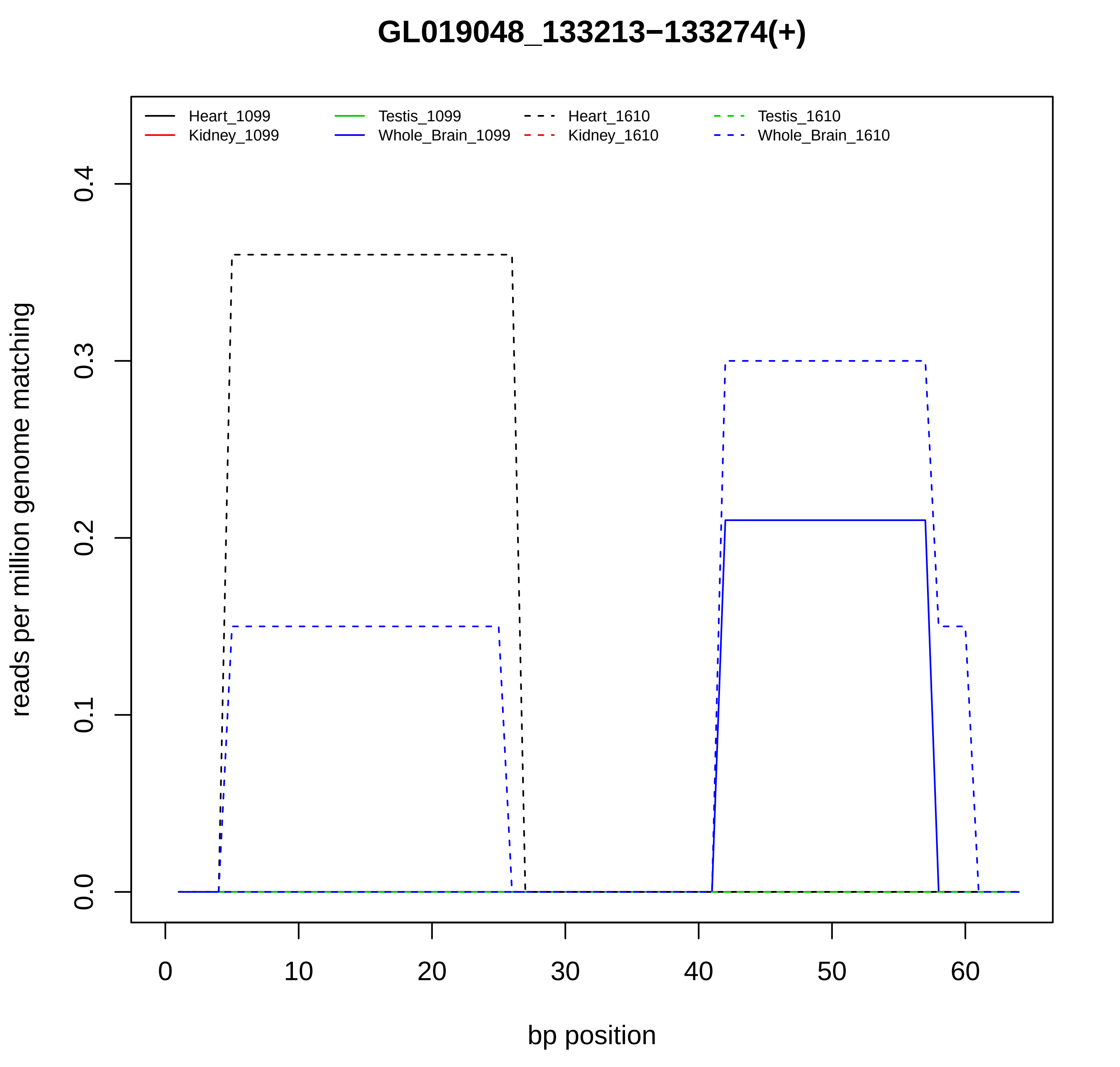

GL019048_133213−133274(+)
Heart_1099
Kidney_1099
Testis_1099
Whole_Brain_1099
Heart_1610
Kidney_1610
Testis_1610
Whole_Brain_1610
reads per million genome matching
0.0
0.1
0.2
0.3
0.4
0
10
20
30
40
50
60
bp position

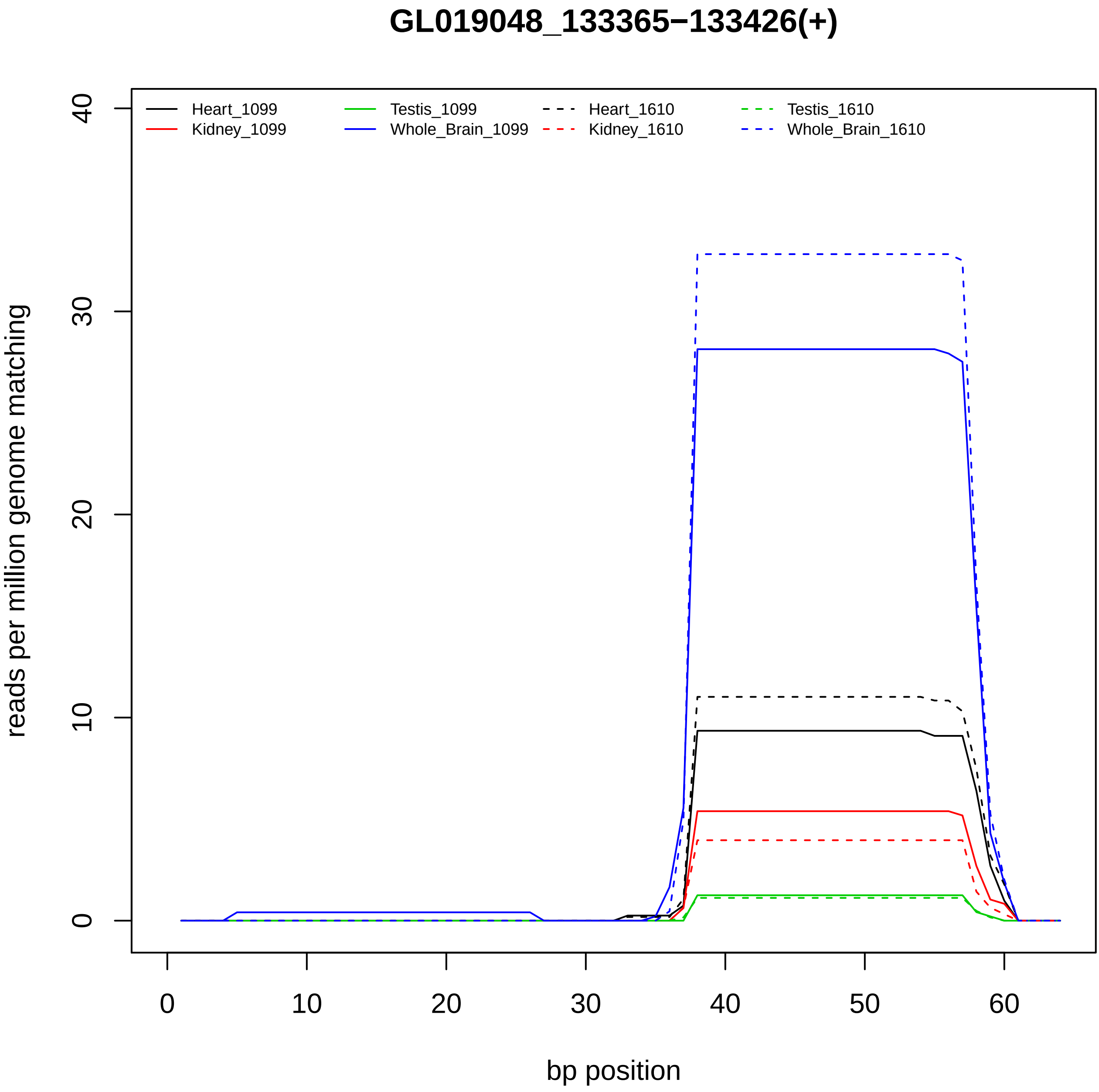

GL019048_133365−133426(+)
Heart_1099
Kidney_1099
Testis_1099
Whole_Brain_1099
Heart_1610
Kidney_1610
Testis_1610
Whole_Brain_1610
reads per million genome matching
bp position
0
10
20
30
40
50
60

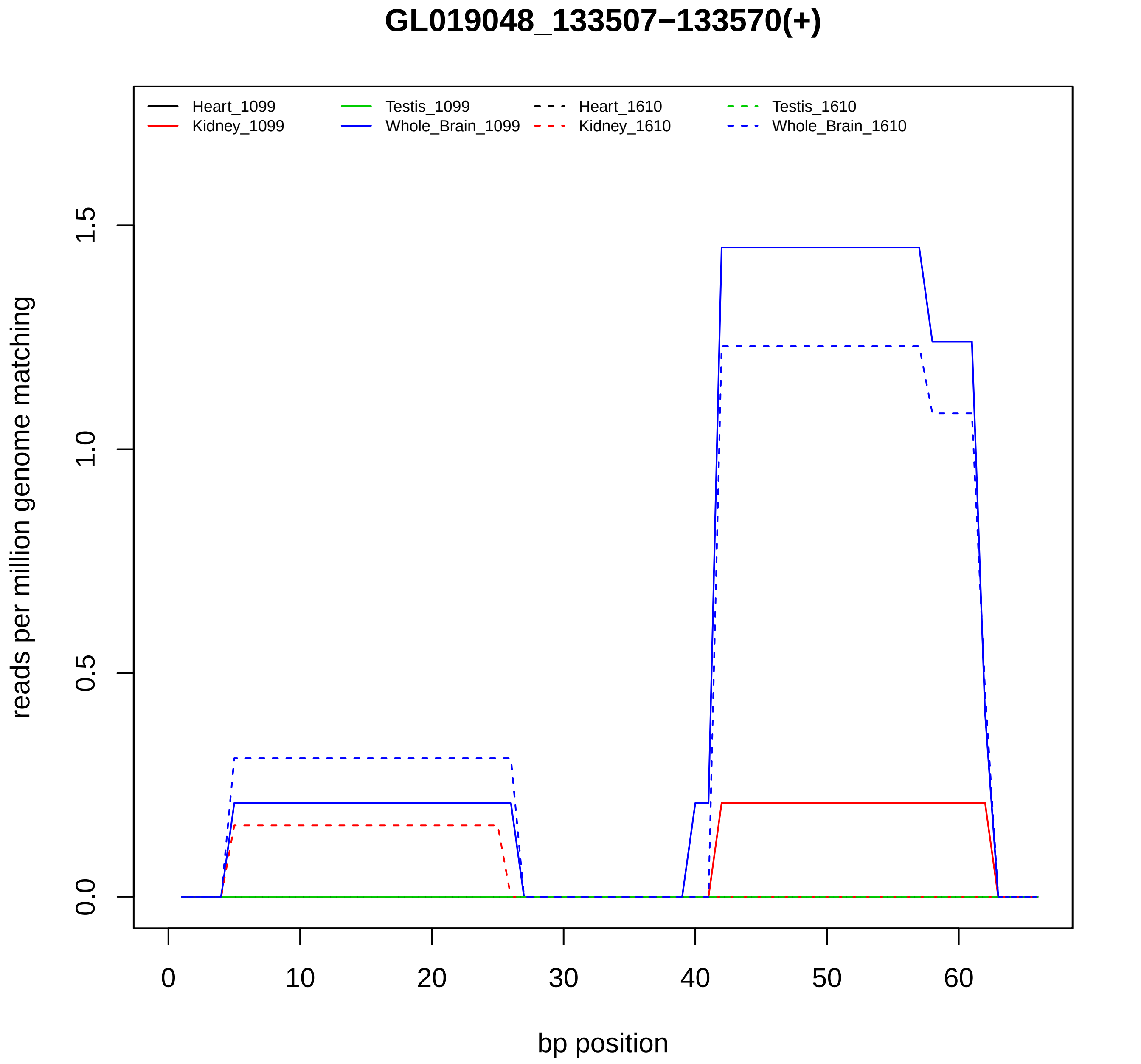

GL019048_133507−133570(+)
Heart_1099
Kidney_1099
Testis_1099
Whole_Brain_1099
Heart_1610
Kidney_1610
Testis_1610
Whole_Brain_1610
reads per million genome matching
0.0
0.5
1.0
1.5
0
10
20
30
40
50
60
bp position

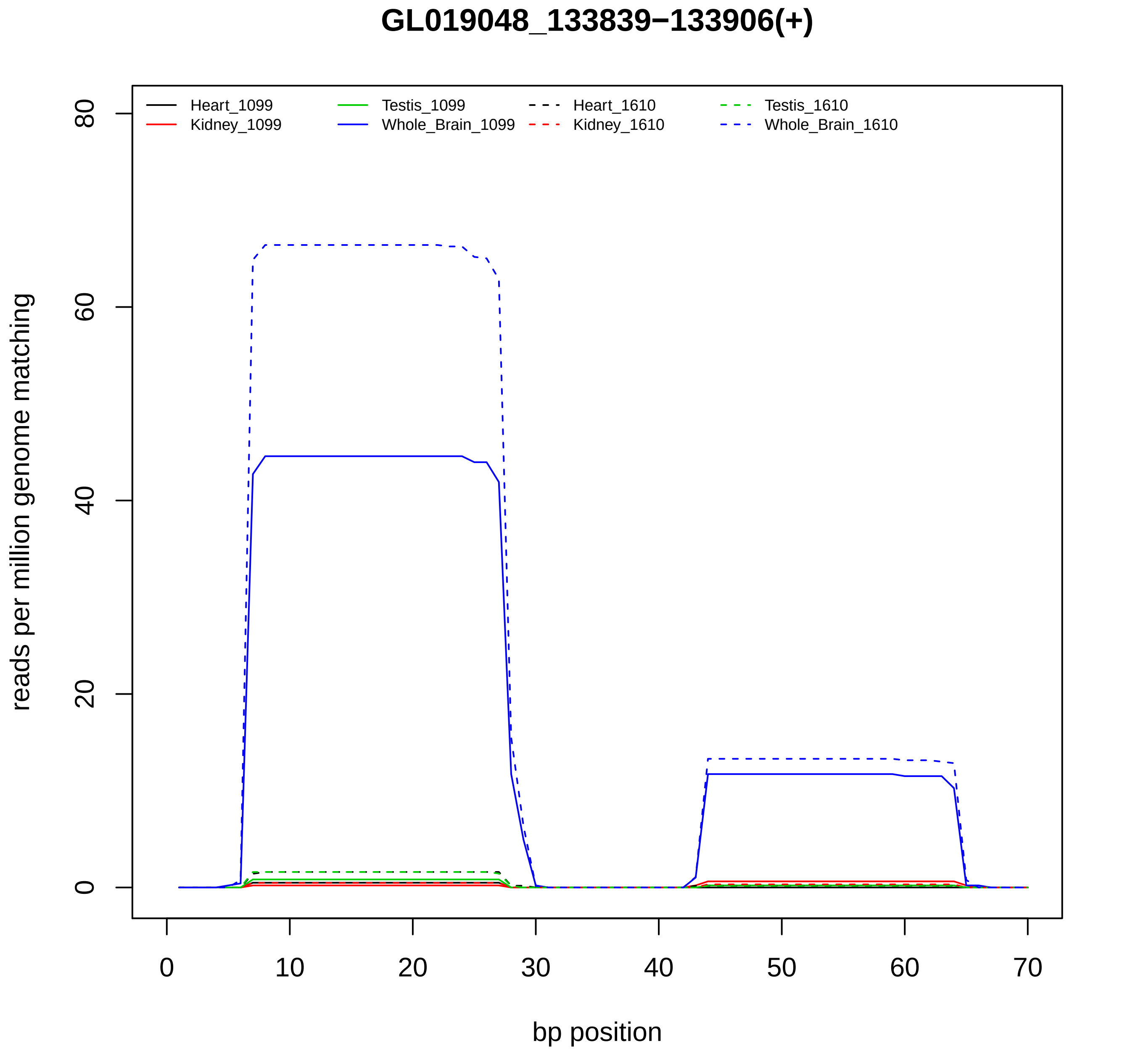

GL019048_133839−133906(+)
Heart_1099
Kidney_1099
Testis_1099
Whole_Brain_1099
Heart_1610
Kidney_1610
Testis_1610
Whole_Brain_1610
reads per million genome matching
0
20
40
60
80
0
10
20
30
40
50
60
70
bp position

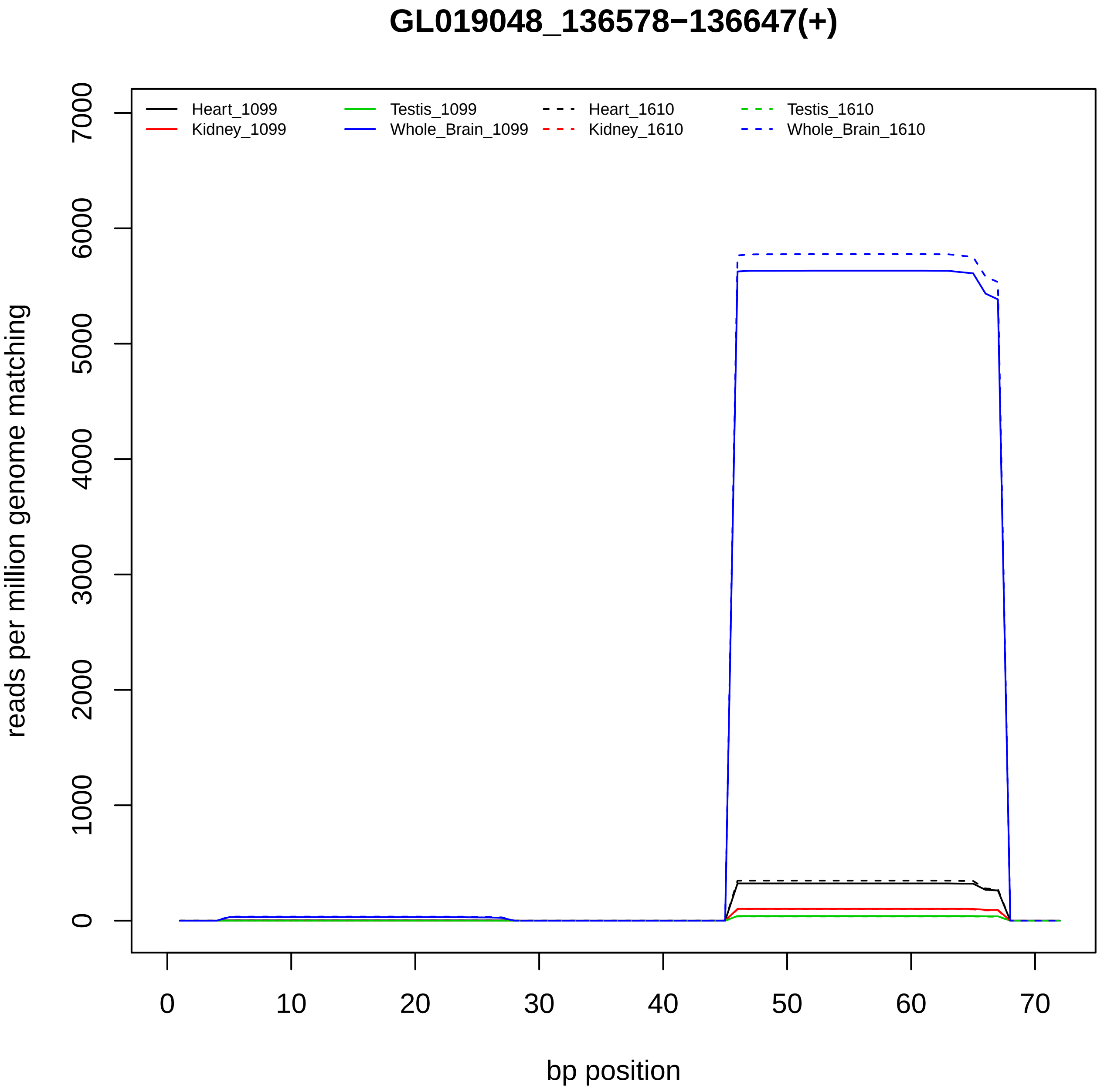

GL019048_136578−136647(+)
Heart_1099
Kidney_1099
Testis_1099
Whole_Brain_1099
Heart_1610
Kidney_1610
Testis_1610
Whole_Brain_1610
reads per million genome matching
0
1000
2000
3000
4000
5000
6000
7000
0
10
20
30
40
50
60
70
bp position

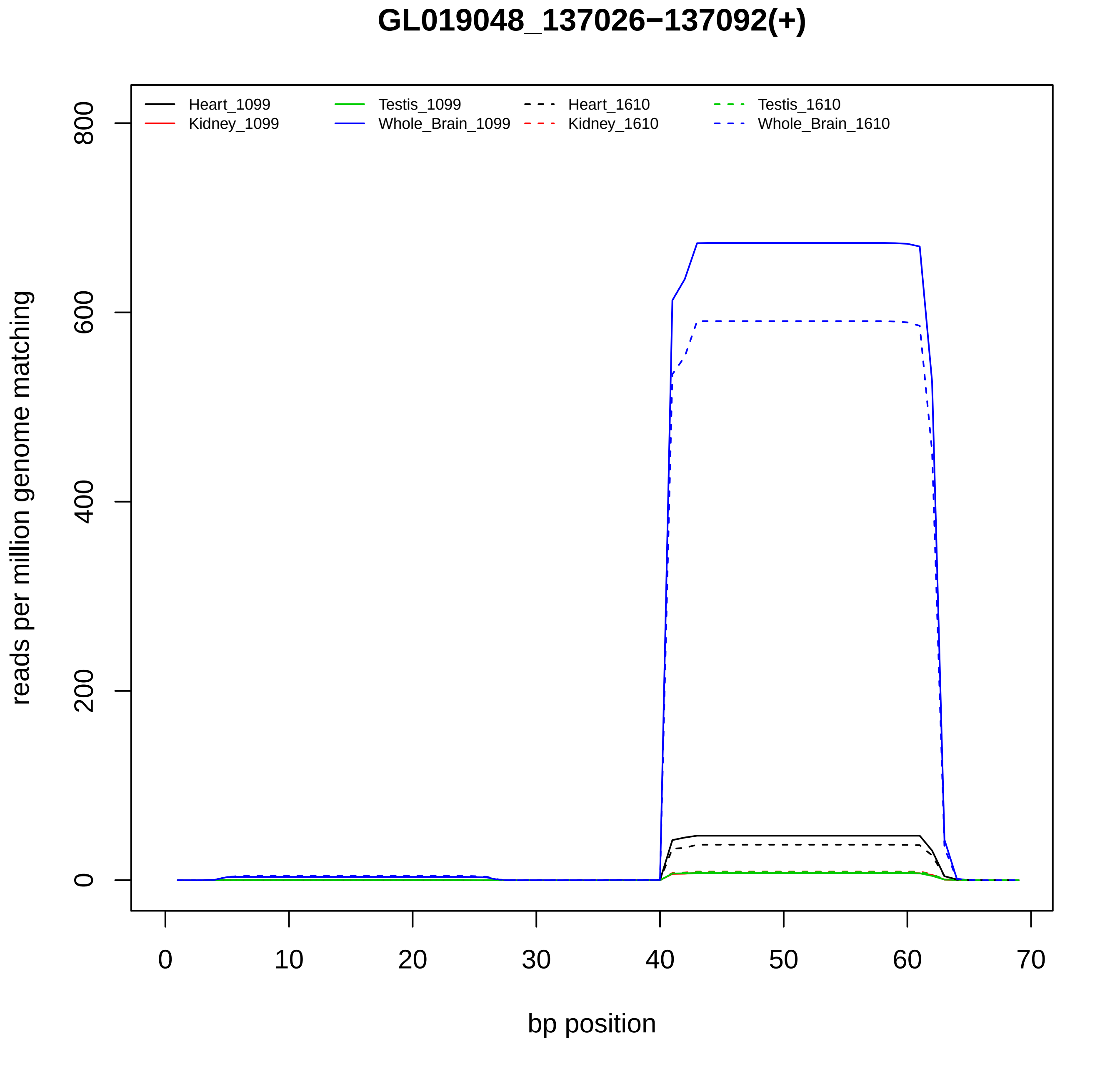

GL019048_137026−137092(+)
Heart_1099
Kidney_1099
Testis_1099
Whole_Brain_1099
Heart_1610
Kidney_1610
Testis_1610
Whole_Brain_1610
reads per million genome matching
0
200
400
600
800
bp position
0
10
20
30
40
50
60
70

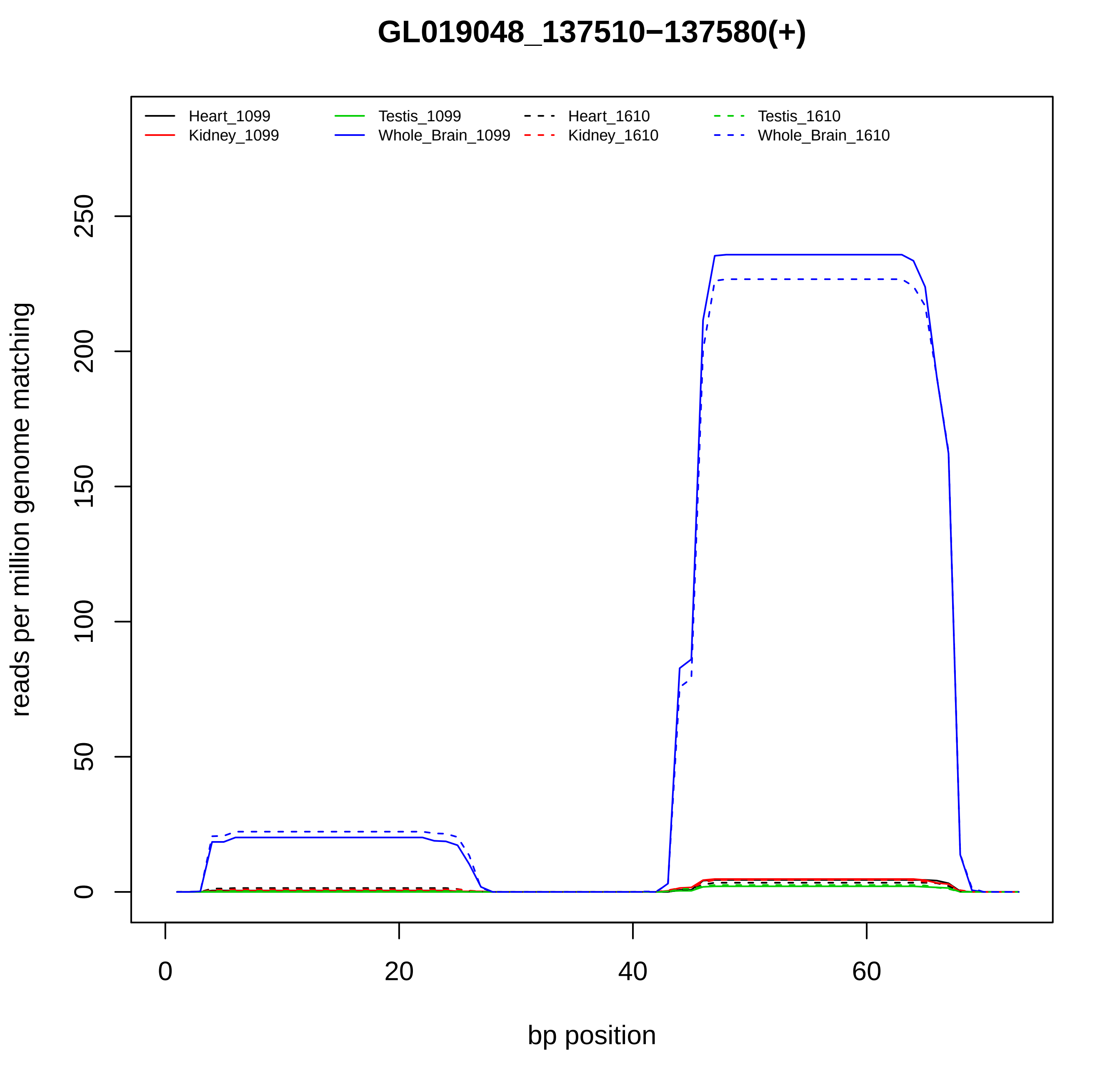

GL019048_137510−137580(+)
Heart_1099
Kidney_1099
Testis_1099
Whole_Brain_1099
Heart_1610
Kidney_1610
Testis_1610
Whole_Brain_1610
reads per million genome matching
0
50
100
150
200
250
0
20
40
60
bp position

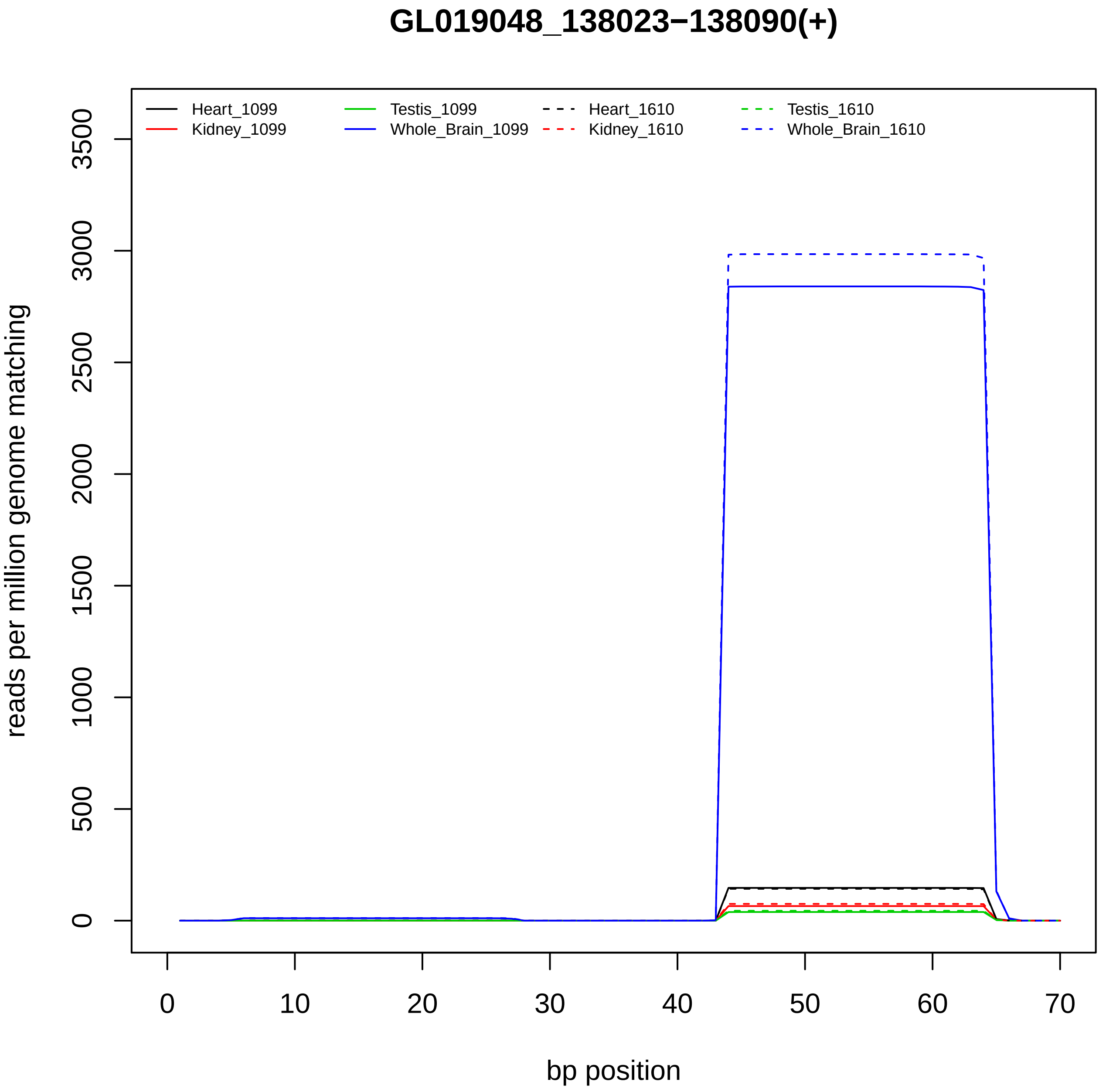

GL019048_138023−138090(+)
Heart_1099
Kidney_1099
Testis_1099
Whole_Brain_1099
Heart_1610
Kidney_1610
Testis_1610
Whole_Brain_1610
reads per million genome matching
0
500
1000
1500
2000
2500
3000
3500
bp position
0
10
20
30
40
50
60
70

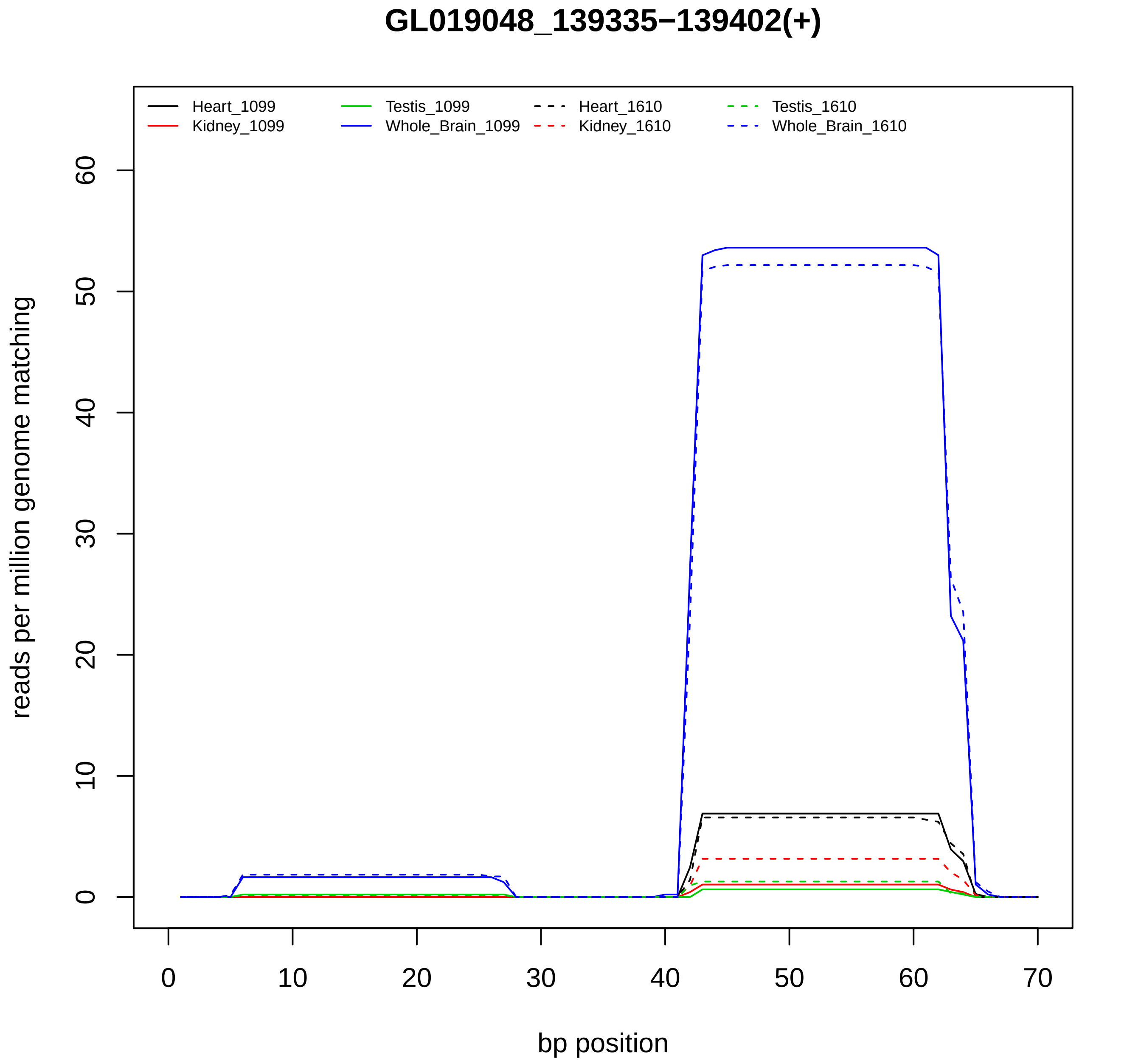

GL019048_139335−139402(+)
Heart_1099
Kidney_1099
Testis_1099
Whole_Brain_1099
Heart_1610
Kidney_1610
Testis_1610
Whole_Brain_1610
reads per million genome matching
bp position
0
10
20
30
40
50
60
70

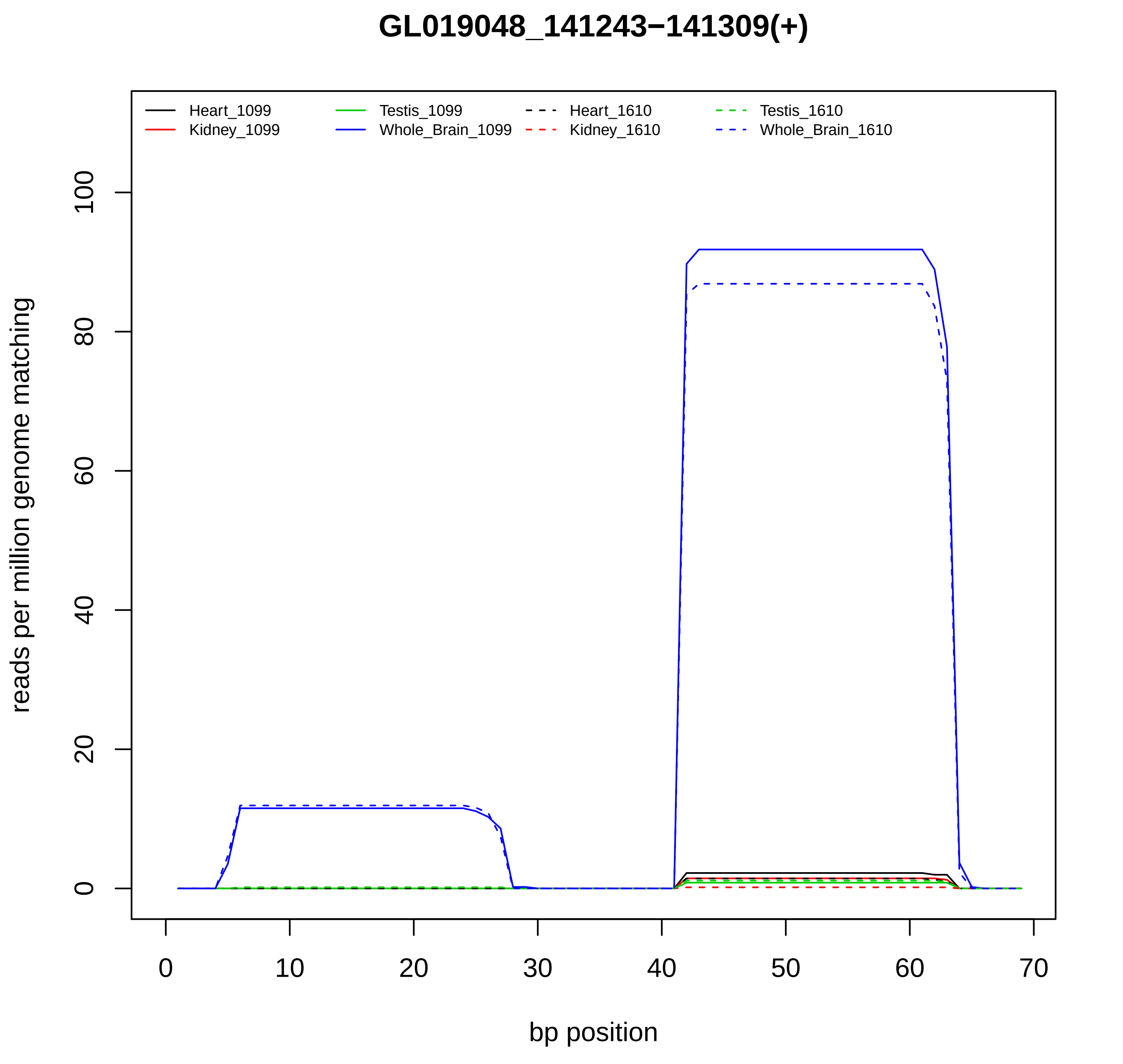

GL019048_141243−141309(+)
Heart_1099
Kidney_1099
Testis_1099
Whole_Brain_1099
Heart_1610
Kidney_1610
Testis_1610
Whole_Brain_1610
reads per million genome matching
0
20
40
60
80
100
0
10
20
30
40
50
60
70
bp position

GL019048_142716−142778(+)

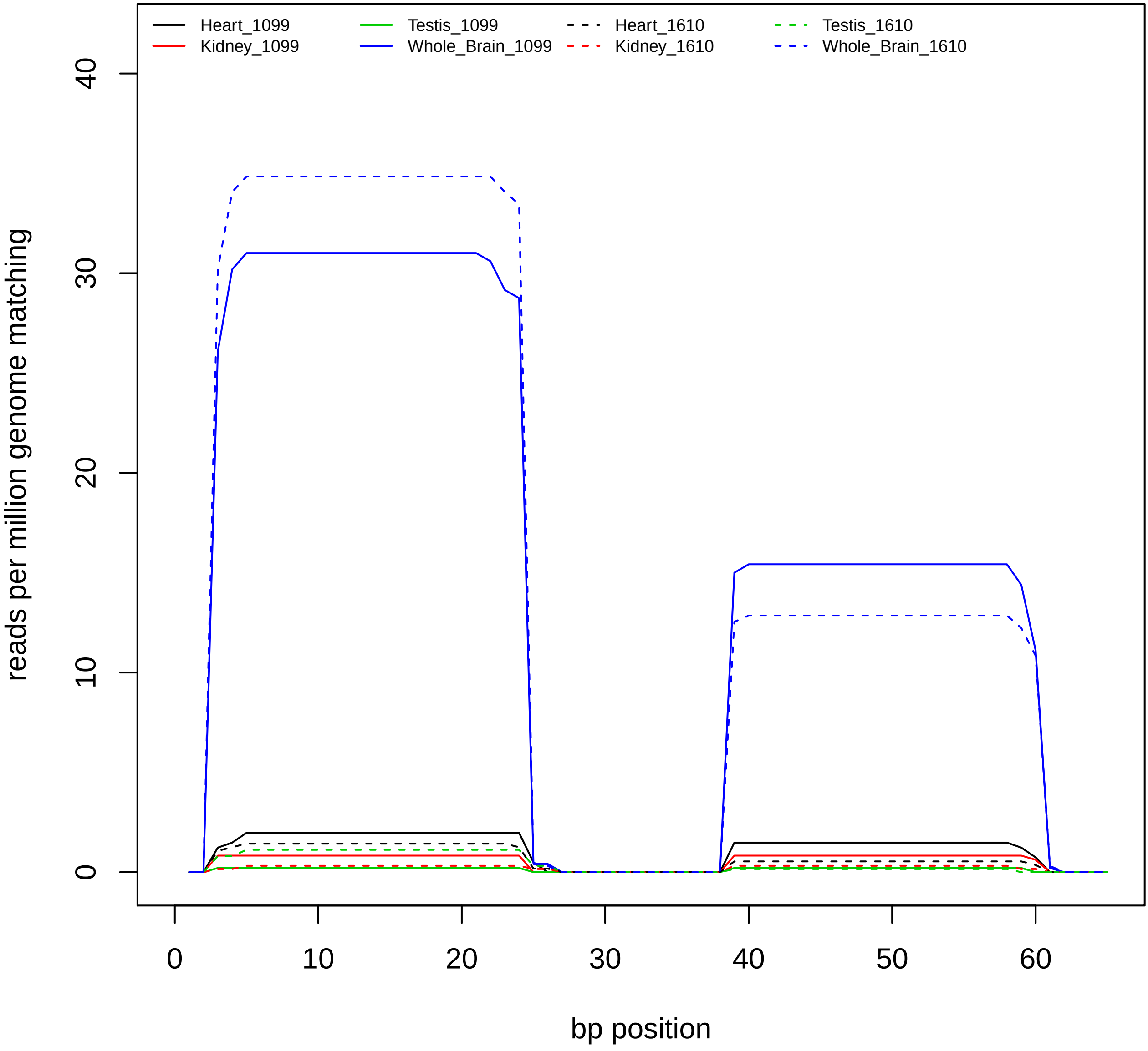

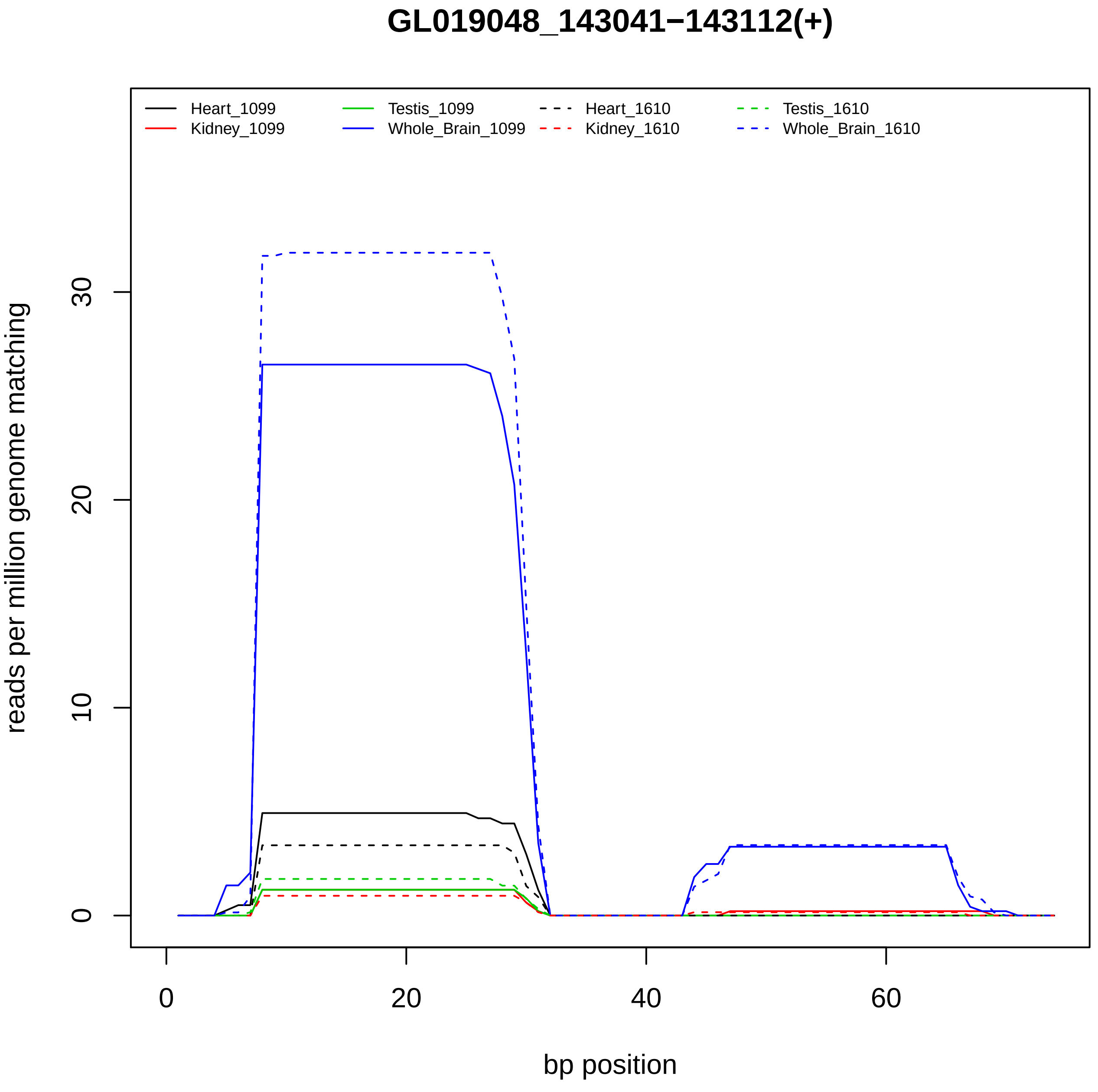

GL019048_143041−143112(+)
Heart_1099
Kidney_1099
Testis_1099
Whole_Brain_1099
Heart_1610
Kidney_1610
Testis_1610
Whole_Brain_1610
reads per million genome matching
0
10
20
30
bp position
0
20
40
60

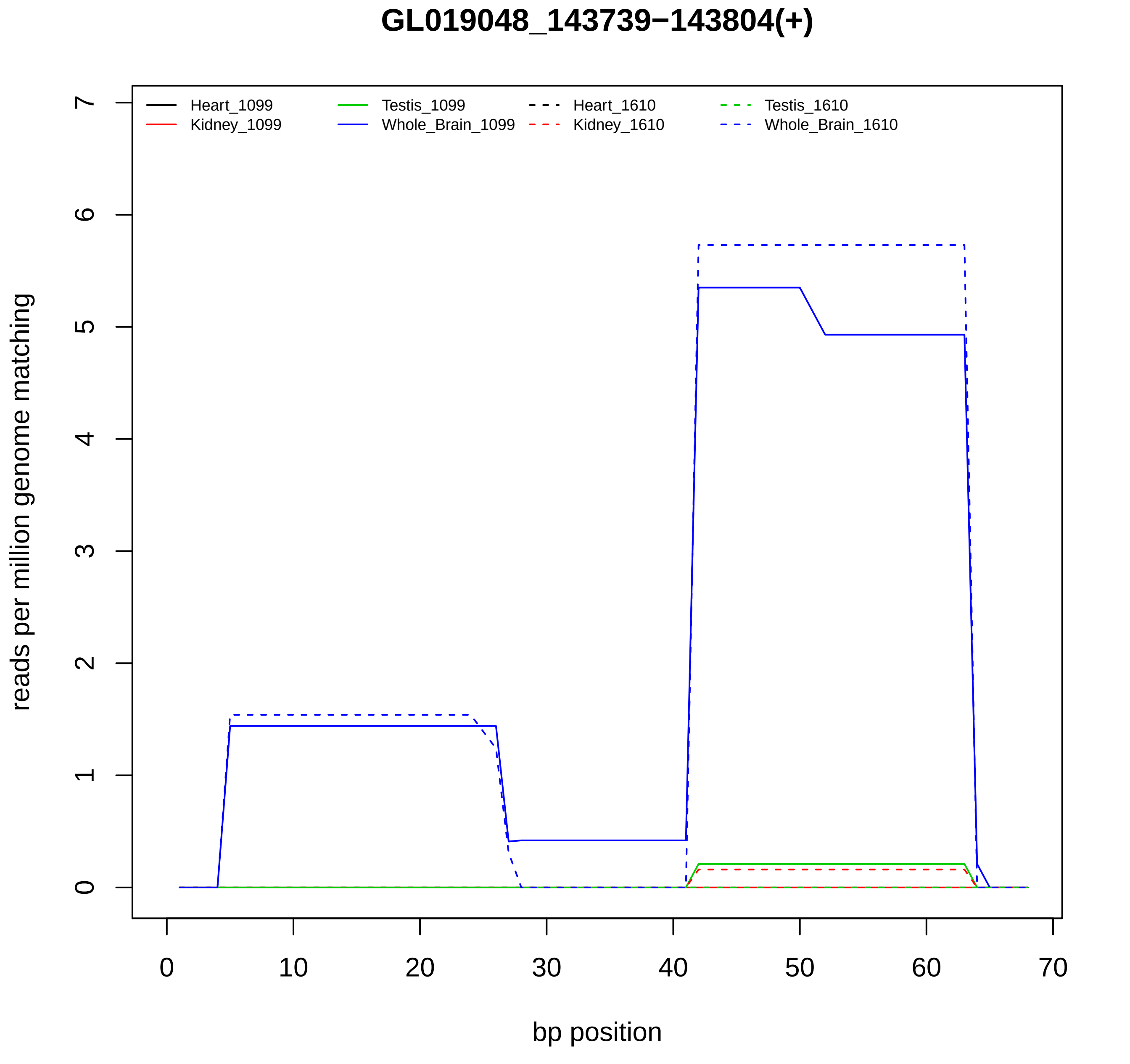

GL019048_143739−143804(+)
Heart_1099
Kidney_1099
Testis_1099
Whole_Brain_1099
Heart_1610
Kidney_1610
Testis_1610
Whole_Brain_1610
reads per million genome matching
bp position
0
1
2
3
4
5
6
7
0
10
20
30
40
50
60
70

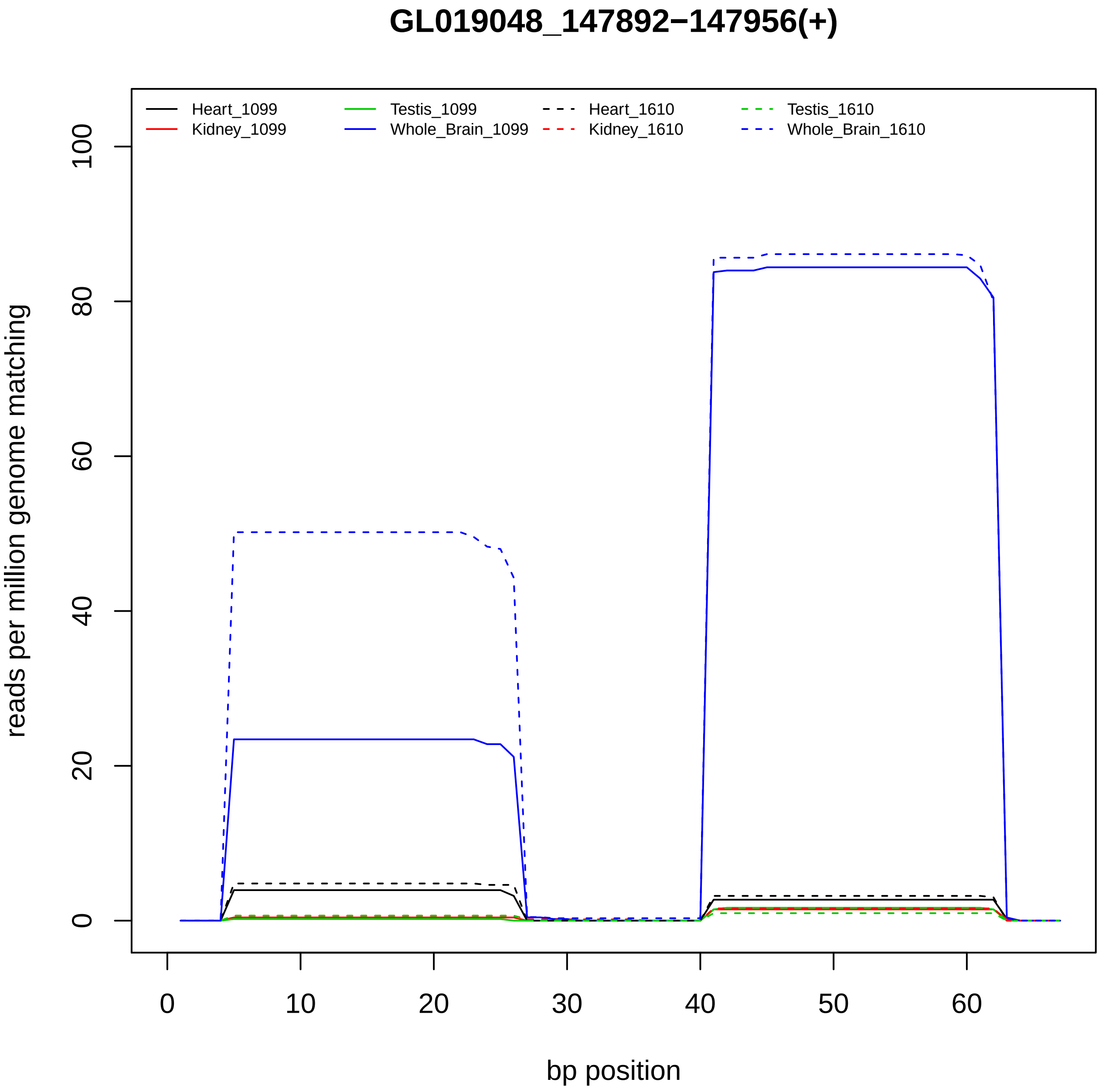

GL019048_147892−147956(+)
Heart_1099
Kidney_1099
Testis_1099
Whole_Brain_1099
Heart_1610
Kidney_1610
Testis_1610
Whole_Brain_1610
reads per million genome matching
0
20
40
60
80
100
0
10
20
30
40
50
60
bp position

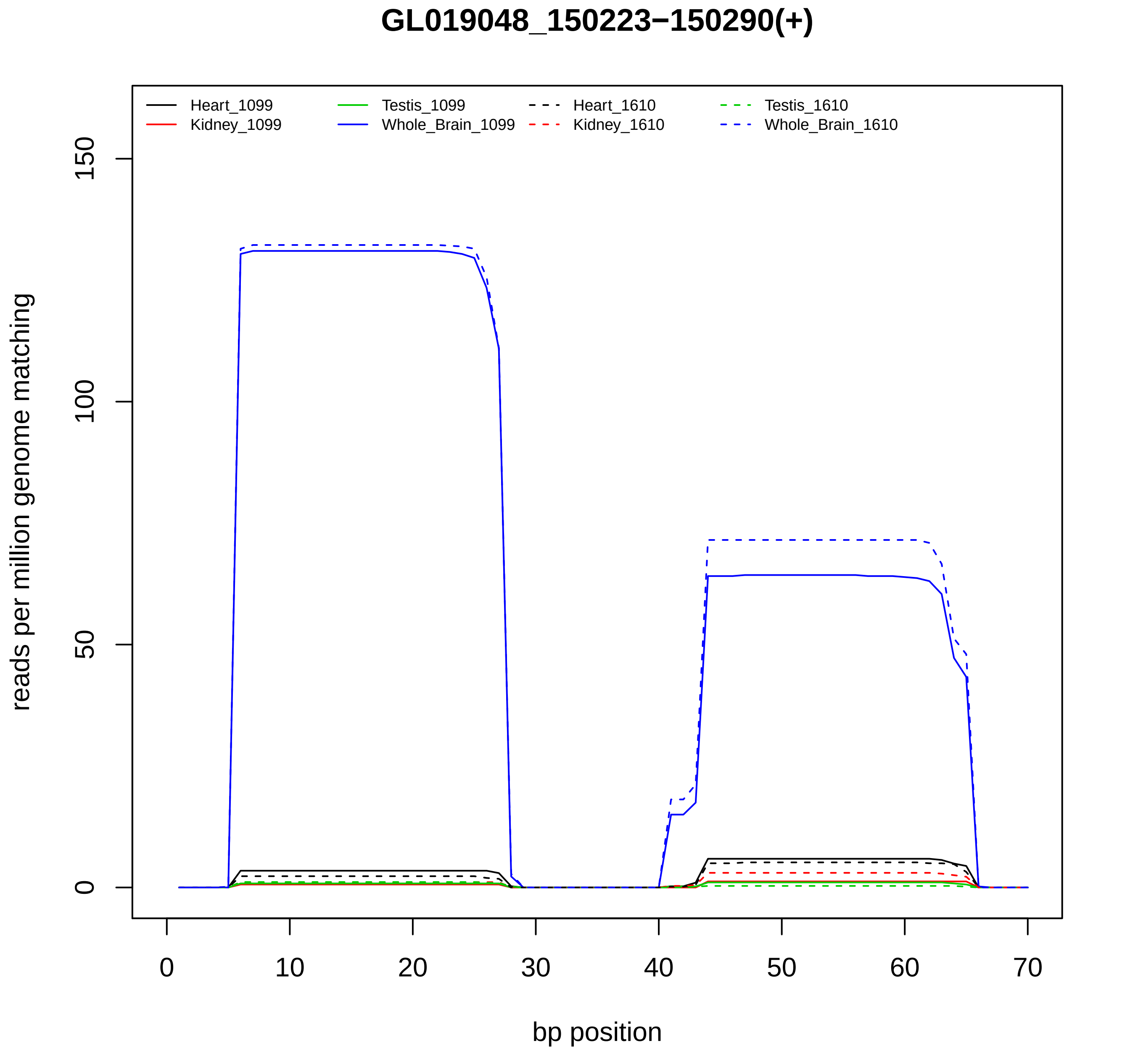

GL019048_150223−150290(+)
Heart_1099
Kidney_1099
Testis_1099
Whole_Brain_1099
Heart_1610
Kidney_1610
Testis_1610
Whole_Brain_1610
reads per million genome matching
0
50
100
150
0
10
20
30
40
50
60
70
bp position

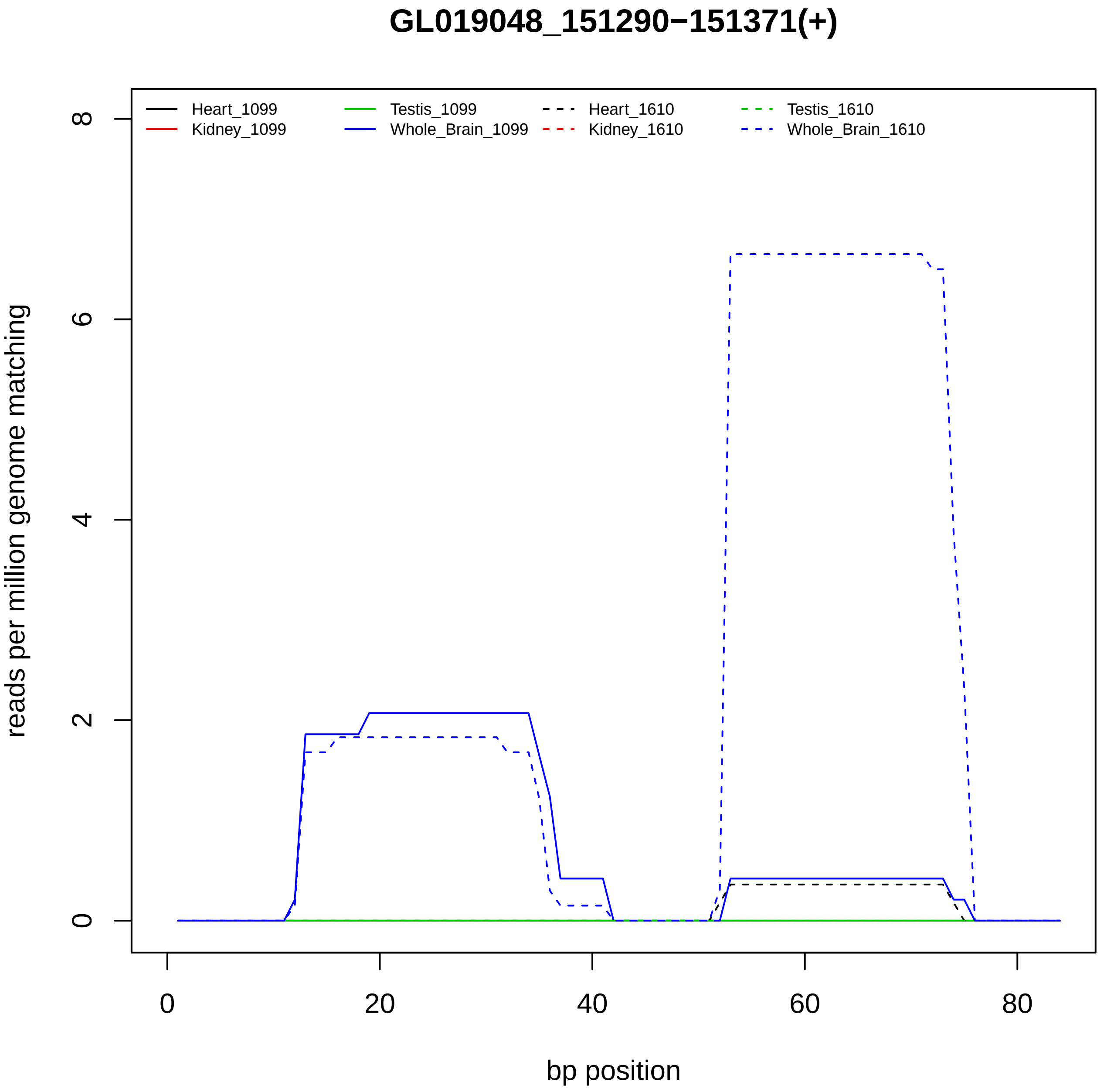

GL019048_151290−151371(+)
Heart_1099
Kidney_1099
Testis_1099
Whole_Brain_1099
Heart_1610
Kidney_1610
Testis_1610
Whole_Brain_1610
reads per million genome matching
bp position
0
2
4
6
8
0
20
40
60
80

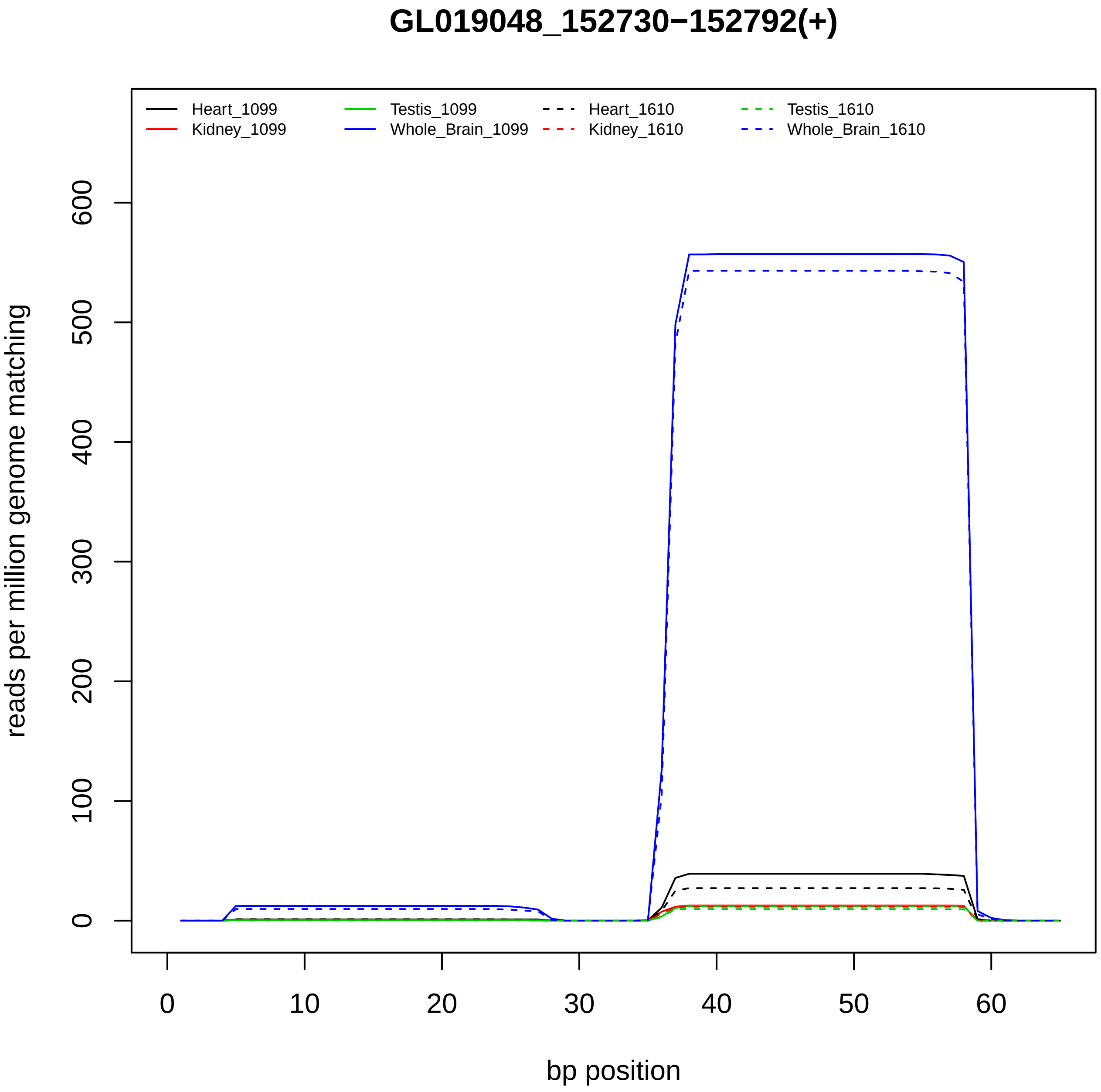

GL019048_152730−152792(+)
Heart_1099
Kidney_1099
Testis_1099
Whole_Brain_1099
Heart_1610
Kidney_1610
Testis_1610
Whole_Brain_1610
reads per million genome matching
0
100
200
300
400
500
600
bp position
0
10
20
30
40
50
60

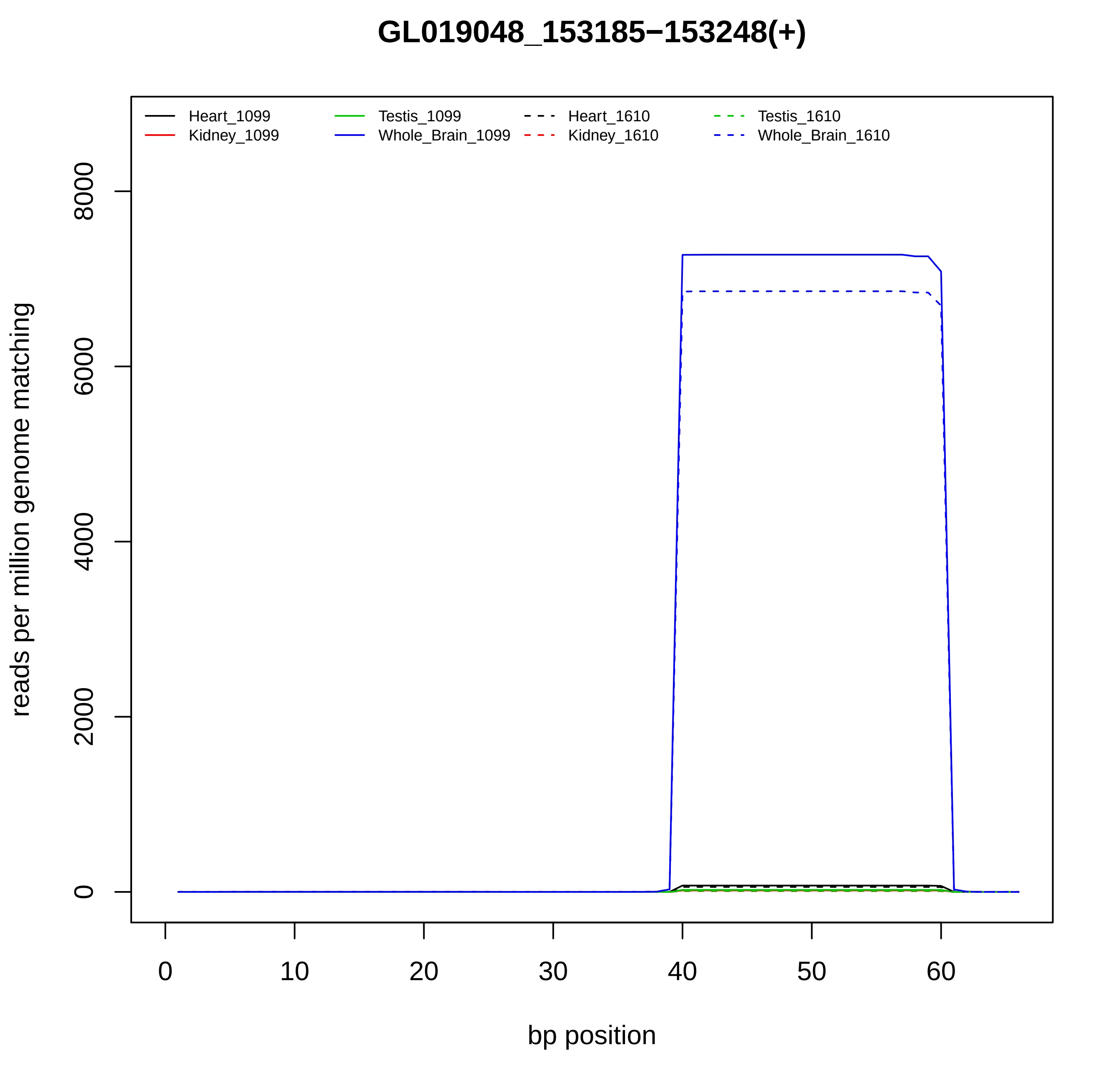

GL019048_153185−153248(+)
Heart_1099
Kidney_1099
Testis_1099
Whole_Brain_1099
Heart_1610
Kidney_1610
Testis_1610
Whole_Brain_1610
reads per million genome matching
0
2000
4000
6000
8000
bp position
0
10
20
30
40
50
60

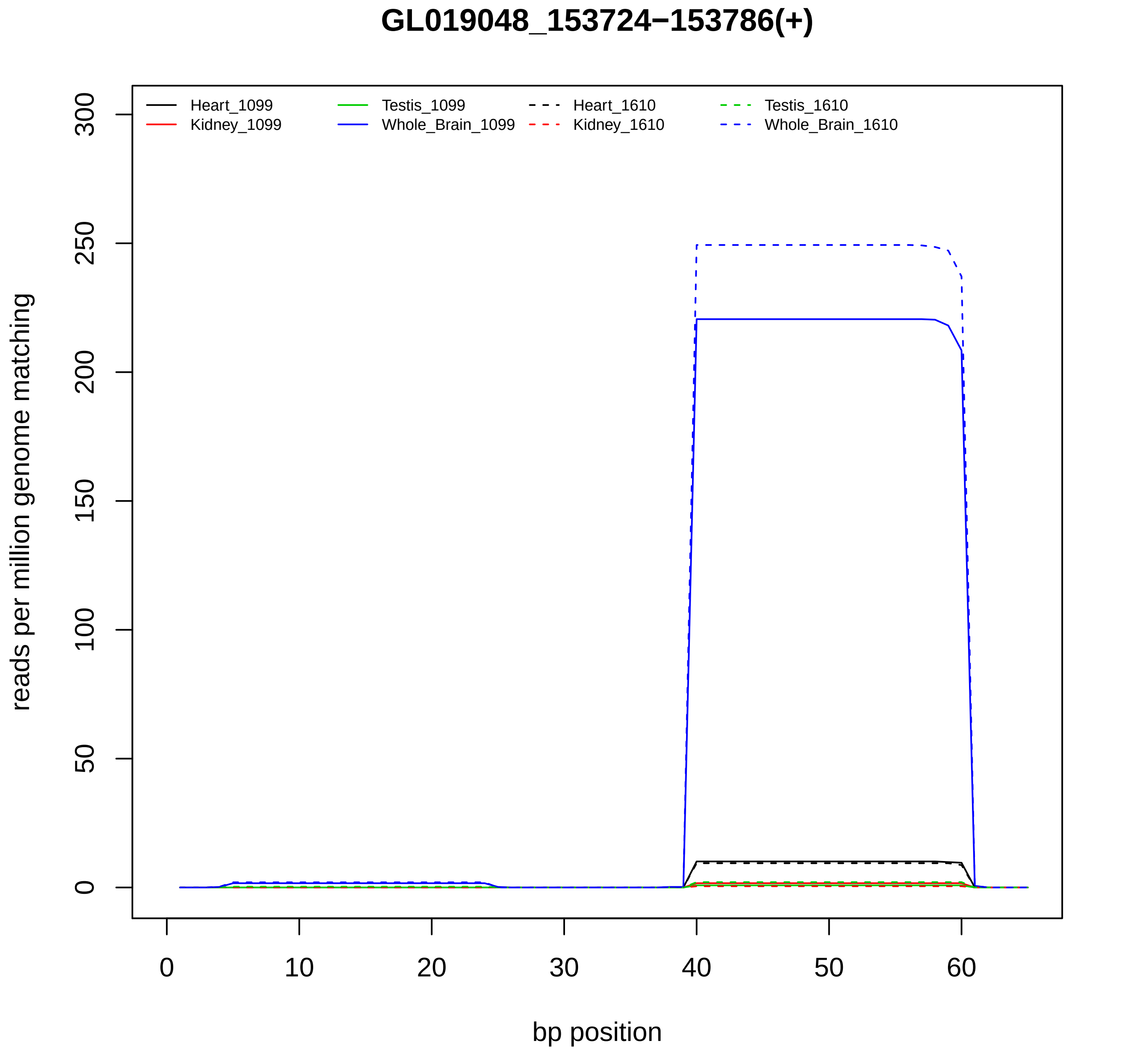

GL019048_153724−153786(+)
Heart_1099
Kidney_1099
Testis_1099
Whole_Brain_1099
Heart_1610
Kidney_1610
Testis_1610
Whole_Brain_1610
reads per million genome matching
bp position
0
50
100
150
200
250
300
0
10
20
30
40
50
60

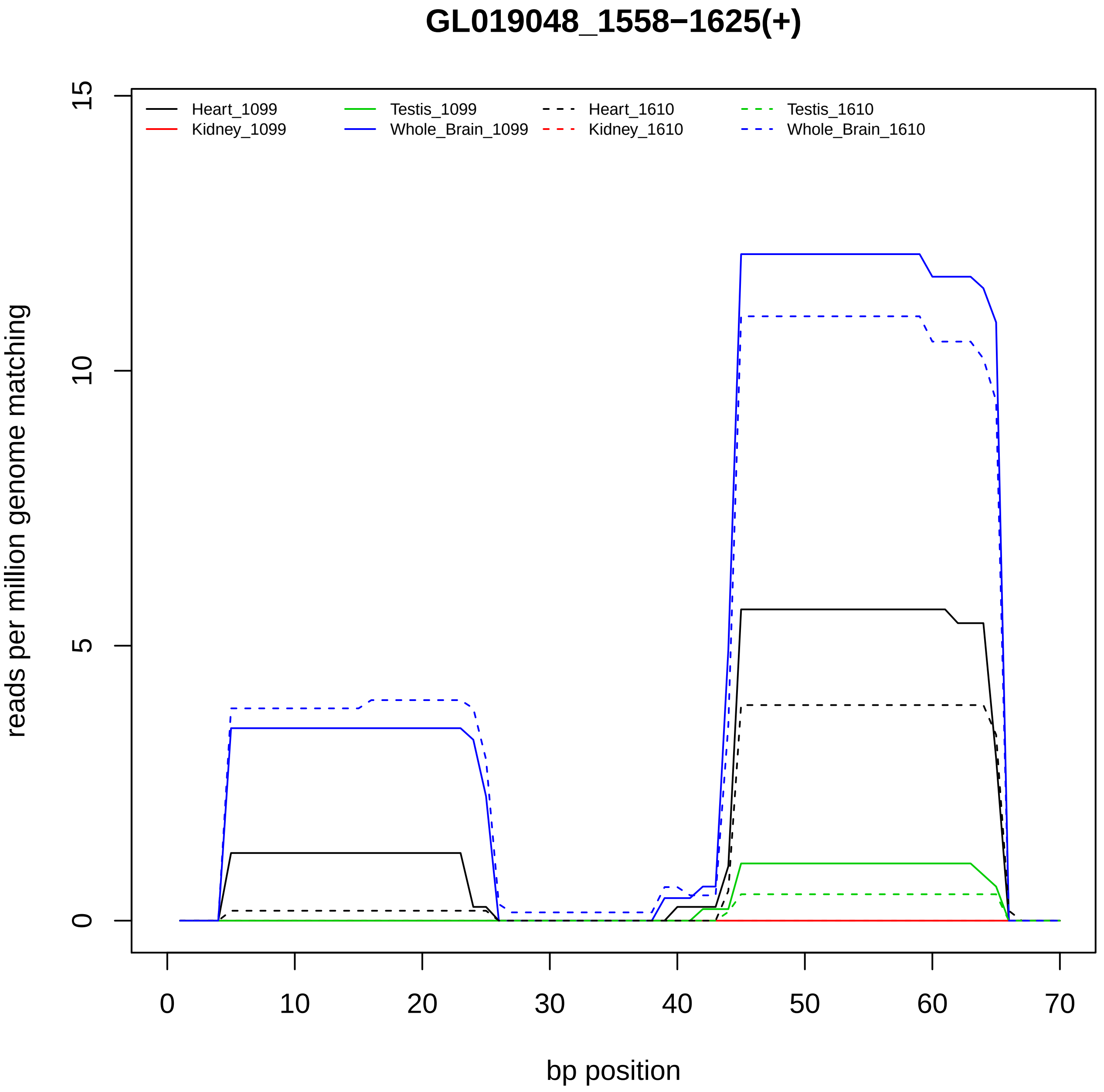
GL019048_1558−1625(+)
Heart_1099
Kidney_1099
Testis_1099
Whole_Brain_1099
Heart_1610
Kidney_1610
Testis_1610
Whole_Brain_1610
reads per million genome matching
0
5
10
15
0
10
20
30
40
50
60
70
bp position

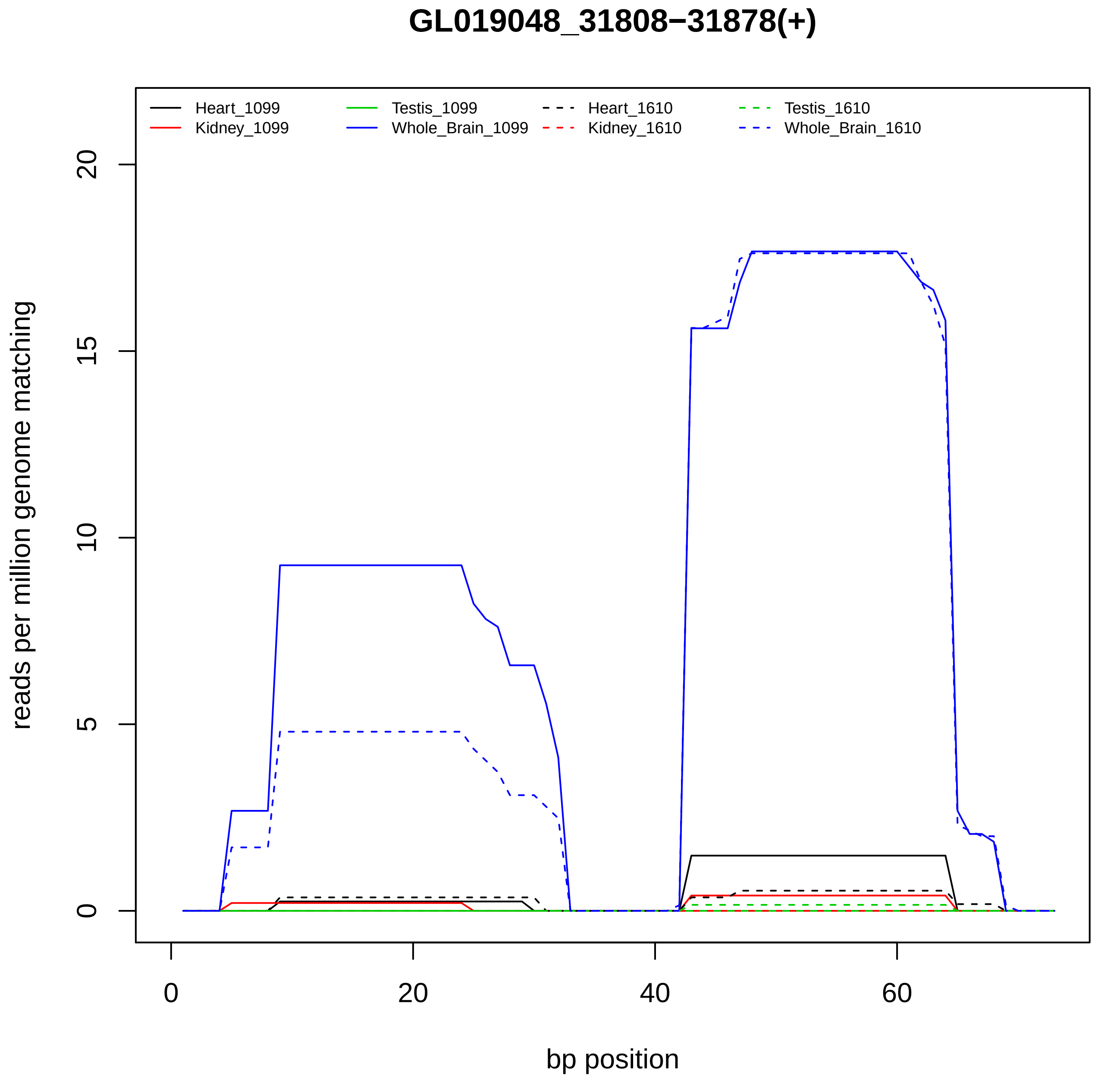

GL019048_31808−31878(+)
Heart_1099
Kidney_1099
Testis_1099
Whole_Brain_1099
Heart_1610
Kidney_1610
Testis_1610
Whole_Brain_1610
reads per million genome matching
0
5
10
15
20
0
20
40
60
bp position

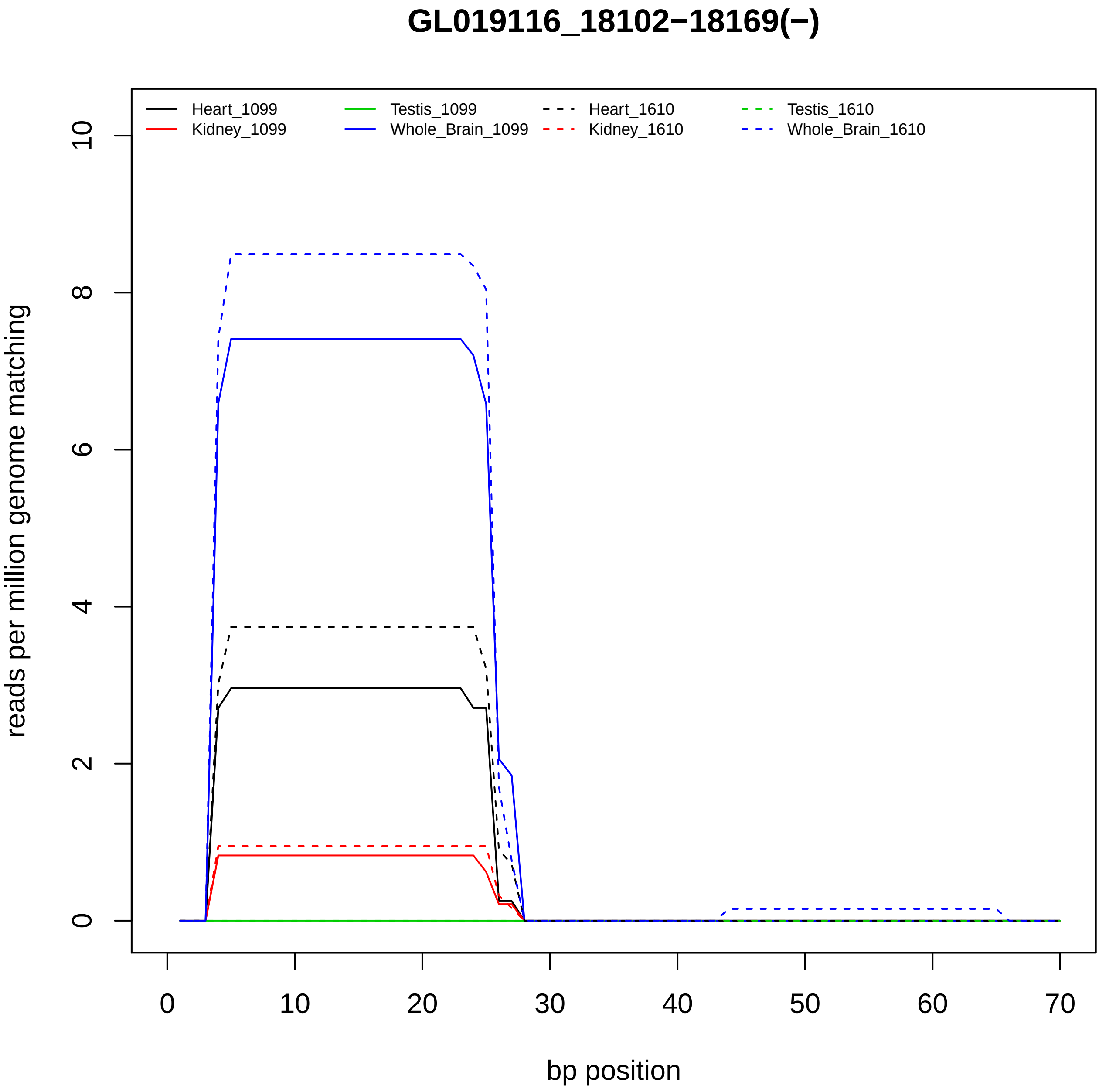

GL019116_18102−18169(−)
Heart_1099
Kidney_1099
Testis_1099
Whole_Brain_1099
Heart_1610
Kidney_1610
Testis_1610
Whole_Brain_1610
reads per million genome matching
0
2
4
6
8
10
0
10
20
30
40
50
60
70
bp position

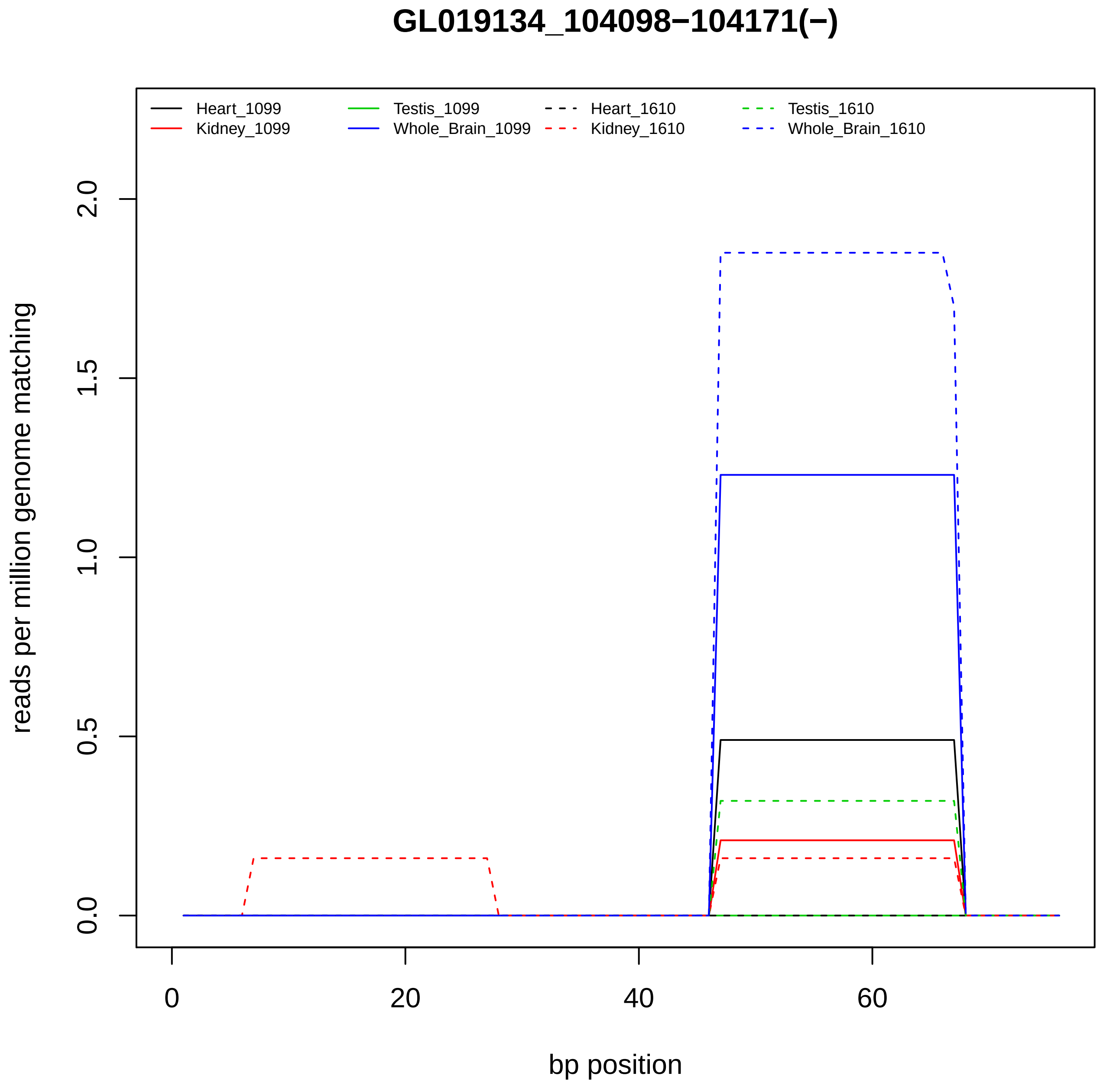
GL019134_104098−104171(−)
Heart_1099
Kidney_1099
Testis_1099
Whole_Brain_1099
Heart_1610
Kidney_1610
Testis_1610
Whole_Brain_1610
reads per million genome matching
0.0
0.5
1.0
1.5
2.0
0
20
40
60
bp position

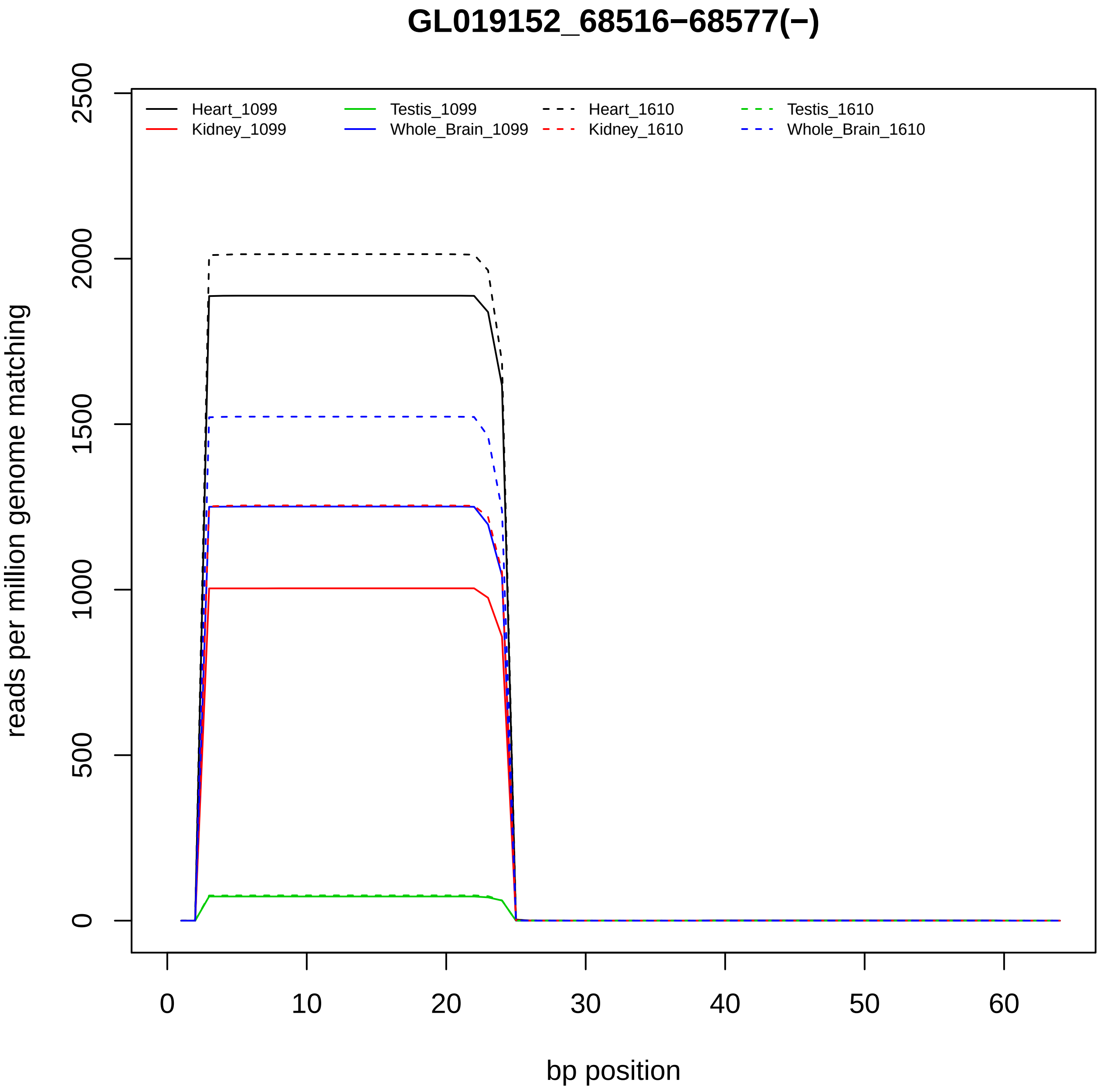

GL019152_68516−68577(−)
Heart_1099
Kidney_1099
Testis_1099
Whole_Brain_1099
Heart_1610
Kidney_1610
Testis_1610
Whole_Brain_1610
reads per million genome matching
bp position
0
500
1000
1500
2000
2500
0
10
20
30
40
50
60

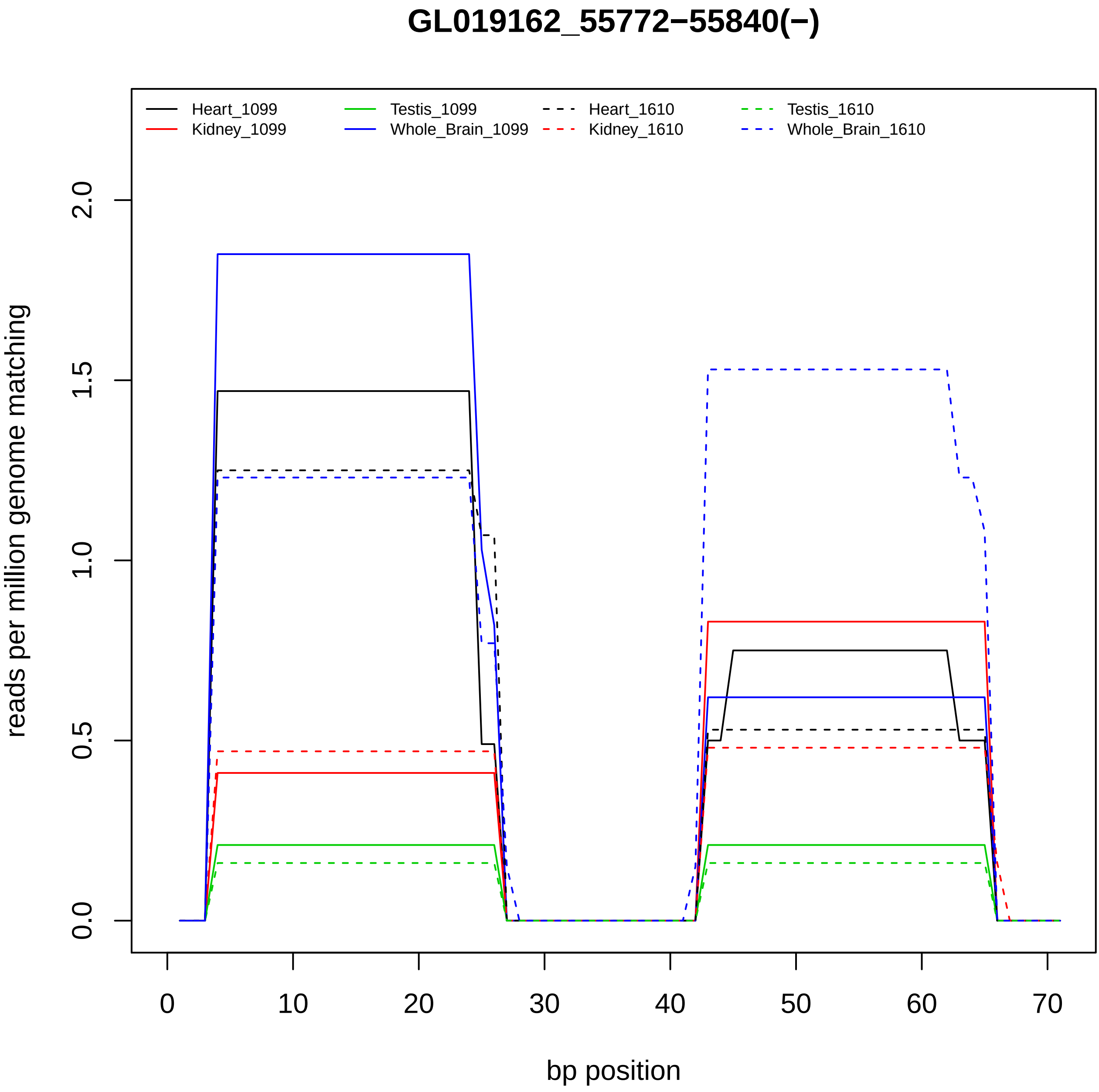
GL019162_55772−55840(−)
Heart_1099
Kidney_1099
Testis_1099
Whole_Brain_1099
Heart_1610
Kidney_1610
Testis_1610
Whole_Brain_1610
reads per million genome matching
0.0
0.5
1.0
1.5
2.0
bp position
0
10
20
30
40
50
60
70

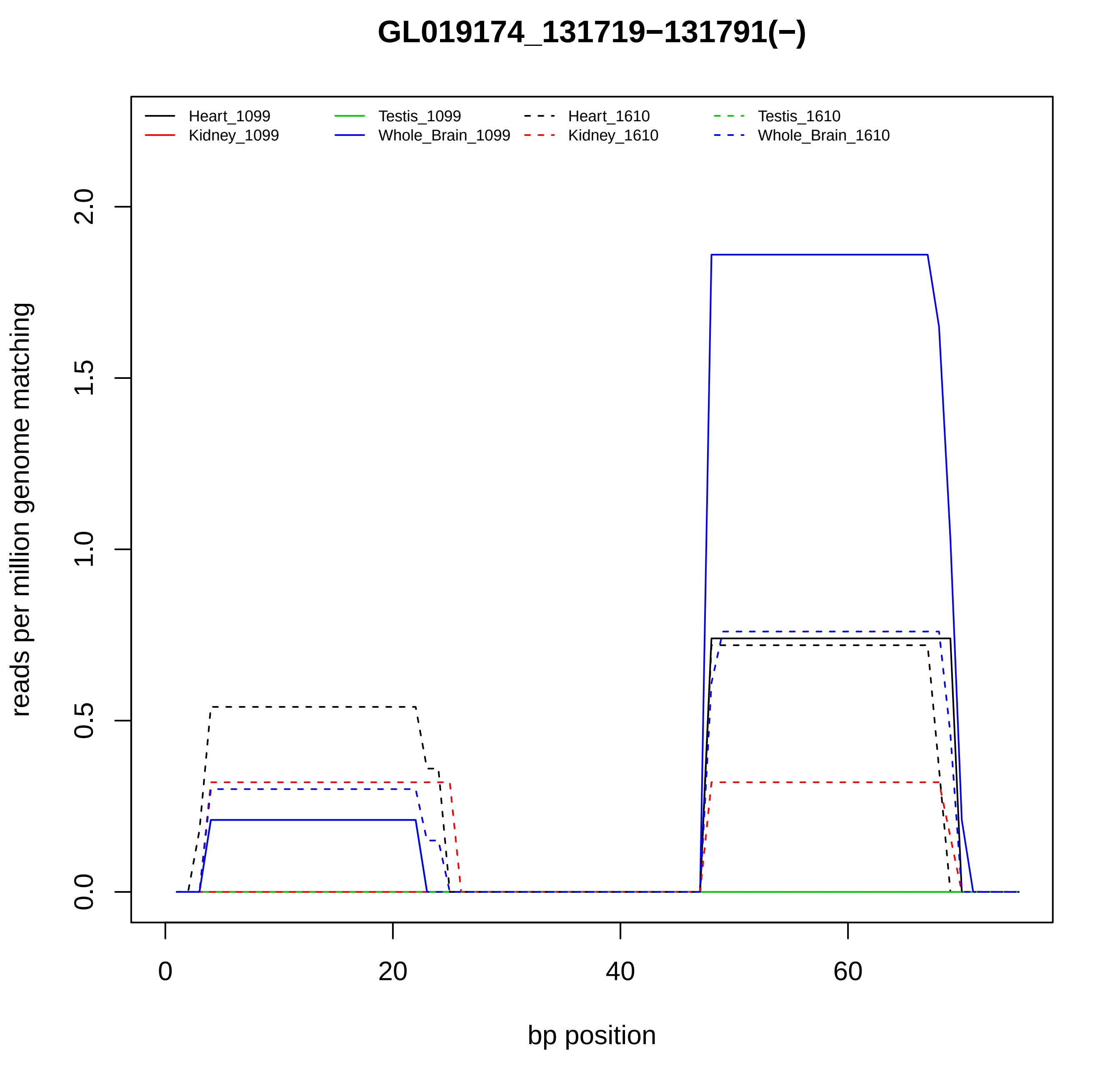
GL019174_131719−131791(−)
Heart_1099
Kidney_1099
Testis_1099
Whole_Brain_1099
Heart_1610
Kidney_1610
Testis_1610
Whole_Brain_1610
reads per million genome matching
0.0
0.5
1.0
1.5
2.0
0
20
40
60
bp position

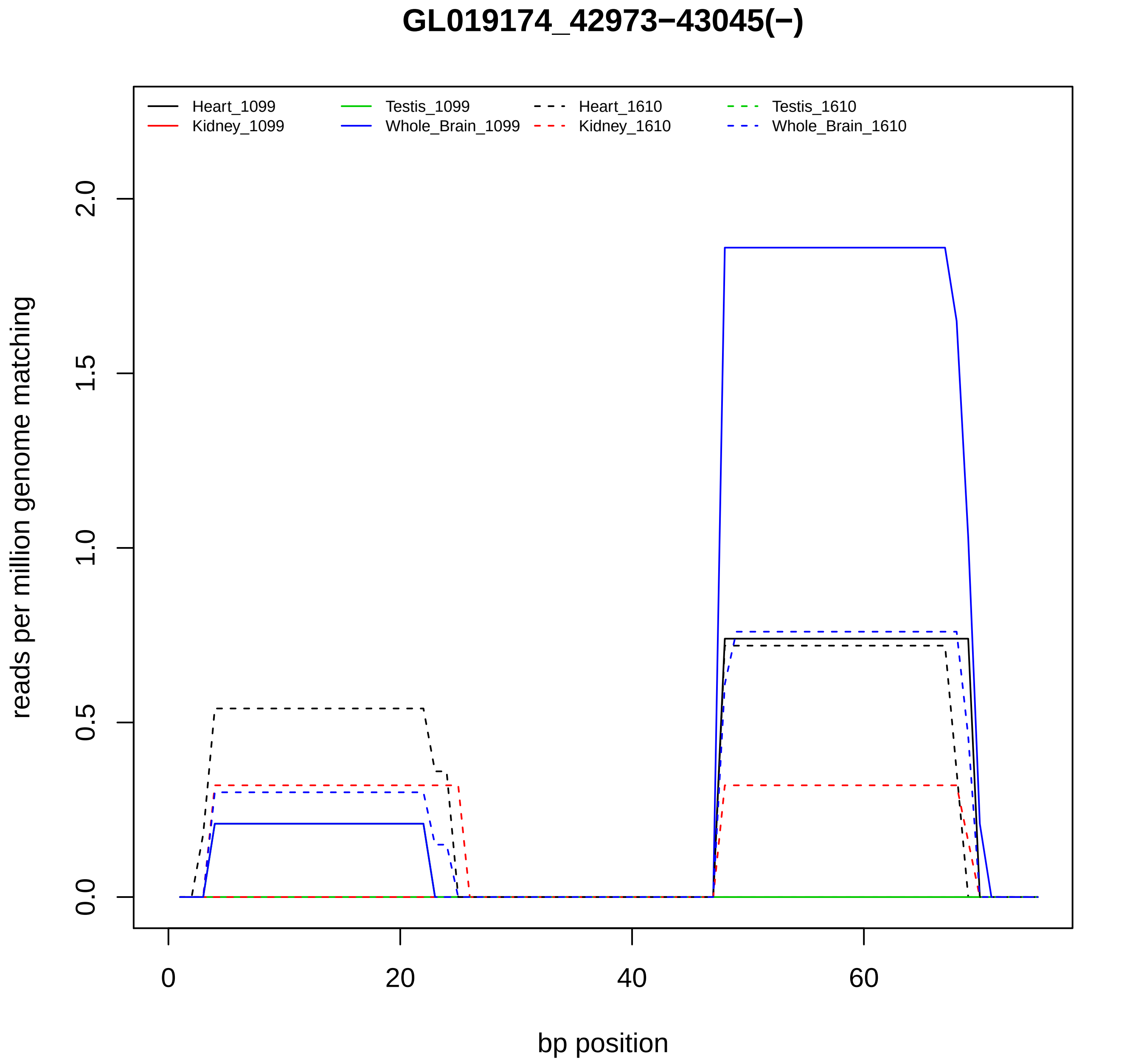

GL019174_42973−43045(−)
Heart_1099
Kidney_1099
Testis_1099
Whole_Brain_1099
Heart_1610
Kidney_1610
Testis_1610
Whole_Brain_1610
reads per million genome matching
0.0
0.5
1.0
1.5
2.0
0
20
40
60
bp position

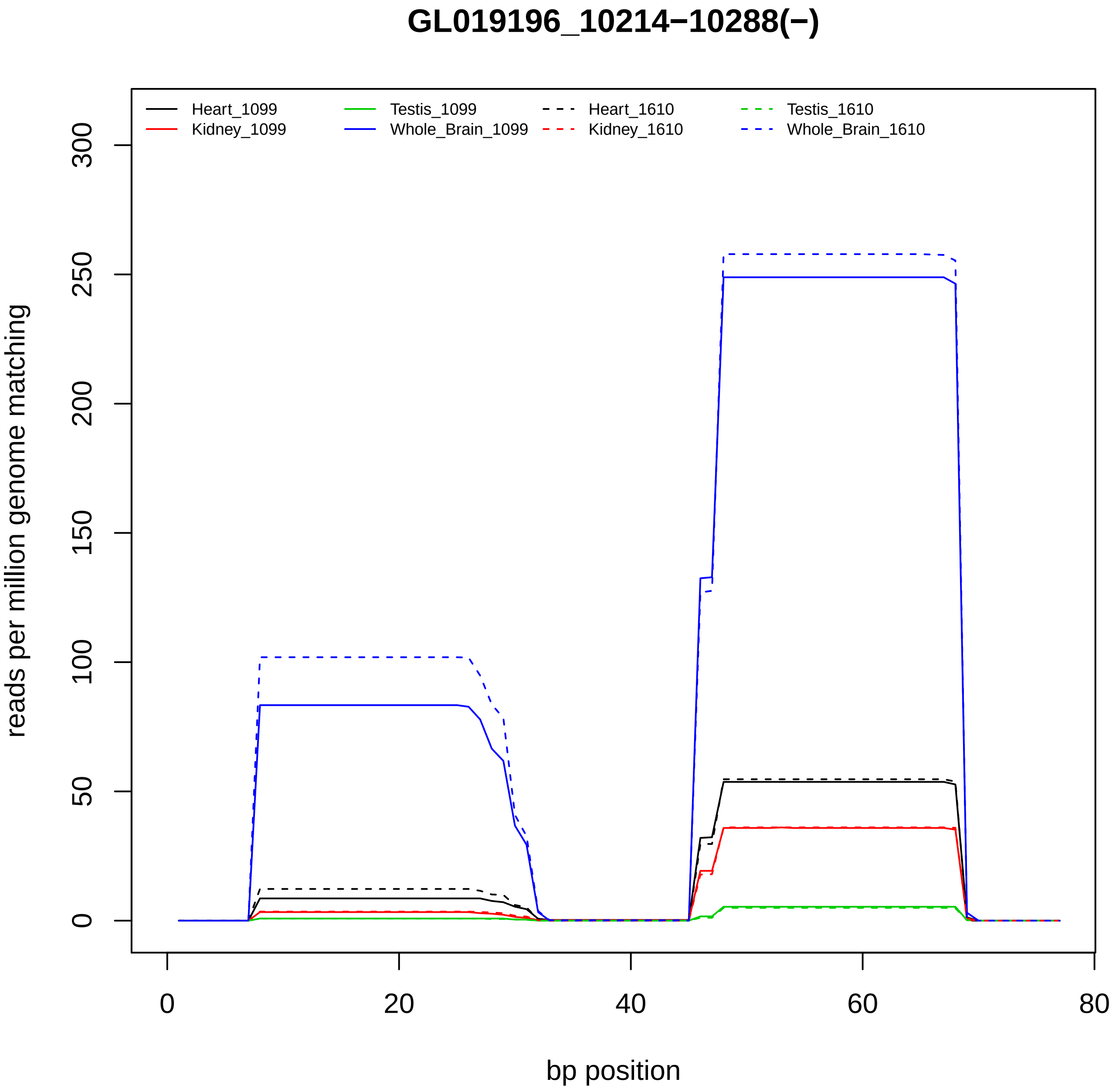

GL019196_10214−10288(−)
Heart_1099
Kidney_1099
Testis_1099
Whole_Brain_1099
Heart_1610
Kidney_1610
Testis_1610
Whole_Brain_1610
reads per million genome matching
bp position
0
50
100
150
200
250
300
0
20
40
60
80

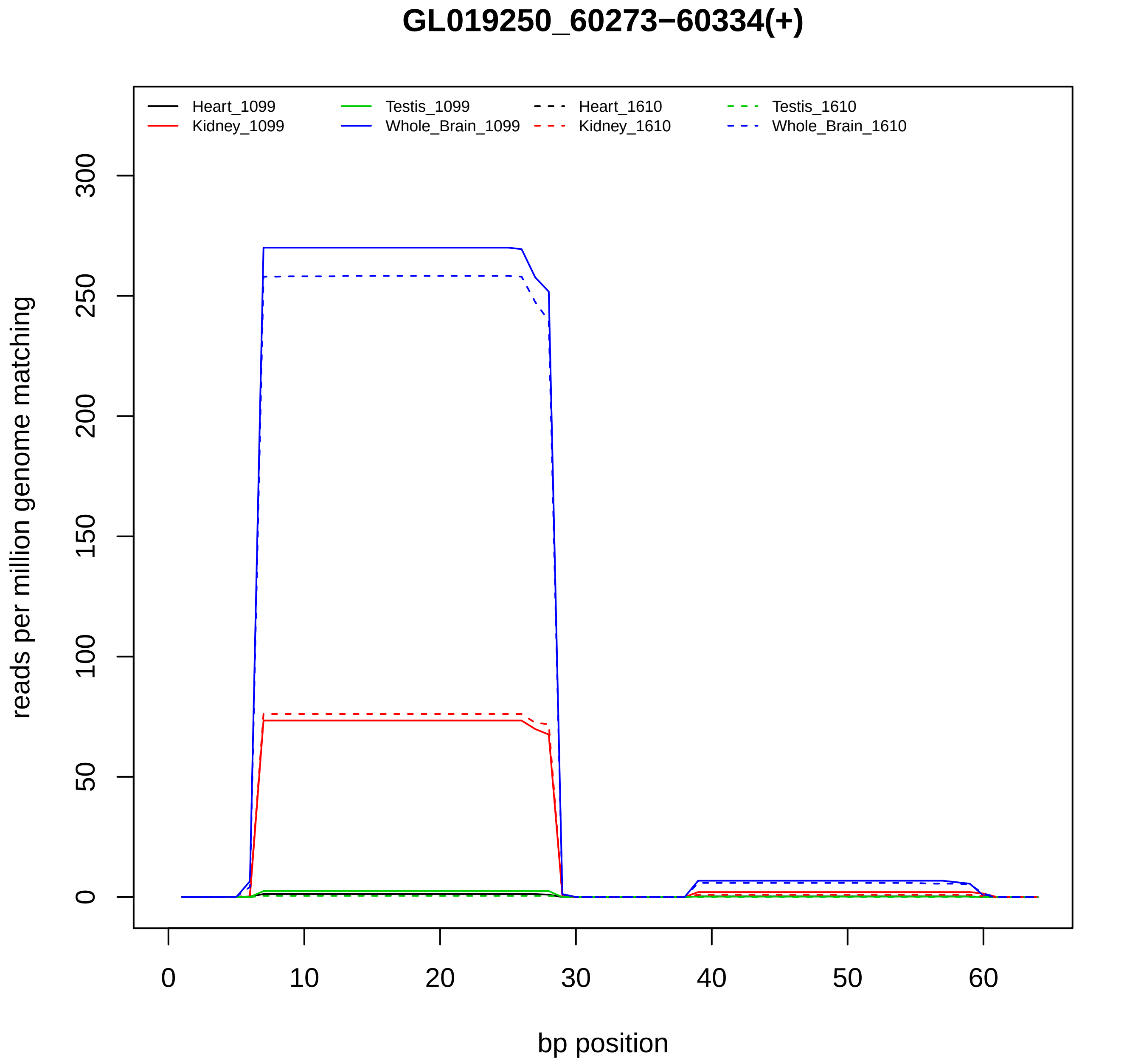
GL019250_60273−60334(+)
Heart_1099
Kidney_1099
Testis_1099
Whole_Brain_1099
Heart_1610
Kidney_1610
Testis_1610
Whole_Brain_1610
reads per million genome matching
bp position
0
50
100
150
200
250
300
0
10
20
30
40
50
60

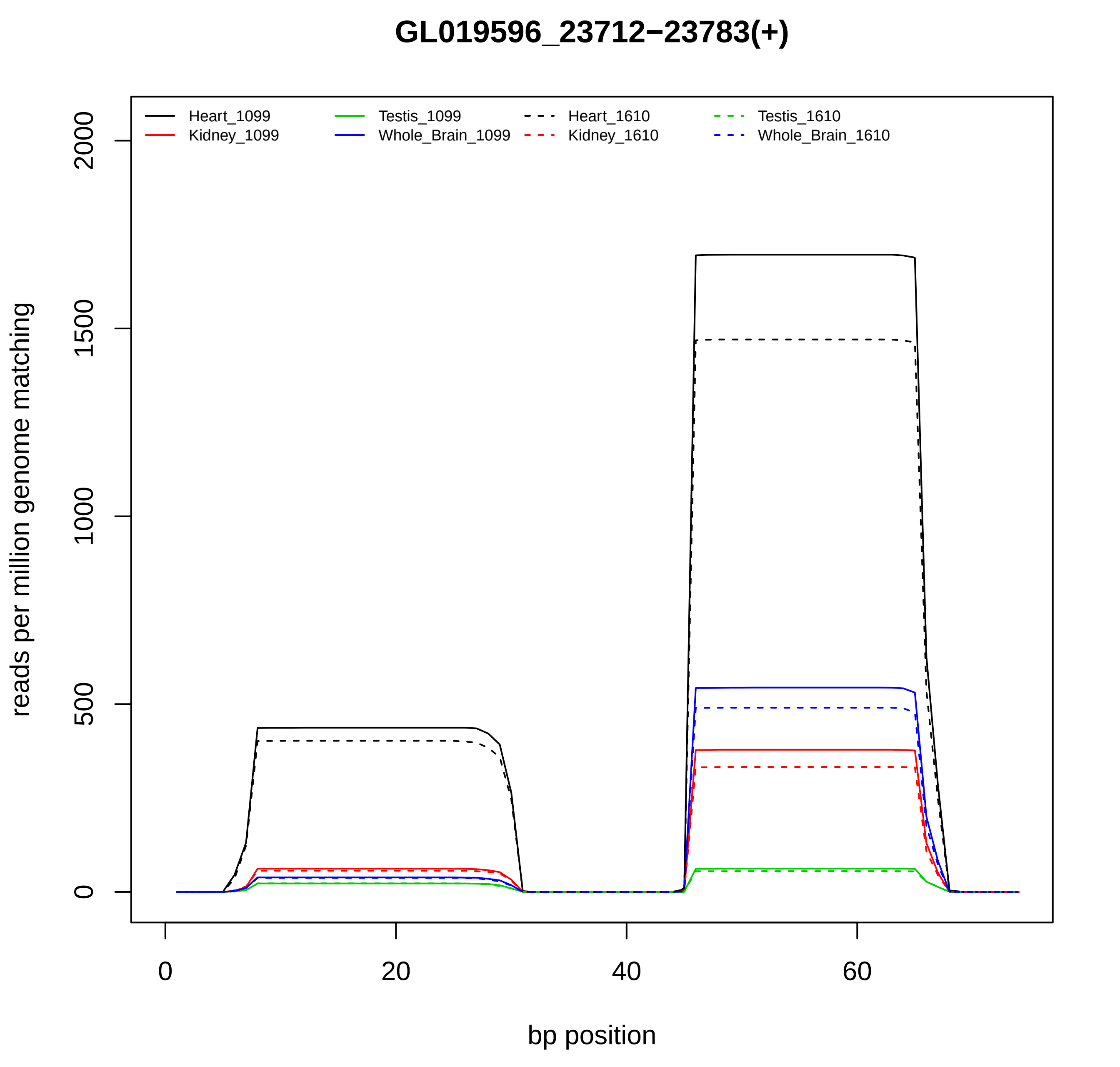

GL019596_23712−23783(+)
Heart_1099
Kidney_1099
Testis_1099
Whole_Brain_1099
Heart_1610
Kidney_1610
Testis_1610
Whole_Brain_1610
reads per million genome matching
bp position
0
500
1000
1500
2000
0
20
40
60

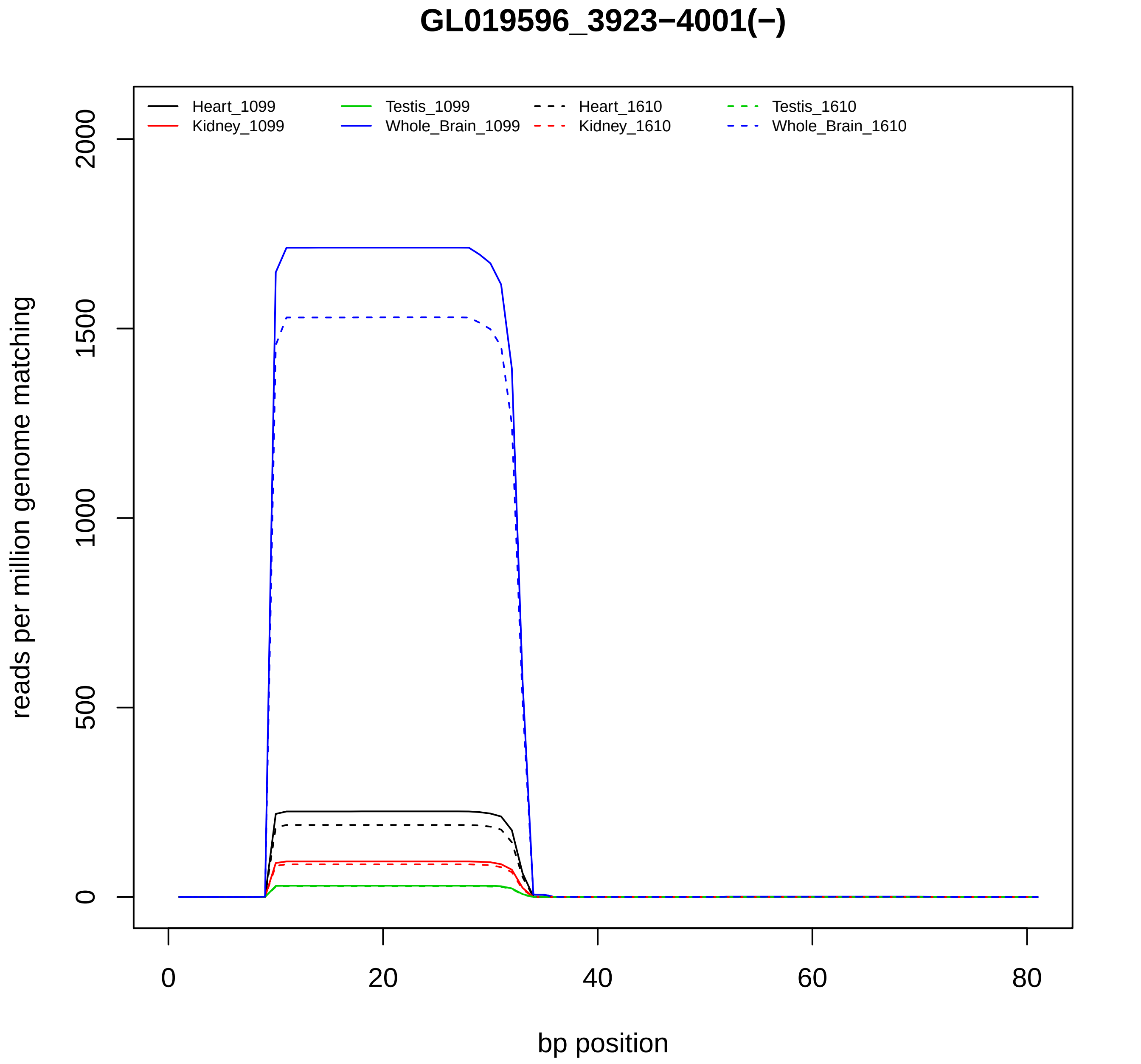

GL019596_3923−4001(−)
Heart_1099
Kidney_1099
Testis_1099
Whole_Brain_1099
Heart_1610
Kidney_1610
Testis_1610
Whole_Brain_1610
reads per million genome matching
0
500
1000
1500
2000
0
20
40
60
80
bp position

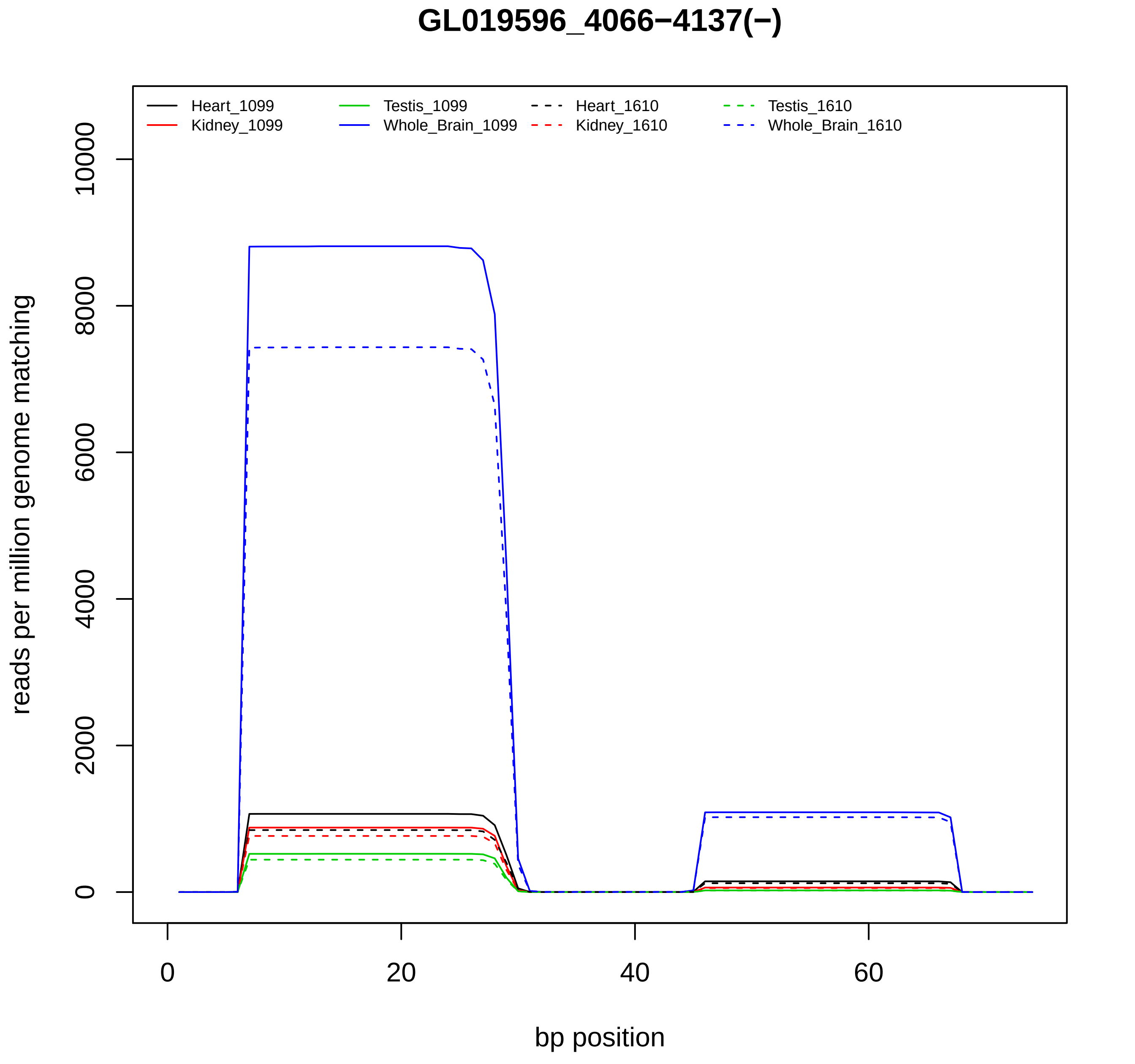
GL019596_4066−4137(−)
Heart_1099
Kidney_1099
Testis_1099
Whole_Brain_1099
Heart_1610
Kidney_1610
Testis_1610
Whole_Brain_1610
reads per million genome matching
0
2000
4000
6000
8000
10000
0
20
40
60
bp position

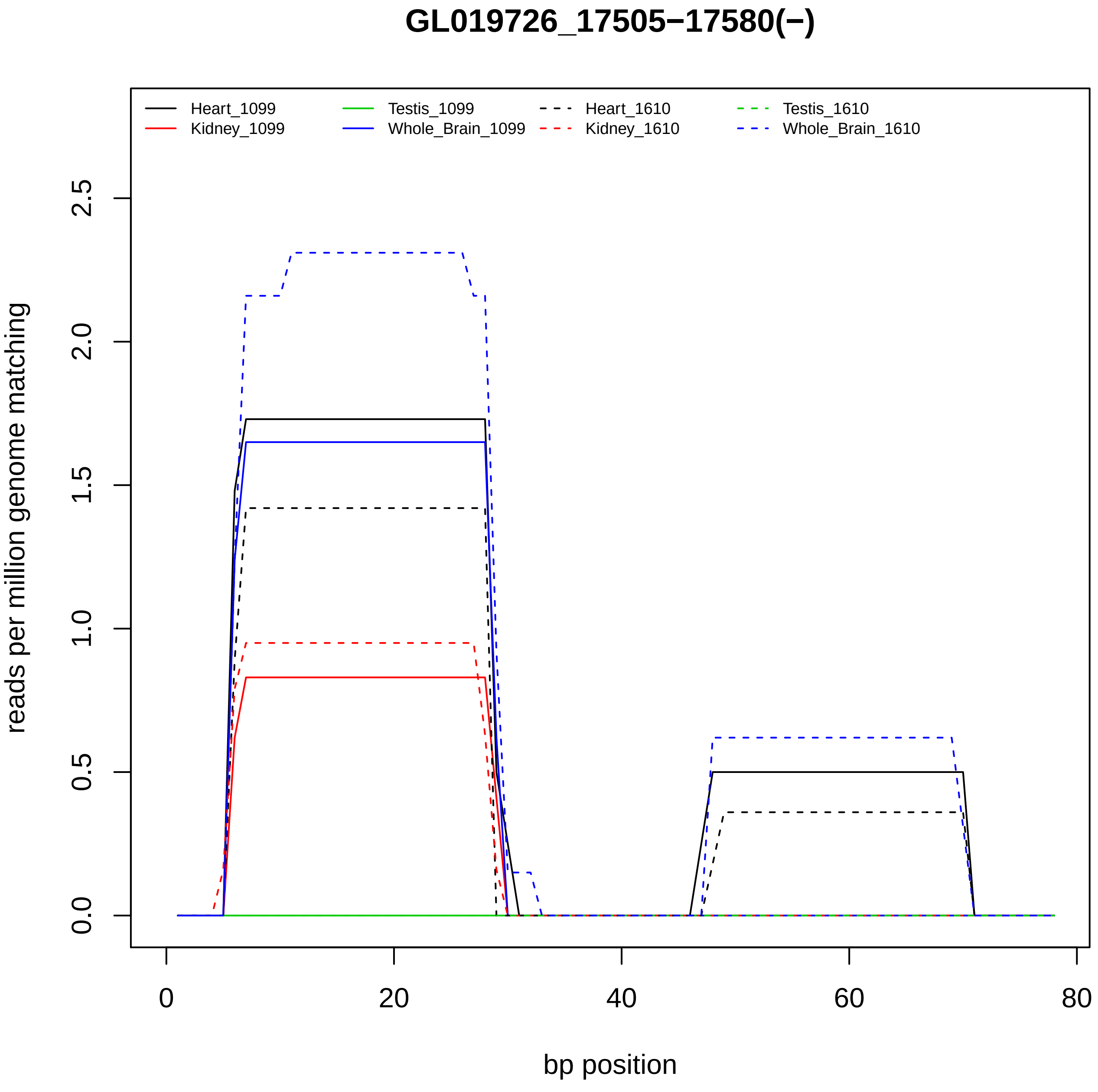

GL019726_17505−17580(−)
Heart_1099
Kidney_1099
Testis_1099
Whole_Brain_1099
Heart_1610
Kidney_1610
Testis_1610
Whole_Brain_1610
reads per million genome matching
bp position
0.0
0.5
1.0
1.5
2.0
2.5
0
20
40
60
80

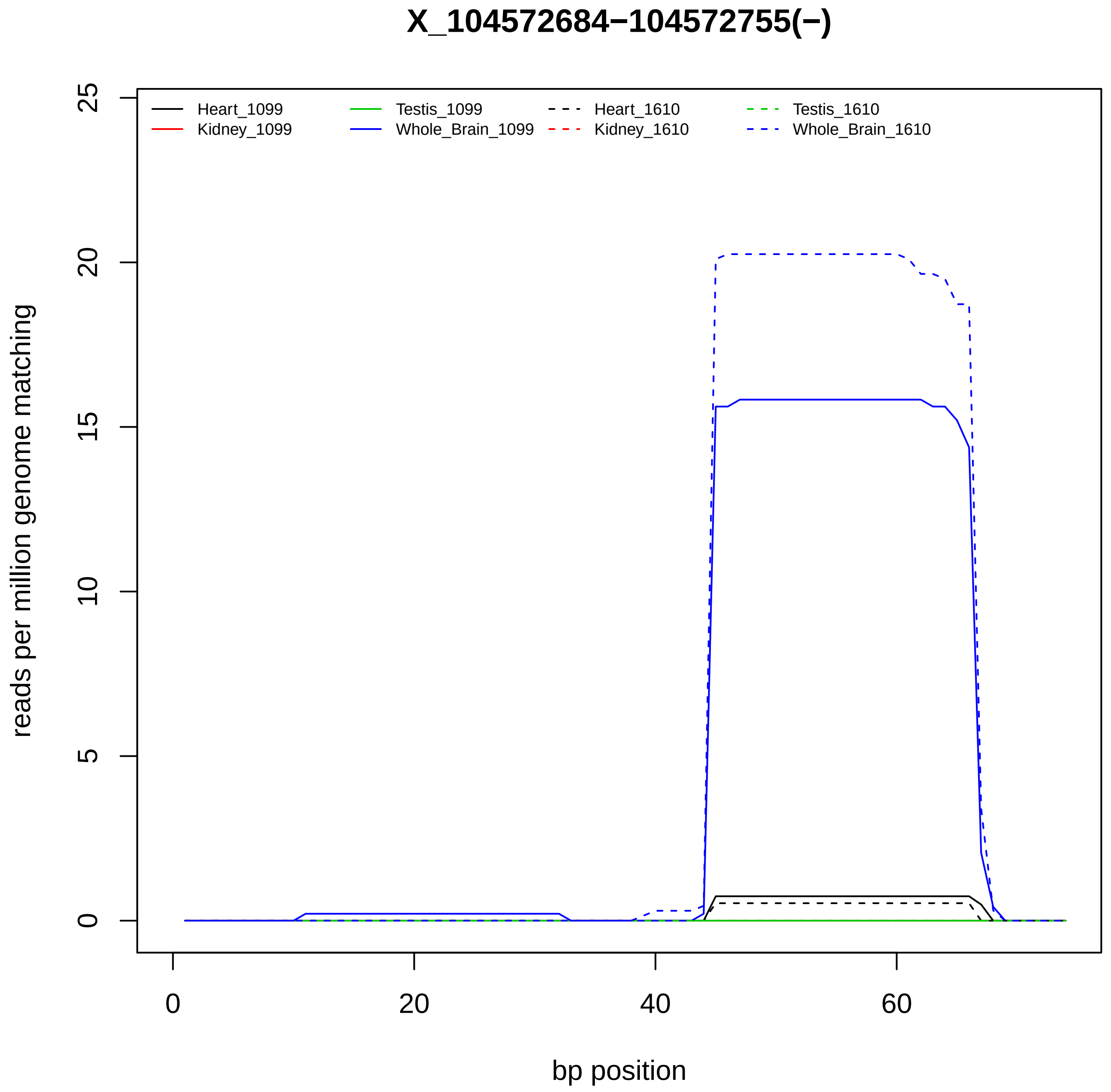

X_104572684−104572755(−)
Heart_1099
Kidney_1099
Testis_1099
Whole_Brain_1099
Heart_1610
Kidney_1610
Testis_1610
Whole_Brain_1610
reads per million genome matching
bp position
0
5
10
15
20
25
0
20
40
60

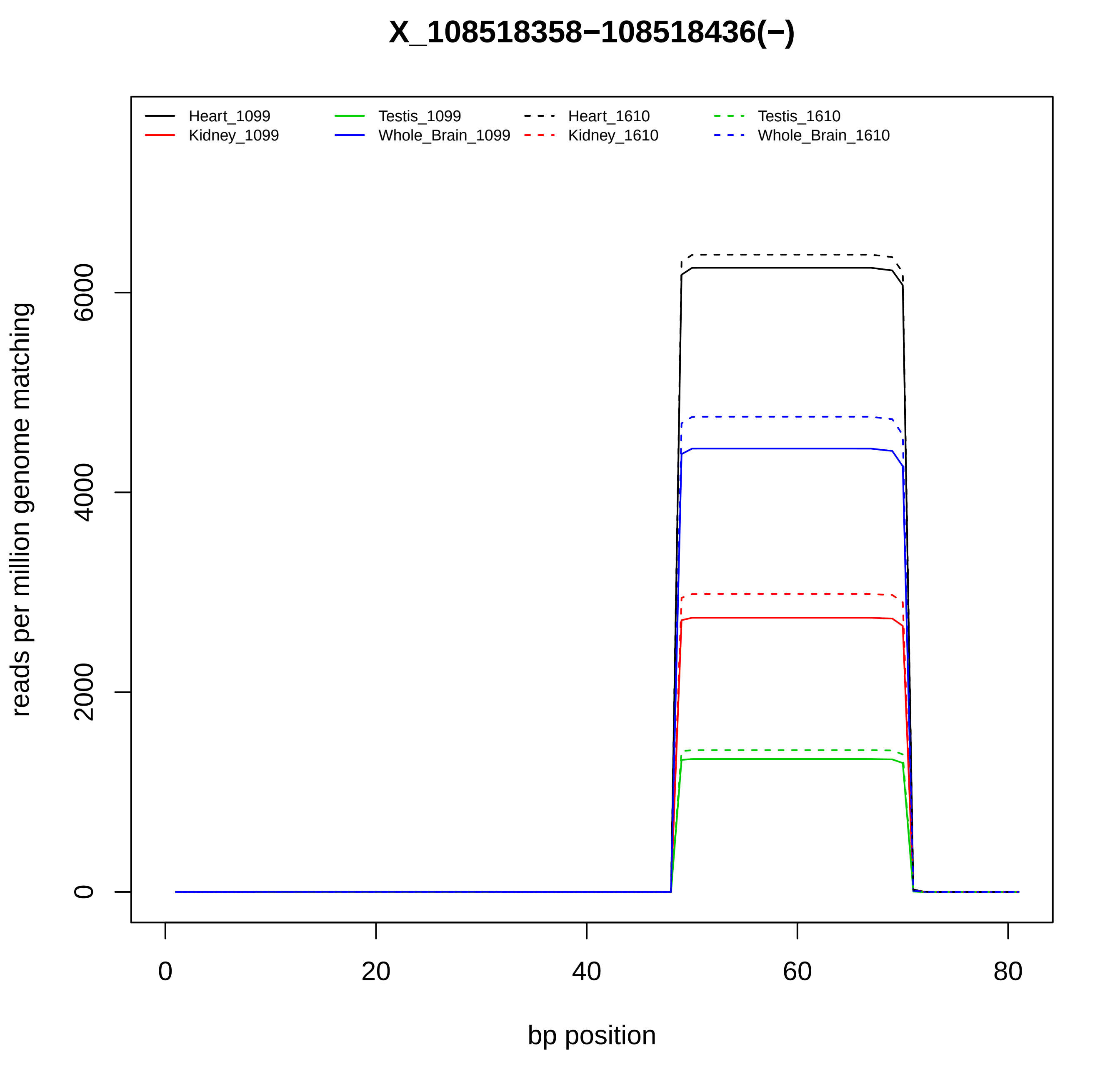

X_108518358−108518436(−)
Heart_1099
Kidney_1099
Testis_1099
Whole_Brain_1099
Heart_1610
Kidney_1610
Testis_1610
Whole_Brain_1610
reads per million genome matching
0
2000
4000
6000
0
20
40
60
80
bp position

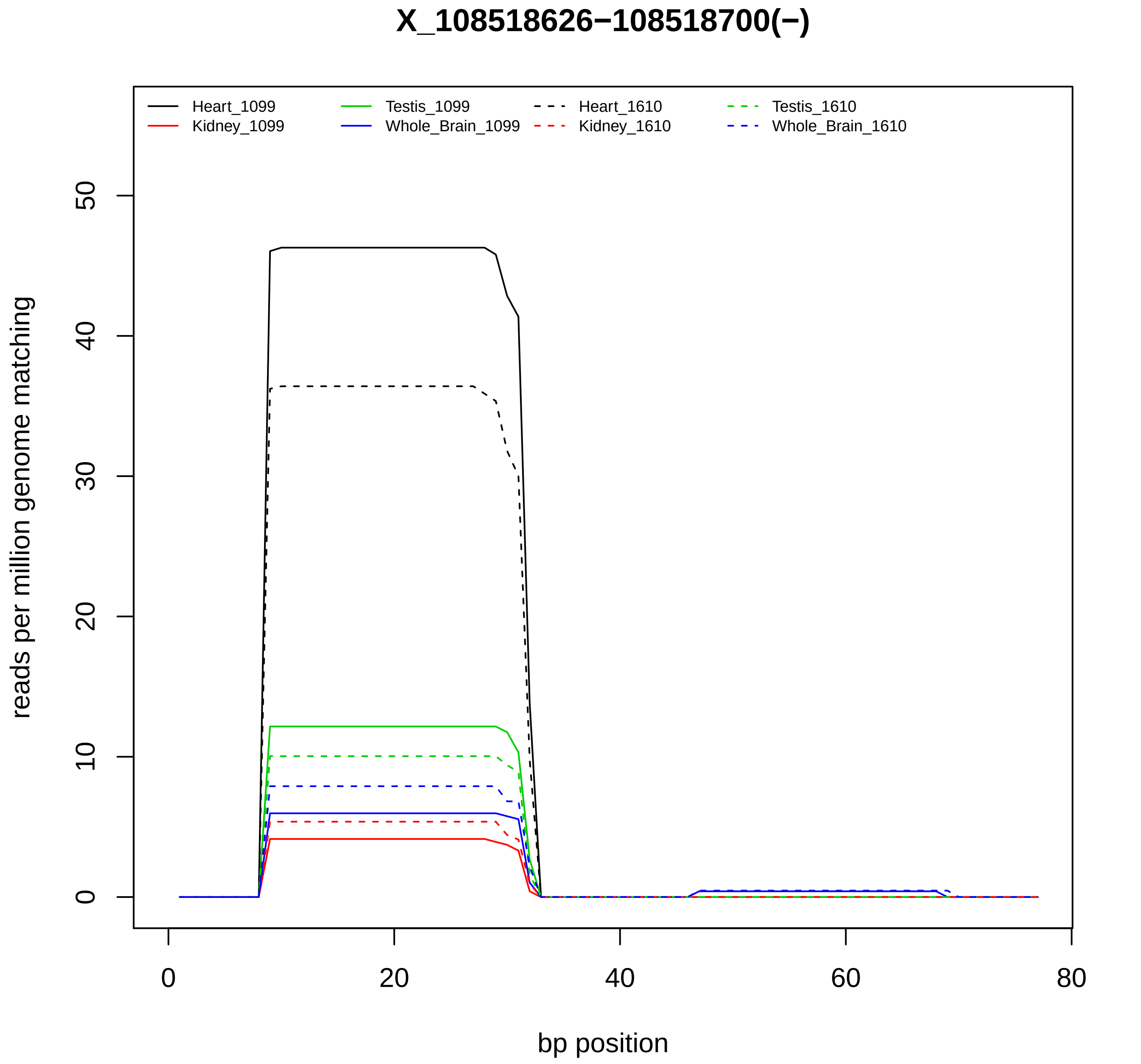

X_108518626−108518700(−)
Heart_1099
Kidney_1099
Testis_1099
Whole_Brain_1099
Heart_1610
Kidney_1610
Testis_1610
Whole_Brain_1610
reads per million genome matching
0
10
20
30
40
50
0
20
40
60
80
bp position

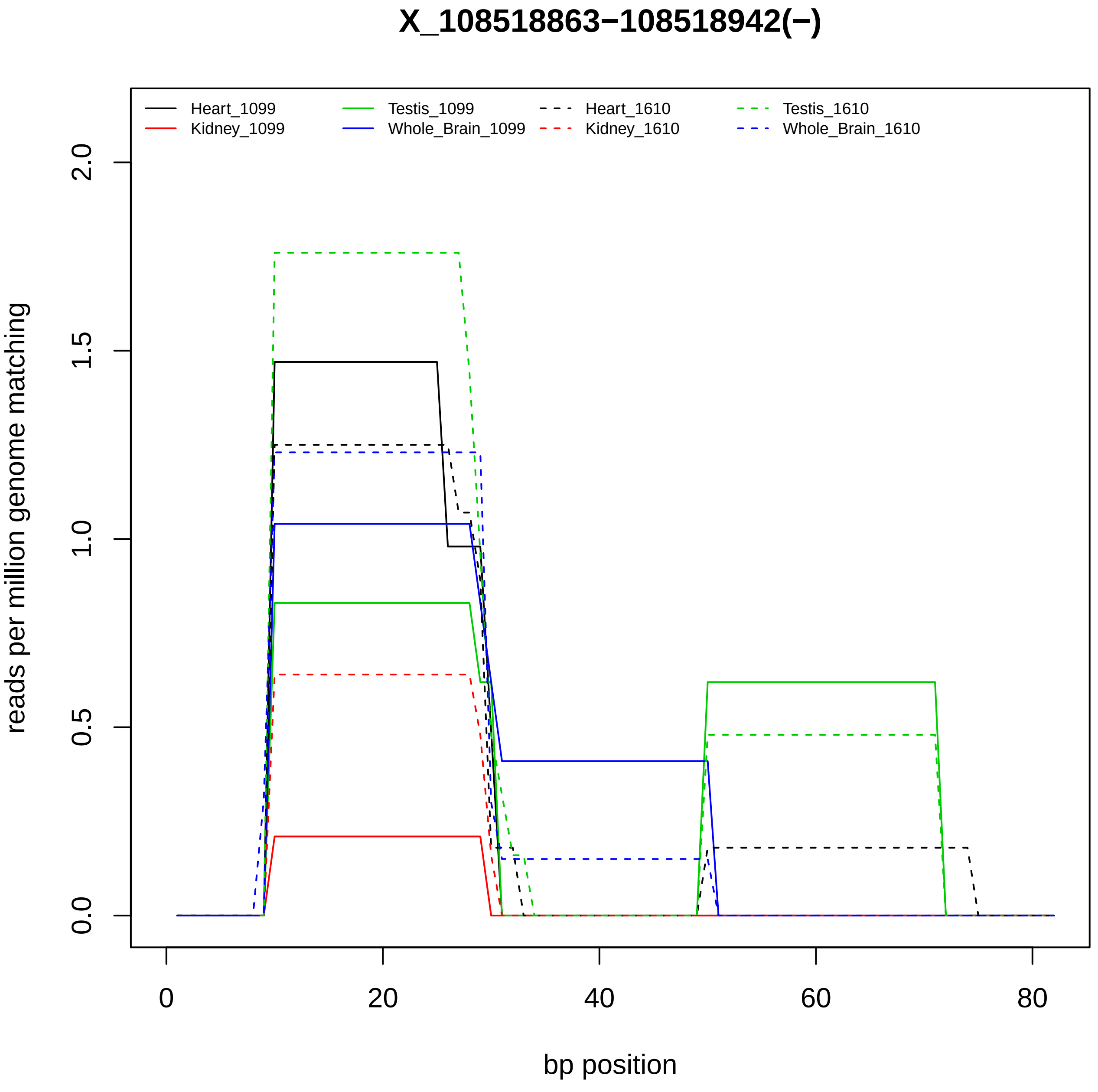
X_108518863−108518942(−)
Heart_1099
Kidney_1099
Testis_1099
Whole_Brain_1099
Heart_1610
Kidney_1610
Testis_1610
Whole_Brain_1610
reads per million genome matching
bp position
0.0
0.5
1.0
1.5
2.0
0
20
40
60
80

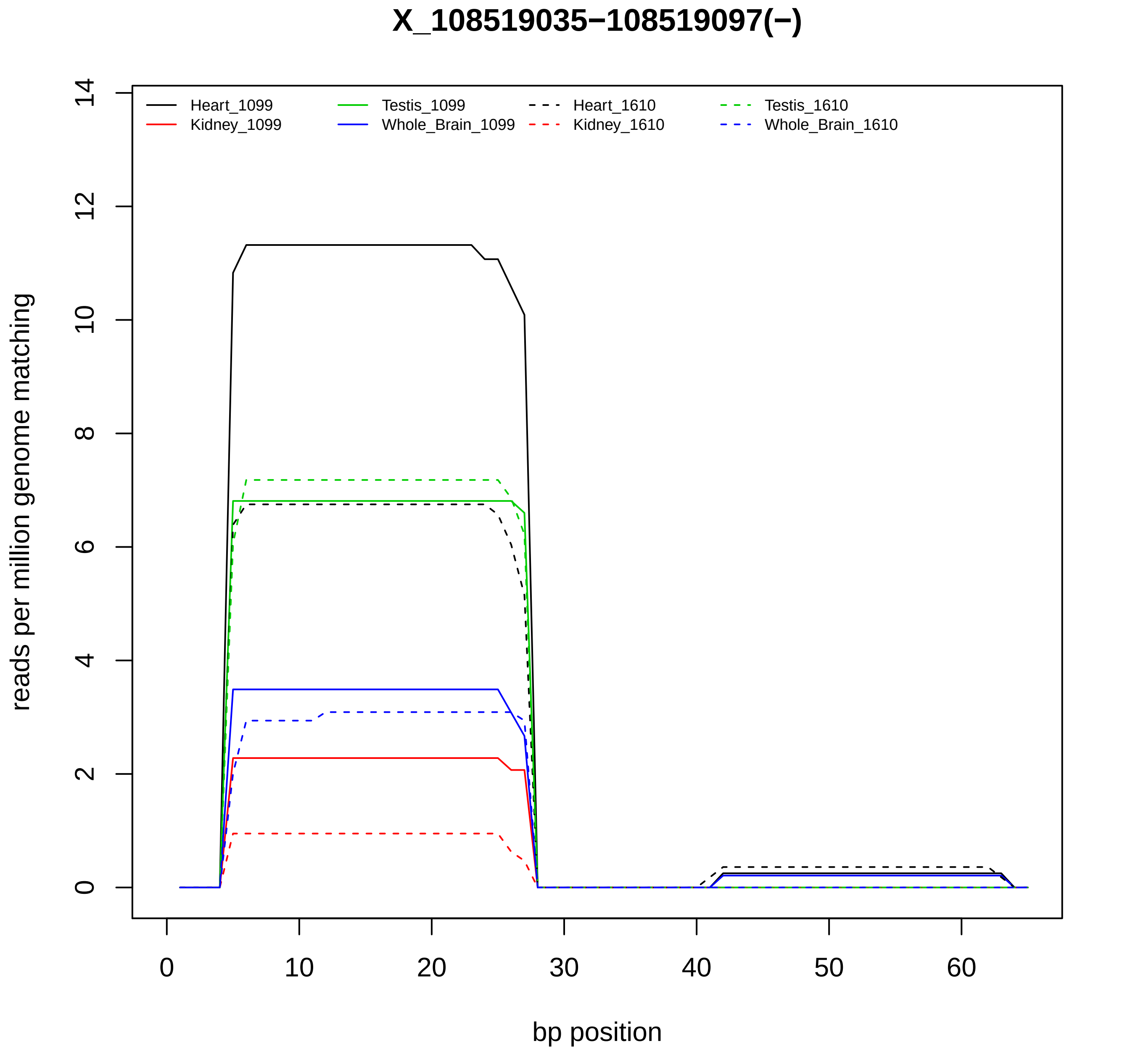

X_108519035−108519097(−)
Heart_1099
Kidney_1099
Testis_1099
Whole_Brain_1099
Heart_1610
Kidney_1610
Testis_1610
Whole_Brain_1610
reads per million genome matching
bp position
0
2
4
6
8
10
12
14
0
10
20
30
40
50
60

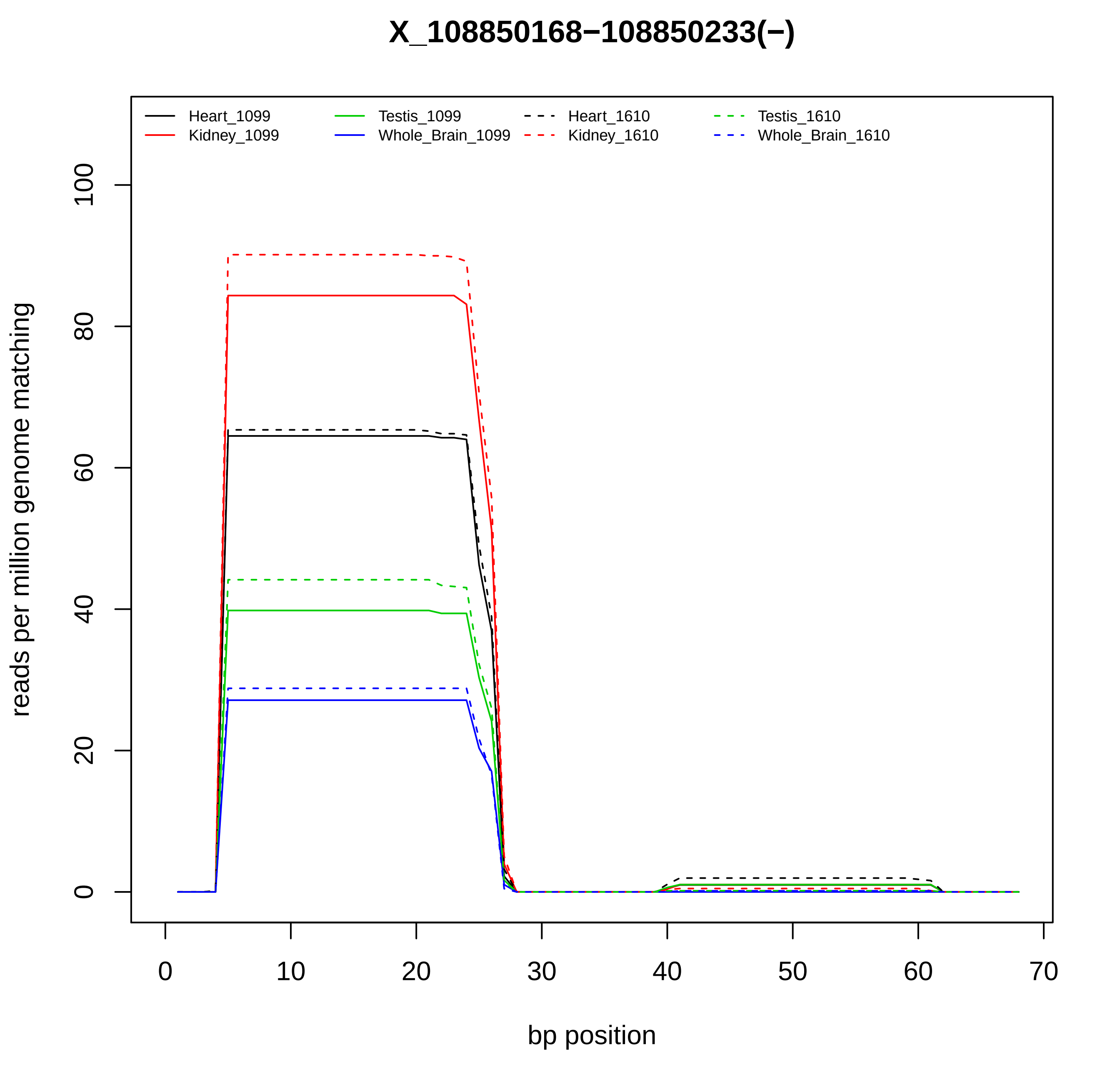

X_108850168−108850233(−)
Heart_1099
Kidney_1099
Testis_1099
Whole_Brain_1099
Heart_1610
Kidney_1610
Testis_1610
Whole_Brain_1610
reads per million genome matching
0
20
40
60
80
100
bp position
0
10
20
30
40
50
60
70

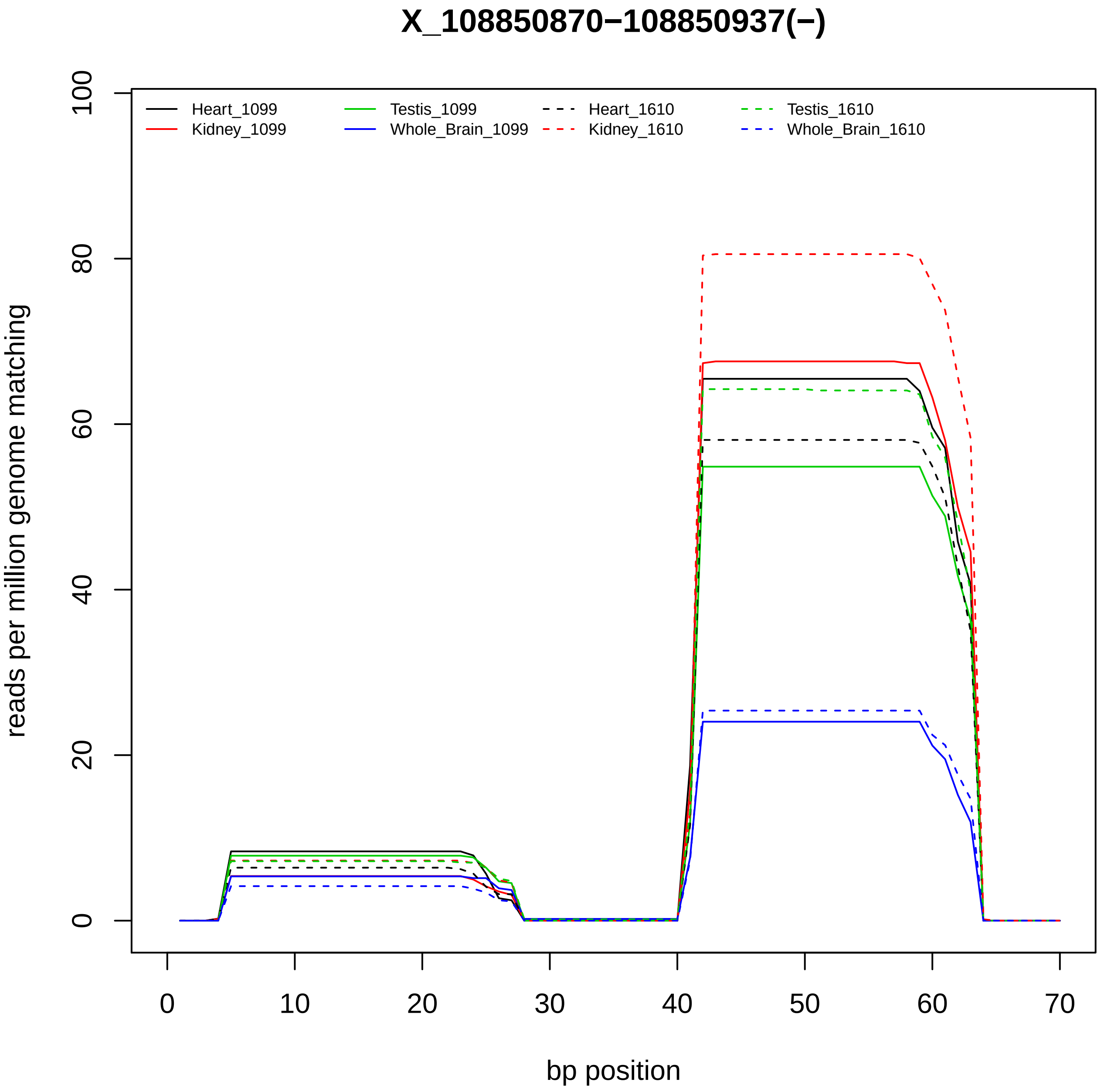
X_108850870−108850937(−)
Heart_1099
Kidney_1099
Testis_1099
Whole_Brain_1099
Heart_1610
Kidney_1610
Testis_1610
Whole_Brain_1610
reads per million genome matching
0
20
40
60
80
100
bp position
0
10
20
30
40
50
60
70

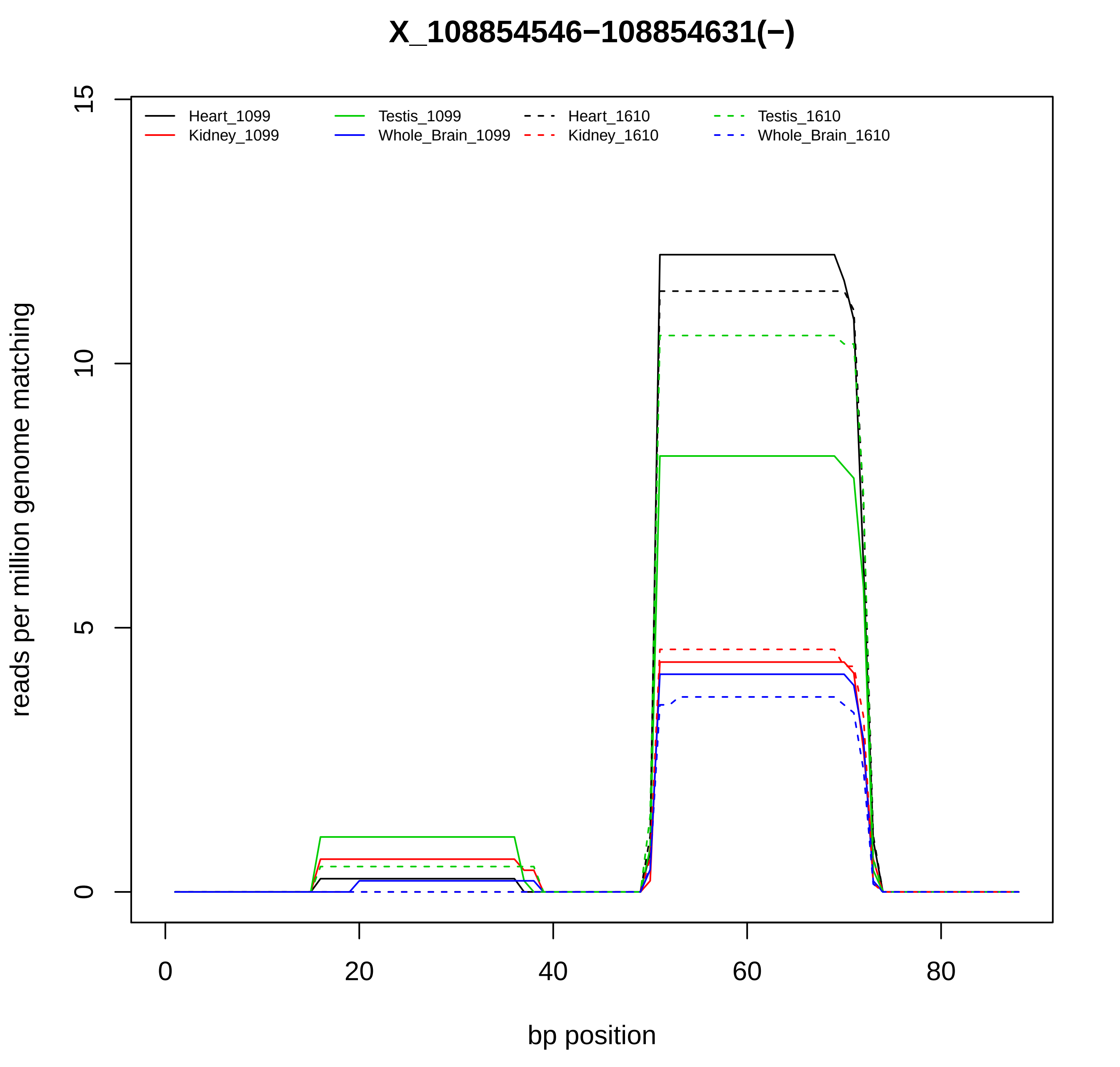

X_108854546−108854631(−)
Heart_1099
Kidney_1099
Testis_1099
Whole_Brain_1099
Heart_1610
Kidney_1610
Testis_1610
Whole_Brain_1610
reads per million genome matching
bp position
0
5
10
15
0
20
40
60
80

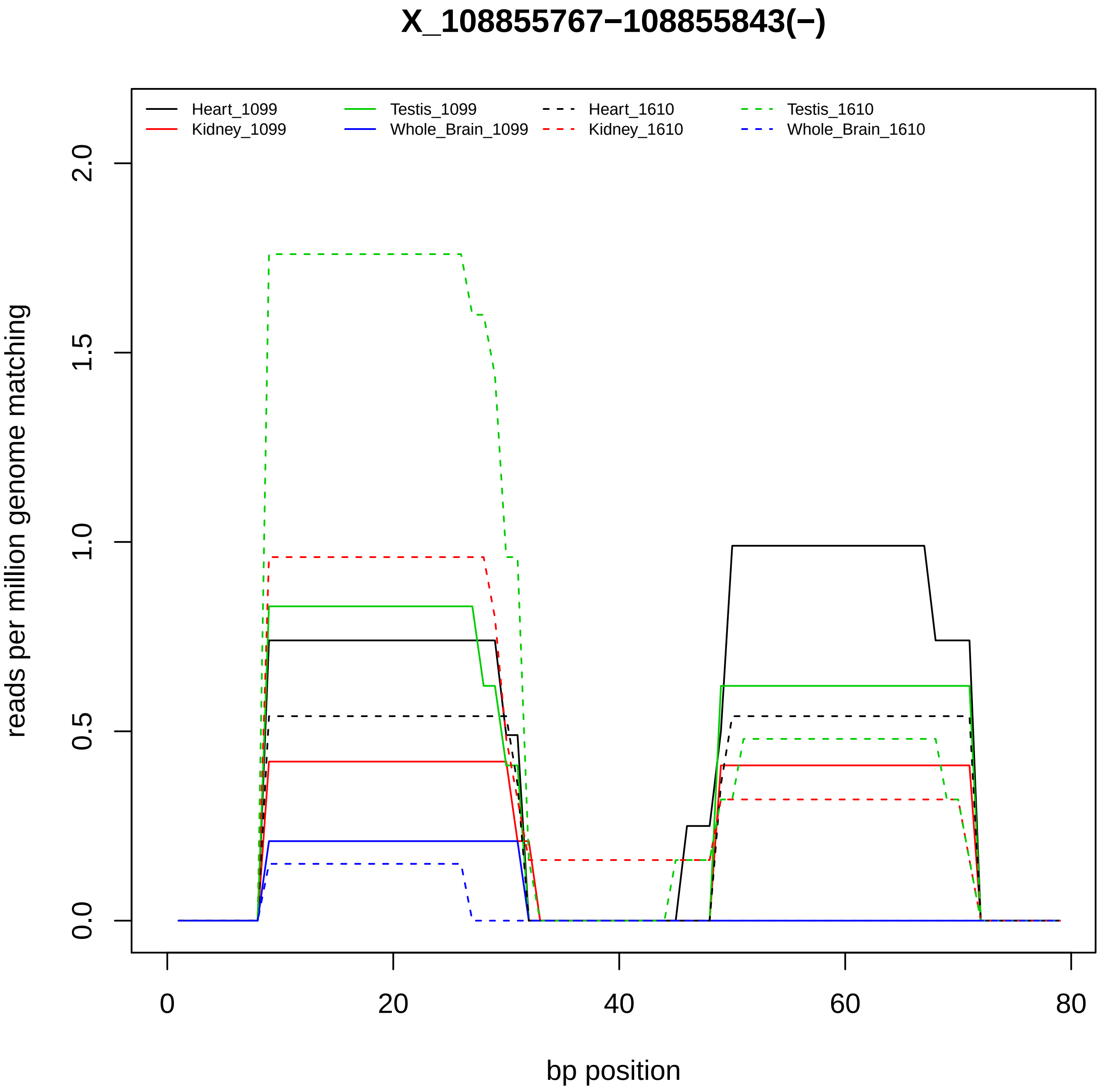

X_108855767−108855843(−)
Heart_1099
Kidney_1099
Testis_1099
Whole_Brain_1099
Heart_1610
Kidney_1610
Testis_1610
Whole_Brain_1610
reads per million genome matching
bp position
0.0
0.5
1.0
1.5
2.0
0
20
40
60
80

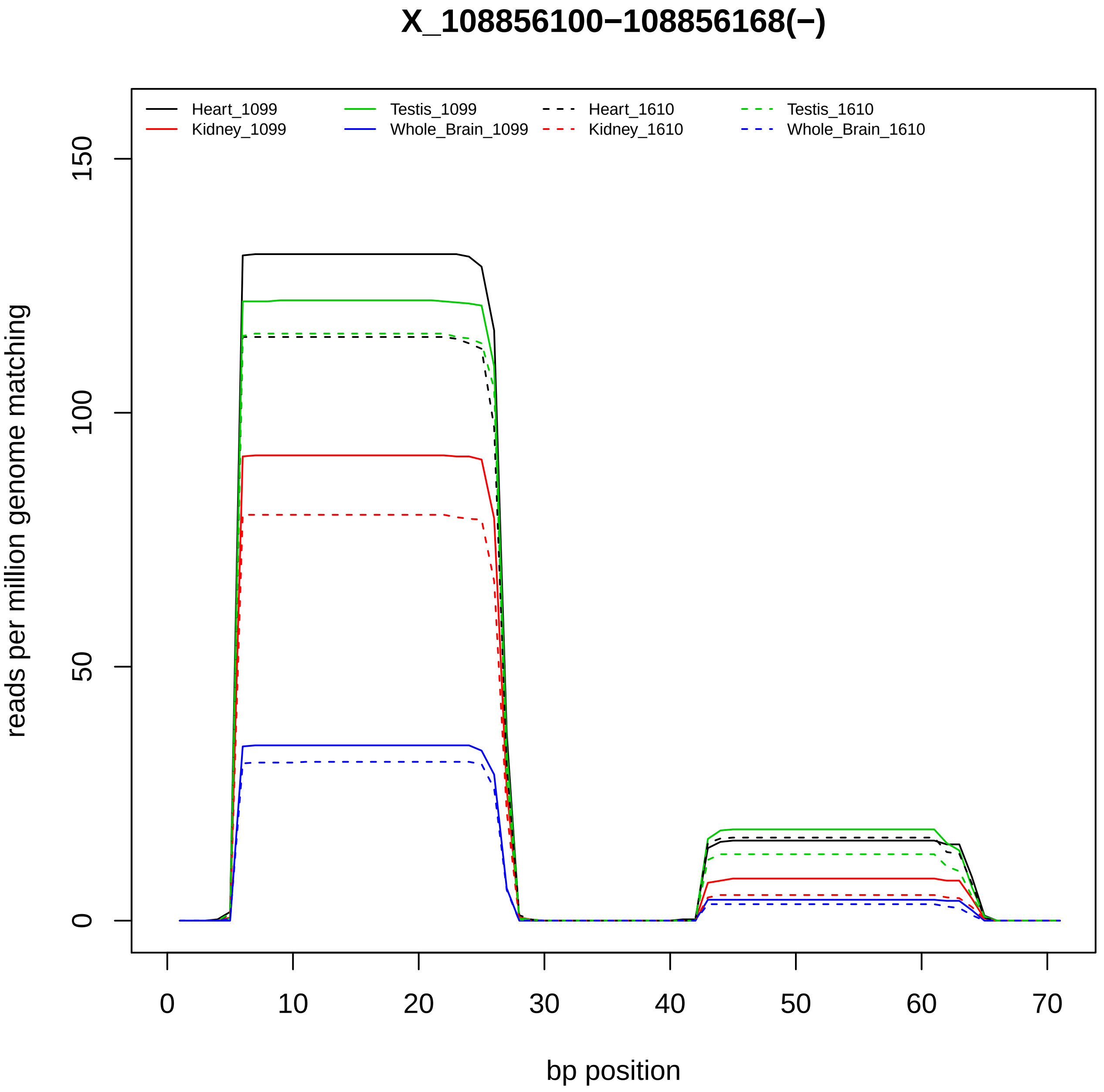
X_108856100−108856168(−)
Heart_1099
Kidney_1099
Testis_1099
Whole_Brain_1099
Heart_1610
Kidney_1610
Testis_1610
Whole_Brain_1610
reads per million genome matching
0
50
100
150
bp position
0
10
20
30
40
50
60
70

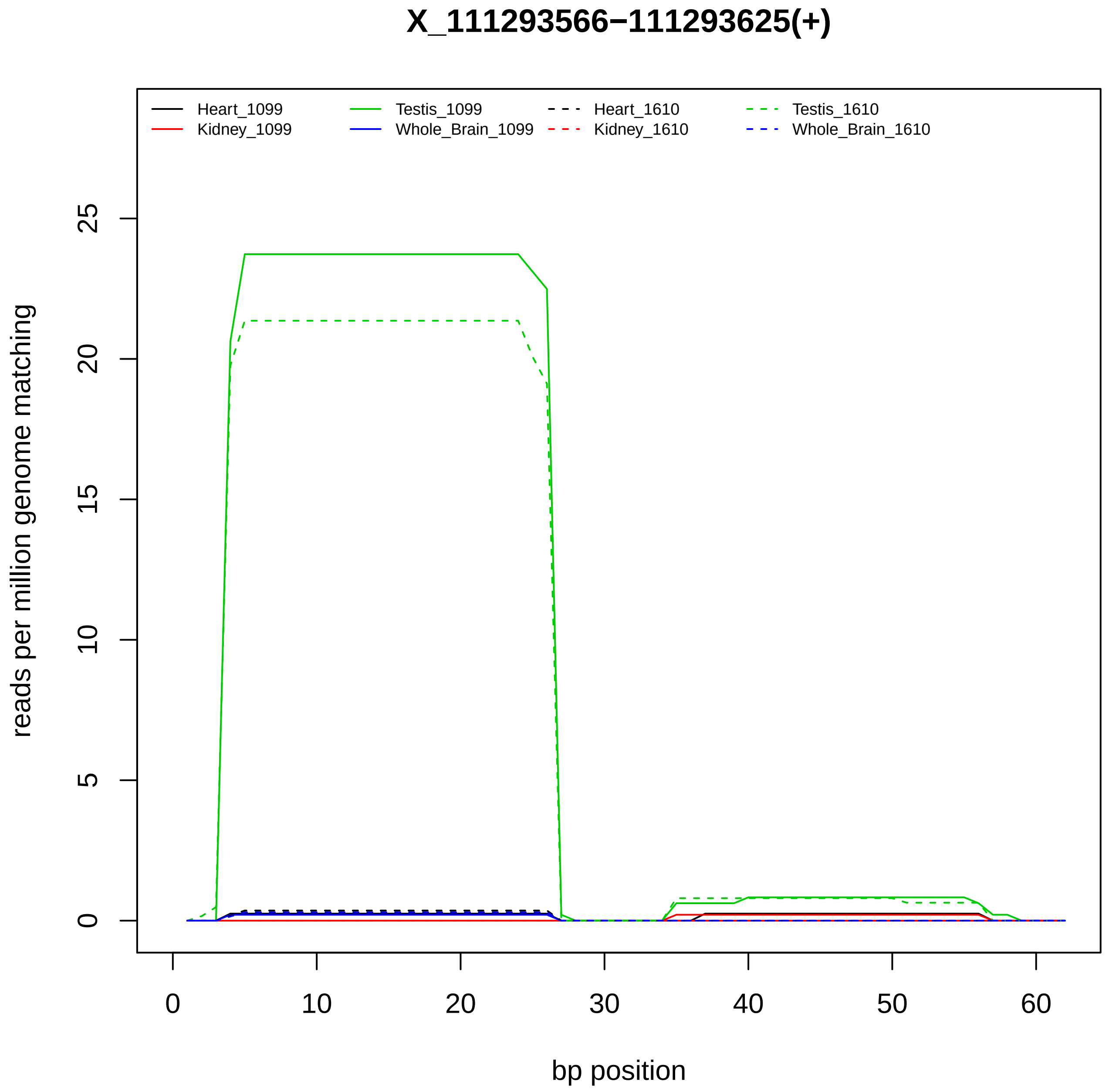

X_111293566−111293625(+)
Heart_1099
Kidney_1099
Testis_1099
Whole_Brain_1099
Heart_1610
Kidney_1610
Testis_1610
Whole_Brain_1610
reads per million genome matching
0
5
10
15
20
25
0
10
20
30
40
50
60
bp position

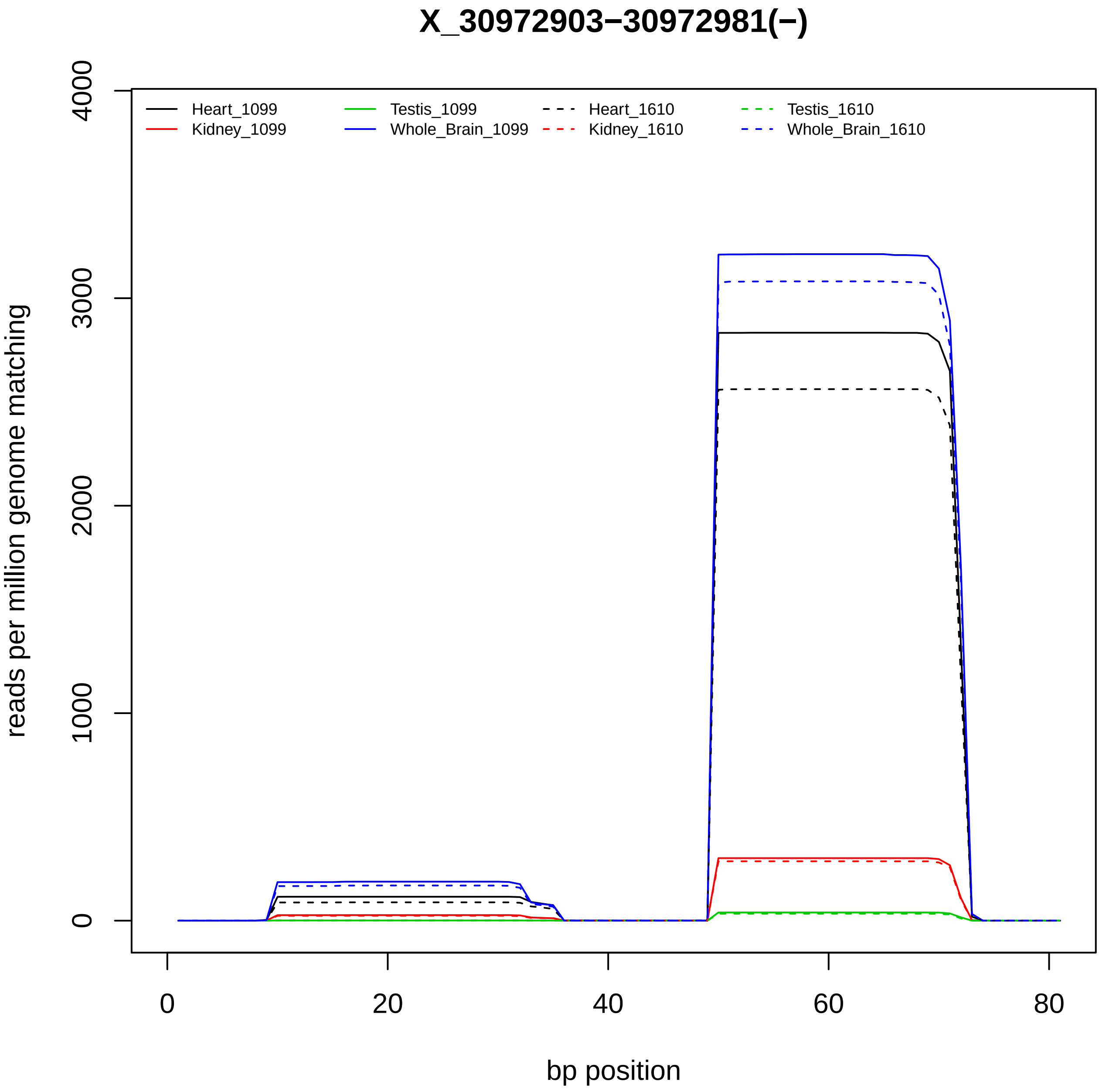
X_30972903−30972981(−)
Heart_1099
Kidney_1099
Testis_1099
Whole_Brain_1099
Heart_1610
Kidney_1610
Testis_1610
Whole_Brain_1610
reads per million genome matching
0
1000
2000
3000
4000
0
20
40
60
80
bp position

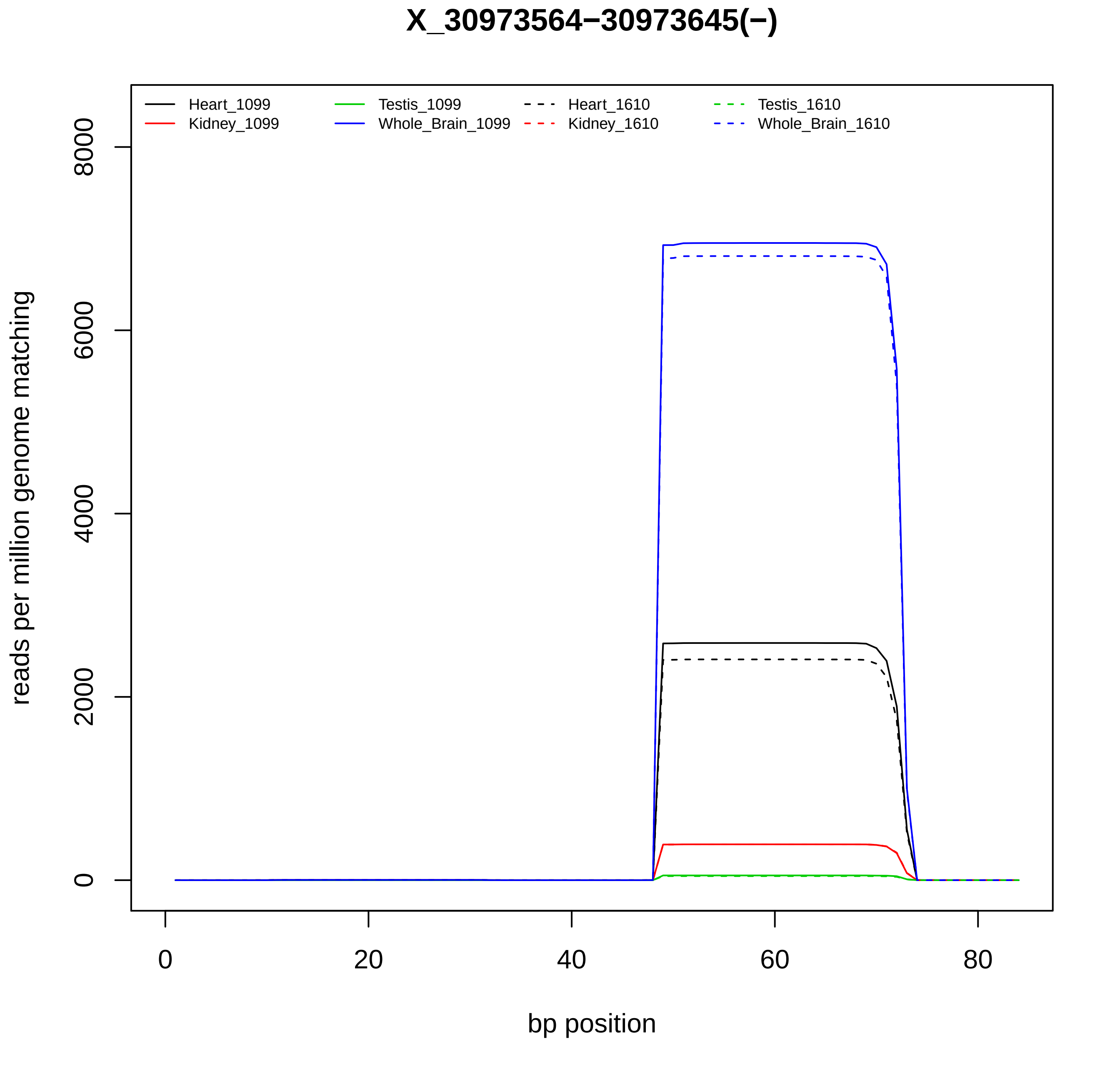

X_30973564−30973645(−)
Heart_1099
Kidney_1099
Testis_1099
Whole_Brain_1099
Heart_1610
Kidney_1610
Testis_1610
Whole_Brain_1610
reads per million genome matching
0
2000
4000
6000
8000
0
20
40
60
80
bp position

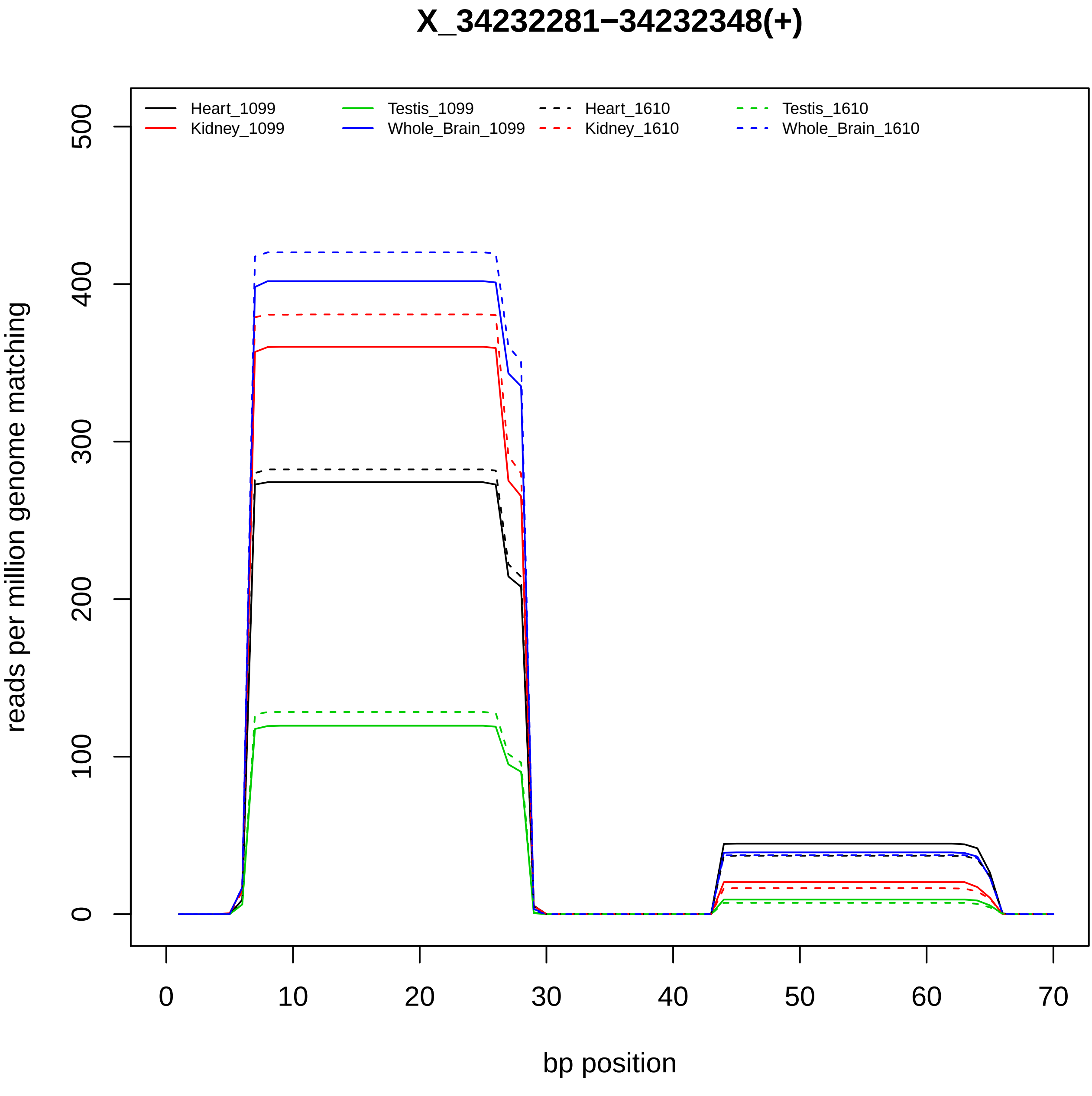
X_34232281−34232348(+)
Heart_1099
Kidney_1099
Testis_1099
Whole_Brain_1099
Heart_1610
Kidney_1610
Testis_1610
Whole_Brain_1610
reads per million genome matching
0
100
200
300
400
500
0
10
20
30
40
50
60
70
bp position

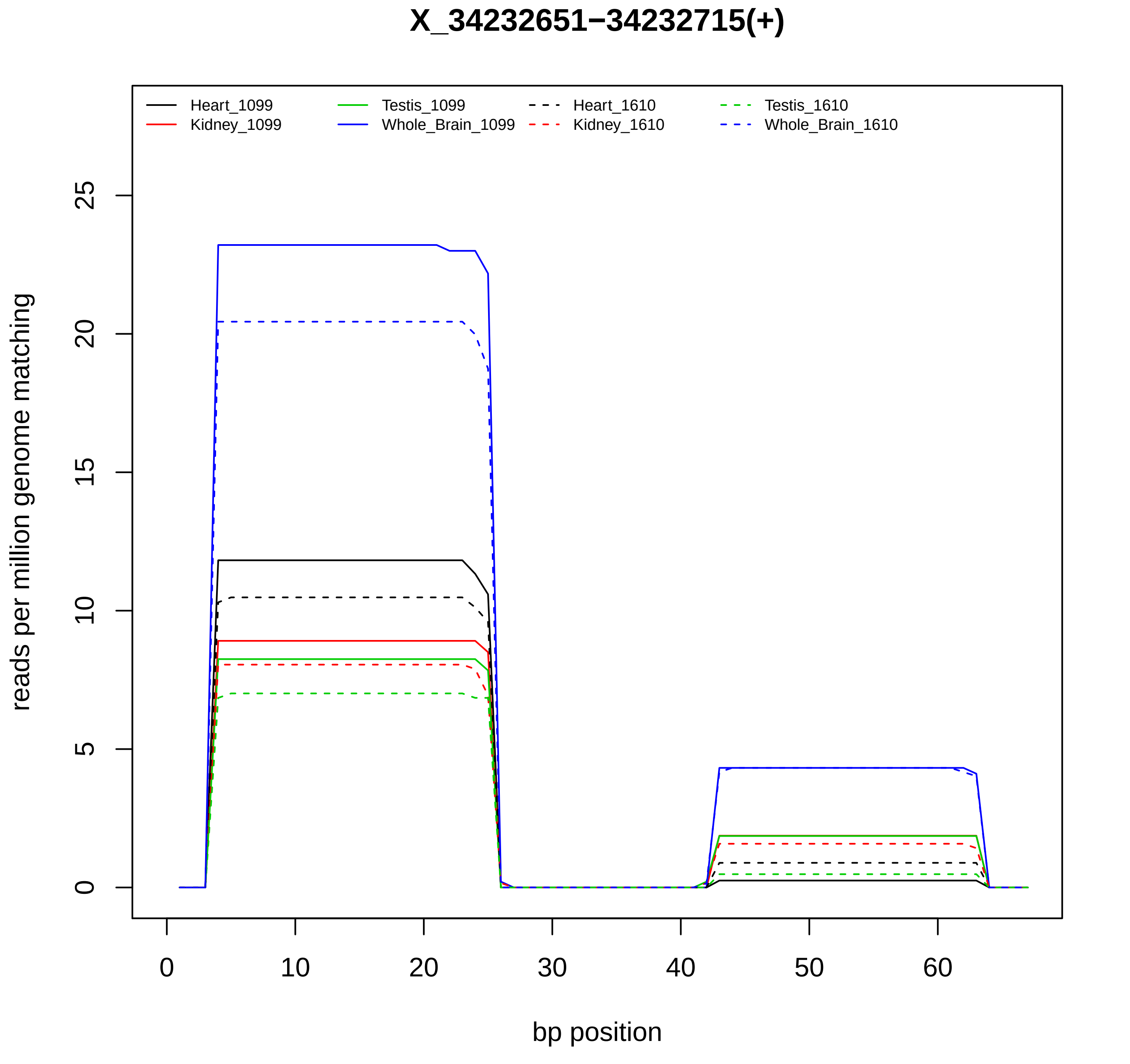

X_34232651−34232715(+)
Heart_1099
Kidney_1099
Testis_1099
Whole_Brain_1099
Heart_1610
Kidney_1610
Testis_1610
Whole_Brain_1610
reads per million genome matching
0
5
10
15
20
25
bp position
0
10
20
30
40
50
60

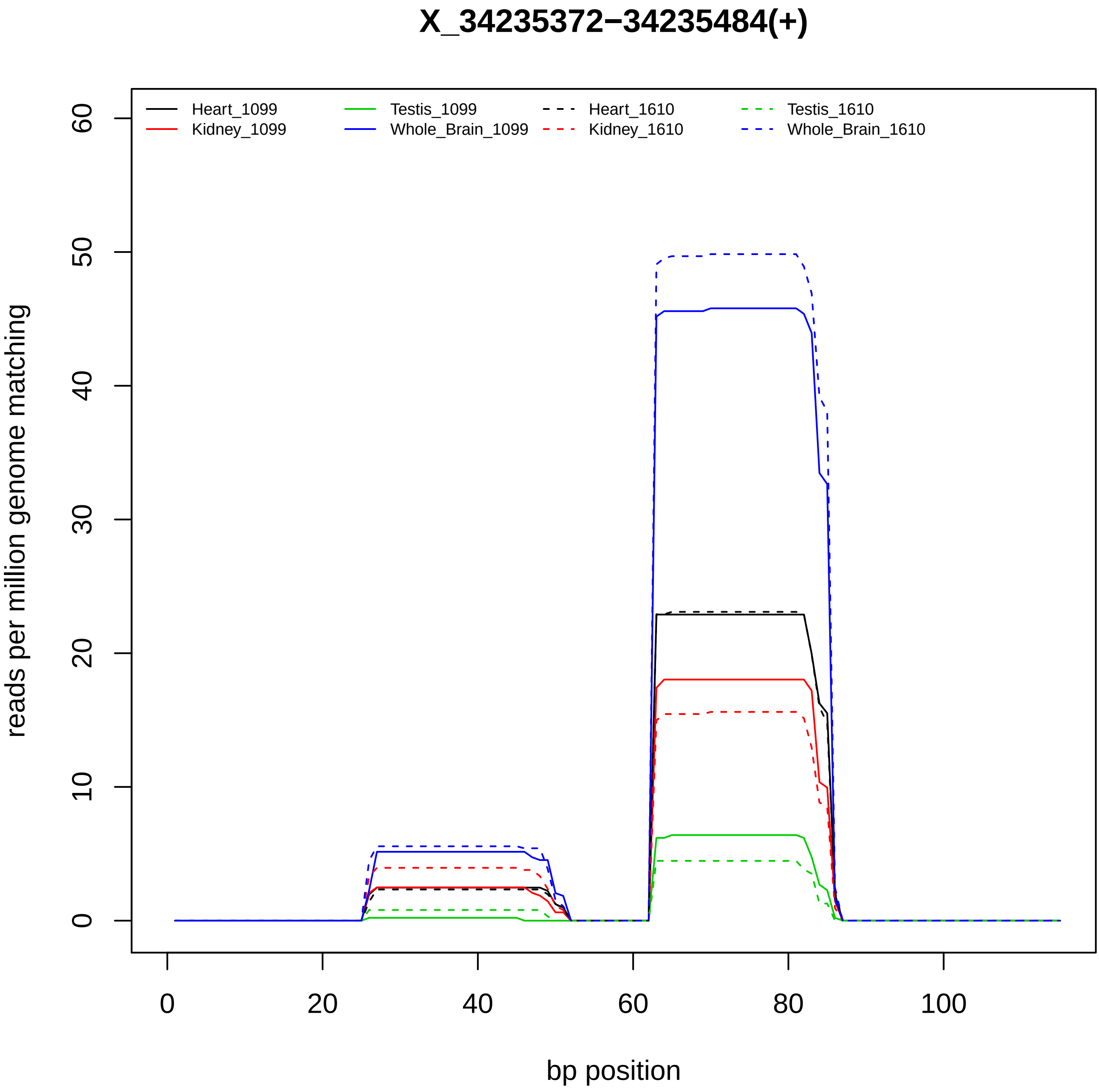
X_34235372−34235484(+)
Heart_1099
Kidney_1099
Testis_1099
Whole_Brain_1099
Heart_1610
Kidney_1610
Testis_1610
Whole_Brain_1610
reads per million genome matching
0
10
20
30
40
50
60
0
20
40
60
80
100
bp position

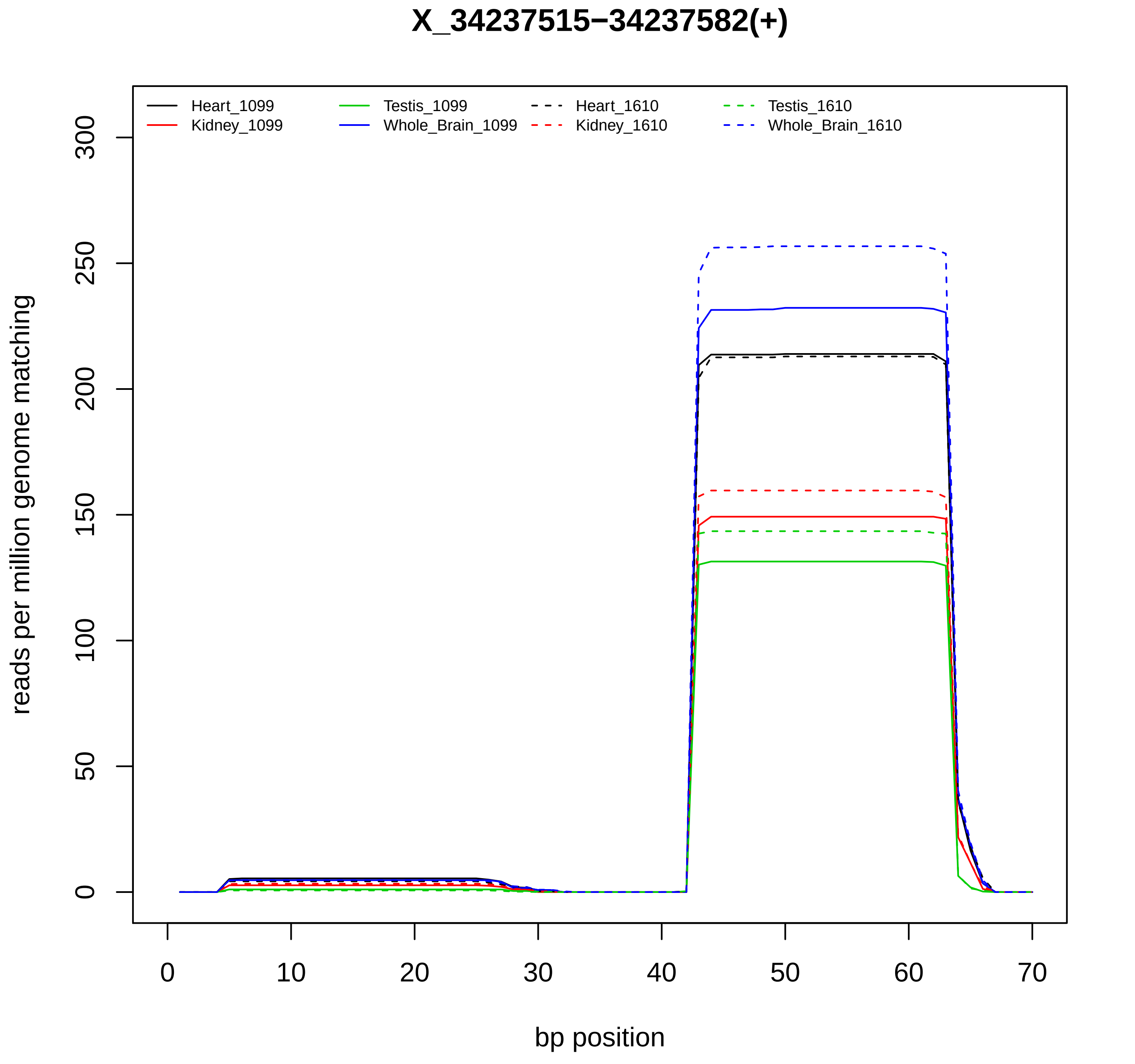

X_34237515−34237582(+)
Heart_1099
Kidney_1099
Testis_1099
Whole_Brain_1099
Heart_1610
Kidney_1610
Testis_1610
Whole_Brain_1610
reads per million genome matching
0
50
100
150
200
250
300
0
10
20
30
40
50
60
70
bp position

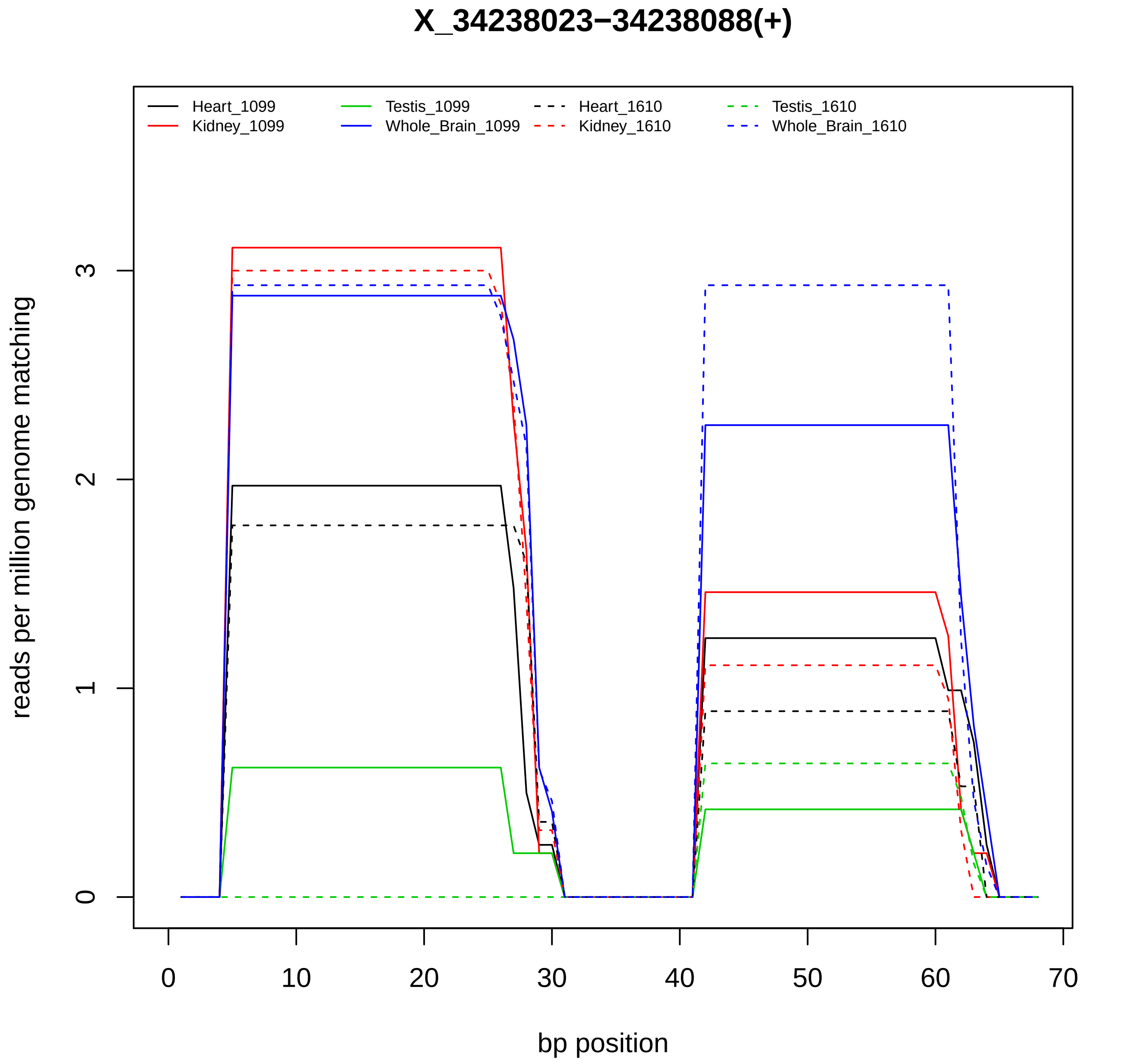

X_34238023−34238088(+)
Heart_1099
Kidney_1099
Testis_1099
Whole_Brain_1099
Heart_1610
Kidney_1610
Testis_1610
Whole_Brain_1610
reads per million genome matching
0
1
2
3
0
10
20
30
40
50
60
70
bp position

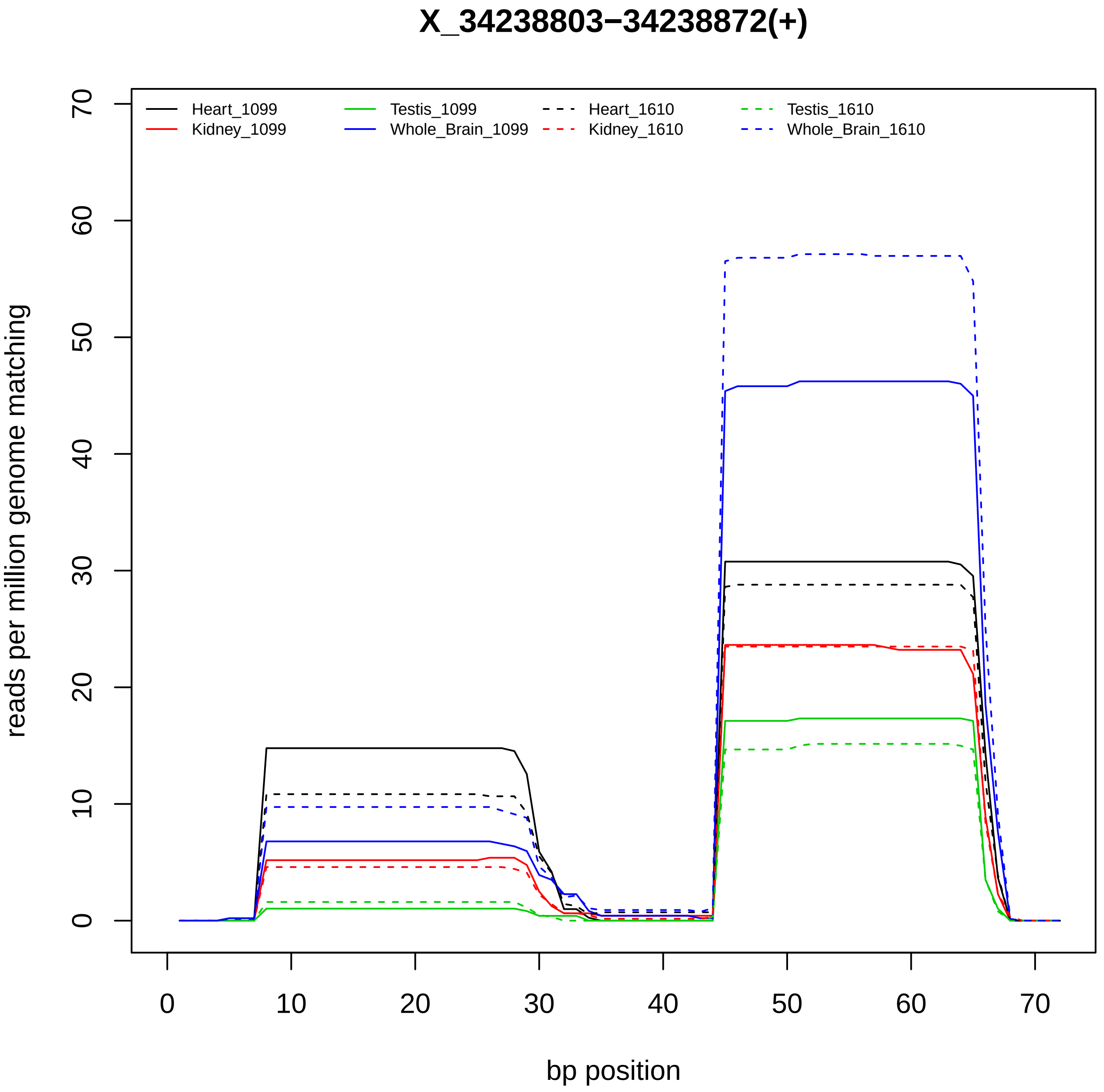
X_34238803−34238872(+)
Heart_1099
Kidney_1099
Testis_1099
Whole_Brain_1099
Heart_1610
Kidney_1610
Testis_1610
Whole_Brain_1610
reads per million genome matching
bp position
0
10
20
30
40
50
60
70

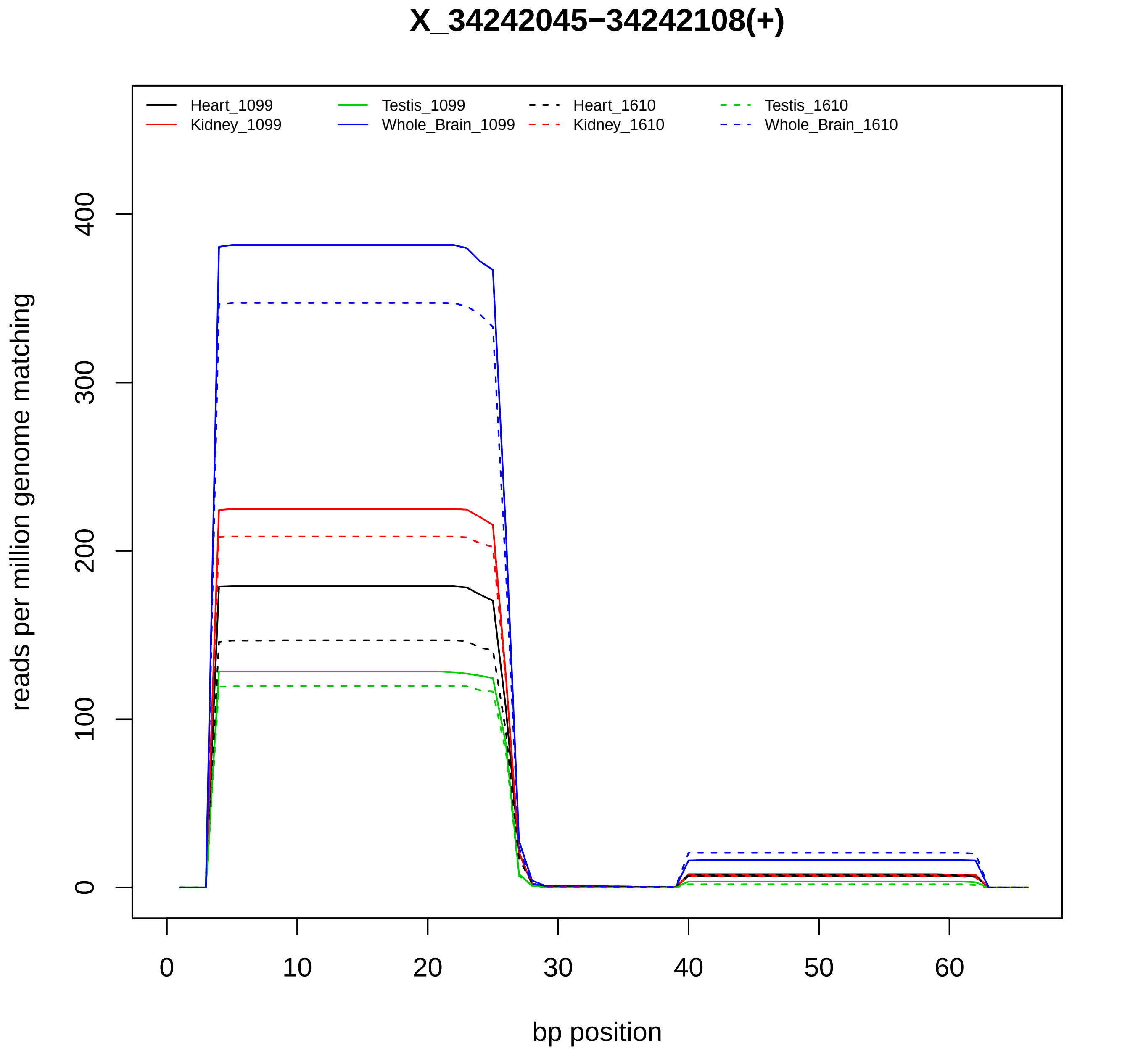
X_34242045−34242108(+)
Heart_1099
Kidney_1099
Testis_1099
Whole_Brain_1099
Heart_1610
Kidney_1610
Testis_1610
Whole_Brain_1610
reads per million genome matching
0
100
200
300
400
bp position
0
10
20
30
40
50
60

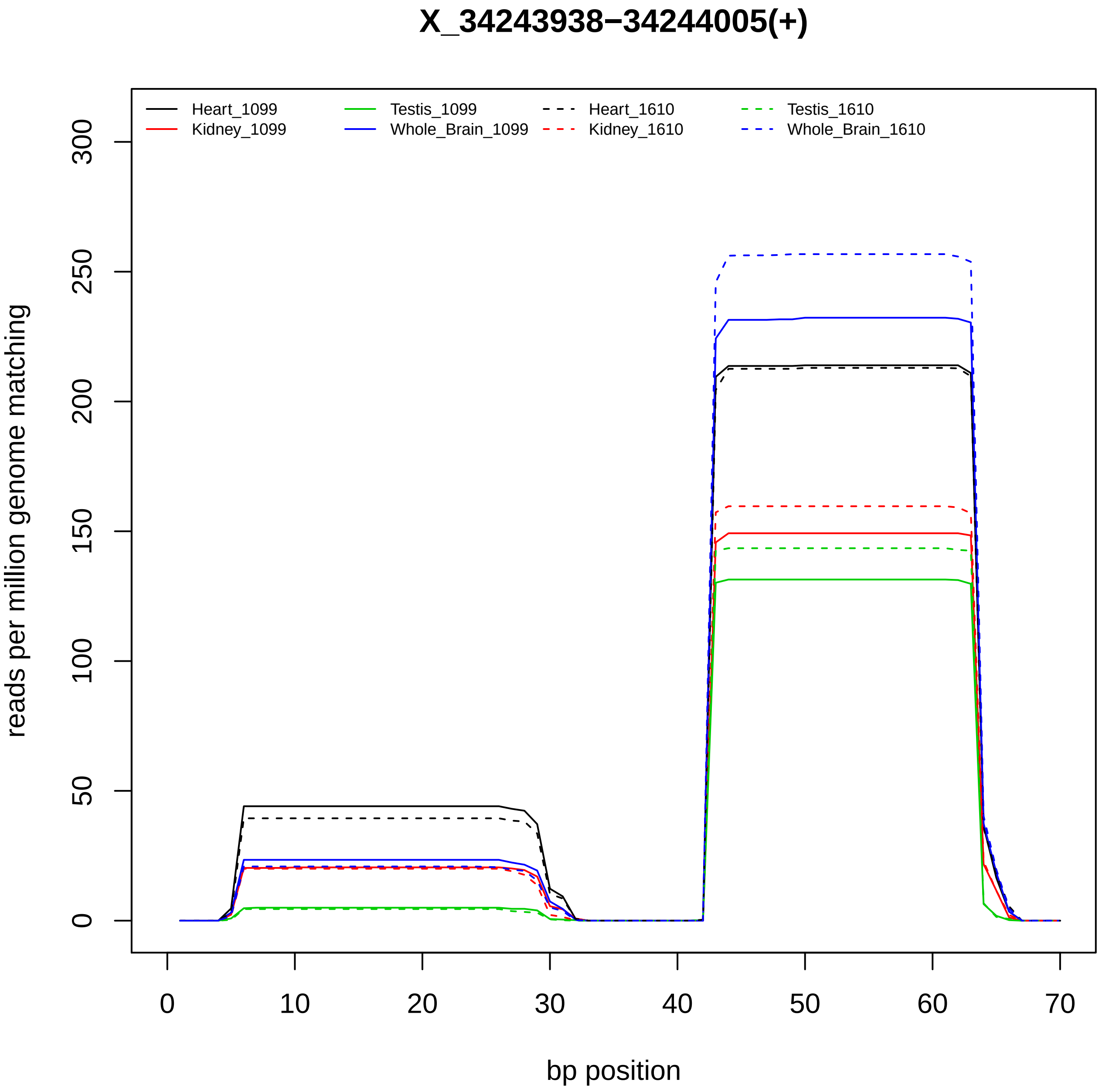

X_34243938−34244005(+)
Heart_1099
Kidney_1099
Testis_1099
Whole_Brain_1099
Heart_1610
Kidney_1610
Testis_1610
Whole_Brain_1610
reads per million genome matching
0
50
100
150
200
250
300
0
10
20
30
40
50
60
70
bp position

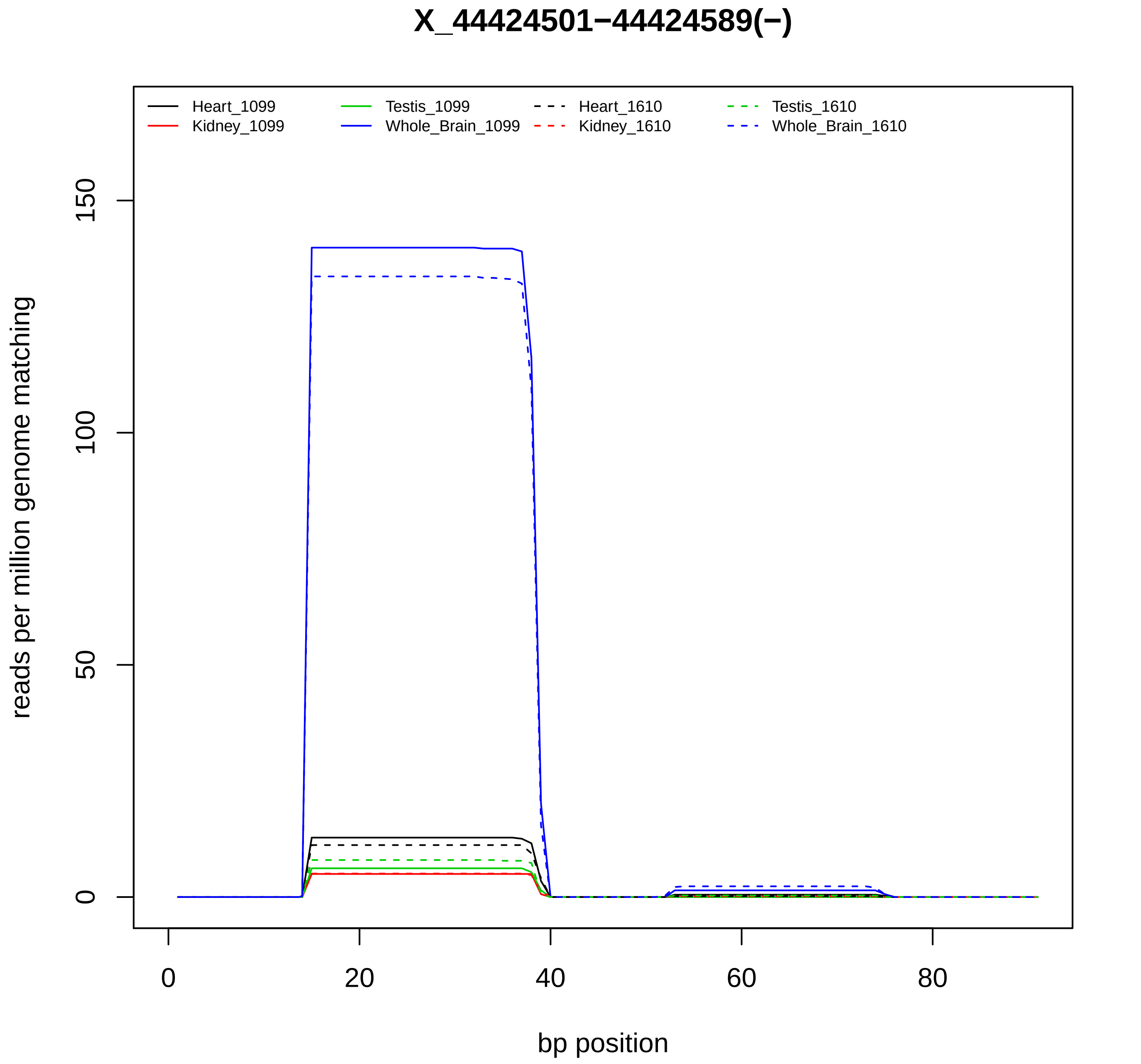

X_44424501−44424589(−)
Heart_1099
Kidney_1099
Testis_1099
Whole_Brain_1099
Heart_1610
Kidney_1610
Testis_1610
Whole_Brain_1610
reads per million genome matching
0
50
100
150
0
20
40
60
80
bp position

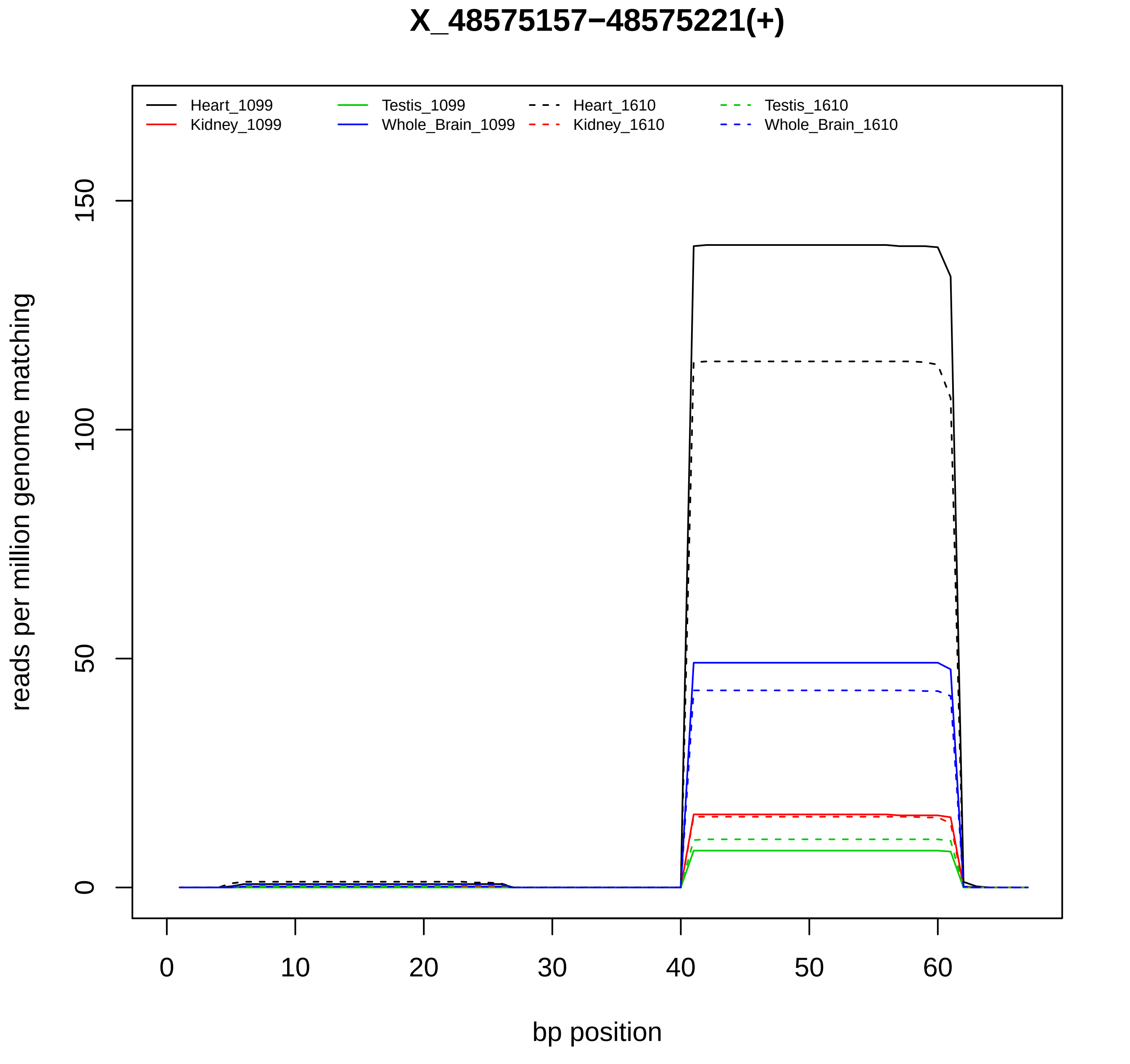
X_48575157−48575221(+)
Heart_1099
Kidney_1099
Testis_1099
Whole_Brain_1099
Heart_1610
Kidney_1610
Testis_1610
Whole_Brain_1610
reads per million genome matching
0
50
100
150
0
10
20
30
40
50
60
bp position

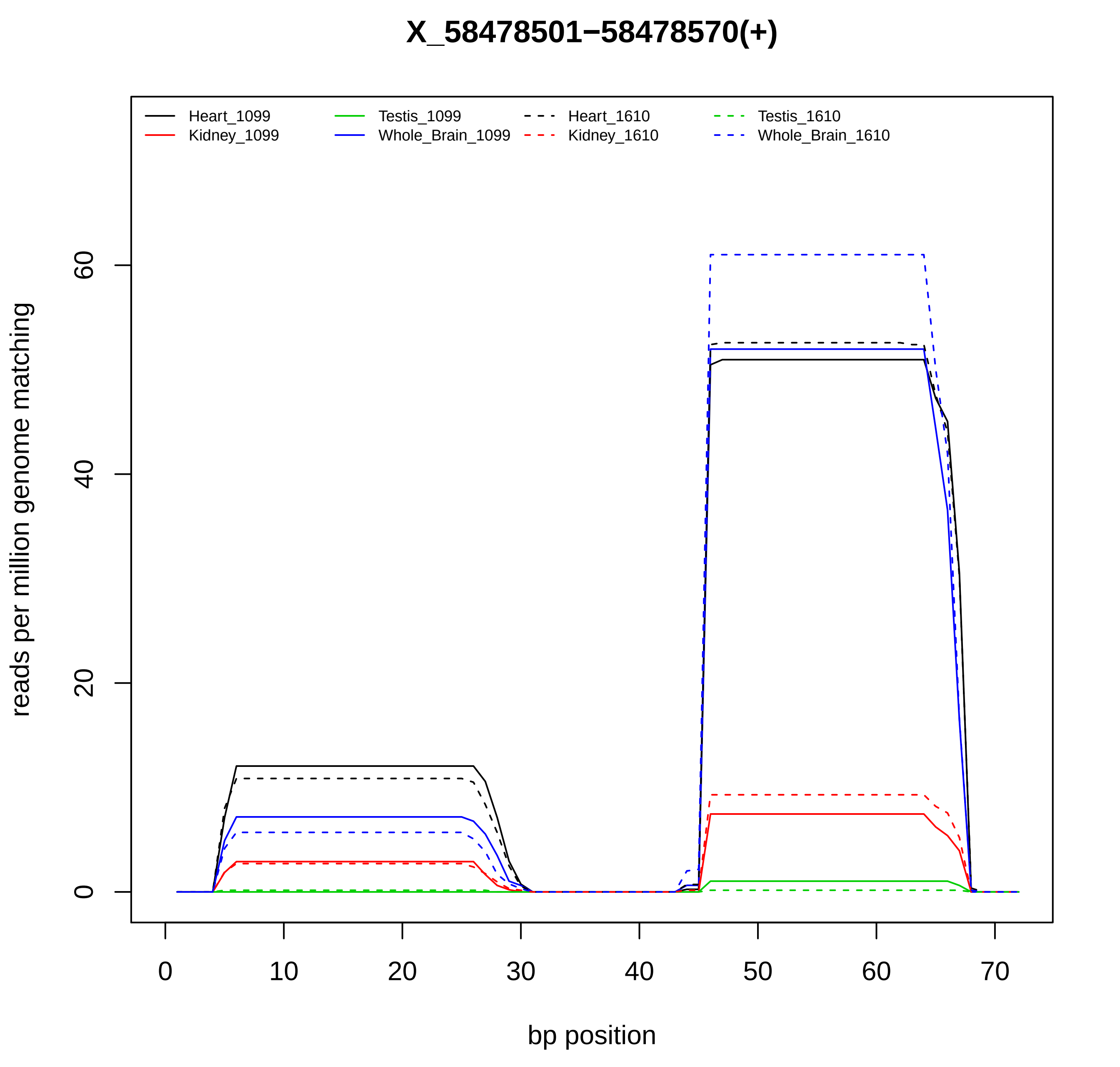
X_58478501−58478570(+)
Heart_1099
Kidney_1099
Testis_1099
Whole_Brain_1099
Heart_1610
Kidney_1610
Testis_1610
Whole_Brain_1610
reads per million genome matching
bp position
0
20
40
60
0
10
20
30
40
50
60
70

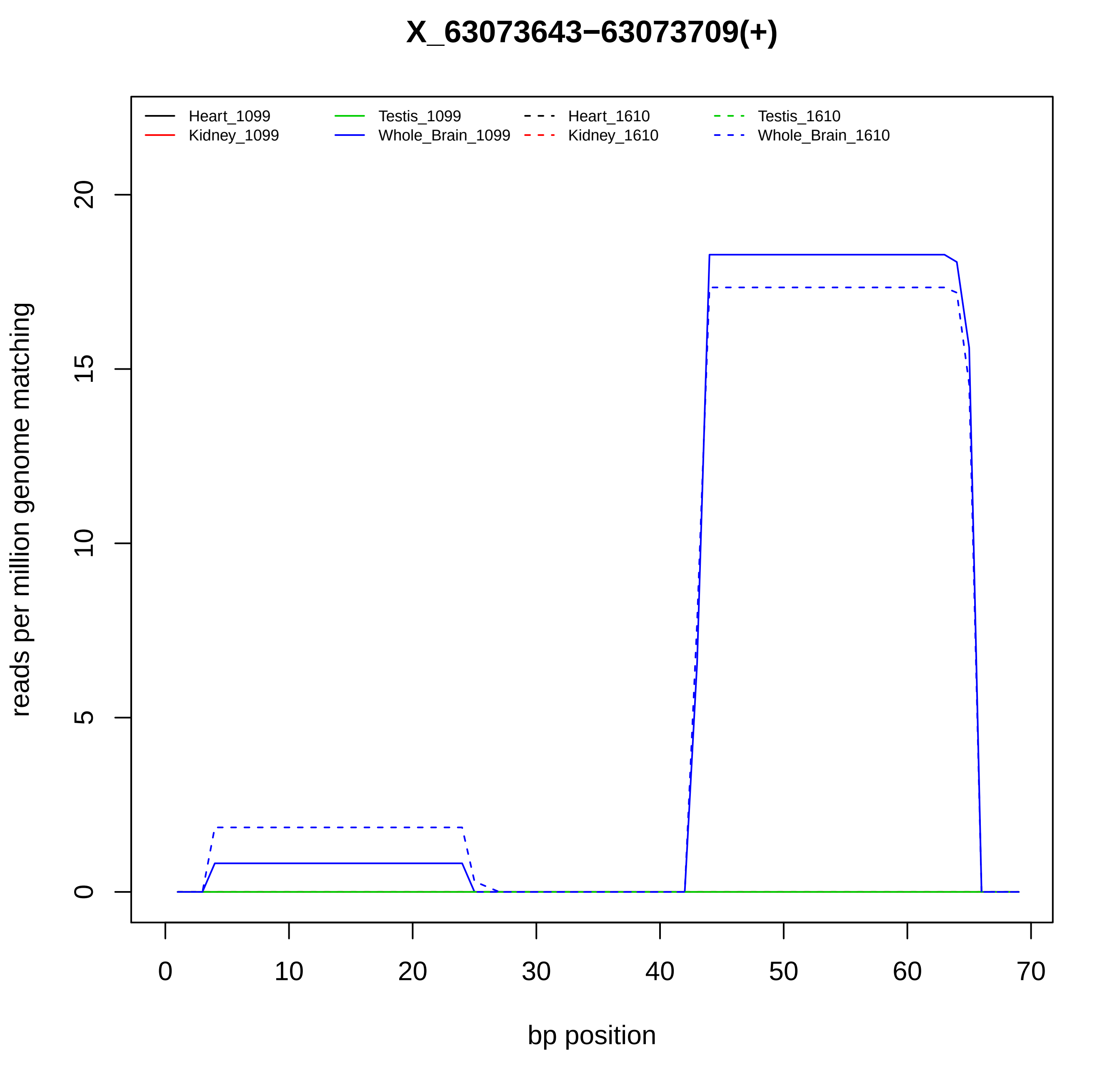
X_63073643−63073709(+)
Heart_1099
Kidney_1099
Testis_1099
Whole_Brain_1099
Heart_1610
Kidney_1610
Testis_1610
Whole_Brain_1610
reads per million genome matching
0
5
10
15
20
0
10
20
30
40
50
60
70
bp position

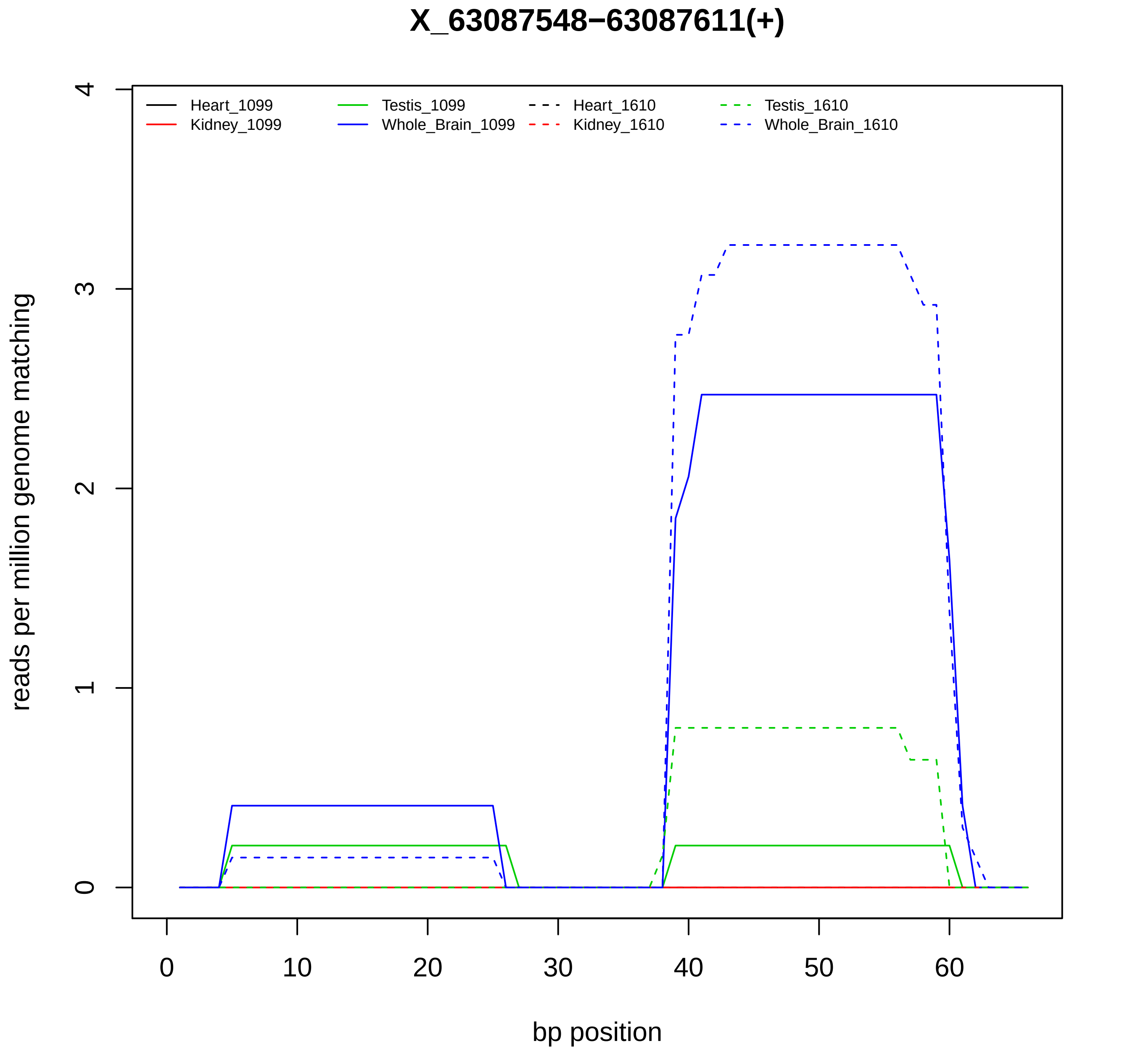
X_63087548−63087611(+)
Heart_1099
Kidney_1099
Testis_1099
Whole_Brain_1099
Heart_1610
Kidney_1610
Testis_1610
Whole_Brain_1610
reads per million genome matching
bp position
0
1
2
3
4
0
10
20
30
40
50
60

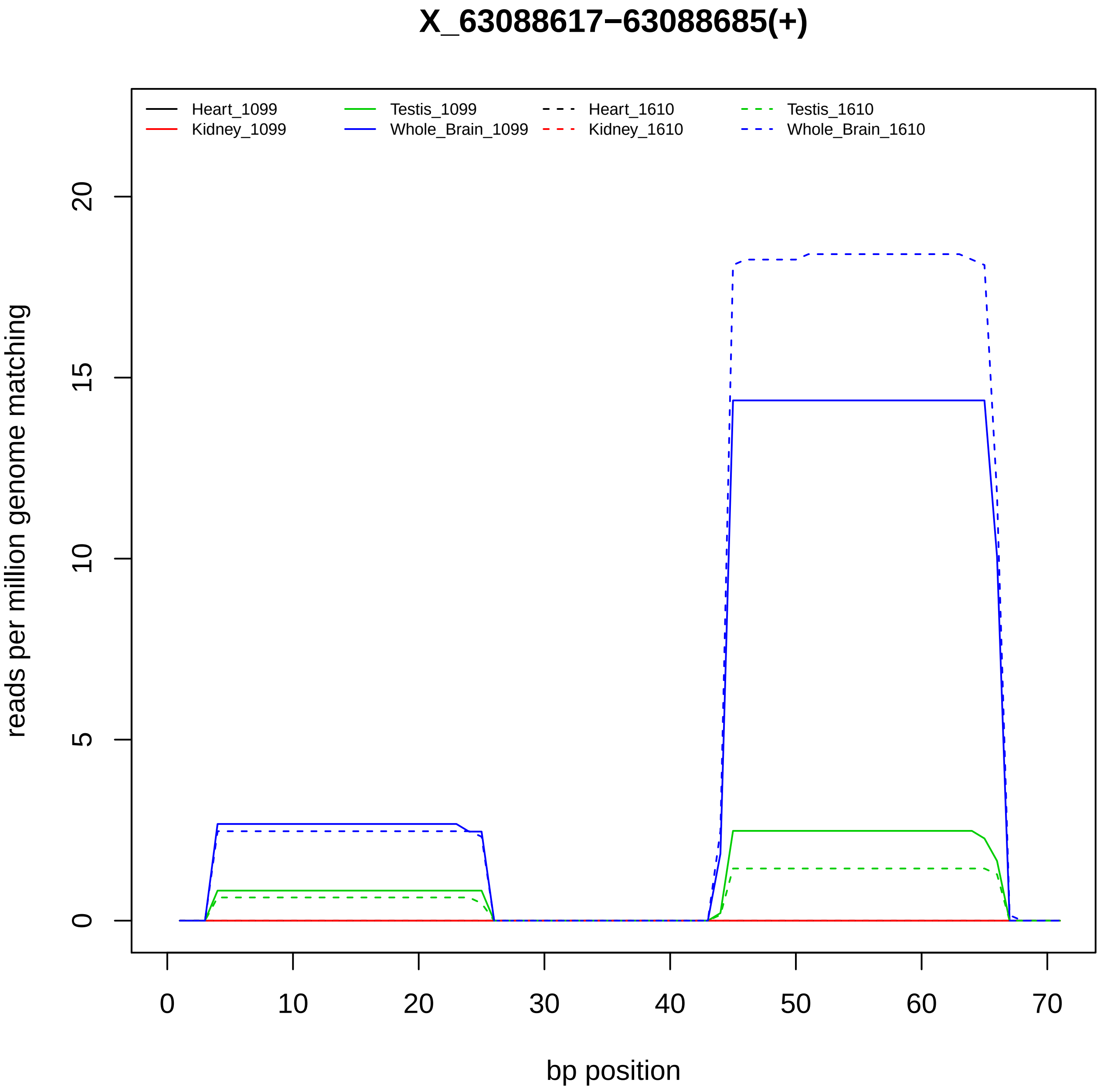

X_63088617−63088685(+)
Heart_1099
Kidney_1099
Testis_1099
Whole_Brain_1099
Heart_1610
Kidney_1610
Testis_1610
Whole_Brain_1610
reads per million genome matching
bp position
0
5
10
15
20
0
10
20
30
40
50
60
70

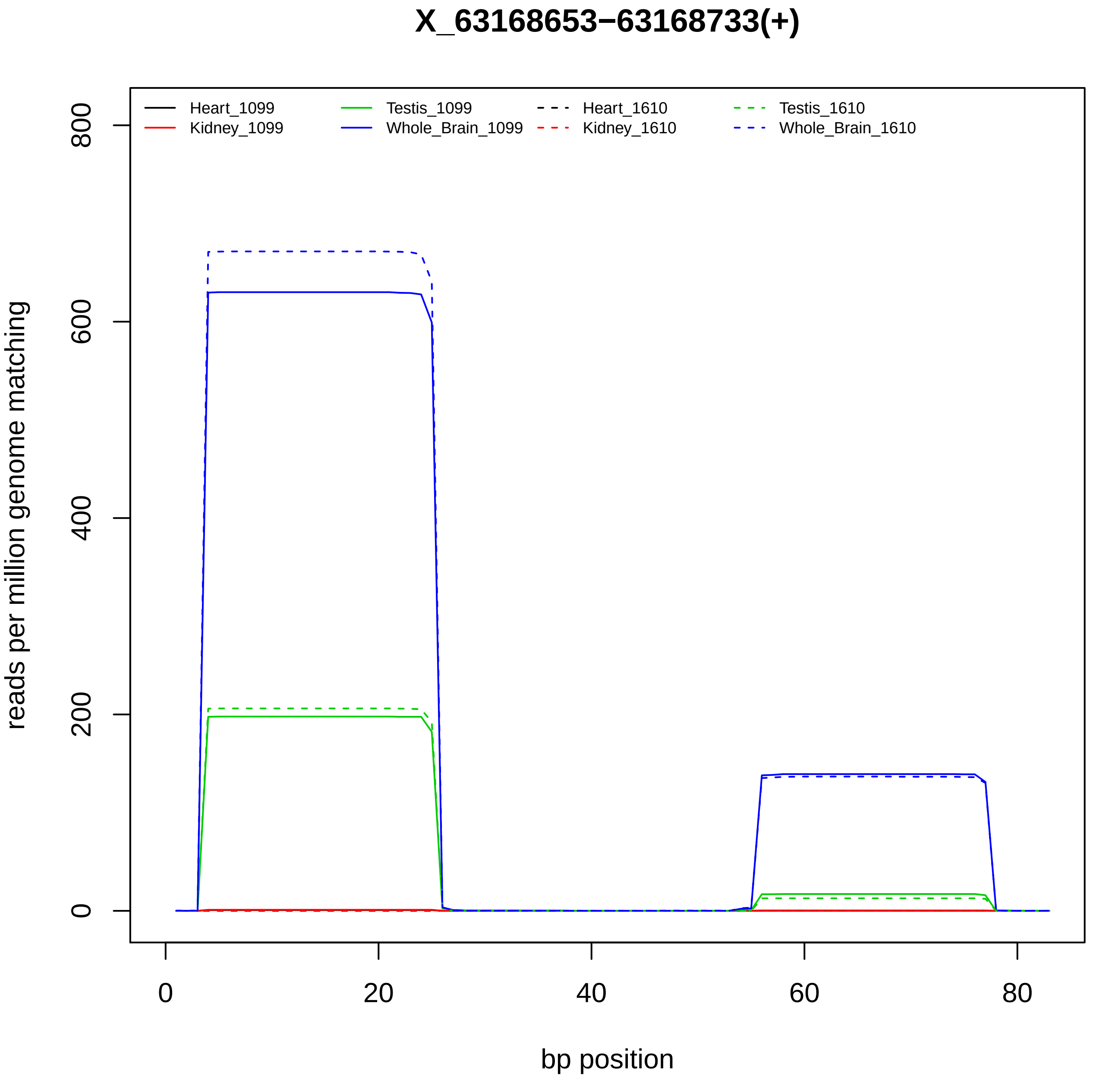

X_63168653−63168733(+)
Heart_1099
Kidney_1099
Testis_1099
Whole_Brain_1099
Heart_1610
Kidney_1610
Testis_1610
Whole_Brain_1610
reads per million genome matching
0
200
400
600
800
0
20
40
60
80
bp position

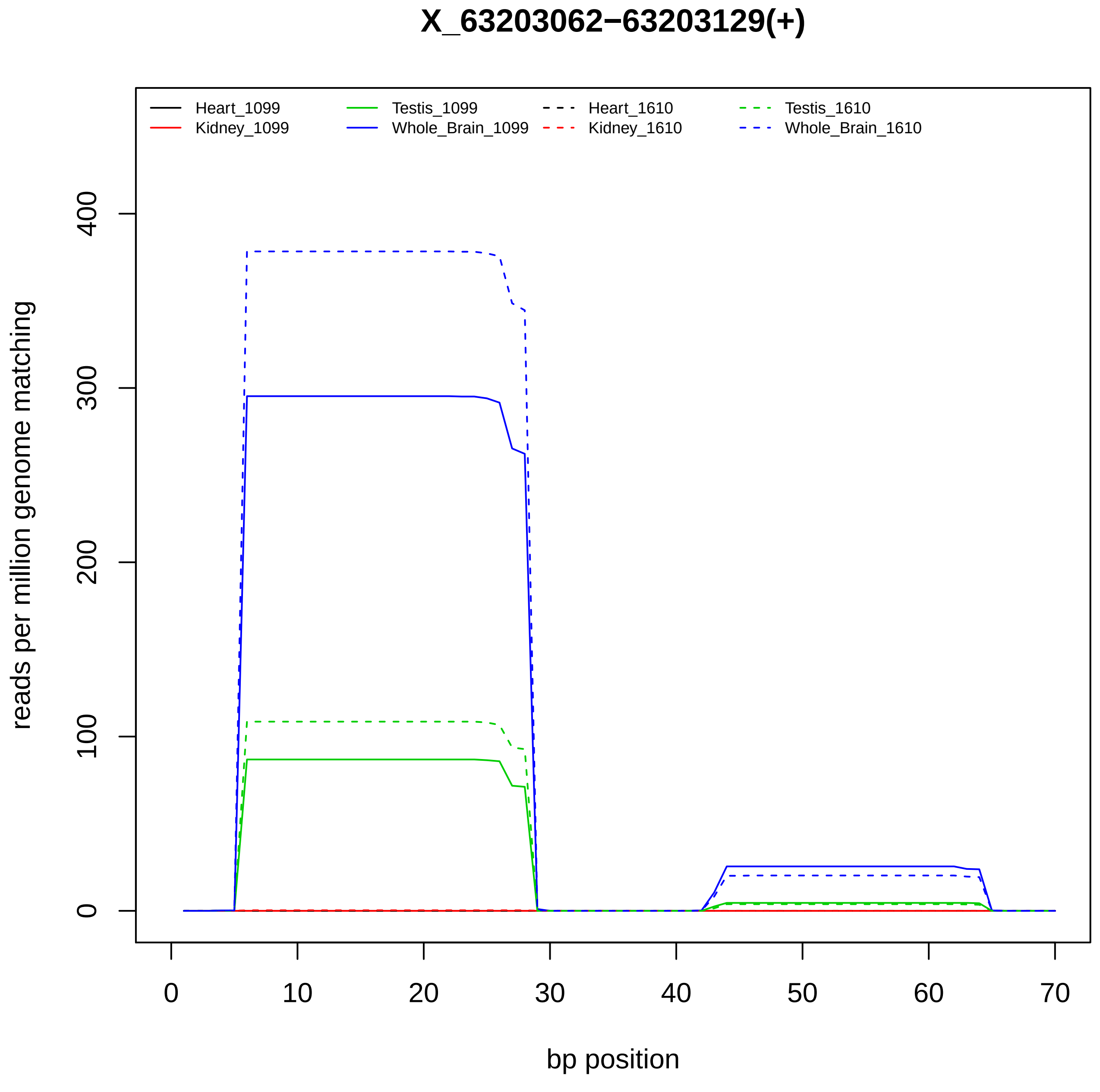
X_63203062−63203129(+)
Heart_1099
Kidney_1099
Testis_1099
Whole_Brain_1099
Heart_1610
Kidney_1610
Testis_1610
Whole_Brain_1610
reads per million genome matching
0
100
200
300
400
0
10
20
30
40
50
60
70
bp position

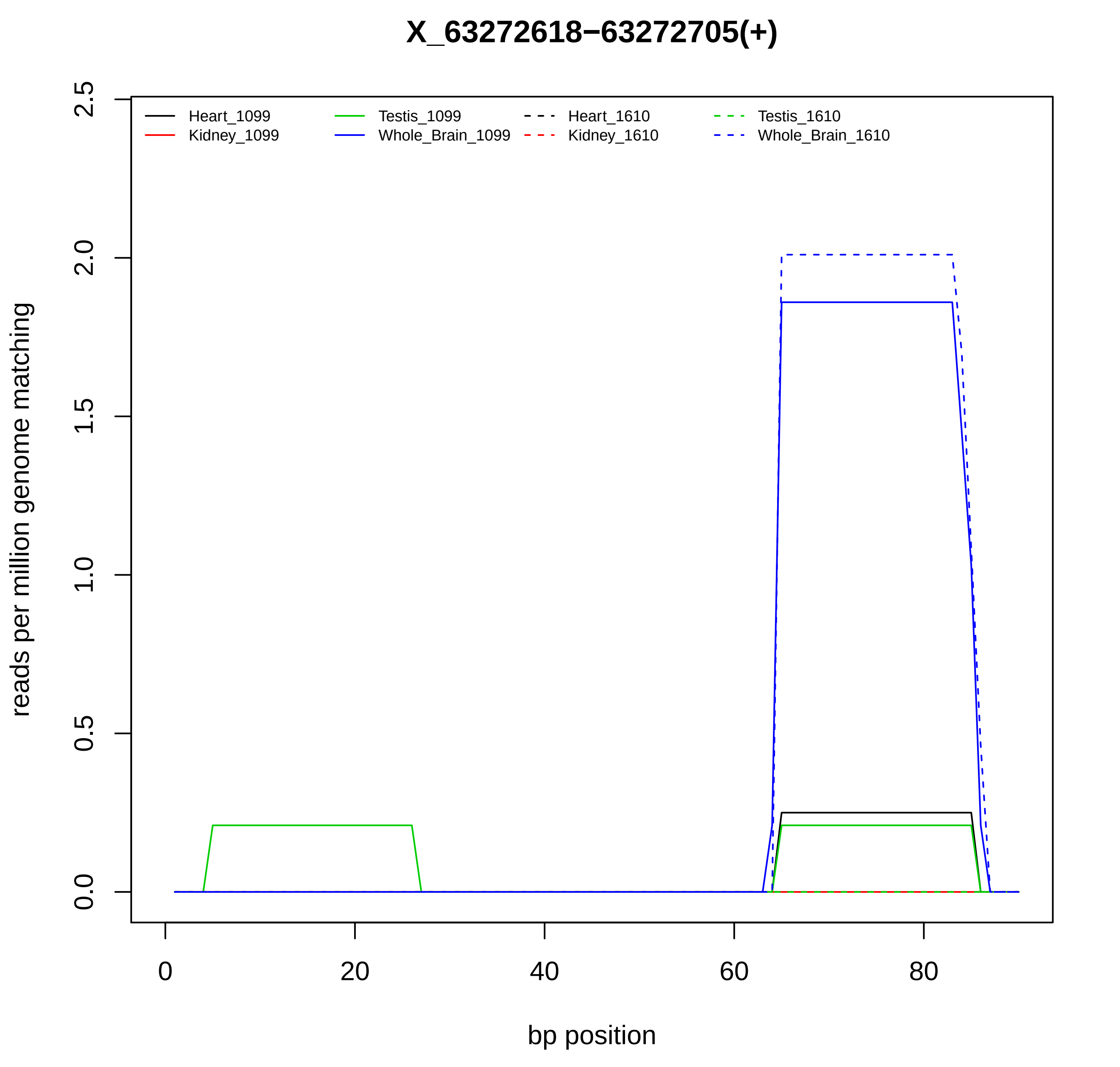

X_63272618−63272705(+)
Heart_1099
Kidney_1099
Testis_1099
Whole_Brain_1099
Heart_1610
Kidney_1610
Testis_1610
Whole_Brain_1610
reads per million genome matching
bp position
0.0
0.5
1.0
1.5
2.0
2.5
0
20
40
60
80

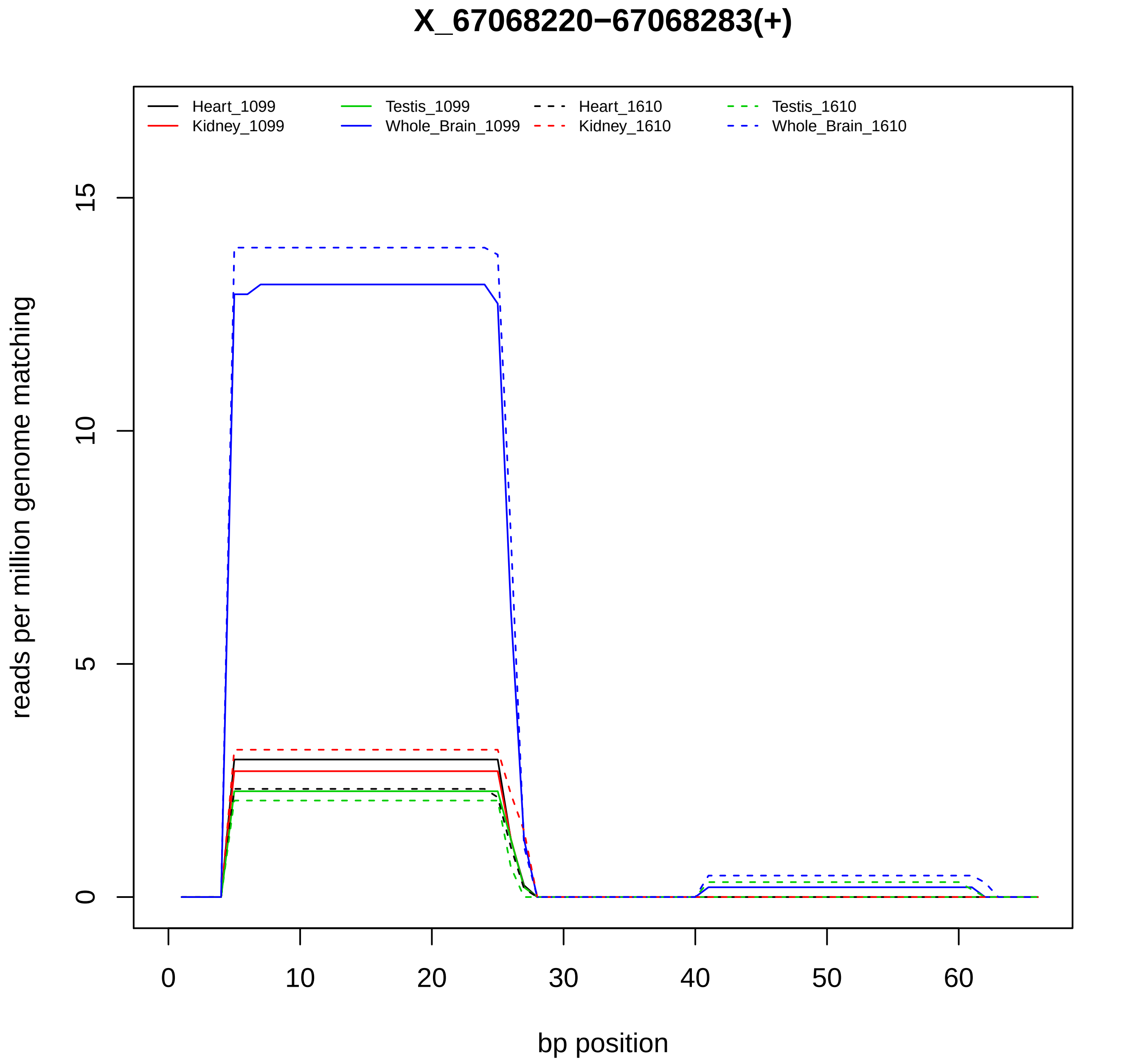
X_67068220−67068283(+)
Heart_1099
Kidney_1099
Testis_1099
Whole_Brain_1099
Heart_1610
Kidney_1610
Testis_1610
Whole_Brain_1610
reads per million genome matching
0
5
10
15
0
10
20
30
40
50
60
bp position

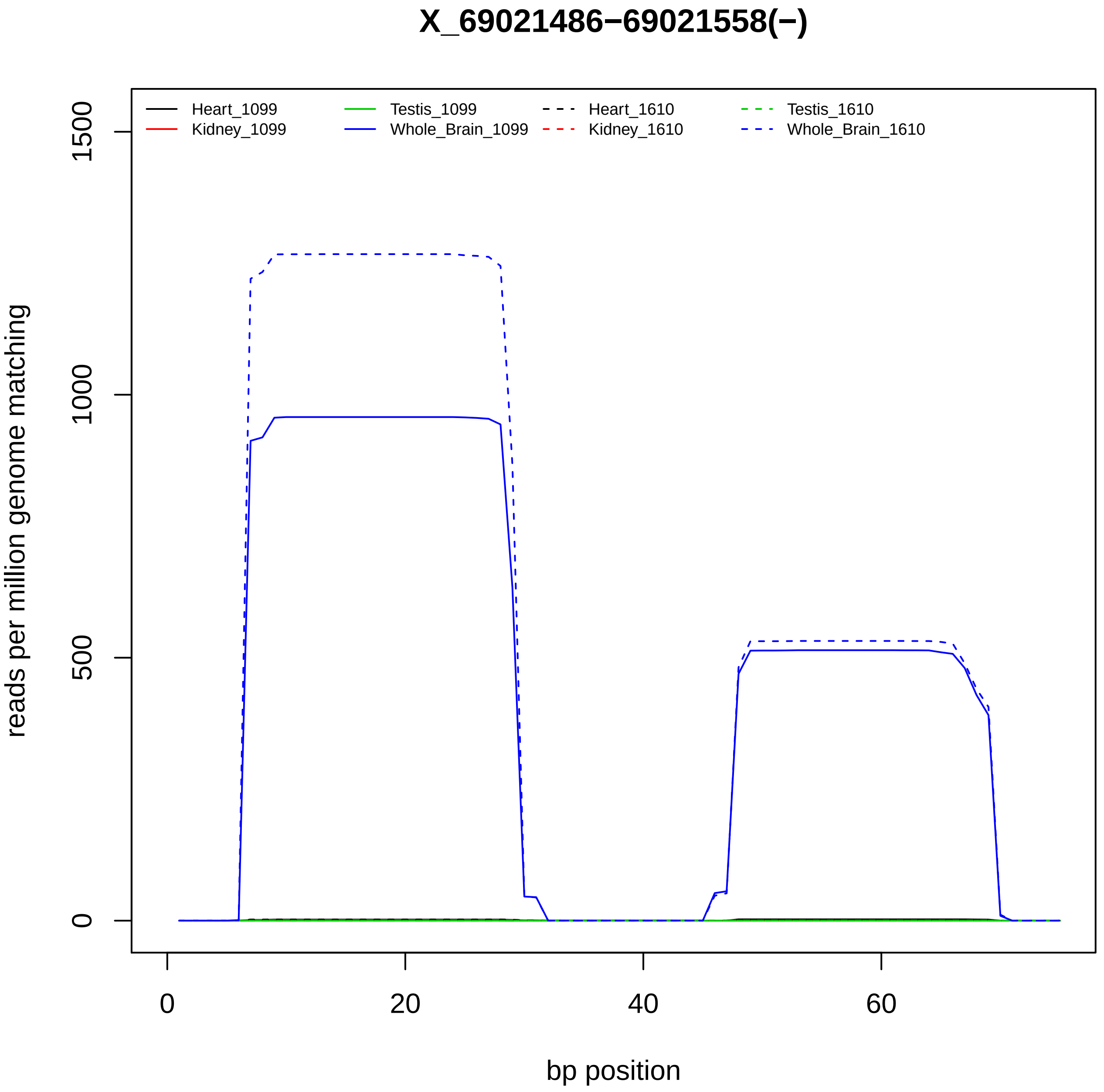

X_69021486−69021558(−)
Heart_1099
Kidney_1099
Testis_1099
Whole_Brain_1099
Heart_1610
Kidney_1610
Testis_1610
Whole_Brain_1610
reads per million genome matching
0
500
1000
1500
0
20
40
60
bp position

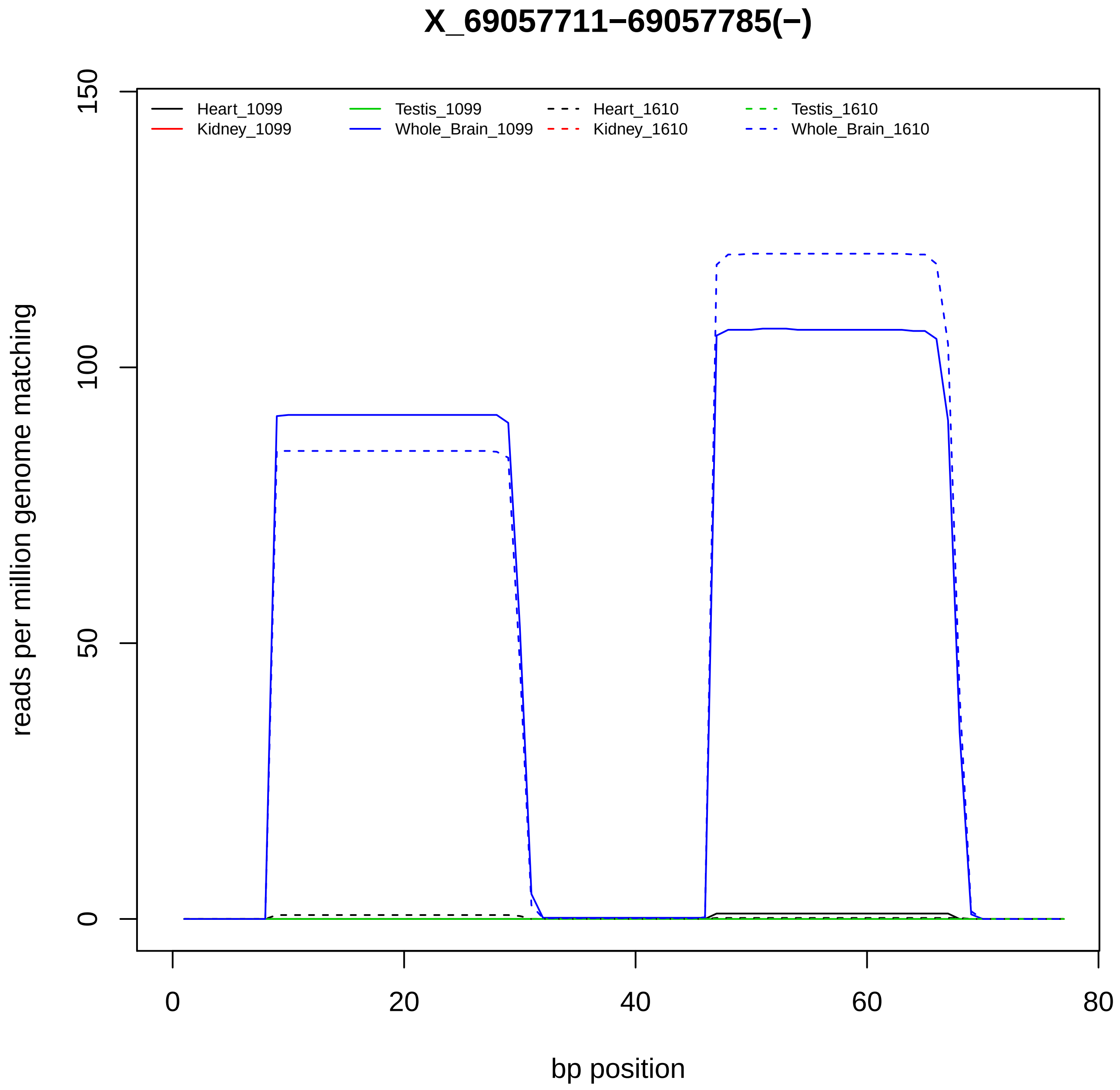

X_69057711−69057785(−)
Heart_1099
Kidney_1099
Testis_1099
Whole_Brain_1099
Heart_1610
Kidney_1610
Testis_1610
Whole_Brain_1610
reads per million genome matching
bp position
0
50
100
150
0
20
40
60
80

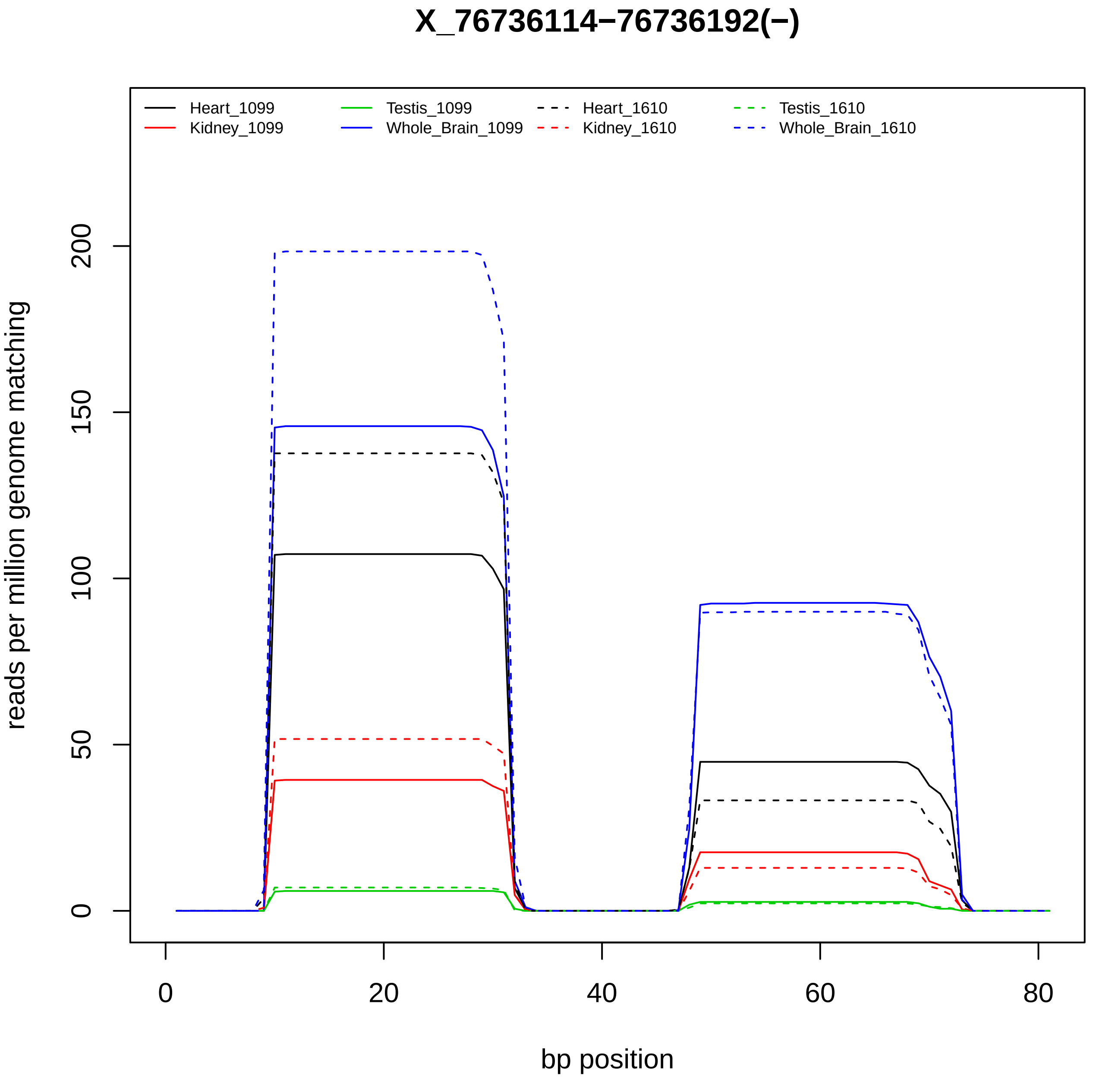

X_76736114−76736192(−)
Heart_1099
Kidney_1099
Testis_1099
Whole_Brain_1099
Heart_1610
Kidney_1610
Testis_1610
Whole_Brain_1610
reads per million genome matching
0
50
100
150
200
0
20
40
60
80
bp position

Fig. S9

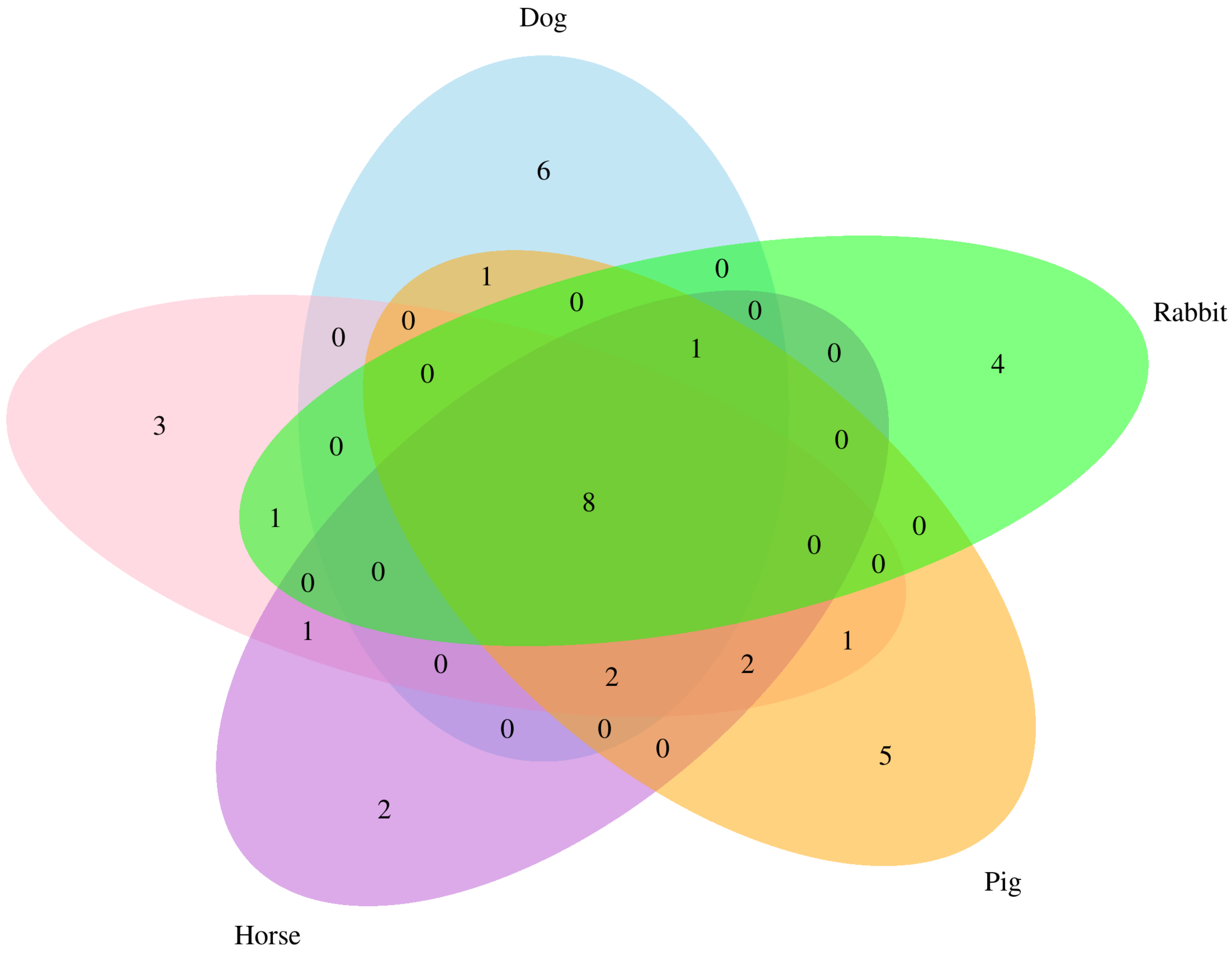

Dog
6
1
0
0
0
0
1
0
0
Rabbit
4
Cow
3
0
0
0
1
8
0
0
0
0
0
0
1
1
0
2
2
0
0
0
5
2
Pig
Horse

Fig. S10

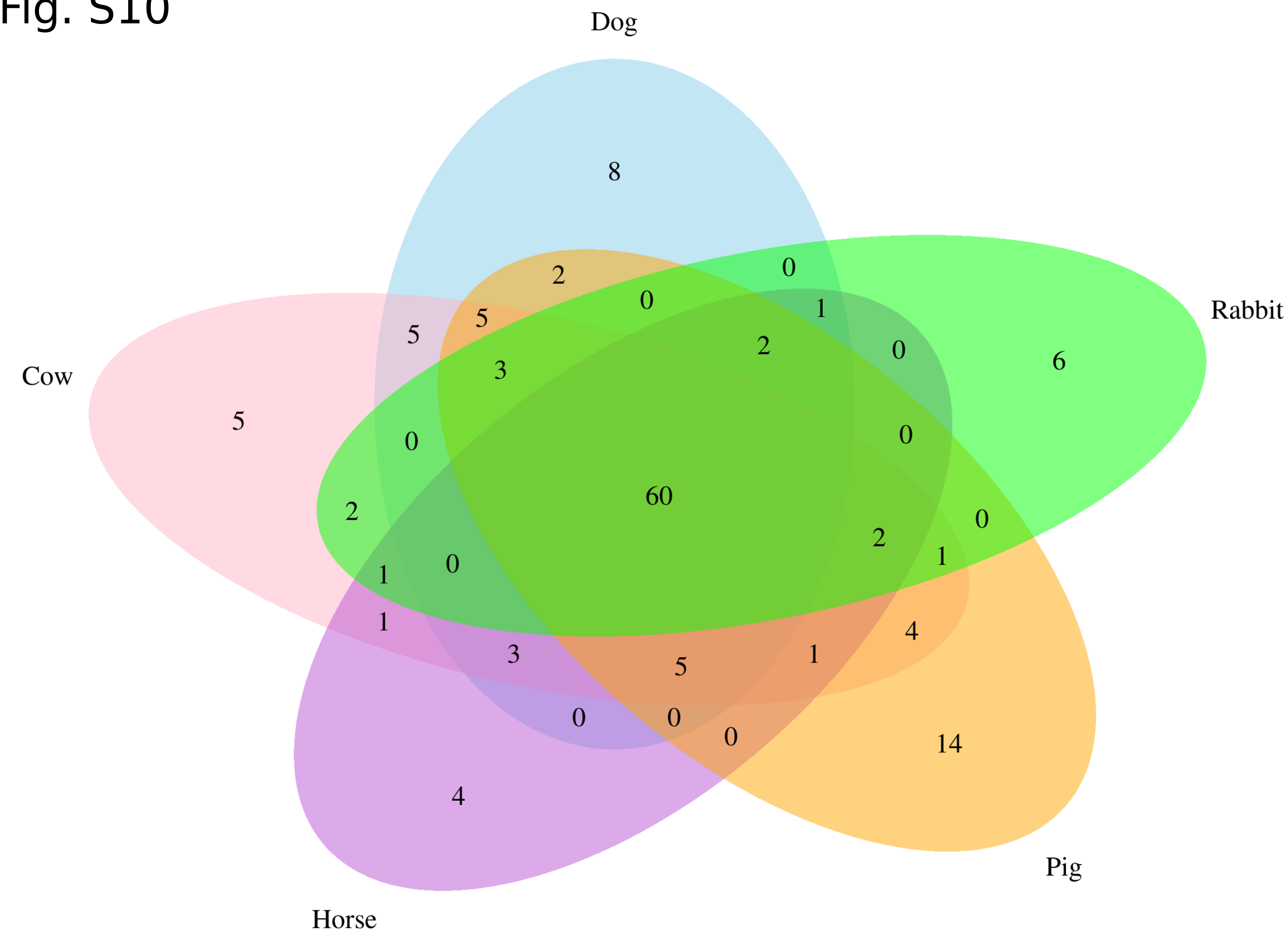

Dog
8
2
0
0
1
5
5
2
0
Rabbit
6
Cow
3
5
0
0
60
2
0
2
1
0
1
1
4
3
1
5
0
0
0
14
4
Pig
Horse

Fig. S11

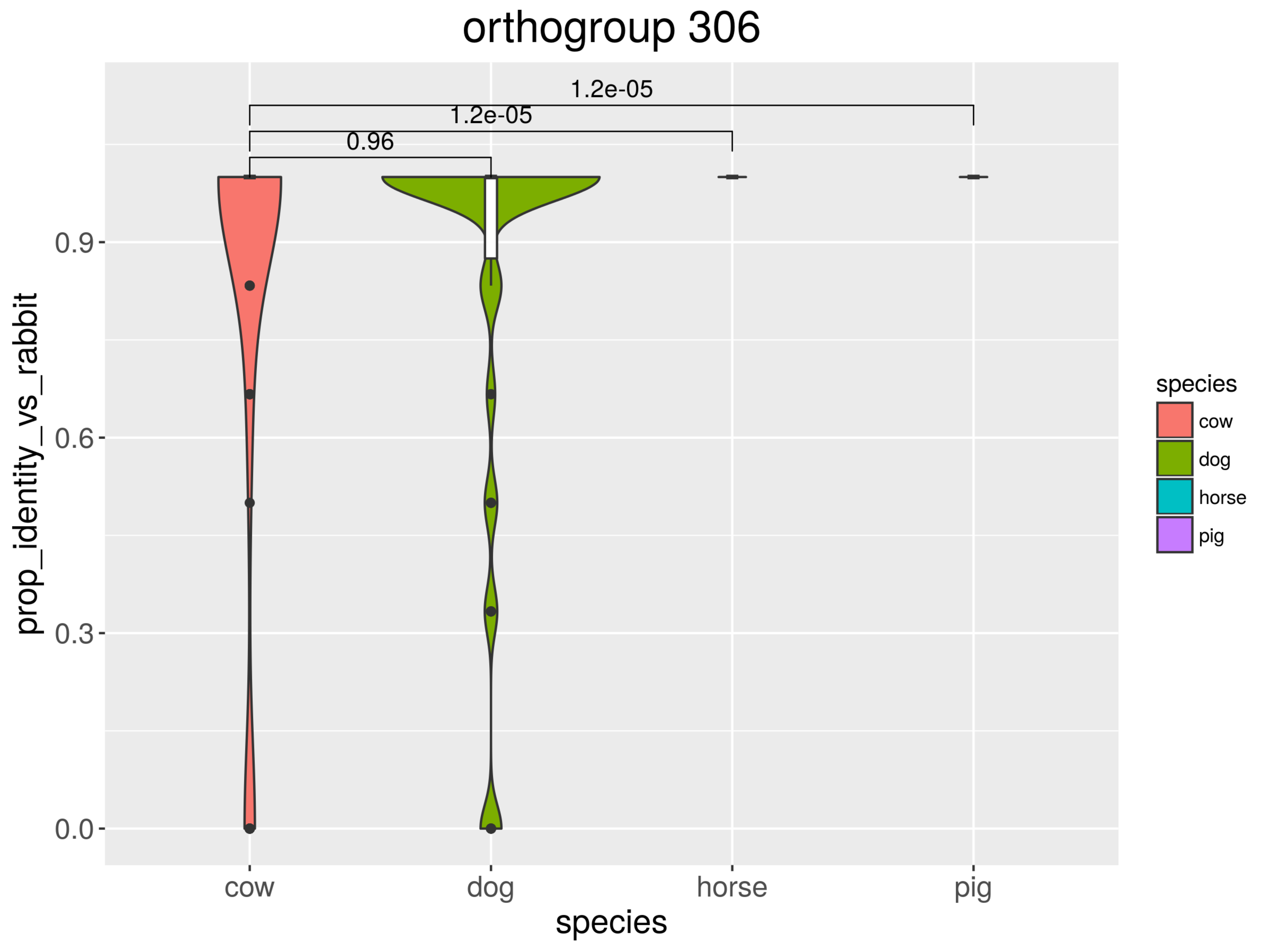

orthogroup 306
1.2e-05
1.2e-05
0.96
0.9
0.6
0.3
0.0
prop_identity_vs_rabbit
cow
dog
horse
pig
species
species
cow
dog
horse
pig

Fig. S12

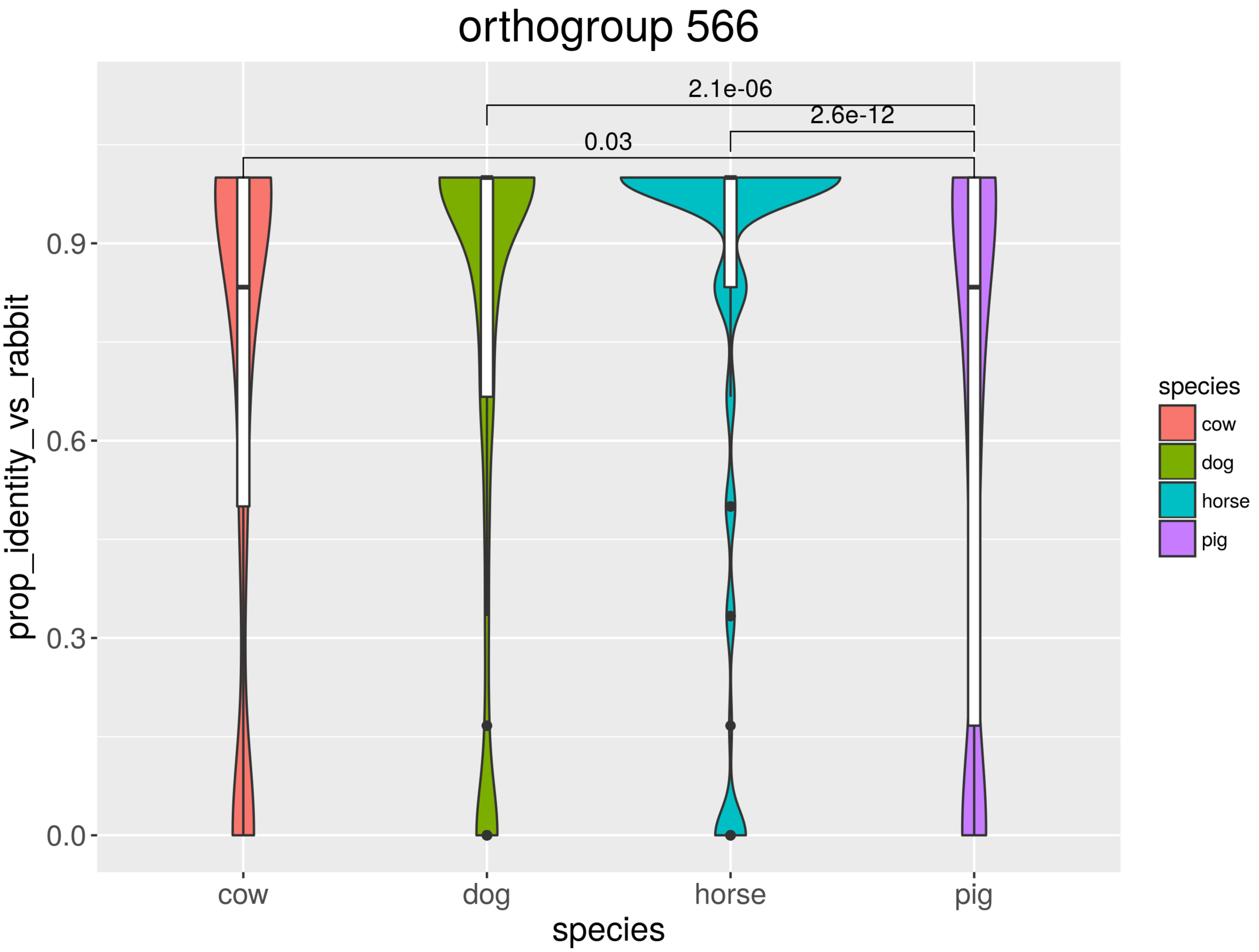

orthogroup 566
2.1e-06
2.6e-12
0.03
0.9
0.6
0.3
0.0
prop_identity_vs_rabbit
cow
dog
horse
pig
species
species
cow
dog
horse
pig

Fig. S13

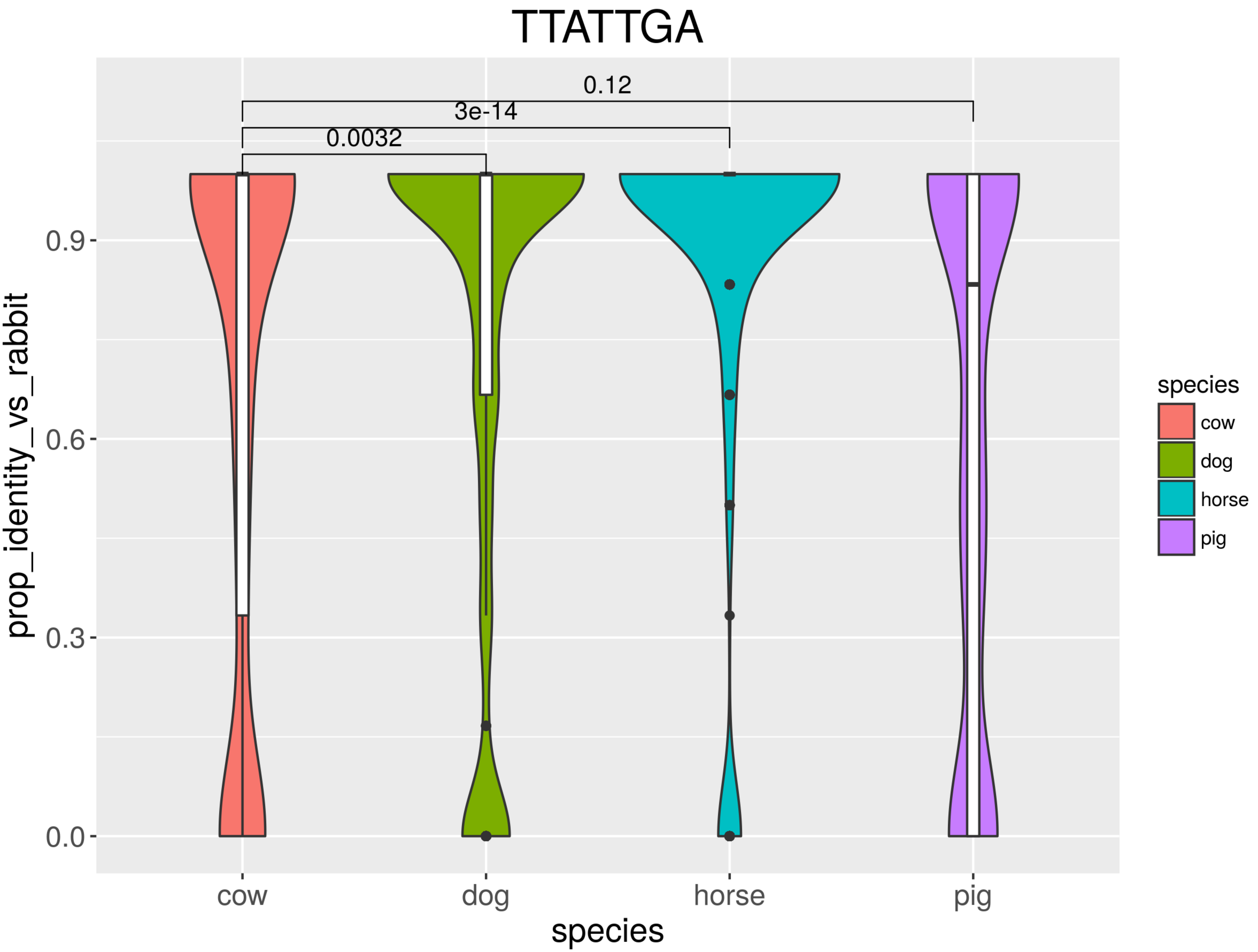

TTATTGA
0.12
3e-14
0.0032
0.9
0.6
0.3
0.0
prop_identity_vs_rabbit
cow
dog
horse
pig
species
species
cow
dog
horse
pig

Fig. S14

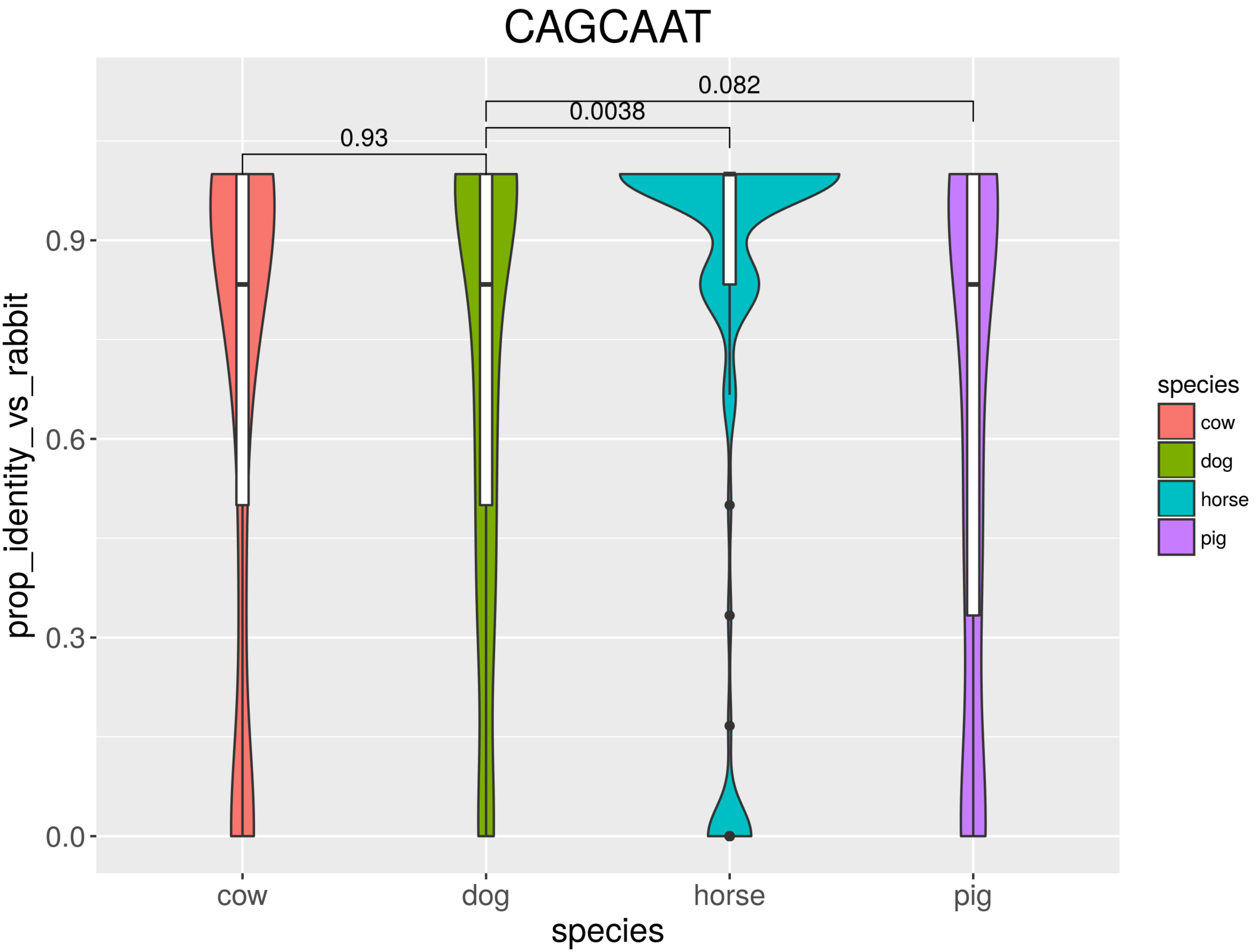

CAGCAAT
0.082
0.0038
0.93
prop_identity_vs_rabbit
0.9
0.6
0.3
0.0
cow
dog
horse
pig
species
species
cow
dog
horse
pig

Fig. S15

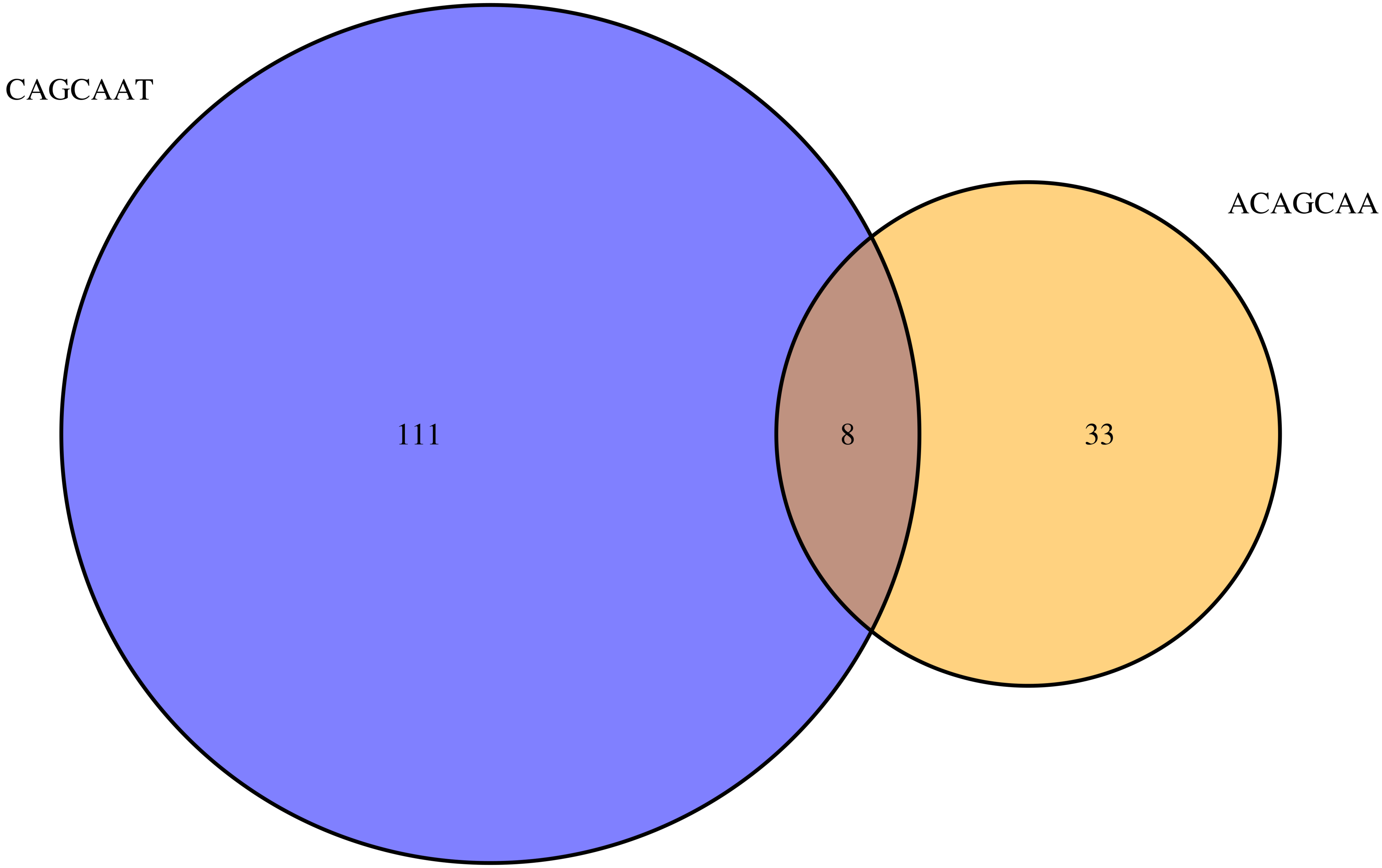

Fig. S16

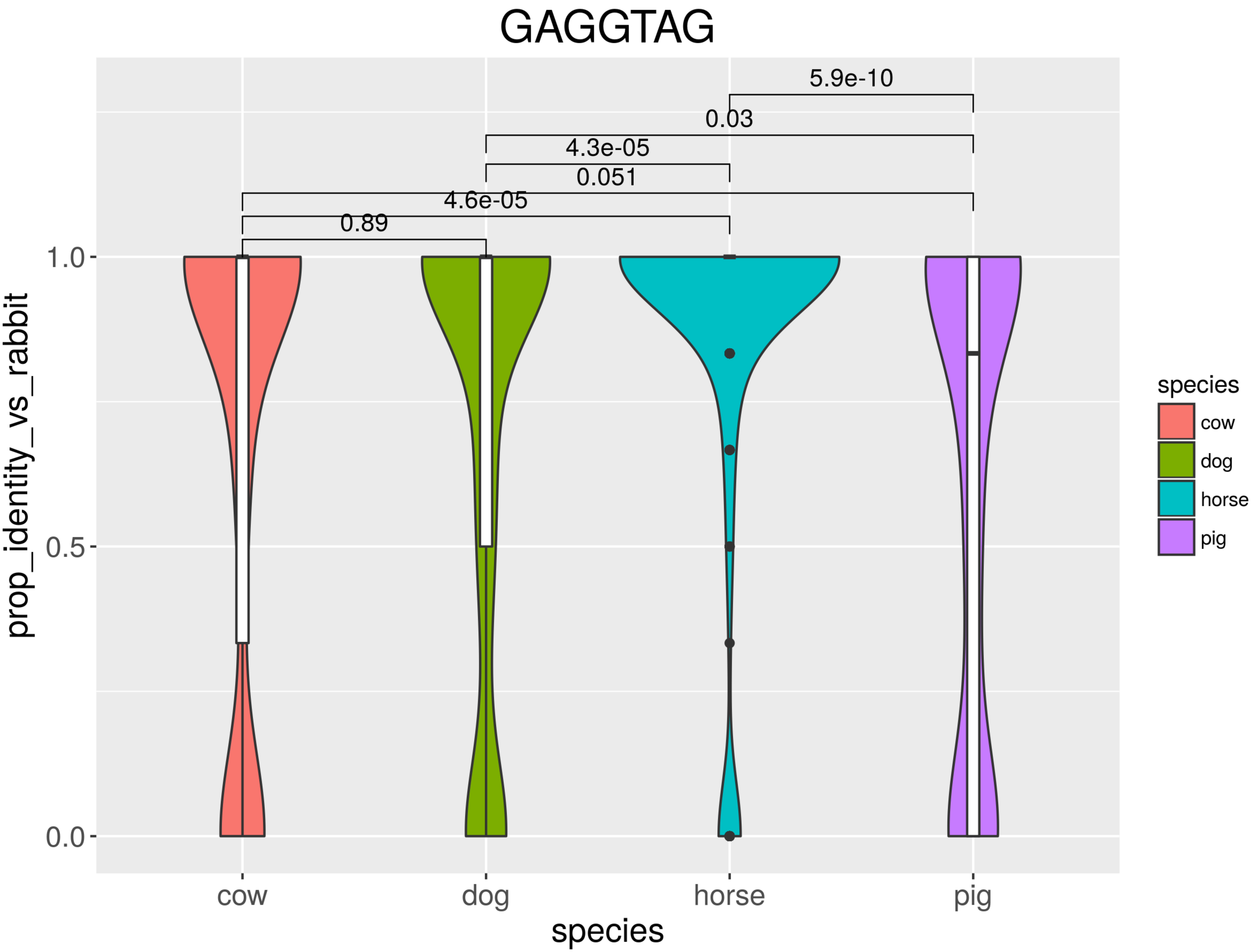

GAGGTAG
5.9e-10
0.03
4.3e-05
0.051
4.6e-05
0.89
1.0
0.5
0.0
prop_identity_vs_rabbit
cow
dog
horse
pig
species
species
cow
dog
horse
pig

File S1

```
10/101213569-101213635(-)_mir_cow_17
GGCCAAGAAGTTCGTTCAGGTTTTTCTGTAAGACGGTATGGAAAACTTGAATGAACCTTTTGGCCAA
..........................................AAACTTGAATGAACCTTTTGGC...  48
....AAGAAGTTCGTTCAGGTTTTTC.........................................  15
.........................................AAAACTTGAATGAACCTTTTGGC...  8
....AAGAAGTTCGTTCAGGTTTTT..........................................  7
...CAAGAAGTTCGTTCAGGTTTTT..........................................  3
.........................................AAAACTTGAATGAACCTTTTGG....  3
..........................................AAACTTGAATGAACCTTTTGG....  2
....AAGAAGTTCGTTCAGGTTTT...........................................  1
.......AAGTTCGTTCAGGTTTTTCTG.......................................  1
........AGTTCGTTCAGGTTTTTCTGTA.....................................  1
.........................................AAAACTTGAATGAACCTTTTG.....  1
..........................................AAACTTGAATGAACCTTTTGGCC..  1
((((((((.(((((((((((((((((.............))))))))))))))))).)))))))).. (-34.82)

10/21340628-21340691(+)_mir_cow_12
AGGGAAGCTTTTTGCTCGCGTTATGTTTCTCATCCGAATATAAGACGAACAAAAGGTTTGTCTG
.......................................ATAAGACGAACAAAAGGTTTGT...     93214
.......................................ATAAGACGAACAAAAGGTTTG....     15911
......................................TATAAGACGAACAAAAGGTTTG....     14275
......................................TATAAGACGAACAAAAGGTTTGT...     8759
........................................TAAGACGAACAAAAGGTTTGT...     3323
........................................TAAGACGAACAAAAGGTTTG....     319
......................................TATAAGACGAACAAAAGGTTT.....     280
.......................................ATAAGACGAACAAAAGGTT......     261
.......................................ATAAGACGAACAAAAGGTTT.....     250
......................................TATAAGACGAACAAAAGGTT......     72
....AAGCTTTTTGCTCGCGTTATGT......................................     38
.......................................ATAAGACGAACAAAAGGT.......     16
........................................TAAGACGAACAAAAGGTT......     13
.............................................CGAACAAAAGGTTTGT...     10
......................................TATAAGACGAACAAAAGGT.......     9
.....................................ATATAAGACGAACAAAAGGTTTG....     7
....AAGCTTTTTGCTCGCGTTATG.......................................     6
........................................TAAGACGAACAAAAGGTTT.....     5
.........................................AAGACGAACAAAAGGTTTGT...     5
.........................................AAGACGAACAAAAGGTTTG....     4
```

```
..........................TTCTCATCCGAATATAAGACGA................     3
......................................TATAAGACGAACAAAAGG........     3
.....................................ATATAAGACGAACAAAAGGTTTGT...     3
....AAGCTTTTTGCTCGCGTTATGTT.....................................     2
.......................................ATAAGACGAACAAAAGG........     2
...........................................GACGAACAAAAGGTTTGT...     2
........................................TAAGACGAACAAAAGGTTTGTC..     2
....AAGCTTTTTGCTCGCGTTAT........................................     1
.......CTTTTTGCTCGCGTTATGT......................................     1
....AAGCTTTTTGCTCGCGTTATGTTT....................................     1
.........................TTTCTCATCCGAATATAAGACG.................     1
..........................TTCTCATCCGAATATAAGACG.................     1
...........................TCTCATCCGAATATAAGACGA................     1
..........................................AGACGAACAAAAGGTTTG....     1
..........................................AGACGAACAAAAGGTTTGT...     1
.......................................ATAAGACGAACAAAAGGTTTGTC..     1
.............................................CGAACAAAAGGTTTGTC..     1
(((.((((((((((.(((..((((((((.......))))))))..))).))))))))))).))). (-20.30)

10/21369597-21369667(-)_mir_Cow_Blast_53
TGACCAACAAGCTTTTTGCTCGTCTTATACGTGAGCATCAGGTATAACCCGGGCCAAAAGCTCGCCCGTCA
........AAGCTTTTTGCTCGTCTTAT...........................................    2
........AAGCTTTTTGCTCGTCTTATAC.........................................    1
...........................................ATAACCCGGGCCAAAAGCTC........    1
...........................................ATAACCCGGGCCAAAAGCTCG.......    1
...........................................ATAACCCGGGCCAAAAGCTCGC......    1
.(((...(.(((((((.(((((..((((((.((.....)).))))))..))))).))))))).)...))). (-27.60)

10/21369601-21369664(+)_mir_cow_13
GGGCGAGCTTTTGGCCCGGGTTATACCTGATGCTCACGTATAAGACGAGCAAAAAGCTTGTTGG
.......................................ATAAGACGAGCAAAAAGCTTGT...     441
.......................................ATAAGACGAGCAAAAAGCTTG....     122
........................................TAAGACGAGCAAAAAGCTTGT...     37
.......................................ATAAGACGAGCAAAAAGCT......     22
....GAGCTTTTGGCCCGGGTTATAC......................................     17
........................................TAAGACGAGCAAAAAGCTTG....     15
.......................................ATAAGACGAGCAAAAAGCTT.....     14
.......................................ATAAGACGAGCAAAAAGC.......     9
```

```
........................................TAAGACGAGCAAAAAGCT......     5
........................................TAAGACGAGCAAAAAGCTT.....     3
......................................TATAAGACGAGCAAAAAGCTTG....     3
....GAGCTTTTGGCCCGGGTTATA.......................................     2
........................................TAAGACGAGCAAAAAGCTTGTT..     2
.............................................CGAGCAAAAAGCTTGT...     1
.(((((((((((.((.((..((((((.((.....)).))))))..)).)).))))))))))).. (-25.90)
```

```
10/30135199-30135277(+)_novel_cow_5
CTCGGTGGGGGTCGGGCGAGAAGCGGGGTGATGTGGGTCGCTCGTGCGTCACCTCCCTCCTCGTCTTAGCCCCGCCCCG
................................................TCACCTCCCTCCTCGTCTTAGC......... 10
................................................TCACCTCCCTCCTCGTCTTAG.......... 2
..........GTCGGGCGAGAAGCGGGGTGA................................................ 1
...............................................GTCACCTCCCTCCTCGTCTTAG.......... 1
...............................................GTCACCTCCCTCCTCGTCTTAGCC........ 1
................................................TCACCTCCCTCCTCGTCTTAGCC........ 1
...(((((((.(.(((((((.((.(((((((((((.(.....).))))))))))).)).))))))).).)))))))... (-46.50)
```

```
10/47321069-47321137(-)_mir_cow_14
TCTGTGTGATATGTTTGATATATTAGGTTGTTATTTAATCCAACTATATATCAAACATATTCCTACAGT
......TGATATGTTTGATATATTAGGTT........................................     416
......TGATATGTTTGATATATTAGGTTG.......................................     336
......TGATATGTTTGATATATTAGGT.........................................     295
..........................................ACTATATATCAAACATATTCCT.....     100
......TGATATGTTTGATATATTAGG..........................................     41
...........................................CTATATATCAAACATATTCCT.....     41
......TGATATGTTTGATATATTAGGTTGT......................................     32
......TGATATGTTTGATATATTA............................................     17
......TGATATGTTTGATATATTAG...........................................     10
......TGATATGTTTGATATA...............................................     5
..........................................ACTATATATCAAACATATTCCTA....     5
......TGATATGTTTGATATAT..............................................     4
......TGATATGTTTGATATATT.............................................     3
.........TATGTTTGATATATTAGGTT........................................     1
...........TGTTTGATATATTAGGTT........................................     1
...........TGTTTGATATATTAGGTTGT......................................     1
......TGATATGTTTGATATATTAGGTTGTT.....................................     1
........ATATGTTTGATATATTAGGTTGTT.....................................     1
```

```
..........................................ACTATATATCAAACATATTCC......     1
.(((((..(((((((((((((((..(((((..........))))))))))))))))))))..).)))). (-24.30)

10/55017701-55017797(+)_mir_Cow_Blast_31
TCCTCATGCTGACATATTTACTAGAGGGTAAAATTAATAACCTTCTAGTAAGAGTGGCAGTCGAAGGGAAGGGCTCATCTGACTTCTGGAAGCCTGG
.....ATGCTGACATATTTACTAGAGG......................................................................1540
...........................................TCTAGTAAGAGTGGCAGTCGAAG...............................218
.....ATGCTGACATATTTACTAGAG.......................................................................204
.....ATGCTGACATATTTACTAGA........................................................................164
..........................................TTCTAGTAAGAGTGGCAGTCGA.................................134
...........................................TCTAGTAAGAGTGGCAGTCGA.................................128
.....ATGCTGACATATTTACTAGAGGGT....................................................................73
..........................................TTCTAGTAAGAGTGGCAGTCG..................................42
.....ATGCTGACATATTTACTAGAGGG.....................................................................39
...........................................TCTAGTAAGAGTGGCAGTCGAA................................32
...........................................TCTAGTAAGAGTGGCAGTCG..................................27
.....ATGCTGACATATTTACTAG.........................................................................22
..................................................................GGAAGGGCTCATCTGACTTC...........11
......TGCTGACATATTTACTAGAGG......................................................................9
..........................................TTCTAGTAAGAGTGGCAGTCGAAG...............................9
..........................................TTCTAGTAAGAGTGGCAGTCGAA................................8
....CATGCTGACATATTTACTAGAGG......................................................................6
.....ATGCTGACATATTTACTAGAGGGTA...................................................................4
......TGCTGACATATTTACTAGAGGG.....................................................................2
......TGCTGACATATTTACTAGAGGGT....................................................................2
.....ATGCTGACATATTTAC............................................................................1
......TGCTGACATATTTACTAGAG.......................................................................1
...........................................TCTAGTAAGAGTGGCAGT....................................1
...........................................TCTAGTAAGAGTGGCAGTC...................................1
..................................................................GGAAGGGCTCATCTGACTTCTGGAAGCCT..1
((((..(((((.(((.(((((((((((((..........))))))))))))).))).))).))..)))).(((((..(((.......)))))))).. (-34.10)

10/85658687-85658751(+)_mir_cow_15
AGTCATGTGAAATTACCAGTATTTGTCTGCTTTGACCTACAAAAACTGGCAGCTTCATGTAATTC
....ATGTGAAATTACCAGTATTTG........................................   212
....ATGTGAAATTACCAGTATTTGT.......................................   174
........................................AAAAACTGGCAGCTTCATGT.....   24
........................................AAAAACTGGCAGCTTCATGTA....   15
```

....AAAAACTGGCAGCTTCATGTAA... 10
....ATGTGAAATTACCAGTATTTGTC...................................... 9
....AAAACTGGCAGCTTCATGTAA... 6
....AAAAACTGGCAGCTTCATGTAAT.. 3
....AAAACTGGCAGCTTCATGT..... 2
......................TTGTCTGCTTTGACCTACAAAAACTG................. 1
....AAAAACTGGCAGCTTCA........ 1
....CAAAAACTGGCAGCTTCATGT..... 1
....AAAACTGGCAGCTTCATGTA.... 1
....AAAACTGGCAGCTTCATGTAAT.. 1
....AAACTGGCAGCTTCATGTAAT.. 1
....(((((((.((.(((((.(((((..(......)..))))).))))).)).))))))).... (-14.20)

10/9091989-9092057(-)_mir_cow_11
GCTTGGCAAAAAAGTTTGTTTGGGTTTTTCTATAACATCTTGCAGGAAAACCTGAACAAACTTTTGGCT
.......AAAAAAGTTTGTTTGGGTTTTT.. 1507
.......AAAAAAGTTTGTTTGGGTTTT... 317
.......AAAAAAGTTTGTTTGGGTTT.. 56
........AAAAAGTTTGTTTGGGTTTTTC....................................... 24
........AAAAAGTTTGTTTGGGTTTTT.. 23
.........AAAAGTTTGTTTGGGTTTTTCT...................................... 22
.........AAAAGTTTGTTTGGGTTTTT.. 21
.......AAAAAAGTTTGTTTGGGTT... 19
.........AAAAGTTTGTTTGGGTTTTTC....................................... 19
........AAAAAGTTTGTTTGGGTTTT... 13
......CAAAAAAGTTTGTTTGGGTTTT... 4
........AAAAAGTTTGTTTGGGTTT.. 3
.......AAAAAAGTTTGTTTGGGT.. 2
......CAAAAAAGTTTGTTTGGGTTTTT.. 2
....AAAACCTGAACAAACTTTT.... 2
.........AAAAGTTTGTTTGGGTTT.. 1
.........AAAAGTTTGTTTGGGTTTT... 1
....GAAAACCTGAACAAACTTTT.... 1
....(((..(((((((((((..(((((((((.(((....))).)))))))))..))))))))))).))) (-28.10)

10/91731796-91731867(+)_mir_cow_16
GAGTTGGCCAAAAAGTTTGTTTGGGTTTTTCCATAACATCTTACAGAAAAACCTGCCCAAACTTTTTGGCCA
.........AAAAAGTTTGTTTGGGTTTTTC... 24

```
.........AAAAAGTTTGTTTGGGTTTTT..........................................  23
..........AAAAGTTTGTTTGGGTTTTT..........................................  21
..........AAAAGTTTGTTTGGGTTTTTC.........................................  19
.........AAAAAGTTTGTTTGGGTTTT...........................................  13
....TGGCCAAAAAGTTTGTTTGGGT..............................................  5
.........AAAAAGTTTGTTTGGGTTT............................................  3
..........AAAAGTTTGTTTGGGTTTTTCC........................................  3
...TTGGCCAAAAAGTTTGTTTGGGT..............................................  2
........CAAAAAGTTTGTTTGGGTTTT...........................................  2
........CAAAAAGTTTGTTTGGGTTTTT..........................................  2
..............................................AAAAACCTGCCCAAACTTTTTG....  2
...............................................AAAACCTGCCCAAACTTTTTGG...  2
..........AAAAGTTTGTTTGGGTTT............................................  1
..........AAAAGTTTGTTTGGGTTTT...........................................  1
........CAAAAAGTTTGTTTGGGTTTTTC.........................................  1
....(((((((((((((((..((((((((((..(((....)))..))))))))))..))))))))))))))) (-33.50)

1/1006019-1006098(-)_mir_cow_1
GGGTTGGCCAAAAAGTTTGTTTGGGTTTTTCCATCGCATCTTACAGGCTAACCTGGATGAACTTTTTGGCCAACCCTATA
.........AAAAAGTTTGTTTGGGTTTTTC.................................................          24
.........AAAAAGTTTGTTTGGGTTTTT..................................................          23
..........AAAAGTTTGTTTGGGTTTTT..................................................          21
..........AAAAGTTTGTTTGGGTTTTTC.................................................          19
.........AAAAAGTTTGTTTGGGTTTT...................................................          13
....TGGCCAAAAAGTTTGTTTGGGT......................................................          5
...............................................CTAACCTGGATGAACTTTTTGG...........          5
.........AAAAAGTTTGTTTGGGTTT....................................................          3
..........AAAAGTTTGTTTGGGTTTTTCC................................................          3
...TTGGCCAAAAAGTTTGTTTGGGT......................................................          2
........CAAAAAGTTTGTTTGGGTTTT...................................................          2
........CAAAAAGTTTGTTTGGGTTTTT..................................................          2
...............................................CTAACCTGGATGAACTTTTTG............          2
..........AAAAGTTTGTTTGGGTTT....................................................          1
..........AAAAGTTTGTTTGGGTTTT...................................................          1
........CAAAAAGTTTGTTTGGGTTTTTC.................................................          1
................................................TAACCTGGATGAACTTTTTGG...........          1
.........................................................TGAACTTTTTGGCCAACC.....          1
........................................................ATGAACTTTTTGGCCAACCC....          1
```

```
..........................................................GAACTTTTTGGCCAACCC....      1
........................................................ATGAACTTTTTGGCCAACCCT...      1
(((((((((((((((((..((..((((...((.............))..))))..))..))))))))))))))))).... (-43.32)

1/107923244-107923319(-)_mir_cow_9
TCCGCTGTAGCAGCACGTAAATATTGGCGTAGTAAAATAAATATTAAACACCAATATTATTGTGCTGCTTTAGCGT
.......TAGCAGCACGTAAATATTGGCG...............................................    37220
.......TAGCAGCACGTAAATATTGGC................................................    5405
.......TAGCAGCACGTAAATATTGGCGT..............................................    1753
.......TAGCAGCACGTAAATATTGG.................................................    344
.......TAGCAGCACGTAAATATTG..................................................    287
.......TAGCAGCACGTAAATAT....................................................    41
.......TAGCAGCACGTAAATATTGGCGTA.............................................    39
.................................................ACCAATATTATTGTGCTGCTTT.....    30
.................................................ACCAATATTATTGTGCTGCTT......    26
.................................................ACCAATATTATTGTGCTGCT.......    15
..................................................CCAATATTATTGTGCTGCTTT.....    13
........AGCAGCACGTAAATATTGGCG...............................................    11
..........CAGCACGTAAATATTGGCG...............................................    10
...........AGCACGTAAATATTGGCG...............................................    6
.............CACGTAAATATTGGCG...............................................    6
.......TAGCAGCACGTAAATATT...................................................    4
............GCACGTAAATATTGGC................................................    3
.........GCAGCACGTAAATATTGGCG...............................................    3
..................................................CCAATATTATTGTGCTGCTTTA....    3
...........AGCACGTAAATATTGGC................................................    2
........AGCAGCACGTAAATATTGGCGT..............................................    2
..................................................CCAATATTATTGTGCTGCTT......    2
.................................................ACCAATATTATTGTGCTGCTTTA....    2
.......TAGCAGCACGTAAATA.....................................................    1
........AGCAGCACGTAAATATTGGC................................................    1
..........CAGCACGTAAATATTGGC................................................    1
......GTAGCAGCACGTAAATATTGGCG...............................................    1
............GCACGTAAATATTGGCG...............................................    1
.......TAGCAGCACGTAAATATTGGCGTAGT...........................................    1
.................................................ACCAATATTATTGTGCT..........    1
.................................................ACCAATATTATTGTGCTG.........    1
................................................CACCAATATTATTGTGCTGCT.......    1
```

```
....................................................AATATTATTGTGCTGCT.......    1
..(((((.(((((((((..((((((((.((((((.......))))..)).))))))))..))))))))).))))). (-31.40)

1/107923394-107923463(-)_mir_cow_10
GTACTGTAGCAGCACATCATGGTTTACATACTACAGTCAAGATGCGAATCATTATTTGCTGCTCTAGAAA
......TAGCAGCACATCATGGTTTACA..........................................    1444
......TAGCAGCACATCATGGTTTAC...........................................    330
............................................CGAATCATTATTTGCTGCTCT.....    64
......TAGCAGCACATCATGGTTT.............................................    49
......TAGCAGCACATCATGGTTTA............................................    41
............................................CGAATCATTATTTGCTGCTCTA....    25
......TAGCAGCACATCATGGTTTACAT.........................................    8
............................TACTACAGTCAAGATG..........................    5
............................................CGAATCATTATTTGCTGCTC......    5
......TAGCAGCACATCATGGTT..............................................    4
......TAGCAGCACATCATGGT...............................................    1
.........................ACATACTACAGTCAAGATG..........................    1
.............................................GAATCATTATTTGCTGCTC......    1
...(((.(((((((.((.((((((..(((.((.......)))))..)))))).)).))))))).)))... (-22.30)

11/104131409-104131481(-)_mir_Cow_Blast_24
TGACAGCGCATTATTACTCACGGTACGAGTTTGAAGTGTCACAGCGCGTACCAAAAGTAATAATGTCCCGTCA
............................................CGCGTACCAAAAGTAATAATGT....... 2
........CATTATTACTCACGGTACGAGTTT......................................... 1
............................................CGCGTACCAAAAGTAATAATGTC...... 1
.(((.(.(((((((((((...((((((.((((((....))).))).))))))...))))))))))).).))). (-28.50)

11/104131411-104131481(+)_mir_cow_25
ACGGGACATTATTACTTTTGGTACGCGCTGTGACACTTCAAACTCGTACCGTGAGTAATAATGCGCTGTCA
......CATTATTACTTTTGGTACGCG............................................   705562
...........................................TCGTACCGTGAGTAATAATGCG......   89052
............................................CGTACCGTGAGTAATAATGCG......   41477
......CATTATTACTTTTGGTACG..............................................   11099
...........................................TCGTACCGTGAGTAATAATG........   9350
...........................................TCGTACCGTGAGTAATAATGC.......   7216
......CATTATTACTTTTGGTACGC.............................................   5821
..........................................CTCGTACCGTGAGTAATAATGCG......   4757
............................................CGTACCGTGAGTAATAATG........   2209
```

```
..........................................CTCGTACCGTGAGTAATAATGC.......   1384
............................................CGTACCGTGAGTAATAATGC.......   1337
......CATTATTACTTTTGGTACGCGC...........................................   1139
...........................................TCGTACCGTGAGTAATAAT.........   950
.......ATTATTACTTTTGGTACGCG............................................   575
......CATTATTACTTTTGGTAC...............................................   381
..........................................CTCGTACCGTGAGTAATAATG........   378
......CATTATTACTTTTGGTACGCGCT..........................................   265
..........................................CTCGTACCGTGAGTAATAAT.........   194
...........................................TCGTACCGTGAGTAATAATGCGC.....   161
...........................................TCGTACCGTGAGTAATAA..........   91
............................................CGTACCGTGAGTAATAAT.........   61
.............................................GTACCGTGAGTAATAATGCG......   53
............................................CGTACCGTGAGTAATAATGCGC.....   46
............................................CGTACCGTGAGTAATAA..........   37
......CATTATTACTTTTGGTA................................................   32
.........TATTACTTTTGGTACGCG............................................   21
........TTATTACTTTTGGTACGCG............................................   17
................................................CCGTGAGTAATAATGCG......   15
..........ATTACTTTTGGTACGCG............................................   14
..........................................CTCGTACCGTGAGTAATAA..........   12
.....ACATTATTACTTTTGGTACGCG............................................   8
.......ATTATTACTTTTGGTACGCGCT..........................................   8
...........................................TCGTACCGTGAGTAATA...........   8
..GGGACATTATTACTTTTGGTACGCG............................................   7
...........TTACTTTTGGTACGCG............................................   7
......CATTATTACTTTTGGTACGCGCTG.........................................   6
.................................................CGTGAGTAATAATGCG......   6
.......ATTATTACTTTTGGTACGCGC...........................................   5
........TTATTACTTTTGGTACGCGCT..........................................   5
..............................................TACCGTGAGTAATAATGCG......   5
......CATTATTACTTTTGGT.................................................   4
.......ATTATTACTTTTGGTACG..............................................   4
..............................................TACCGTGAGTAATAATGCGCTGT..   4
.......ATTATTACTTTTGGTACGC.............................................   3
.....ACATTATTACTTTTGGTACG..............................................   2
.................TTGGTACGCGCTGTGACACTTCAAACT...........................   2
.............................................GTACCGTGAGTAATAAT.........   2
```

```
.............................................GTACCGTGAGTAATAATG........   2
...............................................ACCGTGAGTAATAATGCG......   2
........TTATTACTTTTGGTACG..............................................   1
....GACATTATTACTTTTGGTACGCG............................................   1
......CATTATTACTTTTGGTACGCGCTGT........................................   1
...............TTTTGGTACGCGCTGTGACACTTC................................   1
......CATTATTACTTTTGGTACGCGCTGTGACACTTCA...............................   1
......CATTATTACTTTTGGTACGCGCTGTGACACTTCAAACT...........................   1
...........................CTGTGACACTTCAAACT...........................   1
..........................................CTCGTACCGTGAGTAAT............   1
...........................................TCGTACCGTGAGTAAT............   1
............................................CGTACCGTGAGTAATA...........   1
.............................................GTACCGTGAGTAATAATGC.......   1
...........................CTGTGACACTTCAAACTCGTACCGTGAGTAATAATGCG......   1
...........................................TCGTACCGTGAGTAATAATGCGCT....   1
............................................CGTACCGTGAGTAATAATGCGCT....   1
..............................................TACCGTGAGTAATAATGCGCTG...   1
(((((.(((((((((((.(((((((.(...(((....)))..).))))))).))))))))))).))))).. (-27.00)

1/118508626-118508695(-)_novel_cow_3
ACAAGCCCAAGACCATACATTAGGTGTACTGTCCTAATATACACCTAGTGCATGGTCTTGGGCTTGTGCT
.........................................CACCTAGTGCATGGTCTTGGGC.......    65
.........................................CACCTAGTGCATGGTCTTGGGCT......    8
..........................................ACCTAGTGCATGGTCTTGGGC.......    5
..........................................ACCTAGTGCATGGTCTTGGGCT......    5
.........................................CACCTAGTGCATGGTCTTGGG........    2
.........................................CACCTAGTGCATGGTCTTGGGCTT.....    2
............................................CTAGTGCATGGTCTTGGGCTTGT...    2
.............................................TAGTGCATGGTCTTGGGCTTGT...    2
...AGCCCAAGACCATACATTAGGT.............................................    1
...........................................CCTAGTGCATGGTCTTGGGCT......    1
............................................CTAGTGCATGGTCTTGGGCTTG....    1
((((((((((((((((.(((((((((((.(((....)))))))))))))).)))))))))))))))).... (-45.50)

11/21374966-21375041(-)_mir_cow_19
AGTTGGCAAAAAAGTTTGTTTGGGTTTTTCTGTAATATCTTAAAAACCCAGATGAACTTTTTGGCCAACCCAGTGC
.......AAAAAAGTTTGTTTGGGTTTTT...............................................    1507
.......AAAAAAGTTTGTTTGGGTTTT................................................    317
```

```
.......AAAAAAGTTTGTTTGGGTTT.................................................    56
...........................................AAACCCAGATGAACTTTTTGGC...........    38
........AAAAAGTTTGTTTGGGTTTTTC..............................................    24
........AAAAAGTTTGTTTGGGTTTTT...............................................    23
.........AAAAGTTTGTTTGGGTTTTTCT.............................................    22
.........AAAAGTTTGTTTGGGTTTTT...............................................    21
.......AAAAAAGTTTGTTTGGGTT..................................................    19
.........AAAAGTTTGTTTGGGTTTTTC..............................................    19
........AAAAAGTTTGTTTGGGTTTT................................................    13
......CAAAAAAGTTTGTTTGGGTTTT................................................    4
........AAAAAGTTTGTTTGGGTTT.................................................    3
...........................................AAACCCAGATGAACTTTTTGG............    3
...........................................AAACCCAGATGAACTTTTTGGCC..........    3
.......AAAAAAGTTTGTTTGGGT...................................................    2
......CAAAAAAGTTTGTTTGGGTTTTT...............................................    2
............................................AACCCAGATGAACTTTTTGGCC..........    2
.........AAAAGTTTGTTTGGGTTT.................................................    1
.........AAAAGTTTGTTTGGGTTTT................................................    1
.............................................ACCCAGATGAACTTTTTGGCC..........    1
....................................................TGAACTTTTTGGCCAACC......    1
...................................................ATGAACTTTTTGGCCAACCC.....    1
.....................................................GAACTTTTTGGCCAACCC.....    1
....................................................TGAACTTTTTGGCCAACCCA....    1
.((((((.((((((((..(((((((((((..(......)..)))))))))))..)))))))).)))))).......  (-34.70)

1/130294944-130295019(-)_novel_cow_4
TCACTCCTATGCTGTATTTCACAATTGGTCTAAGACCCAGCAGTTAGACCGATTGTGGAATGCAGCATAGGAGCGC
...............................................ACCGATTGTGGAATGCAGCAT........    53
.........TGCTGTATTTCACAATTGGTCT.............................................    14
.........TGCTGTATTTCACAATTGGTC..............................................    5
...............................................ACCGATTGTGGAATGCAGCA.........    3
.............................................AGACCGATTGTGGAATGCAGCAT........    2
...............................................ACCGATTGTGGAATGCAGCATA.......    2
..........GCTGTATTTCACAATTGGTCT.............................................    1
..............................................GACCGATTGTGGAATGCAGC..........    1
...............................................ACCGATTGTGGAATGCAGC..........    1
.................................................CGATTGTGGAATGCAGCAT........    1
...((((((((((((((((((((((((((((((..........))))))))))))))))))))))))))))))...  (-48.20)
```

```
11/37027748-37027810(-)_novel_cow_7
TCAACCAGGTCCACTGATTTGCGATCATGCTTATATCTCAAATCAGTGGACCTGGTAGATTTA
....................................CTCAAATCAGTGGACCTGGTAGA....      8
.....................................TCAAATCAGTGGACCTGGTAGA....      5
....................................CTCAAATCAGTGGACCTGGTAG.....      3
...ACCAGGTCCACTGATTTGCGAT......................................      1
......................................CAAATCAGTGGACCTGGTAGA....      1
((.((((((((((((((((((.(((.........))).)))))))))))))))))).))....  (-35.30)

11/38482498-38482572(-)_mir_Cow_Blast_5
TGTCGCAGATACTGCATCAGGAACTGATTGGATAAGAATCGGTCACCATCAGTTCCTCATGCATTGCCTTCAGCA
.........TACTGCATCAGGAACTGATTGGA...........................................     5
........ATACTGCATCAGGAACTGATTG.............................................     4
..............................................CATCAGTTCCTCATGCATTGCC.......     4
........ATACTGCATCAGGAACTGATT..............................................     2
.........TACTGCATCAGGAACTGATT..............................................     2
.........TACTGCATCAGGAACTGATTG.............................................     2
..............................................CATCAGTTCCTCATGCATTGC........     2
........ATACTGCATCAGGAACTGATTGG............................................     1
.........TACTGCATCAGGAACTGATTGG............................................     1
..............................................CATCAGTTCCTCATGCATTGCCT......     1
...............................................ATCAGTTCCTCATGCATTGCCT......     1
....((((....(((((.((((((((((.((((........))).).)))))))))).)))))))))........ (-25.40)

11/38494751-38494822(-)_mir_cow_20
TTGGCTTAATCTCAGCTGGCAACTGTGAGATGTTCATACAGTCCTCCACAGTGGTCTCTGGGATTATGCTAA
......TAATCTCAGCTGGCAACTGTGA............................................  21
..............................................CACAGTGGTCTCTGGGATTATG....  12
......TAATCTCAGCTGGCAACTGT..............................................  6
......TAATCTCAGCTGGCAACTGTG.............................................  5
......TAATCTCAGCTGGCAACTG...............................................  1
......TAATCTCAGCTGGCAACTGTGAGA..........................................  1
.((((.(((((((((..(((.((((((((.(((....)))...)).)))))).))).))))))))).)))). (-29.40)

11/4523084-4523153(-)_mir_cow_18
TTGGCCAGAAAGTTTGTTTGGGTTTTTCCGTAAGATGTAACAGGAAAACCTGAAGAGACTTTTTGGCCCA
............................................AAAACCTGAAGAGACTTTTTGG....    14
```

```
......AGAAAGTTTGTTTGGGTTTTTC..........................................    7
......AGAAAGTTTGTTTGGGTTTTT...........................................    6
.......GAAAGTTTGTTTGGGTTTTTCC.........................................    4
......AGAAAGTTTGTTTGGGTTTT............................................    3
.......GAAAGTTTGTTTGGGTTTTT...........................................    2
......AGAAAGTTTGTTTGGGTTTTTCC.........................................    2
.......GAAAGTTTGTTTGGGTTTTTC..........................................    1
..(((((((((((((.((..((((((((.((........)).))))))))..)).))))))))))))).. (-35.30)

1/149720311-149720373(+)_mir_Cow_Blast_39
GCAATCAGTAACAAAGATTCATCCTTGTGTCCATCAAGCAACAAGGAGAATCTTTGTCACTAA
....TCAGTAACAAAGATTCATCCTTG....................................      9
....TCAGTAACAAAGATTCATCCTT.....................................      4
....TCAGTAACAAAGATTCATCCT......................................      3
..........................................AAGGAGAATCTTTGTCACT..      1
......(((.((((((((((.(((((((((.......)).))))))))))))))))).))).. (-24.50)

11/86704766-86704845(+)_novel_cow_6
GAAGGAGGTGTGTCCTTTCTCTAAGTGGCCCAGGGAAAGAGAGACAGTCGGGACCACTGGGCAGAGGACACATCCCCCAC
....................................................ACCACTGGGCAGAGGACACATC......      6
........TGTGTCCTTTCTCTAAGTGGCCC.................................................      2
....................................................ACCACTGGGCAGAGGACACA........      2
........TGTGTCCTTTCTCTAAGTGGC...................................................      1
........TGTGTCCTTTCTCTAAGTGGCC..................................................      1
....................................................ACCACTGGGCAGAGGACACAT.......      1
...((.((((((((((((.((((.(((((((..................))).)))))))).)))))))))))).))... (-34.57)

11/95709442-95709512(+)_mir_cow_21
TCCAAGGAACATTCAACGCTGTCGGTGAGTTTGGGATTTGAAAAAACCACCGACCGTTGACTGTACCTTGG
.......AACATTCAACGCTGTCGGTGAGT.........................................   1365775
.......AACATTCAACGCTGTCGGTGAG..........................................   1103625
.......AACATTCAACGCTGTCGGTGA...........................................   178739
.......AACATTCAACGCTGTCGGTGAGTT........................................   67437
.......AACATTCAACGCTGTCGGTG............................................   18252
.......AACATTCAACGCTGTCGGTGAGTTT.......................................   10662
.......AACATTCAACGCTGTCGGT.............................................   9870
.............................................ACCACCGACCGTTGACTGTACC....   5874
.............................................ACCACCGACCGTTGACTGTAC.....   3591
```

```
........ACATTCAACGCTGTCGGTGAGT.........................................   665
.............................................ACCACCGACCGTTGACTGTA......   369
........ACATTCAACGCTGTCGGTGAG..........................................   341
.............................................ACCACCGACCGTTGACTGTACCT...   288
.......AACATTCAACGCTGTCGG..............................................   259
.............................................ACCACCGACCGTTGACTGT.......   208
.............CAACGCTGTCGGTGAGT.........................................   112
........ACATTCAACGCTGTCGGTGAGTT........................................   94
..........ATTCAACGCTGTCGGTGAGT.........................................   60
............................................AACCACCGACCGTTGACTGTAC.....   60
........ACATTCAACGCTGTCGGTGA...........................................   52
........ACATTCAACGCTGTCGGTGAGTTT.......................................   35
..........ATTCAACGCTGTCGGTGAG..........................................   34
.............CAACGCTGTCGGTGAG..........................................   27
...........TTCAACGCTGTCGGTGAGT.........................................   27
............TCAACGCTGTCGGTGAGT.........................................   27
.......AACATTCAACGCTGTCGGTGAGTTTG......................................   25
............................................AACCACCGACCGTTGACTGTACC....   23
...........TTCAACGCTGTCGGTGAG..........................................   22
.........CATTCAACGCTGTCGGTGAGT.........................................   20
......GAACATTCAACGCTGTCGGTGAG..........................................   19
......GAACATTCAACGCTGTCGGTGA...........................................   14
.......AACATTCAACGCTGTC................................................   13
.............CAACGCTGTCGGTGAGTT........................................   13
..............AACGCTGTCGGTGAGT.........................................   12
............................................AACCACCGACCGTTGACTGT.......   12
..........ATTCAACGCTGTCGGTGA...........................................   11
............................................AACCACCGACCGTTGACTGTA......   9
......GAACATTCAACGCTGTCGGTGAGT.........................................   7
..............................................CCACCGACCGTTGACTGTACC....   7
........ACATTCAACGCTGTCGGTG............................................   6
...........TTCAACGCTGTCGGTGA...........................................   6
.........CATTCAACGCTGTCGGTGAG..........................................   6
..........ATTCAACGCTGTCGGTGAGTT........................................   6
............TCAACGCTGTCGGTGAGTT........................................   6
.......AACATTCAACGCTGTCGGTGAGTTTGGGA...................................   6
.............................................ACCACCGACCGTTGACT.........   6
.......AACATTCAACGCTGTCG...............................................   5
```

........ACATTCAACGCTGTCGGT... 5
............TCAACGCTGTCGGTGAG.. 5
....ACCACCGACCGTTGACTGTACCTT.. 5
..............AACGCTGTCGGTGAGTT.. 3
....CACCGACCGTTGACTGTACCT... 3
.........CATTCAACGCTGTCGGTGAGTT.. 2
...........TTCAACGCTGTCGGTGAGTT.. 2
.........CATTCAACGCTGTCGGTGAGTTT....................................... 2
..........ATTCAACGCTGTCGGTGAGTTT....................................... 2
...............ACGCTGTCGGTGAGTTT....................................... 2
....ACCACCGACCGTTGACTG........ 2
....CCACCGACCGTTGACTGTAC..... 2
....AGGAACATTCAACGCTGTCGGTGAGT... 1
.....GGAACATTCAACGCTGTCGGTGAGT... 1
...........TTCAACGCTGTCGGTGAGTTT....................................... 1
.............CAACGCTGTCGGTGAGTTT....................................... 1
........ACATTCAACGCTGTCGGTGAGTTTG...................................... 1
.......AACATTCAACGCTGTCGGTGAGTTTGGG.................................... 1
.......AACATTCAACGCTGTCGGTGAGTTTGGGAT.................................. 1
....AAAAACCACCGACCGTTGACT......... 1
....AACCACCGACCGTTGACTG........ 1
....AAAAACCACCGACCGTTGACTGT....... 1
....CCACCGACCGTTGACTGT....... 1
......................................TGAAAAAACCACCGACCGTTGACTGTAC..... 1
....................................TTTGAAAAAACCACCGACCGTTGACTGTACC.... 1
......................................TGAAAAAACCACCGACCGTTGACTGTACC.... 1
....GAAAAAACCACCGACCGTTGACTGTACC.... 1
....GACCGTTGACTGTACC.... 1
......................................TGAAAAAACCACCGACCGTTGACTGTACCT... 1
.((((((.(((.((((((..(((((((.((((...........))))))))))))))))).))).)))))) (-33.40)

11/95710635-95710702(+)_mir_cow_22
GCACTCAACATTCATTGCTGTCGGTGGGTTTGACTTTGAATCAACTCACTGATCAATGAATGTAAACT
......AACATTCATTGCTGTCGGTGGGT....................................... 30643
......AACATTCATTGCTGTCGGTGGG.. 28373
......AACATTCATTGCTGTCGGTGGGTT...................................... 16611
......AACATTCATTGCTGTCGGTGG... 2019
......AACATTCATTGCTGTCGGTGGGTTT..................................... 1922

......AACATTCATTGCTGTCGGTG.. 1759
......AACATTCATTGCTGTCGGT... 721
.......ACATTCATTGCTGTCGGTGGGTT...................................... 154
.......ACATTCATTGCTGTCGGTGGGT....................................... 126
.......ACATTCATTGCTGTCGGTGGG.. 68
......AACATTCATTGCTGTCGG.. 47
.......ACATTCATTGCTGTCGGTGGGTTT..................................... 29
.....CAACATTCATTGCTGTCGGTGGGT....................................... 13
....CTCACTGATCAATGAATGTA.... 11
.....CAACATTCATTGCTGTCGGTGGG.. 9
......AACATTCATTGCTGTC.. 7
....CTCACTGATCAATGAATGT..... 7
....CTCACTGATCAATGAATGTAAA.. 7
.....CAACATTCATTGCTGTCGGTGGGTT...................................... 6
..........TTCATTGCTGTCGGTGGGTT...................................... 6
.......ACATTCATTGCTGTCGGTG.. 5
.......ACATTCATTGCTGTCGGTGG... 4
....CTCACTGATCAATGAATGTAA... 4
.........ATTCATTGCTGTCGGTGGGT....................................... 3
....ACTCACTGATCAATGAATGTAA... 3
.......ACATTCATTGCTGTCGGT... 2
.....CAACATTCATTGCTGTCGGTG.. 2
..........TTCATTGCTGTCGGTGGGT....................................... 2
............CATTGCTGTCGGTGGGT....................................... 2
.........ATTCATTGCTGTCGGTGGGTT...................................... 2
.............ATTGCTGTCGGTGGGTT...................................... 2
......AACATTCATTGCTGTCGGTGGGTTTGACT................................. 2
....ACTCACTGATCAATGAATGTA.... 2
.....CAACATTCATTGCTGTCGGTGG... 1
..........TTCATTGCTGTCGGTGG... 1
....TCAACATTCATTGCTGTCGGTGGG.. 1
...........TCATTGCTGTCGGTGGG.. 1
........CATTCATTGCTGTCGGTGGGT....................................... 1
.............ATTGCTGTCGGTGGGT....................................... 1
....TCAACATTCATTGCTGTCGGTGGGTT...................................... 1
........CATTCATTGCTGTCGGTGGGTT...................................... 1
............CATTGCTGTCGGTGGGTT...................................... 1
.....CAACATTCATTGCTGTCGGTGGGTTT..................................... 1

```
..........TTCATTGCTGTCGGTGGGTTT..................................... 1
...........................................ACTCACTGATCAATGAATGT..... 1
.............................................TCACTGATCAATGAATGTAA... 1
.......((((((((((..(((((((((((.((.......))))))))))))))))))))))).....  (-25.30)

1/19881353-19881425(-)_mir_cow_3
TCCTAGTCCCTGAGACCCTAACTTGTGAGGTATTTTAGTAGCATCACAAGTCAGGCTCTTGGGACCTAGGCGG
......TCCCTGAGACCCTAACTTGTGA.............................................  131934
......TCCCTGAGACCCTAACTTGTG..............................................  21541
.............................................ACAAGTCAGGCTCTTGGGACCT......  20176
......TCCCTGAGACCCTAACTTGT...............................................  12124
.............................................ACAAGTCAGGCTCTTGGGACC.......  6205
.............................................ACAAGTCAGGCTCTTGGGAC........  2477
......TCCCTGAGACCCTAACTTG................................................  1629
.............................................ACAAGTCAGGCTCTTGGGACCTA.....  1119
......TCCCTGAGACCCTAACTTGTGAG............................................  536
..............................................CAAGTCAGGCTCTTGGGACCT......  369
......TCCCTGAGACCCTAACT..................................................  275
......TCCCTGAGACCCTAACTT.................................................  268
..........TGAGACCCTAACTTGTGA.............................................  206
........CCTGAGACCCTAACTTGTGA.............................................  141
..............................................CAAGTCAGGCTCTTGGGACC.......  122
.......CCCTGAGACCCTAACTTGTGA.............................................  116
.............................................ACAAGTCAGGCTCTTGGGA.........  115
.........CTGAGACCCTAACTTGTGA.............................................  57
..............................................CAAGTCAGGCTCTTGGGACCTA.....  50
......TCCCTGAGACCCTAAC...................................................  36
..............................................CAAGTCAGGCTCTTGGGAC........  27
........CCTGAGACCCTAACTTGTG..............................................  25
.............................................ACAAGTCAGGCTCTTGGGACCTAG....  23
............................................CACAAGTCAGGCTCTTGGGACCT......  16
..........TGAGACCCTAACTTGTG..............................................  14
..........TGAGACCCTAACTTGT...............................................  13
........CCTGAGACCCTAACTTGT...............................................  11
.............................................ACAAGTCAGGCTCTTGGG..........  10
...........................................TCACAAGTCAGGCTCTTGGGAC........  9
.........CTGAGACCCTAACTTGTG..............................................  8
.........CTGAGACCCTAACTTGT...............................................  6
```

```
.......CCCTGAGACCCTAACTTGTG..............................................  6
.............................................ACAAGTCAGGCTCTTG............  5
...........GAGACCCTAACTTGTGA.............................................  4
...............................................AAGTCAGGCTCTTGGGACCT......  4
.......CCCTGAGACCCTAACTTGT...............................................  3
...........................................TCACAAGTCAGGCTCTTGGGACC.......  3
............................................CACAAGTCAGGCTCTTGGGACC.......  3
..............................................CAAGTCAGGCTCTTGGGACCTAG....  3
.........CTGAGACCCTAACTTG................................................  2
............AGACCCTAACTTGTGA.............................................  2
............................GGTATTTTAGTAGCATC............................  2
.......CCCTGAGACCCTAACTT.................................................  1
..CTAGTCCCTGAGACCCTAACTTGTG..............................................  1
.......CCCTGAGACCCTAACTTGTGAG............................................  1
.........CTGAGACCCTAACTTGTGAG............................................  1
..........TGAGACCCTAACTTGTGAG............................................  1
......TCCCTGAGACCCTAACTTGTGAGG...........................................  1
..........................................ATCACAAGTCAGGCTCTTG............  1
..........................................ATCACAAGTCAGGCTCTTGGGA.........  1
............................................CACAAGTCAGGCTCTTGGGAC........  1
...............................................AAGTCAGGCTCTTGGGAC........  1
...............................................AAGTCAGGCTCTTGGGACC.......  1
................................................AGTCAGGCTCTTGGGACCT......  1
..................................................TCAGGCTCTTGGGACCT......  1
.(((((..((..(((.(((.(((((((((.(((....))).).)))))))).))).)))..))..)))))...  (-34.20)

11/98867676-98867747(-)_mir_cow_23
CGTCTACCCAGTGTTTAGACTATCTGTTCAGGACTCCCAAATTGTACAGTAGTCTGCACATTGGTTAGGCTG
.............................................ACAGTAGTCTGCACATTGGTT......  34856
......CCCAGTGTTTAGACTATCTGTTC...........................................  16797
............................................TACAGTAGTCTGCACATTGGTT......  12926
.............................................ACAGTAGTCTGCACATTGGTTA.....  8597
......CCCAGTGTTTAGACTATCTGTT............................................  2896
.............................................ACAGTAGTCTGCACATTGGT.......  2417
..............................................CAGTAGTCTGCACATTGGTT......  2184
............................................TACAGTAGTCTGCACATTGGT.......  1677
......CCCAGTGTTTAGACTATCTGT.............................................  1398
......CCCAGTGTTTAGACTATCTGTTCA..........................................  1104
```

....TACAGTAGTCTGCACATTGGTTA.....	756
....CAGTAGTCTGCACATTGGTTA.....	681
......CCCAGTGTTTAGACTATCTG..	571
....ACAGTAGTCTGCACATTGG........	268
....TACAGTAGTCTGCACATTGG........	234
....CAGTAGTCTGCACATTGGT.......	224
......CCCAGTGTTTAGACTATCT...	114
....ACAGTAGTCTGCACATTGGTTAG....	78
....ACAGTAGTCTGCACAT...........	59
.......CCAGTGTTTAGACTATCTGTTC...	44
......CCCAGTGTTTAGACTATC..	32
....TACAGTAGTCTGCACAT...........	29
....ACAGTAGTCTGCACATTG.........	17
....TACAGTAGTCTGCACATTGGTTAG....	14
........CAGTGTTTAGACTATCTGTTC...	12
....ACAGTAGTCTGCACATT..........	10
......CCCAGTGTTTAGACTA..	8
....TACAGTAGTCTGCACATTG.........	8
....AGTAGTCTGCACATTGGTT......	8
.......CCAGTGTTTAGACTATCTGTT..	7
....CAGTAGTCTGCACATTGG........	7
....GTAGTCTGCACATTGGTT......	6
.........AGTGTTTAGACTATCTGTTC...	5
..........GTGTTTAGACTATCTGTTC...	5
....TACAGTAGTCTGCACATT..........	5
....CAGTAGTCTGCACATTGGTTAG....	5
.......CCAGTGTTTAGACTATCTG..	4
......CCCAGTGTTTAGACTAT...	3
.........AGTGTTTAGACTATCTGTT..	3
....AGTAGTCTGCACATTGGTTA.....	3
...........TGTTTAGACTATCTGTTC...	2
....TACAGTAGTCTGCACA............	2
.......CCAGTGTTTAGACTATCTGT...	1
........CAGTGTTTAGACTATCTGTT..	1
..........GTGTTTAGACTATCTGTT..	1
.......CCAGTGTTTAGACTATCTGTTCA..	1
........CAGTGTTTAGACTATCTGTTCA..	1
......CCCAGTGTTTAGACTATCTGTTCAG...	1

```
......CCCAGTGTTTAGACTATCTGTTCAGG........................................  1
......CCCAGTGTTTAGACTATCTGTTCAGGACT.....................................  1
...............................................AGTAGTCTGCACATTGGT.......  1
...........................................GTACAGTAGTCTGCACATTGGTTA.....  1
................................................GTAGTCTGCACATTGGTTA.....  1
.(((((.(((((((.(((((((.((((.((((....))....)).))))))))))).))))))).)))))..  (-29.80)

11/98997158-98997234(-)_mir_cow_24
TCGCCACTGATTGTCCAAACGCAATTCTTGTACGAGTCTGCGGCCAACCGAGAATTGTGGCTGGACATCTGTGGCTG
..................................................AGAATTGTGGCTGGACATCTGT.....   14852
.......TGATTGTCCAAACGCAATTC..................................................   4177
.......TGATTGTCCAAACGCAATTCTTG...............................................   3824
.......TGATTGTCCAAACGCAATTCT.................................................   3403
.......TGATTGTCCAAACGCAATTCTT................................................   2049
..................................................AGAATTGTGGCTGGACATCTG......   1715
..................................................AGAATTGTGGCTGGACATCT.......   1124
.................................................GAGAATTGTGGCTGGACATCTG......   986
.................................................GAGAATTGTGGCTGGACATCTGT.....   448
.................................................GAGAATTGTGGCTGGACATCT.......   396
..................................................AGAATTGTGGCTGGACATC........   247
.......TGATTGTCCAAACGCAATT...................................................   157
.......TGATTGTCCAAACGCAAT....................................................   71
..............................TACGAGTCTGCGGCCAACCG...........................   54
..............................TACGAGTCTGCGGCCAACCGA..........................   52
.................................................GAGAATTGTGGCTGGACATC........   45
.......TGATTGTCCAAACGCA......................................................   43
.......TGATTGTCCAAACGCAATTCTTGT..............................................   34
..................................................AGAATTGTGGCTGGACATCTGTG....   30
...................................................GAATTGTGGCTGGACATCTGT.....   22
..................................................AGAATTGTGGCTGGACA..........   21
.....................................................ATTGTGGCTGGACATCTGT.....   18
..........TTGTCCAAACGCAATTCTTG...............................................   17
..................................................AGAATTGTGGCTGGAC...........   11
...........TGTCCAAACGCAATTCTTG...............................................   9
..............................TACGAGTCTGCGGCCAACC............................   9
.........ATTGTCCAAACGCAATTCTTG...............................................   8
.........ATTGTCCAAACGCAATTCTTGT..............................................   8
.......................................................TGTGGCTGGACATCTGT.....   8
```

....AATTGTGGCTGGACATCTGT..... 7
....TTGTGGCTGGACATCTGT..... 7
....CGAGAATTGTGGCTGGACATCT....... 6
..............................TACGAGTCTGCGGCCAAC............................. 5
....GTGGCTGGACATCTGT..... 5
.......TGATTGTCCAAACGCAA... 3
......CTGATTGTCCAAACGCAATTC.. 3
........GATTGTCCAAACGCAATTCTT.. 3
......CTGATTGTCCAAACGCAATTCTTG... 3
....GAGAATTGTGGCTGGACAT......... 3
....AGAATTGTGGCTGGACAT......... 3
....ATTGTGGCTGGACATCTG...... 3
........GATTGTCCAAACGCAATTC.. 2
......CTGATTGTCCAAACGCAATTCT... 2
............GTCCAAACGCAATTCTTG... 2
..............CCAAACGCAATTCTTG... 2
....GAGAATTGTGGCTGGA............ 2
....GAGAATTGTGGCTGGACA.......... 2
....AATTGTGGCTGGACATCT....... 2
.....................................CTGCGGCCAACCGAGAATTGTGGCTGGACATCTGT..... 2
......................................TGCGGCCAACCGAGAATTGTGGCTGGACATCTGT..... 2
....GCCAACCGAGAATTGTGGCTGGACATCTGT..... 2
..........TTGTCCAAACGCAATTC.. 1
........GATTGTCCAAACGCAATTCT... 1
..........TTGTCCAAACGCAATTCT... 1
......CTGATTGTCCAAACGCAATTCTT.. 1
.........ATTGTCCAAACGCAATTCTT.. 1
..........TTGTCCAAACGCAATTCTT.. 1
...........TGTCCAAACGCAATTCTT.. 1
........GATTGTCCAAACGCAATTCTTG... 1
........GATTGTCCAAACGCAATTCTTGT.. 1
..........TTGTCCAAACGCAATTCTTGT.. 1
...........TGTCCAAACGCAATTCTTGT.. 1
..........TTGTCCAAACGCAATTCTTGTA... 1
.......TGATTGTCCAAACGCAATTCTTGTACGAGTCTG..................................... 1
..........TTGTCCAAACGCAATTCTTGTACGAGTCTGCGGCC................................ 1
.........................TCTTGTACGAGTCTGCGGCCA............................... 1
..............................TACGAGTCTGCGGCCA............................... 1

```
..................................AGTCTGCGGCCAACCG...........................   1
..............................TACGAGTCTGCGGCCAACCGAGA........................   1
.......................................GCGGCCAACCGAGAATTGTGGCTGGACATCTG......   1
...............................................CCGAGAATTGTGGCTGGACATCTG......   1
...................................................GAATTGTGGCTGGACATCTG......   1
....................................................AATTGTGGCTGGACATCTG......   1
....................................TCTGCGGCCAACCGAGAATTGTGGCTGGACATCTGT.....   1
...............................................CCGAGAATTGTGGCTGGACATCTGT.....   1
..(((((.(((.(((((..(((((((((((...(.((.....)))...)))))))))))..)))))))).))))).. (-36.10)

11/98997161-98997231(+)_mir_Cow_Blast_13
CCACAGATGTCCAGCCACAATTCTCGGTTGGCCGCAGACTCGTACAAGAATTGCGTTTGGACAATCAGTGG
..............................................AGAATTGCGTTTGGACAATCAGT..   15
.............................................AAGAATTGCGTTTGGACAATC.....   6
...............................................GAATTGCGTTTGGACAATCAGT..   5
..............................................AGAATTGCGTTTGGACA........   2
....AGATGTCCAGCCACAATTCTC..............................................   1
....AGATGTCCAGCCACAATTCTCG.............................................   1
.....GATGTCCAGCCACAATTCTCG.............................................   1
.............................................AAGAATTGCGTTTGGACAAT......   1
...............................................GAATTGCGTTTGGACAAT......   1
................................................AATTGCGTTTGGACAAT......   1
..............................................AGAATTGCGTTTGGACAATC.....   1
..............................................AGAATTGCGTTTGGACAATCAG...   1
((((.(((((((((.(.((((((((((....)))............))))))).).)))))).))).)))) (-24.40)

1/19930460-19930540(-)_mir_cow_4
ATCCGGGTTGAGGTAGTAGGTTGTATGGTTTAGAGTTACACCCTGGGAGTTAACTGTACAACCTTCTAGCTTTCCTTGGAG
........TGAGGTAGTAGGTTGTATGGTT...................................................    108523
........TGAGGTAGTAGGTTGTATGGT....................................................    18966
........TGAGGTAGTAGGTTGTAT.......................................................    7314
........TGAGGTAGTAGGTTGTATGGTTT..................................................    6554
........TGAGGTAGTAGGTTGTATGG.....................................................    2195
........TGAGGTAGTAGGTTGTA........................................................    1031
.......TTGAGGTAGTAGGTTGTATGGTT...................................................    399
........TGAGGTAGTAGGTTGTATG......................................................    356
........TGAGGTAGTAGGTTGT.........................................................    343
.......TTGAGGTAGTAGGTTGTATGGT....................................................    291
```

```
.....................................................CTGTACAACCTTCTAGCTTTCC......      137
.........GAGGTAGTAGGTTGTATGGTT...................................................      73
.......TTGAGGTAGTAGGTTGTATGG.....................................................      38
.............TAGTAGGTTGTATGGTT...................................................      37
..............AGTAGGTTGTATGGTT...................................................      36
........TGAGGTAGTAGGTTGTATGGTTTA.................................................      33
...........GGTAGTAGGTTGTATGGTT...................................................      30
............GTAGTAGGTTGTATGGTT...................................................      21
..........AGGTAGTAGGTTGTATGGTT...................................................      20
.......TTGAGGTAGTAGGTTGTATGGTTT..................................................      16
.....................................................CTGTACAACCTTCTAGCTTTCCT.....      15
.....................................................CTGTACAACCTTCTAGCTTTC.......      10
..............................TAGAGTTACACCCTGGGAGTT..............................      8
......................................................TGTACAACCTTCTAGCTTTCCT.....      8
.......TTGAGGTAGTAGGTTGTAT.......................................................      7
......GTTGAGGTAGTAGGTTGTATGGT....................................................      7
.........GAGGTAGTAGGTTGTATGGT....................................................      5
.............TAGTAGGTTGTATGGT....................................................      4
.............TAGTAGGTTGTATGGTTT..................................................      4
......................................................TGTACAACCTTCTAGCTTTCC......      4
...........GGTAGTAGGTTGTATGGT....................................................      3
............GTAGTAGGTTGTATGGT....................................................      3
...........GGTAGTAGGTTGTATGGTTT..................................................      3
........TGAGGTAGTAGGTTGTATGGTTTAGA...............................................      3
..............................TAGAGTTACACCCTGGGAGT...............................      3
...............................................AGTTAACTGTACAACCTTCTAGCT..........      3
..........AGGTAGTAGGTTGTATGG.....................................................      2
............GTAGTAGGTTGTATGGTTT..................................................      2
........TGAGGTAGTAGGTTGTATGGTTTAG................................................      2
........TGAGGTAGTAGGTTGTATGGTTTAGAGTTACACC.......................................      2
..............................TAGAGTTACACCCTGGGAGTTA.............................      2
.....................................................CTGTACAACCTTCTAGCTTT........      2
...................................................AACTGTACAACCTTCTAGCTTTC.......      2
.......TTGAGGTAGTAGGTTGT.........................................................      1
.......TTGAGGTAGTAGGTTGTA........................................................      1
.........GAGGTAGTAGGTTGTA........................................................      1
.........GAGGTAGTAGGTTGTAT.......................................................      1
..........AGGTAGTAGGTTGTAT.......................................................      1
```

```
...........GGTAGTAGGTTGTATGG.....................................................     1
..........AGGTAGTAGGTTGTATGGT....................................................     1
......GTTGAGGTAGTAGGTTGTATGGTT...................................................     1
.........GAGGTAGTAGGTTGTATGGTTT..................................................     1
..........AGGTAGTAGGTTGTATGGTTT..................................................     1
...............GTAGGTTGTATGGTTT..................................................     1
........TGAGGTAGTAGGTTGTATGGTTTAGAGTT............................................     1
..............AGTAGGTTGTATGGTTTAGAGTTAC..........................................     1
........TGAGGTAGTAGGTTGTATGGTTTAGAGTTACA.........................................     1
........................................CCCTGGGAGTTAACTGTACAACCT.................     1
.............................................GGAGTTAACTGTACAACCTTCT..............     1
.............................................GGAGTTAACTGTACAACCTTCTAGCT..........     1
..............................................GAGTTAACTGTACAACCTTCTAGCT..........     1
.....................................................CTGTACAACCTTCTAGCT..........     1
.................................................TTAACTGTACAACCTTCTAGCTT.........     1
...................................................AACTGTACAACCTTCTAGCTT.........     1
.......................................ACCCTGGGAGTTAACTGTACAACCTTCTAGCTTTCC......     1
..........................................CTGGGAGTTAACTGTACAACCTTCTAGCTTTCC......     1
...........................................TGGGAGTTAACTGTACAACCTTCTAGCTTTCC......     1
..................................................TAACTGTACAACCTTCTAGCTTTCC......     1
...................................................AACTGTACAACCTTCTAGCTTTCC......     1
...........................................TGGGAGTTAACTGTACAACCTTCTAGCTTTCCT.....     1
...................................................AACTGTACAACCTTCTAGCTTTCCT.....     1
.....................................................CTGTACAACCTTCTAGCTTTCCTT....     1
.((((((..(((.(((.(((((((((((((..((.(..(.....)..).))))))))))))))).))).)))..)))))). (-32.40)

1/19931189-19931260(-)_mir_cow_5
TGGCATAAACCCGTAGATCCGATCTTGTGGTGAAGTGGACCGCACAAGCTCGCTTCTATGGGTCTGTGTCAG
.......AACCCGTAGATCCGATCTTGTG...........................................  36750
.......AACCCGTAGATCCGATCTTGT............................................  6624
.......AACCCGTAGATCCGATCTTG.............................................  5357
............................................CAAGCTCGCTTCTATGGGTCTGT.....  3827
.......AACCCGTAGATCCGATCTTGTGG..........................................  3201
...........CGTAGATCCGATCTTGTG...........................................  954
...........CGTAGATCCGATCTTGT............................................  923
............................................CAAGCTCGCTTCTATGGGTCT.......  788
............................................CAAGCTCGCTTCTATGGGTCTG......  726
..........CCGTAGATCCGATCTTGTG...........................................  407
```

......AAACCCGTAGATCCGATCTTGT..	283
....CAAGCTCGCTTCTATGGGTC........	202
............GTAGATCCGATCTTGTG...	192
............GTAGATCCGATCTTGT..	191
......AAACCCGTAGATCCGATCTTG...	144
..........CCGTAGATCCGATCTTGT..	142
.......AACCCGTAGATCCGATCTT..	124
......AAACCCGTAGATCCGATCTTGTG...	122
.......AACCCGTAGATCCGATCT...	72
...........CGTAGATCCGATCTTG...	59
....AAGCTCGCTTCTATGGGTCTGT.....	36
....CAAGCTCGCTTCTATGGGT.........	33
..........CCGTAGATCCGATCTTG...	29
........ACCCGTAGATCCGATCTTGTGG..	21
...........CGTAGATCCGATCTTGTGG..	16
.............TAGATCCGATCTTGTG...	15
.......AACCCGTAGATCCGATC..	9
........ACCCGTAGATCCGATCTTGTG...	6
....ACAAGCTCGCTTCTATGGGTCTGT.....	6
..........CCGTAGATCCGATCTTGTGG..	5
............GTAGATCCGATCTTGTGG..	5
.........CCCGTAGATCCGATCTTGTG...	4
......AAACCCGTAGATCCGATCTTGTGG..	4
....ACAAGCTCGCTTCTATGGGTCT.......	4
.........CCCGTAGATCCGATCTTGTGG..	3
....CAAGCTCGCTTCTATGGG..........	3
....ACAAGCTCGCTTCTATGGGTC........	3
......AAACCCGTAGATCCGATCT...	2
......AAACCCGTAGATCCGATCTT..	2
.........CCCGTAGATCCGATCTTGT..	2
......................................ACCGCACAAGCTCGCTTCTATGGGTCTG......	2
....AAGCTCGCTTCTATGGGTCTG......	2
....AGCTCGCTTCTATGGGTCTGT.....	2
....GCTCGCTTCTATGGGTCTGT.....	2
..........CCGTAGATCCGATCTT..	1
........ACCCGTAGATCCGATCTTG...	1
.........CCCGTAGATCCGATCTTG...	1
.............TAGATCCGATCTTGTGG..	1

..............AGATCCGATCTTGTGG.. 1
.........................TGTGGTGAAGTGGACCGC............................. 1
....CAAGCTCGCTTCTATG............ 1
....AAGCTCGCTTCTATGGGTCT....... 1
.............................GTGAAGTGGACCGCACAAGCTCGCTTCTATGGGTCTG...... 1
....GCACAAGCTCGCTTCTATGGGTCTG...... 1
....CAAGCTCGCTTCTATGGGTCTGTG.... 1
(((((((.(((((((((..(((.((((((.((........))))))))).)))..))))))))).))))))). (-34.90)

12/16533026-16533095(+)_mir_cow_26
TCAGCCAAAAGTTCGTTCGGGTTTTTTGTTTACAGCTCACTAGAAAAACCCAAATGAACTCTTTGGCCAA
......AAAAGTTCGTTCGGGTTTTT.. 328
......AAAAGTTCGTTCGGGTTTTTT... 35
......AAAAGTTCGTTCGGGTTTT... 27
......AAAAGTTCGTTCGGGTTT.. 6
.....CAAAAGTTCGTTCGGGTTTTT.. 5
........AAGTTCGTTCGGGTTTTT.. 5
.........AGTTCGTTCGGGTTTTT.. 2
..........GTTCGTTCGGGTTTTT.. 2
......AAAAGTTCGTTCGGGTT... 1
.......AAAGTTCGTTCGGGTTTTT.. 1
....AAAACCCAAATGAACTCTTTGG.... 1
...((((((((((((((.(((((((((...............))))))))).)))))))).))))))... (-26.46)

12/19542755-19542821(-)_mir_cow_27
GGATTGTTGTACTTTTTTTTTTGTTCGTTGCATTTTTAGGAACAAAAAAAAAAGCCCAACCCTTCAC
....TGTTGTACTTTTTTTTTTGTTC... 289
....TGTTGTACTTTTTTTTTTGTT.. 121
....ACAAAAAAAAAAGCCCAACCCT.... 72
....TGTTGTACTTTTTTTTTTGT... 23
....TGTTGTACTTTTTTTTTTG.. 6
...TTGTTGTACTTTTTTTTTTGTT.. 4
....TGTTGTACTTTTTTTTTTGTTCG.. 3
...TTGTTGTACTTTTTTTTTTGT... 2
....ACAAAAAAAAAAGCCCAACCC..... 1
(((..((((..(((((((((((((((.(.........).)))))))))))))))..))))..))).. (-21.10)

12/19596207-19596281(-)_mir_cow_28

```
GTGCCTTAGCAGCACGTAAATATTGGTGTTAAGATTCTAAAATTATCTCCAGTATTAACTGTGCTGCTGAAGTAA
......TAGCAGCACGTAAATATTGGTG...............................................     26702
......TAGCAGCACGTAAATATTGGT................................................     3027
......TAGCAGCACGTAAATATTGGTGT..............................................     1545
......TAGCAGCACGTAAATATTGG.................................................     344
......TAGCAGCACGTAAATATTG..................................................     287
......TAGCAGCACGTAAATAT....................................................     41
...............................................TCCAGTATTAACTGTGCTGCTG......     26
......TAGCAGCACGTAAATATTGGTGTT.............................................     25
...............................................TCCAGTATTAACTGTGCTG.........     14
...............................................TCCAGTATTAACTGTGCTGCT.......     12
.......AGCAGCACGTAAATATTGGTG...............................................     6
................................................CCAGTATTAACTGTGCTGCTG......     6
........GCAGCACGTAAATATTGGTG...............................................     5
................................................CCAGTATTAACTGTGCTGCT.......     5
......TAGCAGCACGTAAATATT...................................................     4
..............................................CTCCAGTATTAACTGTGCTGCTG......     4
...............................................TCCAGTATTAACTGTGCTGCTGA.....     4
...........GCACGTAAATATTGGTG...............................................     3
..............................................CTCCAGTATTAACTGTGCTGCT.......     3
................................................CCAGTATTAACTGTGCTGCTGA.....     3
...............................................TCCAGTATTAACTGTGCTGCTGAA....     3
................................................CCAGTATTAACTGTGCTGCTGAA....     2
..................................................AGTATTAACTGTGCTGCTGAA....     2
......TAGCAGCACGTAAATA.....................................................     1
.........CAGCACGTAAATATTGGTG...............................................     1
..........AGCACGTAAATATTGGTG...............................................     1
............CACGTAAATATTGGTG...............................................     1
...........GCACGTAAATATTGGTGT..............................................     1
.............ACGTAAATATTGGTGT..............................................     1
............................TTAAGATTCTAAAATTATC............................     1
............................TTAAGATTCTAAAATTATCTCCAGTATTAACTGTGCTG.........     1
.............................................TCTCCAGTATTAACTGTGCTG.........     1
..............................................CTCCAGTATTAACTGTGCTG.........     1
.............................................TCTCCAGTATTAACTGTGCTGCT.......     1
.(((.((((((((((((.((((((((.(.(((..........))).).)))))))).)).)))))))))).))). (-28.50)

12/29660165-29660239(-)_mir_cow_29
```

```
ATAACTGAACACACTGGATTCATTTCTCTGAAAGTGGATTTTGTGGTCTGTCCGGGGAATGGATCCAGCGTGATC
............ACTGGATTCATTTCTCTGAAA..........................................     646
............ACTGGATTCATTTCTCTGAAAG.........................................     223
............ACTGGATTCATTTCTCTGAAAGT........................................     74
............ACTGGATTCATTTCTCTGAA...........................................     52
...........CACTGGATTCATTTCTCTGAAA..........................................     30
............ACTGGATTCATTTCTCTGA............................................     26
.................................................GTCCGGGGAATGGATCCAGCGT....     20
...........CACTGGATTCATTTCTCTGAA...........................................     9
...........CACTGGATTCATTTCTCTGA............................................     5
..................................................TCCGGGGAATGGATCCAGCGT....     4
...........CACTGGATTCATTTCTCTGAAAG.........................................     3
.................................................GTCCGGGGAATGGATCCAGCG.....     3
.................................................GTCCGGGGAATGGATCCAGC......     2
......GAACACACTGGATTCATTTCTCTGAA...........................................     1
........................TCTCTGAAAGTGGATTTTGTG..............................     1
..................................................TCCGGGGAATGGATCCAGCG.....     1
................................................TGTCCGGGGAATGGATCCAGCGT....     1
.........(((.(((((((((((((((.((....(((((....))))).)).))))))))))))))).)))... (-30.60)

12/31820741-31820808(+)_mir_cow_30
ATTCCTTAGCTAGTTTTGCCTCCCTGCTTATGATCACACACAGGGAGACAAAACTAGCTGAGGAATAA
.....TTAGCTAGTTTTGCCTCCCTGC......................................... 47
.....TTAGCTAGTTTTGCCTCCCTG.......................................... 8
..........................................GGGAGACAAAACTAGCTGAGGAA... 5
....CTTAGCTAGTTTTGCCTCCCTG.......................................... 3
.....TTAGCTAGTTTTGCCTCCCTGCT........................................ 3
.........................................AGGGAGACAAAACTAGCTGAGG..... 2
....CTTAGCTAGTTTTGCCTCCCT........................................... 1
.....TTAGCTAGTTTTGCCTCCCT........................................... 1
....CTTAGCTAGTTTTGCCTCCCTGC......................................... 1
......TAGCTAGTTTTGCCTCCCTGC......................................... 1
..........................................GGGAGACAAAACTAGCTGAGG..... 1
..........................................GGGAGACAAAACTAGCTGAGGA.... 1
............................................GAGACAAAACTAGCTGAGGAAT.. 1
((((((((((((((((((.(((((((..............))))))).)))))))))))))))))).. (-39.44)

12/66226562-66226629(+)_mir_cow_31
```

```
AATGTCAAAGTGCTTACAGTGCAGGTAGTGATATGTGCATCTACTGCAGTGAAGGCACTTGTAGCATT
.....CAAAGTGCTTACAGTGCAGGTAG........................................ 2450
.....CAAAGTGCTTACAGTGCAGGTAGT....................................... 475
.....CAAAGTGCTTACAGTGCAGGT.......................................... 357
.....CAAAGTGCTTACAGTGCAGGTA......................................... 295
..........................................ACTGCAGTGAAGGCACTTGTA..... 186
....TCAAAGTGCTTACAGTGCAGGT.......................................... 103
..........................................ACTGCAGTGAAGGCACTTGT...... 95
..........................................ACTGCAGTGAAGGCACTTGTAG.... 81
..........................................ACTGCAGTGAAGGCACTTGTAGC... 62
.....CAAAGTGCTTACAGTGCAGG........................................... 47
....TCAAAGTGCTTACAGTGCAGGTAG........................................ 36
....TCAAAGTGCTTACAGTGCAGGTA......................................... 35
..........................................ACTGCAGTGAAGGCACTTG....... 34
......AAAGTGCTTACAGTGCAGGTAG........................................ 19
...........................................CTGCAGTGAAGGCACTTGTAGC... 19
....TCAAAGTGCTTACAGTGCAGG........................................... 13
.....CAAAGTGCTTACAGTGCAG............................................ 9
......AAAGTGCTTACAGTGCAGGTAGT....................................... 6
....TCAAAGTGCTTACAGTGCAGGTAGT....................................... 4
...........................................CTGCAGTGAAGGCACTTGTA..... 4
...........................................CTGCAGTGAAGGCACTTGTAG.... 4
...........................................CTGCAGTGAAGGCACTTGTAGCA.. 4
....TCAAAGTGCTTACAGTGCAG............................................ 3
.....CAAAGTGCTTACAGTGC.............................................. 2
..........................................ACTGCAGTGAAGGCACT......... 2
.....CAAAGTGCTTACAGTG............................................... 1
.....CAAAGTGCTTACAGTGCA............................................. 1
......AAAGTGCTTACAGTGCAGGTA......................................... 1
.........GTGCTTACAGTGCAGGTAG........................................ 1
.........GTGCTTACAGTGCAGGTAGT....................................... 1
..........TGCTTACAGTGCAGGTAGT....................................... 1
..........................................ACTGCAGTGAAGGCAC.......... 1
.........................................TACTGCAGTGAAGGCACTTGTA..... 1
...............................................AGTGAAGGCACTTGTAGC... 1
(((((..((((((((.((.(((((.(((((.......)).)))))))).)).))))))))...))))) (-25.70)
```

12/66226698-66226768(+)_mir_cow_32

```
GTTCTAAGGTGCATCTAGTGCAGATAGTGAAGTAGATTAGCATCTACTGCCCTAAGTGCTCCTTCTGGCAT
....TAAGGTGCATCTAGTGCAGAT..............................................   175
....TAAGGTGCATCTAGTGCAGATAG............................................   160
....TAAGGTGCATCTAGTGCAGATA.............................................   93
....TAAGGTGCATCTAGTGCAGA...............................................   41
.............................................ACTGCCCTAAGTGCTCCTTCTG....   34
.............................................ACTGCCCTAAGTGCTCCTTCTGG...   25
....TAAGGTGCATCTAGTGCAGATAGT...........................................   12
....TAAGGTGCATCTAGTGCAG................................................   6
.............................................ACTGCCCTAAGTGCTCCTTCTGGC..   5
.............................................ACTGCCCTAAGTGCTCCTTCT.....   4
....TAAGGTGCATCTAGTGCA.................................................   3
....TAAGGTGCATCTAGTGC..................................................   1
............................................TACTGCCCTAAGTGCTCCTTCT.....   1
............................................TACTGCCCTAAGTGCTCCTTCTGG...   1
(((..((((.(((..(((.((((.(((((..(.....)..)).))))))).)))..))).))))..)))..  (-22.00)

12/66226844-66226909(+)_mir_cow_33
CTGTTAGTTTTGCATAGTTGCACTACAAGAAGAATGTAGTTGTGCAAATCTATGCAAAACTGATGG
........................................TGTGCAAATCTATGCAAAACTGA...   1114
........................................TGTGCAAATCTATGCAAAACTG....   990
........................................TGTGCAAATCTATGCAAAACT.....   85
.......................................TTGTGCAAATCTATGCAAAACTG....   78
.......................................TTGTGCAAATCTATGCAAAACTGA...   33
.......................................TTGTGCAAATCTATGCAAAACT.....   28
........................................TGTGCAAATCTATGCAAAAC......   20
........................................TGTGCAAATCTATGCAAAACTGAT..   12
.....AGTTTTGCATAGTTGCACT..........................................   4
.....AGTTTTGCATAGTTGCACTA.........................................   4
....TAGTTTTGCATAGTTGCACT..........................................   2
.......................................TTGTGCAAATCTATGCAAAAC......   2
..........................AAGAAGAATGTAGTTGTGCAAATCTATGCAAA........   1
.........................................GTGCAAATCTATGCAAAACTG....   1
............................................CAAATCTATGCAAAACTG....   1
.........................................GTGCAAATCTATGCAAAACTGA...   1
............................................CAAATCTATGCAAAACTGA...   1
.............................................AAATCTATGCAAAACTGA...   1
(((((((((((((((((((((((((((.......))))...))))))..)))))))))))))))))  (-28.30)
```

```
12/66227007-66227073(+)_mir_cow_34
AGCACTAAAGTGCTTATAGTGCAGGTAGTGTTTAGTTATCTACTGCATTATGAGCACTTAAAGTACT
.....TAAAGTGCTTATAGTGCAGGTAG.......................................  2871
.....TAAAGTGCTTATAGTGCAGGTAGT......................................  415
.....TAAAGTGCTTATAGTGCAGGT.........................................  265
.....TAAAGTGCTTATAGTGCAGGTA........................................  247
.........................................ACTGCATTATGAGCACTTAAAGT...  64
.....TAAAGTGCTTATAGTGCAGG..........................................  37
.........................................ACTGCATTATGAGCACTTAAAG....  16
..........................................CTGCATTATGAGCACTTAAAGT...  14
.....TAAAGTGCTTATAGTGCAG...........................................  8
.....TAAAGTGCTTATAGTG..............................................  2
..........................................CTGCATTATGAGCACTTAAAG....  2
.....TAAAGTGCTTATAGTGCA............................................  1
....CTAAAGTGCTTATAGTGCAGGT.........................................  1
....CTAAAGTGCTTATAGTGCAGGTAG.......................................  1
......AAAGTGCTTATAGTGCAGGTAG.......................................  1
..........TGCTTATAGTGCAGGTAG.......................................  1
.........GTGCTTATAGTGCAGGTAGT......................................  1
..........................................CTGCATTATGAGCACTTA.......  1
..........................................CTGCATTATGAGCACTTAAAGTA..  1
...(((.(((((((((((((((((.(((((......)).))))))))))))))))))))..)))...  (-27.00)

12/66227261-66227328(+)_mir_Cow_Blast_38
ACACAGGTTGGGATCGGTTGCAATGCTGTGTTTCTGTATGGTATTGCACTTGTCCCGGCCTGTTGAGT
.........................................TATTGCACTTGTCCCGGCCTGT..... 94635
.........................................TATTGCACTTGTCCCGGCCTGTT.... 12936
.........................................TATTGCACTTGTCCCGGCCTG...... 5603
..........................................ATTGCACTTGTCCCGGCCTGT..... 1365
.........................................TATTGCACTTGTCCCGGCCT....... 355
..........................................ATTGCACTTGTCCCGGCCTGTT.... 274
.........................................TATTGCACTTGTCCCGGCC........ 250
..........................................ATTGCACTTGTCCCGGCCTG...... 139
.........................................TATTGCACTTGTCCCGGCCTGTTG... 103
.........................................TATTGCACTTGTCCCGGCCTGTTGA.. 49
.........................................TATTGCACTTGTCCCGGC......... 45
....AGGTTGGGATCGGTTGCAATGCT......................................... 36
```

```
.........................................TATTGCACTTGTCCCGG.......... 14
...........................................TTGCACTTGTCCCGGCCTGT..... 14
....AGGTTGGGATCGGTTGCAATGC.......................................... 8
.............................................GCACTTGTCCCGGCCTGT..... 5
.........................................TATTGCACTTGTCCCG........... 4
..........................................ATTGCACTTGTCCCGGCCT....... 3
............................................TGCACTTGTCCCGGCCTGT..... 3
..........................................ATTGCACTTGTCCCGGC......... 2
...............................................ACTTGTCCCGGCCTGT..... 2
..........................................ATTGCACTTGTCCCGGCCTGTTG... 2
..........................................ATTGCACTTGTCCCGGCCTGTTGA.. 2
....AGGTTGGGATCGGTTGCAATG........................................... 1
.....GGTTGGGATCGGTTGCAATGCT......................................... 1
.......TTGGGATCGGTTGCAATGCT......................................... 1
....AGGTTGGGATCGGTTGCAATGCTGTGT..................................... 1
..........................................ATTGCACTTGTCCCGGCC........ 1
...........................................TTGCACTTGTCCCGGCCTGTT.... 1
............................................TGCACTTGTCCCGGCCTGTT.... 1
.............................................GCACTTGTCCCGGCCTGTT.... 1
..((((((((((((.(((.(((((((((((......)))))))))))))).))))))))))))..... (-35.50)

13/13129029-13129099(-)_mir_cow_35
CACACACACACTCAGATAAAGTAGGCACTTTTTGGTTCTTGAAATGCTACCTAGTCTGAGTGAGTGAGTGT
......CACACTCAGATAAAGTAGGCACT..........................................   894
.......ACACTCAGATAAAGTAGGCACTT.........................................   268
......CACACTCAGATAAAGTAGGCAC...........................................   141
...........................................ATGCTACCTAGTCTGAGTGAGT......   118
............................................TGCTACCTAGTCTGAGTGAGTG.....   66
......CACACTCAGATAAAGTAGGCACTT.........................................   52
............................................TGCTACCTAGTCTGAGTGAGT......   39
.......ACACTCAGATAAAGTAGGCACT..........................................   31
......CACACTCAGATAAAGTAGGC.............................................   22
......CACACTCAGATAAAGTAGGCA............................................   10
.......ACACTCAGATAAAGTAGGCACTTT........................................   10
...........................................ATGCTACCTAGTCTGAGTGAG.......   6
.......ACACTCAGATAAAGTAGGCAC...........................................   5
...........................................ATGCTACCTAGTCTGAGTG.........   2
...........................................ATGCTACCTAGTCTGAGTGA........   2
```

```
...........................................TGCTACCTAGTCTGAGTGAG.......    2
...........................................TGCTACCTAGTCTGAGTGAGTGA....    2
......CACACTCAGATAAAGTAGG.............................................    1
......CACACTCAGATAAAGTAGGCACTTT.......................................    1
...........................................TGCTACCTAGTCTGAG...........    1
.........................................AATGCTACCTAGTCTGAGTGAG.......    1
.............................................CTACCTAGTCTGAGTGAGT......    1
..........................................ATGCTACCTAGTCTGAGTGAGTG.....    1
(((.(((.(((((((((...((((.((.((((.......))))))))))...))))))))).))).))). (-25.40)

13/36660314-36660376(+)_mir_cow_36
CCAAATTGTTCATTTGGGTTTTTCCGTAACATCTTATGGAAAAACTTGAGTGAACTTTTGGGT
.......................................AAAAACTTGAGTGAACTTTTGG..      47
...AATTGTTCATTTGGGTTTTTCC......................................      29
...AATTGTTCATTTGGGTTTTTC.......................................      14
.......................................AAAAACTTGAGTGAACTTTT....      5
...AATTGTTCATTTGGGTTTTT........................................      4
.......................................AAAAACTTGAGTGAACTTTTG...      3
.......................................AAAAACTTGAGTGAACTTT.....      2
...AATTGTTCATTTGGGTTTTTCCG.....................................      1
....ATTGTTCATTTGGGTTTTTCCG.....................................      1
.......................................AAAAACTTGAGTGAACT.......      1
(((((..(((((((..(((((((((((((....)))))))))))))..))))))).))))).. (-32.20)

13/51742430-51742497(-)_mir_Cow_Blast_18
TTGTGCTTTCAGCTTCTTTACAGTGCTGCCTTGTAGCATTCAGGTCAAGCAGCATTGTACAGGGCTAT
...............................................AGCAGCATTGTACAGGGCTAT 14341
...............................................AGCAGCATTGTACAGGGCT.. 7437
...............................................AGCAGCATTGTACAGGGC... 1712
...............................................AGCAGCATTGTACAGGGCTA. 1027
...............................................AGCAGCATTGTACAGGG.... 35
...............................................AGCAGCATTGTACAGG..... 8
..........AGCTTCTTTACAGTGCTGCCTTG................................... 7
.................................................CAGCATTGTACAGGGCTAT 5
..........AGCTTCTTTACAGTGCTGCCTTGT.................................. 4
................................................GCAGCATTGTACAGGGCTAT 4
...................................................GCATTGTACAGGGCTAT 4
..........AGCTTCTTTACAGTGCTGCCTT.................................... 3
```

....AGGTCAAGCAGCATTGTACAGGGCT.. 3
....AAGCAGCATTGTACAGGGCT.. 3
....CAGCATTGTACAGGGCTA. 3
........TCAGCTTCTTTACAGTGCTGCCT..................................... 2
..........AGCTTCTTTACAGTGCTGCCT..................................... 2
.............TTCTTTACAGTGCTGCCTTGT.................................. 2
....GCAGCATTGTACAGGGCT.. 2
....AAGCAGCATTGTACAGGGCTAT 2
..............TCTTTACAGTGCTGCC...................................... 1
.................TTACAGTGCTGCCTTG................................... 1
....GGTCAAGCAGCATTGTACAGGGC... 1
....AAGCAGCATTGTACAGGGC... 1
....CAGCATTGTACAGGGCT.. 1
....AGCATTGTACAGGGCT.. 1
....AGCATTGTACAGGGCTA. 1
....GCATTGTACAGGGCTA. 1
....AGCATTGTACAGGGCTAT 1
..........((((.((.(((((((((((((((.......))))....))))))))))).)))))).. (-23.70)

13/54375939-54376008(+)_mir_cow_37
CCTTCAGGACTGTCCAACCTGAGAATGGTGAGCATCCAGGGACAATCTCAGGTTTGTCAGCCCGCAAGGT
.....AGGACTGTCCAACCTGAGAAT.. 1210
.....AGGACTGTCCAACCTGAGAATG... 665
....ATCTCAGGTTTGTCAGCCCGCA.... 111
.....AGGACTGTCCAACCTGAGA.. 110
....ATCTCAGGTTTGTCAGCCCG...... 107
.....AGGACTGTCCAACCTGAGAA... 90
....ATCTCAGGTTTGTCAGCCCGC..... 71
.....AGGACTGTCCAACCTGAGAATGG.. 15
....ATCTCAGGTTTGTCAGCC........ 10
....ATCTCAGGTTTGTCAGCCC....... 7
....ATCTCAGGTTTGTCAGCCCGCAA... 4
....ATCTCAGGTTTGTCAGC......... 3
....ATCTCAGGTTTGTCAGCCCGCAAG.. 2
.....AGGACTGTCCAACCTGAG... 1
....CAGGACTGTCCAACCTGAGAAT.. 1
......GGACTGTCCAACCTGAGAAT.. 1
.....AGGACTGTCCAACCTGAGAATGGT... 1

```
...........................................AATCTCAGGTTTGTCAGCCCGCA....     1
(((((.((.(((.(.(((((((((.((..............)).))))))))).).))))).).))))). (-25.16)

13/54827551-54827622(-)_mir_cow_38
CCTCTGCGTGTTCACAGCGGACCTTGATTTAATGTCTATACAATTAAGGCACGCGGTGAATGCCAAGAGAGG
............................................TAAGGCACGCGGTGAATGCC........  62579
............................................TAAGGCACGCGGTGAATGCCA.......  19991
...........................................TTAAGGCACGCGGTGAATGCC........  15984
...........................................TTAAGGCACGCGGTGAATGCCA.......  5129
............................................TAAGGCACGCGGTGAATGC.........  5040
...........................................TTAAGGCACGCGGTGAATGC.........  2796
............................................TAAGGCACGCGGTGAATGCCAA......  2398
............................................TAAGGCACGCGGTGAATG..........  649
...........................................TTAAGGCACGCGGTGAATG..........  534
...........................................TTAAGGCACGCGGTGAATGCCAA......  418
...........................................TTAAGGCACGCGGTGAAT...........  294
......CGTGTTCACAGCGGACCTTGAT............................................  171
............................................TAAGGCACGCGGTGAATGCCAAG.....  155
.............................................AAGGCACGCGGTGAATGCC........  109
......CGTGTTCACAGCGGACCTTGA.............................................  95
............................................TAAGGCACGCGGTGAATGCCAAGA....  83
.............................................AAGGCACGCGGTGAATGCCA.......  76
......CGTGTTCACAGCGGACCTTGATT...........................................  44
.................................................CACGCGGTGAATGCCA.......  42
............................................TAAGGCACGCGGTGAAT...........  41
............................................TAAGGCACGCGGTGAA............  31
...........................................TTAAGGCACGCGGTGAA............  18
.............................................AAGGCACGCGGTGAATGCCAA......  17
...........................................TTAAGGCACGCGGTGAATGCCAAGA....  14
......CGTGTTCACAGCGGACCT................................................  10
..............................................AGGCACGCGGTGAATGCC........  9
.................................................CACGCGGTGAATGCCAA......  8
...........................................TTAAGGCACGCGGTGAATGCCAAG.....  8
......CGTGTTCACAGCGGACC.................................................  7
......CGTGTTCACAGCGGACCTTG..............................................  7
................................................GCACGCGGTGAATGCCA.......  7
...........................................TTAAGGCACGCGGTGA.............  4
................................................GCACGCGGTGAATGCC........  4
```

....AAGGCACGCGGTGAATGC......... 3
......CGTGTTCACAGCGGACCTT.. 2
........TGTTCACAGCGGACCTTGATT.. 2
....ACGCGGTGAATGCCAA...... 2
....AAGGCACGCGGTGAATGCCAAG..... 2
......CGTGTTCACAGCGGACCTTGATTTAATG..................................... 1
....AAGGCACGCGGTGAATG.......... 1
....CAATTAAGGCACGCGGTGAATGC......... 1
....ATTAAGGCACGCGGTGAATGCC........ 1
....AGGCACGCGGTGAATGCCA....... 1
....AAGGCACGCGGTGAATGCCAAGA.... 1
(((((..((((((((.(((..((((((((...........))))))))..))).))))))))....))))) (-27.60)

13/55227298-55227376(-)_mir_Cow_Blast_6
TGCTTTGCTAAAGCTGGTAAAATGGAACCAAATCAACTGTTCGATGGATTTGGTCCCCTTCAACCAGCTGTAGCTGCGC
....TTTGGTCCCCTTCAACCAGCTGT........ 325991
....TTGGTCCCCTTCAACCAGCTGT........ 230594
....TTTGGTCCCCTTCAACCAGCTG......... 16514
....TTTGGTCCCCTTCAACCAGCT.......... 7743
....TTGGTCCCCTTCAACCAGCTG......... 6175
....TTGGTCCCCTTCAACCAGCTGTA....... 5872
....TTTGGTCCCCTTCAACCAGCTGTA....... 3708
....TGGTCCCCTTCAACCAGCTGT........ 3306
...........AGCTGGTAAAATGGAACCAAAT.. 1466
....TTGGTCCCCTTCAACCAGCT.......... 1326
....TTTGGTCCCCTTCAACCAGC........... 542
...........AGCTGGTAAAATGGAACCAAATC... 240
....TTGGTCCCCTTCAACCAGC........... 228
...........AGCTGGTAAAATGGAACCAAA... 208
....ATTTGGTCCCCTTCAACCAGCTGT........ 201
....TGGTCCCCTTCAACCAGCTGTA....... 141
...........AGCTGGTAAAATGGAACC.. 85
....ATTTGGTCCCCTTCAACCAGCT.......... 85
....TGGTCCCCTTCAACCAGCTG......... 83
....TCCCCTTCAACCAGCTGT........ 62
...........AGCTGGTAAAATGGAACCA... 54
....CCCTTCAACCAGCTGT........ 45
............GCTGGTAAAATGGAACCAAATC... 31

....ATTTGGTCCCCTTCAACCAGCTG......... 31
...........AGCTGGTAAAATGGAACCAA.. 24
....TTTGGTCCCCTTCAACCAG............ 24
....GGTCCCCTTCAACCAGCTGT........ 23
............GCTGGTAAAATGGAACCAAAT.. 22
....TTGGTCCCCTTCAACCAGCTGTAG...... 15
....CCCCTTCAACCAGCTGT........ 14
....TTTGGTCCCCTTCAACC.............. 10
....TTGGTCCCCTTCAACC.............. 10
....TTTGGTCCCCTTCAACCAGCTGTAG...... 8
....CGATGGATTTGGTCCCCTTCAAC............... 7
....GTCCCCTTCAACCAGCTGT........ 7
...........AGCTGGTAAAATGGAACCAAATCAACT... 6
....ATTTGGTCCCCTTCAACCAGC........... 5
....TTTGGTCCCCTTCAAC............... 4
....TTGGTCCCCTTCAACCAG............ 4
....TGGTCCCCTTCAACCAGC........... 4
............GCTGGTAAAATGGAACCAAA... 3
...........AGCTGGTAAAATGGAACCAAATCA.. 3
...........AGCTGGTAAAATGGAAC... 2
............GCTGGTAAAATGGAACC.. 2
.................TAAAATGGAACCAAATCAACTGTT...................................... 2
....TTTGGTCCCCTTCAACCA............. 2
....TGGTCCCCTTCAACCAGCT.......... 2
....TCCCCTTCAACCAGCTG......... 2
.................................CAACTGTTCGATGGATTTGGTCCCCTTCAACCAGCTGT........ 2
....GGTCCCCTTCAACCAGCTGTA....... 2
........TAAAGCTGGTAAAATGGAAC... 1
........TAAAGCTGGTAAAATGGAACC.. 1
.........AAAGCTGGTAAAATGGAACCA... 1
............GCTGGTAAAATGGAACCAAATCAACT... 1
.................TAAAATGGAACCAAATCAACTGT....................................... 1
...................AAATGGAACCAAATCAACTGT....................................... 1
...........AGCTGGTAAAATGGAACCAAATCAACTGTTCGATGGAT.............................. 1
....TTGGTCCCCTTCAACCA............. 1
....ATTTGGTCCCCTTCAACCAG............ 1
....GGTCCCCTTCAACCAGCTG......... 1
....GTCCCCTTCAACCAGCTG......... 1

```
.........................................CGATGGATTTGGTCCCCTTCAACCAGCTGT........ 1
..............................................GATTTGGTCCCCTTCAACCAGCTGT........ 1
...............................................ATTTGGTCCCCTTCAACCAGCTGTA....... 1
....................................................GTCCCCTTCAACCAGCTGTA....... 1
.......................................................CCCTTCAACCAGCTGTA....... 1
..................................................TGGTCCCCTTCAACCAGCTGTAG...... 1
.((...((((.(((((((..((.((.((((((((............)))))))).)).))..))))))).)))).)).. (-31.40)

13/55237542-55237618(-)_mir_Cow_Blast_2
CTTGGGAAACATACTTCTTTATGTGCCCATATGGACCTGCTAAGCTATGGAATGTAAAGAAGTATGTATTTCAGGTC
...............................................TGGAATGTAAAGAAGTATGTAT........ 11903
...............................................TGGAATGTAAAGAAGTATGTA......... 1632
...............................................TGGAATGTAAAGAAGTATGT.......... 1127
...............................................TGGAATGTAAAGAAGTATGTATT....... 851
.................................................GAATGTAAAGAAGTATGTATTT...... 104
.................................................GAATGTAAAGAAGTATGTATT....... 88
...............................................TGGAATGTAAAGAAGTATG........... 32
...............................................TGGAATGTAAAGAAGTATGTATTT...... 20
..............................................ATGGAATGTAAAGAAGTATGT.......... 19
................................................GGAATGTAAAGAAGTATGTAT........ 18
...................................................ATGTAAAGAAGTATGTAT........ 16
..............................................ATGGAATGTAAAGAAGTATGTAT........ 13
...............................................TGGAATGTAAAGAAGTAT............ 9
..................................................AATGTAAAGAAGTATGTAT........ 9
................................................GGAATGTAAAGAAGTATGTATT....... 9
..............................................ATGGAATGTAAAGAAGTATGTA......... 6
...............................................TGGAATGTAAAGAAGTA............. 5
....................................................TGTAAAGAAGTATGTAT........ 5
...............................................TGGAATGTAAAGAAGT.............. 4
.....................................................GTAAAGAAGTATGTAT........ 4
...................................................ATGTAAAGAAGTATGT.......... 3
...................................................ATGTAAAGAAGTATGTA......... 3
.................................................GAATGTAAAGAAGTATGTAT........ 3
........ACATACTTCTTTATGTGCCCATA.............................................. 2
.............................TATGGACCTGCTAAGCTATG............................ 2
..............................................ATGGAATGTAAAGAAGTATGTATT....... 2
..................................................AATGTAAAGAAGTATGTATT....... 2
......................................................TAAAGAAGTATGTATT....... 2
```

```
........ACATACTTCTTTATGTGCCCAT...............................................   1
.................TTTATGTGCCCATATGGACCTGCT....................................   1
.................TTTATGTGCCCATATGGACCTGCTAAGCTA..............................   1
................................GGACCTGCTAAGCTATGGAATGTAAAGAAGT..............   1
.................................................GAATGTAAAGAAGTATGTA.........   1
.............................TATGGACCTGCTAAGCTATGGAATGTAAAGAAGTATGTATT.......   1
...................................................ATGTAAAGAAGTATGTATT.......   1
....................................................TGTAAAGAAGTATGTATT.......   1
.....................................................GTAAAGAAGTATGTATT.......   1
((((..(.((((((((((((((((..((((((((.....)))...))))).)))))))))))))))).)..)))).. (-30.20)

13/58091944-58092006(+)_mir_cow_39
TCCGGAGGGCCCCCCCCAATCCTGTTGTGCTTGGTTCAGAGGGTTGGGCGGAGGCTTTCCTGA
.......................................AGGGTTGGGCGGAGGCTTTCCT..      2346
.......................................AGGGTTGGGCGGAGGCTTTCC...      577
....GAGGGCCCCCCCCAATCCTGT......................................      217
.......................................AGGGTTGGGCGGAGGCTTTC....      100
....GAGGGCCCCCCCCAATCCTG.......................................      36
.......................................AGGGTTGGGCGGAGGCTTT.....      35
....GAGGGCCCCCCCCAATCCT........................................      19
.......................................AGGGTTGGGCGGAGGCTT......      12
.....AGGGCCCCCCCCAATCCTGTTG....................................      7
.......................................AGGGTTGGGCGGAGGCT.......      5
....GAGGGCCCCCCCCAATCCTGTT.....................................      4
....GAGGGCCCCCCCCAATCC.........................................      2
.....AGGGCCCCCCCCAATCCTGT......................................      2
.....AGGGCCCCCCCCAATCCTG.......................................      1
.........CCCCCCCCAATCCTGT......................................      1
.....AGGGCCCCCCCCAATCCTGTT.....................................      1
.....AGGGCCCCCCCCAATCCTGTTGT...................................      1
.......................................AGGGTTGGGCGGAGGC........      1
......................................GAGGGTTGGGCGGAGGCTTTCC...      1
.............................CTTGGTTCAGAGGGTTGGGCGGAGGCTTTCCT..      1
...((((((((.(((((((((((.(((.((...)).))))))))))))).)).))))))))... (-33.40)

13/63487979-63488062(-)_novel_cow_8
TACTGGGTTGGCCAAAAAGTTCATTCGGGCTTTTCCATATCATCTTATAGAAAAACTCGAATGAACATTTTGGCCAACTCAATT
...............................................TAGAAAAACTCGAATGAACATT...............  6
```

```
................AAGTTCATTCGGGCTTTTCC................................................ 2
.................AGTTCATTCGGGCTTTTCCAT.............................................. 1
.................AGTTCATTCGGGCTTTTCCATA............................................. 1
................................................AGAAAAACTCGAATGAACATT............... 1
........................................................TCGAATGAACATTTTGGCCAAC...... 1
...((((((((((((((.(((((((((((.(((((.(((......))).))))).))))))))))).)))))))))))))).... (-44.00)

13/64900488-64900554(+)_mir_cow_40
GGCCGTTAAGACTTGCAGTGATGTTTAACTCCTCTCCACGTGAACATCACAGCAAGTCTGTGCTGCT
.....TTAAGACTTGCAGTGATGTTT......................................... 24882
.....TTAAGACTTGCAGTGATGTT.......................................... 6821
.....TTAAGACTTGCAGTGATGTTTAAC...................................... 6728
.....TTAAGACTTGCAGTGATGTTTA........................................ 6586
.....TTAAGACTTGCAGTGATGTTTAA....................................... 1021
.....TTAAGACTTGCAGTGATGTTTAACT..................................... 732
.....TTAAGACTTGCAGTGATG............................................ 323
.....TTAAGACTTGCAGTGATGT........................................... 295
.........................................GAACATCACAGCAAGTCTG....... 172
......TAAGACTTGCAGTGATGTTTAAC...................................... 170
..........................................AACATCACAGCAAGTCTGTGCT... 98
.........................................GAACATCACAGCAAGTCTGT...... 83
..........................................AACATCACAGCAAGTCTGT...... 69
..........................................AACATCACAGCAAGTCTG....... 56
.....TTAAGACTTGCAGTGATGTTTAACTCCT.................................. 43
.....TTAAGACTTGCAGTGATGTTTAACTCC................................... 38
.....TTAAGACTTGCAGTGATGTTTAACTC.................................... 24
.........................................GAACATCACAGCAAGTCTGTGC.... 22
......TAAGACTTGCAGTGATGTTTAACT..................................... 21
......TAAGACTTGCAGTGATGTTT......................................... 20
........................................TGAACATCACAGCAAGTCTGT...... 19
.........................................GAACATCACAGCAAGTCT........ 12
.....TTAAGACTTGCAGTGAT............................................. 11
.....TTAAGACTTGCAGTGA.............................................. 8
.........................................GAACATCACAGCAAGTCTGTGCT... 8
......TAAGACTTGCAGTGATGTTTA........................................ 7
.......AAGACTTGCAGTGATGTTTAACT..................................... 7
..........................................AACATCACAGCAAGTCTGTGCTG.. 7
......TAAGACTTGCAGTGATGTTTAA....................................... 6
```

```
......TAAGACTTGCAGTGATGTTTAACTCCT..................................  6
..........................................AACATCACAGCAAGTCTGTGC....  6
....GTTAAGACTTGCAGTGATGTT..........................................  4
......TAAGACTTGCAGTGATGTT..........................................  4
......TAAGACTTGCAGTGATGTTTAACTC....................................  4
........................................TGAACATCACAGCAAGTCTGTGC....  4
.......AAGACTTGCAGTGATGTTTAAC......................................  3
........AGACTTGCAGTGATGTTTAACT.....................................  2
......TAAGACTTGCAGTGATGTTTAACTCC...................................  2
.......AAGACTTGCAGTGATGTTTAACTCC...................................  2
.......AAGACTTGCAGTGATGTTTAACTCCT..................................  2
.....TTAAGACTTGCAGTGATGTTTAACTCCTCT................................  2
...........................................ACATCACAGCAAGTCTGTGCTG..  2
.......AAGACTTGCAGTGATGTTT.........................................  1
..........ACTTGCAGTGATGTTT.........................................  1
.......AAGACTTGCAGTGATGTTTAA.......................................  1
...........CTTGCAGTGATGTTTAAC......................................  1
.......AAGACTTGCAGTGATGTTTAACTC....................................  1
......TAAGACTTGCAGTGATGTTTAACTCCTCT................................  1
............TTGCAGTGATGTTTAACTCCTCT................................  1
.....TTAAGACTTGCAGTGATGTTTAACTCCTCTC...............................  1
.....TTAAGACTTGCAGTGATGTTTAACTCCTCTCC..............................  1
.....TTAAGACTTGCAGTGATGTTTAACTCCTCTCCA.............................  1
.....TTAAGACTTGCAGTGATGTTTAACTCCTCTCCACGT..........................  1
......TAAGACTTGCAGTGATGTTTAACTCCTCTCCACGTG.........................  1
...........................ACTCCTCTCCACGTGAACATCA..................  1
......................................CGTGAACATCACAGCAAGTCTGT......  1
...........................................ACATCACAGCAAGTCTGT......  1
.........................................GAACATCACAGCAAGTCTGTG.....  1
.............................TCCTCTCCACGTGAACATCACAGCAAGTCTGTGC....  1
...........................................ACATCACAGCAAGTCTGTGCT...  1
(((.((..((((((((.((((((((((.............)))))))))).))))))))..)).))) (-27.32)

1/3716987-3717060(+)_mir_cow_2
ATTGAGTTGGCCAAAAAGTTCGTTCAAGTTTTCCGTAAGATATGATGAAAAACCTGAATGAACTTTTTGGCCAA
................................................AAAACCTGAATGAACTTTTTGG....2418
.................................................AAACCTGAATGAACTTTTTGG....480
................................................AAAACCTGAATGAACTTTTTG.....124
```

```
.................................................AAACCTGAATGAACTTTTTGGC... 77
................................................AAAACCTGAATGAACTTTTTGGC... 38
...............................................AAAAACCTGAATGAACTTTTTG..... 22
................................................AAAACCTGAATGAACTTTTT...... 21
...............................................AAAAACCTGAATGAACTTTTTGG.... 21
............AAAAAGTTCGTTCAAGTTTTCC........................................ 16
................................................AAAACCTGAATGAACTTTT....... 7
...............................................AAAAACCTGAATGAACTTTTT...... 6
.................................................AAACCTGAATGAACTTTTTG..... 6
..................................................AACCTGAATGAACTTTTTGGCC.. 6
..................................................AACCTGAATGAACTTTTTGG.... 5
...................................................ACCTGAATGAACTTTTTGGCC.. 4
.................................................AAACCTGAATGAACTTTTT...... 3
....................................................CCTGAATGAACTTTTTGGCC.. 3
....................................................CCTGAATGAACTTTTTGGCCA. 3
............AAAAAGTTCGTTCAAGTTTT.......................................... 2
............AAAAAGTTCGTTCAAGTTTTC......................................... 2
..................................................AACCTGAATGAACTTTTTGGC... 2
.................................................AAACCTGAATGAACTTTTTGGCC.. 2
...................................................ACCTGAATGAACTTTTTGGCCA. 2
.............AAAAGTTCGTTCAAGTTTTCC........................................ 1
...............................................AAAAACCTGAATGAACT.......... 1
................................................AAAACCTGAATGAACTTT........ 1
...............................................AAAAACCTGAATGAACTTTT....... 1
.................................................AAACCTGAATGAACTTTT....... 1
......................................................TGAATGAACTTTTTGG.... 1
..................................................AACCTGAATGAACTTTTTGGCCA. 1
...................................................ACCTGAATGAACTTTTTGGCCAA 1
......((((((((((((((((((((.(((((.(((........))).))))).)))))))))))))))))))) (-34.70)

13/73541939-73542004(-)_mir_cow_41
GGCAGAAAAGTTTGTTTGGGTTTTTCTGTCAGATGTTATGGAAAAACCCAAATGAACTTTTTGGTC
.....AAAAGTTTGTTTGGGTTTTTCT.......................................   22
.....AAAAGTTTGTTTGGGTTTTT.........................................   21
.....AAAAGTTTGTTTGGGTTTTTC........................................   19
...AGAAAAGTTTGTTTGGGTTTTT.........................................   10
.....AAAAGTTTGTTTGGGTTT...........................................   1
.....AAAAGTTTGTTTGGGTTTT..........................................   1
```

....AAAAACCCAAATGAACTTTTTG... 1
(((.((((((((..(((((((((((((((........)))))))))))))))..)))))))).))) (-30.90)

14/11010800-11010876(+)_novel_cow_9
GGGTAGGCCAAAAAGTTAGTTCTGATTTTTCCATGAACTGAAAATATCAGAACGAACTTTTTGGCTAACCCAGTGTG
....TCAGAACGAACTTTTTGGCTAAC........ 7
....TCAGAACGAACTTTTTGGCT........... 6
....ATCAGAACGAACTTTTTGGCT........... 5
....TCAGAACGAACTTTTTGGCTAACC....... 3
....AGGCCAAAAAGTTAGTTCTGAT... 2
...TAGGCCAAAAAGTTAGTTCTGATT.. 1
....AGGCCAAAAAGTTAGTTCTGATT.. 1
....ATCAGAACGAACTTTTTGGCTA.......... 1
....CAGAACGAACTTTTTGGCTAACC....... 1
....AGAACGAACTTTTTGGCTAACC....... 1
....CGAACTTTTTGGCTAACCCAGT... 1
((((.((((((((((((.(((((((((((((........))))).)))))))).)))))))))))).)))))...... (-38.00)

14/1566928-1567000(+)_mir_cow_42
GTCCCCTCAGTCCTCTGCTAAAGCCCACCTGTGCTCCCGCCTGGAAAGTTGGGCTTGCAGCAGAGAGTAAGGA
....TTGGGCTTGCAGCAGAGAGT..... 26
....TTGGGCTTGCAGCAGAGAGTAA... 20
........AGTCCTCTGCTAAAGCCCACCT... 6
....TTGGGCTTGCAGCAGAGAGTA.... 6
....TTGGGCTTGCAGCAGAGAG...... 3
....TTGGGCTTGCAGCAGAGAGTAAG.. 2
....CCTCAGTCCTCTGCTAAAGCC.. 1
........AGTCCTCTGCTAAAGCCCACC.. 1
....(((.(.((.((((((.(((((((.((....(((.....))).)).)))))))..)))))))).).))). (-29.60)

14/30815430-30815497(+)_mir_cow_46
CTCCGTGTTCACAGCGGACCTTGATTTAATGTCATACAATTAAGGCACGCGGTGAATGCCAAGAGCGG
....TAAGGCACGCGGTGAATGCC........ 62579
....TAAGGCACGCGGTGAATGCCA....... 19991
....TTAAGGCACGCGGTGAATGCC........ 15984
....TTAAGGCACGCGGTGAATGCCA....... 5129
....TAAGGCACGCGGTGAATGC......... 5040
....TTAAGGCACGCGGTGAATGC......... 2796

....TAAGGCACGCGGTGAATGCCAA...... 2398

....TAAGGCACGCGGTGAATG.......... 649

....TTAAGGCACGCGGTGAATG.......... 534

....TTAAGGCACGCGGTGAATGCCAA...... 418

....TTAAGGCACGCGGTGAAT........... 294

...CGTGTTCACAGCGGACCTTGAT... 171

....TAAGGCACGCGGTGAATGCCAAG..... 155

....AAGGCACGCGGTGAATGCC........ 109

...CGTGTTCACAGCGGACCTTGA.. 95

....TAAGGCACGCGGTGAATGCCAAGA.... 83

....AAGGCACGCGGTGAATGCCA....... 76

...CGTGTTCACAGCGGACCTTGATT.. 44

....CACGCGGTGAATGCCA....... 42

....TAAGGCACGCGGTGAAT........... 41

....TAAGGCACGCGGTGAA............ 31

..CCGTGTTCACAGCGGACCTTG... 20

....TTAAGGCACGCGGTGAA............ 18

....AAGGCACGCGGTGAATGCCAA...... 17

....TTAAGGCACGCGGTGAATGCCAAGA.... 14

..CCGTGTTCACAGCGGACCTTGA.. 13

...CGTGTTCACAGCGGACCT... 10

....AGGCACGCGGTGAATGCC........ 9

....CACGCGGTGAATGCCAA...... 8

....TTAAGGCACGCGGTGAATGCCAAG..... 8

...CGTGTTCACAGCGGACC.. 7

...CGTGTTCACAGCGGACCTTG... 7

....GCACGCGGTGAATGCCA....... 7

....TTAAGGCACGCGGTGA............. 4

....GCACGCGGTGAATGCC........ 4

....AAGGCACGCGGTGAATGC......... 3

...CGTGTTCACAGCGGACCTT.. 2

.....TGTTCACAGCGGACCTTGATT.. 2

....ACGCGGTGAATGCCAA...... 2

....AAGGCACGCGGTGAATGCCAAG..... 2

..CCGTGTTCACAGCGGAC... 1

..CCGTGTTCACAGCGGACC.. 1

..CCGTGTTCACAGCGGACCT... 1

...CGTGTTCACAGCGGACCTTGATTTAATG...................................... 1

```
...CGTGTTCACAGCGGACCTTGATTTAATGTCA.................................. 1
........................TTTAATGTCATACAATTAAGGC...................... 1
.........................................AAGGCACGCGGTGAATG.......... 1
....................................CAATTAAGGCACGCGGTGAATGC......... 1
......................................ATTAAGGCACGCGGTGAATGCC........ 1
..........................................AGGCACGCGGTGAATGCCA....... 1
.........................................AAGGCACGCGGTGAATGCCAAGA.... 1
(((.((((((((.(((..((((((((...........))))))))..))).))))))))...)))... (-23.70)

14/4030321-4030388(+)_mir_cow_43
TGCCCTCGAGGAGCTCACAGTCTAGTACGTCTCATCCCCTACTAGACTGAAGCTCCTTGAGGACAGGG
.....TCGAGGAGCTCACAGTCTAGT.......................................... 111783
.........................................CTAGACTGAAGCTCCTTGAGGA..... 32026
.....TCGAGGAGCTCACAGTCTAGTA......................................... 29037
.........................................CTAGACTGAAGCTCCTTGAGG...... 23696
.....TCGAGGAGCTCACAGTCTAG........................................... 2544
.........................................CTAGACTGAAGCTCCTTGAG....... 2299
.....TCGAGGAGCTCACAGTCT............................................. 1147
........................................ACTAGACTGAAGCTCCTTGAGGA..... 513
........................................ACTAGACTGAAGCTCCTTGAGG...... 486
.........................................CTAGACTGAAGCTCCTTGA........ 437
....CTCGAGGAGCTCACAGTCTAGT.......................................... 286
.......................................TACTAGACTGAAGCTCCTTGAGG...... 190
........................................ACTAGACTGAAGCTCCTTGAG....... 135
.....TCGAGGAGCTCACAGTCTA............................................ 89
.......................................TACTAGACTGAAGCTCCTTGAG....... 89
....CTCGAGGAGCTCACAGTCTAGTA......................................... 84
.....TCGAGGAGCTCACAGTCTAGTAC........................................ 74
.......GAGGAGCTCACAGTCTAGT.......................................... 71
.......................................TACTAGACTGAAGCTCCTTGAGGA..... 65
..........................................TAGACTGAAGCTCCTTGAGGA..... 60
.........................................CTAGACTGAAGCTCCTTGAGGAC.... 45
..........................................TAGACTGAAGCTCCTTGAGG...... 40
.....TCGAGGAGCTCACAGTC.............................................. 38
......CGAGGAGCTCACAGTCTAGT.......................................... 37
....CTCGAGGAGCTCACAGTCT............................................. 28
.......GAGGAGCTCACAGTCTAGTA......................................... 27
.........................................CTAGACTGAAGCTCCTTG......... 21
```

....ACTAGACTGAAGCTCCTTGA........ 21
....TACTAGACTGAAGCTCCTTGA........ 16
....CTAGACTGAAGCTCCT........... 15
........AGGAGCTCACAGTCTAGT.. 13
.....TCGAGGAGCTCACAGT... 11
.....TCGAGGAGCTCACAGTCTAGTACG....................................... 11
..........GAGCTCACAGTCTAGT.. 10
....CTCGAGGAGCTCACAGTCTAG... 7
......CGAGGAGCTCACAGTCTAGTA... 7
....TAGACTGAAGCTCCTTGAG....... 7
....CTAGACTGAAGCTCCTTGAGGACA... 7
.........GGAGCTCACAGTCTAGT.. 3
....CTAGACTGAAGCTCCTT.......... 3
.......GAGGAGCTCACAGTCT... 2
........AGGAGCTCACAGTCTAG... 2
..........GAGCTCACAGTCTAGTA... 2
....ACTAGACTGAAGCTCCT........... 2
...........................CGTCTCATCCCCTACTAGACTGAAGCTCCTTGAGG...... 2
......CGAGGAGCTCACAGTCT... 1
....CTCGAGGAGCTCACAGTCTA.. 1
.........GGAGCTCACAGTCTAGTA... 1
....CTCGAGGAGCTCACAGTCTAGTAC.. 1
.......GAGGAGCTCACAGTCTAGTACG....................................... 1
.....TCGAGGAGCTCACAGTCTAGTACGT...................................... 1
.............................TCTCATCCCCTACTAGACTGAAGC............... 1
....ACTAGACTGAAGCTCCTTG......... 1
.....................................CCTACTAGACTGAAGCTCCTTGA........ 1
....TAGACTGAAGCTCCTTGA........ 1
......................................CTACTAGACTGAAGCTCCTTGAG....... 1
....AGACTGAAGCTCCTTGAG....... 1
..................................TCCCCTACTAGACTGAAGCTCCTTGAGG...... 1
...................................CCCCTACTAGACTGAAGCTCCTTGAGG...... 1
....AGACTGAAGCTCCTTGAGG...... 1
....AGACTGAAGCTCCTTGAGGA..... 1
((.((((((((((((..((((((((((.(.......).)))))))))).)))))))))))).))... (-37.10)

14/8080291-8080362(+)_mir_cow_44
TGTTGTTGTAAACATCCCCGACTGGAAGCTGTACCACACAGCTAAGCTTTCAGTCAGATGTTTGCTGCTACC

......TGTAAACATCCCCGACTGGAAGCT..	425292
......TGTAAACATCCCCGACTGGAAGC...	111546
......TGTAAACATCCCCGACTGGA..	101883
......TGTAAACATCCCCGACTGGAA...	45401
......TGTAAACATCCCCGACTGGAAG..	31294
......TGTAAACATCCCCGAC..	2626
....CTTTCAGTCAGATGTTTGCTGC....	2100
......TGTAAACATCCCCGACTGG...	1805
....TTTCAGTCAGATGTTTGCTGC....	469
....CTTTCAGTCAGATGTTTGCTGCT...	370
....TTTCAGTCAGATGTTTGCTGCT...	302
.......GTAAACATCCCCGACTGGAAGCT..	211
......TGTAAACATCCCCGACTG..	164
....CTTTCAGTCAGATGTTTGCT......	98
....CTTTCAGTCAGATGTTTGCTG.....	77
........TAAACATCCCCGACTGGAAGCT..	45
.......GTAAACATCCCCGACTGGAAGC...	44
......TGTAAACATCCCCGACT...	28
.......GTAAACATCCCCGACTGGA..	27
.......GTAAACATCCCCGACTGGAA...	26
.............ATCCCCGACTGGAAGCT..	20
..............TCCCCGACTGGAAGCT..	20
......TGTAAACATCCCCGACTGGAAGCTGT..	20
............CATCCCCGACTGGAAGCT..	13
...........ACATCCCCGACTGGAAGCT..	12
......TGTAAACATCCCCGACTGGAAGCTG...	12
.......GTAAACATCCCCGACTGGAAG..	11
..........AACATCCCCGACTGGAAGCT..	10
.........AAACATCCCCGACTGGAAGCT..	9
....TTTCAGTCAGATGTTTGCTG.....	9
....CTTTCAGTCAGATGTTTGC.......	8
........TAAACATCCCCGACTGGA..	7
......TGTAAACATCCCCGACTGGAAGCTGTACC.....................................	7
........TAAACATCCCCGACTGGAAGC...	6
....TTTCAGTCAGATGTTTGCT......	6
.........AAACATCCCCGACTGGAAGC...	4
..............TCCCCGACTGGAAGCTGTACC.....................................	4
....TTTCAGTCAGATGTTTGC.......	4

```
................................................TTCAGTCAGATGTTTGCTGC....   4
...............................................TTTCAGTCAGATGTTTGCTGCTA..   4
..............TCCCCGACTGGAAGCTGTACCA....................................   3
..........AACATCCCCGACTGGAAG............................................   2
...........ACATCCCCGACTGGAAGC...........................................   2
............CATCCCCGACTGGAAGC...........................................   2
................CCCGACTGGAAGCTGTACC.....................................   2
......TGTAAACATCCCCGACTGGAAGCTGTACCA....................................   2
.............ATCCCCGACTGGAAGCTGTACCA....................................   2
......TGTAAACATCCCCGACTGGAAGCTGTACCACA..................................   2
....GTTGTAAACATCCCCGACTGGA..............................................   1
.........AAACATCCCCGACTGGA..............................................   1
........TAAACATCCCCGACTGGAA.............................................   1
.....TTGTAAACATCCCCGACTGGAAG............................................   1
............CATCCCCGACTGGAAG............................................   1
..........AACATCCCCGACTGGAAGC...........................................   1
.............ATCCCCGACTGGAAGC...........................................   1
.....TTGTAAACATCCCCGACTGGAAGCT..........................................   1
......TGTAAACATCCCCGACTGGAAGCTGTA.......................................   1
......TGTAAACATCCCCGACTGGAAGCTGTAC......................................   1
.............ATCCCCGACTGGAAGCTGTACC.....................................   1
.................CCGACTGGAAGCTGTACC.....................................   1
......TGTAAACATCCCCGACTGGAAGCTGTACCACACA................................   1
......TGTAAACATCCCCGACTGGAAGCTGTACCACACAGCTAAG..........................   1
......................................CAGCTAAGCTTTCAGTCAGATGTTTGC.......   1
.......................................AGCTAAGCTTTCAGTCAGATGTTTGCTGC....   1
.((.((.(((((((((...(((((((((((..............))))))))))).))))))))).)).)). (-28.34)

14/8084732-8084799(+)_mir_cow_45
GTTCATGTAAACATCCTACACTCAGCTGTAACACACGAGTCGGCTGGGAGGTGGATGTTTACTTCAGC
.....TGTAAACATCCTACACTCAGCT......................................... 80145
.....TGTAAACATCCTACACTCAGC.......................................... 2523
.....TGTAAACATCCTACACTCAGCTG........................................ 407
.....TGTAAACATCCTACACTCAGCTGT....................................... 321
...........................................CTGGGAGGTGGATGTTTACTTC... 84
.....TGTAAACATCCTACACT.............................................. 65
.....TGTAAACATCCTACACTCAG........................................... 46
.....TGTAAACATCCTACACTCA............................................ 43
```

.....TGTAAACATCCTACACTC... 35
....CTGGGAGGTGGATGTTTACTT.... 15
......GTAAACATCCTACACTCAGCT... 13
....CTGGGAGGTGGATGTTTACT..... 12
....ATGTAAACATCCTACACTCAGCT... 5
.......TAAACATCCTACACTCAGCTGT....................................... 3
.....TGTAAACATCCTACACTCAGCTGTA...................................... 3
....CTGGGAGGTGGATGTTTACTTCA.. 3
.....TGTAAACATCCTACAC... 2
.......TAAACATCCTACACTCAGCT... 2
.........AACATCCTACACTCAGCT... 2
...........CATCCTACACTCAGCT... 2
..................................ACGAGTCGGCTGGGAGG................. 2
...........................GTAACACACGAGTCGGCTGGGAGGTGGATGTTTACTTC... 2
........AAACATCCTACACTCAGCT... 1
..........ACATCCTACACTCAGCT... 1
.......TAAACATCCTACACTCAGCTG.. 1
.....TGTAAACATCCTACACTCAGCTGTAAC.................................... 1
...........................GTAACACACGAGTCGG......................... 1
....CTGGGAGGTGGATGTTTA....... 1
....TGGGAGGTGGATGTTTACT..... 1
....TGGGAGGTGGATGTTTACTTC... 1
....GGTGGATGTTTACTTC... 1
......((((((((((.((.((((((((..((......))))))))))..))))))))))))...... (-26.70)

15/22134733-22134800(+)_mir_cow_47
TTTGTAGGCAGTGTAATTAGCTGATTGTACTCTCATGCTTACAATCACTAGTTCCACTGCCATCAAAA
.....AGGCAGTGTAATTAGCTGATTGT.. 267
....AATCACTAGTTCCACTGCCATC.... 98
....CAATCACTAGTTCCACTGCCATC.... 71
....ATCACTAGTTCCACTGCCATCA... 29
....AATCACTAGTTCCACTGCCATCA... 18
....ATCACTAGTTCCACTGCCATC.... 16
....TAGGCAGTGTAATTAGCTGATT.. 12
.....AGGCAGTGTAATTAGCTGATTGTA....................................... 12
....CAATCACTAGTTCCACTGCCAT..... 10
....CAATCACTAGTTCCACTGCCATCA... 10
....CAATCACTAGTTCCACTGCCA...... 7

```
....TAGGCAGTGTAATTAGCTGATTGT........................................ 6
....TAGGCAGTGTAATTAGCTGATTG......................................... 5
.....AGGCAGTGTAATTAGCTGATTG......................................... 5
...........................................ATCACTAGTTCCACTGCCATCAA.. 5
....TAGGCAGTGTAATTAGCTG............................................. 3
..........................................AATCACTAGTTCCACTGCCA...... 3
..........................................AATCACTAGTTCCACTGCCAT..... 2
......GGCAGTGTAATTAGCTGATTGTA....................................... 1
.....AGGCAGTGTAATTAGCTGATTGTAC...................................... 1
.........................................CAATCACTAGTTCCACTGCC....... 1
.........................................CAATCACTAGTTCCACTGCCATCAA.. 1
((((..(((((((.((((((.((((((((..(....)..))))))))))))))).)))))))..)))). (-28.40)

15/22135432-22135496(+)_mir_cow_48
GTTACTAGGCAGTGTAGTTAGCTGATTGCTAATAATACCAATCACTAACCACACGGCCAGGTAAA
......AGGCAGTGTAGTTAGCTGATTGC....................................    5580
......AGGCAGTGTAGTTAGCTGATTGCT...................................    241
......AGGCAGTGTAGTTAGCTGATT......................................    141
.......................................AATCACTAACCACACGGCCAGG....    86
......AGGCAGTGTAGTTAGCTGATTG.....................................    54
.....TAGGCAGTGTAGTTAGCTGATT......................................    22
......AGGCAGTGTAGTTAGCTGAT.......................................    17
........................................ATCACTAACCACACGGCCAGG....    12
......AGGCAGTGTAGTTAGCTGA........................................    6
.......................................AATCACTAACCACACGGCCAG.....    6
.....TAGGCAGTGTAGTTAGCTGATTG.....................................    5
.......GGCAGTGTAGTTAGCTGATTGC....................................    4
......AGGCAGTGTAGTTAGC...........................................    2
.....TAGGCAGTGTAGTTAGCTGA........................................    2
........................................ATCACTAACCACACGGCCAGGT...    2
......AGGCAGTGTAGTTAGCTG.........................................    1
....CTAGGCAGTGTAGTTAGCTGAT.......................................    1
.....TAGGCAGTGTAGTTAGCTGAT.......................................    1
....CTAGGCAGTGTAGTTAGCTGATT......................................    1
.....TAGGCAGTGTAGTTAGCTGATTGC....................................    1
.........CAGTGTAGTTAGCTGATTGC....................................    1
......AGGCAGTGTAGTTAGCTGATTGCTA..................................    1
.......................................AATCACTAACCACACGGCC.......    1
```

```
.......................................AATCACTAACCACACGGCCAGGT...    1
.......................................AATCACTAACCACACGGCCAGGTA..    1
.(((((.(((.((((.(((((.((((((.((....)).))))))))))).)))).))).))))). (-26.00)

15/33298822-33298895(-)_mir_cow_49
CTCTCAATCCCTGAGACCCTAACTTGTGATGTTTACCGTTTAAATCCACGGGTTAGGCTCTTGGGAGCTGCGAG
.......TCCCTGAGACCCTAACTTGTGA.............................................131934
.......TCCCTGAGACCCTAACTTGTG..............................................21541
.......TCCCTGAGACCCTAACTTGT...............................................12124
...............................................ACGGGTTAGGCTCTTGGGAGCT.....2954
.......TCCCTGAGACCCTAACTTG................................................1629
.......TCCCTGAGACCCTAACTTGTGAT............................................507
...............................................ACGGGTTAGGCTCTTGGGAGC......332
.......TCCCTGAGACCCTAACT..................................................275
.......TCCCTGAGACCCTAACTT.................................................268
...........TGAGACCCTAACTTGTGA.............................................206
...............................................ACGGGTTAGGCTCTTGGGAG.......204
.........CCTGAGACCCTAACTTGTGA.............................................141
........CCCTGAGACCCTAACTTGTGA.............................................116
..........CTGAGACCCTAACTTGTGA.............................................57
...............................................ACGGGTTAGGCTCTTGGGA........45
.......TCCCTGAGACCCTAAC...................................................36
.........CCTGAGACCCTAACTTGTG..............................................25
...........TGAGACCCTAACTTGTG..............................................14
...........TGAGACCCTAACTTGT...............................................13
.........CCTGAGACCCTAACTTGT...............................................11
..........CTGAGACCCTAACTTGTG..............................................8
..........CTGAGACCCTAACTTGT...............................................6
........CCCTGAGACCCTAACTTGTG..............................................6
......ATCCCTGAGACCCTAACTTGTGA.............................................4
............GAGACCCTAACTTGTGA.............................................4
...........TGAGACCCTAACTTGTGAT............................................4
.......TCCCTGAGACCCTAACTTGTGATG...........................................4
..............................................CACGGGTTAGGCTCTTGGGA........4
..............................................CACGGGTTAGGCTCTTGGGAGCT.....4
........CCCTGAGACCCTAACTTGT...............................................3
................................................CGGGTTAGGCTCTTGGGAGCT.....3
..........CTGAGACCCTAACTTG................................................2
```

```
......ATCCCTGAGACCCTAACTTGTG..............................................2
.............AGACCCTAACTTGTGA.............................................2
.........CCTGAGACCCTAACTTGTGAT............................................2
..............................................CACGGGTTAGGCTCTTGGGAG.......2
..............................................CACGGGTTAGGCTCTTGGGAGC......2
...............................................ACGGGTTAGGCTCTTGGGAGCTG....2
........CCCTGAGACCCTAACTT.................................................1
......ATCCCTGAGACCCTAACTTGT...............................................1
........CCCTGAGACCCTAACTTGTGAT............................................1
.......TCCCTGAGACCCTAACTTGTGATGTTT........................................1
.......TCCCTGAGACCCTAACTTGTGATGTTTACC.....................................1
.............................TGTTTACCGTTTAAATCC...........................1
...............................................ACGGGTTAGGCTCTTGGG.........1
................................................CGGGTTAGGCTCTTGGGAG.......1
(((.((.((((.((((.(((((((((((..(((((.....))))).))))))))))).))))))))..)).))) (-30.00)

15/33347565-33347645(-)_mir_cow_50
CTCCCAGGTTGAGGTAGTAGGTTGTATAGTTTAGAATTACATCAAGGGAGATAACTGTACAGCCTCCTAGCTTTCCTTGGG
.........TGAGGTAGTAGGTTGTATAGTT..................................................    600031
.........TGAGGTAGTAGGTTGTATAGT...................................................    269183
.........TGAGGTAGTAGGTTGTATAGTTT.................................................    31072
.........TGAGGTAGTAGGTTGTATAG....................................................    29911
.........TGAGGTAGTAGGTTGTAT......................................................    7314
.........TGAGGTAGTAGGTTGTATA.....................................................    4091
.........TGAGGTAGTAGGTTGTA.......................................................    1031
.........TGAGGTAGTAGGTTGT........................................................    343
..............TAGTAGGTTGTATAGTT..................................................    174
..........GAGGTAGTAGGTTGTATAGTT..................................................    169
............GGTAGTAGGTTGTATAGTT..................................................    155
.........TGAGGTAGTAGGTTGTATAGTTTA................................................    137
...............AGTAGGTTGTATAGTT..................................................    114
............GGTAGTAGGTTGTATAGT...................................................    76
..............TAGTAGGTTGTATAGT...................................................    76
..........GAGGTAGTAGGTTGTATAGT...................................................    53
.............GTAGTAGGTTGTATAGTT..................................................    53
........TTGAGGTAGTAGGTTGTATAGTT..................................................    51
........TTGAGGTAGTAGGTTGTATAGT...................................................    49
...........AGGTAGTAGGTTGTATAGTT..................................................    37
```

```
...........AGGTAGTAGGTTGTATAGT...................................................    23
......................................................CTGTACAGCCTCCTAGCTTTCC.....    21
.............GTAGTAGGTTGTATAGT...................................................    17
............GGTAGTAGGTTGTATAG....................................................    9
................GTAGGTTGTATAGTTT.................................................    9
..........GAGGTAGTAGGTTGTATAGTTT.................................................    8
..............TAGTAGGTTGTATAGTTT.................................................    8
........TTGAGGTAGTAGGTTGTAT......................................................    7
...............................TAGAATTACATCAAGGGAGAT.............................    6
......................................................CTGTACAGCCTCCTAGCTTTCCT....    5
........TTGAGGTAGTAGGTTGTATAG....................................................    4
.............GTAGTAGGTTGTATAG....................................................    4
............GGTAGTAGGTTGTATAGTTT.................................................    4
.............GTAGTAGGTTGTATAGTTT.................................................    4
......................................................CTGTACAGCCTCCTAGCTTTC......    4
........TTGAGGTAGTAGGTTGTATA.....................................................    3
..........GAGGTAGTAGGTTGTATAG....................................................    3
...........AGGTAGTAGGTTGTATAGTTT.................................................    3
...............AGTAGGTTGTATAGTTT.................................................    3
...............................TAGAATTACATCAAGGGAGATA............................    2
........TTGAGGTAGTAGGTTGT........................................................    1
........TTGAGGTAGTAGGTTGTA.......................................................    1
..........GAGGTAGTAGGTTGTA.......................................................    1
..........GAGGTAGTAGGTTGTAT......................................................    1
...........AGGTAGTAGGTTGTAT......................................................    1
..........GAGGTAGTAGGTTGTATA.....................................................    1
...........AGGTAGTAGGTTGTATAG....................................................    1
......GGTTGAGGTAGTAGGTTGTATAGTT..................................................    1
........TTGAGGTAGTAGGTTGTATAGTTT.................................................    1
.........TGAGGTAGTAGGTTGTATAGTTTAG...............................................    1
.........TGAGGTAGTAGGTTGTATAGTTTAGAA.............................................    1
.....................................................ACTGTACAGCCTCCTAGCTT........    1
...................................................TAACTGTACAGCCTCCTAGCTTT.......    1
.....................................................ACTGTACAGCCTCCTAGCTTTC......    1
.......................................................TGTACAGCCTCCTAGCTTTCC.....    1
..((((((..(((.(((.(((((((((((((.....(..(.....)..)...))))))))))))).))).)))..))))))  (-33.80)
```

15/33353397-33353467(-)_mir_cow_51

```
GTTGCCACAAACCCGTAGATCCGAACTTGTGGTGGTAGTGCACACAAGCTTGTGTCTATAGGTATGTGTCT
.........AACCCGTAGATCCGAACTTGTG........................................   61412
.........AACCCGTAGATCCGAACTTGT.........................................   22899
.........AACCCGTAGATCCGAACTTG..........................................   11418
..........ACCCGTAGATCCGAACTTGTGG.......................................   4863
.........AACCCGTAGATCCGAACTTGTGG.......................................   3474
..........ACCCGTAGATCCGAACTTGTG........................................   1379
.........AACCCGTAGATCCGAACTT...........................................   436
.............CGTAGATCCGAACTTGT.........................................   345
..........ACCCGTAGATCCGAACTTGT.........................................   335
............CCGTAGATCCGAACTTGT.........................................   276
..........ACCCGTAGATCCGAACTTG..........................................   248
............CCGTAGATCCGAACTTGTG........................................   209
.............CGTAGATCCGAACTTGTG........................................   159
.........AACCCGTAGATCCGAACT............................................   148
............................................CAAGCTTGTGTCTATAGGTATG.....   110
..............GTAGATCCGAACTTGT.........................................   59
............................................CAAGCTTGTGTCTATAGGTAT......   55
..............GTAGATCCGAACTTGTG........................................   33
............CCGTAGATCCGAACTTGTGG.......................................   17
.............CGTAGATCCGAACTTG..........................................   16
.............CGTAGATCCGAACTTGTGG.......................................   16
...........CCCGTAGATCCGAACTTGTG........................................   15
............CCGTAGATCCGAACTTG..........................................   13
............................................CAAGCTTGTGTCTATAGGTATGT....   11
.........AACCCGTAGATCCGAACTTGTGGT......................................   9
...........................................ACAAGCTTGTGTCTATAGGTAT......   6
.........AACCCGTAGATCCGAAC.............................................   5
...........CCCGTAGATCCGAACTTGTGG.......................................   5
............CCGTAGATCCGAACTT...........................................   4
...........CCCGTAGATCCGAACTTGT.........................................   4
............................................CAAGCTTGTGTCTATAGGTA.......   4
..............GTAGATCCGAACTTGTGG.......................................   3
............................................CAAGCTTGTGTCTATAGGT........   3
..........ACCCGTAGATCCGAACTTGTGGT......................................   2
.............................................AAGCTTGTGTCTATAGGTATGT....   2
..........ACCCGTAGATCCGAAC.............................................   1
..........ACCCGTAGATCCGAACT............................................   1
```

```
......ACAAACCCGTAGATCCGAACTTG..........................................   1
...........CCCGTAGATCCGAACTTG..........................................   1
........AAACCCGTAGATCCGAACTTGT.........................................   1
...............TAGATCCGAACTTGTG........................................   1
.........AACCCGTAGATCCGAACTTGTGGTGGTAGTGCAC............................   1
...........................................ACAAGCTTGTGTCTATAGGT........   1
...........................................ACAAGCTTGTGTCTATAGGTA.......   1
...........................................ACAAGCTTGTGTCTATAGGTATG.....   1
.....((((.(((..((((..(((.((((((((......)).)))))).)))..))))..))).))))... (-26.20)

15/39162245-39162317(-)_mir_cow_52
GTTGGCCGAAACATTCACTCGGGTTTTTCCATAGCATCTTAACAGAAAAACTCGAATGAATGTTTTGGCCAAC
...............................................AAACTCGAATGAATGTTTTGGC.... 84
...............................................AAACTCGAATGAATGTTTTGG..... 17
......CGAAACATTCACTCGGGTTTTT............................................. 16
.......GAAACATTCACTCGGGTTTTT............................................. 3
......CGAAACATTCACTCGGGTTT............................................... 2
......CGAAACATTCACTCGGGTTTT.............................................. 2
.......GAAACATTCACTCGGGTTTTTC............................................ 2
........AAACATTCACTCGGGTTTTTC............................................ 1
...........................................AGAAAAACTCGAATGAATGTT......... 1
.............................................AAAAACTCGAATGAATGTTTTG...... 1
................................................AACTCGAATGAATGTTTTGGC.... 1
(((((((((((((((((.(((((((((((...............))))))))))).))))))))))))))))) (-39.46)

15/52956179-52956250(-)_mir_cow_53
GGTGTACTCTACAGTGCACGTGTCTCCAGTGTGGCTCGGAGGCTGGAGACGCGGCCCTGTTGGAGTAACAAC
.......TCTACAGTGCACGTGTCTCCAGT..........................................  5930
.......TCTACAGTGCACGTGTCTCCA............................................  1467
.......TCTACAGTGCACGTGTCTCCAG...........................................  1344
.......TCTACAGTGCACGTGTCTCC.............................................  812
.......TCTACAGTGCACGTGTCTC..............................................  614
.......TCTACAGTGCACGTGTCT...............................................  155
...........................................TGGAGACGCGGCCCTGTTGGAGT......  142
...........................................TGGAGACGCGGCCCTGTTGGAG.......  21
...........................................TGGAGACGCGGCCCTGTTGGA........  14
............................................GGAGACGCGGCCCTGTTGGAGT......  14
.......TCTACAGTGCACGTGTC................................................  2
```

```
......CTCTACAGTGCACGTGTCTC..............................................  2
......CTCTACAGTGCACGTGTCTCC.............................................  2
.........TACAGTGCACGTGTCTCC.............................................  2
......CTCTACAGTGCACGTGTCTCCAGT..........................................  2
........CTACAGTGCACGTGTCTCCAGT..........................................  2
.......TCTACAGTGCACGTGTCTCCAGTG.........................................  2
...........................................TGGAGACGCGGCCCTGTTGG.........  2
......................................GAGGCTGGAGACGCGGCCCTGTTGGAGT......  2
..............................................AGACGCGGCCCTGTTGGAGT......  2
.........TACAGTGCACGTGTCT...............................................  1
.........TACAGTGCACGTGTCTCCA............................................  1
..........ACAGTGCACGTGTCTCCAG...........................................  1
.........TACAGTGCACGTGTCTCCAGT..........................................  1
..........ACAGTGCACGTGTCTCCAGT..........................................  1
...........CAGTGCACGTGTCTCCAGT..........................................  1
.......TCTACAGTGCACGTGTCTCCAGTGTGGCTC...................................  1
.......TCTACAGTGCACGTGTCTCCAGTGTGGCTCG..................................  1
..........................CAGTGTGGCTCGGAGGCTG...........................  1
...........................................TGGAGACGCGGCCCTGT............  1
.............................................GAGACGCGGCCCTGTTGGAG.......  1
..............................................AGACGCGGCCCTGTTGGAG.......  1
.......................................AGGCTGGAGACGCGGCCCTGTTGGAGT......  1
.............................................GAGACGCGGCCCTGTTGGAGT......  1
...............................................GACGCGGCCCTGTTGGAGT......  1
.................................................CGCGGCCCTGTTGGAGT......  1
..................................................GCGGCCCTGTTGGAGT......  1
...........................................TGGAGACGCGGCCCTGTTGGAGTA.....  1
..............................................AGACGCGGCCCTGTTGGAGTAA....  1
.((.((((((((((.((.((((((((((((.(.......).)))))))))))))).))).)))))))))... (-37.70)

15/66264855-66264920(+)_mir_Cow_Blast_27
GTGCTGGGGAGCGGCCCCCGGGCGGGCCTCTGCTCTGGCCCCTCCTGGGGCCCGCACTCTCGCTCC
.........................................CTCCTGGGGCCCGCACTCTCGC...   2758
.........................................CTCCTGGGGCCCGCACTCTCG....   898
.........................................CTCCTGGGGCCCGCACTCTCGCT..   774
.........................................CTCCTGGGGCCCGCACTCT......   82
.........................................CTCCTGGGGCCCGCACTCTC.....   78
........................................CCTCCTGGGGCCCGCACTCTCGC...   64
```

```
........................................CCTCCTGGGGCCCGCACTCTCG....   28
........................................CCTCCTGGGGCCCGCACTCTCGCT..   18
.........................................CTCCTGGGGCCCGCACTC.......   10
....TGGGGAGCGGCCCCCGGGCGGG........................................   9
.........................................CTCCTGGGGCCCGCACT........   7
........................................CCTCCTGGGGCCCGCACTCTC.....   6
........................................CCTCCTGGGGCCCGCACTCT......   5
....TGGGGAGCGGCCCCCGGGCGG.........................................   3
....TGGGGAGCGGCCCCCGGGC...........................................   2
....TGGGGAGCGGCCCCCGGGCG..........................................   2
...........................................CCTGGGGCCCGCACTCTCG....   2
..........................................TCCTGGGGCCCGCACTCTCGC...   2
....TGGGGAGCGGCCCCCGGGCGGGCCTCTGCTCTGGCCC.........................   1
.........................................CTCCTGGGGCCCGCAC.........   1
........................................CCTCCTGGGGCCCGCACTC.......   1
..........................................TCCTGGGGCCCGCACTCT......   1
.............................................TGGGGCCCGCACTCTCGC...   1
............................................CTGGGGCCCGCACTCTCGCT..   1
.............................................TGGGGCCCGCACTCTCGCT..   1
..((.((((.((((.(((((((.(((((........)))))..))))))).)))).)))).))... (-41.70)
```

```
15/74519298-74519367(+)_mir_cow_54
TGACGTCCCTGAGTATATGTGGTGAACCGGAATTTGCCTGGGGTTTCCTCATATTCATTCAGGAGTGTCA
...........................................TTTCCTCATATTCATTCAGGAGTG...     598
...........................................TTTCCTCATATTCATTCAGGAGTGT..     104
...........................................TTTCCTCATATTCATTCAGGAGT....     47
............................................TTCCTCATATTCATTCAGGAGTG...     33
............................................TTCCTCATATTCATTCAGGAGTGT..     12
...........................................TTTCCTCATATTCATTCAGGAG.....     6
...........................................TTTCCTCATATTCATTCAGGA......     3
...........................................TTTCCTCATATTCATTCAGG.......     2
....GTCCCTGAGTATATGTGGTGA.............................................     1
............................................TTCCTCATATTCATTCAGGAGT....     1
.((((..((((((((((((.((.(..((((.......))))..)..)).)))))..)))))))..)))). (-27.10)
```

```
15/74543911-74543981(+)_mir_Cow_Blast_30
GGGAGAGAGGGAAGAGGGAAGGAGGGATAACGTAGGAAAGAGATCTCCCTCCTCCTTCCACCCTGGTCTCC
...............................................CCTCCTCCTTCCACCCTGGTCT..    10
```

..........................ATAACGTAGGAAAGAGATCTCC....................... 3
....GAGAGGGAAGAGGGAAGGAGGGAT... 1
...........................TAACGTAGGAAAGAGATCT......................... 1
....CTCCTCCTTCCACCCTGGTCT.. 1
.(((((.((((..((((((.(((((((..................)))))))))))))..))))..))))) (-32.97)

15/74545195-74545267(+)_mir_cow_55
CGAATCTTTTTGCGGTCTGGGCTTGCTGTACATAACTCAATAGCCGGAAGCCCTTACCCCAAAAAGCATTCGC
.....CTTTTTGCGGTCTGGGCTTGC... 8553
....AAGCCCTTACCCCAAAAAGCAT.... 3669
.....CTTTTTGCGGTCTGGGCTTGCT.. 1052
....AAGCCCTTACCCCAAAAAGC...... 738
.....CTTTTTGCGGTCTGGGCTTG.. 416
....AAGCCCTTACCCCAAAAAGCA..... 411
....AAGCCCTTACCCCAAAAAG....... 94
.....CTTTTTGCGGTCTGGGCTTGCTG... 79
.....CTTTTTGCGGTCTGGGCTTGCTGT.. 73
....AAGCCCTTACCCCAAAAAGCATT... 68
.....CTTTTTGCGGTCTGGGCTT... 52
.....CTTTTTGCGGTCTGGGCT.. 26
......TTTTTGCGGTCTGGGCTTGCT.. 20
......TTTTTGCGGTCTGGGCTTGC... 12
......TTTTTGCGGTCTGGGCTTGCTG... 7
......TTTTTGCGGTCTGGGCTTGCTGT.. 7
....AGCCCTTACCCCAAAAAGCAT.... 7
....AGCCCTTACCCCAAAAAGCATT... 6
.....CTTTTTGCGGTCTGGGC... 4
..........................TGTACATAACTCAATAGCCGGA......................... 4
....GGAAGCCCTTACCCCAAAAAG....... 4
....TCTTTTTGCGGTCTGGGCTTGC... 3
....AAGCCCTTACCCCAAAAA........ 3
....GAAGCCCTTACCCCAAAAAG....... 3
....GGAAGCCCTTACCCCAAAAAGC...... 3
.......TTTTGCGGTCTGGGCTTGC... 2
.......TTTTGCGGTCTGGGCTTGCTG... 2
..........................TGTACATAACTCAATAGCCGG.......................... 2
....AAGCCCTTACCCCAAA.......... 2
......TTTTTGCGGTCTGGGCT.. 1

```
......TTTTTGCGGTCTGGGCTTG................................................ 1
.........TTGCGGTCTGGGCTTG................................................ 1
......TTTTTGCGGTCTGGGCTTGCTGTA........................................... 1
..........................TGTACATAACTCAATAGCCGGAAGCCC.................... 1
...................................................CCTTACCCCAAAAAGC...... 1
..............................................GAAGCCCTTACCCCAAAAAGCA..... 1
((((((((((((.(((..(((((((((((..........)))))...))))))..))).))))))).))))). (-28.90)

15/82197721-82197790(+)_mir_Cow_Blast_28
CGGAGCTCTTTTCACATTGTGCTACTGTCTGCGCCTGTCACTAGCAGTGCAATGTTAAAAGGGCATTGGC
............................................CAGTGCAATGTTAAAAGGGCA.....    6945
............................................CAGTGCAATGTTAAAAGGGC......    6943
............................................CAGTGCAATGTTAAAAGGGCATT...    2525
............................................CAGTGCAATGTTAAAAGGGCAT....    1316
............................................CAGTGCAATGTTAAAAGGG.......    56
....GCTCTTTTCACATTGTGCTACT............................................    18
.............................................AGTGCAATGTTAAAAGGGCATT...    12
.............................................AGTGCAATGTTAAAAGGGCA.....    11
............................................CAGTGCAATGTTAAAAGGGCATTG..    11
............................................CAGTGCAATGTTAAAAGG........    10
...........................................GCAGTGCAATGTTAAAAGGGC......    8
.............................................AGTGCAATGTTAAAAGGGC......    8
................................................GCAATGTTAAAAGGGCATT...    4
...........................................GCAGTGCAATGTTAAAAGGGCA.....    3
.................................................CAATGTTAAAAGGGCAT....    3
............................................CAGTGCAATGTTAAAAG.........    1
..............................................GTGCAATGTTAAAAGGGC......    1
.............................................AGTGCAATGTTAAAAGGGCAT....    1
..............................................GTGCAATGTTAAAAGGGCAT....    1
(((.((((((((.((((((..((.(((...((....))...))).))..)))))).)))))))).))).. (-24.20)

16/19155765-19155835(-)_novel_cow_12
GTTGGCCAGAAAGTTCATTTGGGTTTCTCCGTGCAATCTTGTGAAAAACTGAAATGAACTTTTCGGCTCAT
............................................AAAACTGAAATGAACTTTTCG......   396
............................................AAAACTGAAATGAACTTTTC.......   143
...........................................AAAAACTGAAATGAACTTTTCG......   32
............................................AAAACTGAAATGAACTTTT........   15
...........................................AAAAACTGAAATGAACTTTTC.......   13
```

```
........GAAAGTTCATTTGGGTTTCTC..........................................   9
............................................AAAACTGAAATGAACTTTTCGG.....   8
............................................AAAACTGAAATGAACTTT.........   3
........GAAAGTTCATTTGGGTTTCT...........................................   2
........GAAAGTTCATTTGGGTTT.............................................   1
............................................AAAACTGAAATGAACT...........   1
...........................................AAAAACTGAAATGAACTTTT........   1
.............................................AAACTGAAATGAACTTTTC.......   1
.............................................AAACTGAAATGAACTTTTCG......   1
...((((..(((((((((((.(((((.((((........)).)).))))).)))))))))))..))))... (-27.00)

1/61960482-61960547(-)_novel_cow_2
CCTCTGGCTCAACCATTTGTTCTCACTTGAATCAACAAAGTGGGTGGCAAATGGTGGGTTTGATGA
....TGGCTCAACCATTTGTTCTCACT.......................................  151
....TGGCTCAACCATTTGTTCTCACTT......................................  18
....TGGCTCAACCATTTGTTCTC..........................................  12
....TGGCTCAACCATTTGTTCTCA.........................................  8
....TGGCTCAACCATTTGTTCT...........................................  3
.....GGCTCAACCATTTGTTCTC..........................................  1
....TGGCTCAACCATTTGTTCTCAC........................................  1
...CTGGCTCAACCATTTGTTCTCACT.......................................  1
........................................TGGGTGGCAAATGGTGGGTTT.....  1
........................................TGGGTGGCAAATGGTGGGTTTGA...  1
..((..(((((.((((((((((((((((.........))))))).))))))))))))))..))... (-26.00)

16/20617284-20617347(-)_mir_cow_56
CCAAAAAGTTCATTAGGGTTTTTCTGTGAGATGGGATGGAAAAAAACCGAGTGGACTTTTTGGCC
.......................................AAAAAAACCGAGTGGACTTTTTG...    1924
.......................................AAAAAAACCGAGTGGACTTTTT....    56
........................................AAAAAACCGAGTGGACTTTTTG...    40
...AAAAGTTCATTAGGGTTTTTCT........................................    22
.......................................AAAAAAACCGAGTGGACTTTT.....    12
.......................................AAAAAAACCGAGTGGACTTT......    5
...AAAAGTTCATTAGGGTTTTTC.........................................    2
.......................................AAAAAAACCGAGTGGAC.........    2
.......................................AAAAAAACCGAGTGGACT........    2
...AAAAGTTCATTAGGGTTTTT..........................................    1
.....................................GGAAAAAAACCGAGTGGACTTTT.....    1
```

```
(((((((((((((.((.(((((((((........))))))))).)).))))))))))))).. (-26.00)

16/20712318-20712389(-)_mir_cow_57
GGGTTGGCCAAAAAGTTCATTTGGGTTTTTCCATGTGATCTTACAGAAAAACTCGAACGAACTTTTTAGCTA
................................................AAACTCGAACGAACTTTTTAGC..  72
...............................................AAAACTCGAACGAACTTTTTAG...  23
.........AAAAAGTTCATTTGGGTTTTT..........................................  11
.........AAAAAGTTCATTTGGGTTTT...........................................  6
........CAAAAAGTTCATTTGGGTTTTT..........................................  6
.........AAAAAGTTCATTTGGGTTT............................................  3
................................................AAACTCGAACGAACTTTTTAG...  3
........CAAAAAGTTCATTTGGGTTTT...........................................  2
...TTGGCCAAAAAGTTCATTTGG................................................  1
....TGGCCAAAAAGTTCATTTGG................................................  1
........CAAAAAGTTCATTTGGGTTT............................................  1
.........AAAAAGTTCATTTGGGTTTTTC.........................................  1
..........AAAAGTTCATTTGGGTTTTTC.........................................  1
...........AAAGTTCATTTGGGTTTTTCC........................................  1
...............................................AAAACTCGAACGAACTTTT......  1
..............................................AAAAACTCGAACGAACTTTTT.....  1
...............................................AAAACTCGAACGAACTTTTT.....  1
...............................................AAAACTCGAACGAACTTTTTAGC..  1
....((((.(((((((((.((((((((((((..((((....)))))))))))))))).))))))))).)))) (-27.30)

16/21253052-21253122(+)_mir_cow_58
GGCTGGCCAAAAAGTTTGTTCGGGTTTTCTGTACCATGGGATGGCAAACCCGAATGAACTTTTTGGCCAAA
........AAAAAGTTTGTTCGGGTTTTCT.........................................   44
............................................CAAACCCGAATGAACTTTTTGG.....   12
........AAAAAGTTTGTTCGGGTTTTC..........................................   5
.............................................AAACCCGAATGAACTTTTTGGC....   5
...TGGCCAAAAAGTTTGTTCGGGTTT............................................   2
........AAAAAGTTTGTTCGGGTTTT...........................................   2
...TGGCCAAAAAGTTTGTTCGGGT..............................................   1
............................................CAAACCCGAATGAACTTTTT.......   1
............................................CAAACCCGAATGAACTTTTTG......   1
..............................................AACCCGAATGAACTTTTTGGCC...   1
...(((((((((((((..(((((((((.((((.((...)))))).)))))))))..))))))))))))).. (-40.50)
```

```
16/24305740-24305811(-)_mir_cow_59
CAGGAAAATGACCTATGAATTGACAGACAACGTGACTAAGTCTGTCTGTCATTTCTGTAGGCCAATGTTCTG
.......ATGACCTATGAATTGACAGACA...........................................  1114
.......ATGACCTATGAATTGACAGAC............................................  833
.......ATGACCTATGAATTGACAGA.............................................  213
........TGACCTATGAATTGACAG..............................................  59
.......ATGACCTATGAATTGACAGACAA..........................................  40
........TGACCTATGAATTGACAGACA...........................................  14
........TGACCTATGAATTGACAGA.............................................  11
............................................TCTGTCATTTCTGTAGGCCAAT......  11
.......ATGACCTATGAATTGACAG..............................................  10
............................................TCTGTCATTTCTGTAGGCCAATG.....  9
........TGACCTATGAATTGACAGAC............................................  7
........TGACCTATGAATTGACA...............................................  6
......AATGACCTATGAATTGACAGAC............................................  3
........TGACCTATGAATTGAC................................................  2
........TGACCTATGAATTGACAGACAA..........................................  2
............................................TCTGTCATTTCTGTAGGCC.........  2
............................................TCTGTCATTTCTGTAGGCCAATGT....  2
.......ATGACCTATGAATTGAC................................................  1
.......ATGACCTATGAATTGACA...............................................  1
......AATGACCTATGAATTGACAGA.............................................  1
.......ATGACCTATGAATTGACAGACAACG........................................  1
............................................TCTGTCATTTCTGTAGGCCA........  1
............................................TCTGTCATTTCTGTAGGCCAA.......  1
(((((...((.((((((((.(((((((((((........)).)))))))).))).)))))).))...))))) (-27.70)

16/24306040-24306106(+)_mir_Cow_Blast_43
TCAAAAGTAACAGCATCTCCACTGGAAATTGACGCACAGTCCACATGGAGTTGCTGTTACAATCGAT
..........................................ACATGGAGTTGCTGTTACAATCG..  9
.........................................CACATGGAGTTGCTGTTACAAT....  7
.........................................CACATGGAGTTGCTGTTACA......  6
..........................................ACATGGAGTTGCTGTTACAATC...  4
.........................................CACATGGAGTTGCTGTTACAATC...  3
.........................................CACATGGAGTTGCTGTTAC.......  2
.........................................CACATGGAGTTGCTGTTACAA.....  2
.........................................CACATGGAGTTGCTGTTACAATCG..  2
....AAGTAACAGCATCTCCACTGGA.........................................  1
```

```
......((((((((.(((((.((((...((.....)).))))..))))).))))))))....... (-25.80)

16/24379869-24379945(-)_mir_cow_60
ACATTGAAACAGGCTAGGAGAAATGATTGGATAGAAAATTTTATTCTATTCATTTATCTCCCAGCCTACAAAATGGA
..............................................TATTCATTTATCTCCCAGCCTACA.......   1464
.........CAGGCTAGGAGAAATGATTGGAT.............................................   1040
.........CAGGCTAGGAGAAATGATTGGA..............................................   1033
................................................TTCATTTATCTCCCAGCCTACA.......   483
..............................................TATTCATTTATCTCCCAGCCT..........   424
..............................................TATTCATTTATCTCCCAGCCTAC........   393
........ACAGGCTAGGAGAAATGATTGGAT.............................................   286
........ACAGGCTAGGAGAAATGATTGGA..............................................   249
.................................................TCATTTATCTCCCAGCCTACA.......   154
.........CAGGCTAGGAGAAATGATTGG...............................................   135
........ACAGGCTAGGAGAAATGATTGG...............................................   123
.................................................TCATTTATCTCCCAGCCTACAA......   70
................................................TTCATTTATCTCCCAGCCTAC........   65
..............................................TATTCATTTATCTCCCAGCCTACAA......   64
..............................................TATTCATTTATCTCCCAGCCTA.........   61
................................................TTCATTTATCTCCCAGCCTACAA......   56
.........CAGGCTAGGAGAAATGAT..................................................   22
...............................................ATTCATTTATCTCCCAGCCTACA.......   20
.........CAGGCTAGGAGAAATGATTGGATA............................................   17
.................................................TCATTTATCTCCCAGCCTACAAAA....   14
..............................................TATTCATTTATCTCCCAGCC...........   13
.........CAGGCTAGGAGAAATGATT.................................................   11
.......AACAGGCTAGGAGAAATGATTGG...............................................   11
................................................TTCATTTATCTCCCAGCCT..........   10
...............................................ATTCATTTATCTCCCAGCCTAC........   10
.................................................TCATTTATCTCCCAGCCTACAAA.....   10
..................................................CATTTATCTCCCAGCCTACAAAA....   10
.........CAGGCTAGGAGAAATGATTG................................................   9
.......AACAGGCTAGGAGAAATGATTGGAT.............................................   9
.......AACAGGCTAGGAGAAATGATTGGA..............................................   8
........ACAGGCTAGGAGAAATGAT..................................................   6
................................................TTCATTTATCTCCCAGCCTA.........   6
.................................................TCATTTATCTCCCAGCCTAC........   6
.........CAGGCTAGGAGAAATGATTGGATAGA..........................................   5
```

........ACAGGCTAGGAGAAATGATT... 4
....ATTCATTTATCTCCCAGCCTACAAAA.... 4
........ACAGGCTAGGAGAAATGATTG.. 3
..........AGGCTAGGAGAAATGATTGGA.. 3
.........CAGGCTAGGAGAAATGATTGGATAGAA... 3
.........CAGGCTAGGAGAAATGATTGGATAGAAAA....................................... 3
....TCATTTATCTCCCAGCCT.......... 3
.......AACAGGCTAGGAGAAATGATT... 2
..........AGGCTAGGAGAAATGATTGGAT... 2
.........CAGGCTAGGAGAAATGATTGGATAG... 2
.........CAGGCTAGGAGAAATGATTGGATAGAAA.. 2
....TATTCATTTATCTCCCAGC............ 2
....ATTCATTTATCTCCCAGCCT.......... 2
....ATTCATTTATCTCCCAGCCTACAA...... 2
........ACAGGCTAGGAGAAAT... 1
.......AACAGGCTAGGAGAAATGATTG.. 1
........ACAGGCTAGGAGAAATGATTGGATA.. 1
........ACAGGCTAGGAGAAATGATTGGATAGAAA.. 1
..........AGGCTAGGAGAAATGATTGGATAGAAA.. 1
...................GAAATGATTGGATAGAAAATTTTATTCTA............................. 1
....ATTCATTTATCTCCCAGCCTA......... 1
....CATTTATCTCCCAGCCTACAA...... 1
......................................TTTTATTCTATTCATTTATCTCCCAGCCTACAAA..... 1
....ATTCATTTATCTCCCAGCCTACAAA..... 1
....CATTTATCTCCCAGCCTACAAA..... 1
....TTTATTCTATTCATTTATCTCCCAGCCTACAAAA.... 1
....TCTATTCATTTATCTCCCAGCCTACAAAA.... 1
....TTCATTTATCTCCCAGCCTACAAAA.... 1
....ATTTATCTCCCAGCCTACAAAA.... 1
....TATCTCCCAGCCTACAAAA.... 1
....ATCTCCCAGCCTACAAAA.... 1
.((((.....(((((.(((((..(((.(((((((((.......))))))))).)))))))).))))))....)))).. (-27.10)

16/3031944-3032041(-)_mir_Cow_Blast_29
GCTGTGGCCTATGGCTTTTCATTCCTATGTGATTACTGTTCCAAACTCATGTAGGGCTAAAAGCCATGGGCTACAGTGAGGGGCGCGCGCCTACTCCC
.........TATGGCTTTTCATTCCTATGTGA.. 896
.........TATGGCTTTTCATTCCTATGTG... 595
.........TATGGCTTTTCATTCCTATGT.. 251

```
.........TATGGCTTTTCATTCCTATG.....................................................................    32
.........TATGGCTTTTCATTCCTATGTGAT.................................................................    32
................................................ATGTAGGGCTAAAAGCCATGGG............................    13
.........TATGGCTTTTCATTCCTATGTGATT................................................................    7
.................................................TGTAGGGCTAAAAGCCATGGGC...........................    6
................................................ATGTAGGGCTAAAAGCCATGG.............................    3
.........TATGGCTTTTCATTCCTA.......................................................................    2
.........TATGGCTTTTCATTCCTATGTGATTACTGT...........................................................    2
.........TATGGCTTTTCATTCCTATGTGATTACTGTT..........................................................    2
.................................................TGTAGGGCTAAAAGCCATGGGCT..........................    2
.........TATGGCTTTTCATTCCT........................................................................    1
.........TATGGCTTTTCATTCCTAT......................................................................    1
........CTATGGCTTTTCATTCCTATGTGA..................................................................    1
.........................TATGTGATTACTGTTCCAAACTCATG...............................................    1
................................................ATGTAGGGCTAAAAGCC.................................    1
...........................................AACTCATGTAGGGCTAAAAGCCATGGG............................    1
.................................................TGTAGGGCTAAAAGCCATGGG............................    1
................................................ATGTAGGGCTAAAAGCCATGGGC...........................    1
.......................................................................TACAGTGAGGGGCGCGCGCCT......    1
(((((((((((((((((((...((((((((((..............))))))))))..)))))))))))))))))))(((((((....)))).))).. (-49.14)

16/32621026-32621088(-)_novel_cow_13
CAATAAGTTCATTCGGAGTTGTCCACGCGATCATCTGGAAAACTCTGTATGAACTTTTTGGTC
...TAAGTTCATTCGGAGTTGTCC.......................................    4
.....................................GAAAACTCTGTATGAACTTTTT....    3
..ATAAGTTCATTCGGAGTTGTCC.......................................    2
...TAAGTTCATTCGGAGTTGTCCA......................................    2
......................................AAAACTCTGTATGAACTTTTTG...    2
...TAAGTTCATTCGGAGTTGTC........................................    1
(((.((((((((.(((((((.((((..........)))).))))))).)))))))).)))... (-24.30)

16/33022293-33022362(-)_mir_cow_61
TCAGGGAGTGTACTTCCTGGGGCCTCTGGGCATATAATTCCTGTGGCCTCTGGGTGTGTACCCTCTGTGG
.........................................TGTGGCCTCTGGGTGTGTACCCT......    240
.........................................TGTGGCCTCTGGGTGTGTACC........    107
.........................................TGTGGCCTCTGGGTGTGTACCC.......    99
...........................................TGGCCTCTGGGTGTGTACCCT......    66
...........................................TGGCCTCTGGGTGTGTACC........    10
```

```
......AGTGTACTTCCTGGGGCCTCT...........................................    9
.........................................TGTGGCCTCTGGGTGTGTAC.........    9
...........................................TGGCCTCTGGGTGTGTACCC.......    6
......AGTGTACTTCCTGGGGCCT.............................................    1
......AGTGTACTTCCTGGGGCCTCTGG.........................................    1
......AGTGTACTTCCTGGGGCCTCTGGGC.......................................    1
.........................................TGTGGCCTCTGGGTGTGTA..........    1
..........................................GTGGCCTCTGGGTGTGTACCC.......    1
.........................................TGTGGCCTCTGGGTGTGTACCCTC.....    1
.........................................TGTGGCCTCTGGGTGTGTACCCTCT....    1
.((.((((.((((..((..(((((.(.(((.......))).).))))).)..))...)))).)))).)). (-31.10)

16/40485821-40485893(-)_mir_cow_62
TGTGTCTGCCTGTCTACACTTGCTGTGCAGAACATCCGCTCACCTGTACAGCAGGCACAGACAGGCAGTCACA
...............................................ACAGCAGGCACAGACAGGCAGT.... 466
......TGCCTGTCTACACTTGCTGTGC............................................. 275
..............................................TACAGCAGGCACAGACAGGCAGT.... 216
...............................................ACAGCAGGCACAGACAGGCAG..... 130
..............................................TACAGCAGGCACAGACAGGCAG..... 79
......TGCCTGTCTACACTTGCTGT............................................... 70
..............................................TACAGCAGGCACAGACAGGC....... 44
..............................................TACAGCAGGCACAGACAGGCA...... 31
..............................................TACAGCAGGCACAGACAGG........ 23
...............................................ACAGCAGGCACAGACAGGCA...... 22
.......GCCTGTCTACACTTGCTGTGC............................................. 12
......TGCCTGTCTACACTTGCTGTG.............................................. 11
...............................................ACAGCAGGCACAGACAGG........ 11
......TGCCTGTCTACACTTGCTG................................................ 10
..............................................TACAGCAGGCACAGACAG......... 7
...............................................ACAGCAGGCACAGACAGGC....... 7
...............................................ACAGCAGGCACAGACAG......... 6
.............................................GTACAGCAGGCACAGACAGGCAGT.... 6
.......GCCTGTCTACACTTGCTGTG.............................................. 2
.............................................GTACAGCAGGCACAGACAGG........ 2
.............................................GTACAGCAGGCACAGACAGGCA...... 2
........CCTGTCTACACTTGCTGTGC............................................. 1
.......GCCTGTCTACACTTGCTGTGCA............................................ 1
............................AGAACATCCGCTCACCTGT.......................... 1
```

```
..............................................TACAGCAGGCACAGACA.......... 1
.............................................GTACAGCAGGCACAGACAGGC....... 1
.............................................GTACAGCAGGCACAGACAGGCAG..... 1
.(((.((((((((((...((((((((((((.............))))))))))))...)))))))))).))). (-42.82)

16/40485822-40485889(+)_mir_Cow_Blast_40
GTGACTGCCTGTCTGTGCCTGCTGTACAGGTGAGCGGATGTTCTGCACAGCAAGTGTAGACAGGCAGA
.............................................CACAGCAAGTGTAGACAGGC... 5
.......CCTGTCTGTGCCTGCTGTACA........................................ 4
....CTGCCTGTCTGTGCCTGCTGTA.......................................... 1
............................................GCACAGCAAGTGTAGACAGGC... 1
.............................................CACAGCAAGTGTAGACAGGCA.. 1
....(((((((((((..(.((((((.((((...........)))).)))))).)..))))))))))). (-37.80)

16/40491609-40491681(-)_mir_cow_63
CGTCGCCCCAGTGTTCAGACTACCTGTTCAGGACAATGCCGTTGTACAGTAGTCTGCACATTGGTTAGACTGG
.............................................ACAGTAGTCTGCACATTGGTT....... 34856
............................................TACAGTAGTCTGCACATTGGTT....... 12926
.............................................ACAGTAGTCTGCACATTGGTTA...... 8597
......CCCAGTGTTCAGACTACCTGTTC............................................ 4732
.............................................ACAGTAGTCTGCACATTGGT........ 2417
..............................................CAGTAGTCTGCACATTGGTT....... 2184
......CCCAGTGTTCAGACTACCTGTT............................................. 1891
............................................TACAGTAGTCTGCACATTGGT........ 1677
......CCCAGTGTTCAGACTACCTGT.............................................. 1392
............................................TACAGTAGTCTGCACATTGGTTA...... 756
..............................................CAGTAGTCTGCACATTGGTTA...... 681
......CCCAGTGTTCAGACTACCTGTTCA........................................... 496
......CCCAGTGTTCAGACTACCTG............................................... 467
.............................................ACAGTAGTCTGCACATTGG......... 268
............................................TACAGTAGTCTGCACATTGG......... 234
..............................................CAGTAGTCTGCACATTGGT........ 224
......CCCAGTGTTCAGACTACCT................................................ 219
.............................................ACAGTAGTCTGCACATTGGTTAG..... 78
.............................................ACAGTAGTCTGCACATTGGTTAGA.... 61
.............................................ACAGTAGTCTGCACAT............ 59
......CCCAGTGTTCAGACTACC................................................. 55
............................................TACAGTAGTCTGCACAT............ 29
```

```
......CCCAGTGTTCAGACTA................................................... 22
.........AGTGTTCAGACTACCTGTTC............................................ 22
.............................................ACAGTAGTCTGCACATTG.......... 17
............................................TACAGTAGTCTGCACATTGGTTAG..... 14
.........AGTGTTCAGACTACCTGTT............................................. 13
........CAGTGTTCAGACTACCTGTTC............................................ 12
......CCCAGTGTTCAGACTAC.................................................. 10
.............................................ACAGTAGTCTGCACATT........... 10
............................................TACAGTAGTCTGCACATTG.......... 8
...............................................AGTAGTCTGCACATTGGTT....... 8
..............................................CAGTAGTCTGCACATTGG......... 7
.......CCAGTGTTCAGACTACCTGTTC............................................ 6
................................................GTAGTCTGCACATTGGTT....... 6
..............................................CAGTAGTCTGCACATTGGTTAGA.... 6
............................................TACAGTAGTCTGCACATT........... 5
..............................................CAGTAGTCTGCACATTGGTTAG..... 5
........CAGTGTTCAGACTACCTGTT............................................. 4
..........GTGTTCAGACTACCTGTTC............................................ 3
.............................AGGACAATGCCGTTGTACAGTAGTCTGCACATTGGTTA...... 3
...............................................AGTAGTCTGCACATTGGTTA...... 3
.......CCAGTGTTCAGACTACCTGT.............................................. 2
...........TGTTCAGACTACCTGTTC............................................ 2
............................................TACAGTAGTCTGCACA............. 2
............................................TACAGTAGTCTGCACATTGGTTAGA.... 2
........CAGTGTTCAGACTACC................................................. 1
.........AGTGTTCAGACTACCTGT.............................................. 1
...........TGTTCAGACTACCTGTT............................................. 1
.......CCAGTGTTCAGACTACCTGTTCA........................................... 1
......CCCAGTGTTCAGACTACCTGTTCAG.......................................... 1
......CCCAGTGTTCAGACTACCTGTTCAGGACA...................................... 1
.............................AGGACAATGCCGTTGTACAGTAGTCTGCACAT............ 1
...............................................AGTAGTCTGCACATTGGT........ 1
...........................................GTACAGTAGTCTGCACATTGGTTA...... 1
................................................GTAGTCTGCACATTGGTTA...... 1
.(((...(((((((.((((((((.(((...((......)).....))))))))))).)))))))...)))... (-29.80)

16/45197405-45197478(+)_mir_cow_64
TTTCTTTGGCAGTGTCTTAGCTGGTTGTTGTGAGTAATAATGCAGGAAGCAATCAGCAAGTATACTGCCCTAGA
```

```
......TGGCAGTGTCTTAGCTGGTTGT..............................................1781
......TGGCAGTGTCTTAGCTGGTTGTT.............................................1071
......TGGCAGTGTCTTAGCTGGTTG...............................................105
......TGGCAGTGTCTTAGCTGGTT................................................90
......TGGCAGTGTCTTAGCTGGT.................................................56
.......GGCAGTGTCTTAGCTGGTTGTT.............................................33
......TGGCAGTGTCTTAGCTG...................................................31
.................................................CAATCAGCAAGTATACTGCCCT...26
.....TTGGCAGTGTCTTAGCTGGTT................................................14
.......GGCAGTGTCTTAGCTGGTTGT..............................................11
..................................................AATCAGCAAGTATACTGCCCTA..8
....TTTGGCAGTGTCTTAGCTGGTT................................................6
.....TTGGCAGTGTCTTAGCTGGTTGT..............................................6
......TGGCAGTGTCTTAGCTGGTTGTTG............................................6
..................................................AATCAGCAAGTATACTGCCCT...6
.....TTGGCAGTGTCTTAGCTGGTTGTT.............................................5
......TGGCAGTGTCTTAGCTGGTTGTTGT...........................................5
.....TTGGCAGTGTCTTAGCTGGTTG...............................................4
......TGGCAGTGTCTTAGCTGGTTGTTGTGAGT.......................................3
.................................................CAATCAGCAAGTATACTGCCC....3
.................................................CAATCAGCAAGTATACTGCCCTA..3
.....TTGGCAGTGTCTTAGCTGGT.................................................2
...........GTGTCTTAGCTGGTTGT..............................................2
..........AGTGTCTTAGCTGGTTGTT.............................................2
.............GTCTTAGCTGGTTGTT.............................................2
......TGGCAGTGTCTTAGCT....................................................1
.......GGCAGTGTCTTAGCTG...................................................1
....TTTGGCAGTGTCTTAGCTGGT.................................................1
....TTTGGCAGTGTCTTAGCTGGTTG...............................................1
....TTTGGCAGTGTCTTAGCTGGTTGT..............................................1
.........CAGTGTCTTAGCTGGTTGT..............................................1
..........AGTGTCTTAGCTGGTTGT..............................................1
...........GTGTCTTAGCTGGTTGTT.............................................1
..(((..(((((((((((.((((((((((....(((....)))....))))))))))))).))))))))..))) (-31.30)

16/45760840-45760922(+)_novel_cow_10
TACTGGGTTGGCCAAAATCTTTGTTTCAGTTTTTCCATAACAGTATGCCAAACACTCGAACAAACTTTTTGGCCAACCCAATA
.......................................................TCGAACAAACTTTTTGGCCAAC......   80
```

....TCGAACAAACTTTTTGGCCAACC..... 19
....TCGAACAAACTTTTTGGCCA........ 6
....TCGAACAAACTTTTTGGCC......... 4
....TCGAACAAACTTTTTGGCCAA....... 2
........TGGCCAAAATCTTTGTTTCAGT... 1
....AACACTCGAACAAACTTTTTG............ 1
...((((((((((((((..(((((((.((((((..((((....))))..))).))).)))))))..))))))))))))))... (-34.80)

16/50502451-50502518(+)_mir_cow_65
CCCATGGCACAGGGTCCAGCTGTCGGCCGTGATACCCGATGGGTCGATGATGGTCCCTGTGTTTTGGG
....TCGATGATGGTCCCTGTGTTTT... 26
....TCGATGATGGTCCCTGTGTTTTG.. 14
....TCGATGATGGTCCCTGTGTTT.... 4
....TGGCACAGGGTCCAGCTGTCGG.. 3
....CGATGATGGTCCCTGTGTTTTG.. 2
....TGGCACAGGGTCCAGCTGTCGGC... 1
....TCGATGATGGTCCCTGTGT...... 1
....TCGATGATGGTCCCTGTGTT..... 1
....CGATGATGGTCCCTGTGTTTT... 1
((((..((((((((.(((..((((((((.((.....))...)))))))).))).))))))))..)))) (-37.80)

16/52520095-52520163(-)_mir_cow_66
ATGGGCGTCTTACCAGACACGGTTAGATCTGGTCCTTCTGTCTAATACTGTCTGGTAATGCCGTCCATC
....TAATACTGTCTGGTAATGCCG...... 3147
....TAATACTGTCTGGTAATGCCGT..... 950
....TAATACTGTCTGGTAATGCC....... 234
....TAATACTGTCTGGTAATGC........ 13
....TAATACTGTCTGGTAATGCCGTC.... 5
....AATACTGTCTGGTAATGCCG...... 4
....AATACTGTCTGGTAATGCCGT..... 3
......GTCTTACCAGACACGGTTAGA.. 2
....TAATACTGTCTGGTAATG......... 1
.....................................CTGTCTAATACTGTCTGGTAATGC........ 1
....TCTAATACTGTCTGGTAATGCC....... 1
....CTAATACTGTCTGGTAATGCCG...... 1
....ATACTGTCTGGTAATGCCG...... 1
....ACTGTCTGGTAATGCCG...... 1
(((((((.(((((((((((..(((((((..((....)).)))))))..)))))))))).).))))))). (-31.00)

16/52521331-52521403(-)_mir_cow_67
CTGTGGACATCTTACCGGACAGTGCTGGATTTCTCGGCTCGACTCTAACACTGTCTGGTAACGATGTTCAAAG
....TAACACTGTCTGGTAACGATGT...... 2453
....TAACACTGTCTGGTAACGATGTT..... 2272
....TAACACTGTCTGGTAACGATG....... 1663
....TAACACTGTCTGGTAACGA......... 402
.......CATCTTACCGGACAGTGCTGGA.. 197
....TAACACTGTCTGGTAACGAT........ 97
....TAACACTGTCTGGTAACG.......... 57
....AACACTGTCTGGTAACGATGTT..... 29
.......CATCTTACCGGACAGTGCTGG... 27
.......CATCTTACCGGACAGTGCTGGAT... 9
.........TCTTACCGGACAGTGC.. 6
....TAACACTGTCTGGTAAC........... 5
.......CATCTTACCGGACAGTGCTG.. 4
........ATCTTACCGGACAGTGCTGGA.. 4
........ATCTTACCGGACAGTGCTGGAT... 3
....CACTGTCTGGTAACGATGTT..... 3
........ATCTTACCGGACAGTGCTGGATT.. 2
....CTAACACTGTCTGGTAACGA......... 2
....AACACTGTCTGGTAACGATG....... 2
....CTGTCTGGTAACGATG....... 2
....CTAACACTGTCTGGTAACGATGT...... 2
....AACACTGTCTGGTAACGATGT...... 2
....CTGTCTGGTAACGATGTT..... 2
....TAACACTGTCTGGTAACGATGTTC.... 2
.......CATCTTACCGGACAGTGC.. 1
.......CATCTTACCGGACAGTGCT... 1
......ACATCTTACCGGACAGTGCTGG... 1
.........TCTTACCGGACAGTGCTGGA.. 1
.......CATCTTACCGGACAGTGCTGGATT.. 1
....AACACTGTCTGGTAACGA......... 1
....AACACTGTCTGGTAACGAT........ 1
....CTAACACTGTCTGGTAACGATG....... 1
....CTAACACTGTCTGGTAACGATGTT..... 1
...(((((((((((((((((((((.((((....(((...))).)))).))))))))))))).))))))))... (-34.60)

```
16/52521334-52521400(+)_mir_cow_68
TGAACATCGTTACCAGACAGTGTTAGAGTCGAGCCGAGAAATCCAGCACTGTCCGGTAAGATGTCCA
...........................................CAGCACTGTCCGGTAAGATGTC..  168
...........................................CAGCACTGTCCGGTAAGATGT...  61
....CATCGTTACCAGACAGTGTTAGA........................................  49
...........................................CAGCACTGTCCGGTAAGATG....  23
.....ATCGTTACCAGACAGTGTTAGA........................................  11
..........................................CCAGCACTGTCCGGTAAGATGT...  9
....CATCGTTACCAGACAGTGTTA..........................................  3
...........................................CAGCACTGTCCGGTAAGAT.....  3
..........................................CCAGCACTGTCCGGTAAGATG....  2
....CATCGTTACCAGACAGTGTTAG.........................................  1
.....ATCGTTACCAGACAGTGTTAG.........................................  1
...........................................CAGCACTGTCCGGTAAGA......  1
............................................AGCACTGTCCGGTAAGATGT...  1
.(.(((((.(((((.(((((((((.((.((.......))..)).))))))))).)))))))))).).  (-27.00)

16/52521982-52522058(-)_mir_Cow_Blast_11
GGCCATCGCCATCTTACTGGGCAGCATTGGATGGTGTCTGGTCTCTAATACTGCCTGGTAATGATGACGCTGGGGCC
.............................................TAATACTGCCTGGTAATGATGAC.........   10517
.............................................TAATACTGCCTGGTAATGATGA..........   941
.............................................TAATACTGCCTGGTAATGATG...........   347
..............................................AATACTGCCTGGTAATGATGAC.........   247
.............................................TAATACTGCCTGGTAATGA.............   121
.........CATCTTACTGGGCAGCATTGGA..............................................   95
.............................................TAATACTGCCTGGTAATGAT............   64
.........CATCTTACTGGGCAGCATTGG...............................................   50
.............................................TAATACTGCCTGGTAATG..............   44
.............................................TAATACTGCCTGGTAATGATGACG........   34
.........CATCTTACTGGGCAGCATTGGAT.............................................   14
...............................................ATACTGCCTGGTAATGATGACGC.......   8
............................................CTAATACTGCCTGGTAATGATGAC.........   6
...............................................ATACTGCCTGGTAATGATGACG........   5
..............................................AATACTGCCTGGTAATGATGA..........   4
...............................................ATACTGCCTGGTAATGATGAC.........   3
..............................................AATACTGCCTGGTAATGATGACG........   3
.............................................TAATACTGCCTGGTAA................   2
..............................................AATACTGCCTGGTAATGATG...........   2
```

```
........CCATCTTACTGGGCAGCATTGG..............................................   1
................................................ATACTGCCTGGTAATGATGACGCT......   1
................................................TACTGCCTGGTAATGATGACGCT......   1
((((..((.((((((((..(((((.((((((.((.......)))))))).))))))..)))).)))).))....)))) (-34.20)

1/65453352-65453424(-)_mir_cow_6
TTCTCTTGAGAGATCAGAGGCGCAGAGTGCGTCAATGTCAATGAAGCCTGTGCCTTTTACCTCTTTAAGAGCG
.......GAGAGATCAGAGGCGCAGAGT............................................ 4166
.......GAGAGATCAGAGGCGCAGAG............................................. 162
.......................................CCTGTGCCTTTTACCTCTTTAA..... 49
.......GAGAGATCAGAGGCGCAGAGTGC.......................................... 29
.......................................CCTGTGCCTTTTACCTCTTTA...... 29
.......GAGAGATCAGAGGCGCAGA.............................................. 23
.......GAGAGATCAGAGGCGCAGAGTG........................................... 17
.......GAGAGATCAGAGGCGCAGAGTGCG......................................... 15
.......................................CCTGTGCCTTTTACCTCTTT....... 8
......TGAGAGATCAGAGGCGCAGAGT............................................ 6
.......GAGAGATCAGAGGCGCAGAGTGCGT........................................ 3
.......GAGAGATCAGAGGCGCAG............................................... 1
......TGAGAGATCAGAGGCGCAGA.............................................. 1
......TGAGAGATCAGAGGCGCAGAG............................................. 1
........AGAGATCAGAGGCGCAGAGT............................................ 1
...........................TGCGTCAATGTCAATGAAGCCT........................ 1
..................................ATGTCAATGAAGCCTGTGCCTTTTACCTCTTTAA..... 1
.......................................CCTGTGCCTTTTACCTCTTTAAG.... 1
..((((((((((...((((((((((.((...(((.......))).)))))))))))))...))).)))))))).. (-28.20)

16/54869092-54869155(-)_novel_cow_14
CCAATAAGTTCATTTGGATTTTTCCATAATATGTGGTGGAAAATCCAAATGAACTTTTTTGCCA
...ATAAGTTCATTTGGATTTTT.........................................     6
..AATAAGTTCATTTGGATTTTT.........................................     4
..AATAAGTTCATTTGGATTTTTCC.......................................     1
...ATAAGTTCATTTGGATTTTTCC.......................................     1
...ATAAGTTCATTTGGATTTTTCCA......................................     1
...................................GAAAATCCAAATGAACTTTTTT....     1
.(((.((((((((((((((((((.((((........))))))))))))))))))))))..)))... (-26.50)

16/58628121-58628199(-)_mir_Cow_Blast_14
```

```
CCTACATTAACATGGAGGTCTCTGTCTGGCTTAGGACAGCTGGCTAAGTCTGATCGTTCCCCTCCATACAGCCTTTAAA
.........ACATGGAGGTCTCTGTCTGGCT................................................ 89
..........CATGGAGGTCTCTGTCTGGCT................................................ 30
.........ACATGGAGGTCTCTGTCTGGC................................................. 12
................................................TCTGATCGTTCCCCTCCATACAG........ 12
................................................TCTGATCGTTCCCCTCCATAC.......... 9
................................................TCTGATCGTTCCCCTCCATACA......... 9
.........ACATGGAGGTCTCTGTCTGGCTT............................................... 7
..........CATGGAGGTCTCTGTCTGGCTT............................................... 6
................................................TCTGATCGTTCCCCTCCATACAGC....... 6
.....................CTGTCTGGCTTAGGACAGCTGGCTAAGTC............................. 4
..................................................TGATCGTTCCCCTCCATACAGCC...... 4
.........ACATGGAGGTCTCTGTCTGG.................................................. 3
..........CATGGAGGTCTCTGTCTGGC................................................. 3
...........ATGGAGGTCTCTGTCTGGCT................................................ 2
...........ATGGAGGTCTCTGTCTGGCTT............................................... 2
..........CATGGAGGTCTCTGTCTGGCTTA.............................................. 2
.................................................CTGATCGTTCCCCTCCATACAGCC...... 2
.........ACATGGAGGTCTCTGTCT.................................................... 1
..........CATGGAGGTCTCTGTCTGG.................................................. 1
........AACATGGAGGTCTCTGTCTGGCTT............................................... 1
...........ATGGAGGTCTCTGTCTGGCTTA.............................................. 1
.........ACATGGAGGTCTCTGTCTGGCTTAGGA........................................... 1
................................................TCTGATCGTTCCCCTCCAT............ 1
................................................TCTGATCGTTCCCCTCCATA........... 1
.................................GGACAGCTGGCTAAGTCTGATCGTTCCCCTCCATACAGC....... 1
.......................................CTGGCTAAGTCTGATCGTTCCCCTCCATACAGCC...... 1
.........................................GGCTAAGTCTGATCGTTCCCCTCCATACAGCC...... 1
...............................................GTCTGATCGTTCCCCTCCATACAGCC...... 1
................................................TCTGATCGTTCCCCTCCATACAGCC...... 1
.......................................................GTTCCCCTCCATACAGCC...... 1
...........(((((((...(.(((.(((((((..(....).))))))).))).)....)))))))............ (-20.90)

16/59666108-59666177(-)_mir_cow_69
ATCTCTCCCAGATAATGGCACTCTCAAACAGGTTTCCAGGTTGTTTGAAAGGCTGTTTCTTGGTCAGAAG
............................................TTGAAAGGCTGTTTCTTGGTC.....    520
............................................TTGAAAGGCTGTTTCTTGGT......    78
...........................................TTTGAAAGGCTGTTTCTTGGTC.....    54
```

```
...........................................TTTGAAAGGCTGTTTCTTGGT......    25
............................................TTGAAAGGCTGTTTCTTGGTCA....    18
.............................................TGAAAGGCTGTTTCTTGGTC.....    10
......CCCAGATAATGGCACTCTCAAAC.........................................    5
...........................................TTTGAAAGGCTGTTTCTTGG.......    2
............................................TTGAAAGGCTGTTTCTTGG.......    2
.............................................TGAAAGGCTGTTTCTTGGTCA....    1
.(((..((.(((.((((((.((.((((((((.(.....).)))))))).))))))))))).))..))).. (-21.80)

16/71150562-71150631(+)_novel_cow_11
CTGGTGTCAGTGGCAGAGTGCAGGGACCTCGCGACCAGAGAGGAGGGTCTGTCTCTGCCACTGCCACGGC
......TCAGTGGCAGAGTGCAGGGACCTC........................................    37
......TCAGTGGCAGAGTGCAGGGACCT.........................................    25
......TCAGTGGCAGAGTGCAGGGAC...........................................    9
......TCAGTGGCAGAGTGCAGGGACC..........................................    6
............................................GGGTCTGTCTCTGCCACTGCCA....    6
............................................GGGTCTGTCTCTGCCACTGCC.....    5
...................................................TCTCTGCCACTGCCAC...    1
...(((.(((((((((((.(((((..((((.(.......)..)))).))))))))))))))))).)))... (-37.10)

16/77478038-77478110(+)_mir_cow_70
GGAAGCTGGTTTCACATGGTGGCTTAGATTTTTCCATCTTTGTATCTAGCACCATTTGAAATCAGTGTTTTAG
..............................................TAGCACCATTTGAAATCAGTGTT....  5658
..............................................TAGCACCATTTGAAATCAGTGT.....  975
.............................................CTAGCACCATTTGAAATCAGTG......  437
.............................................CTAGCACCATTTGAAATCAGT.......  308
.............................................CTAGCACCATTTGAAATCAGTGT.....  260
..............................................TAGCACCATTTGAAATCAGT.......  244
..............................................TAGCACCATTTGAAATCAGTG......  202
.....CTGGTTTCACATGGTGGCTTAGA.............................................  132
.....CTGGTTTCACATGGTGGCTTAGAT............................................  84
..............................................TAGCACCATTTGAAATCAGTGTTT...  65
....................................................CATTTGAAATCAGTGTT....  48
..............................................TAGCACCATTTGAAATCAG........  42
.....CTGGTTTCACATGGTGGCTTAG..............................................  30
.............................................CTAGCACCATTTGAAATCAG........  25
...................................................CCATTTGAAATCAGTGTT....  24
..............................................TAGCACCATTTGAAATC..........  18
```

....CTAGCACCATTTGAAATCAGTGTT.... 13
....GCTGGTTTCACATGGTGGCTTAG.. 10
....GCTGGTTTCACATGGTGGCTTAGA... 10
....ATTTGAAATCAGTGTT.... 8
.....CTGGTTTCACATGGTGGCTT.. 7
......TGGTTTCACATGGTGGCTTAGA... 7
....CTAGCACCATTTGAAATC.......... 7
....ACCATTTGAAATCAGTGTT.... 7
....CTAGCACCATTTGAAATCA......... 6
....TAGCACCATTTGAAATCA......... 6
....ACCATTTGAAATCAGTG...... 5
....AGCACCATTTGAAATCAGTGTT.... 4
....GCTGGTTTCACATGGTGGCTT.. 3
......TGGTTTCACATGGTGGCTTAGAT.. 3
....TAGCACCATTTGAAAT........... 3
....CATTTGAAATCAGTGT..... 3
.....CTGGTTTCACATGGTGGCT.. 2
.....CTGGTTTCACATGGTGGCTTA.. 2
.....CTGGTTTCACATGGTGGCTTAGATT.. 2
....CCATTTGAAATCAGTG...... 2
....ACCATTTGAAATCAGTGT..... 2
.....CTGGTTTCACATGGTGGC... 1
....GCTGGTTTCACATGGTGGCT.. 1
......TGGTTTCACATGGTGGCT.. 1
....GCTGGTTTCACATGGTGGCTTA.. 1
......TGGTTTCACATGGTGGCTTA.. 1
......TGGTTTCACATGGTGGCTTAG... 1
............................TTTTTCCATCTTTGTATC........................... 1
....CTAGCACCATTTGAAAT........... 1
....TCTAGCACCATTTGAAATCAGT....... 1
....GCACCATTTGAAATCAGT....... 1
.............................TTTTCCATCTTTGTATCTAGCACCATTTGAAATCAGTG...... 1
....TCTAGCACCATTTGAAATCAGTG...... 1
....TCTAGCACCATTTGAAATCAGTGT..... 1
....GCACCATTTGAAATCAGTGT..... 1
....ATCTAGCACCATTTGAAATCAGTGTT.... 1
....GCACCATTTGAAATCAGTGTT.... 1
....TAGCACCATTTGAAATCAGTGTTTT.. 1

```
....(((((((((.((((((.((.((((............)))))))))))).))))))))).......  (-24.24)

16/77478604-77478672(+)_mir_cow_71
AGGCTGACCGATTTCTCCTGGTGTTCAGAGTCTGTTTTTGTCTAGCACCATTTGAAATCGGTTATGATG
..........................................TAGCACCATTTGAAATCGGTTA.....     11872
..........................................TAGCACCATTTGAAATCGGTT......     10556
..........................................TAGCACCATTTGAAATCGGT.......     3406
..........................................TAGCACCATTTGAAATCGGTTAT....     842
....TGACCGATTTCTCCTGGTGTTCA..........................................     490
....TGACCGATTTCTCCTGGTGTT............................................     409
....TGACCGATTTCTCCTGGTGTTC...........................................     388
......ACCGATTTCTCCTGGTGTTCAGA........................................     252
............................................GCACCATTTGAAATCGGTTATG...     114
..........................................TAGCACCATTTGAAATCGG........     97
......ACCGATTTCTCCTGGTGTTCAG.........................................     86
......ACCGATTTCTCCTGGTGTTCA..........................................     82
....TGACCGATTTCTCCTGGTGTTCAG.........................................     79
..............................................ACCATTTGAAATCGGTT......     66
.....GACCGATTTCTCCTGGTGTT............................................     62
.....GACCGATTTCTCCTGGTGTTCA..........................................     55
..............................................ACCATTTGAAATCGGTTA.....     52
....TGACCGATTTCTCCTGGTGT.............................................     51
...............................................CCATTTGAAATCGGTTA.....     45
...............................................CCATTTGAAATCGGTT......     44
.........................................CTAGCACCATTTGAAATCGGTT......     43
....TGACCGATTTCTCCTGGTGTTCAGA........................................     36
.....GACCGATTTCTCCTGGTGTTCAGA........................................     29
................................................CATTTGAAATCGGTTA.....     28
.....GACCGATTTCTCCTGGTGTTC...........................................     27
.....GACCGATTTCTCCTGGTGTTCAG.........................................     23
..............................................ACCATTTGAAATCGGT.......     20
..........................................TAGCACCATTTGAAATC..........     18
..........................................TAGCACCATTTGAAATCGGTTATG...     18
.........................................CTAGCACCATTTGAAATCGGTTA.....     16
....TGACCGATTTCTCCTGGTG..............................................     15
............................................GCACCATTTGAAATCGGTTATGA..     13
....TGACCGATTTCTCCTGGT...............................................     11
..........................................TAGCACCATTTGAAATCG.........     11
```

```
......ACCGATTTCTCCTGGTGTTC...........................................      10
.......CCGATTTCTCCTGGTGTTCAGA........................................      9
.........................................CTAGCACCATTTGAAATC..........      7
.........................................CTAGCACCATTTGAAATCGGT.......      7
...............................................CCATTTGAAATCGGTTAT....      6
.....GACCGATTTCTCCTGGTGT.............................................      5
....TGACCGATTTCTCCTGGTGTTCAGAG.......................................      5
............................................GCACCATTTGAAATCGGTTA.....      5
............................................GCACCATTTGAAATCGGTTAT....      5
.............................................CACCATTTGAAATCGGTTAT....      5
....TGACCGATTTCTCCTGGTGTTCAGAGT......................................      4
...........................................AGCACCATTTGAAATCGGTTA.....      4
.............................................CACCATTTGAAATCGGTTATGA..      4
.....GACCGATTTCTCCTGGTGTTCAGAG.......................................      3
..........................................TAGCACCATTTGAAAT...........      3
.............................................CACCATTTGAAATCGGTTA.....      3
..........................................TAGCACCATTTGAAATCGGTTATGA..      3
....TGACCGATTTCTCCTGG................................................      2
.....GACCGATTTCTCCTGG................................................      2
.....GACCGATTTCTCCTGGTG..............................................      2
......ACCGATTTCTCCTGGTGTT............................................      2
.........................................CTAGCACCATTTGAAATCGG........      2
.............................................CACCATTTGAAATCGGTT......      2
.........................................CTAGCACCATTTGAAATCGGTTAT....      2
..............................................ACCATTTGAAATCGGTTAT....      2
....TGACCGATTTCTCCTG.................................................      1
.....GACCGATTTCTCCTGGT...............................................      1
.....GACCGATTTCTCCTGGTGTTCAGAGT......................................      1
......ACCGATTTCTCCTGGTGTTCAGAGT......................................      1
....TGACCGATTTCTCCTGGTGTTCAGAGTC.....................................      1
..........ATTTCTCCTGGTGTTCAGAGTC.....................................      1
......ACCGATTTCTCCTGGTGTTCAGAGTCT....................................      1
....TGACCGATTTCTCCTGGTGTTCAGAGTCTG...................................      1
..................................TTTTTGTCTAGCACCATTTGAAA............      1
.........................................CTAGCACCATTTGAAAT...........      1
.........................................CTAGCACCATTTGAAATCG.........      1
...........................................AGCACCATTTGAAATCGGT.......      1
............................................GCACCATTTGAAATCGGT.......      1
```

```
.............................................CACCATTTGAAATCGGT.......      1
...........................................AGCACCATTTGAAATCGGTTAT....      1
................................................CATTTGAAATCGGTTAT....      1
.................................................ATTTGAAATCGGTTAT....      1
....(((((((((((...((((((((((((.....)))))...)))))))...))))))))))).....  (-25.30)

16/79685951-79686025(-)_mir_cow_72
TTCAGTGAACATTCAACGCTGTCGGTGAGTTTGGAATTAAAAATCAAAACCATCGACCGTTGATTGTACCCTATG
.......AACATTCAACGCTGTCGGTGAGT.............................................     1365775
.......AACATTCAACGCTGTCGGTGAG..............................................     1103625
.......AACATTCAACGCTGTCGGTGA...............................................     178739
.......AACATTCAACGCTGTCGGTGAGTT............................................     67437
.......AACATTCAACGCTGTCGGTG................................................     18252
.......AACATTCAACGCTGTCGGTGAGTTT...........................................     10662
.......AACATTCAACGCTGTCGGT.................................................     9870
................................................ACCATCGACCGTTGATTGTACC.....     8959
................................................ACCATCGACCGTTGATTGT........     688
................................................ACCATCGACCGTTGATTGTAC......     682
........ACATTCAACGCTGTCGGTGAGT.............................................     665
........ACATTCAACGCTGTCGGTGAG..............................................     341
.......AACATTCAACGCTGTCGG..................................................     259
................................................ACCATCGACCGTTGATTGTA.......     162
...............................................AACCATCGACCGTTGATTGTACC.....     127
.............CAACGCTGTCGGTGAGT.............................................     112
........ACATTCAACGCTGTCGGTGAGTT............................................     94
..............................................AAACCATCGACCGTTGATTGT........     80
..........ATTCAACGCTGTCGGTGAGT.............................................     60
...............................................AACCATCGACCGTTGATTGT........     59
........ACATTCAACGCTGTCGGTGA...............................................     52
...............................................AACCATCGACCGTTGATTGTAC......     43
.......AACATTCAACGCTGTCGGTGAGTTTGGAA.......................................     36
........ACATTCAACGCTGTCGGTGAGTTT...........................................     35
..........ATTCAACGCTGTCGGTGAG..............................................     34
.............CAACGCTGTCGGTGAG..............................................     27
...........TTCAACGCTGTCGGTGAGT.............................................     27
............TCAACGCTGTCGGTGAGT.............................................     27
.......AACATTCAACGCTGTCGGTGAGTTTG..........................................     25
...........TTCAACGCTGTCGGTGAG..............................................     22
```

.........CATTCAACGCTGTCGGTGAGT...	20
......GAACATTCAACGCTGTCGGTGAG..	19
....AATCAAAACCATCGACCGTTGATTGTACC.....	19
......GAACATTCAACGCTGTCGGTGA...	14
....AACCATCGACCGTTGATTGTA.......	14
.......AACATTCAACGCTGTC..	13
.............CAACGCTGTCGGTGAGTT..	13
..............AACGCTGTCGGTGAGT...	12
....AAAATCAAAACCATCGACCGTTGATTGTACC.....	12
..........ATTCAACGCTGTCGGTGA...	11
......................................AAAAATCAAAACCATCGACCGTTGATTGTACC.....	11
....CCATCGACCGTTGATTGTACC.....	8
......GAACATTCAACGCTGTCGGTGAGT...	7
........ACATTCAACGCTGTCGGTG..	6
...........TTCAACGCTGTCGGTGA...	6
.........CATTCAACGCTGTCGGTGAG..	6
..........ATTCAACGCTGTCGGTGAGTT..	6
............TCAACGCTGTCGGTGAGTT..	6
....TCGACCGTTGATTGTACC.....	6
.......AACATTCAACGCTGTCG...	5
........ACATTCAACGCTGTCGGT...	5
............TCAACGCTGTCGGTGAG..	5
.......AACATTCAACGCTGTCGGTGAGTTTGGAATT.....................................	5
....AAACCATCGACCGTTGATTGTA.......	5
................................GGAATTAAAAATCAAAACCATCGACCGTTGATTGTACC.....	5
....CAAAACCATCGACCGTTGATTGTACC.....	5
....ATCGACCGTTGATTGTACC.....	5
....ACCATCGACCGTTGATTGTACCC....	5
....ACCATCGACCGTTGATTG.........	4
....AAATCAAAACCATCGACCGTTGATTGTACC.....	4
..............AACGCTGTCGGTGAGTT..	3
....AAACCATCGACCGTTGATTGTAC......	3
....ATCAAAACCATCGACCGTTGATTGTACC.....	3
....AAACCATCGACCGTTGATTGTACC.....	3
.........CATTCAACGCTGTCGGTGAGTT..	2
...........TTCAACGCTGTCGGTGAGTT..	2
.........CATTCAACGCTGTCGGTGAGTTT...	2
..........ATTCAACGCTGTCGGTGAGTTT...	2

```
...............ACGCTGTCGGTGAGTTT...........................................     2
.......AACATTCAACGCTGTCGGTGAGTTTGGA........................................     2
........ACATTCAACGCTGTCGGTGAGTTTGGAA.......................................     2
...................................................ATCGACCGTTGATTGTAC......     2
...................................ATTAAAAATCAAAACCATCGACCGTTGATTGTACC.....     2
.....................................TAAAAATCAAAACCATCGACCGTTGATTGTACC.....     2
......................................................GACCGTTGATTGTACC.....     2
...........TTCAACGCTGTCGGTGAGTTT...........................................     1
.............CAACGCTGTCGGTGAGTTT...........................................     1
........ACATTCAACGCTGTCGGTGAGTTTG..........................................     1
.......AACATTCAACGCTGTCGGTGAGTTTGGAATTA....................................     1
.......AACATTCAACGCTGTCGGTGAGTTTGGAATTAAA..................................     1
................................................ACCATCGACCGTTGAT...........     1
...............................................AACCATCGACCGTTGATTG.........     1
.............................................AAAACCATCGACCGTTGATTGT........     1
.................................................CCATCGACCGTTGATTGTA.......     1
......................................AAAAATCAAAACCATCGACCGTTGATTGTAC......     1
.................................................CCATCGACCGTTGATTGTAC......     1
...........................................TCAAAACCATCGACCGTTGATTGTACC.....     1
.............................................AAAACCATCGACCGTTGATTGTACC.....     1
..................................................CATCGACCGTTGATTGTACC.....     1
...((.(.(((.((((((..(((((((.((((.((........)).))))))))))))))))).))).).))... (-22.80)

17/46090328-46090403(-)_mir_cow_73
TCCCTCTCCTTGAGTATTGTCAGAGAGAGCGATTCTGTGCCCACCCTGAGTCGTCTCTCCGCCTGTACTCAGCCAG
.........TTGAGTATTGTCAGAGAGAGCGA............................................    37
.........TTGAGTATTGTCAGAGAGAGCG.............................................    15
..........TGAGTATTGTCAGAGAGAGCGA............................................    11
.........TTGAGTATTGTCAGAGAGAGC..............................................    10
..........TGAGTATTGTCAGAGAGAGCGAT...........................................    10
.........TTGAGTATTGTCAGAGAGAGCGAT...........................................    8
........CTTGAGTATTGTCAGAGAGAGC..............................................    4
.........TTGAGTATTGTCAGAGAGA................................................    3
.........TTGAGTATTGTCAGAGAGAG...............................................    2
......TCCTTGAGTATTGTCAGAGAGAGC..............................................    2
..........TGAGTATTGTCAGAGAGAGCGATT..........................................    2
........CTTGAGTATTGTCAGAGAGAGCGA............................................    1
.....................AGAGAGAGCGATTCTGTG.....................................    1
```

```
...................................................CGTCTCTCCGCCTGTACTC......    1
...................................................CGTCTCTCCGCCTGTACTCAG....    1
.........((((((((.(.(.((((((.((((((.((....))...)))))))))))))).).).))))))))....  (-25.10)

1/75441257-75441323(+)_novel_cow_1
GCGCAGTCCGGTCCCGCGGTGTCTCCGCCTGTTGATGCCGCGCGGCCCGCGGGCTCGGATGCTCGGC
.........................................GCGGCCCGCGGGCTCGGATGCT....  2177
...CAGTCCGGTCCCGCGGTGTCTCC.........................................  527
...CAGTCCGGTCCCGCGGTGTCTCCG........................................  398
...CAGTCCGGTCCCGCGGTGTCTC..........................................  273
...CAGTCCGGTCCCGCGGTGTCT...........................................  261
...CAGTCCGGTCCCGCGGTGTCTCCGC.......................................  230
.........................................GCGGCCCGCGGGCTCGGATGC.....  30
...CAGTCCGGTCCCGCGGTGT.............................................  18
...CAGTCCGGTCCCGCGGTGTC............................................  18
..........................................CGGCCCGCGGGCTCGGATGCT....  13
.........................................GCGGCCCGCGGGCTCGGAT.......  11
...CAGTCCGGTCCCGCGGTG..............................................  2
.........................................GCGGCCCGCGGGCTCGGATG......  2
...CAGTCCGGTCCCGCGG................................................  1
...CAGTCCGGTCCCGCGGT...............................................  1
....AGTCCGGTCCCGCGGTGTCTC..........................................  1
....AGTCCGGTCCCGCGGTGTCTCC.........................................  1
...CAGTCCGGTCCCGCGGTGTCTCCGCCT.....................................  1
(.(((.((((..(((((((.(((..(((..((....)).))).))))))))))..))))))).)...  (-34.10)

17/66961896-66961971(-)_mir_cow_74
CTGCACTGTGCTCACGTGTGTGCCTGCCGAGTGTTGATTCAGTGATCGGTGGCGCCAGCTCTGAGCAGAGTGTTGG
................................................GTGGCGCCAGCTCTGAGCAGAGT.....    165
................................................GTGGCGCCAGCTCTGAGCAGAG......    141
................................................GTGGCGCCAGCTCTGAGCAGA.......    113
................................................GTGGCGCCAGCTCTGAGCAG........    35
......TGTGCTCACGTGTGTGCCTGCCG...............................................    22
................................................GTGGCGCCAGCTCTGAGCAGAGTG....    5
.................................................TGGCGCCAGCTCTGAGCAGAG......    4
.......GTGCTCACGTGTGTGCCTGCCG...............................................    3
.................................................TGGCGCCAGCTCTGAGCAGAGT.....    3
.................................................TGGCGCCAGCTCTGAGCAGA.......    2
```

```
.......GTGCTCACGTGTGTGCCT...................................................    1
......TGTGCTCACGTGTGTGCCTGC.................................................    1
.......GTGCTCACGTGTGTGCCTGC.................................................    1
......TGTGCTCACGTGTGTGCCTGCC................................................    1
......TGTGCTCACGTGTGTGCCTGCCGA..............................................    1
................................................GTGGCGCCAGCTCTGAGC..........    1
................................................GTGGCGCCAGCTCTGAGCA.........    1
.................................................TGGCGCCAGCTCTGAGCAG........    1
...............................................GGTGGCGCCAGCTCTGAGCAGA.......    1
(.(((((.((((((.(.(((((((.(((((.(..((...))..).))))))))))..)).))))))).))))).). (-33.10)

17/67326533-67326616(+)_novel_cow_15
TTTGGTCAAGAATTTTGCCCGGATTTTTCTGTAACATCGTACAGAACATTGTACAGAAAAACCCAAGCAAACTTTTTGGCCAAC
.............TTTGCCCGGATTTTTCTGTAAC.................................................  7
.............TTTGCCCGGATTTTTCTGTAA..................................................  3
.............TTTGCCCGGATTTTTCTGTAACA................................................  3
...................................................TACAGAAAAACCCAAGCAAACT...........  1
.(((((((((((.(((((..((.((((((((((.((..((.....))..)))))))))))).))..))))).))))))))))). (-32.60)

17/74100544-74100614(-)_mir_cow_75
CCTGACACTCTTTCCCTGTTGCACTACTGTGCGCCCCTGGCAAGCAGTGCAATGATGAAAGGGCATCGGTC
......ACTCTTTCCCTGTTGCACTACT...........................................   326
............................................CAGTGCAATGATGAAAGGGCAT.....   325
............................................CAGTGCAATGATGAAAGGGCA......   238
............................................CAGTGCAATGATGAAAGGGC.......   102
......ACTCTTTCCCTGTTGCACTAC............................................   31
......ACTCTTTCCCTGTTGCACTACTG..........................................   12
......ACTCTTTCCCTGTTGCACTA.............................................   9
......ACTCTTTCCCTGTTGCACT..............................................   8
.............................................AGTGCAATGATGAAAGGGCAT.....   8
.............................................AGTGCAATGATGAAAGGGCA......   6
............................................CAGTGCAATGATGAAAGGG........   5
............................................CAGTGCAATGATGAAAGG.........   3
............................................CAGTGCAATGATGAAAGGGCATC....   2
.........CTTTCCCTGTTGCACTACT...........................................   1
.............................................AGTGCAATGATGAAAGG.........   1
.............................................AGTGCAATGATGAAAGGGC.......   1
...............................................TGCAATGATGAAAGGGCA......   1
```

```
.((((..(((((((..(((((((((.((.(((.......))))).)))))))))..)))))))..)))).. (-26.40)

17/74967073-74967136(+)_mir_cow_76
AGGGACTGGAGAGAAAGGCAGTTCCTGATGGTCCCCTCCCCAGGGGCTGGCTTTCCTCCGGCCC
......TGGAGAGAAAGGCAGTTCCTGA....................................    1870
......TGGAGAGAAAGGCAGTTCCTG.....................................    516
......TGGAGAGAAAGGCAGTTCCT......................................    399
......TGGAGAGAAAGGCAGTTCCTGAT...................................    121
.........................................AGGGGCTGGCTTTCCTCCGGC..    90
........................................CAGGGGCTGGCTTTCCTCCGGC..    39
......TGGAGAGAAAGGCAGTTCC.......................................    38
....ACTGGAGAGAAAGGCAGTTCCTG.....................................    13
.........................................AGGGGCTGGCTTTCCTCCGG...    6
......TGGAGAGAAAGGCAGTTC........................................    3
....ACTGGAGAGAAAGGCAGTTCCT......................................    3
......TGGAGAGAAAGGCAGT..........................................    2
.......GGAGAGAAAGGCAGTTCCTG.....................................    2
.........................................AGGGGCTGGCTTTCCTCC.....    2
.....CTGGAGAGAAAGGCAGTTCCT......................................    1
........GAGAGAAAGGCAGTTCCTGA....................................    1
......TGGAGAGAAAGGCAGTTCCTGATG..................................    1
......TGGAGAGAAAGGCAGTTCCTGATGG.................................    1
........................................CAGGGGCTGGCTTTCCTCC.....    1
.(((.((((((.(((((.(((((((((..((......)).))))))))).)))))))))))))) (-39.00)

1/79250536-79250611(-)_mir_cow_7
TTGCCCTCAAGGAGCTCACAGTCTATTGAGTTGCCTTTCTGACTTTCCCACTAGATTGAGAGCTCCTGGAGGGCAG
................................................CACTAGATTGAGAGCTCCTGGA......    17022
........AAGGAGCTCACAGTCTATTGAG..............................................    4019
................................................CACTAGATTGAGAGCTCCTGG.......    3986
........AAGGAGCTCACAGTCTATTG................................................    2982
........AAGGAGCTCACAGTCTATTGA...............................................    2248
.................................................ACTAGATTGAGAGCTCCTGGAG.....    735
................................................CACTAGATTGAGAGCTCCTGGAG.....    448
........AAGGAGCTCACAGTCTATTGAGT.............................................    394
.................................................ACTAGATTGAGAGCTCCTGGA......    120
........AAGGAGCTCACAGTCTATT.................................................    112
................................................CACTAGATTGAGAGCTCCT.........    61
```

```
................................................CACTAGATTGAGAGCTCCTG........    54
................................................CACTAGATTGAGAGCTCC..........    49
.......CAAGGAGCTCACAGTCTATTG................................................    38
..................................................CTAGATTGAGAGCTCCTGGAG.....    36
...............................................CCACTAGATTGAGAGCTCCTGG.......    34
........AAGGAGCTCACAGTCTATTGAGTT............................................    23
........AAGGAGCTCACAGTCTAT..................................................    22
...............................................CCACTAGATTGAGAGCTCCTGGA......    21
.................................................ACTAGATTGAGAGCTCCTGG.......    16
.......CAAGGAGCTCACAGTCTATTGA...............................................    12
................................................CACTAGATTGAGAGCT............    11
.......CAAGGAGCTCACAGTCTATTGAG..............................................    5
.................................................ACTAGATTGAGAGCTCC..........    4
................................................CACTAGATTGAGAGCTCCTGGAGG....    4
........AAGGAGCTCACAGTCTA...................................................    3
...............................................CCACTAGATTGAGAGCTCCTG........    3
....................................................AGATTGAGAGCTCCTGGA......    3
......TCAAGGAGCTCACAGTCT....................................................    2
........AAGGAGCTCACAGTCT....................................................    2
.......CAAGGAGCTCACAGTCTATT.................................................    2
.......CAAGGAGCTCACAGTCTATTGAGT.............................................    2
................................................CACTAGATTGAGAGCTC...........    2
..................................................CTAGATTGAGAGCTCCTGGA......    2
...........GAGCTCACAGTCTATTG................................................    1
...............................................CCACTAGATTGAGAGCTCCT.........    1
.................................................ACTAGATTGAGAGCTCCTGGAGG....    1
.(((((((.((((((((.(((((((.((((((........))))....)).))))))).)))))))).))))))). (-40.30)

1/80871206-80871272(-)_mir_cow_8
ACCTAAAAGTTCATTTGGGTTTTTCTATAACATCTTGCAGAAAAACCGGAATGAACTTTTTGGCCTA
.........................................AAAACCGGAATGAACTTTTTGG....  337
.........................................AAAACCGGAATGAACTTTTTG.....  34
.........................................AAAACCGGAATGAACTTTTT......  16
.........................................AAAACCGGAATGAACTTTT.......  10
.........................................AAAACCGGAATGAACTTTTTGGC...  8
..........................................AAACCGGAATGAACTTTTTGG....  4
.........................................AAAACCGGAATGAACTT.........  3
........................................AAAAACCGGAATGAACTTTTTG.....  3
```

```
........................................AAAAACCGGAATGAACTTTTT......  2
...TAAAAGTTCATTTGGGTTTTT...........................................  1
....AAAAGTTCATTTGGGTTTTTC..........................................  1
...TAAAAGTTCATTTGGGTTTTTCT.........................................  1
........................................AAAAACCGGAATGAACT..........  1
.........................................AAAACCGGAATGAACTTT........  1
...............................................GGAATGAACTTTTTGG....  1
.((.((((((((((((.(((((((((.(((....))).))))))))).)))))))))))).))....  (-28.20)

18/24992699-24992776(+)_mir_cow_78
TGCTGCAGCTGGTGTTGTGAATCAGGCCGACGAGCAGCGCATCCTCTTACCCGGCTATTTCACGACACCAGGGTTGCA
......AGCTGGTGTTGTGAATCAGGCCG.................................................  25155
......AGCTGGTGTTGTGAATCAGGC...................................................  3433
......AGCTGGTGTTGTGAATCAG.....................................................  921
......AGCTGGTGTTGTGAATCAGGCC..................................................  419
......AGCTGGTGTTGTGAATCAGG....................................................  240
.....................................................GCTATTTCACGACACCAGGGT....  150
......AGCTGGTGTTGTGAATC.......................................................  116
......AGCTGGTGTTGTGAATCAGGCCGA................................................  77
.....................................................GCTATTTCACGACACCAGGGTT...  74
.....CAGCTGGTGTTGTGAATCAGGCCG.................................................  69
......AGCTGGTGTTGTGAATCA......................................................  43
...........GTGTTGTGAATCAGGCCG.................................................  38
.............................ACGAGCAGCGCATCCTCTTACCCG.........................  19
......AGCTGGTGTTGTGAAT........................................................  18
.....CAGCTGGTGTTGTGAATCAGGC...................................................  14
.......GCTGGTGTTGTGAATCAGGCCG.................................................  14
............TGTTGTGAATCAGGCCG.................................................  12
..........GGTGTTGTGAATCAGGCCG.................................................  10
......................................................CTATTTCACGACACCAGGGTT...  9
.....................................................GCTATTTCACGACACCAGGGTTG..  8
...........GTGTTGTGAATCAGGC...................................................  6
...........GTGTTGTGAATCAGGCC..................................................  6
.............................ACGAGCAGCGCATCCTCTTACCC..........................  6
.............GTTGTGAATCAGGCCG.................................................  5
..........GGTGTTGTGAATCAGGC...................................................  3
.............................ACGAGCAGCGCATCCTCTTACC...........................  3
..................................................CCGGCTATTTCACGACACCAGGGT....  3
```

```
............TGTTGTGAATCAGGCC..................................................  2
.........TGGTGTTGTGAATCAGGCCG.................................................  2
......AGCTGGTGTTGTGAATCAGGCCGAC...............................................  2
....GCAGCTGGTGTTGTGAATCAGGCCG.................................................  1
......AGCTGGTGTTGTGAATCAGGCCGACG..............................................  1
.....................................................GCTATTTCACGACACCAGG......  1
.................................................CCCGGCTATTTCACGACACCAGGG.....  1
.....................................................GCTATTTCACGACACCAGGG.....  1
.......................................................TATTTCACGACACCAGGGT....  1
........................................................ATTTCACGACACCAGGGTT...  1
.((.((..(((((((((((((...(((((..(((.((......)))))...)))))..))))))))))))).)).)). (-34.60)

1/83546855-83546955(-)_mir_Cow_Blast_25
TGCCCTTGGTGAGGACTCGGGAGGTGGAGGGTGGCGCTGCCAGGGCCAGGGCACTGTCTCAGCTCGCTTCTCCCCCCACCTCCTCTCTCCTCAGGTGCGCT
........GTGAGGACTCGGGAGGTGGAGGG......................................................................  1134
........GTGAGGACTCGGGAGGTGGAG........................................................................  1112
........GTGAGGACTCGGGAGGTGGAGG.......................................................................  822
........GTGAGGACTCGGGAGGTGGA.........................................................................  792
........GTGAGGACTCGGGAGGTGGAGGGT.....................................................................  764
........GTGAGGACTCGGGAGGTGG..........................................................................  395
........GTGAGGACTCGGGAGG.............................................................................  42
........GTGAGGACTCGGGAGGTG...........................................................................  22
.........................................................................CCCCACCTCCTCTCTCCTCAG.......  15
........GTGAGGACTCGGGAGGTGGAGGGTGGC..................................................................  7
.........................................................................CCCCACCTCCTCTCTCCTCA........  5
........GTGAGGACTCGGGAGGT............................................................................  4
........GTGAGGACTCGGGAGGTGGAGGGTGGCGCT...............................................................  2
.........................................................................CCCCACCTCCTCTCTCCT..........  2
.........TGAGGACTCGGGAGGTGGAGGG......................................................................  1
.........TGAGGACTCGGGAGGTGGAGGGT.....................................................................  1
........GTGAGGACTCGGGAGGTGGAGGGTG....................................................................  1
........GTGAGGACTCGGGAGGTGGAGGGTGGCGC................................................................  1
........GTGAGGACTCGGGAGGTGGAGGGTGGCGCTGC.............................................................  1
.........................................................................CCCCACCTCCTCTCTCCTC.........  1
.........................................................................CCCCACCTCCTCTCTCCTCAGG......  1
..........................................................................CCCACCTCCTCTCTCCTCAGG......  1
.(((((....(((((...(((((((((.(((.(((((((.(((.((....)).)))...))))..)))...))).)))))))))...)))))))).))... (-49.90)
```

18/37088146-37088221(+)_mir_cow_79

```
GTCCTGCCAGTGGTTTTACCCTATGGTAGGTTACGTCATGCTGTTCTACCACAGGGTAGAACCACGGACAGGATAC
...............................................ACCACAGGGTAGAACCACGGAC.......    27388
..............................................TACCACAGGGTAGAACCACGGA........    7686
..............................................TACCACAGGGTAGAACCACGGAC.......    7018
...............................................ACCACAGGGTAGAACCACGGACA......    5371
...............................................ACCACAGGGTAGAACCACGGA........    4365
.......CAGTGGTTTTACCCTATGGTAG...............................................    2304
..............................................TACCACAGGGTAGAACCACGGACA......    1453
..............................................TACCACAGGGTAGAACCACGG.........    1378
..............................................TACCACAGGGTAGAACCACG..........    1013
................................................CCACAGGGTAGAACCACGGAC.......    572
..................................................ACAGGGTAGAACCACGGACAGGA...    527
...............................................ACCACAGGGTAGAACCACG..........    480
...............................................ACCACAGGGTAGAACCACGG.........    385
.......CAGTGGTTTTACCCTATGGT.................................................    295
...............................................ACCACAGGGTAGAACC.............    294
................................................CCACAGGGTAGAACCACGGACA......    242
...............................................ACCACAGGGTAGAACCACGGACAG.....    242
.......CAGTGGTTTTACCCTATGGTA................................................    211
..................................................ACAGGGTAGAACCACGGACAGG....    197
................................................CCACAGGGTAGAACCACGGA........    133
..................................................ACAGGGTAGAACCACGGACAG.....    118
..............................................TACCACAGGGTAGAACC.............    114
...............................................ACCACAGGGTAGAACCA............    88
.......CAGTGGTTTTACCCTATGG..................................................    62
..............................................TACCACAGGGTAGAACCAC...........    59
...............................................ACCACAGGGTAGAACCAC...........    47
..................................................ACAGGGTAGAACCACGGAC.......    46
.......CAGTGGTTTTACCCTATGGTAGG..............................................    37
..................................................ACAGGGTAGAACCACGGA........    36
..............................................TACCACAGGGTAGAACCA............    27
.................................................CACAGGGTAGAACCACGGACA......    26
.................................................CACAGGGTAGAACCACGGAC.......    16
................................................CCACAGGGTAGAACCACG..........    15
..............................................TACCACAGGGTAGAACCACGGACAG.....    15
..................................................ACAGGGTAGAACCACGGACAGGAT..    15
................................................CCACAGGGTAGAACCACGGACAG.....    12
```

```
..................................................ACAGGGTAGAACCACGGACA......    11
...................................................CAGGGTAGAACCACGGACAGGA...    10
.......CAGTGGTTTTACCCTATG...................................................    8
.......CAGTGGTTTTACCCTATGGTAGGT.............................................    7
..................................................ACAGGGTAGAACCACGG.........    7
.................................................CACAGGGTAGAACCACGGA........    7
..................................................ACAGGGTAGAACCACG..........    6
.....................................................GGGTAGAACCACGGAC.......    6
.......CAGTGGTTTTACCCTA.....................................................    4
.............................................CTACCACAGGGTAGAACCACGGA........    4
...................................................CAGGGTAGAACCACGGAC.......    4
.............................................CTACCACAGGGTAGAACCACGGACA......    3
.....................................................GGGTAGAACCACGGACA......    3
.................................................CACAGGGTAGAACCACGGACAG.....    3
.................................................CACAGGGTAGAACCACGGACAGG....    3
.................................................CACAGGGTAGAACCACGGACAGGA...    3
......CCAGTGGTTTTACCCTATGGTA................................................    2
................................................CCACAGGGTAGAACCA............    2
................................................CCACAGGGTAGAACCACGG.........    2
...................................................CAGGGTAGAACCACGG.........    2
....TGCCAGTGGTTTTACCCTATGG..................................................    1
........AGTGGTTTTACCCTATGGTAG...............................................    1
........AGTGGTTTTACCCTATGGTAGG..............................................    1
.......CAGTGGTTTTACCCTATGGTAGGTTACGTCATGCTGTTCT.............................    1
................................................CCACAGGGTAGAACCAC...........    1
.............................GTTACGTCATGCTGTTCTACCACAGGGTAGAACCACGGAC.......    1
.........................................TGTTCTACCACAGGGTAGAACCACGGAC.......    1
....................................................AGGGTAGAACCACGGAC.......    1
....................................................AGGGTAGAACCACGGACA......    1
...............................................ACCACAGGGTAGAACCACGGACAGG....    1
...................................................CAGGGTAGAACCACGGACAGG....    1
.................................................CACAGGGTAGAACCACGGACAGGAT..    1
((((((((.(((((((((((((.(((((((..(((......)))))))))).)))))))))))))).)))))))).. (-43.30)

18/53654155-53654229(-)_mir_cow_80
CACTGCCTCTCTGGGCCTGTGTCTTAGGCTCTGCAAGATCAACCGAGCAAAGCACACGGCCTGCAGAGAGGCAGC
.......TCTCTGGGCCTGTGTCTTAGGCT..............................................    1877
.......TCTCTGGGCCTGTGTCTTAGGCTC.............................................    257
```

....CAAAGCACACGGCCTGCAGAG.......	194
....CAAAGCACACGGCCTGCAGA........	177
....CAAAGCACACGGCCTGCAGAGA......	150
....GCAAAGCACACGGCCTGCAGAGA......	96
....GCAAAGCACACGGCCTGCAGA........	71
....CAAAGCACACGGCCTGCAGAGAGG....	71
.......TCTCTGGGCCTGTGTCTTAGGC..	66
....AAAGCACACGGCCTGCAGAGAGG....	38
....CAAAGCACACGGCCTGCAGAGAG.....	35
.......TCTCTGGGCCTGTGTCTTAGG...	34
....GCAAAGCACACGGCCTGCAGAG.......	34
....AAAGCACACGGCCTGCAGAGA......	16
.......TCTCTGGGCCTGTGTCTTAGGCTCT...	14
....AAAGCACACGGCCTGCAGAG.......	14
.......TCTCTGGGCCTGTGTCTTAG..	12
.......TCTCTGGGCCTGTGTCTTA...	8
....GCAAAGCACACGGCCTGCAGAGAGG....	8
.......TCTCTGGGCCTGTGTCTT..	7
....CAAAGCACACGGCCTGCAG.........	6
....AAAGCACACGGCCTGCAGA........	6
....GCAAAGCACACGGCCTGCAGAGAG.....	6
....GCAAAGCACACGGCCTGC...........	4
.........TCTGGGCCTGTGTCTTAGGCT...	3
....GCAAAGCACACGGCCTGCA..........	3
....ACACGGCCTGCAGAGA......	3
....AAAGCACACGGCCTGCAGAGAG.....	3
.........TCTGGGCCTGTGTCTTAGGC..	2
....GCAAAGCACACGGCCTG............	2
....CAAAGCACACGGCCTG............	2
....CAAAGCACACGGCCTGC...........	2
....AAAGCACACGGCCTGC...........	2
.......TCTCTGGGCCTGTGTCT...	1
........CTCTGGGCCTGTGTCTTAGGCT...	1
......CTCTCTGGGCCTGTGTCTTAGGCTC..	1
....CAAAGCACACGGCCTGCA..........	1
....GCAAAGCACACGGCCTGCAG.........	1
....AGCAAAGCACACGGCCTGCAGAGA......	1
....ACACGGCCTGCAGAGAG.....	1

```
.......................................................ACGGCCTGCAGAGAGG....     1
..(((((((((((((((((((.(((..((((.............)))).))))))).))))..))))))))))). (-39.62)

18/53945901-53945968(+)_mir_cow_81
TGACCTGAGACCTCCGGGTTCTGAGCTGTGATGTTGCTTCCCAGCTGGGATCTCTGGGGTCTCGGTTC
.....TGAGACCTCCGGGTTCTGAGCT......................................... 13314
.....TGAGACCTCCGGGTTCTGAGC.......................................... 626
......GAGACCTCCGGGTTCTGAGCT......................................... 284
....CTGAGACCTCCGGGTTCTGAGCT......................................... 67
.....TGAGACCTCCGGGTTCTGAGCTGT....................................... 27
.....TGAGACCTCCGGGTTCTGAGCTG........................................ 25
......GAGACCTCCGGGTTCTGAGCTGT....................................... 18
......GAGACCTCCGGGTTCTGAGCTG........................................ 11
............................................CTGGGATCTCTGGGGTCTCGGT.. 10
...........................................GCTGGGATCTCTGGGGTCTCGG... 8
....CTGAGACCTCCGGGTTCTGAGC........................................... 7
......GAGACCTCCGGGTTCTGAGC........................................... 4
.....TGAGACCTCCGGGTTCTGAG............................................ 3
....CTGAGACCTCCGGGTTCTGAG............................................ 2
..........................................AGCTGGGATCTCTGGGGTCTCGG... 2
............................................CTGGGATCTCTGGGGTCTCGG... 2
....CTGAGACCTCCGGGTTCTGA............................................. 1
.....TGAGACCTCCGGGTTCTGA............................................. 1
.......AGACCTCCGGGTTCTGAGCT......................................... 1
..........CCTCCGGGTTCTGAGCT......................................... 1
...........CTCCGGGTTCTGAGCT......................................... 1
.........ACCTCCGGGTTCTGAGCTGT....................................... 1
........................................CCAGCTGGGATCTCTGGGGTCT...... 1
.........................................CAGCTGGGATCTCTGGGGTCT...... 1
............................................CTGGGATCTCTGGGGTCTCG.... 1
...........................................GCTGGGATCTCTGGGGTCTCGGT.. 1
.((((.((((((((.(((.(((.(((((.((.......)).))))).))).))).)))))))))))). (-35.10)

18/5619316-5619382(+)_mir_cow_77
AGCCACAAAGTTTGTTCGGGTTTTACTGTAGTAGCTCATGGAAAAACCCAAATGAACTTTTTGGGCC
....ACAAAGTTTGTTCGGGTTTTA..........................................  12
....ACAAAGTTTGTTCGGGTTTT...........................................  5
....ACAAAGTTTGTTCGGGTTT............................................  1
```

...CACAAAGTTTGTTCGGGTTTT... 1
....ACAAAGTTTGTTCGGGTTTTAC....................................... 1
....AAAAACCCAAATGAACTTTTTG.... 1
.(((..((((((..((.(((((((.((((......)))).))))))).))..))))))...))). (-23.40)

18/58014868-58014936(+)_mir_cow_82
GGCACCCACCCGTAGAACCGACCTTGCGGGGCCTTCGCCGCACACAAGCTCGTGTCTGTGGGTCCGTGT
......CACCCGTAGAACCGACCTTGCG... 51559
......CACCCGTAGAACCGACCTTGC.. 42218
......CACCCGTAGAACCGACCTTG... 4511
......CACCCGTAGAACCGACCTT.. 1296
......CACCCGTAGAACCGACCT... 743
.......ACCCGTAGAACCGACCTTGCG... 344
.......ACCCGTAGAACCGACCTTGC.. 180
..........CGTAGAACCGACCTTGCG... 158
.........CCGTAGAACCGACCTTGCG... 157
....CAAGCTCGTGTCTGTGGGTCCGT.. 108
....CAAGCTCGTGTCTGTGGGTCCG... 92
....CAAGCTCGTGTCTGTGGGTCC.... 75
..........CGTAGAACCGACCTTGC.. 57
......CACCCGTAGAACCGACCTTGCGG.. 54
........CCCGTAGAACCGACCTTGCG... 49
....CAAGCTCGTGTCTGTGGGTC..... 48
.........CCGTAGAACCGACCTTGC.. 37
........CCCGTAGAACCGACCTTGC.. 32
.....CCACCCGTAGAACCGACCTTGC.. 31
......CACCCGTAGAACCGACC.. 25
.....CCACCCGTAGAACCGACCTTGCG... 25
.......ACCCGTAGAACCGACCTTG... 23
.......ACCCGTAGAACCGACCTT.. 13
....CAAGCTCGTGTCTGTGGGT...... 12
.........CCGTAGAACCGACCTTG... 8
..........CGTAGAACCGACCTTG... 7
......CACCCGTAGAACCGAC... 5
........CCCGTAGAACCGACCTTG... 5
.....CCACCCGTAGAACCGACCTT.. 4
........CCCGTAGAACCGACCTT.. 4
...........GTAGAACCGACCTTGC.. 3

```
...........GTAGAACCGACCTTGCG.........................................      3
......CACCCGTAGAACCGACCTTGCGGGGCCTTCGCCGCACA.........................      3
...........................................ACAAGCTCGTGTCTGTGGGTCC....      2
.............................................AAGCTCGTGTCTGTGGGTCCGT..      2
.....CCACCCGTAGAACCGACC..............................................      1
....CCCACCCGTAGAACCGACCTTGC..........................................      1
............TAGAACCGACCTTGCG.........................................      1
..........CGTAGAACCGACCTTGCGG........................................      1
......CACCCGTAGAACCGACCTTGCGGGGCCTTCG................................      1
............................................CAAGCTCGTGTCTGTGGG.......      1
.((((..(((((((((..(((.((((.(((((....))).).).)))).)))..)))))))))..)))) (-28.40)

18/58015036-58015112(+)_mir_cow_83
CCCGGGCTGAGGTAGGAGGTTGTATAGTTGAGGAGGACACCCAAGGAGATCACTATACGGCCTCCTAGCTTTCCCCA
.......TGAGGTAGGAGGTTGTATAGTT................................................   33022
.......TGAGGTAGGAGGTTGTATAGT.................................................   16078
.......TGAGGTAGGAGGTTGTATAG..................................................   1864
.......TGAGGTAGGAGGTTGTATAGTTG...............................................   1163
.......TGAGGTAGGAGGTTGTAT....................................................   533
....................................................CTATACGGCCTCCTAGCTTTCC...   487
.......TGAGGTAGGAGGTTGTATA...................................................   279
.......TGAGGTAGGAGGTTGTATAGTTGA..............................................   259
....................................................CTATACGGCCTCCTAGCTTTC....   77
.......TGAGGTAGGAGGTTGTA.....................................................   50
.....................................................TATACGGCCTCCTAGCTTTCC...   25
.......TGAGGTAGGAGGTTGT......................................................   20
............TAGGAGGTTGTATAGTT................................................   18
....................................................CTATACGGCCTCCTAGCTTT.....   14
......CTGAGGTAGGAGGTTGTATAGT.................................................   9
............TAGGAGGTTGTATAGT.................................................   8
......CTGAGGTAGGAGGTTGTATAGTT................................................   8
.............AGGAGGTTGTATAGTT................................................   8
..........GGTAGGAGGTTGTATAGTT................................................   7
........GAGGTAGGAGGTTGTATAGTT................................................   6
........GAGGTAGGAGGTTGTATAGTTGA..............................................   6
....................................................CTATACGGCCTCCTAGCT.......   6
....................................................CTATACGGCCTCCTAGCTT......   6
...................................................ACTATACGGCCTCCTAGCTTTC....   6
```

```
...........GTAGGAGGTTGTATAGTT................................................   5
........GAGGTAGGAGGTTGTATAGT.................................................   4
.......TGAGGTAGGAGGTTGTATAGTTGAG.............................................   4
.....................................................TATACGGCCTCCTAGCTTTC....   4
......CTGAGGTAGGAGGTTGTATAG..................................................   3
.........AGGTAGGAGGTTGTATAGTT................................................   3
.............AGGAGGTTGTATAGTTGA..............................................   2
..................................................CACTATACGGCCTCCTAGCT.......   2
..................................................CACTATACGGCCTCCTAGCTTT.....   2
........GAGGTAGGAGGTTGTAT....................................................   1
.........AGGTAGGAGGTTGTATA...................................................   1
....GGCTGAGGTAGGAGGTTGTATAGT.................................................   1
..........GGTAGGAGGTTGTATAGT.................................................   1
.........AGGTAGGAGGTTGTATAGTTG...............................................   1
.............AGGAGGTTGTATAGTTG...............................................   1
..............GGAGGTTGTATAGTTG...............................................   1
.......TGAGGTAGGAGGTTGTATAGTTGAGGAG..........................................   1
............TAGGAGGTTGTATAGTTGAGGAG..........................................   1
.......TGAGGTAGGAGGTTGTATAGTTGAGGAGGAC.......................................   1
.............................GAGGAGGACACCCAAGGAGATCACTATACGGCCTCCTAG.........   1
..................................................CACTATACGGCCTCCTAGCTT......   1
.....................................................TATACGGCCTCCTAGCTT......   1
......................................................ATACGGCCTCCTAGCTTTCC...   1
....................................................CTATACGGCCTCCTAGCTTTCCC..   1
...(((..(((.((((((((((((((((.((...((....)).......)))))))))))))))))).)))..))). (-36.90)

18/58015545-58015611(+)_mir_Cow_Blast_54
TGCGTCCCTGAGACCCTTTAACCTGTGAGGACGTCCAGGGTCACAGGTGAGGTTCTTGGGAGCCTGG
....TCCCTGAGACCCTTTAACCTGT.........................................  144477
....TCCCTGAGACCCTTTAACCTGTGA.......................................  78497
....TCCCTGAGACCCTTTAACCTGTG........................................  58222
....TCCCTGAGACCCTTTAACCTG..........................................  26797
....TCCCTGAGACCCTTTAACCT...........................................  7417
.....CCCTGAGACCCTTTAACCTGTGA.......................................  868
....TCCCTGAGACCCTTTAACC............................................  468
..........................................ACAGGTGAGGTTCTTGGGAGC....  147
.....CCCTGAGACCCTTTAACCTGT.........................................  129
.....CCCTGAGACCCTTTAACCTGTG........................................  111
```

....ACAGGTGAGGTTCTTGGGAGCC... 109
........TGAGACCCTTTAACCTGT... 108
......CCTGAGACCCTTTAACCTGT... 103
......CCTGAGACCCTTTAACCTGTGA....................................... 100
......CCTGAGACCCTTTAACCTGTG.. 58
....TCCCTGAGACCCTTTAAC... 41
....ACAGGTGAGGTTCTTGGGAG..... 39
....ACAGGTGAGGTTCTTGGGAGCCT.. 37
........TGAGACCCTTTAACCTG.. 27
........TGAGACCCTTTAACCTGTG.. 26
.......CTGAGACCCTTTAACCTGT... 25
....TCCCTGAGACCCTTTAACCTGTGAG...................................... 16
.......CTGAGACCCTTTAACCTGTG.. 14
....CAGGTGAGGTTCTTGGGAGCC... 14
.....CCCTGAGACCCTTTAACCTG.. 11
..........AGACCCTTTAACCTGT... 11
......CCTGAGACCCTTTAACCT... 10
......CCTGAGACCCTTTAACCTG.. 10
.........GAGACCCTTTAACCTG.. 8
.....CCCTGAGACCCTTTAACCT... 7
.......CTGAGACCCTTTAACCTG.. 7
.........GAGACCCTTTAACCTGT... 7
.......CTGAGACCCTTTAACCTGTGA....................................... 7
....CAGGTGAGGTTCTTGGGAGC.... 6
....TCCCTGAGACCCTTTAA.. 4
....ACAGGTGAGGTTCTTGGGA...... 4
....CAGGTGAGGTTCTTGGGAG..... 4
........TGAGACCCTTTAACCTGTGA....................................... 3
....CAGGTGAGGTTCTTGGGAGCCT.. 3
.........GAGACCCTTTAACCTGTG.. 2
..........AGACCCTTTAACCTGTG.. 2
...........GACCCTTTAACCTGTG.. 1
............ACCCTTTAACCTGTGA....................................... 1
....TCCCTGAGACCCTTTAACCTGTGAGGAC................................... 1
.........................TGAGGACGTCCAGGGT.......................... 1
....ACAGGTGAGGTTCTTGGG....... 1
...........................AGGACGTCCAGGGTCACAGGTGAGGTTCTTGGGA...... 1
....CACAGGTGAGGTTCTTGGGAGC.... 1

```
............................................AGGTGAGGTTCTTGGGAGCC...  1
.((.((((.((((.((((..((((((((((....))....))))))))))).)))))))))))....  (-28.80)

19/10339865-10339944(-)_mir_Cow_Blast_12
ATCCTAGAACCCTATCGATATTGTCTCTGCTGTGTAAATAGCTCTGAGTAGTGCAATATTGCTTATAGGGTTTTGGTGTT
................................................TAGTGCAATATTGCTTATAGGGT.........      699
................................................TAGTGCAATATTGCTTATAGGGTT........      234
................................................TAGTGCAATATTGCTTATAGGGTTT.......      91
........ACCCTATCGATATTGTCTCTGC..................................................      24
........ACCCTATCGATATTGTCTCTGCT.................................................      14
................................................TAGTGCAATATTGCTTATAGGGTTTT......      14
................................................TAGTGCAATATTGCTTATAGGG..........      13
.................................................AGTGCAATATTGCTTATAGGGTT........      4
.................................................AGTGCAATATTGCTTATAGGGT.........      3
.................................................AGTGCAATATTGCTTATAGGGTTT.......      3
........ACCCTATCGATATTGTCTCTG...................................................      2
........ACCCTATCGATATTGTCTCTGCTGTGT.............................................      2
................................................TAGTGCAATATTGCTTATAGG...........      1
...(((((((((((((((((((((((.(((((....)))))...)))....)))))))))...))))))))))))....  (-30.90)

19/10349510-10349586(-)_mir_Cow_Blast_20
CTAACGAATGCTCTGACTTTATTGCACTACTGTACTTTACAGCTAGCAGTGCAATAGTATTGTCAAAGCATCTGAAA
..............................................CAGTGCAATAGTATTGTCAAAGC........   28417
..............................................CAGTGCAATAGTATTGTCAAAG.........   841
..............................................CAGTGCAATAGTATTGTCAAAGCA.......   167
...............................................AGTGCAATAGTATTGTCAAAGC........   138
..............................................CAGTGCAATAGTATTGTCAAAGCAT......   118
.........GCTCTGACTTTATTGCACTACT..............................................   63
..............................................CAGTGCAATAGTATTGTC.............   55
..............................................CAGTGCAATAGTATTGTCA............   15
...............................................AGTGCAATAGTATTGTCAAAG.........   8
.........GCTCTGACTTTATTGCACTAC...............................................   7
..............................................CAGTGCAATAGTATTGT..............   7
..............................................CAGTGCAATAGTATTGTCAAA..........   6
...............................................AGTGCAATAGTATTGTCAAAGCA.......   6
...............................................AGTGCAATAGTATTGTCAAAGCAT......   6
.........GCTCTGACTTTATTGCACTACTGTACTT........................................   3
..............................................CAGTGCAATAGTATTGTCAA...........   3
```

```
.................................................GCAATAGTATTGTCAAAGC........   3
.........GCTCTGACTTTATTGCACT................................................   2
..........CTCTGACTTTATTGCACTACT.............................................   2
.............................................CAGTGCAATAGTATTG...............   2
...............................................GTGCAATAGTATTGTCAAAGC........   2
........TGCTCTGACTTTATTGCACT................................................   1
.........GCTCTGACTTTATTGCACTA...............................................   1
.........GCTCTGACTTTATTGCACTACTGTACT........................................   1
.............TGACTTTATTGCACTACTGTACT........................................   1
..........CTCTGACTTTATTGCACTACTGTACTT.......................................   1
..............................................AGTGCAATAGTATTGTCAA...........   1
................................................TGCAATAGTATTGTCAAAGC........   1
..................................................CAATAGTATTGTCAAAGC........   1
...................................................AATAGTATTGTCAAAGC........   1
....................................................ATAGTATTGTCAAAGC........   1
....((.(((((.((((.((((((((((.(((((...)))))....))))))))))....)))).))))).))... (-25.00)

19/11033074-11033145(+)_mir_cow_85
TCGGGTAGCTTATCAGACTGATGTTGACTGTTGAATCTCATGGCAACAGCAGTCGATGGGCTGTCTGACATT
.....TAGCTTATCAGACTGATGTTGA.............................................  103042
.....TAGCTTATCAGACTGATGTTGAC............................................  37137
.....TAGCTTATCAGACTGATGTTGACT...........................................  35308
.....TAGCTTATCAGACTGATGTTG..............................................  31212
.....TAGCTTATCAGACTGATGTT...............................................  4133
.....TAGCTTATCAGACTGATGT................................................  952
...........................................CAACAGCAGTCGATGGGCTGT........  609
.....TAGCTTATCAGACTGATG.................................................  107
.............................................ACAGCAGTCGATGGGCTGTCT......  64
......AGCTTATCAGACTGATGTTGA.............................................  62
...........................................CAACAGCAGTCGATGGGCTGTCT......  59
......AGCTTATCAGACTGATGTTGACT...........................................  37
............................................AACAGCAGTCGATGGGCTGTCT......  36
....GTAGCTTATCAGACTGATGTTGA.............................................  35
.....TAGCTTATCAGACTGA...................................................  29
.....TAGCTTATCAGACTGAT..................................................  24
.......GCTTATCAGACTGATGTTGA.............................................  23
...........................................CAACAGCAGTCGATGGGCTGTC.......  19
....GTAGCTTATCAGACTGATGTTG..............................................  18
```

.........TTATCAGACTGATGTTGA...	15
......AGCTTATCAGACTGATGTTGAC..	15
......AGCTTATCAGACTGATGTTG..	13
..........TATCAGACTGATGTTGA...	10
....GTAGCTTATCAGACTGATGTTGAC..	10
........CTTATCAGACTGATGTTGA...	9
..........TATCAGACTGATGTTGAC..	9
.......GCTTATCAGACTGATGTTGACT...	7
.........TTATCAGACTGATGTTGAC..	6
.....TAGCTTATCAGACTGATGTTGACTGTT..	6
....CAACAGCAGTCGATGGGCTG.........	6
...........ATCAGACTGATGTTGAC..	5
....GTAGCTTATCAGACTGATGTT...	4
...........ATCAGACTGATGTTGA...	4
.........TTATCAGACTGATGTTGACT...	4
....CAACAGCAGTCGATGGGCT..........	4
.......GCTTATCAGACTGATGTTG..	3
..........TATCAGACTGATGTTG..	3
....GTAGCTTATCAGACTGATGTTGACT...	3
..........TATCAGACTGATGTTGACT...	3
.....TAGCTTATCAGACTGATGTTGACTGTTGAA.....................................	3
......AGCTTATCAGACTGATGTT...	2
.........TTATCAGACTGATGTTG..	2
...........ATCAGACTGATGTTGACT...	2
.....TAGCTTATCAGACTGATGTTGACTGTTGAATCT..................................	2
....AACAGCAGTCGATGGGC...........	2
....AACAGCAGTCGATGGGCTGT........	2
....ACAGCAGTCGATGGGCTGT........	2
....TGGCAACAGCAGTCGATGGGCTGTCT......	2
.......GCTTATCAGACTGATGTTGAC..	1
.....TAGCTTATCAGACTGATGTTGACTG..	1
.....TAGCTTATCAGACTGATGTTGACTGT...	1
.....TAGCTTATCAGACTGATGTTGACTGTTGA......................................	1
.....TAGCTTATCAGACTGATGTTGACTGTTGAAT....................................	1
...................................TCTCATGGCAACAGCAGTCGATG..............	1
.....................................TCATGGCAACAGCAGTCGATGGGC...........	1
....ACAGCAGTCGATGGGC...........	1
....GGCAACAGCAGTCGATGGGCTGT........	1

```
..........................................GCAACAGCAGTCGATGGGCTGT........  1
..............................TTGAATCTCATGGCAACAGCAGTCGATGGGCTGTC.......  1
.............................................ACAGCAGTCGATGGGCTGTC.......  1
...............................TGAATCTCATGGCAACAGCAGTCGATGGGCTGTCT......  1
......................................CATGGCAACAGCAGTCGATGGGCTGTCT......  1
.......................................ATGGCAACAGCAGTCGATGGGCTGTCT......  1
..........................................GCAACAGCAGTCGATGGGCTGTCT......  1
..................................................AGTCGATGGGCTGTCT......  1
.....................................................CGATGGGCTGTCTGACA..  1
((((..((((((((.(((((.(((((.((((.(.....)))))))))).)))))))))))))..))))....  (-31.90)

19/18824223-18824298(-)_mir_Cow_Blast_26
GAGCTGAGAGCTGGGTCTTTGCGGGCGAGATGAAGGTGTCGGTTCAACTGGCCTACAAAGTCCCAGTCCTCGGCCC
.............................................AACTGGCCTACAAAGTCCCAGT.........    676
...........TGGGTCTTTGCGGGCGAGATGA...........................................    176
...........TGGGTCTTTGCGGGCGAGATG............................................    68
...........TGGGTCTTTGCGGGCGAGAT.............................................    50
.............................................AACTGGCCTACAAAGTCCC............    22
.............................................AACTGGCCTACAAAGTCCCA...........    21
.............................................AACTGGCCTACAAAGTCCCAG..........    18
...........TGGGTCTTTGCGGGCGAGATGAA..........................................    14
.............................................AACTGGCCTACAAAGTCC.............    10
.............................................AACTGGCCTACAAAGTC..............    9
...........TGGGTCTTTGCGGGCGAGA..............................................    5
.............................................AACTGGCCTACAAAGT...............    4
...........TGGGTCTTTGCGGGCGAG...............................................    3
.................................AGGTGTCGGTTCAACTGGCCTACAAAGTCCCAGT.........    2
........AGCTGGGTCTTTGCGGGCGAGA..............................................    1
............GGGTCTTTGCGGGCGAGATGA...........................................    1
...........TGGGTCTTTGCGGGCGAGATGAAGGTGTCGGTTC...............................    1
..............................TGAAGGTGTCGGTTCAACTGGCCTACAAAGTCCCAGTCCT......    1
..((((((..(((((.(((((..(((.((.((((........)))).)).)))..))))).)))))..))))))..  (-42.20)

19/20797030-20797101(-)_mir_cow_86
CCTGACTGGGATATCATCATATACTGTAAGTTTGCAACGAGACACTACAGTATAGATGATGTACTAGTCCGG
.............................................TACAGTATAGATGATGTACT.......  789
........GGATATCATCATATACTGTAAGT.........................................  458
.............................................TACAGTATAGATGATGTA.........  276
```

........GGATATCATCATATACTGTAAG.. 130
....TACAGTATAGATGATG........... 87
......TGGGATATCATCATATACTGT... 54
....TACAGTATAGATGATGT.......... 51
....TACAGTATAGATGATGTAC........ 44
....CTACAGTATAGATGATGTACT....... 27
........GGATATCATCATATACTGT... 22
........GGATATCATCATATACTGTA.. 21
........GGATATCATCATATACTGTAA... 12
.........GATATCATCATATACTGTAAGT... 6
......TGGGATATCATCATATACTG.. 5
....TACAGTATAGATGATGTACTAGT.... 5
....CTACAGTATAGATGATGT.......... 3
........GGATATCATCATATACTG.. 2
.......GGGATATCATCATATACTGT... 2
........GGATATCATCATATACTGTAAGTT.. 2
....CTACAGTATAGATGATGTAC........ 2
....TACAGTATAGATGATGTACTA...... 2
.......GGGATATCATCATATACTG.. 1
.........GATATCATCATATACTGT... 1
..........ATATCATCATATACTGT... 1
......TGGGATATCATCATATACTGTA.. 1
...........TATCATCATATACTGTAAGT... 1
.............TCATCATATACTGTAAGT... 1
.........GATATCATCATATACTGTAAGTT.. 1
....CTACAGTATAGATGATGTA......... 1
....TACAGTATAGATGATGTACTAG..... 1
((.((((((.((((((((.(((((((((.((((......))))..))))))))))))))))).)))))).)) (-31.40)

19/21433034-21433100(-)_mir_cow_87
AGCAGGTGGAGGACACCGACTGTGACTCCTGGGGACTCCGGACAGCAGCCTGTGTTCTCACCTAGCT
....ACAGCAGCCTGTGTTCTCACCT.... 7
....ACAGCAGCCTGTGTTCTCACC..... 2
......TGGAGGACACCGACTGTGACTCC...................................... 1
((((((((.(((((((.(.((((...(((.(....)...)))..)))).).)))))))))))).))) (-29.80)

19/21799495-21799563(+)_mir_cow_88
TAGGCTGAGGGGCAGAGAGCGAGACTTTTCTATTTTCCAAAAGCTCGGTCTGAGGCCCCTCAGTCTTGC

```
.........................................AGCTCGGTCTGAGGCCCCTCAGT.....     18574
.....TGAGGGGCAGAGAGCGAGACTTT.........................................     7303
.....TGAGGGGCAGAGAGCGAGACT...........................................     2343
........................................AAGCTCGGTCTGAGGCCCCTCAGT.....     2204
.....TGAGGGGCAGAGAGCGAGACTTTT........................................     1189
.....TGAGGGGCAGAGAGCGAGACTT..........................................     1144
.........................................AGCTCGGTCTGAGGCCCCTCAG......     761
.........................................AGCTCGGTCTGAGGCCCCT.........     419
.........................................AGCTCGGTCTGAGGCCCCTCA.......     280
........................................AAGCTCGGTCTGAGGCCCCT.........     206
..........................................GCTCGGTCTGAGGCCCCTCAGT.....     134
.....TGAGGGGCAGAGAGCGAGAC............................................     128
.....TGAGGGGCAGAGAGCGAGA.............................................     104
.........................................AGCTCGGTCTGAGGCC............     100
........................................AAGCTCGGTCTGAGGCCCCTCA.......     94
........................................AAGCTCGGTCTGAGGCCCCTCAG......     94
.........................................AGCTCGGTCTGAGGCCC...........     91
.........................................AGCTCGGTCTGAGGCCCCTC........     82
.........................................AGCTCGGTCTGAGGCCCC..........     65
........................................AAGCTCGGTCTGAGGCCCCTC........     40
........................................AAGCTCGGTCTGAGGCCCC..........     37
........................................AAGCTCGGTCTGAGGCCC...........     21
........................................AAGCTCGGTCTGAGGCC............     19
.........................................AGCTCGGTCTGAGGCCCCTCAGTC....     18
.........................................AGCTCGGTCTGAGGCCCCTCAGTCT...     13
.......AGGGGCAGAGAGCGAGACTTTT........................................     12
...........................................CTCGGTCTGAGGCCCCTCAGT.....     10
..........................................GCTCGGTCTGAGGCCCCTCAG......     9
........................................AAGCTCGGTCTGAGGC.............     8
............CAGAGAGCGAGACTTT.........................................     7
.....TGAGGGGCAGAGAGCGAGACTTTTCT......................................     7
........................................AAGCTCGGTCTGAGGCCCCTCAGTC....     5
.....TGAGGGGCAGAGAGCGAG..............................................     4
............................................TCGGTCTGAGGCCCCTCAGT.....     4
.......AGGGGCAGAGAGCGAGACT...........................................     3
......................................AAAAGCTCGGTCTGAGGCCCCTCAGT.....     3
.....TGAGGGGCAGAGAGCGA...............................................     2
...........GCAGAGAGCGAGACTT..........................................     2
```

....CTGAGGGGCAGAGAGCGAGACTTT...	2
......GAGGGGCAGAGAGCGAGACTTT...	2
..........GGCAGAGAGCGAGACTTT...	2
.....TGAGGGGCAGAGAGCGAGACTTTTC.......................................	2
................................TTTTCCAAAAGCTCGGTCTGAGGCCCCTCAGT.....	2
....CTCGGTCTGAGGCCCCTCAGTC....	2
......GAGGGGCAGAGAGCGAGACT...	1
..........GGCAGAGAGCGAGACT...	1
..........GGCAGAGAGCGAGACTT..	1
.......AGGGGCAGAGAGCGAGACTTT...	1
.........GGGCAGAGAGCGAGACTTT...	1
...........GCAGAGAGCGAGACTTT...	1
....CTGAGGGGCAGAGAGCGAGACTTTT..	1
.........GGGCAGAGAGCGAGACTTTT..	1
...........GCAGAGAGCGAGACTTTT..	1
.............AGAGAGCGAGACTTTT..	1
.......AGGGGCAGAGAGCGAGACTTTTCT......................................	1
...........GCAGAGAGCGAGACTTTTCT......................................	1
.....TGAGGGGCAGAGAGCGAGACTTTTCTATT...................................	1
....GCTCGGTCTGAGGCCC...........	1
......................................AAAAGCTCGGTCTGAGGCCCC..........	1
....GCTCGGTCTGAGGCCCC..........	1
....................................CCAAAAGCTCGGTCTGAGGCCCCT.........	1
......................................AAAAGCTCGGTCTGAGGCCCCT.........	1
....AAAGCTCGGTCTGAGGCCCCT.........	1
....GCTCGGTCTGAGGCCCCT.........	1
....AAAGCTCGGTCTGAGGCCCCTC........	1
....GCTCGGTCTGAGGCCCCTC........	1
....AAAGCTCGGTCTGAGGCCCCTCA.......	1
....GCTCGGTCTGAGGCCCCTCA.......	1
....AAAGCTCGGTCTGAGGCCCCTCAG......	1
.............................CTATTTTCCAAAAGCTCGGTCTGAGGCCCCTCAGT.....	1
...............................ATTTTCCAAAAGCTCGGTCTGAGGCCCCTCAGT.....	1
..................................TTCCAAAAGCTCGGTCTGAGGCCCCTCAGT.....	1
...................................TCCAAAAGCTCGGTCTGAGGCCCCTCAGT.....	1
....CGGTCTGAGGCCCCTCAGT.....	1
....AAGCTCGGTCTGAGGCCCCTCAGTCT...	1
....AGCTCGGTCTGAGGCCCCTCAGTCTT..	1

```
.((((((((((((..(((.((((.(((((........))))))))).)))...))))))))))))... (-36.50)

19/23333059-23333131(-)_novel_cow_16
TCCTTGGCTAGGGAGGAATGCTGAACTTTTCATATATGTATTTAAGTTTGGTCTTTTTTCCTTGGCAGACGAT
..............................................TTTGGTCTTTTTTCCTTGGCAG..... 8
...TTGGCTAGGGAGGAATGCTGAACT.............................................. 6
..............................................TTTGGTCTTTTTTCCTTGGCAGA.... 6
...TTGGCTAGGGAGGAATGCTGAAC............................................... 5
....TGGCTAGGGAGGAATGCTGAAC............................................... 5
....TGGCTAGGGAGGAATGCTGAACT.............................................. 4
..............................................TTTGGTCTTTTTTCCTTGGCA...... 4
...TTGGCTAGGGAGGAATGCT.................................................... 1
.....GGCTAGGGAGGAATGCTGAAC............................................... 1
.....GGCTAGGGAGGAATGCTGAACT.............................................. 1
.......CTAGGGAGGAATGCTGAACT.............................................. 1
...TTGGCTAGGGAGGAATGCTGAACTT............................................. 1
....TGGCTAGGGAGGAATGCTGAACTT............................................. 1
.......CTAGGGAGGAATGCTGAACTT............................................. 1
.............................................GTTTGGTCTTTTTTCCTTGGCA...... 1
......((((((((((((.((..(((((...(((....)))..)))))..))..)))))))))))).......  (-23.60)

19/23382437-23382516(-)_mir_cow_89
TGAGCCGCAGTAGTTCTTCAGTGGCAAGCTTTATGTCCTGACCCAGCTAAAGCTGCCAGTTGAAGAACTGTTGCCCTCTG
.................................................AAGCTGCCAGTTGAAGAACTGT.........      1868944
.................................................AAGCTGCCAGTTGAAGAACTG..........      11334
.................................................AAGCTGCCAGTTGAAGAACT...........      8697
..................................................AGCTGCCAGTTGAAGAACTGT.........      4861
.................................................AAGCTGCCAGTTGAAGAACTGTT........      2061
.................................................AAGCTGCCAGTTGAAGAAC............      1256
...........AGTTCTTCAGTGGCAAGCTTT................................................      793
...........AGTTCTTCAGTGGCAAGCTTTA...............................................      679
................................................AAAGCTGCCAGTTGAAGAACTGT.........      201
...........AGTTCTTCAGTGGCAAGCTT.................................................      150
.....................................................TGCCAGTTGAAGAACTGT.........      77
....................................................CTGCCAGTTGAAGAACTGT.........      70
...................................................GCTGCCAGTTGAAGAACTGT.........      66
..................................................AGCTGCCAGTTGAAGAACTGTT........      34
..................................................AGCTGCCAGTTGAAGAACTG..........      30
```

...........AGTTCTTCAGTGGCAAGCT.. 28
....GCCAGTTGAAGAACTGT......... 26
....AGCTGCCAGTTGAAGAACT........... 18
....CCAGTTGAAGAACTGT......... 11
...........AGTTCTTCAGTGGCAAGCTTTAT.. 10
...........AGTTCTTCAGTGGCAAG.. 8
....AAGCTGCCAGTTGAAG............... 8
....AAAGCTGCCAGTTGAAGAACT........... 8
....AAAGCTGCCAGTTGAAGAACTG.......... 6
...........AGTTCTTCAGTGGCAAGC... 5
.................................TGTCCTGACCCAGCTAAAGCTGCCAGTTGAAGAACTGT......... 5
..........TAGTTCTTCAGTGGCAAGCTT... 4
....CTGCCAGTTGAAGAACTG.......... 4
....AAGCTGCCAGTTGAAGAA............. 3
..........TAGTTCTTCAGTGGCAAGCTTT.. 2
..........TAGTTCTTCAGTGGCAAGCTTTA... 2
............GTTCTTCAGTGGCAAGCTTTA... 2
....AAGCTGCCAGTTGAAGA.............. 2
....AAAGCTGCCAGTTGAAGAAC............ 2
....GCTGCCAGTTGAAGAACTGTT........ 2
......GCAGTAGTTCTTCAGTGGCAAGC... 1
............GTTCTTCAGTGGCAAGCTTT.. 1
....TGCCAGTTGAAGAACT........... 1
....GCCAGTTGAAGAACTG.......... 1
......................................TGACCCAGCTAAAGCTGCCAGTTGAAGAACTGT......... 1
....TAAAGCTGCCAGTTGAAGAACTGT......... 1
....TGAAGAACTGTTGCCC.... 1
.(((..((((((((((((((((((((.((((((.((.........))))))))))))).))))))))))))))).))).. (-34.70)

19/23651155-23651226(-)_mir_cow_90
CCAGGGCAACCGTGGCTTTCGATTGTTACTGTGGGAACCGGAGGTAACAGTCTACAGCCATGGTCGCCCCGC
....TAACAGTCTACAGCCATGGTCG...... 30348
....TAACAGTCTACAGCCATGGTC....... 3313
........ACCGTGGCTTTCGATTGTTACT.. 3162
....AACAGTCTACAGCCATGGTCG...... 953
.......AACCGTGGCTTTCGATTGTTA.. 759
........ACCGTGGCTTTCGATTGTTAC... 361
.......AACCGTGGCTTTCGATTGTT... 275

....TAACAGTCTACAGCCATGGTCGC.....	195
....TAACAGTCTACAGCCATGGT........	189
....ACAGTCTACAGCCATGGTCGC.....	158
.......AACCGTGGCTTTCGATTGT..	120
........ACCGTGGCTTTCGATTGTT...	106
.......AACCGTGGCTTTCGATTGTTAC...	95
....TAACAGTCTACAGCCATG..........	93
........ACCGTGGCTTTCGATTGTTA..	90
........ACCGTGGCTTTCGATTGT..	67
....AACAGTCTACAGCCATGGTCGC.....	59
....AACAGTCTACAGCCATGGTC.......	45
.......AACCGTGGCTTTCGATTGTTACT..	44
....TAACAGTCTACAGCCATGG.........	32
....ACAGTCTACAGCCATGGTCG......	20
....TAACAGTCTACAGCCA............	17
....GTCTACAGCCATGGTCG......	13
.......AACCGTGGCTTTCGATTG...	11
........ACCGTGGCTTTCGATTGTTACTGT..	8
...........GTGGCTTTCGATTGTTA..	5
...........GTGGCTTTCGATTGTTACT..	5
...........GTGGCTTTCGATTGTTAC...	4
..............GCTTTCGATTGTTACT..	4
........ACCGTGGCTTTCGATTG...	3
......CAACCGTGGCTTTCGATTGTTA..	3
....ACAGTCTACAGCCATGGTC.......	3
......CAACCGTGGCTTTCGATTGTT...	2
....AACAGTCTACAGCCATGGT........	2
........ACCGTGGCTTTCGATT..	1
......CAACCGTGGCTTTCGATTG...	1
......CAACCGTGGCTTTCGATTGT..	1
............TGGCTTTCGATTGTTA..	1
........ACCGTGGCTTTCGATTGTTACTG...	1
.......AACCGTGGCTTTCGATTGTTACTGT..	1
........ACCGTGGCTTTCGATTGTTACTGTGGG.....................................	1
........ACCGTGGCTTTCGATTGTTACTGTGGGAACCGGAGGT...........................	1
....TAACAGTCTACAGCCAT...........	1
....ACAGTCTACAGCCATGGT........	1
....GTCTACAGCCATGGTC.......	1

....TCTACAGCCATGGTCG...... 1
....TAACAGTCTACAGCCATGGTCGCC.... 1
....ACAGTCTACAGCCATGGTCGCC.... 1
...((((.(((((((((...((((((((((.(((...)))..))))))))))...))))))))).))))... (-38.30)

19/23651582-23651656(-)_mir_cow_91
CGCCGGCACCTTGGCTCTAGACTGCTTACTGCCCGGGCCGCCCTCAGTAACAGTCTCCAGTCACGGCCACCGACG
....TAACAGTCTCCAGTCACGGCC....... 4040
.......ACCTTGGCTCTAGACTGCTTACT... 2478
....TAACAGTCTCCAGTCACGGCCA...... 1575
....TAACAGTCTCCAGTCACGGCCAC..... 727
....TAACAGTCTCCAGTCACGGC........ 485
.......ACCTTGGCTCTAGACTGCTTACTG.. 396
........CCTTGGCTCTAGACTGCTTACT... 253
........CCTTGGCTCTAGACTGCTTACTG.. 196
....TAACAGTCTCCAGTCACGG......... 68
....GTAACAGTCTCCAGTCACGGCC....... 67
.......ACCTTGGCTCTAGACTGCTTAC.. 40
....GTAACAGTCTCCAGTCACGGCCA...... 24
.......ACCTTGGCTCTAGACTGCTT.. 14
....GTAACAGTCTCCAGTCACGGC........ 14
..........TTGGCTCTAGACTGCTTACT... 13
..............CTCTAGACTGCTTACT... 12
....AACAGTCTCCAGTCACGGCC....... 10
....TAACAGTCTCCAGTCACG.......... 9
....GTAACAGTCTCCAGTCACGGCCAC..... 8
......CACCTTGGCTCTAGACTGCTTACT... 5
.......ACCTTGGCTCTAGACTG... 4
........CCTTGGCTCTAGACTGCTTAC.. 4
....AACAGTCTCCAGTCACGGCCA...... 4
....TAACAGTCTCCAGTCACGGCCACC.... 4
.......ACCTTGGCTCTAGACTGCT... 3
.......ACCTTGGCTCTAGACTGCTTA... 2
.........CTTGGCTCTAGACTGCTTACT... 2
.........CTTGGCTCTAGACTGCTTACTG.. 2
..........TTGGCTCTAGACTGCTTACTG.. 2
........CCTTGGCTCTAGACTGC.. 1
......CACCTTGGCTCTAGACTGCTTAC.. 1

..............CTCTAGACTGCTTACTG.. 1
.......ACCTTGGCTCTAGACTGCTTACTGC... 1
....GTAACAGTCTCCAGTCACG.......... 1
....AGTCTCCAGTCACGGCC....... 1
....GTCTCCAGTCACGGCC....... 1
....ACAGTCTCCAGTCACGGCCA...... 1
....CTCAGTAACAGTCTCCAGTCACGGCCAC..... 1
....AACAGTCTCCAGTCACGGCCAC..... 1
((.(((..((.(((((..((((((.((((((...(((...))).))))))))))))..))))).))...))).)) (-29.40)

19/27441351-27441422(-)_mir_cow_92
CTGGCTCTAGCAGCACAGAAATATTGGCACTGGGAAGAAAGCCTGCCAATATTGGCTGTGCTGCTCCAGGCA
.......TAGCAGCACAGAAATATTGGCA... 16808
.......TAGCAGCACAGAAATATTGGC.. 15710
....CCAATATTGGCTGTGCTGCTCC..... 572
.......TAGCAGCACAGAAATATTGG... 365
....CCAATATTGGCTGTGCTGCTCCA.... 185
.......TAGCAGCACAGAAATATTG.. 94
....CCAATATTGGCTGTGCTGCT....... 93
....CCAATATTGGCTGTGCTGCTC...... 80
.......TAGCAGCACAGAAATAT.. 43
.......TAGCAGCACAGAAATATTGGCAC.. 36
............GCACAGAAATATTGGC.. 23
.......TAGCAGCACAGAAATATTGGCACTG.. 21
......CTAGCAGCACAGAAATATTGGCA... 17
......CTAGCAGCACAGAAATATTGGC.. 16
.......TAGCAGCACAGAAATATTGGCACT... 14
.......TAGCAGCACAGAAATATT... 9
........AGCAGCACAGAAATATTGGCA... 9
........AGCAGCACAGAAATATTGGC.. 3
.......TAGCAGCACAGAAATA... 1
.........GCAGCACAGAAATATT... 1
.........GCAGCACAGAAATATTGG... 1
..........CAGCACAGAAATATTGGC.. 1
...........AGCACAGAAATATTGGC.. 1
............GCACAGAAATATTGGCA... 1
.......TAGCAGCACAGAAATATTGGCACTGGGAA.................................... 1
.............................CTGGGAAGAAAGCCTG........................... 1

....CCAATATTGGCTGTGCTGC........ 1
....AATATTGGCTGTGCTGCT....... 1
....CTGCCAATATTGGCTGTGCTGCTC...... 1
....TGCCAATATTGGCTGTGCTGCTC...... 1
....TATTGGCTGTGCTGCTCC..... 1
....CAATATTGGCTGTGCTGCTCCA.... 1
((((....((((((((((.((((((((((..((........))))))))))))..))))))))))))))... (-37.10)

19/27441675-27441747(-)_mir_cow_93
CCCGCCCCAGCAGCACACTGTGGTTTGTACGGCACTGTGGCCACGTCCAAACCACACTGTGGTGTTAGAGCGA
.......CAGCAGCACACTGTGGTTTGT... 7742
.......CAGCAGCACACTGTGGTTTGTA.. 4856
.......CAGCAGCACACTGTGGTTTGTAC... 186
.......CAGCAGCACACTGTGGTTTG.. 150
........AGCAGCACACTGTGGTTTGTAC... 116
........AGCAGCACACTGTGGTTTGT... 61
........AGCAGCACACTGTGGTTTGTA.. 57
.......CAGCAGCACACTGTGGTT.. 6
........AGCAGCACACTGTGGTTTG.. 5
............GCACACTGTGGTTTGT... 4
....TCCAAACCACACTGTGGTGTTAG..... 4
....CCAAACCACACTGTGGTGTTAGA.... 4
....CAAACCACACTGTGGTGTTAGA.... 4
............GCACACTGTGGTTTGTA.. 2
.......CAGCAGCACACTGTGGTTTGTACG.. 2
.............................CGGCACTGTGGCCACGTC.......................... 2
....CCAAACCACACTGTGGTGTTAG..... 2
......CCAGCAGCACACTGTGGTTTGT... 1
.........GCAGCACACTGTGGTTTGT... 1
...........AGCACACTGTGGTTTGT... 1
......CCAGCAGCACACTGTGGTTTGTA.. 1
.............................CGGCACTGTGGCCACGTCCAAACC.................... 1
....CAAACCACACTGTGGTGTTA...... 1
....TCCAAACCACACTGTGGTGTTAGA.... 1
..(((.(.((((.((((.(((((((((.(((((......)))..)).))))))))).)))).)))).).))). (-33.50)

19/27571352-27571418(-)_mir_cow_94
CCTCCCCGCATCCCCTAGGGCATTGGTGTAAAGCTGGAGACCCACTGCCCCAGGTGCTGCTGGGGGT

......CGCATCCCCTAGGGCATTGGTGT...................................... 1005
......CGCATCCCCTAGGGCATTGGTG....................................... 592
....CCACTGCCCCAGGTGCTGCTGG.... 291
....CCACTGCCCCAGGTGCTGCTG..... 145
......CGCATCCCCTAGGGCATTGGT.. 97
......CGCATCCCCTAGGGCATTGGTGTA..................................... 56
......CGCATCCCCTAGGGCATTG.. 54
....ACTGCCCCAGGTGCTGCTGG.... 41
....CCACTGCCCCAGGTGCTG........ 31
....CCACTGCCCCAGGTGCTGCT...... 31
......CGCATCCCCTAGGGCATTGG... 26
....ACTGCCCCAGGTGCTGCTG..... 11
......CGCATCCCCTAGGGCATT... 6
....ACTGCCCCAGGTGCTGCT...... 5
..........................TGTAAAGCTGGAGACCC........................ 4
....ACTGCCCCAGGTGCTGC....... 4
....CACTGCCCCAGGTGCTGCTGG.... 4
......CGCATCCCCTAGGGCAT.. 2
....CACTGCCCCAGGTGCTGCTG..... 2
......CGCATCCCCTAGGGCA... 1
.......GCATCCCCTAGGGCATTGGTGT...................................... 1
.........ATCCCCTAGGGCATTGGTGT...................................... 1
......CGCATCCCCTAGGGCATTGGTGTAA.................................... 1
....ACTGCCCCAGGTGCTG........ 1
....CCACTGCCCCAGGTGCTGC....... 1
....CTGCCCCAGGTGCTGCT...... 1
.............................AAAGCTGGAGACCCACTGCCCCAGGTGCTGCTGG.... 1
.(((((.(((.(.(((.(((((.(((.((..........))))).))))).))).).))).))))). (-30.50)

19/31333520-31333605(+)_mir_cow_95
GGGGTGCGGGGCTAGGGCTAACAGCAGGCTCACTGACGGTTTCCCGGAAACCACGCACATGCTGTTGCCACTAACCTCAACCTTAC
....TGCGGGGCTAGGGCTAACAGC... 4044
....TGCGGGGCTAGGGCTAACAGCA.. 1872
....CTGTTGCCACTAACCTCAACCT... 515
....CTGTTGCCACTAACCTCAACC.... 211
....TGCGGGGCTAGGGCTAACAG.. 172
....CTGTTGCCACTAACCTCAA...... 59
....TGCGGGGCTAGGGCTAACA... 31

```
....TGCGGGGCTAGGGCTAAC................................................................      26
.............................................................CTGTTGCCACTAACCTCAAC.....      14
....TGCGGGGCTAGGGCTA..................................................................      7
.....GCGGGGCTAGGGCTAACAGC.............................................................      5
.....GCGGGGCTAGGGCTAACAGCA............................................................      5
....TGCGGGGCTAGGGCTAACAGCAG...........................................................      4
....TGCGGGGCTAGGGCTAA.................................................................      3
.............................................................CTGTTGCCACTAACCTCA.......      2
..........GCTAGGGCTAACAGCA............................................................      1
....TGCGGGGCTAGGGCTAACAGCAGGCTCACTG...................................................      1
....................................................ACGCACATGCTGTTGCCACTAACC..........      1
.........................................................CATGCTGTTGCCACTAACCTC........      1
..........................................................ATGCTGTTGCCACTAACCTC........      1
..........................................................ATGCTGTTGCCACTAACCTCAA......      1
...........................................................TGCTGTTGCCACTAACCTCAA......      1
...........................................................TGCTGTTGCCACTAACCTCAACC....      1
..............................................................TGTTGCCACTAACCTCAACCT...      1
.............................................................CTGTTGCCACTAACCTCAACCTT..      1
(((((..((((.((((((.(((((((.((.......(((....)))........))...)))))))))).))).)))).)))))..  (-34.36)

19/31973540-31973613(-)_mir_cow_96
CAGGTCTGGGCTCCTGCTGCCCAGTCTGAGGTCTGCACTCTTTGCCTCACTGGACCTAGCAGAGCTCCCACTTG
..............................................TCACTGGACCTAGCAGAGCTCC......36
..............................................TCACTGGACCTAGCAGAGCTCCC.....24
..............................................TCACTGGACCTAGCAGAGCTCCCA....15
..............................................TCACTGGACCTAGCAGAGCT........6
..............................................TCACTGGACCTAGCAGAGCTC.......2
......TGGGCTCCTGCTGCCCAGTCTGAGG...........................................1
..............................................TCACTGGACCTAGCAGAGC.........1
(((((..((((((.(((((.(((((..(((((...........))))))))))...)))))))))))..))))) (-30.00)

19/35249063-35249125(+)_mir_cow_97
CGCGGTGCATTGCTGTTGCATTGCATGTGTGAGGCAGGTGCAGTGCCTCGGCAGTGCAGCCCG
....GTGCATTGCTGTTGCATTG........................................      1346
....GTGCATTGCTGTTGCATTGCA......................................      22
....GTGCATTGCTGTTGCATTGC.......................................      19
........................................CAGTGCCTCGGCAGTGCAGCC..      10
....GTGCATTGCTGTTGCATT.........................................      7
```

```
........ATTGCTGTTGCATTGCA......................................     4
.........TTGCTGTTGCATTGCA......................................     4
....GTGCATTGCTGTTGCAT..........................................     2
.......CATTGCTGTTGCATTG........................................     1
.......CATTGCTGTTGCATTGCA......................................     1
((.((((((((((((..(((((((((.((.....)).)))))))))..)))))))))).))))  (-37.60)

19/38497003-38497067(+)_mir_cow_98
GAATTAGGTAGTTTCATGTTGTTGGGCCTGGATTTCTGAACACAACGACATTAAACCACCCGACT
....TAGGTAGTTTCATGTTGTTGGG.......................................   208
....TAGGTAGTTTCATGTTGTTGG........................................   81
....TAGGTAGTTTCATGTTGTTG.........................................   23
..........................................CAACGACATTAAACCACCCGA..   20
....TAGGTAGTTTCATGTTGT...........................................   4
....TAGGTAGTTTCATGTTGTTGGGC......................................   4
....TAGGTAGTTTCATGTT.............................................   1
....TAGGTAGTTTCATGTTGTT..........................................   1
...(..(((.((((.((((((((((..(.((....)))..).))))))))).)))).)))..)..  (-17.70)

19/38549270-38549340(+)_mir_cow_99
GTATATACCCTGTAGATCCGAATTTGTGTAAGGAATTTTGTGATCACAAATTCGTATCTAGGGGAATATGT
.....TACCCTGTAGATCCGAATTTGT............................................   293386
.....TACCCTGTAGATCCGAATTTGTG...........................................   67256
......ACCCTGTAGATCCGAATTTGTG...........................................   34701
.....TACCCTGTAGATCCGAATTTGTGT..........................................   29870
......ACCCTGTAGATCCGAATTTGT............................................   27567
......ACCCTGTAGATCCGAATTTGTGT..........................................   19846
.....TACCCTGTAGATCCGAATTTG.............................................   15484
....ATACCCTGTAGATCCGAATTTGT............................................   2818
.....TACCCTGTAGATCCGAATTT..............................................   2807
......ACCCTGTAGATCCGAATTTG.............................................   2089
....ATACCCTGTAGATCCGAATTTG.............................................   714
.....TACCCTGTAGATCCGAATT...............................................   651
......ACCCTGTAGATCCGAATTT..............................................   596
......ACCCTGTAGATCCGAATTTGTGTA.........................................   179
.....TACCCTGTAGATCCGAATTTGTGTA.........................................   152
..............................................CAAATTCGTATCTAGGGGAAT....   151
....ATACCCTGTAGATCCGAATTT..............................................   145
```

```
....ATACCCTGTAGATCCGAATTTGTG..........................................   123
.....TACCCTGTAGATCCGAAT...............................................   103
......ACCCTGTAGATCCGAATT..............................................   98
.......CCCTGTAGATCCGAATTTGTGT.........................................   91
.......CCCTGTAGATCCGAATTTGT...........................................   65
....ATACCCTGTAGATCCGAATTTGTGT.........................................   46
.......CCCTGTAGATCCGAATTTGTG..........................................   28
........CCTGTAGATCCGAATTTGT...........................................   25
......ACCCTGTAGATCCGAATTTGTGTAA.......................................   17
..............................................AAATTCGTATCTAGGGGAAT....   15
....ATACCCTGTAGATCCGAATT..............................................   13
..........TGTAGATCCGAATTTGT...........................................   13
........CCTGTAGATCCGAATTTGTG..........................................   13
.....TACCCTGTAGATCCGAATTTGTGTAA.......................................   13
.....TACCCTGTAGATCCGAA................................................   11
.............................................CAAATTCGTATCTAGGGGA......   11
.............................................CAAATTCGTATCTAGGGGAA.....   10
............................................ACAAATTCGTATCTAGGGGAAT....   8
......ACCCTGTAGATCCGAAT...............................................   7
..............................................AAATTCGTATCTAGGGGAATA...   7
........CCTGTAGATCCGAATTTGTGT.........................................   6
.............................................CAAATTCGTATCTAGGGGAATA...   6
........CCTGTAGATCCGAATTTG............................................   5
.....TACCCTGTAGATCCGA.................................................   4
....ATACCCTGTAGATCCGAAT...............................................   4
.....TACCCTGTAGATCCGAATTTGTGTAAGG.....................................   4
.........CTGTAGATCCGAATTTGT...........................................   2
.............................................CAAATTCGTATCTAGGGGAATAT..   2
..............................................AAATTCGTATCTAGGGGAATAT..   2
....ATACCCTGTAGATCCGAA................................................   1
......ACCCTGTAGATCCGAA................................................   1
..........TGTAGATCCGAATTTG............................................   1
..........TGTAGATCCGAATTTGTG..........................................   1
.......CCCTGTAGATCCGAATTTGTGTAA.......................................   1
.....TACCCTGTAGATCCGAATTTGTGTAAGGAA...................................   1
..........................................TCACAAATTCGTATCTAGGGGA......   1
............................................ACAAATTCGTATCTAGGGGA......   1
...........................................CACAAATTCGTATCTAGGGGAA.....   1
```

```
...............................................AAATTCGTATCTAGGGGAA.....    1
.(((((.((((.(((((.((((((((((................)))))))))).))))))))).))))). (-24.49)

19/39081174-39081259(+)_mir_Cow_Blast_50
GGCCCAGGTTCTGTGATACACTCCGACTCGGGCTCTGGAGCAGTCAGTGCATGACAGAACTTGGGCCCGGACGGACCTTCTGCACC
...........................................TCAGTGCATGACAGAACTTGG......................     1322
.....AGGTTCTGTGATACACTCCGACT..........................................................     439
...........................................TCAGTGCATGACAGAACT.........................     216
...........................................TCAGTGCATGACAGAACTT........................     75
...........................................TCAGTGCATGACAGAACTTGGG.....................     55
...........................................TCAGTGCATGACAGAACTTG.......................     43
...........................................TCAGTGCATGACAGAAC..........................     6
.....AGGTTCTGTGATACACTCCGA............................................................     5
...........................................TCAGTGCATGACAGAACTTGGGC....................     4
.....AGGTTCTGTGATACACTCCGAC...........................................................     2
....CAGGTTCTGTGATACACTCCGACT..........................................................     2
.....AGGTTCTGTGATACACTCCGACTC.........................................................     1
.....AGGTTCTGTGATACACTCCGACTCGGGCTCTGGAGCAG...........................................     1
............................................CAGTGCATGACAGAACT.........................     1
..............................................GTGCATGACAGAACTTG.......................     1
............................................CAGTGCATGACAGAACTTGG......................     1
................................................................GCCCGGACGGACCTTCTGCA..     1
((((((((((((((.((.((((..((((...(((....))))))))))).)).)))))))))))))).(..(((.....)))..). (-40.00)

19/44546740-44546810(-)_novel_cow_17
AGTCTGGGGTGGGAGTGGGGAGCCTGGTGCCCTAACAGTAATGCTTGCTCCCCTTCCCCCACCTCATAGTG
.............................................TGCTCCCCTTCCCCCACCTCAT....    6
.............................................TGCTCCCCTTCCCCCACCTCATAG..    4
...CTGGGGTGGGAGTGGGGAGCC...............................................    2
.............................................TGCTCCCCTTCCCCCACCTCA.....    2
...CTGGGGTGGGAGTGGGGAGC................................................    1
..TCTGGGGTGGGAGTGGGGAGCC...............................................    1
...........................TGCCCTAACAGTAATGCTTG........................    1
.............................................TGCTCCCCTTCCCCCACCTC......    1
............................................TTGCTCCCCTTCCCCCACCTCATA...    1
.............................................TGCTCCCCTTCCCCCACCTCATA...    1
...............................................CTCCCCTTCCCCCACCTCATAG..    1
....((((((((.(.(((((((..(((................))).)))))))..).)))))))))..... (-32.74)
```

```
19/49084798-49084861(-)_novel_cow_18
CCTGAGGTCAGACCAGACAGTCTAGCTCAAGTGATTGAGAAGTCTGTCTGTTTGATCTTTGGTA
......................................GAAGTCTGTCTGTTTGATCTTT....     5
.....................................AGAAGTCTGTCTGTTTGATCTTT....     3
...GAGGTCAGACCAGACAGTCTAGCT.....................................     2
......................................GAAGTCTGTCTGTTTGATCTT.....     2
..TGAGGTCAGACCAGACAGTCT.........................................     1
..................................TTGAGAAGTCTGTCTGTTTGATCT......     1
((.((((((((((.((((((.((..(((((....))))).)).))))))))))))))))).)).. (-31.80)

19/50489552-50489632(-)_novel_cow_19
AAACTGTGAGACGTCACATGTGGTGAGTTGGCCTGTGGTCTTAGGTGCTGGCCGCCTCCTGCCTGTGGCGCCCTCCAGGTG
...................................................CCGCCTCCTGCCTGTGGCGCCC........     6
...................................................CCGCCTCCTGCCTGTGGCGCC.........     2
..........ACGTCACATGTGGTGAGTTGGCC................................................     1
...................................................CCGCCTCCTGCCTGTGGCGCCCT.......     1
...(((.(((.(((((((.(..(.(((.(((((.(((.(....).))).))))).))))..).)))))))..))))))... (-35.50)

19/52189636-52189713(-)_mir_Cow_Blast_16
TGCCCTCTTCAACAAAATCACTGATGCTGGAGTTGCGTGTGTCATCACTCAGCACCAGGATATTGTTGGGGAGGACGG
........TCAACAAAATCACTGATGCTGGAGT.............................................  4
........TCAACAAAATCACTGATGCTGGAG..............................................  3
................................................TCAGCACCAGGATATTGTTGGGG.......  2
................................................TCAGCACCAGGATATTGTTGGGGA......  1
...((((((((((((.(((.(((.((((((......(((......))))))))).)))))).)))))))))))).... (-32.60)

19/52189640-52189710(+)_mir_cow_100
CCTCCCCAACAATATCCTGGTGCTGAGTGATGACACACGCAACTCCAGCATCAGTGATTTTGTTGAAGAGG
...........................................TCCAGCATCAGTGATTTTGTT.......   81871
...........................................TCCAGCATCAGTGATTTTGTTG......   34917
...........................................TCCAGCATCAGTGATTTTGT........   8142
.......AACAATATCCTGGTGCTGAGT...........................................   6845
...........................................TCCAGCATCAGTGATTTTGTTGA.....   6071
....CCCAACAATATCCTGGTGCTG..............................................   5430
...........................................TCCAGCATCAGTGATTTTG.........   1980
....CCCAACAATATCCTGGTGCTGA.............................................   573
.......AACAATATCCTGGTGCTGAG............................................   296
```

.....CCAACAATATCCTGGTGCTGA...	290
.......AACAATATCCTGGTGCTGA...	272
.....CCAACAATATCCTGGTGCTG..	257
.....CCAACAATATCCTGGTGCTGAGT...	239
....TCCAGCATCAGTGATTTTGTTGAA....	230
....CTCCAGCATCAGTGATTTTGTT.......	216
....CCCAACAATATCCTGGTGCT...	180
....CCAGCATCAGTGATTTTGTT.......	129
....CTCCAGCATCAGTGATTTTGTTG......	86
....TCAGTGATTTTGTTGA.....	75
....CCAGCATCAGTGATTTTGTTG......	53
....TCCAGCATCAGTGATTTT..........	49
....CCCAACAATATCCTGGTGC..	48
.......AACAATATCCTGGTGCTG..	35
....TCCAGCATCAGTGATTT...........	34
....CCAGCATCAGTGATTTTGTTGA.....	31
....CTCCAGCATCAGTGATTTTG.........	28
.....CCAACAATATCCTGGTGCTGAG..	27
....ATCAGTGATTTTGTTGA.....	27
....ATCAGTGATTTTGTTG......	23
.......AACAATATCCTGGTGCTGAGTGA...	19
.......AACAATATCCTGGTGCTGAGTG..	18
....CTCCAGCATCAGTGATTTTGT........	18
....CCAGCATCAGTGATTTTGT........	16
....AGCATCAGTGATTTTGTTGAAG...	14
....CAGCATCAGTGATTTTGTT.......	13
.......AACAATATCCTGGTGC..	11
....CTCCAGCATCAGTGATTTTGTTGA.....	11
....TCCAGCATCAGTGATT............	8
....AGCATCAGTGATTTTGT........	8
....CCAGCATCAGTGATTTTGTTGAA....	8
.....CCAACAATATCCTGGTGCT...	7
.....CCAACAATATCCTGGTGC..	6
......CAACAATATCCTGGTGCTGAGT...	6
....CATCAGTGATTTTGTT.......	6
....CAGCATCAGTGATTTTGTTG......	6
......CAACAATATCCTGGTGCTG..	5
....GCATCAGTGATTTTGTTGAAG...	5

```
....CCCAACAATATCCTGGTG.................................................. 4
.............................................CAGCATCAGTGATTTTGT........ 4
.............................................CAGCATCAGTGATTTTGTTGA..... 4
........ACAATATCCTGGTGCTG.............................................. 3
......CAACAATATCCTGGTGCTGA............................................. 3
...........................................TCCAGCATCAGTGATTTTGTTGAAG... 3
........ACAATATCCTGGTGCTGAGT.......................................... 2
............................................CCAGCATCAGTGATTTTG......... 2
..............................................AGCATCAGTGATTTTGTT....... 2
...............................................GCATCAGTGATTTTGTT....... 2
...............................................GCATCAGTGATTTTGTTG...... 2
...............................................GCATCAGTGATTTTGTTGA..... 2
..............................................AGCATCAGTGATTTTGTTGAAGA.. 2
...............................................GCATCAGTGATTTTGTTGAAGA.. 2
.......AACAATATCCTGGTGCT............................................... 1
....CCCAACAATATCCTGGTGCTGAG............................................ 1
......CAACAATATCCTGGTGCTGAG............................................ 1
....CCCAACAATATCCTGGTGCTGAGT........................................... 1
.......AACAATATCCTGGTGCTGAGTGAT........................................ 1
..........................................CTCCAGCATCAGTGATTT........... 1
.............................................CAGCATCAGTGATTTTG......... 1
...............................................GCATCAGTGATTTTGT........ 1
.........................................ACTCCAGCATCAGTGATTTTGTT....... 1
..............................................AGCATCAGTGATTTTGTTGA..... 1
................................................CATCAGTGATTTTGTTGA..... 1
.............................................CAGCATCAGTGATTTTGTTGAAG... 1
((((..((((((.(((((((((((((((..((.....))..))).))))))))).))).))))))..)))) (-31.60)

19/52855360-52855442(-)_novel_cow_20
CTTCTCTCACCCAGCCAAGCCTCTCCTAACAGGAATGGAAACTGAAACCCAACTGTTAGGAGGCTTGGCTGCTGTGGGGGTTG
..................................................AACTGTTAGGAGGCTTGGCTGC........... 19
..................................................AACTGTTAGGAGGCTTGGCTGCT.......... 13
.................................................CAACTGTTAGGAGGCTTGGCTGC........... 9
..................................................AACTGTTAGGAGGCTTGGCTG............ 5
.................................................CAACTGTTAGGAGGCTTGGCTGCT.......... 4
..................................................AACTGTTAGGAGGCTTGGCT............. 3
.................................................CAACTGTTAGGAGGCTTGGCTG............ 3
............AGCCAAGCCTCTCCTAACAGGAA................................................ 1
```

```
...(((((((.((((((((((..(((((((((...(((..........))).)))))))))))))))))))..)))))))... (-43.90)

19/55419749-55419821(+)_mir_cow_101
TTGAATTTGCAGTAACAGGTGTGAACATTCTAGCAGCAGTTTGATGATCATGTATGATACTGCAAACAGAACG
..............................................ATCATGTATGATACTGCAAACA..... 118
.....TTTGCAGTAACAGGTGTGAAC............................................... 59
.....TTTGCAGTAACAGGTGTGAACATT............................................ 45
......TTGCAGTAACAGGTGTGAACATT............................................ 26
....ATTTGCAGTAACAGGTGTGAAC............................................... 20
.....TTTGCAGTAACAGGTGTGAACA.............................................. 15
.....TTTGCAGTAACAGGTGTGAACATTC........................................... 15
......TTGCAGTAACAGGTGTGAACATTC........................................... 14
........................................TTGATGATCATGTATGATACTGCAAACAGAA.. 10
...............................................TCATGTATGATACTGCAAACA..... 9
..............................................ATCATGTATGATACTGCAAAC...... 7
......TTGCAGTAACAGGTGTGAAC............................................... 6
......TTGCAGTAACAGGTGTGAACAT............................................. 5
.............................................GATCATGTATGATACTGCAAACA..... 3
...............................................TCATGTATGATACTGCAAACAG.... 3
.....................................AGTTTGATGATCATGTATGATACTGCAAACAGAA.. 3
....ATTTGCAGTAACAGGTGTGAA................................................ 2
..............................................ATCATGTATGATACTGCAAACAG.... 2
..............................................ATCATGTATGATACTGCAAACAGAA.. 2
...............................................TCATGTATGATACTGCAAACAGAA.. 2
....ATTTGCAGTAACAGGTGTG.................................................. 1
....ATTTGCAGTAACAGGTGTGA................................................. 1
....ATTTGCAGTAACAGGTGTGAACA.............................................. 1
.....TTTGCAGTAACAGGTGTGAACAT............................................. 1
.................GGTGTGAACATTCTAGCAGCAGTTTGATG........................... 1
..................GTGTGAACATTCTAGCAGCAGTTTGATGA.......................... 1
..............................................ATCATGTATGATACTGCA......... 1
............................................TGATCATGTATGATACTGCAA........ 1
..............................................ATCATGTATGATACTGCAA........ 1
...............................................TCATGTATGATACTGCAAAC...... 1
...............................................TCATGTATGATACTGCAAACAGA... 1
.......................................TTTGATGATCATGTATGATACTGCAAACAGAA.. 1
.........................................TGATGATCATGTATGATACTGCAAACAGAA.. 1
...........................................ATGATCATGTATGATACTGCAAACAGAA.. 1
```

```
..................................................TGTATGATACTGCAAACAGAA..  1
.....(((((((((.((.(..(((.((((..(((....))).)))).)))..).)).))))))))).......  (-20.80)

19/9527311-9527385(-)_mir_cow_84
GCAGTCACCCATAAAGTAGAAAGCACTACTAACAGCACTGGAGGGTGTAGTGTTTCCTACTTTATGGATGAGTGT
.........CATAAAGTAGAAAGCACTACT.............................................     22781
.......CCCATAAAGTAGAAAGCACT................................................     18969
.......CCCATAAAGTAGAAAGCACTAC..............................................     6945
.......CCCATAAAGTAGAAAGCACTA...............................................     2574
................................................AGTGTTTCCTACTTTATGGATG.....     1852
.........CATAAAGTAGAAAGCACTAC..............................................     1409
.............................................TGTAGTGTTTCCTACTTTATGGA.......     1370
..........ATAAAGTAGAAAGCACTACT.............................................     738
.........CATAAAGTAGAAAGCACTACTA............................................     676
.........CATAAAGTAGAAAGCACT................................................     638
.......CCCATAAAGTAGAAAGCAC.................................................     376
.........CATAAAGTAGAAAGCACTA...............................................     257
.......CCCATAAAGTAGAAAGCACTACT.............................................     172
..............................................GTAGTGTTTCCTACTTTATGGA.......     137
.............................................TGTAGTGTTTCCTACTTTATGG........     113
..............................................GTAGTGTTTCCTACTTTATGGAT......     70
.........CATAAAGTAGAAAGCACTACTAA...........................................     60
.............................................TGTAGTGTTTCCTACTTTATGGAT......     57
.......CCCATAAAGTAGAAAGCA..................................................     41
................................................AGTGTTTCCTACTTTATGGAT......     36
...............................................TAGTGTTTCCTACTTTATGGA.......     30
..............................................GTAGTGTTTCCTACTTTATGG........     26
...............................................TAGTGTTTCCTACTTTATGGAT......     24
........CCATAAAGTAGAAAGCACT................................................     21
..........ATAAAGTAGAAAGCACT................................................     20
.......CCCATAAAGTAGAAAGC...................................................     14
........CCATAAAGTAGAAAGCACTAC..............................................     14
..........ATAAAGTAGAAAGCACTAC..............................................     13
.............................................TGTAGTGTTTCCTACTTTATG.........     13
................................................AGTGTTTCCTACTTTATGGA.......     6
.........CATAAAGTAGAAAGCA..................................................     5
...........TAAAGTAGAAAGCACTACT.............................................     5
...............................................TAGTGTTTCCTACTTTATGG........     4
```

........CCATAAAGTAGAAAGCACTA... 3
..........ATAAAGTAGAAAGCACTA... 3
..........ATAAAGTAGAAAGCACTACTA.. 3
....TGTAGTGTTTCCTACTTTA........... 3
....GTAGTGTTTCCTACTTTATG......... 3
....AGTGTTTCCTACTTTATGGATGA.... 3
......ACCCATAAAGTAGAAAGCACT.. 2
......ACCCATAAAGTAGAAAGCACTA... 2
........CCATAAAGTAGAAAGCACTACT... 2
.........CATAAAGTAGAAAGCACTACTAACAGCACTGGAGGG.............................. 2
....TGTAGTGTTTCCTACT.............. 2
....TGTAGTGTTTCCTACTTTAT.......... 2
....AGTGTTTCCTACTTTATGG........ 2
.......CCCATAAAGTAGAAAG.. 1
........CCATAAAGTAGAAAGC... 1
......ACCCATAAAGTAGAAAGCAC... 1
........CCATAAAGTAGAAAGCAC... 1
.............AAGTAGAAAGCACTAC.. 1
.............AAGTAGAAAGCACTACT... 1
...........TAAAGTAGAAAGCACTACTA.. 1
..........ATAAAGTAGAAAGCACTACTAA... 1
..........ATAAAGTAGAAAGCACTACTAAC.. 1
.........CATAAAGTAGAAAGCACTACTAACAGCACTG................................... 1
..........ATAAAGTAGAAAGCACTACTAACAGCACTGGAGGG.............................. 1
.........CATAAAGTAGAAAGCACTACTAACAGCACTGGAGGGT............................. 1
....GTAGTGTTTCCTACTTTAT.......... 1
....GTGTTTCCTACTTTATGGATG..... 1
(((.(((.(((((((((((.(((((((((...(..(....)..)..))))))))).))))))))))).))).))) (-34.00)

1/99671703-99671780(-)_mir_Cow_Blast_15
TCCTGGCCTAAGAAATCAAGCGTGGGTGAGACCTGGTGCAGAACAGGAAGGCGACCCATACTTGGTTTCAGAGGCTGT
....GCGACCCATACTTGGTTTCAG....... 286
....GCGACCCATACTTGGTTTCA........ 85
....GCGACCCATACTTGGTTTC......... 66
....GCGACCCATACTTGGTTTCAGA...... 24
...........GAAATCAAGCGTGGGTGAGACCT.. 7
...........GAAATCAAGCGTGGGTGAGACC... 6
...........GAAATCAAGCGTGGGTGAGAC.. 2

```
..................................................GCGACCCATACTTGGTTT..........  2
........TAAGAAATCAAGCGTGGGTGAGACC.............................................  1
...........GAAATCAAGCGTGGGTGAGACCTGGT.........................................  1
..................................................GCGACCCATACTTGGT............  1
..................................................GCGACCCATACTTGGTT...........  1
....(((((..(((((((((..((((((...((((........))))....).)))))..)))))))))..)))))..  (-34.90)

20/10187786-10187854(-)_mir_cow_110
GCCAGCTAGGATTTGGAAAGATTGCTTTGAATTTGTAGATCAACGCAATTCTTCAAAATCTTAGCTGGG
......TAGGATTTGGAAAGATTGCTTT.........................................     31
......TAGGATTTGGAAAGATTGCTT..........................................     9
......TAGGATTTGGAAAGATTGCT...........................................     7
......TAGGATTTGGAAAGATTGC............................................     1
..........................................ACGCAATTCTTCAAAATCTTAGC....     1
.(((((((((((((.(((..(((((.((((.........)))).)))))..))).))))))))))))). (-31.90)

20/10188765-10188834(-)_novel_cow_22
GTCAGGGTCTTTTCCAATGAGTTAGTCCTTTGGGCACTGATGCGAAGAACTAACTCATTGGAAGAGACCC
.........TTTTCCAATGAGTTAGTCCTTT.......................................    148
.........TTTTCCAATGAGTTAGTCCTT........................................    23
.............................................AGAACTAACTCATTGGAAGAGA...    17
..........TTTCCAATGAGTTAGTCCTTTG......................................    13
.........TTTTCCAATGAGTTAGTCCTTTG......................................    8
.......TCTTTTCCAATGAGTTAGTCCT.........................................    5
.........TTTTCCAATGAGTTAGTCCT.........................................    3
.......TCTTTTCCAATGAGTTAGTCCTT........................................    3
.............................................AGAACTAACTCATTGGAAGAG....    3
............................................AAGAACTAACTCATTGGAAGAGA...    3
.........TTTTCCAATGAGTTAGTCC..........................................    2
...AGGGTCTTTTCCAATGAG.................................................    1
....GGGTCTTTTCCAATGAGT................................................    1
..........TTTCCAATGAGTTAGTCCTT........................................    1
..........TTTCCAATGAGTTAGTCCTTT.......................................    1
............................................AAGAACTAACTCATTGGAAG......    1
.............................................AGAACTAACTCATTGGAAG......    1
.............................................AGAACTAACTCATTGGAAGA.....    1
..............................................GAACTAACTCATTGGAAGAGA...    1
...............................................AACTAACTCATTGGAAGAGA...    1
```

```
...............................................AACTAACTCATTGGAAGAGAC..     1
...............................................AACTAACTCATTGGAAGAGACC.     1
....((((((((((((((((((((((.(((((.(......).))))).)))))))))))))))))))))) (-41.70)

20/13408717-13408783(-)_mir_cow_111
GGCCAAAAAGTTCATTCAGGTTTTCTGTAAGATGTTAGGGAAGGACCTGAATGAACTTTCTGGCCAA
........................................AAGGACCTGAATGAACTTTCTGG....  214
........................................AAGGACCTGAATGAACTTTCT......  100
........................................AAGGACCTGAATGAACTTTCTG.....  94
.........................................AGGACCTGAATGAACTTTCTGG....  40
........................................AAGGACCTGAATGAACTTTC.......  15
.........................................AGGACCTGAATGAACTTTCTGGC...  12
.........................................AGGACCTGAATGAACTTTCTG.....  6
.........................................AGGACCTGAATGAACTTTCT......  3
....AAAAAGTTCATTCAGGTTTTCT.........................................  2
........................................AAGGACCTGAATGAACTTT........  2
...CAAAAAGTTCATTCAGGTTTTC..........................................  1
.....AAAAGTTCATTCAGGTTTTCT.........................................  1
.......................................GAAGGACCTGAATGAACTTTCT......  1
(((((.((((((((((((((((((((.(((....))).)))).)))))))))))))))).))))).. (-38.40)

20/189853-189928(-)_mir_Cow_Blast_19
CTGCCCTCGGCTTCTTTACAGTGCTGCCTTGTTGCATATGGATCAAGCAGCATTGTACAGGGCTATGAAGGCGCTG
.............................................AGCAGCATTGTACAGGGCTATGA........    81223
.............................................AGCAGCATTGTACAGGGCTAT..........    14341
.............................................AGCAGCATTGTACAGGGCTATG.........    13947
.............................................AGCAGCATTGTACAGGGCT............    7437
.............................................AGCAGCATTGTACAGGGC.............    1712
.............................................AGCAGCATTGTACAGGGCTATGAA.......    1683
.............................................AGCAGCATTGTACAGGGCTA...........    1027
...............................................CAGCATTGTACAGGGCTATGA........    481
.............................................AGCAGCATTGTACAGGG..............    35
...............................................CAGCATTGTACAGGGCTATGAA.......    30
................................................AGCATTGTACAGGGCTATGA........    25
.................................................GCATTGTACAGGGCTATGA........    23
..............................................GCAGCATTGTACAGGGCTATGA........    21
...............................................CAGCATTGTACAGGGCTATG.........    19
...................................................ATTGTACAGGGCTATGA........    11
```

....AGCAGCATTGTACAGG............... 8
....TTGTACAGGGCTATGA........ 8
....AGCATTGTACAGGGCTATG......... 7
....AAGCAGCATTGTACAGGGCTATGA........ 6
....CATTGTACAGGGCTATGA........ 6
....CAGCATTGTACAGGGCTAT.......... 5
....GCAGCATTGTACAGGGCTATG......... 5
....GCAGCATTGTACAGGGCTAT.......... 4
....GCATTGTACAGGGCTAT.......... 4
....AAGCAGCATTGTACAGGGCT............ 3
....CAGCATTGTACAGGGCTA........... 3
....AGCAGCATTGTACAGGGCTATGAAG...... 3
........GGCTTCTTTACAGTGCTGCCT... 2
........GGCTTCTTTACAGTGCTGCCTTG... 2
...........TTCTTTACAGTGCTGCCTTGT.. 2
....GCAGCATTGTACAGGGCT............ 2
....AAGCAGCATTGTACAGGGCTAT.......... 2
....GCATTGTACAGGGCTATG......... 2
............TCTTTACAGTGCTGCC.. 1
...............TTACAGTGCTGCCTTG... 1
....AAGCAGCATTGTACAGGGC............. 1
....GGATCAAGCAGCATTGTACAGGGCT............ 1
....CAGCATTGTACAGGGCT............ 1
....AGCATTGTACAGGGCT............ 1
....AGCATTGTACAGGGCTA........... 1
....GCATTGTACAGGGCTA........... 1
....AGCATTGTACAGGGCTAT.......... 1
....AAGCAGCATTGTACAGGGCTATG......... 1
....ATTGTACAGGGCTATG......... 1
...............................TTGCATATGGATCAAGCAGCATTGTACAGGGCTATGA........ 1
....AGCATTGTACAGGGCTATGAA....... 1
....GCATTGTACAGGGCTATGAA....... 1
....ATTGTACAGGGCTATGAA....... 1
..(((.((((((.((.(((((((((((.(((.(.(....).).))))))))))))).)))))))..)).))).... (-30.30)

20/19588121-19588188(+)_mir_Cow_Blast_10
TTTGGTTACAGTTGTTCAACCAGTTACTAATCTACCTAATTGTAACCAGTTGAACAACTGAACCCAAA
.....TTACAGTTGTTCAACCAGTTAC... 286

```
......TACAGTTGTTCAACCAGTTACT........................................ 273
.....TTACAGTTGTTCAACCAGTTACT........................................ 133
...........................................AACCAGTTGAACAACTGAACCC... 123
..........................................TAACCAGTTGAACAACTGAACC.... 104
..........................................TAACCAGTTGAACAACTGAACCC... 82
.......ACAGTTGTTCAACCAGTTACT........................................ 51
......TACAGTTGTTCAACCAGTTAC......................................... 48
.............................................CCAGTTGAACAACTGAACCCA.. 33
..........................................TAACCAGTTGAACAACTGAAC..... 25
.............................................CCAGTTGAACAACTGAACCC... 20
...........................................AACCAGTTGAACAACTGAACC.... 18
.....TTACAGTTGTTCAACCAGTT........................................... 14
.....TTACAGTTGTTCAACCAGT............................................ 10
.....TTACAGTTGTTCAACCAGTTA.......................................... 9
...........................................AACCAGTTGAACAACTGAAC..... 7
......TACAGTTGTTCAACCAGTTA.......................................... 3
..........................................TAACCAGTTGAACAACTG........ 2
..........................................TAACCAGTTGAACAACTGAA...... 2
.....TTACAGTTGTTCAACCAG............................................. 1
....GTTACAGTTGTTCAACCAGT............................................ 1
....GTTACAGTTGTTCAACCAGTT........................................... 1
......TACAGTTGTTCAACCAGTT........................................... 1
....GTTACAGTTGTTCAACCAGTTAC......................................... 1
.......ACAGTTGTTCAACCAGTTAC......................................... 1
.......................................TTGTAACCAGTTGAACAACT......... 1
...........................................AACCAGTTGAACAACT......... 1
..........................................TAACCAGTTGAACAACTGA....... 1
.........................................GTAACCAGTTGAACAACTGAAC..... 1
.............................................CCAGTTGAACAACTGAACC.... 1
..............................................CAGTTGAACAACTGAACCC... 1
...((((.(((((((((((..(((((................)))))..)))))))))))))))).... (-26.54)

20/23963003-23963075(-)_mir_cow_112
TCAGGTAGGCAGTGCATCTCTAGCTGGCTGTTAAAATTTCTTTTTAACAGTTGCTAGTTGCACTCCCCTCTGT
......AGGCAGTGCATCTCTAGCTGGCTGTT......................................... 23
......AGGCAGTGCATCTCTAGCTGGCT............................................ 8
......AGGCAGTGCATCTCTAGCTGGC............................................. 2
......AGGCAGTGCATCTCTAGCTGGCTGT.......................................... 1
```

......AGGCAGTGCATCTCTAGCTGGCTGTTA.. 1
....CAGTTGCTAGTTGCACTCCCCT.... 1
.((((..((.((((((...(((((.(((((((((((.....)))))))))))))))).))))))))..)))). (-32.90)

20/444308-444389(-)_mir_Cow_Blast_7
GGGCTTTCCTTTGTGCTTGATCTAACCATGTGGTGGAACGATGGAAACGGAACATGGTTCTGTCAAGCACCGCGGAAAGCAC
..........TTGTGCTTGATCTAACCATGT... 12160
..........TTGTGCTTGATCTAACCATG.. 3839
..........TTGTGCTTGATCTAACCATGTG.. 2666
..........TTGTGCTTGATCTAACCATGTGG... 406
..........TTGTGCTTGATCTAACCATGTGGT.. 148
..........TTGTGCTTGATCTAACCAT... 134
..........TTGTGCTTGATCTAACC... 39
..........TTGTGCTTGATCTAACCA.. 28
....CATGGTTCTGTCAAGCACCGCG........ 8
..........TTGTGCTTGATCTAAC.. 4
............GTGCTTGATCTAACCATGT... 3
....CATGGTTCTGTCAAGCACCG.......... 3
........CTTTGTGCTTGATCTAACCATG.. 2
........CTTTGTGCTTGATCTAACCATGT... 2
...........TGTGCTTGATCTAACCATGT... 2
....CATGGTTCTGTCAAGCACCGC......... 2
....ATGGTTCTGTCAAGCACCGCG........ 2
.........TTTGTGCTTGATCTAACCATG.. 1
............GTGCTTGATCTAACCATG.. 1
.........TTTGTGCTTGATCTAACCATGT... 1
..............GCTTGATCTAACCATGT... 1
..........TTGTGCTTGATCTAACCATGTGGTG... 1
...............................GGTGGAACGATGGAAACGGAAC............................. 1
....ATGGTTCTGTCAAGCA............. 1
....CATGGTTCTGTCAAGCACC........... 1
....ATGGTTCTGTCAAGCACCGC......... 1
....ATGGTTCTGTCAAGCACCGCGGA...... 1
..(((((((...(((((((((..((((((((..........((....))..))))))))..)))))))))...))))))).. (-37.30)

2/107133403-107133467(+)_mir_cow_107
CCCAGTTCAAGTAATTCAGGATAGGTTGTGTGCTGTCCAGCCTGTTCTCCATTACTTGGCTCGGG
.....TTCAAGTAATTCAGGATAGGTT...................................... 174213

```
.....TTCAAGTAATTCAGGATAGGT.......................................   40339
.....TTCAAGTAATTCAGGATAGG........................................   3448
.....TTCAAGTAATTCAGGATAG.........................................   653
.....TTCAAGTAATTCAGGATA..........................................   612
.....TTCAAGTAATTCAGGATAGGTTG.....................................   436
........................................CCTGTTCTCCATTACTTGGCTC...   406
........................................CCTGTTCTCCATTACTTGGCT....   192
.....TTCAAGTAATTCAGGAT...........................................   90
......TCAAGTAATTCAGGATAGGTT......................................   69
.....TTCAAGTAATTCAGGATAGGTTGT....................................   46
.......CAAGTAATTCAGGATAGGTT......................................   32
......TCAAGTAATTCAGGATAGGT.......................................   18
........................................CCTGTTCTCCATTACTTGGC.....   17
...........................GTGTGCTGTCCAGCCTGTTCTCCATTACTTGGCTC...   12
.....TTCAAGTAATTCAGGA............................................   11
........AAGTAATTCAGGATAGGTT......................................   11
...........TAATTCAGGATAGGTT......................................   7
......TCAAGTAATTCAGGATAGGTTG.....................................   7
.......CAAGTAATTCAGGATAGGT.......................................   6
........AAGTAATTCAGGATAGGT.......................................   5
.........AGTAATTCAGGATAGGTT......................................   5
......TCAAGTAATTCAGGATAGG........................................   2
.........AGTAATTCAGGATAGGT.......................................   2
..........GTAATTCAGGATAGGTT......................................   2
......TCAAGTAATTCAGGATAGGTTGT....................................   2
.....TTCAAGTAATTCAGGATAGGTTGTGTGCT...............................   2
........................................CCTGTTCTCCATTACTTGG......   2
......................................AGCCTGTTCTCCATTACTTGGC.....   2
........AAGTAATTCAGGATAGG........................................   1
....GTTCAAGTAATTCAGGATAGGTT......................................   1
.......CAAGTAATTCAGGATAGGTTGT....................................   1
...........TAATTCAGGATAGGTTGTGTGCT...............................   1
..............................TGCTGTCCAGCCTGTTCTCCATTACTTGGCTC...   1
.............................................TCTCCATTACTTGGCTC...   1
........................................CCTGTTCTCCATTACTTGGCTCG..   1
(((((.((((((((..((((((((((((.(......)))))))))))))))..)))))))).))) (-28.50)
```

2/107963030-107963103(-)_mir_cow_108

```
TGCCAGCGTCATTTTTGTGATCTGCAGCTAGTATTCTCACTCCAGTTGCATAGTCACAAAAGTGATCACTGGCA
.............................................TTGCATAGTCACAAAAGTGATC.......28108
.............................................TTGCATAGTCACAAAAGTGAT........1611
.............................................TTGCATAGTCACAAAAGTG..........893
.............................................TTGCATAGTCACAAAAGTGATCA......530
.......GTCATTTTTGTGATCTGCAGCT.............................................404
.............................................TTGCATAGTCACAAAAGTGA.........316
..............................................TGCATAGTCACAAAAGTGATC.......251
.............................................TTGCATAGTCACAAAAGT...........79
.............................................TTGCATAGTCACAAAAGTGATCAC.....31
.............................................TTGCATAGTCACAAAAG............30
.......GTCATTTTTGTGATCTGCAGC..............................................14
..............................................TGCATAGTCACAAAAGTGAT........7
..............................................TGCATAGTCACAAAAGTGATCA......6
........TCATTTTTGTGATCTGCAGCT.............................................5
.......GTCATTTTTGTGATCTGCAGCTA............................................4
..............................................TGCATAGTCACAAAAGTGA.........3
..............................................TGCATAGTCACAAAAGTG..........2
...............................................GCATAGTCACAAAAGTGATC.......2
.............................................TTGCATAGTCACAAAAGTGATCACT....2
......CGTCATTTTTGTGATCTGCAGCT.............................................1
.............................................TTGCATAGTCACAAAA.............1
...............................................GCATAGTCACAAAAGTGAT........1
................................................CATAGTCACAAAAGTGATC.......1
.(((((.((((((((((((((.((((((((((......)))..)))))))..))))))))))))).).))))). (-35.00)

21/19989078-19989146(+)_mir_cow_113
CCCATCTGGAAGACTAGTGATTTTGTTGTTGTCTCCTGCGCTCAACAACAAGTCCCAGTCTGCCGCATG
......TGGAAGACTAGTGATTTTGTTGTT.......................................     5009
......TGGAAGACTAGTGATTTTGTTGT........................................     2722
......TGGAAGACTAGTGATTTTGTTG.........................................     169
......TGGAAGACTAGTGATTTTGTT..........................................     62
......TGGAAGACTAGTGATTTTGT...........................................     35
......TGGAAGACTAGTGATTTTGTTGTTG......................................     14
........GAAGACTAGTGATTTTGTTGTT.......................................     11
.............................................CAACAAGTCCCAGTCTGCCGCA..     11
.........AAGACTAGTGATTTTGTTGTT.......................................     8
......TGGAAGACTAGTGATTTTG............................................     6
```

```
........GAAGACTAGTGATTTTGTTGT........................................      6
..........AGACTAGTGATTTTGTTGTT.......................................      4
........GAAGACTAGTGATTTTGTTGTTG......................................      4
.............CTAGTGATTTTGTTGTT.......................................      3
......TGGAAGACTAGTGATTTTGTTGTTGT.....................................      3
......TGGAAGACTAGTGATTTT.............................................      2
.....CTGGAAGACTAGTGATTTTGTTGT........................................      2
.....CTGGAAGACTAGTGATTTTGTTGTT.......................................      2
.......GGAAGACTAGTGATTTTGTTGTT.......................................      2
.............................................CAACAAGTCCCAGTCTGCC.....      2
.............................................CAACAAGTCCCAGTCTGCCG....      2
.............................................CAACAAGTCCCAGTCTGCCGC...      2
......TGGAAGACTAGTGATT...............................................      1
........GAAGACTAGTGATTTTGT...........................................      1
.........AAGACTAGTGATTTTGTT..........................................      1
.......GGAAGACTAGTGATTTTGTTGT........................................      1
...........GACTAGTGATTTTGTTGT........................................      1
............ACTAGTGATTTTGTTGT........................................      1
.............CTAGTGATTTTGTTGT........................................      1
....TCTGGAAGACTAGTGATTTTGTTGTT.......................................      1
...........GACTAGTGATTTTGTTGTT.......................................      1
............ACTAGTGATTTTGTTGTT.......................................      1
..............TAGTGATTTTGTTGTT.......................................      1
......TGGAAGACTAGTGATTTTGTTGTTGTCT...................................      1
............................................ACAACAAGTCCCAGTCTGCC.....      1
..............................................AACAAGTCCCAGTCTGCCGC...      1
............................................ACAACAAGTCCCAGTCTGCCGCA..      1
..............................................AACAAGTCCCAGTCTGCCGCA..      1
..(((.(((.(((((.(.(((((.(((((((...........))))))))))))).))))).))).))) (-21.50)

21/21250110-21250182(-)_mir_Cow_Blast_32
ATTTCTACTTTCGGTTATCTAGCTTTATGACGGCTCTGTGGCACTCATACAGCTAGATAACCAAAGAGAGAAA
.........TTCGGTTATCTAGCTTTATGA........................................... 8
.........TTCGGTTATCTAGCTTTAT............................................. 2
.........TTCGGTTATCTAGCTTTATGAC.......................................... 2
........TTTCGGTTATCTAGCTTTATGA........................................... 1
..............TTATCTAGCTTTATGA........................................... 1
..........TCGGTTATCTAGCTTTATGAC.......................................... 1
```

..........TCGGTTATCTAGCTTTATGACG... 1
....ATACAGCTAGATAACCAAAGA...... 1
.(((((.((((.((((((((((((.(((((..(((....)))..))))).))))))))))))))))))))).. (-33.40)

21/21250110-21250182(+)_mir_Cow_Blast_33
TTTCTCTCTTTGGTTATCTAGCTGTATGAGTGCCACAGAGCCGTCATAAAGCTAGATAACCGAAAGTAGAAAT
......TCTTTGGTTATCTAGCTGTATGA.. 443629
......TCTTTGGTTATCTAGCTGTAT.. 153938
......TCTTTGGTTATCTAGCTGTATG... 133621
.......CTTTGGTTATCTAGCTGTATGA.. 19453
....ATAAAGCTAGATAACCGAAAGT...... 15674
......TCTTTGGTTATCTAGCTGTA... 8675
......TCTTTGGTTATCTAGCTGT.. 6474
....TAAAGCTAGATAACCGAAAGTA..... 3953
....TAAAGCTAGATAACCGAAAGT...... 2830
.......CTTTGGTTATCTAGCTGTATG... 1923
........TTTGGTTATCTAGCTGTATGA.. 1913
....ATAAAGCTAGATAACCGAAAGTA..... 1687
.......CTTTGGTTATCTAGCTGTAT.. 1196
....ATAAAGCTAGATAACCGAAA........ 571
....ATAAAGCTAGATAACCGAAAG....... 519
......TCTTTGGTTATCTAGC... 496
......TCTTTGGTTATCTAGCTGTATGAG... 350
......TCTTTGGTTATCTAGCT.. 264
........TTTGGTTATCTAGCTGTATGAG... 261
....TAAAGCTAGATAACCGAAAG....... 254
......TCTTTGGTTATCTAGCTG... 251
.........TTGGTTATCTAGCTGTATGA.. 155
......TCTTTGGTTATCTAGCTGTATGAGT.. 130
........TTTGGTTATCTAGCTGTATG... 124
....TAAAGCTAGATAACCGAAA........ 116
.......CTTTGGTTATCTAGCTGTA... 101
.....CTCTTTGGTTATCTAGCTGTATGA.. 78
........TTTGGTTATCTAGCTGTATGAGT.. 67
.............TTATCTAGCTGTATGA.. 54
....ATAAAGCTAGATAACCGA.......... 52
.....CTCTTTGGTTATCTAGCTGTAT.. 43
........TTTGGTTATCTAGCTGTAT.. 41

............GTTATCTAGCTGTATGA.. 39
.....CTCTTTGGTTATCTAGCTGTATG... 30
.......CTTTGGTTATCTAGCTGT.. 28
.......CTTTGGTTATCTAGCTGTATGAGT.. 27
....ATAAAGCTAGATAACCGAA......... 26
....TAAAGCTAGATAACCGAAAGTAG.... 26
.......CTTTGGTTATCTAGCTGTATGAG... 24
...........GGTTATCTAGCTGTATGA.. 21
.........TTGGTTATCTAGCTGTATG... 20
.........TTGGTTATCTAGCTGTATGAGT.. 15
.........TTGGTTATCTAGCTGTAT.. 13
..........TGGTTATCTAGCTGTATGA.. 13
....CATAAAGCTAGATAACCGAAA........ 12
.............TTATCTAGCTGTATGAGTGCCACAG................................... 9
....CATAAAGCTAGATAACCGAAAGT...... 9
...........GGTTATCTAGCTGTATG... 8
............GTTATCTAGCTGTATG... 8
........TTTGGTTATCTAGCTGTA... 7
..........TGGTTATCTAGCTGTATG... 7
.........TTGGTTATCTAGCTGTATGAG... 7
....ATAAAGCTAGATAACCGAAAGTAG.... 6
...........GGTTATCTAGCTGTAT.. 5
....ATAAAGCTAGATAACC............ 5
.......CTTTGGTTATCTAGCT.. 4
..........TGGTTATCTAGCTGTAT.. 3
.............TTATCTAGCTGTATGAGTGCCA...................................... 3
....CTAGATAACCGAAAGT...... 3
.....CTCTTTGGTTATCTAGCTGT.. 2
.........TTGGTTATCTAGCTGT.. 2
.....CTCTTTGGTTATCTAGCTGTA... 2
......TCTTTGGTTATCTAGCTGTATGAGTG... 2
......TCTTTGGTTATCTAGCTGTATGAGTGC.. 2
....CGTCATAAAGCTAGATAACCGA.......... 2
....AAGCTAGATAACCGAAAGT...... 2
.......CTTTGGTTATCTAGCTG... 1
........TTTGGTTATCTAGCTG... 1
....TCTCTTTGGTTATCTAGCTGTAT.. 1
.....CTCTTTGGTTATCTAGCTGTATGAG... 1

```
...............ATCTAGCTGTATGAGT.......................................... 1
.........TTGGTTATCTAGCTGTATGAGTG......................................... 1
..........TGGTTATCTAGCTGTATGAGTG......................................... 1
..........TGGTTATCTAGCTGTATGAGTGC........................................ 1
............GTTATCTAGCTGTATGAGTGC........................................ 1
.............TTATCTAGCTGTATGAGTGC........................................ 1
......TCTTTGGTTATCTAGCTGTATGAGTGCC....................................... 1
.............TTATCTAGCTGTATGAGTGCC....................................... 1
......TCTTTGGTTATCTAGCTGTATGAGTGCCA...................................... 1
.............TTATCTAGCTGTATGAGTGCCACA.................................... 1
.............TTATCTAGCTGTATGAGTGCCACAGAGCCGT............................. 1
.............TTATCTAGCTGTATGAGTGCCACAGAGCCGTC............................ 1
..............................................TAAAGCTAGATAACCGA.......... 1
..............................................TAAAGCTAGATAACCGAA......... 1
...............................................AAAGCTAGATAACCGAAAGT...... 1
............................................CATAAAGCTAGATAACCGAAAGTA..... 1
..................................................GCTAGATAACCGAAAGTA..... 1
(((((((.(((((((((((((((.(((((..((......))..))))).))))))))))))))))).))))). (-32.20)

2/122503459-122503524(+)_novel_cow_21
CAGGTGGACCTGGGTGACTTAAGAGAAGTCAAGTCTGTTCTTAAGATCACACAGGTGGACCTGGGT
........................................TTAAGATCACACAGGTGGACCT....   12
........................................TTAAGATCACACAGGTGGACC.....   5
...GTGGACCTGGGTGACTTAAGA..........................................   2
...GTGGACCTGGGTGACTTAAG...........................................   1
....TGGACCTGGGTGACTTAAGA..........................................   1
...GTGGACCTGGGTGACTTAAGAGA........................................   1
....TGGACCTGGGTGACTTAAGAGA........................................   1
............................TCAAGTCTGTTCTTAAGATCACACAGGTGGA.......   1
........................................TTAAGATCACACAGGTGGACCTG...   1
(((((..(((((.((((((((((((.((......)).)))))))).)))).)))))..)))))... (-30.70)

21/2296445-2296535(+)_novel_cow_23
TATAGATGAACTCCACATTGTCCTACTTCTCAGCTGTCTTGACTCCCTAACAAGATAGCAGAGGAGTAGGAGGACATGGAGTTCGTCTCTC
.................TTGTCCTACTTCTCAGCTGTCT....................................................1355
......................................................ATAGCAGAGGAGTAGGAGGAC................71
......................................................ATAGCAGAGGAGTAGGAGGACA...............60
.................TTGTCCTACTTCTCAGCTGTC.....................................................34
```

```
..................TGTCCTACTTCTCAGCTGTCT....................................................18
.................TTGTCCTACTTCTCAGCTGTCTT...................................................16
.................TTGTCCTACTTCTCAGCTGT......................................................12
..................TGTCCTACTTCTCAGCTGTCTT...................................................10
......................................................ATAGCAGAGGAGTAGGAGGA.................7
.................TTGTCCTACTTCTCAGCTGTCTTG..................................................3
..................TGTCCTACTTCTCAGCTGTCTTG..................................................2
......................................................ATAGCAGAGGAGTAGGAGG..................2
.................TTGTCCTACTTCTCAGC.........................................................1
.................TTGTCCTACTTCTCAGCTG.......................................................1
......................................................ATAGCAGAGGAGTAGGAG...................1
....................................................AGATAGCAGAGGAGTAGGAGGA.................1
......................................................ATAGCAGAGGAGTAGGAGGACAT..............1
...((((((((((((..((.(((((((((((.(((((((((.........))))))))).))))))))))).))..))))))))))))... (-51.60)

21/28046433-28046500(-)_mir_cow_114
TGTGGGCTTCCCTTTGTCATCCTTTGCCCAGGGTTCTGAGTGGGGCAGGGACAGCAAAGGGGTGCTCA
.......TTCCCTTTGTCATCCTTTGCCC....................................... 92
.......TTCCCTTTGTCATCCTTTGCC........................................ 17
.......TTCCCTTTGTCATCCT............................................. 2
.......TTCCCTTTGTCATCCTTTG.......................................... 2
......CTTCCCTTTGTCATCCTTTGCCC....................................... 1
.......TTCCCTTTGTCATCCTTTGCCCA...................................... 1
............................................GCAGGGACAGCAAAGGGGTG.... 1
..(((((.(((((((((..(((((.((((.............)))))))))..))))))))).))))) (-33.52)

2/130389238-130389305(-)_mir_cow_109
GGCCAAAAAGTTCATTTGGGTTTTTGTATGGTGTTACAGAAAAACCTGAATGAACTTTTTGGCCTACC
........................................AAAACCTGAATGAACTTTTTGG...... 2418
.........................................AAACCTGAATGAACTTTTTGG...... 480
........................................AAAACCTGAATGAACTTTTTG....... 124
.........................................AAACCTGAATGAACTTTTTGGC..... 77
........................................AAAACCTGAATGAACTTTTTGGC..... 38
.......................................AAAAACCTGAATGAACTTTTTG....... 22
........................................AAAACCTGAATGAACTTTTT........ 21
.......................................AAAAACCTGAATGAACTTTTTGG...... 21
....AAAAAGTTCATTTGGGTTTTT........................................... 11
........................................AAAACCTGAATGAACTTTT......... 7
```

```
....AAAAAGTTCATTTGGGTTTT............................................ 6
...CAAAAAGTTCATTTGGGTTTTT........................................... 6
.......................................AAAAACCTGAATGAACTTTTT........ 6
.........................................AAACCTGAATGAACTTTTTG....... 6
..........................................AACCTGAATGAACTTTTTGGCC.... 6
..........................................AACCTGAATGAACTTTTTGGCCT... 6
..........................................AACCTGAATGAACTTTTTGG...... 5
...........................................ACCTGAATGAACTTTTTGGCC.... 4
....AAAAAGTTCATTTGGGTTT............................................. 3
.........................................AAACCTGAATGAACTTTTT........ 3
............................................CCTGAATGAACTTTTTGGCC.... 3
...CAAAAAGTTCATTTGGGTTTT............................................ 2
..........................................AACCTGAATGAACTTTTTGGC..... 2
.........................................AAACCTGAATGAACTTTTTGGCC.... 2
...CAAAAAGTTCATTTGGGTTT............................................. 1
....AAAAAGTTCATTTGGGTTTTTG.......................................... 1
.......................................AAAAACCTGAATGAACT............ 1
.....................................AGAAAAACCTGAATGAACTTT.......... 1
........................................AAAACCTGAATGAACTTT.......... 1
.......................................AAAAACCTGAATGAACTTTT......... 1
.........................................AAACCTGAATGAACTTTT......... 1
..............................................TGAATGAACTTTTTGG...... 1
((((((((((((((((..((((((((((......))))))..))))..))))))))))))))))....  (-37.90)
```

```
21/43809623-43809687(+)_mir_cow_115
TGTGTACTCGGAATTGTTTGTGTACCTGTTTTCAAATTCCAAGTACACAAACAATTCCAAGTACA
......CTCGGAATTGTTTGTGTACCT......................................    500
.....ACTCGGAATTGTTTGTGTACCT......................................    487
.......TCGGAATTGTTTGTGTACCTG.....................................    333
.......TCGGAATTGTTTGTGTACCT......................................    287
.......TCGGAATTGTTTGTGTACCTGT....................................    275
......CTCGGAATTGTTTGTGTACCTG.....................................    180
.....ACTCGGAATTGTTTGTGTACC.......................................    77
.....ACTCGGAATTGTTTGTGTAC........................................    49
......CTCGGAATTGTTTGTGTACC.......................................    48
......CTCGGAATTGTTTGTGTACCTGT....................................    20
.......TCGGAATTGTTTGTGTACC.......................................    17
......CTCGGAATTGTTTGTGTAC........................................    15
```

```
.........................................AGTACACAAACAATTCCAAGT...    13
.....ACTCGGAATTGTTTGTGTACCTG.....................................    8
........................................AAGTACACAAACAATTCCAAGT...    7
.......TCGGAATTGTTTGTGTACCTGTT...................................    4
.........................................AGTACACAAACAATTCCAAGTA..    4
.......TCGGAATTGTTTGTGTAC........................................    2
....TACTCGGAATTGTTTGTGTACC.......................................    2
.....ACTCGGAATTGTTTGTGTA.........................................    1
........CGGAATTGTTTGTGTACCTGTTT..................................    1
.......TCGGAATTGTTTGTGTACCTGTTTTCAAATT...........................    1
..((((((.((((((((((((((((.((...........)).)))))))))))))))).)))))) (-30.70)

21/55818275-55818342(+)_mir_cow_116
CCCCGGGGTCCTCTTGTCTGAGCCCCAGAAAGAGGAGAGAGTGCTGGGTCACAGAAGAGGGTCTGGGG
............................................TGGGTCACAGAAGAGGGTCTGG.. 27
............................................TGGGTCACAGAAGAGGGTCTG... 9
............................................TGGGTCACAGAAGAGGGTCT.... 3
............................................TGGGTCACAGAAGAGGGTC..... 2
....GGGGTCCTCTTGTCTGAGC............................................. 1
.....GGGTCCTCTTGTCTGAGCCC........................................... 1
.......GTCCTCTTGTCTGAGCCCCAG........................................ 1
...........................................CTGGGTCACAGAAGAGGGTCTG... 1
.(((.((..(((((..((((.(.(((((...............))))).).)))))))))..)).))) (-31.16)

21/66706863-66706934(+)_mir_cow_117
GGCGAGGGGTGCTATCTGTGGTTGAGGACACGGCAAATGAAACTGTCTCACACAGAAATCGCACCCATCTCC
.............................................TCTCACACAGAAATCGCACCCATC...   6567
.............................................TCTCACACAGAAATCGCACCCAT....   6110
.............................................TCTCACACAGAAATCGCACCCATCT..   3011
.............................................TCTCACACAGAAATCGCACCCA.....   2702
.............................................TCTCACACAGAAATCGCACCC......   612
.............................................TCTCACACAGAAATCGCAC........   314
.............................................TCTCACACAGAAATCGCACC.......   229
...............................................TCACACAGAAATCGCACCCATCT..   151
...............................................TCACACAGAAATCGCACCCATC...   45
...............................................TCACACAGAAATCGCACCCAT....   33
....AGGGGTGCTATCTGTGGTTGAGG.............................................   28
....AGGGGTGCTATCTGTGGTTGAGGAC...........................................   27
```

```
..............................................CTCACACAGAAATCGCACCCATC...  23
..............................................CTCACACAGAAATCGCACCCATCT..  19
..............................................CTCACACAGAAATCGCACCCAT....  18
....AGGGGTGCTATCTGTGGTTGAGGA............................................  12
....AGGGGTGCTATCTGTGGTTGAG..............................................  11
....AGGGGTGCTATCTGTGGTTGAGGACA..........................................  10
....AGGGGTGCTATCTGTGGTTGA...............................................  5
.............................................TCTCACACAGAAATCGC..........  4
....AGGGGTGCTATCTGTGGTTG................................................  3
...............................................TCACACAGAAATCGCACCC......  3
....................................................CAGAAATCGCACCCATCT..  3
..............................................CTCACACAGAAATCGCACCCA.....  2
..................................................CACAGAAATCGCACCCATCT..  2
...........................ACACGGCAAATGAAACTGTC.........................  1
.............................ACGGCAAATGAAACTGTCT........................  1
.............................................TCTCACACAGAAATCGCA.........  1
..............................................CTCACACAGAAATCGCAC........  1
..............................................CTCACACAGAAATCGCACC.......  1
..............................................CTCACACAGAAATCGCACCC......  1
.............................ACGGCAAATGAAACTGTCTCACACAGAAATCGCACCCA.....  1
................................................CACACAGAAATCGCACCCATC...  1
.................................................ACACAGAAATCGCACCCATC...  1
...................................................ACAGAAATCGCACCCATC...  1
....................................................CAGAAATCGCACCCATC...  1
((.((.((((((..((((((..((((.(((.(.........))))))))))))))....)))))).)).))  (-26.50)

21/66867089-66867170(+)_mir_cow_118
CAAACCCAGGTCTGCTGACTCCTAGTCCAGTGCTTGTGATGGCTGGTGGGCCCTGAACTAGGGGTCTGGAGGCCTGGGTTTG
..................................................CCCTGAACTAGGGGTCTGGAG...........    450
.............GCTGACTCCTAGTCCAGTGCT................................................    439
..................................................CCCTGAACTAGGGGTCTGGA............    90
..................................................CCCTGAACTAGGGGTCTGG.............    57
...................................................CCTGAACTAGGGGTCTGGAG...........    46
.............GCTGACTCCTAGTCCAGTG..................................................    39
.............GCTGACTCCTAGTCCAGTGCTT...............................................    19
.............GCTGACTCCTAGTCCAGTGC.................................................    9
...................................................CCTGAACTAGGGGTCTGGA............    7
..................................................CCCTGAACTAGGGGTCTGGAGG..........    7
```

```
............TGCTGACTCCTAGTCCAGTGCT................................................   5
.AAACCCAGGTCTGCTGACTCCT...........................................................   3
............................AGTGCTTGTGATGGCTGGTGGGCCCTGAAC........................   2
..................................TGTGATGGCTGGTGGGCCCTGAACTAGGGGTCTGGAG...........   2
....................................................CTGAACTAGGGGTCTGGAG...........   2
...................................................CCTGAACTAGGGGTCTGGAGG..........   2
...ACCCAGGTCTGCTGACTCCTAGT........................................................   1
....CCCAGGTCTGCTGACTCCTAGT........................................................   1
.............GCTGACTCCTAGTCCAGT...................................................   1
............TGCTGACTCCTAGTCCAGTG..................................................   1
..................CTCCTAGTCCAGTGCT................................................   1
................GACTCCTAGTCCAGTGCTT...............................................   1
.................................TTGTGATGGCTGGTGGGCCC.............................   1
.................................TTGTGATGGCTGGTGGGCCCTGAAC........................   1
..................................................CCCTGAACTAGGGGTCTG..............   1
..........................................................TAGGGGTCTGGAGGCCTGGGTT..   1
(((((((((((((.(.((((((((((.(((.(((..(.(....).)..))).))).)))))))))).).))))))))))))) (-51.80)

21/67089273-67089356(-)_novel_cow_25
GCTGCCGGATGAAGTACAGGATGCCCAATGAATTAAATCCATCTAAATACATTTCACTGGGCATCCTCTGCTTTATTGGGCTGC
.....................................................TCACTGGGCATCCTCTGCTTTA.........  181
.....................................................TCACTGGGCATCCTCTGCTTT..........  161
.....................................................TCACTGGGCATCCTCTGCTT...........  106
...........AAGTACAGGATGCCCAATGAAT...................................................  64
.....................................................TCACTGGGCATCCTCTGCTTTAT........  52
....................................................TTCACTGGGCATCCTCTGCTTT..........  44
......................................................CACTGGGCATCCTCTGCTTTA.........  34
....................................................TTCACTGGGCATCCTCTGCTT...........  32
....................................................TTCACTGGGCATCCTCTGCTTTA.........  28
...........AAGTACAGGATGCCCAATGAATT..................................................  22
.....................................................TCACTGGGCATCCTCTGCT............  18
......................................................CACTGGGCATCCTCTGCTTT..........  16
...........AAGTACAGGATGCCCAATGAA....................................................  13
....................................................TTCACTGGGCATCCTCTGCTTTAT........  11
....................................................TTCACTGGGCATCCTCTGCT............  6
...........AAGTACAGGATGCCCAATGA.....................................................  5
......................................................CACTGGGCATCCTCTGCTT...........  4
.......................................................ACTGGGCATCCTCTGCTTTATT.......  4
```

```
..............TACAGGATGCCCAATGAAT...................................................  3
......................................................CACTGGGCATCCTCTGCT............  2
...................................................TTTCACTGGGCATCCTCTGCTTTA.........  2
......................................................CACTGGGCATCCTCTGCTTTAT........  2
...........AAGTACAGGATGCCCAATG......................................................  1
............AGTACAGGATGCCCAATGAAT...................................................  1
.....................................................TCACTGGGCATCCTCTG..............  1
.....................................................TCACTGGGCATCCTCTGC.............  1
...................................................TTTCACTGGGCATCCTCTGCTT...........  1
...................................................TTTCACTGGGCATCCTCTGCTTTAT........  1
...................................................TTTCACTGGGCATCCTCTGCTTTATT.......  1
....................................................TTCACTGGGCATCCTCTGCTTTATT.......  1
.....................................................TCACTGGGCATCCTCTGCTTTATT.......  1
((.(((.(((((((((.((((((((((.((((....................)))).)))))))))).))))))))).))).)) (-40.85)

21/67416614-67416686(+)_mir_cow_119
AGGGCCTTGTACATGGTAGGCTTTCATTCATTCGTTTGCACATTCGGTGAAGGTCTACTGTGTGCCAGGCCCT
......TTGTACATGGTAGGCTTTCATT.............................................  960
...............................................TGAAGGTCTACTGTGTGCCAGG....  539
......TTGTACATGGTAGGCTTTCAT..............................................  111
...............................................TGAAGGTCTACTGTGTGCCAG.....  67
......TTGTACATGGTAGGCTTTCA...............................................  41
...............................................TGAAGGTCTACTGTGTGCC.......  28
...............................................TGAAGGTCTACTGTGTGCCA......  23
......TTGTACATGGTAGGCTTTC................................................  7
...............................................TGAAGGTCTACTGTGTGC........  6
.......TGTACATGGTAGGCTTTCATT.............................................  3
.................................................AAGGTCTACTGTGTGCCAGGCC..  3
.........TACATGGTAGGCTTTCATTCATT.........................................  2
......TTGTACATGGTAGGCT...................................................  1
......TTGTACATGGTAGGCTTT.................................................  1
....CCTTGTACATGGTAGGCTTTCA...............................................  1
.....CTTGTACATGGTAGGCTTTCA...............................................  1
.....CTTGTACATGGTAGGCTTTCATT.............................................  1
............................CATTCGTTTGCACATTCGGTGAAG.....................  1
.................................................AAGGTCTACTGTGTGCCAGG....  1
(((((((.((((((((((((((((((((.....((....))....)))))))))))))))))))).))))))) (-39.60)
```

```
21/67423453-67423517(+)_mir_cow_120
CTTGAGGGGTCTTGGCCTCTGCCCAGGACTCTTTCATGACCAGTAGGCCGAGGCCCCTCACAGGC
......................................ACCAGTAGGCCGAGGCCCCTC......    343
......................................ACCAGTAGGCCGAGGCCCCTCA.....    282
......................................ACCAGTAGGCCGAGGCCCCTCAC....    195
......................................ACCAGTAGGCCGAGGCCCCT.......    113
.......................................CCAGTAGGCCGAGGCCCCTCAC....    74
......................................ACCAGTAGGCCGAGGCCCCTCACA...    29
......................................ACCAGTAGGCCGAGGCCC.........    18
......................................ACCAGTAGGCCGAGGCCCC........    14
.......................................CCAGTAGGCCGAGGCCCCTC......    10
.......................................CCAGTAGGCCGAGGCCCCTCACA...    10
.......................................CCAGTAGGCCGAGGCCCCTCA.....    9
....AGGGGTCTTGGCCTCTGCCC.........................................    7
......................................ACCAGTAGGCCGAGGCC..........    3
......................................ACCAGTAGGCCGAGGCCCCTCACAG..    3
................................TTCATGACCAGTAGGCCGAGGCCC.........    2
.......................................CCAGTAGGCCGAGGCCCCT.......    2
........................................CAGTAGGCCGAGGCCCCT.......    2
....................................TGACCAGTAGGCCGAGGCCCCTCA.....    2
........................AGGACTCTTTCATGACCAGTAG...................    1
................................TTCATGACCAGTAGGCCGAGG............    1
.......................CAGGACTCTTTCATGACCAGTAGGCCGAGGC...........    1
...............................TTTCATGACCAGTAGGCCGAGGCC..........    1
.....................................GACCAGTAGGCCGAGGCCC.........    1
...............................TTTCATGACCAGTAGGCCGAGGCCCC........    1
................................TTCATGACCAGTAGGCCGAGGCCCC........    1
.................................TCATGACCAGTAGGCCGAGGCCCC........    1
................................TTCATGACCAGTAGGCCGAGGCCCCT.......    1
...................................ATGACCAGTAGGCCGAGGCCCCTC......    1
....................................TGACCAGTAGGCCGAGGCCCCTC......    1
(((((((((((((((((((((..((.((.....)).))..))).))))))))))))))))).)).. (-37.80)

21/67457242-67457311(+)_mir_Cow_Blast_41
GAGAAGCCAGGTCACGTCTCTGCAGTTACACCGCTCACGAGTGCCTGCTGGGGTGGAACCTGGTCTGTCT
..........................................GCCTGCTGGGGTGGAACCTGGT......     1810
..........................................GCCTGCTGGGGTGGAACCTGG.......     104
..........................................GCCTGCTGGGGTGGAACCTG........     57
```

```
..........................................GCCTGCTGGGGTGGAACC..........    49
..........................................GCCTGCTGGGGTGGAACCT.........    47
.........................................TGCCTGCTGGGGTGGAACCTGGT......    32
..........................................GCCTGCTGGGGTGGAACCTGGTC.....    31
.......CAGGTCACGTCTCTGCAGTTAC.........................................    29
.......CAGGTCACGTCTCTGCAGTT...........................................    24
.......CAGGTCACGTCTCTGCAGTTACA........................................    22
.............................................TGCTGGGGTGGAACCTGGTCT....    22
..........................................GCCTGCTGGGGTGGAACCTGGTCT....    13
....AGCCAGGTCACGTCTCTGCAGT............................................    12
.......CAGGTCACGTCTCTGCAGTTACAC.......................................    11
............................................CTGCTGGGGTGGAACCTGGTCT....    10
.......CAGGTCACGTCTCTGCAGTTA..........................................    9
...........................................CCTGCTGGGGTGGAACCTGGTC.....    9
......CCAGGTCACGTCTCTGCAGTT...........................................    7
...........................................CCTGCTGGGGTGGAACCTGGT......    4
..............................................GCTGGGGTGGAACCTGGTCT....    4
....AGCCAGGTCACGTCTCTGCAG.............................................    3
..........................................GCCTGCTGGGGTGGAAC...........    3
............................................CTGCTGGGGTGGAACCTGGTC.....    3
.............................................TGCTGGGGTGGAACCTGGTCTGT..    3
.......CAGGTCACGTCTCTGCAGT............................................    2
....AGCCAGGTCACGTCTCTGCAGTT...........................................    2
......CCAGGTCACGTCTCTGCAGTTACA........................................    2
.......CAGGTCACGTCTCTGCAGTTACACC......................................    2
.........................................TGCCTGCTGGGGTGGAACCTGG.......    2
...........................................CCTGCTGGGGTGGAACCTGGTCT....    2
....AGCCAGGTCACGTCTCTGC...............................................    1
......CCAGGTCACGTCTCTGCAGTTAC.........................................    1
.........................................TGCCTGCTGGGGTGGAACC..........    1
.........................................TGCCTGCTGGGGTGGAACCT.........    1
...........................................CCTGCTGGGGTGGAACCTG........    1
..............................................GCTGGGGTGGAACCTGGT......    1
.........................................TGCCTGCTGGGGTGGAACCTGGTC.....    1
............................................CTGCTGGGGTGGAACCTGGTCTGT..    1
...............................................CTGGGGTGGAACCTGGTCTGTCT    1
((.(..((((((..(..(((.((((...(((((....)).))).)))).)))..)..))))))..).)). (-30.70)
```

```
21/67561879-67561945(+)_mir_cow_121
AGAGATGGTAGACTATGGAACGTAGGCTTTGTGATTTTTGACCTATGTAACATGGTCCACTAACTCT
.....TGGTAGACTATGGAACGTAGG.........................................  2242
...........................................TATGTAACATGGTCCACTAAC...  456
.....TGGTAGACTATGGAACGTAG..........................................  254
...........................................TATGTAACATGGTCCACTAACT..  175
.....TGGTAGACTATGGAACGTAGGC........................................  88
...........................................TATGTAACATGGTCCACTAA....  22
...........................................TATGTAACATGGTCCACTA.....  21
....ATGGTAGACTATGGAACGTAGG.........................................  18
.....TGGTAGACTATGGAACGT............................................  10
.....TGGTAGACTATGGAACGTA...........................................  10
.....TGGTAGACTATGGAACGTAGGCT.......................................  7
.....TGGTAGACTATGGAACG.............................................  3
....ATGGTAGACTATGGAACGTAG..........................................  3
..........................CTTTGTGATTTTTGACC........................  3
...........................................TATGTAACATGGTCCACT......  2
.....TGGTAGACTATGGAAC..............................................  1
......GGTAGACTATGGAACGTAGG.........................................  1
....ATGGTAGACTATGGAACGTAGGC........................................  1
((((.((((.(((((((..((((((((............).)))))))..))))))).)))).))))  (-26.60)

21/67563106-67563171(+)_mir_cow_122
GGAGAGATAGTAGACCGTATAGCGTACGCTTTATCTGTGACGTATGTAACACGGTCCACTAACCCT
......ATAGTAGACCGTATAGCGTACG......................................  121714
.......TAGTAGACCGTATAGCGTACG......................................  86494
......ATAGTAGACCGTATAGCGTAC.......................................  9446
.......TAGTAGACCGTATAGCGTAC.......................................  4964
..........................................TATGTAACACGGTCCACTAAC...  2045
......ATAGTAGACCGTATAGCGTA........................................  542
......ATAGTAGACCGTATAGCGT.........................................  350
.......TAGTAGACCGTATAGCGTA........................................  282
..........................................TATGTAACACGGTCCACTAACC..  199
..........................................TATGTAACACGGTCCACTAA....  125
.......TAGTAGACCGTATAGCGTACGCT....................................  90
........AGTAGACCGTATAGCGTACG......................................  79
.......TAGTAGACCGTATAGCGTACGC.....................................  41
.......TAGTAGACCGTATAGCGT.........................................  34
```

.......TAGTAGACCGTATAGCGTACGCTT................................... 33
......ATAGTAGACCGTATAGCG.. 22
........AGTAGACCGTATAGCGTACGCT.................................... 22
....TATGTAACACGGTCCACT...... 21
.....GATAGTAGACCGTATAGCGTACG...................................... 16
.......TAGTAGACCGTATAGCGTACGCTTT.................................. 16
.....GATAGTAGACCGTATAGCGTAC....................................... 12
.........GTAGACCGTATAGCGTACG...................................... 10
.......TAGTAGACCGTATAGCG.. 9
......ATAGTAGACCGTATAGCGTACGC..................................... 8
......ATAGTAGACCGTATAGC... 7
....TATGTAACACGGTCCACTA..... 6
.......TAGTAGACCGTATAGC... 5
......ATAGTAGACCGTATAGCGTACGCTT................................... 5
......ATAGTAGACCGTATAGCGTACGCT.................................... 4
....GTATGTAACACGGTCCACTAAC... 4
........AGTAGACCGTATAGCGTAC....................................... 3
....AGATAGTAGACCGTATAGCGTACG...................................... 2
..........TAGACCGTATAGCGTACG...................................... 2
...........AGACCGTATAGCGTACG...................................... 2
............GACCGTATAGCGTACG...................................... 2
..................ATAGCGTACGCTTTATCTGTGACG........................ 2
....GTATGTAACACGGTCCACTAA.... 2
....AGATAGTAGACCGTATAGCGT... 1
....AGATAGTAGACCGTATAGCGTAC....................................... 1
......ATAGTAGACCGTATAGCGTACGCTTT.................................. 1
.......TAGTAGACCGTATAGCGTACGCTTTATCTGTGAC......................... 1
......ATAGTAGACCGTATAGCGTACGCTTTATCTGTGACG........................ 1
....ACGTATGTAACACGGTCCACT...... 1
....CGTATGTAACACGGTCCACT...... 1
.......((((.((((((.(.((((((((.........).))))))).).)))))).))))..... (-20.60)

21/67563570-67563632(+)_mir_cow_123
AAGAAATGGTTTACCGTCCCACATACATTCTGAATATGTATGTGGGACGGTAAACCGCTTCTT
......................................TATGTGGGACGGTAAACCGCT.... 1382
......................................TATGTGGGACGGTAAACCGCTT... 1179
.....................................GTATGTGGGACGGTAAACCGCT.... 547
......TGGTTTACCGTCCCACATACAT................................... 290

......TGGTTTACCGTCCCACATACA....................................	207
......TGGTTTACCGTCCCACATAC.....................................	201
.....................................GTATGTGGGACGGTAAACCGC.....	105
.....ATGGTTTACCGTCCCACATACA....................................	82
......................................TATGTGGGACGGTAAACCGC.....	74
.....ATGGTTTACCGTCCCACATAC.....................................	48
......TGGTTTACCGTCCCACATA......................................	10
.....................................GTATGTGGGACGGTAAACCGCTT...	10
....ATGTGGGACGGTAAACCGCTT...	9
.....................................GTATGTGGGACGGTAAA.........	7
.....................................GTATGTGGGACGGTAAAC........	6
.....................................GTATGTGGGACGGTAAACCG......	6
......................................TATGTGGGACGGTAAACC.......	5
......TGGTTTACCGTCCCACAT.......................................	4
......TGGTTTACCGTCCCACATACATT..................................	4
.....ATGGTTTACCGTCCCACATA......................................	3
.....................................GTATGTGGGACGGTAAACC.......	3
......................................TATGTGGGACGGTAAACCG......	3
....ATGTGGGACGGTAAACCGCT....	3
....................................TGTATGTGGGACGGTAAACCGCT....	2
....TGTGGGACGGTAAACCGCTT...	2
......................................TATGTGGGACGGTAAACCGCTTC..	2
......TGGTTTACCGTCCCAC...	1
....AATGGTTTACCGTCCCACATACA....................................	1
.....ATGGTTTACCGTCCCACATACAT...................................	1
..........TTACCGTCCCACATACATTCTG...............................	1
...........TACCGTCCCACATACATTCTG...............................	1
.....ATGGTTTACCGTCCCACATACATTCTGAA.............................	1
......TGGTTTACCGTCCCACATACATTCTGAATA...........................	1
...........TACCGTCCCACATACATTCTGAATATGT........................	1
...........TACCGTCCCACATACATTCTGAATATGTAT......................	1
............................TCTGAATATGTATGTGGGACGGT............	1
..............................TGAATATGTATGTGGGACGGT............	1
....................................TGTATGTGGGACGGTAAACCG......	1
....................................TGTATGTGGGACGGTAAACCGC.....	1
....ATGTGGGACGGTAAACCGC.....	1
....TGTGGGACGGTAAACCGCT....	1
....GGGACGGTAAACCGCT....	1

```
.......................................ATGTGGGACGGTAAACCGCTTC..      1
........................................TGTGGGACGGTAAACCGCTTC..      1
(((((.((((((((((((((((((((((.......))))))))))))))))))))))).)))))  (-42.30)

21/67564802-67564865(+)_mir_cow_124
AAAGATGGTTGACCATAGAACATGCGCTGTCTCTATGTCGTATGTAATGTGGTCCACGTCTTCT
........................................TATGTAATGTGGTCCACGTCT...    30574
........................................TATGTAATGTGGTCCACGTC....    195
........................................TATGTAATGTGGTCCACGTCTT..    67
....ATGGTTGACCATAGAACATGC.......................................    9
....ATGGTTGACCATAGAACATGCG......................................    6
........................................TATGTAATGTGGTCCACGT.....    6
........TTGACCATAGAACATGCGCTGTCT................................    4
............................................TAATGTGGTCCACGTCT...    3
........................................TATGTAATGTGGTCCACG......    2
.........................................ATGTAATGTGGTCCACGTCT...    2
.........TGACCATAGAACATGCGCTGT..................................    1
........................................TATGTAATGTGGTCCAC.......    1
.......................................GTATGTAATGTGGTCCACGTCT...    1
.((((((...(((((((..((((((((.........).)))))))..)))))))..))))))..  (-24.30)

21/67564962-67565025(+)_mir_cow_125
AGAGATGGTCGACCATAAAACGTACGTGTGCTTTATGACGTATGTCACATGGTCCACTAATTCT
.....TGGTCGACCATAAAACGTACGT.....................................    124
........................................TATGTCACATGGTCCACTAAT...    66
.....TGGTCGACCATAAAACGTAC.......................................    60
.....TGGTCGACCATAAAACGT.........................................    25
.....TGGTCGACCATAAAACGTACG......................................    17
.......................................GTATGTCACATGGTCCACTAAT...    13
........................................TATGTCACATGGTCCACTAA....    5
........................................TATGTCACATGGTCCACT......    4
.......................................GTATGTCACATGGTCCACTAA....    4
.....TGGTCGACCATAAAACGTA........................................    3
........................................TATGTCACATGGTCCACTAATT..    2
.....TGGTCGACCATAAAACG..........................................    1
....ATGGTCGACCATAAAACGT.........................................    1
....ATGGTCGACCATAAAACGTAC.......................................    1
....ATGGTCGACCATAAAACGTACG......................................    1
```

```
....ATGGTCGACCATAAAACGTACGT....................................    1
........TCGACCATAAAACGTACGTGTGCTT..............................    1
((((.((((.((((((...((((((((.........))))))))...)))))).)))).)))) (-22.10)

21/67565479-67565541(+)_mir_cow_126
AGAGAGGTGGTCCGTGGCGCGTTCGCTTTATTTATGGCGCACATTACACGGTCGACCTCTTTG
.......................................CACATTACACGGTCGACCTCT...     4377
......................................GCACATTACACGGTCGACCTCT...     2096
......................................GCACATTACACGGTCGACCTC....     250
.......................................CACATTACACGGTCGACCT.....     229
.......................................CACATTACACGGTCGACCTC....     166
......................................GCACATTACACGGTCGACCT.....     85
....AGGTGGTCCGTGGCGCGTTCGC.....................................     62
....AGGTGGTCCGTGGCGCGTTCG......................................     48
.......................................CACATTACACGGTCGACC......     21
.......................................CACATTACACGGTCGACCTCTT..     14
....AGGTGGTCCGTGGCGCGTTC.......................................     12
.....................................CGCACATTACACGGTCGACCTCT...     9
......................................GCACATTACACGGTCGACC......     7
....AGGTGGTCCGTGGCGCGTTCGCT....................................     6
........................................ACATTACACGGTCGACCTCT...     6
......................................GCACATTACACGGTCGACCTCTT..     4
......................................GCACATTACACGGTCGAC.......     2
...............................TTATGGCGCACATTACACGGTCG.........     1
.......................................CACATTACACGGTCGAC.......     1
.....................................CGCACATTACACGGTCGACC......     1
.....................................CGCACATTACACGGTCGACCT.....     1
.....................................CGCACATTACACGGTCGACCTC....     1
.........................................CATTACACGGTCGACCTC....     1
((((((((.(.(((((....((.((((........)))).))....))))).).))))))))). (-28.90)

21/67565777-67565842(+)_mir_cow_127
TGAGATGGTTGACCAGAGAGCACACGCTTTATTTGTGCCGTTTGTGACCTGGTCCACTAACCCTCA
........................................TTTGTGACCTGGTCCACTAAC.....   307
........................................TTTGTGACCTGGTCCACTAACC....   15
........................................TTTGTGACCTGGTCCACTAA......   5
........................................TTTGTGACCTGGTCCACTAACCCT..   5
................................TTGTGCCGTTTGTGACCTGGTCCACT........   3
```

```
........................................TTTGTGACCTGGTCCACT........  3
....ATGGTTGACCAGAGAGCACAC.........................................  2
....ATGGTTGACCAGAGAGCACACG........................................  2
.....TGGTTGACCAGAGAGCAC...........................................  1
.....TGGTTGACCAGAGAGCACACG........................................  1
................................TTGTGCCGTTTGTGACCTGGTCC...........  1
...................................TGCCGTTTGTGACCTGGTCCACT........  1
........................................TTTGTGACCTGGTCCACTAACCC...  1
.(((.((((.((((((...(((.((((.........).))).)))...)))))).))))...))). (-20.20)

21/67566510-67566584(+)_mir_cow_128
TACCTGAAGAGAGGTTTTCTGGGTTTCTGTTTCTTTAATGAGGATGAAACACACCTGGTTAACCTCTTTTCCAGT
...............................................AACACACCTGGTTAACCTCTT.......     361
...............................................AACACACCTGGTTAACCTCT........     328
...............................................AACACACCTGGTTAACCTCTTT......     118
...............................................AACACACCTGGTTAACCTC.........     36
..........GAGGTTTTCTGGGTTTCTGTTT...........................................     7
..........GAGGTTTTCTGGGTTTCTGTTTC..........................................     7
................................................ACACACCTGGTTAACCTCTTT......     7
..........GAGGTTTTCTGGGTTTCTGT.............................................     6
..........GAGGTTTTCTGGGTTTCTGTT............................................     6
................................................ACACACCTGGTTAACCTCTTTT.....     5
...............................................AACACACCTGGTTAACCTCTTTT.....     3
..........GAGGTTTTCTGGGTTTCT...............................................     2
..........GAGGTTTTCTGGGTTTCTG..............................................     2
.........AGAGGTTTTCTGGGTTTCTGTTTC..........................................     2
................................................ACACACCTGGTTAACCTC.........     2
....TGAAGAGAGGTTTTCTGGGTTTCT...............................................     1
...........AGGTTTTCTGGGTTTCTGTTT...........................................     1
..........GAGGTTTTCTGGGTTTCTGTTTCT.........................................     1
..........GAGGTTTTCTGGGTTTCTGTTTCTTTAAT....................................     1
.....................................ATGAGGATGAAACACACCTGGTT...............     1
...............................................AACACACCTGGTTAACCT..........     1
................................................ACACACCTGGTTAACCTCT........     1
..................................................ACACCTGGTTAACCTCTTTTCC...     1
.................................................CACACCTGGTTAACCTCTTTTCCA..     1
..................................................ACACCTGGTTAACCTCTTTTCCA..     1
...(((((((((((((..(..(((...((((((.((......)).)))))).)))..)..)))))))))).))). (-28.70)
```

```
21/67566826-67566891(+)_mir_cow_129
GAGGAGAGGTTTTCTAGGTCTCTGTTTCTTTCATGAGGATGAAACACACCTGGTTAACCTTTTTTC
.....GAGGTTTTCTAGGTCTCTGTTTC......................................  195
.....GAGGTTTTCTAGGTCTCTGTTT.......................................  120
.....GAGGTTTTCTAGGTCTCTGTT........................................  31
.....GAGGTTTTCTAGGTCTCTGTTTCT.....................................  29
....AGAGGTTTTCTAGGTCTCTGTT.......................................  15
..........................................AACACACCTGGTTAACCTTTTT..  14
..........................................AACACACCTGGTTAACCTTTT...  13
....AGAGGTTTTCTAGGTCTCTGTTT......................................  12
.....GAGGTTTTCTAGGTCTCTGT.........................................  9
.....GAGGTTTTCTAGGTCTC............................................  2
....AGAGGTTTTCTAGGTCTCTGT.........................................  2
....AGAGGTTTTCTAGGTCTCTGTTTC.....................................  2
..........................................AACACACCTGGTTAACCTTT....  2
....AGAGGTTTTCTAGGTCT.............................................  1
.....GAGGTTTTCTAGGTCT.............................................  1
.....GAGGTTTTCTAGGTCTCT...........................................  1
.....GAGGTTTTCTAGGTCTCTG..........................................  1
............................TTTCATGAGGATGAAACACAC.................  1
............................TTTCATGAGGATGAAACACACCT...............  1
............................TTTCATGAGGATGAAACACACCTG..............  1
............................TTTCATGAGGATGAAACACACCTGGT............  1
................................ATGAGGATGAAACACACCTGGTT...........  1
..........................................AACACACCTGGTTAACCT......  1
(((((((((((..((((((...((((((.(((....))).)))))).))))))..))))))))))) (-27.60)

21/67569686-67569751(+)_mir_cow_130
AAGGAGAGGTTATCCGTGTTGTCTTCTCTTTATTTATGATGAAACATACACGGGAAACCTCTTTTT
.......................................TGAAACATACACGGGAAACCTCT....  553
.......................................TGAAACATACACGGGAAACCT......  57
.......................................TGAAACATACACGGGAAACCTC.....  51
.......................................TGAAACATACACGGGAAACCTCTT...  14
......AGGTTATCCGTGTTGTCTTCT.......................................  10
......AGGTTATCCGTGTTGTCTTCTC......................................  7
.......................................TGAAACATACACGGGAAAC........  4
.......................................TGAAACATACACGGGAAACC.......  4
```

```
.......................................TGAAACATACACGGGAA..........   3
....AGAGGTTATCCGTGTTGTCTTC........................................   1
......AGGTTATCCGTGTTGTCTTC........................................   1
......AGGTTATCCGTGTTGTCTTCTCT.....................................   1
..........................................AACATACACGGGAAACCTCTTT..   1
((((((((((.((((((((((.((((((.........)).)))))).))))))).))))))))))) (-25.90)

21/67571832-67571898(+)_mir_cow_131
AAGGAGAAGTTGCCCGTGTCTTTTTCGCTTTATATGTGACGAAACATTCGCGGTGCACTTCTTTTTC
.........................................AAACATTCGCGGTGCACTTCTT....  896
.........................................AAACATTCGCGGTGCACTTCT.....  472
.........................................AAACATTCGCGGTGCACTTCTTT...  238
......AAGTTGCCCGTGTCTTTTTCG........................................  63
.........................................AAACATTCGCGGTGCACT........  14
.........................................AAACATTCGCGGTGCACTTC......  10
.........................................AAACATTCGCGGTGCACTT.......  9
........................................GAAACATTCGCGGTGCACTTCT.....  7
.........................................AAACATTCGCGGTGCACTTCTTTT..  6
......AAGTTGCCCGTGTCTTTTTC.........................................  5
....AGAAGTTGCCCGTGTCTTTTTCG........................................  4
.........................................AAACATTCGCGGTGCA..........  4
.....GAAGTTGCCCGTGTCTTTTTCG........................................  2
......AAGTTGCCCGTGTCTTTTTCGC.......................................  2
......AAGTTGCCCGTGTCTTTTTCGCT......................................  2
..........................................AACATTCGCGGTGCACTTCTTT...  2
....AGAAGTTGCCCGTGTCTTTTT..........................................  1
......AAGTTGCCCGTGTCTTTTTCGCTT.....................................  1
.........................................AAACATTCGCGGTGCAC.........  1
.......................................CGAAACATTCGCGGTGCACTTC......  1
..........................................AACATTCGCGGTGCACTTCT.....  1
..........................................AACATTCGCGGTGCACTTCTT....  1
((((((((((.(((((((....((((((.........).)))))....))))).)))))))))))). (-22.90)

21/67573244-67573308(+)_mir_cow_132
GAAAGAAGTTGCCCATGTTCTTTTCGCTGATATGTGACGAAACAAACATGGTGCACTTCTTTTCC
.......................................AAACAAACATGGTGCACTTCTT....    2468
.......................................AAACAAACATGGTGCACTTCTTT...    464
.......................................AAACAAACATGGTGCACTTCT.....    269
```

```
........................................AACAAACATGGTGCACTTCTTT...    108
........................................AACAAACATGGTGCACTTCTT....    59
.........................................ACAAACATGGTGCACTTCTTT...    38
.........................................ACAAACATGGTGCACTTCTTTT..    30
.......................................AAACAAACATGGTGCACTTC......    24
........................................AACAAACATGGTGCACTTCT.....    16
....GAAGTTGCCCATGTTCTTTTCG.......................................    12
.......................................AAACAAACATGGTGCACTTCTTTT..    8
.......................................AAACAAACATGGTGCACTT.......    7
........................................AACAAACATGGTGCACTTCTTTT..    7
.........................................ACAAACATGGTGCACTTCTT....    5
.........................................ACAAACATGGTGCACTTCT.....    4
.......................................AAACAAACATGGTGCAC.........    3
.......................................AAACAAACATGGTGCACT........    3
....GAAGTTGCCCATGTTCTTTTC........................................    2
........................................AACAAACATGGTGCACTTC......    2
....GAAGTTGCCCATGTTCTTTTCGCTG....................................    1
.................TTCTTTTCGCTGATATGTGACGAAA.......................    1
........................................AACAAACATGGTGCAC.........    1
(((((((((.(((((((((..((((((........).)))))..))))))).))))))))))).. (-25.90)

21/67575257-67575320(+)_mir_cow_133
AAGGAGGTTGTCCGTGATGTATTTGCTTTATTTGTGGCAGATATTGCACGGTTGATCTCTTTTC
......................................AGATATTGCACGGTTGATCTCT....    28317
......................................AGATATTGCACGGTTGATCTC.....    1519
....AGGTTGTCCGTGATGTATTTGC......................................    72
......................................AGATATTGCACGGTTGATCT......    63
.....................................CAGATATTGCACGGTTGATCTCT....    38
.......................................GATATTGCACGGTTGATCTCT....    36
......................................AGATATTGCACGGTTGATCTCTT...    36
....AGGTTGTCCGTGATGTATTTGCT.....................................    19
......................................AGATATTGCACGGTTGATC.......    18
.........................................TATTGCACGGTTGATCTCT....    15
....AGGTTGTCCGTGATGTATTTG.......................................    13
.....................................CAGATATTGCACGGTTGATCTC.....    11
........................................ATATTGCACGGTTGATCTCT....    6
..........................................ATTGCACGGTTGATCTCT....    6
....AGGTTGTCCGTGATGTATT.........................................    4
```

```
...........................................TTGCACGGTTGATCTCT....     4
....AGGTTGTCCGTGATGTATTT........................................     3
......................................AGATATTGCACGGTTGAT........     3
....AGGTTGTCCGTGATGTATTTGCTTTA..................................     1
.......................................GATATTGCACGGTTGATCTC.....     1
............................................TGCACGGTTGATCTCT....     1
......................................AGATATTGCACGGTTGATCTCTTT..     1
((((((((((.(((((..(((((((((........)))))))))..))))).))))))))))..  (-27.30)

21/67577949-67578015(+)_mir_cow_134
AAAAGGTGGATATTCCTTCTATGTTTACAGGATTGACAGCTAAACATAGAGGAAAATCCACATTTTA
..........................................AACATAGAGGAAAATCCACATT...  363
..........................................AACATAGAGGAAAATCCACAT....  46
....GGTGGATATTCCTTCTATGTTT.........................................  42
....GGTGGATATTCCTTCTATGTTTA........................................  13
.....GTGGATATTCCTTCTATGTTT.........................................  11
..........................................AACATAGAGGAAAATCCACA.....  7
.....GTGGATATTCCTTCTATGTTTA........................................  6
....GGTGGATATTCCTTCTATGTT..........................................  5
..........................................AACATAGAGGAAAATCCACATTT..  5
..........................................AACATAGAGGAAAATCCAC......  2
........................................TAAACATAGAGGAAAATCCACA.....  2
.....GTGGATATTCCTTCTATGTT..........................................  1
......TGGATATTCCTTCTATGTTTAC.......................................  1
..........................................AACATAGAGGAAAATC.........  1
..........................................AACATAGAGGAAAATCC........  1
.........................................AAACATAGAGGAAAATCCACA.....  1
((((.((((((.(((((.((((((((((.(......).).)))))))))))))).)))))).)))).  (-24.70)

21/67578322-67578387(+)_mir_cow_135
AAAAGGTGGATATTCCTTCTATGTTTATGTTATTTGTGGTTAAACATAGAGGAAATTCCACGTTTT
..........................................AACATAGAGGAAATTCCACGT...   526
..........................................AACATAGAGGAAATTCCACG....   52
....GGTGGATATTCCTTCTATGTTT........................................   42
..........................................AACATAGAGGAAATTCCACGTT..   30
....GGTGGATATTCCTTCTATGTTTA.......................................   13
.....GTGGATATTCCTTCTATGTTT........................................   11
.....GTGGATATTCCTTCTATGTTTA.......................................   6
```

```
....GGTGGATATTCCTTCTATGTT.........................................  5
...........................................ACATAGAGGAAATTCCACGTT..  4
.........................................AAACATAGAGGAAATTCCACG....  3
..........................................AACATAGAGGAAATTCCAC.....  2
........................................TAAACATAGAGGAAATTCCACG....  2
.....GTGGATATTCCTTCTATGTT.........................................  1
..........................................AACATAGAGGAAATTCC.......  1
...........................................ACATAGAGGAAATTCCACGT...  1
............................................CATAGAGGAAATTCCACGT...  1
((((.(((((..(((((.(((((((((.............))))))))))))))..))))).)))) (-24.02)

21/67578694-67578759(+)_mir_cow_136
AAAGGTAGATTTTCCTTCTATGATTACGGGTTTGGTGATTAATCATAGAGGAAAATCCACATTTTC
.........................................ATCATAGAGGAAAATCCACAT....  20
.........................................ATCATAGAGGAAAATCCACATT...  8
....GTAGATTTTCCTTCTATGATT.........................................  4
.........................................ATCATAGAGGAAAATCCACA.....  2
.........................................ATCATAGAGGAAAATCCACATTT..  2
....GTAGATTTTCCTTCTATG............................................  1
.........................................ATCATAGAGGAAAATC.........  1
.........................................ATCATAGAGGAAAATCC........  1
....((.(((((((((.(((((((((...(((....))))))))))))))))))))))).))...... (-21.40)

21/67578845-67578916(+)_mir_cow_137
GTGGAAAGGTGGTGGGCTGCAGAACATGTGCTTCCTTCGTGTCATATGTCTGCTGACCATCACCTTTAGAAG
............................................TATGTCTGCTGACCATCACC........  221
............................................TATGTCTGCTGACCATCACCT.......  111
.........TGGTGGGCTGCAGAACATGTGCT........................................  7
............................................TATGTCTGCTGACCATCACCTT......  5
.........TGGTGGGCTGCAGAACATGTGC.........................................  3
............................................TATGTCTGCTGACCATCAC.........  3
.........TGGTGGGCTGCAGAACATGTG..........................................  2
............................................TATGTCTGCTGACCATCA..........  2
....AAAGGTGGTGGGCTGCAGAACAT.............................................  1
......AGGTGGTGGGCTGCAGAACATGT...........................................  1
.........TGGTGGGCTGCAGAACATGTGCTT.......................................  1
.........TGGTGGGCTGCAGAACATGTGCTTCC.....................................  1
............................................TATGTCTGCTGACCATCACCTTT.....  1
```

```
..............................................TGTCTGCTGACCATCACCTTTA....   1
..............................................TGTCTGCTGACCATCACCTTTAG...   1
...............................................GTCTGCTGACCATCACCTTTAGA..   1
....(((((((((((...(((((.(((((((.........).))))))))))))...))))))))))).....  (-31.50)

21/67579075-67579141(+)_mir_cow_138
AAAAGGTGGATATTCCTTCTATGTTTACGTGATTCATAGATAATCATAGAGGAAAATCCATGTTTTC
..........................................ATCATAGAGGAAAATCCATG.....  154
..........................................ATCATAGAGGAAAATCCATGT....  147
..........................................ATCATAGAGGAAAATCCATGTT...  87
....GGTGGATATTCCTTCTATGTTT.........................................  42
....GGTGGATATTCCTTCTATGTTTA........................................  13
.....GTGGATATTCCTTCTATGTTT.........................................  11
.....GTGGATATTCCTTCTATGTTTA........................................  6
........................................TAATCATAGAGGAAAATCCATG.....  6
....GGTGGATATTCCTTCTATGTT..........................................  5
..........................................ATCATAGAGGAAAATCCATGTTT..  3
.....GTGGATATTCCTTCTATGTT..........................................  1
......TGGATATTCCTTCTATGTTTAC.......................................  1
..........................................ATCATAGAGGAAAATC.........  1
..........................................ATCATAGAGGAAAATCC........  1
...........................................TCATAGAGGAAAATCCATG.....  1
...........................................TCATAGAGGAAAATCCATGT....  1
((((.((((((.(((((.(((((.((((..........).))).)))))))))).)))))).)))). (-20.50)

21/67579438-67579504(+)_mir_cow_139
TAAAAGGTAGATTCTCCTTCTATGAGTACATTATTTACGATTAATCATAGAGGAAAATCCACGTTTT
......GTAGATTCTCCTTCTATGAGT........................................  636
......GTAGATTCTCCTTCTATGAGTAC......................................  88
......GTAGATTCTCCTTCTATGAGTA.......................................  76
......GTAGATTCTCCTTCTATGAG.........................................  44
...........................................ATCATAGAGGAAAATCCACGT...  29
......GTAGATTCTCCTTCTATG...........................................  12
.......TAGATTCTCCTTCTATGAGT........................................  11
....AGGTAGATTCTCCTTCTATGAGT........................................  9
.......TAGATTCTCCTTCTATGAGTAC......................................  7
..........................................AATCATAGAGGAAAATCCACGT...  6
......GTAGATTCTCCTTCTATGA..........................................  2
```

```
.....GGTAGATTCTCCTTCTATGAGT........................................  2
...........................................ATCATAGAGGAAAATCCACG....  2
......GTAGATTCTCCTTCTA.............................................  1
.....GGTAGATTCTCCTTCTATGAGTA.......................................  1
.....GGTAGATTCTCCTTCTATGAGTAC......................................  1
......GTAGATTCTCCTTCTATGAGTACA.....................................  1
.............................ATTATTTACGATTAATCATAGA................  1
...........................................ATCATAGAGGAAAATC........  1
...........................................ATCATAGAGGAAAATCC.......  1
..........................................AATCATAGAGGAAAATCCACG....  1
..........................................AATCATAGAGGAAAATCCACGTT..  1
...........................................ATCATAGAGGAAAATCCACGTT..  1
.((((.((.((((.((((.((((((....(((......)))...)))))))))).)))).)).))))  (-16.90)

21/67581380-67581446(+)_mir_cow_140
GAAGAGAGGATACCCTTTGTATGTTCACTTTATTAATGGCGAATATACAGAGGGAGACTCTTATTTG
....AGAGGATACCCTTTGTATGTTC.........................................  1036
..........................................ATATACAGAGGGAGACTCTTAT...  327
....AGAGGATACCCTTTGTATGTT..........................................  156
....AGAGGATACCCTTTGTATGT...........................................  100
..........................................ATATACAGAGGGAGACTCTTA....  53
....AGAGGATACCCTTTGTATG............................................  20
...........................................TATACAGAGGGAGACTCTTAT...  11
....AGAGGATACCCTTTGTATGTTCA........................................  4
...........................................TATACAGAGGGAGACTCT......  4
..........................................ATATACAGAGGGAGACTCTT.....  4
..........................................ATATACAGAGGGAGACTCTTATT..  4
..........................................ATATACAGAGGGAGACT........  3
..........................................ATATACAGAGGGAGACTC.......  3
...........................................TATACAGAGGGAGACTCTTA....  3
....AGAGGATACCCTTTGTAT.............................................  1
.....GAGGATACCCTTTGTATGTTC.........................................  1
....AGAGGATACCCTTTGTATGTTCACTT.....................................  1
..........................................ATATACAGAGGGAGACTCT......  1
.........................................AATATACAGAGGGAGACTCTTA....  1
...........................................TATACAGAGGGAGACTCTTATT..  1
...(((((..(.((((((((((((((.((........)).)))))))))))))))..))))).....  (-28.80)
```

```
21/67583076-67583144(+)_mir_cow_141
TCAGAGCGAGGTTGCCCTTTGTATATTCGGTTTTTTGATGTAGAATATACAAGGGCAAGCTCTCTGTGA
.............................................TATACAAGGGCAAGCTCTCTGT..      4189
............................................ATATACAAGGGCAAGCTCTCTGT..      285
.............................................TATACAAGGGCAAGCTCTCT....      250
............................................ATATACAAGGGCAAGCTCTCT....      106
.............................................TATACAAGGGCAAGCTCTCTG...      37
.............................................TATACAAGGGCAAGCTCT......      22
..............................................ATACAAGGGCAAGCTCTCTGT..      18
......CGAGGTTGCCCTTTGTATATTC.........................................      13
............................................ATATACAAGGGCAAGCTCTCTG...      7
....AGCGAGGTTGCCCTTTGTATATT..........................................      5
.............................................TATACAAGGGCAAGCTCTC.....      3
...........................................AATATACAAGGGCAAGCTCTCT....      3
......CGAGGTTGCCCTTTGTATATT..........................................      2
............................................ATATACAAGGGCAAGCTCT......      2
....AGCGAGGTTGCCCTTTGTATATTC.........................................      1
......CGAGGTTGCCCTTTGTATATTCG........................................      1
............................................ATATACAAGGGCAAGCTCTC.....      1
..............................................ATACAAGGGCAAGCTCTCT....      1
...............................................TACAAGGGCAAGCTCTCTGT..      1
.................................................CAAGGGCAAGCTCTCTGT..      1
(((.((.(((.((((((((.((((((((.(((....)))...))))))))))))))))).))).)).)))  (-32.20)

21/67583565-67583635(+)_mir_cow_142
TTGGAGAGTGGTTATCCCTGTCCTGTTCGTTTTACTCATGTCGAATCGTACAGGGTCATCCACTTTTTCAG
...........................................AATCGTACAGGGTCATCCACTT......    4190
...........................................AATCGTACAGGGTCATCCACT.......    2460
.............................................TCGTACAGGGTCATCCACTTT.....    531
.............................................TCGTACAGGGTCATCCACTT......    279
...........................................AATCGTACAGGGTCATCCACTTT.....    230
............................................ATCGTACAGGGTCATCCACTTT.....    201
.............................................TCGTACAGGGTCATCCACT.......    192
............................................ATCGTACAGGGTCATCCACTT......    110
............................................ATCGTACAGGGTCATCCACT.......    67
.............................................TCGTACAGGGTCATCCACTTTT....    50
...........................................AATCGTACAGGGTCATCCAC........    45
...........................................AATCGTACAGGGTCATCCA.........    20
```

```
......AGTGGTTATCCCTGTCCTGTT............................................ 14
........TGGTTATCCCTGTCCTGTTCG.......................................... 14
.......GTGGTTATCCCTGTCCTGTTCG.......................................... 12
...........................................AATCGTACAGGGTCATCC.......... 7
......AGTGGTTATCCCTGTCCTGTTC........................................... 6
......AGTGGTTATCCCTGTCCTGTTCG.......................................... 6
............TATCCCTGTCCTGTTCGTTTTACT................................... 6
...........................................AATCGTACAGGGTCATC........... 6
......AGTGGTTATCCCTGTCCTGT............................................. 5
.......GTGGTTATCCCTGTCCTGTTC........................................... 5
............................................ATCGTACAGGGTCATCCAC........ 4
...........................................AATCGTACAGGGTCATCCACTTTT.... 3
....AGAGTGGTTATCCCTGTCCTGT............................................. 2
........TGGTTATCCCTGTCCTGTT............................................ 2
............TATCCCTGTCCTGTTCGTTTTACTC.................................. 2
.............ATCCCTGTCCTGTTCGTTTTACTC.................................. 2
.............................................TCGTACAGGGTCATCCACTTTTT... 2
....AGAGTGGTTATCCCTGTCCTG.............................................. 1
.......GTGGTTATCCCTGTCCTGT............................................. 1
.......GTGGTTATCCCTGTCCTGTT............................................ 1
........TGGTTATCCCTGTCCTGTTC........................................... 1
......AGTGGTTATCCCTGTCCTGTTCGT......................................... 1
.............ATCCCTGTCCTGTTCGTTTTACT................................... 1
.......GTGGTTATCCCTGTCCTGTTCGTTTTACTCATGTCG............................ 1
.................................ACTCATGTCGAATCGTACAGGGT............... 1
...................................TCATGTCGAATCGTACAGGGTC.............. 1
......................................TGTCGAATCGTACAGGGTCATC........... 1
............................................ATCGTACAGGGTCATC........... 1
.....................................ATGTCGAATCGTACAGGGTCATCC.......... 1
............................................ATCGTACAGGGTCATCC.......... 1
.............................................TCGTACAGGGTCATCCAC........ 1
...............................................GTACAGGGTCATCCACT....... 1
............................................ATCGTACAGGGTCATCCACTTTT.... 1
..............................................CGTACAGGGTCATCCACTTTTT... 1
.............................................TCGTACAGGGTCATCCACTTTTTC.. 1
..(((((((((....((((((.(.(((((............))))).).)))))))....)))))))))... (-25.00)
```

21/67584375-67584449(+)_mir_cow_143

CTTGAGGAGAAATTATCCTTGGTGTGTTCGCTTTATTTGGGATGAATCATACAAGGACAATTTCTTTCTGAGTAT	
....CATACAAGGACAATTTCTTTCT......	1209
....ATACAAGGACAATTTCTTTCT......	400
....ATCATACAAGGACAATTTCTTTCT......	308
....ATCATACAAGGACAATTTCTTT........	96
....ATACAAGGACAATTTCTTTCTG.....	94
....ATCATACAAGGACAATTTCTT.........	73
.....GGAGAAATTATCCTTGGTGTGTT...	63
.....GGAGAAATTATCCTTGGTGTGT..	27
....TCATACAAGGACAATTTCTTTCT......	24
....ATCATACAAGGACAATTTCTTTC.......	23
....TACAAGGACAATTTCTTTCT......	19
....CATACAAGGACAATTTCTT.........	15
....ATACAAGGACAATTTCTTTCTGA....	12
....ATCATACAAGGACAATTTCT..........	11
....ATACAAGGACAATTTCTTT........	11
....CATACAAGGACAATTTCTTTC.......	11
....ATACAAGGACAATTTCTT.........	10
....ATACAAGGACAATTTCTTTC.......	6
....CATACAAGGACAATTTCTTT........	5
.....GGAGAAATTATCCTTGGTG...	4
.....GGAGAAATTATCCTTGGTGTG...	4
....AGGAGAAATTATCCTTGGTGTGT..	4
....TCATACAAGGACAATTTCTTTC.......	4
....TACAAGGACAATTTCTTTCTG.....	4
......GAGAAATTATCCTTGGTGTGTT...	3
.....GGAGAAATTATCCTTGGTGTGTTC..	3
......GAGAAATTATCCTTGGTGTGTTC..	3
....CATACAAGGACAATTTCT..........	3
....AGGAGAAATTATCCTTGGTG...	2
.....GGAGAAATTATCCTTGGTGT..	2
....ATACAAGGACAATTTCT..........	2
....AATCATACAAGGACAATTTCTT.........	2
.......AGAAATTATCCTTGGTGTG...	1
......GAGAAATTATCCTTGGTGTGT..	1
.......AGAAATTATCCTTGGTGTGTTC..	1
............................CGCTTTATTTGGGATGAAT............................	1
....TCATACAAGGACAATTTCTT.........	1

....AATCATACAAGGACAATTTCTTT........ 1
....TACAAGGACAATTTCTTTCTGA.... 1
....TACAAGGACAATTTCTTTCTGAGT.. 1
((((..((((((((.((((((.(((((((((((.....))).)))).)))))))))).))))))))..)))))... (-25.10)

21/67586365-67586435(+)_mir_cow_144
TCACCTAGGGATCTTGTTAAAAGGCAGATTCTGATTCAGGGACCAAGATTCTGCATTTTTAGCAAGTTCCC
...........TCTTGTTAAAAGGCAGATTCT....................................... 462
....ATTCTGCATTTTTAGCAAGTT... 42
...........TCTTGTTAAAAGGCAGATTC.. 11
....ATTCTGCATTTTTAGCAAGTTC.. 11
.....TAGGGATCTTGTTAAAAGGCAGA... 3
.....TAGGGATCTTGTTAAAAGGCAG.. 2
...........TCTTGTTAAAAGGCAGA... 2
.....TAGGGATCTTGTTAAAAGGCA... 1
....CTAGGGATCTTGTTAAAAGGCAG.. 1
...........TCTTGTTAAAAGGCAGAT.. 1
...........TCTTGTTAAAAGGCAGATT... 1
...........TCTTGTTAAAAGGCAGATTCTGA..................................... 1
............CTTGTTAAAAGGCAGATTCTGAT.................................... 1
............CTTGTTAAAAGGCAGATTCTGATT................................... 1
.................TAAAAGGCAGATTCTGATTCA................................. 1
......................................GGGACCAAGATTCTGCATTTTTAGCAAGTT... 1
....GATTCTGCATTTTTAGCAAGTT... 1
.......((((.((((((((((((((((.(((..((....))...))).))))).)))))))))))).)))) (-27.40)

21/67587333-67587399(+)_mir_cow_145
GAGGAGAGGTTATCCGTGTTATGTTCGCTTGGCTCATCATGAATAATACATGGTTAACCTCTCTTTG
....ATAATACATGGTTAACCTCTCT... 1125
....ATAATACATGGTTAACCTCT..... 42
....ATAATACATGGTTAACCTCTC.... 34
....AGAGGTTATCCGTGTTATGTTC... 5
....ATAATACATGGTTAACCTC...... 5
....ATAATACATGGTTAACCTCTCTT.. 4
....AATAATACATGGTTAACCTCT..... 3
....AGAGGTTATCCGTGTTATGT... 2
....AGAGGTTATCCGTGTTATGTT.. 2
....TAATACATGGTTAACCTCTCT... 2

```
...............GTGTTATGTTCGCTTGGCTCATCATGAATAATACATGG..............  1
..........................................ATAATACATGGTTAACCT.......  1
((((((((((((.((((((..((((((..(((....)))))))))..)))))).)))))))))))). (-28.80)

21/67589482-67589545(+)_mir_cow_146
GTGGATGGTTGATCAGAGAACATACATTTTGTCAATGATGTATGTCAACTGATCCACAGTCCCT
......GGTTGATCAGAGAACATACATT....................................    2268
......................................TGTATGTCAACTGATCCACAGT....    643
......GGTTGATCAGAGAACATACAT.....................................    345
......................................TGTATGTCAACTGATCCACAG.....    98
......GGTTGATCAGAGAACATACA......................................    53
......................................TGTATGTCAACTGATCCACA......    24
......GGTTGATCAGAGAACATAC.......................................    20
........................................TATGTCAACTGATCCACAGTCC..    12
....ATGGTTGATCAGAGAACATACA......................................    8
.....TGGTTGATCAGAGAACATACATT....................................    8
......GGTTGATCAGAGAACATACATTT...................................    7
......GGTTGATCAGAGAACA..........................................    5
........................................TATGTCAACTGATCCACAGT....    5
....ATGGTTGATCAGAGAACATAC.......................................    4
......GGTTGATCAGAGAACATA........................................    3
......................................TGTATGTCAACTGATCCAC.......    3
............................TTGTCAATGATGTATGTCAACTGATCCACAGT....    3
......................................TGTATGTCAACTGATCC.........    2
......GGTTGATCAGAGAACAT.........................................    1
.....TGGTTGATCAGAGAACATACA......................................    1
.....TGGTTGATCAGAGAACATACAT.....................................    1
......GGTTGATCAGAGAACATACATTTTGTCAATGA..........................    1
......................................TGTATGTCAACTGATC..........    1
......................................TGTATGTCAACTGATCCA........    1
.......................................GTATGTCAACTGATCCACAGT....    1
........................................TATGTCAACTGATCCACAGTC...    1
..((((.((.((((((...(((((((((........)))))))))...)))))).)).)))).. (-23.90)

21/67589871-67589936(+)_mir_cow_147
GAAGAGTGGTTATCCCTGCTGTGTTCGCTGTATTTATGACGAATCATACAGGGACATCCAGTTTTT
.........................................AATCATACAGGGACATCCAGTT...   873
.........................................AATCATACAGGGACATCCAGT....   115
```

```
.....GTGGTTATCCCTGCTGTGTTCG.......................................  55
.........................................AATCATACAGGGACATCCAG.....  25
.........................................AATCATACAGGGACATCCAGTTT..  9
.....GTGGTTATCCCTGCTGTGTTC........................................  7
.....GTGGTTATCCCTGCTGTGTT.........................................  6
....AGTGGTTATCCCTGCTGTGTTC........................................  2
.........................................AATCATACAGGGACATCC.......  2
....AGTGGTTATCCCTGCTGTGTTCG.......................................  1
..........................................ATCATACAGGGACATCCAGT....  1
..............................................TACAGGGACATCCAGT....  1
..........................................ATCATACAGGGACATCCAGTT...  1
..........................................ATCATACAGGGACATCCAGTTT..  1
(((((.(((...((((((.((((((((..((....))..)))).))))))))))...))).))))) (-23.60)

21/67592883-67592948(+)_mir_cow_148
GAAGAGAAGTTGTTCGTGGTGGATTCGCTTTACTTATGACGAATCATTCACGGACAACACTTTTTT
.....GAAGTTGTTCGTGGTGGATTCG.......................................  2208
.........................................AATCATTCACGGACAACACTTT...  841
.........................................AATCATTCACGGACAACACTT....  542
......AAGTTGTTCGTGGTGGATTCG.......................................  287
.....GAAGTTGTTCGTGGTGGATT.........................................  94
.........................................AATCATTCACGGACAACACT.....  91
.....GAAGTTGTTCGTGGTGGATTC........................................  79
.......AGTTGTTCGTGGTGGATTCGCT.....................................  42
.....GAAGTTGTTCGTGGTGGAT..........................................  39
..........................................ATCATTCACGGACAACACTTT...  28
.......AGTTGTTCGTGGTGGATTCG.......................................  15
.......AGTTGTTCGTGGTGGATTCGC......................................  14
......AAGTTGTTCGTGGTGGATTC........................................  11
.......................................CGAATCATTCACGGACAACACT.....  7
.........................................AATCATTCACGGACAACACTTTT..  7
..........................................ATCATTCACGGACAACACTT....  6
..........................................ATCATTCACGGACAACACTTTT..  5
......AAGTTGTTCGTGGTGGATT.........................................  4
.....GAAGTTGTTCGTGGTGGA...........................................  2
.........................................AATCATTCACGGACAACA.......  2
.........................................AATCATTCACGGACAACAC......  2
..........................................ATCATTCACGGACAACACT.....  2
```

```
......AAGTTGTTCGTGGTGGAT..........................................  1
....AGAAGTTGTTCGTGGTGGATTC........................................  1
.......AGTTGTTCGTGGTGGATTCGCTT....................................  1
.........................................AATCATTCACGGACAAC........  1
.......................................CGAATCATTCACGGACAACACTT....  1
........................................GAATCATTCACGGACAACACTT....  1
((((((..(((((((((((..(((((((.........).))))))..))))))))))).)))))). (-24.50)

21/67593258-67593328(+)_mir_cow_149
AGGGTGTGTGACTGGTTGACCAGAGTGGCCAGCACTCGGTTCACCCTCTGGGCCACCTAGTCACCAACCCT
....TGTGTGACTGGTTGACCAGAGT.............................................    85
....TGTGTGACTGGTTGACCAGAGTGG...........................................    48
....TGTGTGACTGGTTGACCAGAGTG............................................    21
..............................................TCTGGGCCACCTAGTCACCAACC..    13
..............................................TCTGGGCCACCTAGTCACC......    12
.....GTGTGACTGGTTGACCAGAGTGG...........................................    11
.............................................CTCTGGGCCACCTAGTCACC......    11
....TGTGTGACTGGTTGACCAGAG..............................................    10
......TGTGACTGGTTGACCAGAGTGG...........................................    10
.....GTGTGACTGGTTGACCAGAGT.............................................    9
..............................................TCTGGGCCACCTAGTCACCAAC...    8
..............................................TCTGGGCCACCTAGTCACCA.....    4
....TGTGTGACTGGTTGACCAGA...............................................    3
.....GTGTGACTGGTTGACCAGAGTG............................................    3
.............................................CTCTGGGCCACCTAGTCACCA.....    3
..............................................TCTGGGCCACCTAGTCACCAA....    3
....TGTGTGACTGGTTGACCAG................................................    2
............................................CCTCTGGGCCACCTAGTCACC......    2
.....GTGTGACTGGTTGACCAGA...............................................    1
............................................CCTCTGGGCCACCTAGTCACCAA....    1
.............................................CTCTGGGCCACCTAGTCACCAA....    1
............................................CCTCTGGGCCACCTAGTCACCAAC...    1
.............................................CTCTGGGCCACCTAGTCACCAAC...    1
(((((..((((((((.((.((((((.((..(((.....)))..))))))))..)).))))))))..))))) (-34.70)

21/67593810-67593880(+)_mir_Cow_Blast_36
TAGGGTAAGTGTGTCTCGGGTGAGCATGCAATGAACGTGCTCGCATGTCACTCGGCCCACTACCCAAGATT
.............................................TGTCACTCGGCCCACTACCCA.....    19
```

```
.............................................TGTCACTCGGCCCACTACCC......    17
.............................................TGTCACTCGGCCCACTACCCAA....    11
............................................ATGTCACTCGGCCCACTACC.......    6
............................................ATGTCACTCGGCCCACTACCC......    5
............................................ATGTCACTCGGCCCACTACCCA.....    4
.............................................TGTCACTCGGCCCACTACCCAAGA..    3
.............................................TGTCACTCGGCCCACTACC.......    2
....GTAAGTGTGTCTCGGGTGAGCATG...........................................    1
.............................................TGTCACTCGGCCCACT..........    1
...........................................CATGTCACTCGGCCCACTACCCA.....    1
............................................ATGTCACTCGGCCCACTACCCAA....    1
..(((((...(((..((((((((.(((((.............)))))))))))))..))))))))...... (-28.02)

21/67593959-67594024(+)_mir_cow_150
GAAGAGAGGCTGGCCGTGATGAATTCGATTCATCAAAGCGAGTCATACACGGCTCTCCTCTCTTTT
....AGAGGCTGGCCGTGATGAATTC........................................  657
.........................................GTCATACACGGCTCTCCTCTCT...  434
....AGAGGCTGGCCGTGATGAATTCG.......................................  234
........................................AGTCATACACGGCTCTCCTCTCT...  40
....AGAGGCTGGCCGTGATGAATTCGA......................................  26
....AGAGGCTGGCCGTGATGAAT..........................................  25
....AGAGGCTGGCCGTGATGAATT.........................................  25
.........................................GTCATACACGGCTCTCCTCTC....  13
.......................................GAGTCATACACGGCTCTCCTCTCT...  12
.........................................GTCATACACGGCTCTCCTCT.....  9
.........................................GTCATACACGGCTCTCCT.......  5
....AGAGGCTGGCCGTGATGAA...........................................  4
........................................AGTCATACACGGCTCTCCTCTC....  4
...........................ATTCATCAAAGCGAGTCATAC..................  2
..........................GATTCATCAAAGCGAGTCATACACGGCTCTCCT.......  2
.........................................GTCATACACGGCTCTCCTC......  2
.........................................GTCATACACGGCTCTCCTCTCTT..  2
....AGAGGCTGGCCGTGATG.............................................  1
....AGAGGCTGGCCGTGATGAATTCGAT.....................................  1
....AGAGGCTGGCCGTGATGAATTCGATTCATCAAAGCG..........................  1
...........................ATTCATCAAAGCGAGTCATACA.................  1
................................TCAAAGCGAGTCATACACGGCT............  1
...........................ATTCATCAAAGCGAGTCATACACGGCTCTCC........  1
```

```
...........................ATTCATCAAAGCGAGTCATACACGGCTCTCCTCT.....  1
..................................AAAGCGAGTCATACACGGCTCTCCTCT.....  1
.......................................GAGTCATACACGGCTCTCCTCT.....  1
...........................ATTCATCAAAGCGAGTCATACACGGCTCTCCTCTCT...  1
...............................................CACGGCTCTCCTCTCT...  1
((((((((..(((((((((((.((((..........)))))))).)))))))..))))))))). (-32.00)

21/67598048-67598113(+)_mir_cow_151
GAAGATAGGTTATCCGTGTAGCCTTCGCTTTACTTGTGACGAATCATACACGGTTCACCTATTTTT
.........................................AATCATACACGGTTCACCTATT...  102
.....TAGGTTATCCGTGTAGCCTTC........................................  62
.....TAGGTTATCCGTGTAGCCTTCG.......................................  27
..........................................ATCATACACGGTTCACCTATTT..  13
....ATAGGTTATCCGTGTAGCCT..........................................  10
.........................................AATCATACACGGTTCACCTATTT..  8
.....TAGGTTATCCGTGTAGCC...........................................  7
.....TAGGTTATCCGTGTAGCCT..........................................  6
.........................................AATCATACACGGTTCACCTAT....  4
....ATAGGTTATCCGTGTAGCCTTC........................................  3
....ATAGGTTATCCGTGTAGCCTT.........................................  2
.....TAGGTTATCCGTGTAGCCTT.........................................  1
....ATAGGTTATCCGTGTAGCCTTCG.......................................  1
.....TAGGTTATCCGTGTAGCCTTCGCT.....................................  1
.........................................AATCATACACGGTTCACCT......  1
..........................................ATCATACACGGTTCACCT......  1
.........................................AATCATACACGGTTCACCTA.....  1
..........................................ATCATACACGGTTCACCTATT...  1
.............................................ATACACGGTTCACCTATT...  1
((((((((((...(((((((...(((((.........).))))...)))))))...)))))))))) (-23.70)

21/67598363-67598430(+)_mir_cow_152
GAAGAGAGGTCTTCCATGGTGCATTCGCTTTATTCTTTGACGAATCATACATGGTTGACCTTTTTTTA
....AGAGGTCTTCCATGGTGCATTCG......................................... 2638
....AGAGGTCTTCCATGGTGCATTC.......................................... 296
....AGAGGTCTTCCATGGTGCATT........................................... 178
.....GAGGTCTTCCATGGTGCATTCG......................................... 153
....AGAGGTCTTCCATGGTGCAT............................................ 44
..........................................AATCATACATGGTTGACCTTTTT... 36
```

```
...........................................ATCATACATGGTTGACCTTTTT... 28
...........................................ATCATACATGGTTGACCTTTTTT.. 8
.....GAGGTCTTCCATGGTGCATT........................................... 7
.....GAGGTCTTCCATGGTGCATTC.......................................... 7
..........................................AATCATACATGGTTGACCTTTT.... 6
..........................................AATCATACATGGTTGACCTTTTTT.. 6
....AGAGGTCTTCCATGGTGCA............................................. 4
....AGAGGTCTTCCATGGTGC.............................................. 3
......AGGTCTTCCATGGTGCATTCGC........................................ 2
....AGAGGTCTTCCATGGTG............................................... 1
.....GAGGTCTTCCATGGTGCAT............................................ 1
......AGGTCTTCCATGGTGCATTC.......................................... 1
......AGGTCTTCCATGGTGCATTCG......................................... 1
......AGGTCTTCCATGGTGCATTCGCT....................................... 1
.........TCTTCCATGGTGCATTCGCTTTAT................................... 1
..........................................AATCATACATGGTTGACCTT...... 1
..........................................AATCATACATGGTTGACCTTT..... 1
...........................................ATCATACATGGTTGACCTTT..... 1
...........................................ATCATACATGGTTGACCTTTT.... 1
(((((((((((..((((((((.((((((..........).)))))))).)))))..))))))))))). (-25.90)
```

```
21/67600492-67600557(+)_mir_cow_153
GAGCAGAGGTTGCCCTTGGTGAATTCGCTTTATTTATGTTGAATCACACAAAGGCAACTTTTGTTT
....AGAGGTTGCCCTTGGTGAATTC........................................  88
..........................................ATCACACAAAGGCAACTTTTGT..  56
....AGAGGTTGCCCTTGGTGAATT.........................................  19
....AGAGGTTGCCCTTGGTGAAT..........................................  8
..........................................ATCACACAAAGGCAACTTTTG...  8
....AGAGGTTGCCCTTGGTGAATTCG.......................................  6
.........................................AATCACACAAAGGCAACTTTTGT..  6
..........................................ATCACACAAAGGCAACTTT.....  2
..........................................ATCACACAAAGGCAACTTTT....  2
.........................................AATCACACAAAGGCAACTTT.....  1
.......................................TGAATCACACAAAGGCAACTTTT....  1
.........................................AATCACACAAAGGCAACTTTT....  1
.........................................AATCACACAAAGGCAACTTTTG...  1
((((((((((((((.(((((((.(((((.........).)))))))).))).)))))))))))))) (-26.90)
```

```
21/67603256-67603318(+)_mir_Cow_Blast_44
GGAGAGGTTACCCGAGCAACTTTGCATCTGGACGACGAATGTTGCTCGGTGAACCCCTTTTCG
....................................GAATGTTGCTCGGTGAACCCCT.....      27754
.....................................AATGTTGCTCGGTGAACCCCT.....      5559
...................................CGAATGTTGCTCGGTGAACCCCT.....      5481
....................................GAATGTTGCTCGGTGAACCCCTT....      643
....................................GAATGTTGCTCGGTGAACCCC......      503
...................................CGAATGTTGCTCGGTGAACCCC......      256
....AGGTTACCCGAGCAACTTTGCAT....................................      234
.....................................AATGTTGCTCGGTGAACCCC......      177
...................................CGAATGTTGCTCGGTGAACCCCTT....      146
....................................GAATGTTGCTCGGTGAACCCCTTT...      145
....................................GAATGTTGCTCGGTGAACC........      126
....................................GAATGTTGCTCGGTGAACCC.......      111
....AGGTTACCCGAGCAACTTTGC......................................      105
...................................CGAATGTTGCTCGGTGAACCC.......      80
....AGGTTACCCGAGCAACTTTGCA.....................................      79
.....................................AATGTTGCTCGGTGAACCC.......      72
....................................GAATGTTGCTCGGTGAAC.........      53
.....................................AATGTTGCTCGGTGAACCCCTT....      53
....AGGTTACCCGAGCAACTTTGCATC...................................      44
...................................CGAATGTTGCTCGGTGAACCCCTTT...      32
....AGGTTACCCGAGCAACTTTG.......................................      27
...................................CGAATGTTGCTCGGTGAAC.........      24
...................................CGAATGTTGCTCGGTGAACC........      19
.....................................AATGTTGCTCGGTGAACCCCTTT...      17
....AGGTTACCCGAGCAACTTTGCATCT..................................      13
....................................GAATGTTGCTCGGTGA...........      12
....................................GAATGTTGCTCGGTGAA..........      11
.....................................AATGTTGCTCGGTGAACC........      10
....................................GAATGTTGCTCGGTGAACCCCTTTT..      6
....................TTTGCATCTGGACGACGAATGT.....................      4
....................TTTGCATCTGGACGACGAATGTT....................      4
..................................ACGAATGTTGCTCGGTGAACC........      4
.........................................TTGCTCGGTGAACCCCT.....      4
........................................GTTGCTCGGTGAACCCCT.....      3
...................................CGAATGTTGCTCGGTGAACCCCTTTT..      3
...................................CGAATGTTGCTCGGTGA...........      2
```

```
..........................TCTGGACGACGAATGTTGCTCGGTGAACCCCT.....      2
....AGGTTACCCGAGCAACTTT........................................      1
............CGAGCAACTTTGCATC...................................      1
....AGGTTACCCGAGCAACTTTGCATCTGG................................      1
.....................TTGCATCTGGACGACGAATGTT....................      1
........................CATCTGGACGACGAATGTTGCT.................      1
.....................................AATGTTGCTCGGTGAAC.........      1
....................TTTGCATCTGGACGACGAATGTTGCTCGGTGAACC........      1
...........................CTGGACGACGAATGTTGCTCGGTGAACCCCT.....      1
............................TGGACGACGAATGTTGCTCGGTGAACCCCT.....      1
...............................ACGACGAATGTTGCTCGGTGAACCCCT.....      1
..................................ACGAATGTTGCTCGGTGAACCCCT.....      1
......................................ATGTTGCTCGGTGAACCCCT.....      1
.......................................TGTTGCTCGGTGAACCCCT.....      1
..........................................TGCTCGGTGAACCCCT.....      1
.......................................TGTTGCTCGGTGAACCCCTT....      1
.......................................TGTTGCTCGGTGAACCCCTTT...      1
((((((((((((.((((((((((((........).)))).))))))))))))...))))))). (-25.00)

21/67603399-67603464(+)_mir_Cow_Blast_42
GACGGATGGTCGACCAGTTGGAAAGTAATTGTTTCTAATGTACTTCACCTGGTCCACTAGCTGTCC
......TGGTCGACCAGTTGGAAAGTAAT.....................................  1011
......TGGTCGACCAGTTGGAAAGT........................................  744
......TGGTCGACCAGTTGGAAAGTA.......................................  116
.....ATGGTCGACCAGTTGGAAAGT........................................  86
......TGGTCGACCAGTTGGAAAGTAA......................................  63
..........................................CTTCACCTGGTCCACTAGCTGT..  48
......TGGTCGACCAGTTGGAAAG.........................................  15
.....ATGGTCGACCAGTTGGAAAGTA.......................................  12
..........................................CTTCACCTGGTCCACTAGCTG...  10
..........................................CTTCACCTGGTCCACTAGCT....  8
.....ATGGTCGACCAGTTGGAAAGTAAT.....................................  7
....GATGGTCGACCAGTTGGAAAGT........................................  5
.........................................ACTTCACCTGGTCCACTAGCT....  5
......TGGTCGACCAGTTGGAAAGTAATT....................................  4
......TGGTCGACCAGTTGGAAA..........................................  2
.......................................GTACTTCACCTGGTCCACTAGC.....  2
..........................................CTTCACCTGGTCCACTAGC.....  2
```

```
.....ATGGTCGACCAGTTGGAAAGTAA......................................  1
...........GACCAGTTGGAAAGTAA......................................  1
.....ATGGTCGACCAGTTGGAAAGTAATTGT..................................  1
.....................................ATGTACTTCACCTGGTCCACTAGC.....  1
.......................................GTACTTCACCTGGTCCACTAGCT....  1
...........................................TTCACCTGGTCCACTAGCTGT..  1
(((((.((((.((((((.(((..((((((((....)))).))))))).)))))).)))).))))). (-23.20)

21/67603542-67603606(+)_mir_cow_154
GAAGGGAGATCGACCGTGTTATATTCGCTTTATTGACTTCGAATAATACATGGTTGATCTTTTCT
.........................................AATAATACATGGTTGATCTTT...    3894
......AGATCGACCGTGTTATATTCG......................................    2559
.........................................AATAATACATGGTTGATCTT....    1132
.........................................AATAATACATGGTTGATCT.....    145
......AGATCGACCGTGTTATATTC.......................................    131
......AGATCGACCGTGTTATATTCGC.....................................    87
........................................GAATAATACATGGTTGATCTT....    66
........................................GAATAATACATGGTTGATCTTT...    62
.........................................AATAATACATGGTTGATCTTTT..    54
......AGATCGACCGTGTTATATTCGCT....................................    43
.........................................AATAATACATGGTTGATC......    16
.........................................AATAATACATGGTTGAT.......    11
.....GAGATCGACCGTGTTATATTC.......................................    9
......AGATCGACCGTGTTATATT........................................    6
.....GAGATCGACCGTGTTATATTCG......................................    6
......AGATCGACCGTGTTATATTCGCTT...................................    6
........................................GAATAATACATGGTTGATCT.....    5
..........................................ATAATACATGGTTGATCTTT...    5
.......................................CGAATAATACATGGTTGATCTT....    3
.....GAGATCGACCGTGTTATAT.........................................    2
.....GAGATCGACCGTGTTATATT........................................    2
......AGATCGACCGTGTTATATTCGCTTT..................................    2
.......................................CGAATAATACATGGTTGATCTTT...    2
..........................................ATAATACATGGTTGATCTTTT..    2
......AGATCGACCGTGTTAT...........................................    1
....GGAGATCGACCGTGTTATATTCG......................................    1
........ATCGACCGTGTTATATTCG......................................    1
......AGATCGACCGTGTTATATTCGCTTTATTGACT...........................    1
```

```
.........................................AATAATACATGGTTGA........   1
........................................GAATAATACATGGTTGAT.......   1
...........................CTTTATTGACTTCGAATAATACATGGTTGATCTT....   1
...........................................TAATACATGGTTGATCTTT...   1
...((((((((((((((((..((((((............))))))..))))))))))))))))..  (-24.50)

21/67603874-67603939(+)_mir_cow_155
AGGAGAGGTTGTCTGTGATGAGTTCGCTTTATTAATGACGAATATAACACAGATGGCCTGTTTTCA
........................................AATATAACACAGATGGCCTGT.....   47655
........................................AATATAACACAGATGGCCTG......   2075
........................................AATATAACACAGATGGCCTGTT....   236
........................................AATATAACACAGATGGCC........   115
.......................................GAATATAACACAGATGGCCTGT.....   92
........................................AATATAACACAGATGGCCTGTTT...   16
......................................CGAATATAACACAGATGGCCTGT.....   12
.........................................ATATAACACAGATGGCCTGT.....   11
........................................AATATAACACAGATGGCCT.......   9
.....AGGTTGTCTGTGATGAGTTCG........................................   7
...........................................ATAACACAGATGGCCTGT.....   5
.......................................GAATATAACACAGATGGCCTG......   4
.....AGGTTGTCTGTGATGAGTTC.........................................   3
..........................................TATAACACAGATGGCCTGT.....   3
........................................AATATAACACAGATGGC.........   2
...................................TGACGAATATAACACAGATGGCCTGT.....   2
.....................................ACGAATATAACACAGATGGCCTGT.....   2
.............................................AACACAGATGGCCTGT.....   2
..........................................TATAACACAGATGGCCTGTTT...   2
....GAGGTTGTCTGTGATGAGTTC.........................................   1
.....AGGTTGTCTGTGATGAGTTCGCT......................................   1
....................AGTTCGCTTTATTAATGACG..........................   1
...................................TGACGAATATAACACAGATGGCCT.......   1
..........................................TATAACACAGATGGCCTG......   1
...........................TTTATTAATGACGAATATAACACAGATGGCCTGT.....   1
....................................GACGAATATAACACAGATGGCCTGT.....   1
............................................TAACACAGATGGCCTGT.....   1
......................................CGAATATAACACAGATGGCCTGTT....   1
................................TAATGACGAATATAACACAGATGGCCTGTTT...   1
.........................................ATATAACACAGATGGCCTGTTT...   1
```

....ATAACACAGATGGCCTGTTT...   1
....AATATAACACAGATGGCCTGTTTT..   1
((((.((((..((((((.((.((((((.........).))))).)).))))))..)))).)))).. (-24.20)

21/67604692-67604755(+)_mir_cow_156
AAATAGGTTGCCTGTGAGGTGTTCACTTTTCTAAGCGATGAATATTATACAGTCAACCTCTTTC
....AATATTATACAGTCAACCTCT...   126
....AATATTATACAGTCAACCTC....   9
....AGGTTGCCTGTGAGGTGTTC..   6
....AGGTTGCCTGTGAGGTGTTCA.......................................   1
....AATATTATACAGTCAACC......   1
....AATATTATACAGTCAACCT.....   1
....AATATTATACAGTCAACCTCTT..   1
....((((((.(((((..((((((((((....)))...)))))))..))))).))))))..... (-20.10)

21/68149441-68149509(-)_mir_cow_157
CGGGCGCACCCGTCCCGTGCGTCCCCGGACGTCGCTGTCTGCCCCGGGAACGTCGGGACTGGAGCGCCC
.......ACCCGTCCCGTGCGTCCCCGGAC.......................................   362
.......ACCCGTCCCGTGCGTCCCCGGA..   205
......CACCCGTCCCGTGCGTCCCCGGA..   70
.......ACCCGTCCCGTGCGTCCCCGG...   25
....CGGGAACGTCGGGACTGGAGC....   22
......CACCCGTCCCGTGCGTCCCCGG...   6
......CACCCGTCCCGTGCGTCCCCGGAC.......................................   4
......CACCCGTCCCGTGCGTCCCCG..   2
......CACCCGTCCCGTGCGTCC...   1
......CACCCGTCCCGTGCGTCCCC...   1
.......ACCCGTCCCGTGCGTCCCC...   1
.......ACCCGTCCCGTGCGTCCCCG..   1
.......ACCCGTCCCGTGCGTCCCCGGACG......................................   1
....GGGAACGTCGGGACTGGAGC....   1
.((((((.((.((((((.((((.(((((.....((.....)).))))).)))))))))).)).)))))) (-44.00)

21/70311375-70311437(+)_novel_cow_24
TCGTGGCTGATCCTGAGTCCCGAGGTGGTCCAGATTCCTCGGGGCTGAGTCTGATCACTCCTG
...TGGCTGATCCTGAGTCCCGAGG......................................   13
...TGGCTGATCCTGAGTCCCGAG.......................................   3
...TGGCTGATCCTGAGTCCCGAGGT.....................................   2

```
...TGGCTGATCCTGAGTCCCGA........................................      1
......................................TCGGGGCTGAGTCTGATCACT....      1
....((.(((((((.((((((((((.(((....))))))))))))).))...))))).))..  (-26.90)

2/20797619-20797693(-)_mir_cow_103
TCTATATATACCCTGTAGAACCGAATTTGTGTGGTATCCATGTAGTCACAGATTCGATTCTAGGGGAATATATGG
........TACCCTGTAGAACCGAATTTGT.............................................     3563638
........TACCCTGTAGAACCGAATTTGTG............................................     344620
.........ACCCTGTAGAACCGAATTTGT.............................................     234143
.........ACCCTGTAGAACCGAATTTGTGT...........................................     208840
.........ACCCTGTAGAACCGAATTTGTG............................................     169661
........TACCCTGTAGAACCGAATTTG..............................................     105513
........TACCCTGTAGAACCGAATTTGTGT...........................................     104076
........TACCCTGTAGAACCGAATTT...............................................     23109
.........ACCCTGTAGAACCGAATTTG..............................................     9425
........TACCCTGTAGAACCGAATT................................................     6166
.........ACCCTGTAGAACCGAATTT...............................................     4542
........TACCCTGTAGAACCGAAT.................................................     1670
.........ACCCTGTAGAACCGAATT................................................     1369
...........CCTGTAGAACCGAATTTGT.............................................     480
.........ACCCTGTAGAACCGAATTTGTGTG..........................................     367
..........CCCTGTAGAACCGAATTTGTGT...........................................     267
............CTGTAGAACCGAATTTGT.............................................     199
..........CCCTGTAGAACCGAATTTGT.............................................     193
...............................................ACAGATTCGATTCTAGGGGAAT......     189
................................................CAGATTCGATTCTAGGGGAAT......     154
.................................................AGATTCGATTCTAGGGGAAT......     129
.........ACCCTGTAGAACCGAAT.................................................     112
........TACCCTGTAGAACCGAA..................................................     95
.............TGTAGAACCGAATTTGT.............................................     95
...........CCTGTAGAACCGAATTTGTGT...........................................     93
...........CCTGTAGAACCGAATTTGTG............................................     83
.......ATACCCTGTAGAACCGAATTTGT.............................................     75
........TACCCTGTAGAACCGA...................................................     53
........TACCCTGTAGAACCGAATTTGTGTG..........................................     42
.........ACCCTGTAGAACCGAA..................................................     36
............CTGTAGAACCGAATTTGTG............................................     33
.........ACCCTGTAGAACCGAATTTGTGTGG.........................................     33
```

```
.................................................AGATTCGATTCTAGGGGAATA.....     30
..........CCCTGTAGAACCGAATTTGTG............................................     28
........TACCCTGTAGAACCGAATTTGTGTGG.........................................     23
............CTGTAGAACCGAATTTGTGT...........................................     22
..............GTAGAACCGAATTTGT.............................................     21
.............TGTAGAACCGAATTTGTGT...........................................     20
...........CCTGTAGAACCGAATTTG..............................................     16
.............TGTAGAACCGAATTTGTG............................................     16
........TACCCTGTAGAACCGAATTTGTGTGGT........................................     15
................................................CAGATTCGATTCTAGGGGAA.......     14
...............................................ACAGATTCGATTCTAGGGGAA.......     13
................................................CAGATTCGATTCTAGGGGAATA.....     10
.......ATACCCTGTAGAACCGAATTTG..............................................     9
...............................................ACAGATTCGATTCTAGGGGA........     8
............CTGTAGAACCGAATTTG..............................................     6
........TACCCTGTAGAACCGAATTTGTGTGGTA.......................................     5
........TACCCTGTAGAACCGAATTTGTGTGGTATCCAT..................................     5
..........CCCTGTAGAACCGAATTTG..............................................     4
.........ACCCTGTAGAACCGAATTTGTGTGGT........................................     4
..............................................CACAGATTCGATTCTAGGGGAAT......     4
.................................................AGATTCGATTCTAGGGGAATAT....     4
..............................................CACAGATTCGATTCTAGGGGAA.......     3
.................................................AGATTCGATTCTAGGGGAA.......     3
..........CCCTGTAGAACCGAATTT...............................................     2
......TATACCCTGTAGAACCGAATTTGT.............................................     2
.......ATACCCTGTAGAACCGAATT................................................     1
...........CCTGTAGAACCGAATT................................................     1
.......ATACCCTGTAGAACCGAATTT...............................................     1
...........CCTGTAGAACCGAATTT...............................................     1
.............TGTAGAACCGAATTTG..............................................     1
.......ATACCCTGTAGAACCGAATTTGTG............................................     1
........TACCCTGTAGAACCGAATTTGTGTGGTAT......................................     1
........TACCCTGTAGAACCGAATTTGTGTGGTATCC....................................     1
........TACCCTGTAGAACCGAATTTGTGTGGTATCCA...................................     1
........TACCCTGTAGAACCGAATTTGTGTGGTATCCATGT................................     1
........TACCCTGTAGAACCGAATTTGTGTGGTATCCATGTAGT.............................     1
.........ACCCTGTAGAACCGAATTTGTGTGGTATCCATGTAGTCA...........................     1
..............................GTGGTATCCATGTAGTCAC..........................     1
```

```
...............................................ACAGATTCGATTCTAGGGG.........     1
..............................................CACAGATTCGATTCTAGGGGA........     1
................................................CAGATTCGATTCTAGGGGA........     1
.............................................TCACAGATTCGATTCTAGGGGAATA.....     1
...............................................ACAGATTCGATTCTAGGGGAATA.....     1
.((((((((.((((.(((((.((((((((((.(.(((....))).))))))))))).)))))))))).)))))))) (-29.40)

22/11457910-11457977(+)_mir_cow_159
CCTCGTTCAAGTAATCCAGGATAGGCTGTGCAGGTCCCAAGGGGCCTATTCTCGGTTACTTGCACGCG
.....TTCAAGTAATCCAGGATAGGCT......................................... 3223130
.....TTCAAGTAATCCAGGATAGGC.......................................... 183122
.....TTCAAGTAATCCAGGATAGG........................................... 23183
.....TTCAAGTAATCCAGGATAG............................................ 15670
......TCAAGTAATCCAGGATAGGCT......................................... 5954
.....TTCAAGTAATCCAGGATAGGCTG........................................ 4858
.....TTCAAGTAATCCAGGATA............................................. 2122
.....TTCAAGTAATCCAGGAT.............................................. 801
.....TTCAAGTAATCCAGGATAGGCTGT....................................... 518
.......CAAGTAATCCAGGATAGGCT......................................... 421
......TCAAGTAATCCAGGATAGGC.......................................... 335
........AAGTAATCCAGGATAGGCT......................................... 194
......TCAAGTAATCCAGGATAGGCTG........................................ 188
.........AGTAATCCAGGATAGGCT......................................... 178
.....TTCAAGTAATCCAGGA............................................... 70
............................................CCTATTCTCGGTTACTTGCACG.. 61
......TCAAGTAATCCAGGATAGG........................................... 45
...........TAATCCAGGATAGGCT......................................... 37
......TCAAGTAATCCAGGATAGGCTGT....................................... 37
.........AGTAATCCAGGATAGGC.......................................... 23
......TCAAGTAATCCAGGATAG............................................ 22
.......CAAGTAATCCAGGATAGGC.......................................... 19
........AAGTAATCCAGGATAGGC.......................................... 15
............................................CCTATTCTCGGTTACTTGCAC... 13
..........GTAATCCAGGATAGGCT......................................... 9
....GTTCAAGTAATCCAGGATAGGCT......................................... 5
............................................CCTATTCTCGGTTACTTGCA.... 4
.......CAAGTAATCCAGGATA............................................. 3
.......CAAGTAATCCAGGATAG............................................ 3
```

```
......TCAAGTAATCCAGGATA............................................. 2
........AAGTAATCCAGGATAG............................................ 2
......TCAAGTAATCCAGGAT.............................................. 1
........AAGTAATCCAGGATAGG........................................... 1
.........AGTAATCCAGGATAGG........................................... 1
..........GTAATCCAGGATAGGC.......................................... 1
.............ATCCAGGATAGGCTGT....................................... 1
......TCAAGTAATCCAGGATAGGCTGTGCAGG.................................. 1
..........................TGTGCAGGTCCCAAGGGGC....................... 1
..........................................GGCCTATTCTCGGTTACTTGC..... 1
............................................CCTATTCTCGGTTACTTGC..... 1
...(((.(((((((((.((((((((((.(...(....)..).)))))))))).))))))))).))).. (-29.30)

22/14870587-14870656(+)_mir_cow_160
TGGGGCAGCTGGTGTTGTGAATCAGGCCGTCGCCAATCAGAGAACGGCTACTTCACAACACCAGGGTCAC
......AGCTGGTGTTGTGAATCAGGCCG.........................................    25155
......AGCTGGTGTTGTGAATCAGGC...........................................    3433
......AGCTGGTGTTGTGAATCAG.............................................    921
......AGCTGGTGTTGTGAATCAGGCCGT........................................    787
......AGCTGGTGTTGTGAATCAGGCC..........................................    419
..............................................GCTACTTCACAACACCAGGGT...    244
......AGCTGGTGTTGTGAATCAGG............................................    240
......AGCTGGTGTTGTGAATC...............................................    116
.....CAGCTGGTGTTGTGAATCAGGCCG.........................................    69
......AGCTGGTGTTGTGAATCA..............................................    43
...........GTGTTGTGAATCAGGCCG.........................................    38
......AGCTGGTGTTGTGAAT................................................    18
.....CAGCTGGTGTTGTGAATCAGGC...........................................    14
.......GCTGGTGTTGTGAATCAGGCCG.........................................    14
............TGTTGTGAATCAGGCCG.........................................    12
..........GGTGTTGTGAATCAGGCCG.........................................    10
...........GTGTTGTGAATCAGGC...........................................    6
...........GTGTTGTGAATCAGGCC..........................................    6
.............GTTGTGAATCAGGCCG.........................................    5
......AGCTGGTGTTGTGAATCAGGCCGTC.......................................    5
...........GTGTTGTGAATCAGGCCGT........................................    4
..............................................GCTACTTCACAACACCAGGG....    4
..........GGTGTTGTGAATCAGGC...........................................    3
```

```
............TGTTGTGAATCAGGCC..........................................    2
.........TGGTGTTGTGAATCAGGCCG.........................................    2
.........TGGTGTTGTGAATCAGGCCGT........................................    2
.............GTTGTGAATCAGGCCGT........................................    2
......AGCTGGTGTTGTGAATCAGGCCGTCGCC....................................    2
............................................CGGCTACTTCACAACACCAGGG....    2
....GCAGCTGGTGTTGTGAATCAGGCCG.........................................    1
..........GGTGTTGTGAATCAGGCCGT........................................    1
............TGTTGTGAATCAGGCCGT........................................    1
......AGCTGGTGTTGTGAATCAGGCCGTCGC.....................................    1
.............................TCGCCAATCAGAGAACG........................    1
..............................................GCTACTTCACAACACCAGG.....    1
............................................CGGCTACTTCACAACACCAGGGT...    1
...............................................CTACTTCACAACACCAGGGT...    1
................................................TACTTCACAACACCAGGGT...    1
.................................................ACTTCACAACACCAGGGT...    1
...(((..(((((((((((((...(((((((..........).))))))..))))))))))))).))).. (-33.50)

22/21900469-21900537(-)_mir_cow_161
GGCCAAAAAGTTAGTTTAGGTTTTTCGTAAGATGGTATGGAAAAACCTGAATGAACTTTTTGGCCAGCC
.........................................AAAACCTGAATGAACTTTTTGG......     2418
..........................................AAACCTGAATGAACTTTTTGG......     480
.........................................AAAACCTGAATGAACTTTTTG.......     124
..........................................AAACCTGAATGAACTTTTTGGC.....     77
.........................................AAAACCTGAATGAACTTTTTGGC.....     38
........................................AAAAACCTGAATGAACTTTTTG.......     22
.........................................AAAACCTGAATGAACTTTTT........     21
........................................AAAAACCTGAATGAACTTTTTGG......     21
....AAAAAGTTAGTTTAGGTTTTT............................................     19
....AAAAAGTTAGTTTAGGTTTTTC...........................................     19
.........................................AAAACCTGAATGAACTTTT.........     7
........................................AAAAACCTGAATGAACTTTTT........     6
..........................................AAACCTGAATGAACTTTTTG.......     6
...........................................AACCTGAATGAACTTTTTGGCC....     6
.....AAAAGTTAGTTTAGGTTTTTCG..........................................     5
...........................................AACCTGAATGAACTTTTTGG......     5
............................................ACCTGAATGAACTTTTTGGCC....     4
.....AAAAGTTAGTTTAGGTTTTTC...........................................     3
```

```
..........................................AAACCTGAATGAACTTTTT........      3
.............................................CCTGAATGAACTTTTTGGCC....      3
.............................................CCTGAATGAACTTTTTGGCCA...      3
....................................ATGGAAAAACCTGAATGAACT............      2
...........................................AACCTGAATGAACTTTTTGGC.....      2
..........................................AAACCTGAATGAACTTTTTGGCC....      2
............................................ACCTGAATGAACTTTTTGGCCA...      2
....AAAAAGTTAGTTTAGGTT...............................................      1
....AAAAAGTTAGTTTAGGTTTT.............................................      1
...CAAAAAGTTAGTTTAGGTTTTT............................................      1
.....AAAAGTTAGTTTAGGTTTTT............................................      1
...................................TATGGAAAAACCTGAA..................      1
........................................AAAAACCTGAATGAACT............      1
.....................................TGGAAAAACCTGAATGAACTTT..........      1
......................................GGAAAAACCTGAATGAACTTT..........      1
.........................................AAAACCTGAATGAACTTT..........      1
......................................GGAAAAACCTGAATGAACTTTT.........      1
........................................AAAAACCTGAATGAACTTTT.........      1
..........................................AAACCTGAATGAACTTTT.........      1
...............................................TGAATGAACTTTTTGG......      1
...........................................AACCTGAATGAACTTTTTGGCCA...      1
((((((((((((.((((((((((((((((......))).))))))))))))).)))))))))))).... (-34.30)

22/4264332-4264421(+)_novel_cow_26
GACGGGACTGTGGCCTGTAGGCAGAGATGACAGTCATACTGTTGAATTGCCAGTATGGCAGTGTCACTGACCAACAAGCCTAGACCCCAA
.................TAGGCAGAGATGACAGTCATACTG................................................. 9
.................TAGGCAGAGATGACAGTCATACT.................................................. 8
.....................................................TATGGCAGTGTCACTGACC.................. 1
....................................................GTATGGCAGTGTCACTGACCAAC............... 1
.....................................................TATGGCAGTGTCACTGACCAAC............... 1
...(((.(((.(((.(((.(((((...((((((((((((((.........)))))))))..)))))))).)).))).)))))).)))... (-35.50)

22/49142039-49142107(+)_mir_cow_162
TTCTCTATGGCTTTTTATTCCTATGTGATTCTACTGCTCACTCATATAGGGATTGGAGCCGTGGCGCAC
.....TATGGCTTTTTATTCCTATGTGA..........................................      2731
.....TATGGCTTTTTATTCCTATGTG...........................................      486
.....TATGGCTTTTTATTCCTATGT............................................      203
...........................................ATATAGGGATTGGAGCCGTGGC....      17
```

.....TATGGCTTTTTATTCCTATGTGAT.. 13
.....TATGGCTTTTTATTCCTATG.. 11
....TATAGGGATTGGAGCCGTGGC.... 5
....TATAGGGATTGGAGCCGTGGCGC.. 3
.....TATGGCTTTTTATTCC.. 2
....ATATAGGGATTGGAGCCGTGG..... 2
....CTATGGCTTTTTATTCCTATGT... 1
......ATGGCTTTTTATTCCTATGTG.. 1
....CTATGGCTTTTTATTCCTATGTGA... 1
....ATATAGGGATTGGAGCC......... 1
....((((((((((..((((((((((((.............)))))))))))).)))))))))))..... (-26.42)

22/49189339-49189424(+)_mir_Cow_Blast_49
CAGGCTGAGGTAGTAGTTTGTACAGTTTGAGGGTCTATGATACCACCCGGTACAGGAGATAACTGTACAGGCCACTGCCTTGCCGG
.....TGAGGTAGTAGTTTGTACAGTT... 171611
.....TGAGGTAGTAGTTTGTACAGT.. 38967
.....TGAGGTAGTAGTTTGTACAGTTT.. 5146
.....TGAGGTAGTAGTTTGTACAG... 3687
.....TGAGGTAGTAGTTTGTAC... 2109
.....TGAGGTAGTAGTTTGTACA.. 1546
....CTGAGGTAGTAGTTTGTACAGTT... 790
....CTGTACAGGCCACTGCCTTGC... 356
....CTGAGGTAGTAGTTTGTACAGT.. 284
.....TGAGGTAGTAGTTTGTA.. 67
....CTGAGGTAGTAGTTTGTAC... 54
......GAGGTAGTAGTTTGTACAGTT... 48
.....TGAGGTAGTAGTTTGT... 43
....CTGTACAGGCCACTGCCTTGCC.. 37
....CTGAGGTAGTAGTTTGTACAGTTT.. 27
....ACTGTACAGGCCACTGCCTTGC... 25
..........TAGTAGTTTGTACAGTT... 24
........GGTAGTAGTTTGTACAGTT... 22
...........AGTAGTTTGTACAGTT... 21
....AACTGTACAGGCCACTGCCTTGC... 18
....CTGAGGTAGTAGTTTGTACA.. 16
....CTGAGGTAGTAGTTTGTACAG... 16
....CTGTACAGGCCACTGCCTTG.... 15
..........TAGTAGTTTGTACAGT.. 10

```
......GAGGTAGTAGTTTGTACAGT............................................................     9
.......AGGTAGTAGTTTGTACAGTT...........................................................     9
........GGTAGTAGTTTGTACAGT............................................................     8
...........................TGAGGGTCTATGATACCACCCGGT...................................     7
......GAGGTAGTAGTTTGTACAGTTT..........................................................     5
...........................................................TAACTGTACAGGCCACTGCCTTGC...     4
.......AGGTAGTAGTTTGTACAGT............................................................     3
...........................TGAGGGTCTATGATACCACCCGG....................................     3
............................................................AACTGTACAGGCCACTGCCTTG....     3
.........GTAGTAGTTTGTACAGTT...........................................................     2
.....TGAGGTAGTAGTTTGTACAGTTTGAGGGTCT..................................................     2
..............................................................CTGTACAGGCCACTGCCT......     2
.............................................................ACTGTACAGGCCACTGCCTTG....     2
....CTGAGGTAGTAGTTTGT.................................................................     1
.......AGGTAGTAGTTTGTAC...............................................................     1
.........GTAGTAGTTTGTACAG.............................................................     1
.........GTAGTAGTTTGTACAGT............................................................     1
.......AGGTAGTAGTTTGTACAGTTT..........................................................     1
........GGTAGTAGTTTGTACAGTTT..........................................................     1
.....TGAGGTAGTAGTTTGTACAGTTTG.........................................................     1
....CTGAGGTAGTAGTTTGTACAGTTTGAGG......................................................     1
.....TGAGGTAGTAGTTTGTACAGTTTGAGGGTC...................................................     1
...........................TGAGGGTCTATGATACCACCCGGTA..................................     1
............................................................AACTGTACAGGCCACTGCCT......     1
..............................................................CTGTACAGGCCACTGCCTT.....     1
..(((.((((((((.((((((((((((.....((((.((.((((....))))))..)))))))))))))))).))))))))))).. (-40.50)

22/51543480-51543550(+)_mir_cow_163
CGGGCAACGGAATCCCAAAAGCAGCTGTTGTCTCCAGAGCATTCCAGCTGCGCTTGGATTTCGTTCCCTGC
....CAACGGAATCCCAAAAGCAGCTG............................................   460191
....CAACGGAATCCCAAAAGCAGCTGT...........................................   21127
....CAACGGAATCCCAAAAGCAGCT.............................................   14349
.....AACGGAATCCCAAAAGCAGCTG............................................   12762
....CAACGGAATCCCAAAAGCAG...............................................   5294
....CAACGGAATCCCAAAAGCAGC..............................................   3417
.....AACGGAATCCCAAAAGCAGCTGT...........................................   1714
...............................................CTGCGCTTGGATTTCGTTCCC...   1069
...............................................CTGCGCTTGGATTTCGTTCC....   266
```

```
....CAACGGAATCCCAAAAGCA................................................   135
...............................................CTGCGCTTGGATTTCGTTCCCT..   131
.....AACGGAATCCCAAAAGCAGCT.............................................   115
...............................................CTGCGCTTGGATTTCGTTC.....   81
....CAACGGAATCCCAAAAGCAGCTGTT..........................................   78
..............................................GCTGCGCTTGGATTTCGTTCCC...   60
......ACGGAATCCCAAAAGCAGCTG............................................   40
...........ATCCCAAAAGCAGCTG............................................   38
..............................................GCTGCGCTTGGATTTCGTTCC....   36
.....AACGGAATCCCAAAAGCAGC..............................................   33
.....AACGGAATCCCAAAAGCAG...............................................   29
....CAACGGAATCCCAAAAGC.................................................   25
................................................TGCGCTTGGATTTCGTTCCCT..   24
.........GAATCCCAAAAGCAGCTG............................................   23
..............................................GCTGCGCTTGGATTTCGTTC.....   18
.......CGGAATCCCAAAAGCAGCTG............................................   16
....CAACGGAATCCCAAAAG..................................................   15
..........AATCCCAAAAGCAGCTG............................................   13
...........ATCCCAAAAGCAGCTGT...........................................   10
.....AACGGAATCCCAAAAGCAGCTGTT..........................................   9
................................................TGCGCTTGGATTTCGTTCCC...   9
........GGAATCCCAAAAGCAGCTG............................................   7
......ACGGAATCCCAAAAGCAGCTGT...........................................   7
...............................................CTGCGCTTGGATTTCGTT......   6
..............................................GCTGCGCTTGGATTTCGTTCCCT..   6
..............................................GCTGCGCTTGGATTTCGTT......   4
.............................................AGCTGCGCTTGGATTTCGTTC.....   4
.............................................AGCTGCGCTTGGATTTCGTTCC....   3
................................................TGCGCTTGGATTTCGTTCC....   3
..........AATCCCAAAAGCAGCTGT...........................................   2
......ACGGAATCCCAAAAGCAGCT.............................................   1
.........GAATCCCAAAAGCAGCT.............................................   1
.........GAATCCCAAAAGCAGCTGT...........................................   1
..........AATCCCAAAAGCAGCTGTT..........................................   1
....CAACGGAATCCCAAAAGCAGCTGTTGTCTCCAGAGCATTCC..........................   1
...............................................CTGCGCTTGGATTTCG........   1
.............................................AGCTGCGCTTGGATTTCGTT......   1
.............................................AGCTGCGCTTGGATTTCGTTCCC...   1
```

```
...................................................GCTTGGATTTCGTTCCC...    1
.............................................AGCTGCGCTTGGATTTCGTTCCCT..    1
.(((.((((((((((.((..(((((((..((((....)).))..)))))))..)))))))))))))))... (-33.90)

22/51543961-51544030(+)_mir_Cow_Blast_8
TTGGAATGACACGATCACTCCCGTTGAGTGGGCACCCAAGAAGCCATCGGGAATGTCGTGTCCGCCCAGT
....AATGACACGATCACTCCCGTTG............................................    5314
....AATGACACGATCACTCCCGTTGAGT.........................................    1102
....AATGACACGATCACTCCCGTTGA...........................................    574
....AATGACACGATCACTCCCGTTGAG..........................................    574
.....ATGACACGATCACTCCCGTTGAGT.........................................    572
............................................CATCGGGAATGTCGTGTCCGCC....    489
.....ATGACACGATCACTCCCGTTGAG..........................................    301
............................................CATCGGGAATGTCGTGTCCGC.....    176
.............................................ATCGGGAATGTCGTGTCCGCC....    156
.....ATGACACGATCACTCCCGTTG............................................    135
.............................................ATCGGGAATGTCGTGTCCGCCC...    107
.....ATGACACGATCACTCCCGTTGA...........................................    52
....AATGACACGATCACTCCCGTT.............................................    46
............................................CATCGGGAATGTCGTGTCCGCCC...    40
.............................................ATCGGGAATGTCGTGTCCGC.....    39
............................................CATCGGGAATGTCGTGTCCG......    22
....AATGACACGATCACTCCCGT..............................................    21
............................................CATCGGGAATGTCGTGTCC.......    20
...........................................CCATCGGGAATGTCGTGTCCGC.....    17
....AATGACACGATCACTCCCGTTGAGTG........................................    14
..............................................TCGGGAATGTCGTGTCCGCCC...    14
..............................................TCGGGAATGTCGTGTCCGCC....    13
.............................................ATCGGGAATGTCGTGTCCG......    9
....AATGACACGATCACTCCCGTTGAGTGGGC.....................................    5
..............................................TCGGGAATGTCGTGTCCGC.....    5
............................................CATCGGGAATGTCGTGTCCGCCCA..    4
.............................................ATCGGGAATGTCGTGTCCGCCCA..    4
....AATGACACGATCACTCCC................................................    3
......TGACACGATCACTCCCGTTG............................................    3
......TGACACGATCACTCCCGTTGAGT.........................................    3
....AATGACACGATCACTCCCGTTGAGTGG.......................................    3
.............................................ATCGGGAATGTCGTGTCC.......    3
```

```
....AATGACACGATCACTCCCG...............................................    2
.....ATGACACGATCACTCCCGTT.............................................    2
.....ATGACACGATCACTCCCGTTGAGTGGGC.....................................    2
...........................................CCATCGGGAATGTCGTGTCCG......    2
.....ATGACACGATCACTCCCGT..............................................    1
.......GACACGATCACTCCCGTTG............................................    1
........ACACGATCACTCCCGTTG............................................    1
....AATGACACGATCACTCCCGTTGAGTGGGCACCCAAGA.............................    1
............................................CATCGGGAATGTCGTGTC........    1
.................................................GGAATGTCGTGTCCGC.....    1
...........................................CCATCGGGAATGTCGTGTCCGCC....    1
..................................................GAATGTCGTGTCCGCC....    1
((((...(((((((.((.(((((.((..(((....)))......)).))))).)))))))))...)))). (-27.90)

22/55072945-55073010(-)_mir_cow_164
GCGCTCTCTCCATTACACTACCCTGCCTCTTCTCCACGAGAGGCAGCGGGGTGTAGTGGATAGAGC
........TCCATTACACTACCCTGCCTCT....................................  2220
.......CTCCATTACACTACCCTGCCT......................................  219
........TCCATTACACTACCCTGCCT......................................  92
......TCTCCATTACACTACCCTGCCT......................................  73
.......CTCCATTACACTACCCTGCCTC.....................................  65
........TCCATTACACTACCCTGCCTC.....................................  62
........................................AGGCAGCGGGGTGTAGTGGAT.....  40
.......CTCCATTACACTACCCTGCCTCT....................................  26
........................................AGGCAGCGGGGTGTAGTGGATA....  19
........TCCATTACACTACCCTGCCTCTT...................................  14
........................................AGGCAGCGGGGTGTAGTGGA......  12
........TCCATTACACTACCCTGCC.......................................  6
........................................AGGCAGCGGGGTGTAGT.........  5
.......CTCCATTACACTACCCTGC........................................  4
........................................AGGCAGCGGGGTGTAGTGG.......  4
......TCTCCATTACACTACCCTGCC.......................................  3
..........CATTACACTACCCTGCCTCT....................................  2
........TCCATTACACTACCCTGCCTCTTC..................................  2
........TCCATTACACTACCCTG.........................................  1
......TCTCCATTACACTACCCTGC........................................  1
.........CCATTACACTACCCTGCCTCT....................................  1
..............ACACTACCCTGCCTCT....................................  1
```

```
........TCCATTACACTACCCTGCCTCTTCTC................................ 1
........TCCATTACACTACCCTGCCTCTTCTCC............................... 1
........................................AGGCAGCGGGGTGTAG.......... 1
......................................AGAGGCAGCGGGGTGTAGT......... 1
...........................................CAGCGGGGTGTAGTGGA...... 1
..(((((.(((((((((((.(((((((((((......))))))))).))))))))))))).))))) (-42.30)

2/29141452-29141526(-)_mir_cow_104
GACCAGAAAGTACATTCAAGATTTTCTGTAAGATCTTGCAGGAAAATCTGAATGAACTTTTGGTCAACCCAATAA
..........................................AAAATCTGAATGAACTTTTGGT........... 73
...........................................AAATCTGAATGAACTTTTGGT........... 28
...CAGAAAGTACATTCAAGATTTTCT................................................ 1
...........................................AAATCTGAATGAACTTTTGGTC.......... 1
............................................AATCTGAATGAACTTTTGGTCA......... 1
((((((((.((.((((((.((((((((((((....))))).)))))))))))))).)))))))))).......... (-29.80)

22/9738607-9738675(+)_mir_cow_158
GGAAGGGGGGCCGAGGCACTGTCCGAGAGTGAGTAGCAGGTCTCACAGTGAACCGGTCTCTTTCCCTAC
..........................................TCACAGTGAACCGGTCTCTTT...... 135574
..........................................TCACAGTGAACCGGTCTCTTTC..... 43617
..........................................TCACAGTGAACCGGTCTCTT....... 31623
..........................................TCACAGTGAACCGGTCTCT........ 13335
..........................................TCACAGTGAACCGGTCTC......... 1365
..........................................TCACAGTGAACCGGTCT.......... 1174
..........................................TCACAGTGAACCGGTCTCTTTCC.... 904
..........................................TCACAGTGAACCGGTC........... 180
.........................................CTCACAGTGAACCGGTCTCTTT...... 82
...........................................CACAGTGAACCGGTCTCTTT...... 30
.........................................CTCACAGTGAACCGGTCTCTT....... 24
............................................ACAGTGAACCGGTCTCTTT...... 23
.............................................CAGTGAACCGGTCTCTTTC..... 13
...........................................CACAGTGAACCGGTCTCTT....... 12
.............................................CAGTGAACCGGTCTCTTT...... 12
...............................................GTGAACCGGTCTCTTT...... 12
.........................................CTCACAGTGAACCGGTCTCTTTC..... 12
...........................................CACAGTGAACCGGTCTCTTTC..... 9
.............................................CAGTGAACCGGTCTCTT....... 8
............................................ACAGTGAACCGGTCTCTTTC..... 8
```

```
.....GGGGGCCGAGGCACTGTCCGAGA.........................................      7
...........................................CACAGTGAACCGGTCTCTTTCC....      6
.....GGGGGCCGAGGCACTGTCCGA...........................................      5
.........................................CTCACAGTGAACCGGTCTCT........      4
........................................TCTCACAGTGAACCGGTCTCTTT......      4
...........................................CACAGTGAACCGGTCTCT........      3
............................................ACAGTGAACCGGTCTCTT.......      3
.........................................CTCACAGTGAACCGGTCT..........      2
.........................................CTCACAGTGAACCGGTCTC.........      2
............................................ACAGTGAACCGGTCTCT........      2
........................................TCTCACAGTGAACCGGTCTCTT.......      2
........................................TCTCACAGTGAACCGGTCTCTTTC.....      2
................................................TGAACCGGTCTCTTTC.....      2
.....GGGGGCCGAGGCACTGTCC.............................................      1
......GGGGCCGAGGCACTGTCCGA...........................................      1
.....GGGGGCCGAGGCACTGTCCGAG..........................................      1
....GGGGGGCCGAGGCACTGTCCGAGA.........................................      1
......GGGGCCGAGGCACTGTCCGAGA.........................................      1
.....GGGGGCCGAGGCACTGTCCGAGAGT.......................................      1
.....GGGGGCCGAGGCACTGTCCGAGAGTGAGTAG.................................      1
...............................AGTAGCAGGTCTCACAGTGAAC................      1
.....................................AGGTCTCACAGTGAAC................      1
........................................TCTCACAGTGAACCGGTCTCT........      1
.............................................CAGTGAACCGGTCTCT........      1
..............................................AGTGAACCGGTCTCTTT......      1
...............................................GTGAACCGGTCTCTTTC.....      1
............................................ACAGTGAACCGGTCTCTTTCC....      1
..........................................TCACAGTGAACCGGTCTCTTTCCC...      1
..........................................TCACAGTGAACCGGTCTCTTTCCCT..      1
((.((((((((((...((((((..((((.(........).))))))))))...)))))))))).))... (-28.90)

23/24312336-24312401(+)_mir_cow_166
TCTGGCTGGTCAAACGGAACCAAGTCCGTCTTCCTGAGAGGTTTGGTCCCCTTCAACCAGCTACAG
.........................................TTTGGTCCCCTTCAACCAGCT....   7743
..........................................TTGGTCCCCTTCAACCAGCT....   1326
.........................................TTTGGTCCCCTTCAACCAGC.....   542
..........................................TTGGTCCCCTTCAACCAGC.....   228
.........................................TTTGGTCCCCTTCAACCAGCTA...   41
```

```
.........................................TTTGGTCCCCTTCAACCAG......  24
.........................................TTTGGTCCCCTTCAACC........  10
..........................................TTGGTCCCCTTCAACC........  10
..........................................TTGGTCCCCTTCAACCAGCTA...  9
.........................................TTTGGTCCCCTTCAAC.........  4
..........................................TTGGTCCCCTTCAACCAG......  4
...........................................TGGTCCCCTTCAACCAGC.....  4
.........................................TTTGGTCCCCTTCAACCA.......  2
...........................................TGGTCCCCTTCAACCAGCT....  2
....GCTGGTCAAACGGAACCAAGT.........................................  1
....GCTGGTCAAACGGAACCAAGTC........................................  1
..........................................TTGGTCCCCTTCAACCA.......  1
..........................................TTGGTCCCCTTCAACCAGCTAC..  1
..((((((((..((.((.((((((.((.((((...)))))))))))).)).))..))))))))... (-25.40)

23/30890105-30890179(+)_mir_cow_167
GAGGCATAGCCAGTTGGGGAAGAATGCCCGTGTCTTAAAGTGAGAAGGTTTGTTCTCCAACCTGGCTCTTTATGG
.....ATAGCCAGTTGGGGAAGAATGC................................................     120
......TAGCCAGTTGGGGAAGAATGC................................................     37
................................................TTTGTTCTCCAACCTGGCTCTTT....     14
.....ATAGCCAGTTGGGGAAGAAT..................................................     9
.......AGCCAGTTGGGGAAGAATGC................................................     8
................................................TTTGTTCTCCAACCTGGCTCTT.....     6
.....ATAGCCAGTTGGGGAAGAATG.................................................     5
....CATAGCCAGTTGGGGAAGAATGC................................................     5
......TAGCCAGTTGGGGAAGAAT..................................................     2
................................................TTTGTTCTCCAACCTGGCTC.......     2
......TAGCCAGTTGGGGAAGAATG.................................................     1
.....ATAGCCAGTTGGGGAAGAATGCC...............................................     1
................................................TTTGTTCTCCAACCTGGCT........     1
................................................TTTGTTCTCCAACCTGGCTCT......     1
....................................................TTCTCCAACCTGGCTCTTT....     1
................................................TTTGTTCTCCAACCTGGCTCTTTA...     1
................................................TTTGTTCTCCAACCTGGCTCTTTAT..     1
((((...((((((((((((((.((.(((....((((.....)))).))))).)))))))).))))))))))....  (-26.80)

23/30899890-30899974(-)_mir_Cow_Blast_52
CTATTGAGACATAGCCAGTTGGGGAAGAATGCTGTTACCTTAGAGAGAAGCTGGATTGTTCTCCAACCTGGCTCTTTATGTTGCC
```

```
......................................................ATTGTTCTCCAACCTGGCTCTTT........ 214
..........ATAGCCAGTTGGGGAAGAATGC..................................................... 120
......................................................ATTGTTCTCCAACCTGGCTCTT......... 69
...........TAGCCAGTTGGGGAAGAATGC..................................................... 37
...................................................TGGATTGTTCTCCAACCTGGCTCT.......... 36
...................................................TGGATTGTTCTCCAACCTGGCTC........... 18
..................................................CTGGATTGTTCTCCAACCTGGCT............ 16
..........ATAGCCAGTTGGGGAAGAATGCT.................................................... 14
...................................................TGGATTGTTCTCCAACCTGGCT............ 13
............AGCCAGTTGGGGAAGAATGCTG................................................... 12
......................................................ATTGTTCTCCAACCTGGCTCTTTA....... 11
..........ATAGCCAGTTGGGGAAGAAT....................................................... 9
......................................................ATTGTTCTCCAACCTGGCTCT.......... 9
............AGCCAGTTGGGGAAGAATGC..................................................... 8
............AGCCAGTTGGGGAAGAATGCT.................................................... 8
......................................................ATTGTTCTCCAACCTGGCTC........... 8
...................................................TGGATTGTTCTCCAACCTGGC............. 6
......................................................ATTGTTCTCCAACCTGGCTCTTTAT...... 6
..........ATAGCCAGTTGGGGAAGAATG...................................................... 5
.........CATAGCCAGTTGGGGAAGAATGC..................................................... 5
............AGCCAGTTGGGGAAGAATGCTGT.................................................. 4
...........TAGCCAGTTGGGGAAGAATGCT.................................................... 3
...................................................TGGATTGTTCTCCAACCTGGCTCTT......... 3
...........TAGCCAGTTGGGGAAGAAT....................................................... 2
......................................................ATTGTTCTCCAACCTGGCT............ 2
..................................................CTGGATTGTTCTCCAACCTGGCTC........... 2
........ACATAGCCAGTTGGGGAAGAAT....................................................... 1
...........TAGCCAGTTGGGGAAGAATG...................................................... 1
...........TAGCCAGTTGGGGAAGAATGCTG................................................... 1
...........TAGCCAGTTGGGGAAGAATGCTGTT................................................. 1
...................................TACCTTAGAGAGAAGCTG................................ 1
.............................TGCTGTTACCTTAGAGAGAAGCTGGATTGTT......................... 1
..................................TTACCTTAGAGAGAAGCTGGATTGTTCTCCAACCT................ 1
...................................................TGGATTGTTCTCCAACCTGG.............. 1
..................................................CTGGATTGTTCTCCAACCTGGC............. 1
..................................................CTGGATTGTTCTCCAACCTGGCTCT.......... 1
....................................................GGATTGTTCTCCAACCTGGCTCT.......... 1
..........................................................TTCTCCAACCTGGCTCTTT........ 1
```

```
.......(((((((((((((((((((.(((.(.(((.((.....).).))).).))).)))))))).))))))....)))))...  (-27.80)

23/7335344-7335419(-)_mir_cow_165
CGGCTCCTGATTGTCCAAACGCAATTCTCGAGTCTCTAGCTCTGGCCGAGAGTTGAGTCTGGACGTCCCGAGCCGT
.......TGATTGTCCAAACGCAATTC.................................................    4177
.......TGATTGTCCAAACGCAATTCT................................................    3403
.......TGATTGTCCAAACGCAATT..................................................    157
.......TGATTGTCCAAACGCAAT...................................................    71
.......TGATTGTCCAAACGCA.....................................................    43
.......TGATTGTCCAAACGCAATTCTC...............................................    28
.......TGATTGTCCAAACGCAATTCTCG..............................................    27
................................................AGAGTTGAGTCTGGACGTCCCG......    7
................................................AGAGTTGAGTCTGGACGTCCCGA.....    5
.......TGATTGTCCAAACGCAA....................................................    3
......CTGATTGTCCAAACGCAATTC.................................................    3
........GATTGTCCAAACGCAATTC.................................................    2
......CTGATTGTCCAAACGCAATTCT................................................    2
..........TTGTCCAAACGCAATTC.................................................    1
........GATTGTCCAAACGCAATTCT................................................    1
..........TTGTCCAAACGCAATTCT................................................    1
..............................AGTCTCTAGCTCTGGCCG............................    1
............................................GCCGAGAGTTGAGTCTGGACG...........    1
................................................AGAGTTGAGTCTGGACGTCC........    1
................................................AGAGTTGAGTCTGGACGTCCCGAG....    1
((((((..(((.(((((.((.(((((((((.(((.........))))))))))))).)).))))))))..)))))). (-38.10)

24/20725717-20725782(-)_novel_cow_27
GTGACTGCTTTAAAACCTTTGACAGGTGCTGGGGTGATCTGTCAAAAGTTTTAAAGCAGTCATCAT
...ACTGCTTTAAAACCTTTGACAG.........................................   9
.........................................TCAAAAGTTTTAAAGCAGTCA....   8
...ACTGCTTTAAAACCTTTGACA..........................................   5
...ACTGCTTTAAAACCTTTGAC...........................................   1
.......................................TGTCAAAAGTTTTAAAGCAGTC.....   1
.........................................TCAAAAGTTTTAAAGCAGTC.....   1
((((((((((((((((.((((((((((((....)).)))))))))).))))))))))))))))... (-35.80)

24/23897863-23897929(-)_mir_cow_168
GCCAAAAGGTTCATTTGGGTTTTTCCATAAGATGTTCTAGAAAAACGCAAATGAACTTTTTGGCTAA
```

```
.........................................AAAACGCAAATGAACTTTTTGG....  17
........................................AAAAACGCAAATGAACTTTTTG.....  12
....AAAGGTTCATTTGGGTTTTT...........................................  5
...AAAAGGTTCATTTGGGTTTTTC..........................................  5
...AAAAGGTTCATTTGGGTTTTT...........................................  4
....AAAGGTTCATTTGGGTTTTTCCA........................................  2
......................................AGAAAAACGCAAATGAACTTTT.......  2
...AAAAGGTTCATTTGGGTTTT............................................  1
....AAAGGTTCATTTGGGTTTTTCC.........................................  1
........................................AAAAACGCAAATGAACTTTT.......  1
........................................AAAAACGCAAATGAACTTTTT......  1
..........................................AAACGCAAATGAACTTTTTGG....  1
(((((((((((((((((.(((((((....((.....)).))))))).)))))))))))))))))...  (-32.50)

24/27275573-27275634(-)_mir_Cow_Blast_4
GGCCAAAAAGTTTGTTTGGGTTTCTCCATGGAAAAACCTGAACAAACTTTTTGGCTAACCCA
................................AAAACCTGAACAAACTTTTTGG........ 147
.................................AAACCTGAACAAACTTTTTGG........ 18
...............................AAAAACCTGAACAAACTTTTTG......... 9
................................AAAACCTGAACAAACTTTTTG......... 6
................................AAAACCTGAACAAACTTTTT.......... 5
................................AAAACCTGAACAAACTTTTTGGC....... 5
.................................AAACCTGAACAAACTTTTTGGC....... 5
....AAAAAGTTTGTTTGGGTTT....................................... 3
................................AAAACCTGAACAAACTTTT........... 2
...............................AAAAACCTGAACAAACTTTTT.......... 2
..................................AACCTGAACAAACTTTTTGGCT...... 2
.....AAAAGTTTGTTTGGGTTT....................................... 1
.....AAAAGTTTGTTTGGGTTTC...................................... 1
.....AAAAGTTTGTTTGGGTTTCTC.................................... 1
..............................GAAAAACCTGAACAAACTTTT........... 1
...............................AAAAACCTGAACAAACTTTTTGG........ 1
..................................AACCTGAACAAACTTTTTGG........ 1
...................................ACCTGAACAAACTTTTTGGCT...... 1
((((((((((((((((..(((((.((....)).)))))..))))))))))))))))...... (-34.80)

24/33897674-33897741(+)_mir_cow_169
GCCAAAAAAATTTGTTTGGGTTTTTCTGTAAGATGGTATGGAAAAACCCGAACGAACTTTTGGGCCCAC
```

```
..........................................AAACCCGAACGAACTTTTGGGC.... 153
..........................................AAACCCGAACGAACTTTTG....... 5
..........................................AAACCCGAACGAACTTTTGGG..... 4
..........................................AAACCCGAACGAACTTTTGG...... 3
...AAAAAATTTGTTTGGGTTTTTC........................................... 1
...........................................AACCCGAACGAACTTTTGGGC.... 1
...........................................AACCCGAACGAACTTTTGGGCC... 1
(((.((((.((((((((((((((((((((......)))))))))))))))))))).)))).))).... (-31.00)

24/34841105-34841174(+)_mir_cow_170
GAGTACATACTTCTTTATGTACCCATATGAACATACAATGCTATGGAATGTAAAGAAGTATGTATTTTTG
...........................................TGGAATGTAAAGAAGTATGTAT.....     11903
...........................................TGGAATGTAAAGAAGTATGTA......     1632
...........................................TGGAATGTAAAGAAGTATGT.......     1127
...........................................TGGAATGTAAAGAAGTATGTATT....     851
.............................................GAATGTAAAGAAGTATGTATTT...     104
.............................................GAATGTAAAGAAGTATGTATT....     88
...........................................TGGAATGTAAAGAAGTATG........     32
...........................................TGGAATGTAAAGAAGTATGTATTT...     20
..........................................ATGGAATGTAAAGAAGTATGT.......     19
............................................GGAATGTAAAGAAGTATGTAT.....     18
...............................................ATGTAAAGAAGTATGTAT.....     16
..........................................ATGGAATGTAAAGAAGTATGTAT.....     13
...........................................TGGAATGTAAAGAAGTAT.........     9
..............................................AATGTAAAGAAGTATGTAT.....     9
............................................GGAATGTAAAGAAGTATGTATT....     9
..........................................ATGGAATGTAAAGAAGTATGTA......     6
...........................................TGGAATGTAAAGAAGTA..........     5
................................................TGTAAAGAAGTATGTAT.....     5
...........................................TGGAATGTAAAGAAGT...........     4
.................................................GTAAAGAAGTATGTAT.....     4
...............................................ATGTAAAGAAGTATGT.......     3
...............................................ATGTAAAGAAGTATGTA......     3
.............................................GAATGTAAAGAAGTATGTAT.....     3
..........................................ATGGAATGTAAAGAAGTATGTATT....     2
..............................................AATGTAAAGAAGTATGTATT....     2
..................................................TAAAGAAGTATGTATT....     2
....ACATACTTCTTTATGTACCCAT............................................     1
```

```
....ACATACTTCTTTATGTACCCATA...........................................    1
...........TCTTTATGTACCCATATGAACATACAATGCTA...........................    1
...........................TGAACATACAATGCTA...........................    1
.......................CATATGAACATACAATGCTAT..........................    1
.................................TACAATGCTATGGAATGTAAAGAAGTAT.........    1
.............................................GAATGTAAAGAAGTATGTA......    1
...............................................ATGTAAAGAAGTATGTATT....    1
................................................TGTAAAGAAGTATGTATT....    1
.................................................GTAAAGAAGTATGTATT....    1
.............................................GAATGTAAAGAAGTATGTATTTT..    1
((((((((((((((((((((..(((((..............))))).))))))))))))))))))))... (-24.44)

24/34844423-34844492(+)_mir_cow_171
TGCTAGAGCTGGTAAAATGGAACCAAATCGCCTCTTCAATGGATTTGGTCCCCTTCAACCAGCTGTAGCT
...........................................TTTGGTCCCCTTCAACCAGCTGT....    325991
............................................TTGGTCCCCTTCAACCAGCTGT....    230594
...........................................TTTGGTCCCCTTCAACCAGCTG.....    16514
...........................................TTTGGTCCCCTTCAACCAGCT......    7743
............................................TTGGTCCCCTTCAACCAGCTG.....    6175
............................................TTGGTCCCCTTCAACCAGCTGTA...    5872
...........................................TTTGGTCCCCTTCAACCAGCTGTA...    3708
.............................................TGGTCCCCTTCAACCAGCTGT....    3306
......AGCTGGTAAAATGGAACCAAAT..........................................    1466
............................................TTGGTCCCCTTCAACCAGCT......    1326
...........................................TTTGGTCCCCTTCAACCAGC.......    542
......AGCTGGTAAAATGGAACCAAATC.........................................    240
............................................TTGGTCCCCTTCAACCAGC.......    228
......AGCTGGTAAAATGGAACCAAA...........................................    208
..........................................ATTTGGTCCCCTTCAACCAGCTGT....    201
.............................................TGGTCCCCTTCAACCAGCTGTA...    141
......AGCTGGTAAAATGGAACC..............................................    85
..........................................ATTTGGTCCCCTTCAACCAGCT......    85
.............................................TGGTCCCCTTCAACCAGCTG.....    83
................................................TCCCCTTCAACCAGCTGT....    62
......AGCTGGTAAAATGGAACCA.............................................    54
..................................................CCCTTCAACCAGCTGT....    45
.......GCTGGTAAAATGGAACCAAATC.........................................    31
..........................................ATTTGGTCCCCTTCAACCAGCTG.....    31
```

......AGCTGGTAAAATGGAACCAA..	24
....TTTGGTCCCCTTCAACCAG........	24
....GGTCCCCTTCAACCAGCTGT....	23
.......GCTGGTAAAATGGAACCAAAT..	22
.....GAGCTGGTAAAATGGAACCAAAT..	16
.....GAGCTGGTAAAATGGAACCAAA...	15
....TTGGTCCCCTTCAACCAGCTGTAG..	15
....CCCCTTCAACCAGCTGT....	14
......AGCTGGTAAAATGGAACCAAATCGCCT.....................................	10
....TTTGGTCCCCTTCAACC..........	10
....TTGGTCCCCTTCAACC..........	10
......AGCTGGTAAAATGGAACCAAATCGCC......................................	9
....TTTGGTCCCCTTCAACCAGCTGTAG..	8
....GTCCCCTTCAACCAGCTGT....	7
.......GCTGGTAAAATGGAACCAAATCGCCT.....................................	6
....ATTTGGTCCCCTTCAACCAGC.......	5
....TTTGGTCCCCTTCAAC...........	4
....TTGGTCCCCTTCAACCAG........	4
....TGGTCCCCTTCAACCAGC.......	4
.....GAGCTGGTAAAATGGAACCA...	3
.......GCTGGTAAAATGGAACCAAA...	3
......AGCTGGTAAAATGGAAC...	2
.......GCTGGTAAAATGGAACC..	2
.............AAAATGGAACCAAATCGCC......................................	2
..............AAATGGAACCAAATCGCC......................................	2
......AGCTGGTAAAATGGAACCAAATCGCCTCTTCAATGGA...........................	2
......AGCTGGTAAAATGGAACCAAATCGCCTCTTCAATGGAT..........................	2
...........................TCGCCTCTTCAATGGATT.........................	2
....TTTGGTCCCCTTCAACCA.........	2
....TGGTCCCCTTCAACCAGCT......	2
....TCCCCTTCAACCAGCTG.....	2
............................CGCCTCTTCAATGGATTTGGTCCCCTTCAACCAGCTGT....	2
....GGTCCCCTTCAACCAGCTGTA...	2
....AGAGCTGGTAAAATGGAACCA...	1
....AGAGCTGGTAAAATGGAACCAA..	1
....AGAGCTGGTAAAATGGAACCAAA...	1
.....GAGCTGGTAAAATGGAACCAAATC...	1
.......GCTGGTAAAATGGAACCAAATCGCC......................................	1

```
......AGCTGGTAAAATGGAACCAAATCGCCTC....................................     1
......AGCTGGTAAAATGGAACCAAATCGCCTCT...................................     1
.......GCTGGTAAAATGGAACCAAATCGCCTCTTCAATGGA...........................     1
......AGCTGGTAAAATGGAACCAAATCGCCTCTTCAATGGATT.........................     1
...................................TCAATGGATTTGGTCCCCTTCAAC...........     1
............................................TTGGTCCCCTTCAACCA.........     1
..........................................ATTTGGTCCCCTTCAACCAG........     1
....................................CAATGGATTTGGTCCCCTTCAACCAGC.......     1
..............................................GGTCCCCTTCAACCAGCTG.....     1
...............................................GTCCCCTTCAACCAGCTG.....     1
................................TCTTCAATGGATTTGGTCCCCTTCAACCAGCTGT....     1
.........................................GATTTGGTCCCCTTCAACCAGCTGT....     1
..........................................ATTTGGTCCCCTTCAACCAGCTGTA...     1
...............................................GTCCCCTTCAACCAGCTGTA...     1
..................................................CCCTTCAACCAGCTGTA...     1
.............................................TGGTCCCCTTCAACCAGCTGTAG..     1
.((((.(((((((..((.((.((((((((............)))))))).)).))..))))))).)))). (-31.10)

24/58095652-58095713(+)_mir_cow_172
GCTGTGGAGTGTGACAATGGTGTTTGTGTCCAAACTATCAAACGCCATTATCACACTAAATA
....TGGAGTGTGACAATGGTGTTTG.................................... 34
....TGGAGTGTGACAATGGTGTTTGT................................... 25
....TGGAGTGTGACAATGGTGTTT..................................... 16
....TGGAGTGTGACAATGGTGTT...................................... 3
........................................AACGCCATTATCACACTAAA.. 1
..(((..(((((((.(((((((((((.((....))...))))))))))).)))))))..))) (-25.60)

25/13339491-13339559(+)_mir_cow_174
AGAATCGGGGTTTTGAGGGCGAGATGAGTTTATGTTTTATCCAACTGGCCCACAAAGTCCCGCTTTTGG
..........................................AACTGGCCCACAAAGTCCCGCT.....      4609
..........................................AACTGGCCCACAAAGTCCCGCTT....      1269
..........................................AACTGGCCCACAAAGTCCCGCTTT...      801
..........................................AACTGGCCCACAAAGTCCCGC......      295
...........................................ACTGGCCCACAAAGTCCCGCTTT...      227
...........................................ACTGGCCCACAAAGTCCCGCTT....      183
..........................................AACTGGCCCACAAAGTCCCGCTTTT..      114
...........................................ACTGGCCCACAAAGTCCCGCT.....      60
..........................................AACTGGCCCACAAAGTCCCG.......      58
```

```
.....CGGGGTTTTGAGGGCGAGATGA..........................................     48
...........................................ACTGGCCCACAAAGTCCCGCTTTT..     35
.....CGGGGTTTTGAGGGCGAGATG...........................................     16
..........................................AACTGGCCCACAAAGTCCC........     8
....TCGGGGTTTTGAGGGCGAGATG...........................................     7
..........................................AACTGGCCCACAAAGTCC.........     5
................................................CCCACAAAGTCCCGCT.....     5
....TCGGGGTTTTGAGGGCGAGAT............................................     4
.....CGGGGTTTTGAGGGCGAGAT............................................     3
.........................................CAACTGGCCCACAAAGTCCCGCT.....     3
.....CGGGGTTTTGAGGGCGAGA.............................................     2
....TCGGGGTTTTGAGGGCGAGA.............................................     1
....TCGGGGTTTTGAGGGCGAGATGA..........................................     1
......GGGGTTTTGAGGGCGAGATGA..........................................     1
..........................................AACTGGCCCACAAAGTC..........     1
...........................................ACTGGCCCACAAAGTCCC........     1
...........................................ACTGGCCCACAAAGTCCCG.......     1
.........................................CAACTGGCCCACAAAGTCCCGCTT....     1
.................................................CCACAAAGTCCCGCTTT...     1
.............................................TGGCCCACAAAGTCCCGCTTTT..     1
((((.((((..((((.((((.((.((..(.........)..)).)).)))).))))..)))).)))).. (-25.60)

25/13344243-13344313(+)_mir_Cow_Blast_17
AAAATGAGGGACTTTTGGGGGCAGATGTGTTTGCATTCCACTATCATAATGCCCCTAAAAATCCTTATTGC
..............................................TAATGCCCCTAAAAATCCTTAT...   1513
..............................................TAATGCCCCTAAAAATCCTTATT..   208
..............................................TAATGCCCCTAAAAATCCT......   132
..............................................TAATGCCCCTAAAAATCCTTA....   115
..............................................TAATGCCCCTAAAAATCCTT.....   85
......AGGGACTTTTGGGGGCAGATGTGT.........................................   12
......AGGGACTTTTGGGGGCAGATGT...........................................   11
......AGGGACTTTTGGGGGCAGATGTG..........................................   4
......AGGGACTTTTGGGGGCAGA..............................................   3
...............................................AATGCCCCTAAAAATCCTTATT..   3
....TGAGGGACTTTTGGGGGCAGATGT...........................................   2
...............................................AATGCCCCTAAAAATCCTTAT...   2
................................................ATGCCCCTAAAAATCCTTAT...   2
....TGAGGGACTTTTGGGGGCAGAT.............................................   1
```

```
.......GGGACTTTTGGGGGCAGATGT...........................................   1
......AGGGACTTTTGGGGGCAGATGTGTTTGCATTCCACTATCA.........................   1
............................................CATAATGCCCCTAAAAATCCT......   1
...............................................AATGCCCCTAAAAATCCTT.....   1
..((((((((..(((((((((((((((((.........))).)))....))))))))))).))))))))..  (-25.80)

25/1744656-1744727(-)_mir_cow_173
CTGACGTTGGCTCTGTGAGGTCGGCTCAAGGTGGGTCTGGATGTTGAGCAGGCCTGTCAGGGCGTTGTCACC
......TTGGCTCTGTGAGGTCGGCTCA............................................  482
.......TGGCTCTGTGAGGTCGGCTCA............................................  216
......TTGGCTCTGTGAGGTCGGCT..............................................  84
..............................................AGCAGGCCTGTCAGGGCGTTG.....  46
......TTGGCTCTGTGAGGTCGGCTC.............................................  38
.......TGGCTCTGTGAGGTCGGCT..............................................  22
.......TGGCTCTGTGAGGTCGGCTCAA...........................................  21
......TTGGCTCTGTGAGGTCGGCTCAA...........................................  10
.......TGGCTCTGTGAGGTCGGCTC.............................................  8
.......TGGCTCTGTGAGGTCGGCTCAAG..........................................  5
..............................................AGCAGGCCTGTCAGGGCGTT......  4
........GGCTCTGTGAGGTCGGCTCA............................................  3
............................................TGAGCAGGCCTGTCAGGGCGTT......  3
.............................................GAGCAGGCCTGTCAGGGCGTT......  3
......TTGGCTCTGTGAGGTCGGC...............................................  2
............................................TGAGCAGGCCTGTCAGGGCGT.......  2
......TTGGCTCTGTGAGGTC..................................................  1
.............................................GAGCAGGCCTGTCAGGGC.........  1
.............................................GAGCAGGCCTGTCAGGGCGT.......  1
............................................TGAGCAGGCCTGTCAGGGCGTTG.....  1
.............................................GAGCAGGCCTGTCAGGGCGTTG.....  1
............................................TGAGCAGGCCTGTCAGGGCGTTGT....  1
..............................................AGCAGGCCTGTCAGGGCGTTGT....  1
.(((((...((((((..(((((.((((((.((........)).)))))).)))))..))))))..)))))..  (-32.40)

2/5205390-5205457(+)_mir_cow_102
GAACAAAAAGTTCGTCCAGATTTTTCCATAAGATGTTACAGAAAAACTGGAACGAACTTTTGGGCCAA
...CAAAAAGTTCGTCCAGATTTTT........................................... 550
..........................................AAAACTGGAACGAACTTTTGGGC... 92
...........................................AAACTGGAACGAACTTTTGGGC... 37
```

...CAAAAAGTTCGTCCAGATTTT.. 34
...CAAAAAGTTCGTCCAGATTTTTC.. 9
....AAAACTGGAACGAACTTTTGGG.... 7
....AAAACTGGAACGAACTTTTGG..... 2
....AAACTGGAACGAACTTTTGGG.... 2
...CAAAAAGTTCGTCCAGATTT... 1
....AAAAAGTTCGTCCAGATTTTTC.. 1
....AAACTGGAACGAACTT......... 1
....AAAACTGGAACGAACTTTT....... 1
....AAACTGGAACGAACTTTTGG..... 1
(..(.((((((((((((((.((((((..(((....)))..)))))))))).)))))))))).)..).. (-21.90)

25/34409292-34409364(-)_mir_cow_175
TGCGGCATGCGGGATCTTTAGTTGTGGTGTGTGAATTCTTAGTTGCAGCAACTAAAGATCCCTCAGGCTGCAA
.......TGCGGGATCTTTAGTTGTGGTG.. 1328
....CAGCAACTAAAGATCCCTCAGG...... 470
.......TGCGGGATCTTTAGTTGTGGTGT... 449
.......TGCGGGATCTTTAGTTGTGGT... 335
.......TGCGGGATCTTTAGTTGTGG.. 243
....AGCAACTAAAGATCCCTCAGGC..... 104
......ATGCGGGATCTTTAGTTGTGGT... 48
....CAGCAACTAAAGATCCCTCAG....... 25
......ATGCGGGATCTTTAGTTGTGG.. 21
.......TGCGGGATCTTTAGTTGTG... 14
....CAGCAACTAAAGATCCCTCAGGC..... 13
....CAGCAACTAAAGATCCCT.......... 10
.......TGCGGGATCTTTAGTTG... 8
.......TGCGGGATCTTTAGTTGT.. 7
.......TGCGGGATCTTTAGTTGTGGTGTG.. 7
....CAGCAACTAAAGATCCCTC......... 7
......ATGCGGGATCTTTAGTTGTGGTG.. 6
......ATGCGGGATCTTTAGTTGTGGTGT... 6
.......TGCGGGATCTTTAGTTGTGGTGTGT... 6
....CAGCAACTAAAGATCCCTCA........ 6
....AGCAACTAAAGATCCCTCAGGCT.... 4
....AGCAACTAAAGATCCCTCAGG...... 3
......ATGCGGGATCTTTAGTTGTG... 2
...........GGATCTTTAGTTGTGGTG.. 2

```
.......TGCGGGATCTTTAGTT................................................. 1
......ATGCGGGATCTTTAGTTGT............................................... 1
.........CGGGATCTTTAGTTGTGGT............................................ 1
((((((.((.(((((((((((((((.(((..(((....)))..))).))))))))))))))).)).)))))). (-37.50)

25/34729983-34730047(-)_novel_cow_28
GGCATTCCTGAGGCTGGCTCAGCTGGCTGCCTCCTTTCCTGCTGAGACCCTCCTGGGGAAGCCCT
...ATTCCTGAGGCTGGCTCAGCTG........................................    10
...ATTCCTGAGGCTGGCTCAGCTGG.......................................    6
...ATTCCTGAGGCTGGCTCA............................................    1
...ATTCCTGAGGCTGGCTCAGCT.........................................    1
..CATTCCTGAGGCTGGCTCAGCTG........................................    1
.......................................TGCTGAGACCCTCCTGGGGAAG....    1
(((.(((((.(((..((((((((.((...........)).)))))).))..))).)))))))).. (-30.90)

25/36892052-36892120(+)_mir_cow_176
GGGGCTAAAGTGCTGACAGTGCAGATAGTGGTCCTGTGTGCTACCGCACTGTGGGTACTTGCTGCTCCG
.....TAAAGTGCTGACAGTGCAGAT...........................................      2651
...........................................CCGCACTGTGGGTACTTGCT......      1657
.....TAAAGTGCTGACAGTGCAGATA..........................................      1357
.....TAAAGTGCTGACAGTGCAGA............................................      902
.........................................TACCGCACTGTGGGTACTTGCT......      484
...........................................CCGCACTGTGGGTACTTGCTGCT...      331
...........................................CCGCACTGTGGGTACTTGCTG.....      301
...........................................CCGCACTGTGGGTACTTGCTGC....      142
.....TAAAGTGCTGACAGTGCAG.............................................      125
...........................................CCGCACTGTGGGTACTTGC.......      71
.....TAAAGTGCTGACAGTGCAGATAG.........................................      59
.....TAAAGTGCTGACAGTGCA..............................................      43
..........................................ACCGCACTGTGGGTACTTGCT......      29
.....TAAAGTGCTGACAGTGC...............................................      22
.....TAAAGTGCTGACAGTGCAGATAGT........................................      13
..........................................ACCGCACTGTGGGTACTTGCTG.....      10
.........................................TACCGCACTGTGGGTACT..........      9
.........................................TACCGCACTGTGGGTACTTGC.......      9
...........................................CCGCACTGTGGGTACTTGCTGCTC..      8
....CTAAAGTGCTGACAGTGCAGAT...........................................      6
...........................................CCGCACTGTGGGTACTTG........      4
```

```
..........................................ACCGCACTGTGGGTACTTGCTGC....      4
.....TAAAGTGCTGACAGTG................................................      3
....CTAAAGTGCTGACAGTGCAGA............................................      2
.....TAAAGTGCTGACAGTGCAGATAGTGGTCCT..................................      2
...........................................CCGCACTGTGGGTACT..........      2
..........................................ACCGCACTGTGGGTACTTGC.......      2
.........................................TACCGCACTGTGGGTACTTGCTGC....      2
......AAAGTGCTGACAGTGCAGA............................................      1
.....TAAAGTGCTGACAGTGCAGATAGTGGTCCTGT................................      1
.......................GATAGTGGTCCTGTGTGCTACCGCACTG..................      1
.........................................TACCGCACTGTGGGTACTTG........      1
............................................CGCACTGTGGGTACTTGCTGCTC..      1
(((((..(((((((.(((((((.(.(((((........)))))).))))))).)))))))...))))). (-34.40)

25/36892254-36892323(+)_mir_cow_177
GGCTCCAAAGTGCTGTTCGTGCAGGTAGTGTGATCACCTGACCTACTGCTGAGCCAGCACTTCCCGAGCC
.....CAAAGTGCTGTTCGTGCAGGT............................................    15510
.....CAAAGTGCTGTTCGTGCAGGTAG..........................................    8445
.....CAAAGTGCTGTTCGTGCAGGTA...........................................    3073
.....CAAAGTGCTGTTCGTGCAGGTAGT.........................................    1759
.....CAAAGTGCTGTTCGTGCAGG.............................................    315
.....CAAAGTGCTGTTCGTGCAG..............................................    200
............................................ACTGCTGAGCCAGCACTTCCCGA...    64
......AAAGTGCTGTTCGTGCAGGTAG..........................................    60
......AAAGTGCTGTTCGTGCAGGTAGT.........................................    32
......AAAGTGCTGTTCGTGCAGGT............................................    29
............................................ACTGCTGAGCCAGCACTTCCCG....    20
.....CAAAGTGCTGTTCGTGCAGGTAGTGT.......................................    18
............................................ACTGCTGAGCCAGCACTTCCC.....    16
..........................................CTACTGCTGAGCCAGCACTTCCC.....    11
......AAAGTGCTGTTCGTGCAGGTA...........................................    10
.....CAAAGTGCTGTTCGTGCAGGTAGTGTGATCACCTGACCT..........................    10
.............................................CTGCTGAGCCAGCACTTCCCGA...    10
.....CAAAGTGCTGTTCGTGCA...............................................    9
....CCAAAGTGCTGTTCGTGCAGGT............................................    8
.....CAAAGTGCTGTTCGTGC................................................    7
..........................................CTACTGCTGAGCCAGCACTTCC......    7
......AAAGTGCTGTTCGTGCAGGTAGTGT.......................................    6
```

```
.....CAAAGTGCTGTTCGTGCAGGTAGTG........................................    5
...........................................TACTGCTGAGCCAGCACTTCC......    5
.....CAAAGTGCTGTTCGTG.................................................    4
..........................................CTACTGCTGAGCCAGCACTTC.......    3
...........................................TACTGCTGAGCCAGCACTTCCC.....    3
.............................................CTGCTGAGCCAGCACTTCCCG....    3
...........................................TACTGCTGAGCCAGCACTTCCCGA...    3
.......AAGTGCTGTTCGTGCAGGTAGTGT.......................................    2
............................TGTGATCACCTGACCT..........................    2
..........................................CTACTGCTGAGCCAGCACT.........    2
..........................................CTACTGCTGAGCCAGCACTT........    2
......AAAGTGCTGTTCGTGCAGG.............................................    1
....CCAAAGTGCTGTTCGTGCAGGTA...........................................    1
....CCAAAGTGCTGTTCGTGCAGGTAG..........................................    1
.......AAGTGCTGTTCGTGCAGGTAG..........................................    1
...........GCTGTTCGTGCAGGTAG..........................................    1
.........GTGCTGTTCGTGCAGGTAGT.........................................    1
......AAAGTGCTGTTCGTGCAGGTAGTG........................................    1
.............TGTTCGTGCAGGTAGTGTGATCACCTGAC............................    1
.....CAAAGTGCTGTTCGTGCAGGTAGTGTGATCACCTGACC...........................    1
.........................TAGTGTGATCACCTGACCT..........................    1
......................................TGACCTACTGCTGAGCCAGC............    1
...........................................TACTGCTGAGCCAGCACT.........    1
................................................CTGAGCCAGCACTTCCCG....    1
...............................................GCTGAGCCAGCACTTCCCGA...    1
.............................................CTGCTGAGCCAGCACTTCCCGAG..    1
(((((..((((((((((((.((((.(((.((.(.....).))))))))))))).))))))))...))))) (-30.70)

25/36892458-36892525(+)_mir_cow_178
TGAGAGGCGGAGACTTGGGCAATTGCTGGACGCTGCCCCGGGCATTGCACTTGTCTCGGTCTGACAGT
..........................................CATTGCACTTGTCTCGGTCTGA.... 50444
..........................................CATTGCACTTGTCTCGGTCT...... 12451
..........................................CATTGCACTTGTCTCGGTCTG..... 3090
..........................................CATTGCACTTGTCTCGGTC....... 488
....AGGCGGAGACTTGGGCAATTGCT......................................... 96
...........................................ATTGCACTTGTCTCGGTCTGA.... 54
..........................................CATTGCACTTGTCTCGGT........ 50
..........................................CATTGCACTTGTCTCGGTCTGAC... 27
```

```
....AGGCGGAGACTTGGGCAATTGC.......................................... 19
..........................................CATTGCACTTGTCTCGGTCTGACA.. 16
...........................................ATTGCACTTGTCTCGGTCT...... 14
..........................................CATTGCACTTGTCTCGG......... 8
..............................................GCACTTGTCTCGGTCTGA.... 8
....AGGCGGAGACTTGGGCAATTG........................................... 7
...........................GGACGCTGCCCCGGGCATTGCACTTGTCTCGGTCTGA.... 6
............................................TTGCACTTGTCTCGGTCTGA.... 3
....AGGCGGAGACTTGGGCAATT............................................ 2
....AGGCGGAGACTTGGGCAATTGCTGGACGCTGCCCCGGG.......................... 2
...........................................ATTGCACTTGTCTCGGTCTG..... 2
.........................................GCATTGCACTTGTCTCGGTCTGA.... 2
...............................................CACTTGTCTCGGTCTGA.... 2
.......CGGAGACTTGGGCAATTGCT......................................... 1
..........................................CATTGCACTTGTCTCG.......... 1
.........................................GCATTGCACTTGTCTCGGTC....... 1
..............................................GCACTTGTCTCGGTCT...... 1
..............................................GCACTTGTCTCGGTCTG..... 1
.............................................TGCACTTGTCTCGGTCTGA.... 1
............................................TTGCACTTGTCTCGGTCTGACA.. 1
((..((((.(((((..(.(((((.(((((........)).))))))))).)..))))).))))..)).. (-25.60)

25/42294004-42294070(+)_mir_cow_179
GCCGCTGTCCCTGTCCTCCAGGAGCTCACTTGGTCCGGCCGTGCGCTCCTCGAGGCCAGAGCCCGTG
.......TCCCTGTCCTCCAGGAGCTC........................................ 8845
.......TCCCTGTCCTCCAGGAGCTCACT..................................... 5381
.......TCCCTGTCCTCCAGGAGCTCA....................................... 2121
.......TCCCTGTCCTCCAGGAGCTCAC...................................... 1222
.......TCCCTGTCCTCCAGGAGCT......................................... 286
...........................................CGCTCCTCGAGGCCAGAGCCC... 200
..........................................GCGCTCCTCGAGGCCAGAGCCC... 69
...........................................CGCTCCTCGAGGCCAGAGCC.... 60
...........................................CGCTCCTCGAGGCCAGAGC..... 36
.......TCCCTGTCCTCCAGGAGC.......................................... 31
..........................................GCGCTCCTCGAGGCCAGAGCC.... 26
..........................................GCGCTCCTCGAGGCCAGAGC..... 14
.............................TTGGTCCGGCCGTGCGCTCCTCGAGGCCAGAGCCC... 11
.......TCCCTGTCCTCCAGGAGCTCACTT.................................... 10
```

```
.......TCCCTGTCCTCCAGGAG...........................................  5
........CCCTGTCCTCCAGGAGCTC........................................  5
........CCCTGTCCTCCAGGAGCTCACT.....................................  5
.........CCTGTCCTCCAGGAGCTCACT.....................................  5
.........CCTGTCCTCCAGGAGCTC........................................  3
...........................................CGCTCCTCGAGGCCAGAG......  3
.......TCCCTGTCCTCCAGGAGCTCACTTGGTCCGG.............................  2
............................CTTGGTCCGGCCGTGCGCTCCTCGAGGCCAGAGCCC...  2
...........TGTCCTCCAGGAGCTC........................................  1
....CTGTCCCTGTCCTCCAGGAGCTCA.......................................  1
............GTCCTCCAGGAGCTCA.......................................  1
..........CTGTCCTCCAGGAGCTCACT.....................................  1
.........CCTGTCCTCCAGGAGCTCACTTGGTC................................  1
...........TGTCCTCCAGGAGCTCACTTGGTC................................  1
........CCCTGTCCTCCAGGAGCTCACTTGGTCCGGCC...........................  1
.......TCCCTGTCCTCCAGGAGCTCACTTGGTCCGGCCGTG........................  1
.........................................TGCGCTCCTCGAGGCCAGAGC.....  1
.............................TTGGTCCGGCCGTGCGCTCCTCGAGGCCAGAGCC....  1
...........................................CGCTCCTCGAGGCCAGAGCCCG..  1
..(((.(.(.(((.((((.((((((.(((..((.....))))).)))))).)))).))).).).))) (-28.30)

26/11287997-11288068(-)_mir_cow_180
GCTTTCAGCTTCTTTACAGTGTTGCCTTGTGGCATGGAGTTCAAGCAGCATTGTACAGGGCTATCAAAGCAC
...........................................AGCAGCATTGTACAGGGCTAT........  14341
...........................................AGCAGCATTGTACAGGGCTATC.......  10599
...........................................AGCAGCATTGTACAGGGCT..........   7437
...........................................AGCAGCATTGTACAGGGCTATCA......   3401
...........................................AGCAGCATTGTACAGGGC...........   1712
...........................................AGCAGCATTGTACAGGGCTA.........   1027
...........................................AGCAGCATTGTACAGGGCTATCAA.....   169
......AGCTTCTTTACAGTGTTGCCTTG...........................................   67
...........................................AGCAGCATTGTACAGGG............   35
...........................................AGCAGCATTGTACAGG.............   8
......AGCTTCTTTACAGTGTTGCCTT............................................   7
......AGCTTCTTTACAGTGTTGCCTTGT..........................................   5
.............................................CAGCATTGTACAGGGCTAT........   5
......AGCTTCTTTACAGTGTTGCCT.............................................   4
............................................GCAGCATTGTACAGGGCTAT........   4
```

```
...............................................GCATTGTACAGGGCTAT........   4
...........................................AGCAGCATTGTACAGGGCTATCAAA....   4
..........................................AAGCAGCATTGTACAGGGCT..........   3
.............................................CAGCATTGTACAGGGCTA.........   3
............................................GCAGCATTGTACAGGGCTATC.......   3
..............................................AGCATTGTACAGGGCTATC.......   3
...............................................GCATTGTACAGGGCTATCA......   3
............................................GCAGCATTGTACAGGGCT..........   2
..........................................AAGCAGCATTGTACAGGGCTAT........   2
..........................................AAGCAGCATTGTACAGGGCTATC.......   2
...............................................GCATTGTACAGGGCTATC.......   2
................................................CATTGTACAGGGCTATC.......   2
..............................................AGCATTGTACAGGGCTATCA......   2
......AGCTTCTTTACAGTGTTGC...............................................   1
.........TTCTTTACAGTGTTGCCTTG...........................................   1
..........................................AAGCAGCATTGTACAGGGC...........   1
.............................................CAGCATTGTACAGGGCT..........   1
..............................................AGCATTGTACAGGGCT..........   1
..............................................AGCATTGTACAGGGCTA.........   1
...............................................GCATTGTACAGGGCTA.........   1
..............................................AGCATTGTACAGGGCTAT........   1
.................................................ATTGTACAGGGCTATC.......   1
.............................TGGCATGGAGTTCAAGCAGCATTGTACAGGGCTATCA......   1
............................................GCAGCATTGTACAGGGCTATCA......   1
.............................................CAGCATTGTACAGGGCTATCA......   1
.................................................ATTGTACAGGGCTATCA......   1
..................................................TTGTACAGGGCTATCA......   1
(((((.((((.((.(((((((((((.(((.(((.....))))))))))))))))).))))))...))))).. (-26.80)

2/62007757-62007827(-)_mir_cow_105
GTTGGATTCGGGGCCGTAGCACTGTCTGAGAGGTTTACATTTCTCACAGTGAACCGGTCTCTTTTTCAGCT
...........................................TCACAGTGAACCGGTCTCTTT.......   135574
...........................................TCACAGTGAACCGGTCTCTT........   31623
...........................................TCACAGTGAACCGGTCTCTTTT......   27867
...........................................TCACAGTGAACCGGTCTCT.........   13335
........CGGGGCCGTAGCACTGTCTGA..........................................   1931
...........................................TCACAGTGAACCGGTCTCTTTTT.....   1440
...........................................TCACAGTGAACCGGTCTC..........   1365
```

....TCACAGTGAACCGGTCT........... 1174
........CGGGGCCGTAGCACTGTCTGAGA.. 241
....TCACAGTGAACCGGTC............ 180
........CGGGGCCGTAGCACTGTCTG... 87
....CTCACAGTGAACCGGTCTCTTT....... 82
........CGGGGCCGTAGCACTGTCT.. 51
........CGGGGCCGTAGCACTGTCTGAG... 49
....CACAGTGAACCGGTCTCTTT....... 30
....CTCACAGTGAACCGGTCTCTT........ 24
....ACAGTGAACCGGTCTCTTT....... 23
....CTCACAGTGAACCGGTCTCTTTT...... 15
....CACAGTGAACCGGTCTCTT........ 12
....CAGTGAACCGGTCTCTTT....... 12
....GTGAACCGGTCTCTTT....... 12
....TCACAGTGAACCGGTCTCTTTTTC.... 10
....CAGTGAACCGGTCTCTT........ 8
....CAGTGAACCGGTCTCTTTT...... 8
........CGGGGCCGTAGCACTGTCTGAGAGG...................................... 5
....ACAGTGAACCGGTCTCTTTT...... 5
....CTCACAGTGAACCGGTCTCT......... 4
....TCTCACAGTGAACCGGTCTCTTT....... 4
....CACAGTGAACCGGTCTCT......... 3
....ACAGTGAACCGGTCTCTT........ 3
........CGGGGCCGTAGCACTGTC... 2
.........GGGGCCGTAGCACTGTCTGA.. 2
........CGGGGCCGTAGCACTGTCTGAGAGGT..................................... 2
....CTCACAGTGAACCGGTCT........... 2
....CTCACAGTGAACCGGTCTC.......... 2
....ACAGTGAACCGGTCTCT......... 2
....TCTCACAGTGAACCGGTCTCTT........ 2
....CACAGTGAACCGGTCTCTTTT...... 2
....GTGAACCGGTCTCTTTT...... 2
....CACAGTGAACCGGTCTCTTTTT..... 2
.........GGGGCCGTAGCACTGTCT.. 1
.........GGGGCCGTAGCACTGTCTG... 1
.........GGGGCCGTAGCACTGTCTGAG... 1
......TTCGGGGCCGTAGCACTGTCTGAGAG....................................... 1
........CGGGGCCGTAGCACTGTCTGAGAGGTTTAC................................. 1

```
.........................................TCTCACAGTGAACCGGTCTCT.........   1
..............................................CAGTGAACCGGTCTCT.........   1
...............................................AGTGAACCGGTCTCTTT.......   1
.........................................TCTCACAGTGAACCGGTCTCTTTT......   1
..........................................CTCACAGTGAACCGGTCTCTTTTT.....   1
((((((...(((((((...((((((..(((((((....)))))))))))))...)))))))...)))))). (-30.90)

26/22930909-22930977(+)_mir_cow_181
TGGCTCTGAGAACTGAATTCCATAGGCTGTGAGCTCTAGCAAATGCCCTAGGGACTCAGTTCTGGTGCC
......TGAGAACTGAATTCCATAGGCTGT.......................................     240457
......TGAGAACTGAATTCCATAGGCTG........................................     36639
......TGAGAACTGAATTCCATAGGCT.........................................     10926
......TGAGAACTGAATTCCATAGGC..........................................     1602
.....CTGAGAACTGAATTCCATAGGCTGT.......................................     1030
......TGAGAACTGAATTCCATAGG...........................................     679
.......GAGAACTGAATTCCATAGGCTGT.......................................     146
...........ACTGAATTCCATAGGCTGT.......................................     113
...........................................TGCCCTAGGGACTCAGTTCTGGT...     104
.....CTGAGAACTGAATTCCATAGGCTG........................................     56
...........................................TGCCCTAGGGACTCAGTTCTGG....     54
............................................GCCCTAGGGACTCAGTTCTGGT...     50
...........................................TGCCCTAGGGACTCAGTTCTG.....     43
......TGAGAACTGAATTCCATAG............................................     31
......TGAGAACTGAATTCCATAGGCTGTG......................................     29
...........................................TGCCCTAGGGACTCAGTTCT......     19
.......GAGAACTGAATTCCATAGGCTG........................................     17
.........GAACTGAATTCCATAGGCTGT.......................................     17
..........AACTGAATTCCATAGGCTGT.......................................     17
............CTGAATTCCATAGGCTGT.......................................     15
....TCTGAGAACTGAATTCCATAGGCTGT.......................................     11
...........................................TGCCCTAGGGACTCAGTTC.......     9
.....CTGAGAACTGAATTCCATAGGCT.........................................     8
......TGAGAACTGAATTCCATAGGCTGTGA.....................................     8
............................................GCCCTAGGGACTCAGTTCTGG....     8
...........ACTGAATTCCATAGGCTG........................................     7
........AGAACTGAATTCCATAGGCTGT.......................................     7
.............TGAATTCCATAGGCTGT.......................................     7
..............GAATTCCATAGGCTGT.......................................     7
```

```
......TGAGAACTGAATTCCATAGGCTGTGAGCTCTAGC.............................     5
............................................GCCCTAGGGACTCAGTTCTG.....     5
.....CTGAGAACTGAATTCCATAGGC..........................................     4
..........AACTGAATTCCATAGGCTG........................................     4
......TGAGAACTGAATTCCATA.............................................     3
....TCTGAGAACTGAATTCCATAGGCTG........................................     3
......TGAGAACTGAATTCCATAGGCTGTGAGCTCT................................     3
............................................GCCCTAGGGACTCAGTTCT......     3
......TGAGAACTGAATTCCA...............................................     2
.......GAGAACTGAATTCCATAGGCT.........................................     2
........AGAACTGAATTCCATAGGCTG........................................     2
............CTGAATTCCATAGGCTG........................................     2
.............TGAATTCCATAGGCTG........................................     2
......TGAGAACTGAATTCCATAGGCTGTGAGCTCTAG..............................     2
......TGAGAACTGAATTCCATAGGCTGTGAGCTCTAGCA............................     2
......TGAGAACTGAATTCCATAGGCTGTGAGCTCTAGCAA...........................     2
..............................................CCTAGGGACTCAGTTCTGGTG..     2
.......GAGAACTGAATTCCATAGG...........................................     1
....TCTGAGAACTGAATTCCATAGGC..........................................     1
.........GAACTGAATTCCATAGGCTG........................................     1
......TGAGAACTGAATTCCATAGGCTGTGAGCTCTAGCAAAT.........................     1
...........................TGTGAGCTCTAGCAAATGC.......................     1
.............................................CCCTAGGGACTCAGTTCTGG....     1
..............................................CCTAGGGACTCAGTTCTGGT...     1
.............................................CCCTAGGGACTCAGTTCTGGTG..     1
.(((.((.((((((((.((((.((((..((..((....))..))..)))))))).))))))))).))) (-28.50)

26/24230112-24230188(-)_mir_cow_182
CTACCAATCTCGACCGGACCTCGACCGGCTCGTCTATATTGCCAATCGACTCGGCGTGGCGTCGGTCGTGGTAGATA
.........TCGACCGGACCTCGACCGGCT...............................................   31770
.........TCGACCGGACCTCGACCGGCTCG.............................................   3384
.........TCGACCGGACCTCGACCGGCTC..............................................   3175
................................................ACTCGGCGTGGCGTCGGTCGTGG......   1029
.........TCGACCGGACCTCGACCGG.................................................   889
.................................................CTCGGCGTGGCGTCGGTCGTGG......   506
.........TCGACCGGACCTCGACCGGC................................................   446
................................................ACTCGGCGTGGCGTCGGTCGTG.......   336
................................................ACTCGGCGTGGCGTCGGTCGT........   130
```

```
.................................................CTCGGCGTGGCGTCGGTCGTG.......  129
.........TCGACCGGACCTCGACC...................................................  116
................................................ACTCGGCGTGGCGTCGGTCGTGGT.....  94
.........TCGACCGGACCTCGACCG..................................................  48
................................................ACTCGGCGTGGCGTCGGTCG.........  40
........CTCGACCGGACCTCGACCGGCT...............................................  39
.................................................CTCGGCGTGGCGTCGGTCGTGGT.....  38
.........TCGACCGGACCTCGACCGGCTCGT............................................  36
..........CGACCGGACCTCGACCGGCT...............................................  13
................................................ACTCGGCGTGGCGTCGGTC..........  12
...........GACCGGACCTCGACCGGCT...............................................  9
.........TCGACCGGACCTCGAC....................................................  7
...............GGACCTCGACCGGCTCG.............................................  5
................................................ACTCGGCGTGGCGTCGGT...........  5
................................................ACTCGGCGTGGCGTCGGTCGTGGTA....  4
..........CGACCGGACCTCGACCGGCTC..............................................  3
........CTCGACCGGACCTCGACCGG.................................................  2
........CTCGACCGGACCTCGACCGGCTC..............................................  2
.................................................CTCGGCGTGGCGTCGGTC..........  2
.................................................CTCGGCGTGGCGTCGGTCG.........  2
.................................................CTCGGCGTGGCGTCGGTCGT........  2
..................................................TCGGCGTGGCGTCGGTCGTGG......  2
.................................................CTCGGCGTGGCGTCGGTCGTGGTA....  2
..........CGACCGGACCTCGACCG..................................................  1
......ATCTCGACCGGACCTCGACCGGCT...............................................  1
............ACCGGACCTCGACCGGCT...............................................  1
..............CGGACCTCGACCGGCT...............................................  1
.......TCTCGACCGGACCTCGACCGGCTC..............................................  1
........CTCGACCGGACCTCGACCGGCTCG.............................................  1
..........CGACCGGACCTCGACCGGCTCG.............................................  1
............ACCGGACCTCGACCGGCTCG.............................................  1
....................TCGACCGGCTCGTCTATATTGC...................................  1
....................TCGACCGGCTCGTCTATATTGCC..................................  1
................................................ACTCGGCGTGGCGTCG.............  1
...................................................CGGCGTGGCGTCGGTCG.........  1
..................................................TCGGCGTGGCGTCGGTCGTGGT.....  1
((((((....(((((((.((.((.(((..(((..............)))..))))).))..)))))))))))))... (-30.44)
```

```
26/25988253-25988318(+)_mir_cow_183
CCTTTTTCCTATGCATATACTTCTTTGAGGGTCTGGCCTAAAGAGGTGTAGGGCATGGGAAAATGG
.....TTCCTATGCATATACTTCTTT........................................  5256
.....TTCCTATGCATATACTTCTT.........................................  4086
....TTTCCTATGCATATACTTCTT.........................................  1100
....TTTCCTATGCATATACTTCTTT........................................  840
.....TTCCTATGCATATACTTCTTTG.......................................  733
.....TTCCTATGCATATACTTCT..........................................  635
....TTTCCTATGCATATACTTCT..........................................  275
.........................................AGAGGTGTAGGGCATGGGAAAA...  89
.........................................AGAGGTGTAGGGCATGGGAAA....  68
.........................................AGAGGTGTAGGGCATGGGAA.....  48
......................................TAAAGAGGTGTAGGGCATGGGA......  42
.....TTCCTATGCATATACTTCTTTGA......................................  34
.........................................AGAGGTGTAGGGCATGGGA......  31
........................................AAGAGGTGTAGGGCATGGGAA.....  26
......................................TAAAGAGGTGTAGGGCATGGGAA.....  20
.........................................AGAGGTGTAGGGCATGGGAAAAT..  19
........................................AAGAGGTGTAGGGCATGGGA......  17
........................................AAGAGGTGTAGGGCATGGGAAA....  6
.....TTCCTATGCATATACTTC...........................................  4
.........................................AGAGGTGTAGGGCATG.........  3
.....TTCCTATGCATATACT.............................................  2
.....TTCCTATGCATATACTT............................................  2
....TTTCCTATGCATATACTTC...........................................  2
........CTATGCATATACTTCTTT........................................  2
......TCCTATGCATATACTTCTTTG.......................................  2
......................................TAAAGAGGTGTAGGGCATGGGAAA....  2
........................................AAGAGGTGTAGGGCATGGGAAAA...  2
......TCCTATGCATATACTTCTT.........................................  1
....TTTCCTATGCATATACTTCTTTG.......................................  1
........CTATGCATATACTTCTTTG.......................................  1
.....TTCCTATGCATATACTTCTTTGAG.....................................  1
.....TTCCTATGCATATACTTCTTTGAGGGTCTGGC.............................  1
.....TTCCTATGCATATACTTCTTTGAGGGTCTGGCCTAA.........................  1
.........................................AGAGGTGTAGGGCATGG........  1
......................................TAAAGAGGTGTAGGGCATGGG.......  1
.......................................AAAGAGGTGTAGGGCATGGGA......  1
```

```
.......................................AAAGAGGTGTAGGGCATGGGAAA....   1
..........................GAGGGTCTGGCCTAAAGAGGTGTAGGGCATGGGAAAA...   1
....................................CCTAAAGAGGTGTAGGGCATGGGAAAA...   1
((.(((((((((((.(((((((((((.(((......))))))))))))))..))))))))))).))  (-32.90)

26/31733586-31733654(+)_mir_cow_184
ACCAGTTAGGCCACTCAGAGTAACTATCAGTAACTAACTGGTTACTCTGAGTAACCTAACTGGTATCCT
.........................................TTACTCTGAGTAACCTAACT........      339
.........................................TTACTCTGAGTAACCTAACTG.......      153
.........................................TTACTCTGAGTAACCT............      4
.........................................TTACTCTGAGTAACCTAAC.........      2
....GTTAGGCCACTCAGAGTAAC.............................................      1
..........................................TACTCTGAGTAACCTAACTGG......      1
............................................CTCTGAGTAACCTAACTGGTATC..      1
((((((((((..((((((((((((...((((.....))))))))))))))))..)))))))))).....  (-37.90)

26/37549329-37549395(-)_mir_cow_185
GCCCGGCGCTGGCAGTGTTGGTGGCACTTGCGTGGGGGCGGTGGCACCAGCACTGGCGGTGGCGGTG
.........................................TGGCACCAGCACTGGCGGTGG.....  155
.........................................TGGCACCAGCACTGGCGGTGGC....  55
.........................................TGGCACCAGCACTGGCGGTG......  31
.........................................TGGCACCAGCACTGGCGGT.......  5
......CGCTGGCAGTGTTGGTGGCA.........................................  1
......CGCTGGCAGTGTTGGTGGCAC........................................  1
..............................................CCAGCACTGGCGGTGG.....  1
..............................................CCAGCACTGGCGGTGGC....  1
..(((.(((((.(((((((((((.((((.((......)))))).))))))))))).))))).)))..  (-45.20)

26/39318143-39318212(-)_novel_cow_29
GCCACTAGTCACATGTAACTTACTAAATTCATTTCAAGTGCTTAGTAGTCACACGTGGCCAGTGGCTGCC
...ACTAGTCACATGTAACTTACT..............................................     23
..CACTAGTCACATGTAACTTACT..............................................     5
............................................GTAGTCACACGTGGCCAGTGG.....     1
............................................GTAGTCACACGTGGCCAGTGGC....     1
((((((.(((((.(((.(((.(((((...(((.....))).)))))))).))).))))).))))))....  (-26.40)

26/39599912-39599982(-)_mir_Cow_Blast_37
TACAAATAATTGAAGGCTAATTAGCAGTATAACTAAATAGTAATACTGCCAGTCTCCTTCAGACAAACAGT
```

```
........ATTGAAGGCTAATTAGCAGTAT.........................................   5
..........................................ATACTGCCAGTCTCCTTCAGA........   4
...........................................TACTGCCAGTCTCCTTCAGACA......   4
........ATTGAAGGCTAATTAGCAGT...........................................   2
..........................................ATACTGCCAGTCTCCTTCAGAC.......   2
........ATTGAAGGCTAATTAGCAGTATA........................................   1
.........TTGAAGGCTAATTAGCAGTATA........................................   1
........ATTGAAGGCTAATTAGCAGTATAACTAAAT.................................   1
..........................................ATACTGCCAGTCTCCTTCAG.........   1
...........................................TACTGCCAGTCTCCTTCAGA........   1
...........................................TACTGCCAGTCTCCTTCAGAC.......   1
..................................AAATAGTAATACTGCCAGTCTCCTTCAGACAAAC...   1
..............................................TGCCAGTCTCCTTCAGACAAAC...   1
.........(((((((...(((.(((((((.(((....))).))))))).)))..))))))).........  (-19.60)

26/49743768-49743839(+)_mir_cow_186
TGGATGCGTTCTTACCACTGGCCTGTAGGTGACATGATCCAAGTACAGAGCAGTGGCAAGAACGCATCCAAA
....TGCGTTCTTACCACTGGCCTGT..............................................  5
.............................................CAGAGCAGTGGCAAGAACGC.......  3
.............................................CAGAGCAGTGGCAAGAACGCAT.....  2
...............................................GAGCAGTGGCAAGAACGCATCC...  2
.............................................CAGAGCAGTGGCAAGAACGCATC....  1
..............................................AGAGCAGTGGCAAGAACGCATC....  1
..............................................AGAGCAGTGGCAAGAACGCATCCA..  1
...............................................GAGCAGTGGCAAGAACGCATCCA..  1
(((((((((((((.((((((..(((((..((........))..)))))..)))))).))))))))))))).. (-40.40)

27/1330182-1330250(-)_mir_cow_187
TACAGGTGGCTCATTTAGAAGCAGCCATATGTCGTGTGGGTGGCTGCCTGTGAATGGGCTGCCCTGTAC
......TGGCTCATTTAGAAGCAGC............................................     13
..........................................GCTGCCTGTGAATGGGCTG........     10
...........................................CTGCCTGTGAATGGGCTGCCCT....     7
...........................................CTGCCTGTGAATGGGCTGCCC.....     6
......TGGCTCATTTAGAAGCAGCC...........................................     5
..........................................GCTGCCTGTGAATGGGCTGC.......     4
......TGGCTCATTTAGAAGCAGCCA..........................................     3
........................................TGGCTGCCTGTGAATGGGCTGC.......     2
......TGGCTCATTTAGAAGCAGCCAT.........................................     1
```

```
((((((..(((((((((.(.((((((((..........)))))))).).)))))))))..).))))). (-36.10)

27/18517046-18517110(+)_mir_cow_188
AGCCAAAATGTTCGCTTGGCTTTTTCCATACTACCTTACAGAAAAACCTGAGTGAACTTTTCAGC
.....AAATGTTCGCTTGGCTTTTTCC......................................    61
.....AAATGTTCGCTTGGCTTTTTC.......................................    52
....AAAATGTTCGCTTGGCTTTTTC.......................................    16
.........................................AAAAACCTGAGTGAACTTTTC...    13
.........................................AAAAACCTGAGTGAACTTTTCA..    10
.....AAATGTTCGCTTGGCTTTTT........................................    2
......AATGTTCGCTTGGCTTTTTCC......................................    2
..........................................AAAACCTGAGTGAACTTTTC...    2
..........................................AAAACCTGAGTGAACTTTTCA..    2
......AATGTTCGCTTGGCTTTTTC.......................................    1
.((..(((.(((((((..(.((((((..((......))..)))))).)..))))))).)))..)) (-17.10)

27/21315204-21315273(+)_mir_cow_189
TCCTCAGATCAGAAGGTGATTGTGGCTTTGGGTGGATATCAATCAGCCACAGCACTGCCTGGTCAGAAAG
.....AGATCAGAAGGTGATTGTGGCT...........................................    3100
....CAGATCAGAAGGTGATTGTGGCT...........................................    757
.....AGATCAGAAGGTGATTGTGGCTT..........................................    158
....CAGATCAGAAGGTGATTGTGGCTT..........................................    141
.....AGATCAGAAGGTGATTGTGGC............................................    43
..............................................CCACAGCACTGCCTGGTCAGA...    29
.....AGATCAGAAGGTGATTGTGGCTTT.........................................    28
....CAGATCAGAAGGTGATTGTGGC............................................    15
..............................................CCACAGCACTGCCTGGTCAG....    11
.....AGATCAGAAGGTGATTGTGG.............................................    7
.......ATCAGAAGGTGATTGTGGCT...........................................    3
..........AGAAGGTGATTGTGGCT...........................................    3
....CAGATCAGAAGGTGATTGTGGCTTT.........................................    3
.....AGATCAGAAGGTGATTG................................................    2
........TCAGAAGGTGATTGTGGCT...........................................    2
...........GAAGGTGATTGTGGCT...........................................    2
....CAGATCAGAAGGTGATTG................................................    1
....CAGATCAGAAGGTGATTGT...............................................    1
.....AGATCAGAAGGTGATTGT...............................................    1
....CAGATCAGAAGGTGATTGTG..............................................    1
```

```
....CAGATCAGAAGGTGATTGTGG.............................................     1
..............................................CCACAGCACTGCCTGGTCAGAA..     1
......((((((..((((.((((((((((((.......))))..))))))))))))..))))))...... (-27.20)

27/25707604-25707668(+)_mir_cow_190
CTCACACTTTCTGTAGCTGTTGGAACTATGGTGCATTGTGGTTCTAACAACTACAGAAAGTGTGA
......CTTTCTGTAGCTGTTGGAACT......................................   29
......CTTTCTGTAGCTGTTGGAACTA.....................................   7
.........................................TTCTAACAACTACAGAAAGTGT..   6
......CTTTCTGTAGCTGTTGGAAC.......................................   5
.....ACTTTCTGTAGCTGTTGGAACT......................................   3
.....ACTTTCTGTAGCTGTTGGAA........................................   1
....CACTTTCTGTAGCTGTTGGAAC.......................................   1
.....ACTTTCTGTAGCTGTTGGAAC.......................................   1
.........................................TTCTAACAACTACAGAAAGTG...   1
.((((((((((((((.((((((((((((..(....)..)))))))))))).)))))))))))))) (-39.70)

27/33164738-33164812(-)_mir_cow_191
GAGGGCTGCCCGAGCGCATGTGGCTGAGTGTGGTCCCTTGGCGCCCAGCACAGGCAGCTCGGACTGAAACCCTCC
...........................................CCCAGCACAGGCAGCTCGGACT..........     20
............................................CCAGCACAGGCAGCTCGGACT..........     13
........CCCGAGCGCATGTGGCTGAGTGT............................................     3
............................................CCAGCACAGGCAGCTCGGACTGA........     3
........CCCGAGCGCATGTGGCTGAG...............................................     2
........CCCGAGCGCATGTGGCTGA................................................     1
......TGCCCGAGCGCATGTGGCTGAG...............................................     1
........CCCGAGCGCATGTGGCTGAGT..............................................     1
.............................................CAGCACAGGCAGCTCGGACT..........     1
...........................................CCCAGCACAGGCAGCTCGGACTG.........     1
...........................................CCCAGCACAGGCAGCTCGGACTGA........     1
.............................................CAGCACAGGCAGCTCGGACTGAA.......     1
.............................................CAGCACAGGCAGCTCGGACTGAAA......     1
................................................CACAGGCAGCTCGGACTGAAACC....     1
(((((..(.((((((((.((((.(((.((((.(.....).)))).))))))).)).)))))).).....))))). (-35.20)

27/36261842-36261918(-)_mir_cow_192
CTGACGGGTCCTGTACTGAGCTGCCCCGAGGCCCTTCGCTGTGCCCAGCTCGGGTCAGCTCAGTACCGGGCGCGTCG
........TCCTGTACTGAGCTGCCCCGAG...............................................   484044
```

........TCCTGTACTGAGCTGCCCCGA...	186771
........TCCTGTACTGAGCTGCCCCGAGG...	6555
........TCCTGTACTGAGCTGCCCCG..	2653
..........CTGTACTGAGCTGCCCCGAG..	559
....TCGGGTCAGCTCAGTACCGGGC......	551
........TCCTGTACTGAGCTGCCCCGAGGC..	305
..........CTGTACTGAGCTGCCCCGA...	215
........TCCTGTACTGAGCTGCCCC...	149
........TCCTGTACTGAGCTGCCC..	121
.........CCTGTACTGAGCTGCCCCGAG..	85
....CGGGTCAGCTCAGTACCGGGC......	66
........TCCTGTACTGAGCTGCC...	48
.........CCTGTACTGAGCTGCCCCGA...	31
....TCGGGTCAGCTCAGTACCGGG.......	21
....CGGGTCAGCTCAGTACCGGGCG.....	16
........TCCTGTACTGAGCTGC..	13
........TCCTGTACTGAGCTGCCCCGAGGCCCTTCGCTGTGCCCAGCT............................	12
.........CCTGTACTGAGCTGCCCCGAGG...	11
..........CTGTACTGAGCTGCCCCGAGG...	11
....TCGGGTCAGCTCAGTACCGGGCG.....	8
....CGGGTCAGCTCAGTACCGGG.......	5
......GGTCCTGTACTGAGCTGCCCCGAG..	4
..........CTGTACTGAGCTGCCCCGAGGC..	4
......GGTCCTGTACTGAGCTGCCCCGA...	3
.............TACTGAGCTGCCCCGA...	3
...........TGTACTGAGCTGCCCCGAG..	3
.............TACTGAGCTGCCCCGAG..	3
...........TGTACTGAGCTGCCCCGAGGC..	3
....TCGGGTCAGCTCAGTACCG.........	3
.......GTCCTGTACTGAGCTGCCCCGAG..	2
............GTACTGAGCTGCCCCGAG..	2
..............ACTGAGCTGCCCCGAG..	2
........TCCTGTACTGAGCTGCCCCGAGGCCCTTCGCTGTGCCCAGC.............................	2
....TCGGGTCAGCTCAGTACC..........	2
....TCGGGTCAGCTCAGTACCGG........	2
..........CTGTACTGAGCTGCCCCG..	1
...........TGTACTGAGCTGCCCCGA...	1
.............TACTGAGCTGCCCCGAGG...	1

```
........TCCTGTACTGAGCTGCCCCGAGGCC............................................   1
........TCCTGTACTGAGCTGCCCCGAGGCCC...........................................   1
........TCCTGTACTGAGCTGCCCCGAGGCCCTTCGCTGTGCCC...............................   1
.................................................TCGGGTCAGCTCAGTA............   1
..................................................CGGGTCAGCTCAGTACC..........   1
....................................................GGTCAGCTCAGTACCGGGCG.....   1
.................................................TCGGGTCAGCTCAGTACCGGGCGC....   1
..((((.((((.(((((((((((.((((((((.(......).)))....))))).))))))))))).)))).)))). (-49.00)

27/36261845-36261913(+)_mir_cow_193
CGCGCCCGGTACTGAGCTGACCCGAGCTGGGCACAGCGAAGGGCCTCGGGGCAGCTCAGTACAGGACCC
....CCCGGTACTGAGCTGACCCGAG...........................................     44
....CCCGGTACTGAGCTGACCCGA............................................     13
....CCCGGTACTGAGCTGACCCGAGC..........................................     1
....CCCGGTACTGAGCTGACCCGAGCT.........................................     1
.............................................TCGGGGCAGCTCAGTACAGGAC..     1
.....((.(((((((((((.(((((((((....))))........))))).))))))))))).)).... (-36.50)

28/14208475-14208541(+)_novel_cow_30
GTTGGTCAACGGTCTCTAGGTGCTGGGTGAGGAGAGCCCAGCACCTAGAGACCGTTGGCTATTCTGC
.........................................CACCTAGAGACCGTTGGCTATT....  50
.........................................CACCTAGAGACCGTTGGCTAT.....  26
.......................................AGCACCTAGAGACCGTTGGCTATT....  5
.......................................AGCACCTAGAGACCGTTGGCTAT.....  2
........................................GCACCTAGAGACCGTTGGCTAT.....  2
...GGTCAACGGTCTCTAGGTGC............................................  1
......................................CAGCACCTAGAGACCGTTGGCT.......  1
.........................................CACCTAGAGACCGTTGGCTA......  1
........................................GCACCTAGAGACCGTTGGCTATT....  1
.........................................CACCTAGAGACCGTTGGCTATTC...  1
..........................................ACCTAGAGACCGTTGGCTATTC...  1
..((((((((((((((((((((((((((.......))))))))))))))))))))))))))...... (-52.10)

28/19569099-19569178(-)_mir_Cow_Blast_51
TAACTGGGTTAGGGCCCTGGCTCCATCTCCTTTAGGAAAACCTTCTGTGGGGAGTGGGGCTTTGACCCTAACCTAGCTGG
........TTAGGGCCCTGGCTCCATCTCCT.................................................     1590
........TTAGGGCCCTGGCTCCATCTCC..................................................     993
........TTAGGGCCCTGGCTCCATCT....................................................     263
```

........TTAGGGCCCTGGCTCCATCTC... 114
....AGTGGGGCTTTGACCCTAACC....... 112
........TTAGGGCCCTGGCTCCATCTCCTT.. 27
........TTAGGGCCCTGGCTCCATC... 14
....GAGTGGGGCTTTGACCCTAACC....... 8
....AGTGGGGCTTTGACCCTAACCT...... 6
........TTAGGGCCCTGGCTCCATCTCCTTTAGGA... 5
........TTAGGGCCCTGGCTCCATCTCCTTT... 3
........TTAGGGCCCTGGCTCCATCTCCTTTAGG.. 3
....AGTGGGGCTTTGACCCTAAC........ 3
........TTAGGGCCCTGGCTCCAT.. 2
.........TAGGGCCCTGGCTCCATCTCCT... 2
........TTAGGGCCCTGGCTCCATCTCCTTTAGGAAAA.. 2
........TTAGGGCCCTGGCTCCATCTCCTTTAGGAAAACC...................................... 2
....CTGTGGGGAGTGGGGCTTTGACC............. 2
....AGTGGGGCTTTGACCC............ 2
....AGTGGGGCTTTGACCCTA.......... 2
........TTAGGGCCCTGGCTCCA... 1
.........TAGGGCCCTGGCTCCATCT.. 1
........TTAGGGCCCTGGCTCCATCTCCTTTAG... 1
........TTAGGGCCCTGGCTCCATCTCCTTTAGGAAA... 1
........TTAGGGCCCTGGCTCCATCTCCTTTAGGAAAACCT..................................... 1
....TGTGGGGAGTGGGGCTTTGACC............. 1
....GGGGAGTGGGGCTTTGACCC............ 1
....GAGTGGGGCTTTGACCCTA.......... 1
....GAGTGGGGCTTTGACCCTAAC........ 1
....GGGAGTGGGGCTTTGACCCTAACC....... 1
....GGAGTGGGGCTTTGACCCTAACC....... 1
...(((((((((((.(..((((((((..(((.(((((.....))))).)))..))))))))..).)))))))))))).... (-42.40)

28/33517920-33517983(-)_mir_cow_194
TGCCAAAAAGTTTGTTCAGGTTTCTGTAAGATGGTGCAAAACCCTGAACGAACTTTTTGGCCAA
......................................AAACCCTGAACGAACTTTTTGG.... 157
......................................AAACCCTGAACGAACTTTTTG..... 10
......................................AAACCCTGAACGAACTTTT....... 2
.....................................AAAACCCTGAACGAACTTTTTGG.... 2
....AACCCTGAACGAACTTTTTGGC... 2
....AAAAAGTTTGTTCAGGTTTCT....................................... 1

```
....AAAAAGTTTGTTCAGGTTTCTGT.....................................     1
....AAAAAGTTTGTTCAGGTTTCTGTA....................................     1
......................................AAACCCTGAACGAACTTTTT......     1
........................................ACCCTGAACGAACTTTTTGGCCA.     1
............................................TGAACGAACTTTTTGGCCAA     1
.(((((((((((((((((((.((.((((......)))).)).)))))))))))))))))))...  (-32.70)

28/41329067-41329138(-)_mir_cow_195
TGGGCGTCTGTCTGCCCGCATGCCTGCCTCTCTGTTGCTCTGAAGGAGGCAGGGGCTGGGCTTGCAGCTGCC
........TGTCTGCCCGCATGCCTGCCTCT.........................................  162
........TGTCTGCCCGCATGCCTGCCT...........................................  3
..............................................AGGCAGGGGCTGGGCTTGCAGC....  2
......TCTGTCTGCCCGCATGCCTGCCT...........................................  1
........TGTCTGCCCGCATGCCTGCCTC..........................................  1
.................................GTTGCTCTGAAGGAGGC......................  1
..((((.((((..((((((...((((((((.((..........)))))))))).)).))))..)))).)))) (-38.40)

29/16686245-16686322(+)_mir_cow_196
CCCTCAAGGAGCTTACAATCTAGCTGGGGGTAAACGACTTGCACATGAACGCATCTAGACTGTGAGCTTCTAGAGGGC
...................................................CATCTAGACTGTGAGCTTCTAGA....  14904
.....AAGGAGCTTACAATCTAGCTGGG..................................................  4497
....................................................ATCTAGACTGTGAGCTTCTAGA....  2007
.....AAGGAGCTTACAATCTAGCTGG...................................................  1507
...................................................CATCTAGACTGTGAGCTTCTAG.....  675
.....AAGGAGCTTACAATCTAGCT.....................................................  436
...................................................CATCTAGACTGTGAGCTTCT.......  292
.....................................................TCTAGACTGTGAGCTTCTAGA....  246
.....AAGGAGCTTACAATCTAGCTG....................................................  192
.....AAGGAGCTTACAATCTAGC......................................................  132
...................................................CATCTAGACTGTGAGCTTCTA......  106
....................................................ATCTAGACTGTGAGCTTCTAG.....  91
..........................................ACATGAACGCATCTAGACTGTGAGCTTCTAGA....  44
.....AAGGAGCTTACAATCTAGCTGGGG.................................................  24
...................................................CATCTAGACTGTGAGCTTC........  23
....................................................ATCTAGACTGTGAGCTTCT.......  20
.....AAGGAGCTTACAATCT.........................................................  13
...................................................CATCTAGACTGTGAGCTT.........  12
.....................................................TCTAGACTGTGAGCTTCTAG.....  11
```

```
....................................................ATCTAGACTGTGAGCTTCTA......  9
.....AAGGAGCTTACAATCTAG.......................................................  8
....CAAGGAGCTTACAATCTAGCT.....................................................  6
.....................................................TCTAGACTGTGAGCTTCT.......  4
...........................................CATGAACGCATCTAGACTGTGAGCTTCTAGA....  4
............................................ATGAACGCATCTAGACTGTGAGCTTCTAGA....  4
............................GGTAAACGACTTGCACATGAACG...........................  3
..............................TAAACGACTTGCACATGAACG...........................  3
...................................................CATCTAGACTGTGAGCT..........  3
.....................................................TCTAGACTGTGAGCTTCTAGAG...  3
....CAAGGAGCTTACAATCTAGCTG....................................................  2
....CAAGGAGCTTACAATCTAGCTGG...................................................  2
....CAAGGAGCTTACAATCTAGCTGGG..................................................  2
.....AAGGAGCTTACAATCTAGCTGGGGGTAAACGAC.........................................  2
............................GGTAAACGACTTGCACATGAACGC..........................  2
.............................................TGAACGCATCTAGACTGTGAGCTTCTAGA....  2
.......................................................TAGACTGTGAGCTTCTAGA....  2
..........GCTTACAATCTAGCTG....................................................  1
......AGGAGCTTACAATCTAGCTGGG..................................................  1
..........GCTTACAATCTAGCTGGG..................................................  1
............TTACAATCTAGCTGGG..................................................  1
.....AAGGAGCTTACAATCTAGCTGGGGG................................................  1
.....AAGGAGCTTACAATCTAGCTGGGGGTAAACG...........................................  1
............................GGTAAACGACTTGCACATGAAC............................  1
..........................................ACATGAACGCATCTAGACTGTG..............  1
..........................................ACATGAACGCATCTAGACTGTGAGC...........  1
................................................ACGCATCTAGACTGTGAGCTT.........  1
....................................................ATCTAGACTGTGAGCTTC........  1
..........................................ACATGAACGCATCTAGACTGTGAGCTTCT.......  1
..................................................GCATCTAGACTGTGAGCTTCTAGA....  1
......................................................CTAGACTGTGAGCTTCTAGA....  1
........................................................AGACTGTGAGCTTCTAGA....  1
.....................................................TCTAGACTGTGAGCTTCTAGAGG..  1
(((((.(((((((((((.(((((.((.(.((((.....)))).......).)).))))).)))))))))))).))))). (-34.60)

29/43731469-43731532(+)_mir_cow_197
CCCCCTGTAACAGCAACTCCATGTGGAAGTACCTCTAGTTCCAGTGGGGCTGCTGTTATCTGGG
.....TGTAACAGCAACTCCATGTGGA.....................................    12560
```

```
.....TGTAACAGCAACTCCATGTGGAA....................................    2162
.....TGTAACAGCAACTCCATGTGG......................................    1462
.....TGTAACAGCAACTCCATG.........................................    73
....CTGTAACAGCAACTCCATGTGGA.....................................    44
........................................CCAGTGGGGCTGCTGTTATCT...    25
.....TGTAACAGCAACTCCATGT........................................    16
.....TGTAACAGCAACTCCATGTG.......................................    15
........................................CCAGTGGGGCTGCTGTTATCTG..    5
....CTGTAACAGCAACTCCATGTGG......................................    3
....CTGTAACAGCAACTCCATGTGGAA....................................    3
........................................CCAGTGGGGCTGCTGTTATC....    3
......GTAACAGCAACTCCATGTGGA.....................................    2
........AACAGCAACTCCATGTGGA.....................................    2
.........ACAGCAACTCCATGTGGA.....................................    2
.....TGTAACAGCAACTCCATGTGGAAG...................................    2
.....TGTAACAGCAACTCCA...........................................    1
....CTGTAACAGCAACTCCATGT........................................    1
..........CAGCAACTCCATGTGGA.....................................    1
.........................................CAGTGGGGCTGCTGTTATCTG..    1
.(((..(((((((((.((((((.(((((.((....)).))))))))))).)))))))))..))) (-31.70)

29/43731674-43731745(+)_mir_cow_198
GGGCTCTGACCTATGAATTGACAGCCAGTGCTCTTGTGTCCCCTCTGGCTGCCAATTCCATAGGTCACAGGT
.....CTGACCTATGAATTGACAGCC..............................................  236034
.....CTGACCTATGAATTGACAGCCA.............................................  19174
......TGACCTATGAATTGACAGCCAG............................................  14541
......TGACCTATGAATTGACAGCCAGT...........................................  14021
.....CTGACCTATGAATTGACAGC...............................................  13170
......TGACCTATGAATTGACAGCCA.............................................  13045
......TGACCTATGAATTGACAGCC..............................................  7925
.....CTGACCTATGAATTGACAGCCAGT...........................................  2123
.....CTGACCTATGAATTGACAG................................................  1146
......TGACCTATGAATTGACAGC...............................................  572
.....CTGACCTATGAATTGACAGCCAG............................................  459
....TCTGACCTATGAATTGACAGCC..............................................  336
.....CTGACCTATGAATTGACA.................................................  321
......TGACCTATGAATTGACAG................................................  59
................................................CTGCCAATTCCATAGGTCACAG..  46
```

```
.....CTGACCTATGAATTGAC..................................................  45
................................................CTGCCAATTCCATAGGTCACA...  22
....TCTGACCTATGAATTGACAGCCA.............................................  20
....TCTGACCTATGAATTGACAGC...............................................  11
......TGACCTATGAATTGACA.................................................  6
....TCTGACCTATGAATTGACAGCCAGT...........................................  6
................................................CTGCCAATTCCATAGGTCAC....  6
......TGACCTATGAATTGACAGCCAGTG..........................................  5
....TCTGACCTATGAATTGACA.................................................  3
.......GACCTATGAATTGACAGCCA.............................................  3
....TCTGACCTATGAATTGACAGCCAG............................................  3
.......GACCTATGAATTGACAGCCAG............................................  3
.......GACCTATGAATTGACAGCCAGT...........................................  3
......TGACCTATGAATTGAC..................................................  2
.......GACCTATGAATTGACAGCC..............................................  2
..........CTATGAATTGACAGCC..............................................  2
................................................CTGCCAATTCCATAGGTCA.....  2
...............................................GCTGCCAATTCCATAGGTCACA...  2
...............................................GCTGCCAATTCCATAGGTCACAG..  2
....TCTGACCTATGAATTGACAG................................................  1
.......GACCTATGAATTGACAGC...............................................  1
........ACCTATGAATTGACAGCC..............................................  1
.........CCTATGAATTGACAGCC..............................................  1
..........CTATGAATTGACAGCCA.............................................  1
........ACCTATGAATTGACAGCCAGT...........................................  1
........ACCTATGAATTGACAGCCAGTG..........................................  1
...........................GTGCTCTTGTGTCCCCTCTG.........................  1
...........................................TCTGGCTGCCAATTCCATAGGT.......  1
............................................CTGGCTGCCAATTCCATAGGTC......  1
............................................CTGGCTGCCAATTCCATAGGTCA.....  1
...............................................GCTGCCAATTCCATAGGTCA.....  1
.................................................TGCCAATTCCATAGGTCACA...  1
...((.((((((((((((((.(((((((.((......)).....))))))).))))).))))))))).)).. (-31.90)

29/51025445-51025513(+)_mir_cow_199
GGGCAGCCACTGCCCACCGCACACTGCGCTGCTCCGGACCCACTGTGCGTGTGACAGCGGCTGATCTGT
..........................................CTGTGCGTGTGACAGCGGCTGA.....     1001
..........................................CTGTGCGTGTGACAGCGGCTGAT....     190
```

```
....AGCCACTGCCCACCGCACACTG...........................................     175
..........................................CTGTGCGTGTGACAGCGGCT.......     86
..........................................CTGTGCGTGTGACAGCGGCTG......     62
.........................................ACTGTGCGTGTGACAGCGGCTGA.....     43
.........................................ACTGTGCGTGTGACAGCGGCT.......     41
....AGCCACTGCCCACCGCACACTGC..........................................     27
....AGCCACTGCCCACCGCACACT............................................     21
.........................................ACTGTGCGTGTGACAGCGGCTG......     15
..........................................CTGTGCGTGTGACAGCGGC........     14
.........................................ACTGTGCGTGTGACAGCGGCTGAT....     6
.....GCCACTGCCCACCGCACACTGC..........................................     2
..........................................CTGTGCGTGTGACAGC...........     2
..........................................CTGTGCGTGTGACAGCGG.........     2
....AGCCACTGCCCACCGCACA..............................................     1
.....GCCACTGCCCACCGCACACTG...........................................     1
......CCACTGCCCACCGCACACTG...........................................     1
....AGCCACTGCCCACCGCACACTGCGCTGCTCCGGACCCA...........................     1
.........................................ACTGTGCGTGTGACAGCGG.........     1
.........................................ACTGTGCGTGTGACAGCGGC........     1
...........................................TGTGCGTGTGACAGCGGCTGAT....     1
.........................................ACTGTGCGTGTGACAGCGGCTGATCT..     1
..........................................CTGTGCGTGTGACAGCGGCTGATCT..     1
((((((((.(((..(((((((((.((.((((...))).).)).)))))).))).))).))))).))).. (-29.60)

2/95834555-95834632(-)_mir_cow_106
TGTGTCATTCCCAGATACAATGGACAATATGCTGTTATAATTGTGTGGCATTGTCCTTGCTGTTTGGAGATAATACTG
.................................................ATTGTCCTTGCTGTTTGGAGAT.......  10
.................................................ATTGTCCTTGCTGTTTGGAG.........  3
.................................................ATTGTCCTTGCTGTTTGGAGA........  3
........TCCCAGATACAATGGACAATATG...............................................  2
.................................................ATTGTCCTTGCTGTTTGGA..........  2
................................................CATTGTCCTTGCTGTTTGGAGA........  2
.................................................ATTGTCCTTGCTGTTTGGAGATA......  2
...................................................TGTCCTTGCTGTTTGGAGATAA.....  2
......ATTCCCAGATACAATGGACAAT..................................................  1
.......TTCCCAGATACAATGGACAAT..................................................  1
...........CAGATACAATGGACAAT..................................................  1
........TCCCAGATACAATGGACAATA.................................................  1
```

```
..................................................TTGTCCTTGCTGTTTGGAG.........  1
..................................................TTGTCCTTGCTGTTTGGAGAT.......  1
..................................................TTGTCCTTGCTGTTTGGAGATA......  1
...................................................TGTCCTTGCTGTTTGGAGATAAT....  1
.((((.((..((((((((((.(((((..((((..(.........)..)))))))))))).)))))))..)).))))..  (-25.90)

3/102923771-102923840(+)_mir_cow_211
GGGGAGTCGGTCCTGCCGGCCAGGCCTGCTGGACACTCCTGCCGGCCAGGCCTGCTGGACCGACGCCCGG
.............................................CCAGGCCTGCTGGACCGACGCC...     116
.............................................CCAGGCCTGCTGGACCGACGCCC..     4
..............................................CAGGCCTGCTGGACCGACGCC...     3
....AGTCGGTCCTGCCGGCCAGGCC............................................     1
..............GCCGGCCAGGCCTGCTGGACACTCCTGC............................     1
..........................................CGGCCAGGCCTGCTGGACCG........     1
.(((.((((((((.((.((((.((((.((.((.....)).)).)))).)))).)).)))))))).)))..  (-50.50)

3/106059369-106059445(-)_mir_Cow_Blast_21
GTAGTGTGTGTAAACATCCTACACTCTCAGCTGTGAGCTCGAGGTGGCTGGGAGAGGGTTGTTTACTCCTTCTGCCA
........TGTAAACATCCTACACTCTCAGCT.............................................   108752
........TGTAAACATCCTACACTCTCAGC..............................................   19517
........TGTAAACATCCTACACTCTCA................................................   4017
........TGTAAACATCCTACACTCTCAG...............................................   1599
...............................................CTGGGAGAGGGTTGTTTACTCC........   889
........TGTAAACATCCTACACTCTC.................................................   344
........TGTAAACATCCTACACTCT..................................................   266
........TGTAAACATCCTACACTCTCAGCTGT...........................................   87
...............................................CTGGGAGAGGGTTGTTTACTC.........   81
...............................................CTGGGAGAGGGTTGTTTACT..........   70
........TGTAAACATCCTACACT....................................................   65
................................................TGGGAGAGGGTTGTTTACTCC........   49
........TGTAAACATCCTACACTCTCAGCTG............................................   39
........TGTAAACATCCTACACTC...................................................   35
.........GTAAACATCCTACACTCTCAGCT.............................................   32
...............................................CTGGGAGAGGGTTGTTTACTCCT.......   30
.........GTAAACATCCTACACTCTCAGC..............................................   12
................................................TGGGAGAGGGTTGTTTACTC.........   8
..........TAAACATCCTACACTCTCAGCT.............................................   7
...............................................CTGGGAGAGGGTTGTTTACTCCTT......   5
```

```
...............ATCCTACACTCTCAGCT.............................................   4
............AACATCCTACACTCTCAGCT.............................................   3
........TGTAAACATCCTACAC.....................................................   2
.........GTAAACATCCTACACTCTCA................................................   2
...........AAACATCCTACACTCTCAGC..............................................   2
.............ACATCCTACACTCTCAGC..............................................   2
..............CATCCTACACTCTCAGC..............................................   2
...........AAACATCCTACACTCTCAGCT.............................................   2
..............CATCCTACACTCTCAGCT.............................................   2
...............................................CTGGGAGAGGGTTGTTTAC...........   2
................................................TGGGAGAGGGTTGTTTACT..........   2
...........AAACATCCTACACTCT..................................................   1
..............CATCCTACACTCTCAG...............................................   1
..........TAAACATCCTACACTCTCAGC..............................................   1
.............ACATCCTACACTCTCAGCT.............................................   1
...........AAACATCCTACACTCTCAGCTGT...........................................   1
...............ATCCTACACTCTCAGCTGTGAGC.......................................   1
.................CCTACACTCTCAGCTGTGAGC.......................................   1
........TGTAAACATCCTACACTCTCAGCTGTGAGCT......................................   1
...............ATCCTACACTCTCAGCTGTGAGCT......................................   1
................TCCTACACTCTCAGCTGTGAGCT......................................   1
.................CCTACACTCTCAGCTGTGAGCT......................................   1
...................TACACTCTCAGCTGTGAGCT......................................   1
........TGTAAACATCCTACACTCTCAGCTGTGAGCTC.....................................   1
...............................................CTGGGAGAGGGTTGTT..............   1
...............................................CTGGGAGAGGGTTGTTT.............   1
...............................................CTGGGAGAGGGTTGTTTA............   1
................................GTGAGCTCGAGGTGGCTGGGAGAGGGTTGTTTACTCC........   1
................................................TGGGAGAGGGTTGTTTACTCCTT......   1
((((...(.(((((((.(((...((((((((..(.........)..)))))))).))).))))))).)...)))).. (-28.70)

3/106062499-106062574(-)_mir_cow_212
TTGCTACTGTAAACATCCTTGACTGGAAGCTGTAAGGCGTTGCAAGGAGCTTTCAGTCGGATGTTTACAGCGGCAG
.......TGTAAACATCCTTGACTGGAAGCT.............................................    589014
........GTAAACATCCTTGACTGGAAGCT.............................................    40563
.......TGTAAACATCCTTGACTGGAAGC..............................................    31907
.................................................CTTTCAGTCGGATGTTTACAGC.....    19063
.......TGTAAACATCCTTGACTGGA.................................................    11520
```

.......TGTAAACATCCTTGACTGGAA..	5705
.......TGTAAACATCCTTGACTGG..	5487
.......TGTAAACATCCTTGACTGGAAG...	5428
....CTTTCAGTCGGATGTTTACAG......	3298
....CTTTCAGTCGGATGTTTAC........	1008
.......TGTAAACATCCTTGAC...	946
.........TAAACATCCTTGACTGGAAGCT...	946
....TTTCAGTCGGATGTTTACAGC.....	882
....CTTTCAGTCGGATGTTTACA.......	852
........GTAAACATCCTTGACTGGAAGC..	692
........GTAAACATCCTTGACTGGAAGCTGT...	684
......CTGTAAACATCCTTGACTGGAAGCT...	392
........GTAAACATCCTTGACTGGAAGCTG..	307
.......TGTAAACATCCTTGACT..	162
.......TGTAAACATCCTTGACTG...	142
.........TAAACATCCTTGACTGGAAGCTGT...	132
........GTAAACATCCTTGACTGGAAG...	78
...............TCCTTGACTGGAAGCT...	59
........GTAAACATCCTTGACTGGA...	53
....TTTCAGTCGGATGTTTACAG......	44
........GTAAACATCCTTGACTGGAA..	42
....GCTTTCAGTCGGATGTTTACAG......	42
.......TGTAAACATCCTTGACTGGAAGCTGT...	35
.......TGTAAACATCCTTGACTGGAAGCTG..	33
....TTTCAGTCGGATGTTTACA.......	28
....TTTCAGTCGGATGTTTAC........	27
.............CATCCTTGACTGGAAGCT...	25
.........TAAACATCCTTGACTGGAAGCTG..	25
......CTGTAAACATCCTTGACTGGAAGC..	22
..........AAACATCCTTGACTGGAAGCTGT...	17
..........AAACATCCTTGACTGGAAGCT...	16
..............ATCCTTGACTGGAAGCT...	16
.........TAAACATCCTTGACTGGAAGC..	15
........GTAAACATCCTTGACTGG..	13
......CTGTAAACATCCTTGACTGGA...	13
............ACATCCTTGACTGGAAGCT...	13
....GCTTTCAGTCGGATGTTTACAGC.....	9
...........AACATCCTTGACTGGAAGCT...	7

```
.................................................CTTTCAGTCGGATGTT...........    6
.................................................CTTTCAGTCGGATGTTTACAGCG....    6
......CTGTAAACATCCTTGACTGG..................................................    5
......CTGTAAACATCCTTGACTGGAA................................................    5
.................................................CTTTCAGTCGGATGTTT..........    5
.................................................CTTTCAGTCGGATGTTTA.........    5
................................................GCTTTCAGTCGGATGTTTACA.......    5
........GTAAACATCCTTGACT....................................................    4
................................................GCTTTCAGTCGGATGTTTAC........    4
......CTGTAAACATCCTTGACTGGAAG...............................................    3
.........TAAACATCCTTGACTGGAAG...............................................    2
.............CATCCTTGACTGGAAGC..............................................    2
...............................GTAAGGCGTTGCAAGGAG...........................    2
....................................................TCAGTCGGATGTTTACAGC.....    2
.........TAAACATCCTTGACTGG..................................................    1
.........TAAACATCCTTGACTGGA.................................................    1
..............ATCCTTGACTGGAAGCTGT...........................................    1
........GTAAACATCCTTGACTGGAAGCTGTA..........................................    1
................CCTTGACTGGAAGCTGTAAG........................................    1
................CCTTGACTGGAAGCTGTAAGGC......................................    1
...............................................AGCTTTCAGTCGGATGTTTAC........    1
...........................................AAGGAGCTTTCAGTCGGATGTTTACAGC.....    1
............................................AGGAGCTTTCAGTCGGATGTTTACAGC.....    1
..................................................TTTCAGTCGGATGTTTACAGCG....    1
.(((..((((((((((((..(((((((((((.(...((...))..).)))))))))))))))))))))))..))). (-41.70)

3/120467957-120468025(+)_mir_cow_213
CAGGCTCTGGCTCCGTGTCTTCACTCCCGTGTGTGTCCACCGAGGGAGGGAGGGACGGGGCTGTGCTG
.....TCTGGCTCCGTGTCTTCACTCCC........................................     11930
.....TCTGGCTCCGTGTCTTCACTCC.........................................     3115
.....TCTGGCTCCGTGTCTTCACTC..........................................     1243
.....TCTGGCTCCGTGTCTTCACT...........................................     257
.....TCTGGCTCCGTGTCTTCAC............................................     16
.....TCTGGCTCCGTGTCTTCACTCCCG.......................................     12
.....TCTGGCTCCGTGTCTTCACTCCCGT......................................     7
.....TCTGGCTCCGTGTCTTCA.............................................     5
.....TCTGGCTCCGTGTCTTC..............................................     3
......CTGGCTCCGTGTCTTCACTCCC........................................     3
```

```
.......TGGCTCCGTGTCTTCACTCCC.........................................      3
.............................................GAGGGAGGGACGGGGGCTGT....      3
.............................................GAGGGAGGGACGGGGGCTGTGC..      3
......CTGGCTCCGTGTCTTCACTCC..........................................      2
..........CTCCGTGTCTTCACTCCC.........................................      2
.....TCTGGCTCCGTGTCTT................................................      1
......CTGGCTCCGTGTCTTCACTC...........................................      1
..........CTCCGTGTCTTCACTCC..........................................      1
....CTCTGGCTCCGTGTCTTCACTCCC.........................................      1
........GGCTCCGTGTCTTCACTCCC.........................................      1
.........GCTCCGTGTCTTCACTCCC.........................................      1
...........TCCGTGTCTTCACTCCC.........................................      1
............CCGTGTCTTCACTCCC.........................................      1
.....TCTGGCTCCGTGTCTTCACTCCCGTGTGT...................................      1
..(((.(.(.((((((.(((((.(((((..(((....)))...))))))))))).)))))).).).))). (-35.40)

3/14462433-14462506(-)_mir_Cow_Blast_1
ATTTTTACTTTCGGTTATCTAGCTTTATGAAGACTCCACACCACTCATACAGCTAGATAACCAAAGATAACAAC
.........TTCGGTTATCTAGCTTTATGA............................................8
.........TTCGGTTATCTAGCTTTAT..............................................2
........TTTCGGTTATCTAGCTTTATGA............................................1
..............TTATCTAGCTTTATGA............................................1
.........TTCGGTTATCTAGCTTTATGAA...........................................1
..........TCGGTTATCTAGCTTTATGAA...........................................1
..............................................ATACAGCTAGATAACCAAAGA.......1
...............................................TACAGCTAGATAACCAAAGAT......1
.......((((.((((((((((((.(((((..............))))).))))))))))))))))........ (-24.84)

3/14462435-14462505(+)_mir_cow_202
TGTTATCTTTGGTTATCTAGCTGTATGAGTGGTGTGGAGTCTTCATAAAGCTAGATAACCGAAAGTAAAAA
.....TCTTTGGTTATCTAGCTGTATGA...........................................   443629
.....TCTTTGGTTATCTAGCTGTAT.............................................   153938
.....TCTTTGGTTATCTAGCTGTATG............................................   133621
......CTTTGGTTATCTAGCTGTATGA...........................................   19453
............................................ATAAAGCTAGATAACCGAAAGT.....   15674
.....TCTTTGGTTATCTAGCTGTA..............................................   8675
.....TCTTTGGTTATCTAGCTGT...............................................   6474
.............................................TAAAGCTAGATAACCGAAAGTA....   3953
```

....TAAAGCTAGATAACCGAAAGT.....	2830
......CTTTGGTTATCTAGCTGTATG..	1923
.......TTTGGTTATCTAGCTGTATGA...	1913
....ATAAAGCTAGATAACCGAAAGTA....	1687
......CTTTGGTTATCTAGCTGTAT...	1196
....ATAAAGCTAGATAACCGAAA.......	571
....ATAAAGCTAGATAACCGAAAG......	519
.....TCTTTGGTTATCTAGC..	496
....TAAAGCTAGATAACCGAAAGTAA...	409
.....TCTTTGGTTATCTAGCTGTATGAG..	350
.....TCTTTGGTTATCTAGCT...	264
.......TTTGGTTATCTAGCTGTATGAG..	261
....TAAAGCTAGATAACCGAAAG......	254
.....TCTTTGGTTATCTAGCTG..	251
........TTGGTTATCTAGCTGTATGA...	155
.....TCTTTGGTTATCTAGCTGTATGAGT...	130
.......TTTGGTTATCTAGCTGTATG..	124
....ATAAAGCTAGATAACCGAAAGTAA...	117
....TAAAGCTAGATAACCGAAA.......	116
......CTTTGGTTATCTAGCTGTA..	101
.......TTTGGTTATCTAGCTGTATGAGT...	67
............TTATCTAGCTGTATGA...	54
....ATAAAGCTAGATAACCGA.........	52
.......TTTGGTTATCTAGCTGTAT...	41
...........GTTATCTAGCTGTATGA...	39
......CTTTGGTTATCTAGCTGT...	28
......CTTTGGTTATCTAGCTGTATGAGT...	27
....ATAAAGCTAGATAACCGAA........	26
......CTTTGGTTATCTAGCTGTATGAG..	24
....TAAAGCTAGATAACCGAAAGTAAA..	22
..........GGTTATCTAGCTGTATGA...	21
........TTGGTTATCTAGCTGTATG..	20
........TTGGTTATCTAGCTGTATGAGT...	15
........TTGGTTATCTAGCTGTAT...	13
.........TGGTTATCTAGCTGTATGA...	13
....CATAAAGCTAGATAACCGAAA.......	12
....CATAAAGCTAGATAACCGAAAGT.....	9
..........GGTTATCTAGCTGTATG..	8

```
...........GTTATCTAGCTGTATG............................................   8
.......TTTGGTTATCTAGCTGTA..............................................   7
.........TGGTTATCTAGCTGTATG............................................   7
........TTGGTTATCTAGCTGTATGAG..........................................   7
..........GGTTATCTAGCTGTAT.............................................   5
............................................ATAAAGCTAGATAACC...........   5
............................................ATAAAGCTAGATAACCGAAAGTAAA..   5
......CTTTGGTTATCTAGCT.................................................   4
....ATCTTTGGTTATCTAGCTGTAT.............................................   4
....ATCTTTGGTTATCTAGCTGTATGA...........................................   4
.........TGGTTATCTAGCTGTAT.............................................   3
..................................................CTAGATAACCGAAAGT.....   3
........TTGGTTATCTAGCTGT...............................................   2
.....TCTTTGGTTATCTAGCTGTATGAGTG........................................   2
......CTTTGGTTATCTAGCTGTATGAGTGGTGTGGAGTCTTC...........................   2
...............................................AAGCTAGATAACCGAAAGT.....   2
......CTTTGGTTATCTAGCTG................................................   1
.......TTTGGTTATCTAGCTG................................................   1
....ATCTTTGGTTATCTAGCTGTATG............................................   1
..............ATCTAGCTGTATGAGT.........................................   1
........TTGGTTATCTAGCTGTATGAGTG........................................   1
.........TGGTTATCTAGCTGTATGAGTG........................................   1
.....TCTTTGGTTATCTAGCTGTATGAGTGGTGTGGA.................................   1
.............................................TAAAGCTAGATAACCGA.........   1
.............................................TAAAGCTAGATAACCGAA........   1
..............................................AAAGCTAGATAACCGAAAGT.....   1
...........................................CATAAAGCTAGATAACCGAAAGTA....   1
.................................................GCTAGATAACCGAAAGTA....   1
..(((..(((((((((((((((.((((((.((.......)))))))).)))))))))))))))..)))... (-28.40)

3/15480734-15480804(-)_mir_cow_203
GGGAGGGACGGGACGCGGTGCAGTGTTGTTCTTTCCCCTGCCAATATTGCACTCGTCCCGGCCTCCGGCCC
............................................TATTGCACTCGTCCCGGCCTCC.....   102182
............................................TATTGCACTCGTCCCGGCCTC......   10665
............................................TATTGCACTCGTCCCGGCCT.......   5658
............................................TATTGCACTCGTCCCGGCC........   629
............................................TATTGCACTCGTCCCGGC.........   328
...........................................ATATTGCACTCGTCCCGGCCTCC.....   169
```

```
............................................TATTGCACTCGTCCCGGCCTCCG....   145
.............................................ATTGCACTCGTCCCGGCCTCC.....   115
............................................TATTGCACTCGTCCCGG..........   75
...........................................ATATTGCACTCGTCCCGGCCTC......   14
...AGGGACGGGACGCGGTGCAGTGT.............................................   13
............................................TATTGCACTCGTCCCG...........   13
..................................................ACTCGTCCCGGCCTCC.....   10
...AGGGACGGGACGCGGTGCAGTGTT............................................   9
.............................................ATTGCACTCGTCCCGGCCT.......   8
...........................................ATATTGCACTCGTCCCGGCCT.......   7
.............................................ATTGCACTCGTCCCGGCCTC......   7
..............................................TTGCACTCGTCCCGGCCTCC.....   5
...AGGGACGGGACGCGGTGCAGTGTTG...........................................   4
...AGGGACGGGACGCGGTGCAGT...............................................   3
.............................................ATTGCACTCGTCCCGGCC........   3
...........................................ATATTGCACTCGTCCCGGCC........   2
...AGGGACGGGACGCGGTGCAG................................................   1
..................................CCCCTGCCAATATTGCACTCGTCCCGGCCTCC.....   1
.....................................CTGCCAATATTGCACTCGTCCCGGCCTCC.....   1
...............................................TGCACTCGTCCCGGCCTCC.....   1
.............................................ATTGCACTCGTCCCGGCCTCCG....   1
...................................................CTCGTCCCGGCCTCCG....   1
.(((((..(((((((.((((((((((((.............))))))))))))))))))).))))).....  (-39.22)
```

```
3/2852300-2852363(+)_mir_cow_200
CCAGAAAGTTTGTTGGGGTTTTTCTGTCACATTTAACAGAAAAGCCCAAATGAACTTTTTGGTC
...GAAAGTTTGTTGGGGTTTTTCT........................................     100
...GAAAGTTTGTTGGGGTTTTTC.........................................     71
...GAAAGTTTGTTGGGGTTTTT..........................................     41
...GAAAGTTTGTTGGGGTTTT...........................................     8
..AGAAAGTTTGTTGGGGTTTTTC.........................................     5
...GAAAGTTTGTTGGGGT..............................................     1
...GAAAGTTTGTTGGGGTT.............................................     1
..AGAAAGTTTGTTGGGGTTTTT..........................................     1
..AGAAAGTTTGTTGGGGTTTTTCT........................................     1
...GAAAGTTTGTTGGGGTTTTTCTG.......................................     1
....AAAGTTTGTTGGGGTTTTTCTG.......................................     1
........................................AAAGCCCAAATGAACTTTT.....     1
```

```
.......................................AAAAGCCCAAATGAACTTTTTG...     1
........................................AAAGCCCAAATGAACTTTTTG...     1
((((((((((..((.((((((((((((........)))))))))))).))..))))))))))..  (-33.00)

3/33964968-33965041(-)_mir_cow_204
GCTGTGCCGGGTAGAGAGGGCAGTGGGAGGTAAGAGCTCTTCACCCTTCACCACCTTCTCCACCCAGCAGGGCC
..............................................TTCACCACCTTCTCCACCCAGC......7775
..............................................TTCACCACCTTCTCCACCCAG.......908
..............................................TTCACCACCTTCTCCACCCAGCA.....126
..............................................TTCACCACCTTCTCCACCCA........85
..............................................TTCACCACCTTCTCCACCC.........22
.............................................CTTCACCACCTTCTCCACCCAGC......22
...............................................TCACCACCTTCTCCACCCAGC......18
..............................................TTCACCACCTTCTCCACC..........11
.......CGGGTAGAGAGGGCAGTGGGAGG............................................5
...............................................TCACCACCTTCTCCACCCAGCA.....5
...............................................TCACCACCTTCTCCACCCAG.......4
................................................CACCACCTTCTCCACCCAGC......3
.......CGGGTAGAGAGGGCAGTGGGA..............................................2
..............................................TTCACCACCTTCTCCA............2
..............................................TTCACCACCTTCTCCACCCAGCAG....2
.......CGGGTAGAGAGGGCAGTGG................................................1
......CCGGGTAGAGAGGGCAGTGGGA..............................................1
.......CGGGTAGAGAGGGCAGTGGGAGGT...........................................1
.....................AGTGGGAGGTAAGAGCTCTTCAC..............................1
........................GGGAGGTAAGAGCTCTTCACCCTTCACC......................1
.................................................ACCACCTTCTCCACCCAGC......1
(((.(((.((((.(((((((..((((..((((((....))).)))..))))..))))))).)))).))).))). (-39.20)

3/45448526-45448591(+)_mir_cow_205
GGTGACGGGTATTCTTGGGTGGATAATACGGATTACGTTGTTATTGCTTAAGAATACGCGTAGTCG
........................................TTATTGCTTAAGAATACGCGT.....   1196
........................................TTATTGCTTAAGAATACGCGTAG...   774
........................................TTATTGCTTAAGAATACGCGTA....   243
.........................................TATTGCTTAAGAATACGCGTAGT..   81
........................................TTATTGCTTAAGAATACGCGTAGT..   71
........................................TTATTGCTTAAGAATACGC.......   40
........................................TTATTGCTTAAGAATACGCG......   35
```

```
.........................................TATTGCTTAAGAATACGCGTAG...   33
.........................................TATTGCTTAAGAATACGCGT.....   13
..........................................ATTGCTTAAGAATACGCGTAGT..   10
..........................................ATTGCTTAAGAATACGCGTAG...   9
....ACGGGTATTCTTGGGTGGAT..........................................   6
....ACGGGTATTCTTGGGTGGATAAT.......................................   6
...........................................TTGCTTAAGAATACGCGTAGT..   6
........................................TTATTGCTTAAGAATACG........   4
....ACGGGTATTCTTGGGTGGATAA........................................   2
..........................................ATTGCTTAAGAATACGCG......   2
.........................................TATTGCTTAAGAATACGCGTA....   2
....ACGGGTATTCTTGGGTGGATAATA......................................   1
........................................TTATTGCTTAAGAATA..........   1
.........................................TATTGCTTAAGAATACG........   1
.........................................TATTGCTTAAGAATACGC.......   1
.........................................TATTGCTTAAGAATACGCG......   1
..........................................ATTGCTTAAGAATACGCGT.....   1
((..(((.(((((((((((..(.(((((((.....)).)))))..)))))))))))).)))..)). (-26.60)

3/49797114-49797182(-)_mir_cow_206
GTCGCCCCCCTCAGTCCACCAGAGCCCGGATACCTTAGAAATTCGGCTCTGGGTCTGTGGGGAGCGAAA
...........................................CGGCTCTGGGTCTGTGGGGA......     329
...........................................CGGCTCTGGGTCTGTGGGGAG.....     270
..........................................TCGGCTCTGGGTCTGTGGGGA......     173
..........................................TCGGCTCTGGGTCTGTGGGGAG.....     123
...........................................CGGCTCTGGGTCTGTGGGG.......     55
......CCCCTCAGTCCACCAGAGCCCGGA.......................................     47
..........................................TCGGCTCTGGGTCTGTGGGG.......     31
......CCCCTCAGTCCACCAGAGCCCGG........................................     21
...........................................CGGCTCTGGGTCTGTGGGGAGC....     20
..........................................TCGGCTCTGGGTCTGTGGG........     8
..........................................TCGGCTCTGGGTCTGTGG.........     7
......CCCCTCAGTCCACCAGAGCCC..........................................     3
......CCCCTCAGTCCACCAGAGCCCG.........................................     3
...........CAGTCCACCAGAGCCCGGATACC...................................     3
...........................................CGGCTCTGGGTCTGTGGG........     3
......................................AAATTCGGCTCTGGGTCTGTGGGGA......     3
...........CAGTCCACCAGAGCCCGGATACCT..................................     2
```

```
....................................AGAAATTCGGCTCTGGGTCTGTGGGGA......      2
.......................................AATTCGGCTCTGGGTCTGTGGGGA......      2
..........................................TCGGCTCTGGGTCTGTGGGGAGC....      2
...........CAGTCCACCAGAGCCCGGATA.....................................      1
......CCCCTCAGTCCACCAGAGCCCGGATACC...................................      1
..........TCAGTCCACCAGAGCCCGGATACCT..................................      1
......................................AAATTCGGCTCTGGGT...............      1
...........................................CGGCTCTGGGTCTGTG..........      1
......................................AAATTCGGCTCTGGGTCTGTGGG........      1
......................................AAATTCGGCTCTGGGTCTGTGGGG.......      1
.........................................TTCGGCTCTGGGTCTGTGGGGA......      1
..............................................CTCTGGGTCTGTGGGGA......      1
.((((.((((.(((....((((((((.((((.........))))))))))))..))).)))).)))).. (-36.90)

3/52524777-52524842(-)_mir_cow_207
CCAAAAAGTTCATTTGGGTTTTTCTGTAAGATGTTACAGAAAAATCTGAGTGAACTTTTTGGCCAA
.........................................AAATCTGAGTGAACTTTTTGGC...   23
........................................AAAATCTGAGTGAACTTTTTGG....   19
..AAAAAGTTCATTTGGGTTTTT...........................................   11
.........................................AAATCTGAGTGAACTTTTTGG....   7
..AAAAAGTTCATTTGGGTTTT............................................   6
.CAAAAAGTTCATTTGGGTTTTT...........................................   6
..AAAAAGTTCATTTGGGTTT.............................................   3
.CAAAAAGTTCATTTGGGTTTT............................................   2
.CAAAAAGTTCATTTGGGTTT.............................................   1
..AAAAAGTTCATTTGGGTTTTTC..........................................   1
...AAAAGTTCATTTGGGTTTTTC..........................................   1
....AAAGTTCATTTGGGTTTTTCTG........................................   1
........................................AAAATCTGAGTGAACTTTT.......   1
.........................................AAATCTGAGTGAACTTTT.......   1
.........................................AAATCTGAGTGAACTTTTTG.....   1
..........................................AATCTGAGTGAACTTTTTGG....   1
..........................................AATCTGAGTGAACTTTTTGGC...   1
((((((((((((((..(((((((((((((....)))))))))))))..))))))))))))))).... (-36.70)

3/53564233-53564313(-)_mir_cow_208
ATTGGGTTGGCCAGAAAGTTCATTCAGGTTTTTCTTTAACAGCTTGCTGAAAAACCGGAATGAACTTTTTGGTCAACCCAA
..................................................AAAACCGGAATGAACTTTTTGG.........     337
```

```
..................................................AAAACCGGAATGAACTTTTTG..........     34
..................................................AAAACCGGAATGAACTTTTTGGT........     20
..................................................AAAACCGGAATGAACTTTTT...........     16
............AGAAAGTTCATTCAGGTTTTTC...............................................     10
..................................................AAAACCGGAATGAACTTTT............     10
...................................................AAACCGGAATGAACTTTTTGG.........     4
.............GAAAGTTCATTCAGGTTTTTC...............................................     3
..................................................AAAACCGGAATGAACTT..............     3
.................................................AAAAACCGGAATGAACTTTTTG..........     3
.................................................AAAAACCGGAATGAACTTTTT...........     2
...................................................AAACCGGAATGAACTTTTTGGT........     2
............AGAAAGTTCATTCAGGTTTT.................................................     1
............AGAAAGTTCATTCAGGTTTTT................................................     1
.............GAAAGTTCATTCAGGTTTTT................................................     1
............AGAAAGTTCATTCAGGTTTTTCT..............................................     1
.................................................AAAAACCGGAATGAACT...............     1
..................................................AAAACCGGAATGAACTTT.............     1
........................................................GGAATGAACTTTTTGG.........     1
.((((((((((((((((((((((((.((((((((..(((....)))..)))))))).)))))))))))))))))))))))) (-48.50)

3/74462304-74462373(+)_mir_cow_209
CTTTCCAAAGAATTCTCCTTTTGGGCTTTCTGATTTTATTTTAAGCCCAAAGGTGAATTTTTTGGGAAGT
.....CAAAGAATTCTCCTTTTGGGCT...........................................    202144
.....CAAAGAATTCTCCTTTTGGGCTT..........................................    129696
.....CAAAGAATTCTCCTTTTGGGC............................................    40197
.....CAAAGAATTCTCCTTTTGGGCTTT.........................................    5582
......AAAGAATTCTCCTTTTGGGCTT..........................................    1761
......AAAGAATTCTCCTTTTGGGCT...........................................    1285
.....CAAAGAATTCTCCTTTTGG..............................................    601
.....CAAAGAATTCTCCTTTTGGG.............................................    439
....CCAAAGAATTCTCCTTTTGGGC............................................    271
......AAAGAATTCTCCTTTTGGGC............................................    221
....CCAAAGAATTCTCCTTTTGGGCT...........................................    166
.....CAAAGAATTCTCCTTT.................................................    126
......AAAGAATTCTCCTTTTGGGCTTT.........................................    122
..........AATTCTCCTTTTGGGCT...........................................    48
...........ATTCTCCTTTTGGGCT...........................................    37
....CCAAAGAATTCTCCTTTTGGGCTT..........................................    35
```

```
...........ATTCTCCTTTTGGGCTT..........................................    33
..........AATTCTCCTTTTGGGCTT..........................................    30
.........GAATTCTCCTTTTGGGCTT..........................................    26
.........GAATTCTCCTTTTGGGCT...........................................    18
..........AATTCTCCTTTTGGGC............................................    15
....CCAAAGAATTCTCCTTTTGG..............................................    8
............................................GCCCAAAGGTGAATTTTTTGGG....    8
............TTCTCCTTTTGGGCTT..........................................    7
............................................GCCCAAAGGTGAATTTTTTGG.....    6
..............................................CCAAAGGTGAATTTTTTGGGA...    6
.....CAAAGAATTCTCCTTTT................................................    4
....CCAAAGAATTCTCCTTTTGGG.............................................    4
.......AAGAATTCTCCTTTTGGGCTT..........................................    4
.....CAAAGAATTCTCCTTTTG...............................................    3
......AAAGAATTCTCCTTTTGGG.............................................    3
....CCAAAGAATTCTCCTTTTGGGCTTT.........................................    3
..........AATTCTCCTTTTGGGCTTT.........................................    3
.............TCTCCTTTTGGGCTTT.........................................    3
......AAAGAATTCTCCTTTTGG..............................................    2
.......AAGAATTCTCCTTTTGGGC............................................    1
.........GAATTCTCCTTTTGGGC............................................    1
.......AAGAATTCTCCTTTTGGGCT...........................................    1
.......AAGAATTCTCCTTTTGGGCTTT.........................................    1
...........ATTCTCCTTTTGGGCTTT.........................................    1
.....CAAAGAATTCTCCTTTTGGGCTTTC........................................    1
............................................GCCCAAAGGTGAATTTTTTG......    1
.............................................CCCAAAGGTGAATTTTTTG......    1
................................................AAAGGTGAATTTTTTGG.....    1
............................................GCCCAAAGGTGAATTTTTTGGGA...    1
.............................................CCCAAAGGTGAATTTTTTGGGAA..    1
(((((((((((((((.(((((.((((((...((......)).)))))))))))).))))))))))))))). (-33.90)

3/78601736-78601809(+)_novel_cow_31
GCGCGGGGAGCCGAGGCGGCCAGCGCAGCGGGTTCAGAGCACCGGCCCCGGCCCCGCGAGCTCCCCGCGAGCTG
...............................................CCGGCCCCGCGAGCTCCCCGCGA....13
...CGGGGAGCCGAGGCGGCCAG...................................................2
................................................CGGCCCCGCGAGCTCCCCGCGA....1
.((((((((((((.((.((((.(.((..(((((.....)).))))).).)))))).)).))))))))))..... (-44.50)
```

```
3/7929183-7929249(-)_mir_cow_201
GCCAAAAAGTTCGTTCAGGTTTTTCTGTACCAGCTTCCAGAAGAATCTGGATGAACTTTTTGGCCAA
....AAAAGTTCGTTCAGGTTTTTCT.........................................  487
.........................................AGAATCTGGATGAACTTTTTGG....  152
...AAAAAGTTCGTTCAGGTTTTT...........................................  43
...AAAAAGTTCGTTCAGGTTTTTC..........................................  38
....AAAAGTTCGTTCAGGTTTTTCTG........................................  28
.........................................AGAATCTGGATGAACTTTTTG.....  22
........................................AAGAATCTGGATGAACTTTTTG.....  21
.........................................AGAATCTGGATGAACTTTT.......  14
....AAAAGTTCGTTCAGGTTTTTC..........................................  13
.....AAAGTTCGTTCAGGTTTTTCTG........................................  10
.........................................AGAATCTGGATGAACTTTTTGGC...  9
....AAAAGTTCGTTCAGGTTTTT...........................................  8
.....AAAGTTCGTTCAGGTTTTTCT.........................................  8
........................................AAGAATCTGGATGAACTTTTT......  8
....AAAAGTTCGTTCAGGTTTTTCTGT.......................................  7
...AAAAAGTTCGTTCAGGTTTTTCT.........................................  5
.....AAAGTTCGTTCAGGTTTTTCTGT.......................................  5
...AAAAAGTTCGTTCAGGTTTT............................................  3
....AAAAGTTCGTTCAGGTTTT............................................  3
.......................................GAAGAATCTGGATGAACTTTT.......  3
.........................................AGAATCTGGATGAACTTTTT......  3
.......................................GAAGAATCTGGATGAACTTTTT......  2
...AAAAAGTTCGTTCAGGTTT.............................................  1
......AAGTTCGTTCAGGTTTTTCTG........................................  1
.....AAAGTTCGTTCAGGTTTTTCTGTA......................................  1
.......AGTTCGTTCAGGTTTTTCTGTA......................................  1
.........................................AGAATCTGGATGAACTTT........  1
........................................AAGAATCTGGATGAACTTTT.......  1
(((((((((((((((((((((((((((..........)))))))))))))))))))))))))))...  (-38.30)

3/80666426-80666493(+)_mir_cow_210
TGGCTCAGTTATCACAGTGCTGATGCTGTCCATTCTAAAGGTACAGTACTGTGATAACTGAAGGATGG
........................................GTACAGTACTGTGATAACTGA.......37572
........................................GTACAGTACTGTGATAACT.........24165
.........................................TACAGTACTGTGATAACTGAA......22051
```

....TACAGTACTGTGATAACTGAAG..... 20590
....GTACAGTACTGTGATAACTGAA...... 18462
....TACAGTACTGTGATAACTGA....... 17178
....GTACAGTACTGTGATAACTG........ 10206
....TACAGTACTGTGATAACTG........ 4519
....TACAGTACTGTGATAACT......... 3349
....GTACAGTACTGTGATAAC.......... 1060
.....CAGTTATCACAGTGCTGATGC.. 604
....TACAGTACTGTGATAAC.......... 312
....TCAGTTATCACAGTGCTGATGC.. 300
....GTACAGTACTGTGATAACTGAAG..... 182
....TCAGTTATCACAGTGCTGATG... 106
....GTACAGTACTGTGATA............ 81
.....CAGTTATCACAGTGCTGATG... 62
....TCAGTTATCACAGTGCTGATGCT... 52
.....CAGTTATCACAGTGCTGATGCT... 47
....TCAGTTATCACAGTGCTGAT.. 46
.....CAGTTATCACAGTGCTGAT.. 34
....ACAGTACTGTGATAACTGAAG..... 28
....TCAGTTATCACAGTGCTGA... 26
....TACAGTACTGTGATAACTGAAGG.... 24
....GTACTGTGATAACTGAA...... 19
....GGTACAGTACTGTGATAACT......... 18
....ACAGTACTGTGATAACTGAA...... 17
....ACAGTACTGTGATAACTGA....... 16
....GTACTGTGATAACTGA....... 16
....CAGTACTGTGATAACTGAA...... 12
....GGTACAGTACTGTGATAACTGA....... 11
....GTACTGTGATAACTGAAG..... 11
....TACAGTACTGTGATAACTGAAGGA... 10
....CAGTACTGTGATAACTGAAG..... 9
.....CAGTTATCACAGTGCTGATGCTG.. 6
....GGTACAGTACTGTGATAACTG........ 5
..........................TGTCCATTCTAAAGGTACAGTACTGTGATAACTGAAG..... 5
....AGTACTGTGATAACTGAAG..... 5
....GTACAGTACTGTGATAA........... 4
....TACAGTACTGTGATAA........... 4
....GGTACAGTACTGTGATAAC.......... 4

```
.....................................AAGGTACAGTACTGTGATAACT......... 4
...........................................CAGTACTGTGATAACTGA....... 4
..........................TGTCCATTCTAAAGGTACAGTACTGTGATAACTGAA...... 4
....TCAGTTATCACAGTGCTG.............................................. 3
.....CAGTTATCACAGTGCTGA............................................. 3
..........................TGTCCATTCTAAAGGTACAGT..................... 3
...........................................CAGTACTGTGATAACTG........ 3
...........................................CAGTACTGTGATAACTGAAGGA... 3
....TCAGTTATCACAGTGCT............................................... 2
.....................................AAGGTACAGTACTGTGATAACTG........ 2
..........................................ACAGTACTGTGATAACTG........ 2
............................................AGTACTGTGATAACTGA....... 2
............................................AGTACTGTGATAACTGAA...... 2
.....CAGTTATCACAGTGCT............................................... 1
.......GTTATCACAGTGCTGATGC.......................................... 1
....TCAGTTATCACAGTGCTGATGCTG........................................ 1
..........................TGTCCATTCTAAAGGTACAGTA.................... 1
..........................TGTCCATTCTAAAGGTACAGTACTG................. 1
...............................ATTCTAAAGGTACAGTACTGTGA.............. 1
...................................TAAAGGTACAGTACTGTGATAAC.......... 1
......................................AGGTACAGTACTGTGATAACT......... 1
..........................................ACAGTACTGTGATAACT......... 1
...........................................CAGTACTGTGATAACT......... 1
............................TCCATTCTAAAGGTACAGTACTGTGATAACTGAA...... 1
.......................................GGTACAGTACTGTGATAACTGAA...... 1
.....................................AAGGTACAGTACTGTGATAACTGAAG..... 1
...........................................CAGTACTGTGATAACTGAAGGAT.. 1
....((((((((((((((((((.((((............))))))))))))))))))))))....... (-31.40)

4/101436291-101436357(+)_mir_cow_226
GACACCATGGATCTCCAGGTGGGTCACGTTTAGAGATACACCAACCTGGAGGACTCCATGCTGTTGA
.........................................CAACCTGGAGGACTCCATGCTGT...  3390
.........................................CAACCTGGAGGACTCCATGCTG....  705
.........................................CAACCTGGAGGACTCCATGCT.....  377
........................................CCAACCTGGAGGACTCCATGCT.....  238
....CCATGGATCTCCAGGTGGGT...........................................  183
........................................CCAACCTGGAGGACTCCATGCTG....  147
.......................................ACCAACCTGGAGGACTCCATGCT.....  126
```

```
........................................CCAACCTGGAGGACTCCATGCTGT...  93
.......................................ACCAACCTGGAGGACTCCATGCTG....  63
.........................................CAACCTGGAGGACTCCATGC......  54
........................................CCAACCTGGAGGACTCCATGC......  40
....CCATGGATCTCCAGGTGGGTC..........................................  36
.........................................CAACCTGGAGGACTCCATGCTGTT..  31
......................................CACCAACCTGGAGGACTCCATGCT.....  24
....CCATGGATCTCCAGGTGGGTCA.........................................  23
.......................................ACCAACCTGGAGGACTCCATGC......  20
......................................CACCAACCTGGAGGACTCCATGC......  16
.......................................ACCAACCTGGAGGACTCCATGCTGT...  8
.........................................CAACCTGGAGGACTCC..........  5
.........................................CAACCTGGAGGACTCCA.........  5
.....................................ACACCAACCTGGAGGACTCCATGCT.....  3
....CCATGGATCTCCAGGTG..............................................  2
....CCATGGATCTCCAGGTGG.............................................  2
..........................................AACCTGGAGGACTCCATGCTGT...  2
........................................CCAACCTGGAGGACTCCATGCTGTT..  2
....CCATGGATCTCCAGGTGGG............................................  1
........................................CCAACCTGGAGGACTCC..........  1
....................................TACACCAACCTGGAGGACTCCA.........  1
.....................................ACACCAACCTGGAGGACTCCAT........  1
........................................CCAACCTGGAGGACTCCAT........  1
....................................TACACCAACCTGGAGGACTCCATG.......  1
......................................CACCAACCTGGAGGACTCCATG.......  1
.........................................CAACCTGGAGGACTCCATG.......  1
.....................................ACACCAACCTGGAGGACTCCATGC......  1
..........................................AACCTGGAGGACTCCATGCT.....  1
..................................GATACACCAACCTGGAGGACTCCATGCTG....  1
.....................................ACACCAACCTGGAGGACTCCATGCTG....  1
......................................CACCAACCTGGAGGACTCCATGCTG....  1
...........................................ACCTGGAGGACTCCATGCTGT...  1
............................................CCTGGAGGACTCCATGCTGT...  1
.............................................CTGGAGGACTCCATGCTGT...  1
((((.(((((((((((((((.(((...(((....)))..))).)))))))))..)))))).)))).. (-29.30)

4/10653539-10653610(-)_mir_cow_214
CCTCAGTGTTGAAACAATCTCTGTTGAACCATGTTACAGGAAGTTCACTGGAGTTTGTTTCAACATTGGAGG
```

```
.............................................CACTGGAGTTTGTTTCAACATT.....  622
............................................TCACTGGAGTTTGTTTCAACATT.....  200
......TGTTGAAACAATCTCTGTTGA.............................................  107
......TGTTGAAACAATCTCTGTTGAAC...........................................  105
......TGTTGAAACAATCTCTGTTGAA............................................  73
......TGTTGAAACAATCTCTGTTG..............................................  47
.............................................CACTGGAGTTTGTTTCAACAT......  37
............................................TCACTGGAGTTTGTTTCAACA.......  32
........TTGAAACAATCTCTGTTGAACC..........................................  26
............................................TCACTGGAGTTTGTTTCAACAT......  24
.........TGAAACAATCTCTGTTGAACC..........................................  20
.............................................CACTGGAGTTTGTTTCAACA.......  16
............................................TCACTGGAGTTTGTTTCAAC........  8
...........................................TTCACTGGAGTTTGTTTCAACA.......  8
...........................................TTCACTGGAGTTTGTTTCAAC........  4
......TGTTGAAACAATCTCTGTT...............................................  3
.............................................CACTGGAGTTTGTTTCAAC........  3
...........................................TTCACTGGAGTTTGTTTCAACAT......  3
.........TGAAACAATCTCTGTTGAAC...........................................  2
......TGTTGAAACAATCTCTGTTGAACC..........................................  2
...........................................TTCACTGGAGTTTGTTTCAA.........  2
..............................................ACTGGAGTTTGTTTCAACATT.....  2
......TGTTGAAACAATCTCTGT................................................  1
........TTGAAACAATCTCTGTTGAAC...........................................  1
.........TGAAACAATCTCTGTTGAACCA.........................................  1
............................................TCACTGGAGTTTGTTTCA..........  1
.............................................CACTGGAGTTTGTTTCAACATTG....  1
..............................................ACTGGAGTTTGTTTCAACATTG....  1
(((((((((((((((((.((((..(((((.............)))))..)))).))))))))))))).)))) (-30.32)

4/114451873-114451942(-)_mir_cow_227
CGGGGAGCTGGGGAAAGCAGGAGTGAGGGTCTGCACCCCAGTAAGCCTCGTTCTGCTTTCCCCACAGGCA
.......CTGGGGAAAGCAGGAGTGAGG...........................................    8
.......CTGGGGAAAGCAGGAGTGAG............................................    7
.......CTGGGGAAAGCAGGAGTGA.............................................    6
.......CTGGGGAAAGCAGGAGTGAGGGT.........................................    5
.......CTGGGGAAAGCAGGAGTGAGGG..........................................    4
.............................................CCTCGTTCTGCTTTCCCCACA....    2
```

```
........TGGGGAAAGCAGGAGTGAG...........................................    1
......GCTGGGGAAAGCAGGAGTGAGGGT........................................    1
........TGGGGAAAGCAGGAGTGAGGGT........................................    1
......((((((((((((((((.(((((.(.(((......)))).))))))))))))))))))...))). (-34.80)

4/119142848-119142915(+)_novel_cow_33
AGCTCGGGGCCCAGGGCCGGTGTGTTTGTTGCCTTAGGAACACACGCGTCCTTGGATCCTGACTCGCC
.........................................ACACGCGTCCTTGGATCCTGACT.... 24
.........................................ACACGCGTCCTTGGATCCTGAC..... 9
..........................................CACGCGTCCTTGGATCCTGACT.... 6
..........................................CACGCGTCCTTGGATCCTGAC..... 3
........................................CACACGCGTCCTTGGATCCTG....... 2
.........................................ACACGCGTCCTTGGATCCTG....... 2
...TCGGGGCCCAGGGCCGGTGTG............................................ 1
...TCGGGGCCCAGGGCCGGTGTGT........................................... 1
........................................CACACGCGTCCTTGGATCCTGA...... 1
.........................................ACACGCGTCCTTGGATCCTGA...... 1
.(((((((..((((((.((((((((((.(......).)))))))).)).))))))..)))))...)). (-33.20)

4/119541703-119541776(-)_mir_cow_228
GCCAGTGTCATTTTTGTGATGTTGCAGCTAGTAATATGAGCCCAGTTGCATAGTCACAAAAGTGATCATTGGAA
.............................................TTGCATAGTCACAAAAGTGATC.......28108
.............................................TTGCATAGTCACAAAAGTGAT........1611
.............................................TTGCATAGTCACAAAAGTG..........893
.............................................TTGCATAGTCACAAAAGTGATCAT.....727
.............................................TTGCATAGTCACAAAAGTGATCA......530
.............................................TTGCATAGTCACAAAAGTGA.........316
......GTCATTTTTGTGATGTTGCAGCT.............................................279
..............................................TGCATAGTCACAAAAGTGATC.......251
.............................................TTGCATAGTCACAAAAGT...........79
......GTCATTTTTGTGATGTTGCAG...............................................42
.............................................TTGCATAGTCACAAAAG............30
..............................................TGCATAGTCACAAAAGTGATCAT.....12
......GTCATTTTTGTGATGTTGCAGC..............................................10
..............................................TGCATAGTCACAAAAGTGAT........7
..............................................TGCATAGTCACAAAAGTGATCA......6
.......TCATTTTTGTGATGTTGCAGCT.............................................3
..............................................TGCATAGTCACAAAAGTGA.........3
```

......GTCATTTTTGTGATGTTGCAGCTA..2
....TGCATAGTCACAAAAGTG..........2
....GCATAGTCACAAAAGTGATC.......2
......GTCATTTTTGTGATGTTGC...1
......GTCATTTTTGTGATGTTGCA..1
..........TTTTTGTGATGTTGCAGCT...1
......GTCATTTTTGTGATGTTGCAGCTAG...1
....TTGCATAGTCACAAAA.............1
....GCATAGTCACAAAAGTGAT........1
....CATAGTCACAAAAGTGATC.......1
....TTGCATAGTCACAAAAGTGATCATT....1
.(((((((((((((((((((..(((((((.((.......))..)))))))..)))))))))))))).)))))).. (-33.60)

4/49798044-49798112(+)_mir_cow_215
CCAACAAGTTCGTCTGGGTTTTTCTGTACCATATGGAAAACCTGAATGAACTTTTTGGCCAACCCAGTA
....................................AAAACCTGAATGAACTTTTTGG...........   2418
.....................................AAACCTGAATGAACTTTTTGG...........   480
....................................AAAACCTGAATGAACTTTTTG............   124
.....................................AAACCTGAATGAACTTTTTGGC..........   77
....................................AAAACCTGAATGAACTTTTTGGC..........   38
...................................GAAAACCTGAATGAACTTTTTG............   26
....................................AAAACCTGAATGAACTTTTT.............   21
....................................AAAACCTGAATGAACTTTT..............   7
...................................GAAAACCTGAATGAACTTTTT.............   6
.....................................AAACCTGAATGAACTTTTTG............   6
......................................AACCTGAATGAACTTTTTGGCC.........   6
......................................AACCTGAATGAACTTTTTGG...........   5
....ACCTGAATGAACTTTTTGGCC.........   4
....TGAATGAACTTTTTGGCCAACC.....   4
.....................................AAACCTGAATGAACTTTTT.............   3
....CCTGAATGAACTTTTTGGCC.........   3
....CCTGAATGAACTTTTTGGCCA........   3
....AATGAACTTTTTGGCCAACC.....   3
....AATGAACTTTTTGGCCAACCC....   3
...ACAAGTTCGTCTGGGTTTTTC...   2
......................................AACCTGAATGAACTTTTTGGC..........   2
.....................................AAACCTGAATGAACTTTTTGGCC.........   2
....ACCTGAATGAACTTTTTGGCCA........   2

```
..........................................TGAATGAACTTTTTGGCCAAC......      2
..........................................TGAATGAACTTTTTGGCCAACCC....      2
............................................AATGAACTTTTTGGCCAACCCA...      2
..........................................TGAATGAACTTTTTGGCCAACCCAGT.      2
....................................AAAACCTGAATGAACTTT...............      1
.....................................AAACCTGAATGAACTTTT..............      1
.................................TGGAAAACCTGAATGAACTTTTTGG...........      1
..........................................TGAATGAACTTTTTGG...........      1
......................................AACCTGAATGAACTTTTTGGCCA........      1
.......................................ACCTGAATGAACTTTTTGGCCAA.......      1
.......................................ACCTGAATGAACTTTTTGGCCAAC......      1
..............................................TGAACTTTTTGGCCAACC.....      1
.............................................ATGAACTTTTTGGCCAACCC....      1
...............................................GAACTTTTTGGCCAACCC....      1
..............................................TGAACTTTTTGGCCAACCCA...      1
((((.((((((((..(((((((((..........)))))))))..)))))))).))))........... (-24.10)

4/66346473-66346546(-)_mir_cow_216
CCTCTATGATCGCATCAACACTCGTCTGTTCTTAAATAACATTGAGAACAGGCGGGTGCTAATACGATCATAGA
........ATCGCATCAACACTCGTCTGTT............................................895
........ATCGCATCAACACTCGTCTGT.............................................860
.........TCGCATCAACACTCGTCTGTTC...........................................283
.........TCGCATCAACACTCGTCTGT.............................................217
.........TCGCATCAACACTCGTCTGTT............................................210
.........TCGCATCAACACTCGTCTGTTCT..........................................43
........ATCGCATCAACACTCGTCTG..............................................38
........ATCGCATCAACACTCGTCTGTTC...........................................20
...........GCATCAACACTCGTCTGTTCTT.........................................18
.........TCGCATCAACACTCGTCTG..............................................14
...........GCATCAACACTCGTCTGTTCT..........................................11
........ATCGCATCAACACTCGTC................................................4
..........CGCATCAACACTCGTCTGTTCT..........................................3
........ATCGCATCAACACTCGTCTGTTCT..........................................2
...............................................ACAGGCGGGTGCTAATACGATC.....2
................................................CAGGCGGGTGCTAATACGATC.....2
........ATCGCATCAACACTCGT.................................................1
.........TCGCATCAACACTCGTC................................................1
......TGATCGCATCAACACTCGTCT...............................................1
```

```
........ATCGCATCAACACTCGTCT...............................................1
.......GATCGCATCAACACTCGTCTGT.............................................1
.............ATCAACACTCGTCTGT.............................................1
................................................CAGGCGGGTGCTAATACGAT......1
...............................................ACAGGCGGGTGCTAATACGATCA....1
................................................CAGGCGGGTGCTAATACGATCA....1
..((((((((((.((...(((((((((((((((((......)))))))))))))))))...)).)))))))))) (-38.40)

4/69327771-69327834(+)_mir_cow_217
GATTTAGGTAGTTTCCTGTTGTTGGGATCCACCTTTCTCTCGACAGCACGACACTGCCTTCATT
....TAGGTAGTTTCCTGTTGTTGGG......................................     515
....TAGGTAGTTTCCTGTTGTTGGGA.....................................     448
....TAGGTAGTTTCCTGTTGTTGG.......................................     109
....TAGGTAGTTTCCTGTTGTTG........................................     39
....TAGGTAGTTTCCTGTTGTTGGGAT....................................     35
.......................................TCGACAGCACGACACTGCCTTCA..     6
....TAGGTAGTTTCCTGTTGTT.........................................     4
....TAGGTAGTTTCCTGTTGTTGGGATC...................................     4
....TAGGTAGTTTCCTGTTGT..........................................     3
....TAGGTAGTTTCCTGTTGTTGGGATCC..................................     2
.......................................TCGACAGCACGACACTGCCTTC...     2
.......................................TCGACAGCACGACACTGCCT.....     1
..................................TTCTCTCGACAGCACGACACTGCCTTCA..     1
(((..(((((((.((.(((((((((((..........))))))))))).)).)))))))..))) (-24.80)

4/70414251-70414321(+)_mir_cow_218
GAGGCAAAGTTCTGAGACACTCCGACTCTGAATATGATAGAAGTCAGTGCACTACAGAACTTTGTCTCTAG
...........................................TCAGTGCACTACAGAACTTTGT......   253154
...........................................TCAGTGCACTACAGAACTTTGTC.....   147555
...........................................TCAGTGCACTACAGAACTTTGTCT....   26863
.....AAAGTTCTGAGACACTCCGACT............................................   7223
...........................................TCAGTGCACTACAGAACTTTG.......   3730
...........................................TCAGTGCACTACAGAACTTT........   614
...........................................TCAGTGCACTACAGAACT..........   596
...........................................TCAGTGCACTACAGAACTT.........   306
....CAAAGTTCTGAGACACTCCGACT............................................   211
............................................CAGTGCACTACAGAACTTTGTC.....   177
............................................CAGTGCACTACAGAACTTTGT......   148
```

```
.............................................AGTGCACTACAGAACTTTGT......   146
.............................................AGTGCACTACAGAACTTTGTC.....   93
.....AAAGTTCTGAGACACTCCGA..............................................   88
.....AAAGTTCTGAGACACTCCGAC.............................................   65
............................................CAGTGCACTACAGAACTTTGTCT....   36
...........................................TCAGTGCACTACAGAAC...........   25
.............................................AGTGCACTACAGAACTTTGTCT....   17
.....AAAGTTCTGAGACACTCCGACTCT..........................................   11
..........................................GTCAGTGCACTACAGAACTTTGT......   11
................................................GCACTACAGAACTTTGT......   11
...............................................TGCACTACAGAACTTTGT......   9
...........................................TCAGTGCACTACAGAACTTTGTCTC...   8
..............................................GTGCACTACAGAACTTTGT......   7
..................................................ACTACAGAACTTTGTC.....   7
....CAAAGTTCTGAGACACTCCGA..............................................   5
....CAAAGTTCTGAGACACTCCGAC.............................................   5
..............................................GTGCACTACAGAACTTTGTCT....   5
...................................................CTACAGAACTTTGTCT....   4
.....AAAGTTCTGAGACACTCCG...............................................   3
.................................................CACTACAGAACTTTGT......   3
................................................GCACTACAGAACTTTGTC.....   3
................................................GCACTACAGAACTTTGTCT....   3
..............................................GTGCACTACAGAACTTTGTC.....   2
.....AAAGTTCTGAGACACTC.................................................   1
.....AAAGTTCTGAGACACTCC................................................   1
......AAGTTCTGAGACACTCCGACT............................................   1
.......AGTTCTGAGACACTCCGACT............................................   1
.....AAAGTTCTGAGACACTCCGACTC...........................................   1
..................ACTCCGACTCTGAATATGATAGA..............................   1
...........................CTGAATATGATAGAAG............................   1
...........................................TCAGTGCACTACAGAA............   1
............................................CAGTGCACTACAGAACTT.........   1
.............................................AGTGCACTACAGAACTT.........   1
..........................................GTCAGTGCACTACAGAACTTT........   1
............................................CAGTGCACTACAGAACTTT........   1
............................................CAGTGCACTACAGAACTTTG.......   1
...............................................TGCACTACAGAACTTTGTC.....   1
...............................................TGCACTACAGAACTTTGTCT....   1
```

```
...........................................TCAGTGCACTACAGAACTTTGTCTCT..    1
((((((((((((((((.((((..(((((((.......))).))))))))..)).))))))))))))))...  (-29.40)

4/86669288-86669354(-)_mir_cow_219
GCCAAAAAGTTCATTTGGGCTTTTTCATAAGATGTTACAGAAAAACCCTGATGAACTTTTTGATCAA
.........................................AAAACCCTGATGAACTTTTTGA....  98
.........................................AAAACCCTGATGAACTTTTTG.....  2
...AAAAAGTTCATTTGGGCT..............................................  1
....AAAAGTTCATTTGGGCT..............................................  1
...AAAAAGTTCATTTGGGCTTT............................................  1
..CAAAAAGTTCATTTGGGCTTTT...........................................  1
...AAAAAGTTCATTTGGGCTTTT...........................................  1
........................................AAAAACCCTGATGAACTTTTTG.....  1
........................................AAAAACCCTGATGAACTTTTTGA....  1
..........................................AAACCCTGATGAACTTTTTGAT...  1
..........................................AAACCCTGATGAACTTTTTGATC..  1
..(((((((((((((.(((.((((((.............))))))))).))))))))))))).....  (-23.82)

4/93220888-93220960(+)_mir_cow_220
TGGATCTTTTTGCGGTCTGGGCTTGCTGTTCCTCTCTACAGTAGTCAGGAAGCCCTTACCCCAAAAAGTATCT
.....CTTTTTGCGGTCTGGGCTTGC...............................................  8553
.................................................AAGCCCTTACCCCAAAAAGTAT..  1435
.....CTTTTTGCGGTCTGGGCTTGCT..............................................  1052
.....CTTTTTGCGGTCTGGGCTTG................................................  416
.................................................AAGCCCTTACCCCAAAAAGT....  413
.................................................AAGCCCTTACCCCAAAAAGTA...  261
.................................................AAGCCCTTACCCCAAAAAG.....  94
...............................................GGAAGCCCTTACCCCAAAAAGT....  92
.....CTTTTTGCGGTCTGGGCTTGCTG.............................................  79
.....CTTTTTGCGGTCTGGGCTTGCTGT............................................  73
.....CTTTTTGCGGTCTGGGCTT.................................................  52
................................................GAAGCCCTTACCCCAAAAAGT....  44
.....CTTTTTGCGGTCTGGGCT..................................................  26
................................................GAAGCCCTTACCCCAAAAAGTA...  22
......TTTTTGCGGTCTGGGCTTGCT..............................................  20
...............................................GGAAGCCCTTACCCCAAAAAGTA...  18
......TTTTTGCGGTCTGGGCTTGC...............................................  12
.....CTTTTTGCGGTCTGGGCTTGCTGTT...........................................  9
```

```
......TTTTTGCGGTCTGGGCTTGCTG............................................. 7
......TTTTTGCGGTCTGGGCTTGCTGT............................................ 7
...............................................GGAAGCCCTTACCCCAAAAAGTAT.. 7
.....CTTTTTGCGGTCTGGGC................................................... 4
...............................................GGAAGCCCTTACCCCAAAAAG..... 4
................................................GAAGCCCTTACCCCAAAAAGTAT.. 4
....TCTTTTTGCGGTCTGGGCTTGC............................................... 3
.................................................AAGCCCTTACCCCAAAAA...... 3
................................................GAAGCCCTTACCCCAAAAAG..... 3
.......TTTTGCGGTCTGGGCTTGC............................................... 2
.......TTTTGCGGTCTGGGCTTGCTG............................................. 2
.................................................AAGCCCTTACCCCAAA........ 2
......TTTTTGCGGTCTGGGCT.................................................. 1
......TTTTTGCGGTCTGGGCTTG................................................ 1
.........TTGCGGTCTGGGCTTG................................................ 1
..........................TGTTCCTCTCTACAGTAGTCAGGA....................... 1
...........................GTTCCTCTCTACAGTAGTCAGGA....................... 1
............................TTCCTCTCTACAGTAGTCAGGA....................... 1
......................TTGCTGTTCCTCTCTACAGTAGTCAGGAAGC.................... 1
.(((((((((((.(((..((((((.(((..(..((....))..).))).))))))..))).))))))).)))) (-29.60)

4/94406719-94406792(-)_mir_cow_221
ACCATTTTTGGCAATGGTAGAACTCACACTGGTGAGGTAATGGGATCCGGTGGTTCTAGACTTGCCAACTATGG
......TTTGGCAATGGTAGAACTCACACT............................................1917
......TTTGGCAATGGTAGAACTCACAC.............................................838
......TTTGGCAATGGTAGAACTCACA..............................................699
......TTTGGCAATGGTAGAACTCAC...............................................412
......TTTGGCAATGGTAGAACTCACACTGG..........................................157
......TTTGGCAATGGTAGAACTCACACTG...........................................140
......TTTGGCAATGGTAGAACTCA................................................63
......TTTGGCAATGGTAGAACTCACACTGGT.........................................21
......TTTGGCAATGGTAGAACTC.................................................12
......TTTGGCAATGGTAGAACT..................................................6
.......TTGGCAATGGTAGAACTCACACT............................................3
.......TTGGCAATGGTAGAACTCACA..............................................2
...........CAATGGTAGAACTCACAC.............................................2
.............ATGGTAGAACTCACACT............................................2
......TTTGGCAATGGTAGAAC...................................................1
```

.......TTGGCAATGGTAGAACTCACAC...1
..........GCAATGGTAGAACTCACAC...1
.............ATGGTAGAACTCACAC...1
........TGGCAATGGTAGAACTCACACT..1
...........CAATGGTAGAACTCACACT..1
.......TTGGCAATGGTAGAACTCACACTGG..1
......TTTGGCAATGGTAGAACTCACACTGGTGA.......................................1
....TGGTTCTAGACTTGCCAACT....1
.((((..((((((.(.((((((...((((((................)))))))))))..))))))))..)))) (-26.24)

4/94411430-94411503(-)_mir_cow_222
GTTCTGTGTATGGCACTGGTAGAATTCACTGTGAACAGTCTCGGTCAGTGAATTACCGAAGGGCCATAAACAGA
........TATGGCACTGGTAGAATTCACT..160
........TATGGCACTGGTAGAATTCACTG...84
.........ATGGCACTGGTAGAATTCACTG...50
.........ATGGCACTGGTAGAATTCACTGT..38
.........ATGGCACTGGTAGAATTCACT..33
........TATGGCACTGGTAGAATTCACTGT..28
........TATGGCACTGGTAGAATTCAC...11
.........ATGGCACTGGTAGAATTCAC...4
.............CACTGGTAGAATTCACT..2
....TGAATTACCGAAGGGCCATAAA....2
........TATGGCACTGGTAGAATTCA..1
......TGTATGGCACTGGTAGAATTCAC...1
..............ACTGGTAGAATTCACTG...1
..(((((.((((((..(((((..(((((((((((......)))..)))))))))))))....)))))).))))) (-28.30)

4/95071002-95071066(+)_mir_cow_223
GGGGTCAAGAGCAATAACGAAAAATGTTTGTCATAAACCGTTTTTCATTATTGCTCCTGACCTCC
....TCAAGAGCAATAACGAAAAATG.......................................   9492
....TCAAGAGCAATAACGAAAAATGT......................................   4432
....TTTTTCATTATTGCTCCTGACC...   3787
....TCAAGAGCAATAACGAAAAAT..   3274
....GTTTTTCATTATTGCTCCTGACC...   917
....TTTTTCATTATTGCTCCTGAC....   827
....GTTTTTCATTATTGCTCCTGAC....   367
....TTTTTCATTATTGCTCCTGA.....   123
....TTTCATTATTGCTCCTGACCT..   86

....TCAAGAGCAATAACGAAAAA...	78
....GTTTTTCATTATTGCTCCTGA.....	74
....TTTCATTATTGCTCCTGACC...	62
....TTTTCATTATTGCTCCTGACC...	46
....TCAAGAGCAATAACGAAAAATGTT.....................................	27
....TTTTTCATTATTGCTCCTGACCT..	27
....TTTTCATTATTGCTCCTGAC....	14
....TTTTTCATTATTGCTCCT.......	13
....GTTTTTCATTATTGCTCCTGACCT..	11
....TCAAGAGCAATAACGAAAA..	9
....TCAAGAGCAATAACGAAA...	6
......AAGAGCAATAACGAAAAATGT......................................	6
....TTTCATTATTGCTCCTGAC....	6
....TTTTCATTATTGCTCCTGACCT..	5
.....CAAGAGCAATAACGAAAAATGT......................................	4
....TTTTTCATTATTGCTCC........	4
.....CAAGAGCAATAACGAAAAATG.......................................	3
....TTTTTCATTATTGCTC.........	3
....GTTTTTCATTATTGCTCCT.......	3
....TTTTTCATTATTGCTCCTG......	3
......AAGAGCAATAACGAAAAAT..	2
......AAGAGCAATAACGAAAAATG.......................................	2
....GTTTTTCATTATTGCTCC........	2
....GTTTTTCATTATTGCTCCTG......	2
....TTTTCATTATTGCTCCTGA.....	2
....TCAAGAGCAATAACGAA..	1
.......AGAGCAATAACGAAAAATG.......................................	1
..........GCAATAACGAAAAATG.......................................	1
........GAGCAATAACGAAAAATGT......................................	1
..........GCAATAACGAAAAATGT......................................	1
...........CAATAACGAAAAATGT......................................	1
.......AGAGCAATAACGAAAAATGTTTGTCATAAACCG.........................	1
....GTTTTTCATTATTGCTC.........	1
.....................................CCGTTTTTCATTATTGCTCCT.......	1
.....................................CCGTTTTTCATTATTGCTCCTG......	1
......................................CGTTTTTCATTATTGCTCCTGA.....	1
......................................CGTTTTTCATTATTGCTCCTGAC....	1
......................................CGTTTTTCATTATTGCTCCTGACC...	1

```
.............................................CATTATTGCTCCTGACC...    1
(((((((.(((((((((.((((((((.((......)).)))))))).))))))))).))))))). (-31.00)

4/95112094-95112160(+)_novel_cow_32
AGGAACTTTTGCCCCTAGTAACGGACTGAAGGCATGAGGAAGTCCGTGCAGGAGGCAAAGCAAAGCA
...AACTTTTGCCCCTAGTAACGGACT........................................  1461
....ACTTTTGCCCCTAGTAACGGACT........................................  1189
...AACTTTTGCCCCTAGTAACGGAC.........................................  164
.....CTTTTGCCCCTAGTAACGGACT........................................  28
....ACTTTTGCCCCTAGTAACGGAC.........................................  23
...AACTTTTGCCCCTAGTAACGGA..........................................  17
......TTTTGCCCCTAGTAACGGACT........................................  9
...AACTTTTGCCCCTAGTAACGG...........................................  6
..GAACTTTTGCCCCTAGTAACGGACT........................................  3
....ACTTTTGCCCCTAGTAACGG...........................................  2
..........................................TCCGTGCAGGAGGCAAAGCAA....  2
....ACTTTTGCCCCTAGTAACGGACTGAA.....................................  1
......TTTTGCCCCTAGTAACGGACTGAAGGCATG...............................  1
.....................CGGACTGAAGGCATGAGGAA..........................  1
..................TAACGGACTGAAGGCATGAGGAAGTC.......................  1
....................ACGGACTGAAGGCATGAGGAAGTCC......................  1
.....................CGGACTGAAGGCATGAGGAAGTCC......................  1
........................ACTGAAGGCATGAGGAAGTCCGTGCAGGAGG............  1
.....................CGGACTGAAGGCATGAGGAAGTCCGTGCAGGAGGC...........  1
......((((((((((.(((.((((((.............)))))))))))).)))))))....... (-23.42)

4/95402314-95402385(-)_mir_cow_224
AGGATGACTGATTTCTTTTGGTGTTCAGAGTCAATATAATTTTCTAGCACCATCTGAAATCGGTTATAATGA
............................................TAGCACCATCTGAAATCGGTT.......  35741
............................................TAGCACCATCTGAAATCGGTTA......  22026
............................................TAGCACCATCTGAAATCGGT........  11627
...........................................CTAGCACCATCTGAAATCGGTT.......  7156
............................................TAGCACCATCTGAAATCGGTTAT.....  1824
...........................................CTAGCACCATCTGAAATCGGT........  1743
............................................TAGCACCATCTGAAATCGG.........  1470
...........................................CTAGCACCATCTGAAATCGGTTA......  1074
...........................................CTAGCACCATCTGAAATCGG.........  510
................................................ACCATCTGAAATCGGTT.......  124
```

```
......ACTGATTTCTTTTGGTGTTCAG............................................  101
.................................................CCATCTGAAATCGGTT.......  94
................................................ACCATCTGAAATCGGTTA......  90
......ACTGATTTCTTTTGGTGTTCAGA...........................................  88
.................................................CCATCTGAAATCGGTTA......  55
..................................................CATCTGAAATCGGTTA......  53
......ACTGATTTCTTTTGGTGTTCA.............................................  31
................................................ACCATCTGAAATCGGT........  26
...........................................CTAGCACCATCTGAAATCGGTTAT.....  24
............................................TAGCACCATCTGAAATCG..........  23
........TGATTTCTTTTGGTGTTCAGA...........................................  19
......ACTGATTTCTTTTGGTGTTCAGAG..........................................  15
......ACTGATTTCTTTTGGTGTTC..............................................  14
..........................................TCTAGCACCATCTGAAATCGGT........  14
...........................................CTAGCACCATCTGAAATC...........  13
...............................................CACCATCTGAAATCGGTT.......  13
...........................................CTAGCACCATCTGAAATCG..........  11
........TGATTTCTTTTGGTGTTCAGAGT.........................................  10
........TGATTTCTTTTGGTGTTCAG............................................  9
............................................TAGCACCATCTGAAATC...........  8
.............................................AGCACCATCTGAAATCGGTT.......  8
........TGATTTCTTTTGGTGTTCAGAG..........................................  6
...............................................CACCATCTGAAATCGGTTA......  6
..................................................CATCTGAAATCGGTTAT.....  6
............................................TAGCACCATCTGAAAT............  5
............................................TAGCACCATCTGAAATCGGTTATA....  5
......ACTGATTTCTTTTGGTGTTCAGAGT.........................................  4
.............................................AGCACCATCTGAAATCGGTTA......  4
.................................................CCATCTGAAATCGGTTAT.....  4
......ACTGATTTCTTTTGGTGTT...............................................  3
..........................................TCTAGCACCATCTGAAATCGG.........  3
.............................................AGCACCATCTGAAATCGGT........  3
...................................................ATCTGAAATCGGTTAT.....  3
......ACTGATTTCTTTTGGTGT................................................  2
........TGATTTCTTTTGGTGTTCA.............................................  2
...........................................CTAGCACCATCTGAAAT............  2
.............................................AGCACCATCTGAAATCGG.........  2
................................................ACCATCTGAAATCGGTTAT.....  2
```

.........GATTTCTTTTGGTGTTCA... 1
.......CTGATTTCTTTTGGTGTTCAGAG.. 1
....CTAGCACCATCTGAAA............. 1
....GCACCATCTGAAATCGG......... 1
....GCACCATCTGAAATCGGTT....... 1
....GCACCATCTGAAATCGGTTA...... 1
....CACCATCTGAAATCGGTTAT..... 1
...((((((((((((...(((((((.((((...........)))))))))))...))))))))))))..... (-26.00)

4/95402696-95402771(-)_mir_cow_225
CAGGAAGCTGGTTTCATATGGTGGTTTAGATTTAAATAGTGATTGTCTAGCACCATTTGAAATCAGTGTTCTTGGG
....TAGCACCATTTGAAATCAGTGTT...... 5658
....TAGCACCATTTGAAATCAGTGT....... 975
....CTAGCACCATTTGAAATCAGTG........ 437
....CTAGCACCATTTGAAATCAGT......... 308
....CTAGCACCATTTGAAATCAGTGT....... 260
....TAGCACCATTTGAAATCAGT......... 244
....TAGCACCATTTGAAATCAGTG........ 202
....CATTTGAAATCAGTGTT...... 48
....TAGCACCATTTGAAATCAG.......... 42
....CTAGCACCATTTGAAATCAG.......... 25
....CCATTTGAAATCAGTGTT...... 24
....TAGCACCATTTGAAATC............ 18
....CTAGCACCATTTGAAATCAGTGTT...... 13
......GCTGGTTTCATATGGTGGTTTAG... 11
..............................TTTAAATAGTGATTGTC............................. 9
....ATTTGAAATCAGTGTT...... 8
....CTAGCACCATTTGAAATC............ 7
....ACCATTTGAAATCAGTGTT...... 7
..............................TTTAAATAGTGATTGTCT............................ 6
....CTAGCACCATTTGAAATCA........... 6
....TAGCACCATTTGAAATCA........... 6
....ACCATTTGAAATCAGTG........ 5
....AGCACCATTTGAAATCAGTGTT...... 4
......GCTGGTTTCATATGGTGGTTTAGA.. 3
....TAGCACCATTTGAAAT............. 3
....CATTTGAAATCAGTGT....... 3
......GCTGGTTTCATATGGTGGTTT... 2

.......CTGGTTTCATATGGTGGTTTAGA.. 2
....CCATTTGAAATCAGTG........ 2
....ACCATTTGAAATCAGTGT....... 2
....TAGCACCATTTGAAATCAGTGTTC..... 2
......GCTGGTTTCATATGGTGGTT.. 1
.......CTGGTTTCATATGGTGGTTTAG... 1
.......CTGGTTTCATATGGTGGTTTAGAT... 1
....CTAGCACCATTTGAAAT............. 1
....TCTAGCACCATTTGAAATCAGT......... 1
....GCACCATTTGAAATCAGT......... 1
....TCTAGCACCATTTGAAATCAGTG........ 1
....TCTAGCACCATTTGAAATCAGTGT....... 1
....GCACCATTTGAAATCAGTGT....... 1
....GCACCATTTGAAATCAGTGTT...... 1
....TAGCACCATTTGAAATCAGTGTTCT.... 1
((((((((((((((((.((((((..((((((.............)))))))))))).)))))))))).)))))).. (-29.92)

5/103859009-103859085(-)_mir_cow_239
TGGGTCCATCTTCCAGCACAGTGTTGGATGGTCTAATGGTGAAGCTCCTAACACTGTCTGGTAAAGATGGCCCCCGG
....TAACACTGTCTGGTAAAGATG........ 4263
....TAACACTGTCTGGTAAAGA.......... 96
....TAACACTGTCTGGTAAAGATGG....... 81
....TAACACTGTCTGGTAAAGATGGC...... 56
....CTAACACTGTCTGGTAAAGATG........ 47
....TAACACTGTCTGGTAAAGAT......... 24
....TAACACTGTCTGGTAAAG........... 16
......CATCTTCCAGCACAGTGTTGGA... 2
......CATCTTCCAGCACAGTGTTGG.. 1
....CTAACACTGTCTGGTAAAG........... 1
....CTAACACTGTCTGGTAAAGA.......... 1
....ACACTGTCTGGTAAAGA.......... 1
....CTAACACTGTCTGGTAAAGAT......... 1
....AACACTGTCTGGTAAAGATG........ 1
....CTGTCTGGTAAAGATG........ 1
....CTGTCTGGTAAAGATGG....... 1
....TAACACTGTCTGGTAAAGATGGCCC.... 1
.(((.((((((((((.((((((((((((...((.......))...))).))))))))))))..))))))))))).... (-35.70)

```
5/113009151-113009222(+)_novel_cow_35
GGGCCGTGGGACAGAGTGGAAGGAGGATCCGTGCTCACCAGGCCCTCCATCCCCTCTTGCCCACCAGTCCTG
.............................................TCCATCCCCTCTTGCCCACCAGT....   11
.............................................TCCATCCCCTCTTGCCCACCAG.....   6
...CCGTGGGACAGAGTGGAAGGAG...............................................   5
.............................................TCCATCCCCTCTTGCCCACC.......   4
...............................................CATCCCCTCTTGCCCACCAGT....   3
...CCGTGGGACAGAGTGGAAGGA................................................   2
.............................................TCCATCCCCTCTTGCCCACCA......   2
....CGTGGGACAGAGTGGAAGGA................................................   1
...CCGTGGGACAGAGTGGAAGGAGG..............................................   1
....CGTGGGACAGAGTGGAAGGAGG..............................................   1
.............................................TCCATCCCCTCTTGCCCAC........   1
..............................................CCATCCCCTCTTGCCCACCAGT....   1
((((.(((((..((((.(((.(((((..(.((....))..)..))))).))).))))..)))))..))))..  (-34.50)

5/113410751-113410820(+)_mir_cow_240
TGCGGTGCATTGTAGTTGCATTGCATGTTCTGGCGGTACCCGTGCAATGTTTCCACAGTGCATCACAGAG
....GTGCATTGTAGTTGCATTGC..............................................    427
....GTGCATTGTAGTTGCATTG...............................................    295
....GTGCATTGTAGTTGCATTGCA.............................................    255
........ATTGTAGTTGCATTGCA.............................................    86
............................................CAATGTTTCCACAGTGCATC......    70
............................................CAATGTTTCCACAGTGCATCA.....    68
............................................CAATGTTTCCACAGTGCAT.......    45
.........TTGTAGTTGCATTGCA.............................................    30
....GTGCATTGTAGTTGCATT................................................    28
.......CATTGTAGTTGCATTGCA.............................................    15
.........................TGTTCTGGCGGTACCCGTG..........................    2
....GTGCATTGTAGTTGCA..................................................    1
....GTGCATTGTAGTTGCAT.................................................    1
........ATTGTAGTTGCATTGC..............................................    1
....GTGCATTGTAGTTGCATTGCATGTTCTGGC....................................    1
.........................TGTTCTGGCGGTACCCG............................    1
.........................TGTTCTGGCGGTACCCGTGCAATGTTTCCACAGTGCATCA.....    1
............................................CAATGTTTCCACAGTGCATCACAG..    1
((.((((((((((.(..((((((((((....((.....))))))))))))..).)))))))))).))... (-30.40)
```

```
5/117119380-117119462(+)_mir_Cow_Blast_46
CTTTGGGGTGAGGTAGTAGGTTGTATAGTTTGGGGCTCTGCCCTGCTATGGGATAACTATACAATCTACTGTCTTTCCTGAAG
........TGAGGTAGTAGGTTGTATAGTT.....................................................  600031
........TGAGGTAGTAGGTTGTATAGT......................................................  269183
........TGAGGTAGTAGGTTGTATAGTTT....................................................  31072
........TGAGGTAGTAGGTTGTATAG.......................................................  29911
........TGAGGTAGTAGGTTGTAT.........................................................  7314
........TGAGGTAGTAGGTTGTATA........................................................  4091
........TGAGGTAGTAGGTTGTA..........................................................  1031
........................................................CTATACAATCTACTGTCTTTCC.....  770
.......GTGAGGTAGTAGGTTGTATAGTT.....................................................  761
.......GTGAGGTAGTAGGTTGTATAGT......................................................  706
........................................................CTATACAATCTACTGTCTTTC......  382
........TGAGGTAGTAGGTTGT...........................................................  343
.............TAGTAGGTTGTATAGTT.....................................................  174
.........GAGGTAGTAGGTTGTATAGTT.....................................................  169
.........................................................TATACAATCTACTGTCTTTCCT....  169
...........GGTAGTAGGTTGTATAGTT.....................................................  155
..............AGTAGGTTGTATAGTT.....................................................  114
........................................................CTATACAATCTACTGTCTTTCCT....  97
...........GGTAGTAGGTTGTATAGT......................................................  76
.............TAGTAGGTTGTATAGT......................................................  76
.......GTGAGGTAGTAGGTTGTATAG.......................................................  56
.........GAGGTAGTAGGTTGTATAGT......................................................  53
............GTAGTAGGTTGTATAGTT.....................................................  53
........................................................CTATACAATCTACTGTCTTT.......  53
.........................................................TATACAATCTACTGTCTTTCC.....  40
..........AGGTAGTAGGTTGTATAGTT.....................................................  37
..........AGGTAGTAGGTTGTATAGT......................................................  23
............GTAGTAGGTTGTATAGT......................................................  17
........................................................CTATACAATCTACTGTCTT........  17
.......GTGAGGTAGTAGGTTGTAT.........................................................  15
..........................................................ATACAATCTACTGTCTTTCCT....  15
.......GTGAGGTAGTAGGTTGTATAGTTT....................................................  13
........................................................CTATACAATCTACTGTCT.........  10
...........GGTAGTAGGTTGTATAG.......................................................  9
...............GTAGGTTGTATAGTTT....................................................  9
.........GAGGTAGTAGGTTGTATAGTTT....................................................  8
```

.............TAGTAGGTTGTATAGTTT..   8
........TGAGGTAGTAGGTTGTATAGTTTG...   8
....TATACAATCTACTGTCTTTC......   8
.......GTGAGGTAGTAGGTTGTATA..   5
............GTAGTAGGTTGTATAG...   4
...........GGTAGTAGGTTGTATAGTTT..   4
............GTAGTAGGTTGTATAGTTT..   4
........TGAGGTAGTAGGTTGTATAGTTTGGG...   4
........TGAGGTAGTAGGTTGTATAGTTTGGGGCTCTGC..   4
........TGAGGTAGTAGGTTGTATAGTTTGGGGCTCTGCCCTGCT......................................   4
....ATACAATCTACTGTCTTTCCTG...   4
.........GAGGTAGTAGGTTGTATAG...   3
..........AGGTAGTAGGTTGTATAGTTT..   3
..............AGTAGGTTGTATAGTTT..   3
........TGAGGTAGTAGGTTGTATAGTTTGG..   3
....CTATACAATCTACTGT...........   3
....ACTATACAATCTACTGTCTTTC......   3
....ATACAATCTACTGTCTTTCC.....   3
....GGGGTGAGGTAGTAGGTTGTATAGTT...   2
........TGAGGTAGTAGGTTGTATAGTTTGGGG..   2
........TGAGGTAGTAGGTTGTATAGTTTGGGGCTCT..   2
........TGAGGTAGTAGGTTGTATAGTTTGGGGCTCTGCCCTGC.......................................   2
....GATAACTATACAATCTACTGT...........   2
....GATAACTATACAATCTACTGTC..........   2
....ATAACTATACAATCTACTGTCTTT.......   2
....ATAACTATACAATCTACTGTCTTTC......   2
....ATACAATCTACTGTCTTTCCTGA..   2
.......GTGAGGTAGTAGGTTGT...   1
.......GTGAGGTAGTAGGTTGTA..   1
.........GAGGTAGTAGGTTGTA..   1
.........GAGGTAGTAGGTTGTAT...   1
..........AGGTAGTAGGTTGTAT...   1
.........GAGGTAGTAGGTTGTATA..   1
..........AGGTAGTAGGTTGTATAG...   1
......GGTGAGGTAGTAGGTTGTATAGT..   1
......GGTGAGGTAGTAGGTTGTATAGTT...   1
.............TAGTAGGTTGTATAGTTTGGGG..   1
........TGAGGTAGTAGGTTGTATAGTTTGGGGC...   1

```
........TGAGGTAGTAGGTTGTATAGTTTGGGGCT..............................................   1
........TGAGGTAGTAGGTTGTATAGTTTGGGGCTC.............................................   1
........TGAGGTAGTAGGTTGTATAGTTTGGGGCTCTGCC.........................................   1
........TGAGGTAGTAGGTTGTATAGTTTGGGGCTCTGCCC........................................   1
........TGAGGTAGTAGGTTGTATAGTTTGGGGCTCTGCCCTG......................................   1
.............TAGTAGGTTGTATAGTTTGGGGCTCTGCCCTGCT....................................   1
........TGAGGTAGTAGGTTGTATAGTTTGGGGCTCTGCCCTGCTA...................................   1
..............................TGGGGCTCTGCCCTGCTATGGGA..............................   1
.............................................CTATGGGATAACTATACAATCT................   1
....................................................ATAACTATACAATCTACTGTCT.........   1
.........................................................TATACAATCTACTGTCTTT.......   1
......................................................AACTATACAATCTACTGTCTTTC......   1
..........................................................ATACAATCTACTGTCTTTC......   1
.............................................................CAATCTACTGTCTTTCC.....   1
.........................................................TATACAATCTACTGTCTTTCCTG...   1
...........................................................TACAATCTACTGTCTTTCCTG...   1
...........................................................TACAATCTACTGTCTTTCCTGA..   1
(((..(((.(((..((((((((((((((((((((((...)))))).........)))))))))))))))..))))))..)))) (-38.60)

5/117119642-117119716(+)_mir_cow_241
GAGGGTTGAGGGCAGGACCGTATGAGGTGTCCAAGGAAGGCTCCACTCAGCCGGCCCTTCCCTCCCCTCACGCCA
......TGAGGGCAGGACCGTATGAGGT...............................................     39
......TGAGGGCAGGACCGTATGAGGTGT.............................................     22
......TGAGGGCAGGACCGTATGAGGTG..............................................     15
......TGAGGGCAGGACCGTATGAGG................................................     7
......TGAGGGCAGGACCGTATGAG.................................................     2
......TGAGGGCAGGACCGTAT....................................................     1
......TGAGGGCAGGACCGTATGA..................................................     1
....GTTGAGGGCAGGACCGTATGAGG................................................     1
.....TTGAGGGCAGGACCGTATGAGGT...............................................     1
.............................................CTCAGCCGGCCCTTCCCTCCC.........     1
.............................................CTCAGCCGGCCCTTCCCTCCCC........     1
..............................................TCAGCCGGCCCTTCCCTCCCCTCACGC..     1
(((((..(((((.(((.(((..((((..(((.......)))....))))..))).))).))))))))))...... (-34.50)

5/117120181-117120269(+)_mir_Cow_Blast_45
CCGGCGGGGTGAGGTAGTAGGTTGTGTGGTTTCAGGGTAGTGATGTTGCCCCCTCGGAAGATAACTATACAACCTACTGCCTTCCTCGA
.........TGAGGTAGTAGGTTGTGTGGTT..........................................................  128195
```

```
.........TGAGGTAGTAGGTTGTGTGGT...........................................................  45904
.........TGAGGTAGTAGGTTGTGTGGTTT.........................................................  40599
.........TGAGGTAGTAGGTTGTGTGG............................................................  19365
................................................................CTATACAACCTACTGCCTTCCT...  5832
.........TGAGGTAGTAGGTTGTGT..............................................................  1844
.........TGAGGTAGTAGGTTGTGTG.............................................................  977
................................................................CTATACAACCTACTGCCTTCC....  972
.........TGAGGTAGTAGGTTGTG...............................................................  740
.........TGAGGTAGTAGGTTGT................................................................  343
.........TGAGGTAGTAGGTTGTGTGGTTTC........................................................  58
.................................................................TATACAACCTACTGCCTTCCT...  49
..........GAGGTAGTAGGTTGTGTGGTT..........................................................  43
................................................................CTATACAACCTACTGCCTTC.....  43
..............TAGTAGGTTGTGTGGTT..........................................................  31
............GGTAGTAGGTTGTGTGGTT..........................................................  20
...............AGTAGGTTGTGTGGTT..........................................................  19
...............................TCAGGGTAGTGATGTTGCCCCCTCGGA...............................  16
..............TAGTAGGTTGTGTGGTTT.........................................................  15
.............GTAGTAGGTTGTGTGGTT..........................................................  14
...............................TCAGGGTAGTGATGTTGCCCCCT...................................  13
..........GAGGTAGTAGGTTGTGTGGTTT.........................................................  11
.................................................................TATACAACCTACTGCCTTCC....  10
..........GAGGTAGTAGGTTGTGTGGT...........................................................  9
............GGTAGTAGGTTGTGTGGTTT.........................................................  8
...............................TCAGGGTAGTGATGTTGCCCCCTCGGAAGAT...........................  8
...........AGGTAGTAGGTTGTGTGGTT..........................................................  7
................GTAGGTTGTGTGGTTT.........................................................  7
...............................TCAGGGTAGTGATGTTGCC.......................................  6
................................................................CTATACAACCTACTGCCTT......  6
..................................................................ATACAACCTACTGCCTTCCT...  6
..........GAGGTAGTAGGTTGTGTGG............................................................  5
...............................TCAGGGTAGTGATGTTGCCCCCTCGGAAGA............................  5
...............................TCAGGGTAGTGATGTTGCCCCCTCGGAAGATAA.........................  5
.....................................................TCGGAAGATAACTATACAACCT..............  5
...........AGGTAGTAGGTTGTGTGGTTT.........................................................  4
..............................................................AACTATACAACCTACTGCCTTCC....  4
...............AGTAGGTTGTGTGGTTT.........................................................  3
..................................................CCCTCGGAAGATAACTATACAACCT..............  3
```

....CTCGGAAGATAACTATACAACCT.............. 3
....AGATAACTATACAACCTACTGCCT....... 3
....ATACAACCTACTGCCTTCC.... 3
....AACTATACAACCTACTGCCTTCCT... 3
.............GTAGTAGGTTGTGTGGT... 2
..............TAGTAGGTTGTGTGGT... 2
.....GGGGTGAGGTAGTAGGTTGTGTGGTTT... 2
.........TGAGGTAGTAGGTTGTGTGGTTTCAGGGTAGT.. 2
...............................TCAGGGTAGTGATGTTGCCCCCTCGG................................ 2
...............................TCAGGGTAGTGATGTTGCCCCCTCGGAAGATA.......................... 2
....CCCCTCGGAAGATAACTATACA.................. 2
....CGGAAGATAACTATACAACC............... 2
....TCGGAAGATAACTATACAACCTA............. 2
....CTATACAACCTACTGCC........ 2
....ATAACTATACAACCTACTGCCT....... 2
....CTATACAACCTACTGCCT....... 2
....CTCGGAAGATAACTATACAACCTACTGCCTTCC.... 2
....ATAACTATACAACCTACTGCCTTCC.... 2
........GTGAGGTAGTAGGTTGT.. 1
..........GAGGTAGTAGGTTGTGT.. 1
.......GGTGAGGTAGTAGGTTGTGTGG.. 1
.....GGGGTGAGGTAGTAGGTTGTGTGGT... 1
.....GGGGTGAGGTAGTAGGTTGTGTGGTT.. 1
.......GGTGAGGTAGTAGGTTGTGTGGTT.. 1
........GTGAGGTAGTAGGTTGTGTGGTT.. 1
....CGGGGTGAGGTAGTAGGTTGTGTGGTTT... 1
........GTGAGGTAGTAGGTTGTGTGGTTT... 1
.............GTAGTAGGTTGTGTGGTTT... 1
.........TGAGGTAGTAGGTTGTGTGGTTTCA... 1
..............TAGTAGGTTGTGTGGTTTCAG.. 1
.........TGAGGTAGTAGGTTGTGTGGTTTCAGGGT... 1
.........TGAGGTAGTAGGTTGTGTGGTTTCAGGGTA.. 1
.........TGAGGTAGTAGGTTGTGTGGTTTCAGGGTAGTGA.. 1
...............................TCAGGGTAGTGATGTTGC.. 1
...............................TCAGGGTAGTGATGTTGCCC...................................... 1
...............................TCAGGGTAGTGATGTTGCCCCCTC.................................. 1
...............................TCAGGGTAGTGATGTTGCCCCCTCG................................. 1
...............................TCAGGGTAGTGATGTTGCCCCCTCGGAAG............................. 1

....GATGTTGCCCCCTCGGAAGAT........................... 1
....GTTGCCCCCTCGGAAGAT........................... 1
....TTGCCCCCTCGGAAGATAACTA...................... 1
....CCCCTCGGAAGATAACTA...................... 1
...............................TCAGGGTAGTGATGTTGCCCCCTCGGAAGATAACTATA.................... 1
....CCCCCTCGGAAGATAACTATACA.................. 1
....CCTCGGAAGATAACTATACAAC................ 1
....CCCTCGGAAGATAACTATACAACC............... 1
....CCTCGGAAGATAACTATACAACC............... 1
....TCGGAAGATAACTATACAACCTACT........... 1
....GCCCCCTCGGAAGATAACTATACAACCTACTG.......... 1
....CCCCTCGGAAGATAACTATACAACCTACTGC......... 1
....AGATAACTATACAACCTACTGCCTT...... 1
....ATAACTATACAACCTACTGCCTTC..... 1
....TAACTATACAACCTACTGCCTTC..... 1
....ACTATACAACCTACTGCCTTC..... 1
....CCTCGGAAGATAACTATACAACCTACTGCCTTCC.... 1
....ACTATACAACCTACTGCCTTCC.... 1
....TACAACCTACTGCCTTCC.... 1
....CTCGGAAGATAACTATACAACCTACTGCCTTCCT... 1
....ACTATACAACCTACTGCCTTCCT... 1
....TACAACCTACTGCCTTCCT... 1
....CAACCTACTGCCTTCCT... 1
....CTATACAACCTACTGCCTTCCTC.. 1
....(((((.(((((((((((((((((((((...((((((.....))))))..((....)).)))))))))))))))))))))))))). (-49.20)

5/25109073-25109141(+)_mir_cow_229
GGGTTTGTTCTAGGTATGGTCCCAGGGATCCCAGATCAAACCAGGCCCCTGGGCCTATCCTAGAACCAA
....GCCCCTGGGCCTATCCTAGA.....   1609
....GCCCCTGGGCCTATCCTAGAA....   1526
....GCCCCTGGGCCTATCCTAGAAC...   600
........TCTAGGTATGGTCCCAGGGATC.......................................   179
........TCTAGGTATGGTCCCAGGGAT..   152
........TCTAGGTATGGTCCCAGGGATCC......................................   113
.........CTAGGTATGGTCCCAGGGATC.......................................   87
.........CTAGGTATGGTCCCAGGGAT..   68
.........CTAGGTATGGTCCCAGGGATCC......................................   56
....CCCCTGGGCCTATCCTAGAAC...   55

```
.............................................CCCCTGGGCCTATCCTAGA.....      47
.............................................CCCCTGGGCCTATCCTAGAACC..      44
.......TTCTAGGTATGGTCCCAGGGAT........................................      34
.............................................CCCCTGGGCCTATCCTAGAA....      26
.......TTCTAGGTATGGTCCCAGGGATC.......................................      23
............................................GCCCCTGGGCCTATCCTAG......      17
........TCTAGGTATGGTCCCAGGGA.........................................      15
............................................GCCCCTGGGCCTATCCTAGAACC..      15
........TCTAGGTATGGTCCCAGGGATCCC.....................................      8
.........CTAGGTATGGTCCCAGGGATCCC.....................................      8
............................................GCCCCTGGGCCTATCCT........      7
............................................GCCCCTGGGCCTATCCTA.......      7
.......TTCTAGGTATGGTCCCAGGGATCC......................................      5
.............................................CCCCTGGGCCTATCCTAG......      3
.........CTAGGTATGGTCCCAGGGA.........................................      2
..........TAGGTATGGTCCCAGGGAT........................................      2
..........TAGGTATGGTCCCAGGGATC.......................................      2
............................................GCCCCTGGGCCTATCC.........      2
....TTGTTCTAGGTATGGTCCCAG............................................      1
....TTGTTCTAGGTATGGTCCCAGG...........................................      1
....TTGTTCTAGGTATGGTCCCAGGG..........................................      1
.......TTCTAGGTATGGTCCCAGGG..........................................      1
....TTGTTCTAGGTATGGTCCCAGGGA.........................................      1
.......TTCTAGGTATGGTCCCAGGGA.........................................      1
..........TAGGTATGGTCCCAGGGATCC......................................      1
...........AGGTATGGTCCCAGGGATCC......................................      1
............GGTATGGTCCCAGGGATCC......................................      1
.......TTCTAGGTATGGTCCCAGGGATCCC.....................................      1
.........CTAGGTATGGTCCCAGGGATCCCAGA..................................      1
.........CTAGGTATGGTCCCAGGGATCCCAGATC................................      1
.............................................CCCCTGGGCCTATCCT........      1
...........................................GGCCCCTGGGCCTATCCTAGA.....      1
..............................................CCCTGGGCCTATCCTAGAACC..      1
......((((((((.((((.(((((((..(.............)..))))))).))))))))))))... (-30.72)

5/25849770-25849846(-)_mir_cow_230
TTGAGGTGAAGTTCTGTTATACACTCAGGCTGTGGCTCTCTGAAAGTCAGTGCATCACAGAACTTTGTCTCGAAAGC
..............................................TCAGTGCATCACAGAACTTTGT.........   9905
```

```
.......GAAGTTCTGTTATACACTCAGGCT.............................................. 1514
..............................................TCAGTGCATCACAGAACTTTG.......... 554
.......GAAGTTCTGTTATACACTCAGGC............................................... 444
..............................................TCAGTGCATCACAGAACTTTGTC........ 385
........AAGTTCTGTTATACACTCAGGCT.............................................. 151
..............................................TCAGTGCATCACAGAACTTT........... 144
..............................................TCAGTGCATCACAGAACTT............ 84
.......GAAGTTCTGTTATACACTCAGG................................................ 61
..............................................TCAGTGCATCACAGAACTTTGTCT....... 49
..............................................TCAGTGCATCACAGAACT............. 28
......TGAAGTTCTGTTATACACTCAGGCT.............................................. 18
........AAGTTCTGTTATACACTCAGGC............................................... 14
.......GAAGTTCTGTTATACACTCAG................................................. 13
.......GAAGTTCTGTTATACACTCA.................................................. 7
......TGAAGTTCTGTTATACACTCAGGC............................................... 7
...............................................CAGTGCATCACAGAACTTTGT......... 5
................................................AGTGCATCACAGAACTTTGT......... 5
..............................................TCAGTGCATCACAGAAC.............. 3
......TGAAGTTCTGTTATACACTCAGG................................................ 2
.......GAAGTTCTGTTATACACT.................................................... 1
.......GAAGTTCTGTTATACACTC................................................... 1
.............CTGTTATACACTCAGGC............................................... 1
..........GTTCTGTTATACACTCAGGCT.............................................. 1
............TCTGTTATACACTCAGGCT.............................................. 1
..................................................TGCATCACAGAACTTTGTCTCGA.... 1
((((((..(((((((((.((.((((..((((....(.....)..)))))))).)).)))))))))..))))))....  (-28.80)

5/26157887-26157960(-)_mir_cow_231
CGGGAGGGGGGTCCCCGGTGCTCGGATCTCGAGGGTGCTTATTGTTCGGTCCGAGCCTGGGTCTCCCTCTCCCC
.................................................TCCGAGCCTGGGTCTCCCTCT....42
.................................................TCCGAGCCTGGGTCTCCCTC.....3
.............................................TCGGTCCGAGCCTGGGTCTCCCT......2
......GGGGGTCCCCGGTGCTCGGATCTC............................................1
.................................................TCCGAGCCTGGGTCTCCCT......1
.((((((((((..(((((.((((((((..((((.(.......).)))))))))))))))))..)))))))))). (-48.00)

5/26199807-26199876(-)_mir_cow_232
GTGGCTTAGGTAGTTTCATGTTGTTGGGATTGAGTTTTGAACTCGGCAACAAGAAACTGCCTGAGTTACA
```

```
......TAGGTAGTTTCATGTTGTTGGGA.........................................    288
......TAGGTAGTTTCATGTTGTTGGG..........................................    208
......TAGGTAGTTTCATGTTGTTGG...........................................    81
......TAGGTAGTTTCATGTTGTTG............................................    23
......TAGGTAGTTTCATGTTGTTGGGAT........................................    17
..........................................TCGGCAACAAGAAACTGCCTGAGT....    7
..........................................TCGGCAACAAGAAACTGCCTGA......    5
......TAGGTAGTTTCATGTTGT..............................................    4
..........................................TCGGCAACAAGAAACTGCCTGAG.....    2
......TAGGTAGTTTCATGTT................................................    1
......TAGGTAGTTTCATGTTGTT.............................................    1
.......AGGTAGTTTCATGTTGTTGGGA.........................................    1
...........................................CGGCAACAAGAAACTGCCTGA......    1
(((((((((((((((((.((((((((((.(..(...)..).)))))))))).))))))))))))))))). (-39.50)

5/51209077-51209166(-)_mir_cow_233
CCCTGGCTGAGGTAGTAGTTTGTGCTGTTGGTCGGGTTGTGACATTGCCCGCTGTGGAGATAACTGCGCAAGCTACTGCCTTGCTAGTGC
.......TGAGGTAGTAGTTTGTGCTGTT............................................................. 167661
.......TGAGGTAGTAGTTTGTGCTGT.............................................................. 28558
.......TGAGGTAGTAGTTTGTGCTG............................................................... 3796
.......TGAGGTAGTAGTTTGTGCT................................................................ 1407
.......TGAGGTAGTAGTTTGTGCTGTTG............................................................ 1074
.......TGAGGTAGTAGTTTGTGC................................................................. 994
...............................................................CTGCGCAAGCTACTGCCTTGCT..... 651
...............................................................CTGCGCAAGCTACTGCCTTG....... 432
...............................................................CTGCGCAAGCTACTGCCTTGC...... 364
......CTGAGGTAGTAGTTTGTGCTGTT............................................................. 99
.......TGAGGTAGTAGTTTGT................................................................... 43
.............AGTAGTTTGTGCTGTT............................................................. 39
........GAGGTAGTAGTTTGTGCTGTT............................................................. 37
..........GGTAGTAGTTTGTGCTGTT............................................................. 34
............TAGTAGTTTGTGCTGTT............................................................. 32
..............GTAGTTTGTGCTGTT............................................................. 24
......CTGAGGTAGTAGTTTGTGCTGT.............................................................. 22
...............................................................CTGCGCAAGCTACTGCCT......... 22
.......TGAGGTAGTAGTTTGTG.................................................................. 15
...............................................................CTGCGCAAGCTACTGCCTT........ 11
.........AGGTAGTAGTTTGTGCTGTT............................................................. 9
```

```
............TAGTAGTTTGTGCTGT..............................................................  8
...........GTAGTAGTTTGTGCTGT..............................................................  7
.......TGAGGTAGTAGTTTGTGCTGTTGGTCGGGTTG...................................................  6
........GAGGTAGTAGTTTGTGCTGT..............................................................  5
.......TGAGGTAGTAGTTTGTGCTGTTGG...........................................................  3
...............................................................CTGCGCAAGCTACTGCCTTGCTA....  3
......CTGAGGTAGTAGTTTGTGC.................................................................  2
......CTGAGGTAGTAGTTTGTGCTG...............................................................  2
.........AGGTAGTAGTTTGTGCTGT..............................................................  2
..........GGTAGTAGTTTGTGCTGT..............................................................  2
.............AGTAGTTTGTGCTGTTG............................................................  2
................................................CCGCTGTGGAGATAACTGCGCAAGCTACTGCCTTGC......  2
.................................................CGCTGTGGAGATAACTGCGCAAGCTACTGCCTTGCT.....  2
......CTGAGGTAGTAGTTTGT...................................................................  1
.........AGGTAGTAGTTTGTGC.................................................................  1
......CTGAGGTAGTAGTTTGTGCT................................................................  1
........GAGGTAGTAGTTTGTGCTG...............................................................  1
...........GTAGTAGTTTGTGCTG...............................................................  1
...........GTAGTAGTTTGTGCTGTTG............................................................  1
..............GTAGTTTGTGCTGTTG............................................................  1
.......TGAGGTAGTAGTTTGTGCTGTTGGTC.........................................................  1
.............................GGTCGGGTTGTGACATTGCCCGCTGTGGAGATAA...........................  1
...............................................................CTGCGCAAGCTACTGCC..........  1
................................................................TGCGCAAGCTACTGCCTTG.......  1
.................................................CGCTGTGGAGATAACTGCGCAAGCTACTGCCTTGC......  1
................................................................TGCGCAAGCTACTGCCTTGCT.....  1
..(((((.(((((((((((((((((.(((((.(((((.........)))))))........))).))))))))))))))))))))))...  (-43.00)
```

5/55977927-55977998(+)_mir_cow_234

```
GTGGCTGGATTCAAGTAATCCAGGATAGGCTGTTTCCATCTGTGAGGCCTATTCTTGATTACTTGTTTCTGG
.........TTCAAGTAATCCAGGATAGGCT.........................................  3223130
.........TTCAAGTAATCCAGGATAGGC..........................................  183122
.........TTCAAGTAATCCAGGATAGG...........................................  23183
.........TTCAAGTAATCCAGGATAG............................................  15670
..........TCAAGTAATCCAGGATAGGCT.........................................  5954
.........TTCAAGTAATCCAGGATAGGCTG........................................  4858
.........TTCAAGTAATCCAGGATA.............................................  2122
.........TTCAAGTAATCCAGGAT..............................................  801
```

```
.........TTCAAGTAATCCAGGATAGGCTGT.......................................  518
...........CAAGTAATCCAGGATAGGCT.........................................  421
..........TCAAGTAATCCAGGATAGGC..........................................  335
...............................................CCTATTCTTGATTACTTGTTTC...  230
............AAGTAATCCAGGATAGGCT.........................................  194
..........TCAAGTAATCCAGGATAGGCTG........................................  188
.............AGTAATCCAGGATAGGCT.........................................  178
.........TTCAAGTAATCCAGGA...............................................  70
..........TCAAGTAATCCAGGATAGG...........................................  45
...............................................CCTATTCTTGATTACTTGTTT....  44
...............TAATCCAGGATAGGCT.........................................  37
..........TCAAGTAATCCAGGATAGGCTGT.......................................  37
........ATTCAAGTAATCCAGGATAGGCT.........................................  29
....CTGGATTCAAGTAATCCAGGATAGGCT.........................................  26
.............AGTAATCCAGGATAGGC..........................................  23
.........TTCAAGTAATCCAGGATAGGCTGTT......................................  23
..........TCAAGTAATCCAGGATAG............................................  22
...........CAAGTAATCCAGGATAGGC..........................................  19
.........TTCAAGTAATCCAGGATAGGCTGTTT.....................................  17
............AAGTAATCCAGGATAGGC..........................................  15
..............GTAATCCAGGATAGGCT.........................................  9
...............................................CCTATTCTTGATTACTTGTT.....  6
........ATTCAAGTAATCCAGGATAGGC..........................................  5
...............................................CCTATTCTTGATTACTTGTTTCT..  5
...........CAAGTAATCCAGGATA.............................................  3
...........CAAGTAATCCAGGATAG............................................  3
.......GATTCAAGTAATCCAGGATAGGCT.........................................  3
..........TCAAGTAATCCAGGATAGGCTGTT......................................  3
..........TCAAGTAATCCAGGATA.............................................  2
............AAGTAATCCAGGATAG............................................  2
........ATTCAAGTAATCCAGGATAGG...........................................  2
.....TGGATTCAAGTAATCCAGGATAGGCT.........................................  2
................................................CTATTCTTGATTACTTGTTTC...  2
..........TCAAGTAATCCAGGAT..............................................  1
........ATTCAAGTAATCCAGGATAG............................................  1
............AAGTAATCCAGGATAGG...........................................  1
.............AGTAATCCAGGATAGG...........................................  1
....CTGGATTCAAGTAATCCAGGATAGGC..........................................  1
```

```
..............GTAATCCAGGATAGGC..........................................  1
.................ATCCAGGATAGGCTGT.......................................  1
..........TCAAGTAATCCAGGATAGGCTGTTT.....................................  1
.........TTCAAGTAATCCAGGATAGGCTGTTTC....................................  1
.........TTCAAGTAATCCAGGATAGGCTGTTTCC...................................  1
.........TTCAAGTAATCCAGGATAGGCTGTTTCCATC................................  1
.........TTCAAGTAATCCAGGATAGGCTGTTTCCATCTGTGAGG.........................  1
.......................................CTGTGAGGCCTATTCTTGATTACTTGTT.....  1
..............................................GCCTATTCTTGATTACTTGTT.....  1
.......................................CTGTGAGGCCTATTCTTGATTACTTGTTT....  1
..............................................GCCTATTCTTGATTACTTGTTT....  1
....(..((..(((((((((.((((((((((..............)))))))))).)))))))))..))..) (-30.84)

5/56655695-56655773(-)_mir_cow_235
CTCACCTTCAGGTCATATAAGTGTGGAGTTGGCTAGACTACTGGTAATGACACCCCCACTTGCATGACCCTGAGAGTTG
..................................................CACCCCCACTTGCATGACCCTGA...... 37
........CAGGTCATATAAGTGTGGAGTT................................................. 33
........CAGGTCATATAAGTGTGGAGTTG................................................ 16
........CAGGTCATATAAGTGTGGAGT.................................................. 8
....................................................CCCCCACTTGCATGACCCTGA...... 8
....................................................CCCCCACTTGCATGACCCTGAGA.... 7
..................................................CACCCCCACTTGCATGACCCTG....... 6
........CAGGTCATATAAGTGTGGAGTTGGC.............................................. 3
.........AGGTCATATAAGTGTGGAGTTGGC.............................................. 3
..................................................CACCCCCACTTGCATGACCCT........ 3
...................................................ACCCCCACTTGCATGACCCTGA...... 3
....................................................CCCCCACTTGCATGACCCTGAG..... 3
.......TCAGGTCATATAAGTGTGGAGT.................................................. 2
........CAGGTCATATAAGTGTGGAGTTGG............................................... 2
......TTCAGGTCATATAAGTGTGGAGT.................................................. 1
.......TCAGGTCATATAAGTGTGGAGTT................................................. 1
.........AGGTCATATAAGTGTGGAGTTG................................................ 1
..................................................CACCCCCACTTGCATGAC........... 1
...((.((((((((((.((((((.((.(((.(((((....)))))...))).))..)))))).)))).)))))).)).. (-26.50)

5/62043143-62043205(+)_mir_cow_236
GTGGACTCTAGCTGCCAAAGGCGCTTCTCCTTCTGAACCGAGCGCTTTGCTCAGCCAGTGTAG
....ACTCTAGCTGCCAAAGGCGCT......................................      65
```

```
....ACTCTAGCTGCCAAAGGCGC.......................................      6
....ACTCTAGCTGCCAAAGGCGCTT.....................................      6
........................................AGCGCTTTGCTCAGCCAGTGT..      1
....((.((.((((...(((((((((.((.....))...)))))))))...)))).)).)).. (-19.80)

5/62117727-62117794(+)_mir_cow_237
GCTTTATGGCTTTTTATTCCTATGTGATAGTAATAAAGTCTCATGTAGGGATGGAAGCCATGAAATAC
....TATGGCTTTTTATTCCTATGTGA......................................... 2731
....TATGGCTTTTTATTCCTATGTG.......................................... 486
....TATGGCTTTTTATTCCTATGT........................................... 203
...........................................TGTAGGGATGGAAGCCATGAAA... 28
....TATGGCTTTTTATTCCTATGTGAT........................................ 13
....TATGGCTTTTTATTCCTATG............................................ 11
...........................................TGTAGGGATGGAAGCCATGAA.... 11
...........................................TGTAGGGATGGAAGCCATGA..... 8
..........................................ATGTAGGGATGGAAGCCATGAA.... 6
....TATGGCTTTTTATTCC................................................ 2
..........................................ATGTAGGGATGGAAGCCATGAAA... 2
...........................................TGTAGGGATGGAAGCCATGAAAT.. 2
.....ATGGCTTTTTATTCCTATGTG.......................................... 1
...........................................TGTAGGGATGGAAGCCATG...... 1
..........................................ATGTAGGGATGGAAGCCATGA..... 1
..((((((((((((.((((((((((((.(.(.....).).))))))))))))))))))))))))).... (-26.70)

5/68192485-68192553(+)_mir_cow_238
TAGCCAAAAAGATCATTTGGGTTTTTCTGATGATGTTATGGAAAACTCGAATGAACTCTTTGGCTGACT
..........................................AAACTCGAATGAACTCTTTGGC.....      89
..........................................AAACTCGAATGAACTCTTTGG......      19
....CAAAAAGATCATTTGGGTTTTT...........................................      8
.........................................AAAACTCGAATGAACTCTTTGG......      2
.........................................AAAACTCGAATGAACTCTTTGGC.....      2
.........................................AAAACTCGAATGAACTCTTTGGCT....      2
..........................................AAACTCGAATGAACTCTTTGGCT....      2
.....AAAAAGATCATTTGGGTTTTT...........................................      1
...............................................CGAATGAACTCTTTGGC.....      1
.............................................CTCGAATGAACTCTTTGGCTGA..      1
((((((((.((.(((((((((((((((((((...)))).)))))))))))))))).)).))))))))... (-29.60)
```

```
5/96344472-96344537(+)_novel_cow_34
GTGACAAGTTCGTCTGGGTTTTTCCTTAACGTCTTATGGAAAAACCTGAATGAACTTGTTGACCAA
.......................................AAAAACCTGAATGAACTTGTTGA....   55
.......................................AAAAACCTGAATGAACTTGTTG.....   29
...ACAAGTTCGTCTGGGTTTTTCC.........................................   21
..GACAAGTTCGTCTGGGTTTTTCC.........................................   9
.........................................AAACCTGAATGAACTTGTTGA....   9
........................................AAAACCTGAATGAACTTGTTGA....   5
..GACAAGTTCGTCTGGGTTTTT...........................................   3
..GACAAGTTCGTCTGGGTTTTTC..........................................   3
...ACAAGTTCGTCTGGGTTTTTC..........................................   2
...................................ATGGAAAAACCTGAATGAACT..........   2
..................................TATGGAAAAACCTGAA................   1
.......................................AAAAACCTGAATGAACT..........   1
.......................................AAAAACCTGAATGAACTTGT.......   1
.......................................AAAAACCTGAATGAACTTGTT......   1
........................................AAAACCTGAATGAACTTGTTG.....   1
.........................................AAACCTGAATGAACTTGTTG.....   1
..(((((((((((..((((((((((.(((....))).))))))))))..)))))))))))...... (-29.10)

6/103797774-103797839(+)_mir_cow_246
GGCCTAAAAGTTTGGTTGGGTTTTTCTGTAACATCTGATGGAAAAATCTGAACAAACTTTCCGGCC
....TAAAAGTTTGGTTGGGTTTTTC........................................   203
....TAAAAGTTTGGTTGGGTTTTT.........................................   197
....TAAAAGTTTGGTTGGGTTTT..........................................   36
....TAAAAGTTTGGTTGGGTTTTTCT.......................................   16
.....AAAAGTTTGGTTGGGTTTTTCT.......................................   14
..........................................AAAATCTGAACAAACTTTCCGG..   12
....TAAAAGTTTGGTTGGGTTT...........................................   8
.....AAAAGTTTGGTTGGGTTTTTC........................................   2
....TAAAAGTTTGGTTGGG..............................................   1
....TAAAAGTTTGGTTGGGTT............................................   1
.....AAAAGTTTGGTTGGGTTTT..........................................   1
....TAAAAGTTTGGTTGGGTTTTTCTGT.....................................   1
..........................................AAAATCTGAACAAACT........   1
..........................................AAAATCTGAACAAACTTTCC....   1
((((..((((((((.(..(((((((((((........)))))))))))..).))))))))..)))) (-27.90)
```

```
6/108859150-108859210(-)_novel_cow_36
GGGGACCTGTGCGTGTACACTCATGCCCACGTGCATGAGTGTGCATCCACGGGGACCTGTG
...GACCTGTGCGTGTACACTCATG....................................  12
..GGACCTGTGCGTGTACACTCA......................................  1
...................................TGAGTGTGCATCCACGGGGACC....  1
(((..((((((.((((((((((((((......)))))))))))))).))))))..)))...  (-40.30)

6/115567391-115567465(-)_mir_cow_247
CTGCACCTGCGGATACAACCAAACACGGTTGGAGAATATTTCACAATCTGTGGTTGATTGGATCCGTGGGTGTAG
......CTGCGGATACAACCAAACACGG...............................................     15
.................................................GTGGTTGATTGGATCCGTGGGT....     10
......CTGCGGATACAACCAAACACGGT..............................................     4
......CTGCGGATACAACCAAACACG................................................     1
.......TGCGGATACAACCAAACACGG...............................................     1
......CTGCGGATACAACCAAACACGGTT.............................................     1
.......TGCGGATACAACCAAACACGGTT.............................................     1
................................................TGTGGTTGATTGGATCCGTGG......     1
.................................................GTGGTTGATTGGATCCGTGGG.....     1
................................................TGTGGTTGATTGGATCCGTGGGT....     1
(((((((..(((((.(((.(((.((((((((((((...)))).))).))))).))).))).)))))..))))))) (-36.30)

6/119145910-119145977(-)_mir_cow_248
CGGGCGCTCAATAAATGTTTGTTGAATTGAGATGCGCTAAATTCAACGGGTATTTATTGAGCACCCAC
.........................................TTCAACGGGTATTTATTGAGC...... 1550
.........................................TTCAACGGGTATTTATTGAGCA..... 780
.........................................TTCAACGGGTATTTATTGAG....... 22
.........................................TTCAACGGGTATTTATTGAGCAC.... 9
..........................................TCAACGGGTATTTATTGAGC...... 4
........................................ATTCAACGGGTATTTATTGAGC...... 3
.........................................TTCAACGGGTATTTATTGA........ 2
......CTCAATAAATGTTTGTTG............................................ 1
......CTCAATAAATGTTTGTTGAAT......................................... 1
...........................................CAACGGGTATTTATTGAGC...... 1
...........................................CAACGGGTATTTATTGAGCA..... 1
.(((.((((((((((((..(((((((((.((.....)).)))))))))..))))))))))))).))).. (-35.30)

6/14162442-14162508(+)_mir_cow_242
TTAACTTTAACATGGTAGTACTTTCTGTGACTTTTGAAATAGTAAGTGCTTCCATGTTTTAGTAGAA
```

```
..........................................TAAGTGCTTCCATGTTTTAGT....  33
..........................................TAAGTGCTTCCATGTTTTAG.....  5
..........................................TAAGTGCTTCCATGTTTTAGTAG..  5
..........................................TAAGTGCTTCCATGTTTTAGTA...  3
....CTTTAACATGGTAGTACTTTCT.........................................  1
...........................................AAGTGCTTCCATGTTTTAGT....  1
...(((..(((((((.(((((((.((((..(....)..)))).))))))).)))))))..)))....  (-23.80)

6/14162974-14163040(+)_mir_cow_243
CACCACTTAAACGTGGATGTACTTGCTTTATAGCTAAAAAAGTAAGTGCTTCCATGTTTTAGTGATG
..........................................TAAGTGCTTCCATGTTTTAGT....  33
.......TAAACGTGGATGTACTTGCTTT......................................  21
.......TAAACGTGGATGTACTTGCTT.......................................  16
....ACTTAAACGTGGATGTACTTGCT........................................  11
..........................................TAAGTGCTTCCATGTTTTAGTG...  9
.....CTTAAACGTGGATGTACTTGCT........................................  6
..........................................TAAGTGCTTCCATGTTTTAG.....  5
..........................................TAAGTGCTTCCATGTTTTAGTGA..  5
....ACTTAAACGTGGATGTACTTGC.........................................  3
......TTAAACGTGGATGTACTTGCT........................................  3
......TTAAACGTGGATGTACTTGCTT.......................................  3
....ACTTAAACGTGGATGTACTTG..........................................  1
.....CTTAAACGTGGATGTACTTGCTT.......................................  1
.......TAAACGTGGATGTACTTGCTTTA.....................................  1
...........................................AAGTGCTTCCATGTTTTAGT....  1
...((((.(((((((((.(((((((((((.........))))))))))).))))))))).))))...  (-29.70)

6/14163149-14163218(+)_mir_cow_244
CCTCTACTTTAACATGGAGGCACTTGCTGTGACTTCATAAAAAAATAAGTGCTTCCATGTTTTAGTGCGG
.............................................TAAGTGCTTCCATGTTTTAGT....     33
.............................................TAAGTGCTTCCATGTTTTAGTG...     9
.............................................TAAGTGCTTCCATGTTTTAG.....     5
.............................................TAAGTGCTTCCATGTTTTAGTGC..     4
....TACTTTAACATGGAGGCACTTG............................................     1
....TACTTTAACATGGAGGCACTTGCT..........................................     1
.......TTTAACATGGAGGCACTTGCTG.........................................     1
..............................................AAGTGCTTCCATGTTTTAGT....     1
.((.((((..((((((((((((((((.((((....))))......))))))))))))))))..)))).))  (-28.30)
```

```
6/41545048-41545118(+)_mir_cow_245
TTCTGTTGTGCTTGATCTAACCATGTGGTTGCCAGGTATGAGTAAAACATGGTTCCGTCAAGCACCATGGA
.....TTGTGCTTGATCTAACCATGT.............................................   12160
.....TTGTGCTTGATCTAACCATG..............................................   3839
.....TTGTGCTTGATCTAACCATGTG............................................   2666
.....TTGTGCTTGATCTAACCATGTGG...........................................   406
.....TTGTGCTTGATCTAACCATGTGGT..........................................   148
............................................AAACATGGTTCCGTCAAGCACC.....   143
.....TTGTGCTTGATCTAACCAT...............................................   134
............................................AAACATGGTTCCGTCAAGCAC......   99
.....TTGTGCTTGATCTAACCATGTGGTT.........................................   61
............................................AAACATGGTTCCGTCAAGC........   41
.....TTGTGCTTGATCTAACC.................................................   39
.....TTGTGCTTGATCTAACCA................................................   28
...........................................AAAACATGGTTCCGTCAAGC........   14
...........................................AAAACATGGTTCCGTCAAGCACC.....   10
...........................................AAAACATGGTTCCGTCAAGCAC......   9
...............................................CATGGTTCCGTCAAGCACCATG..   8
.....TTGTGCTTGATCTAACCATGTGGTTGC.......................................   7
...........................................AAAACATGGTTCCGTCAAGCA.......   6
...............................................CATGGTTCCGTCAAGCACCAT...   6
............................................AAACATGGTTCCGTCAAGCA.......   5
...............................................CATGGTTCCGTCAAGCACCA....   5
.....TTGTGCTTGATCTAAC..................................................   4
.....TTGTGCTTGATCTAACCATGTGGTTG........................................   4
....GTTGTGCTTGATCTAACCATG..............................................   3
.......GTGCTTGATCTAACCATGT.............................................   3
.............................................AACATGGTTCCGTCAAGCAC......   3
............................................AAACATGGTTCCGTCAAGCACCA....   3
......TGTGCTTGATCTAACCATGT.............................................   2
...............................................CATGGTTCCGTCAAGCACC.....   2
.......GTGCTTGATCTAACCATG..............................................   1
.........GCTTGATCTAACCATGT.............................................   1
........TGCTTGATCTAACCATGTGGTTG........................................   1
.....TTGTGCTTGATCTAACCATGTGGTTGCCAGG...................................   1
............................................AAACATGGTTCCGTCAAG.........   1
..............................................ACATGGTTCCGTCAAGCAC......   1
```

```
...............................................CATGGTTCCGTCAAGCAC......    1
.............................................AACATGGTTCCGTCAAGCACC.....    1
............................................AAACATGGTTCCGTCAAGCACCAT...    1
................................................ATGGTTCCGTCAAGCACCATG..    1
.(((((.(((((((((..((((((((..((((((....)).)))).))))))))..))))))))).))))) (-31.80)

7/1146997-1147070(+)_mir_cow_249
GTGTGATTATAAAGCAATGAGACTGATTGTCATGTGTCGTTTGTGGGATCCGTCTCAGTTACTTTATAGCCATA
......TTATAAAGCAATGAGACTGATT..............................................19603
......TTATAAAGCAATGAGACTGAT...............................................6181
......TTATAAAGCAATGAGACTGA................................................1314
................................................TCCGTCTCAGTTACTTTATAGCC...611
......TTATAAAGCAATGAGACT..................................................270
................................................TCCGTCTCAGTTACTTTATAGC....130
................................................TCCGTCTCAGTTACTTTATAG.....77
......TTATAAAGCAATGAGACTG.................................................45
.................................................CCGTCTCAGTTACTTTATAGCC...26
......TTATAAAGCAATGAGACTGATTG.............................................23
................................................TCCGTCTCAGTTACTTTATAGCCA..15
......TTATAAAGCAATGAGAC...................................................13
.....ATTATAAAGCAATGAGACTGA................................................9
.......TATAAAGCAATGAGACTGATT..............................................8
.....ATTATAAAGCAATGAGACTGAT...............................................6
...............................................ATCCGTCTCAGTTACTTTATAGCC...6
...............................................ATCCGTCTCAGTTACTTTATAG.....5
.......TATAAAGCAATGAGACTGAT...............................................4
................................................TCCGTCTCAGTTACTTTAT.......3
................................................TCCGTCTCAGTTACTTTATA......3
.................................................CCGTCTCAGTTACTTTATAGC....3
........ATAAAGCAATGAGACTGATT..............................................2
..................................................CGTCTCAGTTACTTTATAGCCA..2
....GATTATAAAGCAATGAGACTG.................................................1
.......TATAAAGCAATGAGACTGA................................................1
........ATAAAGCAATGAGACTGAT...............................................1
...........AAGCAATGAGACTGAT...............................................1
............AGCAATGAGACTGATT..............................................1
......TTATAAAGCAATGAGACTGATTGTCATGTGTCGTTTGTGGGA..........................1
...........................TGTCATGTGTCGTTTGTGGGAT.........................1
```

....TCCGTCTCAGTTACTTT.........1
....CCGTCTCAGTTACTTTATA......1
....ATCCGTCTCAGTTACTTTATAGC....1
....CGTCTCAGTTACTTTATAGCC...1
....CCGTCTCAGTTACTTTATAGCCA..1
.((((..(((((((...((((((.((((.(((((.......))))))))).))))))....)))))))..)))) (-21.30)

7/12915084-12915153(+)_mir_Cow_Blast_34
TGGGGGAACATTCAACCTGTCGGTGAGTTTGGGCAGCTCAGGCAAACCATCGACCGTTGAGTGGACCCCG
......AACATTCAACCTGTCGGTGAGT.. 51895
......AACATTCAACCTGTCGGTGAGTT... 47728
......AACATTCAACCTGTCGGTGAG... 14584
....ACCATCGACCGTTGAGTGGACC... 9177
......AACATTCAACCTGTCGGTGAGTTT.. 5062
......AACATTCAACCTGTCGGTGA.. 3261
....ACCATCGACCGTTGAGTGGAC.... 927
......AACATTCAACCTGTCGGT.. 585
.....GAACATTCAACCTGTCGGTGAGT.. 166
....AACCATCGACCGTTGAGTGGAC.... 134
......AACATTCAACCTGTCGGTG... 74
.....GAACATTCAACCTGTCGGTGAG... 66
.......ACATTCAACCTGTCGGTGAGTT... 61
.....GAACATTCAACCTGTCGGTGAGTT... 58
.....GAACATTCAACCTGTCGGTGA.. 47
....AACCATCGACCGTTGAGTGGACC... 41
....ACCATCGACCGTTGAGTGGACCC.. 33
.......ACATTCAACCTGTCGGTGAGT.. 32
......AACATTCAACCTGTCGGTGAGTTTG....................................... 32
............CAACCTGTCGGTGAGTT... 30
.......ACATTCAACCTGTCGGTGAGTTT.. 30
............CAACCTGTCGGTGAGT.. 20
....AACCATCGACCGTTGAGTGG...... 16
....ACCATCGACCGTTGAGTG....... 15
....ACCATCGACCGTTGAGTGGA..... 13
.......ACATTCAACCTGTCGGTGAG... 11
....ACCATCGACCGTTGAGTGG...... 11
....CCATCGACCGTTGAGTGGACC... 9
......AACATTCAACCTGTCGG... 8

```
............................................AACCATCGACCGTTGAGTGGA.....     6
...........TCAACCTGTCGGTGAGT..........................................     5
.......ACATTCAACCTGTCGGTGAGTTTG.......................................     5
...................................................GACCGTTGAGTGGACC...     5
.........ATTCAACCTGTCGGTGAGT..........................................     4
.........ATTCAACCTGTCGGTGAGTT.........................................     4
........CATTCAACCTGTCGGTGAGTT.........................................     3
.....GAACATTCAACCTGTCGGTGAGTTT........................................     3
.............................................ACCATCGACCGTTGAGT........     3
............................................AACCATCGACCGTTGAGTG.......     3
.....GAACATTCAACCTGTCGGT..............................................     2
.....GAACATTCAACCTGTCGGTG.............................................     2
........CATTCAACCTGTCGGTGAG...........................................     2
..........TTCAACCTGTCGGTGAGT..........................................     2
...........TCAACCTGTCGGTGAGTT.........................................     2
............CAACCTGTCGGTGAGTTT........................................     2
.............................................ACCATCGACCGTTGAG.........     2
............................................AACCATCGACCGTTGAGT........     2
...........................................AAACCATCGACCGTTGAGTGGA.....     2
...........................................AAACCATCGACCGTTGAGTGGAC....     2
..............................................CCATCGACCGTTGAGTGGAC....     2
.......ACATTCAACCTGTCGGTG.............................................     1
....GGAACATTCAACCTGTCGGTGAGT..........................................     1
........CATTCAACCTGTCGGTGAGT..........................................     1
..........TTCAACCTGTCGGTGAGTT.........................................     1
.................................................TCGACCGTTGAGTGGACC...     1
..................................................CGACCGTTGAGTGGACC...     1
..............................................CCATCGACCGTTGAGTGGACCC..     1
...............................................CATCGACCGTTGAGTGGACCC..     1
..((((..((((((((..(((((((.(((((...........)))))))))))).))))))))..)))). (-34.00)

7/12981635-12981711(-)_mir_Cow_Blast_22
CTGCCTCCCGTGCCTACTGAGCTGAAACACAGTTGATTTGTGCACACTGGCTCAGTTCAGCAGGAACAGGAGTCAAG
...............................................TGGCTCAGTTCAGCAGGAAC..........   29631
...............................................TGGCTCAGTTCAGCAGGAACA.........   9452
...............................................TGGCTCAGTTCAGCAGGAACAG........   5133
...............................................TGGCTCAGTTCAGCAGGAA...........   300
...............................................TGGCTCAGTTCAGCAGGAACAGG.......   204
```

.........GTGCCTACTGAGCTGAAACACAGT... 134
.........GTGCCTACTGAGCTGAAACACAG.. 113
....TGGCTCAGTTCAGCAGGA............. 109
....CTGGCTCAGTTCAGCAGGAAC........... 100
........CGTGCCTACTGAGCTGAAACACAGT... 78
.........GTGCCTACTGAGCTGAAACACA... 63
..........TGCCTACTGAGCTGAAACACAGT... 49
.........GTGCCTACTGAGCTGAAACAC.. 47
........CGTGCCTACTGAGCTGAAACACAG.. 35
........CGTGCCTACTGAGCTGAAACACA... 29
....GCTCAGTTCAGCAGGAACAG......... 27
....GCTCAGTTCAGCAGGAACAGG........ 27
....TGGCTCAGTTCAGCAGGAACAGGA....... 27
........CGTGCCTACTGAGCTGAAACAC.. 23
....TGGCTCAGTTCAGCAGG.............. 23
....GGCTCAGTTCAGCAGGAACAG......... 22
....GGCTCAGTTCAGCAGGAAC........... 20
..........TGCCTACTGAGCTGAAACACAG.. 19
....GCTCAGTTCAGCAGGAAC........... 17
....GGCTCAGTTCAGCAGGAACA.......... 17
....GCTCAGTTCAGCAGGAACAGGA....... 16
....CTCAGTTCAGCAGGAAC........... 10
....GGCTCAGTTCAGCAGGAACAGG........ 10
....GCTCAGTTCAGCAGGAACA.......... 8
....CTCAGTTCAGCAGGAACA.......... 7
........CGTGCCTACTGAGCTGAAACA... 6
....CTGGCTCAGTTCAGCAGGAACA.......... 6
.........GTGCCTACTGAGCTGAAAC.. 5
.........GTGCCTACTGAGCTGAAACA... 3
....CTGGCTCAGTTCAGCAGGAACAG......... 3
..........TGCCTACTGAGCTGAAACACA... 2
...........GCCTACTGAGCTGAAACACAGT... 1
............CCTACTGAGCTGAAACACAGT... 1
..........TGCCTACTGAGCTGAAACACAGTT.. 1
....CTGGCTCAGTTCAGCAGGA............. 1
....ACTGGCTCAGTTCAGCAGGAAC........... 1
....TCAGTTCAGCAGGAACA.......... 1
....CTCAGTTCAGCAGGAACAG......... 1

```
....................................................CAGTTCAGCAGGAACAG........   1
................................................GGCTCAGTTCAGCAGGAACAGGA......   1
.((.((((.((.(((.(((((((((....((((((.......)).))))..))))))))).))).)).)))).)).. (-29.60)

7/12981790-12981861(-)_mir_cow_250
GGGAGCAGGGCTTAGCTGCTTGTGAGCAGGTCCACATCAAATCGTGTTCACAGTGGCTAAGTTCCGCCCCCC
..............................................TTCACAGTGGCTAAGTTCCG......  14244
..............................................TTCACAGTGGCTAAGTTCC.......  7648
..............................................TTCACAGTGGCTAAGTTCCGC.....  6755
..............................................TTCACAGTGGCTAAGTTC........  3568
..............................................TTCACAGTGGCTAAGTTCCGCC....  424
..............................................TTCACAGTGGCTAAGTT.........  179
..............................................TTCACAGTGGCTAAGT..........  98
......AGGGCTTAGCTGCTTGTGAGCA............................................  21
...............................................TCACAGTGGCTAAGTTCCG......  8
......AGGGCTTAGCTGCTTGTGAGC.............................................  7
................................................CACAGTGGCTAAGTTCCG......  3
................................................CACAGTGGCTAAGTTCCGC.....  3
.............................................GTTCACAGTGGCTAAGTTC........  2
................................................CACAGTGGCTAAGTTC........  2
...............................................TCACAGTGGCTAAGTTCC.......  2
................................................CACAGTGGCTAAGTTCC.......  2
...............................................TCACAGTGGCTAAGTTCCGC.....  2
......AGGGCTTAGCTGCTTGTGAGCAGGT.........................................  1
............................GGTCCACATCAAATCGT...........................  1
............................................TGTTCACAGTGGCTAAGTTCC.......  1
............................................TGTTCACAGTGGCTAAGTTCCG......  1
.............................................GTTCACAGTGGCTAAGTTCCG......  1
.................................................ACAGTGGCTAAGTTCCG......  1
..................................................CAGTGGCTAAGTTCCG......  1
.............................................GTTCACAGTGGCTAAGTTCCGC.....  1
...................................................AGTGGCTAAGTTCCGC.....  1
(((.((.(((((((((..((.(((((((.(............).)))))))))..))))))))).)).))). (-36.90)

7/12981971-12982041(-)_mir_cow_251
GCCGGCTGGGGTTCCTGGGGATGGGATTTGCTGCCTGTCACAAATCACATTGCCAGGGATTTCCAACCGAC
...........................................ATCACATTGCCAGGGATTTCC.......   10158
...........................................ATCACATTGCCAGGGATTTCCA......   3253
```

```
...........................................ATCACATTGCCAGGGATTTC........   726
...........................................ATCACATTGCCAGGGATT..........   634
...........................................ATCACATTGCCAGGGATTTCCAA.....   536
...........................................ATCACATTGCCAGGGATTT.........   274
...........................................ATCACATTGCCAGGGAT...........   107
..........................................AATCACATTGCCAGGGATTTCC.......   68
...........................................ATCACATTGCCAGGGA............   30
............................................TCACATTGCCAGGGATTTCC.......   19
..........................................AATCACATTGCCAGGGATT..........   15
..........................................AATCACATTGCCAGGGATTTCCA......   14
..............................................ACATTGCCAGGGATTTCC.......   11
..........................................AATCACATTGCCAGGGATTT.........   9
..........................................AATCACATTGCCAGGGAT...........   7
............................................TCACATTGCCAGGGATT..........   5
..........................................AATCACATTGCCAGGGATTTC........   5
...........................................ATCACATTGCCAGGGATTTCCAAC....   4
...............................................CATTGCCAGGGATTTCC.......   3
............................................TCACATTGCCAGGGATTTCCAAC....   3
..........................................AATCACATTGCCAGGGATTTCCAA.....   2
......TGGGGTTCCTGGGGATGGGATT...........................................   1
............................................TCACATTGCCAGGGATTTC........   1
............................................TCACATTGCCAGGGATTTCCA......   1
..............................................ACATTGCCAGGGATTTCCA......   1
...............................................CATTGCCAGGGATTTCCA......   1
............................................TCACATTGCCAGGGATTTCCAA.....   1
..(((.((((((((((((.((((.((((((.((.....)))))))).)))).))))))).))))).))).. (-32.50)

7/13464150-13464217(+)_novel_cow_37
GGTTCTGCATCCGAGGACTCAACCCATTTGATTGGCGGTTGGTTGAATCCACAGATGTGAGACCTGAG
...TCTGCATCCGAGGACTCAACCC........................................... 16
...TCTGCATCCGAGGACTCAACC............................................ 4
..........................................TTGAATCCACAGATGTGAGACC.... 4
..TTCTGCATCCGAGGACTCAACC............................................ 3
...TCTGCATCCGAGGACTCAA.............................................. 2
...TCTGCATCCGAGGACTCAAC............................................. 2
..TTCTGCATCCGAGGACTCAA.............................................. 1
..TTCTGCATCCGAGGACTCAAC............................................. 1
..TTCTGCATCCGAGGACTCAACCC........................................... 1
```

....GTTGAATCCACAGATGTGAGA...... 1
....TTGAATCCACAGATGTGAGAC..... 1
((((..(((((.(.(((.((((((.(((........))).)))))).))).).)))))..)))).... (-24.70)

7/16508924-16509000(-)_mir_cow_252
CCCCGCCAACCCAGTGTTCAGACTACCTGTTCAGGGGGCTCTGAATGTGTACAGTAGTCTGCACATTGGTTAGGCTG
....ACAGTAGTCTGCACATTGGTT...... 34856
....TACAGTAGTCTGCACATTGGTT...... 12926
....ACAGTAGTCTGCACATTGGTTA..... 8597
.........CCCAGTGTTCAGACTACCTGTTC... 4732
....ACAGTAGTCTGCACATTGGT....... 2417
....CAGTAGTCTGCACATTGGTT...... 2184
.........CCCAGTGTTCAGACTACCTGTT.. 1891
....TACAGTAGTCTGCACATTGGT....... 1677
.........CCCAGTGTTCAGACTACCTGT... 1392
....TACAGTAGTCTGCACATTGGTTA..... 756
....CAGTAGTCTGCACATTGGTTA..... 681
.........CCCAGTGTTCAGACTACCTGTTCA.. 496
.........CCCAGTGTTCAGACTACCTG.. 467
....ACAGTAGTCTGCACATTGG........ 268
....TACAGTAGTCTGCACATTGG........ 234
....CAGTAGTCTGCACATTGGT....... 224
.........CCCAGTGTTCAGACTACCT... 219
....ACAGTAGTCTGCACATTGGTTAG.... 78
....ACAGTAGTCTGCACAT........... 59
.........CCCAGTGTTCAGACTACC.. 55
....TACAGTAGTCTGCACAT........... 29
.........CCCAGTGTTCAGACTA.. 22
............AGTGTTCAGACTACCTGTTC... 22
....ACAGTAGTCTGCACATTG......... 17
....TACAGTAGTCTGCACATTGGTTAG.... 14
............AGTGTTCAGACTACCTGTT.. 13
...........CAGTGTTCAGACTACCTGTTC... 12
.........CCCAGTGTTCAGACTAC... 10
....ACAGTAGTCTGCACATT.......... 10
....TACAGTAGTCTGCACATTG......... 8
....AGTAGTCTGCACATTGGTT...... 8
....CAGTAGTCTGCACATTGG........ 7

..........CCAGTGTTCAGACTACCTGTTC... 6
....GTAGTCTGCACATTGGTT...... 6
....TACAGTAGTCTGCACATT.......... 5
....CAGTAGTCTGCACATTGGTTAG.... 5
...........CAGTGTTCAGACTACCTGTT.. 4
.............GTGTTCAGACTACCTGTTC... 3
....AGTAGTCTGCACATTGGTTA..... 3
..........CCAGTGTTCAGACTACCTGT... 2
..............TGTTCAGACTACCTGTTC... 2
....TACAGTAGTCTGCACA............ 2
...........CAGTGTTCAGACTACC.. 1
......CAACCCAGTGTTCAGACTACCT... 1
............AGTGTTCAGACTACCTGT... 1
..............TGTTCAGACTACCTGTT.. 1
..........CCAGTGTTCAGACTACCTGTTCA.. 1
.........CCCAGTGTTCAGACTACCTGTTCAG... 1
....AGTAGTCTGCACATTGGT....... 1
....GTACAGTAGTCTGCACATTGGTTA..... 1
....GTAGTCTGCACATTGGTTA..... 1
....(((...(((((((.((((((((.((((((((....)))))......))))))))))).)))))))...))).. (-32.00)

7/20584145-20584215(-)_mir_cow_253
TGCTGTGTGGAAGACTAGTGATTTTGTTGTTCTGATGTACTATGACAACAAGTCACAGCTTCCTCATAGCG
.......TGGAAGACTAGTGATTTTGTTGTT.. 5009
.......TGGAAGACTAGTGATTTTGTTGT... 2722
.......TGGAAGACTAGTGATTTTGTTG.. 169
....CAACAAGTCACAGCTTCCTCA..... 167
.......TGGAAGACTAGTGATTTTGTT... 62
....CAACAAGTCACAGCTTCCTC...... 46
.......TGGAAGACTAGTGATTTTGT.. 35
....CAACAAGTCACAGCTTCCT....... 24
.........GAAGACTAGTGATTTTGTTGTT.. 11
..........AAGACTAGTGATTTTGTTGTT.. 8
.......TGGAAGACTAGTGATTTTG... 6
.........GAAGACTAGTGATTTTGTTGT... 6
....CAACAAGTCACAGCTTCCTCAT.... 6
...........AGACTAGTGATTTTGTTGTT.. 4
..............CTAGTGATTTTGTTGTT.. 3

```
.......TGGAAGACTAGTGATTTTGTTGTTC.......................................   3
.......TGGAAGACTAGTGATTTT..............................................   2
........GGAAGACTAGTGATTTTGTTGTT........................................   2
..............................................AACAAGTCACAGCTTCCTCA.....   2
.......TGGAAGACTAGTGATT................................................   1
.........GAAGACTAGTGATTTTGT............................................   1
..........AAGACTAGTGATTTTGTT...........................................   1
......GTGGAAGACTAGTGATTTTGTTG..........................................   1
........GGAAGACTAGTGATTTTGTTGT.........................................   1
............GACTAGTGATTTTGTTGT.........................................   1
.............ACTAGTGATTTTGTTGT.........................................   1
..............CTAGTGATTTTGTTGT.........................................   1
............GACTAGTGATTTTGTTGTT........................................   1
.............ACTAGTGATTTTGTTGTT........................................   1
...............TAGTGATTTTGTTGTT........................................   1
.............................................CAACAAGTCACAGCTTCC........   1
..............................................AACAAGTCACAGCTTCCT.......   1
.((((((.(((((.((.(((((((.((((((.((......)).))))))))))))))))))))).)))))). (-31.70)

7/23425296-23425367(-)_mir_cow_254
GCACAGCGCGGCGCTGTTCTGGGAACGCGCTCTCATCACCCAACGTTGCCAACTGCAGAGCCGCGCTGAGCA
.............................................TTGCCAACTGCAGAGCCGCGCT.....  41
.............................................TTGCCAACTGCAGAGCCGCGC......  5
.............................................TTGCCAACTGCAGAGCCGCG.......  2
............................................GTTGCCAACTGCAGAGCCGCGCT.....  2
......CGCGGCGCTGTTCTGGGAAC..............................................  1
.............................................TTGCCAACTGCAGAGCCGC........  1
............................................GTTGCCAACTGCAGAGCCGCGC......  1
..............................................TGCCAACTGCAGAGCCGCGCT.....  1
.............................................TTGCCAACTGCAGAGCCGCGCTG....  1
((.(((((((((.((((..(((.((((................)))).)))...)))).))))))))).)). (-36.99)

7/39194958-39195022(+)_mir_cow_255
AGTGCTTGGCACCTAGTAAGTACTCAGTATATACTTGTTGAGTGCCTGCTATGTGCCAGGCATTG
....CTTGGCACCTAGTAAGTACTC........................................   884
....CTTGGCACCTAGTAAGTACTCA.......................................   310
....CTTGGCACCTAGTAAGTACT.........................................   37
........................................AGTGCCTGCTATGTGCCAGG.....   18
```

```
.........CACCTAGTAAGTACTCA.......................................   15
.....TTGGCACCTAGTAAGTACTC........................................   9
.........CACCTAGTAAGTACTC........................................   9
....CTTGGCACCTAGTAAGTAC..........................................   5
........................................AGTGCCTGCTATGTGCCAG......   5
..........ACCTAGTAAGTACTCA.......................................   2
.....TTGGCACCTAGTAAGTACTCA.......................................   1
........................................AGTGCCTGCTATGTGCCAGGC....   1
........................................AGTGCCTGCTATGTGCCAGGCA...   1
........................................AGTGCCTGCTATGTGCCAGGCAT..   1
((((((((((((.(((((.((((((((((......)))))))))).))))).)))))))))))). (-36.50)

7/50687427-50687497(-)_mir_cow_256
AGCCCTGCGGCCCCACGCACCAGGGTAAGAGAGACTCACTTCCTGCCCTGGCCCGAGGGACCGACTGGCTG
..........................................CTGCCCTGGCCCGAGGGACCGAC......   4328
..........................................CTGCCCTGGCCCGAGGGACCGA.......   3563
..........................................CTGCCCTGGCCCGAGGGACCGACT.....   1769
.......CGGCCCCACGCACCAGGGTAAGA.........................................   913
.......CGGCCCCACGCACCAGGGTAAG..........................................   774
.......CGGCCCCACGCACCAGGGTAA...........................................   583
.......CGGCCCCACGCACCAGGGTA............................................   224
..........................................CTGCCCTGGCCCGAGGGACC.........   138
..........................................CTGCCCTGGCCCGAGGGAC..........   101
..........................................CTGCCCTGGCCCGAGGGACCG........   87
..........................................CTGCCCTGGCCCGAGGGA...........   45
......GCGGCCCCACGCACCAGGGTAA...........................................   44
......GCGGCCCCACGCACCAGGGTA............................................   43
...........................................TGCCCTGGCCCGAGGGACCGAC......   33
......GCGGCCCCACGCACCAGGGTAAGA.........................................   31
...........................................TGCCCTGGCCCGAGGGACCGACT.....   17
.......CGGCCCCACGCACCAGGGT.............................................   13
...........................................TGCCCTGGCCCGAGGGACCGA.......   10
......GCGGCCCCACGCACCAGGGTAAG..........................................   8
..........................................CTGCCCTGGCCCGAGGG............   8
..........................................CTGCCCTGGCCCGAGGGACCGACTG....   8
......GCGGCCCCACGCACCAGGGT.............................................   5
......GCGGCCCCACGCACCAGGG..............................................   2
..........................................CTGCCCTGGCCCGAGG.............   2
```

```
...........................................TGCCCTGGCCCGAGGGACCGACTG....    2
.......CGGCCCCACGCACCAG................................................    1
......GCGGCCCCACGCACCAGG...............................................    1
.......CGGCCCCACGCACCAGGG..............................................    1
.........GCCCCACGCACCAGGGTAA...........................................    1
.........GCCCCACGCACCAGGGTAAG..........................................    1
.......CGGCCCCACGCACCAGGGTAAGAGA.......................................    1
.......CGGCCCCACGCACCAGGGTAAGAGAGACT...................................    1
.......CGGCCCCACGCACCAGGGTAAGAGAGACTC..................................    1
...........CCCACGCACCAGGGTAAGAGAGACTC..................................    1
...........................................TGCCCTGGCCCGAGGGACCG........    1
........................................TCCTGCCCTGGCCCGAGGGACCGA.......    1
.............................................CCCTGGCCCGAGGGACCGACT.....    1
.............................................CCCTGGCCCGAGGGACCGACTG....    1
((((...(((.(((.((..((((((((.(..((.....))..)))))))))..)).))).)))...)))). (-35.50)

7/55523563-55523636(-)_mir_cow_257
TCCGGGCTTGGAGCTCTTGAGGCCTGGCATGTGGTTACCTTGTACCCAGGCACCAGCAGCTCTGCAAGCCCTGC
......CTTGGAGCTCTTGAGGCCTGGC..............................................50
......CTTGGAGCTCTTGAGGCCTGGCA.............................................19
..............................................CAGGCACCAGCAGCTCTGCAAG......15
......CTTGGAGCTCTTGAGGCCTGG...............................................13
..............................................CAGGCACCAGCAGCTCTGCAA.......12
..............................................CAGGCACCAGCAGCTCTGCAAGCC....7
......CTTGGAGCTCTTGAGGCCTGGCAT............................................6
..............................................CAGGCACCAGCAGCTCTGCA........6
...............................................AGGCACCAGCAGCTCTGCAAG......3
.......TTGGAGCTCTTGAGGCCTGGC..............................................1
............................................CCCAGGCACCAGCAGCTCTGCAA.......1
...............................................AGGCACCAGCAGCTCTGCAAGCC....1
...((((((((((((.(((..((((((..((..(.....)..)).))))))..))).)))))..)))))))... (-35.20)

7/62809320-62809388(+)_mir_cow_258
CCTGAGGTGCAGTGCTGCATCTCTGGTCAGTTGGGAGTCTGAGATGAAGCACTGTAGCTCGGGAAGGGA
.......................................TGAGATGAAGCACTGTAGCT..........     1770798
.......................................TGAGATGAAGCACTGTAGCTC.........     1067145
.......................................TGAGATGAAGCACTGTAGCTCG........     107832
.......................................TGAGATGAAGCACTGTAGC...........     57141
```

......................................TGAGATGAAGCACTGTAGCTCGG....... 45760

......................................TGAGATGAAGCACTGTAG............ 17830

.....................................CTGAGATGAAGCACTGTAGCT.......... 10322

.....................................CTGAGATGAAGCACTGTAGCTC......... 5698

....GATGAAGCACTGTAGCT.......... 498

.....................................CTGAGATGAAGCACTGTAGC........... 445

......................................TGAGATGAAGCACTGTA............. 380

....GAGATGAAGCACTGTAGCTC......... 373

....GAGATGAAGCACTGTAGCT.......... 286

......................................TGAGATGAAGCACTGT.............. 225

.....................................CTGAGATGAAGCACTGTAG............ 221

.....GGTGCAGTGCTGCATCTCTGGTC.. 204

....GATGAAGCACTGTAGCTCGGGAA.... 200

....GATGAAGCACTGTAGCTC......... 189

.....................................CTGAGATGAAGCACTGTAGCTCG........ 176

....GATGAAGCACTGTAGCTCGGGA..... 152

....AGATGAAGCACTGTAGCTCGG....... 150

....ATGAAGCACTGTAGCT.......... 132

....................................TCTGAGATGAAGCACTGTAGCTC......... 101

....AGATGAAGCACTGTAGCT.......... 98

....ATGAAGCACTGTAGCTCGGGAA.... 97

....TGAAGCACTGTAGCTC......... 91

....................................TCTGAGATGAAGCACTGTAGCT.......... 77

.....GGTGCAGTGCTGCATCTCTGGT... 65

....ATGAAGCACTGTAGCTC......... 58

....................................TCTGAGATGAAGCACTGTAGC........... 42

....GAGATGAAGCACTGTAGCTCG........ 41

....GATGAAGCACTGTAGCTCGG....... 36

.....................................CTGAGATGAAGCACTGTAGCTCGG....... 34

......................................TGAGATGAAGCACTGTAGCTCGGG...... 30

....AGATGAAGCACTGTAGCTC......... 29

.....GGTGCAGTGCTGCATCTCTGG.. 28

....GATGAAGCACTGTAGCTCG........ 24

....................................TCTGAGATGAAGCACTGTAG............ 23

....GAGATGAAGCACTGTAGCTCGG....... 23

....GATGAAGCACTGTAGC........... 19

....ATGAAGCACTGTAGCTCGGGA..... 15

....TGAAGCACTGTAGCTCGGGAA.... 11

```
........................................GAGATGAAGCACTGTAGC...........     9
.....GGTGCAGTGCTGCATCTC..............................................     7
.........................................AGATGAAGCACTGTAGC...........     7
.........................................AGATGAAGCACTGTAGCTCG........     7
..........................................GATGAAGCACTGTAGCTCGGG......     7
.....GGTGCAGTGCTGCATCTCTGGTCAGTTGGGAGTC..............................     5
............................................TGAAGCACTGTAGCTCG........     5
............................................TGAAGCACTGTAGCTCGGGA.....     4
......................................CTGAGATGAAGCACTGTA.............     3
........................................GAGATGAAGCACTGTAG............     3
............................................TGAAGCACTGTAGCTCGG.......     3
.........................................AGATGAAGCACTGTAGCTCGGG......     3
........................................GAGATGAAGCACTGTA.............     2
..............................TTGGGAGTCTGAGATGAAGCACTGTAGCTC.........     2
....................................GTCTGAGATGAAGCACTGTAGCTC.........     2
...........................................ATGAAGCACTGTAGCTCG........     2
.........................................AGATGAAGCACTGTAGCTCGGGA.....     2
....AGGTGCAGTGCTGCATCTCTG............................................     1
.......TGCAGTGCTGCATCTCTGG...........................................     1
......GTGCAGTGCTGCATCTCTGGT..........................................     1
...................................AGTCTGAGATGAAGCACTGTAG............     1
....................................GTCTGAGATGAAGCACTGTAG............     1
.........................................AGATGAAGCACTGTAG............     1
............................AGTTGGGAGTCTGAGATGAAGCACTGTAGCTC.........     1
.............................................GAAGCACTGTAGCTCG........     1
.....................................TCTGAGATGAAGCACTGTAGCTCGG.......     1
...........................................ATGAAGCACTGTAGCTCGG.......     1
.........................................AGATGAAGCACTGTAGCTCGGGAA....     1
...........................................ATGAAGCACTGTAGCTCGGGAAG...     1
...........................................ATGAAGCACTGTAGCTCGGGAAGG..     1
((((((.(((((((((.((((((.((((......)).)).)))))).))))))))).))))))...... (-37.00)

7/62810740-62810813(+)_mir_cow_259
TCCTCACGGTCCAGTTTTCCCAGGAATCCCTTAGATGCTAAGATGGGGATTCCTGGAAATACTGTTCTTGAGGT
........GTCCAGTTTTCCCAGGAATCCCT...........................................26239
................................................ATTCCTGGAAATACTGTTCTT.....7698
........GTCCAGTTTTCCCAGGAATCCC............................................2816
........GTCCAGTTTTCCCAGGAATCCCTT..........................................1311
```

....ATTCCTGGAAATACTGTTCT......1282
........GTCCAGTTTTCCCAGGAAT...1196
....ATTCCTGGAAATACTGTTC.......1023
........GTCCAGTTTTCCCAGGAATCC...775
....GATTCCTGGAAATACTGTTCTT.....348
........GTCCAGTTTTCCCAGGAATC..205
....ATTCCTGGAAATACTGTTCTTG....126
....GATTCCTGGAAATACTGTTCT......120
.........TCCAGTTTTCCCAGGAATCCCT...97
....GGATTCCTGGAAATACTGTT........50
........GTCCAGTTTTCCCAGGAA..42
....ATTCCTGGAAATACTGTT........41
....GATTCCTGGAAATACTGTTC.......37
....GGATTCCTGGAAATACTGTTCT......36
....GATTCCTGGAAATACTGTT........28
....CCTGGAAATACTGTTCTT.....28
....GGATTCCTGGAAATACTGTTC.......21
........GTCCAGTTTTCCCAGG..19
....CCTGGAAATACTGTTCT......19
....ATTCCTGGAAATACTGTTCTTGA...19
....GGATTCCTGGAAATACTG..........16
........GTCCAGTTTTCCCAGGA...14
............AGTTTTCCCAGGAATCC...13
...........CAGTTTTCCCAGGAATCCCT...13
.........TCCAGTTTTCCCAGGAATCCC..11
....TTCCTGGAAATACTGTTCTT.....10
....TCCTGGAAATACTGTTCTT.....10
..........CCAGTTTTCCCAGGAATCCCT...9
.........TCCAGTTTTCCCAGGAATCCCTT..9
.............GTTTTCCCAGGAATCC...7
............AGTTTTCCCAGGAATCCC..7
........GTCCAGTTTTCCCAGGAATCCCTTA...7
....GGATTCCTGGAAATACTGTTCTT.....7
....TTCCTGGAAATACTGTTCTTG....6
....TTCCTGGAAATACTGTTCTTGA...6
............AGTTTTCCCAGGAATCCCT...5
....GATTCCTGGAAATACTGT.........5
....CCTGGAAATACTGTTC.......5

```
....................................................CTGGAAATACTGTTCTT.....5
.......GGTCCAGTTTTCCCAGGAATCCCT...........................................4
..................................................TCCTGGAAATACTGTTCT......4
.......GGTCCAGTTTTCCCAGGAATCC.............................................3
.........TCCAGTTTTCCCAGGAATCC.............................................3
.................................................TTCCTGGAAATACTGTTC.......3
...............................................GATTCCTGGAAATACTGTTCTTG....3
.......GGTCCAGTTTTCCCAGGAAT...............................................2
...........CAGTTTTCCCAGGAATCC.............................................2
.............GTTTTCCCAGGAATCCCT...........................................2
...............................................GATTCCTGGAAATACTG..........2
................................................ATTCCTGGAAATACTG..........2
..............................................GGATTCCTGGAAATACTGT.........2
..................................................TCCTGGAAATACTGTTC.......2
.................................................TTCCTGGAAATACTGTTCT......2
....................................................CTGGAAATACTGTTCT......2
.........TCCAGTTTTCCCAGGAATC..............................................1
............AGTTTTCCCAGGAATC..............................................1
.......GGTCCAGTTTTCCCAGGAATCCC............................................1
...........CAGTTTTCCCAGGAATCCC............................................1
..............TTTTCCCAGGAATCCCT...........................................1
.......GGTCCAGTTTTCCCAGGAATCCCTT..........................................1
............AGTTTTCCCAGGAATCCCTT..........................................1
........GTCCAGTTTTCCCAGGAATCCCTTAGA.......................................1
...........................................TGGGGATTCCTGGAAATACTGT.........1
................................................ATTCCTGGAAATACTGT.........1
...............................TAGATGCTAAGATGGGGATTCCTGGAAATACTGTT........1
...................................TGCTAAGATGGGGATTCCTGGAAATACTGTT........1
...........................................TGGGGATTCCTGGAAATACTGTTCT......1
.....................................................TGGAAATACTGTTCTT.....1
.(((((.((..((((.((.((((((((((((.............)))))))))))).)).))))..))))))). (-35.22)

7/63067300-63067368(+)_mir_cow_260
CAGGGCTCCTGACTCCAGGTCCTGTGTGTTACCTCGAAATAGCACTGGACTTGGAGTCAGAAGGCCTGA
...........................................ACTGGACTTGGAGTCAGAAGGC....     128296
...........................................ACTGGACTTGGAGTCAGAAGG.....     24419
............................................CTGGACTTGGAGTCAGAAGGC....     6800
...........................................ACTGGACTTGGAGTCAGAAG......     2823
```

```
.....CTCCTGACTCCAGGTCCTGTGT..........................................    1551
...........................................ACTGGACTTGGAGTCAGAAGGCC...    1213
............................................CTGGACTTGGAGTCAGAAGGCC...    1078
............................................CTGGACTTGGAGTCAGAAGG.....    594
..........................................CACTGGACTTGGAGTCAGAAGGC....    387
...........................................ACTGGACTTGGAGTCAGA........    297
............................................CTGGACTTGGAGTCAGAAGGCCT..    280
...........................................ACTGGACTTGGAGTCAGAA.......    268
..........................................CACTGGACTTGGAGTCAGAAGG.....    193
...........................................ACTGGACTTGGAGTCAGAAGGCCT..    166
..........................................CACTGGACTTGGAGTCAGAAG......    116
......TCCTGACTCCAGGTCCTGTGT..........................................    79
.....CTCCTGACTCCAGGTCCTGTG...........................................    72
...........................................ACTGGACTTGGAGTCAG.........    65
............................................CTGGACTTGGAGTCAGAAG......    47
.....CTCCTGACTCCAGGTCCTGT............................................    42
.........................................GCACTGGACTTGGAGTCAGAAG......    29
.............................................TGGACTTGGAGTCAGAAGGC....    22
.......CCTGACTCCAGGTCCTGTGTGTT.......................................    14
.....CTCCTGACTCCAGGTCCTGTGTGT........................................    13
......TCCTGACTCCAGGTCCTGTGTGT........................................    13
.......CCTGACTCCAGGTCCTGTGTGT........................................    8
...........................................ACTGGACTTGGAGTCA..........    8
..........................................CACTGGACTTGGAGTCAGAAGGCC...    8
.....CTCCTGACTCCAGGTCCTG.............................................    7
......TCCTGACTCCAGGTCCTGTGTGTT.......................................    5
..........................................CACTGGACTTGGAGTCAGAA.......    5
............................................CTGGACTTGGAGTCAGAA.......    5
.............................................TGGACTTGGAGTCAGAAGGCC...    5
..........................................CACTGGACTTGGAGTCAGA........    4
................................................ACTTGGAGTCAGAAGGC....    4
.................................................CTTGGAGTCAGAAGGC....    4
.............................................TGGACTTGGAGTCAGAAGGCCT..    4
......TCCTGACTCCAGGTCCTGTGTG.........................................    3
.......CCTGACTCCAGGTCCTGTGTGTTA......................................    3
........................................AGCACTGGACTTGGAGTCAGAAG......    3
....GCTCCTGACTCCAGGTCCTGTGT..........................................    2
..........................................CACTGGACTTGGAGTCAG.........    2
```

....GGACTTGGAGTCAGAAGGC....   2
....GCTCCTGACTCCAGGTCCTGT..   1
....GCTCCTGACTCCAGGTCCTGTG...   1
.......CCTGACTCCAGGTCCTGTGT..   1
.....CTCCTGACTCCAGGTCCTGTGTG...   1
.....CTCCTGACTCCAGGTCCTGTGTGTT.......................................   1
......TCCTGACTCCAGGTCCTGTGTGTTA......................................   1
.................................TCGAAATAGCACTGGACTTGGAGT............   1
....GCACTGGACTTGGAGTCAGA........   1
....CTGGACTTGGAGTCAGA........   1
....AGCACTGGACTTGGAGTCAGAA.......   1
....GCACTGGACTTGGAGTCAGAA.......   1
....TGGACTTGGAGTCAGAAGG.....   1
...........................GTTACCTCGAAATAGCACTGGACTTGGAGTCAGAAGGC....   1
...............................CCTCGAAATAGCACTGGACTTGGAGTCAGAAGGC....   1
.................................TCGAAATAGCACTGGACTTGGAGTCAGAAGGC....   1
....AGCACTGGACTTGGAGTCAGAAGGC....   1
....GCACTGGACTTGGAGTCAGAAGGC....   1
....CACTGGACTTGGAGTCAGAAGGCCT..   1
((((.((.((((((((((((((...((((((........)))))).)))))))))))))).)).)))). (-42.90)

7/74185050-74185114(+)_mir_cow_261
AGCTTTGAGAACTGAATTCCATAGGTTGTGTCAGTGTCAGACCTGTGAAGTTTAGTTCTTTAGCT
.....TGAGAACTGAATTCCATAGGTT......................................   19144
....TTGAGAACTGAATTCCATAGGTT......................................   3954
.....TGAGAACTGAATTCCATAGGT.......................................   1923
....TTGAGAACTGAATTCCATAGGT.......................................   1170
.....TGAGAACTGAATTCCATAGG..   679
....TTGAGAACTGAATTCCATAGG..   331
.....TGAGAACTGAATTCCATAGGTTGT....................................   69
.....TGAGAACTGAATTCCATAGGTTG.....................................   65
.....TGAGAACTGAATTCCATAG...   31
....TTGAGAACTGAATTCCATAG...   13
....TTGAGAACTGAATTCCATAGGTTG.....................................   8
....CCTGTGAAGTTTAGTTCTTTAG..   8
......GAGAACTGAATTCCATAGGTT......................................   6
.....TGAGAACTGAATTCCATA..   3
....TTGAGAACTGAATTCCATAGGTTGT....................................   3

```
.....TGAGAACTGAATTCCA............................................   2
....TTGAGAACTGAATTCCATA..........................................   1
......GAGAACTGAATTCCATAGG........................................   1
.........................................CCTGTGAAGTTTAGTTCTTT....   1
.........................................CCTGTGAAGTTTAGTTCTTTA...   1
((((..((((((((((((.((((((((.((.......)))))))))).)))))))))))).)))) (-27.40)

7/83830834-83830901(-)_mir_cow_262
GCTGCTGAGAGGTCTGTAATAACTAACATTTATGTAGAGGTTGTTAGTTATTAGACCTCTCAGCAGCT
.....TGAGAGGTCTGTAATAACTAACA........................................ 6
...........................................TTAGTTATTAGACCTCTCAGC.... 6
.....TGAGAGGTCTGTAATAACTAAC......................................... 4
((((((((((((((((..((((((((((.............)))))))))))))))))))))))))). (-41.02)

7/90554681-90554757(-)_mir_Cow_Blast_3
GAGTTGTTATCTTTGGTTATCTAGCTGTATGAGTGTATTGGTCTTCATAAAGCTAGATAACCGAAAGTAAAAACTCC
.........TCTTTGGTTATCTAGCTGTATGA.............................................   443629
.........TCTTTGGTTATCTAGCTGTAT...............................................   153938
.........TCTTTGGTTATCTAGCTGTATG..............................................   133621
..........CTTTGGTTATCTAGCTGTATGA.............................................   19453
..............................................ATAAAGCTAGATAACCGAAAGT.........   15674
.........TCTTTGGTTATCTAGCTGTA................................................   8675
.........TCTTTGGTTATCTAGCTGT.................................................   6474
...............................................TAAAGCTAGATAACCGAAAGTA........   3953
...............................................TAAAGCTAGATAACCGAAAGT.........   2830
..........CTTTGGTTATCTAGCTGTATG..............................................   1923
...........TTTGGTTATCTAGCTGTATGA.............................................   1913
..............................................ATAAAGCTAGATAACCGAAAGTA........   1687
..........CTTTGGTTATCTAGCTGTAT...............................................   1196
..............................................ATAAAGCTAGATAACCGAAA...........   571
..............................................ATAAAGCTAGATAACCGAAAG..........   519
.........TCTTTGGTTATCTAGC....................................................   496
...............................................TAAAGCTAGATAACCGAAAGTAA.......   409
.........TCTTTGGTTATCTAGCTGTATGAG............................................   350
.........TCTTTGGTTATCTAGCT...................................................   264
...........TTTGGTTATCTAGCTGTATGAG............................................   261
...............................................TAAAGCTAGATAACCGAAAG..........   254
.........TCTTTGGTTATCTAGCTG..................................................   251
```

............TTGGTTATCTAGCTGTATGA...	155
.........TCTTTGGTTATCTAGCTGTATGAGT...	130
...........TTTGGTTATCTAGCTGTATG..	124
....ATAAAGCTAGATAACCGAAAGTAA.......	117
....TAAAGCTAGATAACCGAAA...........	116
..........CTTTGGTTATCTAGCTGTA..	101
...........TTTGGTTATCTAGCTGTATGAGT...	67
................TTATCTAGCTGTATGA...	54
....ATAAAGCTAGATAACCGA.............	52
...........TTTGGTTATCTAGCTGTAT...	41
...............GTTATCTAGCTGTATGA...	39
..........CTTTGGTTATCTAGCTGT...	28
..........CTTTGGTTATCTAGCTGTATGAGT...	27
....ATAAAGCTAGATAACCGAA............	26
..........CTTTGGTTATCTAGCTGTATGAG..	24
....TAAAGCTAGATAACCGAAAGTAAA......	22
..............GGTTATCTAGCTGTATGA...	21
............TTGGTTATCTAGCTGTATG..	20
............TTGGTTATCTAGCTGTATGAGT...	15
............TTGGTTATCTAGCTGTAT...	13
.............TGGTTATCTAGCTGTATGA...	13
....CATAAAGCTAGATAACCGAAA...........	12
....CATAAAGCTAGATAACCGAAAGT.........	9
..............GGTTATCTAGCTGTATG..	8
...............GTTATCTAGCTGTATG..	8
...........TTTGGTTATCTAGCTGTA..	7
.............TGGTTATCTAGCTGTATG..	7
............TTGGTTATCTAGCTGTATGAG..	7
..............GGTTATCTAGCTGTAT...	5
....ATAAAGCTAGATAACC...............	5
....ATAAAGCTAGATAACCGAAAGTAAA......	5
..........CTTTGGTTATCTAGCT...	4
........ATCTTTGGTTATCTAGCTGTAT...	4
........ATCTTTGGTTATCTAGCTGTATGA...	4
.............TGGTTATCTAGCTGTAT...	3
....CTAGATAACCGAAAGT.........	3
............TTGGTTATCTAGCTGT...	2
.........TCTTTGGTTATCTAGCTGTATGAGTG..	2

```
.................................................AAGCTAGATAACCGAAAGT.........   2
..........CTTTGGTTATCTAGCTG..................................................   1
...........TTTGGTTATCTAGCTG..................................................   1
........ATCTTTGGTTATCTAGCTGTATG..............................................   1
..................ATCTAGCTGTATGAGT...........................................   1
............TTGGTTATCTAGCTGTATGAGTG..........................................   1
.............TGGTTATCTAGCTGTATGAGTG..........................................   1
..............GGTTATCTAGCTGTATGAGTGTA........................................   1
...............................................TAAAGCTAGATAACCGA.............   1
...............................................TAAAGCTAGATAACCGAA............   1
................................................AAAGCTAGATAACCGAAAGT.........   1
.............................................CATAAAGCTAGATAACCGAAAGTA........   1
...................................................GCTAGATAACCGAAAGTA........   1
(((((.(((..(((((((((((((((.((((((..(....)..)))))).)))))))))))))))..))).))))). (-34.30)

7/90554688-90554751(+)_mir_cow_263
TTACTTTCGGTTATCTAGCTTTATGAAGACCAATACACTCATACAGCTAGATAACCAAAGATAA
.....TTCGGTTATCTAGCTTTATGA......................................     8
.....TTCGGTTATCTAGCTTTAT........................................     2
....TTTCGGTTATCTAGCTTTATGA......................................     1
..........TTATCTAGCTTTATGA......................................     1
.....TTCGGTTATCTAGCTTTATGAA.....................................     1
......TCGGTTATCTAGCTTTATGAA.....................................     1
........................................ATACAGCTAGATAACCAAAGA...     1
.........................................TACAGCTAGATAACCAAAGAT..     1
...((((.((((((((((((.(((((............))))).))))))))))))))))....  (-25.00)

8/100305158-100305231(-)_mir_cow_276
GTGGAGGTATTGCACATGACTAAGTTGCATGTTGTCACGGCCTCAGTGCAATTTAGTATGTGTGATATTTTCAC
.......TATTGCACATGACTAAGTTGC..............................................4927
.......TATTGCACATGACTAAGTTG...............................................2630
................................................CAATTTAGTATGTGTGATATT.....481
.......TATTGCACATGACTAAGTTGCA.............................................297
.......TATTGCACATGACTAAGT.................................................39
.......TATTGCACATGACTAAGTT................................................33
......GTATTGCACATGACTAAGTTGC..............................................24
......GTATTGCACATGACTAAGTTG...............................................22
................................................CAATTTAGTATGTGTGATAT......22
```

```
.......TATTGCACATGACTAAG..................................................5
................................................CAATTTAGTATGTGTGAT........4
...............................................GCAATTTAGTATGTGTGATATT.....3
................................................CAATTTAGTATGTGTGATATTT....3
.........TTGCACATGACTAAGTTG...............................................2
.......TATTGCACATGACTAAGTTGCAT............................................2
..............................................TGCAATTTAGTATGTGTGATATT.....2
.......TATTGCACATGACTAA...................................................1
........ATTGCACATGACTAAGTTG...............................................1
........ATTGCACATGACTAAGTTGC..............................................1
......GTATTGCACATGACTAAGTTGCA.............................................1
........ATTGCACATGACTAAGTTGCA.............................................1
.......TATTGCACATGACTAAGTTGCATGTTGTC......................................1
................................................CAATTTAGTATGTGTGATA.......1
...............................................GCAATTTAGTATGTGTGATAT......1
((((((((((..((((((.((((((((((((..((....))..)).))))))))))))))))..)))))))))) (-35.70)
```

```
8/105147057-105147124(+)_mir_cow_277
GAGGGTATGTGCCTTTGGACTACATCGTGGAAGCCAGCACCATGCAGTCCATGGGCATATACACTTGC
.....TATGTGCCTTTGGACTACATCG......................................... 4532
.....TATGTGCCTTTGGACTACATC.......................................... 784
.....TATGTGCCTTTGGACTACATCGT........................................ 629
...........................................GCAGTCCATGGGCATATACACT... 453
...........................................GCAGTCCATGGGCATATACAC.... 364
.....TATGTGCCTTTGGACTACAT........................................... 219
.....TATGTGCCTTTGGACTACA............................................ 185
............................................CAGTCCATGGGCATATACACTT.. 115
.......TGTGCCTTTGGACTACATCGTG....................................... 108
...........................................GCAGTCCATGGGCATATACA..... 105
.......TGTGCCTTTGGACTACATCGT........................................ 101
..........................................TGCAGTCCATGGGCATATACAC.... 83
.....TATGTGCCTTTGGACTAC............................................. 61
....GTATGTGCCTTTGGACTACA............................................ 53
....GTATGTGCCTTTGGACTACAT........................................... 46
..........................................TGCAGTCCATGGGCATATACA..... 41
....GTATGTGCCTTTGGACTAC............................................. 35
............................................CAGTCCATGGGCATATACACT... 35
.........................................ATGCAGTCCATGGGCATATACA..... 34
```

```
...........................................GCAGTCCATGGGCATATAC...... 33
....GTATGTGCCTTTGGACTACATCG......................................... 28
.........................................ATGCAGTCCATGGGCATATACAC.... 26
.......TGTGCCTTTGGACTACATCG......................................... 24
..........................................TGCAGTCCATGGGCATATAC...... 23
.........................................ATGCAGTCCATGGGCATATAC...... 19
.....TATGTGCCTTTGGACTACATCGTG....................................... 18
....GTATGTGCCTTTGGACTACATC.......................................... 13
.........................................ATGCAGTCCATGGGCATATACACT... 13
......ATGTGCCTTTGGACTACATCGT........................................ 12
.......TGTGCCTTTGGACTACATCGTGG...................................... 12
............................................CAGTCCATGGGCATATACAC.... 12
.........................................ATGCAGTCCATGGGCATAT........ 11
.....TATGTGCCTTTGGACT............................................... 8
.......TGTGCCTTTGGACTACATC.......................................... 8
..........................................TGCAGTCCATGGGCATATACACT... 8
....GTATGTGCCTTTGGACTA.............................................. 6
.........................................ATGCAGTCCATGGGCATATA....... 6
...........................................GCAGTCCATGGGCATATA....... 6
....GTATGTGCCTTTGGACT............................................... 5
.........................................ATGCAGTCCATGGGCATA......... 5
........................................CATGCAGTCCATGGGCATATACA..... 5
.....TATGTGCCTTTGGACTA.............................................. 4
......ATGTGCCTTTGGACTACATCG......................................... 4
....GTATGTGCCTTTGGACTACATCGTGGAAGCC................................. 4
............................................CAGTCCATGGGCATATACA..... 4
....GTATGTGCCTTTGGAC................................................ 3
...........................................GCAGTCCATGGGCATA......... 3
........................................CATGCAGTCCATGGGCATAT........ 3
....GTATGTGCCTTTGGACTACATCGT........................................ 2
..........................................TGCAGTCCATGGGCATATA....... 2
............................................CAGTCCATGGGCATATAC...... 2
................................................CCATGGGCATATACACT... 2
.....TATGTGCCTTTGGACTACATCGTGG...................................... 1
........GTGCCTTTGGACTACATCGTGG...................................... 1
..............TTGGACTACATCGTGGAAGCC................................. 1
....GTATGTGCCTTTGGACTACATCGTGGAAGCCAGCA............................. 1
.......................................CCATGCAGTCCATGGGC............ 1
```

```
.........................................TGCAGTCCATGGGCATA......... 1
.......................................CATGCAGTCCATGGGCATATA....... 1
..........................................GCAGTCCATGGGCATATACACTT.. 1
(((.((((((((((.((((((.(((.(((.......))).))).)))))).)))))))))).))).. (-32.60)
```

8/15159565-15159629(+)_mir_cow_266

```
CATTTGCAGGAACTTGTGAGTCTCCTATTGAACATGAACAGGAGGCTGATGAGTTCCCGGGAACA
.....GCAGGAACTTGTGAGTCTCCT.......................................    518
....TGCAGGAACTTGTGAGTCTCCT.......................................    339
.........................................GAGGCTGATGAGTTCCCGGGA...    157
....TGCAGGAACTTGTGAGTCTCC........................................    135
.....GCAGGAACTTGTGAGTCTCC........................................    66
....TGCAGGAACTTGTGAGTCT..........................................    65
.........................................GAGGCTGATGAGTTCCCGGGAA..    62
....TGCAGGAACTTGTGAGTCTC.........................................    33
.....GCAGGAACTTGTGAGTCTC.........................................    16
.....GCAGGAACTTGTGAGTCT..........................................    14
........................................GGAGGCTGATGAGTTCCCGGGA...    5
.........................................GAGGCTGATGAGTTCCCGGG....    4
.....GCAGGAACTTGTGAGTCTCCTA......................................    3
....TGCAGGAACTTGTGAGTC...........................................    1
.....GCAGGAACTTGTGAGTC...........................................    1
....TGCAGGAACTTGTGAGTCTCCTAT.....................................    1
.....GCAGGAACTTGTGAGTCTCCTAT.....................................    1
.........................................GAGGCTGATGAGTTCCCGG.....    1
..(((.(.((((((..(.((((((((.((.......)).)))))))).)..)))))).).))).. (-26.90)
```

8/15183229-15183298(+)_mir_Cow_Blast_9

```
TGCTGTGGATTTCTTTGTGAATCACCATATCTAAGCTAATGTGGTGGTGGTTTACAAAGTAATTCATAGT
............................................TGGTGGTTTACAAAGTAATTC.....    108
.....TGGATTTCTTTGTGAATCACC............................................    95
.......GATTTCTTTGTGAATCACCAT..........................................    20
.....TGGATTTCTTTGTGAATCACCA...........................................    18
............................................TGGTGGTTTACAAAGTAATT......    18
............................................TGGTGGTTTACAAAGTAATTCA....    13
..............................................GTGGTTTACAAAGTAATTC.....    5
.....TGGATTTCTTTGTGAATC...............................................    3
............................................TGGTGGTTTACAAAGTAAT.......    2
```

```
..............................................GTGGTTTACAAAGTAATTCATA..    2
.....TGGATTTCTTTGTGAATCA..............................................    1
....GTGGATTTCTTTGTGAATCACC............................................    1
.........TTTCTTTGTGAATCACC............................................    1
........ATTTCTTTGTGAATCACCAT..........................................    1
.......GATTTCTTTGTGAATCACCATA.........................................    1
..............................................GTGGTTTACAAAGTAATTCA....    1
.((((((((((.((((((((((((((((......((....)).)))))))))))))))).)))))))))) (-32.90)

8/22534817-22534884(+)_mir_cow_267
GAGGAGGCAAGATGCTGGCATAGCTGTTGAACTGCGAACCTGCTATGCCAACATATTGCCATCTCTCT
....AGGCAAGATGCTGGCATAGCTG.......................................... 5978
....AGGCAAGATGCTGGCATAGCTGT......................................... 1849
....AGGCAAGATGCTGGCATAGCT........................................... 1213
....AGGCAAGATGCTGGCATAGC............................................ 87
........................................TGCTATGCCAACATATTGCCATC..... 75
....AGGCAAGATGCTGGCATAGCTGTT........................................ 53
....AGGCAAGATGCTGGCATAG............................................. 33
........................................TGCTATGCCAACATATTGCCAT...... 27
........................................TGCTATGCCAACATATTGCCA....... 22
....AGGCAAGATGCTGGCATAGCTGTTGAACTGCGAACCT........................... 15
........................................TGCTATGCCAACATATTGCC........ 12
........................................TGCTATGCCAACATATTGCCATCT.... 8
.....GGCAAGATGCTGGCATAGCTG.......................................... 4
.........AGATGCTGGCATAGCTG.......................................... 3
.....GGCAAGATGCTGGCATAGCTGT......................................... 3
.....GGCAAGATGCTGGCATAGCTGTTGAACTGCGAACCT........................... 3
...........................................TATGCCAACATATTGCCATCT.... 3
........AAGATGCTGGCATAGCTG.......................................... 2
.......CAAGATGCTGGCATAGCTGT......................................... 2
.........AGATGCTGGCATAGCTGT......................................... 2
........................................TGCTATGCCAACATATTGC......... 2
.........................................GCTATGCCAACATATTGCCATC..... 2
..........................................CTATGCCAACATATTGCCATC..... 2
........AAGATGCTGGCATAGCT........................................... 1
..........GATGCTGGCATAGCTGT......................................... 1
.........AGATGCTGGCATAGCTGTTGAACT................................... 1
....AGGCAAGATGCTGGCATAGCTGTTGAACTGCGAA.............................. 1
```

```
.......................................CTGCTATGCCAACATATTGCC........ 1
.......................................CTGCTATGCCAACATATTGCCA....... 1
..........................TTGAACTGCGAACCTGCTATGCCAACATATTGCCATC..... 1
............................................ATGCCAACATATTGCCATCTCT.. 1
(((..(((((.(((.(((((((((.(((........)))..))))))))).))).)))))..)))... (-32.00)

8/23661694-23661761(-)_mir_cow_268
GCTGGGTAGTGGGGAACCCTTCCATGAGGAGTAGAACACTCCTTATGCAAGATTCCCTTCTACCTGAC
.......AGTGGGGAACCCTTCCATGAGG....................................... 488
.........................................CTTATGCAAGATTCCCTTCTAC..... 110
.......AGTGGGGAACCCTTCCATGAGGA...................................... 39
..........................................TTATGCAAGATTCCCTTCTAC..... 21
.......AGTGGGGAACCCTTCCATG.......................................... 13
.........................................CTTATGCAAGATTCCCTTCT....... 13
.......AGTGGGGAACCCTTCCATGAG........................................ 9
.........TGGGGAACCCTTCCATGAGGAGT.................................... 9
.........................................CTTATGCAAGATTCCCTTCTA...... 6
.......AGTGGGGAACCCTTCCATGA......................................... 2
......TAGTGGGGAACCCTTCCATGAGG....................................... 2
.........TGGGGAACCCTTCCATGAGG....................................... 2
.......AGTGGGGAACCCTTCCATGAGGAG..................................... 2
..........................................TTATGCAAGATTCCCTTCT....... 2
..........................................TTATGCAAGATTCCCTTCTA...... 2
..........................................TTATGCAAGATTCCCTTCTACC.... 2
.......AGTGGGGAACCCTTCC............................................. 1
..........GGGGAACCCTTCCATGAGG....................................... 1
.........TGGGGAACCCTTCCATGAGGA...................................... 1
.......AGTGGGGAACCCTTCCATGAGGAGTAGAACACTC........................... 1
.............................AGTAGAACACTCCTTATGCAAGATTCCCTT......... 1
.......................................TCCTTATGCAAGATTCCCTTC........ 1
.........................................CTTATGCAAGATTCCCTTC........ 1
...((((((.((((((..(((.((((((((((.....)))))))))).))).)))))).))))))... (-34.00)

8/39940834-39940905(-)_mir_cow_269
CCTTTTTCGGTTATCATGGTACCGATGCTGTATATCTGAAAGGTACAGTACTGTGATAACTGAAGAATGGTG
..........................................GTACAGTACTGTGATAACTGA.........  37572
..........................................GTACAGTACTGTGATAACT...........  24165
...........................................TACAGTACTGTGATAACTGAA........  22051
```

```
...........................................TACAGTACTGTGATAACTGAAG.......   20590
..........................................GTACAGTACTGTGATAACTGAA........   18462
...........................................TACAGTACTGTGATAACTGA.........   17178
..........................................GTACAGTACTGTGATAACTG..........   10206
...........................................TACAGTACTGTGATAACTG..........   4519
...........................................TACAGTACTGTGATAACT...........   3349
...........................................TACAGTACTGTGATAACTGAAGA......   3088
..........................................GTACAGTACTGTGATAAC............   1060
...........................................TACAGTACTGTGATAAC............   312
..........................................GTACAGTACTGTGATAACTGAAG.......   182
...........................................TACAGTACTGTGATAACTGAAGAA.....   111
..........................................GTACAGTACTGTGATA..............   81
............................................ACAGTACTGTGATAACTGAAG.......   28
...............................................GTACTGTGATAACTGAA........   19
.........................................GGTACAGTACTGTGATAACT...........   18
............................................ACAGTACTGTGATAACTGAA........   17
............................................ACAGTACTGTGATAACTGA.........   16
...............................................GTACTGTGATAACTGA.........   16
.............................................CAGTACTGTGATAACTGAA........   12
..........................................GTACAGTACTGTGATAACTGAAGA......   12
.........................................GGTACAGTACTGTGATAACTGA.........   11
...............................................GTACTGTGATAACTGAAG.......   11
.............................................CAGTACTGTGATAACTGAAG.......   9
.........................................GGTACAGTACTGTGATAACTG..........   5
..............................................AGTACTGTGATAACTGAAG.......   5
...............................................GTACTGTGATAACTGAAGA......   5
......TCGGTTATCATGGTACCGATGCT...........................................   4
..........................................GTACAGTACTGTGATAA.............   4
...........................................TACAGTACTGTGATAA.............   4
.........................................GGTACAGTACTGTGATAAC............   4
.......................................AAGGTACAGTACTGTGATAACT...........   4
.............................................CAGTACTGTGATAACTGA.........   4
.............................................CAGTACTGTGATAACTGAAGA......   4
.............................................CAGTACTGTGATAACTGAAGAA.....   4
.............................................CAGTACTGTGATAACTG..........   3
.......................................AAGGTACAGTACTGTGATAACTG..........   2
............................................ACAGTACTGTGATAACTG..........   2
..............................................AGTACTGTGATAACTGA.........   2
```

```
..............................................AGTACTGTGATAACTGAA........  2
.......CGGTTATCATGGTACCGATGCT...........................................  1
....................ACCGATGCTGTATATCTGAAAG..............................  1
.......CGGTTATCATGGTACCGATGCTGTATATCTGAAAGG.............................  1
.................GGTACCGATGCTGTATATCTGAAAGG.............................  1
........................................AGGTACAGTACTGTGATAACT...........  1
............................................ACAGTACTGTGATAACT...........  1
.............................................CAGTACTGTGATAACT...........  1
.........................................GGTACAGTACTGTGATAACTGAA........  1
.......................................AAGGTACAGTACTGTGATAACTGAAG.......  1
............................................ACAGTACTGTGATAACTGAAGA......  1
..........................................GTACAGTACTGTGATAACTGAAGAA.....  1
...........................................TACAGTACTGTGATAACTGAAGAAT....  1
(((((((((((((((((((((.(.((((..............))))).))))))))))))))))))).)).. (-28.40)

8/42110907-42110986(-)_mir_cow_270
ATATTGGGTTGGTCAAAAAGTTTATTTGGGTTTTTCATAAGCTATTATCAAAAAACTCAAACAAACTTTTTGGTCAAGCC
....................................................AAACTCAAACAAACTTTTTGGT......      237
..............AAAAAGTTTATTTGGGTTTTT.............................................      112
..............AAAAAGTTTATTTGGGTTTT..............................................      36
.............CAAAAAGTTTATTTGGGTTTTT.............................................      27
...........GTCAAAAAGTTTATTTGGGTTTT..............................................      19
...................................................AAAACTCAAACAAACTTTTTGG.......      15
.............CAAAAAGTTTATTTGGGTTTT..............................................      13
....................................................AAACTCAAACAAACTTTTTGG.......      12
...........GTCAAAAAGTTTATTTGGGTTT...............................................      9
...................................................AAAACTCAAACAAACTTTTTGGT......      8
...............AAAAGTTTATTTGGGTTTTT.............................................      3
............TCAAAAAGTTTATTTGGGTTTT..............................................      2
..............AAAAAGTTTATTTGGGTTTTTC............................................      2
...............AAAAGTTTATTTGGGTTTTTC............................................      2
...........GTCAAAAAGTTTATTTGGGT.................................................      1
.............CAAAAAGTTTATTTGGGTTT...............................................      1
..............AAAAAGTTTATTTGGGTTT...............................................      1
...........GTCAAAAAGTTTATTTGGGTTTTT.............................................      1
...................................................AAAACTCAAACAAACTTT...........      1
...................................................AAAACTCAAACAAACTTTTT.........      1
..................................................AAAAACTCAAACAAACTTTTTG........      1
```

```
.....................................................AACTCAAACAAACTTTTTGGT......      1
....................................................AAACTCAAACAAACTTTTTGGTC.....      1
.....................................................AACTCAAACAAACTTTTTGGTC.....      1
.....((.(((..((((((((((.(((((((((((.((((....))))..))))))))))).))))))))))..))).))  (-29.90)

8/47259549-47259619(-)_mir_cow_271
CGTGGACTTCCCTTTGTCATCCTATGCCTGAGAATATATGAAGGAGGCTGGGAAGGCAAAGGGACGTTCAA
.......TTCCCTTTGTCATCCTATGCCT..........................................   171500
.......TTCCCTTTGTCATCCTATGCCTG.........................................   40361
.......TTCCCTTTGTCATCCTATGCC...........................................   3653
.......TTCCCTTTGTCATCCTATGCCTGA........................................   451
.......TTCCCTTTGTCATCCTATG.............................................   289
.......TTCCCTTTGTCATCCTATGC............................................   289
......CTTCCCTTTGTCATCCTATGCCT..........................................   123
..............................................GCTGGGAAGGCAAAGGGACGT....   84
..............................................GCTGGGAAGGCAAAGGGACG.....   54
........TCCCTTTGTCATCCTATGCCT..........................................   45
..........CCTTTGTCATCCTATGCCT..........................................   20
......CTTCCCTTTGTCATCCTATGCCTG.........................................   19
........TCCCTTTGTCATCCTATGCCTG.........................................   15
...........CTTTGTCATCCTATGCCT..........................................   11
..............................................GCTGGGAAGGCAAAGGGAC......   9
............TTTGTCATCCTATGCCT..........................................   7
...........CTTTGTCATCCTATGCCTG.........................................   7
..............................................GCTGGGAAGGCAAAGGGACGTT...   7
.......TTCCCTTTGTCATCCTA...............................................   6
......CTTCCCTTTGTCATCCTATGCC...........................................   4
.........CCCTTTGTCATCCTATGCCT..........................................   3
............TTTGTCATCCTATGCCTG.........................................   3
.......TTCCCTTTGTCATCCT................................................   2
.............TTGTCATCCTATGCCT..........................................   2
.........CCCTTTGTCATCCTATGCCTG.........................................   2
.......TTCCCTTTGTCATCCTATGCCTGAG.......................................   2
.............................................GGCTGGGAAGGCAAAGGGACG.....   2
.......TTCCCTTTGTCATCCTAT..............................................   1
........TCCCTTTGTCATCCTATGCC...........................................   1
..........CCTTTGTCATCCTATGCC...........................................   1
..........CCTTTGTCATCCTATGCCTG.........................................   1
```

```
.............TTGTCATCCTATGCCTG.........................................   1
..............TGTCATCCTATGCCTG.........................................   1
............TTTGTCATCCTATGCCTGA........................................   1
.......TTCCCTTTGTCATCCTATGCCTGAGA......................................   1
.......TTCCCTTTGTCATCCTATGCCTGAGAATATATGAAGGA..........................   1
..........CCTTTGTCATCCTATGCCTGAGAATATATGAAGGA..........................   1
.......TTCCCTTTGTCATCCTATGCCTGAGAATATATGAAGGAG.........................   1
...............................GAATATATGAAGGAGGCTGGGAAGGCAAAGGGACGT....   1
..(((((.((((((((((.(((((.((((...............))))))))).)))))))))).))))). (-34.06)

8/5672118-5672186(+)_mir_cow_264
TTGGCATAGGAACATGGAAGATTGTCAGTCATCATCTATTTCTGCCAATTTTCCATGTTCCTGTGCCAG
.....ATAGGAACATGGAAGATTGTC...........................................     21
.....ATAGGAACATGGAAGATTGTCA..........................................     14
............................................CCAATTTTCCATGTTCCTGTGC...     12
.............................................CAATTTTCCATGTTCCTGTGCC..     7
.....ATAGGAACATGGAAGATTGT............................................     2
....CATAGGAACATGGAAGATTGTC...........................................     1
.............................................CAATTTTCCATGTTCCTGTGC...     1
.(((((((((((((((((((((((.(((.............))).))))))))))))))))))))))). (-41.12)

8/75656050-75656118(-)_novel_cow_38
GGCTAAAAAGTTTGTGCGGATTTTTCCATCACATCTGGCTAAAAATCCGAATAAACGTTTTGGCCAACA
...TAAAAAGTTTGTGCGGATTTTT............................................     9
..CTAAAAAGTTTGTGCGGATTTTT............................................     6
..........................................AAATCCGAATAAACGTTTTGGCC....     3
..CTAAAAAGTTTGTGCGGATTTT.............................................     2
...TAAAAAGTTTGTGCGGATTTT.............................................     2
..........................................AAATCCGAATAAACGTTTTGGC.....     1
((((((((.((((((.((((((((((((.......)))..))))))))).)))))).))))))))).... (-27.70)

8/78575549-78575624(-)_mir_cow_272
TTCTGTGTGGAAGACTAGTGATTTTGTTGTTTTTAGATAACTGAATCGACAACAAATCACAGTCTGCCATATGGCA
.......TGGAAGACTAGTGATTTTGTTGTT.............................................    5009
.......TGGAAGACTAGTGATTTTGTTGT..............................................    2722
.................................................CAACAAATCACAGTCTGCCATA.....    685
.......TGGAAGACTAGTGATTTTGTTG...............................................    169
..................................................AACAAATCACAGTCTGCCATA.....    167
```

....CAACAAATCACAGTCTGCCAT......	117
....CAACAAATCACAGTCTGCCA.......	116
....CAACAAATCACAGTCTGCC........	87
.......TGGAAGACTAGTGATTTTGTT..	62
.......TGGAAGACTAGTGATTTTGTTGTTT..	48
.......TGGAAGACTAGTGATTTTGT...	35
....CAACAAATCACAGTCTGCCATAT....	28
....AACAAATCACAGTCTGCCAT......	14
.........GAAGACTAGTGATTTTGTTGTT...	11
....AACAAATCACAGTCTGCCA.......	11
....CAACAAATCACAGTCTGC.........	9
..........AAGACTAGTGATTTTGTTGTT...	8
....AACAAATCACAGTCTGCC........	7
.......TGGAAGACTAGTGATTTTG..	6
.........GAAGACTAGTGATTTTGTTGT..	6
.......TGGAAGACTAGTGATTTTGTTGTTTTT..	5
....ACAAATCACAGTCTGCCATAT....	5
...........AGACTAGTGATTTTGTTGTT...	4
.......TGGAAGACTAGTGATTTTGTTGTTTT...	4
....AACAAATCACAGTCTGCCATAT....	4
..............CTAGTGATTTTGTTGTT...	3
...............................TTTAGATAACTGAATCGA...........................	3
.......TGGAAGACTAGTGATTTT...	2
........GGAAGACTAGTGATTTTGTTGTT...	2
.......TGGAAGACTAGTGATT...	1
.........GAAGACTAGTGATTTTGT...	1
..........AAGACTAGTGATTTTGTT..	1
......GTGGAAGACTAGTGATTTTGTTG...	1
........GGAAGACTAGTGATTTTGTTGT..	1
............GACTAGTGATTTTGTTGT..	1
.............ACTAGTGATTTTGTTGT..	1
..............CTAGTGATTTTGTTGT..	1
............GACTAGTGATTTTGTTGTT...	1
.............ACTAGTGATTTTGTTGTT...	1
...............TAGTGATTTTGTTGTT...	1
.........GAAGACTAGTGATTTTGTTGTTT..	1
.......TGGAAGACTAGTGATTTTGTTGTTTTTAGAT......................................	1
...............................TTTAGATAACTGAATCGACA.........................	1

```
.......................................ACTGAATCGACAACAAATCACAG..............    1
.......................................ACTGAATCGACAACAAATCACAGTCT...........    1
...........................................AATCGACAACAAATCACAGTCTGCC........    1
...................................................ACAAATCACAGTCTGCCATA.....    1
..((((((((.(((((.(((((((.(((((((((((....)))))..)))))))))))))))))).))))))))..  (-31.10)

8/83009610-83009677(+)_mir_Cow_Blast_35
TGCTTGGGTTCCTGGCATGCTGATTTGTGACTTAAGATTAAAATCACATTGCCAGGGATTACCACGCA
..........................................ATCACATTGCCAGGGATTACC..... 38664
..........................................ATCACATTGCCAGGGATTACCA.... 26577
..........................................ATCACATTGCCAGGGATTACCAC... 22261
..........................................ATCACATTGCCAGGGATTAC...... 2799
..........................................ATCACATTGCCAGGGATT........ 634
...........................................TCACATTGCCAGGGATTACCAC... 495
...........................................TCACATTGCCAGGGATTACC..... 381
...........................................TCACATTGCCAGGGATTACCA.... 353
..........................................ATCACATTGCCAGGGATTA....... 297
.........................................AATCACATTGCCAGGGATTACC..... 150
..........................................ATCACATTGCCAGGGAT......... 107
..........................................ATCACATTGCCAGGGATTACCACG.. 102
...........................................TCACATTGCCAGGGATTAC...... 58
.........................................AATCACATTGCCAGGGATTACCA.... 57
.........................................AATCACATTGCCAGGGATTACCAC... 47
..........................................ATCACATTGCCAGGGA.......... 30
.............................................ACATTGCCAGGGATTACCAC... 25
.............................................ACATTGCCAGGGATTACC..... 20
.........................................AATCACATTGCCAGGGATT........ 15
.........................................AATCACATTGCCAGGGATTAC...... 11
.............................................ACATTGCCAGGGATTACCA.... 10
.........................................AATCACATTGCCAGGGAT......... 7
...........................................TCACATTGCCAGGGATTACCACG.. 6
...........................................TCACATTGCCAGGGATT........ 5
.........................................AATCACATTGCCAGGGATTA....... 5
............................................CACATTGCCAGGGATTACCAC... 5
.....GGGTTCCTGGCATGCTGATTT.......................................... 4
...........................................TCACATTGCCAGGGATTA....... 3
.....GGGTTCCTGGCATGCTGAT............................................ 2
....TGGGTTCCTGGCATGCTGATTT.......................................... 2
```

....CACATTGCCAGGGATTACC..... 2
....TTGCCAGGGATTACCA.... 2
.....GGGTTCCTGGCATGCTG.. 1
.........TCCTGGCATGCTGATTTGTGACT.................................... 1
.....GGGTTCCTGGCATGCTGATTTGTGACTTA.................................. 1
.....GGGTTCCTGGCATGCTGATTTGTGACTTAAGATTAAAA......................... 1
...................CTGATTTGTGACTTAAGATTAAAATC....................... 1
....ACATTGCCAGGGATTA....... 1
....CACATTGCCAGGGATTAC...... 1
....ACATTGCCAGGGATTAC...... 1
....CATTGCCAGGGATTAC...... 1
....AAATCACATTGCCAGGGATTACC..... 1
....CATTGCCAGGGATTACC..... 1
....ATTGCCAGGGATTACC..... 1
....ATTGCCAGGGATTACCA.... 1
....CATTGCCAGGGATTACCAC... 1
....ATTGCCAGGGATTACCAC... 1
....TTGCCAGGGATTACCAC... 1
....ACATTGCCAGGGATTACCACG.. 1
.((.(((((((((((((((.((((((..............)))))))).))))))))))..))).)). (-26.34)

8/83009836-83009909(+)_mir_cow_273
GGTGCAGAGCTTAGCTGATTGGTGAACAGTGACTGGTTTCCGCTTTGTTCACAGTGGCTAAGTTCTGCACCTGA
....TTCACAGTGGCTAAGTTCTG.......982977
....TTCACAGTGGCTAAGTTCTGC......929381
....TTCACAGTGGCTAAGTTCTGCA.....40476
....TTCACAGTGGCTAAGTTCT........31014
....TTCACAGTGGCTAAGTTC.........3568
....GTTCACAGTGGCTAAGTTCTG.......2234
....TTCACAGTGGCTAAGTTCTGCAC....1636
....GTTCACAGTGGCTAAGTTCTGC......1157
.....AGAGCTTAGCTGATTGGTGAAC...967
....TCACAGTGGCTAAGTTCTG.......537
....CAGTGGCTAAGTTCTG.......504
....TCACAGTGGCTAAGTTCTGC......377
....TTCACAGTGGCTAAGTT..........179
....CACAGTGGCTAAGTTCTG.......154
....CACAGTGGCTAAGTTCTGC......146

```
....CAGAGCTTAGCTGATTGGTGAAC............................................... 142
...............................................TTCACAGTGGCTAAGT........... 98
..................................................ACAGTGGCTAAGTTCTG....... 86
.....AGAGCTTAGCTGATTGGTGAACA.............................................. 78
................................................TCACAGTGGCTAAGTTCTGCAC.... 61
..............................................GTTCACAGTGGCTAAGTTCT........ 60
..............................................GTTCACAGTGGCTAAGTTCTGCA..... 59
................................................TCACAGTGGCTAAGTTCTGCA..... 54
....................................................AGTGGCTAAGTTCTGC...... 41
...................................................CAGTGGCTAAGTTCTGC...... 31
.....AGAGCTTAGCTGATTGGTGAACAGT............................................ 24
..................................................ACAGTGGCTAAGTTCTGC...... 22
.....AGAGCTTAGCTGATTGGTGAACAG............................................. 16
.....AGAGCTTAGCTGATTGGTGA................................................. 14
....CAGAGCTTAGCTGATTGGTGAACA.............................................. 14
................................................TCACAGTGGCTAAGTTCT........ 11
....CAGAGCTTAGCTGATTGGTGA................................................. 9
....CAGAGCTTAGCTGATTGGTGAA................................................ 7
.................................................CACAGTGGCTAAGTTCTGCA..... 6
.....AGAGCTTAGCTGATTGGTGAA................................................ 4
.................................................CACAGTGGCTAAGTTCT........ 4
.............................................TGTTCACAGTGGCTAAGTTCTG....... 4
...............................................TTCACAGTGGCTAAGTTCTGCACC... 4
....CAGAGCTTAGCTGATTGGT................................................... 3
....CAGAGCTTAGCTGATTGGTG.................................................. 3
.....AGAGCTTAGCTGATTGGTG.................................................. 3
.....AGAGCTTAGCTGATTGGTGAACAGTGACT........................................ 3
.................................................CACAGTGGCTAAGTTCTGCAC.... 3
................................................TCACAGTGGCTAAGTTCTGCACC... 3
......GAGCTTAGCTGATTGGTGAAC............................................... 2
....CAGAGCTTAGCTGATTGGTGAACAGTGA.......................................... 2
....CAGAGCTTAGCTGATTGGTGAACAGTGACT........................................ 2
..............................................GTTCACAGTGGCTAAGTTC......... 2
.................................................CACAGTGGCTAAGTTC......... 2
...............................................TTCACAGTGGCTAAGTTCTGCACCT.. 2
....CAGAGCTTAGCTGATTGGTGAACAGTGACTGGTTTCC................................. 1
...........................................TTTGTTCACAGTGGCTAAGTTCT........ 1
..................................................ACAGTGGCTAAGTTCT........ 1
```

....TTGTTCACAGTGGCTAAGTTCTG.......1
....TTGTTCACAGTGGCTAAGTTCTGC......1
....CTTTGTTCACAGTGGCTAAGTTCTGCA.....1
....CAGTGGCTAAGTTCTGCA.....1
....GTTCACAGTGGCTAAGTTCTGCAC....1
....GTGGCTAAGTTCTGCAC....1
....CACAGTGGCTAAGTTCTGCACC...1
....ACAGTGGCTAAGTTCTGCACC...1
....ACAGTGGCTAAGTTCTGCACCT..1
((((((((((((((((....((((((((....(((...)))..))))))))..))))))))))))))))... (-39.80)

8/83010377-83010445(+)_mir_Cow_Blast_23
CTCCGGTGCCTACTGAGCTGATATCAGTTCTCATTTTACACTGGCTCAGTTCAGCAGGAACAGGAGT
....TGGCTCAGTTCAGCAGGAAC...... 29631
....TGGCTCAGTTCAGCAGGAACA..... 9452
....TGGCTCAGTTCAGCAGGAACAG.... 5133
....TGGCTCAGTTCAGCAGGAA....... 300
....TGGCTCAGTTCAGCAGGAACAGG... 204
.....GTGCCTACTGAGCTGATATCAGT....................................... 111
....TGGCTCAGTTCAGCAGGA........ 109
....CTGGCTCAGTTCAGCAGGAAC...... 100
.....GTGCCTACTGAGCTGATATCAG.. 55
.....GTGCCTACTGAGCTGATATC.. 52
.....GTGCCTACTGAGCTGATATCA... 50
....GGTGCCTACTGAGCTGATATCA... 46
......TGCCTACTGAGCTGATATCAGT....................................... 40
....GGTGCCTACTGAGCTGATATC.. 33
....GCTCAGTTCAGCAGGAACAG.... 27
....GCTCAGTTCAGCAGGAACAGG... 27
....TGGCTCAGTTCAGCAGGAACAGGA.. 27
....TGGCTCAGTTCAGCAGG......... 23
....GGCTCAGTTCAGCAGGAACAG.... 22
....GGTGCCTACTGAGCTGATATCAGT....................................... 21
....GGCTCAGTTCAGCAGGAAC...... 20
....GCTCAGTTCAGCAGGAAC...... 17
....GGCTCAGTTCAGCAGGAACA..... 17
......TGCCTACTGAGCTGATATC.. 16
....GGTGCCTACTGAGCTGATATCAG.. 16

```
.............................................GCTCAGTTCAGCAGGAACAGGA..      16
......TGCCTACTGAGCTGATATCA...........................................      15
..............................................CTCAGTTCAGCAGGAAC......      10
............................................GGCTCAGTTCAGCAGGAACAGG...      10
.............................................GCTCAGTTCAGCAGGAACA.....      8
......TGCCTACTGAGCTGATATCAG..........................................      7
..............................................CTCAGTTCAGCAGGAACA.....      7
..........................................CTGGCTCAGTTCAGCAGGAACA.....      6
....GGTGCCTACTGAGCTGATAT.............................................      3
..........................................CTGGCTCAGTTCAGCAGGAACAG....      3
.....GTGCCTACTGAGCTGATAT.............................................      2
....GGTGCCTACTGAGCTGATA..............................................      1
........CCTACTGAGCTGATATCA...........................................      1
...........................TTCTCATTTTACACACTGGCTC....................      1
..........................................CTGGCTCAGTTCAGCAGGA........      1
.........................................ACTGGCTCAGTTCAGCAGGAAC......      1
...............................................TCAGTTCAGCAGGAACA.....      1
..............................................CTCAGTTCAGCAGGAACAG....      1
................................................CAGTTCAGCAGGAACAG....      1
............................................GGCTCAGTTCAGCAGGAACAGGA..      1
((((.((.(((.(((((((((..(((((.............))))).))))))))).))).)).)))). (-26.92)

8/86884872-86884952(+)_mir_Cow_Blast_47
TGGGATGAGGTAGTAGGTTGTATAGTTTTAGGGTCACACCCACCACTGGGAGATAACTATACAATCTACTGTCTTTCCTAA
.....TGAGGTAGTAGGTTGTATAGTT......................................................    600031
.....TGAGGTAGTAGGTTGTATAGT.......................................................    269183
.....TGAGGTAGTAGGTTGTATAGTTT.....................................................    31072
.....TGAGGTAGTAGGTTGTATAG........................................................    29911
.....TGAGGTAGTAGGTTGTAT..........................................................    7314
.....TGAGGTAGTAGGTTGTATA.........................................................    4091
.....TGAGGTAGTAGGTTGTA...........................................................    1031
........................................................CTATACAATCTACTGTCTTTCC...    770
.....TGAGGTAGTAGGTTGTATAGTTTT....................................................    485
........................................................CTATACAATCTACTGTCTTTC....    382
.....TGAGGTAGTAGGTTGT............................................................    343
..........TAGTAGGTTGTATAGTT......................................................    174
......GAGGTAGTAGGTTGTATAGTT......................................................    169
.........................................................TATACAATCTACTGTCTTTCCT..    169
```

........GGTAGTAGGTTGTATAGTT..	155
...........AGTAGGTTGTATAGTT..	114
....CTATACAATCTACTGTCTTTCCT..	97
........GGTAGTAGGTTGTATAGT...	76
..........TAGTAGGTTGTATAGT...	76
......GAGGTAGTAGGTTGTATAGT...	53
.........GTAGTAGGTTGTATAGTT..	53
....CTATACAATCTACTGTCTTT.....	53
....TATACAATCTACTGTCTTTCC...	40
.......AGGTAGTAGGTTGTATAGTT..	37
.......AGGTAGTAGGTTGTATAGT...	23
.........GTAGTAGGTTGTATAGT...	17
....CTATACAATCTACTGTCTT......	17
....ATGAGGTAGTAGGTTGTATAGTT..	15
....ATACAATCTACTGTCTTTCCT..	15
....CTATACAATCTACTGTCT.......	10
........GGTAGTAGGTTGTATAG..	9
....ATGAGGTAGTAGGTTGTATAGT...	9
............GTAGGTTGTATAGTTT...	9
...........................TTAGGGTCACACCCACCACTGGG...............................	9
......GAGGTAGTAGGTTGTATAGTTT...	8
..........TAGTAGGTTGTATAGTTT...	8
....TATACAATCTACTGTCTTTC....	8
...........................TTAGGGTCACACCCACCACTGGGA..............................	7
.....TGAGGTAGTAGGTTGTATAGTTTTA...	6
.........GTAGTAGGTTGTATAG..	4
........GGTAGTAGGTTGTATAGTTT...	4
.........GTAGTAGGTTGTATAGTTT...	4
.....TGAGGTAGTAGGTTGTATAGTTTTAGGGT...	4
......GAGGTAGTAGGTTGTATAG..	3
.......AGGTAGTAGGTTGTATAGTTT...	3
...........AGTAGGTTGTATAGTTT...	3
..........TAGTAGGTTGTATAGTTTTAGG...	3
.....TGAGGTAGTAGGTTGTATAGTTTTAGGGTC..	3
....CTATACAATCTACTGT.........	3
....ACTATACAATCTACTGTCTTTC....	3
....ATACAATCTACTGTCTTTCC...	3
...........................TTAGGGTCACACCCACCACTG.................................	2

...........................TTAGGGTCACACCCACCACTGG................................ 2
....GATAACTATACAATCTACTGT......... 2
....GATAACTATACAATCTACTGTC........ 2
....ATAACTATACAATCTACTGTCTTT..... 2
....ATAACTATACAATCTACTGTCTTTC.... 2
......GAGGTAGTAGGTTGTA... 1
......GAGGTAGTAGGTTGTAT.. 1
.......AGGTAGTAGGTTGTAT.. 1
......GAGGTAGTAGGTTGTATA... 1
....ATGAGGTAGTAGGTTGTATAG.. 1
.......AGGTAGTAGGTTGTATAG.. 1
....ATGAGGTAGTAGGTTGTATAGTTT... 1
........GGTAGTAGGTTGTATAGTTTT.. 1
..........TAGTAGGTTGTATAGTTTT.. 1
...........AGTAGGTTGTATAGTTTT.. 1
.....TGAGGTAGTAGGTTGTATAGTTTTAGG... 1
.....TGAGGTAGTAGGTTGTATAGTTTTAGGG.. 1
.....TGAGGTAGTAGGTTGTATAGTTTTAGGGTCAC.. 1
...........................TTAGGGTCACACCCACCACTGGGAG............................. 1
...........................TTAGGGTCACACCCACCACTGGGAGA............................ 1
....AGATAACTATACAATCTACTGT......... 1
....AGATAACTATACAATCTACTGTC........ 1
....ATAACTATACAATCTACTGTCT....... 1
....TATACAATCTACTGTCTTT..... 1
....AACTATACAATCTACTGTCTTTC.... 1
....ATACAATCTACTGTCTTTC.... 1
....CAATCTACTGTCTTTCC... 1
(((((.(((..((((((((((((((((...(((.....))).((....))....))))))))))))))))..)))))))). (-36.40)

8/86885226-86885312(+)_mir_cow_274
CAGAGTGAGGTAGTAGATTGTATAGTTGTGGGGTAGTGATTTTACCCTGTTCAGGAGATAACTATACAATCTATTGCCTTCCCTGAG
.....TGAGGTAGTAGATTGTATAGTT.. 418867
.....TGAGGTAGTAGATTGTATAGT... 145549
.....TGAGGTAGTAGATTGTATAG.. 11184
.....TGAGGTAGTAGATTGTAT.. 2884
.....TGAGGTAGTAGATTGTATA... 2385
.....TGAGGTAGTAGATTGTATAGTTG... 1315
.....TGAGGTAGTAGATTGTA... 563

```
......GAGGTAGTAGATTGTATAGTT.............................................................    153
..............................................................CTATACAATCTATTGCCTTCC.....    130
.....TGAGGTAGTAGATTGT...................................................................    125
...........AGTAGATTGTATAGTT.............................................................    123
..........TAGTAGATTGTATAGTT.............................................................    102
........GGTAGTAGATTGTATAGTT.............................................................    70
.....TGAGGTAGTAGATTGTATAGTTGT...........................................................    63
......GAGGTAGTAGATTGTATAGT..............................................................    59
.........GTAGTAGATTGTATAGTT.............................................................    53
.......AGGTAGTAGATTGTATAGTTGT...........................................................    48
.......AGGTAGTAGATTGTATAGTT.............................................................    42
........GGTAGTAGATTGTATAGT..............................................................    34
..........TAGTAGATTGTATAGT..............................................................    25
.........GTAGTAGATTGTATAGT..............................................................    20
.......AGGTAGTAGATTGTATAGT..............................................................    10
............................................................AACTATACAATCTATTGCCTTC......    9
.....TGAGGTAGTAGATTGTATAGTTGTGGGG.......................................................    8
.............................................................ACTATACAATCTATTGCCTTCC.....    6
.......AGGTAGTAGATTGTATAGTTG............................................................    5
.....TGAGGTAGTAGATTGTATAGTTGTGGGGTAG....................................................    5
......GAGGTAGTAGATTGTATAG...............................................................    4
.....TGAGGTAGTAGATTGTATAGTTGTGGGGTAGTGA.................................................    4
..............................................................CTATACAATCTATTGCCTTC......    4
.......AGGTAGTAGATTGTATAG...............................................................    3
........GGTAGTAGATTGTATAG...............................................................    3
.........GTAGTAGATTGTATAG...............................................................    3
.....TGAGGTAGTAGATTGTATAGTTGTGGGGT......................................................    3
....GTGAGGTAGTAGATTGTATAGTT.............................................................    2
...........AGTAGATTGTATAGTTG............................................................    2
.....TGAGGTAGTAGATTGTATAGTTGTGG.........................................................    2
.....TGAGGTAGTAGATTGTATAGTTGTGGG........................................................    2
............................................................AACTATACAATCTATTGCCTTCC.....    2
..............................................................CTATACAATCTATTGCCTTCCC....    2
..........TAGTAGATTGTATAGTTGT...........................................................    1
.......AGGTAGTAGATTGTATAGTTGTG..........................................................    1
..........TAGTAGATTGTATAGTTGTGG.........................................................    1
.....TGAGGTAGTAGATTGTATAGTTGTGGGGTA.....................................................    1
.....TGAGGTAGTAGATTGTATAGTTGTGGGGTAGT...................................................    1
```

.....TGAGGTAGTAGATTGTATAGTTGTGGGGTAGTG... 1
.................TTGTATAGTTGTGGGGTAGTGATTTTACCC.. 1
....AACTATACAATCTATTG........... 1
....CTGTTCAGGAGATAACTATACAATCTATTGCCT........ 1
....TAACTATACAATCTATTGCCT........ 1
....AACTATACAATCTATTGCCT........ 1
....CTATACAATCTATTGCCT........ 1
....TATACAATCTATTGCCTTCCCT... 1
(((.(..(((((((((((((((((((((((((((((.....))))))).........))))))))))))))))))))))..)))).. (-42.50)

8/86887433-86887517(+)_mir_cow_275
TAGGAAGAGGTAGTAGGTTGCATAGTTTTCGGGCAGGGATTTTGCCCACAAGGAGGTAACTATACGACCTGCTGCCTTTCTTAGG
.....AGAGGTAGTAGGTTGCATAGTT.. 14345
.....AGAGGTAGTAGGTTGCATAGT... 5500
....CTATACGACCTGCTGCCTTTCT.... 3871
.....AGAGGTAGTAGGTTGCATAGTTT... 771
.....AGAGGTAGTAGGTTGCATAG.. 754
....CTATACGACCTGCTGCCTTTC..... 580
....TATACGACCTGCTGCCTTTCT.... 200
....CTATACGACCTGCTGCCTTT...... 108
.....AGAGGTAGTAGGTTGCAT.. 80
.....AGAGGTAGTAGGTTGCATA... 69
....AAGAGGTAGTAGGTTGCATAGT... 69
....AAGAGGTAGTAGGTTGCATAGTT.. 63
....TATACGACCTGCTGCCTTTC..... 23
.....AGAGGTAGTAGGTTGCA... 21
.....AGAGGTAGTAGGTTGCATAGTTTT.. 20
....CTATACGACCTGCTGCCT........ 16
....CTATACGACCTGCTGCCTT....... 14
......GAGGTAGTAGGTTGCATAGTT.. 12
....CTATACGACCTGCTGCCTTTCTT... 11
.....AGAGGTAGTAGGTTGC.. 8
....AAGAGGTAGTAGGTTGCATAG.. 5
....TATACGACCTGCTGCCTTT...... 5
........GGTAGTAGGTTGCATAGT... 4
........GGTAGTAGGTTGCATAGTT.. 4
..........TAGTAGGTTGCATAGTT.. 4
...........................TTCGGGCAGGGATTTTGCC....................................... 4

```
...........................TTCGGGCAGGGATTTTGCCCACAAGGAGGTA........................... 4
...........................................................CTATACGACCTGCTGCC......... 4
........................................................TAACTATACGACCTGCTGCCT........ 4
.....AGAGGTAGTAGGTTGCATAGTTTTCGGGCAGGGA.............................................. 3
.........................................................AACTATACGACCTGCTGCCT........ 3
.............................................................ATACGACCTGCTGCCTTTCT.... 3
.....AGAGGTAGTAGGTTGCATAGTTTTCGGGC................................................... 2
...........................TTCGGGCAGGGATTTTGCCCACAAGGAGGT............................ 2
...........................TTCGGGCAGGGATTTTGCCCACAAGGAGGTAAC......................... 2
............................................................TATACGACCTGCTGCCTTTCTT... 2
.......AGGTAGTAGGTTGCATA............................................................. 1
.......AGGTAGTAGGTTGCATAG............................................................ 1
........GGTAGTAGGTTGCATAG............................................................ 1
.......AGGTAGTAGGTTGCATAGT........................................................... 1
.......AGGTAGTAGGTTGCATAGTT.......................................................... 1
.........GTAGTAGGTTGCATAGTT.......................................................... 1
...........AGTAGGTTGCATAGTT.......................................................... 1
....AAGAGGTAGTAGGTTGCATAGTTT......................................................... 1
......GAGGTAGTAGGTTGCATAGTTT......................................................... 1
..........TAGTAGGTTGCATAGTTTTCG...................................................... 1
.....AGAGGTAGTAGGTTGCATAGTTTTCGGGCAGG................................................ 1
...........................TTCGGGCAGGGATTTTGCCC...................................... 1
...........................TTCGGGCAGGGATTTTGCCCACA................................... 1
...........................TTCGGGCAGGGATTTTGCCCACAAGGAGGTAA.......................... 1
.............................................................ATACGACCTGCTGCCTTT...... 1
............................................CCCACAAGGAGGTAACTATACGACCTGCTGCCTTTC..... 1
........................................................TAACTATACGACCTGCTGCCTTTC..... 1
(((((.((((((((((((((.((((((.((((((((.....)))))).......)).)))))).))))))))))))))))))).. (-38.41)

8/9153388-9153457(+)_mir_cow_265
CTCTCCGTGTTCACAGCGGACCTTGATTTAAATGTCCATACAATTAAGGCACGCGGTGAATGCCAAGAAT
............................................TAAGGCACGCGGTGAATGCC......     62579
............................................TAAGGCACGCGGTGAATGCCA.....     19991
...........................................TTAAGGCACGCGGTGAATGCC......     15984
...........................................TTAAGGCACGCGGTGAATGCCA.....     5129
............................................TAAGGCACGCGGTGAATGC.......     5040
...........................................TTAAGGCACGCGGTGAATGC.......     2796
............................................TAAGGCACGCGGTGAATGCCAA....     2398
```

```
............................................TAAGGCACGCGGTGAATG........    649
...........................................TTAAGGCACGCGGTGAATG........    534
...........................................TTAAGGCACGCGGTGAATGCCAA....    418
...........................................TTAAGGCACGCGGTGAAT.........    294
.....CGTGTTCACAGCGGACCTTGAT...........................................    171
............................................TAAGGCACGCGGTGAATGCCAAG...    155
.............................................AAGGCACGCGGTGAATGCC......    109
.....CGTGTTCACAGCGGACCTTGA............................................    95
............................................TAAGGCACGCGGTGAATGCCAAGA..    83
.............................................AAGGCACGCGGTGAATGCCA.....    76
.....CGTGTTCACAGCGGACCTTGATT..........................................    44
.................................................CACGCGGTGAATGCCA.....    42
............................................TAAGGCACGCGGTGAAT.........    41
............................................TAAGGCACGCGGTGAA..........    31
....CCGTGTTCACAGCGGACCTTG.............................................    20
...........................................TTAAGGCACGCGGTGAA..........    18
.............................................AAGGCACGCGGTGAATGCCAA....    17
...........................................TTAAGGCACGCGGTGAATGCCAAGA..    14
....CCGTGTTCACAGCGGACCTTGA............................................    13
.....CGTGTTCACAGCGGACCT...............................................    10
..............................................AGGCACGCGGTGAATGCC......    9
.................................................CACGCGGTGAATGCCAA....    8
...........................................TTAAGGCACGCGGTGAATGCCAAG...    8
.....CGTGTTCACAGCGGACC................................................    7
.....CGTGTTCACAGCGGACCTTG.............................................    7
................................................GCACGCGGTGAATGCCA.....    7
...........................................TTAAGGCACGCGGTGA...........    4
................................................GCACGCGGTGAATGCC......    4
.............................................AAGGCACGCGGTGAATGC.......    3
.....CGTGTTCACAGCGGACCTT..............................................    2
.......TGTTCACAGCGGACCTTGATT..........................................    2
.....CGTGTTCACAGCGGACCTTGATTTAAA......................................    2
..................................................ACGCGGTGAATGCCAA....    2
.............................................AAGGCACGCGGTGAATGCCAAG...    2
....CCGTGTTCACAGCGGAC.................................................    1
....CCGTGTTCACAGCGGACC................................................    1
....CCGTGTTCACAGCGGACCT...............................................    1
............................TAAATGTCCATACAATTAAGG.....................    1
```

```
...........................TAAATGTCCATACAATTAAGGC....................     1
............................................AAGGCACGCGGTGAATG........     1
.......................................CAATTAAGGCACGCGGTGAATGC.......     1
.........................................ATTAAGGCACGCGGTGAATGCC......     1
.............................................AGGCACGCGGTGAATGCCA.....     1
............................................AAGGCACGCGGTGAATGCCAAGA..     1
.(((..((((((((.(((..((((((((............))))))))..))).))))))))..))).. (-24.42)

9/10730933-10731006(-)_mir_cow_278
CAGATACTGTAAACACCCTACACTCTCAGCTGTGCACAGTGAGAAAGCTGGGAGAAGGCTGTTTACTCTCTCTG
.......TGTAAACACCCTACACTCTCAGCT...........................................22733
.......TGTAAACACCCTACACTCTCAGC............................................9987
.......TGTAAACACCCTACACTCTCAG.............................................1552
...............................................CTGGGAGAAGGCTGTTTACTCT.....1472
.......TGTAAACACCCTACACTCTCA..............................................1222
.......TGTAAACACCCTACACTCTCAGCTG..........................................237
...............................................CTGGGAGAAGGCTGTTTACT.......217
...............................................CTGGGAGAAGGCTGTTTACTC......173
.......TGTAAACACCCTACACTCTC...............................................98
................................................TGGGAGAAGGCTGTTTACTCT.....57
.......TGTAAACACCCTACACTCT................................................47
.......TGTAAACACCCTACACTCTCAGCTGT.........................................17
...............................................CTGGGAGAAGGCTGTTTAC........14
......CTGTAAACACCCTACACTCTCAGCT...........................................13
......CTGTAAACACCCTACACTCTCAGC............................................10
........GTAAACACCCTACACTCTCAGCT...........................................7
................................................TGGGAGAAGGCTGTTTACT.......6
.......TGTAAACACCCTACACTC.................................................4
...............CCCTACACTCTCAGCT...........................................4
................................................TGGGAGAAGGCTGTTTACTC......4
...............................................CTGGGAGAAGGCTGTTTACTCTC....4
.......TGTAAACACCCTACACT..................................................3
........GTAAACACCCTACACTCTCA..............................................3
........GTAAACACCCTACACTCTCAGC............................................3
..........AAACACCCTACACTCTCAGCT...........................................2
......CTGTAAACACCCTACACTCTCA..............................................1
......CTGTAAACACCCTACACTCTCAG.............................................1
............ACACCCTACACTCTCAGC............................................1
```

..............ACCCTACACTCTCAGC..1
.........TAAACACCCTACACTCTCAGCT...1
..............ACCCTACACTCTCAGCT...1
........GTAAACACCCTACACTCTCAGCTG..1
.......TGTAAACACCCTACACTCTCAGCTGTGCACAG...................................1
...............................GTGCACAGTGAGAAAG...........................1
....CTGGGAGAAGGCTGTTT..........1
....CTGGGAGAAGGCTGTTTA.........1
................................TGCACAGTGAGAAAGCTGGGAGAAGGCTGTTTACTCT.....1
....GAGAAGGCTGTTTACTCT.....1
((((....(((((((.(((...(((((((((...(.....)....))))))))).))).)))))))....)))) (-27.60)

9/10768278-10768354(-)_mir_cow_279
TGAGCGGCTGTAAACATCCTCGACTGGAAGCTGTGAGGCTGCAGAAAGGCTTTCAGTCGGATGTTTGCAGCTGCCGA
........TGTAAACATCCTCGACTGGAAGCT... 747692
........TGTAAACATCCTCGACTGGA... 509061
........TGTAAACATCCTCGACTGGAAGC.. 131329
........TGTAAACATCCTCGACTGGAA.. 106139
........TGTAAACATCCTCGACTGGAAG... 40865
....CTTTCAGTCGGATGTTTGCAGC...... 9692
.........GTAAACATCCTCGACTGGAAGCT... 6207
....CTTTCAGTCGGATGTTTGCAG....... 4770
........TGTAAACATCCTCGACTGG.. 2480
..........TAAACATCCTCGACTGGAAGCT... 2215
........TGTAAACATCCTCGAC... 1930
....TTTCAGTCGGATGTTTGCAGC...... 1079
.........GTAAACATCCTCGACTGGA... 699
.......CTGTAAACATCCTCGACTGGA... 505
....CTTTCAGTCGGATGTTTGCA........ 505
........TGTAAACATCCTCGACT.. 472
.......CTGTAAACATCCTCGACTGGAAGCT... 472
....CTTTCAGTCGGATGTTTGC......... 430
....TTTCAGTCGGATGTTTGCAGCT..... 324
.........GTAAACATCCTCGACTGGAAGC.. 308
....CTTTCAGTCGGATGTTTGCAGCT..... 289
....TTTCAGTCGGATGTTTGCAG....... 177
................TCCTCGACTGGAAGCT... 159
........TGTAAACATCCTCGACTG... 147

.........GTAAACATCCTCGACTGGAA..	110
..........TAAACATCCTCGACTGGAAGCTGT...	84
.......CTGTAAACATCCTCGACTGGAA..	76
.......CTGTAAACATCCTCGACTGGAAGC..	73
..........TAAACATCCTCGACTGGAAGC..	56
.........GTAAACATCCTCGACTGGAAG...	52
...............ATCCTCGACTGGAAGCT...	42
..........TAAACATCCTCGACTGGA...	36
.......CTGTAAACATCCTCGACTGGAAG...	31
.............ACATCCTCGACTGGAAGCT...	25
........TGTAAACATCCTCGACTGGAAGCTG..	22
...........AAACATCCTCGACTGGAAGCT...	20
..............CATCCTCGACTGGAAGCT...	18
............AACATCCTCGACTGGAAGCT...	15
....TTCAGTCGGATGTTTGCAGC......	15
........TGTAAACATCCTCGACTGGAAGCTGT...	13
....CTTTCAGTCGGATGTTTG..........	13
..........TAAACATCCTCGACTGGAAGCTG..	12
....TTTCAGTCGGATGTTTGCA........	11
..........TAAACATCCTCGACTGGAA..	10
..........TAAACATCCTCGACTGGAAG...	10
....TTTCAGTCGGATGTTTGC.........	10
...............ATCCTCGACTGGAAGC..	7
....CTTTCAGTCGGATGTT............	6
...........AAACATCCTCGACTGGAAGC..	5
............AACATCCTCGACTGGAAGC..	5
....CTTTCAGTCGGATGTTT...........	5
..............CATCCTCGACTGGAAGC..	4
...........AAACATCCTCGACTGGAA..	3
...........AAACATCCTCGACTGGAAG...	3
....TCAGTCGGATGTTTGCAGC......	3
.......CTGTAAACATCCTCGAC...	2
......GCTGTAAACATCCTCGACTGGA...	2
...........AAACATCCTCGACTGGA...	2
.............ACATCCTCGACTGGAA..	2
.............ACATCCTCGACTGGAAGC..	2
....CTTTCAGTCGGATGTTTGCAGCTG....	2
....TTTCAGTCGGATGTTTGCAGCTG....	2

```
.......CTGTAAACATCCTCGACTGG..................................................   1
.........GTAAACATCCTCGACTGG..................................................   1
............AACATCCTCGACTGGA.................................................   1
............AACATCCTCGACTGGAA................................................   1
............AACATCCTCGACTGGAAG...............................................   1
.............ACATCCTCGACTGGAAG...............................................   1
.........GTAAACATCCTCGACTGGAAGCTGT...........................................   1
..........TAAACATCCTCGACTGGAAGCTGTGAGGC......................................   1
...............ATCCTCGACTGGAAGCTGTGAGGCTGC...................................   1
........TGTAAACATCCTCGACTGGAAGCTGTGAGGCTGCAGAAAGG............................   1
................................GTGAGGCTGCAGAAAGG............................   1
.......................................TGCAGAAAGGCTTTCAGTCGGATG..............   1
..................................................TTTCAGTCGGATGTTTG..........   1
...............................................GGCTTTCAGTCGGATGTTTGC.........   1
...................................................TTCAGTCGGATGTTTGCAG.......   1
......................................CTGCAGAAAGGCTTTCAGTCGGATGTTTGCAGC......   1
.......................................TGCAGAAAGGCTTTCAGTCGGATGTTTGCAGC......   1
...........................................GAAAGGCTTTCAGTCGGATGTTTGCAGC......   1
.............................................AAGGCTTTCAGTCGGATGTTTGCAGC......   1
.....................................................CAGTCGGATGTTTGCAGC......   1
......................................................AGTCGGATGTTTGCAGC......   1
...................................................TTCAGTCGGATGTTTGCAGCT.....   1
....................................................TCAGTCGGATGTTTGCAGCT.....   1
.(.(((((((((((((..((((((((((((((.((......))....)))))))))))))))))))))))))))).. (-43.80)

9/39435259-39435335(-)_mir_cow_280
CTGACCAACTTTAAGTATACGATGAACTGCAGGGAAGCCTGGCATGCTGCAGTTCATCGTATACTTAAAGTTGGTCA
.........TTTAAGTATACGATGAACTGC...............................................   6
.........TTTAAGTATACGATGAACTGCA..............................................   5
.......ACTTTAAGTATACGATGAACT.................................................   2
......AACTTTAAGTATACGATGAACT.................................................   1
.........TTTAAGTATACGATGAACT.................................................   1
.................................................CAGTTCATCGTATACTTAAAGT......   1
...................................................GTTCATCGTATACTTAAAGTTG....   1
..((((((((((((((((((((((((((((((.(..((...)).).)))))))))))))))))))))))))))))). (-51.90)

9/39435260-39435332(+)_mir_cow_281
GACCAACTTTAAGTATACGATGAACTGCAGCATGCCAGGCTTCCCTGCAGTTCATCGTATACTTAAAGTTGGT
```

```
.......TTTAAGTATACGATGAACTGC............................................. 6
.......TTTAAGTATACGATGAACTGCA............................................ 5
.....ACTTTAAGTATACGATGAACT............................................... 2
....AACTTTAAGTATACGATGAACT............................................... 1
.......TTTAAGTATACGATGAACT............................................... 1
...............................................CAGTTCATCGTATACTTAAAGT.... 1
.................................................GTTCATCGTATACTTAAAGTTG.. 1
.(((((((((((((((((((((((((((((...((...))....))))))))))))))))))))))))))))) (-47.90)

9/61455912-61455985(-)_mir_cow_282
GCGTCGCACAGCTCCAGGGGACGCCGTTCCTGTCCTGGACTATGTGAGTGGTGCTCCCTGGAGCTGAGCAGCGC
......CACAGCTCCAGGGGACGCCGTT..............................................32
......CACAGCTCCAGGGGACGCCGTTC.............................................16
................................................TGGTGCTCCCTGGAGCTGAGC.....14
................................................TGGTGCTCCCTGGAGCTGAGCA....9
...............................................GTGGTGCTCCCTGGAGCTGAGC.....5
......CACAGCTCCAGGGGACGCCG................................................1
......CACAGCTCCAGGGGACGCCGT...............................................1
...............................................GTGGTGCTCCCTGGAGCTGAG......1
................................................TGGTGCTCCCTGGAGCTGAG......1
((((.((.(((((((((((..(((((((((.(((...)))...).))))))))..))))))))))).)).)))) (-45.30)

X/101844106-101844175(+)_mir_cow_329
GTTTCTCCGTTTGCCTGTTTTGCTGATGTTCATTTGACTTATTCTCAGCAAAATAAGCAAATGGAAAATT
....CTCCGTTTGCCTGTTTTGCTGA............................................    75613
....CTCCGTTTGCCTGTTTTGCTG.............................................    5213
....CTCCGTTTGCCTGTTTTGCT..............................................    3056
....CTCCGTTTGCCTGTTTTGC...............................................    417
....CTCCGTTTGCCTGTTTTGCTGAT...........................................    147
.....TCCGTTTGCCTGTTTTGCTGA............................................    74
....CTCCGTTTGCCTGTTTTG................................................    48
.......CGTTTGCCTGTTTTGCTGA............................................    12
........GTTTGCCTGTTTTGCTGA............................................    12
.....TCCGTTTGCCTGTTTTGCTGAT...........................................    11
....CTCCGTTTGCCTGTTTT.................................................    9
....CTCCGTTTGCCTGTTTTGCTGATG..........................................    7
.....TCCGTTTGCCTGTTTTGCTG.............................................    6
..........TTGCCTGTTTTGCTGA............................................    5
```

```
..............................................AGCAAAATAAGCAAATGGAAA...     4
.....TCCGTTTGCCTGTTTTGCTGATG..........................................     3
....CTCCGTTTGCCTGTTT..................................................     2
.....TCCGTTTGCCTGTTTTGCT..............................................     2
......CCGTTTGCCTGTTTTGCTGA............................................     2
.........TTTGCCTGTTTTGCTGA............................................     2
..............................................AGCAAAATAAGCAAATGGAAAA..     2
......CCGTTTGCCTGTTTTGCTG.............................................     1
........GTTTGCCTGTTTTGCTG.............................................     1
....CTCCGTTTGCCTGTTTTGCTGATGT.........................................     1
.....(((((((((.(((((((((((.(((.....)))......))))))))))).))))))))).....  (-25.50)

X/103538165-103538238(+)_mir_cow_330
CAATGGCTCAGTAGCCAGTGTAGATCCTGTCTTTTGTAATCAGTAGCTACATCTGGCTACTGGGTCTCTGATGG
............................................AGCTACATCTGGCTACTGGGTCTC......14914
............................................AGCTACATCTGGCTACTGGGTCTCT.....8352
............................................AGCTACATCTGGCTACTGGGTCT.......6348
............................................AGCTACATCTGGCTACTGGGTC........1100
............................................AGCTACATCTGGCTACTGGGT.........626
............................................AGCTACATCTGGCTACTGGG..........92
.............................................GCTACATCTGGCTACTGGGTCTC......11
..............................................CTACATCTGGCTACTGGGTCTC......11
......CTCAGTAGCCAGTGTAGATCC...............................................8
.............................................GCTACATCTGGCTACTGGGTCTCT.....8
............................................AGCTACATCTGGCTACTG............7
..............................................CTACATCTGGCTACTGGGTCTCT.....7
...............................................TACATCTGGCTACTGGGTCTCT.....7
............................................AGCTACATCTGGCTACTGG...........6
..............................................CTACATCTGGCTACTGGGTCT.......6
.............................................GCTACATCTGGCTACTGGGTCT.......5
...............................................TACATCTGGCTACTGGGTCT.......4
...............................................TACATCTGGCTACTGGGTCTC......4
............................................AGCTACATCTGGCTAC..............3
...........................................TAGCTACATCTGGCTACTGGGTCT.......3
................................................ACATCTGGCTACTGGGTCTC......3
..................................................ATCTGGCTACTGGGTCTC......3
.................................................CATCTGGCTACTGGGTCT.......2
..................................................ATCTGGCTACTGGGTCT.......2
```

```
.................................................CATCTGGCTACTGGGTCTC......2
.....................................................TGGCTACTGGGTCTCT.....2
....GGCTCAGTAGCCAGTGTAGAT.................................................1
........CAGTAGCCAGTGTAGATCC...............................................1
.......TCAGTAGCCAGTGTAGATCCT..............................................1
..........................................GTAGCTACATCTGGCTACTGG...........1
...........................................TAGCTACATCTGGCTACTGGGTC........1
................................................ACATCTGGCTACTGGGTCT.......1
...................................................TCTGGCTACTGGGTCTC......1
....................................................CTGGCTACTGGGTCTC......1
................................................ACATCTGGCTACTGGGTCTCT.....1
.................................................CATCTGGCTACTGGGTCTCT.....1
....................................................CTGGCTACTGGGTCTCT.....1
............................................AGCTACATCTGGCTACTGGGTCTCTG....1
............................................AGCTACATCTGGCTACTGGGTCTCTGAT..1
((..((((((((((((((((((((...(((...........)))...))))).)))))))))))))))..)).... (-34.80)

X/103538854-103538924(+)_mir_cow_331
CATGAACCTGGCATACAATGTAGATTTCTGTGTTTGTTGAGCAACAGCTACATTGTCTGCTGGGTTTCAGG
.............................................AGCTACATTGTCTGCTGGGTTT....   19593
.............................................AGCTACATTGTCTGCTGGGTTTC...   12524
.............................................AGCTACATTGTCTGCTGGGTT.....   5361
.............................................AGCTACATTGTCTGCTGGGT......   1796
.....ACCTGGCATACAATGTAGATTTC...........................................   767
.....ACCTGGCATACAATGTAGATTTCTGT........................................   745
.............................................AGCTACATTGTCTGCTGGGTTTCA..   321
.....ACCTGGCATACAATGTAGATTT............................................   158
.....ACCTGGCATACAATGTAGATTTCTG.........................................   111
.....ACCTGGCATACAATGTAGATTTCT..........................................   102
.............................................AGCTACATTGTCTGCTGGG.......   69
.............................................AGCTACATTGTCTGCT..........   67
.............................................AGCTACATTGTCTGCTGG........   26
.....ACCTGGCATACAATGTAGATT.............................................   23
..............................................GCTACATTGTCTGCTGGGTTT....   21
............................................CAGCTACATTGTCTGCTGGGTTT....   11
.............................................AGCTACATTGTCTGCTG.........   7
..............................................GCTACATTGTCTGCTGGGTT.....   6
............................................CAGCTACATTGTCTGCTGGGTTTC...   5
```

```
..............................................GCTACATTGTCTGCTGGGTTTC...    5
...........CATACAATGTAGATTT............................................    4
....AACCTGGCATACAATGTAGATTTCTGT........................................    4
................................................TACATTGTCTGCTGGGTTT....    4
.....ACCTGGCATACAATGTAGAT..............................................    3
....AACCTGGCATACAATGTAGATTT............................................    3
....AACCTGGCATACAATGTAGATTTC...........................................    3
....AACCTGGCATACAATGTAGATTTCT..........................................    3
...........CATACAATGTAGATTTCTGT........................................    3
.....ACCTGGCATACAATGTAGATTTCTGTG.......................................    3
.....ACCTGGCATACAATGTAGATTTCTGTGTTT....................................    3
...........CATACAATGTAGATTTCT..........................................    2
.....ACCTGGCATACAATGTAGATTTCTGTGT......................................    2
..............................................GCTACATTGTCTGCTGGGT......    2
............................................CAGCTACATTGTCTGCTGGGTT.....    2
..................................................CATTGTCTGCTGGGTT.....    2
................................................TACATTGTCTGCTGGGTTTC...    2
.................................................ACATTGTCTGCTGGGTTTC...    2
....AACCTGGCATACAATGTAGAT..............................................    1
...........CATACAATGTAGATTTC...........................................    1
.............TACAATGTAGATTTCT..........................................    1
............ATACAATGTAGATTTCTG.........................................    1
............ATACAATGTAGATTTCTGT........................................    1
.....ACCTGGCATACAATGTAGATTTCTGTGTTTGT..................................    1
............................................CAGCTACATTGTCTGCTGGGT......    1
................................................TACATTGTCTGCTGGGTT.....    1
.................................................ACATTGTCTGCTGGGTTT....    1
...............................................CTACATTGTCTGCTGGGTTTC...    1
..((((((..(((.(((((((((....((((((((...)))).))))))))))))).)))..)).))))..  (-29.00)

X/117796482-117796549(+)_novel_cow_44
CACTGCTGAAACTCTGTTCAACCCACAACTATAGGAAAGATGGGTTGAAAAGAGTTTCGGTAGTAGCT
...TGCTGAAACTCTGTTCAACCC............................................ 10
...TGCTGAAACTCTGTTCAACCCA........................................... 6
..........................................GGTTGAAAAGAGTTTCGGTAGT.... 3
.((((((((((((((.(((((((((...((.......)).))))))))).)))))))))))))).... (-33.60)

X/137181450-137181514(+)_novel_cow_45
```

```
TCTTGCTCCTGCTTGAACATAGTCTCTTGTGTTGAGGTGGAGACTAGGTCAAGTAGGGCCAGAAA
...TGCTCCTGCTTGAACATAGTCTCT......................................   14
...TGCTCCTGCTTGAACATAGTCT.........................................   7
...TGCTCCTGCTTGAACATAGTCTC........................................   6
..TTGCTCCTGCTTGAACATAGTCT.........................................   3
.....CTCCTGCTTGAACATAGTCTCT......................................   1
........................................AGACTAGGTCAAGTAGGGCCA....   1
(((.((.(((((((((...((((((((...........))))))))..))))))))))).))).. (-30.30)
```

```
X/137199563-137199627(+)_novel_cow_46
TCTTGCTCCTGCTTGAACATAGTCTCTTGTGTTGAGGTGGAGACTAGGTCAAGTAGGGCCAAAAA
...TGCTCCTGCTTGAACATAGTCTCT......................................   14
...TGCTCCTGCTTGAACATAGTCT.........................................   7
...TGCTCCTGCTTGAACATAGTCTC........................................   6
..TTGCTCCTGCTTGAACATAGTCT.........................................   3
.....CTCCTGCTTGAACATAGTCTCT......................................   1
........................................AGACTAGGTCAAGTAGGGCCA....   1
..(((..(((((((((...((((((((...........))))))))..)))))))))..)))... (-27.60)
```

```
X/1632350-1632415(-)_mir_Cow_Blast_55
TGGGCAGAAAGTTCATTTGGGTTTTTCCGTAACATTTAATGGAAAAACCCGAATGAACTTTTTGGC
...........................................AAAACCCGAATGAACTTTTTGG.   8
............................................AAACCCGAATGAACTTTTTGGC   5
..........................................AAAAACCCGAATGAACTTTTTG..   2
......GAAAGTTCATTTGGGTTT..........................................   1
.....AGAAAGTTCATTTGGGTTTT.........................................   1
.....AGAAAGTTCATTTGGGTTTTTC.......................................   1
.......AAAGTTCATTTGGGTTTTTCC......................................   1
...........................................AAAACCCGAATGAACTTTT....   1
..(.((((((((((((((((((((((((((.......))))))))))))))))))))))))))).) (-38.10)
```

```
X/17917700-17917775(-)_mir_cow_288
TGTTGTCGGGTGGATCACGATGCAATTTTGATTAGTATAATAGGAGAAAAATTGCACGGTATCCATCTGCGAACCG
.................................................AATTGCACGGTATCCATCTG.......    406
.................................................AATTGCACGGTATCCATCTGC......    225
.................................................AATTGCACGGTATCCATCTGCG.....    170
..................................................ATTGCACGGTATCCATCTG.......    74
..................................................ATTGCACGGTATCCATCTGC......    45
```

```
.................................................AATTGCACGGTATCCATCTGCGA....    31
..................................................ATTGCACGGTATCCATCTGCG.....    29
..................................................ATTGCACGGTATCCATCTGCGA....    9
.................................................AATTGCACGGTATCCATCT........    3
.................................................AATTGCACGGTATCCATC.........    2
......CGGGTGGATCACGATGCAATT.................................................    1
......CGGGTGGATCACGATGCAATTT................................................    1
..................................................ATTGCACGGTATCCATC.........    1
................................................AAATTGCACGGTATCCATCTG.......    1
...................................................TTGCACGGTATCCATCTG.......    1
..(((.(((((((((..((.(((((((((..(((......)))....)))))))))))..))))))))))))....  (-25.80)

X/17917856-17917929(-)_mir_cow_289
ATTCATGGGTGGGGATTTGTTGCACTGCTTGTGTTATATATAAAGTATTGCACTTGTCCCGGCCTGTGGAAGAA
.............................................TATTGCACTTGTCCCGGCCTGT.......94635
.............................................TATTGCACTTGTCCCGGCCTG........5603
..............................................ATTGCACTTGTCCCGGCCTGT.......1365
.............................................TATTGCACTTGTCCCGGCCTGTG......373
.............................................TATTGCACTTGTCCCGGCCT.........355
.............................................TATTGCACTTGTCCCGGCC..........250
..............................................ATTGCACTTGTCCCGGCCTG........139
.............................................TATTGCACTTGTCCCGGC...........45
.............................................TATTGCACTTGTCCCGGCCTGTGG.....41
.............................................TATTGCACTTGTCCCGG............14
...............................................TTGCACTTGTCCCGGCCTGT.......14
..............................................ATTGCACTTGTCCCGGCCTGTG......7
.................................................GCACTTGTCCCGGCCTGT.......5
.............................................TATTGCACTTGTCCCG.............4
..............................................ATTGCACTTGTCCCGGCCT.........3
................................................TGCACTTGTCCCGGCCTGT.......3
..............................................ATTGCACTTGTCCCGGC...........2
...................................................ACTTGTCCCGGCCTGT.......2
......GGGTGGGGATTTGTTGCACTGCT.............................................1
..............................................ATTGCACTTGTCCCGGCC..........1
..............................................ATTGCACTTGTCCCGGCCTGTGG.....1
.............................................TATTGCACTTGTCCCGGCCTGTGGA....1
.((((((((.(((((..((.(((.((((((((((...)))).))))).)))))..))))).)))))))))....  (-31.20)
```

```
X/17918132-17918202(-)_mir_cow_290
TAGTACCAAAGTGCTCACAGTGCAGGTAGTTTTGGCAGCGCTCTACTGTAGTGTGGGCACTTCCAGTACTC
......CAAAGTGCTCACAGTGCAGGTAG.........................................   126
......CAAAGTGCTCACAGTGCAGGTAGT........................................   45
......CAAAGTGCTCACAGTGCAGGTA..........................................   31
......CAAAGTGCTCACAGTGCAGGT...........................................   29
......CAAAGTGCTCACAGTGCAGG............................................   8
......CAAAGTGCTCACAGTGCA..............................................   1
......CAAAGTGCTCACAGTGCAGGTAGTT.......................................   1
............................................ACTGTAGTGTGGGCACTTCCAG.....   1
...........................................TACTGTAGTGTGGGCACTTCCAGT....   1
............................................ACTGTAGTGTGGGCACTTCCAGT....   1
.(((((..((((((((((.(((((.(((....(((...))))))))))).)))))))))))...))))). (-30.90)

X/17918367-17918441(-)_mir_cow_291
TTGTGTTAAGGTGCATCTAGTGCAGTTAGTGAAGCAGCTCAGAATCTACTGCCCTAAATGCTCCTTCTGGCACAG
......TAAGGTGCATCTAGTGCAGT.................................................     29
......TAAGGTGCATCTAGTGCAGTT................................................     8
......TAAGGTGCATCTAGTGCAGTTA...............................................     8
......TAAGGTGCATCTAGTGCAG..................................................     6
......TAAGGTGCATCTAGTGCA...................................................     3
......TAAGGTGCATCTAGTGCAGTTAGT.............................................     3
......TAAGGTGCATCTAGTGCAGTTAG..............................................     2
......TAAGGTGCATCTAGTGC....................................................     1
..............................................CTACTGCCCTAAATGCTCCTTCT.......     1
...............................................TACTGCCCTAAATGCTCCTTCT.......     1
...............................................TACTGCCCTAAATGCTCCTTCTG......     1
................................................ACTGCCCTAAATGCTCCTTCTG......     1
................................................ACTGCCCTAAATGCTCCTTCTGG.....     1
..................................................TGCCCTAAATGCTCCTTCTGGC....     1
.(((((((((.((((.(((.(((((....((..(......)..)).))))).))).)))).)))..)))))))). (-25.60)

X/17918535-17918601(-)_mir_cow_292
CCATGTAAAAGTGCTTACAGTGCAGGTAGCTTTTTGAGATCTACTGCAATGCAAGCACTTCTTACAT
......AAAAGTGCTTACAGTGCAGGTAG......................................  132
......AAAAGTGCTTACAGTGCAGGTA.......................................  20
.......AAAGTGCTTACAGTGCAGGTAG......................................  19
......AAAAGTGCTTACAGTGCAGGT........................................  13
```

```
.......AAAGTGCTTACAGTGCAGGTAGC.....................................  6
......AAAAGTGCTTACAGTGCAGG.........................................  2
.......AAAGTGCTTACAGTGCAGGTAGCT....................................  2
.........................................TACTGCAATGCAAGCACTTCT.....  2
.......AAAGTGCTTACAGTGCAGGTA.......................................  1
..........GTGCTTACAGTGCAGGTAG......................................  1
..........................................ACTGCAATGCAAGCACTTCT.....  1
..........................................ACTGCAATGCAAGCACTTCTT....  1
..((((((((((((((.((.(((((.(((.((.....)).)))))))).)).)))))))).)))))) (-24.20)

X/18178915-18178984(-)_mir_cow_293
AAATTATTTTTGCAATATGTTCCTGAATATTTAATATAAGTATATTGGGAACATTTTGCATCCATAGTTT
.......TTTTGCAATATGTTCCTGAATAT........................................    18851
.......TTTTGCAATATGTTCCTGAAT..........................................    14064
.......TTTTGCAATATGTTCCTGAATA.........................................    2546
.......TTTTGCAATATGTTCCTGAATATT.......................................    245
......TTTTTGCAATATGTTCCTGAAT..........................................    147
.......TTTTGCAATATGTTCCTGAA...........................................    86
...........................................ATTGGGAACATTTTGCATCC.......    25
........TTTGCAATATGTTCCTGAATAT........................................    24
.......TTTTGCAATATGTTCCTGA............................................    18
......TTTTTGCAATATGTTCCTGAATA.........................................    17
.......TTTTGCAATATGTTCCTGAATATTT......................................    16
......TTTTTGCAATATGTTCCTGAATAT........................................    12
..........................................TATTGGGAACATTTTGCATCC.......    9
........TTTGCAATATGTTCCTGAAT..........................................    8
.......TTTTGCAATATGTTCC...............................................    6
.......TTTTGCAATATGTTCCTG.............................................    6
...........................................ATTGGGAACATTTTGCATCCA......    5
........TTTGCAATATGTTCCTGAATATT.......................................    3
..........................................TATTGGGAACATTTTGCATCCA......    3
............................................TTGGGAACATTTTGCATCC.......    2
............................................TTGGGAACATTTTGCATCCAT.....    2
.........TTGCAATATGTTCCTGAAT..........................................    1
.........TTGCAATATGTTCCTGAATAT........................................    1
..........TGCAATATGTTCCTGAATAT........................................    1
..............ATATGTTCCTGAATAT........................................    1
.......TTTTGCAATATGTTCCTGAATATTTAATAT.................................    1
```

```
.......TTTTGCAATATGTTCCTGAATATTTAATATAAGT.............................    1
.......TTTTGCAATATGTTCCTGAATATTTAATATAAGTAT...........................    1
.........................................ATATTGGGAACATTTTGCATCCA......    1
...........................................ATTGGGAACATTTTGCATCCAT.....    1
...........................................ATTGGGAACATTTTGCATCCATA....    1
(((((((...(((((.(((((((..((((((((...))))))).)..))))))).)))))...))))))) (-23.00)

X/18179086-18179152(-)_mir_cow_294
AACTATTTTTGCGATGTGTTCCTAATATGCAGTATAAATATATTGGGAGCATTTTGCATGCATGGTT
......TTTTGCGATGTGTTCCTAATAT....................................... 4291
......TTTTGCGATGTGTTCCTAAT......................................... 1714
......TTTTGCGATGTGTTCCTAATA........................................ 995
......TTTTGCGATGTGTTCCTAATATG...................................... 603
.......TTTGCGATGTGTTCCTAATATG...................................... 500
......TTTTGCGATGTGTTCCT............................................ 54
.......TTTGCGATGTGTTCCTAATAT....................................... 48
......TTTTGCGATGTGTTCCTAA.......................................... 39
......TTTTGCGATGTGTTCC............................................. 14
.........................................ATTGGGAGCATTTTGCATGCAT.... 10
..........................................TTGGGAGCATTTTGCATGCAT.... 8
.......TTTGCGATGTGTTCCTAATA........................................ 7
......TTTTGCGATGTGTTCCTA........................................... 3
.......TTTGCGATGTGTTCCTAAT......................................... 3
......TTTTGCGATGTGTTCCTAATATGC..................................... 2
.........TGCGATGTGTTCCTAATAT....................................... 1
.........................................ATTGGGAGCATTTTGCATG....... 1
........................................TATTGGGAGCATTTTGCATGCA..... 1
.........................................ATTGGGAGCATTTTGCATGCA..... 1
........................................TATTGGGAGCATTTTGCATGCAT.... 1
((((((...(((((.((((((((((((((.........)))))))))))))).)))))...)))))) (-25.10)

X/18179230-18179297(-)_mir_cow_295
AACTATTTTTGCGATGTGTTCCTAATATGTAATCTAAATGTATTGGGAACATTTTGCATTCATAGTTT
......TTTTGCGATGTGTTCCTAATAT........................................4291
......TTTTGCGATGTGTTCCTAAT..........................................1714
......TTTTGCGATGTGTTCCTAATA.........................................995
......TTTTGCGATGTGTTCCTAATATG.......................................603
.......TTTGCGATGTGTTCCTAATATG.......................................500
```

```
......TTTTGCGATGTGTTCCT............................................. 54
.......TTTGCGATGTGTTCCTAATAT........................................ 48
......TTTTGCGATGTGTTCCTAA........................................... 39
......TTTTGCGATGTGTTCCTAATATGT...................................... 26
......TTTTGCGATGTGTTCC.............................................. 14
.........................................ATTGGGAACATTTTGCATTCAT..... 14
.......TTTGCGATGTGTTCCTAATATGT...................................... 8
.......TTTGCGATGTGTTCCTAATA......................................... 7
......TTTTGCGATGTGTTCCTA............................................ 3
.......TTTGCGATGTGTTCCTAAT.......................................... 3
........................................TATTGGGAACATTTTGCATTC....... 3
..........................................TTGGGAACATTTTGCATTCAT..... 3
......TTTTGCGATGTGTTCCTAATATGTA..................................... 2
........................................TATTGGGAACATTTTGCATTCA...... 2
.........................................ATTGGGAACATTTTGCATTCA...... 2
.........TGCGATGTGTTCCTAATAT........................................ 1
......TTTTGCGATGTGTTCCTAATATGTAA.................................... 1
......TTTTGCGATGTGTTCCTAATATGTAAT................................... 1
........................................TATTGGGAACATTTTGCATT........ 1
.........................................ATTGGGAACATTTTGCATT........ 1
.........................................ATTGGGAACATTTTGCATTC....... 1
..........................................TTGGGAACATTTTGCATTC....... 1
..........................................TTGGGAACATTTTGCATTCATA.... 1
((((((...(((((.(((((((((((((((.......))))))))))))))).)))))...)))))). (-26.80)
```

```
X/18180146-18180217(-)_mir_cow_296
CAGACATCTCGGGGATCATCATGTCACGAGATACCACTGTGCACTTGTGACAGATTGATAACTGAAAGGTCT
.............................................TGTGACAGATTGATAACTGAAA.....  7787
.............................................TGTGACAGATTGATAACTGA.......  1660
.............................................TGTGACAGATTGATAACTGAA......  1453
........TCGGGGATCATCATGTCACGAGA.........................................  857
............................................TTGTGACAGATTGATAACTGA.......  799
............................................TTGTGACAGATTGATAACT.........  794
............................................TTGTGACAGATTGATAACTGAAA.....  537
............................................TTGTGACAGATTGATAACTG........  404
.............................................TGTGACAGATTGATAACTG........  358
........TCGGGGATCATCATGTCACGA...........................................  313
............................................TTGTGACAGATTGATAACTGAA......  307
```

```
........TCGGGGATCATCATGTCACG............................................ 268
.............................................TGTGACAGATTGATAACTGAAAG.... 92
.............................................TGTGACAGATTGATAACT......... 91
........TCGGGGATCATCATGTCACGAG.......................................... 73
.......CTCGGGGATCATCATGTCACG............................................ 63
............................................TTGTGACAGATTGATAAC.......... 53
........TCGGGGATCATCATGTCAC............................................. 43
.............................................TGTGACAGATTGATAAC.......... 24
.......CTCGGGGATCATCATGTCAC............................................. 15
.......CTCGGGGATCATCATGTCACGA........................................... 12
........TCGGGGATCATCATGTCA.............................................. 8
..............................................GTGACAGATTGATAACTGAAA..... 4
........TCGGGGATCATCATGT................................................ 2
........TCGGGGATCATCATGTC............................................... 2
...........................................CTTGTGACAGATTGATAACT......... 2
...........................................CTTGTGACAGATTGATAACTGA....... 2
...............................................TGACAGATTGATAACTGAAAG.... 2
......TCTCGGGGATCATCATGTCACG............................................ 1
.......CTCGGGGATCATCATGTCACGAG.......................................... 1
.........CGGGGATCATCATGTCACGAG.......................................... 1
........TCGGGGATCATCATGTCACGAGAT........................................ 1
............................AGATACCACTGTGCACTTGTGAC..................... 1
.............................................TGTGACAGATTGATAA........... 1
...........................................CTTGTGACAGATTGATAACTGAAA..... 1
................................................GACAGATTGATAACTGAAA..... 1
.((((...((((..((((...(((((((((.(((....)))..)))))))))...))))..))))...)))) (-27.00)

X/18185100-18185173(-)_mir_cow_297
GTGCCCTAGCAGCGGGAACAGTACTGCAGTGGGCAATTGGTGATCTGGAGTATTGTTTCTGCTGCCCGGGCAAG
..............................................GGAGTATTGTTTCTGCTGCCCGG.....117
......TAGCAGCGGGAACAGTACTGCAG.............................................90
...............................................GAGTATTGTTTCTGCTGCCCGG.....78
..............................................GGAGTATTGTTTCTGCTGCCC.......42
......TAGCAGCGGGAACAGTACTG................................................26
......TAGCAGCGGGAACAGTACTGC...............................................26
..............................................GGAGTATTGTTTCTGCTGCC........18
......TAGCAGCGGGAACAGTACTGCA..............................................15
..............................................GGAGTATTGTTTCTGCTGCCCG......15
```

```
......TAGCAGCGGGAACAGTACTGCAGT............................................ 12
......TAGCAGCGGGAACAGTACT................................................. 6
...............................................GAGTATTGTTTCTGCTGCCC....... 5
......TAGCAGCGGGAACAGTAC.................................................. 3
..............................................GGAGTATTGTTTCTGCTGCCCGGG.... 3
.............................TGGGCAATTGGTGATCT............................ 2
..............................................GGAGTATTGTTTCTGCT........... 1
...........................................TCTGGAGTATTGTTTCTGCTG.......... 1
..............................................GGAGTATTGTTTCTGCTG.......... 1
..............................................GGAGTATTGTTTCTGCTGC......... 1
...............................................GAGTATTGTTTCTGCTGC......... 1
...........................................TCTGGAGTATTGTTTCTGCTGCCC....... 1
...............................................GAGTATTGTTTCTGCTGCCCG...... 1
......................................GGTGATCTGGAGTATTGTTTCTGCTGCCCGG..... 1
..........................................ATCTGGAGTATTGTTTCTGCTGCCCGG..... 1
.(((((..(((((((((((((((((.(((...((.....))...))).)))))))))))))))))..)))))..  (-43.00)

X/18185451-18185521(-)_mir_cow_298
GGGGATGCAGCAGCAATTCATGTTTTGAAGTGCTTTAAACGGTTCAAAACGTGAGGCGCTGCTATACCCCC
.......CAGCAGCAATTCATGTTTTGA...........................................    9117
.......CAGCAGCAATTCATGTTTTGAA..........................................    3115
.......CAGCAGCAATTCATGTTTTG............................................    1372
............................................CAAAACGTGAGGCGCTGCTAT......    498
............................................CAAAACGTGAGGCGCTGCTATA.....    320
............................................CAAAACGTGAGGCGCTGCT........    259
.......CAGCAGCAATTCATGTTTT.............................................    253
.......CAGCAGCAATTCATGTTT..............................................    85
............................................CAAAACGTGAGGCGCTGCTA.......    71
.......CAGCAGCAATTCATGTTTTGAAG.........................................    23
.......CAGCAGCAATTCATGTT...............................................    19
.............................................AAAACGTGAGGCGCTGCTATA.....    19
.......CAGCAGCAATTCATGTTTTGAAGT........................................    13
......GCAGCAGCAATTCATGTTTTGA...........................................    12
........AGCAGCAATTCATGTTTTGA...........................................    12
.............................................AAAACGTGAGGCGCTGCT........    10
.......CAGCAGCAATTCATGT................................................    7
.............................................AAAACGTGAGGCGCTGCTAT......    7
.............................................AAAACGTGAGGCGCTGCTATAC....    7
```

```
......GCAGCAGCAATTCATGTTTTG............................................    6
..............................................AAACGTGAGGCGCTGCTATAC....    5
..............................................AAACGTGAGGCGCTGCTATA.....    4
........AGCAGCAATTCATGTTTTGAA..........................................    3
............................................CAAAACGTGAGGCGCTGC.........    3
......GCAGCAGCAATTCATGTTTT.............................................    2
........AGCAGCAATTCATGTTTTG............................................    2
.........GCAGCAATTCATGTTTTG............................................    2
......GCAGCAGCAATTCATGTTTTGAA..........................................    2
.............................................AAAACGTGAGGCGCTGCTA.......    2
............................................CAAAACGTGAGGCGCTGCTATAC....    2
..........CAGCAATTCATGTTTTGAA..........................................    1
............................AGTGCTTTAAACGGTTC..........................    1
............................................CAAAACGTGAGGCGCTG..........    1
..........................................TTCAAAACGTGAGGCGCTGCTA.......    1
..............................................AAACGTGAGGCGCTGCTAT......    1
((((.((.((((((..(((((((((((((.((.......)).)))))))))))))..)))))).)))))). (-35.60)

X/22083697-22083766(-)_mir_cow_299
TGTTGGGAGACCCTGGTCTGCACTCTGTCTGTATGCTTACTGAAGGGAGCGCAGGGCAGGGTTTCCCATA
.......AGACCCTGGTCTGCACTCTGTCT........................................    3156
.......AGACCCTGGTCTGCACTCTGTC.........................................    1789
........GACCCTGGTCTGCACTCTGTC.........................................    1423
........GACCCTGGTCTGCACTCTGTCT........................................    537
.......AGACCCTGGTCTGCACTCTGT..........................................    386
........GACCCTGGTCTGCACTCTGT..........................................    270
.......AGACCCTGGTCTGCACTCTG...........................................    22
........GACCCTGGTCTGCACTCTGTCTG.......................................    13
........GACCCTGGTCTGCACTCTGTCTGT......................................    10
.......AGACCCTGGTCTGCACTC.............................................    4
.......AGACCCTGGTCTGCACTCT............................................    4
........GACCCTGGTCTGCACTCTG...........................................    4
........GACCCTGGTCTGCACTC.............................................    2
.......AGACCCTGGTCTGCACTCTGTCTG.......................................    2
...........................................AGGGAGCGCAGGGCAGGGTTTCC....    2
........GACCCTGGTCTGCACTCT............................................    1
......GAGACCCTGGTCTGCACTCTGT..........................................    1
......GAGACCCTGGTCTGCACTCTGTCT........................................    1
```

```
.........ACCCTGGTCTGCACTCTGTCT.........................................    1
........GACCCTGGTCTGCACTCTGTCTGTATGCT..................................    1
........GACCCTGGTCTGCACTCTGTCTGTATGCTTACT..............................    1
.....................................TACTGAAGGGAGCGCAGGGCAGGGT........    1
............................................GGGAGCGCAGGGCAGGGTTTCC....    1
...((((((((((((.(((((.((((.((.(((....))).))..)))).))))).))))))))))))).. (-42.10)

X/23387300-23387367(-)_mir_cow_300
CAGTGGGGGAGCCAGGAAGTATTGATGTTTCTACCAGTTTAGCGTCAACACTTGCTGGTTTCCTCTCT
..........................................CGTCAACACTTGCTGGTTTCCT.... 343
...........................................GTCAACACTTGCTGGTTTCCT.... 124
...........................................GTCAACACTTGCTGGTTTCCTCT.. 117
..........................................CGTCAACACTTGCTGGTTTCCTC... 110
..........................................CGTCAACACTTGCTGGTTTCCTCT.. 70
...........................................GTCAACACTTGCTGGTTTCCTC... 65
............................................TCAACACTTGCTGGTTTCCTCT.. 64
..........................................CGTCAACACTTGCTGGTTTCC..... 33
............................................TCAACACTTGCTGGTTTCCT.... 33
...........................................GTCAACACTTGCTGGTTTCC..... 22
............................................TCAACACTTGCTGGTTTCCTC... 22
......GGGAGCCAGGAAGTATTGATGTT....................................... 14
..........................................CGTCAACACTTGCTGGTTTC...... 6
............................................TCAACACTTGCTGGTTTCC..... 5
......GGGAGCCAGGAAGTATTGATGT........................................ 3
......GGGAGCCAGGAAGTATTGATGTTT...................................... 3
..........................................CGTCAACACTTGCTGGTTT....... 2
......GGGAGCCAGGAAGTATTGA........................................... 1
.......GGAGCCAGGAAGTATTGATGTT....................................... 1
.........AGCCAGGAAGTATTGATGTT....................................... 1
.............................TCTACCAGTTTAGCGTCAAC................... 1
..............................................AACACTTGCTGGTTTCCTCT.. 1
.((.(((((((((((.((((.((((((((..........)))))))).)))).))))))))))).)) (-32.50)

X/2445310-2445373(+)_mir_cow_283
AAATATATATATATATGTACGTATGTGTATATGAATGTATACGTAGATATATATGTATTTTTGG
...TATATATATATATGTACGTATG.......................................    225
...TATATATATATATGTACGTAT........................................    218
...TATATATATATATGTACGTATGT......................................    40
```

```
...TATATATATATATGTACGT..........................................     32
.......................................TACGTAGATATATATGTATTT....     18
.......................................TACGTAGATATATATGTATTTT...     13
...TATATATATATATGTACGTA.........................................     5
...TATATATATATATGTACGTATGTGT....................................     3
.......................................TACGTAGATATATATGTAT......     3
.....TATATATATATGTACGT..........................................     2
.....TATATATATATGTACGTAT........................................     2
...TATATATATATATGTACG...........................................     1
....ATATATATATATGTACGTATGT......................................     1
.......................................TACGTAGATATATATGT........     1
(((.((((((((((((.((((((((..(......)..)))))))).))))))))))))).)))..  (-22.70)

X/2676187-2676253(+)_mir_cow_284
TTGGCCAAGAGTTTGTTCGGGTTTCTCCATAAGCTGTATAGAAAAACCTCAATGAACTCTTTGGCCA
..........................................AAAACCTCAATGAACTCTTTGG...  829
......AAGAGTTTGTTCGGGTTTCT.........................................  273
.........................................AAAAACCTCAATGAACTCTTTGG...  214
......AAGAGTTTGTTCGGGTTTCTCC.......................................  180
.........................................AAAAACCTCAATGAACTCTTTG....  166
......AAGAGTTTGTTCGGGTTTCTC........................................  139
..........................................AAAACCTCAATGAACTCTTTG....  139
..........................................AAAACCTCAATGAACTCTTTGGC..  31
..........................................AAAACCTCAATGAACTCTTT.....  16
.........................................AAAAACCTCAATGAACTCTTT.....  14
......AAGAGTTTGTTCGGGTTTC..........................................  5
......AAGAGTTTGTTCGGGTTT...........................................  4
.........................................AAAAACCTCAATGAACTCT.......  4
.........................................AAAAACCTCAATGAACTCTT......  4
..........................................AAAACCTCAATGAACTCTT......  4
...........................................AAACCTCAATGAACTCTTTGG...  4
.....CAAGAGTTTGTTCGGGTTTCTC........................................  3
......AAGAGTTTGTTCGGGTTTCTCCA......................................  2
..........................................AAAACCTCAATGAACTCT.......  2
......AAGAGTTTGTTCGGGTT............................................  1
....CCAAGAGTTTGTTCGGGTTTCT.........................................  1
.....CAAGAGTTTGTTCGGGTTTCT.........................................  1
.......AGAGTTTGTTCGGGTTTCT.........................................  1
```

```
.((((((((((((..((.((((((.((.(((.....))).)).)))))).))..))))).))))))) (-28.50)

X/29112797-29112860(+)_novel_cow_39
CCTTACTCCAGAGGGTGTCATTCAGGTAAACTAAAGCATGTCTGTCACCTTCTTGAGTGGAGTA
........................................TCTGTCACCTTCTTGAGTGG....     928
........................................TCTGTCACCTTCTTGAGTGGA...     372
........................................TCTGTCACCTTCTTGAGTG.....     10
........................................TCTGTCACCTTCTTGAG.......     4
...TACTCCAGAGGGTGTCATT..........................................     1
...TACTCCAGAGGGTGTCATTC.........................................     1
........................................TCTGTCACCTTCTTGAGTGGAG..     1
..((((((.((((((((.((..((.((........)).))..)).)))))))).)))))).... (-22.70)

X/29148299-29148362(+)_novel_cow_40
CCTTTCATAGGAAGGTGTCATTCATGTGGTCTACAATTTGAATGCCACTTTTTTGTGAAGAGTA
...TTCATAGGAAGGTGTCATTC.........................................     146
...TTCATAGGAAGGTGTCATTCAT.......................................     54
...TTCATAGGAAGGTGTCATTCA........................................     48
...TTCATAGGAAGGTGTCATTCATG......................................     18
...TTCATAGGAAGGTGTCATT..........................................     10
...TTCATAGGAAGGTGTCATTCATGT.....................................     3
........................................AATGCCACTTTTTTGTGAAG....     3
....TCATAGGAAGGTGTCATTCATG......................................     1
........................TGTGGTCTACAATTTG........................     1
..(((((((((((((((.((((((..((.....))...)))))).)))))))))))))))).... (-24.30)

X/30279403-30279462(+)_novel_cow_41
CCCTGCGCAGACAGGTGCTTTTCACTTAAACTCAGTGAAAGGACCTGCCTGGGTAGAGTA
...................................TGAAAGGACCTGCCTGGGTAG....   34
...................................TGAAAGGACCTGCCTGGGTAGA...   32
...TGCGCAGACAGGTGCTTTTCACT..................................   4
...................................TGAAAGGACCTGCCTGGGTA.....   4
...................................TGAAAGGACCTGCCTGGGT......   2
...TGCGCAGACAGGTGCTTTTC.....................................   1
....................................GAAAGGACCTGCCTGGGTAG....   1
...................................TGAAAGGACCTGCCTGGGTAGAG..   1
..((((.(((.(((((.(((((((((.......)))))))))))))).))).)))).... (-28.30)
```

```
X/30280408-30280470(+)_novel_cow_42
CCCTGCCCAGCCAGGTGCTGTTCACGTAGACTTCCTGTGAGCACACCTGCCTGAGCAGAGTAA
.....................................TGAGCACACCTGCCTGAGCAGA....      130
.....................................TGAGCACACCTGCCTGAGCAG.....      55
.....................................TGAGCACACCTGCCTGAGC.......      36
.....................................TGAGCACACCTGCCTGAGCA......      11
...TGCCCAGCCAGGTGCTGTTCACG.....................................      4
...TGCCCAGCCAGGTGCTGTTCAC......................................      1
..((((.(((.((((((.((((((((.........)))))))))))))).))).)))).....  (-29.90)
```

```
X/30281438-30281501(+)_novel_cow_43
CCCTGCCTGGAGAGGTTCTGTTTACTTCAACTACTGGTAAACAGAGCCTTCCTGAATAGAGTAC
...TGCCTGGAGAGGTTCTGTTTACT......................................     11
.....................................TAAACAGAGCCTTCCTGAATAGA....     7
...TGCCTGGAGAGGTTCTGTTTAC.......................................     3
..(((..(((..((((((((((((((.........))))))))))))))..)))..))).....  (-24.00)
```

```
X/30286984-30287046(+)_mir_cow_301
GCCCTGCCCATTCAGGTGCAGTTCATGTACAATTGGTGTCCTGTGCCTCGAATGGGTATGGTA
......................................TCCTGTGCCTCGAATGGGTATG...      96038
......................................TCCTGTGCCTCGAATGGGTAT....      1788
......................................TCCTGTGCCTCGAATGGGTA.....      850
......................................TCCTGTGCCTCGAATGGGT......      425
.......................................CCTGTGCCTCGAATGGGTATG...      150
......................................TCCTGTGCCTCGAATGGGTATGG..      126
........................................CTGTGCCTCGAATGGGTATG...      104
............................................GCCTCGAATGGGTATG...      70
..........................................GTGCCTCGAATGGGTATG...      65
.........................................TGTGCCTCGAATGGGTATG...      30
.........................TGTACAATTGGTGTCCTGTGCCTCGAATGGGTATG...      13
......................................TCCTGTGCCTCGAATGGG.......      12
...........................................TGCCTCGAATGGGTATG...      10
....TGCCCATTCAGGTGCAGTTC.......................................      1
....TGCCCATTCAGGTGCAGTTCA......................................      1
......................................TCCTGTGCCTCGAATGG........      1
....................................TGTCCTGTGCCTCGAATGGGT......      1
........................................CTGTGCCTCGAATGGGT......      1
.......................................CCTGTGCCTCGAATGGGTA.....      1
```

```
........................................CTGTGCCTCGAATGGGTA.....      1
....................................TGTCCTGTGCCTCGAATGGGTAT....      1
............................ACAATTGGTGTCCTGTGCCTCGAATGGGTATG...      1
................................TTGGTGTCCTGTGCCTCGAATGGGTATG...      1
(((.((((((((((((..(((..(((.........)))..)))..))).))))))))).))). (-32.40)

X/30303210-30303272(+)_mir_cow_302
GCCCTGCCCATTCAGGTGCAGTTCATGTACAATTGGTGTCCTGTGCCTCGAATGGGTATGGTA
......................................TCCTGTGCCTCGAATGGGTATG...      96038
......................................TCCTGTGCCTCGAATGGGTAT....      1788
......................................TCCTGTGCCTCGAATGGGTA.....      850
......................................TCCTGTGCCTCGAATGGGT......      425
.......................................CCTGTGCCTCGAATGGGTATG...      150
......................................TCCTGTGCCTCGAATGGGTATGG..      126
........................................CTGTGCCTCGAATGGGTATG...      104
............................................GCCTCGAATGGGTATG...      70
..........................................GTGCCTCGAATGGGTATG...      65
.........................................TGTGCCTCGAATGGGTATG...      30
.........................TGTACAATTGGTGTCCTGTGCCTCGAATGGGTATG...      13
......................................TCCTGTGCCTCGAATGGG.......      12
...........................................TGCCTCGAATGGGTATG...      10
....TGCCCATTCAGGTGCAGTTC.......................................      1
....TGCCCATTCAGGTGCAGTTCA......................................      1
......................................TCCTGTGCCTCGAATGG........      1
....................................TGTCCTGTGCCTCGAATGGGT......      1
........................................CTGTGCCTCGAATGGGT......      1
.......................................CCTGTGCCTCGAATGGGTA.....      1
........................................CTGTGCCTCGAATGGGTA.....      1
....................................TGTCCTGTGCCTCGAATGGGTAT....      1
............................ACAATTGGTGTCCTGTGCCTCGAATGGGTATG...      1
................................TTGGTGTCCTGTGCCTCGAATGGGTATG...      1
(((.((((((((((((..(((..(((.........)))..)))..))).))))))))).))). (-32.40)

X/30305149-30305211(+)_mir_cow_303
GCCCTGCCCATTCAGGTGCAGTTCATGTACAATTGGTGTCCTGTGCCTCGAATGGGTATGGTA
......................................TCCTGTGCCTCGAATGGGTATG...      96038
......................................TCCTGTGCCTCGAATGGGTAT....      1788
......................................TCCTGTGCCTCGAATGGGTA.....      850
```

```
......................................TCCTGTGCCTCGAATGGGT......      425
.......................................CCTGTGCCTCGAATGGGTATG...      150
......................................TCCTGTGCCTCGAATGGGTATGG..      126
........................................CTGTGCCTCGAATGGGTATG...      104
............................................GCCTCGAATGGGTATG...      70
..........................................GTGCCTCGAATGGGTATG...      65
.........................................TGTGCCTCGAATGGGTATG...      30
.........................TGTACAATTGGTGTCCTGTGCCTCGAATGGGTATG...      13
......................................TCCTGTGCCTCGAATGGG.......      12
...........................................TGCCTCGAATGGGTATG...      10
....TGCCCATTCAGGTGCAGTTC.......................................      1
....TGCCCATTCAGGTGCAGTTCA......................................      1
......................................TCCTGTGCCTCGAATGG........      1
....................................TGTCCTGTGCCTCGAATGGGT......      1
........................................CTGTGCCTCGAATGGGT......      1
.......................................CCTGTGCCTCGAATGGGTA.....      1
........................................CTGTGCCTCGAATGGGTA.....      1
....................................TGTCCTGTGCCTCGAATGGGTAT....      1
............................ACAATTGGTGTCCTGTGCCTCGAATGGGTATG...      1
................................TTGGTGTCCTGTGCCTCGAATGGGTATG...      1
(((.((((((((((((..(((..(((.........)))..)))..))).))))))))).))). (-32.40)

X/30309763-30309825(+)_mir_cow_304
GCCCTGCCCATTCAGGTGCAGTTCACGTACAATTGGTGTACTGTGCCTCGAATGGGTATGGTA
......................................TACTGTGCCTCGAATGGGTATG...      4878
......................................TACTGTGCCTCGAATGGGTA.....      131
......................................TACTGTGCCTCGAATGGGTAT....      126
........................................CTGTGCCTCGAATGGGTATG...      104
............................................GCCTCGAATGGGTATG...      70
..........................................GTGCCTCGAATGGGTATG...      65
.........................................TGTGCCTCGAATGGGTATG...      30
......................................TACTGTGCCTCGAATGGGT......      25
.....................................GTACTGTGCCTCGAATGGGTATG...      24
...........................................TGCCTCGAATGGGTATG...      10
......................................TACTGTGCCTCGAATGGGTATGG..      9
.......................................ACTGTGCCTCGAATGGGTATG...      3
....TGCCCATTCAGGTGCAGTTC.......................................      1
....TGCCCATTCAGGTGCAGTTCA......................................      1
```

```
......................................TACTGTGCCTCGAATGGG.......      1
.....................................GTACTGTGCCTCGAATGGGT......      1
........................................CTGTGCCTCGAATGGGT......      1
.....................................GTACTGTGCCTCGAATGGGTA.....      1
........................................CTGTGCCTCGAATGGGTA.....      1
.....................................GTACTGTGCCTCGAATGGGTAT....      1
(((.((((((((((((..((((.(((.........))).))))..))).))))))))).))). (-34.90)

X/30319607-30319669(+)_mir_cow_305
GCCCTGCCCATTCAGGTGCAGTTCACGTACAATTGGTGTCCTGTGCCTCGAATGGGTATGGTA
......................................TCCTGTGCCTCGAATGGGTATG...      96038
......................................TCCTGTGCCTCGAATGGGTAT....      1788
......................................TCCTGTGCCTCGAATGGGTA.....      850
......................................TCCTGTGCCTCGAATGGGT......      425
.......................................CCTGTGCCTCGAATGGGTATG...      150
......................................TCCTGTGCCTCGAATGGGTATGG..      126
........................................CTGTGCCTCGAATGGGTATG...      104
............................................GCCTCGAATGGGTATG...      70
..........................................GTGCCTCGAATGGGTATG...      65
.........................CGTACAATTGGTGTCCTGTGCCTCGAATGGGTATG...      34
.........................................TGTGCCTCGAATGGGTATG...      30
......................................TCCTGTGCCTCGAATGGG.......      12
...........................................TGCCTCGAATGGGTATG...      10
....TGCCCATTCAGGTGCAGTTC.......................................      1
....TGCCCATTCAGGTGCAGTTCA......................................      1
......................................TCCTGTGCCTCGAATGG........      1
....................................TGTCCTGTGCCTCGAATGGGT......      1
........................................CTGTGCCTCGAATGGGT......      1
.......................................CCTGTGCCTCGAATGGGTA.....      1
........................................CTGTGCCTCGAATGGGTA.....      1
....................................TGTCCTGTGCCTCGAATGGGTAT....      1
............................ACAATTGGTGTCCTGTGCCTCGAATGGGTATG...      1
................................TTGGTGTCCTGTGCCTCGAATGGGTATG...      1
(((.((((((((((((..(((..(((.........)))..)))..))).))))))))).))). (-34.50)

X/30338367-30338429(-)_novel_cow_47
CCCATTCCAAGGCTTGCCATTCATCTGGCTAAACTTGTGATTGGCATGTCTTGGAATGAAGTA
.......................................ATTGGCATGTCTTGGAATGA....      2739
```

```
.......................................ATTGGCATGTCTTGGAATG.....      1146
.....................................TGATTGGCATGTCTTGGAATG.....      706
.....................................TGATTGGCATGTCTTGGAATGA....      290
...ATTCCAAGGCTTGCCATTCATC........................................      137
......................................GATTGGCATGTCTTGGAATG.....      129
.......................................ATTGGCATGTCTTGGAATGAA...      112
........................................TTGGCATGTCTTGGAATGA....      90
...ATTCCAAGGCTTGCCATTCAT.........................................      75
......................................GATTGGCATGTCTTGGAATGA....      71
..CATTCCAAGGCTTGCCATTC..........................................      44
..CATTCCAAGGCTTGCCATTCAT........................................      23
.....................................TGATTGGCATGTCTTGGAAT......      20
..CATTCCAAGGCTTGCCATTCA.........................................      19
...ATTCCAAGGCTTGCCATTCA..........................................      9
.....................................TGATTGGCATGTCTTGGAATGAA...      8
..CATTCCAAGGCTTGCCATT............................................      7
.......................................ATTGGCATGTCTTGGAAT......      7
.......................................ATTGGCATGTCTTGGAA.......      6
...........................................GCATGTCTTGGAATGA....      5
..........................................GGCATGTCTTGGAATGA....      4
.......................................ATTGGCATGTCTTGGAATGAAG..      4
...ATTCCAAGGCTTGCCATTCATCT.......................................      2
..CATTCCAAGGCTTGCCAT.............................................      1
...ATTCCAAGGCTTGCCATTC...........................................      1
..CATTCCAAGGCTTGCCATTCATC.......................................      1
........................................TTGGCATGTCTTGGAA.......      1
......................................GATTGGCATGTCTTGGAAT......      1
................................ACTTGTGATTGGCATGTCTTGGAATGAA...      1
........................................TTGGCATGTCTTGGAATGAA...      1
............................................CATGTCTTGGAATGAA...      1
.......................................ATTGGCATGTCTTGGAATGAAGT.      1
........................................TTGGCATGTCTTGGAATGAAGT.      1
..(((((((((((.(((((.((((..(......)..)))).))))).))))))))))).....  (-30.90)

X/34662670-34662742(-)_mir_cow_306
CGAAAACCTCAGTCAGCCTTGTGGATGTATGTTCTGCAGACCTGACATCTAGAGGACTGACTGAAATTTTCAC
......CCTCAGTCAGCCTTGTGGATGT............................................. 659
.......CTCAGTCAGCCTTGTGGATGTA............................................ 226
```

```
.......CTCAGTCAGCCTTGTGGATGT............................................. 201
..............................................ATCTAGAGGACTGACTGAAATT..... 172
......CCTCAGTCAGCCTTGTGGATG.............................................. 127
......CCTCAGTCAGCCTTGTGGAT............................................... 116
.............................................CATCTAGAGGACTGACTGAAAT...... 89
......CCTCAGTCAGCCTTGTGGATGTA............................................ 66
..............................................ATCTAGAGGACTGACTGAAAT...... 55
.............................................CATCTAGAGGACTGACTGAAA....... 53
.......CTCAGTCAGCCTTGTGGATGTAT........................................... 48
..............................................ATCTAGAGGACTGACTGAAA....... 29
.............................................CATCTAGAGGACTGACTGAAATT..... 20
.......CTCAGTCAGCCTTGTGGAT............................................... 18
.............................................CATCTAGAGGACTGACTGA......... 18
.......CTCAGTCAGCCTTGTGGATG.............................................. 13
.............................................CATCTAGAGGACTGACTGAA........ 11
......CCTCAGTCAGCCTTGTGGA................................................ 6
..............................................ATCTAGAGGACTGACTGAA........ 5
......CCTCAGTCAGCCTTGTGGATGTAT........................................... 4
..............................................ATCTAGAGGACTGACTGA......... 4
...............................................TCTAGAGGACTGACTGAAATT..... 3
..............................................ATCTAGAGGACTGACTGAAATTT.... 3
...........................................GACATCTAGAGGACTGACTGA......... 2
.......CTCAGTCAGCCTTGTGGA................................................ 1
.............................................CATCTAGAGGACTGACTG.......... 1
............................................ACATCTAGAGGACTGACTGAAA....... 1
.............................................CATCTAGAGGACTGACTGAAATTT.... 1
.(((((..((((((((((((.(((((((..(((.....)))...))))))))))).))))))))..))))).. (-30.30)

X/34664591-34664669(-)_mir_cow_307
GGCTTTCAAGTCACTAGTGGTTCCGTTTAGTAGATGGTTGTACATTGTTTCAAAATGGTACCCTAGTGACTACAAAGCC
......CAAGTCACTAGTGGTTCCGTTT................................................... 457
......CAAGTCACTAGTGGTTCCGTTTAG................................................. 219
......CAAGTCACTAGTGGTTCCGTT.................................................... 55
......CAAGTCACTAGTGGTTCCGTTTA.................................................. 54
......CAAGTCACTAGTGGTTCCGTTTAGT................................................ 18
......CAAGTCACTAGTGGTTCC....................................................... 8
......CAAGTCACTAGTGGTTCCGT..................................................... 8
.......AAGTCACTAGTGGTTCCGTTTAG................................................. 5
```

```
.......AAGTCACTAGTGGTTCCGTTT................................................... 3
.....................................................AATGGTACCCTAGTGACTACA..... 2
...................................................AAAATGGTACCCTAGTGACT........ 1
.....................................................AATGGTACCCTAGTGACTAC...... 1
....................................................AAATGGTACCCTAGTGACTACAA.... 1
((((((..(((((((((.(((.((((((.(..((((......))))....).)))))).))))))))))))..)))))) (-34.00)

X/34665630-34665701(-)_mir_cow_308
ACTTACAACTGTTTGCAGAGGAAACTGAGACTTTGTAACTATATCTCAGTCTCATCTGCAAAGAAGTAAGTG
.............................................TCAGTCTCATCTGCAAAGAAGT.....  179
......AACTGTTTGCAGAGGAAACTG.............................................  136
.........TGTTTGCAGAGGAAACTGAGA..........................................  107
......AACTGTTTGCAGAGGAAACTGA............................................  95
.............................................TCAGTCTCATCTGCAAAGAAG......  54
......AACTGTTTGCAGAGGAAACTGAGA..........................................  41
......AACTGTTTGCAGAGGAAACT..............................................  24
......AACTGTTTGCAGAGGAAACTGAG...........................................  22
.........TGTTTGCAGAGGAAACTGA............................................  20
.........TGTTTGCAGAGGAAACTGAG...........................................  15
.........TGTTTGCAGAGGAAACTGAGAC.........................................  10
........CTGTTTGCAGAGGAAACTGAGA..........................................  8
.......ACTGTTTGCAGAGGAAACTG.............................................  6
......AACTGTTTGCAGAGGAAAC...............................................  4
.............................................TCAGTCTCATCTGCAAAGA........  4
.............................................TCAGTCTCATCTGCAAAGAAGTA....  4
.......ACTGTTTGCAGAGGAAACTGA............................................  3
........CTGTTTGCAGAGGAAACTGA............................................  3
.........TGTTTGCAGAGGAAACTGAGACT........................................  3
.........TGTTTGCAGAGGAAAC...............................................  1
.......ACTGTTTGCAGAGGAAACT..............................................  1
.......ACTGTTTGCAGAGGAAACTGAG...........................................  1
........CTGTTTGCAGAGGAAACTGAG...........................................  1
.......ACTGTTTGCAGAGGAAACTGAGA..........................................  1
..........GTTTGCAGAGGAAACTGAGA..........................................  1
..........GTTTGCAGAGGAAACTGAGACT........................................  1
...........TTTGCAGAGGAAACTGAGACT........................................  1
...........TTTGCAGAGGAAACTGAGACTT.......................................  1
............................................CTCAGTCTCATCTGCAAAGAAGT.....  1
```

```
..............................................CAGTCTCATCTGCAAAGAAGT.....  1
((((((...(.((((((((.((.(((((((..(.......)..))))))).)).)))))))).).)))))). (-26.20)

X/35018707-35018776(-)_mir_cow_309
ATCGTAGTCAAATGCTCAGACTCCTGTGGTGGCTGCTCATGCACCACGGATGTTTGAGCATGTGCTACGG
.......TCAAATGCTCAGACTCCTGTGGT........................................    356
...........................................CCACGGATGTTTGAGCATGTGCT....    143
.......TCAAATGCTCAGACTCCTGTGG.........................................    107
.........AAATGCTCAGACTCCTGTGGTG.......................................    87
.............................................ACGGATGTTTGAGCATGTGCT....    80
.......TCAAATGCTCAGACTCCTGTGGTG.......................................    60
.........AAATGCTCAGACTCCTGTGGT........................................    25
............................................CACGGATGTTTGAGCATGTGCT....    20
...........................................CCACGGATGTTTGAGCATGTGC.....    13
.......TCAAATGCTCAGACTCCTGTG..........................................    9
..........AATGCTCAGACTCCTGTGGTG.......................................    9
.........AAATGCTCAGACTCCTGTGG.........................................    7
.......TCAAATGCTCAGACTCCTG............................................    6
...........................................CCACGGATGTTTGAGCATGT.......    5
........CAAATGCTCAGACTCCTGTGGT........................................    4
.......TCAAATGCTCAGACTCCT.............................................    2
.........AAATGCTCAGACTCCTG............................................    2
.......TCAAATGCTCAGACTCCTGT...........................................    2
.........AAATGCTCAGACTCCTGTG..........................................    2
..........AATGCTCAGACTCCTGTGGT........................................    2
.......TCAAATGCTCAGACTCCTGTGGTGG......................................    2
...........................................CCACGGATGTTTGAGCATGTG......    2
.......TCAAATGCTCAGACTCC..............................................    1
......GTCAAATGCTCAGACTCCTGT...........................................    1
......GTCAAATGCTCAGACTCCTGTG..........................................    1
........CAAATGCTCAGACTCCTGTGGTG.......................................    1
.........AAATGCTCAGACTCCTGTGGTGG......................................    1
..........AATGCTCAGACTCCTGTGGTGG......................................    1
.........AAATGCTCAGACTCCTGTGGTGGC.....................................    1
..........AATGCTCAGACTCCTGTGGTGGC.....................................    1
..........AATGCTCAGACTCCTGTGGTGGCT....................................    1
......................................ATGCACCACGGATGTTTGAGCA..........    1
...........................................CCACGGATGTTTGAGCATG.........    1
```

```
..........................................ACCACGGATGTTTGAGCATGT.......     1
..(((((.((.((((((((((..((((((((.(.......)))))))))..)))))))))).))))))). (-37.50)

X/35020238-35020306(-)_mir_cow_310
TTGCTCATGCACCATGGTTGTCTGAGCATGCAGCATGCTTGTCTGCTCATACCCCATGGTTCCTGAGCA
.......TGCACCATGGTTGTCTGAGCATG.......................................     333
.......TGCACCATGGTTGTCTGAGC..........................................     239
.......TGCACCATGGTTGTCTGAGCATGC......................................     134
.......TGCACCATGGTTGTCTGAGCA.........................................     107
.......TGCACCATGGTTGTCTGAGCAT........................................     86
..........................................CTGCTCATACCCCATGGTTCCT.....     12
......ATGCACCATGGTTGTCTGAGC..........................................     6
........GCACCATGGTTGTCTGAGCATGC......................................     6
.........................................TCTGCTCATACCCCATGGTTCC......     5
......ATGCACCATGGTTGTCTGAGCA.........................................     2
...........................................TGCTCATACCCCATGGTTCCT.....     2
...........................................TGCTCATACCCCATGGTTCCTG....     2
.......TGCACCATGGTTGTCTGAG...........................................     1
.......TGCACCATGGTTGTCTGAGCATGCAG....................................     1
.........................................TCTGCTCATACCCCATGGTTCCT.....     1
.((((((.(.(((((((..((.((((((.((((.....)))).)))))).)).))))))).).)))))) (-32.20)

X/35021161-35021228(-)_mir_cow_311
TCGTGGTCAAATGCTCAGACTCCTTGGTGGCTGCTTATGCACCATGGATGTTTGAGCATGTGCTATGG
......TCAAATGCTCAGACTCCTTGGTG....................................... 66
.........................................CCATGGATGTTTGAGCATGTGCT.... 9
......TCAAATGCTCAGACTCCTTGGT........................................ 8
........AAATGCTCAGACTCCTTGGTG....................................... 5
......TCAAATGCTCAGACTCCTTGG......................................... 4
......TCAAATGCTCAGACTCCT............................................ 2
.......CAAATGCTCAGACTCCTTGGTG....................................... 2
......TCAAATGCTCAGACTCC............................................. 1
......TCAAATGCTCAGACTCCTTG.......................................... 1
.........AATGCTCAGACTCCTTGGTG....................................... 1
........AAATGCTCAGACTCCTTGGTGG...................................... 1
.........................................CCATGGATGTTTGAGCATGTGC..... 1
...........................................ATGGATGTTTGAGCATGTGCT.... 1
.(((((.((.(((((((((((((.(((((.(.......)))))).))).)))))))))).))))))). (-32.90)
```

```
X/3998280-3998355(-)_mir_cow_285
ATTTTGAGAGGTAAAAAATTGATTTGACTAGTTCTTTAACACATCTAGCAAATCATTTTTTACTCTCCAAAAAGAA
......AGAGGTAAAAAATTGATTTGACT...............................................    26067
......AGAGGTAAAAAATTGATTTGAC................................................    4645
......AGAGGTAAAAAATTGATTTGA.................................................    234
......AGAGGTAAAAAATTGATTTG..................................................    88
................................................CAAATCATTTTTTACTCTCCA.......    54
................................................CAAATCATTTTTTACTCTCC........    44
.......GAGGTAAAAAATTGATTTGACT...............................................    35
..............................................AGCAAATCATTTTTTACTCTCC........    24
........AGGTAAAAAATTGATTTGACT...............................................    13
......AGAGGTAAAAAATTGATTTGACTAG.............................................    13
.............AAAAATTGATTTGACT...............................................    12
......AGAGGTAAAAAATTGATTTGACTA..............................................    10
................................................CAAATCATTTTTTACTCTCCAA......    10
......AGAGGTAAAAAATTGATTT...................................................    6
.........GGTAAAAAATTGATTTGACT...............................................    6
..........GTAAAAAATTGATTTGACT...............................................    6
...........TAAAAAATTGATTTGACT...............................................    5
......AGAGGTAAAAAATTGATT....................................................    4
...............................................GCAAATCATTTTTTACTCTCC........    4
..............................................AGCAAATCATTTTTTACTCTCCA.......    4
.......GAGGTAAAAAATTGATTTGAC................................................    3
........AGGTAAAAAATTGATTTGAC................................................    2
.........GGTAAAAAATTGATTTGAC................................................    2
........AGGTAAAAAATTGATTTGACTAGT............................................    2
..............................................AGCAAATCATTTTTTACTCT..........    2
............AAAAAATTGATTTGAC................................................    1
........AGGTAAAAAATTGATTTGACTAG.............................................    1
......AGAGGTAAAAAATTGATTTGACTAGT............................................    1
................................................CAAATCATTTTTTACTCTCCAAA.....    1
................................................CAAATCATTTTTTACTCTCCAAAA....    1
.(((((.((((((((((((.((((((.((((.............))))))))))))))))))).)))))))).... (-23.52)

X/62939297-62939367(+)_mir_cow_312
ACTGCACAACCCTAGGAGAGGGTGCCATTCACATAGACTAGAATTGAATGGCGCCACTAGGGTTGTGCAGT
..............................................AATGGCGCCACTAGGGTTGTGC...   1056
```

```
.............................................AATGGCGCCACTAGGGTTGTG....   438
.............................................AATGGCGCCACTAGGGTTG......   372
.....ACAACCCTAGGAGAGGGTGCCAT..........................................   276
.............................................AATGGCGCCACTAGGGTTGT.....   274
.....ACAACCCTAGGAGAGGGTGCCATT.........................................   207
.....ACAACCCTAGGAGAGGGTGCCA...........................................   101
......CAACCCTAGGAGAGGGTGCCATT.........................................   39
.....ACAACCCTAGGAGAGGGTGCCATTC........................................   34
.....ACAACCCTAGGAGAGGGTGCC............................................   32
......CAACCCTAGGAGAGGGTGCCA...........................................   31
.............................................AATGGCGCCACTAGGGTTGTGCA..   27
......CAACCCTAGGAGAGGGTGCCAT..........................................   17
......CAACCCTAGGAGAGGGTGCC............................................   16
.............................................AATGGCGCCACTAGGGTT.......   12
..............................................ATGGCGCCACTAGGGTTGTGC...   11
......CAACCCTAGGAGAGGGTGCCATTC........................................   10
......CAACCCTAGGAGAGGGTGC.............................................   6
.....ACAACCCTAGGAGAGGGTGC.............................................   5
......CAACCCTAGGAGAGGGTGCCATTCA.......................................   5
...........................................TGAATGGCGCCACTAGGGTTGT.....   5
.....ACAACCCTAGGAGAGGGTGCCATTCA.......................................   4
.............................................AATGGCGCCACTAGGGT........   4
............................................GAATGGCGCCACTAGGGTTG......   3
...........................................TGAATGGCGCCACTAGGGTTG......   2
.....ACAACCCTAGGAGAGG.................................................   1
......CAACCCTAGGAGAGGG................................................   1
.....ACAACCCTAGGAGAGGGTG..............................................   1
....CACAACCCTAGGAGAGGGTGCCAT..........................................   1
......CAACCCTAGGAGAGGGTGCCATTCAC......................................   1
.............................................AATGGCGCCACTAGGG.........   1
..............................................ATGGCGCCACTAGGGTTG......   1
............................................GAATGGCGCCACTAGGGTTGT.....   1
..............................................ATGGCGCCACTAGGGTTGT.....   1
..................................................CGCCACTAGGGTTGTG....   1
(((((((((((((((.....(((((((((((............))))))))))).)))))))))))))))  (-45.02)

X/67815920-67815989(+)_mir_cow_313
TAGCAGGTCCTCAATAAGTATTTGTTGCAAGAATAAGAGAACCAACAAGTCTTATTTGAGCACCTGTTAT
```

```
............................................ACAAGTCTTATTTGAGCACCTG....     79
.............................................CAAGTCTTATTTGAGCACCTGT...     51
............................................ACAAGTCTTATTTGAGCACCTGT...     44
.............................................CAAGTCTTATTTGAGCACCTG....     10
....AGGTCCTCAATAAGTATTTGTT............................................     9
....AGGTCCTCAATAAGTATTTGT.............................................     4
............................................ACAAGTCTTATTTGAGCACCT.....     3
.............................................CAAGTCTTATTTGAGCACCTGTT..     2
....AGGTCCTCAATAAGTATTTG..............................................     1
............................................ACAAGTCTTATTTGAGC.........     1
............................................ACAAGTCTTATTTGAGCACC......     1
...........................................AACAAGTCTTATTTGAGCACCTG....     1
((((((((.(((((((((.((((((((...............)))))))))))).))))).)))))))). (-24.76)

X/67896128-67896207(+)_mir_cow_314
AGAGTTCATTCGGCTGTCCAGATGTATCCAAGTACCCTGATATTTGGCAATAAATACATCTGGGCAACTGATTGAACTTT
....TTCATTCGGCTGTCCAGATGTA......................................................      1330
........................................................CATCTGGGCAACTGATTGAACT..      338
....TTCATTCGGCTGTCCAGATGT.......................................................      77
......................................................TACATCTGGGCAACTGATTGAAC...      34
....TTCATTCGGCTGTCCAGATGTATC....................................................      28
....TTCATTCGGCTGTCCAGATGTAT.....................................................      19
........................................................CATCTGGGCAACTGATTGAAC...      16
....TTCATTCGGCTGTCCAGATGTATCCAAGT...............................................      13
....TTCATTCGGCTGTCCAGATG........................................................      10
....TTCATTCGGCTGTCCAGAT.........................................................      8
....TTCATTCGGCTGTCCAGATGTATCC...................................................      7
......................................................TACATCTGGGCAACTGATTGAACT..      6
....TTCATTCGGCTGTCCAGA..........................................................      4
......................................................TACATCTGGGCAACTGATTGA.....      4
.......................................................ACATCTGGGCAACTGATTGAACT..      4
....TTCATTCGGCTGTCCAGATGTATCCAAG................................................      3
........................................................CATCTGGGCAACTGATTGA.....      3
.....TCATTCGGCTGTCCAGATGTA......................................................      2
.......ATTCGGCTGTCCAGATGTATCC...................................................      2
.....TCATTCGGCTGTCCAGATGTATC....................................................      1
......CATTCGGCTGTCCAGATGTATC....................................................      1
.......ATTCGGCTGTCCAGATGTATC....................................................      1
```

```
......CATTCGGCTGTCCAGATGTATCCAAGT...............................................      1
....TTCATTCGGCTGTCCAGATGTATCCAAGTA..............................................      1
....TTCATTCGGCTGTCCAGATGTATCCAAGTACC............................................      1
.............................................GGCAATAAATACATCTGGGCAACT...........      1
...............................................CAATAAATACATCTGGGCAACTGA.........      1
.....................................................ATACATCTGGGCAACTGATTGAACT..      1
.........................................................ATCTGGGCAACTGATTGAACT..      1
..........................................................TCTGGGCAACTGATTGAACT..      1
(((((((((.((((.((((((((((((((((((((.....))))))).....))))))))))))).)))).))))))))) (-37.90)

X/67911312-67911381(-)_mir_cow_315
ATCTGCTGAGTACCGCCATGTCTGTTGGGAATCCACAATCTCCCACCAGGCATTGTGGTCTCTGCTGATG
......TGAGTACCGCCATGTCTGTTGGG.........................................    33
......TGAGTACCGCCATGTCTGTTGGGA........................................    26
......TGAGTACCGCCATGTCTGT.............................................    6
...........................................CACCAGGCATTGTGGTCTCTGCT....    5
......TGAGTACCGCCATGTCTGTTG...........................................    4
......TGAGTACCGCCATGTCTGTTGG..........................................    3
...........................................CACCAGGCATTGTGGTCTCTGC.....    3
......TGAGTACCGCCATGTCTGTTGGGAAT......................................    1
...........................................CACCAGGCATTGTGGTCTCT.......    1
............................................ACCAGGCATTGTGGTCTCTGCT....    1
(((.((.(((.(((((.(((((((.(((((..........))))).))))))).)))))))).)).))). (-33.10)

X/74328422-74328492(+)_mir_cow_316
GGAGCTTATCAGAATCTCCAGGGGTACTTATAATTTGAAAAAGTCCCCCAGGTGTGATTCTGATTTGCTTC
.....TTATCAGAATCTCCAGGGGTAC............................................   5448
............................................CCCCCAGGTGTGATTCTGATTTGC...   857
...........................................TCCCCCAGGTGTGATTCTGATT......   798
.....TTATCAGAATCTCCAGGGGTA.............................................   595
.....TTATCAGAATCTCCAGGGGTACT...........................................   368
...........................................TCCCCCAGGTGTGATTCTGATTT.....   316
...........................................TCCCCCAGGTGTGATTCTGAT.......   244
.....TTATCAGAATCTCCAGGGGT..............................................   238
...........................................TCCCCCAGGTGTGATTCTGATTTG....   209
............................................CCCCCAGGTGTGATTCTGATTTG....   195
............................................CCCCCAGGTGTGATTCTGATT......   121
............................................CCCCCAGGTGTGATTCTGATTT.....   91
```

```
....CTTATCAGAATCTCCAGGGGTAC............................................   70
...........................................TCCCCCAGGTGTGATTCTGATTTGC...   63
............................................CCCCCAGGTGTGATTCTGATTTGCT..   62
...........................................TCCCCCAGGTGTGATTCTGA........   43
.....TTATCAGAATCTCCAGGGG...............................................   39
............................................CCCCCAGGTGTGATTCTGAT.......   31
...........................................TCCCCCAGGTGTGATTCTG.........   23
...........................................TCCCCCAGGTGTGATTCTGATTTGCT..   19
.....TTATCAGAATCTCCAGGG................................................   18
....CTTATCAGAATCTCCAGGGGTA.............................................   8
.....TTATCAGAATCTCCAGGGGTACTT..........................................   8
....CTTATCAGAATCTCCAGGGGTACT...........................................   7
............................................CCCCCAGGTGTGATTCTGA........   5
....CTTATCAGAATCTCCAGGGGT..............................................   3
......TATCAGAATCTCCAGGGGTACT...........................................   3
.............................................CCCCAGGTGTGATTCTGATT......   3
.....TTATCAGAATCTCCAG..................................................   2
........TCAGAATCTCCAGGGGTAC............................................   2
.....TTATCAGAATCTCCAGG.................................................   1
.......ATCAGAATCTCCAGGGGTAC............................................   1
...........................................TCCCCCAGGTGTGATT............   1
............................................CCCCCAGGTGTGATTCTG.........   1
.........................................AGTCCCCCAGGTGTGATTCTGA........   1
..........................................GTCCCCCAGGTGTGATTCTGAT.......   1
...............................................CCAGGTGTGATTCTGATT......   1
................................................CAGGTGTGATTCTGATT......   1
.........................................AGTCCCCCAGGTGTGATTCTGATTT.....   1
.............................................CCCCAGGTGTGATTCTGATTT.....   1
.............................................CCCCAGGTGTGATTCTGATTTG....   1
.............................................CCCCAGGTGTGATTCTGATTTGC...   1
..............................................CCCAGGTGTGATTCTGATTTGC...   1
.................................................AGGTGTGATTCTGATTTGC...   1
.............................................CCCCAGGTGTGATTCTGATTTGCT..   1
(((((..(((((((((.((.((((.((((...........)))).)))).))...)))))))))..))))) (-31.30)

X/7486525-7486590(-)_mir_cow_286
CCAAAAAGGTCGTTCGGGTTTTTCTGTATGATGTTAATGGAAAAACCTGAATAAACTTTTTGGCCA
...AAAAGGTCGTTCGGGTTTTT...........................................   3
```

```
...AAAAGGTCGTTCGGGTTTTTC..........................................  2
...AAAAGGTCGTTCGGGTTTTTCT.........................................  2
..........................................AAACCTGAATAAACTTTTTGG...  2
.........................................AAAACCTGAATAAACTTTTTGG...  1
((((((((...((((((((((((((((.........))))))))))))))))...))))))))... (-26.90)

X/81951229-81951289(+)_mir_cow_318
GGCCATTATAATACAACCTGATAAGTGTTACAGCACTTATCAGGTTGTATTGTAATTGTCT
...................................CTTATCAGGTTGTATTGTAATT....  6345
.....TTATAATACAACCTGATAAGT...................................  3321
.....TTATAATACAACCTGATAAGTG..................................  2668
.....TTATAATACAACCTGATAAGTGT.................................  485
...................................CTTATCAGGTTGTATTGTAAT.....  441
....................................TTATCAGGTTGTATTGTAATT....  100
...................................CTTATCAGGTTGTATTGTAA......  65
.....TTATAATACAACCTGATAAG....................................  61
.....................................TATCAGGTTGTATTGTAATT....  31
...................................CTTATCAGGTTGTATTGTA.......  17
...................................CTTATCAGGTTGTATTGT........  13
..................................ACTTATCAGGTTGTATTGTAAT.....  12
..................................ACTTATCAGGTTGTATTGTAATT....  10
....................................TTATCAGGTTGTATTGTAAT.....  5
.....TTATAATACAACCTGATA......................................  4
.....TTATAATACAACCTGATAA.....................................  4
......TATAATACAACCTGATAAGTGT.................................  4
..................................ACTTATCAGGTTGTATTGTAA......  3
.....TTATAATACAACCTGAT.......................................  2
......TATAATACAACCTGATAAGT...................................  2
....ATTATAATACAACCTGATAAGT...................................  1
.......ATAATACAACCTGATAAGT...................................  1
.......ATAATACAACCTGATAAGTG..................................  1
........TAATACAACCTGATAAGTGT.................................  1
.....TTATAATACAACCTGATAAGTGTT................................  1
.........AATACAACCTGATAAGTGTTAC..............................  1
.........AATACAACCTGATAAGTGTTACAGC...........................  1
....................................TTATCAGGTTGTATTGTAA......  1
.....................................TATCAGGTTGTATTGTAAT.....  1
......................................ATCAGGTTGTATTGTAATT....  1
```

```
........................................CAGGTTGTATTGTAATT....  1
.........................................AGGTTGTATTGTAATT....  1
....................................TTATCAGGTTGTATTGTAATTG...  1
.....................................TATCAGGTTGTATTGTAATTG...  1
.....................................TATCAGGTTGTATTGTAATTGT..  1
(((.((((((((((((((((((((((((....)))))))))))))))))))))))).))). (-37.60)

X/81951230-81951286(-)_mir_cow_317
CAATTACAATACAACCTGATAAGTGCTGTAACACTTATCAGGTTGTATTATAATGGC
...TTACAATACAACCTGATAAGT.................................12
...TTACAATACAACCTGATAAGTG................................6
................................ACTTATCAGGTTGTATTAT......3
...............................CACTTATCAGGTTGTATTAT......1
................................ACTTATCAGGTTGTATTATA.....1
.................................CTTATCAGGTTGTATTATAAT...1
..((((.((((((((((((((((((......)))))))))))))))))).))))... (-28.10)

X/81951391-81951460(+)_mir_cow_319
TAGGCCTCAGTAAATGTTTATTGGATGAATAAATGAATGGCTCATCAACAAACATTTATTGTGTGCCTGC
......TCAGTAAATGTTTATTGGATGA..........................................    96
............................................TCAACAAACATTTATTGTGTGCC...    81
....CCTCAGTAAATGTTTATTGGATG...........................................    60
....CCTCAGTAAATGTTTATTGGAT............................................    57
......TCAGTAAATGTTTATTGGAT............................................    50
............................................TCAACAAACATTTATTGTGTGC....    40
......TCAGTAAATGTTTATTGGATG...........................................    33
............................................TCAACAAACATTTATTGTGTGCCT..    16
...........................................ATCAACAAACATTTATTGTGTGC....    13
.....CTCAGTAAATGTTTATTGGATGA..........................................    7
.....CTCAGTAAATGTTTATTGGAT............................................    6
....CCTCAGTAAATGTTTATTGGA.............................................    3
...........................................ATCAACAAACATTTATTGTGT......    3
...........................................ATCAACAAACATTTATTGTGTG.....    3
......TCAGTAAATGTTTATTGGA.............................................    2
....CCTCAGTAAATGTTTATTGGATGA..........................................    2
......TCAGTAAATGTTTATTGGATGAA.........................................    2
............................................TCAACAAACATTTATTGTGT......    2
....CCTCAGTAAATGTTTATTG...............................................    1
```

```
......TCAGTAAATGTTTATTG...............................................    1
.....CTCAGTAAATGTTTATTGGATGAA..........................................    1
...........................AATAAATGAATGGCTC...........................    1
..........................................CATCAACAAACATTTATTGTGT......    1
..............................................AACAAACATTTATTGTGTGCC...    1
((((((.((((((((((((....(((((.((......))..)))))...)))))))))))).).)))))). (-23.50)

X/82023928-82023987(+)_mir_cow_320
ATGGATATAATACAACCTGCTAAGTGTCCTAGCACTTATCAGGTTGTATTATCATTGTCC
....ATATAATACAACCTGCTAAGT...................................   2755
..................................CTTATCAGGTTGTATTATCATT....   1484
....ATATAATACAACCTGCTAAGTG..................................   1362
....ATATAATACAACCTGCTAAGTGT.................................   702
.......TAATACAACCTGCTAAGTGTCCT..............................   104
.................................ACTTATCAGGTTGTATTATC.......   91
....ATATAATACAACCTGCTAAG....................................   86
...................................TTATCAGGTTGTATTATCATT....   85
..................................CTTATCAGGTTGTATTATCAT.....   56
....ATATAATACAACCTGCTAAGTGTCCT..............................   51
..................................CTTATCAGGTTGTATTATC.......   48
.................................ACTTATCAGGTTGTATTATCAT.....   48
.....TATAATACAACCTGCTAAGTGT.................................   38
.....TATAATACAACCTGCTAAGTGTC................................   30
.....TATAATACAACCTGCTAAGTGTCCT..............................   30
........AATACAACCTGCTAAGTGTCCT..............................   27
..................................CTTATCAGGTTGTATTATCA......   23
......ATAATACAACCTGCTAAGTGTCCT..............................   21
................................CACTTATCAGGTTGTATTATC.......   19
....ATATAATACAACCTGCTAAGTGTC................................   16
.......TAATACAACCTGCTAAGTGTCC...............................   7
....ATATAATACAACCTGCTAAGTGTCCTAGCA..........................   7
....ATATAATACAACCTGCT.......................................   6
.................................ACTTATCAGGTTGTATTATCA......   6
...................................TTATCAGGTTGTATTATCAT.....   5
......ATAATACAACCTGCTAAGTGT.................................   4
.......TAATACAACCTGCTAAGTGTC................................   4
....ATATAATACAACCTGCTAAGTGTCC...............................   4
.....TATAATACAACCTGCTAAGTGTCC...............................   4
```

```
....ATATAATACAACCTGCTAAGTGTCCTAGC...........................   4
.....TATAATACAACCTGCTAAGT...................................   3
......ATAATACAACCTGCTAAGTGTCC...............................   3
....ATATAATACAACCTGCTAAGTGTCCTAGCACTTATC....................   3
.................................ACTTATCAGGTTGTATTAT........   3
...................................TTATCAGGTTGTATTATC.......   3
....ATATAATACAACCTGCTA......................................   2
....ATATAATACAACCTGCTAA.....................................   2
......ATAATACAACCTGCTAAGT...................................   2
......ATAATACAACCTGCTAAGTG..................................   2
........AATACAACCTGCTAAGTGTCC...............................   2
......ATAATACAACCTGCTAAGTGTCCTAG............................   2
.....TATAATACAACCTGCTAAGTGTCCTAGCACTTATCA...................   2
..............................AGCACTTATCAGGTTGTATTATC.......   2
...................................TTATCAGGTTGTATTATCA......   2
..............................AGCACTTATCAGGTTGTATTATCATT....   2
.................................ACTTATCAGGTTGTATTATCATT....   2
.....TATAATACAACCTGCTAAGTG..................................   1
.......TAATACAACCTGCTAAGTGT.................................   1
......ATAATACAACCTGCTAAGTGTC................................   1
.........ATACAACCTGCTAAGTGTCCT..............................   1
..........TACAACCTGCTAAGTGTCCT..............................   1
....ATATAATACAACCTGCTAAGTGTCCTA.............................   1
.....TATAATACAACCTGCTAAGTGTCCTAGC...........................   1
.......TAATACAACCTGCTAAGTGTCCTAGC...........................   1
.......TAATACAACCTGCTAAGTGTCCTAGCACTTATC....................   1
.......TAATACAACCTGCTAAGTGTCCTAGCACTTATCA...................   1
................................CACTTATCAGGTTGTATTAT........   1
......................AGTGTCCTAGCACTTATCAGGTTGTATTATC.......   1
.....................................ATCAGGTTGTATTATC.......   1
................................CACTTATCAGGTTGTATTATCA......   1
....................................TATCAGGTTGTATTATCATT....   1
.................................ACTTATCAGGTTGTATTATCATTG...   1
...................................TTATCAGGTTGTATTATCATTGT..   1
..((((((((((((((((.(((((((....))))))).)))))))))))))....)))) (-27.70)
```

X/82024085-82024155(+)_mir_cow_321
TGTAGGCCTCATTAAATGTTTGTTGAATGAAAAAATGAATCATCAACAGACATTAATTGGGCGCCTGCTCT

```
.........................................ATCAACAGACATTAATTGGGCGC.......   4727
.........................................ATCAACAGACATTAATTGGGC.........   2658
.........................................ATCAACAGACATTAATTGGGCG........   1898
..........................................TCAACAGACATTAATTGGGCGC.......   559
.............................................ACAGACATTAATTGGGCGCCTG....   91
..........................................TCAACAGACATTAATTGGGCG........   79
............................................AACAGACATTAATTGGGCGCCTG....   72
............................................AACAGACATTAATTGGGCGCCT.....   70
..........................................TCAACAGACATTAATTGGGC.........   62
.............................................ACAGACATTAATTGGGCGCCT.....   57
.......CTCATTAAATGTTTGTTGAAT...........................................   53
.......CTCATTAAATGTTTGTTGAATG..........................................   48
...........................................CAACAGACATTAATTGGGCGC.......   31
.......CTCATTAAATGTTTGTTGAATGA.........................................   12
............................................AACAGACATTAATTGGGCGC.......   12
...........................................CAACAGACATTAATTGGGCGCCT.....   7
.........................................ATCAACAGACATTAATTGGG..........   5
......CCTCATTAAATGTTTGTTGAATG..........................................   4
.........................................ATCAACAGACATTAATTGGGCGCC......   4
......CCTCATTAAATGTTTGTTGAAT...........................................   3
...........................................CAACAGACATTAATTGGGC.........   3
............................................AACAGACATTAATTGGGC.........   3
...........................................CAACAGACATTAATTGGGCG........   3
........................................CATCAACAGACATTAATTGGGCGC.......   3
..........................................TCAACAGACATTAATTGGGCGCCT.....   3
....GGCCTCATTAAATGTTTGTTG..............................................   2
.........................................ATCAACAGACATTAATTGG...........   2
........................................CATCAACAGACATTAATTGGGC.........   2
..............................................CAGACATTAATTGGGCGC.......   2
.............................................ACAGACATTAATTGGGCGCC......   2
.........................................ATCAACAGACATTAATTG............   1
........................................CATCAACAGACATTAATTGGGCG........   1
............................................AACAGACATTAATTGGGCG........   1
.............................................ACAGACATTAATTGGGCG........   1
.............................................ACAGACATTAATTGGGCGC.......   1
..........................................TCAACAGACATTAATTGGGCGCC......   1
.........................................ATCAACAGACATTAATTGGGCGCCT.....   1
...........................................CAACAGACATTAATTGGGCGCCTG....   1
```

```
.............................................ACAGACATTAATTGGGCGCCTGCT..    1
.((((((((((...((((((((((((.(((.........)))))))))))))))...)))).))))))...  (-29.60)

X/9189810-9189880(-)_mir_cow_287
AAAAGTTCCGTCAACCATCCAGCTGTTTGAGGTGATGCAAACAAACATCTGGTTGGTTGAGAGAATTTTTT
........CGTCAACCATCCAGCTGTTTGA.........................................   555
......TCCGTCAACCATCCAGCTGTTT...........................................   242
..........................................AAACATCTGGTTGGTTGAGAGA.......   188
........CGTCAACCATCCAGCTGTTTG..........................................   179
........CGTCAACCATCCAGCTGTTT...........................................   109
......TCCGTCAACCATCCAGCTGTTTG..........................................   102
......TCCGTCAACCATCCAGCTGTT............................................   71
...........................................AACATCTGGTTGGTTGAGAGA.......   36
..........................................AAACATCTGGTTGGTTGAGAG........   33
..........................................AAACATCTGGTTGGTTGAGA.........   26
........CGTCAACCATCCAGCTGTT............................................   17
........CGTCAACCATCCAGCTGT.............................................   16
..........................................AAACATCTGGTTGGTTGAGAGAA......   15
............................................ACATCTGGTTGGTTGAGAGAAT.....   12
......TCCGTCAACCATCCAGCTGT.............................................   9
...........................................AACATCTGGTTGGTTGAGAG........   8
..........................................AAACATCTGGTTGGTTGAG..........   5
...........................................AACATCTGGTTGGTTGAGA.........   4
...........................................AACATCTGGTTGGTTGAGAGAA......   3
...........................................AACATCTGGTTGGTTGAGAGAAT.....   3
........CGTCAACCATCCAGCTGTTTGAG........................................   2
............................................ACATCTGGTTGGTTGAGAGA.......   2
......TCCGTCAACCATCCAGCTG..............................................   1
.......CCGTCAACCATCCAGCTGTTT...........................................   1
............AACCATCCAGCTGTTTG..........................................   1
..........................................AAACATCTGGTTGGTTGA...........   1
...........................................AACATCTGGTTGGTTGAG..........   1
............................................ACATCTGGTTGGTTGAGAGAA......   1
...........................................AACATCTGGTTGGTTGAGAGAATT....   1
.............................................CATCTGGTTGGTTGAGAGAATT....   1
((((((((..(((((((.((((.((((((..(.....)...)))))).)))).)))))))..)))))))). (-28.40)

X/92886513-92886580(+)_mir_cow_322
```

```
CTCTTACATGCCTTGAGTGTAGGACCGTTGGCATCTTAATTACCCTCCCACACCCAAGGCTTGCAGGA
......CATGCCTTGAGTGTAGGACCGT........................................ 8836
......CATGCCTTGAGTGTAGGACC.......................................... 1605
......CATGCCTTGAGTGTAGGACCG......................................... 1531
......CATGCCTTGAGTGTAGGACCGTT....................................... 1236
...........................................CCTCCCACACCCAAGGCTTGCA... 549
...........................................CCTCCCACACCCAAGGCTTGC.... 306
.....ACATGCCTTGAGTGTAGGACCGT........................................ 279
.....ACATGCCTTGAGTGTAGGACC.......................................... 237
.....ACATGCCTTGAGTGTAGGACCG......................................... 78
...........................................CCTCCCACACCCAAGGCTTG..... 78
......CATGCCTTGAGTGTAGGAC........................................... 50
.....ACATGCCTTGAGTGTAGGACCGTT....................................... 32
.......ATGCCTTGAGTGTAGGACCGT........................................ 18
......CATGCCTTGAGTGTAGGA............................................ 11
.....ACATGCCTTGAGTGTAGGAC........................................... 11
...........................................CCTCCCACACCCAAGGCTTGCAG.. 10
...........................................CCTCCCACACCCAAGGCT....... 7
.......ATGCCTTGAGTGTAGGACCGTT....................................... 3
.............................................TCCCACACCCAAGGCTTGCA... 3
....TACATGCCTTGAGTGTAGGACC.......................................... 2
.......ATGCCTTGAGTGTAGGACCG......................................... 2
...........................................CCTCCCACACCCAAGGCTT...... 2
............................................CTCCCACACCCAAGGCTTGCA... 2
......CATGCCTTGAGTGTAGG............................................. 1
.......ATGCCTTGAGTGTAGGAC........................................... 1
........TGCCTTGAGTGTAGGACCGT........................................ 1
...............................................CCACACCCAAGGCTTGCA... 1
.((((.((.((((((.((((.(((..((.............))..))).)))).)))))).)).)))) (-21.52)
```

X/92886837-92886902(+)_mir_cow_323

```
CTCACATCCCTTGCATGGTGGAGGGTGAGCTTTCTGAAAACCCCTCCCACATGCAGGGTTTGCAGG
....CATCCCTTGCATGGTGGAGGGT........................................  262
...........................................CTCCCACATGCAGGGTTTGCA..  123
....CATCCCTTGCATGGTGGAGGG.........................................  28
....CATCCCTTGCATGGTGGAGG..........................................  15
....CATCCCTTGCATGGTGGA............................................  5
...........................................CTCCCACATGCAGGGTTTGC...  4
```

```
....CATCCCTTGCATGGTGGAGGGTG.......................................  3
....CATCCCTTGCATGGTGG.............................................  1
....CATCCCTTGCATGGTGGAG...........................................  1
....((..(((((((((..((((((....(.....).....))))))..)))))))))..))....  (-28.60)

X/92897959-92898028(+)_mir_cow_324
TCTCTAATTCTTGCTCCCCAGGTGAGAGTGCTTTGTGAATGCAATCCACCTGGGCAAGGATTCTGAATGT
............................................TCCACCTGGGCAAGGATTCTGA....     237
............................................TCCACCTGGGCAAGGATTCTGAA...     50
....TAATTCTTGCTCCCCAGGTGAGA...........................................     42
....TAATTCTTGCTCCCCAGGTGAGAGT.........................................     42
....TAATTCTTGCTCCCCAGGTGAG............................................     40
..........................................AATCCACCTGGGCAAGGATTCT......     19
..........................................AATCCACCTGGGCAAGGATTCTG.....     14
..........................................AATCCACCTGGGCAAGGATTCTGA....     14
.................................................CTGGGCAAGGATTCTGA....     10
............................................TCCACCTGGGCAAGGATTCTG.....     9
....TAATTCTTGCTCCCCAGGTG..............................................     4
....TAATTCTTGCTCCCCAGGTGA.............................................     4
............................................TCCACCTGGGCAAGGATTCTGAAT..     4
................................................CCTGGGCAAGGATTCTG.....     3
..............................................CACCTGGGCAAGGATTCTGA....     3
...........................................ATCCACCTGGGCAAGGATTCTGAA...     3
....TAATTCTTGCTCCCCAGGTGAGAGTG........................................     2
.....................................AATGCAATCCACCTGGGCAAGGA..........     2
.....................................AATGCAATCCACCTGGGCAAGGAT.........     2
..........................................AATCCACCTGGGCAAGGATT........     2
..........................................AATCCACCTGGGCAAGGATTC.......     2
............................................TCCACCTGGGCAAGGATTCT......     2
.................................................CTGGGCAAGGATTCTG.....     2
...........................................ATCCACCTGGGCAAGGATTCTGA....     2
...............................................ACCTGGGCAAGGATTCTGA....     2
....TAATTCTTGCTCCCCAGGTGAGAG..........................................     1
....TAATTCTTGCTCCCCAGGTGAGAGTGC.......................................     1
....TAATTCTTGCTCCCCAGGTGAGAGTGCTTTG...................................     1
.....................................AATGCAATCCACCTGGGCAAGGATT........     1
...........................................ATCCACCTGGGCAAGGATTCTG.....     1
................................................CCTGGGCAAGGATTCTGA....     1
```

```
...............................................ACCTGGGCAAGGATTCTGAA...     1
................................................CCTGGGCAAGGATTCTGAA...     1
..((.(((((((((...(((((((.((.(((.........))).))))))))))))))))))..))....  (-27.20)

X/92898442-92898507(+)_mir_cow_325
CTCGAATCCTTGGAACCTAGGTGTGAGTGCTGTTCTAGTGCAACACACCTATTCAAGGATTCAAAG
....AATCCTTGGAACCTAGGTGTGAGT......................................  530
....AATCCTTGGAACCTAGGTGTGAG.......................................  194
.........................................AACACACCTATTCAAGGATT.....  125
....AATCCTTGGAACCTAGGTGTGAGTGC....................................  95
....AATCCTTGGAACCTAGGTGTGA........................................  92
.........................................AACACACCTATTCAAGGATTCA...  72
.........................................AACACACCTATTCAAGGATTC....  65
.........................................AACACACCTATTCAAGGAT......  55
....AATCCTTGGAACCTAGGTGTGAGTG.....................................  24
....AATCCTTGGAACCTAGGTGTGAGTGCTG..................................  19
.........................................AACACACCTATTCAAGGATTCAA..  16
....AATCCTTGGAACCTAGGTGTG.........................................  11
....AATCCTTGGAACCTAGGTGT..........................................  5
....AATCCTTGGAACCTAGGTGTGAGTGCT...................................  4
....AATCCTTGGAACCTAGG.............................................  1
....AATCCTTGGAACCTAGGTG...........................................  1
..........................................ACACACCTATTCAAGGATT.....  1
...............................................CCTATTCAAGGATTCA...  1
...(((((((((((...((((((((.(..(((...)))..)..)))))))))))))))))))....  (-30.40)

X/92899706-92899776(+)_mir_cow_326
TCTCTAATCCTTGCTACCTGGGTGAGAGTGCTTTCTGAATGCAATGCACCTGGGCAAGGATTCTGAGAGAG
...........................................ATGCACCTGGGCAAGGATTCTGA.....   11842
...........................................ATGCACCTGGGCAAGGATTCTG......   738
..........................................AATGCACCTGGGCAAGGATTCTGA.....   543
..........................................AATGCACCTGGGCAAGGATTCTG......   333
....TAATCCTTGCTACCTGGGTGAGA............................................   314
....TAATCCTTGCTACCTGGGTGAGAGT..........................................   191
..........................................AATGCACCTGGGCAAGGATTCT.......   180
...........................................ATGCACCTGGGCAAGGATTCT.......   180
...........................................ATGCACCTGGGCAAGGATTC........   94
....TAATCCTTGCTACCTGGGTGAG.............................................   83
```

```
..........................................AATGCACCTGGGCAAGGATTC........   70
...........................................ATGCACCTGGGCAAGGATTCTGAG....   63
............................................TGCACCTGGGCAAGGATTCTGA.....   57
....TAATCCTTGCTACCTGGGTGA..............................................   48
....TAATCCTTGCTACCTGGGTGAGAG...........................................   37
....TAATCCTTGCTACCTGGGTGAGAGTGC........................................   25
....TAATCCTTGCTACCTGGGTGAGAGTG.........................................   12
...........................................ATGCACCTGGGCAAGGATTCTGAGA...   12
.................................................CTGGGCAAGGATTCTGA.....   10
....TAATCCTTGCTACCTG...................................................   3
....TAATCCTTGCTACCTGGGTG...............................................   3
............................................TGCACCTGGGCAAGGATTCTG......   3
................................................CCTGGGCAAGGATTCTG......   3
..............................................CACCTGGGCAAGGATTCTGA.....   3
..........................................AATGCACCTGGGCAAGGAT..........   2
...........................................ATGCACCTGGGCAAGGATT.........   2
................................TTCTGAATGCAATGCACCTGGGCAAGGATTCT.......   2
.................................................CTGGGCAAGGATTCTG......   2
..............................CTTTCTGAATGCAATGCACCTGGGCAAGGATTCTGA.....   2
...............................................ACCTGGGCAAGGATTCTGA.....   2
............................................TGCACCTGGGCAAGGATTCTGAG....   2
....TAATCCTTGCTACCTGGG.................................................   1
....TAATCCTTGCTACCTGGGT................................................   1
........CCTTGCTACCTGGGTGAGAGT..........................................   1
....TAATCCTTGCTACCTGGGTGAGAGTGCT.......................................   1
....TAATCCTTGCTACCTGGGTGAGAGTGCTTTCTGAA................................   1
....TAATCCTTGCTACCTGGGTGAGAGTGCTTTCTGAAT...............................   1
..............TACCTGGGTGAGAGTGCTTTCTGAATGC.............................   1
..........................................AATGCACCTGGGCAAGGATT.........   1
............................................TGCACCTGGGCAAGGATTC........   1
........................AGAGTGCTTTCTGAATGCAATGCACCTGGGCAAGGATTCT.......   1
............................TGCTTTCTGAATGCAATGCACCTGGGCAAGGATTCT.......   1
...............................TTTCTGAATGCAATGCACCTGGGCAAGGATTCT.......   1
.....................................AATGCAATGCACCTGGGCAAGGATTCTG......   1
................................................CCTGGGCAAGGATTCTGA.....   1
...........................................ATGCACCTGGGCAAGGATTCTGAGAG..   1
((((.(((((((((...(..((((....(((.........)))...))))..))))))))))..)))).... (-29.80)
```

```
X/92901860-92901925(+)_mir_cow_327
TCCCGTACCCATTGCATATCGGAGCTGTGAATTCTCAAAGCACCTCCTATGTGCATGGATTACAGG
.....TACCCATTGCATATCGGAGCTG.......................................  70091
.....TACCCATTGCATATCGGAGCT........................................  16217
.....TACCCATTGCATATCGGAGCTGT......................................  10333
.....TACCCATTGCATATCGGAGC.........................................  2638
.....TACCCATTGCATATCGGAG..........................................  443
.....TACCCATTGCATATCGGA...........................................  88
......ACCCATTGCATATCGGAGCTG.......................................  63
.....TACCCATTGCATATCGGAGCTGTG.....................................  36
......ACCCATTGCATATCGGAGCTGT......................................  35
.....TACCCATTGCATATCGGAGCTGTGA....................................  9
......ACCCATTGCATATCGGAGCT........................................  7
..........................................CCTCCTATGTGCATGGATTACA..  7
.........CATTGCATATCGGAGCTG.......................................  6
..........ATTGCATATCGGAGCTG.......................................  4
.........................................ACCTCCTATGTGCATGGATT.....  4
.........CATTGCATATCGGAGCT........................................  3
.......CCCATTGCATATCGGAGCTG.......................................  3
........CCATTGCATATCGGAGCTG.......................................  3
...........TTGCATATCGGAGCTG.......................................  3
.........................................ACCTCCTATGTGCATGGATTA....  3
.....TACCCATTGCATATCGGAGCTGTGAA...................................  2
.........................................ACCTCCTATGTGCATGGAT......  2
......ACCCATTGCATATCGGAG..........................................  1
.........CATTGCATATCGGAGC.........................................  1
.......CCCATTGCATATCGGAGCT........................................  1
........CCATTGCATATCGGAGCT........................................  1
....GTACCCATTGCATATCGGAGCTG.......................................  1
.....TACCCATTGCATATCGGAGCTGTGAAT..................................  1
.....TACCCATTGCATATCGGAGCTGTGAATTCT...............................  1
.....TACCCATTGCATATCGGAGCTGTGAATTCTC..............................  1
.....TACCCATTGCATATCGGAGCTGTGAATTCTCA.............................  1
.........................................ACCTCCTATGTGCATGGATTAC...  1
..........................................CCTCCTATGTGCATGGATTAC...  1
.((.(((.((((.((((((.((((.(((...........))).)))).))))))))))..))).)) (-21.70)

X/96382641-96382731(-)_mir_cow_328
```

```
CTGGGGTGAGGTAGTAAGTTGTATTGTTGTGGGGTAGGGATTTTAGGCCCCAATTTGAAGATAACTATACAACTTACTACTTTCCCTGGTG
......TGAGGTAGTAAGTTGTATTGTT............................................................... 85077
......TGAGGTAGTAAGTTGTATTGT................................................................ 25515
......TGAGGTAGTAAGTTGTATTG................................................................. 1043
......TGAGGTAGTAAGTTGTATT.................................................................. 219
......TGAGGTAGTAAGTTGTAT................................................................... 148
......TGAGGTAGTAAGTTGTATTGTTG.............................................................. 99
................................................................CTATACAACTTACTACTTTCC...... 42
.......GAGGTAGTAAGTTGTATTGTT............................................................... 22
.........GGTAGTAAGTTGTATTGTT............................................................... 18
......TGAGGTAGTAAGTTGTATTGTTGT............................................................. 18
......TGAGGTAGTAAGTTGTA.................................................................... 17
............AGTAAGTTGTATTGTT............................................................... 15
.......GAGGTAGTAAGTTGTATTGT................................................................ 7
......TGAGGTAGTAAGTTGT..................................................................... 6
...........TAGTAAGTTGTATTGTT............................................................... 6
...........TAGTAAGTTGTATTGT................................................................ 4
..........GTAGTAAGTTGTATTGTT............................................................... 4
.................................................................TATACAACTTACTACTTTCC...... 4
.........GGTAGTAAGTTGTATTGT................................................................ 3
........AGGTAGTAAGTTGTATTGTTGT............................................................. 3
................................................................CTATACAACTTACTACTTTC....... 3
.................................................................TATACAACTTACTACTTTCCCT.... 3
............................GTGGGGTAGGGATTTTAGGCC.......................................... 2
................................................................CTATACAACTTACTACTTT........ 2
................................................................CTATACAACTTACTACTTTCCC..... 2
..........GTAGTAAGTTGTATTGT................................................................ 1
.............GTAAGTTGTATTGTTG.............................................................. 1
......TGAGGTAGTAAGTTGTATTGTTGTGG........................................................... 1
......TGAGGTAGTAAGTTGTATTGTTGTGGGG......................................................... 1
......TGAGGTAGTAAGTTGTATTGTTGTGGGGTAGGG.................................................... 1
......TGAGGTAGTAAGTTGTATTGTTGTGGGGTAGGGA................................................... 1
.................................................................TATACAACTTACTACT.......... 1
...............................................................ACTATACAACTTACTACTTTCC...... 1
................................................................CTATACAACTTACTACTTTCCCT.... 1
(..(((.(((((((((((((((((.((((((((((...........)))))).........)))).))))))))))))))))))))..).. (-40.70)
```

X/96383530-96383616(-)_mir_Cow_Blast_48

TCTGTGGGATGAGGTAGTAGATTGTATAGTTTTAGGGTCATACCCCATCTTGGAGATAACTATACAGTCTACTGTCTTTCCCACGGT	
.........TGAGGTAGTAGATTGTATAGTT..	418867
.........TGAGGTAGTAGATTGTATAGT...	145549
.........TGAGGTAGTAGATTGTATAGTTT...	15265
.........TGAGGTAGTAGATTGTATAG..	11184
.........TGAGGTAGTAGATTGTAT..	2884
.........TGAGGTAGTAGATTGTATA...	2385
.........TGAGGTAGTAGATTGTA...	563
..........GAGGTAGTAGATTGTATAGTT..	153
.........TGAGGTAGTAGATTGT..	125
...............AGTAGATTGTATAGTT..	123
.........TGAGGTAGTAGATTGTATAGTTTT..	110
..............TAGTAGATTGTATAGTT..	102
............GGTAGTAGATTGTATAGTT..	70
..........GAGGTAGTAGATTGTATAGT...	59
.............GTAGTAGATTGTATAGTT..	53
....CTATACAGTCTACTGTCTTTC.......	46
...............................TTAGGGTCATACCCCATCTTGGA.................................	43
...........AGGTAGTAGATTGTATAGTT..	42
............GGTAGTAGATTGTATAGT...	34
..............TAGTAGATTGTATAGT...	25
.............GTAGTAGATTGTATAGT...	20
........ATGAGGTAGTAGATTGTATAGTT..	15
........ATGAGGTAGTAGATTGTATAGT...	11
....CTATACAGTCTACTGTCTTT........	11
...........AGGTAGTAGATTGTATAGT...	10
...............................TTAGGGTCATACCCCATCTTGGAG................................	10
....CTATACAGTCTACTGTCT..........	10
...............AGTAGATTGTATAGTTT...	6
.........TGAGGTAGTAGATTGTATAGTTTTAGGGTC..	5
..........GAGGTAGTAGATTGTATAG..	4
............GGTAGTAGATTGTATAGTTT...	4
..............TAGTAGATTGTATAGTTT...	4
................GTAGATTGTATAGTTT...	4
....CTATACAGTCTACTGTCTTTCC......	4
...........AGGTAGTAGATTGTATAG..	3
............GGTAGTAGATTGTATAG..	3
.............GTAGTAGATTGTATAG..	3

```
..........GAGGTAGTAGATTGTATAGTTT.......................................................     3
.........TGAGGTAGTAGATTGTATAGTTTTAGGGT.................................................     3
........ATGAGGTAGTAGATTGTATAGTTT.......................................................     2
.............GTAGTAGATTGTATAGTTT.......................................................     2
.....................................................AGATAACTATACAGTCTACTGTC...........     2
.........................................................AACTATACAGTCTACTGTCT..........     2
...........AGGTAGTAGATTGTATAGTTT.......................................................     1
..........GAGGTAGTAGATTGTATAGTTTT......................................................     1
.........TGAGGTAGTAGATTGTATAGTTTTAG....................................................     1
.........TGAGGTAGTAGATTGTATAGTTTTAGGGTCA...............................................     1
.........TGAGGTAGTAGATTGTATAGTTTTAGGGTCATACCCCA........................................     1
...............................TTAGGGTCATACCCCATCTTGG..................................     1
...............................TTAGGGTCATACCCCATCTTGGAGA...............................     1
..........................................CCCCATCTTGGAGATAACTATAC......................     1
.......................................................ATAACTATACAGTCTA................     1
...........................................................CTATACAGTCTACTGTC...........     1
........................................................TAACTATACAGTCTACTGTCT..........     1
........................................................TAACTATACAGTCTACTGTCTTTC.......     1
.((((((((.(((..((((((((((((((((...(((.....))).(((.....)))))))))))))))))))))..))))))))))). (-43.20)
```

File S2

```
10/1327870-1327932(+)_novel_dog_7
GCGTCTAGCAGGGAAGGTACTGACCAGCTGCTGGGTCTGTTCCCTCCCCTGTCTGCAGTGCTG
.....................................TGTTCCCTCCCCTGTCTGCAGT....  6
...TCTAGCAGGGAAGGTACTGACC.......................................  5
...................................TCTGTTCCCTCCCCTGTCTGCAG.....  5
...TCTAGCAGGGAAGGTACTGA.........................................  4
...................................TCTGTTCCCTCCCCTGTCTGCAGT....  3
..GTCTAGCAGGGAAGGTACTGA.........................................  2
...TCTAGCAGGGAAGGTACTGAC........................................  1
...................................TCTGTTCCCTCCCCTGTCTGC.......  1
...................................TCTGTTCCCTCCCCTGTCTGCA......  1
....................................CTGTTCCCTCCCCTGTCTGCAGT....  1
(((..((((((((.(((.((.((((........)))).))..))).))))).)))...))).. (-23.10)

10/15895194-15895258(+)_novel_Dog_Blast_4
GTCTTGGCGCGACTTCCTCGACTCACTCTCTTTCCCCCGTGAGTCGTGGAACCTCGCCCGAGGGT
.......................................TGAGTCGTGGAACCTCGCCCGA....      83
.........................................AGTCGTGGAACCTCGCCCGAG...      9
.........................................AGTCGTGGAACCTCGCCCGA....      6
.........................................AGTCGTGGAACCTCGCCCGAGG..      5
...TTGGCGCGACTTCCTCGACTC.........................................      4
....TGGCGCGACTTCCTCGACT..........................................      3
....TGGCGCGACTTCCTCGACTCACT......................................      3
.......................................TGAGTCGTGGAACCTCGCCC......      3
.......................................TGAGTCGTGGAACCTCGCCCGAG...      3
....TGGCGCGACTTCCTCGACTCA........................................      2
.......................................TGAGTCGTGGAACCTCGCCCG.....      2
...TTGGCGCGACTTCCTCGACT..........................................      1
....TGGCGCGACTTCCTCGACTC.........................................      1
....TGGCGCGACTTCCTCGACTCAC.......................................      1
...TTGGCGCGACTTCCTCGACTCACT......................................      1
......CGCGACTTCCTCGACTCACT.......................................      1
..............................TTTCCCCCGTGAGTCGTGGAACC............      1
........................................GAGTCGTGGAACCTCGCCCGA....      1
..................................CCCCGTGAGTCGTGGAACCTCGCCCGAGG..      1
...................................CCCGTGAGTCGTGGAACCTCGCCCGAGG..      1
.....................................CGTGAGTCGTGGAACCTCGCCCGAGG..      1
......................................GTGAGTCGTGGAACCTCGCCCGAGGGT      1
.((((((.((((.((((.((((((((............)))))))).)))).)))))))))).. (-31.20)

10/1880513-1880572(-)_mir_dog_1
TTCAAGTAATCCAGGATAGGCTGTTTCCGTCTGTGAGGCCTATTCTTGATTACTTGTTTC
TTCAAGTAATCCAGGATAGGCT......................................     4302424
TTCAAGTAATCCAGGATAGGC.......................................     260057
TTCAAGTAATCCAGGATAG.........................................     33252
TTCAAGTAATCCAGGATAGG........................................     20904
TTCAAGTAATCCAGGATAGGCTG.....................................     18040
TTCAAGTAATCCAGGATA..........................................     3993
.TCAAGTAATCCAGGATAGGCT......................................     3065
TTCAAGTAATCCAGGAT...........................................     1754
TTCAAGTAATCCAGGATAGGCTGT....................................     1232
......................................CCTATTCTTGATTACTTGTTTC     675
..CAAGTAATCCAGGATAGGCT......................................     577
....AGTAATCCAGGATAGGCT......................................     529
...AAGTAATCCAGGATAGGCT......................................     420
.TCAAGTAATCCAGGATAGGC.......................................     227
......................................CCTATTCTTGATTACTTGTTT.     146
TTCAAGTAATCCAGGA............................................     107
......TAATCCAGGATAGGCT......................................     105
.TCAAGTAATCCAGGATAGGCTG.....................................     104
....AGTAATCCAGGATAGGC.......................................     102
..CAAGTAATCCAGGATAGGC.......................................     39
TTCAAGTAATCCAGGATAGGCTGTT...................................     28
.TCAAGTAATCCAGGATAGG........................................     27
```

.....GTAATCCAGGATAGGCT...................................... 25
...AAGTAATCCAGGATAGGC....................................... 22
TTCAAGTAATCCAGGATAGGCTGTTT.................................. 20
......................................CCTATTCTTGATTACTTGTT.. 15
.TCAAGTAATCCAGGATAG... 10
.TCAAGTAATCCAGGATAGGCTGT.................................... 8
......................GTTTCCGTCTGTGAGG...................... 5
.....GTAATCCAGGATAGGC....................................... 4
TTCAAGTAATCCAGGATAGGCTGTTTC................................. 4
.TCAAGTAATCCAGGATA.. 3
..CAAGTAATCCAGGATAGGCTG..................................... 3
TTCAAGTAATCCAGGATAGGCTGTTTCC................................ 3
TTCAAGTAATCCAGGATAGGCTGTTTCCGT.............................. 3
..CAAGTAATCCAGGATAG... 2
...AAGTAATCCAGGATAGGCTG..................................... 2
........ATCCAGGATAGGCTGT.................................... 2
TTCAAGTAATCCAGGATAGGCTGTTTCCGTC............................. 2
...................................AGGCCTATTCTTGATTACTTGTTT. 2
....CTATTCTTGATTACTTGTTTC 2
.TCAAGTAATCCAGGAT... 1
...AAGTAATCCAGGATAG... 1
..CAAGTAATCCAGGATAGG.. 1
...AAGTAATCCAGGATAGG.. 1
..CAAGTAATCCAGGATAGGCTGT.................................... 1
.......AATCCAGGATAGGCTGT.................................... 1
.TCAAGTAATCCAGGATAGGCTGTT................................... 1
........ATCCAGGATAGGCTGTT................................... 1
......TAATCCAGGATAGGCTGTTT.................................. 1
TTCAAGTAATCCAGGATAGGCTGTTTCCG............................... 1
TTCAAGTAATCCAGGATAGGCTGTTTCCGTCTGTG......................... 1
...................................AGGCCTATTCTTGATTACTT..... 1
...................................AGGCCTATTCTTGATTACTTG.... 1
................................GTGAGGCCTATTCTTGATTACTTGTTT. 1
....................................GGCCTATTCTTGATTACTTGTTT. 1
..(((((((((.((((((((((..............)))))))))).)))))))))).... (-24.74)

10/20033388-20033472(-)_mir_dog_2
CGGGGTGAGGTAGTAGGTTGTGTGGTTTCAGGGCAGTGATGTTGCCCCCTCGGAAGATAACTATACAACCTACTGCCTTC
CCCGA
.....TGAGGTAGTAGGTTGTGTGGTT...
..... 134794
.....TGAGGTAGTAGGTTGTGTGGTTT..
..... 34756
.....TGAGGTAGTAGGTTGTGTGGT..
..... 33704
.....TGAGGTAGTAGGTTGTGTGG...
..... 11422
.....TGAGGTAGTAGGTTGTGT...
..... 1463
....CTATACAACCTACTGCCTTC
C.... 941
.....TGAGGTAGTAGGTTGTG..
..... 864
.....TGAGGTAGTAGGTTGTGTG..
..... 693
.....TGAGGTAGTAGGTTGT...
..... 371
..........TAGTAGGTTGTGTGGTT...
..... 69
........GGTAGTAGGTTGTGTGGTT...
..... 51
.........GTAGTAGGTTGTGTGGTT...
..... 39
...........AGTAGGTTGTGTGGTT...

..... 38
.....TGAGGTAGTAGGTTGTGTGGTTTC...
..... 38
......GAGGTAGTAGGTTGTGTGGTT...
..... 36
..........TAGTAGGTTGTGTGGTTT..
..... 19
............GTAGGTTGTGTGGTTT..
..... 19
....CTATACAACCTACTGCCTTC
..... 19
.......AGGTAGTAGGTTGTGTGGTT...
..... 18
........GGTAGTAGGTTGTGTGGTTT..
..... 18
...........AGTAGGTTGTGTGGTTT..
..... 16
...........................TCAGGGCAGTGATGTTGCCCCCTCGGA..........................
..... 14
....AGATAACTATACAACCTACTGCCT..
..... 14
....CTATACAACCTACTGCCTTC
CC... 13
.........GTAGTAGGTTGTGTGGTTT..
..... 12
....TATACAACCTACTGCCTTC
C.... 12
........GGTAGTAGGTTGTGTGGT..
..... 11
....CCCCCTCGGAAGATAACTATAC..............
..... 11
..........TAGTAGGTTGTGTGGT..
..... 10
...........................TCAGGGCAGTGATGTTGCCCCCTCGGAAGAT......................
..... 10
....TCGGAAGATAACTATACAACCT.........
..... 10
....CCCTCGGAAGATAACTATACAACCT.........
..... 9
.........GTAGTAGGTTGTGTGGT..
..... 8
......GAGGTAGTAGGTTGTGTGGTTT..
..... 8
....CTCGGAAGATAACTATACAACCT.........
..... 8
.......AGGTAGTAGGTTGTGTGGTTT..
..... 7
......GAGGTAGTAGGTTGTGTGG...
..... 6
...........................TCAGGGCAGTGATGTTGCCCCCTCGGAAGA.......................
..... 6
....AACTATACAACCTACTGCCTTC
C.... 6
......GAGGTAGTAGGTTGTGTGGT..
..... 5
...GGTGAGGTAGTAGGTTGTGTGGTT...
..... 5
....CCTCGGAAGATAACTATACAACCT.........
..... 5
....TAACTATACAACCTACTGCCTTC
C.... 5
...........................TCAGGGCAGTGATGTTGCCCCCT..............................
..... 4
....AGATAACTATACAACCTACTGCC...

..... 4
....CTATACAACCTACTGCCTT.
..... 4
....GTGAGGTAGTAGGTTGT...
..... 3
....GTTGCCCCCTCGGAAGATAACT..................
..... 3
....CGGAAGATAACTATACAACCT.........
..... 3
....TAACTATACAACCTACTGCCTT.
..... 3
....GTGAGGTAGTAGGTTGTGTGGTT...
..... 2
...........................TCAGGGCAGTGATGTTGCCCCCTCGGAAGATAA.....................
..... 2
....TTGCCCCCTCGGAAGATAACT..................
..... 2
....CCTCGGAAGATAACTATACAAC...........
..... 2
....CCCCTCGGAAGATAACTATACAACCT.........
..... 2
....CTATACAACCTACTGCC...
..... 2
....ATAACTATACAACCTACTGCCT..
..... 2
....GCCCCCTCGGAAGATAACTATACAACCTACTGCCTT.
..... 2
....TAACTATACAACCTACTGCCTTC
..... 2
....ATACAACCTACTGCCTTC
..... 2
......GAGGTAGTAGGTTGTG..
..... 1
........GGTAGTAGGTTGTGTG..
..... 1
.......AGGTAGTAGGTTGTGTGGT..
..... 1
.
GGGGTGAGGTAGTAGGTTGTGTGGTT..
.... 1
.....TGAGGTAGTAGGTTGTGTGGTTTCA..
..... 1
.....TGAGGTAGTAGGTTGTGTGGTTTCAGGGC..
..... 1
.................TTGTGTGGTTTCAGGGCAGT...
..... 1
...........................TCAGGGCAGTGATGTTGCCCCCTC.............................
..... 1
...........................TCAGGGCAGTGATGTTGCCCCCTCGG...........................
..... 1
...........................TCAGGGCAGTGATGTTGCCCCCTCGGAA.........................
..... 1
......................................ATGTTGCCCCCTCGGAAGATA.....................
..... 1
....CCCCTCGGAAGATAACTATAC..............
..... 1
....CCCCCTCGGAAGATAACTATACA.............
..... 1
....CCCCTCGGAAGATAACTATACA.............
..... 1
....CCCTCGGAAGATAACTATACAA............
..... 1
....CCCCTCGGAAGATAACTATACAAC...........
..... 1

```
..............................................CCCTCGGAAGATAACTATACAAC...........
..... 1
............................................CCCCCTCGGAAGATAACTATACAACC..........
..... 1
.............................................CCCCTCGGAAGATAACTATACAACC..........
..... 1
..............................................CCCTCGGAAGATAACTATACAACC..........
..... 1
................................................CTCGGAAGATAACTATACAACCTA........
..... 1
.................................................TCGGAAGATAACTATACAACCTACT......
..... 1
......................................................AGATAACTATACAACCTACTG.....
..... 1
............................................................CTATACAACCTACTGC....
..... 1
.........................................................TAACTATACAACCTACTGCCT..
..... 1
............................................................CTATACAACCTACTGCCT..
..... 1
.......................................................GATAACTATACAACCTACTGCCTT.
..... 1
..............................................................ATACAACCTACTGCCTT.
..... 1
................................................CTCGGAAGATAACTATACAACCTACTGCCTTC
..... 1
..........................................................AACTATACAACCTACTGCCTTC
..... 1
.............................................................TATACAACCTACTGCCTTC
..... 1
........................................................ATAACTATACAACCTACTGCCTTC
C.... 1
..............................................................ATACAACCTACTGCCTTC
C.... 1
(((((.(((((((((((((((((((((...((((((.....)))))))..
((....)).))))))))))))))))))))))))))). (-54.00)

10/20034319-20034387(-)_mir_dog_3
TGAGGTAGTAGGTTGTATAGTTTGGGGCTCTGCCCTGCTCTGGGTAACTATACAATCTACTGTCTTTCC
TGAGGTAGTAGGTTGTATAGTT...............................................  1053826
TGAGGTAGTAGGTTGTATAGT................................................  304275
TGAGGTAGTAGGTTGTATAGTTT..............................................  31038
TGAGGTAGTAGGTTGTATAG.................................................  29969
TGAGGTAGTAGGTTGTAT...................................................  10711
TGAGGTAGTAGGTTGTATA..................................................  6228
...............................................CTATACAATCTACTGTCTTTCC  1561
TGAGGTAGTAGGTTGTA....................................................  1406
.....TAGTAGGTTGTATAGTT...............................................  778
...GGTAGTAGGTTGTATAGTT...............................................  646
......AGTAGGTTGTATAGTT...............................................  542
.GAGGTAGTAGGTTGTATAGTT...............................................  482
...............................................CTATACAATCTACTGTCTTTC.  467
TGAGGTAGTAGGTTGT.....................................................  371
.....TAGTAGGTTGTATAGT................................................  276
....GTAGTAGGTTGTATAGTT...............................................  254
...GGTAGTAGGTTGTATAGT................................................  253
................................................TATACAATCTACTGTCTTTCC  161
..AGGTAGTAGGTTGTATAGTT...............................................  157
.GAGGTAGTAGGTTGTATAGT................................................  108
...............................................CTATACAATCTACTGTCTTT..  106
....GTAGTAGGTTGTATAGT................................................  90
..AGGTAGTAGGTTGTATAGT................................................  61
.....TAGTAGGTTGTATAGTTT..............................................  32
.......GTAGGTTGTATAGTTT..............................................  32
```

```
...............................................CTATACAATCTACTGTCTT...  29
...............................................CTATACAATCTACTGTCT....  27
......AGTAGGTTGTATAGTTT..............................................  20
...GGTAGTAGGTTGTATAG.................................................  18
TGAGGTAGTAGGTTGTATAGTTTG.............................................  17
.GAGGTAGTAGGTTGTATAGTTT..............................................  15
...GGTAGTAGGTTGTATAGTTT..............................................  15
.GAGGTAGTAGGTTGTATAG.................................................  12
.................................................ATACAATCTACTGTCTTTCC  11
...............................................CTATACAATCTACTGT......  10
....GTAGTAGGTTGTATAG.................................................  9
..AGGTAGTAGGTTGTATAGTTT..............................................  9
...........................................GTAACTATACAATCTACTGTCT....  9
................................................TATACAATCTACTGTCTTTC.  9
....GTAGTAGGTTGTATAGTTT..............................................  8
..............................................ACTATACAATCTACTGTCTTTC.  8
..AGGTAGTAGGTTGTATAG.................................................  7
..................................CTGCTCTGGGTAACTATACAATCT...........  6
...................................TGCTCTGGGTAACTATACAATCT...........  6
............................................TAACTATACAATCTACTGTCTT...  5
...........................................GTAACTATACAATCTACTGTCTTTC.  5
TGAGGTAGTAGGTTGTATAGTTTGG............................................  4
................................................TATACAATCTACTGTCTTT..  4
..AGGTAGTAGGTTGTAT...................................................  3
TGAGGTAGTAGGTTGTATAGTTTGGGGCTCTGCCCTGCTCT............................  3
............................................TAACTATACAATCTACTGTCTTT..  3
............................................TAACTATACAATCTACTGTCTTTC.  3
.................................................ATACAATCTACTGTCTTTC.  3
...GGTAGTAGGTTGTATA..................................................  2
TGAGGTAGTAGGTTGTATAGTTTGGGG..........................................  2
......................TGGGGCTCTGCCCTGCTCTGGGT........................  2
..................................CTGCTCTGGGTAACTATACAATC............  2
...............................................CTATACAATCTACTGTC.....  2
...........................................GTAACTATACAATCTACTGTCTTT..  2
.............................................AACTATACAATCTACTGTCTTT..  2
.............................................AACTATACAATCTACTGTCTTTC.  2
....................................................CAATCTACTGTCTTTC.  2
.GAGGTAGTAGGTTGTAT...................................................  1
.GAGGTAGTAGGTTGTATA..................................................  1
TGAGGTAGTAGGTTGTATAGTTTGGG...........................................  1
TGAGGTAGTAGGTTGTATAGTTTGGGGC.........................................  1
TGAGGTAGTAGGTTGTATAGTTTGGGGCTCT......................................  1
........TAGGTTGTATAGTTTGGGGCTCT......................................  1
TGAGGTAGTAGGTTGTATAGTTTGGGGCTCTGCC...................................  1
TGAGGTAGTAGGTTGTATAGTTTGGGGCTCTGCCCT.................................  1
TGAGGTAGTAGGTTGTATAGTTTGGGGCTCTGCCCTGC...............................  1
TGAGGTAGTAGGTTGTATAGTTTGGGGCTCTGCCCTGCTC.............................  1
..................................CTGCTCTGGGTAACTATACAAT.............  1
.....................TTGGGGCTCTGCCCTGCTCTGGGTAACTATACAATC............  1
...................................TGCTCTGGGTAACTATACAATC............  1
.................................CCTGCTCTGGGTAACTATACAATCT...........  1
...................................TGCTCTGGGTAACTATACAATCTACT........  1
...........................................GTAACTATACAATCTACTGT......  1
...................................TGCTCTGGGTAACTATACAATCTACTGTCT....  1
..............................................ACTATACAATCTACTGTCTTT..  1
.(((..((((((((((((((((..((((........))))..)...)))))))))))))))..)))... (-31.90)

10/20818831-20818909(+)_mir_dog_4
TTGCATGGGGAGGAGGGAGGAGAGGGGCCAAGTTCCCTCCGCCTGGAACGCCCTTCCCCCCCTTCTTCACCTGGCGAAC
...............................................ACGCCCTTCCCCCCCTTCTTCA..........
      11664
...............................................ACGCCCTTCCCCCCCTTCTTC...........
      3533
...............................................ACGCCCTTCCCCCCCTTCTT............
```

```
      1373
...............................................ACGCCCTTCCCCCCCTTCT.............
      372
..............................................AACGCCCTTCCCCCCCTTCTTCA..........
      159
..............................................AACGCCCTTCCCCCCCTTCTTC...........
      136
..............................................AACGCCCTTCCCCCCCTTCTT............
      96
..............................................AACGCCCTTCCCCCCCTTCT.............
      55
...............................................ACGCCCTTCCCCCCCTTC..............
      26
...............................................ACGCCCTTCCCCCCCTTCTTCAC.........
      6
..............................................AACGCCCTTCCCCCCCTTC..............
      3
................................................CGCCCTTCCCCCCCTTCTTCA..........
      3
....................................................CTTCCCCCCCTTCTTCA..........
      3
...............................................ACGCCCTTCCCCCCCTT...............
      2
..........AGGAGGGAGGAGAGGGGCC..................................................
      1
..........AGGAGGGAGGAGAGGGGCCAAGTT.............................................
      1
..........AGGAGGGAGGAGAGGGGCCAAGTTC............................................
      1
..............................................AACGCCCTTCCCCCCCT................
      1
...............................................ACGCCCTTCCCCCCCT................
      1
................................................CGCCCTTCCCCCCCTTCTT............
      1
.................................................GCCCTTCCCCCCCTTCTTC...........
      1
..................................................CCCTTCCCCCCCTTCTTCA..........
      1
...............................................ACGCCCTTCCCCCCCTTCTTCACC........
      1
((((..(((((((((((.((.(((((((...(((((........))))))))))))).))))))))))).))..))))..
(-42.70)

10/21022720-21022790(+)_novel_dog_8
GAGCCCTGACAGCACTTAGCCTGGGGCTTGTGAACCGAGACAAGCCCCGGTTCTAAGCGCTGCTCCTGGAT
........ACAGCACTTAGCCTGGGGCT...........................................      12
........ACAGCACTTAGCCTGGGGCTTG.........................................      10
........ACAGCACTTAGCCTGGGGCTT..........................................      4
........ACAGCACTTAGCCTGGGGCTTGT........................................      1
..........AGCACTTAGCCTGGGGCTTGT........................................      1
............................................CCCCGGTTCTAAGCGCTGCTC......      1
....((.((((((.(((((.(((((((((((........)))))))))))..))))).)))).))..)).. (-35.60)

10/22941155-22941217(-)_novel_Dog_Blast_5
GTCTTGGCGCGACTTCCTCGACTCACTCTCTCCCCCGTGAGTCGTGGAACCTCGCCCGAGGGT
.....................................TGAGTCGTGGAACCTCGCCCGA....  83
.......................................AGTCGTGGAACCTCGCCCGAG...  9
.......................................AGTCGTGGAACCTCGCCCGA....  6
.......................................AGTCGTGGAACCTCGCCCGAGG..  5
...TTGGCGCGACTTCCTCGACTC.......................................  4
....TGGCGCGACTTCCTCGACT........................................  3
....TGGCGCGACTTCCTCGACTCACT....................................  3
.....................................TGAGTCGTGGAACCTCGCCC......  3
```

.....................................TGAGTCGTGGAACCTCGCCCGAG... 3
....TGGCGCGACTTCCTCGACTCA...................................... 2
.....................................TGAGTCGTGGAACCTCGCCCG..... 2
...TTGGCGCGACTTCCTCGACT.. 1
....TGGCGCGACTTCCTCGACTC....................................... 1
....TGGCGCGACTTCCTCGACTCAC..................................... 1
...TTGGCGCGACTTCCTCGACTCACT.................................... 1
.......CGCGACTTCCTCGACTCACT.................................... 1
......................................GAGTCGTGGAACCTCGCCCGA.... 1
................................CCCCGTGAGTCGTGGAACCTCGCCCGAGG.. 1
.................................CCCGTGAGTCGTGGAACCTCGCCCGAGG.. 1
...................................CGTGAGTCGTGGAACCTCGCCCGAGG.. 1
....................................GTGAGTCGTGGAACCTCGCCCGAGGGT 1
.((((((.((((.((((.((((((((..........)))))))).))))..)))))))))).. (-31.40)

10/23246288-23246372(+)_mir_dog_5
AGTGTTTCTCTTGGCCTCTGTTCCATGCCTGGGCTGTGCCTGGCCAGTGGGTAGAGCCGCATGGTGTAGGGGCCACAAGG
AACAT
..........TTGGCCTCTGTTCCATGCCTGGGC..
..... 74
..........TTGGCCTCTGTTCCATGCCTGG..
..... 21
..........TTGGCCTCTGTTCCATGCCTGGGCT...
..... 16
..........TTGGCCTCTGTTCCATGCCTGGG...
..... 15
..........TTGGCCTCTGTTCCATGCCT..
..... 9
.........CTTGGCCTCTGTTCCATGCCT..
..... 4
.........CTTGGCCTCTGTTCCATGCCTGGGC..
..... 1
....GCCGCATGGTGTAGGGGCC......
..... 1
.((((((((..(((((((((..((((((...((((.
(((((.......))))).))))))))))..)))))))))..)))))))) (-49.30)

10/23456613-23456673(-)_mir_dog_6
GTGCATTGTAGTTGCATTGCATGTTCTGGCGGTACCCGTGCAATGTTTCCACAGTGCATCA
GTGCATTGTAGTTGCATTGC...   1985
GTGCATTGTAGTTGCATTGCA..   1114
GTGCATTGTAGTTGCATTG..   867
....ATTGTAGTTGCATTGCA..   844
.....TTGTAGTTGCATTGCA..   352
....CAATGTTTCCACAGTGCAT..   214
...CATTGTAGTTGCATTGCA..   168
....CAATGTTTCCACAGTGCATC.   117
....CAATGTTTCCACAGTGCATCA   105
GTGCATTGTAGTTGCATT...   73
....ATTGTAGTTGCATTGC...   19
GTGCATTGTAGTTGCA...   10
...CATTGTAGTTGCATTGC...   8
GTGCATTGTAGTTGCAT..   5
.....................TGTTCTGGCGGTACCCGTG.....................   4
.....................................GTGCAATGTTTCCACAGT......   2
......................................TGCAATGTTTCCACAGTGC....   2
......................................TGCAATGTTTCCACAGTGCATC.   2
.TGCATTGTAGTTGCATTGC...   1
.....................TGTTCTGGCGGTACCC........................   1
.....................................GTGCAATGTTTCCACAGTGC....   1
....AATGTTTCCACAGTGCATC.   1
.......................TTCTGGCGGTACCCGTGCAATGTTTCCACAGTGCATCA   1
((((((((.(..(((((((((((....((.....))))))))))))..).))))))))).. (-25.70)

10/27296054-27296114(+)_mir_dog_7
TGCACCCTGAGAGCTGGAGCAGGTCTCTTACGGGAACCATCTTCAACTCTGTGGATGCAGG
......................................ATCTTCAACTCTGTGGATGCAG.   2
TGCACCCTGAGAGCTGGA...   1
......................................ATCTTCAACTCTGTGGATGCAGG   1
((((.((..((((.(((((..((((((....))).)))..))))).)))).)).)))).. (-22.80)

10/40365583-40365655(-)_mir_dog_8
TGGCTGGGTCTGGACTTCAGGAGACAGCTTGGTCATCCGCACAGAGCTGGCGTTTGAAATCCTGAGCCTGACG
........TCTGGACTTCAGGAGACAGCT..   218
........TCTGGACTTCAGGAGACAGCTT...   152
........TCTGGACTTCAGGAGACAGC...   4
....GCTGGCGTTTGAAATCCTGAGC......   4
....CTGGCGTTTGAAATCCTGAGC......   3
.(...((.((.(((.((((((...((((((.((.....))...))))))...)))))).))).)).))...). (-
24.60)

10/56754566-56754647(-)_mir_dog_9
TTGATGTCGCAGATACTGCATCAGGAACTGATTGGATAAGAATGGGTCACCATCAGTTCCTAATGCATTGCCTTCAGCAT
CT
............ATACTGCATCAGGAACTGATTG..
..   22
............ATACTGCATCAGGAACTGATT...
..   19
.............TACTGCATCAGGAACTGATTGGA..
..   15
.............TACTGCATCAGGAACTGATTGG...
..   14
.............TACTGCATCAGGAACTGATT...
..   5
....CATCAGTTCCTAATGCATTGCC........
..   4
....ATCAGTTCCTAATGCATTGCCT.......
..   3
............ATACTGCATCAGGAACTGA...
..   2
.............TACTGCATCAGGAACTGAT..
..   2
............ATACTGCATCAGGAACTGATTGG...
..   1
....ATCAGTTCCTAATGCATTGCC........
..   1
....CATCAGTTCCTAATGCATTGCCT.......
..   1
..(((((.(.((.((.(((((.(((((((((.
((((........))).).))))))))))).))))).)).)).).))))). (-30.20)

10/56761448-56761524(-)_mir_dog_10
AGTTGGCTTAATCTCAGCTGGCAACTGTGAGATGTTCATACAATCCCCCACAGTGGTCTCTGGGATTATGCTAAACA
........TAATCTCAGCTGGCAACTGTGA...
      37
........TAATCTCAGCTGGCAACTGTG..
      26
....CACAGTGGTCTCTGGGATTATG.......
      21
........TAATCTCAGCTGGCAACTGT...
      10
........TAATCTCAGCTGGCAACTG..
      9
........TAATCTCAGCTGGCAACTGTGAGA...
      5
....CACAGTGGTCTCTGGGATTAT........
      3
.......TTAATCTCAGCTGGCAACTGTG..

```
      2
........TAATCTCAGCTGGCAACT...................................................
      1
...............................................CCACAGTGGTCTCTGGGATT..........
      1
................................................CACAGTGGTCTCTGGGATTA.........
      1
..((((.(((((((((..(((.((((((.(((((....)).)))...)))))).))).))))))))).)))))... (-
28.90)

10/56772853-56772914(-)_mir_dog_11
AAATCTCTGCAGGCAAATGTGATGTCACTAAAGAAATCACACACTTACCCGTAGAGATTCTG
AAATCTCTGCAGGCAAATGTGA........................................   204
AAATCTCTGCAGGCAAATGTG.........................................   34
........................................ACACTTACCCGTAGAGATTCTG   22
AAATCTCTGCAGGCAAATGTGAT.......................................   14
AAATCTCTGCAGGCAAATGT..........................................   6
.........................................CACTTACCCGTAGAGATTCTG   6
.........................................CACTTACCCGTAGAGATTCT.   5
AAATCTCTGCAGGCAAATG...........................................   3
......................................ACACACTTACCCGTAGAGATTC..   2
......................................ACACACTTACCCGTAGAGATTCT.   2
.......................................CACACTTACCCGTAGAGATTCT.   2
.......................................CACACTTACCCGTAGAGATTCTG   2
AAATCTCTGCAGGCAAA.............................................   1
AAATCTCTGCAGGCAAATGTGATG......................................   1
........................................ACACTTACCCGTAGAGATTCT.   1
.((((((((.((....((((((.((......)).))))))......)).)))))))))... (-19.80)

10/69106962-69107030(-)_novel_dog_9
GTGGGCTCCCCGGCCCGGCCGACGGGTGCAGAAATGACCCCGCAGGCCGTCGCGGAGGAGAGCTCGGAG
.........................................GCAGGCCGTCGCGGAGGAGAGCT.....  8
...GGCTCCCCGGCCCGGCCGACGG............................................  3
.........................................GCAGGCCGTCGCGGAGGAGAGC......  3
.........................................GCAGGCCGTCGCGGAGGAGAG.......  1
..........................................CAGGCCGTCGCGGAGGAGAGCT.....  1
.((((((((((.((.(((((..((((..((....))..))))..)))))..)).).)).)))))))... (-40.10)

1/103406119-103406183(+)_novel_dog_1
AAGGACACACAAAGTGGGAGCACTTTCCAGACAGATCGGGAAGTGCCCTCATTTTGTGTATCCCA
....ACACACAAAGTGGGAGCACTTT.......................................      17
.....CACACAAAGTGGGAGCACTTT.......................................      9
.......................................GAAGTGCCCTCATTTTGTGTAT....      4
........................................AAGTGCCCTCATTTTGTGTATC...      3
.....CACACAAAGTGGGAGCACTT........................................      2
....ACACACAAAGTGGGAGCACTT........................................      1
....ACACACAAAGTGGGAGCACTTTC......................................      1
.....CACACAAAGTGGGAGCACTTTC......................................      1
..(((.(((((((((((.(((((((((.((....))))))))))).))))))))))).))).. (-35.10)

1/103406620-103406679(+)_mir_dog_12
ACTCAAAAAATGGCGGCACTTTCCTACCTAGAACAGAAAGTGCCCCCACAGTTTGAGTGG
ACTCAAAAAATGGCGGCACTT.......................................     338
ACTCAAAAAATGGCGGCACTTT......................................     87
.....................................AAGTGCCCCCACAGTTTGAGTG.     26
ACTCAAAAAATGGCGGCACT........................................     22
ACTCAAAAAATGGCGGCACTTTC.....................................     18
..TCAAAAAATGGCGGCACTTTC.....................................     5
..TCAAAAAATGGCGGCACTTTCC....................................     5
.....................................AAGTGCCCCCACAGTTTGAGT..     5
..TCAAAAAATGGCGGCACTTT......................................     4
.....................................AAGTGCCCCCACAGTTTGAGTGG     4
ACTCAAAAAATGGCGGCAC.........................................     2
.....................................AAGTGCCCCCACAGTTTGAG...     2
```

```
....................................AAAGTGCCCCCACAGTTTGAGT..     2
ACTCAAAAAATGGCGGC...........................................     1
ACTCAAAAAATGGCGGCA..........................................     1
......................................AGTGCCCCCACAGTTTGAGTGG     1
(((((((...(((.(((((((((............))))))))).)))...))))))).. (-25.00)

1/103606022-103606086(+)_novel_Dog_Blast_54
AAGGACACACAAAGTGGGAGCACTTTCCAGACAGATCGGGAAGTGCCCTCATTTTGTGTATCCCA
....ACACACAAAGTGGGAGCACTTT.......................................      17
.....CACACAAAGTGGGAGCACTTT.......................................      9
.......................................GAAGTGCCCTCATTTTGTGTAT....      4
........................................AAGTGCCCTCATTTTGTGTATC...      3
.....CACACAAAGTGGGAGCACTT........................................      2
....ACACACAAAGTGGGAGCACTT........................................      1
....ACACACAAAGTGGGAGCACTTTC......................................      1
.....CACACAAAGTGGGAGCACTTTC......................................      1
..(((.((((((((((((.(((((((((.((....))))))))))).)))))))))))).))).. (-35.10)

1/103606523-103606582(+)_mir_dog_13
ACTCAAAAAATGGCGGCACTTTCCTACCTAGAACAGAAAGTGCCCCCACAGTTTGAGTGG
ACTCAAAAAATGGCGGCACTT.......................................     338
ACTCAAAAAATGGCGGCACTTT......................................     87
.....................................AAGTGCCCCCACAGTTTGAGTG.     26
ACTCAAAAAATGGCGGCACT........................................     22
ACTCAAAAAATGGCGGCACTTTC.....................................     18
..TCAAAAAATGGCGGCACTTTC.....................................     5
..TCAAAAAATGGCGGCACTTTCC....................................     5
.....................................AAGTGCCCCCACAGTTTGAGT..     5
..TCAAAAAATGGCGGCACTTT......................................     4
.....................................AAGTGCCCCCACAGTTTGAGTGG     4
ACTCAAAAAATGGCGGCAC.........................................     2
.....................................AAGTGCCCCCACAGTTTGAG...     2
....................................AAAGTGCCCCCACAGTTTGAGT..     2
ACTCAAAAAATGGCGGC...........................................     1
ACTCAAAAAATGGCGGCA..........................................     1
......................................AGTGCCCCCACAGTTTGAGTGG     1
(((((((...(((.(((((((((............))))))))).)))...))))))).. (-25.00)

1/105400273-105400332(-)_mir_dog_14
TCCCTGAGACCCTTTAACCTGTGAGGACATCCAGGGTCACAGGTGAGGTTCTTGGGAGCC
TCCCTGAGACCCTTTAACCTGT......................................     654795
TCCCTGAGACCCTTTAACCTGTGA....................................     285140
TCCCTGAGACCCTTTAACCTGTG.....................................     127633
TCCCTGAGACCCTTTAACCTG.......................................     106210
TCCCTGAGACCCTTTAACCT........................................     19529
.CCCTGAGACCCTTTAACCTGTGA....................................     2116
TCCCTGAGACCCTTTAACC.........................................     1833
....TGAGACCCTTTAACCTGT......................................     598
TCCCTGAGACCCTTTAAC..........................................     504
..CCTGAGACCCTTTAACCTGT......................................     465
......................................ACAGGTGAGGTTCTTGGGAGC.     459
......................................ACAGGTGAGGTTCTTGGGAGCC     374
.CCCTGAGACCCTTTAACCTGT......................................     350
..CCTGAGACCCTTTAACCTGTGA....................................     324
.CCCTGAGACCCTTTAACCTGTG.....................................     204
...CTGAGACCCTTTAACCTGT......................................     135
..CCTGAGACCCTTTAACCTGTG.....................................     119
....TGAGACCCTTTAACCTG.......................................     107
....TGAGACCCTTTAACCTGTG.....................................     100
......................................ACAGGTGAGGTTCTTGGGAG..     84
.....GAGACCCTTTAACCTGT......................................     71
......AGACCCTTTAACCTGT......................................     65
.CCCTGAGACCCTTTAACCTG.......................................     51
TCCCTGAGACCCTTTAACCTGTGAG...................................     51
```

....CAGGTGAGGTTCTTGGGAGCC 45
...CTGAGACCCTTTAACCTGTG..................................... 42
..CCTGAGACCCTTTAACCTG....................................... 40
...CTGAGACCCTTTAACCTGTGA.................................... 40
....TGAGACCCTTTAACCTGTGA.................................... 26
....CAGGTGAGGTTCTTGGGAGC. 24
..CCTGAGACCCTTTAACCT.. 19
...CTGAGACCCTTTAACCTG....................................... 19
.......GACCCTTTAACCTGTG..................................... 15
TCCCTGAGACCCTTTAA... 14
......AGACCCTTTAACCTGTG..................................... 11
....CAGGTGAGGTTCTTGGGAG.. 11
TCCCTGAGACCCTTTA.. 9
......................................ACAGGTGAGGTTCTTGGGA... 9
.....GAGACCCTTTAACCTGTG..................................... 8
.CCCTGAGACCCTTTAACCT.. 6
.....GAGACCCTTTAACCTG....................................... 5
.....................................CACAGGTGAGGTTCTTGGGAGC. 3
.CCCTGAGACCCTTTAACC... 2
....TGAGACCCTTTAACCT.. 2
.....GAGACCCTTTAACCTGTGA.................................... 2
..CCTGAGACCCTTTAACC... 1
...CTGAGACCCTTTAACCT.. 1
......AGACCCTTTAACCTGTGA.................................... 1
................................AGGGTCACAGGTGAGGTTCTTGGGAGCC 1
.....................................CACAGGTGAGGTTCTTGGGAGCC 1
..((..(((.((((..((((((((((....))....)))))))))))).)))..)).... (-26.60)

1/105400740-105400806(-)_mir_dog_15
TGAGGTAGGAGGTTGTATAGTTGAGGAGGACACCCACGGAGATCACTATACGGCCTCCTAGCTTTCC
TGAGGTAGGAGGTTGTATAGTT... 128756
TGAGGTAGGAGGTTGTATAGT.. 54857
TGAGGTAGGAGGTTGTATAG... 5682
TGAGGTAGGAGGTTGTATAGTTG.. 3945
TGAGGTAGGAGGTTGTAT... 2336
....CTATACGGCCTCCTAGCTTTCC 1959
TGAGGTAGGAGGTTGTATA.. 1176
TGAGGTAGGAGGTTGTATAGTTGA... 565
....CTATACGGCCTCCTAGCTTTC. 223
TGAGGTAGGAGGTTGTA.. 155
.....TAGGAGGTTGTATAGTT... 136
....TATACGGCCTCCTAGCTTTCC 109
...GGTAGGAGGTTGTATAGTT... 72
.....TAGGAGGTTGTATAGT.. 64
TGAGGTAGGAGGTTGT... 55
......AGGAGGTTGTATAGTT... 47
....GTAGGAGGTTGTATAGTT... 40
.GAGGTAGGAGGTTGTATAGTT... 32
...GGTAGGAGGTTGTATAGT.. 30
..AGGTAGGAGGTTGTATAGTT... 25
....CTATACGGCCTCCTAGCTTT.. 19
....ACTATACGGCCTCCTAGCTTTC. 16
....ACTATACGGCCTCCTAGCTTTCC 15
.GAGGTAGGAGGTTGTATAGTTGA... 14
....GTAGGAGGTTGTATAGT.. 13
.GAGGTAGGAGGTTGTATAGT.. 12
.GAGGTAGGAGGTTGTATAGTTG.. 11
..AGGTAGGAGGTTGTATAGT.. 10
....CTATACGGCCTCCTAGCT.... 7
....CTATACGGCCTCCTAGCTT... 7
....CACTATACGGCCTCCTAGCTTTC. 7
......AGGAGGTTGTATAGTTG.. 5
.......GGAGGTTGTATAGTTG.. 5
....TATACGGCCTCCTAGCTTTC. 5

```
...........................................CACTATACGGCCTCCTAGCTTT..    4
..........................................TCACTATACGGCCTCCTAGCTTTC.    4
..AGGTAGGAGGTTGTATAGTTGA...........................................    3
......................GAGGAGGACACCCACGGAGATCA......................    3
..........................................TCACTATACGGCCTCCTAGCTTT..    3
.GAGGTAGGAGGTTGTAT.................................................    2
.....TAGGAGGTTGTATAGTTG............................................    2
...........................................CACTATACGGCCTCCTAGCTT...    2
..........................................TCACTATACGGCCTCCTAGCTTTCC    2
...............................................ATACGGCCTCCTAGCTTTCC    2
...GGTAGGAGGTTGTATA................................................    1
..AGGTAGGAGGTTGTATAG...............................................    1
...GGTAGGAGGTTGTATAG...............................................    1
..AGGTAGGAGGTTGTATAGTTG............................................    1
...GGTAGGAGGTTGTATAGTTG............................................    1
.....TAGGAGGTTGTATAGTTGA...........................................    1
......AGGAGGTTGTATAGTTGA...........................................    1
TGAGGTAGGAGGTTGTATAGTTGAG..........................................    1
TGAGGTAGGAGGTTGTATAGTTGAGG.........................................    1
.....TAGGAGGTTGTATAGTTGAGGA........................................    1
.......................AGGAGGACACCCACGGAGAT........................    1
............................................ACTATACGGCCTCCTAGCTT...    1
.(((.((((((((((((((((.((.(.((....)).).....)))))))))))))))))).)))... (-29.20)

1/105400925-105400984(-)_mir_dog_16
CACCCGTAGAACCGACCTTGCGGGGCCTTCGCCGCACACAAGCTCGAGTCTGTGGGTCTG
CACCCGTAGAACCGACCTTGCG......................................    314118
CACCCGTAGAACCGACCTTGC.......................................    215011
CACCCGTAGAACCGACCTTG........................................    25366
......................................CAAGCTCGAGTCTGTGGGTCT.    7304
CACCCGTAGAACCGACCTT.........................................    6730
CACCCGTAGAACCGACCT..........................................    2379
....CGTAGAACCGACCTTGCG......................................    962
...CCGTAGAACCGACCTTGCG......................................    647
.ACCCGTAGAACCGACCTTGCG......................................    610
.ACCCGTAGAACCGACCTTGC.......................................    329
......................................CAAGCTCGAGTCTGTGGGTCTG    322
....CGTAGAACCGACCTTGC.......................................    282
CACCCGTAGAACCGACCTTGCGG.....................................    262
......................................CAAGCTCGAGTCTGTGGGTC..    237
...CCGTAGAACCGACCTTGC.......................................    183
CACCCGTAGAACCGACC...........................................    169
..CCCGTAGAACCGACCTTGCG......................................    165
..CCCGTAGAACCGACCTTGC.......................................    106
.....GTAGAACCGACCTTGCG......................................    75
.ACCCGTAGAACCGACCTTG........................................    54
......................................CAAGCTCGAGTCTGTGGGT...    48
.....................................ACAAGCTCGAGTCTGTGGGTCT.    48
CACCCGTAGAACCGACCTTGCGGGGCCTTCGCCGCACA......................    45
.......................................AAGCTCGAGTCTGTGGGTCT.    34
.....GTAGAACCGACCTTGC.......................................    29
....CGTAGAACCGACCTTG........................................    26
CACCCGTAGAACCGAC............................................    22
...CCGTAGAACCGACCTTG........................................    21
..CCCGTAGAACCGACCTTG........................................    14
.ACCCGTAGAACCGACCTT.........................................    12
...CCGTAGAACCGACCTT.........................................    6
.ACCCGTAGAACCGACCT..........................................    5
......TAGAACCGACCTTGCG......................................    4
CACCCGTAGAACCGACCTTGCGGGGCCTTCGCCGCAC.......................    4
..CCCGTAGAACCGACCTT.........................................    3
.....................................ACAAGCTCGAGTCTGTGGGTC..    3
.......................................AAGCTCGAGTCTGTGGGTC..    3
..CCCGTAGAACCGACCT..........................................    2
```

```
....CGTAGAACCGACCTTGCGG.....................................     2
CACCCGTAGAACCGACCTTGCGGGGCCT................................     2
..................TGCGGGGCCTTCGCCGCACACAAGCTCGAGTCTGTGGGTCTG     2
.ACCCGTAGAACCGACC...........................................     1
...CCGTAGAACCGACCTTGCGG.....................................     1
CACCCGTAGAACCGACCTTGCGGG....................................     1
....CGTAGAACCGACCTTGCGGG....................................     1
CACCCGTAGAACCGACCTTGCGGGG...................................     1
CACCCGTAGAACCGACCTTGCGGGGCCTTCGCCGCA........................     1
...CCGTAGAACCGACCTTGCGGGGCCTTCGCCGCACA......................     1
CACCCGTAGAACCGACCTTGCGGGGCCTTCGCCGCACAC.....................     1
......................................CAAGCTCGAGTCTGTGGG....     1
.......................................AAGCTCGAGTCTGTGGGT...     1
........................................AGCTCGAGTCTGTGGGTCT.     1
..........................................CTCGAGTCTGTGGGTCT.     1
...........................................TCGAGTCTGTGGGTCT.     1
........................................AGCTCGAGTCTGTGGGTCTG     1
.(((((((((..(((.((((.(((((....))).).).)))).)))..)))))))))... (-23.90)

1/106998756-106998812(+)_mir_dog_17
TCTCCCAACCCTTGTACCAGTGCTGTGCCTCAGTCCCTGGTACAGGCATGGGGGGCA
TCTCCCAACCCTTGTACCAGTG...................................  7762
TCTCCCAACCCTTGTACCAGT....................................  2269
TCTCCCAACCCTTGTACCAGTGC..................................  1075
TCTCCCAACCCTTGTACCAG.....................................  379
TCTCCCAACCCTTGTACCA......................................  125
TCTCCCAACCCTTGTACC.......................................  26
TCTCCCAACCCTTGTACCAGTGCT.................................  3
TCTCCCAACCCTTGTAC........................................  2
..TCCCAACCCTTGTACCAGT....................................  1
..TCCCAACCCTTGTACCAGTG...................................  1
....................................CTGGTACAGGCATGGGGGG..  1
(((((((...((((((((((.((((.....))))..))))))))))..))))))).. (-28.40)

1/107507098-107507167(+)_novel_dog_2
GCGTCCTGGGCTGGATGGGACTTGCGTGGCATTCCCTGCCTTCACTAAGTCCCGTTCTGCCCTCCCAGGT
............................................CTAAGTCCCGTTCTGCCCTCCC.... 14
............................................CTAAGTCCCGTTCTGCCCTCCCA... 3
....CCTGGGCTGGATGGGACTTGCG............................................ 2
....CCTGGGCTGGATGGGACTTGCGT........................................... 2
............................................CTAAGTCCCGTTCTGCCCTCC..... 2
...TCCTGGGCTGGATGGGACTTGC............................................. 1
...TCCTGGGCTGGATGGGACTTGCGT........................................... 1
...........................................ACTAAGTCCCGTTCTGCCCTCC..... 1
...........................................ACTAAGTCCCGTTCTGCCCTCCC.... 1
....(((((((.((((((((((((.((((((.....))))...)))))))))))))).))))....))). (-32.70)

1/107628869-107628933(+)_novel_Dog_Blast_6
GTCTTGGGGCGACTTCCTCGACTCACTCTCTTTCCCCCGGGAGTCGTGGAACCTCGCCCGAGGGT
.........................................AGTCGTGGAACCTCGCCCGAG...      9
.........................................AGTCGTGGAACCTCGCCCGA....      6
.........................................AGTCGTGGAACCTCGCCCGAGG..      5
....TGGGGCGACTTCCTCGACTC.........................................      1
........................................GAGTCGTGGAACCTCGCCCGA....      1
.((((.((((((.((((.((((((.((...........)))))))).)))).)))))).)))). (-29.80)

1/109198113-109198185(+)_novel_dog_3
CAGAGGGGGAGGTAGAGTTCTGAGTGATGAGGGGCTCTCAGTGACATCTGGTTCCTCTGCCTCCCCACAGGAA
..............................................TCTGGTTCCTCTGCCTCCCCACA....     17
..............................................TCTGGTTCCTCTGCCTCCCCACAG...     10
...AGGGGGAGGTAGAGTTCTGAGT.................................................     7
..............................................TCTGGTTCCTCTGCCTCCCCAC.....     5
...AGGGGGAGGTAGAGTTCTGAGTG................................................     2
..............................................TCTGGTTCCTCTGCCTCCCC.......     2
```

.....GGGGAGGTAGAGTTCT.. 1
..GAGGGGGAGGTAGAGTTCTGA... 1
..GAGGGGGAGGTAGAGTTCTGAG.. 1
...AGGGGGAGGTAGAGTTCTGAG.. 1
....GGGGGAGGTAGAGTTCTGAGT... 1
....GGGGGAGGTAGAGTTCTGAGTG.. 1
...AGGGGGAGGTAGAGTTCTGAGTGA... 1
.....GGGGAGGTAGAGTTCTGAGTGA... 1
....TCTGGTTCCTCTGCCTCCC........ 1
....TCTGGTTCCTCTGCCTCCCCA...... 1
.....((((((((((((..((..(((.((((.....))))....)))..))...))))))))))))....... (-32.00)

1/109618355-109618435(-)_mir_dog_18
GCTCTGACCTGAGACCTCTGGGTTCTGAGCTGTGATGTTCCTTCCAAGCTGGGATCTCTGGGGTCTTGGTTCAGGGTCGGG
.........TGAGACCTCTGGGTTCTGAGCT.. 204040
.........TGAGACCTCTGGGTTCTGAGC... 11684
........CTGAGACCTCTGGGTTCTGAGCT.. 3174
....CTGGGATCTCTGGGGTCTTGGT........... 2572
....CTGGGATCTCTGGGGTCTTGGTT.......... 1230
........CTGAGACCTCTGGGTTCTGAGC... 391
.........TGAGACCTCTGGGTTCTGA... 207
....CTGGGATCTCTGGGGTCTTGG............ 174
....TGGGATCTCTGGGGTCTTGGTT.......... 126
.........TGAGACCTCTGGGTTCT... 81
.........TGAGACCTCTGGGTTCTGAG.. 71
..........GAGACCTCTGGGTTCTGAGCT.. 69
.............ACCTCTGGGTTCTGAGCT.. 56
..............CCTCTGGGTTCTGAGCT.. 26
...............CTCTGGGTTCTGAGCT.. 25
........CTGAGACCTCTGGGTTCTGAG.. 22
.........TGAGACCTCTGGGTTC.. 20
....CTGGGATCTCTGGGGTCTT.............. 20
....CTGGGATCTCTGGGGTCT............... 16
.........TGAGACCTCTGGGTTCTGAGCTG... 15
.........TGAGACCTCTGGGTTCTGAGCTGT.. 11
....TGGGATCTCTGGGGTCTTGGT........... 11
........CTGAGACCTCTGGGTTCTGA... 8
....CTGGGATCTCTGGGGTCTTG............. 8

............GACCTCTGGGTTCTGAGCT...
.   6
........CTGAGACCTCTGGGTTCT..
.   5
..........GAGACCTCTGGGTTCTGAGC..
.   5
.......CCTGAGACCTCTGGGTTCTGAGCT...
.   5
...........AGACCTCTGGGTTCTGAGCT...
.   5
....TGGGATCTCTGGGGTCTTGG...........
.   3
.........TGAGACCTCTGGGTTCTG...
.   2
..............CCTCTGGGTTCTGAGC..
.   2
....CTGGGATCTCTGGGGTC...............
.   2
....CAAGCTGGGATCTCTGGGGTCT..............
.   2
....CTGGGATCTCTGGGGTCTTGGTTC........
.   2
........CTGAGACCTCTGGGTTC...
.   1
.......CCTGAGACCTCTGGGTTCTGAG...
.   1
.............ACCTCTGGGTTCTGAGC..
.   1
....TGGGATCTCTGGGGTCT..............
.   1
...............................GTGATGTTCCTTCCAAGCTGGGATCTCTGGGGTCTTGGT..........
.   1
....AGCTGGGATCTCTGGGGTCTTGGTT.........
.   1
....ATCTCTGGGGTCTTGGTT.........
.   1
....CTGGGATCTCTGGGGTCTTGGTTCA......
.   1
(((((((.(..(((((((.(((.(((.((((..
((.......))..)))).))).))).)))))))..).))))))).... (-38.60)

1/109922152-109922226(+)_mir_dog_19
GCCTCTCTGGGCCTGTGTCTTAGGCTCTGCAAGATCAACCGAGCAAAGCACACGGCCTGCAGAGAGGCAGCGCTC
...TCTCTGGGCCTGTGTCTTAGGCT...
      24551
...TCTCTGGGCCTGTGTCTTAGGCTC..
      2108
....CAAAGCACACGGCCTGCAGAG...........
      2056
....CAAAGCACACGGCCTGCAGA............
      1633
....CAAAGCACACGGCCTGCAGAGA..........
      1391
...TCTCTGGGCCTGTGTCTTAGGC.. 926
....GCAAAGCACACGGCCTGCAGAGA.......... 753
....GCAAAGCACACGGCCTGCAGA............ 629
....CAAAGCACACGGCCTGCAGAGAGG........ 489
....CAAAGCACACGGCCTGCAGAGAG......... 343
....AAAGCACACGGCCTGCAGAGAGG........ 293
...TCTCTGGGCCTGTGTCTTAGG... 289
...TCTCTGGGCCTGTGTCTTAG.. 211
....GCAAAGCACACGGCCTGCAGAG........... 196
....AAAGCACACGGCCTGCAGAGA.......... 126
...TCTCTGGGCCTGTGTCTTA... 122

....AAAGCACACGGCCTGCAGAG........... 97
...TCTCTGGGCCTGTGTCTT.. 93
....GCAAAGCACACGGCCTGCAGAGAGG........ 88
....AAAGCACACGGCCTGCAGAGAG......... 76
....GCAAAGCACACGGCCTGC............... 43
....CAAAGCACACGGCCTGCAG............. 42
....AAAGCACACGGCCTGCAGA............ 41
...TCTCTGGGCCTGTGTCTTAGGCTCT... 38
.....TCTGGGCCTGTGTCTTAGGCT... 34
....ACACGGCCTGCAGAGA.......... 28
....GCAAAGCACACGGCCTGCA.............. 27
....CAAAGCACACGGCCTGCA.............. 22
....GCAAAGCACACGGCCTGCAG............. 13
...TCTCTGGGCCTGTGTCT... 11
..........GCCTGTGTCTTAGGCT... 11
....CAAAGCACACGGCCTGC............... 11
...TCTCTGGGCCTGTGTC.. 10
..CTCTCTGGGCCTGTGTCTTAGGCT... 9
....GCAAAGCACACGGCCTGCAGAGAG......... 9
....CACGGCCTGCAGAGAGG........ 6
.....TCTGGGCCTGTGTCTTAGGC.. 3
.....TCTGGGCCTGTGTCTTAGGCTC.. 3
....GCAAAGCACACGGCCTG................ 3
....AAAGCACACGGCCTGC............... 3
....ACACGGCCTGCAGAGAGG........ 3
....ACGGCCTGCAGAGAGG........ 3
....CTCTGGGCCTGTGTCTTAGGCT... 2
....ACACGGCCTGCAGAGAG......... 2
.....TCTGGGCCTGTGTCTTAGG... 1
......CTGGGCCTGTGTCTTAGGC.. 1
.......TGGGCCTGTGTCTTAGGCT... 1
.........GGCCTGTGTCTTAGGCT... 1
..CTCTCTGGGCCTGTGTCTTAGGCTC.. 1
...........CCTGTGTCTTAGGCTC.. 1
.................TCTTAGGCTCTGCAAGATCAACCGAG................................ 1
....AGCAAAGCACACGGCCTGC............... 1
....AAAGCACACGGCCTGCA.............. 1
....AAGCACACGGCCTGCAGAGA.......... 1
....GCACACGGCCTGCAGAGA.......... 1
..............................AAGATCAACCGAGCAAAGCACACGGCCTGCAGAGAGG........ 1
....CACACGGCCTGCAGAGAGG........ 1
....GCAAAGCACACGGCCTGCAGAGAGGC....... 1
(((((((((((((((((.(((..((((.............)))).))))))).))))..))))))))))....... (-
36.22)

1/111510949-111511014(+)_novel_dog_4
AGCAGCGGTGGGGCCGGGCTGGGACCGTCCTCTGATGACCTGGTCCCGCGCCCTGTCCCTCTGCAG
....TCCCGCGCCCTGTCCCTCTGCAG 763
....TCCCGCGCCCTGTCCCTCTGCA. 211
....TCCCGCGCCCTGTCCCTCTGC.. 72
..CAGCGGTGGGGCCGGGCTGGGACC.. 4
....TCCCGCGCCCTGTCCCTCTG... 4
....CCGCGCCCTGTCCCTCTGC.. 1
....GTCCCGCGCCCTGTCCCTCTGCAG 1
....CCCGCGCCCTGTCCCTCTGCAG 1
....((((.(((((.((((((((((((((.......)))..))))))).)))).))))).)))).. (-40.20)

11/12753262-12753326(+)_novel_Dog_Blast_7
GTCTTGGCGCGACTTCCTCGACTCACTCTCTTTCCCCCGTGAGTCGTGGAACCTCGCCCGAGGGT
....TGAGTCGTGGAACCTCGCCCGA.... 83
....AGTCGTGGAACCTCGCCCGAG... 9
....AGTCGTGGAACCTCGCCCGA.... 6
....AGTCGTGGAACCTCGCCCGAGG.. 5
...TTGGCGCGACTTCCTCGACTC... 4

....TGGCGCGACTTCCTCGACT...   3
....TGGCGCGACTTCCTCGACTCACT.....................................   3
......................................TGAGTCGTGGAACCTCGCCC......   3
......................................TGAGTCGTGGAACCTCGCCCGAG...   3
....TGGCGCGACTTCCTCGACTCA.......................................   2
......................................TGAGTCGTGGAACCTCGCCCG.....   2
...TTGGCGCGACTTCCTCGACT...   1
....TGGCGCGACTTCCTCGACTC..   1
....TGGCGCGACTTCCTCGACTCAC......................................   1
...TTGGCGCGACTTCCTCGACTCACT.....................................   1
.......CGCGACTTCCTCGACTCACT.....................................   1
..............................TTTCCCCGTGAGTCGTGGAACC............   1
....GAGTCGTGGAACCTCGCCCGA....   1
.................................CCCCGTGAGTCGTGGAACCTCGCCCGAGG..   1
..................................CCCGTGAGTCGTGGAACCTCGCCCGAGG..   1
....................................CGTGAGTCGTGGAACCTCGCCCGAGG..   1
.....................................GTGAGTCGTGGAACCTCGCCCGAGGGT   1
.((((((.((((.((((.((((((((...........)))))))).)))).)))))))))).. (-31.20)

11/12759564-12759628(+)_novel_Dog_Blast_8
GTCTTGGCGCGACTTCCTCGACTCACTCTCTTTCCCCGTGAGTCGTGGAACCTCGCCCGAGGGT
......................................TGAGTCGTGGAACCTCGCCCGA....   83
....AGTCGTGGAACCTCGCCCGAG...   9
....AGTCGTGGAACCTCGCCCGA....   6
....AGTCGTGGAACCTCGCCCGAGG..   5
...TTGGCGCGACTTCCTCGACTC..   4
....TGGCGCGACTTCCTCGACT...   3
....TGGCGCGACTTCCTCGACTCACT.....................................   3
......................................TGAGTCGTGGAACCTCGCCC......   3
......................................TGAGTCGTGGAACCTCGCCCGAG...   3
....TGGCGCGACTTCCTCGACTCA.......................................   2
......................................TGAGTCGTGGAACCTCGCCCG.....   2
...TTGGCGCGACTTCCTCGACT...   1
....TGGCGCGACTTCCTCGACTC..   1
....TGGCGCGACTTCCTCGACTCAC......................................   1
...TTGGCGCGACTTCCTCGACTCACT.....................................   1
.......CGCGACTTCCTCGACTCACT.....................................   1
..............................TTTCCCCGTGAGTCGTGGAACC............   1
....GAGTCGTGGAACCTCGCCCGA....   1
.................................CCCCGTGAGTCGTGGAACCTCGCCCGAGG..   1
..................................CCCGTGAGTCGTGGAACCTCGCCCGAGG..   1
....................................CGTGAGTCGTGGAACCTCGCCCGAGG..   1
.....................................GTGAGTCGTGGAACCTCGCCCGAGGGT   1
.((((((.((((.((((.((((((((...........)))))))).)))).)))))))))).. (-31.20)

1/116556066-116556144(-)_novel_dog_6
ACATAGGACAGGTTTTGAGTCATGGGTTGCTGGGGTAGTTGTGACCTCTGGCTTCTGACTTTCAATCTGTCCCCCACAG
....TCTGACTTTCAATCTGTCCCCC....
      14
....TCTGACTTTCAATCTGTCCCC.....
      3
...TAGGACAGGTTTTGAGTCATGGG...
      1
.....((((((((..((((((..((((...(((((.......))))).))))..))))))..))))))))........
(-30.60)

11/1704590-1704665(+)_mir_dog_20
GATGCGATTATAAAGCAATGAGACTGATTGTCATGTGTCGGTTGTGGGATCCGTCTCAGTTACTTTATAGCCGTGC
.......TTATAAAGCAATGAGACTGATT...
      148467
.......TTATAAAGCAATGAGACTGAT..
      47295
....TCCGTCTCAGTTACTTTATAGCC....
      8917

.......TTATAAAGCAATGAGACTGA...
2893
.......TTATAAAGCAATGAGACT...
1802
....TCCGTCTCAGTTACTTTATAGC.....
1101
....TCCGTCTCAGTTACTTTATAG...... 985
....CCGTCTCAGTTACTTTATAGCC.... 284
.......TTATAAAGCAATGAGACTGATTG.. 158
.......TTATAAAGCAATGAGACTG.. 126
....ATCCGTCTCAGTTACTTTATAGCC.... 88
....ATCCGTCTCAGTTACTTTATAG...... 80
....ATCCGTCTCAGTTACTTTATAGC..... 35
.......TTATAAAGCAATGAGAC.. 33
....CCGTCTCAGTTACTTTATAGC..... 33
........TATAAAGCAATGAGACTGATT... 30
.......TTATAAAGCAATGAGACTGATTGT... 28
....TCCGTCTCAGTTACTTTATA....... 28
......ATTATAAAGCAATGAGACTGAT.. 18
....CGTCTCAGTTACTTTATAGCC.... 18
....CCGTCTCAGTTACTTTATAG...... 16
.......TTATAAAGCAATGAGACTGATTGTCATGT.. 13
......ATTATAAAGCAATGAGACTGA... 11
.........ATAAAGCAATGAGACTGATT... 10
....TCCGTCTCAGTTACTTTAT........ 10
....TCCGTCTCAGTTACTTTATAGCCG... 10
........TATAAAGCAATGAGACTGAT.. 8
............................TGTCATGTGTCGGTTGTGGGAT.......................... 5
......ATTATAAAGCAATGAGACT... 4
.............................GTCATGTGTCGGTTGTGGGA........................... 4
....ATCCGTCTCAGTTACTTTAT........ 4
............AAGCAATGAGACTGATT... 3
....TCCGTCTCAGTTACTTTA......... 3
....ATCCGTCTCAGTTACTTTATA....... 3
............AAGCAATGAGACTGAT.. 2
....ATCCGTCTCAGTTACTTTA......... 2
....CGTCTCAGTTACTTTATAGC..... 2
....GTCTCAGTTACTTTATAGCC.... 2
....CCGTCTCAGTTACTTTATAGCCG... 2
........TATAAAGCAATGAGACT... 1
........TATAAAGCAATGAGACTGA... 1
.........ATAAAGCAATGAGACTGAT.. 1
......ATTATAAAGCAATGAGACTGATT... 1
..........TAAAGCAATGAGACTGATT... 1
...........AAAGCAATGAGACTGATT... 1
.............AGCAATGAGACTGATT... 1
........TATAAAGCAATGAGACTGATTG.. 1
.......TTATAAAGCAATGAGACTGATTGTCAT.. 1
...........................TTGTCATGTGTCGGTTGTGGG............................ 1
...........................TTGTCATGTGTCGGTTGTGGGAT.......................... 1
.............................GTCATGTGTCGGTTGTGGGAT.......................... 1
...........................TTGTCATGTGTCGGTTGTGGGATCCGTCT.................... 1
....TCCGTCTCAGTTACTT........... 1
....ATCCGTCTCAGTTACTTT.......... 1
....CGTCTCAGTTACTTTATAG...... 1
....GTCTCAGTTACTTTATAG...... 1
....TGTGGGATCCGTCTCAGTTACTTTATAGCC.... 1
....CTCAGTTACTTTATAGCC.... 1
....CGTCTCAGTTACTTTATAGCCG... 1
...(((..(((((((...((((((.((((.(((((.......))))))))).))))))....))))))))..))).. (-
22.40)

11/25355355-25355432(-)_mir_dog_21
TTAGCCCTGCGGCCCCACGCACCAGGGTAAGAGAGACTCTCGCTTCCTGCCCTGGCCCGAGGGACCGACTGGCTGGGC

....CTGCCCTGGCCCGAGGGACCGA..........
27787
....CTGCCCTGGCCCGAGGGACCGAC.........
22811
.........CGGCCCCACGCACCAGGGTAAG...
7664
....CTGCCCTGGCCCGAGGGACCGACT........
4851
.........CGGCCCCACGCACCAGGGTAA..
3051
.........CGGCCCCACGCACCAGGGTAAGA..
2748
.........CGGCCCCACGCACCAGGGTA...
1015
....CTGCCCTGGCCCGAGGGACC............
702
....CTGCCCTGGCCCGAGGGACCG...........
612
....CTGCCCTGGCCCGAGGGAC.............
529
....TGCCCTGGCCCGAGGGACCGAC.........
195
....CTGCCCTGGCCCGAGGGA..............
122
....TGCCCTGGCCCGAGGGACCGA..........
76
.........CGGCCCCACGCACCAGGGT..
72
....CTGCCCTGGCCCGAGGGACCGACTG.......
29
....TGCCCTGGCCCGAGGGACCGACT........
27
..........GGCCCCACGCACCAGGGTAAG...
13
....CTGCCCTGGCCCGAGGG...............
8
....TGCCCTGGCCCGAGGGACCG...........
8
....TGCCCTGGCCCGAGGGACCGACTG.......
8
....CTGCCCTGGCCCGAGG................
6
........GCGGCCCCACGCACCAGGGTAAG...
5
........GCGGCCCCACGCACCAGGGTA...
3
........GCGGCCCCACGCACCAGGGTAA..
3
..........GGCCCCACGCACCAGGGTAA..
3
.............CCCACGCACCAGGGTAAGA..
3
..............CCACGCACCAGGGTAAGA..
3
.........CGGCCCCACGCACCAGGGTAAGAG...
3
....CCTGCCCTGGCCCGAGGGAC.............
3
.........CGGCCCCACGCACCAGGG...
2
...........GCCCCACGCACCAGGGTAA..
2
..............CCACGCACCAGGGTAAG...
2

```
...........GCCCCACGCACCAGGGTAAGA..............................................
      2
...............................................TGCCCTGGCCCGAGGGACC............
      2
.........CGGCCCCACGCACCAGG....................................................
      1
.......TGCGGCCCCACGCACCAGGGT..................................................
      1
..........GGCCCCACGCACCAGGGTA.................................................
      1
..............CCACGCACCAGGGTAA................................................
      1
........GCGGCCCCACGCACCAGGGTAAGA..............................................
      1
..........GGCCCCACGCACCAGGGTAAGA..............................................
      1
.........CGGCCCCACGCACCAGGGTAAGAGA............................................
      1
.........CGGCCCCACGCACCAGGGTAAGAGAGACTC.......................................
      1
...............................................TGCCCTGGCCCGAGGGAC.............
      1
.............................................CCTGCCCTGGCCCGAGGGACC............
      1
.............................................CCTGCCCTGGCCCGAGGGACCGA..........
      1
...................................................CTGGCCCGAGGGACCGA..........
      1
................................................GCCCTGGCCCGAGGGACCGAC.........
      1
((((((...(((.(((.((..((((((((.(((.(.....)..))).))))))))..)).))).)))...))))))..
(-37.20)

11/26741534-26741600(+)_mir_dog_22
GTTTTGCACCTCTGAGAGTGGAAGAACTCCTCCTGTGGAGTTGGTCCTAGTCTGGGTGCAAACAGTT
..TTTGCACCTCTGAGAGTGGAAGAACT.......................................    633
..TTTGCACCTCTGAGAGTGGAAG...........................................    232
..TTTGCACCTCTGAGAGTGGAA............................................    199
..TTTGCACCTCTGAGAGTGG..............................................    161
..TTTGCACCTCTGAGAGTGGAAGAAC........................................    158
..TTTGCACCTCTGAGAGTGGA.............................................    157
..TTTGCACCTCTGAGAGTGGAAGA..........................................    126
...TTGCACCTCTGAGAGTGGAAGAACT.......................................    120
..TTTGCACCTCTGAGAGTGGAAGAACTCCTCCT.................................    94
...............................................TAGTCTGGGTGCAAACAGTT    53
........................................TTGGTCCTAGTCTGGGTGCAAAC....    27
...TTGCACCTCTGAGAGTGGAAGAAC........................................    20
..TTTGCACCTCTGAGAGTGGAAGAA.........................................    19
.............................................CCTAGTCTGGGTGCAAACAGTT    15
..............................................CTAGTCTGGGTGCAAACAGTT    14
.TTTTGCACCTCTGAGAGTGGAAGAACTCCTCCT.................................    12
........................................TTGGTCCTAGTCTGGGTGCA.......    12
.TTTTGCACCTCTGAGAGTGGAA............................................    11
..TTTGCACCTCTGAGAG.................................................    10
...TTGCACCTCTGAGAGTGGAAGAACTCCTCCT.................................    10
........................................TTGGTCCTAGTCTGGGTGCAAACA...    10
.TTTTGCACCTCTGAGAGTGGA.............................................    9
.TTTTGCACCTCTGAGAGTGG..............................................    8
.TTTTGCACCTCTGAGAGTGGAAGAACT.......................................    8
..TTTGCACCTCTGAGAGTGGAAGAACTC......................................    8
..TTTGCACCTCTGAGAGTGGAAGAACTCCT....................................    8
........CCTCTGAGAGTGGAAG...........................................    7
........................................TTGGTCCTAGTCTGGGTGCAA......    7
.........................ACTCCTCCTGTGGAGTTGGTCCTAGTCTGGGTGCAAACAGTT    7
```

```
...TTGCACCTCTGAGAGTGGAAG...........................................    6
..TTTGCACCTCTGAGAGTGGAAGAACTCCTCC..................................    6
........................................TTGGTCCTAGTCTGGGTGC........    6
...TTGCACCTCTGAGAGTGG..............................................    5
.TTTTGCACCTCTGAGAGTGGAAG...........................................    4
...TTGCACCTCTGAGAGTGGAAGAA.........................................    4
.........CTCTGAGAGTGGAAGAAC........................................    4
..TTTGCACCTCTGAGAGTGGAAGAACTCC.....................................    4
...........................................GTCCTAGTCTGGGTGCAAACAGTT    4
............................................TCCTAGTCTGGGTGCAAACAGTT    4
...TTGCACCTCTGAGAGTGGAA............................................    3
.......ACCTCTGAGAGTGGAAGAACT.......................................    3
........................................TTGGTCCTAGTCTGGGTGCAAA.....    3
...............................................TAGTCTGGGTGCAAACAGT.    3
........................AACTCCTCCTGTGGAGTTGGTCCTAGTCTGGGTGCAAACAGTT    3
..............................TCCTGTGGAGTTGGTCCTAGTCTGGGTGCAAACAGTT    3
.TTTTGCACCTCTGAGAGTGGAAGAAC........................................    2
........CCTCTGAGAGTGGAAGAAC........................................    2
........CCTCTGAGAGTGGAAGAACT.......................................    2
..........TCTGAGAGTGGAAGAACT.......................................    2
.TTTTGCACCTCTGAGAGTGGAAGAACTC......................................    2
...TTGCACCTCTGAGAGTGGAAGAACTC......................................    2
...TTGCACCTCTGAGAGTGGAAGAACTCC.....................................    2
...TTGCACCTCTGAGAGTGGAAGAACTCCT....................................    2
..TTTGCACCTCTGAGAGTGGAAGAACTCCTC...................................    2
..........................CTCCTCCTGTGGAGTTGGTCCTAGTCTGGGTGCAAACAGTT    2
..........................................GGTCCTAGTCTGGGTGCAAACAGTT    2
...TTGCACCTCTGAGAGTGGA.............................................    1
......CACCTCTGAGAGTGGAA............................................    1
.TTTTGCACCTCTGAGAGTGGAAGA..........................................    1
...TTGCACCTCTGAGAGTGGAAGA..........................................    1
....TGCACCTCTGAGAGTGGAAGAACT.......................................    1
.........CTCTGAGAGTGGAAGAACT.......................................    1
GTTTTGCACCTCTGAGAGTGGAAGAACTC......................................    1
.TTTTGCACCTCTGAGAGTGGAAGAACTCC.....................................    1
..........TCTGAGAGTGGAAGAACTCC.....................................    1
.TTTTGCACCTCTGAGAGTGGAAGAACTCCTC...................................    1
.TTTTGCACCTCTGAGAGTGGAAGAACTCCTCCTG................................    1
..TTTGCACCTCTGAGAGTGGAAGAACTCCTCCTG................................    1
..TTTGCACCTCTGAGAGTGGAAGAACTCCTCCTGT...............................    1
..TTTGCACCTCTGAGAGTGGAAGAACTCCTCCTGTGGA............................    1
..TTTGCACCTCTGAGAGTGGAAGAACTCCTCCTGTGGAGTTGG.......................    1
..............................TCCTGTGGAGTTGGTCC....................    1
..................TGGAAGAACTCCTCCTGTGGAGTTGGTCCTAGTCTGGGTGC........    1
.........................................TGGTCCTAGTCTGGGTGCAAAC....    1
............................CCTCCTGTGGAGTTGGTCCTAGTCTGGGTGCAAACAGTT    1
................................CTGTGGAGTTGGTCCTAGTCTGGGTGCAAACAGTT    1
.................................TGTGGAGTTGGTCCTAGTCTGGGTGCAAACAGTT    1
........................................TTGGTCCTAGTCTGGGTGCAAACAGTT    1
..................................................TCTGGGTGCAAACAGTT    1
..(((((((((...(((.((((..((((((......))))))..))).).))))))))))))..... (-28.50)

11/40300967-40301020(+)_mir_dog_23
GTGGGGAACCCTTCCATGAGGAGTAGAACACTCCTTATGCAAGATTCCCTTCTA
.................................CTTATGCAAGATTCCCTTCT.     35
.TGGGGAACCCTTCCATGAGGAGT..............................     29
.................................CTTATGCAAGATTCCCTTCTA     4
.TGGGGAACCCTTCCATGAGGAG...............................     3
GTGGGGAACCCTTCCATGAGGA................................     2
.TGGGGAACCCTTCCATGAGGA................................     2
..................................TTATGCAAGATTCCCTTCT.     2
GTGGGGAACCCTTCCATGAG..................................     1
.TGGGGAACCCTTCCATGAGG.................................     1
(.((((((..(((.((((((((((.....)))))))))).))).)))))).).. (-23.80)
```

```
11/40904438-40904496(-)_mir_dog_24
AGGCAAGATGCTGGCATAGCTGTTGAACTGAGAACCTGCTATGCCAACATATTGCCATC
AGGCAAGATGCTGGCATAGCTG.....................................9567
AGGCAAGATGCTGGCATAGCTGT....................................2528
AGGCAAGATGCTGGCATAGCT......................................2105
AGGCAAGATGCTGGCATAGC.......................................154
....................................TGCTATGCCAACATATTGCCATC105
AGGCAAGATGCTGGCATAG........................................97
AGGCAAGATGCTGGCATAGCTGTT...................................31
....................................TGCTATGCCAACATATTGCCAT.16
....................................TGCTATGCCAACATATTGCCA..14
AGGCAAGATGCTGGCATA.........................................7
......................TTGAACTGAGAACCTGCTATGCCAACATATTGCCATC6
....................................TGCTATGCCAACATATTGCC...5
....AAGATGCTGGCATAGCTG.....................................3
AGGCAAGATGCTGGCATAGCTGTTGAACTGAGAACC.......................3
....................................TGCTATGCCAACATATTGC....3
.....................................GCTATGCCAACATATTGCCATC3
......................................CTATGCCAACATATTGCCATC3
.GGCAAGATGCTGGCATAGCTG.....................................2
......GATGCTGGCATAGCTG.....................................2
AGGCAAGATGCTGGCATAGCTGTTGAACTGAGAACCT......................2
AGGCAAGATGCTGGCAT..........................................1
.GGCAAGATGCTGGCATAGCT......................................1
...CAAGATGCTGGCATAGCTG.....................................1
.....AGATGCTGGCATAGCTG.....................................1
AGGCAAGATGCTGGCATAGCTGTTGAACTGAGAA.........................1
......................TTGAACTGAGAACCTGCTATGCC..............1
.......................TGAACTGAGAACCTGCTATGCCAACATA........1
....................................TGCTATGCCAACATATTG.....1
...........................................CCAACATATTGCCATC1
.(((((.(((.(((((((((.(((........)))..))))))))).))).)))))... (-27.70)

11/43203256-43203322(+)_novel_dog_10
CTATACTTTCAGGGATCATTTCTATAGTTTGTTACTAGAGAAGTTTCTCTGAATGTGTAGGGTACCC
........................................AAGTTTCTCTGAATGTGTAGGGT....    123
...TACTTTCAGGGATCATTTCT............................................    66
...TACTTTCAGGGATCATTTCTA...........................................    26
........................................AAGTTTCTCTGAATGTGTAGGG.....    24
........................................AAGTTTCTCTGAATGTGTAGG......    23
.........................................AGTTTCTCTGAATGTGTAGGGT....    13
........................................AAGTTTCTCTGAATGTGTAGGGTA...    7
...TACTTTCAGGGATCATTTC.............................................    4
.......................................GAAGTTTCTCTGAATGTGTAGGG.....    4
..ATACTTTCAGGGATCATTTCT............................................    3
.........................................AGTTTCTCTGAATGTGTAGGGTACC.    3
.....................................GAGAAGTTTCTCTGAATGTG..........    2
.......................................GAAGTTTCTCTGAATGTGTAGG......    2
......................................AGAAGTTTCTCTGAATGTGTAGGGT....    2
.........................................AGTTTCTCTGAATGTGTAGGGTA...    2
.........................................AGTTTCTCTGAATGTGTAGGGTACCC    2
CTATACTTTCAGGGAT...................................................    1
.TATACTTTCAGGGATC..................................................    1
.TATACTTTCAGGGATCATTTCT............................................    1
..ATACTTTCAGGGATCATTTCTA...........................................    1
......TTTCAGGGATCATTTCTATAGT.......................................    1
..................TTTCTATAGTTTGTTACT...............................    1
.................ATTTCTATAGTTTGTTACTAGAG...........................    1
..................TTTCTATAGTTTGTTACTAGAG...........................    1
........................TAGTTTGTTACTAGAGAAGTTTCTC..................    1
......................TATAGTTTGTTACTAGAGAAGTTTCTCTGA...............    1
.....................................GAGAAGTTTCTCTGAATGT...........    1
.......................................GAAGTTTCTCTGAATGTGTAG.......    1
```

....AAGTTTCTCTGAATGTGTAG.......   1
.....................................GAGAAGTTTCTCTGAATGTGTAGG......   1
....GAAGTTTCTCTGAATGTGTAGGGT....   1
....AGTTTCTCTGAATGTGTAGGGTAC..   1
....GTTTCTCTGAATGTGTAGGGTACC.   1
.....................................GAGAAGTTTCTCTGAATGTGTAGGGTACCC   1
....TTTCTCTGAATGTGTAGGGTACCC   1
((((((.((((((((..((((((.((((.....)))).)))))).)))))))).)))))))....... (-25.00)

11/46819464-46819544(-)_mir_dog_25
TGAAGTGCTGTGGATTTCTTTGTGAATCACCATATCTAAGCTAATGTGGTGGTGGTTTACAAAGTAATTCATAGTGCTTC
A
....TGGTGGTTTACAAAGTAATTC..........
.   239
..........TGGATTTCTTTGTGAATCACC..
.   187
....TGGTGGTTTACAAAGTAATTCA.........
.   69
....GTGGTTTACAAAGTAATTC..........
.   36
....TGGTGGTTTACAAAGTAATT...........
.   25
....TGGTGGTTTACAAAGTAAT............
.   7
....GTGGTTTACAAAGTAATTCATA.......
.   5
..........TGGATTTCTTTGTGAATCACCA..
.   4
............GATTTCTTTGTGAATCACCAT...
.   3
..............TTTCTTTGTGAATCACC...
.   2
..........TGGATTTCTTTGTGAATCACCAT...
.   2
....GGTGGTTTACAAAGTAATTC..........
.   2
....GTGGTTTACAAAGTAATTCA.........
.   2
..........TGGATTTCTTTGTGAATC..
.   1
..........TGGATTTCTTTGTGAATCAC..
.   1
...............TTCTTTGTGAATCACC...
.   1
................TCTTTGTGAATCACCA..
.   1
....GGTTTACAAAGTAATTC..........
.   1
.((((..(((((((((.((((((((((((((((......
((....)).)))))))))))))))).)))))))))..)))). (-42.70)

11/46838185-46838246(-)_mir_dog_26
TTGCAGGAACTTGTGAGTCTCCTATTGAAAATGAACAGGAGACTGATGAGTTCCCGGGAACA
..GCAGGAACTTGTGAGTCTCCT.......................................   6243
......................................GAGACTGATGAGTTCCCGGGA...   1960
.TGCAGGAACTTGTGAGTCTCCT.......................................   1873
......................................GAGACTGATGAGTTCCCGGGAA..   951
.....................................GGAGACTGATGAGTTCCCGGGA...   805
.TGCAGGAACTTGTGAGTCT..   500
.TGCAGGAACTTGTGAGTCTCC..   452
.TGCAGGAACTTGTGAGTCTC...   343
.....................................GGAGACTGATGAGTTCCCGGGAA..   291
..GCAGGAACTTGTGAGTCTCC..   254
..GCAGGAACTTGTGAGTCT..   191

```
..GCAGGAACTTGTGAGTCTC.........................................   171
..GCAGGAACTTGTGAGTCTCCTA......................................   73
......................................GAGACTGATGAGTTCCCGGG....   45
.......................................AGACTGATGAGTTCCCGGGAA..   22
.TGCAGGAACTTGTGAGTC...........................................   9
..GCAGGAACTTGTGAGTCTCCTAT.....................................   9
..GCAGGAACTTGTGAGTC...........................................   5
...CAGGAACTTGTGAGTCTCCT.......................................   4
......................................GAGACTGATGAGTTCCCGG.....   4
.......................................AGACTGATGAGTTCCCGGGA...   4
...................................CAGGAGACTGATGAGTTCCCGGG....   3
.....................................GGAGACTGATGAGTTCCCGGGAAC.   3
......................................GAGACTGATGAGTTCCCGGGAAC.   3
.....GGAACTTGTGAGTCTC.........................................   2
......GAACTTGTGAGTCTCCT.......................................   1
.TGCAGGAACTTGTGAGTCTCCTATT....................................   1
........................TTGAAAATGAACAGGAGACTGATGAGTTCCCGGGA...   1
...................................CAGGAGACTGATGAGTTCCCGGGA...   1
((.(.((((((..(.((((((((.((.......)).)))))))).)..)))))).).))... (-26.60)

11/55410560-55410627(-)_novel_dog_12
GGGGTAGGGTGGAGAGAACACCAGGCTGGGCTTTGGGAACCCTGGGTTCTGTCTCCACTCTGCCACCA
........................................CCTGGGTTCTGTCTCCACTCTGC.....   30
........................................CCTGGGTTCTGTCTCCACTCT.......   3
........................................CCTGGGTTCTGTCTCCACTCTG......   3
........................................CCTGGGTTCTGTCTCCACTCTGCC....   2
.....AGGGTGGAGAGAACACCAGGCT.........................................   1
........................................CCTGGGTTCTGTCTCCACT.........   1
........................................CCTGGGTTCTGTCTCCACTC........   1
.......................................CCCTGGGTTCTGTCTCCACTCTGC.....   1
(((((((((((((((((((.(((((..(..(.....)..))))))))))..))))))))))))).)). (-39.30)

11/64197130-64197191(-)_mir_dog_27
TATTGCACATTACTAAGTTGCATGTTGTCACGGCCTCAATGCAATTTAGTGTGTGTGATATT
TATTGCACATTACTAAGTTG..........................................   1076
TATTGCACATTACTAAGTTGC.........................................   812
.........................................CAATTTAGTGTGTGTGATATT   101
TATTGCACATTACTAAGTTGCA........................................   54
......................................ATGCAATTTAGTGTGTGTGAT...   26
.......................................TGCAATTTAGTGTGTGTGAT...   13
TATTGCACATTACTAAGTTGCAT.......................................   5
.......................................TGCAATTTAGTGTGTGTGATAT.   5
.........................................CAATTTAGTGTGTGTGATAT.   5
TATTGCACATTACTAAGT............................................   3
TATTGCACATTACTAAGTT...........................................   3
........................................GCAATTTAGTGTGTGTGATATT   3
.ATTGCACATTACTAAGTTG..........................................   2
......................TGTTGTCACGGCCTCAATG.....................   2
.ATTGCACATTACTAAGTTGC.........................................   1
TATTGCACATTACTAAGTTGCATG......................................   1
.ATTGCACATTACTAAGTTGCATG......................................   1
TATTGCACATTACTAAGTTGCATGTTGT..................................   1
TATTGCACATTACTAAGTTGCATGTTGTCACGGC............................   1
TATTGCACATTACTAAGTTGCATGTTGTCACGGCC...........................   1
TATTGCACATTACTAAGTTGCATGTTGTCACGGCCTC.........................   1
........................TTGTCACGGCCTCAATGCAATTTAGTGTGTGTGA....   1
......................................ATGCAATTTAGTGTGTGTGA....   1
.......................................TGCAATTTAGTGTGTGTGA....   1
.......................................TGCAATTTAGTGTGTGTGATA..   1
(((..(((((.(((((((((((((..((....))..).)))))))))))))))))..))).. (-26.20)

11/68461911-68461968(+)_mir_dog_28
TATGTGCCTTTGGACTACATCGTGGAAGCCAGCACCATGCAGTCCATGGGCATATACA
TATGTGCCTTTGGACTACATCG.................................... 1871
```

```
TATGTGCCTTTGGACTACATC..................................... 322
TATGTGCCTTTGGACTACATCGT................................... 231
..TGTGCCTTTGGACTACATCGTG.................................. 65
TATGTGCCTTTGGACTACAT...................................... 63
..TGTGCCTTTGGACTACATCGT................................... 53
TATGTGCCTTTGGACTACA....................................... 44
......................................GCAGTCCATGGGCATATACA 40
TATGTGCCTTTGGACTAC........................................ 29
....................................ATGCAGTCCATGGGCATATACA 26
.....................................TGCAGTCCATGGGCATATACA 16
.....................................TGCAGTCCATGGGCATATAC. 15
......................................GCAGTCCATGGGCATATAC. 14
..TGTGCCTTTGGACTACATCGTGG................................. 12
.ATGTGCCTTTGGACTACATCGT................................... 10
TATGTGCCTTTGGACTACATCGTG.................................. 10
..TGTGCCTTTGGACTACATCG.................................... 9
....................................ATGCAGTCCATGGGCATATAC. 9
..TGTGCCTTTGGACTACATC..................................... 2
.ATGTGCCTTTGGACTACATCG.................................... 2
......................................GCAGTCCATGGGCATATA.. 2
.ATGTGCCTTTGGACTACATC..................................... 1
....TGCCTTTGGACTACATCGT................................... 1
TATGTGCCTTTGGACTACATCGTGG................................. 1
......CCTTTGGACTACATCGTGGAAG.............................. 1
....................................ATGCAGTCCATGGGCATAT... 1
...................................CATGCAGTCCATGGGCATATACA 1
(((((((((.((((((.(((.(((........))).))).)))))).))))))))).. (-27.20)

11/71939403-71939467(+)_novel_Dog_Blast_9
GTCTTGGGGCGACTTCCTCGACTCACTCTCTTTCCCCCGTGAGTCGTGGAACCTCGCCCGAGGGT
.......................................TGAGTCGTGGAACCTCGCCCGA....      83
.........................................AGTCGTGGAACCTCGCCCGAG...      9
.........................................AGTCGTGGAACCTCGCCCGA....      6
.........................................AGTCGTGGAACCTCGCCCGAGG..      5
.......................................TGAGTCGTGGAACCTCGCCC......      3
.......................................TGAGTCGTGGAACCTCGCCCGAG...      3
.......................................TGAGTCGTGGAACCTCGCCCG.....      2
....TGGGGCGACTTCCTCGACTC.........................................      1
..............................TTTCCCCCGTGAGTCGTGGAACC............      1
........................................GAGTCGTGGAACCTCGCCCGA....      1
..................................CCCCGTGAGTCGTGGAACCTCGCCCGAGG..      1
...................................CCCGTGAGTCGTGGAACCTCGCCCGAGG..      1
.....................................CGTGAGTCGTGGAACCTCGCCCGAGG..      1
......................................GTGAGTCGTGGAACCTCGCCCGAGGGT      1
.((((.((((((.((((.((((((((............)))))))).))))..)))))).)))). (-33.70)

11/74256386-74256447(+)_novel_dog_11
ATCAATCCTAAGGTTGGACGGTCTGGGAGGCGTTGCCAGCCGTCGTGCTTTGGGGACATATC
...AATCCTAAGGTTGGACGGTCT......................................   21
....ATCCTAAGGTTGGACGGTCTGG....................................   20
...AATCCTAAGGTTGGACGGTCTGG....................................   10
...AATCCTAAGGTTGGACGGTC.......................................   4
....ATCCTAAGGTTGGACGGTCT......................................   4
...AATCCTAAGGTTGGACGGTCTG.....................................   2
....ATCCTAAGGTTGGACGGTC.......................................   1
....ATCCTAAGGTTGGACGGTCTG.....................................   1
.....................................AGCCGTCGTGCTTTGGGGACAT...   1
.....(((((((((..(((((.((((.((...)).))))))))).)))))))))....... (-25.50)

1/17593858-17593915(-)_mir_dog_29
TGGAGTGTGACAATGGTGTTTGTGTCCAAACTATCAAACGCCATTATCACACTAAATA
TGGAGTGTGACAATGGTGTTTG.................................... 49
TGGAGTGTGACAATGGTGTTT..................................... 32
TGGAGTGTGACAATGGTGTTTGT................................... 31
```

```
....................................AACGCCATTATCACACTAAAT. 4
TGGAGTGTGACAATGGTGTT..................................... 2
...................................AAACGCCATTATCACACTAAAT. 1
...(((((((.((((((((((((.((....))...)))))))))))).))))))).....  (-24.90)

11/9776593-9776657(+)_novel_Dog_Blast_10
GTCTTGGCGCGACTTCCTCGACTCACTCTCTTTCCCCCGTGAGTCGTGGAACCTCGCCCGAGGGT
.......................................TGAGTCGTGGAACCTCGCCCGA....      83
.........................................AGTCGTGGAACCTCGCCCGAG...      9
.........................................AGTCGTGGAACCTCGCCCGA....      6
.........................................AGTCGTGGAACCTCGCCCGAGG..      5
...TTGGCGCGACTTCCTCGACTC..........................................      4
....TGGCGCGACTTCCTCGACT...........................................      3
....TGGCGCGACTTCCTCGACTCACT.......................................      3
.......................................TGAGTCGTGGAACCTCGCCC......      3
.......................................TGAGTCGTGGAACCTCGCCCGAG...      3
....TGGCGCGACTTCCTCGACTCA.........................................      2
.......................................TGAGTCGTGGAACCTCGCCCG.....      2
...TTGGCGCGACTTCCTCGACT...........................................      1
....TGGCGCGACTTCCTCGACTC..........................................      1
....TGGCGCGACTTCCTCGACTCAC........................................      1
...TTGGCGCGACTTCCTCGACTCACT.......................................      1
......CGCGACTTCCTCGACTCACT........................................      1
..............................TTTCCCCCGTGAGTCGTGGAACC............      1
........................................GAGTCGTGGAACCTCGCCCGA....      1
..................................CCCCGTGAGTCGTGGAACCTCGCCCGAGG..      1
...................................CCCGTGAGTCGTGGAACCTCGCCCGAGG..      1
.....................................CGTGAGTCGTGGAACCTCGCCCGAGG..      1
......................................GTGAGTCGTGGAACCTCGCCCGAGGGT      1
.((((((.((((.((((.((((((((...........)))))))).)))))..)))))))))).. (-31.20)

12/13683499-13683566(-)_novel_dog_17
CATCTGGCCGACGGTCCGCTCTGCGCACGGGGTGGTAACAGCGCGGTTCTGAACCGTCGGCCTTATTG
...CTGGCCGACGGTCCGCTCTGCGC.........................................   4
.ATCTGGCCGACGGTCCGCTCTGC...........................................   2
..TCTGGCCGACGGTCCGCTCTGC...........................................   2
..TCTGGCCGACGGTCCGCTCTGCGC.........................................   2
.........................................CGCGGTTCTGAACCGTCGGCCTT....   2
....TGGCCGACGGTCCGCTCTGCGC.........................................   1
....TGGCCGACGGTCCGCTCTGCGCA........................................   1
.........................................CGCGGTTCTGAACCGTCGGCCT.....   1
.....((((((((((.((..((((((...(........).))))))...)).)))))))))))...... (-32.00)

12/25108658-25108722(+)_novel_Dog_Blast_11
GTCTTGGCGCGACTTCCTCGACTCACTCTCTTTCCCCCGTGAGTCGTGGAACCTCGCCCGAGGGT
.......................................TGAGTCGTGGAACCTCGCCCGA....      83
.........................................AGTCGTGGAACCTCGCCCGAG...      9
.........................................AGTCGTGGAACCTCGCCCGA....      6
.........................................AGTCGTGGAACCTCGCCCGAGG..      5
...TTGGCGCGACTTCCTCGACTC..........................................      4
....TGGCGCGACTTCCTCGACT...........................................      3
....TGGCGCGACTTCCTCGACTCACT.......................................      3
.......................................TGAGTCGTGGAACCTCGCCC......      3
.......................................TGAGTCGTGGAACCTCGCCCGAG...      3
....TGGCGCGACTTCCTCGACTCA.........................................      2
.......................................TGAGTCGTGGAACCTCGCCCG.....      2
...TTGGCGCGACTTCCTCGACT...........................................      1
....TGGCGCGACTTCCTCGACTC..........................................      1
....TGGCGCGACTTCCTCGACTCAC........................................      1
...TTGGCGCGACTTCCTCGACTCACT.......................................      1
......CGCGACTTCCTCGACTCACT........................................      1
..............................TTTCCCCCGTGAGTCGTGGAACC............      1
........................................GAGTCGTGGAACCTCGCCCGA....      1
..................................CCCCGTGAGTCGTGGAACCTCGCCCGAGG..      1
```

```
..................................CCCGTGAGTCGTGGAACCTCGCCCGAGG..      1
....................................CGTGAGTCGTGGAACCTCGCCCGAGG..      1
.....................................GTGAGTCGTGGAACCTCGCCCGAGGGT      1
.((((((.((((.((((.((((((((...........)))))))).)))).))))))))))..  (-31.20)

12/2670928-2670990(+)_mir_dog_30
TGATTGTCCAAACGCAATTCTCGAGTCTCTGGCTCTGGCCGAGAGTTGAGTCTGGACGTCCCG
TGATTGTCCAAACGCAATTC...........................................  7879
TGATTGTCCAAACGCAATTCT..........................................  5556
TGATTGTCCAAACGCA...............................................  331
TGATTGTCCAAACGCAATTCTC.........................................  285
TGATTGTCCAAACGCAAT.............................................  272
TGATTGTCCAAACGCAATT............................................  225
.........................................AGAGTTGAGTCTGGACGTCCCG  130
TGATTGTCCAAACGCAATTCTCG........................................  71
TGATTGTCCAAACGCAA..............................................  15
.GATTGTCCAAACGCAATTC...........................................  9
.....GTCCAAACGCAATTCT..........................................  5
....TGTCCAAACGCAATTC...........................................  4
....................TCGAGTCTCTGGCTCTGGCCGA.....................  4
.GATTGTCCAAACGCAATTCT..........................................  3
...TTGTCCAAACGCAATTCTCGAGT.....................................  3
.........................................AGAGTTGAGTCTGGACGTCC..  3
......................................CCGAGAGTTGAGTCTGGACGTC...  2
.......................................CGAGAGTTGAGTCTGGACGTCC..  2
.GATTGTCCAAACGCAAT.............................................  1
...TTGTCCAAACGCAATTC...........................................  1
....TGTCCAAACGCAATTCT..........................................  1
.GATTGTCCAAACGCAATTCTC.........................................  1
....TGTCCAAACGCAATTCTCG........................................  1
....................TCGAGTCTCTGGCTCTGGCCG......................  1
.........................................AGAGTTGAGTCTGGACGTC...  1
.........................................AGAGTTGAGTCTGGACGTCCC.  1
.(((.(((((.((.(((((((((.(((.........)))))))))))).)).))))))))... (-26.20)

12/33757899-33757960(-)_mir_dog_31
TGTAAACATCCTACACTCTCAGCTGTGGAAAGTAAGAAAGCTGGGAGAAGGCTGTTTACTCT
TGTAAACATCCTACACTCTCAGCT......................................   431557
TGTAAACATCCTACACTCTCAGC.......................................   53805
........................................CTGGGAGAAGGCTGTTTACTCT   4523
TGTAAACATCCTACACTCTCA.........................................   2700
TGTAAACATCCTACACTCTCAG........................................   2070
TGTAAACATCCTACACTCT...........................................   1347
........................................CTGGGAGAAGGCTGTTTACT..   715
TGTAAACATCCTACACTCTC..........................................   628
........................................CTGGGAGAAGGCTGTTTACTC.   500
TGTAAACATCCTACACTCTCAGCTGT....................................   252
TGTAAACATCCTACACT.............................................   205
TGTAAACATCCTACACTC............................................   123
.........................................TGGGAGAAGGCTGTTTACTCT   108
TGTAAACATCCTACACTCTCAGCTG.....................................   96
.GTAAACATCCTACACTCTCAGCT......................................   90
........................................CTGGGAGAAGGCTGTTTAC...   40
....AACATCCTACACTCTCAGCT......................................   27
.......ATCCTACACTCTCAGCT......................................   26
........TCCTACACTCTCAGCT......................................   25
......CATCCTACACTCTCAGCT......................................   17
..TAAACATCCTACACTCTCAGCT......................................   15
...AAACATCCTACACTCTCAGCT......................................   14
TGTAAACATCCTACACTCTCAGCTGTGGA.................................   9
.........................................TGGGAGAAGGCTGTTTACT..   9
.GTAAACATCCTACACTCTCAGC.......................................   7
TGTAAACATCCTACACTCTCAGCTGTGGAA................................   7
...AAACATCCTACACTCTCAGC.......................................   6
```

```
.........................................TGGGAGAAGGCTGTTTACTC.   6
.....ACATCCTACACTCTCAGCT......................................   5
..TAAACATCCTACACTCTCAGC.......................................   4
TGTAAACATCCTACACTCTCAGCTGTGGAAAGT.............................   3
........................................CTGGGAGAAGGCTGTTT.....   3
TGTAAACATCCTACAC..............................................   2
........................................CTGGGAGAAGGCTGTTTA....   2
.......................................GCTGGGAGAAGGCTGTTTACTC.   2
.......................................GCTGGGAGAAGGCTGTTTACTCT   2
..........................................GGGAGAAGGCTGTTTACTCT   2
...AAACATCCTACACTCTC..........................................   1
...AAACATCCTACACTCTCAG........................................   1
......CATCCTACACTCTCAGC.......................................   1
.......ATCCTACACTCTCAGC.......................................   1
...AAACATCCTACACTCTCAGCTGT....................................   1
TGTAAACATCCTACACTCTCAGCTGTGG..................................   1
.........CCTACACTCTCAGCTGTGGAA................................   1
.......................TGTGGAAAGTAAGAAAGC.....................   1
........................................CTGGGAGAAGGCTGTT......   1
.....................................AAGCTGGGAGAAGGCTGTTTACT..   1
............................................GAGAAGGCTGTTTACTCT   1
..............................................GAAGGCTGTTTACTCT   1
.(((((((.(((...(((((((((..............))))))))).))).)))))))... (-24.04)

12/33783894-33783956(-)_mir_dog_32
TGTAAACATCCTCGACTGGAAGCTGTGAAGCCACAGATGGGCTTTCAGTCGGATGTTTGCAGC
TGTAAACATCCTCGACTGGAAGCT.......................................  1351240
TGTAAACATCCTCGACTGGA...........................................  815446
TGTAAACATCCTCGACTGGAAGC........................................  290977
TGTAAACATCCTCGACTGGAA..........................................  224913
TGTAAACATCCTCGACTGGAAG.........................................  72059
.........................................CTTTCAGTCGGATGTTTGCAGC  31702
.GTAAACATCCTCGACTGGAAGCT.......................................  21740
..........................................TTTCAGTCGGATGTTTGCAGC  7383
TGTAAACATCCTCGAC...............................................  5261
.........................................CTTTCAGTCGGATGTTTGCAG.  3768
TGTAAACATCCTCGACTGG............................................  3538
.........................................CTTTCAGTCGGATGTTTGC...  1396
.GTAAACATCCTCGACTGGA...........................................  1289
........TCCTCGACTGGAAGCT.......................................  1005
.GTAAACATCCTCGACTGGAAGC........................................  946
TGTAAACATCCTCGACT..............................................  826
.........................................CTTTCAGTCGGATGTTTGCA..  756
..TAAACATCCTCGACTGGAAGCT.......................................  737
..........................................TTTCAGTCGGATGTTTGCAG.  737
.GTAAACATCCTCGACTGGAA..........................................  331
TGTAAACATCCTCGACTG.............................................  261
...........................................TTCAGTCGGATGTTTGCAGC  216
.......ATCCTCGACTGGAAGCT.......................................  203
.GTAAACATCCTCGACTGGAAG.........................................  143
..........................................TTTCAGTCGGATGTTTGC...  93
..........................................TTTCAGTCGGATGTTTGCA..  72
.......ATCCTCGACTGGAAGC........................................  53
......CATCCTCGACTGGAAGCT.......................................  49
...AAACATCCTCGACTGGAAGCT.......................................  43
..TAAACATCCTCGACTGGA...........................................  42
.....ACATCCTCGACTGGAAGCT.......................................  39
TGTAAACATCCTCGACTGGAAGCTGT.....................................  34
....AACATCCTCGACTGGAAGCT.......................................  33
..TAAACATCCTCGACTGGAAGC........................................  22
TGTAAACATCCTCGACTGGAAGCTG......................................  21
............................................TCAGTCGGATGTTTGCAGC  16
..TAAACATCCTCGACTGGAA..........................................  15
...AAACATCCTCGACTGGAAGC........................................  14
```

```
.........................................CTTTCAGTCGGATGTTTG....  14
.........................................CTTTCAGTCGGATGTTT.....  8
......CATCCTCGACTGGAAGC........................................  7
.........................................CTTTCAGTCGGATGTT......  7
..TAAACATCCTCGACTGGAAG.........................................  6
....AACATCCTCGACTGGAAGC........................................  6
..TAAACATCCTCGACTGGAAGCTG......................................  6
...AAACATCCTCGACTGGAAG.........................................  5
.....ACATCCTCGACTGGAAGC........................................  5
TGTAAACATCCTCGACTGGAAGCTGTGAAGCCACAGATGGG......................  5
.GTAAACATCCTCGACT..............................................  4
...AAACATCCTCGACTGGA...........................................  4
...AAACATCCTCGACTGGAA..........................................  4
..TAAACATCCTCGACTGGAAGCTGT.....................................  4
...........................................TTCAGTCGGATGTTTGCAG.  4
.GTAAACATCCTCGACTGG.............................................  3
.GTAAACATCCTCGACTGGAAGCTGT.....................................  3
...............................................GTCGGATGTTTGCAGC  3
.....ACATCCTCGACTGGAA..........................................  2
.GTAAACATCCTCGACTGGAAGCTG......................................  2
TGTAAACATCCTCGACTGGAAGCTGTG....................................  2
..........................................TTTCAGTCGGATGTTT.....  2
..........................................TTTCAGTCGGATGTTTG....  2
.............................................CAGTCGGATGTTTGCAGC  2
..............................................AGTCGGATGTTTGCAGC  2
....AACATCCTCGACTGGAA..........................................  1
....AACATCCTCGACTGGAAG.........................................  1
.....ACATCCTCGACTGGAAG.........................................  1
......CATCCTCGACTGGAAG.........................................  1
...........TCGACTGGAAGCTGTGAAGCCACAGATGGG......................  1
........................................GCTTTCAGTCGGATGTTTGC...  1
...........................................TTCAGTCGGATGTTTGCA..  1
........................GTGAAGCCACAGATGGGCTTTCAGTCGGATGTTTGCAGC  1
((((((((((..(((((((((((((((....))).....)))))))))))))))))))))).. (-31.80)

12/45510231-45510301(+)_novel_dog_14
CCCACGGGTGCTCATTCAGGTGTGATGAGTTGATGTTAGGATATTCAGCGCATCTGGAGCACCTGGGTGGC
............................................TCAGCGCATCTGGAGCACCTGGG....      45
............................................TCAGCGCATCTGGAGCACCTGGGT...      13
.............................................CAGCGCATCTGGAGCACCTGGG....      11
..............................................AGCGCATCTGGAGCACCTGGGT...      7
............................................TCAGCGCATCTGGAGCACCTG......      6
............................................TCAGCGCATCTGGAGCACCT.......      2
............................................TCAGCGCATCTGGAGCACCTGG.....      2
.............................................CAGCGCATCTGGAGCACCTGGGT...      2
...ACGGGTGCTCATTCAGGTGTGA..............................................      1
.((.(((((((((...((((((((.(((((............))))).)))))))))))))))))))).... (-32.10)

12/470419-470490(-)_novel_dog_16
TAGTGACTGGAGAGGAGAGACCTGGGAAGAGGGCATCCTCTGTCTTCACATGTCCTTCTCTCCACAGCCATC
............................................TTCACATGTCCTTCTCTCCACA......     53
............................................TTCACATGTCCTTCTCTCCACAG.....     32
............................................TTCACATGTCCTTCTCTCCA........     12
............................................TTCACATGTCCTTCTCTCCAC.......     8
.....ACTGGAGAGGAGAGACCTGGGA.............................................     5
.............................................TCACATGTCCTTCTCTCCACA......     5
............................................TTCACATGTCCTTCTCTCC.........     4
.....ACTGGAGAGGAGAGACCTGGGAA............................................     3
.............................................TCACATGTCCTTCTCTCCA........     3
......CTGGAGAGGAGAGACCTGGGAAG...........................................     2
.....ACTGGAGAGGAGAGACCTGG...............................................     1
......CTGGAGAGGAGAGACCTGGGA.............................................     1
....GACTGGAGAGGAGAGACCTGGGAAG...........................................     1
.....ACTGGAGAGGAGAGACCTGGGAAG...........................................     1
```

.....ACTGGAGAGGAGAGACCTGGGAAGA..   1
....TCACATGTCCTTCTCTCCAC.......   1
....TCACATGTCCTTCTCTCCACAG.....   1
....CACATGTCCTTCTCTCCACAG.....   1
..((...((((((((((.(((.((.(((((.((......)).))))).)).)))))))))))))..)).... (-
30.30)

12/60675584-60675652(-)_novel_Dog_Blast_52
ATCAGAACCGAATTCTGGAATAGCTTACTAGTGCAGCACTAAGCTATTCCAGAATTCGGTTCTGATTTA
...AGAACCGAATTCTGGAATAGCT.. 39
......ACCGAATTCTGGAATAGCTTACT.. 18
....CTATTCCAGAATTCGGTTCTGA.... 5
.....AACCGAATTCTGGAATAGCTT... 4
......ACCGAATTCTGGAATAGCTTA.. 4
...AGAACCGAATTCTGGAATAGC... 3
......ACCGAATTCTGGAATAGCTT... 3
....TAAGCTATTCCAGAATTCGGTT........ 3
.....AACCGAATTCTGGAATAGCT.. 2
.....AACCGAATTCTGGAATAGCTTA.. 2
...AGAACCGAATTCTGGAATAG.. 1
...AGAACCGAATTCTGGAATAGCTT... 1
......ACCGAATTCTGGAATAGCTTAC... 1
........CGAATTCTGGAATAGCTTACTA....................................... 1
....AGCTATTCCAGAATTCGGTTCT...... 1
....CTATTCCAGAATTCGGTTCTG..... 1
....TATTCCAGAATTCGGTTCTGAT... 1
(((((((((((((((((((((((((...(((((...))))))))))))))))))))))))))))))... (-44.80)

12/60675587-60675655(+)_novel_dog_15
ATCAGAACCGAATTCTGGAATAGCTTAGTGCTGCACTAGTAAGCTATTCCAGAATTCGGTTCTGATTTA
...AGAACCGAATTCTGGAATAGCT.. 39
....CTATTCCAGAATTCGGTTCTGA.... 5
.....AACCGAATTCTGGAATAGCTT... 4
......ACCGAATTCTGGAATAGCTTA.. 4
...AGAACCGAATTCTGGAATAGC... 3
......ACCGAATTCTGGAATAGCTT... 3
....TAAGCTATTCCAGAATTCGGTT........ 3
.....AACCGAATTCTGGAATAGCT.. 2
.....AACCGAATTCTGGAATAGCTTA.. 2
...AGAACCGAATTCTGGAATAG.. 1
...AGAACCGAATTCTGGAATAGCTT... 1
....AGCTATTCCAGAATTCGGTTCT...... 1
....CTATTCCAGAATTCGGTTCTG..... 1
....TATTCCAGAATTCGGTTCTGAT... 1
(((((((((((((((((((((((((((.((......)).)))))))))))))))))))))))))))... (-45.40)

12/8765343-8765410(+)_novel_dog_13
GAATGGGGCTCAGTGGATGGAAGTAGGATTTTCTCCTCTCTTCTTCCTTCCCATTCACTGTGCCCTCC
....CTTCCCATTCACTGTGCCCTCC   54
....CTTCCCATTCACTGTGCCCTC.   26
....TTCCTTCCCATTCACTGTGCCCT..   20
....TTCCTTCCCATTCACTGTGCCCTC.   10
....TCCTTCCCATTCACTGTGCCCT..   9
....TTCCTTCCCATTCACTGTGCCC...   5
....TTCCTTCCCATTCACTGTGC.....   4
....TTCCTTCCCATTCACTGTGCC....   4
....TCCTTCCCATTCACTGTGCCCTC.   4
....TCCTTCCCATTCACTGTGCCC...   3
....CTTCCCATTCACTGTGCCCT..   3
......GGCTCAGTGGATGGAAGTAGGA..   1
....TCCTTCCCATTCACTGTGC.....   1
....(((((.(((((((((((((.((((.............)))).))).)))))))))).))))).. (-29.92)

13/1538931-1539005(-)_mir_dog_33

```
AGTGTGTTTGATAAGCTGACATGGGACAGGGATTCTTTTCACTGTTGTGTCAGTTTATCAAACCCATACCTGGAT
......TTTGATAAGCTGACATGGGAC.................................................  2
............................................TTGTGTCAGTTTATCAAACC............  2
......TTTGATAAGCTGACATGGGACA................................................  1
............................................TTGTGTCAGTTTATCAAAC.............  1
...........................................GTTGTGTCAGTTTATCAAACC............  1
............................................TTGTGTCAGTTTATCAAACCC...........  1
.(((.((((((((((((((((..((((((((.....))).))))))))))))))))))))).)))........ (-
32.40)

13/30906038-30906097(-)_mir_dog_34
TGTAAACATCCTACACTCAGCTGTAGTACATGGATTGGCTGGGAGGTGGATGTTTACTTC
TGTAAACATCCTACACTCAGCT......................................     235289
TGTAAACATCCTACACTCAGC.......................................     4328
TGTAAACATCCTACACTCAGCTG.....................................     492
......................................CTGGGAGGTGGATGTTTACTTC     280
TGTAAACATCCTACACTCAGCTGT....................................     233
TGTAAACATCCTACACT...........................................     205
TGTAAACATCCTACACTC..........................................     123
......................................CTGGGAGGTGGATGTTTACTT.     109
TGTAAACATCCTACACTCAG........................................     84
TGTAAACATCCTACACTCA.........................................     50
......................................CTGGGAGGTGGATGTTTACT..     42
.GTAAACATCCTACACTCAGCT......................................     32
......CATCCTACACTCAGCT......................................     10
....AACATCCTACACTCAGCT......................................     7
TGTAAACATCCTACACTCAGCTGTA...................................     4
...AAACATCCTACACTCAGCT......................................     3
TGTAAACATCCTACAC............................................     2
..TAAACATCCTACACTCAGCT......................................     2
.GTAAACATCCTACACTCAGC.......................................     1
....AACATCCTACACTCAGC.......................................     1
TGTAAACATCCTACACTCAGCTGTAGT.................................     1
TGTAAACATCCTACACTCAGCTGTAGTACATG............................     1
......................................CTGGGAGGTGGATGTTTAC...     1
.......................................TGGGAGGTGGATGTTTACTT.     1
......................GTAGTACATGGATTGGCTGGGAGGTGGATGTTTACTTC     1
.......................................TGGGAGGTGGATGTTTACTTC     1
.((((((((((.((.((((((((............))))))))..))))))))))))... (-24.40)

13/30910306-30910365(-)_mir_dog_35
TGTAAACATCCCCGACTGGAAGCTGGAAGACAGCAAAGCTTTCAGTCAGATGTTTGCTGC
TGTAAACATCCCCGACTGGAAGCT....................................     1245889
TGTAAACATCCCCGACTGGAAGC.....................................     349250
TGTAAACATCCCCGACTGGA........................................     179328
TGTAAACATCCCCGACTGGAA.......................................     126634
TGTAAACATCCCCGACTGGAAG......................................     73581
TGTAAACATCCCCGAC............................................     9510
TGTAAACATCCCCGACTGG.........................................     6111
......................................CTTTCAGTCAGATGTTTGCTGC     5315
.......................................TTTCAGTCAGATGTTTGCTGC     2988
......................................CTTTCAGTCAGATGTTTGCT..     512
.GTAAACATCCCCGACTGGAAGCT....................................     507
TGTAAACATCCCCGACTG..........................................     411
........TCCCCGACTGGAAGCT....................................     262
......................................CTTTCAGTCAGATGTTTGCTG.     195
........................................TTCAGTCAGATGTTTGCTGC     134
.GTAAACATCCCCGACTGGAAGC.....................................     122
..TAAACATCCCCGACTGGAAGCT....................................     101
.......ATCCCCGACTGGAAGCT....................................     96
TGTAAACATCCCCGACTGGAAGCTG...................................     95
.......................................TTTCAGTCAGATGTTTGCTG.     64
TGTAAACATCCCCGACT...........................................     62
......CATCCCCGACTGGAAGCT....................................     58
```

```
......................................CTTTCAGTCAGATGTTTGC...     57
.GTAAACATCCCCGACTGGA........................................     55
.....ACATCCCCGACTGGAAGCT....................................     53
....AACATCCCCGACTGGAAGCT....................................     45
.......................................TTTCAGTCAGATGTTTGCT..     45
...AAACATCCCCGACTGGAAGCT....................................     39
.GTAAACATCCCCGACTGGAA.......................................     33
..TAAACATCCCCGACTGGAAGC.....................................     19
TGTAAACATCCCCGACTGGAAGCTGGA.................................     14
....AACATCCCCGACTGGAAGC.....................................     12
.......ATCCCCGACTGGAAGC.....................................     11
.GTAAACATCCCCGACTGGAAG......................................     10
.......................................TTTCAGTCAGATGTTTGC...     9
..TAAACATCCCCGACTGGAA.......................................     8
.....ACATCCCCGACTGGAAGC.....................................     7
......CATCCCCGACTGGAAGC.....................................     7
..TAAACATCCCCGACTGGA........................................     6
............................................GTCAGATGTTTGCTGC     6
...AAACATCCCCGACTGGAAGC.....................................     5
TGTAAACATCCCCGACTGGAAGCTGGAAGA..............................     5
..TAAACATCCCCGACTGGAAG......................................     4
.....ACATCCCCGACTGGAAG......................................     4
......CATCCCCGACTGGAAG......................................     4
...AAACATCCCCGACTGGAA.......................................     2
......................................CTTTCAGTCAGATGTT......     2
........................................TTCAGTCAGATGTTTGCT..     2
...........................................AGTCAGATGTTTGCTGC     2
.GTAAACATCCCCGACTGG.........................................     1
....AACATCCCCGACTGGA........................................     1
....AACATCCCCGACTGGAAG......................................     1
........TCCCCGACTGGAAGCTGGA.................................     1
TGTAAACATCCCCGACTGGAAGCTGGAA................................     1
TGTAAACATCCCCGACTGGAAGCTGGAAG...............................     1
........TCCCCGACTGGAAGCTGGAAGAC.............................     1
..................................AAAGCTTTCAGTCAGATGTT......     1
......................................CTTTCAGTCAGATGTTT.....     1
........................................TTCAGTCAGATGTTTGCTG.     1
...........................................AGTCAGATGTTTGCTG.     1
.........................................TCAGTCAGATGTTTGCTGC     1
..........................................CAGTCAGATGTTTGCTGC     1
.(((((((((...(((((((((((............))))))))))).)))))))))... (-26.40)

13/35353689-35353744(-)_mir_dog_36
TCGAGGAGCTCACAGTCTAGTATGTCTCAGCCCTACTAGACTGAGGCTCCTTGAGG
TCGAGGAGCTCACAGTCTAGT...................................   373759
...................................CTAGACTGAGGCTCCTTGAGG   242854
TCGAGGAGCTCACAGTCTAGTA..................................   133818
...................................CTAGACTGAGGCTCCTTGAG.   63230
...................................CTAGACTGAGGCTCCTTGA..   22091
TCGAGGAGCTCACAGTCTAG....................................   8089
TCGAGGAGCTCACAGTCT......................................   6439
..................................ACTAGACTGAGGCTCCTTGAGG   5528
.................................TACTAGACTGAGGCTCCTTGAG.   3049
..................................ACTAGACTGAGGCTCCTTGAG.   2359
.................................TACTAGACTGAGGCTCCTTGA..   1520
TCGAGGAGCTCACAGTCTAGTAT.................................   1345
..................................ACTAGACTGAGGCTCCTTGA..   496
TCGAGGAGCTCACAGTCTA.....................................   470
.................................TACTAGACTGAGGCTCCTTGAGG   432
....................................TAGACTGAGGCTCCTTGAGG   406
.CGAGGAGCTCACAGTCTAGT...................................   218
...................................CTAGACTGAGGCTCCTTG...   216
...AGGAGCTCACAGTCTAGT...................................   187
..GAGGAGCTCACAGTCTAGT...................................   186
```

```
TCGAGGAGCTCACAGTC.......................................   147
.CGAGGAGCTCACAGTCTAGTA..................................   135
....................................TAGACTGAGGCTCCTTGAG.   114
...................................CTAGACTGAGGCTCCT.....   110
..GAGGAGCTCACAGTCTAGTA..................................   70
.....GAGCTCACAGTCTAGT...................................   65
...AGGAGCTCACAGTCTAGTA..................................   36
TCGAGGAGCTCACAGT........................................   34
....GGAGCTCACAGTCTAGT...................................   34
....................................TAGACTGAGGCTCCTTGA..   24
...................................CTAGACTGAGGCTCCTT....   23
.................................TACTAGACTGAGGCTCCTTG...   14
.....GAGCTCACAGTCTAGTA..................................   13
....GGAGCTCACAGTCTAGTA..................................   12
...AGGAGCTCACAGTCTAG....................................   10
..................................ACTAGACTGAGGCTCCTTG...   10
.CGAGGAGCTCACAGTCTAGTAT.................................   9
.................................TACTAGACTGAGGCTCCT.....   9
.................................TACTAGACTGAGGCTCCTT....   6
.....................................AGACTGAGGCTCCTTGAGG   6
.....................................AGACTGAGGCTCCTTGAG.   5
.CGAGGAGCTCACAGTCT......................................   4
.CGAGGAGCTCACAGTCTAG....................................   4
..................................ACTAGACTGAGGCTCCT.....   4
................................CTACTAGACTGAGGCTCCTTGA..   4
........................................CTGAGGCTCCTTGAGG   4
....GGAGCTCACAGTCTAG....................................   3
TCGAGGAGCTCACAGTCTAGTATG................................   3
..................................ACTAGACTGAGGCTCCTT....   3
..GAGGAGCTCACAGTCTAG....................................   2
.CGAGGAGCTCACAGTCTAGTATG................................   2
.................................TACTAGACTGAGGCTCC......   2
..GAGGAGCTCACAGTCT......................................   1
......AGCTCACAGTCTAGTA..................................   1
..GAGGAGCTCACAGTCTAGTAT.................................   1
...AGGAGCTCACAGTCTAGTAT.................................   1
.....GAGCTCACAGTCTAGTAT.................................   1
..GAGGAGCTCACAGTCTAGTATG................................   1
.CGAGGAGCTCACAGTCTAGTATGT...............................   1
TCGAGGAGCTCACAGTCTAGTATGTCTC............................   1
..................................ACTAGACTGAGGCTCC......   1
...............................CCTACTAGACTGAGGCTCCTTGA..   1
.....................................AGACTGAGGCTCCTTGA..   1
................................CTACTAGACTGAGGCTCCTTGAG.   1
((((((((((..((((((((((.((....))..)))))))))).)))))))))).. (-29.80)

13/38004855-38004920(-)_novel_dog_18
GCACCCTCCCTGAGCGCTGGGCACCATTCTCCCCACGGGTGCACCTCGCCCTAGGGAGCGTGTCAC
...CCCTCCCTGAGCGCTGGGCACC.........................................     31
...CCCTCCCTGAGCGCTGGGCACCA........................................     10
..ACCCTCCCTGAGCGCTGGGCACC.........................................     3
....CCTCCCTGAGCGCTGGGCACC.........................................     2
.......................................TGCACCTCGCCCTAGGGAGCGTG....     2
...CCCTCCCTGAGCGCTGGGCAC..........................................     1
.......................................TGCACCTCGCCCTAGGGAGC.......     1
.........................................CACCTCGCCCTAGGGAGCGTGT...     1
.......................................TGCACCTCGCCCTAGGGAGCGTGTC..     1
((((.(((((((.(((..(((((((............))))).)).)))..))))))).)))).... (-30.40)

14/18979475-18979550(-)_mir_dog_37
TTCAGTGTTGAAACAATCTCTATTGAACCAGGCTCCAAACCAAGTTCACTGGAGTTTGTTTCAATATTGCAAGAAT
.......TTGAAACAATCTCTATTGAACC............................................... 507
.......TTGAAACAATCTCTATTGAAC................................................ 46
............................................TTCACTGGAGTTTGTTTCAAT........... 32
```

```
............................................TTCACTGGAGTTTGTTTCA............. 23
............................................TTCACTGGAGTTTGTTTC.............. 10
............................................TTCACTGGAGTTTGTTTCAA............ 5
.....TGTTGAAACAATCTCTATTG................................................... 4
.....TGTTGAAACAATCTCTATTGAACC............................................... 2
............................................TTCACTGGAGTTTGTTTCAATA.......... 2
.............................................TCACTGGAGTTTGTTTCAATATT........ 2
.............CAATCTCTATTGAACC............................................... 1
.........................................AAGTTCACTGGAGTTTGTTTC.............. 1
.............................................TCACTGGAGTTTGTTTCA............. 1
..((((((((((((((.(((((.(((((..((.......))..))))).))))).))))))))))))))....... (-
29.20)

14/22936795-22936859(+)_novel_Dog_Blast_12
GTCTTGGCGCGACTTCCTCGACTCACTCTCTTTCCCCGTGAGTCGTGGAACCTCGCCCGAGGGT
.......................................TGAGTCGTGGAACCTCGCCCGA....      83
.........................................AGTCGTGGAACCTCGCCCGAG...      9
.........................................AGTCGTGGAACCTCGCCCGA....      6
.........................................AGTCGTGGAACCTCGCCCGAGG..      5
...TTGGCGCGACTTCCTCGACTC.........................................      4
....TGGCGCGACTTCCTCGACT..........................................      3
....TGGCGCGACTTCCTCGACTCACT......................................      3
.......................................TGAGTCGTGGAACCTCGCCC......      3
.......................................TGAGTCGTGGAACCTCGCCCGAG...      3
....TGGCGCGACTTCCTCGACTCA........................................      2
.......................................TGAGTCGTGGAACCTCGCCCG.....      2
...TTGGCGCGACTTCCTCGACT..........................................      1
....TGGCGCGACTTCCTCGACTC.........................................      1
....TGGCGCGACTTCCTCGACTCAC.......................................      1
...TTGGCGCGACTTCCTCGACTCACT......................................      1
.......CGCGACTTCCTCGACTCACT......................................      1
..............................TTTCCCCCGTGAGTCGTGGAACC............      1
........................................GAGTCGTGGAACCTCGCCCGA....      1
..................................CCCCGTGAGTCGTGGAACCTCGCCCGAGG..      1
...................................CCCGTGAGTCGTGGAACCTCGCCCGAGG..      1
.....................................CGTGAGTCGTGGAACCTCGCCCGAGG..      1
......................................GTGAGTCGTGGAACCTCGCCCGAGGGT      1
.((((((.((((.((((.((((((((............)))))))).))))..))))))))))).. (-31.20)

14/39301847-39301906(-)_mir_dog_38
AAAGTTCTGAGACACTCCGACTCTGAATATGATAGAAGTCAGTGCACTACAGAACTTTGT
......................................TCAGTGCACTACAGAACTTTGT     263949
AAAGTTCTGAGACACTCCGACT......................................     5143
......................................TCAGTGCACTACAGAACTTTG.     2904
......................................TCAGTGCACTACAGAACTTT..     478
......................................TCAGTGCACTACAGAACT....     376
......................................TCAGTGCACTACAGAACTT...     232
........................................AGTGCACTACAGAACTTTGT     127
.......................................CAGTGCACTACAGAACTTTGT     105
AAAGTTCTGAGACACTCCGA........................................     84
AAAGTTCTGAGACACTCCGAC.......................................     70
.....................................GTCAGTGCACTACAGAACTTTGT     21
......................................TCAGTGCACTACAGAAC.....     14
...........................................GCACTACAGAACTTTGT     10
.........................................GTGCACTACAGAACTTTGT     9
AAAGTTCTGAGACACTCCG.........................................     4
......................................TCAGTGCACTACAGAA......     4
........................................AGTGCACTACAGAACTTTG.     4
..........................................TGCACTACAGAACTTTGT     4
...GTTCTGAGACACTCCGACT......................................     2
.....TCTGAGACACTCCGACT......................................     2
.....................................GTCAGTGCACTACAGAACTTTG.     2
............................................CACTACAGAACTTTGT     2
.AAGTTCTGAGACACTCCGACT......................................     1
```

```
....TTCTGAGACACTCCGACT......................................     1
AAAGTTCTGAGACACTCCGACTC.....................................     1
AAAGTTCTGAGACACTCCGACTCTGAAT................................     1
...............TCCGACTCTGAATATGATAGAAGTC....................     1
.....................................GTCAGTGCACTACAGAACT....     1
.......................................CAGTGCACTACAGAACTTTG.     1
(((((((((((.((((..(((((((.......))).))))))))..)).))))))))).. (-17.80)

14/40344005-40344062(-)_mir_dog_39
TAGGTAGTTTCCTGTTGTTGGGATCCACCTTTCTCTCGACAGCACGACACTGCCTTCA
TAGGTAGTTTCCTGTTGTTGGGA................................... 931
TAGGTAGTTTCCTGTTGTTGGG.................................... 742
TAGGTAGTTTCCTGTTGTTGG..................................... 248
TAGGTAGTTTCCTGTTGTTG...................................... 67
TAGGTAGTTTCCTGTTGTTGGGAT.................................. 30
...................................TCGACAGCACGACACTGCCTTC. 7
...................................TCGACAGCACGACACTGCCT... 6
TAGGTAGTTTCCTGTTGT........................................ 4
TAGGTAGTTTCCTGTTGTT....................................... 3
.............................TTTCTCTCGACAGCACGACACTGCCTT.. 2
...................................TCGACAGCACGACACTGCCTTCA 2
TAGGTAGTTTCCTGTTGTTGGGATCC................................ 1
..............................TTCTCTCGACAGCACGACACTGCC.... 1
.(((((((.((.(((((((((((..........))))))))))).)).)))))))... (-24.60)

14/6184457-6184521(+)_mir_dog_40
AGCTGGTTTCATATGGTGGTTTAGATTTAAATAGTGATTGTCTAGCACCATTTGAAATCAGTGTT
..........................................TAGCACCATTTGAAATCAGTGTT      18792
..........................................TAGCACCATTTGAAATCAGTGT.      3743
.........................................CTAGCACCATTTGAAATCAGTG..      1389
.........................................CTAGCACCATTTGAAATCAGT...      1143
.........................................CTAGCACCATTTGAAATCAGTGT.      1118
..........................................TAGCACCATTTGAAATCAGTG..      1004
..........................................TAGCACCATTTGAAATCAGT...      816
...............................................CCATTTGAAATCAGTGTT      246
................................................CATTTGAAATCAGTGTT      220
..........................................TAGCACCATTTGAAATCAG....      174
.................................................ATTTGAAATCAGTGTT      117
.........................................CTAGCACCATTTGAAATCAG....      97
..............................................ACCATTTGAAATCAGTGTT      91
..........................................TAGCACCATTTGAAATC......      49
.GCTGGTTTCATATGGTGGTTT...........................................      48
...............................................CCATTTGAAATCAGTGT.      46
.GCTGGTTTCATATGGTGGTTTAG.........................................      44
...............................................CCATTTGAAATCAGTG..      44
.........................................CTAGCACCATTTGAAATCAGTGTT      43
.........................................CTAGCACCATTTGAAATCA.....      35
..............................................ACCATTTGAAATCAGTG..      34
.GCTGGTTTCATATGGTGGTTTAGA........................................      24
.........................................CTAGCACCATTTGAAATC......      23
................................................CATTTGAAATCAGTGT.      23
..............................................ACCATTTGAAATCAGTGT.      20
..........................................TAGCACCATTTGAAATCA.....      18
..............................................ACCATTTGAAATCAGT...      16
.GCTGGTTTCATATGGTGGTTTA..........................................      12
.GCTGGTTTCATATGGTGGTT............................................      11
...........................................AGCACCATTTGAAATCAGTGTT      11
..........................................TAGCACCATTTGAAAT.......      10
.............................................CACCATTTGAAATCAGTGTT      6
.........................TTTAAATAGTGATTGTCT......................      5
........................................TCTAGCACCATTTGAAATCAGT...      5
.........................................CTAGCACCATTTGAAAT.......      4
..CTGGTTTCATATGGTGGTTTAG.........................................      3
..CTGGTTTCATATGGTGGTTTAGA........................................      2
```

```
.........................TTTAAATAGTGATTGTCTAGC...................      2
.........................................CTAGCACCATTTGAAA........      2
...........................................AGCACCATTTGAAATCAGTGT.      2
.GCTGGTTTCATATGGTGGT.............................................      1
..CTGGTTTCATATGGTGGTTT...........................................      1
..CTGGTTTCATATGGTGGTTTAGAT.......................................      1
.........................TTTAAATAGTGATTGTC.......................      1
.............................................CACCATTTGAAATCAGT...      1
...........................................AGCACCATTTGAAATCAGTG..      1
.............................................CACCATTTGAAATCAGTGT.      1
.((((((((((.((((((..((((((.............)))))))))))).)))))))))).... (-24.22)

14/6184844-6184903(+)_mir_dog_41
ACTGATTTCTTTTGGTGTTCAGAGTCAATATAATTTCTAGCACCATCTGAAATCGGTTA
......................................TAGCACCATCTGAAATCGGTT.     113838
......................................TAGCACCATCTGAAATCGGTTA     71061
......................................TAGCACCATCTGAAATCGGT..     36033
.....................................CTAGCACCATCTGAAATCGGTT.     25701
.....................................CTAGCACCATCTGAAATCGGT..     4901
.....................................CTAGCACCATCTGAAATCGGTTA     3530
......................................TAGCACCATCTGAAATCGG...     3408
.....................................CTAGCACCATCTGAAATCGG...     1490
..........................................ACCATCTGAAATCGGTT.     1292
...........................................CCATCTGAAATCGGTT.     897
..........................................ACCATCTGAAATCGGTTA     785
ACTGATTTCTTTTGGTGTTCAG......................................     593
...........................................CCATCTGAAATCGGTTA     433
ACTGATTTCTTTTGGTGTTCAGA.....................................     415
............................................CATCTGAAATCGGTTA     316
..........................................ACCATCTGAAATCGGT..     249
......................................TAGCACCATCTGAAATCG....     146
ACTGATTTCTTTTGGTGTTCA.......................................     88
.....................................CTAGCACCATCTGAAATC.....     77
.....................................CTAGCACCATCTGAAATCG....     68
......................................TAGCACCATCTGAAATC.....     61
ACTGATTTCTTTTGGTGTTCAGAG....................................     50
.........................................CACCATCTGAAATCGGTT.     50
.........................................CACCATCTGAAATCGGTTA     46
ACTGATTTCTTTTGGTGTTC........................................     36
......................................TAGCACCATCTGAAAT......     34
ACTGATTTCTTTTGGTGTTCAGAGT...................................     26
.......................................AGCACCATCTGAAATCGGTTA     26
.......................................AGCACCATCTGAAATCGGTT.     22
.....................................CTAGCACCATCTGAAAT......     20
.........................................CACCATCTGAAATCGGT..     18
ACTGATTTCTTTTGGTGTT.........................................     13
....................................TCTAGCACCATCTGAAATCGGT..     7
ACTGATTTCTTTTGGTGT..........................................     5
........................................GCACCATCTGAAATCGGTTA     5
..TGATTTCTTTTGGTGTTCAG......................................     4
..TGATTTCTTTTGGTGTTCAGAG....................................     4
.....................................CTAGCACCATCTGAAA.......     4
.......................................AGCACCATCTGAAATCGGT..     4
..TGATTTCTTTTGGTGTTCAGA.....................................     3
.....TTTCTTTTGGTGTTCAG......................................     2
........................................GCACCATCTGAAATCGGTT.     2
....................................TCTAGCACCATCTGAAATCGGTTA     2
ACTGATTTCTTTTGGT............................................     1
....ATTTCTTTTGGTGTTCAG......................................     1
..TGATTTCTTTTGGTGTTCAGAGT...................................     1
......TCTTTTGGTGTTCAGAGT....................................     1
.........................................CACCATCTGAAATCGG...     1
....................................TCTAGCACCATCTGAAATCGGTT.     1
(((((((((...(((((((.((((...........)))))))))))...))))))))).. (-21.60)
```

14/6444861-6444918(-)_mir_dog_42
TCAAGAGCAATAACGAAAAATGTTTGTCATAAACCGTTTTTCATTATTGCTCCTGACC
TCAAGAGCAATAACGAAAAATG.................................... 37681
....................................TTTTTCATTATTGCTCCTGACC 22654
TCAAGAGCAATAACGAAAAATGT................................... 22365
TCAAGAGCAATAACGAAAAAT..................................... 21781
...................................GTTTTTCATTATTGCTCCTGACC 3651
....................................TTTTTCATTATTGCTCCTGAC. 1628
...................................GTTTTTCATTATTGCTCCTGAC. 988
TCAAGAGCAATAACGAAAAA...................................... 317
......................................TTTCATTATTGCTCCTGACC 217
.....................................TTTTCATTATTGCTCCTGACC 200
....................................TTTTTCATTATTGCTCCTGA.. 132
TCAAGAGCAATAACGAAAAATGTT.................................. 96
...................................GTTTTTCATTATTGCTCCTGA.. 86
TCAAGAGCAATAACGAAAA....................................... 24
....................................TTTTTCATTATTGCTCCT.... 23
.....................................TTTTCATTATTGCTCCTGAC. 21
..AAGAGCAATAACGAAAAAT..................................... 19
TCAAGAGCAATAACGAAA.. 17
..AAGAGCAATAACGAAAAATGT................................... 13
.CAAGAGCAATAACGAAAAATG.................................... 10
..AAGAGCAATAACGAAAAATG.................................... 10
...................................GTTTTTCATTATTGCTCCTG... 9
....................................TTTTTCATTATTGCTCCTG... 9
......GCAATAACGAAAAATG.................................... 8
...................................GTTTTTCATTATTGCTCCT.... 8
.CAAGAGCAATAACGAAAAAT..................................... 6
......................................TTTCATTATTGCTCCTGAC. 6
...AGAGCAATAACGAAAAATG.................................... 4
.CAAGAGCAATAACGAAAAATGT................................... 4
....................................TTTTTCATTATTGCTC...... 4
..................................CGTTTTTCATTATTGCTCCTGAC. 4
TCAAGAGCAATAACGAA... 3
......GCAATAACGAAAAATGT................................... 3
....................................TTTTTCATTATTGCTCC..... 3
.....AGCAATAACGAAAAATG.................................... 2
TCAAGAGCAATAACGAAAAATGTTTGTCATAAACC....................... 2
...................................GTTTTTCATTATTGCTC...... 2
..................................CGTTTTTCATTATTGCTCCT.... 2
.................................CCGTTTTTCATTATTGCTCCTG... 2
TCAAGAGCAATAACGA.. 1
...AGAGCAATAACGAAAAATGT................................... 1
....GAGCAATAACGAAAAATGT................................... 1
.......CAATAACGAAAAATGT................................... 1
..AAGAGCAATAACGAAAAATGTT.................................. 1
TCAAGAGCAATAACGAAAAATGTTT................................. 1
...AGAGCAATAACGAAAAATGTTT................................. 1
TCAAGAGCAATAACGAAAAATGTTTGTCATAAACCG...................... 1
.....................................TTTTCATTATTGCTCC..... 1
..................................CGTTTTTCATTATTGCTCCTGA.. 1
.....................................TTTTCATTATTGCTCCTGA.. 1
....TTCATTATTGCTCCTGA.. 1
......................TTTGTCATAAACCGTTTTTCATTATTGCTCCTGACC 1
..........................TCATAAACCGTTTTTCATTATTGCTCCTGACC 1
....CATTATTGCTCCTGACC 1
....ATTATTGCTCCTGACC 1
(((.(((((((((.((((((((.((......)).)))))))).))))))))).))).. (-21.10)

14/7068542-7068603(+)_mir_dog_43
TATGGCACTGGTAGAATTCACTGTGAACAGTCTCGGTCAGTGAATTACCGAAGGGCCATAAA
TATGGCACTGGTAGAATTCACT.. 4745
.ATGGCACTGGTAGAATTCACT.. 2256

```
TATGGCACTGGTAGAATTCACTG.......................................   2200
.ATGGCACTGGTAGAATTCACTG.......................................   1946
TATGGCACTGGTAGAATTCAC.........................................   592
.ATGGCACTGGTAGAATTCACTGT......................................   466
.ATGGCACTGGTAGAATTCAC.........................................   217
TATGGCACTGGTAGAATTCACTGT......................................   168
TATGGCACTGGTAGAATTCA..........................................   96
.......CTGGTAGAATTCACTG.......................................   45
........................................TGAATTACCGAAGGGCCATAA.   43
.ATGGCACTGGTAGAATTCA..........................................   27
........................................TGAATTACCGAAGGGCCATAAA   21
....GCACTGGTAGAATTCACT........................................   18
TATGGCACTGGTAGAATTC...........................................   15
TATGGCACTGGTAGAATT............................................   13
.ATGGCACTGGTAGAATT............................................   10
.....CACTGGTAGAATTCACT........................................   9
TATGGCACTGGTAGAAT.............................................   6
....GCACTGGTAGAATTCACTG.......................................   6
.ATGGCACTGGTAGAAT.............................................   5
......ACTGGTAGAATTCACT........................................   5
......ACTGGTAGAATTCACTG.......................................   5
.....CACTGGTAGAATTCAC.........................................   4
.......CTGGTAGAATTCACTGT......................................   4
..TGGCACTGGTAGAATTCACT........................................   3
..TGGCACTGGTAGAATTCACTG.......................................   2
.ATGGCACTGGTAGAATTCACTGTGAA...................................   2
TATGGCACTGGTAGAATTCACTGTGAACA.................................   2
TATGGCACTGGTAGAATTCACTGTGAACAGT...............................   2
.......................................GTGAATTACCGAAGGGCCATAA.   2
.......................................GTGAATTACCGAAGGGCCATAAA   2
TATGGCACTGGTAGAA..............................................   1
.ATGGCACTGGTAGAATTC...........................................   1
....GCACTGGTAGAATTCA..........................................   1
.....CACTGGTAGAATTCACTG.......................................   1
..TGGCACTGGTAGAATTCACTGT......................................   1
....GCACTGGTAGAATTCACTGT......................................   1
.....CACTGGTAGAATTCACTGT......................................   1
TATGGCACTGGTAGAATTCACTGTG.....................................   1
TATGGCACTGGTAGAATTCACTGTGAA...................................   1
.ATGGCACTGGTAGAATTCACTGTGAAC..................................   1
TATGGCACTGGTAGAATTCACTGTGAACAGTCTCGGTCA.......................   1
.......................................GTGAATTACCGAAGGGCCATA..   1
((((((..(((((..(((((((((((......)))..)))))))))))))....)))))).. (-21.80)

14/7068764-7068832(+)_mir_dog_44
GATTTTGGCACTAGCACATTTTTGCTTGTGTCTCTCCGCTCTGAGCAATCATGTGCAGTGCCAATATGG
...TTTGGCACTAGCACATTTTTGCT...........................................  606
...TTTGGCACTAGCACATTTTTGC............................................  219
...TTTGGCACTAGCACATTTTTG.............................................  214
...TTTGGCACTAGCACATTTTT..............................................  7
..........CTAGCACATTTTTGCT...........................................  3
...TTTGGCACTAGCACATTTT...............................................  2
.............................................CAATCATGTGCAGTGCCAATAT..  2
........CACTAGCACATTTTTGCT...........................................  1
...TTTGGCACTAGCACATTTTTGCTT..........................................  1
....((((((((.((((((..(((((((((......)))...))))))..)))))))))))))).....  (-29.50)

14/7072715-7072788(+)_mir_dog_45
CGCCGTTTTTGGCAATGGTAGAACTCACACTGGTGAGGTAATGGGATCCGGTGGTTCTAGACTTGCCAACTATG
.......TTTGGCAATGGTAGAACTCACACT...........................................
      67438
.......TTTGGCAATGGTAGAACTCACA.............................................
      31228
.......TTTGGCAATGGTAGAACTCACAC............................................
```

```
      25408
.......TTTGGCAATGGTAGAACTCAC...............................................
      16117
.......TTTGGCAATGGTAGAACTCACACTG...........................................
      6051
.......TTTGGCAATGGTAGAACTCACACTGG..........................................
      5726
.......TTTGGCAATGGTAGAACTCA................................................
      4232
.......TTTGGCAATGGTAGAACTC.................................................   433
.......TTTGGCAATGGTAGAACTCACACTGGT.........................................   234
.......TTTGGCAATGGTAGAACT..................................................   201
........TTGGCAATGGTAGAACTCACACT............................................   57
............CAATGGTAGAACTCACACT............................................   23
...........GCAATGGTAGAACTCACACT............................................   22
..............ATGGTAGAACTCACACT............................................   22
........TTGGCAATGGTAGAACTCACACTG...........................................   21
....................................................TGGTTCTAGACTTGCCAACT...   19
........TTGGCAATGGTAGAACTCACAC.............................................   14
............CAATGGTAGAACTCACA..............................................   13
.......TTTGGCAATGGTAGAAC...................................................   11
........TTGGCAATGGTAGAACTCACA..............................................   9
.............AATGGTAGAACTCACACT............................................   9
........TTGGCAATGGTAGAACTCACACTGG..........................................   9
..............ATGGTAGAACTCACAC.............................................   8
........TTGGCAATGGTAGAACTCAC...............................................   7
...........GCAATGGTAGAACTCAC...............................................   7
..........GGCAATGGTAGAACTCACA..............................................   7
...........GCAATGGTAGAACTCACA..............................................   7
.............AATGGTAGAACTCACA..............................................   7
...........GCAATGGTAGAACTCACAC.............................................   6
............CAATGGTAGAACTCACAC.............................................   6
..........GGCAATGGTAGAACTCACACT............................................   6
...................................................GTGGTTCTAGACTTGCCAACT...   5
............CAATGGTAGAACTCAC...............................................   4
..........GGCAATGGTAGAACTCACAC.............................................   4
............CAATGGTAGAACTCACACTG...........................................   4
...........GCAATGGTAGAACTCACACTGG..........................................   4
.......TTTGGCAATGGTAGAACTCACACTGGTGAGGT....................................   4
.......TTTGGCAATGGTAGAACTCACACTGGTGAGGTAAT.................................   4
..........GGCAATGGTAGAACTCACACTG...........................................   3
............CAATGGTAGAACTCACACTGG..........................................   3
..............ATGGTAGAACTCACACTGG..........................................   3
.......TTTGGCAATGGTAGAA....................................................   2
........TTGGCAATGGTAGAACTCA................................................   2
..........GGCAATGGTAGAACTCAC...............................................   2
.............AATGGTAGAACTCACAC.............................................   2
.........TGGCAATGGTAGAACTCACACT............................................   2
........TTGGCAATGGTAGAACTCACACTGGT.........................................   2
..................................................GGTGGTTCTAGACTTGCCAACT...   2
........TTGGCAATGGTAGAACT..................................................   1
...............TGGTAGAACTCACACT............................................   1
...........GCAATGGTAGAACTCACACTG...........................................   1
.............AATGGTAGAACTCACACTG...........................................   1
.............AATGGTAGAACTCACACTGG..........................................   1
.................GTAGAACTCACACTGG..........................................   1
...........GCAATGGTAGAACTCACACTGGT.........................................   1
.......TTTGGCAATGGTAGAACTCACACTGGTG........................................   1
.......TTTGGCAATGGTAGAACTCACACTGGTGAG......................................   1
.......TTTGGCAATGGTAGAACTCACACTGGTGAGG.....................................   1
.((((....))))..((((((..(((((....)))))....(((((((....)))))))...)))))))...... (-
22.50)
```

14/8165110-8165174(-)_mir_dog_46

```
CTTTTTGCGGTCTGGGCTTGCTGTTCCTCTCAACAGTAGTCAGGAAGCCCTTACCCCAAAAAGTA
CTTTTTGCGGTCTGGGCTTGC............................................      54322
CTTTTTGCGGTCTGGGCTTGCT...........................................      3412
CTTTTTGCGGTCTGGGCTTG.............................................      1812
............................................AAGCCCTTACCCCAAAAAGT.      1017
CTTTTTGCGGTCTGGGCTT..............................................      518
............................................AAGCCCTTACCCCAAAAAGTA      499
............................................AAGCCCTTACCCCAAAAAG..      236
..........................................GGAAGCCCTTACCCCAAAAAGT.      198
CTTTTTGCGGTCTGGGCT...............................................      187
...........................................GAAGCCCTTACCCCAAAAAGT.      185
CTTTTTGCGGTCTGGGCTTGCTGT.........................................      166
CTTTTTGCGGTCTGGGCTTGCTG..........................................      126
.TTTTTGCGGTCTGGGCTTGC............................................      52
...........................................GAAGCCCTTACCCCAAAAAGTA      43
.TTTTTGCGGTCTGGGCTTGCT...........................................      36
..........................................GGAAGCCCTTACCCCAAAAAGTA      22
CTTTTTGCGGTCTGGGC................................................      20
.....................TGTTCCTCTCAACAGTAGTCAGG.....................      20
.TTTTTGCGGTCTGGGCTTGCTGT.........................................      18
.....................TGTTCCTCTCAACAGTAGTCAG......................      16
.TTTTTGCGGTCTGGGCTTGCTG..........................................      13
......................GTTCCTCTCAACAGTAGTCAGGA....................      12
............................................AAGCCCTTACCCCAAAAA...      12
CTTTTTGCGGTCTGGGCTTGCTGTT........................................      8
............................................AAGCCCTTACCCCAAAA....      7
....TTGCGGTCTGGGCTTGC............................................      6
.....TGCGGTCTGGGCTTGC............................................      5
...........................................GAAGCCCTTACCCCAAAAAG..      5
CTTTTTGCGGTCTGGG.................................................      3
..TTTTGCGGTCTGGGCTTGCTGT.........................................      3
.....................TGTTCCTCTCAACAGTAGTCAGGA....................      3
.......................TTCCTCTCAACAGTAGTCAGGA....................      3
..........................................GGAAGCCCTTACCCCAAAAAG..      3
..TTTTGCGGTCTGGGCTTGC............................................      2
..TTTTGCGGTCTGGGCTTGCT...........................................      2
....TTGCGGTCTGGGCTTGCT...........................................      2
............................................AAGCCCTTACCCCAAA.....      2
.............................................AGCCCTTACCCCAAAAAGT.      2
.TTTTTGCGGTCTGGGCTT..............................................      1
...TTTGCGGTCTGGGCTT..............................................      1
...TTTGCGGTCTGGGCTTGC............................................      1
..TTTTGCGGTCTGGGCTTGCTG..........................................      1
.TTTTTGCGGTCTGGGCTTGCTGTT........................................      1
.....................TGTTCCTCTCAACAGTAGTC........................      1
..........................................GGAAGCCCTTACCCCAAAAA...      1
...........................................GAAGCCCTTACCCCAAAAA...      1
(((((((.(((..((((((.(((..(..((....))..).))).))))))..))).))))))).. (-26.30)

1/49030637-49030693(-)_mir_dog_47
TAGGCCATGGTAGATAGAGATGGAGTAACCATTTCATCTCATCTGCACTGCCCAGAC
TAGGCCATGGTAGATAGAGATGG..................................  5
TAGGCCATGGTAGATAGAGAT....................................  3
TAGGCCATGGTAGATAGAGATG...................................  2
TAGGCCATGGTAGATAGAGATGGA.................................  2
.AGGCCATGGTAGATAGAGATGGA.................................  2
TAGGCCATGGTAGATAGAG......................................  1
TAGGCCATGGTAGATAGAGATGGAGTAACC...........................  1
...................................ATCTCATCTGCACTGCCCAGA.  1
..(((....((((((.((((((((((....))))))))))))))))...))).....  (-23.10)

1/49034754-49034817(-)_novel_dog_5
AAATTGCCATGATCTGAAGAGGGAGGATTTATTTGTGTCCTCCTCCTATATGAAATGGCCAAAC
...TTGCCATGATCTGAAGAGGGAG....................................... 58
```

...TTGCCATGATCTGAAGAGGGA.. 11
...TTGCCATGATCTGAAGAGGGAGGATTT.................................. 11
...TTGCCATGATCTGAAGAGGGAGG...................................... 9
....TCCTCCTATATGAAATGGCC.... 7
....TGCCATGATCTGAAGAGGGAG....................................... 6
....TGCCATGATCTGAAGAGGGAGGATTT.................................. 6
..ATTGCCATGATCTGAAGAGGGAG....................................... 5
...TTGCCATGATCTGAAGA.. 3
....TGCCATGATCTGAAGAGGGAGGATTTATTTGTGTCCTCCTCCT................. 3
....TCCTCCTATATGAAATGGC..... 3
....TCCTCCTATATGAAATGGCCAA.. 3
....TCCTCCTATATGAAATGGCCA... 2
...TTGCCATGATCTGAAGAG... 1
....TGCCATGATCTGAAGAG... 1
..ATTGCCATGATCTGAAGAGGG... 1
...TTGCCATGATCTGAAGAGGG... 1
....TGCCATGATCTGAAGAGGG... 1
....TGCCATGATCTGAAGAGGGA.. 1
...TTGCCATGATCTGAAGAGGGAGGA..................................... 1
....TGCCATGATCTGAAGAGGGAGGA..................................... 1
....TGCCATGATCTGAAGAGGGAGGAT.................................... 1
............TCTGAAGAGGGAGGATTT.................................. 1
...TTGCCATGATCTGAAGAGGGAGGATTTATTTGTGTCCTCCTCC.................. 1
....CTCCTCCTATATGAAATGGCC.... 1
....CTCCTCCTATATGAAATGGCCA... 1
.....(((((..((((.((..(((((((........)))))))..)).)).)).)))))..... (-22.00)

14/9182247-9182321(+)_mir_dog_48
CCATGACATTGTGTCAATATGCGATGATGTGTGTGATGGCACAGCGTCATCACGTGGTGACGCAACATCATGACG
........TTGTGTCAATATGCGATGATGT...
4202
........TTGTGTCAATATGCGATGATG..
1687
.......ATTGTGTCAATATGCGATGATG.. 249
........TTGTGTCAATATGCGATGAT... 223
.......ATTGTGTCAATATGCGATGATGT... 56
........TTGTGTCAATATGCGATGA.. 21
.......ATTGTGTCAATATGCGATGAT... 13
........TTGTGTCAATATGCGATG... 8
.......ATTGTGTCAATATGCGATGA.. 5
........TTGTGTCAATATGCGATGATGTG.. 4
........TTGTGTCAATATGCGATGATGTGT... 3
......CATTGTGTCAATATGCGATG... 2
......CATTGTGTCAATATGCGATGATG.. 2
....TCATCACGTGGTGACGCAACA........ 2
.......ATTGTGTCAATATGCGATG... 1
......CATTGTGTCAATATGCGATGAT... 1
.........TGTGTCAATATGCGATGATGT... 1
...........TGTCAATATGCGATGATGT... 1
.............TCAATATGCGATGATGT... 1
.......ATTGTGTCAATATGCGATGATGTG.. 1
........TTGTGTCAATATGCGATGATGTGTGTGATGGCACAGC.............................. 1
....TCATCACGTGGTGACGCAACAT....... 1
.(((((..((((((((.((((.((((((((((((....))))).))))))).)))).))))))))..)))))... (-
35.70)

15/13904833-13904906(-)_novel_dog_21
GGGACTGTGGATGCGTAAGCGTGAGGCTGTGAGTGGGGCTCCGCAACACTCCCAGCCTCACGCTTACGCACCCA
..........ATGCGTAAGCGTGAGGCTGTGA.. 24
..........ATGCGTAAGCGTGAGGCTGTG... 2
...........TGCGTAAGCGTGAGGCTGTGA.. 2
...ACTGTGGATGCGTAAGCGTGA.. 1
....CTGTGGATGCGTAAGCGTGAGG.. 1
.........GATGCGTAAGCGTGAGGCTGT.. 1

....TCCCAGCCTCACGCTTACGCAC...   1
.......(((.((((((((((((((((((.(((((..((...))..))))).)))))))))))))))))).))) (-
51.50)

15/16297294-16297359(-)_novel_dog_22
CCCTAGCCAGAGAGGAGTCCTCTGGTTTAAGTGGACCCAAGTGACTCCTTTCCCTGGCCAGACCAA
......................................AAGTGACTCCTTTCCCTGGCCAGA....   8
......................................AAGTGACTCCTTTCCCTGGCC.......   3
......................................AAGTGACTCCTTTCCCTGGCCAG.....   2
...TAGCCAGAGAGGAGTCCTCTG..   1
......................................AAGTGACTCCTTTCCCTGGCCA......   1
..((.((((((((((((((((..(((((....)))))..)).))))))))))..)))).))..... (-28.90)

15/2280050-2280110(-)_mir_dog_49
TGTAAACATCCTACACTCTCAGCTGTGAGCTCAAGGTGGCTGGGAGAGGGTTGTTTACTCC
TGTAAACATCCTACACTCTCAGCT.....................................   431557
TGTAAACATCCTACACTCTCAGC......................................   53805
....CTGGGAGAGGGTTGTTTACTCC   3135
TGTAAACATCCTACACTCTCA..   2700
TGTAAACATCCTACACTCTCAG.......................................   2070
TGTAAACATCCTACACTCT..   1347
TGTAAACATCCTACACTCTC...   628
....CTGGGAGAGGGTTGTTTACTC.   361
....CTGGGAGAGGGTTGTTTACT..   275
TGTAAACATCCTACACTCTCAGCTGT...................................   252
TGTAAACATCCTACACT..   205
....TGGGAGAGGGTTGTTTACTCC   164
TGTAAACATCCTACACTC...   123
TGTAAACATCCTACACTCTCAGCTG....................................   96
.GTAAACATCCTACACTCTCAGCT.....................................   90
....AACATCCTACACTCTCAGCT.....................................   27
.......ATCCTACACTCTCAGCT.....................................   26
........TCCTACACTCTCAGCT.....................................   25
....CTGGGAGAGGGTTGTTTAC...   24
......CATCCTACACTCTCAGCT.....................................   17
....TGGGAGAGGGTTGTTTACTC.   17
..TAAACATCCTACACTCTCAGCT.....................................   15
...AAACATCCTACACTCTCAGCT.....................................   14
TGTAAACATCCTACACTCTCAGCTGTGAGCT..............................   10
.GTAAACATCCTACACTCTCAGC......................................   7
....TGGGAGAGGGTTGTTTACT..   7
...AAACATCCTACACTCTCAGC......................................   6
....AGAGGGTTGTTTACTCC   6
.....ACATCCTACACTCTCAGCT.....................................   5
..TAAACATCCTACACTCTCAGC......................................   4
.........CCTACACTCTCAGCTGTGAGC...............................   4
....GAGGGTTGTTTACTCC   4
TGTAAACATCCTACAC...   2
........TCCTACACTCTCAGCTGTGAGC...............................   2
....................................TGGCTGGGAGAGGGTTGTTTACTC.   2
...AAACATCCTACACTCTC...   1
...AAACATCCTACACTCTCAG.......................................   1
......CATCCTACACTCTCAGC......................................   1
.......ATCCTACACTCTCAGC......................................   1
...AAACATCCTACACTCTCAGCTGT...................................   1
TGTAAACATCCTACACTCTCAGCTGTGA.................................   1
TGTAAACATCCTACACTCTCAGCTGTGAGC...............................   1
.......ATCCTACACTCTCAGCTGTGAGC...............................   1
...........TACACTCTCAGCTGTGAGC...............................   1
........TCCTACACTCTCAGCTGTGAGCT..............................   1
....................AGCTGTGAGCTCAAGGTGGCTGGGAGA..............   1
.................................AGGTGGCTGGGAGAGGGT..........   1
....CTGGGAGAGGGTTGTT......   1
....CTGGGAGAGGGTTGTTT.....   1

```
........................................TGGGAGAGGGTTGTTTAC...     1
..........................................GGAGAGGGTTGTTTACTCC    1
...........................................GAGAGGGTTGTTTACTCC    1
.(((((((.(((...(((((((((.((....))....))))))))).))).)))))))... (-24.60)

15/2283306-2283369(-)_mir_dog_50
TGTAAACATCCTTGACTGGAAGCTGTAAGGTGCTCAGAGGGGCTTTCAGTCGGATGTTTACAGC
TGTAAACATCCTTGACTGGAAGCT........................................ 813503
TGTAAACATCCTTGACTGGAAGC......................................... 64958
..........................................CTTTCAGTCGGATGTTTACAGC 49417
.GTAAACATCCTTGACTGGAAGCT........................................ 38019
TGTAAACATCCTTGACTGGA............................................ 18742
TGTAAACATCCTTGACTGGAA........................................... 12531
TGTAAACATCCTTGACTGGAAG.......................................... 9683
TGTAAACATCCTTGACTGG............................................. 9484
...........................................TTTCAGTCGGATGTTTACAGC 5214
..........................................CTTTCAGTCGGATGTTTAC... 3746
TGTAAACATCCTTGAC................................................ 2879
..........................................CTTTCAGTCGGATGTTTACAG. 1595
..........................................CTTTCAGTCGGATGTTTACA.. 1097
.GTAAACATCCTTGACTGGAAGC......................................... 695
..TAAACATCCTTGACTGGAAGCT........................................ 607
........TCCTTGACTGGAAGCT........................................ 443
TGTAAACATCCTTGACT............................................... 253
TGTAAACATCCTTGACTG.............................................. 239
...........................................TTTCAGTCGGATGTTTAC... 175
...........................................TTTCAGTCGGATGTTTACAG. 115
.......ATCCTTGACTGGAAGCT........................................ 76
.GTAAACATCCTTGACTGGAAG.......................................... 55
...........................................TTTCAGTCGGATGTTTACA.. 55
TGTAAACATCCTTGACTGGAAGCTGT...................................... 48
.GTAAACATCCTTGACTGGA............................................ 41
.GTAAACATCCTTGACTGGAAGCTGT...................................... 41
......CATCCTTGACTGGAAGCT........................................ 29
TGTAAACATCCTTGACTGGAAGCTG....................................... 29
.GTAAACATCCTTGACTGGAAGCTG....................................... 26
.GTAAACATCCTTGACTGGAA........................................... 25
..........................................CTTTCAGTCGGATGTTTA.... 23
.............................................TCAGTCGGATGTTTACAGC 16
................................................GTCGGATGTTTACAGC 16
.GTAAACATCCTTGACTGG............................................. 14
..TAAACATCCTTGACTGGAAGCTGT...................................... 14
.....ACATCCTTGACTGGAAGCT........................................ 13
....AACATCCTTGACTGGAAGCT........................................ 12
............................................TTCAGTCGGATGTTTACAGC 12
...AAACATCCTTGACTGGAAGCT........................................ 8
..........................................CTTTCAGTCGGATGTTT..... 8
..........................................CTTTCAGTCGGATGTT...... 7
..TAAACATCCTTGACTGGAAGC......................................... 4
.......ATCCTTGACTGGAAGC......................................... 4
...........................................TTTCAGTCGGATGTTTA.... 3
......CATCCTTGACTGGAAGC......................................... 2
..TAAACATCCTTGACTGGAAGCTG....................................... 2
........................GTAAGGTGCTCAGAGGGG...................... 2
...........................................TTTCAGTCGGATGTTT..... 2
.........................................GCTTTCAGTCGGATGTTTAC... 2
.........................................GCTTTCAGTCGGATGTTTACAG. 2
...............................................AGTCGGATGTTTACAGC 2
.GTAAACATCCTTGACT............................................... 1
........TCCTTGACTGGAAGCTGTAAG................................... 1
TGTAAACATCCTTGACTGGAAGCTGTAAGG.................................. 1
..TAAACATCCTTGACTGGAAGCTGTAAGG.................................. 1
TGTAAACATCCTTGACTGGAAGCTGTAAGGT................................. 1
.GTAAACATCCTTGACTGGAAGCTGTAAGGTGCTCAGAGGG....................... 1
```

...................................AGAGGGGCTTTCAGTCGGATGT....... 1
..................................CAGAGGGGCTTTCAGTCGGATGTTTAC... 1
....TCAGTCGGATGTTTAC... 1
......................CTGTAAGGTGCTCAGAGGGGCTTTCAGTCGGATGTTTACAGC 1
........................GTAAGGTGCTCAGAGGGGCTTTCAGTCGGATGTTTACAGC 1
.........................TAAGGTGCTCAGAGGGGCTTTCAGTCGGATGTTTACAGC 1
..........................AAGGTGCTCAGAGGGGCTTTCAGTCGGATGTTTACAGC 1
....GGGCTTTCAGTCGGATGTTTACAGC 1
....GCTTTCAGTCGGATGTTTACAGC 1
((((((((((..((((((((((((.(...(....)...).)))))))))))))))))))))).. (-30.80)

15/28107144-28107202(-)_mir_dog_51
CTTTCCTCTCTGCCCCATAGGGTGTAGCTCTAACTACCCTCTAGGGAAGAGAAGGTTGG
.TTTCCTCTCTGCCCCATAGGGTG...................................4819
.TTTCCTCTCTGCCCCATAGGGT....................................1577
.TTTCCTCTCTGCCCCATAGGGTGT..................................1351
.TTTCCTCTCTGCCCCATAGGG.....................................533
.TTTCCTCTCTGCCCCATAGG......................................350
.TTTCCTCTCTGCCCCATAG.......................................45
.TTTCCTCTCTGCCCCATAGGGTGTAGCTCTAACT........................16
.TTTCCTCTCTGCCCCATAGGGTGTAGCTCT............................12
.....................................CCTCTAGGGAAGAGAAGGTTGG12
.TTTCCTCTCTGCCCCATAGGGTGTAGCTC.............................6
.TTTCCTCTCTGCCCCATAGGGTGTAGCTCTA...........................6
..TTCCTCTCTGCCCCATAGGGTG...................................5
.....................................CCTCTAGGGAAGAGAAGGTTG.5
.TTTCCTCTCTGCCCCATAGGGTGTAG................................4
.TTTCCTCTCTGCCCCATAGGGTGTAGCTCTAACTACCCTCT.................4
.TTTCCTCTCTGCCCCATA..3
.TTTCCTCTCTGCCCCATAGGGTGTA.................................3
......TCTCTGCCCCATAGGGTG...................................2
.TTTCCTCTCTGCCCCATAGGGTGTAGCTCTAACTACC.....................2
.TTTCCTCTCTGCCCCAT...1
CTTTCCTCTCTGCCCCATAGGGTG...................................1
...TCCTCTCTGCCCCATAGGGTG...................................1
....CCTCTCTGCCCCATAGGGTG...................................1
.TTTCCTCTCTGCCCCATAGGGTGTAGCT..............................1
........TCTGCCCCATAGGGTGTAGCTCT............................1
.TTTCCTCTCTGCCCCATAGGGTGTAGCTCTAA..........................1
.TTTCCTCTCTGCCCCATAGGGTGTAGCTCTAAC.........................1
.TTTCCTCTCTGCCCCATAGGGTGTAGCTCTAACTACCC....................1
.TTTCCTCTCTGCCCCATAGGGTGTAGCTCTAACTACCCTCTA..............1
....................................CCCTCTAGGGAAGAGAAGGTTG.1
((..(((((((..(((.(((((.((((......)))).)))))))).)))).)))..)) (-23.70)

15/35234770-35234835(+)_mir_dog_52
TTGTTCTAGGTATGGTCCCAGGGATCCCAGATCAAACCAGGCCCCTGGGCCTATCCTAGAACCAAC
....GCCCCTGGGCCTATCCTAGA...... 2830
....GCCCCTGGGCCTATCCTAGAA..... 2678
....GCCCCTGGGCCTATCCTAGAAC.... 1888
....TCTAGGTATGGTCCCAGGGATCC....................................... 552
....TCTAGGTATGGTCCCAGGGAT... 394
....TCTAGGTATGGTCCCAGGGATC.. 378
.....CTAGGTATGGTCCCAGGGATC.. 198
.....CTAGGTATGGTCCCAGGGATCC....................................... 177
.....CTAGGTATGGTCCCAGGGAT... 96
....CCCCTGGGCCTATCCTAGAAC.... 84
...TTCTAGGTATGGTCCCAGGGAT... 72
....CCCCTGGGCCTATCCTAGAACC... 59
....CCCCTGGGCCTATCCTAGA...... 47
...TTCTAGGTATGGTCCCAGGGATC.. 45
....TCTAGGTATGGTCCCAGGGATCCC...................................... 34
....CCCCTGGGCCTATCCTAGAA..... 31
....GCCCCTGGGCCTATCCTAGAACC... 24

```
...TTCTAGGTATGGTCCCAGGGATCC.......................................      23
........................................GCCCCTGGGCCTATCCTAG.......      23
........................................GCCCCTGGGCCTATCCT.........      14
....TCTAGGTATGGTCCCAGGGA..........................................      11
.....CTAGGTATGGTCCCAGGGATCCC......................................      11
........................................GCCCCTGGGCCTATCCTA........      11
...........................................CCTGGGCCTATCCTAGAAC....      9
...TTCTAGGTATGGTCCCAGGGATCCC......................................      8
..........................................CCCTGGGCCTATCCTAGAACC...      7
...TTCTAGGTATGGTCCCAGGGA..........................................      6
............................................CTGGGCCTATCCTAGAA.....      5
....TCTAGGTATGGTCCCAGGG...........................................      3
......TAGGTATGGTCCCAGGGATC........................................      3
........................................GCCCCTGGGCCTATCC..........      3
...........................................CCTGGGCCTATCCTAGA......      3
.........................................CCCCTGGGCCTATCCTAGAACCA..      3
...TTCTAGGTATGGTCCCAGGG...........................................      2
TTGTTCTAGGTATGGTCCCAGGGAT.........................................      2
............................................CTGGGCCTATCCTAGA......      2
............................................CTGGGCCTATCCTAGAAC....      2
TTGTTCTAGGTATGGTCCCAG.............................................      1
TTGTTCTAGGTATGGTCCCAGG............................................      1
.TGTTCTAGGTATGGTCCCAGGGA..........................................      1
.....CTAGGTATGGTCCCAGGGA..........................................      1
........GGTATGGTCCCAGGGA..........................................      1
......TAGGTATGGTCCCAGGGATCC.......................................      1
.......AGGTATGGTCCCAGGGATCC.......................................      1
....TCTAGGTATGGTCCCAGGGATCCCA.....................................      1
.....CTAGGTATGGTCCCAGGGATCCCA.....................................      1
.........................................CCCCTGGGCCTATCCTAG.......      1
..........................................CCCTGGGCCTATCCTAGA......      1
...........................................CCTGGGCCTATCCTAGAA.....      1
..........................................CCCTGGGCCTATCCTAGAAC....      1
.............................................TGGGCCTATCCTAGAAC....      1
............................................CTGGGCCTATCCTAGAACCA..      1
..((((((((.((((.(((((((..(............)..))))))).))))))))))))).... (-30.72)

15/37060840-37060904(+)_novel_Dog_Blast_13
GTCTTGGCGCGACTTCCTCGACTCACTCTCTTTCCCCCGTGAGTCGTGGAACCTCGCCCGAGGGT
.......................................TGAGTCGTGGAACCTCGCCCGA....      83
.........................................AGTCGTGGAACCTCGCCCGAG...      9
.........................................AGTCGTGGAACCTCGCCCGA....      6
.........................................AGTCGTGGAACCTCGCCCGAGG..      5
...TTGGCGCGACTTCCTCGACTC.........................................      4
....TGGCGCGACTTCCTCGACT..........................................      3
....TGGCGCGACTTCCTCGACTCACT......................................      3
.......................................TGAGTCGTGGAACCTCGCCC......      3
.......................................TGAGTCGTGGAACCTCGCCCGAG...      3
....TGGCGCGACTTCCTCGACTCA........................................      2
.......................................TGAGTCGTGGAACCTCGCCCG.....      2
...TTGGCGCGACTTCCTCGACT..........................................      1
....TGGCGCGACTTCCTCGACTC.........................................      1
....TGGCGCGACTTCCTCGACTCAC.......................................      1
...TTGGCGCGACTTCCTCGACTCACT......................................      1
.......CGCGACTTCCTCGACTCACT......................................      1
..............................TTTCCCCCGTGAGTCGTGGAACC............      1
........................................GAGTCGTGGAACCTCGCCCGA....      1
..................................CCCCGTGAGTCGTGGAACCTCGCCCGAGG..      1
...................................CCCGTGAGTCGTGGAACCTCGCCCGAGG..      1
.....................................CGTGAGTCGTGGAACCTCGCCCGAGG..      1
......................................GTGAGTCGTGGAACCTCGCCCGAGGGT      1
.((((((.((((.((((.((((((((...........)))))))).))))..)))))))))).. (-31.20)

15/37111041-37111102(+)_mir_dog_53
```

```
GACTCTAGCTGCCAAAGGCGCTTCTCCTTCTGAACAGAGCGCTTTGCTCAGCCAGTGTAGAC
.ACTCTAGCTGCCAAAGGCGCT........................................   770
.......................................CGCTTTGCTCAGCCAGTGTAG..   44
.ACTCTAGCTGCCAAAGGCGC.........................................   43
.ACTCTAGCTGCCAAAGGCGCTT.......................................   19
.ACTCTAGCTGCCAAAGGCG..........................................   4
.ACTCTAGCTGCCAAAGG............................................   2
.ACTCTAGCTGCCAAAGGCGCTTCT.....................................   2
...TCTAGCTGCCAAAGGCGC.........................................   1
...TCTAGCTGCCAAAGGCGCT........................................   1
.ACTCTAGCTGCCAAAGGCGCTTC......................................   1
.ACTCTAGCTGCCAAAGGCGCTTCTCCT..................................   1
......................TCTCCTTCTGAACAGAG.......................   1
.....................................AGCGCTTTGCTCAGCC.........   1
.....................................AGCGCTTTGCTCAGCCAGTGT....   1
.......................................CGCTTTGCTCAGCCAGTGT....   1
.......................................CGCTTTGCTCAGCCAGTGTAGA.   1
.((.((.((((...(((((((((.((.....))...)))))))))...)))).)).)).... (-19.80)
```

```
15/37181419-37181480(+)_mir_dog_54
TTATGGCTTTTTATTCCTATGTGATAGTAATAAAGTCTCATGTAGGGATGGAAGCCATGAAA
.TATGGCTTTTTATTCCTATGTGA......................................   8347
.TATGGCTTTTTATTCCTATGTG.......................................   908
.TATGGCTTTTTATTCCTATGT........................................   508
........................................TGTAGGGATGGAAGCCATGAAA   215
........................................TGTAGGGATGGAAGCCATGAA.   103
.TATGGCTTTTTATTCCTATG.........................................   60
.TATGGCTTTTTATTCCTATGTGAT.....................................   53
........................................TGTAGGGATGGAAGCCATGA..   38
.......................................ATGTAGGGATGGAAGCCATGA..   25
.......................................ATGTAGGGATGGAAGCCATGAA.   25
.......................................ATGTAGGGATGGAAGCCATGAAA   23
.TATGGCTTTTTATTCC.............................................   17
.TATGGCTTTTTATTCCT............................................   15
.TATGGCTTTTTATTCCTA...........................................   13
.TATGGCTTTTTATTCCTATGTGATAGT..................................   8
.....GCTTTTTATTCCTATGTG.......................................   3
.....GCTTTTTATTCCTATGTGA......................................   3
......CTTTTTATTCCTATGTGA......................................   3
.TATGGCTTTTTATTCCTATGTGATAG...................................   3
.......TTTTTATTCCTATGTGA......................................   2
........................................TGTAGGGATGGAAGCCAT....   2
.TATGGCTTTTTATTCCTAT..........................................   1
.....GCTTTTTATTCCTATGT........................................   1
..ATGGCTTTTTATTCCTATGTGA......................................   1
........................................TGTAGGGATGGAAGCCATG...   1
(((((((((((.((((((((((((.(.(.....).).))))))))))))))))))))))).. (-25.70)
```

```
15/4781487-4781557(+)_novel_dog_19
CTGTGGCCCTGGCTCTGGGGGAATAGGTCTCCCGGTCTGCAGAGCCCTTCCCCCAGTGAGCCGGGACCCTA
.............................................CCTTCCCCCAGTGAGCCGGGAC....      45
.............................................CCTTCCCCCAGTGAGCCGGGACC...      17
......CCCTGGCTCTGGGGGAATAGGTCT.........................................      11
.............................................CCTTCCCCCAGTGAGCCGGGA.....      4
............................................CCCTTCCCCCAGTGAGCCGGGAC....      4
......CCCTGGCTCTGGGGGAATAGGT...........................................      2
...........................................GCCCTTCCCCCAGTGAGCCGGGAC....      2
.............................................CCTTCCCCCAGTGAGCCGGGACCCT.      2
............................................CCCTTCCCCCAGTGAGCCGGGACC...      1
..............................................CTTCCCCCAGTGAGCCGGGACCC..      1
....((.(((((((((((((((..(((.(((...(....).))).))))))))))..)))))))).))... (-36.80)
```

```
15/54704759-54704832(+)_mir_dog_55
CTCATTAAGGTGGGTGGAATAGTATAACAATATGCTAAATGTTGTTATAGTATCCCACCTACCCTGATGTATCT
```

```
............................................TTATAGTATCCCACCTACCCTG........   13
.........................................TTGTTATAGTATCCCACCTACC...........   3
...........................................GTTATAGTATCCCACCTACCC..........   2
............................................TTATAGTATCCCACCTACCCT.........   2
.........GTGGGTGGAATAGTATAAC..............................................   1
.........................................TTGTTATAGTATCCCACCTACCC..........   1
..........................................TGTTATAGTATCCCACCTACCC..........   1
..........................................TGTTATAGTATCCCACCTACCCT.........   1
............................................TTATAGTATCCCACCTACCCTGA.......   1
.............................................TATAGTATCCCACCTACCCTGA.......   1
..(((((.(((((((((.(((.(((((((((((.....))))))))))).))).))))))))).))))).....  (-
35.40)

15/62441310-62441381(-)_novel_dog_23
ATGCATATCTATCTCTGTCTATAGAGATGCCTATACAGACCTATCTGTAGACAGAGATAGATATAGGTACAG
...CATATCTATCTCTGTCTATAG................................................    5
...CATATCTATCTCTGTCTATA.................................................    3
...CATATCTATCTCTGTCTATAGA...............................................    2
..............................................GTAGACAGAGATAGATATAGG.....    1
.............................................TGTAGACAGAGATAGATATAGGT....    1
....(((((((((((((((((((((...(.((....)).)...)))))))))))))))))))))........  (-
31.70)

15/9934107-9934187(+)_novel_dog_20
AGCTGGGCCCAAGGTGGGGCAGGCCAGGCCGGGAGCCTTGTATGATCCCTCTCCAGCCTGTGGCTCCCCACCCAGGCCTG
T
....................................................CCAGCCTGTGGCTCCCCACCCA......
.     27
....................................................CCAGCCTGTGGCTCCCCACCC.......
.     5
...........AGGTGGGGCAGGCCAGGCCGGGA..............................................
.     1
...................................................TCCAGCCTGTGGCTCCCCACCCA......
.     1
.....................................................CAGCCTGTGGCTCCCCACCCAG.....
.     1
....(((((...(((((((.((.((((((.
(((((.............))).)).))))).).)))))))))..))))).. (-41.72)

16/10561412-10561486(-)_novel_dog_24
GTGTCCGGGCCCTTCCTGGTGGCGGCTCGTGCGGGCTCCGGGCACCTGCCACCGGGTGGGGGCCTGGGCCCTGTG
...TCCGGGCCCTTCCTGGTGGCGG..................................................  8
...TCCGGGCCCTTCCTGGTGGCGGCT................................................  3
..............................................TGCCACCGGGTGGGGGCCTGGGC......  2
...TCCGGGCCCTTCCTGGTGGCG...................................................  1
...TCCGGGCCCTTCCTGGTGGCGGC.................................................  1
......................CGGCTCGTGCGGGCTCCGGGCACCTGCCACCGGGTGGGGG.............  1
..........................................CACCTGCCACCGGGTGGGGGCCTGGGC......  1
...............................................GCCACCGGGTGGGGGCCTGGGC......  1
..............................................TGCCACCGGGTGGGGGCCTGGGCC.....  1
...............................................GCCACCGGGTGGGGGCCTGGGCC.....  1
..(((((((((((((((((((((((...((.(((...))).))..))))))))))).))))))))))))...... (-
51.00)

16/11628312-11628376(-)_mir_dog_56
CACCATGGATCTCCAGGTGGGTCACGTTTAGAGATACACCAACCTGGAGGACTCCATGCTGTTGA
.......................................CAACCTGGAGGACTCCATGCTGT...      6243
.......................................CAACCTGGAGGACTCCATGCTG....      1927
.......................................CAACCTGGAGGACTCCATGCT.....      943
.....................................ACCAACCTGGAGGACTCCATGCT.....      574
......................................CCAACCTGGAGGACTCCATGCTG....      462
......................................CCAACCTGGAGGACTCCATGCT.....      345
.....................................ACCAACCTGGAGGACTCCATGCTG....      201
..CCATGGATCTCCAGGTGGGT...........................................      180
```

```
.......................................CAACCTGGAGGACTCCATGC......      120
......................................CCAACCTGGAGGACTCCATGC......      116
......................................CCAACCTGGAGGACTCCATGCTGT...      115
.....................................ACCAACCTGGAGGACTCCATGC......      94
..CCATGGATCTCCAGGTGGGTC..........................................      53
.......................................CAACCTGGAGGACTCCATGCTGTT..      41
....................................CACCAACCTGGAGGACTCCATGCT.....      30
....................................CACCAACCTGGAGGACTCCATGC......      26
..CCATGGATCTCCAGGTGGGTCA.........................................      23
.......................................CAACCTGGAGGACTCCA.........      18
.....................................ACCAACCTGGAGGACTCCATG.......      13
....................................CACCAACCTGGAGGACTCCATG.......      12
........................................AACCTGGAGGACTCCATGCTGT...      12
.......................................CAACCTGGAGGACTCC..........      8
....................................CACCAACCTGGAGGACTCCAT........      8
..................................TACACCAACCTGGAGGACTCCA.........      7
......................................CCAACCTGGAGGACTCCAT........      7
.......................................CAACCTGGAGGACTCCATG.......      7
.......................................CAACCTGGAGGACTCCAT........      6
.....................................ACCAACCTGGAGGACTCCATGCTGT...      6
......................................CCAACCTGGAGGACTCCATG.......      5
..CCATGGATCTCCAGGTGGG............................................      4
..................................TACACCAACCTGGAGGACTCCAT........      4
......................................CCAACCTGGAGGACTCCA.........      2
...................................ACACCAACCTGGAGGACTCCAT........      2
.....................................ACCAACCTGGAGGACTCCAT........      2
...................................ACACCAACCTGGAGGACTCCATG.......      2
....................................CACCAACCTGGAGGACTCCATGCTG....      2
........................................AACCTGGAGGACTCCATGCTG....      2
...........................................CTGGAGGACTCCATGCTGT...      2
......................................CCAACCTGGAGGACTCCATGCTGTT..      2
..................................TACACCAACCTGGAGGACTCC..........      1
......................................CCAACCTGGAGGACTCC..........      1
....................................CACCAACCTGGAGGACTCCA.........      1
.....................................ACCAACCTGGAGGACTCCA.........      1
...................................ACACCAACCTGGAGGACTCCATGCT.....      1
........................................AACCTGGAGGACTCCATGCT.....      1
................................GATACACCAACCTGGAGGACTCCATGCTG....      1
...........................................CTGGAGGACTCCATGCTG....      1
................................GATACACCAACCTGGAGGACTCCATGCTGT...      1
...................................ACACCAACCTGGAGGACTCCATGCTGT...      1
............................................TGGAGGACTCCATGCTGT...      1
.............................................GGAGGACTCCATGCTGT...      1
((.(((((((((((((((.(((...(((....)))..))).)))))))))..)))))).)).... (-26.20)

16/19323162-19323229(-)_novel_dog_25
GAGACTAGTGAGGACTTAAGGAAACTCACATTCTTTGGAGAGTTTTTCCTTGATTCTCACAGCTATCT
.....TAGTGAGGACTTAAGGAAACTCA........................................   278
.....TAGTGAGGACTTAAGGAAACTC.........................................   141
......AGTGAGGACTTAAGGAAACTCA........................................   73
.....TAGTGAGGACTTAAGGAAACT..........................................   55
...ACTAGTGAGGACTTAAGGAAACT..........................................   15
......AGTGAGGACTTAAGGAAACTC.........................................   13
........................................AGTTTTTCCTTGATTCTCACAG......   13
......AGTGAGGACTTAAGGAAACT..........................................   7
....CTAGTGAGGACTTAAGGAAACT..........................................   6
.....TAGTGAGGACTTAAGGAAAC...........................................   2
...ACTAGTGAGGACTTAAGGAAACTC.........................................   2
....CTAGTGAGGACTTAAGGAAACTCA........................................   2
..........................................TTTTTCCTTGATTCTCACAG......   1
...........................................TTTTCCTTGATTCTCACAG......   1
.((.((.(((((((.((((((((((((.((.....))..))))..))))))))))))))))))).... (-23.20)

16/19932378-19932447(-)_mir_dog_57
```

AGTGTCATTTTTGTGATGTTGCAGCTAGTAATATGAGCCCAGTTGCATAGTCACAAAAGTGATCATTGGA
....TTGCATAGTCACAAAAGTGATC...... 70274
....TTGCATAGTCACAAAAGTGAT....... 5508
....TTGCATAGTCACAAAAGTG......... 4475
....TTGCATAGTCACAAAAGTGATCAT.... 2349
....TTGCATAGTCACAAAAGTGATCA..... 1232
...GTCATTTTTGTGATGTTGCAGCT.. 1171
....TTGCATAGTCACAAAAGTGA........ 628
....TGCATAGTCACAAAAGTGATC...... 602
....TTGCATAGTCACAAAAGT.......... 214
...GTCATTTTTGTGATGTTGCAG.. 67
....TGCATAGTCACAAAAGTGATCAT.... 49
....TTGCATAGTCACAAAAG........... 45
...GTCATTTTTGTGATGTTGCAGCTA... 31
...GTCATTTTTGTGATGTTGCAGC... 21
....TGCATAGTCACAAAAGTGAT....... 18
......ATTTTTGTGATGTTGCAGCT.. 17
....TGCATAGTCACAAAAGTG......... 17
....TGCATAGTCACAAAAGTGATCA..... 15
.......TTTTTGTGATGTTGCAGCT.. 9
....TCATTTTTGTGATGTTGCAGCT.. 5
....TGCATAGTCACAAAAGTGA........ 4
....TTGCATAGTCACAAAAGTGATCATT... 4
...GTCATTTTTGTGATGTTGC.. 2
....TTGCATAGTCACAAAA............ 2
....CATAGTCACAAAAGTGATC...... 2
....ATAGTCACAAAAGTGATC...... 2
....TAGTCACAAAAGTGATC...... 2
....CATAGTCACAAAAGTGATCAT.... 2
...GTCATTTTTGTGATGTTGCAGCTAG.. 1
....TGCATAGTCACAAAAG........... 1
....GTTGCATAGTCACAAAAGTGAT....... 1
....GCATAGTCACAAAAGTGATC...... 1
....AGTCACAAAAGTGATCA..... 1
....CATAGTCACAAAAGTGATCATT... 1
(((((((((((((((((..(((((((.((.......))..)))))))..))))))))))))).))))... (-27.10)

16/23958959-23959031(+)_mir_Dog_Blast_5
GAGGGATCCTGTACTGAGCTGCCCCGAGGCCATTCACTGCGCTCAGCTCGGGGCAGCTCAGTACAGGATGCGT
......TCCTGTACTGAGCTGCCCCGAG...
956039
......TCCTGTACTGAGCTGCCCCGA..
771167
......TCCTGTACTGAGCTGCCCCG...
24976
......TCCTGTACTGAGCTGCCCCGAGG..
6914
....CGGGGCAGCTCAGTACAGGATG...
5520
....CGGGGCAGCTCAGTACAGGAT....
4135
........CTGTACTGAGCTGCCCCGAG...
1161
....CGGGGCAGCTCAGTACAGGA.....
1127
........CTGTACTGAGCTGCCCCGA.. 853
......TCCTGTACTGAGCTGCCCC.. 756
......TCCTGTACTGAGCTGCCC... 581
......TCCTGTACTGAGCTGCC.. 292
......TCCTGTACTGAGCTGC... 94
.......CCTGTACTGAGCTGCCCCGAG... 87
.......CCTGTACTGAGCTGCCCCGA.. 63
......TCCTGTACTGAGCTGCCCCGAGGC... 46
........CTGTACTGAGCTGCCCCG... 28

```
........CTGTACTGAGCTGCCCCGAGGC...........................................    25
.........TGTACTGAGCTGCCCCGA..............................................    19
........CTGTACTGAGCTGCCCCGAGG............................................    19
.........TGTACTGAGCTGCCCCGAG.............................................    17
.................................................GGGGCAGCTCAGTACAGGATG...    15
................................................CGGGGCAGCTCAGTACAGG......    14
...............................................TCGGGGCAGCTCAGTACAGGA.....    11
.....ATCCTGTACTGAGCTGCCCCGA..............................................    10
..........GTACTGAGCTGCCCCGA..............................................    7
..........GTACTGAGCTGCCCCGAG.............................................    6
...............................................TCGGGGCAGCTCAGTACAGGAT....    6
....GATCCTGTACTGAGCTGCCCCGA..............................................    5
...........TACTGAGCTGCCCCGA..............................................    5
...........TACTGAGCTGCCCCGAG.............................................    5
............ACTGAGCTGCCCCGAG.............................................    5
......TCCTGTACTGAGCTGCCCCGAGGCC..........................................    4
.................................................GGGGCAGCTCAGTACAGGAT....    4
..................................................GGGCAGCTCAGTACAGGATG...    4
................................................CGGGGCAGCTCAGTACAGGATGC..    4
.......CCTGTACTGAGCTGCCCCG...............................................    3
......TCCTGTACTGAGCTGCCCCGAGGCCA.........................................    3
................................................CGGGGCAGCTCAGTACAG.......    3
.............................................CTCGGGGCAGCTCAGTACAGGAT....    3
...............................................TCGGGGCAGCTCAGTACAGGATG...    3
...................................................GGCAGCTCAGTACAGGATG...    3
.....ATCCTGTACTGAGCTGCCCCGAG.............................................    2
.........TGTACTGAGCTGCCCCGAGG............................................    2
.......CCTGTACTGAGCTGCCCCGAGGC...........................................    2
.............................................CTCGGGGCAGCTCAGTACAGGA.....    2
...................................................GGCAGCTCAGTACAGGAT....    2
.....................................................CAGCTCAGTACAGGAT....    2
....................................................GCAGCTCAGTACAGGATG...    2
......................................................AGCTCAGTACAGGATG...    2
........CTGTACTGAGCTGCCCC................................................    1
....GATCCTGTACTGAGCTGCCCCG...............................................    1
.......CCTGTACTGAGCTGCCCCGAGG............................................    1
........CTGTACTGAGCTGCCCCGAGGCCA.........................................    1
...........TACTGAGCTGCCCCGAGGCCAT........................................    1
.....ATCCTGTACTGAGCTGCCCCGAGGCCATTCA.....................................    1
................................................CGGGGCAGCTCAGTAC.........    1
.............................................CTCGGGGCAGCTCAGTACAGG......    1
.................................................GGGGCAGCTCAGTACAGGA.....    1
...(.(((((((((((((((((((((((((((.....)).))....))))))))))))))))))))))).).. (-
52.00)

16/23958963-23959026(-)_mir_dog_58
TCCTGTACTGAGCTGCCCCGAGCTGAGCGCAGTGAATGGCCTCGGGGCAGCTCAGTACAGGATC
TCCTGTACTGAGCTGCCCCGAG.......................................... 956039
TCCTGTACTGAGCTGCCCCGA........................................... 771167
TCCTGTACTGAGCTGCCCCG............................................ 24976
TCCTGTACTGAGCTGCCCCGAGC......................................... 7125
..........................................CGGGGCAGCTCAGTACAGGAT. 4135
..CTGTACTGAGCTGCCCCGAG.......................................... 1161
..........................................CGGGGCAGCTCAGTACAGGA.. 1127
..CTGTACTGAGCTGCCCCGA........................................... 853
TCCTGTACTGAGCTGCCCC............................................. 756
TCCTGTACTGAGCTGCCC.............................................. 581
TCCTGTACTGAGCTGCCCCGAGCT........................................ 381
TCCTGTACTGAGCTGCC............................................... 292
TCCTGTACTGAGCTGC................................................ 94
.CCTGTACTGAGCTGCCCCGAG.......................................... 87
.CCTGTACTGAGCTGCCCCGA........................................... 63
..........................................CGGGGCAGCTCAGTACAGGATC 51
..CTGTACTGAGCTGCCCCG............................................ 28
```

```
...TGTACTGAGCTGCCCCGA........................................... 19
...TGTACTGAGCTGCCCCGAG.......................................... 17
..........................................CGGGGCAGCTCAGTACAGG... 14
.........................................TCGGGGCAGCTCAGTACAGGA.. 11
....GTACTGAGCTGCCCCGA........................................... 7
....GTACTGAGCTGCCCCGAG.......................................... 6
.........................................TCGGGGCAGCTCAGTACAGGAT. 6
.....TACTGAGCTGCCCCGA........................................... 5
.....TACTGAGCTGCCCCGAG.......................................... 5
......ACTGAGCTGCCCCGAG.......................................... 5
..CTGTACTGAGCTGCCCCGAGC......................................... 5
...........................................GGGGCAGCTCAGTACAGGAT. 4
.CCTGTACTGAGCTGCCCCG............................................ 3
..........................................CGGGGCAGCTCAGTACAG.... 3
........................................CTCGGGGCAGCTCAGTACAGGAT. 3
........................................CTCGGGGCAGCTCAGTACAGGA.. 2
.............................................GGCAGCTCAGTACAGGAT. 2
...............................................CAGCTCAGTACAGGAT. 2
..CTGTACTGAGCTGCCCC............................................. 1
..........................................CGGGGCAGCTCAGTAC...... 1
........................................CTCGGGGCAGCTCAGTACAGG... 1
...........................................GGGGCAGCTCAGTACAGGA.. 1
((((((((((((((((((((((....((.((.....)))))))))))))))))))))))))).. (-49.10)

16/38173722-38173783(-)_mir_dog_59
AGATCAGAAGGTGATTGTGGCTTTGGGTGGATATTAATCAGCCACAGCACTGCCTGGTCAGA
AGATCAGAAGGTGATTGTGGCT........................................   29908
AGATCAGAAGGTGATTGTGGCTT.......................................   1466
AGATCAGAAGGTGATTGTGGC.........................................   221
AGATCAGAAGGTGATTGTGGCTTT......................................   195
.........................................CCACAGCACTGCCTGGTCAGA   145
.........................................CCACAGCACTGCCTGGTCAG.   101
AGATCAGAAGGTGATTGTGG..........................................   48
...TCAGAAGGTGATTGTGGCT........................................   19
AGATCAGAAGGTGATTG.............................................   17
..........................................CACAGCACTGCCTGGTCAGA   15
....CAGAAGGTGATTGTGGCT........................................   13
.....AGAAGGTGATTGTGGCT........................................   12
......GAAGGTGATTGTGGCT........................................   8
AGATCAGAAGGTGATTGTG...........................................   7
AGATCAGAAGGTGATTGT............................................   6
..........................................CACAGCACTGCCTGGTCAG.   4
..ATCAGAAGGTGATTGTGGCT........................................   3
.........................................CCACAGCACTGCCTGGTC...   3
AGATCAGAAGGTGATT..............................................   1
.GATCAGAAGGTGATTGTGGCT........................................   1
...TCAGAAGGTGATTGTGGCTT.......................................   1
.........................................CCACAGCACTGCCTGGTCA..   1
.((((((..((((.((((((((...(((........)))))))))))))))..))))))... (-26.50)

16/55440361-55440434(-)_mir_dog_60
AGTACAGGTGGCTCATTTAGAAGCAGCCATATTTCATACTGTGTGGCTGCCTATAAATGGGTTGCCCTGTACTC
...........................................TGGCTGCCTATAAATGGGTTG..........   77
........TGGCTCATTTAGAAGCAGC...............................................   60
........TGGCTCATTTAGAAGCAGCC..............................................   31
.............................................GCTGCCTATAAATGGGTTG..........   9
..............................................CTGCCTATAAATGGGTTGCCC.......   8
........TGGCTCATTTAGAAGCAGCCA.............................................   6
...........................................TGGCTGCCTATAAATGGGTTGC.........   4
..............................................CTGCCTATAAATGGGTTGCCCT......   4
..............................................CTGCCTATAAATGGGTTGCC........   2
............TCATTTAGAAGCAGCC..............................................   1
........TGGCTCATTTAGAAGCAGCCAT............................................   1
.............................TATTTCATACTGTGTGGCTGCC.......................   1
```

```
....................................TACTGTGTGGCTGCCTATAAAT................   1
...........................................TGGCTGCCTATAAATGGGT............   1
.............................................GCTGCCTATAAATGGGTTGC.........   1
((((((((..(((((((((...((((((((((........))))))))))...)))))))))..).))))))). (-
41.20)

16/8046087-8046151(+)_novel_Dog_Blast_14
GTCTTGGCGCGACTTCCTCGACTCACTCTCTTTCCCCCATGAGTCGTGGAACCTCGCCCGAGGGT
.......................................TGAGTCGTGGAACCTCGCCCGA....      83
.........................................AGTCGTGGAACCTCGCCCGAG...      9
.........................................AGTCGTGGAACCTCGCCCGA....      6
.........................................AGTCGTGGAACCTCGCCCGAGG..      5
...TTGGCGCGACTTCCTCGACTC.........................................      4
....TGGCGCGACTTCCTCGACT..........................................      3
....TGGCGCGACTTCCTCGACTCACT......................................      3
.......................................TGAGTCGTGGAACCTCGCCC......      3
.......................................TGAGTCGTGGAACCTCGCCCGAG...      3
....TGGCGCGACTTCCTCGACTCA........................................      2
.......................................TGAGTCGTGGAACCTCGCCCG.....      2
...TTGGCGCGACTTCCTCGACT..........................................      1
....TGGCGCGACTTCCTCGACTC.........................................      1
....TGGCGCGACTTCCTCGACTCAC.......................................      1
...TTGGCGCGACTTCCTCGACTCACT......................................      1
.......CGCGACTTCCTCGACTCACT......................................      1
........................................GAGTCGTGGAACCTCGCCCGA....      1
.((((((.((((.((((.(((((((..............))))))).))))..)))))))))).. (-27.54)

1/71677608-71677669(-)_mir_dog_61
GGTGCCTACTGAGCTGATATCAGTTCTCATTGTACACACTGGCTCAGTTCAGCAGGAACAGG
.......................................TGGCTCAGTTCAGCAGGAAC...   38477
.......................................TGGCTCAGTTCAGCAGGAACA..   14391
.......................................TGGCTCAGTTCAGCAGGAACAG.   8029
......................................CTGGCTCAGTTCAGCAGGAAC...   3022
.......................................TGGCTCAGTTCAGCAGGAA....   468
.GTGCCTACTGAGCTGATATC.........................................   247
......................................CTGGCTCAGTTCAGCAGGAACA..   229
.......................................TGGCTCAGTTCAGCAGGAACAGG   175
.......................................TGGCTCAGTTCAGCAGGA.....   136
.GTGCCTACTGAGCTGATATCAGT......................................   126
.GTGCCTACTGAGCTGATATCA........................................   83
.GTGCCTACTGAGCTGATATCAG.......................................   71
..TGCCTACTGAGCTGATATCAGT......................................   53
GGTGCCTACTGAGCTGATATCA........................................   36
GGTGCCTACTGAGCTGATATC.........................................   33
..TGCCTACTGAGCTGATATC.........................................   28
.......................................TGGCTCAGTTCAGCAGG......   28
..TGCCTACTGAGCTGATATCA........................................   20
........................................GGCTCAGTTCAGCAGGAACA..   18
........................................GGCTCAGTTCAGCAGGAAC...   17
.........................................GCTCAGTTCAGCAGGAACAG.   16
........................................GGCTCAGTTCAGCAGGAACAG.   14
..........................................CTCAGTTCAGCAGGAAC...   13
..........................................CTCAGTTCAGCAGGAACA..   13
......................................CTGGCTCAGTTCAGCAGGA.....   12
..TGCCTACTGAGCTGATATCAG.......................................   11
.........................................GCTCAGTTCAGCAGGAAC...   11
......................................CTGGCTCAGTTCAGCAGGAACAG.   11
.........................................GCTCAGTTCAGCAGGAACA..   10
GGTGCCTACTGAGCTGATATCAG.......................................   7
.........................................GCTCAGTTCAGCAGGAACAGG   6
........................................GGCTCAGTTCAGCAGGAACAGG   5
GGTGCCTACTGAGCTGATAT..........................................   4
GGTGCCTACTGAGCTGATATCAGT......................................   4
..........................................CTCAGTTCAGCAGGAACAG.   3
```

```
.GTGCCTACTGAGCTGATATCAGTTCTC..................................   2
.......................................TGGCTCAGTTCAGCAG.......   2
..........................................CTCAGTTCAGCAGGAA....   2
...........................................TCAGTTCAGCAGGAACA..   2
............................................CAGTTCAGCAGGAACA..   2
........................TCTCATTGTACACACTGGCTCAGTTCAGCAGGAACAG.   2
............................................CAGTTCAGCAGGAACAG.   2
.GTGCCTACTGAGCTGATA...........................................   1
.GTGCCTACTGAGCTGATAT..........................................   1
....CCTACTGAGCTGATAT..........................................   1
.......ACTGAGCTGATATCAGT.......................................   1
........CTGAGCTGATATCAGT.......................................   1
.............CTGATATCAGTTCTCATTGTAC...........................   1
.......................TTCTCATTGTACACACTGGCTC.................   1
.............................TTGTACACACTGGCTCAG...............   1
......................................CTGGCTCAGTTCAGCAGGAA....   1
........................................GGCTCAGTTCAGCAGGAA....   1
...........................................TCAGTTCAGCAGGAAC...   1
...........................................TCAGTTCAGCAGGAACAG.   1
......................................CTGGCTCAGTTCAGCAGGAACAGG   1
.((.(((.(((((((((..(((((.............))))).))))))))).))).))... (-20.62)

1/71677609-71677673(+)_mir_dog_62
CTGTTCCTGCTGAACTGAGCCAGTGTGTACAATGAGAACTGATATCAGCTCAGTAGGCACCGGAG
CTGTTCCTGCTGAACTGAGCCA...........................................      4
...TTCCTGCTGAACTGAGCCAGT.........................................      3
.TGTTCCTGCTGAACTGAGCCA...........................................      1
.TGTTCCTGCTGAACTGAGCCAG..........................................      1
.......................................TGATATCAGCTCAGTAGGCACCG...      1
(((..((((((((.((((..((((.............))))...)))).))))))))...))).. (-22.42)

1/71678117-71678179(-)_mir_dog_63
AGAGCTTAGCTGATTGGTGAACAGTGACTGGTTTCCGCTTTGTTCACAGTGGCTAAGTTCTGC
..........................................TTCACAGTGGCTAAGTTCTG.  1422415
..........................................TTCACAGTGGCTAAGTTCTGC  1152612
..........................................TTCACAGTGGCTAAGTTCT..  69063
..........................................TTCACAGTGGCTAAGTTC...  7929
.........................................GTTCACAGTGGCTAAGTTCTG.  2852
AGAGCTTAGCTGATTGGTGAAC.........................................  2655
.........................................GTTCACAGTGGCTAAGTTCTGC  1830
..............................................CAGTGGCTAAGTTCTG.  1776
...........................................TCACAGTGGCTAAGTTCTG.  574
.............................................ACAGTGGCTAAGTTCTG.  492
...........................................TCACAGTGGCTAAGTTCTGC  347
............................................CACAGTGGCTAAGTTCTG.  230
...............................................AGTGGCTAAGTTCTGC  219
..........................................TTCACAGTGGCTAAGTT....  179
.........................................GTTCACAGTGGCTAAGTTCT..  175
..........................................TTCACAGTGGCTAAGT.....  150
............................................CACAGTGGCTAAGTTCTGC  149
AGAGCTTAGCTGATTGGTGAACA........................................  93
..............................................CAGTGGCTAAGTTCTGC  51
AGAGCTTAGCTGATTGGTGAACAGT......................................  42
AGAGCTTAGCTGATTGGTGA...........................................  39
.............................................ACAGTGGCTAAGTTCTGC  37
AGAGCTTAGCTGATTGGTG............................................  25
...........................................TCACAGTGGCTAAGTTCT..  23
AGAGCTTAGCTGATTGGTGAACAG.......................................  22
AGAGCTTAGCTGATTGGTGAA..........................................  18
.........................................GTTCACAGTGGCTAAGTTC...  14
............................................CACAGTGGCTAAGTTCT..  11
.......................................TTGTTCACAGTGGCTAAGTTCTG.  5
AGAGCTTAGCTGATTGGTGAACAGTGACT..................................  4
.......................................TTGTTCACAGTGGCTAAGTTCT..  3
```

```
........................................TGTTCACAGTGGCTAAGTTCTG.  3
............................................CACAGTGGCTAAGTTC...  2
AGAGCTTAGCTGATTGG..............................................  1
..AGCTTAGCTGATTGGTGAAC.........................................  1
...GCTTAGCTGATTGGTGAAC.........................................  1
..AGCTTAGCTGATTGGTGAACAGT......................................  1
.....TTAGCTGATTGGTGAACAGTGACT..................................  1
..........TGATTGGTGAACAGTGACT..................................  1
..............................GTTTCCGCTTTGTTCAC................  1
.........................................GTTCACAGTGGCTAAGTT....  1
.......................................TTGTTCACAGTGGCTAAGTTC...  1
...........................................TCACAGTGGCTAAGTTC...  1
......................................TTTGTTCACAGTGGCTAAGTTCT..  1
((((((((((((....((((((((....(((...)))..))))))))..)))))))))))).. (-25.70)

1/71678353-71678406(-)_mir_dog_64
GTTCCTGGCATGCTGATTTGTGACTTAAGATTAAAATCACATTGCCAGGGATTA
...................................ATCACATTGCCAGGGATT.      1077
...................................ATCACATTGCCAGGGATTA      332
...................................ATCACATTGCCAGGGAT..      208
...................................ATCACATTGCCAGGGA...      18
..................................AATCACATTGCCAGGGAT..      18
..................................AATCACATTGCCAGGGATT.      15
....................................TCACATTGCCAGGGATTA      8
....................................TCACATTGCCAGGGATT.      6
....................................TCACATTGCCAGGGAT..      3
GTTCCTGGCATGCTGATTTG..................................      1
.........ATGCTGATTTGTGACTTAAGA........................      1
..................................AATCACATTGCCAGGGATTA      1
((((((((((((.((((((..............)))))))).)))))))))).. (-19.84)

17/19575828-19575902(-)_mir_dog_65
CTTGTGGGGGGTCACTCTGGGCACCGCAACACTGTGCTAGGGATGTTGCAGCTGCCTGGGAGTGACTTCACACAG
.............................................TTGCAGCTGCCTGGGAGTGACT........  341
.............................................TTGCAGCTGCCTGGGAGTGACTTC......  205
.............................................TTGCAGCTGCCTGGGAGTGAC.........  186
.............................................TTGCAGCTGCCTGGGAGTGACTT.......  183
................................................CAGCTGCCTGGGAGTGACTTCA.....  77
..........GTCACTCTGGGCACCGCAACA............................................  68
.............................................TTGCAGCTGCCTGGGAGTGA..........  65
.............................................TTGCAGCTGCCTGGGAGTGACTTCA.....  48
..............................................TGCAGCTGCCTGGGAGTGACTTC......  45
.........GGTCACTCTGGGCACCGCAAC.............................................  41
..............................................TGCAGCTGCCTGGGAGTGACT........  26
..........GTCACTCTGGGCACCGCAAC.............................................  24
.........GGTCACTCTGGGCACCGCAACA............................................  18
..............................................TGCAGCTGCCTGGGAGTGACTTCA.....  14
................................................CAGCTGCCTGGGAGTGACTTC......  12
..............................................TGCAGCTGCCTGGGAGTGACTT.......  10
.........GGTCACTCTGGGCACCGCAA..............................................  9
........GGGTCACTCTGGGCACCGCAA..............................................  8
..............................................TGCAGCTGCCTGGGAGTGA..........  8
..........GTCACTCTGGGCACCGCAA..............................................  7
........GGGTCACTCTGGGCACCGCAAC.............................................  7
..............................................TGCAGCTGCCTGGGAGTGAC.........  7
.............................................TTGCAGCTGCCTGGGAGTG...........  5
................................................CAGCTGCCTGGGAGTGACT........  5
........GGGTCACTCTGGGCACCGCAACA............................................  3
........GGGTCACTCTGGGCACCGC................................................  2
.............................................TTGCAGCTGCCTGGGAGT............  2
.........GGTCACTCTGGGCACCGC................................................  1
.........GGTCACTCTGGGCACCGCA...............................................  1
.......GGGGTCACTCTGGGCACCGCAACACTGTGCT.....................................  1
..........GTCACTCTGGGCACCGCAACACTGTGCT.....................................  1
```

```
......................................AGGGATGTTGCAGCTGCCT..................  1
................................................CAGCTGCCTGGGAGTGACTT.......  1
...............................................GCAGCTGCCTGGGAGTGACTTCA.....  1
..((((.((((((((((..(((((.((((((((.......)).)))))).).))))..).))))))))).)))). (-
36.80)

17/33731860-33731925(+)_novel_Dog_Blast_15
GTCTTGGGGCGACTTCCTCGACTCACTCTCTTTCCCCCCGTGAGTCGTGGAACCTCGCCCGAGGGT
........................................TGAGTCGTGGAACCTCGCCCGA....     83
..........................................AGTCGTGGAACCTCGCCCGAG...     9
..........................................AGTCGTGGAACCTCGCCCGA....     6
..........................................AGTCGTGGAACCTCGCCCGAGG..     5
........................................TGAGTCGTGGAACCTCGCCC......     3
........................................TGAGTCGTGGAACCTCGCCCGAG...     3
........................................TGAGTCGTGGAACCTCGCCCG.....     2
....TGGGGCGACTTCCTCGACTC..........................................     1
.........................................GAGTCGTGGAACCTCGCCCGA....     1
...................................CCCCGTGAGTCGTGGAACCTCGCCCGAGG..     1
....................................CCCGTGAGTCGTGGAACCTCGCCCGAGG..     1
......................................CGTGAGTCGTGGAACCTCGCCCGAGG..     1
.......................................GTGAGTCGTGGAACCTCGCCCGAGGGT     1
.((((.((((((.((((.((((((((.............)))))))).))))..)))))).)))). (-33.62)

1/75509765-75509828(+)_mir_dog_66
TGGAAGACTAGTGATTTTGTTGTTTTTAGATAACTAAATCGACAACAAATCACAGTCTGCCATA
TGGAAGACTAGTGATTTTGTTGTT........................................ 25595
TGGAAGACTAGTGATTTTGTTGT......................................... 23667
TGGAAGACTAGTGATTTTGTTG.......................................... 2344
..........................................CAACAAATCACAGTCTGCCATA 955
TGGAAGACTAGTGATTTTGTT........................................... 749
TGGAAGACTAGTGATTTTGT............................................ 368
..........................................CAACAAATCACAGTCTGCCAT. 279
..........................................CAACAAATCACAGTCTGCCA.. 273
...........................................AACAAATCACAGTCTGCCATA 262
..GAAGACTAGTGATTTTGTTGTT........................................ 260
..........................................CAACAAATCACAGTCTGCC... 237
TGGAAGACTAGTGATTTTGTTGTTT....................................... 210
...AAGACTAGTGATTTTGTTGTT........................................ 185
.......CTAGTGATTTTGTTGTT........................................ 131
..GAAGACTAGTGATTTTGTTGT......................................... 106
....AGACTAGTGATTTTGTTGTT........................................ 76
...AAGACTAGTGATTTTGTTGT......................................... 75
.GGAAGACTAGTGATTTTGTTGTT........................................ 72
......ACTAGTGATTTTGTTGTT........................................ 61
...........................................AACAAATCACAGTCTGCCAT. 47
.......CTAGTGATTTTGTTGT......................................... 44
TGGAAGACTAGTGATTTTG............................................. 38
......ACTAGTGATTTTGTTGT......................................... 36
...........................................AACAAATCACAGTCTGCCA.. 36
TGGAAGACTAGTGATTTT.............................................. 29
....AGACTAGTGATTTTGTTGT......................................... 29
..........................................CAACAAATCACAGTCTGC.... 24
.GGAAGACTAGTGATTTTGTTGT......................................... 21
........TAGTGATTTTGTTGTT........................................ 18
.....GACTAGTGATTTTGTTGT......................................... 12
..GAAGACTAGTGATTTTGTT........................................... 11
..GAAGACTAGTGATTTTGT............................................ 9
TGGAAGACTAGTGATTT............................................... 8
.....GACTAGTGATTTTGTTGTT........................................ 7
TGGAAGACTAGTGATTTTGTTGTTTT...................................... 7
...AAGACTAGTGATTTTGTT........................................... 6
.....GACTAGTGATTTTGTTG.......................................... 6
...........................................AACAAATCACAGTCTGCC... 6
..GAAGACTAGTGATTTTGTTG.......................................... 5
```

```
...AAGACTAGTGATTTTGTTG.......................................... 5
..GAAGACTAGTGATTTTGTTGTTT....................................... 4
TGGAAGACTAGTGATT................................................ 3
....AGACTAGTGATTTTGT............................................ 3
....AGACTAGTGATTTTGTTG.......................................... 3
..GAAGACTAGTGATTTT.............................................. 2
.GGAAGACTAGTGATTTTGTT........................................... 2
.GGAAGACTAGTGATTTTGTTG.......................................... 2
......ACTAGTGATTTTGTTG.......................................... 2
...AAGACTAGTGATTTTGTTGTTT....................................... 2
TGGAAGACTAGTGATTTTGTTGTTTTT..................................... 2
................................ACTAAATCGACAACAAATCACAGT........ 2
......................................TCGACAACAAATCACAGTCTGCC... 2
...AAGACTAGTGATTTTG............................................. 1
.GGAAGACTAGTGATTTTGT............................................ 1
...AAGACTAGTGATTTTGT............................................ 1
....AGACTAGTGATTTTGTT........................................... 1
.....GACTAGTGATTTTGTTGTTT....................................... 1
.......CTAGTGATTTTGTTGTTT....................................... 1
........TAGTGATTTTGTTGTTT....................................... 1
.........AGTGATTTTGTTGTTT....................................... 1
..GAAGACTAGTGATTTTGTTGTTTT...................................... 1
.........AGTGATTTTGTTGTTTTTAGAT................................. 1
TGGAAGACTAGTGATTTTGTTGTTTTTAGATA................................ 1
..............................TAACTAAATCGACAACAAATC............. 1
....................................AATCGACAACAAATCACAGTCTGC.... 1
............................................ACAAATCACAGTCTGCCATA 1
(((.(((((.(((((((.(((((((((((....)))))..)))))))))))))))))).))).. (-25.00)

18/12667547-12667614(+)_novel_dog_26
GTCAAGGGGGGAGGATGAGGGCCAGTTGGCGTGTCGCAGCTTCTGACCTCTCTCCCTCCCTGCAGCTT
.........................................TCTGACCTCTCTCCCTCCCTGCA....   17
.........................................TCTGACCTCTCTCCCTCCCTGC.....   13
.........................................TCTGACCTCTCTCCCTCCCTGCAG...   7
........................................TTCTGACCTCTCTCCCTCCCTGC.....   4
...AAGGGGGGAGGATGAGGGCCAGTTG........................................   1
.........................TTGGCGTGTCGCAGCTTCTGACCTCTCTC..............   1
.........................................TCTGACCTCTCTCCCTCCCT.......   1
.........................................TCTGACCTCTCTCCCTCCCTG......   1
........................................TTCTGACCTCTCTCCCTCCCTGCA....   1
((..(((((((..((.((((..(((..(((........))).))).))))))..)))))))...)).. (-30.50)

18/25675466-25675532(-)_mir_dog_67
GCAGCCACTGCCCATCGCACACTGCGCTGCTCCGGACCCACTGTGCGTGTGACAGCGGCTGATCTGT
........................................CTGTGCGTGTGACAGCGGCTGA.....    1298
..AGCCACTGCCCATCGCACACTG...........................................    896
........................................CTGTGCGTGTGACAGCGGCTGAT....    225
........................................CTGTGCGTGTGACAGCGGCT.......    149
........................................CTGTGCGTGTGACAGCGGCTG......    109
........................................CTGTGCGTGTGACAGCGGC........    29
.......................................ACTGTGCGTGTGACAGCGGCT.......    23
.........................................TGTGCGTGTGACAGCGGCTGA.....    10
.......................................ACTGTGCGTGTGACAGCGGCTG......    7
...GCCACTGCCCATCGCACACTG...........................................    5
.........................................TGTGCGTGTGACAGCGGCTGAT....    5
..AGCCACTGCCCATCGCACACT............................................    4
..AGCCACTGCCCATCGCACAC.............................................    2
.CAGCCACTGCCCATCGCACACTG...........................................    2
........................................CTGTGCGTGTGACAGCG..........    2
.........................................TGTGCGTGTGACAGCGGCT.......    2
.......................................ACTGTGCGTGTGACAGCGGCTGA.....    2
.......CTGCCCATCGCACACTG...........................................    1
..AGCCACTGCCCATCGCACACTGC..........................................    1
........................................CTGTGCGTGTGACAGC...........    1
```

....TGCGTGTGACAGCGGCT....... 1
....ACTGTGCGTGTGACAGCGGCTGAT.... 1
....CTGTGCGTGTGACAGCGGCTGATCT.. 1
.(((((.((((.((.((((((.((.((((...))).).)).)))))))).).))).)))))...... (-25.90)

18/26154534-26154596(-)_mir_dog_68
CTTTTTGCGGTCTGGGCTTGCTGTACATAACTCGATAGCCGGAAGCCCTTACCCCAAAAAGCA
CTTTTTGCGGTCTGGGCTTGC.. 54322
CTTTTTGCGGTCTGGGCTTGCT... 3412
....AAGCCCTTACCCCAAAAAGC. 2294
CTTTTTGCGGTCTGGGCTTG... 1812
....AAGCCCTTACCCCAAAAAGCA 1247
CTTTTTGCGGTCTGGGCTT.. 518
....AAGCCCTTACCCCAAAAAG.. 236
CTTTTTGCGGTCTGGGCT... 187
CTTTTTGCGGTCTGGGCTTGCTGT....................................... 166
CTTTTTGCGGTCTGGGCTTGCTG.. 126
.TTTTTGCGGTCTGGGCTTGC.. 52
.TTTTTGCGGTCTGGGCTTGCT... 36
CTTTTTGCGGTCTGGGC.. 20
.TTTTTGCGGTCTGGGCTTGCTGT....................................... 18
.TTTTTGCGGTCTGGGCTTGCTG.. 13
.....................TGTACATAACTCGATAGCCGG..................... 13
....AAGCCCTTACCCCAAAAA... 12
....GAAGCCCTTACCCCAAAAAGC. 12
....GGAAGCCCTTACCCCAAAAAGC. 9
....AAGCCCTTACCCCAAAA.... 7
....TTGCGGTCTGGGCTTGC.. 6
CTTTTTGCGGTCTGGGCTTGCTGTA...................................... 6
.....TGCGGTCTGGGCTTGC.. 5
.....................TGTACATAACTCGATAGCCGGAA................... 5
....GAAGCCCTTACCCCAAAAAG.. 5
....AGCCCTTACCCCAAAAAGCA 5
CTTTTTGCGGTCTGGG... 3
..TTTTGCGGTCTGGGCTTGCTGT....................................... 3
....GGAAGCCCTTACCCCAAAAAG.. 3
....AGCCCTTACCCCAAAAAGC. 3
....GAAGCCCTTACCCCAAAAAGCA 3
..TTTTGCGGTCTGGGCTTGC.. 2
..TTTTGCGGTCTGGGCTTGCT... 2
....TTGCGGTCTGGGCTTGCT... 2
.....................TGTACATAACTCGATAGCC....................... 2
.....................TGTACATAACTCGATAGCCGGA.................... 2
.....................TGTACATAACTCGATAGCCGGAAGCCCTT............. 2
....AAGCCCTTACCCCAAA..... 2
.TTTTTGCGGTCTGGGCTT.. 1
...TTTGCGGTCTGGGCTT.. 1
...TTTGCGGTCTGGGCTTGC.. 1
..TTTTGCGGTCTGGGCTTGCTG.. 1
..........TCTGGGCTTGCTGTACATAACTC.............................. 1
.....................TGTACATAACTCGATAGCCG...................... 1
.....................TGTACATAACTCGATAGCCGGAAGCC................ 1
.....................TGTACATAACTCGATAGCCGGAAGCCCT.............. 1
.......................TACATAACTCGATAGCCGGAAGCCCT.............. 1
.........................CATAACTCGATAGCCGGAAGCCCT.............. 1
.....................TGTACATAACTCGATAGCCGGAAGCCCTTACCCCA....... 1
....GGAAGCCCTTACCCCAAAAA... 1
....GAAGCCCTTACCCCAAAAA... 1
....CCTTACCCCAAAAAGC. 1
(((((((.(((..(((((((((((.(.......))))))...))))))..))).))))))).. (-23.70)

18/32990114-32990188(-)_mir_dog_69
GCTTCGGTGCTGGGGAGCGGCCCCCGGGCGGGCCTCTGCTCTGGCCCCTCCTGGGGCCCGCACTCTCGCTCCGGG
....CTCCTGGGGCCCGCACTCTCGC......

9504
....CTCCTGGGGCCCGCACTCTCG.......
2233
....CTCCTGGGGCCCGCACTCTCGCT.....
2004
....CTCCTGGGGCCCGCACTCT......... 269
....CTCCTGGGGCCCGCACTCTC........ 226
....CCTCCTGGGGCCCGCACTCTCGC...... 226
....CCTCCTGGGGCCCGCACTCTCG....... 114
....CCTCCTGGGGCCCGCACTCTCGCT..... 81
....CTCCTGGGGCCCGCACTC.......... 74
..........TGGGGAGCGGCCCCCGGGCGGG... 38
..........TGGGGAGCGGCCCCCGGGCGG.. 17
....CCTCCTGGGGCCCGCACTC.......... 10
....TCCTGGGGCCCGCACTCTCGC...... 9
....CCTCCTGGGGCCCGCACTCTC........ 7
....CCTCCTGGGGCCCGCACTCT......... 6
....TCCTGGGGCCCGCACTCTCG....... 6
..........TGGGGAGCGGCCCCCGGGC.. 5
....GGGGCCCGCACTCTCGC...... 5
..........TGGGGAGCGGCCCCCGGGCGGGC.. 3
....CTCCTGGGGCCCGCACT........... 3
..........TGGGGAGCGGCCCCCGGGCG... 2
....CTGGGGCCCGCACTCTCGC...... 2
....TCCTGGGGCCCGCACTCTCGCT..... 2
....GGGGCCCGCACTCTCGCT..... 2
..........TGGGGAGCGGCCCCCGGG... 1
....TGGGGCCCGCACTCTCGC...... 1
....CTGGGGCCCGCACTCTCGCT..... 1
...((((.((.((((.((((.(((((((.(((((........))))).)))))))).)))).)))).)).)))). (-48.50)

18/38543618-38543679(-)_mir_dog_70
GCTCTTTTCACATTGTGCTACTGTCTGCACCTACCACTAGCAGTGCAATGTTAAAAGGGCAT
....CAGTGCAATGTTAAAAGGGC.. 17636
....CAGTGCAATGTTAAAAGGGCA. 16239
....CAGTGCAATGTTAAAAGGGCAT 8102
....CAGTGCAATGTTAAAAGGG... 97
....GCAATGTTAAAAGGGCA. 53
....AGTGCAATGTTAAAAGGGCA. 34
GCTCTTTTCACATTGTGCTACT.. 30
....AGTGCAATGTTAAAAGGGC.. 27
....CAATGTTAAAAGGGCAT 13
....AGTGCAATGTTAAAAGGGCAT 12
....AATGTTAAAAGGGCAT 11
....GCAATGTTAAAAGGGCAT 9
GCTCTTTTCACATTGTGCTAC... 6
....GCAGTGCAATGTTAAAAGGGC.. 6
....GCAATGTTAAAAGGGC.. 5
....GCAGTGCAATGTTAAAAGGGCA. 5
....GTGCAATGTTAAAAGGGCAT 4
GCTCTTTTCACATTGTGCT... 2
GCTCTTTTCACATTGTGCTACTGTCTGCACCT.............................. 2
....GTGCAATGTTAAAAGGGC.. 2
....CAATGTTAAAAGGGCA. 2
....GCAGTGCAATGTTAAAAGGGCAT 2
....TGCAATGTTAAAAGGGCAT 2
GCTCTTTTCACATTGTGCTACTG....................................... 1
....CAGTGCAATGTTAAAAGG.... 1
....GTGCAATGTTAAAAGGGCA. 1
....TGCAATGTTAAAAGGGCA. 1
((((((((.((((((..((.(((..((.......)).))).))..)))))).)))))))).. (-22.70)

18/46298392-46298460(-)_mir_dog_71

GAGGACGGGAGGAAAGGAGGACGTGGTTTCATGCAGGTCCTCACTCCTCCCCTCCCGTCTTCCTCTTCC
.AGGACGGGAGGAAAGGAGGACGTGG... 3
....TCACTCCTCCCCTCCCGTCTTC....... 3
.AGGACGGGAGGAAAGGAGGACGTG.. 1
.AGGACGGGAGGAAAGGAGGACGTGGT.. 1
....TCACTCCTCCCCTCCCGTCTTCC...... 1
((((((((((((...((((((.((((..((.....))..).))))))))))))))))))))))....... (-37.30)

18/46690400-46690478(-)_mir_dog_72
CGGGATCCGGAGTCCGCCTCGCGGTCCAGCGTCGTGGATCCGAGTCGTGGATCGGAGCCGGGGTCCGGAGTCCGCCTCG
....TGGATCGGAGCCGGGGTCCGGA..........
589
....TGGATCGGAGCCGGGGTCCGG...........
85
....TGGATCGGAGCCGGGGTCC.............
13
....TGGATCGGAGCCGGGGTCCG............
12
.......CGGAGTCCGCCTCGCGGTCCAGC...
7
....GTGGATCGGAGCCGGGGTCCGGA..........
6
....TGGATCGGAGCCGGGGTCCGGAG.........
6
....TGGATCGGAGCCGGGGTCCGGAGT........
6
....TGGATCGGAGCCGGGGT...............
4
.......CGGAGTCCGCCTCGCGGTC...
3
.......CGGAGTCCGCCTCGCGGTCCAGCGT...
3
....TGGATCGGAGCCGGGGTC..............
3
.....TCCGGAGTCCGCCTCGCGGTCC..
2
.......CGGAGTCCGCCTCGCGGTCC..
2
.......CGGAGTCCGCCTCGCGGTCCAGCGTCGT..
2
.......CGGAGTCCGCCTCGCGGTCCAGCGTCGTGGATC.......................................
2
..........................CAGCGTCGTGGATCCGAG...................................
2
....TGGATCGGAGCCGGGG................
2
.......CGGAGTCCGCCTCGCGGTCCA...
1
.......CGGAGTCCGCCTCGCGGTCCAGCGTC..
1
....ATCGGAGCCGGGGTCC.............
1
....ATCGGAGCCGGGGTCCGG...........
1
...............................TCGTGGATCCGAGTCGTGGATCGGAGCCGGGGTCCGGA..........
1
....AGTCGTGGATCGGAGCCGGGGTCCGGA..........
1
....GATCGGAGCCGGGGTCCGGA..........
1
....ATCGGAGCCGGGGTCCGGA..........
1
....TCGGAGCCGGGGTCCGGA..........
1

....CGGAGCCGGGGTCCGGA..........
1
(((..((((((.((((.(((.(((((((.((.(.((....)).).)))))))))))).)))).))))))..))).....
(-43.40)

18/46690571-46690649(-)_mir_dog_73
GGGGGTCGGGTTCCGCGCCTGGAGTCCGCCTCGTGGTTCTGAGTCCGGGTCGGATTCCGTGCCTGGAGTCCGCCCCGCG
...........TCCGCGCCTGGAGTCCGCCTCG..
2561
....GTCGGATTCCGTGCCTGGAGT..........
1435
...........TCCGCGCCTGGAGTCCGCCT..
1137
...........TCCGCGCCTGGAGTCCGCCTCGT...
936
....GGTCGGATTCCGTGCCTGGAGT..........
657
...........TCCGCGCCTGGAGTCCGCCTC...
465
....GTCGGATTCCGTGCCTGGAG...........
77
....TCGGATTCCGTGCCTGGAGT..........
54
....GGTCGGATTCCGTGCCTGGAG...........
21
..........TTCCGCGCCTGGAGTCCGCCT..
18
...........TCCGCGCCTGGAGTCCGCC...
15
....GTCGGATTCCGTGCCTGGA............
11
.............CGCGCCTGGAGTCCGCCTCGT...
8
.............CGCGCCTGGAGTCCGCCTCG..
6
..........TTCCGCGCCTGGAGTCCGCCTC...
5
....GGTCGGATTCCGTGCCTGGA............
5
....GTCGGATTCCGTGCCTGG.............
4
....GTCGGATTCCGTGCCTGGAGTC.........
4
...........TCCGCGCCTGGAGTCCG...
2
....TCCGGGTCGGATTCCGTGCC................
2
....TCCGGGTCGGATTCCGTGCCT...............
2
....TCCGGGTCGGATTCCGTGCCTG..............
2
....TCGGATTCCGTGCCTGGAG...........
2
..........TTCCGCGCCTGGAGTCCGC..
1
...........TCCGCGCCTGGAGTCCGC..
1
.............CGCGCCTGGAGTCCGCCT..
1
.................CCTGGAGTCCGCCTCG..
1
...........TCCGCGCCTGGAGTCCGCCTCGTGGTTCT.......................................
1
.............CGCGCCTGGAGTCCGCCTCGTGGTTCTG......................................

```
      1
..............................TCGTGGTTCTGAGTCCGGGTCGGATTCCGTGCC................
      1
.............................................CGGGTCGGATTCCGTGCC................
      1
...............................................GGTCGGATTCCGTGCCTGG.............
      1
..............................................GGGTCGGATTCCGTGCCTGGA............
      1
.....................................................ATTCCGTGCCTGGAGT..........
      1
.................................................GTCGGATTCCGTGCCTGGAGTCC........
      1
.((((.(((..((((.((.(((((((((.((((.((.......)))))).))))))))).)).))))..)))))))...
(-45.60)

18/49798892-49798966(-)_novel_dog_28
TGGATTCCCAGGGGACATGGGCACATGAGAGGCCCTGCCACAGCCCCTCTGCCCACCTCCCCTGCAGAGTTTGTG
.............................................CCTCTGCCCACCTCCCCTGCAG........  14
............................................CCCTCTGCCCACCTCCCCTGCAG........  10
......CCCAGGGGACATGGGCACATGAGA.............................................  4
.......CCAGGGGACATGGGCACATGAGA.............................................  4
............................................CCCTCTGCCCACCTCCCCTGCA.........  4
............................................CCCTCTGCCCACCTCCCCTGC..........  2
.............................................CCTCTGCCCACCTCCCCTGCA.........  2
.......CCAGGGGACATGGGCACATGA...............................................  1
.....TCCCAGGGGACATGGGCACATGAG..............................................  1
.....TCCCAGGGGACATGGGCACATGAGA.............................................  1
.......CCAGGGGACATGGGCACATGAGAG............................................  1
............................................CCCTCTGCCCACCTCCCCT............  1
.............................................CCTCTGCCCACCTCCCCTGC..........  1
...............................................TCTGCCCACCTCCCCTGCAG........  1
............................................CCCTCTGCCCACCTCCCCTGCAGA.......  1
.............................................CCTCTGCCCACCTCCCCTGCAGA.......  1
..............................................CTCTGCCCACCTCCCCTGCAGA.......  1
.((((((.(((((((..((((((...(((.(((.........))).)))))))))..)))))))..))))))... (-
34.70)

18/52260736-52260810(+)_novel_dog_27
GGTAGGAGTAGGACTGGGGACTCAGTTGCTGACTAGACCTGCCTCTCCTGAGACCCTCTTCCTACTCGCAGGGCC
...AGGAGTAGGACTGGGGACTCAGT.................................................  22
..TAGGAGTAGGACTGGGGACTCAGT.................................................  15
..TAGGAGTAGGACTGGGGACTCAG..................................................  11
..TAGGAGTAGGACTGGGGACT.....................................................  9
..TAGGAGTAGGACTGGGGACTC....................................................  6
..TAGGAGTAGGACTGGGGACTCA...................................................  5
...AGGAGTAGGACTGGGGACTCAG..................................................  4
..................................................TGAGACCCTCTTCCTACTCGCAG....  4
....GGAGTAGGACTGGGGACTCAGT.................................................  3
...AGGAGTAGGACTGGGGACT.....................................................  2
...AGGAGTAGGACTGGGGACTCA...................................................  2
...AGGAGTAGGACTGGGGACTCAGTT................................................  2
..................................................TGAGACCCTCTTCCTACTCGCA.....  2
..TAGGAGTAGGACTGGGGA.......................................................  1
..TAGGAGTAGGACTGGGGAC......................................................  1
......AGTAGGACTGGGGACTCAGTTG...............................................  1
........TAGGACTGGGGACTCAGTTGCT.............................................  1
..................................................TGAGACCCTCTTCCTACTCGC......  1
(((.(((((((((..((((.(((((..((...........)).....))))).))))..)))))))).)...))) (-
28.30)

18/52281129-52281193(+)_mir_dog_74
CTGACCTATGAATTGACAGCCAGTGCTCTCATCTCTCCTCTGGCTGCCAATTCCATAGGTCACAG
CTGACCTATGAATTGACAGCC............................................      648522
```

```
.TGACCTATGAATTGACAGCCAGT.........................................      94117
CTGACCTATGAATTGACAGCCA...........................................      56437
.TGACCTATGAATTGACAGCCAG..........................................      50251
.TGACCTATGAATTGACAGCCA...........................................      42069
CTGACCTATGAATTGACAGC.............................................      30323
.TGACCTATGAATTGACAGCC............................................      22556
CTGACCTATGAATTGACAGCCAGT.........................................      6063
.TGACCTATGAATTGACAGC.............................................      2258
CTGACCTATGAATTGACAG..............................................      1701
CTGACCTATGAATTGACA...............................................      1037
CTGACCTATGAATTGACAGCCAG..........................................      942
...........................................CTGCCAATTCCATAGGTCACAG      135
CTGACCTATGAATTGAC................................................      119
.TGACCTATGAATTGACAG..............................................      108
.TGACCTATGAATTGACAGCCAGTG........................................      56
...........................................CTGCCAATTCCATAGGTCACA.      45
..GACCTATGAATTGACAGCCAGT.........................................      31
.TGACCTATGAATTGACA...............................................      21
.TGACCTATGAATTGAC................................................      14
...........................................CTGCCAATTCCATAGGTCAC..      12
..GACCTATGAATTGACAGCCAG..........................................      11
..GACCTATGAATTGACAGCCA...........................................      10
..GACCTATGAATTGACAGCC............................................      5
...........................................CTGCCAATTCCATAGGTCA...      5
...ACCTATGAATTGACAGCCAGTG........................................      4
.......................................CTGGCTGCCAATTCCATAGGTC....      4
.......................................CTGGCTGCCAATTCCATAGGTCA...      3
.........................................GGCTGCCAATTCCATAGGTCACA.      3
CTGACCTATGAATTGA.................................................      2
....CCTATGAATTGACAGCC............................................      2
....CCTATGAATTGACAGCCA...........................................      2
...ACCTATGAATTGACAGCCAGT.........................................      2
CTGACCTATGAATTGACAGCCAGTG........................................      2
..........................................GCTGCCAATTCCATAGGTCACA.      2
..GACCTATGAATTGACAGC.............................................      1
.....CTATGAATTGACAGCC............................................      1
.....CTATGAATTGACAGCCA...........................................      1
......TATGAATTGACAGCCA...........................................      1
....CCTATGAATTGACAGCCAG..........................................      1
.....CTATGAATTGACAGCCAG..........................................      1
......TATGAATTGACAGCCAG..........................................      1
......TATGAATTGACAGCCAGT.........................................      1
........TGAATTGACAGCCAGTGCTCTC...................................      1
....................................CCTCTGGCTGCCAATTCCATAG.......      1
........................................TGGCTGCCAATTCCATAGGTC....      1
...........................................CTGCCAATTCCATAGGTC....      1
........................................TGGCTGCCAATTCCATAGGTCA...      1
........................................TGGCTGCCAATTCCATAGGTCAC..      1
..........................................GCTGCCAATTCCATAGGTCACAG      1
.((((((((((((((.(((((((................))))))).))))).)))))))))... (-29.49)

1/86818494-86818553(+)_mir_dog_75
TTCCCTTTGTCATCCTATGCCTGAGAATATATGAAGGAGGCTGGGAAGGCAAAGGGACGT
TTCCCTTTGTCATCCTATGCCT......................................      203621
TTCCCTTTGTCATCCTATGCCTG.....................................      167700
TTCCCTTTGTCATCCTATGCC.......................................      4922
TTCCCTTTGTCATCCTATGCCTGA....................................      1722
TTCCCTTTGTCATCCTATG.........................................      748
TTCCCTTTGTCATCCTATGC........................................      653
.......................................GCTGGGAAGGCAAAGGGACGT      205
.......................................GCTGGGAAGGCAAAGGGACG.      71
.TCCCTTTGTCATCCTATGCCTG.....................................      50
.TCCCTTTGTCATCCTATGCCT......................................      38
....CTTTGTCATCCTATGCCT......................................      31
```

```
....CTTTGTCATCCTATGCCTG.....................................     25
.....TTTGTCATCCTATGCCT......................................     22
.....TTTGTCATCCTATGCCTG.....................................     21
...CCTTTGTCATCCTATGCCT......................................     19
.......................................GCTGGGAAGGCAAAGGGAC..     19
...CCTTTGTCATCCTATGCCTG.....................................     15
......TTGTCATCCTATGCCTG.....................................     15
TTCCCTTTGTCATCCTA...........................................     8
..CCCTTTGTCATCCTATGCCTG.....................................     8
TTCCCTTTGTCATCCTAT..........................................     7
......TTGTCATCCTATGCCT......................................     6
TTCCCTTTGTCATCCT............................................     5
..CCCTTTGTCATCCTATGCCT......................................     4
.......TGTCATCCTATGCCTG.....................................     3
....CTTTGTCATCCTATGCC.......................................     2
....CTTTGTCATCCTATGCCTGA....................................     2
TTCCCTTTGTCATCCTATGCCTGAGAATATATGAAGGA......................     2
.......................................GCTGGGAAGGCAAAGGGA...     2
.TCCCTTTGTCATCCTATG.........................................     1
.TCCCTTTGTCATCCTATGCCTGA....................................     1
......TTGTCATCCTATGCCTGA....................................     1
TTCCCTTTGTCATCCTATGCCTGAG...................................     1
TTCCCTTTGTCATCCTATGCCTGAGA..................................     1
TTCCCTTTGTCATCCTATGCCTGAGAATATATGAAGGAG.....................     1
....................................GAGGCTGGGAAGGCAAAGG.....     1
....................................GAGGCTGGGAAGGCAAAGGGA...     1
....................................GAGGCTGGGAAGGCAAAGGGACG.     1
......................................GGCTGGGAAGGCAAAGGGACG.     1
.((((((((((.(((((.((((...............))))))))).))))))))))... (-27.76)

1/89974431-89974495(+)_novel_Dog_Blast_16
GTCTTGGCGCGACTTCCTCGACTCACTCTCTTTCCCCCGTGAGTCGTGGAACCTCGCCCGAGGGT
.......................................TGAGTCGTGGAACCTCGCCCGA....      83
.........................................AGTCGTGGAACCTCGCCCGAG...      9
.........................................AGTCGTGGAACCTCGCCCGA....      6
.........................................AGTCGTGGAACCTCGCCCGAGG..      5
...TTGGCGCGACTTCCTCGACTC.........................................      4
....TGGCGCGACTTCCTCGACT..........................................      3
....TGGCGCGACTTCCTCGACTCACT......................................      3
.......................................TGAGTCGTGGAACCTCGCCC......      3
.......................................TGAGTCGTGGAACCTCGCCCGAG...      3
....TGGCGCGACTTCCTCGACTCA........................................      2
.......................................TGAGTCGTGGAACCTCGCCCG.....      2
...TTGGCGCGACTTCCTCGACT..........................................      1
....TGGCGCGACTTCCTCGACTC.........................................      1
....TGGCGCGACTTCCTCGACTCAC.......................................      1
...TTGGCGCGACTTCCTCGACTCACT......................................      1
.......CGCGACTTCCTCGACTCACT......................................      1
..............................TTTCCCCCGTGAGTCGTGGAACC............      1
........................................GAGTCGTGGAACCTCGCCCGA....      1
..................................CCCCGTGAGTCGTGGAACCTCGCCCGAGG..      1
...................................CCCGTGAGTCGTGGAACCTCGCCCGAGG..      1
.....................................CGTGAGTCGTGGAACCTCGCCCGAGG..      1
......................................GTGAGTCGTGGAACCTCGCCCGAGGGT      1
.((((((.((((.((((.((((((((............)))))))).))))..)))))))))).. (-31.20)

1/93194323-93194382(+)_mir_dog_76
TTTTCGGTTATCATGGTACCGATGCTGTATATCTGAAAGGTACAGTACTGTGATAACTGA
.......................................GTACAGTACTGTGATAACTGA     140512
.......................................GTACAGTACTGTGATAACT..     91275
........................................TACAGTACTGTGATAACTGA     48378
.......................................GTACAGTACTGTGATAACTG.     40635
........................................TACAGTACTGTGATAACTG.     10978
........................................TACAGTACTGTGATAACT..     7693
```

```
.......................................GTACAGTACTGTGATAAC...      3223
........................................TACAGTACTGTGATAAC...      634
.......................................GTACAGTACTGTGATA.....      259
............................................GTACTGTGATAACTGA      141
......................................GGTACAGTACTGTGATAACT..      62
.........................................ACAGTACTGTGATAACTGA      40
......................................GGTACAGTACTGTGATAACTG.      25
..........................................CAGTACTGTGATAACTGA      23
.........................................ACAGTACTGTGATAACT..      15
.......................................GTACAGTACTGTGATAA....      12
......................................GGTACAGTACTGTGATAACTGA      10
..........................................CAGTACTGTGATAACTG.      7
...........................................AGTACTGTGATAACTG.      7
........................................TACAGTACTGTGATAA....      6
...........................................AGTACTGTGATAACTGA      5
......................................GGTACAGTACTGTGATAAC...      4
..........................................CAGTACTGTGATAACT..      4
...TCGGTTATCATGGTACCGATGCT...................................      3
.........................................ACAGTACTGTGATAAC...      3
.....................................AGGTACAGTACTGTGATAACT..      3
.........................................ACAGTACTGTGATAACTG.      3
.....................................AGGTACAGTACTGTGATAAC...      2
...................................AAAGGTACAGTACTGTGATAACT..      2
....................................AAGGTACAGTACTGTGATAACT..      2
....CGGTTATCATGGTACCGATGCT...................................      1
....CGGTTATCATGGTACCGATGCTGT.................................      1
............ATGGTACCGATGCTGTATATCT..........................      1
................TACCGATGCTGTATATCTGA........................      1
..............GGTACCGATGCTGTATATCTGAAAGG....................      1
...................................AAAGGTACAGTACTGTGATAACTG.      1
.....................................AGGTACAGTACTGTGATAACTG.      1
...((((((((((((((((.(.((((............))))).)))))))))))))))) (-22.20)

19/38435956-38436011(+)_mir_dog_77
CGGGGCCGTAGCACTGTCTGAGAGGTTTACATTTCTCACAGTGAACCGGTCTCTTT
...................................TCACAGTGAACCGGTCTCTTT   1384570
...................................TCACAGTGAACCGGTCTCTT.   322169
...................................TCACAGTGAACCGGTCTCT..   103266
CGGGGCCGTAGCACTGTCTGA...................................   18256
...................................TCACAGTGAACCGGTCTC...   13571
...................................TCACAGTGAACCGGTCT....   7852
CGGGGCCGTAGCACTGTCTGAGA.................................   1599
...................................TCACAGTGAACCGGTC.....   894
..................................CTCACAGTGAACCGGTCTCTTT   738
CGGGGCCGTAGCACTGTCTGAG..................................   633
CGGGGCCGTAGCACTGTCTG....................................   599
CGGGGCCGTAGCACTGTCT.....................................   409
....................................CACAGTGAACCGGTCTCTTT   250
..................................CTCACAGTGAACCGGTCTCTT.   193
......................................CAGTGAACCGGTCTCTTT   146
.....................................ACAGTGAACCGGTCTCTTT   141
........................................GTGAACCGGTCTCTTT   137
.................................TCTCACAGTGAACCGGTCTCTT.   101
.................................TCTCACAGTGAACCGGTCTCTTT   95
.......................................AGTGAACCGGTCTCTTT   59
..................................CTCACAGTGAACCGGTCTCT..   56
....................................CACAGTGAACCGGTCTCTT.   53
.................................TCTCACAGTGAACCGGTCTCT..   42
......................................CAGTGAACCGGTCTCTT.   38
.....................................ACAGTGAACCGGTCTCTT.   35
....................................CACAGTGAACCGGTCTCT..   33
.......................................AGTGAACCGGTCTCTT.   18
.GGGGCCGTAGCACTGTCTGA...................................   15
..................................CTCACAGTGAACCGGTCT....   13
```

```
......................................CAGTGAACCGGTCTCT..   9
.................................TCTCACAGTGAACCGGTCT....   5
..................................CTCACAGTGAACCGGTCTC...   5
.....................................ACAGTGAACCGGTCTCT..   5
CGGGGCCGTAGCACTGTC......................................   4
....................................CACAGTGAACCGGTCT....   4
.....................................ACAGTGAACCGGTCTC...   4
.GGGGCCGTAGCACTGTCTGAGA.................................   3
.................................TCTCACAGTGAACCGGTCTC...   3
....................................CACAGTGAACCGGTCTC...   3
......CGTAGCACTGTCTGAG..................................   2
...............................TTTCTCACAGTGAACCGGTCTCTT.   2
.GGGGCCGTAGCACTGTCTG....................................   1
.....CCGTAGCACTGTCTGA...................................   1
.GGGGCCGTAGCACTGTCTGAG..................................   1
CGGGGCCGTAGCACTGTCTGAGAG................................   1
CGGGGCCGTAGCACTGTCTGAGAGGT..............................   1
CGGGGCCGTAGCACTGTCTGAGAGGTTT............................   1
CGGGGCCGTAGCACTGTCTGAGAGGTTTAC..........................   1
CGGGGCCGTAGCACTGTCTGAGAGGTTTACATTTCTCACAG...............   1
CGGGGCCGTAGCACTGTCTGAGAGGTTTACATTTCTCACAGT..............   1
..................................CTCACAGTGAACCGGTC.....   1
............................ACATTTCTCACAGTGAACCGGTCT....   1
..............................ATTTCTCACAGTGAACCGGTCTCTT.   1
.(((((((...((((((..((((((((....))))))))))))))...)))))))... (-25.20)

19/45798725-45798792(+)_novel_dog_29
TTCTATAAAGCGTCACAGGCAAATCTGGGGGCTGCACATCAGATGTGCCAGTGACACTTCTTAGAAAC
.....TAAAGCGTCACAGGCAAATCTG.........................................   150
...TATAAAGCGTCACAGGCAAATCT...........................................   11
....ATAAAGCGTCACAGGCAAATCTG.........................................   10
...TATAAAGCGTCACAGGCAAATC............................................   8
.....TAAAGCGTCACAGGCAAATCT...........................................   5
...TATAAAGCGTCACAGGCAAATCTG.........................................   2
......AAAGCGTCACAGGCAAATCTG.........................................   2
.......AAGCGTCACAGGCAAATCTG.........................................   2
...........................................TGTGCCAGTGACACTTCTTAGA...   1
(((((..(((.(((((.((((.(((((..(......)..))))).)))).))))).)))..))))).. (-29.40)

1/97902037-97902126(-)_mir_dog_78
CTAGGAAGAGGTAGTAGGTTGCATAGTTTTAGGGCAGGGATTTTGCCCACAAGGAGGTAACTATACGACCTGCTGCCTTT
CTTAGGGCCT
......AGAGGTAGTAGGTTGCATAGTT....................................................
..........  34216
......AGAGGTAGTAGGTTGCATAGT.....................................................
..........  12439
............................................................CTATACGACCTGCTGCCTTT
CT........  9696
......AGAGGTAGTAGGTTGCATAG......................................................
..........  1531
............................................................CTATACGACCTGCTGCCTTT
C.........  1225
......AGAGGTAGTAGGTTGCATAGTTT...................................................
..........  1137
.............................................................TATACGACCTGCTGCCTTT
CT........  938
............................................................CTATACGACCTGCTGCCTTT
..........  162
......AGAGGTAGTAGGTTGCAT........................................................
..........  149
......AGAGGTAGTAGGTTGCATA.......................................................
..........  146
.............................................................TATACGACCTGCTGCCTTT
C.........  80
```

.......GAGGTAGTAGGTTGCATAGTT..
.... 70
.....AAGAGGTAGTAGGTTGCATAGT...
.... 65
.....AAGAGGTAGTAGGTTGCATAGTT..
.... 61
....CTATACGACCTGCTGCCT..
.... 58
....CTATACGACCTGCTGCCTT.
.... 38
......AGAGGTAGTAGGTTGCA...
.... 37
......AGAGGTAGTAGGTTGC..
.... 34
...........TAGTAGGTTGCATAGTT..
.... 27
.........GGTAGTAGGTTGCATAGTT..
.... 20
......AGAGGTAGTAGGTTGCATAGTTTT..
.... 20
.......GAGGTAGTAGGTTGCATAGT...
.... 15
............AGTAGGTTGCATAGTT..
.... 14
....TATACGACCTGCTGCCTTT
.... 14
....CTATACGACCTGCTGCCTTT
CTT....... 14
.........GGTAGTAGGTTGCATAGT...
.... 13
...........TAGTAGGTTGCATAGT...
.... 13
..........GTAGTAGGTTGCATAGTT..
.... 12
....TATACGACCTGCTGCCTTT
CTT....... 11
....CTATACGACCTGCTGCC...
.... 9
..........GTAGTAGGTTGCATAGT...
.... 8
....TATACGACCTGCTGCCTT.
.... 5
........AGGTAGTAGGTTGCATAGTT..
.... 4
.....AAGAGGTAGTAGGTTGCATAG..
.... 3
........AGGTAGTAGGTTGCATAG..
.... 3
.........GGTAGTAGGTTGCATAG..
.... 3
....TAACTATACGACCTGCTGCCTT.
.... 3
....ATACGACCTGCTGCCTTT
CT........ 3
........AGGTAGTAGGTTGCATAGT...
.... 2
.....AAGAGGTAGTAGGTTGCATAGTTT...
.... 2
............................TTAGGGCAGGGATTTTGC..................................
.... 2
............................TTAGGGCAGGGATTTTGCCCACAAGGAGGT......................
.... 2
....TAACTATACGACCTGCTGCCT..
.... 2

....AACTATACGACCTGCTGCCT..
.... 2
....TAACTATACGACCTGCTGCCTTT
.... 2
..........GTAGTAGGTTGCATAG..
.... 1
.......GAGGTAGTAGGTTGCATAGTTT...
.... 1
......AGAGGTAGTAGGTTGCATAGTTTTAGGGCAGGGA..
.... 1
............................TTAGGGCAGGGATTTTGCCC................................
.... 1
...........................TTTAGGGCAGGGATTTTGCCCACAAGGAGGTAAC...................
.... 1
....CTATACGACCTGCTGC....
.... 1
....GTAACTATACGACCTGCTGCCT..
.... 1
....TATACGACCTGCTGCCT..
.... 1
....AACTATACGACCTGCTGCCTT.
.... 1
....AACTATACGACCTGCTGCCTTT
C......... 1
....ATACGACCTGCTGCCTTT
C......... 1
....GTAACTATACGACCTGCTGCCTTT
CT........ 1
....CGACCTGCTGCCTTT
CT........ 1
....GACCTGCTGCCTTT
CT........ 1
....ATACGACCTGCTGCCTTT
CTT....... 1
....CTATACGACCTGCTGCCTTT
CTTA...... 1
((((((.((((((((((((((.((((((...((((((.....)))))).
(.....)..)))))).))))))))))))))))))))..... (-40.60)

1/97903931-97904008(-)_mir_dog_79
TGAGGTAGTAGATTGTATAGTTGTGGGGTAGTGATTTTACCCTGTTCAGGAGATAACTATACAATCTATTGCCTTCCC
TGAGGTAGTAGATTGTATAGTT..
      975712
TGAGGTAGTAGATTGTATAGT...
      301195
TGAGGTAGTAGATTGTATAG..
      20544
TGAGGTAGTAGATTGTAT..
      7278
TGAGGTAGTAGATTGTATA...
      4931
TGAGGTAGTAGATTGTATAGTTG...
      3025
TGAGGTAGTAGATTGTA...
      1073
......AGTAGATTGTATAGTT..
      600
...GGTAGTAGATTGTATAGTT..
      521
.....TAGTAGATTGTATAGTT..
      481
.GAGGTAGTAGATTGTATAGTT..
      406
TGAGGTAGTAGATTGT..

```
      227
....GTAGTAGATTGTATAGTT........................................................
      214
...GGTAGTAGATTGTATAGT.........................................................
      170
.....TAGTAGATTGTATAGT.........................................................
      130
..AGGTAGTAGATTGTATAGTT........................................................
      130
.GAGGTAGTAGATTGTATAGT.........................................................
      117
..AGGTAGTAGATTGTATAGTTGT......................................................
      115
TGAGGTAGTAGATTGTATAGTTGT......................................................
      100
........................................................CTATACAATCTATTGCCTTCC.
      100
....GTAGTAGATTGTATAGT.........................................................
      77
..AGGTAGTAGATTGTATAGT.........................................................
      56
......................................................AACTATACAATCTATTGCCTTCC.
      14
...GGTAGTAGATTGTATAG..........................................................
      11
TGAGGTAGTAGATTGTATAGTTGTGGGGT.................................................
      11
..AGGTAGTAGATTGTATAGTTG.......................................................
      9
.GAGGTAGTAGATTGTATAG..........................................................
      7
..AGGTAGTAGATTGTATAG..........................................................
      6
....GTAGTAGATTGTATAG..........................................................
      6
......................................................AACTATACAATCTATTGCCTTC..
      6
.......GTAGATTGTATAGTTG.......................................................
      4
........................................................CTATACAATCTATTGCCT....
      4
.GAGGTAGTAGATTGTATAGTTG.......................................................
      3
TGAGGTAGTAGATTGTATAGTTGTG.....................................................
      3
TGAGGTAGTAGATTGTATAGTTGTGG....................................................
      3
TGAGGTAGTAGATTGTATAGTTGTGGGGTAGT..............................................
      3
......................................................AACTATACAATCTATTGCCT....
      3
.......................................................ACTATACAATCTATTGCCTTCC.
      3
..AGGTAGTAGATTGTAT............................................................
      2
...GGTAGTAGATTGTATA...........................................................
      2
...GGTAGTAGATTGTATAGTTG.......................................................
      2
.....TAGTAGATTGTATAGTTG.......................................................
      2
TGAGGTAGTAGATTGTATAGTTGTGGGGTA................................................
      2
..................................................AGATAACTATACAATCTATTGCC.....
```

```
      2
........................................................CTATACAATCTATTGCCTTCCC
      2
....GTAGTAGATTGTATAGTTG.......................................................
      1
TGAGGTAGTAGATTGTATAGTTGTGGG...................................................
      1
.....TAGTAGATTGTATAGTTGTGGG...................................................
      1
.........AGATTGTATAGTTGTGGGGTAGT..............................................
      1
.....TAGTAGATTGTATAGTTGTGGGGTAGTGATTTTACCCTGTTC...............................
      1
............................TAGTGATTTTACCCTGTTCAGGAG..........................
      1
..............................GTGATTTTACCCTGTTCAGGAGATAA......................
      1
.............................................TCAGGAGATAACTATACAATCT...........
      1
........................................................CTATACAATCTATTGCCTT...
      1
.....................................................TAACTATACAATCTATTGCCTTC..
      1
.((((((((((((((((((((((((((((((.....))))))).........)))))))))))))))))))))))...
(-37.90)

1/97904313-97904387(-)_mir_dog_80
ATGAGGTAGTAGGTTGTATAGTTTTAGGGTCACACCCACCACTGGGAGATAACTATACAATCTACTGTCTTTCCT
.TGAGGTAGTAGGTTGTATAGTT....................................................
      1053826
.TGAGGTAGTAGGTTGTATAGT.....................................................
      304275
.TGAGGTAGTAGGTTGTATAGTTT...................................................
      31038
.TGAGGTAGTAGGTTGTATAG......................................................
      29969
.TGAGGTAGTAGGTTGTAT........................................................
      10711
.TGAGGTAGTAGGTTGTATA.......................................................
      6228
....................................................CTATACAATCTACTGTCTTTCC.
      1561
.TGAGGTAGTAGGTTGTA.........................................................
      1406
......TAGTAGGTTGTATAGTT....................................................  778
....GGTAGTAGGTTGTATAGTT....................................................  646
.......AGTAGGTTGTATAGTT....................................................  542
..GAGGTAGTAGGTTGTATAGTT....................................................  482
....................................................CTATACAATCTACTGTCTTTC..  467
.TGAGGTAGTAGGTTGT..........................................................  371
.TGAGGTAGTAGGTTGTATAGTTTT..................................................  316
......TAGTAGGTTGTATAGT.....................................................  276
.....GTAGTAGGTTGTATAGTT....................................................  254
....GGTAGTAGGTTGTATAGT.....................................................  253
.....................................................TATACAATCTACTGTCTTTCCT  243
....................................................CTATACAATCTACTGTCTTTCCT  198
.....................................................TATACAATCTACTGTCTTTCC.  161
...AGGTAGTAGGTTGTATAGTT....................................................  157
..GAGGTAGTAGGTTGTATAGT.....................................................  108
....................................................CTATACAATCTACTGTCTTT...  106
.....GTAGTAGGTTGTATAGT.....................................................  90
...AGGTAGTAGGTTGTATAGT.....................................................  61
ATGAGGTAGTAGGTTGTATAGTT....................................................  50
......................................................ATACAATCTACTGTCTTTCCT  35
```

```
......TAGTAGGTTGTATAGTTT...................................................  32
........GTAGGTTGTATAGTTT...................................................  32
....................................................CTATACAATCTACTGTCTT....  29
....................................................CTATACAATCTACTGTCT.....  27
.......AGTAGGTTGTATAGTTT...................................................  20
....GGTAGTAGGTTGTATAG......................................................  18
ATGAGGTAGTAGGTTGTATAGT.....................................................  17
..GAGGTAGTAGGTTGTATAGTTT...................................................  15
....GGTAGTAGGTTGTATAGTTT...................................................  15
.......................TTAGGGTCACACCCACCACTGGG.............................  13
..GAGGTAGTAGGTTGTATAG......................................................  12
......................................................ATACAATCTACTGTCTTTCC.  11
.......................TTAGGGTCACACCCACCACTGGGA............................  10
....................................................CTATACAATCTACTGT.......  10
.....GTAGTAGGTTGTATAG......................................................  9
...AGGTAGTAGGTTGTATAGTTT...................................................  9
.TGAGGTAGTAGGTTGTATAGTTTTAGGGT.............................................  9
.....................................................TATACAATCTACTGTCTTTC..  9
.....GTAGTAGGTTGTATAGTTT...................................................  8
...................................................ACTATACAATCTACTGTCTTTC..  8
...AGGTAGTAGGTTGTATAG......................................................  7
.................................................TAACTATACAATCTACTGTCTT....  5
...AGGTAGTAGGTTGTATAGTTTT..................................................  4
.TGAGGTAGTAGGTTGTATAGTTTTAGG...............................................  4
.TGAGGTAGTAGGTTGTATAGTTTTAGGGTC............................................  4
...............................................GATAACTATACAATCTACTGTCT.....  4
.....................................................TATACAATCTACTGTCTTT...  4
.......................................................TACAATCTACTGTCTTTCCT  4
...AGGTAGTAGGTTGTAT........................................................  3
.......AGTAGGTTGTATAGTTTT..................................................  3
.................................................TAACTATACAATCTACTGTCTTT...  3
.................................................TAACTATACAATCTACTGTCTTTC..  3
......................................................ATACAATCTACTGTCTTTC..  3
....GGTAGTAGGTTGTATA.......................................................  2
ATGAGGTAGTAGGTTGTATAGTTT...................................................  2
.TGAGGTAGTAGGTTGTATAGTTTTA.................................................  2
.TGAGGTAGTAGGTTGTATAGTTTTAG................................................  2
......TAGTAGGTTGTATAGTTTTAG................................................  2
......TAGTAGGTTGTATAGTTTTAGG...............................................  2
......TAGTAGGTTGTATAGTTTTAGGGT.............................................  2
.......................TTAGGGTCACACCCACCACTG...............................  2
.......................TTAGGGTCACACCCACCACTGG..............................  2
....................................................CTATACAATCTACTGTC......  2
..................................................AACTATACAATCTACTGTCTTT...  2
..................................................AACTATACAATCTACTGTCTTTC..  2
.........................................................CAATCTACTGTCTTTC..  2
.........................................................CAATCTACTGTCTTTCCT  2
ATGAGGTAGTAGGTTGTAT........................................................  1
..GAGGTAGTAGGTTGTAT........................................................  1
ATGAGGTAGTAGGTTGTATA.......................................................  1
..GAGGTAGTAGGTTGTATA.......................................................  1
ATGAGGTAGTAGGTTGTATAG......................................................  1
....GGTAGTAGGTTGTATAGTTTT..................................................  1
......TAGTAGGTTGTATAGTTTT..................................................  1
......TAGTAGGTTGTATAGTTTTA.................................................  1
.......................TTAGGGTCACACCCACCACTGGGAGA..........................  1
.......................TTAGGGTCACACCCACCACTGGGAGAT.........................  1
.......................TTAGGGTCACACCCACCACTGGGAGATAA.......................  1
....................................CACCACTGGGAGATAACTATACAAT..............  1
.......................................CACTGGGAGATAACTATACAAT..............  1
.......................................CACTGGGAGATAACTATACAATCT............  1
.............................................GAGATAACTATACAATCTACTG........  1
..............................................AGATAACTATACAATCTACTGTC......  1
................................................ATAACTATACAATCTACTGTCT.....  1
```

....ATAACTATACAATCTACTGTCTT.... 1
....GATAACTATACAATCTACTGTCTTT... 1
....ACTATACAATCTACTGTCTTT... 1
..(((..((((((((((((((((...(((.....))).((....))....))))))))))))))))..)))..... (-
30.30)

1/99181930-99181993(-)_novel_Dog_Blast_17
GTCTTGGCGCGACTTCCTCGACTCACTCTCTTTCCCCGTGAGTCGTGGAACCTCGCCCGAGGGT
......................................TGAGTCGTGGAACCTCGCCCGA.... 83
....AGTCGTGGAACCTCGCCCGAG... 9
....AGTCGTGGAACCTCGCCCGA.... 6
....AGTCGTGGAACCTCGCCCGAGG.. 5
...TTGGCGCGACTTCCTCGACTC.. 4
....TGGCGCGACTTCCTCGACT... 3
....TGGCGCGACTTCCTCGACTCACT..................................... 3
......................................TGAGTCGTGGAACCTCGCCC...... 3
......................................TGAGTCGTGGAACCTCGCCCGAG... 3
....TGGCGCGACTTCCTCGACTCA....................................... 2
......................................TGAGTCGTGGAACCTCGCCCG..... 2
...TTGGCGCGACTTCCTCGACT... 1
....TGGCGCGACTTCCTCGACTC.. 1
....TGGCGCGACTTCCTCGACTCAC...................................... 1
...TTGGCGCGACTTCCTCGACTCACT..................................... 1
.......CGCGACTTCCTCGACTCACT..................................... 1
....GAGTCGTGGAACCTCGCCCGA.... 1
.................................CCCCGTGAGTCGTGGAACCTCGCCCGAGG.. 1
..................................CCCGTGAGTCGTGGAACCTCGCCCGAGG.. 1
....................................CGTGAGTCGTGGAACCTCGCCCGAGG.. 1
.....................................GTGAGTCGTGGAACCTCGCCCGAGGGT 1
.((((((.((((.((((.((((((((...........)))))))).))))..)))))))))).. (-31.30)

20/16948435-16948505(-)_novel_dog_44
CTCTAAGGTAAAGGGCTCAACTCAACTGATTTTGTCTAAGATGTTGAGTTGAGCCCTTTACCTTAGAGGAA
...TAAGGTAAAGGGCTCAACTCA...   7
...TAAGGTAAAGGGCTCAACT...   6
...TAAGGTAAAGGGCTCAACTC..   4
...TAAGGTAAAGGGCTCAACTCAA..   4
....TTGAGTTGAGCCCTTTACCTT.......   2
...TAAGGTAAAGGGCTCAACTCAAC...   1
.....AGGTAAAGGGCTCAACTCAACT..   1
....TTGAGTTGAGCCCTTTACCT........   1
....TGAGTTGAGCCCTTTACCT........   1
....TGAGTTGAGCCCTTTACCTTA......   1
....TGAGTTGAGCCCTTTACCTTAG.....   1
((((((((((((((((((((((((((...((......))...))))))))))))))))))))))))))... (-47.70)

20/16948438-16948508(+)_novel_dog_35
CTCTAAGGTAAAGGGCTCAACTCAACATCTTAGACAAAATCAGTTGAGTTGAGCCCTTTACCTTAGAGGAG
...TAAGGTAAAGGGCTCAACTCA...   7
...TAAGGTAAAGGGCTCAACT...   6
...TAAGGTAAAGGGCTCAACTC..   4
...TAAGGTAAAGGGCTCAACTCAA..   4
....TTGAGTTGAGCCCTTTACCTT.......   2
...TAAGGTAAAGGGCTCAACTCAAC...   1
....TTGAGTTGAGCCCTTTACCT........   1
....TGAGTTGAGCCCTTTACCT........   1
....TGAGTTGAGCCCTTTACCTTA......   1
....TGAGTTGAGCCCTTTACCTTAG.....   1
((((((((((((((((((((((((((................))))))))))))))))))))))))))... (-46.79)

20/27116590-27116661(+)_novel_dog_36
GACCCAGGCAGGCAGCGGGGAGGTGTGAAGTCAGAAGCGTGCGCCTCTCCCCCCCAGCCTGCCTCAGCCTCC
....TCTCCCCCCCAGCCTGCCTCAGC....   24
....TCTCCCCCCCAGCCTGCCTCA......   3

....TCTCCCCCCCAGCCTGCCTCAG..... 3
...CCAGGCAGGCAGCGGGGAGGT.. 1
....TCTCCCCCCCAGCCTGCCT........ 1
....CCTCTCCCCCCCAGCCTGCCTC....... 1
....TCTCCCCCCCAGCCTGCCTC....... 1
....CTCTCCCCCCCAGCCTGCCTCA...... 1
....CCTCTCCCCCCCAGCCTGCCTCAGC.... 1
....TCCCCCCCAGCCTGCCTCAGCCT.. 1
.....((((((((.(.(((((((.(((..((.....))...))).))))))).)..))))))))........ (-
32.70)

20/3513376-3513445(-)_novel_dog_42
ACAGCATGGCCAGTTGTGTGGTTGGTCGTGTGGACTGCAGAGTGACCAACAGCATGACCGGCAGTGTGGT
......TGGCCAGTTGTGTGGTTGGTC... 3648
......TGGCCAGTTGTGTGGTTGGTCG.. 98
......TGGCCAGTTGTGTGGTTGGT.. 37
......TGGCCAGTTGTGTGGTTGGTCGT... 27
....ACCAACAGCATGACCGGCAGTG.... 17
....CCAACAGCATGACCGGCAGTG.... 4
....ACCAACAGCATGACCGGCAGT..... 3
....ACCAACAGCATGACCGGCAGTGT... 3
....CCAACAGCATGACCGGCAGTGT... 3
.........CCAGTTGTGTGGTTGGTC... 2
...........AGTTGTGTGGTTGGTC... 2
....ACCAACAGCATGACCGGC........ 2
....TGACCAACAGCATGACCGGCAGT..... 2
....CCAACAGCATGACCGGCAGT..... 2
....TGACCAACAGCATGACCGGCAGTG.... 2
....TGACCAACAGCATGACCGGCAGTGT... 2
......TGGCCAGTTGTGTGGT.. 1
......TGGCCAGTTGTGTGGTT... 1
.......GGCCAGTTGTGTGGTTGGTCG.. 1
....TGACCAACAGCATGACCGGCAG...... 1
((.((((.(((.((..(((.(((((((((.((.....)).).)))))))).)))..)).))).)))).)) (-30.40)

20/37461827-37461895(+)_mir_dog_81
GTTCTCTATGGCTTTTTATTCCTATGTGATTTTACTGCTCACTCATATAGGGATTGGAGCCGTGGCGTA
......TATGGCTTTTTATTCCTATGTGA.. 8347
......TATGGCTTTTTATTCCTATGTG... 908
......TATGGCTTTTTATTCCTATGT.. 508
......TATGGCTTTTTATTCCTATG... 60
......TATGGCTTTTTATTCCTATGTGAT....................................... 53
....ATATAGGGATTGGAGCCGTGGC... 35
......TATGGCTTTTTATTCC... 17
......TATGGCTTTTTATTCCT.. 15
....TATAGGGATTGGAGCCGTGGC... 15
......TATGGCTTTTTATTCCTA... 13
....ATATAGGGATTGGAGCCGTGG.... 6
..........GCTTTTTATTCCTATGTG... 3
..........GCTTTTTATTCCTATGTGA.. 3
...........CTTTTTATTCCTATGTGA.. 3
............TTTTTATTCCTATGTGA.. 2
....TATAGGGATTGGAGCCGTGG.... 2
......TATGGCTTTTTATTCCTAT.. 1
..........GCTTTTTATTCCTATGT.. 1
.....CTATGGCTTTTTATTCCTATGTGA.. 1
.......ATGGCTTTTTATTCCTATGTGA.. 1
....ATATAGGGATTGGAGCC........ 1
....ATATAGGGATTGGAGCCG....... 1
....TATAGGGATTGGAGCCGTGGCG.. 1
...(.((((((((((..((((((((((((.............)))))))))))).)))))))))).).. (-26.62)

20/37501074-37501152(+)_mir_dog_82
TGAGGTAGTAGTTTGTACAGTTTGAGGGTCTATGATACCACCCGGTACAGGAGATAACTGTACAGGCCACTGCCTTGCC

TGAGGTAGTAGTTTGTACAGTT...
275591
TGAGGTAGTAGTTTGTACAGT..
69461
TGAGGTAGTAGTTTGTAC...
6975
TGAGGTAGTAGTTTGTACAGTTT..
6072
TGAGGTAGTAGTTTGTACAG...
5920
TGAGGTAGTAGTTTGTACA..
4217
....CTGTACAGGCCACTGCCTTGC.
506
......AGTAGTTTGTACAGTT...
163
....ACTGTACAGGCCACTGCCTTGC.
117
TGAGGTAGTAGTTTGTA..
107
.....TAGTAGTTTGTACAGTT...
104
.GAGGTAGTAGTTTGTACAGTT...
99
....CTGTACAGGCCACTGCCTTGCC
66
...GGTAGTAGTTTGTACAGTT...
61
TGAGGTAGTAGTTTGT...
53
....AACTGTACAGGCCACTGCCTTGC.
44
.....TAGTAGTTTGTACAGT..
35
...GGTAGTAGTTTGTACAGT..
31
..AGGTAGTAGTTTGTACAGTT...
31
....GTAGTAGTTTGTACAGTT...
24
.GAGGTAGTAGTTTGTACAGT..
18
......................TGAGGGTCTATGATACCACCCGGT.................................
15
....CTGTACAGGCCACTGCCTTG..
10
..AGGTAGTAGTTTGTACAGT..
8
....GTAGTAGTTTGTACAGT..
8
....ACTGTACAGGCCACTGCCTTG..
6
....AACTGTACAGGCCACTGCCTTG..
5
...GGTAGTAGTTTGTACAG...
4
...GGTAGTAGTTTGTACAGTTT..
4
TGAGGTAGTAGTTTGTACAGTTTGAGGGTC...
4
.GAGGTAGTAGTTTGTACAGTTT..
3
......AGTAGTTTGTACAGTTT..
3

```
.GAGGTAGTAGTTTGTAC..............................................................
      2
..AGGTAGTAGTTTGTAC..............................................................
      2
....GTAGTAGTTTGTACAG............................................................
      2
TGAGGTAGTAGTTTGTACAGTTTG........................................................
      2
......................TGAGGGTCTATGATACCACCCGGTACAGGAGATAA.......................
      2
..........................................................CTGTACAGGCCACTGCC.....
      2
........................................................AACTGTACAGGCCACTGCCT....
      2
.......................................................TAACTGTACAGGCCACTGCCTTGC.
      2
...GGTAGTAGTTTGTACA.............................................................
      1
..AGGTAGTAGTTTGTACAGTTT.........................................................
      1
.....TAGTAGTTTGTACAGTTT.........................................................
      1
..AGGTAGTAGTTTGTACAGTTTG........................................................
      1
TGAGGTAGTAGTTTGTACAGTTTGAGG.....................................................
      1
TGAGGTAGTAGTTTGTACAGTTTGAGGGT...................................................
      1
......................TGAGGGTCTATGATACCACCCGG...................................
      1
......................TGAGGGTCTATGATACCACCCGGTACAGGAG...........................
      1
......................TGAGGGTCTATGATACCACCCGGTACAGGAGAT.........................
      1
.........................................................ACTGTACAGGCCACTGCCT....
      1
..........................................................CTGTACAGGCCACTGCCT....
      1
......................................................ATAACTGTACAGGCCACTGCCTTGC.
      1
...........................................................TGTACAGGCCACTGCCTTGC.
      1
...........................................................TGTACAGGCCACTGCCTTGCC
      1
.((((((((.((((((((((((.....((((.((.((((....))))))..)))))))))))))))).))))))))...
(-34.70)

20/37620961-37621029(+)_novel_dog_37
TTAGGGCTGTGGCTGGGAGCTTTCTCTGCTTGGAAGGCTGCAGAGGAAGGACTCCAGCCTAATAGCCGG
........GTGGCTGGGAGCTTTCTCTGC........................................  6
.........................................AGAGGAAGGACTCCAGCCTAAT......  3
.........................................AGAGGAAGGACTCCAGCCT.........  2
.........TGGCTGGGAGCTTTCTCTGC........................................  1
....(((((((((((((..((((((((((..........))))))))))...)))))))..)))))).. (-35.70)

20/38443107-38443172(-)_novel_dog_45
GCAGGGTGCTCTTTTTGAGAGGCTTCTGTGATGACAGGCCCAGAGCACTCAAAAAGATGGCTCCTT
......TGCTCTTTTTGAGAGGCTTCTGT.....................................     1194
......TGCTCTTTTTGAGAGGCTTCTG......................................     158
......TGCTCTTTTTGAGAGGCTTCT.......................................     12
.........................................AGAGCACTCAAAAAGATGGCTC...     7
.........................................AGAGCACTCAAAAAGATGGCT....     5
........................................CAGAGCACTCAAAAAGATGGCT....     4
......TGCTCTTTTTGAGAGGCTTC........................................     2
```

```
..........CTTTTTGAGAGGCTTCT.......................................     2
......TGCTCTTTTTGAGAGGCT..........................................     1
......TGCTCTTTTTGAGAGGCTT.........................................     1
.........TCTTTTTGAGAGGCTTCTGT.....................................     1
...........TTTTTGAGAGGCTTCTGTGA...................................     1
.........................................AGAGCACTCAAAAAGATGG......     1
..((((.((((((((((((..((((((((....)))).....)))).))))))))))..)).)))) (-23.60)

20/40144135-40144198(+)_mir_dog_83
CAACGGAATCCCAAAAGCAGCTGTTGTCTCCAGAGCATTCCAGCTGCGCTTGGATTTCGTTCCC
CAACGGAATCCCAAAAGCAGCTG......................................... 1109231
CAACGGAATCCCAAAAGCAGCT.......................................... 45805
CAACGGAATCCCAAAAGCAGCTGT........................................ 45100
.AACGGAATCCCAAAAGCAGCTG......................................... 38226
...........................................CTGCGCTTGGATTTCGTTCCC 10351
CAACGGAATCCCAAAAGCAG............................................ 7821
CAACGGAATCCCAAAAGCAGC........................................... 7482
.AACGGAATCCCAAAAGCAGCTGT........................................ 3587
...........................................CTGCGCTTGGATTTCGTTCC. 2060
..........................................GCTGCGCTTGGATTTCGTTCCC 464
...........................................CTGCGCTTGGATTTCGTTC.. 306
.......ATCCCAAAAGCAGCTG......................................... 297
.AACGGAATCCCAAAAGCAGCT.......................................... 257
CAACGGAATCCCAAAAGCA............................................. 235
..........................................GCTGCGCTTGGATTTCGTTCC. 200
.....GAATCCCAAAAGCAGCTG......................................... 168
CAACGGAATCCCAAAAGCAGCTGTT....................................... 160
..........................................GCTGCGCTTGGATTTCGTTC.. 110
.AACGGAATCCCAAAAGCAGC........................................... 98
..ACGGAATCCCAAAAGCAGCTG......................................... 88
......AATCCCAAAAGCAGCTG......................................... 64
.AACGGAATCCCAAAAGCAG............................................ 60
CAACGGAATCCCAAAAGC.............................................. 58
............................................TGCGCTTGGATTTCGTTCCC 58
....GGAATCCCAAAAGCAGCTG......................................... 43
.......ATCCCAAAAGCAGCTGT........................................ 41
...........................................CTGCGCTTGGATTTCGTT... 31
...CGGAATCCCAAAAGCAGCTG......................................... 18
..ACGGAATCCCAAAAGCAGCTGT........................................ 18
..........................................GCTGCGCTTGGATTTCGTT... 17
......AATCCCAAAAGCAGCTGT........................................ 16
.....GAATCCCAAAAGCAGCTGT........................................ 14
CAACGGAATCCCAAAAG............................................... 11
.AACGGAATCCCAAAAGCAGCTGTT....................................... 10
.........................................AGCTGCGCTTGGATTTCGTTC.. 10
CAACGGAATCCCAAAAGCAGCTGTTGTCTC.................................. 9
............................................TGCGCTTGGATTTCGTTCC. 9
.........................................AGCTGCGCTTGGATTTCGTTCC. 5
.....GAATCCCAAAAGCAGCT.......................................... 3
.........................................AGCTGCGCTTGGATTTCGTT... 3
CAACGGAATCCCAAAA................................................ 2
...........................................CTGCGCTTGGATTTCG..... 2
.AACGGAATCCCAAAAG............................................... 1
..ACGGAATCCCAAAAGCAGC........................................... 1
..ACGGAATCCCAAAAGCAGCT.......................................... 1
...CGGAATCCCAAAAGCAGCT.......................................... 1
........TCCCAAAAGCAGCTGT........................................ 1
CAACGGAATCCCAAAAGCAGCTGTTG...................................... 1
..........................................GCTGCGCTTGGATTTCG..... 1
..........................................GCTGCGCTTGGATTTCGT.... 1
...........................................CTGCGCTTGGATTTCGT.... 1
............................................TGCGCTTGGATTTCGTTC.. 1
......................................TCCAGCTGCGCTTGGATTTCGTTCCC 1
...............................................GCTTGGATTTCGTTCCC 1
```

.((((((((((.((..(((((((..((((....)).))..))))))))..)))))))))))... (-27.10)

20/40144606-40144680(-)_mir_dog_84
GCACTGGGCGGACACGACATTCCCGATGGCTTCTTGGGTGCCCACTCAACGGGAGTGATCGTGTCATTCCAAAGC
....AACGGGAGTGATCGTGTCATTC...... 42
..ACTGGGCGGACACGACATTCCC... 2
....AACGGGAGTGATCGTGTCATT....... 2
........CGGACACGACATTCCCGATGG.. 1
....GAGTGATCGTGTCATTC...... 1
((..((((..((((((((((((((.(((.....(((....))).))).)))))))).))))))).))))..)) (-34.10)

20/40144613-40144675(+)_mir_dog_85
AATGACACGATCACTCCCGTTGAGTGGGCACCCAAGAAGCCATCGGGAATGTCGTGTCCGCCC
AATGACACGATCACTCCCGTTG... 23049
AATGACACGATCACTCCCGTTGAGT...................................... 4075
AATGACACGATCACTCCCGTTGA.. 2355
AATGACACGATCACTCCCGTTGAG....................................... 1756
....CATCGGGAATGTCGTGTCCGCC. 1290
....ATCGGGAATGTCGTGTCCGCC. 450
....ATCGGGAATGTCGTGTCCGCCC 414
....CATCGGGAATGTCGTGTCCGC.. 396
....ATCGGGAATGTCGTGTCCGC.. 182
....CATCGGGAATGTCGTGTCCGCCC 165
AATGACACGATCACTCCCGT... 86
AATGACACGATCACTCCCGTT.. 86
....CCATCGGGAATGTCGTGTCCGC.. 77
....TCGGGAATGTCGTGTCCGCCC 76
....TCGGGAATGTCGTGTCCGCC. 54
.ATGACACGATCACTCCCGTTGAGT...................................... 48
....CATCGGGAATGTCGTGTCCG... 30
AATGACACGATCACTCCC... 29
AATGACACGATCACTCCCGTTGAGTG..................................... 27
AATGACACGATCACTCCCGTTGAGTGGGC.................................. 25
..TGACACGATCACTCCCGTTGAGT...................................... 24
.ATGACACGATCACTCCCGTTGAG....................................... 20
AATGACACGATCACTCCCGTTGAGTGG.................................... 14
....CATCGGGAATGTCGTGTCC.... 13
....ATCGGGAATGTCGTGTCCG... 13
....ACACGATCACTCCCGTTG... 11
....TCGGGAATGTCGTGTCCGC.. 10
AATGACACGATCACTCCCG.. 8
AATGACACGATCACTCC.. 6
.ATGACACGATCACTCCCGTTG... 6
....ATCGGGAATGTCGTGTCC.... 6
.ATGACACGATCACTCCCGTTGA.. 5
....ACACGATCACTCCCGTTGAG....................................... 4
AATGACACGATCACTCCCGTTGAGTGGGCACCC.............................. 4
....GGAATGTCGTGTCCGC.. 4
..TGACACGATCACTCCCGTTG... 3
...GACACGATCACTCCCGTTG... 2
...GACACGATCACTCCCGTTGAG....................................... 2
....CATCGGGAATGTCGTGTC..... 2
....ATCGGGAATGTCGTGTC..... 2
....CCATCGGGAATGTCGTGTCCG... 2
....CCATCGGGAATGTCGTGTCCGCC. 2
....AATGTCGTGTCCGCCC 2
..TGACACGATCACTCCCGT... 1
......ACGATCACTCCCGTTG... 1
..TGACACGATCACTCCCGTTGAG....................................... 1
.........ATCACTCCCGTTGAGT...................................... 1
..TGACACGATCACTCCCGTTGAGTG..................................... 1
..TGACACGATCACTCCCGTTGAGTGGGC.................................. 1
AATGACACGATCACTCCCGTTGAGTGGGCA................................. 1

```
AATGACACGATCACTCCCGTTGAGTGGGCAC................................  1
AATGACACGATCACTCCCGTTGAGTGGGCACCCAAGAA.........................  1
.........................................TCGGGAATGTCGTGTC.....  1
.............................................GAATGTCGTGTCCGCC.  1
......................AGTGGGCACCCAAGAAGCCATCGGGAATGTCGTGTCCGCCC  1
......................................CCATCGGGAATGTCGTGTCCGCCC  1
...(((((((.((.(((((.((..(((....)))......)).))))).))))))))))..... (-24.60)

20/44332273-44332349(-)_mir_dog_86
AAGCACCAGTATGGCTGGATCAGGGGCTCAGGTGCTGCTTTTATCCCTCAGCCCCTGATTCTCTAGCTGGCTCTTCT
...............................................TCAGCCCCTGATTCTCTAGCT.........
      16
..............................................CTCAGCCCCTGATTCTCTAGC..........
      11
...............................................TCAGCCCCTGATTCTCTAGC..........
      9
...........TGGCTGGATCAGGGGCTCAGGT.............................................
      8
..........ATGGCTGGATCAGGGGCTCAGG..............................................
      2
.............................................CCTCAGCCCCTGATTCTCT.............
      1
.............................................CCTCAGCCCCTGATTCTCTAG...........
      1
...............................................TCAGCCCCTGATTCTCTAG...........
      1
.............................................CCTCAGCCCCTGATTCTCTAGC..........
      1
(((..(((((.(((..((((((((((((.(((.............))).))))))))))))).))))))))..))).. (-
33.82)

20/48593158-48593221(-)_mir_dog_87
AACATTCATTGTTGTCGGTGGGTTGTGAGGACGGAGGCCAGACCCACCGAGGGATGAATGTCAC
AACATTCATTGTTGTCGGTGGGTT........................................ 28570
AACATTCATTGTTGTCGGTGGGT......................................... 24988
AACATTCATTGTTGTCGGTGGG.......................................... 10449
AACATTCATTGTTGTCGGTGG........................................... 623
AACATTCATTGTTGTCGGTGGGTTGT...................................... 491
.ACATTCATTGTTGTCGGTGGGT......................................... 455
.ACATTCATTGTTGTCGGTGGGTT........................................ 386
AACATTCATTGTTGTCGGTG............................................ 167
AACATTCATTGTTGTCGGT............................................. 115
.ACATTCATTGTTGTCGGTGGG.......................................... 102
AACATTCATTGTTGTCGGTGGGTTG....................................... 59
AACATTCATTGTTGTCGG.............................................. 15
........TTGTTGTCGGTGGGTTGTGAGGA................................. 13
.........................................CCCACCGAGGGATGAATGTCA. 13
........TTGTTGTCGGTGGGTTGTGAGGAC................................ 7
....TTCATTGTTGTCGGTGGGT......................................... 4
....TTCATTGTTGTCGGTGGGTT........................................ 4
......CATTGTTGTCGGTGGGTT........................................ 4
.........TGTTGTCGGTGGGTTGTGAGGA................................. 4
......CATTGTTGTCGGTGGGT......................................... 3
.ACATTCATTGTTGTCGGTGGGTTG....................................... 3
.ACATTCATTGTTGTCGGTGGGTTGT...................................... 3
........TTGTTGTCGGTGGGTTGTG..................................... 3
..CATTCATTGTTGTCGGTGGGT......................................... 2
...ATTCATTGTTGTCGGTGGGT......................................... 2
.........TGTTGTCGGTGGGTTGTGAGGAC................................ 2
.......................TGTGAGGACGGAGGCCAGAC..................... 2
.................................AGGCCAGACCCACCGA.............. 2
........................................ACCCACCGAGGGATGAATGTCA. 2
AACATTCATTGTTGTC................................................ 1
.ACATTCATTGTTGTCGGTG............................................ 1
```

```
.ACATTCATTGTTGTCGGTGG........................................... 1
..CATTCATTGTTGTCGGTGGG.......................................... 1
...ATTCATTGTTGTCGGTGGG.......................................... 1
.....TCATTGTTGTCGGTGGG.......................................... 1
...ATTCATTGTTGTCGGTGGGTT........................................ 1
........TTGTTGTCGGTGGGTT........................................ 1
....TTCATTGTTGTCGGTGGGTTGT...................................... 1
.........TGTTGTCGGTGGGTTGT...................................... 1
.........TGTTGTCGGTGGGTTGTG..................................... 1
...........TTGTCGGTGGGTTGTG..................................... 1
........TTGTTGTCGGTGGGTTGTGA.................................... 1
.........TGTTGTCGGTGGGTTGTGA.................................... 1
...........TTGTCGGTGGGTTGTGA.................................... 1
.........TGTTGTCGGTGGGTTGTGAGG.................................. 1
........TTGTTGTCGGTGGGTTGTGAGGACGG.............................. 1
AACATTCATTGTTGTCGGTGGGTTGTGAGGACGGAGGCCAG....................... 1
......................TTGTGAGGACGGAGGCCAGACCCACC................ 1
...............................CGGAGGCCAGACCCACCGAGGGATGA....... 1
.........................................ACCCACCGAGGGATGAATGTC.. 1
..........................................CCCACCGAGGGATGAATGTC.. 1
.......................TGTGAGGACGGAGGCCAGACCCACCGAGGGATGAATGTCA. 1
...........................................CCACCGAGGGATGAATGTCA. 1
..........................................CCCACCGAGGGATGAATGTCAC 1
.(((((((((.(..((((((((((....((.(....))).))))))))))).))))))))).. (-28.20)

20/48593343-48593403(-)_mir_dog_88
AACATTCAACCTGTCGGTGAGTTTGGGCAGCTCAGGCAAACCATCGACCGTTGAGTGGACC
AACATTCAACCTGTCGGTGAGT.......................................    480923
AACATTCAACCTGTCGGTGAGTT......................................    406553
AACATTCAACCTGTCGGTGAG........................................    93639
.......................................ACCATCGACCGTTGAGTGGACC    68621
AACATTCAACCTGTCGGTGAGTTT.....................................    35952
AACATTCAACCTGTCGGTGA.........................................    22620
.......................................ACCATCGACCGTTGAGTGGAC.    7411
AACATTCAACCTGTCGGT...........................................    1557
......................................AACCATCGACCGTTGAGTGGAC.    1455
......CAACCTGTCGGTGAGTT......................................    825
AACATTCAACCTGTCGGTG..........................................    544
.ACATTCAACCTGTCGGTGAGTT......................................    534
......CAACCTGTCGGTGAGT.......................................    457
AACATTCAACCTGTCGGTGAGTTTG....................................    315
.ACATTCAACCTGTCGGTGAGT.......................................    297
......................................AACCATCGACCGTTGAGTGGACC    290
.ACATTCAACCTGTCGGTGAGTTT.....................................    183
......CAACCTGTCGGTGAGTTT.....................................    94
.......................................ACCATCGACCGTTGAGTG....    81
......................................AACCATCGACCGTTGAGTGGA..    71
.......................................ACCATCGACCGTTGAGTGG...    70
..........................................ATCGACCGTTGAGTGGACC    70
.............................................GACCGTTGAGTGGACC    70
.......................................ACCATCGACCGTTGAGTGGA..    60
......................................AACCATCGACCGTTGAGTGG...    57
.....TCAACCTGTCGGTGAGT.......................................    53
.....TCAACCTGTCGGTGAGTT......................................    51
.ACATTCAACCTGTCGGTGAG........................................    50
.ACATTCAACCTGTCGGTGAGTTTG....................................    43
........................................CCATCGACCGTTGAGTGGACC    39
..CATTCAACCTGTCGGTGAGTT......................................    35
.ACATTCAACCTGTCGGTGA.........................................    34
AACATTCAACCTGTCGG............................................    31
...ATTCAACCTGTCGGTGAGTT......................................    26
...ATTCAACCTGTCGGTGAGT.......................................    25
......................................AACCATCGACCGTTGAGTG....    25
.....................................AAACCATCGACCGTTGAGTGG...    21
```

```
....TTCAACCTGTCGGTGAGTT....................................    19
.....................................AACCATCGACCGTTGAGT.....    19
......................................ACCATCGACCGTTGAGT.....    17
.....TCAACCTGTCGGTGAGTTT...................................    11
....TTCAACCTGTCGGTGAGT.....................................    10
.......AACCTGTCGGTGAGTT....................................    10
..CATTCAACCTGTCGGTGAGT.....................................    9
....................................AAACCATCGACCGTTGAGTGGA..    9
......................................ACCATCGACCGTTGAG......    8
.......................................CCATCGACCGTTGAGTGGAC.    8
...ATTCAACCTGTCGGTGAGTTT...................................    7
..CATTCAACCTGTCGGTGAGTTTG..................................    7
.........................................ATCGACCGTTGAGTGGA..    7
.........................................ATCGACCGTTGAGTGGAC.    7
........................................CATCGACCGTTGAGTGGACC    7
....TTCAACCTGTCGGTGAG......................................    6
....TTCAACCTGTCGGTGAGTTT...................................    6
....................................AAACCATCGACCGTTGAGT.....    6
...........................................TCGACCGTTGAGTGGACC    6
...ATTCAACCTGTCGGTGA.......................................    5
..CATTCAACCTGTCGGTGAGTTT...................................    5
.ACATTCAACCTGTCGGT.........................................    4
....TTCAACCTGTCGGTGA.......................................    4
....................................AAACCATCGACCGTTGAGTGGAC.    4
..CATTCAACCTGTCGGTGAG......................................    3
...ATTCAACCTGTCGGTGAG......................................    3
....................................AAACCATCGACCGTTGAGTG....    3
........................GGGCAGCTCAGGCAAACCATCGACCGTTGAGTGGACC    3
..CATTCAACCTGTCGGTGA.......................................    2
AACATTCAACCTGTCG...........................................    1
.ACATTCAACCTGTCGGTG........................................    1
.....TCAACCTGTCGGTGAG......................................    1
........ACCTGTCGGTGAGTTT...................................    1
AACATTCAACCTGTCGGTGAGTTTGGGC...............................    1
....................................AAACCATCGACCGTTGAG......    1
.....................................AACCATCGACCGTTGAG......    1
........................................CATCGACCGTTGAGTGGAC.    1
......................TTGGGCAGCTCAGGCAAACCATCGACCGTTGAGTGGACC    1
..((((((((..(((((((.(((((...........)))))))))))).))))))))).... (-23.70)

20/48620330-48620386(+)_mir_dog_89
CTGGGGTTCCTGGGGATGGGATTTGCTGCCTGTCACAAATCACATTGCCAGGGATTT
.....................................ATCACATTGCCAGGGATT.  1077
.....................................ATCACATTGCCAGGGATTT  267
.....................................ATCACATTGCCAGGGAT..  208
.....................................ATCACATTGCCAGGGA...  18
....................................AATCACATTGCCAGGGAT..  18
....................................AATCACATTGCCAGGGATT.  15
......................................TCACATTGCCAGGGATT.  6
..GGGGTTCCTGGGGATGGGATT..................................  3
..GGGGTTCCTGGGGATGGGATTT.................................  3
......................................TCACATTGCCAGGGAT..  3
..GGGGTTCCTGGGGATGGGATTTGCTGCC...........................  1
...((((((((((.((((.((((((.((.....)))))))).)))).)))))))))) (-23.80)

20/48620546-48620615(+)_mir_dog_90
CTGGGGAGCAGGGCTTAGCTGCTTGTGAGCAGAGTCCACACCAAGTCGTGTTCACAGTGGCTAAGTTCCG
.................................................TTCACAGTGGCTAAGTTCCG 28480
.................................................TTCACAGTGGCTAAGTTCC. 19099
.................................................TTCACAGTGGCTAAGTTC.. 7929
.................................................TTCACAGTGGCTAAGTT... 179
.................................................TTCACAGTGGCTAAGT.... 150
.........AGGGCTTAGCTGCTTGTGAGCA....................................... 53
.........AGGGCTTAGCTGCTTGTGAGC........................................ 20
```

```
.................................................GTTCACAGTGGCTAAGTTC.. 14
......................................................CAGTGGCTAAGTTCCG 8
...................................................TCACAGTGGCTAAGTTCC. 6
....................................................CACAGTGGCTAAGTTCCG 6
...................................................TCACAGTGGCTAAGTTCCG 5
.............................................TCGTGTTCACAGTGGCT........ 2
....................................................CACAGTGGCTAAGTTC.. 2
.....................................................ACAGTGGCTAAGTTCCG 2
........CAGGGCTTAGCTGCTTGTGAGCA....................................... 1
.................................................GTTCACAGTGGCTAAGTT... 1
...................................................TCACAGTGGCTAAGTTC.. 1
.................................................GTTCACAGTGGCTAAGTTCC. 1
....................................................CACAGTGGCTAAGTTCC. 1
.................................................GTTCACAGTGGCTAAGTTCCG 1
..........(((((((((..((.(((((((((.(........).)).)))))))))..))))))))).. (-27.70)

20/48620684-48620745(+)_mir_dog_91
CGTGCCTACTGAGCTGAAACACAGTTGATTTGTGCAGACTGGCTCAGTTCAGCAGGAACAGG
.......................................TGGCTCAGTTCAGCAGGAAC...   38477
.......................................TGGCTCAGTTCAGCAGGAACA..   14391
.......................................TGGCTCAGTTCAGCAGGAACAG.   8029
......................................CTGGCTCAGTTCAGCAGGAAC...   3022
..TGCCTACTGAGCTGAAACACAGT.....................................   975
.......................................TGGCTCAGTTCAGCAGGAA....   468
..TGCCTACTGAGCTGAAACACAG......................................   339
.GTGCCTACTGAGCTGAAACACAG......................................   338
.GTGCCTACTGAGCTGAAACACAGT.....................................   286
.GTGCCTACTGAGCTGAAACACA.......................................   243
......................................CTGGCTCAGTTCAGCAGGAACA..   229
.......................................TGGCTCAGTTCAGCAGGAACAGG   175
.GTGCCTACTGAGCTGAAACAC........................................   151
.......................................TGGCTCAGTTCAGCAGGA.....   136
..TGCCTACTGAGCTGAAACACAGTT....................................   82
..TGCCTACTGAGCTGAAACACA.......................................   37
.......................................TGGCTCAGTTCAGCAGG......   28
........................................GGCTCAGTTCAGCAGGAACA..   18
........................................GGCTCAGTTCAGCAGGAAC...   17
.........................................GCTCAGTTCAGCAGGAACAG.   16
CGTGCCTACTGAGCTGAAACACAGT.....................................   15
........................................GGCTCAGTTCAGCAGGAACAG.   14
..TGCCTACTGAGCTGAAACAC........................................   13
..........................................CTCAGTTCAGCAGGAAC...   13
..........................................CTCAGTTCAGCAGGAACA..   13
......................................CTGGCTCAGTTCAGCAGGA.....   12
.........................................GCTCAGTTCAGCAGGAAC...   11
......................................CTGGCTCAGTTCAGCAGGAACAG.   11
.........................................GCTCAGTTCAGCAGGAACA..   10
.GTGCCTACTGAGCTGAAACA.........................................   8
.GTGCCTACTGAGCTGAAAC..........................................   7
.........................................GCTCAGTTCAGCAGGAACAGG   6
........................................GGCTCAGTTCAGCAGGAACAGG   5
CGTGCCTACTGAGCTGAAACACA.......................................   4
CGTGCCTACTGAGCTGAAACA.........................................   3
..TGCCTACTGAGCTGAAACA.........................................   3
......TACTGAGCTGAAACACAGT.....................................   3
..........................................CTCAGTTCAGCAGGAACAG.   3
CGTGCCTACTGAGCTGAAACACAG......................................   2
.......ACTGAGCTGAAACACAGT.....................................   2
.GTGCCTACTGAGCTGAAACACAGTT....................................   2
..................................CAGACTGGCTCAGTTCAGC.........   2
.......................................TGGCTCAGTTCAGCAG.......   2
..........................................CTCAGTTCAGCAGGAA....   2
...........................................TCAGTTCAGCAGGAACA..   2
............................................CAGTTCAGCAGGAACA..   2
```

```
............................................CAGTTCAGCAGGAACAG.   2
..TGCCTACTGAGCTGAAAC..........................................   1
CGTGCCTACTGAGCTGAAACAC........................................   1
...GCCTACTGAGCTGAAACACAGT.....................................   1
...GCCTACTGAGCTGAAACACAGTT....................................   1
......................AGTTGATTTGTGCAGACTGGCT..................   1
.............................TTGTGCAGACTGGCTC.................   1
......................................CTGGCTCAGTTCAGCAGGAA....   1
........................................GGCTCAGTTCAGCAGGAA....   1
...........................................TCAGTTCAGCAGGAAC...   1
...........................................TCAGTTCAGCAGGAACAG.   1
......................................CTGGCTCAGTTCAGCAGGAACAGG   1
.((.(((.(((((((((....(((((..........)))))..))))))))).))).))... (-23.10)

20/50410924-50410984(+)_mir_dog_92
CCAGTGTTCAGACTACCTGTTCAGGGGGCTCTGAATGTGTACAGTAGTCTGCACATTGGTT
........................................ACAGTAGTCTGCACATTGGTT    40697
.......................................TACAGTAGTCTGCACATTGGTT    11488
.........................................CAGTAGTCTGCACATTGGTT    3162
........................................ACAGTAGTCTGCACATTGGT.    2314
.......................................TACAGTAGTCTGCACATTGGT.    976
.........................................CAGTAGTCTGCACATTGGT.    374
........................................ACAGTAGTCTGCACATTGG..    170
.......................................TACAGTAGTCTGCACATTGG..    142
........................................ACAGTAGTCTGCACAT.....    77
...........................................GTAGTCTGCACATTGGTT    38
..AGTGTTCAGACTACCTGTTC.......................................    32
.......................................TACAGTAGTCTGCACAT.....    32
..........................................AGTAGTCTGCACATTGGTT    18
.CAGTGTTCAGACTACCTGTTC.......................................    10
........................................ACAGTAGTCTGCACATT....    9
..AGTGTTCAGACTACCTGTT........................................    7
CCAGTGTTCAGACTACCTGTTC.......................................    7
...........................................GTAGTCTGCACATTGGT.    6
.........................................CAGTAGTCTGCACATTGG..    5
CCAGTGTTCAGACTACCTGTT........................................    4
.......................................TACAGTAGTCTGCACATTG...    4
........................................ACAGTAGTCTGCACATTG...    4
...GTGTTCAGACTACCTGTTC.......................................    2
.......................................TACAGTAGTCTGCACATT....    2
..........................................AGTAGTCTGCACATTGGT.    2
CCAGTGTTCAGACTACCT...........................................    1
.CAGTGTTCAGACTACCT...........................................    1
..AGTGTTCAGACTACCTGT.........................................    1
...GTGTTCAGACTACCTGT.........................................    1
......TTCAGACTACCTGTTCA......................................    1
.......................................TACAGTAGTCTGCACA......    1
..........................................AGTAGTCTGCACATTGG..    1
(((((((.((((((((.((((((((....)))))......))))))))))).))))))).. (-26.40)

20/50760562-50760625(+)_novel_dog_38
GCCTGGGGCGGGGGTCCTGGGCTGGGGCTGGCCGTGCTCCCTGCCTGGACCCCTGCTCACCGCC
........................................CTGCCTGGACCCCTGCTCACC... 372
........................................CTGCCTGGACCCCTGCTCACCGC. 134
........................................CTGCCTGGACCCCTGCTCACCG.. 120
........................................CTGCCTGGACCCCTGCTCAC.... 55
.........................................TGCCTGGACCCCTGCTCACC... 10
........................................CTGCCTGGACCCCTGCTCA..... 7
........................................CTGCCTGGACCCCTGCTC...... 4
.........................................TGCCTGGACCCCTGCTCACCG.. 3
...TGGGGCGGGGGTCCTGGGCTGGG...................................... 2
.......................................CCTGCCTGGACCCCTGCTCACC... 2
.........................................TGCCTGGACCCCTGCTCACCGC. 2
.........................................TGCCTGGACCCCTGCTCAC.... 1
```

```
((...((((((((((((.((((.(((((.......)).))).)))))))))))))))...)). (-43.20)

20/51974329-51974393(+)_novel_Dog_Blast_18
GTCTTGGCGCGACTTCCTCGACTCACTCTCTTTCCCCGTGAGTCGTGGAACCTCGCCCGAGGGT
.......................................TGAGTCGTGGAACCTCGCCCGA....      83
.........................................AGTCGTGGAACCTCGCCCGAG...      9
.........................................AGTCGTGGAACCTCGCCCGA....      6
.........................................AGTCGTGGAACCTCGCCCGAGG..      5
...TTGGCGCGACTTCCTCGACTC.........................................      4
....TGGCGCGACTTCCTCGACT..........................................      3
....TGGCGCGACTTCCTCGACTCACT......................................      3
.......................................TGAGTCGTGGAACCTCGCCC......      3
.......................................TGAGTCGTGGAACCTCGCCCGAG...      3
....TGGCGCGACTTCCTCGACTCA........................................      2
.......................................TGAGTCGTGGAACCTCGCCCG.....      2
...TTGGCGCGACTTCCTCGACT..........................................      1
....TGGCGCGACTTCCTCGACTC.........................................      1
....TGGCGCGACTTCCTCGACTCAC.......................................      1
...TTGGCGCGACTTCCTCGACTCACT......................................      1
.......CGCGACTTCCTCGACTCACT......................................      1
..............................TTTCCCCGTGAGTCGTGGAACC.............      1
........................................GAGTCGTGGAACCTCGCCCGA....      1
..................................CCCCGTGAGTCGTGGAACCTCGCCCGAGG..      1
...................................CCCGTGAGTCGTGGAACCTCGCCCGAGG..      1
.....................................CGTGAGTCGTGGAACCTCGCCCGAGG..      1
......................................GTGAGTCGTGGAACCTCGCCCGAGGGT      1
.((((((.((((.((((.((((((((...........)))))))).))))..)))))))))).. (-31.20)

20/54654166-54654228(+)_novel_dog_39
GGGGCGGGCAGGGCCGGTCTCCCTCCGCTTGCGTCGCTGACCCCCGGCCCTGCCCGCAGGAGA
.....................................TGACCCCCGGCCCTGCCCGCAG....  11
.....................................TGACCCCCGGCCCTGCCCGCA.....  6
.....................................TGACCCCCGGCCCTGCCCGC......  4
...GCGGGCAGGGCCGGTCTC..........................................  1
...GCGGGCAGGGCCGGTCTCCC........................................  1
.....................................TGACCCCCGGCCCTGCCCG.......  1
............................TTGCGTCGCTGACCCCCGGCCCTGCCCGCA.....  1
....................................CTGACCCCCGGCCCTGCCCGCA.....  1
....................................CTGACCCCCGGCCCTGCCCGCAG....  1
......................................GACCCCCGGCCCTGCCCGCAG....  1
...(((((((((((((((...(..((....))..)..))))...)))))))))))))...... (-37.10)

20/54655269-54655337(+)_novel_dog_40
TGATAAGTCCTGCAGGAGGAGAAGCCTGGTTTCACTTGTAGAGCTTCTCCTCCTGCAGGACTTCCCAGG
...TAAGTCCTGCAGGAGGAGAAGC.............................................  5
...........................................TTCTCCTCCTGCAGGACTTCC....  2
..ATAAGTCCTGCAGGAGGAGAA...............................................  1
..ATAAGTCCTGCAGGAGGAGAAGC.............................................  1
...TAAGTCCTGCAGGAGGAGAAGCC............................................  1
...........................................TTCTCCTCCTGCAGGACTTCCC...  1
((..(((((((((((((((((((((((((.........))).))))))))))))))))))))..)).. (-44.40)

20/54993200-54993270(-)_mir_dog_93
TGCTGTGTGGAAGACTAGTGATTTTGTTGTTCTGATGTACTATGACAACAAGTCACAGCTTCCTCATAGCG
.......TGGAAGACTAGTGATTTTGTTGTT........................................
      25595
.......TGGAAGACTAGTGATTTTGTTGT.........................................
      23667
.......TGGAAGACTAGTGATTTTGTTG..........................................
      2344
.......TGGAAGACTAGTGATTTTGTT...........................................      749
.............................................CAACAAGTCACAGCTTCCTCA.....      417
.......TGGAAGACTAGTGATTTTGT............................................      368
.............................................CAACAAGTCACAGCTTCCTC......      310
```

```
.........GAAGACTAGTGATTTTGTTGTT....................................... 260
..........AAGACTAGTGATTTTGTTGTT....................................... 185
............................................CAACAAGTCACAGCTTCCT....... 185
..............CTAGTGATTTTGTTGTT....................................... 131
.........GAAGACTAGTGATTTTGTTGT........................................ 106
...........AGACTAGTGATTTTGTTGTT....................................... 76
..........AAGACTAGTGATTTTGTTGT........................................ 75
........GGAAGACTAGTGATTTTGTTGTT....................................... 72
.............ACTAGTGATTTTGTTGTT....................................... 61
..............CTAGTGATTTTGTTGT........................................ 44
.......TGGAAGACTAGTGATTTTG............................................ 38
.............ACTAGTGATTTTGTTGT........................................ 36
.......TGGAAGACTAGTGATTTT............................................. 29
...........AGACTAGTGATTTTGTTGT........................................ 29
............................................CAACAAGTCACAGCTTCCTCAT.... 28
........GGAAGACTAGTGATTTTGTTGT........................................ 21
.......TGGAAGACTAGTGATTTTGTTGTTC...................................... 20
.....TGTGGAAGACTAGTGATTTTGTT.......................................... 18
...............TAGTGATTTTGTTGTT....................................... 18
............GACTAGTGATTTTGTTGT........................................ 12
.........GAAGACTAGTGATTTTGTT.......................................... 11
.............................................AACAAGTCACAGCTTCCTCA..... 10
.........GAAGACTAGTGATTTTGT........................................... 9
.......TGGAAGACTAGTGATTT.............................................. 8
.....TGTGGAAGACTAGTGATTTTGT........................................... 7
......GTGGAAGACTAGTGATTTTGTTGTT....................................... 7
............GACTAGTGATTTTGTTGTT....................................... 7
............................................CAACAAGTCACAGCTTCC........ 7
..........AAGACTAGTGATTTTGTT.......................................... 6
............GACTAGTGATTTTGTTG......................................... 6
.....TGTGGAAGACTAGTGATTTTGTTG......................................... 5
.........GAAGACTAGTGATTTTGTTG......................................... 5
..........AAGACTAGTGATTTTGTTG......................................... 5
......GTGGAAGACTAGTGATTTTGTTGT........................................ 5
.........GAAGACTAGTGATTTTGTTGTTC...................................... 4
..........AAGACTAGTGATTTTGTTGTTC...................................... 4
............................................CAACAAGTCACAGCTT.......... 4
.............................................AACAAGTCACAGCTTCCTC...... 4
.......TGGAAGACTAGTGATT............................................... 3
...........AGACTAGTGATTTTGT........................................... 3
...........AGACTAGTGATTTTGTTG......................................... 3
.....TGTGGAAGACTAGTGATTTT............................................. 2
.........GAAGACTAGTGATTTT............................................. 2
........GGAAGACTAGTGATTTTGTT.......................................... 2
........GGAAGACTAGTGATTTTGTTG......................................... 2
.............ACTAGTGATTTTGTTG......................................... 2
............................................CAACAAGTCACAGCTTCCTCATA... 2
..........AAGACTAGTGATTTTG............................................ 1
....GTGTGGAAGACTAGTGATTTTGT........................................... 1
........GGAAGACTAGTGATTTTGT........................................... 1
..........AAGACTAGTGATTTTGT........................................... 1
......GTGGAAGACTAGTGATTTTGTT.......................................... 1
...........AGACTAGTGATTTTGTT.......................................... 1
.....TGTGGAAGACTAGTGATTTTGTTGT........................................ 1
........GGAAGACTAGTGATTTTGTTGTTC...................................... 1
...........AGACTAGTGATTTTGTTGTTC...................................... 1
.............ACTAGTGATTTTGTTGTTC...................................... 1
..............CTAGTGATTTTGTTGTTC...................................... 1
................AGTGATTTTGTTGTTC...................................... 1
............................................CAACAAGTCACAGCTTC......... 1
..............................................ACAAGTCACAGCTTCCTCAT.... 1
.((((((.(((((.((.(((((((.((((((.((......)).))))))))))))))))))))).)))))). (-31.70)
```

20/55641195-55641263(-)_novel_dog_46

```
TTCAGGACGTGCTTAGAGATTAGGCTGATAAAACCCTCAGCCTCTCTCTGGGACGCTTCCTAAGAGACC
...AGGACGTGCTTAGAGATTAGGCT...........................................  13
.........................................CTCTCTCTGGGACGCTTCCTAAGA....  2
...((((.(((((((((((..(((((((........))))))).)))))))).))).))))........ (-29.70)

20/56036697-56036762(-)_novel_dog_47
AGCTCGGGGCGGACGGAAGACTTCTCGTGCCCCCGTCCCACTCAGCCCTCTGTCTGCCCCGCAGCT
...TCGGGGCGGACGGAAGACTTCT.........................................     37
...TCGGGGCGGACGGAAGACTTCTC........................................     29
..CTCGGGGCGGACGGAAGACTTCT.........................................     21
........................................CTCAGCCCTCTGTCTGCCCCGC....     12
..CTCGGGGCGGACGGAAGACTTCTC........................................     7
.........................................TCAGCCCTCTGTCTGCCCCGC....     4
........................................CTCAGCCCTCTGTCTGCCCCGCA...     3
...TCGGGGCGGACGGAAGACTTC..........................................     2
....CGGGGCGGACGGAAGACTTCT.........................................     2
....CGGGGCGGACGGAAGACTTCTC........................................     2
..CTCGGGGCGGACGGAAGACTTCTCG.......................................     2
...TCGGGGCGGACGGAAGACTTCTCGT......................................     2
........................................CTCAGCCCTCTGTCTGCCCCG.....     2
...TCGGGGCGGACGGAAGAC.............................................     1
.GCTCGGGGCGGACGGAAGACT............................................     1
..CTCGGGGCGGACGGAAGACT............................................     1
...TCGGGGCGGACGGAAGACT............................................     1
..CTCGGGGCGGACGGAAGACTTC..........................................     1
.GCTCGGGGCGGACGGAAGACTTCTC........................................     1
....CGGGGCGGACGGAAGACTTCTCGT......................................     1
..CTCGGGGCGGACGGAAGACTTCTCGTG.....................................     1
..CTCGGGGCGGACGGAAGACTTCTCGTGC....................................     1
........................................CTCAGCCCTCTGTCTGCCCCGCAG..     1
(((((((((((((((((.(.((....(((.........)))..)).).))))))))))))).)))) (-30.90)

20/56717990-56718056(-)_novel_dog_48
CCCACGGGGTGGCACCTGCCGACGCCGGGCTGTGTGTGCGTCAGCACTCGTGCCGCCCAGGGAGCAG
........................................TCAGCACTCGTGCCGCCCAGGGA....    16
...ACGGGGTGGCACCTGCCGACG...........................................    10
.......................................GTCAGCACTCGTGCCGCCCAGGGA....    9
........................................TCAGCACTCGTGCCGCCCAGGGAG...    8
...ACGGGGTGGCACCTGCCGACGC..........................................    6
........................................TCAGCACTCGTGCCGCCCAGGG.....    4
.......................................GTCAGCACTCGTGCCGCCCAGG......    3
.......................................GTCAGCACTCGTGCCGCCCAGGG.....    3
..CACGGGGTGGCACCTGCCGACG...........................................    2
....CGGGGTGGCACCTGCCGACGC..........................................    2
......................................CGTCAGCACTCGTGCCGCCCAGGG.....    2
........................................TCAGCACTCGTGCCGCCCAGGGAGC..    2
...ACGGGGTGGCACCTGCCGA.............................................    1
...ACGGGGTGGCACCTGCCGAC............................................    1
........................................TCAGCACTCGTGCCGCCCAGG......    1
...........................................AGCACTCGTGCCGCCCAGGGAGC..    1
(((...(((((((((.(((.(((((((.......)).))))).)))...))))))))).))).....  (-35.00)

20/57663957-57664020(+)_novel_dog_41
AGCCCTCCCGGCCTCCCGCTGACCTCTGATCCTGGCGATGAGGGTGCCGGCGGCCGTGAGGGTC
.....TCCCGGCCTCCCGCTGACCTCT..................................... 14
.....TCCCGGCCTCCCGCTGACCT....................................... 3
.....TCCCGGCCTCCCGCTGACCTCTGA................................... 2
........................................AGGGTGCCGGCGGCCGTGAGG... 2
.....TCCCGGCCTCCCGCTGACCTC...................................... 1
.....TCCCGGCCTCCCGCTGACCTCTG.................................... 1
.....TCCCGGCCTCCCGCTGACCTCTGATCCT............................... 1
.((((((.(((((.((.((..(((.((.(((.....))).))))))).)).))))).)))))). (-34.70)

20/7968529-7968602(+)_mir_dog_94
```

CCGCGCTCTCTCCATTACACTACCCTGCCTCTTCTCCATGAGAGGCAGCGGGGTGTAGTGGATAGAGCACGGGT
..........TCCATTACACTACCCTGCCTCT..
40156
.........CTCCATTACACTACCCTGCCT..
3075
.........CTCCATTACACTACCCTGCCTC...
1486
..........TCCATTACACTACCCTGCCT..
1263
..........TCCATTACACTACCCTGCCTC...
1157
....AGGCAGCGGGGTGTAGTGGAT........... 759
.........CTCCATTACACTACCCTGCCTCT.. 391
....AGGCAGCGGGGTGTAGTGGATA.......... 335
....AGGCAGCGGGGTGTAGTGGA............ 144
..........TCCATTACACTACCCTGCCTCTT... 132
....AGGCAGCGGGGTGTAGT............... 92
....AGGCAGCGGGGTGTAGTGG............. 65
............CATTACACTACCCTGCCTCT.. 47
.........CTCCATTACACTACCCTGCC... 45
..........TCCATTACACTACCCTGCC... 44
........TCTCCATTACACTACCCTGCCT.. 27
.........CTCCATTACACTACCCTGC.. 25
...............ACACTACCCTGCCTCT.. 19
.............ATTACACTACCCTGCCTCT.. 10
.........CTCCATTACACTACCCTG... 9
....AGGCAGCGGGGTGTAGTG.............. 9
..........TCCATTACACTACCCTG... 8
..........TCCATTACACTACCCTGC.. 6
.........CTCCATTACACTACCCTGCCTCTT... 6
....GAGGCAGCGGGGTGTAGTGGAT........... 6
.....................................ATGAGAGGCAGCGGGGTGTAGT............... 4
............CATTACACTACCCTGCCT.. 3
....GGCAGCGGGGTGTAGTGGAT........... 3
....GCGGGGTGTAGTGGAT........... 3
....AGGCAGCGGGGTGTAGTGGATAGA........ 3
..............TACACTACCCTGCCTCT.. 2
..........TCCATTACACTACCCTGCCTCTTC.. 2
..........TCCATTACACTACCCTGCCTCTTCT....................................... 2
..............TTACACTACCCTGCCTCTTCTCC..................................... 2
....................................CATGAGAGGCAGCGGGGTGTAG................ 2
....GGCAGCGGGGTGTAGTGGATAGA........ 2
.........CTCCATTACACTACCC... 1
..........TCCATTACACTACCCT.. 1
........TCTCCATTACACTACCCTG... 1
......TCTCTCCATTACACTACCCTGC.. 1
........TCTCCATTACACTACCCTGC.. 1
........TCTCCATTACACTACCCTGCC... 1
........TCTCCATTACACTACCCTGCCTC... 1
..............TTACACTACCCTGCCTCT.. 1
.........CTCCATTACACTACCCTGCCTCTTCTCCATGAGAGGCAGC......................... 1
....AGGCAGCGGGGTGTAG................ 1
....................................CATGAGAGGCAGCGGGGTGTAGT............... 1
......................................TGAGAGGCAGCGGGGTGTAGT............... 1
....GAGGCAGCGGGGTGTAGTGGA............ 1
....GCAGCGGGGTGTAGTGGA............ 1
....GAGGCAGCGGGGTGTAGTGGATA.......... 1
....GGCAGCGGGGTGTAGTGGATA.......... 1
....CAGCGGGGTGTAGTGGATA.......... 1
....AGGCAGCGGGGTGTAGTGGATAG......... 1
....GGCAGCGGGGTGTAGTGGATAG......... 1
....CAGCGGGGTGTAGTGGATAG......... 1
(((.(((((.((((((((((.((((((((((......))))))))).))))))))))))).))))).))).. (-
49.30)

```
20/8334556-8334627(-)_novel_dog_43
ACGATGAGGCACGTGGGCAGCTCCAGGAATAGCAGAGGCGTGACAGGTGCTTCTCACGTTCTTCACAGAGTC
...............................................TGCTTCTCACGTTCTTCACAG....     24
...............................................TGCTTCTCACGTTCTTCACA.....     8
...ATGAGGCACGTGGGCAGCTCC.................................................     4
...ATGAGGCACGTGGGCAGCTC..................................................     3
ACGATGAGGCACGTGGGCAGCTCC.................................................     3
ACGATGAGGCACGTGGGCAGCT...................................................     1
..GATGAGGCACGTGGGCAGCTCCA................................................     1
...............................................TGCTTCTCACGTTCTTCAC......     1
...............................................TGCTTCTCACGTTCTTCACAGA...     1
.(..(((((.(((((((.(((.((.(..((.((....))))..).)).))).))))))).)))))..).... (-
25.40)

21/13305545-13305609(+)_novel_Dog_Blast_19
GTCTTGGCGCGACTTCCTCGACTCACTCTCTTTCCCCCGTGAGTCGTGGAACCTCGCCCGAGGGT
.......................................TGAGTCGTGGAACCTCGCCCGA....      83
.........................................AGTCGTGGAACCTCGCCCGAG...      9
.........................................AGTCGTGGAACCTCGCCCGA....      6
.........................................AGTCGTGGAACCTCGCCCGAGG..      5
...TTGGCGCGACTTCCTCGACTC.........................................      4
....TGGCGCGACTTCCTCGACT..........................................      3
....TGGCGCGACTTCCTCGACTCACT......................................      3
.......................................TGAGTCGTGGAACCTCGCCC......      3
.......................................TGAGTCGTGGAACCTCGCCCGAG...      3
....TGGCGCGACTTCCTCGACTCA........................................      2
.......................................TGAGTCGTGGAACCTCGCCCG.....      2
...TTGGCGCGACTTCCTCGACT..........................................      1
....TGGCGCGACTTCCTCGACTC.........................................      1
....TGGCGCGACTTCCTCGACTCAC.......................................      1
...TTGGCGCGACTTCCTCGACTCACT......................................      1
.......CGCGACTTCCTCGACTCACT......................................      1
..............................TTTCCCCCGTGAGTCGTGGAACC............      1
........................................GAGTCGTGGAACCTCGCCCGA....      1
..................................CCCCGTGAGTCGTGGAACCTCGCCCGAGG..      1
...................................CCCGTGAGTCGTGGAACCTCGCCCGAGG..      1
.....................................CGTGAGTCGTGGAACCTCGCCCGAGG..      1
......................................GTGAGTCGTGGAACCTCGCCCGAGGGT      1
.((((((.((((.((((.((((((((...........)))))))).))))..)))))))))).. (-31.20)

21/17362084-17362148(-)_novel_Dog_Blast_20
GTCTTGGCGCGACTTCCTCGACTCACTCTCTTTCCCCCGTGAGTCGTGGAACCTCACCCGAGGGT
...TTGGCGCGACTTCCTCGACTC.........................................      4
....TGGCGCGACTTCCTCGACT..........................................      3
....TGGCGCGACTTCCTCGACTCACT......................................      3
....TGGCGCGACTTCCTCGACTCA........................................      2
...TTGGCGCGACTTCCTCGACT..........................................      1
....TGGCGCGACTTCCTCGACTC.........................................      1
....TGGCGCGACTTCCTCGACTCAC.......................................      1
...TTGGCGCGACTTCCTCGACTCACT......................................      1
.......CGCGACTTCCTCGACTCACT......................................      1
..............................TTTCCCCCGTGAGTCGTGGAACC............      1
.......................................TGAGTCGTGGAACCTCACCCGA....      1
.((((((.(.((.((((.((((((((...........)))))))).))))..)).))))))).. (-25.80)

21/19542577-19542645(+)_mir_dog_95
AAGGAGCTTACAATCTAGCTGGGGGTGAACGGCTTGCACATGAACGCAACTAGACTGTGAGCTTCTAGA
..............................................CAACTAGACTGTGAGCTTCTAGA  3133
AAGGAGCTTACAATCTAGCTGGG..............................................  2973
AAGGAGCTTACAATCTAGCTGG...............................................  2671
..............................................CAACTAGACTGTGAGCTTCTAG.  2140
..............................................CAACTAGACTGTGAGCTTCT...  1411
AAGGAGCTTACAATCTAGCT.................................................  1245
```

....AACTAGACTGTGAGCTTCTAG. 663
....AACTAGACTGTGAGCTTCTAGA 454
....CAACTAGACTGTGAGCTTCTA.. 418
AAGGAGCTTACAATCTAGCTG.. 320
AAGGAGCTTACAATCTAGC.. 250
....AACTAGACTGTGAGCTTCT... 194
....ACTAGACTGTGAGCTTCTAGA 66
....AACTAGACTGTGAGCTTCTA.. 48
....ACTAGACTGTGAGCTTCTAG. 39
AAGGAGCTTACAATCT... 28
AAGGAGCTTACAATCTAGCTGGGG... 22
....CAACTAGACTGTGAGCTTC.... 16
....CAACTAGACTGTGAGCTT..... 10
.......................GGTGAACGGCTTGCACATGAAC........................ 7
AAGGAGCTTACAATCTAG... 5
.......TTACAATCTAGCTGGG.. 5
.........................TGAACGGCTTGCACATGAACG....................... 5
....CAACTAGACTGTGAGCT...... 5
....AACTAGACTGTGAGCTTC.... 5
.....................................ACATGAACGCAACTAGACTGTGAGCTTCTAGA 5
....AGACTGTGAGCTTCTAGA 5
AAGGAGCTTACAATCTA.. 4
.AGGAGCTTACAATCTAGCTGGG.. 2
..GGAGCTTACAATCTAGCTGGG.. 2
.....GCTTACAATCTAGCTGGG.. 2
....................................CACATGAACGCAACTAGACTGT........... 2
....CGCAACTAGACTGTGAGCTTCT... 2
....ACTAGACTGTGAGCTTCT... 2
....TAGACTGTGAGCTTCTAG. 2
AAGGAGCTTACAATCTAGCTGGGGGT... 1
AAGGAGCTTACAATCTAGCTGGGGGTGA... 1
.........................TGAACGGCTTGCACATGAAC........................ 1
.......................GGTGAACGGCTTGCACATGAACG....................... 1
.......................GGTGAACGGCTTGCACATGAACGC...................... 1
....AACTAGACTGTGAGCT...... 1
....GAACGCAACTAGACTGTGAGCTT..... 1
....AACTAGACTGTGAGCTT..... 1
....ACTAGACTGTGAGCTTCTA.. 1
....GCAACTAGACTGTGAGCTTCTAG. 1
....AGACTGTGAGCTTCTAG. 1
.................................TTGCACATGAACGCAACTAGACTGTGAGCTTCTAGA 1
......................................CATGAACGCAACTAGACTGTGAGCTTCTAGA 1
....AACGCAACTAGACTGTGAGCTTCTAGA 1
.(((((((((((.(((((.((.(.(((.........))).....).)).))))).)))))))))))... (-25.90)

21/20592019-20592076(+)_mir_dog_96
CCACCAGCTGGCGTTCCCTGGCGTGGGAACGAGAGGCAGGGGCTGCTGGCTGGAGGGA
CCACCAGCTGGCGTTCCCTGGC.................................... 1311
....................................CAGGGGCTGCTGGCTGGAGGGA 690
CCACCAGCTGGCGTTCCCTGG..................................... 332
CCACCAGCTGGCGTTCCCTG...................................... 132
.CACCAGCTGGCGTTCCCTGG..................................... 93
....................................CAGGGGCTGCTGGCTGGAGGG. 83
CCACCAGCTGGCGTTCCCT....................................... 62
.CACCAGCTGGCGTTCCCTGGC.................................... 36
....................................CAGGGGCTGCTGGCTGGAGG.. 33
.CACCAGCTGGCGTTCCCTG...................................... 28
CCACCAGCTGGCGTTCCCTGGCGT.................................. 28
CCACCAGCTGGCGTTCCC.. 20
....................................CAGGGGCTGCTGGCTGGAG... 19
.....................................AGGGGCTGCTGGCTGGAGGGA 11
CCACCAGCTGGCGTTCCCTGGCG................................... 8
.....................................AGGGGCTGCTGGCTGGAGG.. 4
...CCAGCTGGCGTTCCCTGG..................................... 3

```
....................................CAGGGGCTGCTGGCTGGA.... 3
......................................GGGGCTGCTGGCTGGAGGGA 3
.....AGCTGGCGTTCCCTGGC..................................... 2
.CACCAGCTGGCGTTCCCTGGCGT.................................. 2
....................................CAGGGGCTGCTGGCTGG..... 2
CCACCAGCTGGCGTTCC......................................... 1
.CACCAGCTGGCGTTCCC........................................ 1
....CAGCTGGCGTTCCCTGG..................................... 1
.CACCAGCTGGCGTTCCCTGGCG................................... 1
..ACCAGCTGGCGTTCCCTGGCGT.................................. 1
.....................................AGGGGCTGCTGGCTGGAGGG. 1
...................................GCAGGGGCTGCTGGCTGGAGGGA 1
((.(((((..(((..(((((.(.((....))...).)))))..)))..))))).)).. (-35.40)

21/25597025-25597081(+)_mir_dog_97
CTACAGTGCACGTGTCTCCAGTGTGGCTCCGAGGCTGGAGACGCGGCCCTGTTGGAA
...................................TGGAGACGCGGCCCTGTTGGA.  142
...................................TGGAGACGCGGCCCTGTTGGAA  80
...................................TGGAGACGCGGCCCTGTTGG..  71
....................................GGAGACGCGGCCCTGTTGGAA  30
....................................GGAGACGCGGCCCTGTTGGA.  12
..ACAGTGCACGTGTCTCCAGT...................................  8
..ACAGTGCACGTGTCTCCA.....................................  7
CTACAGTGCACGTGTCTCCAGT...................................  7
...................................TGGAGACGCGGCCCTGTTG...  6
...CAGTGCACGTGTCTCCAGT...................................  5
.....GTGCACGTGTCTCCAGT...................................  5
......................................AGACGCGGCCCTGTTGGAA  5
..ACAGTGCACGTGTCTCCAG....................................  4
.....GTGCACGTGTCTCCAG....................................  4
CTACAGTGCACGTGTCTC.......................................  3
CTACAGTGCACGTGTCTCC......................................  3
..ACAGTGCACGTGTCTCC......................................  3
...CAGTGCACGTGTCTCC......................................  3
.TACAGTGCACGTGTCTCCAG....................................  3
.TACAGTGCACGTGTCTCCAGT...................................  3
...................................TGGAGACGCGGCCCTGT.....  3
.TACAGTGCACGTGTCTC.......................................  2
.TACAGTGCACGTGTCTCCA.....................................  2
............................CCGAGGCTGGAGACGCGGCCC........  2
..ACAGTGCACGTGTCTC.......................................  1
.TACAGTGCACGTGTCTCC......................................  1
CTACAGTGCACGTGTCTCCA.....................................  1
...CAGTGCACGTGTCTCCA.....................................  1
CTACAGTGCACGTGTCTCCAG....................................  1
...CAGTGCACGTGTCTCCAG....................................  1
....AGTGCACGTGTCTCCAGT...................................  1
......TGCACGTGTCTCCAGT...................................  1
...................AGTGTGGCTCCGAGGCTGGAGAC...............  1
....................................GGAGACGCGGCCCTGT.....  1
............................CCGAGGCTGGAGACGCGGCCCTGTTGGA.  1
......................................AGACGCGGCCCTGTTGGA.  1
...........................TCCGAGGCTGGAGACGCGGCCCTGTTGGAA  1
.....................................GAGACGCGGCCCTGTTGGAA  1
((((((.((.((((((((((((.((....))..)))))))))))))).))).))).. (-31.00)

21/25607357-25607423(+)_novel_dog_50
GGGCTGGGACAGGGCAGCTGGGAGGGCTCTCCGCAGGATCTCACCAGTGCCTTCTGTCCTAGGAGAC
........................................TCACCAGTGCCTTCTGTCCTAG.....    39
........................................TCACCAGTGCCTTCTGTCCT.......    13
........................................TCACCAGTGCCTTCTGTCCTA......    2
...CTGGGACAGGGCAGCTGGGAGGGCT.......................................    1
...(((((((((((((.((((((((..(((....))).)))).))))))))..))))))))))..... (-32.20)
```

```
21/32100953-32101018(+)_mir_dog_98
CCATCGAGGCTAGAGTCACGCTTGGGTATCAGCTATTGCCTTAGTGTGCTAGAGTCCTCGAAGAGT
..ATCGAGGCTAGAGTCACGCTTGG.........................................     1205
.........................................TAGTGTGCTAGAGTCCTCGAAGA..     270
..ATCGAGGCTAGAGTCACGCTT...........................................     191
.........................................TAGTGTGCTAGAGTCCTCGAAG...     136
..ATCGAGGCTAGAGTCACGCTTG..........................................     119
.........................................TAGTGTGCTAGAGTCCTCGAAGAGT     61
..ATCGAGGCTAGAGTCACGCT............................................     30
.........................................TAGTGTGCTAGAGTCCTCGAAGAG.     11
.........................................TAGTGTGCTAGAGTCCTCGAA....     10
.CATCGAGGCTAGAGTCACGCTTG..........................................     9
..ATCGAGGCTAGAGTCACGCTTGGGTATC....................................     9
..ATCGAGGCTAGAGTCACGCTTGGG........................................     7
.........................................TAGTGTGCTAGAGTCCTCGA.....     7
..ATCGAGGCTAGAGTCACGCTTGGGT.......................................     6
..ATCGAGGCTAGAGTCACGCTTGGGTATCAGCT................................     5
.CATCGAGGCTAGAGTC.................................................     4
.CATCGAGGCTAGAGTCACGCTTGG.........................................     4
..ATCGAGGCTAGAGTCACGCTTGGGTA......................................     4
..ATCGAGGCTAGAGTCACGC.............................................     3
.CATCGAGGCTAGAGTCACGCT............................................     3
.CATCGAGGCTAGAGTCACGCTT...........................................     3
.CATCGAGGCTAGAGTCACGCTTGGGTATC....................................     3
....CGAGGCTAGAGTCACGCT............................................     1
....CGAGGCTAGAGTCACGCTTGG.........................................     1
.CATCGAGGCTAGAGTCACGCTTGGGT.......................................     1
.CATCGAGGCTAGAGTCACGCTTGGGTA......................................     1
........................................TTAGTGTGCTAGAGTCCTCGA.....     1
..........................................AGTGTGCTAGAGTCCTCGAAGAGT     1
............................................TGTGCTAGAGTCCTCGAAGAGT     1
...((((((((..((.((((((.(((((........))))).))))))))..)).)))))).... (-28.90)

21/5864108-5864172(-)_novel_Dog_Blast_21
GTCTTGGCGCGACTTCCTCGACTCACTCTCTTTCCCCCGTGAGTCGTGGAACCTCGCCCGAGGGT
.......................................TGAGTCGTGGAACCTCGCCCGA....      83
.........................................AGTCGTGGAACCTCGCCCGAG...      9
.........................................AGTCGTGGAACCTCGCCCGA....      6
.........................................AGTCGTGGAACCTCGCCCGAGG..      5
...TTGGCGCGACTTCCTCGACTC.........................................      4
....TGGCGCGACTTCCTCGACT..........................................      3
....TGGCGCGACTTCCTCGACTCACT......................................      3
.......................................TGAGTCGTGGAACCTCGCCC......      3
.......................................TGAGTCGTGGAACCTCGCCCGAG...      3
....TGGCGCGACTTCCTCGACTCA........................................      2
.......................................TGAGTCGTGGAACCTCGCCCG.....      2
...TTGGCGCGACTTCCTCGACT..........................................      1
....TGGCGCGACTTCCTCGACTC.........................................      1
....TGGCGCGACTTCCTCGACTCAC.......................................      1
...TTGGCGCGACTTCCTCGACTCACT......................................      1
.......CGCGACTTCCTCGACTCACT......................................      1
..............................TTTCCCCCGTGAGTCGTGGAACC............      1
........................................GAGTCGTGGAACCTCGCCCGA....      1
..................................CCCCGTGAGTCGTGGAACCTCGCCCGAGG..      1
...................................CCCGTGAGTCGTGGAACCTCGCCCGAGG..      1
.....................................CGTGAGTCGTGGAACCTCGCCCGAGG..      1
......................................GTGAGTCGTGGAACCTCGCCCGAGGGT      1
.((((((.((((.((((.((((((((............)))))))).))))..)))))))))).. (-31.20)

21/7022767-7022832(+)_novel_dog_49
TATAATGGTCATTACCAAGGCTTTGAGAATGCAGTTTCTCATTTGCTGTGGACATGACCATAAAAA
.........................................TTTGCTGTGGACATGACCATA....     261
...AATGGTCATTACCAAGGCTTTG.........................................     16
.........................................TTTGCTGTGGACATGACCATAAA..     16
```

....TTTGCTGTGGACATGACCAT.....   15
....ATTTGCTGTGGACATGACCATA....   9
....TTTGCTGTGGACATGACCATAA...   9
....ATGGTCATTACCAAGGCTTTGAGAA.....................................   6
....ATGGTCATTACCAAGGCTTTGAGAATG...................................   5
...AATGGTCATTACCAAGGCTTTGAGAA.....................................   4
...AATGGTCATTACCAAGGCTTTGAGAAT....................................   3
....ATGGTCATTACCAAGGCTTTGAGAAT....................................   3
....ATTTGCTGTGGACATGACC.......   3
....TTGCTGTGGACATGACCATA....   3
...AATGGTCATTACCAAGGCTTTGA.......................................   2
....TTTGCTGTGGACATGACCA......   2
...AATGGTCATTACCAAGGCT..   1
...AATGGTCATTACCAAGGCTTT..   1
...AATGGTCATTACCAAGGCTTTGAGAATG...................................   1
.....TGGTCATTACCAAGGCTTTGAGAATG....................................   1
....TTTGCTGTGGACATGACC.......   1
....CATTTGCTGTGGACATGACCATA....   1
....ATTTGCTGTGGACATGACCATAAA..   1
....((((((((..(((.(((..((((((......))))))...))).)))..))))))))..... (-25.30)

22/1846880-1846938(+)_mir_dog_99
TAGCAGCACATAATGGTTTGTGGATTTTGAAAAGGTGCAGGCCATATTGTGCTGCCTCA
TAGCAGCACATAATGGTTTGT......................................23853
TAGCAGCACATAATGGTTTG.......................................9897
TAGCAGCACATAATGGTTTGTG.....................................4813
TAGCAGCACATAATGGTTTGTGG....................................211
.....GCACATAATGGTTTGT......................................72
TAGCAGCACATAATGGTTTGTGGA...................................59
TAGCAGCACATAATGGTT...47
TAGCAGCACATAATGGTTT..31
TAGCAGCACATAATGGTTTGTGGAT..................................22
TAGCAGCACATAATGGT..8
....AGCACATAATGGTTTGT......................................7
.....GCACATAATGGTTTGTG.....................................7
TAGCAGCACATAATGG...5
....AGCACATAATGGTTTG.......................................3
.AGCAGCACATAATGGTTTGT......................................3
......CACATAATGGTTTGTG.....................................3
TAGCAGCACATAATGGTTTGTGGATTTTG..............................3
.....................................CAGGCCATATTGTGCTGCCTCA3
.AGCAGCACATAATGGTTTG.......................................2
..GCAGCACATAATGGTTTGT......................................2
.AGCAGCACATAATGGTTTGTG.....................................2
..GCAGCACATAATGGTTTGTG.....................................2
..GCAGCACATAATGGTTTG.......................................1
...CAGCACATAATGGTTTGT......................................1
TAGCAGCACATAATGGTTTGTGGATTTTGA.............................1
.....................................CAGGCCATATTGTGCTGCC...1
..((((((((..((((((((((..((.....))..))))))))))..)))))))).... (-24.90)

22/1847020-1847084(+)_mir_dog_100
TAGCAGCACGTAAATATTGGCGTTAAGATTCTAAAATTATCTCCAGTATTAACTGTGCTGCTGAA
TAGCAGCACGTAAATATTGGCG...   454301
TAGCAGCACGTAAATATTGGC..   40118
TAGCAGCACGTAAATATTGGCGT..   8476
TAGCAGCACGTAAATATTG..   1060
TAGCAGCACGTAAATATTGG...   656
TAGCAGCACGTAAATATTGGCGTT...   328
.AGCAGCACGTAAATATTGGCG...   124
.....GCACGTAAATATTGGC..   118
TAGCAGCACGTAAATAT..   103
......CACGTAAATATTGGCG...   65
..GCAGCACGTAAATATTGGCG...   46

```
....AGCACGTAAATATTGGCG...........................................      45
...CAGCACGTAAATATTGGCG...........................................      32
.....GCACGTAAATATTGGCG...........................................      30
....AGCACGTAAATATTGGC............................................      25
..........................................CCAGTATTAACTGTGCTGCTGAA      11
.AGCAGCACGTAAATATTGGC............................................      10
.AGCAGCACGTAAATATTGGCGT..........................................      9
TAGCAGCACGTAAATATTGGCGTTA........................................      9
TAGCAGCACGTAAATATT...............................................      7
...CAGCACGTAAATATTGGC............................................      6
..........................................CCAGTATTAACTGTGCTGCTGA.      5
..GCAGCACGTAAATATTGGC............................................      4
........................................CTCCAGTATTAACTGTGCTGCTG..      4
..........................................CCAGTATTAACTGTGCTGCTG..      3
.....GCACGTAAATATTGGCGT..........................................      2
TAGCAGCACGTAAATATTGGCGTTAAGAT....................................      2
TAGCAGCACGTAAATATTGGCGTTAAGATT...................................      2
..........................................CCAGTATTAACTGTGCTGCT...      2
.AGCAGCACGTAAATATTG..............................................      1
.......ACGTAAATATTGGCGTT.........................................      1
TAGCAGCACGTAAATATTGGCGTTAAGA.....................................      1
......................TTAAGATTCTAAAATTATCT.......................      1
.......................................TCTCCAGTATTAACTGTGCTG.....      1
.........................................TCCAGTATTAACTGTGCTG.....      1
((((((((((.(((((((((.(.(((..........))).).))))))))).)).)))))))).. (-24.30)

22/42478013-42478071(+)_mir_dog_101
CAAAGTGCTTACAGTGCAGGTAGTGATATGTGCATCTACTGCAGTGAAGGCACTTGTAG
CAAAGTGCTTACAGTGCAGGTAG....................................3900
CAAAGTGCTTACAGTGCAGGTAGT...................................841
CAAAGTGCTTACAGTGCAGGT......................................709
.....................................ACTGCAGTGAAGGCACTTGTA.611
CAAAGTGCTTACAGTGCAGGTA.....................................426
.....................................ACTGCAGTGAAGGCACTTGT..239
.....................................ACTGCAGTGAAGGCACTTGTAG166
.....................................ACTGCAGTGAAGGCACTTG...94
CAAAGTGCTTACAGTGCAGG.......................................27
......................................CTGCAGTGAAGGCACTTGTA.15
.AAAGTGCTTACAGTGCAGGTAG....................................11
....................................TACTGCAGTGAAGGCACTTGTA.7
......................................CTGCAGTGAAGGCACTTGTAG7
CAAAGTGCTTACAGTGCAG........................................4
.....................................ACTGCAGTGAAGGCACT.....4
......GCTTACAGTGCAGGTAG....................................3
......................................CTGCAGTGAAGGCACTTG...3
CAAAGTGCTTACAGTGCA.........................................2
.....TGCTTACAGTGCAGGTAG....................................2
....GTGCTTACAGTGCAGGTAGT...................................2
.....................................ACTGCAGTGAAGGCACTT....2
CAAAGTGCTTACAGTG...........................................1
CAAAGTGCTTACAGTGC..........................................1
.AAAGTGCTTACAGTGCAGGTAGT...................................1
......................................CTGCAGTGAAGGCACTTGT..1
........................................GCAGTGAAGGCACTTGTA.1
..((((((((.((.(((((.(((((.......)).)))))))).)).))))))))).... (-22.70)

22/42478148-42478220(+)_mir_dog_102
TTCTAAGGTGCATCTAGTGCAGATAGTGAAGTAGATTAGCATCTACTGCCCTAAGTGCTCCTTCTGGCATAAG
...TAAGGTGCATCTAGTGCAGATAG...............................................    283
...TAAGGTGCATCTAGTGCAGAT.................................................    180
...TAAGGTGCATCTAGTGCAGATA................................................    147
...TAAGGTGCATCTAGTGCAGA..................................................    94
............................................ACTGCCCTAAGTGCTCCTTCTG.......    75
...TAAGGTGCATCTAGTGCAGATAGT..............................................    49
```

```
............................................ACTGCCCTAAGTGCTCCTTCTGG......    34
...TAAGGTGCATCTAGTGCAG....................................................    15
...TAAGGTGCATCTAGTGCA.....................................................    9
............................................ACTGCCCTAAGTGCTCCTTCTGGC.....    6
............................................ACTGCCCTAAGTGCTCCTTCT........    3
........TGCATCTAGTGCAGATAG................................................    2
...TAAGGTGCATCTAGTGCAGATAGTGAAGT..........................................    2
.............................................CTGCCCTAAGTGCTCCTTCTG.......    2
...TAAGGTGCATCTAGTGC......................................................    1
...TAAGGTGCATCTAGTGCAGATAGTG..............................................    1
...TAAGGTGCATCTAGTGCAGATAGTGAAG...........................................    1
.............................................CTGCCCTAAGTGCTCCTTCTGGC.....    1
....((((.(((..(((.((((.(((((..(.....)..)).))))))).)))..))).))))......... (-
19.30)

22/42478298-42478355(+)_mir_dog_103
AGTTTTGCATAGTTGCACTACAAGAAGAATGTAGTTGTGCAAATCTATGCAAAACTGA
...................................TGTGCAAATCTATGCAAAACTGA 1599
...................................TGTGCAAATCTATGCAAAACTG. 1254
...................................TGTGCAAATCTATGCAAAACT.. 221
..................................TTGTGCAAATCTATGCAAAACTG. 70
..................................TTGTGCAAATCTATGCAAAACTGA 63
..................................TTGTGCAAATCTATGCAAAACT.. 56
...................................TGTGCAAATCTATGCAAAAC... 41
AGTTTTGCATAGTTGCACTA...................................... 18
AGTTTTGCATAGTTGCACT....................................... 9
..................................TTGTGCAAATCTATGCAAAAC... 9
............................ATGTAGTTGTGCAAATCTATG......... 4
AGTTTTGCATAGTTGCACTAC..................................... 3
.......................................GCAAATCTATGCAAAACTG. 2
........................................CAAATCTATGCAAAACTGA 1
..........................................AATCTATGCAAAACTGA 1
...........................................ATCTATGCAAAACTGA 1
((((((((((((((((((((((.......))))...))))))..))))))))))))).. (-23.00)

22/42478462-42478520(+)_mir_dog_104
TAAAGTGCTTATAGTGCAGGTAGTGTTTCGTTATCTACTGCATTATGAGCACTTAAAGT
TAAAGTGCTTATAGTGCAGGTAG....................................4346
TAAAGTGCTTATAGTGCAGGTAGT...................................775
TAAAGTGCTTATAGTGCAGGTA.....................................524
TAAAGTGCTTATAGTGCAGGT......................................509
.....................................ACTGCATTATGAGCACTTAAAGT82
TAAAGTGCTTATAGTGCAGG.......................................27
.....................................ACTGCATTATGAGCACTTAAAG.16
......................................CTGCATTATGAGCACTTAAAGT9
TAAAGTGCTTATAGTGCAG........................................5
TAAAGTGCTTATAGTG...........................................4
.....................................ACTGCATTATGAGCACTTAAA..3
....GTGCTTATAGTGCAGGTAG....................................2
......GCTTATAGTGCAGGTAG....................................2
.....................................ACTGCATTATGAGCACT......2
.....................................ACTGCATTATGAGCACTTA....2
TAAAGTGCTTATAGTGC..........................................1
TAAAGTGCTTATAGTGCA.........................................1
.AAAGTGCTTATAGTGCAGGTAGT...................................1
......GCTTATAGTGCAGGTAGT...................................1
TAAAGTGCTTATAGTGCAGGTAGTGTTTCGTT...........................1
........................GTTTCGTTATCTACTGCATTATGAGCACT......1
......................................CTGCATTATGAGCACTTAAAG.1
..((((((((((((((((.(((((......)).))))))))))))))))))))..... (-26.60)
```

```
22/42478590-42478656(+)_mir_dog_105
TATGGTTAGTTTTGCAGGTTTGCATCCAGCTGTGTGATATTCTGCTGTGCAAATCCATGCAAAACTG
.............................................TGTGCAAATCCATGCAAAACTG    15859
.............................................TGTGCAAATCCATGCAAAACT.    1592
.............................................TGTGCAAATCCATGCAAAAC..    233
............................................CTGTGCAAATCCATGCAAAACTG    13
.......AGTTTTGCAGGTTTGCATCCAGC.....................................    9
.............................................TGTGCAAATCCATGCAAAA...    9
..............................................GTGCAAATCCATGCAAAACTG    5
...................................................AATCCATGCAAAACTG    5
............................................CTGTGCAAATCCATGCAAAACT.    4
.................................................CAAATCCATGCAAAACTG    3
.............................................TGTGCAAATCCATGCA......    2
................................................GCAAATCCATGCAAAACTG    2
..................................................AAATCCATGCAAAACTG    2
.......AGTTTTGCAGGTTTGCATCC........................................    1
.......AGTTTTGCAGGTTTGCATCCAGCTGT..................................    1
.......................ATCCAGCTGTGTGATATTCTGCT.....................    1
.................................................CAAATCCATGCAAAACT.    1
..................................................AAATCCATGCAAAACT.    1
......((((((((((((((((((..((((.............)))))))))).)).)))))))))) (-24.22)

22/42478714-42478772(+)_mir_dog_106
AGGTTGGGATCGGTTGCAATGCTGTGTTTCTGTATGGTATTGCACTTGTCCCGGCCTGT
.....................................TATTGCACTTGTCCCGGCCTGT250984
.....................................TATTGCACTTGTCCCGGCCTG.10744
......................................ATTGCACTTGTCCCGGCCTGT3300
.....................................TATTGCACTTGTCCCGGCCT..538
.....................................TATTGCACTTGTCCCGGCC...534
......................................ATTGCACTTGTCCCGGCCTG.367
AGGTTGGGATCGGTTGCAATGCT....................................114
.....................................TATTGCACTTGTCCCGGC....52
AGGTTGGGATCGGTTGCAATGC.....................................28
.......................................TTGCACTTGTCCCGGCCTGT28
.........................................GCACTTGTCCCGGCCTGT15
......................................ATTGCACTTGTCCCGGCCT..11
......................................ATTGCACTTGTCCCGGCC...6
........................................TGCACTTGTCCCGGCCTGT5
...........................................ACTTGTCCCGGCCTGT4
AGGTTGGGATCGGTTGCAATG......................................3
..GTTGGGATCGGTTGCAATGCT....................................3
...TTGGGATCGGTTGCAATGCT....................................2
.....................................TATTGCACTTGTCCCGG.....2
AGGTTGGGATCGGTTGCAAT.......................................1
.GGTTGGGATCGGTTGCAATGCT....................................1
AGGTTGGGATCGGTTGCAATGCTGT..................................1
.....................................TATTGCACTTGTCCCG......1
.......................................TTGCACTTGTCCCGGCCTG.1
....................................GTATTGCACTTGTCCCGGCCTGT1
..........................................CACTTGTCCCGGCCTGT1
(((((((((.(((.((((((((((((......))))))))))))))).))))))))).. (-31.50)


23/2222254-2222319(-)_novel_dog_52
CACCTCCATGCTGTGCCACTGTCCCATAGGAAGCAGCGGGACGGAGGAAGACAGCCTGGTGGAAAC
....TCCATGCTGTGCCACTGTCCC.........................................     49
....TCCATGCTGTGCCACTGTCC..........................................     44
....TCCATGCTGTGCCACTGTCCCA........................................     6
.......................................GACGGAGGAAGACAGCCTGGTGG....     6
....TCCATGCTGTGCCACTGT............................................     1
....TCCATGCTGTGCCACTGTC...........................................     1
....TCCATGCTGTGCCACTGTCCCAT.......................................     1
.......................................GACGGAGGAAGACAGCCTGGT......     1
```

```
..((.(((.(((((.((.(((((((...(....)...))))))).))...))))).))).)).... (-29.90)

23/2223136-2223197(-)_mir_dog_107
AGCTGGTGTTGTGAATCAGGCCGTTGCCAATCAGAGAACGGCTACTTCACAACACCAGGGTC
AGCTGGTGTTGTGAATCAGGCCG.......................................   285451
AGCTGGTGTTGTGAATCAGGC.........................................   37641
AGCTGGTGTTGTGAATCAGGCCGT......................................   3030
AGCTGGTGTTGTGAATCAG...........................................   2860
AGCTGGTGTTGTGAATCAGGCC........................................   1688
AGCTGGTGTTGTGAATCAGGCCGTT.....................................   1180
AGCTGGTGTTGTGAATCAGG..........................................   885
.....GTGTTGTGAATCAGGCCG.......................................   807
........................................GCTACTTCACAACACCAGGGT.   508
.GCTGGTGTTGTGAATCAGGCCG.......................................   432
AGCTGGTGTTGTGAATC.............................................   426
......................................CGGCTACTTCACAACACCAGGGT.   190
AGCTGGTGTTGTGAATCA............................................   167
....GGTGTTGTGAATCAGGCCG.......................................   163
.......GTTGTGAATCAGGCCG.......................................   156
......TGTTGTGAATCAGGCCG.......................................   106
.....GTGTTGTGAATCAGGCC........................................   76
.....GTGTTGTGAATCAGGCCGT......................................   64
......................................CGGCTACTTCACAACACCAGGG..   50
....GGTGTTGTGAATCAGGC.........................................   41
AGCTGGTGTTGTGAAT..............................................   33
.....GTGTTGTGAATCAGGCCGTT.....................................   31
.....GTGTTGTGAATCAGGC.........................................   20
...TGGTGTTGTGAATCAGGCCG.......................................   19
...TGGTGTTGTGAATCAGGC.........................................   12
......TGTTGTGAATCAGGCC........................................   11
........................................GCTACTTCACAACACCAGGG..   9
......TGTTGTGAATCAGGCCGT......................................   8
....GGTGTTGTGAATCAGGCC........................................   7
.......GTTGTGAATCAGGCCGT......................................   7
......................................CGGCTACTTCACAACACCAGG...   7
......TGTTGTGAATCAGGCCGTT.....................................   6
.........................................CTACTTCACAACACCAGGGT.   6
....GGTGTTGTGAATCAGGCCGTT.....................................   5
AGCTGGTGTTGTGAATCAGGCCGTTG....................................   5
.GCTGGTGTTGTGAATCAGGC.........................................   4
...TGGTGTTGTGAATCAGGCCGTT.....................................   4
.......GTTGTGAATCAGGCCGTT.....................................   4
..................................AGAACGGCTACTTCACAACACC......   4
..........................................TACTTCACAACACCAGGGT.   4
.GCTGGTGTTGTGAATCAG...........................................   3
.GCTGGTGTTGTGAATCAGGCC........................................   3
....GGTGTTGTGAATCAGGCCGT......................................   3
......................................CGGCTACTTCACAACACCAG....   3
..CTGGTGTTGTGAATCAG...........................................   2
.GCTGGTGTTGTGAATCAGG..........................................   2
...TGGTGTTGTGAATCAGGCC........................................   2
...TGGTGTTGTGAATCAGGCCGT......................................   2
........TTGTGAATCAGGCCGT......................................   2
..CTGGTGTTGTGAATCAGG..........................................   1
....GGTGTTGTGAATCAGG..........................................   1
..CTGGTGTTGTGAATCAGGCCG.......................................   1
.GCTGGTGTTGTGAATCAGGCCGT......................................   1
.GCTGGTGTTGTGAATCAGGCCGTT.....................................   1
........TTGTGAATCAGGCCGTT.....................................   1
...............................CAGAGAACGGCTACTTC..............   1
..................................AGAACGGCTACTTCACAACAC.......   1
......................................CGGCTACTTCACAACACC......   1
......................................CGGCTACTTCACAACACCA.....   1
```

....ACTTCACAACACCAGGGT. 1
....GCTACTTCACAACACCAGGGTC 1
..(((((((((((((...(((((((..(.....)..)))))))..))))))))))))).... (-31.40)

23/3177087-3177151(+)_novel_Dog_Blast_22
GTCTTGGCGCGACTTCCTCGACTCACTCTCTTTCCCCCGTGAGTCGTGGAACCTCGCCCGAGGGT
....TGAGTCGTGGAACCTCGCCCGA.... 83
....AGTCGTGGAACCTCGCCCGAG... 9
....AGTCGTGGAACCTCGCCCGA.... 6
....AGTCGTGGAACCTCGCCCGAGG.. 5
...TTGGCGCGACTTCCTCGACTC... 4
....TGGCGCGACTTCCTCGACT.. 3
....TGGCGCGACTTCCTCGACTCACT...................................... 3
....TGAGTCGTGGAACCTCGCCC...... 3
....TGAGTCGTGGAACCTCGCCCGAG... 3
....TGGCGCGACTTCCTCGACTCA.. 2
....TGAGTCGTGGAACCTCGCCCG..... 2
...TTGGCGCGACTTCCTCGACT.. 1
....TGGCGCGACTTCCTCGACTC... 1
....TGGCGCGACTTCCTCGACTCAC....................................... 1
...TTGGCGCGACTTCCTCGACTCACT...................................... 1
.......CGCGACTTCCTCGACTCACT...................................... 1
..............................TTTCCCCCGTGAGTCGTGGAACC............ 1
....GAGTCGTGGAACCTCGCCCGA.... 1
..................................CCCCGTGAGTCGTGGAACCTCGCCCGAGG.. 1
...................................CCCGTGAGTCGTGGAACCTCGCCCGAGG.. 1
.....................................CGTGAGTCGTGGAACCTCGCCCGAGG.. 1
......................................GTGAGTCGTGGAACCTCGCCCGAGGGT 1
.((((((.((((.((((.((((((((............)))))))).))))..)))))))))).. (-31.20)

23/36551673-36551734(-)_novel_dog_53
TAGCTCAGAGAGACTGCCACATTCCTTGAGCCAAGGAATGTGGCAGTCTCTCTGAGCTCTGC
....................................AATGTGGCAGTCTCTCTGAGC..... 401
....................................AATGTGGCAGTCTCTCTGAGCT.... 370
...................................GAATGTGGCAGTCTCTCTGAGC..... 83
.....................................ATGTGGCAGTCTCTCTGAGCT.... 79
......................................TGTGGCAGTCTCTCTGAGCT.... 71
...CTCAGAGAGACTGCCACATTCC..................................... 64
.....................................ATGTGGCAGTCTCTCTGAGC..... 42
...CTCAGAGAGACTGCCACATTC...................................... 36
......................................TGTGGCAGTCTCTCTGAGC..... 25
......AGAGAGACTGCCACATTCCT.................................... 8
....TCAGAGAGACTGCCACATTCC..................................... 7
...................................GAATGTGGCAGTCTCTCTGA....... 7
.........GAGACTGCCACATTCCTTGAGC............................... 6
.................................AGGAATGTGGCAGTCTCTCT......... 6
.........GAGACTGCCACATTCCTTGAGCC.............................. 5
.................................AGGAATGTGGCAGTCTCTCTG........ 5
...................................GAATGTGGCAGTCTCTCTG........ 5
.................................AGGAATGTGGCAGTCTCTCTGA....... 5
....TGGCAGTCTCTCTGAGCT.... 5
....................................AATGTGGCAGTCTCTCTGA....... 4
...................................GAATGTGGCAGTCTCTCTGAG...... 4
....................................AATGTGGCAGTCTCTCTGAG...... 4
..GCTCAGAGAGACTGCCACATTC...................................... 3
....TCAGAGAGACTGCCACATTC...................................... 3
......AGAGAGACTGCCACATTCC..................................... 3
...CTCAGAGAGACTGCCACATT....................................... 2
......AGAGAGACTGCCACATTCCTTG.................................. 2
.........GAGACTGCCACATTCCTTGAGCCA............................. 2
....TGGCAGTCTCTCTGAGC..... 2
...................................GAATGTGGCAGTCTCTCTGAGCT.... 2
.AGCTCAGAGAGACTGCCACAT.. 1
.AGCTCAGAGAGACTGCCACATT....................................... 1

```
......AGAGAGACTGCCACATTC......................................   1
....TCAGAGAGACTGCCACATTCCT....................................   1
.....CAGAGAGACTGCCACATTCCT....................................   1
.....CAGAGAGACTGCCACATTCCTT...................................   1
........AGAGACTGCCACATTCCTTGAG................................   1
..........AGACTGCCACATTCCTTGAGC...............................   1
..........AGACTGCCACATTCCTTGAGCC..............................   1
..........AGACTGCCACATTCCTTGAGCCA.............................   1
.....................................ATGTGGCAGTCTCTCTGAG......   1
.......................................GTGGCAGTCTCTCTGAGCT....   1
....................................AATGTGGCAGTCTCTCTGAGCTC...   1
......................................TGTGGCAGTCTCTCTGAGCTC...   1
....................................AATGTGGCAGTCTCTCTGAGCTCT..   1
......................................TGTGGCAGTCTCTCTGAGCTCTG.   1
......................................TGTGGCAGTCTCTCTGAGCTCTGC   1
........................................TGGCAGTCTCTCTGAGCTCTGC   1
.(((((((((((((((((((((((((((...))))))))))))))))))))))))))).... (-52.00)

23/36551676-36551737(+)_novel_dog_51
GAGCTCAGAGAGACTGCCACATTCCTTGGCTCAAGGAATGTGGCAGTCTCTCTGAGCTAAAA
....................................AATGTGGCAGTCTCTCTGAGC.....   401
....................................AATGTGGCAGTCTCTCTGAGCT....   370
...................................GAATGTGGCAGTCTCTCTGAGC.....   83
.....................................ATGTGGCAGTCTCTCTGAGCT....   79
......................................TGTGGCAGTCTCTCTGAGCT....   71
...CTCAGAGAGACTGCCACATTCC.....................................   64
.....................................ATGTGGCAGTCTCTCTGAGC.....   42
...CTCAGAGAGACTGCCACATTC......................................   36
......................................TGTGGCAGTCTCTCTGAGC.....   25
......AGAGAGACTGCCACATTCCT....................................   8
....TCAGAGAGACTGCCACATTCC.....................................   7
...................................GAATGTGGCAGTCTCTCTGA.......   7
.....................................ATGTGGCAGTCTCTCTGAGCTA...   7
.................................AGGAATGTGGCAGTCTCTCT.........   6
.................................AGGAATGTGGCAGTCTCTCTG........   5
...................................GAATGTGGCAGTCTCTCTG........   5
.................................AGGAATGTGGCAGTCTCTCTGA.......   5
........................................TGGCAGTCTCTCTGAGCT....   5
......................................TGTGGCAGTCTCTCTGAGCTA...   5
....................................AATGTGGCAGTCTCTCTGA.......   4
...................................GAATGTGGCAGTCTCTCTGAG......   4
....................................AATGTGGCAGTCTCTCTGAG......   4
....................................AATGTGGCAGTCTCTCTGAGCTA...   4
..GCTCAGAGAGACTGCCACATTC......................................   3
....TCAGAGAGACTGCCACATTC......................................   3
......AGAGAGACTGCCACATTCC.....................................   3
.........GAGACTGCCACATTCCTTGGCTC..............................   3
...CTCAGAGAGACTGCCACATT.......................................   2
......AGAGAGACTGCCACATTCCTTG..................................   2
........................................TGGCAGTCTCTCTGAGC.....   2
...................................GAATGTGGCAGTCTCTCTGAGCT....   2
.AGCTCAGAGAGACTGCCACAT........................................   1
.AGCTCAGAGAGACTGCCACATT.......................................   1
......AGAGAGACTGCCACATTC......................................   1
....TCAGAGAGACTGCCACATTCCT....................................   1
.....CAGAGAGACTGCCACATTCCT....................................   1
.....CAGAGAGACTGCCACATTCCTT...................................   1
.........GAGACTGCCACATTCCTTGGCT...............................   1
............ACTGCCACATTCCTTGGCTCAAGGA.........................   1
.....................................ATGTGGCAGTCTCTCTGAG......   1
.......................................GTGGCAGTCTCTCTGAGCT....   1
........................................TGGCAGTCTCTCTGAGCTA...   1
......................................TGTGGCAGTCTCTCTGAGCTAA..   1
........................................TGGCAGTCTCTCTGAGCTAA..   1
```

```
.(((((((((((((((((((((((((...))))))))))))))))))))))))).... (-52.80)

23/5826463-5826520(+)_mir_dog_108
GGGGGCCGATACACTGTACGAGAGTGAGTAGCAGGTCTCACAGTGAACCGGTCTCTTT
.....................................TCACAGTGAACCGGTCTCTTT 1384570
.....................................TCACAGTGAACCGGTCTCTT. 322169
.....................................TCACAGTGAACCGGTCTCT.. 103266
.....................................TCACAGTGAACCGGTCTC... 13571
.....................................TCACAGTGAACCGGTCT.... 7852
GGGGGCCGATACACTGTACGAGA................................... 2715
.....................................TCACAGTGAACCGGTC..... 894
....................................CTCACAGTGAACCGGTCTCTTT 738
GGGGGCCGATACACTGTACGAG.................................... 287
......................................CACAGTGAACCGGTCTCTTT 250
GGGGGCCGATACACTGTACGA..................................... 218
....................................CTCACAGTGAACCGGTCTCTT. 193
........................................CAGTGAACCGGTCTCTTT 146
.......................................ACAGTGAACCGGTCTCTTT 141
..........................................GTGAACCGGTCTCTTT 137
...................................TCTCACAGTGAACCGGTCTCTT. 101
...................................TCTCACAGTGAACCGGTCTCTTT 95
.........................................AGTGAACCGGTCTCTTT 59
....................................CTCACAGTGAACCGGTCTCT.. 56
......................................CACAGTGAACCGGTCTCTT. 53
...................................TCTCACAGTGAACCGGTCTCT.. 42
........................................CAGTGAACCGGTCTCTT. 38
.......................................ACAGTGAACCGGTCTCTT. 35
......................................CACAGTGAACCGGTCTCT.. 33
GGGGGCCGATACACTGTAC....................................... 32
GGGGGCCGATACACTGTACG...................................... 26
.........................................AGTGAACCGGTCTCTT. 18
....................................CTCACAGTGAACCGGTCT.... 13
........................................CAGTGAACCGGTCTCT.. 9
.GGGGCCGATACACTGTACGAGA................................... 8
...................................TCTCACAGTGAACCGGTCT.... 5
....................................CTCACAGTGAACCGGTCTC... 5
.......................................ACAGTGAACCGGTCTCT.. 5
......................................CACAGTGAACCGGTCT.... 4
.......................................ACAGTGAACCGGTCTC... 4
GGGGGCCGATACACTGTACGAGAGT................................. 3
...................................TCTCACAGTGAACCGGTCTC... 3
......................................CACAGTGAACCGGTCTC... 3
GGGGGCCGATACACTGTA........................................ 2
....GCCGATACACTGTACGAGA................................... 2
......CGATACACTGTACGAGA................................... 2
GGGGGCCGATACACTGTACGAGAG.................................. 2
GGGGGCCGATACACTGTACGAGAGTGAGTAGCAGGTC..................... 2
......CGATACACTGTACGAG.................................... 1
GGGGGCCGATACACTGTACGAGAGTGAGTAGCAGGT...................... 1
....................................CTCACAGTGAACCGGTC..... 1
((((((((...((((((..((((.(........).))))))))))...)))))))).. (-22.90)

23/7747073-7747133(+)_mir_dog_109
TTCAAGTAATCCAGGATAGGCTGTGCAGGTCCCAATGGGCCTATTCTTGGTTACTTGCACG
TTCAAGTAATCCAGGATAGGCT.......................................    4302424
TTCAAGTAATCCAGGATAGGC........................................    260057
TTCAAGTAATCCAGGATAG..........................................    33252
TTCAAGTAATCCAGGATAGG.........................................    20904
TTCAAGTAATCCAGGATAGGCTG......................................    18040
TTCAAGTAATCCAGGATA...........................................    3993
.TCAAGTAATCCAGGATAGGCT.......................................    3065
TTCAAGTAATCCAGGAT............................................    1754
TTCAAGTAATCCAGGATAGGCTGT.....................................    1232
..CAAGTAATCCAGGATAGGCT.......................................    577
```

```
....AGTAATCCAGGATAGGCT.......................................   529
...AAGTAATCCAGGATAGGCT.......................................   420
.TCAAGTAATCCAGGATAGGC........................................   227
TTCAAGTAATCCAGGA.............................................   107
......TAATCCAGGATAGGCT.......................................   105
.TCAAGTAATCCAGGATAGGCTG......................................   104
....AGTAATCCAGGATAGGC........................................   102
..CAAGTAATCCAGGATAGGC........................................   39
.......................................CCTATTCTTGGTTACTTGCACG   38
.TCAAGTAATCCAGGATAGG.........................................   27
.....GTAATCCAGGATAGGCT.......................................   25
...AAGTAATCCAGGATAGGC........................................   22
.......................................CCTATTCTTGGTTACTTGCAC.   12
.TCAAGTAATCCAGGATAG..........................................   10
.TCAAGTAATCCAGGATAGGCTGT.....................................   8
.....GTAATCCAGGATAGGC........................................   4
.TCAAGTAATCCAGGATA...........................................   3
..CAAGTAATCCAGGATAGGCTG......................................   3
..CAAGTAATCCAGGATAG..........................................   2
...AAGTAATCCAGGATAGGCTG......................................   2
........ATCCAGGATAGGCTGT.....................................   2
.TCAAGTAATCCAGGAT............................................   1
...AAGTAATCCAGGATAG..........................................   1
..CAAGTAATCCAGGATAGG.........................................   1
...AAGTAATCCAGGATAGG.........................................   1
..CAAGTAATCCAGGATAGGCTGT.....................................   1
.......AATCCAGGATAGGCTGT.....................................   1
.......................................CCTATTCTTGGTTACTTGC...   1
.......................................CCTATTCTTGGTTACTTGCA..   1
..(((((((((.((((((((((.((.......))...)))))))))).))))))))).... (-23.90)

24/17468354-17468422(-)_mir_dog_110
TCAGCTTCTTTACAGTGCTGCCTTGTAGCATTCAGGTCAAGCAGCATTGTACAGGGCTATGAAAGAACC
.......................................AGCAGCATTGTACAGGGCTATGA.......  228945
.......................................AGCAGCATTGTACAGGGCTATG........  43775
.......................................AGCAGCATTGTACAGGGCTAT.........  43533
.......................................AGCAGCATTGTACAGGGCT...........  14052
.......................................AGCAGCATTGTACAGGGCTATGAA......  3037
.......................................AGCAGCATTGTACAGGGC............  2453
.........................................CAGCATTGTACAGGGCTATGA.......  1875
.......................................AGCAGCATTGTACAGGGCTA..........  1724
.......................................AGCAGCATTGTACAGGGCTATGAAA.....  919
...........................................GCATTGTACAGGGCTATGA.......  121
..........................................AGCATTGTACAGGGCTATGA.......  111
.........................................CAGCATTGTACAGGGCTATG........  78
........................................GCAGCATTGTACAGGGCTATGA.......  72
.........................................CAGCATTGTACAGGGCTATGAA......  44
.........................................CAGCATTGTACAGGGCTATGAAA.....  44
..............................................TTGTACAGGGCTATGA.......  42
.............................................ATTGTACAGGGCTATGA.......  39
.......................................AGCAGCATTGTACAGG..............  34
..AGCTTCTTTACAGTGCTGCCTTG............................................  32
...........................................GCATTGTACAGGGCTAT.........  25
..AGCTTCTTTACAGTGCTGCCTTGT...........................................  24
.......................................AGCAGCATTGTACAGGG.............  23
..........................................AGCATTGTACAGGGCTAT.........  22
........................................GCAGCATTGTACAGGGCTATG........  21
............................................CATTGTACAGGGCTATGA.......  18
..........................................AGCATTGTACAGGGCTATG........  16
...........................................GCATTGTACAGGGCTATG........  14
.........................................CAGCATTGTACAGGGCTAT.........  13
........................................GCAGCATTGTACAGGGCTAT.........  9
.........................................CAGCATTGTACAGGGCT...........  8
.............................................ATTGTACAGGGCTATG........  8
```

```
......................................AAGCAGCATTGTACAGGGCTATGA.......  8
.....TTCTTTACAGTGCTGCCTTG............................................  6
.........................................CAGCATTGTACAGGGC............  6
......................................AAGCAGCATTGTACAGGGCTAT.........  6
........................................GCAGCATTGTACAGGGCT...........  5
.................................AGGTCAAGCAGCATTGTACAGGGCT...........  4
.........................................CAGCATTGTACAGGGCTA..........  4
......................................AAGCAGCATTGTACAGGGCT...........  3
......................................AAGCAGCATTGTACAGGGCTATG........  3
..........................................AGCATTGTACAGGGCTATGAA......  3
.......CTTTACAGTGCTGCCTTGT...........................................  2
............................................CATTGTACAGGGCTATG........  2
TCAGCTTCTTTACAGTGCTG.................................................  1
......TCTTTACAGTGCTGCCT..............................................  1
......TCTTTACAGTGCTGCCTTG............................................  1
........TTTACAGTGCTGCCTTG............................................  1
.....TTCTTTACAGTGCTGCCTTGT...........................................  1
......................TTGTAGCATTCAGGTCAAGCAGCA.......................  1
..................................GGTCAAGCAGCATTGTA..................  1
....................................TCAAGCAGCATTGTACAGGGCT...........  1
......................................AAGCAGCATTGTACAGGGCTA..........  1
............................................CATTGTACAGGGCTAT.........  1
.........................TAGCATTCAGGTCAAGCAGCATTGTACAGGGCTATG........  1
...........................................GCATTGTACAGGGCTATGAA......  1
............................................CATTGTACAGGGCTATGAA......  1
.............................................ATTGTACAGGGCTATGAA......  1
...............................................TGTACAGGGCTATGAA......  1
..........................................AGCATTGTACAGGGCTATGAAA.....  1
.............................................ATTGTACAGGGCTATGAAA.....  1
..((((.((.(((((((((((((((.......))))...))))))))))).))))))............ (-23.70)
```

24/18112918-18112982(+)_novel_dog_54

```
AATTTAGGAGATGGTTCTCTTCCCCCGTTTACACCAAGGGAAGGTGGATCTCTCCTCACACATCC
.......................................GAAGGTGGATCTCTCCTCACAC....      7
.......................................GAAGGTGGATCTCTCCTCACA.....      6
.......................................GAAGGTGGATCTCTCCTCAC......      2
........................................AAGGTGGATCTCTCCTCACAC....      2
...TTAGGAGATGGTTCTCTTCCC.........................................      1
.....((((((.((((((((((((..((....))...))))))).)))))))))))......... (-22.30)
```

24/18655386-18655450(-)_novel_Dog_Blast_23

```
GTCTTGGCGCGACTTCCTCGACTCACTCTCTTTCCCCCGTGAGTCGTGGAACCTCGCCCGAGGGT
.......................................TGAGTCGTGGAACCTCGCCCGA....      83
.........................................AGTCGTGGAACCTCGCCCGAG...      9
.........................................AGTCGTGGAACCTCGCCCGA....      6
.........................................AGTCGTGGAACCTCGCCCGAGG..      5
...TTGGCGCGACTTCCTCGACTC.........................................      4
....TGGCGCGACTTCCTCGACT..........................................      3
....TGGCGCGACTTCCTCGACTCACT......................................      3
.......................................TGAGTCGTGGAACCTCGCCC......      3
.......................................TGAGTCGTGGAACCTCGCCCGAG...      3
....TGGCGCGACTTCCTCGACTCA........................................      2
.......................................TGAGTCGTGGAACCTCGCCCG.....      2
...TTGGCGCGACTTCCTCGACT..........................................      1
....TGGCGCGACTTCCTCGACTC.........................................      1
....TGGCGCGACTTCCTCGACTCAC.......................................      1
...TTGGCGCGACTTCCTCGACTCACT......................................      1
.......CGCGACTTCCTCGACTCACT......................................      1
..............................TTTCCCCCGTGAGTCGTGGAACC............      1
........................................GAGTCGTGGAACCTCGCCCGA....      1
..................................CCCCGTGAGTCGTGGAACCTCGCCCGAGG..      1
...................................CCCGTGAGTCGTGGAACCTCGCCCGAGG..      1
.....................................CGTGAGTCGTGGAACCTCGCCCGAGG..      1
......................................GTGAGTCGTGGAACCTCGCCCGAGGGT      1
```

.((((((.((((.((((.((((((((...........)))))))).))))..)))))))))).. (-31.20)

24/24029549-24029607(+)_mir_dog_111
TTAAGACTTGCAGTGATGTTTAACTCCTCTCCACGTGAACATCACAGCAAGTCTGTGCT
TTAAGACTTGCAGTGATGTTT......................................51403
TTAAGACTTGCAGTGATGTT.......................................12581
TTAAGACTTGCAGTGATGTTTA.....................................7847
TTAAGACTTGCAGTGATGTTTAAC...................................3509
TTAAGACTTGCAGTGATGT..854
TTAAGACTTGCAGTGATG...728
.TAAGACTTGCAGTGATGTTTAAC...................................425
TTAAGACTTGCAGTGATGTTTAA....................................371
TTAAGACTTGCAGTGATGTTTAACT..................................332
.....................................AACATCACAGCAAGTCTGTGCT167
....................................GAACATCACAGCAAGTCTGTGC.83
.TAAGACTTGCAGTGATGTTTAACT..................................73
.TAAGACTTGCAGTGATGTTT......................................62
....................................GAACATCACAGCAAGTCTG....48
...................................TGAACATCACAGCAAGTCTGTGC.42
.....................................AACATCACAGCAAGTCTGT...40
....................................GAACATCACAGCAAGTCTGTGCT36
.....................................AACATCACAGCAAGTCTG....32
.TAAGACTTGCAGTGATGTT.......................................28
...................................TGAACATCACAGCAAGTCTGT...24
TTAAGACTTGCAGTGA...22
....................................GAACATCACAGCAAGTCTGT...22
.TAAGACTTGCAGTGATGTTTA.....................................21
TTAAGACTTGCAGTGATGTTTAACTCC................................21
..AAGACTTGCAGTGATGTTTAAC...................................19
TTAAGACTTGCAGTGATGTTTAACTCCT...............................15
TTAAGACTTGCAGTGAT..14
TTAAGACTTGCAGTGATGTTTAACTC.................................14
..AAGACTTGCAGTGATGTTTAACT..................................13
...................................TGAACATCACAGCAAGTCTGTG..13
.TAAGACTTGCAGTGATGTTTAA....................................12
.TAAGACTTGCAGTGATGTTTAACTC.................................11
....................................GAACATCACAGCAAGTCTGTG..11
.....................................AACATCACAGCAAGTCTGTGC.11
TTAAGACTTGCAGTGATGTTTAACTCCTCTCCACGT.......................10
.TAAGACTTGCAGTGATGTTTAACTCC................................7
....................................GAACATCACAGCAAGTCT.....6
..AAGACTTGCAGTGATGTTT......................................5
TTAAGACTTGCAGTGATGTTTAACTCCTCT.............................4
.TAAGACTTGCAGTGATGT..3
......CTTGCAGTGATGTTTA.....................................3
.TAAGACTTGCAGTGATGTTTAACTCCT...............................3
...................................TGAACATCACAGCAAGTCTGTGCT3
TTAAGACTTGCAGTGATGTTTAACTCCTCTC............................2
...................................TGAACATCACAGCAAGTCT.....2
..AAGACTTGCAGTGATGTT.......................................1
.....ACTTGCAGTGATGTTT......................................1
..AAGACTTGCAGTGATGTTTA.....................................1
...AGACTTGCAGTGATGTTTAACTCC................................1
......CTTGCAGTGATGTTTAACTCCTCTC............................1
.......TTGCAGTGATGTTTAACTCCTCTCC...........................1
TTAAGACTTGCAGTGATGTTTAACTCCTCTCCAC.........................1
TTAAGACTTGCAGTGATGTTTAACTCCTCTCCACGTG......................1
..AAGACTTGCAGTGATGTTTAACTCCTCTCCACGTG......................1
...................................TGAACATCACAGCAAGTCTG....1
.......................CTCCTCTCCACGTGAACATCACAGCAAGTCTGTGC.1
...((((((((.((((((((((.............)))))))))).))))))))..... (-23.42)

2/42541959-42542029(-)_mir_dog_112
TGGGTTGGCAGTGTATTGTTAGCTGGTTGAATATATGAATGGCATCAGCTAACATGCAACTGCTATCTTAT

```
.....TGGCAGTGTATTGTTAGCTGGT.............................................
      12480
.....TGGCAGTGTATTGTTAGCTGGTT............................................     681
.....TGGCAGTGTATTGTTAGCTG...............................................     351
.....TGGCAGTGTATTGTTAGCTGG..............................................     287
.....TGGCAGTGTATTGTTAGCT................................................     44
.......................................CAGCTAACATGCAACTGCTATC....     28
.....TGGCAGTGTATTGTTAGC.................................................     22
.....TGGCAGTGTATTGTTAGCTGGTTGAATATA.....................................     15
......................................TCAGCTAACATGCAACTGCTATC....     9
.......................................CAGCTAACATGCAACTGCTATCT...     8
......................................TCAGCTAACATGCAACTGCTAT.....     7
.....TGGCAGTGTATTGTTAGCTGGTTGAATA.......................................     4
......GGCAGTGTATTGTTAGCTGGT.............................................     3
..........GTGTATTGTTAGCTGGT.............................................     3
.....TGGCAGTGTATTGTTAGCTGGTTGAATAT......................................     3
.......................................CAGCTAACATGCAACTGCTAT.....     3
......................................TCAGCTAACATGCAACTGCT.......     2
........CAGTGTATTGTTAGCTGGT.............................................     1
.....TGGCAGTGTATTGTTAGCTGGTTG...........................................     1
......................................TCAGCTAACATGCAACTGCTATCT...     1
((((.((((((((((.(((((((((...((......))...))))))))))))).))))))).)))). (-27.70)

2/42542077-42542150(-)_mir_dog_113
CAGGTAGGCAGTGTATTGTTAGCTGGCTGCTTTGGGTCAATCTGGCAGCCACCACTACCCTGCCACTTGCTTCT
.....AGGCAGTGTATTGTTAGCTGGC.................................................   124
.....AGGCAGTGTATTGTTAGCTGGCTGCT.............................................   122
.....AGGCAGTGTATTGTTAGCTGGCTGC..............................................   81
.....AGGCAGTGTATTGTTAGCTGGCT................................................   43
.......................................CAGCCACCACTACCCTGCCACT.......   36
.....AGGCAGTGTATTGTTAGCTG...................................................   19
.....AGGCAGTGTATTGTTAGCTGGCTG...............................................   6
.....AGGCAGTGTATTGTTAGCTGG..................................................   5
.....AGGCAGTGTATTGTTAGCTGGCTGCTT............................................   4
........................................AGCCACCACTACCCTGCCACT.......   3
........CAGTGTATTGTTAGCTGGCT................................................   1
.........AGTGTATTGTTAGCTGGCT................................................   1
........CAGTGTATTGTTAGCTGGCTGCT.............................................   1
(((((.(((((.(((.((.....((((((((..((.....)).)))))))).)).))).)))))))))).... (-
28.60)

2/43125791-43125855(-)_novel_Dog_Blast_24
GTCTTGGCGCGACTTCCTCGACTCACTCTCTTTCCCCTGTGAGTCGTGGAACCTCGCCCGAGGGT
......................................TGAGTCGTGGAACCTCGCCCGA....      83
........................................AGTCGTGGAACCTCGCCCGAG...      9
........................................AGTCGTGGAACCTCGCCCGA....      6
........................................AGTCGTGGAACCTCGCCCGAGG..      5
...TTGGCGCGACTTCCTCGACTC.........................................      4
....TGGCGCGACTTCCTCGACT..........................................      3
....TGGCGCGACTTCCTCGACTCACT......................................      3
......................................TGAGTCGTGGAACCTCGCCC......      3
......................................TGAGTCGTGGAACCTCGCCCGAG...      3
....TGGCGCGACTTCCTCGACTCA........................................      2
......................................TGAGTCGTGGAACCTCGCCCG.....      2
...TTGGCGCGACTTCCTCGACT..........................................      1
....TGGCGCGACTTCCTCGACTC.........................................      1
....TGGCGCGACTTCCTCGACTCAC.......................................      1
...TTGGCGCGACTTCCTCGACTCACT......................................      1
.......CGCGACTTCCTCGACTCACT......................................      1
.......................................GAGTCGTGGAACCTCGCCCGA....      1
.....................................GTGAGTCGTGGAACCTCGCCCGAGGGT      1
.((((((.((((.((((.((((((((...........)))))))).)))).))))))))))).. (-31.80)

24/46481073-46481141(+)_mir_Dog_Blast_1
```

```
GGGAACATACTTCTTTATATGCCCATATGGACCTGCTAAGCTATGGAATGTAAAGAAGTATGTATTTCA
...........................................TGGAATGTAAAGAAGTATGTAT....  205782
...........................................TGGAATGTAAAGAAGTATGTA.....  5292
...........................................TGGAATGTAAAGAAGTATGT......  4739
...........................................TGGAATGTAAAGAAGTATGTATT...  3842
............................................GGAATGTAAAGAAGTATGTAT....  250
.............................................GAATGTAAAGAAGTATGTATTT..  196
...........................................TGGAATGTAAAGAAGTATG.......  137
.............................................GAATGTAAAGAAGTATGTATT...  101
...........................................TGGAATGTAAAGAAGTA.........  66
...........................................TGGAATGTAAAGAAGTAT........  66
...........................................TGGAATGTAAAGAAGTATGTATTT..  64
..............................................AATGTAAAGAAGTATGTAT....  53
...............................................ATGTAAAGAAGTATGTAT....  38
.................................................GTAAAGAAGTATGTAT....  34
.............................................GAATGTAAAGAAGTATGTAT....  31
....ACATACTTCTTTATATGCCCATA..........................................  29
................................................TGTAAAGAAGTATGTAT....  29
..........................................ATGGAATGTAAAGAAGTATGTAT....  23
............................................GGAATGTAAAGAAGTATGTATT...  20
..........................................ATGGAATGTAAAGAAGTATGT......  17
...............................................ATGTAAAGAAGTATGTATT...  14
............................................GGAATGTAAAGAAGTATGTA.....  13
...........................................TGGAATGTAAAGAAGT..........  10
...............................................ATGTAAAGAAGTATGT......  8
..........................................ATGGAATGTAAAGAAGTATGTA.....  8
....ACATACTTCTTTATATGCCCAT...........................................  7
............................................GGAATGTAAAGAAGTATGTATTT..  7
................................................TGTAAAGAAGTATGTATT...  5
.............................................GAATGTAAAGAAGTATGT......  4
..............................................AATGTAAAGAAGTATGT......  4
..............................................AATGTAAAGAAGTATGTA.....  4
..............................................AATGTAAAGAAGTATGTATT...  4
...........................TGGACCTGCTAAGCTA..........................  3
...............................................ATGTAAAGAAGTATGTA.....  3
.................................................GTAAAGAAGTATGTATT...  3
.........................TATGGACCTGCTAAGCTATG........................  2
.........................................TATGGAATGTAAAGAAGTATGT......  2
.............................................GAATGTAAAGAAGTATGTA.....  2
................................................TGTAAAGAAGTATGTA.....  2
..................................................TAAAGAAGTATGTATT...  2
..............................................AATGTAAAGAAGTATGTATTT..  2
....ACATACTTCTTTATATGCCCATAT.........................................  1
....ACATACTTCTTTATATGCCCATATGGACCTG..................................  1
.........CTTCTTTATATGCCCATATGGACCTGCTAAGCTA..........................  1
..........................................ATGGAATGTAAAGAAGTATG.......  1
.............................................GAATGTAAAGAAGTATG.......  1
........................................CTATGGAATGTAAAGAAGTATGT......  1
............................................GGAATGTAAAGAAGTATGT......  1
........................................CTATGGAATGTAAAGAAGTATGTAT....  1
..........................ATGGACCTGCTAAGCTATGGAATGTAAAGAAGTATGTATT...  1
..........................................ATGGAATGTAAAGAAGTATGTATT...  1
...............................................ATGTAAAGAAGTATGTATTT..  1
..................................................TAAAGAAGTATGTATTT..  1
(..(((((((((((((((((..((((((((.....)))...))))).)))))))))))))))).)..). (-26.30)

24/46490511-46490578(+)_mir_dog_114
TAAAGCTGGTAAAATGGAACCAAATCAACTGTTCAATGGATTTGGTCCCCTTCAACCAGCTGTAGCTG
........................................TTTGGTCCCCTTCAACCAGCTGT.....   440253
.........................................TTGGTCCCCTTCAACCAGCTGT.....   324881
........................................TTTGGTCCCCTTCAACCAGCTG......   27560
.........................................TTGGTCCCCTTCAACCAGCTGTA....   13144
.........................................TTGGTCCCCTTCAACCAGCTG......   9967
........................................TTTGGTCCCCTTCAACCAGCTGTA....   4869
```

```
........................................TTTGGTCCCCTTCAACCAGCT.......   4806
..........................................TGGTCCCCTTCAACCAGCTGT.....   2100
...AGCTGGTAAAATGGAACCAAAT...........................................   1792
........................................TTTGGTCCCCTTCAACCAGC........   977
.........................................TTGGTCCCCTTCAACCAGCT.......   476
...............................................CCCTTCAACCAGCTGT.....   333
.......................................ATTTGGTCCCCTTCAACCAGCTGT.....   273
.........................................TTGGTCCCCTTCAACCAGC........   267
.............................................TCCCCTTCAACCAGCTGT.....   263
...AGCTGGTAAAATGGAACCAAATC..........................................   236
...AGCTGGTAAAATGGAACCAAA............................................   225
..........................................TGGTCCCCTTCAACCAGCTGTA....   119
.........................................TTGGTCCCCTTCAACCAGCTGTAG...   107
....GCTGGTAAAATGGAACCAAATC..........................................   91
..........................................TGGTCCCCTTCAACCAGCTG......   77
..............................................CCCCTTCAACCAGCTGT.....   77
...AGCTGGTAAAATGGAACCA..............................................   61
....GCTGGTAAAATGGAACCAAAT...........................................   61
...AGCTGGTAAAATGGAACC...............................................   55
.......................................ATTTGGTCCCCTTCAACCAGCTG......   54
.......................................ATTTGGTCCCCTTCAACCAGCT.......   43
............................................GTCCCCTTCAACCAGCTGT.....   43
...AGCTGGTAAAATGGAACCAA.............................................   35
........................................TTTGGTCCCCTTCAACCAGCTGTAG...   32
...........................................GGTCCCCTTCAACCAGCTGT.....   31
........................................TTTGGTCCCCTTCAACCAG.........   25
.......................................ATTTGGTCCCCTTCAACCAGC........   18
....GCTGGTAAAATGGAACCAAA............................................   10
........................................TTTGGTCCCCTTCAACC...........   9
.............................................TCCCCTTCAACCAGCTG......   9
.........................................TTGGTCCCCTTCAACC...........   7
.............................................TCCCCTTCAACCAGCTGTA....   7
.........................CAACTGTTCAATGGAT...........................   6
........................TCAACTGTTCAATGGATT..........................   6
............................................GTCCCCTTCAACCAGCTG......   6
.........TAAAATGGAACCAAATCAACTGTT...................................   5
..........................................TGGTCCCCTTCAACCAGCT.......   5
..........AAAATGGAACCAAATCAACTGT....................................   4
........................................TTTGGTCCCCTTCAAC............   4
..........................................TGGTCCCCTTCAACCAGCTGTAG...   4
.........TAAAATGGAACCAAATCAACTGT....................................   3
............................................GTCCCCTTCAACCAGCTGTA....   3
...............................................CCCTTCAACCAGCTGTA....   3
...AGCTGGTAAAATGGAAC................................................   2
...........AAATGGAACCAAATCAACTGTT...................................   2
........................................TTTGGTCCCCTTCAACCA..........   2
....GCTGGTAAAATGGAAC................................................   1
.AAAGCTGGTAAAATGGAACC...............................................   1
....GCTGGTAAAATGGAACC...............................................   1
....GCTGGTAAAATGGAACCA..............................................   1
..AAGCTGGTAAAATGGAACCAAAT...........................................   1
.....CTGGTAAAATGGAACCAAAT...........................................   1
......TGGTAAAATGGAACCAAATC..........................................   1
...AGCTGGTAAAATGGAACCAAATCA.........................................   1
...AGCTGGTAAAATGGAACCAAATCAACT......................................   1
...........AAATGGAACCAAATCAACTG.....................................   1
...AGCTGGTAAAATGGAACCAAATCAACTGT....................................   1
..........AAAATGGAACCAAATCAACTGTT...................................   1
...AGCTGGTAAAATGGAACCAAATCAACTGTTCA.................................   1
................................TCAATGGATTTGGTCCCCTTCAAC............   1
.................................CAATGGATTTGGTCCCCTTCAAC............   1
...................................ATGGATTTGGTCCCCTTCAAC............   1
.........................................TTGGTCCCCTTCAACCA..........   1
.......................................ATTTGGTCCCCTTCAACCAG.........   1
```

....TGGTCCCCTTCAACCAGC........ 1
....TCCCCTTCAACCAGCT....... 1
....CCCCTTCAACCAGCTG...... 1
................................CAATGGATTTGGTCCCCTTCAACCAGCTGT..... 1
....GGTCCCCTTCAACCAGCTGTA.... 1
....CCCCTTCAACCAGCTGTA.... 1
....CCTTCAACCAGCTGTA.... 1
...(((((((..((.((.((((((((...........)))))))).)).))..)))))))....... (-23.00)

24/46916724-46916793(-)_mir_Dog_Blast_2
CTCTTGGCATTCACCGCGTGCCTTAATTGTATGGACATTAAATCAAGGTCCGCTGTGAACACGCAGAGGG
....TCAAGGTCCGCTGTGAACACGC...... 10
....CAAGGTCCGCTGTGAACACG....... 6
....TCAAGGTCCGCTGTGAACACG....... 2
........ATTCACCGCGTGCCTTAATTG... 1
....CAAGGTCCGCTGTGAACACGC...... 1
(((((.((.(((((.(((..((((.(((............))).))))..))).)))))...)).))))) (-22.10)

24/46916732-46916789(+)_mir_dog_115
GTGTTCACAGCGGACCTTGATTTAATGTCCATACAATTAAGGCACGCGGTGAATGCCA
.....................................TAAGGCACGCGGTGAATGCC. 113513
....................................TTAAGGCACGCGGTGAATGCC. 40281
.....................................TAAGGCACGCGGTGAATGCCA 39002
.....................................TAAGGCACGCGGTGAATGC.. 17789
....................................TTAAGGCACGCGGTGAATGCCA 11436
....................................TTAAGGCACGCGGTGAATGC.. 8887
....................................TTAAGGCACGCGGTGAAT.... 3737
....................................TTAAGGCACGCGGTGAATG... 1868
.....................................TAAGGCACGCGGTGAATG... 1753
....CACGCGGTGAATGCCA 410
.....................................TAAGGCACGCGGTGAAT.... 338
......................................AAGGCACGCGGTGAATGCC. 150
......................................AAGGCACGCGGTGAATGCCA 137
.....................................TAAGGCACGCGGTGAA..... 116
....................................TTAAGGCACGCGGTGAA..... 64
....GCACGCGGTGAATGCC. 56
....GCACGCGGTGAATGCCA 24
....................................TTAAGGCACGCGGTGA...... 23
......................................AAGGCACGCGGTGAATGC.. 8
.TGTTCACAGCGGACCTTGATT.................................... 7
....GGCACGCGGTGAATGCC. 5
......................................AAGGCACGCGGTGAATG... 4
....AGGCACGCGGTGAATGCC. 4
....GGCACGCGGTGAATGCCA 4
GTGTTCACAGCGGACCTTGAT..................................... 3
...................................ATTAAGGCACGCGGTGAATGCC. 3
GTGTTCACAGCGGACCTTGATT.................................... 2
......................................AAGGCACGCGGTGAAT.... 2
....GGCACGCGGTGAATGC.. 2
....AGGCACGCGGTGAATGCCA 2
...TTCACAGCGGACCTTGA...................................... 1
.TGTTCACAGCGGACCTTGATTT................................... 1
....TCACAGCGGACCTTGATTTAAT................................ 1
.................................CAATTAAGGCACGCGGTGAAT.... 1
((((((((.(((..((((((((............))))))))..))).)))))))).. (-22.20)

24/47411216-47411276(-)_mir_dog_116
CAGGACTGTCCAACCTGAGAGTGTGTCCCCGCGGTCAGTCTCAGGTTCGTCAGCCCGAGAG
.AGGACTGTCCAACCTGAGAGT....................................... 30151
.AGGACTGTCCAACCTGAGAGTG...................................... 23223
.AGGACTGTCCAACCTGAGAGTGT..................................... 21518
.....................................GTCTCAGGTTCGTCAGCCCGA... 6221
.....................................GTCTCAGGTTCGTCAGCCCGAGA. 4816
.....................................GTCTCAGGTTCGTCAGCCCGAG.. 1508

.AGGACTGTCCAACCTGAGAG.. 698
.AGGACTGTCCAACCTGAGA... 461
......................................TCTCAGGTTCGTCAGCCCGA... 331
......................................TCTCAGGTTCGTCAGCCCGAG.. 140
CAGGACTGTCCAACCTGAGAGT....................................... 93
......................................TCTCAGGTTCGTCAGCCCGAGA. 82
....................................AGTCTCAGGTTCGTCAGCCCGA... 73
.....................................GTCTCAGGTTCGTCAGCCC..... 36
.....................................GTCTCAGGTTCGTCAGCCCG.... 25
.....................................GTCTCAGGTTCGTCAGCC...... 10
....................................AGTCTCAGGTTCGTCAGCCCGAGA. 10
.AGGACTGTCCAACCTGAG.. 7
CAGGACTGTCCAACCTGAGAGTG...................................... 7
....................................AGTCTCAGGTTCGTCAGCCCGAG.. 6
..GGACTGTCCAACCTGAGAGT....................................... 5
CAGGACTGTCCAACCTGAGAGTGT..................................... 4
.AGGACTGTCCAACCTGAGAGTGTG.................................... 3
......................................TCTCAGGTTCGTCAGCCC..... 3
.AGGACTGTCCAACCTGAGAGTGTGT................................... 2
......................................TCTCAGGTTCGTCAGCCCG.... 2
CAGGACTGTCCAACCTGAGAG.. 1
..GGACTGTCCAACCTGAGAG.. 1
......TGTCCAACCTGAGAGT....................................... 1
..GGACTGTCCAACCTGAGAGTG...................................... 1
.....................................GTCTCAGGTTCGTCAGC....... 1
......................................TCTCAGGTTCGTCAGCC...... 1
....................................AGTCTCAGGTTCGTCAGCCCG.... 1
....TCAGGTTCGTCAGCCCGA... 1
....CAGGTTCGTCAGCCCGA... 1
....CAGGTTCGTCAGCCCGAGA. 1
..((.(((.(.(((((((((.((...((....)).)).))))))))).).))).))..... (-25.90)

24/47411216-47411280(+)_mir_dog_117
CTCTCGGGCTGACGAACCTGAGACTGACCGCGGGGACACACTCTCAGGTTGGACAGTCCTGGAGG
....CTCTCAGGTTGGACAGTCCTGGA.. 37
....CGGGCTGACGAACCTGAGACT.. 19
....CTCTCAGGTTGGACAGTCCTGG... 12
....ACTCTCAGGTTGGACAGTCCTG.... 7
....ACTCTCAGGTTGGACAGTCCTGG... 6
....ACTCTCAGGTTGGACAGTCCTGGA.. 6
....TCTCAGGTTGGACAGTCCTGGA.. 6
....CTCTCAGGTTGGACAGTCC...... 4
....CTCTCAGGTTGGACAGTCCTG.... 4
....CTCTCAGGTTGGACAGTCCT..... 3
....TCTCAGGTTGGACAGTCCTGG... 2
....CTCTCAGGTTGGACAGT........ 1
....ACTCTCAGGTTGGACAGTCCT..... 1
((((.((((((.(.(((((((((.((.((....))...)).))))))))).).)))))).)))). (-33.00)

2/46497270-46497350(-)_mir_dog_118
ACTCTTTGGTTACAGTTGTTCAACCAGTTACTAATCTAGCTAATTGTAACCAGTTGAACAACTGAACCCAAAGGGTGCAA
A
.........TTACAGTTGTTCAACCAGTTAC...
. 678
....AACCAGTTGAACAACTGAACCC...........
. 562
....TAACCAGTTGAACAACTGAACCC...........
. 489
..........TACAGTTGTTCAACCAGTTACT..
. 448
....TAACCAGTTGAACAACTGAACC............
. 358
.........TTACAGTTGTTCAACCAGTTACT..
. 234

....TAACCAGTTGAACAACTGAAC.............
.	87
..........TACAGTTGTTCAACCAGTTAC...
.	73
....AACCAGTTGAACAACTGAACC............
.	70
.........TTACAGTTGTTCAACCAGTT...
.	52
...........ACAGTTGTTCAACCAGTTACT..
.	43
.........TTACAGTTGTTCAACCAGT..
.	37
....CCAGTTGAACAACTGAACCC...........
.	31
....CCAGTTGAACAACTGAACCCA..........
.	25
.........TTACAGTTGTTCAACCAGTTA..
.	11
....AACCAGTTGAACAACTGAAC.............
.	8
..........TACAGTTGTTCAACCAGTTA..
.	5
....TTGTAACCAGTTGAACAACT.................
.	3
....TAACCAGTTGAACAACTGA...............
.	3
....CCAGTTGAACAACTGAACC............
.	3
....ACCAGTTGAACAACTGAACCC...........
.	3
....TAACCAGTTGAACAACTGAA..............
.	2
....TTTGGTTACAGTTGTTCAACC...
.	1
.........TTACAGTTGTTCAACCAG...
.	1
..........TACAGTTGTTCAACCAGTT...
.	1
...........ACAGTTGTTCAACCAGTTA..
.	1
...........ACAGTTGTTCAACCAGTTAC...
.	1
..............GTTGTTCAACCAGTTACT..
.	1
...........ACAGTTGTTCAACCAGTTACTA...
.	1
....TTGTAACCAGTTGAACAACTG................
.	1
....TAACCAGTTGAACAACTG................
.	1
....GTAACCAGTTGAACAACTGAAC.............
.	1
....GTTGAACAACTGAACCC...........
.	1
....TAACCAGTTGAACAACTGAACCCA..........
.	1
....ACCAGTTGAACAACTGAACCCA..........
.	1
((((((((...(((((((((((..
(((((..............)))))..)))))))))))...)))))))).....	(-34.04)

25/28188255-28188326(-)_mir_Dog_Blast_3
TTCTTGGCATTCACCGCGTGCCTTAATTGTATGGACATTTAAATCAAGGTCCGCTGTGAACACGGAGAGAGA
....CAAGGTCCGCTGTGAACACGGA......	27

```
.............................................CAAGGTCCGCTGTGAACACG........     6
............................................TCAAGGTCCGCTGTGAACACGG.......     4
............................................TCAAGGTCCGCTGTGAACACG........     2
............................................TCAAGGTCCGCTGTGAACACGGA......     2
........ATTCACCGCGTGCCTTAATTG.............................................     1
.............................................CAAGGTCCGCTGTGAACACGG.......     1
(((((.(..(((((.(((..((((.(((..((....))...))).))))..))).)))))..).)))))... (-
22.40)
```

25/28188264-28188322(+)_mir_dog_119

```
GTGTTCACAGCGGACCTTGATTTAAATGTCCATACAATTAAGGCACGCGGTGAATGCCA
......................................TAAGGCACGCGGTGAATGCC.113513
.....................................TTAAGGCACGCGGTGAATGCC.40281
......................................TAAGGCACGCGGTGAATGCCA39002
......................................TAAGGCACGCGGTGAATGC..17789
.....................................TTAAGGCACGCGGTGAATGCCA11436
.....................................TTAAGGCACGCGGTGAATGC..8887
.....................................TTAAGGCACGCGGTGAAT....3737
.....................................TTAAGGCACGCGGTGAATG...1868
......................................TAAGGCACGCGGTGAATG...1753
...........................................CACGCGGTGAATGCCA410
......................................TAAGGCACGCGGTGAAT....338
.......................................AAGGCACGCGGTGAATGCC.150
.......................................AAGGCACGCGGTGAATGCCA137
......................................TAAGGCACGCGGTGAA.....116
.....................................TTAAGGCACGCGGTGAA.....64
..........................................GCACGCGGTGAATGCC.56
..........................................GCACGCGGTGAATGCCA24
.....................................TTAAGGCACGCGGTGA......23
.......................................AAGGCACGCGGTGAATGC..8
.TGTTCACAGCGGACCTTGATT.....................................7
.........................................GGCACGCGGTGAATGCC.5
.......................................AAGGCACGCGGTGAATG...4
........................................AGGCACGCGGTGAATGCC.4
.........................................GGCACGCGGTGAATGCCA4
GTGTTCACAGCGGACCTTGAT......................................3
.....................TTAAATGTCCATACAATTAAGGC...............3
......................TAAATGTCCATACAATTAAGGC...............3
......................................ATTAAGGCACGCGGTGAATGCC.3
GTGTTCACAGCGGACCTTGATT.....................................2
.....................TTAAATGTCCATACAATTAAGGCA..............2
.......................................AAGGCACGCGGTGAAT....2
.........................................GGCACGCGGTGAATGC..2
........................................AGGCACGCGGTGAATGCCA2
...TTCACAGCGGACCTTGA.......................................1
.TGTTCACAGCGGACCTTGATTT....................................1
...TTCACAGCGGACCTTGATTTAAATG...............................1
......................TAAATGTCCATACAATTAAG.................1
...................................CAATTAAGGCACGCGGTGAAT....1
((((((((.(((..((((((((............))))))))..))).)))))))).. (-22.12)
```

25/30915414-30915490(-)_novel_dog_57

```
CAGTGCTGCTGGTGGCTCTTGGGGACAGCTGCCATCAGAGATCCAGGCTGTCCCTCAGACCTACCTGGAGGGCTGTA
................................................CTGTCCCTCAGACCTACCTGGA........
      14
.................................................TGTCCCTCAGACCTACCTGGA........
      4
........CTGGTGGCTCTTGGGGACAGCT...............................................
      2
........CTGGTGGCTCTTGGGGACAGC................................................
      1
................................................CTGTCCCTCAGACCTACCTGG.........
      1
((((.((.(.(((((.(((.((((((((((...(((...)))...)))))))))).))).))))).).)).)))).. (-
```

```
39.80)

25/34318804-34318876(+)_novel_dog_55
GCCCCGGGCGCCCGGGGTCATGGAGGCGCGCTGGAGGGGGCTGCGCGCGGCCGCCCCGACCTCGGGCGCCTGG
............................................CGCGGCCGCCCCGACCTCGGGC......    8
..........CCCGGGGTCATGGAGGCGCG..........................................    1
...........................................GCGCGGCCGCCCCGACCTCGGGC......    1
............................................CGCGGCCGCCCCGACCTCGGGCG.....    1
............................................CGCGGCCGCCCCGACCTCGGGCGC....    1
...((((((((((((((..((.(((((((.(.((....)).))))).))).))..))))))))))))))) (-
53.80)

25/35016676-35016729(+)_mir_dog_120
GCCTTCTCTTCCCGGTTCTTCCCGGAGTCGGGAAAAGCTGGGTTGAGAGGGCGA
................................AAAAGCTGGGTTGAGAGGGCGA     36398
................................AAAAGCTGGGTTGAGAGGGCG.     5306
................................AAAAGCTGGGTTGAGAGGGC..     2154
.................................AAAGCTGGGTTGAGAGGGCGA     1269
...............................GAAAAGCTGGGTTGAGAGGGCGA     819
...............................GAAAAGCTGGGTTGAGAGGGCG.     536
.................................AAAGCTGGGTTGAGAGGGCG.     223
...............................GAAAAGCTGGGTTGAGAGGGC..     193
................................AAAAGCTGGGTTGAGAGGG...     72
.................................AAAGCTGGGTTGAGAGGGC..     57
..................................AAGCTGGGTTGAGAGGGCGA     57
................................AAAAGCTGGGTTGAGAGG....     42
....................................GCTGGGTTGAGAGGGCGA     15
...............................GAAAAGCTGGGTTGAGAGGG...     8
..................................AAGCTGGGTTGAGAGGGCG.     8
................................AAAAGCTGGGTTGAGAG.....     6
......................................TGGGTTGAGAGGGCGA     5
................................AAAAGCTGGGTTGAGA......     3
..................................AAGCTGGGTTGAGAGGGC..     2
....................................GCTGGGTTGAGAGGGCG.     2
...................................AGCTGGGTTGAGAGGGCGA     2
GCCTTCTCTTCCCGGTTCTTC.................................     1
GCCTTCTCTTCCCGGTTCTTCC................................     1
.CCTTCTCTTCCCGGTTCTTCC................................     1
GCCTTCTCTTCCCGGTTCTTCCC...............................     1
GCCTTCTCTTCCCGGTTCTTCCCGGA............................     1
......................CGGAGTCGGGAAAAGCTGGG............     1
...............................GAAAAGCTGGGTTGAGAGG....     1
.................................AAAGCTGGGTTGAGAGG....     1
.................................AAAGCTGGGTTGAGAGGG...     1
............................CGGGAAAAGCTGGGTTGAGAGGGC..     1
...................................AGCTGGGTTGAGAGGGC..     1
...................................AGCTGGGTTGAGAGGGCG.     1
.....................................CTGGGTTGAGAGGGCGA     1
((((((((..(((((((.((((((....)))))).)))))))..)))))))).. (-36.80)

25/4122347-4122415(-)_novel_dog_56
GAGAGGAGACAGACCCTGGGATCACTTGTTTCCAAGCCCTTGGTGACCCCAAGACCTGCTCCACCCAGG
..........................................TGACCCCAAGACCTGCTCCACC....  67
...AGGAGACAGACCCTGGGATCACT..........................................  42
..........................................TGACCCCAAGACCTGCTCCAC.....  7
...AGGAGACAGACCCTGGGATC.............................................  4
...AGGAGACAGACCCTGGGATCAC...........................................  2
...AGGAGACAGACCCTGGGATCA............................................  1
..........................................TGACCCCAAGACCTGCTCCACCC...  1
....((((.(((..(.((((.(((((.(((....)))....))))).)))).)..))))))).......  (-22.40)

25/44490796-44490868(-)_novel_dog_58
GGGCTGGAAGTCCAAGATTGAGGTCCCGGCAACCTTGGTATCTGATGGGACCGCTGTCTTTGGCTTGCATCCC
......GAAGTCCAAGATTGAGGTCCCG.............................................    20
```

......GAAGTCCAAGATTGAGGTCCC..   3
....GACCGCTGTCTTTGGCTTGC.....   2
(((.((.(((((.(((((...(((((((.((((....))...)).)))))))...))))).))))).)).))) (-
32.60)

25/49330884-49330950(-)_novel_dog_59
GGGTTGGCGCTCACCGGAGCCGAGGTGGTTGAAACCGAGTCCCCGCCTCCGGGGGCACCAGGCTGTC
...TTGGCGCTCACCGGAGCCGAGG..   8
...TTGGCGCTCACCGGAGCCGAGGT...   3
...TTGGCGCTCACCGGAGCCGAG...   2
....TGGCGCTCACCGGAGCCGAGGT...   2
....CCCGCCTCCGGGGGCACCAGGC....   1
((.((((.((((.((((((.((.((((((....))))...)).)).)))))))))).)))).))... (-32.60)

25/50483520-50483602(+)_mir_dog_121
GCCGGCGCCCGGGCTCTGGCTCCGTGTCTTCACTCCCGTGTGTCCGAGGAGGGAGGGAGGGACGGGGGCTGTGCTGGGGC
AGC
..............TCTGGCTCCGTGTCTTCACTCCC...
...   60162
..............TCTGGCTCCGTGTCTTCACTCC..
...   17330
..............TCTGGCTCCGTGTCTTCACTC...
...   7310
..............TCTGGCTCCGTGTCTTCACT..
...   2139
..............TCTGGCTCCGTGTCTTCAC...
...   140
..............TCTGGCTCCGTGTCTTCACTCCCG..
...   129
....................TCCGTGTCTTCACTCCC...
...   39
..............TCTGGCTCCGTGTCTTCACTCCCGT...
...   38
...................CTCCGTGTCTTCACTCCC...
...   36
....GAGGGAGGGACGGGGGCTGTGC......
...   26
....................TCCGTGTCTTCACTCC..
...   19
..............TCTGGCTCCGTGTCTTCA..
...   14
.....................CCGTGTCTTCACTCCC...
...   14
.............CTCTGGCTCCGTGTCTTCACTCCC...
...   9
...................CTCCGTGTCTTCACTCC..
...   7
....GAGGGAGGGACGGGGGCTGT........
...   7
...................CTCCGTGTCTTCACTC...
...   6
..................GCTCCGTGTCTTCACTCC..
...   5
................TGGCTCCGTGTCTTCACTCCC...
...   5
..................GCTCCGTGTCTTCACTCCC...
...   5
................TGGCTCCGTGTCTTCACTC...
...   4
.............CTCTGGCTCCGTGTCTTCACTCC..
...   3
.................GGCTCCGTGTCTTCACT..
...   2
................TGGCTCCGTGTCTTCACTCC..

```
...   2
...............CTGGCTCCGTGTCTTCACTCCC...........................................
...   2
.................GGCTCCGTGTCTTCACTCCC...........................................
...   2
....................................................GAGGGAGGGACGGGGGCTGTG.......
...   2
......................................................GGGAGGGACGGGGGCTGTGC......
...   2
....................................................GAGGGAGGGACGGGGGCTGTGCT.....
...   2
..............TCTGGCTCCGTGTCTTC.................................................
...   1
.........CGGGCTCTGGCTCCGTGTCTTCACTCCC...........................................
...   1
....................................................GAGGGAGGGACGGGGGCTG.........
...   1
.....................................................AGGGAGGGACGGGGGCTGTG.......
...   1
.....................................................AGGGAGGGACGGGGGCTGTGCT.....
...   1
......((((.(((.(.(.((((((.(((((.
(((((.............)))))))))).)))))).).).))).))))... (-44.82)

2/58735283-58735350(-)_novel_dog_32
AGGAGGGCGGGAGTAGTTCAGCAGGAAAGGGCCTAGCCCTTCGTGCACTACTTCTCTGCCCACAGGCT
...AGGGCGGGAGTAGTTCAGCAGGA..........................................   5
...AGGGCGGGAGTAGTTCAGCAGGAA.........................................   5
.........................................CGTGCACTACTTCTCTGCCCACA....   5
...AGGGCGGGAGTAGTTCAGC..............................................   1
........................................TCGTGCACTACTTCTCTGCCCAC.....   1
.........................................CGTGCACTACTTCTCTGCCCAC.....   1
....(((((((((((((...(((.(((.((((...))))))).)))))))).)).)))))).......(-32.10)

2/59454083-59454151(-)_mir_dog_122
AGCTGGTGTTGTGAATCATGCCGACGAGCAGCGCATCCTCTTACCCGGCTATTTCACGACACCAGGGTT
...............................................GCTATTTCACGACACCAGGGT.  562
AGCTGGTGTTGTGAATC....................................................  426
...............................................GCTATTTCACGACACCAGGGTT  211
AGCTGGTGTTGTGAATCA...................................................  167
AGCTGGTGTTGTGAATCATGCCG..............................................  96
.......................ACGAGCAGCGCATCCTCTTACCCG......................  41
AGCTGGTGTTGTGAAT.....................................................  33
.......................ACGAGCAGCGCATCCTCTTACCC.......................  24
................................................CTATTTCACGACACCAGGGTT  23
...............................................GCTATTTCACGACACCAGGG..  18
AGCTGGTGTTGTGAATCATGC................................................  9
.................................................TATTTCACGACACCAGGGTT  4
.......................ACGAGCAGCGCATCCTCTTACC........................  3
AGCTGGTGTTGTGAATCAT..................................................  2
............................................CCGGCTATTTCACGACACCAGGGT.  2
................................................CTATTTCACGACACCAGGGT.  2
.................................................TATTTCACGACACCAGGGT.  2
..................................................ATTTCACGACACCAGGGT.  2
AGCTGGTGTTGTGAATCATGCC...............................................  1
......................GACGAGCAGCGCATCCTCTTACCCGG.....................  1
......................................TCTTACCCGGCTATTTCACGA..........  1
...........................................CCCGGCTATTTCACGACACCAGG...  1
...............................................GCTATTTCACGACACCAGG...  1
.....................................................TCACGACACCAGGGTT  1
..(((((((((((((....((((..(((.((......)))))...))))...))))))))))))).... (-29.30)

2/6117323-6117388(-)_novel_dog_31
GACTATACTTTCAGGGATCATTTCTATAGTTTGTTACTAGAGAAGTTTCTCTGAACGTGTAGGGTA
```

```
..........................................AAGTTTCTCTGAACGTGTAGGGT.     2907
..........................................AAGTTTCTCTGAACGTGTAGGG..     364
...........................................AGTTTCTCTGAACGTGTAGGGT.     335
..........................................AAGTTTCTCTGAACGTGTAGG...     104
..........................................AAGTTTCTCTGAACGTGTAGGGTA     100
.....TACTTTCAGGGATCATTTCT.........................................     66
.........................................GAAGTTTCTCTGAACGTGTAGGG..     29
.....TACTTTCAGGGATCATTTCTA........................................     26
.........................................GAAGTTTCTCTGAACGTGTAGGGT.     26
...........................................AGTTTCTCTGAACGTGTAGGGTA     24
........................................AGAAGTTTCTCTGAACGTGTAGGGT.     16
...........................................AGTTTCTCTGAACGTGTAGGG..     15
.........................................GAAGTTTCTCTGAACGTGTAGG...     11
......................................AGAGAAGTTTCTCTGAACGTGTAGGGT.     11
................................GTTACTAGAGAAGTTTCTCTGAACGTGTAGGGT.     6
.......................................GAGAAGTTTCTCTGAACGTGTAGGGT.     6
........................................AGAAGTTTCTCTGAACGTGTAGGG..     5
.....TACTTTCAGGGATCATTTC..........................................     4
..........................................AAGTTTCTCTGAACGTGTA.....     4
....ATACTTTCAGGGATCATTTCT.........................................     3
..........................................AAGTTTCTCTGAACGTGT......     3
..........................................AAGTTTCTCTGAACGTGTAG....     3
...........................................AGTTTCTCTGAACGTGTAGG...     3
............................................GTTTCTCTGAACGTGTAGGGT.     3
.......................................GAGAAGTTTCTCTGAACGTGT......     2
.......................................GAGAAGTTTCTCTGAACGTGTAGG...     2
..................................TACTAGAGAAGTTTCTCTGAACGTGTAGGGT.     2
...................................ACTAGAGAAGTTTCTCTGAACGTGTAGGGT.     2
..............................................TTCTCTGAACGTGTAGGGT.     2
.........................................GAAGTTTCTCTGAACGTGTAGGGTA     2
..CTATACTTTCAGGGAT................................................     1
...TATACTTTCAGGGATC...............................................     1
...TATACTTTCAGGGATCATTTCT.........................................     1
....ATACTTTCAGGGATCATTTCTA........................................     1
........TTTCAGGGATCATTTCTATAGT....................................     1
....................TTTCTATAGTTTGTTACT............................     1
...................ATTTCTATAGTTTGTTACTAGAG........................     1
....................TTTCTATAGTTTGTTACTAGAG........................     1
..........................TAGTTTGTTACTAGAGAAGTTTCTC...............     1
........................TATAGTTTGTTACTAGAGAAGTTTCTCTGA............     1
........................................AGAAGTTTCTCTGAAC..........     1
...................................ACTAGAGAAGTTTCTCTGAACG.........     1
.......................................GAGAAGTTTCTCTGAACG.........     1
..........................................AAGTTTCTCTGAACGTG.......     1
.........................................GAAGTTTCTCTGAACGTGT......     1
.......................................GAGAAGTTTCTCTGAACGTGTAG....     1
.........................................GAAGTTTCTCTGAACGTGTAG....     1
...................................ACTAGAGAAGTTTCTCTGAACGTGTAGGG..     1
.......................................GAGAAGTTTCTCTGAACGTGTAGGG..     1
.........................ATAGTTTGTTACTAGAGAAGTTTCTCTGAACGTGTAGGGT.     1
...........................AGTTTGTTACTAGAGAAGTTTCTCTGAACGTGTAGGGT.     1
.................................................TCTGAACGTGTAGGGT.     1
............................................GTTTCTCTGAACGTGTAGGGTA     1
...............................................TCTCTGAACGTGTAGGGTA     1
................................................CTCTGAACGTGTAGGGTA     1
..((((((.((((((((..((((((.((((.....)))).)))))).)))))))).))))))....  (-25.50)

26/29296014-29296069(-)_mir_dog_123
TGGAGAGAAAGGCAGTTCCTGATGGTCCCCTCCCCAGGGGCTGGCTTTCCTCTGGT
TGGAGAGAAAGGCAGTTCCTGA..................................   3350
TGGAGAGAAAGGCAGTTCCTGAT.................................   779
TGGAGAGAAAGGCAGTTCCTG...................................   455
..................................CAGGGGCTGGCTTTCCTCTGGT   432
TGGAGAGAAAGGCAGTTCCT....................................   230
```

```
...................................AGGGGCTGGCTTTCCTCTGGT   223
...................................AGGGGCTGGCTTTCCTCTGG.   56
TGGAGAGAAAGGCAGTTCC.....................................   47
..................................CAGGGGCTGGCTTTCCTCTGG.   16
..................................CAGGGGCTGGCTTTCCTC....   6
..................................CAGGGGCTGGCTTTCCTCT...   5
....GAGAAAGGCAGTTCCTG...................................   4
TGGAGAGAAAGGCAGTTCCTGATG................................   4
..................................CAGGGGCTGGCTTTCCTCTG..   4
TGGAGAGAAAGGCAGTTC......................................   2
...................................AGGGGCTGGCTTTCCTCT...   2
...................................AGGGGCTGGCTTTCCTCTG..   2
TGGAGAGAAAGGCAGTT.......................................   1
...AGAGAAAGGCAGTTCCTG...................................   1
...AGAGAAAGGCAGTTCCTGA..................................   1
......GAAAGGCAGTTCCTGA..................................   1
..............................TCCCCAGGGGCTGGCTTTCCTCT...   1
........................................CTGGCTTTCCTCTGGT   1
.((((.(((((.(((((((((..((......)).))))))))).))))))))... (-28.30)

26/30744641-30744709(+)_novel_dog_61
GGCTGCCGGGGGTCAGGTGGACTAGAGCACAGACATAGGGCACCTCTATGTCTCCCTGGCCTCCAGGAC
.....CCGGGGGTCAGGTGGACTAGA...........................................  16
.....CCGGGGGTCAGGTGGACTAGAG..........................................  14
............................................TCTATGTCTCCCTGGCCTCCAG...  14
.....CCGGGGGTCAGGTGGACTAGAGC.........................................  12
.....CCGGGGGTCAGGTGGACTAG............................................  6
............................................TCTATGTCTCCCTGGCCTCCA....  5
......CGGGGGTCAGGTGGACTAGAGC.........................................  4
............................................TCTATGTCTCCCTGGCCTCC.....  4
....GCCGGGGGTCAGGTGGACTAGA...........................................  2
...TGCCGGGGGTCAGGTGGACT..............................................  1
...TGCCGGGGGTCAGGTGGACTA.............................................  1
...TGCCGGGGGTCAGGTGGACTAG............................................  1
....GCCGGGGGTCAGGTGGACTAG............................................  1
....GCCGGGGGTCAGGTGGACTAGAGC.........................................  1
............................................TCTATGTCTCCCTGGCCT.......  1
............................................TCTATGTCTCCCTGGCCTC......  1
.............................................CTATGTCTCCCTGGCCTC......  1
..........................................CCTCTATGTCTCCCTGGCCTCC.....  1
...........................................CTCTATGTCTCCCTGGCCTCCA....  1
.............................................CTATGTCTCCCTGGCCTCCAG...  1
.....((((((((((((..((((((((..(........)....))))).)))..)))))))))).)).. (-31.20)

26/30924103-30924178(+)_mir_dog_124
CCCAGGTGCTCTGACGAGGTTGCACTACTGTGCTCTGAGAAGCAGTGCAATGATATTGTCAAAGCATCGGGGACCA
..........................................CAGTGCAATGATATTGTCAAAGC........... 2834
..........................................CAGTGCAATGATATTGTCAAAG............ 143
...........................................AGTGCAATGATATTGTCAAAGC........... 87
..........................................CAGTGCAATGATATTGTCAAAGCA.......... 11
..........................................CAGTGCAATGATATTGTCAAAGCAT......... 11
...........................................AGTGCAATGATATTGTCAAAG............ 4
.......GCTCTGACGAGGTTGCACTACT............................................... 3
..........................................CAGTGCAATGATATTG.................. 2
.............................................TGCAATGATATTGTCAAAGC........... 2
.......GCTCTGACGAGGTTGCACTACTGTGCTC......................................... 1
..........................................CAGTGCAATGATATTGTCA............... 1
..........................................CAGTGCAATGATATTGTCAAA............. 1
..............................................GCAATGATATTGTCAAAG............ 1
...........................................AGTGCAATGATATTGTCAAAGCA.......... 1
(((.((((((.((((((.((((((((.((...(.....).)).))))))))....)))))).)))))).)))....  (-32.00)
```

```
26/30924432-30924491(+)_mir_dog_125
ACTCTTTCCCTGTTGCACTACTGTGGGCCGCTGGGAAGCAGTGCAATGATGAAAGGGCAT
ACTCTTTCCCTGTTGCACTACT......................................     1979
......................................CAGTGCAATGATGAAAGGGCA.     1270
......................................CAGTGCAATGATGAAAGGGCAT     712
......................................CAGTGCAATGATGAAAGGGC..     287
ACTCTTTCCCTGTTGCACTAC.......................................     187
ACTCTTTCCCTGTTGCACTACTG.....................................     98
ACTCTTTCCCTGTTGCACTA........................................     58
.......................................AGTGCAATGATGAAAGGGCAT     42
ACTCTTTCCCTGTTGCACT.........................................     19
......................................CAGTGCAATGATGAAAGGG...     6
.......................................AGTGCAATGATGAAAGGGCA.     6
..........................................GCAATGATGAAAGGGCA.     4
.....................................GCAGTGCAATGATGAAAGGGCA.     2
ACTCTTTCCCTGTTGCAC..........................................     1
..TCTTTCCCTGTTGCACTACT......................................     1
...CTTTCCCTGTTGCACTACT......................................     1
.......................................AGTGCAATGATGAAAGGGC..     1
..........................................GCAATGATGAAAGGGC..     1
..........................................GCAATGATGAAAGGGCAT     1
...........................................CAATGATGAAAGGGCAT     1
............................................AATGATGAAAGGGCAT     1
.(((((((..(((((((((.((.....((....)).)).)))))))))..)))))))... (-20.60)

2/65300467-65300531(-)_novel_Dog_Blast_25
GTCTTGGCGCGACTTCCTCGACTCACTCTCTTTCCCCCGTGAGTCGTGGAACCTCGCCCGAGGGT
.......................................TGAGTCGTGGAACCTCGCCCGA....      83
.........................................AGTCGTGGAACCTCGCCCGAG...      9
.........................................AGTCGTGGAACCTCGCCCGA....      6
.........................................AGTCGTGGAACCTCGCCCGAGG..      5
...TTGGCGCGACTTCCTCGACTC.........................................      4
....TGGCGCGACTTCCTCGACT..........................................      3
....TGGCGCGACTTCCTCGACTCACT......................................      3
.......................................TGAGTCGTGGAACCTCGCCC......      3
.......................................TGAGTCGTGGAACCTCGCCCGAG...      3
....TGGCGCGACTTCCTCGACTCA........................................      2
.......................................TGAGTCGTGGAACCTCGCCCG.....      2
...TTGGCGCGACTTCCTCGACT..........................................      1
....TGGCGCGACTTCCTCGACTC.........................................      1
....TGGCGCGACTTCCTCGACTCAC.......................................      1
...TTGGCGCGACTTCCTCGACTCACT......................................      1
.......CGCGACTTCCTCGACTCACT......................................      1
..............................TTTCCCCCGTGAGTCGTGGAACC............      1
........................................GAGTCGTGGAACCTCGCCCGA....      1
..................................CCCCGTGAGTCGTGGAACCTCGCCCGAGG..      1
...................................CCCGTGAGTCGTGGAACCTCGCCCGAGG..      1
.....................................CGTGAGTCGTGGAACCTCGCCCGAGG..      1
......................................GTGAGTCGTGGAACCTCGCCCGAGGGT      1
.((((((.((((.((((.((((((((............)))))))).))))..)))))))))).. (-31.20)

2/65306514-65306578(-)_novel_Dog_Blast_26
GTCTTGGCGCGACTTCCTCGACTCACTCTCTTTCCCCCGTGAGTCGTGGAACCTCGCCCGAGGGT
.......................................TGAGTCGTGGAACCTCGCCCGA....      83
.........................................AGTCGTGGAACCTCGCCCGAG...      9
.........................................AGTCGTGGAACCTCGCCCGA....      6
.........................................AGTCGTGGAACCTCGCCCGAGG..      5
...TTGGCGCGACTTCCTCGACTC.........................................      4
....TGGCGCGACTTCCTCGACT..........................................      3
....TGGCGCGACTTCCTCGACTCACT......................................      3
.......................................TGAGTCGTGGAACCTCGCCC......      3
.......................................TGAGTCGTGGAACCTCGCCCGAG...      3
....TGGCGCGACTTCCTCGACTCA........................................      2
.......................................TGAGTCGTGGAACCTCGCCCG.....      2
```

```
...TTGGCGCGACTTCCTCGACT..........................................     1
....TGGCGCGACTTCCTCGACTC.........................................     1
....TGGCGCGACTTCCTCGACTCAC.......................................     1
...TTGGCGCGACTTCCTCGACTCACT......................................     1
.......CGCGACTTCCTCGACTCACT......................................     1
..............................TTTCCCCCGTGAGTCGTGGAACC............     1
........................................GAGTCGTGGAACCTCGCCCGA....     1
..................................CCCCGTGAGTCGTGGAACCTCGCCCGAGG..     1
...................................CCCGTGAGTCGTGGAACCTCGCCCGAGG..     1
.....................................CGTGAGTCGTGGAACCTCGCCCGAGG..     1
......................................GTGAGTCGTGGAACCTCGCCCGAGGGT     1
.((((((.((((.((((.((((((((............)))))))).))))..)))))))))).. (-31.20)

2/65930312-65930378(-)_novel_dog_33
CTGTAGCAGTCTCTGCAGAGGAAAGATGTGGATGGCCAGATACTCTTCCTCAAGGGACTACACCAGT
...TAGCAGTCTCTGCAGAGGAAAGA..........................................    9
...........................................TCTTCCTCAAGGGACTACACC...    3
...TAGCAGTCTCTGCAGAGGA.............................................    2
...TAGCAGTCTCTGCAGAGGAA............................................    1
...TAGCAGTCTCTGCAGAGGAAA...........................................    1
.........................................ACTCTTCCTCAAGGGACTACACC...    1
(((..(.(((((((...(((((((((.(((....))).)).)).))))))).))))))).)..))). (-24.60)

26/774514-774584(+)_novel_dog_60
ACCCACCAGGAGTCTGACGTGGCCTCAGAGAGGACATCGAGTCTCCTGGGCTCATCAGACTCCGTGGTCGA
............................................CCTGGGCTCATCAGACTCCGTGG....      5
.......AGGAGTCTGACGTGGCCTCAGA..........................................      1
.......AGGAGTCTGACGTGGCCTCAGAGA........................................      1
...........................................TCCTGGGCTCATCAGACTCCG.......      1
...........................................TCCTGGGCTCATCAGACTCCGT......      1
...........................................TCCTGGGCTCATCAGACTCCGTG.....      1
...........................................TCCTGGGCTCATCAGACTCCGTGG....      1
.............................................CTGGGCTCATCAGACTCCGTGG....      1
..(.(((((((((((((.(.((((.(((.(((.((.....))))))))))))).))))))))).)))).). (-35.00)

27/1230447-1230519(-)_mir_dog_126
GGAGGGGGGTCCCCGGAGCTCGGTCTCGAGGGTGCTTATTGTTCGGTCCGAGCCCGGGTCTCCCTCTCCCCCC
..............................................TCCGAGCCCGGGTCTCCCTCT......    17
....GGGGGTCCCCGGAGCTCGGTCT...............................................    3
..............................................TCCGAGCCCGGGTCTCCCT........    2
.....GGGGTCCCCGGAGCTCGGTCT...............................................    1
....GGGGGTCCCCGGAGCTCGGTCTCGA............................................    1
............................................GGTCCGAGCCCGGGTCTCCCTCT......    1
(((((((((..(((((.((((((.(.((((.(.......).))))).)))))))))))..))))))))).... (-
46.30)

27/1270718-1270776(-)_mir_dog_127
TAGGTAGTTTCATGTTGTTGGGATTGAGTTTTGAACTCGGCAACAAGAAACTGCCTGAG
TAGGTAGTTTCATGTTGTTGGGA....................................1094
TAGGTAGTTTCATGTTGTTGGG.....................................924
TAGGTAGTTTCATGTTGTTGG......................................399
TAGGTAGTTTCATGTTGTTG.......................................86
....................................TCGGCAACAAGAAACTGCCTGA.62
TAGGTAGTTTCATGTTGTTGGGAT...................................52
TAGGTAGTTTCATGTTGTT........................................11
TAGGTAGTTTCATGTTGT.........................................10
....................................TCGGCAACAAGAAACTGCCTGAG10
TAGGTAGTTTCATGTTGTTGGGATT..................................7
.AGGTAGTTTCATGTTGTTGGGA....................................4
....................................TCGGCAACAAGAAACTGCCT...3
....................................TCGGCAACAAGAAACTGCC....2
.....................................CGGCAACAAGAAACTGCCTGAG2
TAGGTAGTTTCATGTTG..........................................1
.AGGTAGTTTCATGTTGTTG.......................................1
```

```
.AGGTAGTTTCATGTTGTTGGG.....................................1
..GGTAGTTTCATGTTGTTGGG.....................................1
.AGGTAGTTTCATGTTGTTGGGAT...................................1
.....................................CGGCAACAAGAAACTGCCTGA.1
(((((((((((.((((((((((.(..(...)..).)))))))))).))))))))))).. (-28.50)

2/73233563-73233627(+)_novel_Dog_Blast_27
GTCTTGGCGCGACTTCCTCGACTCACTCTCTTTCCCCGTGAGTCGTGGAACCTCGCCCGAGGGT
......................................TGAGTCGTGGAACCTCGCCCGA....      83
........................................AGTCGTGGAACCTCGCCCGAG...      9
........................................AGTCGTGGAACCTCGCCCGA....      6
........................................AGTCGTGGAACCTCGCCCGAGG..      5
...TTGGCGCGACTTCCTCGACTC........................................      4
....TGGCGCGACTTCCTCGACT.........................................      3
....TGGCGCGACTTCCTCGACTCACT.....................................      3
......................................TGAGTCGTGGAACCTCGCCC......      3
......................................TGAGTCGTGGAACCTCGCCCGAG...      3
....TGGCGCGACTTCCTCGACTCA.......................................      2
......................................TGAGTCGTGGAACCTCGCCCG.....      2
...TTGGCGCGACTTCCTCGACT.........................................      1
....TGGCGCGACTTCCTCGACTC........................................      1
....TGGCGCGACTTCCTCGACTCAC......................................      1
...TTGGCGCGACTTCCTCGACTCACT.....................................      1
.......CGCGACTTCCTCGACTCACT.....................................      1
..............................TTTCCCCGTGAGTCGTGGAACC............      1
.......................................GAGTCGTGGAACCTCGCCCGA....      1
.................................CCCCGTGAGTCGTGGAACCTCGCCCGAGG..      1
..................................CCCGTGAGTCGTGGAACCTCGCCCGAGG..      1
....................................CGTGAGTCGTGGAACCTCGCCCGAGG..      1
.....................................GTGAGTCGTGGAACCTCGCCCGAGGGT      1
.((((((.((((.((((.((((((((...........)))))))).))))..)))))))))).. (-31.20)

27/38081668-38081753(-)_mir_dog_128
GCCGGCTCTGGGTCCATCTTCCAGTACAGTGTTGGATGGTCTAGTCACGAAGCTCCTAACACTGTCTGGTAAAGATGGCC
CCCGGG
........................................................TAACACTGTCTGGTAAAGATG...
......       19959
........................................................TAACACTGTCTGGTAAAGATGGC.
......       686
..............CATCTTCCAGTACAGTGTTGGA............................................
......       413
........................................................TAACACTGTCTGGTAAAGA.....
......       388
........................................................TAACACTGTCTGGTAAAGATGG..
......       384
.......................................................CTAACACTGTCTGGTAAAGATG...
......       189
........................................................TAACACTGTCTGGTAAAG......
......       130
........................................................TAACACTGTCTGGTAAAGAT....
......       111
..............CATCTTCCAGTACAGTGTTGGAT...........................................
......       26
..............CATCTTCCAGTACAGTGTTGG.............................................
......       7
.......................................................CTAACACTGTCTGGTAAAGAT....
......       6
.........................................................AACACTGTCTGGTAAAGATG...
......       6
.............................................................CTGTCTGGTAAAGATG...
......       6
..........................................................ACACTGTCTGGTAAAGATG...
......       3
........................................................TAACACTGTCTGGTAAA.......
```

```
......      2
.....................................................TCCTAACACTGTCTGGTAAAG......
......      2
.......................................................CTAACACTGTCTGGTAAAG......
......      2
.............................................................CTGTCTGGTAAAGATGG..
......      2
........................................................TAACACTGTCTGGTAAAGATGGCC
C.....      2
...............ATCTTCCAGTACAGTGTTGGA............................................
......      1
...............ATCTTCCAGTACAGTGTTGGAT...........................................
......      1
........................................................TAACACTGTCTGGTAA........
......      1
.....................................................TCCTAACACTGTCTGGTAAAGA.....
......      1
.......................................................CTAACACTGTCTGGTAAAGA.....
......      1
...........................................................CACTGTCTGGTAAAGATG...
......      1
.........................................................AACACTGTCTGGTAAAGATGGC.
......      1
..............................................................TGTCTGGTAAAGATGGC.
......      1
...............................................................GTCTGGTAAAGATGGC.
......      1
.((((....(((.(((((((((((.(((((((((((...
((.......))...))).))))))))))))..)))))))))))))). (-41.20)

27/38082097-38082159(-)_mir_dog_129
CGTCTTACCCAGCAGTGTTTGGGTGCTGGTTGGGAGTCTCTAATACTGCCGGGTAATGATGGA
........................................TAATACTGCCGGGTAATGATGGA  1136
........................................TAATACTGCCGGGTAATGATGG.  546
........................................TAATACTGCCGGGTAATGA....  64
........................................TAATACTGCCGGGTAATGAT...  57
........................................TAATACTGCCGGGTAATGATG..  40
.......................................CTAATACTGCCGGGTAATGAT...  10
.......................................CTAATACTGCCGGGTAATGATG..  6
.......................................CTAATACTGCCGGGTAATGATGG.  5
.......................................CTAATACTGCCGGGTAATGA....  4
CGTCTTACCCAGCAGTGTTTGG.........................................  2
...........................................TACTGCCGGGTAATGATGG.  2
.......................................CTAATACTGCCGGGTAATGATGGA  2
.GTCTTACCCAGCAGTGTTTGGG.......................................  1
..TCTTACCCAGCAGTGTTTGGG.......................................  1
........................................TAATACTGCCGGGTAA.......  1
.........................................AATACTGCCGGGTAATGATG..  1
..........................................ATACTGCCGGGTAATGATGG.  1
((((((((((.((((((((.(((.(((.......)))))).)))))))).)))))).)))).. (-28.10)

2/7759755-7759820(-)_novel_Dog_Blast_28
GTCTTGGCGCGACTTCCTCGACTCACTCTCTTTCCCCCCGTGAGTCGTGGAACCTCGCCCGAGGGT
........................................TGAGTCGTGGAACCTCGCCCGA....      83
..........................................AGTCGTGGAACCTCGCCCGAG...      9
..........................................AGTCGTGGAACCTCGCCCGA....      6
..........................................AGTCGTGGAACCTCGCCCGAGG..      5
...TTGGCGCGACTTCCTCGACTC...........................................      4
....TGGCGCGACTTCCTCGACT...........................................      3
....TGGCGCGACTTCCTCGACTCACT.......................................      3
........................................TGAGTCGTGGAACCTCGCCC......      3
........................................TGAGTCGTGGAACCTCGCCCGAG...      3
....TGGCGCGACTTCCTCGACTCA.........................................      2
........................................TGAGTCGTGGAACCTCGCCCG.....      2
```

...TTGGCGCGACTTCCTCGACT... 1
....TGGCGCGACTTCCTCGACTC.. 1
....TGGCGCGACTTCCTCGACTCAC.. 1
...TTGGCGCGACTTCCTCGACTCACT....................................... 1
.......CGCGACTTCCTCGACTCACT....................................... 1
....GAGTCGTGGAACCTCGCCCGA.... 1
...................................CCCCGTGAGTCGTGGAACCTCGCCCGAGG.. 1
....................................CCCGTGAGTCGTGGAACCTCGCCCGAGG.. 1
......................................CGTGAGTCGTGGAACCTCGCCCGAGG.. 1
....GTGAGTCGTGGAACCTCGCCCGAGGGT 1
.((((((.((((.((((.((((((((.............)))))))).)))).))))))))))).. (-31.12)

27/975146-975206(-)_mir_dog_130
GAAGTTCTGTTATACACTCAGGCTGTGGCTCTCTGAAAGTCAGTGCATCACAGAACTTTGT
....TCAGTGCATCACAGAACTTTGT 20379
GAAGTTCTGTTATACACTCAGGCT..................................... 5314
GAAGTTCTGTTATACACTCAGGC...................................... 1485
....TCAGTGCATCACAGAACTTTG. 1081
.AAGTTCTGTTATACACTCAGGCT..................................... 932
....TCAGTGCATCACAGAACTTT.. 234
GAAGTTCTGTTATACACTCAGG....................................... 199
....TCAGTGCATCACAGAACTT... 160
....TCAGTGCATCACAGAACT.... 102
.AAGTTCTGTTATACACTCAGGC...................................... 79
GAAGTTCTGTTATACACTCAG.. 29
GAAGTTCTGTTATACACTCA... 28
GAAGTTCTGTTATACACTC.. 21
....AGTGCATCACAGAACTTTGT 13
....TCAGTGCATCACAGAAC..... 9
....CAGTGCATCACAGAACTTTGT 7
.AAGTTCTGTTATACACTCAGG....................................... 6
...GTTCTGTTATACACTCAGGCT..................................... 6
.AAGTTCTGTTATACACTCAG.. 1
.....TCTGTTATACACTCAGGC...................................... 1
..AGTTCTGTTATACACTCAGGCT..................................... 1
......TGTTATACACTCAGGCT...................................... 1
............TACACTCAGGCTGTGGCTCTCT........................... 1
....CAGTGCATCACAGAACTT... 1
....GCATCACAGAACTTTG. 1
....TGCATCACAGAACTTTGT 1
((((((((((.((.((((..((((....(.....)..)))))))).)).)))))))))).. (-18.80)

28/11089077-11089148(-)_mir_dog_131
TGGGGAGGTGCCATTCTGAGGGCCAAGAGTTTGATTATGTGTCACTCTGGCTGCTATGGCCCCCTCCCAGGG
....TCTGGCTGCTATGGCCCCCT....... 475
....TCTGGCTGCTATGGCCCCCTC...... 147
....TCTGGCTGCTATGGCCCCC........ 38
.....AGGTGCCATTCTGAGGGCCAAGAGT.. 37
.....AGGTGCCATTCTGAGGGCCAAG... 12
....TCTGGCTGCTATGGCCCC......... 8
.....AGGTGCCATTCTGAGGGCCAAGAG... 6
.....AGGTGCCATTCTGAGGGCC.. 3
.....AGGTGCCATTCTGAGGGCCAAGA.. 2
.....AGGTGCCATTCTGAGGGCCAAGAGTT... 2
....TCTGGCTGCTATGGCCCCCTCC..... 2
.....AGGTGCCATTCTGAGGGCCAAGAGTTT.. 1
..............TCTGAGGGCCAAGAGTTTGAT..................................... 1
...............CTGAGGGCCAAGAGTTTGATTA................................... 1
..((((((.(((((...(..(((((.((((..(((.....)))))))))))).).)))))..)))))).... (-
31.80)

2/81472850-81472915(+)_novel_dog_30
GGGAGGAGGGCTAAGGCGGCCGGTAAGCAGGGGCACCCAGCCAGGCTCCTTGACCCTCCTCCAGAT
....CAGGCTCCTTGACCCTCCTCC.... 4

```
.........................................CAGGCTCCTTGACCCTCCTCCA...     4
.........................................CAGGCTCCTTGACCCTCCTCCAGA.     2
GGGAGGAGGGCTAAGGCGGCCG............................................     1
.........................................CAGGCTCCTTGACCCTCCT......     1
.......................................GCCAGGCTCCTTGACCCTCCTCCAG..     1
.........................................CAGGCTCCTTGACCCTCCTCCAG..     1
..........................................AGGCTCCTTGACCCTCCTCCAG..     1
............................................GCTCCTTGACCCTCCTCCAGA.     1
.(((((((((.(((((.(((((((......((....)).))).))))))))).))))))))).... (-37.30)

28/14940550-14940610(+)_mir_dog_132
TGAGAACTGAATTCCATAGGCTGTGAGCTTGAGCAAACAGCCTAGGGACTCAGTTCTGGTG
TGAGAACTGAATTCCATAGGCTGT.....................................    124601
TGAGAACTGAATTCCATAGGCTG......................................    24583
TGAGAACTGAATTCCATAGGCT.......................................    3512
TGAGAACTGAATTCCATAGGC........................................    548
.....ACTGAATTCCATAGGCTGT.....................................    272
TGAGAACTGAATTCCATAGG.........................................    109
.GAGAACTGAATTCCATAGGCTGT.....................................    108
TGAGAACTGAATTCCATAGGCTGTGAGCTT...............................    85
.....ACTGAATTCCATAGGCTG......................................    59
......CTGAATTCCATAGGCTGT.....................................    29
......................................AGCCTAGGGACTCAGTTCTGGT.    20
TGAGAACTGAATTCCA.............................................    17
..AGAACTGAATTCCATAGGCTGT.....................................    17
....AACTGAATTCCATAGGCTGT.....................................    14
...GAACTGAATTCCATAGGCTGT.....................................    13
TGAGAACTGAATTCCATAG..........................................    11
........GAATTCCATAGGCTGT.....................................    11
TGAGAACTGAATTCCATAGGCTGTG....................................    11
TGAGAACTGAATTCCATAGGCTGTGA...................................    8
..AGAACTGAATTCCATAGGCTG......................................    7
....AACTGAATTCCATAGGCTG......................................    7
.......TGAATTCCATAGGCTGT.....................................    7
......................................AGCCTAGGGACTCAGTTCTGG..    6
.GAGAACTGAATTCCATAGGCTG......................................    5
......CTGAATTCCATAGGCTG......................................    5
TGAGAACTGAATTCCATAGGCTGTGAGCT................................    3
.GAGAACTGAATTCCATAGGC........................................    2
.......TGAATTCCATAGGCTG......................................    2
TGAGAACTGAATTCCATA...........................................    1
...GAACTGAATTCCATAGGC........................................    1
.GAGAACTGAATTCCATAGGCT.......................................    1
...GAACTGAATTCCATAGGCTG......................................    1
TGAGAACTGAATTCCATAGGCTGTGAGC.................................    1
TGAGAACTGAATTCCATAGGCTGTGAGCTTGAGCAAACAGCC...................    1
..((((((((.((((.((((((((..((....))..)))))))))))).)))))))).... (-32.50)

28/15765927-15765986(-)_mir_dog_133
CGACCGGACCTCGACCGGCTCGTCTATGTTGCCAATCGACTCGGCGTGGCGTCGGTCGTG
......................................ACTCGGCGTGGCGTCGGTCGTG     1318
......................................ACTCGGCGTGGCGTCGGTCGT.     990
.......................................CTCGGCGTGGCGTCGGTCGTG     883
......................................ACTCGGCGTGGCGTCGGTCG..     179
.....GGACCTCGACCGGCTCG......................................     79
.......................................CTCGGCGTGGCGTCGGTCGT.     53
CGACCGGACCTCGACCGGCT........................................     45
......................................ACTCGGCGTGGCGTCGGTC...     42
..ACCGGACCTCGACCGGCTCG......................................     28
.......................................CTCGGCGTGGCGTCGGTCG..     23
CGACCGGACCTCGACCGGCTC.......................................     15
.GACCGGACCTCGACCGGCT........................................     14
....CGGACCTCGACCGGCT........................................     12
......................................ACTCGGCGTGGCGTCGGT....     10
```

..ACCGGACCTCGACCGGCT.. 9
CGACCGGACCTCGACCGG.. 8
CGACCGGACCTCGACCG... 7
.GACCGGACCTCGACCGGCTC....................................... 7
CGACCGGACCTCGACCGGCTCG...................................... 7
.....GGACCTCGACCGGCTC....................................... 5
....CTCGGCGTGGCGTCGGTC... 5
....CGGACCTCGACCGGCTCG...................................... 4
......................................ACTCGGCGTGGCGTCG...... 4
...CCGGACCTCGACCGGCT.. 2
..ACCGGACCTCGACCGGCTC....................................... 2
.....GGACCTCGACCGGCTCGT..................................... 2
CGACCGGACCTCGACC.. 1
.GACCGGACCTCGACCGG.. 1
...CCGGACCTCGACCGGC... 1
...CCGGACCTCGACCGGCTC....................................... 1
....CGGACCTCGACCGGCTC....................................... 1
.GACCGGACCTCGACCGGCTCG...................................... 1
......GACCTCGACCGGCTCG...................................... 1
..........TCGACCGGCTCGTCTATGTTGC............................ 1
.....................GTCTATGTTGCCAATCGA..................... 1
....TCGGCGTGGCGTCGGTCGT. 1
....GGCGTGGCGTCGGTCGT. 1
....TCGGCGTGGCGTCGGTCGTG 1
(((((((.((.((.(((..(((..............)))..))))).))..))))))).. (-23.04)

28/23373851-23373914(+)_mir_dog_134
GTCCACAGGCACAGCCGCTTTGAGCATTTTCACTGAATTGCCAAACCAGTTGTGCCTGTAGACA
....ACAGGCACAGCCGCTTTGAGCA...................................... 44
....ACAGGCACAGCCGCTTTGAGC....................................... 18
....CCAAACCAGTTGTGCCTGTAG... 8
....ACAGGCACAGCCGCTTTGAGCAT..................................... 6
....CCAAACCAGTTGTGCCTGTA.... 2
....CCAAACCAGTTGTGCCTGTAGA.. 2
....ACAGGCACAGCCGCTTTGAGCATT.................................... 1
....................................ATTGCCAAACCAGTTGTGCCTGT..... 1
.....................................TTGCCAAACCAGTTGTGCCTGT..... 1
(((.(((((((((((.(.((((.(((.(((....))).))))))).).))))))))))).))). (-30.60)

28/27372107-27372166(-)_mir_dog_135
[truncated: 464,152 more chars]
